# Genome sequencing and molecular characterisation of *Staphylococcus aureus* ST772-MRSA-V, “Bengal Bay Clone”

**DOI:** 10.1186/1756-0500-6-548

**Published:** 2013-12-20

**Authors:** Stefan Monecke, Vico Baier, Geoffrey W Coombs, Peter Slickers, Albrecht Ziegler, Ralf Ehricht

**Affiliations:** 1 Institute for Medical Microbiology and Hygiene, Technical University of Dresden, Dresden, Germany; 2 Alere Technologies GmbH, Jena, Germany; 3 Australian Collaborating Centre for Enterococcus and Staphylococcus Species (ACCESS) Typing and Research, Curtin University, Perth, Western Australia; 4 Department of Microbiology and Infectious Diseases, PathWest Laboratory Medicine - WA, Royal Perth Hospital, Perth, Western Australia

## Abstract

**Background:**

The PVL-positive ST772-MRSA-V is an emerging community-associated (CA-) MRSA clone that has been named Bengal Bay Clone since most patients have epidemiological connections to the Indian subcontinent. It is found increasingly common in other areas of the world.

**Methods:**

One isolate of ST772-MRSA-V was sequenced using the Illumina Genome Analyzer System. After initial assembling the multiple sequence contigs were analysed using different in-house annotation scripts. Results were compared to microarray hybridisation results of clinical isolates of ST772-MRSA-V, of related strains and to another ST772-MRSA-V genome sequence.

**Results:**

According to MLST e-burst analysis, ST772-MRSA-V belongs to Clonal Complex (CC)1, differing from ST1 only in one MLST allele (*pta*-22). However, there are several additional differences including *agr* alleles (group II rather than III), capsule type (5 rather than 8), the presence of the *egc* enterotoxin gene cluster and of the enterotoxin homologue ORF CM14 as well as the absence of the enterotoxin H gene *seh*. Enterotoxin genes *sec* and *sel* are present. ST772-MRSA-V harbours the genes encoding enterotoxin A (*sea*) and PVL (*lukS/F-PV*). Both are located on the same prophage.

**Conclusions:**

ST772-MRSA-V may have emerged from the same lineage as globally spread CC1 and CC5 strains. It has acquired a variety of virulence factors, and for a CA-MRSA strain it has an unusually high number of genes associated with antibiotic resistance.

## Background

In recent years the epidemiology of methicillin-resistant *Staphylococcus aureus* (MRSA) has changed with the emergence of community associated MRSA (CA-MRSA) strains. Unlike the original healthcare associated MRSA strains, CA-MRSA strains are no longer restricted to the hospital setting and can persist in and be transmitted by healthy individuals in the community. Some of these strains exhibit an enhanced virulence due to the carriage of a number of virulence genes, including the Panton Valentine leukocidin (PVL) *lukF-PV* and *lukS-PV* determinants. PVL is a phage-borne, bi-component toxin [1] associated with chronic/recurrent skin and soft tissue infections and with necrotising pneumonia and fasciitis [2].

ST772-MRSA-V, colloquially known as the Bengal Bay Clone [3], is a multiresistant PVL-positive CA-MRSA initially isolated in India in 2004/2005 [4]. Transmission of Bengal Bay MRSA has subsequently occurred in several countries including England [3], Ireland [5], Germany (H.J. Linde, Regensburg, Germany, pers. communication; [6]), Norway (H. Aamot, pers. communication), Italy [7,8], Abu Dhabi [6,9], Saudi Arabia [10], Hong Kong [6], Malaysia [11], Australia [12] and New Zealand [13]. Many patients had a travel history or family background suggesting an infection in India, Pakistan or Bangladesh ([3,5,12]; H.J. Linde, Regensburg, Germany, pers. communication; author’s unpublished observations), where this strain appears to be increasingly common [14,15].

In order to identify possible factors promoting its recent emergence and spread we have sequenced the ST772-MRSA-V genome.

## Methods

### Strains

One isolate of ST772-MRSA-V (07–17048) was selected for next generation genome sequencing. It was isolated from an Indian healthcare worker in Western Australia as part of standard patient care in 2007 and submitted to the Australian Collaborating Centre for *Enterococcus* and *Staphylococcus* Species for typing. Thirteen related isolates that were previously submitted for typing purposes to the participating institutions were selected. Their microarray hybridisation profiles where compared to isolate 07–17048 especially with regard to genes associated with resistance or virulence (Table 1, Additional file 1).

**Table 1:** Overview on typing and microarray hybridisation data for CC1 reference strains (nr. 1 and 2), CC5 reference strains (nr. 3 and 4), the strain described herein (nr. 5), other Bengal Bay isolates (nr. 6 to 14) and other, related strains (nr. 15 to 18)

**Nr.**	**Isolate**	**Origin**	**Reference**	**RIDOM *spa***	**MLST**	***agr***	**capsule type**	***mecA***	***Delta mecR***	***ccrA/B-1***	***ccrA/B-2***	***ccrAA/C***	***kdp *** **SCC locus**	***blaZ/R/I***	***erm(C)***	***msr(A) + mph(C)***	***aacA-aphD***	***aadD***	***aphA3 + sat***	***fusC *** **(Q6GD50)**	***tet(K)***	***tet(M)***	***tst1***	***sea***	***sea (N315)/sep***	***sec/sel***	***sed/j/r***	***egc (total)***	***seh***	***sek/q***	***ORF CM14***	***lukF/S-PV***	***lukD/E***	***sak***	***chp***	***scn***	***splA + splB***
1	**MW2**	US	[16]	t128	ST1	III	8	+	+	-	+	-	-	+	-	-	-	-	-	-	-	-	-	+	-	+	-	-	+	+	-	+	+	+	-	+	+
2	**Sanger 476**	UK	[17]	t607	ST1	III	8	-	-	+	-	-	-	+	-	-	-	-	-	+	-	-	-	+	-	-	-	-	+	+	-	-	+	+	-	+	+
3	**N315**	Japan	[18]	t002	ST5	II	5	+	+	-	+	-	+	+	-	-	-	+	-	-	-	-	+	-	+	+	-	+	-	-	-	-	+	+	+	+	+
4	**Mu50**	Japan	[18]	t002	ST5	II	5	+	+	-	+	-	+	-	-	-	+	+	-	-	-	+	+	+	-	+	-	+	-	-	-	-	+	+	-	+	+
5	**07-17048**	WA/India	[6,12]	t3387	ST772	II	5	+	-	-	-	+	-	+	-	+	+	-	+	-	-	-	-	+	-	+	-	+	-	-	+	+	-	-	-	+	-
6	**09 V32405**	Saxony	[6]	n.a.	n.a.	II	5	+	-	-	-	+	-	+	+	+	+	-	+	-	-	-	-	+	-	+	-	+	-	-	+	+	-	-	-	+	-
7	**10 V3510**	Saxony/India	[6]	n.a.	n.a.	II	5	+	-	-	-	+	-	+	-	+	+	-	+	-	-	-	-	+	-	+	-	+	-	-	+	+	-	-	-	+	-
8	**11ANR12420**	Saxony	-	n.a.	n.a.	II	5	+	-	-	-	+	-	+	-	+	+	-	+	-	-	-	-	+	-	+	-	+	-	-	+	+	-	-	-	+	-
9	**12ANR75832**	Saxony	-	n.a.	n.a.	II	5	+	-	-	-	+	-	+	-	+	+	-	+	-	-	-	-	+	-	+	-	+	-	-	+	+	-	-	-	+	-
10	**11-18844**	WA	-	t657	n.a.	II	5	+	-	-	-	+	-	+	-	+	-	-	+	-	-	-	-	+	-	+	-	+	-	-	+	+	-	-	-	+	-
11	**11-18907**	WA	-	t657	n.a.	II	5	+	-	-	-	+	-	+	-	+	+	-	+	-	-	-	-	+	-	+	-	+	-	-	+	+	-	-	-	+	-
12	**11-19350**	WA	-	t4599	n.a.	II	5	+	-	-	-	+	-	+	-	-	+	-	-	-	-	-	-	+	-	+	-	+	-	-	+	+	-	-	-	+	-
13	**11-19415**	WA	-	t657	n.a.	II	5	+	-	-	-	+	-	+	-	+	+	-	+	-	-	-	-	+	-	+	-	+	-	-	+	+	-	-	-	+	-
14	**11-19454**	WA	-	t1075	n.a.	II	5	+	-	-	-	+	-	+	+	+	+	-	+	-	-	-	-	+	-	+	-	+	-	-	+	+	-	-	-	+	-
15	**03-16918 (WA-10)**	WA	[6,19]	t5073	ST573	II	5	+	-	-	-	+	-	+	-	-	-	-	-	-	-	-	-	-	-	+	-	+	-	-	+	-	-	-	-	+	-
16	**11-16548**	WA	-	n.a.	ST772	II	5	+	-	-	-	+	-	+	-	+	+	-	+	-	-	-	-	-	-	+	-	+	-	-	+	-	-	-	(+)	+	-
17	**05 V02622**	Saxony	-	n.a.	n.a.	II	5	-	-	-	-	-	-	+	-	-	-	-	-	-	-	-	-	-	-	+	-	+	-	-	+	-	-	-	-	+	-
18	**DP15**	Australia	-	n.a.	ST772	II	5	-	-	-	-	-	-	+	-	+	-	-	+	-	-	-	-	+	-	+	-	+	-	-	+	+	-	-	-	+	-

### Methods

High-throughput *de novo* sequencing was undertaken commercially by Geneservice Source BioScience plc (Nottingham, United Kingdom) using the Illumina Genome Analyzer System (Illumina Hiseq 2000 platform, Illumina, Essex, United Kingdom). The average genome coverage was *ca*. 105. The reads were assembled to contigs using the Velvet *de novo* genome assembler (vers.1.0.15; Illumina). The project was registered with the NCBI BioProject database under the provisional accession number PRJNA207032 and has been deposited at DDBJ/EMBL/GenBank under the accession number AZBT00000000.

Microarray procedures have been previously described in detail [6].

### Analysis

Analysis was performed using automated scripts for full text comparison and BLAST analysis and an in-house database of known, annotated and previously identified *S. aureus* genomes, genes and gene fragments to the query sequence. This allows determination of identity, clonal parentage and (given the constant order of core genomic genes in *S. aureus*) position within the genome of each contig (Additional file 2). In parallel, iterated BLAST searches were used for analysis of individual contigs in order to confirm results (http://blast.ncbi.nlm.nih.gov/Blast.cgi; [20]).

## Results and discussion

In terms of microarray hybridisation patterns, the sequenced strain represents the typical and most common variant of the Bengal Bay Clone (Table 1 and Additional file 1). The isolate 07–17048 was found to belong to the multilocus sequence type (MLST) ST772 (1-1-1-1-22-1-1), *spa* type t3387 (RIDOM nomenclature, [21], repeat sequence 26-16-21-17-34-33-34) and *dru* type dt10ao (5a-4a-0-2d-5b-3a-2 g-3b-4e-3e). According to MLST e-burst analysis, ST772 is considered to belong to CC 1 as it differs from ST1 only in one MLST allele (*pta*-22). However, because of several differences, as shown below, its inclusion into CC1 needs to be re-assessed.

A total of 340 contigs were obtained after initial assembling. Seventy contigs consisting of 2,741,418 base pairs have been analysed (Additional file 2). The overall G/C content was 33%. 1,946 protein coding sequences have been identified (Additional file 3). 1,234 protein coding sequences were completely identical to previously identified genes from other *S. aureus* strains. A total number of 416, 239 and 101 protein coding sequences were completely identical to alleles from CC1 genome sequences (from MSSA476, GenBank accession number BX571857 and MW2-USA400, BA000033), CC5 genome sequences (from Mu50, BA000017; ED98, CP001781 and N315, BA000018) and CC8 genome sequences (from COL, CP000046; Newman, AP009351 and NCTC8325, CP000253) respectively. Three-hundred eighty-three genes were identical only to another ST772-MRSA-V sequence (strain 118, whole genome shotgun project AJGE00000000, [22]). Based on identities to previously published gene sequences, ST772-MRSA-V is most closely related to CC1 and CC5. Genes of CC1 and CC5 backgrounds are scattered across the genome, and no evidence for a distinct part of the genome being affected by a genomic replacement (as observed in, for instance, ST239; [23]) can be found. Theoretically, this may be attributed to a high number of recombination events involving CC1 and CC5 strains, or may indicate a common ancestry for both lineages and subsequent accumulation of random mutations in genes that are essentially orthologs in CC1, CC5 and ST772. The question whether the presence of different capsule types and *agr* groups in otherwise closely related strains (such as ST1 and ST772) can be attributed to recombination or to convergent evolution justifies further study.

Several fundamental differences in the ST772 and CC1 core genomic markers have already been identified by microarray DNA [6] and have been confirmed by sequencing. These differences include *agr* alleles (group II rather than III), the capsule type (5 rather than 8) and the presence of different allelic variants of *hlb, ssl01/set6, bbp, clfB, fnbB, sdrC, sdrD, vwb* and *hsdS.* The *egc* enterotoxin gene cluster*,* Q7A4X2 (a hypothetical protein localised close to *egc*), the metallothiol transferase gene *fosB* and the enterotoxin homologue ORF CM14 are present in ST772 but absent from other CC1 strains. The genes *seh* (encoding enterotoxin H), *lukD/E* (a leukocidin homologue), *splA/B/F* (serine proteases), *ssl11/set2*, *ssl06/set21* (superantigen-like proteins), and Q2FXC0 (hypothetical protein, located next to serine protease operon) are absent in ST772, but present in other CC1 strains. In lieu of *seh*, the enterotoxin homologue ORF CM14 was identified in a similar position, *i.e*., closely downstream of the integration site of the SCC*mec* element. ORF CM14, absent in the related and possibly parental lineages CC1 and CC5 can be found in a number of different lineages including CC12, ST93, CC121, CC395 and CC705. This may indicate a small scale genomic replacement in a region close/downstream to *oriC.* Alternatively, ORF CM14 may have been replaced by *seh* in ancestors of CC1 or entirely deleted in CC5, but retained only in ST772.

Isolate 07–17048 harbours the enterotoxin A and PVL encoding *sea* and *lukF/S-PV* genes. Both genes are located on the same contig, and together with several other phage-associated genes, appear to be on a novel prophage. The phage is integrated into a gene of the putative protein A5IT17 which contains an attachment site of the PVL-carrying phage previously identified in other strains (un-truncated in the CC1 strain MSSA476, BX571857.1: SAS1429; truncated in the CC1 strain MW2-USA400, BA000033.2: MW1377/1443). All phage-associated genes identified are shown in Table 2.

**Table 2:** Genes associated with the PVL prophage in isolate 07-17048

**Designation**	**Explanation**	**Position in contig**	**Length**	**Best match**
*tx_lukF-PV*	Rho-independent terminator of *lukF-PV*	89974…90030	56	MW2-USA400, BA000033.2 [1529274:1529329:r]RC
*lukF-PV*	Panton-Valentine leukocidin F	90081…91059	978	MW2-USA400, BA000033.2 [1529381:1530358:r]RC
*lukS-PV*	Panton-Valentine leukocidin S	91060…91999	939	CIGC128, AHVY01000005.1 [221466:222404:r]RC
*O80065*	Putative protein directly upstream of *lukS-PV* - leader-peptide of lukPV	92106…92239	133	CIGC128, AHVY01000005.1 [222512:222644:r]RC
*tx_amidase1-phiSLT*	Rho-independent terminator of bacteriophage amidase	92327…92383	56	TCH1516, CP000730.1 [1562363:1562418:r]RC
*amidase1-phi12*	Bacteriophage amidase	92388…93843	1455	MW2-USA400, BA000033.2 [1531688:1533142:r]RC
*holin1-phiL54a*	Bacteriophage holin	93853…94156	303	MSSA476, BX571857.1 [1023464:1023766]RC
*sprFG*	Small pathogenicity island RNA F and G	94155…94266	211	ED98, CP001781.1 [892742:892952:r]
*rli28*	Listeria sRNA rli28 homolog, locus 2	94395…94574	179	MW2-USA400, BA000033.2 [2051199:2051377:r]RC
*txpA-phage*	Toxin involved in plasmid maintenance	94400…94535	1662	MW2-USA400 BA000033.2[2051171:2051305:r]RC
*sea*	Enterotoxin A	94652…95426	774	ST772 strain 118, AJGE01000058.1 [57550:58323]RC
*minor-phiNM3*	Bacteriophage minor structural protein	96751…100537	3786	TCH70-ST1, ACHH02000016.1 [79909:83694]RC
*Q8SDK3-phiNM3*	Putative bacteriophagal protein	100552…102037	1485	ST772 strain 118, AJGE01000058.1 [50939:52423]RC
*measure1-phiNM3*	Bacteriophage tail length tape measure protein	102033…106578	4545	04-02981, CP001844.2 [2051791:2056320:r]RC
*tailL-CC5*	Bacteriophage major tail protein	107488…108133	645	N315, BA000018.3 [2024201:2024845:r]RC
*A0EWZ3*	Bacteriophage major capsid protein	109862…111008	1146	Mu50-VRSA, BA000017.4 [2103511:2104656:r]RC
*Q6GF91*	Putative protease	111031…111769	738	A9299, ACKH01000012.1 [7311:8048]RC
*portal1-phiNM3*	Bacteriophage portal protein type 1	111752…112940	1188	Mu50-VRSA, BA000017.4 [2105401:2106588:r]RC
*terminase-phiNM3*	Bacteriophagal terminase	112955…114617	1662	Mu50-VRSA, BA000017.4 [2106604:2108265:r]RC
*rinB-phiPVL*	Bacteriophage transcript activator B	116263…116413	150	TCH1516, CP000730.1 [2111630:2111779:r]RC
*dut-phi*	Phage dUTP pyrophosphatase	117277…117820	543	MRSA252, BX571856.1 [2152883:2153425:r]RC
*sri*	Staphylococcal replication inhibitor	120465…120624	159	AP009351.1 [1110173:1110331]RC
*istB*	Replicative DNA helicase	120617…121397	780	phage, FJ713816.1 [12631:13404]RC and 04–02981, CP001844.2 [886487:887266]RC
*Q4ZAK4*	Putative bacteriophagal protein	121406…122177	771	04-02981, CP001844.2 [885707:886477]RC
*ssb3-phage*	Putative DNA binding protein	123346…123898	552	NCTC8325, CP000253.1 [2065949:2066500:r]RC
*DUF2483-phi80a*	Putative bacteriophagal protein	124707…124929	222	H19, ACSS01000056.1 [6024:6245:r]RC
*ant-phiPV83*	Bacteriophage antirepressor	127131…127929	798	phage, AY508486.1 [26092:26889]RC
*repressor-var3*	Bacteriophage repressor	128338…129067	729	phage, AP001553.1 [2342:3070:r]
*int1-phiPVL01*	Bacteriophage integrase	131132…132338	1206	ST772 strain 118, AJGE01000058.1 [20637:21842:r]

Genes *sea* and *sprFG* are normally associated with haemolysin beta converting phages rather than with PVL phages. In ST772-MRSA-V, haemolysin beta is interrupted, but there is no complete phage integrated into that gene. Only genes encoding the staphylococcal complement inhibitor *scn,* the putative membrane protein Q6GFB6 (usually located next to *scn* on *hlb-*converting phages, *e.g.,* in genomes of USA300 and USA400, N315, Mu50, NCTC8325, MRSA252, MSSA476) and *sprD* (coding for small pathogenicity island RNA D) can be found within *hlb.* A possible assembly error affecting both phage integration sites is highly unlikely. Several other ST772-MRSA-V genome sequences [22,24] also show this association of *lukF/S-PV* with *sea*. Besides, rare naturally occurring variants of ST772-MRSA-V and the related (single locus variant) ST573-MRSA-V (WA MRSA-10, [19]) lack *lukS/F-PV* and *sea* while still harbouring *scn*. Similar constellations can also be observed in ST573/772 MSSA (Table 1). Thus it is more likely that a part of the *hlb*-converting phage was translocated into a PVL phage that is integrated into a different position of the staphylococcal genome.

Other genes that are associated with mobile genetic elements include enterotoxin genes *sec* and *sel.* They are localised in similar position and context as in MW2, where they are also accompanied by phage derived genes and by the gene *ear* encoding the enterotoxin-linked ampicillin resistance protein. For resistance genes *blaZ/I/R, msr*(A)*, mph*(C), *aacA-aphD, aphA3, sat* and *aadE* it was not possible to unambiguously determine with the given set of contigs whether they were situated on the chromosome or on plasmids. The macrolide/clindamycin resistance gene *erm*(C), and *msr*(A)*, mph*(C), *aacA-aphD, aphA3* and *sat* genes have been shown by microarray hybridisation to occur variably in ST772-MRSA-V (Table 1).

The isolate 07–17048 harbours a SCC*mec* V element. Its terminal sequence towards *orfX* was also present in another ST772-MRSA-V sequence (strain 118, [22]) and appears to be unique to ST772-MRSA-V. The SCC*mec* V element consists of a tnpIS431-04, *mvaS-*SCC (a truncated 3-hydroxy-3-methylglutaryl CoA synthase), a putative protein Q5HJW6, *dru* (SCC-associated direct repeat units), *ugpQ* (glycerophosphoryl diester phosphodiesterase), *ydeM* (a putative dehydratase), a bidirectional *rho*-independent terminator of *mecA* followed by *mecA* in an allelic variant identical to GQ902038 and AM990992 [25], a series of genes coding for putative proteins (Q4LAG7, Q3T2N0, Q4LAG4, Q4LAG3, Q3T2M7), a recombinase homologue “*ccrAA*” [26], a SCC*mec* type V recombinase *ccrC*, further genes encoding putative proteins (Q4LAF9, Q7A206, Q7A207, Q9KX75, A9UFT0), a bidirectional rho-independent terminator of *hsdR* and three genes (*hsdR, hsdS, hsdM2*) of a type I restriction-modification system.

## Conclusion

In conclusion, ST772-MRSA-V may have emerged from the same root or lineage as the global CC1 and CC5 strains. It has acquired a variety of virulence factors, and, compared to other CA-MRSA strains, it has an unusually high number of genes associated with antibiotic resistance. Whether it evolved in a hospital setting or acquired these genes in community cannot be decided based on a single sequence. Therefore, more epidemiological data and possibly the sequencing of a number of additional isolates are warranted in order to understand the evolution and spread of this conspicuous strain.

## Competing interests

S. Monecke, V. Baier, P. Slickers, A. Ziegler and R. Ehricht are employees of Alere Technologies. There was no external funding for this study.

## Authors’ contributions

SM, GC and RE wrote the manuscript; GC and SM obtained and characterised isolates; SM, RE and PS analysed the sequence data; VB, PS and AZ wrote software tools used for the study. All authors read and approved the final manuscript.

## Supplementary Material

Additional file 1 Full array hybridisation for isolates from this study. Click here for file

Additional file 2 Raw sequences of 70 contigs of isolate 07–17048. Click here for file

Additional file 3 Annotated draft genome sequence for isolate 07–17048. Click here for file
