# Supplementary material for: Genome sequencing and molecular characterisation of Staphylococcus aureus ST772-MRSA-V, “Bengal Bay Clone”
Source: BMC Res Notes. 2013 Dec 20;6:548. doi: 10.1186/1756-0500-6-548 (PMC3878137; doi:10.1186/1756-0500-6-548)
Supplement: Additional file 1 — Full array hybridisation for isolates from this study. [file 1756-0500-6-548-S1.pdf]

Supplemental table S1: Full array hybridisation for isolates from this study

Target (class)	Target (Description)	Target (Symbol)	CC1 strains for comparison		CC5 strains for comparison			Other ST772-MRSA-V isolates									Related (ST573/772) isolates for comparison			
			MW2-USA400	Sanger 476	N315	MU50	07-17048	09V32405	10V3510	11ANR12420	12ANR75832	11-18844	11-18907	11-19350	11-19415	11-19454	03-16918 (WA10)	11-16548	05V02622	DP15
BACKGROUND & TYPING DATA	geographic origin		US	UK	Japan	Japan	WA / India	Sax	Sax / India	Sax	Sax	WA	WA	WA	WA	WA	WA	WA	Sax	Austr
	spa typing		t128	t607	t002	t002	t3387					t657	t657	t4599	t657	t1075	t5073			
	MLST typing		ST1	ST1	ST5	ST5	ST772										ST573	ST772		
SPECIES MARKER	Domain 1 of 23SrRNA	*rrnD1 (S. aureus)*	POS	POS	POS	POS	POS	POS	POS	POS	POS	POS	POS	AMB	POS	POS	POS	POS	POS	POS
	glyceraldehyde 3-phosphate dehydrogenase, locus 1	*gapA*	POS	POS	POS	POS	POS	POS	POS	POS	POS	POS	POS	POS	POS	POS	POS	POS	POS	POS
	katalase A	*katA*	POS	POS	POS	POS	POS	POS	POS	POS	POS	POS	POS	POS	POS	POS	POS	POS	POS	POS
	coagulase	*coA*	POS	POS	POS	POS	POS	POS	POS	POS	POS	POS	POS	POS	POS	POS	POS	POS	POS	POS
	thermostable extracellular nuclease	*nuc1*	POS	POS	POS	POS	POS	POS	POS	POS	POS	POS	POS	POS	POS	POS	POS	POS	POS	POS
	staphylococcal protein A	*spa*	POS	POS	POS	POS	POS	POS	POS	POS	POS	POS	POS	POS	POS	POS	POS	POS	POS	POS
	IgG-binding protein	*sbi*	POS	POS	POS	POS	POS	POS	POS	POS	POS	POS	POS	POS	POS	POS	POS	POS	POS	POS
REGULATORY GENES	staphylococcal accessory regulator A	*sarA*	POS	POS	POS	POS	POS	POS	POS	POS	POS	POS	POS	POS	POS	POS	POS	POS	POS	POS
	histidine protein kinase, sae locus	*saeS*	POS	POS	POS	POS	POS	POS	POS	POS	POS	POS	POS	POS	POS	POS	POS	POS	POS	POS
	sensor protein	*vraS*	POS	POS	POS	POS	POS	POS	POS	POS	POS	POS	POS	POS	POS	POS	POS	POS	POS	POS
	accessory gene regulator allele I	*agrI (total)*	NEG	NEG	NEG	NEG	NEG	NEG	NEG	NEG	NEG	NEG	NEG	NEG	NEG	NEG	NEG	NEG	NEG	NEG
	accessory gene regulator allele II	*agrII (total)*	NEG	NEG	POS	POS	POS	POS	POS	POS	POS	POS	POS	POS	POS	POS	POS	POS	POS	POS
	accessory gene regulator allele III	*agrIII (total)*	POS	POS	NEG	NEG	NEG	NEG	NEG	NEG	NEG	NEG	NEG	NEG	NEG	NEG	NEG	NEG	NEG	NEG
	accessory gene regulator allele IV	*agrIV (total)*	NEG	NEG	NEG	NEG	NEG	NEG	NEG	NEG	NEG	NEG	NEG	NEG	NEG	NEG	NEG	NEG	NEG	NEG
	haemolysin delta	*hld*	POS	POS	POS	POS	POS	POS	POS	POS	POS	POS	POS	POS	POS	POS	POS	POS	POS	POS
METHICILLIN RESISTANCE AND SCCmec TYPING	alternate penicillin binding protein 2, defining MRSA	*mecA*	POS	NEG	POS	POS	POS	POS	POS	POS	POS	POS	POS	POS	POS	POS	POS	POS	NEG	NEG
	truncated signal transducer protein MecR1	*delta_mecR*	POS	NEG	POS	POS	NEG	NEG	NEG	NEG	NEG	NEG	NEG	NEG	NEG	NEG	NEG	NEG	NEG	NEG
	glycerophosphoryl diester phosphodiesterase	*ugpQ*	POS	NEG	POS	POS	POS	POS	POS	POS	POS	POS	POS	POS	POS	POS	POS	POS	NEG	NEG
	cassette chromosome recombinase genes A/B1	*ccrA-1*	NEG	POS	NEG	NEG	NEG	NEG	NEG	NEG	NEG	NEG	NEG	NEG	NEG	NEG	NEG	NEG	NEG	NEG
		ccrB-1	NEG	POS	NEG	NEG	NEG	NEG	NEG	NEG	NEG	NEG	NEG	NEG	NEG	NEG	NEG	NEG	NEG	NEG
	plasmin-sensitive surface protein	*plsSCC (COL)*	NEG	NEG	NEG	NEG	NEG	NEG	NEG	NEG	NEG	NEG	NEG	NEG	NEG	NEG	NEG	NEG	NEG	NEG
	hypothetical protein from SCCmec elements	*Q9XB68-dcs*	AMB	NEG	POS	POS	NEG	NEG	NEG	NEG	NEG	NEG	NEG	NEG	NEG	NEG	NEG	NEG	NEG	NEG
	cassette chromosome recombinase genes A/B2	*ccrA-2*	POS	NEG	POS	POS	NEG	NEG	NEG	NEG	NEG	NEG	NEG	NEG	NEG	NEG	NEG	NEG	NEG	NEG
		ccrB-2	POS	NEG	POS	POS	NEG	NEG	NEG	NEG	NEG	NEG	NEG	NEG	NEG	NEG	NEG	NEG	NEG	NEG
	potassium-translocating ATPase A, chain 2	*kdpA-SCC*	NEG	NEG	POS	POS	NEG	NEG	NEG	NEG	NEG	NEG	NEG	NEG	NEG	NEG	NEG	POS	NEG	NEG
	potassium-transporting ATPase B, chain 1	*kdpB-SCC*	NEG	NEG	POS	POS	NEG	NEG	NEG	NEG	NEG	NEG	NEG	NEG	NEG	NEG	NEG	NEG	NEG	NEG
	potassium-translocating ATPase C, chain 2	*kdpC-SCC*	NEG	NEG	POS	POS	NEG	NEG	NEG	NEG	NEG	NEG	NEG	NEG	NEG	NEG	NEG	NEG	NEG	NEG
	sensor kinase protein	*kdpD-SCC*	NEG	NEG	POS	POS	NEG	NEG	NEG	NEG	NEG	NEG	NEG	NEG	NEG	NEG	NEG	NEG	NEG	NEG
	KDP operon transcriptional regulatory protein	*kdpE-SCC*	NEG	NEG	POS	POS	NEG	NEG	NEG	NEG	NEG	NEG	NEG	NEG	NEG	NEG	NEG	NEG	NEG	NEG
	methicillinresistance regulatory protein	*mecI*	NEG	NEG	POS	POS	NEG	NEG	NEG	NEG	NEG	NEG	NEG	NEG	NEG	NEG	NEG	NEG	NEG	NEG
	signal transducer protein MecR1	*mecR*	NEG	NEG	POS	POS	NEG	NEG	NEG	NEG	NEG	NEG	NEG	NEG	NEG	NEG	NEG	NEG	NEG	NEG
	homolog of xylose repressor, associated with SCCmec elements	*xylR*	NEG	NEG	POS	POS	NEG	NEG	NEG	NEG	NEG	AMB	AMB	NEG	AMB	NEG	NEG	POS	NEG	NEG
	cassette chromosome recombinase genes A/B3	*ccrA-3*	NEG	NEG	NEG	NEG	NEG	NEG	NEG	NEG	NEG	NEG	NEG	NEG	NEG	NEG	NEG	NEG	NEG	NEG
		ccrB-3	NEG	NEG	NEG	NEG	NEG	NEG	NEG	NEG	NEG	NEG	NEG	NEG	NEG	NEG	NEG	NEG	NEG	NEG
	mercury resistance operon	*merA*	NEG	NEG	NEG	NEG	NEG	NEG	NEG	NEG	NEG	NEG	NEG	NEG	NEG	NEG	NEG	NEG	NEG	NEG
		merB	NEG	NEG	NEG	NEG	NEG	NEG	NEG	NEG	NEG	NEG	NEG	NEG	NEG	NEG	NEG	NEG	NEG	NEG
	cassette chromosome recombinase genes "ccrAA" (hypothetical)	*ccrAA*	NEG	NEG	NEG	NEG	POS	POS	POS	POS	POS	POS	POS	POS	POS	POS	POS	POS	NEG	NEG
	and ccrC	*ccrC (85-2082)*	NEG	NEG	NEG	NEG	POS	POS	POS	POS	POS	POS	POS	POS	POS	POS	POS	POS	NEG	NEG
	cassette chromosome recombinase genes A/B4	*ccrA-4*	NEG	NEG	NEG	NEG	NEG	NEG	NEG	NEG	NEG	NEG	NEG	NEG	NEG	NEG	NEG	NEG	NEG	NEG
		ccrB-4	NEG	NEG	NEG	NEG	NEG	NEG	NEG	NEG	NEG	NEG	NEG	NEG	NEG	NEG	NEG	NEG	NEG	NEG
RESISTANCE : PENICILLINASE	betalactamase	*blaZ*	POS	POS	POS	NEG	POS	POS	POS	POS	POS	POS	POS	POS	POS	POS	POS	POS	POS	POS
	beta lactamase repressor (inhibitor)	*blaI*	POS	POS	POS	NEG	POS	POS	POS	POS	POS	POS	POS	POS	POS	POS	POS	POS	POS	POS
	beta lactamase regulatory protein	*blaR*	POS	POS	POS	NEG	POS	POS	POS	POS	POS	POS	POS	POS	POS	POS	POS	POS	POS	POS
RESISTANCE : MLS-ANTIBIOTICS	rRNA methyltransferase, erythromycin/clindamycin resistance	*erm(A)*	NEG	NEG	POS	POS	NEG	NEG	NEG	NEG	NEG	NEG	NEG	NEG	NEG	NEG	NEG	NEG	NEG	NEG
	erythromycin/clindamycin resistance	*erm(B)*	NEG	NEG	NEG	NEG	NEG	NEG	NEG	NEG	NEG	NEG	NEG	NEG	NEG	NEG		NEG		
		erm(C)	NEG	NEG	NEG	NEG	NEG	POS	NEG	NEG	NEG	NEG	NEG	NEG	NEG	POS	NEG	NEG	NEG	NEG
	lincosamid-nucleotidyltransferase	*lnu(A)*	NEG	NEG	NEG	NEG	NEG	NEG	NEG	NEG	NEG	NEG	NEG	NEG	NEG	NEG	NEG	NEG	NEG	NEG
	energy dependent efflux of erythromycin	*msr(A)*	NEG	NEG	NEG	NEG	POS	POS	POS	POS	POS	POS	POS	NEG	POS	POS	NEG	POS	NEG	POS
	macrolide efflux protein A	*mef(A)*	NEG	NEG	NEG	NEG	NEG	NEG	NEG	NEG	NEG	NEG	NEG	NEG	NEG	NEG	NEG	NEG	NEG	NEG
	probable lysylphosphatidylglycerol synthetase	*mph(C)*	NEG	NEG	NEG	NEG	POS	POS	POS	POS	POS	POS	POS	NEG	POS	POS	NEG	POS	NEG	POS
	virginiamycin A acetyltransferase	*vat(A)*	NEG	NEG	NEG	NEG	NEG	NEG	NEG	NEG	NEG	NEG	NEG	NEG	NEG	NEG	NEG	NEG	NEG	NEG
	acetyltransferase inactivating streptogramin A	*vat(B)*	NEG	NEG	NEG	NEG	NEG	NEG	NEG	NEG	NEG	NEG	NEG	NEG	NEG	NEG	NEG	NEG	NEG	NEG
	ATP binding protein, streptogramin A resistance	*vga(A)*	NEG	NEG	NEG	NEG	NEG	NEG	NEG	NEG	NEG	NEG	NEG	NEG	NEG	NEG	NEG	NEG	NEG	NEG
		vga(A) (BM 3327)	NEG	NEG	NEG	NEG	NEG	NEG	NEG	NEG	NEG	NEG	NEG	NEG	NEG	NEG	NEG	NEG	NEG	NEG
	virginiamycin B hydrolase	*vgB(A)*	NEG	NEG	NEG	NEG	NEG	NEG	NEG	NEG	NEG	NEG	NEG	NEG	NEG	NEG	NEG	NEG	NEG	NEG
RESISTANCE : AMINO-GLYSOSIDES	bifunctional enzyme Aac/Aph, gentamicin resistance	*aacA-aphD*	NEG	NEG	NEG	POS	POS	POS	POS	POS	POS	NEG	POS	POS	POS	POS	NEG	POS	NEG	NEG
	aminoglycoside adenyltransferase,tobramycin resistance	*aadD*	NEG	NEG	POS	POS	NEG	NEG	NEG	NEG	NEG	NEG	NEG	NEG	NEG	NEG	NEG	NEG	NEG	NEG
	3'5'aminoglycoside phosphotransferase	*aphA3*	NEG	NEG	NEG	NEG	POS	POS	POS	POS	POS	POS	POS	NEG	POS	POS	NEG	POS	NEG	POS
RESISTANCE : MISCELLANEOUS GENES	streptothricin acetyltransferase	*sat*	NEG	NEG	NEG	NEG	POS	POS	POS	POS	POS	POS	POS	NEG	POS	POS	NEG	POS	NEG	POS
	dihydrofolate reductase type 1	*dfrS1*	NEG	NEG	NEG	NEG	NEG	NEG	NEG	NEG	NEG	NEG	NEG	NEG	NEG	NEG	NEG	NEG	NEG	NEG
	fusidic acid resistence	*far1*	NEG	NEG	NEG	NEG	NEG	NEG	NEG	NEG	NEG	NEG	NEG	NEG	NEG	NEG	NEG	NEG	NEG	NEG
	hypothetical protein associated with fusidic acid resistance	*fusC (Q6GD50)*	NEG	POS	NEG	NEG	NEG	NEG	NEG	NEG	NEG	NEG	NEG	NEG	NEG	NEG	NEG	NEG	NEG	NEG
	mupirocin resistence protein	*mupA*	NEG	NEG	NEG	NEG	NEG	NEG	NEG	NEG	NEG	NEG	NEG	NEG	NEG	NEG	NEG	NEG	NEG	NEG
	tetrazyklin resistance	*tet(K)*	NEG	AMB	NEG	NEG	NEG	NEG	NEG	NEG	NEG	NEG	NEG	NEG	NEG	NEG	NEG	NEG	NEG	NEG
		tet(M)	NEG	NEG	NEG	POS	NEG	NEG	NEG	NEG	NEG	NEG	NEG	NEG	NEG	NEG	NEG	NEG	NEG	NEG
	chloramphenicol acetyltransferase	*cat (total)*	NEG	NEG	NEG	NEG	NEG	NEG	NEG	NEG	NEG	NEG	NEG	NEG	NEG	NEG	NEG	NEG	NEG	NEG
	23S rRNA methyltransferase	*cfr*	NEG	NEG	NEG	NEG	NEG	NEG	NEG	NEG	NEG	NEG	NEG	NEG	NEG	NEG	NEG	NEG	NEG	NEG
	chloramphenicol/florfenicol exporter	*fexA*	NEG	NEG	NEG	NEG	NEG	NEG	NEG	NEG	NEG	NEG	NEG	NEG	NEG	NEG	NEG	NEG	NEG	NEG
	metallothiol transferase	*fosB*	NEG	NEG	POS	POS	POS	POS	POS	POS	POS	POS	POS	POS	POS	POS	POS	POS	POS	POS
		fosB (plasmid)	NEG	NEG	NEG	NEG	NEG	NEG	NEG	NEG	NEG	NEG	NEG	NEG	NEG	NEG	NEG	AMB	NEG	NEG
RESISTANCE : EFFLUX SYSTEMS	quaternary ammonium compound resistance protein A	*qacA*	NEG	NEG	NEG	POS	NEG	NEG	NEG	NEG	NEG	NEG	NEG	NEG	NEG	NEG	NEG	NEG	NEG	NEG
	quaternary ammonium compound resistance protein C	*qacC (total)*	NEG	NEG	NEG	NEG	NEG	NEG	NEG	NEG	NEG	NEG	NEG	NEG	NEG	NEG	NEG	NEG	NEG	NEG
	transport/efflux protein	*sdrM*	POS	POS	POS	POS	POS	POS	POS	POS	POS	POS	POS	POS	POS	POS	POS	POS	POS	POS
RESISTANCE : GLYCOPEPTIDES	vancomycin resistance gene	*vanA*	NEG	NEG	NEG	NEG	NEG	NEG	NEG	NEG	NEG	NEG	NEG	NEG	NEG	NEG	NEG	NEG	NEG	NEG
	vancomycin resistance gene from enterococci and Clostridium	*vanB*	NEG	NEG	NEG	NEG	NEG	NEG	NEG	NEG	NEG	NEG	NEG	NEG	NEG	NEG	NEG	NEG	NEG	NEG
	teicoplanin resistance gene from enterococci	*vanZ*	NEG	NEG	NEG	NEG	NEG	NEG	NEG	NEG	NEG	NEG	NEG	NEG	NEG	NEG	NEG	NEG	NEG	NEG
VIRULENCE :	toxic shock syndrome toxin 1	*tst1 (consensus)*	NEG	NEG	POS	POS	NEG	NEG	NEG	NEG	NEG	NEG	NEG	NEG	NEG	NEG	NEG	NEG	NEG	NEG
		tst1 ("human" allele)	NEG	NEG	POS	POS	NEG	NEG	NEG	NEG	NEG	NEG	NEG	NEG	NEG	NEG	NEG	NEG	NEG	NEG
		tst1 ("bovine" allele)	NEG	NEG	NEG	NEG	NEG	NEG	NEG	NEG	NEG	NEG	NEG	NEG	NEG	NEG	NEG	NEG	NEG	NEG
VIRULENCE : ENTEROTOXINS	enterotoxin A	*sea*	POS	POS	NEG	POS	POS	POS	POS	POS	POS	POS	POS	POS	POS	POS	NEG	NEG	NEG	POS
	enterotoxin A, allele from strain 320E	*sea (320E)*	NEG	NEG	NEG	NEG	NEG	NEG	NEG	NEG	AMB	AMB	AMB	NEG	NEG	NEG	NEG	NEG	NEG	NEG
	enterotoxin A, allele from strain N315 = enterotoxin P	*sea (N315) / sep*	NEG	NEG	POS	NEG	NEG	NEG	NEG	NEG	NEG	NEG	NEG	NEG	NEG	NEG	NEG	NEG	NEG	NEG
	enterotoxin B	*seb*	NEG	NEG	NEG	NEG	NEG	NEG	NEG	AMB	AMB	NEG	NEG	NEG	NEG	NEG	NEG	AMB	NEG	NEG
	enterotoxin C	*sec*	POS	NEG	POS	POS	POS	POS	POS	POS	POS	POS	POS	POS	POS	POS	POS	POS	POS	POS
	enterotoxin D	*sed*	NEG	NEG	NEG	NEG	NEG	NEG	NEG	NEG	NEG	AMB	NEG	NEG	NEG	NEG	NEG	AMB	NEG	NEG
	enterotoxin E	*see*	NEG	NEG	NEG	NEG	NEG	NEG	NEG	NEG	NEG	NEG	NEG	NEG	NEG	NEG	NEG	NEG	NEG	NEG
	enterotoxin G	*seg*	NEG	NEG	POS	POS	POS	POS	POS	POS	POS	POS	POS	POS	POS	POS	POS	POS	POS	POS
	enterotoxin H	*seh*	POS	POS	NEG	NEG	NEG	NEG	NEG	NEG	NEG	NEG	NEG	NEG	NEG	NEG	NEG	NEG	NEG	NEG
	enterotoxin I	*sei*	NEG	NEG	POS	POS	POS	POS	POS	POS	POS	POS	POS	POS	POS	POS	POS	POS	POS	POS
	enterotoxin J	*sej*	NEG	NEG	NEG	NEG	NEG	NEG	NEG	NEG	NEG	NEG	NEG	NEG	NEG	NEG	NEG	NEG	NEG	NEG
	enterotoxin K	*sek*	POS	POS	NEG	NEG	NEG	NEG	NEG	NEG	NEG	NEG	NEG	NEG	NEG	NEG	NEG	NEG	NEG	NEG
	enterotoxin L	*sel*	POS	NEG	POS	POS	POS	POS	POS	POS	POS	POS	POS	POS	POS	POS	POS	POS	POS	POS
	enterotoxin M	*selm*	NEG	NEG	POS	POS	POS	POS	POS	POS	POS	POS	POS	POS	POS	POS	POS	POS	POS	POS
	enterotoxin N	*seln (consensus)*	NEG	NEG	POS	POS	POS	POS	POS	POS	POS	POS	POS	POS	POS	POS	POS	POS	AMB	POS
		seln (other than RF122)	NEG	NEG	POS	POS	POS	POS	POS	POS	POS	POS	POS	POS	POS	POS	POS	POS	POS	POS
	enterotoxin O	*selo*	NEG	NEG	POS	POS	POS	POS	POS	POS	POS	POS	POS	POS	POS	POS	POS	POS	AMB	POS
	enterotoxin Q	*seq*	POS	POS	NEG	NEG	NEG	NEG	NEG	NEG	NEG	NEG	NEG	NEG	NEG	NEG	NEG	NEG	NEG	NEG
	enterotoxin R	*ser*	NEG	NEG	NEG	NEG	NEG	NEG	NEG	NEG	NEG	NEG	NEG	NEG	NEG	NEG	NEG	NEG	NEG	NEG
	Enterotoxin U and/or Y	*selu/y*	NEG	NEG	POS	POS	POS	POS	POS	POS	POS	POS	POS	POS	POS	POS	POS	POS	POS	POS
	enterotoxinlike protein ORF CM14	*ORF CM14*	NEG	NEG	NEG	NEG	POS	POS	POS	POS	POS	POS	POS	POS	POS	POS	POS	POS	POS	POS
VIRULENCE : HLG AND LEUKOCIDINS	haemolysin gamma / leukocidin, component B	*lukF*	POS	POS	POS	POS	POS	POS	POS	POS	POS	POS	POS	POS	POS	POS	POS	POS	POS	POS
	haemolysin gamma / leukocidin, component C	*lukS*	POS	POS	POS	POS	POS	POS	POS	POS	POS	POS	POS	POS	POS	POS	POS	POS	POS	POS
		lukS (ST22+ST45)	NEG	AMB	NEG	AMB	POS	AMB	AMB	POS	POS	POS	POS	POS	POS	AMB	NEG	POS	POS	AMB
	haemolysin gamma, component A	*hlgA*	POS	POS	POS	POS	POS	POS	POS	POS	POS	POS	POS	POS	POS	POS	POS	POS	POS	POS
	Panton Valentine leukocidin F component	*lukF-PV*	POS	NEG	NEG	NEG	POS	POS	POS	POS	POS	POS	POS	POS	POS	POS	NEG	NEG	NEG	POS
	Panton Valentine leukocidin S component	*lukS-PV*	POS	NEG	NEG	NEG	POS	POS	POS	POS	POS	POS	POS	POS	POS	POS	NEG	NEG	NEG	POS
	F component from hypothetical leukocidin from ruminants	*lukF-PV (P83)*	NEG	NEG	NEG	NEG	NEG	NEG	NEG	NEG	NEG	NEG	NEG	NEG	NEG	NEG	NEG	NEG	NEG	NEG
	S component from hypothetical leukocidin from ruminants	*lukM*	NEG	NEG	NEG	NEG	NEG	NEG	NEG	NEG	NEG	NEG	NEG	NEG	NEG	NEG	NEG	NEG	NEG	NEG
	leukocidin D component	*lukD*	POS	POS	POS	POS	NEG	NEG	NEG	NEG	NEG	NEG	NEG	NEG	NEG	NEG	NEG	NEG	NEG	NEG
	leukocidin E component	*lukE*	POS	POS	POS	POS	NEG	NEG	NEG	NEG	NEG	NEG	NEG	NEG	NEG	NEG	NEG	NEG	NEG	NEG
	leukocidin/ haemolysin toxin family protein	*lukX*	POS	POS	POS	POS	POS	POS	POS	POS	POS	POS	POS	POS	POS	POS	POS	POS	NEG	POS
	leukocidin/haemolysin toxin family protein	*lukY*	POS	POS	POS	POS	POS	POS	POS	POS	POS	POS	POS	POS	POS	POS	POS	POS	POS	POS
		lukY (ST30+ST45)	NEG	NEG	NEG	NEG	NEG	NEG	NEG	NEG	NEG	NEG	NEG	NEG	NEG	NEG	NEG	NEG	NEG	NEG
VIRULENCE : HAEMOLYSINS	putative membrane protein	*hl*	POS	POS	POS	POS	POS	POS	POS	POS	POS	POS	POS	POS	POS	POS	POS	POS	POS	POS
	haemolysin alpha	*hla*	POS	POS	POS	POS	POS	POS	POS	POS	POS	POS	POS	POS	POS	POS	POS	POS	POS	POS
	putative membrane protein	*hlIII (consensus)*	POS	POS	POS	POS	POS	POS	POS	POS	POS	POS	POS	POS	POS	POS	POS	POS	POS	POS
		hlIII (other than RF122)	POS	POS	POS	POS	POS	POS	POS	POS	POS	POS	POS	POS	POS	POS	POS	POS	POS	POS
	haemolysin beta	*hlb_probe 1*	AMB	AMB	AMB	POS	NEG	NEG	NEG	NEG	AMB	NEG	NEG	NEG	NEG	NEG	POS	NEG	POS	NEG
		hlb_probe 2	POS	POS	POS	POS	NEG	NEG	NEG	NEG	NEG	AMB	NEG	NEG	NEG	NEG	POS	NEG	POS	NEG
		hlb_probe 3	AMB	AMB	NEG	POS	AMB	AMB	POS	AMB	POS	POS	POS	AMB	POS	NEG	POS	POS	POS	NEG
		un-disrupted hlb	NEG	NEG	NEG	NEG	NEG	NEG	NEG	AMB	POS	NEG	NEG	NEG	NEG	NEG	NEG	NEG	NEG	AMB
VIRULENCE : HLB-CONV PHAGES	staphylokinase	*sak*	POS	POS	POS	POS	NEG	NEG	NEG	NEG	NEG	NEG	NEG	NEG	NEG	NEG	NEG	NEG	NEG	NEG
	chemotaxisinhibiting protein (CHIPS)	*chp*	NEG	NEG	POS	NEG	NEG	NEG	NEG	NEG	NEG	NEG	NEG	NEG	NEG	NEG	NEG	AMB	NEG	NEG
	staphylococcal complement inhibitor	*scn*	POS	POS	POS	POS	POS	POS	POS	POS	POS	POS	POS	POS	POS	POS	POS	POS	POS	POS
VIRULENCE : EXFOL.TOXINS	exfoliative toxin serotype A	*etA*	NEG	NEG	NEG	NEG	NEG	NEG	NEG	NEG	NEG	NEG	NEG	NEG	NEG	NEG	NEG	NEG	NEG	NEG
	exfoliative toxin serotype B	*etB*	NEG	NEG	NEG	NEG	NEG	NEG	NEG	NEG	NEG	NEG	NEG	NEG	NEG	NEG	NEG	NEG	NEG	NEG
	exfoliative toxin D	*etD*	NEG	NEG	NEG	NEG	NEG	NEG	NEG	NEG	NEG	NEG	NEG	NEG	NEG	NEG	NEG	NEG	NEG	NEG
VIRULENCE : EPIDERMAL CELL DIFF. INHIBITOR	epidermal cell differentiation inhibitor	*edinA*	NEG	NEG	NEG	NEG	NEG	NEG	NEG	NEG	NEG	NEG	NEG	NEG	NEG	NEG	NEG	NEG	NEG	NEG
	epidermal cell differentiation inhibitor B	*edinB*	NEG	NEG	NEG	NEG	NEG	NEG	NEG	NEG	NEG	NEG	NEG	NEG	NEG	NEG	NEG	NEG	NEG	NEG
	epidermal cell differentiation inhibitor C	*edinC*	NEG	NEG	NEG	NEG	NEG	NEG	NEG	NEG	NEG	NEG	NEG	NEG	NEG	NEG	NEG	NEG	NEG	NEG
VIRULENCE : ACME LOCUS	ACME locus	*ACME (total)*	NEG	NEG	NEG	NEG	NEG	NEG	NEG	NEG	NEG	NEG	NEG	NEG	NEG	NEG	NEG	NEG	NEG	NEG
	ACME locus: arginine dihydrolase	*arcA-SCC*	NEG	NEG	NEG	NEG	NEG	NEG	NEG	NEG	NEG	NEG	NEG	NEG	NEG	NEG	NEG	NEG	NEG	NEG
	ACME locus: ornithincarbamoyltransferase	*arcB-SCC*	NEG	NEG	NEG	NEG	NEG	NEG	NEG	NEG	NEG	NEG	NEG	NEG	NEG	NEG	NEG	NEG	NEG	NEG
	ACME locus: carbamatkinase	*arcC-SCC*	NEG	NEG	NEG	NEG	NEG	NEG	NEG	NEG	NEG	NEG	NEG	NEG	NEG	NEG	NEG	NEG	NEG	NEG
	ACME locus: arginine/ornithineantiporter	*arcD-SCC*	NEG	NEG	NEG	NEG	NEG	NEG	NEG	NEG	NEG	NEG	NEG	NEG	NEG	NEG	NEG	AMB	NEG	NEG
VIRULENCE : PROTEASES	aureolysin	*aur (consensus)*	POS	POS	POS	POS	POS	POS	POS	POS	POS	POS	POS	POS	POS	POS	POS	POS	AMB	POS
		aur (other than MRSA252)	POS	POS	POS	POS	POS	POS	POS	POS	POS	POS	POS	POS	POS	POS	POS	POS	POS	POS
		aur (MRSA252)	NEG	NEG	NEG	NEG	NEG	NEG	NEG	NEG	NEG	NEG	NEG	NEG	NEG	NEG	NEG	NEG	NEG	NEG
	serin protease A	*splA*	POS	POS	POS	POS	NEG	NEG	NEG	NEG	NEG	NEG	NEG	NEG	NEG	NEG	NEG	NEG	NEG	NEG
	serin protease B	*splB*	POS	POS	POS	POS	NEG	NEG	NEG	NEG	NEG	NEG	NEG	NEG	NEG	NEG	NEG	NEG	NEG	NEG
	serin protease E	*splE*	NEG	NEG	NEG	NEG	NEG	NEG	NEG	NEG	NEG	NEG	NEG	NEG	NEG	NEG	NEG	NEG	NEG	NEG
	glutamylendopeptidase	*sspA*	POS	POS	POS	POS	POS	POS	POS	POS	POS	POS	POS	POS	POS	POS	POS	POS	POS	POS
	staphopain B, protease	*sspB*	POS	POS	POS	POS	POS	POS	POS	POS	POS	POS	POS	POS	POS	POS	POS	POS	POS	POS
	staphopain A (staphylopain A), protease	*sspP (consensus)*	POS	POS	POS	POS	POS	POS	POS	POS	POS	POS	POS	POS	POS	POS	POS	POS	POS	POS
		sspP (other than ST93)	POS	POS	POS	POS	POS	POS	POS	POS	POS	POS	POS	POS	POS	POS	POS	POS	POS	POS

Supplemental table S1: Full array hybridisation for isolates from this study

Target (class)	Target (Description)	Target (Symbol)	CC1 strains for comparison		CC5 strains for comparison		07-17048	Other ST772-MRSA-V isolates									Related (ST573/772) isolates for comparison			
			MW2-USA400	Sanger 476	N315	MU50		09V32405	10V3510	11ANR12420	12ANR75832	11-18844	11-18907	11-19350	11-19415	11-19454	03-16918 (WA10)	11-16548	05V02622	DP15
**VIRULENCE : STAPHYLOCOCCAL SUPERANTIGEN / ENTEROTOXIN-LIKE GENES (SET/SSL)**	staphyl. exotoxinlike protein	*setC (=selX)*	POS	POS	POS	POS	POS	POS	POS	POS	POS	POS	POS	POS	POS	POS	POS	POS	POS	POS
	staphylococcal superantigenlike protein 1	*ssl01/set6 (COL)*	NEG	NEG	NEG	AMB	POS	POS	POS	POS	POS	POS	POS	POS	POS	POS	POS	AMB	POS	POS
		ssl01/set6 (Mu50+N315)	NEG	NEG	POS	POS	NEG	AMB	AMB	AMB	AMB	AMB	AMB	AMB	AMB	AMB	NEG	POS	NEG	AMB
		ssl01/set6 (MW2+MSSA476)	POS	POS	NEG	NEG	NEG	NEG	NEG	NEG	NEG	NEG	NEG	NEG	NEG	NEG	NEG	NEG	NEG	NEG
		ssl01/set6 (MRSA252)	NEG	NEG	NEG	NEG	NEG	NEG	NEG	NEG	NEG	NEG	NEG	NEG	NEG	NEG	NEG	AMB	NEG	NEG
		ssl01/set6 (RF122)	NEG	NEG	NEG	NEG	NEG	NEG	NEG	NEG	NEG	NEG	NEG	NEG	NEG	NEG	NEG	NEG	NEG	NEG
		ssl01/set6 (other alleles)	NEG	NEG	NEG	NEG	NEG	NEG	NEG	NEG	NEG	NEG	NEG	NEG	NEG	NEG	NEG	NEG	NEG	NEG
	staphylococcal superantigenlike protein 2	*ssl02/set7*	POS	POS	POS	POS	POS	POS	POS	POS	POS	POS	POS	POS	POS	POS	POS	POS	POS	POS
		ssl02/set7 (MRSA252)	NEG	NEG	NEG	NEG	AMB	NEG	AMB	AMB	AMB	AMB	AMB	NEG	AMB	NEG	NEG	POS	NEG	AMB
	staphylococcal superantigenlike protein 3	*ssl03/set8_probe 1*	POS	POS	POS	POS	POS	POS	POS	POS	POS	POS	POS	POS	POS	POS	POS	POS	POS	POS
		ssl03/set8_probe 2	POS	POS	POS	POS	POS	POS	POS	POS	POS	POS	POS	POS	POS	POS	POS	POS	POS	POS
		ssl03/set8 (MRSA252, SAR0424)	NEG	NEG	NEG	NEG	NEG	NEG	NEG	NEG	NEG	NEG	NEG	NEG	NEG	NEG	NEG	NEG	NEG	NEG
	staphylococcal superantigenlike protein 4	*ssl04/set9*	POS	POS	POS	NEG	POS	POS	POS	POS	POS	POS	POS	POS	POS	POS	POS	POS	POS	POS
		ssl04/set9 (MRSA252, SAR0425)	NEG	NEG	NEG	NEG	NEG	NEG	AMB	AMB	AMB	NEG	NEG	NEG	NEG	NEG	NEG	AMB	NEG	NEG
	staphylococcal superantigenlike protein 5	*ssl05/set3_probe 1*	POS	POS	POS	POS	POS	POS	POS	AMB	POS	POS	POS	POS	POS	POS	POS	POS	POS	POS
		ssl05/set3 (RF122, probe-611)	NEG	NEG	NEG	NEG	POS	POS	AMB	AMB	AMB	AMB	AMB	POS	AMB	POS	POS	AMB	POS	POS
		ssl05/set3_probe 2 (612)	POS	POS	POS	POS	POS	AMB	POS	POS	POS	POS	POS	AMB	POS	AMB	POS	POS	AMB	AMB
		ssl05/set3 (MRSA252)	NEG	NEG	NEG	NEG	NEG	NEG	AMB	NEG	NEG	NEG	NEG	NEG	NEG	NEG	NEG	AMB	NEG	NEG
	staphylococcal superantigenlike protein 6	*ssl06/set21*	POS	POS	NEG	NEG	NEG	NEG	NEG	NEG	NEG	NEG	NEG	NEG	NEG	NEG	NEG	NEG	NEG	NEG
		ssl06 (NCTC8325+MW2)	POS	POS	NEG	NEG	NEG	NEG	NEG	NEG	AMB	NEG	NEG	NEG	NEG	NEG	NEG	AMB	NEG	NEG
	staphylococcal superantigenlike protein 7	*ssl07/set1*	POS	POS	POS	POS	POS	POS	POS	POS	POS	POS	POS	POS	POS	POS	POS	POS	POS	POS
		ssl07/set1 (MRSA252)	NEG	NEG	NEG	NEG	NEG	NEG	NEG	NEG	NEG	NEG	NEG	NEG	NEG	NEG	NEG	NEG	NEG	NEG
		ssl07/set1 (AF188836)	NEG	NEG	NEG	NEG	NEG	NEG	AMB	NEG	AMB	NEG	NEG	NEG	NEG	NEG	NEG	NEG	NEG	AMB
	staphylococcal superantigenlike protein 8	*ssl08/set12_probe 1*	POS	POS	POS	POS	POS	POS	POS	POS	POS	POS	POS	POS	POS	POS	POS	POS	POS	POS
		ssl08/set12_probe 2	POS	POS	POS	POS	POS	POS	POS	POS	POS	POS	POS	POS	POS	POS	POS	POS	POS	POS
	staphylococcal superantigenlike protein 9	*ssl09/set5_probe 1*	POS	POS	POS	POS	POS	POS	POS	POS	POS	POS	POS	POS	POS	POS	POS	POS	AMB	POS
		ssl09/set5_probe 2	POS	POS	POS	POS	POS	POS	POS	POS	POS	POS	POS	POS	POS	POS	NEG	POS	NEG	POS
		ssl09/set5 (MRSA252)	NEG	NEG	NEG	NEG	NEG	NEG	NEG	NEG	NEG	NEG	NEG	NEG	NEG	NEG	NEG	NEG	NEG	NEG
	staphylococcal superantigenlike protein 10	*ssl10/set4*	POS	POS	POS	POS	POS	POS	POS	POS	POS	POS	POS	POS	POS	POS	POS	POS	POS	POS
		ssl10 (RF122)	NEG	NEG	NEG	NEG	NEG	NEG	AMB	AMB	AMB	AMB	AMB	NEG	AMB	NEG	NEG	AMB	NEG	NEG
		ssl10/set4 (MRSA252)	NEG	NEG	NEG	NEG	NEG	NEG	AMB	AMB	AMB	AMB	AMB	AMB	AMB	AMB	NEG	POS	NEG	AMB
	staphylococcal superantigenelike protein 11	*ssl11/set2 (COL)*	NEG	NEG	NEG	NEG	NEG	NEG	NEG	NEG	NEG	NEG	NEG	NEG	NEG	NEG	NEG	NEG	NEG	NEG
		ssl11+set2(Mu50+N315)	NEG	NEG	POS	POS	NEG	NEG	NEG	NEG	NEG	NEG	NEG	NEG	NEG	NEG	NEG	NEG	NEG	NEG
		ssl11+set2(MW2+MSSA476)	POS	POS	NEG	NEG	NEG	NEG	NEG	NEG	NEG	NEG	NEG	NEG	NEG	NEG	NEG	NEG	NEG	NEG
		ssl11/set2 (MRSA252)	NEG	NEG	NEG	NEG	NEG	NEG	NEG	NEG	NEG	NEG	NEG	NEG	NEG	NEG	NEG	NEG	NEG	NEG
	staphylococcal exotoxinlike protein, second locus	*setB3*	POS	POS	POS	POS	POS	POS	POS	POS	POS	POS	POS	POS	POS	POS	POS	POS	POS	POS
		setB3 (MRSA252)	NEG	NEG	NEG	NEG	NEG	NEG	NEG	NEG	NEG	AMB	NEG	NEG	NEG	NEG	NEG	NEG	NEG	NEG
		setB2	POS	POS	POS	POS	POS	POS	POS	POS	POS	POS	POS	POS	POS	POS	POS	POS	POS	POS
		setB2 (MRSA252)	NEG	NEG	NEG	NEG	NEG	NEG	NEG	NEG	NEG	NEG	NEG	NEG	NEG	NEG	NEG	NEG	NEG	NEG
		setB1	POS	POS	POS	POS	POS	POS	POS	POS	POS	POS	POS	POS	POS	POS	POS	POS	POS	POS
**CAPSULE- AND BIOFILM-ASSOCIATED GENES**	capsule type 1	*cap 1 (total)*	NEG	NEG	NEG	NEG	NEG	NEG	NEG	NEG	NEG	NEG	NEG	NEG	NEG	NEG	NEG	NEG	NEG	NEG
	capsule type 5	*cap 5 (total)*	NEG	NEG	POS	POS	POS	POS	POS	POS	POS	POS	POS	POS	POS	POS	POS	POS	POS	POS
	capsule type 8	*cap 8 (total)*	POS	POS	NEG	NEG	NEG	NEG	NEG	NEG	NEG	NEG	NEG	NEG	NEG	NEG	NEG	NEG	NEG	NEG
	intercellular adhesion protein A	*icaA*	POS	POS	POS	POS	POS	POS	POS	POS	POS	POS	POS	POS	POS	POS	POS	POS	POS	POS
	intercellular adhesion protein C	*icaC*	POS	POS	POS	POS	POS	POS	POS	POS	POS	POS	POS	POS	POS	POS	POS	POS	POS	POS
	biofilm PIA synthesis protein D	*icaD*	POS	POS	POS	POS	POS	POS	POS	POS	POS	POS	POS	POS	POS	POS	POS	POS	POS	POS
	surface protein involved in biofilm formation	*bap*	NEG	NEG	NEG	NEG	NEG	NEG	NEG	NEG	NEG	NEG	NEG	NEG	NEG	NEG	NEG	NEG	NEG	NEG
**ADHAESION FACTORS / GENES ENCODING MICROBIAL SURFACE COMPONENTS RECOGNIZING ADHESIVE MATRIX MOLECULES (MSCRAMM GENES)**	bone sialoproteinbinding protein	*bbp (total)*	POS	POS	POS	POS	POS	POS	POS	POS	POS	POS	POS	POS	POS	POS	POS	POS	POS	POS
		bbp (consensus)	POS	POS	POS	POS	POS	POS	POS	POS	POS	POS	POS	POS	POS	POS	POS	POS	POS	POS
		bbp (COL+MW2)	POS	POS	NEG	NEG	NEG	NEG	AMB	AMB	AMB	AMB	NEG	NEG	NEG	NEG	NEG	NEG	NEG	NEG
		bbp (MRSA252)	NEG	NEG	NEG	NEG	NEG	NEG	NEG	NEG	NEG	NEG	NEG	NEG	NEG	NEG	NEG	NEG	NEG	NEG
		bbp (Mu50)	NEG	NEG	POS	POS	POS	POS	POS	POS	POS	POS	POS	POS	POS	POS	POS	POS	POS	POS
		bbp (RF122)	NEG	NEG	NEG	NEG	NEG	NEG	NEG	NEG	NEG	NEG	NEG	NEG	NEG	NEG	NEG	NEG	NEG	NEG
		bbp (ST45)	NEG	NEG	NEG	NEG	NEG	NEG	NEG	NEG	NEG	AMB	NEG	NEG	AMB	NEG	NEG	AMB	NEG	NEG
	clumping factor A	*clfA (total)*	POS	POS	POS	POS	POS	POS	POS	POS	POS	POS	POS	POS	POS	POS	POS	POS	POS	POS
		clfA (consensus)	POS	POS	POS	POS	POS	POS	POS	POS	POS	POS	POS	POS	POS	POS	POS	POS	POS	POS
		clfA (COL+RF122)	NEG	NEG	NEG	NEG	NEG	NEG	NEG	NEG	NEG	NEG	NEG	NEG	NEG	NEG	NEG	NEG	NEG	NEG
		clfA (MRSA252)	NEG	NEG	NEG	NEG	NEG	NEG	NEG	NEG	NEG	NEG	NEG	NEG	NEG	NEG	NEG	NEG	NEG	NEG
		clfA (Mu50+MW2)	POS	POS	POS	POS	POS	POS	POS	POS	POS	POS	POS	POS	POS	POS	POS	POS	POS	POS
	clumping factor B	*clfB (total)*	POS	POS	POS	POS	POS	POS	POS	POS	POS	POS	POS	POS	POS	POS	POS	POS	POS	POS
		clfB (consensus)	POS	POS	POS	POS	POS	POS	POS	POS	POS	POS	POS	POS	POS	POS	POS	POS	POS	POS
		clfB (COL+Mu50)	NEG	NEG	POS	POS	NEG	NEG	NEG	NEG	NEG	NEG	NEG	NEG	NEG	NEG	NEG	NEG	NEG	NEG
		clfB (MW2)	POS	POS	NEG	NEG	NEG	NEG	NEG	NEG	NEG	NEG	NEG	NEG	NEG	NEG	NEG	NEG	NEG	NEG
		clfB (RF122)	POS	AMB	NEG	NEG	AMB	NEG	POS	POS	POS	POS	POS	POS	POS	POS	NEG	POS	NEG	NEG
	collagen binding adhesin	*cna*	POS	POS	NEG	NEG	POS	POS	POS	POS	POS	POS	POS	POS	POS	POS	POS	POS	POS	POS
	cell wall associated fibronectinbinding protein	*ebh (consensus)*	POS	POS	POS	POS	POS	POS	POS	POS	POS	POS	POS	POS	POS	POS	POS	POS	POS	POS
	cell surface elastin binding protein	*ebpS (total)*	POS	POS	POS	POS	POS	POS	POS	POS	POS	POS	POS	POS	POS	POS	POS	POS	POS	POS
		ebpS_probe 612	POS	POS	POS	POS	POS	POS	POS	POS	POS	POS	POS	POS	POS	POS	POS	POS	POS	POS
		ebpS_probe 614	POS	POS	POS	POS	POS	POS	POS	POS	POS	POS	POS	POS	POS	POS	POS	POS	POS	POS
		ebpS (01-1111)	NEG	NEG	NEG	NEG	NEG	NEG	NEG	NEG	NEG	NEG	NEG	NEG	NEG	NEG	NEG	NEG	NEG	NEG
		ebpS (COL)	NEG	NEG	NEG	NEG	NEG	NEG	NEG	NEG	NEG	AMB	AMB	NEG	NEG	NEG	NEG	AMB	NEG	NEG
	enolase	*eno*	POS	POS	POS	POS	POS	POS	POS	POS	POS	POS	POS	POS	POS	POS	POS	POS	POS	POS
	fibrinogen binding protein (19 kDa)	*fib*	POS	POS	POS	POS	POS	POS	POS	POS	POS	POS	POS	POS	POS	POS	POS	POS	POS	POS
		fib (MRSA252)	NEG	NEG	NEG	NEG	NEG	NEG	AMB	NEG	NEG	AMB	AMB	NEG	NEG	NEG	AMB	NEG	NEG	NEG
	fibronectinbinding protein A	*fnbA (total)*	POS	POS	POS	POS	POS	POS	POS	POS	POS	POS	POS	POS	POS	POS	POS	POS	POS	POS
		fnbA (consensus)	POS	POS	POS	POS	POS	POS	POS	POS	POS	POS	POS	POS	POS	POS	POS	POS	POS	POS
		fnbA (COL)	NEG	NEG	NEG	NEG	NEG	NEG	NEG	NEG	NEG	NEG	NEG	NEG	NEG	NEG	NEG	NEG	NEG	NEG
		fnbA (MRSA252)	NEG	NEG	NEG	NEG	NEG	NEG	NEG	NEG	NEG	NEG	NEG	NEG	NEG	NEG	NEG	NEG	NEG	NEG
		fnbA (Mu50+MW2)	POS	POS	POS	POS	POS	POS	POS	POS	POS	POS	POS	POS	POS	POS	NEG	POS	NEG	POS
		fnbA (RF122)	NEG	NEG	NEG	NEG	NEG	NEG	NEG	NEG	NEG	NEG	NEG	NEG	NEG	NEG	NEG	NEG	NEG	NEG
	fibronectinbinding protein B	*fnbB (total)*	POS	POS	POS	POS	POS	POS	POS	POS	POS	POS	POS	POS	POS	POS	POS	POS	POS	POS
		fnbB (COL)	NEG	NEG	NEG	NEG	NEG	NEG	NEG	NEG	NEG	NEG	NEG	NEG	NEG	NEG	NEG	NEG	NEG	NEG
		fnbB (COL+Mu50+MW2)	POS	AMB	POS	POS	POS	POS	AMB	AMB	AMB	AMB	AMB	POS	POS	POS	POS	AMB	POS	POS
		fnbB (Mu50)	NEG	NEG	POS	POS	NEG	NEG	AMB	AMB	AMB	AMB	AMB	NEG	AMB	NEG	POS	POS	NEG	NEG
		fnbB (MW2)	POS	POS	NEG	NEG	NEG	NEG	NEG	NEG	NEG	AMB	NEG	NEG	AMB	NEG	NEG	NEG	NEG	NEG
		fnbB (ST15)	NEG	NEG	NEG	NEG	POS	AMB	POS	POS	POS	POS	POS	AMB	AMB	AMB	POS	POS	NEG	AMB
		fnbB (ST45-2)	NEG	NEG	NEG	NEG	NEG	NEG	NEG	NEG	NEG	NEG	NEG	NEG	NEG	NEG	NEG	NEG	NEG	NEG
	Major histocompatibility complex class II analog protein (=Extracellular adherence protein, eap)	*map (total)*	NEG	POS	POS	POS	POS	POS	POS	POS	POS	POS	POS	POS	POS	POS	POS	POS	POS	POS
		map (COL)	AMB	NEG	AMB	POS	NEG	NEG	AMB	POS	POS	AMB	NEG	NEG	NEG	NEG	NEG	AMB	NEG	AMB
		map (MRSA252)	NEG	NEG	NEG	NEG	NEG	NEG	NEG	NEG	AMB	NEG	NEG	NEG	NEG	NEG	NEG	AMB	NEG	NEG
		map (Mu50+MW2)	AMB	POS	POS	POS	POS	POS	POS	POS	POS	POS	POS	POS	POS	POS	POS	POS	POS	POS
	Staphylococcus aureus surface protein G	*sasG (total)*	POS	POS	POS	POS	POS	POS	POS	POS	POS	POS	POS	POS	POS	POS	POS	POS	POS	POS
		sasG (COL+Mu50)	NEG	NEG	POS	POS	NEG	NEG	NEG	NEG	NEG	NEG	NEG	NEG	NEG	NEG	NEG	NEG	NEG	NEG
		sasG (MW2)	POS	POS	NEG	NEG	POS	POS	POS	POS	POS	POS	POS	POS	POS	POS	POS	POS	POS	POS
		sasG (other than MRSA252+RF122)	POS	POS	POS	POS	POS	POS	POS	POS	POS	POS	POS	POS	POS	POS	POS	POS	POS	POS
	SerAsp rich fibrinogen/bone sialoprotein binding protein C	*sdrC (total)*	POS	POS	POS	POS	POS	POS	POS	POS	POS	POS	POS	POS	POS	POS	POS	POS	POS	POS
		sdrC (consensus)	POS	POS	POS	POS	POS	POS	POS	POS	POS	POS	POS	POS	POS	POS	POS	POS	POS	POS
		sdrC (B1)	NEG	NEG	NEG	NEG	NEG	NEG	NEG	NEG	NEG	NEG	NEG	NEG	NEG	NEG	NEG	NEG	NEG	NEG
		sdrC (COL)	NEG	NEG	NEG	NEG	NEG	NEG	NEG	NEG	NEG	NEG	NEG	NEG	NEG	NEG	NEG	NEG	NEG	NEG
		sdrC (Mu50)	NEG	NEG	POS	POS	POS	POS	POS	POS	POS	POS	POS	POS	POS	POS	POS	POS	AMB	POS
		sdrC (MW2+MRSA252+RF122)	POS	POS	NEG	NEG	AMB	AMB	POS	POS	POS	POS	POS	NEG	AMB	NEG	NEG	POS	NEG	POS
		sdrC (other than MRSA252+RF122)	POS	POS	POS	POS	POS	POS	POS	POS	POS	POS	POS	POS	POS	POS	POS	POS	POS	POS
	SerAsp rich fibrinogen/bone sialoprotein binding protein D	*sdrD (total)*	POS	POS	POS	POS	POS	POS	POS	POS	POS	POS	POS	POS	POS	POS	POS	POS	POS	POS
		sdrD (consensus)	POS	POS	POS	POS	POS	POS	POS	POS	POS	POS	POS	POS	POS	POS	POS	POS	POS	POS
		sdrD (COL+MW2)	POS	POS	NEG	NEG	NEG	NEG	NEG	NEG	NEG	NEG	NEG	NEG	NEG	NEG	NEG	NEG	NEG	NEG
		sdrD (Mu50)	NEG	NEG	POS	POS	NEG	NEG	NEG	NEG	NEG	NEG	NEG	NEG	NEG	NEG	NEG	NEG	NEG	NEG
		sdrD (other)	NEG	NEG	NEG	NEG	POS	POS	POS	POS	POS	POS	POS	POS	POS	POS	POS	POS	POS	POS
	van Willebrand factor binding protein	*vwb (total)*	POS	POS	POS	POS	POS	POS	POS	POS	POS	POS	POS	POS	POS	POS	POS	POS	POS	POS
		vwb (consensus)	POS	POS	POS	POS	POS	POS	POS	POS	POS	POS	POS	POS	POS	POS	POS	POS	POS	POS
		vwb (COL+MW2)	POS	POS	NEG	NEG	NEG	NEG	NEG	NEG	NEG	NEG	NEG	NEG	NEG	NEG	NEG	NEG	NEG	NEG
		vwb (MRSA252)	NEG	NEG	NEG	NEG	NEG	NEG	NEG	NEG	NEG	NEG	NEG	NEG	NEG	NEG	NEG	NEG	NEG	NEG
		vwb (Mu50)	NEG	NEG	POS	POS	NEG	NEG	NEG	NEG	NEG	NEG	NEG	NEG	NEG	NEG	NEG	NEG	NEG	NEG
		vwb (RF122)	NEG	NEG	NEG	NEG	NEG	NEG	NEG	NEG	NEG	NEG	NEG	NEG	NEG	NEG	NEG	NEG	NEG	NEG
**TYPE I RESTRICTION-**	type I site-specific deoxyribonuclease subunit, 1st locus	*hsdS1 (RF122)*	NEG	NEG	NEG	NEG	NEG	NEG	NEG	NEG	NEG	NEG	NEG	NEG	NEG	NEG	NEG	NEG	NEG	NEG
	type I site-specific deoxyribonuclease subunit, 2nd locus	*hsdS2 (Mu50+N315+COL+USA300+NCTC8325)*	NEG	NEG	POS	POS	NEG	NEG	NEG	NEG	NEG	NEG	NEG	NEG	NEG	NEG	NEG	NEG	NEG	NEG
		hsdS2 (MW2+MSSA476)	POS	POS	NEG	NEG	NEG	NEG	NEG	NEG	NEG	AMB	NEG	AMB	POS	AMB	NEG	POS	NEG	NEG
		hsdS2 (RF122)	NEG	NEG	NEG	NEG	NEG	NEG	NEG	NEG	NEG	NEG	NEG	NEG	NEG	NEG	NEG	NEG	NEG	NEG
		hsdS2 (MRSA252)	NEG	NEG	NEG	NEG	NEG	NEG	NEG	NEG	NEG	NEG	NEG	NEG	NEG	NEG	NEG	NEG	NEG	NEG
	type I site-specific deoxyribonuclease subunit, 3rd locus	*hsdS3 (all other than RF122+ MRSA252)*	POS	POS	POS	POS	NEG	NEG	AMB	POS	POS	POS	NEG	NEG	NEG	NEG	NEG	AMB	NEG	POS
		hsdS3 (COL+USA300+NCTC8325+MW2+MSSA476+RF122)	POS	POS	NEG	NEG	NEG	NEG	NEG	NEG	NEG	NEG	NEG	NEG	NEG	NEG	NEG	NEG	NEG	NEG
		hsdS3 (Mu50+N315)	NEG	NEG	POS	POS	NEG	NEG	NEG	NEG	NEG	NEG	NEG	NEG	NEG	NEG	NEG	NEG	NEG	NEG
		hsdS3 (CC51+ MRSA252)	NEG	NEG	NEG	NEG	NEG	NEG	NEG	NEG	NEG	NEG	NEG	NEG	NEG	NEG	NEG	NEG	NEG	NEG
		hsdS3 (MRSA252)	NEG	NEG	NEG	NEG	NEG	NEG	NEG	NEG	NEG	NEG	NEG	NEG	NEG	NEG	NEG	NEG	NEG	NEG
	type I site-specific deoxyribonuclease subunit, unknown locus	*hsdSx (CC25)*	POS	POS	POS	POS	NEG	NEG	AMB	AMB	POS	POS	NEG	NEG	NEG	NEG	NEG	AMB	NEG	POS
		hsdSx (CC15)	NEG	NEG	NEG	NEG	NEG	NEG	NEG	NEG	AMB	POS	POS	AMB	AMB	AMB	NEG	POS	NEG	NEG
		hsdSx (etd)	NEG	NEG	NEG	NEG	NEG	NEG	NEG	NEG	NEG	POS	AMB	NEG	NEG	NEG	NEG	NEG	NEG	NEG
**HYALURONATE LYASE**	hyaluronate lyase, first / second locus	*hysA1 (MRSA252)*	NEG	NEG	NEG	NEG	NEG	NEG	NEG	NEG	NEG	NEG	NEG	NEG	NEG	NEG	NEG	NEG	NEG	NEG
		hysA1 (MRSA252+RF122) and/or hysA2 (consensus)	POS	POS	POS	POS	POS	POS	POS	POS	POS	POS	POS	POS	POS	POS	POS	POS	POS	POS
		hysA1 (MRSA252+RF122) and/or hysA2 (COL+USA300)	NEG	NEG	NEG	NEG	NEG	NEG	NEG	NEG	NEG	NEG	NEG	NEG	NEG	NEG	NEG	NEG	NEG	NEG
	hyaluronate lyase, second locus	*hysA2 (all other than MRSA252)*	POS	POS	POS	POS	POS	POS	POS	POS	POS	POS	POS	POS	POS	POS	POS	POS	AMB	POS
		hysA2 (COL+USA300+NCTC8325)	NEG	NEG	NEG	NEG	NEG	NEG	NEG	NEG	NEG	NEG	NEG	NEG	NEG	NEG	NEG	NEG	NEG	NEG
		hysA2 (all other than COL+USA300+NCTC8325)	POS	POS	POS	POS	POS	POS	POS	POS	POS	POS	POS	POS	POS	POS	POS	POS	POS	POS
		hysA2 (all other than COL+USA300+NCTC8325)	POS	POS	POS	POS	POS	POS	POS	POS	POS	POS	POS	POS	POS	POS	POS	POS	POS	POS
		hysA2 (MRSA252)	NEG	NEG	NEG	NEG	NEG	AMB	POS	NEG	NEG	AMB	AMB	NEG	NEG	NEG	NEG	NEG	NEG	NEG
**MISCELLANEOUS GENES**	hypothetical protein next to serine protease operon	*Q2FXC0*	POS	POS	NEG	NEG	NEG	NEG	NEG	NEG	NEG	NEG	NEG	NEG	NEG	NEG	NEG	NEG	NEG	NEG
	unspecific efflux/transporter	*Q2YUB3*	NEG	NEG	NEG	NEG	NEG	NEG	AMB	AMB	AMB	POS	AMB	NEG	AMB	NEG	NEG	POS	NEG	NEG
	hypothetical protein	*Q7A4X2*	NEG	NEG	POS	POS	POS	POS	POS	POS	POS	POS	POS	POS	POS	POS	POS	POS	POS	POS
	immunodominant antigen B	*isaB*	POS	POS	POS	POS	POS	POS	AMB	POS	AMB	AMB	AMB	POS	AMB	POS	POS	AMB	POS	POS
		isaB (MRSA252)	NEG	AMB	POS	AMB	POS	AMB	POS	AMB	POS	POS	POS	AMB	POS	AMB	POS	POS	AMB	AMB
	defensin resistance protein	*mprF (COL+MW2)*	POS	POS	NEG	NEG	POS	POS	POS	POS	AMB	POS	POS	POS	POS	POS	POS	AMB	POS	POS
		mprF (Mu50+MRSA252)	NEG	NEG	POS	POS	NEG	AMB	AMB	AMB	POS	AMB	AMB	AMB	AMB	AMB	POS	POS	AMB	AMB
	transferrin-binding protein	*isdA (consensus)*	POS	POS	POS	POS	POS	POS	POS	POS	POS	POS	POS	POS	POS	POS	POS	POS	POS	POS
		isdA (MRSA252)	NEG	NEG	NEG	NEG	NEG	NEG	AMB	NEG	AMB	AMB	AMB	NEG	NEG	NEG	NEG	AMB	NEG	NEG
		isdA (other than MRSA252)	POS	POS	POS	POS	POS	POS	POS	POS	POS	POS	POS	POS	POS	POS	POS	POS	POS	POS
	hypothetical protein, similar to integral membrane protein LmrP	*lmrP (other than RF122)*	POS	POS	POS	POS	POS	POS	POS	POS	POS	POS	POS	POS	POS	POS	POS	POS	POS	POS
		lmrP (RF122)	NEG	NEG	NEG	NEG	NEG	NEG	NEG	NEG	NEG	NEG	NEG	NEG	NEG	NEG	NEG	NEG	NEG	NEG
