# Supplementary material for: Genome sequencing and molecular characterisation of Staphylococcus aureus ST772-MRSA-V, “Bengal Bay Clone”
Source: BMC Res Notes. 2013 Dec 20;6:548. doi: 10.1186/1756-0500-6-548 (PMC3878137; doi:10.1186/1756-0500-6-548)
Supplement: Additional file 2 — Raw sequences of 70 contigs of isolate 07–17048. [file 1756-0500-6-548-S2.pdf]

>001-contig_254_RC

CAATGATTGTTATTGTCTTAAGCGTAGTTACAACACCAATTATAGGTATTCCAGCTGGTTTATTAGGTGGCGCTTATTATTTAAAAAG
CCGTGAAGAAAAAGGTAAATAAGCACATTCCATCGGCATATAACATTGCTATGACCTTTCAAGTCAGATTTTAATATCAACAAGTA
GCATTTATCGCTGCTTGTTTTTTATGAATTGCTTATAAAATCGTCATGTTTAAACGTTATGTCACTTTGAAAACTAGCAAACACCATA
AAAGACTACAATTTTTTATTTTTATAAACCCCTTTAAATCAACGTTTCTAAGGTTTTTAAAAATCTTTTTAAATTTATTTTCAAAAAAC
ACTGTACATTATGCCAATATGAGCGTATAGTTGGTCTTACGTAATAAAAGCTCGTGAATTAAATTGTAGTGTATTTGTTTAGAATATC
CTCTTTTTTAGTTATGAATTTGTTACAAATATTAAGTGCAAAAGCACATGGAGGTTTTCTATATGAATAACGGTACAGTAAAATGGT
TTAACGCAGAAAAAGGTTTTGGTTTCATCGAACAAGAAAATGGCGGAGACGTATTCGTACATTTCTCAGGTATCGCTAGCGATGGCT
ACAAAACTTTAGAAGAAGGTCAAAAAGTTACTTTCGAAATCACTGAAGGTCAACGTGGAGACCAAGCAGTTAACGTACAAACTGTT
TAATCTTACAAAATAAAACGACTCATTATAAATGAATCGCTTTGATTACCAACAAGGTTCTAAATCTTGTTGGTATTTTTTTATGCTA
TTTTGGATAATATGAGACGATACGATGATGATTTATAAAAACTTATTTGCCATATACATACTAATCAAAGCTATAAGAATGATTGCA
CTAACAAATGCTATGAAAGAAAGTACACTTGTTTTTATATTTCTAGCTTTAGTCGTTTGTACTTCACTAGGATCTTTTCCATTCATAA
AAGCAACAATTCGATCGTAGTTATCCATTTTATGCACTTTTTTAAAATCTTCTATTTTCATAGCTGCATAATAACCTATTCCATATATG
ACTGCCACCGTTATACCCCAAGCCCAATTCCAATAAACAATTAAAGGTCCTGTTAAAATTGCAGCTAATGTCATGAAAATAAGCATC
ACATATGTCCATAAGTTAAGACTTCGTTGCGCTTTAATATCAGGTACAAATGGAATGGTCCCTTTTACTAAATCATCTAAAGTTACAT
TGAACAATTCGCACATCATTAATAAGTTATGTATGTCTGGTAAGCTTTTGTCATTTTCCCAATTAGAAATACTCTGCCTAGATACATA
TAACTTTTCAGCAAGATATTCTTGTGAATAACCATCTCGTTCCCTATATTTTTTAATTTGTTTACTCAGATTCAATTACATACGCCTCC
ATTCAATTAGTACAATCATTACAATCAGACTATCAGATAAAGTTAAATCAAGTCACCTGTATGATTGACTAAATGTTTTGACATACG
ACAACATCGGGGTTCGAAGACTCCCTTAAGACAGACCTGTATGATTACGCACTTGTAATTTTCTCAATGCCAACTTGAATTTCACTT
ATTAATGATGCAACTTTTCCATGCTATTCAATGCTTGCAAAGTTAGTCCTAGTTAAGAGATTACGTTCACACTATTTACTATTCGATT
TTCCAGCTTTCCAACTATGCTTTTCTAATCTTTTTTGTGCTATAAATTCTACTGCTTCAAAAATACATACTGCACTGATGAACCAGCTT
AGGAAACCAACAACAATGCTAATCAACCACATGTGCGTCATATGTAACATCATCCATGTAACTAAAATACAAACTAATACCCCTGT
CATACCAAACACTGCGCCACGACTTACATGCACAGCGACTTTATCACCATATTGCATACCTGTAATAAACATAGACACTAAAAATAC
TGCCGGAAACGTTGCAAATATACCGCCAAATTCTTTCCAAGGTAGTGTTACAGATACAATATAACTTAATAATACTGCAAATCCCCC
CACAAAAAATTTCATTAATGTTAATTTCATGATGTTCTGCCTCCAATAATTTATTACTTAATAACTGAATACAACTAATTATCACGCA
TTTTCCAAACTATAAATGTCTAATACTGAATATTGCGAGGGAAATAACAAACCAACAAACAACCGTGAATATTGCGCCTTTCCGATA
ACCTTTATGCTTAATGTAGAGAGATGTCAAAAATACAGTTAATATACAAGACAGAATACCGACAATTGCTCCAGTACTAAGATTCAT
CGACATCTCCACTAATTGCGTACCACGATGATCTAAAGCTAATGCGATAATAGCCGCAAGAAAGACTGCCGGCATAGTAGCTATAA
TACCTCCTAACTTACCACCTACCTTATCAGCAATAATTGAAGCTAATGCAACAGCGATACCACCAATGACAAAATGAAGAATTGCA
CTTCCAATTGAAAACATCTTCAACGTACCTTTCTATAATTAATTCAATACAAAACGTATCATTCACACAAAGTGTAAAACTTAAATTT
TCTACTGTAAAGGCAACTCAAGCGATGTTCATAAAGCTTTCATGATACTTTCATAAACTAGCAATAAAGGGTTCAAATGAAAGTATA
TCCAGCTCAAATTATTGAAAATTACCATTAACTGTAATTTTCAAATCAACATATAAATATAAATTTTTATGTTTCACGTAAAACAAA
AAAAAGACAAAGCTGTTATGATCTTAGCTTTGTCTTTTATGTGTTAGTCATCATGTATACGACATCCTACTACTAACGTTTATATATT
CGAATTTTTATTTCATAATAATCTTCATGATCTTTGTCTTTATGCTCAACATGTAATCCTGTTTGTTGAATCGCTTGGATACTTTTGCC
TACCTCATCTCGTGCTTGAGTGACATCTTGTGCAAAGCGAAGGTTTTGTGCTTTGACTTTTTCGGGCCCCGTTTTTTGGCGTACTCTAT
CTTCAGTCTGTTTCACATTTAGCTTTTGTGCAATGACTTGCTCAATCAACGCTTCTTGTTCGCTATCAGACAATGATAATACCGCTCTC
GCATGGCGTTCAGTAATTTTACCTTCTCTTAAGCGAAGTAGTACTTTCGGCGCCAACTTCAATAAACGCAACTTATTTGCAATAAAG
CTTTGACTTTTACCTAAACTTTTTGCCAATTCACTTTGCGTTGTATCACCAATTTCCAATAATTTCTTATAGGCTTCCGCTTCTTCAAC
AACAGACAAATTTTCTCTTTGAATATTCTCAATTAATGCCACAACAGCCGTCTCTTCATCATCCATATCACGAATAATAACGTCTGCT
TGAGGTAAATTTAGTGATTGTATTGCTCTAAATCGGCGCTCTCCAGCAATAATTTCAAACATATCTTCTTCAATCGGTCTTACAACAA
TAGGTTGTAGTAAACCATGTTCATGTATTGATTCAGCAAGTTCTTTAATTTTATTTGGTTCAAACACCTGTCTTGGTTGATAACGGTT
GGGAACGATACGTTCAATTTGAATGGATTCAACATTACTATTGCGATCTTCTTCAATATGTCCAATGATGTCATCTTTGTTTTTCAAA
CCAAATAATTTTGAAAAAGGTTTTTTCATTATCCATTCGCTCCTTTTATGTTAGTTGTAAAGGATTATGCATTATTTTTCAAGTAAAG
GAGTCTTATTAGGCGTCCCTGGTTTTCTTGGATACTTTTTCGGCGTCTGTCTTTTTTTATCAATAATGAACATCTGGCGCTCTCCAGCA
TCTTCTGGCAATTCAAAGGTATGTGTTTCTGTAACATTACCACCTAACACACTAATTGCAAATTTTGCTTCTTCTAATTCTTCTTCACC
TTTTGAAGATTTTAATGCAACAAACTGTCCACCTTTTTTAACTAGCGGTAAACACAATTCACTTAACACGGATAATCTAGCTACTGCT
CTTGCAGTAACAACATCATAAGACTCCCTGTAGACACCCTTACCAAATGTTTCTGCTCTATCGTGTATAAAGCTGACATCCTGTAATT
GTAATTCTGACGCTAAATGGTTTAAAAATTGAATACGCTTATTTAATGAATCAACAATCGTCACTTTTAACTGCGGAAACATTATTTT
TAACGGAATACTTGGAAAACCAGCTCCAGCGCCTACATCACATATACTTATAGGCTGATTAAAATCAAAATAAAAACTAGGTGCAA
TGGAATCATAAAAATGTTTCAAATATACATCGTGTTCATCTGTAATACTTGTCAAATTCATCTTTTCATTCCATTCAACAAGTAAACG
ATAATATGTTTGAAACTGTTGTTTTTGAGTCTCAGTTAATTCAATATTATGTTCTTTTAATTGTTCTGCTAACCATTCTACAGTCATTA
GTCACTCACCCTTTGGAGTTTACCTTGTTCTAAGTAAATCAATAATATAGATATGTCTGCTGGATTTACCCCTGATATTCTAGAAGCT
TGTGCAATATTTAAAGGTTTTACTTCTGATAATTTTTCTCGCGCTTCAGTCGCCAAACTATCAATCTTACTATAATCTAAGTCTTCTGG
AATTTTCTTCTCTTCCATACGCTTAACTTTCTCAACTTGTTGTAGTGATTTATTGATATAACCTTCATATTTTGTTTGTATTTCTACTTG
TTCTTCAACATCTGCATTCAATTGATGTTCTTCTTCTAAAATTTCTAAAATTATATCGTAAGTCATTTCAGGTCTGCGTAATAAATCG
ATAGCTAAAATACCATCTTTTAAGCGAGAACCACCATGTTGTTCAATAATCGCTTGCGTATGTTCGTTTGGTTTAATACGAATATCTG
ATAAACGCTTAATTTCCGCATCAATTTGCTGACGTTTTTCATTAAAACGTGCATATCGTTCTTCAGAAATCATACCAAGTTCATATCC
CATATCCGTCAATCTCAAATCAGCATTATCATGACGTAGTAACAAACGATATTCTGCACGTGATGTTAGTAAACGATAAGGTTCATT
AGTACCTTTAGTTACAAGATCATCGATTAAGACACCAATATATGCATCTGAACGACTTAATATCTTTTCGCCTGTGTTTAACACTTTA
CCTGCAGCGTTAATACCTGCCATCAATCCTTGTCCTGCTGCTTCTTCATAACCAGATGTACCATTAATTTGACCTGCAGTATATAAGT
TTTTAATCATTTTCGTTTCAAGTGTAGGCCATAACTGCGTTGGCACAATCGCATCATATTCAATTGCGTAGCCGGCACGCATCATATC
TGCTTTTTCAAGACCTGGTATCGTCTCTAACATTTGACGTTGCACATGTTCAGGAAGACTTGTAGACAATCCTTGCACATATACTTCA
TTTGTATTACGACCTTCAGGCTCTAAGAAAAGTTGATGTCGCGGCTTATCATTAAATCGAACAAATTTATCTTCAATTGAAGGGCAA
TAACGTGGCCCGGTTCCTTTAATCATCCCTGAATACATTGCAGATAGATGTAAATTATCATCGATAACTTTGTGTGTTTCAGCATTAG
TATACGTTAGCCAACATGGCAATTGATCTAATATATATTCTGTTGTTTCAAAGCTGAATGCACGACCTACATCGTCACCTGGTTGTAT
TTCAGTCTTCGAATAGTCAATTGTTTTTGAATTTACACGCGGTGGTGTACCTGTTTTAAAACGAACAATATCAAAACCAAGTTCTCTT
AGATTATCTGATAATGTGATTGATGGTAATTGGTGATTTGGTCCACTTGAATACTTCATATTACCTAAAATGATTTCACCACGTAAAA
ATGTTCCCGTTGTAATAATTACTGCTTTAGATAAATACTCTGTACCAATATTTGTACGTACACCTTTAACTTCATTATCTTCTATAATA
AGTTCGTCTACCATACCTTGCATTATATGCAAATTTTCTTCATCTTCAATCACGCGTTTCATTTCTTGTTGATAAAGTACTTTATCTGC
TTGCGCTCTTAGTGCTCTTACAGCAGGTCCTTTACCAGTATTTAACATTCTCATTTGAATGTGTGTTTTATCGATTGTTTTTGCCATTT

GTCCACCTAAAGCATCAATTTCACGAACAACGATACCTTTAGCTGGTCCACCTACAGATGGGTTACATGGCATAAATGCAATATTAT
CTAAATTTATTGTTAGCATTAATGTTTTAGCACCACGTCTTGCAGATGCTAAACCTGCTTCTACACCTGCATGTCCCGCACCTATAAC
GATTACATCATATTCTTGAACCACAATATAAACCTCCTTATTTGATATCTTACTAGCCTTCTTAAGACGGTATTCCGTCTATTTCAATT
ACTATTTACCTAAGCAGAATTGACTGAATAACTGATCGATGAGTTCATCACTTGCAGTCTCACCAATAATTTCTCCTAATATTTCCCA
AGTTCTAGTTAAATCAATTTGTACCATATCCATAGGCACACCAGATTCTGCTGCATCAATCGCATCTTGTATCGTTTGTCTTGCTTGT
TTTAATAATGAAATATGTCTTGAATTAGAAACATAAGTCATATCTTGATTTTGTACTTCTCCACCAAAGAACAAATCTCGAATTTGTA
TTTCTAATTCATCAATACCTTCTTGTTTTAACATTGAAGTTTGAATTAATGGCGTATCACCTATCATATCTTTAACTTCATTAATATCT
ATGTTTTGCTCTAAATCCATTTTATTAACAATTACGATTACATCTTCATTTTTAACTACTTCATATAATGTGTAATCTTCTTGAGTCAA
TGCTTCGTTATTGTTTAATACAAATAAAATTAAGTCTGCTTGGCTAAGAGCCTTTCTAGAGCGTTCAACACCAATCTTCTCTACTATA
TCTTCTGTCTCACGTATACCAGCAGTATCAACTAATCTTAATGGCACGCCACGAACATTGACGTACTCTTCTAAGACATCTCTAGTAG
TACCTGCTACCTCAGTTACAATCGCTTTATTATCTTGTATTAAATTATTTAACATCGATGATTTACCTACGTTTGGTTTACCAACAATA
ACTGTAGATAAACCTTCACGCATAATTTTACCCTGCGCACCGGTATCTAATAAACGATTAATTTCCTGTTTGATTTCTTTAGACTGCT
CTAAAAGAAATTCAGTAGTCGCATCTTCAACATCATCGTATTCAGGATAATCAATATTCACTTCCACTTGAGCGAGTATCTCTAATA
TAGATTGACGTTGTTTTTTGATTAAGTCACTTAGACGACCTTCAATTTGATTCATCGCAACTTTAGAAGCTCTATCTGTCTTCGAGCG
AATAAAGTCCATAACTGCTTCAGCTTGAGATAAATCAATACGACCATTTAAAAAGGCACGTTTTGTAAATTCACCTGGCTCAGCCAT
TCTAGCGCCATATGTCATAGTAAGTTCCAGCACTCTATTAATCGTTAAAATACCACCATGACAATTAATTTCTATAATATCTTCGCGT
GTAAATGTTTTTGGCGCTCTTAACACAGACACCATAACTTCTTCAACCACTTCTTTAGATTCTGGATCAATAATATGACCGTAATTAA
TCGTATGTGATGGAACATCATTTAAAAGATGTTTTCCTTTATATAATTTGTCAGCAATTTCAACGGCTTGCGGTCCAGACAATCGAAC
AATTCCAATTGCCCCTTCACCCATTGGTGTTGAAATACTCGTAATTGTATCTAAATCCATATTGCTACTCGCCTCCTTCAACGATGTG
AATACATTTTAAAGTAAGTTATTATAACCCTAAGGTTAGTCTTACGTTTGTCTTAGGTAAGACTTCGGGATGTGTTGAGTGGTTAATG
TTTTCCTTCCCCTACCCTATCCTTACTTAATCTTTTTATTAAAAACTTTGGCAATTTTAAGTACGTGCTCAAGACTATTCTGTATTTGT
AAAGTCGTCATATCTTTAGCTGGCTGTCTTGCTATTACAATAATATCTTTGGCCAATATATGCGACTTATGTACTTTGAAATTTTCAC
GTATTGCTCTTTTAATCTTGTTTCTTAACACTGCATTACCTAGTTTTTTAGAAACACTAATACCTAAGCGAAAATGGTCTATTTCTTTA
TTATTACAAGTGTATACAACAAATTGTCTGTTGGCTACAGAATGACCTTTTTTATATATTCTCTGAAAATCTGCATTCTTTTTAATTCG
GTAAGCTTTTTCCAATAACATCACTCGCTTATTTATCGTTTTTATTTGAAGCTATATTTAAACTTCTATTGAGCTTATAACATAAATTT
CTATTTATTCTTAATTTAAACGAAAAAAAAGATCACTGATGAGTCAGTGATCTTATGCAGATAAAACTTTACGGCCTTTACGACGAC
GGCGCGCTAAAACTTTACGGCCATTTTTTGTGCTCATGCGTTTTCTGAAACCATGAACTTTACTATGTTTACGTTTATTTGGTTGATA
AGTACGTTTTACCATGCAAAACACCTCCATCTTTATACAGTTATCTAATATACCAGATTATGCAGACTCTGATCACTTTAATAGATAA
GATTTTGCGTTTCTTAGATTAAAATTCTCGTATTAGCTCATTGATTATCTAGTCATAATTCAAGCAACTACTACAATATAACAAAATC
CTTTTTATAACGCAAGTTCATTTTATACTACTGCTCAATTTTTTTACTTTTATCGATTAAAGATAGAAATACACGATGCGAGCAATCA
AATTTCATAACATCACCGTGAGTTTGGTCCGAAGCATGTGTGTTTACAATGTTTGAATACCTTATACAGTTCTTATACATACTTTATA
AATTATTTCCCAAGCTGTTTTGATACACTCACTAACAGATATTCTATAGAAGGAAAAGTTATCCACTTATGCACATTTATAGTTTTCA
GAATTGTGGATAATTAGAAATTATACACAAAGTTATACTATTTTTAGCAACATATTCACAGGTATTTGACATATAGAGAACTGAAAA
AGTATAATTGTGTGGATAAGTCGTCCAACTCATGATTTTATAAGGATTTATTTATTGATATTTACATAAAAATACTGTGCATAACTAA
TAAGCAGGATAAAGTTATCCACCGATTGTTATTAACTTGTGGATAATTATTAACATGGTGTGTTTAGAAGTTATCCACGGCTGTTATT
TTTGTGTATAACTTAAAAATTTAAGAAAGATGGAGTAAATTTATGTCGGAAAAAGAAATTTGGGAAAAAGTGCTTGAAATTGCTCA
AGAAAAATTATCAGCTGTAAGTTACTCAACTTTCCTAAAAGATACTGAGCTTTACACGATTAAAGATGGTGAAGCTATCGTATTATC
GAGTATTCCTTTTAATGCAAATTGGTTAAATCAACAATATGCTGAAATTATCCAAGCAATCTTATTTGATGTTGTAGGCTATGAAGTT
AAACCTCACTTTATTACTACTGAAGAATTAGCAAATTATAGTAATAATGAAACTGCTACTCCAAAAGAAACAACAAAACCTTCTACT
GAAACAACTGAGGATAATCATGTGCTTGGTAGAGAGCAATTCAATGCCCATAACACATTTGACACTTTTGTAATCGGACCTGGTAAC
CGCTTTCCACATGCAGCAAGTTTAGCTGTAGCCGAAGCACCAGCCAAAGCGTACAATCCATTATTTATCTATGGAGGTGTTGGTTTA
GGAAAAACCCATTTAATGCATGCCATTGGTCATCATGTTTTAGATAATAATCCAGATGCCAAAGTGATTTACACATCAAGTGAAAAA
TTCACAAATGAATTTATTAAATCAATTCGTGATAACGAAGGTGAAGCTTTCAGAGAAAGATATCGTAATATCGACGTCTTATTAATC
GATGATATTCAGTTCATACAAAACAAGGTACAAACACAAGAAGAATTTTTCTATACTTTTAATGAATTGCATCAGAATAACAAGCA
AATAGTTATTTCGAGTGATCGACCACCAAAGGAAATTGCACAATTAGAAGACCGATTACGTTCACGCTTTGAATGGGGGCTAATTGT
TGATATTACGCCACCAGATTATGAAACTCGAATGGCAATTTTGCAGAAGAAAATTGAAGAAGAAAAATTAGATATTCCACCAGAAG
CTTTAAATTATATAGCAAATCAAATTCAATCTAATATTCGTGAATTAGAAGGTGCATTAACACGTTTACTTGCATATTCACAATTATT
AGGAAAACCAATTACAACTGAATTAACTGCTGAAGCTTTAAAAGATATCATTCAAGCACCAAAATCTAAAAAGATTACCATCCAAG
ATATTCAAAAAATTGTAGGCCAGTACTATAATGTTAGAATTGAAGATTTCAGTGCAAAAAAACGTACAAAGTCAATTGCATATCCG
CGTCAAATAGCTATGTACTTGTCTAGAGAGCTTACAGATTTCTCATTACCTAAAATTGGTGAAGAATTTGGTGGGCGTGATCATACG
ACCGTCATTCATGCTCATGAAAAAATATCTAAAGATTTAAAAGAAGATCCTATTTTTAAACAAGAAGTAGAGAATCTTGAAAAAGA
AATAAGAAATGTATAAGTAGGAAACTTTGGGAAATGTAATCTGTTATATAACAGCACTAATGATAACAATCATTTTTTACATTTCTA
TATGCTAATGTGGCAAGATGAGCAAAACTCATTTTGTGGATAATGTTTAAAAGTCATACACACCATACACAAGTTATCAACATGTGT
ATAACTTCGCCAAATCTATGTTTTTAAGACTTATCCACCAATCCACAGCACCTACTACTATTACTAAGAACTTAAAACCTATATAATT
ATATATAAACGACTGGAAGGAGTTTTAATTAATGATGGAATTCACTATTAAAAGAGATTATTTTATTACACAATTAAATGACACATT
AAAAGCTATTTCACCAAGAACAACATTACCTATATTAACTGGTATCAAAATCGATGCGAAAGAACATGAAGTTATATTAACTGGTTC
AGACTCTGAAATTTCAATAGAAATCACTATTCCTAAAACTGTAGATGGCGAAGATATTGTCAATATTTCAGAAACAGGCTCAGTAGT
ACTTCCTGGACGATTCTTTGTTGATATTATAAAAAAATTACCTGGTAAAGATGTTAAATTATCTACAAATGAACAATTCCAGACATT
AATTACATCAGGTCATTCTGAATTTAATTTAAGTGGCTTAGATCCAGATCAATATCCTTTATTACCTCAAGTTTCTAGAGATGACGCA
ATTCAATTGTCGGTAAAAGTGCTTAAAAACGTGATTGCACAAACAAATTTTGCAGTGTCCACCTCAGAAACACGCCCAGTACTAACT
GGTGTGAACTGGCTTATACAAGAAAATGAATTAATATGCACAGCGACTGACTCACACCGCTTGGCTGTAAGAAAGTTGCAGTTAGA
AGATGTTTCTGAAAACAAAAATGTCATCATTCCAGGTAAGGCTTTAGCTGAATTAAATAAAATTATGTCTGACAATGAAGAAGACA
TTGATATCTTCTTTGCTTCAAACCAAGTTTTATTTAAAGTTGGAAATGTGAACTTTATTTCTCGATTATTAGAAGGACATTATCCTGA
TACAACACGTTTATTCCCTGAAAACTATGAAATTAAATTAAGTATAGACAATGGGGAGTTTTATCATGCGATTGATCGTGCCTCTTT
ATTAGCACGTGAAGGTGGTAATAACGTTATTAAATTAAGTACAGGTGATGACGTTGTTGAATTATCTTCTACATCACCAGAAATTGG
TACTGTAAAAGAAGAAGTTGATGCAAACGATGTTGAAGGTGGTAGCCTGAAAATTTCATTCAACTCTAAATATATGATGGATGCTTT
AAAAGCAATCGATAATGATGAGGTTGAAGTTGAATTCTTCGGTACAATGAAACCATTTATTCTAAAACCAAAAGGTGACGACTCGG
TAACGCAATTAATTTTACCAATCAGAACTTACTAAAAATAGATATAAATAAAGGATGACGTGATTAATTAAAACGTCATCCTTTATT
TTTTGGCAAAAATAATTCTAGATGCGTATGTAAAATAAATTTGACAGCATTTTAAACAGCAAATAAAAGACGCCAATTAAATTTATG
ACAAATATATCCAAATTTTAATAAGTGTGCTTATATGCCCTCTAAATTTAAAATTTTAATAGTCAATAACAAGTTGAATATAAAAGT
TAAACGCCGTTAAATAGCGTTAAAAAATTGAAAATGACAGTATTGCCAAAAAATAAGAATTAATTATTTATATGTAAACGGTTTCTA

CCTCTATTTTAAATGAAATTTGTGACAAAAAAAGGTATAATATATTAATGACATACAAAGAAATGGAGTGATTATTTTGGTTCAAGA
AGTTGTAGTAGAAGGAGACATTAATTTAGGTCAATTTCTAAAAACAGAAGGGATTATTGAATCTGGTGGTCAAGCAAAATGGTTCT
TGCAAGACGTTGAAGTATTAATTAATGGAGTGCGTGAAACACGTCGCGGTAAAAAGTTAGAACATCAAGATCGTATAGATATCCCA
GAATTACCTGAAGATGCTGGTTCTTTCTTAATCATTCATCAAGGTGAACAATGAAGTTAAATACACTCCAATTAGAAAATTATCGTA
ACTATGATGAGGTTACGTTGAAATGTCATCCTGACGTGAATATCCTCATTGGAGAAAATGCACAAGGAAAGACAAATTTACTTGAA
TCAATTTATACCTTAGCTTTAGCAAAAAGTCATAGAACGAGTAATGATAAGGAACTCATACGTTTTAATGCTGATTATGCTAAAATA
GAAGGTGAGCTTAGTTATAGACACGGCACGATGCCATTAACAATGTTTATAACTAAAAAAGGTAAACAAGTCAAAGTGAATCACTT
AGAGCAAAGTCGTCTAACTCAATATATTGGACACCTCAATGTGGTTCTATTTGCGCCAGAAGATTTGAATATTGTAAAAGGCTCTCC
TCAAATAAGACGACGCTTTATAGATATGGAGTTGGGCCAAATTTCTGCTGTTTACTTAAATGATTTAGCTCAATACCAACGTATTTTA
AAGCAAAAGAATAATTACTTAAAGCAGTTACAATTAGGCCAAAAAAAGGACTTAACAATGTTGGAAGTATTAAATCAGCAGTTTGC
TGAATATGCAATGAAAGTAACTGATAAACGTGCACATTTTATTCAAGAGCTAGAGTCGTTAGCTAAACCGATTCATGCTGGTATCAC
AAATGATAAAGAAGCGTTGTCGCTGAATTATTTACCTAGTCTTAAATTTGATTATGCTCAAAATGAAGCGGCACGACTTGAAGAAAT
TATGTCTATTCTTAGCGATAATATGCAAAGAGAAAAAGAACGAGGCATTAGCTTATTCGGACCACATCGAGATGATATAAGTTTTG
ATGTGAATGGCATGGATGCTCAAACATATGGTTCTCAAGGACAGCAACGTACAACGGCTTTGTCCATTAAATTAGCTGAAATTGAGT
TAATGAATATCGAAGTTGGGGAATATCCCATCTTATTATTAGACGATGTACTCAGTGAATTAGATGATTCGCGTCAAACGCATTTAT
TAAGTACGATTCAGCATAAAGTACAAACATTTGTCACTACGACATCTGTAGATGGTATTGATCATGAAATCATGAATAACGCTAAAT
TGTATCGTATTAATCAAGGTGAAATTATAAAGTAACAGAAAGCGATGGTGACTGCATTGTCAGATGTAAACAACACGGATAATTAT
GGTGCTGGGCAAATACAAGTATTAGAAGGTTTAGAAGCAGTACGTAAAAGACCAGGTATGTATATAGGATCGACTTCAGAGAGAG
GTTTGCACCATTTAGTGTGGGAAATTGTCGATAATAGTATCGATGAAGCATTAGCTGGTTATGCAAATCAAATTGAAGTTGTTATTG
AAAAAGATAACTGGATTAAAGTAACGGATAACGGACGTGGTATCCCAGTTGATATTCAAGAAAAAATGGGACGTCCAGCTGTCGA
AGTTATTTTAACTGTTTTACATGCTGGTGGTAAATTCGGCGGTGGCGGATACAAAGTATCTGGTGGTTTACATGGTGTTGGTTCATCA
GTTGTAAACGCATTGTCACAAGACTTAGAAGTATATGTACACAGAAATGAGACTATATATCATCAAGCATATAAAAAAGGTGTACC
TCAATTTGACTTAAAAGAAGTTGGCACAACTGATAAGACAGGTACTGTCATTCGTTTTAAAGCAGATGGAGAAATCTTCACAGAGA
CAACTGTATACAACTATGAAACATTACAGCAGCGTATTAGAGAGCTTGCTTTCTTAAACAAAGGAATTCAAATCACATTAAGAGAT
GAACGTGATGAAGAAAACGTTAGAGAAGACTCCTATCACTATGAGGGCGGTATTAAATCGTACGTTGAGTTATTGAACGAAAATAA
AGAACCTATTCATGATGAGCCAATTTATATTCATCAATCTAAAGATGATATTGAAGTAGAAATTGCGATTCAATATAACTCAGGATA
TGCCACAAATCTTTTAACTTACGCAAATAACATTCATACGTATGAAGGTGGTACGCATGAAGACGGATTCAAACGTGCATTAACGC
GTGTCTTAAATAGTTATGGTTTAAGTAGCAAGATTATGAAAGAAGAAAAAGATAGACTTTCTGGTGAAGATACACGTGAAGGTATG
ACAGCAATTATATCTATCAAACATGGTGATCCTCAATTCGAAGGTCAAACGAAGACAAAATTAGGTAATTCTGAAGTGCGTCAAGT
TGTAGATAAATTATTCTCAGAGCACTTTGAACGATTTTTATATGAAAATCCACAAGTCGCACGTACAGTGGTTGAAAAAGGTATTAT
GGCGGCACGTGCACGTGTTGCTGCGAAAAAAGCGCGTGAAGTAACACGTCGTAAATCAGCGTTAGATGTAGCAAGTCTTCCAGGTA
AATTAGCCGATTGCTCTAGTAAAAGTCCTGAAGAATGTGAGATTTTCTTAGTCGAAGGGGACTCTGCCGGGGGGTCTACAAAATCTG
GTCGTGACTCTAGAACGCAGGCGATTTTACCATTACGAGGTAAGATATTAAATGTTGAAAAAGCACGATTAGATAGAATTTTGAAT
AACAATGAAATTCGTCAAATGATCACAGCATTTGGTACAGGAATCGGTGGCGACTTTGATCTAGCGAAAGCAAGATATCACAAAAT
CGTCATTATGACTGATGCCGATGTGGATGGAGCGCATATTAGAACATTGTTATTAACATTCTTCTATCGATTTATGAGACCGTTAATT
GAAGCAGGCTATGTGTATATTGCACAGCCACCGTTGTATAAACTGACACAAGGTAAACAAAAGTATTATGTATACAATGATAGGGA
ACTTGATAAACTTAAATCTGAATTGAATCCAACACCAAAATGGTCTATTGCACGATACAAAGGTCTTGGAGAAATGAATGCAGATC
AATTATGGGAAACAACAATGAACCCTGAGCACCGCGCTCTTTTACAAGTAAAACTTGAAGATGCGATTGAAGCGGACCAAACATTT
GAAATGTTAATGGGTGACGTTGTAGAAAACCGTAGACAATTTATAGAAGATAATGCAGTTTATGCAAACTTAGACTTCTAAGCGCT
GTGAACTGAACTTTTGAAGGAGGAACTCTTGATGGCTGAATTACCTCAATCAAGAATAAATGAACGAAATATTACCAGTGAAATGC
GTGAATCATTTTTAGATTATGCGATGAGTGTTATCGTTGCTCGTGCATTGCCAGATGTTCGTGACGGTTTAAAACCAGTACATCGTCG
TATACTATATGGATTAAATGAACAAGGTATGACACCGGATAAATCATATAAAAAATCAGCACGTATCGTTGGTGACGTAATGGGTA
AATATCACCCTCATGGTGACTTATCTATTTATGAAGCAATGGTACGTATGGCTCAAGATTTCAGTTATCGTTATCCGCTTGTTGATGG
CCAAGGTAACTTTGGTTCAATGGATGGAGATGGCGCAGCAGCAATGCGTTATACTGAAGCGCGTATGACTAAAATCACACTTGAAC
TGTTACGTGATATTAATAAAGATACAATAGATTTTATCGATAACTATGATGGTAATGAAAGAGAGCCGTCAGTCTTACCTGCTCGAT
TCCCTAACTTGTTAGCCAATGGAGCATCAGGTATAGCGGTAGGTATGGCAACGAATATTCCACCACATAACTTAACAGAATTAATCA
ATGGTGTACTTAGCTTAAGTAAGAACCCTGATATTTCAATTGCTGAGTTAATGGAGGATATTGAAGGTCCTGATTTCCCAACTGCTG
GACTTATTTTAGGTAAGAGTGGTATTAGACGTGCATATGAAACAGGTCGTGGTTCAATTCAAATGCGTTCTCGTGCAGTTATTGAAG
AACGTGGAGGCGGACGTCAACGTATTGTTGTCACTGAAATTCCTTTCCAAGTGAATAAGGCTCGTATGATTGAAAAAATTGCAGAG
CTCGTTCGTGACAAGAAAATTGACGGTATCACTGATTTACGTGATGAAACAAGTTTACGTACTGGTGTGCGTGTCGTTATTGATGTG
CGTAAGGATGCAAATGCTAGTGTCATTTTAAATAACTTATACAAACAAACACCTCTTCAAACATCATTTGGTGTGAATATGATTGCA
CTTGTAAATGGTAGACCGAAGCTTATTAATTTAAAAGAAGCGTTGGTACATTATTTAGAGCATCAAAAGACAGTTGTTAGAAGACG
TACGCAATACAACTTACGTAAAGCTAAAGATCGTGCCCACATTTTAGAAGGATTACGTATCGCACTTGACCATATCGATGAAATTAT
TTCAACGATTCGTGAGTCAGATACAGATAAAGTTGCAATGGAAAGCTTGCAACAACGCTTCAAACTTTCTGAAAAACAAGCTCAAG
CTATTTTAGACATGCGTTTAAGACGTCTAACAGGTTTAGAGAGAGACAAAATTGAAGCTGAATATAATGAGTTATTAAATTATATTA
GTGAATTAGAAACAATCTTAGCTGATGAAGAAGTATTACTACAATTAGTTAGAGATGAATTAACAGAAATTCGAGATCGTTTCGGT
GATGATCGTCGTACTGAAATCCAATTAGGTGGATTTGAAGATTTAGAAGATGAAGATCTCATTCCAGAAGAACAAATTGTAATTAC
ACTAAGCCATAATAACTACATTAAACGTTTGCCGGTATCTACATATCGTGCTCAAAACCGTGGTGGTCGTGGTGTTCAAGGTATGAA
TACATTGGAAGAAGATTTTGTCAGTCAATTGGTAACTTTAAGTACACATGACCATGTATTGTTCTTTACTAACAAAGGTCGTGTATA
CAAACTTAAAGGTTATGAAGTGCCTGAGTTATCAAGACAGTCTAAAGGTATTCCTGTAGTGAATGCTATTGAACTTGAAAATGATGA
AGTCATTAGTACAATGATTGCTGTTAAAGACCTTGAAAGTGAAGACAACTTCTTAGTGTTTGCAACTAAACGTGGTGTCGTTAAACG
TTCAGCATTAAGTAACTTCTCAAGAATAAATAGAAATGGTAAGATTGCGATTTCGTTCAGAGAAGATGATGAGTTAATTGCAGTTCG
CTTAACAAGTGGTCAAGAAGATATCTTGATTGGTACATCACATGCATCATTAATTCGATTCCCTGAATCAACATTACGTCCTTTAGG
CCGTACAGCAACGGGTGTGAAAGGTATTACACTTCGTGAAGGTGACGAAGTTGTAGGGCTTGATGTAGCTCATGCAAACAGTGTTG
ATGAAGTATTAGTAGTTACTGAAAATGGTTATGGTAAACGTACGCCAGTTAATGACTATCGTTTATCAAATCGTGGTGGTAAAGGTA
TTAAAACAGCTACGATTACTGAGCGTAATGGTAATGTTGTATGTATCACTACAGTAACTGGTGAAGAAGATTTAATGATTGTTACTA
ATGCAGGTGTCATTATTCGACTAGATGTTGCAGATATTTCTCAAAATGGTCGTGCAGCACAAGGTGTTCGCTTAATTCGCTTAGGTG
ATGATCAATTTGTTTCAACGGTTGCTAAAGTAAAAGAAGATGCAGAAGATGAAACGAATGAAGATGAGCAATCTACTTCAACTGTA
TCTGAAGATGGTACTGAACAACAACGTGAAGCGGTTGTAAATGATGAAACACCAGGAAATGCAATTCATACTGAAGTGATTGATTC
AGAAGAAAATGATGAAGATGGACGTATTGAAGTAAGACAAGATTTCATGGATCGTGTTGAAGAAGATATACAACAATCATCAGAT
GAAGATGAAGAATAATAAAAAAATAAGACTTCCCTATATGTAGGGGAGTCTTATTTTTATGCTAGAAAGTAATGCTGTACTATATTC

AATGATTAGTAATAATTAACTTTCTAATTGTTTCATTGCGTAAGGTATTTCATTGATAAGTCTTGATGGTGGTACCACATACATATCT
TTTGCAAGGTTTTCGCCAATAAAACTATGTGTATATGTGGCACTCATAACCGCTTCTTTTAAGTTATCAAATTGACCGACAAAACTTG
TAATCATACCAGCAAGTGTATCGCCCATACCACCAGTCGCCATTGCTGGGCTACCGATTGTTAATTTAAAGTCTTCATCTTTAAAGA
AAATTTCAGTACCATGTTTTTTAAGTACAACAGTTGCACCTAAACGATCAACTGCTTCACGATTACGCTCATATGTCTGTTCCTCAAT
AGGAATACCACTTAATCGTTCCCATTCTTTGAGGTGTGGAGTAAAGATCACACGACATGTAGGTAATTGCGGTTTCAGTTTACTAAA
GATTGTAATCGCATCGCCGTCTACGATTAAATTTTGATGCGGTTGTATATTTTGTAGTAGGAATGTAATGGCATTATTTCCTTTGAAA
TCAACGCCAAGACCTGGACCAATTAGTATACTGTCAGTCATTTCAATCATTTTCGTCAACATTTTCGTATCATTAATATCAATAACCA
TCGCTTCTGGGCAACGAGAATGTAATGCTGAATGATTTGTTGGATGTGTAGCTACAGTGATTAAACCACTACCGCTAAATACACATG
CACGAGCCGCTAACATAATGGCACCACCTAAGTTAGCAGATCCACCAATTAATAAAATTTTGCCATAATCACCTTTATGTGAATCTT
CTTTACGCTTAGGAATGTTAATAGAATTTAACGTTTCCATAGTGATATAACCTCCCATGTAAAAGCCTTTTCCGAATTTATTCAATTT
TAAAAATATATAGTAACTTTTAACAAAATGTATTATAAATTTCTGAATTCATTATTATTTGTCGTTAAATACAATAGAAAATACTATA
CCTGTATATGCAATTCGTCAATAGATAAATTATTAAATATGCTTACAACAATCTTAATATCCTTTAACGCACTACAATAGTGCTCTGA
TAATAGGTTATAAATGTACGTAAAACCATTGTTTCAATAAAAATGAAAACGTATACTTCAAGAAGGATGGGTTACTTAATATAAAC
AAGGGGGTAACATATATGACTTTATATTTAGATGGTGAAACACTAACAATTGAGGATATTAAATCATTTTTACAACAACAATCAAA
GATTGAAATTATTGATGATGCGTTAGAACGTGTCAAAAAAAGTAGAGCGGTAGTTGAACGTATTATTGAAAATGAGGAAACGGTTT
ACGGTATCACTACAGGTTTTGGGTTATTTAGTGATGTACGTATAGACCCGACGCAATATAATGAATTACAAGTGAATCTGATACGCT
CACATGCCTGTGGACTAGGTGAGCCATTTTCAAAAGAAGTAGCATTAGTCATGATGATTTTACGATTGAATACATTATTAAAAGGTC
ATTCAGGTGCCACTTTAGAATTAGTGAGACAATTACAATTTTTTATAAATGAACGTATTATACCGATAATCCCACAACAAGGCTCTC
TCGGTGCATCAGGAGATTTAGCGCCATTATCACATTTAGCATTAGCATTAATTGGTGAAGGGAAAGTATTGTACAGAGGGGAAGAA
AAGGATAGTGACGATGTATTAAGAGAATTAAATAGACAACCTTTGAACCTTCAGGCTAAAGAGGGTTTAGCATTGATTAATGGTAC
GCAAGCTATGACAGCTCAAGGTGTCATTAGTTATATAGAAGCAGAAGATTTAGGTTACCAATCTGAATGGATTGCTGCATTAACGC
ATCAGTCTCTTAATGGCATTATAGATGCATATCGACATGATGTGCACTCTGTTCGTAATTTTCAAGAACAGATTAATGTGGCAGCGC
GTATGCGTGATTGGTTAGAAGGATCAACATTAACGACGCGACAAGCAGAAATACGTGTACAAGATGCATATACGTTGCGTTGTATA
CCACAAATCCATGGCGCGAGTTTTCAAGTATTCAATTATGTTAAACAGCAATTAGAATTTGAAATGAATGCGGCTAATGATAATCCA
CTTATATTTGAGGAAGCAAATGAAACGTTTGTTATTTCAGGTGGTAACTTCCATGGACAACCTATTGCTTTTGCATTAGATCATCTTA
AATTAGGTGTAAGTGAATTAGCAAACGTATCGGAACGTCGTCTAGAGCGACTAGTAAATCCTCAATTAAATGGTGATTTACCAGCA
TTTCTTAGTCCAGAGCCAGGATTGCAAAGTGGCGCGATGATTATGCAATATGCTGCTGCAAGTCTCGTTTCTGAAAATAAAACTTTA
GCGCATCCAGCGAGTGTTGATTCTATCACTTCATCTGCGAACCAAGAAGATCACGTATCTATGGGAACTACAGCTGCTAGACATGGT
TATCAAATTATTGAAAATGCAAGACGTGTGTTGGCAATCGAATGTGTTATTGCATTACAAGCAGCAGAGTTGAAAGGTGTCGAAGG
ATTATCACCAAAAACACGTCGTAAATATGAGGAGTTTCGAAGTATCGTGCCATCCATTACACATGACCGTCAATTTTATAAAGATAT
TGAAGCGGTTGCACAGTATTTAAAGCAATCAATTTATCAAACGACTGCATGTCACTAAATCGACATGAGTGATGTTTGAAATCGTTA
CTTGAAAAAGCAAATATAGTTTGCTATATTAAAATTAACTTAATAAGACATTGTTCGTAGGACAAGTAATATATAGTGTTCGATATC
AGAGAGCTTGTGGTTAGTGTGAACAAGAATCAACATATATATGAATCTACCTACTTAATTTAAAAGAACAATCGGTGATAACCGTT
ATTTTAGTGAAGTGCAATTTAGGTTTAGTTTATCTTTATAACTTAAATTGTTAAATAGGGTGGCAACGCGTAGACCACGTCCCTTGTA
GGGATGTGGTCTTTTTTTATTTTCTAAAAAATAAAGACGTACGTCATTCCATAATAAATGATAATTATTTGGGAAAGGATGAAGGTT
AATGTTAGACATTAGATTATTCAGAAATGAGCCTGACACAGTTAAGAGCAAAATTGAATTACGTGGAGATGATCCAAAAGTTGTAG
ATGAAATTTTAGAATTGGATGAGCAACGACGTAAATTAATTAGTGCAACAGAAGAAATGAAAGCACGTCGTAATAAAGTAAGCGA
AGAAATCGCATTAAAAAAACGTAATAAAGAAAATGCTGATGATGTGATTGCTGAAATGCGCACATTAGGTGACGATATTAAAGAA
AAAGATAGTCAATTAAATGAAATTGATAATAAAATGACAGGTATCCTTTGTCGTATTCCAAATTTAATAAGTGATGATGTACCTCAA
GGTGAATCTGATGAAGATAACGTTGAAGTTAAAAAGTGGGGTACACCACGTGAGTTTTCATTTGAACCAAAAGCACATTGGGATAT
TGTAGAAGAATTGAAAATGGCTGATTTTGATCGTGCAGCAAAAGTTTCAGGTGCGCGTTTTGTATATTTAACAAATGAAGGTGCGCA
ATTAGAGCGTGCTTTAATGAACTATATGATTACAAAACATACAACACAACATGGTTATACAGAAATGATGGTACCACAGCTTGTGA
ACGCAGATACAATGTATGGTACAGGTCAATTACCTAAATTTGAAGAAGATTTATTTAAAGTAGAAAAAGAAGGATTATATACAATT
CCAACTGCTGAAGTACCATTAACGAATTTCTACCGTAATGAAATTATTCAACCAGGTGTACTTCCTGAAAAATTCACTGGTCAATCT
GCATGTTTCCGTAGTGAAGCAGGATCAGCAGGTAGAGATACAAGAGGATTAATTCGTTTACATCAATTCGATAAAGTGGAAATGGT
ACGTTTTGAACAACCTGAAGATTCATGGAATGCTTTAGAAGAAATGACAACAAATGCAGAAGCAATTCTAGAAGAGTTAGGTTTAC
CATACCGTCGTGTTATTTTATGTACAGGTGATATTGGATTTAGTGCAAGCAAAACATATGATATAGAAGTTTGGTTACCAAGCTACA
ATGATTATAAAGAAATTAGTTCATGCTCAAACTGTACGGATTTCCAAGCGCGTCGTGCTAACATCCGCTTCAAGCGTGACAAAGCAG
CTAAACCAGAATTAGCACATACATTAAATGGTAGTGGTTTAGCAGTTGGACGTACATTTGCTGCTATTGTTGAAAATTACCAAAATG
AAGATGGAACAGTAACAATTCCAGAAGCATTAGTACCATTTATGGGTGGTAAAACACAAATTTCAAAACCAGTTAAATAAAGGCTT
TAGCTACAAGCTTTAAAAAGTATATATTCTACGTATACTTAAAGCAAGGGCAAGATACTTTAAATAATATTTTAAAAAGTGGTGACG
AAGCTGTCGCCACTTTTTTTGTGCTGTAAAAGTATAATAGTGAGCCAATGCACATAACAACAATAAATTAAGTTTGTGGTTTAATGG
GGTGAACGCATTTCATTATAGCAACAATACGGGATAATTATGATGAACTAAAACAATCTAAAACGTAACAATTTTGAGCATCACTA
TTATAGGAAATTAAGTGATAAAAAACTGATTTCGTTGATGTAGTATGAGTAATATCGATGGAGTAGGGTAGGGGGAGAGGATGATG
ATTATAAGGGAGTGTTACATGAATCAATATCCCAGACTCATCATTAGATATAAAAAATTATAAAATTAGTACTTAAAAACAACTAC
AAATCCGTAGAAAATATGGAGGTAGTCTTAAATAAAAATTGAAAATTATCAAAAATAAAAAGTTGATATGAAGATGATTGAAGAC
GCCAGTATGTCTAACCTCAGACAAACTGGTGTCTAATGTTATTGCTTAGGGTATAGAACTGTATTAGACTAGGTATATTATTTTTTCG
TAATTATATAAATATAAAGTGGCAAAGGAGGTAATTGAGATGGCAACACATTTAAGTTTTAGACAAGGCGTACAAGAGTGTATCCC
AACATTATTGGGTTATGCCGGTGTTGGTATTTCATTTGGTATTGTGGCTTCGTCTCAAAACTTTAGTATTTTAGAAATTATCTTGTTAT
GTCTTGTTATATATGCCGGTGCTGCGCAATTTATTATGTGCGCGTTGTTTATAGCAGGTACACCGATATCAGCGATTGTACTAACTGT
ATTTATCGTAAATTCAAGAATGTTCCTTTTAAGTATGTCGCTTGCACCAAACTTCAAGACATATGGGTTTTGGAACCGTGTTGGATTA
GGTTCATTAGTAACTGACGAAACGTTTGGCGTCGCCATTACACCTTATTTAAAAGGAGAAGCTATCAATGATCGTTGGATGCATGGT
CTTAATATCACAGCATATTTATTTTGGGCAATTTCATGTGTAGCTGGGGCTTTATTTGGCGAATATATCTCAAATCCGCAAACGCTAG
GGTTAGATTTTGCTATCACGGCTATGTTTATCTTTTTGGCCATTGCGCAATTTGAATCAATTACTAAATCGCGATTAAGAATTTACAT
AGTACTCATTATTGCCGTCATAGTAATGATGTTATCGCTAAGTATGTTTATGCCTTCATATCTAGCAATATTAATTGCAGCCACAATT
TCAGCAGCGTTAGGAGTGATGATGGAACGATGATAACTCATATGAACATGTTAATACTTATTTTATTGTGTGGTATCGTAACGCTAT
TAATTCGAATTATACCTTTTATCATGATTTCAAAAGTGCAATTGCCTGATGTTGTGGTTCGATGGCTATCATTTATCCCAATCACACT
ATTTACGGCACTTGTCATTGACAGCATTATTCAACAGACGCCTCATGGTGAGGGGTATACATTAAACATCCCTTACATTATCGCGCT
CATTCCGACGGTTATTTTATCTATAATCACGCGTAGTTTAACTATTACAATTATTAGTGGGATTGTTATCATGGCAACATTACGATTT
TTCTTTTAAAAATAACTGAAAATCATTGAACAGATATTTAAACTTAGGTAAACTGTTAGTAATCAGAAAATTCTGATATACAAATCG
TCTATGGTATGACTGGGAATCGTATACACGAAAGCGATTTGGCTAACGTATGACAATGATAACAATATTTTAAATGATAAAAGTAA

TTCATCACTGAATCTCAACTAACACATAACAATTTCATATTTCTTATTGTGAGAAGTTGAGGGACTTGGCCCTGTGATACTTCAGCAA
CCGACTTTATAGCACGGTGCTAAAACCAACGAGTTACTCGAATGATAAGTATAAAGACTTCTTACTTTTCAATAGGGTGAGAAGTTT
TTTTGTTTAAGGAGGAAAGAACAATGACAAATTACACAGTAGATACTTTAAATCTAGGGAAATTTATTACAGAATCTGGGGAAGTC
ATAGATAACTTGCGTTTGAGATATGAGCATGTTGGTTATCATGGACAACCATTAGTTGTAGTTTGTCATGCATTAACTGGCAATCATT
TAACATATGGAACAGATGATTATCCGGGTTGGTGGCGAGAAATTATTGATGGGGGATATATACCCATTCACGATTATCAATTTTTAA
CATTTGATGTTATTGGTAGTCCTTTCGGTTCAAGTTCACCTTTAAACGACCCTCATTTTCCTAAAAAATTAACATTAAGAGATATAGT
CAGAGCGAATGAACGAGGTATACAAGCCCTTGGTTATGATAAGATTAATATTTTAATAGGGGGAAGTCTTGGAGGTATGCAAGCAA
TGGAACTACTTTACAATCAACAGTTTGAAGTAGATAAAGCCATTATCCTTGCTGCAACAAGTCGAACATCATCTTATAGTAGAGCTT
TCAATGAAATTGCAAGGCAAGCCATTCATCTTGGTGGTAAGGAAGGTCTAAGTATTGCACGCCAATTAGGTTTTTTGACATATCGAT
CATCAAAAAGTTATGATGAACGTTTCACGCCGGATGAAGTAGTCGCATACCAACAACATCAAGGTAATAAATTTAAAGAACATTTT
GATTTGAATTGTTATCTGACACTGCTAGATGTATTGGATAGTCACAACATTGACCGAGGTCGCACAGACGTAACGCATATTTTTAAA
AATTTAGAAACGAAAGTCTTAACGATGGGGTTCATAGATGATTTGCTATATCCGGACGATCAAGTTCGTGCATTAGGTGAACGTTTT
AAATATCATCGTCATTTCTTCGTGCCTGATAATGTTGGACATGATGGATTCCTACTAAACTTTAGTACCTGGGCACCTAACTTATATC
ATTTCTTAAATTTAAAGCATTTTAAGCGTAAGTAATAATGTATATATTTTTAAAGAATGAAGCTACTAAATCACAGTTATCATATTCA
AAGACATCTAGAGTACATGAGAGTGGAAATGAATTGTAAATCTAAATCAAGCTTGATTGCTATGTAGTGGGATTAAATAATCATGT
TGAAAATGAAAGCACACAAAAGCGATTCAAATGAGTATGTTAATTACTTATAAAACATGAAAGTTATTATTCAATTAAATGAAATA
GAAGCATACAATCTATAAATTGTTAATTTCCATTAAAGAGGTTAAAATAATAGCTATAGTTAAAAATATGGGAGTGGCTTATGTGTT
TTCAAAAATACAACCTAAAGCAACAATAATTGCAACGATTACGTTGGTATTTGTCGCTTTAGCTTTATATCTAGTGCCTGGTTTGGG
ACTAATATTTGCATTATTTGCAACCATACCAGGTATCGTTTTATGGAATAAATCAATACAATCTTTCGGGGTTAGTGCACTTATTACA
GTAATTATAACAACTGTTTTAGGTAATACTTTCGTTTTAAGTGCCATCATATTAGTCTTAATTGCAAGTTTAATTATTGGTCAATTGCT
CAAAGAAAGAACGTCTAAAGAAAGAATGTTATACGTAACAACAGTAGCGATGAGCTTAATTTCATTAATCGCTTTTATGTTACTACA
AACATTCGGAAGGATTCCACCATCAGCGAGCATAGTAAAACCTTTCAAGCAAACATTACATGAAGCGATTACGATGAGCGGTGCCG
ATGCGAATATGACCCAAATATTAGAAGAAGGGTTTAGACAAGCGACCGTTCAATTACCAGGTTTCATCATTATCATTACATTTTTAA
TTGTCTTAATTAACTTAATCGTTACATTTCCGGTTTTACGAAAATTTAAAATCGCTACACCTGTATTTAAGCCACTTTTCGCGTGGCA
AATGAGCGGTATTTTATTATGGATATACATTATTGTTATCATATGTTTATTATTTACAGGTCAACCGAGTGTGTTCCAGAGCATTCTT
TTAAACTTCCAACTTGTGTTATCATTAGTAATGTATATTCAAGGTTTAAGTGTTATTCATTTCTTTGGTAAAGCGAAAGGTTTGCCGA
ATGCAGTAACGATTTTACTATTGATTATCGGTACAATACTGACACCTACGACACATATTGTAGGACTACTTGGTGTTATCGATTTAA
GTTTGAATTTGAAGCGAATCATGAAAAATAATTCTAAAAAGTGAATAGAGGTGGAATAATGAATCGGCAGTCCACTAAGAAAGCTT
TACTAATACCATTTGTCATCATGATCATCACAGCAATTGTTTTAATGGGTGTATGGTTTATCTTTAATAGTCTTATAGCATTAATTGC
ATCTATCGTTCTTGTCGTGATGATTATTGTTAGCATCATTTTATTCAGACAAGCTTTAATGAAAATGGATAGTTATGTAGATGGTTTG
AGTGCTCAAATTTCAACAACAAATAATAAAGCAATCAAACATTTACCAATTGGTATCATTGTTTTAGATGAAAATGATCACATCGAA
TGGGTTAACCAATTTATGACAAATCATATGGAAGCAAATGTCATTTCTGAATCTGTAAATGAAGTATTTCCAAACATTTTAAAGCAA
TTAGATAGAGTGAAATCCGTTGAAATAGAATATAATCAGTATCATTTCCAAGTACGTTATTCTGAGAATGATCATTGCCTCTATTTCT
TTGATATAACTGAACAAGTACAAACAAATGAACTATATGAAAATTCTAAACCAATCATTGCGACATTATTTTTAGATAACTACGATG
AGATTACTCAAAATATGAATGATACGCAGCGTTCGGAAATCAATTCAATGGTAACGCGTGTTATTAGTCGATGGGCAACTGAGTAT
AATATATTCTTTAAAAGGTATAGTTCCGATCAATTCGTAGCCTATTTAAATCAAAAAATATTAGCTGACTTAGAAGAATCTAAATTT
GATATCTTGAGTCAATTACGTGAAAAAAGTGTTGGTTATCGTGCCCAATTAACATTAAGTATTGGTGTTGGTGAAGGTACTGAAAAT
TTAATCGATTTAGGTGAATTATCACAATCAGGCCTAGACTTAGCATTAGGGCGCGGTGGCGACCAAGTTGCAATTAAAAGTATTAAT
GGTAATGTGCGTTTCTATGGCGGTAAGACTGACCCGATGGAGAAACGTACTCGTGTAAGAGCACGAGTGATCTCACATGCGTTAAA
AGATATCCTTGCAGAGGGTGACAAAGTCATTATCATGGGACATAAACGTCCTGACTTAGATGCAATTGGTGCAGCAATCGGTGTGT
CTAGATTTGCAATGATGAATAATTTAGAAGCATACATCGTATTAAATGAGACTGACATTGATCCAACATTACGACGCGTGATGAAC
GAAATTGATAAAAAGCCAGAGTTAAGAGAGCGATTTATAACATCAGATGATGCTTGGGATATGATGACATCTAAGACAACCGTAGT
GATTGTTGATACGCATAAACCGGAACTGGTTTTAGATGAAAATGTCTTAAATAAAGCAAACCGTAAAGTTGTTATCGATCATCATAG
ACGTGGTGAAAGCTTCATCTCTAATCCATTGTTGATATATATGGAACCATACGCAAGTTCAACAGCTGAATTGGTAACAGAGTTACT
GGAATATCAACCAACAGAACAACGTTTAACACGTCTTGAATCAACAGTGATGTATGCAGGTATTATTGTAGATACAAGAAACTTTA
CATTACGAACAGGATCAAGAACATTCGATGCAGCGAGTTATTTACGTGCACATGGTGCAGATACGATTTTAACGCAACATTTCTTAA
AAGATGATGTGGATACTTACATTAATCGATCTGAATTAATTCGAACTGTAAAAGTTGAAGATAATGGCATAGCCATTGCGCATGGTT
CAGACGATAAAATTTATCATCCAGTAACAGTTGCACAAGCAGCAGATGAACTGTTAAGTTTAGAAGGTATTGAAGCATCATATGTT
GTTGCGAGACGTGAAGATAATCTGATTGGTATATCTGCGCGTTCACTCGGTTCAGTAAATGTTCAGTTAACAATGGAAGCACTTGGT
GGCGGTGGACATTTAACCAATGCTGCAACACAACTTAAAGGTGTGACAGTCGAAGAGGCGATAGCACAATTACAACAAGCAATTAC
AGAACAATTAAGTAGGAGTGAAGATGCATGAAAGTAATTTTTACACAAGATGTTAAAGGTAAAGGTAAAAAAGGTGAAGTTAAAG
AAGTACCAGTAGGTTATGCAAATAACTTCTTATTGAAAAAGAATTATGCTGTAGAAGCAACACCAGGTAACCTTAAACAATTAGAG
TTACAGAAAAAACGTGCAAAACAAGAACGCCAACAAGAAATTGAAGATGCTAAAGCATTAAAAGAAACGTTATCAAACATTGAAG
TTGAAGTATCAGCAAAAACTGGTGAAGGTGGTAAATTGTTTGGATCAGTAAGTACAAAACAAATTGCCGAAGCACTAAAAGCACAA
CATGATATTAAAATTGATAAACGTAAAATGGATTTACCAAATGGAATTCATTCCCTAGGATATACGAATGTACCTGTTAAATTAGAT
AAAGAAGTTGAAGGTACAATTCGCGTACACACAGTTGAACAATAAAGTTGGATTGAAATAAGAGGTGTAACCATTCATGGATAGAA
TGTATGAGCAAAATCAAATGCCACATAACAATGAAGCTGAACAGTCTGTCTTAGGTTCAATTATTATAGATCCAGAATTGATTAATA
CTACTCAGGAAGTTTTGCTTCCTGAGTCGTTTTATAGGGGTGCCCATCAACATATTTTCCGTGCAATGATGCACTTAAATGAAGATA
ATAAAGAAATTGATGTTGTAACATTGATGGATCAATTATCGACGGAAGGTACGTTGAATGAAGCGGGTGGCCCGCAATATCTTGCA
GAGTTATCTACAAATGTACCAACGACGCGAAATGTTCAGTATTATACTGATATCGTTTCTAAGCATGCATTAAAACGTAGATTGATT
CAAACTGCAGATAGTATTGCCAATGATGGATATAATGATGAACTTGAACTAGATGCGATTTTAAGTGATGCAGAACGTCGAATTTTA
GAGCTGTCATCTTCTCGTGAAAGCGATGGCTTTAAAGACATTCGAGACGTCTTAGGACAAGTGTATGAAACAGCTGAAGAGCTTGA
TCAAAATAGTGGTCAAACACCAGGTATACCTACAGGGTATCGAGATTTAGACCAAATGACAGCAGGGTTCAACCGAAATGATTTAA
TTATCCTTGCAGCGCGTCCATCTGTAGGTAAGACTGCGTTCGCACTTAATATTGCACAAAAAGTTGCAACGCATGAAGATATGTATA
CAGTTGGTATTTTCTCACTAGAGATGGGTGCTGATCAGTTAGCCACACGTATGATTTGTAGTTCTGGTAATGTTGACTCAAACCGCTT
AAGAACGGGTACTATGACTGAGGAAGATTGGAGTCGTTTTACTATAGCGGTAGGTAAATTATCACGTACGAAGATTTTTATTGATGA
TACACCGGGTATTCGAATTAATGATTTACGTTCTAAATGTCGTCGATTAAAGCAAGAACATGGCTTAGACATGATTGTGATTGACTA
CTTACAGTTGATTCAAGGTAGTGGTTCACGTGCGTCCGATAACAGACAACAGGAAGTTTCTGAAATCTCTCGTACATTAAAAGCATT
AGCCCGTGAATTAGAATGTCCAGTTATCGCATTAAGTCAGTTATCTCGTGGTGTTGAACAACGACAAGATAAACGTCCAATGATGA
GTGATATTCGTGAATCTGGTTCGATTGAGCAAGATGCTGATATCGTTGCGTTCTTATACCGTGATGATTACTATAACCGTGGTGGCG
ATGAAGATGATGACGATGATGGTGGTTTCGAGCCACAAACGAATGATGAAAACGGTGAAATTGAAATTATCATTGCTAAGCAACGT

AACGGTCCAACAGGCACAGTTAAGTTACACTTTATGAAACAATATAATAAATTTACCGATATCGATTATGCACATGCAGATATGATG
TAAAAAAAGTTTTTCCGTACAATAATCATTATGATGATAAAATTGTACGGTTTTTATTTTATTCTGAACGGGTTGATATATGTTAAGT
TTGTGTATTGAAAGTGATAAATTAGTACTGTCAACGCCTCTGTTAAAGGGTTTTTAGGACGTTGAAGACGATTTGTTAAAATGATTTT
TCTTTTAAAAAGGTCGAAAATCAATGTTCGATTTTTATTTGCATTATGGTCTCGATATTGGTAGAATATCAAATGGTTAAATGAGAA
AAACTTGGAGGTGCTCACATGTCATCAATCGTAGTAGTTGGGACACAATGGGGAGACGAAGGAAAAGGAAAAATAACGGATTTCTT
GGCAGAACAGTCAGATGTTATCGCGCGTTTTTCAGGTGGTAATAATGCAGGCCATACCATTCAATTTGGCGGAGAAACATATAAATT
ACATTTAGTACCATCTGGTATCTTTTACAAAGACAAATTAGCGGTAATCGGTAACGGTGTCGTTGTTGATCCAGTTGCACTATTGAA
AGAATTAGACGGATTAAATGAACGTGGCATTCCTACAAGTAATTTACGTATATCTAATCGTGCACAAGTGATTTTACCATATCACTT
AGCACAAGATGAATATGAAGAACGTTTACGCGGTGACAATAAGATTGGTACAACTAAAAAAGGTATCGGTCCAGCATATGTAGAC
AAAGTTCAACGTATCGGTATTCGTATGGCAGATTTACTTGAAAAAGAAACATTCGAAAGATTATTAAAATCAAACATTGAATATAA
ACAAGCATATTTCAAAGGTATGTTTAACGAAACATGTCCATCATTTGATGATATCTTTGAAGAATACTATGCTGCAGGTCAACGTTT
AAAAGAATTTGTAACAGACACATCAAAAATCTTAGACGATGCATTTGTAGCAGATGAAAAGGTACTTTTCGAAGGTGCGCAAGGTG
TAATGTTAGATATCGACCATGGTACATACCCATTCGTTACATCAAGTAATCCAATTGCAGGTAACGTTACTGTTGGTACAGGTGTAG
GTCCTACATTCGTTTCAAAGGTAATTGGTGTATGTAAAGCTTATACATCACGTGTTGGTGATGGTCCATTCCCTACTGAATTATTCGA
TGAAGATGGACATCATATTAGAGAGGTTGGTCGTGAATACGGTACAACAACAGGACGTCCACGTCGTGTAGGTTGGTTTGATTCAG
TTGTATTACGTCACTCTCGTCGTGTAAGTGGTATTACAGATTTATCTATTAACTCAATCGATGTTTTAACAGGCCTAGACACAGTGAA
AATCTGTACAGCTTATGAATTAGACGGTAAAGAAATTACTGAGTACCCAGCAAACTTAGATCAATTAAAACGTTGTAAACCAATCTT
TGAAGAGTTACCAGGTTGGACAGAAGACGTAACAAGTGTGCGTACTTTAGAAGAATTACCTGAAAATGCACGTAAATATTTAGAGC
GTATTTCAGAATTATGTAATGTACAAATTTCTATCTTCTCAGTTGGTCCAGATAGAGAACAAACAAACCTATTAAAAGAATTGTGGT
AGAACTTTATATAAGTCATACGCAATGATTATAAATACATGAGCCTTCTATCTTTATTGGTAGGAGGCTTTTGCTTTGTTTGCTTCTG
TATCGATTCGATTATTTAGATAAAAATTACTAACGTAAAGGTGATATTTGCTAGTCATAATTTAAAAGATTAGATGATATTTAACGA
AATTAAGAAGAAATACTTGAATGTAATAAGTCTGATGTCGAAAATAGCTATTAAAATAGAGTAGACGTAAGTGTAAATGAAAGTAC
CTAAAATAGAAAAATTTCAAAAATAGCGTAATTATTATAATAAATAGACTGCCAATAAAATGCAATTTTTCACTTATAACATTCTTC
AAAAAATAATAGCAAAATTATGTAAAAAATATCTTGTCATGGCAAGATTGGCTGTGCTATAATCTATCTTGTGCTTAAGAACGGCTC
CTTGGTCAAGCGGTTAAGACACCGCCCTTTCACGGCGGTAACACGGGTTCGAGTCCCGTAGGAGTCACCATTTTTTAGGTCTCGTAG
TGTAGCGGTTAACACGCCTGCCTGTCACGCAGGAGATCGCGGGTTCGATTCCCGTCGAGACCGTACAAATGCCTATCCAAGAGGAT
AGGCATTTTTTTGCGTTTAATATTATATTAATAAAAGATATATGGACGAATGATAATCATATTGATTTATCTGTTCGTCCATTTTCTTT
ATAATGTATGAACCTCAAGTAACTTAGTGGTTGGATATGAAAGATAAACGTAGACAATAAAATCTTTATTAGACGTACAAACATAT
GCTACTGTCAACATATTTCTTCGTTGTGATATGCCACCAATCCTCCATAACATCAATTGTTAAAGTAACGAATAATGATATTTATTTT
CTGAGCAATGACGTGCAACTAGAAGTTGCCATTACCCTAATTTTATTATTGGAATAGAGACCTCATCATTGTGTTAAATATCATTGTC
ACAATCCGCCGTGAGAAACTAATAAAAAATATTAATATATAAGTTTATATTGGAAAATAGAATTAATAGCTTATAAATGGTAAATT
ATATAATAGGTTACTATACGTTATAAGACGGAAAATGCGCACAATAACAAAAATAGTAAGCGACATCCTGTGATTTTTTACACAAA
CATAAACGATAAAGAACAAAAAATGATAAAATAATATTAATGATTTAAGAAAAGAGGTTTATGCAAATGGCTAGAAAAGTTGTTGT
AGTTGATGATGAAAAACCGATTGCTGATATTTTAGAATTTAACTTAAAAAAAGAAGGATACGATGTGTACTGTGCATACGATGGTA
ATGATGCAGTCGACTTAATTTATGAAGAAGAACCAGACATCGTATTATTAGATATCATGTTACCTGGTCGTGATGGTATGGAAGTAT
GTCGTGAAGTGCGCAAAAAATACGAAATGCCAATTATAATGCTTACTGCTAAAGATTCAGAAATTGATAAAGTGCTTGGTTTAGAA
CTAGGTGCAGATGACTATGTAACGAAACCGTTTAGTACGCGTGAATTAATCGCACGTGTGAAAGCGAACTTACGTCGTCATTACTCA
CAACCAGCACAAGACACTGGAAATGTAACGAATGAAATCACAATTAAAGATATTGTGATTTATCCAGACGCATATTCTATTAAAAA
ACGTGGCGAAGATATTGAATTAACACATCGTGAATTTGAATTGTTCCATTATTTATCAAAACATATGGGACAAGTAATGACACGTGA
ACATTTATTACAAACAGTATGGGGCTATGATTACTTTGGCGATGTACGTACGGTCGATGTAACGATTCGTCGTTTACGTGAAAAGAT
TGAAGATGATCCGTCACACCCTGAATATATTGTGACGCGTAGAGGCGTTGGATATTTCCTCCAACAACATGAGTAGAGGTCGAAAC
GAATGAAGTGGCTAAAACAACTACAATCCCTTCATACTAAACTTGTAATTGTTTATGTATTACTGATTATCATTGGTATGCAAATTAT
CGGGCTGTATTTTACAAATAACCTTGAAAAAGAGCTGCTTGATAATTTTAAGAAGAATATTACGCAGTACGCGAAACAATTAGAAA
TTAGTATTGAAAAAGTATATGACGAAAAGGGCTCCGTAAATGCACAAAAAGATATTCAAAATTTATTAAGTGAGTATGCCAACCGT
CAAGAAATTGGAGAAATTCGTTTTATAGATAAAGACCAAATTATTATTGCGACGACGAAGCAGTCTAACCGGAGTCTAATCAATCA
AAAAGCGAATGATAGTTCTGTCCAAAAAGCACTATCACTAGGACAATCAAACGATCATTTAATTTTAAAAGATTATGGCGGTGGTA
AGGACCGTGTCTGGGTATATAATATCCCAGTTAAAGTCGATAAAAAGGTAATTGGTAATATTTATATCGAATCAAAAATTAATGAC
GTTTATAACCAATTAAATAATATAAATCAAATATTCATTGTTGGTACAGCTATTTCATTATTAATCACAGTCATCCTAGGATTCTTTA
TAGCGCGAACGATTACCAAACCAATCACCGATATGCGTAACCAGACGGTTGAAATGTCCAGAGGTAACTATACGCAACGTGTGAAG
ATTTATGGTAATGATGAAATTGGCGAATTAGCTTTAGCATTTAATAACTTGTCTAAACGTGTACAAGAAGCGCAGGCTAATACTGAA
AGTGAGAAACGTAGACTGGACTCAGTTATCACCCATATGAGTGATGGTATTATTGCAACAGACCGCCGTGGACGTATTCGTATCGTC
AATGATATGGCACTTAAGATGCTTGGTATGGCGAAAGAAGACATCATTGGATATTACATGTTAAGTGTATTAAGTCTTGAAGATGA
ATTTAAACTTGAAGAAATTCAAGAGAATAATGATAGTTTCTTATTAGATTTAAATGAAGAAGAAGGTCTAATCGCACGTGTTAACTT
TAGTACGATTGTGCAGGAAACAGGATTTGTAACTGGTTATATCGCTGTGTTACATGACGTTACTGAACAACAACAAGTTGAACGTGA
ACGTCGTGAATTTGTTGCCAATGTATCACATGAGTTACGTACACCTTTAACTTCTATGAATAGTTACATTGAAGCACTTGAAGAAGG
TGCATGGAAAGATGAGGAACTTGCGCCACAATTTTTATCTGTTACCCGTGAAGAAACAGAACGAATGATTCGACTGGTCAATGACT
TGCTACAGTTATCTAAAATGGATAATGAGTCTGATCAAATCAACAAAGAAATTATCGACTTTAACATGTTCATTAATAAAATTATTA
ATCGACATGAAATGTCTGCGAAAGATACAACATTTATTCGAGATATTCCGAAAAAGACGATTTTCACAGAATTTGATCCTGATAAA
ATGACGCAAGTATTTGATAATGTCATTACAAATGCGATGAAATATTCTAGAGGCGATAAACGTGTCGAGTTCCACGTGAAACAAAA
TCCACTTTATAATCGAATGACGATTCGTATTAAAGATAATGGCATCGGTATTCCTATCAATAAAGTCGATAAGATATTCGACCGATT
CTATCGTGTAGATAAGGCGCGTACGCGTAAAATGGGTGGTACTGGATTAGGACTAGCCATTTCGAAAGAGATTGTGGAAGCGCACA
ATGGTCGTATTTGGGCAAACAGTGTAGAAGGTCAAGGTACATCTATCTTTATCACACTTCCATGTGAAGTCATTGAAGACGGTGATT
GGGATGAATAATAAGGAGCATATTAAATCTGTCATTTTAGCGCTACTTGTCTTGATGAGTGTCGTATTGACATATATGGTATGGAAC
TTTTCTCCTGATATTGCAAATGTCGACAATACAGATAGTAAGAAGAGTGAAACGAAACCTTTAACGACACCTATGACAGCCAAAAT
GGATACAACTATTACGCCATTTCAGATTATTCATTCGAAAAATGATCATCCAGAAGGTACGATCGCGACGGTTTCTAATGTGAATAA
GCTGACGAAACCTTTAAAAAATAAAGAAGTGAAGTCCGTGGAACATGTTCGTCGTGATCATAACTTGATGATTCCTGATTTGAGCA
GTGATTTTACATTATTCGATTTTACGTATGATTTACCGTTATCAACATATCTTGGTCAAGTACTGAACATGAATGCGAAAGTACCAAA
TCATTTCAATTTCAATCGTTTGGTCATAGATCATGATGCTGATGATAATATCGTGCTTTATGCTATAAGCAAAGATCGCCACGATTAC
GTAAAATTAACAACTACAACGAAAAATGATCATTTTTTAGATGCATTAGCAGCAGTGAAAAAAGATATGCAACCATACACAGATAT
CATCACAAACAAAGATACAATTGATCGTACGACGCATGTTTTTGCACCAAGTAAACCTGAAAAGTTAAAAACATATCGCATGGTAT
TTAACACGATTAGTGTTGAGAAAATGAATGCTATACTATTTGACGATTCAACCATCGTTCGTAGTTCAAAGAGTGGTGTTACTACTT

ATAACAATAATACAGGTGTCGCAAACTATAACGATAAAAATGAAAAATACCATTATAAAAACCTGTCCGAAGATGAAGCAAGTTCC
AGCAAAATGGAAGAAACGATTCCAGGAACCTTTGATTTTATTAATGGTCATGGTGGTTTCTTAAACGAAGACTTCAGATTGTTTAGT
ACGAATAATCAGTCAGGCGAGTTAACATATCAACGTTTCCTTAACGGTTATCCAACGTTTAATAAAGAAGGCTCTAATCAAATTCAA
GTCACTTGGGGTGAAAAAGGCGTCTTTGACTATCGTCGTTCGTTATTGCGCACCGACGTTGTGTTAAATAGTGAGGATAATAAAACG
TTGCCGAAATTAGAGTCTGTACGTTCAAGCTTAGCGAACAATAGTGATATTAATTTTGAAAAAGTAACAAACATCGCTATCGGTTAC
GAAATGCAGGATAACTCAGATCATAATCACATTGAAGTGCAGATTAACAGTGAACTCGTACCGCGTTGGTATGTAGAATATGATGG
CGAATGGTATGTTTATAACGATGGGAGGCTTGAATAAATGAACTGGAAACTGACAAAGACACTTTTCATTTTCGTGTTTATTCTTGT
CAACATCGTGTTAGTATCGATTTATGTTAATAAAGTCAATCGCTCACACATTAATGAAGTCGAGAGTAACAATGAAGTGAATTTTCA
ACAAGAAGAAATTAGGGTGCCAGCCAGTATTTTAAATAAATCTGTTAAAGGCATACAATTAGAACAAATTACTGGACGATCAAAAG
ACTTTAGTTCAAAAGCTAAAGGTGATTCTGATTTATCCACATCAGATAGTGGAAAATTATTAAATGCGAACATCAGCCAATCGGTAA
AAGTTAGTGATAATAACTTAAAAGATTTAAAAGATTACGTTAACAAACGCGTATTTAAAGGTGCTGAATATCAATTAAGCGAGATT
AGTTCAGATTCTGTAAAATATGAACAAACGTATGATGATTTTCCAATTTTAAATAACAGCAAAGCGATGTTGAACTTTAAAATAGAA
GATAACAAAGCAACTAGTTATAAACAGTCAATGATGGATGACATTAAGCCGACTGATGGTGCAGATAAGAAGCATCAAGTTATTAG
TGTGAGAAAAGCAATCGAGGCATTATATTATAATCGTTACTTGAAAAAAGGTGATGAAGTCATTAATGCTAGACTCGGTTACTACTC
AGTCGTGAATGAAACGAATGTTCAATTGTTACAACCAAACTGGGAAATTAAAGTGAAGCATGACGGTAAGGACAAAACGAATACTT
ACTATGTCGAAGCGACAAATAATAACCCTAAAATTATTAATCATTAATATGAATCGTAATAAGCTAGCATTGCAAGCTCATCATATG
TGAGAAGCGGTGCTAGCTTTTTGCTGGTACGGTTTATTATGGCTGACAGGCGACATTTCTGCGTCTCTAACGTGCGCATTTATTCATA
TTTTAAGTAGAACCGCATTGTAAAATTAACGTAACTGCTATTTTAAAAACTTTAGTATTTGTCTAATCATTGTTATAATAATTAAGAA
ATTCATTGCACGTGATTATCAAAATTTAATTATAAGAGACCGGTCGATGAACTAAAGTTACATAATAGGAAAGGTATACAAAACAG
CTAATATACTGATAGTTTCTGTAGGGAAAATCGTATATTTGTACTGATGTATATTGTAGTCATATAGAGAGATTGACTGCTTAAATA
GAAAGGATGAGCCGCTTGATACGCATGAGTGTATTAGCAAGTGGTAGTACAGGTAACGCCACTTTTGTAGAAAATGAAAAAGGTAG
TCTATTAGTTGATGTTGGTTTGACTGGCAAGAAAATGGAAGAATTGTTTAGTCAAATTGACCGTAATATTCAAGATTTAAATGGTAT
TTTAGTAACCCATGAACATATTGATCATATTAAAGGATTAGGTGTTTTGGCGCGTAAATATCAATTGCCAATTTATGCGAATGAAAA
GACTTGGCAGGCAATTGAAAAGAAAGATAGTCGCATCCCTATGGATCAGAAATTCATTTTTAATCCTTATGAAACGAAATCTATTGC
AGGTTTCGATGTTGAATCGTTTAACGTGTCACATGATGCAATAGATCCGCAATTTTATATTTCCATAATAACTATAAGAAGTTTACG
ATTTTAACGGATACGGGTTACGTGTCTGATCGTATGAAAGGTATGATACGTGGCAGCGATGCGTTTATTTTTGAGAGTAATCATGAC
GTCGATATGTTGAGAATGTGTCGTTATCCATGGAAGACGAAACAACGTATTTTAGGCGATATGGGTCATGTATCTAATGAGGATGCG
GGTCATGCAATGACAGACGTGATTACAGGTAACACGAAACGTATTTACTTATCACATTTATCACAAGATAATAATATGAAAGATTTG
GCGCGTATGAGTGTTGGTCAAGTATTGAACGAACACGATATTGATACGGAAAAAGAAGTATTGCTATGTGATACGGATAAAGCTAT
TCCAACGCCAATATATACAATATAAATGAGAGTACCCTATAAAGTTCGGCACTGCTGTGAGACGACTTTATCGGGTGCTTTTTTATG
TTGTTGGTGGAAAATGGCTGTTGTTGAGATTAAGGGGTCTAGTTGAAATGTGGAAAATAATTCGATATTAAATGTAATTTATAAATA
ATTTACATAAAATCAATCATTTTAATATAAGGATTATGATAATATATTGGTGTATGACAGTTAATGGAGGGAACAAAATGAAAGCTT
TATTACTTAAAACAAGTGTATGGCTCGTTTTGCTTTTTAGTGTAATGGGATTATACCATGTCTCGAACGCGGCTGAGCAGCATACAC
CAATGAAAGCACATGCAGTAACAACGATAGACCAAGCAGCAACAGATAAGCAACTAGTAACGCCAACAAAGGAAGCGGCTCATTA
TTATGGTGAAGAAGCGGCAACCAACGTATCAGCATCAGCGCAGGGAACAGCTGATGATACAAACAACAAAGTAACATCCAACGAA
CCATCTAATAAACCATCTACAGCAGTTTCAACAACAGTAAACGAAACGCGCGATGTAGATACACAACAAGCCTCAACACAAAAACC
AACTCACACAGCAACATTCACATTATCAAATGCTAAAACAGCATCACTTTCACCACGAATGTTTGCTGCCAATGTACCACAAACAAC
AACACATAAAATATTACATACAAATGATATCCATGGCCGACTAGCCGAAGAAAAAGGGCGTGTCATCGGTATGGCTAAATTAAAAA
CAGTAAAAGAACAAGAAAAGCCTGATTTAATGTTAGATGCAGGAGACGCCTTCCAAGGTTTACCACTTTCAAACCAGTCTAAAGGT
GAAGAAATGGCTAAAGCAATGAATGCAGTAGGTTATGATGCTATGGCAGTCGGTAACCACGAATTTGACTTTGGATACGACCAGTT
GAAAAAGTTAGAGGGTATGTTAGACTTCCCGATGCTAAGCACTAACGTTTATAAAGATGGGAAACGCGCGTTTAAACCATCAACGA
TTGTAACAAAAAATGGTATTCGTTTTGGAATTATTGGCGTAACGACACCAGAAACAAAGACGAAAACAAGACCTGAAGGCATTAAA
GGTGTTGAATTTAGAGATCCATTACAAAGTGTGACAGCAGAAATGATGCGTATTTATAAAGACGTAGATACATTTGTTGTTATATCA
CATTTAGGGATTGATCCTTCAACGCAAGAAACATGGCGTGGTGATTACTTAGTGAAACAATTAAGTCAAAATCCACAATTGAAGAA
ACGTATTACAGTTATTGATGGTCATTCACATACCGTACTTCAAAATGGTCAAATTTATAACAATGATGCATTAGCACAAACAGGTAC
AGCACTTGCGAATATCGGTAAGATTACATTTAATTACCGCAATGGAGAGGTATCGAATATTAAGCCGTCATTGATTAATGTTAAAGA
CGTTGAAAATGTGACACCGAACAAAGCATTAGCTGAACAAATTAATCAAGCTGATCAAACATTTAGAGCACAAACTGCAGAGGTAA
TTATTCCAAACAATACCATTGATTTCAAAGGAGAAAGAGATGACGTTAGAACGCGTGAAACAAATTTAGGAAACGCGATTGCAGAT
GCTATGGAAGCGTATGGCGTTAAGAATTTCTCTAAAAAGACTGACTTTGCCGTGACAAATGGTGGAGGTATTCGTGCCTCTATCGCA
AAAGGTAAGGTGACACGCTATGATTTAATCTCAGTATTACCATTTGGAAATACGATTGCGCAAATTGATGTAAAAGGTTCAGACGTC
TGGACAGCTTTTGAACATAGTTTAGGCGCACCAACAACACAAAAAGATGGTAAGACAGTATTAACAGCGAATGGTGGTTTACTACA
TATCTCTGATTCAATCCGTGTTTACTATGATATGAATAAACCATCTGGCAAACGAATTAACGCTATTCAAATTTTAAATAAAGAGAC
AGGTAAGTTTGAAAATATTGATTTAAAACGTGTATATCACGTAACGATGAATGACTTCACAGCATCAGGTGGCGACGGATATAGTA
TGTTCGGTGGCCCTAGAGAAGAAGGTATTTCATTAGATCAAGTACTAGCAAGTTATTTAAAAACAGCTAACTTAGCTAAGTATGATA
CGACAGAACCACAACGTATGTTATTAGGTAAACCAGCAGTAAGTGAACAACCAGCTAAAGGACAACAAGGTAGCAAAGGTAGTGA
GTCTGGTAAAGATGCACAACCAATTGGTGACGACAAAGTGATGGATCCAGCGAAACAAACAGCTTCAGGTAAAGTTGTGTTGTTAC
CAGCGTATAGAGGAACTGTTAGTAGTGGTAGAGAAGGTTCTGATCGCGCATTGGAAGGAACTGCTGTATCAAGTAAGAGTGGGAAA
CAATTGGCTAACAAGTCAGCGCCTAAAGGTAGCGCACATGAGAAACAGTTACCAAAAACTGGAACTGATCAAAGTTCAAGCCCAG
CAGCGATGTTTGTATTAGTAGCAGGTATAGGTTTAATTGCGACTGTACGACGTAGAAAAGCTAGCTAAAATATATTGAAAACAATA
CTACTGTATTTCTTAAATAAGAGGTACGGTAGTGTTTTTTTATGAAAAAAAGCGATAACCGTTGATAAATATGGGATATAAAAACGG
GGATAAGTAATAAGACATCAAGGTGTTTATCCACAGAAATGGGGATAGTTATCCAGGATTGTGTACAATTTAAAGAGAAATACCCA
CAATGCCCACAGAGTTATCCACAAATACACAGGTTATACACTAAAAATCGGGCATAAATGTCAGGAAAATATCAAAAACTGCAAAA
AATATTGGTATAATAAGAGGGAACAGTGTGAACAAGTTAATAACTTGTGGATAACTGGAAAGTTGATAACAATTTGGAGGACCAAA
CGACATGAAAATCACCATTTTAGCTGTAGGGAAACTAAAAGAGAAATACTGGAAGCAAGCCATAGCAGAATATGAAAAACGTTTA
GGCCCATACACCAAGATAGACATCATAGAAGTTCCAGACGAAAAAGCACCAGAAAATATGAGCGACAAAGAAATCGAGCAAGTAA
AAGAAAAAGAAGGCCAACGAATACTAGCCAAAATCAAACCACAATCCACAGTCATTACATTAGAAATACAAGGAAAGATGCTATC
TTCCGAAGGATTGGCCCAAGAATTGAACCAACGCATGACCCAAGGGCAAAGCGACTTTGTATTCGTTATTGGCGGATCAAACGGCC
TGCACAAGGACGTCTTACAACGCAGTAACTACGCACTATCATTCAGCAAAATGACATTCCCACATCAAATGATGCGGGTTGTGTTAA
TTGAACAAGTGTATAGAGCATTTAAGATTATGCGAGGAGAAGCGTATCACAAATAAAACTAAAAAATAGATTGTGTATAATATAAA
AGGAAGGGATTTATATTAAAATTTTGAATTCAAAAATTATTGAAAGGGAAGCTACCTTAGAAATTGAATCTATGGCCACTAATACAT
TGAAAATAAACCCAGACATTAATTCTTACTATACAGAAATGTCTTTCGATGGAGAATTGGAAGTGTATGATCCTGAAAATTTGAATA

AAAAATTTCGTTGGAAAAATACAAGTTCAAGTTAAAGGAAAAGAAGTAGCTAAAAGAGGAGGTAAGATTATTCGTCGAAGTAATG
GGTTCTGTTGCAAAGTAAAAAAATATAGCTAACCACTAATTTATCATGTCAGTGTTCGCTTAACTTGCTAGCATGATGCTAATTTCGT
GGCATGGCGAAAATCCG

>002-contig_329_RC

AATAGTTCATACAGAAGACTCCTTTTTGTTAAAATTATACTATAAATTCAACTTTGCAACAGAACCGTATTATGGAATAGAGATGTT
GGTAACATTTATACAGGATCATTATACTTAAGTTTAATTTCGTTATTACAGAACCACACATTCCAACCAGAAGAGAAAGTATGTCTA
TTTAGTTATGGTTCAGGAGCAGTAGGAGAAATCTTTAGTGGTTCAATCGTTAAAGGATATGACAAAGCATTAGATAAAGAGAAACA
CTTAAATATGCTAGAATCTAGAGAGCAATTATCAGTCGAAGAATACGAAACATTCTTTAACAGATTTGATAATCAAGAATTTGATTT
CGAACGTGAATTGACACAAGATCCATATTCAAAAGTATACTTATACAGTATAGAAGACCATATCAGAACATATAAGATAGAGAAAT
AAACTAGTGGCCGATTGTGCTTGATGAGCTTGGGACATAAATCCTAACTCGAAATAAATAAGCATATCACTAAACTGATTTTTTAAA
GTTTACAGTGATATGCTTATTTTTTTATCTTACGATTTTGTACGTGCATGCTTGCCTAGGGGTATGGCTCGAGCCA

>003-contig_276

GGCTGGAGCCATTAGTCTCTCGCACATACTATTCCCTCAGGCGTCAGCACTTACAAAATCGGTTGTAATTTTCATTTTTATACGCATT
CTTACTGAGATTATACTAATAAGAGGAATAGTAAAAGCAATTCTAAGTAAAATTGCAGATAAGAGGTTTGTTAAAAGCAGTTCTCA
GTAAAATTACAGATAAGAGGTACGTTAAAAGCAGTTCTAAGTAAAATTGCAGATAAGAGGTTTGTTAAAAGCAGTTCTAAGTAAAA
TTGCAGATAAGAGGTACGTTAAAAGCAATTCCATGCAAAATTGCTGATAAGGGGTAAGTTAAAAGCAGTTCTCAGTAAAATTGCAG
ATAAGAGGTACGTTAAAAGCAGTTCTAGGCAAAATTGCAGATAAGAGGTGCGTTAAAAGCAGTTCTCAGTAAAATTGCTGATAAGG
GGTAAGTTAAAAGCAATCCTAAGTAAAATTGCAGATAAGAGGTAAGTTAAAAGCAATCCTAAGTAAAATTGCAGATAAGGGGTAC
AGAAAAACTAGACTTGATTACAAAATGGAGCTTGGGACATAAATGATTTTTTAAAAATGAGATGAGACGTAGATTAACTCCATAAT
CAATACGAATCTATCGACTTCTTTATTTATGATATTCATCTCTTTTTAATGGAAATAAAAGTGCGATTAATGTGATAATACAGTTACG
TTAATTAAAAAAATAAAAATGCAAGGAGAGGTAATATGCTAACTGTATATGGACATAGAGGATTACCTAGTAAAGCTCCGGAAAAT
ACAATTGCATCATTTAAAGCTGCTTCAGAAGTAGAAGGTATAAACTGGTTGGAGTTAGATGTTGCAATTACAAAAGATGAACAACT
GATTATCATTCATGATGATTATTTAGAACGGACTACAAATATGTCCGGGGAAATAACTGAATTGAATTATGATGAAATTAAAGATGC
TTCTGCAGGATCTTGGTTTGGTGAAAAATTCAAAGATGAACATTTGCCAACTTTCGATGATGTAGTAAAAATAGCAAATGAATATAA
TATGAATTTAAATGTAGAATTAAAAGGTATTACTGGACCGAATGGACTAGCACTTTCTAAAAGTATGGTTAAGCAAGTGGAAGAAC
AATTAACAAACTTAAATCAGAATCAAGAAGTGCTCATTTCAAGCTTTAATGTTGTGCTTGTTAAACTTGCAGAAGAAATCATGCCAC
AATATAACAGAGCAGTTATATTCCATACAACTTCGTTTCGTGAAGACTGGAGAACACTTTTAGATTACTGTAATGCTAAAATAGTAA
ACACTGAAGATGCCAAACTTACTAAAGCAAAAGTAAAAATGGTAAAAGAAGCGGGTTATGAATTGAACGTATGGACTGTAAACAA
ACCAGCACGTGCAAACCAACTTGCTAATTGGGGAGTTGATGGTATCTTTACAGACAATGCAGATAAAATGGTGCATTTGTCTCAATA
GAAAGTTAGAGGTGAGTCTTACGTTTCAGTGACGGTAGACTTACCTTTAACATGTTACATACTAAAAAATTAATTTGAATAAGAAAG
AGAGACATATATGAAATACGATGATTTTATAGTAGGAGAAACATTCAAAACAAAAAGCCTTCATATTACAGAAGAAGAAATTATCC
AATTTGCAACAACTTTTGATCCTCAATATATGCATATAGATAAAGAAAAAGCAGAACAAAGTAGATTTAAAGGTATCATTGCATCT
GGCATGCATACACTTTCAATATCATTTAAATTATGGGTAGAAGAAGGTAAATACGGAGAAGAAGTTGTAGCAGGAACACAAATGAA
TAACGTTAAATTTATTAAACCTGTATACCCAGGTAATACATTGTACGTTATCGCTGAAATTACAAATAAGAAATCCATAAAAAAAGA
AAATGGACTCGTTACAGTGTCACTTTCAACATACAATGAAAATGAAGAAATTGTATTTAAGGGAGAAGTAACAGCACTTATTAATA
ATTCATAATAAAACAGTGAAGCAACCATCGTTACGGATTGCTTCACTGTTTTGTTATTCATCTATATCGTATTTTTTATTACCGTTCTC
ATATAGCTCATCATACACTTTACCTGAGATTTTGGCATTGTAGCTAGCCATTCCTTTATCTTGTACATCTTTAACATTAATAGCCATC
ATCATGTTTGGATTATCTTTATCATATGATATAAACCACCCAATTTGTCTGCCAGTTTCTCCTTGTTTCATTTTGAGTTCTGCAGTACC
GGATTTGCCAATTAAGTTTGCATAAGATCTATAAATATCTTCTTTATGTGTTTTATTTACGACTTGTTGCATACCATCAGTTAATAGA
TTGATATTTTCTTTGGAAATAATATTTTTCTTCCAAACTTTGTTTTTCGTGTCTTTTAATAAGTGAGGTGCGTTAATATTGCCATTATTT
TCTAATGCGCTATAGATTGAAAGGATCTGTACTGGGTTAATCAGTATTTCACCTTGTCCGTAACCTGAATCAGCTAATAATATTTCAT
TATCTAAATTTTTGTTTGAAATTTGAGCATTATAAAATGGATAATCACTTGGTATATCTTCACCAACACCTAGTTTTTTCATGCCTTTT
TCAAATTTCTTACTGCCTAATTCGAGTGCTACTCTAGCAAAGAAAATGTTATCTGATGATTCTATTGCTTGTTTTAAGTCGATATTAC
CATTTACCACTTCATATCTTGTAACGTTGTAACCACCCCAAGATTTATCTTTTTGCCAACCTTTACCATCGATTTTATAACTTGTTTTA
TCGTCTAATGTTTTGTTATTTAACCCAATCATTGCTGTTAATATTTTTTGAGTTGAACCTGGTGAAGTTGTAATCTGGAACTTGTTGAG
CAGAGGTTCTTTTTTATCTTCGGTTAATTTATTATATTCTTCGTTACTCATGCCATACATAAATGGATAGACGTCATATGAAGGTGTG
CTTACAAGTGCTAATAATTCACCTGTTTGAGGGTGGATAGCAGTACCTGAGCCATAATCATTTTTCATGTTGTTATAAATACTCTTTT
GAACTTTAGCATCAATAGTTAGTTGAATATCTTTGCCATCTTTTTTCTTTTTCTCTATTAATGTATGTGCGATTGTATTGCTATTATCG
TCAACGATTGTGACACGATAGCCATCTTCATGTTGGAGCTTTTTATCGTAAAGTTTTTCGAGTCCCTTTTTACCAATAACTGCATCAT
CTTTATAGCCTTTATATTCTTTTTGTTTTAATTCTTCAGAGTTAATGGGACCAACATAACCTAATAGATGTGAAGTCGCTTTTCCTAGA
GGATAGTTACGACTTTCTGTTTCATTAGTTGTAAGATGAAATTTTTTTGCGAAATCTCTTAAATATTCATCCATTTTTTTAACGGTTTT
AAGTGGAACGAAGGTATCATCTTGTACCCAATTTTGATCCATTTGTTGTTTGATATAGTCTTCAGAAATACTTAGTTCTTTAGCGATT
GCTTTATAATCTTTTTTAGATACATTCTTTGGAACGATGCCTATCTCATATGCTGTTCCTGTATTGGCCAATTCCACATTGTTTCGGTC
TAAAATTTTACCACGTTCTGATTTTAAATTTTCAATATGTATGCTTTGGTCTTTCTGCATTCCTGGAATAATGACGCTATGATCCCAAT
CTAACTTCCACATACCATCTTCTTTAACAAAATTAAATTGAACGTTGCGATCAATGTTACCGTAGTTTGTTTTAATTTTATATTGAGC
ATCTACTCGTTTTTTATTTTTAGATACTTTTTTTATTTTACGATCCTGAATGTTTATATCTTTAACGCCTAAACTATTATATATTTTTAT
CGGACGTTCAGTCATTTCTACTTCACCATTATCGCTTTTAGAAATATAACTGCTATCTTTATAAACTTGTTTGAAATTTTTATCTTCAA
TTGCATCAATAGTATTATTAATTTCTTTATCTTTTGAAGCATAAAAATATATACCAAACCCGACAACTACAACTATTAAAATAAGTG
GAACAATTTTTATCTTTTTCATCAATATCCTCCTTATATAAGACTACATTTGTAATATATTACAAATGTAGTATTTATGTCAAAATAA
TGTTATAATTTTTGTGATATGGAGGTGTAGAAGGTGTTATCATCTTTTTTAATGTTAAGTATAATCAGTTCATTGCTCACGATATGTG
TAATTTTTTTAGTGAGAATGCTCTATATAAAATATCCGGTTCTGTTGCAAAGTTGAATTTATAGTATAATTTTAACAAAAAGGAGTCT
TCTGTATGA

>004-contig_253_RC

TAAAAGGTATTGAATGTATTTACGGTCTATATAAAAAGAACCGCAGGTCTCTTCAGATCTGCGGATTTTCGCCATGCCATGAAATTA
GCATCATGCTAACAAGTTAACACAAAGTATTATTTTAAAATTGAGATTAGACATTTATTTTTCAACTTTGCAGCAGAACCTAATTTTT

TCTCTTTGTAAATAAATGCAAAGAGATACCATAAGGATCTCTTACATAACCATAACCTTCAGTATAGAATTCTGGACTAAATGTTTT
CAATACCTCACTGCCTTTTTCTATTAACTGGTCATATACATGTTTAGTTTCTTCTACTTGGTCAAAAGTGAGACAAAGAGATATATTA
TTACCTTGTGTTATGGGCAAACCTTCAGTGTCATCTGCGATCATAATTTTTATATCTCCAAATTGAAGTACACATTGATCAATTTTAT
TTAAATCATTTTCGTCAATATTAAGTTGCTTATCTATAGGTCTATCTTTAATACGTTGAATATACAGTGTTTTAGCGCCAAACAGCTC
TTCATACAACTTTTTTAAACCCTCTGCATTTTGAGTGATTAAAAAATATGGACTTACTTGAAATTTCATGTTTTTTCCTCCTTAGATTT
GTTATAAATAGAGTATATACTTAATTAGTGTCATCTATTGACACTAAAAGGAGAATAATAATGAAAAAATCTGTTAGATTATATAAT
ATGATTGAATATTGTAATGAAAATAGGAACTTCAAATTAAATGATTTAATGTCAGAATTTAATATTTCTCGTAGTACCGCTTTAAGG
GATATAAAAGAAATTGAAGCATTAGGAGTACCTTTATATAGTAATCCAGGGAAAAATGGTGGTTATACGATCATAGGTAATCGAGA
CCAAACGAAAATAGCAATCTCAGATGAAGAGTTGAAAGCTTTAGTATTTACACTTTCGAGTATTTCAAATGTGAGTAATCTACCTTT
TCAAACAGAATATCAAGAAATATTAAAAAAATTATATAATAACTCGAATAAAAAAGAGTTAATAAACCAATATAATGATCTATTTC
AATATTTTAATGAAGATAAGTATCAGTTCAAAAGTTATAAGTTATTTAATGAAATCATTAGATTGATAATTGAGAATAAGTCTTTTG
AAACCTGTTATTCACAAAACTATATTAAAGAACAATATAAAGGTATTGGCATTATGTATAAAAATCATCAATGGTATTTTGTTGTAG
CTAATATAGAATCAAAGTTAGTGAATCTATTAAATATTTCGAAAATAAAAGAACTATATGAAATGGGAGAGACTCAAGAGTGTAAT
GATATAACTATGCAGAATTTTCAACAGTTCATGGTTAAAAATGAAACAGCTATTGATATTCTTATTAGAAGCAATGTTATGGGATTG
AATATCTTGAAAGGCTACCTGTGGAGTGACTATATGATTGAAAATATTGACGAAGAGACATATTTATTTAAATCAAAAGTGAACGC
GAAAGATATAGATTTTATAGCTAAGTTAATTGTCACAGGTGGTGTCAATGTAAAAGTAGAGACCCCTAATAGTTTGAAAAATGCTGT
TAAAGTTGAATTAACTAAAATAATAAACATGTATTAATAGTAATTTAATCATAATTGTAATAACAAAAAGATGACAACATTAAACA
ATTTAAATTTATACAAAAAAGCATTACCAAATTTAAAATTGGGTAGTGGTAGTGCTACAGCATACATAAATAGTTATAATTAAATAA
AGATGGGTCAATGAAGGTGAATTAAGTGAATAAAAATATTACAGCTCTAAAATCTACTGAAAATACTACATATATGCAGTATACGG
TTCATATCGATGATCAAATTATAAATAATATAAAAGAGTGTACCAAAAAGTTTAAAATTTTGCCTATGGAAGATAAAATTCCATTAT
CACCGTTGTTACAACCAGAATATGCAGGAGAGGTACAAGATTTTATTAGTACATATGAGCAGTTTATGATTAATTTTGGTAAAGTAA
TATTGGATAGTCAAGGCATAAAAATACAGTTTGAAAGTGAATCATTAAGTAGTATTCAACGAGGCATTCAAGAACATTGTTACTTA
AATGAGCGAACAAATGACATTGATGTGACTAAAGAATGGTATTTATGCAAATTTTCAAATCAAACATTAGAAGAGGATAAAAATCA
ATTGTATAATGCTTTAAAGCAACTCATGAATGATTCTAAAAATAAAAAGCAAGCTTTTCAAGAGGTGTTTAAGGTTCATATTGATAT
TTATCTCTACAATAAAGAAAAATCTGAATATAAGTATCAAGTATCTAGTTATTTCAATCTTGTAAAAGAAAATCCTAAAATAACTTA
TAAAAAAAGACATTTACAAGAGAAACAAGGTGTCAAGGGTACAACTTTCACGAACAAATTAAAATTATTGAACAAAATGTATGGTG
TAGATGTTGCAAAATATCAGCCATTTTATAATTCTAATAATCCTGAATATGAAAGAGGTCAATTTGGTGAAAGATACATTTCTCAAA
GATCTAACTATGAATTTAATAGACTTCAATATCAAATTATAGATATGTTATCGAAAATACTTGATAAGCATCCATTACCAAAGTCAG
ATAATAATTATAAACATATTCCAACTATTGAAAAAGCAATATTAAGTGGGGATTCTCATAGTTTTTATGAGTATTTTGAAGATATAA
TGAAAGAAATTATAAGTATGGAAAATTCTTCTTTAAAGGAAAAATTATTGACTGATTTTACATATCAATCTCAATGTAGATGGTACT
CTGAAAGTGAAAAATTAAAATTGCAACTTGAAAGTTTTATGCATAAAGTTTTGGAGAGCAATTATTATGAGGGTAACAAATTATAT
AGAATGTTGTCTCATGCAATTGAAGAAACAATCAATGAGGCTGATGAAGATAAGGTTCATTTCAATTATTTTAAAGATTATTTTTTG
ACAGACGGTGGGGTGAAGAATTGGGAGCAGATTAGCGAGAAAATTACTGAATTCAATGGTAAAGTTATTAACGATATCCAAAATG
AATATAATAAGATTCAATTTAATAACGCAAATCGAAATCAGAAACTAAACTTTAATTATTTATACCATTGCTTTGAGTTTAGTAATA
ACTTAGTAAAAGCAAGGGTAAATGGAAATAGAGGTTACATTTTATATTTTGTAGATAAATATAAAATAAAAATGCCCTTTTTGGATC
AATTATTAATCAATTATTTGAATCAAGAAATAGGTCAGGAAAGTATTAATTATAATATGCAAACATTGTTTGAAAAAGAAAGATAT
GATAGAAGTAGTACCATTGAAAAATTAGTAGCAACAAGCAAATTTAAGTATGAAAAAGATGATTCAGATTTATTCAAACAACTTTT
CAATGATGTTGAAAATTCAATAGACAGATTAGGTATTTACTTACTAAATAATGGTATAAATTCGAATGATGAAAATGCAAGATATTA
TAGATCGTTTTTAAAGGAACTTAGTAGAATAAAAAGTAAATTAACGCCATTTTCTCTTGAAATAAGTAAGTCTAGCGGAAGAGAGC
AACATTATCCTGATGATGCTATTGATGATAAAGATGAGAGAAGAAAAAATAAAGAAGAAACATATCATGCTTTTGATGATAAAAGC
GATATTGACTCCAAATTAAAGAATAAAATAAATGTTTCTATCGATAATTTATTGTCAGTTAAAATTAAGAGCAATTTGTATGATATT
AGTCCAAATACAAAAAAACAATAACTATAATGAGCCAACTCAAGAATGAGTTGGCTTTTTAATTGTAATTTTACGCTTCCATTACAG
GAATCAATGTTATGGAAGCCATTAAGCAATTAATTTGAGTCATTTATAGTATAGGTACAACGTTGTGATGTTCACATGATAATCAGC
CCGGGAGATTATTGTGTAATAACTTATAGAAGGAGCAAATCACCATGTATCAAGCAAATATACGTGATTTAATTACAAAATTGCCTC
AAAGCAATAAAACAGAACACTTTTTAATGAACAAATTTTCAAATCAAGATAAAGTTCAGCAGCTACAAAGACAAATTAGCCAACAG
CTAGATCAACAATATAATGAGCTTTTGGCTAATGAAAAAGCTAAGCTAGACCAATACGTGGAAGTACACCATAATTTAGAACCATT
AAAGAAAGAGATTGAATCAGAATCTATTAACCTTGATACCGATAAATTACCTGATATCAAAGCGACAATGCTTGAAAAGGCTAAGA
ACGATGAACATTTTGATAAAATCGAACAGCTATTTGATAGATTAGATCAGTCATTAAATGGTACGAATCGATTATATACGCAATTAT
CGTTGATTGGCACACGAACACATCGGATCACAACGAAAAGATTTAATGTTCAAGGCTTGCCTAAATTAGTCCAACAAATGATTTTAC
CTTCGCAATTTAAAAAGGTTTATACAATAGATTTTAAATCATTCGAACCATCAGTTGCTGCGTATATGACACAAGACGAACAACTGA
TTGACTACTTGAATCATGAAGAAGGGTTATACGATGCATTACTGAGAGACTTATCTTTGTCAAAAGAGAAGCGCGTGAGTGTGAAA
CGTGCATTTATAGGGTCATTTCTTTTTGGCGGTCGTTATAGTAGCTCTAAATTCAAAATCAATCAAGAGGTTAGTGAAATTAACTGG
CTACAAGTAATGAGCAAATTCAAGAAGGTCATTGAATTTAAGGAGCAAGTCGAAAAATATAAAACAATGCCTACGCCTTACGGCAT
TGAACATGATATGAGCGCATTTCAAGGTAGTAGTATTATGGCAATTTATGTACAAACGGTAGCAAGCTATATTTTCAAGCACATTTT
GTTGGAAGTGTACAAAGCACAGTGCGAAAAAAAAAACGTTCAAGATTATAGTGCCGATACACGATGCGATTATGATTGAATGTAATG
ATAAGGGGATTGCACAAAATGTAGCACAGCTCATGAAAGATACAGCTAATCAACTGTTTAATGGTGAATTTGCACATGTGACAGTG
GAAGCTTTAGGAGGTATAGACAATGAATAATGATAGAGGAAAAAGTCTTCAAATTCCCCAAAGTACATTGTTAAAAGAAGGATCTA
TATACGTTGCTACGTTACATTCTGTGTACGAGAAGAACTTCTCAGGTGATATTAAACATCAGTTTACGTATGAAGTAGAACTTAACC
AAGAAACGCACTATGTAAATCGCAATATTACCGTAAAATCTATGAGTCACCAATTATCAATTGCTGATTGGATTAAACGTCACAGTA
ACTATAACGTAAACCACATTAATTATGATCCATACATTGATCGAAAACATTTGGTCCTTGTAGGGCAATATAACGGAAATTATTATA
TTCAAGATGTAGCACCATTAGATGAATTTGGAGGGGTATTGTAATGAATCATATTTTAGAAATGTTAATAAAATTATTAAAAGTGGG
TATGGAGGCAATCGACCGTAAAGGTCTGATTGCCATCCTAACAAGTAGTATTGGAAATGATGAAATGGATGATTCTGAACAAGCTG
TAATGGTGTATAACGAGCTTATCGATAAGCTACAGCTTAACATTCCTAAAGATGTCGACTATAGACCTAACATATACAGTTATTTTG
GTATTCAAAAAAAGCCAAATGACACAATATTAGTGGAAATGATGATATGTATTTTTCATATCAAGCGTTTTGATTCAGAGTTGTTTG
TTTTCAAAGATAAAGGTTGGCAAAAGGTAAGTGAAGATGAATTGCAAGGGTTGATATCTAAAATGATACAAGTGTTGCTAGTTGAT
TATAAACCTTCACTAAGTACTTTGAAAAACGTAGTAGATGGCATACAGAAATCAACGGACATAGAAAAACTTGTTGAGCATAGACA
GTACATTGGTTGTGGACGAAATATGTTCAATCTAAAGACCTTTAAAGTGGTTGATAATGACCTTGAAATATTCCCTAAAACACGCTT
AGATTTGGAATTAGATATAAATGACACGATTACGGACAAGATACCCCCGAATTTCAAACAATATATGTTAGAGTTGGCGAATTATG
ACCATGATTTACAATATTTCCTTTTCCAACATATGGCAGTGTTATTGACGGCAGATACTAAACTACGTCGTGGACTTTTTTTGTATGG
AACTGCAAAAAATGGGAAATCGGTCTATATTAAATTAGTTAAGTCATTCTTTTATAGTAATGATATCGTATCTAAAACACTTAATGA
ACTTGGCGGGCGTTTCGATAAGGAAAGTCTAATTGGTAAACGAATTATGGCAAGTGATGAAGTGGGGAAAGCTAATATTGATGAAG

CAACTGTGAATGATTTCAAAAAATTACTATCTGTTGAACCAATTCATGCTGACCGTAAAGGAAGAACGCAAGTAGAAGTTACTTTA
GATTTAAAACTCATTTTTAATACGAATGCTGTACTCAATTTTCCATCATCACATGCAAAAGCATTAGAGCGTAGAATTGCTGTTATTC
CATGTGAATATTATGTTGAAAAAGCTGACCCTGACTTAATTGAAAAGTTACAGGATGAAAAGAAAGAAATCTTTCTTTACTTGATGT
ATGTGTATAAGCAAATTGTAAAAAATGATATCGAGTACCTCCAAAATGATCGTGTTACTGAAATTTCTCATGATTGGTTAAATTTTG
GATATGAATTTGTTTCTAGTAAATCAGCAAATATTGCACATCAGAAAGCGTGTATTAATTTACTCAGAAAACTTATAGAAATCAAAC
CAGGGTCACGTATCAAAGTGTCTAGGCTAAATGAGGTTATTAGAGATGAAATTAAAGTAAGCTCTCAAGTTATTAATGATTTGGTTC
AAGCTAACTTTAATGTACAAAGTAGACTAAATAATGGTTATAAGTATTGGGTCGATTTAGGATGGAAAGAAACTGATAAAAAAGAT
GACATGATTTCATTCGATAAAAATGAGAATGTAACAGATGATGAATTCTTATACGAAGATGATTTGAACTTAGGTTGGGAGGACTTT
GACGATGAATAATGAACAAATTGAAGCATTTGTAGAAGTGCTTGTGCCTATCATAGAAGAACGTATCAATAAAGGTAAGTAATCTA
ATTACGTACTACAGGCAGTTGCCTGTAGTACTCATATGATTAAGTGGTAAAAGTGATAAAAATGAAACGAAATTATAAATATATATT
ATCTATATGTTGTTACAAGACCGATGGTCTGTAGCAATAATCTAATAAAAGGAGCGGTATGATATGAAGGGTAAAATTGCACTTTAT
TCACGTGTTAGTACGTCTGAGCAGTCAGAACATGGTTATTCTGAAAAGGAGCAGGAACAACTACTCATCAAAGAAGTTATGAAAAA
TTTCCCAGGTTATGACTATGAGACATATACTGACTCAGGCATTTCAGGTAAAAATATTGAAGGTCGTCCGGCAATGAAACGTCTATT
ACAAGATGTTAAGGATAATAAAATCGAAATGGTATTAAGTTGGAAATTGAATCGTATTTCTCGCTCAATGAGAGACGTGTTTAATAT
TATTCATGAATTCAAAGAACATGACGTAGGGTATAAATCGATTTCTGAGAATATTGATACATCCAATGCTTCTGGAGAAGTACTCGT
TACAATGTTTGGACTAATAGGATCTATAGAACGCCAGACTTTGATTTCCAATGTGAAACTTTCTATGAATGCTAAGGCAAGGAGCGG
AGAGGCAATCACCGGTCGTGTTTTAGGCTACAAATTATCACTTAATCCATTGACACAGAAAAATGATTTAGTTATTGATGAAAATGA
AGCTCATATTGTACGGGAAATCTTTGATTTATATTTGAATCACAATAAAGGATTTAAAGCAATCACGACAATTCTAAATCAAAAAGG
ATATCGTACCATTAATCAAAAACCATTTTCAGTGTTTGGCGTGAAATACATTTTGAATAATCCAGTCTATAAAGGCTATGTCAGATT
CAATAATCATCAAAATTGGGCTGTTCAGCGAAGAAGTGGTAAAAGTGATAAAAATGATGTGATATTGGTCAAAGGTAAGCATGAAG
CCATTATAAGTGAAGATGTATTTGATCAAGTTCATGAGAAACTAGCTTCTAAAAGTTTTAAACCGGGTCGACCTATTGGTGGAGATT
TCTACTTACGTGGCCTTATTAAATGCCCAGAATGCGGAAATAATATGGTATGTCGACGGACGTATTATAAAACGAAAAAGTCCAAA
GAACGCACAATCAAACGCTATTACATTTGTTCATTATTCAATCGCTCAGGAAGTTCTGCCTGTCATAGTAATGCGATTAATGCTGAA
GTCGTCGAACGCGTAATCAATGTTCATTTGAATCGTATTCTTTCACAACCTAATGTTATTAAGCAGATTGCGTCAAGTGTGATAGAA
GAACTGAAACAAAAGCATAGTAAACAAACAGAAATAAAATATGATATTAATAGTCTAGAAAAACAAAAAGCAAAAGTTAAAACAC
AACAAGAACGATTATTGGAATTGTTCTTAGATGATGAAATGGATAGCGAAATGTTAAAAGCTAAACAAAGTGAAATGAATCAACAG
TTAGAAGTATTAGACCAACAAATTAAAGAAGCAAAACAAGCAAATCAATCACAGGATGATATACCCAATTTTGATAAGTTAAAAGC
ACGACTCATTTTGATGATAACACGATTCAGTGTGTACTTAAGAAAGGCTACACCCGAAGCTAAAAATCAACTTATGAAAATGTTAAT
TGATTCAATTGAAATTACGAAAGATAAACAAGTGAAACTCGTAAGGTATAAAATTGACGAAAGTCTTATCCCTCAATCTTTGAAAA
AAGATTGGGGGTCTTTTTTTATACCTAAATTTAACTTTGTGATAAATGTCACAAAGAAAAATAGGATTGAAAATTTATCACTTTTACC
ACTTTTTTAGAGTGACAAAAGTGGAGGAGTTTTGAAATATTTTATAAATATATATTTTATTTATGGAGTACACATTATTAATTAAGGA
GGTCATTATAATGACGCTAAGCAAACAACTTAAAACGTATATCACTGAACGATTTAAATTAAATTATCAAGAAACTTGGGCTTGTGA
AACCATAGATGCGGTGGCTGAAGATGTATTACCTGAAAAATATATTAAAAATAGTCCACTTGAACATAAAATTTTAAATACTTTTAC
CTATTACAATGATGAATTACATGAAATCAGCATTTACCCTTTTTTATGTTATCTAGATAAGGAATTAGTAGCAATAGGTTATTTAGAT
AATTTTGATTTAGACTTTATATTTTTAAATGACACTCATCAAGTTATTATTGATGAACGCTACTTGTTACAAAAAGGGGGCCAGTAAT
TATGAATTGGATCAAGGTCGCTCAACTATCTGTCACAGTTATCAATGAAGTCATTGAGATCATGAAAGAAAAACAGAGTGGAGGAA
AATAGTATGAACATCAATCGATACATCACAAGAGGAATTAATGAAAGTATTCCACTAGACCTTCAAATCTTACTTTGGCACATGGTA
GAAGAAAAAGATAACCAGCCTCATACCGATTACCTACACATTTTCAAACTACAAGAAGATGATAATATGTTGTCAATTAAACATGA
ACAAGAACAGCCCGCATACAAGTTAGAATATCACTATACAAACTATGAAAAAAATCAAAATGCATTACCTAAGAAAGTCTACGTCA
TTCGTGAAGATGGCGTAGACGCTTTTTATTATGTGATGCTTTTACCAGAAGAATACTAAAGGAAGTGATTATATATATGAATACAAT
CAAAAATACGATATACACAGAAGCTATATTTAGCAAGGATGAAAAACACCGCTATTTACTCAAGAAAACATGGGATGAAAAGAAA
CCTGCTTGTACAGTGATAACGATGTACCCTCATTTAGATGGTGTATTATCACTCGATCTCACAACTGTTCTTATTCTTAACCAATTAG
CGAATTCTGAACAATATGGTTCTGTATATCTTGTAAATCTATTCTCTAATATTAAAACACCAGAGAACCTTAAACATATTAAAGAAC
CTTATGATGAGCACACAGATATACACTTAATGAAAGCGATAAGTGAAAGTGACACAGTGATTTTAGCCTATGGTGTTTATGCAAAG
CGTCCCGTTGTTGTCGAACGTGTTGAGCAAGTGATGGAAATGTTAAAACCTCATAAAAAGAAAGTAAAAAGGCTCATAAATCCAGC
AACGAATGATATTATGCATCCGCTTAATCCTAAAGCGCGTCAAAAATGGACATTGAAATAAAGGAGGATTATCTATGAACCATGAA
ACTAAACAATCAGATTGGCATACGGTTGCTAATTGTTTAGAATCGCAAAATTATACATCGATCGTAAAAGGATTAGTACATCATTTC
ACAGCGATTGAAGATGAAGAAATACTTGATAAAATCTATGATGATTTTATGAATGATGACTCTATAACAACGGTACTTAACAATGA
TTTACAGATAATTATTAATCATTACCTATCAAAATGAACATTATATTACTTACAATAAAAATAGAGCATCCTTCACTTTGCGGTGAG
GGATGCTCTTTTAGTTTATTTTTTTATTGGTTACTCGCCATAAGCTCTGCTTGTTCTACGACTTGCTCTACAGCCATTTTTTGTAAATCT
GGTGGATAGCCATATTTTTTAAGCAATCTTCTAACAGCTACGCGCATTTTAGCTTTTGCGCTATCACGTTTAGACCAATCAACACCCA
TGTTTTCTTTCACTGTTTTAGTTAGCTCATGAGCAATCGCACGTAGTTCTTTATCTCCCATGGCTTCTTTTGCTGTTTCATGTGAAGCT
AAAGCATCGTAAAACGCAATCTCATCTGAATTCAGGCCTAATTCTTTTCCTCGTTGTTGTTCTTGTTTGATATCTTTAGCGAGTTGAA
TAAGTTCTTCAATCACTTTAGATGTTTCAATGGAACGACTATTATATTTATTAATCGAGTTTCCTAACATTTCAGAGAAACGCTTAGA
TACTGTCGCGTTCGTTTTCATTAATGATTTGACTTGCCCTTTGAGTAATCGATTTAATAATTCTACTGCAACATTTTTTTGTTTCAATC
CTTCCACGTCTTTTAAGAAATCATCTGATAGGATTGATAAATCGGGTTGTTCAAGACCTAGCGTTTGATAAACATCAATGACATCTT
CAGTCACAACAGATTGTGACACAAGTTGATTAATCTCTGCTTCAACTTCTGCAGGTGTTTTACGTATTTTTCCTTCTTTTGGCGGTTGT
AACAATTTAACAAGTCCTGCTTTAACCGCTTTAAAGAAGGCAATTTCATCATTGAGTTCTTGGGCTGTTGGTTCAGTCGCACAAAGA
GCAAAGGCTTTACCTAACTCTGTGACCGTTTTAATAAAACGTTGACGTTCATCTTCACCTAAACCAATCACATAATCCATCGTATTCG
ATATTGTATAATAACGTTCCAATTTTTTATTTGAATTAAATTTAGAATAATCAAGATTATATAACATATCTTGAATTACATCATACTT
CAATAACATCAATTCAACCGCTTTATCTGTATCTATCGCTGTTTGAGCTTGATCAGATTCTGTATACTCTTTAAGTGCTTCTTTCAAAC
TTTCGGCAATACCCACATAATCGACAATCAATCCACCCGGTTTATCTTTAAACACTCGATTAACTCGGGCAATTGCTTGCATCAAATT
ATGGCCTTTCATTGGTTTATCAATATACATCGTATGCATAGAAGGAACATCAAACCCTGTCAGCCACATATCTCGAACAATCACGAG
TTGTAATTCATCATTCACATCTTTCATACGTTTTTCTAATAAATTACGACGTTTTTTAGGACCAATATGTCTTTGGAAAGAAGCTGGG
TCACTAGAGGAGCCCGTCATTACCACTTTAATGACCCCTTTATCATCATCATCTGAATGCCATTCTGGTTTTAGACGAATGATTTCAT
CATATAAATCAACAGCAATTCGACGACTCATCGTTACAATCATTCCTTTGCCTTTCATCGCTTGTTGACGTGTTTCAAAGTGTTGGAT
GATATCTTTGGCTAGGGCTTCGATACGAGGTTTTGCACCTGCTAAGGCTTCAATTCGTGACCATTTTGATTTTAAACGCTGTTTTACA
TCCTCTTCTTGGTCTTCTGTAATGTCATTATATGCTTCATCTAAATCTAAATTTTGGGGTAGATTTAATGGAATTACACGACTTTCATA
GTAAATTTTAACTGTACTTCCATCAGCTACAGCTTGTGTCATGTCATAAACATCGATATAGTTTCCGAAAACCATTTGTGTATTTTTA
TCCGTTGAAGCTACGGGTGTGCCCGTAAATCCGACGAATGTTGCATTCGGTAAAGCATCTCTTAAATATTTAGCATAACCATATTTA
ATGCCTTCACCTTTATCATTATACTTTGCATTAAAGCCATATTGTGTACGATGAGCCTCATCTGCCATAACAATCACATTTTTACGTT

CTGTTAGGGCAGCCATGGTCGTTTCATTTTGTTCAGGTTCAAATTTTTGCATTGTTGTAAATACAATACCACCCGACTCAACAGATAA
TAACGATTTTAATTCTTTACGTGTTTCAGCTTGTTTTGGCGTTTGTCTTAATAATCCTTTACCAGAACGTCCTTTTGATTTAACAAATG
TACTGTATAGTTGGTTATCTAAATCATTACGATCTGTAACAACGACTAAGGTAGGATTATTCAGCATTTGAATTAATTTTCCAGAGA
AAAAGACCATGGTTAAACTTTTACCAGACCCTTGGGTATGCCAAATAACGCCGCCTTTACCATCACCCGGCCCAGATGAAGCTAATA
AAGCTCTATCAACAGCTTTATTAACAGCATAGTATTGATGATATGCTGCTAGAATTTTACTGATATGCCCTTTACCATCATCTTGGAA
TAATACAAAATATCGAATTAAATCAAGTAGAGTATTAGGATTTAACATCCCATGAATCAGTACGTCTAAGCTAGCTAAACTTGACG
AAGATTCTGTTTCTCCATCTTTAGAACGCCAAGTCATAAAACGATCATAGTTCGCAGTTAGTGAACTGGCTTTAGTATTAATACCATC
ACTTGTAACAAGCACTTCATTAAATGTAAATAATTGTGGAATACGCATCTTATACGTTTCTAATTGATGATAACCATCTTCGACGCCT
ACGGTTTCATTGGTTGAATTTTTAAGTTCGATCACAACGATAGGCAAGCCATTGATAAAGAGGACAATATCGGGACGTTTTGTATAG
TCTCCGTTAATGACAGTGAATTGATTGACTGCTAAAAAATCATTGTTTTGTGGATGTTCAAAATCAACGATTTTAACAATTTCTACTA
CCGATTGTCCTTCGTCATCATAGTCTTCGATTTCAATACCATTGATCAAATTTTCATGAAAGGTAAGGTTATTTTCTAAAAGATTGGG
CGACTTCTCTAAAGTTAGTTCATGTATGGCTTTTTCGATAAAACGATGATGAATATCTGAATTAATCTTTCTTAATGATTTTTCTAATC
GTTCATGAAGGACAACATCTTTATCACTTTTACGTTCTGGCGCTAGACCTGTCATACTAATCTCATTACCTTTTTTGTAGTCATAGCC
CAGTGATTGTAGCCATTCTAATGCGACTTGTTCTAAATCATCTTCACTAAATTGAAAGCTCATCTTCATTCACCTCGATATCATCAGG
TATCTCTATTTCCCCCGACATTAATTTTGGTAGTAAAGTATCTCTTAATTGTGTTAGTTTTTGGTTTTCTATATTTTTTTGGTCCATCAT
TTTTAAAATATGATTATTTTCCTTTTGATATGTTGATAGCAAAGTTTCATTAGTTGGCATTGCTACTTTAGTGTTCTTAATATCTTTTT
GAGATAATAGAGGTTGTGTAGAACCTCTATTCAATGATATATAGTCTATACTTTTAAGTACTTGATATATAATGCTTTCAAAATCACT
GATTATTACAAAAGTATTATCTGATGGCCATGTTCTTGTAGAAAATCTTTGGATTACACCATGAGTTCCTACTCTTCCAATTATAATA
ATTTTTTCATTATATAAATAATCATTGGTATAACCCATTATTTTACTAGCACCAATTATTGGCACCACATTTTCTATATCTTTTTTATC
AACTTTATTTTTAGGTCTTTTACCACTTTTTATTTTTATATAATTTCCTAATTCATCAACTTTCCAACCGCTAGGTATCTCACCTAGTTC
ACTATCAATCATCTCGCCACCACTTGATTTGTACGGATTTCCATTTTCATCTGGGAATTCAAAATCAACAAACCAACGTTTGAACAGT
GTTTGTGATAGTTCCTCTAGGTTTGCTATGATTCTTTTATTAAGTTCTATTTTTTTATCTAAAATTTTTAAAATAGAACTTATAATAGT
TTCTTTTCTTTTGCTTTTAGGAGAATAAATTTCTAAATTATCTAGTATAGGTTTTGTGAGATATTTAGTAGCACTACCTATTGATTGCG
AACGTAAAAAATTCAATTTAAATTTAAGATAATAATATAAATAATCAATATCTATGACGTCAAATTTAGGTTTTATAATATATGTCC
TTTGATAAGCGTCAAATTTTCCATTGAATCTATTTATATGGAAAATTCCATTAGCATTATTGCCAGCAAGTAAGATTACATCATCATC
ATATGAATAAGAATTAATTGTTAAAGGATTAGGAGCACAAGTGAAAAATTTATATTTGCCATTTTCAATCGCAGCATTAGAATTAAG
TCGACCTGTCTTATAATCGCTTATTTCAGATAAAAGATATTTTTTAAACTCCATAGCCTAATCCCTCCAATGATTTGCGGATTTGGTT
TTCAAGTTCTTTTGATTTCGCAAATTGTTCGCTTAATTCAGAAGTAATGCGTTCCATTTTTTGCTCAAACGGTTCTTTATCTTCTTCAA
CATCAGTTAAACCTACATAACGTCCTGGCGTTAAAATATATTCATTATTCTTAATTTCTTCAAGGTTAGCTACTTTACAAAAACCAGC
TATATCTTCATAGGATTTGTCGTTTGTACCTTTCCACGCATGATACGTTTGTGCTACTTTTTGAATATCTTCATCTGAAAATTCTTTTA
ATGTTCTAGATACCATATGACCGATTTCACGAGCATCAATAAATAAAATTTCATTTTTACGTTCTTTTTTACCATTTTGACCTTTATTA
TTACTAATGAACCAAAGACATACAGGTATTTGTGTTGAATAAAAGAGTTGACCTGGTAAGGTAACAATACATTCCACTAAATCTTGT
TCGATAAGATTTTTTCGGATTTCTAATTCATCTTTTCCACTTGTAGACATTGAACCGTTGGCTAATACAAATCCTGCTGTACCATTAG
GTGCTAATTTTGAAATCATATGTTCAATCCATGCATAGTTGGCATTACCTTTTGGCGGAATACCAAATTGCCAACGGTAATCATCAA
GTAATCGTTCTTGACCCCAATCACTTGCGTTAAAAGGAGGATTGGCTAATATGTAATCTGCTTTTAATCCTTTATGTAAATCGTTATG
GAATGTATCAGCATTTCGTTCACCTAAGTCATTATCAATACCACGAATCGCTAAGTTCATTTTAGCTAATTTCCAAGTTGTAGGATTA
GATTCTTGCCCATAAATCGCAATATCGTCTAATCGACCTTGATGTCTTTCAACGAAGCGTTCACTTTGTACAAACATCCCACCTGAAC
CACAGCATGGATCGTAGATACGACCTTTATAAGGTTCAATCATCTCAACCAATAATTTTACAATTGATGAGGGGGTATAGAACTCTC
CGGCATTTTTCCCTTCAGCGCTTGCAAACTTAGCAATAAAATACTCATACACTCGGCCTAATACATCTTGCTTACGACTTTCAGTATC
GCCTACCTTAAAAGTAAATAAATCAATAATATCGCCTAATTTTTCTTTATCTAACGCAGGGCGCGCATATTCTTTAGGTAACACACCT
TTTAATGATTCATTTTCTTTTTCAATCGCAATCATGGCTTTATCAATAATTTGTCCAATTTCTGGTTTTTTCGCATTATCATTAATATAT
TGCCATCTTGCTTCTTTTGGCACCCAGAAAATATTTTCTGCTAAATATTCATCTTGATCCTCTTCATCAGCATAAGGATCTTGCTTCAA
TTCTTCATACTTTTCTTCAAAAGAATCTGATACATATTTTAAAAAGATTAAACCTAATGCTACGTTCTTATATTCAGCAGCATCCATA
CTTCCTCTTAATTTATCGGCAGCTTGCCATAATTTTTCTTCAAATCCGATTGTCGCCATTTATATTACACCTCTATATTATCCTTTATA
CAAATATAGCAAATATTAAATCTTTATGTGACTTTTCTTGTGATTTTCTTTGTGAAAAATATTGTTTGTTGTATTATTTAAGTAATATT
AAGTAGCTAATTATAAAAGTACAGATTATTATATAGTTAAACTGAGTATGAATGGGGGAGCAAATTTGTTATTTACTGAAGAACAA
TTAAAATTATATTCTAAACCATTGTCAGAATCTGAAAAAGAAAAGTGTGAAAATGCAATAAGAATTATTCAAGAATCTCTGGAGTC
ATTAGGATATGAAATAAAAAAGGGTATACATAGAAACAATGAAGATACGCTATCATATCAAATTAAAATGACTAATTCATCGAAAG
ATTATGAACTAAGTATATTTGTGAAAGGTTCGTATGCAACAAATACCAATGTAAGACAAAATAGTGACGTTGATATTGCAGTGGTA
AAAGAAAGTGAGTTTTTTGATAAATATAGAGAAGGTAAAACTAGAGAAAATTATAAATTTATTTCTAGTAATAAGCCTCCGTATTAT
TTTAAAGATGAAGTAGAAGAAGCTTTGATTGAAAAATTTGGAAGAAGTGAGGTAAGAAGAGGTAATAAAGCAATTAGAATCAATG
GCAATACTTACCGTAAAGAAACAGATTGTGTACCTTGTTTTAGATATAGAGATTATAGTAATGATTATATGGATGATCCAAATAATT
TCATTGGAGGAATCACAATTTATTCAGATAAAGGTGAACGAATTATAAATTATCCGGAACAGCATATAAATAATAGTGTTATAAAA
AATAACAATACAAATTATAAATATAAAAAGATGGTTAGAATAATAAAGGAAATAAGATATCAATTAATAGATAGTAAAAATAGAA
ACGCGGAACAAACTTCTTCATTTGGAGTTGAAGGTTTGTTTTGGAACATACCGGATTACAAATATAGCAATGATGAAATGTTAGGTG
ATACATTTAATGCATTAATTGCATTTTTAATAGATAATATAGATAAATTAAGTGAATTTAAAGAACCTAATGACAGATAAAGTCCGT
ATAATTGTGTA

>005-contig_59

ACACACAATCAATTGCAGAAGCACAAGCACTACTAGATGAATTTGAAGCCCAAATGGACGAAGACGTTAAAATTGAATTATAGATG
TAATAAGGGGAAAGGAGGAATTTAAGTTTCCTCCTTTTTTGTTTGTTTTAAAGACTAATGTACCTACTTTTTAAAGAAGTAATTAAAA
AAAGCAACTTTTCCAAGTCGCTTTAATTCATTAAAATATTTTTATTTACTTTAATCTAACCATATATTATTATTGATAAGTTGCTCTAT
GTCTACTTTTTAGTTAAGTGCACTTCTATTTCCGTTTTGGCTGATTCAACTGTTTCATTACCTCGATATATCATCAAATATTTAGACTG
AGTAAAGCCAGGGTCTGGCATCATATCATACCAATAATATCGATTACCATTTTCTATAAATTTAATGTATCCCGTTTCATATGAAGTG
CCACCTAAATGGTATAAATTGATTTTAGAAATTAATTTATTTCTCACTTTATAATCTAACTCTTGAATAGTTACAGTCTCTTTATCAAT
ATTGATATCAAAAGAAAGAGTAACACTGTCATTCTCGTAAACTTTTACAACAATAGGCTGAATTCTATTATTGGTATTGTTCTCATTT
AATGTTATACCACCATACATACAAGTTTTTTTACTTCCAGCTCCTTGATTACTATCACACTGTATATTTTCATTTGAAAAATAGCATT
GATCAAAATAATTGACGCCAAAAATATCGACTTCTTTCCCATCAAATGAACTAGCCATTGTACTGTCTTTTAACTCAGTTTTAACATA
GTCATAATGACCAGGGTTATTTATTGGAACAATGAAGTCATGACTCAAAAATTTGTCACTTAGCTTTATTTTTTTAGCATTAACTGGA
TCGCTATTATATAAATACCATATATTATGCCAAGAACCCGTATATTGACTAGATTTGTTTAATTCACTTATATTTGGATCATTTTGTGT

CTCTGCTCTAGCTTCCAACAAAGAATAGCTACTGTAACCAAATAACAAAGTGATTAGTATAAATACTTTTCTCATATTACTCCTTTTG
AATATATAATTAATTTAGTTGACCTTTATCAACCATCGCTATTTTATAACATATATTCATTTAATGTAACTATAATTTTTTAAATTTAA
TTATTATATTACAACAAATTGCACTTTAATAATCCTCTTATATTTTAATGACTGTCCCTACGTCAACTTTTAATATAGTATATATGCTT
TTTTATAATTATCTTGAAATTCACTTTAAACGATACGGAAATAGAATTTATATCCTTCCTCTTCAACGAAAGCTTCAGCAATTAAAAA
AGCAACTTTTCCAAGTCGCTTTAATTCATCAAAAGTTTTGACACCAAGCATATTCAAATTTAATAAATATTCGTCTTGCTGTATTCCA
TCATATTTCATTATTTAAAAGCGATTTTACGAATAAGTTCATTTGTAATTTCTTTCTCATTATTAATTGCATGAAGAATTTGTATTTGC
AGTTCTCGTGTAACATACATACCTTCAAGTGAAAGGTTTTTAGCGATTTGTTTATTAATCTTTGAATCAATAGTAATCATGATATCGC
CTCCTATATATCACTTTTGTATCTTTTATCACTTAACAGGGTTATTTATGCCTTACCTAATAATCTTATTTAAAAATTGGTCATACAAC
TTTAAAATTAACTTTCACAAATTGCAATTACAGTTATACGACTAATCACTTTTAAGACTTACGTATCTATTTCGCTAATAAACTTTTG
AAAATGATGATTTAACAATGCACACTTTTGATTGTTTAAACATGTCTATTTTAAAATTATTTTTAGATATAAATGCGAATTTTGAAGT
TGCTACTCTTTAAGGTTTACTAAAATAAAAGTTTATATAAAGAAACTTGAACATGAGAACAAATAAAACTAAATTTATGACAATCTT
AAACCTCTAAAAAACAACATTTTGAAACCACTAATCATAATTAATATCTTTATAATTTTAAATCTTTAATATTATTTTAGTAAAAATA
TATCATTAACTATCCCATTCAACTGTTTTTTAATAATAGTAAAATCACAGTTCAAATCTAATGTCTTAATAATGATTTGACTACCACT
CATATACTCACGTTTTATAAAATCATGAGCTTCCAAATTAGAATTTAACCATATATTGGAAATTTTTTCTATTGATTCATAATCTAAA
TTGTCTAATTTCTTTATCACTATATTCAACTCTTATTTAAACCATTTCAAAACATTATTAAACCTTAATCAATCATACCTGACTTAATC
CATTCATCAAATTCTGTAAATACAATATTTCCATGGTAATATGTACCGTTTTGGTAATCATGATTTTTTTTCCACCAATTTTTTGAATT
TTCATTCTCTTCAACATCAATATAATTAGTGTAAAAAGTTCCTGACATTACAATGGGACAAATGCCCTCTTTATTACCAAGATCTGCT
AAATTTGACGCTGTTGTTACTGCTTTTCCAATCCATACAAAATCATTTATACCTGAACTCTTTCTACCTGCCTTCACTGCCAAATTTTT
ATCTGCAGCCAAACCAATACCTGCTTTGATATTTGGAAGATTTCTTTCATCTAATAATTTATTCAACATTTTTAAATAGGTATTAATG
TAAACTGCACGTTGGTAGACATCATATATTTCTTCTTTGCTAGCAGTTGAATATACAGCAAATACGCAATCTCCCCTAATACCAATTT
CTTTCAATTCATTATCTTGAGTATCCTTTTGTAAAATCTCTATAACTTCAGACGTAAAGCCTCTAATCACCTTGGCAACATCTACTTC
ACTATTTTCTGTGAATAATTTAGTAGAATTTCTTAAATCTATAAAAATTGCACTAAGCCAAGCTTTATAACCATTCTCATATGTAAAA
TTACCATTATTAGGGAATCTTTTCACTTCATTTATAAAATTAGTATTCTCTAAGATTTCTTCAACTTTTTTCTTTCTTGACTTGTAATCA
TAGCCTCTTATTTTCATATTTAAAAACCTCCCGCTTTAACTAAAATAATACCGATAGAATGCAAAGTTAAAATTCCTAAAATCCCCC
AAAGTACCAATGCTAATCCTTTCTTATAAAAATGATATTTTTTTGTAGATATTTTTGAATTTATATAAATCTGTGATAAAATATCATG
GATTTCTTCTTCTGATGTTCTATTAATTCTTTGCTTTTTATACTCAGAATAAGTCTTGCTTGAAATGCTTTCAAAATAATATAAGGAGT
CATTTTCTATTCCGTTATATGCCTTAACAGATTTTCTTAACTGGGGAAAAAGCACCCTAATTATATTAAATACTCCATCAAAAAATAA
AAAATAGACCGCTCCTTCAAATATTAAATAAATTAAATTCGAAAAATTTATATTGTTCAATGCGTTTAATACTATTTCTTTTTGACTA
TCTAAGAATAGTTTAGAAGAAATAGTAACAATAAAAACTCCTAACATTGCTAAAACAATTGAAGCTTTTGTATCACAACTCTTTATC
CACTCTAAATGCCTATCTAACTCGTTTAAAAGCTCATTTTTTAAATTGTTGTTTTCCATCAGATTAATCATCCTTTTGAATTATAAAAA
ATTAAAAACTAATATTTTCATAATTTAAATTTCTATTTTACAATCTACTTTATTATGAAAACTCTTATTACAAATAATTATAACAAAT
CAAGAACTTACGTTCTCTATATATTCATAATTCTTAAGATATAAATATTTAAAAATGAGAAATGGATTATTTGATGAACAGCAATTG
GAGACGTGGTTGCAAGCAATAATTGGAAATATGGATACGAAAGATGTAAAAGATGTGAATCGTCTACACTTAATTGGACAAATTCT
ATGAGAATAGATATTGTTAATTTAAGAAAGTAGGCGGTTTTATTATGACAAGAGAAATAAGATCATTTAGTTCAGAGTTTAAGTTAC
AAATGGGTAGATTATATAAAAATGGTAAACCTAGGAAATCGATAAAGCAACATCAAAACACTGGTTTATTCAATCATCAAGATAAC
CTATCAGATGAAGAAAAAGAGCTGATTAAATTACGCAAAGAAGTCCAACATTTAAGGATAGAGAACGATATTTTAAAGCAAGAAG
CGCTGATCATGGGACGAAAAAAGTAGTCATTCTACTAATGAAACACCTAATAATGAAAATCAATCGAATCAAATTCAAAATAATAG
TCAAGATCAATCTAAAACAGAAGAAGATACAAATGATGAACAAAAAGTAAGTAGAGATAATGTTTTTGATTACGCTATTGACATGA
AGTAGGAGATGCTGATTTATTAAAGTTCATAAAACCGGAATACAATGGTTCAAAATGGACAATTAATGTAAATAATAAATCAGGTA
TTGAGGCTAATACCATCGTAGTTAAAGATGATGAAACAGTTCAAATATGGAATGGATTTAAAACATCTATGAATCATGAAACTAAA
ATTGAATTGTAGAAAGAATTAATGAGACATCAAGTATACTAACTAATAACACATGCGGTCTATATAAATATATGATAAGAATCAAA
GAATTAATACTATAAACAGCGCATTTCCTACTTTTTGGAGTAGGAAATGCTCTTAAAATTATATTTTTTTGATTGTTATTTACATTTTT
TAATGCTAAATACTTAATTTAATAGTTGATTGTTAAACTGATCCTTAAATTCTTTTCTTTTTGTTAAATCATCAGAATAAAAGCATAA
TCCTACTAATCTAACATCATTATTTTCAGCAAGTACTTCAATATCATGTCTTTTATTCAATGATAACATAAATTGCTCTTTCCATTTAT
CATTTAATAATAAATGTTGACCTTTAGGTTCTACAAATACTTGATATGTGTATTCATTATCTTTTAAGTACAACAAGAAGTCAGGCAT
AAAACCTCTCACACCATCTATTTCTCTAAACTTAATTTTACGTTCGTTACGTATTAAATAAACTTCTTCGTATTTATTTTGTAGATCTT
CCATCATGTTATTGATTAAGTCAATCAAATCACTCTCAAGACCATTTACAATAGCTTTATCATAAATGTACCAATCATGTTGTCCCAT
ATTTTTAGGTCTCTTCTGATCATCTAAGTCTGTTACATCTCTATTGACATTATTCACTTCGACAACATAATCATCGATTAATTCTGAAA
GTTTCATACCTTCAAAAATAGTTGTACCTTTGACTTTCAGATAATTATTTTTAATTTTTGTTTCTAAATTACTTAAATAATCATTCAAT
ACAGACAACTTTAGTTTAGGTGTGATATCACTAATATCCATCTCTTTTGGTATAGTTACACTGATATCTAAATCTCCTAAAAAAATCTT
TTGACTTTATAAATGTCTGCATACTTGTGATACTAGGTACATACTCTTTTAAATTAGAATATCTAAAGAATGGATTACTTCTTAAAGC
TTTTTGTAATAATGGTTTATTAATATTTAATCTTATTTCTTTACGATCTTCAGCTATAGCTTTAATACCTTGAGTTAAATTTGACTCAC
TAACCTTGAATAGCTCCTTTTGATATTCCCTAGATGCGTTATAATTTTCTAATTTCTTGTAGTCCTCAGCAGTTGTTGGCACTGTTTTG
TTAACATATATTTTTCCAAATTTGAAAAGGTCAGTTTTTTTAAATTTATTTTTCACTTTTCCTTCGTGTATCTCGCTTATATCAGCATTT
ATTTGAACTTTTGCTTCTGTTAAAGATTGGTGTAAATTTTTAATATAAGAAGAGTCATTAATCGTATGATAATGTAACGTTTCGATTG
ATTTTAAATCACTAAATTCATTATCAAATCTACGCTTATACGATTTTTCTTCTTCATAAATAAATGGATAATATCTAGCTCCTCGACC
GATGAGCTGCGCTTCTGCATTCGTCGCGTTTTTGTTGACCTTTTTCTTCTCATCTATTTTGACAATATCGAAAAGATTTAAGACGTCCC
AACCTTCATTAAGTTTGTACACTGCAAAGATAGCTCTTATAGGGTTATCTGGATCTTCTAGATTATTCAGTAATTGAGCATTTTTCTC
TTTTTCTTTATCATTATTTACATTTAATGTCGTTTGTTCATTAAAATCCCATTTCAAGTCATCTAACACTTTTACTATATTTGCTTGCTT
ATAAAAACTGAAGACCTTACTCCAAATACTATTTTGACCACTGTAAAATTTAAAACCATTATCAATTATCGTTTCTAATTGCTCATAA
GTTAATTGTTCTAAACGGTCAAGCATCATTTGATGTATTTCTTGTGAGCCTTTAATAGAAGTCGATTGAAACATGATAACTGGCTTAA
GCGTTATATGGTTTTTATCAGCTATATAATTTTTGTATTCACTCATTAATAAAGCATGTAATATTTTATGTTCATTTTCTTCACTCGCA
TGCAAAAGTGTCACGTTTTTTGAATAACCTTGTTGCATAAATTCCTTTAAATCATAGCGATAAAGAATTCTATCTTTATATTTTTCAA
AAATATCATCTTCTAACTGTACCGTAGCTGTAAATTCTAACAATTTATTCTTTTTGTTTTGCTTCATAATCCTATCAACAGTTACTTCC
CAGTTAGTTTCTTCAATTTGCTGAGTAGTTTTTTTGCCTTTTTTCTTTGTGCTGACATTTAGATGATGAGCTTCATCAGCTAATAACAC
AATAGGAAATTGAGCTAAATCTTCATAAGTAATTTCATTTTCTCTTGGATAATTTAATTTATCATGCAATGCATTAATCGTTGTTAAT
TTAAGGTAAATCGTATTTTCATCAGGTAAAACGGGATAATTATCTACTACTTGAATATTTATCAATTTACCATCAATGACAATGCCTT
CTGGATTAAACAAATACTTACTAGATGACGTGTTGGTTAAATTGTCATACGTTTTATTGACAATCGCAGTACTATTAACAAAAAACA
AGAAATTTTGATAACCTTTTTCTTTAAATAAATATAATACTGATGCAGCTAAAACAAGTGTTTTACCAGAACCAGTTGCCATATTAA
ACATCAATTTGTTGTATAAATTACCTTCATCTTGCTCTTCAAGATACATTAATCTTTGCACAGCTTCTTCTTGATATGGTCTTAAATGA
TGATTTAAATTATCAATAATATAATTAGGTATATCTGGATACTGTAAAAACAGTTGATTTTCTATACGATCATGTAGCATTTCTGACA

TAATTACTCACCTCCGTAGAAGCTATGGTTAAATGCTATTTCATCTTCTGTGAAATTATATGCTGAATCTTCAATGTCAGAGTAATTA
ACATATAATTGATTATTATCTAACAATCTAATTAGCACTTCTTTTTGGTCTTCAAGTTCTAATTCATGAAATTCTTTTTTAGTACTTGT
AAACTTATCAAAATCTACTTCATAATTAAGAACTCCATCATTTAATAATTTATCGATTCGTTGACTTAATTCTTCTTTTGTCCCACAAT
TTATAAGACTTTCAACGATTTCTTGATTTTCTTTTGCCAATTCAACATATACAAAAGAACCACCACCTTGCCAATCTAAATCAGTTGA
AACACCGCCTTGCTCACCCTCAATAACTTTTTGTAACCTTGGGATAGATACAGTATTAATGTAGTCCATTTGTTCAACACCAATATAT
TGACGATTCATTTTATGTGCTACCGCTTGAGTAGTGGCAGAACCCATAAAGAAATCTAATACAATATCATTTTGTGAAGAAGTAATA
TCTACTAAACTTTTAATTAGTTTTTCAGGCTTAGCAAAATTAAATACTCTCTTATTAAAAAGTTTATTTATTTCATTAGCTCCTCTTTG
ATTAGTTCCAAATTCTATACCCCAAAAATCATTCGGTATCTGTCCTTTAGTATCATCTAGATATATTTTTACATAAGGTTGGCCATCA
CCTCTTGTTGTCCAATATATTTTGCCTTCATTATCTAATTTCTCTATTTCTTCCTTTGTTTTCATCCAC

>006-contig_274

TTCCCAATGTGCATCTACTTTACTAATGCGTTTGATAAAAACTTAAATAATATTAATTCAGTCATCAGTGGCGTTAAATCTTTTATCA
TTTTTAGTTATAGTTGATAAATTTATATTTATAAGCATATATGGATATTTCATCAAAAATTTTTATTCATATAAATCCGAACTGCATA
CATATTTGTTGAAATAAGAGGTATTATTTTTCGGGAAATTGCTGTCTGAGTTATAAGTATTAGTTTTATAAAATGAGTTGAACTATAG
CAAAAACGATTAAAATACTGATAATCCATTTTTGTATTATGTTAGGGACTTTTTTACTTAATTTTAACCCTATTGGAGCAAATATAAT
ACTCCCTATTATAAGGAATAAGGCGTCATATAAAGGGATATAACCTTGAATAAGTTTGATGACAAAAGCACCAATTGAAGATATAA
AAGCAATTACTATACTATTAGCGACTACAGTATTCATTGGTAATTTGAATAAAACCAATAATATAGGAATAATAATGAAGGCACCA
CCTGCACCTACTATACCTGAAATAATACCAATGAAAAGGCCAATGATAACTAATAAATATTTATTAAATGAAGACTTTTCGGAACTA
GGTTTCACTTTAATAAACATTAATGTTAATGCAAGTAAAGCAATAATGATATATACCGTATTTACAAATGTAGCATCAAATAAATTT
GCTAGAAATGCACCTAACATACTCCCTATAATCATGCCGCCACCCATATAAAGAACTAATTGTGGCGAGAACTCTGTTTTTTTTCGA
GCTTTTAATGAGCCACTTAATGTACTGAAAAAGACTTGGCTAGAAGTAAGACCTGATGCGATATATGCGCTATATGCAGGGGCTCC
GAATAATGGTGGTAATAATAAAATAGCTGGATAAATAATGATAGCACCACCTACGCCTACTAGACCAGATATGAACCCGCCGAATA
CCCCAATGAGTAACATGATAACTATATTAACAATATCCATTACTTACTTTTCACCAATAAGTTTACAGCTTCATTAATTAACTCTTGG
GAGCTTTCTTCATCATCCGCAGCTGCTTTTACACATTCTATTAAATTCTCACTAATAATGATACCCATCAAGCGTTGGAGTGAACTCT
TTGATGCACTTATTTGTGTAATGACATCTTTACAGTCTTTTCCTTCCTCCATCATTTTAATAATTCCATTTAGTTGCCCTTGTATTCTAT
TAATACGATTAATCATTTTTTTATCATAATTCATAGTCATACCTCCACTTTTAATTGAATAAAAATATATTAATAGATAAACACAAAT
GTGTCAAATACCCCTAGAGGTATTTGACAAGTTCCATTCAACTGTTTAGAATACCCCTACAGGTATTTTTAGGGAGGTTATTATGAA
ACAATACGGAGAAAAGTTTATCGATGAATTTAGTAAAGCAGAATTGGAAAAACTAGCCAAGCAAGGGCAATTAATTGATGTTAGA
ACAGAAGAGGAGTATGCATTAGGACATATCAATGGTTCCATACTTCATCCTGTTGATGAGATTGAGTCATTCAATAAAGAAAAAAA
TAAAACCTATTATGTAATCTGTAGAAGTGGTAACAGAAGTGCTAATGCTAGTAAATATTTAGCTAAACAAGGTTATAACGTTATAAA
TCTTGATGGTGGTTATAAAGCTTATGAAGAAGAAAACGATAGTTATGATACACAAGAAGAATATAAAAGTATAGAAATTAAAGCAG
ATCGTAAACAATTTAACTATCGTGGTCTTCAATGTCCAGGGCCAATTGTAAAAATTAGTCAAGAAATGAAGAATATTGAAGTAGGT
GACCAAATTGAAGTCAAAGTCACAGACCCTGGATTCCCTAGTGACATTAAAAGTTGGGTGAAACAAACAAGGCATACTTTAGTTAA
GCTTGATGAAAATAACAATGGAATTAATGCGATTATTCAAAAAGAAAAAGCAAAAGATTTAGATATAAATTATTCTGCTAAAGGTA
CTACAATTGTATTATTTAGTGGAGAATTAGACAAAGCTGTAGCAGCATTGATTATTGCAAATGGCGCTAGAGCTGCTGGAAAAGAT
GTAACTATCTTCTTTACTTTTTGGGGGCTTAATGCATTAAAAAAAGTGCAAACAGTTAATGTTAAAAAGCAAGGTATTGCAAAAATG
TTTGATTTAATGTTGCCCAAAAAGAATATACGAATGCCTCTTTCCAAAATGAATATGTTTGGTTTAGGAAATATGATGATGCGCTAC
GTAATGAAAAAGAAAAATGTTGATTCATTACCAACACTTATCAATCAAGCTATTGAGCAAAATATCAAATTAATCGCTTGTACGATG
AGTATGGATGTCATGGGTATTCAGAAAGAAGAACTTAGAGATGAAGTTGAGTACGGTGGTGTAGGCACTTATATTGGTGCTACTGA
AAATGCGAATCATAATTTATTTATCTAATTAAATCTATTAATAAAAGGAGTTGTTATCATGTTTTTTAAACAGTTTTACGATAATCAT
TTATCTCAAGCATCATATTTAGTGGGTTGTCAACGTACAGGAGAGGCAATAATAATAGACCCTGTTCGTGATTTATCGAAATATATA
GAAGTTGCAGATTCTGAAGGTTTAACAATTACACAAGCTACAGAAACACATATTCATGCTGATTTTGCTTCAGGAATTCGTGATGTG
GCTAAACGTTTAAATGCAAATATATATGTGTCTGGCGAAGGTGAAGATGCATTAGGGTATAAAAATATGCCATCAAAAACACAATT
TGTTAAACATGGAGATATCATTCAAGTAGGCAATGTTAAATTAGAAGTTCTGCATACTCCAGGGCACACGCCTGAAAGTATTAGCTT
TTTACTCACTGATTTAGGCGGTGGTTCAAGTGTTCCGATGGGATTATTTAGTGGTGACTTTATTTTTGTTGGTGATATAGGTAGACCT
GATTTATTAGAAAAATCTGTTCAAATAAAGGGTTCTACAGAAATTAGCGCGAAACAAATGTATGAGTCCGTTCAAAATATTAAAAA
TTTACCAGACTATGTTCAAATCTGGCCGGGTCATGGTGCTGGAAGCCCTTGTGGTAAAGCATTAGGTGCCATACCTATATCTACAAT
AGGTTATGAGAAAATTAATAACTGGGCATTTAATGAAATTGATGAGACTAAATTTATTGAATCATTAACATCAAATCAACCAGCAC
CACCGCATCATTTTGCACAAATGAAACAAGTTAATCAGTTTGGTATGAATTTATATCAATCATATGATGTTTATCCTAGTTTAGATAA
TAAGAGAGTAGCATTTGATCTTCGTAGCAAAGAGGCCTTTCACGGTGGCCACACAAAAGGAACAATCAATATACCATACAACAAAA
ACTTTATTAATCAAATTGGTTGGTACTTAGATTTTGAAAAAGATATAGATTTAATTGGAGATAAATCTACTGTTGAGAAAGCGAAAC
ACACTTTACAATTAATTGGGTTTGATAAGGTAGCAGGCTATCGTTTGCCAAAATCAGGCATTTCAACCCAGTCCGTTCATAGCGCTG
ATATGACAGGTAAAGAAGAACATGTATTAGACGTACGTAATGATGAAGAGTGGAATAATGGACACTTAGATCAAGCAGTTAATATT
CCACATGGTAAATTATTAAATGAAAATATTCCTTTTAATAAAGAGGATAAAATATATGTACATTGTCAGTCAGGTGTTAGAAGTTCA
ATTGCAGTGGGTATATTGGAAAGCAAAGGTTTTGAAAATGTGGTGAATATTAGAGAAGGCTATCAAGATTTTCCAGAATCATTAAA
ATAATTTAAGGATGTGGAAAAAATGAATAAGCATTATCAAATTGTTATTATTGGTGGCGGTACAGCAGGTGTTACCGTAGCATCAA
GACTATTAAGAAAAAATCAAAACTTAAAAGAGAAAATAGCAATTATAGACCCAGCAGACCATCATTACTATCAACCATTATGGACG
TTGGTTGGTGCAGGGGTATCTAGTTTGAAAAGTTCTCGTAAAGATATGGAAAGTGTTATACCTGAAGGTGCTCACTGGATAAAACA
GGCTGTTTCAAGTTTTCAACCTGAAAATAATAGCGTTATTTTAGGAGATAATACAGTCGTTTATTATGATTTTTTAGTAGTAGCTCCA
GGATTACAGATTAATTGGTCTTCAATTAAAGGACTAAAAGAAAATATAGGTAAAAATGGTGTTTGCTCTAACTATTCACCTGACTAT
GTTAACGAAACTTGGAACCAAATTTCTAATTTTAAACAAGGAAATGCCATTTTTACGCATCCAAACACCCCTATAAAGTGTGGAGGT
GCGCCTATGAAAATTATGTATTTAGCTGAAGATTATTTTAGGAAACATAAAATCCGTTCTAACGCTAATGTGATATATGCAACGCCA
AAAGATGCTTTATTTGACGTAGGAAAATATAATAAAGAATTAGAGAGGATTGTTGAAGAAAGAAATATAACAGTCAATTATAATTA
TAACCTTGTTGAAATCGACGGTGACAAAAAAGTGGCTACATTCGAACATATCAAAACATACGATAGAAAAACAATAAGTTATGATA
TGTTACATGTAACACCACCTATGGGTCCCTTAGATGTAGTAAAAGAAAGTACACTTTCAGATAGTGAGGGTTGGGTAGATGTTAACC
CAACCACATTACAGCATAAAAGCTACTCTAATGTATTTGCACTTGGTGATGCTTCAAATGTACCTACTTCAAAAACAGGCGCAGCTA
TTCGTAAGCAAGCACCTATCGTCGCTAATAATTTATTGCAAGTGATGAATAATCAAATGTTAACGCATCATTATGATGGTTATACTT
CATGTCCTATTGTTACTGGATATAATAGGTTAATACTTGCAGAGTTTGATTATAATAAAAATACTAAAGAAACAATGCCGTTTAATC
AGGCCAAAGAACGTAGAAGTATGTATATATTTAAGAAAGATTTATTACCTAAAATGTATTGGTACGGCATGCTAAAAGGATTAATA
TAATAAAGTACAGAAAAAAATAAATTTTTAATGAAAAATCTTTTACTATAAAATATTAAGTATTTAAATGACGTGTCAGTGTTGTGT

TTATATGTCGTGAATTTTTAGCTCTAAATAGTAAAAGATTGAAAAAGTTGTTACTGTTTTAAATGATCGCGATGAAGTCATTCAATA
AGAATGATTATGAAAATAGAAACAGCAGTAAGATGTTTTCTAATTGAAAATCATCTCACTGCTGTTTTTTTAAAGGTTTATACCTCA
TCCTCTAAATTATTTAATAATAATTAATGGTATTTGAGCAAGTTTAGCGACTTTATGACTGACATTACCAATTTCCATTTCTTGCCAG
ATATTCAAACCACGTGTACTCAAAATGATAGCTTGGTATGTACCTCCAATAGTAATTTCAATAACTTTGTCTGTTGAACACTAAGAG
CAATTTTAATATCATAATGTGTTGTAAACATTTTTTTGATTGGAGTTTTTTTCTGAGTTAAACGATATCCTGATGTATTTTTAATTTTG
CACCATTTCCAAAAGGATAAGTGACATAAGTAAAAAGGCATCATCGGGAGTTATCCTATCAGGAAAACCAAGATAATACCTAAGTA
GAAAGTGTTCAATCCGTGTTAAATTGGGAAATATCATCCATAACCTTTATTACACCTATAATTCTATTTTAACGTCTTCGTCCATTTG
GGCTTCAAATTCATCGAGTAGTGCTCGTGCTTCTGCAATTGATTGTGTGTTCATCAATTGATGGCGAAGTTCGCTAGCGCCTCTTATG
CCACGCACATAGATTTTAAAGAATCTACGCAAGCTCTTGAATTGTCGTATCTCATCTTTCTCATATTTGTTAAACAATGATAGATGCA
ATCTCAACAGATCTAATAGTTCTTTGCTTGTGTGTTCACGTGGTTCTTTTTCAAAAGCGAATGGATTGTGGAAAATGCCTCTACCAAT
CATGACGCCATCAATGCCATATTTTTCTGCCAGTTCAAGTCCTGTTTTTCTATCGGGAATATCACCGTTAATTGTTAACAATGTATTT
GGTGCAATTTCGTCACGTAAATTTTTAATAGCTTCGATTAATTCCCAATGTGCATCTACTTTACTCATTTCTTTACGTGTACGAAGAT
GAATAGATAAATTGGCAATGTCTTGTTCGAAGACGTGCTTCAACCAATCTTTCCATTCATCGATTTCATAGTAGCCAAGGCGTGTTTT
AACACTTACCGGAAGCCCACCTGCTTTAGTCGCTTGAATAATTTCGGCAGCAACGTCAGGTCTTAAGATTAAGCCGGAACCCTTACC
CTTTTTAGCAACATTTGCTACAGGACATCCCATATTTAAGTCTATGCCTTTAAAGCCCATTTTAGCTAGTTGAATACTCGTTTCACGG
AACTGTTCTGGCTTATCTCCCCATATATGAGCGACCATCGGCTGTTCATCTTCGCTAAAAGTTAAGCGTCCACGCACACTATGTATGC
CTTCAGGGTGGCAAAAGCTTTCAGTATTTGTAAATTCAGTGAAAAACACATCCGGTCTAGCTGCTTCACTTACAACGTGTCGAAAGA
CGATATCTGTAACGTCTTCCATTGGCGCCAAAATAAAAAATGGACGTGGTAATTCACTCCAAAAATTTTCTTTCATAATATATTTATA
CCCTCTTTATAATTAGTATCTCGATTTTTTATGCATGATGATATTACCACAAAAACCTAACTTATACAAAAGTAATTTTAGTTGGTAT
AACCATCCGAAAGGGAAGTCTACGAGTAGTCTAAAATGAATGTTGTGGTAAGTTGATCAGTATATAAATCAAGGATTATCGTATTA
GATAGTTCATTATTAATGATACACTACTTATGAATATGATTCAGAATTTTCTTTGGCTACTTTTACAGTAAAGCGATTTTTTAGTTATC
TTATAACAAAGACAAATTTATAAAGGTGATATTATGGAAGATTTAAAGCATTCTTTAAAAAGTTTAGGTTGGTGGGATTTATTTTTT
GCGATACCTATGTTTCTACTATTCGCATACCTTCCAAACTATAATTTTATAACGATATTTCTTAACATTGTTATCATTATTTTCTTTTC
CATAGGTTTGATTTTAACTACGCATATAATTATAGATAAAATTAAGAGCAACACGAAATGAATCATTAATACGAATGTGATTAAAC
ATAAAACTGAAGGAGCGATTACAATGGCGACTAAGAAAGATGTACATGATTTATTTTTAAATCATGTGAATTCAAATGCCGTTAAA
ACTAGAAAGATGATGGGAGAATATATTATTTATTATGATGGTGTTGTTATAGGTGGTTTGTATGATAATAGATTATTGGTCAAGGCG
ACTAAAAGTGCACGTCATCAATTTCAAGATAATACATTAGTATCGCCGTATCCTGGTGCTAAAGAGATGATATTAATTCCAGATTTT
GACGAAGCAACAAATCTCACTGATTTATTTAAGACCATAAAAAATGATTTGAAAAACTAATCATTAATTGCCATACTCAACTATTTT
GAAAATCATCAATTTCATATTGTCAATTTATTGAGTATCTCGATTTATTGTTATACACAAACAGTATTTTTGTTATCATAGAGTTAAT
AAATATAATAAATTGAATAAGTAACTTTACAAACCTAGAATGATTTAATTCAATCACAATTATTTGAAAATAGAGGTGAGCAGGTG
AACGATATGTTAATTAGTCTTGTAATCCCAGTTTTTGCTTTGTTAGTTATTGGTGGTATTATTTGGATGATTATAGAAGGTATAGTAC
ATATTTCAAAAAAGAATAAAGCAATTGATAACTTTTTTAACCAAGTTAATAAAGTAAGTGAGACATATAAATTCGCTACTACTTTTT
TATTTCTAATCTTGGCTACGGCTGGTATTTCTCAATTTTATCTATATTATATAGTATCAGCGTTTCTTTTTTGGCTTGTCATACTTACCT
TTGGCATTGCAGGTATCATTTTTTTAATGCCATATGGATTATGTTTTCTACCGTTTTATAAGCAAAAAAAGAAAAAACAGACATTTA
AAAAATACATGGTTTACACTACGATTGGTTTGTCAATTTGTCTAGGCTTATCTCTAGTTTTGGTTCACACTACGAAAATTTATATGGA
CGAAGGTGGCGTAAGATACTATTACGGTAGTTTTGTAATGAAACAAGCGGGCGGTTATGCTTATTTAGCTTTAGCGGTACTTTCAAC
GTTGTTAATTGTTGCGAAAAAAGCTACAAATAAAAATAAAGAAATCGAAACCGTCGACAATACAAATATAACGGAAAGATAATTA
AGGGAGTGCTCATTCAGGAGTGCTCTTTTTTGATGTCCAAATTTAGTTGCAAATGAAGGCATAGAAGAAATTTTTATAGTCATTTATT
GGATAATGAATTATGGTCATATCGATTAATTTTATTAGTGAGGATTTTACAAACATTTGTTGAATAAATATGGTAATGATAATATAT
GAATATTTTTGAAAAAACACTAATATGATTAAATTATTTGTTAAAATAGCATTAAGTAAAAAACAAACAGGGAACAAGGTGGGATT
TCATGAGTCAATTGCTAAATGATACGTTATCGGCTTGGTTGTTAATTGAATCTTTAAGTGCAGGAGAAGTAAATTTTACAGCGGAAG
ATATACTCTCAGCTGAACATTTTAAAAATGGTACAAAGCAAGCACAGCTTCAAAGTTTTGATGAATATTTTGAAATATGGAACGATG
AACGTTTTATTGTGTCAGAAGAAAAATCAGAGACTGGGGAACTTATATTTAAATTTTATAGACATTGCTTCCGCTATAATGAAATTA
ATTTGAAAATTCAAGATATTTTTGAAAATCACTCTGATATTCATAATCCAAATGGGACACACTGTTATGGTTACACATTTAACACAG
ATAAACACGGCAAAGTGATAGTTGATTCTATACATATTCCGATGATTATGAGTGCATTAAAAGAAATTGAAAAGAACAAAAATGCC
AATATAGAAGAAAAATTTAATGATTCTGTTGAAAAATTTGTTCAAAAAGTAAAAGAAATTTTAGCAGATGAACCAATTAATGAATT
TAAATTGAAGAAGATGGACAAAGCTTATGATGAGTACTTTTCTGTATTAAATTCAAAGAAAGATGGATTATTTGGACATTATGTAGC
AATAGAATATGTGAAAGATAGTGATTTACCACAGCCGGAATTTAACAGTTTCTTCATAAGTGATATTGAGAAAGCAAGAAAATCTC
CCAACCAAACTTTAATTGATTACATTGAAGGTGTAGAAGAAAGTCAGCGCATAGAAGTAGATGAAAATAAAGAAATGTTTGACAAA
TTTTTACATCCTTCACGTTTGCCTGATGGACGATGGCCATCACAGACTGAGTTTAGATTGTCTTTAATGCAACAACTTGCTGTAAACC
AAATTACGAGTGGTAATGAAAGAATAAGTTCAGTTAATGGGCCACCAGGGACAGGTAAGACTACTTTATTAAAAGATATATTTGCT
CATCTAGTAGTTGAAAGAGGTAAAGAGTTAGCTAAACTAAATAATCCTAAAGATGCATTTGTCAAAACAAAAATTCATGAAACGGA
TGATAAATACGTATACTTACTAAAGGAATCTATTGCCAAATATAAGATGGTAGTCGCATCTAGTAATAATGGAGCTGTTGAAAATAT
ATCTAAAGATTTACCGAAAATTGAAGAAATTATAAGAAATCCCGAAAAATGTAAATTCCCTAAATATGAACAGAATTATGCAAATT
TAGCACATGAATTAAAAGATTTTGCTGAAATAGCTGAAGGTTTGATTGGTGAAAGTGCCTGGGGCTTATTTTCTGGAGTATTTGGAA
AAAGCAGTAACATTAATAAAGTATTGGAGCACATGCTAAAAAAAGATGGGAATGATATTGGCTTTGCTAAATTACTACAAAATGAG
AATAATCGTATGAGTTATAACGAGTTAATGAGTGAATGGCAATCACATCAACGTGCATTTTTAGAAGAGTTGAGGCATGTTGAAAT
GTTAAAAGAAGAATCTATTAGAGCATATGATGTTTATAAAAATTGTGAGTCTTTCTCTAAGATTGAACAGGTTATTAATAGTGAAAA
AACAAGTATTGAAGAACAGGTATATCATTTAGATAATGAAACGTTACGAGACAATAAAGAAATAGAAGATTTGGATAATCGAATTA
ATTATATTGTTAAGCAAATAGAAACTTTAAATGAGTTAATTAAATCCATCAAAGAAAGCAACAAAGGTTTTATTAACAAACTGAAA
GCGATGTTTAATTCAGAAGAAGATGAAAGCTATAAAGATCATAATAAAGAGAAGCAACAATTATTAACACAACAGTTAGAGTTAG
AGAAATGTAAAAAAAAACAAACATGAAGACCTTGTTAGCAAACTAAAAGAAAAAGAGAAATTAATTAAACAATTAACTAAAGTACA
GTTGCAATTAGACGAGTTAAATTCACAGTTACAAGAGTTAGAAGCATATCGTATTGAGTCAAAAATTACAATTCCAGAAAAAGATT
TTTGGAGTGACAACAATTATGATGAGCGCCAAGTTACTAATCTGTGGACGAGTGACGAACTTCAATACAGACGTGCCATGCTCTTTT
TAAGAGCAATGATATTGCATAAATTATTATTGATTGCTAATAATACAACTATTTATTATGCGATTAATGATTTTAAAGATAGAAGGA
AATTAATTGATGCAAATCCAGATAAAGTACACAACGCATGGAATGTGATGCATTTAATATTTCCAGTAGTTAGTACGACGTTTGCAA
GTTTTAAATCTATGTATGGGGGCATACCAAAAGATTTCATAGACTACTTATTTATTGATGAAGCAGGACAAGCAATACCTCAAGCAG
CTGTGGGAGCATTATATCGTTCAAAAAAAGTTGTAGCTGTAGGTGATCCGATTCAAATAGAACCGGTTGTGACTTTAGAAAGTCATT
TAATTGATAACATTCGTAAAAATTATCATGTTCCGGAATATCTAGTTTCTAAAGAAGCTTCTGTGCAGTCTGTTGCAGACAACGCCA
ATCAATATGGTTTTTGGAAATCTGATGCTACTGATAGTAATCAAAAAACCTGGATAGGCATACCTTTATGGGTGCACAGACGATGTT
TAAAACCTATGTTCACGATAGCTAACCAAATCGCTTATAATAATAAAATGGTGTTGCCAAGTAATATTACAAAAGTAGGTAAAACA

GGTTGGTATGACGTTAAAGGAAACGCAGTTCAAAAACAATTTGTGAAAGAGCATGGTGAAAAAGTAGTGGGATTATTAGCTGATGA
TTGGATTGAAGCAATTAAGGAAGGTAAAAATGAACCGAGCTCATTTGTAATATCGCCTTTTTCAGCAGTACAGCAACAGATTAAAC
GTATGTTAAAGCAACAACTACCGACTAGAATTGATATTGAACGTACAAAAATTAATCAATGGGTCGATAAATCCATTGGTACTGTTC
ATACTTTTCAAGGTAAAGAGGCTCAGAAGGTGTATTTTGTAATAGGTACTGATAATACCCAAGATGGTGCTGTGAACTGGTCATGCG
AAAAACCAAACTTGTTAAACGTTGCAGTGACAAGAGCTAAGAAAGAGTTTTATGTAATTGGCGACATGCAAAGAATACAGATGAA
ACCATTTTATGAGACGATTTTTAAAGAAAGAAATGTAAAATAACATACAAAAAAGTATATAGAGGAAGTTATACATTTTAAAAGGA
GCAAAATTGAATAATGGAGAAATTTAACAACTGGATATTAAATGCAATAAGTGGATCTCAAACAGACAAGAATGGAACAACTAAA
GAATTAAAAGGGGCAAAATTTATCATTTTATATGCATATTCAATGCTCGTTTTGCTTGCGTTAGTAATTTCTAACATATTCATTCACA
TTTTGGAGCCTAAACTATCAATCACCACTCAAATCATCATCGTTTTGATTTTAATTGAAGCACTAATTGGACTGCGTTTCTTGAAAGC
GTACGATGTTAAGCGTGGCAAAGATAAAGAAAATAAGAAAAATAGTAAGGATTTCGTTAAACTAAAATCAATTTTAGTAGCAATTT
TATTTACATCATTGGCGCTGACAGCAGGTACTGTAGCTGATATATACGGTTTCACTGACTTAGGAAATACTAGAAGTGATTTAATCG
TTTGGAGCATAGGTGGTATTATATTTGGCCTCGTATGTTACACAATGGAAGATAAAAGATAACGATAAGGAGCTGGCGATTATAAA
GCTAGCTCCTTTTTTAACTTATATATGTAAAGAACTATCCTAAGGGTTTTTAATCATATGTCAATAATTTCTATAATACATTATTAAA
ACATCAATTAAATAAGTTTTAAAATTTTACACATATTTTTATTTAAAAAAGATGTATAATTAATGTATTAAATATAGAAAGAAGTTG
ATATTATGAAAAAGTGTATTAAGACTTTGTTTTTAAGTATCATTTTAGTAGTGATGAGTGGTTGGTATCATTCAGCACATGCGTCAGA
TTCGTTGAGTAAAAGTCCAGAAAATTGGATGAGTAAACTTGATGATGGAAAACATTTAACTGAGATTAATATACCGGGTTCACATG
ATAGTGGCTCATTCACTTTAAAGGATCCATTAAAATCAGTTTGGGCAAAGACTCAAGATAAAGATTACCTTACCCAAATGAAGTCG
GGAGTCAGGTTTTTTGATATTAGAGGTAGAGCAAGTGCTGATAATATGATTTCAGTTCATCACGGCATGGTTTATTTGCATCATGAA
TTAGGAAAATTTCTCGATGATGCTAAATATTACTTGAGTGCTTATCCAAACGAAACAATTGTGATGTCTATGAAAAAGGACTACGAT
AGCGATTCTAAAGTTACGAAGACATTTGAAGAAATTTTTAGAGAATATTATTATAATAACCCGCAATATCAGAATCTTTTTTACACA
GGAAGTAATGCGAATCCTACTTTAAAAGAAACGAAAGGTAAAATTGTCCTATTCAATAGAATGGGGGGTACGTACATAAAAAGTGG
TTATGGTGCTGACACGTCAGGTATTCAATGGGCAGACAATGCGACATTTGAAACGAAAATTAATAATGGTAGCTTAAATTTAAAAG
TACAAGATGAGTATAAAGATTACTATGATAAAAAAGTTGAAGCTGTTAAAAATTTATTGGCTAAAGCTAAAACGGATAGTAACAAA
GACAATGTATATGTGAATTTCTTGAGTGTAGCGTCTGGAGGCAGCGCATTTAATAGTACTTATAACTATGCATCACATATAAATCCT
GAAATTGCAAAAACGATTAAAGCAAATGGGAAAGCTAGAACGGGTTGGCTGATTGTTGACTATGCAGGATATACGTGGCCTGGATA
TGATGATATCGTAAGTGAAATTATAGATAGTAATAAATAAGGATTCAATAATGATATTAAGACGAGTATGAAAATAGTTAGATTCT
AATTATTTTCACTACTCGTTTTTATTTTGAAAATAAGTAATAATTCAACAATATTATAAATTGAACAGATTGTTTATGGAATTTTTGA
TAATATTAAAGTGAAAAAGTGTTATAAATTGATAAATATATGTAATTAACAAAAACAAATCATTTTAAAAAGAAGAGAGTTGTAAG
ATGATGAAACGATTAAACAAATTAGTGTTAGGCATTATTTTTCTGTTTTTAGTCATTAGTATCACTGCTGGTTGTGGCATAGGTAAAG
AAGCGGAAATAAAGAAAAGCTTTGAAAAAACATTGAGTATGTACCCTATTAAAAATCTAGAGGATTTATACGATAAGGAAGGCTAT
CGTGATGATCAGTTTGATAAAAATGATAAAGGTACATGGATTATAAATTCTGAAATGGTTATTCAACCTAATAATGAAGATATGGTA
GCTAAAGGCATGGTTCTATATATGAATAGAAATACCAAAACAACAAATGGTTACTACTATGTCGATGTGACTAAGGACGAGGATGA
AGGAAAACCGCACGACAATGAAAAAAGATATCCGGTTAAAATGGTCGATAATAAAATCATTCCAACAAAAGAAATTAAAGATGAA
AAAATAAAAAAAGAAATCGAAAACTTTAAGTTCTTTGTTCAATATGGCGACTTTAAAGATTTGTCGAAGTATAAAGATGGAGACAT
TTCATATAATCCAGAGGTGCCGAGTTATTCAGCCAAATATCAATTAACTAATGATGATTATAATGTAAAACAATTACGTAAAAGATA
TGATATACCAACAAATAACGCGCCGAAGTTGTTGTTGAAAGGTACAGGGAATTTAAAAGGTTCATCAGTTGGATATAAAGATATTG
AATTTACGTTTGTAGAGAAAAAAGAAGAAAACATTTACTTTAGTGATGGGTTAATTTTTAAACCAAGTGAGGATAAATAATCATGA
CTCGATTAAAATATGAAATCAATCCAGGAAAGATAATAATAAGAATAGGATTGTGAAATTATGAAACGATTAAACAAATTGGTATT
GTACATTAGTTTTTTGATATTAATCATTAGTATCACTGCTGGTTGTGGCATAGGTAAAGAAGCGGAAGTTAAGAAAAGCTTTGAAAA
AACATTGAGTATGTACCCTATTAAAAATCTAGAGGATTTATACGATAAAGAAGGTTATCGTGATGACGAATTTGATAAAAATGATA
AAGGTACATGGATTATTGGTTCTGAAATGGCAACTCAAAATAAGGGGGAAGCTCTGAAAGTTAAAGGTATGGTCTTATATATGAAT
AGAAATATCAAAACAACAAAAGGATTTTACTATGTTAATGCAGTAAAGAAAGATGAAGATGGTAGACCTCAGGATAATAAAATAG
AATATCCGGTGAAAATGGTAGACAATAAAATCATTCCAACTAAAGGTATCAAAGACGAAAACATAAAAAAAGAAATCGAAAATTT
TAAGTTCTTCGCGCAATATGGCAGCTTTAAAGATTTGTCGAAGTACAAAGATGGAGATATTTCGTACAATCCAGAGGTACCGAGTTA
TTCAGCTAAATATCAATTAACTAATGATGATTATAATGTAAAACAATTACGTAAAAGATATAATATACCAACGAATAAAGCGCCAA
AGTTATTGTTGAAAGGTTCAGGTAACTTAAAAGGTTCATCAGTTGGATACAAAAAAATTGAATTTACTTTTGTAGAGAAAAAAGGG
GAAAACACATACTTTACTACTAACCTACATTTTAAACCGAGTAAGGATGAATAATCGTGACTAAAAATGAGTATAAAATCGATCCC
GGAAAAATAACTAGTAATACAGAAGCAACTAGCGCAATAGCTAATATTAGTTATGAAATTGAAAATGCAAATGATAATGGTTTAGA
AAAAGAAAAAAATTAATGGGCAGATTAATAGTTTGAAAAATGATGGGGATTTTCCTAAAAACTTAGACTACATAGATAGTTACACAG
ATCCCAGTACGGGGACCACGGCAACGGCATTTTTAAATAAAGATACAGGTAAAGTTACTGTTGGAATGGCTGGTACAAACTTTCAC
GGCGATCAACTTAAAAGAGTAGCACTTAGTTCGATGTCTCCGTTATTATTCCCACCCTCTAAGCAAGATATGAGAGATGTACGAGGC
ACTATGAAGGATGGTGTAGCGGATTTAGCCATTGGAGTAGGAATGGTCAATTATAAAGGAAAACATTTTGCAAATACACAGCAGTT
TATTGAGAATTTACAAAAAAAGTACGAAATTGATACAGTTACTGGTCATTCTTTAGGAGGGAGAGATGCTATCTTTCTTGGGCTTCG
TTATAACATCAAAAATGTTGTGGCATACAATCCTGCTCCGTTAGAAGTAAAAAGCATTCGTGATAAATTTGGTGGTCAATTGTTTAG
AAATACGACATTTCCAGATGAAAAATATTTAAAAGAGTTAATGGATAATTATGATGGAGATATTACTAAGGTTATAACTCAAAAGG
ATGGATTAGACTATTTAGTGAAGCGTACAGATCATTTAACTTGTGGTGATGTGTTACGTATAAACAATGGTCAGGGCCATGCGATGG
AGAATTTTTTAGGAGAAAAAGAACAACGTGAAATAATCGAAGAACTAATGTTAGTAAAAGGATATCGCGATGCAAATGACAAAGC
ATTTAAGGCTTTGAAAAAAAATACTGAGAAAAAACTCGGTAAAATAGATGAAATCAAATCGAAGTTACTGCAAACGAACGGTGGC
GCCCTCTCGTCTTCTCAACAAAAATTGCTTGAAACTTTAGTGGCATTTTCGGTAGCTGAAGGACTAAGTAAAATGGTTGACCAAGAA
CTTCAGCAGTTGAAAAATATGTTCAATCTTATGGATGAGAAATTTGAAGCAAATTGGAAAGACGCACAAGAAGCTAGTGATATTGT
AGGTAAACATCTTTCATACCCAGAAAAGGTAAGTGCATTAGATAACGGTGGTGTCAATGAAAGTAAACTTGCTACCGAACCACATA
ATGAAATAAAAGACAAACTAAATAAAATTACGGACTTATCTAAGAAATATAATTCGTATTTACAGCAAATCGAAAAAAGTATTAAT
GAAATAGTTGCTAAGGATCAACAATTGGCAGGGCAAATAGGTGATTTAATATGATGTTTGAAGAATACAAGAAAATAGATGATCTT
GAAAATGCATATGAAATAGAGTTGAAACGTATTGAAAGGGATATACAAAATCTTAGTGATTTAAAATATCACCTTAGAAGAGAAAA
TGAACAAAGTTATGATGCATTTTTGTATTTAAAGAATAAAATGAACTATAGTGAAGAGTCAAATGCTAAGGTGAGACGACTAGTTG
AAGAATTTGATTATGAAGCAGATACTTATATTAGACAAAAAGAGTTAAAGTTAGAAGATTATAAAGAAGAAATAAGAAGAGAATA
TATACAGCAATCTGAAAAAATAATGGAGGCGAAGTAGCTTATGGGAATCCCTGATCCGTTATCTAATCCTATGTCTGATCCTTTACT
AGGAGATAGTTGGATGGTAGGTGATTTGAAAAAAAGACAGGAAAAAAAACTAATCACAACAGTCATCGCACCTGAACATGATGAA
AAGCATCGTGAAAATAAAAGTAAAATGAAAAAATCTGAAAAGCAATTGATCAATCAGTTAATTAATCATGGTCATACATTTAAGAG
TGATTTTAAAAATGTAGCTAAAGGTGATTGGGTTAATAAAGCCATGCAAGACTTAGATAAATTAGGCAATGAGCTTAAAAAAATAA
TGAATTAGTTGAATTGTCACATACTTTTTCAAACCAACAACGAACTAAAAAATCTATATAAATATGATTAAAAGAAGTCATAACCAT

TCGTAAAGGATGGTTATTTTTAATAGTAAACAAAATTGCTGCTAACTTACATCTTGAACCAAGTGAGGATAAATAACCTCCTGCCTC
CTACACGATTCAACACGAATTTCAAATATATTGCTGTAAAATAATGTAAAATAAACGTATTCATATTACTGAATTGAGGGATAGTAT
GCAACGTGATTATTTAATTCGAGTAGAAACTGAGAGTATGTCAGATTTCAAAAGGCTCAATGGTTTAATGATTGGTTTTGTTATTAA
AGGTGAGGCGCATATTTATGATGAAAATAATATGACGCAATACAACAGTGGCGACATTTTCATCATTAACCACCGCGACTTGTATCG
ATTTCAACTTCAACAAGAGGGCATCATATGTTATATCCAATTCCAAATGAAATATTTAGCAGACAAGTTTGATGATGCGCATTGTCT
ATATTTTCACTTAACAGATGCGACCACAACCAAGAATATACATCAACTGAGAAATATAATGGCAAGACTGGTTTCAACACACATTC
GACATAATGAGTTGTCTAAATTGACTGAGCAACAACTTGTGATTCAGTTGCTTATGCATATGATTCATTATGTCCCGCGTACATATCA
TTCGAACCAAAGTATCTTAAATGATGATAAAGTGAATCAAGTATGCGACTATATCGAGTTACATTTTCATGAAGATTTAAGCCTTTC
AGAATTAAGCGAATACGTTGGGTGGTCAGAGAGCCATCTGTCTAAAAAGTTTACAGAATCGCTAGGTGTAGGATTCCAACATTTCTT
AAATACGACGCGAATTGAGCATGCGAAACTCGATTTAACATACACAGATGAAACGATTACTGATATTGCATTGCAAAATGGCTTTT
CAAGTGCAGCGAGCTTTGCGAGAACATTTAAACACTTTACGCATCAAACGCCTAAACAATATCGAGGTGATCGTCCAGCAATCACT
GAAAACCAGCAATCGTTACAACATAATTATCACGACCGTGAATTGATATTACTTTTAAATGACTACATTGAAGAAATGAATCATTTC
ATTGAAGATATTGAAAAGGTGAACTATAAAGAGATTGCCTTTAAACCAACTAATCAACAACTAAATCAATTTAATCATATTATTCAA
GTGGGCTATTTGAGGAATTTGCTCAATACACAGTATCAATCACAGTTACTTACATGCCATCATGATTTTCATGTCAATGAAGTATTA
GCATATGATGTGATGCCATATATTATGAAAAAGCTCAATGCGCCATTCACGTATGATGCAGAGATTTCGAATATATTTTATGATATC
GATTTGTGTTTAGACTTTTTATTAGATCATAACTTTAGTTTGACAATGCATTTGGATCAGTATGACTCACGTGATTATATCGATGCAT
TCAAAGTATTTATCCATCACGTTGCCCTGCATGTCAGTCATAGAAAAGATTTGAAGTTCAACTTGTATGTGACGACATTGCACAATT
CTTTGATTGAAATGATTGATTATTTTAAAGCATTATTCCCCAATGGTGGCTTGTACGTTCACTTAGATCAAGCTACGGAAAGACATCT
ACCATTGTTGAAACGACTTGAGCCACACATCGACCATTTTGTATTTGATGCCAATTCAAATGACGCTGTTGATTTTAATAAAATGAA
TGATGATGAATTTAAAACCGCAAGTCAAATGATTATTAATAAAACGAATTACCTTATCGACTTAATGCATCGCCATAACCTAAAGCG
TCCACTCATTTTACTCAATTGGAATACATTGACGGGTGATACATTTATTACAAACGGCGAATATTTTAGAGGTGGTATCATCATTGA
GCAATTATTAAAATTAAGTTCTAAAGTAGAGGGTATCGGGTATTGGTTGAATTATGATTTGCACGTCAGTCATTGTAGAAATGAACG
GGATTATATGAATTCTATTGAACTGTTTCATCAATATAATGGAAAACGTCCGGTCTATTTCACGGCATTGCTATTTAATAAATTAACA
AGTAATATTTTATATTCTGATGATACATGTATTGTCACGGGAACTGATTCAAATTTTCAAATATTGTTATATGATGCAAAGCATTTTA
ATCCGTACTTAGCGTTGGACAATCAAATGAATATGCGTGCAACGGAAATGATCCATTTGAACATTAATGCCTTGGAAGAAGGTATG
TATAAGATTAAACATTTTACCTTAGATAAAGAAAATGGTGCGTTATTTAATCTTTGGCGCAAACATCATACGATACATGGCATGGAC
AAGGACTCTATAGATTACGTTAATCGAATGAGTTTTCCGAAATTAGAAGTATATGATATAGATATCACGGACACACTGGCATTAAAC
ATTAAAATGATTACGAATGGGATTCACTTAATTGAAGTAAAACGTTACCCAAGTTCATAAAATGATCACAAATCACAAATTTTGATA
TACATAATTTGTGATTTTTTATATTCAAAGCTAAAATTGCAAAAAATTAATGGTTAACATCTCTGTTGTTGGCAATATAAATAATGAT
TAATCATTTATGATGTAACTAAGGAGATGAAGGATATGAATCAACAATTAATTGAAACTTTAAAATCTAAAGAAGGCAAAATGATT
GAGATCAGACGTTATTTACATCAGCATCCAGAATTATCTTTTCATGAAGATGAAACGGCGAAATACATCGCTGAATTTTACAAAGGT
AAAGATGTGGAAGTAGAAACGAATATCGGACCACGTGGAATTAAAGTAACGATTGATTCAGGGAAACCTGGTAAAACATTAGCAA
TCCGTGCAGACTTTGATGCATTACCTATTACTGAAGATACAGGATTATCTTTTGCATCACAAAATAAAGGTGTTATGCACGCATGTG
GTCACGATGCACATACAGCATACATGCTTGTATTAGCAGAGACGCTTGCTGAAATGAAAGATAGTTTTACAGGAAAAGTCGTTGTG
ATACATCAACCAGCTGAAGAAGTACCACCAGGTGGTGCTAAAGCAATGATTGAAAATGGTGTATTAGACGGTGTTGATCATGTATT
AGGTGTACACGTCATGAGCACAATGAAAACAGGTAATGTGTATTACAGACCTGGTTATGTTCAAACAGGACGCGCATTTTTCAAATT
GAAAGTTCAAGGTAAAGGTGGTCATGGTTCATCACCCCATATGGCCAATGATGCCATTGTTGCAGGTAGCTACTTCGTCACAGCGTT
ACAAACAGTTGTATCTAGACGACTAAGTCCATTTGAAACCGGTGTTGTCACAATCGGTTCATTTGACGGTAAAGGTCAATTCAATGT
CATTAAAGATGTTGTTGAAATTGAAGGTGATGTACGTGGATTAACAGATGCTACAAAAGCAACAATTGAAACAGAAATTAAACGTT
TATCAAAAGGATTAGAAGCATTGTATGGTGTAACTTGCACATTAGAATATAACGATGATTATCCTGCATTATATAATGATCCAGAGT
TTACTGAGTACGTGGCTAAGACGTTAAAAGAAGCAAACCTTGATTTTGGTGTCGAAATATGTGAACCACAACCACCTTCAGAAGAC
TTTGCATACTATGCTAAAGAACGTCCAAGTGCCTTTATTTATACAGGTGCAGCTGTGGAAGATGGTGAAATTTACCCACATCATCAT
CCTAAATTTAACATTTCAGAAAAATCATTACTTATTTCGGCAGAAGCTGTAGGGACAGTTGTTTTAGATTACCTTAAAGGAGATAAC
TAACATGAATGAAACGTATCGCGGGGGCAACAAGTTAATCTTAGGTATTGTATTAGGTGTTATTACATTTTGGTTGTTTGCACAATC
ACTTGTAAATGTTGTACCAAATTTACAACAAAGTTTTGGTGCAGACATGGGAACAATTAGTATTGCGGTAAGTCTAACCGCACTATT
TTCAGGCATGTTTGTTGTTGGAGCAGGCGGCCTAGCAGATAAAATTGGGCGCGTGAAAATGACGAATATCGGTTTATTGTTAAGTAT
TATTGGTTCAGCATTAATTATTATTACGAATTTACCGGCATTATTAATTTTAGGTCGTATTATACAAGGCGTATCAGCAGCGTGTATT
ATGCCTTCCACATTGGCCATTATGAAAACTTATTATGAGGGTGCTGAACGTCAGCGCGCCTTAAGCTACTGGTCTATCGGTTCTTGG
GGTGGAAGTGGTATCTGTTCACTCTTCGGTGGGGCAGTTGCGACAACTATGGGTTGGAGATGGATTTTCATCTTCTCAATTATCGTTG
CCGTACTTTCAATGTTACTCATCAAAGGGACGCCTGAAACGAAATCAGAAGTTACCAATACACATAAATTTGACGTTGCAGGGCTA
ATTGTTCTAGTAGTTATGTTGCTAAGTTTAAACGTTGTCATTACTAAAGGTGCAGCACTTGGTTACACATCATTATGGTTCTTTGGTT
TAATTGCAATCGTAATTGTAGCATTCTTTATTTTCTTAAAAGTTGAGAAAAAAGTAGATAATCCGCTTATTGATTTTAAATTATTTGA
AAATAAACCATATACAGGTGCAACGATTTCGAACTTCTTATTAAACGGTGTTGCAGGTACATTAATTGTAGCGAATACATTCGTGCA
ACAAGGTTTAGGTTATACAGCATTGCAGGCAGGATACTTATCAATTACTTATTTAATCATGGTGTTATTGATGATTCGAGTTGGTGA
AAAATTATTACAAAAAATGGGTTCTAAGCGACCAATGTTATTAGGTACATTCATTGTGGTCATTGGTATTGCACTTATTTCATTAGTA
TTCTTACCAGGCATATTTTATGTTATCAGTTGTGTCGTAGGATATTTATGTTTCGGACTAGGCTTAGGTATTTATGCAACACCTTCTAC
AGATACAGCTATTTCAAATGCACCGTTAGATAAAGTTGGCGTTGCTTCAGGTATTTATAAAATGGCTTCATCACTTGGTGGTGCATT
CGGTGTCGCAATTAGTGGTGCAGTATATGCCGGTGCAGTTGCTGCAACGAGCATTCATACAGGTGCGATGATTGCACTTTGGGTTAA
CGTATTAATGGGAATCATGGCATTTATCGCAATTTTATTTGCGATTCCTAATGATGATAAACGTGTCAAAGATGCGAAATAATAACG
ATGACACGCCCAGTGTTTTGATAATGATTTAAATGAAAGCAAAGCAAATTAAATGAGGAAATGTATAGAGCAGTGCATATAGATAA
ATCTATGATTGGATTAACACTAATATAAATATAAATGAAAAACCATCCACTTAAACTTTTGATAAGTTAGGTGGATGGTTATTTATT
GCGTTGTTCTAAAAAATTTGGACATTTATATGGAAAGAACTATATATAATGTGAAGGTTATGGCAATTCGCTTCAATGAAGGCGACGT
GTAAAAGTGTATATTAAATGATTCGAGGTCAGACTCCGAGAACAGTGTCCGTCATGCTTACCGCATACTAATGAAGCATCATTATAT
CAGTGTTTATAGCCGATTTAAGATAATAAAGTTTGCTAAATATAAAAGAGCTTGGGACATTCAATAAAAATCGAATTATCATCAATA
AGATTTTGCTTTAAACATTGACTATGAAACTGGATAAATAAAGATTCAATTAATGCATCAGTATTAGGATT

>007-contig_191

TCAATACGAAGTATTGTATAAATATAGAACAGCAGTAAGATATCTTCTAATTGAAAATTATCTTACTGCTGTTTTTTAGGGATTTATG
TCCCAGCCTGTTTTACATGCATCTGAATCTCTAATTTTAAAAAAATATGAATATAAATAAGACAGTAAAAATTAAATTTCAGTTGTT
GCAATTTCTTCATCTGTAGGTACATCATCGTTAAGGCCAACAAGTGCTTCAGAAACATTTCGTGAATGATAACCGATACGTTCAAGA

ACACCAATCATATCGATATATAGTAATCCGCCTTTTGTTGTACATTCACCACGATTAAGGCGTTTAATATGACCTTTGCGTAGTTTAT
GTTCAATATTAAATGATTCTCTACTACGTTCTACAATTTCATCTTTTTTCGTTTTGTCATAAACATCTAACATGTCGATGGCTTTATCA
AATGACTCAGCAACATGGTTGAATAATTTATCCATACCGCGTTGTGCATCTTCTGTAATGCGAATATCTTCATCATGTTGGCGTTTTA
ATTGAGCGACATACTCTTCTGTTAGCTCTGCTACTTTTAAAATAGAGCGATTGACATCAAACATAACTGCTAAACGCTCAACGTCAG
CCTTCGTTATGGCTTTTGTAGAAATTCTAACTAAATAATTTCGAATGCTATCATTGATTGTTTCAACAGCTTGGTGCTTTTGTTCAAGC
TTTTTGATCAATTTTTTATCGTCTTTTGTAATTTCGCGAATGTCTTCAAACATTGATAAGACAATCTGACCAACATTTTGTAATTCTTT
TTGAGTTTCTTGTAATGCAACACCAGGTGCGTGATAAACAAGATCTTTGTTTAAGTGCTGAGGTTTATAGTCATCAGCAATATCTTTA
CCTGGGACAAGCTTTGTAACAATCCATGCTAAACCTGCTACAAATGGTAATTGAATCAAAGTATTTGTTATGTTGAAGATACCATGT
GATACTGCAATCGTCATCGCTGGTTTTAAGTGCCATACATCTTGTAACAAACTAATCAAATGAATCACAACTGGCAAGAAAATTGTG
AAGATAATTACCCCGATTAAGTTAAAGATGACGTGTACAAGCGCCGCACGTTTTGCAGCGATTGAGCCGGCTAAACTAGCTAAGAT
AGCTGTAATCGTTGTACCAATATTATCGCCTAGTAACACAGGGATTGCTGCGTTTAAGCTAATTAAATCTTGTTGATAAAATTCTTGT
AAAATACCAATCGTCGCACTTGAACTTTGAACTAGTGCTGTTAACCCTGCGCCGACAATGACAGCAAGTATTGGATTTGTAGACATA
TCAAGCATTAATTGCTTAAATCCATCTAATGATGCTAAAGGTTTAACGGCATCACCCATAAATTCTAGACCGAAGAATAGAGAACC
GAAACCGAATAGTATGCGGCCAATGTTATTGATTTTAGAGCGTTTAAAGAAAAAGATTAAAAATGCACCTAATGCTAAAATTGGCA
TTGCATATTCGCCTAAATCTATACCGATAATAAATGCGGTTACCGTTGTTCCGATATTGGCACCCATTATCACTCCAATAGCTTGTTT
TAACGTCATAAATCCAGCTGTTACCAGTCCGATTGTGATAACGGTCGTACCTGAACTACTTTGTATTAAAATAGTTACAACGATACC
TGCAATAACACCTAATACTGGATTTGATGTAAATTTGTTTAAAATATCTCGTAGCCTGTCTCCTGCTGATGCTTGAAGCCCGTCTCCC
ATGATTTTTAAGCCGTAAAGGAAAATACCTAAACCACCTAAAAAGGAGAAAATGACTTCTGTAACCGACATTTCCATTATTTCACCT
CAAATAAGCTTTATATTTAGATTATCGCTTATAATTGTAAATTTAATGTTAAGATTAGGTAAAATTATTTAACAATATATGTTATTTG
TATATGACTTGTAAAATATCGTCACTTATTATGTAAATTTTCAGTGTGAAATGGCGGGTTTGCAATCACTTGTTTAACAAAATGATGC
AATCAATCATGTAATTATGTTTCATCAAAAAAATCATGTGAGTGGGATAACGAAATAAAGTTTGTGAACATATCACTTCTATCCCAC
TCCATGATTTGAAATCACCATATAGACAGATTTAATGTGCCTCGTTATAACAATTTGTGTTCTTTTAATAATGTCTCAATGTACGTAC
CTTTTACCTTTTTAAGGAATCCAGCTAATGCGAGTTTTTGTATTTTCGAATCTTTAGTAATCTCGCGCAAATCTTGATGGTCATTCAGT
TCGTATACGGCATCCATTAAGACGCGAAGATCAAATGTACTATTGATGACCTCTGGAATACCACGATCTATATTTAGTAATTGATAA
ACAGCTTCCATGGCAGTACGAACCGAATATTCTGTTGTAAATACAGTGTCTCGCTCTGTTTCTGCAAAGTTACCAATAAATGCTAAA
TTTTGTGATTGATGCGGAACGACTAAAGGTCTGTCGCCGATAGCACGCGTCATGAAATAAGAGGTAATATATGGCATATAAACAGG
AATCGTATTAGATGCATGTTTTGCTAAGTCTTCAATTTTGTCAGTTGGTACACCTAAGTGATATAGCCATTCTTGACATATTTCATTA
CCACTACATTCTGTGATTGGCTTTTTAATATAATCGCCGTTTACATCTGAATATAAAGCATAAATCCACGTAGATATTTCATTTTTAG
GTTGGTCTTTAAACTGTTGCTGACGATTGATTGTAAAACTGATTTGCCATGCAGAATCATTGATTGTAATAATTCCGCCAGTAACCGT
TTTGCCTGCAAGAGGGTCACGTTTACAAATGCTTTCTATTGTATCGATAATATCTTTATTGTTTGTTGTCGATGTTGCAGAAACAAAC
CAACTCTTTTGAGGAATATTTTGGCAAAACTTATCAGGATTACCAAATTCAGGACTTTGTCGCGCTAAATTTTTCCATAGTGTCCAAC
TACCACCTAATTCGTCAGTTGGTGGTGCTGGTGTATCATTATCACCATACGTAGAGCTTTCCGTAATACTACCGTTTGTCACAAAGAC
AAGATCATCTACAGTCAGTTTAATTGACTCTGCTTTGCCATGTCGGTGAATTAATATTTCTCGGGCAATTTTTTGACTTGTCGTAACA
TCTACTTTAATATCTTCTACTTTTACATCGTATTCAAATTGAACCCCATGCGATTTTAAATATTCAACCATAGGTAATACTAAAGATT
CATATTGATTATATTTAGTGAATTTTAAAGCTGAAAAGTCTGCGAGACCACCAATATGATGAACGAATCGCATTAGATAGCGACGC
ATTTCCATAGCAGAATGCCACGGTTCAAATGCAAACATCGTTTTCCAGTAAATACAAAAGTTTGAATTAAAGAAGTCATCGGAAAA
TACATCTGTTATTTTGACATCATCTAAATCTTCTTCATTCGTTAAGCATAAATCTAAAATTTCTTTAATCGCTTTTTTGGTCAAAGTGA
AGTCTCCATCGGTAACTAAACGTTGACCCTGTTTTTCAATAACTCGACAGCGAGAATAGTTAGGGTCTTCTTTGTTTAGCCAATAGA
ACTCATCTAATACAGACGCGTTATCGATTTCTAATGAAGGGATAGATCTGAATAAGTCCCACAAACATTCAAAGTGGTTCTCCATTT
CACGACCACCTCGGACAACATAGCCTTTTAAAGGCATATTTTCACCATCAAGACTACCACCTGCTTTAGGTAACTCTTCTAAAATAT
GAATCTTCGAACCTTCCATTTGACCATCCCTAATTAAGAAACAAGCTGCCGCAAGTGAAGCTAACCCAGACCCGATTAAATAAGCA
GATTTGTTTTCTACATTTTCAGGTTTTTTAGGGCGCGCAAATGCTTCATAATTTCCATAACTGTAATACATATCCACCAAGTCCTTTCG
TATTAGAATACACTCAGACTATACCCCTTTGTGAATTGCAAATATTAGTATTTATATTGGTAAATTTGACGAAATTAGATCATTTTTA
TATTTTTGATTTTTAAAATTTTATCACTCGAATGTTTTTGGATTCTGAGGTGCGGGACTTGGAATAATTCGAATCATTATGTGGCATG
TTAAACAGCTAAATATAAGCTATACACATTTAACAAGCGGATACTCCTGACGCAATTATAGTATCATTACATATTGTTTAATCGTTT
GGGGTAATGTAATTAAAAGGTAAAATAGGCAGAGTGTTATACACAAAATGTAAAGAAAGAGGCGATAGAATGTCTAAAATCAGGT
CTTTTACAATATTAAGTCTACTTATTTACTTAGCTATGATGTGCTATACAGTAGTGACCTATTCAAAATTACCAACCAAAGTACCTAT
TCATTATAATTTAGCAGGGGATGCTGATAACTTTGCTGATAAATGGGTACTGCTTTTGATAAATAGCGCATTTATAGTGATTTGGCTT
ATATTTTTCATTGCAGGTAGATACTATGAACGATTTGCCAAATGGTCACATTATAATCATACACCACGTGAAATTCGAGCGATTAAA
TTATTTTTAAGTACGTTAAATTTAGAGATTATGAGCTATATGTCTATCTTCACAGTATTAGAAATTTGGCAAATACAACACCATCATC
AATTAAATTTACTATGGTTTAATATGATATTTATCATTATCATTGGTTTGACGCTTGTCATATTTTGTTTACTTCCTACAATTCACAAA
ATGAGAGATTCTCAATAAAAATATAAACTGTCATGATGCATTTTTGAAGCGGATTCCTGAGATTATTATAAATAATATCTAGTATTT
ATAATTAAAATGATATATTATTTATTATAGTTAAATATTGTGTATATTTTGTGAACTTTTTGTAAAAATTCGATTGCCTGTCACATAT
AGGAGTGTTACATTTTAAATATGTGATCATCGCAAAATATAAGTTGAAATAGGTTGTAGATTAATCAGAATGATAAATATTTTATAT
AAAGAGAGGGAGTCATTATGACACTACTTACTGTAAATCCATTCGATAATGTTGGATTATCAGCCTTAGTTGCAGCAGTACCTATTA
TTTTATTTTTATTATGCTTAACCGTTTTTAAAATGAAAGGCATTTATGCAGCATTGACAACTTTGGTTGTTACATTGATTGTGGCTTTA
TTTGTATTTGAATTACCAGCGCGTGTATCAGCAGGTGCGATTACAGAAGGCGTTGTTGCCGGTATTTTCCCAATAGGATATATCGTTT
TAATGGCAGTTTGGTTATATAAAGTTTCTATTAAAACAGGACAATTTTCTATTATTCAAGATAGTATTGCAAGTATTTCAGTGGACCA
AAGAATCCAACTATTATTAATTGGATTTTGTTTCAACGCATTTTTAGAAGGTGCAGCAGGATTTGGTGTGCCAATTGCGATTTGTGCA
GTATTATTAATTCAACTTGGATTTGAACCATTAAAAGCAGCGATGTTATGTTTAATTGCTAATGGTGCGGCGGGTGCCTTTGGTGCA
ATTGGTTTACCAGTTAGTATTATTGATACGTTTAACTTAAGTGGAGGCGTTACAACATTAGATGTTGCGAGATACTCAGCATTAACA
CTTCCAATTTTAAACTTTATTATTCCATTTGTTTTAGTATTCATTGTAGATGGTATGAAAGGTATTAAAGAAATTTTACCTGTCATTTT
AACAGTGAGTGGTACATATACTGGATTACAATTATTATTAACAATATTCCATGGTCCAGAACTAGCAGACATTATTCCATCACTAGC
AACAATGGTGGTGTTAGCATTTGTTTGTCGTAAATTTAAACCGAAAAACATTTTCAGATTGGAAGCGTCTGAACATAAAATTCAAAA
ACGAACGCCTAAAGAAATTGTCTTTGCTTGGAGTCCGTTCGTAATTTTAACTGCCTTTGTATTAGTATGGAGTGCACCATTCTTCAAA
AAATTATTCCAACCTGGAGGTGCACTTGAAAGTTTAGTAATAAAATTGCCAATTCCAAATACTGTGAGTGATTTATCGCCTAAAGGA
ATTGCGTTGCGTCTCGATTTAATTGGTGCAACTGGGACAGCGATTTTATTAACAGTAATTATTACAATTTTAATTACGAAGTTAAAAT
GGAAAAGTGCAGGTGCTTTATTGGTCGAAGCAATTAAAGAATTATGGTTACCGATCCTTACAATTTCAGCTATCCTAGCTATTGCTA
AAGTTATGACATACGGTGGTTTGACTGTAGCAATTGGACAAGGTATTGCTAAAGCGGGAGCAATTTTCCCATTATTCTCTCCAGTAT
TAGGTTGGATTGGTGTGTTTATGACGGGTTCAGTTGTAAATAACAATACTTTATTCGCACCTATTCAAGCGACAGTGGCACAACAAA
TTTCAACAAGCGGTTCATTACTTGTGGCAGCTAACACTGCAGGTGGTGTAGCAGCGAAACTTATTTCACCACAATCAATTGCCATTG

CGACTGCAGCTGTTAAAAAAGTTGGTGAAGAATCTGCATTATTAAAAATGACGCTAAAATACAGTATTATATTTGTTGCTTTTATTT
GTGTTTGGACGTTTATACTAACGTTAATATTCTAAATATAAATAATGTTGTCACTTGGATTCAAATGACATTTTAAATCTAATTATTC
ATGAATCGAACTAGTACGAAATGCATTGAGCATCTTGTCTAGTTCGATTTTTTAATGTCTAAAAATGTCGTATATGTAATTAGAGTA
GAAAGTGTTGAGGCGTTTCAGAAGTTGTTTAGAAAAGTAAGTAAAATAAAAAATGCACTGAGCAACAAAAGATGTTGCTAGTGCAT
TTAGATGATTCTTATCATTTCAAATAAGAATGTGTTAATCAACGTATATAAGTTAAAATTGGTTTGGATAAAATGATATCTATCGTTG
TGTATTGTTTGTTTTTATAGTTCGCGACGACGTCCAGCTAATAACGCTGCACCTAACGCTAATGATAATCCACCAAATACAGTTGTAC
CGATGAATGGATTTTCTTCACCAGTTTCTGGTAATGCTTGAGCTTTGTTAGCATCTGCATGGTTTGCTGGTTGCTTCTTATCAACAAC
AAGTTCTTGACCAGGTTTGATCATGTTTTTATCAGCTAATTTGTTATCTGCAGCAATTTTGTCAGCAGTAGTGCCGTTTGCTTTTGCAA
TGTCATTTACTGTATCACCAGGTTTAACGACATGTACTCCGTTGCCGTCTTCTTTACCAGGTTTTTTGTTGTCTTCTTTGCCAGGCTTG
TTGCCATCTTCTTTACCAGGTTTTTTGTTGTCTTCTTTACCAGGCTTGTTGCCGTCTTCTTTGCCAGGCTTGTTGTTGTCTTCTTTACCA
GGTTTGTTGCCGTCTTCTTTACCAGGTTTTTTGTTGTCTTCCTCTTTTGGTGCTTGAGCATCGTTTAGCTTTTTAGCTTCTGCTAAAATT
TCTTTGCTCACTGAAGGATCGTCTTTAAGGCTTTGGATGAAGCCGTTACGTTGTTCTTCAGTTAAGTTAGGTAAATGTAAAATTTCAT
AGAAAGCATTTTGTTGTTCTTTGTTGAAATTGTTGTCAGCTTTTGGTGCTTGTGCATCATTTAGCTTTTTAGCTTCTGCTAAAAGGTTA
GCGCTTTGGCTTGGGTCATCTTTTAAGCTTTGGATGAAACCATTGCGTTGTTCTTCGTTTAAGTTAGGTAAATGTAAGATTTCATAGA
AAGCATTTTGTTGTTCTTTGTTGAATTTGTTATCCGCTTTCGGTGCTTGAGATTCATTTAACTTTTTAGCTTCTGACAATAGGTTAGCA
CTTTGACTTGGGTCATCTTTTAAGCTTTGGATGAAACCATTGCGTTGTTCTTCGTTCAAGTTAGGCATGTTCAAGATTTCATAGAAAG
CATTTTGTTGTTCTTTGTTGAAATTGTTGTCAGCTTTCGGTGCTTGAGATTCGTTTAATTTTTTAGCTTCACCTAAAACGTTAGTGCTT
TGGCTTGGATCGTCTTTAAGACTTTGAATGAAACCATTGCGTTGCGCTTCGTTTAAGTTAGGCATGTTCAAAATTTCATAGAAGGCG
CTTTGTTGATCTTTGTTGAAGTTATTTTGTTGCGCATCAGCTTTTGGAGCTTGAGAGTCATTAAGTTTTTTAGCTTCACCTAAAACGTT
AGCACTTTGGCTTGGATCATCTTTAAGGCTTTGGATAAAACCATTGCGTTGATCAGCATTTAAGTTAGGCATATTTAAGACTTGATA
AAAAGCATTTTGTTGAGCTTCATCGTGTTGCGCAGCATTTGCAGCAGGTGTTACGCCACCAGATATAAGTAATGTACCTAAAGTTAC
AGATGCAATACCTACACCTAGTTTACGAATTGAATAAATGTTTTTCTTTTTCAAATTAATACCCCCTGTATGTATTTGTAAAGTCATC
ATAATATAACGAATTATGTATTGCAATACTAAAATCTATATTTATAATTAAATTTAAAGGTAAGTTTTACAACTTATAAAATAAATA
TTTTGCTGAAAGATTTATTCAGGGAGTAAAAGGCTTAAAACCGCAAAATCACGCTATTTCGATTAATAAAAACAGATAAAATATAG
AGATATTTTTTATAATGAAAGCGGTTTAATACATATGAGTTGAAAAATATTTTTAAGAATAAAGTGGGGCTTTGAATGTGCTGAGGT
TTAATTTCGGAAATGGTTTTAAGTGATATAGAAAATAGAAAGTTGTTCATTATGTGCTGAGGTTTTATTAAAAAATAGAAAACACAA
GTGCACTTTAGAATACAGCACACTTGCGTAATAGAGATATTATTCAAAAACAAGATGTAAATGATCTTTATCTGCTAATAATTGATT
CACTTGAGCTAATAATTGTTCAGCATGGTCTTGCTGCGCGTCATCCATATGAATTAAAATTTTTCTTTCATCTTCAGTTGAGCGTTCTT
TTATTAGATAGCCTTGCTTTTTTAAATTATTGAGAGCTCTAACAGTTTGAGGGTATTTATGGTGGATTGTTTCAATTAAATCTTTAAG
AAGAACGATGTTTTTATTTTGAGAAGTGATAATAGCTAGAATTGTGAATTCTACAAAACTTAATGTTAGATGTTTTTTGATAATATTC
TTGAAATACATTGTATACATCATCAAGTTTAGAAATTCTTTACTATCTTTTGGTATCATCTGTGATTCACTTTGATCTGCAAGGTTAA
ATTGTTTAATGATTTGATCAAACAATGTAACACGTTCTGCAATTTTCTCTCGTTGTTCTTCAGATATTGAAATGTAAGTATTACGCTC
ATCAATTTTACTTCGAACTTTACTAATATATGAATGTTTCACAAGTACTTTTATATGCTGTACTAAATCCGATTGTTTATAACATAAA
TCTGAAACAATCTTCTTAAATGGAAGTGTGTTTTCTTGCTGATGAAATAAATAAGTCAGTAATATAAATTCTTTTATAGTCATATCGA
CTTCAGGCTTGACTTTTTTCTTAAAACGAAACATATATGCTTCAATGATTATAAAATCTCTAATTTTGTCATGGTTATTATATTTCATT
GTTTTATCTCCTTGTATATGCACTTTATTTTAATATAACACAATATAATAAGAAAAATAATCCATAATTTACAATTAAAAATAATATG
ATATTTCTATTAAAATTTGTGTTTTTGAATAAATGACATTATCAAACAATATATTTAAATAAGAAATAGTTATCGATATTTGAAAAAT
CGAAGAATATTGATTGATATTATAGAATATGAGTCGGTGTTATTTGTATATAAAGTATTAATATGTAATTAAGAAAGTCGACGCTTG
TTAACAATAACAACAACTATATGTATAAAAAAACCTCGCAACTGTTAGTTAACTGAATCATTTAGCTAACTTCGTTGTGAGGTCATT
TTGTTTTAATAATATCGTTATAACTTTTTCACGGTTAATAATAAGTATATGAAGAATGGGGCACCAAAAGCAGCAATAAATACACCT
GCTGGCACTTCTTTAGGCAAGAATAAGGTACGCCCAATTAAGTCTGCAATAACAATTGATATGGCACCAATCATTGCTGACATTAGT
AACTTTTTAGCATAACTTCCGCGAACGATTGTTTTCGCGATATGTGGTGCGATTAAACCGACAAACCCAATGTTACCTACTAAACTG
ATTGCCATAGATACGAGTATAGTAGAAGTGATTAATTGGATTAGTTTCATACGTTGTACATGTAAGCCTAAGCCAATCGCTACAGGG
TCATCAAGTATAGATATTTTCATTTTTGGTATAACAAGAAATAACAACGGCACAACAGCTAAAATAACCATACCCAAAATGATTGTA
TCTTTAAACGTAGCACCGTAAAGACTTCCGACTAGCCATGTATAAGCTTTGGCAGCAGATAATTGCTTCGTTGTAATGAGTAATCCT
TGGACAAGCGCAATAAACAACGTTTGCATCGAAATACCGATGATTATGAGTGTTGTCGGGCGTATTTGTCCTTTCGTTTGAAACACT
AATAGTATCATCATTGCAACTGCGCCACCTAATACTGCAAATAGTGAAAGTAAATGTATCGTTAAATGGCTGAAAAATGCAATAAA
GACAACAGCACTTAAGCTAGCACCACCTGTGATACCGATAATATCAGGTGAGGCAATTGGATTTTTTAATACATTTTGCAACATTAA
ACCACTCATTCCTAGTGCGGCACCTGCTAAAATCGCAAGTGTAATGCGAGGTAAGCGTAATACTTCTAAAGTGAATTGATCCATACT
GTCATTTGGATTTATAAAGTACATCAGTACGCGTTGTAATGGTATAAAGCTTGAACCAATCATCATACTTACCACTGAAACGATGGC
TAAAAAGATTAACGCGAAGATGAGATGGTAATTGTCTTTTTTATTAATCTTTTCGGTCATAAGCGTTGACGTCCTTTCTTCATAATAT
AGATTAAGACAATAGCGCCAATGACAGCGGTAACGACACCGATAGGCAACTCTAGTGGCTTAATTATTATACGAGCAACAATGTCT
GAAATGATCATTAGGATTGCTCCAGCTAATGCAGTAAAAGGAATTAAATACTTATAGTTTGGTGGTAATAATCGTTTGCTAATATTC
GGTACGATAAGACCCACAAAGACGATTGATCCAGCTACGGCTACCGAAATACCGGCTAACATACTGATGAGCATAATAATCATCCA
TTTGATTAATTTTATGTTTTGACCGAGGCCGGTTGCAATGTCGTCACTTGTCATCAAGATGTTGATGTGTGCAGCCATGCTAAATGCA
ATTAAAATAAGTATCAATACAAGCGGAATAATCCATGGGATATCCCAAATATTACGTAATGAAACGGAGCCACTTAACCAAAATAA
TAGGCCTTGTAAGTCTGTTTCGTTCATAATAAGTATGCCTTGAGTAAAGGCTGTAAATAGCATCGCAATCGCAGCACCTGCCAAAAT
GACACGGTGAGGTGAGAATAGTGTTTGTCTAAACATACCTAGCGCAACAACTAATATAGTAACAACAATGGCACCTAAAAATGCAA
TAACTACAATCATTTTAAAAGATTGAATTTGGATAAATGTAATACTAAAAATGACAAAAAATACTGCGCCTGCATTGACACCGAAA
AGCCCTGGTGAGGCTATTGGGTTTCGTGTAAGTGCTTGCATCAACAAACCTGAGACAGCAAGGGCAGCACCAGTCAATAACGCAAT
GATTGTTCTCGACGCCCGTGCACCAGTGACAACATCATGTAAATCGTTTTCACTATCAAAGTTGAATAACGCCTGTATCACCGTACC
TGGTGACACAAGCGTATTTCCAATCATTAAACTTAAGATAGCTACTATTGCAAGACATAAACCAGCAATAACGATTTGGTATTTTGG
TTTAAGTAGCATCGTAAAACTCCTTAATTATTTTGATTGTTTTTCAATATTTAACTTTTCATATAAATCGTCAATAAGTTTTAATGAAG
ATTTATATCCGCCAGCTAAGTTCCAAGTGATTTCATCTAAATCATCAGATACTTGGTTGTTTTTAACTGCGTCTAAATTTTTCCACTCT
TTACTTGAAGTCCATTCGCTTTCAGTCTTTTTAACTAATGCAGCATCTTTCGCATTTGGATCTGATTTTACTACAAAAATATGATCAG
CGTTCATTAATGGAATGCTTTCTTTAGATGTAAGTTGGATAATATCTTTACCATTATCAACTTGTTTTTGTAAGTCTTTATTACGTTTG
AATCCTAAATCATTTAAGATTTCACCAGCATATCCACCAGCATAAATTCTTGTATGATCAGCACGGAAGTTAACAACTGAAGCTTTC
AATGGCCATGCATCTTTATACTTTGCTTTTGCATCTTTTTGGAATGCAGCTACTTTATCATCGTACTTTTTAAGTAAATCTTCAGCTTC
TTTTTCTTTCCCTAAAGCTTTCCCCATTAACTTAGTTGTATCTTTGAATTTGAAAACTGTATCAGTAGAAACTGTTGGTGCAATTTTAG
ATAATTGATCGTAAACTTTTTCGTTTCTAACTTTTGACGCGACAATTAAGTCCGGTTTTAATTTAGAGATTTCCTCTAAGTTAGGTGC
AGGTTCTTGACCTACAATCTTAGTATCTTTTAAATCATTTTTTATGTATTCGAATTTCGGTTTTTGTGTCCATGATTCTACAGCACCTA

CAGGTTTAACACCTAAAGATACAGCGACGTCAGTGGCACCTTGATATAGCGTAACAACACGCTTTGGTTTCCCTTTAATTTCAGTTG
TACCCATTGCATGTTTAATTGAAGTTGTTTCCTTATCTTTGTTATCAGATGATTGCTTATTTGAATTCTCACTACATCCTGCTAAAACA
AGTAGGAAAGCAAGCGTAACAACAAGCATTTTAATTACTTTATTCATTGACTAATTAGCCTCCTTCGTGATGTATGACAATGAGAAT
CATTATCACGGTTTAGTATGAATTAAATTTTTTCCTAAGTCAATAAAATATTTATGATTTACATGCAACTTATAATTATTTGACATAT
AAATGCATAAAAAATATAATCCTAATTACTTGATAGTGAGAATCATTATCAATTAGGTAACACACAATATTATAGAATTTTAAATTT
GAGGAGGAAGCGCTTTTGATTGAAAAAAGTCAAGCATGTCACGATTCATTGTTAGATTCTGTAGGGCAAACACCTATGGTTCAACTT
CATCAACTATTTCCGAAACATGAAGTGTTTGCAAAGTTAGAGTATATGAATCCTGGAGGCAGCATGAAAGATCGACCTGCCAAGTA
CATCATTGAACATGGTATTAAACATGGTTTAATCACTGAGAATACACATTTAATTGAAAGTACTTCTGGTAATTTAGGCATTGCGTT
GGCAATGATATCTAAAATCAAGGGATTAAAACTCACGTGTGTTGTTGATCCTAAAATATCACCAACAAATTTGAAAATTATTAAAA
GTTATGGTGCCAATGTAGAAATGGTTGAAGAACCTGATGCACATGGGGGTTATTTAATGACTCGTATTGCAAAGGTGCAAGAAATG
TTAGCCACTATTGACGATGCATATTGGATTAATCAATATGCGAATGAGTTAAATTGGCAATCCCATTATCATGGTGCAGGCACAGAG
ATTGTTGAAACAATTAAGCAACCTATAGATTATTTTGTCGCGCCAGTCAGCACGACAGGTAGCATTATGGGTATGAGTAGAAAAAT
AAAAGAAGTGCATCCAAACGCACAAATTGTTGCTGTTGATGCGAAAGGGTCAGTCATTTTTGGTGACAAACCTATTAATAGAGAAT
TACCTGGTATCGGTGCTAGTCGTGTACCCGAAATATTGAATAGATCAGAAATTAATCAAGTGATCCATGTAGATGATTATCAATCTG
CTTTGGGCTGTCGAAAACTGATTGATTATGAAGGCATATTTGCCGGAGGTTCAACAGGCTCGATTATTGCAGCGATTGAGCAGTTGA
TAACGTCAATTGAAGAAGGTGCAACAATTGTCACGATTTTACCAGATCGAGGCGATCGTTACTTAGATTTAGTTTATTCAGATACAT
GGTTAGAAAAAATGAAATCAAGACAAGGAGTTAAATCAGAATGAATAGAGAGATGTTGTATTTAAATAGATCAGATATTGAACAA
GCGGGAGGTAATCATTCACAAGTTTATGTGGACGCATTAACAGAAGCATTAACAGCCCATGCGCACAATGATTTTGTACAACCGCTT
AAGCCGTATTTAAGACAGGATCCTGAAAATGGACACATCGCAGATCGAATTATTGCAATGCCAAGTCATATCGGTGGTGAACACGC
AATTTCAGGTATTAAGTGGATAGGTAGTAAGCACGACAATCCATCGAAACGTAATATGGAGCGTGCAAGTGGTGTCATTATTTTGA
ATGATCCAGAAACGAATTATCCAATTGCAGTTATGGAAGCAAGTTTAATTAGTAGTATGCGTACTGCAGCAGTTTCAGTGATTGCAG
CTAAGCATTTGGCTAAAAAAGGATTTAAAGACTTAACAATCATTGGATGCGGGCTAATCGGAGACAAGCAATTACAAAGTATGTTA
GAGCAATTCGATCATATTGAACGCGTGTTTGTTTACGATCAATTCTCTGAAGCATGTGCACGCTTTGTTGATAGATGGCAACAACAG
CGTCCGGAAATTAATTTTATTGCGACAGAAAATGCTAAAGAAGCAGTATCAAATGGTGAAGTAGTCATTACATGTACCGTAACGGA
TCAACCATACATTGAATATGATTGGTTACAAAAGGGTGCATTTATTAGCAACATTTCTATCATGGATGTGCATAAAGAAGTCTTTAT
TAAAGCTGACAAAGTCGTAGTAGATGACTGGTCACAATGTAATCGAGAAAAGAAAACTATTAACCAATTGGTGTTAGAAGGTAAAT
TCAGCAAAGAAGCACTTCATGCTGAACTAGGACAACTTGTGACAGGTGACATACCAGGACGTGAAGACGATGATGAAATCATATTA
CTTAATCCGATGGGTATGGCTATCGAAGATATTTCAAGTGCTTACTTTATTTATCAACAGGCACAACAACAAAATATTGGGACAACA
TTGAACCTATATTAAGAATGCGAGGTGTCTGAACATTGCAGAATCATACAGCAGTCAATACAGCACAAACGATAATATTAAGAGAT
TTAGTTGATGCATTATTATTTGAAGATATAGCCGGTATTGTATCGAATAGTGAGATTACTAAAGAAAATGGACAAACCATTTTGATA
TACAAACGTGAAACACAGCAAATAAAGATACCTGTTTATTTTAGTGCTTTAAATATGTTTCGTTATGAAAGTTCACAACCAATTACG
ATAGAGGGAAGGCTGTCTAAGCAACCTTTAACGGCAGCTGAATTTTGGCAAACAATTGCTAATATGAATTGTGATTTAAGTCATGA
ATGGGAAGTGGCTCGCGTTGAAGAAGGACTGACTACTGCTGCCACACAGCTTGCTAAACAATTATCAGAATTAGATTTAGCGTCAC
ATCCTTTTGTGATGTCAGAGCAGTTTGCAAGTTTAAAAGATCGTCCATTTCATCCATTAGCTAAAGAAAAAAGAGGATTAAGAGAA
GTGGATTATCAAGTGTATCAAGCTGAATTAAATCAATCATTTCCTTTAATGGTTGCAGCAGTTAAAAAGACACATATGATTCATGGC
GATACTGCAAATATCGATGAATTAGAAAATTTGACAGCACCTATAAAAGAACAAGCGACAGACATGTTAAATGATCAAGGGTTATC
AATAGATGACTATGTACTATTTCCAGTACATCCTTGGCAATATCAGCATATTCTGCCGAACGTCTTTGCGAAAGAGATTAGTGAAAA
GTTGGTTGTACTATTACCGTTAAAATTTGGAGATTATCTGTCGTCTTCAAGTATGCGTTCATTAATTGATATTGGCGCACCGTATAAC
CATGTCAAAGTACCATTTGCAATGCAGTCATTAGGCGCATTAAGGCTAACGCCTACGCGTTACATGAAAAACGGAGAACAAGCAGA
ACAATTATTACGTCAGCTTATAGAAAAAGATGAAGCACTAGCTAAGTATGTCATGGTTTGTGATGAAACAGCTTGGTGGTCATATAT
GGGTCAAGATAATGATATTTTCAAAGATCAATTAGGTCATCTAACTGTTCAGCTAAGAAAATATCCCGAAGTGCTAGCCAAAAATG
ATACGCAACAGCTAGTGTCAATGGCAGCACTCGCGGCAAATGATCGCACTTTATATCAAATGATTTGTGGAAAAGATAATATTTCTA
AAAATGATGTCATGACGTTATTTGAAGATATCGCACAAGTCTTTTTAAAGGTAACACTATCATTTATGCAATACGGCGCATTACCAG
AGTTGCATGGTCAAAATATATTGTTGTCATTTGAAGATGGACGTGTACAAAAAATGCGTGTTACGTGATCATGATACTGTCAGAATTT
ATAAACCATGGCTAACAGCACATCAGCTTTCATTGCCGAAGTATGTCGTCAGAGAAGATACACCTAATACGCTAATTAATGAAGAT
TTGGAAACATTCTTTGCTTATTTTCAAACATTAGCTGTATCGGTAAATCTATATGCCATTATTGATGCAATTCAAGATTTATTTGGTG
TAAGTGAGCATGAACTTATGTCGTTGTTAAAACAAATTTTAAAAAATGAAGTGGCAACTATTTCCTGGGTTATAACTGATCAGCTAG
CTATCAGACACATTTTATTTGATAAACAGACGTGGCCATTCAAACAAATTTTATTACCATTGCTATATCAACGTGATAGTGGTGGAG
GTAGTATGCCTTCAGGTTTAACTACCGTACCAAATCCAATGGTGACATATGATTAATCAGTCTATATGGCGCAGTAACTTTCGCATTT
TATGGCTCAGTCAGTTTATAGCGATTGCTGGACTGACAGTACTTGTGCCATTATTGCCAATTTATATGGCATCACTACAAAATCTATC
AGTCGTAGAAATACAGTTGTGGAGTGGTATAGCGATTGCTGCTCCAGCTGTAACGACGATGATAGCTTCGCCGATATGGGGGAAGC
TAGGTGATAAGATCAGCCGAAAATGGATGGTGTTAAGAGCGTTACTTGGTTTGGCGGTATGCTTATTTTTAATGGCATTGTGTACGA
CACCATTACAGTTTGTACTTGTGAGGTTATTGCAGGGACTATTTGGTGGTGTTGTTGATGCATCAAGTGCGTTTGCGAGTGCAGAGG
CGCCAGCTGAAGATCGTGGAAAGGTATTAGGAAGACTGCAAAGTTCAGTCAGCGCAGGGTCTCTTGTGGGGCCATTAATTGGCGGT
GTTACAGCTTCGATATTAGGTTTTAGTGCGTTACTGATGAGTATTGCCGTTATTACTTTTATTGTCTGTATTTTCGGTGCATTAAAATT
GATTGAAACGACACATATGCCAAAATCACAAACACCAAATATTAATAAAGGTATTCGCCGTTCATTTCAATGTCTATTATGCACACA
ACAAACATGTCGATTTATTATCGTTGGCGTTTTAGCAAACTTTGCTATGTATGGCATGCTAACTGCATTATCACCACTTGCTTCATCA
GTGAATCATACAGCGATAGATGACCGTAGTGTGATTGGATTTTTACAGTCCGCATTTTGGACGGCTTCGATATTAAGCGCGCCTTTA
TGGGGACGCTTTAATGATAAATCATATGTTAAATCAGTATATATATTTGCCACGATTGCATGTGGTTGTAGTGCGATACTGCAAGGT
TTAGCGACGAATATAGAGTTTTTAATGGCTGCAAGAATACTTCAAGGATTAACATATAGTGCATTGATTCAAAGTGTCATGTTTGTT
GTCGTGAATGCGTGTCATCAACAACTTAAAGGCACATTTGTTGGAACGACGAACAGTATGTTAGTTGTTGGTCAAATTATTGGCAGT
CTTAGTGGCGCTGCCATTACAAGTTATACTACACCAGCTACTACGTTTATTGTTATGGGCGTAGTATTTGCAGTAAGTAGTTTATTTT
TAATTTGTTCAACCATCACTAATCAAATCAACGATCACACATTAATGAAATTATGGGAGTTGAAACAAAAAAGTGCAAAATAAAGA
ATTAATACAACATGCAGCGTATGCGGCTATCGAACGCATTTTAAATGAATATTTTAGAGAAGAAAATTTATATCAAGTACCACCTCA
AAATCATCAATGGTCTATACAATTATCAGAGCTCGAAACTTTAACGGGTGAATTTCGCTATTGGTCTGCGATGGGGCATCATATGTA
TCATCCAGAGGTATGGCTTATCGATGGAAAAAGTAAAAAAATAACAACTTATAAAGAAGCAATTGCGCGTATTTTGCAACATATGG
CTCAAATTGCAGATAATCAAACGGCAGTGCAACAACATATGGCGCAAATTATGTCTGACATCGATAATAGCATTCATCGCACGGCG
CGTTATTTGCAAAGTAACACAATAGACTACACAGAAGATCGCTATATCGTTTCAGAACATTCTTTATACTTAGGTCATCCATTTCATC
CGACTCCTAAGAGTGCAAGTGGGTTTTCAAAAGCAGATTTAGAGAAATATGCACCCGAATGTCATACATCATTCCAATTGCATTATT
TAGCTGTGCATAAAGATGTTCTGCTCACGCGCTATGTAGAAGGTAAAGAAGATCAGGTTGAGAAAGTGTTGTATCAATTAGCAGAT
ATAGACATATCAGAGATACCCAAAGATTTTATTTTATTACCAATACATCCTTATCAAATCAATGTGTTGCGACAGCATCCACAGTAT

ATGCAATATAGTGAACAAGGTTTAATAAAAGACCTTGGCATTTCCGGTGATTCAGTGTACCCGACGTCTTCGGTTAGAACTGTATTT
TCAAAAGCATTAAACATTTATTTAAAATTACCGATACACGTTAAAATTACTAATTTTATACGTACGAATGATCTTGAACAGATTGAA
CGGACAATCGATGCCGCGCAAGTTATTGCATCAGTCAAAGATGAGGTTGAAACACCCCATTTTAAATTGATGTTTGAAGAAGGATA
TCGTGCATTGTTACCGAATCCGTTAGGGCAAACAGTTGAACCTGAAATGGATTTATTAACAAATAGTGCCATGATTGTTCGTGAAGG
GATACCGAATTACCATGCTGATAAAGATATTCATGTATTGGCGTCATTATTTGAAACGATGCCTGATTCACCGACCTCTAAGTTATC
ACAAGTGATTGAGCAAAGTGGTTTAGCACCAGAAGCATGGCTTGAATGTTATTTGGATCGTACATTATTGCCGATATTAAAGCTGTT
TAGTAACACAGGCATTAGTCTAGAAGCACATGTACAAAATACATTAATTGAATTAAAAGATGGCATACCCGACGTGTGCTATGTCA
GAGATTTAGAAGGCATTTGTCTATCTAGAACGATTGCTACTGAAAAACAGCTTGTGCCAAATGTTGTGGCAGCATCAAGCCCTGTTG
TATATGCACATGATGAAGCATGGCATCGTCTTAAATATTACGTTGTAGTAAATCACTTAGGACATTTAGTATCAACTATTGGTAAAG
CGACTAGAAATGAAGTTGTGTTATGGCAAATTGTAGCGCATCGTCTTATGACATGGAAAAAAGAATACGCGAATAACGCAGTATTT
GTTGACTGTGTAGAAGATTTATATCAAACGCCGACCATTGCGGCTAAAGCGAATTTGATGAGTAAATTGAATGATTGTGGTGCAAA
CCCTATTTATACACATATACCAAATCCAATTTGTCATAACAAGGAGGTATCGTATTGTGAATCAAACAATTCTTAATCGTGTAAAGA
CTAGAGTGATGCACCAACTGGTATCATCACTTATTTATGAGAATATTGTTGTGTATAAAGCGTCATATCAAGACGGTGTCGGTCATT
TTACAATAGAAGGACATGATTCAGAGTATCGTTTTACTGCTGAAAAGACACATAGCTTTGATCGTATACGTATCACATCACCAATTG
AGCGTGTCGTAGGAGATGAGGCAGATACAACAACAGACTATACACAATTATTGAGAGAGGCTGTATTTACATTTCCTAAAAATGAT
GAAAAGCTAGAACAATTTATTGTTGAGTTATTACAGACAGAATTAAAAGATACACAAAGTATGCAGTATCGAGAATCAAACCCACC
AGCAACACCTGAGACATTTAACGACTATGAATTTTATGCGATGGAAGGGCATCAGTATCATCCAAGTTACAAATCACGTTTAGGATT
TACGTTGAGTGATAATTTGAAATTTGGTCCTGATTTTGTACCAAACATTAAACTGCAGTGGTTAGCTATCGACAAAGGTAAAGTAGA
AACGACGGTATCAAGAAATGTTGTAGTTAACGAAATGTTACGTCAACAAGTTGGCGATAAGACTTATGAACATTTTGTACAGCAAA
TTGAAGCGTCTGGCAAACATGTAAATGATGTTGAGATGATACCTGTACACCCATGGCAGTTTGAACATGTCATCCAAGTTGATTTGG
CTGAAGAAAGGCTTAATGGCACAGTACTATGGTTAGGGGAAAGTGATGAGCTATATCACCCTCAACAATCGATTCGTACGATGTCG
CCAATAGACACGACAAAATATTATTTAAAGGTACCAATAAGTATAACGAACACTTCAACGAAACGAGTGTTGGCGCCTCATACAAT
TGAAAATGCAGCGCAAATTACGGATTGGTTAAAGCAGATACAGCAACAAGATACGTATTTAAAAGATGAATTAAAGACAGCTTTTC
TAGGGGAAGTCTTAGGACAGTCTTATTTAAATACACAACTTTCGCCTTATAAACAAACTCAAGTTTATGGTGCGTTAGGTGTTATAT
GGCGTGAAAATATATATCACATGTTAATCGATGAAGAGGATGCGATACCATTTAATGCACTTTATGCAAGTGATAAGGATGGTGTA
CCATTCATTAAAAATTGGATTAAACAATATGGTTCTGAAGCTTGGACAAAGCAATTTTTAGCTGTAGCGATTCGTCCAATGATTCAT
ATGCTTTATTATCACGGTATTGCCTTTGAATCGCATGCACAAAATATGATGCTCATTCATGAAAATGGTTGGCCTACACGTATTGCCT
TAAAAGATTTCCATGATGGTGTTCGTTTTAAGCGTGAGCATTTAAGTGAAGCTGCTTCACACCTGACATTAAAGCCAATGCCAGAAG
CACATAAAAAAGTGAATAGTAATTCATTTATTGAAACAGATGACGAACGTTTAGTACGCGACTTTTTACACGATGCATTTTTCTTTA
TTAATATCGCCGAAATCATCTTATTTATTGAAAAGCAATATGGTATCGATGAGCAGCGACAATGGCAATGGGTTAAAGGCATCATC
GAGGCGTATCAAGAAGCATTTCCAGAGTTGAATAACTATCAACATTTCGATTTGTTTGAACCTACGATTCAAGTTGAAAAGTTAACG
ACACGTCGATTATTAAGTGACTCCGAGTTAAGAATTCATCATGTTACAAATCCATTAGGTGTAGGAGGTATCAATGATGCAACAACT
ATCTCTGAAACATAGATTAAACAATGGTGATTCAGTTTATGGCATTTTTAATTCTATACCGGACCCATTGATGATCGAGGTTATCGC
AGCAAGCGGGTATGACTTTGTTGTGATTGATACAGAACACGTGGCGATTAATGATGAGACACTAGCGCATTTAATTCGTGCAGCTG
AAGCAGCGCATATTATACCAATTGTACGTGTCACTGCAGTGATAGATAGAGATATCATTAAAGTGTTGGATATGGGTGCGAGAGGT
ATTATTGTGCCACACGTTAAAGATCGTGAGACAGTTGAGCATATTGTGAAATTAAGTCGTTATTACCCGCAAGGATTAAGAAGTTTG
AATGGTGGTCGCATGGCAAGATTTGGACGTACACCATTACTTGATGCAATGGAGATGGCTAATGAGCATATTATGGTGATTGCCATG
ATAGAAGATGTTGAAGGGGTTATGGCCATTGACGATATAGCTCAAGTCGAAGGTTTAGACATGATAGTCGAAGGTGCCGCAGATTT
ATCGCAGTCACTTGGCATACCATGGCAAACGCGTGATGATCAAGTAACATCACATGTTCAACATATTTTTGAAGTTGTGAATGCACA
TGGTAAACATTTTTGTGCATTACCACGTGAAGATGAAGATATTGCAAAATGGCAGGCACAAGGTGTACAAACATTTATTTTAGGTGA
TGATCGCGGAAAAATATATCGCCATTTAAGTGCATCTCTAGCGACGTCTAAACAGAAAGGGGATGAAGGCTAATGCGTATAGTTCA
ACCTGTTATTGAACAATTAAAAGCACAATCTCATCCAGTTTGTCATTATATCTATGATTTAGTCGGACTGGAACATCATTTGCAACAT
ATTACATCGTCATTGCCGAGTAATTGTCAAATGTACTATGCAATGAAAGCAAATAGTGAACGAAAAATCCTAGATACAATTAGTCA
GTATGTTGAAGGATTCGAAGTTGCATCTCAAGGTGAAATAGCAAAAGGTCTTGCTTTTAAACCAGCAAATCATATTATTTTTGGTGG
CCCTGGTAAGACAGACGAGGAACTAAGATATGCAGTAAGTGAAGGTGTTCAGCGTATTCATGTTGAAAGTATGCATGAATTACAAC
GGCTAAATGCCATCTTAGAACATGAAGATAAGACACAACACATTTTATTGCGTGTTAATTTAGCAGGACCATTTCCCAATGCAACGT
TGCATATGGCAGGACGCCCAACACAATTTGGTATTTCTGAAGACGAAGTTGATGATGTCATTGAAGCTGCGCTCGCAATGCCAAAG
ATTCATCTAGATGGATTTCATTTTCATTCTATTTCTAACAATTTAGACTCGAATTTACATGTCGATGTAGTGAAACTTTATTTTAAAA
AAGCAAAGGCATGGTCTGAAAAACATCGATTTCCACTCAAACATATCAATCTTGGTGGTGGCATAGGCGTTAACTATGCAGATTTA
ACTAACCAATTTGAATGGGATAATTTTGTAGAAAATTTTAAAACACTTATCGTTGAGCAAGAAATGGAAGATGTGACATTGAACTTT
GAATGTGGGCGCTTTATTGTGGCACATATTGGTTACTATGTGACAGAAGTGCTAGATATTAAGAAAGTGCATGGTGCTTGGTATGCC
ATTTTAAGAGGAGGTACGCAACAATTTAGACTGCCGGTATCTTGGCAGCATAACCATCCTTTTGAAATTTATCGTTATAAGGACAAT
CCATATTCATTTGAAAAAGTTTCAATTTCGAGACAGGACACAACGTTAGTCGGTCAATTATGTACACCGAAAGATGTCTTTGCTAGA
GAAGTGCAGATAGACTCAATCAGTACAGGCGACGTTATTGTTTTCAAATATGCAGGTGCATACGGATGGTCTATTTCACATCACGAT
TTCTTAAGCCATCCACATCCTGAATTTATTTATTTAACGCAAACAAAGGAGGATGAATAACTATTGAATCATATTCATGAACATTTA
AAATTGGTACCAGTAGATAAGATTGATCTTCACGAAACATTCGAACCTTTAAGATTGGAAAAGACGAAAAGTAGTATTGAAGCAGA
TGATTTTATACGTCATCCTATTTTAGTGACAGCGATGCAACATGGTAGATATATGGTTATAGATGGTGTGCATCGGTATACAAGTTT
GAAAGCGTTAGGATGTAAGAAAGTTCCAGTGCAAGAAATCCATGAAACACAATATTCAATTAGTACATGGCAACATAAAGTTCCAT
TTGGTGTGTGGTGGGAAACGTTACAACAAGAACATCGCTTGCCATGGACTACTGAGACAAGACAAGAAGCGCCATTTATTACGATG
TGTCATGGTGATACAGAACAATATTTGTATACAAAAGATTTAGGCGAAGCACATTTTCAAGTATGGGAAAAGGTTGTCGCAAGTTA
TAGTGGTTGTTGTTCTGTAGAGAGAATTGCACAAGGTACATATCCTTGTCTTTCTCAACAAGATGTACTCATGAAGTATCAGCCATT
GAGTTATAAGGAAATTGAAGCGGTTGTTCATAAAGGGGAAACTGTGCCAGCAGGTGTGACACGCTTTAATATTTCAGGACGATGTC
TTAATCTTCAAGTACCACTGGCATTACTTAAACAAGATGATGATGTTGAACAACTGCGCAATTGGAAGCAGTTTTTAGCAGATAAGT
TTGCCAATATGAGATGCTATACTGAAAAAGTATACTTGGTGGAGCAATAGTTTTACTGTGATGTTGAGGGAAATATGATGATTTAGC
GTATTGATAGCGAAAATATAATAAAACAATATAGTGTGGAGAACTTTTGATATTTTATAAATATTGAAGTTCTCCATTTTTGTATTTT
GCATATAAAAATTAAATAAAATAAGGTATATTAAGGTAAAGTATAAATTTTAAATAAATGGGGAATGAGTATGAGCTCAATTATAG
GAAAAATAGCAATTTGGATAGGCATCGTAGCTCAAATATATTTTAGTGTCGTTTTTGTTAGGATGATATCTATTAATATTGCTGGAG
GATCTGATTACGAAACAATTTTTTTATTAGGATTAATATTGGCTCTTTTCACTGTTTTACCAACCATCTTTACTGCGATTTATATGGAA
AGTTACTCTGTAATCGGAGGTGCACTTTTTATTGTTTATGCTATTATTGCACTGTGTTTATATAATTTCCTTTCGTCAATTTTATGGCT
GATTGGTGGTATTTTGCTGATTTGGAATAAATACTCAAAAGATGAATCGACAGACGAAAATGAAAAAGTTGATATTGAAAGTACAG
AGAATCAATTTGAATCTAAAGATAAAATCACTAAAGAATAAAGAGAATATTTAAGGCGGAGTATAAATATTAAATAAATGGGGAA

TAGACATGGAAAAAAATGTAGAAAAATCATTCATAAAGATAGGTTTATATTTTCAAATAGCTTATATAGTACTCATGGCTATAACTT
TATGTGGGTTTGTAATTTGCTATGGACTAATTTTCGGCCTTTTCTATTTATTATCAGGTAGCAAAGTTGATTATTTAATAGTAACAAT
AGTTATATCGGCAATAATTTCTATATTTGTAATTATACTTTCAATCGTACCTGTCATCGTATTGGCATCTGACTTATTTAAAGAAAGG
ATTTCAAAAGGTGTCATATTAATTGTATTGGCTATTATTGCTTTAGTATTATGCAACTTTGTATCTGCAATACTCTGGTTTGTTTCAGC
CATATCTATTTTAGGTAGAAAAAAATTAGTAGCTGCAGCAGATACTACCACTATTCAAAAAAGTAAAGGGAACGCAAATCAAGCAT
CACATAAAGACACGTGTAAAAAGGAACTTGATAGTCAAGACATGATGGAACATCCTGAGGTTAAAAATCCCACGACTAAAAACCTT
GAAGGATTTAACGAAGAAATACATAAAGATGAAGCTACAACTAAAGTTGTCAGTGATAACACGGAACCGCCGATTGAATCAAAAG
ACCATGTCTCGAAAAAAGATTGATGACAAACTAATCGAGAGACTTAAAAAAATAATATTCAACATAAGAACTTTTAAAACGACATT
TAAACGCATTGCCAATCACTAATGGTAGTGCGTTTAACTATACCTTAAATATCTGAATATTTTGTTAAATGGAGCTACCTTTGTTGTA
CTATTCAAATGAAGAGGAGTAAAATGTAATTAAAGGAAAGAAATTTGAGGAGTGATCTTTATGACAAACAACAAAGTAGCATTAGT
AACTGGCGGAGCACAAGGGATTGGTTTTAAAATTGCAGAACGTTTAGTGGAAGATGGTTTCAAAGTAGCAGTTGTTGATTTCAATG
AAGAAGGGGCAAAAGCAGCTGCACTTAAATTATCAAGTGATGGTACAAAAGCTATTGCTATCAAAGCAGATGTATCAAACCGTGAT
GATGTATTTAACGCAGTAAGACAAACTGCCGCGCAATTTGGCGATTTCCATGTCATGGTTAACAATGCCGGCCTTGGACCAACAACA
CCAATCGATACAATTACTGAAGAACAGTTTAAAACAGTATATGGCGTGAACGTTGCAGGTGTGCTATGGGGTATTCAAGCCGCACA
TGAACAATTTAAAAAATTCAATCATGGCGGTAAAATTATCAATGCAACATCTCAAGCAGGCGTTGAGGGTAACCCAGGCTTGTCTTT
ATATTGCAGTACAAAATTCGCAGTGCGAGGTTTAACACAAGTAGCCGCACAAGATTTAGCGTCTGAAGGTATTACTGTGAATGCATT
CGCACCTGGTATCGTTCAAACACCAATGATGGAAAGTATCGCAGTGGCAACAGCCGAAGAAGCAGGTAAACCTGAAGCATGGGGT
TGGGAACAATTTACAAGTCAGATTGCTTTGGGCAGAGTTTCTCAACCAGAAGATGTTTCAAATGTAGTGAGCTTCTTAGCTGGTAAA
GACTCTGATTACATTACTGGACAAACAATTATTGTAGATGGTGGTATGAGATTCCGTTAATAATCATCCACTAATGATAAATAAATC
CTTATTGTTAAGTTTAATCACTTAGCAGTAAGGATTTTTTAGTGCACTTAGAAGGGGGTGTATTGGTAGAAAATTAATAAGCGAAGT
TCTTAAGTGAGTTATGATGTCACAGTCTAATGCATCAGTTGAAAGCATTATTAGTATTAACACACCCAAGATATTATAAAACATCAC
AAAAACACCACTATCTAATTTATCTCAATAAAAATTCACAAAGTTATCTCATTTTATTTTTATAAATAAAAAATATCGATAAAAAGC
TTACAATACTTTATGTTTTTATGATATATTTTTAATGTATAAATGAGGTGGAAGATTTGGAAAGAGTTTTGATAACTGGTGGGGCTGG
TTTTATTGGGTCGCATTTAGTAGATGATTTACAACAAGATTATGATGTTTATGTTCTAGATAACTATAGAACAGGTAAACGAGAAAA
TATTAAAAGTTTGGCTGACGATCATGTGTTTGAATTAGATATTCGTGAATATGATGCAGTTGAACAAATCATGAAGACATATCAATT
TGATTATGTTATTCATTTAGCAGCATTAGTTAGTGTTGCTGAGTCGGTTGAGAAACCTATCTTATCTCAAGAAATAAACGTCGTAGC
AACATTAAGATTGTTAGAAATCATTAAAAAATATAATAGTCATATAAAACGTTTTATCTTTGCTTCGTCAGCAGCTGTTTATGGTGAT
CTTCCTGATTTGCCTAAAAGTGATCAATCATTAATCTTACCATTATCACCATATGCAATAGATAAATATTACGGCGAACGGACGACA
TTAAATTATTGTTCGTTATATAACATACCAACAGCGGTTGTTAAATTTTTTAATGTATTTGGGCCAAGACAGGATCCTAAGTCACAAT
ATTCAGGTGTGATTTCAAAGATGTTCGATTCATTTGAGCATAACAAGCCATTTACATTTTTTGGTGACGGACTGCAAACTAGAGATT
TTGTATATGTATATGATGTTGTTCAATCTGTACGCTTAATTATGGAACACAAAGATGCAATTGGACACGGTTATAACATTGGTACAG
GCACTTTTACTAATTTATTAGAGGTTTATCGTATTATTGGTGAATTATATGGAAAATCAGTCGAGCATGAATTTAAAGAAGCACGAA
AAGGAGATATTAAGCATTCTTATGCAGATATTTCTAACTTAAAGGCATTAGGATTTGTTCCTAAATATACAGTAGAAACAGGTTTAA
AGGATTACTTTAATTTTGAGGTAGATAATATTGAAGAAGTTACAGCTAAAGAAGTGGAAATGTCGTGAAAATGACATTGAAGCTGT
CCATAATAATAAGGGTTATGCCTATCAAAGAAAATTAGACAAACTAGAAGAAGTGAGAAAAAGCTATTACCCAATTAAACGTGCG
ATTGACTTAATTTTAAGCATTGTTTTATTATTTTTAACTTTACCGATTATGGTTATATTCGCCATTGCTATCGTCATAGATTCGCCAGG
AAACCCTATTTATAGTCAGGTTAGAGTTGGGAAGATGGGTAAATTAATTAAAATATACAAATTACGTTCGATGTGCAAAAACGCAG
AGAAAAACGGTGCGCAATGGGCTGATAAAGATGATGATCGTATAACAAATGTCGGGAAGTTTATTCGTAAAACACGCATTGATGAA
TTACCACAACTAATTAATGTTGTTAAAGGGGAAATGAGTTTTATTGGACCACGCCCGGAACGTCCGGAATTTGTAGAATTATTTAGT
TCAGAAGTGATAGGTTTCGAGCAAAGATGTCTTGTTACACCAGGGTTAACAGGACTTGCGCAAATTCAAGGTGGATATGACTTAAC
ACCGCAACAAAAACTGAAATATGACATGAAATATATACATAAAGGTAGTTTAATGATGGAACTATATATATCAATTAGAACATTGA
TGGTTGTTATTACAGGGGAAGGCTCAAGGTAGTCTTAATTTACTTAATAAGTTCAAATAAAAGTTATATTTTAAAGATTGTGACCAA
TTGTTACAGTATAACGAGGAATCCCTTGAGACAGTATCAAATGGCATTAAGAAATATGTGCCATCATTGATTTGCATGGCTATAAAT
ACTATTCATCTGATGAGATAGCCATGTTAAGAAATTGAAAGTATAGCATTAAAGGGGTTTGTAACAGTTGAAAATTATATATTGTAT
TACTAAAGCAGACAATGGTGGTGCACAAACACATCTCATTCAACTTGCCAACCATTTTTGCGTACACCATGATGTTTATGTCATTGT
AGGCAATCGTGGACCAATGATTGAACAACTAGATGCAAGAGTTAATGTAATTATTCTCGAACATTTAGTAGGTCCAATTGACTTTAA
ACAAGATATTTTAGCTGTCAAAGTGTTAGCACAGTTATTCTCGAAAATTAAGCCTGATGTTATCCATTTACATTCTTCTAAAGCTGGA
ACGGTCGGACGAATTGCGAAGTTCATTTCGAAATCGAAAGACACACGTGTAGTTTTTACTGCGCATGGATGGGCTTTTACAGAGGGT
GTTAAACCAGCTAAAAAATTTCTATATCTAGTTATCGAAAAATTAATGTCACGTATTACAGATAGCATTATTTGTGTTTCAGATTTCG
ATAAACAGTTAGCGTTAAAATATCGATTTAATCGATTGAAATTAACCACAATACATAATGGTATTGCAGATGTTCCCGCTGTTAAGC
AAACGCTAAAAAGCCAATCACATAACAATATTGGCGAAGTAGTTGGAATGTTGCCTAATAAACAAGATTTACAGATTAATGCCCCG
ACAAAGCATCAATTTGTTATGATTGCAAGATTTGCTTATCCAAAATTGCCACAAAATCTAATCGCGGCAATAGAGATATTGAAATTA
CATAACAGTAATCATGCGCATTTTACATTTATAGGCGATGGACCTACATTAAATGATTGTCAGCAACAAGTTGTACAAGCTGGGTTA
GAAAATGATGTCACATTTTTGGGCAATGTCATTAATGCGAGTCATTTATTATCACAATACGATACGTTTATTTTAATAAGTAAGCAT
GAAGGTTTGCCAATTAGCATTATAGAAGCTATGGCTACAGGTTTGCCTGTTATAGCCAGTCATGTTGGCGGTATTTCAGAATTAGTA
GCTGATAATGGTATATGTATGATGAACAACCAACCCGAAACTATTGCTAAAGTCCTGGAAAAATATTTAATAGACAGTGATTACAT
CAAAATGAGTAATCAATCTAGAAAACGTTATTTAGAATGTTTTACTGAGGAGAAAATGATTAAAGAAGTGGAAGACGTTTATAATG
GAAAATCAACACAATAGTAAATTACTAACATTGTTACTTATCGGTTTAGCGGTTTTTATTCAGCAATCTTCGGTTATTGCCGGTGTGA
ATGTTTCTATAGCTGACTTTATCACATTACTAATATTAGTTTATTTACTGTTTTTCGCTAACCATTTATTAAAGGCAAATCATTTTTTA
CAGTTTTTCATTATTTTGTATACATATCGTATGATTATTACGCTTTGTTTGCTATTTTTTGATGATTTGATATTTATTACGGTTAAGGA
AGTCCTTGCATCTACAGTTAAATATGCATTTGTAGTCATTTATTTCTATTTAGGGATGATCATCTTTAAGTTAGGTAATAGCAAAAAA
GTGATCGTTACCTCTTATATTATAAGCAGTGTGACTATAGGTCTATTTTGTATTATAGCTGGTTTGAACAAGTCCCCTTTACTAATGA
AATTGTTATATTTTGATGAAATACGTTCAAAAGGATTAATGAATGACCCTAACTATTTCGCGATGACACAGATTATTACATTGGTAC
TTGCTTACAAGTATATTCATAATTACATATTCAAGGTCCTTGCATGTGGTATTTTGCTATGGTCTTTAACTACAACGGGGTCTAAGAC
TGCGTTTATCATATTAATCGTCTTAGCCATTTATTTCTTTATTAAAAAGTTATTTAGTAGAAATGCGGTAAGTGTTGTGAGTATGTTA
GTGATTATGCTGATATTACTTTGTTTTACCTTTTATAATATCAACTACTATTTATTCCAATTAAGCGACCTTGATGCCTTACCGTCATT
AGATCGAATGGCGTCTATTTTTGAAGAGGGGTTTGCATCATTAAATGATAGTGGATCTGAGCGAAGTGTTGTATGGATAAATGCCAT
TTCAGTAATTAAATATACATTAGGTTTTGGTGTTGGCTTAGTGGATTATGTACATATTGGCTCGCAAATTAATGGTATTTTACTTGTT
GCCCATAATACATATTTGCAAATTTTTGCGGAATGGGGCATTTTATTCGGTGCGTTATTTATCATATTTATGCTTTATTTACTGTTTGA
ATTATTTAGATTTAACATTTCTGGGAAAAATGTAACAGCAATTGTTGTAATGTTGACGATGCTGATTTACTTTTTAACAGTATCATTT
AATAACTCAAGATATGTCGCTTTTATTTTAGGAATTATCGTCTTTATTGTTCAATATGAAAAGATGGAAAGGGATCGTAATGAAGAG

TGATTCACTAAAAGAAAATATTATTTATCAAGGGCTATACCAATTGATTAGAACGATGACACCACTGATTACAATACCCATTATTTC
ACGTGCATTTGGTCCCAGTGGTGTGGGTATTGTTTCATTTTCTTTCAATATCGTGCAATACTTTTTGATGATTGCAAGTGTTGGCGTTC
AGTTATATTTTAATAGAGTTATCGCGAAGTCCGTTAACGACAAACGGCAATTGTCACAGCAGTTTTGGGATATCTTTGTCAGTAAAT
TATTTTTAGCGTTAACAGTTTTTGCGATGTATATGGTCGTAATTACTATATTTATTGATGATTACTATCTTATTTTCCTACTACAAGGA
ATCTATATTATAGGTGCAGCACTCGATATTTCATGGTTTTATGCTGGAACTGAAAAGTTTAAAATTCCTAGCCTCAGTAATATTGTTG
CGTCTGGTATTGTATTAAGTGTAGTTGTTATCTTTGTCAAAGATCAATCAGATTTATCATTGTATGTATTTACTATTGCTATTGTGACG
GTATTAAACCAATTACCTTTGTTTATCTATTTAAAACGATACATTAGCTTTGTTTCGGTTAATTGGATACACGTCTGGCAATTGTTTC
GTTCGTCATTAGCATACTTATTACCAAATGGACAGCTCAACTTATATACTAGTATTTCTTGCGTTGTTCTTGGTTTAGTAGGCACATA
CCAACAAGTTGGTATCTTTTCTAACGCATTTAATATTTTAACGGTCGCAATCATAATGATTAATACATTTGATCTTGTAATGATTCCG
CGTATTACCAAAATGTCTATCCAGCAATCACATAGTTTAACTAAAACGTTAGCTAATAATATGAATATTCAATTGATATTAACAATA
CCTATGGTCTTTGGTTTAATTGCAATTATGCCATCATTTTATTTATGGTTCTTTGGTGAGGAATTCGCATCAACTGTCCCATTGATGAC
CATTTTAGCGATACTTGTATTAATCATTCCTTTAAATATGTTGATAAGCAGGCAATATTTATTAATAGTGAATAAAATAAGGTTATAT
AATGCGTCAATTACTATTGGTGCGGTAATGAATTTAGTATTATGTCTTGTTTTGATATATTTTTATGGAATTTACGGTGCTGCTATTGC
GCGTTTAATTACAGAGTTTTTCTTGCTCATTTGGCGATTTATTGATATTACTAAAATCAATGTGAAGTTGAATATTGTAAGTACGATT
CAATGTGTCATTGCTGCCGTTATGATGTTTATTGTGCTTGGTGTGGTCAATCATTATTTGCCCCCTACAATGTACGCTACGCTGCTATT
AATTGCGATTGGTATAGTAGTTTATCTTTTATTAATGATGACTATGAAAAATCAATACGTATGGCAAATATTGAGGCATCTTCGACA
TAAAACAATTTAAGTATCGGTAACGCTATACTTTAGAAATTTAAGATTAAGAAGAAAAGGCAATTTCTTATTGAAAAATGGAAGTT
GTCTTTTTAATTCTCTTTAAAAGCGGGAAACAAAAGCAGTTAAATGCCTTTTTGCATTCAATATTAAGTATTATATCAATTTCGAATA
TTTAAATTTTATATAATTGGATATAACAAATAAATAATAATTATTGCAAAACACACCCGAAATTAATTATTATAAAAGTATATTCAT
AAAAGGAGGAATATACTTATGGCATTTAAATTACCAAATTTACCATATGCATATGATGCATTGGAACCATATATAGATCAAAGAAC
AATGGAGTTTCATCACGACAAACATCACAATACGTACGTGACGAAATTAAACGCAACAGTTGAAGGAACAGAGTTAGAGCATCAAT
CATTAGCGGATATGATTGCTAACTTAGACAAGGTACCGGAAGCGATGAGGATGTCAGTCCGTAATAATGGCGGTGGTCATTTTAAC
CATTCATTATTCTGGGAAATACTATCACCTAATTCTGAAGAAAAAGGTGGCGTAATAGATGACATCAAAGCGCAGTGGGGCACTTT
AGATGAATTTAAAAATGAATTTGCAAATAAAGCAACAACATTATTTGGATCAGGTTGGACTTGGTTAGTTGTTAATGATGGCAAATT
AGAAATTGTGACAACGCCAAACCAAGATAATCCATTAACAGAAGGCAAAACACCAATCTTACTATTTGATGTTTGGGAGCATGCCT
ACTATCTGAAATATCAAAATAAACGTCCAGACTATATGACTGCATTTTGGAATATTGTTAACTGGAAAAAAGTTGATGAATTATACC
AAGCAGCAAAATAATATAACATAATATAATTGAGGTGGAGCATCTACAAGGTGTTCCACTTTTTTGTACTTAATATCTTTACTTATTT
GTTTTTGTGTAATGGGATAGCACGTAAAAGTGGCAAAATAATAAGATAAATTTCAAACTCGAAATACCTTATTATTGTTGACTAAAA
CGATGACTTAAATGTTATATACATTAGACAACACTATTAATTAATCAAGATATTGTCATTAAATAAATTGACAAAATAAGTAATTAT
TATGAATCTAAAGTGAAATTTTTATAAAAAAATTGTAATGATTCAAAAATTTTGTTGCATTTCTTTTGTAATCATATGATAATGTAAAT
GTAATCAAATTGTAATATAAGGGGAAAAGACAATGAAAAAAATTAGCAACAGTAGGTTCTTTAATTGTAACAAGCACTTTAGTATTC
TCAAGTATGCCTTTTCAAAATGCGCATGCCGACACAACTTCAATGAATGTGTCGAATAAACAAAGCCAAAATGTACAAAATCATCG
TCCTTATGGCGGAGTAGTACCACAAGGAATGACGCAAGCACAATATACTGAATTAGAGAAAGCTTTACCCCAATTAAGCGCTGACA
GTAATATGCAAGACTATAATATGAAATTGTATGATGCGACGCAAAATATTGCTGATAAATACAATGTGATAATTACAACTAATGTA
GGGGTATTTAAACCACATGCTGTTAGAGATATGAATGGCCATGCGTTACCTTTAACAAAAGATGGCAATTTTTATCAAACGAATGTA
GATGCAAATGGTATTAATCATGGTGGTAGTGAAATGGTGCAAAATAAAACAGGTCATATGAGTCAACAAGGCCATATGAATCAGAA
CACACACATGAACCAACAGCCACACATGCAACAAGGTCATATGCAATCATCAAACCATCAAATGATGAGTCCAAAAGCAAATATGC
ATTCATCAAATCATCAAATGAACCAAAGTAACAAAAAAGTTTTACCAGCTGCTGGTGAAAGTATGACATCAAGTATTCTTACTGCA
AGTATTGCCGCACTACTATTAGTATCTGGGTTATTCTTAGCATTTAGACGACGTTCAACAAATAAATAAACATAATACGATTAATAA
TAGAAAAATCGTGTGATTATCTGAGCGAGCCTAGGACATAAATCAATGTCCTAGGCTCGCTAATGTTATATTGGCAGTAGTTGACTG
AATGAAATTGCGCTTGTAACAAGCTTTTCCATTTCTTGCTCACTTCCTAAATAGTCATCACAAAATTTCTTATATTTCAATAATTTTAA
TAATCCGATTGTCTTATACGTGTCAGTGTTAATTCAGAGATTTCCTGTGGAATATACCACTTATTAATCATAATTGGATAAGGTGTTT
GTGCGTACAATGTTTCAATAATCAGCCAACAATGTGTATCACCATCAAACACGTGACTATGATTTTTGAAGTGGGGCGCTTTGGTAA
TAGACATTTTTAAATCTGATTGATATGCATTGTTATAAATCGTTTGCTCAACGAACGTCTTCATGTCGTCTTCGTTTTGTGTATTCACT
TTAAATGTGTCAATGACATTTAACGGTATAAAGGTAAAACAAAATGCATCAGCTTGCTTAGAATGATTGTCCTTTTTTTGATAATAG
CGTTCCATTGCAATGACGGCAGAAGGATGGTTTGCAAACAAATGATTTGTATATTCACTTTCTAAATCAACACGATAATTAATTGAT
GACATAGATACGCGAGCTAGCAATATTTGATCAAGTGGATGCTTAAATTGATCCATACTTGAAGCGTGTTGGGCATTTGTTTGTGGA
ATAACAAAGTGTCCCTTCCCTCTTGTACTCTCTACGATGCCATCTTCGGCTAACAATTTTATAGCTTGGCGCAAAGTCATGCGACTGA
CATCAAAGCGCGCACAAAGTTCCTTTTCAGTAGGTAATGCATGGCCACTCGGATATTTTCCTATTTGAATTTCTTTATATAACGTATT
ATAAATCGTTAAAAATTTTGGTTGTGTTTGCGTCACGTAGACAACCTCCATAAAGTTACTTAATCACTCTCATCATACAATAATTTTT
ACTCGAATTGGAAAAATTATAAAAATTAAATATAGATAGGCTTTGAAAATTAGTTTTATACAAGGTTAGAAGCTATAACTGTAAAA
TGTTCTTAATATTGTCAAAATGTAATGCTTGAAAGCGCTTTTAAAAAATATTATTATATACATGGTTAGACAAATAGACAAATCACT
ATACAAATATTGGGAGGAATATTTTATGAAATCAACACCACACATTAAACCAATGAATGACGTCGAAATTGCAGAAACGGTTCTAT
TGCCAGGAGATCCGTTAAGAGCTAAGTTCATTGCAGAAACTTATTTGGATGATGTGGAACAGTTCAATACAGTGCGAAACATGTTTG
GTTTTACCGGAACATATAAAGGTAAAAAAGTTTCTGTCATGGGTTCCGGTATGGGTATGCCATCTATTGGCATTTACTCTTATGAATT
AATTCATACATTTGGTTGTAAAAAATTAATTCGCGTTGGCTCTTGTGGCGCGATGCAAGAAAACATTGATTTATATGATGTGATTATT
GCACAAGGTGCCTCTACTGATTCAAATTACGTTCAACAATATCAATTACCAGGTCATTTTGCGCCAATTGCTTCTTATCAATTATTAG
AAAAAGCAGTTGAAACAGCACGTGACAAAGGTGTACGTCATCATGTAGGTAATGTGTTATCAAGTGATATTTTCTATAACGCGGAT
ACAACAGCGAGTGAACGTTGGATGCGTATGGGTATTTTAGGTGTAGAAATGGAATCAGCTGCATTATACATGAATGCAATTTACGC
TGGTGTCGAAGCATTAGGTGTGTTCACAGTGAGCGATCATTTAATTCATGAAACGTCAACAACACCTGAGGAAAGGGAACGTGCAT
TTACAGATATGATTGAAATTGCACTGTCATTGGTGTAGATGATTATGAATGTTGAATATTCTAAAATAAAGAAAGCAGTACCTATTT
TATTATTCTTATTTGTATTCAGTTTGGTTATAGACAACTCATTTAAATTGATTTCTGTAGCCATTGCTGATGACTTAAACATATCTGTA
ACGACAGTAAGTTGGCAAGCGACATTAGCCGGTTTAGTAATTGGTATTGGCGCTGTAGTATACGCTTCATTATCTGATGCCATTAGT
ATACGCACACTATTTATTTATGGCGTGATATTAATCATTATCGGATCAATTATTGGTTACATTTTCCAACATCAATTCCCATTACTTTT
AGTTGGACGTATTATTCAAACTGCCGGTTTAGCTGCTGCAGAGACATTATATGTGATATATGTTGCAAAGTATCTTTCTAAAGAGGA
CCAGAAGACTTACCTTGGCTTAAGTACGAGCAGTTATTCCTTGTCATTAGTTATCGGTACATTATCAGGTGGATTTATTTCTACGTAT
TTACACTGGACAAATATGTTTTTAATTGCATTAATCGTAGTATTTACGTTGCCATTCCTATTTAAATTATTACCAAAAGAAAATAATA
CGAATAAAGCTCATTTAGATTTTGTTGGCTTAATTCTAGTGGCAACTATTGCTACAACAGTCATGCTGTTTATTACGAACTTTAATTG
GTTATATATGATTGGTGCCTTAATTGCGATTATCGTTTTTGCGCTATATATTAAAAATGCGCAACGTCCATTAGTAAATAAATCATTT
TTCCAAAATAAACGTTATGCTTCATTTTTATTTATAGTATTTGTAATGTATGCTATCCAATTGAGTTATATTTTTACGTTCCCATTCAT
AATGGAGCAAATTTATCATCTGCAACTAGACACAACATCACTGTTATTAGTACCGGGTTATATAGTAGCAGTCATTGTTGGTGCACT

AAGTGGTAAAATCGGCGAATATCTGAATTCAAAACAAGCGATTATCACAGCAATTATTTTAATAGCACTGAGCTTGATTTTACCTGC
ATTTGCAGTAGGTAATCACATTTCAATCTTCGTCATTTCTATGATATTCTTTGCAGGTAGCTTTGCTTTAATGTATGCACCTTTACTTA
ACGAAGCCATTAAAACAATAGATCTTAATATGACAGGTGTGGCTATTGGTTTTTATAATTTAATTATTAATGTGGCGGTATCTGTAG
GTATTGCGATTGCTGCGGCTCTAATCGATTTTAAAGCATTAAATTTCCCAGGCAATGATGCATTAAGTTCACATTTCGGTATTATTTT
AATTATTTTAGGTTTAATGAGTATTGTCGGATTAGTTTTATTCGTCAGCTTAAATCGTTGGACACAATCTGAAAAATAAATAGATATA
AATTCGCGAGATATATTCGTATTTATAGTAAAATTAAATAAAGAGATTATATAACACGAGGAGTAGTAAGTATGAAATTTGAGAAA
TATATAGATCACACTTTATTGAAGCCTGAGTCAACACGTACGCAAATCGATCAAATCATCGATGAAGCGAAAGCATACAATTTTAA
ATCTGTATGTGTGAATCCAACGCATGTTAAATATGCAGCAGAGCGACTAGCTGATTCAGAGGTGCTCGTTTGTACGGTAATAGGATT
CCCATTAGGTGCGTCGACAACTGCAACGAAAGCATTTGAAACAGAAGATGCAATTCAAAATGGTGCAGATGAAATTGACATGGTCA
TCAACATCGGCGCATTAAAAGATGGACGTTTTGATGATGTACAACAAGACATTGAAGCAGTGGTGAAAGCTGCGAAAGGTCACACA
GTAAAAGTGATTATTGAGACGGTATTGTTGGACCATGACGAAATCGTAAAAGCGAGTGAATTAACAAAAGTGGCTGGTGCGGACTT
CGTTAAAACTTCAACAGGTTTTGCAGGTGGCGGTGCGACTGCAGAAGACGTTAAATTAATGAAAGATACAGTAGGTGCTGATATAG
AAGTAAAAGCATCAGGTGGCGTACGTAATTTAGAAGATTTCAATAAAATGGTTGAAGCAGGTGCGACACGTATTGGTGCGAGCGCA
GGCGTTCAAATTATGCAAGGTTTAGAAGCAGATTCAGATTACTAAT

>008-contig_256

AGCAGATTCAGATTACTAATATATGTAAATTTTGGGAGTGATAGCTATGACAAGACCATTTAATCGTGTACATTTAATCGTAATGGA
TTCAGTAGGTATTGGTGAAGCGCCAGACGCAGCTGATTTTAAAGATGAAGGTTCACATACTTTAAGACATACCTTAGAAGGTTTCGA
TCAAACTTTACCAAACCTTGAAAAGTTAGGTCTAGGGAACATCGATAAATTACCAGTAGTAAATGCAGTTGAACAACCAGAAGCAT
ACTATACTAAATTGAGTGAAGCTTCAGTTGGTAAAGATACAATGACTGGTCACTGGGAAATTATGGGATTAAATATTATGCAACCTT
TTAAAGTATACCCTAATGGATTCCCTGAAGAGTTAATTCAACAAATTGAAGAAATGACAGGTCGTAAAGTTGTTGCTAACAAACCG
GCATCGGGTACGCAAATTATCGATGAGTGGGGCGAGCACCAAATGAAAACTGGTGACTTAATTGTTTATACAAGTGCAGACCCAGT
ATTGCAAATTGCTGCACATGAAGACATTATCCCATTAGAAGAGTTATATGATATTTGTGAAAAGGTTCGTGAGTTGACAAAAGACCC
TAAATATTTAATTGGTCGTATTATCGCACGTCCATATGTTGGTGAACCAGGAAACTTTACACGTACATCTAATCGACATGACTATGC
GTTAAAACCATTTGGTAAAACTGTCTTAGATCATTTGAAAGACGGTGGTTATGATGTTATTGCCATCGGTAAAATTAATGACATTTA
TGATGGTGAAGGTGTAACAGAAGCGGTTCGTACGAAGAGTAACATGGACGGTATGGATCAATTGATGAAAATTGTTAAGAAAGATT
TCACAGGTATTAGCTTCTTAAACTTAGTAGACTTTGATGCATTATACGGTCATCGTCGTGATAAACCAGGTTATGCACAAGCAATTA
AAGATTTCGATGATCGCTTGCCAGAACTGTTTAGCAACTTAAAAGAAGACGATTTAGTAATTATTACAGCAGACCATGGTAATGACC
CGACAGCGCCAGGTACGGACCATACGAGAGAATATATCCCAGTAATTATGTACAGTCCGAAATTTAAAGGTGGTCATGCACTAGAA
AGTGATACTACATTCAGTTCTATCGGTGCAACTATAGCAGATAATTTCAACGTAACATTACCAGAGTTCGGTAAAAGTTATTTAAAG
GAATTGAAATAGAATAAATTTAGATATAATAAAAACAGCAGTGAAGTTAACTATAACAATAGTTTTCTTCACTGCTGTTTTTATTAT
AATAGAGAAACGTAAGACGGTAGGACCTCTTATTTAGGAGTATCCTGATTTAATGTTAAACAATACGTTTTCGGATTGAACCGGAA
ATTAAATCTACAATTGCGACCATTAGTACTAAACCGATTAATATAATACCTACACGGTCCCAAGAACGTGTTTGAATGGCAAATATG
AGTGGTGTCCCGATACCACCAGCCCCAATTAGCCCCAGTATAGAAGCTGAACGTAAGTTTAGTTCAAAGCGATAAAGTATGAGAGA
TAGAAAGGCAGGCATAATTTGTGGTATGACTGCAAATACGAGTGTTTTAATCTTATTTGCACCACTGGCCTTTAATGATTCTATAGC
ACTGAAATCTAGACCTTCAATATCTTCAGCTAAAAGTTTCCCAAGCATACCTACAGAATGGATACCTAAAGCTAATACACCTGAAAA
TGAACCTGGGCCAACAGCTTTGATAAATATAAGTGCCATTACAATTTCTGGGAAGACACGTATAACACTTAAAATAAATTTGCTAAC
ACCTGAAACCGGGCGTAACTTTACCATATTATTTGCACCTAGAAATGCTAATGGAATACAGATAATTGCGGCGATGAAAGTACCTA
CAACGGCTATCGCAAAGGTTTCAAGTAAACCACGTAATAAGTCTTCGCCATCTGGTATATAGATATAGCTGATGTCAGGATGGAAT
AATCCGCTGAATATGGATTTTAAGATTTCTAATGATTTACTTTTAAGTTCTAAACTTGGTACACCTGCAAATGCCCAGATGATAATAG
CTAAGACGACAATTGCAATAAGCCATCTTTTAATCAATTTTCGTTTGTGTGCTTTTGTGTGAACATTATATTTTGCTATTTGCTGTGTC
ATGCGAGATGTGCCCTCACTTTCGTACTGATGTAATCAATGACGACGACGATAACTAAAGTAAATAAAATAATCGTTGCTGTTTTTG
GATATTGAAATAAACCAAGTGTTTGATCATAAAACAATCCAATACCGCCAGCGCCGACTAATCCAAGAACAGCTGAAGCACGTATA
TTTACTTCAAATGCATATAATACGTATGACATAAATGACGATATGGCTTGTGGTACAACACCGAAAACAATCCATTTTATTTTATTA
GCACCAACAGCCGTCATTGCTTCCATTGGACCTGGATCTATCGTTTCCAATGATTCATATAATAATTTTCCAATAATACAGATAGTTA
AAATAAACAATGCTAATATCCCTGGAATTTGACCGATTCCAAATACCGCCACAAAGATTGCTGCTAATAGCAAATCCGGTATAGTA
CGAACTATATTTAAAATAAAACGCGAGGGTATTGAAATCCACTTTTGATGAACGATATTGCTAGCACATAATAACGCAATTGGTATT
GAAACGATGCTACCTAATACTGTACTTACGATAGCCATTCGAATGGTATCTAACATTGGCGTTGTAATTTGTTGTAAATACTCGAAA
TCAGGTGGAATCATTTGTTTGAATAGATCACCTATTTGAGGTATTCCTATCATTAAATCTCCAAAATTAAACCCCGTATAAATGAAG
CTCCAAATGATAAGCACGATGATTAACATGAAGGTAAAACTCGTTTTTAAAGAAACCTTTTTCTTTAAAAGGGAGTCATACTTTGTA
GGTGTTTCTAAAGGCATGTTAGTTCACTCCTAGCTTTTCATCTTCTTTAATTGTACGTCCATATATTTCACTAAATACGTCATCTGTTG
CTTCAGATGCAGGACCATCATAGACAACTTCACCATCACGTAAACCAATGATGCGTGTGCCATATTCTTTTGCCAAGTCAACAAAAT
GTAAATTAATTAAAATTGTAATGCCTAATTCTTGGTTGATTTTTCTTAAATCATCCATAACCTGTTTCGTTGTTAATGGGTCTAATGA
AGCAACTGGTTCATCTGCAAGAATAATTTCGGATTCTTGGCATAGCGCACGTGCAATAGATATACGTTGTTGTTGGCCACCTGATAA
TTCATCAGAGCGTTGATTATATTTATCTAAGATGTTGACCCGTTCTAGTGCATCCATTGCTTTAATTTTGTCTTCTTTTGGAAATAAAC
CTAATACCATTTTCCAAGTAGGGTGATAACCTACACGTCCACTTAGTACATTTCGTAATACACTTGACCGTTTAACTAAATTAAAAT
GTTGGAAAATCATACCTATATTTCGGCGCATTTCTAATAATGCTTTACCATGGGCTTTAGTGATTGATTTACCTTGGATGAAAATTTC
ACCTGACGTGATATTATGCAAACGATTTACAGATCTTAATAACGTGGATTTCCCAGCACCAGATAGTCCGACAATAACTGCAAATTC
ACCTTTTTCAATATTTAAGTTAATATTTTTCAAGCCTACATGACCGTTAGGATAGACTTTACTGACGTTTTTAAATTCGATTTGACTCA
TGTTTGACACCTTTCTTTAATAAAGAAAAGCCAAGGTAAACTGAAATATCATAGACATTGGGTTTCTGTGTACAAATGGTTTCAAAT
GTACAGCGACACCTTTCTATATCTGTTTCAGTTTAGCGCCTAGCTTTTCAATATTAATCTAGAATATATCTATTGAACGAAAGCTTTT
AATACCAAATTCGCTAATGATTCATTGGTTAAATAATGATTATTTCATATCTTTAACTAATTTTTCGTACTCTCTTACAATGTCGAAA
TTTGAATCTTTCGTTTCTGTGTATCCTTCATGTGAATAAACTTCGCTAATAATTTTGTGACCTTCTTTTGATTTAGCAATGTCTATAAA
AGCTTTTTTCAATTTTTCTTGAAAATCTTTATCCATATCTGGTCTTACAGAAATTGTGTCATTCGGAATAGCTTGTGTTAATTTTAAAA
TTCGTGTGTCTTTAAATACATTTGGTTGGTCTTTTTTCACAGTATTACGTGCATCGTTAAATACAGCTGCAGCATCTACATCTCCATTT
AATAATGAGATAACTGCTTGGTCATGACCTTTAACATTCACAATTTTCATATCTTTAGTTGCATTAATACCTGCTTCGTTTTTTAACAT
CGCAAGTGGGAATGTATATCCAGCAGTTGATGTTACATCTTGTAAGGCAATTTTCTTACCTTTTAAATCTTTCAAGCTTTTAATTTTT
GAGTCTTTTTTAACAAGAATTTCTGATTTATAGCTATCTACAAGTTCTTTACTTGCTGAACCATCTTCTTTTACACCGAAACGTTGTGC
TTGTAATAATAAATCAGCTGCTTTTTGATCATGTGCTAATGTGTATGCCGTTGGTGGTAAGAAACCAACATCAACTTTTTTAGACTTC
ATAGCTTCAACAATTGTATTGTAGTTAGTTGATACAGACACTTTAACTGGAATCCCTAATTCTTTAGATAGTAATTTTTCTAATGGTT

TTGCTTTAGCTTCTAATGTTCCAGCATTTTGCGAAGGTACAAATTGAACGGTTAATTCTTTAGGTTTGTATCCTCCTGATTTAGAATC
CGAATCATTACTAGCGTTCTTTTGATTATCTAAAGAACTTGAATTTCCACATGCTGCTGCAAAAACAATGACTGCTAACATTAATAC
AAATAAACACTTAAAATTTTTCATTTGATAACTGTCCCCTTCACATTCTTAATCGTGTAATATGTATGCAATGAAATTACACTTATAG
CGTACCACATTTAAATGAGAAAAAGTATTTTATAAAAAATGAATTTTAATAACTTTCTTTAAGAGTTTGATAAGGTTTTGTAAATTTT
TCGTTAAATTGCCCATTTCAAACATATTTTTATCCTATAATTCAAGAACAAATTTATTTTAAAAGGAGCCTCACAAAATGAAAAAAA
TATATAAGTCATTAACTGTCTCTGCAATTGTTGCAACGGTATCATTAAGTGCTTTACCGCAATCTTTAGCTATAACGCATGAATCGCA
ACCTACAAAGCAACAGCGAACGGTATTATTCGATCGTTCTCATGGTCAAACAGCTGGTGCTGCAGATTGGGTTAGTGATGGTGCATT
TTCAGATTATGCGGATTCAATACAAAAACAAGGTTATGACGTTAAAGCTATTGATGGTCATTCGAACATAACAGAAGCAAGTTTGA
AAATTTCCAAAATATTTGTAATTCCTGAGGCTAACATTCCTTTCAAAGAATCAGAACAGGCAGCAATTGTTAACTATGTGAAACAAG
GTGGCAATGTTGTCTTTATTTCAGATCATTACAATGCTGACCGAAATTTAAATCGTATTGATTCATCAGAGGCAATGAATGGTTATC
GACGTGGAGCGTATGAAGATATGTCGAAAGGTATGAATGCAGAAGAAAAAAGTTCTACTACAATGCAAGGTGTGAAAAGTTCAGA
TTGGTTATCTACAAACTTTGGCGTACGTTTTCGATATAATGCACTAGGTGATTTAAATACGAGCAATATTGTTTCTTCAAAAGAAAGT
TTCGGTATTACTGAAGGTGTGAAATCTGTCTCTATGCATGCCGGATCGACATTAGCAATTACTAATCCAGAGAAAGCAAAAGGTATT
GTGTATACACCAGAACAATTGCCAGCGAAAAGTAAATGGTCACATGCTGTAGATCAAGGTATTTATAATGGGGGCGGTAAAGCAGA
AGGCCCCTATGTAGCAATTTCTAAAGTTGGAAAAGGTAAAGCAGCATTTATCGGTGATTCATCACTTGTGGAAGATAGTTCGCCCAA
ATATGTAAGAGAAGATAATGGAGAAAAGAAGAAAACATATGATGGTTTTAAAGAACAAGACAACGGTAAGCTATTAAATAATATA
ACGGCTTGGATGTCTAAAGATAATGATGGGAAATCACTTAAGGCGAGTGGCCTAACATTAGATACAAAGACTAAGTTGCTTGATTT
TGAACGACCAGAGCGTTCAACTGAGCCTGAAAAAGAGCCATGGTCACAACCGCCGAGTGGTTATAAATGGTATGATCCAACAACAT
TTAAAGCAGGTAGTTATGGCAGCGAAAAAGGCGCAGATCCTCAGCCAAACACACCAGATGATCATACACCACCAAATCAGAACGA
AAAAGTAACATTTGATATCCCGCAAAATGTTTCTGTAAATGAGCCATTTGAAATGACAATACATTTAAAAGGATTTGAAGCAAATC
AAACACTTGAAAATCTTAGAGTTGGTATTTACAAAGAAGGCGGACGTCAAATCGGACAATTTTCAAGTAAAGATAACGATTATAAC
CCACCAGGTTACAGTACTTTGCCAACAGTTAAAGCAGATGAAAACGGAAATGTCACAATTAAGGTCAATGCTAAAGTACTTGAAAG
TATGGAAGGTTCAAAGATTCGTTTAAAACTCGGTGACAAAACCTTGATTACAACAGACTTCAAATAAATATATAATAGAAATAAGA
AAGATGTTTGTGATTGAAGGAGTGAGTGAGGATGTCAAACATAGCATTTTATGTCGTGAGTGACGTACATGGTTATATTTCCCAAC
AGATTTTACGAGTAGAAATCAATATCAACCTATGGGATTGTTACTAGCGAATCATGTTATAGAACAAGACAGAAGGCAGTATGACC
AAAGTTTTAAAATAGATAATGGTGATTTTTTGCAAGGGTCACCATTTTGTAATTACTTAATCGCGCATAGCGGCAGTAGCCAGCCTT
TAGTTGATTTTTATAATCGAATGGCATTCAACTTTGGTACGCTTGGTAATCATGAATTTAATTATGGATTACCATACTTAAAAGACAC
TTTACGCAGACTCAATTATCCAGTTTTGTGCGCTAATATTTATGAAAATGATAGTACATTGACTGATAACGGTGTGCAGTATTTTCAG
GTTGGAGATCAAACGGTTGGTGTGATAGGTTTAACGACACAATTTATTCCCCATTGGGAACAACCAGAGCATATTCAGTCACTTACG
TTTCATAGTGCTTTTGAAACACTTCAACAACACTTACCTGAAATGAAGCGACATGCAGATATCATTGTGGTTTGTTACCATGGTGGA
TTTGAAAAGGATTTAGAAAGTGGTATGCCGACCGAAGTGTTAACGGGTGAAAATGAAGGATATGCCATGTTAGAAGCATTTTCTAA
AGATATAGATATCTTTATTACGGGTCACCAACATCGACAAATTGCTGAAAGATTTAAGCAAACGGCTGTGATTCAACCTGGTACGA
GAGGTACAACTGTAGGCAAAGTTGTCTTGAGTACTGATGAATATGAAAATGTATCCGTTGAATCATGTGAATTACTTCCTGTTATAG
ATGATTCCACATTTACTATTGATGAAGATGACCAACATTTACGAAAGCAGTTAGAGGACTGGTTAGATTACGAAATTACTACATTGC
CATATGATATGACGATTAATCATGCATTTGAGGCACGTGTGGCACCGCATCCTTTTACTAATTTTATGAATTACGCTTTATTAGAAAA
AAGTGGTGCAGATGTTGCCTGTACAGCTTTGTTTGATTCTGCTAGTGGTTTCAAGCAAGTCGTGACGATGCGAGATGTTATTAACAA
TTACCCATTTCCAAATACATTTAAAGTTTTAGCTGTAAGTGGTGCCAAACTTAAAGAAGCCATTGAACGATCAGCAGAATATTTTGA
CGTGAAAAATGATGAAGTAAGTGTGAGCGCAGACTTCCTTGAACCCAAACCCCAACACTTTAATTATGATATATATGGTGGCGTAA
GTTATAACATTCATGTTGGAAGGCCAAAGGGACAACGTGTGAGCAATATGATGATCCAAGGGCACGCAGTTGATTTAAAGCAGACA
TATACAATTTGTGTAAATAATTATCGTGCAGTTGGCGGTGGTCAGTATGATATGTATATCGACGCGCCAGTTGTAAAAGATATTCAA
GTTGAAGGCGCACAATTACTTATTGATTTTTTATCAAATAATAATTTGATGCGCATCCCGCAAGTTGTTGATTTTAAAGTTGAAAAGT
GACGGATATATGTAAAACTTACTTGATTTTAATTGGGTAATTTTTTGTTTTTGTTTAAACTAACCACTCTTAAACGTCAATATACTTTA
AGAAAAATGTTGATTTTACAAAAAATGAGGCTTATATTTTATATAACTTAATATTATGAAGTGATTAATTTGGAAACAATTAAACAA
GAAGTAAGAGAATCTATTGTTAAAGGAATTATCGCGGGATATAAGGACTATGTTAATGAGAGAAATGAAGTCAAGAAACGTATGGT
GATTAGTGATGCCTATGCCTTTACTAAATCTAATCATATTGAAAGTCAAGTTGCTAAGCATTTGGAAAATTTTGTTAAGTATATTAAA
GAAAATGCTGGACCGTCATGGAAGTATTTAAAGTTTATATTTAATATGGATGAAGAGAAAAATAATATCATGTTTATATTGAAAAAT
GAAGATTACTTTGATGAAAAAAATGTAAGCGTAGGGAAGAGTTTAGTTGCCGATAAAAATCAAAAAAGTAAAAACTATCTAGAGG
GACTTATGGCTAAGAATAGGGACATTAATTTTGATACTGTTGAAGAAGATTTTAAAATTAGGCATCAAATGACTGCAGATAGTATAT
TATTTAATATAAAAGAAGGCAGTAATAGAGATATCAATTCTTATTTTTTAATTATTACATATCGTATTGATAAAGAATCAAAGCAAT
TAGCTGCAATCAAACAATGGTTACCAAATCCAGAAACAAATTCTGCAATTATGGTTGATGATTTGATAGAGTTGATAGAAAAAGTA
ATAGTAGAAAGAGAAGATTATCATATTGACGAAGAAGAGTTAGAAGTACTTAAAAACGATGGAGAGTTAGAACTCATAGATGTGG
AACATGCATTTGGTATCAGTATCGATGAAGGTAACGATATGATTGAAAGTGAAAGGTGAAGTTAATGTTTATTGGCAAGAATTTAG
AATACGTGAGAAAGCTTAATGCTCTAAGTAGAAAAGAATTATCAGAAAAAATAAATGTGAGTGAACAAGCAATTTGGCAATATGA
AACAAAAAATATGATGCCAGAAATTAGTAAAATTTATGATATGACATCTATTTTTAATGTTAAATCTAGCTACTTTATTAGCGAGCA
GCCCGAAGAGCTCCTAATAAATTCTGTCGATAAACATAGTATAGCTTTTAGAGCAAAAAATTATAAAGTATCTACGAAACTATTAA
ATAAGCAATATTATCAAGCAATGTATTTATCTAATTTAACTAACTACTTATTTTCCTTTGTAAAGATACCGGATAATATTATTTTATC
GTTAATAAATAATTTAGATGACTTGTTGAATGGCAATTTAGAATCACTAAATAAAAAAGAAAGCATAAAAGAAACTGCTAAAGTTG
TGAGGGCTAAAATTCTTCAAGACGAAAGCAATGAAGCACTATTATTTATGCTTGAAAAAGCGGGTATAGTTATATACGAAAAGCGA
ATTAATGATAGCATAGACGCATATAGTTTTTGGAGTAAAGATCTGACACCTTTTATTATACTGGGTACAAATAAGGGGGTAGCAGTT
AGAAGGAATTTTGATCTTGCACATGAGCTGGGACATCTCGTTCTACATCGTCATATTCAATTCGACTTATTATCTCCTGAAGAATATA
AGACAATTGAACATGAGGCAGATATTTTCGCCTCTGAATTTTTACTACCTGAAGAAGCGTTTAAAAAAGACTTTGATCAAATGACCA
AAAAATCAAATCCTGACTATTTAGCTGTATTGAAAGAAAAATGGTATGTTTCTATACAAGCAATTGCAATGAGAGCGTATTATCTAG
GCCTAATGTCTAGTACTCAGTACAGATATTTTTGGGCATCATTAAATAAAAAAGGCTATAAAAGTAAAGAGCCTTTAGACGACGTG
ATTGAAATGTCACGTCCTGTCAAAATGAATAGTTTATTAAAGTTGTATTTTGATAGAAATTTACTGACACCACAAAAATTATTGAAT
TATTTAAAAGTCGATGAAACATTTTTGAATCATCTAGCAGGTATTAATTTAAAACTCTTTAAGGATTATGTTAATGAAAATAGAGAA
TATAATATAACGAATCTATATAAATAAGGCACCAATATTTAAAACTCGAGCTGAAAAGATCATCATTTCAGTTCGAGTTTTTTTGTG
TAACATCTGTTGATAGGTAGCGACAAGGACCTTTTAATGTTGTGTTTTGTGCGATATAACAAGCTTTTTAGGCTATTAAAGAATATTC
AGAATTAAATATATAATTATGAATAAATTATGTCATTGAAAAAATTATGATTATAAAATCATTCTAAAAACACCGAAATAACAATG
ATTTCATGAAAACATTTATTTTAAAATTTGATATTTGTTCAAATAATATTCGAAATTAAACTTTTTTGTATAGAATTTTCTTTATATCC
TGAGAGACATGTACTATAATGTTTGTGAAATAATTCACAAAGTATAAAGGAGTGGTTGTATATGTTAACTATACCTGAAAAAGAAA
ATCGTGGATCGAAAGAACAAGAAGTGGCAATTATGATTGATGCTCTAGCTGACAAAGGGAAAAAAGCATTAGAAGCATTATCTAA

AAAGTCACAAGAAGAAATTGATCATATTGTTCATCAAATGAGCTTAGCAGCTGTTGATCAACATATGGTGCTAGCAAAATTAGCAC
ATGAAGAAACTGGAAGAGGTATATACGAAGATAAAGCGATTAAAAATTTATACGCTTCTGAATATATATGGAATTCAATAAAAGAC
AATAAGACAGTAGGGATTATTGGTGAAGATAAAGAAAAAGGATTAACGTATGTAGCGGAACCAATTGGTGTTATTTGTGGTGTTAC
GCCAACAACAAATCCTACGTCGACAACTATTTTTAAAGCGATGATTGCAATTAAGACAGGAAATCCAATCATTTTTGCATTCCATCC
AAGTGCACAAGAATCGTCGAAGCGTGCAGCAGAAGTTGTATTAGAAGCGGCAATGAAGGCAGGTGCACCTAAAGATATTATTCAGT
GGATTGAAGTGCCTTCTATCGAAGCAACAAAACAATTAATGAATCACAAAGGTATTGCATTAGTTCTAGCAACAGGTGGTTCGGGC
ATGGTTAAGTCTGCATATTCAACTGGCAAACCGGCATTAGGTGTGGGACCAGGTAACGTGCCGTCTTACATTGAAAAAACAGCACA
CATTAAACGTGCAGTAAATGATATCATTGGTTCAAAAACATTTGATAATGGTATGATTTGTGCTTCTGAACAAGTTGTAGTCATTGA
TAAAGAAATTTATAAAGACGTTACTAATGAATTTAAAGCACATCAAGCATATTTTGTTAAAAAAGATGAATTACAACGCTTAGAAA
ATGCAATTATGAATGAACAAAAAACAGGTATTAAGCCTGATATTGTCGGTAAATCTGCAGTTGAAATAGCTGAATTAGCAGGTATA
CCTGTCCCCGAAAATACAAAACTTATCATAGCCGAAATTAGCGGTGTAGGTTCAGACTATCCGTTATCTCGTGAAAAATTATCTCCA
GTATTAGCCTTAGTAAAAGCCCAATCTACAAAACAAGCATTTCAAATTTGTGAAGACACACTACATTTTGGTGGATTAGGACACACA
GCCGTTATCCATACAGAAGATGAAACATTACAAAAAGATTTTGGACTAAGAATGAAAGCTTGTCGTGTACTTGTAAATACACCATC
AGCGGTTGGAGGTATTGGTGATATGTATAACGAATTGATTCCGTCTTTAACATTAGGTTGTGGTTCCTACGGTAGAAACTCAATTTC
ACATAATGTTAGTGCGACAGATTTATTAAACATTAAAACGATTGCTAAACGACGTAATAATACTCAAATTTTCAAGGTGCCTGCTCA
AATTTATTTTGAAGAAAATGCAATCATGAGTCTAACAACAATGGACAAGATTGAAAAAGTGATGATTGTCTGTGACCCTGGTATGG
TAGAATTCGGTTATACAAAAACAGTTGAGAATGTATTAAGACAAAGAACGGAACAGCCTCAAATTAAAATATTTAGCGAAGTCGAA
CCGAACCCATCAACTAATACAGTATATAAAGGTCTGGAAATGATGGTTGATTTCCAACCGGATACAATCATTGCACTTGGTGGTGGT
TCAGCGATGGATGCTGCAAAAGCAATGTGGATGTTCTTTGAACACCCTGAGACATCATTCTTCGGTGCTAAACAAAAGTTCCTAGAC
ATCGGTAAACGTACTTATAAAATAGGCATGCCTGAAAATGCGACGTTCATTTGTATCCCTACGACATCAGGTACAGGTTCAGAAGTA
ACACCATTTGCAGTTATCACAGATAGTGAAACAAATGTAAAATATCCGTTGGCTGATTTTGCTTTAACACCTGACGTTGCAATTATT
GACCCTCAATTTGTGATGAGTGTGCCAAAAAGCGTTACAGCAGATACAGGAATGGATGTACTAACGCATGCAATGGAATCATATGT
ATCTGTAATGGCTTCAGACTATACAAGAGGTTTGAGTCTACAAGCGATTAAATTGACGTTCGAATATTTAAAATCATCTGTTGAAAA
GGGTGATAAAGTTTCAAGAGAGAAAATGCATAACGCATCAACTTTGGCTGGTATGGCATTTGCAAATGCATTCTTAGGTATTGCACA
CTCAATTGCACATAAAATTGGTGGCGAATATGGTATTCCGCATGGTAGAGCGAATGCGATATTACTACCGCATATTATCCGTTATAA
TGCCAAAGACCCGCAAAAACATGCATTATTCCCTAAATATGAGTTCTTCAGAGCAGATACAGATTATGCAGATATTGCCAAATTCTT
AGGATTAAAAGGGAATACGACAGAAGCACTCGTAGAATCATTAGCTAAAGCTGTCTACGAATTAGGTCAATCAGTCGGAATTGAAA
TGAATTTGAAATCACAAGGTGTGTCTGAAGAAGAATTAAATGAGTCAATTGATAGAATGGCAGAGCTCGCATTTGAAGATCAATGT
ACAACTGCTAATCCTAAAGAAGCACTAATCAGTGAAATCAAAGATATCATTCAAACATCATATGATTATAAGCAATAATCTATCTG
ATAATAATCATATAACTCACCTGAAATTACAAAAGTAAAAAATGCCACATAAACTTTAAGTCGATAATCATTATACGGTTATCGGCT
TTTATTTATTGCCAAATCTTCAGAGAGATACAAACTAGACAATCATTTTTTTAAATAAAGAAAATATTAAGATTGATACTCATTTCAC
AAACTATTACTACTTTAGAGTATAATTATTTTTAATTTCATATAAATAAAAAGGCGAAAATAATGCGGTTTAAAAGTAATTAATTGT
TTAAACGATATGTAATATGTAAATACTATATATATAATACCAATTTTAATGAAAATTTTTAAGGGAGGTAAATAATGGAAAGTACAT
TAGAATTAACAAAAATTAAAGAAGTATTACAAAAAAACTTGAAGATTTTAATTATTTTACCGCTATTATTTTTAATTATTAGCGCTAT
TGTTACATTTTTCGTCTTATCACCTAAATATCAAGCTAATACTCAAATCTTAGTGAATCAAACTAAGGGTGACAATCCTCAGTTTATG
GCACAAGAGGTTCAAAGTAATATTCAACTTGTAAATACGTATAAAGAAATTGTTAAAAGTCCTAGAATTTTAGATGAGGTGTCAAA
GGACTTAAATGATAAGTATTCACCATCTAAATTGTCGAGTATGTTGACAATTACAAACCAAGAAAATACGCAACTTATCAACATCCA
AGTTAAAAGTGGTCATAAACAAGATTCGGAAAAAATTGCGAATAGCTTCGCTAAAGTTACAAGTAAACAAATTCCGAAGATTATGA
GTGTGGATAACGTATCAATTTTATCTAAAGCAGACGGTACAGCAGTTAAAGTCGCACCAAAAACTGTAGTGAATCTAATCGGTGCA
TTCTTTTTAGGATTAGTTGTCGCGCTTATATATATCTTCTTCAAAGTAATTTTCGATAAGCGAATTAAAGATGAAGAAGATGTAGAG
AAAGAATTAGGATTGCCTGTATTGGGTTCAATTCAAAAATTTAATTAAGGATGGTTGCTACTTATGTCAAAAAAGGAAAATACGAC
AACAACACTATTTGTATATGAAAAACCAAAATCAACAATTAGTGAAAAGTTTCGAGGTATACGTTCAAACATCATGTTTTCAAAAG
CAAATGGTGAAGTAAAGCGCTTATTGGTTACTTCTGAAAAGCCTGGTGCAGGTAAAAGTACAGTTGTATCGAATGTAGCGATTACTT
ATGCACAAGCAGGCTATAAGACATTAGTTATTGATGGCGATATGCGTAAGCCAACACAAAACTATATTTTTAATGAGCAAAATAAT
AATGGACTATCAAGCTTAATCATTGGTCGAACGACTATGTCAGAAGCAATTACGTCGACAGAAATTGAAAATTTAGATTTGCTAAC
AGCTGGCCCTGTACCTCCAAATCCATCTGAGTTAATTGGGTCTGAAAGGTTCAAAGAATTAGTTGATCTGTTTAATAAACGTTACGA
CATTATTATTGTCGATACACCGCCAGTTAATACTGTGACTGATGCACAACTATATGCGCGTGCTATTAAAGATAGTCTGTTAGTAATT
GATAGTGAAAAAAATGATAAAAATGAAGTTAAAAAAGCAAAAGCACTTATGGAAAAAGCAGGCAGTAACATTCTAGGTGTCATTT
TGAACAAGACAAAGGTCGATAAATCTTCTAGTTATTATCACTATTATGGAGATGAATAAGTATGATTGATATTCATAACCATATATT
GCCTAATATCGATGACGGTCCGACAAATGAAACAGAGATGCTGGATCTTTTAAAACAAGCGACAACACAAGGTGTTACAGAAATCA
TTGTAACATCACATCACTTACATCCTCGATATACCACACCTATAGAAAAAGTGAAATCATGTTTAAACCATATTGAAAGCTTAGAGG
AAGTACAAGCACTAAATCTAAAGTTTTATTATGGTCAGGAAATAAGAATTACCGATCAAATTCTTAATGATATTGATCGAAAAGTTA
TTACCGGTATTAATGATTCACGCTATTTACTAATAGAATTTCCATCAAATGAAGTTCCACACTATACTGATCAATTATTTTTCGAATT
ACAGAGTAAAGGCTTTGTACCGATTATTGCACATCCAGAGCGGAATAAAGCAATAAGTCAAAACCTTGACATACTATACGATTTAA
TTAACAAAGGTGCTTTAAGTCAAGTGACAACGGCGTCATTAGCGGGTATTTCCGGTAAAAAAATTAGAAAATTAGCAATTCAAATG
ATTGAAAACAATCTGACACATTTCATCGGTTCAGATGCGCATAACACAGAAATCAGACCGTTCTTAATGAAAGACTTATTTAATGAT
AAGAAATTACGTGATTATTATGAAGATATGAACGGATTTATTAGTAATGCGAAGTTAGTTGTTGATGATAAAAAAATTCCTAAACG
AATGCCACAACAAGATTATAAACAGAAAAGATGGTTTGGGTTATAAACAGCAAATGAGGGGTTTTATGGCACATTTATCTGTGAAA
TTGCGGCTTTTAATACTAGTATTAATCGATTCACTGATAGTGACATTTTCAGTATTCGTAAGTTATTACATTTTAGAACCGTATTTCA
AAACATATTCTGTCAAATTATTAATATTGGCAGCTATATCACTATTCATATCGCATCATATTTCAGCATTTATTTTTAATATGTATCAT
CGAGCGTGGGAATATGCCAGTGTGAGTGAATTGATTTTAATTGTTAAAGCTGTGACGACATCTATCGTTATTACGATGGTGGTCGTG
ACAATTGTTACAGGCAATAGACCGTTTTTTAGATTGTATTTAATTACTTGGATGATGCACTTGATTTTAATAGGTGGCTCAAGGTTAT
TTTGGCGTATTTATCGGAAATACCTTGGAGGTAAGTCATTTAATAAGAAGCCAACTTTAGTTGTTGGTGCTGGTCAAGCAGGTTCAA
TGCTGATTAGACAAATGTTGAAAAGTGACGAAATGAAACTTGAACCGGTATTAGCAGTCGATGATGACGAACATAAACGCAATATC
ACAATTACTGAGGGTGTAAAAGTCCAAGGTAAAATTGCGGATATTCCAGAACTAGTGAGGAAATATAAGATTAAAAAAATCATCAT
TGCAATTCCAACTATTGGTCAAGAGCGTTTGAAAGAAATTAATAATATTTGCCATATGGATGGCGTTGAGTTATTGAAAATGCCAAA
TATAGAAGACGTCATGTCTGGTGAGTTAGAAGTGAACCAACTTAAAAAAGTTGAAGTAGAAGATTTACTAGGCAGAGATCCTGTTG
AATTAGATATGGATATGATATCAAATGAATTGACGAATAAAACTATTTTAGTTACGGGTGCAGGTGGTTCAATAGGATCAGAAATTT
GTAGACAAGTTTGTAATTTCTATCCAGAACGTATTATTCTACTTGGCCATGGTGAAAACAGTATTTATTTAATCAATCGTGAATTGCG
AAATCGCTTCGGAAAAAATGTTGATATCGTTCCTATTATAGCGGATGTGCAAAATAGAGCGCGTATGTTTGAAATTATGGAAACGTA
TAAACCATACGCAGTTTATCATGCAGCAGCACACAAGCACGTGCCGTTAATGGAAGACAACCCTGAAGAAGCAGTACGTAATAATA

TTTTAGGTACGAAAAATACTGCTGAAGCTGCTAAAAATGCAGAGGTAAAGAAATTCGTTATGATTTCTACGGATAAAGCCGTTAAT
CCGCCTAATGTCATGGGCGCTTCAAAGCGAATTGCAGAAATGATTATTCAAAGTTTAAATGATGAAACGCATCGAACAAATTTTGTT
GCAGTGAGATTTGGTAATGTACTTGGATCGAGAGGATCTGTGATTCCACTTTTCAAAAGTCAAATTGAAGAAGGTGGGCCAGTTACT
GTGACACATCCTGAAATGACACGTTACTTTATGACAATTCCTGAAGCTTCTAGACTAGTTTTGCAGGCAGGGGCATTAGCAGAAGGT
GGCGAAGTATTTGTGCTAGATATGGGAGAACCAGTGAAAATTGTTGATTTGGCACGTAATTTAATTAAGCTAAGTGGTAAAAAAGA
GGACGACATACGCATTACTTATACAGGGATTAGACCCGGCGAAAAAATGTTTGAAGAGCTTATGAATAAAGATGAAGTTCATCCTG
AACAAGTATTTGAAAAAATTTATCGTGGCAAAGTACAACATATGAAATGTAATGAAGTTGAAGCCATTATTCAAGACATCGTCAAT
GACTTTAGTAAAGAAAAAATTATTAACTATGCCAATGGCAAAAAGGGAGATAATTATGTTCGATGACAAAATTTTATTAATTACTG
GGGGCACAGGATCATTCGGTAATGCTGTTATGAAACGGTTTTTAGATTCTAATATTAAAGAAATTCGTATTTTTTCACGCGATGAGA
AAAAACAAGATGACATTCGAAAAAAATATAATAATTCAAAATTAAAGTTCTACATTGGTGATGTGCGTGATAGTCAAAGTGTAGAA
ACAGCAATGCGAGATGTTGATTACGTATTCCATGCAGCAGCTTTAAAACAAGTGCCGTCATGTGAATTCTTTCCAGTTGAGGCAGTG
AAGACAAATATTATTGGTACAGAAAATGTCTTACAAAGTGCTATTCATCAAAATGTTAAAAAAGTCATATGTTTATCTACAGATAAG
GCAGCGTATCCTATTAATGCTATGGGTATTTCAAAAGCAATGATGGAAAAAGTATTCGTAGCCAAATCAAGAAATATTCGTAGTGA
ACAAACGCTTATTTGTGGTACAAGATACGGTAATGTGATGGCTTCAAGAGGATCAGTAATACCTTTGTTTATCGACAAAATCAAAGC
TGGAGAACCTTTAACGATTACAGATCCTGATATGACAAGATTTTTAATGAGCTTAGAAGATGCGGTAGAACTAGTTGTTCATGCATT
TAAGCATGCAGAGACAGGAGATATTATGGTTCAAAAAGCACCAAGCTCAACGGTAGGGGATCTTGCGACCGCATTATTAGAATTGT
TTGAAGCTGATAATGCAATTGAAATCATTGGTACGCGACATGGAGAGAAAAAAGCAGAAACATTGTTGACGAGAGAAGAATACGC
ACAATGTGAAGATATGGGTGATTATTTTAGAGTGCCGGCAGACTCCAGAGATTTAAATTATAGTAATTATGTTGAACCCGGTAACGA
AAAGATTACGCAATCTTATGAATATAACTCCGATAATACACATATTTTAACGGTGGAAGAGATAAAAGAAAAACTTTTAACACTAG
AATATGTTAGAAACGAATTGAATGATTATAAAGCTTCAATGAGATAGGAGAGATTGACGTTGAATATTGTAATTACAGGAGCAAAA
GGTTTTGTAGGAAAAAACTTGAAAGCAGATTTAACATCAACGACAGATCATCATATTTTCGAAGTACATCGACAAACTAAAGAGGA
AGAATTAGAGTCAGCATTGTTGAAAGCAGACTTTGTCGTGCATTTAGCGGGTGTTAATCGACCTGAACATGACAAAGAATTCAGCTT
AGGAAACGTGAGTTATTTAGATCATGTACTTGATATATTAACTAGAAATACGAAAAAGCCAGCGATATTATTATTGTCTTCAATACA
AGCAACACAAGATAATCCTTATGGTGAGAGTAAGTTGCAAGGGGAACAGCTATTAAGAGAGTATGCCGAAGAGTATGGCAATACG
GTTTATATTTATCGCTGGCCAAATTTATTCGGCAAGTGGTGTAAGCCGAATTATAACTCAGTGATAGCAACATTTTGTTACAAAATT
GCACGTAACGAAGAGATTCAAGTTAATGATCGGAATGTTGAACTAACGCTAAACTACGTGGATGATATCGTCGCTGAAATAAAGCG
TGCTATTGAAGGAACTCCAACGATTGAAAATGGTGTACCTACAGTACCAAACGTATTTAAAGTGACATTGGGAGAAATTGTAGATT
TATTATACAAGTTCAAACAGTCACGTCTCGATCGAACATTGCCGAAATTAGATAACTTGTTTGAAAAAGATTTGTATAGTACGTATT
TAAGCTATCTACCTAGTACAGACTTTAGTTATCCCTTACTTATGAATGTGGATGATAGGGGTTCTTTTACAGAATTTATAAAAACACC
GGATCGTGGTCAAGTTTCTGTAAATATTTCTAAGCCAGGTATTACTAAAGGTAATCACTGGCATCATACTAAAAACGAAAAATTTCT
AGTCGTATCAGGTAAAGGGGTAATTCGTTTTAGACATGTTAATGATGATGAAATCATTGAGTATTACGTTTCTGGCGACAAATTAGA
AGTTGTAGACATACCAGTAGGATACACACATAATATTGAAAATTTAGGTGACACAGATATGGTAACTATTATGTGGGTGAATGAAA
TGTTTGATCCAAATCAGCCAGATACGTATTTCTTGGAGGTATAGCGCATGGAAAAACTAAAATTAATGACAATAGTTGGTACAAGG
CCTGAAATCATTCGTTTATCATCAACGATTAAAGCATGTGATCAATATTTTAATCAGATATTAGTACACACTGGTCAAAATTATGATT
ATACATTGAATCAAATTTTCTTTGATGATTTGGAATTGAGACAACCGGACCACTACTTAGAGGCAGTTGGAAGTAACCTTGGAGAAA
CGATGGGGAATATTATTGCGAAGACATATGACGTTTTATTACGCGAACAACCAGATGCACTTTTAATTCTTGGTGATACAAATAGTT
GTTTAGCAGCAGTATCTGCTAAACGATTAAAGATTCCTGTGTTCCACATGGAAGCGGGTAATAGATGCTTTGATCAGAATGTACCTG
AAGAAATCAATCGTAAAATTGTTGACCATGTCAGTGATGTGAATCTACCTTATACGGAACATAGCAGACGTTATTTATTAGATGAAG
GCTTCAATAAAGCGAATATCTTTGTGACAGGATCACCGATGACAGAAGTGATAGAAGCGCATCGAGATAAAATTAATCACAGTGAC
GTTTTAAATAAACTAGGATTAGAACCGCAACAATACATTTTAGTATCTGCGCATAGAGAAGAGAATATCGATAATGAAAAGAATTT
TAAATCATTAATGAATGCGATAAATGATATTGCCAAAAAGTATAAAATGCCTGTGATTTATTCAACGCATCCAAGAAGTTGGAAGA
AAATTGAAGAAAGTAAATTTGAATTTGATCCATTAGTTAAACAGTTAAAGCCATTTGGTTTCTTTGATTATAATGCATTGCAAAAAG
ATGCATTTGTTGTGCTATCAGATAGTGGAACATTGTCAGAAGAGTCGTCTATTTTGAAGTTCCCTGGTGTCCTTATTCGAACTTCCAC
AGAAAGACCGGAAGTACTAGATAAAGGTACGGTTATTGTAGGTGGTATTACCTATAACAATCTAATCCAATCCGTTGAACTAGCAA
GAGAGATGCAAAACAATAACGAACCGATGATTGATGCTATTGATTATAAAGACACTAACGTTTCGACAAAGGTAGTTAAAATTATT
CAAAGCTATAAAGATATTATCAATCGAAATACTTGGAGGAAATGACGATGAGGATAGCGATTGAAAAGATAATTGGTTTGCTGAAA
AACCAGTCCTCTAAAGAATCGAATGTTAAGATTCATCGCTTGGCGTATATTACAAACTCAAAATTTGATGGCAATAACTATATAGAT
AGATGGTGTAAAATCAGGAATTCTCACATTGGTGAATACAGTTATATTGGATTTGGTAGTGATTTTAATAATGTAGAAGTAGGAAGA
TATTGTTCGATATCTTCGGATGTAAAAATTGGGTTAGGAAAACATCCTACACACTTTTTTAGCTCATCACCGATTTTTTATTCTAATA
ATAATCCATTTAACATAAAGCAAAAGTTTATAGACTTTAATGACCAACCAAGCCGTACAACAATTAAAAATGATGTGTGGATTGGT
GCAAATGTAATTATTATGGATGGTTTAACAATAAATACTGGTGCAGTCATAGCAGCCGGCTCAGTTGTTACTAAAAATGTAGGAGC
ATATGAGGTTGTTGGTGGGGTTCCTGCAAAAGTGATTAAGAAGCGATTTGACAATAAAACAATTGAAAAACTTTTGGAAAGCAAGT
GGTGGGAGAAAACGCCTGATAAACTAAAAGGATTTTCGGTTGAATATTTAAATAAAAAGGATACTTAATGATATGAGAATTTTAAA
TATTGTATCGAGTAATATTGTTCAAGACCCAAGGGTACTTAAACAAATGGAAACAATTAAAGACGTTACGGATGATTATAAAATTG
TTGGAATGAATAATTCACAAGCTACTAATAAGCGATTGGAAAATTTAGATTGTAATTATCGTTTGTTAGGTAGCAAGGTAGATCCAA
AAAATATTCTTTCTAAATTAATTAAGCGTATAAGATTTGCAACAGGTGTTATCCGAGAAATTAAAGCTTATAAACCTGACGTGATTC
ATGCAAATGATTTCGACGTATTATTAATGGTCTATTTAAGCAATTATAAAAAAGCTAATATTGTTTATGATGCGCATGAAATATATG
CGAAAAATGCCTTTATTAATAAAGTTCCACTTATTTCAAAGTTTGTAGAAAGTATAGAAAAACACATAGTAAAACATCGTGTTAATG
CCTTCGTAACAGTAAGTCATGCAGCAAAAGAATATTATCAATCTAAAGGATATAAGAAGGAAGCGAATGTTATTACGAATGCACCT
ATTTTAAATGATAGCAGAGAATTTAAAGAAATCGAAAACTTTAAAGAAATTGTATATCAAGGTCAAATTGTAATGGACAGAGGATA
TGAAGAGTTTATTATTGCTTCATCAGCTTTTAAACAAAATGCTCCTTCATTCATAATTCGAGGGTTTGGTCCGCATGAAGAAGTGATA
AAAGAACTGATTAGTTATAACTCGGAAAATATTAGGTTGGATAAACCAGTTGAAGTAAAAGAATTGGTTGATAAGTTAGCAGAAAG
TAATGTTGGTGTTATCTTGACGAAACCTGTATCTATTAATTTTGAATATACAGTATCTAATAAAATTTTTGAATGTATACATGCTGGT
TTACCAGTAATTTTATCTCCTGTCAAAGAGCATATTTATCTCAATGAAAAATATAAATTTGGCATTGTTTTAAAGGAAGTTACGCCGT
TAGAAATTGAAAAGGCGGTTAGAAAATTAAGAGATAATCACGATTTGTTTAATCATTTACGTCAAAATGCAATTAAGGCGTCTAAA
ATTTTGAATTGGCAAATAGAAAGTGAACGATTAGTAGAATTATATAAATTTTAAAGAGAGGTAAACTATGAAATTTTTTGTACTTTG
TGCAATTATCAGCATGAACATATTTATAGTAATCTCTACATTTACTAAAGAAGTATTAGGGTTCCCTATAGAGCCGGTGTATTACTCT
ACCATGGTTGGTATAGCATTAATTACTACGGTGTTTGCTATTTATAAGATAATTGTCACGCAAGAAATTCCGCGAGGGTTAATATTA
TTAATTGCTATATGTTTGCTTTATCTAGCTTTTTATTATTTTTCACCAGATAAGGAAGAGAAACTAGCTAAAAATAATATTCTATTCTT
TTTAACATGGGCAGTTCCAGCGGCAATTAGTGGTATTTATATTAAATATATAAACAAGGCTACGGTAGAAAGATTTTTTAAATTAGT
ATTTTTCATATTTTCTATTTCATTTATTTTTGTAATTTTAATACCAAAACTTACAGGTGAGATACCTAGCTATATCAATTTTGGACTTA

TGAACTATCAAAACGCTTCGTACCTTTCAGCATTTACTGCCGGATTAGGCATTTATTTCATTATGAAAGGTTCAGTGAAACATAAGT
GGATATATGTTCTATTTACAATAATTGATATCCCTATTGTGTTTATACCAGGAGGGCGTGGAGGTGCTATTTTATTAATTCTTTACGG
CTTATTTGCATTTATACTTATTACGTTTAAAAGAGGAATACCTATTGCAGTAAAAAGCATTATGTATATTTTTGCATTAAGCATATCT
AGTGTATTGATTTACTTTCTTTTTACAAAAGGTTCGAATACTAGAACATTTTCATATCTACAAGGTGGAACACTTAATTTAGAAGGTA
CTTCTGGAAGAGGACCGATTTATGAAAAAGGTATTTACTTTATTCAACAAAGTCCGTTATTAGGCTATGGGCCATTTAACTATTATA
AACTAATCGGAAATATACCACATAACATCATTATTGAGTTGATTCTATCATTTGGCTTATTAGGGTTTTTTATCATAATGATTTGCAT
TTTGCTACTAGTTTATAAAATGATTAGGAACTATGATCCAAACACTATAGATTTACTCGTTATGTTTATAGCAATCTATCCAATCACA
TTATTAATGTTTAGTTCAAATTATTTAGTTGTAAGTGAATTTTGGTTTGTGTTGTTCTATTTTATTACAAAAGGACGGCGTCATCATGG
TTAAGAAAGTTTTTATTATGGATAGCGTAAAGACAATAATTGGTACGTTGCTTATAGCTTTAGGATTACAATTTTTAGCTTATCCAAT
TATTAATCAACGAGTAGGTAATGAAGCGTTTGGTTCTATTTTAACGATTTATACAATAATAACAATCACGAGTGTTGTATTAGGCAA
TACGCTTAACAATATACGATTAATTAATATGAATCTATACAAATCCAATCATTACTACTGGAAATTTGTGTCGATACTTTTAATTTCA
ATTCTGATTGAGAGTATAGCTTTAATTATTGTATTTCTTTACTTTTTTAATTTGAACACCATCGATATTATCTTTTTAATTCTACTTAAT
ATTTTAATGTGTTTAAGGATTTATCTGAATGTATTTTTTAGGATGACTTTAAAATATAATCAGATTTTGTATATTGCTCTTATTCAATT
TTTAGGTTTGCTGATAGGACTATTTCTATATTATTTAATCCAAAACTGGATTGTTTGTTTTATTACCAGTGAATTGTTTGCAACGATAT
ATACATTGGTTAAATTACGGGGATTAACTATAGGCGAGTATCAAAGTGAAGATAATAATGTGGTCAAAGATTATGTGATGCTACTG
AGTACAAATAGCCTTAATAATTTGAATCTCTACTTAGATAGATTAATCTTATTACCAATTATAGGTGGAACAGCTGTAACTATATCA
TTTCTTTCAACATTTATTGGGAAAATGTTAGCTACATTTCTGTATCCGATTAATAATGTAGTACTTTCATATATTTCTGTAAATGAAA
GCGACAATATAAAGAAGCAATATTTGAAAACTAATCTATTTGCTATAGCTGCACTATGTTTAGTCATGATTATATGTTATCCAATTA
CATTAATTATTGTCTCTTTACTGTATAACATTGATTCAAGTTTATATTCGAAGTTTATTATTTTAGGTAATATAGGTGTTTTATTCAAT
GCAGTGAGTATTATGATCCAAACTTTAAATACAAAACACGCATCAATAACATTACAAGCGAATTATATGACGCTTCACACGATTAC
ATTTATATTCATAACTATTTTAATGACAATTGCGTTTGGTCTAAATGGATTCTTTTGGACAACGCTGTTCAGCAACATTATTAAGTAT
GTGATTTTAAATATTATAGGTTTAAAGTCTAAATTCATTAATAAAAAGGACGTCGATTAGATGAGTGAAAAAAAGATTTTGATTTTA
TGTCAGTATTTTTATCCGGAATATGTATCTTCTGCGACGTTACCAACTCAATTGGCGGAAGATTTAATTGCGAATCACATTAATGTCG
ATGTCATGTGTGGATGGCCATATGAATATAGTAATCATAAACAGGTTTCTAAAACCGAGATGCATCGTGGTATTCGCATTCGACGTC
TCAAGTATTCGAGGTTTAATAACAAAAGTAAGGTTGGAAGGATCATCAATTTCTTTAGTTTATTTTCAAAATTCGTGATTAATATACC
TAAAATGTTGAAATATGATCAGATTCTTGTTTACTCTAATCCACCAATCTTGCCATTAATACCAGACGTTTTACACAGACTGCTTAAG
AAAAAATATTCTTTTGTGGTGTATGATATAGCACCTGATAATGCGATTAAGACAGGTGCAACTCGTCCAGGTAGCATGATTGATAAG
CTGATGCGTTACATTAATAGACATGTCTACAAGAATGCTGAAAATGTCATTGTCCTTGGTACGGAAATGAAAAACTACTTACTAAAT
CATCAAATTTCTAAAAATGCTGACAATATCCATGTGATTCCTAACTGGTATGACATGCGTCAATTACAAGACAATCGTATCTATAAT
GACACATTTAAAGCTTACCGTGAGCAATACGACAAAATTTTATTGTATAGCGGTAATATGGGGCAGTTACAGGATATGGAGACACT
TATCTCATTTTTAAAATTAAATAAGGATCAGCCTCAAACGTTAACAATACTTTGTGGTCATGGTAAGAAATTTGCAGATGTCAAAAC
GGCAATAGAAGACCATCGTATTGAAAATGTTAAAATGTTTGAGTTTTTAACAGGTACAGACTATGCTGACGTATTAAAAATTGCGG
ATGTATGTATTGCATCGCTGATTAAAGAAGGCGTCGGTTTAGGCGTGCCGAGCAAGAATTATGGCTACCTTGCAGCTAAGAAACCG
TTGGTACTCATCATGGATAAGCAATCTGATATCGTTCAACATGTTGAACAATATGATGCGGGTATCCAAATTGATAATGGCGATGCA
CATGCCATTTATAACTTCATCAACACTCACTCGAGTAAGGAATTGCACGAGATGGGTGAGCGCGCACATCAACTGTTTAAAGATAA
ATATACGAGAGAAATTAATACTATGAAGTATTACAATCTGTTGAAGTGAGGAGATAATTATGAAGCGATTATTCGATGTAGTGAGT
TCAATATATGGTTTAGTAGTTTTAAGTCCGATTCTGTTAATTACAGCATTACTAATTAAAATGGAATCACCTGGACCAGCCATTTTCA
AACAAAAAAGACCGACGATTAATAATGAATTGTTTAATATTTATAAGTTTAGATCAATGAAAATAGACACACCTAATGTTGCAACT
GATTTAATGGATTCAACATCGTATATAACAAAGACAGGGAAGGTCATTCGTAAGACCTCTATTGATGAATTGCCACAATTATTGAAT
GTTTTAAAAGGAGAAATGTCAATTGTAGGTCCTAGACCAGCGCTTTATAATCAATACGAATTAATCGAAAAACGTACAAAAGCGAA
CGTGCATACGATTAGACCAGGTGTGACAGGACTAGCTCAAGTGATGGGGAGAGATGATATTACTGATGATCAAAAAGTAGCGTATG
ATCATTATTACTTAACACATCAATCTATGATGCTTGATATGTATATCATATATAAAACAATTAAAAATATCGTTACTTCAGAAGGTGT
GCATCACTAATGAGAAAAAATATTTTAATTACAGGCGTTCATGGATATATCGGTAATGCTTTAAAAGATAAGCTTATTGAACAAGG
ACATCAAGTAGATCAAATTAATGTTAGGAATCAATTATGGAAGTCGACCTCGTTCAAAGATTATGATGTTTTAATTCATACAGCAGC
TTTGGTTCACAACAATTCACCTCAAGCAAGGCTATCTGATTATATGCAAGTGAATATGTTGCTGACGAAACAATTGGCACAAAAGGC
TAAAGCTGAAGACGTTAAACAATTTATTTTTATGAGTACTATGGCAGTTTATGGAAAAGAAGGTCAGGTTGGTAAATCAGATCAAA
TTGATACACAAACACCAATGAACCCTACGACCAACTATGGTATTTCCAAAAAGTTCGCTGAACAAGCATTACAAGAGTTGATTAGT
GATTCGTTTAAAGTAGCAATTGTGAGACCACCAATGATTTATGGTGCACATTGCCCAGGAAATTTCCAACGGTTAATACAATTGTCA
AAGCGACTGCCAATCATTCCCAATATTAACAATCAGCGCAGTGCATTATATATTACACATCTGACAGCATTTATTGATCAATTAATA
TCATTAGAAGTGACAGGCGTGTATCATCCTCAAGATAGTTTTTACTTTGATACATCGTCAGTAATGTATGAAATACGTCGCCAATCA
CATCGTAAAACGGTATTGATCAACATGCCTTCAGTGTTAAATAAGTATTTTAATAAGTTGTCGGTCTTTAGAAAATTATTTGGCAATT
TAATATACAGCAATACGTTATATGATAATAATAATGCACTTGAAGTTATTCCTGGAAAAATGTCACTTGTTATTGCGGACATCATGG
ATGAAACGACAACCAAAGATAAGGCATAAGTCATCTATTAAATAAAATCAACATACAAATCGTTTTATTTGGAGGTTATAGTATGA
AGTTAACAGTAGTTGGCTTAGGTTATATTGGTTTACCAACATCAATTATGTTTGCAAAACATGGCGTCGATGTGCTTGGTGTTGATAT
TAATCAGCAAACGATTGATAAGTTACAAAGTGGTCAAATTAGTATTGAAGAACCTGGATTACAAGAGGTTTATGAAGAGGTACTGT
CATCGGGAAAATTGAAGGTATCTACAACGCCAGAAGCATCTGATGTTTTTATCATTGCCGTTCCGACGCCGAATAATGATGATCAGT
ACCGGTCATGTGACATTTCGCTAGTTATGCGTGCATTAGATAGTATTTTATCATTTTTAGAAAAAGGAAATACTATTATTGTAGAGTC
GACAATTGCGCCTAAAACGATGGATGATTTTGTAAAACCAGTCATCGAAAATTTAGGATTTACAATAGGTGAAGATATTTATTTAGT
GCATTGTCCAGAACGTGTACTGCCAGGAAAAATTTTAGAAGAATTAGTTCATAACAATCGTATCATTGGCGGTGTGACTAAAGCTTG
TATTGAAGCGGGTAAATGTGTCTATCGCACATTCGTTCAGGGAGAAATGATTGAAACAGATGCACGTACTGCTGAAATGAGTAAGC
TAATGGAAAACACATATAGAGACGTGAACATTGCTTTAGCTAATGAATTAACAAAAATTTGCAACAACTTAAATATTAATGTATTA
GATGTGATTGAAATGGCAAACAAACATCCGCGTGTTAATATCCATCAACCTGGTCCAGGTGTAGGCGGTCATTGTTTAGCTGTTGAT
CCGTACTTTATTATTGCTAAAGACCCTGAAAATGCAAAGTTAATTCAAACTGGACGTGAAATTAATAATTCAATGCCGGCCTATGTT
GTTGATACAACGAAGCAAATCATCAAAGCGTTGAGCGGGAATAAAGTCACAGTCTTTGGTTTAACTTATAAAGGTGATGTTGATGA
TATAAGAGAATCGCCAGCATTTGATATTTATGAGCTATTAAATCAAGAACCAGACATAGAAGTATGTGCTTATGATCCACATGTTGA
ATTAGATTTTGTGGAACATGATATGTCACATGCTGTCAAAGACGCATCGCTAGTATTGATTTTAAGTGACCACTCAGAATTTAAAAA
TTTATCGGACAGTCATTTTGATAAAATGAAGCATAAAGTGATTTTTGATACAAAAAATGTTGTGAAATCATCATTTGAAGATGTATC
GTATTATAATTATGGCAATATATTTAATTTTATCGACAAATAAAATGTGTCAAACTAGGGCATACATGATTAAGGAAAGATAAGCTG
TCATGTGTTTGAACTTCAGAGAGGATAATGTTATGAAAAAAATTATGGTTATTTTCGGTACGAGACCCGAAGCAATAAAAATGGCA
CCATTAGTAAAAGAAATTGATCATAATGGGAACTTTGAAGCGAACATTGTGATTACAGCACAACATAGAGATATGTTAGATAGTGT
GTTAAGTATATTTGATATTCAAGCTGATCATGATTTAAATATTATGCAAGATCAACAAACGTTAGCGGGCCTTACGGCGAATGCGCT

TGCTAAACTTGATAGCATCATTAATGAGGAACAACCGGATATGATTTTAGTACATGGTGATACTACAACGACTTTTGTAGGAAGTTT
GGCAGCATTTTATCATCAAATTCCGGTCGGACATGTAGAAGCTGGACTTCGAACACATCAGAAATACTCACCATTTCCTGAAGAGTT
AAATCGAGTCATGGTAAGTAATATTGCTGAATTGAATTTTGCGCCAACAGTAATTGCAGCTAAAAATTTACTTTTTGAAAACAAAGA
CAAAGAGCGTATCTTTATTACTGGAAATACAGTTATTGACGCATTGTCAACAACAGTTCAAAATGATTTTGTTTCAACGATTATTAA
TAAACATAAAGGCAAGAAAGTTATTTTACTAACAGCGCATCGTCGTGAAAATATTGGGGAACCGATGCATCAGATTTTTAAAGCAG
TAAGAGATTTGGCAGATGAATATAAAGATGTTGTCTTCATTTATCCAATGCATCGTAATCCAAAGGTAAGAGCGATTGCCGAAAAA
TATTTATCTGGGAGAAATCGGATTGAATTAATTGAGCCATTAGATGCGATTGAGTTCCATAATTTTACAAATCAATCGTACCTCGTG
CTGACAGATTCTGGTGGTATTCAAGAGGAGGCTCCTACATTTGGAAAACCTGTGTTGGTATTAAGGAATCATACAGAGCGTCCCGA
AGGCGTTGAGGCGGGAACATCGAGAGTAATTGGCACAGATTATGACAATATTGTTCGAAATGTGAAACAATTGATTGAGGATGATG
AAGCGTATCAACGTATGAGTCAAGCGAATAATCCATATGGTGATGGACAAGCATCACGACGTATTTGTGAAGCAATAGAATATTAT
TTTGGATTGCGCACAGACAAGCCGGATGAATTCGTACCTTTACGTCACAAATAATAAAAAACCCCTAATCATGAAGTTGGTTTAGAC
AACCAGCGGTGACTAGGGGTTTTTAATATATTTATTTTTGATAGTGGTAGCCAATATCATATTTGAATACTTTATTTGATAATATTGG
ACTTTGCTGTCCATCGTCATCACTTTTTAAACGTACATTTTTATGAGCTTCTTTAAATACATCGGAATTCAACCAATTATTAAAGCTA
TCTTCAGATTCCCAAATAGTTAAGATTTTAACTTCGTCTGTATCCTCGGTATTTAATGTTTTAGTGACAAACATTTGTTGGAAGCCTT
CAATAGTTTCAATACCTTGTCTATTGTAAAAACGTTCAATCGTTTCTTCCGCACTGCCTTTTTGTAATTGTAATCTATTTTCTGCCATA
AACATGGGCAATCACTCCTCTATTTTATGATTTGATTTGGGTAATGTTTTTACAAATGTAAAGAGTACAGCGGTTTGTATGATAACCA
TTATGATTAATCCTACACGGACTGCAAGAACATCCACCATATAAATTGAAAAACCTATTACAATGTACAAGCTAATTAAAATTTTAA
TTTTCTGTTGTAGCGTGTAGCCTCGATGTAAATAAAAGTTTTCTACATATTCTTTATAAATTTTTTGATTAATAAGCCAATTGTAAAA
GCGATCTGAACTTCGAGCAAAACAAAAAACTGCTACGAGTAAAAAAGGGGTCGTTGGCAGTAAAGGTAATACGGCACCTGCAATA
CCAAGCGCTGTAAATATTAAGCCAATGACGATTAAAATAAGTCGCATTGAAAAAACTCCATTCTAGTACTAATGCGCATGTAATATT
GTTTTAGTAATATAACTCATGCTAAATATAATGTGTATGATAAGTGCAATGACTCAGTAAAATGAAACGTTGTTGAATTATTCTTGT
CCCATTAACGCATTTTAAGCGCGACTTTCATAACAACCAAACTATTTAATGAGAATTATTCTCAAGTATTATAGTTATATTATGTGTT
TTATTTTTGAAAAGTGCAATATGTTTTCGAAAATAAGATTATTTTTATGTGCAAAAACGACGCAAAAGTTTTAAAAATGAGACTTCT
GTGAGCTGATTATTTTATAAAATGTAAACGCTTACTATATAATGTGAATCATATCGTTTAAAAGCATTATTAAATATGATGCTAAGA
GATTTATATTATAGCCAATAAACAAAGGAGAGATAATATGGCAGTAAACGTTCGAGATTATATAGCAGAGAATTATGGTTTATTTAT
CAATGGGGAATTTGTTAAAGGTAGCAGTGACGAAACAATCGAAGTGACTAATCCAGCAACTGGAGAAACACTATCACATATTACAA
GAGCAAAAGATAAAGATGTCGATCATGCAGTCAAAGTGGCGCAAGAGGCATTTGAATCATGGTCATTAACTTCTAAATCAGAACGT
GCACAAATGTTGCGTGATATTGGTGATAAATTAATGGCACAAAAAGATAAAATTGCAATGATTGAAACATTAAATAATGGTAAACC
GATTCGTGAGACAACAGCAATTGATATTCCATTTGCTGCAAGACATTTCCATTATTTCGCAAGTGTTATTGAAACAGAAGAAGGTAC
AGTAAATGATATCGATAAAGACACAATGAGTATCGTACGACATGAGCCGATTGGCGTCGTAGGTGCTGTTGTTGCTTGGAACTTCCC
AATGCTATTAGCTGCATGGAAGATTGCGCCAGCCATTGCTGCAGGTAATACAATTGTGATTCAACCTTCGTCTTCAACACCATTAAG
TTTATTGGAAGTTGCTAAAATTTTCCAAGAGGTATTACCTAAAGGTGTTGTCAATATACTAACGGGTAAAGGTTCAGAATCAGGTAA
TGCAATTTTCAATCATGATGGTGTAGATAAATTATCATTTACGGGCTCAACTGATGTAGGTTATCAAGTTGCCGAAGCTGCAGCAAA
ACATCTAGTACCCGCTACATTAGAGCTTGGTGGTAAAAGCGCCAATATCATATTAGATGATGCTAATTTAGACCTTGCAGTTGAAGG
TATTCAGTTAGGTATTTTATTCAACCAAGGTGAAGTATGTAGTGCAGGTTCTCGATTATTAGTTCATGAAAAAATTTATGATCAATTG
GTGCCACGTTTACAAGAGGCATTTTCAAATATTAAAGTTGGAGATCCACAAGATGAAGCTACACAAATGGGTAGTCAAACTGGTAA
GGATCAATTAGATAAAATTCAATCATATATTGATGCAGCAAAAGAATCAGATGCACAAATTTTAGCAGGCGGTCATCGCTTAACTG
AAAATGGATTAGATAAAGGGTTCTTCTTTGAGCCGACATTAATTGCTGTGCCAGACAATCATCACAAATTAGCACAAGAAGAAATA
TTTGGACCAGTGTTAACAGTTATTAAAGTGAAGGACGATAAAGAAGCAATTGATATAGCTAATGATTCTGAGTATGGTTTAGCAGG
CGGTGTATTTTCTCAAAATATCACACGTGCATTAAATATTGCTAAAGCTGTACGTACAGGACGTATTTGGATTAACACTTACAACCA
AGTACCAGAAGGCGCACCATTTGGTGGTTATAAAAAATCAGGTATCGGTCGAGAAACTTATAAAGGTGCGTTAAGTAACTATCAAC
AAGTTAAAAATATTTATATTGATACAAGCAATGCTTTAAAAGGTTTGTACTAGAATAAATATCGTTTCTGAAGCGTGTTTGTAGGCA
GTCTAGCGGTAAGTCTTAGCATGTTTACGGAGTTGTTTAGATTTTAAGCGAAACAAAATATATAGGAACACGTATCATGATATTAGG
ATATAATGACTAAAATAATAGCAGTAGGATGGTTTTTAATTGCAAATCATCTTACTGCTGTTTTTAATTATGCTAATTTGCGATGCGG
TTATTATAAGGACAGAGTTGTTTATTAATTATGGTGATTTAGAAATATGAAGTTCGATATGCAAAGTCATCGTTTGTTTTAATATGTG
GAACAATCATTAAAGTTATTGTGATTTTTTGAACTTAATGAAACTAAACAATAAATTTGAGATACTTTTTTGTCATTTTTATGTAACT
AACACAATAATCTCGTACATTATTAAAATTTTCTATATGATAGGGATAAAGCAAAGCGCGAGTGTGCTGTAAAAGTTTTTCCAAGGT
GATATTACATAAAGATATAAAGGGTAAAGATTAATGAGTTGTCATGTAAATGACGATGATGTATAAATCATGGTTAATTACGGAAG
CATTAATATTAACCTGAGAAGCTATAAAGAATTATTTTTAAAAGCGACAATATTAAATACGACGCATTTATTTAGGAGTGGCAAACG
TATGAATGGGAAAAAGGCGAATACGATAAACAGATACAAATATTTTCATCATGTCAATCATCAAAAAAATTCAACAAAGTTCTAAAA
AGACGCTGTGGGCATCACTTATCATCACATTGTTATTTACGGTGATTGAATTTGTCGGAGGTTTAGTATCTAATTCATTGGCATTACT
GTCAGATTCATTTCATATGCTTAGTGATGTATTAGCACTTGGTTTATCTATGTTGGCCATTTATTTTGCAAGTAAGAAGCCGACTGCA
CGATACACATTTGGATATTTAAGATTTGAGATATTAGCTGCATTTTTAAATGGTTTAGCATTAATTGTAATTTCAATCTGGATTTTAT
ATGAAGCTATTGTACGTATTATTTATCCGCAACCAATTGAAAGTGGCATTATGTTTATGATTGCTAGTATTGGTTTACTCGTTAATAT
TATTTTGACAATTATCCTTGTAAGGTCTTTAAAACAAGAAGACAATATCAATATTCAAAGTGCATTATGGCATTTCATGGGAGACTT
ATTGAACTCTATTGGTGTCATCGTTGCAGTTGTATTGATTTACTTTACAGGATGGCGCATCATCGACCCAATCATTAGTATTGTAATT
TCACTCATCATTTTACGTGGTGGTTATAAAATTACGCGTAATGCGTGGTTAATTTTAATGGAAAGTGTGCCTCAACATTTGGATACTG
ATCAAATTATGGCAGATATTAAAAACATAGATGGCATATTAGATGTACATGAATTTCATTTGTGGAGTATTACAACAGAGCATTATT
CATTAAGTGCCCATGTTGTGTTAGATAAAAAATATGAGGGTGATGATTATCAAGCGATTGATCAAGTATCATCATTGTTGAAAGAAA
AATATGGCATTGCACATTCAACGTTGCAAATTGAAAACTTGCAATTGAATCCATTAGATGAGCCATACTTCGACAAATTAACATAAA
TAAAACATTGTAGCGCCTAAAACATTAATCTATGTCATAGGCGCACGTTTCGTTTTATACTTATGTTGCATCATTTAAATGATTTTCG
TCAATTTCTTTGATGCTATCTACATCTAACACGACATCTTTAGGTTTCAAAATATGAATATGTTTTTCATCATTTGTATGTAAAATGC
GTTCTATGATGTACCTTTGACCGGCCATTGTTTCTACAGCAATCTTTTTGTTTCTAGCTAAACTTGCTACGACAGATTCTTTATCCATA
ATGATAGCCCCCTATATATATGTTTATTTACTTATACCCTAACATGATTTTTATACTCTTTGAAAATATATTTTACAGAATTTTATCTA
AATATTTAAAAAAATAGCTTAATATCCTTGTAATCCGATAAGAATTATAGTAATATTTTTTCAACCATTGTTATAGGAGGTCTTATTA
ATGACATTATTTTTATTAGAAGCTAACAATCTTGATTTTGCATCAACGAAAGAAGAACTAGAAGCAAAGGCAGCATCACTATCTACG
AAGACAATTCCAACATTAATTGAAGTACAAGCTACTGAAAATTTAACTCATGGTTATTTTATTGTGGAAGCAAATGACGAAGCAGA
AGCTAAACAATTTTTAACAGAAGCAGATATTAGTATTCAATTAGTCAAAGAAGTACGCTTAGTTGGTAAAGATTTAGATGAAGTTA
AAAATGGTGATGCACATGTTGATTACCTTGTAACTTGGAACATTCCGGAAGGCATTACGATGGATCAATATTTAGCACGTAAAAAG
AAAAATTCTGTTCATTATGAAGAAGTGCCAGAAGTTGAATTTAAACGCACATATGTATGTGAAGATATGTCTAAATGTATTTGTTTA
TACAACGCACCTGATGAAGAAGCGGTACGTCGCGCGCGAAAAGCAGTTGATACACCGATTGATGGCATCGAAAAACTTTAATAAGA

CAACAAGTTGATGAGATATATGTATATAGGTTTGACGTGGATTTCGATTGCAGTTAATTAGAATAGCTCAATGCTATAAATGTAAGT
GGTTGATATGACGAAACTAATGAACTAAATGCAAGTATTGTCTAAAACAATCATTTTATTGAAATTTAGTAGAGCTGAAATTAATAT
AACGTCGTTAATTGAATAACGCTTATGTTATAAGAGCACTCATACCAAACCATAATCATCTATAGATATAACAATTCACGATATAAG
GGCTGTGTTTGGCATAGCCCTTTAGATATACACTTAATTCCTATTAAAATAGTAGGGATTAAAAGGGGGCTTGTCATGATTAAAATT
CAACAATTACAACATCACTTTGGATCACATAAAGTAATTCATAACTTTAATTTGGACATTAGCAAGGGAGAAATAGTCACTTTCATA
GGGAAAAGTGGTTGCGGAAAGTCTACTTTACTCAATATTATCGGTGGATTTATTCATCCATCGTCTGGTCGTGTCATTATTGATAACG
AAATTAAACAACAGCCATCTCCAGATTGTTTAATGTTATTTCAACATCATAATTTGCTGCCATGGAAAACGATTAATGACAACATTA
GGATTGGATTTCAACAGAAAATTAGTGATGAAGAGATTAACGCACAGCTTAAATTAGTTGATTTAGAAGACAGGGGAAAGCATTTT
CCCGAGCAACTGTCCGGGGGTATGAAACAACGTGTGGCACTATGTCGAGCGCATGTGCATAAGCCTAACGTTATATTGATGGATGA
GCCATTAGGTGCATTAGATGCATTTACACGTTATAAACTTCAGGATCAACTAGTGCAACTAAAACATAAAACGCAATCAACTATTAT
TTTAGTGACGCATGACATTGATGAAGCTATTTATCTTTCTGACCGCATTGTTCTGTTAGGTGAAGGGTGCAATATTATTTCTCAATAT
GAAATTACAGCATCACATCCACGCAGTCGTAATGATAGCCACCTACTTAAGATTCGTAATGAAATTATGGAAACATTTGCATTGAAT
CATCATCAAGTTGAACCTGAATATTATTTATAAGGAGTGAGTGACGATGAAAAGGTTAAGCATAATCGTCATCATTGGAATCTTTAT
AATTACAGGATGTGATTGGCAAAGGACGTCTAAAGAACCGTCTAAAAATGCCCAAAATCAGCAAGTGATTAAAATTGGATATTTGC
CGATTACACATTCAGCTAATTTGATGATGACTAAAAAATTATTATCACAATACAATCATCCGAAATATAAACTAGAATTAGTTAAAT
TCAATAATTGGCCAGATTTAATGGACGCATTAAACAGTGGTCGTATTGATGGTGCATCAACTTTAATAGAGCTAGCGATGAAATCAA
AACAGAAGGGCTCAAATATAAAGGCTGTGGCATTGGGCCATCATGAAGGCAATGTCATTATGGGACAAAAAGGTATGCACTTAAAT
GAATTTAATAATAATGGCGATGATTACCATTTTGGTATACCACATCGTTATTCAACACATTATCTTTTACTTGAGGAATTACGTAAAC
AATTAAAGATTAAACCGGGGCATTTTAGCTATCATGAAATGTCGCCAGCAGAAATGCCAGCCGCATTGAGTGAACACAGAATTACA
GGGTATTCTGTAGCCGAACCATTCGGTGCACTGGGTGAAAAGTTAGGCAAAGGTAAGACTTTGAAACATGGTGATGACGTTATACC
TGATGCGTATTGCTGTGTGCTAGTACTGAGAGGGGAATTGCTTGATCAACACAAGGATGTAGCGCAAGCATTTGTACAAGATTATA
AAAAGTCTGGCTTTAAAATGAATGATCGCAAGCAAAGTGTAGACATTATGACGCATCATTTTAAACAAAGTCGTGACGTTTTAACA
CAGTCAGCGGCATGGACATCCTATGGTGATTTAACAATTAAGCCATCCGGCTATCAAGAAATTACGACATTGGTAAAACAACATCA
TTTGTTTAATCCACCTGCATATGATGACTTTGTTGAACCGTCATTGTATAAGGAGGCATCGCGTTCATGACACGTCCCACACATAAC
AAATTTATATTACCTATTATCACATTTATTATTTTCTTAGGCATTTGGGAAATGGTCATTATTATTGGGCATTACCAACCTGTATTGTT
ACCTGGTCCTGCTCTTGTAGGAAAAAGTATATGGACTTTCATTGTTACTGGAGAAATTTTCCAACATTTAGCAATTAGTTTATGGAG
ATTTGTAGCGGGCTTTGTTGTCGCATTGTTGGTTGCTATTCCATTGGGCTTCTTACTTGGAAGGAATCGTTGGCTATACAACGCTATC
GAACCGCTATTTCAATTGATTAGACCGATATCTCCGATAGCATGGGCACCATTTGTTGTTCTATGGTTTGGTATTGGTAGTTTGCCAG
CGATTGCGATTATTTTTATCGCTGCTTTTTTCCCAATTGTGTTCAATACTATTAAAGGCGTTAGAGACATTGAACCTCAATATTTAAA
AATAGCGGCAAATTTAAATTTAACTGGGTGGTCATTGTATCGCAATATATTATTTCCCGGGGCATTTAAACAAATCATGGCTGGGAT
ACATATGGCGGTAGGAACAAGTTGGATATTTTTAGTTTCTGGTGAAATGATTGGTGCACAATCGGGATTAGGTTTTTTAATCGTTGA
TGCACGAAATATGTTGAACTTAGAAGATGTTTTAGCAGCAATATTCTTTATCGGATTATTTGGTTTTATTATTGATCGATTCATTAGT
TATATTGAGCAGTTTATACTTAGAAGATTTGGTGAATAAGGAGAGATGATGATGACTTTAGAAACGCTTATCAAAGAACAATTAGA
TCCTCATTTAGTAGAAGTTGATGAAGGGACGTATTATCCGAGAACATTTATTCAACAATTATTTGTAGATGGTTATTTCGGTGAGGC
GGCATTGAGAAAAAATGCTGAAGTAATCGAAGCTGTATCGCAGTCTTGTTTGACAACAGGATTTTGTTTATGGTGCCAATTAGCTTT
TTCAACGTATTTAGAAAATGCCACGCAGCCACATTTAAATAATGACTTACAACAGCAATTGTTATCTGGAGAAATATTAGGTGCTAC
CGGATTGTCTAATCCGATGAAGTCATTTAATGATTTAGAAAAGTTGAACCTTGAACACACTTATGCTGATGGACAATTGGTTGTCAG
TGGACGTATGCCAGCTGTAAGTAATATTCAAGAAGACCATTATTTGGTGCGATTTCGAAACATGAATCATCAGATGAATTTGTCAT
GTTCATTCTACGTGCCAATCAAGATGGTATCACTCTCGTTGAAAAAACAAACTTTTTAGGAGTAAATGGCTCGGCTACGTATCAAAT
TACTTTGAATCAAGTCGTAGTGCCACAATCACAAATTATCACGCATGATGCGAAGCAGTTTGCGGCAACTATTCGCCCACAATTTAT
TGCTTATCAAATTCCAATAGGATTAGGCTCAATTAAAAGTTCTTTAGAGTTAATTGATGCATTTTCAAATGCGCAAAACGGAATAAA
TCAATATTTAGAGTATGATGTTGAAGCTTTTAAAAAACGTTATCGTCAACTTAGAGAGGAATATTATGCAATATTAGATGACGGTAA
CTTAACTTCACATTTAAGTGAATTAATATCATTGAAGAAGGACATTGGCTATTTATTGCTAGATGTAAATCAAGCTTCTGTTGTCAAT
GGTGGTTCAAGAGCGTACACACCATATTCGCCACAAGTTCGCAAGTTAAAAGAAGGATTCTTCTTTGCAGCATTGACACCAACATTA
AGACATTTAGGTAAACTTGAAGCAGAGTTGAAGGGGTAAGTGTGATAAGCTGATTTTTTGTTTAGATGCGTTTGTTGAAACATTTTT
TTAAAATAATATAAATCTTAGTTTATAAACATTTTCTGTTAATTTGTTATATCCTTTTAACTAGGAAAATATACATTTCGTAATAATA
ATAATCGTTATCATTGAAAAAGTGTTAATAAGGTGTATAATGAAAATGTGAACAATTAATGAACTTCTTATTTTAAAGAAGGTGAAT
ACTATAGATACGCATACTAAAGAACAACAATTCTCGAATCTAGTAAGATCTTATCGTAAAGAATACGTGGGTAAAGGACCCAATAG
TATTCGAGTGTCGTTTAAAGATAATTGGGCGATTGCACATATGACAGGTGTTTTGAGTAAAGTTGAGAGTTTTTACCTAAACGACAA
ACGCAATGAATCGATGCTCCATTATACACGCACAGAGAAGATTAAACAGATGTATAAAGAAATAGATGTAAATGAGATGGAAAGT
CTTGTAGGCGCTAAGTTTGTAAAATTATTTACAGATATTGATTTGAATGATGATGAAGTCATTTCAATATTTGTTTTCGATAAGTCAA
TAGAATAAGTGTTGCTGGTGTAAGGTACACGGTGCTGTTTGCTAACTTCGCTTTGAATTTAACAATAATTCAAGGGGGTGGTATGTC
AAACGGTGCCGTTTTTTTGTCATATTTTTAAAACAAGCAACATGCAACACGTACTTTAAGGAAGTCAAAATTTATCATTTAGGAGAG
ATGGATATGAAAATCGTAGCGTTATTTCCAGAAGCAGTAGAAGGTCAAGAAAATCAATTACTTAATACTAAAAAAGCATTAGGATT
AAAAACATTTTTAGAGGAAAGAGGACATGAGTTCATTATATTAGCAGATAATGGTGAAGACTTAGATAAACATTTACCAGATATGG
ATGTGATTATTAGTGCGCCATTTTATCCTGCATATATGACTCGTGAACGTATTGAAAAAGCACCGAACTTGAAATTAGCAATTACAG
CAGGTGTAGGATCTGACCATGTAGATTTAGCGGCAGCAAGTGAACACAATATTGGTGTCGTTGAAGTTACAGGAAGTAATACAGTT
AGTGTGGCAGAACATGCGGTTATGGATTTATTAATACTTCTTAGAAACTATGAAGAAGGTCATCGTCAATCAGTAGAAGGTGAATG
GAACTTGTCTCAAGTAGGTAATCATGCGCATGAATTACAACACAAAACAATTGGTATTTTTGGATTTGGTCGAATTGGACAACTTGT
TGCTGAAAGATTAGCGCCATTTAATGTAACATTACAACACTATGATCCAATCAATCAACAAGACCATAAATTGTCTGAATTTGTAAG
CTTTGATGAACTTGTTTCAACAAGTGATGCGATTACAATTCATGCACCATTAACACCAGAAACTGATAACTTATTTGATAAAGATGT
TTTAAGTCGTATGAAAAAACACAGTTATTTAGTGAATACTGCACGTGGTAAAATTGTAAATCGCGATGCGTTAGTTGAAGCGTTAGC
ATCCGAGCATTTACAAGGATATGCTGGTGATGTTTGGTATCCACAACCTGCACCTGCTGATCATCCATGGAGAACAATGCCTAGAAA
TGCTATGACGGTTCACTATTCAGGTATGACTTTAGAAGCACAAAAACGTATTGAAGATGGAGTTAAAGATATTTTAGAGCGTTTCTT
CAATCATGAACCTTTCCAAGATAAAGATATTATTGTTGCAAGTGGTCGTATTGCTAGTAAAAGTTATACAGCTAAATAGAATAAGGA
TGCTGGGCTAGCGATTAACGCTTTCAATTTTATATAAATGAATCATATAAGCACTACTGCTGTTGTAAAGATGGCAGTAGTTTTTTTA
TGATTACATCTAAGTATAGTCACGGCTATGTTAGGACAATGATTTAACATTTACGCACATATGTGTTCACTTACGCAATTATTGATAA
ATTTCATTCATTTGGAATAATATATAGTCATATAGTGCTAATTTTTTTGAGGCATTATATGCAAGATGCATTGTAAAGTCGATGCATT
TGTTTGTTAAATAACTTTGTATAACTAAAAATTCTTTGTTTCAACGTATAATCTAATAATAGATTTTATATAAATGGTAGTGTGTATA
TATGTGGAAAGGGGTGTAGTTATTAATGAAACGCTTAAGTACGACTTTGAAAGTACGATTGATTAGCAATTTTTTACAGCTAATTAT
TACGACAGCATTTATACCGTTTATAGCACTATATTTAACAGATATGTTAAGTCAATCAATTGTCGGTATATATCTTGTTGGTTTAGTG

GTTCTAAAATTTCCATTGTCCATTATATCTGGTTACCTTATTGAGATATTTCCGAAAAAGTTGCTAGTACTTATTTATCAAGCGACGA
TGGTGATAATGCTTGTGTTCATGGGCGTATTTGGGTCACATCAATTGTGGCAAATTATTGGTTTTTGTGTTGCATATGCCATATTTAC
AATCGTTTGGGGATTACAATTTCCAGTTATGGACACATTAATTATGGATGCAATTACCGAAGACGTGGAACATTATATTTACAAGAT
TAGCTATTGGATGACAAACTTATCGGTAGCTATTGGGGCATTGTTAGGTGGCTTGATGTATGGCTACAGTATGTTACTACTTTTCTTA
ATAGCAGCTTGTATATTTTTAATTGTACTCTTTATTTTATATATTTGGTTACCTCAAGACCGAAATCAAGTAAAGCAAAGTGATGACA
AGAGGCATGCAAGTCGTTATCAAAAATTACAAATAATGAATATATTTCGCAGTTATAAATTAGTTTTGAAAGACCGTAATTATATGT
TATTGATTTCGGGGTTCAGTATCATCATGATGGGTGAATTTTCAATCTCCTCATATATTGCTATTAGACTAAAGGATCAGTTTGAAAC
AATAAGTATAGGTTCATATGATATTACAGGTGCTAAGATGTTAGCAATCTTGCTAATGATTAATACGGTCGTCGTCATTTTACTCAC
GTATTCAATCTCGAAAGTTGTATTGAAAATAGATTTTAAAAAAGCTTTAATCACTGGTTTGCTGATTTATATTGTTGGCTATAGTGGT
CTAACCTATCTTAATCAGTTTGGCTTATTAGTTGTTTTTATGATAATTGCGACTGTAGGTGAAATTATTTATTCGCCTATAGTTTCAGA
ACAACGCTTTAAAATTATTCCTAAAGCTAAAAGAGGAACATATAGTGCAGTTAATGCATTAGGTATTCATTTTTCAGAAACACTAGC
TAGGTTAGGGATTGTGTTGGGTGTTTTCTTAACGTCATTACAAATGGGACTGTATATGTTTATCGTTTTAACAATTGGTGCTAGCATG
CTTGTTGCTGGTGTATTTGGGGGACAAAAACAAGTGAATACAAATTGAATTTATTTAAATATTTATTTTATGCATCTTTTTATATGGA
AAACCTATTAATTTGGTCGTAATTAAAATAGAGATAAATTTATTAAGAATTTGTTAAAATTTAAGGATAACAATTGTTTTATGCGAG
TTGTTTGTTTAATATAAACTTGCTTCATGGATATTTTGTAACAACAATATTGAGTTTGCAATGAGATGTGAACAACAGAAAGTGATT
AACGAGTTGTAGTGTGCTATTTATCGAAGATGTATGTAATTTACATTGAAAAATATACCAATTGTAACGATGATACAAGATGTATTT
TAGGGTAGAATATATCGCTCATATGTTGCGAATGGTGTTTTATATTAATCATTACATAACGAGTTAATTAACATTTCGTCGGTTATCA
TATCATCATGCAATTTCAGTAAGTAATGAGTATTATGATATGAAAGAAGGACTTTTTATGATTATGGGTAATTTGAGATTTCAACAG
GAATATTTTCGTATATACAAAAATAATACAGAATCAACGACACACCGTAATGCGTATTGGGTTAAACTCGCTAAAAATGTTGAAGC
TACTAAAATGATGTATGCATTATCGACAATTGTGCAACAACATGCATCTATAAGACATTTTTTTGATGTTACTACCGATGACAATTTA
ACAATGATACTTCATGAATTTCTGCCTTTTATTGAGATAAAACAAGTTCCATCTTCTTCCGCAAACTATGATTTAGAAGCTTTTTTTA
AGCAAGAATTAAGTACTTACCATTTTAATGATTCACCTTTATTCAAAGTTAAATTGTTTCAGTTCGCTGATGCTGCATATATACTATT
AGATTTTCATGTGTCCATTTTCGATGATAGTCAAATTGATATTTTTCTTGATGATTTATGCAATGCATATCGTGGCAATACTGTTATTA
ACAATACTCGACAGCATGCACATATAAATAGAAATGATGATAAAGACAATCAAGATGCATCGCATATAGCATTAGACTCAAACTAT
TTTCGGTTAGAGAATAACTCTGACATCCATATTGATAGTTATTTTCCAATTAAGCATCCATTTGAACAAGCTTTATATCAAACGTATT
TGATTGATGATATGACATCAATAGATATGGCATCGTTGGCTGTTAGTGTGTATTTAGCTAATCATATAATGAGTCAACAACATGATG
TCACATTAGGTATACATGTACCATCACATTTACCAAATGATTTACACGGAAATATTGTGCCGTTAACGTTAACAATCGATGCAAAAG
ATGTATGTCAACGTTTTACAACAGATTTTAATAAATGTGTGTTGCAAAATATGTCGCAATTACAGTGCGCGAAGTCTTCGCTTTCACT
AGAGACTATTTTTCATTGTTATCATCATATGATGTCTTGTTGTAATGATGTTATTGAGGATGTACATCAAATACATGATGCACATACA
TCTTTAGCGGATATTGAAATTTTTCCACATCAACACGGGTTCAAAATTATATATAACAGTGCAGCATATGATTTGCTCTCAATCGAG
ACGCTGAGTGACTTAGTTCGAAATATTTATTTGCAAATTACTGAAGAAAATGGAAATAAACGAACAACTGTAGATGAACTTAATTT
GATGACAGAACGTGATATTCAATTATATGACGATATCAATTTAAGTTTGCCTGAGATAGATGATGCGCAAACAGTTGTTACCTTATT
TGAGCAACAAGTTGAAGCAACGCCGAATCATGTCGCTGTGCAATTTGACGGAGTGTTTATAACATATCAAACATTGAATGCACGCG
CGAATGATTTAGCACACCGTTTGAGAAACCAGTATGGTGTTGAACCTAATGATCGTGTCGCTGTCATAGCTGAAAAAAGTATTGAG
ATGATAATAGCGATGATAGGTGTGTTGAAAGCTGGTGGGGCTTACGTGCCAATTGATCCGAACTATCCAAGTGATCGTCAGGAGTA
CATTTTAAAAGATGCAACGCCTAAAGTTGTAATAACGTACCAAGCTTTATATGAAAATGGTAAACAAAATATTAATCACATTGATTT
GAATAAGATAGCGTGGAAAAATATTGATAATCTTTCTAAATGTAACACGTTAGAAGATCATGCTTATGTTATTTACACGTCGGGGAC
AACTGGTAACCCTAAAGGGACATTAATTCCGCACCGAGGTATTGTTCGCTTGGTCCATCAAAATCATTATGTACCATTAAATGAAGA
GACGACGATTTTGTTATCAGGAACTATAGCCTTTGATGCTGCAACATTTGAAATATATGGTGCATTGCTCAATGGTGGAAAGTTGAT
TGTTGCTAAAAAAGAACAATTATTAAATCCAATAGCGTTAGAACAATTAATCAATGAAAATGACGTTAATACTATGTGGTTAACCTC
CTCATTATTTAATCAGATTGCTAGTGAACGAATAGAAGTATTGGTACCGTTAAAGTATTTATTAATTGGTGGAGAAGTATTGAATGC
TAAGTGGGTGGATTTGCTTAATCAAAAACCGAAGCATCCTCAAATTATTAATGGTTATGGACCAACTGAAAATACAACATTTACAAC
GACTTATAATATACCTAACAAAGTTCCAAATTGTATTCCTATTGGTAAACCGATTCTTGGTACTCATGTTTATATCATGCAAGGCGAG
CGTCGATGTGGCGTTGGTATTCCTGGAGAATTATGTACAAGTGGCTTTGGGTTAGCTGCAGGTTATTTAAATCAGCCAGAATTGACA
GCAGATAAATTTATCAAAGATTCAAATATAAATCAGCTGATGTATAGAAGTGGTGATATCGTTCGTTTGTTACCCGATGGCAACATA
GATTATTTATATCGAAAGGACAAACAAGTTAAGATTCGAGGGTTTAGGATTGAGTTGTCAGAGGTTGAGCATGCGCTCGAGCGTAT
ACAAGGTATTAATAAAGCAGTTGTTATTGTTCAAAATCATGATCAAGATCAGTATATCGTTGCTTATTATGAAGCGATGCATACATT
ATCACATAATAAGATTAAATCACAATTACGTATGACCTTACCGGAGTACATGATACCAGTTAATTTCATGCATATTGAGCAAATTCC
TATTACTATTAATGGGAAATTAGATAAGAAGGCATTGCCTATCATGGACTATGTCGATACGGATGCCTATGTAGCACCGAGTACAG
ATACCGAACACTTGCTATGCCAAATTTTTGCAGATATTTTACATGTGAATCAAGTAGGTATTCATGATAATTTCTTTGAATTAGGTGG
CCATTCATTAAAAGCAACGTTAGTGGTGAATCGGATAGAGGCATCTACTGGGAAACGATTACAAATTGGTGATTTATTACAAAAGC
CAACTGTATTTGAACTAGCACAAGCGATTGCTAAGGTTCAAGAACAAAACTATGAAGTGATTCCAGAAACTATAGTTAAAGATGAT
TATGTGCTGAGCTCTGCACAAAAGCGTATGTATTTATTATGGAAATCAAACCATAAAGATACGGTGTATAACGTACCTTTTTTATGG
CGGTTATCATCAGAACTTAATGTAGCTCAATTGCGACAAGCAGTGCAGCGTTTGATAGCGCGACATGAGATTTTACGAACACAATAT
ATTGTTGTAGATGATGAGGTTCGACAACGTATTGTGGCAGATGTTGCAGTTGACTTTGAAGAAGTTAACACGCATTTTACGGATGAA
CAAGAAATCATGCGCCAATTTGTAGCACCTTTTAATTTGGAAAAGCCAAGTCAAATTAGAGTGAGATACATTAGAAGTCCCTTACAT
GCATACCTCTTTATAGATACACATCATATCATTAATGACGGTATGAGTAATATACAATTAATGAATGATCTTAACGCACTTTATCAA
CATAAATTATTGTTACCACTTAAATTGCAATATAAAGACTATAGTGAGTGGATGTCGCATCGTGATATGACGAAACATAGACAATAT
TGGTTATCTCAATTCAAAGATGAAGTACCTATTTTAAGCTTACCGACAGACTATGTTAGACCAAATATTAAAACGACAAATGGAGCA
ATGATGTCATTTACAATGAATCAACAAATGAGACAGCTACTTCAAAAGTATGTAGAAAAGCATCAAATTACTGATTTTATGTTCTTT
ATGAGTGTGGTCATGACGTTGTTAAGCAGATATGCTCGAAAAGATGATGTTGTTGTCGGTAGTGTGATGAGTGCGCGTATGCATAAA
GGCACGGAGCAAATGCTAGGCATGTTTGCTAATACGTTGGTATATAGAGGGCAACCGTCACCTGATAAAATGTGGACACAGTTTTT
ACAAGAGGTTAAGGAAATGAGTTTGGGTGCATACGAGCATCAAGAATACCCATTCGAATGTTTAGTAAATGACTTAGATCAATCAC
ATGATGCCTCACGGAATCCATTATTTGATGTCATGTTAGTACTACAAAACAATGAAACGAATCATGCTCATTTTGGGCATAGTAAAT
TAACACACATTCAACCCAAATCAGTGACGGCGAAATTTGATTTATCTTTCATCATTGAAGAAGATCGCGATGACTATACAATCAATA
TCGAGTATAATACCGATTTATATCACTCAGAAACAGTTCGTCACATGGGTAATCAATGTATGATTATGATTGATTATATTTTGAAGC
ATCAAGATACACTACAAATTTGTGATATACCAAACGGTACGGAGGAACTTCTAAATTGGGTCAATACGCATGTTAACGATCGAATG
CTTAATGTCCCGGGAAATAAATCTATCATAAGTTACTTTAATGAAGCTGTCTCACGACAAGGTAATCATGTTGCGCTAGTCATGAAT
GATTTGACAATGACGTATGAAACATTACGCAACTATGTGGATGCCATTGCGCACATGCTCCTATCAAATGGTGTGGGCAATGGTCAA
CGGGTTGCCTTGTTTACAGAACGTAGTTTTGAAATGATTGCGGCGATGTTGGCGACAGTTAAAGTAGGTGCATCTTATATACCTATC
GATATTGATTTTCCGAATAAACGACAAGGTGCAATTTTGGAGGATGCTAAAGTAACTGCAGTCATGTCTTACGGCGTTGAAATTGAA

ACGACATTACCAGTCATTCAATTGGAAAATGCTAAAGGCTTTGTTGAATCAAAGGAAAATGAACAATATGATGATTTACATGGCAA
TCAACTTGAAAACACAGCGATGTTAGATAATGAGATGTATGCTATTTACACATCTGGTACGACCGGGATGCCTAAAGGGGTTGCCA
TACGACAACGAAATTTGTTGAATTTAGTGCATGCATGGTCAACTGAATTGCAATTAGGCGACAATGAAGTATTTTTGCAACATGCAA
ATATTGTTTTTGATGCATCAGTTATGGAGATTTATTGTTGTTTGTTAAATGGTCATACGCTTGTGATTCCAGATAGAGAGGAACGTGT
TAATCCAGAACAGTTACAACAACTCATTAATAAGCATCGTGTGACGGTTGCGTCGATTCCGTTACAGATGTGTAGTGTTATGGAAGA
CTTTTATATTGAAAAGTTGATTACAGGCGGGGCAACTAGTACGGCATCCTTTGTTAAATATATTGAGAAGCATTGTGGCACGTATTT
CAATGCCTATGGACCATCTGAGTCAACAGTCATCACATCGTATTGGTCACATCATTGTGGTGATTTGATACCTGAGACGATTCCAAT
TGGCAAACCCTTATCTAACATCCAAGTGTATATTATGTCAGATGGTTTGTTATGCGGTATTGGTATGCCAGGCGAGTTGTGTATTGCA
GGTGATAGTTTAGCGATAGGATATATTAATCGTCCAGAATTAATGGCTGATAAATGGCAAAATAATCCATTTGGTAAAGGAAAGTT
GTATCATAGTGGTGATTTAGCACGTTATACATCTGATGGTCAAATTGAATTTTTAGGAAGAATAGATAAACAAGTGAAAGTTAACG
GGTACCGTATTGAACTTGATGAAATTGAAAATGCAATATTAGCTATTCGTGGTATATCTGATTGTGTTGTAACAGTAAGTCACTTTG
ATACGCATGATATATTGAATGCTTATTATGTTGGAGAGCAACAAGTGGAGCAGGATTTGAAGCAATATTTAAATGATCAGCTGCCTA
AGTATATGATTCCTAAGACTATAACGCATATCGATTGTATGCCATTAACCACGAATGACAAGGTGGATACTACGCGTTTGCCAAATC
CATCACCTATACAACAGTCTAATAAAGTGTATAGCGAACCCTCTAATGAAATTGAGCAGACATTTGTTGATGTATTTGGAGAGGTAT
TGAAACAAAATGATGTCGGTGTTGACGATGATTTCTTTGAACTTGGTGGTAACTCATTAGAGGCGATGTTAGTTGTCTCGCATTTAA
AACGATTTGGCCATCATATTTCAATGCAGACATTATACCAATATAAAACCGTGCGACAGATTGTTAATTATATGTACCAAAATCAAC
AATCATTAGTTGCATTACCGGATAATCTTTCGGAATTACAAAAGATTGTTATGTCTCGTTATAACTTGGGTATTTTAGAGGATAGTCT
AAGTCATCGACCTCTAGGAAATACACTATTGACTGGCGCGACAGGTTTTTTAGGTGCTTATCTGATTGAAGCACTACAAGGATACAG
TCATCGCATTTATTGTTTCATACGTGCTGATAATGAGGAAATAGCATGGTATAAGTTGATGACGAATTTAAATGATTATTTTTCAGA
AGAGACGGTTGAAATGATGTTATCAAACATTGAAGTCATTGTTGGTGATTTCGAGTGTATGGATGATGTTGTTTTACCAGAAAACAT
GGATACGATTATTCATGCAGGTGCTCGTACAGATCACTTTGGTGATGATGATGAATTTGAAAAAGTAAATGTTCAAGGTACTGTTGA
TGTCATACGTTTGGCACAACAACATCATGCAAGGTTAATATATGTGTCTACGATAAGTGTGGGAACTTATTTTGATATAGACACAGA
AGATGTGACATTTTCAGAAGCGGATGTCTATAAAGGGCAACTACTAACATCACCATATACACGGAGCAAATTTTATAGTGAATTAA
AAGTATTAGAAGCTGTAAATAATGGCTTAGATGGTCGGATTGTACGTGTTGGTAATTTGACGAGTCCTTACAATGGAAGATGGCATA
TGAGAAATATAAAGACTAACCGTTTTTCAATGGTAATGAATGATTTGTTACAACTGGATTGTATCGGGGTTAGCATGGCTGAAATGC
CTGTAGATTTTTCTTTTGTGGATACGACTGCAAGACAAATTGTCGCATTAGCACAGGTCAACACACCACAAATCATTTACCATGTGC
TATCACCTAATAAAATGCCGGTGAAATCTTTGTTAGAATGCGTTAAGCGCAAAGAAATTGAACTCGTCAGCGATGAATCATTTAATG
AAATTTTACAGAAACAAGACATGTACGAAACGATTGGATTAACTAGTGTTGACCGTGAACAACAACTAGCAATGATAGATACAACA
TTAACATTAAAAATAATGAATCACATCAGTGAAAAATGGCCAACGATAACTAACAATTGGCTGTATCATTGGGCACAATATATCAA
AACAATATTCAATAAGTAAGTAGGGAAAGTTATGACAGTATTTGTAATGCAATTACAGAGTAACTTGAAAAGTATTGAAGAATTAA
TATCACAAAGTCGTTGGTCATATAAAAAACCGCGTACAGTCAACTATAGATACAATCAAGATAAACTCATGCACAGATTGGGAGAT
ATTTTAGTGCAATATGGAATTCAACATGACACAGGTTTATTACCACATGAATGGCATTATCACATTTCGCCACGAGGTAAGGCAGAT
ATTGTTCAACACAATCGTGATGGACAGCCCATCTATGTGAGCTTATCATATAGTTATCCTTATATCGTGTGTGTTGTCGATAAAGAAC
CAGTTGGTATTGATATCGAAAAGATATCACAACGTTTAGACTGGCGTACGTTAGTGACGTGTTTCTCTACAAACGAAGCACATCAAA
TATGTAGTTTAAATGATTTTTATCAAATATGGACACAAAAAGAAAGTTTTACAAAATTGATTGGTGAAGGTTTAATCAAAGGATTGG
ATATTTATGATATGACACAATCACACTTTTATCAATCACGTGAAGTGAAGTTCAAACAATTTATTTTTGATCAGTTTATGGTACAGGT
ATGTTTCTTAGGAGAGGCACCCTGGGGTTATAAAAAAGTGTCTGTATTTCAGTTATTGAGTAGTTAATATGACGTCTGACAGTATCA
TTGCGCAGTGATTTTAAACTATACTGCCTAAATGTTTACTGTTAGATGTTTTTTTATTGGAAAAACGAAAGGTGAATACACGATGGC
ATCATTCAAAATCATGACGAGATTAAAGTAAGTGATAAGGTGAAAATATACTGAGCAATATTGAAATTACCGAAAATAATAGTTTG
TTGCGACGCATTTGAGACTTTAATGCTCACGTAAGTTGGCTGGAATCATATCATGTCAGTTAATAAAATAACGTGAGCATCAATCAT
CATACTCAAACGATGATTTAAAACTCACGTTAATGTTAGTCAGTGTATTAATTTAATTTATAGCGATAGACAAAATGTTGTCGCACT
AATTCAATTGTCATCATCCAGACGATATGGCCAAAGAATTCTGACAGATGCTCTTGGAATGGTTGATCCCACACAGCAGGTACAGTA
TGCATGATTGGCATAATGATAAGGTGGAATAATACCCAAATAGCAATACCAAAAACAGCACCTTGTCCCATTGCTAAGTAAGCGTA
TTTTTTAACTAATATGCAGTAAATAATTGCAATGACGATAGAAAAACTAAAGTGGACAATAAAGCTTACCCAAGGCAATTCCATATT
TGAAAATGTATATGTTTGATGCGTAAACTCACTACTAAATCCTAATTGTTGCAATAACTCTTGAGGTGGGTTCGTTGCATTACGTTCT
GGTGTGCGAGGTGGAAACATGACCTCCCAACCTAATTTTACAATTCCAGATAACAAGCCACCGATAATTCCAGCATAAATGAATAT
ACCCATTTGTCGTTTCGTCAACTTAAGGACTCCTCTCTATTTACTATCCTCATTATAGTATTTTGTGAAAATAATCACAATAAAAAGC
TTTGCAAAACTTAAAATATTTTGATAAACAAATTTGTAAAAGGTTATTAGTTTTTTACATCTCATATTGTATCGTACTGTATTCAAAA
TTGTTAACGAGATGAACGGTAAATAAATGTTTTAGCGCAATGGATAATCGAACTGGAATCATTGCGTGTCAAAACTTGTTAGGCCTT
AATTTCATAGTTATGAATTAAGGATTGTTGTGCCAACAAAATCATTATTGTAAATAGATTCAATGATATTTGGCTTGTTTCCTGATGC
AATGATAACTTTAGGACAGCCATTTTCAATCGCATTTTTGGCATCTAGCACTTTGGGAATCATACCTCCATAAATATCACCATGTTCA
ATATATTGATGAATATCGACTAATGGCAATTGAGGTATAACCATATCATTGATGAGTACACCTGCAATATTACTTAATACATAAATA
GGCGCTTTTAATGATGATGCAATAAAATAGGCAAGCGTGTCAGCATTAATATTGTAAAATTCTCCATCATGGTTATTGAAACCAATC
GAATTGATGATAGGTACAAATTTAGTACATATATACTGTAAAGCATCCTTATTTAAAGCGGTCGGGACACCGACATATCCATATTGT
TGATCAAAACGTTTAATTTCAAATAATTGAGCATCCAAACCACATAAGCCTATCGCAGAACATTGGTGCTGGTTAAATTGAGCTACT
AATGCAGTGTTAACGTCTGCAATGAGCGTGTGTTTAGTAATGGTCATGGTTGCTTTATCAGTCACTCTTAGGCCATTAACAAAGTGT
GGCTCGATTTGCTGGTTTGATAATGCATCATTAATAAATGGGCCACCGCCATGAACGATAATAGGGTAGATGTTGTTTAATCGTAAA
TGCTTAATATTGTTAATAATTGATGGATGCATGTCACTAAGTGTACTGCCACCAATTTTAATGACAATAAATTTCATCTAACCAACAC
CACCTTATGTTCGATATGATGCGTTGATACGCACATAATCATAGGATAAATCACAACCGTATGCAGTCGCTGCAGCGTTACCTAAAC
CAAGCTGAACGTCAATTGTGACATTTTCATGAGTTAATGTATTCGACATAGCTTGCTCATCAAATAGTACAGCCATACCTTTATCAA
CGACAGGTATTTGGTTCAGTTGAACATATGTGCAGTTAGGATCAATTTCACATCCGCTGTAGCCAATAGCTGTAATGATTCGACCAA
AATTGGCATCTTCGCCAAAAATAGCTGATTTTACTAGATTTGAACTTACGATAGTTTTACCGATTTTTCTTGCATCTGATATTGATTT
AGCGCCTGACACATTGACGCTGATTAACTTTGTTGCGCCTTCGCCATCTCTGGCTATAGCTTTAGCTAAAAATGTACAGACAAAATT
GAATGCATCAACAAATGTTTCCCATTGTGGATGGTCTTGACTAAGTATTTGGTGTTCAACTTGGTGATTTGCCATGACTAATACCATG
TCATTTGTACTTGTATCGCCATCAACAGTAATCATATTAAATGTATGGTCAGTCGAAGATTTTAATAATTGATGAAGTGTATTCGATT
CAATCGATGCATCGGTTGTTATAAAAGCAAGCATGGTAGCCATATTTGGGTGAATCATACCTGAACCTTTGGTGCTACCACCAATTG
TAACGGTTTTACCATCGATTTTTAGTGATACAGCGATATGTTTTGTACAGGTATCAGTTGTTAAAATTGCCTCGTTAAACGCACCTGG
CGTTGCAAAATTAGCATCCTTAATATGTTCGGTCCCAGTCTTAATTTTATCCATAGGCAAATATTCACCAATGACCCCAGTTGAAGC
AACAGCAACATGCTCAGATGGTATTTGAAGTTGTTGAGCAACCCATGTTTGTGTTTGTCGTGCATCATCTATGCCTTGTTGACCGGTA
CAAGAATTTGCATTAGCTGAATTAACAACAAGTGCTTGTAATTTTCCTTTAGACTTTTGTAAAGTGTCTTCAGTGACAATAAGTGGTG
CAGCTTTAAACTGATTTAAAGTATATACGGCAGCTGCACTTGCCAAAGACGATGAGTAAATCCACCCAAAGTCTTTTTTGTTAGCGC

GTAAACCGATGTGCATACCACCAGCCGTGAAGCCTTGAGGTGTACTGATATCGCCATGTTTAATAATTGAAAAGTTATATTGTTGTG
ATGTCGTTTCTTGATGTTTCATTCTAACACCCCTTATGGATAAACTGGTGATTGATTTAGGCCAGTCGTCACTTCAAAATCATATAAT
ATATTTAAATTTTGAATGGCTTGCCCACTTGCGCCTTTGACAAGGTTATCAATCACTGATACTAAAATTGCTGTTTGCGTTGTTTCAT
CTACATAGATGCCGATATCGCAGTAGTTACTACCGAGTACTTCTTTTGTGGTTGGAAAAGTCCCAATATCTCTAATTCTGACAAATG
GCTGATTAGCATAATAAGAGGTCATTAATTTATGTAATGATTCAGTCGTATATTCAGATGATAATTTGACATATATTGTTGATAAAA
TACCTCGTGTCATTGGTACGAGATGTGGTGTAAATATGACTGATACATCTTGACCCGCAATGATAGATAAATATTGCTCGATTTCCG
GTTTGTGTTTATGGTTTCCGATTGCATAAGCGCTTAGATTTTCATTCATTTCTGAAAAATGAACACGTTGTGATAATGAACGACCAGC
ACCTGACACGCCGGTCTTAGCATCAATAATAATAGATGACAAATCTACTATTTTTTCGCTAATAAGTGGATGTAATGCTAATAATGT
TGCTGTAGGGAAACAGCCAGGATTAGAAATGAGCTTCGTTCCATTGTTATCAAACGATTGCCATTCTGAAATGCTGTAAATAGCATG
ATTCAAATCATCTTGTGCTGCAGCAGTTTCTTTGTAATATGCTTCATATATTTCACGATTCTTAATTCTAAATGCGCCAGATAAATCG
ATAACATGAATACCTTTTTCTACTAAGGGAGGGATACATGTTTTACTTACGGGTGCTGGTGTCGCAAAGAAAATCACATCACAGTCA
TTATTGTCCACTGTAAGTGCTTCGAAATGTTGCATAATATGTTGTAAATGTGGAAATGTTAATTTCAACGGTTCATCTACTTTTGAAT
GTGAGTAGATGTGTGCAATCGTTACATGAGGATGTGTTTGTAACAATCGAATTAATTCAATTGCGCCATAACCGCTACCGCCAACGA
TACCTACTTTAATCATCATGAATCCCTCCTATATCATATATAAATGATTAATTGTTAATTTTTAAAAACGTCTTGAAAAGCTGCAACA
ATTTGATGGATTTCCTCTTTATCAATGACTAGAGGTGGTGACAATCGAATGATAGTACGATGCGTGTCTTTGCACAAGATTCCACGT
TGAATCAGTTGATCCACAAAAGGTGCAGCATCTGTGTTAAGCTCTATGCCTATAAATAAACCACGACCTCTAATTTCTTTAATACTA
GGATGTTTAAGTTGTAGCAACGCTTTTAATAAAAATGAACCTAAGCGTTCTGATCGTTCAACCAGTTGTTCATCTTTAAGTACATCA
AGCGCTGCCGTCGATATTGCAATGGCTAAAGGGTTACCACCAAATGTCGAACCATGTGTACCTGGTGTTAGAACACGCATGACATC
ATTATTTGCAAGTACAGCAGATACAGGGTATAAGCCGCCACCCAATGCCTTACCTAAAATATAAATGTCTGGAACAACTTGCTCCCA
TTCCATCGCAAACCATTTCCCAGTTCTACCAAGACCAACTTGAATTTCATCTGCAATCAATAATATTTGATATTTATCACATAGATGA
CGCACAGCTTGAATATATCCTTTCGGTGGTATATTAACGCCACCTTCACCTTGAATTGGTTCCAAAATAATTGCTGCTGTATTTGGTG
AAATAGCTTGTGTTAATTGTTCAATGTCTCCAAAATCTACAGTTGTAGTGCCTTGAAGTAGGGGGTGAAATCCTGCTTTATATGCGTC
GTGGTTAGATAGTGATAATGAGCCAAGTGTACGACCGTGAAAATTGTTATTCATAGCGATGATTTCAACTTGTCCGTCAGTAATGCC
TTTAACTTCAGAGCCCCATTTTCTAGCAATTTTAATGGCTGCTTCAACAGCTTCAGTACCAGAGTTAAGGGGGAGTACTTTGTCTTTC
TTAGCAAGATGACAAATTTTTTCTTCCCATTTCCCGAGATTGTCACTATAAAGGACACGTGAAATGATAGACAACTTTGAAGCTTGT
TCTGTCATCGCTTTAACAATTGTTGGATGACAATGGCCTTGGTTTGCAACTGAAAAACCCGAAATGCAATCTATATATTGTTTGCCAT
CAGTATCCCAAACTTTGACACCTTTACCTTTAGAAATGACAAGCTTAAGTGGTGCATAATTATTAGAGCTATAATAATCAGTTAATT
CAATGATTGAATTCATCGGTCTCCCTCCTTTATATTTATACAATTTAAAGAATTAATATTAATATATTACAGTTGTTCAATATAAGTG
TCAAATGTAAATTTAATAATATAAAGATAAGGTTGTGTTTGTATTAATGTGATTTGTAAAATGAAAGTACTAATATTTTTTGTGGATA
ATCATTGATATGTATGTATACGATTGCAGTTTAATGTAGTATGAATAAAAAATGCCACAAGCATATGTTTAACATATGACTGTGGCA
TAGTATTATGCTTGTGGAATTTTACGATGCTTAATTTTATAAATAATGAAGCCGATAATGAAACCAATCAAACTGAGAACAACCCAG
CCCATACCAATGTCTGATAATGGTAAATATTTTTGGCTGAAATTAATCAAAGTTTGTGAGAATGATGTGCTTGAAATGAACTCTGGA
CTAGCTTTTAATCCATCTACTAATGCAGCAATCATTGTAAAGAAAATGGTACATTGATAAATAAGTTTTGAATGATGGAATTTGCTA
CTAAATAATGTTAGTACAATCAAGGCAATTGCTAATGGATATAAGAACATTAACACTGGGACTGAGTACATAATAATCTTAGTTAA
ACCAACATTCGCGAATAAGAACGAAATAAAGCTTACAACTGTTGCAATCGCTAGGTAATTCATTTTAGGGAAAAGGTGTTCGAATG
TTTCTGAAAATGCCGTAATCAAACCGATGGCTGTTTTTAAACAAGCAACCATAACGATAAGTGACAACAGGACGATACCGTAGTTA
CCTAAGTAGTATTGAGTAATTTGCGCTAAGGCAATACCACCATTTTCACTAAGTTTGAAATGACCAATACTTAATGTACCCATGATT
GCTAGTAGGGTATAAATGATCCCCATCATAATGATACTGATAGTACCAGACTTAATTGTTTCTTTAGCGATATCAGTTGGATTTTCGA
TACCTAACTTTTTAATCGTTGCAACAATGATAATACCAAATGCCAATGACGCTAGCGCATCTAAGGTATTGTATCCATCTAAAAAGC
CGTTAAATAAGGCATGTGATTGATATTGTTTACTAATAGGTGCATCAGATATGCCACCTAATGGATGGATAAAAGCAAATAATAAA
ATAATTGCTAATAATACTAAGAATACCGGATTTAAAAATTTACCGATATATTCTAAAATTCTTGATGGCTTTCTCGCAAAAAACCAT
GCAATCACAAAGAAGACGAAGCTAAAAATAAATAAATATAAAGTGATTTGCTTTGGTGATAAAAATGGCGAAAATGCAATTTCAA
ATGATGTCGTTGCCAGTCTAGGTAAGGCGAAAAATGGTCCGATAACTAGATATAAGGCAATCGTGAAAATGTAAGCATATGTTTTA
TTAACACGCGATGCAATTTCAAATAAACCAGATGTCTTTGAAATGCCAATAGCAATGATACCTAGAAATGGTAAGCCAATTGCTGT
AATTAAAAATCCTAAGTTAGCGATAAAAACGTTAGAACCAGCAGCTTGACCCAAGTGTATTGGGAAGATAAGATTGCCGGCACCAA
AGAATAAACCAAATAACATAGAACCTATAAACATGTTTTCTTTAAATGTTAGTTTCTTCTTCATATATGTATTTGTTCTCCTTTTTAGA
ACTTATAAAAATCTACTTAATTAAGATAATATCAAAAATTAAACATAAAGTGATACATAATTTTGCAATAAATACTAAATTTTTTGA
AAATTCCGGAATAAATACTGTTTTTTATGTAGTAGGGGCGTAAGTTAAGAACGATTTAACAGATATAGAATAAGTTTATAGATAGG
AGGTTAGACAACCCGACGTATTAATACTTTGTGAAATAAAGATCCCCCTCAACATGATCGAAGGGGGGGAGTATATGTTTAGTTCA
ATATATTTAGTAATTCGCTTGTGGTTACTGTTTGCCCCATTATAGGGAATACATTATCTATTGGAAATTGATGTAGCGTTTCGTTTTGT
GCACTCATCATATCTGTAACAAAAAACTGATTGTAGTTTAATTGATAGGCATCTCGCGCTGTCGTATCTACGCCAATATGCGTTGCG
ACACCACCAAGAACAATCGTATCAATTCCTCGACGTCGCAATTGTAAGTCCAAATCTGTTCCTACAAATGCACTAAAATGTCGTTTG
TCTATGACAAAATCGTCATCTCTCTTGTCTAATAAATGATGGAAACGACTGTAGTCGTCGCCTTCTTTTGGTGGTAATGAGATCATTG
CATTTGGTTGCAATACATCTTTACCATCATAGAAATTCACGCGAACAAAAGCGATAAAGCCATTGTTTTTTCTAAAAACATCTATTA
ATTTATTAGCGTTTTGAACGACATTTTCAGCTGTATGTGGCGCATAATCCATCTTAAGTATACCTTCTTGCAGGTCGATTAACACTAA
AGCGGTTTTATTAAAATTAATCATCTTATATCAGAGTCCTTTTCGTTTAATAAAGTATTCGCTTAAACTTATACCCTGTTTACGATGA
AATTATTTATTTTGTTGTGAAAAAGCTTTAGCGATATCGATGAGTTTCTTCGGTGCGTCTTCGACAGACATTTTGACTTCGACAAAAT
GCATCACATCGGGATGACCATTAATTGCATTAAACGTGTCTTGTAAATCTTTTGATGATTCAACGTCATGAATTTCAACATTTTTACC
ACCAAATACAGCTGGTAAAGCTTTATAATCCCACATGTGAATTTCATTATAAGGTTCATACATGCCGTGAATAAGTCGTTCTACCGT
ATAGCCGTCATTATTAATCACAAATAATACCGGTTTAATATGCTGTCTAATCATAGTTGAAATAGCTTGAACAGTTAGTTGCAATGA
GCCATCACCAATTAATAATAAGTTACGACGATCTTTGTCTGCTAATTGTGAACCTAATGTTGCAGGTAATGTATAGCCGATAGAACC
CCATAACGGTTGCCCTATAAAAGTATTGTTTTTGTATAATGCTAAATCATAAGCACCAAAGAATGATGTACCTTGATCAGCAATAAT
GACATCATTTGGTTTTAAGAAATTTTGCATCATTTTAAAATAAGTTTGTTGTGTTAATGGTTCTGTGCCAACTGTATAATCGGGTGAT
GTTGGACGATGATACGCAGGGAACGTTGCGTTATTCGTATGTGAAATATTGGATAACTGTTTTAACAATGATGGTAGAGATATTTCA
TCATTTGTAACATCGTCAATTTTGATATTGTGATGATTTAACATAACGACATCATCGATATTGAATTGGTATGAAAAACCTGCTGTTG
CTGAATCTGTTAATTTGGCTCCAATATTTAAAATTAAATCGCTGTTGTCCACATAATCTCGTATTTTATCTTCGGCAATTTTCCCATCG
TAAATACCCATATAATATGGATTTTCCTCATTAAAAGCACCTTTTCCTAATGAAAGTTGTGCTACTGGTATCTGTGTTTGATTTACAA
AATCTTCTAATTCTTGATGAAGGTGAAAACTGTTAATTTCATGTCCAGTAATGATGATAGGCTGCTTCGATTGATGCAATTTAGATGT
TAATAACTCTATATATGTTGATGCATCCGTATCTTTGGCTGCCGTTACTTCAAATGGTGTCGGTATCTCAATTTCAGAGATTGCGACA
TCGATTGGTAAATGTAAATGAACTGGGCGTCTTTCGGCGATTGCTGTATTAATTAAACGTGGTATTTCGGTTGTTGCATTTTCAGGTG
TGATATAACCTTGTGCAACGGTTATATGTGCAAACATTTTTCGGTAGTCGTCAAATGTACCTTCACCAAGTGAGTGATGTACATATTT

ACCGGCATGTTCAACAGCACGTGTCGGCGCACCTGTAATCGCAATGACAGGTATGCGTTCAGCATATGAACCTGCGATACCGTTGA
CGGCACTTAATTCGCCAACACCAAATGTAGTAACTAATGCAGCGAGTCCATTAAGACGGGCATAACCGTCCGCTGCGTAACTTGCG
TTTAATTCATTTGTATTTCCTACCCAATCTACATTGGGATTGCTGATAATATCGTCTAGAAAAGCGAGATTAAAATCACCAGGAACA
CCAAAAATTTTATCGACGCCTGCTCGATGAATAGCGTCAATTAAGTAAGCTCCAATGCGTTGTTTCATAAAAAAACCACCTTTTCTC
TATATTTACACTTAAAATGATAGCCTCGAATATAGTGTAAGGTCAAGGAAGTTGACTTTGTTGTGAAAACGCTAACAATAGTGAACA
ATTAATATTTTTTAGGAAATGAGTATGAAGGGGGGCTTTAATTGATGATCTTAAAATTCAAAATTTAAGGGGAGAGCGAACTATCA
ATGATGCTGCAAAATACAATAGCAAAGAAAAAACAGGCGCGTCACATTATGAAGGTATACGCACCTGTTTATAGTAAGCATTATTT
AGCTTCAAATAATTGATCGCCAAATGAAATGTTGCCATGTTCACCTTGTTTAAAATCAAGGTTTGTAATGTTTCCTTGTGTCACGATA
ATAGGCGTAATATCACTCTTTGCATGATTGCGGATGTAGTCTAAATCAAAGTTGATTAATAAATCACCTTGTTTAACTTCTTGACCTT
CCTCAACATGTAAAGTAAAGCCTTCTCCGTTTAATTTAACAGTGTCTAAACCGATGTGGATTAATAGTTCTAAACCACTATCTGATA
CAAGACCAATTGCATGTTTTGTTGGGAAAATCATTTGTACTTTACCGTTGAATGGTGCACGAACTTCACCTTGTGAAGGTTTGATAG
CGATACCGTCACCCATCATTTTTTCGCTGAACACTTGATCAGGCACTTCTGATAAAGGTGTTACTTCACCAGTTAATGGTGCATGAAC
GATATGGCTCAATTCGCTTGTTGCAGATTTATCTTCTGCAACAACAACAGTTTCGTCTTTATCGTCTTCCATAGTAGTAGGGTTTTCT
ACTACTTGACCATTCATAATCTGTTGCATTTCATGTTTGATTTGGTCAGATTTAGGACCAAAAATTGCTTGCATATTATTGCCGACTT
CTAATACACCAGATGCGCCTAAATCTTTCAAACCAGGAACATCAACTTTAGATTTGTCGTTAACTTCAACACGTAGACGTGTGATAC
AAGCGTCTAAATGTTTAATGTTTGCTTTGCCACCCATAGCTTCTAATACTGCATATGGTAATTCAGTTGCTGAAGCAGTAGCCGCTTG
TGATTGTTTATCTTCACGACCTGGTGTTTTGTATTTTAATTTTACAATTAAGAATCGGAATACGAAGTAGTAAATAACTGCGTATACA
AGACCTACAGGAATGACTAACCACCATTGTGTCTTATTAGGTAGTATACCGAGTAAGAAGTAGTCGATGAAACCACCTGAGAATGT
ATAACCTAGATGAAGATCTAATAAGTACAATGTTAAGAATGATAAACCATCAAGTACTGCGTGAATAAAGAATAATAATGGTGCTA
CAAATAAGAATGAGAATTCTAATGGTTCTGTAATACCAGTTAAGAATGATGTTAAAGCAGCAGAACCCATTAAACCTGCTACCACT
TTCTTATTTTCAGGTTTAGCTGTGTGATAAATTGCTAAAGCTGCTGCAGGTAAACCGAACATCATAACAGGGAATTCACCTTGCATG
AATTTACCAGCTGTCAAATGTGCGCCTTCACGAATTTGTTCGATAAAGATACGTTGGTCACCGTGAATAATTTCACCAGCTGCATTTT
TCCATGAACCAAACTCGAACCAGAACGGTGCGTGGAAAATGTGATGTAGACCGAATGGAATTAATAAACGCTTGATGAAACCAAAT
AAGAATACGGCAACACCAGTATTTGAATCTAATAATCCTGTACTGAATGCATTTAATCCTGATTGAATCGTTGGCCAAATTAATGCC
ATTGGGAATGCTAAAATAAATGATGTTGTAGCCATCATAATAGGTACGAAACGCTTACCAGCAAAGAAACCTAAATAAGATGGTAA
GTTAATGTTATAGAACTTGTTATAACACCAAGCTGCCAGGGCCCCGATAATAATACCGCCGAACACACCTGTTTGTAATGTTGGAAT
ACCTAAAATGCTAGCGTAACCACTCGCTGGATCAGTAACATTTTTAGGTGTAACTTGTAAAAAGTCGCCCATTGTTTTGTTCATGATT
ATGTAACCGACGAATGCTGCGATAGCTGCTACGCCATCACCGCCAGCTAATCCGATTGCGACACCTAATGCGAAAATCATAGGTAA
GTTTTCAAAAATAATACTACCGGCAGCTGTCATTAATTTAGCGACATTTTGAACGCCACCATTTTGTATAAACGGCAAGTAGTGTTG
TAATGCTTCGCCTTGTATAGCTGTACCGATAGCTAATAACAGACCCGCTGCTGGTAAAATTGCAACAGGTAACATTAGCGCTTTACC
AATACGTTGCAATTGACCGAAAAGTTTCTTCCTCACTTGTCCAACCTCCAAAGTTGTATGTAATATTTAAGATTCAATAAAAAAGAC
ACGAGCAAATAGACTGTGTTGGTAACCATCACAGCTTAATTTAACTCATGCCTAATCTTACTTAGTAACACGTTGGTGTATGTAATT
AATGTAAAAGAAGCAATTGACTAAGTAGTATATAAAACTGATACGTTAGTTTATCTAGCTTACCATCACATCTTATTGAATCTTATTT
GTTTGCGACTCCTATTTTAGCATGGTGAAGTATGCGTTTTCAATACAATTATTAAATTCAATTTCAAATCTCTTAAATGTTCACAGAT
TATTATTAAAACATAATATAAGATAATCCTGAATAAAATGTATAAAAGTTGTTGTAATAATCCAGCAGTAGCAGCTATTATCAGCAC
TTAAAAGAAAGGATAATCTTTGATGGTGTAATAATTAACTTGTGACAGAAATATGTAAGATTATTACAATGAACAGGCTTTAACTGA
TTAATATTGTAATGTGTTTTCTCTTATAGTAAAATGTAAGCGATTACACAACTCAACTATATTTTCCTAATAATTATATTGTTAAGGA
GGGCTACTTTGACAGGCTTTTCAGTGTATTTAGGACAACCTTTAGATGAAGCGTATATTAAGCGAATGATTAAACAAGGTTACCAAA
TGATTTTTACATCTGTACAAATACCAGAAGAAGATGACGAGACAAAATATCATTATTTCACAAAACTACTCAATTTATTAAAACATG
AACAAGTGACTTACCTCATAGATGCTAATCCATCTATTTTAACACCATCTTTTTATGAGCATCTTCGACAATATGATGCACAATTTAT
GATTCGTATCGATCATAGTACATCAATTGAGGCAATCGAAGCGATAATGGCACAGGGTTTAAAGTGCTGTTTGAATGCAAGTATTAT
TTCCCGGGAATTGTTAACAAGCTTACATCAACAATTGAATGATTTTACATTACTTTCATTTTGTCATAACTATTATCCAAGACCAGAT
ACGGGATTATCTGTTGACTTGGTCAATAAGAAAAATGAACTCATTTATCAATTTAATCCAAAGGCACAAATATATGGTTTTATTGTA
GGGAGTGATTTGCGAGGTCCTTTGCATAAAGGCTTGCCAACAATTGAAGCAACGAGACATAGTCATCCTGTCGTTGCAGCTAAATTA
TTACAAGAAACTGGTGTATCTGAAGTGTTAGTTGGAGACTCATTGATTGAAATGAGGCAGGCAAAACAACTTATAGATTTTTGCAA
GCATAGGCATTTCACGTTATGTATTGAAGAAGTGTTTGATACGACAGTGACTTACCTTTTCGATATGTGTCATAAAGTACGTCCGGA
TAATCCGGAAAATGTCATTCGTTCGGAAACGTCAAGACAAATATGTCCGCATTCGATTCAACCACAGTTTACGACGCAACGACGCA
TTGGTTCAGTAACCGTTGATAATTTGAATAACGGACGTTATCAAGGTGAAATGCAAATTGTGAGACAAACGCTTAGTGCACATGAC
AATGTGAATGTTGTTGCACAAATTATTAAAGAAGACTTACCACTGTTAAGTTGTATCGAGCCTAATGATACATTTGATTTTCAAAAA
ACTAGGGAGTGTAAGAAGTGATGGAAAATAGTACGACCGAAGCACGTAATGAAGCGACGATGCATCTTGATGAAATGACTGTGGA
AGAGGCTTTAATTACGATGAATAAAGAAGATCAGCAAGTCCCGTTAGCAGTTCGAAAGGCAATACCACAATTGACAAAAGTAATTA
AAAAAACAATTGCACAGTATAAAAAGGGTGGACGATTGATCTATATCGGTGCAGGTACAAGTGGAAGATTGGGTGTCTTAGATGCA
GCGGAGTGTGTACCTACATTCAATACTGACCCTCATGAAATTATAGGTATTATTGCTGGTGGACAACATGCTATGACGATGGCTGTA
GAAGGTGCGGAAGATCACAAAAAATTAGCGGAAGAAGATTTGAAAAATATAGATTTAACATCAAAAGATGTCGTTATAGGAATTG
CCGCGAGTGGCAAAACGCCATATGTTATAGGCGGTTTAACATTTGCTAACACAATCGGTGCTACAACAGTATCTATTTCATGCAATG
AACATGCAGTTATAAGTGAAATTGCGCAGTATCCAGTAGAAGTTAAAGTTGGTCCAGAAGTATTAACTGGTTCAACGCGTTTAAAG
TCTGGTACAGCACAAAAATTAATTTTAAATATGATTTCAACCATCACAATGGTTGGTGTCGGAAAAGTTTATGATAACCTCATGATT
GATGTTAAAGCAACCAATCAAAAACTGATCGACCGTTCAGTGCGTATTATTCAAGAAATATGTGCTATCACATATGATGAAGCAAT
GGCGTTATATCAGGTATCTGAGCATGATGTGAAAGTTGCGACAGTTATGGGTATGTGTGGCATTTCTAAGGAAGAAGCAACAAGAC
GGTTATTAAACAATGGTGACATTGTTAAACGAGCAATCAGAGATAGACAACCTTAGGAGGGATTTAAATGACCAAAGAACAACAA
CTTGCAGAACGAATTATTGCTGCAGTAGGTGGTATGGATAATATAGATAGTGTCATGAACTGTATGACACGTGTGCGTATTAAAGTA
TTAGATGAAAATAAAGTAGATGACCAAGAACTAAGGCATATTGATGGTGTCATGGGTGTTATACACGATGAACGCATTCAAGTTGT
GGTTGGACCTGGTACAGTCAATAAAGTGGCTAATCATATGGCGGAATTAAGTGGTGTTAAACTAGGTGACCCAATACCACACCATC
ACAATGATAGTGAAAAAATGGACTATAAATCATATGCAGCTGATAAAGCAAAGGCGAATAAAGAAGCACATAAAGCAAAACAAAA
GAATGGTAAGTTGAATAAAGTATTGAAATCAATTGCCAATATCTTTATACCGTTGATTCCTGCATTTATTGGAGCTGGATTAATTGGT
GGTATTGCAGCAGTACTGAGTAACTTAATGGTGGCAGGCTATATTTCAGGTGCTTGGATTACGCAACTTATAACAGTATTCAATGTC
ATTAAAGACGGTATGTTAGCATACTTAGCTATTTTTACTGGTATTAATGCGGCTAAAGAATTTGGTGCGACACCAGGACTTGGTGGC
GTGATTGGTGGTACAACGTTATTAACGGGTATTGCTGGTAAAAATATTTTAATGAATGTCTTCACTGGAGAACCATTGCAACCTGGA
CAAGGTGGAATTATTGGCGTTATTTTTGCCGTTTGGATTTTAAGTATTGTCGAAAAGAGATTGCATAAAATTGTGCCAAATGCGATT
GATATTATTGTAACGCCGACTATTGCATTGTTGATTGTAGGACTATTAACTATCTTTATCTTTATGCCATTAGCAGGTTTTGTTTCAGA
CAGTTTAGTTTCAGTAGTTAATGGAATTATTAGTATTGGTGGCGTATTTAGTGGATTTATCATTGGAGCAAGCTTCCTACCATTAGTT

ATGTTAGGGCTTCATCATATTTTTACGCCAATTCATATAGAAATGATTAACCAATCTGGTGCTACTTACTTATTGCCAATTGCAGCGA
TGGCTGGTGCTGGACAAGTAGGTGCCGCATTAGCACTTTGGGTAAGATGTAAACGCAACACAACATTACGTAATACTTTAAAAGGT
GCATTGCCAGTTGGTTTCCTAGGTATCGGAGAACCATTAATCTATGGTGTGACTTTGCCATTAGGTCGACCTTTCTTAACTGCTTGTA
TTGGCGGTGGTATTGGTGGCGCTGTAATAGGTGGAATTGGACATATTGGTGCCAAAGCAATAGGCCCAAGTGGTGTGTCACTATTAC
CATTAATCTCAGATAATATGTATTTAGGTTATATTGCAGGATTACTTGCTGCGTATGCTGGTGGATTCGTTTGTACATATTTATTTGG
AACGACAAAGGCGATGCGACAGACAGATTTGTTGGGTGATTAATGATGACAAATATTTTATATCGCATTGATAAGCAGTTGAGTGA
TTTTACGAAGACAGAAAAGATAATCGCTGATTACATTTTAAAGAATCCACATAAAATCATTGATATGACTGTGAATGATTTGGCAGA
TGTTACGAATGTTAGTACAGCATCAATTGTTAGATTTAGTCGGAAAATGACACATCAAGGTTTTCAAGAGCTAAAGATTGCGATATC
TCGATACTTACCCGAAGATATTGCAACCAATCCACATTTAGAATTGATTGAAAATGAATCTGTAGAAACTTTGAAAAATAAAATGAT
TGCTAGAGCAACGAATACGATGCGATTTGTAGCTACTAATATTATGGATGCGCAAATTGATGCAATTTGTGATGTGTTGAAAAATGC
CAGGACAATATTTTTATTTGGATTTGGCGCATCGAGTTTGACTATTGGTGATCTTTTTCAAAAGTTATCTCGTATTGGCTTAAATGTC
AGGTTATTACATGAAACGCATTTACTTGTGTCAACATTTGCGACGCATGATGATAGAGATTGCATGATTTTTGTGACGAATCAAGGT
AGTCATAGTGAATTGCAGTCAATTGCACAGGTGGCCACACATTACAGTATTCCCATCATAACTATATCTAGTACAGCTAATAATCCA
GTGGCTCAAATTGCAGACTATGCATTGATTTATGGCAGAACTGATGAAAATGAAATGCGTATGGCGGCTACAACGTCACTATTTGCA
CAGTTATTCACGGTAGATATATTGTACTATCGATTTGTAGCATTAAATTATCATGCGATTCTAGATTGTATAACCCAATCGAAAATG
GCACTTGATAATTACAGGAAGCATCTTGCGACGATAGATTTTAAACATTAGATTAATATAAAGTTAGTTGCAACGTAAACAATTGAA
GTTTTTGATGTGATTCAGTTGCTTGATGAAAAATTGTTGCTACAAGTTTGTAAAACTGGCATAGAGGCTATTTTGAAAATGATAAAG
GCGTGACAAATTAAAGCAAAGTTCAGATTTGAGCTTTGCTTTTTTCATAGATGGAAATTAAATGCTAAAGAATAATTTTAATGCTAA
ATGAACTAATACGAAGGTGCTAAAAGCAATTATTATCAAATTAGTGATATGAATGAAATGTTTAAATCGTTTGCGTTGATTGTATTC
GGGACTTTTATAGAGTAGGTAGTAGTACATAAAGAGTATGGCTGCGAACATGCCGAATATTGAAGTGAAGTTACCTACAGTGACTG
CCATGTAATGATTTAAAGTTAAATAGTAACTTAGACTAATAATAAAAAATAAAATGATTTGATAGGTAATTAACACCATTCAAGTCC
CTCCATTAATCGTAGACAAAAAATTTATCAATGATTTGATTATTTGTATTCAAATTTTAGTATACGACTTACCTCAAAGACAAATATT
AAAAAATAAGACAATAAAATTAGAATAAATTTACGTTTGAAGATAAAAAACAAACATTTTTCATTAAAGTTTATGATATATTTAGA
GCAATAGAAAGTGTATGGAAGGGGATATGAATGGCATACCAAAGTGAATACGCATTAGAAAATGAAATGATGAATCAACTTGAAC
AATTGGGTTACGAAAGAGTAACAATACGTGATAATAAGCAATTGCTTGATAATTTTAGAACGATTTTAAATGAGCGTCATGCGGAC
AAATTAGAAGGCAATCCCTTAACAGATAAAGAATTTCAACGTCTGTTAACGATGATTGATGGGAAAAGTATTTTCGAGAGTGCCCG
TATTTTACGTGATAAATTACCACTTAGACGTGATGATGAGTCTGAGGTTTATTTGTCGTTTTTAGATACGAAAAGTTGGTGTAAAAAT
AAGTTTCAAGTGACGAATCAAGTATCTGTCGAGGATACATATAAAGCACGTTATGATGTAACGATATTAATCAACGGACTACCCCTT
GTCCAAGTTGAATTGAAACGTCGAGGTATTGATATTAATGAGGCGTTTAACCAAGTAAAACGTTACCGCAAACAAAATTACACAGG
CTTATTCCGCTACATACAAATGTTTATCATTAGTAATGGTGTTGAAACGCGATACTTTTCTAATAATGATAGCGAACTATTGAAGAG
TCACATGTTTTATTGGAGTGATAAACAGAATAACCGAATCAATACATTACAATCGTTTGCTGAGTCGTTTATGAGACCTTGTCAATT
AGCTAAGATGATATCGCGCTATATGATTATTAATGAAACAGATAGAATACTGATGGCAATGCGTCCGTATCAAGTGTATGCGGTAG
AAGCACTTATTCAACAAGCGACTGAGACAGGGAATAATGGATATGTATGGCATACAACTGGAAGTGGTAAGACATTGACTTCTTTT
AAAGCGAGTCAGATTTTATCACAGCAAGATGACATTAAGAAAGTTATCTTTTTGGTTGACCGTAAAGACTTGGATAGTCAAACAGA
AGAGGAATTTAATAAATTTGCTAAGGGTGCTGTAGACAAAACTTTTAATACCTCGCAACTGGTACGCCAACTAAATGATAAAAGTTT
GCCACTTATTGTAACGACGATACAAAAAATGGCTAAAGCGATTCAAGGTAATGCCCATTTATTAGAACAGTATAAAACGAATAAAG
TTGTATTTATTATTGATGAGTGTCATCGCAGTCAATTTGGTGACATGCATCGTCTAGTTAAACAACATTTCAAAAATGCCCAATACTT
TGGATTCACTGGTACGCCACGTTTTCCAGAAAATAGTAGTCAAGATGGTAGAACAACTGCAGATATTTTCGGTAGATGCTTACATAC
GTATTTAATTAGAGATGCCATTCATGATGGTAATGTACTTGGTTTCTCAGTTGACTATATTAATACTTTTAAAAATAAAGCTTTAAAA
GCAGAAGATAACAGCATGGTTGAAGCAATTGATACGGAAGAAGTATGGTTAGCGGATAAACGTGTGGAATTAGTAACACGACATA
TCATCAATAATCATGATAAATATACACGTAATCGTCAATATTCAAGTATATTTACAGTCCAAAGTATTCACGCGCTTATTAAATATT
ATGAGACATTTAAGCGACTTAACAAAAAGTTGGAACAACCGTTAACGATAGCTGGTATATTTACGTTTAAACCTAATGAAGATGAT
CGTGATGGTGAAGTGCCATATCATTCACGTGAAAAATTAGAGATAATGATTAGCGATTATAATAAAAAGTTCGAGACGAATTTTTC
AACAGACACAACTAATGAGTATTTTAATCATATTTCAAAAAACGTTAAAAAGGGCGTTAAAGATAGTAAAATTGATATCTTAATCG
TTGTTAATATGTTCTTAACTGGTTTTGATAGTAAAGTACTGAACACTTTATATGTTGATAAGAATTTAATGTATCATGATTTAATTCA
AGCGTATTCACGTACAAATAGGGTTGAAAAAGAATCAAAGCCATTTGGTAAAATTGTAAACTATCGTGACTTGAAAAAAGAGACAG
ACGATGCACTGAGAGTATTCTCACAAACAAATGATACGGATACAATTTTAATGCGCAGTTATGAAGAGTATAAAAAAGAATTTATG
GACGCTTATCGTGAGCTTAAAATGATTGTGCCGACACCACACATGGTTGATGACATTCAAGATGAAGAAGAGCTAAAGCGCTTTGT
TGAAGCTTATCGTTTATTAGCTAAAATAATATTACGTTTAAAAGCATTTGACGAGTTTGAGTTTACAATTGATGAAATTGGAATGGA
TGAACAAGAGAATGAAGACTATAAAAGTAAATATTTAGCTGTGTACGATCAAGTAAAAAGAGCGACGGCTGAGAAAAATAAAGTA
TCCATTTTAAATGATATTGATTTCGAAATAGAAATGATGCGTAATGATACGATTAATGTGAATTATATTATGAATATATTGAGACAA
ATTGATCTTGAAGACAAAGCGGAACAACGTCGTAACCAAGAACAAATTAGACGCATTTTAGATCATGCAGATGATCCGACATTGAG
GTTAAAACGAGATCTAATTAGAGAATTCATCGACAATGTTGTACCTTCTTTAAATAAGGATGATGATATCGATCAAGAATATGTTAA
TTTCGAAAGTATTAAAAAAGAAGCGGAGTTCAAAGGATTTGCTGGAGAGAGATCTATCGATGAACAAGCCCTAAAAACAATTTCAA
ATGACTACCAGTATAGTGGTGTTGTAAACCCACATCACCTTAAAAAAATGATTGGTGATTTGCCATTGAAAGAAAAGCGTAAAGCA
AGAAAAGCCATTGAATCTTTCGTGGCAGAAACAACTGAAAAATACGGTGTGTAATGATTCAGCCCCCTCGCTAGATTAGTGTAGGG
GGCATTATTATTTTCTATCTTTTGTAAGTTATCATGTATTTGGCAATCATCACTTGGTTTCAATACAATTTTATCATCGTATGCGTATA
GAAATAATGTCTATAGGCATTTAAAATAGTTTATAATATTAACAGGATAAGTATTAAATGTATGTTGAAGGAGGTGCGTAGCTATGG
ATAATAGAAATATGATTAATCGTGTTTTTAGTCAAAAGATATTACATCAAATTGCAATCAAAAATAAAAGTGATGTTGTTGATGAGG
CATATGATTTTTATATACAGGGACCTAAAAATATCAATGTAATACAGAAGATGAAATCTTTATATAATTATCTTAAAAAGTCTTATC
GTAACGAATATTTTTACAAAAACACAATACTTAATAAACTCCTTCTAGGACGACATTCTATTAATACAACTACTGCACTTTCTGAGA
TGCCCATAGGGAAAAGTATTGCTGATTTTATATTGTTAAACGGCAAAGGCGTTGTCTATGAGATTAAAACAGAATTAGATAAGCTG
GATAGATTAGATAATCAAATTAATGATTATTATGAAGTGTTTAATTATGTAGTAGTTATTACAAATGACAAACATCTGAATAAAGTT
ATGGCTAGATACAAAGATACAACAGTTGGAATTTTAGTGTTAACAACTAGAAATACACTGAGTGAAGTTCAAAAACCTAAAGAAAA
CAATAGTCTCTTAAACACAAAAGCGATGTATAACTTTTTACGAAAAGAAGAAAGAAAAAGAGTTATTGCACAAAATCATATGGATG
TGCCAACTTATAATGATTTCACAGAGTATGATGTGTTATTTGACGTGTTTAAAGAAATACCAATGACGAAACTGCATAACAATATGA
TTTCTGAGTTGAAAAAAAGAGGCAACATGAAAGAATACAAAGATGAATTTTTAGCAGCGCCGGCTGAAATTAAGTTCTTGTTATAT
TTCGCAAAAATGACAAAGAAAGATAAAAATAAACTATATCATTTTCTTAAGGAGGATTAATATGTATTATCCTTATTTGCGTGGGAA
ACAAAATGAACTATTTGCAATTAGAGAATTGTTAGAGAAAGGTTTGATTGGTGATTGTATTCAACCTATAATTGAACCGATTAAGTA
TACAACCACGTTTAAAAATGTTTTGAAATATTGTGGTGAAAAAGAATTTCGTATAAATTTAGTAGTAAATTCGAAGTTAACTGAAGA
AGAGATTAGTAACGAAACTGTTGAAGACTTAAGCGAAATAATAACAAAAAACAAAAGTGTTATTCAAAAAGCTTACTTGGGTCCTT

CTGATGAAGGCGATGATAGGTTGAAACAGCAATTTTCAAGTAATAGTTTAGCTATTTTAACAAGTGTAGATGACTGGGAAATGTTTG
GAGATAAAAATAAACTTGAAATGGTTTTTGTACCAGATGATAGACACATTAAACGTAAATTGCGTAATATTCCAAACAAAGGCATC
ATTATGGATCCTTTTAATAAACTAAGTCGTAATGTTGATTATTTAGATAATGATGACGAGTTTTATAGCGACGATCACCTTTATTATA
AGGAAGATGGATACGTAGCATTTTCAGACTATTCTGTTATAGGTGGAGAATATGTAGACGGTGGCTTTTCGCCATTAGCTATTGCGA
TACATATTGTCTATTTTGATGAGGCTAATGAGTTAAGAGTTAAACATTTTGTCTCTGATTCTAATAACGATAGATTAAATCCAGCTAA
AAAGTTTTTTGAGGCTGTAGATAAATTAGTAACTTGGTCTAAAAACTTAGATAATAAAAATAGATCTTATGCAATCGGACAATTTGA
AGAATTAAATGAAAATAATAAGTATCCAGGATTAGGTTTAATTAAAAGGTTATCTATCATGCATCATCTAGAAATTATGAATAGATA
CTTGGAGTCTCAAAATGAAAATATGTGAAAAATGTTTTAATAATACTGAAATCGTAGAAATCATTGTAAATGATAATAGCAAATTTG
ACAATTGTGATATTGATAACGATCATCTTGGTGTTAAAATATTTGATACGACTCGACACATAGATAAATTAGAGCAGATTAGAGATT
ATTTAAGACCAGCGTTAGAATTATATGATATTAGTATAAATTTACCAGATACTTTTAGCCAAAAAGAAGGTAAAAAAATTGAAACA
GCATTAAAAGATGATTGGAGTATATTTAATGTTGAAGAAGCTCAAATAAGTTGTATTTTAAATGAACTTTTTAAAGATGATGAAAAT
ATAGATAGACGGGTGTTGGAAGGTTTAGTAAGCGCTAAAATCATTAAAGATAAAAAATATACAAATGAAAATCTAATTGTAGCAAA
TAATGACTGGGATGGATTTTGTGAGAGCTTGAAGTATAAAAATAGATTTCATAATAATATGATCAATTTAGAGAATTTAGCGTTCTT
TCTCGATATCACTACTAATTACTATAGTATTGAGGAATTTAGAGAAAGATTTGAACCATTATATAGATCTAGAATAGTTAAGTTCAT
TACAGAGAAAAGTGAACTAATGTCACCACCAAAGGGATTTGCAACGGCAGGAAGATTGAATTCTAAATGGATAAGCGTATTATACC
TTAGTACAAAAGAACAGGTCAGTATAGAAGAAGTTAAACCTAAGCATAATGACATTATTTATATAGGTAAGGTTAAGTTACAAAAA
AACGTTACAAAAGAGAAATTGAAAATTGCGAATTTAACTAATTTAACTAGTAATGCAATCAAAGCTGGCGATGATGGATTTAGAAA
GTATTTTGTAAATCACCAAACATTAAAAAAGATTCATAAAGGAATAACAAATCCAAGCGATGAACAAGGAATAGATTATTTGCCAT
TTCAATATTTAGCTGATTATATTCGAAGCCTTAAATATGACGGGATAATGTATGAGAGCATATTGCAAGATGGTACTTTTAACTTTGT
GTTTTTTGATCAAAATTTATTTGAATGTGTTAATTATGAAAGAAAAAGAGTAAGTGATGTTAAATATACATTGACTAAGCTAAGTTG
ACACACTCGGAAATCTGAATATTCTAATAAAATATAAATAATCCCCCGCACTTCACATAGTGGCGGGGGATTTATTCTGGCGTTAAA
ATGCCCAAAGTATGGGGGTGACTCTTAAATCATGGCAAAGCACCTAAAACTTGATATGGTGCCGAGCCCTAACTTAGCACAATATA
GTTAGTATGACAAAGTCATCTAACATAACACATCAGAGATTGCCTGATTTATTCCAGACCATACAGTGATACAGTTGCACAACGCAT
GACTTTTATTTAGCAATAACTGCTACTTCTGAAATAAGTTGCTTTGCATAGTCTGATTGCGGATGTTTGATAATATCTTCTGTGTTATT
CAGTTCAACGATTTCGCCATTTTTCATAACTGCTACGCGATCACATATTTCATTGATAACACCCATGTCATGTGTGATGAATAAATAA
GTGATGCCGAAGTCTAACTGTAATTGTTTTAATAACTCGATGATATCTTTTTGAATTGAAACGTCTAAAGCGGACACTGCCTCGTCG
CAAACAATCACTTTAGGTTCTACAGCAAGTGCTCTCGCGATACTTACACGCTGACGTTGCCCACCAGATAATTCGTGTGGATAGCGA
TATAAGAAACTTTGATCTAGGCCAACTTTTTCTAACAACGATACGACAGTTTTAATAATGTCATCATTATCTTTGACTTTCCCATGAA
TGATTAGTGGTCGTTTAATCACATCAATGACTTTAAATCTTGGATTAATAGATGCGAATGGATCTTGAAAAATCATTTGTATCTCTTG
TCGTAAAGATTTCAATTCATCATCTTTAAATAAACTTAATGGTAATTCGTTATACCAAATAAAGCCTTCTGACACTTCCTTTAGACCG
ACGACCGTCTTAGCTAATGTCGATTTCCCTGACCCTGATTCACCGACAATGCCTAATGTTTCGCCTTTTCTAATACCCAAGTTAATAT
CATTAACTGCTCGGTATAGGCTGCCACTCGGTGATGTGTAATCCACGCTCACGCGATCGAATTTTAATAAAATATCATTGTTTAACG
GTCTTGGCGGACGCGTTTGATGAATATCAGGAATCGCATCTATTAAGCGTTTTGTATAGGTATGTTGTGGCGATTTAAAAATACTTTC
AACCGTGCCACTTTCAACGACACTTCCATCTTTCATTACAATCACATCGTCGCAAAATTGATACACAGCGCCTAAATCGTGAGTGAT
AAAAATAATAGATGTTTCTGTGTACTCATAAAGGGACTTCATTAACTGCAGTAATTGATTTTGTGTACTGGCATCTAATGCCGTTGTT
GGTTCATCTGCGATTAAAATTTGTGGCTTTAAAATCAATGCCATTGCTATCATGACACGTTGACGCATACCACCAGAAAGTTCATGT
GGATAAGCATCAAATTGTCGAGTTGCATGTTTTATACCTACTTTTTCTAAAATGTCTATTGTCATCGACTTTGCTTCAGATTTAGATA
CACGTTTATGTTGAAATAGTACTTCTGTAATTTGTTTGCCAATCGTTAATCTTGGATTCAACGAAGAGAGTGGATCTTGAAAAATCAT
TGAAATATCCTTACCTCGAATTTGTTGTAACGCTGAAGTTGATAAATTATTTAACGATTGCCCATTAAAAATAATTTCTCCTGTTAAT
GTGTGATCTGGATAATCTGGTAGTAGCCCTAAAATAGATTTAGCAGTAATACTTTTTCCTGATCCTGATTCACCAACAATACCTAGG
ATATGTTTTTTTCGTAATTCGAAAGAGACATTTTTTACCGCTTGAACTGTAGTTTCATCATAATTGAATTGTACATTCAGACTGTTGA
CTTCTAATAAATTTGACATGATGTTGTGCCTCCCTATTCACAAATGTGTAAATTTATTCTATACTTTTGTATATCATAGTAATCTACTC
AAGATTATTTTACAAGGAGTGATTATCATAATTACTTATTTAGAATCTTTTAAGTCGTTGTATCACAAGGCATTTTACAAGTTTGTAT
TATCGGTATTAAGCTTGCCAATATTTTTATATGCAGTGATAAAGTTTTTCTTTTCTGCTAAGAGAAAAAATTTTTACGCTAATAATTC
TGAAATTTCAGAAATTGAACGAGCGTTACATCAAAAATATAAATATTTATCGCAGCAAAAGTCATCCACACAAATACATAAAGAAG
CATTAAAAATATTCAAGGCACAAAGTTCTAATACGAGTTCAAAGAATATTGAACAAGCACATTTTTCAACATACTTTGAAAATTTAT
TATTTCATAAGTTCATCATGATCAAAGTAATATTGGCCTTGCCGATGTTCATCTTATTGACTATTTATTTACAGCCATTAGTTAGATA
TATTTTTGAGAGAATTGTCATGGCTGTGATTGTCATCATTGGTGTTATTGTCAGTGTGTTTACCATTCTGTATTTTTCACCGCTTGATG
CGGCTTATAGCATACTGGGACAAAATGCAACAAAGGCACAGATACATCAATTCAATGTATTACATCATCTTAACGAACCTTATTTTA
TTCAATTGTGGGATACCATCAAGGGTGTTTTTACCTTTGACTTAGGTACGACTTACAAAGGGAATGAGGTTGTGACTAAAGCAGTTG
GCGAAAGAATTCCAATTACAATAATTGTCGCAGTATTAGCGCTAATTGTGGCATTAATTATTGCAATACCAATTGGTATTATCAGTG
CGATGAAGCGAAATAGTTGGCTTGATATCACGTTAATGATAATTGTATTAATTGGTTTATCTATTCCAAGTTTCTGGCAAGGGCTATT
ATTCATTTTAGCGTTCTCATTGAAATTGGATATTTTGCCACCATCTTATATGCCAGAACATCCAATATCGTTGATTTTACCTGTACTTG
TCATTGGAACAAGTATTGCTGCTTCTATCACGCGTATGACAAGGTCTTCTGTACTTGAAGTAATGCGCAGCGATTATGTTTTAACTGC
TTATGCAAAAGGATTATCGACGACACAAGTTGTTATTAAACATATTTTGAAAAATGCCATTATTCCAATTGTAACGTTAGTTGGTCTT
CTAGTGGCAGAGTTACTAGGCGGTTCAGCAGTGACGGAACAAGTATTTAACATTAATGGTATCGGGCGTTATATCGTCCAAAAACA
ACTAATACCTGATATCCCAGCAGTCATGGGTGGGGTCGTATATATATCAATTGTAATATCTTTAGCAAACTTAATTATTGATATATTT
TATGCTTTAATCGATCCAAAATTACGTAGTGAAATTAACGAAAGGAAGTGAGGCATATGGTAAAACTTACAACAAAGATAGCTTCC
TTAAAACTATTCGCAAGTTATGCTATAGCAACTTATATTTTAGTTATATTAACGAGTGCATTAAATCTTTTTAAAGGTTATGTGGCCG
ATACGTTCTATATTGCTGAAACATTGCTAATCGTTTTAACCATCATTTTAATTATCATTTTAACAACGGAACAAACATGGAAGCATCA
TGACTTATGGCGACGTATCGTCGAAGTGTTGTTATTGTTGATGACATTAACAGGCAACGTATTTACATTATTAATGTTTGTAAGTATT
AGACGTTACCAACGTACATCGCAAATACATAGTTATAACGGGTGGGAATCGTTTATACGAAAAACTACTAGACATCGTATTGCGAT
TATCGGGTTACTTATTTTAGTCTACATGCTGACATTATCAATTGTGTCACAATTTACATTTGATACGACATTGGCTACTAAAAATCAG
TTCAATGCACTGTTACATGGACCGAGTCTAGCCTATCCGTTTGGTACTGATGATTTCGGTAGAGACTTATTTACACGCGTAGTTGTAG
GAACGAAGCTGACATTTTCAATTTCAATTATTTCAGTAGTTATTGCAGTTATTTTTGGTGTGTTACTAGGCACTATCGCAGGTTATTT
TAATCATATTGATAATTTAATAATGCGAATTTTAGATGTAGTGTTTGCAATTCCATCATTATTGTTAGCGGTGGCAATTATTGCATCA
TTTGGAGCAAGTATTCCAAATTTAATTATTGCTTTAAGTATCGGTAATATACCATCATTTGCACGGACAATGCGTGCCAGTGTTTTAG
AAATTAAACGCATGGAATATGTAGATGCAGCACGTATCACTGGTGAAAACACTTGGAATATCATATGGCGTTATATTTTACCGAATG
CGATTGCGCCTATGATTGTACGTTTTTCATTAAATATAGGTGTGGTTGTATTAACAACAAGTAGTTTAAGTTTCCTAGGACTTGGTGT
TGCACCTGATGTAGCTGAATGGGGCAACATTTTACGTACCGGTAGTAACTACTTGGAAACGCACAGTAATTTAGCTATTGTACCTGG
TGTTTGTATTATGTTCGTCGTTTTAGCATTTAATTTTATAGGTGATGCAGTGCGTGATGCACTAGATCCAAGAATTCATTAAAAAGGT

AGGGATAGATGTGAAGAAAATCATTAGTATCGCAATTATAGTTTTAGCGTTGGTATTAAGTGGTTGTGGTGTCCCTACGAAATCAGA
AGTGGCTCAAAAGTCATCGAAAGTTGAAGTGAAAGGCGAGCGACCAACAATACATTTCCTAGGACAAGCAAGTTATGAAAATGAT
ATGAATATCGTTAAAGATCAATTGGAAAATGCAGGATTTAACGTGAAGATGAATATCCAACCAGATTATGGTAGCTATCGTACACA
ACGTCAAGCCGGCAATTATGATATCCAAATTGATGACTGGATGACAGTGTTTGGTGACCCGAACTATGCTATGACGGCATTATTTAG
TTCTACAGGATCAAATAGTTTATTGAAAGATAAACATGTAGACCAGTTGTTAAATAAAGCTTCTACTCAAAATGAAGCAGATGTTAA
ACAAACATATAAGCAAATTGAAGATGAAGTTGTATTTGATAAAGGGTATATGGCGCCTTTATATGGATCAAAAAAGAATTTAGTAT
ATGACAATAAAGTGTTAGATAAAAATAGTGTTGGATTGCCAAATTCACGTGCATTAATATGGCAACAATTTGATTACAACAATAGT
AGAGAACGAGATACGCGGCCACTTGTGATGACACAACAAGATGGTGAAATTCCTACATTGGATCCAATACGTTCAATTGCGCCGTC
AGTATATTCAATTAATATGAATATGTACACAAGGTTATTATTATTAGATGAAAATGATCACTTAACAACGAAAGGTTCGTTAAGTCG
TGATTATGCTGTGAATAAGGACAATAAAGCATTTTATTTCTTGTTAAGAGATGATGATTATTTTGTGAAAGTGGTCAATGGACAAGC
ACGTAATACTGGAGAGCGTGTATCGGCTGAAGATGTTAAGTTTTCTTTAGATAGAGCACGTGATAAAAAGTCTGTGCCTAACAATA
ATACTTACAATATGCACAAACATATAAATGACATCAAGATATTAAAAGATGAGGACATCGATCAGTTGCGTAAAGAGAAAGACAA
GGACGATAAATCAATCTATGATAAGTTGATTAAAGCTTATAACGTCAAATCGTTAACGACAGATGGTCAAAAAGTAAATAATAAAG
ACGGTATTTATCAAATTGTTAAAATTACGACAGATCAATCGATGCCTCGAGAGGTAAATTACTTAACACACTCTTCGGCAGGCATTT
TATCTAAAAAATTTGTTAATCAAGTAAATCAAGAATATCCAAAAGGATATGGGGATAGCAGTACAATTCCTGCAAATTCAGATGGG
AAAAATGCGCTGTATGCAAGTGGCGCATACATTATGACACAGAAAAATGCATATCAAGCAACGTTTCAACGTAATCCAGGATTCAA
CGAAACAGAAAAAGGTAGTTATGGACCAGCTAAAATTAAAAATATTACATTGAAGTTTAATGGTGACCCGAATAATGCATTGTCAG
AACTTAGAAATCATTCAATTGATATGTTGGCAGATGTGAATCAAAAACATTTTGATTTAATTAAGTCGGATAAAAATTTAAGCATTA
TTCGCAAAAATGGACGCAAGTCAGTCTTTTTAATGCTAAATATTAAAAAAGGTATATTTAAGACGCATCCAAACTTGAGACAAGCA
GTAGTTAATGCGATAGATCAGGATCAATTTATTAAGTTTTATCGTGGCGATAAATTTAAAATTGCATCACCGATTACACCACTTGTC
GATACTGGTAACGAGCAACGTCAAGATATAGAAAAAGTAGAAAAAGCCATTAATCAATAATGTTTTATATATTTAACAGAAAGTAG
GAGGATATAGTATGGTCATTAACTTAAATGACAAACAGACAAAAACATCTAAAGAAGGGTTAATTTCCGTATCACATCCTCTTGCG
GCTAAAATTGGTAAGGATGTATTAGATCAAGGTGGCAACGCCATGGATGCAGTGATTGCAATTCAACTGGCATTGAATGTGGTAGA
ACCATTTGCATCAGGTATTGGTGGTGGCGGGTATTTGCTATATTATGAGCAAAGTACTGGCAGTATAACTGCTTTTGATGCGCGTGA
GACAGCCCCTGCACATGTAGACAAACAATTTTATCTAGATGATTCAGGCGAATATAAATCATTTTTTGATATGACTACACATGGTAA
AACTGTCGCTGTGCCAGCAATTCCAAAGCTGTTTGATTATATTCACAAGCGTTATGCTAAGTTGTCATTGGAAGATTTAATTAATCCT
GCAATTGAACTAGCTATTGAAGGTCATGCAGCCAATTGGGCTACTGAAAAATATTCGCGCCAGCAACACGCACGATTGACAAAGTA
TCATGAAACGGCACAAGTATTTACGCATGAAAATCAATATTGGCGTGAAGGTGATTGGATTGTACAACCCGAATTAGGTAAGACAT
TTCAAATATTAAGAGAACAAGGGTTTAATGCATTTTATAAAGGTGACATTGCGAAACAATTAGTCAATGTTGTTAAAGCATGTGGTG
GGACAATCACTTTAGAGGATCTAGCCAATTATGACATTCAGATTAAAGCGCCAATCAGTGCGACATTTAAAGACTATGACATTTATT
CAATGGGACCATCTAGTTCTGGCGGTATCACGGTCATTCAAATATTGAAGTTATTAGAACATGTCGATTTACCATCTATGGGTCCAA
GATCTGTCGATTACTTGCATCATTTGATACAAGCGATGCATTTAGCATATAGTGATCGCGCGCAATACTTGGCGGATGATAATTTTC
ATGAGGTGCCTGTACAGTCATTAATTGATGACGATTATTTAAAAGCACGCAGTACGCTCATTGATAGCAATAAAGCAAATATTGATA
TAGAGCATGGTGTTGTGTCTGATTGCATTAGTCATACAGATGTTGAAGAAAATCATACCGAAACAACTCATTTTTGTGTGATTGATA
AGGAAGGTAATATTGCTTCATTTACGACATCAATCGGTATGATTTACGGATCGGGTATTACGATTCCAGGGTACGGCGTGTTATTGA
ATACCACAATGGATGGTTTTGATGTAGTAGATGGTGGTATTAACGAAATTGCACCATATAAACGACCACTAAGTAACATGGCTCCA
ACGATTGTGATGCATCACGGGAAGCCGATATTAACAGTAGGTGCACCTGGTGCCATAAGTATCATTGCTAGTGTTGCGCAAACATTA
ATCAATGTATTAGTGTTTGGCATGGATATTCAGCAGGCTATAGATGAACCTAGAATTTATAGTAGCCATCCTAATCGCATTGAATGG
GAGCCTCAATTTTCACAATCTACAATATTAGCATTGATTGCACGTGGACATGCAATGGAACATAAACCAGATGCCTATATTGGAGAT
GTACATGGGTTACAGGTTGACTTGAATACACGTGACGCGTCGGGAGGTGCTGATGATACGAGGGAAGGCACAGTGATAGGTGGCG
ATGTATTATCAATTAGAAAACAACCATTACCTAGTCCGAAAATATACGATAATGACACCCATCGAGTATATTTCAATGATATGCAGT
TGCCATTATATGCTGAACAAGTGCGGTGGATGCATGACAAATATTGGGTTGATGAAAGTGTTATTAGAATCATTTTCCCTGAAGTTA
GTGTGCATATTGAAGATTTAAGAAGTTATGAAATCGCCGGAAAAAATTATATAGATATTGCCTGGTTGGCACGGAAGAAAGGTTAT
CAAGTTACCTTAAAGGATGACAGTTTATACTTAACTGATGAAACATATCATTCAGTGAAAGCAAACACAAATGCATACTATAGATA
TGATCGAGATAGTATCACAAGATAATTTATGTATTTGATATATAGTTTGAAGTGGATTTTGATTCTTGTTGAACGAAAGGGTTCGATT
TCAAACTTTAGAGTGATGCTTGAGTGTAGTAATGTTGAAGTTGTTATAAGAAACGCTAGTCGCCTTTGTAAAATATAAAACGCCCCA
CAAATTCGTTTTTGATGTACACCACAATAGTTAGTCTAAAATCTAACTTGATGTGATTCGGTACAATTAAATGAATGATGTGAGGCG
TTTTTATAATGTGTGATTTTAATAAATGATTTGGTTATTTCTATAATTACATCATAAGTATTAATGTTCACTTAATTTATTATAAGTAA
GGCTTTCTTTGTATATAATTTGGTCTTTTCCAGGACTTGAAAAATAAAAATAAAGTTTCTGATCTTGATGATTATTGCCTTTTAAATC
ACTCATTCCCTTTAATTCCACCTTTGTTGATGCATTTCTGGGAATATTATATCTACGTTTTAATTGTTTAACGTTTGGATTATCATTCG
ACAACTTACCAGATAAATTATAATAATCAGTGGTAGGATCATGTGTGACTTTCAAATCATTGCTATTTAAAATGCTTGTTAAATCAC
CACTTTGAATCAAAAATTGATTGTTTTCGATTTTTTGTTTCAGCGCGGGATCTTTTACGTCTTTTGTGAAAACGATTTTATTATTAACT
ACTTTTACTGGATAACTTTTGTATGTCGAGTCAGTAGCATTTTTTCTATCGTTTGTAGTTGTGTCATATTCACCAGTTATTTTATGTGT
GTTCTTATCTACCTTTAACAACATACGGTCTTCTTTTAAAAGCTCATCTGATCCAACAACTGAATAAGAGGATTCTATATACCATGTG
TCTTGATCATTATTTTCATAATGGGGATTATCGTGACCATCAATTTCATAAAGCGTTTCTAAGTTTTTAATAGGATACGTACTTAGTA
CTTTTTTAAGACCATCTTTCAAATGAATTTGTTCCCACTTCATTGCCAAAAACATACCGCCACTGACTACAATTGAAATAATAATAAT
TGCTGCTAAGTTTAACCAGAAAATTTTATGTGCTTTCATACATTCCCACCGTTTCTCAAAATACTTCATTAACACTATAATAATATAT
TTTGAAAAATATTTACATCAGTATTAAAGTGAATATCAAATTTTAAATTTATGAAAATAATAGATATTTATAAAAAGCGGAAAAGA
GATACAATAAAAAACTGCATGACGTTGAGGAGTCACACAGTGTAATTAAAAATTTAAAATGTAGTTGCTAATTTTTCAGCATTATTA
ATACTAGTTGCTTTAATTTCTTCAGTCTTATGAGGTTCAGCATTGTGTCCTTCAATAATGATTGTTTCATATGATGGCACACCTAAGA
ATGTCATAATTGTTCTTAAATAACGGTCACCCATTTCAAAATCAGCAGCAGGTCCTTCAGTATAATATCCACCACGTGATTGAATGT
GTAATACTTTTTTGTCAGTTAACAAACCTTGAGGTCCTTCAGCAGAATATTTAAAAGTTTTACCTGCAATTGAAATAGCATCAATATA
TGCTTTAACTACAGGTGGGAAAGAAAGGTTCCACATAGGCGTTACAAATACATATTTATCTGCACTTAAAAATTCTTCTAAAATGTC
ACTCAATCTTGAAACTTTCATTTGTTCATCATCAGTTAACGTTTCGCCATTACTCATTTTTCCCCAACCAGTTAATACATCTTTGTCAA
TAACTGGAATATAAGTTTCAAATAAATCAATATGTTTCACTTCATCATCAGGATGTTGTTGTTGATATGTTTCGATAAATGCTTTACC
AGCCGCCATAGAATTTGATACCAGTTCATTAAAAGGGTGTGCTGTAATATATAATACTTTTGCCATTTGAAAATTCTCCTCTGTTTCT
GTTATTTTCTTGAGTATAATTATTATATTAGATATAAAATTTGATATCAATAAAAATATTCAAATTACCATCATTTTCTTCATCTATAT
TTGGCAGTACTACTAAAGTATGAGTGCATTTAATTATGAAATAGTTGATTTAGAATATATACTTAATACCCAAAATATATGAAGGAT
GGATGCCACTATGACAAAGCGACCAAAACGTATTTTGGCAACAATTATCATTTTTCTTTCACTATTATTTACGATTATTTATATAGAT
GACATTCAAAAATGGTTTAACCAATATACCGATAAATTGACACAAAATCATAAAGGACAAGGACACTCAAAATGGGAAGACTTTTT
TAGAGGGAGTAGGATTACTGAGACTTTTGGCAAATATCAACATTCACCATTTGATGGTAAGCATTATGGCATTGATTTTGCATTGCC

AAAAGGTACACCAATTAAAGCGCCGACGAATGGTAAAGTAACACGTATCTTTAATAATGAATTAGGCGGCAAGGTATTACAGATTG
CCGAAGACAATGGAGAATATCACCAGTGGTATCTACACTTAGACAAATATAATGTCAAAGTAGGTGATCGAGTTAAAGCAGGTGAT
ATTATTGCATATTCAGGCAATACAGGTAAACAAACGACAGGCGCACATTTACATTTTCAAAGAATGAAGGGTGGCGTAGGTAATGC
ATATGCAGAAGATCCTAAACCGTTTATCGATCAGTTACCTGATGGGGAACGTAGCCTATATGATTTGTAGTTATAGAAGAGTGCCCG
CAGTCTAAAAAATTAAGCAATCATTGTGTGAGTATGATACTTACATAATGGTTGCTTTTTTCAATGAAAATCGTAATGCTAAGTCAT
ACTTGTTTGATTTAGATATTACTTAAAATGTAAGACAAGGTTGTTTGCATTGGCAGTGAAATATCGCACATAAAAAAACATTATTGT
CACACTAGAAAATAGTTGTGCACTATATCAATTTTCTGTATAAAAGTTTAATTCTGACAGTAATGTAAACGTTTACAATTTATGATTG
ACATTAATAATGACTGAATATATGATTTATGTAAGTATTTGTGCAAACGTTTTCACAAAGTGTATTGCACAATCAAACTGTAAACAA
AGTATGGGAGGCATAACATGGCAGAACTAAAGTTAGAGCATATTAAAAAGACGTATGATAACAACAATACTGTAGTGAAAGATTTT
AATCTACATATTACTGACAAAGAATTCATTGTATTTGTTGGACCATCGGGATGTGGTAAATCAACAACATTACGAATGGTTGCTGGA
CTAGAGTCTATCACATCTGGAGATTTTTATATTGATGGGGAACGCATGAACGATGTTGAACCAAAGAATAGAGATATTGCGATGGT
ATTTCAAAACTATGCATTATATCCACATATGACTGTTTTTGAAAATATGGCATTTGGGCTAAAGCTACGTAAAGTAAATAAAAAAGA
GATTGAACAAAAAGTTAATGAAGCAGCTGAAATATTAGGATTAACTGAGTATCTTGGTCGTAAACCAAAAGCGTTATCTGGCGGAC
AGCGTCAACGTGTTGCTTTGGGCAGAGCTATTGTTAGGGATGCGAAAGTCTTTTTAATGGATGAACCATTATCGAATCTTGATGCGA
AGCTTCGAGTACAAATGCGCACAGAAATATTGAAATTACATAAGCGACTTAATACTACGACAATTTATGTTACACATGATCAAACT
GAAGCATTGACGATGGCTAGTCGAATTGTTGTTTTGAAAGATGGCGACATTATGCAAGTCGGCACACCTAGAGAAATATATGATGC
CCCTAATTGCATATTTGTGGCGCAATTTATCGGCTCACCAGCAATGAATATGTTGAATGCTACAGTTGAAATGGACGGATTGAAGGT
AGGAACACACCATTTTAAATTACATAATAAAAAATTTGAAAAGTTAAAAGCTGCTGGCTACTTAGACAAGGAAATTATTTTAGGTA
TTCGAGCTGAAGACATTCATGAAGAACCAATATTTATTCAAACTTCTCCAGAGACACAATTTGAATCTGAAGTAGTTGTATCCGAAC
TGTTAGGTTCAGAAATTATGGTACATAGCACATTCCAAGGAATGGAATTGATTTCTAAATTAGATTCAAGAACACAAGTGATGGCG
AACGACAAGATTACACTAGCATTTGATATGAATAAGTGTCACTTTTTTGATGAAAAAACAGGAAATCGTATCGTCTAAGGGGGAGT
ATTCATGTCTAAAATTTTAAAATGTGTCACGTTAGCCGTGGTAATGTTATTAATCGTAACTGCATGTGGCCCTAATCGTTCGAAAGA
AGATATTGATAAAGCATTGAATAAAGATAATTCTAAAGACAAGCCTAACCAACTTACGATGTGGGTGGATGGCGACAAGCAAATGG
CGTTTTATAAAAAAATTACGGATCAATATACTAAAAAAACTGGCATCAAAGTAAAGCTTGTAAATATTGGTCAAAATGATCAACTA
GAAAATATTTCGCTAGACGCTCCTGCAGGAAAAGGTCCAGATATCTTTTTCTTAGCACATGATAATACTGGAAGTGCCTATCTACAA
GGCTTAGCTGCTGAAATCAAATTATCAAAAGATGAGTTGAAAGGTTTCAATAAGCAAGCACTTAAAGCGATGAATTATGACAATAA
GCAACTAGCATTGCCAGCTATCGTAGAAACAACCGCACTTTTCTATAATAAAAAATTAGTGAAAAATGCACCGCAAACGTTAGAAG
AAGTTGAAGCTAATGCTGCCAAGCTAACTGATAGTAAAAAGAAACAATACGGTATGTTATTTGATGCTAAAAATTTCTATTTTAATT
ATCCGTTTTTATTCGGCAATGATGATTATATTTTCAAGAAAAATGGCAGTGAATATGATATTCATCAGCTAGGACTAAATTCAAAAC
ATGTCGTCAAAAATGCTGAACGATTACAAAAATGGTACGACAAAGGGTATCTTCCTAAGGCAGCAACACATGATGTCATGATTGGT
CTTTTTAAAGAAGGAAAAGTAGGACAATTTGTCACTGGACCGTGGAACATTAATGAATATCAAGAAACGTTTGGTAAAGATTTAGG
AGTAACAACATTACCTACAGATGGTGGCAAACCTATGAAACCATTTCTCGGTGTACGTGGTTGGTATTTATCTGAATATAGTAAACA
TAAGTATTGGGCTAAAGATTTAATGCTGTATATCACTAGTAAAGATACATTACAAAAATATACAGATGAAATGAGCGAAATTACTG
GACGTGTTGACGTGAAATCATCTAATCCAAATTTAAAAGTGTTTGAAAAGCAAGCACGTCATGCTGAACCGATGCCTAATATTCCTG
AAATGCGACAAGTTTGGGAACCGATGGGCAATGCAAGCATATTTATCTCAAATGGTAAGAATCCTAAACAAGCGTTAGATGAGGCG
ACGAATGATATAACGCAAAATATTAAGATTCTTCATCCATCACAAAATGATAAGAAAGGAGATTAGTTATGACGAAACGTAACCCT
AAATTAGCGGCATTATTATCTGTTATACCTGGTTTGGGACAGTTTTATAATAAAAGACCCATTAAAGGTACGATATTTTTTATCTTTT
TCATCAGTTTTATTTCTGTATTTTATAGCTTTTTAAATATTGGTTTTTGGGGATTGTTCACATTAGGGACAGTACCTAAGTTAGACGAT
TCTCGTGTCTTACTTGCACAAGGTATTATTTCTATCTTACTCGTTGTTTTTGCAATCATGCTATATATCATTAATATTTTAGATGCATA
TCGTAATGCTGAACGATTTAATCGCAATGAAGAAATAAAGGATCCGAAGGCACGTATGGTTGCAACATGGGACAAGACGTTCCCAT
ACTTATTAATCTCACCAGGTACATTCTTATTGATATTTGTAGTTGTATTTCCATTAATATTTATGTTTGGAGTAGCATTTACAAATTAC
AATTTATACAACGCGCCTCCGAGACACACATTAGAATGGGTTGGTTTAGATAACTTTAAAACGTTATTCACAATTGGCGTTTGGCGT
AAAACATTTTTCAGTGTTATTACTTGGACATTAGTATGGACGCTTGTTGCAACGACACTTCAAATTGCATTAGGGCTGTTTTTGGCAA
TTATTGTAAATCACCCTGTCGTCAAAGGTAAGAAATTTATCCGTACTGTGTTAATCCTACCTTGGGCTGTACCATCATTTGTGACAAT
TTTAATATTTGTAGCGTTATTTAATGATGAATTTGGTGCGATAAATAATGATATTTTGCAACCTTTATTAGGTGTAGCACCAGCATGG
TTAAGTGATCCGTTTTGGGCAAAAGTGGCATTAATCGGCATTCAAGTATGGCTTGGATTCCCATTTGTCTTTGCACTGTTCACTGGAG
TACTGCAAAGTATTTCATCAGATTGGTACGAAGCAGCAGATATGGATGGTGCGTCTAGTTGGCAAAAGTTTAGAAACATCACATTCC
CGCATGTCATTTACGCCACAGCGCCATTGTTAATTATGCAATATGCAGGTAATTTCAATAATTTTAATCTTATTTATCTATTTAATAA
AGGCGGTCCACCAGTGTCAGGGCAGAATGCTGGTAGTACAGATATCTTGATATCTTGGGTGTATAATCTGACATTTGAGTTTAACAA
CTTCAACATGGGTGCAGTTGTGTCATTAATTATTGGATTTATTGTTGCTATTGTCGCATTTATTCAATTCAGACGTACAAGTACGTTT
AAAGATGAGGGAGGTTTATAAGATGACAAAGAAGAAAAACATATTAAAAGCAATCGGTATTTACGGTTTTATAGCGATGATGTTTG
TCATCATTTTATATCCACTACTGTGGACATTTGGCATTTCCCTTAATCCAGGTACGAACTTGTATGGTGCCAAAATGATACCAGACAA
TGCAACATTTAAAAATTATGCATTCTTACTATTCGATGACAGTAGTCAATACCTGACTTGGTATAAAAATACGCTTATCGTAGCATCT
GCAAATGCACTGTTTAGTGTGATATTTGTCACGTTAACAGCATATGCTTTTTCTAGATATCGCTTTGTTGGTCGTAAATACGGGCTGA
TTACATTTTTGATTTTACAAATGTTCCCTGTATTAATGGCAATGGTCGCAACCTATATTTTGCTAAATACAATTGGATTATTAGATTCT
TTATTTGGACTAACACTGGTATATATTGGTGGATCAATACCGATGAATGCCTTTTTAGTGAAAGGTTACTTCGATACGATTCCAAAA
GAACTTGATGAATCTGCCAAAATTGATGGTGCAGGGCATATGCGTATTTTCTTACAAATTATGCTTCCATTAGCTAAGCCGATTTTA
GCAGTTGTTGCTTTGTTCAATTTTATGGGGCCATTTATGGACTTTATATTACCTAAAATACTATTAAGAAGTCCTGAAAAATTCACAT
TAGCAGTTGGATTGTTCAACTTTATTAATGATAAGTATGCAAATAATTTCACAGTGTTTGCAGCAGGGGCAATTATGATTGCAGTAC
CTATAGCAATCGTATTCTTGTTCTTGCAACGCTATTTAGTATCAGGTTTAACAACAGGTGCGACAAAAGGTTAGTTTGAAATTAGGA
GCGGGACAGAAATGATAAAGAACCACTAATGAATTTATTGTGTAGTGGCTCTTGGGAGTGGGACAGAAATGATATTTTCGCAAAAT
TTATTTCGTCGTCCCACCCCAACTTGCATTGTCTGTAGAAATTGGGAATCCAATTTCTCTATGTTGGGGCCCCGCCAACTTGCACATT
ATTGTAAGCTGACTTTTCGCCAGCTTCTGTATTGGGGCCCCGCCAATATAACACTGTATAGCCTAGAACGTTGATTGATGTTTAAGG
CTCGACATACGCACTAATGACGATAAAGATTAAAAGGAGGACGTTATGATGACGATTAAAGTTGGAATCATTGGGTGTGGTGGTAT
TGCGAATGGCAAGCACATGCCAAGTTTACAAAAAGTTGAAAATGTTGAAATGATCGCATTTTGTGACGTAGACATTTCGAAAGCAG
CGAGTGCGGCAGAAGCATACGGAACTGACAATGCAAAGGTTTATGATGATTACAAAGCATTGTTAAAAGATGACACGATTGATGTT
ATCCATGTTTGTACGCCAAATGACTCGCATTGTGAAATTACTGTAGCAGGGTTGCATGCCGGTAAACATGTGATGTGTGAAAAACCA
ATGGCTAAAACGACAGCAGAAGCTCAAAAAATGATAGATACAGCTAAATCAACAGGTAAAAAATTAACAATAGGTTATCAAAATC
GCTTCAGACCAGATAGTCAATTTTTATATAAATCAGCGCAACGTGGCGACTTAGGAGACATTTACTTCGGAAAGGCACATGCCATTC
GTCGTCGAGCAGTACCAACATGGGGTGTCTTTCTAGACGAAGAAGCTCAAGGTGGAGGACCATTAATCGATATCGGTACACACGCT
TTAGATTTAACGTTATGGATGATGGATAATTATGAGCCAGAATCAGTGATGGGTTCAACATTCCATAAATTAAATAAACAGCATCAT

GCGGCAAACGCTTGGGGTTCATGGAATCCAGATGAATTTACAGTTGAAGATTCTGCGTTTGGATTTATTAAAATGAAGAATGGAGC
GACGATCATTTTAGAATCCGCTTGGGCGATTAATTCTTTAGAAGTGGATGAGGCAAAATGTTCATTATCAGGAACTAAAGCAGGTGC
TGATATGAAAGATGGTCTACGTATTCATGGTGAAGACATGGGTACACTTTATACCAAACACGTTGAATTGGAAAACAAAGGCGTCG
ACTTTTATGAAGGTAATGAAGTGGATGAAGCTGAAGAAGAAGCAAAAGCTTGGATTGATGCAGTTGTATATGATACTGAACCAGTT
GTGAAACCGGAACAAGCAATGGTAGTTACAAAAATTCTTGAAGCGATTTATCAGTCTGCAAAATCAGGCAAAGCAATTTACTTTGA
ATAACATCATACGGTAAGGAGGCACATCATGACAAAATTAAAAGTTGGTGTGATAGGTGTTGGTGGTATTGCACAAGACCGTCATA
TTCCAGCATTGCTGAAACTCAAAGACACAGTCTCATTAGTTGCAGTACAAGATATTAATACAGTGCAGATGATTGATGTTGCGAAGC
GCTTTAATATACCTCATGCAGTTGAGACACCTAGCGAGCTGTTTAAACTTGTTGATGCGGTGGTCATTTGTACACCCAATAAATTCC
ATGCTGATCTTTCTATAGAAGCATTGAACCATGGTGTCCATGTTTTATGCGAAAAGCCAATGGCAATGACGACGGAAGAGTGTGAC
CGCATGATTGAAGCGGCTAATAAAAATCACAAATTACTAACTGTCGCTTATCATTATCGCCACACAGATGTGGCAATTACTGCTAAA
AAAGCAATTGAATCAGGTGTGGTTGGTAAACCTTTAGTAGCACGTGTACAAGCGATGCGTAGGCGTAAAGTACCTGGGTGGGGCGT
TTTTACCAATAAAGCGTTGCAAGGTGGCGGTAGTTTAATCGATTATGGTTGCCACTTGTTAGACTTATCTTTGTGGCTATTAGGCAAA
GATATGGTGCCGCATGAAGTGCTAGGAAAAACATATAATCAATTGAGTAAACAACCGAATCAAATTAATGATTGGGGAACATTTGA
TCATACTAAATTTGATGTCGATGATCATGTTACTAGTTATATGACATTTGCCAATCGAGCAAGCATGCAGTTTGAATGTTCGTGGTCT
GCAAATATAAAGGAAGATAAGGTACACGTTAGTTTATCAGGCGAAGATGGCGGTATCAATTTATTTCCATTTGAAATATATGAGCC
CCGCTTTGGAACTATTTTTGAAAACAAAGCTAATGTTGAGCATAACGAAGACATTGCTGGTGAGAGACAGGCGCGTAACTTTGTCA
ATGCGTGTTTAGGGATAGAAGAGATTGTGGTGAAACCGGAAGAAGCACGCAATGTAAATGCCCTTATAGAAGCGATTTATCGTAGC
GATCTTGATAACAAGAGCATACAACTTTAATGATTATTATATATAATACAAAATTCTCAATATAAAAAGAAGGAGTGCTTTTCAATG
AAAATAGGTGTATTTTCAGTATTATTTTACGATAAAAATTTTGAAGATATGTTAGATTATGTCGCAGAATCTGGATTGGATATGATT
GAAGTTGGAACAGGTGGTAACCCAGGAGATAAATTTTGTAAGTTAGATGAGTTGTTAGAAAATGAAGACAAGCGACAAGCATTTAT
GAAGTCAATCACAGACAGAGGCTTACAAATAAGTGGTTTCAGTTGTCATAACAATCCAATTTCTCCAGATCCGATAGAAGCGAAAG
AAGCCGATGAAACGTTACGTAAAACAATCCGTTTAGCAAATCTATTAGACGTGCCAGTTGTTAATACATTTTCTGGCATTGCAGGGT
CAGATGATACCGCTAAAAAGCCTAATTGGCCTGTTACACCTTGGCCAACAGCCTACTCTGAAATTTATGATTATCAGTGGAATGAAA
AGTTGATACCATATTGGCAAGATTTAGCTGAGTTTGCAAAAGAGCAAGATGTAAAAATTGCCATAGAGTTACATGCAGGATTTTTA
GTTCATACACCATATACGATGTTAAAGTTACGTGAGGCTACAAATGAATATATCGGTGCTAACTTAGATCCTAGTCATTTATGGTGG
CAAGGTATTGATCCAATTGCTGCGATTCGCATATTAGGCCAAGCAAATGCAATTCATCACTTCCATGCTAAAGATACGTATATTAAT
CAAGAAAATGTAAATATGTATGGTCTAACTGATATGCAACCATATGGTAACGTTGCGACAAGAGCATGGACATTCCGTACAGTTGG
TTATGGACATAGTCCATATGTATGGGCAGATATCATAAGTCAACTTATTATTAATGGATATGATTATGTATTAAGTATTGAACATGA
AGATCCTATTATGTCAGTAGAAGAAGGTTTCCAAAAAGCTTGTCAAACTTTGAAATCTGTTAATATTTACGACAAGCCAGCAGACAT
GTGGTGGGCATAATACGAACTCGAGGTTAGTCTGAAGTTTGTCTGAAGTAAGACTGGTGGCAGTGTTGAATAAATGCATATGTCGC
CAAGCCATTGCCAAAAATTTCACACCTTAAATCATGTCATTGTTTGTAAAGAAGGTGTACTTTATATAAGTGTTTAGCGATGGTCAT
ACCCATTCACAGTAACAATCCTCACCATTGAAAAGAGTATATAACCTTTTCGATAGTGAGGTATATGATAATAAAAAAAGCCTGTTG
TCACAATGGTCATAGACACGACATACTTTAAAGGCTTCTGAATATAATATTTCAGAACGCACTTTAAAGATGGACGTCGATGTCGAC
TAATGTGATGACAGGCTTTCATCTTTTTAAATATTCATTAATTTCTCTTCTTGTTTAATACGTACATATAAGAAATACGCATACGGTA
CTAATAAAATAGTTGTATATGTTGCGTGTGTTAATAATAATACACCGATTAATTCAGGAATGATGTTTAAGAAGTAATTTGGGTGTT
TTGTAATTTTATATAATCCAGATTTAATAATAGGATGGTTAGGTAAAATGAATAATTTTAATGTCCAAATACCACCTAAAGTTTTAAT
AACCATAAATAACATGATATAAGCAAAGATTAATATAACTAAGCCAATACCATTTGCAAAGCTAAATGTATCTTTATTAATAAATGC
CTCTACACCAGCCAATACATAAATTAAAACGTGTGTTATTGCTAAAAACTTCGAATTTTTAACGCCATATTCAACTGCACCGTCTGCT
TTTAATTGTTTTGAGTGATTAATAGATATCTTTAAGCTGACAAGTCTGATACAGAAAAAGATAAGTAATATAGATAGAATCATGATG
TCCTCCGTCATTATGTCATATGTATAAGCGTTGATTTTGACAACATAAAGTATTTTATAGATAAAACTTGTCAAATACTATTAACTAT
TTATTAATTTTAGTACATAAATATGTTTCTAAGTATGTGTTTATGTTCAGTATTTTGGATAATTTAATAATTTTAAGGATATTAAGCGC
TTACACCGACGTGATATATTTGGCTTAACGAAAATGATTGAGGTGACAGAGATGAACTTTTTTGATATCCATAAGATTCCGAACAAA
GGCATTCCATTATCGGTACAACGTAAATTATGGCTTAGAAACTTCATGCAAGCTTTCTTTGTAGTGTTCTTTGTTTATATGGCTATGT
ATTTAATTCGAAACAACTTTAAGGCGGCCCAACCGTTTTTAAAAGAGGAAATTGGATTATCTACATTAGAACTTGGTTATATCGGAT
TAGCATTTAGTATCACGTACGGTTTAGGGAAAACATTACTTGGATATTTTGTCGATGGACGTAACACAAAACGTATTATCTCGTTCTT
ACTTATCTTATCTGCGATTACAGTTTTAATTATGGGATTTGTTTTAAGTTACTTTGGTTCTGTAATGGGATTATTAATTGTACTTTGGG
GACTTAACGGGGTGTTCCAATCAGTTGGTGGACCTGCAAGTTATTCAACGATTTCAAGATGGGCGCCAAGAACGAAACGTGGCCGA
TACTTAGGATTTTGGAATACATCACATAATATCGGTGGTGCCATTGCAGGTGGTGTTGCACTTTGGGGTGCTAATGTATTCTTCCATG
GAAATGTTATAGGGATGTTCATTTTCCCATCGGTGATTGCATTACTTATTGGTATCGCAACATTATTTATCGGAAAAGATGATCCGG
AAGAATTAGGATGGAATCGTGCTGAAGAAATTTGGGAAGAGCCGGTCGATAAAGAAAATATTGATTCTCAAGGTATGACGAAATG
GGAGATCTTTAAAAAATATATCCTGGGAAATCCTGTTATATGGATTCTATGTGTTTCAAACGTCTTTGTATACATTGTACGAATCGGT
ATTGATAACTGGGCACCGTTATATGTGTCAGAGCATTTACACTTTAGTAAAGGCGATGCAGTTAATACGATATTCTACTTTGAAATT
GGTGCATTAGTTGCAAGTTTATTATGGGGCTACGTATCAGACTTATTAAAAGGTCGTCGTGCAATTGTAGCTATTGGCTGTATGTTTA
TGATTACATTTGTTGTCTTATTCTACACAAATGCTACAAGTGTCATGATGGTTAACATTTCATTGTTTGCATTAGGTGCGTTAATCTTT
GGTCCGCAATTATTAATTGGTGTATCATTGACTGGTTTTGTTCCTAAAAATGCCATCAGTGTAGCAAACGGAATGACAGGTTCATTC
GCGTATCTATTCGGTGACTCAATGGCGAAAGTTGGTTTGGCGGCTATTGCTGATCCAACACGTAACGGTTTAAACATCTTTGGATAT
ACATTAAGTGGATGGACAGATGTTTTCATCGTCTTCTATGTTGCATTATTCCTAGGCATGATTCTATTAGGAATCGTTGCTTTCTATG
AAGAAAAGAAAATTAGAAGTTTAAAAATCTAATATCAATCGGATTAAAAGTATCGCCAATTTATTGCAGTATAGTTGGCAATCCTG
CCCCGACGGCATGTGCGTGAAGAGATGAAAGATACTGCTTCTACCCTTGCAAATATATCATCTCTATGTCTCGGGGCAGATCATAAT
TCCCTGTTATGAAGTATCCTTATTTGCCCGACTTAGGGTGACTCAATGAATTTACTCCTTACAATAAAGACATATAGCGGTGTCAATA
TTGTAGGGAGTATTGTTTTATATTTAAACTCTCTAAAAAGCGGACTGAAAGAAAAGTGAAAACTTCTCTATCAGTCCGCTTTTTCAT
AGAACAAAATGTAGGCGCCATAATCATTAGTTATGTGCTAATCTATTTTGCTTGCTTACAATAATCACTTGGCGACATTTGTAAATAT
TTTTTAAAATGATAGCTAAACATTTTATACTCTGAAAAGCCTACTTTGTCTGCAATTTCATAGTGTTTGTAATGTCGATCTAACAATT
GCAGAGATTGTAAAATACGATAGCGATTTAAATAATCGACAATTGTAATACCAACATGATCTTTAAATGTTCGCATCGCATACGATT
CACTAACATCGATATGTTGAATTAAATCTGAAACAGTCACTTTCGTTTGATAAGATTGCTTAATTTGATCAACAATCTGATTTACATA
ATAATCATCGTATTCTACTTTTAATAGTGGTTGGAAGGCATCATGACAAGATGCTAAGCTACGGCCGTTCTGTGATTGTTGCTCTAAT
AAGGTACGGACAAGTCTTCCTAAAATAACTTCTAATTGTGCATGGTCTACTGGTTTTAATAAATAATCAAGAACATGATGTTGAATA
CCGGCTTTCATATATTCAAAGTCATCGTAACTCGATAATATGATGACATTACAATCTAGATGCGCAATATCATTGAGTAAATCGACG
CCATTCTTACGTGGCATACGAATATCAGTAATTACTAATTCTGGCTGATGTTGTTGAATTAGTGATAATGCTTCAACACCATCTTTAG
CAGTGTATATTGTATTGAAATGATAGTCTCCCCACGGAATGATTTGCTTTAAACCTTCTCGAATAATACGCTCATCATCACAAATAA
CTACCTTAAACATCTACATTCCCCCTTGAAAGTGGTATTTTATAACAAATTAACGTACCTTGATTACGCTTTGAAAAAATATGGAGTC

GTGCATGTGAACCATATTGAATCATTGCTTTATTATGTAAATGATTTAATCCCAAATGCTTAGTATCAAATACATCATTATTAAGAGA
TTGGCGTACATATTGCAGGCGAGATGACGACATCCCAATACCATTGTCGCAAACTAAAACATGTAAATTCTGACGTGCCAATGTCA
GGCGTATAGTAATGTCCAATGACTCAGTATCTCTACCATGTTTAATAGCATTTTCTATGAGTGGCTGAAGCATCATTTTACCAATTGT
CTGGTGACGCGCTTCTTCAGAACTTTCGATATGGAGCTTAATCATGTCATCAAAACGGATGTTTTGTATCGCAACATACTGTTCAATG
TAGTTCAACTCTTCGTTTAATTCCACTGTATGTGAGTTTGTACGTAATGAGTAACGTAACATTTGCGATAATTGTTGGACCACAGTTT
GTGCTAATTTAGGAGATAACGTAATTAAATATTGTATTGTTTGCATCGTATTGAATAGGAAATGAGGTTGGAATTGGCGCTCTATTT
CCTTTAACTGAATATCACGCAAGCGACGTTCTGTATGCTCGATAGAATGGATTAGTTGCTCATTTGATTCAAATAAATCGTAAATAT
AATTATTAATTTCTTCTAGTTCACTGTTGTTTTTTAAAGGCGTATATGTACCTAGATGACGATTTTTGGCATAGTAAATTTTTTGAATA
ATCGTTTCGATATCTTTTGTTTGTCGTTTAGCCATATTATCTGCGCTAATGAAACCAAATATTACTAGTAAAACAAGAACTACGGCCA
TAACAATTAACAACGTGATACCATCTTCAATGTTTTCATGTATATCTTTATAAATAATGAGACGATGGTCAGCATGGTTTAATTTTAC
AGATTCATTCATAAATCCGAATTGTTGCGGTCTATACTTTTCACCTATAGTAAAACGGTCATCGTTGGCGTATAAAATATTGTCATAT
TGATCAACGATAAGTGCGAATTGTCGGCTATCTTTCTTAATTTCACTTAAACGTGGGGTGTTAGCCATATAAATTTTAAGCATATATG
TACTATTATTGAATTTAAGCTGATGCGTTGAAAATAAATACATATTTTTAGTGTTTAAATGTTCATAATTATTGGTTATAAACTGATT
TGGTCCAGATAATTCATAATAAAGTGTTGCGGGCTGTTGGTGTATTAATTTTAATAATTCACGTTTTGTAGCGGTAACATCATGATGA
TTTGTTAAATCGAGCTCTTGAAACGAATTATTATGCTGTGTAATAAATGTCTGAATCTGCTTTTCAGTGTGATGTAAAGATGACTGAC
TTTCATCAACATGTTGATGAATCGTACGATGCTCAATCCAAATATAGATGGCATAGAAGCTTACTAGTCCAATAATAATGACTAAAA
ATACTGGAAAAATAGTAGACGCAAATAACGATCGTCTCAATTGATGTCTATAAGGTTTGTATGCCGTCATTGAATCATCTCCAAAAA
TTTATGATGTGGAATGTCCGGTAATTTAGATTTCGGTATTAAAGGTATGTTCTTAAGATTTTCGATAGACTGATCGCTTTGTTCACTA
ACATCCTTTCGAATTGACTTGGCATCGAACTCTGCAACTAATCGTTGTTGTACTGAGCGGCTTGTTAAATATTGCACTAACTTTTTAC
GCTTAGGATGAGGGTGTGCATTTTTAACTAAAGCAATACCATCAACATTTAACATTGTTCCTTCAATTGGATAAACGATTGATACAG
GATAACCTTTGTTTTTCCATGTGCGTGCATCTTGTTCGTAGCTTAGACCTGCGTAATATTTACCTTTTGCAACATCTTCAATGACTTTA
GACGTCTTTGACAGTTGCATCGCATGGTTTTGGAATTGATGCACATCACTTACTCGATGATGCATGCTATAAATAGCGCGCATATGT
TGATAGCCTGTCGTTGTTGTATTTGGATTTGAGTACGCAATTTTACCTTTAAGTATAGGTTGTAATAAATCTTGATAACCTCGAATCT
TAATATCTCCTTGTAAATCTGAATTCACTACTATAACTGTTGGCATTAATAGAAAACTAGTAACATATTTATTGTTCGAGCGATAATC
CTCTAATTGCTGTGTTACAGATGTATCTTGATAGGGAACAAAATCTTCTGGATGATCAATTGTTTCTGATAACACACCACCCATAAA
GACGTCACCACGCTCCGAAAAATCTTCGTTATGCAAGTTTGAAAGCAGTACTTGAGTAGATCCATGTTTGATTTCAATTTTGACATG
CTCTTGTTTTTCAAATTCATTTAAAATTGGACGAATCAAGTTTGATTGATACGGAGAATAAACTGTTAATACATTTTTATCTGATTCA
GAGTGACGCGTATTAGCGCATGCTGATAAAAAAAATGAGAAATAATAGCAAGATATAAATTTTTGATTTCATGATATCCCATCAATTC
TATGTATATTTTAATACAATAATTTTAGCAATAAATGACGCATAAGTAATGTTAAATATTTAGAAATGTTTATAGATGACTTGTTAA
GACGTTGCAAATGTTGTGATAGCACAAAATTTTTGTTTGTCAAGACGATTTACCGAGGCTGTAAAATCAAACTGTTATATTTTATTTG
TAGCTGTTATATAAAAATCGGCAAGATATTGAACGGTTCAAAAGTGAATTTTTACGTCAATAAAAGTATTTAATCCAGTCTCTTCAT
ATATAAAAGTAAATCTTTCTAAGTGTTGATTTAACGCTTATCAACAATCATTTTTTATAAACAAATATATACTCCTAAATTAACTTTT
AAAGCAATGAAAATAGTGAACATTATAACTGTTGTGTAACAGAATGCAATTAGCATATTACTGTTACACAAATTAGTACAGTTTCTA
TGTTTTGACATACATTTGATGAAAATTGTACATAATTTATGTGAAAAAAATCACAACAAACATGCTACAATGACTATGAAAACGTTA
ACATAGCATTTCAAATTCACAACATTATACAGATGGAGGCGTTTAGTATGTTAGAAACAAATAAAAATCATGCAACAGCTTGGCAA
GGATTTAAAAATGGAAGATGGAACAGACACGTAGATGTAAGAGAGTTTATCCAATTAAACTACACTCTTTATGAAGGTAATGATTC
ATTTTTAGCAGGACCAACAGAAGCAACTTCTAAACTTTGGGAACAAGTAATGCAGTTATCGAAAGAAGAACGTGAACGTGGCGGCA
TGTGGGATATGGACACGAAAGTAGCTTCAACAATCACATCTCATGATGCTGGTTATTTAGACAAAGATTTAGAAACAATTGTAGGT
GTACAAACTGAAAAGCCATTCAAACGTTCAATGCAACCATTCGGTGGTATTCGTATGGCGAAAGCAGCTTGTGAAGCTTACGGTTA
CGAATTAGACGAAGAAACTGAAAAAATCTTTACAGATTATCGTAAAACACATAACCAAGGTGTATTCGATGCATATTCTAGAGAAA
TGTTGAACTGCCGTAAAGCAGGTGTAATCACTGGTTTACCTGATGCATACGGACGTGGACGTATTATCGGTGACTATCGTCGTGTAG
CTTTATATGGTGTAGATTTCTTAATGGAAGAAAAAATGCACGACTTCAACACGATGTCTACAGAAATGTCAGAAGATGTAATTCGTT
TACGTGAAGAATTATCAGAACAATATCGTGCATTAAAAGAATTAAAAGAACTTGGACAAAAATATGGTTTCGATTTAAGCCGTCCA
GCAGAAAACTTCAAAGAAGCAGTTCAATGGTTATACTTAGCATACCTTGCTGCAATTAAAGAACAAAACGGTGCAGCAATGAGTTT
AGGTCGTACATCAACATTCTTAGATATCTATGCTGAACGTGACCTTAAAGCAGGCGTTATTACTGAAAGCGAAGTTCAAGAAATTAT
TGACCACTTCATCATGAAATTACGTATTGTTAAATTTGCTCGTACACCTGATTACAATGAATTATTCTCTGGAGACCCAACTTGGGTA
ACTGAATCTATCGGTGGTGTAGGTATTGACGGACGTCCACTTGTTACGAAAAACTCATTCCGTTTCTTACACTCATTAGATAACTTAG
GTCCAGCACCAGAACCAAACTTAACAGTATTATGGTCAGTACGTTTACCTGACAACTTCAAAACATACTGTGCAAAAATGAGTATTA
AAACAAGTTCTATCCAATATGAAAATGATGACATTATGCGTGAAAGCTATGGCGATGACTATGGTATCGCATGTTGTGTATCAGCGA
TGACAATTGGTAAACAAATGCAATTCTTCGGTGCACGTGCGAACTTAGCTAAAACATTACTTTACGCTATCAATGGTGGTAAAGATG
AAAAATCTGGTGCACAAGTTGGTCCAAACTTCGAAGGTATTAACAGCGAAGTATTAGAATATGACGAAGTATTCAAGAAATTTGAT
CAAATGATGGATTGGCTAGCAGGTGTTTACATTAACTCATTAAATGTTATTCACTACATGCACGATAAATACAGCTATGAACGTATT
GAAATGGCATTACATGATACAGAAATTGTACGTACAATGGCAACAGGTATCGCTGGTTTATCAGTAGCAGCTGACTCATTATCTGCA
ATTAAATATGCACAAGTTAAACCAATTCGTAACGAAGAAGGTCTTGTAGTAGACTTTGAAATCGAAGGCGACTTCCCTAAATACGG
TAACAATGACGACCGTGTAGATGATATCGCAGTTGATTTAGTAGAACGCTTCATGACTAAATTACGTAGTCATAAAACATATCGTGA
TTCAGAACATACAATGAGTGTATTAACAATTACTTCAAACGTTGTATACGGTAAGAAAACTGGTAACACACCAGACGGACGTAAAG
CTGGCGAACCATTTGCACCAGGTGCAAACCCAATGCATGGCCGTGACCAAAAAGGTGCATTATCTTCATTAAGTTCTGTAGCTAAGA
TCCCTTACGATTGCTGTAAAGATGGTATTTCAAATACATTCAGTATCGTACCAAAATCATTAGGTAAAGAACCAGAAGATCAAAACC
GTAACTTAACTAGTATGTTAGATGGTTACGCAATGCAATGTGGTCACCACTTAAATATTAACGTATTTAACCGTGAAACATTAATAG
ATGCAATGGAACATCCAGAAGAATATCCACAGTTAACAATCCGTGTATCTGGTTACGCTGTTAACTTCATTAAATTAACACGTGAAC
AACAATTAGATGTAATTTCTCGTACATTCCATGAAAGTATGTAACAAAATTTAAGGTGGGAGCACTATGCTTAAGGGACACTTACAT
TCTGTCGAAAGTTTAGGTACTGTCGATGGACCGGGATTAAGATATATATTATTTACACAAGGATGCTTACTTAGATGCTTGTATTGC
CATAATCCAGATACTTGGAAAATTAGTGAGCCATCAAGAGAAGTCACAGTTGATGAAATGGTGAATGAAATATTACCATACAAACC
ATACTTTGATGCATCGGGTGGCGGTGTAACAGTCAGTGGTGGCGAACCATTGTTACAAATGCCATTCTTAGAAAAATTATTTGCAGA
ATTAAAAGAAAATGGTGTGCACACTTGCTTAGACACATCGGCTGGATGTGCTAATGATACAAAAGCATTTCAAAGGCATTTTGAAG
AATTACAAAAACATACAGACTTGATATTATTAGATATAAAACATATTGATAATGACAAACATATTAGATTGACAGGAAAGCCTAAT
ACACACATCCTTAACTTCGCGCGCAAACTGTCAGATATGAAACAACCTGTATGGATTCGACATGTCCTTGTGCCTGGTTATTCTGAT
GATAAAGACGATTTAATTAAACTAGGGGAATTTATTAATTCTCTTGATAACGTCGAAAAGTTTGAAATTCTGCCATATCATCAGTTA
GGTGTTCATAAGTGGAAAACATTGGGCATTGCATATGAATTAGAAGATGTCGAAGCGCCCGATGATGAAGCTGTTAAAGCAGCCTA
CCGTTATGTTAACTTCAAAGGGAAAATTCCCGTTGAATTATAAATACAATTCAGACCGAAAAGAAAGCATATGCAACTTCAAGAGT
GAAGGGGCATATGCTTCTTTTTCAATTGAGTATTGAGTATTAGCAAGACGTAGTAAGTATATGAGACAACTTCTACAATGGTTGAAG

GAAGACGTTTTTGTAAGTAGCTATGCTGATAAAGAATGTGATGTCTTGTTAAAGGTGGGGTTCCAATATCATCATTTAGCTGATGTT
GAATGGGTTATTATTTGCTACTTGCATATGAATATGAGTCTTTTCAAATTTTTATTGACCCTGAGTAATGAAAAATATTAAGATGAAA
CTTAATATTAAAGCAATGCGGAGCGTGATTATGAAGAGAATTAGTAAAGATATATGGGCAGTATTTAAATTACTGTATCAAAATAA
AGGGCGTTTTAGCATTAATGCCTTACTATTGCAGTTAATCATGATTTTTATTAGTAGTACATACTTAATTTTACTATTTAATATGATGT
TAAAAGTAGCTGGGCAAAGCCAACTTACGATTAACAATTGGACGGAAATCATAAGTCATCCTGCCAGTGTGATACTTCTTATTATAT
TCATATTAAGTGTTGCTTTTCTGATTTATGTAGAGTTTTCATTGTTAGTTTATATGGTTTATGCCGGCTTTGATCGACAGATTATTACA
TTTAAATCCATTTTTAAAAATGCCTTTGTAAATGTGCGTAAACTCATAGGTGTACCAGTTATTTTCTTTGTCATTTATTTAATGTTAAT
GATACCCATTGCCAACCTAGGACTAAGTTCAGTATTAACAAAAAATATTTACATACCTAAATTTTTAACGGAAGAACTTATGAAAAC
GACGAAAGGTATAATCATTTACGGTACCTTTATGATTGCTGTATTTATATTAAACTTTAAATTAATATTTACCTTACCGTTAACGATT
TTAAACCGTCAGTCGTTATTTAAAAATATGAGACTAAGTTGGCAAATTACGAAGCGAAATAAGTTTCGACTTGTTATAGAAATAGTT
ATATTGGAACTCATCATTGGTGCGATTTTAACATTAATTATTTCAGGAGCAACATATCTTGCTATTTGTGTAGATGAAGAAGGAGAT
AAGTTTTTAGTCTCATCAATTTTATTTGTTGTATTGAAAAGCGCATTGTTCTTCTATTATTTATTTACGAAATTATCATTAATCAGTGT
GTTAGTACTGCACTTAAAACAAGAGAATGTATTAGACCAACCGGGCTTAGAATTTAAATACCCAAAACCGAAACGGAAGTCTAGGT
TCTTTATAATTTCAATGGTGCTTGCAGTGACATGTTTTATCGGTTATAACATGTACTTACTTTACAATAATACTATCAATACAAATAT
CTCCATTATTGGCCATCGTGGTTTCGAAGATAAAGGCGTTGAAAATTCTATTCCGTCATTGAAAGCTGCTGCAAAAGCGAATGTCGA
ATACGTTGAGTTAGATACAATTATGACGAAAGATAAACAATTTGTTGTTAGTCATGATAACAATTTGAAACGTTTAACAGGTGTTAA
TAAAAACATTTCTGAATCTAATTTCAAAGATGTCGTCGGTTTGAAAATGCGTCAAAATGGACATGAAGCAAAACTTGTATCCTTAGA
CGAATTTATTGAAACGGCTAAACAATCAAATGTGAAGCTATTAGTAGAGTTAAAGCCACATGGTAAAGAACCAGCAAATTATACAC
AACGTGTTATTGATATTTTGAAAAAGCATGGTGTTGAACATCAATATCGTGTGATGTCATTGGATTATGATGTGATGACTAAGTTGA
AAAAAGAAGCGCCATATCTCAAGTGTGGTTATATCATTCCGTTGCAGTTTGGTCATTTTAAAGAAACATCATTAGATTTCTTTGTCAT
CGAAGATTTTTCTTATTCGCCAAGACTTGTTAATCAAGCGCACTTGGAAAATAAAGAAGTCTATACTTGGACTATTAACGGCGAAGA
AGATTTAACGAAATACTTACAAACCAATGTTGATGGTATTATCACAGATGACCCAGCATTAGCTGATCAGATTAAAGAAGAAAAGA
AAGACGAAACATACTTCGATCGTTCTATAAGAATTTTGTTTGAATAATATAAACAAAGACCTCTAAAGTTATCAAGATGATACCTTC
AGAGGTCTTTTTAACGTTGCCATCTATGGGATAGGCAATCGTTTCATTCGTTTATATTCATATGACAAGTATTTGTATGGCAATTTGG
CGTCACAAACACTTACATGATTTATTGGTGAATTATTAATTGTTTTGTGAATGCAAAGGGTTAGAAATTGAATCGTAAATACTTTCTA
ATCTATGTTTCGCTTTAGTCATTTGATCCAAATTTTTAGTGCGTATAACGGATTTTGCAATATAATGTGCAGCTAAAATATCGCGTTT
TTGATAAGCGTCTAAATTTAGGTACGATAATTTATTTAAGTCAGTGTTTGCTATTAATTCATGTAATTGATCTACAAGCGCTTGATGT
TGATACGTATGTGATGTAGTTTCAGATTTGCTTGCTAATTTAATACCAGTCGTATCAAGGAGCGCCGCTTTAATACCAGCAACTAAA
TATGTTTTGATTTTCATTTGTGTTGTCATGCTTTGTTACTCCTTTGATGTACATTAATCAAAAAAATTATACACTATTGTATATTGCAA
AGCTAATTAACTATAACAAAAAGATAGTTAATGCTTTGTTTATTCTAGTTAATATATAGTTAATGTCTTTTAATATTTTGTTTCTTTAA
TGTAGATTGGGCAATTACATTTTGGAGGAATTAAAAAATTATGAAAAAGCAAATAATTTCGCTAGGCGCATTAGCAGTTGCATCTA
GCTTATTTACATGGGATAACAAAGCAGATGCGATAGTAACAAAGGATTATAGTGGGAAATCACAAGTTAATGCTGGGAGTAAAAAT
GGGACACCGATTTCTAATGGTTACTTTTGGGGGAAAATCGATAGTCTAGAATCACAATTTTCTAAAGCATTAGCAATAATTGAAGAG
TATCAATATGGTGAGAAAGAATATAAAGATGCAAAAGATAAGTTTATGGATAGAATTTTGTCAGAAGATCAATATCTTTTGGAGAA
AAAGAAAGCTCTGTATGAGAAATATAAAGAGTGGTATAAAAAGCACAAAGAGATAAACCCAACTTATCCAAAAATGCAAACATTC
CATGAATTTAGTGTATATAATCTAACTATGGAAGAATACAATGAGATTTCTAAATCTTTGAAAGATGCGGAAGAAGAGTTTCGTAA
AAATGTAAGAGAAGTTCAAGAGAAAGAAGTCAAATTAAAAACATATAGCGAAGATGAAGAAGAAAAGGCAACTAAAGAAGTATA
CGATCTTGTAGCCGAAGTTGATACTATATATGTATCTTATTTTGGACATGATAAACATGGTCAAAGTGCTAAAGAATTGCGTGCGAA
ATTAGATTTAATAATTGGAGAGGAAAAAAACGCCTTTCGTATTACTAATGAACGTATAAAAAATGAAATGATTTATGACTTAAATTC
TATTATTGACGATTTCTTTATGGAAACTAACCAAAATAGACCGGAAAATATAACAAAATTTGATTGGGATAAGCATGATTATAGAA
ATAAACCAGAAAACAAAGCAAATTTTGAGGCTCTAGTTAAAGAAACTAAAGGTGCGGTCGATAAGGCAGATGATTCTTGGAAAACT
AAAACTGTCAAAAAATTACGGTGAATCTGAAACAAAATCGCCAGTAGTAAAAGAAGAGAAGAAAGTTGAAGAACCTCAAGCACCTA
AAGTTGATAACCAACAAGAAGTTAAAACTACTGCTGGTAAAGCTGAAGAAACAACACAACCAGTGGCACAGCCATTAGTAAAAAT
TCCACAAGGAACAATCTATGGTGAAACTGTAAAAGGTCCAGACTATCCAACTATGGAAAATAAAACGTTACAAGGTGTAATTGTTC
AAGGTCCAGATTTCCCAACAATGGAACAAAGCGGCCCATCATTAAGCAATAATTATACAAACCCACCGTTAACGAACCCTATTTTA
GAAGGTCTTGAAGGTAGCTCATCTAAACTTGAAATAAAACCACAAGGTACTGAATCAACGTTAAAAGGTACTCAAGGAGAATCAAG
TGATATTGAAGTTAAACCTCAAGCATCTGAAACAACAGAAGCATCACATTATCCAGCGAGACCTCAATTTAACAAAACACCTAAAT
ATGTTAAATATAGAGATGCTGGTACAGGTATCCGTGAATACAACGATGGAACATTTGGATATGAAGCGAGACCAAGATTCAATAAG
CCATCAGAAACAAACGCATACAACGTAACGACAAATCAAGATGGCACAGTAACATATGGCGCTCGTCCAACACAAAACAAGGCAA
GCAAAACAAACGCATATAACGTAACAACACATGCAAACGGTCAAGTATCATACGGAGCTCGTCCGACATACAAGAAGCCAAGCAA
AACAAATGCATACAACGTAACAACACATGCAAATGGTCAAGTATCATATGGCGCTCGCCCGACACAAAACAAGCCAAGCAAAACA
AATGCATATAACGTAACAACACATGCAAATGGTCAAGTATCATACGGAGCTCGCCCGACATACAAGAAGCCAAGCGAAACAAATG
CATACAACGTAACAACACATGCAAATGGTCAAGTATCATATGGCGCTCGCCCGACACAAAAAAAGCCAAGCGAAACAAACGCATA
TAACGTAACAACACATGCAGATGGTACTGCGACATATGGGCCTAGAGTAACAAAATAAATTTATAACTCTATCCAAAGACATACAG
TCAATACAAAGAATTATGTATCTATACAACAGTAATCATGCATTCTATGATGCTTCTAACTGAATTAAAGCATCGAACAATCGGAAG
CATATTTCTAAATTATTTATTCATTATAGTCTTAAACATAACATGACCTAATATATTACTAACCTATTAAAATAAACCACGCACATCT
AAGTGATATACGACAATCACAGCAATAATAATTGCTTTAGAAAGTCGTACCGAACTGGAACTTACAAGTCTAGTTCGAACACACAC
TGATGTGAGTGGTTTTCTTTATTTTAAACATGAACAATCAGATAAGTTACTAGCATTAGCAAATATTATTAAATCAAAGGGCTTCGA
TTCATAAAATTTAAAACAATGATTAAAATTAGACGTGTAAATGTTAAATTCTAAAACGGAAATAACCACCATCCCATTAAACCACTT
TTTTGTTCAATCACTATATTTCACACAGCTTCATTAATAAAACGAAATTGCTTCAACCCGCTTCAACTTCAGCCTACTTCATTCAATA
ACCTAACGAATCCGCTTCATCCAAAATCAACCATTCTAACGCACATACTCAAATATAGCAGCTGCACCCATGCCGACACCAATACAC
ATCGTAACCATGCCGTAACGGCTATCGGGACGTCTACCCATTTCATTAAGTAAACGCGCGGTTAACATTGCCCCTGTAGCACCTAAT
GGATGACCTAAAGCAATAGCGCCACCATTCACATTCGTACGTGATATATCTAGACCTACTTCTTTAATAGATGCAATCGTTTGAGAA
GCAAATGCTTCGTTCAATTCGATCAAATCAATGTCTTCAACAGATAGATTGCTGAGTGACAATACTTCAGGAATCGCATATGCAGGC
CCAATACCCATAATTTTCGGGTCAACGCCTACTGCCTTATAACCAACGAATCGTGCAATAGGTGTCACGCCTAGTTCTTTCACTTTAT
CTCCAGACATTAAAACTACAAATCCTGCACCATCAGAAAGTGGGGCAGATGTTCCCGCAGTCACAGTGCCGTCAGCTTTAAATACT
GTACGTAATTTGGCTAATGCCTCCATCGTGGTGTCAGGGCGTATAAATTCATCTTGGTCAAAGATATTTGTGTGTACTTTTGGTCCTG
CGTTTGTATATTCAACTGAGTTTACCCGTATTGGAATAATTTCATCTTTGAACCGACCATCACGTTGTGCGTCATAGGCACGTTGATG
ACTTCTGACAGCATAAGCATCTTGATCTTCGCGTGATACGTCAAATTGGGATGCTACATTTTCAGCAGTTAAACCCATAGGATATGA
CGCACCTATATCATCATATTGTAAGGTTGGATTGTTTGTGGGCTCGTTGCCACCCATTGGTACGGCACTCATCAATTCAACGCCACCA
GCTACAAGTATATCTCCTTGACCAGCCATAATTTGATTGGCTGCAATCGCGATGGTTTGTAATCCTGATGAGCAGTAGCGATTCACT

GTTTGACCCGGTACCGTGTCAGATAATCCCGCACGCAATGCAATCGTTCGTGCAATGTTTTGTCCTTGTAATCCTTCTGGAAAAGCC
GTACCAACAATGACATCTTCAATCATATTCTTATTGAATTTTCCGTCAATACGTTTCAATACGCCTTGTAATACTTTGGCTGCGACAT
CATCAGGTCTTTCGTGGAATAATGCGCCTTGCTTTGCTTTCGCTGCGGCTGAACGCCCATAAGCTACAATGTATGCTTCTTGCATGGT
TATCATCCTCTCTTAATGACTATCTTTTAATTACGTAATGGCTTACCAGTTTTTAACATATGTGCAATTCTTTCATATGATTTTTTAGA
TTTTAGTAAGTCAATAAAGCCAATTTTCTCCAACGATTGAATGTAACGTTGATTTATAAATGTATTTCTTGGTAAATCACCACCCGCT
AAAATTGTGGCGATATTTAAGGCGATATGATAATCATGGTCGCTAATAAAATGCCCCCGTCTTTGCGCATCTAATTGTCCTTGGATC
AATGCTTTGAAGTCTTCACCTAAAGCGATATATTGATGTCTAGGATTCGGAATATAGTTTGTTTCTGCTTCATATTTCGCACGTTTGA
GCGCAACTTCGACACGTTGTGCTGTATTGAAAATAATCGTATCTGTATCACGTAAATAACCATAACGACGTGCCTCAAAGGCATTTG
TAGAGACTTTCGCAAATGCGATATTCGTCAGTACTTTTGTCATGGAAGCTTGTTTGTCATCAAACTTATGCGATGTGCGTAATATGCG
ATCAGCCATTTCTGCAAGGCCACCGCCACTCGGTAATAAGCCAACGCCTGCTTCAACAAGACCTATATATGTTTCACTTGCAGCGAC
AACAATAGGTGAGTAAAGTACAAGCTCACAGCCACCGCCTAAGGCACGACCTTGAACAGCTGTGACTACTGGTTTCAAACTATACT
TCAAACGATTAAAGCTATAATGTAATTTATCAATTGATTGTGCAACGACATCATCTACAAGACCGTCTTCATGCGCCTTTTTCATTAA
GAAAAGGTTAGCACCCACACTGAAATTATTACCATCTGCATAAATGACCATACTTGTGTAATGGTCATTTTCCAGTAAATCAATGGC
ATCAACTAACGCATCGTTGAATTCATCGGTAATGACATTATTTTTACTTTGTAATTTCAGTAACAGTTGATCATCATGAGTTACGGAA
AGTTTGGCATCACCTTTATCCCAAAGTTCATCTTTTACGAAGTGAGAAACAGGTGTTGCATATTCGATGGTCTCATCTTGTTTATAAA
AGCCACCATCTAAATCACTAATCCATTGTGGTAAGTCTCCAATTTCGTCTTCCATACGTGTTTTAACACGTTCATATCCCATTGCATC
CCATAATTGGAATGGACCAAGTTTCCAGTTGAACCCCCAGACAAGCGCACGGTCTATGTCTCGGAAATCATCGGTAGCTTTAGGTAC
ATTGATAGCAGAGTAATAGAAATTATTACGTAATGTCTCCCATAAAAATAGTCCCGCTTCGTCTTGCGCATTGAATATGACATCAAG
GTTATGCACTAAGTCTTTATTAAATTCATTTAAAATTGGTAATTGTGGTTGCGATACAGGTACATAATCTTGTTTTTCAACATCGTAA
ACAAGTCGAGCTTTAGTTTCTTTATCCTTTTTGTAAAATCCTTGTTTCGTTTTACGTCCGAGTGCGCCATTGTCAAACAACGTATTTAC
AATTTTGACATCATGAAAATAAGGCGTTTCTTCAGGTACTTGTTGCATGCCTTTAATTACAGACACTGCAATATCTAAACCGACTAG
GTCAGATAGGGCATATGTACCTGTTTTAGGACGACCAATCGCTTGCCCAGTTAAAGCATCCACATCTACAATGCTTAACTTGTGTTG
CTCGGCGCGATACATAATATCATTCATTGTTTGCGTGCCGACTCTATTTGCGACAAAGCCAGGCACATCATTGACGACAATGACACC
TTTACCTAACACATTTTGCGCGAAATTTTTTACATCTAATATGATAGATTCCTTCGTGTGTGACGTAGGTATTAACTCCACTAATTTC
ATAATACGTGGTGGGTTAAAGAAATGTAGACCAAAGAATCGTTCTTGATCCTTCTCGTTAAATGTTTGAGCAATCGCATTAATTGGA
ATACCTGATGTATTTGTAGCGAATAAAGCATCTTCTTTAGCATGTTGTAGAACTTGTTGCCAAACAGCATGCTTAATTTCAATATCTT
CTTTAACTGCTTCGATATATAAATCAGCATCATCATTTACCAAGTCATCATCAAAATTACCATATGTTAAATGACTCGCTAGATTTAA
GTCGAATAGTAGCGGCCGTTTCTTATCTGTAATTTTATCGTAAGATTTTTTCGCAATGAGATTTGGATCGTTTTTGTCCACTACAATA
TCTAATAGTTTTACTTTAAGTCCAGCATTCACAAAGAGTGCTGCCAGTTGAGCGCCCATCGTGCCTGCGCCAAGAACGGTTACTTTA
TTAATTGTCATAGTGATTCCTCCAATTTAGTTGAGGATAAGATAACCATTAAGATAATTGGAATAACGTTGCTATTTTATAAAATTA
ATTAAGTATCTTTGACAGTCATCTTAGCCTCTTATTTAAGGAAAAAGCTTTATGCTTAAAATAAGTCTTTTTTTAGTGATATTACTGC
ATCTCATATAATTCTTTGCTATTTATACGAAAGCAGAATCTCCAGTCAAAGCGCGTCCAATTACTAAGGCATTAATTTCATGTGTACC
TTCGTACGTGTAAATCGCTTCTGCATCAGAGAAGAAACGTGCAATATCATAATCGTCAGCTAGTATGCCATTACCACCTGTAATACC
ACGGCCCATAGCTACTGTCTCACGCAAACGTAAGGCATTCATCATCTTCGCCGTTGAAGTTGCAACCTCGTCATATTCACCATGTGC
TTGCATATTAGCTAATTGAGCACATGTTGCCATTGCTTGAGCTAAATTACCTTGCATCATTGCTAGCTTTTCTTGTATTAACTGATATT
TACTAATTGGTTTGCCGAATTGCTTACGCTCAGTGACATAATCTAATGTGGCACGTAAAGCGCCAGCCATACCACCTGTAGCCATAT
AAGCAACGCCTGCTCTCGTTGAATAAAGAATTTTGGCAATATCTTTAAAGCTTGTTATGTTTTGTAAGCGATCCGCTTCATCTACTTT
GACATTAGTTAATTTAATTAGGGCGTTAGGAACAATGCGAAGTGCGATTTTATTATCAATGACTTCAATATCGACGCCATCTTGTTCT
GGTCTGACTACAAAGCAATGGGGTTTGCCAGTTTCTTTATTTACTGCGAATACTGGAATGACATCAGATACATGTGCACCACCAATC
CATTTCTTTTCACCATTGATAACCCAAGTATCGCCCTGGCGTTCAGCGACTGTTTCAAGACCTCCCGCAACGTCCGAACCGTGTTCTG
GTTCAGTTAAAGCAAAGCATGTACGCAGTTCATGTGACTGTAATTTAGGTACATATTTCGCAATTTGTTCTTTGCTACCTCCGAAATA
GAAAGTGTTATGCCCTAAACCTTGGTGAACACCGAGTAGGGTAGCTAAGGAAATATCAAATCGCGCGAGTAGGTAAGACATGAAA
AACTGAAATAGTTGACTAGGCATTTTGGCGTTTGGACGATCCTTGTAAAGTAATGGATTGTTAAAATAATTTAATTCTCCCAGATCTT
TAAAATAGTCCTCGGGTACAGTAGCGTCTATCCAATGTTGATTAATATTTTCACGGTACTTACTTTCTAGCAATGAATCTACTTGTTG
TAAAAATTCGACTTCACCGTCTGTTAAACCTTTAGCAATACTAAGTACATCTTCAGGAAATAATGTTTTTAAGACCGTTTCTTTTTCA
AATGTCATATAAATTCCTCCTAAAAATAATATGAATACTAATGTGAAATGCATTTAATTCAAAAACAACACGCTTTATTTGTAAACG
CTTACACTAAATGTCAAAAATTTTTATCACCTTTAAAGTGTTTGTGAGACTTTGTCATTCATCATTTGTCGAATCGCAAGTTTATCTG
GTTTCTGCGTACTGTTTAACGGCATATGTGTCACTGGTACATACATTCTTGGGACTTTATAACCTGCTAAACGACTTCGCATATGTTG
ATCTAAAATTTCAGCGTAATGAGGTTCATCTTCGCGAAGTATAATGGCTGCAGCAATTGATTCACCATATTTTGGATGATCATAGCC
AACGACCACACACCGGTCTACTAGTGGATGCTCAGCTAAAGCATTTTCGACTTCGGATGGTAAGACATTTTCGCCACCAGTTATGAT
TAATTCTTTTTTGCGGTCAATAATAAATATATCGCCATCGTCGTCCATCTTCGCTAAGTCACCAGTTAATAAATAACGACCATGAAAT
GCTTTGGCAGTCTCTGCTGGTTTATTCCAATATCCTGGCGTGACATTTTTAGCCTTAATTGCAAGTTCGCCAATCTCACCAGTAGGTA
CTTCCTCACCGTTATCATCAAGGATACGTGCATCAACGAACATGACTGCTTTACCAATACTCATTGGCTTACGTTTTGAATTTTCCGG
TGTATTAACAAGTACAAGAGGTGCTTCAGTTAAACCATAGCCGTTAATAATGTTTATGCCATATTGTTTAAAAGCTGCTTGGATACT
TGGTAATGGTTGTGAACCACCTTGGATGATATAATCCATAGCTTTAAAATTTTCAGGATTAAAATTACTAGCACGTAGCGTACTATA
ATACATTGTCGGAATCATGATAATAAATGTAGGGTGATATTGTGCAATCATGTCATTCAATTCTTCGCCGTTAAAGTAACGTTGAAG
AATAAGTGTGCCACCTGACATCAATACTGGTAATACAGTATCGTTAAACCCTAAAACATGGAACATTGGTGTTGATACAATCGTAAT
ATAGTTTGAATTGAACTTATACGTCAGCTCTAAGTTTGCACCGTTATGAACAAATGATTCATATGAGAACATCACACCTTTAGGTGA
TCCGGTTGTACCACTTGTATAAATTAATGCTGCAAGATCTTGTGGTTCGACAGGTGTTGCTTGAAAAGGTTGGTGATAATCTGGATTT
ACGATTTCATCATATTGCGCTACATCAATATCCATATGCAATAAGTTTTGGTCAATATCGGTGAGTGAACTTAAATGTTTTTCAGCAT
AGAAGAGCAGTTTTAATTGTGCATCTTCCACAATGGCTGCAATTTCTTTTGGGTTAAGCCGCCAATTCAATGGTAAAAAAACCGCAC
CTGTTTTAAAACAAGCAAACAATAAATCTAATATTGCAATATCATTTGGCGCAAAAATACCGATAACATCGCCTTTTTTAACACCTT
GAGATGTTAAATAATGTGCCATATTATCAGCGCGTGCATTGAGTTGTTGGTATGTCCAAGATGTTTGTTTTGCGTGATCAATAACGG
CAGGCTTGTCATCATCGAAGTCTGAACGCGTTTTTATCCAATCGAAATTCATTAGTATACCCCCTTTAGCTTCACTTTCATACTTTAT
GAATTGATTGTTTAAGTTGTCCCCATTTTTCTTTGTAAATGCTGGTATCAATTAATTTTAAATGATCAGCAATAATTGGTTTAAAAGC
CATTTGATTCAAAATATCTTTATGCAAATCAAGACCTGGTGCAATTTCAATTAGTTTCAAGCCTTGATTGGTGAGTTCGAATACTGCA
CGCTCAGTAACAAAATAGACTTCTTGCTCGAGTGATTGTGAATATTGTGCATTAAAGTCGATATGGCTCACATCTGATACAAATTTC
TGGTTTTGTCCTTCAGTTTCAATGTTTAATCGTTGATTATGGCATGAGACATGACTGCCAGCTACAAAAGTACCTGAAAAGATAATTT
TATTTACAGATTGCGTAATGTCTATAAAGCCACCACATCCATTTAGTCGGTCATTGAAGTAAGACACGTTGACATTGCCGTATTGAT
CAACCTCAGCAAAGCTAAGATAGGCAACTGATACACCATTGTTATAAATAAAATCCCATGCTCGATCATGAGGCATGCGCACATCT
GCATTGTAATTCATACCAAAATGTTCACGACTCCCAACGAATCCACCGAAAATGCCAACATCTAAAATCGGTTGCACATCATGTTCA

ACACATTCTTCGTGCAATAAATTAGAGAGTTCATTATTGATGCCATAACCGATGCTAATTGTATCGCCATAAGTTAAAAACTGAGCA
GCACGTCGGAGAATCAATTTGCGACTATTAAAAGGTAATGCGGGTTCAGGTATTCCATCAATTCGTTCTTCTCCAGACAAGGCTGGT
AAATAATGACTCTGAATTACTTGGCGGTGATTCTTTTCATCTTCTGTGACGTATACATAATCGACAAGATTTCCTGGGATAACAACTT
CATTCGGTTTTAGTTGATATCCGTCAACTAAAGCTTTAACTTGTACAATAACTTTCCCATGATTGGCTTTCGCGTTTAATGCGACATG
ATAACACTCGCTCAAGTACGCTTCTTGAGTTAAATAAATGTTACCTTGTTGATCTGCGTATGTTCCTCTCAGTAGTGCCACATCAACG
CTAGGGAATGTGTAATGTAAGTATGTTTCATCGTTGATGGTTACTAATGAAACTAAATCATCAGTTGTTCGTGTATTTACTTTACCGC
CACCGTATCTAGGATCAACAGCTGTGTTTAATCCGATTTTAGTAATAACTCCAGGTAATAATTGATTACTCTGACGATAATGAGTTG
CAATGATACCTTGTGGTAAAAAATAAGCTTCAATGTCATTATTTTTCATTGCTTGTGCCGTTTTGGAAGAAGCCGTTAAAATGCTCAT
AATGACACGTTTAATCATGCGACGTTCTATAAAATCATCTAAATCCGGTGCGGCACCTAAACTATGGATATCATTCGCTAATATAAA
CGTTAAATCTTTGGGCGTATGATATGTGTCATGTTGCGCTAACACAGCACGTAGAACTTCGGCGGGTAAGTTGGCTACAGCTAATGC
TGGTAAACCAATCACATCACCATCTTTAATGATATGTTGTAAGTCGTGCCATGTGATTTGTTTCAAGCAAGTCACCTCCATCACATTT
GATAAAATATAGCGTTTTTACACTTTGTGTAAACCCTTACAAGAAATATAACATAACGACGTTTAAAATCAATTAGAAATATCTTTT
TATTCTGATAATAGACACAGTATAGACACATTTTGATGGTTGATAACAATTGTAATATCAAGGGTTTGTAATGAATTGAATATCATT
AAAATACTTATATAAAAATATTGCTCGGAATATAAAAAGTTAAATAGGTTTTGATTTTTAAATATGAAATACAAAGCGCCCAATCGA
ACAAAGTATTTATATTAAAATATGGAAAATCCATCAATATTAAATTAAAATAGTTTTATTATGAAAAGTGAAAGTAGGTAAGTCTAT
GGAAGGTCTTAATCATCGAAGAAATACAGAAAAAGAAGAGACAACACAAACGCAATCAGTTGCACCTAATACAGGTGAAGAGGGG
ATGTCATCAGCAAGTACACAATCAACTAAGACGTCCGACATACATAATGAATCTATCAATAAACAAATGGAAGCCAAAGCGCATGA
AACAGCGCAAAATGCAGATTTAAAAACCGAAGCAAGAAGTTTATTTGATAATGCAACCAAATCAATCGGTAGACTAGCCGGCAATG
ATGAAAGCTTAAATCTTAATTTAAAAGATATGTTTTCTGAAGTATTTAAGCCGCATACTAAAAACGAAGCAGATGAAATATTTATAG
CGGGTACTGCTAAAACTACGCCAGCAATTTGTGACATATCAGAAGAATGGGGGAAGCCATGGCTCTTTTCTCGAGTATTCATCGCTT
TCACAGTAACATTTATTGGATTATGGGTCATGGCAGCGATTTTTAATAACACTAACGCGATTCCGGGTCTCATTTTTATAGGGGCTTT
AACAGTACCATTATCGGGTTTGTTCTTCTTTTATGAATCAAATGCGTTTAAAAATATTAGCATTTTTGAAGTTATTATCATGTTCTTTA
TTGGCGGCGTATTTTCATTACTAAGTACGATGGTATTATATAGATTTGTCGTTTTTAGTGATCAATTCGAAAGGTTTGGTTCTTTAAC
ATTTTTCGATGCATTTTTAGTAGGATTAGTTGAAGAAACTGGAAAAGCACTCATTATTGTTTATTTCGTCAATAAATTGAAAACAAA
TAAGATTTTGAATGGATTATTAATCGGTGCTGCTATTGGTGCAGGGTTCGCAGTTTTTGAATCAGCAGGTTATATTTTGAATTTCGCT
TTAGGGGAAAATGTCCCATTATTAGATATTGTCTTCACACGTGCGTGGACTGCGATTGGTGGTCATTTAGTTTGGTCAGCGATTGTTG
GTGCTGCAATAGTTATTGCGAAAGAACAGCATGGCTTTGAATTCAAAGATATTTTTGATAAACGCTTTTTAATATTCTTTTTATCAGC
CGTTGGTTTACATGGCATTTGGGATACATCTTTAACTGTACTTGGCAGTGATACGTTGAAAATATTTATTTTAATCGTTATTGTGTGG
ATACTTGTATTCATTTTAATGGGGGCAGGTTTAAAACAAGTGAATTTACTGCAGAAAGAATTTAAAGAACAACAGAAAAAAGTAGG
CGAATAATAATTAAAGCTTATGTTGCTCATATGTTTGTGACATAAGCTATTTTTATAATTTGTCTTTAAAAGAGTGGAATAGGAATAC
TTTTCGGAGTTAAAAAAGTGTTTCACGTTAAACAAATAGTGACAATTAGGTTTATATAAAATGAACATGATTCACTGAAAGTATGTA
ATAATCATTTTATTGAAAATCATCAAATAGAAATTAATACAATCATATAAGCAAATTAAACCACGCCATAATCATATTGGATGATTT
CGGCGTGGTTTTTATAGTTGAAGCAGGGCTGAGACATAAATCAATGTCCCACACTCCCTTATCGTTCAATCGTTGTTCGATAATCGA
TTAAATAGATACCTTCAGGTGTTACTTTATAATTTTTAACCTTAGAGTTAGCAGCGACTATTTGATCGTTGTAAGCAATATAACTGTT
TGGTACATCTCGACTTGATAATTTAATAATATCATTAGAAATATTATGACGTTCCTTAACATCTACAGTATGATTCAATTGATTAATT
AAATCATCAACGTTGCTATTATTGTAGTCTCCTTTATTAATAGCACCATCTTTTTTATATGCTTGATTAAAGAAATAGCCAGTATCTC
CACGAGGAATTGTTCCGAAACTATACATCGTTGCATCCCATGCAGAACGGTCTTTTAAGTAACCTTCTATGTCATCAACACTTTTAAT
GTCGATTTCAATATTTGCTTTTTTAGCATCTGATTGTAATACTTGCGCAATTTTCGATAGCTCTGGACGACCGTCATACGTAATTAAC
TTAATTTTTAAAGGGTGTTCTTTTGTATAACCATCTTTAGCTAATAACGTTTTTGCTTGTTCGATATTTTGTTTGCTTAACTTAGGTTCT
TTAATATATGGAATTTTATCATTAAATGGACTCGTTGCAGGTTTCGCATAACCTTGATAAATATGATCTGCAATACCTTGTCTATCAA
TGATATGATCTAATGCTTCACGAACGGATTTAGTCATTTTTTTATTAGTATGATTATACATAAGTAAAGAAGTTCTAAATCCAGATTC
TTTTGACACTTTTAAATTTTGATTATTTTCTATGTCACGAACTTTATTAACTGGGACATCAGTTATTAAATCATCTTTTTGAGATTCTA
AATTTCTGACGCGATTATTGCCGTCTTCTTGGTACGTCACAGTAATATGATCAAGTTTCGGTTTACCTTGCCAATAGTCCTTAAAATT
CGACAATGATATTTTTCGAGATTGCTTATAATCTTTTATTTGGTAAGGGCCTGTACCAACAGGAGTTTGATTAACATCTGATTTAGCA
TCTGTATCATAAATTGCCATAAAAGGATTAGCTAATTCAGATACAAGTTCAGGGTAAGCGGAGTTGGTTTTAATTGTCAGTTTTTGA
CCTTTAGCGGTAATTGATGATATTGGTAATGAATATTTGACCAAGTCGCTTTTTTTCATGCTATTTTCAAGGCTAGATTTCACTTTTTC
TGCAGTCAATTTTTGACCGTTTTGAAATTTAATATTATCTTTTAATTCTATATCTAACGTTGTATCATTTGGTTGATGATACGATTTCA
CTAATGCTTTTTCTATTTTTCCTTGATCATTTGTTTTAAATAATGATTCTGCAGCACCAATCTTAACTGGTACATCTGTTTCATAAGGT
GCAATAGACTTTGTTTTTAACGGTAACGAAATATTTAAGTCTTTGCCAGATGAATGCATTGAGCCACATCCTGATAACACTAATACT
GCTGAAAATATAGTTGCTAGTCTTTTAAACTTCATTTCATTAACACTCTCTTTCTAATTACTATGTAAAACCCAACAATTAATATTTT
AAAACTTTATTTTGTTAAAGTAAAATATTGTTCAAGTTTAGTAATCATTAAATTTCAATTAATTGTAGTAATTATGCTTTTTAAAAAT
AAATATTAGAAATGAAGTTAGCAACATTTATAGTGATTCACGATAAACATATATACCTAAGTATTGAGCAACTGCTGTAGTACTACA
GCTTGGTTATGTTTAGTATCTTTTGCTGCATATAACAATAGAACATGATTATGCTGATTTACAATATCCTTTAATTTTTCAAAAGCAT
CTTTTTGCGCATCCTGATCACGTAATTCTTTTTCATATTTTTCTTTAAAAGCTCCAAAAAGTTTAGGATCATGTTGGAACCATTGTCGC
AACTCAGTAGAAGGGGCAATGTCTTTTAACCAATAATCTAGGTTAGCAGTTCTTTTCGAAATACCTCTCGGCCAGACTCTATCGACT
AGGATACGAATAGCGTCGGTATTATCTTTATTGTCATAAATCCGTCCAATATCTACGGTCATCTTGTGAACTCCTTTCTTATGAAATT
CAGTGAGCATACATCAATGCATGTTGTGGTGGGACGACCAAATAAATTTTGCGAAAATATCATCTCTGTCCTACTCCCAATTAAAAA
ACAGCCATGACAAAGTAAAGTCATAGCTGTTTTGGTATAGATGTCATTTATTTTTACGTTTAGTTAAATACTTCAAACCAACTGCAA
AGACCGTGTACCCGGCTATGGTTTGTATCAATGTTTTAATTAAATTATTATTTTTAACAATAATATTTGCAGTAACAATACTTACGAA
ATATAATGCAAATACTTTTACGTAACGTTGATTAAGTTTCATATGAGCACTAAACCTCTTTTCTCAATTATACTGCAACGCTTAGTCT
TGGAATAAATGTTTCGTAGTGTACGCGATCCATATCGTAATTTAAAGATTTAAGTGCTTCGATCATAGATTGTAAGAATTTTGTACC
ACCACAGATATAAATTTCAGGTTTATTTGCTAAAAATACTTGTAATTCTTCAGCGCCAATATAGCCTTGTTTATCTTTTAAGTGAGTA
TATAACTTAGCGTTGTCATGATGGCTTGCGATACTGTTGAAGTTGTCTTTGAAAGGTAAATGTTGTTCATTTTCAGCAACTTGAACCA
TCTGTGTATCTAAACCTTTGGCAGAGGCAGCTTCATACATAGCTACTAAAGGTGTAACACCAATACCTGAACCTAAGAAAAGTTGTG
GTTCAGTCGTATTCTCTAATACGAATCCACCTACAGGTGCAACTAAATTAATCATATCGCCTTCTTTAATCTCATCGTGTAAAATTGT
TGAAACTTCGCCTTCATGTTCTGTTGTGACATCACGTTTAACGCCAAAAGTTAAATGGTTTTTATCACCTGATACGATAGAATAGTGA
CGTTTAGCTCTATATGGAAGTTTATCACTAGAAACATCAACTGTGATGTATTGGCCTGGTATAAATTCACTAAAGTCATATTCTTCAG
TTTCAACTGTAAATGATTTAATGTCTTCAGATTCTTGTTTAATATTGGTAATTTTGAATGGTTTAAAACCAATCCACATCATTTGATC
ATAAATTTCTTTTTCAATTTGGATGAACACGTCCGCAATAACGCCATATGCTTTCGCCCAAGCTTGAATGACAGGGTCATTTTCTTCT
AATCCTGTCACATCTTGAATGGCTTTTAATAAATTTTCCCCCACAATTGGATAATGTTCAGCATAAACTTGTAGTGCGCAGTGTTTAT
ATGCGACTGGCATAATGACTGGTTTAATAACACTTAAATTATCGATATTAACCGCTGCAGCCATTACAGCTTGTGCTAATGCTGAAG

ATTGCATGCCTCGTTTTTGGTTCGTTTGATTAAACATGTTTAAAAGTTCAGGATGCGCTTTAAACATTTTTGGATAAAAGATTGACGT
AATTTCTGTCCCTTTCTCTTTAAGTAAAGGCACCGTTTGTTTGATAATGTCTTTCTCTTGTTCTGTAATCATGATACTCCTCCTTTAATC
TGTCTATTTTGATTATTCTACTAAAAATTTCGATATTCAATTAATTGGTTTGAGAAAATATAAATAAAATGGCAAATTTGATAATTGT
ATGACATTTTTGATTTTTTAAATACTTATCAATAAGCATTGTGTACAATTGTCTGTTTGCACACCGACGATTGCGCGCATTTATTTGA
CTAATTCAAAAAACATTGTTGTTTTCCTAGAAAAAAGTAAACATGATAATAAAAATGTGAAAGTGTAAATAATCACTGGCGAAGTA
CGAAGACTAAAGACATCTAAGATGTAATCGTATACAAATTAAAAAGGTGTAAAAATTAAAATAAAATGTGAAATAAATCACAATTT
AATATTGACCCAGTACTTAATGCATGTTACATTTTATATGTGAAATAAATCACAAACTTAAAAGCGGATGACACATGACCTTTTAAG
TTATGCGTTGAAAATAAAAGAGATGTTTATTTGCTTTTGTATCGTCAATAAGCAGCATTAAACTAACATATTTGAAGCTACACGTAT
GTTAGTGAATTAATCATAAGGGAGTTTTTGTAATGAACAAATTTAAAGGGAACAAAGTTGTATTAATAGGTAATGGTGCAGTAGGT
TCAAGCTACGCATTTTCATTAGTGAACCAAAGCATTGTTGATGAATTAGTCATCATTGATTTAGACACTGAAAAAGTTCGAGGAGAT
GTTATGGATTTAAAACATGCTACACCATATTCTCCAACAACAGTTCGTGTGAAAGCTGGCGAATACAGTGATTGTCATGATGCGGAT
CTAGTTGTCATCTGTGCTGGTGCTGCACAAAAACCTGGAGAAACACGTTTAGATTTAGTATCTAAAAACTTGAAAATATTCAAATCA
ATTGTTGGTGAAGTAATGGCATCAAAATTTGATGGTATTTTCTTGGTAGCTACAAATCCTGTTGATATTTTAGCGTATGCAACATGGA
AATTCTCTGGTTTACCTAAAGAACGTGTTATAGGTTCTGGTACAATTTTAGACTCTGCACGCTTTAGATTATTGTTAAGCGAAGCGTT
CGATGTTGCGCCACGTAGCGTCGATGCTCAAATTATTGGTGAACATGGTGACACTGAATTACCAGTATGGTCACACGCTAATATTGC
GGGTCAACCTTTGAAGACATTACTTGAACAACGTCCTGAGGGCAAAGCGCAAATTGAACAAATTTTTGTTCAAACACGTGATGCAG
CATATGACATTATTCAAGCTAAAGGTGCCACTTATTATGGTGTTGCAATGGGATTAGCTAGAATTACTGAAGCGATTTTCAGAAATG
AAGATGCCGTATTGACTGTATCAGCATTATTAGAAGGCGAATATGATGAAGAAGATGTTTATATTGGTGTTCCAGCAGTCATCAATA
GAAACGGTATTCGCAACGTCGTAGAAATCCCATTAAACGACGAAGAACAAAGCAAGTTCGCACATTCAGCTAAAACATTAAAAGAT
ATTATGGCTGAAGCAGAAGAACTTAAATAACTTTTTATAAAATCTATACCATTCCAAAAATTGTAAAACCTTACCCCAAAAAATTGT
ATAAAGGGCTATTTGATACAACTATATGTGTCGAGTAGCCCTATTTTTAATGTAGTGAAAGTCGTTGTTGAAATTAAATTCAAATCT
GGCACTTAAGCTTTAATCATAGCATTAAGATGATTGTCTAGCAGAGGCGATTTGCGGGCTCACTACAGTGTATGATGAACTTAATGC
TTCAAATGTAACATTAAAAATAAAAGCAACGATGTCACTTCTTACTTCGTACATCGTTGCCATTAGTTCTGTTGATATTTCGGTTAGT
CTTAATCCCCGAGCAATTCTTCAATCTCATTTTTGATAACTGTAACGTGAGGCCCATAAATTACTTGCACACCAGTGCCTTGCTGGAT
TACACCTTTGGCACCAGTACTTTCGAGTAATACTTTATCGACTTTGTCATTTTGATGAAGTGTGACGCGTAGTCTCGTTGCACAACAG
TCAACGATTTCAATGTTATCCTTGCCTCCCAAACCAGCAACAATAGTTTGTGCTCTTTCAGTAGCCTCAACTTGTTGTGCTGCAGCTT
TATCTTCTCGACCAGGTGTTTTGAAATTAAATTTCGTAATTAAGAATCTGAAAACGATGTAATACAAACAGAACCACACAATTCCAA
TAGGTATGACGTATAGGTAGTTTGTTTTACTATTACCTTGTAGCACACCAAAAAGTAAGAAATCGATAAAGCCTCCACTGAAGGTTT
GACCAATTGTAATGTTGAAAATGTCTGCCATCATAAATGCTAATCCATCAAAGAAGGCATGGATTACATAAAGAATAGGTGCGACA
AACAAGAAACTAAACTCTAAAGGTTCGGTAATACCTGTTAAAAATGAAGTGAGTGCAGCGGATAACATTAAACCGCCGACAACTTT
TTTATGTTCAGGTTTAGCTGTGTGATAAATTGCAAGTGCGGCACCACATAAGCCGAACATCATCGTAATAAAACGGCCTGACATAAA
GCGTGACACACCTGAATAATACTTCGTCACATCTGGATCACCAAGTTGAGCAAAGAAGATGTTCTGCGTACCTTGAACTAAGTGCCC
TTTGACTTCTAAAGTACCACCAAGTGCCGTCTGCCAAAACGGTAAGTAAAAAATATGGTGTAAACCGAGTGGACCTAACAATCTTA
AGATGAAGCCATAAACAAAAGTACCGATGGCACCTGTTTTCGTTACAAATCCACCAACATGATAAATGCCGGCTTGTATGCTTGGCC
AAATGAAAAACATCAATACACCTAAAAAGATTGCGGCAAATGCTGTGACAATAGGGACAAATCTAGAGCCACCAAAGAAACCTAA
ATACGGTGGTAATACCACTTTGTGATATTTGTTGTGAAGTATTGCGGTCATAATACCTGTGATAATCCCGCCAAAAACACCGGTTTC
AACCGTTTGTATACCGAGCACCATGCCTTGTCCATTTTGTGCAAGCTGATCTTTTGCCAATGTGCCCGTGATAGTTAATAAGCCATTC
ATAGTTACGTTCATAATTAAGAAACCGAGCAGCGCAGCTAAACCTGCAGTACCTTTATCGCTTCTAGATAATCCGATTGCGACACCA
ATGGCAAAGATGACCGGTAAATTTTGGAAAACAATACTACCTGCAGCTGACATTAATGTAAAAATATTTTGTAATAAGGTAATATCT
AAAATAGGGTATGCTTTAACGGTGTTTGGATTACTTAATGCACCACCGATACCCAACAATAGACCTGCAGCTGGTAAGATTGCGATA
GGTAGCATAAAGGACTTGCCGAACTGCTGTGCTTTTTCAAATAAAGATTTCATCAACATCCCTCCTAATTATTCTCAATATAGCTTTT
GAGAAATTTAATATCAATATATATTCTGTGTATGAAAATATTTTTCATAAAAATTGTTTGAATCATGTAACAATCATATAAATTGCTG
TTTATATTGTTGTGAAAATGAGTTGACAAAAGTCGGTATAGATATGTAGACATCCTATTTTTAGCGAGGTAGGTTGATAAGGCATAT
CGGATAAATTTATAAGCCATAAAATAGATAAATAGTGTTTGTATCCAAAAATATGAGAAAGTTAAAACTATTTTTCAAAATAAACA
TGATTCACCACATAAATAAATATACATAAGCTATCGATAACAACTTAAGAAAGGTGGATATATAAATGAAAAGAAAGATTATTATG
GATTGTGATCCAGGACACGATGATGCAATAGCATTAATTTTAGCGGGGGCAATTGACAGTCCACTAGAGATATTAGCTGTAACAAC
AGTCGCAGGTAATCAATCAGTTGACAAGAATACGACAAACGCCTTGAACGTATTGGATATTATGGGACGCCAAGATATAGCAGTAG
CGAAAGGTGCGGATAGGCCGTTAATTAAACCAGCTGCCTTTGCTTCTGAAATACATGGGGAATCTGGATTAGATGGTCCGAAACTA
CCGTCGACACCATCACGTCAAGCAGTTGCAATGCCAGCATCAGATGTGATTATAAACAAAGTGATGACGAGTGATACACCTGTAAC
AATTGTAGCGACAGGTCCTCTTACGAATGTAGCAACGGCATTGATTCGTGAGCCAAGAATCGCTGAGCATATTGAATCTATTACTTT
GATGGGTGGTGGTACATTTGGAAATTGGACGCCTACAGCAGAATTCAATATTTGGGTAGATGCTGAAGCAGCGAAGCGTGTTTTTG
AAAGTGGGATTACTATAAATGTGTTTGGTCTAGATGTAACACATCAAGTTTTAGCCGACAATCACGTGATTGAACGCTTTGAAAGTA
TCAATAATCCTGTTGCACAGTTCGTCGTAGAATTATTGCAATTCTTTAAGAAGACATACAAGACTCACTTTAATATGGATGGTGGTC
CAATACATGATGCTTGTACAATTTTGTATTTGTTACAACCAGAATTGTTTACAATGGTACCCGTTAATATCGACATTGAACATCAAA
GTCCACTAACTTATGGCACTATGGCTGTCGATTTAAATCATGTTACAGGTAAGCCTGTCAATGCTTATTTTGCTACAGCAGTTGATGT
TGAAGAAGTGTGGAACTTGATAGATTATAAGTTACGTACTTACGAATAATAATACTTAATTAAATAGATACAGTTAGCCCTAAGGC
GCCTGATATAAGCGTCCTTAGGGTTTTTGTATTGGATTACATTGGTCATGACAATTTGATAATGATTGAGATATGTATGTTAAAAATG
CTTCAAAAAACATAAAACACAAAAACATATGTAAAAATTTCAATTGCTTTATTTTAAAAGATTAAAATCACAAAAAATATAGATAT
AATTGAAATGTTGTTAAAACATCACAATGATGTACATGCTCATTTTGAAAATATGTGTTCAAAGTGTGTACATCGGTGTGACGATTG
ATGATAAATTGAGCTCAGATGCTCAAAGGAGGATCGAACATGAACGGGGATAATCAGCAAATACTCAGAGAAATTGTATTGAATCC
TACTATTCATGGTAAAGAACTTGAATCGATATTTGGTTTGTCTCGTAGACAACTAGGATATCGCATTCAAAAAATCAATTTGTGGCT
TGAACAAGAGGGTTATCCAAAACTTGAAAGAACAAGCCAAGGAAATTTTATTGTAAGTTCTGAAATCATGACGTTATTCAAGCGAG
ATGTATCGGAGCAGCAAATGTTAAACGGCAACAATGTCATTTTTAGCATAGAAACACGTCGTTATTATTTAATGCTCATGCTTTTTA
GTAAGGAAAATGCAATGTCTCTAAACCATTTTTCAATTGATTTACAAGTCAGTAAAAATACTGTCATTCACGATATAAATCATGTGA
AAGAGCAATTGGAAGATCATGGTTTGTCACTTAAGTATTCTCGAAAGCATGGTTATGAAATTGTTGGTGATGAATTTGAAGTTCGCC
GTTTCTTCATTAAGTTGATTGATCAAAGGTTGAATCATGATATTACTAAAAGTGAAGTTTTAAAGGCGCTCAACTTAACATTCGAAG
ATATCGCATATCAAAAAGACAAGATCAAACAGGTAGAACAATTTTTGAAGAGTCGCTTTATAGACAAATCACTTAGTTCATTGCCTT
ATGTCCTTTGTGTGATTCGTAGACGAATTCAAAGTGGTCATGTGATGAATCCATTAAATATTAATTATCAGTATTTGAGGGATACGA
AAGAATATCAAGCAACGGAGATTATGACGCAACATGAGCCGGATTTGCCAGAAGCGGAAAAGTTATATTTGACATTACACTTACTT
TCAACAAGTGTGCAATGGACTGATTTGCAGGAATCAGATAACATATCGAATTTAACGATGGCCATCGCTCAAATGATTCACCATTTT
GAACAAATCACTTTTATTAACATTGAAGATAAGGAGAAATTATCACAGCAACTCTTGTTACATTTAACGCCTGCTTTTTATAGGATT

AAATATAACTTAACGGATCGTGATGAATTAATAAATCCTTTACAAGGAAATTATCAATCCTTATTTCATATGGTGAAACAATCATGT
CAATCGTTAACTGAATATTTCGGAAAATCGTTGCCTGATAATGAAATAGCATATTTAACCATGTTGTTCGGAGGTAGTTTGAGACGT
CAAGATGAAAACTTCGATGGCAAGATAAAAGCTATTATCGTGTGTACACAAGGCACGTCAGTATCACAAATGATGTTATACGAGTT
GCGAAACTTATTTCCAGAAATTATTTTCTTAGATGCGATTTCACTTAGAACATTTGAAAATTACACATTAGATTATGACATCGTCTTT
TCACCAATGTTTGTCCTAACACATAAAAAATTATTTATCACAAAAGTAGCTTTATCTGAAAATGAGCAACGAAAGTTACGTAAAGAT
GTGATGAAGTACATTAATAAGGAATCGGCTGACATTGATAAGGAAATAAACAAGTTAATGGCATTAATTGAACGCACAACGACAGT
TAATGACATTACAGAACTACGTGATGGTTTAGAAGATTTTATTGCCAATTATAATTCAATTTCAACCATTAATGGATCGATTGTCAC
ACAAAATAAGACATTAGATTTAGCTGACTTGATACCGGCAAGGCACGTGAAAAGAATGCATCATGTTGAAAATATTGATGAAGCTA
TTGCTAAAGCAAGTGATGTGTTAGTTGCTAATCATTTTATTGATATTAAATATATTCATGAGATGCAACAGGTATTTGATGATTCGTA
TATGGTTATCATGCAAAATATTGCTATTCCACATGCATACTCTGAAAAGCATGTACATAAAACAGCGATGAGTATGTTGATATTACA
AGAACCAATATACATGTCAGATGGCACAGCAATCCATATTATTGTACCTATTGCTGCTGTTGATAAAGTGACACACTTAAGAGCGTT
ACTACAATTGAGAGATGTGGCGCAAGACAATGACGCAATTAAGCGCATCATACAAAGTCGCAAAAATTCTGATGTAAATGAGATTT
TAAAAAATTATTCAAATAAAGAAGCGAGGGAAAATGGATGGGACAGCAATTAGTGCATAAAGAAAATATAATGCTCAATTTGTCG
GCAACTGATAAAGAATCCGTATTGTCACAAATGTCAGATGTGTTATTTCAAAATGGGTTCGTGAAGTCAACGTTTAAAGATGCAGTC
ATCGACAGAGAAAAAGAATTTGCTACTGGTTTACCAACGCATCTATGTTCGGTCGCTATACCGCATACAGATGTCGAACATATTAAC
CATAGAACGATAGGTGTGGCTGTTCTAGAAAAAGAAGTGCCGTTTATTGAAATGGGAACACTTGATCAACAGACAGAAGTGAAAAT
CGTTTTTATGTTAGCAATGGATAAAGTAGATGATCAACTTAAGCTGTTACAACAGTTGATGCAAATTTTTCAAAGTGAAGAAAAATT
GGAGCAGATTCTACGAACGAAAGATGAAACAATTTTAGCAACACTAATCAATGATTATTTGGAATATAACTAAAAATTAATTGGAG
GAATTGAAAATGAAACAAGTATTAGTAGCGTGTGGTGCAGGTATTGCAACGTCAACAGTAGTAAATAATGCAATTGAAGAAATGGC
AAAGGAACACAATATTAAAGTAGATATTAAACAAATCAAAATTACAGAAGTTGGACCTTATGAAGACACTGCAGATTTATTAGTTA
CAACTGCAATGACAAAAAAAGAATATAAATTCCCAGTTATCAACGCACGTAATTTTTTAACTGGTATTGGTATTGAAGAAACAAAA
CAACAAATCTTAACAGAGTTACAAAAATAACGGAATTGATATGTAACGTGGGTCAATAACATATAAATATATGCAAATTGCATATA
AATGACTATCACGACAAATGATTGTTGATGACATAGAAACGCAGTGACTGTAATTCAAAGGGAGTGCAGTCGAATGTAGCAAGGGT
TGTCGTTGAAATGACCGTAACGTCAAATGTAATCACAATCGGAAAGTATCGTGACAATGCATATATAACAGGGAGGGTTTAAATAT
GAGTTACTTCACTGATTTTGTAAGGGGATTTTTAGATTTAGGTGCAACTGTTATTTTACCGGTTGTCATATTCTTGCTTGGCCTATTCT
TTAGGCAGAAAATTGGAGCGGCATTTAGGTCTGGTTTAACAATAGGTGTGGCTTTTGTAGGGATTTTCTTAGTCATCGATTTATTAGT
TAAAAATTTAGGGCCAGCAGCACAAGCGATGGTTAAAAATTTAGGCGTCAGTCTGAATGTGATTGATGTAGGTTGGCCAGCAACAT
CATCTATCGCTTGGGCATCATCTGTCGCAGCATTTATTATTCCACTCGGAATCATAGTTAACGTTGTATTGCTAGTAACTAAAGTGAC
AAAGACGATGAATGTAGATATTTGGAATTTTTGGCATTATACGTTTACAGCAGCAATGGTTTATGCCGTATCAGGCAGTATTTGGCA
AGCGTTATTAGCAGCAGTTATTTTCCAAGTTATCTGTTTGAAAGTAGCAGATTGGACAGCACCGATGATGAGTGAGTTCTTTGATTT
ACCAGGTGTATCGATTGCTACAGGAAGTACAATTTCTTATGCACCAGGTATTTACTTAGTTAAATTGTTACAAAAAGTACCCGGTCT
GAATAAGTTAGATGCTGATCCTGAAACAATTCAAAAACGTTTTGGCGCATTTGGAGAGTCTATCTTTGTCGGCTTAATTTTAGGTTTA
GGTATTGGTGTGTTAGCAGGTTACAAACCTGGAGACATCATTAATTTAGGAATGTCAATGGCTGCAGTAATGGTATTAATGCCTAGA
ATGGTAAAAATCTTAATGGAAGGTTTAATGCCAGTTTCAGAGTCTGCAAGAACATGGCTAAATAAACGTTTTGGCGAACGTGAAAT
TTATATTGGTTTGGATGCAGCAGTAGCATTAGGTCATCCAGCGGTTATTTCGACAGCATTAATTTTAGTACCTATCACTGTTTTATTA
GCCGTTATTTTACCAGGAAACCAAGTACTACCTTTTGGTGACTTAGCAACGATACCATTTGTTGTCGCGTTTATTGTTGGTGCAGCAA
GAGGAAACATTATTCATTCTGTCATTGTGGGTACGATTATGATTGCAATTTCACTATATATTGCAACAGACGTAGCACCTATTTTCAC
AGATATGGCGAAAGGTACGAATGTACAAATGCCAAAAGGTTCATCTGAAATTTCAAGTATTGATCAAGGTGGTAATATCGTTAACT
ATCTTATCTTTAAACTATTTAGTCTATTCAATTAAAAATCGAGGTGTTTGTGGTGAAAGCTTTAGTAAAAACAAGAGAAGGACATGG
CAACTTAGAACTTCTTGATAAAGAAGTTGCAACACCGCTAGATGATAAAGTAAAGATTAAAGTACATTATGCAGGAATTTGTGGCA
CAGATATTCATACTTATGAAGGTCATTATAAAGTTAATTTCCAGTGACATTAGGTCATGAATTTTCTGGTGAAATCGTTGAAGTTGG
AGCAGACGTTAAAGATTTTAAAGTTGGTGACCGTGTCACTTCTGAAACGACATTCTATGTTTGTAATGAGTGTGAATACTGTAAATC
AAAAGACTATAATTTATGCAACCATCGAAAAGGTATTGGAACACAAGTTGATGGCGCATTTACTAATTATGTCATTGCACGTGAAG
AAAGTTTGCATCATATTCCAGATGAAGTATCGTATCAGTCTGCAGCTATGACAGAACCATTAGCATGTGCACATCATGGCGTTTCTA
AGATTCAAGTCAATTCAGGCGATGTAGCAGTTGTAATGGGACCTGGGCCAATCGGATTACTTGTAGCACAAGTGTTAAAAAGTAAA
GGCGCAACTGTTGTGGTAACTGGATTGGACAATGACAAAGTCAGATTAGATAAAGCAGAAGCATTGCACATGGATTATGTAGTCAA
TTTACAACAAACAGACTTAAAAACGTATATCAATGGAATTACAGACGGTTACGGTGCAGATGTTGTTGTTGAATGTTCAGGTGCAGT
TCCAGCAGCACGACAAGGTTTGGATATTTTACGCAAAAAAGGTTTCTACAGTCAAATAGGTATTTTTAAGGATGCTGAAATTCCATT
TGATATGGAAAAAGTGATTCAAAAAGAAATAACAGTTGTTGGTAGTAGAAGTCAAAAGCCAGCAGATTGGGAACCTTCATTGCAAC
TTATGGCGGATGGTTTAGTAAATGCTGAAGCTTTGGTGACAAAAAATATATGATATTTCGAAATGGGACGAGGCGTATCAACATTTAA
AATCCGGCGAAGGTATTAAAGCATTACTTAAGCCGCTCGATTTAGATGAAAATGAAGGAGAGAATTAATATGGTAGAATCAATGCT
AACTTTTATGCTTGGGCCATTAAGACAAATCACTGATTTTTATATGGAACATTTACTCGTAAGTAATTCCATTGTCATTGCAGGTTAT
TTTGCGACAGGTATTTTTAAAAAGAAAAAAGTTGTGAATTAAATCAAATTTGAGGTGATTTACAAGTGAAAGCATTGAAATTATATG
GCGTGGAAGATTTACGGTATGAGGATAACGAAAAGCCAGTCATTGAAAGTGCGAATGACGTTATTATTAAAGTACGAGCGACTGGC
ATATGTGGTTCAGACACGTCACGATACAAAAAAATGGGGCCATACATTAAAGGTATGCCATTTGGTCATGAATTTTCAGGTGTAGTA
GATGCCATTGGAAGTGATGTTACGCATGTTAATGTGGGCGACAAAGTGACAGGTTGCCCAGCAATACCTTGTTATCAATGCGAGTAT
TGTTTAAAAGGTGAATATGCACGATGTGAAAAGTTATTCGTCATTGGCTCATATGAACCTGGATCGTTCGCGGAATATGTCAAATTG
CCAGCGCAAAATGTTTTAAAGGTTCCAGACAATGTTGATTACATTGAAGCAGCAATGGTTGAGCCATCAGCCGTTGTTGCGCATGGG
TTTTATAAATCGAATATACAACCTGGTATGACTGTTGCAGTAATGGGGTGTGGCAGTATAGGTTTGTTAGCTATTCAATGGGCACGA
ATATTTGGTGCTGCACATATCATCGCTATAGATATAGATGCGCATAAACTAGATATTGCAACATCATTGGGCGCACATCAAACAATC
AATTCAAAAGAAGAAAATCTTGAGAAATTCATCGAAAATCATTACGCCAATCAAATCGATTTAGCTATAGAATCATCAGGTGCTAA
AGTTACGATTGGTCAAATATTGACGCTACCTAAAAAAGGTGGCGAGGTGGTATTACTCGGAATACCATATGATGATATTGAGATTG
ATCGCGTTCATTTTGAAAAAATTCTGCGTAACGAGTTGACAGTATGTGGCTCTTGGAACTGTTTGTCCAGTAATTTTCCGGGCAAAG
AGTGGACGGCAACCTTACATTATATGAAGACGAAAGATATTAATGTAAAGCCTATTATTTCTCATTTTTTACCGTTAGAAAAAGGCC
CGGAGACATTTGATAAATTAGTTAACAAGAAAGAACGATTTGATAAAGTCATGTTTACGATTTATTAGTATGCACCTTTGAGGACGA
AAACACTGGTATAGTTATAGCTATGAAAGTGCGAATGCCGTCTGGTCTACAGATACTATCGAAATAACTCATCTTCGAATATACGTT
GCTAAATAGCCGGTTTACTTGTGTGAAATATGCTTGTGAATCGGTTGTTTTGCATTTTGTATACTTAAAATGAGATGGCAATATTTGA
TAATTTTTAAAGTGAAAATCAAGTGCAGCCACTTAATAAGATAAATTTATTATAATATATGGTAAAATGATGGCAGTAATAATGAAT
TTGAAAAAGAGTAAACATTAATACCTTTAACAATTTAATATTGTCAGTGTTAATGATTAACTGCATGGCAAAACAACTTAGAATGGT
CAGTTACAAAAATACATTTTTATAAAAAATTATCACACTATTGTGACAACTATCTTTGGATTAATAAAAGAGGCAAGTGAGCAATAG
GTTAGGCTTATGTGCGGACATAGGTCAGTAATGTATAAATGGAAATGATGTAATGACAGAATGGAGGACAACATGATTTATGCAGG

TATTTTAGCAGGAGGTATTGGTTCGAGAATGGGGAACGTGCCATTACCAAAACAATTTTTAGATATTGATAATAAACCGATTTTAAT
CCATACAATTGAGAAGTTCATTTTAGTGAGTGAATTTAATGAGATTATTATCGCAACGCCAGCACAGTGGATTTCCCATACACAGGA
TATTTTAAAAAAATATAACATTACAGATCAACGTGTCAAAGTAGTTGCAGGTGGTACGGATCGAAACGAAACAATTATGAACATTA
TCGACCATATTCGCAATGTAAATGGAATTAATAATGATGATGTGATTGTAACTCATGATGCCGTAAGACCATTTTTAACTCAACGTA
TTATTAAAGAGAACATTGAAGTAGCAGCAAAATATGGTGCAGTAGATACAGTCATTGAAGCAATTGATACGATTGTAATGTCTAAA
GATAAACAGAACATACACAGTATCCCTGTAAGGAATGAAATGTATCAAGGCCAAACACCACAATCATTTAATATTAAATTATTACA
AGATAGTTATCGCGCCTTAAGTAGTGAACAAAAAGAAATCTTATCAGATGCATGTAAAATCATTGTCGAATCTGGATATGCAGTTA
AATTGGTACGTGGAGAACTATACAACATTAAAGTGACAACACCGTATGATTTAAAAGTAGCAAATGCCATTATTCAAGGTGATATT
GCCGATGATTAATCAAGTATATCAACTCGTTGCACCGAGACAGTTCGACGTCACATATAATAATGTTGATATTTATGGTAATCATGT
CATCGTAAGACCTTTATACTTGTCTATTTGTGCAGCTGATCAAAGGTATTACACAGGTCGAAGAGATGAAAATGTACTGCGCAAAAA
ATTGCCAATGTCATTAGTTCATGAAGCTGTTGGTGAAGTTGTATTCGATAGTAAAGGCGTATTTGAAAAAGGTACGAAAGTAGTAAT
GGTGCCGAATACACCTACAGAGCAACATCATATTATTGCGGAGAATTACTTAGCCTCTAGTTATTTTAGATCTAGTGGTTATGATGG
TTTTATGCAAGACTACGTTGTGATGGCACATGATCGTATCGTTCCGCTGCCTAATGCCATTGATTTGAGTACGATTTCATACACAGAG
TTAGTGTCAGTAAGTTATCATGCTATACAACGATTTGAACGTAAATCTATACCTTTGAAAACCAGCTTTGGTATTTGGGGTGATGGT
AACTTAAGTTATATTACTGCTATTTTGCTACGTAAGTTGTACCCAGAAGCTAAAATTTATGTATTTGGTAAGACAGACTATAAATTA
AGTCATTTTTCATTTGTAGATGACATCTTTACAGTAAATCAAATACCAGATGATCTTAAAATTAATCATGCATTTGAATGTGTTGGAG
GTAAAGGAAGTCAAGTTGCACTTCAACAAATAGTTGAACATATTTCACCAGAAGGCAGTATTGCTTTGTTAGGCGTAAGTGAATTAC
CCGTGGAAGTGAATACACGATTAGTACTTGAAAAAGGATTAACATTGATTGGTAGTAGTCGAAGCGGCTCTAAAGATTTTGAGCAA
GTTGTTGATTTATATCGTAAGTACCCAGACATTGTTGAAAAGTTAGCATTATTAAAAGGACATGAAATTAATGTATGTACGATGCAA
GATATCGTCCAAGCGTTTGAAATGGATTTATCGACATCTTGGGGAAAAACAGTATTGAAATGGACGATTTAATAAACCGATGGGGG
AATAACAATGACAAAAACGAAACAAGCAATACATATTGATAACATATACTGGGAACGTGTTCAGTTATATATTGAAGGACATAGTG
AAGGTGTCGATTTAACATCAGGACAATTTGTTCTGAGGAATTTAACCGAAACAAAAACATTAGAAGCAAATGAAATGAAAATAGAC
GGTAATACATTTATATGTAGATTCAACGTCGCAATATTAGACGATGGGTATTATTTACCAATGGATAAATATTTATTTGTTTATCATG
ACCAGTTAGAGTATATTGGACAACTTAATCCAAATATTATTGATCAAGCTTATGCGGCATTAAATGAAGAGCAAATTGAAGAATAC
AATGAGCTGACTACACAAAATGGAAAAGTGAACTATTTATTAGCGTATGATGCTAAAGTTTTCCGTAAAGGTGGCGTATCACAACA
TACGGTCTATACCATTACTCCGGAAATAGCAAGTGACGTTAACGAATTTGTATTTGATATTGAAATCACCTTACCTCAAGAGAAATC
AGGGGTCATTGCGACAAGCGCACACTGGCTTCATAAACAAGGACATAAAGCTTCATTTGAAAGTAGAAGTTTCTTATTTAAAGCTAT
TTTTAATATTACAAAGTTACTACATATTAAAAGAAGCAAAACAATATTATTCACATCAGATTCGCGTCCGAATTTATCAGGGAATTT
CAAGTATGTATATGATGAGTTACTTCGCCAAAAAGTAGATTTTGATTATGATATTAAAACGGTATTTAAGGCGAATATTACGGATAG
ACGTAAATGGAGAGACAAGTTTAGATTGCCATATTTACTTGGTAAGGCAGATTATATTTTTGTTGATGATTTCCATCCATTAATTTAT
ACGGTTCGCTTTAGACCATCACAAGAAATTATTCAAGTGTGGCATGCCGTTGGTGCTTTTAAAACAGTTGGCTTTAGTCGTACAGGA
AAAAAAGGTGGTCCGTTTATCGATTCATTAAACCATCGTAGTTACACGAAAGCATATGTTTCATCAGAAACCGATATTCCATTTTAT
GCTGAAGCATTTGGAATTAGAGAAGAAAATGTTGTACCAACAGGTGTACCACGTACTGATGTACTATTTGATGAAGCTTATGCAAC
ACAAATTAAACAAGAGATGGAAGATGAATTGCCAATTATAAAAGGTAAGAAAGTTATTCTATTCGCACCGACATTTAGAGGTAATG
GTCACGGTACGGCACATTATCCATTTTTTAAAATTGATTTTGAACGTTTAGCAAGATACTGCGAGAAGCATAATGCAGTTGTGTTAT
TCAAAATGCATCCGTTCGTAAAAAATAGACTTAATATTTCACGTGAACATAGACAATACTTTATCGATGTGTCAGATCATCGTGAAG
TTAACGATATTCTCTTTGTTACAGACTTGTTGATTAGTGATTATTCATCTTTAATATATGAATATGCAGTATTTAAAAAGCCGATGAT
TTTCTATGCATTTGACTTAGAAGATTACATTACGACGCGTGATTTCTATGAACCATATGAATCATTTGTTCCAGGTAAAATTGTACAA
TCCTTTGATGCATTAATGGATGCTTTGGACAATGAAGATTATGAGGTTGAAAAAGTTGTGCCATTCTTAGATAAACATTTTAAATAT
CAAGATGGTCGCTCAAGTGAACGTTTAGTCAAAGATTTGTTTAGACGCTAATGTTGGCATATATTACTTGCCATGCAAATGAGTCAT
AAGGCATAGTTTTCAAGAAGGGTTCTGACAATGAAGTGAGCAACATGTCATGATGAGAAGCAGGACTATACAATGAGAATAACCTT
TTAATTTTTCATGCATAAGGCGATTCAATCAAAAGCAATCACCTTCCAACTGAATTGTCATTTTGTAAAATAAAATAATCGATCCAA
TCGTTATCTAATTCATAAATGTGTAAACACATACGTTATGAATGGATAACGATTTTTTGTTATGTTAAAGTGGTACATTAATCATGTA
TTTCGTATGATAATTAACGACAAGTGTAATGGTTAAATGTATTTTATGATGAAATGCTATAATAGGCATGGTTACAATGAGCTTGCT
CATACATATTAATATAATTACAAAAACACGTCGGAGGTACGACATGATTAAAAATACAATTAAAAAATTGATAGAACATAGTATAT
ATACGACTTTTAAATTACTATCAAAATTGCCAAACAAGAATCTAATTTATTTTGAAAGCTTTCATGGTAAACAATACAGCGACAACC
CCAAAGCATTATATGAATACTTAACTGAACATAGTGATGCCCAATTAATATGGGGTGTGAAAAAAGGATATGAACACATATTCCAA
CAGCACAATGTACCATATGTTACAAAGTTTTCAATGAAATGGTTTTTAGCGATGCCAAGAGCGAAAGCATGGATGATTAACACACG
TACACCAGATTGGTTATATAAATCACCGCGAACGACGTACTTACAAACATGGCATGGCACGCCATTGAAAAAGATTGGTTTGGATA
TTAGTAACGTTAAAATGCTAGGAACAAATACTCAAAATTACCAAGATGGTTTTAAAAAAGAAAGCCAACGTTGGGATTATCTAGTG
TCACCTAATCCATATTCGACATCGATATTTCAACATGCATTTCATGTTAGTCGAGATAAGATTTTGGAAACAGGTTATCCAAGAAAT
GATAAATTATCACATAAACGCAATGATACTGAATATATTAACGGTATTAAGACAAAATTAAATATTCCATTAGATAAAAAAGTGAT
TATGTACGCGCCAACTTGGCGTGACGATGAAGCGATTCGAGAAGGTTCATATCAATTTAATGTTAACTTTGATATAGAAGCTTTGCG
TCAAGCGCTGGATGATGATTATGTTATTTTATTACGCATGCATTATTTAGTTGTGACACGTATTGATGAACATGATGATTTTGTGAAA
GACGTTTCAGATTATGAAGATATTTCGGATTTATACTTAATCAGCGATGCGTTAGTTACCGACTACTCATCTGTCATGTTCGACTTCG
GTGTATTAAAGCGTCCGCAAATTTTCTATGCGTATGACTTAGATAAATATGGCGATGAGCTTAGAGGTTTTTACATGGATTATAAAA
AAGAGTTGCCAGGTCCAATTGTTGAAAATCAAACAGCACTCATTGATGCATTAAAACATATCGATGAGACTGCAAATGAGTATATT
GAAGCACGAACGGTATTTTATCAAAAATTCTGTTCATTAGAAGATGGACACGCGTCACAACGAATTTGCCAAACGATTTTTAAGTGA
TAACTTAAAAACAATAAAAAATTATAAATTAATTAGTTAAGTGATATAAATAATAAACGAAATGTTTGCTTGTATGTTATTATTTGT
GTATGAAAACGTCTGATATAATGTAATATAGGTTTAAACAAAATATGCTAAAATATAAGCAAAATATGTATAATTTTAAGTAATAC
ACAGACATTTAGAGTTTTGATTTTAAAATGAGTTTTCAATTGGAAAATGCAACGAAATTTAAATAATTAAATATAGATATAGTTGAA
TGGAGGAAGTATTTTATGAAATACGCTGGTATTCTAGCTGGAGGTATAGGCTCAAGAATGGGTAACGTACCTTTACCTAAACAATTT
TTAGATTTAGACAACAAACCGATTTTAATCCATACATTAGAAAAATTTATTTTAATTAATGATTTTGAAAAAATTATTATCGCGACG
CCACAACAATGGATGACGCATACGAAAGATACACTTAGAAAATTCAAAATTTCTGATGAAAGAATTGAAGTCATTCAAGGTGGTAG
CGATCGTAACGATACAATTATGAATATCGTTAAACATATTGAATCAACAAATGGTATTAACGATGACGATGTTATTGTGACACATGA
TGCAGTTAGACCATTTTTAACGCATCGTATTATTAAAGAAAATATTCAAGCTGCTTTAGAGTACGGTGCAGTAGATACAGTGATTGA
TGCTATAGATACGATTGTTACATCTAAAGATAATCAAACGATTGATGCAATTCCAGTGCGTAATGAAATGTACCAAGGTCAAACAC
CTCAATCGTTTAATATTAATTTATTAAAAGAAAGCTATGCACAGTTGAGTGATGAGCAAAAGAGTATTTTATCTGATGCTTGTAAGA
TTATTGTAGAAACAAACAAACCGGTTCGACTTGTAAAAGGTGAGTTATATAACATTAAAGTAACAACACCTTACGATTTAAAAGTA
GCGAATGCTATTATTCGAGGTGGTATTGCCGATGATTAATCAAGTATATCAATTAGTTGCACCTAGACAATTTGAAGTTACGTATAA
CAACGTAGATATTTACAGTGACTATGTCATTGTACGTCCTTTATATATGTCAATTTGTGCTGCCGATCAAAGATATTATACTGGTAGC

CGTGATGAGAATGTCTTATCTCAGAAATTGCCAATGTCTTTAATTCATGAAGGTGTTGGTGAGGTCGTATTTGACAGTAAAGGTGTG
TTTAATAAAGGTACAAAAGTAGTTATGGTACCGAATACGCCGACAGAAAAAGACGATGTCATTGCTGAAAACTATTTAAAATCGAG
CTACTTCAGATCAAGTGGACATGATGGGTTTATGCAAGATTTTGTGTTGCTAAATCATGATAGAGCTGTACCACTACCTGATGATAT
TGATTTAAGTATTATTTCATATACAGAGCTTGTAACAGTAAGTTTGCATGCTATTCGTCGTTTTGAAAAGAAATCTATTTCAAATAAA
AATACATTTGGTATTTGGGGTGATGGTAACTTAGGTTACATTACAGCCATTTTATTACGTAAATTATATCCAGAGTCTAAAATATATG
TCTTTGGTAAAACAGATTATAAATTGAGTCACTTCTCATTTGTTGATGATGTCTTCTTTATTAATAAAATACCTGAAGGCTTAACATT
TGATCATGCATTTGAGTGTGTGGGTGGTCGCGGTAGTCAATCAGCCATAAATCAAATGATCGATTACATTTCACCAGAAGGAAGCAT
TGCACTGTTAGGTGTAAGTGAGTTCCCAGTAGAAGTTAATACACGTCTAGTATTGGAAAAAGGACTAACGTTGATTGGTAGTAGTCG
AAGTGGTTCAAAAGATTTCCAAGATGTTGTAGACTTATACATTCAATACCCAGATATTGTAGATAAATTAGCGTTGTTAAAAGGTCA
AGAATTTGAAATTGCAACAATTAATGATCTTACAGAAGCTTTTGAAGCAGACCTGTCTACATCTTGGGGTAAAACAGTATTAAAATG
GATTATGTAAATAGAAAAGGATGAATTAACGTTGGTTAAAAGTAAGATATATATAGATAAAATCTATTGGGAACGTGTTCAGTTAT
TCGTTGAAGGACATAGTGAAAACCTAGATTTAGAAGATAGTAATTTTGTATTAAGAAATTTAACTGAGACACGTACAATGAAGGCG
AATGATGTCAAAATAGATGGGAATCAATTCGTTTGTCGTTTCAATGTAGCTATCTTAGATAATGGTTATTACTTACCTGAAGATAAG
TACTTATTAGTGAATGAGCAAGAACTTGATTATATTGCACAGTTAAACCCAGATGTGATTAATGATGCATATCAAAATCTAAAGCCA
GAACAAGAAGAAGAATACAACGAATTAGAAACACAAAATGGTAAAATCAATTTCTTATTGCAGACTTACCTAAAAGAATTTAGAA
AAGGCGGCATTTCAAAGAAAACGGTTTATACTGTTACACCTGAAATTTCTAGCGATGTTAATGAATTTGTCCTTGATGTTGTTGTAA
CGACTCCGGAAGTTAAAAGTATTTATATCGTTCGTAAATATAAAGAATTACGTAAGTATTTCCGCAAACAATCATTTAATACAAGAC
AATTTATTTTTAAAGCGATATTTAATACGACGAAATTTTTCCACTTGAAAAAAGGGAATACGGTGTTGTTCACATCAGACTCTAGAC
CAACGATGTCTGGAAACTTTGAATACATCTATAACGAAATGTTACGTCAAAATTTAGATAAAAAGTATGATATTCACACTGTTTTTA
AAGCGAATATTACAGATAGACGTGGCATCATCGACAAGTTTAGATTGCCATATTTACTTGGGAAGGCAGACTACATCTTTGTTGATG
ACTTTCACCCATTGATTTATACAGTGCGTTTTAGACGTTCTCAAGAAGTTATTCAAGTATGGCATGCCGTTGGTGCCTTTAAAACAGT
TGGCTTTAGTCGTACTGGTAAAAAGGGTGGACCATTTATTGATTCATTAAATCATCGTAGCTATACAAAAGCTTATGTATCATCTGA
AACCGATATTCCATTCTACGCTGAAGCATTTGGTATTAAAGAGAAAAATGTAGTGCCTACAGGTGTTCCACGTACTGATGTACTATT
TGATGAAACTTATGCGACACAGATCAAACAAGAGATGGAAGATGAATTACCAATTATTAAAGGTAAGAAAGTCATTCTTTTCGCAC
CAACATTTAGAGGTAGTGGTCATGGTACAGCACATTACCCATTTTTCAAAATTGATTTCGAACGTTTAGCAAGATATTGCGAAAAAA
ATAACGCGGTTGTATTATTTAAAATGCATCCATTTGTGAAAAATAGACTTAATATTGCAGACAAACATAAACAATATTTTGTTGACG
TTTCTGACTTTAGAGAAGTTAATGATATACTGTTCATAACAGATTTATTAATTAGTGACTATTCATCTTTAATATATGAATATGCAGT
ATTTAAAAAGCCAATGATTTTCTATGCATTTGATTTAGAAGATTATATTACGACGCGTGATTTTTATGAACCATATGAATCATTTGTT
CCAGGTAAAATTGTGCAATCATTTGACGCATTAATGGACGCCTTGGACAATGAAGATTATGAAGGAGAAAAAGTCATTCCATTCTT
AGATAAACATTTTAAATATCAAGATGGCCGATCAAGTGAGCGTTTAGTCAGAAATTTATTTGGTAGCTAAGTTTATATAGTAGTCAA
AGTGGGAGAGGTATAATGATGAAATTTTCAGTAATAGTTCCAACATACAATTCAGAAAAGTATATAACAGAATTACTTAATAGCCT
TGCGAAACAAGATTTTCCGAAAACTGAATTTGAAGTGGTTGTAGTTGATGACTGTTCAACAGATCAAACGTTACAAATAGTTGAAA
AGTATCGTAATAAATTGAACTTGAAAGTAAGTCAACTCGAAACAAATTCTGGTGGTCCAGGTAAACCTAGAAATGTGGCGTTAAAA
CAAGCAGAAGGTGAATTTGTATTATTTGTGGACTCCGATGACTATATAAACAAAGAGACTTTAAAGGATGCAGCAGCATTTATTGAT
GAACATCACTCAGATGTCTTGTTGATTAAAATGAAAGGTGTTAATGGTCGTGGTGTACCACAATCTATGTTTAAAGAAACAGCACCT
GAAGTTACTTTGTTAAATTCAAGAATTATCTATACTTTAAGCCCAACTAAAATCTATAGAACAGCATTACTAAAAGATAATGACATT
TATTTTCCAGAAGAATTAAAGAGTGCAGAAGATCAATTATTTACAATGAAAGCATATTTGAATGCAAATCGAATCAGTGTGTTAAGT
GATAAAGCGTATTATTATGCTACAAAGCGTGAAGGTGAACATATGAGTAGTGCGTATGTTTCACCTGAAGACTTTTACGAAGTCATG
AGATTGATTGCTGTAGAAATATTAAATGCAGATTTAGAAGAAGCTCATAAAGATCAAATCTTAGCAGAATTTTTAAATCGTCATTTT
AGTTTTTCTCGTACGAATGGCTTCTCACTTAAAGTTAAACTAGAAGATCAACCGCAATGGATTAATGCTCTAGGAGACTTTATACAA
GCAGTTCCAGAACGTGTAGATGCATTGGTGATGAGTAAATTACGACCATTGTTGCACTACGCGAGAGCGAAAGATATAGACAACTA
TAGAACTGTAGAAGAAAGTTACCGTCAAGGTCAATACTACCGTTTTGATATTGTAGATGGTAAATTAAACATTCAATTCAATGAAGG
CGAACCATACTTTGAAGGCATTGATATCGCTAAGCCAAAAGTGAAAATGACAGCATTTAAATTTGATAATCATAAAATTGTTACAG
AGCTAACGTTAAATGAATTTATGATTGGCGAAGGACATTATGATGTCAGACTTAAATTACATTCACGAAACAAGAAGCACACAATG
TATGTACCTTTAAGTGTCAATGCGAATAAACAATATCGTTTTAACATTATGTTAGAAGATATTAAAGCGTATTTACCTAAAGAAAAA
ATTTGGGATGTTTTCTTAGAAGTCCAAATAGGTACGGAAGTATTTGAAGTGCGTGTTGGTAATCAACGTAATAAATATGCATATACT
GCAGAAACAAGTGCATTAATTCATTTGAATAATGATTTTTATAGATTAACACCGTATTTCACAAAAGACTTTAATAACATTTCGTTAT
ACTTTACAGCTATTACATTAACGGATTCAATCTCAATGAAGTTAAAAGGTAAAAACAAAATCATTTTAACTGGTCTGGATCGTGGTT
ATGTATTTGAAGAAGGTATGGCTAGTGTCGTGCTAAAAGACGACATGATTATGGGAATGTTAAGCCAAACGTCAGAAAACGAAGTG
GAAATCTTACTTAGTAAAGATATTAAGAAGCGAGACTTCAAAAATATTGTTAAGTTAAACACTGCACATATGACTTACTCGCTAAAA
TAAATAATAAATGCCCTCAAATCTATGTGAACTAACATGATTTGAGGGTTTTATTTTACTATTTATGAAATGATTATGACATTTCCCT
GATTTTCATTTTCATATACATTAAATTGTATACACTGAAAATGAGGAGGTCGTCTATAATGATAAATAAAAATGACATAGTAGCAGA
TGTAGTAACAGATTATCCGAAAGCAGCAGATATTTTTAGAAGCGTAGGTATAGACTTTTGTTGCGGTGGACAAGTAAGTATAGAAG
CAGCATCCTTAGAAAAAGAAATATGTAGATTTGAACGAATTATTACAGCGTCTCAATGACGTTGAACAAACGAATACACCAGGTTCG
CTTAACCCTAAATTTTTAAATGTTTCGTCACTTATTCAATATATTCAAGCAGCATATCATGAACCTCTTAGAGAAGAATTTAAAAATT
TAACACCTTATGTGACGAAATTATCGAAAGTACATGGACCTAACCATCCATATTTAGTCGAGTTAAAAGAAACATATGATACATTTA
AAAATGGCATGTTAGAGCATATGCAAAAAGAAGATGATGTTGATTTTCCAAAACTAATTAAATATGAACAAGGTGAAGTAGTAGAC
GATATTAATACAGTGATTGATGATTTAGTATCTGATCACATTGCAACAGGACAATTGTTAGTGAAAATGAGCGATTTAACATCTAGC
TATGAACCACCGATAGAGGCATGTGGTACGTGGCGACTCGTTTATCAGAGATTAAAAGCACTTGAAGTGTTAACACATGAGCATGT
TCATTTAGAGAATCATGTTTTATTTAAAAAAGTATCATAAATAACGCGATTAGAAACTGTTGGCAAAGTAATGTCAGCAGTTTTTCG
CTATACTTAACAGAAATTTAGTGACATGAACAGCATTATTTGAAAAGAAAAATGGTCAACTTAGCATAAAAATTGATATGAAAATT
TAATGGTATAGATAATTAAATAGTAGCGTGTTTTTTTAATAATTTATTCATGAATTTTACATGCACTATTATGATAAAATAAACATAA
TTATAATTCACTGAGGTGCTATCGTGCTATCGCTAACAATGTTATTACTTGAGCGTGTAGGTTTAATTATTATTTTGGCCTATGTGTT
GATGAATATTCCATATTTTAAAAACTTAATGAATCGTCGACGTACATGGAAAGCACGTTGGCAATTATGTATTATTTTCAGTTTGTTT
GCCTTAATGTCTAATTTAACTGGTATCGTCATCGATCATCAACATAGTTTGTCAGGAAGTGTGTACTTCCGTTTAGATGATGATGTAT
CTTTAGCTAACACACGTGTATTAACGATAGGTGTCGCAGGATTAGTTGGTGGTCCTTTTGTAGGTCTATTTGTTGGCGTTATTTCAGG
TATTTTCAGAGTGTATATGGGTGGGGCGGATGCACAAGTTTATCTTATCTCATCTATATTTATCGGTATAATTGCTGGTTATTTTGGC
TTACAAGCTCAAAGACGCAAGCGTTACCCGAGTATTGCGAAAAGTGCCATGATTGGAATTGTTATGGAAATGATTCAAATGTTGAG
CATTTTAACATTTTCCCACGACAAAGCATATGCGGTTGACCTCATATCATTAATTGCACTACCAATGATTATTGTTAATAGCGTTGGT
ACGGCGATTTTTATGTCTATTATCATTTCAACATTAAAGCAAGAAGAGCAAATGAAGGCTGTTCAAACACATGACGTACTGCAATTG
ATGAACCAGACATTGCCGTATTTTAAAGAAGGATTGAATAGAGAATCGGCACAGCAAATTGCGATGATTATTAAAAATTTAATGAA

AGTATCTGCCGTAGCAATTACAAGCAAAAATGAAATCTTATCGCATGTAGGTGCAGGTAGTGATCATCACATACCAACAAATGAAA
TATTAACAAGTCTGTCTAAAGATGTATTGAAATCAGGAAAGTTGAAAGAAGTTCATACTAAAGAAGAGATTGGTTGTAGTCATCCG
AATTGCCCGCTTAGAGCAGCTATCGTGATACCACTTGAGATGCATGGTTCTATCGTCGGTACATTGAAGATGTATTTTACAAACCCT
AATGATTTAACTTTTGTGGAACGTCAACTTGCAGAAGGATTGGCAAATATTTTTAGTAGCCAAATTGAACTTGGTGAAGCCGAAACG
CAAAGTAAGTTATTGAAAGATGCTGAGATTAAGTCATTACAGGCACAAGTGAGTCCACATTTTTTCTTCAATTCAATTAACACGATT
TCAGCTTTAGTTAGAATAAATAGCGAAAAGGCACGAGAGTTACTATTAGAATTGAGTTATTTTTTCAGAGCGAATTTACAAGGCTCA
AAGCAACATACGATTACTTTAGATAAAGAGTTAAGTCAAGTGCGTGCATACTTATCACTCGAACAAGCACGTTATCCAGGAAGATT
TAATATCAATATTAATGTTGAAGACAAATATCGCGATGTGCTTGTACCACCATTTTTAATTCAAATTTTAGTTGAAAATGCCATCAA
ACATGCGTTTACGAATCGAAAGCAAGGTAACGATATTGACGTGTCAGTGATTAAAGAAACTGCAACACATGTACGTATTATTGTAC
AAGATAATGGTCAGGGTATTTCTAAAGATAAAATGCATTTGTTGGGAGAAACATCTGTAGAATCAGAATCTGGAACTGGTAGTGCT
TTAGAAAATTTAAACTTACGCCTAAAAGGATTATTTGGAAAATCCGCAGCATTACAATTTGAATCGACATCGAGCGGTACCACTTTT
TGGTGTGTACTTCCTTATGAAAGACAAGAGGAGGAATAAATATGAAAGCATTAATCATAGATGATGAGCCATTAGCACGTAATGAA
TTAACATATTTATTAAATGAAATTGGTGGTTTTGAAGAAATTAATGAGGCAGAAAATGTAAAAGAAACATTGGAAGCACTACTGAT
CAATCAATATGACATTATATTTTTAGATGTCAATTTAATGGATGAAAATGGGATCGAATTAGGAGCTAAGATTCAAAAGATGAAAG
AGCCACCTGCGATTATTTTTGCAACTGCACATGACCAATACGCAGTACAGGCATTTGAATTAAATGCGACAGACTATATTTTGAAAC
CGTTTGGTCAAAAACGTATTGAACAAGCAGTCAATAAAGTGCGTGCGACTAAAGCCAAAGATGATAATAACGCAAGTGCAATTGCG
AATGATATGTCGGCGAATTTTGATCAAAGTTTACCTGTTGAAATTGACGATAAAATTCACATGTTAAAGCAACAAAATATTATTGGG
ATTGGCACACATAATGGTATTACAACCATACATACAACGAATCATAAATACGAAACAACAGAGCCATTGAATCGTTATGAAAAACG
ATTGAATCCCACTTATTTTATACGTATTCATCGTTCATATATTATTAACACTAAACACATTAAAGAAGTGCAACAATGGTTTAACTAC
ACTTATATGGTAATATTGACAAATGGTGTCAAGATGCAAGTTGGACGTTCATTTATGAAAGATTTTAAAGCGTCGATAGGATTACTT
TAACAGTAATCCTTTTTTTTATGCATTTTACCTATGATATTTTGTATTTCGGACTAAAAATCACGCAAATCGAAGTGAGCCATCTATA
CTTTAGTTAAATCAAACGTAGGAGGCAATGGTCGTGAAACAACAAAAAGACGCATCAAAACCAGCACACTTTTTTCACCAAGTCAT
TGTAATTGCTTTAGTACTCTTTGTATCGAAAATAATTGAATCATTTATGCCAATTCCTATGCCTGCATCAGTAATCGGTTTAGTATTA
TTATTTGTATTATTATGTACTGGTGCTGTTAAGTTAGGCGAAGTCGAAAAAGTAGGAACGACACTAACAAATAACATTGGCTTACTC
TTCGTACCAGCCGGTATCTCAGTTGTTAACTCTTTAGGTGTCATTAGCCAAGCACCATTTTTAATCATTGGACTAATAATCGTCTCAA
CAATACTATTACTTATTTGTACTGGCTATGTCACACAAATTATTATGAAAGTTACTTCGAGATCTAAAGGTGACAAAGTCACAAAAA
AGATCAAAATAGAGGAGGCACAAGCTCATGATTAACCACTTAGCACTAAACACACCTTACTTCGGAATACTGTTATCCGTTATACCA
TTTTTCTTAGCGACCATATTATTTGAAAAAACTAATCGTTTCTTCTTATTCGCACCGCTATTTGTCAGTATGGTATTTGGTGTGGCCTT
CCTCTATTTAACAGGCATTCCGTATAAGACTTACAAAATAGGTGGAGACATTATTTATTTCTTCTTAGAACCGGCAACAATCTGTTTT
GCGATTCCGTTATATAAAAAGCGTGAAGTGCTTGTTAAACATTGGCATCGTATCATCGGAGGTATTGGCATCGGTACAGTTGTAGCG
TTATTAATTATTTTAACTTTTGCGAAGTTAGCACAATTTGCCAATGATGTTATTTTATCAATGTTACCTCAAGCAGCAACTACAGCGA
TTGCGTTACCAGTATCAGCTGGTATCGGTGGTATAAAAGAATTAACATCATTAGCAGTTATTTTAAATGGTGTCATTATTTATGCCCT
AGGTAATAAATTCTTGAAGCTTTTCCGAATTACTAACCCTATTGCCCGAGGATTAGCACTTGGAACAAGTGGTCACACATTAGGTGT
AGCACCAGCCAAAGAATTAGGACCTGTAGAAGAATCAATGGCAAGTATAGCTTTAGTGTTAGTTGGTGTAGTTGTTGTAGCAGTTGT
GCCTGTCTTTGTAGCAATATTCTTCTAAAACGAAAAACCTAAGCAAGATAATAGCAATTTGAGCCATTGTTATTATCGTAAAAAAAC
GTCTATACTCCGGTTTATAACTGGGATATAGACGTTTTTATGTATGTATTACTTTTTACTAGGAATATGGTATTTAGCCGTTCGCTTTA
TGTATTGCCGCTTTTGAAAAATGATAGAACGAATTAATTTAAACAAGAATTAAAATGTAAGTTATATCATTTGTGAAATTTCAATTA
AATATTTTTCGACAAATTCTTGAAATTACTTAATGTCATCAACACTTTATAAAAAAACGGCGTATGCTTATATGTAAGAATGCAATT
AAGAGGAGATTTTATAAATGAGTATGCAAAGTTCATATAAACGTTTAGTTAAAGTGCTACTAACGATTTTAGTGATTGTGTTTTCAA
TTTTACTAGTAGGTGGTTGGTATATTTTTAAAAATGAAGCACCTCGTCCAACAAAGATAGTAGATCAACAAGGTCATACGCTTGTTA
CAAAAGACGAGCTGATCAGTGGGCAAGCGATATATGAGAAATATGGGTTAACAGATTATGGCTCCTACCTAGGCAATGGTTCTTAC
TTAGGCCCAGATTATACAGCAGAGGCATTACATCATTACCTGATTGGTATGCGTAAATATTATGCACAAGATATTTATCATAAGCAA
CTTCATAAATTAAATCATGCAGAGTTAGCCGTAGTTAAAGATAAAGTGATGAAAGAAATACGGATTAATCGTTATTCTGAAAAAGA
AGATCAACTTGTATTAACGCCAGGACAAGTATACGGTCTAAAATATTTGCAAGATTATTATAAAGAAGAGTTTGTTAATAACCCTAA
ACAAGTAGGGTTAAATGAAAATATGATTAAACAGTTTCAAAATGATGATTATATGGTCGCAGGAAATAAAGTTGAACATTTAAGTC
AATTCTTTTTCTGGGGTGCTTGGCTATCGTCAACAGATAGACCTGGAAAAACATTTTCATATACAAATAATTGGCCATACGATGTGG
ATGCAGGAAATACGTTGCCTAGTGCAGGGATTTTATGGACTGCTATATCTGTAACATTACTGATTGCAGGCTTGGCATCCATTATAT
ATATTCAAAAGCGTTATCAATTTGACATGAAGCCGACGTATGAGTCGGAACGGGAACTACCAAAGAGTGAAGTGGATCGTAAAATC
ACTGATAGTCAACGAAAAGTTGGGAAATATTTAGTCGTTGTCATGTTACTATTTTTGGTTCAAATATTATTAGGCGAATTACTGGCA
CATTATTATGTAGAGAATAAATTTTTTGGTATAGAAATACAAAGGCTCTTTCCATTTAATATTGCGAAGACATGGCATGTGCAATTA
GTTATTTTCTGGGTTGCTACGACTTGGTTAGCAGCAGGAATATACATTGTACCTAAAGTATTGGGCAAAGAACCAAAGAAACAAGG
CGTATTAGTAGATATATTGTTTTGGGCCTTAATCATAGTTGTGATGGGCAGCATGATTGGAGAATGGGGTCATATTTTAGGTTGGAT
TGATTCTCATTGGTGGCTATTCGGGCACTTTGGTTGGGAATATATAGAATTAGGTAAATTTTGGCAAATATTATTTATAGTAGGTATG
ATCTTATGGGTTATCATTCTTTGTCGAGGTTTTTTACCAGCTATTCGCAACAAAGTACAAGTGAATCATGATAAACGTCACTTATTGA
CGCTATTGTTTGTTGGAGCAATTGCAATTCCGTTATTTTATCTTGCATCATTATTTATAATGCCGAATTCACATGTGACGTTTGCCGAT
TACTGGCGATGGTGGATTGTTCACTTATGGGTTGAAGGTATATTTGAAGCATTTGCCGTAGTGTTGATTGGTTTCTTAATGGTTAATA
TGAAATTAACTACCATTGAAACGACGATTCGTGCATTATATTTTCAATTAATCTTATTACTAGGTACAGGTATTGTTGGTATGGGTCA
TCATTACTATTGGCAAGGTGATCATTCCATATGGCTAGCACTGGGTTCTTGTTTTTCAGCATTAGAAGTCGTGCCGCTTTGTTTATTA
ATTTGGGAAGCATACACACACTATCGCTTATATAAGAATAGTGACATCGCTTTTCCTTACAAAGGTACATTTATTTTTCTTGCTTCAA
CAGGATTGTGGAATGCAATTGGTGCTGGAGCATTAGGCTTTTTAATTAACACACCAGCAGTTAACTACTTTGAACATGGTACGCAAT
GGACTGCAGCACATGCCCATGGTTCTATGGCTGGCGTGTATGGTATGTTCTCAATTGCAATTGCGTTGTATGTTTTTAAAAATATTAC
TAAAAAAGAATTTTGGACACCGAAAGTTGAAAAATGGATAAAAATATCATGTTGGGCTTTAAATATTGGCTTGGCAGGTATGGTTTT
CGTTACATTAATGCCAGTAGGTTATATCCAACTTAAAGATGCATTAGAACATGGTTATTGGCATTCAAGACTAACGTCATTTTATGA
ACAACCACTTGTAAAAGCAATCATGTGGGGGAGAATGCCTTGGGACATTATATTCACAGTAGGTGTCATTATATTAGTAGTTATTTT
TATAAGGGGATACAGACACTTAAAACCAAAATACAGAGTATAGATCATCATATTACCGAGTGATTTTAGTGTGTTTTAACGTTTGAG
GGGAAGAGATATCATACAATAGGATTCAAACTATAAGTTCTTAGATCATGTAAACATAAAGAACAGTATAAAGTAATTTTCTGATT
AAAAATGTACTTCTCACTGTTTTTATAGAAATTATGTCTTATAAAACGTCTATACTCCGGTTTATAACTGGAATATAGACGTTTTGAT
GTATGTACTACTTTTTACTTGGAATATAAAACTGTGCATGACGATAATGAAATACGATGTCAGACGAATCAAAAGGTTTGCCAGTCA
TTGTATAAAACGTTTGGTGATAACGTAAACATGGTTCGCCTGCAGATAATTGTAGTAATGTCGCTTCACTTGAAGTGAGTTTATCTA
CATTAAAGAAAATATCTGAAAAACCAATACGAAGTTTCATGTTTGATTCTAAATAATCGAAGATAGAGCCCTTAGCAATATCATCAT
TTAAATATTTCACGATTTCTTTATGATAATAAGAATATTCGATACATAAAACATCATCGTCCACGAATCTTAATCGCTCTAAATAGTA

GACGGTATCATCTGCATTTAATTGGAGTTCATCTTGTACAGATTTAGGTGGCGTTGCCATCTCCTTAAAAACAAGTACCTTACTTGTC
ATTCGATGTTCACCTAAACTTTTAGAGAAACCATTAGTCTTAAAGACGTTGATACGATTGGCATCTGCAATATTTCTCACATAAATA
CCACTGCCTTGTGCTTGATAGATCAAACCATCTTGTTCCAATAAGCCTAATGCTTTAATGATAGTACTCTTACTTACTTGATAACGTT
CTTTTAATTGCGTCACGCTTGGCAATTTATCACCGGGTTTGAAATTAGATTGATGTATAAACGCATTAAGTTGCTTAGCAATATGTTC
ATACTTTAACAATATTCTGTCCTCGTTTCACTTTGTCTGTAATTATTATAGCACAAAATATTATAATTGTATCGGCGTTTACAAATTG
AACCGGTACAATTATAATTGGAGGTAAGTAAATACATTTTCATTCTACTATCAAGTTGAGGGGGTAATATTTATGAGCAATAAATAT
AAAGAACAAGCCCAAGACATTCTTACAGCTGTAGGTGGTGTCGAAAACATTGTTGATGCAACGTATGATACGAAGTGCATTACAAT
TCATATGCAACATACAATTCCTTCTACAGCAAATGAAGTGAAACAAATAGTTGATGTGACATCTGTAGCAGAAAATGATGCGCAGT
TAGTTATAAAATTAAATGGAAATGTTGATGAAGTGTATCAGCAATTACAGCGATTAATTAAGAATGCTAATGTCGAAGAGAGTGAG
AATACTGACAATATTAATAGCCAAGATACAAGTTATACACCTCAAGTAAAAGTAACAACACCAATTTTAGTGAAAGCACCAATCGC
TGGTCGTCGTATTTTACTTAAAGAAGTAAGAGATTCAATTTTTAGAGAGAAAATGGTAGGCGAAGGCTTGGCAATCAAAGCTCATG
AAGAATCCAAAGTAATCGCACCGTTCAATGGTTTAGTATCTATGATTGTACCAACTAAGCATGCAGTTGGTATTCAATCAGAAGACG
GTGTGGACATAGTCATTCATATTGGCGTGAATACAGTTGACTTGGAAGGTAAAGGGTTCAAGTGCTTTGTAAAGCAAAATGATCGT
GTTGAAGCAGGGCAAACGTTGTTACAATTCGACCAGCAATATATACAACAACAAGGCTACAATGCTGACGTTATTGTCGTTATTAGC
AACTCTGCCGATTTAGGAAAAGTAGAACTGACAATGAATGAAATCATTACGACTGAAGATGTTATTTTTAAAATATTTAAAAACTA
GGAGTGTGTTGTAATAATGACAAAATTACCGCAAAATTTCATGTGGGGTGGCGCACTTGCCGCAAATCAATTTGAAGGTGGATATG
ATAAAGGTGGTAAAGGGTTAAGTGTAATTGATGTTATGACGAGTGGTGCACATGGCAAAGCACGTCAGATTACAGAATTTATAGAT
CCCAATCACTATTATCCAAATCATGAAGGTATTGATTTTTATCATCGTTATAAGGAAGATATTGCCTTGTTTAAAGAAATGGGATTG
AAATGTTTACGTACGTCGATTGCGTGGACACGTATCTTTCCGAATGGGGATGAAGATGTGCCAAACGAAGAAGGACTCGCCTTTTAT
GATCGTATCTTTGATGAATTAATTGCACAAGGTATTGAACCTGTTGTGACGTTATCACATTTTGAGATGCCACTTCATTTAGCGAAAC
ATTATGGTGGATTTAGAAATAGAGAAGTTGTCGATTATTTTGTGCATTTTGCGCGTGTTGTATTTGAAAGATATAAAGATAAAGTTA
CATATTGGATGACGTTTAATGAAATTAATAATCAGATGGACACATCAAATCCTATCTTTTTATGGACGAATTCTGGGGTAGCATTGA
CAGAAAATGATAATCCTGAAGAAGTCTTGTATCAAGTAGCACATCATGAACTTTTAGCCAGTGCTTTAGCAGTTCGTCTTGGTAAAG
AGATTAATCCGAAGTTTAAGATTGGAACAATGATTTCACATGTACCCATTTATCCATATTCGTGTCATCCGAAAGATATGATGGAAG
CACAAATTGCGAATCGCTTACGTTTCTTTTTCCCGGATGTCCAAGTGAGAGGTTATTATCCAAGCTATGCTAAAAAAATGTTGGCAC
GAAAAGGATATGATGTTGGATGGCAAGAAGGGGACGACAGTATTTTACAGCAGGGCACGGTTGATTATATTGGCTTTAGTTATTAC
ATGTCTACGGCTGTAAAACATGATGTTGATACTACAGTTGAAAACAACATCGTCAACGGTGGTTTGAATCATTCTGTGGAGAATCCG
CATATCGCAACGAGTGATTGGGGTTGGGCGATTGATCCAGATGGCTTAAGATATACATTGAATGTGTTATATGATCGTTATCAGTTA
CCACTTTTTATTGTGGAAAATGGTTTTGGTGCAGTTGATGAAGTGGTAGATGGACATATTCATGATGATTATCGCATTGAATATTTAA
AAGCACATATTACAGCAGCGATAGAAGCAGTTGATCAAGATGGTGTAGATTTAATCGGTTATACACCGTGGGGAATCATTGATATT
GTTTCATTTACAACCGGTGAAATGAAGAAACGCTATGGTTTAATATATGTTGATCGAGATAATGATGGTCATGGCACGATGGAACG
CTTGAAAAAAGATTCGTTCTATTGGTATCAACAAGTGATAGCATCAAATGGAGATAAATTATAAAGGTATATTATAAGTATTTTAGG
GTTAGAGCCCGAGACATAAATTAATATAGTAGGACCTACAGTGTTATATTGGCGGGCCCCAACACAAAGAATTTCGAAAAGAAATT
CTACAGGTAATGCAAGTTGGCGGGGCCCCAACATAGAAGCTGGCGGAAAGTCAGCATACAATAATGTGCAAGTTGGCGGGGCCCC
AACACAGAGAATTTCGAAAAGAAATTCTACAGGTAATGCAAGTTGGGGAAGGACAGAAATAAATTTTGCGAAAATATCATTTTTGT
CCTACTCCCTTTAACATTTCTGTTCCAATCCCGTGATGTTAAAATTTTAAGAGAAAAATAATGCCACTAACTAAGTATGTATAGTGTT
AATGTTTGAGTGTTTATGAAAGTCTTTAACCAAAATTGATGACTTACTAATCAACGATAACGATATTGTTAAGTAGATGATTAGTGT
CACAAAGAAATTAAAGTAGCAGTCAAACATAGATACAAAGTACAGTTAGTGGCATTATATTTAGTGCTCTTTTTTAGCGACAAAAG
TAATATAATTCATATCTTTACGCAATTTAGTCATCGTTTTAAACATTTTACAAAACATTGGTCGATTTTCTTTTTTTAAAGCATTGTTG
ATAATCTTTATAGTTCCAACAATACCTTCGTCATAAATTAAACCTTTTGGTGTCATTAAACTCATTGGACCAGTATGATAATGCACAT
GATTAAAACCAGCTTGATTATATAAATCTAACCAGCCAAGTTTCGTCTGCGGTGAGACATTGACATTAATTGCTGCAGATAATGATT
TAACTACATGTGTGGCATGTGATTCATTAACGATGACAATATCATGTGTTAACAAGATACCCCCAGGCTTTAAGACTCGGTAGTACT
CGCGTAATGCTTTTTCCTTTATGGCGATGGGTAACATTGTTAACATTGCTTCATTTAAAACGATATCGAATTGATTGTCATCAAAGGG
CAATTTAACAGCATTCGCTTGTTGAACTTGAATATATGATTCAAGACCTGCTGCTGAAATATTTTCCTGTGCTTTTTCTAATGCTTTCT
TATTTATATCAACGCCTTGAATGTGACAGCCATATGTATGAGCTAGATAAATAGATGTTGTGCACATATTACATGCCACTTCTAACA
CTTTTTTATCTTGTGAAAATGCCCCTTGTTGTATTAACCAATCTGTTGCTGCTTTACCACCGGGGCGTAGACGAGTTTTTCCTAATTTA
GCTAAAAATGTATGACCAGCTTCTTTGGACATAGCATAACCTCCTTGTAATTGATAATTATTATCATTAATATAGCATATTTATATGC
GATAATTTGGAAGTTAGAAAGACTACAAAAAATTGTCACTTCATTTATAACTAAACATGAATCATCAAGCATAGGTTTAGTGGAAA
ATTATTTCTAATAGATGATGTTTTATAAAGATAAAAAAGCACAGCCATGATATACGAATGTTGCAAGTTATATGCTAAACCTTCGTA
ACATAGCTGTGTTTGATTCATTTACACTTGATTTACTTCTTCTAGTAGAGGAATAGATGCTTGCGCGCCGTGTTTTTGTACAGTGAGT
GAGCTCGCTTTATTACCAAAATCAATAGCATCTGCTAAGTTATCTTGCGACTTGTTTAAGCGACTGACAAATGCACCAATAAATGTG
TCGCCTGCAGCAGTTGTATCAATCGCATTTACTTTATAAGCTTCGATGTGTTGGCTTTGATTTTTAGTAGCAAAATATGTACCTTGCT
TACCTAGCGTAATCAAAACAGTCTTAATGCCTAAAGATAAAAAGTAATTGGCATTGTCTTTCATAGATTGTTCATTAGTTACTTTAAT
CCCAGATAACAATTCGGCTTCTGTTTCGTTTGGCACAATAATATCGATTAATGATAATAATTCATTAGGTAATGCTTTCGCTGGTGCA
GGATTTAATACTGTCGTCACACCATGTGCCTTGGCAATTTCAAATGCAGATATAATAGCCGGGATGGGTACTTCTAATTGTGCAACG
ACAAAGTCTGCATTGATTATAGCGTCTTTTGCGTTAATAACATCTTCAGGTGTCATCGTCATATTCGCACCACCATAAACATAGATG
GTATTTTGTCCTTCTGCATTCACAGTGATAAAGGCTTGGCCCGTTTTTGCTTCAGTTGATTTGATAATATATGATGTATCAATATGAG
CTGCTTTAAAATCTTCTAAGATGAAATCAGCAACGCCATCAGTGCCAATTTTAGTAATAAATGTTGTGTCTGCTTGCATGCGTGCAG
TGGCAATAGCCTGGTTGGCACCTTTACCTCCGCCGAATGCTTTTTGTGCTTCTTCAACATGTAATGTTTCGCCTGGTTGTGCATATCTT
TCAACTGTTAAAAATTGATCGACATTCGTTGAACCTAAAATAACAACTTTGTTGGTCATGTGTACGCTCCTTTCAAGTTATAACTTTT
AAAAAGTAACATTCGATTCTAATGCAATATTAGAGTAGGGCGTTGTTTCACCAGTACGAATATTACCTTTATTTAATGGGTGAGCTA
AGTTACTTTTCATTTCTTCGTGAGGAATGAAAATGATTTCGATTTCCGATGAAATCAATTGTTTAATTTGTTGCAATTGTGTAGGGTT
ATGTTCTTTTATTTCTTCTGCTAAGTATATTTTTTGTATTTCCATTTCTTCTAACACTGTAGCTAAGACATCAATAAAGCGTGGTAAGT
TTTTAGTTACAGCTAGGTCGATACGACGATGATCATTTGGAATTGGCATGCCAGCGTCATTAATCGTTAATAGATCAAAATGACCAA
TTGTCGCGATTGCTTTTGAAATATGTTCATTTAAAACAGCTGTTTTTTTCATGACATCTACACTCCTTATTTTATAAATACTGTAACAG
AAGCGGCTACTAAAATGAGTACTAAGCCGATGATTGTAATAACCATTTCTTTTGACGTTTTATGTTGTTTTAAGAAATAAATACCAG
TTAATGTAGCAAGCACAACGGATGTTTGAGAAAGAATAAATCCAGTTGCTAAACCATTCATATTAGGTTGTGCTGAAATAAGATAT
GTTAAGGCACCAAATGCAAAGAAGAAACCTGAAATAATTTGTAACCACGTAATTTTATTACGGAATGGATTCTCTGCTTTCATATTC
ATAAAGCCATAAATGACTGCAACAATTACCATACCCATTGCTTGAGGTAAAAAGGCAGTTAGGCCATCAATAGAAGTTGCTTGCGG
TGCAGCTGAATATAACCAGTATCCAAATTCACCAATTAACAGAAGTACCACTGCACGACGTAAATTTTTGGCGTTACTTGCTTCTTT
GCGTTCACTCCAAACTGTCATACGCGCACCAATTAGAATAACGACTAAAGCTGTAAATCCAATGATTTTATGACCAATGCCTGGCCA

ATTTCCTAATGCAAAGACACCCCATAAAGATGCGCCTAATAATTGGAATGCTGTTGTGACTGGCATGGCACGAGATGAGCCGACTA
ATTCGAACGCTTTAAATGTAATGATTTGTCCGAATCCCCATCCTGCACCTGATAATAAGGCGAATAGCAAATTGGTTCCAGTAGGGA
AGCCACTTGATGTGACTACGGCTAATATAATAGCGAAGATTAACGTACCTACAGTAGCACCGATAATTTGATGTACAGGTTTACCAC
CAAACTTTGAAGCGACTGTTGGGAAGAAGCCCCAGCCAATTAAGGGGCCTAACCCGATAAGTAATGCAACAATGCTCATGTTATAC
ACCTCACGATTTGTTTTTAGTAAAACGTTTTACCAATGCCATCGTACCATTTTTATTTGCAAAATGCATGCGTTTTCATAAAAACTGA
AAACGTTTAAATCATATTTAAAAATTCATACATTATTATTTTAATTTCTTAGTGAAATAATTATGATTAAAAACCCTCAGCATTAAAG
CAACGCTAAGGGCCTAACAATGATGAGTATATTTCGGAAGATACGTAGTTAGTTTGAAAGATGATAGCCAGTTGTTGCACGAATTTT
TAAAGTCGTTGGTAATTCAATCATATCAATGGATTTATCTAAGTGCTGTAATCGTTGAAGTAATAAGGTTAAAGATGTTTTGCCAAT
ATCAGTTATAGGTTGTGCCACAGTAGTTAAAGGTGGCGAGACGTACGCTGCATAATCAATGTCGTCATAACCTATTAATGAGATATC
TTTCGGAATACTGATGCCATGTTCAATTAGTCCTCGTAAAATGCCAATAGCGAGTTCATCGTTAATAGCGAAGATTGCAGTGGCAGA
TTGAACCATGATGTCATCAACAATGGTTAGCCCACCGCGCTTAGATAATTCAGTATGGACGATTTGTGGTTCTGGCAATTGATTCGC
GCGCAAAGTATCAACAAATCCAGCGACACGAGTCGACATATTCGCCATCATGTCATATGGTGCAACAATCATCATATTGTTGTGACC
GAGTTCTATTAAATGTTGTGCTGCAAGTTGTCCGCCTTGATATTCATTTGTCCGAACAAAATCTGTATAGCCTTGATGGTCATTTTGA
TCCAGTACGACATAAGGTACATGATGTTTCTTTAGATAGTTATTTAGGGCGTCCGGGGATGAAATGTATTGTGCGATAATTAATCCG
TCAATACCTCGATCAATTAAATGTTTAATATTGTCATACAAATCAGTTGCTGTAGATGTTAAAAAGCATAAATCAACATCAGATGGT
TTATGGTCATGAATACTTTGCATCAGTGCTGAGAAAAACGGATTTGTTAAGCTAGGCAAAATGACGCCAATAGTTTGAATTTTACTG
CCGCGCAATTGTTTTGCATGTTTATTAGGGGCATAGCCTAAACGTTCTGAAACAGCATGTACGTTTTTTATCGTTGTTGCGGAAAAAC
GACTATCATTATGATTTAAAATATGTGACACAGTTGTAACTGATACACCAGCTTCTCTAGCAACATCTTTAATTGACACTTTTTTCAT
ATTATATTCCTTCTCTTTATTGTGTAATTAATCATACAGTTATATACCCGATTATTATATTCATCATATCATGATAAACATTTGATGAC
ATATGAAGACGTGAATGACTTTTATTGTCTACAAACAGACGTCTGAAGTATACGATTAAGGTTGAGACGAGAGAATATATTATTAA
GGTTAATACCTTTTAATGATTATGATAACAAGAATTTTAAAATGTTTATTGTGATTAAAAATACAAAAGTCATTATTTTAAAGAATA
AAATATTTGTTTTTGAACAAAGTATTAAAAAACATAACGAAATGATGTATATTGAACTATGTGATTGAAAAAATTTTTAATTAAGGC
TTGTTAAAACTTAAGAGAGGATGTTTTTAAATGCAATTCAAATTAAAAGAAGAAGAGATTATTAGTTTTTTAGAATTGAAATATCCA
GAAAAAGAGTTCGAATATGGTCGTTTGTTAGTTGGACAACATAAACGTGATGATTTAGATGTTTATTACTTTGGTGATACGTTTTTA
ATGTGCACGATTATTTCATTCAAGACATTTGAAATTAAAGAAACAGTAGAATTATCATATGATGCTGTTAATCGTATTGTGTTAAAA
GATGGATGGTTATTTAGAAAAATGAGAATAGAAACAATGCAAAAAGTGTTAAAATACGGTACATCTAAATTAATGTTAACTGATTT
TCAAAAAGAGAATTATAATAAATATATTCAAGGTCAGAAACAACGCGTGATATTTGAAAATGGCCATTTTGTCTAATTGATAGTGA
ATATAATTAGAGTAAGCGAAGAACAGAGAAAAACGTTCTTCGCTTATTTTTCTTGAATTTCTTGTATATCATTTTAAAGTGATAAAG
ATATTTCGAAACATAATGCTTCTATTATATTTAAGATTTAACTGTTTTTGGAATGATCATGTATGCAGACAATGAGCCAAGGATCATC
AATACAATGCTGACTATAAATGTTACGGTTGCAGCTACACTAGGCGTATAGTTTAGTTGTAACATACTGAAAACTGTAGTACTTATT
GCTATACCAAAGGCGCCACCTAGTGTGCCACTCATTTTATATAATCCTGTAGCTAAACCAACTTTTTCATTTGGCATACTGAAAATTG
CAATTGTAAGGCCGGGTGTTGCGACTAAACCATTTCCAATTGCGCAAATGACAAACCCAATAACAACAGCAATGATATATTGTGAT
GGTTGAAGTGATGTCATGCTAATAATAGTGATGCCGATGACAGGGAACAACGGACCAATGATGAGCATCAATTTGCCACCGAAACG
TAATGTTGCTTTTTCACCTAAACGAATCATCGCAACTGCCACAATGGCATATGGCAATGTAACAAGTCCAGATTGCGCAGCTGATAA
ACCAAGGTGTGTTTGAGCAAATATGAAAAAGACCACTGTTACGCCTAGACCACTATTTAAAACAAAGTTATTTAAAAATGCACCAA
TGAACGGACGGTTGCGTAATACTGAGAAATCAATAAAAGGTACTTCATGTCGACGTTCGATGATGATGAATATTAATGTAGTGATG
ATAAAAATGCTCAGACAAATGAATGAAAATGTACTAAACCAACCTTGTTCGAATCCTTGTGTTAACAATAATGTAAAGCTACCAAT
CATAACAGCGAAAATCGACATACCTTTGTAATCGAATGGATGACGGTGGCTATGTTGACTTACTTTTTCAGGTGTGCCTTTTAGAAG
CAATATGGCAATGAAAGCAATGACTATACTAATGATGAAATTCGTTTGCCATCCGAAATTTGTGGCAATTAAACCGCCGATAACAC
CAGCTAGGCCGATGCCACCAACAGTACTAATCATTAGATAACTAATCGCTCGTCTTAAATGATCTCCTTTAAATTGATTATTTAAAA
CGCCAACTGTTGAAGGTAACAAGATAGCTGCTGATAGACCTTGTAAAATTCTACCGATGATGAGCAGTGCAGTGATGTCCGATATA
ATTAATAGAAGAGATGCAAACATACTGATTATGAGACCCATGTATGTCATTCTCAGTTGTCCTATTTTATCAGCAATATCACCTGCA
GCCACCATGAAGATACCTGTGGCGAAGGAAGTTAAACTAATAGATAAATTTAACACGGCAGGAGAGGTTTGATACGTTTGACCAAC
GAGAGGTCCTATATTAATAAATGATTGTGCAAACAACCAATATGTTAATGCAGACAACATAATCGCAATAATAATATTACTGCGTG
GTGAAGATTGTGTGTTATTCACAGATGTCACTCCTTGTAAAAATTAAATATAGAAAGTCATGAGATGAAGTGAACGGATGTATAGA
TGATCGTTCACTCACACATAAGTCATGATTTCCTTACATATCATACCACAGTTATTGAGAATGAATATCAATTAATTGCTTGTATAAT
TGATTTTTTATAGTATGTTTAAGTGAAACAGCTTTATTTGAAAGTGATTAAAGTAAATGAAGGTACAGAGGAGTGAGAACAATGTGC
ACAGGATTCACAATACAAACTTTAAATAATCAAGTACTTCTTGGACGCACGATGGATTATGATTATCCATTAGATGGTTCGCCAGCA
GTGACGCCTAGAAATTATCGTTGGAAATCTCGCACTGGCACGACAGGCCAAACGCAATATGGCTTTATTGGCACAGGAACAGATAT
GGAAGGTTTTATTTATGGTGATGGTGTTAATGAACATGGCGTTGCCATTTCAACACAATATTTCCGAGGTTATAGTTCATATGGATC
AACACACAAAGCGGACGCGATGAATATTACGCAAAATGAAATTGTGACATGGATTTTGGGATATACAACAAGCATTGAAGATATGA
AACAACAAGCATCCCAAATACATGTTGTAGCTGTATATTTAAATGACATCGGTGAAGTTCCGCCATTGCATTATCATGTTTCCGATG
CAACTGGACATTCAGTCGAAGTTTCATTTAAAGAGGGTGAAGTGGTTATAAAAGATAATCCTATTGGTGTCTTAACAAATCATCCAG
ACTTAGATTGGCATTATAGTAATTTAAGACAATATATCAATATTACTCCTTATCCAGCAACAGCAAAGTTATTGGAAGGTGTAACGA
TTGAACCTTTAGGCAATGAAGCAGGTACATTTGGATTGCCAGGTGGATTTACTTCAACTGAGCGCTTTGTGAGAATGGCATTTATGA
AAGCAAACATTGCTCAAAACAATGATAAAGAAATGGATTTAATGAATGCATTTTATTTATTAGATGCGGTAAATATACCGATTGGA
ATTGTACGTCCGCATGATGCTGACAATCACTATACGATGTATCAGACCGTAATAAATTTAACTACAAGAACGTTATATATTAAGTAT
TATGGCAGCAATGAATTAGTAGCATTAAAGCTCACAGATGATTTAATTAATAGAAAAGATATGACGATTTTTAAGCCTGAGAAGCA
TATCACTGTTAGAAAGTTGAATGACAATCAATAGCGATTGAGAATGGAATTTGGTTGATATGATTTGATGGATGATTACTATCATAC
ATTAGCGTTGAAGCGCGAACATGGACGAATGATTAGTTGTTAGTTTTGAAAATTTATAAAAGGTTAAACGAATGCAGTGAGAGATG
TCATTTTAATATCAATGTATGGCTATTTTGTAATGACAATGTAATGAGTTTAGTAAAACATTTCGGGAATATTAAATAGTTGGAAAA
TGAGAATTAAATCCTTTACTCAGTTGTCTAATTCTTTTAGTATGTGCAGTACAGTTTAATTGAAAACTAACTTTAACTTTAATGGAGG
ATGTTTTATACATGAAAAAATTAACAGCAGCAGCGATTGCAACGATGGGCTTCGCTACATTTACAATGGCGCATCAAGCAGATGCA
GCAGAAACGACAAACACCCAACAAGCACATACACAAATGTCAACACAATCACAAGACGTATCTTATGGTACTTATTATACAATTGA
TTCTAATGGGGATTATCATCACACACCTGATGGTAACTGGAATCAAGCAATGTTTGATAATAAAGAATATAGCTATACATTCGTAGA
TGCTCAAGGACATACGCATTATTTTTATAACTGTTATCCAAAAAATGCAAATGCCAATGGAAGCGGCCAAACATATGTGAATCCAG
CAACAGCAGGAGATAACAATGACTACACAGCGAGTCAAAGCCAACAGCATATTAATCAATATGGCTATCAATCAAATGTAGGTCCA
GACGCGAGCTATTATTCACATAGTAACAACAACCAAGCGTATAACAGCCATGATGGTAATGGAAAGGTCAATTCCCCTAATGGCAC
GTCTAATCAAAATGGCGGATCAGCAAGTAAAGCGACAGCTAGTGGTCATGCGAAAGACGCAAGCTGGTTAACAAGTCGTAAACAA
CTACAACCATATGGACAATATCACGGTGGTGGTGCGCATTACGGTGTCGACTATGCAATGCCTGAAAATTCACCAGTTTACTCATTA
ACTGATGGTACAGTAGTACAAGCAGGTTGGAGTAACTATGGTGGTGGTAATCAAGTAACGATTAAAGAAGCGAACAGTAATAACTA

CCAATGGTATATGCATAATAATCGTTTAACTGTTTCAGCTGGTGATAAAGTCAAAGCTGGTGACCAAATTGCATATTCAGGTAGTAC
GGGTAATTCAACAGCGCCTCACGTACACTTCCAACGTATGTCTGGTGGCATCGGTAATCAATATGCAGTAGACCCAATGTCATACTT
GCAAAGTAGATAATACAGAAAATCCCAAGTTGCGATATCATACGCAGCTTGGGATTTTTTCGTTTTAATAATAGGTATAAGTCCATT
GTTTGTCCTCTAAAACATGTTGATAAAAAGGATCTTGGCCAATTAACTTGATATCATCTGCAAGTGCTTCAACTTCATCTAAATGATG
GGTAGTTAATATAATTAAACATTTAGATTTCATAATGTTAAGTAGTTGGTGGATGTCATGTCTAGATTTTAAATCAATACCAACTGTC
GGTTCATCTAAAATGAGAATTCGAGGTTTACCTAGTAAACCTACTAATATATTAATTTTACGTTTATTCCCACCGGACAATGTAGAT
ACTTTGGCAGACGTATCATCAAAGTTTAATTGCTGTAAATATTCGTTGATAGTTGTATCGTTAATTGGATTTTTACAAAGTGATTTAA
AAAAATTAATGTTTTCAGCCACTGTCATGTGTTCAAATAACGCAATGTCTTGTGGCACATAACCGATGTGATTTTGTATTTGTCTTTG
ATTCCATTTTTCGCCGAAATAGTTGATAGTTCCATCATTAGCTTTTTCAATACCAGCGATCATACGAAGTAATGTTGATTTTCCAGCG
CCATTATCACCAAGTAATACGGTTAAACGATTACTATCAAAGGACATAGTTAAATGATTGAAAATCTGTTTGTTACGGTAACGCTTT
GAAAGATTATTAATTTCTATCATCAGTTACGCTCCTTTACATGAAAATAATCAAGTATACGATACCCATAGCAAGTGCATATATAAA
TGTCATGAATAATCGATGACTTATTGTTTGAATATGGAATAATATAAAGACGATACCTATCTCATAAATCAATATAAGTAACAGTGA
TTTTAAGTAAAATATTAAGCTGAGTGGTTGAGACAAATATAGACTAACTGCCAATAGTACCAACAATAACAAGATCGTATGTGTCA
TTACATAAGTACTATATAGTTTGAAACGGCTTAAATGATATTGTGATAATCGTTGCAATGCTGCTTGTTGGTTTAAACGATAATGAA
TTACTACTTGAACAGCGCTAACAAATAAAATCACCGCAAAGATTAAGCTAATTGAAATAGAGTGTTGTGCTTGTTTAGTAAGCGAC
ACAAATTTGATTTTAGATTCAGGTGTATGTTTATGATAGGACTTGTTGATAGCATCGATGGATTGATGCTGTTTCATATCCTCAAGGT
GTTCATAAATAATGTTAGGAATTTGCTGTTCATATAATGAACTACTAACAATTTCTACAGCAATACCACCTATAAAGTCATCTCTACC
ATATAACTGTATCGTTTCTTTTAAACGGTTCTCTTTTAATTTTTGAGAGAAACCTTTAGGAATTTGCATACTTAAAATAGCTTCCTTTT
TAGTAACCTCATCTTCAATATAGCTTTCATCTTCATCGACTTTTTTAATAGTTACATAGTCAGATTGTTTAATTTTATTGACGAATGAT
TTTGATGCAGTGGTTTGGTCTAAATCTTGAATGGTAATCGGTATTTTGAAGTTGTCATGTGCTACACGGTAACCGATACCAATAAGT
ACGAGTGCGATGACAATGGTTGTTACGAGCAAGATGTATTGTAACCATTGCTTGAACACAACAAGTTGTATATAAGGCTTCATTGAC
GATACCTCCAAACCAATACAGTTAAATTAATTATCAAAAGTGCGATGGAGCTAAGATAGAAACTAGGGTGTAGTTCTAAAATGTAG
TTGTTTAAAATAATTTCTAACAATTGATTTGTTACAACTGCGAACGGTTGAATATTGAAAACGCCATTTGCTATATGTTGTAAAAAA
ATCGTAGGTATTGTTAAACCAGATAACACCAGGATGACAATAGCTAATATGACTTTACTAATACTATTCAATAAGCCTGTTGTTAAA
AGTTCGATGAGTAATAACCACAGTATTAAAAAGGTAACATAATAGCTTAAATGAATGGCTAACGTTGGCCAATTATATAATTCAAA
GGTATTCGGAATACTGAACACAATCCAAACTACACCAACGATACTCCATAACATAGTATAAAACCATGTAATCAACGCACGAATGA
TTAACAAACGCTCTTTAGAAAAATGAAACATTTTCAATCGCGCTTTCAATACAGTATCTTGATTCATTTTCAAAACTGTAAATAAAG
ATAGTGCAAAGATGAATACCGTTGCTAAAAATCCTGTAATTGCATAATAACTGCCCGTATCGTATAAATGAATCGGTTCTAAGTTAA
ATGCACCTGAACGGTTTAATCCTGTAATCAGCAAATCAGTCATAACATTGATACTGTCAGAATGTGATGCTTTCGGTGCTAAGTCTT
GAAAAGCTAAGATGCCACCCATTGATCGCATAAGACGTTGGTAAACAGAATCTGTTAGCTGAGATAGCACGACACTTTTCATGGAT
TGTTGATCATATGTATATACTGAAATTGGTAGTTCGCCTTGTTTATAAAATGCCTTGGTCATACCTTTATCAAAAACAAAATAGCCTT
GAAGTTTCTGCTGTTTTAGCAAATCGTGTGCTTTCTTTTCATCATAACCTTTAATGCTCACATTTTTACCTAGGTTACTCCCTTTACCA
ATAGAGTTTAAGATCAATTTCGTTTCACTTGATTGATCTTTATCTACGACACCTATATTAAAATGATTGTCATCTTCTGTTACATGTTG
GATCGTCGTTAATGTAATAAGGAGTGCCGCTAATATAAATAGTAAATAGATAATCAAATACCACTTTTTCAATAAAAAAGAGTGGT
AGATGCGAAACAAATGTATTGTTTTCATTTAACCACTCCTCCAATGATAAGATTGAAAGGCAAGATGACCTTCCAATCTTATTTTTAT
ATTTTAGTTATTTAGATGCCTTTTTTAAAATTGATTCAAACATTTTGCCGCCATTTTTTTCGATTTCTTTTTCAAGTTTTTCACGGTCTT
TTGAAGATAGACTATTGAAATCTTTCGCACCACTATCATCAAAATCAATATCTGCTTTCAATTTTGTGCTAGATTTTAAAATGAAATT
AATTGGTTCTTCAGCATATTTGATGCCGATATTTAACGTAGATTTCTGAGTGTTATTTTTTACGTCAGAATCTATATTATTTTCAAAAG
TGAATTCATTTTCGTCGCTATATTTATCTAATGCGACAGTAATTTTACCTTTATCTTGACGTTTTGTGCCATCTACTTTCTCTTGGTTAT
CTAATTTGATTTTTGATTCATCATATTCTGTCTTTTTACCAAATTCGTATTTATCACTGTATTTATTGTCTTTTTCCTTAGAAGATACGC
CTTTAATTGTATATTTCGCTTCAGCATACGTGTATTTATCTTGATCGAAATCAAGTGCGTAATCTAGTTTTAACTTATCGTCTTCTAAA
GTATTAGTACCTTTGATTTTAGTTTTATTATTTTCTTTGTCTGTAATAGTAATTTCTCGTTTTACAATCGTATGTTTTTCGGTATAAATT
TTAGATTGAATTTTAGCAAATTCATCCTTTTTAGTTTCTTTGACATCGTCAATTGCTTTTTTGATGTCTTTTTCAAAGTCTTTTGTAGCA
CCTTGTTCTTCCATTAATTTTTTAAGGTCTTTATCCTTTTTAGCTTCTTCTAATACAGCTAATGTAATTTTTTTAGTGTCAGCTCTGCTA
AGTGTTAACGTGACAGGTCTAACTTTGTACTTTTCACCATTAACCTTAATTTCTTCTTTTTTACCTTTATCAAAATTATCGTCATCTAA
TTTGTCGACAATAAGTTCGGAATATTTTTCGGCAATTTTGCTGTAGTCACTTTGTTGTGCTTGAGCATTACTAAAAAGAGTATTTAAA
TTTAGTTGTTGGTTTGTAATACCATTTTCTTTTGCTGTTTCTTCATCTTCACCTGTAAGTTTTGAATAAGTTGATAATAAATCAGAATT
ATTAACACTATATTTCCCTTTAAATAATGGTGATTCGAAATAATGCTTATCTTTATCTGCAGCTAACTGGAATTTCCCTAATGCAGAG
TCTGCGATTGTTGGTTCAAGATTAATCATTGATTTCTCTTTTTTAGGATCATGTCCATATGACATTTTAATTTTCGATGCATTAACAAC
AGATTTAGGAATGCCAAGCCCTTTAACAATTTCATCTGATGCATCTGCGCTTAATTCTAATGAAGATAAAAACGAATTATCTTTCAT
CTTTTCTTGGAACTTCACTTCATTTTCAAAACGGTCATTAAAATAATCTTTATACATTTTTGCAGTTTGTTGTTCACTTTTTAAGTATG
TATTTTTCGGTGTGTTTGCAAAAAATGCATAAACTCCCCATGCGATTCCACCTATTAATAATAAGACAATAATAATAGGAATTATAA
TTTTTAACTTTTTAGACATTTTGCTTCCTCCCAATTATTTATGAGTTAATCATATCAGAACAATACATTTTATTAAATGGTTTATTTTA
AATTTTGTTTAAATTAATAGAATTTTAGTTATAATAATGTTTAATAAGTTATTGAAAATCTAATAAACTACAAAAAATATTTTGATTA
CATAATGATTATTTAAAAATGTCGGTTAACTTTAAGGTGACAAGTTATTGCAATTTTGAAGGTAGACATCATATTTTATAATCGAAT
AACATACAAAATAAAGCGACCAATTTTGCAGTGTTACGAATTGTAAACTGCGAACTGGTCGCTTTTTATTGATGTGATGATTAATTT
AATGGATGTAATTATATGATGAAACTTCTGAAGCAGAGATGGTTCTTGATGAAACGATATATTCACCGATCCAATTCATTTCTGAAA
TTAGAATGCTTCCATCAATATTAACTTTTTCAACGTAGGCTACATGACCAAATGGACCGTTTACTGTTTGTAAAATTGAACCTCGTGT
TGGGTGTCTATCTACTTTGAAGCCATTGCTTGAAGCTTGGCCTGCCCAGTTTTTAGCATCTCCCCAAAATGTACTAATCGTGTGTCCA
TCTTTGGCACGTTTATCAAAGACATACCATGTACATTGTCCAGCAGTATATAAGTTGTTCTTACTTGTGATAAGAGGCTGATCAATG
ATTTTACCGTTACCTAATGCTAAAGGTTTACCGTCAGCAGTCTTTGCTTTGTCATTAAATTCGGCGATTTGTAATTCGTCATACAATT
CGTCTAAATCGATGCCTGTAATAAGCCCTTTGTTATCTTTTGAAAAAGCGTTATTTAATTCATCGTCATTGTCTTCGACATTCGGTATT
GCTGGTGTCAAAGGATTGCTTGGTGACGTTTGAGGCGGTGTGTGTGAATCAATTGCGTCATTAATGTGCGTATACTGACCACTTAAT
GAAGAATGGTACTGATTGTTGTTAAGATCACGTTGATTTGCGTGTTGATGATTGTCGTTTGTACGTGACTGGTTTTGATGATTGTTGT
TTGGCGTGTTGTTTTTGTCATATGTATAAGTATACGCGCCGGTGTCTTTATTCACTTTGAACTGTGCGTTTGGGTGTGCTTTCTTTGCT
TCTTCTAATGTTTTGCTATCATTCGTATATGCTTGAGCCGAGTTAGGCGACATACTAAATAAAGTAAGAGTTGTCATCGTCAGTAAA
ATTGTTTTCTTCATAATAACCATTTAATCCTTTATGTATTTAATTTAATTTTAGTATACACATTTATATTACAAAAATGAATGGTTAAT
TAAAAATATATGGGTATATTCAATATATTTATTTAAAAAAAGCTAAAAATACTTAAAAAACTATATACATATACTAATAATTTATAT
ATTATTTGAGTAAGGAGCACTTTTTCAAAAAATAGTGTCCCTAAAAAGTTTTGATAAACTTAAAATATTCAGGAGGTTTCTAGTTAT
GGCAATGATTAAGATGAGTCCAGAGGAAATCAGAGCAAAATCGCAATCTTACGGGCAAGGTTCAGACCAAATCCGTCAAATTTTAT
CTGATTTAACACGTGCACAAGGTGAAATTGCAGCGAACTGGGAAGGTCAAGCTTTCAGCCGTTTCGAAGAGCAATTCCAACAACTT

AGTCCTAAAGTAGAAAAATTTGCACAATTATTAGAAGAAATTAAACAACAATTGAATAGCACTGCTGATGCCGTTCAAGAACAAGA
CCAACAACTTTCTAATAATTTCGGTTTGCAATAAGCATTCTGAAATTGGCAAAGTCACATTTTCTAATGTGGCTTTGCTTATCATTTT
TTAAGAAAACAACTGAAAGGAAATAAGCATGAAAAAGAAAAATTGGATTTATGCATTAATTGTCACTTTAATTATTATAATTGCCA
TAGTTAGTATGATATTTTTTGTTCAAACAAAATATGGAGATCAATCAGAAAAAGGATCCCAAAGTGTAAGTAATAAAAATAATAAA
ATACATATCGCAATTGTTAACGAGGATCAACCAACGACATATAACGGTAAAAAAGTTGAGCTGGGTCAAGCATTTATTAAAAGGTT
AGCAAATGAGAAAAACTATAAATTTGAAACAGTAACAAGAAACGTTGCTGAGTCTGGTTTGAAAAATGGTGGATACCAAGTCATGA
TTGTTATCCCAGAAAACTTTTCAAAATTGGCAATGCAATTAGACGCTAAAACACCATCGAAAATATCGCTACAGTATAAAACAGCT
GTAGGACAAAAAGAAGAAGTAGCTAAAAACACAGAAAAAGTTGTAAGTAATGTACTTAACGACTTTAACAAAAACTTAGTCGAAA
TTTATTTAACAAGCATCATTGATAATTTACATAATGCACAAAAAAATGTTGGCGCTATTATGACGCGTGAACATGGTGTGAATAGTA
AATTCTCGAATTACTTATTAAATCCAATTAACGACTTCCCGGAATTATTTACAGATACGCTTGTAAATTCAATTTCTGCAAACAAAG
ACATTACAAAATGGTTCCAAACATACAATAAATCATTATTGAGTGCGAATTCAGATACGTTCAGGGTGAACACAGATTATAATGTTT
CGACTTTAATTGAAAAACAAAATTCATTATTTGACGAGCACAATACAGCGATGGATAAAATGTTACAAGATTATAAATCGCAAAAA
GATAGTGTGGAACTTGATAACTATATCAATGCATTAAAACAGATGGACAGCCAAATTGATCAACAATCAAGTATGCAAGATACAGG
TAAAGAAGAATATAAACAAACTGTTAAAGAAAACTTAGATAAATTAAGAGAAATCATTCAATCACAAGAGTCACCATTTTCAAAAG
GTATGATTGAAGACTATCGTAAGCAATTAACAGAATCACTGCAAGATGAGCTTGCAAATAACAAAGACTTACAAGATGCGCTAAAC
AGTATTAAAATGAACAATGCTCAATTTGCTGAAAATCTAGAAAAACAGCTACATGATGATATCGTTAAAGAACCAGATAAAGATAC
AACATTTATCTATAACATGTCTCAACAAGACTTTATAGCTGCAGGTTTAAATGAGGATGAAGCTAATAAATACGAAGCAATTGTCAA
AGAAGCAAAACGTTATAAAAACGAATATAATTTGAAAAAACCGTTAGCAGAACACATTAATTTAACAGATTACGATAACCAAGTTG
CGCAAGACACAAGTAGTTTGATTAATGATGGTGTCAAAGTGCAACGTACTGAAACGATTAAAAGTAATGATATTAATCAATTAACT
GTTGCAACAGATCCTCATTTTAATTTTGAAGGCGACATTAAAATTAATGGTAAAAAATATGACATTAAGGATCAAAGTGTTCAACTC
GATACATCTAACAAGGAATATAAAGTTGAAGTCAATGGCGTTGCTAAATTGAAAAAGGATGCTGAGAAAGATTTCTTAAAAGATAA
AACAATGCATTTACAATTGTTATTTGGACAAGCAAATCGTCAAGAAGAACCAAATGATAAGAAAGCAACGAGTGTTGTGGACGTAA
CATTGAATCATAACTTTGATGATCGCTTATCGAAAGATGCATTAAGCCAGCAATTGAGTGCGTTATCTAGGTTTGATGCACATTATC
AAATGTACACAGATACAAAAGGCAGAGAAGATAAACCATTCGATAACAAACGTTTAATTGATATGATGGTTGACCAAGTTATCAAT
GACATGGAAAGTTTCAAAGACGATAAAGTAGCTGTGTTACATCAAATTGATTCAATGGAAGAAAACTCAGACAAACTGATTGATGA
CATTTTAAATAACAAAAAGAATACAACAAAAAATAAAGAAGATATTTCTAAGCTGATTGATCAGTTAGAAAACGTTAAAAAGACAT
TTGCTGAAGAGCCACAAGAACCAAAAATTGATAAAGGCAAAAATGATGAATTTAATACGATGTCTTCAAATTTAGATAAAGAAATT
AGTAGAATTTCTGAGAAAAGTACGCAATTGCTATCAGATACACAAGAATCAAAAACAATTGCAGATTCAGTTAGTGGACAATTAAA
TCAATTAGATAATAATGTGAATAAACTACATGCGACAGGTCGAGCATTAGGCGTAAGAGCGAATGATTTGAACCGTCAAATGGCTA
AAAACGATAAAGATAATGAGTTGTTCGCTAAAGAGTTTAAAAAAGTATTACAAAATTCTAAAGATGGCGACAGACAAAACCAAGC
ATTAAAAGCATTTATGAGTAATCCGGTTCAAAAGAAAAACTTAGAAAATGTTTTAGCTAATAATGGTAATACAGACGTGATTTCACC
GACATTATTCGTATTATTGATGTATTTACTATCAATGATTACAGCATATATTTTCTATAGTTATGAACGTGCCAAAGGACAAATGAAT
TTCATTAAAGATGATTATAGTAGTAAAAACCATCTTTGGAATAATGTCATTACGTCAGGTGTTATTGGTACAACTGGTTTGGTAGAA
GGATTAATTGTCGGTTTAATTGCAATGAATAAGTTCCATGTATTAGCTGGCTATAGAGCGAAATTCATCTTAATGGTGATTTTAACTA
TGATGGTCTTCGTACTTATTAATACGTATTTACTAAGACAGGTAAAATCTATCGGTATGTTCTTAATGATTGCTGCATTGGGTCTATA
CTTTGTAGCTATGAATAATTTGAAAGCGGCTGGACAAGGTGTGACTAATAAAATTTCACCATTGTCTTATATCGATAACATGTTCTTC
AATTATTTAAATGCAGAGCATCCTATAGGCTTGGCGCTAGTAATATTAACAGTACTTGTGATTATTGGCTTTGTACTGAACATGTTTA
TAAAACACTTTAAGAAAGAGAGATTAATCTAATGTTGATGAATAGCGTGATTGCTTTAACTTTTTTAACAGCATCTAGCAATAATGG
CGGACTTAATATTGACGTGCAACAAGAAGAGGAAAAGCGAATCAATAATGATTTAAATCAATATGATACAACGCTATTTAATAAAG
ACAGCAAAGCGGTTAATGATGCGATTGCTAAGCAGAAAAAAGAACGACAACAACAAATAAAAAATGATATGTTTCAAAATCAAGC
GAGTCACTCGACTCGCTTGAATGAAACTAAAAAAGTGTTATTTTCCAAATCTAACTTAGAAAAGACTTCGGAGAGTGATAAAAGCC
CCTATATTCAAAACAAGCAGGAGAAAAAAATATTCCCGTACATTTTGATGTCTGTAGGGGCTTTTTTGACTTTAGGATTTGTCATTTT
TTCAATTCATAAAGGGAGACGAACGAAAAATGAATCAGCACGTAAAAGTAACATTTGATTTTACTAATTATAATTACGGCACATAT
GACTTAGCAGTACCAGCATATTTACCGATAAAAAATTTAATAGCTTTAGTATTGGATAGTTTGGACATTTCAATATTTGATGTCAAT
ACACAAATTAAAGTGATGACGAAAGGTCAATTACTTGTTGAAAATGATCGACTCATTGATTATCAAATCGCTGATGGAGATATTTTG
AAGTTACTATAGGAGGAAAAATAGATGGTTAAAAATCATAACCCTAAAAATGAAATGCAAGATATGTTAACGCCTTTAGATGCTGA
AGAAGCAGCTAAAACAAAATTACGCTTAGATATGAGAGAGATTCCTAAGTCTTCAATTAAACCAGAACATTTTCATTTAATGTACTT
ATTAGAACAACATTCTCCATATTTTATAGATGCTGAATTAACTGAACTACGTGATAGTTTCCAAATACATTATGACATTAATGACAA
TCATACACCTTTTGATAATATTAAATCATTTACTAAAAATGAAAAATTACGTTACTTACTCAATATCAAAAATTTAGAAGAAGTAAA
TCGTACACGCTACACATTTGTGTTGGCACCAGATGAATTATTTTTCACAAGAGATGGATTACCAATTGCTAAAACAAGAGGTTTACA
AAATGTTGTTGACCCATTACCGGTGTCAGAAGCTGAATTTTTAACAAGATATAAAGCACTGGTTATCTGTGCATTCAATGAGAAACA
ATCATTTGATGCTTTAGTTGAAGGAAACTTAGAATTACATAAAGGAACGCCATTTGAAACTAAAGTTATTGAAGCGGCAACGTTAG
ATTTACTAACGGCATTTTTAGATGAACAGTATCAGAAACAAGAACAAGATTATAGTCAAAATTATGCATATGTACGCAAAGTAGGG
CATACCGTTTTTAAATGGGTTGCTATCGGTATGACAACGTTAAGTGTTTTATTAATTGCATTCTTAGCCTTTTTATATTTTTCAGTAAT
GAAGCATAATGAGCGCATTGAAAAAGGATACCAAGCATTTGTAAAGGATGATTATACGCAAGTACTAAATACGTATGATGATTTAG
ATGGTAAAAAATTAGATAAAGAGGCACTTTACATTTATGCCAAAAGTTATATCCAAACAAATAAACAAGGTTTAGAAAAAGATAAG
AAAGAAAATTTACTTAATAACGTGACACCAAATTCAAATAAAGACTACTTATTATATTGGATGGAATTAGGACAAGGACATCTTGA
TGAAGCGATTAATATTGCCACTTATTTAGATGATAACGATATTACAAAGTTAGCGTTGATTAATAAGTTAAATGAGATTAAAAATAA
CGGAGATTTATCGAATGATAAACGTTCTGAAGAAACGAAAAAGTATAACGATAAATTGCAAGATATTTTAGACAAAGAAAAACAA
GTTAAAGATGAAAAAGCGAAATCTGAAGAAGAGAAAGCAAAAGCGAAAGATGAGAAATTAAAGCAACAAGAAGAGAACGAAAA
GAAACAAAAAGAACAAGCACAAAAAGATAAAGAAAAACGCCAAGAAGCTGAAAGAAAAAAATAGTATAGGACTGAGGCAAAGA
CAATGCATAAATTGATTATAAAATATAACAAACAATTGAAAATGCTCAATTTGCGAGATGGTAAGACATATACTATTAGCGAAGAC
GAGCGTGCAGATATTACGTTGAAATCGTTAGGCGAAGTCATTCATTTAGAACAAAATAATCAAGGTACTTGGCAAGCGAATCATAC
TTCTATTAATAAGGTGCTTGTTAGAAAAGGTGACCTTGATGACATTACATTACAACTTTATACAGAAGCTGATTATGCATCATTTGCT
TATCCTTCAATTCAAGATACGATGACAATTGGGCCAAATGCGTATGATGATATGGTTATTCAAAGCTTGATGAATGCCATCATTATT
AAAGATTTTCAATCAATACAAGAATCACAATACGTACGCATTGTGCACGATAAAAATACAGATGTGTATATTAACTATGAACTACA
AGAGCAATTAACAAACAAAGCTTACATTGGTGATCATATTTATGTTGAAGGGATATGGCTCGAAGTACAAGCTGATGGTTTAAATG
TATTGAGTCAGAATACAGTGGCATCGTCATTAATTCGCTTAACACAAGAGATGCCACATGCACAGGCAGATGATTACAATACGTAC
CATCGTTCGCCAAGGATTATTCACCGTGAACCGACCGATGATATTAAGATTGAAAGACCACCACAGCCAATACAGAAGAACAATAC
AGTGATATGGCGTTCCATTATACCGCCATTAGTAATGATTGCTTTAACTGTTGTCATCTTTTTAGTGAGACCAATTGGTATTTATATTT
TAATGATGATTGGTATGAGTACAGTAACGATAGTATTTGGTATTACAACGTATTTCTCTGAAAAGAAAAAGTATAACAAAGATGTTG

AAAAACGAGAGAAAGATTACAAAGCTTATTTGGATAATAAATCTAAAGAAATTAATAAAGCGATTAAAGCACAACGTTTTAGTTTG
AATTACCATTATCCAACGGTTGCTGAAATTAAAGATATCGTTGAAACGAAAGCACCAAGAATATATGAAAAAACATCGCATCATCA
CGATTTCTTACATTATAAGTTAGGTATTGCGAATGTAGAAAAGTCATTCAAATTAGATTACCAAGAAGAAGAATTTAACCAACGTCG
TGATGAACTATTCGACGATGCTAAAGAATTGTATGAATTTTACACAGATGTAGAACAAGCACCATTAATCAATGATTTAAATCATGG
ACCAATTGCATATATTGGTGCACGACATCTCATTTTAGAAGAATTGGAGAAAATGCTAATCCAATTGTCAACATTCCATAGTTATCA
TGATTTAGAGTTTCTATTTGTGACACGTGAAGATGAAGTTGAAACATTGAAATGGGCACGTTGGTTGCCACATATGACATTGAGAGG
GCAAAACATTAGAGGATTTGTTTACAATCAACGAACACGTGACCAAATTTTAACGTCAATCTATAGCATGATCAAAGAACGTATCC
AAGCTGTGCGTGAACGCAGCAGAAGTAATGAGCAAATTATTTTCACACCGCAATTAGTGTTTGTCATTACAGATATGTCATTAATTA
TTGATCACGTCATTTTAGAATATGTAAACCAAGATTTATCAGAATATGGTATTTCATTAATCTTTGTTGAAGATGTGATTGAAAGTTT
GCCAGAGCATGTAGATACCATTATTGATATCAAGTCTCGTACTGAAGGCGAACTGATTACGAAAGAAAAAGAATTAGTTCAATTGA
AATTTACACCGGAAAATATTGATAACGTCGATAAAGAATATATCGCGCGACGTTTGGCGAATTTGATACACGTCGAACATTTGAAA
AATGCAATTCCTGATAGTATTACATTTTTAGAGATGTATAACGTGAAAGAAGTAGATCAGCTTGATGTAGTTAATCGATGGAGACAA
AACGAAACATACAAAACGATGGCAGTACCTTTAGGCGTAAGAGGTAAAGATGATATTTTATCATTGAACTTACATGAAAAAGCACA
CGGGCCACATGGTTTAGTTGCTGGTACCACTGGTTCAGGGAAATCTGAGATTATCCAATCATACATTTTATCTTTAGCTATTAATTTT
CACCCTCATGAAGTTGCATTCCTATTGATTGACTATAAAGGTGGGGGTATGGCGAACTTATTTAAAGATTTAGTCCATCTAGTTGGT
ACGATTACAAACTTAGATGGCGATGAAGCGATGCGTGCCTTAACATCAATCAAAGCCGAATTGAGAAAACGTCAACGTTTATTCGG
AGAGCATGATGTTAACCATATTAATCAATACCATAAGTTATTTAAAGAAGGTGTTGCGACAGAACCAATGCCACATTTATTCATTAT
TTCCGATGAGTTTGCCGAATTAAAATCAGAACAACCTGATTTTATGAAAGAACTTGTATCAACGGCACGTATTGGACGTTCGTTAGG
TATTCATTTAATACTTGCGACACAAAAACCATCGGGTGTTGTTGATGACCAAATTTGGTCTAACTCTAAATTTAAGTTGGCATTAAA
AGTACAAGATAGACAAGACAGTAATGAAATTTTAAAAACACCAGATGCAGCAGACATTACATTACCAGGTCGTGCGTATTTACAAG
TTGGTAATAATGAAATTTATGAATTGTTCCAATCTGCATGGAGTGGAGCAACATATGACATCGAAGGCGATAAATTAGAAGTTGAA
GATAAGACGATTTACATGATTAATGACTATGGTCAACTGCAAGCGATCAACAAAGACTTGAGTGGACTTGAAGATGAAGAAACGAA
AGAAAATCAAACTGAGTTAGAAGCGGTTATAGATCATATCGAATCTATTACAACACGATTAGAAATTGAAGAAGTTAAGCGTCCAT
GGCTACCACCATTGCCAGAAAATGTGTATCAAGAAGATTTAGTAGAAACAGATTTTAGAAAATTATGGTCAGATGATGCAAAAGAA
GTGGAATTAACATTAGGACTTAAAGACGTACCAGAAGAACAATATCAAGGACCGATGGTATTGCAATTGAAAAAAGCTGGGCACA
TCGCGTTAATCGGAAGTCCAGGATATGGTAGAACAACGTTCTTACACAACATTATTTTCGATGTTGCAAGACACCATCGTCCTGATC
AAGCACACATGTACTTGTTCGATTTCGGTACCAATGGTTTGATGCCAGTCACAGATATACCACATGTCGCTGATTACTTTACAGTAG
ATCAAGAAGACAAGATTGCTAAGGCGATACGGAAGATACATGATATTATTTCTGAGCGAAAAAGACTATTAAGTCAAGAGAGAGT
AGTTAATATAGAGCAATATAATAAAGAAACTGGAAATTCAATTCCAAATGTTTTCTTAATTATCGATAACTACGACACGGTGAAAG
AATCACCATTTATGGAAGAATATGAAGAAATGATGTCTAAGGTGACACGTGAAGGTTTAGCATTAGGGGTATACATCATTCTTTCAG
GATCAAGATCAAGTGCAATAAAATCAGCTATATTTACTAACATTAAAACAAGAGTTGCACTATACCTCTTTGAAAATAATGAATTAA
CAAATATTATTGGTTCTTATAAAAAAGGTGTGAAAGATGTTAAAGGTAGAGCTGCAATAAATGATGATAACTTCACACAGTTCCAA
ATTGCGCAACCATTTGAATTAGCAGAAGGACAAACATATAACGAGCGCATTAAAAATGAAGTAGCACAAATGAAAGAATTCTATGT
AGGAGATTATCCAAAACATATCCCAATGATGCCAGATAAGGTATTAATGGATGATATTCAAGAAACATATGATTTGGAAAAAATCA
TTCATGAAGAACACAAATTACCATTAGGATTAGACTTTGAGGATGTGGAACTAGTTAGCTTAGATCTGACAAGTTCTTCGATTGTAA
CAGCAATTAAACCAACTGAGATGGAAAAAATGAATGATGTTATAATGTCTAGTTTATCAGTATATAGTAAAAACCAATTTGTTATCT
TGGTTGATGCAGAAGATAATATGAGCCAGTATGCAGAAGATGTAACCTCTTATTATTCAGCACCATCAGATTTGAGCAACATTAGAT
TAGGATTTAAGCAAGAAATAGAAGCAAGGAAAAATGGCGAGAAAAGTATAGAGGAATGCAAGATTGTATTTATAAATAATATTAA
ACGATTCAATCAATTAACTGGAATGACAGAAGATGAAATTCGTGTGTTATTTAATGAAGGTCAAAAAGTTAATATTATCATTATTGC
AAGTGGACTATACTCAGATACAATTGGTGCATTTGATAGAGAAAGTAAAATGATGGTAAGAACTATAAATCAAGCTTTGATTAGTC
ATAAGATATCGGAACAAGAATTTATAAGAGTTAAAGATCGATTTGGTGAACCAGAATTAAAAGTGGGAGAAATGTATTACATTAAT
AATCAAGAATATCAAAAAAATAAAACTAATGGAGGGATAGAGTTGATAAAGTTAAACCAAGCCTCTGTGAGCAAAGAAATTTCAAG
CATTCGAACAAATGGACAAGGCTTAAAACAATCAAATGGGAATGTAAATTTAAGTAAAACGAATCTCGTTACTTTTAAAGAATATG
TAAATATGTTTGAAGATTATCAATCAGCTCTATCTAATTATGAAAACATAATTGAACAAGATACGACGGCTATGGATACAACGGTA
ACAGAAATTGTTGAAAACGATAGAGAAATAGCAGGACAAATTAATAAATAATTAAACAAGACGGGTGAGAAAAATGAGTAATAAG
GGAGAAATAAGAAGGCAAATTGCTAATAAAGAAAGAGAAAAAGCAAGTAAAGAAGCACAACTAACAGATTTAAAAGAGGATTTA
AGAAGGTTGAAAGACGCTTCGAAAAAATTGGATACAGCAGGAGAAGATTTTAACAAAGGACAGAGTAGTTATAACAAAGTAGAAA
TTAGTACTTCAGATTGGAAAGGAGAAAGAAGGACTAAAAGTGACAGCAAAAAGAAAGATGTCGATTCTGAATTAAAAAAAGTTGA
ACAAGACTTTGATGATGCGAAGAAGGCTATTAAAAAAGATATTCAGGATAAAGAAGGAGAAATCAAGGGAGTCGAAGGAGAAATC
AGTACTATAAATGCAGCTATAGATGCACTTAAATCAAAATTATAATAATAGAGAGGGAAATTTATTATGGGGTACAAAGTTGATAT
GTCTGAAGTGCACAATATGCAAAAGTCTATTGATTCAAGCTTAAGTGCAATAAATACAAAGGTTAGCACGCTTAGCAGTAGCATAA
ACAACCTTATAAATACTGAAGGATTCGAAGGTCAGACAGCAAGTAGTGTCAAAAACTATAGTAAGACATTTCATTTACAATCAATA
AAAAAGATTGAAACAATAAACAAAGATTTTAAATCAGATATTGCGAAATCTATACGTAAATTCCAAAGTGAAGTTGATAATAATGC
TTCAGCTATATTGGATGAAGACGAAATAAAAAAATACAAAAAAGATATTGATGATGCATTAAAAGATGTTTTCAAATCTTCTAAAG
ATGCAAACGGAGCTATTTCAGATGTTTCAGATTTAACAACAGCCAAAAAAATAAATACAGAAAACCTGGCCAATAAAATGGGAGA
CTTTAATAAAGATATTGATCAAACGGTGGAAAGATTAACGAACTTCGACGCCAACAATTCTATAGATGGTGATAAGACAGATAACT
TAATAACTGAATTAAGTGGTGTGAATTCATATGTCAAAAACATGAAGCCAAACCGTGCAAGAATTAGTTCTACAAGTGGAAAAATT
GAAGGTGCAATTGCAAGGCATAAAACAAGTGAAGAGTTAGTAAGATGGCAAACTTATATGGAAACGTTAAGTGAAAATCTTTATAA
AACACCAGGTCTAAGTAAAGCAGCTTCTGACATAATTACCCAAGCTGGACGCGAGTACTATGCTATCAAAGCTGTAGGGAATGGCA
GTGCTTTAAGAGGATATAAAGAATATCTGAAAACACGAGATATTAATAAATTAATTAACAAAATGAGTAAAAACCAATTAAAAAG
AATGTCATTGGTATTAAATACAGATAGAGGTAATATTAAAATAAAAAATGCAGTAAAAATGGCAAGTGAATTTGCCCGTAATAATC
CATTTAAAAAAGGTAACTTGGTAAATTGGATGAGCAAAGTCCAAGGGTATCAAAGTGTAAGTGCCCAAGTATTAAAAAAAGGGTTG
CAAGATAAGAACTTTAAATATACATTTGGAGATGCAAAGAAATTTTTTGATACAGCTGAAATGAAAAAAGCAGCAGTTACAGAATT
TAAAAATACCTTTGTACCTCAAAACATAAGGGAAACATTCTTTAAGAAAGAAAATATAACATCCAAGACAGCATTAAAGAAAAATA
TGAGCAAATTCTGGAATGAAGATATCAAGGGAGGAATCAAAGAGTTTAAATTAGACTTTGCTAACAAAAATATCATTGGTAAACTT
GGTAAAAATGTTGAAACTAGGAGGGAAAGCTCTAAAGCCACTAGCATTTGTTACTGCAATTACAGATAACATAGGAAAGAAATCTTT
GCAAGAACAATTAGTGGGTATGGGTGTAGATTTAGGTGCTATTGGTGCTAGTGCAGCTGCCGGTGCAGCTATTGGAACGGCAATAC
CATTACCTGTAGTAGGTACTTTAGTAGGAGCATTAGCTGGAACAGCAGCAGGTATACTTTTAGATAGTAAAATAGCAGGTATGAGT
AAATCACTAACCGAACATGCTAAAGACGGAATAAACTCGGGTATTAATAAAGTTAAAAGTGGATTCGGAAAAATAACTTCAGGATT
TAAATCAGTATTTGGTTAAGAGAGGACTATTATAATGAATTTAAGAGAAATTGTAGGAAATATAAAAAGACCGTATTTAACACCAC
TAATAATATTTACGATTTTAATCTCGCTTTTTTTTGATGCAATTATGTTTTTTAACAGTAAGCTATATGATAAGTTACCTATATATTTA

GTCATATTTTTAGTTTTTGGAATATTAGTGTCAGTGTTGCTGTATATACAAGAAAAAGGTGAAAAGATAAAAGTTGAGAAAAAAAA
TGTGAATTGGTATTTGACATTAAATGTTATAGCAGGTTATTCAATGCCATTATTTATTACATCTGGTTATGTAGTTGGAGCAACAGTT
TATGGGACAGATGTTTTAAATTATTGGTGTGGAATAATTTTGACATTATTTGTTTCGTGGTTTGCATTATTTTTATTTTATAAAAATGA
GTTTGATAGTGAAAATCCAAACAAAGCAGTTAATGTCATAGCAATTATTATAAAGTTATCTGCTTTAGGATTAATATTTTATATTAGT
ATTATTGCACCTAGTGTTGAAGATGAAAAAAACTTTATTTTTGTAAGTATTTTAATTAATATAGCTGTCGATGCATTACTTGTTAGAT
CATATTTTAACTATGCATTATATAAGAGTGTAAAAAAAGATATCGAAAACGGGGTGGATGATTAATGGAAGTAGGAATGTTAATTA
TGCCGTTTTGTGTGAAGATTACATTTGAAATTGATAAAAATGATAATTGGTTAAATGCATTAGATACAGTAAATGAATTTTTTATGG
ATAAAGATATATATCCTACTGGACCTATAATTTTCCAAAGAGAATTAGCAGGTTTGGGTGAATATAAATATACAGCATATATAGCTC
TTAATGATGAACTCCAAGATATACCTGAATTGAACATTAAATATATTGATTGTTTAGAGGTAGGACCTACGTTATCAACTAAGTGTT
TTGAAGAAGAGGAATTTGAAAGAGCCTATAAAGAAATTGAATATGTAGCTAACGAAAATAATATTAAGATTCTAAATCAACCATAT
TATCATGTAATGGTGGATTATTTTGGTGGTACGGCTTTTGAAATTTATGCTCAAGTAGATTTGGATGAGAGTGAAGTATATGGATAA
AAATGGATACTCATATATTATGATGAAAAATGTAGCTTATAAAACTTATTTTGATGTGACTATGGAAGACTATGAAGATATATTAAA
AGACTTTACAAATCTTGCAATTTCTAAAGGTAAAACAATTACAGGGCCATTAACCTTTGCAGTCACGCAAATAGACTTGCAAAAAA
GGATGAATATTGATTTGTTTATTTCAGTTGAAAAATGTTTTAAATCAAATGAAGAACTATCATATAGAACATATTTTTGTCTAGATAA
CATGTTACATGGTCGTATCACTTCAAACAATTTTATAATAGATGAAATAGCGTTACTTGAGGATATGAACGAATTTGCTTCTGACAA
CAATTTAACGTTTACTTCGCCTTATTATCACACAATTAGAAAAAATTTTAGCGGTGAACAAGGTTGGATTGATGTAAAGTCAAAAGT
ATATGAAAATGATTAATTAATAATCAATAGAGGATAAAATATGATAATTTTTATTTTAATAACAATATTTGCTATTTATTATATAGCT
ATGATTGCTAGTTTGTTTAAAAGTGAAGGTTTTTCAATAATAGGTTTAATATTGGATATAGTTATTTTGACAACGTTAATTTTTTATT
ATTTTGTAGGTGCGCGTTTTGTTGATCATGATTTAAGCAACTTTTTAGCATTTATGCATTTGGGTTCATTTGTGTATATTTACTTTGCA
ATTAAGTGTTTATGGGTGAAACCTAAGGTAGTAAATTATTTGATTGCAAAAGAATTGGATGAATCTAAAGAGGTCATTGAAGAGCA
GGAATTAGATTTACAGACATCAAAGATAAGAGGTATCTACTTTTTCATTATTGCAGTAGTGATGTTAATTATTGCTAAATTAAGGAT
GCAACCAGAATTACAAGTAGATGCGTTATCAATGAATCCAGTTTTTATTTTTATAGGAGTTATTATCATCTTAATTTGGCTAGTACTG
GATATATATCGTAAGAAAAAATACGGTATATTCTTATTCAAGACGATAGTGCCATTAGTTGTTACGACTTGGATTATTATAGCTACT
ATTGTACTTTCGTAAGAAATTTTCAATATTTTTTATTTTGGTTATAATTGTTGATGGATATTAATTAATCGATAGAAAAATCTTAATG
GTTTTCAAAAAAAATACGTAAAAAGTATATACTATTAATATCTTTTTTACTATAATTATAAATAGTTAAATAAATTGAAAGACAAAAT
AATGAGTAGGAGTTAATGAGATGGAAAACCAAAAACAAGGCAATGGCTTAAAAATTGCAACATGGGTATTTATTGTATTAACAGTA
GTTACACCGCTATTTGGTATTGGAAGTATTGTTTGTAGTATTAATTACAAAAAATACGATGCAGAAAAAGGTTCGAAGTTATTGAAA
ATTGCAATTATCGTAACAATAATTGTTTTTGTTTTAAATTTATTAGCATATTTAGGTTTAAGATAAGTAAAACGAATTTGAAGAAGCA
TTAAGCGACATTTGGGTGTTGTTTAATGCTTTTTATTTAACGGAAATAAGTGTAGAGAATAAATTAATAGTTTAATCAAGAGATATT
TCGAACACATAGGGGAGATAATATGACTTTCGAAGAGAAGTTAAGTCAAATGTACAACGAGATTGCGAATAAAATTAGTAGCATGA
TACCAGTAGAATGGGAAAAGGTATATACAATGGCTTATATAGATGATGGAGGAGGTGAAGTATTCTTTAATTATACTAAAATAAAC
AGCGATGAATTGAATTATTACACCGATATACCTAAGGAGTATAACATTTCTGTGCAAGTATTTGATGATTTATGGATGGATTTATAT
GATTTGTTTGAG

>009-contig_245_RC

TCATGTGAATTTGACTTTACAAGAGACGGCAAATTGAATGTTTCATTTGATTATATTGATTGGGCGAATTCAGAGTTTGGACAAATG
GGAAGAGAACATTATTACATGTATAAAAAATTTGGAATTTGGCCTGAAAAAGAATATGCCATAAATTGGGTAAAAAAAATAAAAG
ATTATGTTAAAGAGCAAGATGAAGCTGAACTATAGGGGCGATAATATGACTTTCGAAGAAAAGCTAAGTCAAATGTACAATGAAAT
TGCAGATGAAATCAGTGGAATGATACCAGTTGAATGGGAAAATATATATACAATTGCCTATGTAACTGATCAAGGTGGAGAGGTCA
TTTTTAATTATACTAAACCAGGTAGCGATGAATTGAATTATTACACGGATATCCCTAGAGAGTATAATGTCTCTGAAAAAGTATTTT
ATGATTTGTGGACGGATTTATATAGATTGTTTAAGAAGTTAAGAGAAACTTTTAAAGAAGAAGGGCTTGAACCATGGACATCAAGT
GAATTTGACTTTACAAGCGAAGGTAAATTAAAAGTTTCATTTGATTATATTGATTGGATAAATACAGAGTTTGATCAATTAGGCCGT
GAAAACTATTATATGTATAAAAAGTTTGGTGTTTTACCAGAAATGGAATACGAAATGGAAGAAGTTAAAGAAATCGAGCAATATAT
TAAAGAGCAAGATGAAGCTGAACTATAGGGGCGATAACATGACTTTCGAAGAAAAGCTAAGTCAAATGTACAATGAAATTGCAAA
TGAAATCAGTGGAATGATACCAGTAGAATGGGAAAAAGTATATACAATTGCCTACGTAGATGATGAAGGTGGAGAGGTTGTTTTTA
ATTATACTAAACCAGGAAGTGAAGATTTGAATTATTATTCAGATATTCCTAAAGATTGCAATGTCTCAAAAGATATTTTTAAGAATT
CATGGTTTAAAGTTTATCGAATGTTTGATGAGTTAAGAGAAACTTTTAAAAAAGAAGATTTAGAACCGTGGACATCATGTGAATTTG
ACTTTACAAGAAAGGGAAATTTAAAAGTATCATTTGATTATATAGATTGGATTAAATTAGGTTTTGGCCCATCAGGAAAGGAAAAC
TACTATATGTACAAAAAAAATTGGTATTTTACCAGATATGGAATATGAAATGGAAGAAATTCGAGAAGTAGAGAAGTATGTTAAAGA
CCAAGAGTAGCATATATGTTATAGAAGGCTGTGCAAAATCACCCTCGTTTTACATTTGACTCAAAGAAGAAGGTAAACGATAAGAT
TATTTGCAACTTAAAAAGTCAATTAGCTTATCGGTATCCATACATCATGGATAAATGAGTTTAACTAATTAATAAATCACGATATAA
ATTTAAAATTGCCATTAATTGAAAAGGTTATTGTAGTGTATTTTATAAAGTTGATGCACATCGACTTATTTTTTAAAACTTATATCAA
AAAAATATGAGAAAAGTATATACTATTAAAATGTTTTTTACTATAATTATAAATAGTTAAATATTTGGGAATTAAATAAATATATAG
GAGGCAATTTTTAGAGATACATAGCAATGTAAAGGTATCAAATAGTTATTGCATAATTTATTATGATGACATAATTTTCAGTAGATA
GTTGTAAATTAACACTGAAATAGATGTTTATATTAAGAAGACTTTAATATCATAATTAATCAAAATCTTGCGTCAAACATTTAAACG
AATTTTAAGTTGATAACAATTGGCAAAACATTGTTCAAACATCACAATGATAAAGCGTATTATCAGTATTGTAGTGTGTGGAAAATG
ACAGCCATCTAAGGAGAAAAATGATGAAAAGAATATTGGTAGTATTTTTAATGTTAGCAATTATATTGGCAGGTTGTTCTAATAAAG
GTGAAAAGTATCAAAAAGATATTGATAAAGTGTACAAAGAACAGAATCAAATGAATAAAATTGCCTCGAAAGTACAAAACACTAT
TAAAACAGACATTAAACAAGAAGACAGTAATACACATGTTTATAAAGATGGTAAAGTCATTGTTATTGGTATTCAATTATATAAAG
ATCGTGAAAAAATGTATTATTTCGCATATGAAATAAAAGATGGTAAGGCAGAAATCAATAGAGAAATAGACCCAATTAAGTATATG
AAAGACCATAAAGCAGATTATGAAGATGAAAATGTAGAAGTGGAAAAAGATTAACGTATGCTTTCTTTAAAACTTTTAACCAGCTT
CAAAAAATAAATGAGCTTTAGCGACAATAATTTTTAAAATAAACACGATATAAATTTAAACATTTATATATAAGAACACAAACGTA
AGTTTGTAAATAATAATAAAGGAGTAAGTCAATGGAAAAATCGATCAAAATAATGACAATAATAGGAATTGTGATTCAGGGTTTAG
CAACGGTATTTAGTTTACTATTGATGGTTTTAGCAGCATCAGGTGTAATGACTACAGATGTGTCAACAACAGTTAATGGTGAGGTTG
ACCCAGTTGATGCAGAAACAGCAGCAGCAATTTTCACTGTATTATTCTTATCCCTATTCATATTTGGAATCATTTCAATTATTTTAGG
TGCAATCGGTATGTTTAAAGCATCTAAAAACAAAAAAATGAGTGGTATATTGTTGATTATTGGAGCTGTAATAAGTGGTAACATAAT
TACATTTGCTTTATGGTTAGTCAGTGGTATTAAACTCCTTACTAATAACAAGCCTAAAGATGAAATAAGCGACTTATCATAAACATC
GTATATTGAAATTTCAAGATTTCTTAAGTAATTAAATAAAGTGCTCTCTTAGAAGTAGATTTTCAGTCAATAACTGCTTTTAAGAGA
GCATTTTGTATTTGATAATGCTCATAGCATCGGATAAAAACCATTGACGAACTTAAAAAAATACGTTAGACAATGCACTTCTAAATA
TTAAAGGCATCGACTAACGCATTGTTCAAATATAGTTAATTATTTTTATAAAATTGATGATGATCATTCAAGTAAGCATAGAATAAA

CCTATAATGAGTCCGCCTCCAATATAGTTACCGATAAAAGCCGCAGCGATATTCGAAATAGCTGGTATGAAGTGCAATGTATCAACT
TGATAAATTAAACCACCCATAAATAAGCAACTGTTGTAAACGACATGTTCATAACCCATAAAGGCGAATATGGTAACACCGAACAT
CATGACAAACATTTTTGCGAGTACATCGTCAATTTGCATGGCAATAACTAATGAAATATTGATAAAGAAATTGGCGAATATCGCTTT
CATTAATATACTTACAAAACCAGTAGACAACGTTTTATGCTCTATAACTGCTGATAACTGATTTAACATATCTGGCGTCATTACATTT
GAAAAACGCATGAAACTAAATAAAATAGCAGCACCTAAAATATTTCCTGCAAAGCACAATAAAAATATTTTCAATACTCTAGTCGG
TTTAATTACTTTATAATACAGGCCTACAGTAAAGTACATGAAGTTACTGGTTAGTAGTTCGGAGTTTGTAAATAAAATGAGTACTAA
CGCAAAACTGAATGTAATGGCGCTGGCCATATTCACAATGCCTGGCGGTAAATCTGGTTCGTGTGTTGCTTTAACTGATAATACGAA
GACCGTAATAATCCCGATAATAAATCCTGCCATCATAGCGCGTAATAAATAACGTTTTAAATAAACGCTTTGTAATATATCTTTCGT
TCTTATCGTTTCGACTACGTTATTTACCCAGTCGTCCCCATAAAAAATCTTATCCCATTTAATATGTTTCTCCTTCACAATGACATCCT
CGCTTTTCATTTGTTAATCAGAGTATACTATGAATCGTCTTATTTGTGAATAATTTCACAAAATTAATTTTAAAAACTAATATTTTTG
AATAACAGCTAGGATTTTTTAACAAATCATTTATTTTAAAATAATTAATCGTCAGTTAGTATCAATGAATAAATACAGATTTTCATGC
AAAAAAACCTAGTAATGATGCGACGATCATTACTAGGAGGTTATGCGTCTATTAAGTTGTCGCTTGTTTCGGACGGCGTATAACTAC
ATCGATGATGAAACCTATGATTGCCAAAAGAATGAATGGTACAAGCCAAGCTAAATCGATATCTGCTAAAGGTAACATCATAAACC
ATTTCAAAATAACACCGTGTAATAAGTTGAAACTATTTAGTATTTGTAAAATTGAAATAATCAATGTAATAACAGTTGCGAGTCGAT
AGGCCCAACTGAATCTGAATGTGCTAAACATGTTAGCAAATGATATCAGTACAAGTGCAATAGACACGGGATATATTAATGTCAAC
AATGGTACAGCAATTTTTAAAATCATTTCTAAACCAAGTGTTGTAAATAAGAACCCTATGATAGAGAAAATAAGTGCGAATATTTTA
TAAGAAAACTTAGGTACGTGTTTCTTAGTAAATGTGGCGCAAGCATTGACGAGTCCTATACATGTTGTTAAACATGCAAGTATCACT
GTCATACCAAATACTATGTTGCCAAATGAACCAAATACGCGTAATGAATTGTACGTCAATATATCAGTACCATCTTTAAAGTTTCCT
GGAGCTGTTGACGCACCTACATATGCCAACGCAAAGTAAATCATACCAAGTAATATAGCTGCAATAAATCCTGAAAAGCAGACATA
CTTTAATATTTTCATGCGATCTGTGAGGCCTTTAAACTTATAGCCATTGACAATGACTACGGAAAAAGCTAACGCAGCAACAAGATC
CATTGTAAAATAGCCTTCCAAACTTCCTGAAATGAAAGGATGTGTTATATATTTATCCTTAGGTGCACTTAGTGCAGATTCAGGGTT
GAAAATGACAGCAATACTTAATAGAGCGACCATTAATAGTAATAACGGTGTTAATAATTTACCTAAATTATCAACGATTTTCGATGG
ATTTAAACTAATCCAGTAAACGATGGCAAAAAAGACTGCTGCGAATATAATTAAAGTCCATTGGTTGTGCACAGGTAAAATGTGTC
TTGTACCAATTTCGTACGCGACATTTGCAGCACGTGGAATACCGTAAAATGCTCCGATAGACATGTAAATCACGACAGCAAAAATA
AACCCGAACCATGGATGTATACGATTGCCTACACTTTCAACACCTTCATCATAAAATGCAACAACAATAACAGTAATAAAGGGGAG
TAATATGCCTGTAAGGGCAAAGCCTAACATACCAATCCACATATTTGACCTGCTGTATGGCCAAGCATGGGCGGGAATATTAAATT
TCCGGCTCCAAAAAAATAGTGAAAATAACATGAGGCCCGAAATAATAACTTGTTTTTTCAATGTAATTTCCTTCCTAAAACTAGATAT
TCATAATAATTTAAAAAATCTGAAAAATAAAAACGTTCTTACTTTATCTTTAAATGACAATACTATGATTCTATTATTTTATAAAAAA
GATTGCAATAATAACGTCTTGCTATTATTGGATAATGACAATTAAAGTGTCGTTTTTATGAATTAAAATAGGTGGAAGTAAGTTGGA
AATACGTTGGAGCGTTGTGATTTTTATGAGAAATTGTAAGAGTGAGGACTGCTATTTATAAAAGGTAAACTATGGATGAGTTTATGG
TTGTTCCAATAGGTAAATAAGAGATAGCACACTGTACACATAATGATACGTGGATGAGTGAATATGTCTGATAGGAAGGCCTAGTC
ACTGAAATGAATTCAAGTTTATTACGTGATAAATCACAAATCTCTCTCATGTGATAGGTCTCCCATTAAATCATGCTTATATGAAAT
GTTCACATATTTGTTAGCTTTTCAAGAAATAATATTAAATATCTTTTCATAGTATAGCAATATTAAATAAATGCATTTATAATTTTAA
CTGTATTTTAAATATTAATCATGAGGTGATAAGATGAATAAAATTTCAAAGTATATTGCAATAGCATCATTATCGGTAGCGGTTACA
GTTTCGGCACCACAAACGACAAATTCTACAGCGTTTGCCAAAAGTTCTGCTGAAGTTCAACAAACACAACAAGCTTCTATACCAGC
ATCACAAAAGGCGAATCTTGGTAATCAAAATATTATGGCAGTGGCTTGGTATCAAAATTCAGCTGAAGCAAAAGCATTATATTTAC
AAGGTTATAACAGTGCAAAGACTCAGTTAGATAAAGAGATTAAAAAGAATAAAGGTAAACATAAGTTAGCTATTGCTTTGGATTTA
GATGAAACAGTTTTAGATAATTCTCCATATCAAGGCTATGCATCAATACATAATAAACCTTTCCCAGAAGGTTGGCATGAATGGGTA
CAAGCTGCTAAAGCTAAACCTGTCTATGGCGCAAAAGAATTCTTGAAATATGCTGACAAAAAAGGTGTCGATATCTACTATATTTCT
GATAGAGATAAAGAAAAAGATTTAAAGGCAACACAAAAGAACTTAAAACAACAAGGTATCCCTCAAGCTAAGAAGAGTCATATTT
TACTAAAAGGTAAAGATGATAAGAGTAAAGAATCACGCAGACAAATGGTTCAAAAGGATCATAAACTTGTCATGCTATTTGGAGAT
AATTTATTAGACTTTACAGATCCAAAAGAAGCTACAGCTGAATCTCGTGAAGCATTAATTGAAAAACATAAAGACGATTTCGGTAA
GAAATATATCATTTTCCCTAACCCAATGTATGGTAGTTGGGAAGCTACAATTTACAACAATAACTATAAAGCAAGTGACAAAGCAA
AAGATAAATTACGTAAAAATGCTATTAAGCAATTCGATCCTAAAACAGGCGAAGTTAAATAATATATGAATTGGACGTCTACATGT
ACTTAAAGGTATATGTAGGCGTTTTTAAATTGATCCTTAAATTTTAAATGTACCGTACAAAAATAATAGACAATTAATTGCATTTTCC
ATAAATTTGCTTTTAATATAAAAAGTTCGGGTATAATGAAGGATAAGTGTATGTATAACATTACAGAGCGCAACACGACGTACGTG
CATCTAAGAATATAAACAATGTTTAAATCATAGTAGTAGGGAGAGAAATGCATGTTTTTAGCTTGGAATGAAATACGGCGCAACAA
ATTGAAGTTTGGACTAATTATTGGTGTGTTAACGATGATTAGTTACTTGCTATTTTTATTATCTGGATTGGCGAATGGTCTTATCAAT
ATGAATAAAGAAGGCATTGATAAGTGGCAAGCAGATGCCATTGTTCTAAATAAAGATGCCAATCAAACTGTGCAACAATCTGTTTT
TAACAAGAAAGATATTGAAAATAAATACAAGAAGCAAGCTACTTTGAAGCAAACAGGGGAAATTGTGTCTAATGGCCATCAAAAA
GACAATGTTTTAGTGTTCGGTGTTGAAAAGTCATCATTTTTAGTTCCGAGTTTAATAGAAGGGCATAAAGCGACTAAAGATAATGAA
GTGTTAGCTGATGAAACACTTAAAAATAAAGGATTTAAAATTGGCGACACATTATCACTATCTCAATCAGATGAAAAATTGCATATC
GTAGGTTTTACAGAAAGTGCAAAATATAATGCGTCACCAGTCATTTTCACGAATGACGCTACCATTGCCAAGATCAATCCTAGATTG
ACTGGAGATAAAATTAATGCAGTTGTTGTACGTGATTCAAATTGGAAAGACAAAAAATTAAACCAAGAGCTTGAAGCGGTAAGTAT
TAATGACTTTATTGAAAATTTACCAGGTTATAAACCGCAGAACTTAACATTAAACTTTATGATTTCATTCTTATTTGTCATTTCAGCT
ACAGTTATAGGCATTTTCTTATATGTCATGACATTACAAAAAACAAGTTTATTTGGCATATTAAAGGCTCAAGGATTTACGAATGGC
TATTTAGCGAACGTAGTTATTTCGCAGACGCTCATATTAGCATTATTTGGTACGGCATTTGGCTTACTGTTAACAGGCGTTACAGGTG
CATTTTTACCTGATGCAGTACCTGTCAAATTCGATGTACTAACATTGCTCGTATTTGCAATTGTGTTAATGATTGTCTCTGTATTAGG
AAGTTTATTCTCCATTTTAACAATTAGAAAAATAGATCCGTTAAAGGCGATTGGGTAGGAGGTGTAGCAAATGTTGAAATTTGAAA
ATGTAACAAAGTCATTTAAAGATGGGAATCGTAACATTGAAGCGGTTAAAGATACAAATTTTGAGATAAATAAAGGTGATATTATA
GCATTGGTTGGACCTTCTGGCTCTGGTAAAAGTACATTTCTAACTATGGCAGGTGCTTTACAAACGCCGACATCTGGGCACATTTTA
ATCAATAACCAAGATATTACGACAATGAAGCAAAAAGCATTGGCAAAAGTTAGAATGTCTGAAATAGGTTTTATTTTACAAGCTAC
AAACCTTGTGCCATTTTTAACGGTAAAGCAACAATTTACATTATTGAAAAAGAAAAATAAGAATGTTATGTCTAATGAAGACTATCA
GCAACTTATGTCACAATTAGGGCTAACTTCATTGCTTAATAAGTTACCTTCAGAAATTTCAGGTGGTCAGAAACAACGTGTGGCGAT
AGCTAAAGCGTTATATACGAATCCGTCGATTATTTTAGCGGATGAACCTACCGCGGCGTTAGATACTGAAAATGCGATTGAAGTCAT
TAAAATTCTACGTGATCAAGCCAAACAAAGAAAGAAAGCATGTATTATTGTTACACATGATGAACGACTTAAAGCATATTGTGATC
GTTCATATCATATGAAAGATGGCGTCCTTAATCTTGAAAATGAAACAGTAGAATAGTTTTATTAAGCCGGTACATCATGTGCCGGTA
TTTTTATGTTTATGTATTATTTGAATAAACTTTCACATTCAATTAATAATAATTATTATCGAAAATTAGAAATATTCCGTGAAATATA
ATATTTTTTGTAGTAAAATGGCCTCTAAGTATTCAATATTTAAATATGGGGATTGAATATAAAATTATCGTAATGGGGGTCAATGGT
TATGGATTTATTGATAGGTACTTTATTTTTATTTTTGGTCTTAGTGATTTTTACATTATTTACATATAAAGCACCTAATGGTATGCGTG
CCATGGGAGCATTAGCTAATGCAGCAATCGCAACATTTTTAGTAGAAGCATTTAATAAATATGTTGGTGGCGAAGTATTCGGTATTA

AATTTTTAGAAGAGCTAGGAGACGCTGCGGGAGGTCTAGGTGGTGTCGCTGCCGCTGGATTAACAGCATTAGCTATCGGTGTGTCA
CCAGTATATGCATTAGTTATAGCAGCCGCGTGCGGTGGTATGGATTTATTACCAGGTTTCTTTGCGGGTTATATGATTGGATATGTGA
TGAAATATACAGAGAAATATGTGCCGGATGGTGTCGACTTAATTGGATCGATTGTCATCTTAGCGCCATTAGCTCGTCTTATTGCAG
TATTATTAACGCCAGTAGTGAATAGTACATTGATTCGAATTGGTGATATTATCCAAAGTAGTACGAATACGAATCCAATTATCATGG
GTATCATTTTAGGTGGTATTATTACGGTTGTCGGCACAGCGCCATTGAGTTCAATGGCATTGACAGCATTATTAGGTTTAACGGGTG
TACCTATGGCTATTGGTGCCATGGCAGCATTTAGTTCGGCATTTATGAATGGGACGCTATTCCATCGCTTAAAATTAGGTGATCGTA
AGTCTACGATTGCAGTAAGTATTGAACCTTTATCACAAGCAGATATTGTATCAGCCAATCCAATTCCAATCTATATTACAAATTTCTT
TGGTGGTGCGATTGCTGGTTTAATTATTGCTATGTCAGGTTTAATTAACGATGCGACAGGTACAGCTACACCGATTGCAGGATTTTT
AGTTATGTTTGGATTTAATCATCCGACGACAATTGTGATTTATGGTGTAGTAATGGCGATTGTAGGTGCGCTTGCAGGTTATCTTGGT
TCAATTGTATTTAAAAAATATCCAATTGTTACTAAGCAAGACATGATTAATCGAGGTGCAGTAGACGCATAGCATCATCATTTTGAA
TAGTAAAAACAAATAAAACATAGTAACGTGATTCAGTCGATGTAACAGTCGATAATGAGTCACGTTTTTTTATAGAAAAATATAAG
ACATAAAAATGTCATAATTTATAGTCGACAAATATCATACTGTATAAACATTTATCATTTTCTCGGGTGCCTTTTACGCGATGGAATG
AACTTACTTTTTACGAAATTATGCGTATTTTATAAACAAATATCATTGATATAACGGTAAATGTAAGCGTTTACAACAGAAATAACT
GCATGCTACGATATTTTTGTAAATTCACTGATTCAAGTATTTTAAGTCAATATGAGGAGGGATGTTATGAGCGATTCTGAGAAAGAA
ATTTTAAAAAGAATTAAAGATAATCCGTTTATTTCACAACGTGAACTTGCTGAGGCAATTGGATTATCTAGACCCAGCGTAGCAAAC
ATTATTTCAGGATTAATACAAAAGGAATATGTTATGGGAAAGGCATATGTTTTAAATGAAGATTATCCTATTGTTTGTATTGGCGCA
GCGAATGTAGATCGTAAGTTTTATGTGCATAAAAAATTTAGTTGCAGAAACATCAAATCCTGTAACGTCAACACGCTCTATTGGTGGC
GTAGCAAGAAATATTGCTGAGAACTTAGGTAGGCTTGGCGAAACGGTCGCTTTTTTATCTGCTAGTGGACAAGATAGTGAATGGGA
AATGATTAAACGATTGTCCACACCATTTATGAATTTGGATCATGTTCAACAATTTGAAAATGCGAGTACAGGTTCATATACAGCTTT
AATTAGTAAAGAAGGCGACATGACATATGGCTTAGCAGATATGGAAGTATTTGACTACATTACGCCTGAATTTTTAATTAAGCGTTC
ACACTTATTGAAAAAGGCTAAGTGCATTATTGTCGATTTGAATTTAGGCAAAGAGGCATTAAACTTCTTATGTGCCTATACCACGAA
ACATCAAATCAAATTAGTTATCACCACGGTTTCTTCCCCAAAAATGAAAAATATGCCTGATTCATTACATGCTATTGATTGGATTATC
ACGAATAAAGATGAAACAGAAACATACTTAAATTTAAAAATAGAATCTACTGATGATATAAAAATAGCTGCTAAACGCTGGAATGA
TTTAGGTGTTAAAAATGTTATTGTGACAAATGGCGTGAAAGAACTCATTTATCGAAGTGGTGAGGAAGAAATCATCAAGTCAGTTA
TGCCATCAAATAGTGTGAAAGATGTTACAGGTGCAGGTGATTCATTCTGTGCTGCAGTAGTGTATAGCTGGTTAAATGGGATGTCTA
CTGAAGATATATTAATTGCTGGTATGGTTAACGCAAAGAAAACGATAGAAACGAAATATACAGTTAGGCAAAACCTAGATCAACAG
CAACTTTATCACGATATGGAGGATTATAAAAATGGCAAATTTACAAAAGTATATTGAGTATTCTCGAGAAGTTCAGCAAGCACGGG
AGAACAATCAACCGATTGTAGCATTAGAATCAACAATTATTTCGCATGGTATGCCGTACCCACAAAATGTTGAAATGGCAACAACA
GTAGAGCAAATTATCAGGAATAATGGTGCCATTCCAGCAACCATAGCCATTATAGATGGCAAAATTAAAATTGGTTTAGAAAGCGA
AGATTTAGAAATACTGGCAACTAGTAAAGACGTTGCTAAAGTATCTAGAAGGGATTTAGCAGAAGTTGTTGCGATGAAGTGTATTG
GTGCTACTACTGTAGCGACGACGATGATATGTGCTGCAATGGCTGGTATTCAATTTTTTGTTACAGGAGGTATTGGGGGCGTCCATA
AAGGTGCAGAACATACGATGGACATTTCAGCAGACTTAGAAGAACTGTCTAAAACAAATGTCACTGTTATCTGTGCAGGTGCCAAA
TCAATTTTAGACTTACCTAAGACGATGGAGTATTTAGAAACAAAAGGCGTTCCAGTTATTGGATATCAAACGAATGAATTGCCAGC
ATTCTTCACTCGCGAAAGCGGTGTTAAGTTAACAAGTTCGGTTGAAACGCCAGAACGACTTGCTGACATTCATTTAACAAAACAGCA
GTTAAATCTTGAAGGCGGCATTGTTGTTGCTAATCCAATTCCATATGAGCATGCCTTATCAAAAGCATATATTGAGGCAATCATAAA
TGAAGCTGTTGTTGAAGCGGAAAATCAAGGTATTAAAGGTAAGGACGCCACACCGTTCTTGTTAGGGAAAATTGTAGAAAAAACGA
ATGGTAAAAGTTTAGCAGCAAATATAAAACTTGTTGAAAACAATGCGGCGTTGGGTGCTAAAATTGCTGTCGCTGTTAATAAATTAT
TGTAGGTGATGATACATGAATATTTTATTCGCTATCACAGGGATAGCATTTGCACTATTTGTTGCGTTTTTATTCAGTTTTGATCGTA
AAAACATAGACTTCAAAAAGACGTTAATAATGATATTTATTCAAGTGTTGATTGTGTTATTTATGATGAACACAACGATTGGTTTAA
CAATCTTAACTGCATTAGGCTCATTTTTTGAAGGGTTAATAAATGTTAGTAAAGCAGGTATAAACTTTGTTTTTGGAGATATACAAA
ATAAAAATGGCTTTACGTTCTTTTTAAATGTGTTGCTGCCATTAGTGTTCATTTCTGTATTAATAGGCATTTTTAATTATATTAAGGTA
TTACCATTTATTATCAAATATGTAGGTATCGCTATTAATAAAATAACGAGAATGGGGCGCTTAGAAAGTTACTTTGCTATTTCAACA
GCAATGTTTGGACAACCAGAAGTATATTTAACAATAAAAGATATTATTCCAAGATTATCTAGAGCGAAATTATATACAATTGCGAC
GTCTGGTATGAGTGCTGTTAGTATGGCAATGTTGGGTTCATATATGCAGATGATTGAGCCCAAGTTCGTAGTTACAGCTGTAATGTT
AAATATTTTTAGTGCGCTTATCATCGCCAGTGTAATCAATCCCTATAAATCTGATGATAGTGATGTTGAAATTGATAACTTAACTAA
ATCAACGGAAACGAAATCAGTGAATGGAAAAACAGGAAAACCTAAGAAAGTTGCCTTTTTCCAAATGATTGGTGATAGTGCGATGG
ATGGGTTTAAAATCGCTGTTGTAGTAGCCGTGATGTTGTTAGCGTTTATTTCATTAATGGAAGCAATCAATATCATTTTTGGTAGTGT
TGGTTTGAATTTTAAACAGTTAATTGGTTATTTGTTTGCACCTATCGCATTCTTAATGGGGATTCCTTGGAGTGAAGCTGTTCCAGCT
GGCTCTTTAATGGCTACAAAATTAATTACAAATGAGTTTGTAGCAATGCTAGATTTTAAAAATGTTTTGGGTGACGTATCAGCTAGA
ACACAAGGTATTATCTCAGTTTACTTAGTGAGTTTTGCTAATTTCGGTACTGTTGGTATCATCGTTGGTTCAATTAAAGGAATTAGTG
ATAAGCAAGGAGAAAAAGTTGCATCCTTTGCAATGAGGTTGCTACTTGGTTCAACTCTAGCTTCAATCATTTCAGGATCAATTATTG
GCTTAGTATTGTAAATTAATCGAAGTATCTAAATTAAATTCATGGCAAAGCTAAACCCCGTCACCAAGTTGGCGCAACAGCGCATCA
TAACTTAGTGACGGGGTTTTATCATAACAATCTACTTTTTCGTAGCCGTTTTTGAAATGTATGTTGATGGTTTATCTTTTTCAAAAATT
GTTAATCCCGTTATATCTTTTTTATGTTTTGAAGGGACAATGAAGCTAAGTATATAAGCAAAGACAAAAGCAACTGTAAATGAAATG
GTAGATACATAGAAAGGTGAGTTACCTTTGCCAACACCATTATAGATATAAGCAAAGATGATACCCAATATTAATCCACAAATAAC
ACCGAATGTATTCGTACGTTTAGTGAAAATACCAACTGCAAATACACCAGCTAATGGAACACCGAATAATCCAGTTACAAACAAGA
ATAAATCCCATAAGTCATTTGAATTAGAAGCAATTAAGTATAGTGACATTCCAAAACCGAAAATACCTGCAATGATGATAATCAAA
CGTGCAAAGTTAACTTCGTGTCGCTCGCTACCTTTTCCGAAGAAGCGTTGCTTAATGTCGATTGAAATACAAGCAGATATAGAATTT
AAACTAGATGAAATGGTAGACTGTGCAGCGGCGAAAATGGCTGCAATAAGTAATCCTGCTACAAATGGTGGCATCTCAGTCAAAAT
GAAATATGGCACTACAGATGATGTATTGAAGCCTTTTGGTAAAACAGCTTCATGTGTATAAAATGAATACAGCATTGTACCCATACC
ATAAAATAAGGGTGCTGAAATTAAAGCTAGGATACCATTTGTCCATAACGATTTATTTGTTTCTTTTAAACTATCAGAAGCTTGATA
ACGCTGCACGACGTCTTGACTCGCTGTGTATTGATACAAGTTGTTGAAAATATTTCCTAGGAAAATAATTGGAATGGCAGCTGCCGC
AGTGTTTAGCTTCCAATTATCTGCACTAATTAATTTTTTGTGCTCAATCGCATCTGCAAAGACAGTGCCAAAACCGCCTTTAATGTTC
ATAACACCTAGAATAATAATAACTAAAGCGCCGCCTAATAAAATGACGCCTTGAATGAAATCACTCCAAACCACACCTTCGAATCC
ACCTAAAAATGTATATAAAATACATAGTAAACCAACGAGTGATGCAACGATATAAGGGTTCATGTCTGATACAGATGTGATTGCTA
ATGTTGGTAAGTAGATAACGATTGCAACACGCCCTAAATGGTAAACGACAAATAATAATGAGCCAATAACACGTATGCTAGGGCCG
AATCTAGCTTCTAAATATTCATATGCAGATGTTACCTTTAACTTTTTAAAGAATGGAACATAGAAATAAATGAGTAATGGAATAATT
GCTACGATAGCGATGTTACCTGCAATATATGACCAATCTGTTAAAAATGCTTTCTCTGGTGTAGACATAAACGTAATTGCACTTAAT
GTAGTAGCATAAATGGAAAAACCAACTACCCAAGATGGCAAGCGACCACTTGCGGTAAAGAAACTATTCGTACTTTGACTCGCGCG
CTTGGTAAAATAAACGCCAATGAGTAACATAGCTAGTAGATAAATGATAACGGCAACCCAGTTTAGTGTGCCAAATCCAACTTCTTT
CATGGGCAACATCCCCTTTACAATGTATTGATTCTTTGATGTCTATAAATCGTATTTTGCAATGAGTTGATCTAATGTTTGTCGATGT

GCTTCGTTAAAAGGTTTGAAAGGTCTTTTCGGTAATCCTGCATCAATGCCACGATGACGTAATATTTCTTTCAATGTTGGATAAATCC
CCATTGATAACACTGTTTCGATAATGTCGTTTGAATCATGTTGTAGTTGGTAAGCTTCTTGAATTTGACCTTGTCTTGCTAAGTCGAA
GATTTTTCTAGCGCGGCGACCATTAACGTTATATGTAGAACCAATTGCACCATCTACGCCAGAAATCGTAGCTTGAACTAACATTTC
ATCAAAGCCAGATAAGATTAATTTGTCTGGGAATGCTTTTCTAATACGTTCGAGTAGGAAGAAGTTTGGCGCTGTATATTTAACACC
AACAATTTTTTCATGATTAAATAGCTCGCTGAATTGTTCAATAGAAATATTCACACCTGTTAAATCTGGTATTGCATAAATAATCATA
TTGTTCTGAGTTGCTTCGATAATATCGAAATAGTAATCTCTAATTTCTTCAAAAGTAAATGGATAGTAGAATGGTGTTACGGCGGAA
AGTGCATCATAACCGAGTTCTGTGGCATATTTTCCAAGTTCAATGGCTTCATTTAAATCTAACGAACCTACTTGAGCAATCAATTTCA
CTTTATCCCCAACTGCCTCTTTGGCAACTTTGAAAACTTGCTTCTTCTGCTCTGTATTTAATAAAAAGTTTTCGCCTGAGCTACCATTT
ACATAAAGACCGTCTAATTCTTCAGTTTCAATGGCATTTTGAGCAATTTGTTTGAGTCCTTGTTCATTTACTTGACCATTTTCATCAAA
AGGAACGAGTAACGCTGCATATAAACCTTTTAAATCTTTGTTCATTATGAAGTCCCTCCAAAAATCATTTGATAATATAGTTTACAG
CTATAATTGTAAACGCTATCATAAAATGTAACAATATCTTTTTGAAAATTGTAGTCATATTTATGTATAATTAATGAAAATGTTTTTC
AAAATCAATAGAAATGGAGTGAGTAAGGTGTATTACATCGCAATCGATATTGGAGGCACTCAAATTAAATCGGCAGTTATTGATAA
GCAATTGAATATGTTTGACTATCAACAAATATCAACGCCGGACAACAAAAGTGAGCTTATTACTGACAAAGTATATGAGATTGTAA
CAGGATATATGAAGCAATATCAGTTGATCCAACCTGTCATAGGTATTTCATCAGCAGGCGTTGTTGATGAACAAAAAGGCGAAATT
GTATACGCAGGGCCAACCATTCCGAATTATAAAGGTACTAATTTTAAGCGATTATTAAAATCACTGTCTCCTTATGTCAAAGTAAAA
AATGATGTAAACGCTGCATTACTAGGCGAATTGAAATTACATCAATATCAAGCAGAACGGATCTTTTGTATGACGCTTGGTACAGGC
ATTGGGGGTGCGTACAAGAATAATCAAGGTCATATTGATAATGGTGAGCTTCATAAGGCAAATGAAGTTGGGTATTTATTGTATCGT
CCAACTGAAAATACAACGTTTGAGCAACGTGCTGCAACGAGTGCATTGAAAAAGCGCATGATTGCCGGAGGATTTACGAGAAGCAC
ACATGTGCCAGTATTGTTTGAAGCAGCTGAAGAAGGTGATGATATTGCAAAACAAATATTGAATGAGTGGGCAGAAGATGTAGCAG
AAGGGATTGCCCAAATACAGGTCATGTATGATCCAGGGCTTATATTAATTGGGGGCGGTATATCTGAACAAGGAGATAATCTCATT
AAATATATCGAGCCGAAAGTTGCACACTATTTACCAAAAGACTATGTTTATGCACCAATACAAACGACTAAGAGTAAAAATGATGC
AGCATTATATGGCTGTTTGCAATGATAGTTGAAAGAAGGAGTCATTCTAAAATAGAATTTGAAACCGTTACGAGAGATGAGAGCTG
TTGTTAGTTCCACACATCACACTCTATCTAGGACCAATCTAAACTATATCAACCAACAGTGTGCCACAGACATATTAAATTGAAGAA
GCTGAGATATTAAAATTTTAGAAAATGAAAAAAATATTTGGTATTGAAATTAAAAAAGCACCTAGCAACTCGTTGGGACAATCACG
ATGATTGTCTACAGTTGCAGGTGGATTTGAATATACTACTAGTTATTTGTTGTCTAGGATAATAGATTTAGTATGTTGATAAGTTTGA
CTCAGATTCGTATTTTCTAATAAATGATAACTCACGATATCGATTAAAAAGAGTGTCGCAATTTGTGTGTTGATAAATTGATGGTCG
GTATTACGCGATTGATCCGTTGTTAAAAGTACTAAATCTGCACAATCTGTAAGTTTACTACCTTCAAAATTTGTGATGGCAACGACA
TATGCACCATGAGATTTGGCGACTTCCGCTGCAGAAATTAATTCCGAAGTATTACCACTATTTGACATAGCAATAAACATATCCGAA
TGAGATAGTAGGGATGCCGATATTTTCATTAAATGTGAATCGGTAGTAACATTACCTTTTAGCCCCATACGAATCATACGATAATAA
AATTCAGTCGCTGATAAACCAGAGCTACCTAGTCCAGCAAAGAGTATATGTCGACTTGATTGAAGTTTGTCGATAAAGGTTTGGATA
ATGTCGTTATCAATAAATTCACCAGTTTGTTGAATGATTTGTTGATGATATTTATGAATTCTTTGAATAATTGGGCTATTTTCAATAA
CTGTCTCTGTCATTTCTTGTTGAATATTAAATTTTAAATCTTGGAAATTCTCATAATCCAGCTTATGACTAAAGCGTGTCATCGTTGCT
GGTGATGTACCAATCGCATGGGCTAAGGAGTTAATCGTTGAAAAGGCATCGCTATAACCATTTTGTCTTATATAATTGACGATGCGT
TTATCAGTTTTTGTAAATAAATGTTGATAACGTTGAACACGATTCTCAAATTTCATTGTGTCACCCCTTCATCTTAATGATTACTATTA
TATATGAAAAATATTTTCAAGATAGTAAAAAGCATTGATAAAAATTATCTTAATGATATATTGTAAATGACTTTACGTGAAAAAACG
ACTTATGGAGTGAGGAATAATGTTACCACATGGATTAATAGTATCTTGTCAGGCACTACCAGATGAACCATTGCATTCATCTTTTAT
TATGTCGAAAATGGCATTAGCTGCGTATGAAGGTGGTGCTGTTGGTATTCGCGCAAATACTAAGGAAGACATTTTAGCAATTAAAG
AAACGGTAGATTTACCAGTTATTGGCATTGTGAAACGTGACTATGATCACTCAGATGTTTTCATTACTGCAACGTCAAAAGAAGTTG
ATGAACTGATAGAAAGCCAATGTGAAGTCATTGCATTGGATGCAACGTTACAGCAACGTCCGAAAGAAACGTTAGACGAATTAGTA
TCATATATTAGAACACATGCACCGAACGTTGAAATCATGGCTGATATCGCGACCGTTGAAGAAGCTAAAAATGCCGCACGACTTGG
CTTTGATTATATTGGCACGACGTTACATGGCTATACTAGTTATACGCAAGGACAATTACTTTATCAGAATGACTTCCAATTTCTAAAA
GATGTACTACAAAGTGTTGATGCAAAAGTTATTGCGGAAGGTAATGTCATTACACCGGATATGTATAAACGTGTGATGGACTTAGG
CGTTCATTGTTCAGTCGTTGGTGGTGCGATAACACGACCAAAAGAAATTACGAAACGTTTTGTTCAAATTATGGAAGATTAAATGAT
AACGATAAAAAAACGAGATGACCATCATTAATTAAAGGCACCTAATTATCTTAGGTGGCTGAATGAATGTAATGGGTTCATCTCGTT
TTGTTTGTTTATGATAGTGATTTTATTTTCAACTTTATCCAAAAATAAGTAAAGCGACGGGGATGGTGATTAATAGCGACAACGCCA
CGCGTAAAAACCAAATGATGATGAGTTTCCAGACAGGTATTTTAATTTCAGTTGCTAGTATACATGGCACTAATGCTGAGAAAAAG
ATAATGGCTGATACGCTTACTACACCGACGACAAATTTAGTACTCATTGCAGCTTTAGTTACTAACAAAGATGGTAGAAACATTTCT
ACAATAGAAATCGCTGACGCTTTTGCAAGTACAGCCTGATCAGCAATTGGGAAGATATAAATAAATGGATAGAATATATAGCCAAG
CCAATCAATGAATGGTGTATAGTTCGCTACAATCAGTCCTAAAAAACCAATCGATAATATAGAAGGTAAAATACCAACAGTCATTT
CTAAACCGTCTTTCAAATTGTCCCAAACGTTCTTCACGAGAGATGGTGTTAATGCATTTTGTATCATCGCCTCTGCATATGCAGTTTT
CAGTCTGCTTCCTTCAATAGCAACTTCTTGTTCTCCTTCTTGTCCGTTATAATATTCTGTTGATTCATTGCTGATTGGCGGTAGCCATG
CAGTAATTGCAGTCACGACAAATGTGATGACTAAAGTTATCCAAAAGTATAAATTCCAATGCGGCATTAATCCTAAAGTTTTAGCAA
CGATAATCATAAAAGTTGCTGAAACTGTTGAAAAGCCAGTCGCAATAATCGTGGCTTCTCGTTTGTTGTACATCCCTTGCTTATAGA
CACGATTAGTAATCAATAATCCTAAGGAATAACTGCCGACAAACGAAGCCACTGCATCGACAGCGGATTTTCCTGGTGTTTTAAAA
ATAGGTCTCATAATAGGCTCCATATAAACACCGACAAATTCTAATAAGCCATAGCCCACTAATAAAGAAAGCGCAATTGCACCTAC
TGGAATTAAGATACTTAATGGCATCATTAATTTTTCAAACAAAAACGGACCATAGTTAGCTTTAAATAGTATTGATGGACCGATTTT
AAATACATACATTATACCAATCATTGCACCTGCAACTTTAAATAATGTAATGACCAAGTTTGTGATTGAAGTCATAAAAGTACGTCT
CACTATTGGTAACGCTGTACCAATTAAAATCATAATCAGTGCAACATAGGGCATAAGTGGACCTATGATTGAGCGAATGGCTAGAT
GAACATGATCGACGAAAATAGTGTTGTTACCATTAATCGTAAAAGGAATAAAGAAACATAGTATGCCCACTAAACTATAGACAAAA
AAACGCCATGCACTTGGTTGTTGTGCATTAGAATGATATTGATTCATTAAAGCAACCCCTTTGTTTAAATGAATACACAAAACTGTA
TGATGCATCTTCCCCTTAATGAGATGAATCATTATTTTAATTTAGAAAAATCTGAAAACTTACTATAATTGTATAGTTTGAATTATTT
TCATACCAATACAAATTAACTAATTATATATAGATTGAAACTATATTACTTAATAAAATATTTATCTTAAATGTTGTTGTGTTGATTC
AACGCCACAACTAAAAGTGTTTATAAATTATTTGGAAATGCACATATTTGTAAATGATTAGTATCGATTTAATATCGTATTATTAAA
TTTTTATTAATTTTGTAGTCTTAATCAAAAAATAATATATGTCATGTTATATTGAAGGTGCAGTTGTTTTTCATTCTCAAGAGGGGGT
CAAAAAAATACTTTTGAGGTGATTATATGTTAAGAGGACAAGAAGAAAGAAAGTATAGTATTAGAAAGTATTCAATAGGCGTGGTG
TCAGTGTTAGCGGCTACAATGTTTGTTGTGTCATCACATGAAGCACAAGCCTCGGAAAAAACACCAACTAATGCAGCGGCACAAAA
AGAAACACTAAATCAACCGGGAGAACAAGGGAATGCGATAACGTCACATCAAATGCAGTCAGGAAAGCAATTAGACGATATGCAT
AAAGAGAATGGTAAAAGTGGAACAGTGACAGAAGGTAAAGATACGCTTCAATCATCGAAGCATCAATCAACACAAAATAGTAAAA
CAATCAGAACGCAAAATGATAATCAAGTAAAGCAAGATTCTGAACGACAAGGTTCTAAACAGTCACACCAAAACAATGCGACTAA
CAAAACTGAACGTCAAAATGATCAGGTTCAAAATACCCATCATGCTGAACGTAATGGATCACAATCGACAACGTCACAATCGAATG
ATGTTGATAAATCACAACCATCCATTCCGGCACAAAAGGTAATACCCAATCATGATAAAGCAGCACCAACTTCAACTACAACCCCG

TCTAATGATAAAACTGCACCTAAATCAACAAAAGCACAAGATGCAACCACGGACAAACATCCAAATCAACAAGATACACATCAAC
CCGCGCATCAAATCATAGATGCAAAGCAAGATGATACTGTTCGCCAAAGTGAACAGAAACCACAAGTTGGCGATTTAAGTAAACAT
ATCGATGGTCAAAATTCCCCAGAGAAACCGACAGATAAAAATACTGATAATAAACAACTAATCAAAGATGCGCTTCAAGCGCCTAA
AACACGTTCGACTACAAATGCAGCAGCAGATGCTAAAAAGGTTCGACCACTTAAAGCGAATCAAGTACAACCACTTAACAAATATC
CAGTTGTTTTTGTACATGGATTTTTAGGATTAGTAGGCGATAATGCACCTGCTTTATATCCAAATTATTGGGGTGGAAATAAATTTAA
AGTTATCGAAGAATTGAGAAATCAAGGCTATAATGTACATCAAGCAAGTGTTAGTGCATTTGGTAGCAACTATGATCGTGCTGTAG
AACTTTATTATTACATCAAAGGTGGTCGCGTAGATTATGGTGCAGCACATGCAGCTAAATACGGACATGAGCGCTATGGTAAGACA
TATAAAGGAATCATGCCTAATTGGGAACCTGGTAAAAAGGTACATCTTGTAGGGCATAGTATGGGTGGTCAAACAATTCGTTTAAT
GGAAGAGTTTTTAAGAAATGGTAACAAAGAAGAAATTGCCTATCATAAAGCGCATGGTGGAGAAATATCACCATTATTCACTGGTG
GTCATAACAATATGGTTGCATCAATCACAACATTAGCAACACCACATAATGGTTCACAAGCAGCTGATAAGTTTGGAAATACAGAA
GCTGTTAGAAAAATCATGTTCGCTTTAAATCGATTTATGGGTAACAAGTATTCGAATATCGATTTAGGATTAACGCAATGGGGCTTT
AAACAATTACCAAATGAGAGTTACATTGACTATATAAAACGCGTTAGTAAAAGCAAAATTTGGACATCAGACGACAATGCTGCCTA
TGATTTAACGTTAGATGGCTCTGCAAAATTGAACAACATGACAAGTATGAATCCTAATATTACGTATACGACTTATACAGGTGTATC
ATCTCATACTGGTCCATTAGGTTATGAAAATCCTGATTTAGGTACATTTTTCTTAATGGATACAACGAGTAGAATTATTGGTCATGAT
GCAAGAGAAGAATGGCGTAAAAATGATGGTGTCGTACCAGTAATTTCGTCATTACATCCGTCCAATCAACCATTTGTTAATGTTACG
AATGATGAACCTGCCACACGAAGAGGTATCTGGCAAGTTAAACCAATCATACAAGGATGGGATCATGTCGATTTTATCGGTGTAGA
TTTCCTAGATTTCAAGCGTAAAGGTGCAGAACTCGCCAACTTCTATACAGGTATTATAAATGACTTGTTGCGTGTTGAAGCGACTGA
AAGTAAAGGAACACAATTGAAAGCAAGTTAAATTCATATTCGTAATTTAATATGCTATGTATTTCGTGCTACTAACATGGCACAGCA
GATATAAGTAGCATCACAGTGTTGAATTTAAAAATAGTAAAGTGAAATAAAGCGCCTGTCTCATTAGAGAAAACTAAAGGGACAGG
CGTATCTGTTTATGAGCTTAATAAATTGTATGAATAATATGGTTGATCGAATAACTGTTTATCATGATGATAAATTGAGTTTTTTAAA
ATAATGATATATTACATCATTGTTATAGCGTTTAAGAAATCAACAACTTTACGATAAATAGTGATTGCTTCGTCATTAGGTCTACGAT
CAAAATCATGCTCGTTTTTATTCACGCGTTCAAATGTTGAATGTGGAACATGATTCATGATATGTTCGCTTTCCTCAACGGGAACATC
ATAATCGCCATTACAATGCGTAATGAACACCGGGGGAAGTGTTTTAAGTTCATATGGTGCAATATTATATTTTGAATCAGTATAATC
AGCAATGTTAATCATATTTATCCATTTACCTGTGCCACGTGCATAAACGTAGATTAAAAAACGTTGTGCGATTTGATCTTGAACAAC
CGGTGTTGGTGAAGTGAGTTGTGCAATCATTGTTTCGTTTACGCTTTGAGCTATTTTTGCGTAATAACTATTAGTTGTTTTAAAAGGT
TCAGTGTTGATGCGACTATAACCATAAAAATCAATAACACCATCAATATCTCTGTCTCGTGCAATTAATAGACTTAAATATGCACCT
GATGATCTGCCAAAGGTAAAAATAGGGCAATTAGAATATTGTGATTGAATCGCATCGAATGATGCGTAGACATCCTCAATAATGCA
ATCGAGACTTACTTCTGGTAATAAACGATAACTTAGTTGAATTAAATCGTAATGTTCCGTAAGGATATCGATATACTGTGGGATAA
ATCGTTAGCTTTACCGAACATTAATCCACCACCGTGGATGTAGACAATAGCGCCTTTTGTTGGTTGATTTTTTGCTTTAATAATTGTG
TAAGGTAATGCAAATGCATCTTTAGTAATTACTTTATCTTTAATTTCAGTCACGATTTAATAGGCTCCTTATTTTTGATATTGATGTCA
TTATAACACTGTCTTAAATTTCCATGAAAAATAGTCTTAAGACGATGAGTCATGATAATTCTGTTCCAATTGACGTAAAGCGTCACG
GGTATGCTTCTTTAGACCTTCCCCATAATCCATCATTTTAACAATATCTTTAAAAGCAGCATGTGGAATGGCTAAATCTTCTAAATCT
GCCATAGAAAATTCAAGATTGATATCATGTGGTCGCTGTTCAGCAAGTTTATGCACAAAGTCAGGTTCTGTGACAAAAGGCGAAGA
CATGCCGACCATATCTGCATGTTGTAAAGCATCTAAAGCAGACTCTGGAGAATTAATCCCGCCACTTGCAATTAAAGGGATACGAC
CTGCTAAATGTTCATAGACAATTTGGTTAACTGGTCGACCGAAATGATCACCTGGTGTACGAGACGTATTTTGATAAATATGTCGAC
CCCAGCTAGCGATTGCTAAGTATTGGATGTTTGAAACGTCCATGACCCAATCGATTAATTGGTTGAACTCGTCAATGGTATATCCTA
AATCACTGCCTCTGGTTTCTTCTGGCGTTGCTCGAAATCCTAAAATAAAATTGTCAGGTGCTTCTTTATCAATCACTTCTTGTACCGC
ACGCATAACTTCTAAACATAATCTTGCACGATTTTTTAATGAGTCGGCACCGTAATGGTCTGTACGTTTATTCGAAAAAGTTGAGAA
AAATGTTTGAATCAGCAAACGTTGTGCAATCGAAATTTCCACACCATCAAAACCTGCTTTAATCGCGCGTAATGTAGCATCGCGATA
CTGCTGAATGATGCTATTGATTTTCTCATGAGACATGGCGATAACATCGTGTTCAATCGGTGAATGCAAAGTCATAGGACTTGGTCC
ATACACCTTTCCAAAATTTAAAATGGCTTGATTTGAAAAACGACCAGCATGCGCTAGCTGGATAATAGCGAGGCTACCATGTTGTTT
CATCGTAGATGCCATGTTAGTTAATCCAGGGATACAAGCATCATGATCAATATTAAAGCCATATTCAAACAATTGACCATAAGGTTC
AATGTAAGCAGCGCCGGTGACTTGCATTCCAGCTGAATTAGAGCGACGTGCAGCATAAGCCAAGTCTGCTTTTGTAATATAGCCTTC
TTTTGTTGATGTGTTTACGGTCATTGGTGATAATACAAAGCGATTCGAAATTTTGATGCCATTAGGTAAGTGGATTGATTGTAAAAG
TGGTTTGTATCGGTACATACTATGATTCCTTTTCTATTCAATATTGTTTTCAAAGTACCATGGAAAGAATGAATAATCAATGATGAAC
AGTCTTGATAGAATAGAATTGGTACATGGAAAACATTTTTAAAATTGAACTAATGAATGGCATTTGTAGGTCTGAAAATATGAATAT
GGAAAAGAAAAATAAAGGCGAAAAGATATAAAAGTTAATTGAAAAACGTTATCATATACGTGGGTATATGAAGAGGGAATGGTAT
TAAGAACGCTAAAATGTTATGTCGATTTGACATGACAGGATAAGTTTGGAGATGACGGATTGGTTAAATTAAGCGTATTAGACTAT
GCCTTAATAGATGAAGGTAAGGATGCACAAAAGGCATTGCAAGATTCAGTGACACTTGCAAAATTAGCAGATCGACTTGGCTTTAA
GCGAATTTGGTTTACGGAACATCATAATGTACCAGCGTTTGCGTGTAGTAGTCCAGAACTTTTGATGATGCATACATTGGCGCAGAC
AAATCACATACGAGTTGGCTCTGGTGGTGTGATGCTGCCGCACTATCGACCTTATAAAATTGCTGAGCATTTTAGAATGATGGCAGC
GTTATATCCAAATCGTATTGATTTAGGTATTGGTAATAATCCAGGTACTACTATGGTAAAGCAAGCTTTAGATGGAATAAATCCTAC
ATATGATAGTTACGATGAATCGATTTCGTTATTACGTGATTATCTTACAATAAAGGATAAACCAAGTGCGCATACGTTAGGTGTCCA
ACCACACATTGATCATTTTCCAGAAATGTGGTTATTAAGTAGTAGCGCAACATCTGCCAAAATGGCTGCCGAACTAGGTATAGGGCT
TTCTGTTGGAACATTTTTGCTACCAGATATAAATGCGATACATGCAGCGAAGGATAACATTGATATTTACAAAAAACATTTCCAAGC
ATCAACGATTAAAATGGACGCAAAGGTGATGGCATCTGTATTTGTCATTGTAGCTGATAACGAAGCGGAAGTAGCAGCATTACAAC
ATGCCTTAGATGTTTGGTTATTAGGTAAATTACAATTTGCAGAATTTGAAGATTTTCCTTCAGTAGACACAGCACAAAAGTATAAGC
TTAATGATCGAGACAAAGAGATGATTCAAGCACATCAAGCACGCATCATTGCAGGTACACAAGAACAGGTTAAAGCACAATTAGAT
GATTTCATTGCTACGTTTGAAGTTGATGAGGTGTTAGTAGCACCGCTTATTCCAGGTATTGAACAGCGTTGTAAAACATTAAAATTA
CTCGCGGAAATTTATTTGTAGCATTTTAAATAGAAGATAAAGGATGAAGATAAGATGAAAAAGTTAGCCAATTATTTATGGGTAGA
AAAAGTAGGAGATTTGTATGTGTTTAGTATGACACCTGAATTGCAAGATGATATTGGGACAGTAGGTTATGTTGAATTCGTAAGTCC
AGATGAAGTTAAAGTGGATGATGAAATTGTGAGTATCGAAGCATCGAAAACGGTCATTGATGTGCAAACGCCATTGTCAGGAACGA
TTATTGAGCGAAATACAAAAGCGGAAGAAGAACCGACAATTTTAAACTCTGAAAAAACAGAAGAAAATTGGTTGTTCAAATTGGAT
GATGTCGATAAAGAAGCATTCCTAGCATTACCGGAGGCTTAAATGGAAACGTTAAAATCAAATAAAGCGAGACTTGAATATTTAAT
CAATGATATGCGTCGAGAGAGAAATGACAATGACGTATTGGTAATGCCATCTTCATTTGAAGATTTGTGGGAATTATATCGAGGCTT
AGCAAATGTCAGACCGGCATTACCTGTAAGTGATGAATATTTAGCTGTACAAGATGCTATGTTAAGTGATTTGAATCGTCAACATGT
TACGGATTTGAAGGATTTGAAGCCGATAAAAGGTGACAATATCTTTGTTTGGCAAGGTGATATCACGACGTTAAAAATCGATGCTAT
TGTTAATGCTGCAAATAGTCGTTTTCTAGGATGTATGCAAGCTAATCATGACTGCATTGATAATATTATTCATACAAAAGCGGGTGT
TCAAGTTCGACTTGATTGTGCAGAGATCATTCGACAACAAGGGCGCAATGAAGGTGTAGGTAAAGCCAAAATAACACGTGGATATA
ATTTGCCAGCAAAGTATATAATTCATACGGTTGGTCCGCAAATACGTCGATTGCCTGTTTCAAAGATGAATCAGGACTTGTTAGCTA
AATGTTATCTTAGCTGTCTTAAATTGGCTGATCAACATAGTTTAAATCATGTCGCTTTTTGCTGTATATCTACAGGTGTATTTGCTTTT

CCTCAAGATGAAGCAGCAGAAATTGCTGTTCGAACAGTAGAAAGCTATCTCAAAGAAACAAATTCAACATTGAAAGTCGTGTTCAA
TGTATTTACAGATAAGGATTTACAACTGTATAAGGAGGCATTTAACCGTGATGCAGAGTAGTAATTGGAATGCAATGTCTCTGTTAA
TGGATGACAAGACAAAGCAGGCTGAAGTATTGCGTACTGCGATTGATGAAGCAGATGCGATAGTGATTGGAATTGGTGCAGGCATG
TCTGCATCTGACGGATTTACATATGTAGGAGAGCGTTTTACGGAAAATTTCCCAGATTTTATTGAAAAATATCGCTTCTTTGATATGT
TGCAAGCGAGTTTACATCCTTATGGCAGTTGGCAAGAATATTGGGCATTTGAGAGTCGTTTTATTACATTAAACTATTTAGATCAAC
CTGTAGGTCAGTCTTACCTCGCTTTAAAATCCTTGGTGGAAGGTAAACAGTACCACATTATAACTACGAATGCAGATAATGCTTTCG
ATGTAGCTGATTATGATATGACTCATGTATTTCATATACAAGGGGAGTATATACTGCAACAGTGTAGTCAGCATTGTCATGCTCAAA
CGTATCGCAATGATGATTTAATTCGTAAAATGGTTGTTGCGCAACAAGATATGCTTATACCTTGGGAGATGATTCCAAGATGTCCAA
AATGTGATGCCCCAATGGAAGTGAATAAACGTAAAGCGGAAGTTGGGATGGTTGAAGATGCTGAATTTCATGCGCAACTACATCGT
TATAATGCTTTTCTAGAGCAACATCAAGATGATAAAGTGTTGTATTTGGAAATTGGAATTGGTTATACTACACCACAATTTGTGAAG
CATCCTTTTCAGCGTATGACACGTAAAAATGAAAATGCACTTTATATGACGATGAATAAAAAGGCATATCGCATTCCGAATTCAATT
CAAGAACGTACCATACATTTAACTGAGGATATCTCAACATTGATTACAACAGCACTCCGGAACGATAGCACAACGCAAAATAACAA
CATTGGAGAGACAGAAGATGTACTTAATAGAACCGATTAGGAATGGAGAATATATTACTGATGGTGCGATTGCACTCGCTATGCAA
GTTTATGTTAATCAGCATATCTTTTTAGATGAAGATATTTTATTCCCTTATTATTGTGATCCAAAAGTGGAAATTGGACGTTTTCAAA
ATACTGCTATAGAAGTGAATCAAGATTATATAGATAAACACAGTATTCAAGTAGTTCGCCGAGATACTGGTGGTGGCGCTGTGTAT
GTTGATAAAGGTGCCGTTAATATGTGTTGTATTTTAGAACAAGACACTTCAATTTATGGTGATTTTCAACGATTTTATCAGCCAGCTA
TAAAGGCGTTGCATACATTAGGTGCAACAGATGTGATACAAAGCGGTAGAAATGATTTAACATTGAATGGTAAAAAAGTGTCAGGC
GCCGCAATGACATTAATGAACAATCGTATTTATGGCGGTTATTCGCTATTACTTGATGTTAATTATGAAGCAATGGATAAAGTGTTA
AAGCCTAATCGCAAAAAGATTGCATCGAAAGGGATTAAATCTGTGCGTGCACGTGTTGGTCATCTTAGAGAAGCACTGGATGAAAA
GTATCGTGATATAACAATTGAAGAATTTAAAAATTTAATGGTGACGCAGATTTTGGGAATCGATGACATTAAAGAGGCAAAACGAT
ACGAATTATCTGATGCAGATTGGGAAGCGATTGAAGAATTGGCGAGTAAAAAGTATAAAAATTGGGATTGGAATTATGGGAAGTCA
CCTAAATATGAATATAATCGAAGTGAAAGATTATCATCAGGTACGGTAGACATAACAATTTCTGTTGAACAAAATCGTATCGCAGA
TTGTCGTATTTATGGGGATTTCTTTGGACAAGGTGATATAAAAGATGTGGAAGAAGCATTACAAGGAACAAAAATGACAAGAGAAG
ATTTAACGCACCAGTTAAAGCAATTAGACATCGTTTATTATTTTGGCAATGTTACGGTAGAATCATTGGTTGAGATGATTTTAAGTTA
ATATTGTTTAGGCGTTAATGTCACACCCTCAATGAATATGTGTATTTGCTTTTAAAATGGTGACTATGTAGTTATGTAATTTGATCAT
ACGATTGAAAATGAATTTAGTTAATTAGCAAAGACATCATATTTATAGCGTTTGTGTTTATTAGTGATTGGGTATTTGAAAATGGTT
AATGATAGTTTTTAAAGGAATAAATTCATTTTCATTTATATAAGTAAGATTAAATACTAGCTACTAAAGTCTATTTATATACGTCAAA
ATATAATGACTGCTATAATGAGTAATCAATAGACACAAAGAGGAGATTATGTGATGAATAATAAAGTATTAGTAACCGGTGGTACA
GGGTTTGTTGGCATGCGAATTATTTCACGATTATTAGAACAAGGTTATGAGGTACAAACAACGATACGTGATTTAAGTAAAGCTGAT
AAAGTAATTAAAACAATGCAAGACAATGGCATTTCCACAGAGCGATTAATGTTTGTCGAAGCGGATTTATCACAAGATGAACATTG
GGATGAAGCAATGAAAGATTGTAAATATGTCTTGAGTGTAGCATCTCCGGTGTTTTTCGGTAAAACAGACGATGCAGAAGTGATGG
CGAAGCCTGCCATTGAAGGCATACAACGTATTTTAAGAGCTGCAGAACATGCTGGCGTGAAGCGTGTGGTGATGACTGCAAACTTT
GGTGCAGTTGGTTTTAGCAATAAAGATAAAAATTCAATCACAAATGAAAGTCATTGGACAAATGAAGATGAACCAGGCTTATCAGT
ATATGAAAAATCAAAATTGTTAGCTGAAAAGGCAGCGTGGGATTTTGTTGAGAATGAAAATACAACAGTAGAATTTGCCACAATCA
ATCCAGTTGCAATTTTTGGGCCATCATTAGATGCACACGTTTCAGGAAGCTTTCATTTATTAGAAAATTTATTGAATGGTTCAATGAA
ACGTGTACCGCAAATTCCATTGAATGTTGTTGATGTGAGAGACGTAGCTGAACTACACATTTTGGCAATGACAAATGAACAGGCTA
ATGGCAAGCGATTTATTGCTACAGCTGATGGACAAATTAATTTGTTGGAAATTGCAAAATTAATTAAAGAAAAGAGACCTGAAATA
GCTCAAAAAGTTTCTACTAAAAAATTACCAGACTTTGTTTTGAGTCTAGGTGCTAAATTTAATCATCAAGCTAAAGAAGGTAAACTT
TTATTAGATATGAATCGAAATGTAAGTAACGAACGAGCAAAAATACTTCTTGGTTGGGAACCGATTGCGACACAAAAAGAAGCAAT
TTTAGCAGCTGTCGATAGTATGGCTAAGTATCATTTAATATAATACAAAACACCGTCCATAAGTAAGTGGCTTTCCTCTTGATAAAA
GAGGCATTCACTCAATATGTGACGGTGTTTTATATATTTACGTTAACACTATTGCTGTGGCTTTTGACGGCCTTTTAATAAAATTGCT
GTCGCACCTACGATAATAATAAATAGAATCGCACCAAATAATCCCATATATTTTACTGCGTTACCGAACACGATACCGACAGTTAAA
AAGTCTGTATCTGAGAATGTTGTTGCAGCACCACCTAATTCGCCTAAAAATGGCAAGAATAATAATGGTAAAAACGTGATTAGGAT
ACCATTTAGAGCGGCGCCAGCAACAGCACCTTTAATACCGCCTCTTGCATTACCGAATACAGCAGCCGTTGCACCTAAGAAGAAGT
GTGCAACTACGCCAGGTAAAATGACGACGCCACCAAATAAGAATAAGATAAACATACCGATGACACCTGTAATAAAGCTGACAAA
GAATCCAATTAATACTGCATTTTGTGCATAAGGGAACACAATAGGGCAGTCTAATGCAGGTTTAGAATTTGGTACAAGCTTTTCAGA
AATTCCTTTAAATGCTGGGACGATTTCAGCTAAGATTAAACGAACGCCCGTTAAAATAATAAATACACCAGCAGCAAATGTCACAC
CTTGAATTAAAGAAAAGACAATAAAGTTTTGACCATCACTAATAGATTCGTGTACATAACTAACGCCCGCAAATAAGCATGCGATG
AAGTAAAGTAATGCCATCGTAATCGAGATACTAATTGTACTTTCTCGTAAGAAACTTAAGCCTTTTGGAAATTTAATCTCTTCCGTTG
ATTTAGACTTACCTTTGAATAATTGACCTACAGCACCTGCGGCAAAGTAACTGATTGAGCCAAAATGACCTAACGCTACTTGGTCAT
TCCCTGTAATTTTTCGCATTGTAGGTTGGAGTAATGCAGGTAAGACTGCCATGATTAATCCTAATACGAGTGCGCCGATAACAATCG
TTAGCCAGCCTTTAATATGACTGACTGTTAAAATGATTGCTAAAAACGCAGCCATGTAAAATGTATGATGACCTGTTAAAAAGATAT
ATTTTAAATTAGTGAAGCGGGCAATTAAAATATTAACAATCATGCCACAGACCATGATGAGTGCAGCTGTTGTTCCAAAATCTTTTA
AGGCTAGTGAGACGATAGCTTCGTTGTTAGGTACGATACCTTGCACACCAAATGCGTGTTGGAATATTTTGCCGAATGGTTCAAGAG
ATCGAACGACGACATCAGCACCTGCACTTAAAATTAAGAAGCCTAATATCGTTTTAATGGTTCCTGAAGTGATCGTTGCGGCAGGTT
TTTTCTGAACGATTAAACCTATAAAGGCAATCAGTGCAACAAGAATGGCTGGTTGACTTAAAATATCGACTATAAAATTAAGGATT
GCTTGCATAGGTCGTACCTCCTTTAAATCATGTTAAGTTGTTGTAATTTTTCTGAGAGCTTTTGTTGTAATTCAGCTTTGTCTAAAATA
TTATCAAGAACTAAGACATCCCCTAGACGTTCGGCATTTTCAGCTAAATCTCTACCACAAATAAACAAGTCAGCCATCTCTGGACTT
GCTGTCATAATGTCACTATGTTCAACTTCGATATCAGATGGTGCATTAAGTTGCCTAAGTGCTTCTTGTGCGTTCATTTCTACCATAA
AACTACTTCCTAAACCGTGGCCACATACTACTAAAATTTTCATATTAATCATGCTCCTTTAAAATGTTTTTAATGTCTTGTGCGTTTGT
TGCAGTTAATAGTTGCTGGACTGTTTGGTTATCGCCCAGTATGGTTGCTAAATTTTGTAATACAGATAAGTGTGAATGATTGTCGATG
GCACTCAATACAAAAATGAGTGATGCGTAGTGATCTTCATCACAGAATGCCACATGTTGGTTCAACTTTAATAGACTTAAACCAACT
TGATGTACGTCATTGTTCGGTCTTGCATGTGCAATTGCAATTTCAGGTGCGATAACGATATAAGGTCCAAGTTCATTAACGCTATCA
ATCATTGCTTGAACATAGCCTTGTTCAATAATTTGTTCTTGTAGTAATGGCTGAGAAGCTATAGTTATAGCTTCAGTCCAATCATTTA
CTTGTTCTTTTACAATGATGCGTGTTGTTGACAAAATGTCTAATGACACGTTATTAAGCCTCCTTTGTCATAGTTAAAGCAATGTGTT
GTTTAATTTTAAAAATATTCCCATCTAAGAAATCTTGTCGATATAAGTCGTTGCTTAAGCATTCGCTTAACTGTCCCAATGCCTTTAA
ATGTGCATTGGGGTGGTCCGTTGCTAATGTAATTACAAGGTGAACGGGATCGTTAGCTTTACTACCAAAGATAATCCCTTCAGTGAA
ATATGTTAGTGCGAAACCTACACCATTCTGTACATAATCAGTACCAGCGTGAATAAGTGCAATATGTGGACTAATGACCATATATGA
CCCGAATTGTTCAAATTGTTTTAAAATTGCAGCTGTATAATTTGAATAGACAATGCCATCATTGATTAAAGGTTGCACAGCCACTGC
AATTGCGGATTCAATTGATAATGGTTGTTTATTTATAATGATGCGATGTTCAGGCAATAAATCTGCGAGTGACTTGCCATCAGTTGC
CATTTTCATGACTCGTTGTTCTCTTGAGTCATTGATAATTTGATTCAATTTTTGACGAGATTGTTGATTGATAAATGGATCGACATGA

ATAACTGGTACAGCTGATATTTCACAAGGTACTGTTGAAATGACATAATCAATGTTATCTTGCAATAATCGACTTTCTTCCAATTGAT
AAATGGAATAGGCATCCCAAATGTGAAACTCAGGATACAGGTGATTTAGTTTTGATTTTAAAAGTTGCGACGTGCCTATACCAGAA
CCACATAGTAAGACAACCTTAATCATTGATTGTTTATGTGTTGCAACACGCTCTATACTTGATGCGAAGTGAATTGTAATGTATGTTA
ATTCATCTTCGTTGAAGCGAATAGCAGCATCTTGTTCAATTGGGCTAATATGCTTGCTAACGGCTTCAATGATTTGAGGATAGCGAC
GCATAACTTCTTGCCTCAAAGGATTAGGTTGTAGCATATCATATTTAATACGATGTATAGCTGGTTTGATATGTGTGATCAGACTGGT
ATGTAACTTGTTGTCTTTTGACATATCAATGCCTAATTCTTGGCTAACACAAGTGATTAATTCATGTATATTTTGCGATAAATCATGG
TATTCAAAGGCAATTGAAGACGCTGTATGTTCGGTCATTTTAGAGCCTAGTAAATGTAACGTGATAAAGATAATTTCAGACTCTGGA
AATGTGACATTACAACTACGTTCTAAGTTTTCTATCATTTTTGAAGCAATAGCATACTGATTAGTATGTCGCCATTTATCAATTTCAT
TGATAGGTATATCGAACGAAAAATTTTCATTTAGACGCTGAATGGCAATGAGTATATGATAGATTAAGCCATCGATAGCCGACTGA
ACTAAATGATAATTTTCACTATTTAATGTCTTAATAATGGCACGGCGAACCAATGCGATTGATTCTGAATTAAAGATATCCGCCTCT
ATAAAAGGTGCAGCTTGTTTCATATATTGATGTATAAAGTGTGCATACGCTTTACGATAGTGATCTTCCTCACCAATAATATTGAATC
CTTTATTGTGGACATAATTTAACTTTAAATGGTATTGATCTAGTTGGGCTTGAATCATTTTAATATCATCTGCAATTGTCCGACGCGA
AACATTAGCATCTTGCGCAAGTTGCTTTGTTGAAACAGGATCGGTTGTTTCGAATAACTTTAAAGCGATATGTGTGAGTCGTTCATCT
TTTGAAAAATGAATTTGATTTGTTAGATTGTGCTCTAATTCATTTAATAGTGTCGCGTGAGCTGTTGTTACTTTGATGCCTGCAGCTTT
ATTACGGCTGACTTGGTAATGATAAGTTTCAGCATATTGCTCAATATATGCTATATCATATTGAATGGTACGAGGTGATACACCAAG
TTGATTAGCAATGGTATTGATTGGAATAAACGTTTGCTCATGAATTAAAAGATACAAAATTTCGATTTGTCTATAACTTAACAACGT
AATATCCTCCTATTTGTAATTGTAAGCGATTTCTTAAAAACGTAGATATGCAATCTCTTTCATATTTTAATCCGAAAAATTGCATATC
AAAATGTTTATGGCGCAAGATTTTATAGGAACTTTTAAAATAAATTAGATATTCATGTTGACAATTTAAAAATGTCGCAGTATATTT
AGTTAGACATCTAACGAAATGGTGGTGCAATAAATGGAATTCACTTATTCGTATTTATTTAGAATGATTAGTCATGAGATGAAACAA
AAGGCTGATCAAAAGTTAGAGCAATTTGATATTACAAATGAGCAAGGTCATACGTTAGGTTATCTTTATGCACATCAACAAGATGG
ACTGACACAAAATGATATTGCTAAAGCATTACAACGAACAGGTCCAACTGTCAGTAATTTATTAAGGAACCTTGAACGTAAAAAGC
TGATCTATCGCTATGTCGATGCACAAGATACGAGAAGAAAGAATATAGGGCTGACTACCTCTGGGATTAAACTCGTAGAAGCATTC
ACTTCGATATTTGATGAAATGGAACAAACACTCGTATCGCAGTTATCTGAAGAAGAAAATGAACAAATGAAAGCAAACTTAACTAA
AATGTTATCTAGTTTACAATAAATGATAAGTGTGACTGGTAGAAATCAGTCACTTTGTCTTTAATATTATAGTTAGATATCTAATTGT
TAGTAAGCTAATTATTGGAAAAGACAAGGAGTATTGAACAATGAAAGACGAACAATTATATTATTTTGAGAAATCGCCAGTATTTA
AAGCGATGATGCATTTCTCATTGCCAATGATGATAGGGACTTTATTAAGCGTTATTTATGGCATATTAAATATTTACTTTATAGGATT
TTTAGAAGATAGCCACATGATTTCTGCTATCTCTCTAACACTGCCAGTATTTGCTATCTTAATGGGGTTAGGTAATTTATTTGGCGTT
GGTGCAGGAACTTATATTTCACGTTTATTAGGTGCGAAAGACTATAGTAAGAGTAAATTTGTAAGTAGTTTCTCTATTTATGGTGGT
ATTGCACTAGGACTTATCGTGATTTTAGTTACTTTACCATTCAGTGATCAAATCGCAGCAATTTTAGGGGCGAGAGGTGAAACGTTA
GCTTTAACAAGTAATTATTTGAAAGTAATGTTTTTAAGTGCACCTTTTGTAATTTTGTTCTTCATATTAGAACAATTTGCACGTGCAA
TTGGGGCACCAATGGTTTCTATGATTGGTATGTTAGCTAGTGTAGGCTTAAATATTATTTTAGATCCAATTTTAATTTTTGGTTTTGAT
TTAAACGTTGTTGGTGCAGCTTTGGGTACTGCAATCAGTAATGTTGCTGCTGCTCTGTTCTTTATCATTTATTTTATGAAAAATAGTG
ACGTTGTGTCAGTTAATATTAAACTTGCGAAACCTAATAAAGAAATGCTTTCTGAAATCTTTAAAATAGGTATTCCTGCATTTTTAAT
GAGTATCTTAATGGGATTCACAGGATTAGTTTTAAATTTATTTTTAGCACATTATGGAAACTTCGCGATTGCAAGTTATGGTATCTCA
TTTAGACTTGTGCAATTTCCAGAACTTATTATCATGGGATTATGTGAAGGTGTTGTACCACTAATTGCATATAACTTTATGGCAAATA
AAGGTCGTATGAAAGACGTTATCAAAGCAGTTATCATGTCTATCGGTGTTATCTTTGTTGTATGTATGATTGCTGTATTTACAATTGG
ACATCATATGGTTGGACTATTTACTACTGATCAAGCCATTGTTGAGATGGCGACATTTATTTTGAAAGTAACAATGACATCATTATT
ATTAAATGGTATAGGTTTCTTGTTTACTGGTATGCTTCAAGCAACTGGTCAAGGTCGTGGAGCTACAATTATGGCTATTTTACAAGGT
GCGATTATCATTCCAGTATTATTTATTATGAATGCTTTGTTTGGACTAACAGGTGTCATTTGGTCATTATTAATTGCTGAGTCACTTTG
TGCATTTGCTGCAATGTTAATTGTCTATTTATTACGTAATCGTTTGACAGTTGATACATCTGAATTAATAGAAGGTTAAATATTTCGT
ACACTTCTGACTGAGTATATTTCGGTCGGAAGTGTATTTTTCGAAAAAAATAAATATATGATACGATTATGAAAAAATAAAGTGAG
AATGGCATATGTATAAATCTAAAATACTGTTGAAATATATTTTTAGTGAAGAATCAGAAGTTAAAGACTTAACTGAAGAAAAATAT
AATCAAGATTACGAAGCATTAACATTTAGCTTTAAAGAGGAAACATATCAAAGTAGGTTAGCTAAGAAAACACCGACTAAAACAG
GGTATTTTGTTACATGTTGGACAAAAGACGAGAACAATTGCAATCAACCATACTCAAAAGAGGCATTTGCAGATTACTTAATGATTA
TAGTTATTGATGAAGAATTAAGCGGTTATTTCTTATTTCCTAGGGAATTATTGGTAGAAAAAGGCATCTTAACTACATTTGAACATA
AAGGTAAGATAGCTTTTAGAGTTTATCCTAAGTGGTGTAATCAATTGAATAAAACAGCAGGGCAAACACAAAAGTGGCAATGTAAA
TATTTTTTTGAATACTAATAAAAAGTCATATGGTTGAACTTATAAGAGATGAATATATCATCTTTCGATAAGATAACCCTATGACTTT
ATTTTTTATCATTTAATGATGAACGGTTTCTTGTCCTACTTTATTCCAAGTGAGGATAAAGCTCAACATTGCAAACACACTGATTGCT
GTTAATAAAATAAAACCGACATCCCATCCGAATTTATCAACTACAGCACCTAAGACGATGTTGGCCATTACAGCACCAAACAGATA
ACCAAATAATCCTGTTAATCCAGCTGCTGTGCCAGCTGCTTTTTTAGGTACATAATCTAATGCTTGTAAACCAATTAACATAACTGGT
CCATATATTAAGAAACCAATGGCAATTAATGAGACATTGTCTAACCAAGCATTGCCTGGAGGATTTAACCAATAAATTAATACAAA
TACTGTGACACCTAACATAAAGAAGAAACCTGCAGGTCCACGACGACCTTTGAATAATTTATCAGAAATGTAACCACATAATAATG
TACCAGGAATTCCAGCCCATTCGTATAAGAAGTATGCCCAACCTGATGCTTTTAAGTCGAAATGTTTTTCTTCACTTAAGTAGACTG
GCGCCCAATCAAGTACACCATAACGCACGAAATAAACAAATATATTTGCAAAGGCAATTGCCCATACCCATTTATTGTTCAGTACAT
ATTTAAATAAAATTTCTTTTGTAGTTAATTCTGTTTCTAATGTTTTCTTATCGCTTGTAGCAAAGTCATTTTTATAAATTTCGATTGGA
GGTAAACCTTGAGATTGAGGTGTGTCTCTAATCAATACGTATGAAATTGCGGCAATGATAAGTGCTAAGAGTGCAGGGTAAATGAA
TACACCTTCGAAACCTTTTAAATAACCAAAGTTGATAAATGCTGTTGTTGTAATACCCCAAGCAGCAATAGGTGCCATAATACCTCC
ACCAACATTATGCGCAACGTTCCAAAGGGCAGTCTTACTTCCGCGTTCACTTACACTAAACCAGTGAACGAGAACACGGCCTGAAG
GTGGCCAGCCCATACCTTGAAACCATCCATTTAAGAATAATAGGACAAACATAATACCGATACCTGATGTAAAGAACGGTACAAAT
CCCATTAACAAATTGACGATAGCAGTGAGTGCTAATCCAAGAACTAAGAATATCCGAGCATTGCTCCGATCACTTACAGTACCCAT
AAAGAACTTACTAAATCCATATGCGATGGAAACAGCAGAAAGTGCAAAACCTAGTTCCGCTTTTGTAAAACCTTGCTCTTGCAATGC
CGGCATCGCTAATGAAAAGTTTTTACGTAATAAATAGTACCCAGCGTAACCGATGAAAATACCAAGAAATACTTGGAGACGTAATC
GTTTATAGGTATCATCTATCTGATTTTCTGGCAAAGGCTTAATATGCTTTGCAGGTTTAAGAAAATTCATAAAATCCTCCTTAATATG
TATTTATATGCATTTTGTGCGATAAACTTATATTAGACATGTATGAAAGCGTTGTCAATATACTTTTGTGAAATCTTGCATATTTAGT
TATTGAAGTATTTTATAAACATTTAGAAAAAATAAATTGCTACACATAAATTTATAATTATGCAATTATTATGTCGTTATACATACTA
GCTGTGTATCTACGAATGAGTACAAACATATTTTTATTTGCAGAAAAGGGGTAAATGGCATATAACTATCTTTTTTATGTAAGCTGG
TATAAAATTTATACTAACAGGAGGGATAGTATGAATATAGTAGGGCATCATCACATATCCATGTATACAAAAGATGCAAAACGTAA
TAAGGATTTTTACACAAATGTCCTTGGATTACGATTAGTTGAAAAGTCGGTTAATCAAGACAATCCTTCAATGTATCATTTGTTTTAT
GGGGACGAAGTAGGTACAGCCGGAACAATTTTAAGCTTTTTTGAAATTCCCAATGCGGGTCATAAGCAGCCAGGTACTGAAACGAT
TTATCGATTTTCATTATTAGTACCAAATCAAGCGGCACTTCATTATTTTGAAAAACGTCTTGAGAATAATGGTATTACGTCTGAACGT
TTGTACTATCTTGGACAAGAAGGTGTTGTCTTTAAAGATGAAGACGACTTAGAAATCATATTGCTTGTTAATGATAGTTTTGAAGTA

CCACATCAATGGCAACATAACGCTTATAGTGAAATACCTCAAGCATATCAAATTTTAGGAATAGGGCCAGTCGAATTAAGAGTTAG
AAATGCAGCGCGTACGGTAGAATTTTTGGAAAATGTCTTAGGTTATCGCAAAAGAGATAATAAATCATTCGATGTGCTGACATTAG
CACCACAAGGTTTATATTCGGATTTTGTAGTTATTGAGCAACAGGGACAACGTGAAAGACCTGGACGAGGTTATATCCATCATATTG
CAGTTAATACACCACAAATGAGTGACTTAGAGGCAATTTACAAGAAATTACAACAACAAACACAAAGTAATTCAGGTATAATTGAT
CGCTATTTCTTTAAATCATTATACTATCGCCATAATTCAATTATGTATGAATTTGCGACTGAAGCGCCTGGATTTACTATTGATACAC
CTCTTGAACAATTAGGAAGTCAATTGAACTTGCCTGACTTTTTAGAAGCAGAACGTGAACAAATTGAAAGTAAGTTACACGAAATA
TAAAGGAGAATGTTTAATGGCCAAATTAGAAATGAATAAAAATACGCCTCTTGAGTTTGGTTTGTATTCCTTAGGTGATCATTTATT
GAATCCATTGAAAGGTGAAAAAGTTAGTTATGAGCAACGTATTAATGAAATTATTGAAGCAAGTAAATTAGCAGATGAAGCAGGTA
TTGATGTTTTTGCAGTTGGTGAAAGTCATCAGGAGCATTTTACAACACAGGCACATACGGTTGTGTTAGGTGCAATTGCCCAAGCGA
CAAAGCATATTAAAGTTTCAAGTTCTTCAACGATTATTAATGCAACAGATCCTGTAAGAGTATTTGAAGACTTCGCGACATTAGATT
TGATTTCTCATGGTAGAGCCGAAATTGTAGCTGGCAGAGCATCAAGAACAGGTATTTTTGACTTGTTTGGCTATGATTTAAAAGACT
ATGATGAATTGTTTGAAGAAAAATTAGGTTTACTTTTAGAGTTAAATAAAACTGAGCGTATTACTTGGTCTGGAAAATATCGTCCAG
AACTTAGAAATATGAAAATATTCCCAAGACCAATCGATAATATATTGCCAATATGGCGTGCTGTTGGTGGTCCACCTGCAAGTGCTA
TTAAAGCGGGAAAACAAGGTGTGCCAATGATGATTACAACCCTTGGTGGCCCAGCAATGAACTTTAAAGGTTCTATAGATGCTTAT
CGTCAAGCGGCAACTGAAGCAGGTTTCGATGCTTCGCCTAAGTCTTTACCAGTAAGTACAGCGAGTCTGTTTTATACAGCTGAAACA
ACTCAGGATGCTATGAGAGAATTTTATCCACATTTGAATACAGGGATGTCATTTATTCGTGGTGTTGGTTATCCGAAACAGCAATTT
GCTAATTCGTCAGATTATCGAGAAGCGCTAATGGTTGGAAGCCCGCAACAAATTATTGAAAAGATATTGTATCAACACGAGTTGTA
TGGTCATCAACGTTTTATGGCACAGCTTGATTTTGGCGGTGTGCCATTTGAAAATGTTATGAAGAATATTGAGTTAATTGGCAACGA
CATTATACCGGCGATTAAAAAGCATTTATCAAAATAGGAGGGGCGTCATCATGAATATTGTATTATTGTCAGGTTCCACAGTAGGTT
CTAAAACGAGAATTGCTATGGATGATTTAAAAAATGAACTAGAAGTCATCAATGAGGGACATCAAATAGAGTTGATGGATTTACGA
GAACTTGAATTAGAATTTAGCGTTGGAAAGAATTATCTAGATACTACAGGAGATGTATATAAATTAACGACGTCGTTAATGCAGGC
TGATGTGATTTTTATTGGTTTTCCAATTTTTCAAGCTTCCATCCCTGGTGCTTTGAAAAATGTGTTTGATCTACTTCCAGTCAATGCGT
TTCGTGACAAGGTAATAGGACTTGTAGCGACAGCAGGTTCTAGTAAACATTATTTAATTCCTGAAATGCATTTAAAACCAATATTGA
GTTACATGAAAGCACATACGATGCAAACGTATGTATTTATTGAAGAGAAAGATTTTTCAAATCAACAAATTGTCAATGATGATGTTG
TATTTCGGTTAAAAGCGTTGGCACAATCCACAATGCGAACTGCCAAAGTACAACAACAAGTGTTTGAAGAAGAAAACAACCAATAC
GACTTTTAAAGTATAAAAATAAGACGCTCGGCACACTAAATTTGTAAGTGTTTGAGCGTCTTTTCATATTAACTATATAGCCAATGA
ACGACGATAAAGGCAAGTGATGACAAGCATATTGAGGTAATAATGATTGTCATAAGCGGTTTAAGTGCGCGATTTTTAAGATCTTT
AAATGCCACATTTAACCCTAAAGCAACCATGGCCATTAATAAGCAAATTGTTGATACAGTATTTAAAATATTTAGCAATGCTGACGG
AATAGTTACATATGTATTCACTAAGGCCATAATGACAAATCCAATTAAAAAGTATGGAATGCTTATTCGACCCTTGCTAGATGATTC
TGATGAACGGAAACGCATAATTAAAATAAGTACGATGGTTAATGGAATCAGTAAGAATACTCTACCAAGTTTGCCCAGAAGTGCAA
TTTTAAGTGCATCACTACCACCAAAGCCACCAGCTAAGACAACGTGTGCAATTTCATGAAGACTAACACCAGACCAAGCGCCATAA
ACATTTGTCGTCATTGAAAAGATAGCGTAGATAGCTGTATATATAAGTGAGAATATCGTACCAATCAATGCGATGATACCGATACTA
ATAGCTGTATCCTTTTCACGTGATTTGAATATTGGAGCTACTGCAGCAATAGCGGCAGCACCACAAACGCCTGTACCTACACCTAGT
AATAATGCGATGTTTTTGTCACCATGTAACAGTTTGTTGACAAAGAGCATCATTACAATACTAAAAATAACGACACCTACATCGATG
GCTAATAGTCTACTACCTTGACCGATAATATCGAATATATTGAGTTTAAGTCCATATAGGATGATTGCAAATCTTAATAAATATTTA
GATGAAAACGTAATACCTGAGCTATATTGTTCAGGATATCCTCTAAAGTGACGATATAGAATAGCGATTAATATCGCGATAGTTAAT
GCGCCAACCTTATCTAAGATTGGCAATTTAGCTGCTAAAAAGCTAAATAATGCGACTATAAATGTTAATGATAGTCCAATCATAAAA
TGCTTATTTTTCAATGATGCCATGAGCAGTGCCTCCTTTAATAGCATTTTAGCACTGTTTTGTCGTATTTTTAAATATAAATTTGGAAT
GAATAATAAAGTAGTGATTAAATTAAGTTGTGTGATAGGAAACTTGGACATCAATCAAAGTAATAGGCACTACAACGCTTATTGGC
GGGGCCCCAACAAAGAAGCTGACGAAAAGTCAGCTTACAATAATGTGCAAGTTGGGGATGGGCCCCAACAAAGAGAAATTGGAAC
CACAATTTCTACAGACAATGCAAGTTGGCGGGGCCCCAACAAAGAGAAATTCGAAAAGAAATTCTACAAGCAATGCAAGTTGGCG
GGGCCCCAACAAAGAGAATTTCGAAAAGAAATTCTACAAGCAATGCAAGTTGGGGAAGGACAACAAATTTAAGATACAATGCGTA
ACATTAATATGTTATTATAATGATAATTTACAGAATTATATGAAAAATGAATGAGGATGTGATGGTATGTTTGGAATGAAAGTGAAT
GAACAAATAACATTAAAAATTTTAGAAGCTCATGACACAGAAGCGCTTTTCAATTTAGTCAATCGTTCAAGAAATTCACTTAGGGA
ATGGTTACCTTGGGTAGATGCAACTGAGCAACCATCAGATACGCGTGCATTTATCAAAAGAGGACTTTTGCAATTTGCTGATGGTAA
TGGATTTCAGTGTGGCATTTGGTATGAAGGAACGTTAGTTGGTGTCATCGGTTTACATGAAATTAATCACATGCACAGAAAAACTTC
ATTAGGGTACTATTTAGATAAAGAATTTGAGGGTCATGGGATTATGACACAAGCAGTTGAGGCATTGATAAAGTATTGTTTCGAAG
AGCTTGACTTAAACCGAATTGAGATTAGTGCCGCAGTTAATAATGAAAAAAGCCGGGCTATTCCTGAAAGGCTGGGATTTACTAGA
GAAGGTATGTTACGTGACAATGAATTACTAAATGGTATTTATTCAGCGAGTTACATCTATAGTTTATTAAAATCAGAATACGACCAA
AAATGACAAATTAGACTTACAAAAGAGTGATGACATTTAAAATGGCAGCGCTCTTTTATTTAATTTTTGAAAATAAAAGGTTGTTGA
CAGTATTATTTTATAACAATATAATGATTTTGATAATTATTATCAACTAGATAATGTTCATGGGAGGATGCCTTAAAATCATCATTTT
AAGAGCCACGTATTATCTTGGCATTTAGGGAGTGTGAGATAGTACGAATGAGGGCATGTCTATGGGGATAATAGAATTTCTATAAT
GAGGTGTCAAAATGAAAAAGTTAACAACGCTATTATTAGCATCAACGTTATTAATTGCTGCATGTGGGAACGACGATAGTAAGAAG
GATGATTCAAAGACATTGAAAAAAGATGATGGTGTTAAAGCAGAATTAAAACAAGCAACAAAAGCATATGATAAATATACTGATG
AACAGTTAAATGAATTTTTAAAAGGTACAGAAAAAATTTGTTAAAGCGATTGAAAATAATGATATGGCCCAAGCAAAAGCGTTATAT
CCAAAAGTTCGTATGTATTATGAACGCTCTGAACCAGTTGCAGAAGCATTTGGAGATTTAGATCCTAAAATTGATGCACGTCTTGCA
GATATGAAAGAAGAGAAAAAGGAAAAAGAATGGTCAGGATATCATAAGATTGAAAAAGCATTATACGAAGATAAGAAAATTGAT
GATGTGACTAAAAAAGATGCACAACAATTATTGAAAGATGCAAAAGAATTGCATGCCAAAGCTGATACATTAGATATCACACCAAA
ATTAATGTTACAAGGTTCTGTTGACCTATTAAATGAAGTTGCAACTTCTAAAATCACAGGTGAAGAAGAAATTTATTCACATACAGA
TTTATATGATTTTAAAGCGAACGTTGAAGGCGCACAAAAAAATTTATGACTTATTTAAACCTATTTTAGAGAAAAAAGATAAAAAATT
AAGTGATGATATTCAAATGAACTTCGATAAAGTGAATCAATTATTGGATAAATATAAAGATAACAACGGCGGTTATGAGTCATTTG
AAAAAGTATCGAAGAAAGACCGTAAAGCATTTGCGGATGCTGTTAATGCATTAGGAGAGCCACTAAGTAAAATGGCTGTGATTACT
GAATGACAAATTATGAACAAGTTAACGATAGTACGCAATTTTCAAGACGTACATTTTTGAAAATGTTAGGTATTGGCGGTGCCGGTG
TTGCAATTGGCGCAAGTGGTGTTGGTAGCATGTGGTCTTTCAAATCAATGTTCAATACACCAGAAGATCCGGAAAAAGATGCGTAT
GAATTTTATGGTAAAGTGCAACCAGGCATTACCACACCCACGCAAAAAACATGCAATTTCGTTGCGTTAGATTTGAAGTCAAAAGA
TAGAGATGCAATTAAGGCAATGTTTAAAAAGTGGACGGTTATGGCTGATCGTATGATGGATGGTGATACAGTTGGCAAGCCGAGTA
ACAATCCTTTAATGCCACCAGTAGATACCGGTGAATCGATAGGATTAGGTGCAAGCAAGTTAACGATTACCTTTGGGATTAGTAAGT
CTTTGATGAAGAAAATTGGGTTATCTAGTAAAATTCCCGATGCCTTTAAAGATTTACCGCATTTTCCGAATGATCAGTTAATAGACG
ATTACAGCGATGGTGATATTATGATTCAAGCATGCTCAAATGATTCGCAAGTATCCTTTCATGCGGTTCATAATTTAGTTCGTCCATT
TCGAGATATTGTTAAGGTACGTTGGGCGCAATCTGGTTTTATCTCTGCTAAAGGTAAGGAAACACCTAGAAATTTAATGGCATTTAA
AGATGGAACAATTAATCCTAGAAAAAGTAATCAACTTAAAGATTATGTGTTTATTGATGACGGATGGGCGAAACATGGAACTTATT

GTGTTGTCAGACGTATTCAAATACACATTGAAACGTGGGATCGTACTGCACTGGAAGAACAAGAGGCTACATTTGGTCGGAAACGA
CATAGTGGTGCGCCGTTAACAGGTGGGAAAGAGTTTGATGAAATTGACTTAAAAGCGAAAGATAGTCATGGCGAGTATATTATTGA
TAAAGATGCCCATACGAGACTAGCGAAAGAAGCAAATACGTCAATTTTACGTAGAGCCTTTAATTATGTTGATGGTACGGATGACC
GCACAGGTAACTTCGAAACAGGCTTGTTGTTCATTGCTTTTCAAAAAGCGACAAAACAATTTATCGATATACAAAATAATTTAGGTA
GTAATGATAAATTAAATGAATATATTACACATAGAGGTTCTGCTTCATTTTTAGTATTACCAGGTGTTAGTAAGGGAGGATACCTTG
GTGAAACATTATTTGACTAAATTTGTAGCAATGCTAATAACTGCTGCTATGGTGTGTAGCTTTGGGTTACTGAAAAGTCAGGCAGCA
GAACAACAAAGTATTAGTGATGTATATAGTGTGATAACGGATGCGAAATCTGCACTTTCTAATAATTCGATATCGAATGACAATAA
GCAGAAAGCAATTGAGCAAGTGGTAAGTGCAGTTAAGAAATTATCGCTTGAAGATAATAGTGAAAGTAATGCTGTCAAATCAGATG
TGAGAAAGCTTGAAGATGCAAAAGCGAATGATAATCAAAAAGATACACTTTCGCAATTAACGAAGTCATTAATTGCTTATGAAGAG
AAATTGGCTAGTAAAGATGCGGGTTCTAAAATTAAACTATTGCAACAGCAAGTCGATGCCAAAGATGCAGTAATGACAAAAGCGAT
TAAAGATAAAAATAAAGCAGAGCTAGAATCGCTGAACAATAGTTTGAATCAGATTTGGACAAGTAATGAAACGGTAATTCGCAATT
ATGACGCAAATCAATATGGACAAATTGAAGTCGCATTACTGCAACTTAGAATCGCAATTCATAAGTCGCCATTAGATACAGCGAAA
GTGTCACATGCTTGGACAACTTTTAAATCAAATATTGATCATGTCGATAAGAAAAGTGATACGTCTGCAAATGATCAATACCGTGTA
TCACAATTAAATGATGAGTTAGAGAAGGCGATTAAAGCTATCGACGACAATCAATTGTCGGATGCTGATGCTGCGCTTACACATTTT
ATAGAAATTTGGCCGTATGTTGAAGGTCAAATTCAAACTAAAGACGGTGCTTTGTATACGAAAATTGAAGATAAAATACCATATTA
TCAAAGTGTATTAGACGAACATAATAAAGCACATGTGAAAGATGGTTTAGTAGATTTAAATAACCAAATTAAAGAGGTTGTTGGCC
ATAGTTATAGCTTCGTCGATGTGATGATTATCTTTTTACGTGAAGGGCTAGAAGTGTTGTTAATTGTAATGACATTGACTACCATGAC
GCGTAATGTAAAAGATAAGAAAGGGACTGCAAGTGTGATTGGTGGTGCAATTGCCGGACTTGTACTGAGTATTATCTTAGCAATTA
CGTTTGTAGAAACTTTAGGGAATAGTGGCATTCTTCGTGAAAGTATGGAAGCGGGATTAGGTATCGTTGCGGTCATATTAATGTTTA
TCGTTGGTGTTTGGATGCACAAACGTTCAAATGCAAAACGTTGGAATGACATGATTAAAAATATGTATGCTAATGCGATTAGTAATG
GTAATTTGGTATTGTTAGCGACGATTGGTTTAATATCTGTGTTGCGTGAAGGTGTTGAGGTTATCATTTTCTATATGGGGATGATAGG
TGAGCTAGCGACAAAAGATTTTATTATTGGTATTGCTTTAGCTATCGTTATATTAATTATCTTTGCGTTATTATTTAGATTTATCGTCA
GATTGATACCTATATTCTATATATTTAGAGTATTGTCGATCTTTATTTTTATTATGGGATTCAAAATGCTTGGCGTAAGTATTCAAAA
ATTACAATTATTAGGTGCGATGCCGAGACATGTTATCGAAGGATTCCCAACGATTAACTGGTTAGGATTCTATCCAAGTTATGAACC
ATTGATAGCACAAGCTGCCTATATTATGGTAGTTGCTATCTTAATCTTTAAATTTAAAAAATAAAAAACAGGCCGAGTGCCTGTTTT
TTTGTTGCGATGTTGGAAATATTCGGTATTGCAGTATAACGATAATCACAGCATTAATTCTTATAAGATTAATGTGTTGGCGGTTTGC
CTCGGTATGTAAATTTAACGATGAACGTACTGAACTCAAAGAGCAATATGAGTGGCAATGTGAGTAATATATTTAATGTTAAATCG
GGTGGTGCAATGATACTTGCTAATACAAAGCAAGCGAAATAAATATATTTTCGATAATGCTTCAATGATGTGGTATCTATAAGACCG
AATTTTGCAAGTCCTATGAATAATATTGGCAATTGGAATAAAATGCCAAAAGTGAAAAGCCAACGGATAAGTTCAACTAAATACGC
TTTAAAGCCAATGACAGGCGAAATGTTTAAAGTTAATGATAATTTTAACGCGAATTGAATGATCATTGGAAAGCTAACATAAAATG
CAAAAGCGACGCCAGCACAGAATAATAACACGCTGAAAAAACTATATTTATAAATAAATTGGCGCTCATTATTATGCAAACCAGGC
GCAACAAACGCCCACAATTGATAAAACATAACCGGTGAAATGAGACAAAACGCGATGAAAAATATAATCATCACGTATATTTGGAT
CATTTCTGTGAATGAAAATGCATGTAAGGACACATGTGCACGGGTAATATACGTTATGAATGGTGTCATCCACCAAAATGATGAAA
CATATACGACGATGACCGTAATGACGAACGACAATAAAATTTTTACTAACCGATGGCGTAGTTCGCTAAAGTGAACCATTAATGTA
TCATCAGTACTATTGCTCTCGCTGTTGCTTCGATTCCTTACTGGGTGTATCGTGAGACTCTTTATCTAAATCTTCTGTTGCAGATTTAA
ATTCTTTTAAAGTAGAACCGATAGCACGACCAAATTGTGGTAATTTTTTCGGACCAAAAATAATTAAAGCGATAATGCTAATGACGA
CAAGACTTGTTGGACCTGTGATGCCTAAAATAAAAGTGTTAGTTATCATGATAATCAACCTCACTCATAAGTAGTATATAGTATTTA
CTTTATACCGAACGATAATGATTATCAACAAATTTTATATAATAATTAAAATATTGCTATAAACACAACTTACTGAGGTATTAATTCT
GAAATAATAGCTTGTTCTTGCAAGTCGATATAAGTACAAGTACCAGATTGAATATCGATATGCCATTTATAAATGCCAGCTTCAGCC
ATTTCATCACAAAATGTTTCGAAATCTGTTTGCCCTTGTTGATGTCTTGTTAAGACGTCTTGAACTATTGTTTTGTTTGATTTTTGAGC
AACAGGAATCGTACTTTTCACAGATGACGTAACGATATCATCTTCTGATTGATGTACGTATGTTGCAGTGCCATCTTGAATGTTGAC
GATATTGTAAGTCATCCCCATATCTTTAAAAGCTTTGAATAGTTTTGGAAAGTCAACACCAGTAAATTGTTGATGTGCTTGTTGAATT
GCAGATAATGTAAATGCCATAATAATCTCCTTTGAATGGTTTGATTTAGTGAAAATGCGTGCATCATACTAAAATTAGAATGCTGTT
AATTATGCAGGAAGGTGTAACGAAAGTAAACAATTATGACTTGTAGGCGTTGAGGTGTTGTTTCAAAATGTCATACATAGGTGGAC
AATCATATTTTTTTGTGTAGATTTGACAAATATAGTTGTCAAAAAGACAAATATCATTTACAGTAGGACTAAGGAGTTGATGCGATG
TGCGTAATCGATTGAAAGAGTTACGAGCACGAGATGGCTTAAATCAAACGCAGCTTGCCAAACTAGCTGGTGTTTCAAGACAAACC
ATTTCGCTAATTGAGCGAAACAATTTTATGCCATCAGTATTAACGGCAATAAAGATTGCTCGCATTTTCAATGAAACGGTGGAAACG
GTTTTTATTATTGAGGAGGATGAGGCATGAAAATACTAAGATATATCGGATATCTTTTACTGGGTGGACTTGTAGGTGGTATCATAG
GTGGAATTTTAGGTAACTTTGATGGATTGGGTATTGAGAACTTGACGTTTGCGACATATAACAATGTCGTTGTAATATCGATTGTTG
CGACTATTATTATCATATTGGTAGAAGCCATTGTTTTGATGAATCAAAGACGTGCATTGAAGTATAAGCGACTTGTAGATGAAGAGG
TAGATATCGATGCAACAGATCAATATGAATTGCTTGCGAATCGTTATGCTTTAAATGGAAGTATATTAAGTATTCTGCAGACAGTTA
TTGCTTTTTTAGTATTGCTTATTTTTGTGGTAGGGCACGCTGAAGCAAATGGAATACTATTCTTTTTAATACCATTTTTTGTTAGTGCT
ATTTTCAATACACAATTTACACTGTTTAATAGAAGGTTTGATGACAGAATACCAAAAATTGCAGATAAGAATTACACTGAAAAGCG
ATTGGAAATATTGGATGAAGGTGAACGCCATATAGAATTAATTGCATTATTTAAAACATATGCGATCAACTTATCAATATTGATACT
AGCCATTGTAGTAATTGGGCTTTATTCAATTACTACTGGAATTAATCAAAGCTTTAGTTTGCTACTTATCATTGCTATTTTCATATATA
ACGTCTTTAGTTATTTATTGAAGAGAAGACGTTTTTATTAAAATTAAACAAAGAGGAGCATGGATATGACAACATTGTTAAACGTAG
ATAGTGTGAACAAACAATACAAAGATTCGGATTTTAAATTGCAAGATGCATCTTTAACGATTTCTACTAATGAGACAGTTGGATTAA
TTGGGAAAAATGGCTCAGGTAAATCGACATTAATTAATATTCTAGTAGGCAATCGACATAAAGATAACGGTAGTATCACATTTTTTG
GAGAAGAACATACCGTGGATGATGTCGAATATAAAGAACACATAGGTGTAGTGTTTGATGATTTGAGAGTGCCTAATAAATTGACT
ATTAAAGATATTGATAAAGTATTTCAATCTATTTATATGACTTGGAATAGTCAAAAATTCTTTGATTTAATCAAATATTTCGAGTTAC
CACTACAAACTAAAATTAAAACTTTTTCAAGAGGGATGCGAATGAAGATAGCTTTAACAATTGCGCTTTCTCATGATGTGAAGTTAT
TAATCTTAGATGAAGCAACTGCAGGTATGGATGTTTCTGGACGAGAAGAAGTAATGGAACTATTAGAAGATTTTGTCGCTCAAGGT
GGAGGCATCTTAATATCATCGCATATTTCTGAAGATATAGAACATTTAGCTGATAAATTAGTGTTTATGAAAGATGGACGAATGATT
TTAACTGAACAGAAAGATATACTTTTAGCACAATATGGAATTGTTACGACAGGAGATAAAGATGTTGAAATTCCTAAGCATTTAATC
ATTGCTTCTAGATTGTCAAAGGGGAAATATCAAATTTTAGTTAAAGATTATGCAGAAATTAAAAATGCAGAACCTTTAAAACACATT
GATGACGCTACGAAAATCATAATGCGAGGTGAAGTATAATGAAAGGTATGTTCCTAAGTAGTTTTTATGCAACGAGAAAGCAAACA
TATATTTATTTTATAGTCGCTATCATAGCTGCGGGATACTTTGCAGTATTTAATCCGTTGATGAGTTCGGCAATGGCTGGGGTTATGT
TAATCACACCCATTACTGATAATATTAAACATGAAAAAGACTCAAGATGGATGTATTATGTATCTACTTTACCGGTTAAACGTAATG
ATTATATTAAGTCATACTTTGCCTTTTATTTAATCTTATTCGGTGCAAGTTTAATGATTGGATTAGTTGTGACTACAATCGTGACCCA
AAGTGTGATGATTGGTATTATGTCAGGTTTAATGAGTTTTGGTATCATAGGGGCATACTCTATCATTTTCCCATTGACATTTAAATTT
GGCGCTGAAAACTCTAATGTCATTATGATATGTGCATCTATACTACTACTTATTTCTTTCGTGGTCTTTTTCTTTATATATGGTATGGT

TAGTGGTGCATCTGCATTAGAATTTGAAAAAATTAGCACTGAAGGATGGCTAGTTGTCATAGCATATGCGGTCATTGGTATAGTTAT
AACGAGCGTTTCTTATATATTGTCTATTAAAATTTTTAACAAACAAGAACTATAATCAATATTCTTGGTGTGAAGCTAGTCCTTGAAA
TAATATTTAGAGCAGGTGATAAATCAATACGATTATCATCTGCTCTTTTTGGTGTGGAATGAAATGCAGGGTGATAAGTATAGGTGA
CATATCTAGATTGATTCATTTGTTTTGAGGTGGTTATGTTGTGTGGGAATTATTTCCTTTTAGATAGTGGGGATTAGAGGATATATGT
TATTTATAAGTATCATTTGATGATTGTATAGGCTAACGATTTCCTCGGAAATATTTAAAAACCTCGATCATGTAGCATAACTGAAGC
TTGTCACAAAAGTATAATGTGAAGTTCGACACTTTTGGATTCAGTTCAAATACTTTGACCGAGGTGAATACTATTTATTCATGTTTAT
TACGTAGACGTTGATTTTTAAAATAAATCAGCATGATGCAGAAAGTCGCAACGGATACCGAGATAATCATTACTTGGTCATGCCCCT
TAAAGATAAGGCTGATGATATATGATACTACAAGTATGCCTAGTGAAGAAGAAATATATTTTGCGTTTGTCAGTTCATTATGGAAAT
AAGGCGTGATTAACCATAATCCAATATAGAATATTAAAACACTGATATACATCATATTAATTTCAAACAAGTCATTTAGTTTATTGT
TATTACTAAAAACAATTGCAGCATTAATCACACCTAAAGCGATATTGATTAATAGATGCGTATACGATAAACGGAAACCGATAGAT
GACAATTTATGATTAATGTAATTTTCAGTAATGATCCAATATACACCGAAAAGACTAATTAAAATCATAAATTGGAATATATAAATG
TAACTAAAATGATCAATGCTAAATGATGACGAAGCTAAACCAACCAGTACCTCGCCAAAGATAATAATTGTTAGTAACGAAAAACG
TTCTACTAAATGCATCATATTAACAGGTGATAATACAAGATATTTCTGAAATGGAATAAGTCCTGTCGCTGCAATGAATACGCCTAA
AAATCCAGGGATGTAATGGATACTTTGTGGTAGTACTAATGATAGAAATGATAAAAATGAAATCACAAAGGCTACGCTCGCAAAAG
CTTGACATGTACGCTTATCGCCATAATCTAACCCTGTACGTATATGTAATAAATACTGTAATCCGATACTTAAATACATAATTGCCAC
GCATAAGAAGAATGGGAAGAATGTCTTTTCAAAGTCCGGATATAGGCTGTTAGATAGGAAGACCATGATGAACATATTAAACATCA
TAAACGAGACGTCTTTGAATGTAACTTGACCAAATCGATTTGTAAAAAAATGTTTGATGAGACCACATTAACCATAAGAACAAACTC
ATGACGATGTATTTGAAAAATAAATCAGCTGAAATGGAACCGTTTTGTGTTGTTAAAATCACATGTGCAATTTTTTGAATGGCATAG
ACGAAAATTAAATCAAAGAACAACTCATGGAATCCTGCACGCTTTTCAGCTAAATGTTTTGGTGTTAATGCATTAACCATAAAATTT
TAACTCCTTTAAGATGTGTAATTAATTTACTAAGTATACTATTTATTTTTTCTAGTGAATAGGGGCAGATTTGGCGATGAAGTGGAAG
GAGAGGTGACTGCATGGTAATTGCGGAATTAACAATCATCAGCGATTTAATATTTGACTAGAGACGTAATGGTAATAAAAAATTGA
TGAGAAATTGATGGTGAAACCAGCTGTGAATAGCGATGCAATGATAGATAGAATTTAATTAGAGTCATTACGCGAAATGATTAATG
ATAATTTGTGGTAAATCAAAGCATAATTTTGTACTATAGATGAGGATGATAGAGCATATTTAAGAGTGTGAAATGTTAAAGTGAAA
CCGTTTACGTTTCCGATTGCCCAAACAAATTACATCATTGTATAATATGATTTGTTAAATGCATAACAAGAATGAAAATGTAACATA
CGTAGCAATTGGTTTCATAAATTGGATGTTAGTGGCGTATTGGTTCATTAGACGTATTAGTAATAAAATTGTATATATCATAAGGAG
ATGAATATGACATGACGAGAGTCGTATTAGCAGCAGCATACAGGACACCTATTGGCGTTTTTGGAGGTGCGTTTAAAGACGTGCCA
GCCTATGATTTAGGTGCGACTTTAATAGAACATATTATTAAAGAGACGGGTTTGAATCCAAGTGAGATTGATGAAGTTATCATCGGT
AACGTACTACAAGCAGGACAAGGACAAAATCCAGCACGAATTGCTGCTATGAAAGGTGGCTTGCCAGAAACAGTACCTGCATTTAC
AGTGAATAAAGTATGTGGTTCTGGGTTAAAGTCGATTCAATTAGCATATCAATCTATTGTGACTGGTGAAAATGACATCGTGCTAGC
TGGCGGTATGGAGAATATGTCTCAGTCACCAATGCTTGTCAACAACAGTCGCTTCGGTTTTAAAATGGGACATCAATCAATGGTTGA
TAGCATGGTATATGATGGTTTAACAGATGTATTTAATCAATATCATATGGGTATTACTGCTGAAAATTTAGTGGAGCAATATGGTAT
TTCAAGAGAAGAACAAGATACATTTGCTGTAAACTCACAACAAAAAGCAGTACGTGCACAGCAAAATGGTGAATTTGATAGTGAA
ATAGTTCCAGTATCGATTCCTCAACGTAAAGGTGAACCAATCTTAGTCACTAAGGATGAAGGTGTACGTGAAAATGTATCAGTCGA
AAAATTAAGTCGCTTAAGACCAGCTTTCAAAAAAGACGGGACAGTTACAGCAGGTAATGCATCAGGAATCAATGATGGTGCTGCGA
TGATGTTAGTCATGTCAGAAGACAAAGCTAAAGAATTAAATATCGAACCATTGGCAGTGCTTGATGGCTTTGGAAGTCATGGCGTA
GATCCTTCTATTATGGGTATTGCACCAGTTGGCGCTGTAGAAAAGGCTTTGAAACGTAGTAAAAAAGAATTAAGCGATATTGATGTA
TTTGAATTAAATGAAGCATTTGCAGCACAATCATTAGCTGTTGATCGTGAATTAAAATTACCTCCTGAAAAGGTGAATGTTAAAGGT
GGCGCTATTGCATTAGGACACCCTATTGGTGCATCTGGTGCTAGAGTTTTAGTGACATTATTGCATCAACTGAATGATGAAGTTGAA
ACTGGTTTAACATCATTGTGTATTGGTGGCGGTCAAGCTATCGCTGCAGTTGTATCAAAGTATAAATAATAAGAAAACAGGTTATCA
CAACAGTATTAATTACATGTTGGCATAACCTGTTTTTATTTGTTTATGGATTTATTGGGTAATATTAGTCATTTGATGGTTTAATTGCA
AATGCTCTAACAGGGAACCCAGGTGCATCTTTTGGTTTAGGGCTGATAGCGTAAATGATGGCGCCACGAGTTGGTAATTGATCTAAA
TTAGTTAATAACTCGACTTGGTATTTATCCTGACCAAGAATATAACGTTCGCCAACTAAATCACCATTTTTTACAACGTCCACAGATG
CATCGGTATCGAATGTTTCATGACCAACAGCTTCAACACGACGTTCTTCAATTAAGTACTTCAAAGCATCTAATCCCCAACCCGGTG
CATGTTGTTGTCCGTTCGCATCTTTGTTTTCAAACTTTTCAATATTAGGCCAACGTTTTGACCAATCGGTACGAAGTGCAACAAAAGT
GCCAGGTTCAATAGTACCATGCTCTTTTTCCCATGCTTCTATATGCGCACGTGTTACAATGAAATCATTGTTGTTCGCTACTTCTGTTG
AAAAGTCTAATACAATTAACGGCAATACCAATTCTTTTAAATCAATGTCTTCTAAATAACGTTTATTCTCGACAAAGTGAATTGGTG
CATCAATGTGAGTACCATATTGCGTTACAATATTCCAACGTTGCACATAGAAACCATGATCTTTAACCGTGAATAAAGTTGAAACTT
CGCCTTTTTCAAACTCACTAAAACGTGGTATTTCCGGATCAAATGTATGCGTTAAATCAACCCAAGTTGCTTGTTTTAAAGTATTTAA
TTGTTGCCATAAAGGATATTGTGTCATAAAATCACCCGTTTTTAGTTTATTATATGATAAATGCTGCGATTATTCTTGGCGTTTAGCT
TTAACAGCATTCACAAGCACAGTCAATGCATCTTTAACTTCTTCTTCTTTTCGCGTTTTCAAACCACAGTCAGGGTTTACCCAGAATA
ATGAGCGGTCGATTTGTTGTAGTGAACGATTGATTGCTGTAGTAATTTCTTCTTTTGTTGGAATACGTGGACTATGAATATCATACAC
ACCTAGACCAATACCTAAATCATAATTAATATCTTCAAAGTCTTTAATTAAATCACCATGGCTACGAGATGTTTCAATTGAAATAAC
ATCAGCATCTAAGTCATGAATAGCATGAATGATTTGACCGAATTGAGAATAACACATATGTGTATGGATTTGAGTTTCATCACGAAC
TGAAGACGTTGCAAGTTTAAATGATAAAACAGCATCTTTAAGATATTGTTCGTGATATTCAGAGCGTAATGGTAAGCCTTCACGTAA
TGCAGGTTCGTCAACTTGGATAACTTTGATTCCTGCAGCTTCAAGTGCTAATACTTCTTCGTTGATTGCTAAAGCAATTTGATCTTGA
ACGACTTTACGTGGTAAATCAACACGTTCAAATGACCAGTTTAGAATTGTTACAGGTCCAGTTAACATACCTTTAACTGGTTTATCT
GTTAAGCTTTGTGCATAAACTGTTTCATCAACAGTTAAAGGCGCTGTCCATTTTACATCACCATAAATGATTGGTGGTTTTACGGCAC
GTGAACCATATGATTGCACCCAACCGAATTTAGTTACTAAGAAACCTTGTAATTTTTCTCCGAAGAATTCAACCATGTCATTACGTTC
AAATTCACCGTGAACTAATACATCTAAGCCAATGTCTTCTTGAATTTTAATCCATCGAGCAATTTCATTTTTTAAGAATGTTTCATAT
GCTTCGTCTGTAATGCGTTTGTTCTTCCAATCTGCACGGTATTTTCGAACTTCTCGGCTTTGTGGGAATGATCCAATAGTTGTTGTTGG
TAAATCCGGTAAGTTCAAACGTTTTTGTTGTTGTTCAATACGTTGCGCGAATGGTGATTGTCTTGAAGTACGCACGCTTTCGAAATCA
TAATCTAAGTTTTTGAATGATTGATTTTGGAAACGCTCATAACGTGCTTTTAATTTATCATATTTAACACTATCGTTTTGATTAAATA
GGCGACGCAATGCATCTAATTCGTCTAATTTTTCAGTTGCAAAGCTTAAGCCTTCGCCAACACTTGTATCTAATGTTTCATCATCTAA
AGATACTGGAACATGTAATAATGAAGATGATGGTTGAATGACAAGTTCATTAGTGTGTGCTAACAATTTATCGATTAAGACTTTTTT
AGCTTCAATGTCACTTGCCCACACATTACGACCATCAATAATTCCAGCGTATAATGTTTTTGATTTATCAAAATCTCCAGCTTCAATT
TGTTTAAGGTTATAGCCATTATCATGGACAAAGTCTAAACCTAAACCACCAACAGGTAAAGAACTTAAGAATTTAAGATGTGCACG
TTCAAAGTATGTTTGAATGACTAATTTTTTAGCAACACCAGCTTTTTCGAAATAGTCATAAGCTTCACGTGTAATATTTTCATAGCTT
TCGCTGTCGTCTGTAACTAAGATTGGCTCATCAACTTGAATGTACTCAGCACCTGCATCAATTAATGATTCAAACACTTCTTTATAAA
GTGGTAATAACGTTTTAACTTTTTCTTCAAAAGTTTGGTGACCGCCTTTTGATAATTTAACAAAAGTAATCGGACCAACAATGACAG
GGTGAGCGTTAACGTTTAAAGATTGGGCATATTTAAAGCGATCTAATAATACATTGCGACTCACTTTAGGCTCAACATTGTCCCATT
CAGGTACGATGTAATGATAGTTAGTGTTAAACCATTTTATAAGTGCACTTGCAACATGGTCTTTATTACCGCGAGCAATATCAAATA

ATAAATCATCATCAATAGTTCTTCCTTGGAAACGTTCAGGGATGATGTTGAATAATAATGACGTATCTAATATATGGTCATATAAAG
AGAAATCACCAACTGGGATGCTATCTAAGTGATAGTACTTTTGTAATAATAAATTTTCTTTATGTAGATCAGTTAATGTTTGATCTAA
TTCTTCTTTAGAAATCTTCTTTGCCCAATAACTTTCGATGGCTTTTTCCATTCTCTTTTTCTACCTAATCTTGGGAATCCTAAGTTTGA
TGTTTTAATTGTTGTCATAATATTGCCTCCTTGTGAGCAGTAATAGATTTTGAGTATGCTGCAAGTTCTAATGAATCTTCGACATTTT
GAAACGGTGTGATAATGTATAAACCATTAAAATATTCATGAACAGTATCGATTAAATCCTTTGAAAGCTTAAGACTTAGTTCTCGTG
TTTTGGCTTTATCATCTTTAACTGCTTCAAATTGTTGTAAAATTTCATCTGACATCTTGATTCCTGGCACTTCATTATGCAAAAAGAGT
GCGTTTTTGTAACTTGCGATAGGCATAATGCCTATGAAAAATGGTTTGTTCAAGTGCTTAGTGGCATGGTAAATTTCAATGATTTTCT
CTTTGCTGTACACGGGTTGTGTTATAAAATAAGACATTCCGCTTTCTATCTTTTTCTCTAATCTTTTGACGGCACCATCTAATTTACGA
ACATTAGGGTTAAAGGCGCCAGCGATGTTGAAGTGTGTACGTTTCTTCAGCGCATCACCGTCAGTGTTAATACCTTGATTAAATCTT
AGAGCGAGTTCAGTTAATCCTTTAGAATTAACATCATAGACATTGGTTGCACCTGGTAAGTGACCAACTTTTGAAGGATCACCAGTT
ATGGCTAATATTTCGTTAACGCCAATGAGCGATAATCCAAGTAAATGGGACTGCAAGCCGATTAAGTTTCGGTCTCGACATGTAATA
TGTACGAGTGGTTCAATATTGTAATATTGCTTAATTAAGCTAGCAGCAGCAATATTGCTAATTCTGACAGTTGCCAATGAATTATCT
GCGAGTGTTACCGCATCTACATTAGCTTTATCAAGTTTAGCGATATTTTCAAAAAATCTATCCGTGTCTAAATGTTTCGGTGTATCCA
ATTCGATAATAACGGTTGGACGTTCTTGAACCTTAGATGTTAATGATTGTCTAACTTTATTTTGAGATGGATTGAAAAGTGCTTTCGT
TGGTATCGGAATCACTTTTTTGTCATTAACAGGTTTAAGTGTCTGAATAGATTCTTTAATAAATTTGATGTGCTCTGGCGTTGTACCA
CAGCAACCACCAATTAAACGAACACCTTCGCGAATTAGATTTTGAGCAACTTGACCGAAATATTGTGCATTGTCACTATACTTAAAT
TCACTATTTTCAATATCTAATAAGCTGGCATTTGGATAACAAGATAAGAATGCGTGCTCTGGTAATTCAATATGTGTGAAAGACTCT
TGCATATGGTGCGGGCCATGATGACAATTGAGTCCCACGATGTTTGCACCACATTGAACGAGTTGTTTTAATCCTTCATTGATTGCCT
GACCATTAACTAAGTAATTTGTGTTTGAAGCGGTTAATTGAGCAATGATTGGAATGTCGTATTTCTTTCTCGTTCGTGAAATGACATT
TGTTAACTCTTCTAGGTCGTAATACGTTTCGAAAAGTAGCGCGTCAACGCCTTCTTCAATTAAGGTGTCTATTTGAATTTCAGTATGA
TAAAGAATAGTTTGTAAGCTGATATCCTCTTGTTTGATACCTCTAAACCCACCAACTGTGCCTAATATATACGTATCTTTATTTGCTG
CTTTTTTTGCGATGCGAACGGCGGCTTGATGTATTGCTTTAACTTTATCTTCAAGACCGAATCGTTTTAACTTTTCAAAATTTGCACC
ATAAGTATTGGTTTGAATGACATCAGCACCGGCTTCAATATATGAACGATGGATGCGTTCAACTTTATCTGGATGGCTAAGATTATA
TGCTTCTGGACAGGTGTCTAATCCTTCAGAGTATAAAATGGTTCCTATAGCGCCATCAGCTACTAAAACATTATCTTTCAATTGTGTG
AGGAATTGACTCATTGAATGCCTCCTTTAATGCGTATTTGATGTCTGCAATGAGTTCATCAGGATCTTCGAGACCAACACTTAATCG
GAATAGACCGAAAGTGATACCACGTTCTTGCCTCACTTCTTCAGGTAGTGTTGCGTGAGACATTGTTGCTGGGTGCGAAAGTATTGT
TTCCACACCACCTAGACTCACTGAAACGAGTGGTAATGTCAGTGCATCGACAAATTGTTGTGCTTTAGACTCATCAGCTAAACGTAA
GCCAATAACGGCACCGCCATTTTTAGCTTGTTCTAAATGAGATGCAGTAAGTCCCGGATAATAAACTTCTGAAATTTCATCTTGCTTT
ATTAAAAATGACACGATTTTTTGCGCGTTTTCGACAGATTGTTTAAATCTGATTGGAAATGTTTTTAAATGTTTAGCAAGTGTCCAGC
TATCTTGAGCAGATAACATATTGCCTGTACCATTTTGTATTAAATAAAGAGCTTCACTAATTGCCTCATTATTAGTAATGACAGCACC
AGCAATTAAATCGCTATGTCCACTTAAAAATTTTGTAGCACTATGAATGACAATATCAGCGCCAAGTAATAAAGGTGATTGACCTAA
CGGTGTCATGAATGTATTGTCCACAGCTACCAGTAGTTCATGCTTTTCGGCTATTTTAGAAACAGCTTTGATATCAGTAATTTTAAAA
CAGGGATTCGATGGTGTTTCGATATAAATTAATTTTGTGTTTGATTGAATGGCACCCTCGATTTGTTCGAGCTTGGTAGTATCTACGG
TTGTAAATTCAATATTAAATCGATTCAAAATTTGCTCAGTGAGGCGAAAAGTACCGCCATATACATCATCGGGTAAGATGACATGAT
CACCAGATTTGAAAGTCAAAAGTACTGCTGAAATCGCAGCAATACCTGATGCAAAAGCAAAAGCGAATTTCCCCTGTTCTAATCGT
GCTAACTTCTCTTCTAAAAGTTCACGGTTAGGGTTGCCACTTCGTGCATAATCATATTTAACATCGCCACCAAGACTTGTTTGATGGA
ATGTTGAAGAATCATAGAGTGGTGGGTTAGCTGAATGATATTCCACACCTCTACGCCAATCGAATATCACTTCTGTCTCTTTTGAAA
GTGTCATACAATCTCTCCAATCTGAGCTTTATCTAATGCTTGGATGATATCGCGTTCGATGTCTTCATAATTTTCAACACCTAGTGAT
AAGCGGATTAAATACTCATCAATGCCACGTTTATCTTTTTCAGCATCTGGCATATCAACATGTGTTTGGGTGTAAGGGAAGGTCACT
AATGTTTCAGTACCTCCTAAACTTTCTGCAAAAATGCAAATGTCTAAATTTTTTAATAATTTAGCGACGCTATAGGCCTTGTTAAGTC
TTAAACTAAGCATGCCAGTTTGCCCGCTATATAGTACTTCGTCAATTGCTTGAAGTGACTGACATTTTTTAGCAAGTTTTCTAGCGTT
TGATTGCGCACGCTCAATGCGTAAATGCAAAGTTTTAAGTCCACGTAACAACAAATAACTATCTATTGGTGAAAGTGTTGCGCCAGT
CATGTTGTGAAAATCAAACAACTGTTGCGCGAGTGATTCACCTTTGACGGTTACGACACCTGCTAGTACATCATTATGTCCACCAAT
ATATTTCGTAGCTGAATGTAAGACTATATCAGCACCTTCTGCTAATGGTGTTGAAAGATAAGGTGTTAAAAATGTATTGTCGATAAT
TGACAATAAGCCTTTAGCTTTACAAAGTTGATAGTACGGCTTTACATCAATAGCAATCATTTGTGGGTTAGATATTGGTTCAATGAA
TAATGCAACTGTTTTATCAGTGATTTCTTTTTCAACTTGTTCATAATCTGTAAAATCAACGTACTTAAATTTGATATTGTATTGTTGCT
CATAAAATTCAAATAATCTAAATGTGCCGCCGTATAAATCGAATGAAACTAAAATTTCATCGTGTGGTTTAAATAAATTACATATTA
ATTGAATGGCTGACATTCCACTTGATGTAGCGAATGATGCAATACCATGTTCGAGTTTGGCAAAACAGGTTTCAAATGTTGAGCGTG
TAGGATTTTTAGTACGTGTATAATCAAAACCTGTCGATTGTCCTAGTTTTGGATGCTTGTAGGCAGTAGATAAATGGATTGGATTCG
CTATAGCACCGGTTGAATCATCGGTTAATGTGATTTGGGCTAACTGTGTATCCTTCATATTAAGACCCTCCTATAAGAAAAAATAAA
AAAAGCTTCCGTCCTTCGTACCCGAATGAATCGGATAAAAAGGACGAAAGCTTATGTTTCGCGGTACCACCTTTATTTGTTATTCCA
TCGCTGAAATAACCTTATTCAGTACGCATTAAAAGTAAATATGCTTACTGAACAATTATCACAATTAAAGTCAGTAAGTAAGGATAT
AGTAATGTGCTATCCCATACTTATTAACAAAAAATCGTGCGTAAAGAATCCAGTACGCCATTTAACATCAATGTTAATACTGTATCG
CTATAACGGGCGAACCCGTAGACACCTCATATTGGCATCAACACTCCAAGGCCATTTTCAAACACGCTTTCAAAATCTTCTCTCAGC
TACTAAAGACTCTCTGTATAAGCAGGGTGTGTTTTACTTTCCTCTTTATTGTGTTTACGTTTCATTAAACTGTTATAAGATATTAATTA
GCTTACAGAGTAAAAAAAGATTTGTCAACAATTATTCAGAAAATTTTGATTTAAAAGTTAATTTGTTTGTGAAATTGTAATTGGTAT
CTTGAAGTTGAAAAATGAATTATTTTTTAAATAAAGTGTGGTTAATGGTTGTCTGACTCATTTAGAATACATAAAATATATTTAACTG
TTGTTATCAAATAAAAAGTGATGTGAGTGAATTGTCAAAAAGTGAAGATCAACGTATTACTAAAACAAAAGATGAACAAATTAAGC
AAATAGATATATCGGATATCAAACCGAATCCGTATCAGCCCCGAAAAACTTTCGATGAAAATCATTTAAATGATTTGGCAGATTCA
ATTAAGCAATATGGAATTTTGCAACCAATTGTGCTTAGAAAAACAGTTCAAGGTTATTACATTGTAGTTGGTGAAAGAAGGTTTAGA
GCTTCGAAAATTGCTGGTCTAAAATACGTATCAGCGATTATCAAAGATTTAACAGATGAAGATATGATGGAACTGGCGGTCATCGA
AAATTTACAACGAGAAGACTTAAATGCGATTGAAGAAGCTGAAAGTTATCAACGTTTGATGACAGATTTGAAAATTACACAACAAG
AAGTAGCGAAGCGATTGAGTAAGTCGCGCCCGTATATAGCGAATATGTTGAGGTTATTACATTTGCCGAAAAAGATTGCTGACATG
GTAAAAGATGGGCGACTGACAAGTGCACATGGACGAACGTTATTGGCAATTAAAGATGAACAACAAATGCTTAGGTTAGCGAAAC
GGGTTGTTAAAGAAAAGTGGAGTGTTAGATATTTAGAAAACCATGTTAATGAATTAAAAAATGTTTCGTCAAAGTCGGAAACAGAC
AAAGTAGATATAACTAAGCCTAAATTTATAAAGCAACAAGAACGACAGTTGCGAGAACAGTATGGTACCAAAGTAGATATATCAAT
AAAAAAATCGGTTGGTAAAATCTCATTTGAGTTTGATTCACAAGAAGATTTTCTAAGAATAATTGAACAATTAAATCGTAGGTATGG
TAAATAGTTACACAATTTTATATAATAACTCTTTGTGCAAGTGTAAATAAATTGTAATCAGTGAACATTTGATTCTAGATATATTGAG
ACTTTCGTAGGTTGGAAGTATATAAAAATATGAATACAATATTGATATATCATATAGAAGGGAGTAACGCTTATCATGAATCAAGTC
ATGAATATTATTTCATCTCTATTTGAGCCATTAACAAAAATAGAAACATATGAAAACATTGCAACTAAAATCGCTATGATTGTTATT
TATATTATCGTAGCCCTCATAGTTATTAAAATACTGAATAAAATGATTGAACAGGGATTTAAGATTCAAAATAAGAGTAAAAAGAG

TAACAAAAAGCGCTCTAAAACTTTAATATCTCTTGTTCAAAATGTAGTGAAGTATATCGTTTGGTTTATAGTTATTACGACGATTTTA
AGTAAATTTGGCATTAGTGTTGAAGGTGTTATTGCAAGTGCTGGTGTCGTAGGTTTAGCAGTAGGTTTTGGTGCTCAAACCATTGTT
AAAGACGTTATTACTGGATTTTTCATTATATTTGAAAGTCAATTTGATGTAGGTGATTATGTTAAAATAAACAATGGTGGTACGACT
GTGGCAGAGGGAACGGTTAAATCAATAGGACTTCGTTCAACACGAATCAATACAATTTCAGGAGAATTAACAATTTTACCAAATAG
TAGTATGGGTGAAATAACGAACTACTCAATTACAAATGGTACAGCTATCGTTAAAATTCCAGTGTCTGTCGAAGAAAACATTGATA
ATGTTGATAAAAAACTAAACAAACTATTTACTTCTTTACGTAGTAAATATTACTTATTTGTTAGTGATCCGGTTGTTATTGGTATTGA
TGCTATTGAAGATACAAGAGTAATATTGAGAATATCTGCAGAAACAATTCCAGGTGAAGGATTTGCTGGAGCTCGAATTATTCGCA
AAGAAGTACAAAAAATGTTTTTACAAGAAGGTATTAAAACACCTCAACCAATTATGACTGCTTATAATCATAGTGAAAACGGTGTT
TAGTAGTTTATAATACATGGAGGTCATATTTAATGGCGTCAAAATATGGAATAAATGATATAGTAGAAATGAAAAAACAACATGCG
TGTGGAACAAACCGTTTTAAGATTATTAGAATGGGTGCAGACATAAGAATTAAATGTGAAAATTGTCAAAGAAGTATTATGATTCC
ACGTCAAACGTTTGATAAAAAACTTAAAAAAATCATCGAATCTCATGATGATACACAAAGATAGGAGAATGATTAATGGCTTTAAC
AGCAGGTATCGTTGGATTGCCAAACGTTGGTAAATCAACATTATTTAATGCAATAACAAAAGCAGGTGCTTTAGCAGCGAACTATC
CATTCGCTACGATTGATCCTAATGTAGGGATAGTAGAAGTGCCAGATGCTAGATTACTTAAATTAGAAGAAATGGTTCAACCTAAA
AAGACATTGCCGACTACATTTGAATTTACAGATATCGCTGGTATTGTGAAAGGTGCTTCAAAGGGAGAAGGGTTAGGTAATAAATT
CTTATCACATATTAGAGAAGTAGATGCGATTTGTCAGGTCGTTCGTGCATTTGATGATGATAACGTAACTCATGTTGCTGGTCGAGT
AGACCCTATTGATGATATTGAAGTTATTAATATGGAATTAGTACTAGCGGACTTAGAATCTGTTGAGAAACGTTTGCCTAGAATTGA
AAAATTAGCACGTCAAAAAGATAAGACTGCTGAAATGGAAGTACGTATTTTAACAACTATTAAAGAAGCTTTAGAAAATGGTAAAC
CCGCTCGTAGTATTGACTTTAATGAAGAAGATCAAAAATGGGTGAATCAAGCGCAATTACTGACTTCTAAAAAAATGCTTTATATCG
CTAATGTTGGTGAAGATGAAATTGGTGATGATGATAATGATAAAGTAAAAGCGATTCGTGAATATGCAGCGCAAGAAGACTCTGAA
GTGATTGTTATTAGTGCAAAAATTGAAGAAGAAATTGCTACATTAGATGATGAAGATAAAGAAATGTTCTTAGAAGATTTAGGTAT
CGAAGAACCAGGATTAGATCGATTAATTAGAACAACTTATGAATTATTAGGATTATCAACATATTTTACTGCTGGTGTGCAAGAAGT
ACGTGCTTGGACATTTAAACAAGGTATGACTGCACCTCAATGTGCTGGTATCATTCATACTGATTTTGAACGTGGATTTATCCGTGCC
GAAGTAACAAGTTATGACGACTATGTAAAATATGGTGGCGAAAGTGGCGCTAAAGAAGCGGGCAGACAACGATTAGAAGGTAAAG
AATATATTATGCAAGATGGCGATATCGTTCATTTCAGATTTAATGTATAAACGATAGAGTGAAGTTAATTAAATAGTATATATGGAG
AAGAGGCGGAATCAATTGTTCGCCTCTTTTAATTATGCGTATAATTTATTAAAAGAATGGAATAATTTTACTCGCGTTAATAATATCT
TGAGTGCTGAAAAATTGTTTGCCTTCGCCAGTATAAGCAGGCTCTAAAACAAGATTAGCCTTTGCACAATAAAGCCATTCAGGATGA
ATGCCACTATTAAGTATCTCTTGGAATTCTTGAAAATCTTTAGACCAATCAATATTTAAATTCATTCCTTACCACCTCACAACTATTA
TAGAACACGTGTTCGCTTTTGTGAAGTGTATTTTTAAAAATATCATAGAAAATTTCAAATGAATTAATGTCAAAAATGATATGAAGA
GTAGTTTGAATTATTATTGTAAAAAGTAATGGCGCATGATATAATTCTTTATTGTGAGTAATGAAAATTATTCCTTGCTTATCTGTTT
TAAGATTGATAAGCCGTATAGACCACAAGGAGGTGCAAATATAAAATGAGAACATATGAAGTTATGTACATCGTACGCCCAAACAT
TGAGGAAGATGCTAAAAAAGCGTTAGTTGAACGTTTCAACGGCATCTTAGCTACTGAAGGTGCAGAAGTTTTAGAAGCAAAAGACT
GGGGTAAACGTCGCCTAGCTTATGAAATCAATGATTTCAAAGATGGCTTCTACAACATCGTACGTGTTAAATCTGATAACAACAAA
GCTACTGACGAATTCCAGCGTCTAGCTAAAATCAGTGACGATATCATTCGTTACATGGTTATTCGTGAAGACGAAGACAAGTAATA
ATTAGAGGGGGCGTTTAAATGCTAAATAGAGTTGTATTAGTAGGTCGTTTAACGAAAGATCCGGAATACAGAACCACTCCCTCAGG
TGTGAGTGTAGCGACATTCACTCTTGCAGTAAATCGTACGTTCACGAATGCTCAAGGGGAGCGCGAAGCAGATTTTATTAACTGTGT
TGTTTTTAGAAGACAAGCAGATAATGTAAATAACTATTTATCTAAAGGTAGTTTAGCTGGTGTAGATGGTCGCTTACAATCCCGTAA
TTATGAAAATCAAGAAGGTCGTCGTGTGTTTGTTACTGAAGTTGTATGTGATAGCGTTCAATTCCTTGAACCTAAAAATGCGCAACA
AAATGGTGGCCAACGTCAACAAAATGAATTCCAAGATTACGGTCAAGGATTCGGTGGTCAACAATCAGGACAAAACAATTCGTACA
ATAATTCATCAAACACGAAACAATCTGATAATCCATTTGCAAATGCAAACGGACCGATTGATATAAGTGATGATGACTTACCATTCT
AATAAAAATTAACGAAATTAAAGCGAAAAAATTATCAAAGGAGGCACACAATCATGGCAGGTGGACCAAGAAGAGGCGGACGTCG
TCGTAAAAAAGTATGCTATTTCACAGCAAATGGTATTACACATATCGACTACAAAGACACTGAATTATTAAAACGTTTTATCTCAGA
ACGCGGTAAAATTTTACCACGTCGTGTAACTGGTACTTCAGCTAAATATCAACGTATGTTGACTACAGCTATCAAACGTTCTCGTCA
TATGGCATTATTACCATATGTTAAAGAAGAACAATAATATATAATTTATTGTCAAACCCCGTAGGCATAGGCTTACGGGGCTTTTTG
TGTTTTGGGGTATAGAAAAAGGGCAAAAAAGGATGATGTGAATGTTTTGTGTTCGGAATTTGCACAAAGATATGTTTATATTGCAAA
AATAATATGAATTTAGATGCATAAAAAAAGAACTACGCATTTTAAATAAAATGCATAGCTCTTCTTTTTCTTGCATACGAATTAAAA
TAACTCGCGAGACCTATAAGTCTCTTTCCTCACTAGATAGTTTATACTTTTGGTCTGTTGAAGTCAATAATTTTATCTAAAGCTATAA
AAAATCTTTTGATAGCTAATGCATTATTATATAATAGCTTTCGTTTCTTTTATATCGCTTTTGAAGTTGGTCCAAATCGTGATATCTTGCT
TGGATAATTGCATTACTACAAACTTGATTATGTAATTCTAGCGTAGCGAAAGTATCTATGAAATTTTTTATTCCGAACATGTTTCTAG
ATATGCCTATATTATTCCCTTTTTCAAATAAATATTGAGGAGATCTACTGTCATAATTTAGATTTGCTATGATGGGTTGGTTATGAGC
TGATTTGTTTCTTATATTTTTAACTAAAGGCATTAAAATATTAGCAACTCTCAATTCTTCGTCATTGTACTTCTTGTAATAGAAGTTGA
GAAACGAAACGAATTGACCTAGTTGCATGAATTCAATGCAAACTCATGCGGGTGGATTTTGATAGTATTTATTCAACTTCTCGGGTA
GTTGTCCTCGTTTATTCATATGCTTGAATATTTCGTTTTTATTTTTGATTTTGGTTTCCATAACTTCTTCTGGTGTTCTTGAATTTGTGT
CAAAATTTGAATTGCTATATGATTTATCAATACATAAGAACTCATCTATTATTTTATAACCATCTTCTTGGTTATTTTCTGTTATTAGT
TTTAAGACTAGACACTTTAAACTATGTTCAATATCTAAAGTTAAATGCAACATTGTGTATCTTAATTTCATATCTATAGTTGCTAAAT
CTGATAAATAAGCAAATTCTATGAAATAGCCGCCATTCTTTTTTTCGAAATTTTTTCGGAAATAAGCTAGTTTGAAGAAGTAATTATT
TTTTCTAAGAATTTCATTTGCTTTTTCGGTGTCAATAATATTAAAAAATATATTCATCTGTTTTAATTTCGCTATTTGCTCATCAAAAT
TGAGCATAGGCTTAATTTCTGCTAGTGTATCATCTTTTTCCAATTTTAACTCCCCAATCGTTCAAATTTATTCATCATATCTTTCGCCA
TCTGATTAGTAACATGTGTGTATATCTCTAGATTTTTTTTATAATCTGAATGACCTACATGCTCTTGCATTGCTTTTAAGTTAATTCCT
AATTGAGCAAGTGTAGATATATGCGAATGATGTAATGTATGCGTCGTTATAGGTTTCTTAATAGAACTAATATCAGCGCCCCCCTTT
AATAATGTGGCTAATTTTGTTCGAGTCGATAGGGCTACCAGCCGTATTTGTGAATATGTACTCTCTATCAATAAACTTATCATTCCAA
GCATAAGTGTTCTTAGTAAGTCGATGCTTTGGGTAGTGAGCCCTATGGCCTTATAGCTATTACTTCTTTCAGTTGTCTCCTTTACTCCG
AATGCTCCCGTCTTTTTTCAGTTATCCAATTAATTGTACCATCGATATCTAGCGTTTTATCTTCATAGTTTATATTTTCTCTCTTTATTG
CAAGTAGCTCACCGATACGCATGTCATTAGCAATTTGAAACTGTACCATAGCTTTTACCATTTTATAATTACGTTTTGTCGTTGGATA
TTTTTATACTTAATTACATAGTCGAAACAATCCAGTAACTCCTTATCTTCATTATCTTCTAAAGTGTTATTACGTTTAGCTAGTAACGC
ATCACTATAGGGATATCTATTTTATCTATTACACATATAACTTTGAATTGCTTACTATTTTAAATTAACAAATTCTATTCTCTTAGATT
TAGTCCAATTATGTGTAGACGATTTATAGTTATTAAATTCAGAGTGGTAGCAAATTAAAGTTAATCAAGAGTTAAGATGAATTTAAT
TCATGAATACGTCTATTATTTTTATAATTGTAGCAAATAAAGCTTTACATCAAGGAGGTAATTAAATATGTTCAAAAAACATCATTC
TAAAAATTCAATTGTATTAAAATCTATTCTATCGCTAGGTATCATCTATGGGGGAACATTTGGAATATATCCAAAAGGAGACGCGTC
AACACAAAATTCCTCAAGTGTACAAGATAAACAATTCCAAAAAGTTGAAGAAGTACCAAATAATTCAGAAAAAGCTTTGGTTAAAA
AACTTTACGATAGATACAGCCAAAATACAATAAACGGAAAATCTAATAAATCTAGGAATTGGGTTTATTCAGAGAGACCTTTAAAT
GAAAATCAAGTTCGCATACATTTAGAAGGTACATACACAGTTGCTGGCAGAGTGTATACACCTAAGAGGAACATTACTCTTAATAA

AGAAGTTGTCACTTTAAAAGAATTGGATCATATCATAAGATTTGCTCATATTTCTTATGGCTTATATATGGGAGAACATTTGCCTAA
AGGTAACATCGTCATAAATACAAAAGATGGCGGTAAATATACATTAGAGTCGCATAAAGAGCTACAAAAAGATAGGGAAAATGTA
GAAATTAATACTGATGATATAAAAAATGTAACTTTCGAACTTGTGAAAAGTGTTAATGACATTGAACAAGTTTGAAATTAAGTTAA
ATTAGTATATATAGTGTTTTATCGCTAATACTTTGAAAGTTAGGTATCCGAAGATGCCTAACTTTCTTTGTTATGATTAGCACCATCA
TATAGAAAATTCTTTAATGATGTTTATGCCGAAATCTACAAAAATAATTTCTCTTTTATGGTTAATCAATATGTTTTTATATTCGACA
ACCTTAAAATTAACTTCTTTCAATTCCATAATAAAGTCTCTATAAAATAACTTAGTTTAAAAACGATTCGTATCTTTCAGATTCAAAT
ACCATCATTTTCTCCTAATACTTACACTTTAATTACAATTATGTAAGTTGTTTTCAGAGGCATCCTATTTTATATTTTGTCAGAGGTGA
TCTTTTGAAAACGATTTTAAAAACAATAACATATCTTGCACTTACTATCATTGGCGCTTATGCTGCTTTATTCATTTTAAAAACAATA
GACTCTCATGGTATAACAGATCAATTTAACCCATTAGTAAAGGAAGATGATTCTTATGTTAAAACGACAGAGGTGTCTACTAGAATG
GATGATCAACTCCGAAGTTATACTCAAAGTGCTTTTAATAAAGAAGGGAAAGAAACGCAATTAATGTATACTGCTACATTTGATGTT
AAACCGCATAGATACTTGAAAATTACACATAAAGGTCATCATGTAGAAACTTTTGAAGAAGTTGAAAAGGAAGAAGTACCTAAAA
AAGCATTAGACAAACTGAGTCGATAATAGCATGCTTATATTACATGGTTCAATTATAAAATGAGTACGAACGAAAGTAACACATGA
TGATTAATTTAAAAATATTTGTAATAATTATGAATAAAATTAAAAACAAGGGGTAATACAATCTATATAGCATATAAGCTTTTGTTA
TGAGTTTCAAAAATAGGAAGAGAGAGTGATATTATGAAATTAAAATCATTAGCAGTGTTATCAATGTCAGCGGTGGTGCTTACTGC
ATGTGGCAATGATACTCCAAAAGATGAAACAAAATCAACAGAGTCAAATACTAATCAAGACACTAATACAACAAAAGATGTTATTT
CTTTAAAAGATGTTAAAACAAGCCCAGAAGATGCTGTGAAAAAAGCTGAAGAAACTTACAAAGGCCAAAAGTTGAAAGGAATTTC
ATTTGAAAATTCTAATGGTGAATGGGCTTATAAAGTGACGCAACAAAAATCTGGTGAAGAGTCAGAAGTACTTGTTGCTGATAAAA
ATAAAAAAGTGATTAATAAAAAGACTGAAAAAGAAGATACAGTGAATGAAAATGATAACTTTAAATATAGCGATGCTATAGATTA
CAAAAAAGCCATTAAAGAAGGACAAAAAGAATTTGATGGTGATATTAAAGAATGGTCACTTGAAAAAGATGATGGCAAACTTGTTT
ACAATATCGATTTGAAAAAAGGTAATAAAAAACAAGAAGTTACTGTTGATGCTAAGAACGGTAAAGTATTAAAGAGTGAGCAAGA
TCACTAAAAAGATTCGTTGCATTAACGCTTGTAAAATGTCTTAATTCATATTAATTAATAAAATACCCTCACAACGTAATGTAATAT
ATCCAGTATAGATTTGAAAGGATATATGCCAAACGTGTTGTGAGGGTTTATTTGCATCTATTTATCCATGCGAATATCGACTTCTTCT
AAATGTTTCTGATATTCTTTAACCTTACTTTCTAAAAACATTTCATATGGTGCATCAAAGAAGTCAGCTAAATGCATGGCATCTTTAA
ATTTAGGTTCATGGTGATGATTCTCCCATTCCCAAATTTGATGTGCTTCATATTTAGTACCATATTTCTCATTTAATTGCTGTGCTAAT
TCGTCAATTTCTAAATTATGTTTAGTTCGTAAGTTATATAAAATATGCATATTCATTGTTCATTAACCTCGCTTCTTGATTTAAAATTC
AAAGATGCTCTAATAATATGTGTATATTCTAGTCCTATAACATAAAATAATCAAGCTATGAATTCGGCAGAAAAAATAATGTATATG
ATATTGCCGGTACAGAGACTTTAAATAACGATAGCTACATTGAATAAAATTGATATTCAATTACTACTTTTAAAAATATTTGGATAA
AAATAATTTGAATTTGTTTTAGAATTGTAAATAAGGGGTACTACTTAGAATAACGCAAAATAAATAGAAAAAGGAGACTGAAAATT
ATGTTTGGATTTATTGGAATGTTAATTATCGGTGGCTTAATTGGATGGGCTGCTGGTGCTATTATGGGTAAAGATATCCCAGGTGGT
ATTTTAGGCAATATTATCGCAGGTATTATTGGATCATGGGTAGGTGGCAAACTATTCGGACAATGGGGTCCTGAATTAGGAAGTATT
TACATCTTGCCAGCATTAATTGGTTCAATTATCTTAATTGCAATCGTAACGTTAATTTTAAGAGCTGTTCGTAAAAAATAAAATATTC
AACTTAGGTTGAATTGAAATAAAGATGGCATTCAGTTGTCATCTTTTTTATTTGCGTGAAAATGGTCAATATGTATTATAATATTTGA
AAATGTAGATAAGATAGGATTAAAAAGTAATAAATTAATATGAGGAGAGGTTGGAAATAAAAAAGCAAGTATTTGATAAATAAAA
AAAGGATGACGTTGGGTTGCGTCATCCAGGAGTATTATGTTCATGAATATAAAAGACTGTGTAGTCATTTAAGAACTACACCCATAT
AGTAACACATCACTTGATTAAAGACAATACTATTTTTAGAGTCATTAATTTAAAATATGAATTAATTATTTTTTTAGAATAGAATATT
AAGATTGATATCGAATTAGTAGTCAAAGTGTTATGGTAGATATGAAATACATAAGGTGAGGAGTAATATTATGACGATTTATTTAGT
TAGACATGGCGAATCAAAATCGAATTATGATAATAAACATTTTAGATCTTATTTTTGTGGACAATTAGATGTGCCGTTAACGGATAC
TGGCACAAAAAGTGCGGACGATTTATGTGATTATTTTAAAGAGAAACAGATTAAACATGTATATGTTTCAGACTTATTAAGAACAC
AGCAAACGTTTGAACATATTTTTCCATATGACATTGCATCAACGACTACGCCTCTATTAAGAGAACGTTCACTTGGCGTATTTGAGG
GTGAATATAAAGATGAAATCAGTGCGAATCCGAAATATGAAAAATATTTCAATGATTCAAACTTTAAAGACTTTCGTCATAGTTTTT
CACAAAAAGCGCCTGAAGGAGAAAGTTATGAAGATGTATATCAACGCGTAGAACATTTTATGAATCATGTTGTCAATGAAGATACA
CAAAAAGATGATATTGTCATTGTTGCACATCAAGTTGTCATTCGTTGTTTAATGGTTTATTTTAATAACGTTTCACGGGAAGAAGCTG
TGGATTTAAAAGTTGAAAATTGCAAACCATATATCATTGAATAGAAGTATTGAGGTTCTGAAAAGCTGTGTTTTATAGCGGCCTTCA
GAACCTTTTTTGTATTTGGAATTTAATGGGAATCATTCTTATCAACAGACTTACCACCAATAGCTAATGTGATAATTGTGCTTGCGAA
CAAAATAATTGAAGTTATAAATGACGCTAATGTAAAAGGTGTAAATTGTGTGAGCGATAATATCAATGATAAAAAGAACCAAGAG
ACAGCCCCTAGAAATTGTAAAATAGCAGAAGAAATTCTAAAACTCATATAATCATATATAGCTAAAACAAGTGAAGGTAATGCGAC
TAATACCATTATAATGATGTTGAGGGTGAATAAATATGGCTGTTCAAAAGTTACTGTGTTTGGTCCTGTTGGATGCATGGCTGCTAA
GAACAAGCAAACAATCGTCGATAAAATTGCTAAAATAATAAAAGTAATTCGAACTTTCATCATGATCATCCTTTGTTTATAGAGTCA
ATATAAGTATGGAATATGTTAGGGATATAGTCAAATGCGTCAACTAATGGGTGATTTTGGCATAGATAGAGAATTTAAGGCAATTA
AAGAAGCATCAAACAGTAATATGCTGCTTGATGCCCAAATGATGACTTTAGCTAAATTGATTAGTCACTTTTAAAGATAAAGAATTG
TCATGAATTAAAGCACATGTAATGATGTGTTACATGTCGCAATGATGGCTTTCAGTTATTTATCGATAACATCACTCTTGATACCTTT
AGATTTTAAGAAATCTTTAATTTTATCTTGTTGCTTTTTATTAACATCACCGGCATATTTTGTTGGCACGTCGACAACATTGATTTTAT
TTTGCGGTTGATAGCTAAGCTTTTCAATATCTTCATCAACATTGGCGATTGTACTATTTAAAGCTTTGAAGTAATTCATCATTAATTC
AACGGGTTTCTTATATTCTTTAGGAATATTGTTTTCAGTGACAAATTTCTTGAAATGCAAATCGTTTTTAACAGCTAAGTTAGATAAG
TGGCTAAGTGTTTCTGCTTGTTTTTCAGTCACTTTTGTTTGACTGTCAATTTGTTTATCTAGTTTATGTTGCATAATATATTTGTTATCA
AGTATATCGCTATTTACAGACAAATACTTTTCTATAGCTTGCTTCATCTCTGCATCACTAATATCACTATTTTTCTTATCTGAGTTAAA
GATATCTTTTGTTTCTAATTTTTTAGCGCTTTTAGGTGCATGGATGCCAGTACTTGTATGATGATCTTCGTTATCAGATTGATCGGACG
CGCAACCTGTAAGAATTAATGTCGATGCTAAAAATGTACTTAGTAGTAATCTCTTTTTCATAATGTAATATAACTCCTTAGTTTATCT
TTAATTGAAAAAATATGTATTCATGTTTAATAGAGTAACATTGAATTAGTTTGGAATGTCACGATGACCTTGCAATGACCATAGACG
TAAATGATTACGTGCATGAGTTACTTTTTCTATCAATAATGCGTCGTTTTGAACATTGTTAAGGATAGCATTATCTATAAATAACTGC
ATAATTGGTTGTATCAATTTAGAGGTAGGTATCGTACGTAAAAGCATAATGATTTCGTTCACATACTTTTCCTTCTCAATATCATTTT
TCATATTGATTTGTTTGCGAGAGGTACATACTTTAAGCATTATCGCACACCTCGTTGTATATATTAAGTTTATCATAACATGATTTTA
TGTCGGGATAAAAAAATAACAGCATCTTAACAAATGTAAGATACTGTCAGTGAAATGAATGAAACTTTAGTTTCTGATAATATAGT
CAAAGGCATTTAATGCTGCATTTGCACCAGCGCCCATTGAAATAATAATTTGTTTGTTCTTCTGATCTGTGACATCGCCAGCAGCAA
ATATTCCAGGTACGTCAGTATTATTGTTACGATCAATAACAATTTCACCACGTTCGTTTAATTCAACAGCATCTTTTAACCATGATGT
GTTTGGAAGTAAACCAATTTGAACAAAGATACCATCTAAGTTAAGTAGATGTTCTTCGCCGGTGCTCATGTCTTCGTAACGTATACC
TGTAACATGGTCTTCTCCGACAACTTCAGTAGTTTTGGCATTTGTTTTGATATCAACATTTGATAAAGAACGTAAACGATCTTGTAAC
ACGTTGTCTGCTTTTAATTCGCTAGCGAATTCGAATAATGTAACATGATTAACGATACCAGCAAGGTCAATTGCTGCTTCAACCCCA
GAGTTACCGCCACCGATAACTGCTACGTCTTTATTTTCAAATAGAGGTCCGTCACAGTGAGGGCAGAATGCAACACCTTTATTAATC
AATTGCTCTTCACCTGGAATGTTTAGCTTACGCCAACCTGCACCAGTAGCAATAATGACTGTTTTACTTTCTAAGACAGCACCGTTTT
CTAACGTAACTTTAATTGCTTCGTCAGTCTTTTCGATATCTGTAGCACGTATACCTGTCATTGCATCAATGTCATATTGATCAATGTG

CGCTGCTAAGTTAGAAGAAAATTCAGAACCAGTTGTTTCTTTAACAGTAATGAAGTTCTCAATACCAGCAGTATCATTAACTTGGCC
ACCGATACGATCAGCAACTATACCAGTACGTAAACCTTTACGTGCTGTGTAAATCGCTGCACTACCACTAGCAGGACCACCACCAA
CGATTAAGACATCATAAGGTTCTTTATTTTCAAACTCAGATGCATCTGCCGTACTGCCTAGTTTCGAAAGAATATCTTGGATTGTCAT
ACGACCATTGCCAAATTCTTCGCCGTTTAAAAAGACAGCAGGGACTGCCATGATGTTTTCAGATTCTTCACGGAACACTGCACCATC
AATCATAGAATGCGTGATGTTAGGGTTGATCACACTCATTAAGTTAAGTGCTTGAACGACATCAGGACATTTTTGACACGTTAAACT
AATGAATGTTTCAAAATGGAATGAACCTTCTAAGTTTTTAATTTGGTCAATAATTGACTGTTTTTCTTTAGGTGCACGACCACTAACC
TGTAAAATTGCTAAAACAAGTGAGTTAAACTCGTGACCTAATGGAATTCCTGCAAATGTTACACCTGTTTCTTCGCCAGGACGGTTG
ACTGAGAAACTTGGTGTACGTTTTAAAGACTTTTCAGAAAGTGAAAGTCTAGGTGACATATCAGAAATTTCTGTTAATAATTCTTTG
AGTTCCTTAGATTTTTCATCTGAACCAAGGCTGGCAACGAATTCAACGTTGCCCTCCATTAGTTCTAATAATTGTTTAAGTTGTTGTT
TTAAATCAGCATTAAGCATGGTTGTAATGCCTCCTTAGATTTTACCTACTAAATCTAAACCAGGTTGCAATGTTTTAGCGCCTTCTTC
CCATTTAGCTGGGCATACTTCGCCAGGGTTTTTACGAACATATTGAGCTGCTTTGATTTTGTGAGCTAATGTACTAGCGTCACGGCCA
ATTCCGTCAGCGTTAATTTCAGATGCTTGTACAACACCGTCTGGGTCGATAATGAATGTACCACGTTGAGCTAAACCAGTAGCTTCA
TCTAATACATCAAAATTACGAGTGATTGTTTGTGATGGGTCACCAATCATAGTGTAAGTGATTTTGCTAATTGCATCTGAATGGTCAT
GCCATGCTTTGTGTACGAAGTGAGTATCAGTTGATACTGAGAATACATTTACGCCTAATTTTTGTAATTCTTCATATTGGTTTTGTAA
GTCTTCTAATTCAGTTGGACAAACGAATGAGAAGTCAGCAGGATAGAAGCATACTACGCTCCAAGAACCTTTTAAATCTTCTTGTGT
AACTTCTTTAAATTGATCTTTTTTTGGATCGAAAGCTTGCGCTGTAAATGGTAAGATTTCTTTGTTAATTAATGACATAAATATCTTC
CTCCTAAGAATTTAAGTATGAATTAGAACTATCAATTGATTGTGCTTAATTATAATAATTCTAATCTCTTAGTTAGCATTATTACATT
TTGATTCAGAATAGTCAACTGGATAACTTTGTAAAGTGAATGATTACTTTTAAAATAGAGAAAGATAATATAAAGTGCTTTGATAAT
GGATTTTGTAGTTGATGATTTAAAAGGTTGTGTCTATATTTAATATCTTGATTTTAATGTAAAAAATGTAAAAAAAGAAGAGTTGTA
TTCTCAACTAAGTCAACCTTATTGATAATGGTGTGAGAATATTTGTTCGAGATGGATGAAGGTGATGCATGAGAAACTGGATTTTTC
AAGTGCGAGACAATATTTTTAAAAGTTCAATTATTAACTTATAAGCAAATAATTGCTATAAAAAAGTTTGTACGTGTACAATTGCAA
TATGAAGATTTTAAATTAATTGTAAAGTATCGAGGAGTGGGTAACGTGTCAGAACATGTATATAATCTTGTGAAAAAGCATCATTCT
GTTAGAAAATTTAAGAATAAACCTTTAAGTGAAGACGTTGTTAAGAAATTGGTAGAAGCTGGACAAAGCGCTTCGACGTCAAGTTT
CCTGCAAGCATACTCAATTATTGGTATCGACGATGAGAAGATTAAAGAAAATTTACGAGAAGTTTCTGGACAACCTTATGTTGTAGA
AAATGGCTATTTATTCGTCTTTGTTATTGATTATTATCGTCATCATTTAGTTGATCAACATGCTGAAACTGATATGGAAAATGCATAT
GGTTCAACGGAAGGTTTGCTAGTAGGTGCAATCGATGCAGCATTAGTTGCCGAAAATATTGCGGTAACTGCTGAAGATATGGGGTA
TGGCATTGTCTTTTTAGGATCATTAAGAAATGATGTTGAACGCGTTCGAGAAATTTTAGACTTACCTGACTATGTCTTCCCGGTATTT
GGTATGGCAGTAGGGGAACCCGCAGATGACGAAAATGGTGCAGCCAAGCCACGCTTACCATTTGACCATGTCTTCCATCATAATAA
GTATCATGCTGATAAGGAAACACAGTATGCACAAATGGCAGATTACGACCAGACAATCAGCGAGTACTATGATCAACGTACAAACG
GGAATCGCAAAGAAACATGGTCGCAGCAAATTGAGATGTTCCTAGGAAACAAAGCAAGATTAGATATGTTAGAACAATTGCAAAA
ATCAGGCTTAATACAGCGATAGCAAGATACCAAAATAACCCGCCCCCCTCTAGCTTAAAATGATAAGTATAGCTAGAGGGGGCGGG
TATTTCTTGCAATGAATTAGTGTGAAGTTAATGCAGCATTATCATTTGAATCGAAAGTATCTTTATCCCAATGTTTAGTTAACTTGGC
GGTACCTGTACCAGCTAGCATTGAATCGTTCACGTTTAATGCTGTTCTACCCATGTCAATCAATGGTTCAACGGAGATGAGCACGCC
GGCTAAAGCGACTGGCAAGTTTAACGTTGACAACACCAATATGGATGCAAATGTAGCCCCGCCACCGACGCCAGCAACGCCGAATG
AACTAATAATCACGACAGCGATTAACGTTACAATAAATTGTAAATCAATTTCTACATTAGCGACGGGTGCGACCATAATTGCAAGC
ATGGCAGGGTAAATGCCTGCACAACCATTTTGTCCAATCGACAATCCAAATGTCGCAGCGAAATTGGCAATACCTTCTGGCACGCCT
AGACGTCTTGTTTGTGTTTGTACATTCAATGGTAAGGCACCCGCGCTTGAGCGTGATGTGAATGCAAAGATTAATACTTCCAAAGTC
TTTTTAACATAGCGAATTGGGCTAATACCTAACAGGCTTAAAATAATTAAGTGAATGATATACATCGTAATTAATGCAGCGTACGAT
GCGATTAAGAATTTTCCTAAAGTCCAAATGGCGCCAAAGTCACTTGTCGATAATGTGTTGGCCATAATTGCTAATACACCGTATGGC
GTTAAACGTAAGACGAACGTCACAATCGCCATTACTAGTGAATAGATAGCGTCAATCGCACGCTTAAGCAATTCACCATGATCAGG
TTGTTTGCGTGCTACGCGTAAATAAGCAAATCCTATAAACGAAGCAAATATCACGACAGCAATCGTGGAAGTTGCACGTTGTCCAG
TGAAATCTAAGAATGGATTTTTAGGCAATAATTCCAAAATTTGTTGTGGTAACGTATGTGCTGTTAAATCTTTCGCTTGTTTAGCAAT
TTCGCTTCCACGTGCTTGTTCAGCGTTACCAAGGTTAATTGTTGATGCATCTAAACCAAACACCAAGGCATACACAACACCAACAAT
CGCAGCAATGGTGACAGTGCCAATTAAAAAGATAAAAATGAGACTACCAATTTTAGCAAACTTTTCTCCGATTTGAATTTTAGTGAA
TGCAGCTACAATAGAAATGAAAATTAAAGGCATAACAATCATTTGCAACAATGCAACGTAACCTTGTCCGACAATGTTGAACCAGT
CACTTGTTGATGTAATAACATTCGAATGTGTGCCATAAATAAGATGCAATAACACACCGAATACTATACCAATCCCTAAAGCTGTAA
ACACACGTTTCGCAAAAGATATATGTTTGCGAGCCATCATGTGCAATATTACGATGAAAATCACCAATACAATAATATTAATCAGTG
TAAGAAAAGCATTCATGAACGTCACTCCTCAAATTTTTGAATATAATTCCGACTAGTATGCTAAGAATAATATTAATTTTAAAATAT
TTCAAGAGGGCATTTGCAATTAAATGTAAACATGATGAAATTCGACCGTTGGCATAAGGGGATTTGTAAACAAATCGTTTTGAATTC
AGTAGTGATAGGTGGAAAGGGTAAATAGTGTGATGAGCTAGTCGATTAAAGTTAATATTTGAAAATGAAGCAGGGCATTAAATGCA
ATAAATTAAATAAGTTGTCATTAAAGCATTAATAATAGAAATGATTTTAACAGGAAAAAAGTGATGAATATTTGGAAAAGATATAT
ATCGTGCACTGTCATGAGAGATAAGATATGAGAAAACAAATTTATCCTTGAATCGAGATGTGGCTATGGCGGTTTGATAAAAATTG
CTAACCTCATATTTATTGAATGAAAAGATAATAAATGATCAACCTTGAAAAGACGCTTCGAACAAACACGTTGTAAAAAATTCTGTC
GAATCAAAATTAGCATCTTTTGAGTGTTCGTGTGTGCAGGTGGTAGTTCAAAAGCTACCGTAAACAAATATTTTTGTTTGAGCAAAA
AGGCGTTTGTAGTAAATGTGAATATGAGTGTGCACTTGTGCGATTCGTCAGTTACTAACACAGCCAAACATTTAAATTGAATGGACT
TATTAAGATGAATCTATTTAGAAGTACACAGAAGCCATCCCATTTAAAAGTAACATACTATAAAAATTTATTCAAAATTCAATATTA
ATCATAATGAATAATTAAAAAACCTGAAACAAATTAGTTTGATATGCTCTCTTCAAAGTAGATATTGAATGGAGTATATCAGCTTGT
TTCAGGTTTTATGAATTGAGTATTAAATTTGAAAGTAAATTACAGTTCTATAGGTTTATAAAAATCAGTTTTTAAATCAAAGCTATAT
ATGAATTGAGTATTAAATTTGTGTTCAAATCACAAGTTTACAGGTTGAATAAACGTGCGAAGAAACCTTTTTTCTCTTCTTGAGGTTT
TGGTTCTGACATTTCTGACTGTGAAGCTGTGCTGTCATTTTCTGCTTTCGCTTCCAATGAATCGTTCGAAGTAGCTTGTTTAGAAGCTT
CTGCATGGGCTTCTGCTACTTGTTGTTCAGCATCATGTTGCGCTTCTGCATCTACTGTAGCTTCTTTATTTGAAGTCTCAGCTGCATTG
TCATTTTTAGACTGCTCAACGCTGGCTTTTTCAATTGCTTCAGTTGCAGAAATTTGATCCTCTTTATCTACTTTAGTAGCAGTGCCATC
GCCTGCATTTTCTGCTTTAGTATCCGTCTTAGCGTCTGTTGATGTATTCACTCCAGCAACCTTCGAATCTTTACTGTCAGCTTTATCTT
CAGTAACAGCATTCTTTTCAACTTTATTTGTTGATTCAGCTAATGCTTTTGCTTGAGAGGCTTGCGCTTCTTGGATAGCCTCTTGTGAT
GTTTGGATGGCTTTTAAGGATTCGCTATTTGAATCGATTTTAGATGTAAGTTGATTTTGAAGTTCTTTTAATTCTTGTTGTTGCTGATG
TACTTGATTCATCATTTGACCAAGTAGGTGACGTTCTTCGCGCATTTTCTGGATTTCTAGGCGTAAATCTTCTACTAGTTGAGCGACA
TTTTGATTAGTAGGTAAGTTTTTGTCATCGTTTTTGACAATGACTTGCAGGAAGTCTTTTTCTTTTTCTAATTCATCAAACGCTAGATC
ATAACTATTTGTTTGTTTTACTTTGTCGGCAATGTCTTTGAAAAGCTCGATATCTTCTTCTTTGAAATCAGTTGCTTCACGACCACGAT
ATTCAGTCTTGCTTAGTTGGTAACCACGTTCTTCTAAATGTTGAACAATTTTACGTACTTGCTTCTCACTTAGTTCTACGCGTTGTGCA
AATTCTTTAGTTAACATACTTTAAAGTCCTTTCTGCATATACTTTATGTATACTGACGTTTGTCTACGGGCAGTGAAACGTCAGTCTT
GCTTCATACATAGTTTAAACGTTATTGTTAGTGATTTCAAGTTTGTTTAAAATGGTTTACGTAAATCCATTTCTTCTAATAAAGCATA

AATAATTTCACGTGCGATACCATTGGCAAATACATACTTCAAAATATAATTTGATTTAACACTTGTACCGTAGATATCCTTTGTCAGT
TTAATATTTTTCTTTAAAAATAAGTCCACTTTTTTAGTGTGACCATCATTAGTTGGATGAATGTAATCAATTTCAGTTTGTCTTTCAAA
TTGATGTTCGTATAAAAACTGTCTACGGTGTTCAATAATGAGGCCGTTATCTTGTGTTAAATCTGCAGGTTCTTTAGGGACTTCCATC
ATTAAAAAGTTAATACTTCTAGAATGAACAAGGTTTGATAATAAGTTTAGTTCATGTTCAATTTGATGTAATGTTTGCGTCATTAACT
CATCATGGTTGGGTAGGTCTTTTGCGAATAAATAAATTAAAAATGCTGAATTGGTTGTGGCTTCATAATGTGCAGTTGCTAGACTGA
CAACAACATCGTTTTCTAAGCCTACGATAAAGACATAATCGTTTTCGGTTTTGTTGTTTTCTAATGAGCGTTTGATGATACGTTTATC
TTCAGTTAAAGATAAATTTAACTTAGTATCGTAAAGTTTAAGTGCTTCGTTGTAATGTGGATCTTTGACAGATTGAATGGTTTTAAAT
TCCATAAGAACACCTCCCCAATTTAAATAATATTATAGCATAATCGCCTGCTGTAAAAGACTGTTCATAAACTTTTAAATGGTATAA
AAAACTGTACTATCTTAAATTAGACAGTACAGTAATCTCATTTTGAATTCAGTGTGATAACTAAGCTTTGGGACCTTTAGATGCTTCA
GCAAAATGTGTAATATCAATCTCTTCATAAGCTGAATTATTTTCATGCACTTCTTGATGTGATGATTTGTCACGAACCGCTACAACTA
ACATTTTATCGTCTAAAATAAGTTGTTTATATTTTTCTAATTCATCAGGCGCTAAGTTGTAGCGTGATAAAACTGCATGTTCACCATC
TTCTCCTGTTAACAGTTTAGTCATTCTATCACTAAATGTTCCACTTGTTGAGATAAGGGAGATTTCAGAGTCGTGTAAGTCATTTAGG
TGTAATTTACTTTTACTAATAATTGTTAGCTCTGATTCTAAATAACCTTCAGATTTCTTTTGATTGATTACGTTGTATAATTCGCCAGT
GTCATTTACTACAGTAATATCTGCCATAGTTGTCGCCCCTTTAAAAATTTGTTTATTTAATCTTTTACCCTTCTTATTATAAAGTAAAA
CCCTTACATTATTAAGTTATAAGTCTTCATTCGCATTAAACGTCTCTGTACATTTATAAAACTATTAAAAGATTACCTAAGCAGTTAT
TGAAAAAATGCCGAAAATTTGCTATTATCGTTAAATAATTTACATAAACTCATATAATCTAAAGAATATGGCTTTAGAAGTTTCTAC
CATGTTGCCTTGAACGACATGACTATGAGTAACAACACAATACTAGGAGTAGCTTCAGCCATTAAATTGTAACCATGGTGGGTGATT
TATATCATTTTATATGATGGTCACAGTTTATTTGATGAAACTTCTTTTACATTGATTGCATGACAAATTCGTTATGCATGTTCGTTCAC
TCATAAACCCTGAAACTATTATTTAGTTTGGGGATTTTTTTGTATCTAGCACCAATTTAAGAGCAAAATGTTTCACACAAATCTGAGG
AGGTTTTAAGAGTGGAGTTACTAGGACAAAAAGTAAAGGAAGACGGCGTTGTCATTGATGAGAAGATTTTAAAAGTCGATGGATTT
TTAAATCATCAAATTGATGCAAAGTTAATGAATGAAGTTGGTCGCACTTTTTACGAGCAATTTAAAGATAAAGGGATTACTAAAATC
TTAACCATTGAAGCTTCCGGTATCGCACCTGCAATCATGGCTGCACTGCATTTTGATGTGCCATGTTTATTTGCGAAAAAAGCAAAA
CCTAGCACTTTGACGGATGGTTATTATGAAACATCTATTCATTCATTTACTAAAAATAAAACAAGTACGGTCATTGTTTCAAAAGAG
TTTTTATCAGAAGAAGATACTGTACTTATCATCGATGACTTTGTAGCAAATGGTGATGCTTCATTAGGATTATACGATATCGCACAG
CAAGCGAATGCTAAGACAGCTGGTATTGGTATTGTTGTTGAAAAGAGTTTCCAAAATGGGCATCAACGTTTAGAAGAAGCAGGTTT
AACAGTTTCTTCTCTCTGCAAGGTTGCTTCACTAGAAGGAAACAAAGTGACATTGGTGGGAGAAGAATAATGAAAAATTTAATCCT
AAGTGTTCAACATCTTTTAGCTATGTACGCAGGTGCTATCTTAGTTCCAATCATTGTTGGTACAAGTTTGAAGTTTACACCTGAACAA
ATCGCTTACTTAGTTACAGTAGATATATTTATGTGTGGGGTTGCCACATTTTTACAAGCCAATAAAGTAACAGGAACAGGATTACCA
ATCGTTCTTGGATGTACATTCACGGCTGTTGCGCCCATGATTTTAATTGGTCAAACGAAAGGAATAGATGTACTTTATGGTTCGCTAT
TTTTATCAGGGATATTAGTTATTATCATCGCGCCTTTCTTTTCACATCTTGTAAAATTCTTCCCACCAGTAGTAACGGGTAGTGTTGTT
ACTATCATTGGTATCAATTTAATGCCAGTAGCAATGAATTACTTAGCTGGAGGTCAAGGTGCAAAGGACTATGGAGATGTTAAGAA
CATTTTGTTAGGTTTAATGACATTAATCATTATTCTTGTTTTACAAAGATTCACAACTGGATTTATTAAGAGTATTGCCATATTAATT
GGACTCGTTTTAGGAACGATAGGTGCTGGCTTACTTGGGATGGTCGATATTAATCAAGTCAATCATGCCGGTTGGTTAGGCATCCCA
GTGCCGTTTAGATTCTCAGGATTTAGCTTTGATGTGACATCGACGTTAGTGTTCTTTATTGTAGCTATCGTTAGTTTAATTGAGTCGA
CAGGTGTCTATCATGCGTTAAGTGAAATTACCGGTAAGAAGTTAGAAAGAAAAGATTTTCGTAAAGGTTATACTGCGGAAGGTCTA
GCGATAGTGTTAGGTTCTATATTCAATTCATTTCCGTATACAGCCTATTCGCAAAATGTAGGACTTGTTTCTTTATCCGGCGCTAAGA
AAAACAATGTTATATACGGCATGGTCGTGTTATTACTTATATGTGGTTGTATACCTAAGCTTGGCGCATTAGCAAATATCATACCGC
TACCTGTGTTAGGCGGTGCGATGATAGCTATGTTTGGCATGGTAATGGCATATGGTGTTAGTATATTAGGACATATCGATTTTAAAA
ATCAAAACAATTTATTAATTATCGCTGTATCAGTAGGATTAGGTACTGGTATAAGCGCTGTACCACAAGCATTTAAAGGTTTAGGTG
AACAATTTGCATGGTTGACTCAAAACGGAATTGTTTTAGGCGCAATCTCTGCAATTATTCTTAATTTCTTTTTTAATGGAATAAAGTA
TAAACAAACGGAAGAAAATGTGAAATAATATAACTAATTAATTTGAAAAATGGAGGCTGTTTTTAATGTGGGAAAGTAAATTTGCA
AAAGAATCATTAACGTTTGATGATGTGTTATTAATTCCAGCACAATCTGATATTTTACCGAAAGACGTTGATTTAAGCGTACAATTA
TCAGACAAAGTTAAATTAAATATTCCAGTTATTTCTGCTGGTATGGATACTGTAACTGAATCTAAAATGGCGATTGCTATGGCTCGT
CAAGGTGGTTTAGGTGTTATTCATAAAAATATGGGCGTTGAAGAACAAGCGGACGAAGTTCAAAAAGTAAAACGCTCAGAAAATG
GTGTCATTTCAAACCCATTTTTCTTAACGCCAGAAGAAAGCGTTTATGAAGCAGAAGCATTAATGGGTAAATACCGTATTTCAGGTG
TACCAATTGTTGATAATAAAGAAGATCGCAACTTAGTAGGTATTTTAACAAACCGTGACTTACGTTTTATTGAAGACTTCTCGATTA
AAATTGTAGATGTAATGACGCAAGAAAATTTAATTACAGCTCCAGTGAATACAACACTTGAAGAAGCAGAAAAAATTCTCCAAAAA
CATAAGATTGAAAAGTTACCATTAGTTAAAGACGGACGTCTAGAAGGTCTTATTACTATTAAAGATATTGAAAAAGTTATCGAATTC
CCTAATGCAGCAAAAGATGAACATGGTCGTCTACTTGTAGCCGCAGCAATTGGTATTTCAAAAGATACTGATATTCGTGCTCAAAAA
TTAGTCGAAGCAGGTGTGGATGTCTTAGTTATCGATACAGCACATGGTCACTCTAAAGGCGTTATCGATCAAGTGAAACATATTAAG
AAGACTTACCCAGAAATCACATTAGTAGCTGGTAACGTAGCAACTGCAGAAGCAACAAAAGATTTATTTGAAGCGGGTGCAGATAT
TGTTAAAGTTGGTATTGGCCCAGGTTCAATTTGTACGACGCGTGTTGTAGCAGGTGTTGGTGTACCACAAATTACAGCAATTTATGA
TTGTGCAACTGAAGCACGCAAACATGGTAAAGCTATCATTGCTGATGGCGGTATTAAATTCTCAGGAGATATCATTAAAGCATTAGC
TGCTGGTGGACATGCGGTTATGTTAGGTAGCTTATTAGCAGGTACTGAAGAAAGTCCAGGTGCTACAGAAATTTTCCAAGGTAGAC
AATACAAAGTATATCGCGGTATGGGTTCTTTAGGTGCGATGGAAAAAGGTTCAAACGACCGTTACTTCCAAGAAGACAAAGCGCCT
AAGAAATTTGTACCTGAAGGTATCGAAGGACGTACAGCATATAAAGGTGCTTTACAAGATACAATTTACCAATTAATGGGCGGAGT
GCGTGCTGGTATGGGTTATACTGGTTCACACGATTTAAGAGAATTACGCGAAGAAGCACAATTCACACGTATGGGTCCTGCTGGTTT
AGCAGAAAGCCACCCACATAATATTCAAATTACGAAAGAATCACCGAACTACTCATTCTAATTAAGATAAAGGAGAACGACAAATA
TGGAAATGGCAAAAGAACAAGAGTTAATCCTTGTCTTAGACTTTGGTAGCCAATACAACCAATTAATTACACGCCGAATTCGTGAA
ATGGGCGTTTATAGTGAATTACACGATCATGAAATTTCAATTGAAGAAATTAAGAAAATGAATCCAAAAGGTATTATCTTATCAGGT
GGTCCAAATTCAGTTTATGAAGAAGGTTCATTTACAATTGATCCGGAAATATATAATTTAGGAATTCCAGTACTTGGTATTTGTTAC
GGCATGCAATTAACTACTAAATTATTAGGTGGTAAAGTTGAACGTGCCAATGAACGTGAATACGGTAAAGCAATCATTAATGCGAA
GTCAGATGAGTTATTCGCTGGCTTACCAGCAGAACAAACTGTTTGGATGAGTCATTCTGATAAAGTTATTGAAATTCCAGAAGGCTT
TGAAGTTATCGCTGATAGCCCAAGTACAGACTATGCAGCAATCGAAGATAAGAAACGTCGCATTTATGGTGTTCAATTCCATCCAG
AAGTACGTCATACAGAATATGGTAATGATTTATTAAATAATTTTGTCCGTCGTGTTTGTGATTGTAAAGGTCAATGGACAATGGAAA
ACTTTATCGAAATCGAAATTGAAAAGATTCGTCAACGAGTAGGAGACCGTCGTGTATTATGTGCGATGAGTGGCGGCGTAGATTCA
TCTGTTGTTGCTGTATTATTGCATAAAGCAATTGGGGATCAACTAACATGTATCTTTGTAGACCATGGCTTACTTCGTAAAGGTGAA
GGCGACATGGTTATGGAACAATTCGGTGAAGGGTTCAACATGAATATTATTCGTGTTAATGCGAAAGATCGTTTTATGAATAAATTA
AAAGGTGTTTCAGATCCTGAACAAAAACGTAAAATCATTGGTAATGAATTTGTATATGTATTTGATGATGAAGCATCAAAACTGAA
AGGTGTAGACTTCCTTGCGCAAGGAACACTTTATACAGACGTTATCGAATCAGGTACCAAGACAGCACAAACAATCAAATCACATC
ACAATGTTGGTGGATTACCAGAAGACATGGAATTCGAATTAATCGAACCAATCAATACATTGTTTAAAGATGAGGTACGTAAATTA

GGTATTGAATTAGGTATTCCAGAACATTTAGTATGGAGACAACCATTCCCAGGACCTGGTCTTGGTATTCGTGTACTTGGAGAAATT
ACTGAAGATAAACTAGAAATCGTTAGAGAATCAGACGCAATTTTACGCCAAGTGATTAGAGAAGAAGGTCTTGAAAGAGAAATTTG
GCAATACTTCACAGTGTTACCAAACATTCAATCAGTAGGTGTTATGGGAGACTACCGTACGTATGATCACACAGTAGGTATCCGTGC
AGTAACATCTATCGACGGCATGACAAGTGATTTTGCACGCATCGATTGGGAAGTCTTACAAAAGATTTCAAGTCGTATCGTAAACG
AAGTAGACCACGTTAACCGCGTAGTCTATGACATTACATCAAAACCACCAAGCACAATTGAGTGGGAATAATTATATATAGCAAAT
GATAGGCTGGAGTTACCGTTACTATGCGGTTTCCAGCCTTTTTGGTGTGCTTGGCATGGGCAACATCATGACGTGAAATAATGGGAA
TCATCTCTTTCTTATATTTTGAGACGGAGATGATGAGAAGTTAGACAAAAATGTAGAAGAAGTATTTAACGACAATCCTAGTCATAC
AATGATTGTAGATGATATCCGTTAGAGGAGTGAGAGAAAATTTATTTGTTACTAAGAGACAAGAGTTTTAAATAAATATCTTTTGTA
CTACATAAAAGAAAGTGGTCGTTATCATAGAATATTTGGCGATAAGGTAAATCTGATTATGTTAGAACCTATATTTATTGATGATTT
AGTGAGGTTTGAGAAGACAAGAATGATTGAATTACAATTGACAATTGACACCCCTTAGATTAATATTAATTTATTAGAAGTCAACTA
ACGCCTCTTAGCAATGCGAAACACAGTTGGCCTTTTTTTTGTAATAGGAGAATTTATAGTTATGGATAAATCACTTGAAATTAACTT
AAAAGAATTAGAAGGCATAATGTATAAAGAAAAAGTAATCGACGTTAAAGAGTATGATAATTTAAAAGTAACTTTTGAGACACTAA
ACGATGCTCCTAAAAGTGAAATACCCACTCCCTTTACAATAGAACAAATGTTATATAAATTAGAAACTAAAAATTTATATTTTCCTG
AAATTAAGTCAAAAGAAAAATGTGTGGAGCTTTTACATAGTGTTGGCTATTATAATTTGAAGCATTTTATGTATAATGAATTTGGTA
AAGATGAGCCGAAAAATTTTGATGAAGTATATATACTTTATAAATTTGATAGATATTTATTGAAACAATTATTTAGTTTAGTAAATA
TGTTGGAAACTCATATAAGAAATATTATTTTAGAAGTATATATGTTAGAAATTGAGTATAATAACAAGCCTTCGACACTGTTCTATT
TAGATAAAGATTTATATTTTGAGAAAGTTAATGATGAGTATAAATATTCTGCCAAAAAGCTAAAAGAGTTCAATAGGTTACAAAAT
GTTTTTTGGAGAGCTATAGAAAAAAAGAAAAGTAACGATAACGTTAAACATAATATTAAAAAATACAATATTATACCAGCTTGGGT
TTTATTCCAAAATTTTAGTTTTGGAGATTTGTCTACTTTTTATAGAACTACATTACCAACTTATAGAAATAAAGTAAGTAAGCGAATT
GAAAATTTAATAGAAAAAAATACTGGTATAAGCATAAAACTTCCAGAAAAGTTATTGTGTGCATGGTTGAATAGTATCAGGTTTTTG
AGAAATAGAATAGCTCACACAGATATAATTTATGGAATTAATTTTACAAACACCTGTGCAAAACATCATAGTGATGAGGAAATGTA
TGTAAATATAGAAAAGTATAAATATCAGCAAAGGTTAGTTACATTTTTATTAGCTATGAAGAAAATATTTATGAGTATGCCAGAAA
ATAATATTATTGAATGGAATGAGACTCTAACTAAGATAGAGAATAAATGTTCAGAACATAACTTTATTAAACTTTCTAGGTTAGGAG
TTATTGAAAATAACCTTTCTTACTTTAAAATAACTAAATAAAAAATAGCGCACACTAATAACTAAGTGATTGTCTTTTTACGCTGAA
TAATGCCCTGTGTTGTAGTTAGAAAGATTTTCAAATAACACTATATTTATAAAAATGTTGAATTATAATATGGTCGGTTTATTTTATA
GGAGTAATAAAAAGCATTTATCTTTTATCCGCCACAAATAATGAGTAAATGTACCTTGCTTTCAATGTTGATATTTTACAACCTTGTA
GAGTTGATTTGATATATCATCTCATACGTTGTAGGCAATGAATACGTAAATTATGCAGTGACATGAACTGTTGTGAAGTATAGAGCA
TTAAAAATCTTGATATATCAATAGTTATAAACTGGTACAAATTAACGAAAAGTACTCTTGTCATCGAGTGGGAATAATTATATATAG
CAAATGATAGGCTAGAAGTACCGTTATTACGCGGTTTATAGCCTTTTTTGGTGCGATTGGTATGGGCAACATCATGTGATGAAATGA
TGGGAGTTATATCTGTAGAAATTGGCGTACCAATTTCTTTATGTTGGGGCCCCGCAGTCAACTACTGCCAATATAAGATTGTAGAGC
CTAGGATTTTGATTTTATGTCCTGGGCTCGTTTCCTATTCTTATTAAATAGTATTTAAAAATCTATACTATTTATATTGAAATTTTTAT
TTATGGTTATGCCATAACTACTTTGTATTTCCGGTACTAAACTTTGCAATATTGGGTAATAGTTATTAGCGAAACCAATAATAATGGA
ATTAATCACATTGATACTAAGTTCTTTATCTTCAGGTAATGCATTTTCTATTATTTTGATTAAGTCTATATATTTTATTTCATCAGAAT
TTTTATGTGCTAAATACTTCATAAAATCAGTTTGCACTTTTTCGAAAACGCTATATCTTAAGTCCATTTGTACAAATATTAAGCTGAC
AGTTTTAGTTTCGAATTGACTTTGACCCAAAACTGAAGCAAGTTCTTCTATTGATTGATATAGCAATCTGATATCGCCGCTATCTTCA
AGCATATGAAACACTCCTTTATCATTTTAGAATAACCATGCAATGGCTTCTTTGACTACAGTTGAGATTATTCCAGCATCACCTATAC
TTAGACCGGCATTTATTGCGCCTCGTTTAACCGCATCACCGACAGCGTTAGCTGGTACTTCTGACCATTTTAGCAATGGTTTCAATTC
TTTTACAATACCTGAATACCCTTTATCAAAAATAGTTGCAGCTGTCGATGGAAGTCTAACCGATGGAGCCACTTTCTGCTAATATTGT
AGGAATGATACCATGAAAATATTATTTAATAAATGGGTTTGTTTGTTCTTATTTTTAGTAATAATATCATGGGTGATAATTAGTATAA
AATTTGATAATATCAGCACGATGCAAATATTAATTTCTAAACTGTTTATATTTATTGCTTATATACTATATTTAGTTAAGTTCATTCGT
TTCAAAAAAACAGTGGCGATCATTAAACAAGTATAAAGAAAGTCGATTTTGTCTTTCTCTCGAACATATATAAGTTTATATAGATGT
AGAACTAATTTTTATTTGTAAATTGTTTTGTGATTGGAAATGAGTATGAAAATGTTCTCCAATCAACAGAGTATGTCTTGGAAGAAA
TCTATTATACTTATGTGAATAAGTCATAATGTCAGACATACATAATAAGACGAATAGATTGTAAATAGCTTTTTACTTCTAATTGTTT
AGGCGGCCTTTGTTGGTCGCCATTTTTATAAAATAATATAAATGACCAAACCACAGCACCTATTAATTTAAGAGTGAGGTCATTTTT
AAAGGTAAAAAAGGAGTAATTTTATATATTAAAATGATATATAAAACAAGTAGTATTAGTATGTTGTGAATTGTTATAGAATTTGAC
TTGTTAGCAATTTCGTAGCTATTGTAATAGCAAGTACTGTATGGTTGTATTTTTTAAAGGTAGTGGAATGATATTGGATTTTGAAGTG
GAATATTTGTAGGTGGTTACAACGAAGCATATTAGAATTACAAAAGGAACGATGAAATTAAATGTCCATCATTCCTTATGCCTAGTC
GTTTTTTAGTTAATATATTTAATTGCTAACTTATTATTAGGGTCTCTTTCAAGAATTTTATTTGCGACCAATTGAATATTCTCATGTGA
AATCATATCAAAGTCTGGTTCTCCAAGGATTTCTAAAAATTCGTATAATAAATTAGCATTATTAAATTGCGAAATCTCTAACGATTG
CCAATAATGATAGTATGCTAAATCATAGGCATTATCTATGTAATTGAAATGAAAGTGATAGATGCTTGCGACCATTTCATGATATTC
AATTGTATTTATTTGATTCGCAAGATTTGATAAATATGATACTAATTTTTCATCTGAAATTTCGTTCGACATAAAGTATGTTAGAATA
TTCTCCATATCTTTATTCTCTAACATATTTTTTAATTCAGTTGGTAATAAATTTAATAGAATGTTAACGCTTTTCATTTATTTAACCCA
TCCATTCGATATATATTGCCATTTTTTCGTGTATAAGTTATGACTACAGTTCTACCACTCATAACAAGTGTTTTTTGGTATGCGGACTT
TTTGTATCGGCTTTGCTTTCCATACCTCATAACATGCGACATTACTTTAGAAACATCTTTCCAATTGTTTTTAGTAACTTTATTCCAAT
TATGTTTACTAGCAAGAATATGTTTTCTTTTATTAGCTCCTAGATTGGCTACAGAAGGTAAAACTTTACCAAAACTCTTTGTTTTGAT
AGCGGTTTGAGTTGCTTTTTTTCCATATGTTTTAATTGCATAGTTTATTCCGTATCTAGCTACAGCAGCTAATATTACTAATGGCAAT
GCAGAAGATTTAGCTTCAATTGAGTTGAATTTTATTATTTCGCCAGTTTCTTTGTCGATAAATGTAGCTTCATATTCTGTATTTTCAAA
TTGCTTTACGAAAACGTCATAATCTTTATTTATTAGTTTTCCATTTTCATCGACGTATCTGCCCTTAACATCCATGGAGTTAGTATTTA
AATCATATTGAATATTTGCTTTTAAATCAATATCTTCTTTAGTTAATTCAATTGTTGGTTTCGAATTGGTACTTGTTTTATCAATAATT
TTTACTTCATTGGGATTTTGTAAATTATTTTTATGTAAATCTTCTTGTGTTATTTCTTTATAAGAAGTCTCGCTTGCATTAACATTTCTA
GTTTCAATCACTCCTGTCACAGAAGTAATGGTTAATACTAAGACTAGTATTCGAATAAAGTTTTTAAGTGTATAATTTTTCATTTACT
ATCTCCATTTGTGACTTTTATTTTGAAATCTTTGAAATTATTAGAATAAGAATTTAAGGTATATGAATTTAATTTCTTATTTGACTGCT
TCATTTTGATAATTTCATTTGTGGTTAAAGCTGTGGCGAAAGAATTTCCGCTAAATAATTCTCTTTTTCCTTGATTATTAATGGACTCT
ATATTTTCGCTTAAATTATATATGTCTATTTTTCCTGAAGAACTATATCTCGATATTTTGAAATCTTTGCTTAAACCACCAACAGATAT
AATATTTGATTTTGAAGCTGGGAAGTCACTTTTTCCGCCTAAATTGTTGCCAGAAGAAGCGATAACAATTGTTCCATTTTCAATTAAT
TTATCTAACTTTTTAGAAATTTTGTAATTATATTTAGGAAACCCAAAGCTTATATTCACAATATCTACGTTGTGTTTAATACACCAAT
CAATAGCCTGTTCAACATTAGAGGGTGTACTTTCGCCATTATTATCTAATACTTTTAAATGATAGAGATTTGCTTTAGTAATGTTCTG
GATTACTAAATTACTGATTGCAGTACCGTGATTATGACTATCTTTTGTATGTTTATCAGCAGAAAAGTTTATTGTTGTTATATTTTGTT
TGATGTGCTCAGTATTGACCCCACTATCTAAAATTGCGACATTAATCTCTTTATTATTTTTCTCAATTGTAAAATATACCAATATACT
ACTTACTAAAATAAATGAGAAAATCCAGTTTTTCAAAATTCCTCCTCCTTATATATAATATAAATATAACAAATTAATGAATTTATA
ACTATATAATTTTAACATTTATGAAATTTTCATTGAACAAGAGAGAGTAATATTGAAGTTAGTTAATATGTAATTTGATGAAAAAAC

TAAAAAGTGTAATAATCATTCTGACTTAAATGAAGGCTGATGTCTTAATATTTTCAACAAAAAGAAACGTTGTTTTAATCTAATTAT
CATTTCACACGTTGCAAGTAGTAAATACGCTAGTATAAAATGATGTGAATTTATGAGAAATATAGAACATTAAAAATCTTGATATAT
CAATAGTTATAAACTGATACAAATTAAATAAAAGCACTCATGTTATCGAGTGGGAATAATTATAAAATAAAACTACCTGTTTACAC
GGATGGTTTTTAATTTGAGTAGTAGGGATAATAAAATTGAATGTGTGAATTTATGTTTGTTATTGCTTCTTATTTACCCAAAATATAA
TTTAATATATTATTTACATAACGATATAGCCCTCATTCTATATGCGGCATGGCTATACTTTTATAGGAATCAACATAAAGGATAGATT
AATTGAGGTGAACCATGAGATCAGACAATGATATTATTAATACAATTTTAAGTGTTGCCAAAAATAATGAAAATATAAGACTAGTA
TGTATGAATGGGTCTAGAGTCAATAATAAAGTTGATTATGATCAGTACCAAGACTTTGATATTGCTTATATTGTTAATGATTTAAAA
GAACTAACACATAATTTGTCCTGGATCAATAAATTCGGAAACATTATAATGATGCAGTTGCCAGATAATCAGGATTTATATGAAAAT
CAATCAGTCGATAGTTTTCATATCTTAATGCTATTTGATGATTATAAAAGAATAGATTTGACTTTAATCACTAAAAAGTATCTATCAG
AGTATTTGAGTAGCGATTCATTATTAAAAATTTTATTAGATAAAGATAATGTAGTTGATAATAATTTTTCGCCAGACGATTCAAAAT
ACTGGTTGAAAGAACCATATCAAAAATTATTTGATGAATGTATAAACGAGTTTTATTGGGTTTCTACATACGTGATGAAAGGATTAT
GGAGAAATCAGTTACTGTATGCTTTTGACCATTTAAATATTTGTAGGGAAATGTTACTTCTAATGCTTGCATGGGACAAAGGACATC
TATTAGATTATAAAGTAAATTTTGGTAAGAATTATAAGTATTTAACAAATCACATGTCTAAATCTGAAGAAAACAATCTTATTTCAA
CATACCCCACTTTAAATAGTAGTGAGATTAAAAAATCTTTATATAAAATGATTATATTCTTTGATGATATTACCAAATCAGTTAGTG
ATAAGTGTGACTTAATTTACGATGGAAAGCAATATTTAGAAGTGAAAAAGTATATAGGATTTGACTATATTAATTAGATTTAAAAAT
AGAAGAAAGAAACTTATTCTAAGTTCTGATTTATTTATAAGACGGATTTAAAATACAAGAAGCTGGAAAGTCTACTCCTCTCTATAA
CTATAAAAAAATAGCTATTAAAAATCTATCGTCATAGATTTCTTCATAGCAAACCTTAGCTAATTAAAGGTCCTGAGGAATAAATTTT
ATCAAATAATTGGAATGAAAAATTAAATATGATACGATTTCGTCGGGATGTTAATAAGGGATTGTTTAATTTCTGAATAAGGAGTTT
TACAATGTACATGATTAAAAATTCAAGAATAATAAAAGGTTCGCTTGCTTTTGCTCTTGTTTCTGGCGTGATTGTATGTTCAGGGTTA
ACTAGTGAGCATTCTTATGCAAAAGAAAATAATGTCATGCATGCATCTGCAAATAGTTCAGGTGAAATCGTTAAATTGAATGATAAT
ACCGCTAGAATTGATTTGAAAAAAGGTATTGATTATAGTGTTGACAAAAATGGAGTGGCAACACTAAAAGATGTAAAGACTGGTAA
AAAGGAAATTTTGCCATCAACTGCAAAAGATAAAAATGGTAAAAATGTTACGTTAATTTATTTTGAAAAAGACGGTAAATTAGGTT
TTTATGTGCAGAAAACACAAAAAGAACGTGGAGTAGGGAAATGCGTTTCTGGTATAGCGGGTGGCGCAGTGACAGGAGGCACTAC
TTTAGGTCTTGCAGGTGCAGGAGTAGGAACAGTTACTATTCCAGTAATTGGGACAGTTAGTGGAGGCGTAGTTGGAGCTGTTGGTG
GTGCTGTCGGCGGTGGTCTAACCGGTGGAGCCACATTCTGCTAATAGTGTAGGAGTGATACCATGGAAAAATTATTTAATATATGGG
TGTGTTTGTTCTTATTTTTAGTAATAATATCATGGGTGATAATTAGTATAAAATTTGGTAATATCAGCACGACGCAAATATTAATTTC
AAATCTGTTTATATTTATCGCTTATATACTATATTTAGTTAAGTTCATTCGTTTCAAAAAAAGATGAAAATAGGTTTTTTGACCAAAA
CCTAATTGTGATAGCATATGAAAAATGAGTAGTTTATCATTTTTTAGTCTAATATACTACAAAAATTTTACTTATGAAGCGAAAATA
ACGTGCAAGTAACGTGCAAATAAAAAAAACGACCTAAATCCCTTTAAAAACAAGGATTAGGTCGTATTTATTTGATACTGAAATTG
TCTAACGAAAATAACATGCAAGTAACGTGCAAATAAAAATCAAGAGTAATATGATATATATTATATGCCTAGGTAAAGTTTACGAA
CCTACAGATTTGAAATATTCTTCAGCAACTCCGTCTTTTATAATTCTTGAAATAACTGAGGTTTTTATTTTTACGTTGTTTTGAATTGT
CTAAAATAGTCATACATAATCTGTTTAACCCCTTACTTAAGCTTGTAAAATGCGGAAATAAGTTTTCCTTTTTTAGTTATTTTTTCTTG
GTATATTTTAAATCCACAATTATTGCAATAAAATTCTTTCAAATTCTTATTATCATACCAATCAACACAAACATGTCTTAGTGCAGTA
CGATCATAAATTTCAGAAGCAATAGATAAGGCATAGTCCATTACAATTTCTCCTGGTATATGCTTTTTGTGTTTATCGCTCCTACCTA
ATTTAGTTATTAATAATGCTTGAAATTCTCCATTCTTTTTAGCATCAGACGAGATTTTTCTTTTTTCGTTTTTCCACTAACGTCTTTTTC
AAGCTTTATATTTACAATTTTTAAGAAGAAGTATCCCATTATTTCAAGGTCTGATTTATCTATAATTAAATACGTACGTAAGGAATTT
TGATCGTTCATTGTTATTGCTTTATGTTTTAGGGAATGTGCTATTGAAGGGTTTAAAGATGTTTGAAATGTCTTGGTTTCTTGACTTAT
ATAGTTATGAAAGAGGGCGTTGTCCTTTATAGCATTTCTGAGATTAGAAATGCTATAAATAAAGTGATTAAGATTTTTTGTTTTCATG
TAATTTAAGAAATGGATTTGTTTTTTGTGGGTTATTTTTAATTCTATTCTTAAATGGTTTGAATTTTGCGATTGCAAATATGATAGCTG
CTTCTTTTGACAAAGTAATAGAACCTTTTTCTTCTGGTACCATGATAATCACTCCTTGTGCAATTGGATTTAAATATTATAAAATAAT
TTTACTACACAAAATATATTGTGTAAATAGTTTCTTATTGTTACCAAATAATATGTTTGGAAGCGTTATGTAATGCCAATCAACACCG
AAGTTCTCATGTCCTCTTTCCTTTTACGACTACATATATTGTTAATAATATTGAAATAATAAACTGATAGTGATAATATTTATTTAAC
TGATCCATGAGAGTAGGAACCCCATCATACGGGAATTTCGAAATCATGGGTTTTTTGTTTTAAAGGATTGATGCAATAGATAAAGTA
GCCATTCTAATATATGGAGTATTTATCAGCTGAAAATTAAATCAGATATGGGTTTTCTTCATTAAATAATTTTTCTATAAGGAAACAG
AGGCAACGCTACTGTCACCTCTGCTTGAAATAATTAGGATGAAATACTCTGAGTCTAAAGTCCACACACAATTCGCTCAAATCCTCC
AAACCTCCGACTACATCTCCAACCCCCCAAACACCAATACTTACCAAAGCATTGATATGAGTGCCAATACTGGCAATGTGCCTTGTT
TGAAAAAGATACTAATATTGCTTGAAAGGCCACCATAAATAGCAACGCCAATGATATACACTAAAATAGCTGCGCATATTTCTTTTG
GATTACTGCTGATAAACAAACCGTATATTAGCAAAACTCCGATTAAACCGTTATATACGCCTTGGTTCTTCAAAAGTAGGTTAATAT
TTTTGTCTTTCAATTTATCGACGCTTATATTAAATGTCTCGCTAGTCTTTTTGGAAGTTGTAGCAATCGTTTCAAGGTACATAATATAG
AAAAACTCTAATGCCACAAATATGATTAAAATTGTTGAGATGATATTCACTATAACGCTCCTTTATTATTAAATATTTTCTTGTAAAA
ATGATTGCAGTGTCTGTGGTTGATCATTGACTAATTGTTGGAAATCATTGGATTCTTGGTCTAATAGTCCTCTTGCTCCTGCGTCGTA
CATTGATGCCAATAATGCACCAAAGCCTTTAGGTTCATCGTACATTTCTGCAAATGTCTCTAATGAAACGGGCTCATATTTAATTTCT
GTGCCTGATGCCTCAGATAAAATTGCAGCAAGTTCTTTCATATCATAACTGTAGCCTGATAATAAGTAGCGTTTGCCCCAAGTATCT
GGATTTTTAATAATAGCAATGACACCTCTAGCAATATCATTTCTAGTAATATAATTAATACGACCATCGCCAGCTGGATAAATCAGT
TTATGCATATTCATCAATTCTGGTAAATATGGTTTAAGTGGATCCATGTACATTGCCATTCTTACATACGTATAGTCAATGCCACTTG
TTGCCAATAGACGTGCTGCATAACCAAAATAAGGACTCATATGGAATGGATTATTATGCTGATCTGCGTAATAACCTATGAAAATG
ATATGAGCAACGCCGCTCTGCTTTGCCGCATATACTAAATTTTCCACTTCAGGAATACGTTTGAATGATGGATGGATAATACTTGGA
ATAAACACAACGGTATCCATTCCTTTAAATGCTTCTACCATGCTTTCTTGATTAAAATAATCTAATTGTCGAACAGGAACTTTTCCGC
GCCAATCTTCTGGAACTTTCTCAACATTTCTAACACCAATGTGAAAATGATCTATGTGATTTGCAATGGCTTGATTTGTAATATGTGT
GCCTAAATGACCTGTAGCACCTGTTAACATAATATTCATTCACTTCATCTCCTAATCTTTATATACATAACATAATACTTATTTGATG
GTTTTCAAAACATTTGATTTTATAAAAAATTCTAATCTGTATTTATTGTCGACGTGTATAGTAAATACGTAAATATTATTAATGTTGA
AAATGCCGTAATGACGCGTTTTAGTTGATGTGTATCACTAATATCATTGAAAATTTTAATCAGGTACTACGACAATATGATGTCTGTT
TTGTGTCTGAAAGTTTTACAGTTTTTAAAATAAAAATGGTATAAAGTGTGATTTGTATAAAAAAGAGTCTCGACGGATAAGAATTGA
TTAATAACAGTTAGCATTTTATTAATTACCTTAACAATGATTCAAGTTTAGTTAAATGAGGTTTAATTTGAAAGGGGATAGCGCCTC
AATATAATGTAGGTAGATTGTTCATATTACGTAATTGAAAAATCAAATTTAAATAGATTGGGGCTAAAAATTATGAAATTTAAAGCG
ATAGCAAAAGCAAGTTTAGCATTGGGAATGTTAGCAACAGGTGTAATTACATCGAATGTACAATCAGTACAAGCGAAAGCAGAAGT
TAAACAACAAAGTGAATCAGAGTTAAAACACTATTATAATAAACCAATTTTAGAGCGTAAAAATGTGACTGGATTTAAATATACTG
ATGAGGGTAAACACTATTTAGAAGTCACAGTAGGGCAACAGCATTCTCGAATCACTTTACTTGGATCTGATAAAGATAAATTTAAA
GACGGAGAAAACTCAAATATAGATGTGTTTATCCTTAGAGAAGGTGACAGTAGACAAGCAACAAATTACTCAATTGGTGGCGTTAC
AAAATCAAATAGTGTGCAGTATATTGATTATATCAATACGCCAATTTTAGAAATCAAGAAAGATAATGAAGATGTACTTAAAGATT
TTTACTACATTTCAAAAGAAGACATCTCATTAAAAGAACTTGATTATAGATTAAGAGAACGTGCGATTAAACAACACGGCTTGTATT

CAAATGGTCTTAAACAAGGTCAAATTACAATTACAATGAATGATGGCACAACACATACAATCGATTTAAGTCAAAAACTTGAAAAA
GAACGTATGGGTGAGTCAATCGACGGCACTAAGATTAATAAAATTCTAGTAGAAATCAAATAATACTTTCTAACAACAAAGCGCTA
TGTTGAATAGTGCTTGTTATGGAAATATATGGAAGTTAAGCGACGTACTGTTGCTTAGCTTCTTTTTTTGAGGGGAAAAGTTACAAA
ACTCACACAAACAGTCGCACCACGCATTATCTTTTGCTTAAATAACTTAATCATATTTTATGAATAGTTAAAAGCAGGTTAATGTGA
ATATCTGAATACAGCTCCTATAATATGGGTGTATGATTCAAATTACGTAATAAAACAATCTAATTATTATAGATTGGAGCATACAAC
TATGAAAATGAAATCAATTGCAAAAATAAGTTTGTTATTAGGAATATTAGCAACAGGTGTAAACACTACAACGGAAAAACCAGTTC
ATGCCGAAAAGAAACCTATTGTAATAAGTGAAAATAGCAAAAAATTAAAAGCTTATTATAATCAACCTAGTATTGAATATAAAAAT
GTGACAGGTTATATCAGTTTCATTCAACCAAGTATTAAATTTATGAATATCATAGATGGTAATTCTGTTAATAATATTGCTTTAATTG
GCAAAGATAAGCAACATTATCATACGGGTGTACATCGTAATCTTAATATATTTTACGTTAATGAGGATAAGAGATTTGAAGGTGCA
AAGTACTCTATTGGGGGTATCACGAGTGCAAACGATAAAGCTGTCGACCTAATAGCAGAAGCAAGAGTTATTAAAGAAGATCATAC
TGGTGAATATGATTATGACTTTTTCCCATTTAAAATAGATAAAGAAGCGATGTCATTGAAAGAGATTGATTTTAAATTAAGAAAATA
CCTTATTGATAATTATGGTCTTTACGGTGAAATGAGTACAGGAAAAATTACAGTCAAAAAGAAATACTATGGAAAGTATACATTTG
AATTGGATAAAAAGTTACAAGAAGACCGTATGTCCGATGTTATCAATGTCACAGATATTGATAGAATTGAAATCAAAGTTATAAAA
GCATAACACATATACTTGATGACGAAATAAGTTGAAATTGAAATAGAGAGGTTAAGTGACGATCAAACGTTGCTTAACTTCTTTTTA
ATGCTTAAAAATCATTTCAAAGGCACATAGAAACGCTATATTAACCTCATAATCACTCATTATTTTTTGCTTAAATTACTTAATAATA
CTTCAATAATTGTTAAAAGGGGTTTAATGTGATTATCTTAGAATGCCATCTATAATGATGTTGTATGATTCAAATTACGTAAAAAGA
CAATCGAATATAATATAGATTGGAGCATACAATTATGAAAATGAGAACAATTGCTAAAACCAGTTTAGCACTAGGGCTTTTAACAA
CAGGCGCAATTACAGTAACGACGCAATCGGTCAAAGCAGAAAAAGTACAATCAACTATAGTTGACAAAGTACCAACGCTTAAAGC
AGAGCGATCAGCAATGATAAACATAACAACAGGTGCAAATACAGGGGCAACGCAAGCAGCTAACACAAGACAAGAACGCATGCCT
AAACTCGAAAAGGCACCAAATACTAATGAGGAAAAAACTTCAATTTCCAAAATAGAAAAAATATCACAACCTAAACAAGAAGCGC
AGAAATCGCTTAATATATCCACAACGCCAGCGCCTAAACAAGAACATTCACAAACGACAACCGAATCCACAACGCAGCAAACTAA
AGTGACAACACCTCCATCAACAAACACGCAGCAAACTAAAGTGACAACACCTCCATCAACAAACACGCCACAACCAATGCAATCTA
CTAAATCAGACACACCACAATCTCCAACCATAAAACAAGCACAAACAGATATGACTCCTAAATATGAAGATTTAAGAGCGTATTAC
ACGAAACCGAGTTTTGAATTTGAAAAGCAGTTTGGATTTATGCTCAAACCATGGACGACGGTTAGGTTTATGAATGTTATTCCAAAT
AGGTTCATCTATAAAATAGCTTTAGTTGGAAAAGATGAGAAAAAATATAAAGATGGACCTTACGATAATATCGATGTATTTATCGTT
TTAGAAGACAATAAATATCAATTAAAAAAATATTCTGTCGGTGGCATCACGAAGACTAATAGTAAAAAAGTTGATCACAAAGCAGA
ATTAAGCGTTACTAAAAAAGATAATCAAGGTATGATTTCACATGATGTTTCAGAATACATGATTACTAAGGAAGAGATTTCCTTGAA
AGAGCTTGATTTTAAATTGAGAAAGCAACTCATTGAAAAACATAATCTTTACGGTAACATGGGTTCAGGAACAATCGTTATTAAAAT
GAAAAACGGTGGGAAGTATACGTTTGAATTACACAAAAAACTGCAAGAGCATCGTATGGCAGATGTCATAGAAGGTACAAACATT
GATAAAATTGAAGTGAATATAAAATAATCATGACGTTCTCTAAATAGAAGCTGTCATCGGAAAAACAAGAAGTTAAGTGACAACGG
TTTACATGTTGCTTAGCTTCTTTTATTATGCGCAATGATGTAAAAAGACGAATATTCATTTGTTTGTAAAAGTGGCATTTCTATGTCTT
AAAAGTGACGAAACTTCAAATGTGCCAAGTGTTGAATCACATCAAAATCATTTTTATTTAACGAACATTATGGATTTCTTAATTTAC
TTAACGATGATTCAAATATAGTTAAACAAGGTTTAATGTGAATGGAGCAATACGCCATCTATAATAAAGCTGTATGATTCAATGAAT
GTAATCGAACAAATCTAATAATTACGAATGGAGCATACAACTATGAAAATAACAACGATTGCTAAAACAAGTTTAGCACTAGGCCT
TTTAACAACAGGTGTAATCACAACGACAACGCAAGCAGCAAAAGCGTCAACACCATCTTCCACTAAAGTGGAAGCACCACAATCAA
CACCGCCCTCAACTAAAGTAGAAGCACCGCAATCAAAACCAAACGCGACAACACCATCTTCCACTAAAGTGGAAGCACCGCAACA
AACAGCAAATGCGACAACACCTCCATCAACAAACACGCCACAACCAATGCAATCTACTAAATCAGACACACCACAATCGCCAACCA
CAAAACAAGTACCAACAGAAATAAATCCTAAATTTAAAGATTTAAGAGCGTATTATACGAAACCAAGTTTAGAATTTAAAAATGAA
ATTGGTATTATTTTAAAAAAATGGACGACAATAAGATTTATGAATATTGTTCCAGATTATTTCATATATAAAATTGCTTTAGTTGGTA
AAGACGATAAAAAATATGGTGAAGGAGTACATAGGAATGTCGATGTATTTGTCGTTTTAGAAGAAAATAATTACAATCTGGAAAAA
TATTCTGTCGGTGGTATCACAAAGAGTAATAGTAAAAAAGTTGATCACAAAGCAGGAGTAAGAATTACTAAGGAAGATAATAAAG
GTATAATCTCTCATGATGTTTCAGAATTCAAGATTACTAAAGAGCAGATTTCCTTGAAAGAACTTGATTTTAAATTGAGAAAACAAC
TTATTGAAAAAAATAATCTGTACGGTAACGTTGGTTCAGGTAAAATTGTTATTAAAATGAAAAACGGTGGAAAGTACACGTTTGAA
TTGCACAAAAAATTACAAGAAAATCGCATGGCAGATGTCATAGATGGCACTAATATTGATAACATTGAAGTGAATATAAAATAATC
ATGACATTCTCTAAATAGAAGCTGTCATCGGAAAAACAAGAAGTTAAGTGACAACGGCCTACATGTTGCTTAGCTTCTTTTGTTATG
TTCGATGATTTGAGAACCCGAATTTTCGATGGGTCCAAATATGACGTGGAAGAGTCCTGAATTTATCTGTAAATCCCTATCTATCGG
GTGTGGATCACAACGGAATCAGTTTTATTTAACGAACATTATAGATTCCTTAATTTACTTAATAATGATTCAATGATTATTAAACATG
GTTTAATGTGAAAGGTCAAATACGCCAACTATAATAAAGCTGTATGATTCAATAGACGTAAGCGAACAAATCTAATAATTACGAAT
GGAGCATACAACTATGAAAATGACAGCAATTGCGAAAGCAAGTTTAGCACTAGGTATTTTAGCAACAGGAACAATAACATCAACGC
ATCAAACTGTAAATGCGAGTGAACATGAAGCAAAATATGAAAATGTGACAAAAGATATTTTTGACTTAAGAGATTACTATAGTGGC
GCAAGTAAGGAACTTAAAAATGTTACTGGTTATCGTTATAGCAAAGGTGGCAAGCATTACCTTATCTTTGATAAAAATAGAAAATTC
ACAAGAGTACAGATATTTGGTAAAGATATTGAAAGATTTAAAGCACGCAAAAATCCGGGATTAGACATATTTGTTGTTAAAGAAGC
GGAAAACCGTAATGGCACAGTGTTTTCATATGGTGGTGTCACTAAGAAAAATCAAGACGCTTATTATGATTATATAAACGCACCAA
GATTTCAAATCAAGAGAGATGAAGGTGACGGTATTGCTACGTACGGTAGAGTACACTACATTTATAAAGAAGAGATTTCACTTAAA
GAACTCGACTTTAAATTGAGACAGTATTTAATTCAAAATTTTGATCTGTATAAAAAGTTTCCTAAAGATAGTAAGATAAAAGTGATA
ATGAAAGATGGCGGCTATTATACGTTTGAACTTAATAAAAAATTACAAACAAATCGCATGAGTGATGTCATTGACGGTAGAAATAT
TGAAAAAATAGAAGCCAACATTAGATAATTCAATGAAATATGGATAATAGTAAAATATGGATAGTATAGAGGAGTTAGGCAACAT
AAGTTGCTTAGCTTCTTTTTTGTGTTGGAGAGATGAAAATGAAGCGGGTTCATGATCAAAACTTGTGGAAATAGTTGATACTTATAG
ATGCGTGATGTCGCTTTAGTGACATGAAACAATGTGGAAAACATAATTAAATTGAGGGAAAGTGTGAATAGTTAAAAAATTAGCAT
TGTGTTATAAAAAATAATTAATACTGTTAGGATTTCATTAACTAACTTAACGTTGGTTCAAAAATAGTTAAAAAGAGGTTAATTCAT
AGCGCAGTATCTCGCTTATATAATGATAGTAGATTGTTCGTATTACGTAATTGAATTAATCATATAAAAATATATTAAGACAAAATT
TATAAATAGATTGGGAGAATAGTACTATGAAATTAAAAACGTTAGCTAAAGCAACATTGGCATTAGGCTTATTAACTACTGGTGTG
ATTACATCAGAAGGCCAAGCAGTTCAAGCAAAAGAAAAGCAAGAGAGAGTACAACATTTATATGATATTAAAGACTTACATCGAT
ACTACTCATCAGAAAGTTTTGAATTCAGTAATATTAGAGGTAAGGTTGAAAATTATAACGGTTCTAACGTTGTACGCTTTAACCAAG
ATGGTCAAAATCACCAATTATTCTTATTAGGAGAAGATAAAGCTAAATATAAACAAGGACTTCAAGGTCAAGATGTCTTTGTGGTA
AAAGAATTAATTGATCCAAACGGTAGACTATCTACTGTTGGTGGTGTAACGAAGAAAAATAACCAATCTTCTGAAACTAATACACC
TTTAGTAGTTAATAAAGTGTATGGAGGAGATTTACATGCATCAATTGACTCATTTTCAATTAATAAAGAAGAAGTTTCACTGAAAGA
ACTTGATTTCAAAATTAGAAAGCAATTAGTTGAAAAATATGGTTTATATAAAGGTACGACTAAATACGGTAAGATCACTATCAATTT
GAAAGACGAGAAAAAGGAAGTAATTGATTTAGGTGATAAACTGCAATTCGAGCGCATGGGTGATGTGTTGAATAGTAAGGATATTC
AAAATATAGCAGTGACTATTAATCAAATTTAAATTAAACAATCAATGACTTTAAAGTAATAAATTTGAAGCAGCTTAACGATGAAA
TGTTGAATAAATACGTACATCCTCCAAAAAGGGGCGTATCTAAATCAACAGTGTCGTTAGGCTGTTTTTATGTTTTATAACGCAGGG

TATGAGCGTACTAAAAATTCACATTACTTCTGAAAGTGATGTCCATTGAATATTAATTAGTTCTTCATTAACCATGATTTAATTTTAA
TTAAACGAGTGTTAATGTCAGTCTGTCTCAATGCCCTTTATAATAAATGTGTATTATTCAAATTACGTAATAAAAGCAATCCAATAT
ATTAAGATTGGAGCATATGAATATGAAATTTACAGCGATAGCTAAAGCGATATTTGTATTAGGAATATTAACAACAAGTGTAATGA
TAACAGAAAATCAATCGGTTAATGCAAAAGGAAAGTATGAAAAAATGAACCGTTTATATGATACAAACAAGTTACATCAATACTAT
TCAGGACCTAGTTATGAGTTAACAAATGTTAGTGGCCAAAGTCAAGGTTATTATGACTCTAACGTTTTGCTTTTTAACCAACAAAAT
CAAAAGTTCCAAGTATTTTTATTGGGAAAAGATGAAAATAAATACAAAGAAAAAACACATGGTTTAGATGTCTTTGCGGTACCAGA
ATTAGTAGATTTAGATGGAAGAATATTTAGTGTTAGTGGTGTAACGAAGAAAAACGTAAAATCAATATTTGAGTCTCTAAGAACGC
CGAACTTACTAGTTAAAAAAATAGACGATAAAGACGGTTTTTCTATTGATGAATTTTTCTTTATTCAAAAGGAAGAAGTGTCATTGA
AGGAACTTGATTTTAAAATAAGAAAACTGTTGATTAAAAAATACAAACTGTATGAAGGGTCAGCTGATAAAGGTAGAATTGTTATT
AATATGAAAGATGAAAATAAGTATGAAATTGATTTAAGTGATAAATTAGATTTCGAGCGTATGGCAGATGTCATTAATAGTGAACA
AATTAAGAACATCGAAGTGAATTTGAAATAATCAATGATATATAGAATAAAAGCTTAAGAAGCGGTTTAATAATCCCATGTTTAAT
GATTTTGATACGTGTTTTAATAATAAAAACATATCGAACATTGACTACGTTATTAAGCTGCTTTTTTGTACACTTTGTATCGAATAAC
TTAAGATCTAAAACTAATCGGAAAGAACAATGATTCCCCCAAAAAAATTTATGTTGCTATTAAAAATCAGTTAATACGAATGTTAA
AATACGTTTGATTTTCATTAATAATGATTCAAGTTTATTTAAATGAGCGTTAATGTCAGTCTGTTTTGATGCACCTTATAATAAAGAC
AGATAGTTCAAATTACGTAATCATAACAATCCAATATATCAAGATTGGAGCAAATAAATATGAAATTGACTGCATTAGCAAAAGCA
ACATTAGCATTAGGAATATTAACTACAGGTGTGTTTACAGCAGAAAGTAAAGCTGTTCACGCGAAAGTAGAACTTGATGAGACACA
ACGCAAATATTATATCAATATGCTACATCAATACTATTCTGAAGAAAGTTTTGAATCAACAAACATTAGCGTTAAAAGTGAAGATTA
CTATGGTTCTAACGTTTTAAACTTTAACCAACGAAATAAAACTTTCAAAGTGTTTCTGCTTGGTGACGATAAAAATAAATATAAAGA
AAAAACACATGGCCTTGATGTCTTTGCAGTACCTGAATTAATAGATATAAAAGGTGGCATATATAGCGTTGGCGGTATAACAAAGA
AAAATGTGAGATCAGTGTTTGGATTTGTAAGTAATCCAAGTCTACAAGTTAAAAAAGTTGATGCTAAACATGGCTTTTCGATAAATG
AGTTGTTCTTTATTCAAAAGGAAGAAGTATCGTTGAAGGAACTGGATTTTAAAATAAGAAAAATGTTAGTCGAAAAATATAGATTG
TATAAAGGCGCGTCAGATAAAGGTAGAATCGTTATTAATATGAAAGACGAAAAGAAATATGTAATTGATTTAAGTGAAAAATTAAG
TTTTGATCGTATGTTTGATGTAATGGATAGTAAGCAAATTAAAAATATTGAAGTGAATTTGAATTAGTTTGAGTTAATAGCATAATA
GCTTAAGAAGCGACTTAACGACAAAATGTGAATTGACATCTGTGTCCTTATATAAGGAACTGTGTTAAATACATTACTGTTGTTAAG
TTGTTTTTGTAATTCAAAGAGCAGAACAGAGTAACATCATCAGTTGTAGTAAACGATAATCCAGTAAAACAACTAAATGAAATAAT
GAAAGTCATTTAACCTGAACATTAAAATATATTTGTTTTTCATTAAGAATAATTCAAGTATATTTAAATCGAGGTTAATTATCGTATG
AAACGATGCACGTTATAATAAAAATGTATGATTCAAATTACGTAATGAAAACAATCCAATATATTAAGATTGGAGCAAATAAATAT
GAAATTAACAGCGATAGCTAAAGCTGCATTAGCTTTAGGAATTTTAACAACAGGAACTTTAACAACAGAAGTTCATTCAGGTCATG
CAAAACAAAATCAAAAGTCAGTAAATAAACATGACAAGGAAGCATTATACCGATACTACACTGGAAAGACTATGGAAATGAAAAA
TATTAGTGCTTTGAAACATGGTAAAAACAACTTACGTTTTAAGTTTAGAGGTATTAAGATTCAAGTTTTACTGCCTGGAAATGATAA
AAGTAAATTTCAACAGCGTAGTTATGAGGGGTTAGATGTTTTCTTTGTTCAAGAAAAAAGAGATAAGCACGATATATTTTATACTGT
TGGTGGTGTAATACAGAATAATAAAACATCTGGAGTTGTCAGTGCACCAATATTAAATGTTACAAAGGAAAAAGGTGAAGATGCTT
TTGTGAAAGGGTACCCTTATTATATTAAAAAAGAAAAAATAACATTAAAAGAGTTAGATTTTAAATTGAGAAAGCATCTAATCGAA
AAATATGGACTTTATAAAACAATCTCAAAAGATGGTAGGGTTAAAATTAGCTTGAAAGATGGCAGTTTTTATAACCTTGATTTAAGA
TCTAAATTAAAATTTAAATATATGGGGGAAGTCATAGAAAGCAAACAAATTAAAGATATTGAAGTTAACTTAAAGTAAATAATTAC
GAATAATTAAAAGTAATTGAAGCGGCTTAACGGTGAAATGTAAATTGGTGCGCATAGCTTATACAAAAAGGAGGCATCAATCGATA
TCGTCGTTAAGCCGTTTTGGTTTGCGTTTCATAAATCCTATTCCAATCTCTATGAATATAAAAATTTCCACCACCAACATCAAA

>010-contig_255

TTTAAGCGATGCCCAAGTCGAACGTATTATAGACGCATATAAGCGTAAAGAAACGATTGATAAATATAGCTACAGCGCGACATTAC
AAGAGATCGCCGATAACGATTACAACTTAAACATACCGCGATATGTTGATACATTCGAAGAAGAAGCGCCAATTGATTTAGATCAA
GTCCAACAAGATTTGAAAAATATCGATAAAGAAATCGCAGAAGTTGAACAAGAAATCAATGCATACCTGAAAGAACTTGGGGTGTT
GAAAGATGAGTAATACACAAAAGAAAAAATGTGCCAAAATTGAGATTCCCAGGGTTTGAAGGCGAATGGGAAGAGAAGCAATTTGC
TGATTTTACTAAAATAAATCAAGGATTACAGATTGCTATTAATGAACGTAAAACTGAATATTCTCCAGAGTTGTATTTTTATATAAC
AAATGAATTTTTAAGACCAAATAGTCAAACTAAATATTTTATCGAAAATCCCCCTCAATCAGTAATTGCAAATAAAGAAGATATTTT
AATGACTAGAACAGGTAATACTGGAAAAGTAGTAACTAATGTATTTGGAGCGTTTCATAATAATTTTTTAAAATTAAATTTGATAA
AAATCTGTATGATAGATTGTTTTTAGTAGAGGTTTTAAATTCATCTAAGATACAAAATAAAATACTATCTTTAGCAGGATCTTCGAC
GATACCAGATTTAAACCATAGTGATTTTTATAGTATTAGTTCTTCTTATCCGCTGCTTAGAGAACAGCAAAAAATAGGTAAATTCTTC
AGCAAACTCGACCGACAAATTGAATTAGAAGAACAAAAGCTTGAATTACTTCAACAACAGAAAAAAGGCTATATGCAGAAAATCT
TCTCACAGGAATTGCGATTTAAGGACGAGAATGGAAATGATTATCCGGATTGGGAGAAAAAGAAACTAAAAGAAATAGCTTGTGTT
TATACAGGAAACACGCCAAGTAAAAAAGAAAATATATATTGGAATAAGGGTGAATACGTTTGGGTTACACCTACTGACATTAATAA
TAGTAAAAATATTTATGAAAGTGAAAACAAATTAACCCAAGAAGGCTATAAAAAAGCAAGACAATTACCAGAAAATACACTATTG
GTTACGTGTATAGCTAGTATAGGAAAAAACGCAATATTGAGAAAACAGGGCTCGTGTAATCAACAAATAAATGCAGTAGTTCCATT
TGAAAATATAAATATAGATTATCTTTATTATATTTCTGATTCATTATCAACGTTCATGAAATCTATTGCAGGAAAAACGGCTACACA
AATAGTTAATAAAAACACTTTCGAAAATTTGGAAATTTATTTAGCTCCTTTTGAAGAACAGAATAAAATAGCAGATTTAATTAGCTC
ACTAGAAGAATTAATTGAAAAGCAAGCATCGAAGTTAATTAAAATGAAGAGTCGTAAACAAGGAATGCTTCAAATAATGTTTATTT
AAGAGTAAGAAGAATTAACTATTTTGATAAGGCGAATTGATTTTAATGATTGCGTCGAGTTAAAAATATAGTAAAAATAAATCGAA
ATAAATACGAACATTAAATTTAGACACTGTGATAGTACGGTGTCTTTTTTGTGTCGAAATTGTGTACAGAATAAGTAGTTAAATAAA
GATTAAGTTGAGATAAAGTGTTATTCGTAAATAAAAGAGAGTAGATCGATAGGAATTGAATGATATTAGTTAACTATTTATTAAATT
ACTTAATAATGATTAATTTTTAGTTAAAGTAAGTTTAATGTGAAGCACGACCATTGCTCATTATAATGAATGAGGATTGTTCGTATTG
CGTAATAGAATAAATCAAATAGACTAAAAATTGGGAGCATAGAATTATGAAATTAAAAAATATTGCTAAAGCAAGTTTAGCACTAG
GGATTTTAACAACAGAGATGATTACAACTACTGCTCAGCCAGTAAAAGCAGACGAAGCTAGTAGCAGATTATCAGTTACTTCAAAA
GATACACAAAATTTAAAGACGTATTATACTGGAGCAAGTTTTGATCTTAGAGGATTAAGCGGATATAAAGAAGGAGATAAAGTAAT
ATTTCCTCAAAATGGTCAACAAATTGATGTTACATTAACAGGTAAAGAGGGAGACGTGGTTCAATCTAATGACGACGTTACAAATG
TTGATGTGTTTGCTGTTCCTGAAGGTACAGGTCGTTCTGCCATAATGACTACAATTGGTGGGATTACTACACCAAACACTCAAAGTT
ATAAAGACACTACAAATAATGTGAACTTAAGAGTATCAAAGAGTACAAGTCCCAATACCATATCTGTGTCTGTAAAAAATTATGAT
ATTTTTAAAGAAAAAATTTCATTAAAAGAACTTGATTTCAAATTAAGAAAGCATTTAATTGATAATCATGATCTTTATAAAACAGAA
CCTAAAGACAGTAAAATTAGAATTAATATGAAAGATGGCGGCTACTATACGTTTGAATTAAATAAAAAGTTACAAACTCATCGTAT
GGGTGACGTAATTGATGGTACTAAAATAAAAGAAATTAATGTTGAATTAAACTAAAAATTCGAGGGAGCATATCATGAGGGAAAAT
TTTAAGTTACGTAAAATAAAAGTCGGTTTAGTATCAGTTGCAATTACAATGTTATATATCATAACAAACGGACAAGCAGAAGCATCT

GAAAATCAAAATATAGAATCTAAAAATTCTTCTAGTAATATAGCATCACAGCCAAAACAAATTAATGAGAATTATACAGGACATAA
AGAAAATGAAAAAGGTGAAGATCAAAATAAACCTGCTAACTTTGTAAAATTAGGAAGTGTTAATCCTGGAGATACATCTGTCAAAG
GTACAACATTACCACATCAAATTATACTTTTAAATATTGATAAACAGAGTGTAGAACCAGTGGAAGATAATAATGGTGGATTTGTA
ATGTCGGATGAAAAAGGAGAATTTGAATATAAACTTAAAGATCGTAAAATAGTTCATAACCAAGAAATAGAAGTTTCATCATCTTC
CTTAGATGGATTAGAAGAGGATGACGAAGAGGAAGAAATTGAAAAAAAATCTTCGGATAACAAAGGTGAAAGTATAGAATCACCT
ATAGAGAAAAATGAAGAAGTTGAAGAGTCAAGTAATGATGCTACAGCTACATACACTACACAAAGATACGAAGGTGCGTATAAGA
TTCCTGATAAGCAATTAGAAAAGAAAGGCGATCATCACCAGATTTTAGTGGAACCTATTACTGAATGGACAGGCATAATTAAAGGA
CATACTTCAGTGAAAGGAAAAGTTGCATTATCAATTAATAATAAATTTATTAATTTTGAAGAAAGTGGTAAAAGCAAAAAAACTTT
AACTGAAGAAGAAGGTAAATCAAGAATTGAAGGTATCTGGAAGCACATTGATGATAAAGGATATTTTGATTTTGATTTCAAAAAGA
AAAGATTTGATAATTTAAACTTAAAGAAAGATGATATTGTTTCTTTGACATTTTTACCAGAAGATGAAGATGAGGCGTTAAAACCAA
TTATTTTCAAAACAAAAGTAACAAGCTTTGATAACATTGCTTCTGCATATACAGAATATAATCCTGAAAAGGTAGAAAAAGTAAAA
ACTCTAAATAATGGTTTAGAGGACTTGAATGTCAAAGATATTTATGGTTTTGTATACGAGAGTGAGCGTGGTATTGGTATTCCGCAA
GCAAATAATAACGCCACTAAAGTAATTGAAGGAAAAACGAAATTTGCCAATGCTGTCGTAAAAGTTTACTCAAGTTTAGGGGATGG
GCAAGAATTCCCTGATTTACAAGTAAATGAAAAAGGAGAATTCAGCTTCGACTCTTTTGATGCAGGTTATAGGTTATACAATGGTGA
GAATTTAAACTTTACTGTTGTTGATCCTGTTACTGGTAAACTATTAAGCAAATTGGTAACTAAAGAAATTGATATTTATGAATCACCT
GAACAAAAAGCAGACCGTGAATTTGATGAAAGACTTGAAAATACACCTGCTTACTACAAGTTATATGGTGATAAAATAGTTGGTTT
CGATACTAATGATTTCCCAATTACTTGGTTCTATCCATTAGGTGAAAAGAAAGTTGAACGTAAGGCACCAAAATTAGAAAAATAATT
AATAAAATAAAACAGCTTAATGATACGATGAAACTAGTGACTTAATCACTGACTTCAACGTCATTGAGCTGTTTTTTGTGCTTTGTT
ACAAAGCATTATTGAATTTATTTTACGTGTTCATATTTTGAAACATCAAAGCCGTCTTGCTTAGCTTTGTTGATAATATCTTTGATTG
AATGTAGTCCTTTATCGGCGAAGTATGATCTTAAGTTGTCTTTTGTAGCTTGGTCAGCATTCTTATCTAATAACACATCGATATAACT
TAACTCGTGTTCTAAGAAGTTTGCGTCATCATGTAGTACGAGTCCATTTTGAGAATACACTTTCGCATCTGCTTGATTACCATATCCA
ACAACGCCAGTTGCTAAAACACCTACCATTGCCGTAGCTACTAAAACCTTTTTAAATTTCATATCTATCACTCCTCTAAAAATTGTAA
CTCTATCATAACACTGAATATTAAGAAAATTACGTTTATTAAGTCGATTTAATAATTTTTAATAAATAGTTAAAGTGGCAAATATTG
CTAAAAAGCAATTAATCTTTAGTGCGATGCTTGTGTTCCTTAGTATTGAGTAGTAGATTTCGAAGATATTTTAATTCAAATGAAACA
ATAATAAAAAAATGATGCAACATAATAATAAGTGCAAATTTAATTAATAAATTAAATTGATTGTACATGTATATTTGGTAACGTAA
AAGAGAAATATACAAAATAATTAATTATTTATATGAAAAGAGAATATAAATGAAGTATAAAACAGAGAGACGTGAAGCGATGGGA
TATTTAAAAAGGTTTACATTGTACATAAGCGTTATGATTTTAATATTTGCGATAGCAGGTTGTGGCAAAGGTAATG

>011-contig_288_RC

AATGGCGACGTGTCAATAAATTGGAATGTTCCAAGTTATGACGTGGAATATAAAATGAGCAATAAAGATGAAAATGTTAAGCAATT
AAGAAGTCGTTATAACATTCCTACTGATAAAGCTCCAATGTTAAAAATGCATATTGACGGTGACTTAAAAGGTAGTTCTGTTGGATA
TAAAAGGTTAGAAATAGACTTTTCAAAAGAAGATAGGGATATTTCAGTCATTGATTATTTAAGTTATAAGCCAGCGAAAAAATAGT
GTTGATAATTAAATATTAGGGTGTGAAATGATGAGATATTTAAAAAGAGTTGTACTGTACATAATTGTTATGGTTTTGAGTGTTTTTA
TAATAGGTTGTGATAAATCAAGCGATACTTCAGAAAAGCCAAAAGAAGATTCAAAAGAAGCACAAATTAAAAAGAGTTTTGCAAA
AACGTTAGACATGTATCCTACTGAAAATCTAGAAGACTTTTATGACAAAGAGGGATATCGAGATGGAGAATTTAAAAAAGGTGACA
AAGGGACTTGGCTAATTAGATCTGAAATAGTTAAACAGCCAAAGGGCAAAGTGATGAAAACAAGAGGTATGCAATTATATATTAAT
AGAAATACCAAAACAGCCAAAGGTTTCTTTGTTTTGAAAGAAATAAGTGAAAATAATAATCGTGTAAATAAAGATAAGGAGGAAA
AATACGAAGTGAAAATGGTAGGAAATAAAATTATTCCTACTGAACAAATTAATGATGAGAAAATAAAAAAAGAAATTGAAAACTT
CAAGTTTTTTGTGCAATATGGAAACTTTAAAAATTTCGAAAAATACAACAATGGTGAGTTTTCATATAATCCTGAAGCACCAATCTA
TTCGGCTAAATATCAATTACACAACGACGATTACAATGTAAGGCAACTACGTAAAAGATATGACATTTCAACAAAAGAAACACCGA
AGTTACTTTTGAAAGGTGGAGGAGATTTAAAAAATTCCTCAGTTGGTCAAAACGATATTGAATTTACTTTTGTTGAAAGAAAAGGTG
AGAATATTTATTTTAACGATAGTGTTGAATTCATACCAAGTAAGTAAAGTTAAATTAAAATGTGTGGTTAGATGAAATGATCAAATA
TCAAAAAATGTGCAAAGAGATGTGACAAGATGAAGTTTTTTCAGAAATTATACATATTTATATTAATTTTAATCGTATTAATGGCAG
GATGCGAAAGTAATAAGATCACTGGAGATTCGAAAGAAACACAGATCAAAAAGAGCTTTGCGAAAACATTAGATGTATACCCTAC
GAAAAATCTAGAAGGTTTTTATGACAAAGAAGGATATCGAGATGGCGAATTTAAAAAGGGTGACAAAGGGAAGTGGGTTATTAGA
TCTGAAATGACAACAGAACTGAAAAATGAAAATATGGTATCTAAAGGTATGGTCATACGTTTAAATAGAAATAGTAGAACATGCAC
TGGTGAATATTTTGTCAGGATAGTTAAAGAAGACAGTGAGGGCAAGGTATATACTGATGAACGAAAATATCCAGTGAAAATGGAA
AATAATAAAATCATTCCATTAAAACCAATCGATGATGAAAAAGTAAAAAAAGAAATTGAAGAATTTAAATTCTTTGTACAATACGG
GAATTTCAAAGAATTGGAAAACTATAAAGACGGAGAAGTGACATATAACCCAGAAGCACCAATATACTCTGCACAATATCAATTGA
AAAATAGTGATTATAATGTAGAACAACTACGTAAGCGATATAATATACCGACGAAAAAAGCGCCTAAATTATTATTGAAAGGGTCA
GGTAATCTAAAAGGCTCAACAGTCGGATATAAAAATATTGAATTTACCTTTGTTGAAAATAAGGAAGAAAAT

>012-contig_282_RC

TATAAATCCTCGAGATTCTTAATAGGATACATATCTAACGTTTTTGCAAAGCCCTTTTTGATTTGGTCTTCTTTTGAGTCTTCTTTTGA
GTCTTCTTTTGCTTTTTCAGCAGTAACACTAGATTTATCACAGCCTATTATAAAAATACTCAAAGCCATAATGCTTATGTACAATACG
TCTCTCTTTAAATTTCTCATTATTCCACATCTTTCTTGTCTGATTTTTTATTGAATCTCTACTGATTATCTTTTGATGGGTTAAAATTTA
AAAAATCACTGACAGAAATTTGGTTATCTTTTTGCTTTGAAAAGATATATTCCACTTTTTTATATCCTATCGAGCTACCTTTTATATCC
CCATCTATATGAAATTTTAAAGTTGGCGATTCACTGGTTGTAATTGGATAAACATTTCTAAGTTTTTTTACATTTCTATCACTATTTTC
TATTTTGTACTCTGCTTCATAATATGGAACATTTTCATTGCTCGATATTTTCCCGTTTTGATAATTTTTCAAGTCTTTAAAATCTGCAT
ATTGCCCAAAAAAATTTAAAATTTTCTATTTTATGTTTGAGTTTTTCATCTTCTACTTTATCTAAAAGGACTATTTTATTATTTTTTAGTT
CAATACGATAATCTTTCCTTTGCGGCATCTTATCCTCTTCGTTATAAGTTTTTCTTACAAAGTAATATCCTTTAGCCGTCCGCGTATTT
CTATTCAAATGTAATACCATACCTTCACTATCCAATTCACCCGCTTTATTACTTTTGGCAAAATCTGTATATATCGTCCACGTCCCCTT
ATCACCTTTTTCAAATTCGCCATCTCGATATCCTTCTTTGTCATATAAATCTTCAAGATTTTTGATTGGGTAAATATCTAATGTTTTCG
CAAAGCTCTTTTTAATTTGGCCTTCTTTTGAGTCTTCTTTTGATTTTTTTGCATTCTCGCTTGAACTATTACAACCAATTATAAAAATA
CTCAACACCATAATGCTTATGTACAATACTACTCTTTTGAATCCCTCATTTTTTCACTTCTCTCTATCATGTTAATGTTTGTTGAGTA
CTATCAACCTTTGTGTTATTTTTAGTCACTTGGAATTAAGTTTAATCCATCTGAAAAATATATATTTCTTTTTTATTTTCTATAAATA
TAAATTCAATTTCCTTATATCCAACTGAAGACCCTTTCAAATCCCCCGAACCTTTCAACAACAATTTAGGCGCCTTTTCTGTTGGAAT
ATTATATCTTTTTCGTAATTCTTTAACATTATAATCATCATTTTTCAGTTTATATTTCGCTGAATAAATAGGAGCTTCAGAATTATAGG
AAATGTCACCATTTTCATAATTCTCTATTCCTTTAAAACTGCCATATTGCACAAAAAACTTAAAGTTTTCAATTTCCTTTTTCAACTTT

TCATCTTTTATCTCCTCAGTTGGAATAATTTTATTGTTTATCATTTTCACAGGATACTTTTTTTCTGTTTCTCTTTCATCAAGGCTTTTT
CTTGAATCTATTTCTTCTATTGAAAAATAACCGGTTGTGGTCCTAGTATTTCTATTCATGTATAATATCATGCCTCTTGAAGTTAAACT
TTTACCTTTTGGTTGAATTATCATTTCAGATCTAACAACCCACGTCCCTTTGTCTCCCTTTTTAAATTCTCCATCTCGATATCCTTCTTT
GTCATATAAGTCCTCGAGATTCTTAATTGGGTACATATCTAACGTTTTCGCAAAGCTCTTTTTGATTTGTGTTTCTTTTGAATCTTCAG
GAGTGTCAAATACACCACTGCAACCTGACACTAATAGAATTAAGACTGACGTACCTATACACAACGCGTATTGCCTAAAATTATTCA
TTGTTCCGTATCCCCCCTTTTTTCATCATAGTTAATAGTCCTCACTTGGGGTAAACTCCAAACTATCAGTAAAAAATACATTCTCTTCT
TGATTCTCAACGAAGGTAAACTCAATGTCCTTATGTCCGATTGATGAACCTTTTAAATCTCCAGTACCTTTCAACAACAATTTAGGAC
CTTTTTTGGTTTGAATGTCATACATTTTACGTAATTGTTTTACATTATAATCATCATTACTTAATTGAAATTTAGCAGAATAAGTAGGT
GCTTCTGAGTTATATGAATAATCACCATTTTGATAATTCTTTAATCCTTTAAAATCACCATATTGTGAGAAAAACTTAAAGTTTTCAA
TTTCTTTTTTTACACCTTTATCATTAATTGGATCAATAGGTACTATTTTATTATTCACCATCTTTACTGGATAACGTTTTTCTTTATCCT
CTGTCTTTTTTAAAGTATCATTACTTGTTTCATCTACAATAAAATATCCTTTTGCGGTTCTTGTATTTCTATTTAAAAATAGATACATC
CCTCTTGATTTCATAATTTCACCTTTTGGCTGTTTAACCATTACTGAACTGATAACCCATGTCCCTTTGTCACCTTTTTTAAATTCGCC
ATCTCGATATCCTTCTTTGTCATATAAGTCCTCGAGATTCTTAATTGGGTACATATCTAATGTTTTCGCAAAGCTCTTTTTGATTTGTA
TTTCTTTTGAATCTTCTTTTGTTTCATTACCTTTGCCACAACCTGCTACAATAACTATTAAAATCAAGATACTTATGTACAATGCAAA
ACTTTTTAAATTTCCCATCATTTCGCGTCTCTCTGTTTTAGAATTATTTCTACATCTAATAAACTTTAGCTATCTTCATCAGACGGCCC
AAAATTCACAAAATCTCTTACAGCTGTTTCTTGGTCTTTTACTTTTGAAAAATTATATTCTATATTTTTATAACCAACTGAACTTCCTT
TTATGTCGCCATCTATATGTAATTTTAATACTGGCGACTTTTTGGTTTTGATTGGATAAACTTCTCTAAGTTTTTTTACATTTTTATCC
GTATTACTTATTTGATATTCTGCATCGTAGCTTGGCACATTATCTGTACTTGATATATTTCCATTTTTATAATTTTTAAGATTTCTAAA
ATCAGCATACTGACTAAAGAATTTAAAATTTTCTATTTTATTTTTGAGATTTGGATCTTCTACTTTATCCAAAAGTACTATCTTATTAT
TTTTTAATTCAACATGATATTTTTTTTGTTGATCTTTTTCATTAAATTCATCATGAACTTTGCTTATGTAATAATATCCTGTTGCTTTCT
TTGTATTTCTGTTAAGAAATAAAACCATACCTTCGTCATCTATCTCGCCTGGTTTGTTACTTTTAGCAAAACTTGTGAGTAAAGTCCA
TGTCCCTTTATCGCCCTTTTTAAATTCGCCATCTCGATATCCTTCTTTATCATATAAATCTTCAAGATTTTTGATTGGGTACATATCTA
ACGTTTTCGCAAAGCTCTTTTTGATTTGTGTTTCTTTTGAATCTTCTTTTATTTTATTATCTTTACCACAACCTACTATCGCAAATATTA
AAATCATAACGCTTATGTACAATGCAAAATTTTTTAAATTTCCCACCATTTCACGTCTCTCTGTTTTAGATTTATTTCTACATCTAATA
AACTTTAGCTATCTTCATCAGACGGCCCAAAATTCAAATAATCTCTTAATGTTGTCTCTTGATCTTTAACTTTTGAAAATTTATATTCT
ATTTTTTTGTATCCAACTGAACTTCCTTTTATATCACCATCTATATGTAACTTTAATGTTGGAGCCTTTTTCGTTGTAATTGGATAAAT
ATCTCTAAGTTTTTTTACATTTGTATCACTATTATTCAATTTGTATTCTGCTTCATAGCTCGGTACATTTTCATTAGTTGTTATACTTCC
ATCTTGATAATTTTTTAAATCTTTAAAATCGGCATACTGACTAAAAAAATTTAAAATTTTCAATTTTTTGTTTAAGTTTTTCGTCTTCTA
CATTATCTAAAAGAACAATCTTATTATTTTTAAGTTCAACACGGTATTTTTTCTCATTCTGATTTTTGCTAATATCATCATAAATTTTA
TTTACAAAATAATAACCTGTTGCCTTTTTGGTATTTCTATTTAGATATAAAACCATGCCTTCGTCATCTATTTCACCCGGTTTGTTACT
TTTTGAAAAACTTGTGAGTAAAGTCCAAGTCCCCTTATCGCCCTTTTTAAATTCACCATCTCTATATCCTTCTTTGTC

>013-contig_317_RC

CAGATTTTGCTTTCAATAATAAACCTGGAGAATTAAAAAATGAAGGGATGGTTTTAAATCTAGATAGAAATAATAGAAAAGCTGAA
GGATACTATTTTGTAGATACTTTATATGATAATAATGAAAAAAATAAGAGCAAAAAATATAGAGTTGAGATGAAAAATAATAAAAT
TATTTTATTAGATAAGGTAGAGAATGAGGCACTAAGACAAAAAATAGAGAACTTTAAATTTTTTAGCCAATATGCAAATTTTAAGG
ATTTAAAAAGTTATAATAATGCTAGAATAACAAGTAATGAAAATGTACCCACTTATGATGCAGAATATAGACTTGGTAATAAAGAT
TATAATGTCAAGCAATTGAGAAGTAGATATAATATAGTAACTGATAAAAAACCTTTATTGAAAATGCATGTTGATGGAAATCTTAA
GGGAAGTTCTACAGGCTATAAAAAGATAGAATATGACTTTTCAAAAGAAGAAAACAGTGAGTTGTCTGTGGTTGACTCATTAACAT
ATAATACTTCAAAGGAGTATAATAATGGCAGATAAGTATAAAATAAATCCTGGGGAGCTTAATAAAAGCACTGAAGAAACAACAG
CTGTTTCTAAAATTAGTTATGAAATCGAAAATGCAAATATAAATGATCTAAGCAATAGAAGCATAACAAGACAAATGAATATTATC
AGGAAAAATGGTAGATTTCCTTCAAATCTTGAATATTTAGATAGTCACACAGATAAAAAGTACGGTGTGACAGCATCAGCTTTCCTA
AATAAAGATACTGGTAAAGTAGTTATTGGCATGACTGGTACAAATTTCAAAAAGGAAGCTCTTGCAAAGCGATATTCGGGAAACGG
AGACAAACAAGATAAAATAGACTCAGATGAGACTGAAAAGGATTTAATTTCCGATTTCGAAATTGGAATATCTCCTCCGTCTGATA
AAAGCAAACATTATGAAGAAACACAAGAGTTCATCAAAAAAAATAAAAAATAAGTATGACATTGATTTTATTACTGGGCATTCACTA
GGTGGTAGAGAAGCGGTAATATTAGGAATGAGTAATGGTATTCCGAATATTGTTGTTTATAATCCAGCTCCTATTTCAATATTTAAT
TTGAACCCTAATTCCCCGGAAGGAAAACGTTTATTAGAATTATATAAGAATTATAAAGGTAATATTACTAGGTTTGTTGCAGAAAAT
GATGAATTGACAGAAAATCTGAAGAAATATAAGCATTTTATGTTTTTTGGTAATGATATAGTTCTTAAGAATGGTAAAGGTCATTCA
ATGGATGGATTCTTAACTGAACAAGAACAAAAAGCCATTAAAAAAGAACTTAAAAAAGTACAAGGTTATGTAGAAGAAAATAATA
AGTCGTTCGTAAAAAAATTCCAATAATGCTATGTCTAAATTAGCTAGTATAGAATTACTTAGAGCCAATATGATGACTGCGAATGGTG
GTTGGTTATCTTCTTCACAACAGAAAGTTTTAGAAAGTTTAACAGCTTTAACAATTGCACAGTCATTCAATCAACTGATAGAAGACG
AAATTAATCAAATCAAAAAAAATGTATAATGAAAAGAAAAAGAAATTTGAAAAAAAATTGGGAAGACGCACAAAAAGCTGGGAACG
CTGTAGGAAAAGATATAACCGTGAGTGAAGTGCTAGAAGCTTTAAATGAAGGTCAAGTGAATGAAAGTAGTATGGTAGGAGATCCT
GAACAAATGATATCTCTAAATGAAGGCAACTTTCAATGATAGAATCTTCTATATCAAATTATATTCTAAGAGTTAGATTTAGTATT
AATGAAATCGTTGATAAAGATCAAGTGCTTGCATCTCAAATAGGTGGATTACTATGATGTTTAATCAAATTAATAATAAAAATGAAT
TAGAAGAATCATATAATTCTGAGAAAAAACGTATAGAGAATGAACTGCAAAATTTGAATGAACTTAGGCATAGAGCTCGAAAAGA
AAATGAACGTAGTTATGATGTTTTTCAATATTTGAAACGCGAAATGAATTATAGTGAAGATGCACAAAGGAAAATGACGAGAAATA
TAGAAGCGTATGAGCAAGAAATCAATGAGATAATTAGAAAGCAAGAATGGAAATTAGAAGAATATAAAGAAGACTTAAAAAAATC
TTATGAAAACCAGTTAGATAAACTAAGTGATTGATAATTTGGAGGAGATTATATGAGTCCAATGATAGGACCAGTTATAGGACCGG
ATATAGTACCGGACATAGAACCGACTATAGTACCAGACATAGGACTGGATATTTCGTTAAGGAAAAAAGATGCTCAAAAAAAAAC
ATTCGCACAGTTATTGCACCTGAACATAAGCATAAATACAAAGATATTGAAAACGGATTAAAAGGTGAAGAAAAAGTATTAATTGA
ACAAATGGCGCAGCATTGCGAAGCTTTTAAAGCTAATTTTAAAGGCGCAGCTCAAGGAGATTGGGTTAAAAGTGCTATGTCTGAGA
TTGACAGCATTAAGGATGACCTGAAAAAAATTAATAGCTAAACTACATATCATATTAATAAATAAAGCTCCCTTCAAAATTTTCATT
TTTTCAATGTCTACTTTGGAGGGAGCGCTTCAGTGTATTCACAACTGACACTAGGCCGCTTCTTTTTAATTTATTTTATTCCCATGTAA
AGTAATTCGAACCGAATGGACATATCACACTCTCAATTGTAAAAACACAACATAAATTAATGTCCCGCATTAAACATATCCACATTC
CAAACAGCAACAATCAAAATCCTCAAGTACCAAAAGCTATGACCACTTTATCAAAATCAACGCAAGTCAACATCAACCGAACTACT
TGCCATAAATCATCTTCACCTTACAAACCATAAAGCGCTGCAGTTAAGTATTGAATGGCATAGCCAATTGAAATGAAAATAGCAAT
CGTTATAATAGCACTACCAATGGTATATAACGTATGTTTCGTAGATAATCCTTCAGTCTTTGAACCAAAAACACTGAGTAGAATAAA
TGCAATGCCAATGACATATAAGGCTAAAATGAAAAATAACATGTTTATACACCTCGATTCTCTCTCGTGATATATATATCTTTGTCTC
CATTATAAAGTAAAGTGCTCAATAATTTAATTAAGTATACTATTTTTTTATTATCGAAGATTGCGAAAGAGGAAACGCTCCTATATA

ATGTAAAACGTAATTATTCCTATTTACAAATAAATTGAAGTGAGGGTATATATATGGCTAAAATTCCAGTTACGGTATTAAGTGGTT
ATTTAGGCTCGGGGAAGACAACGTTGTTAAATCATATTTTACAAAATCGAGAAGGTCGACGTATCGCGGTAATTGTAAATGATATG
AGTGAAGTCAATATCGATAAAGATCTTGTCGCAGATGGTGGGGGACTATCGCGTACAGATGAAAAATTAGTCGAACTTTCTAATGG
TTGTATCTGTTGTACACTTAGAGACGATTTATTAAAAGAAGTTGAGCGTTTAGTGAAAAAAGGTGGCATCGATCAAATTGTTATTGA
GTCAACAGGGATTTCAGAGCCAGTACCTGTTGCACAAACTTTCTCATATATTGATGATGAACTTGGCATTGATCTTACAGCGATTTG
CCGTTTAGATACAATGGTTACAGTTGTGGATGCTAACCGCTTCGTACATGACATCAACTCAGAAGATTTATTGATGGATCGTGATCA
AAGCGTTGATGAAACAGATGAGCGTTCGATTGCTGATTTATTAATTGACCAAGTTGAATTTTGTGATGTATTGATTATTAATAAAAT
TGATTTAATTAGTGAAGAAGAACTAGCGAAGTTAGAAAAAGTGTTAAGTGCATTGCAACCGACTGCTAAAATTATTAAGACAACAA
ATTCTGAAGTAGATTTAAAAGAAGTCTTAAATACGCAGCGTTTTGATTTTGAAAAAGCGAGCGAGTCAGCAGGATGGATCAAAGAA
CTTGAGTCTGGTGGTCATGCATCGCATACGCCTGAAACAGAAGAATATGGCATATCATCATTTGTATATAAACGTCGTCTACCTTTC
CATGCTAAAAGGTTCAATGATTGGTTAGAAAGCATGCCAAATAATGTCGTTCGATCAAAAGGTATCGTATGGTTAGCACAATACAA
TCATGTAGCATGTTTATTATCTCAAGCAGGGTCATCTTGCAATATTCATCCAGTTACATATTGGGTGGCTAGTATGTCTGAAGCGCAA
CAAACACAAATATTAGCAGAACGTCAAGATGTCGCAGCTGAATGGGATCCAGAATATGGCGATCGTCATACACAATTTGTCATTAT
TGGTACAGAATTAGATGAAGAAAAATTAACAAAAGAACTCGACGCATGCTTAGTCAATGCGCAAGAAATTGATGCAGATTGGCAA
CAATTTGAAGATCCATATCAATGGCAAATTAGACCAGCACGATAAGTTGAATGAAGTATAATCATTTTTGAATTGTGGCTTAATTGT
TTGCAATTTATGACAATTAATAAAAAGTTAAGGTGTTCTTAGGATCTTTTTGTTTTCAATCTATCTAAAGTGTGATAGTTTTGATAAA
GCAGAAATTTGCATTTTATCCATAGGGCTAGGACATGTATGTGTCTTAGTCCTTTTTATATTTACGTTGATGAAAAATGGCAAAACC
ATCGTACCAACTGTTAATTGAAGGTACAATCATTGTTTAACAGTCATAAAAATGAGTGAAGTAAATGCAGCTATCAATGTACTATTA
ACACTTACGGTCACACGAGTGTTTCAGTCTAAACGTGCAACGCAATTCCGCACGTGGCCAATATATCTTGCGCTAGATCATGAAAAT
CATTAAGATTTCGCACGCCAAATCATGAATGTCTCAGACCTCGAGTATCAAACATAAAAGACGCCACAAAAATATAACGCGTCAGA
CTACAAAGATCACGAATATTCCGTACGTCCCACGAAGAACATCCTATACAAAAAAACAAGACCATTTATTTGCAACAAACTAACAA
GACTAACCTCACATCAATAAATCAACACAAAGCAAAGCCACCATCCCTATTGGCATAGTGGCCTGAGAATTTTAAGTATTCAATTCG
CTTAAATTATTTTGCGAAAATGTCGATAATTGCTTTGATGATTTTAATGATAGTACCTACAATAGCCATCGTTTTGTCCTCCTGTATG
TTGATTTAACATAGTGATGAGTTGTTGATCGTTAATGTTTGAGATTAGTTGTTACCTAAAAATTTACCAAGTAAATCTTTAAAGAATT
TGAATAATTTTGCTACGAATTCCATGTGAATGGCCCCCTTCAAATAAGATGTTCATATCTATGAGATTTTTATAACTTACTTACCAGT
GAATTTCTCAATTAATCCTTTAATGAATTTAATGATTCCTGCAATGATACCCATGTGAAAGACCTCCTTTGTTTGTTATGAAATCTTA
TTTACCAGTGAATTGTTCGATTAAGCTTTTGATAACTTTAATGATGCCAGCGATGATACCCATTAAGATTACCTCCTTTGCTTATGAG
TTAACTTCATTGTACATAGTTATCTTGTGCGTAATTGATTTTTTTCCTAACTTGAATTGTTATAATCATAACTGCGAATTTTTTATTTT
AAAAGGACGGGAAATACTGCCTAACTGTACAGGTGCATTTACAAAATCTTTACAATCATTGGTTTAATAGAGGTTAAGCTCATGAAT
TTGACATGAAGCAGAAATTTAATTATATTTTATGTTATAAGTTTAAAACGAATAACACGTTAGGTCTCGTCTAGGCAAAGCATATTT
ATGAAGATGAATTGAACAGACAGAAGTAACATTTTATATACATATGTGCATGGGAGCACGCTTAAAGAGAACACTGCAACAAGGTA
TATCAACGTACCGTGATGCAGTGTCTAGTTAAACTGAAACCTGCAATAGTAAATTTTCTGCCATTATGTACAGAATCTACTATTGTA
GGTTTTTATTTTTATACATAAGCAATTATGCGAGAGATTAGAAATAAGGAGGTTATGCAGTGTTAAGTTTTCAATTGCTATTTTCACT
GTTTGTTATTGCGCTTATCATTGCATTGATAAGTGGCTTGTTGTTTTTAGCACCAGTTATGCCAATGAGATATATTAAATTACATTTAT
ACATACTAGTCATGCCAGTATTATTTGCAGTCATTGGCTTTTTCGGTATTCATGGTCAACATGTCTTAGGTCCATTTAAAATAGATCG
TTTATCTTGGTTATTAGCTGGCTTTGTAATGGCGCTTGGTTTTATTATTCAAAAGTTTTCAATGCGATATTTACTAGGTGATCATCATT
ATAGACATTACTTTCCATTGTTCACTGCGATTACGTCGTTTGCATCTTTAGCATGGATGTCTGAAGACTTAAGACTGATGGCACTCTG
CTGGGGTATGACATTATTATGTTTAACATTGCTGATGAACGTTAATCGTTTTTGGAAAGTGCCACGTGAGTCTGCGAAATTATCAAG
TATGACATTTTTATGTGGTTGGCTTGCATTCGTTGGAGCAATTGTAACTATTTATATTGCGACTGGCGAGTGGCGCGTGCCTCAACAT
ATGTCTAATTCGACATGGTCATTGTTGACGAATGTACTACTTGTATTAGCTGTCATGATACCGGCAGCACAATTTCCTTTTCATCGAT
GGTTGATTGAATCTGTAACGGCACCAACGCCAGTATCGGCAATTATGCATGCAGGAATTGTGAATGCAGGTGGTGTTATTCTAACTC
GTTTTGCGCCGATATTTGATAATGGATTTGCGTTATCATTATTACTTATCTTTTCTAGTATTTCTGTATTGTTAGGATCGGGTATTAGC
TTAGTTCAAGTTGACTATAAACGCCAATTAGTGGGCTCTACGATGAGTCAAATGGGCTTTATGTTAGTTCAATGTGCATTGGGTGTA
TATTCAGCAGCGATTATTCATTTAATATTGCACGGTATTTTTAAAGCAACATTATTTTTACAATCAGGTTCTATCGTGAAGCGATTCA
ATATTCCAAAACAAGCATCTGCTAAAGACGCTTATGGCTGGATTGTCATGGGACGTGTATTAGCTATTATCGTGGCATTTCTGTTTTG
GATGAGTAGTGACAGAAGTGCATATGAAGTGTTAAGTGCACTCATTCTAGCTTGGTCATTACTTGTATCTTGGAATCAAATGGTAGC
CTTTAGTAAAGGACGCATGGCACGTCTTGTTGGTATGATTTTGATTGCAATTGTGACATTTATCTACATCATCACACATAATTATTTT
TACGATGTATTGCGAAATATAACAACACATGCGACAACACCGCCTACAGTGAGTGTCATCATTAGTGTTGTCATTTTAATCTTTGGT
AGTTTATTAAGTATTTGGGTGGCGCGTCATCGATACTCTAAGGGTTTTGCGGTATTGTACGTGTGGTTAGTTAATCTAGGTGAAGCA
CGCTCGAAAGCGATAGAAAGTCATCCGAATTATTTGAAGAAGTATTTATAGGAGGTGAAAGGTATGACAACACAGTTAAATATCAA
TTCAGTCATTGAAAATGCGAAACGTGTTATTACACCATTATCACCGATTTCGATTTTTGCAGCACGAAATCCATGGGAAGGATTAGA
AGCGGATACGTTTGAAGATGTTGCAAAATGGTTACGTGACGTTCGAGATGTGGATATTTTCCCAAATAAAGCATTAATAGAAAGCG
CTGTGGCACGTGGTGAATTAGATGAAAGTGTCTTTAATCAACTTGTTACTGATATGTTACTTGAACATTACTACAATATCCCGCAAC
ACTACATCAATCTTTATATTGATAACATTAAAACATTAAAAGACGTACCTGCATCATATATGGATCATTCAAATGTTGATGTTGTTGC
TGATCTACTATTAGAAAAATCAAAACGTGATATGGCTGAATCATATCATCACTATGATGTACGTCCGATGAGTGATGCAATAATAGA
TGAACAAGGTGAGCCACTTAGCGAACAAGTGAATCGTCAAATGATTAAATGGACGAAACTTTATATCGATCAATTTCTATCGAGTT
GGACAATGCCGAAGCGTGAGCAAAGTTTTTACCATGCATGGTTGCATTTAGCGCAACATGACCATAGTTTTACTAAAGCACAGCGC
CAAGTGATTAAAGGCTTACCCAATGATCCTGAAATGACGATAGAGTCAGTATTAACTCATTTTTCAATAGATCAGGAAGACTACCA
AGCTTATGTTGAAGGACATCTTTTGGCGTTACCGGGTTGGGCAGGTATGTTATATTACCGTTCACAACAGCATCACTTTGAACAACA
TTTGTTAACGGATTATTTGGCAATTCGGTTAGTTGTCGAACAATTGCTAGTTGGTGATGAGTTTAAGTCAGTCACTAAAGATTGTGA
AAGTAGATCGGAAAATTGGTTTAAGCAAACTGTTGCATCATGGTGTTACTACAGTGATATGCCTAGCGATGTATTACTACAACATGA
CGTCCATGAAATTCAAACATTTATTCATTTTGCAGCAATTATGAATAAAAATGTATTTAAAAATTTATGGCTAATTGCCTGGGAAAT
GACATACGAATCTCAGTTAAAACAAAAAATTAAAGCAGGTCATGAAAGTGTGGCGGGCGCATTAGATGTAAACCAAGTAAATGTCT
CAGAAAATGATAACGTTAATCAGCCACATTCAGTATTGTTAAATGACACACAAGCAGTTGATGAAAATAATAGCGAGCTAAATCAG
GTGGGCACATCAACGAAAGCGCAAATTGCATTTTGTATAGATGTTCGTTCAGAACCATTTCGTAGACATATCGAAGCAGCAGGGCC
CTTTGAAACGATTGGTATTGCAGGCTTCTTTGGATTACCTATTCAAAAAGATGCCGTCGACGAACAATTCAAACATGATTCATTACC
TGTCATGGTAACGCCGGCATATCGCATTAAAGAATTTGCAGACCGCTACGATATGAATGTTTATCGACAACAGCAACAGACAATGT
CATCGATGTTTTACACATTTAAATTGATGAAAAATAATGTTATGCCTAGTCTGTTATTGCCTGAATTAAGTGGGCCATTTTTAAGCTT
GAGTACCATTGTCAATTCGATTATGCCTAGAAAAAGTCGCGCGTCTTTACAAAAAATAAAACAAAAATGGTTGAAAAAGCCTGAAA
CAAAGTTAACGATTGATCGTGAGTTTGACCGAACATCAGATTTACCTGTTGGATTTACTGAGCAAGAGCAAATTGATTTCGCGTTAC
AAGCGTTGAAATTGATGGATTTAACCGAAGCATTTGCGCCGTTCGTTGTGTTAGCAGGTCATGCTAGTCATTCTCACAATAATCCAC

ATCATGCATCACTTGAATGTGGGGCTTGTGGTGGCGCATCAAGCGGTTTTAATGCTAAGTTATTAGCGATGATATGTAATCGTCCAA
ATGTCAGACAAGGATTAAAACAAGCAGGCGTGTATATTCCAGAGACAACTGTTTTTGCGGCAGCAGAACATCATACATCTACTGAT
ACGTTGGCATGGGTATATGTGCCAGACACATTATCAGCTTTAGCTCTAGATGCATATGAATCATTGAATGACGCGATGCCGATGATT
TCTGAACAATCGAATCGCGAACGTTTGGACAAACTGCCAACGATTGGTCGTGTGAATCATCCAGTGGAAGAAGCGCAGCGGTTTGC
GAGTGATTGGAGTGAGGTACGTCCAGAATGGGGCTTGGCTAAAAATGCATCATTTATAATTGGACGACGCCAATTAACAAAAGGTA
TTGATTTAGAAGGGCGGACATTTTTACACAATTATGATTGGCGTAAAGATAAAGATGGCACATTATTAAATACCATCATTTCTGGTC
CAGCGCTTGTGGCACAATGGATTAATTTACAATATTATGCGTCGACAGTTGCGCCGCATTTTTACGGAAGTGGGAATAAAGCGACAC
AAACCGTCACGTCAGGTGTTGGTGTCATGCAAGGTAATGCGAGTGATCTGATGTATGGCTTATCATGGCAATCTGTTATGGCTGCTG
ATCGAACGATGTATCATTCGCCAATTCGTTTGCTTGTTGTTATTCAGGCACCCGACTATGTTGTAGCTAGACTACTCGCGAATAATGA
GCATTTCGCTAGGAAGGTGTCTAATCATTGGCTGCGTTTAATGAGCGTTAATGAGGAAGGGCGTTTTAAAAGTTGGATTTAACGATG
AAATGTTGCGATGCTAAGTAACTAATATATCATCATATCTACAATGTTCTAGCTTCTTAAAATATTCTAAAAATGATAAACTTAATGT
AATGGTTGATGTCGTGATAACGATACAAAGATGAAGTAATCAATTTTAATATTATTGGAGTTAGTGATATGAAAAGAACGAAAGGT
GAAATCGAAGCTGAAATCAGTAAAGCCATTACGCAATGGGAAAAAGATTTCCTTGGCAGAGGTTCCTTGTCGGTTAAATCAGACAT
TTTAAGAGATATGGTGATTATTAGTTTACAAGGTATCTTAACGCCAGCAGAATATCGTGTGTGTAGTACGAATGAAGGATTACTAAA
TATTAAACGAACACGTTCTGAATTAGTTGAATCCGGTGAGCAAGATTTGAATGATATCATTCTTAAAATTACAGGTATCAAAGTGAT
GAGCTTCCACAGTGATTTAAGTACAGTTACAGGTGAACGTATTATCGTATTCAAACTTGAGGATAATTTGGAAAAGCATATTTAAGA
AGGTTAGGTATGCTTTTGTTGGCTTATTAAAGCTATAGAAGTATGTTTGGTTAAACAGCGCAACGCCATATATTTGGGTGTGCTGTTT
TTCTATTATATGATGTGTTTATTACGTGGATGAATGCGTGGGTCTGAATATAGCCCGTGAAAGGCGTATGTCATAAAATTTGCGAAT
GGTGACAAGTCGACGCAAGCATATTTGTTGCGAGACATGCCCACTATCCTTTTACATAAAGAGTATATGTGTGACGTAGGCATATAA
TCGATAAAGTATTCCTAAAAAATTAGGTATAATGACCATTGTTGCAATAAGTTTTTCGGCCTTGAAACCGATAACGCATAGTATAAT
AGGAATAAAATATAATACAAATATCCAGTAAATCAAATTTGAAAACGATGGATGAATAGGGGTAAAGAATCTAACTAATGTAATG
ATTAACGCTATATAGACAAAAATAACTCTATTGATACGCCTTACCCCCTCTGTATAATAAATATAGATCGTACTAAATTGAAAATAG
CAAAATATTGTTATTTCAATTATACAATGTTTTATTTGCAATATACATAACAATATGAACTTTATAATTTTGATATTGTCGGTAAAGT
GAAATTTGTGGCACATTGATATTATGGATAACATTTGTAAAATGAAAATGATGATACGAGGAGATTGTAAGATGATAGATAAAAAA
TTAACATCACCGAAAATGACAGTGCCTTTATTTTTAATCGCGCTGATTGTATTTATAGGTATGTTTTACAGTGTAGTGACAAATCAAG
AATGGCTTAAAAATATAGATATGGGATCATTAACATGGTTTACAGATTATTTCGGTGAGCCACAACGTCAGTATGTTAACAATTTAT
TTAATTACTATATGACGTTTAGTGCGGAAATTGGAGATGTCAAAGGTGTCGTGTTGATTTCCATTATCGTCACAATCGTACTGTTTAT
TAAACAGAGGCATTTAGCGGTTTGGTTTGTGACATATTTGGTTTCAGGCGTCATCATGAACAAATTAATTAAAGATACTGTTTTACG
TCCAAGACCATATAATCATTTAGCCGTTGATACAGGCTTTTCATTTCCAAGTGGACATTCCAACGCCAGCACATTATTATATTTCGCC
TTAATGATCATAATTATTTCACTTGCTGCTAAGACAATAACAAAAGTGTTGAGTGCGCTAGTTATGGGAATATTATGGCTTAGCATA
TTATTTTGTCGCCTTTATTTTCATGCGCATTACTTTTCAGATGTCATTGGCGGCACGTCACTAGCAATCATTTGGGTAGCGTTATTCTT
AATGGTATACCCATACTTTATTAATCATCGACGACAACGCGTTTAGCAAACATCTTTAGACATAGTGGAGGTATATAGAAAAATGA
GAATTAAAACACCGAGTCCATCGTATTTAAAAGGCACAAATGGACATGCGATATTATTATTACATTCATTTACAGGTACAAATCGGG
ATGTGAAGCATCTTGCAGCTGAGTTAAATGACCAAGGATTTAGTTGTTATGCACCGAATTATCCAGGTCATGGTTTATTGTTGAAAG
ATTTCATGACATATAATGTAGATGATTGGTGGGAAGAAGTTGAGAAAGCTTACCAATTTTTAGTCAATGAAGGTTATGAATCTATCA
GTGCAACGGGTGTGTCTTTAGGTGGATTAATGACATTAAAATTGGCACAACACTATCCTTTGAAACGTATCGCTGTCATGTCAGCAC
CAAAGGAAAAGAGTGATGATGGTTTAATAGAACATTTAGTTTATTATAGTCAACGCATGTCGGATATTTTAAATTTAGATCAGCAAG
CATCGAGTGCGCAATTAGCAGCAATTGATGATTATGAAGGTGAAATTACGAAGTTTCAACATTTTATTGATGATATCATGACAAATT
TAAATGTTATTAAAATGCCAGCTAATATATTATTTGGTGGTAAAGATGCGCCATCCTATGAAACAAGTGCACACTTTATTTATGAAC
ATTTAGGATCAGTAGACAAAGAATTAAATGGTCTGAAGGATTCGCATCATTTAATGACGCATGGAGAAGGCAGAGATATTTTAGAA
GAAAATGTTATTCGCTTTTTCAATGCTTTAACATAATTGAAGATTTTAAAAATCAACACTTGAGTGACGATGTTATTATATTTGTCAT
TTGAGTGTTTTTTGTGGCGTGATAGTATAGGGAACTTAGCTAAAGTTAATTTGTAGTGTAATGTCGTGGAAATATGATGGAGTTATA
CCAGAAGTTGATTTGTATTGTAATGTCGTGGAAATTTTATGGGCATTGCACCTGAAGTTGTTATGTGGCGAGATGTTGGGGATATAG
ATGGTGTTGTATGTGGTGAGATGTTGGCGCACGTATACAGCGTTGAACCAGAAATTTTAATGTAGCGTAATGACAACACATTACAA
GAGTTGACTATATTATGATAAATACAGGCGCTACCGTATTAATGAAACAATTTTGATGCAGGTGTATTGCATGTTATTTACATACAT
GTTAGAGTGTTACTTAAACACGTAGACGCTTTAACGCTTTAAAGGAGATGTGAATGATGAAAAGACAACAATCACAATGGAAGTCA
TCAACTGGATTTATTTTAGCTAGTGCGGGTTCTGCAATCGGTCTTGGTGCCATGTGGAAATTCCCATATATGGCAGGGATTTATGGC
GGCGGTGCCTTTCTAGCTATGTTCTTAATATTCACCATTTTTGTTGGGTTGCCATTACTCATTATGGAATTCACTGTTGGGAAAATGG
GACGGACATATACAACACAAATATATAGTAAATTAACTGGTAAAAAATGGCTCAATATCATTGGCTGGAACGGTAATTTGGCAGTG
TTTATTTTATTTGGCTTCTATAGTGTTATCGGTGGTTGGATTGTCATTTACATCGGACAAGTATTATGGCAATTAGTTATATTTCAACG
CATCAATCATCTCCAAGAAATGAATTTTGAAGCGGTAATATCAAATCCTTGGTTAACCGTTCTAGGGCAAGGTATATTCATATTCGC
TACGATGATTATTGTCATGTTAGGTGTTGAAAAAGGATTAGAAAAGGCATCGAAAGTTATGATGCCATTGCTGTTTGTCTTTTTAAT
CGTCATTGTGATTAAGTCTTTAACATTAGATGGCGCCTTAGAAGGTGTGAAATTTATTTTACAACCAAGAGTATCAGAGATTACTGC
TGATGGCATCTTGTTTGCGCTAGGTCAATCATTCTTTACGTTATCATTAGGAACTACAGGTATGATTACTTATGCGAGTTATGCCTCT
AAAGACATGACGATTAAGTCATCAGCTATTTCTATCGTTGTTATGAATATCTTTGTATCTGTATTGGCAGGTCTAGCTATATTTCCGG
CTTTACATAGTTTTGGCTATGAACCACAAGAAGGGCCTGGATTATTATTTAAAGTACTGCCAATGGTCTTTAGTCAAATGCATCTAG
GCACATTATTCTATTTGGGATTCTTAGTGCTGTTCTTATTTGCGGCTTTAACGTCATCTATTTCTTTATTAGAATTAAATGTTTCTAAC
TTCACGAAGAATGACAATACAAAACGTAAAAAAGTCGCAGTGATCGGTAGTATTTTAGTATTTATCATTAGTATTCCAGCAACCTTA
TCTTTTGGTATCTTAAAAGATGTAAGATTCGGTGCGGGAACGATTTTTGATAATATGGATTTCACCGTTTCGAATGTATTGATGCCAT
TAGGCGCATTAGGTACTACGCTTGTCGTAGGACAATTATTAGATAAAAAATTATTACAACAATATTTGGTAAAGATCGATTTAGAT
TATTCAGTGGTTGGTATTACTTAATTAAGTATGCGATGCCTGTCGTTATTATTTTAGTCTTTATCGTGCAATTATTTAGTTAATATATA
GAAGCATCTGACACACAAGTATTTGTGTTGCTCAGGTGTTTTTTGTTTGGAATTGAAAATTTAGTACTGGTTCTGAATTAATCGAAAC
TTAGATAATTGCAAAATAACAAAATGTAGTTTATAATTCTTATCTGAAAAGTAAGGATTAAAATTGAGACATGAAGAAAGGTTTCA
TTCATTTGATCGGCATGTCAGATGAGGGTGCATCTATGATTACTTATGATTTAATTGGCAATACGCCATTAGTACTGTTAGAACATTA
TAGTGATGATAAAGTTAAAATTTATGCCAAGCTTGAACAATGGAATCCTGGAGGCAGTGTTAAAGACAGACTCGGGAAATATTTAG
TAGAGATGGCAATTCAAGAAGGGCGTGTGCGTGCAGGTCAAACTATTGTTGAAGCGACTGCTGGCAATACAGGCATAGGGTTAGCT
ATTGCAGCAAATAGACATCATTTGAAATGTAAGATCTTTGCGCCGTATGGTTTTTCAGAAGAAAAGATTAATATTATGATAGCGCTT
GGTGCAGATGTTTCAAGAACGAGTCAGTCTGAAGGTATGCATGGGGCACAATTAGCTGCACGTTCCTATGCTGAAAAATATGGTGC
CATTTATATGAATCAATTTGAATCCGAACATAATCCGGATACATATTTTCATACATTGGGGCCCGAATTGACTTCAGCATTACAGCA
AATTGATTATTTTGTGGCTGGTATTGGCTCAGGCGGTACATTTACAGGTACCGCACGTTATTTAAAGCAACATCACGTGCAATGTTA
TGCCGTTGAGCCAGAAGGGTCCGTGTTAAATGGAGGGCCAGCTCATGCACATGACACTGAAGGTATCGGTTCTGAGAAATGGCCGA

TATTTCTAGAGAGACGTCTTGTAGATGGTATATTTACGATTAAAGATCAAGATGCCTTTCGAAATGTCAAAAGTTTGGCTATAAATG
AAGGGTTGTTAGTAGGCAGTTCTTCAGGTGCAGCATTACAAGGTGCATTGAATTTAAAAGCGCAATTATCTGAAGGTACGATTGTTG
TCGTATTTCCAGATGGTAGTGATCGCTATATGTCTAAGCAAATATTTAATTATGAGGAGAATAATAATGAACAAGAAAACTAAATTA
ATTCATGGTGGGCACACAACAGACGATTATACAGGTGCCGTTACAACACCAATTTATCAAACAAGTACATATTTACAAGATGATATT
GGTGATTTACGTCAAGGATATGAATATTCTCGTACTGCGAATCCAACAAGAAGTTCTGTAGAAAGCGTTATTGCGACATTAGAAAAT
GGCAAACATGGCTTTGCATTTAGTTCAGGTGTTGCAGCAATCAGTGCAGTTGTTATGCTGTTGGACAAAGGAGATCATATTATTTTA
AATTCAGATGTATACGGCGGTACTTATCGCGCATTGACAAAAGTATTTACACGATTTGGCATTGAAGTGGATTTTGTAGATACAACG
CATACAGATTCAATTGTACAAGCGATACGCCCAACAACAAAGATGTTGTTTATTGAAACACCTTCTAATCCATTATTACGTGTTACT
GACATTAAAAAGTCTGCTGAAATTGCGAAAGAACACGGTTTGATTTCAGTTGTTGATAACACATTTATGACACCTTATTATCAGAAT
CCATTAGATTTAGGTATCGATATTGTCTTACATTCTGCAACGAAATATTTAGGTGGACATAGTGATGTCGTTGCTGGTTTAGTTGCAA
CATCGGATGACAAGCTTGCAGAACGTTTAGCATTTATTTCAAATTCAACAGGTGGCATTTTAGGACCTCAAGATAGCTATTTACTTG
TGAGGGGTATTAAAACATTAGGTTTACGTATGGAACAAATTAATCGCAGTGTTATTGAAATTATTAAAATGTTACAAGCACATCCAG
CTGTGCAACAAGTGTTCCATCCAAGTATTGAAAGTCATTTAAATCATGATGTCCATATGGCTCAAGCGGATGGCCATACAGGTGTGA
TTGCATTTGAAGTGAAAAATACAGAAAGTGCCAAACAATTGATTAAAGCAACATCGTATTACACATTAGCTGAAAGTTTAGGTGCA
GTGGAAAGTTTAATTTCAGTACCTGCATTGATGACACATGCATCCATTCCAGCAGATATTCGAGCTAAAGAAGGTATTACAGACGG
ACTTGTAAGAATTTCTGTAGGTATTGAAGATACTGAAGATTTAGTCGATGATTTAAAACAAGCACTAGATACGTTATAAATAATAGC
AGCACTGGCATATATTTTGAGTTCAACGTTTGTTGTTCTTGAATATGCTAGTGCTTTTTTGTGCGATGTTAATATTTAGTAATTGCGTT
GTGGAGGATTGTTAGACATTTTTATTCATTTAATTCGATAAATGTCATAGTAGTCTCACAATTCGTCATTGTCACATGAATTGCTTAT
TTTTTAATTTTTTAGAAAATTCGGCAAATTGTATTGACGATATATGCAATTTTACATATAATGCTCTTATTCCTAGTGGATTAATAAG
AATTTGTAGGAGGGGCGATGATGATTGAGTTTCGACAAGTTAGTAAATCATTTCATAAGAAAAGGCAAACAATAGATGCTTTGAAG
GACATATCATTTACGGTCAATCGCAATGATATTTTTGGTGTGATTGGATATAGTGGTGCAGGAAAAAGTACGTTGGTAAGACTCGTG
AATCATCTTGAAGCTGCCTCGAATGGACAAGTGATTGTAGATGGACATGATATTACGAATTATAGCGAAAAAGGCATGCGAGAAAT
TAAGAAAGATATCGGTATGATATTTCAGCATTTCAATTTATTAAATTCAGCTACCGTATTTAAAAAATGTAGCAATGCCACTCATTTTA
AGTAAGAAAAGCAAAACAGAAATTAAGCAACGAGTAACAGAAATGCTTGAATTTGTAGGATTGAGTGATAAAAAAGACCAATTTC
CTGATGAATTATCTGGTGGGCAGAAGCAAAGGGTGGCTATTGCAAGAGCGCTTGTTACTAATCCGAAAATACTCCTATGCGATGAA
GCAACAAGCGCATTGGATCCAGCAACGACTGCTTCGATATTGACGTTATTAAAGAATGTCAATCAAACCTTTGGCATTACAATTATG
ATGATTACACATGAAATGCGCGTTATTAAAGACATTTGTAATCGTGTTGCTGTAATGGAAAAGGGGCAAGTGGTTGAAACAGGAAC
TGTTAAAGAGGTGTTTAGTCATCCTAAAACGACGATTGCTCAAAATTTTGTGTCTACAGTTATACAGACTGAGCCAAGTACATCATT
GATTCGTCGATTGAATGACGAACAAGTTGACGATTTTAAAGATTATAAAATCTTCGTCGAGGAAACTCAGGTGACACAACCGATTA
TAAATGACTTGATTCAAATTTGTGGCAGAGAGGTTAAAATTTTATTTTCATCTATGTCAGAAATACAAGGTAACACCGTATGTTATA
TGTGGCTTCGATTTAATATAGATCAACAATTTGATGACACGGCAATAAATCAATATTTCAAAGAGAAAAATATTCAATTTGAGGAG
GTGCATTAACATATGTTTGGTTCTGATTTAGACAGTGCAAAGTTATTACAAGCATTGTACGAAACGTTATATATGGTATCTATTGCTT
TATTTTTAGGAGCAGTGATTGGTATTCCATTAGGTGTCTTATTGGTAATTACTCGAAAACAAGGCATATGGCCCAATATAGTGATAC
ATCAAGTTTTAAATCCTTTAATCAATATTTTAAGGTCACTACCATTTATTATTTTGTTAATTGCGATTGTGCCATTCACAAAATTAGTA
GTAGGTACTTCAATTGGTACGACTGCTGCCATCGTGCCTTTAACAGTATATGTGGCACCTTACATTGCAAGACTTGTTGAAAACTCA
TTATTGGAAGTAGACGAGGGGATTATTGAAGCAGCGAAAGCGATGGGCGCTTCACCACTACAAATCATTAGATATTTTTTAATTCCT
GAAGCTTTAGGTTCGTTAGTATTAGCAATTACCACTGCGATTATTGGACTTATTGGAAGTACGGCGATGGCAGGAGCTGTTGGCGGT
GGTGGTATAGGAGACTTAGCTTTAGTGTATGGATATCAAAGATTTGATACGACGGTCATTATTATTACCGTTATTGTATTAGTCATTA
TTGTCCAAGTGATTCAAACGCTAGGGAATGTTCTAGCTAGATTCATACGTAGACATTAATGATATATAGTGAAGATTTTGAAAGGAA
TTGATAGAATGAAAAGATTGATTGGGTTAGTTATCGTAGCACTTGTATTATTAGCAGCGTGTGGTAGTAACAATGATAAAAAAGTA
ACAATTGGTGTCGCATCAAATGACACTAAGGCTTGGGAGAAGGTTAAAGAATTAGCTAAAAAAGATGATATTGATGTGGAGATTAA
GCACTTCTCCGATTACAATTTACCGAATAAAGCATTAAACGATGGTGATATTGATATGAATGCATTCCAACATTTTGCATTTTTAGAT
CAGTATAAGAAGGCACATAAAGGAACAAAGATTTCAGCATTGAGTACAACAGTTTTAGCACCGTTGGGCATTTACTCAGATAAAAT
TAAAGATATCAAAAAGGTTAAAGATGGTGCTAAAGTTGTCATTCCAAATGATGTATCAAACCAAGCACGTGCACTTAAACTATTAG
AAGCAGCTGGTTTAATAAAACTGAAAAAAGATTTCGGATTAGCAGGTACAGTGAAAGATATAACGTCAAATCCAAAACATTTAAAA
ATTACTGCAGTAGATGCACAACAAACTGCACGTGCTTTATCTGATGTCGATATTGCAGTTATTAATAACGGTGTAGCAACTAAAGCG
GGGAAAGACCCTAAAAATGATCCGATATTTTTAGAAAAATCAAATTCAGATGCTGTAAAGCCATATATTAATATTGTTGCAGTTAAT
GACAAAGACTTGGATAACAAAACATATGCTAAAATCGTAGAATTGTATCATTCAAAAGAAGCTCAAAAAGCGTTGCAGGAAGATGT
CAAAGATGGAGAGAAACCTGTTAATTTATCTAAAGATGAGATTAAGGCAATAGAAACGTCATTAGCAAAATAAATTATATTGCGTC
CTACAAGCAAAGTTCATGCTTATGTTTGTAGGGCGTTATTGTTGGAGAATAAAATTATTTCCAATAGAGAAAGGGATTGTAATCATT
TTATAGTGAAATATTATGAAATTGTAATAATTTAGATATTGTAAAATCTAATAAGTTGTAATAATTTTAAGGGGTAATTATAAAATT
TGATGATACAGTATATGATTTTTTTGTAATCATAATGTCATCAAACATCAACCTATTATACATAATAAAATCGTATAATGATGTAGTA
TTCATAAATTCGGATAAAAGAATGTTAGGAAAGTTAAGCAAGAGGAGGATTTTAAAGTGCAAAAAAAAGTAATTGCAGCTATTATT
GGGACAAGCGCGATTAGCGCTGTTGCGGCAACTCAAGCAAATGCGGCTACAACTCACACAGTAAAACCGGGTGAATCAGTGTGGG
CAATTTCAAATAAGTATGGGATTTCGATTGCTAAATTAAAGTCATTAAACAATTTAACATCTAATCTAATTTTCCCAAACCAAGTAC
TAAAAGTATCTGGCTCAAGTAATTCTACGAGTAATAGTAGCCGTCCATCAACGAACTCAGGTGGCGGATCATACTACACAGTACAA
GCAGGCGACTCATTATCATTAATCGCATCAAAATATGGTACAACTTACCAAAATATTATGCGTCTTAATGGTTTAAATAATTTCTTTA
TTTATCCAGGTCAAAAATTAAAAGTATCAGGTACTGCTAGCTCAAGTAACGCTGCGAGCAATAGTAGCCGTCCGTCAACGAACTCA
GGTGGCGGATCATACTATACAGTACAAGCAGGTGACTCATTGTCATTAATCGCATCAAAATATGGTACAACTTATCAAAAAATTATG
AGCTTAAATGGCTTAAATAATTTCTTTATATATCCGGGTCAAAAATTAAAAGTAACTGGTAATGCAACATCTTCAAACTCAGGATCT
GCAACAACGACAAATAGAGGTTACAATACACCAGTATTTAGTCACCAAAACTTATATACATGGGGTCAATGTACATATCATGTATTT
AATCGTCGTGCTGAAATTGGTAAAGGTATTAGTACTTATTGGTGGAATGCTAATAACTGGGATAACGCAGCGGCAGCAGATGGTTA
CACTATCGACAATAGACCTACTGTAGGTTCTATCGCTCAAACAGATGTAGGTTACTATGGTCATGTTATGTTTGTAGAACGTGTAAA
TAACGATGGTAGTATTTTAGTTTCAGAAATGAACTATTCAGCTGCACCAGGTATTTTAACTTACAGAACGGTACCAGCTTACCAAGT
AAATAATTATAGATATATTCACTAAAGTCTTACGTATATAAATATATAATGAATTCCTATTACATTTCACAAGCTGAAAAGTTGGAT
TGAATCCTAAACTTTATGGCGGGGCTATCAAAGTCGTGAATGTTGTAATAGGAATTTTTGTATAAGAAAAAGGATTGGTCGAATGA
ACTTCATGTTCTATGTTCGACCAATCCTTTTCTGAATTAGTATTCATGCTTTACTTTGCGAATGCTTGTAAATAATCTAGCACCGTTTG
TTATTAAAGTAACAACTGCCACTGCTTTTTTGAACTTACGTGGTGACTTAATTGAAATGTAAAAGTCTAACACGGTTCCTACAGTGCT
TAAACCTAATGAAAGATATACTAATTTTTTATTTTTAGCATGATATTTATAGCCATTGTAGCCGTCGACTATGAAACCTGCGACATTT
AGTAAACTTGATAAACGTTGTGATTTGGAACGTTTTGCCATAATAATAATTCCCCCTAATATCTTCTGTTTATATGAAAAGTTGGGTG
CGCTGAATTGCTAACGTTTTGCGCTATAACTACTCATATATGATAACATAATTGTACAGTATAATTTGAAAAATTGATTTCACAAAG

TTGGGGTGTCAAAGATGATTAAATGTGTCTGTTTAGTTGAAGAAACAGCTGATAAAATATTGCTTGTTCAAGTAAGGAATCGCGAA
AAGTATTATTTCCCAGGTGGTAAAATAGAAGAAGGGGAATCACAAGTACACGCGCTGTTAAGAGAAGTAAAAGAAGAATTAAATT
TAACATTAACAATGGATGAAATTGAATATATCGGGACAATTGTAGGTCCTGCATATCCACAACAGGATATGTTAACTGAGTTAAAT
GGATTTCGCGCATTAACCAAAATCGATTGGGAAAACGTAACTATCAATAATGAAATTACGGATATACGCTGGATTGATAAAGATAA
TGATGCGTTGATTGCGCCTGCTGTCAAAGTTTGGATTGAAACGTATGGTGGTAAACATGACAAATAATGACACCATCATGTTACGAC
ATTATGTCCCACAAGATTATTCGATGTTAGAAGCTTTTCAATTAAGTGAAAGTGATTTGAAGTTTGTTAAAACGCCAGAGGAAAATA
TTACAGCTGCAATGTCTGATAATGAAAGGTATCCCATCGTTGTAATGGATGGCAGGCAATGTGTGGCCTTTTTTACATTACATCGTG
GAAAAGGGGTCGCACCATTTAGCGATAACCAAGATGCAGTATTTTTCAGGTCATTTAGTGTTGATCAACGTTATCGTAATAGAGGAA
TAGGTAAAGTGGTAATGGAAAAATTGGCGTCATTTATCACTTCAACATTTCAGGATATTAATGAGATTGTGTTAACGGTTAATACTG
ACAATCCACATGCCATGGCACTTTATAGCCAACAAGGATATCAATATATGGGAGATAGTATGTTCATCGGAAGACCTGTTCATATTA
TGGCGTTAACTATAAAATAAATTAAATTTAAAAGCATCTTTACTCATCGTCGACCACAAAAATTAATGATGAATAAAGGTGCTTTTT
GTTATAGATCATCGGACAATTTACTATAGTAAAAAGCGACCTAGTGAACAATTGACATATATCCACAGGTCGCTTAACTTAAGTTAT
ATTGCTAGTTGCGATTAATTGATAGACTCATCATTTTTGCGCTGTCGAGATGGTCTTTTTATTAAAAATGCCGTAATCCAAGCCGTAA
TCGGAATACTGATTGCAACGGCAATACCGCCTAAAATAATAGAAATAAATTCTTGGGCAAATATTTTCGAGTTTATAATATGACCAA
ATGAATATTTAAGTTTGAAAAACCAAATAAATAAAGCAAGTTGGCCACCAAAAAAGGCAAGGTAAATCGTGTTCGCAGATGTCGCT
AAAATTTCTCTACCAACACGCATGCCAGATTGGAATAATTCGTATTGTGTAAGTGTTGGATTTACTTGATGCAATTCATAAATGGGT
GAACTAATGGTAATCGTTAAATCTATCACAGCTGCAATAACAGCAAGAATAATAGTGAACACCATAAATTGAACCATATCAATGCC
AATATTCATTGAATACACATATGTTTCATCTTGTTGTTCGGTTGAAAAGCCTTGTAGATGACCGAAGTAGACCGATAAATAAATGAG
TGTAATCAACAATATTGTTGTAACGATAGTGCTGATAAATGCAGCTTGTGTTTTAACATTATAACTATTGAGTACGAATAAATTACA
AGCGCCAATAATAATGCAGAAAAAGAATGTGACGACATAAATCGGTACGCCAAAAATAATCAATACAATACTAATAATTAAAATA
GCAAAATTTAAAAATAGGGTTAAATAAGAGATGAATCCCTTTTTACCTCCGAAAATTATCATCAGAAAGAGGAGCAATAACGCCAA
TATAAATACAGCATTCATTGTTTCGCCCTCCTTAATGTTTCAAATATTTCCATAAACAATATTGTGATAGGAATTGTAAGTACGATAC
CTATACCGCCTGTTAGTGCGCGCGCAATTTCTAACGACCAATTCATCGAAATAGTATAAGTCACTGTATTGGCATTTTTTAAAAAGA
TTAAAAACATAGGTAGTGCACCGGATAAATATGAGAATAATAAGATGTTAGTCATTGTTCCCATAATATCTTGGCCGATGTTTCGCC
CAGCAAGCGCCCATCTCCTCATTGAAATGTGCGGCGTACGCTGTAAAATTTCATGCATACCACTAGCAATTGTAATTGCAACATCCA
TAATAGCGCCAAGTGAACCTATTAACACTGAGGCTAGGAAGATATCTTTCGGTGGTAATGATAAAAAATTCATCGTTTCATATTTAA
TACCTTTACCGTCTGTCATATATATGATTAATTCTGTTAAACCTATACTCAAAAAAGTTCCGATAATTGTACTGGCTATGGTAATGAG
TGTACGCATATGCCAGCCTGTAACGAGCAATAAAGTGAGTATTGTTGAACAGATCATGGCAATGGTCATGAGTAAGAATAAATTAA
TATTGCTATGTTGAATATGAATGTAAATTGCGATTAATATGGCAATAGAATTCAAGATTAACGATAAAATCGATTGCAGTCCGACTT
TGCGACCAACCAATAATACAGTTAATAAGAACAAACCAGTGATGATAACCGTTAAGGTATCACGCTTCTTTTCTATAATATAAGCAT
CACTTGGCTTGTTAGAAATATGTAATAATACTTTTTCGTGTGTGCGAAATGCCTCAGAATCTGCTTGCGATTTGACGTACTGATGATT
AATCGTCGTCGTTTCTCCAGCAAATTGACCATTTAATATTTTGACTTTTAATTGATTTTTATATTTAATATCACGATTATTTTGTGCAT
CTTTTGTAGGTGTCGAAGAAACATGTTTGACATCTATAATTTGACCAATTGGTTTGTTGTAAAAGTTCTCATTATTGAATGTAAATAA
AATAGCACCAATGAATGCGATGCAGAACAAACCTAAAATTATATTAAATGGCTTTGTAAATAAATTTCTATATTTCAAAAACAAAA
CCCCAATTCTATGAATGAATTAATATGGTGATTATACGCCCTTAATTTTTTATTTTCAAAGATATTACTGCTAAGTGTAAAACGAAAA
TCATCATTGATAGCATCGAATTACTTAATGGAATGTAGACGTTTTAGTCATTAATTGCTGAATGAGTGTTAATAAGATACCAATATC
ACTCTTCGTATAAGGTTCTTTCGTAATAGCACATATCGTTCTTTTTAATTCAGTATGATCTAATTTTATATCTATCCATGATTTAGATT
CTGGTAAATGTATATTTTGTGATGAAATGATGTAACCTTCTTTTTGACGAAGGAGATACTGCGCAAGTGGTTGGCTACTGATTGTGT
ATACATCTGATTTAGTAATCTTGCGCAATTGTTTTTTTACAGTTTCGGCAAATGGTGCCAAGCAATAAATATGACTATGCTCAAACTG
AATTAATGGTGGGTGTGTCGCCATCGTAATTGGATCGTCTGAAGGCGCATATAAATGATAGTGCTCTTCGAATAAAGGTAGCATATG
TAATTGTTTGTGTTTACGTATTTCTGGTGTAAGTTCCGTGAAACCAATGTCTATATTCCCATTTAATACGCTATTTATAATTGTGTCAT
GTTCTAATAAGCTCGGTATGACATGTGTATCATTTTGTAAATGAAACGTTTGGATAAGTGGTAGTAACATGTGGGATACGTCACTCT
CATCATAGCCAATGTAGATACTTTTATTTTTAGTTAATCCATGGCTTTGAAATTGTTCAATCGTGCTATCTAAATGTTCAATAATACG
CAGAGCTTCATTAAATAATAATTTCCCTTCAGATGTGAGCGTAATATTGCGTCCTTGCTTTTTAAATAAAGACACATTAAGTTCTTGT
TCTAATAATGTAATTTGACGGCTTATCGCTGATTGAGCAATGTTTAGTTCAAGTGCTGTTTCAGAGATATGTTCTCTTTTAGCGACCT
CGATAAAATATCTTAATTGTTTAATTTCCATAGCGATATAGGCACCTCCGAAAATGAGTGTTTTGTAACTATTATAGCAATATTATTG
ATGAATGTTCTATTTTTTAGATGAATATCTTCTATTTTATATATTGAACAGATAAATTTTTTAGATTATAGTAATTATCATTAATAACT
AATATCAGAATATTCTAAAAAAGGGGTGTGCATCATGCACAATGAGAAATTAATTAAAGGCTTATATGACTATCGTGAGGAACATG
ATGCGTGTGGTATTGGTTTTTATGCGAATATGGATAATAAAAGGTCTCACGACATCATTGATAAATCGCTTGAAATGTTGCGACGCT
TAGATCATAGGGGCGGGGTCGGCGCAGATGGCATCACTGGTGATGGCGCAGGTATTATGACTGAAATACCTTTTGCATTTTTCAAAC
AACATGTAACGGACTTTGATATCCCAGGTGAAGGTGAATATGCCGTGGGGTTATTTTTTTCCAAAGAACGCGTTTTAGGTTCTGAAC
ATGAAGCAGTTTTTAAAAAAAAATTTTGAAGGCGTAGGGTTATCAATTCTTGGTTATCGTAATGTACCAGTTAATAAAGATGCCATTG
CTAAACATGTAGCAGATACGATGCCAGTCATTCAACAAGTGTTTATTGATATTAGGGACATTGAAGATGTTGAAAAGCATTTGTTTT
TAGCGAGAAAACAATTAGAGTTCTATTCGACTCAGTGCGATTTAGAATTGTATTTTACGAGCTTATCACGCAAAACAATTGTATATA
AAGGTTGGTTACGATCAGACCAAATTAAAAAACTATATACAGATTTATCGGATGATTTATATCAATCAAAGCTAGGGTTAGTGCATT
CGAGATTTAGTACGAATACATTCCCGAGTTGGAAAAGGGCACATCCTAACCGTATGTTAATGCATAATGGTGAGATTAACACGATT
AAAGGTAATGTAAACTGGATGCGAGCACGCCAACATAAATTAATCGAAACATTATTTGGCGAGGATCAACATAAAGTGTTTCAAAT
TGTCGATGAGGATGGTAGTGACTCTGCTATTGTAGATAATGCGCTAGAGTTCTTATCGTTAGCCATGGAGCCAGAAAAGGCAGCGA
TGCTACTCATACCTGAACCTTGGTTATATAATGAAGCGAATGATGCAAATGTACGTGCGTTTTATGAATTTTATAGTTATTTAATGGA
ACCGTGGGATGGTCCTACAATGATTTCGTTCTGTAACGGTGACAAACTTGGCGCGCTTACAGATAGAAATGGATTACGTCCAGGTCG
TTATACGATTACTAAAGATAACTTTATTGTCTTTTCATCTGAAGTGGGTGTTGTGGACGTACCTGAAAGTAATGTTGCTTTTAAAGGT
CAATTGAATCCTGGAAAGTTATTGCTTGTTGATTTTAAACAGAATAAAGTCATTGAAAATAATGATTTAAAAGGTGCGATTGCTGGA
GAATTACCATATAAAGCGTGGATTGATAACCATAAAGTTGACTTTGATTTTGAAAATATACAATATCAAGATTCGCAATGGAAAGA
TGAGACGTTATTTAAATTACAACGTCAGTTTGCATACACGAAAGAAGAGATTCATAAATATATTCAGGAACTTGTAGAAGGTAAGA
AGGATCCTATCGGTGCAATGGGATATGATGCGCCAATTGCAGTGTTGAACGAGCGACCAGAATCACTATTTAATTACTTTAAACAGC
TGTTTGCACAAGTTACGAATCCACCAATTGATGCGTATCGTGAAAAAATCGTAACGAGTGAACTTTCTTATTTAGGTGGCGAAGGTA
ACTTACTAGCACCTGACGAAACGGTTTTAGATCGTATTCAATTGAAAAGGCCGGTATTGAATGAATCACACTTAGCAGCGATTGATC
AGGAACATTTTAAATTAACTTATTTATCAACGGTATATGAAGGGGATTTGGAAGATGCGTTAGAAGCATTAGGACGAGAAGCAGTG
GATGCTGTAAAGCAAGGCGCTCAAATTCTAGTGTTAGATGATAGTGGATTAGTTGATAGCAATGGCTTTGCGATGCCGATGTTACTC
GCAATAAGTCATGTGCATCAATTACTTATTAAAGCAGATTTACGTATGTCTACAAGTTTAGTCGCTAAATCTGGTGAGACACGAGAA
GTGCATCATGTTGCTTGTTTACTCGCATATGGCGCGAATGCAATTGTGCCATACCTAGCGCAACGTACAGTTGAACAACTGACATTG

ACAGAAGGGTTACAAGGCACCGTTGTCGATAATGTTAAGACATATACGGATGTATTGTCCGAAGGTGTCATTAAAGTAATGGCTAA
GATGGGAATTTCGACAGTGCAAAGTTATCAAGGTGCACAAATATTTGAAGCGATTGGCTTGTCTCATGATGTGATTGATCGTTATTT
TACTGGGACACAGTCTAAGTTATCGGGTATTTCGATTGATCAAATTGATGCTGAAAATAAAGCACGTCAACAAAGTGATGATAATT
ATCTTGCATCAGGTAGTACATTCCAATGGAGACAACAAGGTCAACATCATGCTTTTAATCCGGAATCTATTTTCTTATTGCAGCACG
CATGTAAAGAAAATGACTATGCGCAATTTAAAGCATACTCTGAAGCGGTGAACAAAAATAGAACAGATCACATTAGACATTTACTT
GAATTTAAAGCATGTACACCGATTGACATCGACCAAGTTGAACCGGTAAGTGACATTGTCAAACGCTTTAATACAGGGGCGATGAG
TTATGGATCGATTTCAGCGGAAGCACATGAAACGTTAGCACAAGCCATGAACCAATTAGGTGGAAAGAGTAATAGTGGTGAAGGTG
GCGAAGATGCAAAACGTTATGAAGTACAAGTTGATGGAAGCAACAAAGTAAGTGCGATTAAACAAGTTGCTTCTGGGCGTTTTGGT
GTAACTAGTGATTATTTACAACATGCCAAAGAAATTCAAATTAAAGTTGCGCAAGGTGCAAAGCCTGGTGAAGGTGGTCAATTACC
TGGTACTAAGGTATATCCGTGGATTGCGAAGACAAGAGGGTCAACGCCAGGTATCGGTCTGATTTCACCACCGCCACATCATGATA
TTTATTCAATAGAAGATTTAGCGCAACTGATACATGATTTGAAAAATGCGAATAAAGATGCGGATATCGCGGTAAAATTAGTTTCG
AAAACAGGTGTTGGTACCATTGCATCTGGGGTGGCAAAAGCATTTGCAGATAAAATTGTCATCAGTGGTTACGATGGTGGTACAGG
GGCTTCACCTAAAACGAGTATTCAGCATGCCGGTGTTCCTTGGGAGATTGGTTTAGCAGAAACACATCAAACATTAAAACTAAATG
ACTTAAGAAGTCGTGTTAAGTTAGAAACAGACGGTAAGTTATTAACTGGTAAAGATGTAGCGTACGCATGTGCGCTTGGAGCGGAA
GAATTTGGATTTGCAACTGCACCATTAGTGGTGTTGGGCTGTATTATGATGCGTGTATGCCATAAAGATACATGTCCAGTAGGAGTT
GCAACTCAAAACAAAGATTTACGTGCTTTATATAGAGGTAAAGCACATCATGTTGTTAATTTTATGCATTTTATTGCACAAGAATTA
AGAGAAATTTTAGCATCTTTAGGTTTGAAACGTGTAGAAGACTTAGTTGGAAGAACTGATTTATTACAACGATCATCAACATTAAAA
GCGAATAGCAAAGCGGCTAGTATTGATGTTGAAAAACTGTTATGTCCTTTCGATGGGCCAAACACAAAAGAAATTCAACAAAATCA
TAATCTTGAGCATGGATTTGATTTAACAAATTTATATGAAGTAACGAAGCCATATATTGCTGAAGGGCGTCGCTATACAGGTAGCTT
TACAGTAAATAATGAACAACGTGATGTAGGGGTTATTACAGGTAGTGAGATTTCGAAACAATATGGAGAAGCAGGACTTCCTGAAA
ATACAATTAATGTTTATACGAATGGTCATGCTGGTCAAAGTCTTGCAGCATATGCACCGAAAGGCTTAATGATTCATCATACTGGAG
ATGCGAATGACTATGTTGGTAAAGGATTATCTGGTGGTACGGTCATTGTCAAAGCACCTTTTGAAGAACGACAAAATGAAATTATTG
CTGGTAACGTCTCATTCTATGGTGCGACAGGTGGTAAGGCATTTATTAACGGTAGTGCAGGAGAAAGATTCTGTATTAGAAATAGTG
GTGTAGATGTTGTCGTTGAAGGTATCGGCGACCATGGATTAGAGTATATGACTGGTGGACATGTCATTAATTTAGGTGATGTAGGTA
AGAACTTCGGTCAAGGTATGAGTGGTGGTATTGCTTACGTTATCCCGTCTGATGTAGAAGCTTTTGTTGAAAATAATCAACTAGATA
CGCTTTCGTTTACAAAGATTAAACACCAAGAAGAAAAAGCATTCATTAAGCAAATGCTGGAAGAACATGTGTCACACACGAATAGT
ACGAGAGCGATTCATGTGTTAAAACATTTTGATCGCATTGAAGATGTCGTCGTTAAAGTTATTCCTAAAGATTATCAATTAATGATG
CAAAAAATTCATTTGCACAAATCATTACATGACAATGAAGATGAAGCGATGTTAGCTGCATTTTACGATGACAGTAAAACAATCGA
CGCTAAACATAAACCAGCCGTTGTGTATTAAGGAAAGGGGGAGATACGATGGGTGAATTTAAAGGATTTATGAAGTATGACAAACA
GTACTTAGGTGAATTATCACTGGTAGACCGTTTGAAGCATCATAAAGCATATCAACAACGATTTACTAAAGAAGATGCCTCTATCCA
AGGTGCGCGATGTATGGATTGTGGAACGCCGTTTTGTCAAACCGGACAACAGTATGGTAGGGAAACAATAGGTTGTCCAATTGGAA
ACTACATTCCTGAATGGAACGACTTAGTGTATCATCAAGATTTTAAAACTGCTTATGAACGCTTAAGCGAAACAAATAACTTTCCTG
ACTTTACAGGGCGTGTATGTCCTGCACCATGCGAAAGTGCTTGTGTGATGAAGATTAATAGAGAATCGATTGCGATTAAAGGTATTG
AACGCACGATTATTGATGAAGCTTTTGAAAATGGTTGGGTTGAGCCGAAAATTCCAAGTCATCGTAGAAATGAAAAAGTTGCAATC
ATTGGTAGTGGTCCTGCAGGATTGACTGCCGCTGAAGAACTGAATTTATTAGGGTACCAAGTGACAATTTATGAGCGTGCTAGAGA
ATCAGGCGGTTTATTAATGTATGGTATTCCAAATATGAAGCTTGATAAATATGTCGTTCGTCGTCGTATTAAATTAATGGAAGAAGC
GGGCATAACTTTCATTAACGGCGTTGAAGTAGGTGTTGATATTGATAAAGAAACTTTAGAATCTGAGTATGACGCTATTATATTATG
TACTGGGGCACAAAAAGGTAGGGATTTACCTTTGGAAGGTCGCATGGGTGAAGGTATACATTTTGCTATGGACTATCTGACTGAAC
AAACACAACTTTTAAATGGTGAAATTGATGATATCACTATTACAGCAAAGGATAAAAATGTCATCATTATTGGTGCTGGTGATACAG
GAGCAGACTGTGTAGCAACGGCATTAAGAGAAAACTGTAAATCGATTGTTCAATTTAATAAATATACGAAATTGCCAGAAGCAATT
ACATTTACAGAAAATGCATCATGGCCTTTAGCAATGCCGGTGTTTAAAATGGACTATGCGCACCAAGAGTACGAAGCTAAGTTTGG
TAAGGAACCACGTGCATATGGTGTTCAAACAATGCGTTACGATGTTGACGATAAAGGACACATACGTGGTTTGTATACTCAAATTTT
AGAGCAAGGCGAAAATGGTATGGTCATGAAAGAAGGACCTGAAAGATTTTGGCCTGCTGACCTTGTATTATTATCAATCGGCTTCG
AAGGTACAGAACCAACTGTACCGAATGCTTTTAACATTAAAACGGATAGAAATCGAATCGTGGCGGATGATACAAACTATCAAACT
AATAATGAAAAAGTATTTGCTGCTGGAGATGCTAGACGTGGTCAAAGTTTAGTTGTATGGGCGATTAAAGAAGGTAGAGGCGTAGC
GAAAGCAGTAGATCAGTATTTAGCGAGTAAAGTTTGTGTATAATCTTTGTATGGAAATGGTGGTTACGTTGACGTTGTGGCATGCTG
AATCGAGTTTGAAATAATCTAGTATCTATCAACGTCACATGCTATCTTTGTAACCTAAAAACAAAGGTTTGTAAGACAACAAATAGA
TTAATTATAAGTAGTGATTTTTTACATTCGTTTATAGGTCAACTGTAGTGGAAGACAATGATTTGTGGTAATCATGTAATGCTTAAAA
ACAATATTGACTTTTACAGAACGTTCATATATGATAAATATTGTGTTTAGGAGGAATACCCAAGTCCGGCTGA

>014-contig_260_RC

CATATCAAAAAAGCTGATTTCTATCAAATAATTAATAGAAATCAGCTTTTTTACATTGCCTAAGAACTTAATGTCCCAAGCCCTTTTT
TATTTGTCTTTGAATGGCTCGTAATTTTTGATAATAGAAATGATAAGGCATTGAGATTGGAAGGGCATTTGGCTTGTGCAATATACA
TAGCTAAATGTCTTTTTTGTTTTGTGAAATATGATGGATGGCTTGTGTGGACAAGTTTGCTATTTATAGATATGCATTTTTCAATTTAG
GAGTTGGCCATGCATCTACACTTTATAATGGTGAGAGCGTGGTGAGGTATTGTTAATAACGCAATTGTAGCGAGGAGTTATTGCTAC
ATATGTCGTTATGGCTCATTGATTTTCTGAAATGGCTACCCCAGATAATTGTGACAAAATAAAAATATTTCTTTGAAAGCCTTTACAT
AACTTGTCTAGACAAGTTATACTCATTTTAAGACATTAAGGGAGTGAAATATATGGCTGTAAAAAGAGAAGATGTAAAAGCCATCG
TAACCGCTATTGGGGGAAAAGAAAATCTTGAAGCTGCAACGCATTGTGTAACACGATTACGTTTAGTGCTGAAGGATGAAAGTAAA
GTTGATAAAGACGCATTAAGTAATAACGCGTTGGTCAAGGGGCAGTTTAAAGCAGACCATCAATATCAAATTGTCATTGGTCCAGG
AACAGTCGATGAAGTGTATAAGCAGTTTATTGATGAAACAGGTGCTCAAGAAGCTTCGAAAGATGAAGCGAAACAAGCAGCTGCG
CAAAAAGGGAATCCAGTACAACGTTTGATCAAATTATTGGGGGATATTTTTATACCAATATTACCTGCGATTGTGACAGCTGGTTTG
TTAATGGGAATCAATAATTTACTAACAATGAAAGGTTTATTTGGTCCGAAAGCACTTATTGAGATGTATCCACAAATTGCTGATATT
TCAAACATCATTAATGTGATTGCGAGTACGGCATTTATTTTCTTACCAGCATTAATTGGTTGGAGTAGTATGCGTGTATTTGGTGGTA
GTCCGATTCTAGGCATAGTCTTAGGTTTGATTTTAATGCATCCGCAATTAGTATCTCAGTATGATTTGGCAAAAGGGAATATTCCGA
CATGGAACTTATTTGGCTTAGAGATTAAGCAGTTGAATTACCAAGGTCAAGTGTTGCCAGTTTTAATTGCAGCTTATGTTCTAGCTA
AAATTGAAAAAGGATTAAATAAAGTCGTTCACGATTCGATAAAAATGTTGGTCGTTGGCCCCGTAGCGCTTTTAGTTACTGGATTTT
TAGCATTTATTATCATTGGACCAGTTGCATTATTGATTGGTACAGGAATTACATCTGGTGTTACATTTATATTCCAACATGCAGGATG
GCTTGGCGGAGCAATATATGGATTGTTATATGCACCACTTGTAATTACAGGACTACACCATATGTTTTTAGCAGTAGATTTCCAATT
GATGGGTAGCAGCTTAGGTGGTACGTATTTATGGCCAATCGTTGCGATTTCCAATATTTGTCAGGGCTCTGCAGCATTTGGAGCATG
GTTTGTCTATAAACGTCGTAAAATGGTTAAAGAAGAAGGCTTGGCATTAACATCTTGTATTTCTGGTATGTTAGGTGTTACTGAACC

AGCTATGTTTGGTGTGAACTTACCTCTGAAATATCCATTTATCGCTGCGATATCAACGTCTTGTGTATTGGGGGCAATCGTTGGTATG
AATAACGTGCTTGGAAAAGTTGGTGTTGGTGGCGTGCCAGCATTCATTTCAATTCAAAAAGAATTTTGGCCAGTATATCTTATTGTG
ACAGCTATTGCTATTGTTGTACCATGTATACTAACAATTGTGATGTCTCATTTTAGTAAACAAAAAGCGAAAGAAATTGTTGAAGAT
TAATAAAATAAAAAAGGGGCGTTCGTTATTTGGACGTCCTTTATTACGTTATAAGGTGGTAATTGTGTGTCGAAAGAAATAGATTGG
AGAAAATCCGTTGTATATCAAATTTATCCTAAGTCGTTTAATGATACGACGGGGAATGGTATAGGAGATATCAATGGAATTATAGA
AAAATTGGATTATATCAAGTTATTGGGTGTTGATTATATTTGGTTAACACCAGTGTATGAATCACCGATGAATGATAATGGCTATGA
TATCAGCAATTATTTAGAAATCAATGAAGACTTTGGAACGATGGATGATTTTGAAAAGTTAATCAAAGTTGCGCATCAAAAAGACT
TGAAAGTGATGTTAGATATTGTCATTAATCATACGTCGACGGAGCATGAATGGTTTAAAGAAGCCCGTAAATCTAAAGATAACCCTT
ATAGAGATTATTACTTTTTCAGATCATCTGAAGACGGGCCGCCAACAAATTGGCATTCTAAATTCGGTGGTAATGCATGGAAGTATG
ATTCTGAGACAGATGAATATTATTTACATTTATTTGATGTCAGTCAAGCTGATTTAAATTGGGATAATCCGGAAGTACGTCAATCGT
TATATCGCATAGTCAATCATTGGATAGACTTCGGCGTTGATGGTTTTCGATTTGATGTCATTAACTTAATTTCTAAAGGTGAATTTAA
GGACTCTGACAAAATAGGTAAAGAATTTTATACGGATGGTTCTAGAGTGCATGAGTTTCTGCATGAATTAAATCGTCAAACGTTTGG
TAACACTGACATGATGACTGTAGGAGAAATGTCTTCGACGACGATTGAAAATTGTATTAAGTATACACAACCAGAACGCCAAGAAT
TGAATAGTGTTTTTAATTTTCATCATCTAAAGGTTGATTATGTTGATGGTGAAAAGTGGACAAATGCGAAGCTTGATTTCCATAAGTT
AAAGAAAATTCTGATGCAATGGCAACGAGGTATTTATGACGGTGGTGGATGGAACGCGATTTTCTGGTGTAATCATGATCAGCCAC
GGGTAGTGTCTAGATTTGGTGATGATACGTCGGAAGAGATGAGGATACAAAGTGCTAAAATGTTAGCTATCGCACTGCATATGTTG
CAAGGGACGCCATATATTTACCAAGGGGAAGAAATTGGTATGACGGACCCACATTTTACATCAATAGCACAATATCGCGATGTTGA
ATCGATTAATGCCTACCATCAGTTGTTAAGTGAAGGGCATGCTGAAGCGGATGTGTTAGCGATTTTAGGACAGAAGTCACGAGACA
ATTCGAGAACGCCTATGCAATGGAGTGATGATGTTAATGCTGGATTTACAGCTGGTAAGCCTTGGATTGATATTTCGGAAAATTATC
ATCAGGTCAACGTTAGACAAGCACTTCAGAATAAAGAGTCTATTTTCTATACGTATCAAAAATTAATACAATTAAGACATACGCATG
ATATTATTACGTATGGAGACATTGTGCCACGTTTTATGGATCATGATCATTTATTTGTTTATGAACGTCATTATAAGAATCAACAATG
GCTAGTAATTGCGAATTTCTCAGCATCGGCTGTTGATTTGCCAGAAGGATTGGCTAGAGAAGGTCGTGTTGTGATTCAAACAGGCAC
AGTGGAAAATAATACGATAAGCGGGTTTGGTGCAATTGTAATCGAAACAAACGCGTAAAATAAATTGAGTGGATGCGTTTATATGG
CGAAACAAAAAAAGTTTATGAAGATTTATGAGGCGTTGAAAGAAGATATATTAAATGGGCAGATTCAATATGGTGAACAAATTCCA
TCTGAACATGATTTGGTGCAATTGTACCAGTCATCTCGAGAGACCGTGCGTAAGGCATTAGATTTGTTGGCATTAGACGGCATGATT
CAAAAGATTCATGGTAAAGGGTCACTTGTCATTTATCAGGAGGTTACAGAGTTTCCATTTTCTGAACTTGTTAGTTTTAAAGAAATG
CAAGAAGAAATGGGCGTCGCATATTTAACTGAAGTTGTTGTGAATGAGGTTGTTGAAGCGCATGAAGTTCCAGAAGTTCAACATGC
TTTAAACATCAATTCTAGTGAATCACTCATTCATATTGTTAGAACTCGTCGGCTTAACCAACATGTGAAGATTGTTGATGAAGATTAT
TTTCTAAAGTCGATTGTTTCAGATATAGGTAATGATGTTGCGAGTGATTCTATTTATGATTATTTGGAAAAGGTATTAAATCTTAATA
TTAGTTATTCAAGTAAGTCTATTACTTTTGAACCGTTTGATGAACAAGCATATCAATTGTTTGGTGATGTATCGGTGGCTTATTCAGC
AACAGTTCGAAGTATTGTGTATTTAGAAAATACAATGCCGTTTCAATATAATATTTCAAAACATCTTGCAAATGAATTTAAATTTAA
TGACTTCTCAAGACGTCGTACAAAGTAAACAATGATATAAATGATTTATACTTGCAATTAACTATTAAAATATAGTAATATATATCT
TGCCGTGCTAGGTGGGGAGGTAGCGGTTCCCTGTACTCGAAATCCGCTTTATGCGAGGCTTAATTCCTTTGTTGAGGCCGTATTTTTG
CGAAGTCTGCCCAAAGCACGTAGTGTTTGAAGATTTCGGTCCTATGCAATATGAACCCATGAACCATGTCAGGTCCTGACGGAAGC
AGCATTAAGTGGATCATCATATGTGCCGTAGGGTAGCCGAGATTTAGCTAACGACTTTGGTTACGTTCGTGAATTACGTTCGATGCT
TAGGTGCACGGTTTTTTATTTTTTAAATATTAAACCGATTATTAAGAGTTGAAAATATATATTTATAGAAGCTACTTTCTTGAAGACA
ATTCAGCGTATTATACGTGGAACATGTTTGTGGGAAGTAGCTTTTTTATATGTGAAGTTTGATTCAAGTGAACTCGATGCGCAGTTTG
AATGATTTTTGTGTCAATGAAAAGTAAGAAGTTATTATTTGATGATAAAGAAATGATGGTGAAATGAGGGGGAGTATCTTACAATA
GAATTATTAATGAGATACGTTATGATTATTGACAATCAAATGCCTACGGAGGACATATGCAAATATATTTAAGTACTTTAACAGAGT
TAGATTATGATAAATCTTTAAATAGTATTGAAGAAAGTTTTGATGATAATCCTGAAACGAGTTGGCAAGCACGTGCGAAAGTAAAA
CATTTAAGAAAATCTCCTTGCTATAATTTTGAATTAGAGGTAATAGCGAAAAATGAAAATAACGATGTCGTTGGACACGTTTTATTA
ATTGAAGTAGAAATTAATAGTGATGATAAGACGTATTATGGTTTGGCGATTGCCTCTTTATCAGTTCATCCTGAATTACGTGGACAA
AAATTAGGTCGTGGCTTGGTTCAAGCAGTAGAAGAGCGTGCCAAAGCACAAGAGTATAGTACGGTTGTTGTAGACCATTGTTTTGA
CTACTTTGAAAAGTTGGGTTATCAAAATGCTGCTGAGCATGACATTAAATTAGAATCTGGTGATGCACCGTTACTTGTAAAATATTT
ATGGGATAATTTGACGGATGCACCACACGGAATCGTAAAATTTCCAGAACATTTTTATTAATTGTTCAATTAAGAAGTAAAGGTATT
ATCATGCTATAATGAGAGGTAATTGTTTATGGAGGTGCTAACTTGAATTATCAAGCCTTATATCGTATGTACAGACCCCAAAGTTTC
GACGATGTCGTCGGACAAGAACATGTCACGAAGACATTGCGCAATGCGATTTCGAAAGAAAAACAGTCGCATGCTTATATTTTTAG
TGGTCCGAGAGGTACGGGGAAAACGAGTATTGCCAAAGTGTTTGCTAAAGCAATCAACTGTCTAAATAGCACTGATGGAGAACCTT
GTAATGAATGTCATATTTGTAAAGGCATTACGCAGGGGACTAATTCAGATGTGATAGAAATTGATGCTGCTAGTAATAATGGCGTTG
ATGAAATAAGAAATATTAGAGACAAAGTTAAATATGCACCAAGTGAATCGAAATATAAAGTTTATATTATAGATGAGGTGCACATG
CTAACAACAGGTGCTTTTAATGCCCTTTTAAAGACGTTAGAAGAACCTCCAGCACACGCTATTTTTATATTGGCAACGACAGAACCA
CATAAAATCCCTCCAACAATCATTTCTAGGGCACAACGTTTTGATTTTAAAGCAATTAGCCTAGATCAAATTGTTGAACGTTTAAAA
TTTGTAGCAGATGCACAACAAATTGAATGTGAAGATGAAGCCTTGGCATTTATCGCTAAAGCGTCTGAAGGGGGTATGCGTGATGC
ATTAAGTATTATGGATCAGGCTATTGCATTTGGTGATGGTACGTTAACATTGCAAGATGCGTTGAATGTCACAGGTAGCGTACATGA
TGAAGCGTTGGATCACTTGTTTGATGATATTGTACAAGGTGACGTACAAGCATCTTTTAAAAAATACCATCAGTTTATAACAGAAGG
TAAAGAAGTGAATCGCCTAATAAATGATATGATTTATTTTGTCAGAGATACGATTATGAATAAAACATCTGAGAAAGATACTGAGT
ATCGAGCACTGATGAACTTAGAATTAGATATGTTATATCAAATGATTGATCTTATTAATGATACATTAGTGTCGATTCGTTTTAGTGT
GAATCAAAACGTTCATTTTGAAGTGTTGTTAGTAAAATTAGCTGAGCAGATTAAGGGTCAACCACAAGTGATTGCGAATGTAGCTG
AACCAGCACAAATTGCTTCATCGCCAAACACAGATGTATTGTTGCAACGTATGGAACAGTTAGAGCAAGAACTAAAAACACTAAAA
GCACAAGGAGTGAGTGTCGCTCCTGTTCAAAAATCTTCGAAAAAGCCTGCGAGAGGCATACAAAAATCTAAAAATGCATTTTCAAT
GCAACAAATTGCAAAAGTGCTAGATAAAGCGAATAAGGCAGATATCAAATTGTTGAAAGATCATTGGCAAGAAGTGATTGATCATG
CCAAAAATAATGATAAAAAATCACTCGTTAGTTTATTGCAAAATTCGGAACCTGTGGCGGCAAGTGAAGATCACGTACTTGTGAAA
TTTGAGGAAGAGATCCATTGTGAAATCGTCAATAAAGACGACGAGAAACGTAGTAGTATAGAAAGTGTTGTATGTAATATCGTTAA
TAAAAACGTTAAAGTTGTTGGTGTACCATCAGATCAATGGCAAAGAGTTCGAACGGAATATTTACAAAATCGTAAAAACGAAGGCG
ATGATATGCCAAAGCAACAAGCACAACAAACAGATATTGCTCAAAAAGCAAAAGATCTTTTCGGTGAAGAAACTGTACATGTGATA
GATGAAGAGTGATACATGACAAGCGATATAATCGTATGTATAATGAAAGAAACATCATTTTATTGATAAATATTTATTGATTTTCAA
GGAGGAAATGGAATATGCGCGGTGGCGGAAACATGCAACAAATGATGAAACAAATGCAAAAAATGCAAAAGAAAATGGCTCAAG
AACAAGAAAAACTTAAAGAAGAGCGTATTGTAGGAACAGCTGGCGGTGGCATGGTTGCAGTTACTGTAACTGGTCATAAAGAAGTT
GTCGACGTTGAAATCAAAGAAGAAGCTGTAGACCCAGACGATATTGAAATGCTACAAGACTTAGTGTTAGCAGCTACTAATGAAGC
GATGAATAAAGCTGATGAGCTTACTCAAGAACGTTTAGGTAAACATACTCAAGGCTTAAACATCCCTGGAATGTGATCATAGATGC
ATTATCCAGAACCTATATCAAAACTTATTGATAGCTTTATGAAATTGCCAGGCATTGGTCCAAAGACAGCCCAACGTCTGGCTTTTC

ATACCTTAGATATGAAAGAAGACGATGTTGTTCAGTTTGCCAAAGCATTAGTAGATGTTAAGAGAGAATTAACATATTGTAGCGTAT
GTGGTCACATTACTGAAAATGATCCATGTTATATTTGTGAAGATAAGCAAAGAGATCGTTCAGTTATTTGTGTTGTGGAAGATGACA
AAGATGTCATAGCTATGGAAAAAATGAGAGAATACAAAGGTTTATATCACGTTTTACATGGGTCTATTTCGCCTATGGATGGCATTG
GACCAGAAGATATTAATATTCCTTCATTGATTGAACGCTTGAAAAACGATGAAGTTAGCGAATTAATCTTAGCTATGAACCCGAACT
TAGAGGGGGAATCTACAGCCATGTATATTTCTAGATTAGTTAAGCCTATAGGTATCAAAGTGACGAGATTAGCACAAGGGTTATCG
GTAGGTGGCGATTTAGAGTATGCTGACGAAGTAACATTATCTAAAGCAATCGCAGGTAGAACAGAAATGTAATGTCTTCTATTAAA
CATTTTTGATTTTAATACTATAGTAAGAAAAGTCACAGTGTAATCATTGTGGCTTTTTTTATGGTGTGGTGTGATGTACTACTTTATTT
GCGGTGTGGCGGTGGTATGGTTTACCTAGTTTTACTGAGGGATGGGTAATCTTTAGGAAGCAAGCCGTTGGTTGTGATTTGTTACTT
CTAATAGTAATGATGTGAATTGGATTATCGAAGTAGGAATACCGTTGTGGTGTGTTGATGTTATTAATTTGATAAATGCGGTTGATG
GTTAATGACTATGCAAATGAAATTCTTTTGTAATTGAAATGATAGATGCTGGCTTAGTAGTTGTACTTCTTTGGTCTAAAGCTTATTA
AATCAGCCTGTATAGCGGTGTTTTGAGAGGTTATTTAAAACTTGTAAATTTATTTTTAATTTCTGGTAAAAAAATAAAGTTCTGTTTT
GCGGTTTTTTTGATTGATATGGTTAGAGAAAAATCTGTTTCTTGTTCTAAAAAACGTACTATTTATAAGTAGGAATTTTTTAAGTTCG
ATTTTTAGGATAAGGGCGTTCAGTATAGATAACAAAGGTGTAATTTTTACTGTTGTTAAGCAGTTTGAAAGCCTGTATAGTATTTATT
TGTTGAGGCAAACAAAACAACTCAACTTAAGAAATAACTTGAATTACTAACGAAAATTAATTTTAAAAAGTTGTTGACTTAAATGTT
AATAAAATGT

>015-contig_316_RC

TAAATGAGTAAGCGAGAGCCGAAGAAGAGGAAAGAAGCAAGCGATTGTCACAAGTCAAGAAAGGTTCTTAGCGAGGATGGTAGCT
AACTTACGTTCCGCTAGAGTAGAACGTTGCTAGGCAAGAAATGAATGCGATGAGCCGCATTGAGTGTGAAATTGATATTTTAATAA
TGTGCACTTTTGATTATTTAAATCTGTGACTAGAAGTATAAGTGAAGACATTCAGAAGTATTATAAAAAGTGAACAGCAGTAAGAT
AGTTTTTAATCATAAATCATCTTACTGCTGTTTTTAGATTTTATGTCTAATATCTTTTAAATCGAAGTACAAAAAGGAAATTAATTAT
TATACAATAGACAAGCTATTGCATAAGTAACACTAACTTTTGTCAAAGAAGTGTTACTATATAATTAATACTTTTGAAAGTAACTAA
TTCCAAAACAGTGTAAATAAAGGAAGCGTATATCATGAAGCAACCTATTTTAAATAAATTAGAAAGTTTAAATCAAGAAGAAGCGA
TTTCTTTGCATGTTCCGGGTCATAAAAATATGACTATTGGTCATTTATCTCAATTATCAATGACAATGGATAAAACTGAAATACCTGG
ATTAGATGATTTACATCATCCTGAAGAAGTCATTTTGGAGAGTATGAAGCAGGTGGAGAAGCATTCAGATTATGATGCTTATTTCTT
AGTGAATGGCACCACTTCAGGAATATTATCTGTCATCCAGTCTTTTTCACAGAAAAAAGGCGATATCTTAATGGCAAGAAATGTACA
TAAATCTGTATTACATGCGCTCGATATTAGCCAACAAGAAGGGCATTTTATTGAAACGCATCAAAGTCCGTTAACGAATCATTATAA
TAAAGTTAATTTAAGCCGTTTGAATAATGACGGTCACAAACTTGCTGTGTTGACTTATCCTAACTATTACGGTGAAACATTTAATGT
AGAAGAGGTTATCAAATCTTTGCACCAATTAAATATTCCTGTACTCATTGACGAAGCACACGGCGCGCACTTTGGATTGCAAGGATT
TCCAGATTCTACATTAAATTATCAAGCTGACTATGTTGTTCAATCTTTTCATAAAACGTTACCAGCTTTAACGATGGGCTCGGTACTT
TATATTCATAAAAATGCACCTTATAGAGAAACTATTATAGAATATCTAAGCTACTTCCAAACATCTAGTCCTTCGTATTTGATTATGG
CTAGTTTAGAGTCAGCTGCCGAGTTCTATAAAACATATGATAGTACCGTGTTTTTTGATAAGAGAGCGCAATTAATCGAATGTTTGG
AGAAGAAGGGTTTTGAAATGCTTCAAGTTGATGATCCGTTGAAGTTGCTGATAAAATATGAAGGTTTTACAGGTCATGATATTCAAA
ATTGGTTTATGAATGCACATATCTATTTAGAATTAGCGGACGACTATCAAGCATTAGCGATATTGCCGTTATGGCATCATGATGATA
CGTATTTATTTGATTCGCTTTTACGTAAAATTGAAGATATGATTTTACCGAAAAAATCAGTTTCTAAAGTTAAACAAACACAACTTTT
AACAACTGAAGGTAACTATAAACCAAAACGCTTTGAATATGTTACTTGGTGTGATTTGAAAAAGGCAAAAGGTAAAGTTCTGGCGC
GACATATTGTCCCGTATCCGCCAGGGATTCCTATTATTTTCAAAGGAGAAACAATAACTGAAAATATGATAGAATTGGTAAATGAAT
ATCTGGAAACTGGAATGATAGTTGAAGGAATTAAAAATAATAAAATTTTAGTTGAGGATGAATAAAATGTCAGCTTTTATAACTTTT
GAGGGCCCAGAAGGCTCTGGAAAAACAACTGTAATTAATGAAGTTTACCATAGATTAGTAAAAGATTATGATGTCATTATGACAAG
AGAACCGGGCGGCGTTCCTACTGGTGAAGAAATACGTAAAATTGTATTAGAAGGCAATGATATGGACATTAGAACTGAAGCAATGT
TATTTGCTGCATCTAGAAGAGAACATCTTGTATTAAAGGTCATACCAGCTTTAAAAGAAGGTAAGGTTGTGTTGTGTGATCGCTATA
TCGATAGTTCATTAGCTTATCAAGGTTATGCTAGAGGGATTGGCGTTGAAGAAGTAAGAGCATTAAACGAATTTGCAATAAATGGA
TTATATCCAGACTTGACGATTTATTTGAATGTTAGTGCTGAAGTAGGCCGCGAACGTATTATTAAAAATTCAAGAGATCAAAATAGA
TTAGATCAAGAAGATTTAAAGTTTCACGAAAAAGTAATTGAAGGTTACCAAGAAATCATTCATAATGAATCACAACGGTTCAAAAG
CGTTAATGCAGATCAACCTCTTGAAAATGTTGTTGAAGACACGTATCAAACTATCATCAAATATTTAGAAAAGATATGATATAATTG
TTAGAAGAGGTGTTATAAAATGAAAATGATTATAGCGATCGTACAAGATCAAGATAGTCAGGAACTTGCAGATCAACTTGTTAAAA
ATAACTTTAGAGCAACAAAATTGGCAACAACAGGTGGGTTTTTAAGAGCGGGTAATACAACATTCTTATGTGGTGTCAATGATGAC
CGTGTAGATGAAATATTGTCTGTGATTAATCAAACGTGTGGTAATAGAGAACAGTTGGTTTCACCTATTACACCTATGGGAGGCAGT
GCGGATTCGTACATTCCATATCCAGTTGAAGTTGAAGTTGGCGGTGCTACTGTATTTGTTATGCCAGTTGATGCATTCCATCAATTTT
AATTCTATAATACAATCATCAATTAGAGATACTTAAAATAGTGTATTAATAAGTTATTAACAATTTTGGGTTGCTTGACTGCGACTA
GTTCAGATGCCAATAGATTTGATTTTTGTGGTTCTAAAAATAATCACAAATCATGGTCGCTATTGTTGCAGTAATTAGTTGCTCTTTG
GCAACCTTTTTATATAAAAGCAAAAGGGAGTTTGTAATGAATGGATGAACAGCAACAATTGACGAATGCATATCATTCAAATAAAT
TATCGCATGCCTATTTATTTGAAGGTGATGATGCACAAACGATGAAACAAGTTGCGATTAATTTTGCAAAGCTGATTTTATGTCAAA
CAGATAGTCAATGTGAAACAAAGGTTAGTACATATAATCATCCAGACTTTATGTATATATCAACAACTGAGAATGCAATTAAGAAA
GAACAAGTTGAACAACTTGTGCGTCATATGAATCAACTTCCTATAGAAAGCACAAATAAAGTGTACATCATTGAAGACTTTGAAAA
GTTAACTGTTCAAGGGGAAAACAGTATCTTGAAATTTCTTGAAGAACCACCGGACAATACGATTGCTATTTTATTGTCTACAAAACC
TGAGCAAATTTTAGACACAATCCATTCAAGGTGTCAGCATGTGTATTTCAAGCCTATTGATAAAGAAAAGTTTATAAATAGATTAGT
TGAACAAAACATGTCTAAGCCAGTAGCTGAAATGATTAGTACTTATACTACGCAAATAGATAATGCAATAGCTTTAAATGAAGAAT
TTGATTTATTAGCATTAAGGAAATCAGTTATACGTTGGTGTGAATTGTTGCTTACTAACAAGCCAATGGCACTTATAGGTATTATTGA
TTTATTGAAACAGGCTAAAAATAAAAAACTGCAATCTTTAACTATTGCAGCTGTGAATGGTTTCTTCGAAGATATCATACATACAAA
GGTAAATGTAGAGGATAAACAAATATATAGTGATTTAAAAAATGATATTGATCAATATGCGCAAAAGTTGTCGTTTAATCAATTAA
TTTTGATGTTTGATCAACTGACGGAAGCACATAAGAAATTGAATCAAAATGTAAATCCAACGCTTGTATTTGAACAAATCGTAATTA
AGGGTGTGAGTTAGATGCCAAATGTAATAGGTGTTCAGTTTCAAAAAGCGGGAAAATTAGAATATTATACACCTAATGATATACAA
GTAGATATAGAAGACTGGGTAGTTGTCGAATCTAAAAGAGGCATAGAGATAGGTATTGTTAAAAATCCATTAATGGATATTGCTGA
AGAGGATGTTGTGTTACCTCTTAAAAATATTATTCGCATTGCTGATGACAAAGATATTGATAAATTTAATTGTAATGAACGAGATGC
TGAAAATGCATTAATACTATGTAAAGACATTGTAAGAGAACAAGGTTTGGACATGCGTTTAGTCAATTGCGAATATACATTAGATA
AATCGAAAGTTATTTTTAATTTTACGGCGGATGATCGTATTGATTTTAGAAAATTAGTAAAAATATTAGCGCAACATTTAAAAACAC
GTATCGAGTTGAGACAAATTGGTGTAAGGGATGAAGCCAAATTGCTTGGCGGTATCGGACCTTGTGGTAGGTCGTTATGTTGTTCTA
CATTTTTAGGGGATTTTGAACCAGTATCGATTAAGATGGCTAAGGATCAAAATTTATCATTAAATCCAACTAAAATTTCTGGTGCAT
GTGGTCGTTTGATGTGTTGTTTAAAATATGAAAATGACTATTATGAGGAAGTACGTGCACAATTACCTGATATTGGTGAAGCAATTG

AAACGCCTGATGGTAACGGGAAAGTAGTTGCTTTAAATATATTAGACATTTCTATGCAGGTGAAGCTTGAGGGACATGAACAGCCA
CTTGAATATAAATTAGAAGAAATAGAAACTATGCATTAAGGAGGCATTATTACATTTGGATCGCAATGAAATATTTGAAAAAATAA
TGCGTTTAGAAATGAATGTCAATCAACTTTCAAAGGAAACTTCAGAATTAAAGGCACTTGCAGTTGAATTAGTAGAAGAAAATGTA
GCGCTTCAACTTGAAAATGATAATTTGAAAAAGGTGTTGGGCAATGATGAACCAACTACTATTGATACTGCGAATTCAAAACCAGC
AAAAGCTGTGAAAAAGCCATTACCAAGTAAAGATAATTTGGCTATATTGTATGGAGAAGGATTTCATATTTGTAAAGGCGAATTAT
TTGGAAAACATCGACATGGTGAAGATTGTCTGTTCTGTTTAGAAGTTTTAAGTGATTAATCAAGCACACTCAAATAGTGTTATAATT
ATAAATGAATATGGTTTGGATAAGTCTGAGACAATGCATGTTTCAGGCTTTAATTGTGTATAAAGTTTTGGTGATTGCATAAGAGAT
GGCGGTACTAAATGTTATTATTAAGTGTGCACGCAGTATCATTAGTTATAAAATGTAGCTGTTAAAAGTCAAAAATACATCGAATGT
AGTTAGGCATATAATATAAAAAGAGTTTTCAATTACTCAATAGAAAAAGGTTGTCTTCATAGGAGTTAAAAATGTTAAAAGAGAAT
GAACGATTTGATCAACTAATCAAAGAAGATTTTAGTATTATTCAAAATGATGATGTTTTTTCATTTTCAACGGATGCTTTGTTGTTAG
GGCATTTTACAAAACCTAGAACAAAAGATATTGTGTTGGACTTATGTTCAGGCAATGGGGTGATACCCTTGTTATTGTTTGCGAAAC
ATCCACGACATATAGAAGGTGTTGAGATTCAAAAAACACTTGTCGATATGGCGCGACGCACATTTCAATTCAATGATGTTGATGAAT
ATTTAACAATGCATCACATGGATTTGAAAAACGTTACTAAAGTATTTAAACCTTCACAATATACTTTAGTAACGTGTAATCCGCCTT
ATTTTAAAGAGAATCAGCAACACCAACATCAAAAAGAAGCACATAAGATAGCGAGACATGAGATTATGTGTACACTTGAAGATTGC
ATGATTGCAGCCCGTCATTTATTAAAAGAAGGTGGCAGGCTAAACATGGTACATCGTGCAGAGAGACTAATGGATGTCTTGTTTGA
AATGAGAAAAGTGAATATTGAACCTAAGAAAGTCGTTTTTATATATAGTAAAGTAGGGAAATCAGCACAAACGATAGTAGTAGAA
GGTCGAAAAGGTGGAAATCAAGGTTTAGAAATCATGCCCCCATTTTATATTTATAATGAAGATGGTAATTATAACGAAGAAATGAA
GGAAGTATATTATGGATAGTCATTTTGTATATATTGTAAAATGTAGTGATGGAAGTTTATATACAGGATACGCTAAAGACGTTAATG
CACGTGTTGAAAAACATAACCGAGGTCAAGGAGCCAAATATACGAAAGTAAGACGTCCGGTGCATTTAGTTTATCAAGAAATGTAT
GAGACAAAGTCTGAAGCATTGAAGCGTGAATATGAAATTAAAACTTATACCAGACAAAAGAAATTGCGATTAATTAAGGAGCGAT
AGTATGGTTGTATTATATTTAGTGGGCACACCAATTGGTAATTTAGCAGATATTACTTATAGAGCAGTTGATGTATTGAAACGTGTT
GATATGATTGCTTGTGAAGACACTAGAGTAACTAGTAAGCTGTGTAATCATTATGATATTCCAACTCCATTAAAGTCATATCACGAA
CATAACAAGGATAAGCAGACTGCTTTTATCATTGAACAGTTAGAATTAGGTCTTGACGTTGCGCTCGTATCTGATGCTGGATTGCCC
TTGATTAGTGATCCTGGATACGAATTAGTAGTGGCAGCCAGAGAAGCTAATATTAAAGTAGAGACTGTGCCTGGACCTAATGCTGG
GCTGACGGCTTTGATGGCTAGTGGATTACCTTCATATGTATATACATTTTTAGGATTTTTGCCACGAAAAGAGAAAGAAAAAAGTGC
TGTATTAGAGCAACGTATGCATGAAAATAGCACATTAATTATATACGAATCACCGCATCGTGTGACAGATACATTAAAAACAATTG
CAAAGATAGATGCAACACGACAAGTATCACTAGGGCGTGAATTGACTAAGAAGTTCGAACAAATTGTAACTGATGATGTAACACAA
TTACAAGCATTGATTCAGCAAGGCGATGTACCATTGAAAGGCGAATTCGTTATCTTGATTGAGGGTGCTAAAGCGAACAATGAGAT
ATCGTGGTTTGATGATTTATCTATCAATGAGCATGTTGATCATTATATTCAAACTTCACAGATGAAACCAAAACAAGCTATTAAAAA
AGTTGCTGAAGAACGACAACTTAAAACGAATGAAGTATATAATATTTATCATCAAATAAATTAATTACTTTATCGATTATATGAAAT
TTTAAACGATTTTATAAACGCAAGCTGTAATTTTAAATGGTAAGTTATCATTTTGCATTGATACTGATAAAATGATGTTGACTATGAT
AAAAAAATGATGACATCGACGTTTTTTAATGTAAAATAAATACATTGAAAGTAATAAATACCTTAACATTGAATAAGATGAAAATG
AGATGACGAGATAAATGTTCGCGTCCGTTGAAATGCATAGAAATCTTAGATATTATTTGAAGTGAGACATTACGAGGAGGAACAGT
TATGGCTAAAGAAACATTTTATATAACAACCCCAATATACTATCCTAGTGGGAATTTACATATAGGACATGCATATTCTACAGTGGC
TGGAGATGTTATTGCAAGATATAAGAGAATGCAAGGATATGATGTTCGTTATTTGACTGGAACGGATGAACACGGTCAAAAAATTC
AAGAAAAAGCTCAAAAAGCTGGTAAGACAGAAATTGAATATTTGGATGAGATGATTGCTGGAATTAAACAATTGTGGGCTAAGCTT
GAAATTTCAAATGATGATTTTATCAGAACAACTGAAGAACGTCATAAACATGTCGTTGAGCAAGTGTTTGAACGTTTATTAAAGCAA
GGTGATATCTATTTAGGTGAATATGAAGGTTGGTATTCTGTTCCGGATGAAACATACTATACAGAGTCACAATTAGTAGACCCACAA
TACGAAAACGGTAAAATTATTGGTGGCAAAAGTCCAGATTCTGGACACGAAGTTGAACTAGTTAAAGAAGAAAGTTATTTCTTTAA
TATTAGTAAATATACAGACCGTCTATTAGAATTCTATGACCAAAATCCAGATTTTATACAACCACCATCAAGAAAAAATGAAATGAT
TAACAACTTCATTAAACCAGGACTTGCTGATTTGGCTGTTTCTCGTACATCATTTAACTGGGGTGTCCATGTTCCGTCTAATCCAAAA
CATGTTGTTTATGTTTGGATTGATGCGTTAGTTAACTATATTTCAGCATTAGGCTATTTATCAGATGATGAGTCACTATTTAACAAAT
ACTGGCCAGCAGATATTCATTTAATGGCTAAGGAAATTGTGCGATTCCACTCAATTATTTGGCCTATTTTATTGATGGCATTAGACTT
ACCGTTACCTAAAAAAGTCTTCGCACATGGTTGGATTTTGATGAAAGATGGAAAAATGAGTAAATCTAAAGGTAATGTCGTAGACC
CTAATATTTTAATTGATCGCTATGGTTTAGATGCTACACGTTATTATCTAATGCGTGAATTACCATTTGGTTCAGATGGCGTATTTAC
ACCTGAAGCATTTGTTGAGCGTACAAATTTCGATCTAGCAAATGACTTAGGTAACTTAGTAAACCGTACGATTTCTATGGTTAATAA
GTACTTTGATGGCGAATTACCAGCGTATCAAGGTCCACTTCATGAATTAGATGAAGAAATGGAAGCTATGGCTTTAGAAACAGTGA
AAAGCTACACTGAAAGCATGGAAAGTTTGCAATTTTCTGTGGCATTATCTACGGTATGGAAGTTTATTAGTAGAACTAATAAGTATA
TTGACGAAACAACGCCTTGGGTATTAGCTAAGGACGATAGCCAAAAAGATATGTTAGGCAATGTAATGGCTCACTTAGTTGAAAAT
ATTCGTTATGCAGCTGTATTATTACGTCCATTCTTAACACATGCGCCGAAAGAGATTTTTGAACAATTGAACATTAACAATCCTCAAT
TTATGGAATTTAGTAGTTTAGCGCAATATGGTGTGCTTACTGAGACAATTATGGTTACTGGGCAACCTAAACCTATTTTCCCAAGATT
GGATAGCGAAGCGGAAATTGCATATATCAAAGAATCAATGCAACCGCCTGCTACTGAAGAGGAAAAAGAAGAGATTCCTAGCAAA
CCTCAAATTGATATTAAAGACTTTGATAAAGTTGAAATTAAGGCAGCAACGATTATTGATGCTGAACATGTTAAGAAGTCAGATAA
GCTTTTAAAAATTCAAGTAGACTTAGATTCTGAACAAAGACAAATTGTATCAGGAATTGCCAAATTCTATACACCAGATGATATTAT
TGGTAAAAAAGTAGCAGTTGTTACTAACCTGAAACCAGCTAAATTAATGGGACAAAAATCTGAAGGTATGATATTATCTGCTGAAA
AAGATGGTGTATTAACCTTAGTAAGTTTACCAAGTGCAATTCCAAATGGTGCAGTGATTAAATAACTGTATTTTTAAAAATTAGGAG
AGATAATTATGTTAATCGATACACATGTCCATTTAAATGATGAGCAATACGATGATGATTTGAGTGAAGTGATTACACGTGCTAGAG
AAGCAGGTGTTGATCGTATGTTTGTAGTTGGTTTTAACAAATCGACAATTGAACGCGCGATGAAATTAATCGATGAGTATGATTTTT
TATATGGCATTATCGGTTGGCATCCAGTTGACGCAATTGATTTTACAGAAGAACACTTGGAATGGATTGAATCTTTAGCTCAGCATC
CAAAAGTGATTGGTATTGGTGAAATGGGATTAGATTATCACTGGGATAAATCTCCTGCAGATGTTCAAAAGGAAGTTTTTAGAAAG
CAAATTGCTTTAGCTAAGCGTTTGAAGTTACCAATTATCATTCATAACCGTGAAGCAACACAAGACTGTATCGATATCTTATTGGAG
GAGCATGCTGAAGAGGTAGGCGGGATTATGCATAGCTTTAGTGGTTCTCCAGAAATTGCAGATATTGTAACTAATAAGCTGAATTTT
TATATTTCATTAGGTGGACCTGTGACATTTAAAAATGCTAAACAGCCTAAAGAAGTTGCTAAGCATGTGTCAATGGAGCGTTTGCTA
GTTGAAACCGATGCACCGTATCTTTCGCCACATCCGTATAGAGGGAAGCGAAATGAACCGGCGAGAGTAACTTTAGTAGCTGAACA
AATTGCTGAATTAAAAGGCTTATCTTATGAAGAAGTGTGCGAGCAAACAACTAAAAATGCAGAGAAATTGTTTAATTTAAATTCAT
AAAGTTAAAAGTGAGAAAGATCACCGCCATAAATGTAAACGATGCTATATTCGTTTAATATGCTATGGTTCTTTCTCGCTTTTTTAA
ATTAAAATATCGTGCATGTGCAATACGTACGATAGAGATGGTTAGAGCTTTGAAATTAAGAATTGTAGGAAGGCGTTTTAAATGAA
AATCAATGAGTTTATAGTTGTAGAAGGACGAGATGATACTGAGCGTGTTAAACGAGCTGTTGAATGTGATACGATTGAAACGAATG
GTAGTGCCATCAACGAACAAACTTTAGAAGTAATTAGAAATGCTCAACAAAGTCGAGGCGTTATTGTATTAACAGATCCAGATTTC
CCAGGAGATAAAATTAGAAGTACAATTACTGAACATGTCAAAGGTGTTAAACATGCGTATATTGATAGAGAAAAAGCTAAAAATA
AAAAAGGGGAAATTGGTGTTGAACATGCCGACTTAATTGATATTAAAGAAGCGTTAATGCATGTTAGTTCACCCTTTGATGAAGCTT

ATGAATCAATTGATAAATCTGTGCTTATAGAGTTGGGGTTAATTGTTGGGAAAGATGCAAGGCGCCGTAGAGAAATTTTAAGTAGA
AAATTGCGAATCGGCCATTCCAATGGTAAGCAGTTATTGAAAAAGTTAAATGCATTTGGTTATACCGAAGCGGATGTAAGGCAAGC
TTTAGAAGATGAATGAGGAAGTGAAAATGTTGGATAATAAAGATATTGCAACACCATCAAGAACGCGAGCGTTGTTAGATAAATAT
GGCTTTAATTTTAAAAAAAGTTTAGGACAGAACTTTTTGATAGATGTGAATATCATTAATAATATCATTGATGCAAGTGATATTGAT
GCACAAACTGGGGTGATTGAAATTGGTCCAGGTATGGGGTCGTTGACAGAACAATTGGCCAGACATGCTAAAAGAGTATTGGCATT
TGAAATTGATCAACGTTTAATACCTGTATTAAATGATACACTATCACCTTATGATAATGTGACGGTGATTAATGAAGATATTTTAAA
AGCGAATATTAAAGAAGCTGTTGAAAATCATTTACAAGATTGTGAAAAAATAATGGTTGTTGCAAACCTGCCGTACTATATTACGA
CGCCAATTTTATTAAATTTAATGCAACAAGATATACCAATTGATGGCTACGTGGTGATGATGCAAAAAGAAGTGGGCGAACGCTTA
AATGCTGAAGTAGGTTCAAAAGCATATGGTTCGTTATCAATTGTCGTACAATACTATACAGAGACTAGTAAAGTATTAACGGTACCT
AAATCTGTATTTATGCCACCACCTAATGTTGATTCAATAGTTGTAAAACTGATGCAGAGAACTGAACCGTTAGTAACAGTAGATAAC
GAGGAAGCATTCTTTAAGTTAGCAAAAGCAGCATTTGCACAAAGAAGAAAGACAATTAACAATAACTATCAAAATTATTTTAAAGA
TGGTAAACAACACAAAGAAGTGATTTTACAATGGTTGGAACAAGCAGGTATTGATCCAAGACGTCGCGGTGAAACGCTATCTATTC
AAGATTTTGCTAAATTGTATGAAGAAAAGAAAAAATTCCCTCAATTAGAAAATTAAATGATTGACAAAGCAAAGCACTATTGTTAA
AATTTAAATTTTGTTTGACGAAAACGCTGCAAATATGGTATTATGTAACTTGTAGCGAGGTGGAGCAATATGCCAAAATCAATTTTG
GACATCAAAAATTCTATTGATTGTCATGTAGGAAATCGTATTGTACTGAAAGCCAATGGAGGCCGTAAGAAAACAATAAAACGTTC
TGGAATTTTAAAAGAAACATATCCGTCAGTTTTCATTGTTGAGTTAGATCAAGACAAACACAACTTTGAGAGAGTATCTTATACATA
CACTGATGTGTTAACTGAAAATGTTCAAGTTTCATTTGAAGAGGATAATCATCACGAATCAATTGCACACTAAATAAGACATATAGA
GATGTTAGACGTTTCTTAGTATAAGAAGTAAATATTATGATAATTATTTGAGTGTTGGGCATTATGTTCAATACTCTTTTTATTTACA
AAATGTTTAACACTGATGTTTCGCTTATAGATTTTTCAGTAAATGGATACTTGTATTTATAAACACAAATACAAGTAAATACTAAGT
AATTAGATGGAAAAAATTACTTTTTTATTAAAAAAACACTAAAAAACAAATTAAAATGTCAAATATTAATTCTCTTTATGTTAAAAT
CATCATATTAAGATAACGAAAAGAGGGCGGAAAATGATATATGAAACGGCACCAGCCAAAATTAATTTTACGCTCGATACACTTTT
TAAAAGAAATGATGGCTATCATGAGATTGAAATGATAATGACAACAGTTGATTTAAATGATCGTTTAACTTTTCATAAAAGAAAAG
ATCGAAAGATAGTTGTTGAGATTGAACATAATTATGTGCCTTCTAATCATAAAAATCTCGCATATCGTGCAGCGCAACTATTTATTG
AGCAATATCAACTAAAGCAAGGTGTAACAATTTCTATCGATAAAGAAATACCTGTTTCTGCTGGCTTAGCTGGAGGTTCGGCTGATG
CAGCAGCAACGTTAAGAGGATTGAATCGACTTTTTGATATAGGGGCGAGTTTGGAAGAATTGGCTCTACTAGGCAGTAAAATCGGG
ACAGATATTCCGTTTTGTATTTATAATAAAACTGCACTATGTACTGGAAGAGGAGAGAAAATCGAGTTTTTAAATAAACCACCTTCA
GCTTGGGTGATTCTTGCTAAACCAAACTTAGGCATATCATCACCAGATATATTTAAGTTGATCAATTTAGATAAGCGTTACGACGTA
CATACGAAAATGTGTTATGAGGCCTTAGAAAATCGAGATTATCAACAATTATGTCAAAGTTTGTCTAATCGATTAGAGCCAATTTCT
GTTTCAAAACACCCACAAATCGATAAATTAAAAAATAATATGTTGAAAAGTGGTGCAGATGGTGCGTTAATGAGTGGAAGCGGACC
GACTGTGTATGGGCTAGCACGAAAAGAAAGCCAAGCAAAAAATATTTATAATGCAGTTAACGGTTGTTGTAATGAAGTGTACTTAG
TTAGACTATTAGGATAGAAGGGTTGAAAAGATGAGATATAAACGAAGCGAGAGAATTGTTTTTATGACGCAATATTTGATGAACCA
TCCGAATAAATTGATTCCATTAACTTTTTTTGTGAAAAAATTTAAACAGGCGAAGTCTTCAATAAGTGAAGATGTCCAAATTATAAA
AAATACATTCCAAAAAGAAAAGTTAGGTACAGTAATTACTACTGCTGGCGCAAGTGGTGGTGTTACGTATAAACCAATGATGAGTA
AAGAAGAGGCGACTGAAGTTGTTAATGAGGTCATTACTCTATTAGAAGAGAAAGAACGTTTGTTACCTGGCGGGTATTTATTTTTAT
CAGATTTGGTAGGTAATCCATCGCTACTAAACAAAGTTGGTAAGTTAATTGCCAGTATTTACATGGAAGAAAAATTAGATGCTGTTG
TTACCATTGCGACAAAAGGTATTTCATTGGCAAATGCGGTTGCTAATATTTTAAATTTACCAGTAGTAGTGATTAGAAAAGACAACA
AGGTGACTGAAGGTTCTACAGTTTCAATTAATTACGTTTCAGGATCTTCAAGAAAAATAGAGACAATGGTACTTTCGAAGAGAACTT
TAGCAGAAAATTCAAATGTTTTAGTTGTCGATGATTTTATGAGGGCTGGTGGCTCTATTAATGGTGTTATGAATTTAATGAATGAGTT
TAAAGCCCATGTAAAAGGGGTATCAGTACTTGTAGAATCAAAAGAAGTTAAACAAAGATTGATTGAAGATTATACTTCCTTAGTGA
AATTATCTGATGTAGATGAATATAATCAAGAGTTTAACGTAGAACCTGGCAACAGTTTATCTAAGTTTTCATAAAAGGAGTTTTAGT
ATTATGAAAATCATTAACACAACAAGATTACCGGAAGCACTTGGACCATATTCGCATGCAACAGTTGTGAATGGTATGGTTTATACT
TCTGGTCAGATTCCATTGAATGTTGATGGGGAAATCGTAAGCGCTGATGTTCAAGCACAGACAAAACAAGTTTTAGAAAATTTAAA
GGTTGTTTTGGAAGAAGCAGGATCTGATTTGAATTCTGTTGCGAAAGCGACCATTTTCATTAAAGATATGAATGATTTCCAAAAAAT
AAATGAAGTGTATGGTCAATATTTTAATGAACACAAGCCAGCGCGTAGTTGTGTAGAGGTTGCGCGTTTGCCAAAAGATGTGAAAG
TAGAAATTGAATTAGTAAGTAAAATTAAGGAATTATAATTTTCGATTAATATGTTTAATCAAGCTTCTAAATAAAACAGAGAGATAT
ATACTATAGGGGGGCTCACTACATGAAAGTGACAGATGTAAGACTTAGAAAAATACAAACAGATGGACGAATGAAAGCACTCGTT
TCCATTACATTAGATGAAGCTTTCGTAATTCATGATTTACGTGTAATTGAAGGAAACTCTGGCTTGTTCGTTGCAATGCCAAGTAAA
CGTACACCAGATGGTGAATTCCGCGACATCGCGCATCCTATTAATTCAGATATGAGACAAGAAATTCAAGATGCAGTGATGAAAGT
ATATGATGAAACAGATGAAGTAGTACCAGATAAAAACGCTACATCAGAAGATTCAGAAGAAGCTTAATCAATTTTATATTTAGCGA
TGTAATACATTTGCAATAAGTTGATTTGATACTGTCGATAAAGCATAAAGCTTTGTCGGCAGTTTTTTAGTTTGTATTAATGTTTTTT
TATTTTTAATGAAAGGCTAATAAATATATACGTTAACAGATTATGATGATATGAAAATTATTGATTCATTGCTGATAAAAATTGTGT
TTGAATGATGCTCGTATTTTTGAAGTAAGAAAAAAGTTGTTTTTAAAATTACAACGAATTAAAAACAATGCCTTTTATATGTTGAAA
GAGTATTGCAGATTAAATTATAATAATGACGAAGTGTAAAATTTAATGGGGGTTAATGTTCATGCGAAGACACGCGATAATTTTGG
CAGCAGGTAAAGGCACAAGAATGAAATCTAAAAAGTATAAAGTGCTACACGAGGTTGCTGGGAAACCTATGGTCGAACATGTATT
GGAAAGTGTGAAAGGCTCTGGTGTCGATCAAGTTGTAACCATCGTAGGACATGGTGCTGAAAGTGTAAAAGGACATTTAGGCGAGC
GTTCTTTATACAGTTTTCAAGAGGAACAACTCGGTACTGCGCATGCAGTGCAAATGGCGAAATCACACTTAGAAGACAAGGAAGGT
ACGACGATCGTTGTATGTGGTGACACACCGCTCATCACAAAGGAAACATTAGAAACATTGATTGCGCATCATGAGGATGCTAATGC
TCAAGCAACTGTATTATCTGCATCGATTCAACAACCATATGGATACGGAAGAATCGTTCGAAATGCGTCAGGTCGTTTAGAACGCAT
AGTTGAAGAGAAAGATGCAACGCAAGCTGAAAAGGATATTAATGAAATTAGTTCAGGTATTTTTGCGTTTAATAATAAAACGTTGT
TTGAAAAATTAACACAAGTGAAAAATGATAATGCGCAAGGTGAATATTACCTCCCTGATGTATTGTCGTTAATTTTAAATGATGGCG
GCATCGTAGAAGTCTATCGTACCAATGATGTTGAAGAAATCATGGGTGTAAATGATCGTGTAATGCTTAGTCAGGCTGAGAAGGCG
ATGCAACGTCGTACGAATCATTATCACATGCTAAATGGTGTGACAATCATCGATCCTGACAGCACTTATATTGGTCCAGACGTTACA
ATTGGTAGTGATACAGTCATTGAACCAGGCGTACGAATTAATGGTCGTACAGAAATTGGCGAAGATGTTGTTATTGGTCAGTACTCT
GAAATTAACAATAGTACGATTGAAAATGGTGCATGTATTCAACAGTCTGTTGTTAATGATGCTAGCGTAGGAGCGAATACTAAGGT
CGGACCGTTTGCGCAATTGAGACCAGGCGCGCAATTAGGTGCAGATGTTAAGGTTGGAAATTTTGTAGAAATTAAAAAAGCAGATC
TTAAAGATGGTGCCAAGGTTTCACATTTAAGTTATATTGGCGATGCTGTAATTGGTGAACGTACTAATATTGGTTGCGGAACGATTA
CAGTTAACTATGATGGTGAAAATAAATTTAAAACTATCGTCGGCAAAGATTCATTTGTAGGTTGCAATGTTAATTTAGTAGCACCTG
TAACAATTGGTGATGATGTATTGGTGGCAGCTGGTTCCACAATCACAGATGACGTACCAAATGACAGTTTAGCTGTGGCAAGAGCA
AGACAAACAACAAAAGAAGGATATAGGAAATAATCATTTACGTATTTAAAATGACTAGGATAAAAGGATAATCCTATGTAATATTA
ATGTAATCTTTATGATTTAATGATTCGCATAGTAATGGAGTTACATTTTATATATAATAGTAATTGCGTAAGTAAATAATTGGAGGA
CTATAAATGTTAAATAATGAATATAAGAATTCGTCATTAAAGATTTTTTCATTGAAAGGAAACGAAGCATTAGCGCAAGAAGTTGCT

GACCAAGTAGGAATTGAACTAGGTAAATGTTCAGTTAAACGTTTTAGTGATGGAGAAATTCAAATTAATATCGAAGAGAGTATTCG
TGGTTGTGACGTATTTATTATTCAACCAACATCATATCCTGTGAATCTACATTTAATGGAATTATTAATTATGATTGACGCTTGTAAA
CGTGCTTCTGCAGCAACAATCAATATTGTAGTGCCATATTATGGATATGCAAGACAAGATAGAAAAGCCCGTAGCCGTGAGCCAAT
CACAGCTAAATTAGTTGCAAACTTAATCGAAACAGCTGGCGCAACTCGTATGATTGCGTTAGACTTACATGCACCACAAATTCAAG
GATTCTTTGATATTCCAATTGACCACTTAATGGGTGTGCCAATTCTTGCTAAACATTTCAAAGATGATCCGAATATTAACCCAGAAG
AATGTGTCGTTGTTTCACCAGACCATGGCGGTGTTACACGTGCACGTAAATTAGCTGACATTTTAAAAACTCCAATTGCAATTATAG
ATAAACGTCGTCCTAGACCAAATGTTGCTGAAGTGATGAACATTGTTGGTGAGATTGAAGGACGTACGGCAATTATTATTGACGAT
ATTATTGATACAGCAGGTACAATCACTTTAGCTGCACAAGCATTAAAAGATAAAGGTGCTAAAGAAGTATATGCTTGTTGTACACA
CCCTGTTTTATCAGGACCGGCTAAAGAACGTATCGAAAATTCTGCTATAAAAGAATTAATCGTAACAAACTCAATTCATTTAGATGA
AGATCGCAAACCATCTAACACTAAAGAATTATCTGTTGCTGGTTTAATCGCACAAGCTATCATTCGTGTATACGAAAGAGAATCAGT
TAGCGTATTATTTGACTAATATTTAAAAGGCGTTTGACGAACATATCCCAAACGTGTATAATAGTTTCGTTCGTGATTATACGAATA
AATAAACACTTGCAAGCAACGATTATGTTGATGGGTAAGTGAGGTGCTCGTTTTGAGCAAAAATGAAAGGTGGAAATGAGAATGGC
TTCATTAAAGTCAATCATCCGTCAAGGTAAGCAAACACGTTCAGATCTTAAACAATTAAGAAAATCTGGTAAAGTACCAGCAGTAG
TATACGGTTACGGTACTAAAAACGTGTCAGTTAAAGTTGATGAAGTAGAATTCATCAAAGTTATCCGTGAAGTAGGTCGTAACGGT
GTTATCGAATTAGGCGTTGGTTCTAAAACTATCAAAGTTATGGTTGCAGACTACCAATTCGATCCACTTAAAAACCAAATTACTCAC
ATTGACTTCTTAGCAATCAATATGAGTGAAGAACGTACTGTTGAAGTACCAGTTCAATTAGTTGGTGAAGCAGTAGGCGCTAAAGA
AGGCGGCGTAGTTGAACAACCATTATTCAACTTAGAAGTAACTGCTACTCCAGACAATATTCCAGAAGCAATCGAAGTAGACATTA
CTGAATTAAACATTAACGACAGCTTAACTGTTGCTGATGTTAAAGTAACTGGCGACTTCAAAATCGAAAATGATTCAGCTGAATCAG
TAGTAACAGTAGTTGCTCCAACTGAAGAACCAACTGAAGAAGAAATCGAAGCTATGGAAGGCGAACAACAAACTGAAGAACCAGA
AGTTGTTGGCGAAAGCAAAGAAGACGAAGAAAAAACTGAAGAGTAATTTTAATCTGTTACATTAAAGTTTTTATACTTTGTTTAACA
AGCACTGTGCTTATTTTAATATAAGCATGGTGCTTTTTGTGTTATTATAAAGTTTAATTAAACTTTATTACTTTGTACTAAAGTTTAAT
TAATTTTAGTGAGTAAAAGACATTAAACTCAACAATGATACATCATAAAAATTTTAATGTACTTGATTTTAAAATACATACTTACTA
AGCTAAAGAATAATGATAATTGATGGCAATGGCGGAAAATGGATGTTGTCATTATAATAATAAATGAAACAATTATGTTGGAGGTA
AACACGCATGAAATGTATTGTAGGTCTAGGTAATATAGGTAAACGTTTTGAACTTACAAGACATAATATCGGCTTTGAAGTCGTTGA
TTATATTTTAGAGAAAAATAATTTTTCATTAGATAAACAAAAGTTTAAAGGTGCATATACAATTGAACGAATGAACGGCGATAAAG
TGTTATTTATCGAACCAATGACAATGATGAATTTGTCAGGTGAAGCAGTTGCACCGATTATGGATTATTACAATGTTAATCCAGAAG
ATTTAATTGTCTTATATGATGATTTAGATTTAGAACAAGGACAAGTTCGCTTAAGACAAAAAGGAAGTGCGGGCGGTCACAATGGT
ATGAAATCAATTATTAAAATGCTTGGTACAAACCAATTTAAACGTATTCGTATTGGTGTGGGAAGACCAACGAATGGTATGACGGT
ACCTGATTATGTTTTACAACGCTTTTCAAATGATGAAATGGTAACGATGGAAAAAGTTATCGAACACGCAGCACGCGCAATTGAAA
AGTTTGTTGAAACATCACGATTTGACCATGTTATGAATGAATTTAATGGTGAAGTGAAATAATGACAATATTGACAACGCTTATAAA
AGAAGATAATCATTTTCAAGACCTTAATCAGGTATTTGGACAAGCAAACACACTAGTAACTGGTCTTTCCCCGTCAGCTAAAGTGAC
GATGATTGCTGAAAAATATGCACAAAGTAATCAACAGTTATTATTAATTACCAATAATTTATACCAAGCAGATAAATTAGAAACAG
ATTTACTTCAATTTATAGATGCTGAAGAATTGTATAAGTATCCTGTGCAAGATATTATGACCGAAGAGTTTTCAACACAAAGCCCTC
AACTGATGAGTGAACGTATTAGAACTTTAACTGCGTTAGCTCAAGGTAAGAAAGGGTTATTTATCGTTCCTTTAAATGGTTTGAAAA
AGTGGTTAACTCCTGTTGAAATGTGGCAAAATCACCAAATGACATTACGTGTTGGTGAGGGTATCGATGTGGACCAATTTCTTAACA
AATTAGTTAATATGGGGTACAAACGGGAATCCGTGGTATCGCATATTGGTGAATTCTCATTGCGAGGAGGTATTATCGATATCTTTC
CGCTAATTGGGGAACCAATCAGAATTGAGCTATTTGATACCGAAATTGATTCTATTCGGGATTTTGATGTTGAAACGCAGCGTTCCA
AAGATAATATTGAAGAAGTCGATATCACAACTGCAAGTGATTATATCATTACTGAAGAAGTAATCAGCCATCTTAAAGAAGAGTTA
AAAACTGCATATGAAAATACAAGACCCAAAATAGATAAATCAGTGCGCAATGATCTGAAAGAAACGTATGAAAGCTTTAAATTATT
CGAAAGTACATACTTTGATCATCAAATACTACGTCGTTTAGTAGCGTTTATGTATGAAACACCTTCGACAATTATTGATTATTTCCAA
AAAGATGCAATCATTGCAGTTGATGAATTTAATCGTATTAAAGAAACTGAAGAAAGTTTAACAGTAGAGTCTGATTCGTTTATTAGC
AATATTATTGAAAGTGGTAATGGATTTATAGGACAAAGTTTTATAAAATATGATGATTTTGAAACATTGATTGAAGGCTATCCTGTC
ACTTATTTTTCATTATTCGCTACAACAATGCCGATAAAACTAAATCATATTATTAAATTTTCATGTAAACCTGTCCAACAATTTTATG
GGCAATATGACATTATGCGTTCTGAATTTCAACGATATGTTAATCAAAACTATCATATCGTGGTTTTGGTCGAAACCGAAACTAAAG
TTGAACGTATGCAAGCGATGTTAAGTGAAATGCATATTCCATCAATAACAAAATTGCATCGCTCAATGTCATCGGGGCAAGCAGTG
ATTATTGAAGGCAGTTTATCTGAAGGATTTGAACTACCTGATATGGGATTAGTTGTCATTACTGAGCGTGAGCTTTTTAAATCAAAA
CAGAAAAAGCAACGAAAACGTACGAAAGCTATCTCAAATGCTGAAAAAATTAAGTCTTACCAAGATTTAAATGTGGGAGATTATAT
TGTTCATGTGCATCATGGTGTTGGTAGATATTTAGGTGTTGAGACGCTCGAAGTGGGGCAAACGCATCGTGATTATATTAAATTGCA
ATATAAAGGTACGGATCAACTATTTGTTCCAGTAGATCAAATGGATCAAGTTCAAAAATATGTAGCTTCGGAAGATAAGACGCCAA
AATTAAATAAACTCGGTGGCAGTGAATGGAAAAAAACAAAAGCTAAAGTTCAACAAAGTGTTGAAGATATTGCTGAAGAGTTGATT
GATTTATATAAAGAAAGAGAAATGGCAGAAGGTTATCAATATGGGGAAGACACAGCTGAGCAAACAACATTTGAATTAGATTTTCC
ATATGAACTTACGCCTGACCAAGCTAAATCTATCGATGAAATTAAAGATGACATGCAAAAATCGCGTCCAATGGATCGCTTGCTAT
GTGGTGATGTTGGTTATGGTAAAACTGAAGTTGCAGTGAGAGCAGCATTCAAAGCTGTAATGGAAGGAAAGCAGGTTGCATTTTTA
GTTCCTACAACTATTTTAGCTCAGCAACATTATGAGACGTTAATTGAGCGTATGCAAGATTTTCCTGTTGAAATTCAATTAATGAGTC
GTTTTAGAACGCCTAAAGAGATAAAACAAACTAAGGAAGGACTTAAAACTGGATTTGTTGACATAGTTGTTGGTACACACAAATTA
CTTAGTAAAGATATACAGTATAAAGATTTAGGGCTGTTGATTGTAGATGAAGAACAACGATTTGGTGTACGCCATAAAGAGCGTAT
TAAAACATTAAAACATAATGTAGATGTACTAACATTGACTGCAACCCCAATACCTAGAACATTGCATATGAGTATGCTAGGTGTGC
GCGATTTGTCAGTGATTGAAACGCCGCCAGAAAATCGTTTCCCAGTTCAAACATATGTATTAGAACAGAACATGAGTTTTATCAAAG
AAGCTTTAGAAAGAGAACTATCCCGTGATGGCCAAGTGTTTTATCTTTATAATAAAGTGCAATCCATTTATGAAAAACGAGAACAA
CTCCAGATGTTAATGCCAGATGCTAACATTGCAGTTGCTCATGGACAAATGACAGAGCGCGATTTAGAAGAAACGATGTTAAGTTTT
ATCAATAATGAATATGATATTTTAGTAACGACGACGATTATTGAAACAGGTGTCGATGTCCCAAATGCAAATACTTTGATCATTGAA
GATGCAGATCGCTTTGGATTGAGTCAGTTGTATCAATTAAGAGGTCGTGTTGGTCGTTCAAGTCGTATTGGTTATGCATACTTCTTAC
ATCCAGCAAATAAGGTACTAACTGAGACTGCAGAAGATCGATTACAAGCGATTAAAGAATTTACGGAGTTAGGCTCAGGATTTAAG
ATTGCGATGCGTGATTTGAACATTCGTGGTGCTGGTAATTTGTTAGGTAAACAACAGCACGGCTTTATTGATACAGTTGGATTTGAT
TTGTACAGTCAAATGTTAGAAGAAGCTGTAAATGAAAAACGTGGTATTAAGGAACCAGAATCTGAGGTGCCAGAAGTCGAAGTTGA
TTTAAACTTGGATGCATATTTGCCGACAGAATATATTGCAAATGAACAAGCTAAAATTGAAATTTATAAAAAGCTACGAAAAACTG
AAACATTTGATCAAATTATCGACATTAAAGATGAATTAATTGATCGTTTTAATGATTATCCTGTTGAAGTAGCACGTTTGCTTGATAT
AGTGGAAATAAAAGTACACGCATTACATTCAGGTATCACGTTGATTAAAGATAAAGGGAAAATAATTGATATTCATTTATCTGTAA
AAGCCACTGAAAATATTGATGGCGAAGTGCTGTTCAAAGCAACACAACCTTTAGGTAGAACAATGAAGGTTGGTGTTCAAAATAAT
GCAATGACAATTACTTTAACGAAACAAAATCAATGGCTTGATAGTTTGAAGTTTTTAGTTAAGTGCATTGAAGAAAGTATGAGAATC
AGTGATGAAGCATAAAGAAGCATTTAATGGCGTTGTCGTGTTAACTGCTGCATTAATTGTCATTAAAATTCTGAGTGCTGTATATCG

AATTCCATATCAAAATATATTAGGTGATACAGGTTTGTATGCATATCAACAAGTGTATCCAATTGTAGCATTAGGAATGATATTATC
GATGAATGCCATTCCTAGTGCAATTACACAAAATATAGGGAAGTATCATAGTGACGAAGCATATGCAAAAGCGGTCGCTTATATAC
AATTAGTTGGTATATTATTATTTATTGCTATTTTTGTGTTTGCGAACAATATTGCACATATGATGGGTGATAGCCATTTAACACCAAT
GATTCAAGCAGCAAGTTTAAGCTTTATATTTATAGGTATGCTTGGCGTGTTAAGAGGTTATTATCAATCTGCAAATAATATGACAGT
TCCGGCTATTTCCCAGGTTATAGAACAAGTTATACGAGTAGGTATTATCATTGTTACTATTGTTATTTTTGTAGACAGAGGTTGGACG
ATATATGAAGCGGGAACAATTGCTATTTTAGCATCAACGATAGGTTTTTTAGGTTCTTCAATTTATTTAGTAGCGCACCGACCTTTTA
AGTTTAAAATGGTAAATAACACTGCAAAGATTGTTTGGAAACAGTTCGCACTTTCGGTTTTGATTTTCGCTATCAGTCAATTAATCGT
AATTTTATGGCAAGTGATTGATAGTGTTACTATTATTAAGTCACTTCAAGCGATACGCGTGCCATTCGATGTTGCCATAACTGAAAA
AGGAGTCTATGACCGTGGTGCATCATTTATTCAGATGGGATTGATTGTAACTACAACATTTAGTTTTGCGCTCATTCCTCTGTTAAGT
GACGCAATCAAAATGAATAATCAGGTACTTATGAATCGTTATGCAAATGCGTCATTAAAGATTACGATTTTAATAAGTACAGCAGC
GGGAATAGGATTAATTAATTTATTGCCTTTAATGAACGGTGTGTTTTTTAAGACGAATGATTTAACCTTAACGTTAAGTGTTTATATG
ATTACGGTCATTTGTGTATCGTTAATTATGATGGATATGGCTTTATTACAAGCGCAACATGCTGTGAGACCTATTTTTGTTGGTATGA
CGGCAGGATTGGTTATTAAATTTATACTTAATATCATTTTGATTCGTTTAAGTGGCATTATTGGTGCGAGCATTAGTACTGTTGTATC
ATTAATTATATTCGGTACGATTATCCATATTGCTGTCACGAGAAAATACCACTTACATGCGATGAGACGATTTTTTATCAATGTTGTT
TTAGGTATGGTATTTATGTCGATTGTTGTTCAATGCGTGTTAAACATAGTGACAACACACGGTAGATTCACTGGACTCATTGAATTAT
TATGTGCAGCAGTATTAGGTATCATTGCATTGTTTTTCTATATTTTTAGATTTAATGTTTTGACATATAAAGAGTTAACTTATTTACCA
TTTGGTTCAAAGTTGTATCAAATTAAGAAAGGAAGACGTTGATGGCACATACCATTACGATTGTTGGCTTAGGAAACTATGGCATTG
ATGATTTGCCGCTAGGGATATATAAATTTTTAAAGACACAAGATAAAGTTTATGCAAGAACGTTAGATCATCCAGTTATAGAATCAT
TGCAAGATGAATTAACATTTCAGAGTTTTGACCATGTTTATGAAACACATGACCAATTTGAAGATGTCTATAATGATATTGTGGCGC
AATTGGTTGAAGCTGCTAATGAAAAAGATATTGTCTATGCGGTTCCGGGTCATCCTAGAGTTGCTGAGACAACTACAGTGAAATTAC
TGGCTTTAGCAAAGGACAATACTGATATAGATGTGAAAGTTTTAGGTGGTAAAAGCTTTATTGATGATGTGTTTGAAGCAGTTAATG
TAGATCCAAATGATGGCTTCACACTGTTAGATGCGACATCATTACAAGAAGTAACACTTAATGTTAGAACGCATACATTGATTACGC
AAGTTTATAGTGCAATGGTTGCTGCTAATTTGAAAATCACTTTAATGGAACGATATCCTGATGATTACCCTGTTCAAATTGTCACTGG
TGCACGAAGCGATGGTGCGGATAACGTTGTGACATGCCCATTATATGAATTGGATCATGATGAAAATGCATTCAATAATTTGACGA
GTGTATTCGTACCAAAAATCATAACATCGACATATTTGTATCATGACTTTGATTTTGCAACGGAAGTGATTGATACTTTAGTTGATGA
AGATAAAGGTTGTCCATGGGATAAAGTGCAAACGCATGAAACGCTTAAGCGTTATTTACTTGAAGAAACATTTGAATTGTTCGAAG
CTATTGACAATGAAGATGATTGGCATATGATTGAAGAACTAGGAGATATTTTATTACAAGTGTTATTGCATACTAGTATTGGTAAAA
AAGAAGGGTATATCGACATTAAAGAAGTGATTACAAGTCTTAATGCTAAAATGATTCGTAGACACCCACACATATTTGGTGATGCC
AATGCTGAAACTATCGATGACTTAAAAGAAATTTGGTCTAAGGCGAAAGATGCTGAAGGTAAACAGCCAAGAGTTAAATTTGAAAA
AGTATTTGCAGAGCATTTTTTAAATTTATATGAGAAGACGAAGGATAAGTCATTTGATGAGGCCGCGTTAAAGCAGTGGCTAGAAA
AAGGGGAGAGTAATACATGAGATTAGATAAATATTTAAAAGTATCACGGTTAATAAAGCGACGTACGCTAGCAAAAGAAGTAAGT
GATCAAGGTAGAATTACAATAAATGGTAATGTTGCTAAAGCTGGATCGGATGTTAAAGTTGAAGATGTGCTGACGATTCGCTTTGGT
CAAAAATTAGTAACAGTTAAAGTAACTGCATTAAATGAACATGCATCTAAAGATAACGCGAAGGGCATGTATGAAATCGTTGAAGA
GCGTCGACTTGAAGAAGCGTAAATTGGAGGTGACAAGCAATGAAAAATAAAGTAGAACATATAGAAAATCAGTACACGTCGCAAG
AGAACAAGAAAAAACAACGTCAAAAAATGAAAATGCGTGTTGTTCGTAGGCGTATTACAGTATTTGCGGGCGTATTACTTGCGATA
ATTGTTGTTTTATCAATCTTGCTTGTTGTCCAAAAACATCGCAATGATATCGATGCACAGGAGCGAAAAGCGAAAGAAGCACAGTTT
CAAAAGCAACAAAATGAAGAAATTGCGTTAAAAGAAAAGTTGAATAATCTGAATGACAAAGATTATATTGAAAAAATTGCGCGTG
ATGATTATTACTTAAGCAACAAAGGTGAAGTGATTTTTAGGTTGCCAGAAGACAAAGATTCGTCTAGCTCAAAATCTTCGAAAAAA
TAAATCCAAATTGATTCAAAATTATCCGAGTATAGACATTGTGAAAAAATCCAAACAAGGATATAATAAGGGAAAATCGAATCAAA
TCGGGAGGATTTATTTAACATATGTCAATCGAAGTTGGAAATAAGCTTAAAGGTAAAGTCACTGGTATTAAAAAGTTTGGTGCATTC
GTAGAATTACCTGAAGGAAAAAGTGGTTTAGTTCACATTAGTGAAGTCGCAGATAATTATGTTGAAAACGTAGAAGAGCACCTTTC
TGTTGGTGATGAAGTAGACGTAAAAGTATTATCTATTGCTGATGATGGAAAAATTAGTCTTTCAATTAAGAAAGCTAAAGACCGTCC
ACGTAGACAACATACGAGTAAACCAAGTCATCAAAAACCAGTGCAAAAAGCCGAAGATTTTGAAAAGAAATTAAGCAATTTCTTA
AAAGATAGTGAAGATAAATTAACTTCAATCAAACGTCAAACAGAATCTAGACGCGGTGGCAAAGGTTCAAGACGTTAATTAAAATA
AATAAAGACTGTTTCGATAAGGAATATATTTAGAATGATGCGTATCGAATAATCGATTGCAGCGTTAGACAATCTAAGACTGTTTCT
TAAATAAGGAGCAGTCTCTTTTATTTGTAATGATATAACTAAGACTTATACCATTTTTGAAAATTGTAAAAGTGAGGTGATGTTATG
CAGTTAAATAGTAATGGTTGGCATGTTGATGACCATATTGTTGTCGCTGTTTCTACAGGTATTGATAGTATGTGTTTATTGTATCAAC
TACTAAATGATTATAAAGATAGTTATAGAAAACTAACATGCTTACATGTCAATCATGGCGTTAGGTCAGCTTCAATCGAGGAAGCC
AGATTTTTAGAAGTATACTGCGAACGTCATCACATCGATTTACATATCAAAAAGTTAGATTTGTCGCATAGTCTCGACCGAAATAAC
AGCATTCAGAATGAAGCTCGAATTAAACGTTACGAATGGTTTGATGAAATGATGAATGTATTAGAAGCGGATGTATTGCTAACGGC
GCATCATTTGGACGATCAATTAGAAACTATTATGTATCGTATTTTTAATGGGAAATCGACGCGTAATAAACTAGGATTTGATGAGTT
ATCGAAGCGAAAAGGTTATCAGATTTATCGACCACTTTTAGCTGTCTCTAAAAAAAGAAATAAAACAATTCCAAGAGAGATATCATA
TTCCATATTTTGAAGATGAATCTAATAAAGATAACAAATATGTTAGAAATGATATTCGTAATAGAATTATTCCAGCTATTGATGAAA
ATAATCAACTTAAAGTATCGCATTTATTAAAATTAAAACAATGGCATGATGAACAATATGATATTTTGCAATATTCAGCTAAACAAT
TTATTCAAGAATTTGTGAAGTTTGATGAACAGTCAAAATATTTAGAGGTTTCTAGACAAGCTTTTAATAACTTACCAAACTCATTAA
AGATGGTTGTGTTGGATTGCCTATTATCAAAGTATTATGAGTTGTTTAATATTAGTGCTAAAACATACGAAGAGTGGTTTAAACAAT
TTAGTAGTAAGAAAGCACAATTCAGTATTAATCTCACGGATAAATGGATAATTCAAATTGCATATGGTAAATTAATAATAATGGCTA
AAAATAATGGCGATACATATTTTAGAGTTCAAACTATTGAAAAGCCAGGTAATTATATTTTTAACAAATATCGATTAGAGATACATT
CTAATTTACCAAAATGTTTATTTCCGCTTACAGTGAGAACACGACAAAGTGGCGATACATTTAAACTGAATGGGCGCGATGGTTATA
AGAAAGTGAATCGCCTGTTTATAGATTGTAAAGTGCAACAGTGGGTTCGGGATCAAATGCCAATCGTATTGGATAAACAACAGCGC
ATTATTGCGGTAGGAGATTTATATCAACAACAAACAATAAAACAATGGATTATAATTAGTAAAAATGGAGATGAATAGCGTTATGC
ATAATGATTTGAAAGAAGTATTGTTAACTGAAGAAGATATTCAAAATATCTGTAAGGAATTGGGAGCACAATTAACAAAGGATTAT
CAAGGTAAACCATTAGTATGCGTGGGTATCTTAAAAGGCTCAGCAATGTTTATGTCAGATTTAATTAAACGAATTGATACCCATTTA
TCAATTGATTTCATGGATGTTTCTAGTTATCACGGAGGCACTGAGTCAACTGGTGAAGTTCAAATCATTAAAGATTTAGGTTCTTCTA
TTGAAAATAAAGACGTATTAATTATTGAAGATATCTTAGAGACTGGTACTACACTTAAGTCAATTACTGAATTATTACAATCTAGAA
AAGTTAATTCATTAGAAATAGTTACTTTATTAGATAAACCAAACCGTCGTAAAGCGGACATTGAAGCTAAGTATGTAGGTAAAAAA
ATACCAGATGAATTTGTTGTTGGTTACGGTTTAGATTATCGTGAATTATACCGAAACTTACCATATATCGGTACGTTAAAACCTGAA
GTGTATTCAAATTAATTTTTTAATCAATTTCAGTTATTATTACTATGCGTTTGAGAAATAATAGTGTAGACTCAAAAATATGAAAAAT
GTATTTCATATATATTTAATTTAGACAAGACATATGTCTTGAAAAGTTGAAAAATATAGAGATTGATAAAACTAATACGGATGTGA
ATGACATTGATGTTAAGCTCAATTACTAGCTTATAAAACATGTCATATGTTACAATTTTTGTTAGTTTTATTATGGGAAGTAGGAGGA
AATGACGCATGCAGAAAGCTTTTCGCAATGTGCTAGTTATCGTAATAATAGGCGTTATTATTTTTGGTCTATTTTCATATTTAAACGG

TAATGGAAATATGCCGAAACAGCTTACATATAATCAATTTACTGAGAAGTTGGAAAAAGGTGACCTTAAAACTTTAGAAATCCAAC
CACAACAAAATGTCTATATGGTAAGTGGTAAAACGAAAAATGATGAAGACTATTCATCAACTATTTTATATAACAACGAAAAAGAA
TTACAAAAAATTACTGATGCTGCTAAAAAGCAAAACGGTGTAAAATTAACGATTAAAGAAGAAGAAAAACAAAGTGTCTTTGTGA
GTATACTTTCAACATTAATTCCAGTTGTAGTCATAGCGTTATTATTTATTTTCTTCCTAAGCCAAGCACAAGGTGGCGGTAGTGGCGG
TCGTATGATGAACTTTGGTAAATCTAAAGCAAAAATGTACGATAATAATAAACGTCGTGTTCGTTTCTCTGATGTAGCAGGGGCAGA
TGAAGAAAAACAAGAATTAATTGAAATTGTTGATTTCTTGAAAGATAATAAAAAATTCAAAGAAATGGGATCTAGGATTCCTAAAG
GTGTCTTACTTGTTGGACCTCCAGGTACTGGTAAAACATTACTTGCTAGAGCGGTTGCAGGTGAAGCTGGCGCACCATTCTTCTCTAT
TAGTGGTTCAGACTTTGTAGAGATGTTTGTTGGTGTTGGTGCGAGCCGTGTTCGTGACTTATTCGATAATGCTAAGAAAAACGCGCC
TTGTATCATCTTTATCGATGAGATTGATGCTGTTGGTCGTCAACGTGGTGCAGGTGTTGGTGGCGGTCATGATGAACGTGAACAAAC
CCTAAACCAATTATTAGTTGAAATGGATGGTTTCGGTGAAAATGAAGGTATCATTATGATAGCTGCTACAAACCGTCCTGATATCCT
TGACCCAGCCTTATTACGTCCAGGTCGTTTTGATAGACAAATTCAAGTTGGTCGTCCAGATGTGAAAGGCCGTGAAGCAATTCTTCA
TGTTCATGCTAAAAACAAACCACTTGATGAAACGGTTGATTTAAAAGCAATTTCACAACGTACACCTGGTTTCTCAGGTGCTGATTT
AGAGAACTTATTAAATGAAGCATCTTTAATTGCTGTACGTGAAGGTAAAAAGAAAATTGACATGAGAGATATCGAAGAGGCAACG
GATAGAGTTATAGCCGGACCTGCTAAGAAATCTCGAGTTATTTCTAAGAAAGAACGTAATATTGTTGCTCATCACGAAGCTGGTCAT
ACAATTATCGGTATGGTACTTGATGAGGCAGAAGTAGTGCATAAAGTTACTATTGTTCCACGTGGACAAGCAGGTGGTTATGCAAT
GATGCTACCTAAACAAGATCGTTTCTTAATGACTGAACAAGAGTTATTAGATAAAATCTGTGGTTTACTTGGTGGACGTGTATCAGA
AGATATTAACTTTAACGAAGTATCAACAGGTGCTTCAAATGACTTCGAACGTGCAACACAAATCGCACGCTCAATGGTTACGCAAT
ATGGTATGAGTAAAAAATTAGGACCATTACAGTTCGGTCATAGCAATGGTCAAGTATTCTTAGGTAAAGATATGCAAGGTGAGCCT
AATTATTCAAGCCAAATCGCATATGAAATTGATAAAGAAGTTCAACGAATCGTTAAAGAACAATACGAACGTTGTAAACAAATTTT
ATTAGAGCACAAAGAACAATTAATTTTAATTGCTGAAACATTATTAACAGAAGAAACATTAGTTGCTGAACAAATTCAATCATTATT
CTACGAAGGTAAATTACCTGAAATTGATTATGATGCAGCTAAAGTTGTTAAAGATGAAGATTCTGAATTTAATGATGGTAAATTCGG
TAAATCTTATGAAGAGATTCGTAAAGAGCAATTAGAAGATGGACAACGTGACGAAAGTGAAGATCGTAAAGAAGAAAAAGATATT
GCTGAGGATAAAAAAGAAGCTGATAAATCTGATGAAAAAGATGAACCAGCACATCGACAAGCCCCAAATATCGAAAAACCTTACG
ATCCAAATCACCCAGACAATAAATAATCGATTATATTCAGTACCTCTTTCTATGATAAAGTTATAGAAAGAGGTACTTTTATCGTTTT
TGAAAATACGTATTAGATTTTAAGTCGTTGAATTGTTATAGCAGAAAATAATTGTAAAACAAGTTACTTCATTATTTAGAATGATGG
GTGTAGAATAAGTACAATTGTTGCATTTTATGAAGTAAAGTAATTTTTTAAATATAGAGTAATAGAGGAGATTGAAATAATGACAC
ACGATTATATTGTTAAAGCATTAGCATTTGATGGGGAGATTAGGGCTTATGCTGCTTTGACAACTGAAACTGTTCAAGAAGCACAAA
CGAGACATTATACATGGCCGACAGCATCTGCTGCAATGGGAAGAACAATGACAGCAACAGCTATGATGGGCGCAATGTTGAAAGG
TGATCAAAAATTAACTGTCACTGTAGATGGCCAAGGACCTATTGGACGAATTATTGCCGATGCAAATGCTAAAGGCGAGGTGCGTG
CTTATGTAGACCATCCACAAACTCATTTTCCATTAAATGAGCAAGGTAAACTTGATGTAAGACGAGCAGTAGGGACAAATGGATCT
ATTATGGTTGTTAAAGACGTTGGAATGAAAGACTATTTCTCTGGAGCAAGTCCAATTGTTTCAGGAGAACTTGGTGAAGATTTTACT
TATTATTATGCTACAAGTGAACAAACACCTTCATCGGTAGGTCTTGGTGTATTGGTAAATCCTGATAATACGATTAAAGCAGCAGGA
GGATTTATCATTCAAGTTATGCCAGGTGCCAAAGATGAAACAATTTCAAAATTAGAAAAAGCAATTAGTGAAATGACACCAGTTTC
TAAATTAATTGAACAAGGATTAACGCCAGAAGGATTACTAAACGAAATCTTAGGTGAAGACCATGTGCAAATTTTAGAGAAAATAC
CTGTTCAATTTGAATGTAATTGTAGTCATGAGAAATTTTTAAATGCTATTAAAGGATTGGGCGAGGCTGAGATTCAAAATATGATTA
AAGAAGATCATGGTGCTGAAGCAGTATGTCATTTCTGTGGAAATAAATATAAATATACTGAAGAAGAATTAAACGTGTTGCTAGAA
AGTTTAGCGTAATTTAATTTAAATCAATACGCTAAAATGTTTATTTTTAGCGGTTTAGTGAAATGTAGAACTAAATAGTTGTATAATC
CTTAGTGATTTTGTTTGCTTTCTAGAATTTATTTGATAAAATAATTCTATATCCGATAAATAAACTAAGATTTCAACAACTAACTAAA
AAGGAGTGTTCTTAATGGCACAAAAACCAGTAGATAATATTACTCAAATTATTGGCGGTACACCGGTAGTCAAATTGAGAAATGTA
GTAGATGACAATGCAGCAGATGTTTATGTAAAATTGGAATATCAAAATCCAGGTGGTTCTGTAAAGGATAGAATTGCTTTAGCAAT
GATTGAAAAAGCAGAGCGAGAAGGCAAAATTAAACCTGGCGATACAATTGTAGAACCAACAAGTGGTAATACAGGTATCGGTTTA
GCATTTGTATGTGCTGCTAAAGGATATAAAGCAGTATTTACTATGCCCGAAACAATGAGCCAAGAGCGTCGTAATTTATTAAAAGC
ATACGGTGCGGAATTAGTTTTAACGCCTGGATCAGAAGCGATGAAAGGTGCAATTAAAAAAGCTAAAGAATTGAAAGAAGAACAT
GGTTACTTCGAGCCACAACAATTTGAAAACCCTGCGAACCCTGAAGTTCATGAGTTAACTACAGGTCCTGAGTTATTACAACAATTT
GAAGGGAAAACTATCGATGCGTTCCTAGCTGGTGTTGGTACTGGTGGTACGTTATCTGGTGTAGGTAAAGTTCTGAAAAAAGAATA
TCCTAACATCGAAATTGTTGCTATAGAGCCTGAGGCTTCTCCAGTATTGAGCGGTGGTGAGCCAGGTCCACATAAATTACAAGGTTT
AGGTGCTGGATTTATTCCAGGCACTTTGAATACAGAAATCTATGACAGTATTATTAAAGTAGGAAATGATACAGCGATGGAAATGT
CTCGTCGAGTTGCTAAAGAGGAAGGTATTTTAGCAGGTATTTCATCAGGTGCTGCGATTTATGCTGCCATTCAAAAAGCAAAAGAAT
TAGGAAAAGGTAAAACAGTAGTAACAGTATTGCCGAGTAATGGTGAACGCTACTTATCAACACCTTTATATTCATTCGATGACTAAT
TAATGTCATTTAAAAGAGTGAGTTATCTTTTTGAGATAACTTGCTCTTTTTTTCTACCATGTATAATTTTAAAAATATGAGCGTTAAA
TTAAACATTTTTCTGATAAAAACATCCAGTGAATGATAAGATAATAAACGTACATACTAATAACTAGTAAATAGCAGGAGTAAATT
TTATTAGAGTTAAACAATACATAATTAAAGGGTGGTTAACATGACTAAAACAAAAATTATGGGCATATTAAACGTCACACCAGATT
CATTCTCAGATGGTGGAAAATTTAATAATGTTGAATCAGCTATAAATAGAGTGAAAGCCATGATAGATGAAGGTGCTGACATTATA
GATGTTGGAGGTGTTTCAACGAGACCCGGTCATGAAATGGTTTCATTAGAAGAAGAGATGAACAGAGTATTACCTGTTGTTGAAGC
TATTGTCGGTTTTGATGTAAAAATTTCAGTCGATACATTTCGAAGTGAGGTTGCTGAAGCATGTTTAAAATTAGGCGTTGATATGATT
AATGATCAATGGGCGGGTCTGTATGATCATCGTATGTTCCAAATTGTAGCTAAATATGACGCGGAAATTATTTTAATGCATAATGGA
AATGGTAATCGTGATGAACCGGTTGTCGAAGAAATGTTAACATCTTTGTTAGCACAAGCACATCAAGCTAAAATAGCTGGTATACCT
TCAAATAAAATTTGGCTAGATCCAGGTATAGGTTTCGCTAAAACTAGAAATGAAGAAGCCGAAGTTATGGCAAGACTGGATGAACT
TGTTGCAACAGAATATCCAGTTTTATTAGCGACAAGCCGGAAACGTTTCACTAAAGAGATGATGGGTTATGATACAACACCGGTTG
AAAGAGATGAAGTAACTGCAGCTACGACTGCATATGGTATTATGAAAGGCGTTAGAGCAGTACGCGTTCATAATGTCGAGTTGAAT
GCTAAATTAGCTAAAGGTATAGATTTTTTAAAGGAGAATGAAAATGCAAGACACAATCTTTCTTAAAGGTATGCGCTTTTATGGATA
TCATGGTGCTTTATCAGCTGAAAATGAAATAGGGCAAATTTTCAAAGTGGATGTAACTTTGAAAGTAGACTTAGCTGAAGCTGGGC
GTACTGATAATGTTATTGATACAGTTCATTATGGTGAAGTGTTCGAAGAGGTTAAATCAATTATGGAAGGTAAGGCCGTTAATTTAC
TTGAGCATCTAGCTGAACGTATTGCAAATCGTATAAATTCACAATATAATCGTGTAATGGAAACGAAAGTGAGAATCACTAAAGAA
AACCCACCGATTCCGGGTCATTATGATGGAGTAGGTATCGAAATAGTGAGGGAGAATAAATGATTCAAGCATACTTAGGATTAGGT
AGTAATATTGGTGATAGAGAAAGCCAGTTAAACGATGCTATAAAGATTTTGAATGAATATGATGGTATTAACGTATCTAATATTTCT
CCGATTTATGAAACAGCACCAGTTGGGTATACTGAGCAACCTAACTTTTTAAATTTGTGTGTTGAAATTCAAACAACACTCACAGTA
TTACAACTGTTGGAATGTTGTTTGAAGACAGAAGAATGTTTACACCGTATTAGAAAGGAACGATGGGGTCCTAGAACTTTAGATGT
GGATATTTTGTTGTATGGAGAAGAAATGATAGATTTACCAAAACTGTCGGTGCCACATCCGAGAATGAATGAACGTGCATTTGTTTT
AATCCCATTAAATGATATAGCAGCAAATGTCGTAGAACCACGTTCGAAATTGAAAGTGAAAGATTTAGTTTTTGTCGATGACAGTGT
AAAGAGATATAAATAATGCATTGTTGAGAACATTCATATTTATTGGGAATAGATGATTGATTATTGGAGATGTTTCCGCATTTTGAT

TATCTCTAAATGCATTTGATTTCGAATTAGTATATGATGATTTAGTATGTGTTAGAATTAAGTTATTTCATAAAATCAAAGTAAGCAA
TATTTGATAAATTGCTGTAATTTATTTGGGCTTTTGTTGAAGTTTGTGGAAGATAATTTTTATACATTTACTTAGGGTTGTATTAATGA
TAATCGTATTTTAAATGAATAAGTAATTCATAAACGAAAACAGAAATTGCTTTATTACATTTACAAGCTCTTGTAAAATTAAAAAAG
TGATAAAATTGATGTTATGAATATAAGAAAACATGCCACATCATAACTTTAAGGTGTAATGGTTAATGATAAAGTATTAGAAACAT
CGAAAGAGATGTATGTTGAGCAAAAATGTCTGATATTTTATAAAACTTTAAAGGAAAATGTTTGAGTGTACCAGTTGGAATACTAA
AGGATTACAACAAGTTAAAGGAGAGAAAGTTATGTCAGAAGAAATGAATGACCAAATGTTGGTTCGACGTCAAAAATTACAAGAA
TTATATGATCTTGGTATAGACCCGTTTGGTTCTAAATTTGACCGTTCAGGTTTATCTAGTGATTTGAAAGAAGAGTGGGACCAGTATT
CTAAAGAAGAATTGGTAGAAAAAGAAGCGGATAGTCATGTCGCTATAGCTGGACGATTAATGACTAAGCGTGGTAAAGGTAAAGC
AGGATTTGCACACGTTCAGGACTTAGCTGGACAAATTCAAATTTACGTTCGTAAAGATCAAGTTGGCGATGACGAATTTGATTTATG
GAAAAATGCTGATTTAGGCGATATCGTTGGTGTTGAAGGTGTAATGTTCAAAACAAATACTGGCGAATTATCGGTTAAAGCGAAGA
AATTCACGCTACTAACTAAATCATTGCGACCATTACCGGATAAATTCCACGGTTTACAGGATATTGAACAGAGATATCGTCAAAGAT
ATTTAGATTTAATTACGAACGAAGATAGCACTCGTACATTTATTAATCGTAGTAAAATCATTCAAGAAATGCGTAATTATTTAAATA
ATAAAGGTTTCTTGGAAGTAGAAACACCTATGATGCACCAAATTGCTGGTGGCGCAGCTGCTAGACCATTTGTAACACATCATAATG
CATTAGATGCAACGTTATACATGCGTATTGCTATTGAGTTGCATTTAAAACGTTTAATTGTCGGTGGACTTGAAAAAGTATATGAAA
TTGGTAGAGTATTCCGTAATGAAGGTGTATCAACTAGACATAACCCTGAATTCACAATGATTGAATTATATGAAGCATATGCAGATT
ATCATGACATTATGGATTTAACAGAATCTATGGTGAGACATATTGCCAATGAAGTATTAGGTTCTGCAAAAGTACAATACAATGGG
GAAACGATTGATTTAGAATTTGCTTGGACTCGTTTGCATATTGTTGATGCTGTAAAAGAAGCTACTGGTGTAGATTTTTATGAAGTTA
AAAGTGATGAAGAAGCTAAAGCTTTAGCTAAAGAACATGGTATTGAAATTAAAGATACAATGAAATATGGTCATATTTTAAATGAA
TTCTTTGAGCAAAAAGTTGAAGAAACACTTATTCAGCCAACGTTTATCTATGGTCATCCGACTGAAATTTCACCTTTAGCGAAGAAA
AATCCTGAAGATCCTAGATTTACTGATCGTTTCGAATTGTTCATTGTAGGTAGAGAGCATGCAAATGCATTTACTGAATTAAATGAT
CCTATTGATCAAAAAGGTCGTTTTGAAGCGCAACTTGTTGAAAAAGCGCAAGGTAATGATGAAGCGCATGAAATGGATGAAGATTA
CATTGAAGCGTTAGAATATGGTATGCCTCCGACAGGTGGTCTTGGTATCGGTATTGACAGATTGGTTATGTTATTAACTGACTCTCC
ATCAATCAGAGACGTGTTATTATTCCCTTATATGAGACAAAAATAAATGACGTTGATTGTTAGTAAGAGCTCTCGTGTATACAACAT
GTGTATGCGAGGGTTTTCTTAATTATGATAATTAGTTCGTGTTTGAATGTTTTTGATAGTAATAGTTAACGATAGTGGTGCTATTTTT
GACTGTTAAACAAGGTAGTTGGCTGATAGATGAAAATGACGATATAGTTCGGATGAGAAATGTTAACGATAGACATTGAGATATCT
TATAACAAAAATTGCTATATTAGTATAATTTATCTTAATCAGCTATAAAAGTACTTTAAAATTGTATAGAATGTGTATGGTTTTGTAC
ATATGTGTATGATAGAATACTGAAAGTGTTATGAATTAGAGAGCACGAGAAATGTCAGTTTTGAAGAATAAAAAAGTTGATTAAAA
GTGTTGACTTTATCAATTGAATGAAGTAATATATAAAAGTCGTCAAAAACAGACGAAACACACTAAAAGCTGATGTGACAAAGTTC
ACAACAAAGTGTAAAATTAACTATTGCACCTTATTAATTAAGCGTGTATCATAAATAAGTAAGTTATTTGTCTGGTGACTATAGCA
AGGAGG

>016-contig_250

CTTACGTTCCGCTAGAGTAGAACGTTGCCAGGCAAAAATGGATGCGATGAGCCGCATTGAGACCGTGAGGTCTCTTTTTTTATGTCT
AAAACGTCAAAATAAAAGTTAACACAAAGAAAATGGCTTGACGAAGTGAAAACGTTTGAATCTGACGAAACGAGAAAAGAGCGCA
ACGAGTTTAGTAGAGCTAAATGAGTAAGCGAGAGCCGAAGGAGAGGAAAGAAGCAAGCGATTGTCACAAGTCAAGAAAGGTTCTT
AGCGAGGATGGTAGCCAACTTACGTTCCGCTAGAGTAGAACGTTGCCAGGCAAAAAATGGATGCGATGAGCCGCATTGAATTTTAG
AAATAATAATTTAATAATGTGCACTTTTGATTGTCTAAGTATGTACAACTTTAATTTTGTGTTTATATAAATTTAAAATGATATCATC
GAAAACAAAATATTGTATAAATAGAGAAGAGCAGTAAGACGGTATCTAATTGAAAATGATCTTACTGTTCTTTTATATACTTTATTG
AAATACAAAAAGGAAATTAATTATTATACAATAGACAAGCTATTGCATAAGTAACACTAACTTTTATCAAAGAAGTGTTACTTTATA
ATTAATGATTTTATTAGAGCGTCTACATGCGGTTTTAAAGCATCATCGTCTATACCGCCAAAGCCTAATATAAATTTAGGGGTTTTCT
TATAGTCTTGATCATCATCAAAATTATAAACTTGTAATTTTAACTTTACTTTGTTTGCTCTATCAAGACACTCTTGTAATGTTAATCCA
TTTTTTACTGTAATTGTAAAATGCATACCCGTTTCAGCACCTTGAATATCAAGCTGCTCTTTGTAAGGTTTCAATCTTTTTAAAATATA
GGTTAGTTTTCTACGATAAATTCGTCTCATTTTATTTAAATGCCTTTCAAAACCACCGGAAGATATAAACGTTGCAATAAGGTTTTGC
ATATGAACAGGTACAGTGTTGCCTTCAATGTGATTTTGAGAATGATATTTTTTCATTATAGAATAGGGTAACACCATATATGCAACT
CGACAGCTAGGAAAAATAGACTTTGAAAATGTACTGATATAAATCACTTTTTCTCCTCTTGAATATAGACCTTGAATTGCTGGAATG
GGTTTGCCGAAATATCTAAACTCGGAATCATAATCATCTTCTATAATAAATCGTTCTTCTTTTTCTTGAGCCCATTGTATTAATTGAG
TTCGCTTTTTTAAGTCCATCACATATCCAGTTGGAAATTGATGGGAAGGCGTTATATATACTATATTTTTTTGTGATTTAATAACTTC
ATCTACGTTTATTCCATTATCTTCAACTTCAATTTGTTCATATTCAACTTGTTTTTTATCTAAAATATTTTTGATTGGTGGATAACTAG
GTTTTTCGATAATAAATGTTGAAGTATAAAGTAAATCGACTAATTGATTTACTAATTGTTCGGTAGATGAGCCAATTATAATTTGATT
AGGATCACAAATTACACCACGATTAGTAAATAAATAAAATGCCAGTTGAAATCTTAAATGTAATTCTCCTTGAAAATGTCCTCTACG
TAATTGATTTAAATGATTTGTATCATAAAGATCTTTGGAATACTTTCTGAAAAGTTCTATAGGGAAATGTTTCGTATCTATTTCATCC
AAATTAAAAGCATAATCATAAGCTTCATCACTCGCTTTTGGTTTATATGAATCATCATCAAAAAGAGAGGGGATAGGTTGATTGTTT
AAAATTGTTAAAGATTCAATTTCGGACACAAAATATCCAGAGCGAGGTCTTGAATAAATGTAACCTTCGTCTAATAGAAGTTGATAT
GCATGCTCTACGGTTGTTTGGCTGATAGACAAATGTTTGCTTAATTGTCTTTTAGAATAAAATTTATCGCCTTCTTTAAATTGACCTTC
AATTATTTGTTTTTTTAATTTTTCATAAAGTTGATGGTATAAAGTGTTTTTCAATTTTATAACTGACCTCCTAAATTTATCTTATTTTGT
ACCTTTTTAAATATCAGTTTATACATTACAATGTATTTAATCAACTTGAAAAGGGGTTTTATGTATAATGAGTAAAATTATTGGATCA
GACAGAGTCAAAAGAGGTATGGCTGAAATGCAAAAAGGCGGCGTTATTATGGATGTCGTTAATGCTGAGCAAGCAAGAATTGCAG
AAGAAGCTGGCGCGGTAGCAGTTATGGCATTAGAACGAGTACCTTCTGATATTAGAGCTGCTGGTGGCGTTGCACGTATGGCAAAC
CCTAAAATTGTAGAAGAAGTAATGAATGCTGTTTCTATTCCAGTCATGGCTAAAGCACGTATTGGTCATATCACTGAAGCAAGAGTA
TTAGAGGCGATGGGTGTTGACTATATTGATGAATCAGAAGTGTTAACACCAGCAGATGAGGAATATCACTTAAGAAAAGATCAATT
TACAGTACCATTTGTATGTGGATGTCGTAATTTAGGTGAAGCTGCGCGTAGAATTGGTGAAGGTGCTGCTATGTTACGTACTAAAGG
TGAACCAGGTACAGGTAATATTGTTGAAGCTGTAAGACATATGAGACAAGTTAATTCAGAAGTTAGTCGTTTGACTGTAATGAATG
ATGATGAGATTATGACTTTTGCGAAAGATATCGGTGCGCCTTATGAAATTTTAAAACAAATTAAAGACAATGGTCGTTTACCGGTAG
TTAACTTTGCAGCTGGTGGCGTTGCGACTCCTCAAGATGCTGCTTTAATGATGGAATTAGGTGCCGACGGTGTATTCGTTGGATCAG
GTATTTTTAAATCAGAAGATCCAGAAAAATTTGCTAAAGCAATTGTTCAAGCAACAACACATTACCAAGACTATGAGCTAATTGGA
AGATTAGCAAGTGAACTTGGCACTGCTATGAAAGGTTTAGATATCAATCAATTATCATTAGAAGAACGTATGCAAGAGCGTGGTTG
GTAAGATATGAAAATAGGTGTATTAGCATTACAAGGTGCAGTACGTGAACATATTAGACATATTGAATTAAGTGGTCATGAAGGTA
TTGCAGTTAAAAAAGTTGAACAATTAGAAGAAATCGAGGGCTTAATATTACCTGGTGGCGAGTCTACAACGTTACGTCGATTAATG
AATTTATATGGATTTAAAGAGGCTTTACAAAATTCAACTTTACCTATGTTTGGTACATGCGCAGGATTAATAGTTCTAGCGCAAGAT
ATAGTTGGTGAAGAAGGATACCTTAACAAGTTGAATATTACTGTACAACGAAACTCATTCGGTAGACAAGTTGACAGCTTTGAAAC

AGAATTAGATATTAAATGTATCGCTACAGATATTGAAGGTGTCTTTATAAGAGCGCCACATATTGAAAAAGTAGGCCAAGGCGTAG
ATATCCTATGTAAGGTTAATGAGAAAATTGTAGCCGTCCAGCAAGGTAAATATTTAGGCGTATCATTCCATCCTGAATTAACAGATG
ACTATAGAGTAACTGATTACTTTATTAATCATATTGTAAAAAAAGCATAGCTTAATGTATGCTAAATCAACGAATTATTGATATTTA
TAGATTTGTTGAGAAGACAATATCTCCTTCAAACTTAGCTTTGGAGGAGCTATTTTTTTATGTCAAAATTAAAAATGATAAAAAAATA
AAGATATACATAAAAAAACCCTTCAAAGAGACTGAGAATAGTCAAAATTTTGAAGGGGTTAATTCGATGTTGATGTATTTGTTAAA
TAAAGAATCCAGCGATTGCAGCTGAAATGAAAGATACTAGTGTTGCACCGAATAATAGTTTCAAACCAAAGCGGGCAACTGTATCT
CCTTTTTTGTCATTAAGTGATTTAATCGCACCTGAAATAATACCAATAGAGCTAAAGTTAGCAAATGATACTAAGAATACAGATGTA
ACACCTTTTGCGTGTTCAGATAAATCACTAAGTTTACCAAGTGCTTGCATTGCTACAAATTCGTTAGATAATAGTTTTGTCGCCATAA
CTGAACCGGCTTGAACTGCATCTTGCCATGGCACACCGACTAAGAATGCAAATGGTGCAAAGACAAAACCAATTAATGTTTGGAAA
TCCCAAGAAATAGCGCCACCTGAAACTGTACTAAAGATATTGCTTACAATTCCATTTAATAGAGCGATAATAGCAATGTATCCGATT
AACATTGCGCCTACAATGACAGCTACTTTAAATCCATCTAAAATATATTCTCCTAGCATTTCGAAGAATGATTGTTGTCTTTCTTCAG
TTTCTTCAACTAATAATTTGTCATCTTCTTCATTAACTTTATAAGGGTTAATAATTGAAGCGATGATGAAACCACCAAATAAGTTTAA
GACAACAGCCGTTACAACATATTTAGGTTCAATTAAGGTAAAGTATGCACCGATAATTGAAGCAGAAACAGTCGACATTGCTGAAG
CTGTTAATGTGTATAAACGTTGCTTAGGTATGTATGGTAATTGTTTTTTAATTGAAATAAATACTTCAGATTGTCCCAAAATTGCTGC
AGCAACTGCATTGTATGATTCTAAACGTCCCATACCATTAATTTTAGAAATTAAGAATCCTAAAACATTAATGATTAAAGGTAAAAT
CTTTGTGTATTGAAGGATACCGATAATCGCTGAAATAAATACGATAGGTAATAATACACTGAAGAAGAATGGTGGTTGCTTAGGAT
CGATATATTGAATACCACCGAATACAAAGTTAACACCATCTGCTGCTTTTAATAATAAGTAGTTAAAACCGTTTGAAATACCACCAA
TAACCTTGATTCCCATTGTAGTTTTAAGCAAGATAAATGCAAAGATAAGCTGAATTGCAAGTAAAATTCCTACATATTTCCAGCGAA
TATTTTTCCTGTCTGAGCTAAATAGAAACGCAAGTGCTAAAAAGAAGATAATTCCGATAATCCCAATTAGAATATGCATATATTTCT
CATTCCTTTAGTTTTTTCTACAATCTATCATACAATAAAATGGAAGGGCTAACATCATAAATTTTTGAAAATATAAAAACAAATTAA
TTGAAAAAGGTCAAAATAGGTCATATAATATAGTCAAAGAAGGTCAAAAAGGGGTGATATACATGCACAATATGTCTGACATCATA
GAACAATACATCAAACGTTTATTTGAAGAGTCGAATGAAGATGTCGTTGAAATTCAGAGAGCGAATATCGCACAGCGTTTTGATTG
TGTACCATCACAATTAAATTATGTAATCAAAACACGATTCACTAATGAACATGGTTATGAAATCGAAAGTAAACGTGGTGGTGGTG
GTTACATCCGAATCACTAAAATTGAAAATAAAGATGCAACAGGTTATATTAATCATTTGCTTCAGCTGATTGGACCTTCTATTTCTCA
ACAACAAGCTTATTATATTATTGATGGGCTTTTAGATAAAATGTTAATAAATGAACGTGAAGCTAAAATGATTCAAGCAGTTATTGA
TAGAGAAACGCTATCAATGGATATGGTTTCTAGAGATATTATTAGAGCAAATATTTTAAAACGTTTGTTACCAGTTATAAATTATTA
CTAAATGAAATGAGGTGTTGAAGTGCTTTGTGAAAATTGTCAACTTAATGAAGCGGAATTAAAAGTTAAAGTTACAAGTAAAAATA
AAACAGAAGAAAAAATGGTGTGTCAAACTTGTGCTGAGGGGCACCATCCGTGGAATCAAGCTAATGAACAACCTGAATATCAAGA
ACATCAAGATAATTTCGAAGAAGCATTTGTTGTTAAGCAAATTTTACAACATTTAGCTACGAAACATGGCATTAATTTTCAAGAAGT
AGCGTTTAAAGAAGAAAAACGTTGCCCATCATGTCATATGACTTTGAAAGATATTGCACATGTTGGTAAATTTGGGTGTGCTAATTG
TTATGCAACATTTAAAGATGACATCATTGATATCGTCCGCAGAGTTCAAGGTGGACAATTTGAGCACGTTGGAAAGACACCACATTC
TTCACATAAAAAGATAGCTTTAAAGCGAAAAATCGAAGAAAAGAATGAATATTTGAAAAAACTTATTGAAATCCAAGATTTTGAGG
AAGCAGCCATTGTTAGAGATGAAATTAAAGCACTAAAAGCTGAGAGTGAGGTGCAACATGATGACGCATAATATTCATGATAATAT
CAGCCAATGGATGAAAAGTAATGAAGAAACACCAATTGTTATGTCTTCTAGAATTCGGTTAGCGCGTAATTTAGAAAATCATGTGC
ATCCACTAATGTATGCTACTGAAAATGATGGATTTAGAGTTATAAATGAGGTACAAGATGCCTTGCCAAATTTTGAATTAATGCGTC
TTGATCAAATGGATCAACAAAGTAAAATGAAAATGGTTGCAAAGCATTTGATTAGTCTTGAACTAATAAAACAACCAGCAGCCGCA
GTATTAGTGAATGATGATGAATCTTTAAGTGTCATGATAAATGAAGAGGACCATATTCGTATTCAAGCTATGGGAACTGACACGAC
ATTACAGGCTTTATATAATCAAGCTTCATCAATTGATGATGAATTAGATCGAAGCCTTGATATAAGTTATGATGAACAACTTGGTTA
TTTAACTACATGTCCTACCAATATAGGTACTGGTATGAGAGCAAGCGTGATGCTACATTTACCAGGTCTATCTATTATGAAAAGAAT
GACACGGATTGCTCAAACCATTAATCGTTTTGGATATACAATCAGAGGTATTTACGGTGAAGGTTCGCAAGTTTATGGACATACTTA
TCAAGTATCCAACCAACTTACACTTGGTAAATCTGAGTTAGAAATCATAGAAACATTAACAGAAGTTGTTAATCAAATCATTCATGA
AGAAAAACAAATACGACAAAAGTTAGACACTTATAATCAATTAGAAACACAAGACCGTGTTTTTCGCTCGCTAGGTATTTTACAAA
ACTGTAGAATGATAACTATGGAAGAGGCTTCTTATAGATTAAGCGAAGTTAAACTTGGTATAGATTTAAATTACATTGAATTACAAA
ACTTTAAATTTAATGAATTGATGGTAGCTATACAGTCACCATTTTTATTAGATGAAGAAGATGACAAATCTGTAAAAGAAAAACGA
GCAGATATACTAAGAGAACATATAAAGTAGGAGGTCATTATTTATGTTATTTGGTAGATTAACTGAGCGTGCACAGCGCGTATTAGC
ACATGCACAAGAAGAAGCAATTCGTTTAAATCATTCAAATATAGGAACAGAACACCTATTATTGGGACTAATGAAAGAACCTGAAG
GAATTGCTGCAAAAGTATTAGAAAGTTTTAATATCACTGAAGATAAAGTAATTGAAGAAGTTGAAAAATTAATCGGACATGGTCAA
GATCATGTTGGTACATTGCATTATACACCTAGAGCTAAAAAAGTTATTGAATTATCGATGGATGAAGCTAGAAAATTACATCACAAT
TTTGTTGGAACGGAACATATTTTATTAGGCTTGATTCGTGAAAATGAAGGTGTTGCAGCAAGAGTTTTTGCAAATCTAGATTTAAAT
ATTACTAAAGCACGTGCACAAGTTGTGAAAGCTTTAGGAAACCCTGAAATGAGTAATAAAAATGCACAAGCTAGTAAGTCAAATAA
TACTCCAACTTTAGATAGTTTAGCTCGTGACTTAACAGTCATTGCCAAAGACGGTACATTAGATCCTGTTATAGGACGTGATAAAGA
AATTACACGTGTAATTGAAGTATTAAGTAGACGTACGAAAAACAATCCTGTACTTATTGGAGAGCCAGGTGTTGGTAAAACTGCTA
TTGCTGAAGGTTTAGCGCAAGCCATAGTGAATAATGAGGTACCAGAGACATTAAAAGATAAGCGTGTTATGTCTTTAGATATGGGA
ACAGTAGTTGCAGGTACTAAATATCGTGGTGAATTTGAAGAGCGTCTGAAAAAGGTTATGGAAGAAATCCAACAAGCAGGTAATGT
CATCCTATTTATTGATGAGTTGCATACTTTAGTTGGTGCTGGTGGTGCTGAAGGTGCTATCGATGCTTCGAATATTTTGAAACCGGCA
TTAGCACGTGGTGAATTACAATGTATTGGTGCTACTACATTAGATGAATATCGCAAAAATATTGAAAAAGACGCGGCTTTAGAACG
TCGTTTCCAACCTGTACAAGTTGATGAACCTTCAGTAGTAGATACAGTTGCTATTTTAAAAGGATTAAGAGATCGTTACGAAGCACA
CCATCGTATTAATATTTCAGACGAAGCTATTGAAGCAGCTGTTAAATTAAGTAACAGATACGTTTCAGATCGTTTCTTACCAGATAA
AGCAATTGATTTAATTGATGAAGCAAGTTCTAAAGTAAGACTTAAGAGTCATACGACACCTAATAATTTAAAAGAAATTGAACAAG
AAATTGAAAAAGTTAAAAATGAAAAAGATGCCGCAGTACATGCTCAAGAGTTTGAAAATGCTGCTAACCTGCGTGATAAACAAAC
AAAACTTGAAAAGCAATATGAAGAAGCTAAAAATGAATGGAAGAATGCACAAAATGGCATGTCAACTTCATTGTCAGAAGAAGAT
ATTGCTGAAGTTATTGCAGGATGGACAGGTATCCCATTAACTAAAATCAATGAAACAGAATCTGAAAAACTTCTTAGTCTAGAAGA
TACATTACATGAGAGAGTTATTGGGCAAAAAGATGCTGTTAATTCAATCAGTAAAGCGGTTAGACGTGCCCGTGCAGGGTTAAAAG
ATCCTAAACGACCAATTGGTAGCTTTATCTTCCTTGGACCAACTGGTGTTGGTAAAACTGAATTAGCTAGAGCTTTAGCTGAATCAA
TGTTTGGCGATGATGATGCGATGATCCGTGTAGACATGAGTGAATTTATGGAAAAACACGCAGTGAGCCGATTAGTTGGTGCTCCTC
CAGGATATGTTGGTCATGATGATGGTGGACAATTAACTGAAAAAGTTAGACGTAAACCATATTCTGTAATTTTATTTGATGAAATTG
AAAAAGCTCATCCAGATGTATTTAATATTCTATTACAAGTTTTAGATGATGGACATTTGACAGATACAAAAGGACGTACAGTTGATT
TCAGAAATACAATTATCATAATGACATCAAACGTTGGGGCACAAGAATTACAAGATCAACGATTTGCTGGATTCGGTGGTTCAAGT
GATGGACAAGATTATGAAACAATTCGAAAAACGATGTTAAAAGAATTAAAAAATTCATTCCGTCCAGAATTTTTAAACCGTGTAGA
TGATATCATTGTATTCCATAAACTAACAAAAGAAGAATTAAAAGAAATTGTAACAATGATGGTTAATAAATTAACAAATCGATTAT
CTGAACAAAACATAAATATTATTGTTACTGATAAAGCGAAAGACAAAATCGCAGAAGAAGGATATGATCCAGAATATGGTGCAAG

ACCATTAATTAGAGCGATACAAAAAACTATCGAAGATAATTTAAGTGAATTAATATTAGATGGTAATCAAATTGAAGGTAAGAAAG
TTACAGTAGATCATGATGGTAAAGAGTTTAAATATGACATTGCTGAACAAACTTCAGAAACTAAAACACCATCGCAACCATAATTA
TAAAACAGTCCAAAACAAATTAAAGTTTTGGGCTGTTTTTTTAGTAGCATTGAACTATAGAAATTCGTGAAAGTATCCATCAACGAA
ACAATCTATAAAACAATCATCAAAGGATAGTTAAGAATTATATGTAACAAGTTAATGAGCCTACAGCGATAGCATAAAGGTATGAA
TTTTTATAAAGGTTTTTTGTTTGAAGATACTAGCAACTTACGTCAAAATAAAATAGGTCTATATAATATTGTGATAAATCTAGAGAA
AAGAGTTTACTAAGAAATTATTAAGATTTTATCTTTGAAAGATACCGACTATCAACATGATGTAAGTTTATTTTATAATATTCATAAA
AAATAAATCTGGTAAAACAGTTTGCGTTAGTAGTCATGTTAAAATACAAACAAATGTAACTCACATTTAATTTGTCATAATGGGAAT
GTGCGTTTAAAATAGATTTGTTCAAAGGAAAGTGGAGGTGCAATTTTGGCCAAGAAAAAAGTGATTTTTGAATGTATGGCTTGTGGT
TATCAATCTCCTAAATGGATGGGGAAATGTCCTAATTGTGGCGCTTGGAATCAAATGGAGGAAATTGTTGAAAAAGCAGCCAATCC
TAAACATGGAGTTAAAACCAAGGAATTAGCAGGTAAAGTACAAAAATTAAATAGTATTAAACATGAAACAACGCCGAGAGTGTTA
ACAGATTCAGCAGAATTCAACCGTGTATTAGGTGGAGGTATTGTGAGCGGATCGTTAGTACTTATTGGTGGGGATCCAGGTATTGGT
AAGTCAACGTTACTTTTACAAATTTGTGCATCGTTATCTCAAAAGAAAAAAGTACTATATATTACTGGAGAAGAATCGCTTAGTCAG
ACTAAATTACGTGCAGAGCGATTAGATGAAGATTCAAGTGAATTGCAAGTATTAGCTGAAACAGATCTTGAAGTTATTTATCAAAC
AGTAAAAGAAGAACAACCTGATTTATTAGTAGTGGATTCGATTCAAACAATATATCATCCTGAAATCAGCTCTGCGCCAGGTTCTGT
TTCACAAGTTCGTGAAAGTACACAAAGTTTAATGAATATTGCTAAACAAATGAACATTGCAACTTTTATAGTGGGTCATGTAACGAA
AGAAGGTCAAATTGCTGGCCCAAGATTGCTAGAACACATGGTTGATACTGTGCTTTATTTTGAAGGCGATGAACACCACGCATATCG
AATTTTGCGAGCTGTTAAAAACCGTTTTGGTTCAACGAATGAAATGGGAATCTTCGAAATGAAGCAAAGTGGATTAAAAGGTGTAA
ATAATCCATCTGAAATGTTTTTAGAAGAACGTTCAACAAATGTTCCAGGTTCAACAATTGTTGCAACCATGGAGGGAACCAGACCA
CTTTTAATAGAAGTTCAAGCGCTGGTAACTCCAACGACTTTTAACAATCCGAGACGAATGGCAACAGGGATTGATCATAATCGATTA
AGTTTGTTGATGGCTGTTTTGGAAAAGAAAGAAAATTATCTATTACAACAACAAGATGCTTATATCAAAGTAGCTGGCGGTGTAAA
GTTAACGGAGCCAGCAGTTGATTTAAGTGTAATTGTAGCAACTGCATCTAGCTTTAAAGATAAAGCTGTCGACGGATTAGATTGCTA
TATTGGAGAAGTTGGTTTAACGGGCGAGGTACGTCGTGTATCTCGGATAGAACAACGCGTGCAAGAGGCTGCAAAACTAGGTTTCA
AACGTGTAATTATTCCTAAAAATAATATAGGCGGATGGACATATCCTGAAGGTATACAAGTAATAGGTGTAACTACTGTACATGAA
GCATTGTCATTTGCTCTTCATTCATAAAACATCAAGAAAGGAGGACATTGTGTGAATATCGTTAAACTAATGGTTATTATTATTTACT
TAATTATTGGGAGCGCATTAGGAATAATTATTATTCCTGAAATTGCAAATGATCTTGGATTACAAAACTCCAGCTTTTTAAAAAATC
ACTATGTAGATGGCATTATCGGTAGTATTTTTATGTTCTTAATTTTTGGTGTATTTATTAGACGAGTTACTAACGCTATAAAAGGTTT
AGAACATTTTATTATGCGTAGAAGTGCTGTTGAAATACTATTCGCAACAATAGGTTTAATAATCGGATTACTTATTTCTGTTATGGTG
TCGTTTATATTAGAATCAATTGGTAACTCTATTTTTAATCATTTCATTCCTGTCATAATTACGATATTACTATGTTATTTCGGTTTCCA
ATTTGGCCTTAAAAAACGAGATGAAATGTTAATGTTTTTACCTGAGAATATAGCGCGTTCCATGTCACAACATACTAAAAGTGCTAC
GCCAAAAATTATCGACACAAGCGCAATTATTGATGGTCGTATTTTAGAAGTCATTCGTTGCGGTTTTATCGATGGCAATATTTTAATT
CCACAAGGTGTTATTAATGAATTACAAATTGTTGCAGATTCAAATGACAGTGTTAAACGTGAAAAGGGTAAAAGAGGCTTAGATAT
TTTAAATGAATTGTATGATTTAGATTATCCTACAAAGGTTATACATCCAACTAAAACACATAGTGATATTGATACAATGCTATTAAA
GCTAGCTAAACAATATCATGCAAGTATTATTACGACAGATTTCAACCTAAATAAAGTTTGTCATGTACATGGTATCAAAGCATTAAA
TGTTAATGATTTATCAGAAGCAATCAAACCTAATGTACATCAAGGTGATCAACTGCATATTTTACTGACAAAAATGGGTAAAGAGC
CTGGTCAGGCAGTAGGATATCTAGATGATGGTACGATGGTGGTTGTTGATAATGCTAAAAATCTTATTGGCAGTCATGTCAATTTAG
AAGTAGTCAGCTTATTGCAAACATCTTCAGGAAGAATTGTTTTTGCTAAAAAAATCGAAGATACAGTATCATTATAAAAACTGTAGT
TGACGAAAACAAAATAGATATTAAAAGGTATATGAAATGGCGAAGTTTGTAATTTGAAGTATAACTATAAATAAATGCAAAACATA
TTAGTTATCAAAAAATACAAATAGTGCTAATATATCATTGAAACAAGTTCATTAAGTGTTAAATTTTAGATATTAATAAAGTTATTC
TTATAGCAATCAAGACATACAAAATTAGAATCAATTTTAACGTATTTGAATAGTTAATTTGAAGTTTAATTTCACAGTACATAATAT
TCATTTTTAAGTCGTTGAAATGTTTATGTAGTGCAGTCTTGATTCTTAGGGATAGCTTTTTTAAAGTGTTGAAAAAACAACTGCTTTC
TATTTAAAATTTAAGCATATTACCCAGGGAATTATGTTATGATTAAACAATGAAATTATTTTAAGGTCATGTTAAAAAGCATTGTTTC
GTTTTAACAATAAGACGAGTTAGACATTTGTTATGACGGTCGAAGATAGCAAATGAACAATTTATATGAACTAACTTGAAAATCTG
ATATTTAATTTAGGAGTGAAAAAAATGAGCGATCGTATAAGAGTAAGATATGCACCAAGTCCAACTGGTTATCTTCATATTGGTAAT
GCAAGAACAGCATTATTCAATTACTTGTATGCTAAACATTACAACGGAGATTTTGTGATTCGAATTGAAGATACTGATAAAAAACGT
AATTTAGAAGATGGAGAAACATCACAATTTGATAATCTTAAATGGTTAGGATTAGATTGGGATGAGTCTGTAGATAAAGACAATGG
CTACGGACCATATCGTCAATCTGAACGTCAACATATCTACCAACCATTAATAGATCAGTTACTAGCAGAAGATAAAGCATATAAAT
GCTATATGACAGAAGAAGAATTAGAAGCTGAACGTGAAGCGCAAATCGCTCGTGGTGAAATGCCTCGCTATGGTGGTCAACATGCG
CATTTGACTGAAGAACAACGTCAACAATTTGAAGCAGAAGGACGCCAACCATCAATTCGTTTCCGAGTACCTCAAAACCAAACGTA
TTCATTTGATGATATGGTAAAAGGAAATATTTCATTTGATTCAAATGGTATTGGTGACTGGGTTATCGTAAAAAAAGATGGCATTCC
AACGTACAATTTTGCAGTAGCTATAGATGATCATTACATGCAAATTTCAGATGTAATTCGTGGTGATGATCATATTTCAAACACGCC
TAAACAAATTATGATTTATGAAGCATTTGGCTGGGAGCCACCTCGTTTTGGTCATATGTCATTAATTGTTAATGAAGAACGTAAAAA
GTTAAGTAAACGTGATGGGCAAATTTTACAATTTATTGAGCAATATCGTGACTTAGGTTATTTACCTGAAGCGTTATTTAATTTTATT
GCGTTATTAGGTTGGTCTCCTGAAGGTGAAGAAGAAATCTTTTCTAAAGAAGAATTTATCAAAATCTTTGATGAAAAGCGTTTGTCA
AAATCACCAGCATTTTTCGATAAGCAAAAATTAGCATGGGTTAATAACCAATATATGAAACAAAAAGATACTGAAACAGTATTCCA
ATTAGCATTACCTCATTTAATTAAAGCAAATTTGATTCCTGAGGTGCCGTCAGAAGAGGATTTATCTTGGGGACGCAAATTAATTGC
GCTTTATCAAAAAGAAATGGGTTATGCCGGTGAAATTGTACCTTTATCAGAAATGTTCTTTAAAGAAATGCCAGCTCTTGGTGAAGA
AGAACAACAAGTGATTAATGGAGAGCAAGTACCAGAGTTAATGACGCACTTATTCAGTAAATTAGAAGCACTTGAACCATTTGAAG
CGGCTGAAATTAAAAAGACAATTAAAGAAGTTCAAAAAGAAACAGGAATAAAAGGCAAGCAATTATTTATGCCTATTCGTGTTGCT
GTAACAGGCCAAATGCATGGTCCTGAATTACCAAATACAATTGAAGTACTTGGTAAAGAAAAAGTGCTAAACCGTTTAAAACAATA
TAAGTAATGAAATAGACAAGATCAAGGTAGTATATACTTGAATTTTATAACGTAACCACATATGATAAAGTTGTATTAACAAATTAC
AACAATATGAATAAAGTATTTTATATAAAACGAAGGATTAGTAATTAAATTTATACGATGCAGAGAGTGTACGGTTGCTGTGAGTA
CAACGTAGAAATTAATGAATGCACCTTCGTAAAATGAATTAAATATATAATGAGAGTGATGAGCATTAAGTTGACTTAGTTTCCTTG
ATAATTTGGAAGCGCCCGCAATATTATTAATGTTATTCGCTAAATTCAGAGTGGAACCGTGCGGAAGCGCCTCTAACAATACAATTT
GTATGTTAGTGGTGCTTTTTTGATATTTAATTTCGCAGGTACTTCAATTATGTATTTACGTAGTTCAATCATTGAGGAGGAAATGATC
TTGTTAAAAAGAATGAGAGACGATATAAAAATGGTATTTGAGCAGGATCCAGCGGCACGTTCAACATTAGAAGTCATTACAACGTA
TGCAGGTTTACATGCAGTTTGGAGTCATTTGATTGCACATAAGTTATACAACCAAAAAAAATATGTTGCAGCACGCGCGATATCTCA
AATTTCAAGATTTTTCACAGGTATAGAAATCCATCCAGGTGCTAAAATTGGAAAGCGTCTATTTATAGATCATGGTATGGGCGTTGT
AATAGGAGAAACATGTACAATTGGTGATAATGTGACAATCTATCAAGGCGTGACACTTGGTGGGACAGGGAAAGAAAGAGGGAAA
AGACACCCAGATATAGGAGACAATGTTTTAATAGCAGCGGGTGCGAAAGTTTTAGGAAATATTAAAATAAATTCAAATGTAAATAT
TGGTGCAAATTCAGTTGTTTTACAATCAGTTCCAAGCTATTCAACGGTTGTTGGTATACCAGGACATATTGTTAAGCAAGATGGTGT
TCGAGTTGGAAAAACATTTGATCATCGCCATCTACCTGATCCAATTTATGAACAAATTAAGCATTTAGAACGACAACTTGAAAAGAC

TAGGAATGGAGAGATTCAAGATGATTACATTATATAATACGCTTACACGTCAAAAAGAAGTGTTCAAGCCTATAGAACCAGGAAAA
GTAAAAATGTATGTATGTGGTCCTACTGTATATAACTACATTCATATTGGTAACGCAAGACCAGCAATTAATTATGACGTAGTGAGA
CGTTACTTTGAATACCAAGGATATAATGTAGAATATGTATCAAATTTTACAGACGTAGATGATAAATTAATTAAACGTTCTCAAGAA
TTAAATCAGTCTGTTCCCGAAATTGCAGAAAAATATATCGCAGCTTTTCATGAAGATGTTGGTGCGTTAAATGTTAGAAAAGCGACT
TCAAATCCAAGGGTAATGGACCATATGGATGACATTATTCAATTTATTAAAGAGTTGGTGGATCAAGGTTATGCATATGAAAGTGGT
GGCGATGTTTACTTTAGAACACGTAAATTTGAAGGTTATGGTAAATTAAGTCATCAATCCATAGATGACTTAAAAGTGGGTGCTCGT
ATAGATGCAGGAGAGCATAAAGAAGATGCACTTGATTTTACATTGTGGAAAAAAGCGAAGCCTGGCGAGATTAGTTGGGATAGCCC
ATTTGGTGAAGGTAGACCAGGATGGCATATAGAATGTTCTGTAATGGCATTTCATGAGCTAGGACCTACAATTGATATACATGCGG
GTGGTTCAGATTTACAATTTCCACATCATGAAAATGAAATAGCACAATCAGAAGCACATAATCATGCGCCATTTGCTAATTATTGGA
TGCATAATGGTTTCATTAATATTGATAATGAAAAAATGAGTAAATCACTAGGCAACTTTATTTTAGTTCACGATATTATTAAAGAAG
TTGATCCAGATGTACTAAGATTCTTTATGATTAGCGTACATTATAGAAGCCCAATTAACTATAATCTAGAATTGGTAGAATCAGCAC
GTAGTGGACTAGAGCGTATTCGCAATAGTTATCAATTAATTGAAGAGCGCGCACAAATTGCTACTAATATTGAAAATCAACAGACA
TATATTGATCAAATTGATGCGATTTTAAATCGTTTTGAAACAGTTATGAATGATGATTTTAATACAGCTAATGCAATTACAGCTTGGT
ATGATTTAGCAAAACTTGCGAATAAATATGTACTAGAGAACACAACATCAACAGAAGTAATTGATAAATTTAAAGCAGTTTATCAA
ATTTTCAGCGATGTTTTAGGTGTACCGTTAAAATCTAAAAATGCAGATGAATTATTGGATGAAGATGTTGAAAAATTAATCGAAGAG
CGTAATGAAGCAAGGAAAAACAAAGATTTTGCACGAGCAGATGAAATTCGAGACATGCTGAAATCACAAAACATTATATTAGAAG
ACACACCTCAAGGGGTTAGATTTAAACGTGGATAATCAACAAGATAATCACATTAAATTATTGAATCCATTGACCTTAGCATATATG
GGAGACGCAGTCTTAGATCAATATGTACGTACCTATATCGTTTTAAAGCTTAAAAGTAAGCCTAATAAACTACATCAAATGTCTAAA
AAATATGTATCTGCCAAAAGTCAGGCGCAAACGTTAGAATATTTAATGGAGCAAGAATGGTTTACAGACGAAGAAATGGATATTTT
GAAGCGAGGGCGTAACGCGAAAAGTCATACTAAAGCTAAAAACACTGATGTTCAAACATATCGTAAAAGTTCAGCGATAGAAGCA
GTGATAGGTTTTCTTTATTTAGAAAAAAGAGAAGAACGATTAGAGGCATTATTAAATAAAATAATAACAATAGTAAACGAAAGGTA
GTGACGATGTGGAAGATACGGTTATTGTTGGTAGGCATGCTGTTAGAGAAGCGATTATTACTGGGCATCCGATAAATAAGATATTG
ATTCAAGAAGGTATTAAAAAGCAACAAATTAATGAAATTTTAAAAAATGCAAAAGATCAAAAAATCATTGTTCAAACTGTACCAAA
ATCTAAATTAGATTTTTTAGCAAATGCACCACATCAGGGTGTTGCAGCGCTTATTGCACCATATGAATATGCTGACTTCGATCAATTT
TTAAAACAGCAAAAAGAAAAAGAAGGTTTATCGACAGTACTTATATTAGACGGCTTAGAAGACCCACATAACTTGGGATCAATTTT
AAGAACAGCCGATGCAACGGGAGTTGATGGTGTTATTATTCCTAAACGTCGTTCAGTTACACTAACGCAAACAGTTGCAAAAGCCT
CAACAGGTGCAATTGAACATGTACCAGTTATTCGAGTGACAAATTTAGCTAAAACTATCGATGAACTAAAAGATAATGGCTTTTGG
GTAGCTGGCACTGAAGCTAATAATGCAACAGATTATAGAAATCTAGAAGCGGACATGTCATTGGCTATTGTAATTGGTAGCGAAGG
ACAGGGTATGAGTCGCCTAGTAAGTGATAAATGCGATTTTTATATTAAGATTCCAATGGTTGGACATGTAAACAGTTTGAATGCTTC
GGTTGCAGCAAGTTTAATGATGTACGAAGTATTTCGAAAAAGACATGATGTTGGAGAAATATAATGAAAGAACGTTACTTAATCAT
TGATGGATACAATATGATAGGACAATCACCAACGCTAAGCGCCATTGCAAAAGAGAATTTAGAAGAAGCTAGAATGCAATTAATA
GATGCAATTGCAAATTATAATGCAGTTATTTCAGATGAAATTATTTGTGTTTTCGATGCTTATGACCAATCGGGTGTTGAAAGAGAA
TACATGTATCATGGCGTTAAAACGATTTTTACCAAGGAAAAAGAAACAGCTGATAGTTTCATAGAACGTTATGTTTATGAACTTTAT
GACAAGCATACTAAGCATATTACAGTTGTAACAAGTGATATGAGTGAGCAACATGCTATCTTTGGATCAGGTGCATATAGAATATC
ATCTCGCGAAATGTGGAGAGATTTAAAAGAAAATGAAATTGATGTGAGTAAATCATTAGATGATATAAGTGAAAACAAGCCAAGA
ACTCGAATTCCGTTATCTTCTGAAATCCTTGCAGAATTTGAAAAAATACGAAGAGGACATCATAAGAAATGACATTTCCGTCATCTT
GAAATTTTGAATGTAACAATATTAAACTAACCTTAAATTTTAGATAGAAGGGGTTAGTTTAATACTTGAAATACGATTTGACAACTC
AAGACAGTACAATCAAACGTAACAATGCAATAAATGATAAAGACTTCGAAAAGTTAGTAATGGATCTAAAACCATTAATTATTCGA
CGCATCAAAACATTTGGATTTAATCATTATGATTTAGAAGACTTATATCAAGAAATACTTATACGGATGTATAGGTCGGTCCAAACA
TTTGATTTTAGTGGAGAGCAGCCTTTCACAAATTATGTTCAATGTTTAATTACGTCTGTAAAGTATGATTATTTGAGAAAATATTTAG
CTACAAATAAAAGAATGGATAATTTGATTAATGAATATAGAGTTACGTATCCATGTGCAATAAAGCGTTATGATGTTGAAAACAAT
TATTTGAATCAATTAGCAATTAAAGAGTTGATTCGTCAGTTTAAGTATTTGAGTGCATTTGAAAAAGATGTCATGTATTTAATGTGTG
AACAATATAAGCCGAGAGAAATTGCTCAACTGATGCATGTAAAAGAGAAAGTGATTTATAATGCCATACAACGATGTAAAAATAA
AATAAAACGTTATTTCAAAATGATTTGAAAAGCGCCTTAGGACGTGAATTGAATTAGAACGTGTTACTTACTGATGGTTTGACATTT
GTTATAAATTTTATGTATAGTATACTGGTATTATAATGAATAAAGGTGAATTATTGTGAGAAAAATACCTTTAAATTGTGAAGCTTG
TGGCAATAGAAATTATAATGTTCCTAAGCAAGAAGGCTCGGCAACAAGATTAACCTTAAAGAAATATTGTCCAAAATGTAACGCGC
ACACAATTCATAAAGAATCGAAATAAATACATTCGAAATAATACTTTGATAATATGTTCAAAGGATTTGGAGGTTGAGCAGATGGC
TAAAAAAGAAAGTTTCTTTAAAGGCGTTAAGTCTGAAATGGAAAAAACAAGTTGGCCGACGAAAGAAGAGCTATTTAAATATACTG
TAATTGTAGTTTCTACTGTTATATTCTTCTTAGTCTTTTTCTATGCCTTAGATTTAGGAATTACAGCATTGAAAAATTTATTATTTGGT
TAGAGGAGTGAAGACATGTCTGAAGAAGTTGGCGCAAAGCGTTGGTATGCAGTGCATACATATTCTGGATATGAAAATAAAGTTAA
AAAGAATTTAGAAAAAAGAGTAGAATCTATGAATATGACTGAACAAATCTTTAGAGTAGTCATACCGGAAGAAGAAGAAACTCAA
GTAAAAGATGGCAAAGCTAAAACGACTGTTAAAAAAACATTCCCTGGATATGTTTTAGTGGAATTAATCATGACAGATGAATCATG
GTATGTGGTAAGAAATACACCAGGTGTTACTGGTTTTGTAGGTTCTGCAGGTGCAGGGTCTAAGCCAAATCCATTGTTACCAGAAGA
AGTTCGCTTCATCTTAAAACAAATGGGTCTTAAAGAAAAGACTATCGATGTTGAACTCGAAGTTGGCGAGCAAGTTCGTATTAAATC
AGGTCCATTTGCGAATCAAGTTGGTGAAGTTCAAGAAATTGAAACAGATAAGTTTAAGCTAACAGTATTAGTAGATATGTTTGGCC
GAGAAACACCAGTAGAAGTTGAATTCGATCAAATAGAAAAGCTTTAATTAACAATTAAAGTTATTAAACTAACCAAAAGATAAAAA
AGAGTATTGATTTTTTAATTAGAAAAGTGTTAAAATTATGTGGTCGCGCTTTTAGAGCGCCCATTTCGTCACGAAATGTTAAGAGTG
GGAGGGCAAAACTGAGCCCTGTGACCACATCACGATATCAAGGAGGTGCACATCGTGGCTAAAAAAGTAGATAAAGTTGTTAAATT
ACAAATTCCTGCAGGTAAAGCGAATCCAGCACCACCAGTTGGTCCAGCATTAGGTCAAGCAGGTGTGAACATCATGGGATTCTGTA
AAGAGTTCAATGCACGTACTCAAGATCAAGCAGGTTTAATTATTCCGGTAGAAATCAGTGTTTATGAAGATCGTTCATTTACATTTA
TTACAAAAACTCCACCGGCTCCAGTATTACTTAAAAAAGCAGCTGGTATTGAAAAAGGTTCAGGCGAACCAAACAAAACTAAAGTT
GCTACAGTAACTAAAGATCAAGTACGCGAAATTGCTAACAGCAAAATGCAAGACTTAAACGCTGCTGACGAAGAAGCAGCTATGC
GTATTATCGAAGGTACTGCACGTAGTATGGGTATCGTTGTAGAATAATTTTACGAATATTAAATTTGATTACATGATTTAAACGATG
AAGCAGATAACAGAGATAATAATGATGAATTATAAATATAATCTGAATGACTAGATTAATGATTGATTTATTCATAAGATTAATTCT
TCTGTTGTCTGCTCTTAACTTGCATATAGCAAGTAATGTGGGAGGAAATTCCGCTAAAACCACTAAAGGAGGAACTATAAATGGCTA
AAAAAGGTAAAAAGTATCAAGAAGCAGCTAGTAAAGTTGACCGTACTCAGCACTACAGTGTTGAAGAAGCAATTAAATTAGCTAA
AGAAACAAGTATTGCTAACTTTGACGCTTCTGTTGAAGTTGCATTCCGTTTAGGAATTGATACACGTAAAAATGACCAACAAATCCG
TGGTGCAGTTGTATTACCAAACGGAACTGGTAAATCACAAAGTGTATTAGTATTCGCTAAAGGTGACAAAATTGCTGAAGCTGAAG
CAGCAGGTGCTGACTATGTAGGTGAAGCAGAATACGTTCAAAAAATCCAACAAGGTTGGTTCGACTTCGATGTAGTAGTTGCTACA
CCAGACATGATGGGTGAAGTTGGTAAATTAGGTCGTGTATTAGGACCAAAAGGTTTAATGCCAAACCCTAAAACTGGAACTGTAAC
AATGGATGTTAAAAAAGCTGTTGAAGAAATCAAAGCTGGTAAAGTAGAATACCGTGCTGAAAAAGCTGGTATCGTACATGCATCAA

TTGGTAAAGTTTCATTTACTGATGAACAATTAATTGAAAACTTCAATACTTTACAAGATGTATTAGCTAAAGCTAAACCATCATCTG
CTAAAGGTACATACTTCAAATCTGTTGCTGTAACTACAACAATGGGTCCTGGAGTTAAAATTGATACTGCAAGTTTCAAATAATAAA
TGATATAAACAATTACAGGCTGAAAGAAATATCTTTCAGTCTGTAAAAATATATTGACAATAAGTAATTTCCAAGTTATATTACTTA
TTGTGATTATTTTACCTAAGACAGTAGGAGTTATTTATAACTTAAAACTTATCCTGCCGAGGCTAAAATTGACTTGAACGTGATGAT
CTATGATCTTTCAAGCACTTTTTGCCGTGGGTAGAAAGTGCTTTTTTTATTAATTTTAAAAAAAGCACCAAAAATTTAAATGGAGGT
GTCTGAATGTCTGCTATCATTGAAGCTAAAAAACAACTAGTTGATGAAATTGCTGAGGTACTATCAAATTCAGTTTCAACAGTAATC
GTTGACTACCGTGGATTAACAGTAGCTGAAGTTACTGACTTACGTTCACAATTACGTGAAGCTGGTGTTGAGTATAAAGTATACAAA
AACACTATGGTACGTCGTGCAGCTGAAAAAGCTGGTATCGAAGGCTTAGATGAATTCTTAACAGGTCCTACTGCTATTGCAACTTCA
AGTGAAGATGCTGTAGCTGCAGCGAAAGTAATTTCTGGATTTGCTAAAGATCATGAAGCATTAGAAATTAAATCAGGCGTTATGGA
AGGCAATGTTATTACAGCAGAAGAAGTTAAAACTGTTGGTTCATTACCTTCACACGATGGTCTTGTATCTATGCTTTTATCAGTATTA
CAAGCTCCTGTACGCAACTTCGCTTATGCGGTTAAAGCTATTGGAGAACAAAAAGAAGAAAGCGCTGAATAATTTTTAGCGTAAAA
AAATTAAAAATAATGGAGGAATTATAAAATGGCTAATCATGAACAAATCATTGAAGCGATTAAAGAAATGTCAGTATTAGAATTAA
ACGACTTAGTAAAAGCAATTGAAGAAGAATTTGGTGTAACTGCAGCTGCTCCAGTAGCAGTAGCAGGTGCAGCTGGTGGCGCTGAC
GCTGCAGCAGAAAAAACTGAATTTGACGTTGAGTTAACTTCAGCTGGTTCATCTAAAATCAAAGTTGTTAAAGCTGTTAAAGAAGC
AACTGGTTTAGGATTAAAAGATGCTAAAGAATTAGTAGACGGAGCTCCTAAAGTAATCAAAGAAGCTTTACCTAAAGAAGAAGCTG
AAAAACTTAAAGAACAATTAGAAGAAGTTGGAGCTACTGTAGAATTAAAATAATTCAAGTATCTTAAACTTAATAATCAAAGTTTT
ATAGCAAGTATTGCTATAATATAATGATTCTTTGAGAAGTTAAAACCCCGTTATTTTGATAACGGGGTTTTATTCATTTAAAGACTGA
GTGAAATGTTATAATTATAATGACGAGTTACAAAGTGAAGATGAGGTGGAATAATGAGTCATTATTACGATGAAGATCCAAGTGTA
ATTAGCAATGAACAACGTATTCAATATCAATTAAACCATCATAAAATTGATTTAATAACTGATAATGGAGTGTTTTCGAAAGATAAA
GTAGATTATGGTTCAGATGTTCTTGTTCAAACTTTTTTAAAAGCGCATCCACCTGGTCCAAGTAAGCGAATTGCCGATGTTGGTTGTG
GTTACGGACCAATTGGTTTGATGATTGCTAAAGTATCACCACATCATTCAATTACAATGCTAGATGTTAATCACAGAGCGCTAGCCT
TAGTTGAAAAAAACAAAAAAATAAATGGTATTGATAATGCGATCGTAAAGGAAAGTGATGCTTTGTCTGCTGTGGAAGACAAAAGT
TTTGATTTTATTTTAACCAATCCACCAATAAGAGCAGGGAAAGAAACCGTGCATCGTATATTCGAGCAAGCATTACATAGATTAGAC
TCGAACGGTGAACTATTCGTTGTAATTCAGAAGAAGCAAGGTATGCCATCTGCAAAGAAAAGAATGAATGAACTTTTTGGAAATGT
AGAAGTGGTAAATAAAGATAAAGGATATTACATTCTGAGAAGTATAAAAGCTTGAAATGAAATGGATATTCTGTTATAGTTATATA
ATGTAAAAATTTATGTTCAATAAGTGTGTACTTTTACGTTAAATAGATAAGTTAATTAAGAATAAATATAGAATCGAAAATGGTGTC
ATCATTAGTGTTGCCGTTTTCTTTTTGTCTTTTTATTAATATGCTTATGATATTTAGCTAAAAGCGGATCACATAATTTTTGAGGGGTG
AATCTGTTTGGCAGGTCAAGTTGTCCAATATGGAAGACATCGTAAACGTAGAAACTACGCGAGAATTTCAGAAGTATTAGAATTAC
CAAACTTAATAGAAATTCAAACTAAATCTTACGAGTGGTTCCTAAGAGAAGGTTTAATCGAAATGTTTAGAGACATTTCTCCAATTG
AAGATTTTACTGGTAATTTGTCATTAGAGTTTGTGGATTACCGTTTAGGAGAACCAAAATATGATTTAGAAGAATCTAAAAACCGTG
ACGCTACTTATGCTGCACCTCTTCGTGTAAAAGTGCGTCTAATCATTAAAGAAACAGGAGAAGTTAAAGAACAAGAAGTCTTTATG
GGTGATTTCCCATTAATGACTGATACAGGTACGTTCGTTATCAATGGTGCAGAACGTGTAATCGTATCTCAATTAGTTCGTTCACCAT
CCGTTTATTTCAATGAAAAAATCGACAAAAATGGTCGTGAAAACTATGATGCAACAATTATTCCAAACCGTGGTGCATGGTTAGAA
TATGAAACAGATGCTAAAGATGTTGTATACGTACGTATTGATAGAACACGTAAACTACCATTAACAGTATTGTTACGTGCATTAGGT
TTCTCAAGTGACCAAGAAATTGTTGACCTTTTAGGTGACAATGAATATTTACGTAATACTTTAGAGAAAGACGGCACTGAAAACACT
GAACAAGCGTTATTAGAAATCTATGAACGTTTACGTCCAGGTGAACCACCAACTGTTGAAAATGCTAAAAGTCTATTGTATTCACGT
TTCTTTGATCCAAAACGCTATGACTTAGCAAGCGTGGGTCGTTATAAAACAAACAAAAAATTACATTTAAAACATCGTTTATTTAAT
CAAAAATTAGCTGAGCCAATTGTAAATACTGAAACTGGTGAAATTGTAGTTGAAGAAGGTACAGTGCTTGATCGTCGTAAAATCGA
CGAAATCATGGATGTACTTGAATCAAATGCAAACAGCGAAGTGTTTGAATTGCATGGTAGCGTTATAGACGAGCCAGTAGAAATTC
AATCAATTAAAGTATATGTTCCTAACGATGATGAAGGTCGTACGACAACTGTAATTGGTAATGCTTTCCCTGACTCAGAAGTTAAAT
GCATTACACCAGCAGATATCATTGCTTCAATGAGTTACTTCTTTAACTTATTAAGCGGTATTGGATATACAGATGATATTGACCATTT
AGGTAACCGTCGTTTACGTTCTGTAGGTGAATTACTACAAAACCAATTCCGTATCGGTTTATCAAGAATGGAAAGAGTTGTACGTGA
AAGAATGTCAATTCAAGATACTGAGTCTATCACACCTCAACAATTAATTAATATTCGACCTGTTATTGCATCTATTAAAGAATTCTTT
GGTAGCTCTCAATTATCACAATTCATGGACCAAGCAAATCCATTAGCTGAGTTAACGCATAAACGTCGTCTATCAGCATTAGGACCT
GGTGGTTTAACACGTGAACGTGCTCAAATGGAAGTACGTGACGTTCACTACTCTCACTATGGCCGTATGTGTCCAATTGAAACACCT
GAGGGACCAAACATTGGATTGATTAACTCATTATCAAGTTATGCACGTGTAAATGAATTCGGCTTTATTGAAACACCATATCGTAAA
GTTGATTTAGATACACATGCTATCACTGATCAAATTGACTATTTAACAGCTGACGAAGAAGATAGCTATGTTGTAGCACAAGCAAAC
TCTAAATTAGATGAAAATGGTCGTTTCATGGATGATGAAGTTGTATGTCGTTTCCGTGGTAACAACACAGTTATGGCTAAAGAAAAA
ATGGATTATATGGATGTATCGCCGAAGCAAGTTGTTTCAGCAGCGACAGCATGTATTCCATTCTTAGAAAATGATGACTCAAACCGT
GCATTGATGGGTGCGAACATGCAACGTCAAGCAGTGCCTTTGATGAATCCAGAAGCACCATTTGTTGGAACAGGTATGGAACACGT
TGCAGCACGTGATTCTGGTGCAGCTATTACAGCTAAGCACAGAGGTCGTGTTGAACATGTTGAATCTAATGAAATTCTTGTACGTCG
TCTAGTTGAAGAGAACGGCGTTGAGCATGAAGGTGAATTAGATCGCTATCCATTAGCTAAATTTAAACGTTCAAACTCAGGTACAT
GTTACAACCAACGTCCAATCGTTGCAGTTGGAGATGTTGTTGAGTATAACGAGATTTTAGCAGATGGACCATCTATGGAATTAGGAG
AAATGGCATTAGGTAGAAACGTAGTAGTTGGTTTCATGACTTGGGACGGTTACAACTATGAGGATGCCGTTATCATGAGTGAAAGA
CTTGTGAAAGATGACGTGTATACTTCTATTCATATTGAAGAGTATGAATCAGAAGCACGTGATACTAAGTTAGGACCTGAAGAAAT
CACAAGAGATATTCCTAATGTTTCTGAAAGTGCACTTAAGAACTTAGACGATCGTGGTATCGTTTATATTGGTGCAGAAGTAAAAGA
TGGAGATATTTTAGTTGGTAAAGTAACGCCTAAAGGTGTAACTGAGTTAACTGCCGAAGAAAGATTGTTACATGCAATCTTTGGTGA
AAAAGCACGTGAAGTTAGAGATACTTCATTACGTGTACCTCACGGCGCTGGCGGTATCGTTCTTGATGTAAAAGTATTCAATCGCGA
AGAAGGCGACGATACATTATCACCTGGTGTAAACCAATTAGTACGTGTATACATCGTTCAAAAACGTAAAATTCATGTTGGTGATA
AGATGTGTGGTCGACATGGTAACAAAGGTGTCATTTCTAAGATTGTTCCTGAAGAAGATATGCCTTACTTACCAGACGGACGTCCGA
TCGATATCATGTTAAACCCTCTTGGTGTACCATCTCGTATGAACATCGGACAAGTATTAGAGCTACACTTAGGTATGGCTGCTAAAA
ATCTTGGTATTCACGTTGCATCACCAGTATTTGACGGTGCAAACGATGACGATGTATGGTCAACAATTGAAGAAGCTGGTATGGCTC
GTGATGGTAAAACTGTACTTTATGATGGACGTACAGGTGAACCATTCGATAACCGTATTTCAGTAGGTGTAATGTACATGTTGAAAC
TTGCGCACATGGTTGATGATAAATTACATGCGCGTTCAACAGGACCATATTCACTTGTTACACAACAACCACTTGGCGGTAAAGCGC
AATTCGGTGGACAACGTTTTGGTGAGATGGAGGTATGGGCACTTGAAGCATATGGTGCTGCATACACATTACAAGAAATCTTAACTT
ACAAATCCGATGATACAGTAGGACGTGTGAAAACATACGAGGCTATTGTTAAAGGTGAAAACATCTCTAGACCAAGTGTTCCAGAA
TCATTCCGAGTATTGATGAAAGAATTACAAAGTTTAGGTTTAGATGTAAAAGTTATGGATGAGCAAGATAATGAAATCGAAATGAC
AGACGTTGATGACGATGATGTTGTAGAACGCAAAGTAGATTTACAACAAAATGATGCTCCTGAAACACAAAAAGAAGTTACTGATT
AATACGCAATTTACAAAACAGGCAAAAAGATACTAAGCTGAATTTTATTGATGATTCAGTTTAGTACTTTAAGCCATTTTAAATAAA
TGCAAATCAATCAAATAGCACAGCTAATCTAAATTGAAGGAGGTAGGCTCCTTGATTGATGTAAATAATTTCCATTATATGAAAATA
GGATTGGCTTCACCTGAAAAAATCCGTTCTTGGTCTTTTGGTGAAGTTAAAAAACCTGAAACAATCAACTACCGTACATTAAAACCT

GAAAAAGATGGTCTATTCTGTGAAAGAATTTTCGGACCTACAAAAGACTGGGAATGTAGTTGTGGTAAATACAAACGTGTTCGCTA
CAAAGGCATGGTCTGTGACAGATGTGGAGTTGAAGTAACTAAATCTAAAGTACGTCGTGAAAGAATGGGTCACATTGAACTTGCTG
CTCCAGTTTCTCACATTTGGTATTTCAAAGGTATACCAAGTCGTATGGGATTATTACTTGACATGTCACCAAGAGCATTAGAAGAAG
TTATTTACTTTGCTTCTTATGTTGTTGTAGATCCAGGTCCAACTGGTTTAGAAAAGAAAACTTTATTATCTGAAGCTGAATTCAGAGA
TTATTATGATAAATACCCAGGTCAATTCGTTGCAAAAATGGGTGCAGAAGGTATTAAAGATTTACTTGAAGAGATTGATCTTGACGA
AGAACTTAAATTGTTACGCGATGAGTTGGAATCAGCTACTGGTCAAAGACTTACTCGTGCAATTAAACGTTTAGAAGTTGTTGAATC
ATTCCGTAATTCAGGTAACAAACCTTCATGGATGATTTTAGATGTACTTCCAATCATCCCACCAGAAATTCGTCCAATGGTTCAATTA
GATGGTGGACGATTTGCAACAAGTGACTTAAACGACTTATACCGTCGTGTAATTAATCGAAATAATCGTTTGAAACGTTTATTAGAT
TTAGGTGCACCTGGTATCATCGTTCAAAACGAAAAACGTATGTTACAAGAAGCCGTTGACGCTTTAATTGATAATGGTCGTCGTGGT
CGTCCAGTTACTGGCCCAGGTAACCGTCCATTAAAATCTTTATCTCATATGTTAAAAGGTAAACAAGGTCGTTTCCGTCAAAACTTA
CTTGGTAAACGTGTTGACTATTCAGGACGTTCAGTTATTGCAGTAGGTCCAAGCTTGAAAATGTACCAATGTGGTTTACCAAAAGAA
ATGGCACTTGAACTATTTAAACCATTCGTAATGAAAGAATTAGTTCAACGTGAAATTGCAACTAACATTAAAAATGCGAAGAGTAA
AATCGAACGTATGGATGATGAAGTTTGGGACGTATTGGAAGAAGTAATTAGAGAACATCCTGTATTACTTAACCGTGCACCAACAC
TTCATAGACTTGGTATTCAAGCATTTGAACCAACTTTAGTTGAAGGTCGTGCGATTCGTCTACATCCACTTGTAACAACAGCTTATAA
CGCTGACTTTGACGGTGACCAAATGGCGGTTCACGTTCCTTTATCAAAAGAGGCACAAGCTGAAGCAAGAATGTTGATGTTAGCAG
CACAAAACATCTTGAACCCTAAAGATGGTAAACCTGTAGTTACACCATCACAAGATATGGTACTTGGTAACTATTACCTTACTTTAG
AAAGAAAAGATGCAGTAAATACAGGCGCAATCTTTAATAATACAAATGAAGTATTAAAAGCATATGCAAATGGCTTTGTACATTTA
CACACTAGAATTGGTGTACATGCAAGTTCGTTCAATAATCCAACATTTACTGAAGAACAAAACAAAAAGATTCTTGCTACGTCAGTA
GGTAAAATTATATTCAATGAAATCATTCCAGATTCATTTGCTTATATTAATGAACCTACGCAAGAAAACTTAGAAAGAAAGACACC
AAACAGATATTTCATCGATCCTACAACTTTAGGTGAAGGTGGATTAAAAGAATACTTTGAAAATGAAGAATTAATTGAACCTTTCAA
CAAAAAATTCTTAGGTAATATTATTGCAGAAGTATTCAACAGATTTAGCATCACTGATACATCAATGATGTTAGACCGTATGAAAGA
CTTAGGATTCAAATTCTCATCTAAAGCTGGTATTACAGTAGGTGTTGCTGATATCGTAGTATTACCTGATAAGCAACAAATACTTGA
TGAGCATGAAAAATTAGTCGACAGAATTACAAAACAATTCAACCGTGGTTTAATCACTGAAGAAGAAAGATATAATGCAGTTGTTG
AAATTTGGACAGATGCAAAAGATCAAATTCAAGGTGAATTGATGCAATCACTTGATAAAACTAACCCAATCTTCATGATGAGTGAT
TCAGGTGCCCGTGGTAACGCATCTAACTTTACACAGTTAGCAGGTATGCGTGGATTGATGGCCGCACCATCTGGTAAGATTATCGAA
TTACCAATCACATCTTCATTCCGTGAAGGTTTAACAGTACTTGAATACTTCATCTCAACTCACGGTGCACGTAAAGGTCTTGCCGATA
CAGCACTTAAAACAGCTGACTCAGGATATCTTACTCGTCGTCTTGTTGACGTGGCACAAGATGTTATTGTTCGTGAAGAAGACTGTG
GTACTGATAGAGGTTTATTAGTTTCTGATATTAAAGAAGGTACAGAAATGATTGAACCATTTATCGAACGTATTGAAGGTCGTTATT
CTAAAGAAACAATTCGTCATCCTGAAACTGATGAAATAATCATTCGTCCTGATGAATTAATTACACCTGAAATTGCTAAGAAAATTA
CAGATGCTGGTATTGAACAAATGTATATTCGCTCAGCATTTACTTGTAACGCACGACATGGTGTTTGTGAAAAATGTTACGGTAAAA
ACCTTGCTACTGGTGAAAAAGTTGAAGTTGGTGAAGCAGTTGGTACAATTGCAGCCCAATCTATCGGTGAACCAGGTACACAGCTT
ACAATGCGTACATTCCATACAGGTGGGGTAGCAGGTAGCGATATCACACAAGGTCTTCCTCGTATTCAAGAGATTTTCGAAGCACGT
AACCCTAAAGGTCAAGCGGTAATTACGGAAATCGAAGGTGTCGTAGAAGATATTAAATTAGCAAAAGATAGACAACAAGAAATTG
TTGTTAAAGGTGCTAATGAAACAAGATCATACCTTGCTTCAGGTACTTCAAGAATTATTGTAGAAATCGGTCAACCAGTTCAACGTG
GTGAAGTATTAACTGAAGGTTCTATTGAACCTAAGAATTACTTATCTGTTGCTGGATTAAACGCGACTGAAAGCTACTTATTAAAAG
AAGTACAAAAAGTTTACCGTATGCAAGGTGTAGAAATCGACGATAAACACGTTGAGGTTATGGTTCGACAAATGTTACGTAAAGTT
AGAATTATCGAAGCAGGTGATACGAAGTTATTACCAGGTTCATTAGTTGATATTCATAACTTTACAGATGCAAATAGAGAAGCATTT
AAACACCGTAAGCGTCCTGCAACAGCTAAACCAGTATTACTTGGTATTACTAAAGCATCACTTGAAACAGAAAGTTTCTTATCTGCA
GCATCATTCCAAGAAACAACAAGAGTTCTTACAGATGCAGCAATTAAAGGTAAGCGTGATGACTTATTAGGTCTTAAAGAAAACGT
AATTATTGGTAAGTTAATTCCAGCTGGTACTGGTATGAGACGTTATAGCGACGTAAAATACGAAAAAACAGCTAAACCAGTTGCAG
AAGTTGAATCTCAAACTGAAGTAACGGAATAACAAGTATATAACAGAGGCTAAGTCTTTAGCCTCTTGTTATTTTTATGTAAATTAT
TTGATTTAATGTTGACGAATTCTCTTGTTCAATGTTAATATATTAAAGGTTGATGCAAGCAGAACTTTGGAGGATAAATTATTGTCTA
AGGAAAAAGTTGCACGCTTTAACAAACAACATTTTGTAGTTGGTCTTAAAGAAACGCTTAAAGCGTTAAAGAAAGATCAAGTTACA
TCTTTGATTATTGCTGAAGACGTTGAAGTATATTTAATGACTCGCGTGTTAAGCCAAATCAATCAGAAAAATATACCTGTATCTTTTT
TCAAAAGCAAACATGCTTTGGGTAAACATGTAGGTATTAACGTCAATGCGACAATAGTAGCATTGATTAAATGAGAATTAGTAAGT
GTTTTACTTACTAAATTTTATTTAACCTAAAAATGAACCACCTGGATGTGTGGGATTAAAAAGTGAAGAGAGGAGGACATATCACAT
GCCAACTATTAACCAATTAGTACGTAAACCAAGACAAAGCAAAATCAAAAAATCAGATTCTCCAGCTTTAAATAAAGGTTTCAACA
GTAAAAAGAAAAAATTTACTGACTTAAACTCACCACAAAAACGTGGTGTATGTACTCGTGTAGGTACAATGACACCTAAAAAACCT
AACTCAGCGTTACGTAAATATGCACGTGTGCGTTTATCAAACAACATCGAAATTAACGCATACATCCCTGGTATCGGACATAACTTA
CAAGAACACAGTGTTGTACTTGTACGTGGTGGACGTGTAAAAGACTTACCAGGTGTGCGTTACCATATTGTACGTGGAGCACTTGAT
ACTTCAGGTGTTGACGGACGTAGACAAGGTCGTTCATTATACGGAACTAAGAAACCTAAAAACTAAGAATTTAGTTTTTAATTAAAT
CTTAAACTTAAAATATTTAATATAAGGAAGGGAGGATTTACATTATGCCTCGTAAAGGATCAGTACCTAAAAGAGACGTATTACCA
GATCCAATTCATAACTCTAAGTTAGTAACTAAATTAATTAACAAAATTATGTTAGATGGTAAACGTGGAACAGCACAAAGAATTCTT
TATTCAGCATTCGACCTAGTTGAACAACGCAGTGGTCGTGATGCATTAGAAGTATTCGAAGAAGCAATCAACAACATTATGCCAGT
ATTAGAAGTTAAAGCTCGTCGTGTAGGTGGTTCTAACTATCAAGTACCAGTAGAAGTTCGTCCAGAGCGTCGTACTACTTTAGGTTT
ACGTTGGTTAGTTAACTATGCACGTCTTCGTGGTGAAAAAACGATGGAAGATCGTTTAGCTAACGAAATTTTAGATGCAGCAAATA
ATACAGGTGGTGCCGTTAAGAAACGTGAGGACACTCACAAAATGGCTGAAGCAAACAAAGCATTTGCTCACTACCGTTGGTAAGAT
AAAAGCTTTTACCCTGAGTGTGTTCTATATTAATGAATTTTCATTAAGCGTTCATGCTTAGGGCATCGCCATATCTATCGTATTTATT
CAGTAATATAAACTGGAAGGAGAAAAAATACATGGCTAGAGAATTTTCATTAGAAAAAACTCGTAATATCGGTATCATGGCTCACA
TTGATGCTGGTAAAACGACTACGACTGAACGTATTCTTTATTACACTGGCCGTATCCACAAAATTGGTGAAACACACGAAGGTGCTT
CACAAATGGACTGGATGGAGCAAGAACAAGACCGTGGTATTACTATCACATCTGCTGCAACAACAGCAGCTTGGGAAGGTCACCGT
GTAAACATTATCGATACACCTGGACACGTAGACTTCACTGTAGAAGTTGAACGTTCATTACGTGTACTTGACGGAGCAGTTACAGTA
CTTGATGCACAATCAGGTGTTGAACCTCAAACTGAAACAGTTTGGCGTCAGGCTACAACTTATGGTGTTCCACGTATCGTATTTGTA
AACAAAATGGACAAATTAGGTGCTAACTTCGAATACTCTGTAAGTACATTACATGATCGTTTACAAGCTAACGCTGCTCCAATCCAA
TTACCAATTGGTGCGGAAGACGAATTCGAAGCAATCATTGACTTAGTTGAAATGAAATGTTTCAAATATACAAATGATTTAGGTACT
GAAATTGAAGAAATTGAAATTCCTGAAGACCACTTAGATAGAGCTGAAGAAGCTCGTGCTAGCTTAATCGAAGCAGTTGCAGAAAC
TAGCGACGAATTAATGGAAAAATATCTTGGTGACGAAGAAATTTCAGTTTCTGAATTAAAAGAAGCTATCCGCCAAGCTACTACTA
ACGTAGAATTCTACCCAGTACTTTGTGGTACAGCTTTCAAAAACAAAGGTGTTCAATTAATGCTTGACGCTGTAATTGATTACTTAC
CTTCACCACTAGACGTTAAACCAATTATTGGTCACCGTGCTAGCAACCCTGAAGAAGAAGTAATCGCGAAAGCAGACGATTCAGCT
GAATTCGCTGCATTAGCGTTCAAAGTTATGACTGACCCTTATGTTGGTAAATTGACATTCTTCCGTGTGTATTCAGGTACAATGACAT
CTGGTTCATACGTTAAGAACTCTACTAAAGGTAAACGTGAACGTGTAGGTCGTTTATTACAAATGCACGCTAACTCACGTCAAGAAA

TCGATACTGTATACTCTGGAGATATCGCTGCTGCGGTAGGTCTTAAAGATACAGGTACTGGTGATACTTTATGTGGTGAGAAAAATG
ACATTATCTTGGAATCAATGGAATTCCCAGAGCCAGTTATTCACTTATCAGTAGAGCCAAAATCTAAAGCTGACCAAGATAAAATG
ACTCAAGCTTTAGTTAAATTACAAGAAGAAGACCCAACATTCCATGCACACACTGACGAAGAAACTGGACAAGTTATCATCGGTGG
TATGGGTGAGCTTCACTTAGACATCTTAGTAGACCGTATGAAGAAAGAATTCAACGTTGAATGTAACGTAGGTGCTCCAATGGTTTC
ATATCGTGAAACATTCAAATCATCTGCACAAGTTCAAGGTAAATTCTCTCGTCAATCTGGTGGTCGTGGTCAATACGGTGATGTTCA
CATTGAATTCACACCAAACGAAACAGGCGCAGGTTTCGAATTCGAAAACGCTATCGTTGGTGGTGTAGTTCCTCGTGAATACATTCC
ATCAGTAGAAGCTGGTCTTAAAGATGCTATGGAAAATGGTGTCTTAGCAGGTTATCCTTTAATTGATGTTAAAGCTAAATTATATGA
TGGTTCATACCATGATGTCGATTCATCTGAAATGGCCTTCAAAATTGCTGCATCATTAGCACTTAAAGAAGCTGCTAAAAAATGTGA
TCCTGTAATCTTAGAACCAATGATGAAAGTAACTATTGAAATGCCTGAAGAGTACATGGGTGATATCATGGGTGACGTAACATCTC
GTCGTGGACGTGTTGATGGTATGGAACCTCGTGGTAATGCACAAGTTGTTAATGCTTATGTACCACTTTCAGAAATGTTCGGTTATG
CAACATCATTACGTTCAAACACTCAAGGTCGCGGTACTTACACTATGTACTTCGATCACTATGCTGAAGTTCCAAAATCAATCGCTG
AAGATATTATCAAGAAAAATAAAGGTGAATAATATAACTTGTTTTGACTAGCTAGCCTAGGTTAAAATACAAGGTGAGCTTAAATG
TAAGCTATCATCTTTATAGTTTGATTTTTTGGGGTGAATGCATTATAAAAGAATTGTAAAATTCTTTTTGCATCGCTATAAATAATTT
CTCATGATGGTGAGAAACTATCATGAGAGATAAATTTAAATATTATTTTTTATTAGAATAGGAGAGATTTTATAATGGCAAAAGAAA
AATTCGATCGTTCTAAAGAACATGCCAATATCGGTACTATCGGTCACGTTGACCATGGTAAAACAACATTAACAGCAGCAATCGCT
ACTGTATTAGCAAAAAATGGTGACTCAGTTGCACAATCATATGACATGATTGACAACGCTCCAGAAGAAAAAGAACGTGGTATCAC
AATCAATACTTCTCACATTGAGTACCAAACTGACAAACGTCACTACGCTCACGTTGACTGCCCAGGACACGCTGACTACGTTAAAAA
CATGATCACTGGTGCTGCTCAAATGGACGGCGGTATCTTAGTAGTATCTGCTGCTGACGGTCCAATGCCACAAACTCGTGAACACAT
TCTTTTATCACGTAACGTTGGTGTACCAGCATTAGTAGTATTCTTAAACAAAGTTGACATGGTTGACGATGAAGAATTATTAGAATT
AGTAGAAATGGAAGTTCGTGACTTATTAAGCGAATATGACTTCCCAGGTGACGATGTACCTGTAATCGCTGGTTCAGCATTAAAAGC
TTTAGAAGGCGATGCTCAATACGAAGAAAAAATCTTAGAATTAATGGAAGCTGTAGATACTTACATTCCAACTCCAGAACGTGATT
CTGACAAACCATTCATGATGCCAGTTGAGGACGTATTCTCAATCACTGGTCGTGGTACTGTTGCTACAGGCCGTGTTGAACGTGGTC
AAATCAAAGTTGGTGAAGAAGTTGAAATCATCGGTTTACATGACACATCTAAAACAACTGTTACAGGTGTTGAAATGTTCCGTAAA
TTATTAGACTACGCTGAAGCTGGTGACAACATTGGTGCATTATTACGTGGTGTTGCTCGTGAAGACGTACAACGTGGTCAAGTATTA
GCTGCTCCTGGTTCAATTACACCACATACTGAATTCAAAGCAGAAGTATACGTATTATCAAAAGACGAAGGCGGACGTCACACTCC
ATTCTTCTCAAACTATCGTCCACAATTCTATTTCCGTACTACTGACGTAACTGGTGTTGTTCACTTACCAGAAGGTACTGAAATGGTA
ATGCCTGGTGATAACGTTGAAATGACAGTAGAATTAATCGCTCCAATCGCGATTGAAGACGGTACTCGTTTCTCAATCCGTGAAGGT
GGACGTACTGTAGGATCAGGCGTTGTTACTGAAATCATTAAATAATTTCTAATTTCTTAGATTTTATATAAAAAGAAGATCCCTCAA
TCGAGGGGTCTTTTTTTAATGTGTAAATTTTGTAATGGCTATTCGATTTAGAAGAACAATAATTGATGAAAGATTGACTAATAAAAC
TTATAACTGATAATACTGTTTAAATAAAATTGTTGAGTCTTGGACATTGTAAAATGCTCCCTTCAAAGTTTTCATTTTTTCAATGTCT
ACTTTGAAGAGAGCATTTCATTAGTTTATGTCTCAGATTCATATCTTTCAATTAATTTAAATGCTTAATTTGTTTTAAATACTTGCTCT
AATTCAATGATTTTTAAAAATACAGCTACAGCGTATTTTAATGATTTTTCATCAATATCAAATTTGGGATTATGGTGTGGCGCTGTAA
TACCTTTACTTTCATTACCACAACCAGTCAGAAAGAATGCACCTGGTCGTACTTTCAAATAATGTGAAAAATCTTCTCCAATCATCAT
TAAATCTGATTCATTAAAGCGTACATGTAAGTCATTTGTTGCTTCTTTAATAACTTGATATGCTTTCTCGTTATTATGGACAGGCAAA
TACCCTTTAATATAATTCAAATCATAGTTAATATCATTTGCTATTGCTAAACCTTGTAGAAGCTTATCCATTTTGTCCATTACATGATT
CTGTATATCTGAATCGAAAGTTCTAACTGTACCTTTACAAAATGCTTGATCAGGAATAACGCTATCTGTGGTGCCTGCTTGAATCATT
CCAAATGAAAGTACAGCTTGTTTAACTGGATCGATCGTACGTGAAATTATTTTTTGTGCACTTAAAATGAACTCTGCCATGATTACT
ATTGGGTCAATGGTTTCATGAGGTTTGGCACCATGACCACCACGACCTTTAATTGTGACGCTAAATTCATCTGGAGAGGCCATGATT
GCCCCCGCACGTGAATGAATAGTTCCAGTAGGATAACCACTCCATAAATGTGTACCGTAAATTCTATCTACATTTTCCAGACATCCA
GCATCTATCATTTCTTGAGAACCACCTGGCATGATTTCTTCACCGTACTGGAATATTAATGCAACATTACCTTCTAATAAATGTTTAT
GTTCATCTAAAATCTCTGCTACAGTAAGTAAAATTGCTGTATGACCATCATGCCCACACGCATGCATACATCCTGGATTTTTAGACTT
ATAAGGCACATCGTTTAATTCCTCGACAGGTAACGCATCAAAGTCAGCTCTTAATGCAATGGTAGGTCCTGTGCCCAAGCCTTTAAA
TGTGGCTTTGATACCATTGCGGCCGATAGGAGTTTCAATATCACAAGATAACTGGCTTAATTGGTTAACAATATAATCATGTGTTTG
AAATTCTTCAAAAGATAACTCAGGATATTGGTGTAAATAACGTCTGAGTTGAATTGTTTTATTTTCTTTATTATTTGCTAGTTGGAAC
CAATCTAACACCCTTATCACTACTTTCTAAAATAATGTTTATAGTATAACATTTTATGAAATTATCGTACTAAATGATTGCTTTGAGA
TATTTTATCTATGAATGATAAGGCTTTCAAGTTATGTAGAATTACTGTATGATAAAGGTATTACCAAACAATACTTAAGGGGGATTA
TATACTGTGGTTCAATCATTACATGAGTTTTTAGAGGAAAATATAAATTATCTAAAAGAAAATGGTTTGTATAATGAAATAGATACA
ATTGAAGGTGCAAACGGACCAGAAATCAAAATCAATGGGAAATCATACATTAACTTATCTTCAAATAATTATTTAGGACTAGCAAC
AAATGAAGATTTGAAATCAGCTGCAAAAGCAGCTATTGATACACATGGTGTAGGTGCAGGCGCTGTTCGTACAATCAATGGTACAT
TAGATTTACACGACGAATTAGAAGAAACACTAGCAAAATTTAAAGGAACAGAAGCTGCAATAGCTTATCAATCAGGATTTAATTGT
AATATGGCTGCTATTTCAGCTGTCATGAATAAAAATGATGCTATTTTATCAGATGAGCTTAATCATGCATCAATTATTGATGGATGTC
GCTTATCTAAAGCTAAAATTATTCGAGTTAACCATTCAGACATGGATGATTTACGTGCGAAAGCAAAAGAAGCAGTTGAATCAGGT
CAATACAATAAAGTGATGTATATCACTGATGGCGTTTTTAGTATGGATGGTGATGTGGCTAAATTACCTGAAATTGTAGAAATTGCA
GAAGAATTTGGTTTATTAACTTATGTTGACGACGCTCATGGTTCAGGTGTTATGGGTAAAGGCGCTGGTACGGTTAAACATTTTGGT
TTACAAGATAAAATCGATTTCCAAATAGGTACGCTTTCTAAAGCAATTGGTGTCGTTGGCGGTTATGTAGCAGGTACAAAAGAGTTA
ATAGATTGGTTAAAAGCACAATCACGACCATTCTTATTCTCTACATCATTAGCACCTGGGGATACCAAAGCAATAACTGAAGCAGTT
AAAAAGTTAATGGATTCAACTGAATTACATGATAAATTATGGAACAATGCACAATATTTAAAAAATGGATTGTCAAAATTAGGATA
TGATACAGGTGAGTCAGAAACTCCAATTACACCAGTAATTATTGGTGATGAAAAAACAACTCAAGAATTTAGTAAGCGTTTAAAAG
ACGAAGGTGTCTATGTGAAATCTATCGTTTTCCCAACAGTACCAAGAGGTACAGGACGTGTAAGAAATATGCCTACAGCTGCACAT
ACAAAAGACATGTTAGATGAAGCAATTGCGGCTTATGAAAAAGTAGGAAAAGAAATGAAGTTGATTTAATATTTATTTATTCCCAC
GGCAAATATTGTCGTGGACTTTTTTTAATGTTTAGTTTATTAACAGTAAGTTCTTATAACAATGTTTAGTGCTCCCCAAAATTGAAGT
TTGAATTTTAAAAGCATCTTGTAGAATTTAGTTGTATTTTTTACAAAGAAATTCATTTTGATTATTTTTGATAATGAGCATTTTAATAG
TAATACATGTTTATAGTGTGTAGTATATGTCTATACTAGTAGTAACTATATAGAGAAAGTAGGAATAAACTATGTCACAAGATGTAA
ATGAATTAAGTAAGCAACCAACGCCAGATAAAGCAGAAGATAACGCATTTTTCCCATCACCATATTCCCTTAGTCAATATACAGCA
CCTAAAACAGATTTTGATGGTGTTGAACACAAAGGTGCCTATAAAGATGGTAAATGGAAAGTATTGATGATTGCTGCTGAAGAGAG
ATATGTATTATTGGAAAATGGAAAAATGTTCTCTACGGGTAATCATCCTGTTGAAATGTTATTACCTTTACATCATTTAATGGAAGC
AGGTTTTGACGTTGATGTTGCGACATTATCTGGTTATCCAGTTAAATTAGAATTATGGGCTATGCCAACTGAAGACGAGGCAGTTAT
AAGTACTTATAATAAATTGAAAGAAAAATTAAAACAGCCAAAAAAATTAGCAGATGTGATTAAAAATGAATTAGGACCTGATTCAG
ACTATTTATCTGTCTTTATCCCAGGCGGACATGCTGCAGTTGTTGGTATTTCTGAAAGTGAGGACGTTCAACAAACATTAGATTGGG
CATTAGACAATGACCGCTTTATAGTTACATTATGTCATGGACCAGCAGCACTACTTTCAGCAGGGCTTAACAGAGAAAAATCTCCAT
TAGAAGGATACTCTGTTTGTGTCTTCCCTGACTCATTAGATGAAGGTGCAAATATTGAAATAGGTTATTTACCTGGACGCTTGAAAT

GGTTAGTTGCTGATTTATTAACTAAACAAGGATTAAAAGTAGTTAACGACGATATGACAGGAAGAACGTTAAAAGATCGTAAATTA
TTAACAGGTGACAGTCCTTTAGCTTCAAATGAGTTAGGAAAATTAGCAGTTAATGAAATGTTAAATGCAATACAAAATAAATAATT
AAATATTAATTAGAGGAGCCCCATATGTAAATGTATGAGGGCTCTTTTTTTGGCAAAATTTAAGTGATACTTGTAAAATAGAACCTA
TTATGAGTATGATTTAAGAAAACGCTTGCAAAACTAATAACCGCAACTAGCGATATGGAGGAAACATGATGTCTTATAGCATTGGA
ATTGATTATGGAACAGCTTCAGGCCGTGTGTTTTTAATTAATACAACTAACGGTCAAGTAGTATCAAAATTTGTGAAACCATATACA
CATGGTGTCATTGAGAGTGAATTAAATGGTTTGAAAATACCACATACATATGCACTTCAAAATAGTAATGATTATTTAGAAATTATG
GAAGAAGGAATATCATATATAGTACGTGAATCAAAAATAGATCCAGTCAATATAGTAGGTATTGGTATAGACTTTACTTCATCTACT
ATTATTTTTACTGACGAAAACCTTAACCCGGTACATAATTTAAAACAATTTAAAAACAATCCACATGCGTATGTGAAACTTTGGAAA
CATCATGGTGCATATAAAGAAGCAGAGAAATTATATCAAACTGCTATTGAAAATAATAATAAGTGGTTAGGCCATTATGGATATAA
TGTTAGTAGTGAATGGATGATTCCCAAAATAATGGAAGTCATGAATCGAGCACCAGAAATTATGGAAAAAACGGCTTATATTATGG
AAGCGGGCGATTGGATTGTAAATAAATTAACTAATAAAAATGTACGCTCGAATTGTGGATTAGGTTTCAAAGCATTTTGGGAAGAA
GAAACAGGGTTTCATTATGATTTATTTGATAAAATAGACCCCAAATTATCAAAAGTAATTCAAGATAAAGTATCTGCACCGGTTGTT
AATATTGGTGAAGTAGTAGGGAAACTGGACGATAAAATGGCACAGAAATTAGGATTATCAAAAGAGACTATGGTAAGTCCTTTTAT
TATTGATGCCCATGCTAGTTTATTAGGTATTGGGTCTGAAAAAGATAAAGAAATGACTATGGTGATGGGAACAAGCACATGCCATC
TTATGTTAAATGAAAAGCAACATCAAGTGCCAGGTATATCAGGTTCTGTAAAAGGAGCAATTATTCCAGAATTATTTGCTTATGAAG
CGGGGCAATCAGCAGTAGGTGATTTGTTTGAGTATGTCGCTAAGCAAGCACCAAAGTCATATGTAGATGAAGCAGCAAATAGAAAT
ATGACTGTATTTGAATTAATGAATGAAAAGATAAAACATCAAATGCCAGGTGAAAGTGGGCTCATTGCTCTTGATTGGCATAATGG
AAATCGAAGTGTATTAAGTGATAGCAATTTAACAGGTTGTATCTTTGGATTAACTTTACAAACTAAGCATGAGGATATTTATAGAGC
ATATTTAGAAGCTACAGCATTTGGTACTAAGATGATTATGCAACAGTATCAAGATTGGCATATGGAAGTAGAAAAGGTATTTGCAT
GTGGCGGTATACCTAAAAAGAATGCTGTTATGATGGATATCTATGCGAATGTACTGAATAAAAAACTAATTGTTATGGATAGTGAG
TATGCACCAGCAATAGGCGCAGCAATATTAGGTGCAGTCAGTGGTGGCGCACATAATTCAATTAATGATGCAGTTGATGCTATGAA
AGAGCCAATTTTATACGAAATTAATCCAGAAGCGGAAAAAGTACAAAGGTATGAAACATTATTTAAAGCTTATAAGGCTTTACATG
ATATCCATGGTTATAAAAAAGCTAATATAATGAAAGATATCCAGAGTTTAAGAGTTGAGGGATAAAAAATTTAATTTGACAGTAAT
TAGCAATAATAAACGCTATAAATTACTAAAATCAATTAATACAACTAGAATTTCCATTTACAGAAATATCAGCAACATGTACATGTC
ATATATACAAGAAAGCGCTTTTATATTATAATTATTATGAAAATGAATATTATGTCCGCTTGTGATGGACTATAGCTATATAGAAGA
GACAAAGGAGATATTTCTATGAAAAAAATTATGATTACTGGTGCATTAGGACAAATTGGTACAGAATTAGTTGTTAAGTGCAGAGA
AATTTATGGGACAGATAATGTTCTTGCTACAGATATTAGGGAACCTGAAGCAGACTCACCTGTACAAAATGGACCATTTGAAATTTT
AGACGTAACAGATCGTGACCGCATGTTTGAGTTAGTTAGGGACTTTGAAGCGGATAGTCTAATGCATATGGCAGCATTATTATCAGC
AACTGCAGAGAAAAATCCAATTCTAGCTTGGGATTTAAATATGGGTGGATTAATGAATGCATTAGAAGCTGCAAGAACTTATAATT
TGCACTTTTTCACACCAAGTTCAATTGGTGCATTTGGAGACTCAACTCCTAAAGTTAATACGCCACAAGTAACGATTCAGCAACCTA
CGACAATGTATGGTGTAAATAAAGTAGCTGGAGAATTATTGTGTCAATACTATTTCAAACGTTTTGGTGTAGATACAAGAAGTGTTA
GATTCCCAGGTTTAATCTCGCATGTTAAAGAGCCAGGTGGCGGTACTACAGACTATGCTGTTGAAATATACTTCAAAGCAGTAAGA
GAAGGTCATTATACAAGCTTCATAGATAAAGGCACGTATATGGATATGATGTATATGGATGATGCAATTGAAGCAATTATTAAACTT
ATGGAAGCAGACGACGCTAAATTAGAAACTAGAAACGGTTATAATTTGAGCGCAATGAGTTTTGATCCAGAGATGGTAAAAGAAG
CAATTCAAGAATACTATCCCAATTTTACATTAGATTACGATGTTGATCCTATTAGACAAGGTATCGCTAATAGTTGGCCGGATTCTAT
TGATACAAGCTGTTCACGTGGCGAATGGGGATTTGATCCTAAATATGATTTAGCGAGCATGACTAAATTAATGTTAGAAGCTATTGA
ACAAAAAGATACTGTTAAAAATAATAACTAATCATTTCCATTCACTTTAATACACGGAATGATATTTTAAATTACTCTTTATTTTAAT
AAACTAGTGCATGAATTCTAATATTATTCATTATACATATTGAATTCGCGAGCTTAGTTCATTTTAAAGTAAAGAGTTTTTTGTATGT
AAAGTATATTAGTAAAACACAGATTACTTGTCTCTGTGAAATTAATACGTTTATACTAAAAGATATAGTCTTTCTAGAAAATCTGTT
AATTAATTTTGAATTTTTAGAAAATTTATTGAACAGCAAAATATGGATTGTTATAATTTAAGTTAAACAAATTCTTATACAATATTAT
TAGGAGGCAATCACCATGTCACAAGCAGTTAAAGTTGAACGACGAGAAACATTAAAACAAAAACCAAATACATCTCAACTAGGTTT
TGGTAAATATTTTACTGATTATATGTTGAGTTATGATTATGATGCAGATAAAGGATGGCATGATTTGAAGATAGTACCTTATGGTCC
TATTGAAATTTCACCTGCTGCACAAGGTGTTCATTATGGTCAATCGGTATTCGAAGGATTAAAAGCATATAAAAGAGATGGGGAAG
TTGCACTTTTCCGTCCTGAAGAAAATTTTAAGCGTCTTAATAACTCGTTAGCACGATTAGAAATGCCTCAAGTAGACGAAGCAGAAT
TGTTAGAGGGGCTAAAACAATTAGTTGATATAGAAAGAGATTGGATTCCTGAAGGGGAAGGTCAATCATTATATATTCGTCCATTTG
TTTTTGCAACAGAAGGGGCACTTGGCGTTGGTGCATCACATCAGTATAAATTATTAATTATTTTATCTCCTTCAGGTGCATATTATGG
TGGTGAGACTTTAAAACCAACTAAAATCTATGTAGAAGATGAATATGTGCGTGCTGTTCGTGGCGGTGTAGGCTTTGCAAAAGTTGC
AGGTAACTATGCGGCAAGTTTATTAGCACAAACAAATGCAAATAAATTAGGTTATGACCAAGTATTATGGCTTGATGGTGTTGAAC
AGAAATATATCGAAGAAGTTGGTAGCATGAATATTTTCTTCGTTGAAAATGGAAAAGTAATTACACCAGAGTTGAATGGCAGTATT
TTACCTGGTATTACACGTAAATCTATTATCGAATTAGCTAAAAACTTAGGATATGAAGTCGAAGAGCGCCGCGTTTCAATCGATGAA
TTATTCGAATCATATGATAAAGGTGAGTTAACAGAAGTATTTGGTAGTGGTACTGCAGCAGTTATTTCACCTGTGGGTACATTGAGA
TACGAAGATCGTGAAATCGTTATTAATAATAATGAGACTGGTGAAATTACTCAAAAATTATACGACGTCTATACTGGTATTCAAAAT
GGTACTTTAGAAGATAAAAATGGTTGGAGAGTCGTTGTACCAAAATATTAATAAAAATTGAATATAATCATGAAAACAATATGTAA
AAGTTAATCATGACTAAAATCCCTTTCAATGAAGCGAAGCATGGTAATAAATTAAGTTTACAATGTTTATTGTTAAGTATTGAAGGG
GATTTCACTTATTATTATATTTAATTCAATATTTAAAAAGAACAATACTACGAGTTCATTTTCAGGGAAATAACGAACATCATAGAA
TCAGCATAATACGAAATAAATAAATAGGAGTATTGATATTAATGGAATGGATATTATTTGATAAAGATGGCACGTTAATTGAATTTG
ATAGAAGTTGGGAAAAAAATAGGGGTACGATTTGTACAATCATTGCTTGAGACTTTCCCAGTACATAATAAAGAAGCTGCTTTAAGA
CAACTCGGTGTCATTAAAGAATCTATTGATCCAAAATCAGTGATGGGTTCAGGATCTTTACAACAAATTATCCAAGCATTTAATGAT
GTGACGGGACAAGATACAACCGACTGGTCCAAGTCAACAAGTCAAAAGCTGGTAGATGAACGTATTCCTGAAATTAATTGGGTAGA
AGGTGTTAAAGAAGCACTTATCGATTTGAAAGCAAAAGGCTATCAACTTGGTATTGTTACGAGTGATACTAAAAAAGGTGTAGAAC
AATTTTTAGCACATACCAATGCTACCTCGTTGTTCGATTTGATCATTTCTACCGAAGCGGATGCCTATGAGAAGCCAAATCCTAAAG
TATTATCGCCTTTATTTGAGCAATATAATGTAGATCCTCAGAAAGTAGCTATAGTAGGAGACACTGCTAATGATATGAAGACAGCAA
GTAATGCAAATTTAGGTATGGCAATAGGTGTATTAACAGGTATTGCAACAAAAGAAGAATTACATGAAGCTGATATTATTTTAAAT
AGTGCGGCAGATATTTTAGAAGCTTTAAATTAAAAAGAAAGACATAGTGTATATGTGAATTAATGTATTCGATAGAATTGATAAAA
TAATCGATAATATCTATGGAATATACATTGAATATAAACATATACGCTATGTCTTTAGTCTTTTATTGTGTATCTACTTGTCGATATG
TTTGAATAATTCGAGCAATTTTGTTTATCATAGGATTTAAAGATTCGGGGTCCTTATGGATATCATATTCATTAATATTGATACGTAC
AACTGGACATGCATTAAAGCTATTAATCCAATCGTCATAGCGTTTAAATAGCTTTTTCCAGTATTCAGGGTCTGTATTAATTTCCATT
TCGCGACCACGTTCAATAATACGATCAATGACCTCATCATAGTTACATTCTAAATAAATCATTATATCAGGTTTAGGAAAATAAGGT
GTCATGACCATGGCATTAAATAAGTCTGAATATGTTTTGAAATCTTCTTTACTCATTGTGCCTTCTTCTTCATGCATTTTTGCAAAAAT
ATCAACATCTTCATAAATTGATCGATCTTGGACAAAGCCACCACCATATTCAAACATACGCTTTTGTTCTTTAAAACGTTCAGCTAA
GAAGTAAATTTGCAAATGGAAACTCCATCGTTCAAAATCGCTGTAAAATTTATCTAAATATGGATTATGTTCGACATTTTCAAAAGA

CGTTTTAAAGTTTAATTTATCTGCAAGTGCTTGCGTTAGTGTTGATTTTCCAACACCAACTGTACCTGCAATGGTTATAATGGCATTT
TGTGGAATACCGTAATTATTCATTGGTAATATCTCCTATCATAGGTAATATAATATGTAATATATCTTCGTAATCTTGTTCATTTTTAA
GAAAATCAATAGAAGTTGTATCGATTAAAACTACATTTGAACCATTACTTTGTAAGGACTCATAATACTCACGATAATCTTTTTTTA
ACTTTAACAGATATTCATCTTCTATTTGATGCTCAAAACTACGGTTACGTTTAGCAATTCTAGATTTTAACACATCAAGGTCTGCATC
TAAAAAGATAATCATATTCGGCATAATCATATCTTCAGTTAAAATATCATAAATTTTACTGAATTTCTGAAATTCAACAGAACTCAA
AGTATTTTTAGCAAATATCTTATTTTTATGTATATGATAATCACTAACTACACCTTGATTTAGTTGTGTTACATCTTGAAATTGCTTAT
ATCTATTGCATAAAAAGAACATTTCAGTTTGAAAACTCCATTTAGAGATATCTTCATAAAAGTCTGATAAAAATGGATTTTCTGTGA
TGATTTCTTTTTCTTCATAAAAATCTAAAGTTTGACTTAATTTGTGTGCAAGTGAAGATTTACCTACGCCAATAGGACCTTCAATTGC
TATAAAAGGTTTGTTCATATTCACATCTCCAAATGTTAAAATAGACATTGAGTATTGTACCATAAGTGAGGTATCTGGACATAAATG
ACAAATGATATATATTTTATGACATTAGCGATTGAAGAAGCTAAAAAAGCAGCTCAACTAGGCGAAGTACCTATAGGTGCTATCAT
CACTAAAGATGATGAAGTTATCGCTAGAGCACATAATTTAAGAGAAACACTACAACAACCAACGGCGCATGCTGAACATATTGCAA
TTGAACGTGCAGCCAAAGTGTTAGGTAGTTGGCGTTTAGAAGGTTGCACATTATATGTAACCTTAGAACCATGTGTCATGTGCGCAG
GAACAATTGTAATGAGTCGCATTCCAAGAGTCGTCTATGGCGCAGATGATCCTAAAGGTGGTTGTAGTGGCAGTTTAATGAATTTAT
TGCAACAATCTAATTTTAATCATCGTGCAATTGTTGATAAAGGTGTACTTAAAGAAGCATGTAGCACATTATTAACAACATTTTTTA
AAAACTTAAGAGCCAATAAGAAATCCACCAATTAGACGATAGTACATACTAATTAAAATTTGTTAGAATTACATTATATGATAAATT
AATGACAATATAAATGTTAATAGAAAAGTATGTGCGCTGAGTATATATTCTTATTTAAGAACAAGATTTTAAATTTACGAAACGAGG
TAACATAATGATAAAACTAATAGCCACTGATATGGATGGCACGCTACTTAATGCAGCACATGAAATTTCTCAACCTAATATTGATGC
GATTAAATACGCTCAAGAACAAGGGATAACGGTTGTTATCGCGACAGGTCGAGCATTTTATGAAGCACAAGCACCAGTTGCTGACA
CAGATTTAACAGTACCATATATTTGTTTGAATGGTGCTGAAGTACGTGATGAAACTTTCAATGTAATGAGCACTTCACACCTTAATA
AATCGTTAGTACACAAAATTACAAATGTTTTAAAAGATGCAGGTATTTATTATCAAGTATACACGAGTCGTGCGATTTATACTGAAG
ATCCACAAAGAGATTTAGACATTTACATAGATATTGCTGAGCGTGCAGGTCAACATGCAAACGTTGAGCGTATTAAAAATGGTATT
CAAAGACGCATAGATAATGGTACGTTGAAAGTTGTTGATAATTATGATGCTATTGAAAACATACCTGGTGAATTAATTATGAAAAT
ATTAGCATTTGATGGAAATTTAGAAAAAATTGACAAAGCTAGTAAAATTTTAGCTGAATCTCCGAATTTAGCTATATCATCATCTTC
GAGAGGAAATATAGAAATAACGCATTCAGATGCACAAAAAGGTATTGCGCTAGAAACAATTGCCGAAAGATTAGGGATTGAAATG
AAAGAAGTCATGGCAATAGGTGACAATTTAAATGACTTATCAATGTTAGAGAAAGTTGGCTATCCAGTTGCGATGGAAAATGGTGC
AGAAGAAGTTAAAAAAATAGCGAAATATGTCACAGATACGAATGAAAATAGTGGTGTTGGAAAAGCTATTATGAAATTATTACGTG
AACAACAAGTTTAATAAAAAAAGAGGGGTCAAATATGAAAGGATTAATTATTATTGGCAGTGCACAAGTGAATTCACATACAAGTG
CACTAGCAAGATACTTAACTGAGCATTTTAAAACACATGATATTGAAGCGGAAATATTCGATTTAGCAGAAAAGCCGTTAAATCAA
TTAGATTTTTCAGGAACAACACCGTCTATTGATGAAATCAAACAAAATATGAAAGATTTAAAAGAGAAAGCAATGGCGGCGGACTT
TTTAATATTAGGAACGCCAAACTATCATGGTTCATATTCTGGAATATTGAAAAATGCATTAGATCATCTAAATATGGATTATTTTAA
AATGAAACCTGTAGGCTTAATAGGAAATAGTGGTGGTATTGTTAGTTCAGAGCCATTGTCACATTTAAGAGTAATCGTCAGAAGTTT
ACTAGGCATTGCTGTACCAACTCAAATAGCAACACATGATTCTGATTTTGCTAAAAATGAAGATGGTTCATATTACTTAAATGATAG
TGAATTCCAATTACGAGCAAGATTATTTGTCGATCAAATTGTATCTTTTGTGAATAATAGTCCATATGAACATTTAAAATAATATTAA
AAAATATGTAAATATACATTAAAAATAGGTATGTAGCTTAAGGTATTTCATTGAGAATGCTTTTAAGTCACATGCCTATTTTTTGTTC
TAATGCACCAATAGTAAGCCTTTGAGATTACTATTGTGAGCAATGTTATTAATTAAAATAAGATGTAATAGCGAATTGAAAACAAG
CTTAGATTAAAAGTGAGTTTTAAAAATATAGATAAATAATTTAAAGCAATTAAAGAAAAAAAGTATTAAAAATTGCAATTCTTAAA
CTGTGATTCATATTAATATTTGTAGCAAATAACATTGTAAAATAAAGAAAAATAATTAAAGTATTGCACTTTATTGAAATTTATATT
ACGATAGTAATGCAGAAATTTATATATGCAAAATATTATATTTATCAAATTTTGATATTTTAAAGGAGTATTATTAAATGAATAATA
AAAAGACAGCAACAAATAGAAAAGGCATGATACCAAATCGATTAAACAAATTTTCGATAAGAAAGTATTCTGTAGGTACTGCTTCA
ATTTTAGTAGGGACAACATTGATTTTTGGGTTAAGTGGTCATGAAGCTAAAGCGGCAGAACATACGAATGGAGAATTAAATCAATC
AAAAAATGAAACGACAGCCCCAAGTGAGAATAAAACAACTGAAAAAGTTGATAGTCGTCAACTAAAAGACAATACGCAAACTGCA
ACTGCAGATCAGCCTAAAGTGACAATGAGTGATAGTGCAACAGTTAAAGAAACTAGTAGTAACATGCAATCACCACAAAACGCTAC
AGCTAGTCAATCTACTACACAAACTAGCAATGTAACAACAAATGATAAATCATCAACTACATATAGTAATGAAACTGATAAAAGTA
ATTTAACACAAGCAAAAAACGTTTCAACTACACCTAAAACAACGACTATTAAACCAAGAACATTAAACCGTATGGCTGTGAACACA
GTAGCAGCACCACAACAAGGTACAAATGTTAATGATAAAGTTCATTTTTCAAATATTGATATTGCTATTGATAAAGGACATGTAAAT
TCAACAACTGGCAAAACTGAATTTTGGGCGACTTCTAGTGATGTTTTAAAATTAAAAGCGAATTACAAAATCGATGATTCTGTTAAA
GAGGGCGATACATTTACATTTAAATATGGACAGTATTTCCGTCCGGGTTCTGTAAGATTACCTTCACAAACTCAAAATTTATATAAT
GCCCAAGGTAATATTATTGCAAAAGGTATTTATGATAGTACAACAAACACAACAACATATACTTTTACGAACTATGTGGATCAATAT
ACAAATGTTAGCGGTAGCTTTGAACAAGTTGCATTTGCGAAACGTGAAAATGCAACAACTGATAAAACAGCTTATAAAATGGAAGT
AACTTTAGGTAATGATACATATAGCGAAGAAGTCATCGTCGATTATGGTAATAAAAAAGCACAACCGCTTATTTCAAGTACAAACT
ATATCAATAATGAAGATTTATCACGAAATATGACTGTTTATGTAAATCAACCTAAAAATACATATACAAAAGAAACATTAGTAACA
AATTTAACTGGTTATAAATTTAATCCAGATGCTAAAAACTTTAAAATTTATGAAGTAACTAACCAAAATCAGTTTGTGGATAGTTTC
ACCCCAGATACTTCAAAACTTACAGATGTCACTGATAAATTTAAAATTACATACAGTAATGATAATAAGACGGCGACAGTTGATTTA
TTGAATGGTCAATCTAGTAGTGATAAACAGTACATTATTCAACAAGTTGCTTATCCAGATAATAGTTCAACAGATAATGGGAAAATT
GATTATACTTTAGAAACACAAAATGGAAAAAGTAGTTGGTCAAACAGTTATTCAAATGTGAATGGTTCATCAACTGCAAATGGCGA
CCAAAAGAAATATAATCTAGGTGACTATGTATGGGAAGATACAAATAAAGATGGTAAACAAGATGCCAATGAAAAAGGGATTAAA
GGTGTTTATGTCATTCTTAAAGATAGTAACGGTAAAGAATTAGATCGTACGACAACAGATGAAAATGGTAAATATCAGTTCACTGG
TTTAAGCAATGGAACTTATAGTGTAGAGTTTTCAACACCAGCCGGTTATACACCGACAACTGCAAATGCAGGAACTGATGATGCTGT
TGATTCAGATGGTTTAACAACAACAGGTGTCATTAAAGACGCTGACAACATGACATTAGATAGTGGATTCTATAAAACACCAAAAT
ATAGTTTAGGTGATTATGTTTGGTACGACAGTAATAAAGATGGTAAACAAGATTCGACTGAAAAAGGAATTAAAGGTGTTAAAGTT
ACTTTGCAAAACGAAAAAGGCGAAGTAATTGGTACAACTGAAACAGATGAAAATGGTAAATATCGTTTCGATAATTTAGATAGCGG
TA

>017-contig_194_RC

ATTTCACACTTGATAACGGATACTTCGAAGAAGATACATCAGATAGCGACTCAGATTCAGATAGCGATTCAGATTCAGATAGCGAC
TCAGACTCAGACAGCGACTCAGATTCAGACAGCGACTCAGACTCGGATAGCGATTCAGATTCAGACAGTGATTCAGATTCAGACAG
CGATTCGGATTCAGACAGCGACTCAGACTCGGATAGCGACTCAGACTCAGATAGCGACTCAGATTCGGATAGCGACTCAGACTCAG
ACAGCGACTCAGATTCAGACAGCGACTCAGACACAGATAGCGACTCAGACTCAGACAGCGACTCAGACAGTGATTCAGACTCAGAT
AGCGATTCAGATTCAGACAGCGACTCAGACTCAGATAGTGACTCAGATTCAGATAGTGACTCAGACTCAGACAGTGATTCAGACTC
AGACAGTGATTCAGATTCAGATAGCGACTCAGACTCAGATAGCGACTCAGATTCAGACAGCGATTCAGACTCAGATAGTGACTCAG

ATTCGGACAGCGATTCAGACTCAGATAGCGACTCAGATTCAGACAGTGATTCAGACTCAGATGCAGGTAAGCACACACCTGTTAAA
CCAATGAGTACTACTAAAGACCATCACAATAAAGCAAAAGCATTACCAGAAACAGGTAATGAAAATAGCGGCTCAAATAACGCAA
CGTTATTTGGCGGATTATTCGCAGCATTAGGATCATTATTGTTATTCGGTCGTCGTAAAAAACAAAATAAATAATACAGCTTATACC
AGGTCCGTGAGGCCTGGTTTTTTATTTCAAAATTTATGAAATGTATCTTATGTTTGAGATTAAATAGTGCAATTTTTTAAAAAATATA
ACATAAATCAACATAAATTTAATTCATCTTTACATTAAAAGTACAAACGAATAAGAAAAGCAATTAAACACAGTATCAAACCATTT
AAAACAATTGATATAAAAGACAAACGAATAATTAAAAATTACTAGAATTAATTTATTCAAACCAAAGTGTAAAATAACAGTCTTAG
ATAAATAAATTTATTTAAAGTATTGTGCTTTATCTAAAAATGTATTACGATGGGAATACAAATTTTTATATGTAAAAATATTAATTTT
TTGCAAATATTGATATTTTAAAGGAGATTTATATGATTAACAGGGATAATAAAAAGGCAATAACAAAAAAGGGTATGATTTCAAAT
CGCTTAAACAAATTTTCGATTAGAAAGTATACTGTAGGAACTGCATCGATTTTAGTAGGTACGACATTGATTTTTGGTCTAGGGAAC
CAAGAAGCTAAAGCTGCTGAAAACACTAGTACAGAAAATGCAAAACAAGATGATGCAACGACTAGTGATAATAAAGAAGTAGTGT
CGGAAACTGAAAATAATTCGACAACAGAAAATAATTCAACAAATCCAATTAAGAAAGAAACAAATACTGATTCACAACCAGAAGC
TAAAAAAGAATCAACTTCATCAAGTACTCAAAAACAGCAAAATAACGTTACAGCTACAACTGAAACTAAGCCTCAAAACATTGAAA
AAGAAAATGTTAAACCTTCAACTGATAAAACTGCGACAGAAGATACATCTGTTATTTTAGAAGAGAAGAAAGCACCAAATAATACA
AATAACGATGTAACTACAAAACCATCTACAAGTGAAATTCAAACAAAACCAACTACACCTCAAGAATCTACAAATATTGAAAATTC
ACAATCGCAACCAACGCCTTCAAAAGTAGACAATCAAGTTACAGATGCAACTAATCCAAAACAAACGGTAAATGTATCAAAAGAA
GATCTTAAAAATGATCCTGAAAAATTAAAAGAATTAGTTAGAAGTGAAAATAACACTAATCATTCAACTAAACCAGTTGCTACAGC
ACCAACAAGTGTTGCACCAAAACGATTAAATGCGAAAATGCGTTTTGCAGTTGCACAACCAGCAGCAGTTGCGTCAAATAATGTAA
ATGATTTAATTACAGTGACGAAACAGACGATCAAAGTTGGCGATGGTAAAGATAATGTGGCAGCAGCGCATGACGGTAAAGATATT
GAATATGATACAGAGTTTACAATTGACAATAAAGTCAAAAAAGGCGATACAATGACGATTAATTATGATAAGAATGTAATTCCTTC
GGATTTAACAGATAAAAATGATCCTATCGATATTACTGATCCATCAGGAGAGGTTATTGCCAAAGGAACATTTGATAAAGCGACTA
AGCAAATCACATATACATTTACAGATTATGTAGATAAATATGAAGATATAAAAGCACGTTTAACTTTATACTCATATATTGATAAGC
AAGCAGTACCTAATGAAACTAGTTTGAATTTAACGTTTGCAACAGCAGGTAAAGAAACTAGCCAAAACGTTTCTGTTGATTATCAA
GACCCAATGGTTCATGGTGATTCAAACATTCAATCTATCTTTACAAAGTTAGATGAAAACAAACAAACTATTGAACAACAAATTTAT
GTTAATCCTTTGAAAAAAACAGCAACTAACACTAAAGTTGATATAGCTGGTAGTCAAGTAGATGATTATGGAAATATTAAACTAGG
AAATGGTAGTACCATTATTGACCAAAATACAGAAATAAAAGTTTATAAAGTTAACCCTAATCAACAATTGCCTCAAAGTAATAGAA
TCTATGATTTTAGTCAATACGAAGATGTAACAAGTCAATTTGATAATAAAAAATCATTTAGTAATAATGTAGCAACATTGGATTTTG
GTGATATTAATTCAGCCTATATTATCAAAGTTGTTAGTAAATATACACCTACATCAGATGGCGAACTAGATATTGCTCAAGGTACTA
GTATGAGAACAACTGATAAATATGGTTATTATAATTATGCAGGATATTCAAACTTCATCGTAACTTCTAATGACTCTGGCGGTGGTG
ACGGTACTGTTAAACCTGAAGAAAAGTTATACAAAATTGGTGACTATGTATGGGAAGACGTTGATAAAGACGGTGTCCAAGGTACA
GATTCGAAAGAAAAGCCAATGGCAAACGTTTTAGTTACATTAACTTACCCAGACGGTACTACAAAATCAGTAAGAACAGATGCTAA
CGGTCATTATGAATTCGGTGGTTTGAAAGACGGAGAAACTTATACAGTTAAATTCGAAACGCCAGCTGGATATCTTCCAACAAAAG
TAAATGGAACAACTGATGGTGAAAAAGACTCAAATGGTAGTTCTGTAACTGTTAAAATTAATGGTAAAGATGATATGTCTTTAGAC
ACTGGTTTTTATAAAGAACCTAAATATAATCTTGGTGACTATGTATGGGAAGATACAAATAAAGATGGTATCCAAGATGCTAATGA
ACCTGGTATCAAAGATGTTAAGGTTACATTAAAAGATAGTACTGGAAAAGTTATTGGTACAACTACTACTGATGCCTCGGGTAAAT
ATAAATTTACAGATTTAGATAATGGTAACTATACAGTAGAATTTGAAACACCAGCAGGTTACACGCCAACGGTTAAAAATACTACA
GCTGATGATAAAGATTCTAATGGTTTAACAACAACAGGTGTCATTAAAGATGCAGATAATATGACATTAGACAGTGGTTTCTATAA
AACACCAAAATACAGTTTAGGTGATTATGTTTGGTAC

>018-contig_252

ACACCTGTTAAACCAATGAGTACTACTAAAGACCATCACAATAAAGCAAAAGCATTACCAGAAACAGGTAGTGAAAATAACGGCT
CAAATAACGCAACGTTATTTGGTGGATTATTTGCAGCATTAGGTTCATTATTGTTATTCGGTCGTCGCAAAAAACAAAACAAATAAT
ACAATATGACCCAGGTCCTTGTGGCCTGGTTTTTTTATAATTACACATGCAATAGATGTATTTTTCATATAAAATAAACAATAAAGA
TACGGAATAAAACTTATATGAGGCGATAATATGAATTACATTTTAGGAACAATTTTAGAAAGTAAAATTACAGGTGTAGAAAAAGC
GCAAATAAATAGATTGAAGTTGTTCAAACAACACGGCATATCTTCAAAATGTGTATATGTTAAATGGAATCCTTATTCATACACATA
TGCGAAGCAACATCAGATTGAAAATGATGTATTTACAATGTATGACTATTTTCAAAAAGCAATCAATTATAAAAAGACAAAGCAAG
TTAACTGGATACAGTATTGGGAAAAGTCATGTAGGTACACATTGAAATTTGTGGAAAATTCAAATGATGTCAGAATATATGATGAA
GAGCAATTTATAATGTATGCTCATTTTTTAGATAAACAGTATCATCATTTAAACTATGTGAATTATTTTGATCATAAAAGAAGAAAA
GTAAAACGCGAATTGTATGATGGAAGAGGCTTTTTAAGTTGTTCTCGAATTTTAGGTGAAGGACAACGGATTGTACTCGAAAATTAC
TATACACCTAATGGGGAAATCGTCATCCAAAAATATTTCGACGATATAAAAGGGAAAAACACGCTCACAAAGGTTATCTTAAATGA
AGATCAGCATCAACAATTTTTTGATACAGAAGATGAATTAGTTCAATATTTTCTCCATCAATTATGTAAAAATAATGATCAAATCAT
ATTAGATCGTCCTCATGAATTAGGAAATGTTATAGCGGGATTAAATCAAAGTATTCCAGTTGTTGTTGTGCTCCATAGTACACATTT
ATTCGGTACCGGTAATGGTATAAAAAGTTTTTATAAAACAGTATTTAATAATTTAACACGTTATAAAGCGATTGTTGTATCAACAGA
AAAGCAATGCCAAGATATTTCACAATATATTGAAAATAAAATACCAGTTATCAATATTCCGGTTGGCTACGTGGCAAATTTAAAGTA
TCAATTTGACATCAATCAAAAGGAGAAAAATCATATCATATCAATTGCTCGCCTCGTTGAAAATAAACAAATTAAACATCAAATTG
AAGTGATCAAGCAATTAGTAACAAAACATCCCAATATTCAATTGAATATTTATGGACATGGAAATGGTTTGTCAGAATATCGACAA
CTTGTAGAAGATTATCATTTATCGGAACATGTTAAATTTCATGGTTTTAAGACGCATATTAATGAAGAGATTGCTAAAGCAGAACTG
ATGTTATCGACAAGTAAAATGGAAGGTTTTGGCTTAGCAATTTTAGAGTCGCTTTCAGTAGGTACACCAGTGATCAGTTATGATGTA
GATTATGGTCCATCAGAACTGATTCAAGATGGATTTAATGGCTATTTAGTACCTCAAGGTGACATCAATCAAATGGTTGAAAAGGTC
GACCAATTACTAAATAATACTCAAAAATTGCAACAGTTTTCAATTAATAGCATAGAATCTGCACAACAGTACAATGCAACTACTATC
AGTACAAAGTGGCAAAATATTTTAAACTAAGTCAAAAGAAAAGAGCATTTTCCTAGAATCATCTATTTAACAAACCTCGTTCATACA
AACTTACATGTTTAGTTAAATGACATAGGAAAACGCTTTTTTTTATAAAAATAAAGGTGAAGTACATTTATATAAGACGAACAAATT
GGTCCCATTGTTTAATTAACGACGCTTTACTATATTGTTGTGCTTTTGCCAAACTACCTTTTGACAGTCGTTGCTGTACTTCAGGATG
ATCAATCACATATTTTACTTTATCAAATAGGGCATCTTCATCATTTTTAGTAATTAAATAACCATTGAAATCTGAAGTAATCAGTTCG
TTAGGTCCATATTTAATATCATAACTAATAACTGGAACACCATGTGCTAAAGATTCAAGTAGCGCTAAAGAGAAACCTTCCATGTTA
CTTGTTATTAAACTCAAATAGGCATCGCTATATTCTTGGTCTAGATTGCTTAAAAAGCCGCGTAAGTAAACATGATTTTCCAATCCAT
ATTTTTGTATCAATTCATTTAATTTTTTACTTTCAGAACCAAAACCATACATATGAAGCTCTATTTTTGGGACATACGATACTAAGCG
TTTAATTAATTCAATTTGTTGATGTAATTGTTTTTCAGGTGAATAACGAGCAACGGAAATTAATTTAACACTGCGCTGATCTAATGTT
TGGACTGGTGTATCAATTGTTTCACTATAGCCGACAGGAATATTAACAACTGGAATAGTATGGTTAATACGTTTTTCAACATCTAAT
TTTTGCTGCTCAGTAGAAACGATAATTGCACGATATCGAGATAAATTTTCAAACATCGCTTTATATACATTTTTAAATGGCGATGAA
TCTAATGCATCAATATTTTTAATGTGTGTACTGTGAAGCACAGCTACTACTGGGATTGACTCAGGCGTTAAGTTGAAAATAGGTGCT

GTGTACACATTACGATCACTGAAAAATAAATCCCCATGTTGATATAGTTGTTTAATGAAAAATGCGCCTAATTCCGTTTCATTATTA
AAGAAATATTGTTTGTTAGCATAGTAAACAATAATTTTTTGTACTTCTGGTTTGCCATCCTTGTAAGAAAAATACTTTTCTAATTTTG
TGTCACCTTCTGGATTATAGAAAAATTCACATAATGTTTGTTGTTTATCAACAAGAATCCTACTACAACTTAAAAAGCCACGCACAT
CATAAAAATCACGTTTTACTTTTCGTCTTTGACTATCAAAATGATTCACATAATCTAATATACGATATTTAGGATCTTGAAAATGGGC
ATACATTAAGAAACGCTCTTGATCATATATTCTAAAGTCATGACTATTTTCAACATGTTTAAAGTATAATGACATTCATCAGTCCAA
TACGACAACCAGTCAAATGGTTCATTGCGTTCTAAATATGTTGCTTCTTGGAAGAAATCATACATATTAATATAGTCAGAACTAGTA
ATATAATTTTGGGCATTTCTATATAAATATCTATTCCATGACAGAAATACACATTGCGCTGGTCTTCCCATTTCTTTAAATAAATTTA
AACGATTAATAATTGCTTTCTCTATCCCAGTTAAATTAACACCTAAACTATTACCTACAAAATAATTCATTTACAACACCACTTATAT
CTATTTTTTATAATTATATCACATAATATTTAATTACTTCTTTTAACTGGAAGATGTGTTTATTTATAAAACAACAAATTTTGATATTT
ATAATGATAGTAGTTATTCAATCAACTACGACCAATATATCATTGTAGAGCTTAGGATATTGATTTATGACTCAAGCACATCAAATG
AGAAGATTTATAAAAGAGATATACAACTCTAGAAGGTATAATAAAAACGCGCAACTAATGTTACGCGTTTGAATTAATCATATGAT
ATTATTTGCGATACTTTAATTTAGCGAAAGCATCATGTTGATGGATAGACTCTTCATTACGACATTCGATATCGAAACCGTCTAACC
AATCAAATTCAACTAAGTCCGCGGCAATTAAACGAATTAAGTCTTCGACAAAACGTGGATTTTCATATGCACGCTCTGTCACACGTT
TTTCATCAGGACGTTTTAAAATAGGGTATAGAATTGAACTTGCATTAGCTTCCATTGCATCTAAAATTTTATTTTTATAGTCATCAAC
TATGTCTTGATCTTTATTAATATATGTTTTAACAGTGACAACACCACGTTGGTTGTGCGCTGAATACTCACTTATTTCTTTTGAACAA
GGGCATAGCGTTGTGACAGTTGCTTCAATAGTAAGTTCTTTACGTGTAACTTTATCACCGTCAATTGCTAATCCATAAGTGACATCG
GCATTGCCAACTGCTTTAATATTTGTGGTTGGACTATAGCGATCAAAGAACCATTTCCCAGAAACATCAACGCCTGCCGCATTTTGT
TTCATATTCGTTTGTAAAGTGCGTAACACCTGATAAAGTGTATTAAATTCAAGTTCAATACCATTATCATAGTGCTTTTCAACACTTT
CAATTATACGGCTCATATTAATACCTTTTTCGTCTTTTGTTAAACTTGTTGAAAAACTAAATGTGCCAGCTGTTTGATACTGGTCAAC
AAGTACAGGGTACACTAAGTTTTTAATACCAACTTCTTCTATTTCAAATAAAAAATCTTTATGTGTACTTTGTAAATCTGTCATTTCG
TTCTTAGTAGTAGGTTTCGTGCCTTCAATAGGATCTACGGAACCAAAGTGTTTCCAACGACCTTCTCGTGTCGATAAATCAAATTCA
GTCATTTTTTTCCTCCGTTAAGATTTAAAGTGATATGTCCAATATGGTTCGACTGTTAAAAAGCTGTGTTGTTTACCATCGATTTCAG
GACTTGCTAATTGTTTTAAAAATGGACCTGTTTGAGAAGCATGTGCTTCAAATGCCTTAATTTTAAGTTCTTTAAAATCTGTAATATC
ATTTTGAATATCAGGTTCTCCAAGAGCTTCGGTTGCATCATTACTGAACGCAACTAAAGTTAAACGAGGGCGTTCTTCTTTAGGCAT
GCGTTCAACCGTTCGAATTACAGCGTCTGCTGTTGCTTCGTGATCAGGATGTACTGCATATCCAGGATAAAATGAAATAATCAATGA
TGGATTTGTATCATCGATTAAAGATTTAATCATACCATCTATATGTTCATAGGGTTCAAATTCGACAGTTTTGTCACGTAAACCCATT
TTTCTTAAATCAGTAATACCGATAACTTTACAAGCTTCTTCTAGTTCACGCTCACGAATACTTGGTAATGATTCGCGTGTTGCAAATG
GGGGATTACCTAAATTTCTGCCCATTTGTCCTAGGGTTAAACATGCATATGTTACAGGTATGCCTTTTTGGATATAATTTGCTAATGT
GCCTGCAGATGAGAAGGTTTCATCATCAGGATGTGGAAATATTACTAATACATGTCTTTCGTCAGTCATGTTGATGCCTCCTCTATA
AATTAAATGGTCGCTCACTAATTTGAAGTGCTGCAGCGAGTTGACCTTCGTAATTAAAACCTGCAATTAAAAATTCATCATGTTCAT
TGACCTCAAAATGCGTTAGACCTTGTACATAAACCCAACCACCATTTGATAGTTTAAGACCAATGCGATAAGGTTCTTTATTACCAC
CTTTTAGTTGTGCATGCGTATATGTTATTTGTATGTTTCTTAAAAAAGTACCAGCATTAAAAACACGTTGATCGAAATGGTTCGCATA
GGCCCCATTTGTCGTTTCAACATGCAGATACACAGGTTTATGTTCAAAAGAAGCAAGTAAATCTATAACTTCTTGTTCTTTAATTGGT
TCCAACACGTTCACTCCTTACACTATCAATGTGTTTATCTTTCTATTTTACTAAAAACTATTCAATAATTGTATACGATTGCTCAATTA
TTTATAAATTAATTTTCATGAAGGGTAATTACTCAGGATTACGCAATCATACAGCATTAGTTTTTTACTTTTAAAAATCAAAAATTTG
TTGGAATTTGAAAAGTGTTAAACATTAAAAATGATGCTATATTAATGGTGTATGAATGAATTCATAAGTTTTTAAAATGTATTAAAT
TTGTGGAGGCATGTAAACAATGAAAGTATTAAACTTAGGATCGAAAAAACAAGCATCATTCTATGTTGCATGTGAGTTATATAAAG
AGATGGCATTTAATCAGCACTGTAAACTTGGTTTAGCAACTGGTGGTACAATGACAGATTTGTATGAACAACTTGTTAAGTTATTAA
ATAAAAATCAGTTAAACGTAGACAATGTATCCACGTTTAATTTAGACGAATATGTAGGTTTAACTGCATCACATCCGCAAAGTTATC
ACTATTATATGGATGACATGCTTTTCAAACAATATCCTTATTTTAATAGAAAGAACATTCATATTCCAAATGGAGATGCCGATGATA
TGAATGCGGAAGCGTCAAAATATAATGACGTTTTAGAACAACAAGGTCAACGTGATATTCAAATTTTAGGTATTGGAGAAAATGGT
CATATTGGATTTAATGAACCTGGTACGCCGTTTGATAGCGTTACTCATATCGTTGATTTGACTGAAAGTACTATTAAGGCTAATAGTC
GATATTTTGAAAACGAAGATGATGTTCCAAAGCAAGCCATTTCGATGGGACTTGCTAATATTCTTCAAGCCAAACGTATCATTTTAC
TCGCATTTGGTGAAAAGAAACGTGCTGCTATTACACATTTATTAAATCAGGAAATTTCTGTTGATGTTCCAGCCACATTACTTCACA
AACACCCGAATGTTGAGATATATTTAGACGACGAAGCTTGCCCGAAAAATGTTGCGAAAATTCATGTCGATGAAATGGATTGATTG
CAATGTTTAATTAAGAAATGCCTCGGGAAAGGTTCCAATAGAAAGATAAAAAGCATTGGAAGGATGATTTTTAGTGGAATTACAAT
TAGCAATTGATTTATTAAACAAAGAAGACGCGGCTGAGTTAGCAAATAAAGTAAAAGATTATGTAGATATCGTAGAAATCGGTACG
CCAATCATTTACAACGAAGGTTTACCAGCAGTTAAACATATGGCAGACAACATTAGTAATGTAAAAGTATTAGCAGACATGAAAAT
TATGGATGCAGCTGATTATGAAGTTAGCCAAGCAATTAAATTTGGCGCGGATGTAATTACAATACTAGGTGTTGCAGAAGATGCAT
CAATTAAAGCAGCTATTGAAGAAGCTCATAAAAATAATAAACAATTACTAGTTGATATGATTGCTGTTCAAGATTTAGAAAAACGT
GCAAAAGAACTAGATGAAATGGGTGCGGATTATATTGCAGTACACACTGGTTATGATTTACAAGCAGAAGGGCAATCACCATTAGA
AAGTTTAAGAACCGTTAAATCTGTTATTAAAAATTCTAAAGTTGCAGTAGCAGGTGGAATTAAACCAGATACAATTAAAGATATTGT
CGCTGAAAGTCCTGATCTTGTTATTGTTGGTGGCGGAATCGCAAATGCAGATGATCCAGTAGAAGCTGCAAAACAATGTCGCGCTG
CAATCGAAGGTAAGTAATATGGCTAAATTTAGTGACTATCAATTAATTCTAGATGAATTAAAGATGACTTTGTCACATGTTGAAGCG
GATGAGTTTTCAACTTTTGCATCCAAAATACTACATGCTGAACATATATTTGTAGCTGGCAAAGGACGTTCAGGATTCGTGGCGAAT
AGTTTTGCAATGCGCTTAAATCAGCTCGGCAAACAGGCACATGTTGTTGGAGAATCAACGACACCTGCGATTAAGTCGAATGATGT
ATTTGTAATTATCTCTGGTTCAGGTTCCACGGAACATTTAAGATTATTAGCAGACAAAGCAAAATCAGTAGGTGCTGACATCGTATT
AATTACTACAAATAAAGATTCTGCAATAGGCAATCTAGCTGGGACGAACATCGTTTTGCCTGCAGGTACAAAATATGATGAACAAG
GCTCGGCACAACCATTAGGAAGTTTGTTTGAACAAGCATCTCAATTATTTTTAGATAGTGTTGTAATGGGATTGATGACTGAAATGA
ATGTTACGGAACAAACGATGCAACAAAATCATGCTAATTTAGAATAAAATAAAGATAGTCGATAATATGATGCCTAGGCAGAAATA
TTATCGATTATTTTTTTATTTAAATAATAAATTATAGTATAATATAAATAATAAACGAATAGGGGTGTTAATATTGAAGTTTGACAAT
TATATTTTTGATTTTGATGGTACGTTGGCAGACACGAAAAAATGTGGTGAAGTAGCAACACAAAGTGCATTTAAAGCATGTGGCTTA
ACGGAACCATCATCTAAAGAAATAACGCATTATATGGGAATACCTATTGAAGAATCATTTTTAAAATTAGCAGACCGACCATTAGA
TGAAGTAGCATTAGCAAAGTTAATCGATACATTTAGACATACATATCAATCTATTGAAAAGGACTATATTTATGAATTTGCGGGTAT
AACTGAAGCCATTACAAGTTTGTATAACCAAGGGAAAAAACTTTTCGTGGTGTCTAGTAAGAAGAGTGATGTATTAGAAAGAAATT
TATCGGCTATTGGATTAAATCACTTGATTACCGAAGCTGTTGGATCCGATCAAGTAAGTGCATATAAACCAAATCCTGAAGGCATAC
ACACAATTGTGCAACGCTACAATTTAAATAGCCAACAAACGGTGTATATTGGTGATTCAACGTTTGATGTTGAGATGGCACAACGTG
CTGGTATGCAATCTGCAGCTGTCACTTGGGGTGCACATGATGCAAGGTCATTACTTCATTCAAATCCGGATTTTATTATTAATGATCC
ATCAGAAATTAATACCGTATTATAAAACTTGTTAAAACAGAGAATACCATGGTTAAAATGCATATTCATAAATATTAGATTATACTT
AGAAATATTTCGCTTTAGATTAGGAATTTAAAATAAATATTTATTAAACATTATGAATTTTTAAAGAGTAATGTCTGACTCGTTGATA
ATTTATTTTTGTAAAAATAAATTAAAGTAATGACAAAGTTATTGAAGTAAATTGAGTATAAACATTTAAATACGATGTCGAAAATGG

CGATAGCATATCACTTACATGAAGTTGTGTGCTATCGCTATTTTTAGTTATAATTCCAAAAAGTTAATCGTTCGATGATTTAAGAATT
ATTATTGTTTAATTCAAATGTATGAGGGTATAAAATCATTGAATTTAATTCGATAAAGCGAAATTTTTGAACAAACATACTTTTGTAT
TTATATAAAAGTTTAAATTCTTATAAATTTGACAAAACTAATTAACTCCGTATAATTATGAAACATACAAGAGGGAGTGTATGAATT
CATGAATTTTAATAAAGAGAATATTAACATGGTGGATGCAAAGAAAGCTAAAAAAACCGTTGTTGCAACCGGTATCGGTAATGCAA
TGGAATGGTTCGATTTTGGTGTCTATGCATATACAACTGCGTACATTGGAGCGAACTTCTTCTCTCCAGTAGAGAATGCAGACATTC
GACAAATGTTGACTTTCGCAGCATTAGCCATTGCGTTTTTATTAAGACCAATTGGTGGTGTCGTATTTGGTATTATTGGTGACAAATA
TGGACGTAAAGTTGTATTAACATCTACAATTATTTTAATGGCATTTTCAACATTAACCATTGGATTATTGCCAAGCTATGATCAAATT
GGACTTTGGGCACCAATACTATTATTGCTTGCAAGAGTACTACAAGGGTTTTCAACAGGTGGAGAGTATGCGGGGGCAATGACATA
TGTTGCCGAATCATCTCCAGATAAGCGTCGTAACTCATTAGGTAGTGGACTAGAAATTGGGACATTATCAGGTTACATAGCTGCTTC
AATTATGATTGCTGTATTAACATTCTTTTTAACAGATGAACAAATGGCATCATTTGGTTGGAGAATCCCATTCTTACTCGGTTTATTC
CTAGGATTATTTGGCTTATATTTACGTCGTAAGCTGGAAGAATCACCAGTTTTCGAAAATGATGTTGCAACACAACCAGAAAGAGAT
AACATTAACTTTTTACAAATCATCAGATTTTATTACAAAGATATATTTGTATGTTTTGTAGCTGTTGTATTCTTCAATGTTACAAACTA
TATGGTAACTGCATATTTACCAACCTATTTAGAACAAGTTATTAAATTAGATGCAACGACAACAAGTGTATTAATTACTTGTGTCAT
GGCAATAATGATTCCATTAGCATTAATGTTTGGTAAGTTAGCGGATAAAATAGGTGAAAAGAAAGTATTTCTAATTGGTACTGGTGG
GCTAACATTATTTAGTATCATCGCATTTATGTTATTACATTCACAATCATTTGTTGTAATAGTAATCGGTATATTTATATTAGGATTTT
TCTTATCAACTTACGAAGCGACAATGCCAGGGTCGTTACCAACGATGTTTTACAGTCATATAAGATATCGAACTTTATCAGTAACAT
TTAATATCTCTGTTTCGATATTTGGTGGTACGACGCCATTAGTTGCAACATGGTTAGTTACGAAAACTGGAGATCCATTAGCACCTG
CGTATTATTTAACAGCAATCAGTGTTATTGGCTTTTTAGTTATTACATTCTTACATTTAAGTACAGCAGGAAAATCTCTAAAAGGTTC
GTATCCAAATGTAGATAACGAGCAAGATAGAGCTTATTATGCAGAACATCCAAAAGAAGCATTATGGTGGGTTAAAGAACGTAAG
AATTAGAGATTTTAATAAAAAGTATAAATCAATCGTATATAAGCACTTTAAAGCTAGTAGGTTCTGCTAACTTTAAAGTGCTTTTTA
AATTGAGAACTGTAATTAGCCGTAATAAAGTTTTTGTATATACATAAACCCCCACTGCAATGATTATCGCAATGGGGGAAAGAGGG
GACTTAAAGCATATGTTTAGCTTTGAATACTTAAAATTCTCTTGCTATTGAAATGTTAGGATGTAAATATGTCTTAGAGTATTTTGTC
CAACGCAATTAATATTGAGACTCTAACCTTCAATATTATTATAGAGAACACAAACTTAAATAGATTGGGTGACTTATTTGTGTCAGT
TATTGCGATTGCGATAACTTCTTTTCTCTATATACATATAGTAACGTCTTATCTAATAAAAAACATGGTACTACAGTATCAAATTTAT
CTAGGGCTTAAGTTTGATTTTTATAATAGGCAGGTTTACCTGATAAAAATACTTATTCATTATATAATGTTAACAATATGTATTTTAA
AGTTTACATTGAGTGAGGGATATTGATGAACGTAATTTTAGAACAGTTGAAAACACATACTCAAAATAAACCTAATGACATAGCAT
TACATATAGATGATGAAACAATTACATATAGTCAACTAAATGCCCGCATCACTAGCGCAGTTGAATCTTTGCAGAAATATTCACTTA
ACCCTGTCGTTGCTATTAATATGAAATCATCGGTTCAAAGTATTATTTGTTATTTAGCTTTGCATCGTGTACATAAAGTGCCTATGAT
GATAGAAGGTAAATGGCAAAGTACTATACATCGTCAATTGATTGAAAAATATGGTATTAAAGATGTAATTGAAGATACAGGTCTCA
TGCAGAGTATAGACTCACCGATGTTTATTGATGCAACGCAATTACAGCACTACCCCAATTTATTACATATTGGTTTTACTTCAGGGA
CAACTGGACTGCCAAAAGCATATTATCGTGATGAAGATTCATGGTTGGCTTCTTTTGAAGTTAATGAAATGTTGATGTTAAAAAATG
AAAATGCAATAGCAGCCCCTGGACCACTATCGCACTCGTTAACATTATATGCGTTATTGTTTGCTTTAAGTTCCGGTCGTACTTTTAT
AGGACAGACCACTTTTCATCCTGAAAAGTTACTTAATCAATGTCGTAAAATATCATCATACAAAGTTGCTATGTTTCTTGTTCCAACG
ATGATTAAATCATTATTGTTAGTTTACAACAATGAACATACAATCCAATCATTTTTTAGCAGTGGAGATAAGCTGCATTCTTCTATTT
TTAAAAAGATAAAAAATCAAGCAAATGACATAAATTTGATTGAATTTTTTGGTACATCGGAAACCAGTTTTATCAGCTATAACTTGA
ATCAGCAAGCACCAGTTGAATCAGTAGGTGTGCTATTTCCAAATGTGGAATTGAAAACAACGAATCACGATCACAATGGTATAGGA
ACTATTTGTATAAAAAGTAATATGATGTTTAGTGGCTATGTAAGTGAACAATGTATAAATAATGATGAATGGTTTGTTACTAATGAT
AATGGCTATGTAAAAGAGCAGTATTTATATTTAACGGGACGTCAACAGGATATGTTAATTATTGGTGGTCAAAATATATATCCAGCA
CATGTTGAACGCCTTTTAACGCAATCTTCGAGCATTGATGAAGCAATTATCATCGGTATTCCAAATGAGCGTTTTGGTCAAATAGGC
GTATTGCTTTATTCTGGTGATGTGACACTTACACATAAAAATGTAAAACAATTTTTAAAAAAGAAAGTGAAACGCTATGAAATTCCA
TCGATGATTCATCATGTAGAAAAGATGTATTACACTGCAAGTGGTAAAATTGCTAGAGAAAAAATGATGTCGATGTATTTGAGAGG
TGAATTATAATATGAATCAAGCAGTCATAGTTGCAGCTAAACGAACTGCATTTGGGAAATATGGTGGCACTTTAAAACATTTAGAG
CCAGAACAATTGCTTAAACCTTTATTCCAACATTTTAAAGAGAAGTATCCAGAGGTAATATCTAAAATAGATGATGTAGTTTTAGGT
AATGTTGTTGGGAATGGTGGCAATATTGCAAGAAAAGCATTGCTTGAAGCGGGGCTTAAAGATTCAATACCTGGCGTCACAATCGA
TCGGCAATGTGGGTCTGGACTTGAAAGTGTTCAATATGCATGTCGCATGATCCAAGCCGGAGCTGGCAAGGTATATATTGCAGGTG
GTGTTGAAAGTACAAGTCGAGCACCTTGGAAAATCAAACGACCGCATTCTGTGTACGAAACAGCATTACCTGAGTTTTATGAGCGT
GCATCATTTGCACCTGAAATGAGCGACCCATCAATGATTCAAGGTGCTGAAAATGTGGCCAAGATGTATGATGTTTCAAGAGAATT
ACAAGATGAATTTGCTTATCGAAGTCATCAATTGACAGCGGAAAATGTAAAGAATGGAAATATTTCTCAGGAAATATTACCTATAA
CCGTTAAAGGAGAAATATTCAACACTGATGAAAGTCTAAAATCACATATTCCGAAAGATAACTTTGGCCGATTTAAGCCCGTGATC
AAAGGTGGGACCGTTACCGCTGCGAATAGTTGTATGAAAAATGATGGTGCAGTTTTATTGCTTATTATGGAAAAAGATATGGCATA
CGAATTAGGTTTCGAGCATGGTTTATTATTTAAAGATGGTGTTACGGTAGGTGTTGATTCTAATTTTCCTGGCATTGGTCCAGTACCA
GCCATTTCCAACTTACTAAAAAGAAATCAATTAACGATAGAAAATATTGAAGTCATTGAAATTAACGAAGCGTTCAGTGCACAGGT
AGTTGCCTGCCAACAAGCTTTAAATATTTCAAATACGCAATTAAATATATGGGGTGGTGCATTAGCATCAGGTCATCCATACGGTGC
AAGCGGTGCCCAATTAGTGACTCGATTATTTTATATGTTTGACAAAGAGACTATGATTGCATCTATGGGGATAGGGGGAGGTCTAGG
AAATGCAGCATTATTTACTCGATTCTAACCAGCGATTAAATGTGTCATTTTCTAAGGATAGTGTGGCTGCATATTATCAGTGTTTTAA
CCAACCTTATAGAAAAGAAGTACCACCATTAATGTGTGCGTCATTATGGCCAAAATTTGATTTATTTAAAAAATATGCAAATAGCGA
ACTGATTTTAACAAAATCAGCAATTAATCAAACTCAAAAGATAGAAGTAGACACAATATATGTAGGGCATTTAGAAGATATTGAAT
GCCGACAGACTCGCAATATCACACGTTATACAATGGCTTTAACATTAACTAAAAATGATCAACATGTCATAACGGTTACACAAACTT
TTATTAAGGCGATGAAGTAGAGATGAAGTTTAATGAGATATGGATAAATGAATATTTGGCGCTCGTAAATGATGATAATCCAATAC
ATAATGAGATTGTGCCAGGACAATTAGTGAGTCAAATGATGCTGATGGCTATGTCATTAGAGACAAACCAGTGTCAAATTAACTAC
GTTAAACCTATTTTAATAAATGAAAATATCGAATTCATTGAACAACACGAACACGAAATTATAGCAATTAATGACGATGGAGAGAT
TAAAATAAAAATTTCTTTGAGCACAAAAAAATAACCGATATTAGCTGCATGAACGCATATTAATTAGGAGATGAAAGGACAGCTAA
TATCAGTTATGTATTGTTATTATTATTGGGAACAGAGATGAATATAGGTTACGTTTCTTTCTTTGCACGGGGATGCATTAATCTAAAA
TAATAATAACAACTATATCAATGTTTAATAAATTCTGGATTATTGGAACGATTAGTCAATTTAACTAACTTTCATATGATCTATATCG
TCTTGTAATAAAGAGAGCAATTTGAATATTTCAGTATCACTAAATGAATCGTCACATTTAATTGAAACATGCTGAAACGTTTTGGTT
ATAATTTCATAAACTGGTGCGCCTTCATGGTGATACTGTCGATAAATAATCATAACCTATATTACCTCCTTTGCTACTCTATGGTTAT
ATTATAAATAACATTTTTATGTGTGACATCAACCTTAAGTATCAACTTTTTATCAGACATAGAACGTATGATTTACTAAGACTATTTA
TGTATAAAAGTTCTAAATAAATATATATTTATAGAGTCGCCTGGCAGTCATTTGGGAAATATAACATATATGATTAGAGAGGCATCT
ATCGCAAAAGAATGATAATGATAGAGGTATTGAGCATATAGATGAGTTTAAGTTCATCTTGAAAATAAAGGGTTATTTAGTCATAG
ATGTAGATGTATAGGAAATATTTGTATGTATTGTTCGATATGTATGAAATTTTCAATAAAAGCTAATAACGCTTATATGTAACTTTCA
AATTTAAATTATATACAGAGCATGATGATTATAAAAAAATAACCACATCACATAAATTGAGTTCATACCCAATTTAAGTGGTGTGGA

TAATAATGTTGATTTATAGATGAACCGCCTAATCGTTAAACCTCTGTTACTTCAACATCGATATGTTCAATACGGTTGTATGCACCGT
GATCCACAGGACCAACAAAATCATTCATTTTCCAACCGTTTTTAATAGCAGAAGCGACGAAAGCTTTCGCGCTAATCACAGCTTCTT
TCGGTGACTTACCGTTAGCTAAATATGCAGTTGTTGCCGCAGCAAATGTACAACCAGCACCATGGTTATAACTTTGTTGGAACATGT
CTGTTGTTAGTTGATAAAATGTTTGACCATCATAGTATAAGTCATACGATTTATCTTGATCTAAAGCTTTGCCACCTTTAATGATGAC
ATGCTGTGCGCCTTTATCAAAGATAATTGTTGCAGCCTTTTTCATATCTTCAATTGAATTTAATTTACCTAATCCTGATAATTGACCC
GCTTCAAATAAGTTTGGTGTCACTACCGTTGCTTTAGGTAGTAAATATTTAATCATCGCCTCAGTATTTCCAGGATTAAGCACTTCAT
CTTCGCCTTTACAAACCATGACAGGATCTACTACAAAATATTGTGCATTAGATGCCTCATATACTTCTCCAGCACGTTTGATTATCTC
CTCAGTACCTAACATACCTGTTTTAATAGCATCAGGTCCGATTGATAAAGCCGTTTCAAGTTGTTTTTCAAATACATCCATTGGTAAT
GGTGTAACATCGTGTGACCATGTATCTTTATCCATAGTAACGATGGCAGTTAAAGCGACCATGCCATACGTATCTAATTCTTGGAAC
GTTTTCAAATCTGCTTGCATACCTGCGCCAGCACTTGTGTCAGAACCGGCAATTGTTAAAACTTTCTTTAAAGCCATTGAGCTTCACT
CCTACATAATAATATTGTATTCATCATATCATTTTTAACCTAATTGAAAAATATTAAGCATTCAATATTTGATGATTGTTGAAATGAA
TCATTCATACTATTGTAACTTTTGAAAATGTCATTCACTTTAGATAAGTGTGATATGTTAAAATATGTCCTGAGGTGAGATTGAATGG
AATGGTCGCAAATTTTTCATGACATAACAACGAAACATGACTTTAAAGCTATGTATGATTTTTTAGAAAAAGAATATTCGACTGCAA
TCGTATACCCTGATAGGGAAAATATATATCAAGCGTTTGATTTAACACCGTTTGAAAATATCAAAGTTGTTATATTAGGACAAGACC
CGTATCATGGTCCAAACCAAGCACATGGATTAGCATTTTCAGTGCAACCTAACGCAAAATTCCCTCCATCTTTACGTAATATGTATA
AAGAATTAGCAGATGATATTGGATGCGTTAGACAAACACCGCATTTACAAGATTGGGCAAGAGAAGGCGTCTTGTTATTGAATACA
GTTTTAACCGTAAGACAGGGTGAAGCAAATTCTCATCGTGATATTGGTTGGGAAACATTTACTGATGAAATTATTAAAGCAGTGTCT
GATTATAAAGAACATGTTGTCTTTATTTTGTGGGGGAAACCTGCACAGCAAAAAATAAAGCTTATCGATACATCTAAACATTGTATT
ATAAAATCAGTGCATCCTAGTCCACTGTCTGCATATAGAGGATTCTTTGGATCAAAACCGTATTCCAAAGCGAATGCCTATTTAGAG
TCAGTAGGAAAATCACCAATTAATTGGTGTGAAAGTGAGGCGTAGATGTTGAATAGAGAAACTTTAATAGCACGAATTGAGCAAGA
ATTAGTACAAGCAGAGCAGGCACAGCATGACCATGACTTTGAAAAACATATGTATGCCATACATATATTAACATCTTTATATGCTTC
AACATCAAATACACCACATATTGGTGAACAACAAATGAATCGTCGTATTGCTAACCATAATCAAATGCCACAATCACAAATAACGC
AGCCAACTCATCAAGTGACAGCTGCTGAAATTGAAGCGATGGGTGGTAAAGTAAATACGCATTCAGCACATCATCATAATAAGTCA
TATTCACAACCTTCAAACCAACAACAAAGATTAGCGACAGATGATGACATTGGCAATGGTGAATCCATATTTGATTTTTAAAAAGC
AACAATGAAACATAATTACTTAATAGCTTGTTAAGTATGTAGGTTAATAATCAAGACGCATATACTTATATTCGAGTGTTCGGATTT
AAACATTTATTAATACTGAATTATATAAGGAGAGGTAGCAATGAAATTATTTATTATTTTAGGTGCATTAAACGCGATGATGGCTGT
CGGAACAGGTGCATTTGGTGCGCATGGTTTACAAGGAAAAATAAGTGATCACTATTTATCAGTATGGGAAAAAGCAACGACGTATC
AAATGTACCATGGCTTAGCATTATTAATTATAGGTGTAATTAGTGGTACAACTTCAATCAATGTTAACTGGGCTGGCTGGTTAATAT
TTGCTGGTATTATTTTCTTTAGTGGATCATTATATATTTTAGTATTAACTCAAATTAAAGTTTTAGGTGCGATTACGCCAATTGGTGG
CGTATTGTTCATCATTGGATGGATAATGTTAATCATTGCGACATTCAAATTTGCTGGTTAAATTTTAAAACTTTAGATTACCTATGTA
ACTAAACATTAAATTTTTAATAAAAATAATCAAGAAAAAGAGTTACAAACTCATCTTTTGGGTATAGAATACCTTCGAGGTGAGTTT
TTATTTATGGAAAAAAAGAATAAGCAAATAGATAGAGGCGATTTAAAACAAAACCTATCTGAAAAGTTTGTATGGGCGATTGCATA
TGGTTCATGTATCGGATGGGGCGCATTCATCTTACCAGGAGACTGGATTAAGCAGTCAGGTCCGATTGCAGCATCAATTGGTATTGT
TATTGGTGCATTATTAATGATATTAATTGCGGTTAGTTATGGCGCATTAGTAGAGAGATTTCCAGTATCAGGGGGCGCGTTTGCCTTT
AGTTTCTTAAGTTTCGGCAGATATGTGAGTTTCTTCTCATCATGGTTTTTAACTTTTGGTTATGTCTGTGTCGTTGCTTTAAATGCGAC
CGCATTCAGTTTACTAGTTAAATTCTTATTGCCAGATGTCTTAAATAATGGGAAACTATACACCATTGCGGGCTGGGACGTTTATATT
ACGGAAATCATTATTGCGACCGTATTACTACTTGTATTCATGCTAGTAACGATTCGTGGCGCAAGTGTATCTGGATCATTACAATATT
ATTTCTGTGTGGCGATGGTAATCGTCGTATTATTGATGTTCTTTGGTTCATTCTTTGGTAATAATTTTGCACTTGAAAATTTACAACCG
TTAGCTGAACCTAGCAAAGGATGGTTAGTGTCTATTGTGGTTATTGTATCCGTGGCACCATGGGCATATGTTGGATTTGATAATATTC
CACAAACAGCAGAAGAGTTTAACTTTGCACCAAACAAGACATTTAAGCTTATCGTGTACAGTTTATTAGCAGCATCATTAACTTATG
TTGTCATGATTTTATACACTGGTTGGTTATCAACAAGTCATCAAAGTTTAAATGGGCAGTTGTGGTTAACAGGTGCTGTTACACAAA
CAGCATTTGGTTATATTGGATTAGGTGTATTAGCAATTGCAATTATGATGGGTATATTTACTGGTTTAAATGGATTCTTGATGAGTTC
AAGTCGCTTGTTATTTTCTATGGGACGTTCAGGTATTATGCCAACAATGTTTAGTAAATTACATAGTAAATACAAAACACCATATGT
CGCAATCATATTCCTAGTAGGTGTGTCGTTAATTGCACCTTGGCTAGGAAGAACTGCATTGACTTGGATTGTAGATATGTCATCTAC
AGGTGTATCCATTGCCTACTTTATTACATGTTTGTCTGCTGCGAAATTATTCAGTTATAACAAACAAAGTAATACGTATGCACCGGTT
TACAAAACGTTTGCTATTATCGGCTCATTTGTATCATTCATTTTCTTAGCGTTGTTATTAGTGCCAGGTTCTCCTGCAGCACTGACTGC
ACCGTCTTATATTGCATTACTTGGATGGTTAATCATCGGTTTAATATTCTTTGTGATTCGATATCCTAAATTGAAAAATATGGATAAT
GATGAATTAAGTCGCTTGATTTTAAATAGAAGTGAAAATGAAGTTGATGATATGATTGAAGAACCTGAAAAAGAAAAAACTAAATA
ATAAAAGAATCGCACAATATACCTTCTTCATTCGGAGGCGTATCGTGCGATTTTTTGTATTATAAATTGACATTTAAAACGAGGCAG
CTGAACCTTATATATAATTGCTAAGAGTTTGGGCTGAGCCATTTCTAACAATATTTATAATCGTTTAAAAGATTTCACGAACCCAGA
AACAATTAATTTGGAAATTTGGTCGGCGAATAATAAACCTAATGCGATGGCTCCTGCAATAAGTGTAACCTCTAGCATGGTATTGAT
TGCTGTACTGAAATTTAATAATACTAAATTTTTCGTTGCATCGTAAGCTAATCCACCTGGTACTAATGGGATGATACCCGTTACCATA
AAAATGATGGCAGGTTCTTTTTGTTTACGAGCCATATAATGACTTAACAAGCCTAATGCTAAACTACCAAAGAAACTAGAGTATATA
GTGTGCACATTAAAGCCGTTGAAGAATAAGGTGTAAACCATCCATCCACATGTACCAACGAAACCACATGATAGATATAATTTTCT
AGGTGCATCAAAAATGACGCAGAAGAACATTGAAGCTAAAAAGCTAAAGATAAAGTTTAAGATCCAAAACATAGTCTGATACTCCT
ATACTAAAATTAATACGCTACCAACGCCAGCACCGATGCCAAACGCAGTAACCAATGCTTCTAATGATTTCGTTGTGAACATCAACA
TGTGTCCACCAAATAAATCTTGTATTGCGTTTGTTATTAATACACCAGGAACAATAGGCATGACTGCCGCAATGATAATAGTTGCCA
AGTCACCTGTTGGAATAAGTGTATGTCCAATAACGGCGATAATCCCAATAACTAATGAACCAATGAATTCTGGGATAAACTGTGCG
TGTAACTTACGATCTAAAATCTCAGTGACTAGGTATCCTAGACTACCTGCTAATATCGCAGTTAAAACATCAATCAATCTACCACCT
TGTAAATATAAGAAACTCATTGCAATCATTGCTGCAGCAAAACCTTTAAAGGGAAGACTGCTGTCACGCTTAGCAACATATATTTTT
TCAAGTTGCGTTTTTGCTTCGGCTAAAGAAATTTCATTATTTGTAATTTGACGCGAAATTTTATTAGCTTGCGAAATTTTTATTAAGTT
TGTATCTCGAGAGGTAATTCTAAATATTCTAGGAAACGATTCCGAATGTAACGTAAACTGGATGACAGTGTTTGTAACAAAGCTGTT
ACTTTCACTGTAACCAAGTTTTTTTGCAATACGTGTCATGGTATCTTCTACACGCGTACCTTCTGCACCAGATTCTAATAGTATGCGA
GCAGCAAGCATGACAACGTCTTTGATAAGTACCTCTTGTTTGTATTCTTCTGAATTTATGTCCATTTCATCACCATTGTTTATAAGAA
TTTAATACTCATTATAGTTTATACACTATAAAATAACCACATGAGCTTTTTGATAAGTTGTTATTGATTTTAAAAAACTAATATTTGA
TACTATTGTAATAATAATCATCGGAATTTATAGTAGTTAAAATTCTCTTGAAGTATAAAAAGGAAAAATTATTGAAAAAATTCGACA
AAAATATGTTTCAAATATTTCAAATAGGTTAAAATAATATTAGTTAACCATTACAAAAATTGTATAGAGTAGCGACTGTATAATTTC
TATTGAGGTTAACGTTTATATGTAGTGATAGTAGTTAAAGTTCTCCCAAGGAAGACTACTCGGGTACACTTTGCTATGAGCAAAGTG
TACTTTGTTATTGATAATACATTAGCACATATATATAAGTTTAAACATACAGATTTCAACTATTTACCAAATCACGTCTTATGTATAC
GGGAATATACTGAAGATAAACGAAAAATTCCAAGCTTAAACCGATAAGCTTGGAATTTTTTTATTAATTTATAAACGTACCAATGTA
TTAAGAAATCGCAAAGAATTGATCGAATTCGTTTGTGTTAATAATATGTCCTACAAAGAAACTACCGAATTCACCGTATCGTGCTGT

TGTTTCATCAAAGCGCATTTCGTATACAATTTTTTTGAATTGTAATACGTCATCTGAGAACAATGTTACGCCCCATTCGAAATCATCA
AACCCTACAGAACCAGTAATAAATTGTTTGATTTTGCCAGCATATTTTCTACCAATCATACCATGGTCATACATTAATTTTTGGCGTT
CTTCCATAGTTAACATGTACCAGTTATAAGTTTCATTACGACGTTTGTTCATTGGATAGAAACAAATATAATCAGAATGTGGTAATT
CTGGGTATAATCTTGCTTTGATATGAGGGTTCTCATAAGGATCTTCATCAGATTTACCAGCTAAATAATTGCTCAATTCAATGACTGA
TACATATGAATATGTAGGGATTAAGAAGTCAGCAATGCGCAATTTGTTAAATTCATTTTCAATATGATTTAAAGACTTCATTTCAGG
ACGTAAGAACCATAATAACAAATCTGCTTTTTGACCAGTTATATTATAAATAGCTTGATCACCAGATTTTGATGATCTTACAGTTGCT
GTATTTTCTAAAAATGATTGAAATTCAGTGACAAGTGCATCGCGTTCGTCCTTTGGAACTATACGTAATGATGCCCAATCAACTGCA
TAAAATAAATGTAGACTATACCAACCATCTAATGTTTCGGCTGCTTGACTCATGTTTATCGCTCCTTTTCAAAAATCATCAGTTCTTT
ATAAATTTTATCATAAAGCAAAGGGGTAACGATAAAATTATGATTACAAATTGGTGACGTGGCATTATGAAATAAAATGGCGTATA
ATTATACCGTGAATGATTAATAAGATTTATATTACAGGAGGACATTATGGCTGATTTATTAAATGTATTAAAAGACAAACTTTCTGG
TAAAAACGTTAAAATCGTATTACCTGAAGGAGAGGACGAACGTGTTCTAACAGCTGCAACACAATTACAAGCAACAGATTATGTTA
CACCAATCGTGTTAGGTGATGAGACTAAGGTTCAATCTTTAGCGCAAAAACTTGATCTTGATATTTCTAATATTGAATTAATTAATCC
TGCGACAAGTGAATTGAAAGCTGAATTAGTTCAATCATTTGTTGAACGACGTAAAGGTAAAGCGACTGAAGAACAAGCACAAGAAT
TATTAAACAATGTGAACTACTTCGGTACAATGCTTGTTTATGCTGGTAAAGCAGATGGTTTAGTTAGTGGTGCAGCACATTCAACAG
GAGACACTGTGCGTCCAGCTTTACAAATCATCAAAACGAAACCAGGTGTATCAAGAACATCAGGTATCTTCTTTATGATTAAAGGTG
ATGAACAATACATCTTTGGCGATTGTGCAATCAATCCAGAACTTGATTCACAAGGACTTGCAGAAATTGCAGTAGAAAGTGCAAAA
TCAGCATTAAGCTTTGGCATGGATCCAAAAGTTGCAATGTTAAGCTTTTCAACAAAAGGGTCTGCTAAATCAGACGACGTGACAAA
AGTTCAAGAAGCTGTCAAATTAGCACAACAAAAAGCTGAAGAAGAAAAATTAGAAGCAATCATTGATGGCGAATTCCAATTTGATG
CTGCGATTGTACCAGGTGTTGCTGAGAAAAAAGCGCCAGGTGCTAAATTACAAGGTGATGCAAATGTCTTTGTATTCCCAAGTTTAG
AAGCTGGTAATATTGGTTACAAAATTGCACAACGTTTAGGTGGATATGATGCAGTTGGTCCAGTATTACAAGGTTTAAATTCTCCAG
TAAATGACTTATCACGTGGCTGCTCAATTGAAGATGTATACAATCTTTCAATTATCACAGCAGCGCAAGCCTTACAATAACGATGGA
TTTAGCGAGTAAATATTTTAATGGCGTCAACTGGCGATATATCGATCATTCTTCTGGATTAGAACCTATGCAATCTTTCGCATTCGAT
GATACATTTTGCGAAAGTGTGGGCAAAGATATATCAGATAATGTTGTGCGTACTTGGATTCATCAACATACTGTTATTCTTGGTATTC
ATGATTCAAGATTGCCGTTTTTAAAAGATGGCATTGATTATTTAACGAATGAGATTGGTTATAATGCCATTGTTAGAAATTCTGGTG
GCTTAGGTGTCGTTCTAGATCAAGGTGTATTAAATATATCGCTGATGTTCAAAGGACAAACAGAAACAACGATTGATGAAGCGTTT
ACTGTGATGTACCTCTTAATTAGCAAAATGTTCGAAAATGAGAATGTTGATATTGATACGATGGAAATTGAACATTCTTATTGCCCA
GGAAAATTTGACTTAAGTATCGATGGTAAGAAATTTGCAGGCATATCGCAACGAAGAGTTAGAGGCGGTATTGCTGTACAAATTTA
TCTTTGTGTTGAAGGCTCTGGTTCAGAACGTGCATTGATGATGCAAACATTTTATGAACATGCTTTAAAAGGTGAAGTGACTAAATT
TAAATATCCTGAAATTGAACCATCTTGTATGGCCTCATTAGAGACATTGCTTAACAAAACGATTACTGTTCAAGATGTAATGTTTTTA
CTATTATATGCAATCAAAGATCTTGGCGGTGTATTAAATATGACGCCAATTACTCAAGAAGAATGGCAAAGATACGATACGTATTTT
GATAAAATGATTGAAAGAAACAAGAAAATGATAGATCAAATGCAATAGTTAAATATTTAACAGCCCCTTTCAAGTTATCGTTTGGG
GCTGTTATTTTTGAAAGTATCCATAAAAAGCATAAATATGTTATTGACCTAATATTTAAATTGAACATCATTAAATTAAAAGTAATG
ATATGATGGAAATAATAAATTTAATAAGAAAATTTCATGTTTTACGTATTAAATAGATTGAGAATAGGGTATATATAATGCGTATGA
CTATGAAAGAAACGACCTTCTATTGAAATGTTCTAATACTTTAGTAGTAAATCTATATATTCCAATTATTATTTGTAGTATTATAGTT
AGGGTGGAAAATTATAAATTGTAGGGCTATTTTATAAAAAGATAAAGAATCTGAAATATCACTCGATAAGATTTAAACTTTGAAGT
AAACGAAAGTTAGTTTTATACTAATCAAGTTAAGTCAAATATAAATTCAAAATAAAGATTATACATAATATGAATAATGTGAGATA
AAGACTGCAATGAAATTTAACGCGAATGCGTATGCAACTTAGAATATGATGTCTTTTACTAATAAATAAAAATAATGAATCAAGTA
TAGGATAAATATAAAAGGTGATATTGACATGACAAGAAAAGGATATGGGGAATCGACAGGTAAGATTATTTTAATAGGAGAACAT
GCTGTTACATTTGGAGAGCCTGCTATTGCAGTACCGTTTAACGCAGGTAAAATCAAAGTTTTAATAGAAGCCTTAGAGAGCGGGAA
CTATTCGTCTATTAAAAGCGATGTTTACGATGGTATGTTATATGATGCGCCTGACCATCTTAAGTCTTTGGTGAACCGTTTTGTAGAA
TTAAATAATATTACAGAGCCGCTAGCAGTAACGATCCAAACGAATTTACCACCATCACGTGGATTAGGATCGAGTGCAGCTGTCGC
GGTTGCTTTTGTTCGTGCAAGTTATGATTTTTTAGGGAAATCATTAACGAAAGAAGAACTCATTGAAAAGGCTAATTGGGCAGAGCA
AATTGCACATGGTAAACCAAGTGGTATTGATACGCAAACGATTGTATCAGGCAAACCAGTTTGGTTCCAAAAAGGTCATGCTGAAA
CGTTGAAAACGTTAAGTTTAGACGGCTATATGGTTGTTATAGATACTGGTGTGAAAGGTTCAACAAGACAAGCAGTAGAAGATGTT
CATAAACTTTGTGAGGACCCTCAGTACATGTCACATGTAAAACATATCGGTAAGTTAGTTTTACGTGCGAGTGATGTGATTGAACAT
CATAACTTTGAAGCCTTAGCGGATATTTTTAATGAATGTCATGCGGATTTAAAGGCGTTGACAGTTAGTCATGATAAAATAGAACAA
TTAATGAAAATTGGTAAAGAAAATGGTGCGATTGCTGGAAAACTTACTGGCGCTGGTCGTGGTGGAAGTATGTTATTGCTTGCCAA
AGATTTACCAACAGCGAAAAATATTGTAAAAGCTGTAGAAAAAGCTGGTGCAGCACATACTTGGATTGAGAATTTAGGAGGTTAAT
GCGTTGATTAAAAGTGGCAAAGCACGTGCACATACGAATATTGCACTTATAAAATATTGGGGTAAAAAAGATGAAGCACTAATCAT
TCCAATGAATAATAGCATATCTGTTACATTAGAAAAATTTTACACTGAAACGAAAGTCACTTTTAACGACCAGTTAACACAGGATCA
ATTTTGGTTGAATGGTGAAAAGGTTAGTGGCAAAGAATTAGAGAAAATTTCAAAATATATGGATATTGTCAGAAATAGAGCTGGCA
TCGATTGGTATGCAGAAATTGAAAGCGACAATTTTGTACCAACAGCAGCAGGGTTGGCTTCATCGGCAAGCGCATATGCAGCTTTA
GCAGCAGCTTGTAATCAAGCGCTAGACATGCAGCTGTCAGATAAGGATTTATCGAGATTGGCGCGAATTGGTTCGGGTTCTGCGTCG
CGTAGTATTTATGGTGGATTTGCAGAATGGGAAAAAGGGTATAGTGATGAGACGTCATATGCCGTTCCACTTGAATCGAATCATTTT
GAAGATGACCTTGCCATGATATTTGTTGTGATTAATCAACATTCTAAAAAGGTACCTAGTCGATATGGTATGTCATTGACACGAAAC
ACATCAAGGTTTTATCAATATTGGTTAGATCATATTGATGAAGATTTAGCTGAAGCAAAAGCAGCGATTCAAGACAAAGATTTTAA
ACGCCTTGGTGAAGTAATTGAAGAAAATGGTTTGCGTATGCATGCCACGAATCTAGGATCAACACCGCCGTTCACATATCTTGTGCA
AGAAAGTTATGATGTCATGGCGCTTGTTCACGAATGCCGAGAAGCGGGGTATCCGTGTTATTTTACAATGGATGCGGGACCTAATGT
GAAAATACTTGTAGAAAAGAAAAACAAGCAACAGATTATAGATAAATTATTAACACAGTTTGATAATAACCAAATTATTGATAGTG
ACATTATTGCCACAGGAATTGAAATAATTGAGTAAGGAAGAGATAAAATGATTCAGGTCAAAGCACCCGGAAAACTTTATATTGCT
GGAGAATATGCTGTAACAGAACCAGGATATAAATCTGTACTTATTGCGTTAGATCGTTTTGTAACTGCTACTATTGAAGAAGCAGAC
CAATATAAAGGTACCATTCATTCAAAAGCATTACATCATAACCCAGTTACATTTAGTAGAGATGAAGATAGTATTGTCATTTCAGAT
CCACATGCAGCAAAACAATTAAATTATGTGGTCACAGCTATTGAAATATTTGAACAATACGCGAAAAGTTGCGATATAGCGATGAA
GCATTTTCATCTGACTATTGATAGTAATTTAGATGATTCAAATGGTCATAAATATGGATTAGGTTCAAGTGCAGCAGTACTTGTGTC
AGTTATAAAAGTATTAAATGAATTTTATGATATGAAGTTATCTAATTTATACATTTATAAACTAGCAGTGATTGCAAATATGAAGTT
ACAAAGTTTAAGTTCATGCGGAGATATTGCTGTGAGTGTATATAGTGGATGGCTAGCGTATAGTACTTTTGATCATGAATGGGTTAA
GCATCAAATTGAAGATACTACGGTTGAAGAAGTTTTAATCAAAAACTGGCCTGGATTGCACATCGAACCATTACAAGCACCTGAAA
ATATGGAAGTACTTATCGGTTGGACTGGCTCACCGGCGTCATCACCACACTTTGTTAGCGAAGTGAAACGTTTGAAATCAGATCCTT
CATTTTACGGTGACTTCTTAGAAGATTCACATCGTTGTGTTGAAAAACTTATTCATGCTTTTAAAACAAATAACATTAAAGGTGTGC
AAAAGATGGTGCGTCAGAATCGTACAATTATTCAACGTATGGATAAAGAAGCTACAGTTGATATAGAAACTGAAAAGCTAAAATAT
TTGTGTGATATTGCTGAAAAGTATCACGGCGCATCTAAAACATCAGGCGCTGGTGGTGGAGACTGTGGTATTACAATTATCAATAAA

GATGTAGATAAAGAAAAAATTTATGATGAATGGACAAAACATGGTATTAAACCATTAAAATTTAATATTTATCATGGGCAATAAAT
GATTTGATAGGGGAGCAATCATCTAATTTAATTAGAAGATAAATGCTCCATTTTTTATTGTCAGCCGTGTTACGTATCTTGAAAAAA
ATATTAAAAAGAGTAAAATAGATAGAGTTAACGAAAAAATTAATGAAATCGACGATATAGATATGATAAAGAAAGGTGGGTAGCA
ATATGAAAAATACATTCCTTATTTGTGATGAATGTCAGGCAGTCAATATAAGAACGTTACAAAAGAAGTTGGAAAAATTAGATCCC
GATGCTGAAATCGTGATAGGTTGTCAATCTTATTGTGGACCTGGACGCCGAAAAACATTCACTTTTGTTAATAACCGCCCACTGGCT
GCGCTTACTGAAGAAGAATTAATCGAAAAAGTTTCTCAACAATTAAAGAAACCACGTGATCCTGAAGAAGAAGAGCGTTTAAGAA
AACGACATGAAGAACGTAAACGTCGTAAAGAAGAACAAGATAGAAAGCTTAAAGAAAAATTAGAAAAGCGAAAAGCACAACAAT
AAAACCTGATGGCAGCATCATTCAATGCGTGCCACCAGGTTTTTATGTTTTGTCTAGAAATTAAATAAATCATTAAATGATTCGGCC
ATCGTAGGATGCGTATAAATATTATCTCGTAATACGGTATATGGAATGTTTTGATCAATCGCAAGTTTAATTATATTAATTAATTCTT
CAGATTGCTTACCATATAATGTAGCACCTAAAATCATATTATTTTCATTATTAATGACTACTTTAAATAAACCTCTTGGATCATTGTT
AATTTTGTGACGAGGTATAGCACTTACTAAAAGTTGATGTTCAGTGTAATCATAATGTTGAGCGGCAGCTTCTTTACCAGTTAATCC
AACACGTGATAATGGTGGATCTATAAATACTGTATAAGGCACGCTGCCTCTATTGTCAGTCGTACGTGACTGATTACCATATAACGC
TGATTTGATAATTCGATAATCATCTAAAGATATATACGTAAATTGAAGTCCGCCTTTAACATCACCTGCAGCATAAATATGCGGCAC
AGTTGTTTGAAGATGAGCATTGACTTTAATTTCGCCTCTGTCGCCTAATTCGATATCAGTATTTTCTAAAGCTAAATCCGTATTCGGT
TTGCGACCGATAGCCAAAAGTACTGCATCAGCCTCAAAGTTACCAACGTTAGTATGGACTGTTGTATGATGATTGTCAGATGACAAT
TCAGTCGTTTCAACATTTGTATACAATGCAATGCCTTTATTTTCTAAGTCAGTAATAGCATGTGCAACGACATCTTGATCTTCACGTG
GCATAAATGATTCACCACGTTCTAATACTGTAACCTTACTACCTAAATTCGCAAACATTGAAGCAAATTCTAAGGCGATATAACCGC
CACCTACAATAACGAGGTGCTTAGGTTGATAGCTAATGTTTAATAAACCTGTCGAATCGAAGACGTGTTTAGCTTGATCAAGGCCTT
TAATGTTAGGAATGACAGAGGTAGCACCGGTATTAATAATGATATGAGGTGCAGTAATACTATCGACGATATCGTCATGTTGATCTA
ATAAATTCACTTCAGTATTAGATTTAAACTGCGCTTTAAAATCCAGTACATCAATGTTGTTATCGTCTGCTAATAAGTGGTAATTTTT
ATTGTTTAGCGCATTGACAACATCGTTTTTACGGTTATAACTTGCTTCAAAAGATTTGCCTTCTAATCCATCATGTACAAGTGTCTTC
GAAGGTATACATCCTATATTTATACAAGTGCCTCCATACATTTTCGAAGATTGTTCGATAACTGCGACGTGTTGACCTGTTGATGCA
GCGTATTTCGCTAAAGTTTTACCAGCTTTCCCAAATCCTATTACAATTAAATCATATGTTTTCATGACATAAATCCTCCTTTTGAATGT
CTTCAATGACATCTTTGATTGTTTTTCCATTATAAAAATTAATAATGATATTCTGTTCGTCTTGCTGATAATGTGACATGGTAGTTGC
AATATTACGAGCAATTTGACAGTGACTGCCTTCGTCGCCAGTAAATAGACGTGTGTGGTGTTCTTTCTCTAAGACAAAATGTTTATA
TAATGTTGCTAGAGAGACATCAGCACTTTGATCATTTGCTAAATAACCGCCATCTTTACCTCGTATTGTGTCAATCATTTTTAAATCG
ACAAGTTGAGTCGTCACGCGTCGTAATTGAACAGGATTTAAACAAGTTAATTCTGCTAATGAACTACTATTGAATTTTTCTGAATGA
TGCTTAGTTAAAAAAGCTAATACATGCACGGCAATGTTAAATTCTAAATTCAATGTTTCTCACCTCATAATTGTAACTGTATTAGTTG
CAATTAAAAATCATTTGCTCAATGTGTCAATACTTATGTTTGAAAAGTGTTGAATTAGGTGTGGTTATTGTGATGATTTATTGTTTG
AAAGTTGATGCATTAACGGTGAGATTGCACAATTGAAATTTTAATTATGTATGAACTGATAGATATGAAATGAATATTGAAAATGA
GAGCAATAAAATTTTGAATGATGATGTTGTGTGATTGTATAAACTTATTCGTATTTTAGATACGTTTTGATATTTCGCATGATGAGGA
AATACTTTTAACAACTGATGACATATTTTAATAGTGAATATATTTTGAGATATTAATATATTTAATGTTAAGGATGTTGTCGCAATAA
AATTAAAATATTGAAAGATAATTTTTATTGATTAATTGCGAATATACAAAGACACCATTATATCCATATGCTAAAATAAGAAAAAA
GGCGACATGTTATCTCAATTTACCTGCATCTAGACTCCTTTACATAAGAAATAATCATCGCCGAGTTTTAAAATTTTACAACTTTGTA
GATGTAAAAAGCATTTGATTTTAAATAGTTTGAAAATCATATATTTTTGAAAAAGGAGCATGCCGATGAGTATTGACATGTATTTAG
ACAGATCTCGAAACCAAGCTTCAAGTGTGGGGAATTTGAGTCAAACAATGAATTCAAATTATGATGCGTTGGAAAAAGCAATTACT
CAATTTATTAATGATGATGCGCTTAAAGGGAAAGCGTATACGTCAGCTAAGCAATTTTTTAGTACGGTGTTAATTCCATTATCAACA
AGTATGAAAACATTGAGTGATTTAACGAAGCAAGCTTGCGATAATTTTGTGTCACGTTATACGAGTGAGGTTGATAGCATATCTTTA
AAAGAATCAGAGCTTGAAGAAGATATCAGATCATTAAGTCAACAAATTACGCGATATGAAAATTTGAATAACAATTTGAAAAAGCA
TGCTTCCGATAATCAGCAAGCCATTTCATCGAACCAACAAATAATACGAACATTAGGTCAACAAAAACATGAATTAGAAGAGAAGC
TACGCAAATTGCGTGAGTTTAATCAAAAATCACCAGAAATATTTAAAGAAGTTGAAGAATTTCAAAAAATTGTCCAACAAGGACTT
ACCCAAGCGCAGAATTTTTGGAACTTTTCAACAAATCAATTTAATATACCTTCAGGTAAAGAACTTGATTGGGCTAAAGCAAGTCAT
GAAAAATATTTGAAAGTTGCTATGGGGAAAATTGAACATAAAGCAGAGAAAGAAACTTTAAATAAAGCAGACTTTGCTGTTATAAA
GGCATATGCCAAAGAGCATCCAGAAGACGATATCCCGAAAAGTATACTGAAATATATAAATGACAATAAAGACAGTATTAAAAGA
GATATAGGATTAGATATCACTTCAACACTTTTAGAGCAAGGCGGTATAAATGCAAGTAAATTCGGTGTATTTATCAATACAGCAGGT
GGAGTGAAAGGCCCAGCAGGTCCAAATTCATTTGTGGAAGTCAAACGTACATCAGGTAATGTGTTTATAGAAAATGGTAGTAAATT
TGCAAAAGGCGGAAAATACCTAGGTAAAGGTGTTGCTGGTGTAGGATTTGGTATAGGTATGTATGATGACCTTGCAAATGATGATA
AAACATTTGGAGAGGCATTATCGCATAATGGTATGACGCTTGCAGCTGGATCTGCAATGGGGACTATTGTGTCTGTAGGTATAACTA
TGCTGGCAAGTAATCCAGTAGGATGGGCGGTTTTAGGAGGTTTTGCTGCAGCAACAGGTTTTGCAATAGCGGCTGATTGGGCATATG
AAAATAATTTGTGGGGATTAAAAGATGAAACAGACTGGGTAGGACATAAAATAGATGACGGTATCGATGTTGTAAAAAAATCTAC
AAAAAAGGCTGTTGATAGTGTAGAAAAAGCTGTTGGTGAAGCTACAAAAAGTATATCTAATCATATAAATCCTATGAAATGGAGCT
GGTAAAAAGTGCTATGCGAATCTAAAATTATCAATAAAAACCCAAAATATCGAATTATTAAATATAATGGTGAATACTTAATGGTC
GATATAATAAGCACTTGGATTAGTTTATTTTTTCCTTTTATTAATTGGTTCATCCCCAAAAGATACGTCAAAATTAGTAGAGAAGAGT
TTGAAAATTTAAATATTGTTAAACCTGCTAAAAAGAATGTTTTTTGGCCAGTTGCAGGTATCTCTACTTTATTCGCAGTTACATTAAG
AAAGTATACGCATTTACTTGACACTCAACTTGATAAAAAAATTAGTTATTGCCATATGTTGTATCACATTTATAGGGATTTTAATATTT
TATGTACGCCTAATTAAAAAATCATCTTTAAATATTTATAATACTAAAAATAAAAGGTCGAAAATCATCTTAATACCAACACTAAAA
AATTTTTGTTTCACATTGTTCGGATATGTTTTTTTAATTTTTTGGACATTGATTTTCTCATATGCTCTATTATCAATGAGTTATCAAAA
CATAATAGTATATTTTGCTTGGATTACAGCAATAATGGGTTTTTTTCTAGTGAATATAGCTTTAATTATAGATAAAAACATTCATGTC
ATACTTAAAAATTAAGTGAAAGTACTAAATTCAGATTAAAAATATGAAAATATCAGTTAATAAAACCTTTGATGACAGTAACTTGA
TAGTATTTGAATGTCTTTTGAAGTATCGAAAAATGTATTACACATAAAATTAAAGGGATGTGGAGACATTGCTTTGCGAATCTAAAG
TTATTAATAAAAACCCTAAATATCGAATAATCAAATACGATAGTGAATATTTAATGATTGATTTGGCAAGTAATTGGATTGTCTTCT
TCTTTCCATTTATTAACTGGCTCATACCGAAAACATATGTCAAAATCACTAAGAATGATTATGAAAAATTAAATATTGTCAAACCAG
TTAAAAATAAATCGATAGGATGGACCATATTCGCGGGTATTGTGTTACTTGGTGGTACTGTAAGAAGAAATACTTATTTATTTGATT
TTCAATTAGAAGAACTAATTGTTTGGAGCAGCTGTTTCATTGGGTTTTTAGAGATTATATTTTTTTATTGTTATCTAAATAAGAAATT
AACATTAAATATTTATAATGAAAGTAAAAATAATGAACTTAAATTAAGATTATTACCCTCCTTTAAAAATATTTGTTTCACAATTTTT
TATTACCTATTTACTGGTTTCATGTCTTATGGGGCATTTTACTTGTTGGTATTTGAAAATGTGCAAAATTTAATCTTATATGTTTCTTG
GCTTTTCATGACTATGCTATTTATGTTTATGAATATGCATTCAATTATAGATAAAAAAGTACATATATTCTTAAAGTCTAATAAATAG
TTACAAATTTAGTTAGTTTTCAATTGTTAATTAGGGGTGGTAAACAGTGCTTTGTGAATCTAGACAAATTTATAAAAATCCTAAATAT
CGAGTTATTAGATATAATAATGAATATTTCATGGTCGATTTAGTAAGTACTTGGATTACTTATTTTTTCCCTATGATTAATTGGTTTTT
GCCCAAAAAATACGCAAAAATTAGCGAAAATGAATTTGAAAGGTTAAATATAGTCGAGCCTGTTAAAAATAATGTTTTTTGGCCGG
TTGCAGGAAGTTCAGTTCTATTTGGAATTATATTGAGAAAGTACGGTAACTTCTTTAATGTTCAGTTTGAAAAACAACTAGCAATCA

CTGTATTTTTTATCATGTTAATAGGGATGTTAATTTTTTATTTTTATCTAAATAAAAAATTAACATTAAAAATTTTTAATACCAACGTG
GTTAATAAGAATAGAGTTGTATTAATACCGACTTTCAAACAAGGATTGTTAATAGTTTTTGCGTACTTTTTTTTAGGAAGCGCTTCAA
TATTTACTTTAACCATTCTTTTGACGACAGAGTCACAAAATATAATAATATTTTTAACTTGGGTTATTATCACGATGTTTTTCTTTTTA
GTGAATATGGCTTCGATAGGTAATAAAAATGTTCATGTTATTTTAAGAATAAACGAGTAGTAAAAATGATCGAAATTGGTAGTATC
GCATATTTAAACGGTGGTTCAAAAAAATATAATCATATCTTAAATCAGGAAAATAGATAGTAATGTTTGAATATACCAAGTGAAAA
TGCCCATTGTGATTGATAAAGGAAAAAACTGTTTTAATTTAAAAATGAAATATAGAGCGTGTTATTTTGTATGATAGCTCTTATAAC
ATTGGCGTTTTTAATCCAATAGTATGGACGGTTTTAGCTGGTTTTGTTGCGGAATCTGTTTAGACAATTCTTAAAAATTGGACTTTTC
ATACTAATAAATTTGGATTAAGAGATAAAACTAATTGGATAAGACATTAAATAGACGCTGTTTAAAAATACCACAGAAAAAGCTGT
AGATAGTGTTGGAACGATAGTTGGTGAAGGTACAAAAAGTATTGGGGATCATTTAAAACTAATGAAACGGGAGTGGTAAATGTTGC
TTTGCGATGTCAGAGTCATTTATAAAAATCCGAAATACAAAGTCATTCAACATAACGGTGAATACTTATTAGTCGATTTAGTAAGCA
CTTGGTTCGTGTACTTTTTTCCTTTCATTAATTGGTTCATTCCAAAAAAGTACGCGATAATTAGCGAAGAAGAATTTGAAAATTTAAA
TGTTGTTAAACCAAATAAAAATAATGTTTTCTGGTCAGTTATAGGAAGTTCGGTTTTGTTTGGAGTTACTTTAAGGAAATACATACAT
GTTTTTGATGTTCAATTAGATAAGCTAGTTGTAATGATATTGTGTGCTCTCGCTTTAATTTGTGTTATAGTTTTTTATTTTAACTTAAA
TAGAAAGCTTAAGTTAAAAGTGTTTGATACAAATATTGAAAAAAATAAGAGAGTTATATTAATACCAACGTTTAAACTTGGCTGTTT
TTTAGTTTTCGGATATATTTTCGCTGGAAGTTTTTCAATATTTTCATTAATTGCCCTTATGACAATCGAACCTCAAAATATAATAATAT
TTATTTATTGGATTATGATGACAATGCTTTTCTTTTTGTTAAATATGACTTCGATAGGTAATGAAAAAGTTCGCGTTATAATGAAAAA
TAATTGATTACATTTAAAATATTCTAAATGTTGTCGACACAATCCTTTTAAGACGCTAGTAGAATTTAAATGACTTCTAATGTATATG
AAAGTGTATCAATATAAAACCAATTGAAAAGAAGTGGAGACATTGCTTTGTGAAACTGAAAATATTAATAAGAATCCCAAATATAG
AATTATCAAATACAAAGATGAATATTTGATGATTGATTTAGTAAGTACATGGTTAGCACTCTTTTTCCCAATGATTAATTGGCTGATT
CCAAAAAAGTACGTCAAAATCAGCGAAAAAGATTTTGAAACTTTAAACATTGTGAAGACAACTAAAATCAATTCTTTTTGGCCAGT
GGCAGGAAGTACGGTCTTATTTGGTGTTATGTTAAGAAGGTATTCCCATTTATTTATCGTTAAATATGAATATAGTATAGTAATTTTA
ATTTGTTGCATCATAATACTAGGTATTTTTCTGTTTTTTTTATATTTAAATCAAAAGTTAAAGTTACAAATCTATAATGAAAACAAAA
ATAAAAGCAATAAGATAATCATATTTCCCACTTTAAAGAGTCTTTGTTTATCAATAGTTTTATATATTTATTTAGGTGGTGGTTCATT
CTTTACTATTTATATGTTATTGACGATTGAAGTTCAAAATATAATATTATTTATAACTTTGTTTGTAATATTTTTGTTTTTCTTATTTTT
AAATATGTGCTCACTATATGACAATAAAGTTCATGTATTATTTAAATCAAATGGAATTGAAAAGTTTTAATAGGAATAATAAACAAA
GGAATGGTAGTGATAAATATTGCTATGTGAATCTAAAGTTATCAACAAAAATCCTAAGTATAGAGTTATTAAATATGGTGATGAAT
ATTTAATGATTGATTTAGTAAGTACCTGGTTAACTTTATTTCTTCCTATGATTAATTGGTTAATTCCAAAAAAAATATGTCAAAATCAG
TAAAAAAGAATTTGACGATTTAAACATTGTCAAACCTGTTAAAAATAAAGCTTTTTGGCCAGTTGCAGGTAGTACTATTTTGTTCGG
AGTTACGTTTAGAAAATATATTCCTTCACTTAATATTCAATTAGAGAAAAACATGGTGATTGTAATATGTTGTGCAATATTTCTGGGT
GTTTTAATACTTTTTTTATTTCTGAATCGTAAGCTAAGGTTGGAAATTTATAATAATAACTCTAGTAAAGGGAAAATAATTTTATTTC
CTTCATTAAAAAACTTTTGTTTCACAATATTTTATTATTTTTTATTTGGCGGTCTTTCAATAATGGCTCTAAGTATGTTATTAACTTTA
AATCCTCAAAATATAATAGGCTTTATTGGTTGGTTGGTAATGACTGCAGGTTTCTTTCTGTTAAACATGTCATCGATTATTGACAAAA
AAATTTATGTATTATCTAAAACTAACACGGTGGAAAAATGATGGTTTAGCTGGATTTACTGCAGGTTCTATTTCGGCAATACTTGTA
TATTGGACCAATCAAAAAAATGAATTTGGAATAAAAGATAAAAACGATTGGATAGGACATAAACTAGACGTTGGTATAGATGCTGT
AGAAAAATCTGCAGAAAAAACAGTAGATGGTGTTGAAAATGTCATGGTGAAGCTTCAAAAAGTATTTCTAATCATATAAGCCCTAA
GAAATGGAGCTGGTAAATGTTGCTATGCGAATCTAAAATCATCAATAAAAACCCAAAATATAGAATTATTAAATATAATGATGAAT
ACTTAATGGTCGATATAATAAGCACTTGGATTAGTTTATTTTTCCTTTTATTAATTGGTTCATCCCAAAAGAATACGTCAAAATTAGT
AGAGAAGAGTTTGAAAACTTAAATATTGTTAAACCTGCTAAAAAGAATGTTTTTTGGCCAGTTGCAGGTAGCTCTGCTTTGCTGGGA
GTTGCATTAAGAAAATATACACATTTACTTGACATTCAACTTGATAAAAAAATTAGTTATTGCCATATGTTGCATCACATTTATAGGG
ATTTTAATATTTTATGTACGCCTAATTAAAAAATCATCTTTAAATATTTATAATACTAAAAATAAAAGGTCAAAAATTTTTTTAATAC
CTACACTAAAAAATGTTTGTTTCACATTATTTGGATATATTTTATTTGGCGGATTGACTATGCTATTCCTAGATGCACTATTATCAAT
GAGTTATCAAAACATAATAGTATATTTTGTTTGGATTGCAGTTATAATGGGTTTTTTTCTAGTTAATATAGCTTTAATTATAGATAAA
AACATTCATGTCATACTTAAAAACCAATAGAAATTACCAAATTAAATTTGCAATGGCTTTAATATTGTCGTTCTTAAATGTTTTTCAA
TTTCTGATTCAAAAGTACATGTAATTTAAAGGTCAACGAGCAATCGCGCTATATGCATATCAATTTAAGCAATACTTATTTTAACTG
AGTTTTATATAACGTTTTCCTATTACACCGCTTAAATTCAAGCTCTAATTCTAACATTTGGAAGTTGTATTAAATCATGATAGTGTGA
TAGTGGAAAAGTTATAAAGATAAGGAGCTGGGTCTAATGAAGCAATTTGTAAACTTAGGTAAATCTGATGTTGAAGTGTTTCCAAT
CGCACTTGGGACGAACGCAGTAGGTGGGCATAATTTATATCCGAACTTAGATGAAGAACAAGGAAAAGATGTTGTTCGTCAAGCCA
TTAATCATGGTATTAATTTATTAGATACGGCATATATTTATGGGCCAGAACGATCAGAAGAATTGGTTGGAGAAGTTGTTAAAGAAT
ATCCGCGAGAGCAAATTAAAATTGCTACGAAAGGGTCTCATGAATTTGATGAAAATCAAGAAGTACATCAGAACAATCAACCGGA
ATATTTAAAACAACAAGTTGAGAATAGTTTGAAACGTCTACAAACTGATTATATCGATTTATATTATATATTCATTTTCCGGATAACAA
CACTCCGAAAGATCAAGCAGTTGCAGCATTACAAGAGCTTAAGGAACAAGGGAAGATTAAAGCAATTGGTGTATCAAATTTCACAT
TAGATCAACTTAAAGAAGCAAATAAAGATGGTTACGTTGATGTTGTACAGTTAGAATATAATTTATTGCATCGCGAAAATGAGGCA
GTATTACAATATTGTGTTGATCACCAAATCACATTTATTCCATATTTCCCATTAGCATCCGGTATTTTAGCTGGAAAATATGATGAGA
ACACTAAATTTAGTGACCATCGTACTACACGTCGTGATTTTAAACCAGGTGTATTTGAAGAAAATGTGCGTCGCGTAAAAGCTTTGG
AAAGCATAGCTGCAGCACATCAAACTTCAATTGCGAACATTGTATTAGCATTTTATTTAACGAGACCAGCTATCGATGTGATTATTC
CTGGTGCAAAACGTGCAGAACAAGTCGTTGAAAATATTAAAGCTGCAGATATCGTTTTATCAGATGATGAGATTCAATATATCGAT
GAACTGTTTCCGATTGAAGACTAAATGAATTGTATTTAATGGTGATATATGTATCTAATTTCATCATGAATTAGCCCCGTATCACAA
ACGACGTTATATAAGGCATGACGAAATTTCGTCAAATTTATATAAAAGTTGTAAGCGTTTTACTATTCGGTAGGCAAAGCTTATGAT
AGTATAATTAGGTAACGAAAAACACAGATATTTATTAATCAAAAGTGAGGTTAGGCTAAAATGATTACTGTTTTGTTTGGTGGGAGT
AGACCAAACGGTAATACTGCACAATTAACAAAATTCGCTTTGCAAGATTTAGAGTATCAATGGATTGACGTGACACAACATCAGTT
TAAACCGATACGTGACGTGAGACATACAGCAGAGACTATTACTTCATATGACGATGACTATTTGCCGATTCTAGATAAAATATTGGC
TAGTGATACAATTATTTTTGCATCACCAGTGTATTGGTATAGCATTTCAGCACCATTGAAAGCATTTATCGAACATTGGTCAGAAAC
ATTACAAGATAAACGATATCCTAATTTTAAGGCACAAATGGCCGAAAAGGATTTTAGAGTTATTTAGTTGGTGGAGATTGTCCAAA
AATAAAAGCGAAGCCAGCAATTACGCAAATGAAATATAGTTTAGACTTTTTAGGTGCCACTTTAAATGGTTATATTATTGGAACTGC
TGAAAAGCCTGGTGACATCATGAAAGACAACTATGCCTTAGCACGTGCAACTGAGTGGAATAGTATATTGCAATAAGACATACCTC
ATACTAAACGAAGTGATTTTTTGCTTCGTTATTTTTTATTTTTAATTTTAACGGAAAATATAAATATATAAATTGAATAGACAATCAT
TGTTTATTAAGTATTGAGCAAGTTGTGGTATAATGCCGTTATTAAAACTTGTTAATAAGGGGAGAAAGTCATGTTCAAGGTAAGACA
AGCAACTGAAAAAGATGTTGTTCAAATTAGAGATGTCGCAACTAAAGCTTGGTTTAATACATACTTAAATATATACGCTGCGACAA
CAGTTAATCACTTGTTAGAAGCTTCATATAATGAACATCATTTAAAGAAAAGACTTCAAGAACAATTATTCTTAGTCGTTGAAGAAG
GTAATGACATCGTTGGCTTTGCTAACTTTATTTACGGTGAAGAATTATATTTATCAGCTCATTATGTTAAACCAGAATCGCAACATAC
AGGTTATGGTACAGCATTGTTAAATGAAGGATTATCACGTTTTGAAGATAAATTTGAAGGTGTTTACTTAGAAGTAGATAATAAAAA

TGAAGAAGCAGTAGCTTACTATAAAGAGCAAGGTTTTACAATCTTACGCTCTTATCAGCCAGAAATGTATGGCGAAAAGTTAGACT
TAGCACTTATGTACAAAGCATTTTAAGTAAATTAAATATTATATTAAACGAAAAGGAGGGGAATAATGACTAACGCATATGTAGGT
TTAAAATTAGTAGAAGAAAAAGTTTTTAAAGACCCGATACATCGATATATTCATGTTGAAGATCAATTGATATGGGATTTAATTAAA
ACTAAGGAATTCCAAAGGTTACGTCGAATTAGACAACTAGGAACACTGTACCTATCTTTTCACACAGCAGAACATAGTCGCTTTGGA
CATTCTTTAGGTGTGTATGAAATAGTTAGACGATTAATTGATGAGTCATTTATTGGTCATGATGCATGGGACAATAAAGATAGACCG
TTGGCATTATGTGCTGCATTATTACATGATTTAGGACATGGTCCATTTTCACATAGTTTTGAAAAAATATTTAATACAGACCATGAAG
CATACACACAAGCGATTATTACTGGAGATACTGAGGTGAATGCTGTATTACGTAAAGTGTCGCCTGAGTTTCCAAGAGAAGTTGCG
GAAGTAATTAATAAAACGCATCATAATAAATTGGTCATTTCGATGATTTCGTCACAAATCGATGCGGATAGAATGGATTATTTACAA
CGTGATGCGTATTTCACAGGTGTATCATATGGTGCTTTTGATATGGAACGTATTTTAAGATTAATGCGACCTTCTAAAGATGAAGTA
CTAATTAAAGAAAGTGGTATGCATGCAGTTGAAAACTTTATTATGAGTCGTTATCAAATGTATTGGCAAATTTATTTCCACCCAGTT
AGTCGTGGTGGAGAAGTGCTGCTTAATAATTGTTTGAAACGCGCAAAACAGCTTTATAATGAAGGCTATGAATTTAAGTTGCATCCA
CATGATTTTATTCCATTTTTTGAAGAGACAGTTACGATTGAACAATATGTTGAACTCGATGAAGCGGTAGTTACGTATTATTTGGAA
AAATGGACAAAAGAAGATGATGCCATTTTAAGTGATTTAGCAAGTCGATTTATTAATCGAGACTTATTTAAATATATTCCATTTGAT
GGCTCAATTATTACAATATCAGAACTGCAAGAACTGTTTGAAGCAGGTGGTATTAATCCAGATTATTATTTTGTGAGTGAAGCATTT
TCGGATTTGCCATATGACTATGATCGACCTGGGTCAAATCGCAAACCGATTCATTTATTAAGACAAGATGGTACGATTAGAGAAATA
AGCAATCAATCTTTAGTCATTCATAGTATTACAGGCATTAATCGCCAAGACTATAAATTATATTATCCTAGAGAAATGGTTGCAAAG
ATTAAAGATAAGACAATTAGAGAAGCTATTGAAAATTTGATTAATGAGCTTAATTAAAAAGGGCTAAAATTGTTATCGTTAAATAT
GGAGGTTATATCATTGTCTGAGAAAAAAGGCTTTAATTTTAATATCATAAAAAATGACCCTCTAGATGGTCATAAAGGTACAAATAT
TGGTTCAATTAGCTTAGACAATATTGCACCAGTTTTTATCGATGTTGCTAACAAAGAAGCATTTATTGATATTGGAGGCATGCATGC
TCGCGCCAAAGTTGAAAAAGGCGTGAAATGGATTACTGATAAAGCTGCTGTTGAAGGCGATGAAGCTAAAGAATATTGGTTGTGTT
GGGTAACAACAGAACGTAATGAACAAGGACCATATTATGCTGGTTTAACAGCGTGCTATTTATTAGTGAATAAAGCAATTCGTCGT
GGTTATAAAAGTATGCCTGAACATGTTAATATGATGGATAAATCAATGAAACATCATATTATCATAGATCAAATTGGTGACGAGAA
TAAAGCTATTTTAAAAGACTTTTTAATGAACCATGATGAAGGTATGTGGAAGCATTCTTCTGATGCTTTACATCAAGCATTTAATTA
AATATTAGAAACTAAAATTTCCCAATTAATCTATAAAGATATGATTCATTTCTCAATGACGACGTAAATCGTGAGGTGAAAATAATG
TCTTAGATTGATTGGGAGTTTTTTTTAATTTTTTTGAAATTAAATTAATCTGTAGTTAATAAAAAATTTGAATAACTGACACATTTTTT
TGATCATAGCTATATACTTTGTGAATTAATTCACATTATAATAAGAGTGAAGATAAGAGTATTATAAATTATCTTTAAATAAATATA
TGTGAAGTAAAAATTACACGTTAGCATATCGATTATGTCATTTCTTTTAACATATTAACTAGGGAAACGTTAAAAGTTAACGGTTGA
TATCTAACTAAAAACAAGGTCACAGTAGTATGTTTTAATCTGGCGTCTATTACAAATAAAAATTACATCTATAATTATTCGTTTTCTT
TTTTGAAAGTAATAGCCAATTAATATCATACATACTGGAGTGACTATAAGGAGGACATTATTATGAGAGCAGCAGTTGTAACGAAA
GATCACAAAGTAAGTATTGAGGACAAAAAGTTAAGAGCTTTAAAACCTGGTGAAGCGTTGGTACAAACGGAATATTGTGGCGTTTG
TCATACCGATTTACATGTTAAGAATGCTGATTTTGGTGATGTTACAGGCGTTACTTTAGGTCATGAAGGTATTGGTAAAGTCATCGA
AGTTGCGGAAGATGTAAAATCATTAAAAATTGGAGACCGTGTGTCTATCGCTTGGATGTTCGAAAGCTGTGGAAGATGTGAATATT
GTACAACAGGTCGTGAAACACTTTGCCGTAGTGTGAAAAATGCTGGTTATACAGTAGATGGTGCAATGGCTGAACAAGTTATTGTT
ACTGCAGACTATGCTGTGAAAGTACCTGAAAAATTAGATCCAGCAGCAGCGTCTTCTATTACATGCGCAGGTGTGACAACTTATAA
AGCTGTAAAAGTAAGTAATGTAAAACCTGGACAATGGTTAGGTGTTTTTGGTATAGGTGGTTTAGGTAACCTAGCTTTACAATATGC
TAAAAACGTTATGGGGGCTAAAATTGTTGCCTTCGACATCAATGATGATAAATTAACATTCGCGAAAGAATTAGGTGCAGATGCTA
TTATTAATTCTAAAGATGTTGATCCAGTTGCAGAAGTTATGAAATTAACTGATAACAAAGGATTAGATGCAACAGTGGTAACTTCAG
TTGCTAAGACGCCATTTAACCAAGCGGTTGATGTTGTAAAAGCTGGTGCAAGAGTTGTTGCCGTTGGTTTACCTGTTGATAAAATGA
ACTTAGATATCCCAAGATTAGTGCTTGACGGTATTGAAGTAGTAGGTTCACTTGTTGGTACAAGACAAGACTTACGTGAAGCGTTTG
AATTTGCTGCTGAAAATAAAGTAACACCTAAAGTTCAATTAAGAAAATTAGAAGAAATTAATGATATTTTTGAAGAAATGGAAAAT
GGTACTATAACTGGTAGAATGGTTATTAAATTTTAAAAATATCAACTGACTATATAGATAAAGAAGGTAGTGCTCTGAACACTATCA
TTATAATCAAACACGAGGTTTTCATGAAAGATAGTGAAAATCTCGTGTTTTTTTGGTTTTGAGGTGTTGTTTGTATTTTATAAAATGG
CTTACATATATGAAGCGTTGATTAAGTATGGAATTGTTAATTAATTGAACCTATTTAGCTTTAAGAAGGCATAACAAGATGACCTTA
TTTTATGCTATAATATTTCTATTATGCGAAGATTAAGGTGAGTAGTAAATTGGATAAAAAAGTAAGTATTCAAACAAAGCAAGTGTT
GAAACAGCACAACGAAAAAGAAAAATTTGAATTTACTACTGAAGGAACTTGGCAACAAAGGCAATCTAACTTTATTCGGTATGTAG
AACAAATTGAGGATGCAACAGTTAATGTTACAATAAAAGTGGATGATGATAGCGTTAAGTTGATTCGTAAAGGCGACATTAATATG
AATTTGCATTTTGTTGAAGGACAAACGACAACAACTTTTTACGATATATCGGCTGGACGAATTCCACTAGAAGTTAAAACATTACGC
ATTTTACATTTCGTAAGTGGAGACGGTGGCAAGCTAAAGATTCATTATGAATTATATCAAGATAATGAAAAAATGGGTTCTTATCAA
TATGAAATTAACTATAAGGAGATAGGCGAATGAATATTATTGATCAAGTGAAACAAACATTAGTAGAAGAAATTGCAGCAAGTATT
AACAAAGCAGGATTAGCAGATGAGATTCCTGATATTAAAATTGAAGTTCCTAAAGATACAAAAAATGGAGATTATGCTACTAATAT
TGCGATGGTACTGACTAAGATTGCAAAGCGTAATCCTCGTGAAATTGCTCAAGCGATTGTTGATAACTTAGATACTGAAAAAGCAC
ATGTAAAACAAATTGACATTGCTGGTCCAGGATTCATTAATTTTTACTTAGATAATCAGTATTTAACAGCAATTATTCCTGAAGCAA
TTGAAAAAGGTGATCAATTTGGACATGTAAATGAATCAAAAGGGCAAAATGTATTGCTTGAGTATGTTTCAGCTAACCCTACAGGA
GATTTACATATTGGTCATGCTAGAAATGCAGCAGTTGGTGATGCTTTAGCTAATATTTTAACTGCAGCTGGCTATAATGTAACACGT
GAATATTATATTAATGATGCTGGTAATCAAATTACTAACTTAGCGCGTTCGATTGAAACACGTTTCTTTGAAGCTTTAGGTGACAAT
AGTTATTCAATGCCAGAAGATGGCTATAATGGTAAAGATATTATTGAAATAGGTAAAGATTTAGCAGAGAAACATCCTGAAATTAA
AGATTATTCTGAAGAAGCACGTTTGAAAGAATTTAGAAAATTAGGCGTAGAATACGAAATGGCTAAACTGAAAAATGATTTAGCAG
AGTTCAATACGCATTTTGATAATTGGTTTAGTGAAACATCTTTATATGAAAAAGGCGAAATTCTTGAAGTTTTAGCAAAAATGAAAG
AATTAGGTTATACGTATGAAGCTGATGGCGCTACATGGTTACGTACAACTGATTTTAAAGACGACAAAGACAGAGTATTAATTAAA
AATGACGGTACATATACGTATTTCTTACCAGATATTGCGTACCACTTCGATAAAGTTAAACGTGGTAATGACATTTTAATCGATTTAT
TTGGTGCTGATCATCATGGTTATATTAATCGTTTGAAAGCATCTCTTGAAACGTTTGGTGTAGATAGTAATCGTTTAGAAATTCAAAT
CATGCAAATGGTTCGTTTAATGGAAAATGGTAAAGAAGTGAAGATGAGTAAACGTACTGGTAATGCGATTACATTAAGAGAAATTA
TGGACGAAGTTGGCGTTGACGCTGCACGTTATTTCTTAACTATGCGTAGTCCTGATAGTCACTTTGATTTTGATATGGAATTAGCGAA
AGAGCAATCTCAAGACAATCCAGTTTACTATGCTCAATATGCACATGCGCGTATTTGTTCAATTTTAAAACAAGCGAAAGAGCAAG
GAATTGAGGTAGCTGCTGCGAATGATTTTACAACGATTACAAATGAAAAAGCGATTGAATTGTTGAAAAAAGTAGCTGATTTCGAA
CCTACAATTGAAAGTGCAGCTGAGCATAGATCAGCACATAGAATTACTAATTATATTCAAGATTTAGCTTCTCATTTCCATAAATTC
TATAATGCTGAAAAAGTGTTAACAGATGATATTGAAAAAACAAAAGCACATGTTGCTATGATTGAAGCGGTCAGAATTACATTGAA
AAATGCATTGGCAATGGTCGGTGTAAGCGCACCTGAATCAATGTAAGAACATTTATATACACTCCAACGTAGAGTTTCTCGAAAGA
TACTTTGTGTTGGAGTGTTTTTTTATTAGGTATGTGACATATTGAGGAATGCTTAGTATGTGAATAAGGTTAAGAGGAACACAGTTG
GATGCTCTGCACAACTGCATAAGAGAGCCTGAGACATAAATCAATGTTCTATGCTCTACAAAGTTATAATGGCAGTAGTTGACTGA
ACGAAAATTCGCTTGTAACAAGCTTTTTTCAAT

>019-contig_265

ACAGACAATGCAAGTTGGGGTGGGACGACGAAATAAATTTTACGATAATATCATTTCTGTCCCACTCCCTCTAAAATGGAGGGTGT
AAATGTTAGGAACTGATGAATTATATAAAGTTTATATGAACATCTCGGACCACAATTTTGGTGGCCTGCTGATAATGACATTGAAA
TGATGTTAGGTGCAATTTTAGTTCAAAATACTAGATGGCGAAATGCAGAAATTGCATTGAATCAGATTAAAGAACATACGCATTTTA
ATCCAAATCATATATTAGAACTACCTATTGAAACGTTACAATCATTGATACATTCAAGTGGCTTTTATAAAAGTAAATCACTGACGA
TTAAAACATTATTAACATGGTTAGCACGACATCATTTCAATTATCAAGAGATTAATGAGCGATATAAAGGTGGATTAAGAAAAGAA
TTATTATCTTTGAAAGGTATTGGAAGTGAAACAGCAGATGTCTTACTTGTTTATATATTCGGACGTATTGAATTTATTCCAGATAGCT
ATACAAGAAAAATATATGATAAATTAGGATATGAAAACACTAAAAATTATGATCAATTAAAAAAAGTAGTCACATTACCAAATCAT
TTTACAAATCAAGATGCTAATGAATTTCATGCTCTGTTAGATGTATTTGGTAAACATTACTTTAGAGACAAAGATATAAAGAATTAT
GATTTTTTAGAACCTTACTTTAAAAAGTAAACGCTGTGAAGTTAGATAGATGAGTTTATATGAAATATAAAAAATAATTTACTATTT
TCTTTTAGTATGTGGACTTATATAATAAATAGAAGCATATAAAGAAAAAAACAGTTGTTTGTTTGTGCAGCAACTGCATAAAAGCCT
CTAATCACTAAAGGTGAAGAGGAACGCAGTTGGATGCTAAGGCACAACTGCATAAAAGCCTCTAATCGCTAAAGATGAAGAGGAA
CGCAGTTGGATGCTACCGCACAACTGCATAAATCCCTCTAATCGCTAAAGCGAAAAGTGGGATTAAAAAGGAGATGTGATAGTGTG
AAGAAATCGTTAATTGCTTTTATTTTGATTTTTATGCTTGTCCTGAGTGGCTGTGGTATGAAAGATAATGATAAACAAGGTAGCAAT
GATAATGGCTCGTCTAAATCGCCGTACCATAGAATTGTTTCGTTAATGCCTAGTAATACTGAAATTTTATATGAATTAGGATTAGGT
AAATACATAGTTGGTGTTTCAACGGTTGATGATTATCCAAAAGATGTGAAAAAGGGTAAGAAACAATTTGATGCTTTGAATCTAAA
TAAAGAGGAACTTTTAAAGGCAAAGCCAGATCTAATTCTTGCGCATGAGTCGCAAAAGGCAACTGCTAATAAAGTATTGTCATCAT
TAGAGAAACAAGGCATCAAAGTAGTGTATGTTAAAGATGCACAATCAATTGATGAAACTTACAACACATTTAAGCAAATTGGGAAA
TTAACGCATCATGATAAGCAGGCTGAACAACTTGTTGAGGAAACTAAAGATAATATCGATAAAGTCATAGATTCAATTCCTGCTCAT
CATAAAAAATCAAAAGTATTTATTGAGGTTTCATCAAAGCCTGAAATATATACAGCAGGGAAGCATACATTTTTTAATGATATGTTA
GAAAAATTAGAAGCCCAAAATGTGTATAGTGACATTAATGGTTGGAACCCTGTAACGAAGGAAAGTATTATTAAAAAGAACCCAG
ATATATTAATTTCGACGGAAGCTAAGACAAGATCAGATTATATGGATATCATCAAAAAAAGAGGTGGATTCAATAAAATTAATGCT
GTCAAGAATACACGTATTGAAGTTGTAAATGGTGATGAAGTATCAAGACCAGGTCCACGTATTGATGAAGGATTAAAAGAATTAAG
AGATGCAATTTATAGAAAATAAACCATTCTAATTATGCCCCTTATTGCTACATGTAAAAAATACATGTTTGAGATAAGGGGTTTTTA
AAATATATTTAGTGAATGATAGCAACGCGAGTATGTGATTGCTATAATGAATGTAATTATCGATGAAAGAAAAGAGAATGCTATGA
CATTTAATAAAGTATTATTGAGCTGGATAGTCATATTGATTATAACAACTAGCATATATCTATTTTGGCAGTTGGGCGATATCAATG
ATGTATTTAACCAGTCTATTCTAATCAATGTTAGATTACCGAGATTATTAGAAGCATTGTTGACAGGTATGATATTAACTGTTGCAG
GCCTTATATTTCAAACAGTTTTAAATAATGCATTGGCAGATAGCTTTACATTAGGATTGGCAAGCGGCGCTACATTTGGTTCAGGAT
TAGCATTATTTTTAGGTTTAACAACGTTATGGATTCCTGTATTTTCAATAACATTTAGTTTCATAACATTAATAACTGTATTAGTCATC
ACGTCGGTATTGAGCCAAGGCTATCCAGTTAGAATCTTAATATTAAGTGGTTTAATGATTGGTGCATTATTCAATTCACTTCTATATT
TTTTGATTTTATTAAAACCTCGCAAATTAAATACAATTGCCAATTATTTGTTTGGTGGCTTTGGTGATGCAGAATACTCAAATGTATC
TATAATAGCAATCACATTTATCATTGCACTGTTTGGTATATTTATCATTCTTAATCAACTAAAGTTATTGCAATTAGGAGAACTAAAA
AGTCAGTCACTAGGCTTAAATGTTCAATTGATTACATATATCGCGTTATGTATAGCTTCTATGATAACGGCGATAAATGTCGCATAT
GTTGGCATCATTGGATTCATTGGTATGGTGATACCACAACTCATTAGAAAATGGCAGTGGAAACAATCATTAGGAAGACAATTGGC
TTTGAATATTGTAATTGGAGGACAAATAATGGTTATGGCAGATTTTATTGGTAGCCATATACTGTCACCAGTACAAATACCGGCAAG
CATTATTATTACATTAATTGGTATACCAGTGTTATTTTACATGCTAATATCTCAGTCGAAACGGTTACACTAGCACATGACATTTGCT
AAAATAAAAATAACTATAAACATAAAGAGGGCATAAGCGATGGATTTGAATCAAATTAAAGCAGTTGTATTTGATTTAGAAGGTAC
GTTGTTGGACAGAGTTAAATCTCGAGAGAAATTTATCGAAGAGCAATATGAACGATTTCATGACTACTTAATTCATGTTCAACTGGC
AGATTTTAAAAAAGCATTTATTGAGCTAGATGACGATGAAGATAATGATAAACCTGATTTATATAAAGAAATCATTAAACGTTTCCA
TGTAGATAGGTTAACTTGGAAAGACTTATTTAATGATTTTGAAATGCATTTTTATCGTTATGTATTTCCTTATTACGATACTTTGTATA
CACTAGAAAAGCTATCGCAAAAAGGCTTTCAAATTGGTGTTATCGCAAATGGTAAATCTAAGATTAAACAATTTCGATTACATTCAC
TTGGTTTGATGCATGTTATTAATTATTTATCAACATCAGAAACAGTTGGTTTTCGTAAACCACATCCTAAAATTTTTGAAGATATGAT
TGATCAACTAGGGGTATTACCTGAGCAAATTATGTATGTTGGCGATGATGCGTTAAATGATGTAGCTCCAGCACGAGCTATGGGCAT
GGTTAGTGTATGGTATAAACAAGAAGATGCTGAAATTGAACCACTCGAAGAAGAAGTTGATTTTACAATTACAACAGTGGAAGAAT
TATTAACCATTTTACCAATAAAAAATGATAATAAAGGAGAAAATTATGGATCTATTTACTAGAAAAGATGGAACATCGATACATTA
CAGTACATTAGGTGAAGGCTATCCTATCGTATTGATTCATACTGTACTTGATAATTATTCTGTGTTTAATAAATTAGCAGCAGAATTA
GCAAAATCATTTCAAGTTGTGTTAATTGATTTACGTGGACATGGCTATTCTGATAAACCTCGTCACATTGAAATAAAAGATTTTTCTG
ATGACATTGTTGAATTACTTAAATATTTATATATTGAAGAAGTTGCATTTGTATGCCATGAAATGGGTGGAATCATTGGTGCGGATA
TTTCAGTACGTTATCCTGAATTTACATCATCACTTACGTTGGTAAATCCAACATCTATTGAAGGTGAATTACCGGAAGAACGTTTATT
TAGAAAATATGCCCATATTATTCGAAACTGGGATCCTGAAAAACAAGATAAATTTTTAAATAAGCGTAAGTATTATCGTCCGAGAA
AAATGAATCGATTCCTCAAACATGTCGTAGATACAAATGAAATATCAACTAAAGAAGAAATTCAAGCAGTTAAAGAGGTATTCAAA
AACGCTGATATTTCTCAAACTTATAGAAATGTCGTAGTACCGACAAAAATTATTGCAGGAGAATTCGGTGAAAGAACAACAAGATT
AGAAGCTAAAGAAGTAGCTGATTTAATCCAAAATGCGGACTTTGAAGTATATCAAGAATCAAGTGCATTCCCATTTGTTGAAGAGC
AAGAAAGATTCGTCGAAGATACAGCTTCATTTATCAACAAACATCACGATGAAAAGCATGTTTAATTTTTAGTGATAAGTGAAATAT
AAAAGTAGTAATTATTATTTGTAATATAATTGTAATATGACTGTTGTTTTAGAAATGATTGTTGTATAATGGTTGTGAGCACAAGAC
GATATAAATAAAAGGAGAGAGTGCTTATGAAAAAGCTACTAACGGCAAGTATAATTGCATGTTCTGTTTTAATGGGAGTAGGCATA
GCGAACACTAATGCCGAAGCAGCAAGTGGCAACTCTATTGATACTGTTAAACAATTAATTAAGGGTGATCAGTCATTAGAAAATGT
GAAAATTGGCGAATCTATTAAAGATGTTTTAACTAAGTACAAAAATCCGATGTATTCTTACAATGAAGATGGAACTGAACATTATTA
CGAATTCCATACTAAAAAAGGTATGTTATTAGTAACTACTGATGGTAAGAAAAACAATGGTAAAGTAACTCATATTTCAATGATGT
ATAATGATGCTAATGGTCCAACATATCAAGCTGTTAAAAATTATGTTGGCAAAGCAGTAACACATACGGAATATAGCAAAGTTGCT
GGTAATTTTGGATATATTGAAAAAGGCAAAACGACTTATCAATTTGCCTCAGCACCAAAAGATAAAAACATAAAATTATATCGTAT
TGATTTGGAAAAATAATAATTAAATTAATCGTGCTTAACCGACAAGTTAAATCGCAATACTTGTCGGTTATTTTATTACATGGCATA
ATACAGTGAATATTAATTTTTATGATTTGCGAAGGGGATATTAAACCAATACAATAAATAAAGATGTTTCATTTTAAAGGAGTACTA
CCAATGCGTATTAACGATAAAATTTTACTAGAAAATATAGAAGATTACTTTAATCATAAAGGTTTATCACCGCATTTGATTGATGAT
ATTAAAGAGAAAGTAATTACTGATATAAAAAATTCTGAAAAGAAAGATCAAGATTATATTGAATATAAAAGAAAATCTCCAGCACA
AATCATATTAATGATTCAAAGGAATTTATTTGCTTTACAAATGAATCCAGTTATTTCTTTATTATAAACTTCATTCTCATATCCTATT
TATATGATAAACAGTATGTTCAGTTTCAAGCTATTACTGGAATGAGTCTATTTTATTGTTTAGTGATTTTTCCAATGACTATTGTTGTA
TACCTGAGGGTGTCTCAAAAGAATTACTTGCGTAGTAATAAAATAGAAATGATTATGGGTACAATTATCGCTATCATATCCTTGTTA
TTAATTATATTACAAGCATTTAATATTACTTGGGGCGTTATACCAATTACAAATTTTGGACATCAATTTTTCTTTTTCATTGGTATTAT
TTTAGTAATTGCCGGCATATTTTATAAGCGACTTGAGTTTTCGGGAATCGGCTTATTATTTTGTCAAAAAACCGTCGATGCAATGATT

CATAATCCACAATCAGCCCAGATTTTTTCATTAATTATATGGATATTATTAGTAGTTCTAGTTATATATTTCACAATTAGATTATCTTC
ACGTACAAGATTATAAATATGATAAAACTATTCACTTGATTAATTGTATTAATTGAGATGAATAGTTTTTTTATTGTTGGAATAACTT
TTGGTAATTTATAAATAATTTAAAAAAATTGTTTATAAAATGGAAGCGTATATAGAATGAAGGTTGGGTATATAGTTTATTGAGGGA
GGTGTCACAATGAATAAAGTCACAATTAATCCTCAAATCCAATTAACTTATCAAATTGAAGGTAAAGGGGATCCTATAATATTACTT
CATGGATTGGATGGTAATTTAGCTGGATTTGAAGATTTGCAACATCAACTAGCATCATCATATAAAGTACTTACTTACGATTTAAGA
GGTCATGGCAAGTCTTCTAAAAGTGAATCATACGATTTAAACGATCACGTTGAGGATTTAAAAATTCTAATGGAGAAGTTAAATATT
CATGAGGCACATATTCTAGGACATGATTTAGGTGGGGTAGTTGCTAAGTTATTTACAGATAAATATGCTTATCGTGTAAAATCATTA
ACTACCATTGCATCGAAGAAAGATGACTTAATACACAGCTTTACTCAATTGTTAATACAATATCAAGATGATATAGCGGGTTTTAAT
AAGTCTGAAGCGTATATTCTTTTATTTTCTAAATTGTTTAGAAATCAAGAGAAGACGATGAAATGGTATCAAAAACAAAGAATATAT
AGCATTAAGTCTGAGGATGATAGTGCGGTGGCTATTCGTTCATTAATTTTGCATAAAGATGAACCTATGTATTTAAAAAAACGTACA
TGTGTACCTACTTTGTTAATTAATGGGGAACATGATCCTTTGATTAAAGATAAAAATCATTTTAAATTGGAAGCGCATTTTTTAAATG
TTACGAAAAAAATCTTCGAACATTCAGGACATGCACCGCATATTGAAGAACCAGAAGCATTTATGAATTATTATTTAAATTTTTTAA
AAAGCGTATCATAATATGTGATATATAAACCTAGGGTATAAAGTCCTTAGGCAATGTGAAAAAGCTGATTACTATTCATTATTTGAT
AGAAATCAGCTTTTTTTGAAATGTATTTGATATATACTGCTCGTTATGCGGCTATCTTCCTTATATTAAGTGCCATTAGTGCAAAACC
TCTTAACAATTAGGTAAAAAGAGCATAAAAAAAGGAAGTTTAATAGAATGTATCATCTATCAAACTTCACCAAATTGCGCTAAACA
AAATTATAGTTCAATTTCGTTGTTTGCTTCAGTGATTCGTTTATTTACTCGACTCAATAATGATTCGATTTTTTTACGTTGTTGTGCAT
TAACAAGAATTAATACAGTTCTTTCATCATGCTCATTACGTTTTTTATCGAAGTAATCTTCTTGAGATAAAATTTTAACTGCTTTAAC
AACTTGTGGTTGTTTGTAGTTTAAATGATTAATAATATCTTTAAGATAGTATTCTTTCTCTTTGTTTTCGCTGATGTATGTCAATACAG
CGAATTCTTCAAAGCTAATTGAAAATTCCTTTTTAATTAAACTTTTTAATTTGTCAGCATAAGTGACCATTGATAACAACTCAAAGCA
ATCATTGATTTTTGTAATTGCCATGTTTAAAACCTCCCTATTTGATGCATCTTGCTCGATACATTTGCCCGATAATATATTGATATCTA
ATCTTTATTTATTATAGATATGTTAGTCATAATTTTGCATTAAATAAGTTTTATTAAATATATTTAATGCTCTATTATTTAGTTAATTA
TAACTAATTAAAAATGAGAAGTAAACAAAAAAGTGTTTATAAAACAAATTATTCATTAGACACAGTGATTGTATTTCTGGGTTAGC
ATTTGGTTTAGTCAAAAATATCAGCATTTTTTATAATTTACCTTAATTTAATCGACAATACAAGGTAATTTATTTAATGAAACGATTT
AGCGCAATTAAATGTTTCGATATTCAATGTGTGTCTAAAGAGATTCTTTGTTATAGCTTAACTCTTCCAAGTTAACTTCTTCTACTATT
TATACCCATTTTTCAGAATTTTATCACTAAAAATGAAAAATTTTTATAATTTTATTAATTTAGTAATTCTCAGTGCAGTTTTAATGATA
CGAAAAACACATTAATTTAGTGATTAAAACCAATCTGTAGCACGTTAAAGTGTCTATACGTTATTCCGATTGATAACGGGTAACAAA
TAATAGAAAGTACGATAATTGATCATGCATTAAAGCACCTTTATTTTTCAAACTCGACTCAATGAATTATAAAAAGTTGTGCATTTT
GACTTATTTTTAATTAATTGAATAACCAACTATGAAATATTAGCACGAATAACATAATTTTTGCTATTAATTTGGTGTAAATTATTAA
AAAATAACATTAATTTAGGTCTTAAGAATGAGTTGACTATCGAATTTGCTAAATTAAAATTTGTTGTGTCAAAGCATACTACCCAAA
TCAACGCTTTTCTAAAGATTTTTAGACCACAAAAAAAATAGCTTATCACTTTTAAAAATAATTACAAGTTAAAATGATAAGCTAAAAG
TTAATTTAAAATAATCTTAAAAGTATAATGCCTGTCAAAATACATAATAGACCAATAGTTTTTCTGGATGTCATTGCTATTTTAGGTG
AACCAAATAATCCAAAGTGATCTATCAATATGCCCATTAGAATCTGGCCAAACATCCCAATAAGTGTTGTTAATGCTGCACCCATAT
GAGGCATTAAGATAATGTTAGCTGTTACAAAAGCCATGCCAAGTATACCGCCAGTAAAATAGATAGGCTTTAATTTACCGAATTTTA
AATGACTTGTTTTTAGTTTTAAAGAACGATTAAAAATAGCGGTTAAAATCAATAGCGCTATTGACCCAATTGTAAATGATACTAATG
ATGCAAAGGCTGGTGAATGAGTATGACTTGCTAAAGCACTATTAATTGTCGTTTGAATAGGTGGAAAGAAACCAAAAATAAATCCT
AAGAGAAGCCAAAACAGTAAATACTTTTGATCAGTTAGTAATAAATTATTCTTGTTAAATTGATTCATTATGACGATGCCGATAATG
AGTAACAATACTCCAATTGCTTTAATTAAATTAAAATCATGAATTGTAGCGCCAAATAAGCCCAATGTATCAATAATGACACCCATA
ATAATTTGACCCGCAACTGTTGCAATTACAGTTAATGTTGCACCTAATTTTGGCAATAACAATAAATTGCCAGTTAAAAAGCTAACC
CCAAGCAAACCACCGACTACCCATGTGTAGTTAAATGATTGATTATTGTAAAAGTGAATAGTAAATACTTCTGGATTGATAATGATA
TTTAAAATAATTAAACAAATTGTTCCAACTGAAAATGAAATGAATGAAGTATAGAAAGGCGATTTAGTATACAGTGATAGTCTTGA
ATTGACAGATGTTTGGATAGGAATAAGCATTCCAACTAGGACACCAATGATATAGAAAAGAAGCATATGAATAAAGTCTCACTTTC
CAAAGTTAATAATAAAATCATATATTTACATTATCATTATAGAGAAAATATACAACTCATTTACAATAATGTTGAAAAAAACAGGA
CATTATTACAACAAAATCACCCTAGACAATCATGATGAAAGTCTAGGGTGTTAGTATTTATACTAAATTAATTGCGCTTTCTTTATAT
CTATTTCTTATCTTCAAATAAAACAGTTGTTGTTTTATCAATAATTTGAGCAGAATGTACTTTGGTGATAGTACCACTATTTGACTTT
ATAATATTTAAATCTACTAATGCATTCATACTATCACGTGCAACTTGCTCGGTCACTACGTTGTTTAATTGTGATAATTGCAGTTTTA
CAGGCTTGTCTAGTGAAGATTTAAAACTTAATTCAAGTGTTTTAGTCATGAATATGTCCTCCTGATTAAATTGATAAAGATTTGATGA
GTTCGATATTGCTATATGTTTCTCCAGTAAGTCGCTCAATAAGTTTGCTGAATGTTTTAATTTGGTCGTTTGATGCATCAGGGTTAAT
GTTAGCGAATCGACGCTTAAATTCTGTTTGTTTGCCGTTAGCGTCTACTTTAGTAAATGATAATACAATAGTGATGTGGTTTATTTTA
CTCATATTTTAAAACCTCCTTTCACACTATATATCGAAACAAAATAATAAAATGGCTAATTTTATTTTCTATGTTTAAAATCTATAAA
AAAGGCAATAGATATATGTAACTAAAATATAATATAGCTATGATTAAGACACTTAAAGCAAGGGGAGGTTGAGTAATGAATAAAGT
AGAAGCGATTAAATTTAATGATGATATTGTTAAAATGTATGAAGCGCTCAAGATAAAATCTGAACGTGACTATTTATTCTTTAAGTT
AGCTATACATAGTGGATTGAAAGTATCAGAATTATTAACAATTACAGTCTCTCAAGTTAAGAGACTAATTGAAAAGTGTACGTTATC
AGAAATGTGTAAAGCACATTTTCATTCGTTGATTAAAATTAGGTTACCAGAAACATTATCGAAAGAACTACTTCAATATATAGAGGA
CAGGAGTCTTTCGAATGAAGACGTTCTTTTTCAATCACTACGAACAAATCAAGTATTATCTAGACAGCAAGCATATCGAATAATTCA
CCAAGCATCAATTGAAGCTGGTATAGATAATGTAGGACTAACGACATTGCGTAAGACATTTGCATATCATGCTTATCAAAAAGGTA
TACCTATACCAGTCATTCAAAAGTATTTAGGGCATCAATCTGCTATTGAAACACTAAATTTTATCGGTTTAGAAAATGAGTGTGAAC
ATAGTATTTATATTTCATTACAATTATAGAAACAAAGGAGGCTAATAATGAGTTTGGTTTATTTATTAATTGCTATACTTGTGATTAT
GGCGATGATACTTCTAATGTCTAAACGTAGAGCATTGGCTAAATATGCCGGGTACATAGCGTTGGTTGCACCTGTAATTTCATCTAT
CTATTTTTTGATTCAAATACCATCAGTAGCTAAACTGCAATATCTTTCTACCTCTATTCCATGGATTAAGACATTAGATATTAATTTA
GATTTACGTTTAGATGGTTTAAGTTTAATGTTTTCTCTTATTATTTCACTTATTGGAATTGCAGTATTCTTCTATGCAACTCAATATTT
ATCCTCTCGAAAAGACAATTTACCAAGGTTTTATTTTTATTTAACGTTATTTATGTTCAGTATGATTGGTATTGTATTATCAGACAAT
ACGATATTGATGTACATTTTTTGGGAATTAACGAGTGTATCATCATTTTTATTGATTTCATATTGGTATAACAACGGAGATAGTCAAT
TTGGTGCGATGCAATCATTTATGATTACAGTATTTGGTGGTTTGGCATTATTAGTTGGTTTTATCATGTTGTATATTATGACAGGAAC
GAATAACATCACAGAGATATTAGGACAAGCAGATTATATTAAGAATCATGGATTGTTTATCCCTATGATTTTTATGTTTTTATTAGGT
GCATTTACAAAATCAGCACAATTTCCATTTCATATTTGGCTACCTAGAGCAATGGCTGCACCTACACCTGTAAGTGCTTATTTACATT
CAGCCACGATGGTAAAAGCTGGTATCTTTTTATTACTTCGATTTACACCATTATTAGGTCTTAGCAATATGTACGTATATATCGTTAC
GTTTGTTGGTTTAATAACAATGTTATTTGGTTCAATTACAGCTTTAAAACAATGGGATTTAAAAGGTATCCTAGCGTACTCTACAATC
AGTCAACTTGGGATGATTATGGCTATGGTGGGTATAGGTGGCGGATATGCTCAACACCAACAAGACGCAATAGCATCTATTTATGT
ATTTGTATTATTTGGTGCGCTATTTCATCTAATGAATCATGCCATCTTTAAATGTGCGCTTTTCATGGGAGTAGGTATTTTAGATCAT
GAAGCAGGTTCAAGGGATATACGAATTTTAAGTGGAATGCGTCAACTATTTCCTAAAATGAATCTAGTCATGACGATAGCGGCTCT
ATCTATGGCTGGAGTACCATTTTTAAATGGATTTTTAAGTAAAGAAATGTTTTTAGATGCATTAACACAAACTGGACAATTATCCCA

ATTTAGTTTGATTTCAATGATAGCTATCGTGTTTGTTGGTGTTATTGCGAGTGTTTTTACATTCACATATGCACTATACATGGTAAAA
GAAGTATTTTGGACAAAATATGATTCTAAGGTTTTTACTAAAAAAAATATCCACGAACCATGGTTGTTTAGTTTACCATCTCTTATAT
TAATGGTGCTAGTACCTGTAATCTTTTTTGTACCAAATATATTTGGGAAGGGGATTATCGTTCTAGCATTAAGAGCTGTATCAGGTG
GTAATCATCAAATTGATCAATTGGCACCACATGTTTCGCAATGGCATGGATTTAACATACCGCTTCTTTTAACCATCATCATTATTTT
ATTGGGTAGTGTACTAGCAATCAAAGTAGATTGGAAAAAAGTGTTCACAGGTAAAATTAGACAGATTTCAGTTTCAAAAAGCTATG
AGATGGTATATCGACATTTTGAAAAGTTTGCTACGAAGCGATTTAAACGTGTTATGCAAGATCGTTTAAACCAATACATTATTATGA
CCTTAGGCATATTTATGATTATCATTGGATATGGTTATATTCGAATTGGACTTCCTAAAGTACATCAGTTACATGTTTCTGAATTTGG
GGCATTAGAAATTATATTAGCAATCGTAACTGTCACAATTGGTATTTCTTTAATTTTTATACGTCAACGACTGACAATGGTCATTTTA
AATGGAGTCATCGGATTTGTTGTGACCTTATTCTTTATAGCAATGAAAGCCCCTGATCTAGCATTGACTCAGCTAGTAGTTGAAACA
ATAACGACGATACTATTTATTGTCAGTTTTTCAAGATTACCAAACGTGCCAAGATCTAACGCTAACAAAAAAAGAGAAATAATTAA
AATTTCTGTATCACTCTTGATGGCACTTATTGTTGTATCATTAATTTTTATTACACAACAAACAGATGGTTTATCATCAATATCAGAC
TTTTATTTAAAAGCTGACAAACTAACAGGTGGTAAAAATATTGTAAATGCGATACTTGGTGACTTTAGAGCATTAGATACATTATTT
GAAGGATTAGTGTTAATTATTACTGGGCTAGGTATTTACACATTATTAAATTATCAAGATCGGAGGGGACAAGATGAAAGAGAATG
ATGTCGTGTTAAGAACGGTCACGAAACTTGTTGTATTTATTTTATTGACTTTCGGATTCTATGTCTTCTTCGCAGGTCATAATAATCCT
GGTGGTGGGTTTATTGGTGGTTTAATATTTAGTTCAGCGTTTATTTTAATGTTTCTGGCTTTTAATGTTGAAGAGGTTTTAGAAAGTTT
ACCGATTGATTTTAGAATTTTAATGATTATTGGAGCATTGGTATCATCTATTACTGCGATAATACCTATGTTTTTTGGAAAACCATTT
TTGTCTCAATATGAAACAACTTGGATACTTCCAATTTTAGGACAAATTCATGTAAGTACAATAACACTTTTTGAATTAGGTATTTTAT
TCTCAGTTGTTGGTGTTATTGTCACAGTGATGTTGTCGCTTAGCGGAGGTCGATCATGAATTTAATATTATTACTAGTTATAGGATTT
TTAGTGTTTATAGGAACATATATGATTTTATCAATCAATTTAATTCGTATTGTAATCGGAATTTCAATATATACTCATGCTGGTAATC
TCATTATTATGAGTATGGGAACGTATGGTTCTAGTAGATCAGAACCACTAATAACTGGTGGAAACCAATTGTTTGTTGATCCCTTGT
TACAAGCTATTGTACTAACTGCAATAGTTATAGGGTTTGGGATGACTGCGTTTTTACTTGTACTTGTTTATAGAACTTATAAAGTAAC
AAAAGAAGATGAAATTGAAGGCCTAAGGGGGGAAGATGATGCTAAGTAACTTATTGATTTTACCAATGTTATTACCATTCCTTTGTG
CCTTAATCCTTGTATTTTTAAAAAATAATGATCGTATTTCTAAATATTTATACTTAGGTACAATGACTATCACCACAATTATTTCATT
AATGCTATTAATTTATGTTCAGCGTCACCGTCCAATTACGCTAGACTTTGGAGGATGGTCAGCGCCCTTTGGTATACAGTTTTTAGGA
GATTCTTTAAGTTTAATTATGGTTACAACCGCTTCGTTTGTGATTACTTTAATTATGGCATACGGATTTGGGCGTGGCGAACATAAAG
CAAATCGTTATCACTTGCCATCGTTCATATTATTTTTAAGTGTTGGCGTGATAGGCTCTTTTCTAACATCAGATTTATTTAATTTATAC
GTCATGTTTGAAATTATGTTACTAGCGTCATTTGTACTCATTACACTTGGACAATCTGTAGAACAATTACGTGCTGCAATTATTTATG
TTGTCTTGAATATTATTGGTTCATGGCTATTCTTATTAGGTATAGGTTTACTTTATAAAACAGTAGGTACATTAAACTTTTCACATATT
GCAATGCGTTTGAATGACATGGGAGATAATCGCACTGTTACAATGATTTCATTAATCTTCTTAGTCGCATTTAGTGCGAAAGCAGCG
CTGGTCCTTTTTATGTGGCTACCCAAAGCCTACGCTGTGTTAAATACTGAGCTTGCAGCATTATTTGCAGCGTTAATGACCAAAGTA
GGGGCCTATGCATTAATTCGATTCTTCACTTTACTATTTGATCAACATAATGATCTCATACATCCATTGCTAGCAACTATGGCTGCTA
TAACTATGGTCATCGGCGCTATAGGTGTCATTGCTTATAAAGATATTAAAAAGATTGCAGCTTACCAAGTCATAATCTCAATAGGAT
TTATCATTTTAGGTTTAGGAACAAACACGTTTGCAGGTATTAATGGTGCAATATTTTATTTGGTAAATGACATTGTTGTAAAAACATT
GCTATTTTTTATTATTGGTAGTTTAGTTTACATTACAGGCTATCGACAATATCAATATTTGAATGGCTTAGCTAAAAAAGAACCTTTA
TTTGGAGTTGCGTTTATTATAATGATTTTTGCTATTGGCGGCGTGCCTCCATTTAGTGGCTTTCCGGGGAAAGTACTTATTTTCCAAG
GTGCATTGCAAAATGGCAATTATATTGGACTAGCGTTAATGATTATTACTAGTCTAATTGCAATGTACAGTTTATTTAGGATACTTTT
TTATATGTATTTTGGAGATAAAGATGGGGAGGAAGTTAATTTTAAGAAAATCCCGCTATATCGAAAAAGAATTTTAAGTATTTTAGT
AGTTGTGGTTATCGCAATCGGAATTGCTGCACCTGTTGTGTTAAATGTTACAAGTGATGCAACTGAGTTGAACACGAGTGATCAATT
ATATCAAAAACTTGTAAATCCGCATTTGAAAGGAGAGGACTAAATGAATCAAATAGTTTTAAATATTATCATTGCATTCTTATGGGT
ATTATTTCAAGATGAAGATCATTTTAAATTCTCGACTTTCTTTTCTGGATATCTAATTGGTTTAATTGTCATTTATATATTACACAGGT
TTTTCAGCGATGATTTTTATGTTAGAAAAATATGGGTAGCTATTAAATTTTTAGGTGTTTATTTATATCAATTAATAACATCTAGCAT
TAGCACGATTAATTATATTCTTTTTAAAACAAAAGATATGAACCCTGGATTACTTTCATATGAAACAAGACTAACAAGTGATTGGTC
AATAACATTTTTAACAATTTTAATTATTATAACTCCAGGGTCTACAGTAATACGAATTTCTCAAGACTCTAAAAAGTTTTTTATTCAT
AGTATCGACGTGTCAGAAAAAGAAAAAGATAGTTTGTTAAGAAGTATTAAGCATTATGAAGACTTAATATTGGAGGTGTCGCGATG
ATACAAACAATAACACATATTATGATTATTAGTTCACTCATTATTTTTGGAATTGCATTAATCATCTGTTTATTTAGATTAATCAAGG
GACCTACAACAGCAGATCGTGTCGTTACATTTGATACAACAAGTGCTGTCGTAATGTCAATTGTGGGTGTGTTAAGTGTACTTATGG
GCACCGTTTCTTTCTTAGATTCAATCATGCTCATTGCCATTATATCTTTTGTAAGTTCTGTTTCAATATCACGCTTTATTGGTGGGGGG
CATGTGTTTAATGGAAATAACAAAAGAAATCTTTAGTCTTATTGCTGCTGTGATGTTGTTGTTAGGTAGTTTTATTGCTCTTATTAGT
GCAATAGGTATCGTGAAATTCCAAGATGTTTTCTTAAGAAGTCACGCTGCGACAAAAAGTTCAACTTTATCCGTGTTATTAACTTTA
ATCGGTGTATTAATTTATTTTATTGTGAATACAGGATTTTTCAGTGTGCGTTTATTACTGTCACTTGTTTTTATTAATTTAACTTCACC
AGTCGGCATGCACTTAGTCGCTCGCGCTGCTTATCGCAACGGCGCTTATATGTATCGAAAAAATGATGCTCACACACATGCATCAAT
ATTATTAAGTTCAAATGAACAAAACTCTACAGAAGCATTACAATTACGTGCTGAAAAACGAGAAGAGCATCGTAAGAAATGGTATC
AAAACGATTGAAGTGTTGGCTAAATTGTTAAGCATAACAATGCTTTTGAAAATCTGTTTTCAAAATTAATCATTAAAAGAGTACTTT
GAAATTGGTTAGTCGATTTTAAGGTGCTCATTTTTTTGTATTATAAGTTAATGTTGTTATGTGAAAGCAAGTTGTTTATTTATACAAAC
AATTAGTGATAAAATAAAATTAAATAATTGCAAATTTAAGTACTGCAATCGTAGACATATATCTATATTAAAGTGCTTTTGTGGTCG
CTAACTCAATTTATCCTCTAAAGTATAATTACAACGAGATGTGATACTTAATTTTCAATTAAACAATACTTTTTAGAGAGGTGAAAG
TTTGGAAATATTTGAAACAATTCTTATATTTATAGCTGTTGTGATACTAAGTTCGTTTGTCCATACTTTCATACCTAAAGTACCCCTA
GCATTTATACAAATTTTCTTGGGCATGTTACTATTTATTACCCCAATCCCTGTTCAATTTAATTTTGATTCTGAATTGTTTATGGTAAC
AATGATTGCGCCTTTGTTATTTGTAGAAGGTGTTAATGTTTCTAGAGTCCATTTAAGGAAATATATTAAGCCAGTGATGATGATGGC
ATTAGGATTAGTCATTACTACTGTGATAGGTGTAGGTTTATTTATTCATTGGATTTGGCCAGATTTACCTATTGGAGCAGCATTTGCA
ATTGCTGCCATTCTTTGTCCTACTGATGCAGTAGCAGTGCAAGCAATCACTAAAGGAAAGGTCTTGCCAAAAGGAGCAATGACAAT
TCTTGAAGGTGAGTCATTATTGAATGATGCTGCTGGTATTATTTCATTTAAAATAGCTGTTGGAGTATTAGTTACAGGTGCTTTTTCA
CTTGTTGATGCTGTTCAGTTGTTTTTAATTGCATCAATTGGTGGCGCAGTGGTTGGTTTACTTATAGGTATGGCATTAGTAAGGTTCC
GATTAACATTGATGCGTCGAGGATATGAAAACATTAATATGTTTACAATTATTCAATTGTTAACACCATTTGTTACGTATTTAATTGC
TGAATTGTTTCACGCATCAGGAATCATTGCAGCAGTAGTTGCAGGACTTGTACATGGTTTCGAACGTGACAGAATTATGCAAGTACG
TACACAACTGCAAATGAGTTACAATCATACATGGAATATACTAGGTTATGTTTTAAATGGCTTTGTTTTTTCAATATTAGGATTTTTA
GTACCTGAAGTTATTATTAAAATTATCAAAACAGAACCGCACAATTTAATCTTTTTAATAGGCATCACTATTGTTGTTGCTTTAGCTG
TCTATCTATTTAGATTTGTTTGGGTTTATGTCTTATATCCTTATTTTTATTTAGCCATCAGTCCATTCCAAAAAATGATGACTAAAAAT
GATGATGATAATCCAACGACTGAGAAACCACCAAAGCGAAGTTTATACGCTTTAATTATGACGTTATGTGGTGTGCATGGAACAAT
TTCTTTAGCAATCGCATTAACGTTACCGTATTTTTTAGCAGGGCATCATGCTTTTACGTATAGAAACGACTTATTATTTATTGCATCT
GGTATGGTTATTATTAGTTTGGTAGTTGCGCAAGTATTATTGCCATTATTAACGAAACCTGCACCTAAAACAGTAATTGGCAATATG

TCGTTTAAAGTTGCTAGAATTTATATATTAGAACAAGTTATTGATTATCTAAATCAAAAATCTACTTTCGAAACAAGTTTTAAATATG
GTAACGTGATTAAAGAATATCATGATAAATTAGCATTTTTAAAAACTGTAGAGAAAGATGATGAAAACTCTAAAGAATTAGAACGT
CTACAAAAAATTGCTTTTAATGTAGAAACAAAAACATTAGAGTCTTTAGTAGATGAAGGACAAATAACGAATAGTGTACTTGAAAA
CTATATGCGTTATGCTGAAAGAACACAGGTATATAGACAAGCATCATTAATAAGAAGAATGATTGTATTATTACGAGGTGCTTTATT
AAAACGAAGAGTACAAACGAGAGTGAACTCCGCATCTTCACTTAGTGTTACGGATAACTTAATGGAATTAAATAAAATTAATAAAT
TAGTCCATTATAATGTGGTTAGTCGTTTGTCTAAGGAAACAACAAAAGATAATACACTTGAAGTTGGAATGGTTTGTGACGGTTATT
TAATGCGAATTGAAAACTTAACACCATCAAATTTCTTCAACTCAGCAAGTGAAGATACGATTACTAAAATTAAATTAAATGCATTGA
GAGAACAACGTCGCATTTTACGTGAGTTGATTGATACAGATGAAGTATCAGAAGGTACAGCGTTAAAACTAAGAGAAGCCATCAAT
TACGATGAAATGGTTATTGTAGATAGTATGACGTAGTTCCTAATTATGCTAAAAGGGATTGATGAAAAACTGAAGGGCTTTTCATCA
ATCCCTTTTATTTTATGGGAATTGAATAGATAGTTTTAAACTATACGAATTATTAATATTTGAGATTTAATTGAAATAAGTTTTAAAA
ATTGGAGGAGATAGATTAAGCGAAGTCATTTAAAGGTGAAGTTAAGTGTATTCACAAAAAATAGCCACACTCATATGACATCGGAT
GAGTGTGGCTTAAGGATCTATGGGGGGAGGAAACCATAGATGTTTACTTTGATAGGCCAGATTAAATATCAAAGTATGCGATTATTT
ATAGCTTGATGCAAAAGTGGTATGCCTATTAAAAGTTACTGCACATAGCTTTTAATATTCCGTTCAAAGGAAAGGGGCATACAATTG
AACAATCTGTAATAGTACTTTTAACCAGCTATGCTAAAAGTCTAGTAGGGAGAACAGTTGTCCAATCACATAAGAACCTCTAACTTC
GTTAGTACGATTAAGAAAAGCTTTTTAGTTAGTATGTAATACAATTTATTGACGCGCGTGAATCTCTTTTATAAGAGTGTGTAGGGA
ATGGCGTTGTATAAATTGTATTAGAAGAACTTCTAACGCATCTCTGTGGTTAAAAGAGATGAAGGGAACGACAGTTTAATTAAAAC
TGCATAAGAACTTCTAGCTTTTCTCTCTCGTTCAAAGAGAAGCAGCTGTTCGCAGTTTAATCAAAACCACATAAAGCTTTTAAC

>020-contig_263 RC

CCACATAAAGCTTTTAACTTTACTCTTTGATTTAAAGAGTGACAAATGTTTACAGTTTAATTAAAACTGCATAAGAACTTCTAACTTT
TCTCTTTCGTTCAAAGAGAAGTTCTAATACCACCATATCGTGCGATCGGGAACGGTATATATATTAATAGGAGGGTAATATATATTT
AACGCACGATATGGGACTATTAGCCTTCGACTTTGTTATGTTGATGTGTGGCCTAAAATATTGGAGATACCAATATTTTAGGTTGCA
TCAACATCAATTCATCTTACTTCATTAAAACACAGCGTGTTATTTCATGCTTCCGTGTACAGTTTCAATATTTGATTTCATCATTTTGT
AGTAAGAGTCACCTTTAGTGCCTTCTTTACCGATTGAATCTGTGTACACTTCACCAAAGATATCTTTCTTCGTTTCTTCAGATAAACT
TTCCATTGCTTTCTTATCAACACTTGTTTCTACTAATAAGTGTTTTAATTTGTGCTTTTTAACAAACTCAATAGCTTGTCTCATTTGTTC
AGGTGTACCTTGTTTTTCAGTGTTAATTTCCCAAATATAACCTGGTGTAATACCGTATTGTTTTGAGAAGTACTTGAAGGCACCTTCA
CTTGTAATCATGGCACGTTGTTCTTTTGGAATGTCATTAAATTTGTCTTTACTGTCATTATTTAATTTTTCCAATTGAGCAATGTATTT
GTTACCTTGCTTTTCATAATCTGCTTTATGTTTTTTGTCGTTATCGATAAATGTTTGTTGAATTGTTTTTACGTATTTAATACCGTTATC
TAAACTTAACCATGCGTGTGGATCTTGTTTATCTTTGTTGCCTTCTTCACCGTTTAAATAGATAGGTTTAACATCTTTTGATACTGCGA
TAACTTTTTTATCTTTTAATGATTTACCAGCCTGTTCTAAGGCTTTTTCAAACCAACCGTTACCAGTCTCTAAATTTAATCCGTTGTAT
AAAATAACGTCAGCGTCAGTTAACTTTTTAATATCTTTAGGTTTAACTTCATATTCATGAGGATCTTGACCAACAGGTACAATACTAT
GAATATCGACGTTGTCTCCACCAACATTTTTAGCCATATCATATAAAATTGAATTCGTCGTTACTACTTTTAATTTGCCATTTGACTT
ATCACTGCTTTGTTTACCACCAGTACCACATGCAGCAACTAGAAGTAATAAGGCTAATAATAAAGGTACTAATTTTTTCATGTTAAA
CTTCCTCGTTTCTTTCTATTCGTAAATTTTGTGAAAAATAATGTGATGATATAAATTACAAACGTACAAAGTACGATTGTCGCACCAC
TAGGAATGTTGTAAATATAGCTGTAATAAAGTCCGACAATTGAACTTATGACACTTATTAAACTTGCTATAATCATCATTGAGTATA
GTTTTTTACTAATTAAAAATGCTGTAGATGCAGGTGTAATTAATAATGCAACTACAAGAATAATACCTACCGTTTGAATACTTGCTA
CTGTTACTAATGAGAGTAACAACATCACAAAGTAATGTAATAACGTCGTATTTAGACCACTCATTCTACTAAACGTTGGATCGAATG
TAGAAATCATTAATGGACGATAGAAAATAATGATTAGAATAAGGACGATTGAACCAATCACAATAGTTGTTAAAAATGCACTATTT
GTGATTGCCAGTAAATTACCAAACAGAATATGGTACAAATCTGTCGTAGTGTTTATTAAGCTAATAATAATAATCCCCGAAGCTAAG
AAAGCGGTAAAACTAATTCCAATAGCGGCGTCAGGTTTCGTTTTACTACTAGATGTGATATAACCGATAAAAATACTTGCGATCATA
CCAGTTATAAGTGCGCCTACAAACATTGGAATACCAAATAAGAATGATAGGGCAACACCAGGTAATACTGCGTGACTCATTGCATC
TCCCATTAATGAAAGACCACGTAATACAATTAAACTACCAACTGTACCACAAACTATCCCTACAATAATTGAAGTTATCAATGCTCG
ATTCAAGAATTGATATGTAAATAAATGTTCGACAAACTCTAACATGTTATATTGCTCCTTTGACTAGGGTCACTACAGTCAGTGCTA
CTCATAAATGTTTCGTTTAAGCGAGTGACACTCATAGCCTCTTCACTATCACCAAAGTATCGTAATGTTTGATTTAATAGAATAATGC
GATCAAAGTATTGCTTTGCTTTTGATAGATCATGGTGGATGATAAGAATAAGTTTTCCTTGTTGTTTTAAGTTCTCGATTTTTGTCATG
ATTAATTTTTCGCTACTAAAATCAATTCCGACAAACGGCTCATCTAGAAAATAAACTTCACTTTCGGACATCAATGTTCTTGCTACTA
GCACACGTTGTAATTGTCCACCACTTAATTCTGAAATTTGTCGATGACGTAAAGATTCTAATTCTAAATCGCTTAATAACTGTTTGAG
TTTATCCCTTGCTGATTTATTAGGTCGTCTAAACCATCCAATTTCTTTGTAGCAACCTGATAAAATCACTTGTTCCACACTTATAGGA
AAATCTAAATCAATATGTGCTTTTTGTGGAATATATGTAATATGTTGCAGTTGTTGTTGTATAGGTTTGTTATATAACAATTTAGTAC
CGGTAGCATTAAATTCACCAATTAAAGACTTGATAAGGGAAGATTTACCAGCACCATTCGGGCCCATGATACCAATTATTTCGCCGC
GTACTGGTATCGATAAGGAAATGTTTTTAAGTACATGCTTATTACCTAAAAACAGATTTAAATCTTTTGTTTCTAACAAACGTTTATA
CCTCCTAATTAAAAGTTTAGGCTAACCTAATTAACTGTATAATAAACTGAGAATATTTATCATGTCAAGTAAATTCGTGATATAATA
TAGACAATGTATGTGAGGTGAAAGTATGTTAACTGAAGAAAAAGAGGACTATTTAAAGGCAATCCTTACGAATAATGGCGATAAAA
ACTTTGTGACAAATAAAATCTTATCTCAATTTTTAAATATTAAGCCTCCATCTGTAAGTGAAATGGTAGGACGTCTTGAAAAAGCAG
GCTATGTTGAAACAAAACCATACAAAGGTGTTAGATTAACAGAGGATGGTTTAACGCATACGCTTGATATCATTAAGAGACATCGA
CTATTAGAATTATTTTTAATAGAAATATTGAAATATAATTGGGAAGAAGTACATCAAGAAGCAGAAATTTTAGAACATCGAATTTCA
GATTTATTTGTTGAAAGGCTGGATAGCTTGTTAAATTTCCCAGAAACTTGCCCGCACGGCGGTGTGATTCCTAGAAATAATGAATAT
AAAGAGAAATATATAACAACGATATTGAATTATGAACCTGGCGATATCGTTACAATCAAACGTGTGAGAGATAAGACCGATTTGCT
AATATATTTGTCTAGTAAAGATATTTCTATTGGTAATGAAGTGGAAATTGTATCGAAAGATGAAATGAATAAAGTAATTATCATTAA
ACGTAATGATAATGTAATTATTGTCAGTTACGAAAATGCAATGAACATGTTTGCTGAAAAATAAAATAAAGAAGCCATAAAGATAT
CCATGATTGAACTGATAAAGACATATGGATAATTGCTTTAGGCTTCTTTTTTATTAGTTAATTTATCAAGTGAGTATATTTGAGTAAA
ATATTCACTGCATAAAGATTGAAGATAATCCAGATTGTACTATAAATGAAGATAGGTACATGACTGAGTTCTTTAAGTGCACTACCA
TCCCACTGTGGACTCGGACGCTGGAAAGTCAATTTAGCAATCGTCCAACTAGATTGTAGAACTTCGCCTAATAATACACCTAAAATA
TATTGATAACTCATTGTGACAAGTAGTTGAATTTCTACTATATTTTCATCTTTTAATATAAAATACAACATGATAGAAATTAAAGTTA
TAACAACAATGGGTGAGCCTTTTCTAGATGTTAAAATTAAAAAATAAATAAATATCAATAAATAGGTAAATATAAAGAAACTAGGT
ATCTGATAATGGCTCGACGCTAAACCTATCAATAACATAATAGGAGGCATAAAATAACCACCAATCGTTGTAAGCCATTGGCCTGC
TAGATGTCTAGATTGTGTAATTGCGAATCCTTGTTGTAATGTCTGTTGTCGCTCTCGTGGACTTGTTACAATGACTAAATCTTTTGCA
CGGCCACCAGCGAGTTTATTAAACAGTACATGACCAAATTCATGTGTTAAAACAGGGATATAGTTTAAAATGACATCTAAATAGTTC
AAAACAGGCTTATGTCTATATTGATGAATAGCAATATAACAAGCTGCAACAATAACGATAATGTATATATTAAGTTGAATTGTCGTA
TTAAAAAAGTTTGATAAATAATTCATTGTTAACCTCATATAAGATATTAATTTAAAGTTTGCTTAGCACTTATTATAAATGATATTGG

CATCAATAGCGTTAGACTTTAGACTTACCTTAGTTAAACTAATTTTAATTTTTGAAAAGGTGAATATGTGTTAAAATAAAGCAAAAT
CATTTCGATATAAATAGGATGAATATAAATACTGTTAATATTGATTACACTAACATAATAATGAAATAAGATAGGAGATTCCTGTTA
TGACTGTTGAAGAAAGATCCAATACAGCCAAAGTTGACATTTTAGGGGTCGATTTTGATAATACAACAATGTTGCAAATGGTTGAA
AATATTAAAACCTTTTTTGCAAATCAATCAACGAATAATCTTTTTATAGTAACAGCCAACCCTGAAATAGTGAATTACGCGACGACA
CATCAAGCGTATTTAGAGTTAATAAATCAAGCGAGCTATATTGTTGCTGATGGGACAGGAGTAGTCAAAGCTTCGCATCGTTTAAAG
CAACCTCTAGCGCATCGTATACCTGGTATTGAGTTGATGGATGAATGTTTGAAAATTGCTCATGTAAATCATCAAAAAGTATTTTTG
CTAGGGGCAACTAATGAAGTTGTAGAAGCGGCACAATATGCATTGCAACAAAGATATCCAAACATATCGTTTGCACATCATCACGG
TTATATTGATTTAGAAGATGAGACAGTAGTAAAACGAATTAAACTGTTTAAACCTGATTACATATTTGTAGGTATGGGATTCCCTAA
ACAAGAAGAATGGATTATGACACATGAAAACCAATTTGAATCTACAGTGATGATGGGCGTAGGTGGTTCTCTTGAAGTATTTGCTG
GGGCTAAAAAGAGAGCGCCTTATATCTTTAGAAAATTAAACATTGAATGGATATATAGAGCATTAATAGATTGGAAACGTATTGGT
AGATTAAAGAGTATTCCTATATTTATGTATAAAATAGCCAAAGCAAAAAGAAAAATAAAAAAGGCGAAATAATCATGATGACAAA
AATAAAACCGAGGAAATCCTTAAATGGAGATTCTCGGTTTTTTCGGTTTATTTAATAACGAAGCGGGACTCATCGAGTTTGTTTCTA
AATTCTTTTTGTTCGGCTTTGGATTTCTTTTTAAAATCGTTAAGGAAAGCTTCATATTTAGGTAATACATCATCAAGTTCACCGTAAT
CTTTTAACTTTCCGCCTTCAATCCAAGCAATCTTAGTACAAAATTGTCTCACTTGTCCTAAGTTATGACTAACGAAAAAGATGGTTTT
GTTTTGCTCTTTAAACTCGTAAATTTTATCTAAACATTTTTGTGCAAAAGTTTGGTCACCTACAGATAAAGCTTCGTCAATGACTAAG
ATATCTGGATTAACTGTGATATTAATTGAAAAACCAAGTTTTGCACGCATACCACTTGAATACTTTTTAACTGGTTGATAAATAAAC
TCACCAAGTTCACTAAATTCAATAATCTTAGGTGTCATCGCTTTAATTTCTTTTCGCTTAAAGCCCATACATAACATTTTAAATTCGA
TATTTTCAATCCCTGTAAGTTGTCCACTCAAGCCAGCACTAATTGCGATAACGCTGACTTCACCATTACGATCCACTTTGCCAACAGT
AGGCGACAAAGAACCGCCAATGATATTGCTCAACGTTGATTTGCCGGAACCATTGATGCCAACAAGCCCTATGACGTCGCCTTCAT
ATGCTTTTAAACTAATGTCATCTAAAGCGAAAAATGTTTTGTTTTTATGTTTGGGAATGAGCGCATCTTTCATACGTTCTTTATTTGTA
CGATAAATACGATATTCTTTTGTTACATTTTTAATGTTTACCGAAACGTTCATTTGTAGACCTTCCTTATTCACATTTATCTAGATTAT
AATATACTACTCAACAGTTGTTAAATTTTAAAACCTGTTGTAAAGTGTATAGAAGATTTTGTTATTATCAGAGTGGGTGTTTTGACAC
AAAATGTTAATCATCAATGATAACAATGATATTTAAAAACTAAACTTATTTCAACTTACATGATTGTATACTATAATGTATTTGTAAT
AAACTAATATTTTAAAGAACTAGACAATAATTTTGATAGCATCCATGTATAGTGATAGTATTTACAACAATTATTATAATACTATTT
AGTTAAGTAGAGAAATAGTTAAACATTTGAAAGTGTGGTTTAATGGAATGTCAGCAATAGGAACAGTTTTTAAAGAACATGTAAAG
AACTTTTATTTAATTCAAAGACTGGCTCAGTTTCAAGTTAAAATTATCAATCATAGTAACTATTTAGGTGTGGCTTGGGAATTAATTA
ACCCTGTTATGCAAATTATGGTTTACTGGATGGTTTTTGGATTAGGAATAAGAAGTAATGCACCAATTCATGGTGTACCTTTTGTTTA
TTGGTTATTGGTTGGTATCAGTATGTGGTTCTTCATCAACCAAGGTATTTTAGAAGGTACTAAAGCAATTACACAAAAGTTTAATCA
AGTATCGAAAATGAACTTCCCGTTATCGATAATACCGACATATATTGTGACAAGTAGATTTTATGGACATTTAGGCTTACTTTTACTT
GTGATAATTGCATGTATGTTTACTGGTATTTATCCATCAATACATATCATTCAATTATTGATATATGTACCGTTTTGTTTTTTCTTAAC
TGCCTCGGTGACGTTATTAACATCAACACTCGGTGTGTTAGTTAGAGATACACAAATGTTAATGCAAGCAATATTAAGAATATTATT
TTACTTTTCACCAATTTTGTGGCTACCAAAGAACCATGGTATCAGTGGTTTAATTCATGAAATGATGAAATATAATCCAGTTTACTTT
ATTGCTGAATCATACCGTGCAGCAATTTTATATCACGAATGGTATTTCATGGATCATTGGAAATTAATGTTATACAATTTCGGTATTG
TTGCCATTTTCTTTGCAATTGGTGCGTACTTACACATGAAATATAGAGATCAATTTGCAGACTTCTTGTAATATATTTATATGACGAA
ACCCCGCTAACCATTAATAAATGGAAGTGGGGTTCATTTTTGTTTATAATTTAAGTAAATAACATATTAAGTTGGTGTATTATGAAC
GTTTTAATAAAGAAATTTTATCATTTGGTAGTTCGAATACTTTCTAAAATGATTACGCCTCAAGTGATTGATAAACCGCATATCGTAT
TTATGATGACTTTTCCAGAAGATATTAAGCCTATCATCAAAGCATTAAATAATTCGTCGTATCAGAAAACTGTTTTAACAACACCAA
AACAAGCGCCTTATTTATCTGAACTTAGCGACGATGTTGATGTGATAGAAATGACTAATCGAACATTGGTAAAACAAATTAAGGCTT
TGAAAAGCGCGCAGATGATTATTATCGATAATTATTACCTATTACTAGGTGGATATAATAAGACTTCTAATCAACACATTGTTCAAA
CGTGGCATGCAAGTGGTGCATTAAAAAACTTTGGCTTAACAGATCATCAAGTCGATGTGTCTGACAAGGCAATGGTTCAGCAGTAC
CGTAAAGTTTATCAAGCGACGGATTTTTACTTAGTGGGTTGTGAACAAATGTCACAATGTTTTAAACAGTCTTTAGGTGCAACAGAA
GAGCAAATGCTGTATTTTGGGCTTCCGAGAATTAATAAATATTACACAGCTGATAGAGCAACGGTTAAGGCAGAGTTAAAGGATAA
ATATGGAATTACAAATAAGTTGGTATTATATGTACCAACATATAGAGAAGATAAAGCAGATAATAGGGCTATTGATAAAGCTTATT
TTGAAAAATGTTTACCAGGATATACACTGATTAATAAATTACATCCATCAATTGAAGATTCAGACATTGATGACGTATCTTCAATCG
ACACGTCTACATTAATGCTAATGTCAGATATAATTATTAGCGACTATAGTTCGCTGCCAATAGAAGCTAGCTTGTTAGATATTCCAA
CTATATTTTATGTGTATGATGAAGGAACATATGATAAAGTGAGAGGCCTGAATCAATTTTACAAAGCAATACCGGATAGCTACAAA
GTGTATACTGAAGAAGATTTAATAATGACGATACAAGAAAAAGAACATCTATTAAGTCCGTTATTTAAAGATTGGCATAAGTATAA
TACTGATAAAAGTTTACATCAGCTCACAGAATATATAGATAAGATGGTGACAAAATGAGGCTTACGATAATCATACCTACATGTAA
TAATGAGGCAACAATTCGACAATTGTTAATATCTATTGAGAGTAAAGAACACTATAGAATCCTTTGTATTGATGGTGGTTCTACTGA
TCAAACAATTCCTATGATTGAACGGTTACAAAGAGAACTCAAGCATATTTCATTAATACAATTACAAAATGCTTCGATAGCTACGTG
TATTAATAAAGGTTTGATGGATATCAAAATGACAGATCCACATGATAGTGACGCATTTATGGTCATAAATCCAACATCAATCGTATT
GCCAGGTAAATTAGATAGGTTAACTGCAGCTTTCAAAAATAATGATAATATTGATATGGTAATAGGGCAGCGAGCTTACAATTACC
ATGGTGAATGGAAATTGAAAAGTGCTGATGAGTTTATTAAAGATAATCGAATCGTTACATTAACGGAACAACCAGATTTGTTATCA
ATGATGTCTTTTGACGGAAAGTTATTCAGTGCTAAATTTGTTGAATTACAGTGTGACGAAACTTTAGCTAACACATACAATCACGCA
ATACTTGTCAAGGCGATGCAAAAAGCTACGGATATACATTTAGTTTCACAGATGATTGTCGGAGATAACGATATAGATACACATGC
TACAAGTAACGATGAAGATTTTAATAGATATATCATAGAAATTATGAAAATAAGACAACGAGTCATGGAAATGTTACTATTACCTG
AACAAAGGCTATTATATAGTGATATGGTTGATCGTATTTTATTCAATAATTCATTAAAATATTATATGAATGAACACCCAGCAGTAA
CGCACACGACAATTCAACTCGTAAAAGACTATATTATGTCTATGCAGCATTCTGATTATGTATCGCAAAACATGTTTGACATTATAA
ATACAGTTGAATTTATTGGTGAGAATTGGGATAGAGAAATATACGAATTGTGGCGACAAACATTAATTCAAGTGGGCATTAATAGG
CCGACTTATAAAAGATTCTTGATACAACTTAAAGGGAGAAAGTTTGCACATCGAACAAAATCAATGTTAAAACGATAACGTGTACA
TTGATGACCATAAACTGCAATCCTATGATGTGACAATATGAGGAGGATAACTTAATGAAACGTGTAATAACATATGGCACATATGA
CTTACTTCACTATGGTCATATCGAATTGCTTCGTCGTGCAAGAGAGATGGGCGATTATTTAATAGTAGCATTATCAACAGATGAATT
TAATCAAATTAAACATAAAAAATCTTATTATGATTATGAACAACGAAAAATGATGCTTGAATCAATACGCTATGTCGATTTAGTCAT
TCCAGAAAAGGGCTGGGGACAAAAAGAAGACGATGTCGAAAAATTTGATGTAGATGTTTTTGTTATGGGACATGACTGGGAAGGTG
AATTCGACTTCTTAAAGGATAAATGTGAAGTCATTTATTTAAAACGTACAGAAGGCATTTCGACGACTAAAATCAAACAAGAATTA
TATGGTAAAGATGCTAAATAAATTATATAGAACTATCGATACTAAACGATAAATTAACTTAGGTTATTATAAAATAAATATAAAAC
GGACAAGTTTCGCAGCTTTATAATGTGCAACTTGTCCGTTTTTAGTATGTTTTATTTTCTTTTTCTAAATAAACGATTGATTATCATAT
GAACAATTAGTGCTAATCCAGCGACAAGGCATGCACCACCAATGATAGTGAATAATGGATGTTCTGCCCACATACTTTTAGCAACA
GTATTTGCCTTTTGAATAATTGGCTGATGAACTTCTACAGTTGGAGGTCCATAATCTTTATTAATAAATTCTCTTGGATAGTCCGCGT
GTACTTTACCATCTTCGACTACAAGTTTATAATCTTTTTTACTAAAATCACTTGGTAAAACATCGTAAAGATCGTTTTCAACATAATA
TTTCTTACCATTTATCCTTTGCTCACCTTTAGACAATATTTTTACATATTTATACTGATCAAATGAGCGTTCCATTAATGCATTCCCCA

TCATATTACGTTGCTTCTCGCCACCAAGGTTTTTATAGTCTCCCGCACCCATGATAACTTGATTAATTCTAAATTTACCTCGTTTGGTA
GTAATCGTATGGTTGTAATTTGCTGTATCACTTGATCCAGTTTTTAAACCATCTGTACCAGTCAAACTCATTTTTGCACCTTCCAATG
AAAAGTTGAATGTGTAATACGTAACTGCATGCGTTGTTGGTGCTAACTGCTTTGTAAAGTCTAATATTTTAGGTGTCTCTTTAATCAC
GTGTAAATCTAAAATGGCGTAGTCTCTAGCAGTCGTTACAGTACGTTCTTGGTCTTTATACTTTGTTGGTGCAAATGTACGTAATCTT
GAATTTTCAGCACCCGTTGGATTGACGAAATGTGTATTTTTCATTCCGATAGCTTTAGCTTTGTTATTCATTAAATCAACGAAATCGC
TGGTGTTTTTTGAAACCTTCTTAGCTAAAATTAATGCCGCGGCATTACTAGAATTAGATACTGTAATTTGTAATAGGTCTGCGATTGT
CCATACTTGTCCAGGATATAGTTTCGTATTACTCAACTCAGGTAGTGTAGACATAATATATTCTTTGTTCGTCATTGTGACAGTGTCA
TCAAGTGAAAGCTGCCCCTTATTTACAGCTTCCAATGTTAAGTACATTGTCATTAATTTAGTCATAGACGCTGGATTCCACTTAGTAT
CGATATTGTATTGATACAGTAATTGTCCAGTTTGACTTACATTAACAGCACTCGTCGGTTCGTATGCAGCTGACAAACCTGCATAAC
CATATTGATTTGCTGCTTGTACAGGGGTTACGTCACTGTTAGCAGCTTGTGCATATGGTGTCATAATACTTAATGTTAAACATAAAAT
GATGATAATAGATATTAAATTTTTCATAAAGCGTTAATCTTCCCTTTTCCAATTCTTAAATATTCCCTAAAAGCAATGGTTATTCCTA
CTTACGGAAATCATTGCTAATTCACTTCACCTTAATTAAATTGTTGAAAATAAAGTTTTCTGCAGTTAATTTGAAAAATAATGCAAAT
ATATTACGTGTGTAGCTAAAGGTGTTATAATGTTTGTACGAAGAGCAAACTTACTCAAAAGCGATTAATTTTCATGTTTTAATATAA
AGACTTTGAGAAGTTATTACAAAAAATGCAATAGAAATATTCTATCATATAAATGTTATGAGCGGTATTTTGGGGCAACACTTTATT
TGATTTTTAAAGTTTTGTTGGGAGAAAGTATATGATAGAAATGCATGTATCTATCTAAATGAATTAACTATAAATTTCAAACAGAAG
AGGTAAAACTATGAAACGAGAAAATCCATTGTTTTTCTTATTTAAAAAACTATCATGGCCAGTGGGTCTTATCGTTGCAGCTATCAC
TATTTCATCACTAGGGAGCTTAAGTGGACTATTAGTGCCACTGTTTACTGGTCGAATTGTAGATAAATTTTCCGTGAGCCATATCAAT
TGGAATCTAATCGCATTATTTGGTGGTATCTTTGTTATCAATGCTTTATTAAGCGGATTAGGTTTATATTTATTAAGTAAAATTGGTG
AAAAAATTATTTATGCGATACGCTCAGTTTTATGGGAGCATATCATACAATTAAAAATGCCATTCTTTGACAAAAATGAAAGTGGTC
AATTAATGAGTCGATTAACTGACGATACGAAAGTGATAAATGAATTTATTTCACAAAAGCTACCTAACTTATTACCATCAATCGTTA
CATTAGTTGGGTCACTAATCATGTTATTTATTTTAGATTGGAAAATGACATTATTAACATTTATAACGATACCGATATTCGTTTTAAT
TATGATTCCTCTAGGTCGTATTATGCAAAAGATATCGACAAGTACACAATCTGAAATTGCAAACTTCAGTGGTTTGTTAGGGCGTGT
CCTAACTGAAATGCGTCTTGTTAAAATATCAAATACAGAGCGTCTTGAATTAGATAATGCACATAAAAATTTGAATGAAATATATAA
ATTAGGTTTAAAACAGGCTAAAATTGCGGCAGTTGTACAACCAATTTCAGGTATAGTTATGTTGCTAACAATTGCAATTATTTTAGG
TTTTGGTGCATTAGAAATTGCGACTGGTGCAATCACTGCAGGTACATTAATTGCAATGATATTTTATGTTATTCAGTTATCTATGCCT
TTAATCAATCTTTCAACGTTAGTTACAGATTATAAAAAGGCAGTCGGTGCAAGTAGTAGAATATACGAAATCATGCAAGAACCTATT
GAACCGACAGAAGCTCTTGAAGATTCTGAAAATGTATTAATTGATGACGGTGTATTGTCATTTGAACATGTAGACTTTAAATATGAT
GTGAAGAAAATATTAGATGATGTGTCGTTCCAAATCCCACAAGGTCAAGTGAGTGCTTTTGTAGGTCCTTCTGGGTCTGGTAAAAGT
ACGATATTTAATCTGATAGAACGTATGTATGAAATTGAGTCAGGTGATATTAAATATGGCCTTGAAAGTGTCTATAATATCCCGTTA
TCTAAGTGGCGACGCAAAATTGGATATGTTATGCAATCAAATTCGATGATGAGTGGTACAATTAGAGACAATATTTTATACGGAATT
AATCGTCATGTTTCAGATGAAGAACTTATTAATTATGCTAAATTAGCGAACTGTCATGATTTTATCATGCAATTTGATGAAGGATAT
GACACGCTTGTAGGTGAACGAGGATTGAAACTGTCTGGCGGACAACGTCAACGTATTGATATTGCTAGAAGTTTTGTTAAAAATCCT
GATATTTTGTTACTTGATGAAGCAACAGCTAATCTCGATAGTGAAAGTGAATTGAAAATTCAAGAAGCTTTAGAAACATTGATGGA
AGGTAGAACAACGATTGTCATTGCGCATCGTTTGTCTACAATTAAAAAAGCCGGTCAAATTATATTCTTAGACAAAGGACAGGTAA
CAGGTAAAGGTACGCATTCAGAACTGATGGCATCACATGCGAAGTATAAAAACTTTGTAGTGTCTCAAAAATTAACAGATTAATTTT
ATATATAAGTAAGCTTGGAGCAAATACACATATACCATCGAGGAAATTAAAGTGTGGCACATTGATGGATATAGATGTTAATAAAT
TGCTTCAAGCTTTTGTCTATTTTAAATCATTTGAGAAGTTACGACATAATAATTCTTAAATTAATGAAATCGATATTTTAAGAAAAAA
ATGCTCATGGTATAATACAAGTTATAAGCAAACATACATATATTAAATACTGTAGCCACGAGTCATAATTCTTCATATTTTACATAG
CAATTTAACTGATTTTAGAGTCCATGGTACAGAAGTTTGATATTTCAATGTTTCTAAATTTTTAAAAAATTAAATCATAGGTGGGTGC
CAAATGTTTTTATTAATCAACATTATTGGTCTAATTGTATTTCTTGGTATTGCGGTATTATTTTCAAGAGATCGCAAAAATATCCAAT
GGCAATCAATTGGGATCTTAGTTGTTTTAAACCTGTTTTTAGCATGGTTCTTTATTTATTTTGATTGGGGTCAAAAAGCAGTAAGAGG
AGCAGCCAATGGTATCGCTTGGGTAGTTCAGTCAGCGCATGCTGGTACAGGTTTTGCATTTGCAAGTTTGACAAATGTTAAAATGAT
GGATATGGCTGTTGCAGCCTTATTCCCAATATTATTAATAGTGCCATTATTTGATATCTTAATGTACTTTAATATTTTACCGAAAATT
ATTGGAGGTATTGGTTGGTTACTAGCTAAAGTAACAAGACAACCTAAATTCGAGTCATTCTTTGGGATAGAAATGATGTTCTTAGGA
AATACTGAAGCATTAGCCGTATCAAGTGAGCAACTAAAACGTATGAATGAAATGCGTGTATTAACAATCGCAATGATGTCAATGAG
CTCTGTATCCGGAGCTATTGTAGGTGCGTATGTACAAATGGTACCAGGAGAACTGGTACTAACGGCAATTCCACTAAATATCGTTAA
CGCGATTATTGTGTCATGCTTGTTGAATCCAGTAAGTGTTGAAGAGAAAGAAGATATTATTTACAGTCTTAAAAACAATGAAGTTGA
ACGTCAACCATTCTTCTCATTCCTTGGAGATTCTGTATTAGCAGCAGGTAAATTAGTATTAATCATCATCGCATTTGTTATTAGTTTT
GTAGCGTTAGCTGATCTATTTGATCGTTTTATCAATTTGATTACAGGATTGATAGCAGGATGGATAGGCATTAAAGGTAGTTTCGGT
TTAAACCAAATTTTAGGTGTGTTTATGTATCCATTTGCGCTATTACTCGGTTTACCTTATGATGAAGCGTGGTTGGTAGCACAACAAA
TGGCTAAGAAAATTGTTACAAATGAATTTGTTGTTATGGGTGAAATTTCTAAAGATATTGCATCTTATACACCACACCATCGTGCGG
TTATTACAACATTCTTAATTTCATTTGCAAACTTCTCAACGATTGGTATGATTATCGGTACATTGAAAGGCATTGTTGATAAAAAGAC
ATCAGACTTTGTATCTAAATATGTACCTATGATGCTATTATCAGGTATCCTAGTTTCATTATTAACAGCAGCTTTCGTTGGTTTATTTG
CATGGTAATATGTCGAAGAGTGACTATGATAATACATTTTAACTAATAAATATGTCCAGGCATGTCGTCTATTGATATAGGTGAGAT
GCTTGGACTTTTTTATTATTGATATAAAGGTATTTAAATATTTTTAAAGTTACCGAAATTGAAGCATTATAAAAACCAAGTGCACAT
GGTAATACACTTGGCTTTTATGGGAAATGAATATTATTGTACATATGACAGTAAGGACTAGGTACAGTCATAGTACTTCGAGCAAAA
TTTGTTTTGTTATTATAAACAACACAAAGGAGATAACTTCTCTATTGAAGAAGTTAAAAACATTATAGCAGACAATGAAATGAAAGT
AAATTAAAAATTCAGAATATTTTTAATTATTATATTGTGAGTGATATTTATTAGGGAAAGCTATTCTTCATATAAATTAGTTAAATAG
TAAATCTTTGTTAGAACTCTTTCCATAGAGTTGTGACAGCATTTTTGTTGTGCTACTATTTTTATAGTCTAATAAATATATAAAGGGG
ATGGTTTCGTGAATAAAACGGTTAAAGATTTAATACTAGTTGTCTTAGGTTCATTTATCTTTGCTGCAGGTGTAAATGCATTTATTAT
TTCTGGTAACTTAGGTGAAGGCGGGGTTACAGGTTTAGCAATTATTTTATATTATGCGTTTCATATTTCACCAGCCATCACTAACTTC
TTGGTCAACGCAGTATTGATTGCCATTGGTTATAAATTTTTGAGTAAGAGAAGTATGTACTTAACTATTCTTGTAACAATTCTTATTT
CAATATTTTTGAGTTTAACAGAATCATGGCAAGTAGAAACTGGAAACAGCATTGTGAATGCCATTTTTGGTGGTGTAAGCGTTGGAC
TAGGAATCGGAGTAATTATCCTTGCAGGCGGTACAACAGCAGGTACAACAATTTTGGCAAGAATTGCAACGAAATACCTCGATGTA
AGCACGCCATATGCTTTGCTTTTCTTCGATATGATCGTTGTTGCAATTTCACTTACAGTTATTCCACTTGATAAAGTATTAGTAACAG
TAATATCACTTTATATAGGAACAAAAGTGATGGAATATGTCATAGAAGGTTTAAACACTAAAAAAGCTATGACGATTATTTCAACT
AATCCCGACAAACTTGCCAAAGCAATAGACGAGCAAATTGGAAGAGGTTTAACCATTTTAAACGGACATGGCTATTATACGCGTGA
AGAAAAAGATGTCTTATACGTTGTTATTTCTAAAACACAAGTTTCAAAAGCAAAGCGATTAATTAAACAAATCGATAAAGATGCAT
TCCTCGTAATTCATGATGTAAGAGATGTCTATGGTAATGGCTTTCTTGCAGATGAATAAATAAATGGTATGAGCACACATACTTAAA
TAGAAGTCCACGGACAAGTTTTTGAACTATGAAGACTTATCTGTGGGCGTTTTTTATTTTATAAAAGTAATATACAAGACATGACAA
ATCGAGCTATCCAATTTAAAAAGTAATGTTAGTCAATAAGATTGAAAAATGTTATAATGATGTTCATGATAATCATTATCAATTGGG

ATGCCTTTGAAAATTGATAATTTAAAAATAGAAATTATTTTTATAAACAGAAAGAATTTTATTGAAAGTAGGGAAATTATGAATCG
TTTGCATGGACAACAAGTTAAAATTGGTTACGGGGATAACACGATTATAAATAAATTAGATGTTGAAATACCAGATGGCAAAGTGA
CGTCAATCATTGGTCCTAACGGCTGCGGGAAATCTACTTTGCTAAAGGCATTGTCACGTTTATTGGCAGTTAAAGAAGGCGAAGTAT
TTTTAGATGGTGAAAATATTCATACACAATCTACGAAAGAGATTGCAAAAAAAATAGCCATTTTACCTCAATCACCTGAAGTAGCA
GATGGCTTAACTGTTGGGGAATTAGTTTCATATGGTCGTTTTCCACATCAAAAAGGATTTGGTAGATTAACTGCTGAGGATAAGAAA
GAAATTGATTGGGCAATGGAAGTTACAGGAACTGATACATTCCGACACCGTTCAATCAATGATTTAAGTGGTGGTCAAAGACAACG
TGTTTGGATTGCAATGGCATTAGCACAAAGAACTGATATTATCTTTTTAGACGAACCAACAACATATTTAGATATCTGTCATCAATT
AGAAATACTAGAATTAGTTCAGAAGCTAAATCAGGAACAAGGTTGTACAATTGTCATGGTTCTTCATGATATCAACCAAGCGATTC
GTTTCTCAGATCATCTTATTGCGATGAAAGAAGGGGATATCATCGCTACAGGTTCAACAGAAGACGTATTAACACAGGAAATATTA
GAAAAAGTTTTTAATATTGATGTTGTTTTAAGTAAAGATCCTAAAACTGGAAAACCTTTACTGGTAACTTATGACTTATGTCGCAGA
GCTTATTCTTAATTAAGTAAGTTAATATGATAAAAAGGACAATTAACATGACAAATAGAGAGAACCCAACGCCATTGAAGTTTTTAT
CCTATATTATAGGTTTAAGTATGATACTACTAATCACACTATTTATTTCTACATTAATAGGTGACGCCAAAATTCAAGCCTCTACAAT
TATAGAGGCTATTTTTAATTATAATCCTAGCAATCAACAGCAAAACATCATCAATGAGATTAGGATTCCCAGAAATATAGCAGCAGT
AATTGTAGGTATGGCGCTTGCAGTTTCTGGTGCGATTATACAAGGTGTTACTCGTAATGGTCTTGCTGATCCGGCGCTCATTGGTTTA
AATTCAGGTGCTTCATTTGCTTTAGCATTAACATATGCAGTTTTACCAAACACTTCATTTTTAATATTGATGTTTGTTGGATTTTTAGG
TGCTATTCTAGGAGGTGCTATTGTATTAATGATAGGCCGATCTAGACGTGATGGATTTAATCCGATGCGTATTATTTTAGCGGGTGC
AGCAGTAAGTGCTATGTTAACAGCGCTAAGTCAAGGTATTGCATTAGCTTTTAGACTAAATCAAACAGTAACATTTTGGACTGCTGG
AGGCGTTTCAGGCACAACATGGTCACACCTTAAGTGGGCAATTCCATTAATTGGTATTGCGTTATTCATTATATTAACAATTAGTAA
ACAACTTACCATTTTAAATCTTGGTGAATCATTAGCTAAAGGTTTAGGTCAAAATGTAACAATGATCAGAGGCATATGTTTAATTAT
TGCTATGATTCTAGCAGGTATTGCAGTTGCTATCGCTGGACAAGTTGCATTTGTAGGTTTGATGGTACCTCATATAGCAAGATTTTTA
ATTGGAACTGATTATGCTAAAATTCTACCATTAACAGCCTTGTTAGGTGGGATACTCGTGCTTGTTGCCGATGTGATAGCACGATAT
TTAGGAGAAGCGCCTGTTGGTGCAATCATTTCATTTATCGGTGTTCCTTACTTTTTATATTTAGTTAAAAAAGGAGGACGCTCAATAT
GATTAGTTCAAATAATAAACGCAGACAATGGATTGCACTGGCTGTTTTTAGCATTCTACTATTTCTAGGTTGTACTTGGAGTATTACC
TCAGGTGAATACAACATACCTGTTGAAAGATTTTTCAAAACTTTAATTGGACAAGGTGATGCCATTGATGAGTTAATCTTATTAGAT
TTCAGATTACCTCGGATGATGATTACTATTTTGGCTGGCGCAGCGCTTAGTATTAGTGGTGCAATAGTGCAAAGTGTCACGAAAAAT
CCAATAGCTGAACCCGGTATATTAGGTATTAACGCAGGTGGCGGATTTGCAATCGCATTATTTATTGCAATTGGTAAAATTAATGCT
GACAACTTTGTTTATGTACTGCCGTTAATAAGTATACTAGGTGGTATCGCCACTGCATTGATTATTTTTATTTTCAGTTTTAATAAAA
ATGAAGGTGTTACACCTGCGAGTATGGTATTAATAGGTGTAGGTTTACAAACAGCATTATATGGTGGCTCAATTACAATTATGTCAA
AATTTGATGATAAGCAATCTGATTTCATCGCTGCTTGGTTTGCAGGTAATATTTGGGGTGACGAATGGCCATTTGTCATTGCATTTTT
ACCGTGGGTGTTGATTATTATTCCTTACTTACTATTTAAATCGAATACACTAAATATTATTCATACGGGTGATAATATTGCACGAGGT
CTAGGTGTAAGGTTAAGCAGAGAACGTTTAATATTATTCTTTATCGCAGTGATGTTATCATCTGCTGCTGTAGCAGTAGCAGGTTCG
ATTTCGTTTATCGGATTAATGGGTCCGCATATTGCCAAACGTATCGTTGGACCACGTCACCAGTTGTTTTTACCAATTGCCATTTTAG
TAGGGGCATGTTTACTTGTTATAGCTGATACAATTGGCAAAATTGTATTACAACCAGGTGGGGTTCCAGCAGGTATTGTCGTAGCAA
TTATTGGTGCACCGTATTTCTTATATTTAATGTACAAAACGAAAAATGTATAGTGTCAAAGGACACAACTTATTGCTATGAAAGGCA
CTTTATTATAAGGCTTTTCATAGCATTTTTTATTTAATGAGCCACTCAAGACTATTTATTTTTTCAATAATGAACCATTAAGTTATCAA
GAGGATCTTATCAAAAATATATTTGATAACGGTATCAGGTTAATTCTTTATGATAGCGCATTCATTTATTCTGTTTTATACTATGACT
GATAATACCAAGGAGGTACAACATGATGAAAAAGTTAATCAATAAAAAAGAAACATTTTTAACTGATATGCTTGAAGGATTGTTAA
TTGCGCACCCAGAGTTAGATCTGATTGCTAATACAGTTATTGTAAAAAAAGCTAAGAAAGAACATGGTGTAGCAATAGTCTCTGGA
GGTGGAAGCGGACATGAACCTGCGCATGCCGGTTTTGTTGCAGAAGGTATGCTAGATGCAGCGGTTTGTGGCGAAGTATTTACATC
ACCTACACCTGATAAAATATTAGAAGCTATTAAAGCAGTAGATACTGGTGATGGTGTATTACTAGTTGTAAAAAACTATGCAGGTG
ACGTGATGAATTTCGAAATGGCACAAGAGCTTGCAGAAATGGAAGGTATAAATGTTCAAACTGTTATTGTTCGTGACGACATTGCT
GTGACAAACGAAGTACAACGTCGTGGTGTTGCAGGAACAGTGTTTGTTCATAAGCTTGCCGGTTATCTTGCTGAAAAAGGTTATTCA
TTAACAGAGATAAAATCGCGTGTAGAAGCGTTGTTACCTGAAATTAAAAGTATTGGTATGGCAATTGAGCCACCGCTTGTTCCAACT
ACTGGAAAATATGGCTTTGATATTGAAGACGACAAAATGGAAATCGGTATTGGTATACATGGTGAAAAAGGTATTCATAGGGAAGA
AGTAAAGGATATTGATCATATTGTTGGAACATTGTTAGACGAATTGTATAAAGAAGTTACTGCCAATGATGTCATATTAATGGTAAA
TGGTATGGGTGGTACGCCGTTATCTGAATTAAATATCGTAACTAAATATATTCAACAAAATTTAGCTGCAAGAACGGTTAATGTTGC
TAAATGGTTTGTTGGTGATTATATGACATCTTTAGACATGCAAGGTTTTTCTATAACTATCGTGCCTAATAAACCAGAATATTTGGAA
GCATTTTTAGCACCAACAACAAGTCAATACTTTAAATAAGAAGTGAATATGAATAAATATACATTTATGAGGTGGCACAAATGAAA
GTGAATGATATGAAAGCACGTTTATTAAATTTAGAAGAAACGTTTAAAAAACATGAATCTGAATTAACTGAATTAGATCGAGCAAT
TGGTGATGGTGACCACGGGGTTAACATGGTTCGTGGGTTTAGTAGTCTTAAAGACAAACTTGATGATAGCTCAATGCAATCATTGTT
CAAATCAACTGGTATGGCATTGATGTCAAATGTTGGGGGTGCATCTGGACCACTGTATGGCTTTAGCTTTGTTAAAATGTCTGCAGT
CACCAAAGATGATATGGATAATCAAGATTTCATTACACTAATTCAGGCATTTGCCGAAGCGGTTGAATCACGTGGTAAAGTTACTTT
AAATGAAAAGACAATGTATGATGTAATAGCGCGAGCAGCAGAGAAGCTTAAAAATGGTGAAACTTTAACATTCAATGATTTACAGC
AATTAGCAGATAATACAAAAGATATGGTAGCAACGAAAGGTAGAGCTGCATATTTTGGAGAAGAATCAAAAGGTTATATTGATCCA
GGTGCTCAAAGTATGGTTTATATTTTAAACGCTTTGATTGGAGATGAAGATAATGCCTAAAATTATACTTGTTAGCCACAGTAAAGA
AATTGCAAGTGGTACAAAATCTTTGTTAAAGCAAATGGCAGGTGACGTTGATATTATACCAATCGGGGGATTACCAGATGGTTCAA
TTGGAACTTCATTTGATATCATCCAAGAAGTTTTGACTAAATTAGAGGATGATGCATTGTGTTTTTACGATATTGGATCTTCAGAAAT
GAATGTAGATATGGCAATTGAAATGTATGATGGTAATCATCGTGTGTTAAAAGTTGATGCACCAATTGTTGAAGGCAGTTTTATCGC
AGCAGTAAAGCTATCAATCGGCGGTTCAATTGATGATGCATTAGCAGAAATGAAACAATCATTTTAGTTAAAATTTACTAATAATGA
AAAATGTAAACCTTTTTCAAATGAAACTTTATAAAAAATATGATAGTATATATGTAAATGTTTAATAAAATCTGGAGAAATAGGAG
GACATTGCCATGCAACACCTTATAAAAAAACATGTATTGAATGGCGAGTTTGATTTAGTACGACAATTGATGTCCGAAACAGATTTT
ATGGAATTTGAAGAAGCATATATTTCAAGTGCGCATGAAGTAGAAAGTATGATGTTTTATACATGTATTTTAGATATGATTAAGTAC
GAAGAATCATCTGAAATGCATGACTTAGCATTTTTATTGCTTGTGTATCCACTAAGTGAATATGAAGGTGCTTTGGATTCTGCTTATT
ATCATGCAGACGCTTCCATAAAACTTACTGACGGCAAAGAAGTTAAAAGTTTGTTACAAATGTTATTATTGCATGCGATACCAACAC
CTGTTATTTCAGATAAGAAGGCTTTTGATATCGCCAAGCAAATTTTAAAATTAGATCCTAATAATAATGTTGCTCGTAACGTCTTAA
AAGACACTGCCAAACGTATGGACAACGTTGTTGTTGATATAAATGAATTACACCAACGTAATGCACGTTAATTACATTTCAATTATA
TTAGCTTAATAATAGTTTTAACATTTGGTTGGGTTGGGACATATGTTCCAGCCTTTTTTAATACTTAAAAACTAACGAAGTATACTTG
TGTGCACAAATGGTTTTTATACAACATTTTATAAATTTATACATTTTAATAAAGAACATACGATAGATGGTTTAAACCTTGTTAACTG
AGAAATTTTGATATGTATTCTTCGAAATTTAACTAAATATACGAAATTCAAGAAGCACAATAATTAATCATTTTTCCTATACAAAAG
TTCGTATGACTGCATTATAAAAGCATAAATTTATAATTTTTTTAAATGTCATTGAACGTGATAATGTGAATGGATTGAGCAATTTTGA
AAAAGTGAAAAATAACCTATGCGACTTGCAATTAATTTTCAGTACGTTATAATGCACACTGTGCAAAATTAAGGAGGTCTATTATTC

ACATGATGATGAATAAAGAAGCAACAAAAATTGGATTTGCCTACGTCGGCATTGTAGTGGGCGCAGGATTTTCAACAGGACAAGAA
GTTATGCAATTTTTCACTAAATATGGCTTGTGGGCTTATTTAGGTGTCATTATATCTGGTTTTATTTTAGCTTTTATTGGGCGCCAAGT
AGCAAAAATTGGTACTGCCTTTGAAGCGACAAACCATGAATCAACATTACAATACGTATTCGGTGAAAAGTTTAGTAAAGTCTTTG
ATTATATTTTAATCTTCTTCTTATTTGGTATAGCTGTAACCATGATAGCTGGTGCAGGCGCAACATTTGAAGAAAGTTATAACATACC
TACATGGCTAGGTGCTTTAATTATGACATTAGCGATTTATATTACGTTGCTATTAGACTTTAATAAAATAGTACGTGCACTTGGTATC
GTTACACCATTTTTAATTGTTTTAGTTGTATTAATCGCTGGCGTTTATTTATTTAAAGGTCATGTTTCATTAGCAGAAGTTAACCAAGT
AGTGCCTGAGGCAAGTATTTGGAAGGGAATCTGGTTTGGTACAATATATGGTGGATTAGCTTTTTCTGTAGGTTTTAGTACCATCGT
AGCAATCGGTGGGGATACTGAAAAGCGTACAGTGTCAGGTGCAGGCGCGATGTATGGTGGTATTATCTATACTGTATTACTAGCATT
GATCAACTTTGCATTGCAAAGTGAATATCCAACTATTAAAAATGCCTCAATTCCAACATTGACATTAGCAAATAATATCCATCCTTT
AATAGCAACAGTGTTATCTGTTATTATGCTGGCGGTTATGTATAATACTATTCTAGGACTAATGTATTCATTTGCAGCACGTTTTACA
GAACCATACAGTAAAAATTATCATATCTTTATTATTATAATGATGGTAGCAGGTTATTTATTAAGTTTCGTAGGATTTGCTGAATTAA
TTAATAAGTTATATACAATTATGGGATATGTAGGCTTATTTATTGTAGTAGCTGTAATTATTAAATATTTCAAACGTAAAAATGCGG
ATAAAAAACATATTGCTTAATATCATATGATGGATATCCGAAACTTTACAATTGAATCACTTTGTTTTAACCTTAAAAGCAATTCGTC
TCTACTCTTATCGGGCGAATTGCTTTTTATATTTATTCAGTCTATTAATATGAGCGTCTAACAAATAGAGAGGTACGATGTAATGAAT
AAAGATAATAAATGGACGATGATAACTGCGCTTTTTATATCTGTAATCAGTGTATTGTTAGCATTTCATCTGAAACAACATTATGAC
CAAATTACAAATGAGAACCATGCTAATAAAGACAAAATTAATATTAAAAATAAAAATGTGCGCATTTATCAAAACCTTACATACAA
TAGAGTTTTCCCTAACAGTAAATTAGATATTATTACACCTGTTGATATGTCTTCTAATGCCAAACTGCCAGTTATTTTTTGGATGCAC
GGTGGTGGTTATATTGCGGGTGATAAGCAGTATAAAAACCCATTATTAGCGAAAATTGCTGAACAAGGGTACATTGTTGTGAATGT
AAATTATGCATTGGCGCCACAATATAAATATCCCACACCATTAATTCAAATGAATCAAGCAACTCAATTCATTAAAGAAAATAAAA
TGAATTTACCTATTGATTTTAATCAAGTAATTATTGGCGGTGATTCTGCAGGTGCTCAATTAGCTAGCCAATTTACGGCAATACAGA
CGAATGATCGCTTAAGAGAAGCTATGAAATTTGATCAGTCATTCAAACCATCGCAAATTAAAGGTGCTATACTATTTGGTGGTTTTT
ATAATATGCAAACAGTTAGAGAAACTGAGTTTCCAAGAATACAGTTATTTATGAAAAGTTATACTGGCGAAGAAGATTGGGAAAAG
AGCTTTAAAAATATTTCACAAATGTCGACAGTAAAACAATCGACAAAAAATTATCCACCAACATTTTTATCTGTTGGAGATAGCGAT
CCATTCGAAAGTCAAAATATAGAATTCAGTAAGAAATTACAAGAATTGAATGTACCAGTAGATACTTTGTTTTATGATGGTACGCAT
CATTTACATCATCAGTATCAATTTCACCTTAATAAACCTGAATCGATAGATAATATCAAAAAAGTGTTACTTTTCTTAAGTCGTAATA
CATCTTCTAGTGGTATTCAAACTGAAGAGAAACCACAAATAGAAAATCCGAGTAATGAATTACCGTTAAATCCTTTAAACTAATGAT
AAACAGTAGTAATTTATTACTTAAGCAACATTTAAGATCTTCAAATTGAAAACGAAGAATTTAAAACATGTGGGGCTAATGTGTAA
GAGTCTGAGATATAATAAATTGTTAGCGGTTCTTTATCATTTCTATCTCACCCTATTATGATGTGACATTTATTTTACAAACTAATTTG
TTTTGAACCTGAAAATAACTTTTTACAACTAAATTGTCGAAAAACAGTGTGTACATGATATAATAAATTTATAAATTGAAAAGAATT
CAAAAGAAAAAATAAAATATAGATTGAGCACGAAGTGATTTGAAATAAGGTTGTGAAAGGGAATGACAAGGTCAGCATTAAAACC
ATTTAAAAATAAACGCGTTATGGTTACTGGACGTATACAACGTGTTTTGTTTAAAAATTATTTAGATAGACATAGCACATTTAAGCC
GAATGTAAGGATATTATTAAAAGATGTATTTGTTTCAGGTGTATCAATAGATCATTTATGGTTATATGAGACAAATAAATACTATGC
ATTGGCAATGGAACTTATTCATCAACGAGTAAAATTTAGTGCGAATGTTGTACCATATTACAAAATAAATAGAAATAATAATTTATT
CGTACAAGATTATGGAATTAAGCGTAAAGGTAGGTTAATTACTGAAGAAGCTTACAATCAAAACAATCAGTATCAGGATAAGATAT
ATGAAAAATTACCGGATATAGATTTTAGACTCGAAGATTTTTATAGTAAGGAAAACTAAACAACATATATAAAGCACCACTAATCG
TAGTTGATAGTTGATCAATTACACATTAGTGGTGCTTTATTTTGAATTTAAATTTGATAAATGAATAAGAGTATACGCATTCATTTAA
GATTAGATGACATTGATAAGTATGAGTAATGTATTAGGTAACCATTAAATTCGTTGTTATGCGATTCTGATACAAGATTATGATAAA
ATAGCTTTAATTAAGAATTTTAGCCATCATATAGTCATCGTAATATTTACCATCGATAAATAACTTATCTTTTAAAACGCCTTCGATT
TGAAAATCGGCACTTTTAAAAAGCTCGAGGGCAGGTTGGTTATTGAGTGGTACATTTGCTTCAATTCGGTGTATTTGATTGTTTAAA
CACCAAGCCATAATGGCATCAAGAAGTGCTTGGCCAATTCCACGATGTTGATATAATTTCTTTACACCTAAATCAATTTTAGCAACA
TGTTTAATGCGTTGAAATGGTGTCGTATTAACAAAGGCAAAGCCAACGAGTTGTTCATCACTTTCAGCAACAAAGATGACTTTATGT
GGAGAAGTGATATATTCTTCTAATTGTTTACTAGCCGATGTGACGCTAGGATCATATTCTCCTGGTGTGTAGAACATATACGGAGAT
TCGTCGTATATTTTCGCTAACATTGAAATGAAATTTTCTACATCTTTGATACTAACTCTACGTATAATATGGGCCATAAAAAGCCTCC
AGTATTTTGAAATTTTAAAACATTTGCTACTATTATAATATATATGTAACTAAAAGGTGGAGTAATATGTTTTTTGGTTACAGTTTAA
AGGAGTATTTTAGATGAAACCTAAAGTTTTATTAGCAGGTGGAACAGGATATATTGGTAAGTATTTAAGTGAAGTGATTGAAAATG
ATGCTGAACTTTTTACTATATCAAAATATCCAGACAATAAAAAAACAGATGATGTTGAAATGACTTGGATTCAGTGTGATATATTTC
ATTACGAACAGGTTGTTGCAGCAATGAATCAAATAGATATTGCTGTATTCTTTATCGACCCAACAAAGAATTCTGCCAAAATAACAC
AATCATCAGCAAGAGATTTAACATTAATCGCAGCAGATAATTTTGGTCGAGCAGCGGCTATTAATCAAGTAAAAAAAGTAATCTAC
ATACCTGGGAGTCGTTATGATAATGAAACAATTGAACGCCTAGGTGCATATGGTACTACTGTAGAAACAACAAATTTAGTTTTTAAA
CGTTCTTTAGTTAATGTAGAATTACAAGTTTCAAAGTATGATGATGTTAGATCAACGATGAAGGTAGTTTTACCAAAGGGATGGACA
TTAAAGAACGTTGTAAACCATTTTATTGCATGGATGGGTTACACTAAAGGAACTTTTGTGAAAACAGAAAAATCACATGATCAATTT
AAGATATATATTAAGAATAAGGTGCGACCGCTCGCAGTATTTAAAATAGAAGAAACAGCTGACGGAATAATAACTTTAATTTTATT
GAGTGGAAGTTTAGTGAAAAAATACACAGTTAATCAAGGGAAGTTAGAATTTAGATTAATCAAAGAGTCGGCAGTCGTTTATATAC
ATCTATACGATTATATCCCTCGATTATTTTGGCCGATTTATTACTTTATACAAGCACCAATGCAAAAAATGATGATTCATGGCTTTGA
AGTTGACTGCCGGATTAAAGATTTTCAAAGTCGATTAAAATCAGGAGAAAATATGAAATATACTAAATGATATTGGGTGATATGGA
TGCAAATACTACTAGTAGAAGATGACAATACTTTGTTTCAAGAATTGAAAAAAGAATTAGAACAATGGGATTTTAATGTTGCTGGT
ATTGAAGATTTCGGCAAAGTAATGGATACATTTGAAAGTTTTAATCCTGAAATTGTTATATTGGATGTTCAATTACCTAAATATGAT
GGGTTTTATTGGTGCAGAAAAATGAGAGAAGTTTCCAACGTACCAATATTATTTTTATCATCTCGTGATAATCCAATGGATCAAGTG
ATGAGTATGGAACTTGGCGCAGATGATTATATGCAAAAACCGTTCTATACCAATGTATTAATTGCTAAATTACAAGCGATTTATCGT
CGTGTCTATGAGTTTACAGCTGAAGAAAAACGTACATTGACTTGGCAAGATGCTGTCGTTGATCTATCAAAAGATAGTATACAAAA
AGGTGACGATACGATTTTTCTATCCAAAACAGAAATGATTATATTAGAAATTCTTATTACCAAAAAAAATCAAATCGTTTCGAGAGA
TACAATTATCACTGCATTATGGGATGATGAAGCATTTGTTAGTGATAATACGTTAACAGTAAATGTGAATCGTTTACGAAAAAAATT
ATCTGAAATAAGTATGGATAGTGCAATCGAAACAAAAGTAGGAAAAGGATATATGGCTCATGAATAATTTGAAATGGGTAGCTTAT
TTTTTGAAATCTCGCATGAACTGGATATTTTGGATATTGTTTTTAAACTTGCTTATGTTAGGCATTAGTCTAATCGATTATGATTTTCC
AATAGACAGTTTATTTTATATTGTTTCTTTGAATTTAAGTTTAACAATGATTTTTCTTATATTGACATATTTTAAAGAAGTAAAATTAT
ATAAGCATTTTGACAAAGATAAAGAAATAGAAGAAATTAAACATAAAGATTTAGCGGAAACGCCATTTCAACGTCATACAGTTGAT
TATTTATATCGTCAAATCTCAGCGCACAAAGAAAAGGTTGTTGAGCAACAGTTACAATTGAACATGCATGAACAAACCATTACAGA
ATTTGTGCACGACATAAAAACACCTGTGACAGCCATGAAATTATTAATTGATCAAGAAAAAAATCAAGAAAGAAAACAGGCATTAC
TATATGAATGGTCTCGTATAAACTCGATGCTGGATACACAGCTGTATATTACTAGATTAGAATCTCAACGCAAAGATATGTATTTTG
ATTACGTGTCACTTAAACGCATGGTCATTGATGAAATACAATTAACAAGACATATTAGTCAGGTTAAAGGTATTGGTTTTGATGTTG
ACTTTAAAGTGGATGATTATGTTTATACAGATACAAAATGGTGTCGTATGATTATTAGACAGATTTTGTCAAACGCATTGAAATATA

GTGAGAATTTTAATATTGAAATTGGGACAGAATTAAATGATCAACATGTTTCGTTATATATTAAAGACTATGGCAGAGGTATTAGTA
AAAAAGATATGCCGCGAATATTTGAACGAGGATTTACGTCAACGGCTAACAGAAATGAAACGACGTCTTCAGGTATGGGTCTATAT
TTAGTAAATAGTGTAAAGGATCAATTAGGTATTCACCTGCAAGTCACGTCGACTGTTGGTAAGGGGACAACTGTCAGATTGATTTTC
CCATTACAAAATGAAATTGTTGAACGCATGTCGGAAGTGACAAATTTGTCATTTTAAACATGCGTTTTGTTACTTAGAATTGATACA
TCAATGCAGCTTCAACGTTATAATAAGATAGATGTTAGTCATATGTTAAATTGAAGATACAAGTGCCAAAGCCTAAAGGAAATGAA
GTTAAGATAAATTATAGGAGTGTTAAAGTGGCAATTTTAGAAGTAAAACAATTAACAAAAATATATGGAACTAAAAAAATGGCACA
AGAAGTGTTGCGAGATATCAATATGTCTATTGAAGAAGGCGAGTTTATTGCTATTATGGGTCCCTCTGGATCTGGGAAAACGACATT
ATTAAATGTTTTAAGTTCAATTGATTATATTTCACAAGGTTCTATTACATTAAAAGGAAAAAAATTAGAAAAGCTTTCAAACAAGGA
ATTATCTGATATACGCAAGCATGATATTGGTTTTATTTTTCAAGAGTATAATTTACTGCATACATTGACTGTTAAAGAAAACATAATG
TTACCACTAACGGTTCAGAAGTTAGATAAAGAACATATGTTAAATCGTTATGAAAAAGTAGCAGAAGCATTAAATATATTGGATAT
TAGTGATAAATACCCTTCTGAATTGTCTGGTGGACAAAGACAACGAACATCTGCTGCAAGAGCGTTTATTACATTACCTTCTATTAT
ATTTGCTGACGAACCAACAGGTGCACTGGATTCTAAAAGTACTCAAGATTTATTAAAACGATTAACAAGAATGAATGAAGCATTTA
AGTCTACAATTATTATGGTAACGCATGATCCTGTTGCAGCAAGTTATGCCAATAGAGTAGTGATGCTAAAAGATGGTCAAATTTTCA
CTGAATTATACCAAGGGGATGACGATAAACATACCTTTTTCAAAGAAATAATACGTGTACAAAGTGTTTTAGGTGGCGTTAATTATG
ACCTTTAACGAGATAATATTTAAAAATTTCCGTCAAAATTTATCACATTATGCCATCTATCTTTTTTCATTAATTACGAGTGTAGTAT
TGTATTTTAGCTTTGTAGCATTAAAATACGCGCATAAACTAAACATGACAGAGTCATATCCAATTATAAAGGAAGGCTCACAAGTCG
GAAGCTACTTTCTATTTTTCATCATAATTGCATTTTTGTTATATGCCAATGTGTTATTTATTAAACGACGAAGTTATGAGCTTGCATTA
TATCAAACATTAGGTTTATCTAAATTCAACATTATTTATATACTAATGCTCGAACAATTACTAATATTTATAATTACGGCAATATTAG
GTATTATTATTGGTATTTTTGGTTCGAAACTGTTATTAATGATTGTCTTTACATTATTAGGAATTAAAGAAAAGGTTCCAATTATTTTT
AGTTTGAGGGCGGTATTTGAAACATTAATGTTAATCGGTGTCGCTTATTTTTTAACATCTGCTCAAAATTTTATATTAGTGTTCAAAC
AATCTATTTCACAGATGTCAAAGAATAACCAGGTTAAAGAAACAAATCATAATAAAATTACATTTGAAGAGGTTGTTTTAGGCATCT
TAGGTATAGTATTGATTACCACAGGATACTATCTATCTTTGAACATTGTTCAATATTATGATTCTATCGGTACACTTATGTTTATTTTA
TTGTCAACTGTGATTGGGGCATACTTATTTTTTAAAAGCTCTGTTTCTCTAGTTTTTAAAATGGTGAAGAAGTTTAGAAAAGATGTTA
TAAGTGTAAATGATGTCATGTTCTCATCATCTATTATGTATCGTATTAAGAAAAATGCTTTTTCACTTACGGTCATGGCAATCATTTC
AGCGATTACTGTTTCAGTTCTTTGCTTTGCTGCTATAAGTAGAGCGTCCTTATCAAGTGAAATAAAATATACTGCACCACACGACGTT
ACAATTAAAGACCAACAAAAAGCTAATCAGTTAGCAAGTGAATTAAACAATCAAAAAATTCCTCATTTTTATAATTATAAAGAAGT
AATTCATACGAAATTGTATAAAGATAATTTATTTGATGTAAAAGCGAAAGAACCATACAATGTAACAATTACTAGTGATAAATACA
TCCCTAATACTGATTTGAAACGTGGGCAAGCTGATTTATTTGTAGCGGAAGGTTCTATCAAAGATTTAGTGAAACATAAGAAGCATG
GTAAAGCAATTATAGGAACGAAAAAACATCATGTTAATATTAAGTTACGTAAAGATATTAATAAAATCTATTTTATGACAGATGTTG
ATTTAGGTGGACCAACGTTTGTCTTAAATGACAAAGACTATCAAGAAATAAGAAAGTATACAAAGGCAAAGCATATCGTCTCTCAA
TTTGGATTCGATTTGAAACATAAAAAAGATGCTTTAGCATTAGAAAAAGCGAAAAAATAAAGTTGATAAATCTATTGAAACAAGAAG
TGAAGCGATAAGCTCAATATCAAGTTTAACCGGAATATTATTATTTGTAACATCATTTTTAGGTATTACATTCTTGATTGCTGTATGT
TGCATTATATACATAAAGCAAATAGATGAAACCGAAGATGAGTTAGAGAATTATAGTATTTTGAGAAAGCTTGGATTTACACAAAA
AGATATGGCAAGGGGACTAAAGTTTAAAATTATGTTTAATTTTGGGTTACCTTTAGTTATTGCACTATCACATGCATATTTTACATCA
TTAGCATATATGAAATTAATGGGTACAACGAATCAAATACCGGTTTTCATAGTAATGGGATTATACATTTGTATGTATGCTGTTTTTG
CAGTGACGGCTTATAATCATTCCAAGCGAACAATTAGACATTCCATATAAAATATACAGATGGCTTTCAGTAGAGTAGTGGATTCGA
ATTCACGAACTATACTGGAAGCTTTTTATTATAAATGAAGAGAAGTTATATTTTTAGCATGTATAGTTGAATACTGGGTTAAAATAC
CATATTAATAATGAAGTAAAGGTATGAGTGATTATGAAAGTGTTTTGAATGAAATATATTTAATTGGTGATGCTTTTAATTGAAAAG
ATTAACAGGATTCAACTTTGTAAATTGTATTAAATGTGAGAAAATAAAAGTATATTCATTGAGAGATATATGAGTCAATGATCGTTT
TAAACAAGATAAGTGTATTTTAATATGTAAAAGTTATGTAATAAATATTGTATCGTTGCAAATTTCCCAACTATATTCATTTACAATT
TTTAGAGTTGTATAGATAAAAAATTGCCTATACAATAACTTTTTCAAAACAAATGCCTACTACTATTTAAATTTTATGTAAAAGTTAA
GTTTCGGAATTGTTAAAAATTTGTAAATTGAATTGTGGGATTGTAAATTTTGTGCTATTTTCATATAAGGGCAAATACAGTAAATTGT
TTACTGTGAGGCATTTTTGAAATTAATATCAGTACACTAAAATTATACTGACGATTGATAATAACAAATTTGTATCATTAGTTTGTAA
ATTCACTTGTCTTAATTTGAAACAAATTATAACTGAATGTGATTGGTGACAATCGCTTAAATGGAGGATTTTAAATGTTTAGTAAGA
AAAAAGATAAGTTTATGGTTCAATTAGAAGAGATGGTTTTCAATCTGGATCGTGCTGCTATTGAATTCGGTAAAATGGATTTCAATA
CTCATTTAGATTTAAAAGCATACTCAGACAACATTAAAACTTATGAGTCACATGGTGACGAATTAGTACATCAAGTAATTACTGATT
TAAATCAAACATTTATCACACCAATTGAACGTGAAGATATTTTATCATTATGTGATGCAATTGATGATGTTTTAGATGCAATTGAAG
AAACGGCAGCTATGTTTGAAATGTATTCAATCGAATACACAGATGAATATATGGCTGAGTTTGTTGATAACATTCAAAAAGCAGTTG
CAGAAATGAAACTTGCTGTCGGCTTATTAGTCGATAAAAAATTATCACATATGCGTATTCATTCAATTAATATTAAAGAATTTGAAA
CAAACTGTGATGGTATTTTAAGACAGTCAATTAAACATATTTTCAATAGCGAAACAGATCCAATCACATTAATTAAAATAAAAGAT
ATTTATGAAAGCATGGAAGAAATTGCTGATAAATGTCAAATCGTAGCAAATAATTTTGAAACTATTATTATGAAAAATAGCTAAGG
GGAGTATATATTTATGTCATATATAATCATCGTCACTATAGCTGTAGTTATTTTCTCGCTGATATTTGACTTTATCAATGGATTCCATG
ATACAGCCAATGCAGTAGCTACTGCTGTATCTACTAGAGCGTTAACGCCTAAAACGGCAATTTTAATGGCAGCAGTGATGAACTTTA
TAGGTGCTTTAACATTTACGGGCGTTGCAGGCACCATTACTAAAGACATTGTCGATCCATTTAAATTGGAAAATGGATTAGTTGTTG
TGTTAGCTGCAATACTTGCGGCTATTATTTGGAATTTAGCTACTTGGTTTTACGGAATTCCAAGTTCGTCTTCACATGCACTTATAGG
TTCAATTGCGGGTGCAGCAATCGCATCTGAAGGCTCATTTGGAGTGTTACATTACCAAGGTTTCACAAAAATTATTATTGTATTAAT
CGTTTCACCGATTATCGCATTTTGTGTTGGTTTCTTGATGTATTCAATTTTTAAAGTTATCTTTAAAAATGCAAATTTAACAAGAGCG
AATCGTAACTTTAGATTTTTCCAAATTTTCACAGCAGCGTTACAATCATTCTCTCACGGTACGAATGATGCGCAAAAGTCAATGGGT
ATTATTACGTTGGCATTAATTGTTGCTAATGTACAGAATGATGGCAGCGTTGAACCACAGTTATGGGTAAAATTTGCCTGTGCGACA
GCAATGGGGCTTGGTACTGCAATTGGTGGCTGGAAAATTATCAAAACTGTAGGTGGTAATATTATGAAAATACGTCCAGCAAATGG
TGCTGCGGCCGATTTATCATCTGCATTAACAATTTTTGTTGCATCATCGCTACATTTCCCATTATCAACAACTCACGTTGTGTCATCAT
CAATCTTAGGTGTTGGTGCTTCTAACCGAGCTAAAGGTGTAAAATGGAGCACTGCGCAACGAATGATCATTACATGGGTGATTACAT
TACCTATTTCAGCATTGTTAGCAGGTTTACTATTCTATATACTTAACTTATTTTTCTAATTGAAAATAAAACTAAACTGAACTTCAGT
ATCACAAACATATGGTGGTATTGAAGTTCAGTTTTTTATGTAAGTAGATAACATATTTTATAGAATTTACGTAGAATGACCAAGACA
AAGCATTTTAATTAGGAGGCTAAGTTATAAATGTATTCCATGGAACATAAACAGTTAGGTCATTAAAATATAAGTAAGCGTAAAAT
AATGATTTAATAGTTTATATTCTTCAATGAAGGAATAATTAAATAGTTATCAACTTGTTTAATTAACCTAAATTGTATTGGACTTTAT
TTAATATAAATTTGTATATGTAGACGAAAAAGCGTAAAATACTGGTATAACAAATTAAATCATTATATAGTCAACTTAGTTGCATTA
TTTTGATTTTTAATATATTATGCATTTAATTTAATCAAATAGGTAGGTTATTATTTTTTACCTAACTACCATTCATACAAAATAAAAAT
AAGTATGATAGGGAAGGGTGAGATAATTTTTTGTACCAAAATGAAAATTTTTTTTCAAAAATAAAAAACTAGCAATCTCACATGAT
GTGAATTGCTAGTATATATCAGTACAATTTATTTAATTAATGGATGAATGCATAGCTAGAAACTTCTGAAGCTGGAATTGTACGGTA
GTTCATATTGTATGGACCATATGTGTAATTCATTTCAGAAATCAAGATACTACCATCACCATTGACACGTTCAACATAAGCAACATG

ACCATATGGACCAGGTGTGCTTTGCATAATTGAACCAACTGATGGTGTGTTGTTTACTTGGTAACCATCATTAGCTGCGTTACCAGC
CCAATACTTAGCGTCTGACCAATATGTGCTAATTGGACTACCAGCTTGAGCACGACGGTCAAATACGTACCATGTACATTGACCAGC
AGTGTATAAATTTTGGTGATTAAAAGATGATGCATTGCCATTGCTACCTGTTGTAGCTGTTGGTGTTGTACCACCTGATCCACCATTA
GGAATTTGTAATGTTTGGTTAGGCATAATTAAATAACCACGTAAGTTATTGGCTGCCATTAATTGATCAACTGAAACACCATATCTG
CTAGCAATGATATTTAATGATTCACCAGCTTGTACAGTATGAGATGATGCTGAACCAGCTTGTGGAGAAGTGTTTGACGTATTTTGT
GCATCACTTCCACCTACTGAGATAACTTGACCAGGGAATACCAAGTTGTTATCTAATTGGTTATTTTGTTTAATACTCTCTACTGAAG
TGTTGTATTTTTGAGCAATACTCCATAATGATTCACCAGATTGTACTGTATGTTGTGTAGAAGCTTGTGCATCATGATGCGTTAAAAA
TGCAGCTGCACCAGATGTTGCTGTTATTGCAAATGCTAATTTTTTCAAAGGGACTCCTCCTTAAAATTAAGTTTTACTACTTTTAAAA
ATTATTAAGACAATTTATATAGTAAATTAAGTTTTCGTTTTATATTACGATTTCCATATTATCAAAAATAAGTAGCTTTGTGTGGTTT
GTTATTATCTTGTAATATTATTAACATTTTAGGATATCGGTTCATATTTTTATAAATGCTGATTTGATAGTGTATAAAAGCGTTTTAGT
GATTTTAACTAATTATAAAAAATTATATTTTGTAACATTGTAAAATGTAAAATTACTATACTTATTAAAGGTTATTTCTTTTGATGAA
GCAAAATTTACTGTAAAATTTGACGCATAAAACGACAGTAGAAGGAGGAATTATTTTGTCGCAAAATACAAATCATTCATATTATC
ATCAAAACCAGCATGCTCAATCAATAAGTAAAGTGTGGCTTTATTTTATGTATTATTGGATTATATTTGGCATAGGATGCTATCTAG
GTCAGTTTTTACCATTAAGTTGGCGACAACCCTTGTCATTTGGATTACTGATTATTATTTTAGCAACACTTGTTTTTGAAAGAGCGAG
ACGGTTCGGTTTAATTATTTCACATATTTACGCTGTAGTGATCGGCTTATTGTCATACGCAACGTTTACCACGTATTTACAAAATTTA
GGACCAGATATTTTCTATAAAAATATCGCATTAGCAACTTTTGCATTTATAGCATTTGGTATTATTGGTTATTTCTTCGTTGGAGATG
CATCGAGTATAGGCAAATATTTATTCGTTACATTAATAACATTAATTATTGCGAGTCTAATTGGTATTTTTCTTCAAAATCCTATTTTT
TACACTATTATTACCGTCGTTAGTTTGTTGTTATTTCTACTTTATACTTTGTATGATTTTAATCGTTTAAAAAGAGGTGACTATTCACC
AAGAGAAATGGGATTTAATCTATTTATTAATTTGTTGAATATTATTAAGGATATACTTTATCTTGCTAATATGTTCAGAAGATAAACA
GTTAAATTAATAAAGAAATTTGTATAAAGTTTTGGAATAATCAATAATGAATATCAACCTTAAACTGGAGCGATTGTCATAAGTTAG
AAACTTAAACTGAGATTTTACTGATAAAGCAATAAAAATATATTACCTTAATTGTAAAGATATTATTAATCATTGAAATGATTAACT
GCTATAGTTCTAAAGTAAACAAATCTTAGAAGATAAACTTTAAACAGGTGTACTTGCCTTTCTAATTACACTTTTATTAGAAAAGTA
AAGTATAGCTACGTATTAATTTTAAAATCGAAACATTAATAGGAAGAAGTATAGATAATATAAACAATTTAATGTTAGTTTGAGCTC
ATCTCAAAAGTAGAATGTGATTTAAATAAGTGTTTATTTTTAAATTGAAAATGAATATATAACTAACAAAAGATAATAGTAATACGT
TGTAGATGTGGTCATTTAAAATTATTTTGAAAATACTAAAAAGTAGTGTGTTTTCAATTGGATAGTTTAATTAAACTGACTACATCTA
TTTTTGTAATATTTAAATGATTGATATATAAGTTTAAAAATTAACCATTATATAGTTTTATTTATTTTTGATTTGGTATTCGTATTGTG
TTAAATTTACGTTTAATTAAATAAGTTAAATATTATAGGGAATGATAAAAGTGGTACTTAAATATAGAAAGAGGTAAAGTTATGGC
AAAATCATGCTTGCATATACTTACTAATAATGAATATGCGACAACGCGTTGCCAAGATGGCATAGTCTTATTTTGGCCAATTGACGG
GGAAATCGAACTACAAAAATTTCGTAAAAGTAAAATAATTGAAGATGATATATATATTATTAATCATCTGGATGTATTTAGTATTAA
GAATAATAAAAAAACGATCATGTTGTATTTGAGTAGCGATTGGTTTGCGGAATTAGGCTTTACTTTCTTTAATTACCACTATACAGC
AAAGTTGATTAAATCATCCTATAATTTGAAATGTCTACTATTAAAATTGACATATCGATACCTTGATAATCAGCCTCTTAATGACGCT
GATATTAGAAAATTACAGGATATTATTAAAATCATTGCAAAAGAAGCAAGTATGGATAAAAAGATTGCACAAAATCAATATCGATA
TGCCTATTATGGTGATTTGCGTGACGAGCTCGAATATATTTATCAAAATGTAAATCAACGATTGACATTAAAAAGTGTCGCTGATAA
ATTATTTGTCTCAAAGTCAAATTTGTCATCACAATTCCACTTACTTATGGGCATGGGTTTTAAAAAAATATATTGATACTTTGAAAATT
GGTAAATCGATTGAAATTCTACTTACTACTGATAGTACTATTAGCAACATAAGTGAACATTTAGGTTTTAGTAGTAGCTCCACTTACT
CTAAAATGTTTAAAAGTTATATGGATATAACACCGAATGAATATCGTAATTTATCAAAATATAATAAATGTTTAATGCTAAAGCCAG
AACCACTAGTAGGCAAAATGGTACAAGAAGTAAAAGAAATCATATTGAATTATATTGAACATTATAAAAACCACCTAACTGATGTT
ATACATATTGATGAAGACAAATTTGAAACACCTAAATTGTTTCAAACGGTTATTCAAATAAATACTTATACAGAAATGAAATTAGTT
TTCTTAGAAGGAATCTTTAAAACCTTATTGAATAAGAACAGTCAAGTTGTCTTTTTCATCATGCCATCGATTCTAAAAAGTAAAAAT
ACCATGTCCGAAGAAGAAAAATTCACAATCATTAAAACAATAATTGAAAGTGATCTAAAGATAGCATTTAATATAAATGATATTGA
AACAACTTATTTTGTTGAAGAAGCTTTTATGAGTGTTTTCAGACAAATATCTCCAAACGAATTAAGTAATCATAATAATTACGAAGT
GCATTTTGTTTTTGATTTATCATTGATGGAAATTAGAACAATTTATCGAATGATATTAAAATTACATAACATCATGTTGAATGTGAAA
TTAGGATTGAACATTACCTGTTTATTTGAAAAACCTTCAGTTTTTAAATCATTAGTATCACAAATAAAGCGACTTAAATTCGATTCGT
TAATAATAGATAATGCAAATTTAAGTAGCCCTTATTTGATGGGGGAAAGTGATGAGTTACTATTGAAAAATATTTTGCATTTTAAAA
ATTTAAAACAAGTAATTAATGAATTGGATATTGAACAAGAAAAGCTTATTTTTCTAAATGTTGAAAATCATAAACTGCTTAATAATA
AAGAACGAGATTTAAGTAATAGTGCTCCATTAATTTATAAGACATTAAGTGCGCTGTATCACAACTTTGATGGCTTTGGATTAAACA
TTTTTGATAATCATCATACATTTAATGCGATGCATCTATATGATAAAAATGGATTTAAAACAACACTAGGTCTTATATTGGAAAAAT
TTATCGAATATGTCTCGAAACCAAAATACGAAAACAGTTATTATTCTATTTTTGATATAGAGAATTATTATTGCCTGGTTATTTATGA
TTGGCGAGTGATTGAGAGCGAGACAATTATGAGCAATTTTGAGGATAGTCAAGTTTATATAAATTTCAAAAATAATGTTTTAAACGA
TAAATATCTAATTGTAATAGAAACATTGGACGAAAATAGTGGCAACATTAATCATTTGATTTCTAAAGAATTAAGAGATAAATATG
AATGGAACCCTAGTTTACTATCTAAAATTGACAACTACCTTAAACCAGCAATCGAGATTAAAGAGCATAATTTTAGTGATAATTCCT
TGAATATTAACGTTACTTTTAATGCGTTATACATAATTAAAATAGGAAAAAAATAACACCTTGATATGTATTGCAAAATTTAATTTG
CATTTGTTGGTAATTTTGACAACGTATTAAAAACAAATGAGAAAGACAATGGCAAAAAAATTTAAGATAATAATGACAGAAGCATT
GTCTTTATATATTTGGGGGTGCAACATTTTGAATACTGAGAAATTAGAAACATTGCTTGGCTTCTATAAACAATATAAAGCATTATC
TGAATATATTGATAAAAAATATAAGTTGTCGCTAAATGATTTAGCAGTCTTAGATTTAACGATGAAGCATTGCAAAGATGAAAAAG
TACTTATGCAATCATTTTTAAAAACTGCAATGGATGAGCTAGATTTAAGTAGGACAAAATTATTAGTTTCTATAAGAAGACTAATTG
AAAAAGAAAGACTTAGTAAAGTTAGATCATCTAAAGATGAGCGTAAAATTTATATTTATTTAAATAATGATGATATATCTAAATTTA
ATGCTTTATTTGAAGATGTAGAACAATTTTTAAATATTTAATTGAAATTGAGTGTCGAAAGCATAGAATTTGCTTATCGGCACATTTT
TAATTTATACATATTTTAAAACTAAGTAACAGTTTGAAGAAATCGTAGTGCAATAATGTTAATTGTGAAAATGTATATAAACATAAA
AAAATCATGTATAATATATGTTGTTAATTAAACAGTTCGAAAGCGAGATGACATTATGGGACGTAAATGGAATAACATTAAAGAAA
AAAAGGCCCAAAAAGATAAAAACACAAGTAGAATATATGCGAAATTTGGTAAGGAGATTTATGTTGCAGCAAAATCTGGTGAACC
CAATCCAGAATCTAACCAAGCTTTAAGGTTGGTGCTTGAACGCGCTAAGACATATTCAGTGCCGAATCATATTATTGAAAAAGCAAT
AGATAAAGCTAAGGGTGCTGGAGACGAAAACTTTGATCACCTAAGATATGAAGGATTTGGCCCAAGCGGATCAATGCTAATTGTTG
ATGCGTTAACAAATAATGTAAATCGTACTGCCTCTGATGTGCGAGCTGCTTTTGGTAAAAACGGCGGTAATATGGGTGTATCTGGAT
CAGTTGCTTATATGTTTGATCATGTGGCAACATTTGGTATTGAAGGAAAGTCTGTTGACGAAATACTTGAAACATTAATGGAACAAG
ATGTAGATGTAAATGATGTGATTGACGATAATGGATTGACAATAGTCTATGCTGAACCAGATCAATTTGCAGTCGTTCAAGATGCGC
TTCGTGCAGCAGGTGTCGAAGAATTTAAAGTTGCTGAATTTGAAATGTTACCTCAAACAGATATTGAACTTTCTGAAGCGGACCAAG
TAACATTTGAAAAATTAATCGATGCATTAGAAGATTTAGAAGATGTACAAAACGTATTCCATAATGTGGATTTGAAATAATGAAAT
CAGCAGAACAATGGATTGATGAATTGCAACTTGAATCACATCCTGAAGGTGGTTTCTATAGAGAGACAATTCGAGAAGTATTGAAA
GATGGACGCAGAGCGCCGTTTAGTAGTATTTATTTTTTACTTACAGATGACAATATTTCGCATTTTCATCGAATTGATGCTGATGAAG
TATGGTACTATCATGCTGGCGATTCTCTAACAATTCATATGATAAATCCGGATGGGGAATATACGACTGCAACATTGGGTACTGATA

TCCAAAATGGAGATGTATTGCAATATGTAGTGCCTAAAGGAACAATTTTTGCTTCTTCAATCGAAATATCAAATACTTTTAGTTTAGT
AGGTTGTATGTGTCAACCGGCATTTGAGTTTAAGCAATTTGAATTGTTTAAGCAATCTGAATTAATTACGCAATATCCGCATCTTAA
ATCAGTAATTGAAAAATATGCTTTAAAATAAAAATGATCAATGAAGTGGTTTGAAGGTTGTTAATAAACCTTTGAGTCACTTCATTT
TTATATGTATTCTTGATTGAATCAGAATAGATTTGATGCTTCAGCTGTTTTTAATGAAAATAGCATTAAATGATTTTGAAAACGATAA
GAGTGTGTTATTTATATTTTTGAAAAATCACTTTTATGAAGAAATGTGTGTACTATCTAATTAATATTGTCTATTTTAAGTAAATTGA
AGCGAGTTGTAGTAAAATAATAATAAATATTTTCATATGATTAACAAAAACTATAAAACTGTATCATGACATGACATCATTTATGTA
GTTTTATATAATAATGACAAGGAGTTGGTGACTGTGAAAGTAAAGTATATAGATAAACGTCACTGGCGTCGCCTAATTGATAGGGA
ATACACAGAGGTAAAAGTTAATAATAATAGGTTTAAGGGTATTATAGGCTTAGTCACGATGAAAAAGGTTCGTGATCCTTTAGAGG
TGACGGTAGTTGGACAAAATATCATTGTCGCAGATGACAATTATAAATGGTTGCAAATACTACCTGAAAAGAAACGTTATAGTATA
ACTGTAATGTTTGATAATAAAGGCAATCCATTAGAATATTATTTTGATATAAATATCAAAAATATAACGCAAAAAGGTAATGCGCGT
ACAGTAGATTTATGTTTAGATGTTTTAGCATTACCAAGTGGTGAATATGAGTTGGTAGATGAAGATGATTTAATGTTTGCATTAGAA
AGTGAGCAAATTACAAAAAAGCAATTTCATGAAGCATATATGATTGCACATCAAATTATGGCAGAGTTAGAAAATGATTTTAAAGG
ATTCCAAAAGAAAATCATGTACTGCTTTAATAAAATTAATGCAAAGGCTCAAAAAAATCATCAAAAGCCACAAAATAAAACTAATA
TTGAAAAAAGCAAACAAATAAAGCCTAAGCAATATAATCAAACTAAAAATCACCAACAACAAAAGAAAAACTAAGTAATTCAAGC
TGCAGCCATGCCAATAAAATTGGTGAAATTATTCGTTAGTAATGAACTCATGAATGCTTGTAGCCATGAAAGTTCAATAATTGAATA
ATTTATGGGGGGAAATTATGAAGATTGAAGACTATCGTTTACTAATAACATTAGACGAAACGAAAACGTTACGTAAAGCGGCTGAA
ATTTTATATATATCTCAACCTGCTGTTACACAAAGACTAAAAGCTATTGAAAATGCTTTTGGAGTAGATATTTTTATCAGAACAAAA
AAACAATTGATTACAACAACTGAAGGAACAATGATTATTGAGCATGCCCGTGACATGTTGAAAAGAGAGCGATTATTTTTTGACAA
AATGCAGGCACATATTGGTGAAGTGAATGGAACAATATCAATCGGGTGTTCTTCTTTGATTGGACAAACCTTACTTCCTGAAGTTTT
GAGCCTATATAATGCCCAATTTCCTAATGTTGAAATACAAGTGCAAGTTGGTTCAACTGAACAAATTAAAGCAAATCATAGAGATT
ATCATGTTATGATAACTCGTGGAAATAAGGTAATGAATTTAGCTAACACACATTTATTTAATGATGATCATTATTTTATTTTTCCAAA
AAATAGAAGAGATGATGTTACAAAGTTACCATTTATAGAGTTTCAAGCTGATCCGATTTATATAAATCAAATAAAACAATGGTATA
ACGATAATTTAGAACAAGATTACCATGCAACTATTACAGTGGATCAAGTAGCAACTTGCAAAGAAATGTTGATTAGTGGTGTAGGT
GTTACAATCTTGCCAGAAATTATGATGAAAAATATCAGCAAAGAACAATTTGAGTTTGAAAAAGTAGAAATTGATAATGAACCGCT
GATTCGTTCGACATTTATGAGTTATGATCCGAGCATGTTGCAATTGCCACAAGTTGATTCTTTTGTAAATCTCATGGCGAGCTTTGTT
GAACAACCAAAGGCGTAGTTTTAGACTAATTTAAGGTTTGTATTTAATTTTAAACTATTCGGTTAAATTGAACGTAGTTGGTTGCTA
ATGCACCAACAGCAAAAGAGCCCCTAATTAATAAATTAAAAGGGGACAAAGGAACGCAGTTGATTGCTAATGCACCAACAGCAAA
AG

>021-contig_267_RC

AAGAGCCCCTAATTAATAAATTAAAAGGGGACAAAGGAATACAGTTGGATGCTAACGCACCAACAGCAAAAGAGCCCCTAATTAA
TAAATTAAAAGGGGCTCTAAGAAGCGGGGTTATGGATATGTTTGCAGCGTTATTACAAATAAAGAATTATAAACTCTTTGTTGCTAA
TATGTTTCTACTAGGTATGGGTATTGCGGTTACGGTCCCATATCTTGTTCTTTTTGCAACTAAAGATTTAGGTATGACAACAAATCAG
TATGGATTACTTCTAGCATCTGCAGCGATTAGCCAGTTTACAGTAAATTCAATTATTGCTAGATTTTCGGATACGCATCACTTTAATA
GAAAAATTATTATTATTCTCGCATTATTAATGGGTGCGCTTGGTTTTTCAATATACTTTTTTGTAGATACAATCTGGTTATTCATATTA
CTATATGCGATTTTCCAAGGATTATTTGCACCAGCAATGCCCCAACTTTACGCATCTGCTAGAGAATCTATCAATGTTTCAAGCTCTA
AAGATAGAGCTCAATTTGCCAACACAGTATTACGTTCAATGTTCTCATTGGGCTTTTTATTTGGTCCATTTATTGGTGCCCAATTAAT
CGGATTAAAAGGCTATGCTGGATTGTTTGGTGGAACAATAAGTATCATTTTATTTACTTTAGTACTTCAAGTGTTTTTCTATAAGGAT
TTAAACATTAAACACCCTATTAGTACGCAACAACATGTTGAAAAAATTGCTCCTAATATGTTTAAAGACAAAACGCTTTTATTACCA
TTTATTGCATTTATTTTATTACACATTGGACAATGGATGTATACGATGAATATGCCTTTATTTGTTACTGATTATTTAAAAGAAAATG
AGCAACATGTCGGTTATTTAGCTAGTTTATGTGCTGGTTTAGAAGTGCCATTTATGATCATTCTTGGCGTTTTATCATCTAGATTACA
GACTCGAACATTGTTGATTTATGGAGCGATTTTTGGTGGTTTATTCTACTTCAGCATTGGGGTATTTAAAAACTTCTATATGATGTTA
GCAGGACAGGTGTTTTTAGCTATTTTCTTAGCGGTTCTTTTAGGAATTGGTATTAGTTATTTCCAAGATATCTTACCAGATTTTCCAG
GATACGCCTCAACACTATTTTCTAATGCAATGGTTATTGGACAGTTAGGCGGTAACCTATTAGGTGGTGCTATGAGTCACTGGGTAG
GTTTGGAAAATGTATTTTTTGTATCAGCAGCATCAATCATGTTAGGTATGATACTTATATTCTTTACTAAAAATCAAAAAATTACAAA
AGAGGATGTGATATCAACATGACAATTATTTTATGGCTACTTATCATCGCTGCCTTCATGTTAGCATTTGTTGGGTTGATTAAGCCGA
TTATTCCTTCTGTTTTAGTATTATGGGTTGGCTTTCTAATCTATCAATTTGGCTTTCATAATCAGCATTTATCATGGGTGTTTTATGTAT
CTATGACATTGCTAACAATACTAATTTTATGTGCGGACTTTTTAGCTAATAAATATTTTGTGAATCGCTTCGGTGGTTCTAAGTTTGG
AGAGTATGCAGCTTTAATTGGTGTGGTTATTGGATGTTTTGTTTTACCGCCATTTGGAATTATTATTATACCTTTTATTTTGGTATTCA
TAGTTGAATTAATACAAGGCTATTCATTTGAAAGAGCAGTTAAAGTAAGTATAGGTTCAATCGTAGCATTTTTAACAAGTAGTATAG
CTCAAGCAATCATTATGTTTATAATGATTGTATGGTTCTTTATAGATGCTTTATTGATTAATTAATAAAAAGCTTATTGCAAAATATG
TTTTTCGGTAACTGTAATTTAGTGATTTTATCATTAACAGTACCAAATTCGCATATGATGTAATAAGCTTTTTGTTTATAAAAATGTA
TGAGAATGTTTTTTCGAAATATTTCTTTCAATGCGTAATCCAATAGGTATAACTATAAATGAAAATATAATAAATACCCAATTGCTT
ACCTGAATATATGGTCCAAAGGCAAGTAATGATTGTGGAATAAAGAATACATAGAAAAATCCTACAATTAGTAGAATGATTGCTAA
ATAGGTGAATATCCATATCCAATTTGAATTGTTAAAACATTGCAGTTGTATTGTTTTAAAACTAAACCATATAAATAAAAAGACAAT
AAAGAATCCGACGACTACTGAAAACGGGAATGAAACAAAATATAAATTACTTCCATTTTTTTCCATGAAAAATCCTAAAAATCCTTT
GAGAAAACTAACAATCCCAATTAATAGAACGATGTGTTGATAGATATATTTAAAAATATTTTTAAATGTTTCATTAGGCATCGCTTT
TAGTTCTTTTATTGCATGTGCTTTTGGGTCGTGATTGAAAAAATCTAAGGCTAATAAACCATGTTGTTCTGCGCTTAATAATTGTTTG
AGTATACGGTTAATAATTAACTCTGTATCATGAGGGTTGACGCGAAAGTCAGAGCGCATATAAGTCATATAATTCTCGAAGATTTCT
CTATCAGTATTGCTTAATCTTAATGATTTAACATTATTTTCTTTTGTTAATTGCGCAGTACTTTTCATTGTTACTTAAGCGCTCCTTTA
AAAATGTTTAATTCCAAATTAAAATGGAAATGATTTTATAGTATTAGTAAGGTCAATCATATCATATTAAACGCATAAATATAATGA
TTAATATTGGAGAGGAAAATGAGGACACTTAATAAAGATGAACATAATTATATCAAGCAAATAGCTAATATACATGAGACATTATT
GTCGCAAGTAGAATCCAACTATAAATGTACTAAACTGAGTATTGCTCTTAGGTACGAGATGATATGTTCAAGATTAGAACATACAA
ATGATAAAATTTATATATATGAAAATGAAGGTCAATTAATAGCGTTTATTTGGGGACATTTTAGTAATGAAAAAAGTATGGTTAACA
TTGAACTGCTATATGTTGAACCACAATTTCGCAAACTAGGAATAGCTACGCAACTGAAGATTGCGCTTGAAAAATGGGCAAAAACT
ATGAATGCAAAGCAAATAAGCAGTACAATTCATAAAAATAATTTGCCAATGATATCTTTGAATAAAGATTTAGGTTATCAAGTGAG
TCATGTGAAAATGTATAAAGATATTGATTAGAATTAGGATTATGTTGCTAATTCATGTTAAAATTAAAAAAGATTTAATGACGTTAA
GGAGTTTTATATGAAGAAATTAATCATCAGTATTATGGCGGTCATGCTATTTTTAACAGGTTGTGGTAAAAGTCAAGAGAAAGCCAC
TCTGGAAAAGGATATCGATAATTTACAAAAAGAAAATAAAGAATTAAAAGACAAAAAAGAAAAGCTTCAACAAGAAAAAGAAAA
ATTAGCAGATAAGCAAAAAGACCTTGAAAAAGAAGTGAAAGATTTAAAACCATCAAAAGAAGATAACAAGGATGATAAAAAAGA

CGAAGACAAAAATAAAGACAAAGATAAAGATAAAGAGGCATCACAAGATAAGCAATCAAAAGATCAAACTAAGTCATCGGATAA
AGATAATCACAAAAAGCCTACATCAGCAGATAAAGATCAAAAAGCTAATGACAAACACCAATCATAATCGAATTGCTTACTTGTTA
TAGATGAAAGGTACAGCGTTTTAAACCTTATTTTAAGGGTATGTATTAATTAAAATGTGGTCATGATTGAAACAGAACGTAAAAATA
GACAACGTAATTAATAAAGGAGAGAAACGGCATGCATGAACAAGATTTTAGAATTTTAGAGGGTCAAGATATTACTTTGCCAGAAT
TAGGTAGAGAATTAGAAAATATTACAGGACATACGATTGCTGATTCTACTGGCGAAATTAAGCGTGTAATTGCACATTTACCAAACT
TTGAGTCCGATACAGATACTTTTGTTGCTACATATCGTTTAAACCATCAACAAGATTTTATAGATGCAACTTTTACTGCGCTGAAATC
AGATAGAGCACGTTTAAAAGAAGTGCCAGTTCATGTTGAACTTATAAGTTATATTTCTAAATCAAAATAAACTGCTATCTAAAACGC
AAAGTTGATCAAAATATCGATTTTGTGTTTTTTATTGAGAAATTATATAGGAGTGTCAATCGATGATTTATTGTGAAACAGAGCGTTT
AATATTAAGAGACTGGCATGAAGATGATCTGTTACCTTTTTAAAAAAATGAATGCGAATTATGACGTACGTAAATATTTTCCAAGTTT
ATTGAGTTATCGTCGTTCAGAATTAGATATGAGAACTATGGATGCGGTTATTAAAGATTATGGCATTGGATTATTTGCTGTAGAAGA
TAAAGAGTCACATCAATGGATAGGCTTTATAGGTTTGAATTATATTCCAGAAACAAGCGATTATCCATTTAAAGAATTACCGCTTTA
TGAAATAGGTTGGCGCTTGTTGCCAGAATTTTGGGGAAAAGGATTAGCAACTGAAGGCGCAAAGGCAACTTTGAAGTTAGCAGAAG
AACATCAAATATACGATGTCTATAGTTTTACAGCAGAAGCAAATAAAGCTTCACAACGTGTAATGGAAAAAATTGGCATGACAGTG
TATGATCATTTCGAATTACCCAATCTAAGTAAGTATCATTTATTAAAAAGGCAAGTACGCTATTACATTAATCTTCGAAAGTGAAAA
ATTTATACATAAGCGTAACAAACACCCCTAACATTGTTTAGCTGATGATAACAATATTAGGGGTGTTTGTTTATTCTTTAACCTTAGA
ATGATTAATCGTATGAACGAGTACCCAGAGGTTTGAAATTTAATATTGATTCTATTAATGATTCCTTAGTGTCGCATAACGGTGCAA
GAGCACGATACTTAGGATCAATAAAACCTTCCTCAATCATATGGTCAATCATTGTTTGTAGTGGATTGAAAAAGCCATTAATATTAT
AAATGGCAATAGGCTTTTCATGGATACCTATTTGAGCCCAACTATACATTTCGAAAAATTCTTCTAGTGAACCTGCGCCACCAGGAG
CCATGACAAATGCATCTGCAAGTTCTGCCATTTTATTTTTACGTTCATGCATAGAATCAACTAAAATTAATTCAGTTAAACGTTGGCT
TGTGATTTCATGTTCATCTAACATTTTAGGCATGACGCCAATAGCTTTGCCGCCATGATCTAATACACCATCTTGAATGGCACCCATA
ATGCCAATTGACCCTGCACCAAATACTAATTCATAACCTTGTTCAGCAAAATATTTACCTAAATCGTATGCTTTTTGTACATATGAAG
GGTCATGACCTTTGCTTGCACCACAATAAACTGCGATTCGTTTCATGTTAATCCAGCTCCTTAATTCGATGAATGACTTTTAATAGTG
ATTGTTCAAACACTTTTTGATCTTGCTTTGTAAAAGGTGGGGGACCTTTGTGGCGACCACCTTGTTTTCTAATTTGTGCATTCATATAT
CGTTTATCTAATAGTTGTTGAATATTTTTGGAATTGTATATCTTCCCATTATGATGCATGACAATTAAGACTTTGTCGACTAATAAAC
TTGCGAGTCCATAATCTTGAGTGACTACGATATCATCCTTCGTTGATAATTGAACAATTTTGTAATCAACTGCATCTGGTCCATCATC
AACATATAATGTTGATACATGTGGAGGATATAATTGGTTCGAAAAATGGCTGAAGCTCCGAATAATTGTCACAAAAATGCCTGTCTC
AGTTGTTAAATCTATAATAGAATCAACAACAGGACAAGCATCTCCATCAATAATAATATGTGTCACAATTATGCCTCTGTATTGTTT
TCTTTATTTTGTTGAGAGGCGCTTTTGGCAACATAATCTTTATATTTTTTAAATGACTTGATGCGTGCTTTATCAGCTTCTTGTTGGCG
TTTTTGTTCTTCTTTGTGTCGTTTTTCAATATTTTTTTGTAACTTTTTATTCATTTTAGCGATTTCTTTGCGATTTTTTTCAGCTAGTTTA
TCGCCTTTTTTCTCAGTTTTCTCATCTAATTTATTAGGTGTTAAGCCTGCTTTTTCTTCGTATTTTTGTGATTTTTTCATATCTTTAATAC
GTTGTATTTCATTCTTTTCGCGGGCTTTTTGCTCTTCTTTATGACGCTTTTCAATATTTTTTTGAAGTATTTTATTCATTTTATCAGCGT
CTTTACGATTTTGTTTAGCTAATTTTTCGCCTTTTTTCTCAATATAGGCAGGATCATGTTCTCTAGCAAACTTTTTAAGTTCACGTTTA
TTTTCAAAATCTTGTTTTTTATCGCCGACATATTCTTTAACATCACTCGCTGTGTTACTGATTGCTGCAGATGTTTTTGAAGCAACTTT
ACTTGTAGCATCTGTAACTTTTTGTACGTCCGGATGTTGTTTGATACGTTTACGTTCAACAATTAACGGTACCAATACAATTGGTAAT
ACATTAATCATAAATTTGATGACTTTTTTCTTATCCATAGATCTTGCCTCCATAATTACTTTATTAATTTTACATACCCTATGATACAT
CAATATAAACGATGATAGTAGTGAATCACTATTAAGTATTTCAGATGTTTTTTAAAAGAAGACAATAAAAACTGCCAATCAAGTGA
TTCCTTAATTGACAGTCTATATTTTAAAAGGAAATTAAATACCTTTACCAATGCCAAATCCGAAGTAAAGTATAGCAATAAAGATTA
CTAATACAATTCTGTAAATGGCAAATGGAATTAGTTTGATTTTGTTAATTAGATGCAAGAATGTTTTGATTGCAATTAGTCCAACAG
TAAATGCAGCTAAAAAGCCTAAAATATAAAAAGGTATATCAGCAATCTGAATATCTTGATAATGTTTTAATAAAGATAAACCACTA
GCTGCTAACATAATTGGAACAGCCATAATAAATGTAAAGTCCGATGCTGCTTTATGATTTAATTTCATTAATACCCCAGTTGAAATT
GTTGAGCCTGAACGGCTGAAACCAGGCCACATAGCTACTGCTTGAGAAATACCAATTACAAATGCTTGGAAATAACTGATTTGATC
TACTGTTTGTGGGTTTTTAACTTTAGCTGAGTATTTATCAGCAATAATCATATAGATAGCACCTACGAATAAGCCAATCATAACAGTT
GGCACACTAAATAAATGTTCTTCGATGAAATCATCAAATAGTAAGCCTAAAATACCTGCTGGCACCATACCCACTAATACATGTAAT
AAATTTAAACGTCTTGGCTTTGAACGTCTTTGTTGATCGTTATCTCCTTCAACATGTTTGTGTTTACCAATATGTAAAATCTCTAAGA
AGCGTTCGCGGAACACCCATGCTGCTGCAAAGACGGATCCTAATTGGATGACGATTTTAAATGTAAATGCTGACTGAGAACCTAAA
AATTCAGATGATTTTAACCACATATCATCAACTAGGATCATATGTCCAGTAGAGGAAACAGGTGCAAATTCTGTTAATCCTTCGACG
ACCCCTAAGATAATACCTTTTATTAATTCAATGATAAACATAATGTACCCACTTTCATTACTCAATTTAATTTATTTAAATATCAAAA
TTACCATATCATGATAGCATATTCATTTAAAGACATGCTAGTTATAGTTATAATACTAGACTAAAGATGTATATATTCATTTTCTTTT
ACATGTAAAACTACAATATTTTATTGAGCTATTTAATTTGATTTTAAGGAAAACCTTTTATAATAGGTTTAGGTGATATAATTGTGAA
AAAATTAACAACAATACTGTTTCAATATAAAATTTTTCCGGTACTCATGTTCTTGGTCAGTACTGGTCTCGGCATACTCGTTATAACG
CAAAATATTTTAATAGCAGATTTTTTAGCTAAAATTATAAGACATCAATTTCAAGGTTTATGGATTGTATTATTTATTTTATTAGGTG
TTTTACTTTTAAGAGCAACTGTGCAATTTCTAAATCAATGGTTAGGTGATACATTAGCATTTAAAGTTAAGCATATGCTTAGACAGC
GGGTTATTTATAAAAATAATGGTCATCCAATCGGTGAACAAATGACTATACTCACAGAAAACATTGATGGTCTAGCACCTTTTTATA
AGAGTTATTTGCCTCAAGTGTTCAAATCAATGATGGTTCCGCTCATCATAATCATTGCAATGTTTTTCATCCATTTCAATACCGCATT
AATTATGTTAATAACTGCACCATTTATTCCTTTGTTTTATATTATTTTCGGTTTGAAAACGCGAGATGAGTCAAAAGATCAAATGACT
TATTTGAATCAATTTAGTCAACGGTTTTTAAATATTGCTAAAGGTTTAGTGACGTTAAAGCTATTTAATCGTACAGAGCAAACAGAG
AAGCATATTTACGACGATAGTACTCAGTTTAGAACTTTAACAATGCGCATTTTACGCAGTGCTTTTTTATCGGGATTAATGCTCGAAT
TTATAAGTATGTTAGGTATTGGATTGGTTGCATTGGAAGCAACGCTAAGCTTAGTAGTATTTCATAATATTGATTTTAAAACTGCGG
CAATTGCGATTATTTTAGCGCCTGAATTTTATAATGCAATTAAGGACTTAGGGCAAGCGTTCCATACTGGAAAACAAAGTGAAGGTG
CCAGTGACGTTGTGTTTGAGTTTTTAGAACAACCGAACTATAATAATGAATTTCTATTAAAGTATGAGGAAAACCAAAAGCCATTTA
TTCAGTTAACAGACATATCATTTCGATATGATGATTCTGATAGATTGGTATTAAATGATTTAAATTTGGAAATATTTAAAGGTGATC
AAATTGCACTTGTAGGTCCAAGCGGGGCAGGTAAATCCACTTTGACACATCTTATTGCAGGTGTTTATCAGCCAACAATAGGTACTA
TAAGTACAAACCAGCGTGATTTAAATATAGGAATACTTAGTCAACAGCCATATATTTTCAGTGCTTCTATAAAAGAGAATATTACGA
TGTTTAAAGATATAGAAAATAATACTATTGAAGAAGTGCTAGACGAAGTAGGTTTATTAGACAAAGTGCAATCTTTCACAAAAGGC
ATTAACACAATAATAGGTGAAGGAGGCGAAATGTTATCTGGTGGACAGATGAGACGCATAGAACTTTGCCGTCTTTTAGTTATGAA
GCCAGATCTCGTTATATTTGATGAGCCTGCAACTGGTTTAGATATTCAAACAGAACACATGATTCAGAACGTTCTGTTTCAACATTTT
AAAGATACAACGATGATTGTCATTGCACATAGAGATAATACAATTCGCCATTTACAACGACGCTTGTATATAGAAAATGGAAGACT
GATTGCTGATGATCGCAATATTTCAGTAAATATAACAGAAAATGGTGATGACTTATGAAAACACGACTAAAATTTCAAGTAGATAA
GGATTTATTGTTAGCTATAGTTGTTGGTGTTTGTGGAAGTTTAGTTGCGCTCGCCATGTTTTTCTTAAGTGGTTATATGGTGACACAA
AGTGCACTTGGTGCGCCACTATACGCTCTGATGATTTTAGTCGTAACAGTAAAATTGTTTGGGTTTTTAAGAGCTATTACTCGATACG
TAGAGCGCCTTATTTCTCATAAAGCTACATTTACAATGCTACGTGATATTCGGGTACAGTTTTTCGGTAAATTAGTAAATGTCATTCC

TAATGTTTACCGTAAACTGAGTTCTAGTGATTTAATTTCACGTATGATTAGTCGTGTTGAGGCATTACAAAATATATATTTACGTGTT
TATTATCCACCAGTCGTCATCGGTTTGACAGCGCTAGTTACAGTCATAGTTTTGGCGTTCATTTCAATCGGCCATGCGCTATTGATTA
TGGTTAGCATGTTGTTTACTTTACTCATTGTTCCTTGGTTAAGCTCAAAAAAAGCACGTACTTTAAAGAAACATGCAGCTAATGAAC
AGGCCCGATTTTTAAATCATTTTTATGATTATAAAGCTGGTATGGATGAACTACGTCGATTTAATCAAATTAATCATTATCGAGATA
ATTTGATGGCTAAATTAAATCATTTTGATAAATTACAACTTAAAGAGCAACGCTTTTTAACGATTTATGATTTTATATTAAATATTAT
TGCTATGCTTTCGATTTTTGGTAGTTTAGTTCTAGGATTAATTCAAATTAATGCAGGCCAACTAAATATTATTTATATGACGAGTATA
GTTTTAATGGTCTTAACTTTATTTGAACAAGCTGTACCAATGACAAATGTCGCGTATTATAAAGCGGATACTGACCAAGCATTGCAC
GATATTAATGAAGTGATATCTGTACCTTCTACTAATGGAAAAAAACGTCTTAATGATAAGTATGATGCAACGAACATTTATGAAGTT
AAGGATGCTAGTTTTAAGTATTGGAATCAGCAAACGTATGTGTTGTCGGATATTAATTTTAATGTTAATAGAGGCGAAAAGATTGCG
ATTGTGGGTCCTTCTGGTTCAGGAAAAAGTACATTACTACAAATTATGGCAGGGTTATATCAATTAGATAGTGGCTCTGTTCGTTTC
GAAAATATGGATATGTTTGAAATAGATGACAAAGATAAGTTTGAATCGTTAAATGTCTTGCTACAATCTCAACAATTATTTGATGGT
ACAATACGTCAAAATTTATTTACCGATGAAAAAGATGAAGCGGTGCAAGCAATATTTAAGCAATTAGATTTAGAACATTTGGCACT
AGAACGTCAAATTGACTTAGATGGTCATACATTATCTGGCGGAGAAATTCAGCGTTTAGCGATTACGAGGATGTTATTAAAAGATA
CTGCATCAACATGGATTTTAGATGAACCAACAACTGCATTAGATAAACAAAATAGTTTAAAAGTTATGGATTTAATTGAAGCACAT
GCAGAAACATTAATTGTTGCTACACACGATTTAACTTTATTGTCACGTTTTGAGACCATCATTGTGATGATAAATGGTAAAATAGTT
GAAAAGGGAAACTATCAACAATTACTCGCTAATCAAGGTGCTTTATGGAATATGATTCAATATAATGCATAAAAAAACTGCTTGAT
AGGCTAAGAAACCTTTCAAGCAGTTTTTAACTAACAAGCTTACGCGTGCTAATTGTTTTTCTATTCTTAATAAATTCTAAACATTAC
TTTAATTGTCATGACAAAAGTTAATTATTTTTCCTTTGTTTCATCAAATGCATGAATGACTTTACCTAATAAGCGATTAAGTTCTTTA
ACTTCATCTTGAGATAAAGAAGAAGCTGAAGCGACTTTGTCAGATGCATTACTTAATTCTGGTCTAATAGTTTCACTTTTGTCAGTCA
AGTGAATAAATACTTCACGTTGATCGACTTCGGAACGTTCACGCTTAATTAAGTCTACTTGTTCCATTCGTTTTAATAATGGTGATAC
TGTACCAGTATCGAGTGCTAATTCAGTTACGACTTTCTTGACGTTTACAGGAGATTCATCCCATAAAATTGTTAAGACAAGAAATTG
TGGGTATGTTAGATTGTACTTCTTAAAAACTTTGTTAGAGTAGTAGCGATTAACTTGTCTTTGAGCATTGTACAAACTAAAGCATAG
CTGTTCTTTTAAATTATGTTGATCAGACATTAAAGTTCTCCTCCAGACATACTATCCGTTTTTTTCTCTTTTCGGATTGGTAATCATTA
AAAAGTTGATTGTTTATTAATTCACAACTTTCTTTGATTCAATGCCATGCTAAAATTAAAGTATGTTTAAAGTTTAGAAGATATTTTT
GATTAAATCAAGCAAAAAGATAATTTAATATATATGTTATCATTTTTAAAAATAACTGTAATAGAAAAGAGAATATAAAATGAAAA
ATAATAAAGATGAAAAAATAAGAATATCCATAATTAACGGATTTTTGGGTAGTGGTAAAACCACGTTACTGACACATTATATTAGT
GAATTATTAAAAAATGATGAGAAAATTAAAATCATCATGAATGAATTCGGTACTTTTGATATTGATAGCAATAGTATTTCAAATGAA
ATTGAAGTCCATTCATTGATTAATGGTTGTGTTTGTTGCGATCTTAAACAAGAACTTGTCTATGAACTAAAAGCCATTGCTTTAAAAG
GGGACGTTAATCATGTCATCATAGAAGCGACAGGCATTGCGCATCCTTTGGAATTACTAGTTGCATGTCAAGATCCGCAAATCGTTA
ATTTCTTTGAAAAGCCGATTATTTATGGTGTATTAGATGCGACTCGATTTTTAGAACGTCATCAATATACCGAAAATACAGTTTCGCT
GATGGAAGATCAGTTGAAACTAAGTGACATGATTATTATTAATAAAATTGATCTTATAACTGATGACAGTCTTGAGAAAATTGATAA
GCAATTAGGTATGATTTGTGCAAGTATTCCAACTTATAAAACAACCTATGGAAAAGTTTCGTTGGAAGAATTGGACTTAACTGTTAA
AGACAGAGAGATATCGTCTCATCATCACCATCATCATGGGATTAAAAGTATGACTTACACGTTTACAGGTCCGATTGATCGTCAATT
GTTTTATCAATTTATAATGAAATTACCGGAATCTGTTCTACGTTTGAAAGGTTATGTGTCATTTAGAGATCAACCAAATGCAATTTAT
GAATTTCAATATGCATATGGTTTACCAGACTATGGAATAATTGGCATGCAATTACCATTAACGATTGTTATTATTGGTGAAACTTTA
GATACAAATCACATACGTAATCAATTGGATATGCTACAATTTACGTAAATCAGAAAATGTGTTGTTTCAATTTAACGGAAATAGCAC
ATTAATTTCTTAATACTTTTATTGTGAATATTTTGTGACATTGAGGGATGGAGTGGATGAGATATGGAACAAATAATGATTAATCAC
TATGTTCATTTTTCTAGGCTTGTACAAGGTTTTTGGCGTGCAAATGAATGGAAGATGACTGCGAAAGAGTTAAATTATTTTATAAAT
GAATTAGTTGAACGTGGAATTACAACGATGGATCATGCTGATATTTATGGGGATTATCAATGTGAATCACTGTTTGGTAATGCTTTG
GATTTATCACCCGAATTAAGAAATAAAATTCAAATTGTTACGAAATGTGGTATCATTTTGCCTTCTAAGCAATTTGATTTTACAAATG
GACATCGTTATGATTTGAGTAGTAAGCACATCGTGAAATCTGTTGAACAGTCATTAATCAATTTGAATGTAGATTATTTAGATAGTC
TACTCATTCATCGTCCTTCACCATTGATGGATCCAGAACAAGTTGCTGATGCATTAACTAAACTTGTTAAACAAGGTAAGTTGAAGT
CATTCGGGGTGTCGAATTTTAATCATTCACAATACCAATTGTTAAATCAATATATTATGAAAGAAAGACTACATATTAGCATCAATC
AATTAGAATTATCGCCATATCACGTTGATAGTTTACAAGATGGAACAATGGATTCAATGTATCAAAACCATGTTCAAATTATGGCTT
GGAGTCCTTTTGCAGGCGGTAAAATTTTCGACAAGGAAGATATTAAAGCGCAACGTATTATGAAAGTTGTTCAATCAATAGCTGAC
AAATATGGTGTGAGTGACACAGCTGTGATGATAGCATGGTTAGTAAAAATACCGCATCGTATCATGCCGATACTTGGAACAAGTCA
GTTAAAGCGTATTGATCAAGCAATCGAAGGGCTACAACTTAATTTAGATGATCAGTCGTGGTTTGACATTTACACCGCTATTATCGG
ACAAGATATTCCGTAAACTTATTTACTTTTAAATCATAAAGGAGCATATCATGACAAACGAAGATAAACGTTTCGAACAATTAAGAT
TTGAACGCAAATTTATAGTTATTCCGTATTTAATTTATGCAGTCATTGTATTACTATTAAATATTTTCTATTCTGATTTGAAAATAACA
ATGACATTATTCGGACTTTTCTTTGCGTATAATGTAGTCATTTTGTTCATAGCATTTATTAAACATTATAAACGCACATTGTTACTAA
GTCTTATATTAACAGTGCTTAGTGGCGCGGCATTCTTTGGAATTATTTATGTTTATGGCATTAATCATTTTTAAATATTAAAAAGAGA
CTATCTACAATAATCTCATTTGCATTTTATCTAACTAAGTTTAACTTTTGCTTAGAAAAATGGTGCTATGAGGATATTCAGTAGATAG
TTTTTTTATGATCTAATATTTAAACTTAGATATCGTTTTGTAATTAACCGAGATAACTCATCACCTAGCAATATTATTTACAAAATAT
TTCGATAAAATTGTGTATTTTGCATAAATTAAAAATGTATGTTTTAAAAAATTGTTATAATTAAATTAAGATTAAACAAAGGGGTGA
CTGTTATGTCAGAAGAAAAACATGTAGTTGAACATGAACAACAAAAGAAAGAAAAGACAAAAAAGCAATACAAGCCATTTTGGAT
TGTCATGAGTTTTATAATACTTATAGTTGTACTATTACTCCCGGCACCTTCAAGTCTGCCGATAATGGCTAAGGCAGTACTAGCTATT
TTAGCTTTTGCAGTTATTATGTGGGTAACGGAAGCTGTATCATATCCGGTGTCAGCAACTTTAATTATCGGCTTAATGATATTACTTT
TAGGATTTAGCCCGGTTCAAAATTTAGGGGAGAAGCTAGGTAATCCGAAAAGTGGCAGTGCTGTTTTAGCTGGAAGTGACCTTCTA
GGAACTAATCATGCATTATCATTAGCGTTTAGTGGATTTGCAACTTCAGCTGTAGCTCTCGTTGCAGCCGCATTATTTTTGGCTGCTG
CTATGCAAGAAACGAATTTGCATAAAAGACTAGCTCTTTTAGTGTTATCAATTGTTGGTAATAAAACTAGAAATATAGTTATTGGAG
CAATTATCGTTTCAATTGTACTTGCATTTTTCGTTCCTTCTGCAACAGCTAGAGCAGGGGCAGTTGTACCAATCTTGCTGGGTATGAT
TACGGCATTTAAAGTTTCCAAAGATAGCAAGTTAGCGTCTTTATTAATAATTACTTCAGTACAAGCTGTGTCAATTTGGAATATTGGT
ATCAAAACGGCGGCAGCACAAAATATCGTAGCGATTAATTTTATAAACCATCAATTAGGATTTGATGTTTCATGGGGCGAGTGGTTC
TTATATGCAGCGCCTTGGTCCATAGTTATGTCCATAGCTTTATATTTCATCATGATTAAAGTGATGCCTCCAGAAATTAATACAATAG
AAGGTGGTAAAGATTTAATAAAAGAAGAATTGCATAAACTTGGCCCCGTTAGCGCACGTGAATGGCGTTTAATTGTTATATCGATGT
TATTATTACTGTTTTGGTCAACTGAAAAAGTATTACATCCGATTGACTCTGCATCCATTACTATTATTGCTTTAGGTGTTATGTTAATG
CCGAAAATTGGTGTCATGACATGGAAACATGTTGAAAATAAAATACCATGGGGAACAATTATCGTGTTTGGTGTAGGTATTTCACTA
GGTAACGTTCTTTTGAAAACAGGTGCAGCTCAATGGTTAAGTGATCAAACTTTTGGTGTTTTAGGTTTAAAACATTTACCTATTATCG
CGACAATTGCACTTATCACGCTTTTTAATATATTGATTCATTTGGGCTTTGCGAGTGCAACAAGTTTATCATCAGCGTTAATACCTGT
TTTTATTTCGCTAACCTCTACGTTACACTTAGGAGACCAGTCTATAGGATTTGTTTTAATTCAACAATTTGTTATTAGTTTTGGTTTCT
TATTACCTGTTAGTGCACCTCAAAATATGTTGGCTTATGGCACTGGTACTTTTACGGTTAAAGATTTCTTGAAGGCAGGTATACCATT

GACAATTGTAGGGTATATTCTAGTGATAGTTTTTAGCATGACTTATTGGAAATGGTTAGGTTTGCTTTAATTAAAAATATAAATAAG
AATCTAGGTTATTTTAAAGTGACAAAAAGCTTAATAAAATAAAAAGATAATTGAAGGGTGTTTTGTTTATGGCAATTGCTGTGTTAT
TAAATCGAATGTTTCGAATGGAACACAATCCATTATTTGAATATATTTATCAACAAAAAGAAGACATTGATGCATGTTATTTTATCA
TTCCGGAAGAGGACATGTCTTCAGCTTCTGATTTGAAAGCACAGTTTTATCGCGGTACTTTGCAGCGCTTTTACCAATCGTTGCACGC
AGAAAAGCTTACACCTTATGTTATGTCTTATGACGATATCATTTCATTTTGTAAAGAAAACAATATCTCTGAAGTAGTGACTGCGGG
TGATATTATGAGTTATCATCTTGAAGAATATGATATTTTACATCAACGTTCTTTATTCAATGAAGCACGCATTGCCGTTACTTTGATA
CGTGGGAATCATTACTTTAAAGCGAGTAAAACAATGAATCAACAAGGGGAGCCATACAAAGTTTTTACTAGTTTCTATAAAAAATG
GCGACCTTACTTGAGGCATAGAGACGTATATCACTATGATTTAAAATCATTCGAAGACTTCGTCATTGCATCACCTGATGATTTAGT
GTTTGATGACATAGCGTTTGGATCCTCACAAATAATTGAACAGAATAAATGGCAACATTTTTTAGATCAAGATATACAGAATTACGA
AAGCGGAAGAGACTATTTACCTGAAGTGTTAACAAGTCAGCTAAGTGTTGCTTTAGCATATGGATTATTAGATATTATTGAAATTTT
TAATGATTTATTGGCGCGTTATGATCAAGATGAGGCAAACTATGAAGCATTTATACGTGAACTCATTTTTAGAGAATTTTATTATGT
GTTAATGACACAGTATCCAGAAACCTCATACCAAGCTTTCAAACCTAAATATCGACAGATAAAATGGTCGCAAAATGAAGCGGATT
TTAATGCATGGTGCGAAGGGCAAACAGGATTTCCAATCATTGATGCAGCAATAATGGAATTGACACAAACTGGTTTTATGCATAAT
CGAATGAGAATGGTTGTGTCGCAATTTTTAACCAAAGATTTATTTATAGATTGGACATGGGGAGAAAAATTCTTTAGAAAGCACCTT
ATTGACTATGATGCAGCATCAAATATTCATGGATGGCAATGGTCTGCATCTACTGGTACGGATGCGGTGCCGTATTTTAGAATGTTT
AATCCAATTAGGCAGAGTGAACGCTTTGATCCTAAAACATTATATATCAAAACATATCTTCCAGTTTTAAATCAAATTGATGCAAAG
TATTTACATGACACACATAGAAATGAATCTGAATTATTTAAACAAGGGATTGAGTTAGGTCGTCATTATCCAAAACAAATAGTGGAT
CATCAAGAAAAGAGATCTCAAGTTTTAGCGACATTTAAAGCATTAGACTAATTCGGCTCAATTGATAGATCAGGCATGAGTAAAAT
CTGGAGCAGAATATAAAGTTCTTAAGCAATATAAATAAGCCAATTTCTATTATTGATGTGATAGAAATTGGCTTTCCTCAAATATAT
AAGTTGTATTTAGTTTTCTTATTAATTTGATGTTATCTTAGTATGTCCGTAAATAAAGTGAGGTATAGTACGACATACTCTAAAAACG
TAGTGAGATAAATATATTTCAATCTAACTTTTATGTTTTGAGGCACTTGCCATTTAGGATATTGTCGTTCGTAATACGACACTTGTTG
TATAAATACACCTAGTCCAAATGGCAGCATCATGAGTAAGATACTTCTTAAATAACTTAAACCAATATCATACCATATGTGTCCAAT
AATCAATTGAAAGCCAATGATAGATACTATTAAAACGATTATATTTATTGTCACTTGTTCAAACGCACTCCTTTTCCAAATAATAGA
ATTGCTGCTTGCATCACAACCATAAAACATACAAACATAGCAGTTTTAAGCGTTAGACTTTCTAGAATGTGATTTAAAACATGTAAG
GGCTCATTAAAGAAATAAACGGAATGTAAGCGTAAGAAACGACCAATATAAATTCCGAATCCATTTAAAAACATTAGCACGACAAC
AATTAATCTATTAAGCCAACGGTGAGAAGTCAATGTTAGTATTTCAAAATAGATTAAAATCATCACATAAACCGCTAAGAAGACAC
CAAGCAGTAAATAGGTAAAGTATTTCCACTCACTTAAATTTAGTCCTGCGTAAAAGTTGAATTGAAATTGGTTCAAATGGATTAAAT
CAGTTACCATATAAAATGTATTTGGTAAAAGTAACACAAATATAAAACTATATATGATAAATAGTGGCCATTCATACTTTTTATTCG
GTTTGAATAATCGTAATAATAGACTAAGCTCAAAAGGTATATATGCTAAAAACAAATTTAAAGTCATAAATTGAAAAATTTTAGTCT
CAAAAAGTGAGACGATAAATAAAATTAAAAAGTAAATTCTAGCGATGTATCGAGATTGCATCAATAATAAACACTACTTTCTAATG
AAAAAGTTATTCATTCAAATGTGATAGTTATAAACGCATTCAATTCCTAATTAAAAATTTAATTTAACTCGTTGAAGTATATTAATA
AACGAGAATGCATTTATGATTAAGCACTTATGATAACTTGTAATAAAATTTGATTCAACTAAAAAGTAAAGTGCTATTGAGGATCAA
GTAAATTTAAACCGACTGCATCTTTGAATGAACGTTGAATGTCCAAATCAGTTACAGTAAGAATGATTTCAGCAGTTGCAGAAACA
ATTGCTTTAGCGAGATGTCCGCTAAAAGTTTCATATGATTGAGTACCGAAAGTAGCATGCAAATGTGCAAAATGACCATTGTCTAGA
CGAGAAATATTACCTAATAAGCTCGTCAATTCCAGTGGCTCAGTAATATGTTTTTCTTCGTATTGTTTCGTTGTTAAATTGAAAAATT
TTAATACAACGTCATCACATGCACCAATGCCGCTGACAGATGTAAATGTTAAGTCTTGGTCATCTGCAAAGGTTGTTATACATTCAA
CGATATCTTCTCCTTTTTCCAACACTAGTAGTATAGTATGATTACTTTTTTGCAATTTCATATGATCAATCCCCTTTATTTTAATATGT
CATTAATTATACAATTAAATGGAAAATAGTGATAATTACAAAGAAAAAATATTGTCAAATGTAGCAATGTTGTAATACAATATAGA
AACTTTTTACGAATATTTAGCATGAATTGCAATCTGTTGTGGAAAAGAAGAATAACAGCTTTAAGCATGACATGGAGAAAAAAGAG
GTGAGCATATGAATAAACAGATTTTTGTCTTATATTTTAATATTTTCTTGATTTTTTTAGGTATCGGTTTAGTAATACCAGTCTTACCT
GTTTATTTAAAAGATTTGGGATTAACTGGTAGTGATTTAGGATTACTAGTTGCTGCTTTTGCGTTATCTCAAATGATTATATCGCCGT
TTGGTGGTACGCTAGCTGACAAATTAGGGAAGAAATTAATTATATGTATAGGATTAATTTTGTTTTCAGTGTCAGAATTTATGTTTGC
AGTTGGCCACAATTTTTCGGTATTGATGTTATCGAGAGTGATTGGTGGTATGAGTGCTGGTATGGTAATGCCTGGTGTGACAGGTTT
AATAGCTGACATTTCACCAAGCCATCAAAAAGCAAAAAACTTTGGCTACATGTCAGCGATTATCAATTCTGGATTCATTTTAGGACC
AGGGATTGGTGGATTTATGGCAGAAGTTTCACATCGTATGCCATTTTACTTTGCAGGAGCATTAGGTATTCTAGCATTTATAATGTCA
ATTGTATTGATTCACGATCCGAAAAAGTCTACGACAAGTGGTTTCCAAAAGTTAGAGCCACAATTGCTAACGAAAATTAACTGGAA
AGTGTTTATTACACCAGTTATTTTAACACTTGTATTATCGTTTGGTTTATCTGCATTTGAAACATTGTATTCACTATACACAGCTGACA
AGGTAAATTATTCACCTAAAGATATTTCGATTGCTATTACGGGTGGCGGTATATTTGGGGCACTTTTCCAAATCTATTTCTTCGATAA
ATTTATGAAGTATTTCTCAGAGTTAACATTTATAGCTTGGTCATTATTATATTCAGTTGTTGTCTTAATATTATTAGTTTTTGCTAATG
GCTATTGGTCAATAATGTTAATCAGTTTTGTTGTCTTCATAGGTTTCGATATGATACGACCAGCCATTACAAATTATTTTTCTAATATT
GCTGGAGAAAGGCAAGGCTTTGCAGGCGGATTGAACTCGACATTCACTAGTATGGGTAATTTCATAGGTCCTTTAATCGCAGGTGC
GTTATTTGATGTACACATTGAAGCACCAATTTATATGGCTATAGGTGTTTCATTAGCAGGTGTTGTTATTGTTTTAATTGAAAAGCAA
CATAGAGCAAAATTGAAAGAACAAAATATGTAGCATAAGTATTTTGGTGTATATTGATATAAAGTAAAGCGTAATATTATGAATGA
TTAGCATCGTTTTTCTTATGAATTTTATTAAGAAAATTCGATGCTTTACATTTAAAAAGATTCGATTGACTAAATGTTTTACTCTTTAT
ATTTAAATGTTATATGTAACAAAAAATGATTTTGAGTAATAAACATGTTACAAATATTACATTCTTTTTAAATTGCAATCCACATACC
TAATTCATTAACGTTAATGTGTTAAGATGATAAAAAATGAGTAAGGAAATGTGGGTAAGGGGATGACAGTAAAAAATTTATTTTTA
GGCTTTGTTGCTGTAATATTAACCGTTTGTTTAATTGGTTTATTAATATTAGCAACAAATGAAGATGCGCTTGCTAAGGTACATAAAA
CAATTAATACGCTTAACGCGATAAATGTATCAACTGAAGATACTTATAAAAAGAAAATGGATATTCTCAATATTCATACTGCTAAAG
CATCTGAAGTGAATGAAAATGTGAAAAAGCAAAATCATTTTAAACATCGTGTGAATGCAAATAAATCAAATTCTTTTAACGAACAA
GAGTGCCAAGTTATTGCTGATCGTTATGCAGATAAGCATATCAATGATAATTATGGTTTAGAAAGAATTTCTAAGACAAATCATGGA
TATAATTATGTGTATTCCAATGATAATTCAACTAGTAAGCAACATGTAAGTATTTCAAATCAAGGCATAATAACGAAATAATAGATG
GAACAGTGTATTCTAATTGGATATACTGTTTTTATTTTGCAATAATTTAATTTAAAAAGGTGAATTCAACTTATAAAATGATGTAAAT
GTTATGTCAAAATCAACCAATCCGTAATGTATTTTAAAATGTTAATATAGTTCTGAAGAAGTATAAATGAGGTGTTGAAATGGCTAA
AAATAAGAAAACGAACGCGATGCGTATGCTTGATCGTGCAAAAATTAAATACGAAGTTCATAGCTTTGAGGTACCAGAAGAACATT
TATCTGGTCAAGAAGTCGCAGAACTCATACAAGCAAATGTTAAAACAGTATTTAAAACGCTTGTTCTAGAAAATACAAAACATGAA
CATTTTGTATTTGTTATCCCAGTAAGTGAAACTTTAGATATGAAAAAGGCAGCTGCTTTGGTTGGAGAGAAGAAATTGCAGCTTATG
CCTTTAGATAATTTGAAAAATGTAACGGGGTACATTCGTGGTGGGTGTTCGCCTGTTGGTATGAAAACATTGTTTCCAACAGTCGTT
GACAAATCGTGTGAAAATTATAGTCATATCAGTGTGAGTGGTGGGCTTCGAACAATGCAAATCACAATAGCTGTTGAGGATTTGATT
ACAATAACTAAAGGCAAAATTGGAGCAGTTATCCATGAATGATTAATAACAACAAAAAGTATGGGGCAAGATTAGGAGTGTGACA
GAGATGAAATTTTTATAAAATTCATTTCTGCCACACTCCTTTTTGATTGAATTAGCATTTTACGATCATAAACAGTCATTATAATTGA
GTATTTGAACATAAAAATGTAATTTTATCGTCACAATTTGAGTGTTTGTGATTGTTTTTGGTAATTTATGATTGAAAAGTGAAAGCGT

ACTCATTATAATACAGAGTGAGATGGGGTGATGATGATAATTACAGAAAAAAGACACGAGTTAATTTTAGAAGAACTTTCGCACAA
AGATTTTTTGACTTTACAAGAATTAATAGATCGAACTGGGTGCAGTGCTTCAACAATACGAAGAGATTTATCTAAACTACAACAATT
AGGGAAATTGCAACGTGTGCATGGTGGTGCAATGTTAAAAGAAAATCGTATGGTTGAGGCGAATTTAACTGAAAAATTAGCAACGA
ATCTTGATGAAAAGAAAATGATTGCTAAAATAGCAGCTAATCAAATCAACGATAATGAATGCTTATTTATCGATGCTGGTTCATCTA
CATTGGAGCTAATTAAATATATTCAAGCGAAAGATATCATTGTGGTAACCAATGGTTTAACACATGTAGAAGCTTTACTTAAAAAAG
GTATTAAAACAATTATGCTAGGTGGTCAAGTTAAAGAAAATACACTTGCTACGATTGGTTCTAGTGCCATGGAGATATTAAGACGAT
ATTGTTTCGATAAAGCTTTTATCGGGATGAATGGATTAGATATTGAACTTGGATTAACTACTCCCGATGAGCAAGAGGCATTAGTTA
AACAAACAGCAATGTCATTAGCCAATCAATCATTTGTACTTATTGATCATTCTAAGTTTAATAAAGTATATTTTGCTCGTGTACCTTT
GCTAGAAAGTACGACAATCATCACATCTGAAAAAGCATTAAATCAAGAATCGTTAAAAGAATACCAACAAAAGTATCACTTTATAG
GAGGGACTTTATGATTTATACAGTGACTTTCAATCCTTCAATTGACTATGTCATTTTTACGAATGATTTTAAAATTGATGGTTTGAAC
AGAGCAACAGCAACATATAAATTCGCTGGGGGGAAAGGTATTAATGTCTCGCGCGTCTTAAAGACATTGGATGTTGAGTCAACTGC
CTTGGGATTTGCAGGTGGATTTCCTGGAGAATTCATTATAGATACATTAAATAACAGTGCAATTCAATCGAATTTTATTGAAGTTGA
TGAAGATACACGTATTAATGTGAAATTAAAAACAGGACAAGAAACAGAAATCAATGCACCGGGTCCTCATATAACGTCAACACAAT
TTGAACAACTGTTACAACAAATTAAAAATACAACAAGCAAAGATATAGTTATTGTTGCTGGAAGTGTACCAAGTAGTATTCCAAGC
GATGCGTATGCGCAAATTGCACAAATTACAGCACAGACAGGTGCTAAATTAGTAGTCGACGCTGAAAAAGAATTGGCTGAAAGCGT
TTTACCATATCACCCACTATTTATTAAACCTAATAAAGATGAATTAGAAGTGATGTTTAATACAACAGTGAACTCAGACGCAGATGT
TATTAAATATGGTCGTTTGTTAGTTGATAAAGGTGCGCAATCTGTTATTGTCTCGCTTGGCGGTGATGGTGCTATTTATATTGATAAA
GAAATCAGTATTAAAGCAGTTAATCCACAAGGGAAAGTGGTTAATACAGTTGGCTCTGGTGATAGTACAGTTGCAGGCATGGTGGC
TGGAATTGCTTCAGGTTTAACGATTGAAAAAGCATTCCAACAAGCAGTCGCATGCGGTACTGCCACGGCATTTGATGAGGACTTAG
CAACACGGGACGCTATAGAAAAAATAAAATCACAAGTTACGATTAGCGTACTTGATGGGGAGTGAAAATAATGAGAGTAACAGAG
TTATTAACAAAAGATACGATAGCAATGGATTTAATGGCAAATGACAAAAATGGTGTTATTGATGAGTTAGTAAATCAATTAGACAA
AGCAGGTAAATTAAGTGATGTCGCGTCATTTAAGGAAGCGATTCACAATCGAGAATCACAAAGTACAACTGGTATCGGCGAAGGTA
TTGCCATTCCACATGCCAAAGTGGCCGCAGTTAAGTCACCAGCTATTGCGTTTGGTAAATCTAAAGCAGGCGTAGATTATCAAAGTT
TGGATATGCAACCAGCACACTTATTCTTTATGATTGCAGCGCCAGAAGGTGGCGCCCAAACACATCTAGATGCTTTAGCTAAGTTGT
CTGGTATTTTAATGGATGAAAATGTACGTGAGAAATTATTACATGCTTCATCACCTGAAGAAGTACTAGCGATCATAGATGAGGCTG
ATGATGAAGTGACAAAAGAAGAAGAGGCAGAAGCTGAAGCACAACAAGTTGCAACTGCAGAACAATCATCTAAACAATCTAATGA
GCCATATGTGTTAGCAGTAACTGCTTGTCCAACAGGTATTGCACACACATATATGGCACGTGATGCATTGAAAAAGCAAGCGGATA
AAATGGGTATTAAAATTAAAGTTGAAACGAACGGTTCAAGCGGCATTAAAAACCATTTAACTGAACAAGATATTGAAAATGCAACA
GGTATCATTGTTGCTGCTGATGTTCATGTTGAGACGGATCGCTTCGATGGTAAAAATGTCGTAGAAGTACCAGTAGCAGATGGTATT
AAACGCCCAGAAGAATTAATTAATAAAGCATTAGATACAAGTCGTAAACCTTTTGTTGCCCGTGATGGTCAAAGAAAAGGTAACTC
AAATGACAGTCAAGAAAAATTAAGCCCAGGTAAAGCTTTCTATAAACACTTAATGAACGGTGTTTCTAACATGTTGCCACTTGTAAT
ATCTGGTGGTATTTTAATGGCAATTGTATTTTTATTTGGAGCAAATTCATTTAATCCAAAAAGCTCAGAGTACAATGCGTTTGCAGA
GCAGCTTTGGAACATAGGTAGTAAAAGTGCATTCGCGTTAATCATTCCAATTTTATCTGGATTCATTGCACGTAGTATTGCGGATAA
ACCTGGTTTCGCTTCAGGTCTTGTAGGTGGTATGTTAGCAATTTCAGGTGGTTCAGGATTTATTGGTGGTATTATTGCAGGTTTCTTA
GCAGGTTACTTAACACAAGGTGTTAAAGCGATGACACGTAAGTTACCACAAGCATTAGAGGGATTAAAACCAACATTAATTTACCC
ACTATTGACAGTAACAGCTACAGGCTTATTGATGATTTATGCCTTTAATCCACCAGCATCTTGGTTAAATCATTTGTTATTAGATGGA
TTAAACAATTTATCAGGTTCTAATATTGTATTATTAGGTTTAGTTATTGGCGCTATGATGGCGATTGATATGGGCGGTCCATTCAACA
AAGCGGCATATGTTTTTGCAACAGGTGCGTTGATTGAAGGTAATGCAGCACCAATTACAGCTGCAATGATTGGTGGTATGATTCCAC
CGTTAGCAATTGCGACAGCGATGTTAATTTTCAGACGTAAATTTACAAAAGAACAACGTGGTTCAATTATCCCTAACTATGTGATGG
GTATGTCCTTTATTACAGAAGGTGCGATTCCATTTGCAGCTGCCGATCCATTACGTGTTATTCCTTCAATGATGATTGGTTCAGGTAT
AGGTGGCGCAATTGCTTTAGGCTTAGGTTCACGAATTACTGCGCCACATGGTGGTATTATTGTAATTGTTGGTACGGATGGTGCACA
CTTACTTCAAACTCTTATTGCACTTCTAGTTGGTACATTAGTTTCAGCATTAATTTACGGTTTAATCAAACCCAAGTTAACTGAAACA
GAAATCGAAGCTTCAAAATCAATGGACGAGTAGTTTTAATGATGTAAAATGATTGTTAGCAAAGAGCTTCATATTAACTTGTAAGTT
CAATGAATATATGTTAGTTTTATACATCGTGTTAACGGTAGCTTATACAAAGCTGTAAAAACACTTTCTATTAATTCAGTTTTTATGA
ATTGATATGAAGGTGTTTTTATTTTTAGATAAATGAATGAAGAAATAGACACCACAAATGTATAGACTTTTTTAATATTTTGCAAAA
AGTTATGCCAAACGAAGCAGATATAGTAAAATATGAGTGTCTTAAAGTGAAAATTTATAAATAAAGAAGGGTTTATACGTGTCAGA
ATTAATTATATATAACGGCAAAGTTTATACTGAAGATGGCAAAATCGATAATGGTTACATTCATGTGAAAGATGGACAGATTGTTGC
AATTGGAGAAGGGGATGATAAAGCAGTAATTGATAATGATACGACAAATAAAATTCAAGTGATTGATGCTAAAGGTCATCATGTAT
TACCAGGTTTTATTGATATACATATTCATGGTGGTTATGGTCAAGATGCAATGGATGGGTCATACGATGGCTTAAAATATCTATCCG
AAAATTTGTTATCTGAAGGGACGACATCATACTTGGCCACTACAATGACGCAATCTACTGATAAAATAGATAAAGCACTTACAAAT
ATTGCTAAATATGAAGCGGAGCAAGATGTTCACAATGCAGCGGAAATTGTAGGTATACATTTAGAAGGACCATTTATATCTGAAAA
TAAAGTTGGTGCTCAACATCCGCAATACGTTGTACGCCCATTTATCGATAAAATTAAACATTTTCAAGAGACTGCTAACAGATTAAT
AAAGATTATGACGTTTGCACCTGAAGTTGAAGGTGCAAAAGAAGCGCTTGAAACGTATAAAGATGACATTATTTTTTCAATTGGTCA
TACAGTGGCAACATACGAAGAAGCAGTCGAAGCTGTTGAGCGAGGAGCTAAACATGTCACGCATTTATATAATGCAGCGACGCCAT
TCCAGCATAGAGAACCAGGTGTTTTTGGAGCAGCATGGTTGAATGATGCTCTACATACCGAAATGATTGTTGATGGCACACATTCTC
ATCCGGCATCGGTTGCTATTGCTTACCGTATGAAAGGTAATGAACGTTTTTATTTAATTACCGATGCAATGCGTGCAAAAGGTATGC
CTGAAGGAGAATATGATTTGGGTGGACAAAAAGTAACTGTTCAATCGCAACAAGCACGTCTTGCAAATGGTGCGCTTGCTGGTAGT
ATTTTAAAAATGAATCATGGGTTACGTAACTTAATATCATTTACAGGTGATACATTAGATCATTTATGGCGAGTAACAAGTTTAAAT
CAAGCCATTGCATTAGGTATCGATGATAGAAAAGGTAGTATTAAAGTAAATAAGGATGCAGATCTTGTTATTCTAGATGATGATAT
GAATGTAAAATCTACAATAAAACAAGGTAAGGTTCACACATTTAGCTAATAAATAATCATAATTAAATGTATGCAATAGATTTAAT
CTGTTAACATAAGCACTTTATATTATGATAAAATAGAAGCAATAACATTTTTTTCTGGGGGTGTCTAAATGGGAAGGCGATAACATG
TAGTTGTAATTTAAGTCATAGTGATAAATTTGAATGCGTGTTACCCATGAGTGACACATATAACATGGAGGTGAATCCCTAGAAATA
GGGAATTAATTGGAAACTTCGACCATAATTAGTTTGATTATATTTATTCTATTAATTGCATTAACCACTGTATTTGTTGGTTCAGAAT
TTGCATTAGTAAAAATTAGAGCAACAAGAATTGAACAGCTAGCAGATGAAGGAAATAAACCTGCTAAAATAGTAAAAAAGATGAT
TGCTAATCTAGATTATTATCTTTCTGCTTGTCAGTTAGGTATAACAGTAACATCTTTAGGGTTAGGTTGGCTTGGTGAACCAACGTTT
GAAAAGCTATTACACCCAATATTTGAAGCAATCAATTTACCAACTGCATTAACGACGACGATTTCGTTTGCAGTGTCATTTATAATC
GTTACGTATTTGCATGTAGTACTTGGTGAATTAGCGCCTAAATCTATAGCGATTCAACATACTGAAAAGCTTGCTTTAGTATATGCA
AGACCATTGTTCTATTTCGGTAACATTATGAAACCATTGATTTGGCTGATGAATGGTTCTGCACGTGTTATTATTAGAATGTTTGGTG
TAAATCCTGATGCCCAAACTGATGCAATGTCAGAAGAAGAAATCAAAATTATTATTAACAATAGTTATAATGGTGGAGAAATCAAC
CAAACTGAATTGGCATATATGCAAAATATCTTTTCATTCGATGAAAGACATGCAAAAGATATAATGGTACCTAGAACTCAAATGATT
ACACTAAATGAACCTTTTAATGTAGACGAATTACTAGAAACAATAAAAGAACATCAATTTACGCGTTATCCAATTACTGATGATGGT

GATAAAGACCACATTAAAGGATTTATTAACGTCAAAGAATTTTTAACTGAATACGCTTCTGGAAAAACGATTAAAATAGCAAACTA
TATACATGAGTTGCCAATGATTTCAGAGACAACACGTATCAGTGATGCATTAATTAGAATGCAACGTGAACATGTACATATGAGTCT
TATTATAGATGAATATGGTGGAACGGCAGGTATTTTAACGATGGAAGATATTTTAGAAGAAATCGTTGGGGAAATTCGTGATGAAT
TTGATGATGATGAAGTGAATGATATCGTTAAAATTGATAATAAGACATTCCAAGTAAATGGTAGAGTACTATTGGATGATTTAACTG
AAGAGTTCGGTATAGAATTTGATGACTCTGAGGATATTGATACGATAGGTGGATGGTTACAATCTCGTAATACCAATTTACAAAAA
GATGATTACGTGGATACAACTTATGATCGCTGGGTTGTTTCAGAAATCGATAACCACCAAATTATTTGGGTGATATTAAACTATGAA
TTTAATGAAGCGAGACCTACTATCGGACAGTCTGATGAAGATGAAAAATCAGAATAGATATTAATATATAAACCAACTAAGAATGA
TTTAATTCATTTTTGGTTGGTTATTTTTTTGACTAAAATTAATGAAAAGTGAAAATAGTATTGGAACTTAATATCTTTAATGATTTAA
TGAATATTTTTTATTGAAAGCGATAATTCGTATTAATTGAGTTTGTTGAAAAATTTAGGGTAATGTAAAGATATAAAAGATACATAG
ATTGGAGAGGTATAAAGATGTTGAATGAGATACAAATATTAAATAATGGATACCCGATGCCTTCAGTTGGGTTAGGTGTTTATAAA
ATCTCTGACGAAGATATGACTAAAGTTGTAAATGCTGCAATTGACGCAGGGTATAGAGCGTTTGATACAGCATACTTTTATGATAAT
GAGGCTTCACTAGGACGAGCATTAAAGGATAATGGCGTCGATAGAGAAGATTTGTTTATAACAACGAAGTTATGGAATGACTATCA
AGGTTATGAAAAAACATTCGAATATTTCAACAAATCGATTGAAAATTTACAAACTGATTATCTTGATTTATTTCTAATACATTGGCCT
TGTGAAGCAGATGGTCTATTTTTAGAAACATATAAAGCTATGGAAGAACTTTACGAGCAAGGTAAGGTAAAAGCAATAGGTGTATG
TAATTTTAATGTTCATCATCTAGAAAAATTAATGGCTCAATCAAGTATCAAACCAATGGTGAATCAAATTGAGGTACATCCATATTT
TAACCAACAAGAATTACAAGAATTTTGTGATCGTCACGATATTAAAGTGACTGCATGGATGCCTTTGATGAGAAATAGAGGACTAC
TAGACAACCCTGTCATTGTTAAAATTGCTGAAAAATATCATAAAACACCAGCACAAGTTGTATTACGTTGGCATTTAGCACACAATA
GAATTATTATTCCAAAATCTCAGACACCTAAACGCATTCAAGAAAATATAGATATTTTAGATTTTAATTTAGAATTAACAGAAGTAG
CTGAAATTGATGCTTTAAATAGAAATGCAAGACAAGGTAAAAATCCAGATGATGTGAAAATTGGGGATTTAAAATAACTGGATGTT
AAATTTTACGTTTATGAATGCCTTTTAATGTGTACATTAAAATAAATGAGTTGGTTTTTACTATTTGATAAAACAATACTCAGGTACA
TTCAAAATCTTTTAAATAAAAAGGATGGACATAGATGAAAATTAGAGTCGTCATTCCTTGTTTTAATGAAGGGGAAGTCATTACACA
AACACATCAACAATTAACTGAAATACTTTCACAAGATAGTAGTGTGAAAGGCTATGATTATAATATGCTTTTCATAGATGATGGTAG
TACGGATACCACTATAGATGAAATGCAACATCTTGCCACAATAGATAGGCATGTCAGCTTTATTTCTTTTAGTAGAAATTTTGGAAA
AGAAGCAGCTATGATTGCAGGTTACCAGCATAGTACTGAATTTGATGCAGTCATCATGATAGATTGTGATTTGCAACATCCACCTGA
ATATATTCCGAAAATGGTTGAAGGTTTTATGGAAGGCTATGATCAAGTGATTGCAAAGCGTGATAGAAGTGGTGAAAATTTTAGTC
GCAAAACATTAAGCCATTTGTATTATAAGTTAGTTAATTGCTTTGTAGAAGAAGTACAATTTGATGATGGTGTTGGTGATTTTAGAC
TTTTAAGCCAAAGAGCTGTTAAATCCATTGCATCACTTGAAGAATATAATCGATTTTCAAAAGGGTTATTTGAATGGATAGGCTATA
ATACTAAAGTGTTTACGTATCAAAATGTTGAGAGACAAAAAGGAGAATCTAAGTGGTCCTTTAAAAAGTTATTTAATTATGGTATTG
ATGGATTGATTTCCTTTAATAGTAAACCTTTGAGAATGATGATTTATCTTGGCTTGTTTATCTTTTCAATAAGCGTGCTATATATTATC
TATTTATTCATCAATATTATGATATCTGGTGTTAATATTCCGGGATATTTTTCAACGATTGCAGCTATTTTATTATTAGGCGGCATAC
AGTTAATTTCAATTGGTGTTGTAGGTGAATATATTGGCAGGATATATTATGAAGTTAAGGCACGTCCTAAATATATTATTCAAGCTA
CAAATCTTTCAAGTATTGAAAATGATGAGAAGGATACCCATAAAGTTTATTCTAAATAAACAAAAAAAGAAGCCCTCATTAATGGG
AGCTTCTTTTTAGTCTTTGCATTTTATTTTATAAATAAATCGGATTATGACGTAATGTCTAATTTGTGTAATGTTACAGTCATCGTAGT
TCCTACATCTATATCACTGCTTACACTGATTTTTGCGTTATTTTGTTGCGCGAGTTCATTAGCTATATATAAGCCTAATCCAGAACCA
CCCGTTTTTGTATTACGAGAGTTTTCTACTCTGAATGTACGTTCGAATATACGTTCTTGTAGTTCTGGTATAATGCCAATACCTTCATC
GCTAATAGCAATGTCGATAGTATCTTGATCTTTGTTTTCACTAATATTAATATCAATGCGACTACCAACATTTGAAAATTTTAGCGCA
TTATCAAGTAAGTTTGTTAAAATACGCTCAAGTGGCGTTCGATATTGATAAAATGCATCAATTTCGCTACAGAAATTCACTTCTAAT
GTGCGGTTTTCATGTTTGATACGTTGCTCATATGGTTGCAATATTGATACAAGTAATTGGTCTAGTTGTATTAATTCTGGGGGATATG
TTTTACCTGTATTTAAAGTGATAATATGAGTCATATCATCAAATAATGTTGATAATCTGTTTGCTTGTTTAATTAATATGTCGTATGA
CTCTTTAATCTCATGATCCTTAGTGATTATACCATCACGTAGTCCTTCAGAATATGAAATAATGCTTGCTAAAGGTGTTTTTAAATCA
TGGGCTAAGTTTTGAATCAGTTCTGTTTTTTCTTGTTGTTCGGATTTAATTTGATTCATTTGTTGCGTAATTTCAGAAGCCATTTTATT
AAAAGATTGATTTAATTCATAAATTTCTTTTGGTGAATTAAACGTTTTATCATTGCTTGCGTAATTTCCGTTAGCAAATTGCTTAGTTT
TTATATTAAACTGCTTAATTTTTTGTATAAGTGGATTAATAAAAATACTACATATTAATAAGGTTAAACAGCTTGTAATTATTGTCGT
TAAGGTCAAAGTTAGTGTCATATGGCCGTTAAACCACATTAAAATATATGCAATTGCTAAAATAGTTGAAGTTAATAGTATACTCGA
TACGACGCCAATAATGATTTGACTTCTAATTGATAACACCATTATCGGCTCCTTTCAAATTTATATCCTAATCCCCATACAGTTGTGA
TGGTATATGTTGTAAAGCTCTCTTTTTCTAATTTTTCTCTAATACGGTGTATATGGACATTCACGGTATTAGCATCTTCGTAATAGTCA
TATCCCCAAACTTTTTCAAGTAATTCTGATTTAGAAATAACTTCATTTTCTCTAGAAGCTAAATACCACAATAACTCAAATTCCTTAA
TACGCATAGGGACTTCGTGACCATTTACAGTCACAACTTTACTTAAGTTAATAAGTGTTAATTCATCAAACGACAGTTGTTCAACTG
GTTGATGATGGTATTTCTTCATTCTTGTAAGTAAATTATTAATACGTAAAACGAGTTCCCTTGGACTAAATGGTTTTTTGACATAGTC
ATCTGCACCTAAAGTTAAGGCGTAAATGGTATCATGTTCTTGTGTTTTGGCAGTTAAATAGATAAAGGGGATATCTAATTTTTGCCTT
TTCATTTCTTTGACAATGTCGTAACCATTAACTTCTGGCATCATGATATCAAGTACCATGATATCAATATCATTTGATAGTAAAGAAA
TTGCTTCTTTACCGCTAGTTGTCGTTGTTACTTTGTAACCTTCATATTCAAAATAGGTTTGACAAATGTCTACAATGTCTTGTTCATCA
TCCACGATCAGTAAGTGGGTCATCTATTTTTTCACCTCTGTTCTTACGACCTCTAAAGTAATTAATGATTTCTTTAAGTGAAATCTGTT
TTAACAATGAGTGACTCATTAGTAAAAGGATAAAGAAAGTTAATTGAAGAGGATACGTAAATATCATATCTGCTAAGATATAATTT
ATCATAACAAAGGCTCCAAAGAAACTAGCAGCATATGCAAAAACTCCAAAAATTAAACCTAATCCAATTGCAATCTCTCCGAGTGG
GACAACAATATCAAATAATGACGTCGTATGTGCAACTATATTTGCGAAAAACCACTTATACCACTCTGGTGAATCAGTATTGTTAGC
GATGACTGGTACTAAACCTTTCAGCGTAAATCCGCCCGTTAATTTTTCGTAGCCTTGCATTAACATAACAATACCTGAACCCACACG
AATGATAAATGTAACGAGTAGCAATAATTTATTCATGATGTTCACATTCTTTCTATTTATTGTGTGTAATTTATATAAACATAAGATT
AAGCAACATAATGCGATTTGTAGTGTTATGTGAACAGGAAGTGTTTGAGCTCATGGGTTGCTTAAGCTAAACAAACCTCAAGCTCTA
AAAAAATTTACTTATGTGCTCTGCAATCTTATGGGATTTAATAGTTGTATATATATTGAAAAAAGGAAAGTATGATTTCTACAAGCT
AGGGGGGCTGTGAAATCATACGCTTTCCATTGAGTAATGAGTGTAATGTATATTTTTAAAATTTGTAATGTATAGTAATAGTATGTT
GTAAATAAAAGTTTAACTCAGAAATTGATTATTTTAATTTAGCGCCGCCGAAGATGACGTGAGCTTTTTTACAATAGATTGTTACTTC
ATCATATTTGCTTAAATCTACATTTTTAAGATCAAATGTTTGTTTTTCTTTATCGTAGTCAACCATTGCGATTTCTTTACCGTTTTTAAT
GTCGCCATTTTTTGTTAGGTAGACGTATAAATCTGGACCTTTTGATGATTTGTAGTTAGTAAGCATTAATTTACCATTTTTAATCTCA
GCTTTACCTTCAACAGTTTCACCGTTTTTAGAACTGAATGTACCTGTTAGGTGTTTTGTTTTATCAGTTTTAACATTGCTATCTTCTGA
CTTTGTTTTTTGTTCAGTTTTGTTACCTTGATCTTGTGAATTAGAATTACCACAAGCGCCTAAAGTTAATACAGAGGCAACAGCACCA
ACTGCTAAAAAATATTTTGTATTCATGCTAACTCCTCATTTCTTCAATTTGATAAGTTAAGTTTAAAATGAAGGCAAATAATATGCCA
TTAACTAATTCTTAACTTCGTTTAAATATCGCTTAACTAATATTGAAGAAAGAATAATTTGAAAATGAAAATAATTGTCTGATTTAA
ATAAATAGGAGTCGAAATAAGAGGAGGGCATTTTTTTAGGTGAGAAAATATTTATCTGAACTAGATAACACTATAAAACACTTCTG
ATATTTTGAACTAAGAATATCAGAAGTGTTTTATAGTTTTGGTATTAAAATTTATAAACCTTTTGCCACAATAAAAACAACAAGAAA
ATAAGATAGACGATTAAATACAAGTACCAATATGGTGTTAAAACCATCATAACGATAAATGAAATGTTAATAATGATAAGTCCTTT

AATCATTAGTTTCTTTATTTTGTTATTATTTTCAGCAGTTTCATCTAAGCTATTAAATGTTGAAATAAACAACATAATACCACCAATG
ATGAAAATGGCAGGGGCTAAAAATGGAATAGACATATATATTGATTGTGTACTTGAAAAGTCGGTAACACGTTGCGTTAAACTGAC
AACAATCGTTAATATGATAGCAATAAATTTTTGGTCTGTAATGTATACAGGTTTTAATTTTTTATCAATGTAAAACTGTAGTATCGTC
CAAATGATAAACATCGTTATAGTACACAGAGTAATAAAGTTATATTTATCAATTATGAATGTATTTATAAAGGCGTTTATCGTAATA
GATAACAACATTGCACTTAATGATAAATGTTGCATTTGATGCCTATATAAATTGAATCCAGTTATAAGTACGACGATAGCAATATTA
ATTAATATATATACAATCATGATGGACTCCTGTTCGTTATACTTCAATCAACTATTATATCAATTAATATATTGTTATTCATTAGATA
TGACTTGTAAAAAGTAAAATAAGTTATCCTTTATACACCTTTTTTATTGCTCCAAAGTAATGTATGAAGTTGTGGTAACACATAAAC
GTGATTCATATCATTACTTTGCATAACTAAATCCACCAACTGCTCGTAGCGTTCTAACAACTTTTCGGTATGATTATCTACGCTGTCT
GATAAATATGGGTTACCAACTTGTAAATAGAAGGGAATATCTGGATAACGGTGGTGTATCATTTTGGCAAAATCATAATCTTTATCG
TCGAATACAACTACTTTTAAGTTTAATGAGGAAGGTACGCATTGTGTAATCACTTCATCTAACTTTTTTAAATCAGGTGTCATAGTTG
AACTTGGTGGTTTTGGACTAATCGTTAAATCATCAATTTGTGTCATCCAAGGTTGGAATTTACTGCCTTGTGTCTCCAGTGCGCTGAA
AATACCTTTATCTTGAAATAAGTCAACTAACTCTTGGATACCTTTAATTAATGCTGGGTTACCACCAGAAATTGTAACGTGATTAAA
TAAATCGCCACCAATTCGTTTTAATTCATCATAAATTTCTTCAGCGGTCATGAGTTTTATATCGCCTTTAGCACTACCATCCCAAGTA
AATGCAGAATCACACCAGCTACAGCGATAATCACATCCAGCTGTTCTCACAAACATCGTTTTTCTACCGATTACTCTTCCTTCACCCT
GAATGGTTGGGCCGAATATTTCGAGTACAGGAATTTTAGCCATTAGTTACACCTGTTCCTTTGGTCTAAATACGACATAACTTGTTG
GTGTTTCTCTTACAAATACTTGAATACATTTTGGTTGGTGTTCGAGCGATGCCAAATTTTCTTTAACAATTTGATAAATTGTTTCCGCT
ACGATTTCAGTTGAAGGGATTTTGTTTTTAAAAGCAGGTAAGTTATTTAACAGTTGATGGTCAAATTTACCGTGTATCATCTTTTTTA
AATGGCTAAAGTTCACTAAAAAGCCAGTGTCATCTAGTTTATCACCGACAATTGTTAAATTAACAAAGTAAGTATGACCATGGACAT
TTTGACAAATACCTGCTTCTTCACACGGAATGTGATGTGCAGCTGAAAAATTAAAATCTTTATTTAATTCGAATTGATATGGATGCG
TTGTACTAGGATAGATTTGTTGTAACATTTTAAAGCGCTCCTTTACTTTCAAGATATTGATTTAGTCCACGTTGACGTAAATGACAAG
CTGGACATTCACCACAGCCATCCCCAATGATACCGTTATAGCATGTTAATGTTTTTGTACGAATATAATCTAAAACTTCGAGTTCATC
ACTTAATTTCCACGTTTCTGCTTTGTTTAACCACATTAAAGGAGTATGAATGACAAAATCTTTGTCCATAGCTAGGCTTAATGTTACG
TTCATTGATTTTATAAAACTATCGCGACAGTCTGGGTAGCCTGAAAAGTCTGTTTCACATACGCCTGTAATAATATGCTTAGCCCCA
ATTTGATAAGCTAGAGCGCCTGCAAACGACAAGAAAAGTAAATTTCTAGCTGGAACAAATGTATTAGGTATACCATCTTCATTATTA
GTAATTTCCATATCATGTTGTGTTAATGCGTTTGGAGTAAGTTGTGATAATAATGACATATCTAAAACGTGATGTTTCATTCCTTGAT
CTTGTGCAATTTGTTTTGCGACTTCAATTTCAGTATCATGTCTTTGGCCATAATTAAACGTTACGAGTTCAACTTCTTTGAAATGTTTT
TTTGCATAAAAGAGACATGTTGTACTGTCTTGACCACCACTAAAGACAACGATGGCTTTTTCATTATTTAATACACTTTCCATTTTGT
AATTGCTCCTATCATTAATAATATTAATAAAGAGGTTAATGGCATTGATAAGCCCGTTTTTAATTTATAAAATAAAAAAAGCCTATC
TCCATAAAAGATAGACGAAAGAAATGGGTTGCTCCTATAATATATATTAAAGGTTTACCAACGAATGTTTGAAATTTCAATGTCTAG
TTTTTTATAGAGGGTTTCAGCTAGGAACCTCTGATATTCATTTATGTACGATGAATAGAATATACCATATATTAGTTAAAATCAAGGT
CAGGATAATGACAATGCCTTTAAAGTTTTAGCTACATCCAATACATATTATAAAAATAAAACACATTTAGAACGGAGTATACAATG
ATTCTAGTCATAGATAATAATGATTCATTTACATATAATTTAATAGACTATATTAAGACTCAAACGAAACTAACAGTTCAAGTTGTT
GGTATTGATAATCTGCTGATAGAAGACGTCATTAATATGAAGCCAAAAGCAATTGTTATTTCACCTGGGCCGGGTAATCCGGATGAT
TATCCTATCTTGAATGAAGTGTTAGAACAATTTTATCAGCGTGTACCTATACTAGGTGTATGTTTAGGATTTCAATGTATCGTGTCTT
ATTTTGGTGGAAATATCATTCACGGCTATCATCCTGTACACGGACATACTACACAGTTACGCCATACCAATGAAGGTATTTTTCAAG
GACTGCCTCAAAATTTCAATGTAATGCGTTATCATTCATTAATTGCTGACGGAGCGACTTTTCCAAATTGCTTAAAGATTACAGCAA
AAAACGATGAAGCGATTATTATGGCATTTGAGCATATTAGATTTCCGGTTTTTGGTGTGCAATATCATCCTGAATCTATTTTGAGTGA
ATACGGTTATCGACAAGTTGAATTATTTTTATCGAAGGTAGGTGATTACTGTGAGAATAGAATATAATTATCGCTACTATTTAACTG
AAAATGAATATAAGCAATACCATATTCAATTAAAGGGATTTATAAAGAAGTATGTTGCTACTAAGTTGGCTGATGTGGGAGAAGTG
ATACACTTTGCACAAGCGCAGCAACGACAAGGTAGATATGTCTCGTTATATTTAAGTTACGAAGCGGCAAAGTATTTTAATCATGTT
ATGTGTACACATTCATTAGCTAAAGATGATATTTATGCAGTAGCTTATAGTTTTGAAAAAGCGGAAAGCATAAATTCAACATATGAA
CATCAAACTTCTTATGTATCAAAGCATCATTTTTCATTTGTTGAATCTTCTGAGGTTATGATGACTAATATTAACCGTGTCCAACAAG
CAATTGTTGAAGGCGAAACGTATCAAGTGAACTATACGGCGCGCTTAACAGATAACATTTATTATCCTATTAGTACTTTATATGAAC
GATTAACTCAATTTAGTAATGGTAATTATACTGCGTTATTACAAACAGATGAAATCCAAGTAGCGTCTATCTCACCAGAATTATTTTT
TCAAAAAGGACAATTTAACAATGTCGATAACGTTATCATAAGCAAACCGATGAAAGGGACAATGCCTAGAGGTAAAACGGAAGCT
GAAGATCAACAGTATTATAAAACATTGCAAACTTCTTCGAAAGATCGTGCAGAAAATGTCATGATTGTTGATTTACTAAGAAACGA
TATAGGGAGAATATCACAGAGTGGCTCAATTAAGGTGTATAAACTATTTTTTATTGAGGCATATAAAACTGTATTTCAAATGACTTC
GATGGTAAGTGGAACTTTAAAAAAATAATACAGACTTAACTCAAATTTTAACATCGTTATTTCCTTGTGGTTCGATTACAGGTGCACC
GAAACTGAATACAATGAAATATATTAAACAATTAGAAAGTTCACCTCGTGGTATATACTGCGGAGCAATTGGACTATTACTTCCAAC
TGAAGATGATAAAATGATTTTTAATATTCCGATTCGCACTATTGAGTATAAATATGGACAAGCGATTTATGGAGTCGGAGCAGGTAT
TACAATTGATTCTAAGCCAAAAGATGAAGTGAATGAATTTTACGCAAAAACCAAGATTTTGGAGATGTTATAATGCAATTATTTGAA
ACAATGAAAATTGATAATGGACATATCCCTAGACTTACTTATCATACTAATCGCATAAAAATGTTCTTCTGAGCGATTAAACTTTAAA
TTTGATGAACATGCATGGCGAAATGAATTAAACGATGTAACAACAAAGTATCACAGTGGTCAATATAGACTTAAAATCGTATTAAA
TGCTGAAAGCAAATTTGAAACGATAGTGTCACCTTTACCTGAGAAAAGTAGTTTTACAGCAAAATTTCAAGTGTTGCCCAAAGTAGT
TAATCCAACTTTTATAAAAAAATAAAACGACAGAACGAAAGCATTTAGCACACAATCATGAAACAGATTTAATATTGCTAACTTCAG
AGGACGGCAAGGTCCTTGAATTTGATATTGGCAACATTGTCATTGAAGAGGATGGAAAATGGTACACACCAAGTTATAAAGATGAT
TTCTTAAAAGGATGCATGCGTGATTATTTAATAGATAGTGACAAACTTGTTGAAAAAGACTTTAATAAAAACGAATTGATTTATAAA
TATCATAACAATGAGATACGTTTATTTTTGATAAATAGTTTACGAGAGGTTGCCGATGTCCACCTTTGCCTTTAAAATTAGTATGATA
ATAAAGTAGAAAATGATGTGAAAACAAATTTAAAAATTAAATGGGGCCAAATATGATTATTGTTTATATTGTGCTGTTGTTAATTCT
TGTATACGTAAATTATCGATTAGTGAATCGATTGCTATCTGAAAATAGAATATATGTTGTTCGTTTGATAGCAACAATTACTACTGTT
ATAAGCTTTATCCTTGTATACGCATTAATTCACGAACTTATGCCTTTTGTTGTGCGGGCAATGGATTTAATGTACCACCAGTAAATTT
AGTGAGTAGGTAAAATGAACTATTCAATCGATTCTATAATTAAAAATGAAAGTAAAGTAACCAAATATTGAAGAGGTGTACAAATG
AAAATATATAGTCAAGGTGACCAAGCCATTGTAGTCGCAATTGAAAAAGAAGTATCTAAAAGTTTAACCGAAGATTTATTAACACT
TCGCTCATATTTAATTGAACAAAATTATCCATTTATTATAGAAATTGTGCCATCAGAATCAGACATGATGATTGTCTATGACGCAAG
GGATATGATTAAACACCATAATATACAATCACCTTTTTTATACATGAAAGCACTAATAGAATCGATTCATTTAAACATAAAACATGA
TTTTAACCAGCAAGATTTGATTGAAATACCAATTGTGTATGGTTCGAAATATGGTCCGGATTTAGAATCACTTTTAAAACATTACAA
AATCAAGCTAGAAACTTTTATTGAATTACATTCTAAGGCGCAATATTTTGTTTCGATGATGGGATATTCACCTGGGTTTCCTTATTTA
ACTGGATTAAATAAGAAATTGTATATTAATCACACGAGTAAACAGAAAAAATTCATTCCAGCTGGTTCTGTAGTACTTGAAGGGAA
AAAATGCGGTATTGTAACTACGGATACAATTAATGATTGGTTAGTTATTGGTTATACACCATTATCACTTTTTAATCCGAAAGAATC
AGATTTCGCACGCTTAAAGTTAGGCGATAATATTAAATTTAGACCTATCAATGAAAATGAATTAGAAGTAGGAGCGTTTAAAGATG
TCAATCATAATTGAAAAAAGTGGCTTATTCAGTAGCTTTCAGGACTTTGGCAGAAGGGGATATGAACATGATGGTGTAATTCCATGT

GGTGCACTTGATACTTTAGCACATGAAATTGCTAATCGATTAGTTGCAAATGACAAGAATGAAGCAACTTTGGAAATGACTAATAA
AATGGCAACGATTCGTTTTACAGAACCTACGCTGATTGCATTAGCAGGGGGTAATGTCAAAGCTTACACTGAGCATATGACTATATC
TCCATATAAATTGTATTTGTTAGATAAAGGCGATGTTTTAAAGTTTAGAGAAACAAGTTATACATCGCGAGTGTATTTAGCTGTGGG
AGGCGGATTTGAATTAGATGCATGGTTAGGATCTAACTCAACCGACTTTAATGTAAAAATTGGTGGTTTTAAAGGTAGAACATTACA
AGATGGCGATGAAATAAAGCTTAAGAGAGATTATACAGCTCGTCATCATAAGTTATTTGAAAACCTTGCTCACACGAAACAAACAG
ATTGGGGTATTGATGGATACGCCTTGTCATTTAATTATATGTCTGATGTATTTCATGTCGTTAAAAATAAAGGTACGGAAGATTTTAA
AGAAGATGCCATTCAAAGATTTGTGAAACATGATTATAAAGTAACGAGCAAAGCAAATCGCATGGGGATGATGCTTGAAGGTGAA
AAAATCAAAGCTTTTTATGAAGATATGCCACCGTATCAGACTGTCAAAAAAGGAACGATACAAATTAAGCGTGATGGCACACCTAT
TATCCTATTAAATGATCATTATACGCTAGGTAGCTACCCGCAAATCGGTACAATCGCAAGTTATCATTTAACGAAATTAGCACAAAA
ACCGCAAGGATCACGTTTGAAATTTCAATTTATAGATATTTTAACGGCTGAAAAGAACCTTGTTAAGTATAGTAACTGGTTAAACCA
ATTATTCCATGGAATAGAATATAGAATGCAATTAGAAATGATGAAATAATATTTGGTACGTAGCTCATACTCGAGTCCGATGCAAAT
AATTTCCTCCTAATGTATAATGAAATAATACTGTGTTTTATCTGCGAAATGTATCATTTTCTAATTCGTTTCACAGTAAAATGAAAAG
ATAAAGTGTGTTTTTACTTGAATTTTGACTAAAATTACTCTATATTTATTAATTGAGCTATGCTTATTATTACAATTTGATTACAAATT
TTAAATTTGTTAATTGAATGATAATATTAAATAAAGAAACTTACACAAGCAAATATGAGTTGTAGCCCAAAATACTTGTTAAATCAA
AGTTGAAAGCTACAAATAATGAAAATTATAAACTTGAATCTGAAAGTAATTACTATAATTATGACAATGTTAACTTTTAAACGCACT
TATTAATTAACTACATAATGTTAATATCTAATTTATTCAAGTACTTTCGCAAGATTTATTATCTAAATAACGGGGGAAAGAATCATG
AGTTCACAAAAAAAGAAAATTAGTCTTTTTGCGTTCTTCTTATTAACCGTAATAACGATTACCTTGAAGACGTATTTTTCTTATTATG
TTGATTTTTCTTTAGGTGTTAAAGGTTTAGTACAAAACTTAATATTATTGATGAATCCTTATAGTTTAGTAGCACTGGTTTTAAGTGT
GTTCCTATTCTTTAAAGGCAAAAAAGCATTTTGGTTCATGTTCATAGGCGGCTTCTTATTGACGTTCCTATTATATGCCAATGTTGTG
TACTTTAGATTCTTCTCTGATTTTTTAACGTTTAGTACTTTAAACCAAGTAGGTAACGTAGAATCTATGGGTGGTGCGGTTAGTGCAT
CATTCAAATGGTATGACTTTGTTTATTTCATTGATACGTTAGTTTACTTATTCATTTTAATATTTAAAACAAAATGGTTAGACACAAA
AGCATTTAGTAAGAAATTTGTTCCTGTCGTAATGGCGGCTTCAGTAGCATTATTCTTCTTAAACTTAGCTTTTGCTGAAACTGACAGA
CCAGAATTATTAACACGTACATTTGACCATAAATATTTAGTGAAATATTTAGGACCTTATAACTTTACAGTATACGATGGTGTTAAA
ACTATCGAAAATAATCAACAAAAAGCGCTAGCATCTGAAGATGACTTAACAAAAGTATTAAATTATACGAAACAACGTCAAACAG
AGCCTAACCCAGAATATTATGGGGTGGCAAAGAAGAAAAATATTATTAAGATTCATTTAGAAAGTTTCCAAACCTTCTTAATTAATA
AAAAGGTTAATGGTAAAGAAGTAACACCGTTTTTAAACAAATTATCAAGTGGGAAAGAGCAATTCACATACTTCCCTAACTTTTTCC
ATCAAACAGGTCAAGGTAAAACATCTGACTCTGAATTTACAATGGATAACAGTTTATACGGTTTACCGCAAGGTTCTGCCTTTTCAT
TAAAAGGAGATAATACGTATCAGTCATTACCAGCAATTTTAGATCAAAAGCAAGGCTACAAATCTGATGTCATGCACGGTGACTAT
AAAACATTCTGGAACAGAGACCAAGTATATAAACACTTTGGTATCGATAAATTCTATGATGCAACATACTATGACATGTCAGATAA
AAACGTTGTAAACTTAGGCTTGAAAGACAAAATTTTCTTTAAAGATTCTGCTAATTATCAAGCTAAGATGAAATCACCATTCTATTC
TCATTTAATTACATTGACTAACCACTATCCATTCACATTAGATGAAAAGGATGCAACTATTGAGAAATCAAACACAGGTGATGCAAC
AGTTGATGGTTATATTCAAACAGCACGTTATTTAGACGAAGCATTAGAAGAATATATTAATGACTTGAAGAAAAAAGGATTATATG
ACAATTCAGTGATTATGATTTATGGTGACCACTATGGTATCTCTGAAAACCATAACAATGCCATGGAAAAACTATTAGGTGAAAAA
ATCACACCGGCTAAATTTACAGATTTAAACAGAACTGGTTTCTGGATTAAAATCCCTGGTAAATCTGGTGGTATCAATAATGAATAT
GCTGGTCAAGTCGATGTAATGCCAACAATTTTACATTTGGCTGGTATAGATACGAAGAACTATTTAATGTTCGGTACTGATTTATTCT
CTAAAGGTCATAATCAAGTAGTTCCATTCAGAAATGGTGACTTTATAACAAAAGATTATAAATATGTTAATGGTAAGATTTATTCTA
ATAAAAATAATGAACTCATAACTACTCAACCAGCTGATTTCGAAAAGAATAAAAAGCAAGTTGAAAAGGATCTCGAAATGAGTGA
CAACGTGCTTAATGGTGATTTGTTTAGATTCTACAAAAATCCAGACTTCAAAAAGGTAAATCCTTCGAAGTATAAATATGAAACAGG
ACCTAAAGCAAACTCTAAAAAATAATATCTAAACACGAACTCGGATTGATAAAATATCAATCCGGGTTTTTATTATGTTCTTTTATA
TTATGTTTTACATTATATGTGTTGATAAAAAGGTATATTATAAAGATCATTAGCATAGTCATCAAAGCCTTAACAAGTTTTATTTATA
GTTGAAGCAACAATATATTAAATTATGACACTTAACGGTATCGATTTCTTTTGATTTCAAAAAGGTGTATAATGTATAGAACTGATT
TAAGTACGTAGAGATTTCAACAGAAGAAGGAAGATGGGTATGGAAGCATATAAAATTGAACATTTAAATAAATCTTATGCCGATAA
GACTATATTCGATAACCTAGATTTATCAATTTCAGAAGGTGAAAAAATAGGTTTAGTAGGCATAAATGGTACAGGGAAAAGTACGT
TGTTAAAAGTAATTGGTGGTATTGATGATGATTTTACAGCCAATGTTATGCATCCAAATCAATATCGAATTCGATATTCGTCTCAGA
AACAGGACCTTAATGAAGATATGACAGTTTTTGATGCAGTATTAAGTTCTGATACAACAACTTTACGCATCATCAAGCAATATGAGC
AGGCAGTACAAGCTTATGCGGATGACCAAAGTGATAAATTGTTCAAGCGAATGATGGATGCGCAAGATGCTATGGATCAACATGAT
GCTTGGGACTATAACGCTGAAATTAAAACAATCCTCTCAAAACTAGGTATACATGATACTACTAAATACATTAAAGAATTATCCGGC
GGACAACAAAAACGTGTTGTACTTGCTAAAACATTAATAGAACAACCAGATTTATTGTTATTAGATGAACCTACGAACCATTTAGAC
TTCGAATCAATCAGCTGGTTGATCAATTATGTGAAGCAATATCCTCATACTGTTTTATTCGTAACCCATGATCGATATTTTTTAAATG
AAGTTTCCACTAGAATTATTGAACTAAACAGAGGTAAGTTAGCGTCATATCCTGGTAACTATGAATCTTATATTGAAATGCGCGCTG
AAAGAGAAGTAACACTTCAAAAGCAACAACAAAAGCAACGAGCTTTATATAAGGAAGAACTTGCTTGGATGAGGGCTGGAGCTAA
GGCTCGTACTACAAAGCAACAAGCTAGAATTAATCGATTTAATGACCTAGAAAATGAAGTTAACCAGCAATATAAAGACGATAAAG
GTGAATTGAATCTTGCTTATTCAAGATTAGGTAAGCAAGTGTTCGAATTAGAAGACTTATCAAAGGCTATTAATGATAAAGTATTAT
TTGAACATCTGACGGAAATTATTCAAAAAGGTGAGCGTATTGGTGTTGTTGGGCCAAATGGAGCTGGTAAAACAACACTCTTAAAT
ATTTTGAGTGGAGAAGACCAACAATTCGAAGGTAAATTGAAGACTGGGCAGACGGTTAAAGTAGCTTATTTTAAGCAAACAGATGA
GACCCTGGATAGAGATATTCGTATGATTGATTATTTAAGAGAAGAAAGTGAGATCGCAAAAGAAAAAGATGGAACCTCGGTATCTA
TTACACAACTTCTTGAACGATTTTTATTTCCAAGTGCAACTCATGGTAAAAAAGTTTATAAATTATCTGGTGGAGAGCAAAAGCGTT
TGTATTTATTACGTCTACTCGTACACCAGCCAAATGTTCTGTTGTTAGATGAACCGACAAATGATTTAGATACTGAGACTTTAACAAT
ACTTGAAGATTATATTCATACTTTCGGTGGTACAGTGATTACCGTAAGCCATGATCGCTACTTCTTAAATAAAGTTGCACAGTCATAT
TGGTTTATTCATGATGGTCAGATGGAAAAGATTATCGGAACTTTTGAAGATTATGAAAGTTATAAAAAATCATTAGATAAAAATAA
ATCCACATTGAAGCAACAATCTAAATCTTCTACAACTGTACGTAAGAAAAATGGTTTATCATATAAAGAAAAATTAGAATATGAAC
AATTGATGAAACGCATAGAACAAGCGGAAGTAAGAATGGAAGAAATTGATGTGCTCATGATTGAGGCAAGTGCAGATTATGGGAA
AATTAAAGAATTAAACGAAGAAAAAGAACAACTTGAAATTCAATATGATTTAGACATCACAAGATGGAGTGAGTTAGAAGAAATT
AAAGAACAACAATAAGGGGTCAATTTATGATGCAACAAACATTATCGCATTACTTTGGGTATGAAACGTTTCGACCAGGACAAGAA
GAAATTATTAGCAAAGTATTAGACCATCGTAATGTGCTTGGTGTCTTACCAACTGGTGGAGGTAAGTCTATATGCTATCAAGTACCA
GGTTTATTGTTAGGTGGTACAACAATTGTAATAAGTCCACTAATATCATTAATGAAAGATCAAGTGGATCAATTAAAAGCGATGGG
AATTCAAGCTGCTTTTTTAAATAGTAGTTTGACTCAAAAAGAGCAACAACGTATTGAAAAAGCATTATCAAATGGAGAAATTCAATT
TTTGTATGTTGCACCAGAACGATTTGAAAACCGATATTTTTTAAATATGCTTCAGCGTATAAAGATTCACTTAGTCGCGTTTGATGAA
GCGCATTGTATTTCTAAATGGGGTCATGATTTCAGGCCGAGTTACCAAAATGTTATTTCAAAAGTATTTACGTTACCTCAAGATTTTA
CAATAATAGCGTTGACAGCAACTGCCACGGTTGAAGTACAGCAAGATATTAGAGAAAAGTTAAATATCGCTCAAACTGATCAAATT
AAAACGAGTACTAAGCGTAGAAACTTAATTTTTAAAGTAATCCTACTTATCAACGTCAAAAATTTATATTGGATTATATTAAAACA

CACGATGAAGATGCAGGTATTATTTATTGTTCTACACGTAAGCAAGTTGAAGAGCTTCAAGAAGCCTTAGAAAGTCAGAAAATTGA
AAGTGTTATATATCATGCAGGTTTGAGCAATAAAGAAAGAGAAGAAGCGCAGAATGATTTCTTATTTGATCGTGTTAAAGTAGTCG
TTGCTACAAATGCTTTTGGTATGGGTATTGATAAATCCAATGTACGCTTTGTTATTCATTATAATATGCCTGGAGATTTAGAATCTTA
TTATCAAGAAGCGGGTCGTGCAGGTCGTGACGGGTTGAAAAGTGAATGTATTTTGTTATTTAGCGAACGCGATATCAATTTACACGA
GTATTTTATAACAGTCTCTCAAGCTGATGATGACTATAAAGATAAAATGGGCGAAAAGTTAACTAAAATGATTCAATATACAAAAA
CAAAAAAATGTCTAGAAGCAACAATTGTCCATTATTTTGAACCGAATGAAAAATTAGAAGAATGTGAACAATGTAGTAATTGTGTT
CAACAAGATAAATCATATAATATGACACAAGAAGCTAAGATGATTATTAGTTGCATCGCTCGTATGAAACAACAAGAGAGTTATAG
TGTTATCATTCAAGTGTTAAGAGGAGAGTCAACAGATTATATTAAGTATAAAGGTTATGACCAAATTTCAACCCATGGTTTAATGAA
AGGTTACACAACATCAGAATTAAGTCACTTAATAGATGAATTAAGATTCAAAGGGTTCTTAAATGAAAATGACGAAATATTAATGT
GTGATACTTCAATTAAAAAATTACTCAGTAATGAAGTAGAAGTATTCACAACACCATTTAAGCAAAAAGCGACTGAAAAAGTATTT
ATAAATACGGTTGAAGGGGTTGACCGAGTATTATTCAGTCAGTTGGTAGAAGTTCGTAAAAAGTTAAGTGACAAATTAACGATAGC
ACCTGTAAGTATATTTTCTGATTACACGTTGGAGGAATTTGCTAAACGTAAGCCTGCTTCGAAACAAGATATGATTAATATTGATGG
CGTAGGTAGTTACAAATTAAAACATTATTGTCCAGCATTTTTAGAAACGATTCAAAATTATAAAGCCAAAGTATAGTGAAGACACCT
AGTAAGCATTCCTTGTTTTACTGGGTGTTTTTTTGAATTCAAGGTAAAATAACACTATTTCTTAACTGGAGATTACCTATTATTCATT
ATTTTATTAAAAAAGTTCAAGGATAAATCATTTAAATTTGAAAATTGTAATAAACGTGGTTAAATAGGGAATACATACATATAATCT
CGGTTTTATGCTATGAGAAAAAAAAGAGGTGGCAAAGTGATTAAGTTTAAAAATGTAACTAAGCGTTATGGCAAACATGTTGCTGTC
GATAACATTAGTTTCAATATTAATGAGGGTGAATTTTTTGTGCTAATTGGACCTTCAGGTTGTGGAAAAACTACGACATTAAAAATG
ATTAATCGACTCATTCACTTAAGTGAAGGTTATATTTATTTTAAAGATAAACCAATAAGTGATTATCCAGTATACGAAATGCGTTGG
GATATTGGATACGTATTGCAGCAGATTGCATTATTCCCACATATGACAATCAAAGAAAATATTGCACAAGTGCCACAAATGAAAAA
GTGGAAAGAAAAAGATATAGATAAAAGAGTAGATGAATTACTTGAAATGGTTGGATTAGAACCTGAAAAATATAAAAACAGAAAA
CCTGATGAATTGTCAGGGGGGCAACGACAACGTGTAGGAGTTATACGTGCGTTAGCAGCTGATCCACCAGTTATTTTAATGGATGA
ACCGTTTAGTGCATTAGACCCAATCAGCCGAGAAAAACTTCAAGATGATTTAATTGAATTACAAACTAAAATTAAGAAGACAATCA
TATTTGTTACACATGATATTCAAGAGGCGATGAAACTTGGTGATAAGATTTGTCTTTTGAATGAAGGGCATATTGAACAAATTGACA
CACCAGAAGGATTTAAAAATAATCCTCAAAGTGAATTTGTTAAACAATTTATGGGTAGTCATTTAGAAGATGATGCGCCATGTGTTG
AAGAGAACGCAATTATCCGTGACTTGGATATTATGAAACCAATCGATGAGGTTACATCTATGAGCGCTTATCCAATTGTTTATGACA
ATCAACCAATTGAAGTATTGTATCAACTTTTATCAGAGAGCGAGCGTGTCATTGTCATGCAAGAAGATAGCGTAGGTCAATATGTTA
TTGATAGGAAAGATATCTTCAAATATTTGTCCCAGAAAAAGGAGGTAGCTCAACATGACTAACTTTTTCGACATATTGAGTGAACGT
AAGGGGCAACTCTTTTCGACAATGATAGAACATATTCAAATATCATTTATCGCATTATTGATTGCAACTGCTATTGCGGTACCATTA
GGTATTTTATTAACGAAGACTAAAACGATATCTGAAATCGTAATGAATATTGCGGCAATTCTTCAAACCATACCATCGTTGGCATTA
TTAGGTTTAATGATTCCTTTATTTGGTATCGGTCGTGTGCCAGCAATTATTGCACTTGTAGTGTATGCGTTGTTACCAATTTTAAGGA
ATACGTATACTGGAATTAAAGAAGTTGATCCATCACTCATTGAAGCGGCTAAAGGTATAGGTATGAAACCATTTAGACGTTTAACTA
AAGTCGAACTTCCGATAGCAATGCCTGTTATAATGGCTGGTGTAAGAACGGCTATGGTATTAATTATAGGTACAGCAACACTAGCA
GCATTAATTGGTGCAGGCGGACTAGGAGATTTAATTTTATTAGGTATAGACCGTAACAATGCATCGTTGATATTATTAGGTGCAATT
CCAGCAGCCTTATTGGCAATTATATTTGATTTAATTTTAAGATTTATGGCTAAATTATCTTATAAAAAGTTATTGATGACGTTAGGTG
TTATAGTGATGATTATTATACTGGCTATCGCTATTCCTATGTTTGCACAAAAAGGTGATAAAATTACGTTAGCTGGAAAGCTTGGCT
CCGAGCCTTCGATTATTACAAATATGTATAAAATTTTAATAGAAGAAGAGACCAAAAATACTGTAGAAGTGAAAGATGGTATGGGG
AAAACAGCATTTTTATTTAATGCTTTAAAATCTGATGATATAGATGGGTATTTAGAGTTTACTGGAACAGTTTTAGGTGAATTAACA
AAAGAACCATTGAAGTCAAAAGAAGAGAAAAAAGTTTATGAACAAGCTAAGCAAAGTCTTGAAAAGAAATATCAAATGACTATGT
TAAAACCAATGAAGTATAACAATACGTATGCTTTAGCTGTAAAACGTGATTTTGCTAAACAACATAATATACGTACAATTGGTGATT
TAAATAAGGTTAAAGATCAACTTAAACCAGGATTTACATTGGAATTTAATGATCGTCCAGATGGTTACAAAGCTGTTCAAAAGGCTT
ATAATTTAAATTTAGATAACATACGTACAATGGAACCTAAGTTGAGATATCAAGCGATCAATAAAGGTAATATTAATTTAATAGAT
GCATATTCAACTGACGCTGAATTAAAACAATATGATATGGTTGTGTTAAAAGATGATAAGCACGTATTTCCACCATATCAAGGAGC
ACCATTATTTAAAGAAAGCTTTTTAAAGAAACATCCAGAAATTAAGAAACCGTTAAACAAACTAGAAAACAAAATATCTGATGAAG
ATATGCAAATGATGAACTATAAAGTAACAGTTAAAAATGAAGACCCATATACAGTTGCGAAAGATTATTTAAAAGCAAAAGGGTTA
ATCAAATAACGACCAACGCCACATAAGATGCGTAACACCAAATTATATCTTATGTGGCGTTGTTATATTTAAATCTATAATTATGTT
CAATTTAAACATGCAATAATGATTAAAAAATATGACATGTTAAACACAATGTAAGCTATTATGATGTGAAAATAGTAGCATTGCATT
TTAGAAACATAGAGCGATATAATGAATATAAGTTTTTTAAAATTTCAGTTAATTCTAAGGAGGTTGTTTTTATTATGAAAGAACAAC
TTAATCAACTATCAGCATATCAGCCTGGTTTATCTCCAAGGGCATTGAAAGAAAAGTATGGCATTGAAGGAGATTTATATAAACTTG
CATCAAATGAAAATTTGTATGGACCATCGCCTAAAGTTAAAGAAGCGATATCAGCACACTTAGATGAGTTATATTATTATCCTGAAA
CAGGATCACCGACATTAAAAGCGGCGATTAGTAAACATTTAAATGTAGATCAATCACGCATTTTATTTGGTGCGGGATTAGATGAA
GTTATATTAATGATTTCTAGAGCTGTATTAACGCCAGGGGATACTATTGTTACAAGTGAAGCGACATTCGGTCAATATTATCACAAT
GCGATTGTTGAATCAGCTAATGTGATACAAGTACCTTTAAAAGGTGGTGGCTTCGATTTAGAAGGTATTTTAAAAGAAGTTAATGAA
GATACGTCATTGGTATGGTTATGTAATCCAAATAATCCTACAGGTACATATTTTAATCATGAGAGCTTAGATTCGTTTTTATCTCAAG
TACCTCCACATGTACCAGTAATTATAGATGAAGCTTATTTTGAATTTGTGACAGCAGAGGACTACCCGGATACACTTGCTTTGCAAC
AAAAATATGACAATGCTTTCTTATTACGTACATTTTCAAAGGCGTATGGATTAGCGGGTTTACGTGTGGGATATGTGGTAGCAAGTG
AACATGCGATTGAAAAATGGAACATCATTAGACCACCATTTAATGTGACACGTATATCTGAATACGCAGCAGTTGCAGCACTTGAA
GATCAACAATATTTAAAAGAGGTAACACATAAAAATAGTGTTGAACGCGAAAGATTTTATCAATTACCTCAAAGTGAGTATTTCTTG
CCAAGTCAAACGAATTTTATATTTGTAAAAACAAAGCGGGTAAATGAACTTTATGAAGCACTTTTAAATGTAGGGTGTATTACGCGA
CCATTTCCAACTGGTGTTAGAATTACAATTGGTTTTAAAGAACAAAATGATAAAATGTTAGAAGTTTTATCAAATTTTAAATACGAA
TAGTAAGTGGGGAGTGGGACATAAATGATATTTTCTCAAAATTTATTTCGTCGTCCCACCCTGGCAAGGTTGACTAGAATTGAAAAA
AGCTTGTTACAAGCGCATT

>022-contig_262

AAAAAGCTTGTTACAAGCGCATTTTCGTTCAGTCAACTACTGCCAATATAACTTTGTAGAGCATTGAACATTGATTTATGTCTCAAG
CTCAATGCAGTGTGAATGATGAGGTGAGAGTATTCAGTGTAAAAAGCAACAATAGATGATATTGTTTTGTATCAATTGCTTTTTTGC
TATACTGAATCAATACTGATATTTTGAGGAGAAGATTAAAATGACCCGTAAATCAATCGCGATTGATATGGATGAAGTATTGGCAG
ATACATTAGGAGAAATCATTGATGCTGTCAATTTTAGAGCGGATTTAGGTATTAAAATGGAAGCTTTGAATGGTCAAAAACTTAAAC
ATGTTATTCCTGAACATGATGGATTAATTACAGAAGTATTGAGAGAACCAGGCTTCTTCAGACATCTTAAAGTGATGCCGTATGCAC
AAGAAGTTGTGAAAAAATTAACTGAACATTATGATGTATATATTGCTACAGCAGCAATGGATGTACCAACATCATTTAGTGATAAA
TATGAATGGTTACTAGAGTTCTTTCCATTTTTAGATCCTCAGCATTTTGTTTTTTGTGGTAGAAAAAACATCGTTAAAGCTGATTATTT

AATAGATGACAATCCTAGGCAGCTTGAAATTTTTACTGGTACACCGATTATGTTTACAGCAGTGCATAATATTAATGATGATCGATT
TGAACGCGTAAATAGCTGGAAAGATGTAGAACAGTATTTTTTAGATAATATTGAGAAATAAAAATATATCACTTGAAAAATTTCAT
GTAGAAAAGATGATGGATAGGCTATAAAGTAATTGTGACTGAGATGAACTTTTATGTCTTAGACACTACAACACTATATTGGCAGT
AGTTGACTGCGGGGCCCCAACATAGAGAAATTGGATTCCCAATTTCTACAGACAATGCAAGTTGGGGTGGGCCCCAACATAAAGAA
ATACTTTTTCTTTAGAAATTAGTATTTCTTATGCATGAGTGTAACTCATGCATTCATATTTTTAAGTACACATTAGCTGTGACTAATG
ATAAAGAATCGCTACATAATCAATCATTAGTCGTTCTTTATCATTTCCGTCCCGCTCTCAATAAATGTTAGTCTATCTCATTATTATA
AATCGGATGAATGTGTTAATCTATGGCAGATTACACGTCATCCGATTTTTTATAGAATTTGAAAAAGACGCATAAACCACTATGATT
TAAAATACAACATCAATCATTTTAGTGGCATGCGCCAAAATTATATGTCTGTTTTTGAAACAGGGTAATAGCTTAAAGCTAATAAAA
ACGAATATAAGGTGCGTTGAATCTTATGATTATACTCCAAACCTAATATAATATCGGGTTAAGATCATTCCGGATGCTTACAAATCA
TTGACAGTAAGTAACTGAATGGCATTTGGTATAACCTCAATATCAATAGGTGTTTCTAATGAAATTTCGCCATCAATATCAACTTTC
ATTGCTGGATCTGTTGTAAGTGAAATCTTTTTACCAGGTATATGCTCAATACCTTGAGTAATTTCATTCCAATTCATGCTATCACGCT
TTTTAAAAATATCATTTAAAATACTGAAACTTTGTTCATTAAAAATGAAAGTGTTCAGTTCACCATCTTGAGGAGACAAATCAGTCA
ATGGTATACGACTACCACCAATGAATGGACCATTTGCTGTTAGTATCATGGTCGTTTCGCCAGAATATGTCTTATCATCTATTGATAA
TTGATAATTAAATTGTGTTGGATTTAGCAGTGTTTTGACAGTTGATCCAATATAACTCAATTTACCAAATATATCTTTTGAACCATCT
TGTACGTTTTCAGCGTTTTGAACAATGAGACCTAAGCCAACAAAGTTGAGTGCATATTGATTATTTATTTTAATTACATCGTATGTAC
CAACTTGTGCAGAAATCATTTGTTCACTAGCTTGTTTATGATTAGGTGCTATATTTAGCGTTTTTGTAAAATCATTAAAAGTACCGCC
TGGTAAAATGCCAATAGGGAGTTGAAGGTCATGTGACATAACACCATTTATAAGTTCGTTAACCGTGCCATCACCGCCAAGAATAA
ATAATATATCTACATCTTTTGCATAGTTTTTAGTTTTGATTTCTTGGCAATATTTAATAATGTCACCTTCGTTTTCACTCAATTGAATA
GAAAGATGCTTACAAATTGAACTTAATGCTGTTGTAACTTCCCCAATACCTTGATTAATATTTTTTAATCCACTGTGTTCATGGTAAA
AGAGGACACCATGTGTATATTTATTTTCCATAGTTTAGCCTACTTTCTAAAAATTGGTTCATTAAATATATATACCCACTTTTAATTG
TTAATACCAAAAATATGTTTTAAATAGAGAAAATGGTAATAACTGACATTGATGTCTATAGAGTGGGACGAGAAAATATAGTTATA
GCTGTCTATATCGAGCATATTAAGCTTTTATTTATACTGATATCTTGAATTTAATTAATAGAAACCTATAAAAAAACAGTAAGCCATT
TAAATGACTTACTGTTTTTTAATTTAGGCCAACAATATTAACGAATACCTTTCATTGCTTTAATAATTAAAGGTGAGAATGCTAATAC
AATTGTTGTAACAATAATTGCAACAACACCTAGGAAAATAAAGTAATTTGTTTGACCTAGTGGTTCTATTAACTTAACTAAAGTACC
ATTGATTGCTTGTGCAGAAGCGTTAGTTAAGTACCAAATACTCATCATTTGGGCATTAAATGCTTTAGGTGCTAACTTAACAGCAGC
ACTATTACCCGTTGGTGATAAGCATAGCTCACCGATAACACAAATAATGTACGATAAAATAACCCAGTTAACTGAGAAGTTTGATG
AACCTGATGCATAACCTACAATACCAATTAGTATGTATGACGCACCTGCTAAGAACGTACCAATTGCAAATTTTACTGGCAGGCTAG
GTTGTTTAGTTCCAAGCTTTTGCCATAAAAGTGAAATAATTGGAGCTAGTAATAAAATAAATAATGGGTTAATTGATTGGAAGATCG
CTTCACCAAAGTTTGTTTTCCAACCAAATAAGTTTAATTTCATATCTGAATGTTCAATTCCATATATGTTTAATACATTAGACCCTTGT
TCTTGAATAGCCCAGAACACCATTCCAAGAATAAATAATGGAATAAATGCTTTAACACGAGAACGTTCAGTATCAGTGACATCTTTA
CTTCTAATAATTAAAGTGAAGTAAATGATTGGTAATGCAATACCTAATACTAAAACAGTATTACTAACTAAGTTAAATGATAATGAA
TTAGTTAATGCACCAATAACGATAATTAATACAATTGCTAAGACAACACTTCCGATAATAAGACCATACTTTTTCTTTTCAGCTGGT
GTCAATGGGTTAGTAGGTTTCATACCAACGCTACCTAAGTTTTTGCGGTTGAAAAGTACATACCATACTAAACCTAATGCCATACCA
ACTGCTGCAATCAAGAATCCGCCGTGGAAGTTTTTAACATTAACAAAGTGTTGCAAAATAATAGGTGATAATAATGCACCCATATTA
ACTGACATATAGAAAATAACAAAACCTGCATCCATACGTCTATCATTTTCAGGATATAAACGGCCAACGATATTTGAAATGTTAGGC
TTCATTAAACCTGAACCAATAATGATGAAGAACATTGATGTGAATAAGCCGATTAATGCAAATGGTAAGCTTAAACAATATGTCC
GATAATAATAAAGACTGCACCTAATAAAGTAGCGCCTCTAGTACCTGTAATTCTGTCAGCAATCCATCCGCCTGGTATTGATGTCAT
ATAGATTAATGAACCATAAACTGACATAATTGACATAGCTGTTGTTTTATCAATTCCAAGGCCATTATCTGTTACGGCAAAGTACAT
GTAGAAAATGAGTAGGGCACGCATGCCATAATAACTAAACCTTTCCCAGAACTCTACAAAGAAGAGTACGCCTAGTCCTCGAGGAT
GCCCGAAAAATCCTGTTTGAGGTATGTCTTGAATTTGATTTCCATGGGAGTTTTGTTGTGTCATGTATACATCCCATCCTTTCTTCCCC
TAATACAATAGTTTAAGTAAATTTGATTGTGCCTTATCAACTAAATACCCCAACAAATCACAAAAAAATAAAAATATTGAGAATATT
CTGTTATTAAGCGCGCACAATTAATTAGAGATAATATAAAAATATACTTATTGACGTTAAAGTGCAATAGGTTTCTATAAAAATGTT
GTTTAATTCGTGTACTAATATTCTTTTGGTGTACTTTGGTATAAAATGTTATTTTAATGTAACCTATTATGTATGATAAATGAAAAAC
ACACCAATAAGTTAGGAAAATCAACTTATTGGTGTGTTTATTAAATGATACATTTAACGATTATCTATTTTTTCGGGATATAAATCAT
GATTCATCAAACGATGCTCAGCCATTTTTTCATATTTAGAATTTGGACGTCCATAGTTTGTATAAGGATCAATAGAAATTCCACCAC
GTGGTGTGAACTTGCCCCAGACTTCAATATAATGTGGGTCCATAAGCTCTATCAAATCATTCATAATAATATTCATACAATCTTCGTG
AAAATCACCGTGATTTCTGAAACTAAATAAGTATAATTTCAAAGATTTTGATTCAACCATTTTAACATTTGGAATATATGAAATATA
GATAGTTGCAAAATCTGGTTGCCCAGTAATTGGACATAATGATGTAAATTCTGGACAGTTGAATTTTACGAAATAGTCACGACCTTG
ATGCTTATTATCAAACGATTCTAATACATCAGGACGGTAGTCGAAATTGTAAGTATTGTCTTGATTTCCTAATAAAGTTATATCTTGT
AATTCATCTTGTTGACGGCCATGTGCCATATAAAGCGCTCCTTTAAATTTATTTTTTTATTATTTTGGCGTCTCGGCGTGCTTTTTCAA
ACATGTAATAACTTGCACCGATAATAACGACGTAACCTAATGTTGCATAGAAATCTGGAGATTCTCCGAATAGAATAAATCCAAGT
ATTGCTGTGAAAATTATAGATGCATACGTAAAAATAGAAATATCTTTTGCGGCTGCAAAACTATATGCTAAAGTAACACCAATTTGA
CCCACAGCGGCAGCTAAGCCAGCCCCTAATAGATAAAGTATTTGCATCTGACTCATTGGTTCATAAGTATATGCAGTGAAAGGTATT
AAAACGATGACAGAAAATAAGGAGAAGTAAAATACTATAGTATATGGTGCTTCTCTTGTACTAAGTGCTCGAACACATGTATATGC
TGATGCTGCAAAAATACCTGAGAATAAGCCAGCTAATGATGGAATCATAGATGATGAAAATTCAGGTTTCACTATTAAAAGCATAC
CTAAAATAGCAATTATCATTGCTGTTATTTGATACTTCCTTACTTTTTCATGTAAGAAAACAATGCTTAATAAAATCGTCCAGAAAGG
ATTGAGTTTCATTAATGAATCGGCATCACTAAGTACCATATGATCAATGGCATAAATATTTAACAATACACCAATAAGTCCAAGTGT
TGATCGTGTTATTAATAAGGGTTGACTTGAAAGTCTGCCAAACATTGGCTGATGGTATTTATATATAAAAAATAATGGAATAAACAT
TGCTACTAAGTTTCGTGCTAATGATTTTTGAAAAACAGGAAGGTCACCTGCAAGTCTGAAAAACACTGACATAAAACTGAAACCAA
TAGCCGAAATTAAAATGGCAATGATACCTTTTACTTTAGGATTCAATTTTATCCCCTCTTTTATATAAAATTAACGTATTTATATTAG
CATAAAACAACATGTTGTGCATAAATAGTTGAAATTTACTATAAAAAGACTATAATAGACTGTACCGAACAAACGTTCTGTATTTAT
TTGTCGGAATAATAGGGCATTACACTTTTATGAATGTTTGTGTTATTACATAAAACAAATATCAATTCAGTATCAAGCTAATAAGCT
TTTTCTTGATTTCTGTTGATACAATTGAGATTGACACAGATTTAAAAAAATCAAGTGATATCTACTAAAAAATTTTTTTAAATTTGTT
CAAGTTTTTCTAATTTAGTATTGGTGCCTAGTTGGAACGTTTTACGAACATTCGATTAGAAAATGGCACTTTAAATCATAGTGTGTCT
TATGTATAATGAAACACATAATATAGTGTTGGTGAAACGAAAAAGACACAATATCTTGTGTTTTGTATGCAAATGCTTTATTTATGA
AGAAATTACATTTAAAAGTAATTTAACACAGAAATTTAATAGTTATTATCAATTAATAGTCATATTTTTAGAAAATGTACTGAGCAA
ATGGAAGATATCCAATGATGTAAACACTACATATAGTGATTTTTATACATTCAACCCATATAAGCTACTATTTTCTCAAATATAAAT
CTATGCAATTGGTTTACATTTGAGAAAATAAGTAGCTTCATTATAGTTAATACAATGCTGAGATAACCATAGTAACCATGTTGTTAA
AGCATTTTTTAATTGGAATGACTACTTTATTTAAAAGGGTTGAAGAAAGAAGGTGATCCAATGAAAATAATATATTTTTCATTTACT
GGAAATGTCCGTCGTTTTATTAAGAGAACAGAACTTGAAAATACGCTTGAGATTACAGCAGAAAATTGTATGGAACCAGTTCATGA
ACCGTTTATTATCGTTACTGGCACTATTGGATTTGGAGAAGTACCAGAACCCGTTCAATCTTTTTTAGAAGTTAATCATCAATACATC

AGAGGTGTGGCAGCTAGCGGTAATCGAAATTGGGGACTAAATTTCGCAAAAGCGGGTCGCACGATATCAGAAGAGTATAATGTCCC
TTTATTAATGAAGTTTGAGTTACATGGAAAAAACAAAGACGTTATTGAATTTAAGAACAAGGTGGGTAATTTTAATGAAAACCATG
GAAGAGAAAAAGTACAATCATATTGAATTAAATAATGAGGTCACTAAACGAAGAGAAGATGGATTCTTTAGTTTAGAAAAAGACC
AAGAAGCTTTAGTAGCTTATTTAGAAGAAGTAAAAGACAAAACAATCTTCTTCGATACTGAAATCGAGCGTTTACGTTATTTAGTAG
ACAACGATTTTTATTTCAATGTGTTTGATATTTATAGTGAAGCGGATCTAATTGAAATCACTGATTATGCAAAATCAATCCCGTTTAA
TTTTGCAAGTTATATGTCAGCTAGTAAATTTTTCAAAGATTACGCTTTGAAAACAAATGATAAAAGTCAATACTTAGAAGACTATAA
TCAACACGTTGCCATTGTTGCTTTATACCTAGCAAATGGTAATAAAGCACAAGCTAAACAATTTATTTCTGCTATGGTTGAACAAAG
ATATCAACCAGCGACACCAACATTTTTAAACGCAGGCCGTGCGCGTCGTGGTGAGCTAGTGTCATGTTTCTTATTAGAAGTGGATGA
CAGCTTAAATTCAATTAACTTTATTGATTCAACTGCAAAACAATTAAGTAAAATTGGGGGCGGCGTTGCAATTAACTTATCTAAATT
GCGTGCACGTGGTGAAGCAATTAAAGGAATTAAAGGCGTAGCGAAAGGCGTTTTACCTATTGCTAAGTCACTTGAAGGTGGCTTTA
GCTATGCAGATCAACTTGGTCAACGCCCTGGTGCTGGTGCTGTGTACTTAAATATCTTCCATTATGATGTAGAAGAATTTTTAGATAC
TAAAAAAGTAAATGCGGATGAAGATTTACGTTTATCTACAATATCAACTGGTTTAATTGTTCCATCTAAATTCTTCGATTTAGCTAAA
GAAGGTAAGGACTTTTATATGTTTGCACCTCATACAGTTAAAGAAGAATATGGTGTGACATTAGACGATATCGATTTAGAAAAATAT
TATGATGACATGGTTGCAAACCCAAATGTTGAGAAAAAGAAAAAGAATGCGCGTGAAATGTTGAATTTAATTGCGCAAACACAATT
ACAATCAGGTTATCCATATTTAATGTTTAAAGATAATGCTAACAGAGTGCATCCGAATTCAAACATTGGACAAATTAAAATGAGTA
ACTTATGTACGGAAATTTTCCAACTACAAGAAACTTCAATTATTAATGACTATGGTATTGAAGACGAAATTAAACGTGATATTTCTT
GTAACTTGGGCTCATTAAATATTGTTAATGTAATGGAAAGCGGAAAATTCAGAGATTCAGTTCACTCTGGTATGGACGCATTAACTG
TTGTGAGTGATGTAGCAAATATTCAAAATGCACCAGGAGTTAGAAAAGCTAACAGTGAATTACATTCAGTTGGTCTTGGTGTGATG
AATTTACACGGTTACCTAGCAAAAAATAAAATTGGTTATGAGTCAGAAGAAGCAAAAGATTTTGCAAATATCTTCTTTATGATGATG
AATTTCTACTCAATCGAACGTTCAATGGAAATCGCTAAAGAGCGTGGTATCAAATATCAAGACTTTGAAAAGTCTGATTATGCTAAT
GGCAAATATTTCGAGTTCTATACAACTCAAGAATTTGAACCTCAATTCGAAAAAGTACGTGAATTATTCGATGGTATGGCTATTCCT
ACTTCTGAGGATTGGAAGAAACTACAACAAGATGTTGAACAATATGGTTTATATCATGCATATAGATTAGCAATTGCTCCAACACA
AAGTATTTCTTATGTTCAAAATGCAACAAGTTCTGTAATGCCAATCGTTGACCAAATTGAACGTCGTACTTATGGTAATGCGGAAAC
ATTTTACCCTATGCCATTCTTATCACCACAAACAATGTGGTACTACAAATCAGCATTCAATACTGATCAGATGAAATTAATCGATTT
AATTGCGACAATTCAAACGCATATTGACCAAGGTATCTCAACGATCCTTTATGTTAATTCTGAAATTTCTACACGTGAGTTAGCAAG
ATTATATGTATATGCGCACTATAAAGGATTAAAATCACTTTACTATACTAGAAATAAATTATTAAGTGTAGAAGAATGTACAAGTTG
TTCTATCTAACAATTAAATGTTGAAAATGACAAACAGCTAATCATCTGGTCTGAATTAGCAGATGATTAGACTGCTATGTCTGTATT
TGTCAATTATTGAGTAACATTACAGGAGGAAATTATATTCATGATAGCTGTTAATTGGAACACACAAGAAGATATGACGAATATGTT
TTGGAGACAAAATATATCTCAAATGTGGGTTGAAACAGAATTTAAAGTATCAAAAGACATTGCAAGTTGGAAGACTTTATCTGAAG
CTGAACAAGACACATTTAAAAAAGCATTAGCTGGTTTAACAGGCTTAGATACACATCAAGCAGATGATGGCATGCCTTTAGTTATG
CTACATACGACTGACTTAAGGAAAAAAGCAGTTTATTCATTTATGGCGATGATGGAGCAAATACACGCGAAAAGCTATTCACATAT
TTTCACAACACTATTACCATCTAGTGAAACAAACTACCTATTAGATGAATGGGTTTTAGAGGAACCCCATTTAAAATATAAATCTGA
TAAAATTGTTGCTAATTATCACAAACTTTGGGGTAAAGAAGCTTCGATATACGACCAATATATGGCCAGAGTTACGAGTGTATTTTT
AGAAACATTCTTATTCTTCTCAGGTTTCTATTATCCACTATATCTTGCTGGTCAAGGGAAAATGACGACATCAGGTGAAATCATTCGT
AAAATTCTTTTAGATGAATCTATTCATGGTGTATTTACCGGTTTAGATGCACAGCATTTACGAAATGAACTATCTGAAAGTGAGAAA
CAAAAAGCAGATCAAGAAATGTATAAATTGCTAAATGACTTGTATTTAAATGAAGAGTCATACACAAAAAATGTTATACGATGATCT
TGGAATCACTGAAGATGTGCTAAACTATGTTAAATATAATGGAAACAAAGCACTTTCAAACTTAGGCTTTGAACCTTATTTTGAGGA
ACGTGAATTTAACCCAATCATTGAGAATGCCTTAGATACAACAACTAAAAACCATGACTTCTTCTCAGTAAAAGGTGATGGTTATGT
ATTAGCATTAAACGTAGAAGCATTACAAGATGATGACTTTGTATTTGACAACAAATAACAATTAAATTAAAAGACCTTCACATGTA
AAGGGAAATAGCGATTCGTTTCGTCTTGCCTCCTACATGTTGAAGGTCTTTTCTCTATGCTTAAAAGCGTATGCATATTTTGAAAAAG
TAAAAATTGTATTTAATGATGTCAATGAGGCTAGAAAGTATACAAGATATTGCATTACAAAAGGGTGTTCTATTTTACTTTAAGCAA
ACAAAATACACATTCCGAAAAATTAAATTTCAGTTTAATTGCAAATATCAATAAAATTGACACTAAATTATTTGAAAGGCTATTGAA
ATTATGGTCAAAAAACGCTACTATTAATGAGAAATATTATCAATGATAATGATTATCATTAATTTAAAGGGAGAAAAATTTGTAATG
AAGTATTTATTAAAGGGAAATATTTTGCTTCTATTACTAATATTGTTGACAATTATTTCGTTGTTCATAGGTGTGAGTGAACTATCAA
TTAAAGATTTACTACATTTAACTGAGTCACAGCGGAATATTTTATTCTCAAGCCGAATACCAAGGACGATGAGTATTTTAATTGCTG
GAAGTTCGTTGGCTTTAGCAGGCTTGATAATGCAACAAATGATGCAAAATAAGTTTGTTAGTCCGACTACAGCTGGAACGATGGAA
TGGGCTAAACTAGGTATTTTAATTGCTTTATTGTTCTTTCCAACCGGTCATATTTTATTAAAACTAGTATTTGCTGTTATTTGCAGTAT
TTGCGGTACGTTTTTATTTGTTAAAATCATTGATTTTATAAAAGTGAAAGATGTCATTTTTGTACCGCTTTTAGGAATTATGATGGGT
GGGATTGTTGCAAGTTTCACAACCTTCATCTCATTGCGCACGAATGCTGTTCAAAGCATTGGTAACTGGCTTAACGGGAACTTTGCC
ATTATCACAAGTGGACGCTATGAAATTTTATATTTAAGTATTCCTCTTTTAGCATTGACATATCTTTTTGCTAATCATTTCACGATTGT
AGGAATGGGTAAAGACTTTACTAATAATTTAGGTTTGAGTTACGAAAAATTAATTAACATCGCATTGTTTATTACTGCAACTATTAC
AGCATTGGTAGTGGTGACTGTTGGAACATTACCGTTCTTAGGACTAGTAATACCAAATATTATTTCAATTTATCGAGGTGATCATTTG
AAAAATGCTATCCCTCATACGATGATGTTAGGTGCCATCTTTGTATTATTTTCTGATATAGTTGGCAGAATTGTTGTTTATCCATATG
AAATAAATATTGGTTTAACAATAGGTGTATTTGGAACAATCATTTTCCTTATCTTGCTTATGAAAGGTAGGAAAAATTATGCGCAAC
AATAATAAAAAAATAATGCTTTTAATTGCAGTAACGTTATTAATTAGTATGCTGTACTTATTTGTAGGTATTGATTTTGAAATATTTG
AATATCAATTTTCAAGTCGTTTAAGAAAGTTCATATTAATTATTTTAGTAGGTGCTGCCATTGCAACTTCAGTGGTGATTTTTCAAGC
GATTACAAATAACCGTCTATTGACACCATCAATAATGGGGTTAGATGCAGTTTATTTATTTATCAAAGTATTGCCAGTCTTTTTATTT
GGAATTCAATCGGTATGGGTTACTAATGTATATTTGAACTTTATATTAACACTTATAACGATGGTGTTATTCGCACTAATCCTATTCC
AAGGTATCTTTAAAATTGGACATTTTTCAATTTATTTTATCTTACTTATTGGTGTCCTTTTAGGAACATTTTTTAGAAGCATAACAGGT
TTTATTCAACTGATTATGGATCCTGAGTCATTTTTAGCAATACAAAGTAGTATGTTTGCTAATTTTAATGCTTCTAATTCGAATTTAGT
TACTTTCTCAGCAGTGCTATTAGTAATCTTATTAGTCATTACAATTTTACTATTGCCTTATTTAGATGTATTGCTTTTAGGTCGTGCTG
AAGCAATTAATCTTGGGATATCGTATGAAAAATTAACGCGAATTCTACTTGTAATAGTCTCAGTTTTAGTTTCTGTGTCAACTGCATT
AGTAGGACCAATTACATTTTTAGGTTTATTAACTGTAAATCTAGCGCATGAACTAATGAAGACGTATGAACATAAGTATATTTTAAT
TGCGACAATTTGCTTGAGTTGGATTAGTTTATTTAGTGCGCAATGGGTAGTTGAAAATGTGTTTGAAGCTACGACAGAAATGAGTAT
ACTTATTGATTTGATTGGTGGAAGTTATTTCATTTATCTATTAGTTAGAAGGAGAAATGCGCAATGATTCAAGTTGAAAATTTAACT
AAAACTATAAATAATCAAATGATATTGGAAGATATTAGCATAGATATCGAAAAAGGTAAATTGACTTCTTTAATTGGACCTAATGG
TGCGGGTAAGAGTACTTTACTTTCAGCGATATGTAGGTTAATTCGTTTTGATAACGGTGAAGTGAAAATAGATGGACAGCTCATGTC
TGATTATAAAAATAATGACTTGTCGAAAAAAATATCTATATTAAACAAACAAACCATACTGAAATGAATATTACGGTAGAGCAGT
TGGTAAACTTTGGACGATTCCCTTATTCTAAAGGTCGTTTGACGAAAGAGGATCATGATATTGTCAATGATGCGCTAGATTTGTTGC
AACTACAAGATATCAGAAATCGTAATATTAAGTCATTATCTGGTGGACAACGTCAGCGTGCATACATTGCAATGACAATAGCACAA
GATACTGAATATATTTTGCTAGATGAACCATTAAATAATTTAGATATGAAGCATGCTGTTCAAATTATGCAAACGTTAAAAATGTTA

GCGCATAAAATGAATAAAGCGATTGTCATTGTGTTACATGATATTAACTTTGCGTCCTGTTATTCAGATCAGATTGTAGCATTGAAA
AACGGACAACTAGTTAAGTCAGATTTGAAAGATAATGTCATTCAAAGTAGTGTTTTAAGTGATTTATATGACATGAATATTCAAATT
GAACATATAAGAAATCAAAGGATTTGTTTATATTTTAAGGATTGATAATTTGGAGACACTTTAAAGGGGTGATGCGCCAATTAAAG
AAGGGTTAAACGTAAAGCATTTATTTATATTTCACATCAAGCACACAGATTAAGCCAAAAGAGGAGAATATTATATTATGAAGAAA
ACAGTCTTATATTTAGTATTAGCAGTAATGTTTTTATTAGCGGCATGCGGTAACAATTCTGATAAAGAACAATCAAAATCAGAAACT
AAAGGTTCTAAAGATACAGTAAAAATTGAAAATAACTATAAAATGCGTGGCGAGAAAAAAGATGGTAGTGACGCTAAAAAAGTTA
AAGAAACTGTTGAAGTACCAAAAAATCCTAAAAATGCAGTTGTGTTAGACTATGGCGCATTAGATGTAATGAAAGAAATGGGCTTA
TCAGATAAAGTAAAAGCATTACCTAAAGGGGAAGGCGGTAAGTCATTACCGAATTTCTTAGAATCATTTAAAGATGATAAATATAC
AAACGTTGGTAATTTAAAAGAAGTGAATTTTGATAAAATTGCTGCGACGAAACCCGAAGTAATCTTTATCTCTGGACGTACAGCTAA
TCAAAAGAATTTAGATGAATTCAAAAAAGCTGCACCTAAAGCGAAAATTGTTTATGTTGGTGCAGATGAAAAGAACTTAATTGGTT
CAATGAAACAAAACACTGAAAATATCGGTAAAATTTACGATAAAGAAGATAAAGCTAAAGAATTAAATAAAGATTTAGATAACAA
AATTGCTTCAATGAAAGATAAAACGAAAAACTTCAATAAAACTGTTATGTATTTACTAGTTAACGAAGGTGAATTATCAACATTTGG
ACCTAAAGGTCGTTTTGGTGGATTAGTTTACGATACATTAGGATTCAATGCAGTTGATAAAAAAGTAAGTAATAGCAATCATGGAC
AAAATGTTTCTAACGAATATGTTAATAAAGAAAATCCAGATGTTATTTTAGCGATGGATAGAGGTCAAGCGATAAGTGGTAAATCA
ACTGCGAAACAAGCATTAAATAATCCTGTATTAAAAAATGTTAAAGCAATTAAAGAAGACAAAGTATATAATTTAGATCCTAAATT
ATGGTACTTTGCAGCTGGATCAACTACAACTACAATTAAACAAATTGAGGAACTTGATAAAGTTGTAAAATAATTTTAAAAGAGGG
GAACAATGGTTAAAGGTCTTAATCATTGCTCCCCTCTTTTCTTTAAAAAAGGAAATCTGGGACGTCAATCAATGTCCTAGACTCTAA
AATGTTCTGTTGTCAGTCGTTGGTTGAATGAACATGTACTTGTAACAAGTTCATTTCAATACCAGTGGGCTCCAAACATAGAGAAAT
TTGATTTTCAATTTCTACTGACAATGCAAGTTGGCGGGGCCCAAACATAGAGAATTTCAAAAAGGAATTCTACAGAAGTGGTGCTTT
ATCATGTCTGATCCACTCCCTATAATGTTTTGACTATGTTGTTTAAATTTCAAAATAAATATGATAGTGATATTTACAGCGATTGTTA
AACCGAGATTGGCAATTTGGACAACGCTCTACCATCATATATTCATTGATTGTTAATTCGTGTTTGCATACACCGCATAAGATTGCTT
TTTCGTTAAATGAAGGCTCAGACCAACGCTTAATGGCGTGCTTTTCAAACTCATTATGGCACTTATAGCATGGATAGTATTTATTACA
ACATTTAAATTTAATAGCAATAATATCTTCTTCGGTAAAATAATGGCGACAGCGTGTTTCAGTATCGATTAATGAACCATAAACTTT
AGGCATAGACAAAGCTCCTTAACTTACGATTCCTTTGGATGTTCACCAATAATGCGAACTTCACGATTTAATTCAATGCCAAATTTTT
CTTTGACGGTCTTTTGTACATAATGAATAAGGTTTTCATAATCTGTAGCAGTTCCATTGTCTACATTTACCATAAAACCAGCGTGTTT
AGTTGAAACTTCAACGCCGCCAATACGGTGACCTTGCAAATTAGAATCTTGTATCAATTTACCTGCAAAATGACCAGGCGGTCTTTG
GAATACACTACCACATGAAGGATACTCTAAAGGTTGTTTAGATTCTCTACGTTCTGTTAAATCATCCATTTTAGCTTGTATTTCAGTC
ATTTTACCAGGAGCTAAAGTAAATGCAGCTTCTAATACAACTAAGTGTTCTTTTTGAATAATGCTATTACGATAATCTAACTCTAATT
CTTTTGTTGTAAGTTTAATTAACGAGCCTTGTTCGTTTACGCAAAGCGCATAGTCTATACAATCTTTAACTTCGCCACCATAAGCGCC
AGCATTCATATACACTGCACCACCGATTGAACCTGGAATACCACATGCAAATTCAAGGCCAGTAAGTGCGTAATCACGAGCAACAC
GTGAGACATCAATAATTGCAGCGCCGCTACCGGCTATTATCGCATCATCAGATACTTCGATATGATCTAGTGATAATAAACTAATTA
CAATGCCACGAATACCACCTTCACGGATAATAATATTTGAGCCATTTCCTAAATATGTAACAGGAATCTCATTTTGATAGGCATATT
TAACAACTGCTTGTACTTCTTCATTTTTAGTAGGGGTAATATAAAAGTCGGCATTACCACCTGTTTTAGTATAAGTGTATCGTTTTAA
AGGTTCATCAACTTTAATTTTTTCATTTGGGATAAGTTGTTGTAAAGCTTGATAGATGTCTTTATTTATCACTTCTCAGTACATCCTTT
CTCATGTCTTTAATATCGTATAGTATTATACCAATTTTAAAATTCATTTGCGAAAATTGAAAAGAAAGTATTAGAATTAGTATAATTA
TAAAATACGGCATTATTGTCGTTATAAGTATTTTTTACATAGTTTTTCAAAGTATTGTTGCTTTTGCATCTCATATTGTCTAATTGTTA
AGCTATGTTGCAATATTTGGTGCTTTTTTGTATGGAATTGCAAAGCAATATCATCATTAGTTGATAAGAGGTAATCAAGTGCAAGAT
AAGATTCAAATGTTTGGGTATTCATTTGAATGATATGTAGACGCACCTGTTGTTTTAGTTCATGAAAATTGTTAAACTTCGCCATCAT
AACTTTCTTATTATATTTATGATGCAAGCGATAAAACCCAACATAATTTAAACGTTTTTCATCTAAGGAAGTAATATCATGCAAATTT
TCTACACCTACTAAAATATCTAAAATCGGCTCTGTTGAATATTTAAAATGATGCGTACCGCCAATATGTTTTGTATATTTTACTGGGC
TGTCTAAGAGGTTGAATAATAATGATTCAATTTCAGTGTATTGTGATTGAAAACAATTAGTTAAATCACTATTAATGAATGGTTGAA
CATTTGAATACATGATAAACTCCTTTGATATTGAAATTTAATTTAATCACGATAAAGTCTGGAATACTATAACATAATTCATTTTCAT
AATAAACATGTTTTTGTATAATGAATCTGTTAAGGAGTGCAATCATGAAAAAAATTGTTATTATCGCTGTTTTAGCGATTTTATTTGT
AGTAATAAGTGCTTGTGGTAATAAAGAAAAAGAGGCACAACATCAATTTACTAAGCAATTTAAAGATGTTGAGCAAAAACAAAAA
GAATTACAACATGTCATGGATAATATACATTTGAAAGAAATTGATCATCTAAGTAAAACTGATACAACTGATAAAAATAGTAAAGA
ATTTAAGGCACTACAAGAAGATGTTAAAAACCATCTCATACCTAAATTTGAAGCATATTATAAGTCAGCAAAAAATTTGCCTGATG
ATACAATGAAAGTTAAGAAATTAAAAAAAGAATATATGACGCTTGCAAATGAGAAGAAGGATGCGATATATCAATTAAAAAAATT
CATAGGTTTATGTAATCAATCTATCAAGTATAACGAAGACATTTTAGATTATACGAAACAATTTGAAAAAAATAGATACAAAGTTG
AATCAGAAATTAAATTAGCTGATAATAAAAGTGAAGCAACTAATCTTACGACAAAATTAGAACATAATAATAAAGCGTTAAGAGAT
ACTGCGAAGAAGAACCTAGATGATAGTAAAGAAAATGAAGTAAAAGGCGCGATTAAAAATCACATTATGCCAATGATTGAAAAAC
AAATTACCGATATTAACCAAACTAATATTAGTGATAAGCATGTTAATAATGCAAGGAAAAACGCAATAGAAATGTATTACAGTCTG
CAGAACTATTATAATACACGTATTGAAACAATAAAGGTTAGTGAGAAGTTATCAAAAGTCGATGTAGATAAGTTGCCGAAAAAGGG
TATAGATATAACTCACGGCGATAAAGCCTTTGAAAAAAAGCTTGAAAAATTAGAAGAAAAATAACTATAATCATTTTTCAAAGTTA
AAAATTTTGAATTTATGGTTAACATGTCAACTTACTATGTGTATAATGGTAAACATTGATATTAACTATATGTATAAAAATGTCACG
CAGATGCTATTTAAATGTGATAAATATTTTTAGAGGTGAATAGAGTGGCTATAAAGCTAAGTTCAATTGACCAATTTGAACAGGTTA
TTGAGGAAAATAAATATGTTTTTGTATTAAAACATAGTGAAACTTGTCCAATATCGGCAAATGCGTACGATCAATTTAATAAATTTT
TATATGAACGCGATATGGACGGTTATTATTTGATTGTCCAACAAGAACGCGATTTGTCAGATTATATTGCTAAAAAAACGAACGTTA
AACATGAATCACCTCAAGCATTTTATTTTGTAAATGGTGAAATGGTTTGGAATCGAGACCACGGTGATATCAATGTGTCGTCATTAG
CACAAGCAGAAGAATAATGAAACTATAGGGTTGGAACATTTTGCCTTACACTACTAGACGTGAATAGCACAACTTAAATTCGTGTG
AATCAGAGTAGTTTGGCTATAATGATGTTCTGACCTTTTATTTTATGTCACCTTTAGAAGCAGTTAAGTTAGTACTTTTTTACAAACA
TATGTATAATGTATTCGAGTATTTTTATTGAAAATATTTTGGAAAACGACGAATCCAATAAGAAAATTTAAACATGATTTGTAAGTT
AGTTTAATAGGAAATATATGCTAAACCAAAAGAAGCATACTGTTATTTACTGGAATAATTAATAATCATGTCATGTTAAATGTTCGC
ATATAATCACGAGATAAAATCTAAAATTTAAGATTAATCTTTTATGAATAAAAAACGTATCACAACAAATAATAAAGTAAGGTGGT
CAAGGTTATGAAAGTATTAGTAGCCATGGATGAGTTTCATGGAATTATTTCGAGTTATCAAGCTAATAGATATGTTGAAGAGGCAGT
TGCAAGCCAAATTGAAACTGCGGATGTAGTTCAAGTACCATTGTTTAATGGAAGACATGAATTATTAGATTCTGTATTTTTATGGCA
ATCTGGGCAAAAGTATCGTATACCAGTACATGATGCAGATATGAATGAAGTTGAAGGTGTTTACGGACAAACTGATACAGGGATGA
CCGTTATCGAAGGGAATTTATTTTTAAAAGGTAAAAAACCAATTGTTGAACGAACAAGTTATGGTTTAGGAGAAATGATTAAACAT
GCATTAGATAACGACGCAAAACATGTTGTAATTTCACTAGGTGGGATTGATAGTTTTGATGCTGGTGCAGGTATGTTACAAGCATTA
GGTGCTCAATTCTATGATGACGAAGGGCGTGTCGTAGATATGAGACAAGGTGCTGGTGTAATTAAATATATTCGTCGTATGGATATG
TCGAACTTACACCCTAAAATGGAAACAGCAAGAATTCAAGTAATGTCGGATTTTTCAAGTCGATTATATGGTAAGCAAAGTGAAAT
CATGCAAACTTATGATGCGCATCAGTTGAATCATAATCAAGCAGCAGAAATCGATAATTTAATTTGGTATTTTAGTGAGTTATTTAA

AAGTGAATTGAAAATTGCAATTGGTCCAGTTGAACGTGGTGGTGCTGGTGGTGGAATTGCAGCAGTCTTGAATGGACTGTATCAAG
CTGAAATATTAACCAGTCATGCATTAGTAGACCAACTAACACATTTAGAAAATTTAGTTGAACAAGCGGATTTAATTATTTTTGGAG
AAGGATTAAATGAAAATGATCAGTTGCTAGAAACGACAACATTGCGTATTGCAGAACTTTGTCACAAACATCAAAAGGTTGCCATT
GCAATTTGTGCAACTGCTGAAAAGTTTGATTTATTTGAATCACAAGGGGTTACAGCAATGTTTAATACATTTATCGATATGCCAGAA
ACTTATACTGACTTTAAAATGGGATTACAAATTAGGCATTATACGGTTCAGTCTTTAAAACTGTTGAAAACACATTTTAATGTTGAG
GTTTAGTAAAGAAGGACTAAATTGGTGATGCTGTCATGATGGTTAATAACATTTATGATGGTTAGCAAAACGAATTAGAAGATCGA
AAGTATACGTAAAAAATATGAAAAATCACGCTATCATTGCACTGAATGTTAGCGTGATTTTTTTATTAATTAAGCCTGAGTTGAACT
AGTATATAATCGTTGGTTTTTAGTGATTTTCAGCGATATCTTCTACAATTCCAATGATTACTTGTACTGCTTTTTCCATAACATCAATG
GATGCATATTCATATGGGCCGTGGAAGTTACCGCAACCTGTAAAGATGTTTGGAGTTGGTAACCCCATAAATGACAATTGTGAACC
ATCTGTACCACCACGAATAGGTTCAGTGTTTGCTGGAATATCTAATTTGGCAAAGACACGTTTAGGTATATCAATAATATGAGGCAA
TGGTAATATTTTTTCTGCCATATTGAAATATTGATCCGATATATCAACTTTAACTGGATAATTTTCAAAATGGGAATTGATATCGTCA
CGTATTTCTAAAATACGTTTCTTACGCAATTCGAATTGTTTTTTATCATGATCACGAATAATGTATTGCAAAGTTGCTTTTTCAACAG
TTCCTTCAAAGTTCATTAAGTGATAAAAGCCTTCGTATCCTTCTGTTCGCTCCGGAACTTCACTATCAGGTAGCAAACTATCGAATTG
TTCACCTAAACGTATTGCGTTTACCATTGCATTTTTAGCTGAACCAGGATGAACATTTACACCGTGGCATGTAATAACCGCTTCAGC
AGCGTTAAAGCTTTCATATTGTAATTCTCCATATTGACTACCATCCATAGTATAAGCAAAATCAGCATTGAAGCGGTCAACATCAAA
TTTATGTGGACCACGACCGATTTCTTCGTCTGGTGTAAATCCAATGCGAATGGTACCATGTTTAATTTCTGGATGTTCTTGTAAATAA
CAAATAGCTTCCATAATTTCCACAATACCCGCTTTATCGTCTGCACCTAGTAACGATGTACCATCAGTTACCATTAATGTGTGACCAA
CTAAACTGTTAAGTTCTGGAAATACTTTAGGATCTAAGACACGTTTAGTATTGCCTAGTTTGTATGGCTTACCATCATAGTTTTCAAT
AATTTGCGGTTTAACATTTGAAGCATTGAAATCAGGTGATGTATCAACATGCGCCAAAAATCCAACTGTTGGGACGTCGACATCGAT
GTTACTTTCTAATGTAGCAAATAAGTAGCCATTTTCATCTAAATCAGTTGGCAATCCTAATTGTTGTAATTCTTTTTCTAATAAATGT
AACAAATCCCATTGCTTTTCAGTTGAAGGTGTTGTTGTAGATTTTGGATCAGATTGCGTATCAATTGTCGTATATCTTGTTAATCTAT
CTATCAATTGGTTCTTCATTATATTCGACCCCTTAAACTCTATTATTCATGTTGTAAGATTTTTTATATGTCTTACCTTTGATTTTACCA
TACAGTTGTTTGATACGTGTGTATAGGTAATATAGAATTTCAGAAACTAATATACCGAAAGCAATCGCACCTGAAATCAGTGTAACT
TCTAAAAATGTATTTACAGCACTTGTATAATCATTCGATACTAAAAAACGAGTCGCTTGATAAGCTGCACCACCAGGTACTAATGGT
ATAATGCCTGGCACTATGAATATAATTACCGGTCGTTTATATCTGCGACTCATAGTATGACTCATTAAGCCTAAAATTAAGCTTCCC
AAAAATGAAGCGCCAACTTTTCCAAACTCTAAATCTACCGTTAATTGGTAAATCGTCCATGCAATGGCACCCACAAATCCACATGCT
ACTAAGAGGCGTTTGGGTGCATTGAAAATGATAGAGAAAAGTACTGTTGATATAAAGCTGATTGTAAAATGAAATAAATAAAATAG
CATGCTTTAACAGTCCTTCCTTAAATGATTAATAAAACGATTGCGACACCAGCACCGATTGCGAATGCTGTTAATGCAGCTTCAACA
CCACGAGACATACCTGCAAGTAATTCACCCGCTAATAAATCTCGAATGGCATTGGTAATTAATATACCAGGGACAAGTGGCATGAC
ACTGGCTATAGTAATGATATCTTGATTGGTTGCAATGCCTAATTTAGTAAATGTGGCTGCAATGGATATGACCACAGCGGCTGCAAC
AAACTCTGAGAAAAATTTAATTTGTATATAGCGTTGCACAAAGCTGAATGTTAAAAATGCGGATCCGCCAGCAATGACTGCAATCC
AACAATCTGATGCGACACCACCAAACATAAATAGGAAGAAGCCACATGCAATGGCAGCTGCAAAGAAATTCGTTAAAAAAGAATA
TTGTAATGATGCATGCTGTAAATGAATAAATTCAGATTTAGCTTCATCAATTGTGAGTTCTTTATTTGATATTTTACGTGAAAGACTA
TTCGTTAAAGCAATTTTCTCTAAATCTGTTGTACGCTCTTGTACACGAATTAATCTTGTACTTGTTCGATCATTTAATGAAAAAATAA
TTGCAGTTGAACTGACAAAACTATATGTATTATGAAGACCATAACTATGTGCGATACGGTTCATTGTATCTTCAACTCGATATGTTTC
AGCACCTGATTCAAGTAAAATTCTACCTGCAATTAATACAACATCAATCACTTTGTTTTCATCTATAATTGTGATTGAATCTGGCATA
TCAATTCACCTCCAATGATATGTGTTATTTATTTGAACAATTGAAGTTTACAACTTGTTGTTGCAACTTTCAATAGTGAGACTTTGTG
TTAGTATGATGAACTTGTATGGTTCAAATTTAAATAAGAAAAACTGTTAATCTTTGCTATTATACTATGATTTAATAATAGCAAAGG
ATTAACAGTTTTGTCGTTGTTATAAATTGATAATAGGGTTAAACATTACTTTGTTTCGCCCTTGATTTTTGGCTACATGCACCATATC
GTCTGCATCTTTAAACACTTTACGCTGTGATTTTGGATCGTCATCTGTTAAATAACCAACACCGATAGACACTGACAATTTAATAACT
TCTTTGTTTGGTAAATGGAATGATGATTTTTCAACACCCGAACGAATATTTTCAGCCAATTTAACACTTTGATCAAGTGAATAATTGT
GAATGACAACTGAGAACTCTTCGCCACCATTTCTAAAAATTTTAAATTGATTCGGCACATAGTTTTTAAGTAATTGAGACATTTGTTT
TAATACAGCATCACCTGATTTGTGTGAGTAGGTATCATTGACATCTTTAAATCCATCGATATCGATTAATAATAATGCGATACTTTGA
TGTTCTTTTTCAGCTTTTCGTGAAATTTCATTTAAATGTCTATCAAATTCTTTTACATTACCTAAGCCTGTTAAGTAATCATATTTATC
TTCGTTTTCATAACGATTTACGAGTGAGAAGAAATGCCAAATATCGACAAATGTTATCGCTGAAGCTAAAGTGATAATTAATGAAAT
TGGTATTAAAATGATAACTTCCGATAGTGTGTAAATAGGACTCACTAACGCGACACCAAATAAAATGATTATTGTAACAACATTAA
GTATTAATAATGATAGCACATCATTTTGTTTTAAAAATGGTCCAATAGCACTTGTTACTGCAGCAATAACAATCAACGTAACACCGT
ACATAATCGAGTTGTTAAATACTACAATTTCAACAATTGCTACAATTACTGTGGCAGATAATGTATAGACCATATTTGTAAATCTAC
CTAAAAACAATAAAGGAACGAATGTTAAGTGAATTAAATAATCTTCACGATAAGGGATAGGGTAGACAGATAATAATAATGATAC
GATTGTCATTAAAACAGTGACATAAGCCTTAGAAAAAACCATACGTTTGTTTTCTGAATACTGTAAGCGATGGAATAAATAGATTCC
AGCGACTATAACAGATATATTGTATATAAATGCTTCGAACATGTCTGAATCGACTCCTTTAATTGACCACTAGCTATTGTAAGTGAA
AACTTACAATTTGTCATTAGTTTACATATAAAATTAATGTATGATATAGACTTTGATGTTAAAATGTTGCCTTAAATGATATGATGAA
AAAATGAATAATAGGCGATATATAAGAAATGAATCGTATAGTTGTAAATATGATATCATTGATTGAACGAATTAATTTATAATAAA
GCTATAAGATATACGTAGAAAATAGATATATCATTCTATAAAGACAATATTAAATAAATATAACGTTAAAAACAATTAATATCGAT
GAAGGTGAATAAATGGTTACATTATTACTAGTTGCAGTAACAATGATTGTCAGTTTGACGATAACACCAATTGTTATTGCAATATCG
AAAAGATTAAATTTAGTTGATAAACCAAATTTTAGAAAAGTACACACTAAACCTATTTCAGTTATGGGTGGTACAGTGATTCTCTTT
TCATTTTTAATAGGTATTTGGATTGGTCATCCTATTGAAACAGAAATCAAACCACTTATTATTGGTGCGATTATTATGTACGTACTTG
GGCTTGTAGATGATATCTACGATTTGAAACCGTATATAAAATTGGCTGGTCAAATTGCCGCTGCCTTAGTAGTTGCTTTTTATGGTGT
GACTATTGATTTTATTTCGTTGCCAATGGGTACAACGATTCATTTTGGATTTCTTAGTATTCCAATTACTGTGATTTGGATTGTTGCTA
TTACAAATGCAATTAACTTAATTGATGGACTCGATGGTTTGGCGTCGGGTGTTTCTGCAATCGGACTCATTACAATAGGGTTCATTG
CAATTTTACAAGCTAATATTTTCATAACGATGATTTGTTGTGTTTTATTAGGCTCTTTAATTGGGTTTTTATTTTACAATTTCCATCCT
GCCAAAATATTTTTAGGTGATAGTGGGGCTTTAATGATTGGATTTATCATCGGATTCCTTTCTTTACTCGGATTCAAAAATATTACAA
TTATTGCATTGTTCTTCCCAATTGTTATCTTAGCAGTTCCATTCATTGATACTTTGTTCGCAATGATTCGACGTGTGAAAAAAGGGCA
GCATATAATGCAAGCTGATAAATCGCATTTGCATCATAAACTATTAGCTTTAGGCTACACACATAGACAAACAGTATTATTAATCTA
TTCAATCTCTATTTTATTTAGTCTTTCGAGCATTATTTTGTATGTATCGCCACCATTAGGTGTTGTATTAATGTTTGTATTAATCATATT
TAGTATTGAATTAATTGTTGAATTTACAGGATTAATAGATAACAACTACCGACCAATATTAAATTTAATTAGTCGTAAGTCATCTCA
TAAAGAGGAATAGGGAATGAAAGCATAGCTGTATGGGATAATTTGTATTATATGGCTTTACTCTTTACAATTTTTTTGTATTAAATTT
CAAATATAAAAAGCACTGCCATAAACGTGTACTTCAATTGTCGTTTAATAATACGCAATTGATATTTACCGTCTTATGATAGTGCTTT
TTATTTTTATTCAGTTGGTATATCGAAAGGTAACTGCTTTGGAGTTTCTTCAGTCAAATCGAAATTTCCTGCAGTCATTTGATTTAAA
AAGTTAATAAACGCTTCATAGTCACTTTTAACGACATCGATATAGTAGCTTACCTTATCAGTGTAAGTTTGGTTTCTTAACATAAAAT
GAGTTGAAGCTAATTCATATTCAAATTTACCAGTTTGATCATAATTCAGTGTTACTATACATGGTACTGCTTCTCGTAGTTCGACACG

CCCGATATCATAAATGACGTCTCTAACAGCACCGCTATAGGCGCGAATTAAACCGCCACCACCTAATTTAATACCACCAAAATATCT
TGTTACTACGACACACGCATTATGAACATCGAGCTTTTTAATATGTCTAACATTGGGACACCGGCAGTTCCTGTCGGTTCACCATCA
TCATTCGCTTTTTGAATATTCATTTCAGGTCCAATAGTATATGCAGAACAATTATGAGTGGCATCTTTATGTTCTTTTTTTATTGCAGC
AATAAATGCTTTAGCTTCATCTTCATTTTGAACAGGTTTGATATGAGCAATGAATCTTGATTTACTAATCACATTTTCAATAATGTGT
TCTTTTTTAACAGTAATGATATTTTGTGTCATAATAACTCCTTAATTCATAAGCTTAAGATTATTTAATCTTCATTATACACTGAAAAT
GACATGACTATAAATCGTTTGATTGCCATTTTCTTTTTAACTGAAATATTGTATCATTGCTATGAGTATATTTTAGGAGGACGACTAT
GAAAATTGCTGTGATGACCGATTCTACAAGTTATCTGTCGCAGGACTTAATCGATAAATATAATATTCAAATAGCGCCATTAAGTGT
GACTTTTGATGATGGGAAGAACTTTACAGAAAGTAATGAAATAGCAATTGAAGAATTTTATAATAAAATGGCATCGTCTCAAACGA
TTCCAACAACAAGCCAACCAGCAATTGGCGAATGGATTACTAAATATGAAATGTTAAGAGATCAAGGTTACACAGATATCATTGTC
ATTTGCTTATCAAGTGGGATTAGTGGAAGTTATCAATCTAGTTATCAAGCAGGGGAAATGGTTGAAGGTGTTAATGTACATGCATTT
GATAGTAAGCTTGCAGCAATGATTGAAGGATGTTATGTATTACGTGCTATTGAAATGGTTGAAGAAGGATACGAGCCACAGCAAAT
TATTGATGATTTAACTAATATGCGTGAACACACAGGTGCATATTTAATTGTTGATGACTTAAAGAATTTACAAAAAAGTGGTCGAAT
TACTGGTGCTCAAGCATGGGTTGGAACATTATTGAAAATGAAGCCAGTTCTTAAGTTTGAAGATGGCAAGATTATACCAGAAGAAA
AAGTTCGTACTAAAAAGCGTGCCATTCAAACATTAGAAAAGAAAGTATTAGATATTGTAAAAGACTTTGAAGAAGTAACTTTATTT
GTCATAAATGGAGATCATTTCGAAGATGGTCAAGCGTTATACAAAAAGTTACAAGATGATTGTCCTTCAGCTTATCAAGTAGCATAC
TCTGAGTTTGGTCCAGTTGTTGCAGCACATTTAGGTTCTGGTGGATTAGGTTTAGGCTATGTTGGCAGAAAAATAAGATTAACATAA
TTATAAAATTTTAATAAAAGAGTCTATATTGTAATTGGAAATTATCTCTCGTATACATGGCTTTAAATGTTCATCATTTGAAAGCCAA
AATGCTAAAGATATAAGAAAATCATTATAATATTAGGCTCTTTTTTACGTTGAAATGAGGTTTTAAGCATTAAACATTACGGGAAAT
TAATTCATCCTCATACTTCACTTACTAATGAAAAAATTAAAAAAGAAGTAACAGGTGTCATCAAACAAAATTCAAACTATTATTGTG
TTCAATGTGAAAGTACAAATCCAAAGCATTTTTATCAGTATGATTCCTCAGTACATTCCAAGAAAATTGTATATTGCAGAAATTGTA
TATCACTGGGTCGAATGGATAATGTAACAAGATATAAAATAACAGAGAGTTCGCAAAGTTCATCACAAGCATATTATCATCTCTCAT
TTGAATTGTCGGAACAGCAGTCTTATGCCTCAGAACATATTGTTCGAGCCATTAGAAAGAGACAAACGATTTTGTTATATGCCGTAA
CAGGTGCAGGTAAGACAGAAATGATGTTTCAAGGCATTCAATATGCAAGAATACAGGGAGATAATATAGCTATTGTGTCACCACGT
GTAGATGTTGTTGTAGAAATTAGTAAACGTATTAAAGACGCATTTCTTAATGAAGATATAGACATACTACACCAGCAATCAAGACA
ACAATTTGAAGGGCATTTTGTTGTATGCACAGTGCATCAACTTTACCGATTCAAACAGCACTTTGATACTATTTTTATTGATGAAGTC
GATGCCTTTCCTTTATCAATGGATAAAAATCTACAACAAGCATTGAAGTCATCTTCTAAAGTTGAACATGCAACAATTTATATGACA
GCAACACCACCGAAACAACTTCTGTCAGAGATTCCCCACGAAAATATAATTAAATTGCCAGCTCGCTTTCATAAAAAATCACTTCCA
GTTCCTAAATATCGTTATTTCAAACTTAATAATAAGAAGATTCAGAAAATGTTATACCGAATTTTACAAGATCAAATTAATAATCAA
CGTTATACACTGGTGTTTTTAACAATATAGAAACAATGATTAAAACATTTTCGGTTTATAAGCAGAAAATTACTAAATTAACATAC
GTCCATAGCGAGGATGTTTTTCGCTTTGAAAAAGTTGAACAATTAAGGAATGGACATTTCGATGTCATTTTTACTACGACAATATTA
GAACGTGGATTTACAATGGCAAATTTGGATGTTGTTGTTATCGATGCACATCAATATACTCAAGAGGCTTTAATACAAATTGCTGGA
CGTGTTGGACGAAAATTAGAATGTCCTACTGGAAAAGTATTGTTTTTTCATGAAGGGGTAAGTATGAATATGATTCAAGCTAAAAA
AGAGATTCAAAGGATGAACAAATTAGCATTAAAAAGAGGTTGGATTGATGAATAATTGTTTGAGTTGTGGTGCTAAGTTATATGAA
AATATAACCATTTATAATTTGTTCAAGAAACCTAATAGATTATGTGACAGATGCAAAGAGAATTGGGACAATATTAAACTTGATATT
AAAGCAAGGCGATGTTCAAGGTGCTTAAAACACTTAAATCAAGATGAAGCGTATTGTTTAGACTGCAAGTTTCTATCGGCACACTTT
AATTTAATGGAACAATTATATTGTCAATTTCAATATGACGGTTTAATGAAAGAGATGATACATCAGTATAAATTTTTGAAAGACTAT
TATTTATGTGAATTATTGGCACATTTGATTGAAATACCACAAACATCTTATGACTATATTGTGCCAATTCCTTCTTCGCCGGCACATG
ATTTATCTAGAACATTTAACCCGGTAGAAGCAGTACTAAAAGCTAAAGGGATTCGCTTTGATAAGATTTTAAAGATGTCAAATAGA
CCAAAACAGTCTCATTTAACTAAGAAAGAGCGTCTGGCAGATGAAAATCCATTTATTATTGATACGGAATTAGATTTAAATGGCAA
GGAAATATTACTCGTTGACGATATTTATACAACTGGATTAACAATTCATCGTGCAGGGTGTAAATTATATGCTAAAAATATCAGAAA
ATTCAAAGTGTTTGCGTTTGCACGATAGCGTAAAAATGTTAAAATATAATAAAGAGTTACCAATAAAGAGGTTTAAGGAGAGATTA
CTATGATTAGATTTGAAATTCATGGAGATAACCTCACTATCACAGATGCTATTCGCAACTATATTGAGGAAAAAATTGGTAAGTTGG
AACGTTATTTTAATGACGTACCAAATGCAGTGGCGCATGTTAAAGTTAAAACTTATTCAAATTCAGCTACTAAAATTGAAGTAACAA
TTCCATTGAAAAATGTTACGTTAAGAGCTGAAGAGCGAAACGATGATTTATACGCAGGTATTGATTTAATTAATAATAAACTTGAAA
GACAAGTTCGAAAATATAAAACACGTATTAATCGTAAGAGCCGTGATCGAGGAGATCAAGAAGTGTTTGTTGCCGAATTACAAGAA
ATGCAAGAAACACAAGTTGATAATGACGCTTACGATGATAACGAGATAGAAATTATTCGTTCAAAAGAATTCAGCTTAAAACCAAT
GGATTCAGAAGAAGCGGTATTACAAATGAATCTATTAGGTCATGACTTCTTTGTATTCACAGACAGAGAAACTGATGGAACAAGTA
TCGTTTACCGCCGTAAAGACGGTAAATATGGCTTGATTCAAACTAGTGAACAATAAATTAAGTTTAAAGCACTTGTGTTTTTGCACA
AGTGCTTTTTTATACTCCAAAAGCAATTTCTGACTAATTCATAGTTCGATAATGTAATTTGTTGAATGAAACATAGTGACTATGCTAA
TGTTAATGGATGTATATATTTGAATGTTAAGTTAATAATAGTATGTCAGTCTATTGTATAGTCCGAGTCGAAAATCGTAAAATATTTA
TAATATAATTTATTAGGAAGTATAATTGCGTATTGAGAATATATTTATTAGTGATAAACTTGTTGACAACAGAATGTGAATGAAGTA
TGTCATAAATATATTTATATTGATTCTACAAATGAGTAAATAAGTATAATTTTCTAACTATAAATGATAAGATATATTGTTGTAGGCC
AAACAGTTTTTTAGCTAAAGGAGCGAACGAAATGGGATTTTTATCAAAAATTCTTGATGGCAATAATAAAGAAATTAAACAGTTAG
GTAAACTTGCTGATAAAGTAATCGCTTTAGAAGAAAAAACGGCAATTTTAACTGATGAAGAAATTCGTAATAAAACGAAACAATTC
CAAACAGAATTAGCTGACATTGATAATGTCAAAAAGCAAAATGATTATTTAGATAAAATTTTACCAGAAGCATATGCACTTGTTAG
AGAAGGCTCTAAACGTGTATTCAATATGACACCATATAAAGTTCAAATTATGGGTGGTATTGCAATTCATAAAGGTGATATCGCTGA
GATGAGAACAGGTGAAGGTAAAACATTAACAGCGACAATGCCAACATACTTAAATGCATTAGCTGGTAGAGGTGTTCACGTTATTA
CAGTCAATGAATACTTATCAAGTGTTCAAAGTGAAGAAATGGCTGAGTTATATAACTTCTTAGGTTTGACTGTCGGATTAAACTTAA
ACAGTAAGACGACAGAAGAAAAACGTGAAGCATACGCACAAGACATTACTTACAGTACTAATAATGAGCTAGGTTTTGATTACTTA
CGAGATAACATGGTGAATTATTCTGAAGATAGAGTAATGCGTCCATTACATTTTGCAATCATTGATGAGGTTGACTCAATTTTAATC
GACGAGGCACGTACGCCATTAATTATTTCTGGTGAAGCTGAAAAGTCAACATCACTTTATACACAAGCAAATGTTTTTGCGAAAATG
TTAAAACAGGACGAAGATTATAAATACGATGAAAAAACGAAAGCCGTACATTTAACAGAACAAGGTGCGGATAAAGCTGAACGTA
TGTTCAAAGTTGAAAACTTATATGATGTACAAAATGTTGATGTTATTAGTCATATCAACACAGCTTTACGTGCGCACGTTACATTAC
AACGTGACGTAGACTATATGGTTGTTGATGGCGAAGTACTAATTGTCGATCAATTTACAGGACGTACAATGCCAGGCCGTCGTTTCT
CAGAAGGTTTACACCAAGCTATTGAAGCGAAGGAAGGCGTTCAAATTCAAAATGAATCTAAAACTATGGCGTCTATTACATTCCAA
AACTATTTCAGAATGTACAATAAACTTGCGGGTATGACAGGTACAGCTAAAACTGAAGAAGAAGAATTTAGAAATATTTATAACAT
GACAGTAACTCAAATTCCGACAAATAAACCTGTGCAACGTAACGATAAGTCTGATTTAATTTACATTAGCCAAAAAGGTAAATTTG
ATGCAGTAGTAGAAGATGTTGTTGAAAAACACAAGGCAGGGCAACCAGTGCTATTAGGTACTGTTGCAGTTGAGACTTCTGAATAT
ATTTCAAATTTACTTAAAAAACGTGGTATCCGTCATGATGTGTTAAATGCGAAAAATCATGAACGTGAAGCTGAAATTGTTGCAGGC
GCTGGACAAAAAGGTGCCGTTACTATTGCCACTAACATGGCTGGTCGTGGTACAGATATCAAATTAGGTGAAGGCGTAGAGGAATT
AGGCGGTTTAGCAGTAATAGGTACAGAACGACATGAATCTCGTCGTATTGATGACCAGTTACGTGGTCGTTCTGGACGTCAAGGTG

ATAAAGGGGATAGTCGCTTCTATTTATCATTACAAGATGAATTAATGATTCGTTTTGGTTCTGAACGTTTACAGAAAATGATGAGCC
GACTAGGTTTAGATGACTCTACACCAATTGAATCAAAAATGGTATCAAGAGCTGTAGAATCAGCACAAAAACGTGTAGAAGGTAAT
AACTTCGACGCGCGTAAACGTATCTTAGAATACGATGAAGTATTACGTAAACAACGTGAAATTATCTATAACGAAAGAAATAGTAT
TATTGATGAAGAAGACAGCTCTCAAGTTGTAGATGCAATGCTACGTTCAACGTTACAACGTAGTATCAATTACTATATTAATACAGC
AGATGACGAGCCTGAATATCAACCATTCATCGACTACATTAATGACATCTTCTTACAAGAAGGTGACATTACAGAGGATGATATCA
AAGGTAAAGATGCTGAAGATATTTTCGAAGTCGTTTGGGCTAAGATTGAAGCAGCATATCAAAGTCAAAAAGATATCTTAGAAGAA
CAAATGAATGAGTTTGAGCGTATGATTTTACTTCGTTCTATTGATAGCCATTGGACTGATCATATCGACACAATGGATCAATTACGT
CAAGGTATTCACTTACGTTCTTATGCACAACAAAATCCATTACGTGACTATCAAAATGAAGGTCATGAATTATTTGATATCATGATG
CAAAATATTGAAGAAGATACTTGTAAATTCATTTTAAAATCTGTAGTACAAGTTGAAGATAATATTGAACGTGAAAAAACAACAGA
GTTTGGTGAAGCGAAGCACGTTTCAGCTGAAGATGGTAAAGAAAAAGTGAAACCGAAACCAATCGTTAAAGGCGATCAAGTTGGT
CGTAACGATGATTGTCCATGTGGTAGTGGTAAAAAATTCAAAAATTGCCATGGAAAATAAATGATATAAAATAACTTCTTCCAATTA
AACACCTATAGTTTGTGTTATGGGAGGAGTCTTTTTATTTTACAAGCGTTAAATACTTTAAAAAATGTGAAGAAGTTGTTAAACGTT
GTTATGTACTTAGTTTTAAAAAATCGGTTTAGGCATATGTCGATGATAAATGTACTGATTTTTAACAATAAATGCATAAACTAATTGT
CAGTGTGCTTATATTTCTTAACATTGTTATTTAACAAAATTATGTTAAAATTTAGCATTATAAAAGATGCAAATCAATGACTTGAATT
GAAATATAAATAGGAGCGAATGCTATGGAATTATCAGAAATCAAACGAAATATAGATAAGTATAATCAAGATTTAACACAAATTAG
GGGGTCTCTTTGACTTAGAGAACAAAGAAACTAATATTCAAGAATATGAAGAAATGATGGCAGAACCTAATTTTTGGGATAACCAA
ACGAAAGCGCAAGATATTATAGATAAAAATAATGCGTTAAAAGCAATAGTTAATGGTTATAAAACACTACAAGCAGAAGTAGATG
ACATGGATGCTACTTGGGATTTATTACAAGAAGAATTTGATGAAGAAATGAAAGAAGACTTAGAGCAAGAGGTCATTAATTTTAAG
GCTAAAGTGGATGAATACGAATTGCAATTATTATTAGATGGGCCTCACGATGCCAATAACGCAATTCTAGAGTTACATCCTGGTGCA
GGTGGCACGGAGTCTCAAGATTGGGCTAATATGCTATTTAGAATGTATCAACGTTATTGTGAGAAGAAAGGCTTTAAAGTTGAAAC
TGTTGATTATCTACCTGGGGATGAAGCAGGGATTAAAAGTGTAACATTGCTCATCAAAGGGCATAATGCTTATGGTTATTTAAAAGC
TGAAAAAGGTGTACACCGACTAGTACGAATTTCTCCATTTGATTCATCAGGACGTCGTCATACATCATTTGCATCATGTGACGTGAT
TCCAGATTTTAATAATGATGAAATAGAGATTGAAATCAATCCGGATGATATTACAGTTGATACATTTAGAGCTTCTGGTGCAGGTGG
TCAGCATATTAACAAAACTGAATCAGCAATACGAATTACCCACCACCCCTCAGGTATAGTTGTTAATAACCAAAATGAACGTTCTCA
AATTAAAAACCGTGAAGCAGCTATGAAAATGTTAAAGTCTAAATTATATCAATTAAAATTGGAAGAGCAGGCACGTGAAATGGCTG
AAATTCGTGGCGAACAAAAAGAAATTGGCTGGGGAAGCCAAATTAGATCATATGTTTTCCATCCATACTCAATGGTGAAAGATCAT
CGTACGAACGAAGAAACAGGTAAGGTTGATGCAGTGATGGATGGAGACATTGGACCATTTATCGAATCATATTTAAGACAGACAAT
GTCGCACGATTAATATATATTTTAAAACCGAGGCTCTAAAAGGGCGTCGGTTTTTGGTTTTTTTAAAGGTAGCTAAATAAATTGTAA
ATTAGATTTTGGAATATGATTTGTTTATGAATATTTAAGTACAATTCGGTAGATAGAGTTAGAATATATTTTTTAAAAGTTGTGTTTG
TTAAAACAACATATGCAGTGTGCATTTAGTAATATTACCTATGGCGATTTTCAAGGTATTGAATTAATTATTGAAAACGTTCTCAATT
ACATGGTATGAATACATTTTACACTATGATAAAAGGTTGTATTCTTTTTATATTGTTAACCATTTGATTACATCGTTATAACAATAGC
TTTTGACAAAATGTATTGTGCTATAGTATTTGCATACTTAAAATACTAACAGCAAAGGAATGACAGCAAGATGAAAAAATCTCTTAC
AGTGACGGTTTCGTCAGTGTTAGCTTTTTTAGCTTTAAATAATGCAGCACATGCACAACAACATGGCACACAAGTAAAAACACCTGT
TCAACATAATTATGTATCAAATGTTCAAGCACAAACGCAATCACCGACAACTTATACAGTAGTTGCTGGCGATTCATTATATAAGAT
TGCTTTAGAGCATCACTTAACGTTGAATCAATTATATTCATACAATCCTGGTGTAACACCTTTAATTTTTCCTGGTGACGTGATTTCA
CTTGTGCCTCAAAATAAAGTGAAACAAACTAAAGCGGTTAAATCACCAGTAAGAAAAGCAAGCCAAGCTAAAAAGGTAGTAAAAC
AACCTGTACAACAAGCATCTAAAAAAGTAGTAGTTAAGCAAGCACCTAAGCAAGCAGTAACTAAGACAGTTAATGTAGCATACAA
ACCTGCTCAAGTACAAAAATCAGTACCAACTGTACCTGTTGCACATAACTACAATAAATCAGTTGCTAACAGAGGAAACTTATATG
CTTATGGAAACTGCACATATTATGCTTTCGATCGTCGTGCACAATTAGGTAGAAGTATAGGAAGTTTATGGGGCAATGCAAATAACT
GGAATTACGCAGCAAAAGTTGCAGGATTTAAAGTAGATAAAACACCAGAAGTTGGCGCTATTTTCCAAACAGCTGCTGGCCCATAT
GGACATGTTGGTGTTGTTGAATCTGTAAACCCTAATGGAACAATTACTGTTTCTGAAATGAACTATGCTGGATTTAATGTTAAATCTT
CAAGAACAATTTTAAATCCAGGAAAATATAATTACATCCACTAAGTAATATATCAAGACAAGACTATCCTCTTAGCCTGTTTAAGTA
ACAGGTTGAGAGGATTTTTTGGTATCATTTAATCAGAGTTATATAAAGAAGATATTTAAATGATATTTAATTGATTGATATGTAAAA
AGAAAGTATAATTTATGATTAATTAATGGAGGAGGTAATTGAAATGGGTGTACATCAATATTTTAAAAGATTATCAGATATGGAAA
GACTTATAAGATTACCTGGAAAATTTAAATATTTTGAACACAATGTTGCAGCACACTCCTTTAAAGTAACTAAAATTGCTCAATATC
TAGCAACAGTTGAAGAATATCATGGACGAAAGATTAATTGGAAAAGCTTATATGAAAAAGCATTAAATCATGATTTCGCCGAAGTG
TTTACTGGTGATATAAAAACACCTGTTAAATATGCGAGTAGTGAATTAAAAAAATTATTTTCGCAAGTTGAAGAAGAAATGGTAGA
GACCTTTATTGAAGAAGAAATTCCATTACAATATAGAGATGTTTATAAGCAACGACTGCAAGAAGGTAAAGATGATTCATTAGAAG
GCCAAATACTTTCAGTTGCTGATAAAATTGATTTGCTTTATGAAACATTTGGAGAAATACAAAAACGTAATCCCGAAGAATTATTTT
TCGAAATTTATGAAATGAGTCTAGAAACAATTATTCAATTTGACCATTTAGCATCTGTACAAGATTTTATTAATAATATCATTCCAGA
AATGTTGACTGAAAACTTTATACCTAGAACAGAATTAAGAGAAACAACCATGAACATTTTAAATAAAAGAAAAGAGGAAAATGAA
TGATATGGTATTTTAGCGCAGCATTCTTTCCATGTGTCTTGGTAGTATTATTTAGTGTAATAACAAGAAGTAAATGGGTCGGTACTAT
TCTGACATTAATTTTAATTGGTGCCTCAATCTATAAAGAGTATTTCCATAACGAGTGGATTATTTTTATTGATGTAGTGTCATTATTA
GCTGGTTATTTAATTATAGATCAACTCGAATTTCATAAACATCAAGATGAAGATCGCTAAGATTAACTTTAAAATAATGTTTCAAAC
AAATTTGTTGAAACAAAATGATGATTAATATAATGTGTATTTACATACTAAAAATAACAAGATAAACGATTTGATTTAAGGCAAGC
ATAGTTAGCACAACTATGTTTGTTTTTCTTTGTTCGACATTTTTACGAACAAACGTTTGCTTTTTGTGTGACTACTTTGCTAAAATATG
TAATGAGAAAGCAAAGAGTTGAGCGGAAATATAAATAATACGTTGAAAGAGGAGGCATATGTGACAATGGTTGAACATTATCCTTT
TAAAATACATTCTGATTTTGAGCCTCAAGGTGACCAACCGCAAGCAATTAAAGAAATCGTGGAAGGTATTAAAGCGGGGAAAAGAC
ATCAAACTTTATTAGGTGCTACTGGCACAGGGAAAACATTTACGATGAGTAATGTTATTAAAGAAGTTGGGAAACCAACGTTAATT
ATCGCGCATAACAAAACATTAGCAGGACAATTATATAGTGAGTTTAAAGAATTTTTTCCTGAAAACAGGGTGGAATACTTTGTAAGT
TACTATGATTATTATCAACCAGAGGCATACGTACCGTCTACTGACACTTTTATTGAAAAAGATGCCTCAATCAATGATGAAATTGAT
CAACTACGACATTCTGCTACAAGTGCATTATTTGAACGCGATGATGTAATTATTATTGCTAGTGTAAGTTGTATATATGGTTTAGGTA
ATCCTGAAGAATATAAAGATTTAGTAGTAAGTGTTCGAGTTGGTATGGAAATGGATAGAAGTGAATTACTTAGAAAACTTGTAGAT
GTGCAATATACACGAAATGACATCGATTTCCAACGAGGAACGTTTCGAGTGCGTGGTGATGTAGTGGAAATATTCCCAGCCTCTAA
AGAAGAACTTTGTATAAGGGTTGAGTTTTTCGGCGATGAGATTGACCGTATCCGAGAAGTTAACTACCTAACAGGTGAAGTGTTGA
AAGAAAGAGAACATTTTGCGATATTCCCAGCTTCTCACTTCGTAACACGTGAAGAAAAGTTGAAAGTTGCGATTGAACGTATTGAA
AAAGAATTGGAAGAACGATTGAAAGAATTACGAGATGAGAATAAATTACTAGAAGCGCAAAGGTTAGAACAGCGTACCAACTATG
ATTTAGAAATGATGCGAGAGATGGGATTCTGTTCAGGAATTGAAAACTATTCCGTACATTTAACTTTGCGACCACTGGGTTCGACAC
CATATACTTTATTGGATTACTTTGGCGATGATTGGTTAGTAATGATTGATGAATCACATGTGACATTACCGCAAGTTCGAGGCATGT
ATAACGGAGACAGAGCGCGTAAACAAGTTTTGGTGGATCATGGGTTTAGATTACCGAGTGCATTAGATAACCGTCCACTTAAATTT
GAAGAATTTGAAGAAAAGACAAAACAACTTGTGTATGTATCTGCAACGCCTGGACCATACGAAATTGAACATACGGATAAGATGGT

TGAACAAATTATTCGTCCTACTGGTTTACTGGATCCTAAGATTGAGGTTAGACCTACTGAAAATCAAATTGACGATTTATTAAGTGA
AATTCAAACAAGAGTTGAGCGTAATGAACGCGTACTTGTTACAACGCTCACTAAAAAGATGAGTGAAGATTTAACCACATACATGA
AAGAAGCGGGTATTAAAGTTAATTATCTGCATTCAGAAATCAAGACATTAGAACGAATCGAAATAATTAGAGACTTACGAATGGGT
ACATATGATGTTATCGTAGGTATTAATTTATTAAGAGAGGGTATTGATATACCAGAAGTTTCTCTAGTTGTCATATTAGATGCAGAT
AAAGAAGGGTTTTTACGTTCTAACCGCTCATTAATTCAAACAATAGGTAGAGCTGCGCGTAACGATAAAGGTGAAGTCATTATGTAT
GCCGATAAAATGACTGATTCGATGAAGTATGCAATTGATGAGACACAACGTCGTCGAGAAATACAGATGAAACATAATGAAAAAC
ATGGTATTACACCTAAAACAATTAATAAAAAAATACATGATTTAATTAGTGCTACTGTTGAAAATGACGAAAATAATGACAAAGCA
CAAACTGTGATACCTAAGAAGATGACGAAAAAAGAACGTCAAAAGACAATCGACAATATAGAAAAAGAAATGAAACAAGCAGCG
AAAGATTTAGATTTCGAGAAAGCTACAGAATTAAGAGATATGTTATTTGAATTAAAAGCAGAAGGGTGACAAGTAAATGAAAGAA
CCATCCATAGTAGTAAAAGGTGCTCGTGCGCATAACTTGAAAGATATTGATATCGAACTACCTAAAAATAAATTAATTGTTATGACA
GGTTTATCTGGGTCAGGTAAATCGTCATTAGCATTCGATACTATATATGCTGAAGGACAACGACGTTATGTTGAATCATTAAGTGCC
TATGCGCGTCAATTTTTAGGCCAAATGGACAAACCAGATGTTGATACAATTGAAGGATTATCGCCAGCAATTTCAATAGATCAAAA
AACAACAAGTAAAAATCCAAGATCAACTGTAGCAACAGTAACAGAAATATATGATTATATACGTTTGTTATATGCACGTGTTGGTA
AACCTTACTGTCCAAATCACAATATAGAAATTGAATCGCAAACAGTACAACAAATGGTTGACCGCATTATGGAATTAGAGGCACGT
ACAAAGATTCAATTATTAGCACCTGTCATCGCTCATCGTAAAGGTAGTCATGAAAAGCTAATCGAAGATATTGGTAAAAAAGGTTA
TGTACGTTTAAGAATCGATGGCGAAATTGTTGATGTAAATGATGTACCTACTTTAGATAAGAACAAGAATCATACAATAGAAGTTGT
TGTAGACCGATTAGTTGTTAAAGATGGAATTGAAACACGACTAGCTGACTCTATAGAAACTGCGTTAGAGCTTTCAGAAGGACAAT
TAACAGTTGATGTCATTGACGGGGAAGACCTTAAGTTTTCAGAAAGCCATGCTTGTCCTATATGTGGATTTTCAATCGGAGAGTTAG
AACCAAGAATGTTTAGCTTTAACAGTCCTTTTGGTGCTTGTCCGACATGTGATGGCTTAGGCCAAAAGTTAACAGTCGATGTAGACT
TGGTTGTTCCCGACAAAGATAAGACGCTAAACGAAGGTGCAATAGAACCTTGGATACCGACGAGTTCTGATTTTTATCCAACATTGT
TAAAACGTGTTTGTGAAGTTTATAAAATCAATATGGATAAACCTTTTAAAAAGTTAACAGAACGTCAACGTGATATATTATTGTATG
GTTCGGGTGACAAAGAAATTGAATTTACATTTACACAACGTCAAGGTGGTACTAGAAAACGAACAATGGTTTTCGAGGGTGTAGTT
CCTAATATAAGTAGACGATTCCATGAATCTCCTTCAGAATATACACGTGAAATGATGAGTAAATATATGACCGAACTACCTTGTGAA
ACTTGTCATGGAAAGCGATTGAGTCGTGAAGCTTTATCTGTTTATGTAGGTGGTTTAAATATTGGTGAAGTAGTCGAATACTCAATC
AGTCAAGCGCTGAACTATTATAAAAACATTGATTTGTCAGAACAAGATCAAGCGATTGCAAATCAAATATTGAAAGAAATTATTTC
CCGACTCACTTTTTTAAATAATGTGGGACTTGAATATTTAACGCTAAACAGAGCTTCAGGTACACTTTCAGGTGGTGAAGCACAACG
TATTCGATTGGCAACGCAAATTGGGTCGCGTTTGACTGGTGTCTTATATGTATTAGATGAGCCATCAATTGGACTGCATCAAAGAGA
TAATGATCGATTAATTAATACACTTAAAGAAATGAGAGATTTAGGAAATACTTTAATTGTAGTTGAACACGATGATGATACAATGC
GTGCGGCTGATTACTTAGTGGACATAGGGCCTGGTGCTGGTGAACATGGAGGGCAGATTGTGTCTAGTGGGACACCTCAAAAGGTA
ATGAAAGATAAAAAATCATTAACAGGACAATACTTGAGTGGTAAGAAACGTATTGAAGTACCTGAATATCGCAGACCGGCTTCAGA
TCGTAAAATTTCTATACGTGGAGCTAGAAGCAACAATCTTAAAGGGGTTGATGTGGACATACCACTATCAATCATGACGGTTGTTAC
AGGTGTATCAGGTTCTGGTAAAAGCTCATTAGTAAATGAAGTATTATACAAATCATTAGCTCAAAAAATTAATAAATCTAAAGTAA
AGCCAGGATTGTACGATAAGATTGAAGGTATTGATCAACTTGATAAAATTATTGATATTGATCAATCACCGATAGGTAGAACGCCA
CGCTCTAATCCAGCAACATATACTGGTGTGTTTGATGATATACGTGATGTGTTTGCGCAAACAAATGAAGCTAAAATTCGAGGATAT
CAAAAAGGGCGTTTTAGTTTTAATGTAAAAGGTGGACGCTGTGAAGCTTGTAAAGGTGACGGTATTATTAAAATTGAAATGCATTTT
TTACCTGATGTTTATGTTCCTTGTGAAGTGTGTGATGGTAAACGATATAATCGTGAGACACTAGAGGTTACTTACAAAGGTAAAAAT
ATTGCTGACATTTTAGAAATGACTGTTGAAGAAGCAACACAATTTTTTGAAAATATTCCTAAGATTAAGCGCAAGTTACAAACACTA
GTTGATGTTGGTCTTGGATACGTCACATTAGGTCAACAAGCTACAACGTTATCAGGTGGTGAGGCTCAACGTGTGAAACTTGCATCT
GAACTTCATAAACGTTCAACTGGTAAATCTATTTATATCCTAGATGAACCGACAACAGGGTTACATGTTGACGATATTAGTAGATTA
TTAAAAGTATTAAACCGATTAGTTGAAAATGGTGATACTGTTGTAATTATTGAACATAACCTAGATGTTATCAAAACAGCAGACTAT
ATTATAGACTTAGGTCCTGAAGGTGGTAGTGGCGGTGGTACTATTGTTGCGACTGGCACACCCGAAGATATTGCTCAGACAAAGTC
ATCATATACAGGAAAGTATTTAAAAGAAGTACTTGAACGAGATAAACAAAATACTGAAGATAAATAAGATTAAAAGAAGTGAAGG
ATGTTATAAATTTATCCTTCGCTTCTTTTTATTAATTTAGTAATGAATAGTAGAAAGAAAAGATGCGTAAAAAGAATTATGTTAAGA
TAGGGTCAATCTAGAGTTGCTAAACATAAAGACTTGACTAATCGTAGGAATGGGAGTGGGACAGAAATGATAAAGAATCACTAAC
GATTTATTATGTAGTGGTTCTTTGTCATTAGCCACAGCTATTATGTACTTAAAAATAGGAATGGGAGTGCAATTCATGCATAAGAAA
TACTAATTTCTAAAGAAAAAGTATTTCTTTATGTTGGGGCCCACCCCAACTTGCATTGTTTGTAGAATTTCTTTTCGAAATTCTCTGT
GTTGGGGCCCCGCCACCTAATTCCAATATATCATTGTAGAGCTTAGGTCATTGATTTATGGCTCGGACTTTTATGGCGATATGAACC
ATGTAAATTAAGCAAGCAATAAATTAATGATTGATATTGACTTGTAAAATAATAACAATAATGAACAATTAATATTTATTTTAGCTT
TTCAATGTAGATTGGTGTTATATTTTTGATATGATAAGAAGAGAGATGTAAGAGTAGGGATAAATACAATTGAGGTGAACCCATGTTAA
CGACAGAAAAACTAGTTGAAACATTAAAGTTAGATTTAATCGCTGGTGAAGAAGGACTATCGAAGCCAATTAAAAATGCTGATATA
TCAAGACCGGGCTTAGAGATGGCAGGTTATTTTTCACATTATGCGTCAGATAGAATACAACTATTAGGAACAACGGAACTATCGTTT
TACAATTTATTACCAGATAAGGATCGCGCAGGTCGTATGCGTAAACTATGCAGACCAGAAACGCCTGCAATTATTGTGACGCGTGG
ATTGCAGCCACCAGAAGAATTAGTTGAAGCTGCAAAAGAATTAAATACCCCACTTATAGTTGCTAAAGATGCGACTACAAGTTTAA
TGAGTCGCTTAACAACGTTTTTAGAGCATGCACTTGCAAAGACGACATCTTTACATGGTGTTTTAGTAGATGTTTACGGTGTTGGTGT
ACTAATTACCGGTGATTCAGGAATAGGTAAAAGTGAGACTGCGTTGGAATTAGTTAAACGTGGGCATAGATTAGTAGCAGATGATA
ATGTAGAAATACGTCAAATTAATAAAGATGAACTAATAGGGAAACCACCAAAGTTAATAGAACATCTATTAGAAATACGTGGACTA
GGTATTATCAATGTTATGACTTTATTTGGCGCGGGTTCAATATTAACTGAAAAACGAATTAGATTAAATATTAATTTGGAAAACTGG
AACAAGCAAAAGTTATATGACCGCGTAGGTCTTAATGAAGAGACGCTAAGTATTTTAGATACTGAAATCACTAAAAAAACAATACC
TGTAAGACCTGGTAGAAATGTTGCGGTAATTATTGAGGTCGCTGCAATGAACTATCGATTAAATATCATGGGCATTAACACTGCCGA
AGAATTTAGTGAAAGATTAAATGAAGAAATTATCAAGAACAGTCATAAGAGTGAGGAGTAGGTTGAATGGGTATTGTATTTAACTA
TATAGATCCTGTGGCATTTAACTTAGGACCACTGAGTGTACGATGGTATGGAATTATCATTGCTGTCGGAATATTACTTGGTTACTTT
GTTGCACAACGTGCACTAGTTAAAGCAGGATTACATAAAGATACTTTAGTAGATATTATTTTTTATAGTGCACTATTTGGATTTATCG
CGGCACGAATCTATTTTGTGATTTTCCAATGGCCATATTACGTGGAAAATCCAAGTGAAATTATTAAAATATGGCATGGTGGAATAG
CAATACATGGTGGTTTAATAGGTGGCTTTATTGCTGGTGTTATTGTATGTAAAGTGAAAAATTTAAACCCATTTCAAATTGGTGATAT
CGTTGCGCCAAGTATAATTTTAGCGCAAGGAATTGGACGCTGGGGTAACTTTATGAATCACGAGGCACATGGTGGACCTGTGTCAC
GCGCTTTTTTAGAACAATTACATTTGCCTAATTTTATAATAGAAAATATGTATATTAACGGCCAATATTATCATCCAACATTCTTATA
TGAATCCATTTGGGATGTCGCTGGATTTATTATTTTAGTTAATATTCGTAAACATTTAAAATTAGGAGAAACATTCTTTTTATATTTA
ACTTGGTATTCAATTGGTCGATTCTTTATAGAAGGATTACGTACAGATAGCTTAATGCTCACAAGTAATATTAGAGTTGCACAATTA
GTATCAATTCTTTTAATTTTAATAAGTATAAGTTTAATTGTATATAGAAGGATTAAGTATAATCCACCGTTGTATAGCAAAGTTGGG
GCGCTTCCATGGCCAACAAAAAAAGTGAAGTAGTGATATTTTGAGAAAATTTTTATCAAAAACACATCATCACACAAACCCTTTATG
GCGTGTATACCGTCTTGTTAAATTTTCGAAAGTTTTTAAGAATGTAATTATCATTGAATTTTCGAAATTTATTCCAAGTATGGTACTG

AAAAGACATATATATATAAACAACTTTTAAATATTAATATCGGTAATCAATCGTCGATAGCTTATAAAGTAATGTTAGATATTTTTT
ACCCAGAACTGATTACGATTGGTAGTAACAGTGTTATTGGTTACAATGTAACAATTTTGACGCATGAAGCATTAGTTGATGAATTTC
GTTATGGACCAGTGACGATAGGATCTAACACTTTGATTGGTGCAAATGCTACCATTTTACCCGGTATAACGATTGGTGACAATGTAA
AAGTTGCAGCTGGTACGGTTGTTTCAAAAGATATACCGGATAATGGATTTGCATATGGCAACCCTATGTATATAAAAATGATTAGGA
GGTGACAATTTTATGGCGCAAAAGAATAATAATGTAATTCCAATGACTTTTGATGATGCATTTTATCGTAAAATGGCTAAACAGAAG
TTTAAACAAAGAGAATATAAACGAGCTGCTGAATACTTTGAAAAAGTGTTAGAATTGTCACCTGATGATCTGGAAATTCAAATTGA
TTATGCACAATGTCTAGTGCAACTTGGTATTGCTAAAAAAGCAGAACATTTATTTTATGACAATATTATTTATAATAGGCATCTAGA
AGATAGCTTTTATGAATTGAGTCAGCTCAACATTGAAGTTAACGAACCAAACAAGGCATTCTTGTTTGGTATTAATTATGTTATTGTT
AGCGACGACCAAGATTATAGAGATGAATTAGATCAAATGTTTGATGTGAAATATCAAAGCGAAGAACAAATTGAACTTGAAGCTCA
ATTGTTTGTAGTTCAAATACTATTCCAATATCTTTTTTCTCAAGGTCGATTAAAAGATGCAAAGAATTATGTCTTACATCAACCACAA
GAAGTTCAAGATCATCGTGTCGTACGTAATTTATTGGCAATGTGTTATTTATATCTCGGTGAATATGATACGGCTAAAGCATTGTAC
GAAGCACTATTACAAGAGGATAGTACAGATATATATGCATTATGCCATTATACTTTGCTACTTTATAACACTAAGGAAAATGAACAA
TATCAAAAATATTTAAAAATATTAAACAAAGTTGTACCTATGAATGACGATGAAAGTTTTAAATTAGGTATTGTATTAAGTTATTTA
AAGCAGTATCGTGCATCACAACAATTGTTGTACCCTTTATATAAAAAAGGGAAATTTTTATCAATTCAAATGTACAATGCTTTAGCA
TATAATTATTATTATTTAGGTGAAGAAGACGAAAGTCATTACTACTGGGATAAATTGAAGCAAATTTCTAAAGTGGAAATTGGACAT
GCGCCTTGGGTAATTGAAAATAGCAAAGAAGTTTTTGACCAACATATTTTGCCATTACTTCAAAGTGATGACAGTCATTATCGTTTA
TATGGTATTTTTTTATTGGATCAATTAAATGGTAAAGAAATTGTGATGACGGAAAGTATTTGGCAGGTTCTGGAAAATCTAAATAAT
TATGAGAAATTGTATTTAACGTATTTAGTTCAAGGTTTAACGCTCAATAAATTAGACTTCATTCATCGCGGCTTGTTAACGCTTTACC
AAAATGAATTATTTGTAAGTGAAAATGATTTAATGGTTGCATGGATTAATCAAGGTGAACTCATAATTGCTGAAAAAGTAGATTTAA
CTGATGTTGAGCCATATATCGGTGCGTTTATTTATTTGTATTTTAAAAATCAACCTCGAAACGTTACAAAGAAGCAAATTACAACAT
GGTTAGGCATAACACAATATAAACTGAACAAAATGATTGAATTTCTCTTGAGCATATAGATTTATGAAAAGTTAGATTTATTATATA
ATGCGCATAATGATTAATAATGAGGAGGCGTTAATAAAATGACTGAAATAGATTTTGATATAGCAATTATCGGTGCAGGTCCAGCT
GGTATGACTGCTGCAGTATACGCATCACGTGCTAATTTAAAAACAGTTATGATTGAAAGAGGTATTCCAGGCGGTCAAATGGCTAA
TACAGAAGAAGTAGAGAACTTCCCTGGTTTCGAAATGATTACAGGTCCAGATTTATCTACAAAAATGTTTGAACACGCTAAAAAGT
TTGGTGCAGTTTATCAATATGGAGATATTAAATCTGTAGAAGATAAAGGCGAATATAAAGTGATTAACTTTGGTAATAAAGAATTA
ACAGCGAAAGCGGTCATTATTGCTACAGGTGCAGAATACAAGAAAATTGGTGTTCCGGGTGAACAAGAACTTGGTGGACGCGGTGT
AAGTTATTGTGCAGTATGTGATGGTGCATTCTTTAAAAATAAACGCCTATTCGTTATCGGTGGTGGTGACTCAGCAGTAGAAGAGGG
AACATTCTTAACTAAATTTGCTGACAAAGTAACAATCGTTCACCGTCGTGATGAGTTACGTGCACAACGTATTTTACAAGATAGAGC
ATTCAAAAATGATAAAATCGACTTTATTTGGAGTCATACTTTGAAATCAATTAATGAAAAAGACGGCAAAGTGGGTTCTGTGACATT
AACGTCTACAAAAGATGGTTCAGAAGAAACACACGAGGCTGATGGTGTATTCATCTATATTGGTATGAAACCATTAACAGCGCCAT
TTAAAGACTTAGGTATTACAAATGATGTTGGTTATATTGTAACAAAAGATGATATGACAACATCAGTACCAGGTATTTTTGCAGCAG
GAGATGTTCGCGACAAAGGTTTACGCCAAATTGTCACTGCTACTGGTGATGGCAGTATTGCAGCGCAAAGTGCAGCGGAATATATT
GAACATTTAAACGATCAAGCTTAATTCGAGGTCGAATTAAGATGTTGAGTTGTAAGTTATTTGGATATTTATTTTAATAGTGTCATCA
CATTGTTAAAATAATGTCTTACTTTTAAATTAAAGCAAATTATATAGAAAACTAGAACTTAGTACGTATCATTTGTGCGTTTCAATGA
GTTCTAGTTTTTTTATATGTTATATTAAACTTATAACTTTATGGGAGTGGGACAGAAATGATAAAGAGCCACTAATGATTTATTATGT
AGTGGTTCTTACACATTAGCCACAGCTAATGTGTA

>023-contig_244_RC

TCTGTGTTGGGGCCCCTGACTAGAGTTGAAAAAAGCTTGTTGCAAGCGCGTTTTCATTCAGTCAACTACTAGCAATATAAAATTATA
GACCCTAGGACATTGATTTATGTCCCAAGCTCCTTTTAAATGATGTATATTTTTAGAAATTTAATCTAGACATAGTTGGAAATAAATA
TAAAACATCGTTGCTTAATTTTGTCATAGAACATTTAAATTAACATCATGAAATTCGTTTTGGCGGTGAAAAAATAATGGATAATAA
TGAAAAAGAAAAAAGTAAAAGTGAACTATTAGTTGTAACAGGTTTATCTGGCGCAGGTAAATCTTTGGTTATTCAATGTTTAGAAG
ACATGGGATATTTTTGTGTAGATAATCTACCACCAGTGTTATTGCCTAAATTTGTAGAGTTGATGGAACAAGGAAATCCATCCTTAA
GAAAAGTGGCAATTGCAATTGATTTAAGAGGTAAGGAACTATTTAATTCATTAGTTGCAGTAGTGGATAAAGTTAAAAGTGAAAGT
GACGTCATCATTGATGTTATGTTTTTAGAAGCAAGTACTGAAAAATTAATTTCAAGATATAAGGAAACGCGTCGTGCACATCCTTTG
ATGGAACAAGGTAAAAGATCCTTAATCAATGCAATTAATGATGAGCGAGAGCATTTGTCTCAAATTAGAAGTATAGCTAATTTTGTT
ATAGATACTACAAAGTTATCACCTAAAGAATTAAAAGAACGCATTCGTCGATACTATGAAGATGAAGAGTTTGAAACTTTTACAAT
TAATGTCACAAGTTTCGGTTTTAAACATGGGATTCAGATGGATGCAGATTTAGTATTTGATGTACGATTTTTACCAAATCCATATTAT
GTAGTAGATTTAAGACCTTTAACAGGATTAGATAAAGACGTTTATAATTATGTTATGAAATGGAAAGAGACGGAGATTTTCTTTGAA
AAATTAACTGATTTGTTAGATTTTATGATACCCGGGTATAAAAAAGAAGGGAAATCTCAATTAGTAATTGCCATCGGTTGTACGGGT
GGACAACATCGATCTGTAGCATTAGCAGAACGACTAGGTAATTATCTAAATGAAGTATTTGAATATAATGTTTATGTGCATCATAGG
GACGCACATATTGAAAGTGGCGAGAAAAAATGAGACAAATAAAAGTTGTACTTATCGGTGGTGGCACTGGCTTATCAGTTATGGCT
AGGGGATTAAGAGAATTCCCAATTGATATTACAGCGATTGTAACAGTTGCTGATAATGGTGGGAGTACAGGGAAAATCAGAGATGA
AATGGATATACCAGCACCAGGAGACATCAGAAATGTGATTGCAGCTTTAAGTGATTCTGAGTCAGTTTTAAGCCAACTTTTTCAGTA
TCGCTTTGAAGAAAATCAAATTAGCGGTCACTCATTAGGTAATTTATTAATCGCAGGTATGACTAATATTACGAATGATTTCGGACA
TGCCATTAAAGCATTAAGTAAAATTTTAAATATTAAAGGCAGAGTCATTCCATCTACAAATACAAGTGTGCAATTAAATGCTGTTAT
GGAAGATGGAGAAATTGTTTTTGGAGAAACAAATATTCCTAAAAAACATAAAAAAATTGATCGTGTGTTTTTAGAACCTAACGATG
TGCAACCAATGGAAGAAGCAATCGATGCTTTAAGGGAAGCAGATTTAATCGTTCTTGGACCAGGGTCATTATATACGAGCGTTATTT
CTAACTTATGTGTGAATGGTATTTCAGATGCGTTAATTCATTCTGATGCGCCTAAGCTATATGTTTCTAATGTGATGACGCAACCTGG
GGAAACAGATGGTTATAGCGTGAAAGATCATATCGACGCGATTCATAGACAAGCTGGACAACCGTTTATTGATTATGTCATTTGTAG
TACACAAACTTTCAATGCTCAAGTTTTGAAAAAATATGAAGAAAAACATTCTAAACCAGTTGAAGTTAATAAGGCTGAACTTGAAA
AAGAAAGCATAAATGTAAAAACATCTTCAAATTTAGTTGAAATTTCTGAAAATCATTTAGTAAGACATAATACTAAAGTGTTATCGA
CAATGATTTATGACATAGCTTTAGAATTAATTAGTACTATTCCTTTCGTACCAAGTGATAAACGTAAATAATATAGAACGTAATTAT
ATTATGATATGATAATAGAGCTGTGAAAAAAATGAAAATAGACAGTGGTTCTAAGGTGAATCATGTTTTAAATAAGAAAGGAATGA
CTGTACGATGAGCTTTGCATCAGAAATGAAAAATGAATTAACTAGAATAGACGTCGATGAAATGAATGCAAAAGCAGAGCTCAGTG
CACTGATTCGAATGAATGGTGCACTTAGTCTTTCAAATCAACAATTTGTTATAAATGTTCAAACGGAAAATGCAACAACGGCAAGA
CGTATTTATTCGTTGATTAAACGTGTCTTTAATGTGGAAGTTGAAATATTAGTCCGTAAAAAAATGAAACTTAAAAAAAATAATATT
TATATTTGTCGTACAAAGATGAAAGCGAAAGAAATTCTTGATGAATTAGGAATTTTAAAAGACGGCATTTTTACGCATGAAATTGAT
CATTCAATGATTCAAGATGACGAAATGAGACGCAGTTACTTGAGAGGAGCTTTTCTGGCAGGTGGCTCAGTGAATAACCCTGAAAC
ATCTTCGTATCATTTGGAAATTTTTTCTCAAAATGAGAGTCATGCAGAAGGCTTAACGAAACTAATGAATAGTTATGAGTTGAATGC

CAAACATTTAGAGCGAAAAAAAGGAAGTATTACGTATTTAAAAGAAGCGGAAAAGATTTCGGATTTTCTTAGTTTGATAGGTGGCT
ATCAAGCGTTATTAAAATTTGAAGACGTACGTATTGTAAGAGATATGCGTAATTCTGTTAACCGACTCGTTAATTGTGAAACGGCAA
ATCTAAATAAAACAGTTAGTGCTGCGATGAAACAAGTTGAGAGCATTAAATTGATTGATAAAGAAATTGGTATTGAAAATTTACCA
GACAGGTTGAGAGAGATTGCTAGAATTCGAGTAGAACATCAAGAAATTTCGTTGAAAGAGCTTGGAGAAATGGTATCAACTGGTCC
AATTTCAAAATCAGGTGTAAATCACCGATTAAGAAAACTGAATGATTTAGCCGATAAGATTAGAAATGGTGAACAAATAGAATTAT
AAGTAAGAAGGTGTTTTTGGTAGTGAATTATCAAAAATACCTTTTTTATGATTAAAAAGTGTTTAATGAATAATAGTCGACTAACTA
TTAAATTGAGCTGGAAAGTTTAATGAAGGAAATTCAATAACCAAGCGTTACCTAATTTTCTCATTAAGTAGCATCATATGTATCAAT
TTGGTCATAAGAAAATATGAATGTGATAATTAATTGAGTCACTGAAAGTCCCTGATCATAGACAAAAACAAAATCAGCATAAATAA
GAATCCCGACATTGCGGGATTCTCAGTATTGAAAAGTATTGTATTTTATTAAAACAGCCTCCTTGAAGGGAATTGAACCCCTATCTT
AAGAACCGGAATCTTACGTGTTATCCATTACACCACAAGGAGCAAATATGTAATCATATCTCTGTACTATGAGAAGTAAAATAAGT
ACAATCTAAAATAGATATTATCAGTTTACAAAGGAAAGAGAAAAGCGTCAAACAATGTAACTATTTAAAGTCAAAGTGTTTGACCA
AATTTGACTTAATATGTAAAATAATGAGTAACAGTTATTACAAGGAGGAAATATAGATGAATTTAATTCCTACAGTTATTGAAACAA
CAAACCGCGGTGAACGTGCATATGATATATACTCACGTTTATTAAAAGACCGTATTATTATGTTAGGTTCACAAATTGATGACAACG
TAGCAAATTCAATCGTATCACAGTTATTATTCTTACAAGCGCAAGACTCAGAGAAAGATATTTATTTATACATTAATTCACCAGGTG
GAAGTGTAACAGCTGGTTTTGCGATTTATGATACAATTCAACACATTAAACCTGATGTTCAAACAATTTGTATCGGTATGGCTGCAT
CAATGGGATCATTCTTATTAGCAGCTGGTGCAAAAGGTAAACGTTTCGCGTTACCAAATGCAGAAGTAATGATTCACCAACCATTAG
GTGGTGCTCAAGGACAAGCAACTGAAATCGAAATTGCTGCAAATCACATTTTAAAAACACGTGAAAAATTAAACCGCATTTTATCA
GAGCGTACTGGTCAAAGTATTGAAAAAATACAAAAAGACACAGATCGTGATAACTTCTTAACTGCAGAAGAAGCTAAAGAATATG
GCTTAATTGATGAAGTGATGGTACCTGAAACAAAATAATTCAAAGTAAAGAGTAGACTAAGCAGTCTGCTCTTTTTGTATGAGTAA
ACCGAGGTGTCAATAAATTGTTTACTATACTTTGAGCGGAAATATGATTGAATGAAGCTAGTTGAACCGTAACTATATGAAATGTTC
CCTTCAAAGTAGACATTGAAAGGAACATTTCAATCCTTTGTTTGTAAGTCGCTCTAGACATTACATTTAGTACATATGTTGTTTCTAA
TGCTCATTAATGGTATTGATTATTCTTTGATTAAATCTTCAAGTGCCATTTTTAAATTACTATATTTAAATTGGAATCCCAATGCTTGA
ATTTTATTAGGTAATACTTTTTGAGTATCCAATACTACTGTTGACATTTGACCAAGTATGAGACGCATTGCAAGACTTGGTGCCCAA
GTTTCATGGGGCTTATGCATAGCTCTTGCTAAAGTGTAGCCAAATAAATTTTGACGCTCAGGTATAGGTGCAGTTAAATTAAACGGA
CCACTAGCTGACTCGTTATTTATTAAAAATAAAATAGCTTGAATTAAATCATTGATATGAATCCATGAATACCATTGTTGACCAGAA
CCTAATTTACCACCAATGTAATATTTGTATGGTAGTTTCATTGTTTGTAACGCACCGCCTTCATTCGATAAAATCATACCGAAACGAC
CGATGACAACTCGCGTACCTAATTGTTCAAATTGTTGTGCGAAACGTTCCCATTGATACACAATATCTGATAAGAAATCAAATGGTA
AAGTTTTATAAACTTCTGTGTAACTCATAAATAAATCAGGAGGATAGTAACCAGTGGCACTAGCATTAAATAAAACTTTAGGTGCTT
TATTACGAGATTTAAACAATTCATATAAAGCTTGCGTAGATTGAACTCTACTTAGCATTAGCGTTTGTTTATATTCCGGTGTCCATCG
TTTATTCAGTGTAGCACCTGCTAAGTTGATGACCACATCGATATTTTGAGGAACTTTGTGTTCCCACCCAGATTTAGCCCAGTTGACA
TATGAAATTTTCTTATCATTTGAAATTTGGTCGTGTCGCGTTAATATCGTGATATGTGAATCTGATTTTTTAATTTCATTAACTAATTG
AGATCCAACCATACCAGTCCCACCAGTAATTAAGTATTGTTTCATTATCATTCACCCCATGTAATTTTGTATTTAGTTACTATTTAAT
AATCATTATTGTTGTTCAAAGGTTATACATTATTTAGACAATAATATGTCAATAACTTTTTTTGAATTGTATTTTAATAAAAATGATA
TAAGTTATAATAAAGGTGTACCTTCGATAACGAATAAACATCTCTTAAAAGTATGTGTAAAACGCTGCATGATACAAACGAAGGTA
AAAATTTGACTCCCTTTAGTAGTGGACCCGTACGTTAATCGTGCGGGTCGTTTTTATTTATTTCTTATTCCCATTATACATCAATTTAA
AGCATTAATTTTTTAAACAAATTTAAGAATACATAGTAATATAACAATCTAAACATAAAAACTTTTAACACAACACTTAAACCAATG
CTTTAATTTTCAATACGTAGCTATAATTTGTTGTAAAATCAAAAAGGTTAAAATGTTAATTTTCAAAAAAAGGCTCAAAATATGTTT
GATTTAGTTATTAAATGTTAAGATATATAAGACTACTATTTCTTTGTAAAAATGAATCCGATTTACGAGTGAGTAATAGTGAAGGCA
GTTTTAAGTTGAAGAAGGCAAAAAGAGTAAATGTTTATTAATATTTGTAGAAACTAGGTAAGCAAATTAGTTGTGAAAATGTTAAT
GGTTGCGTGATAATTTCTATATTTAAATTAGTTTGAAGTGAGGGAGAGTATGTCGAATCAAAATTACGACTACAATAAAAATGAAG
ATGGAAGTAAGAAGAAAATGAGTACAACAGCGAAAGTAGTTAGCATTGCGACGGTATTGCTATTACTCGGAGGATTAGTATTTGCA
ATTTTTGCATATGTAGATCATTCGAATAAAGCTAAAGAACGTATGTTGAACGAACAAAAGCAGGAACAAAAAGAAAAGCGTCAAA
AAGAAAATGCAGAAAAAGAGAGAAAGAAAAAGCAACAAGAGGAAAAAGAGCAGAATGAGCTAGATTCACAAGCAAACCAATATC
AGCAATTGCCACAGCAGAATCAATATCAATATGTGCCACCTCAGCAACAAGCACCTACAAAGCAACGTCCTGCTAAAGAAGAGAAT
GATGATAAAGCATCAAAGGATGAGTCGAAAGATAAGGATGACAATGCATCTCAAGATAAATCAGATGATAATCAGAAGAAAACTG
ATGATAATAAACAACCAGCTCAGCCTAAACCACAGCCGCAACAACCAACACCAAAGCCAAATAATAATCAACAAAATAATCAATC
AAATCAGCAAGCAAAACCACAAGCACCACAACAAAATAGCCAATCAACAACAAATAAACAAAATAATGCTAATGATAAGTAGTAT
TTAGTCAAACAAAAATGAACCAGTATGACAGACAACACAATTAATTAGGTTGTCTCGAATATTGGTTCTTATTTTTATAATTGTTAA
TTAGGGGAGAGATGATACTTAAATAGTTAGTTGTTTATTTTACGGATAGTGAAATTTATTTTGAGTGAGGTGGGACAGAAATGATAT
TTTCGCAAAATTTATTTCGTCGTCTGACCCCAACTTCCACATTATTGTAAGCTGACTTTCCGTCAGCTTCTGTGTTGGGGCCCCGCCA
ACTTGCACATTATTGTAAGCTGACTTTCCGCCAGCTTCTGTGTTGGGGCCCCTGACTTTAATTGAAAAATGCTTGTTACAAGTGCATT
TTCGTTCGGTCAACTACTACTAATGTGACTTTTCGGATTCTAGAGCATTGATTTATGTCTTAGTTCAAATAATAAGCAAATGATTAGC
GAGTCAGTATAAGGGTATACATTTGACTACAGCGAATAAAATAAAACGTTTTGTTGAATAACAAATCGAAAATATATTGCAAGCGC
TTTATCAAATTATTTAGAAAATTTAAGTTTTATGCTTGCAATTTTTGAAATAGAATAGTACTATTGCAAGTGTAAAGAGGTTAATTTT
TGTCCCACGCGGGACTTAAAAAGGCAACCACTGGTTGTGACATATCCTTATTTACATTTATAAATATAAGGAGGAGGTAGTAGTGA
AAGACTTATTGCAAGCACAGCAAAAGCTTATACCGGATCTCATAGATAAAATGTATAAACGTTTTTCTATTCTTACTACTATCTCAA
AAAATCAGCCTGTCGGACGTCGAAGTTTAAGCGAACATATGGATATGACTGAACGTGTACTGCGTTCTGAAACAGATATGCTTAAG
AAACAAGATTTGATAAAAGTTAAGCCTACCGGAATGGAAATTACAGCTGAAGGTGAGCAACTGATTTCGCAATTGAAAGGTTACTT
TGATATCTATGCAGATGATAATCGTCTGTCAGAAGGTATTAAGAATAAATTTCAAATTAAGGAAGTTCATGTTGTTCCTGGTGATGC
TGATAATAGTCAATCTGTTAAAACAGAATTAGGTAGACAAGCAGGTCAATTACTTGAAGGCATATTACAAGAAGACGCGATAGTTG
CTGTAACTGGCGGATCCACGATGGCATGTGTTAGTGAAGCAATTCATTTATTACCATATAATGTATTCTTCGTACCAGCCAGAGGTG
GACTAGGCGAAAATGTTGTCTTTCAGGCAAACACAATTGCAGCCAGTATGGCACAACAAGCTGGCGGTTATTATACGACGATGTAT
GTACCTGATAATGTCAGTGAAACAACATATAACACATTGTTGTTAGAGCCATCAGTCATAAACACTTTAGACAAAATTAAACAAGC
AAACGTTATATTACACGGCATTGGTGATGCGCTGAAGATGGCGCATCGACGTCAATCACCTGAAAAGGTCATTGAACAACTTCAAC
ATCATCAAGCTGTCGGAGAGGCATTTGGTTATTATTTTGATACACAAGGTCAAATTGTCCATAAGGTTAAAACAATTGGACTTCAAT
TAGAAGACCTTGAATCAAAAGACTTTATTTTTGCAGTTGCAGGAGGCAAATCGAAAGGTGAAGCAATTAAAGCATACTTGACGATT
GCACCCAAGAATACAGTGTTAATCACTGATGAAGCCGCAGCAAAGATAATACTTGAATAAGAGATAAAAAGTTTAATACTTTTTAA
ATATCATTTTAAAGGAGGCCATTATAATGGCAGTAAAAGTAGCAATTAATGGTTTTGGTAGAATTGGTCGTTTAGCATTCAGAAGAA
TTCAAGAAGTAGAAGGTCTTGAAGTTGTAGCAGTAAACGACTTAACAGATGACGACATGTTAGCGCATTTATTAAAATATGACACT
ATGCAAGGTCGTTTCACAGGTGAAGTAGAGGTAGTTGATGGTGGTTTCCGCGTAAATGGTAAAGAAGTTAAATCATTCAGTGAACC
AGATGCAAGCAAATTACCTTGGAAAGACTTAAATATCGATGTAGTGTTAGAATGTACTGGTTTCTACACTGATAAAGATAAAGCAC

AAGCTCATATTGAAGCAGGCGCTAAAAAAGTATTAATCTCAGCACCAGCTACTGGTGACTTAAAAACAATCGTATTCAACACTAAC
CACCAAGAGTTAGACGGTTCTGAAACAGTTGTTTCAGGTGCTTCATGTACTACAAACTCATTAGCACCAGTTGCTAAAGTTTTAAAC
GATGACTTTGGTTTAGTTGAAGGTTTAATGACTACAATTCACGCTTACACAGGTGATCAAAATACACAAGACGCACCTCACAGAAA
AGGTGACAAACGTCGTGCTCGTGCAGCGGCAGAAAACATCATCCCTAACTCAACAGGTGCTGCTAAAGCTATCGGTAAAGTTATTC
CTGAAATCGATGGTAAATTAGATGGTGGTGCACAACGTGTTCCTGTAGCTACAGGTTCATTAACTGAATTAACAGTAGTATTAGAAA
AACAAGACGTAACAGTTGAACAAGTTAACGAAGCTATGAAAAATGCTTCAAACGAATCATTCGGTTACACTGAAGACGAAATCGTT
TCTTCAGACGTTGTAGGTATGACTTACGGTTCATTATTCGACGCTACACAAACTCGTGTAATGTCAGTTGGCGACCGTCAATTAGTTA
AAGTTGCAGCTTGGTATGATAACGAAATGTCATATACTGCACAATTAGTTCGTACATTAGCATACTTAGCTGAACTTTCTAAATAAT
TTTAGTATAGTTTTTATTCAAATACGCTAGTGCTCAGAACTATTTAGCATTAATTAAAGCTTATGAGTAAGCGGGGAGCACAAACGC
TTCTCCGCTTATTTTTATATAAAATTTCCTAATTACAAGGAGGAAACACCATGGCTAAAAAAATTGTTTCTGATTTAGATCTTAAAGG
TAAAACAGTCCTAGTACGTGCTGATTTTAACGTACCTTTAAAAGACGGTGAAATTACTAATGACAACCGTATCGTTCAAGCTTTACC
TACAATTCAATACATCATCGAACAAGGTGGTAAAATCGTACTATTTTCACATTTAGGTAAAGTGAAAGAAGAAAGTGATAAAGCAA
AATTAACTTTACGTCCAGTTGCTGAAGACTTATCTAAGAAATTAGATAAAGAAGTTGTTTTCGTACCAGAAACACGCGGCGAAAAA
CTTGAAGCTGCTATTAAAGACCTTAAAGAAGGCGACGTATTATTAGTTGAAAATACACGTTATGAAGATTTAGACGGTAAAAAAGA
ATCTAAAAATGATCCAGAATTAGGTAAATACTGGGCATCTTTAGGTGATGTGTTTGTAAATGATGCTTTTGGTACTGCGCATCGTGA
GCATGCATCTAATGTTGGTATTTCTACACATTTAGAAACTGCAGCTGGATTCTTAATGGATAAAGAAATTAAGTTTATTGGTGGCGT
AGTTAACGATCCACATAAACCAGTTGTTGCTATTTTAGGTGGAGCAAAAGTATCTGACAAAATTAATGTCATCAAAAACTTAGTTAA
CATAGCTGATAAAATTATCATCGGCGGAGGTATGGCTTATACTTTCTTAAAAGCGCAAGGTAAAGAAATTGGTATTTCATTATTAGA
AGAAGATAAAATCGACTTCGCAAAAGATTTATTAGAAAAACATGGTGATAAAATTGTATTACCAGTAGACACTAAAGTTGCTAAAG
AATTTTCTAATGATGCCAAAATTACTGTAGTACCATCTGATTCAATTCCAGCAGACCAAGAAGGTATGGATATTGGACCAAACACTG
TAAAATTATTTGCAGATGAATTAGAAGGTGCGCACACTGTTGTATGGAATGGACCTATGGGTGTATTCGAGTTCAGTAACTTTGCAC
AAGGTACAATTGGTGTATGTAAAGCAATTGCAAACCTTAAAGATGCAATTACGATTATCGGTGGCGGTGATTCAGCTGCAGCAGCA
ATCTCTTTAGGTTTTGAAAATGACTTCACTCATATTTCAACTGGTGGCGGTGCGTCATTAGAGTACCTAGAAGGTAAAGAATTGCCT
GGTATCAAAGCAATCAATAATAAATAATAAAGTGATAGTTTAAAGTGATGTGGCATGTTTGTTTAACATTGTTACGGGAAAACAGT
CACAAGATGACATCGTGTTTCATCACTTTTCAAAAATATTTACAAAACAAGGAGTGTCTTTAATGAGAACACCAATTATAGCTGGTA
ACTGGAAAATGAACAAAACAGTACAAGAAGCAAAAGACTTCGTCAATGCATTGCCAACATTACCAGATTCAAAAGAAGTAGAATC
AGTAATTTGTGCACCAGCAATTCAATTAGATGCATTAACTACTGCAGTTAAAGAAGGAAAAGCACAAGGTTTAGAAATCGGTGCTC
AAAATACGTATTTCGAAGATAATGGTGCGTTCACAGGTGAAACGTCTCCAGTTGCATTAGCAGATTTAGGCGTTAAATACGTTGTTA
TCGGTCATTCTGAACGTCGTGAATTATTCCACGAAACAGATGAAGAAATTAACAAAAAAGCGCACGCTATTTTCAAACATGGAATG
ACTCCAATTATTTGTGTTGGTGAAACAGACGAAGAGCGTGAAAGTGGTAAAGCTAACGATGTTGTAGGTGAGCAAGTTAAGAAAGC
TGTTGCAGGTTTATCTGAAGATCAACTTAAATCAGTTGTAATTGCTTATGAGCCAATCTGGGCAATCGGAACTGGTAAATCATCAAC
ATCTGAAGATGCAAATGAAATGTGTGCATTTGTACGTCAAACTATTGCTGACTTATCAAGCAAAGAAGTATCAGAAGCAACTCGTA
TTCAATATGGTGGTAGTGTTAAACCTAACAACATTAAAGAATACATGGCACAAACTGATATTGATGGGGCATTAGTAGGTGGCGCA
TCACTTAAAGTTGAAGATTTCGTACAATTGTTAGAAGGTGCAAAATAATCATGGCTAAGAAACCAACTGCGTTAATTATTTTAGATG
GTTTTGCGAACCGCGAAAGCGAACATGGTAATGCGGTAAAATTAGCAAACAAGCCTAATTTTGATCGTTATTACAACAAATATCCA
ACGACTCAAATCGAAGCGAGTGGCTTAGATGTTGGACTACCTGAAGGACAAATGGGTAACTCAGAAGTTGGTCATATGAATATCGG
TGCAGGACGTATCGTTTATCAAAGTTTAACTCGAATCAATAAATCAATTGAAGACGGTGATTTCTTTGAAAATGATGTTTTAAATAA
TGCAATTGCACACGTGAATTCACATGATTCAGCGTTACACATCTTTGGTTTATTGTCTGACGGTGGTGTGCACAGTCATTACAAACAT
TTATTTGCTTTGTTAGAACTTGCTAAAAAACAAGGTGTTGAAAAAGTTTACGTACACGCATTTTTAGATGGTCGTGACGTAGATCAA
AAATCCGCTTTGAAATACATCGAAGAGACTGAAGCTAAATTCAATGAATTAGGCATTGGTCAATTTGCATCTGTGTCTGGTCGTTAT
TATGCAATGGACCGTGACAAACGTTGGGAACGTGAAGAAAAAGCTTACAATGCTATTCGTAATTTTGATGCCCCAACTTATGCAACT
GCCAAAGAAGGTGTAGAAGCAAGCTATAATGAGGGCTTAACTGACGAATTCGTAGTACCATTCATCGTTGAGAATCAAAATGACGG
TGTTAATGATGGAGATGCAGTGATCTTCTATAATTTCCGACCTGATAGAGCAGCACAATTATCGGAAATTTTTGCGAACAGAGCATT
CGAAGGCTTTAAAGTTGAACAAGTTAAAGACTTATTCTATGCAACATTCACTAAGTATAACGACAATATCGATGCGGCTATCGTTTT
CGAAAAAGTTGATTTAAATAATACAATTGGTGAAATTGCACAAAATAACAATTTAACACAATTACGTATTGCAGAAACTGAAAAAT
ATCCTCACGTTACTTACTTTATGAGTGGTGGACGTAACGAGGAATTTAAAGGTGAACGCCGTCGTTTAATTGATTCACCTAAAGTTG
CAACGTATGACTTGAAACCAGAAATGAGTGCTTATGAAGTTAAAGATGCATTATTAGAAGAGTTAAATAAAGGTGACTTGGACTTA
ATTATTTTAAACTTTGCTAACCCTGATATGGTTGGACATAGTGGTATGCTTGAGCCGACAATCAAAGCAATCGAAGCGGTTGATGAA
TGTTTAGGTGAAGTCGTTGATAAGATTTTAGACATGGACGGTTATGCAATTATTACTGCTGACCATGGTAACTCTGATCAAGTATTG
ACGGATGATGATCAACCAATGACTACGCATACAACGAACCCAGTACCAGTGATTGTAACAAAAGAAGGCGTTACACTTCGAGAAAC
TGGTCGCTTAGGTGACTTAGCACCTACATTATTAGATTTATTAAATGTAGAACAACCTGAAGACATGACAGGTGAATCTTTAATTAA
ACACTAATATTGTAAAAGATGTTAAGTAAACGCTTAATGACACTTATTTTTTGAAAATAATAGTAATATCATTTTGTTAAATGAAAG
AATAAAGCTATAATAATTATAGAATAACTATTTAAAGGAGATTATAAACATGCCAATTATTACAGATGTTTACGCTCGCGAAGTCTT
AGACTCTCGTGGTAACCCAACTGTTGAAGTAGAAGTATTAACTGAAAGTGGCGCATTTGGTCGTGCATTAGTACCATCAGGTGCTTC
AACTGGTGAACACGAAGCTGTTGAATTACGTGATGGAGACAAATCACGTTATTTAGGTAAAGGTGTTACTAAAGCAGTTGAAAACG
TTAATGAAATCATCGCACCAGAAATTATTGAAGGTGAATTTTCAGTATTAGATCAAGTATCTATTGATAAAATGATGATCGCATTAG
ACGGTACTCCAAACAAAGGTAAATTAGGTGCAAATGCTATTTTAGGTGTATCTATCGCAGTAGCACGTGCAGCAGCTGACTTATTAG
GTCAACCACTTTACAAATATTTAGGTGGATTTAATGGTAAGCAGTTACCAGTACCAATGATGAACATCGTTAATGGTGGTTCTCACT
CAGATGCTCCAATTGCATTCCAAGAATTCATGATTTTACCTGTAGGTGCTACAACGTTCAAAGAATCATTACGTTGGGGTACTGAAA
TTTTCCACAACTTAAAATCAATTTTAAGCAAACGTGGTTTAGAAACTGCAGTAGGTGACGAAGGTGGTTTCGCTCCTAAATTTGAAG
GTACTGAAGATGCTGTTGAAACAATTATCCAAGCAATCGAAGCAGCTGGTTACAAACCAGGTGAAGAAGTATTCTTAGGATTTGAC
TGTGCATCATCAGAATTCTATGAAAATGGTGTATATGACTACAGTAAGTTCGAAGGCGAACACGGTGCAAAACGTACAGCTGCAGA
ACAAGTTGACTACTTAGAACAATTAGTAGACAAATATCCTATCATTACAATTGAAGACGGTATGGACGAAAACGACTGGGATGGTT
GGAAACAACTTACAGAACGTATCGGTGACCGTGTACAATTAGTAGGTGACGATTTATTCGTAACAAACACTGAAATTTTAGCAAAA
GGTATTGAAAACGGAATTGGTAACTCAATCTTAATTAAAGTTAACCAAATCGGTACATTAACTGAAACATTTGATGCAATCGAAAT
GGCTCAAAAAGCTGGTTACACAGCAGTAGTTTCTCACCGTTCAGGTGAAACAGAAGATACAACAATTGCTGATATTGCTGTTGCTAC
AAACGCTGGTCAAATTAAAACTGGTTCATTATCACGTACTGACCGTATTGCTAAATACAATCAATTATTACGTATCGAAGATGAATT
ATTTGAAACTGCTAAATATGACGGTATCAAATCATTCTATAACTTAGATAAATAATTTTCTTTATAATCAAATGCTGACATAATTTTA
GTTGAGGATTATTATGACGGTATAAATTAAATAAAGATTTTGAGTTCACGCTTAAATAAGTTCACGCTTAAATTTATAGCCTGCCAC
AGAGTTGAGACTGTGGTAGGTTTTTTATTTTGAAGTATTAATCATAACAGACTAATAATCATGAGGTAACTAATAACACATATTTAA
CTTGTATTCTTAAACTGGTATAATAAATTTATGTTGAAATGAATATTGTATGACAGGGTATTCACTTTTATTAAAAGGTAAAATTAAA

TAAAGGTTTTATAGAACGTATTTAAATATATGAGGAGTAAACAAATGGCTGATAGAACGAATAAAGAAATTAAAACAGGACGCTTT
ATTGCAACTGCATCAATCGTATTCTCAATATTATTGATTATTCATTACTTTGTTTCGTTGGATAATGCGACTGCCAAAGCATTACTTA
ATTTAACGAATCAAAACACTTCAGATAAAGCGATTGATTACATTTTAAACAGCTTTAGATTCACTGGTATTATGTATATTTTGGCTTA
TCTAGCAGGCTTCATCACTTTTTGGAATCGACATACTTATGTGTGGTGGTTTATGTTTGCAGTTTATGTATCAAATAGTTTGTTTACGT
TGATTAATTTATCAATCACAATTCAAGCAATAAAAGCTGCACACGGTGCGTACTTAACATTGCCAATTTTAATTGTTATTATAGGTTC
GGTTGCATTAGCGATTTATATGCTTGTTGTTTCTATCAAACGTAAAAGTACATTTAATCGCTAGAAAATTGATTTTAACAATAAAAAT
ATGATATACTACTTGTCGTATATAAGGAACGGAGGACAATTTATGCATACATTTTTAATCGTATTATTAATCATTGATTGTATTGCAT
TAATAACTGTTGTACTACTCCAAGAAGGTAAAAGCAGTGGACTTTCAGGTGCCATCAGTGGTGGTGCTGAGCAGTTATTCGGTAAAC
AAAAACAACGTGGCGTCGATTTATTCTTAAATAGATTAACAATTATTTTATCAATATTATTTTTTGTACTTATGATTTGCATAAGTTA
TCTTGGTATGTAAGGTCCGGCGATGTAAATGTCGGGCTTTTTTATTTATAATTAAGAATGTAATAGTTTAACAATAAGCTATGTAAA
ATATATAGCCTAGTTAAGTATGCAAAGGGAGCGTTAGATTTATGCAGATAAAATTACCAAAACCTTTCTTTTTTGAGGAAGGTAAAC
GTGCCGTGTTATTACTACATGGTTTTACAGGCAATTCGTCTGATGTTCGTCAATTAGGTCGATTTTTACAAAAGAAAGGTTATACATC
ATATGCACCGCAATATGAAGGCCATGCAGCACCGCCAGAAGAGATACTTCAATCAAGTCCTTTTGTTTGGTTTAAAGATGCCTTAGA
TGGCTATGACTACCTTGTTGAACAAGGTTATGATGAAATCGTTGTTGCTGGCTTGTCATTAGGTGGCGATTTTGCATTAAAATTAAGC
TTAAATAGAGATGTAAAGGGTATTGTAACGATGTGTGCACCTATGGGTGGCAAAACTGAAGGTGCCATTTATGAAGGCTTTTTAGA
ATATGCACGCAATTTTAAAAAGTATGAGGGTAAAGATCAAGAAACGATTGATAACGAAATGTATCATTTCAAACCAACTGAAACTT
TAAAAGAATTAAGTGAAGCATTAGATACGATTAAAGATCAAGTTGATGAGGTGTTTGACCCAATTTTAGTGATTCAAGCAGAAAAC
GACAATATGATTGATCCACAATCCGCAAATTATATATATGAACATGTAGACTCTGACGATAAAAATATCAAGTGGTACAGTGAATC
TGGACATGTTATTACGATTGATAAAGAAAAAGAACAAGTATTTGAAGATATTTATCAATTTTTAGAGTCATTAGACTGGTCAGAATA
AAAAGAGATTTTAACATTAGAAAGGAGGGGCATAATGAATTTAAAGCAATCTATAGAAGAAATTATTAATCAACCTGAATATGAAC
CTATGTCGGTATCAGATTTTCAAGATGCATTAGGTTTAAGCAGTGCCGACTCGTTTAGAGATTTAATTAAGGTGCTTGTGGAGTTAG
AACAATCAGGATTAATCGAACGTTCGAAAACAGACAGATACCAAAAAAAGCATAGTTCTAGAGGTCAATCAAAATTGATAAAAGG
AACGTTAAGTCAAAATAAAAAAGGCTTTGCATTCTTAAGACCTGAAGATGAGGATATGGAAGATATATTTATTCCCCCGACGAAAA
TTAATCGTGCCTTGGATGGAGATACTGTTATTGTAGAAATCCATCAATCAAAAGGTGAACATAAAGGTAAAATCGAAGGGGAAGTT
AAGTCGATTGAGAAGCATTCTGTAACTCAAGTTGTTGGTACGTATAGTGAAGCTAGACATTTTGGCTTTGTTATTCCGGATGATAAA
CGTATTATGCAAGATATTTTCATTCCTAAAGGTCAAAGTTTAGGCGCAGTCGATGGTCATAAGGTACTTGTACAAATTACTAAGTAT
GCTGATGGTTCAGATAATCCTGAAGGGCATATTTCAGCAATATTAGGACATAAAAATGATCCAGGTGTAGATATTTTATCTATTATT
TATCAACATGGTATTGAAATTGAGTTTCCTGATGTAGTATTACAAGAAGCTGAAGCAGTACCTGATCATATTGAGAATACTGAAATT
AAAGGCCGTCATGATTTACGTGATGAATTGACAATCACAATTGATGGTGCTGATGCTAAAGACTTAGATGACGCAATTAGTGTTAA
AAAGTTAGCGAACGGTAATACGCAATTAACTGTAAGTATTGCTGATGTCAGCTATTATGTAACAGAAGGTTCTGCATTGGATAAAG
AGGCATATGATAGAGCGACAAGTGTATATCTTGTTGACCGTGTAATTCCAATGATTCCACATCGATTAAGTAATGGTATTTGTTCAT
TGAATCCTAATGTTGATCGTTTAACTTTAAGCTGTCGCATGGAAATCGATGCTAGTGGTCGCGTTGTTAAACATGAAATTTTTGATAG
TGTTATACATTCTGATTATCGAATGACGTATGATGCGGTAAATCAGATTATTACTGAAAAGGATCCTAACATTCGCGAACAATATAA
AGAAATTACGCCTATGTTAGATTTAGCACAAGATTTATCTAATCGTTTGATTCAAATGAGAAAACGACGTGGTGAAATCGATTTTGA
TATTAGTGAAGCAAAAGTATTAGTTAACGAAGACGGTATACCAACAGATGTTCAATTAAGACAACGTGGCGAGGGTGAACGTCTAA
TTGAATCATTTATGTTAATTGCAAATGAAACAGTTGCTGAACATTTTAGTAAGTTAGATGTACCTTTTATTTACCGAGTGCATGAGCA
ACCTAAATCAGATCGCTTAAGACAATTCTTTGATTTTATTACAAACTTTGGCATCATGATTAAGGGTACTGGCGAAGATATTCATCC
AACAACACTTCAAAAGGTTCAAGAAGAAGTAGAAGGTCGACCTGAACAAATGGTCATTTCAACAATGATGTTACGTTCAATGCAAC
AAGCGCATTATGATGATGTGAACTTGGGACATTTTGGCTTATCAGCTGAATATTATACGCATTTTACATCACCAATTAGACGTTATCC
TGATTTAACAGTTCATCGTTTAATCCGTAAGTATTTAATTGAGAAATCAATGGATAACAAAGAAGTGAAGCGTTGGGAAGACAAAT
TGCCTGAGTTAGCTGAACATACTTCTAAACGTGAACGTCGTGCTATTGAGGCAGAACGTGATACTGATGAATTGAAAAAAGCAGAA
TATATGATTCAACATATTGGTGATGAATTTGAAGGTATTGTCAGCTCAGTAGCTAACTTCGGTATGTTCATTGAATTGCCAAATACG
ATAGAAGGTATGGTTCATATTGCGAATATGACTGATGATTATTACCGTTTTGAAGAGCGTCAAATGGCATTAATTGGTGAGCGTCAA
GCTAAAGTATTTAGAATTGGTGACACAGTTAAGGTTAAAGTGACGCATGTTGATGTAGATGAACGATTAATTGATTTTCAAATTGTA
GGTATGCCTTTACCGAAAAATGATCGATCACAGCGCCCAGCGCGAGGTAAGACAATTCAAGCTAAAACGCGTGGTAAATCATTAGA
TAAATCGAAATCTGATGATAAAGGTCATAAGAAAAAAGGTAAGCAACGTAAAGGTAAAAATGAACGTAAAAATGACAAATCAGGA
AACAGTAAACATAAGCCATTTTATAAAGATAAAAGTGTGAAAAAGAAAGCACGTCGTAAGAAAAAATAAGCAGCAATGAGGTGAG
TATGAATGGCTAAGAAGAAATCACCAGGTACATTAGCGGAAAATCGTAAAGCTAGACATGATTATAATATTGAAGACACGATTGAA
GCAGGAATTGTATTGCAAGGCACAGAAATAAAATCAATTCGCCGAGGTAGTGCTAACCTTAAAGATAGTTATGCGCAAGTTAAAAA
CGGTGAAATGTATTTGAATAATATGCATATAGCACCATACGAAGAAGGGAATCGTTTTAATCACGATCCTCTTCGTTCTCGAAAATT
ATTATTGCACAAGCGTGAAATCATTAAATTGGGTGATCAAACACGTGAGATTGGTTATTCGATTGTGCCGTTAAAGCTTTATTTGAA
GCATGGACATTGTAAAGTATTACTTGGTGTTGCACGAGGTAAGAAAAAATATGATAAACGTCAAGCTTTGAAAGAAAAAGCAGTCA
AACGAGATGTTGCGCGCGATATGAAAGCCCGTTATTAAGCGATTTAGTTGCTTAATCGGGCTATATTTGATATAGTTATATGTGCTTT
TGTAAATTACAAAAGTATGATTTGTTTGATTTATTATTTCGGGGACGTTCATGGATTCGACAGGGGTCCCCCGAGCTCATTAAGCGT
GTCGGAGGGTTGTCTTCGTCATCAACACACACAGTTTATAATAACTGGCAAATCAAACAATAATTTCGCAGTAGCTGCCTAATCGCA
CTCTGCATCGCCTAACAGCATTTCCTATGTGCTGTTAACGCGATTCAACCTTAATAGGATATGCTAAACACTGCCGTTTGAAGTCTGT
TTAGAAGAAACTTAATCAAGCTAGCATCATGTTGGTTGTTTATCACTTTTCATGATGCGAAACTTTTCGATAAACTACACACGTAGA
AAGATGTGTATCAGGACCTCTGGACGCGGGTTCAAATCCCGCCGTCTCCATTATATAGTCTGCAACCTTAGTGGTTGTAGGCTTTTTG
TTTTTGAGTGCACAGATAGTGCACAGAAAATGCACAGGATACAAAAAGACACCACCAATTTTAGTTGGTAGTGCCTTATAAGTATTT
AACTATTTCTTTCTGTGTACTAGGATATAAATGACCATATCGGTTATATACTTCAGTCGTATCGCTGTGACCTAAACGCTGAGCTATA
ATCATTACACTGGCCCCTAGATTAACTAACATAGATGCATGACTATGTCTTAACTCGTGTATAACGATTCTAGGGAAATTTTGTCCAT
TTGGTAATTGCTCATCCAACACTTTTAATGCGTTAGTAAACCAACGATCAATGGTAGACTCGCTGTAAGCTTTATAGAATGTACCAA
ATAATACATAATCATCTTTAAATATGTTATTCTCTTTATACCAATTTAAATAACCTTTGATGTCATCCATCATGTGAGTAGGCAAATA
TATATCGCGTATGGCTGATTTCGTTTTAGGGGCTGTCACTTCACCGTGATAGTCCGTTTTGTTTATATGGATAAAATCATCATCAAAG
TTAATATCGCGCCATGTGAGCGCTCTGATTTCGCCTTTGCGTGCTCCAGAGTAGAACAATAGTTTAAAGAATAACTTTTGTTGTTGTG
TCACTAGCGCACCATAAAATTGATTGAATTGTTCTAATGTCCAATAATTCAATCGTTTCTGTGATTCTATTTCAAAATTCCCTACAAG
AGATGCAACATTTTGTTTTAACTCATGAAACTTCATTGCATGATTCAGTAATGATACTAAATATACATGCATCTTCTTTAAGTAGTCT
CCAGAATGCCCCTCTTTTAACTTCTTATTCTGAAATTTCATCACATCTTGTGTAGTCATTTTAAATACATCCATAGATTTAAAATAAG
GTAGCAAGTGGTTATTTGTATGTGTTTTTAATGCTTTTACACTTGATGTCTTTCGACGTGCTGAATACCAGTCTATATATTCTTCAACG
AGCTTGTCAAAAGGTAATCTATTAAGGTGTCCGACTCCCTCTAATTCGTCCATCATTTCGTTACATTTCTTAACTGCGTCTTTACGTTG
TTTAAACCCCTTACGTGTTATGTACTTACGAGTATTGCTCTTGTCATAGTAAGTGATACGGAAATAATAAGTACCGCGTTTAGTGTCT

TTATATATGTTATGGGATAGGTTTAAGTTATGTGTCATGAGTATCGCCTTGTTTTTTAAAATATTTTTATTGAGTAACTTTTCTTTCA
TAACATCGATTTTATTCATTAATTTTTGGTATTCTTTAGAGTCTATAAATTCTGCTATTTTACTTCTTCTAGTATGATTGTGTAATATTT
CATGCTTAGTTGAAATTTCTTGTATATTACTTTTGAGGTCGTCAAATTGCGACAACAAGTTTAAAAAGTAGACTTCAGGATATTTTAA
TGAAATTTGGATTAATAAATGATAAATAAATTCAGTGAGATATAATTGTACAGGTAATTTTTTGAAATCTTCGTTTAGCAATAAATA
AACACCGTGATGGTAAAATGTCATGTTCTCATTTGAGATGAATGAATACATTTGTTCTATTATACAATCTATATTTGAATCTGTATGA
TCATTAATAATTGTAATTAAACTAGCTACTTTAAAATAATAAGTATTTTGCTCACTAGAGGGCATATAATTAACGATAAAATCAATT
AACTTGTCTTGAATATTGTTTTTCATATTCACTGATTTTATGTTTTGATAAATATCTTCGTATGAAATCTCATTTCCATATAGTAAAAA
ATTAATAGACACATTACCATATTCTGCAATAGCTTTTAACCTTTTGGCATTGGGGATTGACACCCCTTTTTCCCATCGACTAACTATA
CTATCCGAAATATATTTTTTTTCTTTTGTTAATTTTGATATTTCTTCGCCAAATTCTCTTAAATTCAATCCTTTAGCAAGTCTGATACTT
TTGATGCGTAAACCAATATCTGTTTTTTTATCCATATTTTCACTTCCTATCTAATGAGAATATCTATAAAAACTTTCGTAAACCGACG
TTTTTGTGTTGCTTTTAGTTTTCGTGTCTGTTATATTATACATACAGACGAGAACTTACGTCTAGGGAAGAGGTGATAATAAGTGAAT
AAAGTACTGGGTTATAGGAAAATGTTGAACTTCACACAACAACAAATGGCAAAAGAGCTTAAAATATCAGAACAGTCATACAGAA
ATAAAGAAAAAGGAAAAACGGAATTTAAGAAAGAAGAAATGTTAACTTTTAAAAACATACTTATTTCAAAGGGAATGACCAATAT
AACTTTAGATGATATTTTTTTTAATTAAAATCCGACGTAAAACTACGTAAAAGAGGTGCTCAAATGACAAAACTAGCTTATCCAATG
TTATACATTACTAGAAAAGAAAAAGGTGACACACAAAAGAAAGTTGCTAGCAAACTTGGTATTAGTCCACAACGTTACCAGTTAAA
GGAAAGTGGTAAAGCAATATTTAATTTAAATGAGTGTCAGATTCTTTCAGAAATGTATGATATGCCAATTGATGAGTTATTTTCATC
AAAAATTAAAGTAGGAGAATAGGAGGCAAGGCAATGAAAATGTACTTAACTTATATCTGCTTAGTTTCATTGTTAACAATTTTATTA
CTAGCAATATCTAACATGTATGTTGCTTTTAGCGTTTATGCTTGGCTAATAACTTTAGGATGTAATTTAACAGGAGGATTAGAAAAT
GAATAATGAACAAAAAGAAGTAATAGAACACGTGGTTTATCAACTTGAGTTAAGTGTCATGAATAACTTGGAAAGTTATGAACACA
CAGAATATGTTAATGGTATTGAAGTGGTTTCAGAGATCAGTCGTGAAAAGCACTTAGAATTGATAATGAAATGGTGCGCACAAGAA
TTAAAGAATAATTTTCAATTAGAGAAAGGAGAATAAAAATGAATTGGGAAATTAAAGATTTAATGTGTGACATTGAAGTGATAAAA
CAAAAAATTAATGATGTAGCTACCAAACATGCTTGGTTTGTTGAAGATAGATTTGTAAAAAATGAATTAGAAACAAAACGGGAACA
TATTAATTTTTCTGCTAGCTATTTAGAACATCGTATACAAAATGAACATACAGTTGAGTTATTACATGTGTACTTAAAAGAATTCGGT
GAACTTATACAAAAATTTCATGAAATAGAAAAAGCATCATCTGAGAACTTTGGCGAGGTATCAGATGACGCACAAAAATTAAAAAT
CACAGAGTAATTTAGAAATTACACATGTTTATTATAACATTTTTTACTCTGTGAATCACTAGAGGTGCAAAAAATGAATGAAATTAA
ATTAGAATATGACACACATGTTTCAGTGGTACATTATGAAAGTTTAGACTCACGTTCATTTAATAGCTTTTCAAAAATTAATTGGAG
TAAGTTGGTTAATAAACTGTCTGTACCTATAGAAGCAAATTATAAGTATGCACGTGGTGTTGCTGTTTACGGTGATATTAAAAACGG
TGCAAATGATCATGGTGAAATTATCAAAAAGCATCGCAATGACGTTAATGTCGTATACAGAGATGTGATTGTACTTGATTACGATGA
AATAAATGATTTAAAGCAATTACATGAAGCAATCAGCTCAGCTTTAAGCAATGTTGCATGGTTTTGGCACACAAGTTACTCGCACAG
AACTGAACAAGCTAGAATACGCCTGTATATCCCTCTAAATGAGCGAATAAGTGCAGATGATTATCGTAATTATACAAAAGTATTAG
CGAATAAAATTGGCCACAAAGTTGATGAAGGTTCATATCAGCCAAGTAGATGTTTTGCGTTACCAGTTATTCAAAAAGGACACATA
TTTATTAAACGAGTGAATGACTGTCCGATTATGAATGTTGATATGCTCGAGCAGTGGTCAAAGGGGTATAAACAATCAAATGATAG
TCCTAATATCAAAGGGTACACACGACGTGATAGTGCGTATTGGCGAGATATAGCTTTTGGTGTAAGTGAGGGAGAGCGCAATTCAA
CATTGGCTTCAATTACAGGTTATCTTTTGCGTAGGTATGTAGATCCAAACTTAGTTTATGGGTTAGTGAGTGCGTGGGCAAGTGTAT
GCAAACCACCTATTAATCAAAGTGAAGTAAACAATACTTTTAAAAGTATTTTGAAAAAAGATAGTAAATACAGTTAGAAATGGAGG
TTTTTGTTTGGAAGATGTTACAAACGAAGAAGTATTTGAAATGATTGATAGCAGAACCGGTGTTTTAAATGCTAATGATTGGAAAAG
TCAATTAAGGCGTTCTGCTACTACACAAGCATTGAAAAAAACGACTACAAATGCTGAAATCATATTGTGTAATGATGAGAGTTTAA
AAGGGCTAGTACAATATGACGCTTTTGAAAAAGTAACCAAACTGAAACGTCTACCGTATTGGAGGTCAAAAGGGGATGCGAATTAT
TATTGGGCTGATATAGATACCACACATGTGATTTCACATATTGATAGATTGTATAATGTGCAGTTTAGCCGTGATCTTATTGATACTG
TGATTGAAAAGGAAGCTTATCAAAATAGATTCCACCCTATTAAATCGATGATTGAATCTAAATCATGGGACGGAATCAAAAGAATT
GAAACGCTCTTCATTGATTATTTAGGTGCTGAAGATAACCATTACAATAGAGAAGTTACAAAAAAATGGATGATGGGTGCAGTTGC
TAGAATCTATCAGCCAGGTATTAAATATGATTCCATGATTATTTTATATGGTGGTCAAGGTGTTGGGAAATCTACGGCAGTGAGTAA
ATTGGGAGGTCATTGGTATAACCAAAGTATTAAAACGTTTAAAGGTGATGAGGTCTATAAGAAATTGCAGGGTTCTTGGATATGTG
AAATTGAAGAACTATCGGCATTTCAAAAGTCTACTATTGAAGATATTAAGGGTTTTATAAGTGCCATTGTAGATATTTATAGAGCTT
CGTATGGTAAACGCACAGAGCGTCATCCTAGACAGTGTGTGTTTGTAGGGACAACCAATAACTATGAGTTTTTAAAAGACCAAACA
GGCAATCGTCGTTTTTTCCCTATTACGACAGATAAAAATAAAGCAACTAAAAGCCCATTTGACGATCTAACACCAGATGTTGTGCAA
CAAATGTTTGCCGAAGCTAAAGTATATTTTGATGAGAATCCGACGGATAAAGCATTGTTGCTAGATAAAGAAGCGAGTGAAATGGC
TTTAAAAGTCCAAGAAGCTCATTCTGAAAAAGATGCTTTAGTTGGAGAAATAGAAGAATTTCTTGAACGTCCTATTCCGTCAGACTA
CTGGTATAGAACGTTAGAAGAAAAAAGAGTGTCTGCGCATGGTGTTATAGACCAAGACTATATTAAATTATATGGTGATGGTAAAT
TGATTGAATTACCGAATACAAAACCAGGTGCTTATGTATGGCGTGACAAGGTATGTAGCATGGAAATTTGGAAAGTGATGATGAAA
CGAGATGACCAACCACAACAACACCATTTAAGAAAAATTGATAAAGCGTTAAGAAATACAAATTATTGTGACATTGTGAAAAAGCA
AACGCGATATGGTGAAGGTATTGGTAAGCAATATGGCTTTAGTGTAGATTTAGCTTCTTATTATCAGAAACTTAAAGTTTAGACATC
TTATTTTTAGGACAGTAAGACACTTATAAGACAAGTTTAAGACACCCGCAATCCCTTGTGGCAGTATATGCCATGCTATAAGTGTCT
TGGTGTCTTGATAGTTTTTAGGGTAAAGTTTTATAAAAATTATTTACACAATATACAAAATATATAAATGTAGGTCGTAAACAGTGA
GACAGTGAGACAGATTAAGTGAAGCCCTTGAGGGAGTAAGTGTAAAAAGAAGTTCATAAGTGTCTTGAATTGCTATTCGAATAAGA
CAGTGAGGCACCTATCAAAAATTAGGAGGAAGAAAATGAATAAAAATCAATTAAAGTCAGAAATTTTAGAATATATAAAGGCGCA
TGCTGGTACATCATTTGTAGAAATAGAACGTGTATTTGAAGAAAATAATTTTGATTATAAAGGTGACGGCGCATATACAAGTGGTCA
ACATCCCAATGTTGTGTTTTGGATTGGGTGGAGTCAAGAAGCGTTTGATGTTATCGCTGAACTTAAAAAAGACAGACGTATTGAGAT
GGATATTTGTGAGCCAATTGTTTATATGGTTGATGGTAAAGGTTTGGATTTGCCTATTGTAAGGTCGAAAAATATTAAAACAGATCA
TTGGCTACCTGTCACGTTTACTATTAGTAAGAAAGAAACGGAGTGTGTCTAATATGAATGACAAAGAGAAAATTTATAATCAACTTC
ATCATGATGCACCAATTCAAAATATACCAGCACCCGAAAATTTATTTGTCGAATATATAGAAGCTGATGAAGTATGGTATTCACCAG
TTGTATGTATGGCTTTAAGTAAAGCGCATAATATTAATTTTTATGATAGTGATGATGTGGGGTGCATTGATAAAGCAGCCACATGTA
GCATTAAAAAATTTAATCCTGAGACAGGTGAGTTTGAACAATTCAGCAAAATGGCTCAAAAGGAGATAACGCAATGAACATAGAA
ACTGTAGTAAATGAATTTGAAACACAAGCAGGCACGTTACTAAGGTACTACACTGGATTATTAGAACATAGTAAAGTACAACCGTA
TTGCTTTAAGTTATACAATGATCCGTTTGATATGGTTTATGTGATGATGAACGGGAAGTTATTCGGTCATGTATATATTAAAGATTGT
AAAGTAAGGCAATCATTTGAATTAGCGTCACCTAAGCACACTGAGGGGCTTATAAGAAGTATAGAAGGTCATTATGTAGGTTATGA
ATTACATGACGGTAAACAGCTTTCTATTAGTGATATGATGGCCAGTCAATTATTTGAAGATGAGTATTTTATGTATGGATTACAAAC
ATATGCAGAATCAAATAATAGTGATGTGTTTGAGTACCTAGAAAATGGATTTGATACCGATACACTTGAGGGCATTCAATCGAGTA
ATACTGATGTGATATCGAATATTGAAATGTTGTATCAGATAGCTACGGGAATCAATGAACCAGCACCAGAGTTAGTTGAGGGGTTG
AGATTAGTAACTGAGTTTGTACAAGATGAGAAGGCTGCACAAGAGGATTACACGGGGTTAGAACGTAAATTGAATGATCTAAAAGC
GTCTTATTATAGTATAAGTAAATAATGTTATGAGGGAGTCGCATGTAGTATGTGACTCCTTATAAAAAAACGGCAAGGTTTGTACAA

GGTATAGAAGTTTAAAATGGTAAGGTTTTCGGAAGGTGTTGGCTTTTAAAACCCAAAAGTTTCCCAAAGGTGCGCAGACTCTGAGA
ACAATGAAATAGGAAGGTGTACAAGGTTAAAAAAAGCGAGGTACAGAACTTTAAAATAGTAATTTTGAGGTAGAAAGAAAACAAT
GTTTTATACCAGGGTTGCAAAATGGTGAGAAAAGGGATAAATATGAGCAATTGAATCAAGAAGAAGTGAAAACCCCAAGCTTTTCC
GTAGGTTATAAGAATAGTGCGATTTTAGATAGGTACGAAGAATTGCGTCAAGAAGGAAACTAAAGGTAAGTGGTTTTTGATATTGA
TAAAAGTAATAAGGTTTTAATAAGGTATAAAAAATTTAAAAAGATAATATATGTTAATGTTTGTTATTTTTGAAGAGGGCAAAAGTT
TTGTTGTTCGTATTTTGTTCGTGTAAAAAACGGGAACTTAAGTTCTGTAGAAACTACTAAGTTTTACTACGGAATTTCTTTTGTTTATT
GTTTTAAGAGGGATAGGGGTGATGCTAAACACCTGATATAATGCGATTTATAGCGAACATAAGTTTGATTTAGATGTGTAGAAATG
GTATAATTAGAGTAAGCAAACAACAGAAAGCATGGTGAGACAATGAGTGAATTTGAAGTAAAAGAAAAGACGTACAACTTACCGA
ATGAACACCGCCAAGTACTCAATGTGATAAGAAATACGTCTAATAAATATATTACTAAAACAAAGCTGCTTAATCAATTGGGATAT
GAAGTGAATAAGGCTAACAATAGATGGTTAACACAAGTCATTACAAGCTTAATCATTAATTATCATTATCCTATCGGATATAGCTAT
AAAAAAGATACTAGAGGCTATTACATCATTAAAACACAAGCTGATAAGATAGAAGCTATCAAAAGTATTAAGGGCTTAATTGAGGG
CAGTCAGAACCGTTTAAAAGCCCTAGAAGAAATTGAAGTGTAACATGATAACTATGAAATGAAAGAGGGTAACATAAATGAAAAC
AAATCATTTATTTGAAAAATATTCCGATGAAGTAAAAGGCTACAAAGAAGAAATTAATAATTTAGAATCTAAAATTGAAAATACTA
CAAAAACTATTGAAGATCTATCTTCCAAATATAAAGAGTATATAAAAGTTGGCAACGATAATGAGGCTGATAAGACGTTTAATAAG
ATTTCAAAATTAGAAGATGAGAAAGCAAAAGATAATAAAAGATTTGAAATTAAAAAGGAATTATTTAACAGTATTAAGCGCGAAA
AACTCATAGACTTATTGTTGAATAGGAAGAATATTCCTGAGTTATATCAAGAGGAAGCACAAATTTTAGCGCGTGAATTAGAAGGT
ACAATCAAACAGTTTAATAATGTTATTGATAAAATCAACAATATGAATGAAGAATATCGAGAAGATATGTATAAGTTCGATTCCTTG
ATAGATCAAAATGAAATGAAAAAAGATAATTTATTTAGACAACGATATGGCGAAGTGATTGTACTTTACCTCAACAACTTTCTTATT
AATACAAAATCAATTCGTTTCAATGAACACAAAAAATTGGAGGTTAAGAAATAATGCAATTTAGTAAAACATTAGAAGCAATAAAC
AATGCTGAATTAGATGAAAACCAACGCTTATCTATTTTAGAAGCACTTAAAGAAGATGTGAACGATGAGGAAAAAAGAAACGTAA
GAGATGTTCCAACTATTTCAGATTTAGCTGATAAAGTAAATATTAGAAAAAACAAATAGACAAAAGCCAAGCCTTAATTGGTTTGG
CTTTATTAAGTAGTGGGGTGGATAAATGAAACTGAGTAAATCTAAGAACGTTTTATATTATCGTAATGTCGACAATAAACTATCTGA
GTATCAACTATTAACGCAATTTAACCCAGCATTTATTAATAAAAAAATTAAGATGTGTGAATTCCAAATTGAAAGTATGTACCATAT
GAGTGCGTCGACCACAACATGTGATGAAATAATGGGGGTCGTGTCTGTCTCATATCCGATTGAAAAATTAGTTATCAAAATTATTGA
AACAAAAGCAGGGTTACAAAACTATAAAAATAGATCTATAAATAATATGGCGTTGTTGAAAAAGGTACTAAATCATTATACAGAAA
AAGAGCAGAAGCAAGTTGTAAAATATATGCGTTCAAATGGACGATATAAGCCCTACAACGTCATTGAACGCTTACAAGTTGATTTG
TATCAAGCAAGTATTAAACAACGTTCAGAACGTCAAAAACAAAGAAATACAGCAATTGAAAACAGTAAGATTGCACGAGTAAATG
AATATCACCAATCTTCATATGTAAAAGTGGTGTAACAATGGATAAAAAGCAAATAAAAGGCTTCGTGTGTGATTATCATAAGCGAA
CTAGAAGTGATGTATTAATAGATGATGATATAAATACTGATGAATTCTTTTCAATAGGTGATGAAAATTCTAATGAATGGATGACAG
ACGATAATGTTGATGATCATATTATAAAGAATCACTTAGAAATGATTGTTGACCGAGTAGCTAATGATAAAGAGTTTTATATTTTCG
ATTCTTTAATACAAGGACGTAGTTATCAAGATATTAGTAGTGTCTTAGATTGTTCTGAACAATCTGTAAGATTATGGTATGAAACCTT
ATTAGATAAAATTGTGGAGGTGATAGAATGAGTGAGTTAACGGCAAAGCAAGCGCGTTTTGTGAATGAGTATATAAGAACACTTAA
TGTGACACAAAGTGCCATAAAAGCAGGTTATAGCGCAAATAGTGCACATGTGACAGGGTGTAGGTTATTAAAGAAGCCACACATCA
AGCAATATATACAAGAACAAAAAGATAAGATTATAGATGAGAATGTATTAACTGCAAAAGAGTTACTACATGTGCTTACGAATGCG
GCAGTCGGTGACGAAACAGAAACGAAAGAAGTTGTGGTCAAGCGTGGGGAATATAAAGAGAATCCACAAAGTGGCAAAGTACAGT
TAGTCTATAATGAACATGTTGAACTGATAGAGGTGCCAATTAAGCCAAGTGATCGTTTAAAAGCTCGTGATATGTTGGGTAAATACC
ATAAGTTATTTACAGATAAGCATGATATCAACGGGGATGTTCCTATATTCATTAACATTGGTGAATGGGACGGAGACGATGAGGAA
TTAGATAAAACTGTAAAAGATGTATCTAACGCTAATCCTAACCATACTGTGATTGTGGATGATATACCGTTAGAGGATTGAAGAAA
ATGAAGCTATGCTATTTATAAATTAATACTAATTAGTTTGATACCATAGCTTATTTACTGAGAAAGTAGACTTAAATGTAACAACAC
CAGTGTTTATTGATAATATTGGTGGGTTTGAGGAGTAGCAATAAAATAAAGGAGGTAATTGTGTAAAATATCTCTTTTTGTTATTTCT
TATTTATTTACAACCGATAAAATTAAATGTATTATATATATAACGATCTAGCCATAACTCTATTCGGGTTATGGCTACTTTTATAGGG
GTAAATTTATGAAGCCATTTGAAAGTCATAATAAACAATTGAAAATTCTAAGAAGAAGAGGAATGGAAGTACCGAGTAGTGCTAAA
AGAGATTTAGAAAATGAAAATTATTATAATATCATAAATGGTTATAAAGATTTATTTTTAGAACTAGATGTTAATGGTAATTTTTTG
GTTCCTGATAAATATAAGCAAGGTACTCATTTTAAAGAAGTCTTTTCTTTATACAAACTAGATAGAAAATTTAGGAATGTTTTATTA
GAGTATTTGTTAGTATTTGAAACTCATATTAAATCAAGAATTTCATATTATTTTAGCGAAAAATATAGAGAACCACATTCATATTTAT
ACTTTAAAAATTATTCATCTGACACAAGTAAGACAGATAGCATCGTGAAAATGGTTGCTACATTTAGCTCGGTTATGAGTAATAGAA
AAAATAAACCATTAAAACATTATATTAATACTCATAATGGAGTGCCACTATGGATATTGGTGAATTATTTAACTTTAGGTAATGTTT
CAAAAATGTATTCCAATTTGGATGATGATCTTCGATTGGAAGTTGCTAAAGACTATAAAAGGAAATTGGAAAGAGATTATAAAACA
CGTGTTCAAATAACTCCATCAGATGTAGACAGTATACTACAACAAGCACATATGTTTCGTAACGTGTGTGCGCATGAAGAAAGATT
GTATGATTATAAAATAGACAGGGCTAAAAGTAGAGCTAATATATTCGCCAATTATAACAAAATATACGATAAAGAATACGTTCCTA
CAATGAATGGTAGTTATGTATTCGATTTGTTGATTTCACTATGTCTATTTTTGAATAAACATGATTACATAAAATTGATGAAAAATAT
GGATAAACTAATAAGTAATTATTCACATTCTTTCTATACAATTACTATAGATGACCTATATACAAAAATGAATTTTCCAGATCAAAC
AAAAATACTGGATATGTTATAAAAGATATTTTTTAATGTCACTTACGAGTGGCGTTTTTTTACGCTGAGAAACGCCCTGTGTTGCAGT
AAACGATGAGTGTGTATATAGAAATAGATAAAGTATAGATATAGATTTAGATATTATAAATTAATATTTGAGATGCTGCCTCTTTAA
AAAAAGACAAGTTACATTAAAAGTAACCTGTCTTTATCTATAACTAGTTTATAGTTATTTTATCTATGTCTTTCTTGTATAACATATA
ATTCTTATGCTCAGTAAAATCTTGTCCAACTATTCCAGAGATTTTAGGATCTTTTTGGTCTTTACTATGAATGGTTACTTTATCACCAT
CTTTAATGATTTTCTGTCCTTTTAATTTGTTAGTTAAATTCTTCCATGTATTATTAGCATCAATTCTTTCGTTGAAATTACTAATATTTA
CTTTTTTGATATTAATAGTAGTTAAACTTTCAATATCTATATTTTGTTTCGAAAAATCTCCATTAGAGTTCCCATTTGCTGAAATTTTA
TTTTCGCTACCATTTTTCAATTTATAATTAACATCTATTGTTTCATTTTCATTTCCATCAATTATATTAGCCTCTTTCAAAGCATCTCTT
ACATTTTTCCACAACTGTCCGTCTGTTGTTTCAGCAGCTTTTGCAACGTTATTAATACCATTATAATTTGAAGAAGAATGAAAACCTG
AACCTACTGTTGTTAAAACTAATGCACTTGCTATCAATGTTTTTGTTAATAGTTTTTTATTCATTTTATTTTCTCCTATAACTTATTTGC
AATCGATTACAAAGTAAATTTACAATAATTATTTAAGTAAATCAATTAAATAATTATTAACAAATCTATAATATTTTATCATTAAAA
TATAATAATTTTGAGCTAGAAATATTCGTCATTTATGCTATAATCTTTTTAGACACAGCAATGTGTTCAAATTTTCATCTATTCGTAA
GTTAACCTTCGGGCTGACTTTTTATTTCCATTATTCACATGTTAATCTTGTTGTTATTTAGGCAGGTACTTCGGTACTTGCCTATTATT
ATATCTCTATACGATAGGAATCGACTATATGACTTACTAAGTTTTATAGCAAATTAGACAATTAACACATAAGGCATTTAATATTGA
GTTGTTATAGTAGTTGTATAATATATAGCTAGTTCCTTATAACAGCAAAAAAATAATTTTGACTATAAGATTAAATATAAGAATATA
AAATTAACAGTAGAAACCAATTTTAGAATTTGAAAAATTGAATGCATTAATTATAAGAGTGTGAATATATATACAATGTTATTAATT
CAAGATGCTTAGAAATCCTCTGTTTCTCCTTGAGAAAGAGTTTTGTATATAAGTTCAGATGAAGTATAGTTAATTAATTTTCAAAGC
ATAACTTAATTCTTAGAAATAACGTGAAATCATTAAATATAATTAATTTTCTTTTAATATTTTTTTAATTGAATATTTAAGATTATAA
CATATATTTAAAGTGTATCTAGATACTTTTTGGGAATGTTGGATGAAGGAGATAAAAATGAATAAGAGTCGATTTATTTCATGCGTA
ATTTTGATATTCGCACTTATACTAGTTCTTTTTACACCCAACGTATTAGCAGAGAGCCAACCAGACCCTACGCCAGATGAGTTGCAC

AAATCAAGTGAGTTTACTGGTACGATGGGTAATATGAAATATTTATATGATGATCATTATGTATCAGCAACTAAAGTTATGTCTGTA
GATAAATTTTTGGCACATGATTTAATTTATAACATTAGTGATAAAAAACTAAAAAATTATGACAAAGTGAAAACAGAGTTATTAAA
TGAAGATTTAGCAAAGAAGTACAAAGATGAAGTAGTTGATGTGTATGGATCAAATTACTATGTAAACTGCTATTTTTCATCCAAAGA
TAATGTAGGTAAAGTTACAGGTGGTAAAACTTGTATGTATGGAGGAATAACAAAACATGAAGGAAACCACTTTGATAATGGGAACT
TACAAAATGTACTTATAAGAGTTTATGAAAATAAAAGAAACACAATTTCTTTTGAAGTGCAAACTGATAAGAAAAGTGTAACAGCT
CAAGAACTAGACATAAAAGCTAGGAATTTTTTAATTAATAAAAAAAATTTGTATGAGTTTAACAGTTCACCATATGAAACAGGATA
TATAAAATTTATTGAAAATAACGGCAATACTTTTTGGTATGATATGATGCCTGCACCAGGCGATAAGTTTGACCAATCTAAATATTT
AATGATGTACAACGACAATAAAACGGTTGATTCTAAAAGTGTGAAGATAGAAGTCCACCTTACAACAAAGAATGGATAATGTTAAT
CCGATTTTGATATAAAAAGTGAAAGTATTAGATATATTCGAAAGGTAAGTACTTCGGTGCTTGCCTTTTTAGGATGCATATATATAG
ATTAAACCGCACTTCTATATTAATAGAAAGTGCGGTTATTTATACAGTGAATCTAAACTATAATAATTGGAATCATCTTTTTGAAATT
TCGACATCTAGATGAAATTGTGTTGAACCAACAGTTTTATTATCTTTGTAAATTTTTAAGAAGCTTTCTGGAAGACCGTATCCTGTGT
AAAATAAATCATACGAGAAATTAGAACCATCATTCATATGAAATACTACACTCCCCTTATCAAAACCGCTATAAAATTTTGAAGAA
GTGCCGTATTCTTTACCTTTACCAGTATCATTGTGTCCATAAATATTAAATTCATCTTGCAAGTATTTTCTTAGTTTGACATCTATTTC
TTGTGCGGTAACCATTTTTTTATCTGTAGAAACACTTTTAGTTGTAACTGTTTGATGCTTGCCATTGATAATTAAATTTATAGGTATTA
TTCTTGGCGAATCTAATTTATTGTTTTCTGCTTTAGTAACACCACCATATATAGTACGAGAGTTAGAACCATATTTATAGCTTATACC
AAAAACATCGACATTTTTCCCTTTAGATCACTAGTTATATATTCATTGTCAAATTCAGCATACAATGTATCATTTTTATACGAGTATT
CTAAGCGGTGTGATTCTGGTGAATTTTTGTCTTTAACATTCTTTAAATTCACATATTCATATTTAGTATAAAAATTTCTTAGGTTTCCT
GTACCTACATCGCCGTTAGATAAGACTGTATGATTAGAAGAAAAAATAAATAAAGTAATAACAATTACAAATAAGTAGTTCAGTTT
TGGAGTACAAAATGACAAAATGTACTTCAAAATGGACTACTTTTTTCATTTTAACTTACATACCAAATTACATACAAAATGATTTAT
AAAATGCAATTGAAAATGATAATTGAATGAAAATTTGTGATTTTGTGGTATTATGATAATATAACTTATAGAAAAAATTGCTTATTC
AACTAACGGGCAGGATAATGAGAAAAGGTTTTTTGATAGAAATAAGAAATTAGATGGTAAATTAGTTAAAAAGGCTGTGTTATAAA
TGAAAGTTTCTTTGATTGCTGCGATGGATAAGAATAGAGTGATTGGCAAAGAGAATGACATTCCTTGGAGGATTCCCAAGGACTGG
GAATATGTTAAAAATACTACAAAGGGACATCCGATAATATTAGGTAGGAAGAACCTTGAATCAATCGGAAGAGCCTTACCTGACAG
AAGAAATATTATTCTGACGAGAGATAAGGGGTTTACCTTTAATGGTTGTGAAATTGTTCATTCAATAGAAGATGTTTTTGAGTTATG
TAAAAACGAAGAAGAAATTTTTATTTTCGGAGGAGAACAGATTTATAATTTGTTTTTCCCTTATGTTGAGAAAATGTACATCACAAA
AATACATCATGAATTCGAAGGAGATACTTTTTTTCCAGAAGTGAATTATGAGGAATGGAATGAGGTATTTGCCCAAAAAGGGATAA
AGAATGATAAAAATCCGTATAACTACTATTTTCATGTATATGAAAGAAAAAACTTATTGAGTTAACGGGTGCGTTAATTGAAGAAG
AACAGTAAAGATAAAGCACAAGAGCTTGGGATTACTCCAAGCTCTTTTTGTCATTTGAAATAAATTATATCTTTCCAATCAAATTGT
TTATCTGTAATCCCTAATCTTTGAGCAGGTGTCAAAACCTTTTTATCCGCTGATTTAAAAGGGCGACAAAAGTTGTAGTAAGTTCTTA
GAATTGTAATCGCAAATTGAGCATACTTCGGATTGAAGTTAGCGTAAATATAGCTCTTCTTATCACCACGAGCTGTTGTTAATGGTC
TTTCAAGAGATGATATATAACGTCTTATCTGTTGAATAAAGCTATTGGTCGAATGGTCATTAACATTTAATAGCATCTTTGCGATTTC
GTTTGGTTCCAGAGCAGATAAATCCGTTCTACAATCAACTGAATAAAATCCTTTATCCTTAGTGGCTAATGGATGTTTGATAGGGTT
ATCGGCATATTTGCGATATCTTTCTCCATCTTTACTAATAGCCTCTTCGTGGAATTGATGACTTGTTAGTAATTCAGTTAGATAATGA
GATGCCAAAGTCCGTAGAAACTTTGTTTTGAATCCTCTGATATCTCCCCAATCGAGCAGTTCTGCTTTTGCTTGTCCGAATTCTTTTA
AGCATTGTTTTCTGCTTTTCGTCTTGTTAACTTGGCAAATAAAATGATGTGCATCGCTCAACCTTAATTCTTTCGTGAATACTCTATAA
AAGGCGTTTCTAATAGAGAAGTCATCATCACTAATCATTCGCCATTCGTCGGCATTAACCATTTGTTTAATTAACCAATAGTGAGCA
GTTGTAGTATAAGGAGCATCAACATTTAAACCATCGACAAATCTACTCCTATTAGTAATTTTCCCTAATTCGTGAAGATAAGCATTC
CTATTTTCGCTATCGTTAGCGGATGGTTCTTGTGGATAATAAGACCAATCTAACCTATCATTTTTACGACTAAAAGTATTGAGATGGT
CTTCTTTGAATTTTCTTGTATCTTCATTTAATTCATCCATTGAAATATTCCAATCATAGGCAACATCAGAACGGAACACATATCTTGA
TAAGACCTCAGCAGATACAACTATATACGTTTGCATATTAAGGTCTTCAAACCCCGTATATTTCTTTGAACCTTGTCCTTTCTTACGG
ACGTTATTTAGGTAGTAGTGCATCTTGTCCGTATTAAGCCACATCTCGTTAAATTTCTTTGTTTGAAGGGGTTGTGTCTCATATCGTTC
TAAAAACTCCAAACAACGCCTATACAGCCACTCCAGCTTATGATAGTAGGTTCCAACTCCAATTCCTAAAATATCACAAGTACGACT
GACTGAAACCCTTCCAACAACCATCTTAGCGAACATTGGAAGAATGGTGTTCTTTTGTTGGTGATAAGTAGTGGTTTCTTCTCTTTTT
GGTAGAACATTAGTGAACTTCTTACACGCCTTACATTGATAGCGTTGAGATTTGCCCCTACTTTTCCCCCTTTTGTAAAATTGCTTCG
GCTCATTGAATGGGGTTGATTCTTCTTCCGAGCAGCCTTCTTTATGAAATTGATAATCTGGCTCAACATCTTGGATGCTATTGATTTC
TATAAGGCGTTTAATTTCTTCAACAACAGACCAATTAGATAAAGGGGTTACTGTACAGTTTTGCGATACACCTCTGCCAATAGGATT
GTCATTGCAGTACATACCTTTATGACCTTTATCTTTGGAACTACCAGTCATTTTATATCGACTTGGTTTCCCTTTTACATCTTTGAATT
TGTGCTGTTCTTTCCCAAAATTACAACAAAACGGATTGATGCAGTAATTATATTGAATGGGATATTCAGAACCGTTCCATTTAAAAG
ACGTTGGCAAGAACAACATTTTGTTATACCTTGTTGCAAATCGTTTTGGAGTTAATAAATCATATTGTTTAGCCCTATCTTTTAATTC
TTCGTTTGAAATCGGAATTTTAACATCTACAATTTCATCTTCCTTCGTGGCTAATCTTTTAAGCTTGGTCATTGTCTATCAACCCACTC
TATCTTTGATACCCATTTTTTCAACAATCCGCTTATTACTTCGCTTGTTGGCAATCCATTCAATAAACTTCTTATCGTCTAAAATGTTC
AATAAGAATTCATCCACTGCTTCGCTTTCAGTACGCTCTGCATATTCAGCGTATTGCTTAACTATCACCCTTACTTGCTCTGATATTTC
CCAATCCACAGACTTAGCGTTCTTATTTTTTGGCTCAAGAAATTGCATAGGAAAATGCACCCCTTTTTGCTTAGTAAATTGTAACCAT
TTTAACACACCAATTTGCATTCCTACAATAATTATTTCACGTTCGCATTTTTATATACATTTTAACTTACATTTTGTGGTGTAAATTAA
CAACAAACGTTGATTTAACAATAAAAAACGAGTATCAAATTCAGTTGCAATTTGATACTCATTTAGGTTTACATTTTCACTTTTTGTA
CTCCAAAACTGAACTACTTA

>024-contig_320

TCTTTTTTTCATATTAGGCACTTCCTTTTCATTATATTTATAATAACATTATAAATATAAGTTGAATAATAAATTTTAGGAAATTCATA
AAAAATACAAAAGGTTATAAAGGAAGAGTTATAGAAAAATATGTATCATCCCACACGTTGCAGGCAGTGAATACGTATTTGAATAC
GTTAATTATGAGGTGATGTTGGGTGCACAAATTTATATAGTTTTATCAATTTTAACATTTTACACACAACTGATTAGTCAATTTAAAC
GTTGATATGACAATGCTTATAGCGAGTTATACATGAATAGATAAACGCTTTAATGAACTCCCGCCGTCTCCATATTTGTAGCCTACA
ACCTTTGTGGATGTGGGCTTTTTTATTATGTGTTTTTCAGGGGATAATGCATTGTAGAAATAGTTGTGAGTATTGAAATAGCAGTGTT
TGTATAGGTGTTTATTTGATGGAGGAAAGAGTAATAAGTGATTATGAATTAGTTTTTGAGATATAAGAGGAAAGGGATGTGTGTCA
AATAAGTGTCAAAAAAAGTTGAGCTTAGAGTTTTAGAATGTAAATTTTATTTGCAAAATTAATTAAGCTAAAGAAGTATCATAAATAA
GAGAATTAAATATATAGTTTAGAATTAGAATCAAGATTTTTATAAATATTATATTTTTCTTTTAATTGACTTAAATTTGATATATTGTT
TAAATGTAATGAGGAAAAGTTGTGATAGTTTCATATGGTAAATTAAAGGTTTTAATATAGCTGTTTTTTAATCGATTTTTAAGGATG
ATATCGTTTGCGAGTGAACGTATCTATTTAGTGAAATAGTAATTATAGATGGGGGCGTAATCAATGAATGACTTGAGTTTATCTTCA
TTTTTGAAACGCAGTAACAAATTTATGCAATTCAATTGTTTTATTTGTTTGATTCTAATAATAGTATTTTACATAATTGGTATGAATAT
ACAGGACTTTAGTGATTTTCCTAGTAAAGATTTAAATCATAAAGTGACTTATAATTTTAATGGGTTTTTGGAGATATTTGTTAATAAT

GCTTTTATAGTTCCTTTGGTGTCACTTATATTATCGATAATACCAATACCATATTTGTATTTTATTCCTACAATTTCTACAATTTATTC
ATTGTCGGTTATTATTGGGGTTACATTTTCATATAAATTAAACGAAGGGATAGCTATTTTTATTGGTATTTTGCCTCATGGTATTTTA
GAAATATACTTAACAAGCATTGAACTATCAATGTTATTCTTATTAAATGCGTATATTAGAAAAAATTCAATGAATTTATTTAGAAAA
AGAAAAGAGACATTGCCAAAATTTTTTGTTTTATTAAAATCAATAGTAAAGTGTTACCTGCTAATATTTTTACCTGTGGCATTTTTAT
GCGCTTTAATTGAAATTACTGTTACACCAACTGTTTACAAATTTTTAACTAATATAATATAGAACTTTTAAAAGGGCATAGCTAACT
ATGTGTATAGGTTTATTTTTGATCGATAAGTATTGTTTGAAAAATGAGTTCACGAAAAATGTTGCATGGTATTCGTGAAATTATGATC
GAGTCATTTCAAAAATGACCATTTCAAAACAACAAAAAAGAGTAGGCGAGCTACTCTTTTTTGTTATATTTAACAATTAAATTAGTA
TGTCATTATGAGTGTAAGTAAGCTAATCTATAATGACAATAATAACAGTAAAGATTATAACGTTGAATGCGAGCATTCATAAATTTA
TACTTCATCTAAACCACTGTGGTCGTCATCTTTTTGCTTTTCTTTTTCTTTCTCTCGTTCTTGTTCTTTTTTGTACTCTTCTTCAAATTCT
TTTTCTTTCTTTTCTACTTCTTCTCTTGTTTCCGCTCTATGAGAAAAAATCTTCGGTTTTAAGTTTACTAAATTTGAATGATTTAGAATCA
ACTGTTTTATCTTCTGAGTATTTATGGACATTTAAATTAATATTTCCATCACCTCTTAACTCATAGATGAACATGGCTTGTGCAGTTTT
GTCTTTTTTAATTTGATCTTGGTTATGTTCTGTCCAATCTTTATATTTTTTATCACTTAAAAGATAACCATCTCTTAATTTATTTACTGT
ATTTTTATCATCTTGAGTAATATTAATATAGTCATGAGAAATAGAAGATGGATTTAAATCTTTATCGTCTTTTTTAGCAGTAATTTCC
ATTTTAAAAGCGATATATTTCTTTTTCTCATCTTTTTCATTGATGATAAACGGTTCTTTTATTTTAGCTTCAAATTTGTCACTAACAAT
AGTATCGCCTTTAATTTTTATATCCATATTTTTTTTGCTTTTAAATTCTTTAAGTTCTTCATTTAATTCTTCATTGTCATTTTCTTTCTTT
TTGTGACTAGTGCTCTCTTTTTTTGCACTATCTTGATGATGTCCACAAGCACCTAAGATAAGTGTACTTGCTAATAATATCCCCATTA
CTTTTTTCATTTAACATGTCTCCTTTATTTCGCAAAAGTTTATTTTAAAAACTCTAAATGACTTATCATTTTGAGTAATTAAACAAAGT
TGATATTTTGTGAGATTCTAAGATGATATTAAATAATTCTTGTAATAATGATCCTATGTATTGTTGCAATAAATTAATGAAACTATAA
TTACTAATATTATATTACTTTTATTGATAGAAATATATTACTTTTTTAAAAAACTTGTAATATATCGAAAGATTTAAATGTAAAATTT
TGATTTGTTAAGAAATTACGTTTGTAAAAATAAAAAAATCAACTTATTTGTATGAGATAAATATGTATTGAAGAAGAAGTGTTATTA
ATTTGGAAAATACTGGTCAAAGATGGGAGCTCTCAAAAGCGTTATTGTATTTTTTAGTCAATACAAATAGATTGCCGTAATAATAAT
CGTACTTGATGGTTAAAAAATTACTTAAGGCTATAAAGCAAAACTTTTTATATGAGCAGTCGAATATAACGTTTAAAATGATTGTTT
TTGGATATAAACGATTAAGTAAAATGCTTTTTCAGTTTGAAATTAATCATATAAATTTCTTATGGGAGGGTTGATATCTTAATGATTA
ACATTATTTCAGCTATAGGATCTATTGGAACATTTATTATGGCTTTATTTTATTTTGTATCAGTTTCAGTTCAACTTTATCAAATGAAA
ATTAGCTTTCTGCCAGCTTTAGGTTTTAACCAAATTTTATTAGAAAGGGAGGAGGATCAACTTAATATAATGAATTCGGCAACAGAA
GAGCATCATCATAAAGATTATATTAAACTATATAATTTAGGTGGCGGTGCTGCTAAAAAAAATTGCAATAGAGGTTTTATTGGGTAAT
GATAAAGTCATTCAGAAAAAAATACGTGAATATTTTACCTAGTAAAGAAGGGGACATGTTACCAATTAATAAAAATGTGTACGAAGA
ATTAGAAAGAACGATTGAGAACAATGGTTATGAAGCTGATTTGAATGTACGTATGACTTATTATCATAATGTAAGTCGCAAACAAC
AGGAAGTTATATTAAAAGGTCAAATCGACCGTTTTAATACTTATAATAATAAAGAAATTTATGATTTGCAGTTTATCTAAAAATTGA
TTTAAGAGGGTAGTTGTTTATTGCGAAAAATATCATTCAATTTTAATGAAATAATGGCGTCATTACTATAAAATATTACTTTATGTTG
TAATGCATTTTTCTATAAGATAGAACTAAAAGGAGGGGCAAAGATGCAAATTAGACAAATACATCAACATGACTTTGCTCAAGTTG
ACCAGTTAATTAGAACGGCATTTGAAAATAGTGAACATGGTTATGGTAATGAATCAGAGCTAGTAGACCAAATTCGTCTAAGTGAT
ACGTATGACAATAACTTAGAAATAGTAGCTGTTCTTCAAAACGAAGTTGTAGGGCACGGTTTACTAAGTGAAGTTTATCTTGATAAC
GGAGCACAACGGGAAATTGGATTAGTGTTAGCACCTGTATCTGTTGATATTCATCATCAAAATAAAGGTATTGGGAAGCGATTGATT
CAAGCATTAGAACGAGAAGCAATATTAAAAGGATATAATTTTATCAGTGTATTAGGATGGCCGACGTATTATGCCAATCTAGGATA
TCAACGCGCAAGTATGTACGACATTTATCCACCATATGATGGTATACCAGACGAAGCGTTTTTAATTAAAGAATTAAAAGTGAACA
GTTTAGCGGGAAAAACAGGTACCATAAATTACACATCTGCTTTTGAAAAAATATGATTTCAAGCTAGGATTACATTAGGCAGAGTTC
ATATTAATAATACAAATTGTTTGCAATGAAATCGTACGTTGTCGTTTGCAATTTATAAAATAGCAATAAATAAAATGTTTGTTAGGA
AAGTATTATTGTGGATAATAAAAAATCGATACAAATTAATTGCTATAATGCAATTTTAGTGTATAATTGCATTAATGGAGATTAAAT
ATATCTTTAAAGGGTATACAGTTAATATAAAATGACTTTTTAAAAAGAGGGAATAAAATGAATATGAAGAAAAAAGAAAAACACG
CAATTCGGAAAAAAATCGATTGGCGTGGCTTCAGTGCTTGTAGGTACGTTAATCGGTTTTGGACTACTCAGCAGTAAAGAAGCAGAT
GCAAGTGAAAATAGTGTTACGCAATCTGATAGCGCAAGTAACGAAAGCAAAAGTAATGATTCAAGTAGCATTAATGCTGCACCTAA
AACAGACAACACAAACGTGAGTGATACTAAAACAACGTCAAACACTAATAATGGCGAAACGAGTGTGGCGCAAAATCCAGCACAA
CAGGAAACGACACAATCATCATCAACAAATGCAACTGCGGAAGAAACGCCGGTAACTGGTGAAGCTACTACTACGACAACGAATC
AAGCTAATACACCGGCAACAACTCAATCAAGCAATACAAATGCGGAGGAATTAGTGAATCAAACAAGTAATGAAACGACTTCTAA
TGATACTAATACAGTATCATCTGTAAATTCACCTCAAAATTCTACAAATGCGGAAAAAGTTTCAACAACGCAAGATACTTCAACTGA
AGCAACACCTTCAAACAATGAATCAGCTCCACAGAGTACAGATGCAAGTAATAAAGATGTAGTTAATCAAGCGGTTAATACAAGTG
CGCCTAGAATGAGAGCATTTAGTTTAGCGGCAGTAGCTGCAGATGCACCGGCAGCTGGCACAGATATTACGAATCAGTTGACGAAT
GTGACAGTTGGTATTGACTCTGGTACGACTGTGTATCCGCACCAAGCAGGTTATGTCAAACTGAATTATGGTTTTTCAGTGCCTAATT
CTGCTGTTAAAGGTGACACATTCAAAATAACTGTACCTAAAGAATTAAACTTAAATGGTGTAACTTCAACTGCTAAAGTGCCACCAA
TTATGGCTGGAGATCAAGTATTGGCAAATGGTGTAATCGATAGTGATGGTAATGTTATTTATACATTTACAGACTATGTAAATACTA
AAGATGATGTAAAAGCAACTTTGACCATGCCCGCTTATATTGACCCTGAAAATGTTACAAAGACAGGTAATGTGACATTGGCTACT
GGCATAGGAAGCAATACTGCTAGTAAGACAGTTTTAATCGACTATGAGAAATATGGACAATTCCATAATTTATCAATTAAAGGTAC
GATTGATCAAATCGACAAAACAAACAATACGTATCGTCAAACGATTTATGTCAATCCAAGTGGAGACAATGTTGTATTACCAGTGTT
AACTGGTAATCTAATTCCTAAGAGTAATAGTAATGCTTTAATAGATGCCAACAATACTAATATTAAAGTTTATAAAGTGGATAATGC
TGATGATTTGTCACATAGTTATTATGTGAATCCAAATGATTTTGAAGATGTAACAGATCAGGTCAGAATTTCATATCCTAATGATAA
TCAATATAAAGTAGAGTTTCCAACAGACGATGATCAAATTACAACACCGTATATTGTAGTTGTTAATGGTCATATTGATCCGAATAG
CAAAGGTGATTTAGCTTTACGTTCAACTTTATATGGATATGACTCAAGGTTTGTATGGAGATCTATGTCATGGGACAACGAAGTAGC
ATTTAATAACGGATCAGGTTCTGGTGACGGTATCGATAAACCAGTTGTTCCTGAACAACCTGATGAGCCTGGTGAAATTGAACCAAT
TCCAGAGGATTCAGATTCTGACCCAGGTTCAGATTCTGGCAGCGATTCTAATTCAGATAGCGGTTCAGATTCGGGTAGTGATTCTAC
ATCAGATAGTGGTTCAGATTCAGCGAGTGATTCAGATTCAGCAAGTGATTCAGACTCAGCGAGTGATTCAGATTCAGCAAGTGATTC
AGATTCAGCAAGTGATTCAGACTCAGCAAGTGATTCAGATTCAGCAAGCGATTCAGATTCAGCGAGTGATTCCGACTCAGCGAGCG
ATTCAGACTCAGGTAGTGACTCAGATTCCGATAGCGATTCCGACTCAGATAGCGACTCAGATTCAGACAGCGATTCTGACTCAGAC
AGTGACTCAGATTCCGATAGCGATTCCGACTCAGACAGTGACTCAGATTCCGATAGCGATTCCGACTCAGACAGTGACTCAGATTCA
GATAGCGATTCAGATTCCGACAGTGATTCCGACTCAGATAGCGATTCCGACTCAGATAGCGACTCAGATTCAGACAGCGATTCAGA
TTCAGACAGCGATTCAGATTCAGATAGCGATTCAGATTCCGACAGTGACTCGGATTCAGATAGCGATTCAGATTCCGACAGTGACTC
AGATTCCGACAGTGACTCAGACTCAGACAGTGATTCGGATTCAGATAATGATTCGGATTCAGATAGTGATTCCGACTCCGACAGTG
ACTCGGATTCAGATAGCGATTCCGACTCAGACAGTGACTCGGATTCAGACAGCGATTCAGACTCAGATAGCGACTCAGAATCAGAC
AGCGACTCAGATTCAGATAGCGACTCAGACTCGGATAGCGATTCGGACTCAGATAGCGATTCAAACTCAGACAGCGATTCAGATTC
AGACAGCGACTCAGACAGTGACTCAGATTCAGATAGTGACTCGGATTCAGATAGCGATTCAGATTCCGACAGCGACTCAGATTCAG
ATTCAGCGAGTGATTCCGACTCAGAAAGTGATTCAAATAGCGATTCCGAGTCAGGTTCTAACAATAATGTAGTGCCGCCTAATTCAC

CTAAAAATGGTACAAATGCTTCTAATAAAAATGAGGCTAAAGATAGTAAAGAACCATTACCAGATACAGGTTCTGAAGATGAAGCG
AATACGTCACTAATTTGGGGATTATTAGCATCATTAGGTTCATTACTACTTTTCAGAAGAAAAAAAGAAAATAAAGATAAGAAATA
AGTAATAATAATATTAAATTAATCGTATGATTCATGAAGAAGCCACCTTAAAAGGTGGCTTTTTTACTTGGATTTTCCAAATATATTG
TTTGAATATAATTAATAATTAATTCATCAACAGTTAATTATTTTAAAAAGGTAGATGTTATATAATTTGGCTTGGCGAAAAAAATAGG
GTGTAAGGTAGGTTGTTAATTAGGGAAAATTAAGGAGAAAATACAGTTGAAAAATAAATTGCTAGTTTTATCATTGGGAGCATTAT
GTGTATCACAAATTTGGGAAAGTAATCGTGCGTGTGCAGTGGTTTCTGGGGAGAAGAATCTATATGTATCTGAGTCATTGAAACTAA
ATGATAACAAAAGTAAAAGCTTATCATTAGAAAAGTATAAAGAGAGTCTTAGAAGTATAATGTGTACAAAAGAAATCAACAAAAA
TGATGGATATGATGAGCCTGAATATAAAGAAGCACTGAATACTTACAGAAAAAAATTATTTGCTGAATTAGATGCACTTAATAAAT
TTTTAGATGAAGAGAGAAAAAATAGCCAGTTACATTAAAAAAAATATGGAAGTGCCAAGTAATATTTTAGGATTAACACACGGTAGG
TATACAGCAATATATAATGCAATTAAAGAAAATAAAAATGAATTCGAAAAAAATGTAGAAAATATTGAAAGTAAGCATCTGGATTT
AAAAAGGTTTGATGAAGACAAAGATTATGAAGCAAGAGTTAAATTAAATGAATTAGAAAATAAAGTTTTAATGTTAGGTCAAGCTT
TTCCTGATAAAGTAGATGCTAGAGAAAGTCTTTATAATAAATTGGATATGATTGTTGGTTTAAGTAATGATGAAATTGAAGAAAGAC
ATCCTCAAAATGAAAGGTTATTAAAAGAACGAGTTGAAGATTTAGAAACAATTATCGATGAATTCTTTAATGACATAGGTGAAAAT
AGACCGGAGAATATATCGCCATTAACTAACAATGAATATAAAAATAAGGAAATGATTACAAAGCTAAAATCTGACACTGAAGCAG
CTAAAAATGACGAAAACAAGAGATGTAATCGAAGTAAAAGAAGTTTAGATTCTCAAAATTATAAATCTGCTTCACAAGAAGTAACT
GCAGAGCAAAAAGCAGAATATGAAAAAAGAGCTGAAGAAAGAAAAGCGAGATTTTTGGATAATCAAAAAATTAAGAAAACACCT
GTAGTGTCATTAGAATATGATTTTGAGCATAAACAACGTATTGACAACGAAAACGACAAGAAACTTGTGGTTTCTGCACCAACAAA
GAAACCAACATCACCGACTACATATACTGAAACAACGACACAGGTACCAATGCCTACAGTTGAGCGTCAAACTCAGCAACAAATTA
TTTATAATGCACCAAAACAATTGGCTGGATTAAATGGTGAAAGTCATGATTTCACAACAACGCATCAATCACCAACAACTTCAAATC
ACACGCATAATAATGTTGTTGAATTTGAAGAAACGTCTGCTTTACCTGGTAGAAAATCAGGATCACTGGTTGGTATAAGTCAAATTG
ATTCTTCTCATCTAACTGAACGTGAGAAGCGTGTAATTAAGCGTGAACACGTTAGAGAAGCTCAAAAGTTAGTTGATAATTATAAA
GATACACATAGTTATAAAGACCGATTAAATGCACAACAAAAAGTAAATACTTTAAGTGAAAGTCATCAAAAACGTTTTAATAAACA
AATCAATAAAGTATATAATGGCAAATAATTAATGCATGGCTGCAAAGCAAATAATGAGTTTGTCGTAAAAATAACAACATTTTAAA
CTAGCAATAAATAATATCAAAGTCATCATTTCAATGATGCAATCTAGTATAGTCCACATTCTAAACAGGTGTGGACTATTACTTTTTT
CACTTTATATTACGAAAAAATTATTATGCTTAACTATCAATATCAATAATTAATTTTAAGCTGAAAAACAATAAAAATGTTAAGACA
ACGTTTACTTCAAGTTAATTATTATACTGAAAATTCTGGTATATAATGCTGTTAGTGAATATAACAGGGAAATTATATTGGTTATAAT
ATTGAGTCTATATAAAGGAGAAATAACAGATGAAAAAGAAATTATTAGTTTTAACTATGAGCACGCTATTTGCTACACAACTTATCA
ATTCAAATCACGCTAAAGCATCAGTGACAGATAGTGTTGACAAAAAATTTGTAGTTCCAGAATCAGGAATTAATAAAATTATTCCA
GCTTACGATGAATTTAAGAATTCGCCAAAAGTAAATGTTAGTAATTTAACTGACAATAAAAACTTTGTAGTTTCTGAAGACAAATTG
AATAAGATTGTAGATTCATCGGCAGCTAGTAAAATTGTAGATAAAAACTTTGCCGTACCAGAATCAAAGTTAGGAAACATTGTACC
AGAGTACAAAGAAATCAATAATCGCGTGAATGTAGCAACAAACAATCCAGCTTCACAACAAGTTGATAAGCATTTTGTTGCTAAAG
GCCCAGAAGTAAATAGATTTATTACGCAAAACAAAGTAAACCACCACTTCATTACTACGCAAACCCACTACAAGAAAGTTATTACT
TCATACAAATCAACACATGTACATAAACATGTAAATCATGCAAAGGATTCTATTAATAAACACTTTATTGTTAAACCATCAGAATCG
CCTAGATATACACATCCATCTCAATCTTTAATTATCAAGCATCATTTTGCAGTTCCTGGATATCACGCGCATAAATTTGTAACACCTG
GGCATGCTAGCATTAAAATTAATCACTTTTGTGTTGTGCCACAAATAAATAGTTTCAAGGTAATTCCACCATATGGTCACAATTCAC
ATCGTATGCATGTACCAAGTTTCCAAAATAACACAACAGCAACACATCAAAATGCTAAAGTAAATAAAGCATATGACTATAAATAC
TTCTATTCTTATAAAGTAGTTAAAGGTGTGAAGAAATATTTCTCATTTTCACAATCAAATGGTTATAAAATTGGGAAACCATCATTA
AATATCAAAAATGTAAATTATCAATATGCTGTTCCAAGTTATAGCCCTACACACTACGTTCCTGAATTTAAGGGTAGCTTACCAGCA
CCACGAGTATAAAAATTGGCACTAAGTTTACGAGATATGATAAATACCTATTATTTTAAATATAGTCTACAATCTATGTGGTTGTAG
GCTGTATTTTTTGCAGTTTATCAATAAACACCCATCAACAAATTATACCGTTTTTCTACTTTGAAAGTTGGAAGTAACATAATCTTAA
TTAAATATATTATTAATTAAGATAAATATAATACTCAAGATTATTGTTAATAGTTTGATCATCGCAAGTTAATTATTGTTTCTAAAAT
ATTGGTATATAATTTTCAATGGCGAAGAAAACAGGGTGAAAAAGTCGGTTTTTATATCAAAGCAAATAAGGGAGCATAAACAAATG
AAAAGGAAAGTATTAGTACTAACAATGGGTGTAATTTGTGCAACTCAATTATGGCATTCTAATCAAGCAAACGCATTAGTGACAGA
GAGTGTTGAAACTAATTTTGTTGTAAAAGATTCGGATAATAAAAATATTTTACAACCCCATACTGAAATTACTACTGAGGAGAAATT
TTCAATAGTAGAAAAGAGTCCATTAAATAAATTAAAATCACTATCTAATGACAACTACATAGAATATGATTTACATACTAATCAAAC
AGGCATTAAAAAAGGTTGGTTTTATGGTTATAGTGAAATTGACTCATCACATTTCACAGACCGTGATAAACGTGTTATTAGACGTGA
TCATGTTAAAGAAGCACAAAGCTTAATTAACGATTATAAAGATACACAAAGCTATGAAGATCTCGCTAAGGCAACTGCAAAAGTAA
GTACACTTAGTCAGTCTCACCAAAATTATTTAAATAAACAAATTGATAAAGTGAATAATAAGATAGAGAAAACTGAAAAACGCTAA
TCCAAAGTAAATTATAAGTTATACATCTCGTTTTTAAATGACAATTTATCCCCGTAAATATTATAAATAATCTTTTCAAATTCCGCAT
AGATATAGAGACGCTAATAAACCTCTTTGTCTCGATATGATAGTCTGCAACGATTCATGTTGTAGGCTTTTTAATTTTACAAATAAG
GCTAAATATATAAGTTCTGGCACCTAAAATATAGAAAATACATAAAAGTAAGTATAGTTATTTTATTATAATTATTAAATTTTTATTA
ATTAATTGTAAAAATGTAGAATTACAATTAATTAACGTTTAATATTAAAATTAACTAAAAAGAAAGAGGTGTTAGTTATGACAGAA
TACTTATTAAGTGCTGGCATATGTATGGCAATTGTTTCAATATTACTTATAGGGATGGCTATCAGTAATGTTTCGAAAGGGCAATAC
GCAAAGAGGTTTTTCTTTTTCGCTACTAGTTGCTTAGTGTTAACTTTAGTTGTAGTTTCAAGTCTAAGTAGCTCAGCAAATGCATCAC
AAACAGATAACGGCGTAAATAGAAGTGGTTCTGAACATCCAACAGTATATAGTGCAACTTCAACTAAAAAATTACATAAAGAACCT
GCGACATTAATTAAAGCGATTGATGGTGATACGGTTAAATTAATGTACAAAGGTCAACCAATGACATTTAGACTATTATTGGTTGAT
ACACCTGAAACAAAGCATCCTAAAAAAGGTGTAGAGAAATATGGTCCTGAAGCAAGTGCATTTACGAAAAAAATGGTAGAAAATG
CAAAGAAAATTGAAGTCGAGTTTGACAAAGGCCAAAGAACTGATAAATATGGACGTGGCTTAGCGTATATTTATGCTGATGGAAAA
ATGGTAAACGAAGCTTTAGTTCGTCAAGGCTTGGCTAAAGTTGCTTATGTTTATAAACCTAACAATACACATGAACAACTTTTAAGA
AAAAGTGAAGCACAAGCGAAAAAAGAGAAATTAAATATTTGGAGCGAAGACAACGCTGATTCAGGTCAATAATGCTCATTGTAAA
AGTGTCACTGCTGCTAGTGGCACTTTTATAATTTTTGGATCACGATATGATTTATTATCAATTCAGAATTAAAAAAGTAAATAGTATC
AAAAGCAAGTGTATTTAATATTAGAAAATAAAAATTTTAAATTTAGTATTAAAATGGAATATTACTATATAGTTCAATGTGTATTAT
CACAGAAAATAAAATAATGCTTTACTTCTATATTTAAAAGTGTATAATGAAAGTTAAGTAATAAAGAGCGTGAAGAAAAATGTGAG
TTATTTATATAGAATATTCTCCTTTTCATTTATGAATTTGTTACAAAATATTTAGTGCAAAAGCACGACGGAGGTATTCAATATGAAT
AACGGTACAGTTAAATGGTTTAATGCAGAAAAAGGTTTTGGTTTCATCGAAAGAGAAGATGGTAGCGACGTATTCGTACATTTCTCA
GCAATCGCTGAAGATGGATACAAATCATTAGAAGAAGGCCAAAAAGTTGAATTCGACATCGTTGAAGGCGACCGTGGCGAGCAAG
CTGCAAACGTAGTTAAAATGTAATTTTAACTTATTCAAACAGCCCTTACTATAGGGCTGTTTTTTTATGCTTTAAATCGATAACAGTT
GGTGTGGTAAAAGCACTAGCTGTTATTTTTTTGTCCAATAAATATAGGTGGAGATCTAACAATATATAATGGTTCTATAATAAATCG
AATTGATGGAAAAGTTTTTTACTTTTCATCTGTCCGACTTTTGATTTTGAATATAAAAAAGCGCCAATACAGAACTTTAATAATGACG
AGAATTAAAGTCTGTATATGGCGATAACAAGAAGTAATGTTAAACACTCAAAATGTTTAACAATAATAGGATACCACATCGCATAA
TATCTTACTACTTAATTAATAATTTAACTAATCAACTTTTTGTTAATTTTTTATTAAGACTGATTAATTATTGAGAATATTTATTGTTT

TTAAAATCTCATAATAATTCAGTAATCTTGTTTTCATTTAAAAGGTGGAACATTAAAATAATTAAATAAAAATATTGCGTTTAATTTA
CAGCGTCAAATATACTTATTTCTAATGCTTTGGGGTCTACTGAAACAAGTAAAGAATGATCGATGTTACTAATATTGCCATTCTCCA
AATTTATTTCTGTGAGTATTTGGAAGCTACCATTAGGCAATGGTTTAACAATAGACAATTGCTTTTCCGCTTGTTGTATTAAAAAAGG
TTTTGTAGATTGATTATTAATATGCCATTCACTCATGTATGTTTTTCACTCCTGCTTTAAAATAGGGTTAAAAAGTTTATAGTTGAGA
CATTCATGTTCAACCAAAATTTTGTTCAAATTCAATAAATGTCTTGTTTAAAATAGAAATATTGTAAATGTTATCGTCCAAAATTTCA
CCAGTTAAGTATTTGTTTTGAATTAAAATTTGGCAGTTAGTTAAGAAGTCTTGATAATCACGATCGCAAAAATAGTTTTCACGTGCA
TCTTTAGCATCGCCAAAAAAGTTAGCGACTGTTTCTGTTTCTCCATTATTCGAACGTTCAATATATAATTTGTAAAATTTAGCTATTG
TATACTTTTGTTCTTTAGTTAGTTCATTCAAAATATTGGGCCTCCTGAAATATCATTTGTAATCTATACCCAATTTATTGCAAAACAC
AAACTAATTTAACTATTTGATGAAACTGTGTTAATAAGCTTTAACAAGCCTTAGTTTGTATGGATCTATAAAATTATCTTTAATTGCA
TAGGGTGAAATAATATGTAGTCCATAACTTTTAACTGATTTTTCACTTACACCAAATTTATAAGCTTGGTAGATAATTTTAGTACAAT
ACGTAAATTTTTTGCTGTTCAAATTTAATGTAACTAGATAACGATGATTTGTATTCTCATAGTTTTTCTTAACCCAGTCAGCCGCTTTT
TTACCTGCACCAGGATAGCTGCAACGATAAACTTTCATCCAATCATTTTTGCCACTTGCATAATTATATTTAAAAGATTCGAAGGATT
GTGTAGTTGGTTTGTCGCCAGGCCCCTCAATTTGTAAAATCGTTTTATCATCAATCGCGATACTACAATGACCAAAAAATCCCCACA
TGACAGGGCCTTTTGTAACAATAATATCACCAGGTTGTAATTGGAATTTGTCATCTTGAATTTCTGAATACTTATTATCTGCAATTGT
TTTTGGTGAGTTTATAGGGGATACGACAACGAATAATATAAGTAAAATTATCGTTCGTTTAATATAGTTCACTTAAAAGCTCCTTGTT
GAAGAAATATATGTAAATAGTCTTAAATTAGAATTGTAATCTTTAATAAGCTTGTAAGACTAAAACATATCTTAAATATTAAAGTAT
GAGAGTGTGAAATGTCTATTAAGAATAAAAAACAGTCTGAAACATCATTGAGACGTTTCAGACTGGATATAAAATGAATTTCATTT
ATAGCACACCAAATATAAATGTGTATATTAATATAGCGAATCCAAAAATATAGAGAATGACAGTGAAACTTAAATAGGACTCTTTT
TTAGATTCTTTGCCAGTTTTTTTCATTAATACGAGTATAAACGTAGCGGCAACTAAGAAAATTAATGCTAGCCAAAACAGAATTGCA
AAATGTAAAGACATGTGAAACCCTCCTTATATACCGTAATTATTAACCTATAAAACTATAAAAAAATAGGATAAATGTAATAGAGAA
CGAGTACGATATTTATAAAAAATAATATTTCACTTAACCAGTTTTTAGTTATCATTGCAATGGTAAAGGATACGATGAGTATCACAC
CACAAATGATAATACCAGGCAGGAGCCAACATAAATCATCTAAATCTTTATTATATGTGATTAAAATATTAAAGATAACAAAAGTG
GTAGTAATAACTATATTGATAGCATTTAACAAAATGTTATTCATGACTGACACCTACTAGTATAAAAATAGCTTCTTAATAAATATG
ATAACACTATTTTCCAATAGGTAAATAAAAAGTATAGTTATTTTTAAAATATTATTTGTAGAATGCTAAAGTTTATTAATTCAAACA
AGTGAGCATATATAGGGGTGTATAGTATTAATATGAAAAGAATATTTACATACTAAAAACTCTTAAAGTTGTATAATTCGTTAATAT
TTTTAGAATCAGATATGAAAAAAAATGTGCAAAATCACTTTTGCACATTTACAAATACTAGTTTTCTGATAAGATTAATAATAACACT
TTAAATAGCGCTTAATAAATGAAGGGGGCAAGTCTTATGACGTTTTACAATTTCATCATGGGTTTTCAAAATGATAACACACCATTT
GGTATATTGGCCGAACACGTTAGTGAAGATAAAGCATTCCCTCGATTAGAAGAAAGACACCAAGTAATTAGAGCATATGTGATGTC
TAATTACACAAATCATCAATTAATTGAAACTACAAATAGAGCTATTAGCTTATATATGGCAAATTAATTTGAGTAGTACCAATTATG
ATGTATTAGTGCATCCCAAATATCTTTTGTTTTAAAGTTTATTTCATCATTTCTTATCGAAAATGGTGTAATAATGTCTTTATCTAACC
AAGTGTTGATAAGTTCATTTGGTACACCATCTAACAACATTTCACTTTTACTAATTATAAAACATTCCCAGTCAAGTGAAACATTTTG
TGGATTCACATAATTACATTGATTATGATTATCCATAAACACTCACTCCTTTAAAATTCTGTACTCTTCATTGCGTTTTACCCCGTCAC
ATTATCTTTTAAACTAAAATCATCATTACTTATGAAAAAAATGTACATCAAAAGCAAAGGTTTTCGCTACCGAAAAAGTTTAAATAA
TGGTTTAATATATTTGGTACTCATTTTAATAAAAAGAGAATACATTTTGAGCTATCAATACTTTTTATTGAAGAGGTGTTATTGTTGG
CTAAAACGTTATATTTAATGCGCCACGGACAAACTTTGTTTAATTTTAAGGGACTAATTCAGGGATTTGGAGATTCGCCGCTAACAG
AACTTGGAATTGCTCAAGCTCAAAAGGCACGTAGTTATTATGAAACTAAGGGGATAAACTTCGATTTATATGCATCATCAACGCAA
GAACGCGCAAGTGACACACTTGAAAATGTTGCACCTAACCAATCGTATCAACGTTTTAAGGGACTGAAGGAATGGCATTTTGGATT
ATTTGAAGGTGAGTCAGTCTATCTATTTGATAATCTATACAAGCCTGAAGACATATTCGGAGATCGAATTGTTCCTTTCAAAGGAGA
GGCAAGGCAACAAGTTGAAGATCGCATTGTGAAAACTTTACATGACATTATGTCTCAAACAAAGAATAATGCATTAGTCGTGAGTC
ATGGAACAATAATGGGAGTATTTTTAAGATATTGCCTTAAACTAGATGAAGCATTAAAGCATAATATCGGTAATTGTAATATCCTGA
AATTTGAATATGACAATGGAACATTTAAATTTGTTGAGTTAATTGATCCAAATTTATAATAAATAAAGCGTTATACATAGACACAAG
CATGTCTTTTGTATAACGCTTTTAAAATACAACTATATAACGCTTTAGAATAATAATTGAATAAGTTTTTGTAATATCATCAGAGCAA
CAATAATAATTATTATGCTTGATAATTTATTGATTATTGTTAATAATTTTCCGGTTTTATCAATTGATCCAACCATTTTTCCTAAAATT
GCGAGTAAAAAGAACCATAACCATGAAACGCTAATACAAGCGATTGTAAACGCAATTTTATTGCTGCCACTATATAATGCAGCACT
ACTACCAATTACACCAATTGTATCTAAAATAGCATGTGGATTGAGTAATGAAACTGATAAAGCAAAACTTACTTGTTTCATTGGAGA
CATAATTTGAGCTTCTCCATCTGTTGAGGGTTTATCATGCCAAATGGTCCAAGCCATATACATCAAGAAAATTAAACCAACTATATA
AATAATTGCTTGAAGTACAGGTAAAGACATAATAATGATAGATACTCCTACCACTGCAATAATAATAAGTAAGCTGTCTGACAACC
CGGCTGTAATTATTGCAGGCAATACATATCTATATTTTGGTTGATTAGCTCCTTGGTTAAAAATAAATACATTTTGTGCACCTAAAGG
TAAAATTAGACCAATCGCTAAGATAAATCCATGAATAATTGCGGTTACCATTAAGTTTTGACACCCCTAAAACATAGAATATTCTGA
AAAAACTTATTGATATCATACCAAATAAAAAACAACTACTCAATAGTAGAGTAGTTGTATGAAAAATAGAACCTATATGATTTGAA
TTTATGAGAAATGTAGAATATATAAAACTGTATTACTTATTAATCAAATCATAAAAAAGCCAATGTTCATCGAAATAGGTATTTCCG
ATTTTAATTGCATTTTTCTCAAAACCAAGAATGTCGAATCCTAGTGCACTATAGAAAACTTTAGCACCTATGTTGTTTGACACAATTG
ATGTAAGTAAAGATTCGTAATTGTTCTGCCGTGCATAATTAATAATGAAATTAATGAGCTCGCTATTAATCGATTTATCGTTATTAGT
AACAAAGTTGTATTTAATTAATGATTTATGTTCTTTTCCAACGTATCGAATTTGTTCTAAGGCAGCTGTTGCGATAAGTGTTTCGTTTT
CATAACAGCCAAATACAATACATTTCGGTGAACCTTGATTTAAAATGTCATGTATTAAGGTGTCAGTCATTGTTTGACTATAATGTG
AATCTTGAGTGAATTCTTCATTGACTGTCGAAACAAGTCTCTTATACTCATTAAAATCATTATTCGTTAATGTTTTAATTTCAATCATT
ACTGACCTCCTTAATCATTATTAAGAAAGATTTTGTACTAAAAAAATTTCTTAATTACATATTAAAATGTTTATTAAAATTTTGCAAT
CACTGATTTATATGCACATTCTTGTAAATGGATTATCAATTTAATTATATTTATTTTTAAGTTCAGGAAATTATGGAATTGGTTATTG
GTCGACACTGGACTTTTATTTGTAGACGGTATGCGAAGAAGTCAAGCCAGTATTGGAACCGGTCGCATTTATGATGACTAAGGTACA
ATCTTTTTCAGTTAAATATTGCCAGAAATTTCAATCACGAATAGAAAAAATAACCAACCAAAAATTTTTGGTTGGCAAATTTGAATT
TTATGTGAATTTTATTAATAACGTTGAATAATCTCTTTAAGTTAATGCTAATCTACAACATTTACATTTAAATCGACATTAATATTTC
CTTGAGTCGCTTTTGAATATGGACAAAATTCATGAGCCATTTGTAAATATTTTTCAGCTTCTTCTTGAGATATAACATTTTTAATTGTC
GCATCAATTGAAACACTTAATTTAGGACTTTCTGAGTCTGAATCATCTTCTAGTCTCACTGTTAGTGTTACTTCTGGATGAGCATCAC
GCACTTTGTTTTGCTTTAAAATTAGGTCGAAAGCACCGTTGAAGCAAGATGCATAACCTGCTGCAAATAATTGTTCGGGGTTAGTAG
CTTTACCATCTGCTTGAGCAGGCGGAACGATATCAATATCTAATGCTCGATCATCAGTATAAACATGTCCTTTACGTCCGCCAACAT
TCGTCGCTTTAGTTTCATAATGTATTGCCATACTTATAACCTCCTAATTTAAAATACATTTATGCTTTACCCATTCGAAAATACCTTAA
TCATTTTCATTTATAGCATTGTTTGATTGAAGGATAAAAAGTTGTTGTTTACAATAAAAATAATGAGTATCTGAAATGAGGGATTCA
CTATGACACATGTGGAAGTAGTAGCGACTATCGCGCCACAATTATCTATCGAAGAAACTTTAATTCAAAAAATTAATCATCGTATTG
ATGCAATAGACGTATTAGAATTACGAATTGATCAAATTGAAAATGTCACAGTTGATCAAGTGGCAGAAATGATTACAAAGCTGAAG
GTTATGCAAGATTCATTCAAATTATTAGTTACGTATCGTACAAAGTTACAAGGTGGCTATGGGCAATTTACAAATGACTCGTATCTT
AATTTAATATCAGACTTAGCAAATATCAATGGCATAGATATGATTGATATAGAATGGCAAGCAGATATTGACATTGAAAAACATCA

ACGAATCATTACACATTTGCAACAGTATAATAAAGAGGTGGTTATATCACATCATAATTTCGAAAGTACGCCTCCATTAGATGAATT
GCAATTTATATTTTTTAAAATGCAAAAATTCAACCCAGAATACGTTAAATTAGCAGTAATGCCACATAATAAAAATGATGTGTTAAA
TTTATTGCAGGCAATGTCTACATTTTCAGATACTATGGACTGCAAAGTTGTTGGTATTTCAATGTCTAAACTTGGACTAATAAGTAGA
ACGGCTCAAGGCGTTTTTGGTGGTGCATTGACTTATGGTTGTATCGGAGAACCACAAGCTCCAGGACAGATTGATGTTACTGATTTA
AAAGCACAAGTGACTTTATACTAATAAAATGAATTAGGTTGCTTTTGTGTAAGTGCTGTCTTATAATTGAGAAGTATTATCATTGTTC
GTGGAGGAGCGATTTTAGATGGAATTACAACAAGCAATAGCTAATAGAAGAAGTGTGAAAAAATTTAAAAGAGATATGCACATAG
ATGACGCATTGCTATATCAAGCAATTGAGAAAGCTGCTGATGCTCCAAATCACGGAATGAGGGAACCATGGAGAGTTGTGCATGTT
CCGAAAGACAGATTAGGAGATATGAGTAAGGATATTTCTAAATTTGCATTTCCTAATGAATTAGATAAGCAACAATGTCATTATGAT
GCAGTTACGAAACTAGGTGGCATGTTATTGCTTATTTTAAAAACAGATCCAAGACAACGTCAAAATGATGAAAACTACTTTGCATTT
GGTGCATATGCACAAAATCTTATGTTGTTACTTTATGAAGCGGGAATAGGTACATGTTGGAAATCGCCATTATATATCTATGATCCT
AAAGTAAGAAAAACACTTGGTATAAAGAAAGATGAAGTTCTTGCTGGATTCTTATATTTAACGGATTTAGAAGAAGATATGCCTAA
AGCACCGCGTAAAAATAGAAACTTAATTACATTATATTAATATGTATAATTATAGAAACATTAATAAAAGCTGAGTCATGAATTGAT
GGACATCTATCGAGTTAGAGATTTAATCTAACTTACTAGAGTCGGTACAATCACAGTCTCAGCTTTTTATTGTGCAGTATATACACAT
TTTTATTTTAGTATTTATTTAAAAGTTTCTGCTAAAAAATGATTCAACTTGCTCAGGTGACTTAGCATTTGCTGAATGAAGGTGTGCAA
TTTTGTCGCCGTTTTTAAATACTAGCAAGCTAGGGATACCCATAACTTCATTTTCAACAACTACATCTTCTAATTCATCACGATTAAC
AGTATACCATTGGTAATCATTATATTGTTCTACGATTGGGTCAATCCATAAATCCATAGCACGACAGTCTGGGCACCATCCTGCCTC
AAATTTAACAATTACAGGTGTATCGCTATTAATTACAGATTTAAATGATTCATTACTTTTGATTGATTGCATTGTAACAACTCCTCTA
GATAGTTTAATAATTTTTATTATAGCTAAATTTATATCATAATAAAAAATTTTAGCTTCAAAATGAAAGCCTTTTGCTATGGAAAATG
ATATATTTATTTTAAATACATAAAGGAGGTTGCAGTCGTATGATTAAATTTTACCAATATAAGAATTGTACAACTTGTAAAAAGGCA
GCAAAGTTTTTAGATGAATATGGCGTAAGTTATGAACCAATTGATATCGTTCAACATACACCTACAATAAATGAATTTAAAACAATA
ATTGCAAATACAGGCGTAGAAATTAATAAATTGTTTAATACACACGGCGCGAAATATCGTGAGCTTGATTTGAAAAATAAATTACA
AACTTTATCAGATGATGAAAAGTTAGAGTTGTTATCATCTGATGGTATGTTAGTAAAGCGTCCTCTAGCAGTAATGGGCGATAAGAT
AACATTAGGATTTAAAGAAGATCAATATAAAGAGACTTGGTTAGCGTAAGTGAAATGTAAGCGTTTACTAAATATCTCGATATTTA
GATTCATTACATGTAAAATGAAATAAGCTATACAATTGTTAATTTTTATAAATATAGTTGAATAGCATCTAGCCTTATGGCATCATTA
ATGATGTAAAGATTAATTAGGAGGGGATTCTCTTGGCAGTACCAAATGAATTGAAATATTCAAAAGAGCATGAATGGGTTAAAGTT
GAAGGTAATGTAGCAACAATTGGAATCACAGAATACGCACAAAGCGAGTTAGGTGATATTGTTTTCGTTGAATTACCAGAAACAGA
TGATGAAATTAATGAAGGGGATACGTTTGGTAGCGTAGAATCAGTTAAAACTGTATCAGAATTATATGCACCAATCTCTGGTAAAG
TAGTTGAAGTCAACGAAGAACTAGAAGATAGTCCCGAATTTGTAAATGAATCTCCATACGAAAAAGCATGGATGGTAAAAGTAGA
AATTAGTGATGAAAGTCAGATTGAAGCTTTATTAACAGCTGAAAAATATTCAGAAATGATTGGTGAATAATCACCGTGTAACTCCTT
AATCTAAGATTGAGGAGTTTATTTTTAGTCTGAGTAAGTAAATAATATGAAAGAAAATTTATTAGGTACTATTATTTGGAGTATTGC
TACATTTTATTATTCAAGAATGATGAAAATAATGAATTTAGCTATTTTAAAAATAAAAATTGGGGGAAGTTAATATGCTAAACATTC
AAGACGTTAGTCATCTTTCTAAAAAGGAGCAAAAAGCATATAACCGTTTCGTAGAATCTGTAGAAAACGGTAATTTACCAGTACTA
CCATGTATTGAAATGGATCTAAAAGAGATGAAAGAAGAAACATTAAACCAGAGTAAGATTGGTGGAATGCCATTTTTAAAATCTTT
TAAAGATATACCATTAGATGAAAATAATGTACCAATGGTATTGTTAGCACAGATTAATTTGGATAATCTTCCAGAACAACAAGAATT
ATTTCCTGTAAATGAAGGGATATTGCAGTTTTGGATTAGTTCAGAAGATCAAATGTATGGTATGACTGAAAATTTAAAGGGAAACA
ATATAAACTCAAGGCTTGTTTATATAAAAGAGCCAATTACAGATTTATCACTCGAAAATATTCAAGCGCATTTGAAGTCATTAGATG
CTGATAATGAGGATATCCCGTTCAGTGGAGCATTTTCTATAAAATTTAGATTGTCGAAACAAACTATTACATGTACTGATTATAAGT
ACGATGAGGACGTGCTTGCATTGTGGAATAAAGTCAATCCATCCTTCGCGCTAAAATCAATGTTTGGTGGTTATGATGAATTGATGG
AACCTGTGTGTAACACATTTACTGCTAAGGAACCATTTAATCAACTTGGTGGTTATCCATATTTTGACCAAATAGATCCAAGAACGA
ACGATCAAGAACTGAAAATGTATGATAGAGTCTTACTGCAAATTGATTCTACAAGAGATGGTAATTCTTCGATTATATGGGGTGATT
TAGGTATTGCCAATATCTTAGTAAAATCTACTGACCTTGAGGCTATGAAGTTTGATGATTACATGTATTCATGGGATTGCAGCTAAG
AGAATCATAAAAATATATTACTTTAATAAGTGTAGTATCTATAGATCAATCTAGGGAGTGGGACAGAAATGATATTTTCGCAAAATT
TATTTCGTCGTCCCACCCCAACTTGCATTGTCTGTAGAAATTGAGAATCCAATTTCTCTTTGTTGGGG

>025-contig_192_RC

GCCCCTGACTAGAATTGAAAAAAGCTTGTTACAAGCGCATTTTCGTTCAGTCAACTACTGCCAATATAACTTCGTAGAGCATAGAAC
ATTGATTTATGTCCCAGCCTGTTTTCATAAGCCACAGCTAATGTGTATTTAAAAATAGGAATACATGAGTAAAACTCATGCATANGA
AATACTAATTTCTAAAGAAAAAGTATTTCTTTATGTTGGGGCCCCGCCAACTTGCACATTATTGTAAGCTGACTTTCCGTCAGCTTCT
GTGTTGGGGCCCCGCTATAATTGAAAAGTTTGTTATAGGTGTATTTTCTTTTGGTTAACTATTGGTAATATAACATTGTAGATTTTAG
GATGTTGATTTTTGACTTAGCCTTTGTCTGCTTATGTTAAATGTTACAGCTAGTCAAACATTGAGTTTGTAATTATTATATTAAGCTA
GTTTAAATTTTTTGTGATTTATGTTTTATTCATACCAATTTTGTGAAATGATACAGTGACAGATGTTTATTGATTTTAAAGGGTGATG
ATGATGGCTATTGTAAATAAAGTGATAATTGTTGAAGGAAAATCTGATAAAAAAAGGGTGCAACAGGTTATTGCAGAACCAGTCAA
TATTATTTGTACTCATGGAACAATGAGTATAGATAAGCTTGATGATATGATAGAATCACTGTATGATAAACAAGTTTTTGTATTAGC
CGATTCTGATGACGAAGGAGATCGAATTAGAAATTGGTTTAAACGTTATTTGAGTGAAAGTGAACATATATTTATTGATAAAACTTA
CTGTCAAGTTTCGAATTGCCCCAAACAATATTTGGCGCATGTACTTTCAAAACATGGCTTTACTTGTAAGAAAGAAACACCTCTTTT
ACCGAATATAAATAATGAAAGGTTAGTTTTAGTAAATGAATAATTCATTAGACATCAAAGATGTAACTACATTTTATGAGGAAGAC
AAACATTTAATCTTTGGTTATACACCAACGTGTGGTACTTGTAAGGTTTCAGAAAGAATGTTAGACATTGCTAATGAAATATTGCAG
TTGCCATTATTGAAAATAGATTTAAACTTTTATCCTCAGTTTTGTAAAGATATGCAAATCATGTCTACACCGATTTTATTGTTGATGA
ATAAAGATAAAGAAGTAAAACGAATTTATGCATTTAAATCGGTGACTGATTTGTTAGAAAATTTAAAATAGTTATTGACGAAATTTT
ACTAAGGTGTTAGGATTAATACAAATTAAATGATGCGTAAACTCTTATCGAGAGTGGTGGAGGGATGTGCCCTACGAAGCCCGGCA
ACCGTCTTATATAGAAATGGTGCCAATTCACATAAAGTTTTAACTTTTGAAGATGAGAGAAACAATACTACTATTGCTTTCTCAATTT
TTCTATCGATATTGAGAAAGCATTTTTTATTTTATTAAGCAACACAGGGAGGAATCAACGTGATTGAATTAAAAGAAGTTGTTAAAG
AATATCGGACTAAAAATAAAGAAGTCCTTGCTGTAGATCACGTTAATTTATCGATTCGAGCAGGATCGATTTATGGCGTCATTGGTT
TTTCTGGAGCAGGAAAAAGTACTTTGATTCGAATGTTTAATCATTTAGAAGCGCCTACATCAGGTGAAGTTATTATAGATGGAGACC
ATATAGGTCAATTGTCCAAAAAATGGATTAAGAGCAAAAAGACAAAAAGTAAGTATGATCTTCCAACATTTTAATTTGTTATGGTCA
AGGACTGTGTTAAAAAATATTATGTTTCCGCTTGAAATTGCAGGTGTCCCTAGAAGGAGAGCTAAGCAAAAAGCATTAGAACTTGT
CGAACTCGTCGGTTTAAAAGGTAGAGAAAAGGCTTATCCATCAGAGTTATCAGGTGGACAAAAGCAACGTGTTGGGATTGCACGAG
CGTTAGCTAATGATCCAACGGTCTTGCTTTGTGATGAGGCAACAAGTGCACTTGATCCGCAAACAACAGATGAAATTTTAGATCTAC
TACTAAAAATTAGAGAACAACAAAATTTAACAATTGTACTAATTACGCATGAAATGCATGTCATTCGTCGTATTTGTGATGAAGTTG
CAGTTATGGAAAGTGGTAAAGTGATAGAACAAGGACCGGTGACACAGGTTTTTGAAAATCCGCAACACACTGTGACAAAGCGATTT

GTGAAAGACGATTTAAATGATGATTTCGAAACATCTTTAACAGAATTAGAGCCATTAGAAAAAGATGCTTATATCGTTAGATTAGTT
TTCGCTGGTTCAACAACAACCGAGCCTATTGTATCGAGTCTATCAACTGCCTATGATATTAAAATTAATATTTTAGAAGCAAATATT
AAAAATACAAAAAATGGAACAGTCGGCTTTTTAGTTCTGCATATTCCATATATTTCAAGTGTAGATTTTGGAAAATTCGAAAAAGAG
TTAATTGAGCGACAAGTTAAAATGGAGGTGTTAAGACATGGGTAAATCATTTAGTGAAATTATAAATGAAATGATTACAATGCCTA
ATATTCAGTGGCCAGAAGTTTGGACTGCAATAGTCGAAACACTATACATGACAGTCGTCTCAACTATATTTGCATTTATACTCGGTC
TTATTTTAGGTGTGTTATTATTCTTGTCTGCTAAAGGTAAGTCTATCGGTGCAAGGTTATTTTATTCTATCGTTTCTTTCATTGTTAAC
TTATTTAGAGCGATACCATTTATTATTTTAATTTTATTATTAATTCCATTTACAAGTTTGATACTTGGAACGATAAGTGGTCCGACAG
GTGCGTTACCAGCCTTGATTATTGGCGCAGCACCGTTTTATGCAAGGCTCGTAGAAATTGCTTTTAAAGAAATTGATAAAGGTGTCA
TCGAAGCGGCCTGGTCAATGGGTGCCAATACTTGGACAGTAATTCGTAAAGTCCTCTTACCTGAAGCCATGCCAGCGCTAGTGTCTG
GCATTACAGTTACAGCAATCGCTTTAGTTGGTTCAACAGCAGTTGCAGGTGTAATTGGTGCCGGTGGTTTAGGAAATTTAGCATACT
TAACAGGTTTCACTCGAAATCAAAATGATGTCATTTTAGTATCAACTGTTTTTATTTTAATTATTGTATTTATAATCCAATTCATTGGG
GATTGGCTTACAAATAAACTTGATAAACGATAAATTGGGGGGTCATTTTATGAAAAAATTATTTGGTCTTATTTTAGTATTAACATTT
GCAGTTGTATTAGCAGCTTGCGGTAATGGAAACAAAAGTGGCAGTGACGATAAGAAAATAACAGTAGGTGCTTCACCAGCACCACA
TGCTGAAATTTTAGAAAAAGCAAAACCATTATTAGAGAAAAAAGGTTATGAACTAGATATTAAAACAATTAACGATTACACTACGC
CTAATAAATTACTAGATAAAGGTGAAATTGACGCAAACTATTTCCAACATACACCATATTTAAACACAGAGAAAAAGGATAAAGGT
TACAAAATCGTAAGTGCCGGTGATGTTCACTTAGAACCTATGGCTGTATACTCTAAAAAGTATAAAAGTTTAAAAGAATTACCAAA
AGGTGCGACAGTTTATGTGTCTAATAATCCAGCTGAACAAGGACGCTTCTTAAAATTCTTCGTTGATGCAGGTTTAATTAAGATCAA
AAAAGGCGTAAAAATTGAAGATGCTAAGTTTAGTGATATTACAGAGAATAAAAAAGATATTAAGTTTAATAATAAACAATCAGCAG
AATTCTTACCTAAAATTTATCAAAATGAAGACGCTGATGCTGTTATCATTAATTCAAACTTTGCAATCGAACAAAAACTAAATCCTA
AAAAAGATTCTATTGCTGTAGAAAGTGCGAAAGATAATCCTTATGCAAACTTAATTGCTGTTAAAGAAGGACATCAAGATGATAAG
AAAATCAAAGCATTAATTGAAGTATTACAATCTAAAGATATTCAAGACTTCATTAATGAAAAATACAACGGTGCAGTTATTCCTGCT
AAATAAATCATATTAATGGCTCAGTAACGTGATGTTGCTGGGCTTTTTAATTTAAACAGGTATTTATATGATATTTAGGAATGAGAT
GCTATTACGGATAAAGTAAAATCCTATATGCATGCGTGCTACTCATTTATTCTTATTTTTAATTTCACATGGATAGTGACTACTACAT
CATAAATCATTAGTGATTCTTTATTATTTCAGTCCCACTCCCTCAAGTTAATGATGAATCTATTTCGCTAGTTATAAAATGACGTCAA
ATCAATATCAAAAATTATTTAAGTAAAATGTTTAGATAATTTTTCAGTGGGTAAGTATTATATATAACATTTAATTATTCCGAGGAG
GCATTTATTATGGCAGACGAAAGTAAATTTGAACAAGCAAAAGGTAATGTTAAAGAAACAGTAGGTAATGTTACTGATAATAAAAA
TTTAGAAAACGAAGGTAAAGAAGATAAAGCTTCTGGTAAAGCGAAAGAATTCGTTGAAAATGCAAAAGAAAAAGCAACTGATTTT
ATTGATAAAGTAAAAGGTAACAAAGGCGAGTAATTTTACTTTTTTATTCCTAATACACTGCACATGCCACTTAGTCGGTATAACCAT
CGGCTAGGTGGTTTTTGTATTAAAAAAGTGGATACCAAAATTTATTAATAATTATTTTAATGTTAGAAAAAAACTAAATAAAAACTC
GCTAATGATATCCAATAATATGTATACAAAACGAGACATATATTGCATATGATTAACGAGATACTGAATATATTTTATCACCCCTAA
AATGATTATTCATTTTCAGCGGTAATTCGACCTAAAGTCAAACTTACAATAAAACCGATGATAAATACTACTAATGAAACGAACCAC
ATCACGATATTAGTTGGTAAACCTGGAAATACTGCAAAGAGGGAGCCAACAACAAAACCAATGATTAATGCAAAAGTCATTAGTTT
ATGATGTGTTAGGAAATACTGGATAATTTTGCTTGAAATAATGAATCCAGCAAGCACCCCAAATCCGACTGCAAGTAATATAGGAA
GACCGGTAAAGTTAAGTTTAACAACTTCAGATATTGCTAGCATGACCGTACCATAGACGCCAAATACTAATAACATAAATGACCCT
GAAATACCTGGGAGTAACATAGCACTAGATGCACACATACCTGCAATAAAATATTTAATAATAAGACTAGTTGATAGAGTAAGTGT
TTCTCCAGCATGTTTATCACCATTATTCATTAATGTAATAACAATTAAGATAGCGATACCAGCTATAACCATCATGTAATGTTTAGTT
GTAAATGACGTTTTATAGTTAGAAATTTTCAATAAATATGGAACGATACCAATGATTAATCCACCAAAGAAAAACATAGTTGGAAT
ATGGTGTTGGCTTAATAAATAATTAAAAAGATTACTTAGTGATCCCATTGCCAGTAACATTCCAATTATAATGGGGATTAAAAATGT
AAAACTTGGCCAAAAACGTCGTGAGAATATGCCGCTAATTGAAGCGATAAATTGATTGTAAATACCTAACAATAATGCGATAGTCC
CACCGCTAACACCAGGTACCAAGTCACTCGTTCCCATAGCAAAACCTTTTAGAATATTAATCCATTTAAATTGTTGCATGAATAACT
CCTTTCAAACGATTGGAATAAAATCATAAATAGCATCATACCATATTACAAATGTCCTAGTGAAATGATAACATATTTTAAATTCAT
AAAATCCATTGAGAAATTATGTGCACTTATTATCATTTATATTTTTAAAGAGAGCGGATTAGAGTTGTACTTTAGAGTTATTAAAAA
TAATAAAAAGGGTTTAAGTTGTTTATATTAAGGTATAAGTAAGTTATAATTAACTGAACGCATTATTACAAAGTCTTTTTGACTACA
AATTAAAATTATTATAAACTAGTTAAGAAAACTTTATATTTTACGGAGGGAATATAAAATGGCATCAACATTAGAAATCAAAGACC
TACATGTGTCTATTGAGGATAAAGAAATCTTAAAAGGTGTTAACTTGACAATTAACACTGATGAAATACATGCGATTATGGGACCA
AACGGGACAGGTAAATCAACTTTATCATCTGCAATTATGGGACACCCAAGCTATGAAGTAACTAAAGGAGAAGTACTTTTAGACGG
TGTAAATATTTTAGAATTAGAAGTTGATGAAAGAGCAAAAGCAGGATTATTCTTGGCAATGCAATATCCATCAGAAATTACAGGTG
TTACAAATGCTGATTTCATGCGTTCAGCAATCAATGCGAAACGTGAAGAAGGACAAGAAATCAACTTAATGCAATTTATTAAGAAA
TTAGATAAAAACATGGATTTTCTAGACATAGATAAAGACATGGCACAACGTTATTTAAATGAAGGTTTCTCAGGTGGAGAGAAGAA
ACGTAACGAAATCTTACAATTAATGATGTTAGAACCTAAGTTTGCAATCTTAGATGAAATCGATTCAGGGTTAGACATCGATGCATT
AAAAGTTGTATCTAAAGGTATTAACCAAATGCGTGGGGAAAACTTTGGTGCATTAATGATTACACACTATCAACGATTATTAAATTA
CATTACTCCTGATAAAGTACATGTAATGTATGCTGGTAAAGTCGTTAAATCTGGTGGTCCAGAATTAGCAAAACGTCTTGAAGAAGA
AGGATATGAATGGGTTAAAGAAGAGTTCGGTTCAGCTGAATAATCTTAATTAATACAGTATCCATGAGATGTTCATCTATATATGAT
GAAAATGAACATTTATACGAAATAGTAAATTTCATCAAGTAGGAGGAAAAAGTTATGACAACTGATATTTTGAACATTTCTGAAGA
ACAACTTGTTGATTATTCTAAAGCCCACAATGAACCTTCTTGGATGACAGAATTACGTAAAAAAGCTTTGAAATTAACAGAAACTTT
AGAAATGCCAAAACCTGATAAAACAAAATTAAGAAAATGGGATTTTGATTCTTTTAAACAACACGATGTAAAAGGTGATGTTTATC
AATCTTTATCACAATTACCTGAGTCAGTAAGAGAAATTATTGACGTAGATCATTCTAAAAACTTAGTAATTCAACATAATAATACGA
TTGCGTACACACAAGTTGATGATAATGCATCTAAAGATGGCGTTATCGTTGAAGGTTTAGCAGACGCTCTTATGAACCATAGTGATT
TAGTACAAAAGTACTTTATGAAAGATGCAGTAACAGTAGATGAACATCGTATCACAGCGCTACACACGGCATTAGTTAATGGTGGC
GTATTTGTTTATGTTCCTAAAAATGTAGTTGTAGAACATCCAGTACAATACGTTGTGTTGCACGACGACAAAAATGCAAGCTTTTAT
AACCATGTTATCATCGTTACTGAAGAAAGCGCCGAAGTCACATATGTTGAAAATTACTTATCAAATGCATCTGGTGAAGGAAATCA
ATTAAATATTATTTCTGAAGTGATTGCTGGTGCAAATTCAAATATCACATATGGCTCAGTGGACTATATGGATAAAGGCTTTACAGG
TCATATCATTCGACGTGGTATTACTGAAGCGGATGCCTCAATTAATTGGGCACTAGGTTTAATGAATGAGGGTAGCCAAATTATTGA
TAATACAACAAATTTATTTGGTGATCGTTCAACAAGTTCACTTAAATCAGTAGTTGTAGGTACAGGCGAACAAAAAATTAATCTAAC
ATCTAAAATCGTACAATATGGTAAAGAAACAGATGGTTATATCCTTAAACATGGTGTTATGAAAGAACATGCATCGTCTGTATTTAA
TGGTATCGGCTACATTAAGCATGGTGGAACTAAATCAATTGCTAATCAGGAATCACGTGTATTAATGTTATCTGAACATGCTCGTGG
TGACGCGAATCCTATTTTATTAATTGATGAAGATGATGTACAAGCTGGTCATGCTGCATCAGTAGGTCGTGTTGATCCAGATCAACT
TTACTATTTAATGAGTCGTGGTATTTCTCAAAGAGAAGCGGAACGTCTTGTTATACATGGTTTCTTAGATCCAGTAGTACGTGAATTA
CCTATCGAAGACGTTAAACGTCAATTGAGAGAAGTAATTGAACGCAAAGTTTCTAAATAATATTTTGAAAATAAAAGTTTGTAATA
GATATAGACTGTCGATATTGGTATAAGACTAATACAACGTCAGTATTTAAATGATTAGGATTTTTATTTAAGAAAGGTCGTGAATGA
AGTGGCCGAACACTCATTTGACGTTAATGAAGTAATCAAGGATTTTCCGATATTAGATCAAAAAGTCAATGGCAAACGTTTAGCAT

ATCTTGATTCAACAGCGACAAGTCAAACGCCTGTGCAAGTGTTAAATGTTTTAGAGGATTACTACAAGCGTTATAATTCAAACGTTC
ATCGTGGTGTTCATACATTAGGATCATTGGCAACTGATGGTTATGAAAATGCCCGTGAAACTGTTCGTCGTTTTATTAATGCGAAGT
ATTTTGAAGAAATCATTTTCACACGCGGAACAACTGCGTCGATTAACCTTGTAGCACATAGCTATGGTGATGCAAATGTTGAAGAGG
GCGATGAAATTGTTGTCACTGAAATGGAACATCATGCCAATATTGTTCCTTGGCAACAGTTAGCAAAGCGTAAAAATGCGACATTG
AAATTTATACCAATGACAGCTGACGGTGAATTAAACATCGAAGATATTAAGCAAACGATTAATGATAAAACAAAGATCGTTGCTAT
TGCACATATTTCTAATGTACTCGGTACAATTAATGATGTTAAAACCATTGCAGAAATAGCTCATCAACATGGTGCAATTATCAGTGT
TGATGGGGCGCAAGCAGCACCACATATGAAACTTGATATGCAAGAAATGAATGCTGATTTTTATAGTTTTAGTGGTCATAAAATGCT
TGGACCAACAGGTATTGGCGTATTATTTGGTAAACGTGAGTTACTACAAAAAATGGAACCGATTGAGTTCGGTGGCGACATGATTG
ATTTTGTAAGTAAGTATGATGCAACATGGGCTGATTTACCTACTAAATTTGAGGCGGGTACTCCATTAATTGCACAAGCAATTGGGC
TTGCAGAAGCTATTCGCTATTTAGAACGCATAGGTTTTGATGCAATTCATAAATATGAACAAGAATTAACGATATATGCTTATGAGC
AAATGTCTGCAATTGAAGGAATTGAAATTTATGGCCCGCCAAAGGATCGTCGTGCAGGTGTAATAACGTTTAATTTACAAGATGTAC
ATCCACACGATGTTGCTACAGCCGTAGATACAGAAGGTGTAGCGGTTAGAGCTGGGCATCATTGTGCGCAACCGTTAATGAAATGG
TTAAATGTGTCTTCAACAGCTAGAGCGAGTTTTTATATATACAACACGAAAGAAGACATTGATCAGTTAATAAATGCCTTGAAACAA
ACGAAGGAGTTTTTCTCTTATGAATTTTAATAATCTAGATCAATTATATAGATCTGTCATTATGGATCATTATAAAAATCCTAGAAAT
AAAGGTGTATTAGATAACGGGTCTATGACAGTAGATATGAATAACCCGACATGCGGTGACCGTATACGACTAACATTTGATATAGA
AGACGGCATTATAAAAGATGCTAAGTTTGAAGGTGAAGGTTGTTCGATTTCAATGGCAAGTGCATCGATGATGACACAAGCTGTTA
AAGGTCATTCACTTGGAGAAGCAATGCAAATGAGCCAAGAATTTACGAAAATGATGCTTGGTGAAGACTATGTGATTACAGAAGAA
ATGGGAGATATTGAAGCATTGCAAGGTGTATCTCAATTCCCAGCTCGTATTAAATGTGCCACATTAGCTTGGAAAGCATTGGAAAA
AGGTACTGTTGCTAAAGAAGGTAAAGCAGAAGGTACGACTGAAGAAGAATAGAATGCTGTTAATCATAGATAATTTTGATATTAGA
CATATAAAAGTATAAAAATTTTTATAAGATGTCATGTCATTGTTATAATATGGTTTACATCATGAATTAAAAACTTACGCACGCCGT
TGTAAATATATTTTTAAGGAGTGATTGAAATGGCTAAAAAAGCACCTGATGTTGGGGATTATAAATATGGATTCCACGACGATGAT
GTGTCCATTTTCAGATCAGAACGTGGTTTAACTGAGAATATCGTTAGAGAAATTTCTAACATGAAAAATGAGCCGGAATGGATGTTA
GATTTCCGTCTTAAATCATTAAAATTGTTTTATAAAATGCCAATGCCTCAATGGGGTGGCGACTTATCAGAATTGAATTTCGATGAC
ATTACTTACTATGTAAAGCCTTCAGAACAAGCTGAACGTTCATGGGATGAAGTGCCAGAAGAAATTAAAAGAACTTTCGATAAATT
AGGAATTCCTGAAGCTGAACAAAAATATTTAGCTGGTGTTTCTGCTCAATATGAATCTGAAGTTGTTTACCATAATATGGAAAAAGA
ACTTGAAGAAAAAGGTATTATCTTTAAAGATACAGATAGTGCTTTACAAGAAAATGAAGAATTATTCAAAAAATACTTTGCTTCTGT
AGTACCTGCAGCAGATAACAAATTTGCGGCGTTAAACTCAGCAGTATGGTCAGGTGGTTCGTTCATTTATGTACCTAAAAATATCAA
ACTAGATACGCCACTACAAGCTTATTTCCGTATTAACTCTGAGAACATGGGTCAATTTGAACGTACATTAATCATTGCTGATGAAGG
TGCTTCTGTACATTACGTAGAAGGTTGTACTGCACCAGTTTATACAACTAGTTCTTTACACTCTGCTGTTGTGGAAATCATTGTGCAT
AAAGATGCGCACGTTCGTTATACTACGATTCAAAACTGGGCGAACAATGTATACAATTTAGTTACAAAACGTACTTTTGTTTATGAA
AACGGAAATATGGAATGGGTAGATGGTAACTTAGGTTCTAAGTTAACGATGAAATATCCAAACTGTGTTCTTTTAGGTGAAGGTGC
AAAAGGTAGTACATTATCTATTGCATTTGCTGGTAAAGGACAAGTTCAAGATGCCGGTGCTAAAATGATTCATAAAGCACCAAATA
CATCTTCAACGATTGTTTCTAAATCTATTTCTAAAAATGGTGGTAAAGTTATTTATCGTGGTATTGTACATTTTGGACGTAAAGCAAA
AGGCGCTCGTTCAAATATTGAGTGTGATACATTAATCTTAGATAACGAATCAACATCAGATACAATTCCATATAACGAAGTATTCAA
CGATCAAATATCATTAGAACATGAAGCAAAGGTTTCAAAAGTTTCTGAAGAACAATTATTCTATCTAATGAGTCGTGGTATTTCTGA
AGAAGAAGCGACAGAAATGATTGTTATGGGATTCATCGAACCATTTACAAAAGAACTTCCAATGGAATACGCGGTCGAAATGAACC
GTTTAATCAAGTTCGAAATGGAAGGTAGTATTGGATAGCTTTAAACCGCGATGTTAAGCTATTCTTGACTTCCGGAAATGGCTATTG
ATACCATTTTGATACTGAATATAACAAAAAGCCACATTACTGTGGCTTTTTTGTTTTATAACTAAATCGGATTGATAGATAAGCTTTG
TACTTATTTATATTAGTCCGATTTTTTGATTATTGTAAAAAATAATCATTGATGGTGGATAAAGCGACAACACAAATACAACATGAT
TGTGGCATTAGAGTGCTGGTCTTTATTAAATTAATTGAAAACTACATCAAATATTCTTTAAAGATAATTCGATATTAGTTCGATTAAG
ATTCGTTGTATAACTGAGTTAAAATAAGAAAACTATTAATAATATTAAGTTCACTACAGATGTTGCTAATGGACCGTAAGTTTTAAA
GACATCTTTACTTTTATAACCAACAATCGCATCTAAAAATTGAACTAAAATCATTGCAATGGATATAGTTATCAAAAATATAGCACT
ATGAATGACTAAAGAAAAAATAGCTAATAAAAATAAAGGTAAGCTTCGACTAAGTGCATAATATGCATTTATATTATGGCTAGATG
AACATGCTTGAATTGAATAACCTAAACTTACACTGGCACTGATTATTGTAAATATTGCTAAAACAAAATACATGTTAATCCTTCTTTC
TATATTTGGATATAAACAAGTACTTGTCTAAAGTTATTTAAAAGATAATTAGAATAAATTTATGAGAAACTTATTGTTATCATTATA
ATGGTTTCAATTCATTATAACTATGTCATAAACTGAATTTGTTAAAATTTTTCATTATGTAAATTTATTAATAACAAACAGCTCGAAC
TATAGCATCATTTTACTAATGAATGCATTAAAGTAACTATGACTAAAAATGCATATTAATTATCATCATTAAGACTATTATATATAAT
GAATTTTAACTGGTTTATTAAACGAGAACGTCGGGAATTAAGTAATTACAATAAAAATAAGATATGACAATAAGGAGACTACACGC
GTGATCATTGCCATAATTATATTGATATTTATTTCGTTTTTCTTTTCAGGAAGCGAGACGGCATTAACGGCTGCCAATAAAACAAAAT
TTAAAACTGAAGCTGACAAAGGTGATAAAAAAGCAAAAGGCATTGTAAAGTTACTTGAAAAACCAAGTGAGTTTATTACAACGATT
CTAATTGGGAATAATGTCGCGAATATTTTATTACCAACACTTGTTACAATTATGGCTTTACGTTGGGGGATTAGCGTTGGTATTGCAT
CAGCTGTTTTAACAGTTGTTATCATTTTGATTTCCGAAGTGATTCCCAAGTCTGTCGCTGCAACATTTCCAGATAAAATAACAAGGCT
TGTATATCCAGTTATTAATATTTGTGTCATTGTGTTTCGCCCTATCACATTACTTTTAAATAAGTTGACGGACAGTATTAATCGAAGT
TTATCTAAGGGCCAACCTCAAGAACATCAATTTTCAAAAGAAGAATTTAAAACAATGTTAGCAATTGCTGGACATGAAGGTGCTTT
AAATGAAATTGAGACGAGTAGGTTGGAAGGTGTCATTAATTTTGAAAATTTAAAAGTAAAAGATGTAGATACAACACCTAGAATTA
ATGTGACGGCATTTGCTTCAAATGCGACATACGAAGAAGTTTATGAAACGGTTATGAATAAGCCATACACTAGATATCCAGTGTAC
GAGGGAGATATTGATAACATTATTGGAGTGTTTCATTCTAAATATCTGTTGGCTTGGAGTAATAAAAAAGAAAATCAAATTACAAA
CTATTCAGCTAAGCCATTATTTGTGAATGAACACAATAAAGCTGAATGGGTATTACGTAAGATGACTATTTCTAGAAAACATTTAGC
AATTGTGTTGGACGAATTTGGTGGTACCGAAGCGATAGTGTCACATGAAGACTTAATTGAAGAATTATTAGGTATGGAAATTGAAG
ATGAGATGGATAAAAAGGAAAAAGAAAAACTTTCTCAACAGCAAATTCAATTTCAACAACGGAAAAATCGCAACGTATCTATATA
AGGAGCGAACAGCTATGTGGAATAAGAATCGACTTACTCAAATGTTAAGTATTGAATATCCAATTATACAAGCAGGTATGGCAGGA
AGTACGACACCGAAATTAGTTGCATCAGTAAGTAACAGTGGTGGATTAGGCACAATAGGCGCAGGTTACTTTAATACGCAGCAATT
AGAAGATGAAATAGATTATGTACGCCAATTAACGTCAAATTCTTTTGGTGTAAATGTCTTTGTACCAAGTCAACAATCATATACCAG
TAGTCAAATTGAAAATATGAATGCATGGTTAAAACCTTATCGACGCGCATTACATTTAGAAGAGCCGGTTGTAAAAATTACCGAAG
AACAACAATTTAAGTGTCATATTGATACGATAATTAAAAAGCAAGTGCCTGTATGTTGTTTTACTTTTGGAATTCCAAGCGAACAGA
TTATAAGCAGGTTGAAAGCAGCGAATGTCAAACTTATAGGTACAGCAACAAGTGTTGATGAAGCTATTGCGAATGAAAAAGCGGGT
ATGGATGCTATCGTTGCTCAAGGTAGTGAAGCAGGTGGACATCGTGGTTCATTTTTAAAACCTAAAAATCAATTACCTATGGTTGGA
ACAATATCTTTAGTGCCACAAATTGTAGATGTCGTTTCAATTCCGGTCATTGCCGCTGGTGGAATTATGGACGGTAGAGGAGTTTTG
GCAAGTATTGTCTTAGGTGCAGAAGGGGTACAAATGGGCACTGCATTTTTAACATCACAAGATAGTAATGCATCAGAACTACTACG
AGACGCAATTATAAATAGTAAAGAAACAGATACAGTCATTACAAAAGCGTTTAGTGGAAAGCTTGCACGCGGTATCAACAATAGGT
TTATCGAAGAAATGTCCCAATACGAAGGCGATATCCCAGATTATCCAATACAAAATGAGCTAACAAGTAGCATAAGAAAAGCCGCA

GCAAACATCGGCGACAAAGAGTTAACACATATGTGGAGTGGACAAAGCCCGCGACTAGCAACAACGCATCCCGCCAACACCATCA
TGTCCAATATAATCAATCAAATTAATCAAATCATGCAATATAAATAATCGACCGCAATCCACAAAAGCACAAGCACCCCCAAACAT
TATTTTAGTGCTTGCCATTTTTGTGGATTGCGTTTCTATTTTACCAATCTAGTCAAGCGAAATCATCAAACGAAAGTTCACTGAAATG
ATTATGATCAAACGAAATTATCAAACTAAAGTTCGCTGAAATGATTATAGTCAAACGAAAACATCAAACTAAAGTTCGCTGAAATG
ATTATGATAAAAGTTATATGGTATGATGACATTGGTGATATATATGATAAACATCGGATTAACAGGTTGGGGTGATCACTATTCATT
ATATGAAGATTTAGAACGCCAAACCGATAAACTTAAAACATATGCTGGACATTTTCCGGTTGTCGAATTAGATGCGACATACTATGC
GATACAACCGGAAAGAAATATATTGAAATGGATAAAAGAAACGCCTGATACATTTGAATTTGTGGTCAAAATTCATCAAGCACTCA
CATTGCATGCAGACTACAAAACATTTGCAGATACAAGGCAAGAACTATTTGATCAATTTAAGAATATGTTAGAGCCCTTACATACAC
AGAAAAAATTAGCAATGGTATTGGTTCAATTTCCGCCATGGTTTGACTGCAATGCACAAAATATCAAATATATTTTGTATGTAAGAC
AGCAATTACAAGCATTTCCAATGTGTGTAGAATTTAGGCATCAATCATGGTTTAGTGATGCATTTAAAGAACAAACATTGGCATTTT
TAACAGAACATCAAATCATTCATGCAGTAGTTGATGAACCACAAGTGAAAGATGGCAGTGTACCTTTAGTCAATCGAATCACAAAT
GAAATTGCGTTTGTACGTTATCATGGACGTAATCATTACGGTTGGACTAAGAAAGATATGTCAGATCAAGAATGGCGAGATGTACG
CTATTTATATGATTATAATGAGCAAGAATTAATAGACTTGGCACAAAAGGCACAAATATTAGCACAAAAAGCTAAGAAAGTTTACG
TCATATTTAACAATAATTCTGGTGGTCATGCAGCAAATAATGCCAAAACATATCAGCGATTATTGAATATAGAATATGAAGGGTTAG
CACCACAACAATTAAAATTATTTTAAGAGGCGACGACTATGTTATTAACAATTACATTATTGGTTTTAATCGGAGGTTTGTCAGCGA
TTATAGGGTCAATCGTAGGCATTGGAGGCGGTATTATTATCGTTCCAACAATGGTTTACCTCGGTGTTGAACATGGATTACTACATA
ATATTACAACACAAGTAGCGATAGGGACGTCTTCAGTCATTCTAATTGTGACAGGACTTTCTTCATCACTTGGATATTTAAAAACAA
AACAAGTTGATATTAAAAATGGTTCCATCTTTTTATTTGGACTATTACCAGGTTCATTGCTTGGGTCCTTCATTAGTAGATATTTAAC
ATTTGAGTCATTTAATTTATATTTTGGTATCTTTTTAATTTTCGTAGCTATTTTATTAATGGTAAGAAATAAGATTAAACCGTTTAAAA
TTTTCGATAAACCCAAGTATGAAAAGACTTATGTAGACGCTAAAGGTAAAACATATCATTATAGTGTGCCACCATTGTTTGCTTTTA
TTACAACGTTTTTAATTGGTATATTGACAGGTTTATTTGGTATTGGAGGTGGCGCACTAATGACGCCACTAATGCTTATTGTATTTAG
ATTTCCACCTCATGTAGCTGTTGGAACAAGTATGATGATGATTTTCTTTTCAAGTGTCATGAGTTCTATAGGGCACATTGCTCAAGGT
CACGTAGCTTGGGGTTATGCAATCATTTTAATTATTTCTAGTTATTTTGGTGCGAAAATCGGTGTCAAAGTGAATCAATCAATTAAGT
CAGATACGGTAGTAACATTATTGAGAACAGTAATGTTGTTAATGGGTATATATTTAATTATTCGTGCGTTGATTTAATACAACTTTAA
AAGGAGGACGTCAATTTGAGGCTTACAATTTATCATACGAACGATATTCATAGTCATTTACATGAATACGAACGCATTAAAGCATAT
ATGGCAGAACAACGGCCACGACTTAATCATCCTTCTTTATATGTTGATCTAGGTGATCATGTAGATTTATCCGCACCTATAACTGAA
GCAACTTTAGGTAAAAAGAATGTGGCATTACTAAATGAAGCAAAATGTGATGTTGCAACAATCGGTAATAATGAAGGGATGACCAT
TTCACACGAAGCTTTAAATCACCTTTACGACGAAGCAAAATTTATAGTGACATGTAGCAATGTTATAGATGAATCAGGTCATTTACC
AAATAATATCGTTTCTTCTTATATTAAGGATATAGACGGTGTGAAAATACTATTCGTTGCAGCGACAGCACCTTTTACCCCATTTTAT
CGTGCACTAAATTGGATTGTTACCGATCCACTTGAATCTATAAAAGAAGAAATTGAACTTCAACGAGGTAAATTTGATGTATTAATC
GTGCTAAGTCATTGTGGCATTTTCTTCGATGAAACATTATGCCAAGAATTGCCTGAAATTGATGTCATTTTTGGTAGTCATACGCATC
ATTATTTTGAACATGGTGAAATCAATAATGGTGTACTGATGGCGGCAGCTGGAAAGTATGGTAATTATCTTGGAGAGGTTAATTTAA
CTTTTGAGGCACATAAAGTAGTACATAAAACTGCAAAGATTATTCCTTTAGAAACATTACCTGAAGTTGAAACTTCATTTGAAGAAG
AAGGAAAAACGTTAATGTCCAATTCAGTAATTCAACATCCAGTAGTGCTTAAGCGTAGTATGAATCACATAACTGAAGCTGCATAC
TTATTAGCTCAAAGTGTTTGTGAGTATACACATGCACAATGTGCCATCATCAATGCTGGCTTACTCGTTAAAGATATTGTAAAAGAT
GAAGTGACAGAATATGACATTCATCAAATGTTACCGCATCCGATTAATATGGTAAGGGTTAGACTTTCTGGTGTGAAATTAAAAGA
GATTATAGCTAAAAGTAATAAACAAGAATATATGTATGAACATGCACAAGGTTTGGGTTTCAGAGGGAATATATTTGGAGGATATA
TTCTTTATAATTTAGGGTACATTCATTCTACAGGGCGTTACTATCTGAATGGAGAAGAAATCGAAGACGACAAAGAATATGTATTAG
GTACGATAGACATGTATACGTTCGGTCGTTATTTCCCAACACTGAAAGAATTACCAAAAGAGTATTTAATGCCAGAGTTTTTAAGAG
ATATATTTAAAGAAAAATTATTGGAATATTAAAAAGTAAGATTATTAGATTTTCATTTGTCATGAATTTCGATATAATGTTTAAAGA
TACACTTAACAGGAGGGTATGTGTTGTTATGGCGACAAAAAACGAGGAAATATTACGTAAACCGGATTGGTTGAAAATAAAATTAA
ATACCAACGAAAACTATACAGGACTTAAGAAGATGATGAGGGAAAAAAATCTTAATACTGTATGTGAAGAAGCTAAATGTCCTAAT
ATACATGAATGTTGGGGTGCACGTCGTACAGCGACATTTATGATTTTAGGTGCCGTATGTACAAGAGCTTGTCGTTTTTGTGCGGTT
AAGACAGGTTTACCTAATGAACTTGATTTAAATGAGCCTGAACGTGTAGCTGAATCAGTTGAATTAATGAACTTGAAACACGTTGTT
ATCACTGCTGTTGCGCGTGATGATTTAAGAGATGCTGGTTCAAATGTTTATGCTGAGACAGTACGTAAAGTTAGAGAAAGAAATCC
ATTTACAACGATTGAAATTTTACCATCAGATATGGGCGGGGACTATGATGCGTTAGAAACATTAATGGCTTCAAGACCTGACATTTT
AAACCATAATATTGAAACTGTTCGTCGATTAACACCGAGAGTTCGTGCGCGTGCGACTTACGACAGAACATTAGAGTTTTTACGTCG
TTCAAAAGAATTACAACCGGATATCCCAACTAAATCAAGTATTATGGTTGGATTAGGTGAAACTATAGAAGAAATTTATGAAACGA
TGGATGATTTACGTGCGAATGATGTAGATATTTTAACGATTGGTCAATATTTACAACCTTCACGTAAACATTTAAAGGTTCAAAAAT
ATTACACGCCTTTAGAGTTTGGTAAATTAAGAAAAGTGGCAATGGATAAAGGGTTTAAACATTGCCAAGCTGGACCTTTAGTACGT
AGTTCTTATCATGCGGATGAGCAAGTAAATGAAGCTGCTAAAGAAAAGCAACGCCAAGGTGAGGCACAGTTAAATAGTTAATATTT
AACAATTAATAAGGCATAAAGGCTTAGTTTGTACAAAACGAACGTGTCATAGAAGTAATCATTATTTTTATGAAACACTAAAAGTA
TAAACTGAGCCTTTCATTGTCATAGATAGGTGAAGAATTTGATAAAAGTAGATCAACATTACTTTGAATTAATAGAAAATTATCGCG
AATGTTTTAATGAAGAACAATTTATTGCTAGGTATTCAGATATTTTAGATAAATATGATTACATTGTTGGTGACTATGGTTACGATCA
ATTACGATTAAAAGGCTTTTACAAAGATTCTAATAAAAAAGCAGAGATGAGTAAACGTTTTTCAAATATTCAAGATTACATATTTGA
ATATTGTAACTTTGGTTGTCCTTACTTTGTATTAAGACATTTGTCTAAACAAGAGGTTAAAAAGTTAATCGAAGAAGTTCATCCGTCT
GATGTGATAGATGACGACAATAAACTTCAAGATGTGAAGATTAAGCCAACCATTCAAGATACTGAACATTAATAAAACCCTTAGCT
AGATTGAAAATGGGAATCATGCAATTCAAGCATGGACCTGTAATCTAGTTAGGGGTTTTTATCTTTAATGAATGACTTCATTTAAAT
ACTCAGTAATTTCATCGCCTTCTTCAGCATTTACACCTAAAATATGAGCGATATAGCCTTCTTCTTTTAAATCATCAGTACCGATAAT
ACCGAATTTATTTGTTTGCATATTAAGTACGAGTGTCTTACCATAATGTCTATTTGTATGGACTAACATCAAATCATATCGACTATGC
TCGCCAACAAAACCAACAAACTGAACTTGACTCTCTTCGTTGTCATCATATAAATACATATCAATCATTTTGTAGCGACTCCTTTTAA
AAGTAGTAAAGTTAGTATAACGACAAATGAAGTATACTGCAAAATTATGATAATATATAAGTGAGAGGTGACAAGGAATGTATTTT
GTAGACAAAGATAAACTAACTCAGAAATTAGCCTATTTACAAGCATTAACTGATGATTATCATGAGAGCAAGCACAATCATTATGC
ATTTGAACGCATTGCTCAAATGTTGATAGAATCATCGGTAGATATAGGGAATATGATTATCGATGCATTTATTTTAAGGGATCCTGG
TAATTATAAAGATGTGATTGATATATTAGAACTAGAAAATGTTATTACTAAAGAAACACAGCAGGCGATTAATAAAACTGTCGGTA
TTCGTAAACAATTTACATATGATTACACAGCCTTAGATATTAAGATTATCATGCCAATGTTTGATGACGCATTACCTTATTACAAACA
ATTTATTACAGAAGTAACGACATTTTTACATCAAGAAAATGTACCAGTAACAGCTTTTGGTAAAGGAGAAAATCAATAATGAAACA
GTATAAAGCGTATTTAATCGATTTAGATGGCACAATGTATATGGGAACAGATGAGATTGATGGAGCAAAACAATTCATCGATTATTT
AAATGTAAAAGGCATTCCTCATTTATACGTAACTAATAATTCAACAAAAACACCTGAGCAAGTAACTGAAAAATTACGTGAAATGC
ACATTGATGCTAAACCAGAAGAGGTTGTAACGTCAGCGTTAGCCACTGCTGATTATATTTCAGAACAATCACCAGGAGCATCAGTA
TATATGTTAGGTGGGAGTGGTTTAAATACTGCGTTAACCGAAGCGGGACTTGTCATTAAAAATGACGAGCATGTTGATTATGTAGTT

ATTGGACTTGACGAACAAGTTACATATGAAAAGCTTGCGATTGCAACGTTAGGTGTAAGAAATGGTGCAACATTTATTTCTACAAAT
CCTGATGTATCAATTCCTAAAGAGCGTGGTTTATTACCTGGTAATGGTGCTATTACAAGTGTTGTAAGTGTATCGACAGGTGTATCG
CCACAATTTATTGGTAAACCAGAACCGATTATTATGGTTAAAGCATTAGAAATTTTAGGATTAGATAAATCCGAAGTTGCTATGGTA
GGCGATTTGTACGATACCGATATTATGTCTGGTATTAACGTAGGTATGGATACGATTCATGTACAAACAGGTGTATCTACGTTAGAA
GATGTGCAAAATAAAAATGTGCCACCAACGTATTCTTTTAAAGATTTAAATGAAGCAATAGCTGAATTAGAAAAATAGATATAGTC
ATTTTATAAAGTAGGTGAATTGATTTGGTAAAAATAGTTGTTTCGAGGAAAATCCCAGATAAATTTTATCAACAATTAAGTAAACTT
GGTGACGTTGTTATGTGGCAAAAATCATTAGTGCCTATGCCTAAAGATCAATTTGTGACAGCACTTCGTGACGCAGATGCTTGTTTT
ATTACATTAAGTGAACAGATCGATGCAGAAATTTTAGCGCAATCACCAAATTTAAAAGTAATTGCGAATATGGCTGTAGGATATGA
CAACATCGATGTTGAAAGTGCAACAGCGAATAACGTGGTTGTCACGAATACACCAAATGTACTTACTGAAACAACTGCAGAATTAG
GATTTACATTAATGCTTGCTATAGCACGCCGTATTGTAGAAGCTGAAAAATATGTAGAAGCAGATGCATGGCAAAGCTGGGGTCCT
TATTTATTGTCAGGTAAAGATGTCTTCAATTCAACTATTGGAATATATGGTATGGGAGATATTGGTAAAGCTTTTGCAAGAAGGTTG
CAAGGGTTTAATACTAATATTCTTTATCATAATCGATCAAGACATAAAGATGCAGAGGCGGACTTTAATGCAACATATGTTTCTTTT
GAAACGTTGTTAGCAGAAAGTGATTTTATCATCTGTACAGCGCCACTTACAAAAGAAACACATCATAAATTTAATGCTGAAGCATTT
GAACAAATGAAAAATGATGCAATTTTTATTAATATCGGTAGAGGACAAATTGTAGATGAAACAGCATTAATCGATGCACTAGACAA
TAAAGAAATTTTAGCATGTGGTTTAGATGTATTAGCAAATGAACCGATTGATCATACACATCCATTAATGGGACGTGATAATGTTCT
GATTACACCACACATTGGTAGCGCATCAGTAACAACACGGGACAATATGATTCAATTATGTATTAATAATATAGAAGCGGTTATGA
CAAATCAGGTACCACATACTCCAGTAAATTGAAAATAAGTATGGTTTTTAATCATTGGTAAAACAAGCAAAGCAATTGTTAAATAT
GTTATGCTTGACTTGCGAATGTATATATTTGCGCTGATGATAATTCAATAACATTATAAACTCGTCATATTAATGTGATGAGTTTATT
TGATTTTGACGTATATAACAAAATTGTTATATAGACTGTAATTGTTTATTGTTTAAATAACCTTTAAAATGAATGATGTTCTCAACAA
AGTGTATCATTTACAATAATTGAGTGATGACAACCAATTACTATGTTGAATCTATAAAATTAAAATTGAATTTCATTATGAATATCG
ATATAATGAATTGTAAAAAACAAATTTTAAAACGTATTTTATATTGAACGGTCTTTATAAAGGCGTTTTTACTAAGAACTTTAAGAG
GTGCAATATGAAATCTAAAAGTAAACAGCCACCTAATAAATATGTTGAAGCATTCAAACCATATTTATTAACACTATTGTATTTGGC
AATATTTATTACTTTATATTTAATTTATGGCAGTGGCGACACACACAATAACTTCATTTATAATGAGTTCTAATGAGGGAGACTTAAT
ATGACAGATATTATTAACAAGCTGCAAGCGTTTGCGGATGCAAATCCACAAAGCATTGCTGTTAGACACACAACTGATGAATTAAC
TTATCAACAGTTAATGGATGAGTCTAGTAAATTAGCACATCGATTACAAGGTAGTAAGAAACCGATGATTTTATTCGGTCACATGTC
ACCATATATGATTGTTGGGATGATTGGTGCCATTAAAGCAGGATGTGGATATGTACCTGTAGACACTTCAATTCCTGAAGACCGTAT
TAAAATGATTATTAACAAGGTTCAACCAGAGTTTGTATTTAATACGACTGATGAATCATTTGAAAGTTTAGAAGGCGAAGTATTTAC
AATAGAAGATATTAAAACATCTCAAGACCCAGTAATTTTTGATAGTCAGATTAAAGATAACGACACAGTATACACAATCTTTACATC
TGGTTCTACTGGGGAACCTAAAGGTGTTCAAATTGAATATGCAAGTTTAGTTCAATTTACTGAGTGGATGTTAGAACTTAATAAATC
AGGAAATGAACAACAATGGCTTAACCAAGCGCCATTTTCATTTGATTTATCTGTAATGGCTATTTATCCATGTTTAGCATCAGGCGG
TACATTAAATCTTGTAGATAAAAACATGATTAATAAACCTAAATTATTAAATGAAATGCTAACAGCAACACCGATTAACATTTGGGT
ATCAACACCATCATTTATGGAAATGTGTTTATTATTACCAACGCTTAATGAAGAACAATATGGTAGTCTTAATGAATTCTTCTTCTGT
GGTGAAATTCTACCTCACAGAGCAGCAAAAGCGTTAGTAAACCGTTTCCCAAGTGCGACGATTTACAACACATATGGTCCAACTGA
AGCTACGGTAGCAGTTACAAGTATTCAAATTACACAAGAAATCTTAGATCAATATCCGACATTACCTGTTGGCGTTGAAAGACCAG
GCGCAAGATTATCTACTACAGATGAAGGTGAACTTGTTATCGAAGGTCAAAGTGTAAGTTTAGGATACTTAAAAAATGACCAAAAA
ACAGCTGAAGTATTTAATTTCGATGACGGTATTCGTACATATCACACTGGTGATAAAGCGAAGTTTGAAAATGGTCAATGGTTCATT
CAAGGTCGTATTGATTTCCAAATCAAATTGAATGGCTACAGAATGGAATTAGAAGAAATTGAAACACAATTACGCCAGTCTGAGTT
CGTAAAAGAAGCGATTGTTGTACCTGTATATAAAAATGATAAAGTTATTCATTTAATTGGTGCAATTGTGCCAACGACTGAAGTTAC
GGACAATGCAGAAATGACTAAAAATATTAAAAATGACTTGAAATCACGCTTACCAGAGTATATGATTCCTAGAAAGTTTGAATGGA
TGGAACAATTGCCATTGACTTCAAATGGTAAAATTGACAGAAAGAAAATTGCAGAGGTAATTAACGGATGATTCCATATGGTGATT
TTACATTCTTCTTAATTGCTTTAATTGCATTATTACCAGTCATTATACTTGGATTTTTAGGTAAGCGAAGTTACATTTATAATGGCGTA
GTTACAGCATTTATGATTGTGTTAATCTTTTCTTCTGATAAACATAATCTGTTTGACCAAAAGTATTTAAGTGTTCAATTAATTAGTTT
TATTATTTACGTCGTATGGCAAGTTTTATTGATAATGTTTTATTATCATTCAAAACCAAAAAATAATTCATTTTCAAAATTTGTAACT
GTAATGGTTTTATCAATATTGCCATTAGCACTTGTGAAAGTGTTACAAAGTACATGGTTAGGTGGACATCAGATTCACTTCCATGAA
AGTAAATTAATTGAATTTGTTGGTTTCTTAGGAATTTCTTATGTTACATTCAAAAGTGTGCAGTTAATTATGGAAATTCGTGATGGTT
CTATCAAAGAAATTAAAGTATGGAAATTAATTCAATTTATTTCATTCTTCCCAACGATTTCATCTGGACCAATCGATCGTTACAAAC
GTTTCGTTAAAGACGATAAAAAAGTACCAACAGGCAATGAATATCGTGAATTAGTATTAAAAGCAATTCACATGATTATGCTTGGTT
TCTTGTATAAATATATTGTTGCTTACTTTATTAACACATATGCAATCATGCCGTTACAATTAGACTTACATGGCTTTGTCAATTTGTG
GTTATATATGTACGCATACAGCTTATATTTATTCTTTGACTTTGCAGGTTATAGTTTATTTGCGATAGCATTTAGTTATTTATTCGGTA
TTAAAACACCACCAAACTTCGATAAACCTTTCAAAGCGAAAAATATTAAAGATTTCTGGAATAGATGGCATATGACATTATCATTCT
GGTTCAGAGATTGTATTTACATGAGATCTTTATTCTACATGTCTCGTAAAAAATTATTGAAGAGTCAATTTGCAATGTCTAACGTGGC
ATTCTTAATCAACTTCTTCATAATGGGAATTTGGCATGGTATCGAAGTGTATTACATTGTTTATGGTTTATACCATGCAGCATTGTTT
ATAGGTTATGGCTATTATGAACGTTGGCGTAAGAAACATCCGCCACGTTGGCAAAATGGTTTCACAACAGCACTTAGCATTGTGATT
ACATTCCACTTTGTAACATTTGGCTTTTTAATCTTCTCAGGTAAACTTATATAATAAAGGAGAATTTAATTATGGAATTTAGAGAACA
AGTATTAAATTTATTAGCAGAAGTAGCAGAAAATGATATTGTAAAAGAAAATCCAGACGTAGAAATTTTTGAAGAAGGTATTATTG
ATTCTTTCCAAACAGTTGGATTATTATTAGAGATTCAAAATAAACTTGATATCGAAGTATCTATTATGGACTTTGATAGAGATGAGT
GGGCAACACCAAATAAAATCGTTGAAGCATTAGAAGAGTTACGATGAAATTAAAACCTTTTTTACCCATTTTAATTAGTGGAGCGGT
ATTCATTGTCTTTCTATTATTACCTGCTAGTTGGTTTACAGGATTAGTAAATGAAAAGACTGTAGAAGATAATAGAACTTCATTGAC
AGATCAAGTACTAAAAGGCACACTCATTCAAGATAAGTTATACGAATCAAACAAGTATTATCCTATATACGGCTCTAGTGAATTAG
GTAAAGATGACCCATTTAATCCTGCAATTGCATTAAATAAGCATAACGCCAACAAAAAAGCATTCTTATTAGGTGCTGGTGGTTCTA
CAGACTTAATTAACGCAGTTGAACTTGCATCACAGTATGATAAATTAAAAGGTAAGAAATTAACATTTATTATTTCACCACAATGGT
TTACAAACCATGGTTTAACGAATCAAAACTTTGATGCTCGTATGTCTCAAACTCAAATTAATCAAATGTTCCAGCAGAAAAACATGT
CTACTGAATTAAAACGTCGTTATGCACAACGTTTATTACAGTTTCCACATGTACACAATAAAGAATACTTGAAATCTTATGCTAAAA
ACCCTAAAGAAACTAAAGATAGTTATATTTCTGGTTTTAAAGAGAATCAATTGATTAAAATAGAAGCGATTAAATCATTGTTTGCAA
TGGATAAATCTCCATTAGAACATGTTAAACCTGCTACAAAACCAGACGCTTCTTGGGATGAGATGAAACAAAAAGCAGTTGAAATT
GGTAAAGCTGATACTACATCGAATAAATTTGGTATTAGAGATCAATACTGGAAATTAATTCAAGAAAGTAAGCGTAAAGTTAGACG
TGACTACGAATTCAATGTTAATTCTCCAGAATTCCAAGATTTAGAATTACTTGTAAAAACAATGCGTGCTGCTGGTGCAGATGTTCA
ATATGTAAGTATTCCATCAAACGGTGTATGGTATGACCACATTGGTATCGATAAAGAACGTCGTCAAGCAGTTTATAAAAAAATCC
ATTCTACTGTTGTAGATAATGGTGGTAAAATTTACGATATGACTGATAAAGATTATGAAAAATATGTTATCAGTGATGCCGTACACA
TCGGTTGGAAAGGTTGGGTTTATATGGATGAGCAAATTGCGAAACATATGAAAGGTGAACCACAACCTGAAGTAGATAAACCTAAA
AATTAAAATACAAATAGCACATAACTCAACGATTTTGATTGAGCGTATGTGCTATTTTTATATTTTAAATTTCATAGAATAGAATAG

TAATATGTGCTTGGATATGTGGCAATAATAAAATAATTAATCAGATAAATAGTATAAAATAACTTTCCCATCAGTCCAATTTGACAG
CGAAAAAAGACAGGTAATAACTGATTATAAATAATTCAGTATTCCTGTCTTTGTTGTTATTCATAATATGTTCTGTTAACTTAATATC
TTTATATTAGAATACTTGTTCTACTTCTATTACACCAGGCACTTCTTCGTGTAATGCACGCTCAATACCAGCTTTAAGAGTGATTGTA
GAACTTGGGCATGTACCACATGCACCATGTAATTGTAATTTAACAATACCGTCTTCCACGTCAATCAATGAGCAGTCGCCACCATCA
CGTAATAAAAATGGACGAAGACGTTCAATAACTTCTGCTACTTGATCAAACATCGTTGTATCTTCAGTAGGCATCAGGCAAGTCTCC
TTTCGAGAGATGAATTTGATAATCTTTATTAGTATATTATAATAGATAGTGTAATAAAAATCTATCAAACCAATGGGGGATAAATAT
ATGGAGCACGTGAGTGTGGTAGTATATGGGGCAGATGTTATATGTGCAAGTTGCGTTAATGCGCCAACATCGAAAGATATTTATGA
CTGGCTACAGCCGCTATTAAAAAGAAAATACCCAAATATATCATTTAAATATACGTATATAGATATTACAAAAGATAATGACAACT
TAACAGATCATGATTTACAATTTATTGAAAGAATAGAACAAGATGAACTATTTTATCCATTAATTACAATGAACGATGAATATGTAG
CAGATGGTTATATACAAACAAAGCAAATCACTCGATTTATAGACCAAAAGCTTGTGAATGAATAATATACACATAACTAAAAGCAA
TGTCAGTAATAGTGTGAACTGACATTGCTTTTTAATTTTAACCATTATGATATTTATATAACCAAAGTACGCCGGATTTTAAAATAGA
AGCGAGTCGTCCTGTTACAGTGCGGTCCATGATATATGCAAAACCTTGCTTATCTCCTAATGACCCAACGATACCTTGTACCTTTAGT
TCCGGCATTTTGTCAGGTAATGGTTCATTTAGCCATTGCTTTTTAAGCACATCGGCAATTTGATCACCTTGAACTTCGGCTAACTGAG
CACTTGGCGCATGTGGTAAATCAGCACAATCACCAACTACATAGACGTTACGATATGTTGGTACTTGATGATACTGGTTAACTATCA
CGCGTCCATTACTATTTATATCAATCGGCAAGTTACGAACAACTTCAACAGGTTGAATTCCTGCTGTCCATACAACTAAATCAATAT
CTTTAGGTTCATCACAGTTATATATTTTACCAGGTTCAACTTTATTAATATTTGAATTTGGAACAACGGTAACATTATTTTTGGCGAA
CCATTTCGCAACATACTTACTTAATTTTTCTGGAAAATTTCTTAAAATTCGCGGCCCACGGTCATAAAGATATATTTCCAAGTCTGAT
CTACTTTCTCTTAATTCGCTGGCAAGTTCTATGCCGCTTAATCCAGCACCAACGATACCGACTTTAGCACCTTCTGGTAGTTCACTAA
TACTATGGAAAGTATCCCGAGCCTTTGAGAGTGTTTGAATACTATGTGTATATTCTTCGGCTCCTGGAACGTTATGATATTTATCTTC
ACATCCTAAACCAATGATTAGCTCATCATAATCAATTTTAGAATTACCGACTGAGACAATTTGAGCATCTAAATCTATGTCGTTAAT
TTCACCATAAACTGTATTCACTTGTGGATGATTAGGGAATTTCATACGAACATCTTTATCTGATTTCGTGCCCGCAGCTAAAGCATAA
AATTCTGGTTTCAATCCATGAAATGGCATACGATCAACTAATGTGACTGTATAATCTTGTGGTAAAGAAGTAGTTAAAATGCGTGAC
ATGATACGCATATTACCATATCCGCCGCCTAACAAAACTAAGTTTTTCATAATGAAGTACCCCTTTTATATGTTAATATTTACTTTAA
TTTTGAAAAGATCAAAGTGCATAGATATATTTGTTAATGAACAGTTATAAAATGCAAGTGTCTTTGATTCATCAATAACCCCTAAAA
AAGTAAATATGTATTTATATGCTTATTATATATTTTTTTGAAAATGACTACAAATAAATATTTGTACTAACTAAGGCACTATTACATA
ACTGGGATAAGAAACATCAGTTGTTGCTAATTGAGCACATCATGTTAAAATACCAGTTTTAAAATGCTATAATATTGTAACAAATGA
AATAAGAAGGTGAAGTATGAATCCGATCGTAGAATTTTGTCTCTCTAACATGGCAAAAGGTGGAGATTATGTTTTTAATCAACTGGA
AAATGACCCAGATGTCGATGTGTTAGAGTATGGTTGCCTGACACATTGTGGTATATGTTCAGCCGGGTTGTATGCTTTAGTAAATGG
TGATATTGTTGAAGGTGATTCGCCGGAAGAATTATTACAAAATATATATGCACATATAAAAGAAACTTGGATTTTTTAAGGAGGAGT
TATCATGCCAACAGTTATATTAACAGAAGCAGCTGCTTACGAAGTAAAAGATATGCTTAAAGCAAATGAAATGCCAGATGGCTATT
TAAAAATTAAAGTGAATGGTGGCGGGTGCACTGGTTTAACATACGGTATGGGTGCAGAAGAAGCGCCTGGTGAAAATGATGAAGTC
TTAGAATACTTTGGATTAAAAGTATTAGTAGACAAAAAAGATGCACCCGTATTAAATGGTACGACTATTGATTTTAAGCAATCATTA
ATGGGTGGCGGTTTCCAAATCGACAATCCTAATGCAATTGCTTCATGTGGCTGTGGTAGTTCATTTAGAACTGCAAAAGTTGCAGGT
AATCCTGAAAATTGCTAATAAAATAACTTTTTAATGGTTAACCCAATTTTTGGGATACAAATTGATATCAATAACGATTAAGGACAT
ATTTGATTATGTCCTATTTTTGCGTAAAGATATTTTGTTTTATTAAAAAGCTTATTGAAAATTAAGAATCTGACTATAAATAGTATTT
TAAATGATCGTGATAGCGGATACATTTTTAATCGAAATCAGGCTACGAAAAAATTAATTAATTTTTTCACAATCTGACTGAATCTTG
CTTGAAAATATAACATAAAAGCTATAGAATTAGTATTGGATTAAAAATGTCTTTTTTGTGAACATCACAGGTTTTTACATAATTTAA
AAGACTTAAATAACAAAATGATGATAAACTAGTATACATTGTGGTAAAAGTAGTAATGACCAGTTGTTACTAGTAATTATCAGCATT
TGGTTAAAAAAATATGAAAGCTTAGGTGAAATTAAATGGCTCAAGATCGTAAAAAAGTACTTGTACTTGGTGCTGGTTATGCAGGT
TTACAAACTGTAACTAAATTGCAAAAAGCGATATCAACAGAAGAAGCAGAAATTACGCTTATTAATAAAAATGAATATCACTATGA
AGCAACATGGTTACATGAAGCATCAGCAGGTACACTAAACTATGAAGATGTATTATATCCTGTGGAAAGTGTCTTGAAGAAAGACA
AAGTGAACTTTGTTCAAGCAGAAGTAACAAAAATTGACCGTGATGCTAAAAAGGTAGAAACAAATCAAGGTATTTATGACTTTGAT
ATTTTAGTAGTAGCATTAGGTTTCGTTAGTGAAACATTCGGCATCGAAGGTATGAAAGATCATGCTTTCCAAATTGAAAATGTTATC
ACAGCACGTGAATTATCACGTCATATCGAAGACAAATTTGCTAACTATGCAGCATCAAAAGAAAAAGATGATAACGATTTATCTAT
CTTAGTTGGTGGTGCTGGATTCACTGGTGTTGAATTCTTAGGTGAATTAACAGACAGAATTCCTGAATTATGTAGCAAATATGGTGT
GGATCAAAATAAAGTTAAAATCACTTGTGTTGAAGCAGCACCTAAAATGTTACCAATGTTCTCAGAAGAATTAGTTAACCACGCAG
TTAGCTACTTAGAAGACCGCGGTGTTGAATTTAAAATTGCTACACCAATCGTTGCTTGTAACGAAAAAGGTTTTGTAGTTGAAGTAG
ATGGTGAAAAACAACAATTAAATGCAGGTACTTCAGTATGGGCAGCTGGTGTACGTGGTAGTAAATTAATGGAAGAATCATTTGAA
GGCGTTAAACGTGGACGTATCGTTACAAAGCAAGATTTAACAATCAATGGTTACGACAACATTTTTGTTATTGGTGACTGTTCAGCG
TTTATCCCAGCTGGAAAAGAACGTCCATTACCAACTACAGCACAAATTGCAATGCAACAAGGTGAAAGTGTTGCTAAAAACATTAA
ACGCATCTTAAACGGTGAATCAACTGAAGAATTCGAATACGTTGATCGTGGAACTGTTTGTTCTTTAGGTTCACATGACGGTGTAGG
TATGGTATTTGGTAAACCTATCGCTGGTAAAAAAGCAGCATTCATGAAAAAAGTGATTGATACACGTGCGGTATTCAAAATCGGTG
GTATCGGTTTAGCATTCAAAAAAGGTAAATTCTAGGCTTTAAATAAAGTTTCAGCTAAACACATAAAATTAAATTATTCATTTATAA
AGTCCTTCAGTGTCACTGAAGGGCTTTATTGTTGTACAATTTGAAGTGAGTAACATATCAAATCTAAGGAGGTACATTATGAATTTT
AAATTAAATAACACACTAAGCAACGAAATAAATACATTGATTATTGGTATACCAGAACATTTAAATCAGTTAGAGCGCATTAGTTTT
AATCATATCGATATTACAGAATCACTTGAAAGACTAAAACATCAACATATTATTGGTAGTAAAGTTGGGAAGATTTATACAACTGC
ATTTGATGTACAAGATCAAACATATCGTTTAATTACAGTTGGTTTAGGAAACTTAAAGACACGTAGTTATCAAGATATGTTGAAAAT
ATGGGGACATCTTTTCCAATACATAAAGTCAGAACACATTGAAGATACGTATTTACTTATGGATTCATTTATTTCAAAATATGATCA
GTTATCAGATGTATTAATGGCATGCGGTATTCAAAGTGAGCGTGCAACATATGAATTCGATCATTATAAATCAAGTAAGAAGGCAC
CGTTTAAGACGAATTTAAACCTTATTAGTGAATCATTAATTGAATTAGATTTTATTCATGAGGGTATCAGTATTGGCCAATCCATTAA
TTTGGCAAGAGACTTTAGTAATATGCCACCGAATGTATTAACACCACAAACATTTGCAGAAGATATTGTTAATCATTTTAAAAATAC
AAAGGTCAAAGTAGATGTTAAAGATTATGACACTTTAGTTTCTGAAGGATTCGGACTTTTACAAGCAGTAGGTAAAGGTAGTAAGC
ATAAACCGAGATTAGTAACCATCACATATAATGGCAAAGACAAAGATGAAGCACCAATTGCCTTAGTTGGTAAAGGTATAACGTAT
GATTCTGGTGGTTATAGTATTAAAACGAAGAATGGCATGGCTACAATGAAGTTTGACATGTGTGGCGCTGCGAATGTCGTTGGTATC
ATTGAAGCGGCTAGTCGTTTACAACTGCCTGTAAATATTGTCGGAGTGCTTGCGTGTGCTGAAAATATGATAAATGAAGCATCAATG
AAGCCAGATGATGTATTTACAGCATTAAGTGGTGAAACTGTAGAAGTAATGAATACAGACGCTGAAGGTAGATTAGTCCTTGCAGA
TGCTGTGTTTTATGCAAATCAATATCAGCCTAGTGTGATTATGGACTTTGCTACATTAACGGGTGCAGCAATTGTTGCACTAGGCGA
TGATAAAGCTGCTGCATTTGAATCGAATAGTAAAGTGATATTAAACGATATATTACAAATAAGTTCTGAAGTCGATGAAATGGTATT
TGAATTACCGATTACTGCAACCGAACGTGCAAGTATTAAACACAGTGATATCGCTGATTTAGTTAACCATACGAATGGACAAGGTA
AAGCGCTATTTGCGGCAAGTTTTGTAACACATTTTAGTGGTCAAACACCTCACATTCATTTCGATATTGCAGGTCCAGCAACGACTA
ATAAAGCTTCATATAATGGTCCAAAAGGGCCAACAGGATTTATGATTCCGACGATAGTACAATGGTTAAAACAACAATAATTTAAT

TATTTGCCTGATACAACTTGTGTCTTGATATACATTTCTAATTTAACTCTTGAAAGTATAAAGGATCGCATTGTATCAGGCATTTTCA
TTGTTGTAAGAAAATATAAATCGCATATCCTTGTGAGCATAAATCAACTTGACTAGCATTGCAAAAAAATATTATAAGCATATGTTC
ATTCAGTCAACGACTACTAATATTGCATTTTAGAACTTAGGATTTTGATTGGTGTTCCAAGCTTGTAAAATTGTTTAATTTAATGAAA
GCTAAAATTATTTTAAGAAAGACAATTAACTAAAAAATAAAGATTATTGGTATAGTTTGCTATTGACAGGTGTTAAAGCTACTGATA
CTATGTAGTCTATAAGCGCTTTAGTTCGATAGAGTGATAAAGGATGGTTGTCATATGATAAATGCAGTAGTAATAGCAGTAATTTTA
ATGATTGTGCTATGTTTATGTCGATTAAACGTAGTTATAAGCTTATTTATCAGTGCGCTAGTTGGTGGCTTAATTTCAGGCATGAGCA
TTGAAAAAGTTATAAATGTATTTGGGAAAAATATAGTCGATGGTGCTGAGGTAGCATTAAGCTATGCTTTATTAGGTGGATTTGCAG
CATTAATTTCATACAGTGGTATCACAGACTATTTAGTAGGAAAAATTATAAATGCAATTCACGCTGAAAATAGTCGATGGTCAAGA
GTTAAAGTCAAAGTGACAATAATCATTGCATTATTAGCTATGAGTATCATGAGTCAAAACTTAATTCCTGTACATATTGCATTCATTC
CAATTGTCATCCCACCATTGTTAAGTCTGTTTAATGACTTAAAAATAGATAGACGTTTAATCGGTTTGATTATCGGTTTTGGTTTATG
TTTCCCGTATGTGTTATTACCATATGGATTCGGTCAAATTTTCCAGCAAATTATTCAAAGTGGCTTTGCAAAGGCGAATCACCCAATT
GAGTTTAATATGATTTGGAAAGCAATGCTTATTCCTTCAATGGGGTATATTGTTGGCTTACTTATCGGTTTATATGTATATCGTAAAC
CACGTGAATATGAAACACGTAAAATTTCAGATAGTGACAATGTTACAGAGTTAAAACCATATATCTTAATAGTAACAATTGTAGCA
ATACTAGCTACATTTTTAGTACAAACATTTACAGATTCAATGATTTTTGGTGCACTGGCAGGGGTACTCGTATTCTTTATTTCACGTG
CATATAATTGGTATGAATTAGATGCTAAGTTTGTTGAAGGTATTAAAATTATGGCTTATATTGGTGTAGTTATTTTAACAGCAAATG
GATTTGCTGGTGTAATGAATGCTACTGGTGATATAGATGAATTAGTTAAAACTTTAACAAGTATTACTGGTGATAATAAATTATTTA
GCATTATCATGATGTATGTGATAGGTTTAATTGTCACTTTAGGTATTGGATCATCATTTGCAACAATTCCTATTATCGCATCATTATT
CATTCCTTTTGGAGCGTCAATTGGACTAGATACAATGGCATTAATCGCATTGATTGGAACAGCGAGTGCATTAGGTGACTCAGGTTC
ACCTGCAAGTGATTCAACATTAGGACCAACTGCGGGATTAAATGTTGATGGCCAACATGATCATATACGCGATACATGTGTACCAA
ACTTCTTGTTTTATAATATTCCTTTAATGATTTTCGGTACTATTGCTGCTATGGTACTATAAGCATAAAGAGGTGAAAATATGACTCA
TTTATTAGAGACATTTGAGATGTCAATAGATCACCAGGAAGATGGTTTAGTTGTTATTTCTATGCCTGTTACTGATAAAGTAAAACA
ACCATTTGGATATTTACATGGTGGTGCTTCGATTGCTTTAGGTGAAACAGCATGTTCATTAGGATCTGCTAATCTAATTGATACAACC
AAATTTATTCCATTAGGTTTAGAGATGAATGCCAATCATATTCATTCTGCTAAAGATGGTCATGTTACTGCGACAGCTGAAATTATTC
ATCAAGGTAAGTCGACACATGTATGGGATATAAAAATTAAGAATGACAAAGAACAATTAATTACAGTTATGCGTGGTACAGTTGCT
ATTAAGCCTTTAAAATAAAAGAACTGCTAGCTGAAATGTTATGAGATATTCATAACTACGGCTAGCAGTTTTTTATGCGCTATATT
GTTGTAATTTTAGAAATGCTTGTTCAATGCGTTCGGCAGCTTTACGGCCACCCATAACATTTCTACCAAATGGTCCTAATTCTAAGTC
TGCAAAGCATCCTGCGACAAATAGATTTGGTATCCATTCTAATTTTTCGGAAATAACAGGGTAATTACATTCGTTGATAGGTGCATC
ATAATTTTGTATTAATTGCTTAATAAGTGGTTGTGACATAAAATCTTGTTCAAAACCAGTTGCAACCATAATCTGTTGATATGGAAC
AGAATTATTTTCAGTGTTAATTAAACCATCACTAATTTGAGTGATAGGTGTTTTATGCACATTTATACGACCATTTTTAATATGTTTTT
TAAGGCGTAAGTACAGTTCGTGAGGCATTGATCCTTTATGACGTTCACGTTGTACAATGGCATTTCTTTCAGGCATGCTTTTAGTACT
TAAAAATGTAGACATATTCTTCGGACCTAACCAACCAGGATCAGCATCAAAGTCATGTATTTCAATATCTTTATTTAGCCATAAATG
AATCTTTTTATCGTTATCATGATTTAACAATTTAAGTGCAAGATGTGCAGCCGTAATGCCACTACCAACGATATGATCGGTCTTATCA
TATACTAGTTGATCATGTTCTTTCTCGAAGATATGATTTACATTCTGTTTGTCTTTTAAAATGTCAGGCATAAACGGAATATTTGTAC
TACCTATTGCAATAACGACGCAATCTGTAGTGATAATTTGTCCATCTTCTAACTTGATATGCCATTTGTCTTCTTGTTTATCTAAAGTT
TGAACTAAACCTTGAACCAAGCAATCCTCTAATTGATATTGTTTAGAAGCATGTGCAATATGATCCATAAACATTGTCAATTCAGGT
CGTTGATAAGGACCATAAAAAGCATTTGTATATTGGTGCTGTTTAGCGAATTGTTTTAGATGGAACGGTTGTGGATGTACGTGATGT
ACAATCGGTGATCTTAAATAAGGCATTTCTATTCGATTTGTATATGAGTTAAACCTTTGGCAAAAAGTTTCGTGTGGGTCAATGATT
GTTAATCGGTCTGTTGTTAATCCGCTTGATAATAGTTTTTGTGCGATTGCAGTTCCCTGTATGCCACCGCCGATAATTGTCCAATGCA
TAATAAAACCTCTCTCTTTTTAAAACGTAATCGTTACGATTTATAATTATTATTATCATAATACATAACGACATGAAAGGCAATTAA
ATTAAAGAGATATATGTAGATAGGGCGAAGCTGTAGTCAAAGAAAAAATCATTGAAAAAGAGGTAACAATGTCAAAAGAAAACAG
CAGTAAAATCATTCCTAATTTGGAATCATCTTACTGCTGTTTGTTGTTGATTTATATTCATGATTTTGTTATATAATCTACAATTTTGT
GTCTTTTAAGTCTTCCGAAATTTCATCGACTTTAGTCTTTTTAGTATAAGGCGTTTTAATATTATATGCTGCTTTCATAATCATATGAC
TTGAAAGAGGACCTGTAATTAATACAAAGATAATCGCAACGATTAATTGCATATTTACAAAACCTTGAGTAGCAATAAAATATAAA
AACGTACCAAATAGTAATGACATTGCACCTAATGTTGATGCTTTTCCGGCAGCATGTGCACGTGAATATACATCTTCAAGTCTCAAT
AATCCTATAGCTGCTAGGGCGCTAATTAAAGCACCGATGATAACAAAGATAAGTGCAAGACTAATCAGTATGATTTTGATCATGTTC
AATCACCTTACCTTTGTCCATAAATTTAGAGAATACTGCAGTACCTAAAAAAGCTAATATACCAATCATCATAATAACGACAATCAT
GTATTTAATATTTAATAAAATACTGAATAATGCTATAACTGCCATTAATTGAAGACCAATCGCATCTAATGCGACAACACGATCGGC
AAGTGATGGGCCTAGCACAACGCGAATGAGCATAGCTAACATAGAAATGACAACTATGATTAATGCAATAACGATAATAACATTAT
GATTCATTATATTTCGCCCACCTCTCTTACAATTTTCTCTAATGATGTTTTAATACTTTCTACTTCTTGCTCTTTAGTTGAAAAATCTAT
GGCATGAATATAAATTTTTGTACGATCGTCACTTACACCAAGCACTACAGTACCAGGTGTTAATGTAATTAAATTAGACAGCAAGAC
AATTTGCCAATCTTTTTTTAAATCTGTGTGATAAACAAAGAATCCTGGTTCATTTTTAATCGAAGGTTTAATAATAATTTTCAAAACA
TCAAAATTAGCTTTAATCAGTTCGATTAAGAAAATAATAACTAATTTAATAATACGATATAGCGTGATGACATAAAATCTACCTGGT
AACACCCTGTGTAAGAGGTAAACAAGAACTAGGCCAAAGATGAAACCTAACACAAAGTTATTTGTTGTGTAACTATTTGTCACAAA
CAACCAAAACACTGCGATAATAAAGTTTAATACTAATTGTACAGCCATGTTATTTACCTCCTAATACAGCTTTAACATAGGTTGATG
GATTGTAGAATGTTTCTGCACCAGCTTTTACCATTGGATATAAGTAATCTGCTGACAATCCATATAAAACAGTTATCACAACTGCAA
CGATTGCAATCGTAGTTAAATAATTGACGTCGACTTTGTTATTAAGATCATATCCTTTTGGTTGACCGAAAAAGCCTTGTAGGAATA
TGCGAATGACAGAATATAATACGACTAAACTTGATAATAAGACAATGACACCACTTAAATAAAATCCTCTTTCAAATGTTGATTGG
ACAATGAAAAATTTTCCATAAAAGCCACTGAGTGGGGGAATGCCAGCTAAACTTAATGCTGCGATAAAGAATGACCAACCAAGTAC
AGGATATCGTTTAATTAAGCCACCAAATTGTCTTAAATCAGCAGTGCCTGTAATTTTAATCATAATTCCGATAAGCAAGAATAATGC
AAGTTTTACTAACATGTCGTGCAATGTATAGTAAATAGCCCCAATCATACCTGACTCTGTCATCATTGCAACGCCGACTAAGATCAC
ACCTACAGCAATCATGACATTGTATAGGATGATTTTTTTAATGTTGGCATATGCAACAGCACCGACACAACCAAAGATGATCGTTAA
TAATGCTAAGAATAAAATGACATAATGTGAAAAGCTTACATTATCACTAAAGAATAGGCTCAATGTTCTAGCGATTGCATAAACAC
CAACTTTTGTTAACAAAGCACCAAAGAATGCAATGATTGGAATTGGTGGTGCATAGTATGCACTAGGTAACCAAACAAACATTGGG
AATACGCCAGCTTTTGTAGCAAAAACAAAGATAAATAGTATGAAAACGATATTGACTAAGCCACTGTCATGCGCTGAAAGGTTAGC
TAATTTATTGCTTATATCTGCTAGATTCAATGTTCCTACTACTGAATATAAAATCGCTACACCCATTACGAAGAAGGATGACGATAC
AACGTTAACAAGAACATATTTGATTGTTTCTTGTAGTTGAATTTTTGTAGAACCAATTACTAATAAGAAATAAGATGACATTAAAAA
TACTTCGAAAAATACGAATAGGTTGAAAATGTCACCAGTTGTGAATGCACCAATGATACCTATTAACATAAATAGTACTGAAAAAT
AATAATAATATCTTTCACGTTCAATACCAATTGTTTGGTATGAATATAAAATCACAATAGCTGTAATAATAATACTAGTAATTATTA
GTAGGGCACTGAATATGTCTAATACAAAGACAATACTGTATGGTGCTTTCCATGAACCTAGCTCTACGCGTATTGGTCCATGTTTAA
CAACATTTGCTAAATTGATAATTGCCACGACCAAGGTTAATAATGTACCGCCTAGTGCGACATAACGCTTTATAATAGGACGCTTTC
CAATAAAGACAAGTAATATGGCTGTAATTACTGGAATAACTAGCGTTAACACAAGCATATTACTTTCAATCATCTTCTGGAACTCCT

TTCATACTCTCAACGTTATCTGTGCCTAATTCTTTATATGTTCTAAATGCTAATACTAAGAAAAAGGCTGTTGTCGCAAAGGCGATAA
CGATTGCTGTTAAAATAAGTGCTTGCGGGATAGGATCAACATAGCTTTTTACGTTCGCTTCATAAATTGGAACAGTACCATGTTTAA
GTCCGCCCATAGTTATTAAAAATAAATTTGCTGCATGTGTTAATAGTGTAGTTCCCATAACAATTCGTATCAGACTTTTAGACAAAA
CGAGATAGACACTAATTGCTGTGAGAATACCACTAACAAAAATCATAATAATTTCCACTATTCGTTCTCTCCAATCGAAATAATAAT
TGTCATGACAGTACCAACTACTGCACATAAAACACCGAAATCAAAGAATACTGCTGTTGTCATATGAACAGGTTCTAATATAAATA
ACGGTATATCAAATGTGACATGCGTAAAGAAATTTTTGCCTAAAAACCAACTTGCGATAGGCGTCGCAATACAAAAAACTAATCCG
ATACCTATCAAGATTTTAAAATCTAATGGGAAAATTTTACGCATTGTTTCTATATCAAATGCAATCGTAATGATAACAAGTGAACTT
GCGAATAATAATCCGCCGACGAATCCGCCACCAGGTGTATAATGTCCTGCTAAGAAAAGTGAAAAACCAAAGACCATTACCATGAA
AAAGATAATAACTGCAGCAAATTGCAAAATTAGATCATTTTGTTGTCTATTCATGATTTTTCACCTCGTTACCTTGCGTTTGACGCTT
TTTACGTAATTTAATCATTGTATATACAGCTAATCCTGCGATACCAAGCACAGATGACTCGAATAAAGTATCCATACCACGGAAATC
AACAAGTATGACGTTTACCATGTTTTTACCGTGAGCTAAATCATAAACGTGCTCTTGATAAAACTTAGATATCGATTCAAAATGTCT
ATTTCCGTATGCAATTAAACCGATAATAATGACGGACAAACCAACACCACCAGCAATTAAAGCATTAGTAAGCTGGAATGAGCGCT
TTTCATTATAACGATTTAAATTTGGTAAGTGGTAGAAGCATAATAAGAACAATGCTGTTGAAATAGATTCAACGACAAACTGTGTCA
ATGCTAAGTCGGGTGCTTTAAAGAATATAAACAATACAGACACAGCATATCCAACTGCACTTAACATAATGATGCTAAATAATCTT
GATTTAGCGAAAAGAATTAAAAAGGCAGCACTTAATAATAAAATTACGATACAAACTTCGAAAATTCTAATCGGACTAACGTCTTT
AAAATTAATGTTGAAAGGTACTGAGAATATAGTGACAAATGTTAATAAAATTAATGCACCAAAAATGATAACTAAATTATTACGTG
AATAATCGGTAACATAGCTATTCGTCATCTTTTCAGAGTAGTTTGGAATAACATTTGCACTTCTGTTGTACCAATAATTGAATGTTAG
TTTACCAGGTTGTCGTTGCAACAATTTCACCCAATAACTAAATGTCACAATTAGTAAGATACCTAAAATATAAATCACTAATGTTGA
TAAAAAGGCAGGCGTTAATCCATGGAACATATGGAATTCAACATCATCAATTACCGTATGATTAATCGAAGATGTAGCTGGTTCAA
TAATCGAATTAGTTAAAATGCCAGGGAATAAACCGAATACAATTACTAATGTAGCTAAAATAGCTGGTGATAAAAGCATTAATATT
GATACTTCGTGTGCTTTTTTAGGTAATTGTTCAGGTTTATATTGTCCGAAAAATATATGCATTATAAATTTAATTGAATATACAAATG
TGAAGACACTGCCCACTATACCAATGATTGGGAATAGATAGCCTAATGTATCAACACTGAATAAATTTGCTTGGCTTGCTGTAAATG
TTGTTTCTAAAAATGATTCTTTTGATAAGAAACCATTGAACGGTGGTACACCAGCCATACTTAATGCTGTAATAACAGTGATTGTAA
ATGAAATAGGCATAATTGTTAGTAAGCCACCTAATTTCTTAACATCACGTGTACCAGTAGAATGATCCACTGCACCTGTAATCATAA
ATAGGGCACCTTTAAATGTTGCATGGTTGATTAAATGGAATATTGCAGCCGTAAATGCAGCAGCATATATTTTGCTATCATCGCCTT
GATAGTGATAACTAATGGCACCGATTCCAAGCATCGCCATAATCATACCTAATTGGGATACTGTTGAAAATGCCAGTATACCTTTCA
AGTCTTGTTGTTTTGTTGCGTTTAGCGAAGCCCAGAATAACGTAATTAAACCAACGAGTGTGACAGTCCATACCCAACCTTGCGATG
CTGCGAAGATTGGTGTCATTCGAGCGATTAAATATAACCCTGCTTTAACCATTGTTGCTGAATGAAGATAAGCACTGACTGGTGTAG
GTGCTTCCATTGCATCTGGTAGCCAAATATAAAATGGAAACTGAGCAGATTTTGTAAAAGCACCAATCATGATTAAAATCATCGCA
AAAATGAAGAATGGGCTATTTTGAATTTCAGAAGCATGTTGAATCATGTACTGAATGCTAAATGATTGTGTTGGTATAGCGAGTAAG
ATGATACCACCTAATAATGATAGACCACCAAATACTGTGATTATGAGCGATTTTTGAGCACCATATATAGATGCTTGTCGTTCGCGC
CAGAATGAAATAAGTAAAAAACTAGAAAATGACGTTAGCTCCCAGAATAAATATAGAATAATAACATTATCTGAAAGTACGACAC
CTAACATTGCACCCATAAATAGTAATAAATAACAATAAAAATTCCCTAGTTGTTCTGACTTACTTAAGTAGCCGATTGAATATAATA
CTACTAAACTGCCGATTCCTGAAATAAGCAAACTAAAGAGTAAACCTAAGCCATCAAGATATAAATCAAAGTTCATACCAAAATGA
GGCATCCAATTTAAGGTTTTCATTACAGTATTACCTGACATCGTCGTTTTAATTAATGTAAGCATATAAATAAATATGACGATAGGG
ACAGGTAATACGAACCATCCTAAATGTATACGTTTAAAAAATCTATACAGGATAGGAATAATGAGTGCGAATATTAACGGTAATAT
CACCGCAATATGTAACAAACTCACTATGTTGTCCTCCTTTAAAAAATATTTATGTTATTCATTATACATGAATGATATAGTTCTGAAA
AACGTACACACTCCTTGTTGTGCTTTATTTTCAGAAGTATTTAAATAAGAAGAAACACGTCATTTTTTATTTAAAATTTTCTTTGTATT
GAAGTGAATAATCTTCTTTTAAGCGTGCTAAACTAGCTAAAGACATTTCAGCATGTTTTGTTTGCTGAGCTTTAAGTTTAGTTTCTAA
ATCTGTAATTGCTTGTTGAAGTGAATCTTCATAGCGCAATACATCAACATTGAAGTCGCGTAATTGTGAACGTTTCGTATAGCGTTTT
TCAAAATGGCTTAATGCTTTGCGTTCATGGAAAAATACACCTTCAGTTTCAGTAGGGTTATGTAAATCACCTTGTTTCGGGTGTTTGA
TAACTTGTTCAACTTTAACAAGGACATCGTCTCCATTTTCTTCAACAATCGTGACACCATAGCTACCTGTTTTGTGTGAAAATCGATA
TAGCTTCATGCTATTTTCCTCCCTTAAAAGTATGTTAATATATATGTATCATAACATGAATGGAGAATATAAATGGCTAACTATCCAC
AGTTAAACAAAGAAGTACAACAAGGTGAAATTAAAGTGGTTATGCACACAAATAAAGGTGACATGACATTCAAATTATTTCCAAAT
ATTGCACCAAAAACAGTTGAAAATTTTGTGATACATGCAAAAAATGGTTATTATGATGGAATAACATTCCACCGTGTCATTAATGAC
TTCATGATTCAAGGTGGCGATCCAACAGCTACTGGTATGGGTGGCGAAAGTATTTATGGCGGTGCTTTTGAAGATGAATTTTCATTA
AATGCATTTAACTTATATGGCGCATTATCAATGGCTAACTCAGGACCTAATACTAATGGTTCACAATTTTTCATTGTTCAAATGAAA
GAAGTACCTCAAAATATGTTAAGTCAACTTGCAGATGGTGGTTGGCCACAACCAATCGTTGATGCATATGGCGAGAAGGGTGGTAC
ACCATGGTTAGATCAAAAACATACAGTATTCGGTCAAATCATTGATGGTGAAACTACATTAGAAGATATTGCAAATACAAAAGTGG
GACCACAAGATAAACCACTTCATGATGTTGTAATTGAATCTATTGATGTTGAAGAATAATATCTAAACATAATTAACTACCAACATT
TTAAACTCGGATAAAGCTAATTTATGAATGGATTAGTATATATTCCAACGAAAATAAATAAACTAATATGATGAGCAATCTCAATAT
ATTTATCAAGAAAGCACAGTTTTTAAATAGATGTGTATTTTAAAGATAATAGTTGAGGTTGCTTTTTATGTTTTTACAGAGAATTGCT
ATTCAAATAGTAAATAAATTGAAAACAAAGTAGCTGGATATCATATTGATTTAGATAGGAATTTGTTGCTAATTTTATTTGTAAATC
CAAGTTTGTAGAATTCTTATTCATTTATAAAATAATATTCGTATGATTTGATGTTTTAATTAGTCCACCATTTCGATTTGTGCTATGAT
AATAGTGTTAAGTAAACGAAATAAGGGGTTATTAAGTTGAATAACTACAAAATTGGCCAACATATCAAGGTGCGTGTAACTGGTAT
TCAACCATACGGTGCGTTTGTTGAGACCCCTAATCATACTGAAGGACTGATTCATATATCAGAAATTATGGATGACTACGTTCATAA
TTTGAAGAAATTTCTATCAGAAGGCCAAATTGTTAAAGCTAAAATTTTGTCTATAGATGATGAAGGAAAGCTTAATCTATCATTAAA
GGATAATGATTACTTCAAAAATTATGAGCGTAAGAAGGAAAAACAATCAGTATTAGATGAAATCAGAGAAACAGAAAAATATGGG
TTTCAAACACTTAAAGAACGCTTACCAATCTGGATAAAACAGTCAAAGCGAGCAATTCGAAACGACTAAAGGAACAGATAAATCGT
ACCGAAAATCATACAAAGGGTCTGAAATGAAAGTTTCTTAGACTATAAAAGAGATTAGTATCTATTAAATTTTATTAGATACTAATC
TCTTTTTGTCTACGATAACGTAATATGATTGATTCTATTTACACGTACAAATGGTTTAAGGTGACATATCCATTATCTTTGTTAGATA
GAATCGTTGATTTGCAATATTGTATGTGGATTTGTTTTTTTTATTTATTTTAGAAATGAGAACTACAACTTAAAGTATTAAACGAATT
GCAACTATATAAACAGATAATTGGAGAATGAAAAAATTACATGTTATAGTCAACTCAATAATTTTAAGGAGGAATTAAGTAATGAA
AAGTAAATACGAACCATTGTTTGATAAAGTAGAATTACCAAATGGAGTAGAGTTGAGAAATCGATTTGTGTTAGCCCCTTTAACAC
ATATTTCTTCAAATGATGATGGTACTATTTCAGATGTAGAACTTCCTTATATTGAAAAGCGTTCACAAGATGTTGGTATTACAATTAA
TGCTGCGAGTAATGTGAGTGATGTCGGAAAAGCATTTTCAGGACAGCCATCAATCGCGCATGACAGTAATATTGAAGGACTAAAAC
GATTAGCTACAGCAATGAAGAAAAACGGTGCCAAAGCACTCGTACAAATACATCATGGCGGTGCACAAGCATTGCCTGAATTAACA
CCTGGTGGAGACGTCGTAGCACCAAGTCCAATTTCTTTAAAAAGTTTCGGTCAGAAACAAGAACATAGTGCTAGAGAAATGACGAA
TGAAGAGATTGAACAAGCAATCAAGGATTTTGGTGAAGCAACGCGACGTGCAATTGAAGCAGGATTTGATGGTGTTGAAATACATG
GCGCGAATCATTACTTAATTCATCAATTTGTATCACCATACTATAATAGAAGAAATGATGTATGGGCAAATCAATATAAATTCCCGG
TTGCTGTGATTGAAGAAGTGCTTAAAGCGAAAGAAGTGTATGGCAATAAAGACTTTATTGTTGGATATAGATTGTCTCCGGAAGAA

GCGGAGTCTCCAGGAATCACAATGGAAATTACAGAGGAACTCGTTAATAAAATTAGCCATATGCCAATCGACTATATTCATGTTTCA
ATGATGGATACGCATGCAACGACACGTGAAGGTAAATACGCTGGACAAGAAAGACTGCCTTTAATTCACAAATGGATAAATGGTCG
TATGCCACTTATCGGTATTGGTTCAATTTTCACAGCTGACGAAGCTTTAGATGCAGTTGAAAATGTTGGTGTTGACTTAGTAGCCATT
GGTAGAGAGCTACTATTGGATTATCAATTTGTTGAAAAAATTAAAGATGGACGGGAAGATGAAATTATTAATTACTTTGATCCAGA
GAGAGAAGATAATCATCACTTAACTCCTAATTTATGGCATCAATTTAATGAAGGATTCTATCCATTACCACGTAAAGATAAATAAGT
GTTTAACTGTTACCACTAATGATATTGTGTATGCAATTTGTTAGTGGTTTTCAATATTTATAAATAAAAATATTCTTAGTAAAATATA
CTTTTGATTAAGTGTAAAATTATCAATTCTTTTGCTTGTAATCATTTAAGTAATTATGTCATTATAAATGTAAGGGTTTTCAAAATAG
ACAAATTTAATGAAACTTAGTGATTATTTCATCTTTTTTGGTGGAAAAGTATCTGCTTTGAAAACTAATGTAATTATTTGGATAATGT
TTCATGTATAATAGTTATAAATGATGAAAAGGGGAGATATGATGACTAAATCTGAAAAAATTATTGAGTTAACAAATCATTACGGA
GCACATAATTATTTACCATTGCCAATTGTCATTTCAGAAGCTGAAGGGGTATGGGTTAAAGATCCTGAAGGCAATAAATATATGGAT
ATGTTATCTGCATATTCCGCTGTTAACCAAGGTCATAGACATCCGAAAATTATTCAAGCATTAAAAGATCAAGCTGATAAAGTGACT
CTAGTTTCACGTGCTTTTCATAGTGATAACTTAGGTGAATGGTACGAAAAAATTTGTAAACTGGCAGGTAAAGATAAAGCTTTACCA
ATGAATACAGGTGCTGAAGCAGTAGAAACAGCTTTGAAAGCAGCACGACGCTGGGCATACGATGTTAAAGGAATTGAGCCAAATA
AAGCAGAAATCATTGCATTTAATGGTAACTTCCATGGTCGAACAATGGCGCCAGTTTCATTATCTTCAGAAGCAGAATACCAACGTG
GTTATGGTCCGTTATTAGATGGATTTAGAAAAGTTGATTTTGGAGATGTAGATGCATTGAAAGCTGCAATTAATGAAAATACTGCAG
CAGTTTTAGTAGAACCAATTCAAGGTGAAGCGGGTATAAATATACCGCCAGAAGGATATTTGAAAGCAATTAGAGAATTATGTGAT
GAACATAATGTCTTATTTATTGCTGACGAAATCCAAGCAGGATTAGGTCGTTCGGGTAAATTATTTGCTACGGATTGGGATAATGTA
AAACCTGATGTCTATATTTTAGGTAAAGCACTAGGTGGTGGTGTCTTCCCAATTTCTGTTGTATTAGCAGATAAAGAAGTATTAGAT
GTCTTTACACCTGGCTCACATGGTTCAACATTTGGTGGTAATCCACTTGCTTGTGCTGCATCAATTGCTGCATTAGATGTTATCGTTG
ATGAGGATTTACCAGGGCGCTCTTTAGAATTAGGAGATTATTTTAAAGAACAATTAAAGCAAATTGATCATCCATCAATTAAAGAA
GTCCGTGGACGTGGTTTGTTTATAGGTGTGGAACTTAATGAAAGTGCTAGACCATATTGTGAAGCTTTGAAAGAAGAAGGTTTATTA
TGTAAAGAAACGCATGATACTGTCATTCGTTTTGCACCACCATTAATTATTACTAAAGAAGAATTGGACCTTGCACTTGAAAAAATA
AGACATGTATTTCAATAATGTCAAAAAGTTGTAACAATCAGTTTGTAAGGGCTTTAAAAAGGAATACGTTATGTTACAATATGTTTG
AAAGCGAAATCATTAGGTAAAGAAGAGGCGAAAAGGATCATGACTGAGAACAATAATTTAGTAACTTCTACTCAAGGAATTATTAA
AGAAGCATTGCATAAATTAGGATTTGACGAAGGAATGTACGATTTAATTAAAGAACCTTTAAGAATGTTACAAGTGCGTATCCCTGT
ACGAATGGATGATGGCACTGTTAAAACATTCACAGGTTACCGTGCGCAACATAATGATGCTGTTGGACCAACAAAAGGGGGCGTGC
GTTTCCACCCAGATGTTGATGAAGAAGAAGTAAAAGCATTATCAATGTGGATGACTTTGAAATGTGGCATTGTAAACTTACCATACG
GTGGTGGTAAGGGTGGTATCGTTTGTGATCCACGTCAAATGAGCATTCATGAAGTTGAACGTTTATCACGCGGATATGTAAGAGCA
ATTTCACAATTCGTAGGTCCGAACAAAGATATTCCAGCACCAGATGTATTTACAAACTCACAAATTATGGCTTGGATGATGGATGAA
TATAGTGCATTAGATAAATTTAATTCACCAGGTTTCATCACAGGTAAACCAATTGTATTGGGTGGTTCTCATGGACGCGACAGATCA
ACTGCACTAGGTGTAGTTATTGCAATTGAACAAGCTGCAAAACGTCGTAATATGCAAATTGAAGGTGCCAAGGTTGTTATTCAAGGT
TTCGGTAATGCCGGAAGTTTCTTAGCTAAATTCTTATATGATTTAGGTGCAAAAATTGTAGGTATCTCTGATGCTTACGGTGCATTAC
ACGATCCAAATGGCTTAGATATAGATTATTTATTAGACCGTCGTGATAGTTTTGGTACGGTAACAAATTTATTTGAAGAAACAATCT
CAAATAAAGAATTGTTTGAATTAGATTGTGACATTTTAGTACCAGCGGCTATTTCAAACCAAATTACAGAAGACAATGCACATGATA
TTAAAGCTAGTATCGTTGTTGAAGCTGCTAATGGACCTACAACACCAGAAGCAACACGTATTTTAACTGAACGTGGTATATTATTAG
TTCCAGACGTATTAGCAAGTGCTGGTGGTGTAACGGTTTCTTACTTCGAATGGGTACAAAATAATCAAGGTTATTATTGGTCTGAAG
AAGAAGTTAATGAAAAGCTACGTGAAAAATTAGAAGCGGCATTTGATACGATTTACGAATTGTCTCAAAACCGAAAAATAGATATG
AGACTTGCAGCATATATCATAGGTATTAAACGTACAGCAGAAGCAGCTAGATATCGTGGTTGGGCATAATTAATTATCATATGTGAT
TTAACGAGCTTGGGACAGAAAACAAAGCCCTAAGCTCGTTAATTTTATTTTAGCAGTAGTTGACTGTAAAACAATGCCCGTGTAACA
CGCTCTTTTCAAAAATAGTCGGGGCCCCAACACAGAGGCTGGTGGAAAGTCAGCTTACAATAGTGTGCAAGTTGGCGGGGCCCCAA
CACAGAGGCTGGCGGAAAGTCAGCTTACAATAATGTGCAAGTTGGGGTGGAGCCCCGACACAGAGAAATTAGCTCCTCAATTTCTA
CAGACAATGCAAGTTGGCGAGGCCCCAACACTGAGAATTTCTAATTAAACGCTCAACTATATATAGTTGATCAAAAAGACAGCAGT
AGGATAATTTCAATTTGGAAATATCTTACTGCTGTTTTTTATAGTAATAGCTATATTTTTGTATTTATGTTTTCTAGTTTAACAATACT
ACTTAATTACTTCTTTATATTTATCAGCGAAATTTGTAAAGACACCATCAACGCCATATTTATTTAATCGTAACATATCAGCTTGTTC
ATTCACTGTATAAGGATGTACTATAAATCCTAAGTCTTTTAAATGATGGGTATTTTGTTCAGTTAAATCTGTATAATCAGGACCTAAT
CCAATCGCATAAGAGCGTATCTCTTTTAAGCGTTGGTCGTTAAATTGTTGTAGTTCACCTTTATCAACTAATTTTACTAATGGCACAT
GCTTATTTTGACGATGAATTTTCTTTAAACTTTCGTCAGAAAATGATTGAATCATTACATGTCCATTTTTTAATTTATTGTTATTTAAA
AGGTGATGCTTTTTCAATGAAGCTAATAATTGTTCTTCCATTCCTGGGTATACATCAGGTGACTTTGTTTCAATATAATAGTTTGCAT
TCGGGCCATAACGTTCTAAAATTTCATCTAAAGTGGGTACTTTAGCATTTTTATAACTTGCTCTTGCGTATTTTGGATATTTTTTATTA
AACCAACTTCCTGCATCTAACTGTTTTAATTCATCAAGGGTATAATCCTCAACTTTACCGTGTCCATTTGTTGTACGGTTAACAGTTT
CATCATGCATAGCAACTAAATGGCCATCTTTGGTACGTTGTAAATCAATTTCGATATAAGATGCTTTTAACTCATTATGACTCTTATC
ATATGCTTGAAACGTATGCTCGGGTGCATAGCCACTTGCGCCACGATGTGCGATAGTAGTGAATCGCTCATTCGTTAAATTTGTATG
CCATTGAATAGCCTGAGGTTTATTTGCAATTTGATTTGTTTGTTCAGCGCCAGCAGTAGGTACTGATAAAAATCCCATAGTAAAAAC
AGCAGAAGCAGCCATAAATTTAGTGAAGCTTTTCGAAGAGTTAGTCATAGTCTTTATCCTTTCATCTCTTTATTTGTAGTCTAACAAT
TTTAAAATACTACAAAAACCTGCTTGTGAAAACGCTTTACACATTTCTTTTACAATTTATAACATTTTTGTAATATAAATTTAAAAGA
ATTATCAAACATTTTAGGAATCTTAAAAATGTGATGTTCAACAAGACGAAGAGAGCATTAAAATACCCACAATGTAATTTTAATAG
ACATTGTGGGTAGGTAGATTAACGTATTTATTGTGATAGTAATTGTTTAGCAACATCAAGTTGTTGTTTGACCGATGATTGACCTGTT
GAACCGTAACTTTGACGTCGTTTTAAACAATTTTCAGGCTGCAAATAATCGTAAATATCGGCATCAATACTAGAATGATGTTGTTGA
TATGTTGCTAAAGGAACATCTAATAAATAATGACCTTGTTGTATACATTCTAAGACAATTTTTCCTACAATTTCATGTGCAGTTCTAA
ATGGAATATTTTTAGTTACTAAATAATCTGCTAGTTCCGTTGCATTTGAAAAATCTTCTTTAACAGTTTGATTGAGTCGTTCTTTATTA
ATTGTCATCGTTTGAATCATACCTTCGAAAATACGTAAAGAACCTTTAATTGTATGGACAGCATCGAATAAACCTTCTTTATCTTCCT
GCATATCTTTGTTATATGCTAGAGGTAATCCTTTTAAAGTCATAAGCATGCTCATTAAATGACCAGTCGTTCGACCAACTTTACCTCT
AATTAATTCTGCCATATCAGGATTTTTCTTTTGTGGCATAATAGATGAGCCAGTTGAAAATGCATCTGATAATGTAATGAATTTAGCT
TCGTCTGTGGACCAGAAAATAATTTCCTCTGCAAAGCGTGATAAGTGAACCATCGTTAAAGAAATATTATGCAATATTTCAATAATA
TAGTCTCTGTCACTAACAGCATCTAGGCTATTCTCATAGAGACTGCCAAAGTTCAACAATGCTGTTGTCTCGTGTCTATCGATAGGGT
ATGTGGTACCACTTAAGGCTGCTGCACCTAAAGGATTAATATCGATTCGTTTTAAACTATCTTCAAATCGTTGTTGGTCTCGTTGTAA
CATCCAAAAATAAGTCATAATATGATGTGCAAATGAAATTGGCTGTGCACGCTGTAAATGAGTATAACCAGGCATAATTGTATCAA
CATTATTGGAAGCGATGTCTACAATTACACTTTGTAACGACTTAATTAATGCGATGATATCTTGCACTTGTTTCTTAGTGTACAAGTG
CATGTCTGTTGCAACTTGATCGTTTCTACTGCGTCCAGTATGCAACTTACCACCAGCATCACCAATACGTTTAATTAATTCATGTTCA
ATATTTAAATGAATATCTTCTAATGATGCACTAAATTGAATTTGATCTTGATGATAATCATGTTGAATAGATTTTAGTCCTTGTATAA
TTTGTTCGCTGTCTTGTTGACTAATAATTCCTTGATTCGCAAGCATAGTTGCATGTGCAATGCTGCCTTCGATATCTTGATCTATGAG

CGTTTGATCAAAAGTAATGGATGCGTTAAAGTCGTCAACCCACTCTTCAGGTTGTACTTCAAATCTACCGCCCCAAGCTTTATTGCTC
ATTGCTATAGCCTCCATGTAGCATCGCATTTACTTGAGTAGGTAAACCATAGATATCGATAAAGCCAACAGCAGCGTCTTGATTAAA
TGCATCTTCTTTTGTATAAGTTGCTAATTTTTCATCATATAATGTGTAAGGTGATTTTCTACCATTTACGATGGCATTACCTTTGAATA
ATTTAATTCTGACATCACCACTTACGTATTGCTGAGTACTATCAATAAATAATTTTAAGCTATCAGTTAAAGGTGAGAACCAAAGTC
CATTGTATAGTTGTTCAGCAAATTGCTTCTCGATGATTGGTTTAAAGTGTGCGACATCTTTCGTTAACGTAATCGTTTCTAATGCTTT
ATGCGCTTTTAAAATAACTTCTGCAGCAGGTGCCTCATAAATTTCTCTTGATTTGATACCTACAAGTCTATTTTCTACATGGTCAATT
CTTCCGATACCATGCTTACCAGCTAATGCATTCAACGTTAAAATTAAATCGTCTAATTCATATGTTTTGCCATCAATTTGAACTGGGA
TGCCTTTATCAAACGTTAAAATGATTTCATCAGCAGTATCTGGTGTTTCTTCTAAAGCATTTGTTAGATCGAACGCATCCTCTGGTGG
CGCAGCATAAGGATCTTCTAAAATACCACATTCATTCGCTCTGCCCCATAGATTTTGATCGATAGAATAAGGTGAATCATGGTTGAT
TGATACAGGGATATTATGTTTAATTGCATAATCGATTTCTTCTTCACGACTCCATGCCCACTCACGTACAGGTGCGAATGCTTTCAAT
GATGGGTTTAATGCTTTAATGGCAACTTCGAAACGTACTTGGTCATTCCCTTTACCAGTACAACCATGTGCAATACCTACTGAATTTG
TTTTCTCAGCAATCTCTACTAATTTTTTAGCGATTAATGGTCTTGATAAAGCTGAAACTAATGGATATGCATTTTCATACATTAAATT
TCCTTTGATTGCATAACTTACATACTCATCACTAAATTCTTTTGTTGCATCAATAATATGACATTCAACTGCTCCCATATCTAAAGCTT
TTTTATAAACGATGTCTAAATCTTTACCTTCACCAACATCTAGGCAACAAGCTACAACTTCGTATCCTTTGTCGATAAGCCATTGAAC
GGCCACACTTGTATCTAGTCCTCCTGAATATGCTAAAACAATTTTCTCTTTCATAAAATTCACCTCATTGTAGTTATCTGAATATTTC
GTATGAACACATGATAGCAAACTTTTAATGAAAATAAAACCATTATTTTACATATTTATTCATAAAATGCGATAGAGATAGTTAGAG
TTGTAAGTATAAATGCTTATTTATCAATGTTTAAAAGTATTTTAAAAAACATGCAAATTGTATAAAAAATGTTTGAGTGTTTGACGC
CAGTTTGAAATGTTTGTTGAAAAATAGGTGTGAAACGTCACATAAATATCAATTTTATTGATTAAGTACAAACTTGTGAATTGAATC
GCAAACGTAAACTTAGTAAAATTAAGATATTAAATGAAACAGGAGGCTTTTATAGATGACTCATATTCAATTAGATTTTAGTAAAAC
GTTAGAATTTTTCGGTGAACACGAATTAAAACAACAACAAGAAATTGTTAAATCAATTCACAAAACAATTCATGAAGGTACTGGTG
CAGGTAGTGACTTCTTAGGCTGGGTTGATTTACCAGTTGATTACGACAAAGAAGAATTTTCAAGAATTGTTGAAGCATCAAAACGCA
TTAAAGAAAATTCTGATGTTTTAGTAGTCATCGGTATTGGTGGTTCTTACTTAGGTGCACGTGCAGCAATCGAAATGTTAACGTCAT
CATTTAGAAACAGCAATGAATACCCTGAAATTGTATTTGTTGGTAATCACTTATCATCAACATATACGAAAGAGTTAGTTGATTATT
TAGCAGACAAAGATTTCTCTGTAAACGTTATTTCTAAATCTGGTACAACTACAGAACCAGCAGTTGCATTTAGATTGTTCAAACAAT
TAGTTGAAGAAAGATACGGTAAAGAAGAAGCACAAAAACGTATATTTGCAACAACGGATAAAGAAAAAGGTGCTTTAAAACAGTT
GGCTACAAACGAAGGTTATGAAACGTTTATCGTACCTGATGATGTAGGTGGAAGATATTCTGTTTTAACAGCAGTAGGATTATTACC
AATTGCAACAGCTGGAATTAACATCGAAGCTATGATGATTGGTGCTGCAAAAGCACGTGAAGAATTATCTTCAGATAAATTAGAAG
ACAACATTGCATACCAATATGCGACAATTCGAAACATTTTATATGCAAAAGGTTATACAACAGAAATGTTGATTAACTATGAACCAT
CTATGCAATACTTTAATGAATGGTGGAAACAATTATTTGGTGAATCAGAAGGTAAAGACTTCAAAGGTATCTATCCTTCAAGTGCCA
ACTACACAACTGATTTACATTCTTTAGGTCAATATGTACAAGAAGGCCGTCGTTTCTTATTCGAAACAGTGGTAAAAGTAAATCATC
CTAAATATGATATTACTATTGAAAAAGATAGTGATGATCTAGACGGATTAAATTATTTGGCTGGTAAAACAATCGACGAAGTTAAC
ACAAAAGCATTCGAAGGTACATTATTAGCGCATACTGATGGTGGTGTTCCTAACATGGTAGTGAACATTCCACAATTAGATGAAGA
AACTTTCGGTTATGTCGTATACTTCTTCGAACTTGCTTGTGCAATGAGTGGATACCAATTAGGTGTAAATCCATTTAACCAACCTGGT
GTAGAAGCATATAAACAAAACATGTTCGCATTATTAGGTAAACCTGGTTTTGAAGACTTGAAAAAAGAATTAGAAGAGCGTTTATA
AAATACATTACTTCAATGATTAGTGAAGTTTGAAAAGATAGAACTAGACGTTAACTATTTAAAGCATATTTTCGAGGTTGTCATTAC
AAATGTAAAAATGTAATGACAACCTCGTTTTTATTTATATGCAAGAACTAGGTTACTAGCTAATGTGACAAGATGTTAAGAGAAAAT
TAAAGAAAAAATAACATCTGTCATACAATAATATTGTTATACTACTAGAGACTGATTTATTAGCATGATTACATGTTAATGTTTCTTT
ACTTAGTAATTAACTTTATAATGTAAGAATAATTATCTTCAACCAAAGAAAGGGATTGATGATTTGTCGTTTCATCAAGTAGAAGAA
TGGTTTGAGATATTTCGACAGTTTGGTTATTTACCTGGATTTATATTGTTATATATTAGAGCGATAATTCCAGTATTTCCTTTAGCACT
CTATATTTTAATTAACATTCAAGCTTATGGACCTATTTTAGGTATATTGATTAGTTGGCTTGGATTAATTTCTGGAACATTTACAGTCT
ATTTGATCTGTAAACGATTGGTGAACACTGAGAGGATGCAGCGAATTAAACAACGTACTGCTGTTCAACGCTTGATTAGTTTTATTG
ATCGCCAAGGATTAATCCCATTGTTTATTTTACTTTGTTTTCCTTTTACGCCAAATACATTAATAAATTTTGTAGCGAGTCTATCTCAT
ATTAGACCTAAATATTATTTCATTGTTTTGGCATCATCAAAGTTAGTTTCAACAATTATTTTAGGTTATTTAGGTAAGGAAATTACTA
CAATTTTAACGCATCCTTTAAGAGGGATATTAATGTTAGTTGTGTTGGTTGTATTTTGGATTGTTGGAAAAAAGTTAGAACAGCATTT
TATGGGATCGAAAAAGGAGTGACATCGTGAAAAAAGTTGTAAAATATTTGATTTCATTGATACTTGCTATTATCATTGTACTGTTCG
TACAAACTTTTGTAATAGTTGGTCATGTCATTCCGAATAATGATATGTCACCAAGCCTTAACAAAGGGGATCGTGTTATTGTAAATA
AAATTAAAGTTACATTTAATCAATTGAATAATGGTGATATCATTACATATAGGCGTGGTAACGAGATATATACTAGTCGAATTATTG
CCAAACCTGGTCAATCAATGGCGTTTCGTCAGGGACAATTATACCGTGATGACCGACCGGTTGACGCATCTTATGCCAAGAACAGA
AAAATTAAAGATTTTAGTTTGCGCAATTTTAAAGAATTAGATGGAGATATTATACCGCCTAACAATTTTGTTGTGCTAAATGATCAT
GATAACAATCAGCATGATTCAAGACAATTTGGTTTAATTGATAAAAAGGATATTATTGGTAATATAAGTTTGAGATATTATCCTTTT
TCAAAATGGACGATTCAGTTCAAATCTTAAAAAGAGGTGTCAAAATTGAAAAAAGAATTATTGGAATGGATTATTTCAATTGCAGT
CGCTTTTGTCATTTTATTTATAGTAGGTAAATTTATTGTTACACCATATACGATTAAAGGTGAATCAATGGATCCAACTTTGAAAGAT
GGCGAGCGAGTAGCTGTAAACATTATTGGATATAAAACAGGTGGTTTGGAAAAAGGTAATGTAGTTGTCTTCCATGCAAACAAAAA
TGATGACTATGTTAAACGTGTCATCGGTGTTCCTGGCGATAAAGTAGAATATAAAAATGATACATTATATGTCAATGGTAAAAAAC
AAGATGAACCATATTTAAACTACAATTTAAAACATAAACAAGGTGATTACATTACTGGGACTTTCCAAGTTAAAGATTTACCGAATG
CGAATCCTAAATCAAATGTCATTCCAAAAGGTAAATATTTAGTGCTTGGAGATAATCGTGAAGTAAGTAAAGATAGCCGTGCGTTT
GGCCTCATTGATGAAGACCAAATTGTTGGTAAAGTTTCATTTAGATTCTGGCCATTTAGTGAATTTAAACATAATTTCAATCCTGAA
AATACTAAAAATTAATATGAAACAAATACAACATCGTTTGTCGGTTTTAATACTGATAAACGATGTTTTATTTTGTTAGTACCACAA
TAAAAGCTAAGTTCGAAATGAACTTATAATAAATCAATCACAATCACTTTGTGTTAAAATATGTGTCAAAGGAAGTGAGGGTTTGTC
ATGACATTACATGCTTATTTAGGTAGAGCGGGAACAGGTAAGTCTACGAAAATGTTGACCGAAATAAAACAAAAAATGAAAGCAG
ATCCGCTTGGAGATCCAATCATTTTAATTGCGCCAACTCAAAGTACATTTCAATTAGAACAAGCCTTTGTCAATGATCCGGAATTAA
ATGGTAGTTTAAGAACAGAAGTGTTGCATTTTGAACGATTAAGTCATCGTATTTTCCAAGAAGTTGGTAGTTATAGCGAACAAAAGT
TATCTAAAGCTGCAACGGAAATGATGATTTATAACATTGTTCAAGAACAACAAAAGTATTTAAAACTTTATCAATCACAAGCAAAA
TATTATGGGTTTAGTGAAAAATTAACAGAACAAATTCAAGATTTTAAAAAATATGCAGTAACGCCTGAACATTTAGAACACTTTATT
GCTGATAAAAATATGCAAACTCGAACTAAAAATAAGTTAGAGGATATTGCTTTAATATACCGTGAGTTCGAACAACGCATTCAAAA
CGAGTTTATTACGGGTGAGGATTCATTACAATATTTTATTGATTGTATGCCGAAATCAGAGTGGCTAAAACGTGCTGATATATATAT
TGATGGTTTTCACAACTTTTCAACGATTGAGTATTTAATAATCAAAGGATTAATTAAATATTCGAAGAGTGTCACAATTATATTGAC
GACAGATGGTAACCACGATCAATTTAGTTTATTTAGAAAACCATCGGAAGTGTTACGACATATTGAAGAAATAGCAAATGAACTCA
ATATTTCTATTGAACGTCAATATTTCAAGCAATTATATCGCTTCAATAATCAAGATTTAAAGCATCTTGAACAAGAATTTGATGCACT
TCAAATCAATCGAGTGGCATGTCAAGGTCATATCAATATTTTAGAATCTGCGACTATGAGAGAGGAAATAAATGAAATTGCGCGAC
GTATCATCGTTGATATTCGTGATAAGCAATTACGATATCAAGATATTGCAATTTTATATCGTGACGAGTCTTATGCTTATTTATTTGA

TTCCATATTACCGCTTTATAATATTCCTTATAACATTGATACAAAGCGTTCGATGACACATCATCCGGTCATGGAAATGATTCGTTCA
TTGATTGAAGTTATTCAATCTAATTGGCAAGTGAATCCAATGCTACGCTTATTGAAGACTGATGTGTTAACGGCATCATATCTAAAA
AGTGCATACTTAGTTGATTTACTTGAAAATTTTGTACTTGAACGTGGTATATACGGTAAACGTTGGTTAGATGATGAGCTATTTAATG
TCGAACATTTTAGCAAAATGGGGCGTAAAGCGCATAAACTGACCGAAGATGAACGTAACACATTTGAACAAGTCGTTAAGTTAAAG
AAAGATGTCATTGATAAAATTTTACATTTTGAAAAGCAAATGTCACAAGCGGAAACTGTAAAAGATTTTGCAACTGCTTTTTATGAA
AGTATGGAATATTTCGAACTGCCAAATCAATTGATGACAGAGCGAGATGAACTTGATTTAAATGGTAATCATGAAAAGGCGGAGGA
AATTGATCAAATATGGAATGGCTTAATTCAAATCCTTGATGACTTAGTTCTAGTATTTGGAGATGAACCAATGTCGATGGAACGTTT
CTTAGAAGTATTTGATATTGGTTTAGAACAATTAGAATTTGTTATGATTCCGCAAACATTGGACCAAGTAAGTATTGGTACGATGGA
TTTGGCTAAAGTCGATAATAAGCAACATGTTTACTTAGTAGGTATGAATGACGGCACCATGCCACAACCAGTAACTGCATCAAGTTT
AATTACTGATGAAGAAAAGAAATATTTTGAACAACAAGCAAATGTAGAGTTGAGTCCTACATCAGATATTTTACAGATGGATGAAG
CATTTGTTTGCTATGTTGCTATGACTAGAGCTAAGGGAGATGTTACATTTTCTTACAGTCTAATGGGATCAAGTGGTGATGATAAGG
AGATCAGCCCATTTTTAAATCAAATTCAATCATTGTTCAACCAATTGGAAATTACTAACATTCCTCAATACCATGAAGTTAACCCATT
GTCACTAATGCAACATGCTAAGCAAACCAAAATTACATTATTTGAAGCATTGCGTGCTTGGTTAGATGATGAAATTGTGGCTGATAG
TTGGTTAGATGCTTATCAAGTAATTAGAGATAGCGATCATTTAAATCAAGGTTTAGATTATTTAATGTCAGCATTAACGTTTGACAA
TGAAACTGTAAAATTAGGTGAAACGTTGTCTAAAGATTTATATGGTAAGGAAATCAATGCCAGTGTATCTCGATTTGAAGGTTATCA
ACAATGCCCATTTAAACACTATGCGTCACATGGTCTGAAACTAAATGAACGAACGAAGTATGAACTTCAAAACTTTGATTTAGGTG
ATATTTTCCATTCTGTTTTAAAATATATATCTGAACGTATTAATGGCGATTTTAAACAATTAGACCTGAAAAAAATAAGACAATTAA
CGAATGAAGCATTGGAAGAAATTTTACCTAAAGTTCAGTTTAATTTATTAAATTCTTCAGCTTACTATCGTTATTTATCAAGACGCAT
TGGCGCTATTGTAGAAACAACACTAAGCGCATTAAAATATCAAGGCACGTATTCAAAGTTTATGCCAAAACATTTTGAGACAAGTTT
TAGAAGGAAACCAAGAACAAATGACGAATTAATTGCACAAACATTAACGACAACTCAAGGTATTCCAATTAATATTAGAGGGCAA
ATTGATCGTATCGATACGTATACAAAGAATGATACAAGTTTTGTTAATATCATTGACTATAAATCCTCTGAAGGTAGTGCGACACTT
GATTTAACGAAAGTATATTATGGTATGCAAATGCAAATGATGACATACATGGATATCGTTTTACAAAATAAACAACGCCTTGGATTA
ACAGATATTGTGAAACCAGGTGGATTATTATACTTCCATGTACATGAACCTAGAATTAAATTTAAATCGTGGTCTGATATTGATGAA
GATAAACTAGAACAAGATTTAATTAAAAAGTTTAAGCTGAGTGGTTTAGTGAATGCAGACCAAACTGTTATTGATGCATTGGATATT
CGTTTAGAACCTAAATTCACTTCAGATATTGTACCAGTTGGTTTGAATAAAGATGGCTCTTTGAGTAAACGAGGCAGCCAAGTGGCA
GATGAAGCAACAATTTATAAATTCATTCAGCATAACAAAGAGAATTTTATAGAAACAGCTTCAAATATTATGGATGGACATACTGA
AGTTGCACCATTAAAGTACAAACAAAAATTACCATGTGCTTTTTGTAGTTATCAATCGGTATGTCATGTAGATGGCATGATTGATAG
TAAGCGATATCGAACTGTAGATGAAACAATAAATCCAATTGAAGCAATTCAAAATATTAACATTAATGATGAATTTGGGGGTGAGC
AATAGATGACAATTCCAGAGAAACCACAAGGCGTGATTTGGACTGACGCGCAATGGCAAAGTATTTACGCAACTGGACAAGATGTA
CTTGTTGCAGCCGCGGCAGGTTCAGGTAAAACAGCTGTACTAGTTGAGCGTATTATCCAAAAGATTTTACGTGATGGCATTGATGTC
GATCGACTTTTAGTCGTAACGTTTACAAACTTAAGCGCACGTGAAATGAAGCATCGTGTAGACCAACGTATTCAAGAGGCATCGATT
GCTGATCCTGCAAATGCACACTTGAAAAACCAACGCATCAAAATTCATCAAGCACAAATATCTACACTTCATAGTTTTTGCTTGAAA
TTAATTCAACAGCATTATGATGTATTAAATATTGACCCGAACTTTAGAACAAGCAGTGAAGCTGAAAATATTTTATTATTAGAACAA
ACGATAGATGAAGTTATAGAACAACATTACGATATCCTTGATCCTGCTTTTATTGAATTAACAGAGCAATTGTCTTCAGATAGAAGT
GATGATCAGTTTCGAATGATTATTAAACAATTGTATTTCTTTAGCGTTGCAAATCCAAATCCTACAAATTGGTTGGATCAATTGGTGA
CACCATACGAAGAAGAAGCACAACAAGCGCAACTTATTCAACTACTAACAGACTTATCTAAAGTATTTATCACAGCTGCTTATGAT
GCTTTAAATAAGGCGTATGATTTGTTTAGTATGATGGATAGCGTCGATAAACATTTAGCTGTTATAGAAGATGAACGACGTTTAATG
GGGCGTGTTTTAGAAGGTGGCTTTATTGATATACCTTATTTAACTGGTCACGAATTTGGCGCGCGTTTGCCTAATGTAACAGCGAAA
ATTAAAGAAGCAAATGAAATGATGGTCGATGCCTTAGAAGATGCTAAACTTCAGTATAAAAAATATAAATCATTAATTGATAAAGT
GAAGAGTGATTACTTTTCAAGAGAAGCTGATGATTTGAAAGCTGATATGCAACAATTGGCGCCACGAGTAAAGTACCTTGCGCGTA
TTGTGAAAGATGTTATGTCAGAATTCAATCGAAAAAAGCGTAGCAAAAATATTTTGGATTTTTCTGATTATGAACATTTTGCATTAC
AAATTTTAACTAATGAGGATGGTTCGCCTTCAGAAATTGCCGAATCATACCGTCAACACTTCCAAGAAATATTGGTCGATGAGTATC
AAGATACGAACCGAGTTCAAGAGAAAATACTATCTTGCATCAAAACGGGTGATGAACATAATGGTAATTTATTTATGGTTGGAGAT
GTTAAGCAATCCATTTATAAATTTAGACAAGCTGATCCAAGTTTATTTATTGAAAAGTATCAACGCTTTACTATAGATGGAGATGGC
ACTGGACGTCGAATTGATTTGTCGCAAAACTTCCGTTCTCGAAAAGAAGTACTGTCAACGACTAACTATATATTCAAACATATGATG
GATGAACAAGTCGGTGAAGTAAAATATGATGAAGCGGCACAGTTGTATTATGGTGCACCATATGATGAATCGGACCATCCAGTAAA
CTTAAAAGTCCTTGTTGAAGCGGATCAAGAACATAGTGATTTAACTGGTAGTGAACAAGAAGCGCATTTTATAGTAGAACAAGTTA
AAGATATCTTAGAACATCAAAAAGTTTATGATATGAAAACAGGAAGCTATAGAAGTGCGACATACAAGGATATCGTTATTCTAGAA
CGCAGCTTTGGACAAGCTCGCAATTTACAACAAGCCTTTAAAAATGAAGATATTCCATTCCATGTGAATAGTCGTGAAGGTTACTTT
GAACAAACAGAAGTCCGCTTAGTATTATCATTTTTAAGAGCGATAGATAATCCATTACAAGATATTTATTTAGTTGGGTTAATGCGC
TCCGTTATATATCAGTTCAAAGAAGACGAATTAGTTCAAATTAGAATATTGAGTCCAAATGATGACTACTTCTATCAATCGATTGTA
AATTACATTAATGACGAAGCAGCAGATGCAATTTTAGTTGATAAATTAAAAATGTTTTTATCAGATATTCAAAGTTACCAACAATAT
AGTAAAGATCATCCGGTGTATCAGTTAATTGATAAATTTTATAATGATCATTATGTTATTCAATACTTTAGTGGACTTATTGGTGGAC
GTGGACGACGTGCAAATCTTTATGGTTTATTTAATAAAGCTATCGAGTTTGAGAATTCAAGTTTTAGAGGTTTATATCAATTTATTCG
TTTTATCGATGAATTGATTGAAAGAGGCAAAGATTTTGGTGAGGAAAATGTAGTTGGTCCAAACGATAATGTCGTTAGAATGATGA
CAATTCATAGTAGTAAAGGTCTAGAGTTTCCATTTGTCATTTATTCTGGATTGTCAAAAGATTTTAATAAACGTGATTTGAAACAACC
AGTTATTTTAAATCAGCAATTTGGTCTCGGAATTGATTATTTTGATGTGGATAAAGAAATGGCATTTCCATCTTTAGCTTCGGTTGCA
TATAGAGCTGTTGCCGAAAAAGAACTTGTGTCAGAAGAAATGCGATTAGTCTATGTAGCATTAACAAGAGCGAAAGAACAACTTTA
TTTAATTGGTAGAGTGAAAAATGATAAATCATTACTAGAACTAGAGCAATTGTCTATTTCTGGTGAGCACATTGCTGTCAATGAACG
ATTAACTTCACCAAATCCGTTCCATCTTATTTATAGTATTTTATCTAAACATCAATCTGCGTCAATTCCAGATGATTTAAAATTTGAA
AAAGATATAGCACAAATTGAAGATAGTAGTCGTCCGAATGTAAATATTTCAATTGTGTACTTTGAAGATGTGTCTACAGAAACCATT
TTAGATAATGATGAATATCGTTCGGTTAATCAATTAGAAACTATGCAAAATGGTAATGAAGATGTTAAAGCACAAATTAAACACCA
ACTTGATTATCGATATCCATATGTAAATGATACTAAAAAGCCCTCAAAACAATCTGTTTCTGAATTGAAAAGACAATATGAAACAG
AAGAAAGTGGCACAAGTTACGAACGAGTAAGGCAATATCGTATCGGTTTTTCAACGTATGAACGACCTAAATTTCTATGTGAACAA
GGTAAACGAAAAGCGAATGAAATTGGTACGTTAATGCATACAGTGATGCAACATTTACCATTCAAAAAAGAGCGCATATCTGAAGT
TGAGTTACATCAGTATATCGATGGATTAATCGATAAACATATTATCGAAGCAGATGCGAAAAAAGATATCCGTATGGATGAAATAA
TGACATTTATCAATAGTGAGTTATATTCGATTATTGCTGAAGCAGAGCAAGTTTATCGTGAATTACCGTTTGTAGTTAACCAAGCATT
AGTTGACCAATTGCCACAAGGAGACGAAGACGTCTCAATTATTCAAGGTATGATTGACTTGATCTTTGTTAAAGACGGTGTGCATTA
TTTTGTAGACTATAAAACCGATGCATTTAATCGTCGTCGTGGGATGACAGATGAAGAAATTGGTACACAATTAAAAAATAAATATA
AGATACAGATGAAATATTATCAAAATACGCTTCAAACGATTCTTAATAAAGAAGTTAAAGGTTATTTATACTTCTTCAAATTTGGTA
CATTGCAACTGTAGTATTTTGATTTTCAAAAGAATAAAAAATAATTTCGATTAAGTGCAAAGCCCTTGTAGCAGAATGAACATAACT

CATTTTCAAAATTGTCTTACTTATTTATTTGTTATTTGATAACGAAAAAAGTTATAATGTGAATTAAGATAAAGATGAGGAGTTGAG
AATGAATGAAATTCTTATCATTCAAGTATAATGACAAAACTTCATATGGCGTTAAAGTAAAACGCGAAGATGCTGTATGGGATTTA
ACACAAGTATTTGCTGACTTTGCAGAAGGAGATTTCCATCCTAAAACATTGTTAGCTGGTTTACAACAAAATCATACTTTAGATTTTC
AAGAACAAGTACGTAAAGCAGTTGTAGCAGCAGAAGATAGCGGCAAAGCTGAAGACTATAAAATTTCATTTAATGACATTGAATTC
TTACCACCAGTAACACCTCCGAATAATGTGATTGCTTTTGGTAGAAATTACAAAGATCATGCGAACGAATTAAATCATGAAGTAGA
AAAATTATATGTATTTACAAAAGCAGCGTCATCTTTAACAGGAGATAATGCAACAATTCCAAATCATAAAGATATTACTGATCAATT
AGATTATGAAGGTGAATTAGGTATTGTTATTGGTAAGTCTGGTGAAAAGATTCCAAAAGCATTAGCTTTAGATTATGTTTACGGCTA
TACAATTATTAACGATATCACTGATCGCAAAGCACAAAGTGAACAAGATCAAGCATTTTTATCAAAAAGTTTAACTGGCGGTTGCCC
AATGGGTCCTTATATCGTTACTAAAGACGAACTACCATTACCTGAAAATGTAAATATTGTTACAAAAGTTAACAATGAAATTAGACA
AGATGGTAACACTGGCGAAATGATTCTTAAAATTGATGAATTAATAGAAGAAATTTCAAAATATGTTGCACTACATCCGGGAGATA
TTATTGCAACTGGTACACCAGCAGGCGTTGGTGCAGGTATGCAACCACCTAAATTTTTACAACCAGGTGATGAAGTTAAAGTGACTA
TTGATAATATTGGAACGCTGACAACTTATATCGCTAAATAATTATCATTTAAAAAGCTAACCAGTCTTTATATAGATTGGTTAGTTTT
TTCTTGCTTTTCTAAAAAGGTGTTAAAGATAAATTATTTATAATGTTACCATTTTGAGATGAAAGTGAAATATTGATATTAAGAAGT
AGTTGATTATTTTACAGCAGATTCACAATATTCTAATAAGGAATAAAATGTCATGTTCTTCCTCTCAAATATAGAAGTGTGGTAGAA
TATATATTCGTGTATAATCAAATCATGATTAAATTACAAGCAAGTGGGTATTAATCCCAAGAAGCTTTAATTTAATAATATAAATAA
TTAAAGTGGGGGAATCAGTATGTTACATTTACATATATTAAGTTGGGTATTAGCGATTATTTTATTTATCGCTACATACTTAAACATT
TCAAAAAATCAAGGCGGATCACCATTTTTCAAACCGTTGCACATGATTTTACGCTTATTTATGCTGTTGACGTTAATTTCAGGATTTT
GGATATTAATTCAGTCATTTATGAATGGCGGGGCAAATCATATGTTGCTTACATTGAAAATGCTGTGTGGTGTTGCAGTAGTTGGAT
TGATGGAAGTGTCGATTGCTAAAAGAAAGAGACATGAACAAAGTCACACAATGTTTTGGATAACAATGGCATTAATTATCATCACA
ATGGTATTAGGTGTCATTCTACCGTTAGGGCCTATATCAAAATTATTCGGTATTGGCTAATTGATAAGACATATAGTATATGACATA
AGGCATGGACAATTATTAGTCCAAGACCTTATGTCTTTTTTGATTCTAAAAATGTTTAAACATAAAAAAAGGACTGGATAGTAGTAC
CAGTCCAACATAATTCTAACCTAGACAATTAAGATTAGATTGAATCGCATGATTCATTTATTTAGCTTTGTAACCAATCATATTGATT
AAATCTTTAGGGTGGCTATATGGTGGTGCATAAGCCACTTCAAACTCAGTTAACTCATCTACAGTTAGCTGGTTCATCATTGCCATC
GATAGTACATCAATACGTTTATCTGCACCTTCTTTTCCTACTGCAGCTGCTCTTAAAATCTGACGGTTTGAAGTGTCATAATATACCC
TTAAGTGTAAAGGGGAATTTCCTGGGTAATAATTCGCGTGTGCACCTTGAGTGACTTCTACCATTTTATAGTCAAATTGCTTTAGTTC
ATTTGGTTTAACGCCGACACTCGCAAATGTATAATCAAAGAACTTCACAATATTGTTGCCTAAGAAGCCTTTGAATTCAATAGTGTC
ATTTCCAGCAATTTGTTCGGCAACAATACTTGCTGCACGGTGAGCGCCCCAAGCTAAAGGAACACTAGCCGGTAGATCGACATGTC
GATAATGTGATGTTGCAATATCGCCTATTGCATAAATGTTTGGAACATTTGTTTCAAATTTATCGTTTACCGGTATGAAACCTTTTCG
ATCAAGTTTGATATTTGAACTTTCGATAAATTTTGAATTGGGGTGAGTACCGACACCTTCAATAATCATATCGTAATGTTCAACTTTT
CCTGATTTAAATGTAATTTCATTTCCATTGATAGCATCAATTTCCTCATTTAAACGGTATGGAATCTCCCGCTTATCTAATTCATCAA
GTATAGGTTGATTCATGTCAGCATCCATTAATTTATTTATCTTATCAGATCGATGAATTAAAGTAGGGTGTAAACCACGTTCGTAAA
GATTTTCAAGAACTTCTAATGAAACATACCCTGCACCTACAACTAATACTTTATCAACTTGATTTGCTTTGATGAATTGATCGATAGC
ATCAGTGTCTTCTAAATTTCTAAGTGTAAATGTAATATCACTTTCAAAGCCAAGGCTATTTGCACTTGCACCAGGGCTTAAAATGAG
TTTATCGTAAGATTCTTCAAATTGTTCGTTGGTCTTTCTATTTAATACAGTTACAGTTTGTCTTTCATCATTGATTGCAATAACTTCAT
GATAAGTTTTTACTGTAATTTGCTTTCTATCATAAAATTTTTCAGGTGTATACGCTAAAGCATATTTTCTATCTTCAACAACTTCGCCA
ATGACATAAGGCAATGCACAATTAGCAAAGCTCATATCACGATCTTTTTCAAAAATAATAATGTCACTTTCTTTATCTAAACGTCGA
ATTTGGCTGGCACATGTTGCACCGCCAGCGACTGCTCCGACTACGACTATTTTGGGCATCGTAACTTACCTCCTATTGTTTTAAAATA
TTATAAAATGATTAGTTATAAATTAACAGTAATATCTAATATTTAAATTAAAGAAATCATTCAAATATCGACCAATGCCATCTTCAT
TATTGTTGAATGTAATATTGTTCGCTACATCTTTAAGTTCTTGCAAACCATTTTCCATAGCAACACCATGGCGGGCGTACTCAATCAT
TTCAATATCATTATCTTCATCACCGAATGCAATAATATTATTTCGGTCAATATTTAAAAATTGTCTAACTTGCTCAATGCCTCTTGCTT
TATTAATACCAAGTTTTACAATTTCAATGACAGGGAATGGTGCGCCCCAGCGTCGATGCTCAATATGATCGGCATAAAAATGAGTA
AGCATATTTTTGATTTCAGGTATTTTACTTTCTTCGGCTTCAATTAAAATTGAGGTAGGGGATTCTTTCAAGTGGACAAGTAAATTAC
CAGTTTGAATTCTTGGATTACCCATTGAAAAACCTTCAAATAATCTTGGATCATGATTGTTAATGAAAACATAATCTTTCACTTCTGC
TATAATATTCGATACTTGATATTGTTGTAATCCTTGAATAATGTTTTGTGCGATGCCTAAATCTAAAATTTCATGGCAAGTTTTGAAG
TTTTTATCTTTAGGGTGATGTACGTAAGCGCCATTAAAATTAACAATTGGTGTCGTTAAATTTAATTCATGATAATACATTTGACTTG
CACGATAAGGTCTGCCAGTCGCAATCATAATTTGGTGTCCACGTTGTTGTAATTCATTTAATACTTGTTTAGTATATGATGAAATTTC
TTTGTTATCGTTTAATAATGTTCCGTCTAAGTCTAGACATATTAAATGTGGTTGCATATCTCTCTCCTCATAAGTTGCTTAGTTCATAT
AGATACATCATAGCATTTTATTGGCGTTTTGTCGGATATTTGATATATTATGAATAATATGACATTAAAGAGGTGATATGGATGGAA
GAGGCATTGAAAGATAGTATCTTAGGTGCATTAGAAATGGTAATTGACCCTGAATTAGGAATTGATATCGTTAATTTAGGTTTAGTA
TACAAAGTGAATGTTGATGATGAAGGCGTATGTACAGTTGATATGACTTTAACATCAATGGGATGTCCAATGGGACCTCAAATTATT
GATCAAGTTAAAACAGTATTAGCAGAGATTCCTGAAATTCAGGATACTGAAGTGAATATCGTATGGAGTCCACCTTGGACAAAAGA
TATGATGTCACGTTACGCTAAGATTGCACTTGGTGTGAGCTAACATAAACGTGCATTGCCTGTAGAATTTCTTTTAGAAATTCTCTGT
GTTGGGTCCCCGCAGTCAATATAACATTGTAGAGTATAATTCATTGTGAATAAGCTCCCTTCAAAGTAGACATTGAAAAATGAAAAT
TTTGAAGGGAGCCTTATTTATGTTCTAGGTTCTGATTTCATTTTGAAAATTTATATCTAACTTGTTTCAAAAAATATGAATTAAGACA
GTGACTTGCACTTCCAAGAGTGCACAATTCATTTTCTAGTTGAATATTTTCTTGAAAATTTTTATAATAAATTGAATTTATATTACAG
TTATATTACAATACATAATACTTCATAGTTTTTAAGTTGCCGAGTTGCATTACAAATTGGATTTCGAAGTAAGAATCATCATTGGTAA
TTTTTTTAATTTAACAGTATTTTAGTCAATATGATTTTGATGATGTCGTAATTCAATAATATACATATGCATTTCAATATTCTTTTAAA
CAGCATAACTAAATTTAATTAAGCATCTAAGAAGAACAGTTTTAAAAGGGAAGAATGCAAAAATGAACAAAACAAAGGGTTTTAC
AAAGTATAAGAAAATGAGATATATGCCAGGGCTCGATGGTTTGAGGGCAATCGCTGTTCTAGGAATTATTATTTACCACTTAAACA
AGCAATGGTTGACAGGTGGCTTTTTAGGTGTGGATACATTTTTTGTGATCTCTGGTTATTTAATTACAAGCTTATTACTCAAAGAGTA
TGATGACACAGGTATCATTAAATTGAAAAGCTTTTGGATACGTCGTTTAAAACGTTTATTACCAGCAGTCATAGTTTTATTAATGGTT
GTAGGGACAGCAACCTTATTATTAAAATCAGATAATATCATTAGGGTTAAACATGATATTATTGCTGCGATATTTTATGTATCAAAC
TGGTGGTATATAGCAAAAGATGTTAATTATTTTGAGCAATTTTCATTTATGCCATTAAAGCATTTATGGTCTTTAGCAATTGAAGAAC
AGTTTTACATATTTTTCCCAGTTATTTTGGTTACATTATTGTTAACAATTAAAAAGCGATACAAAATAGGATTTATTTTTTTGGGAGT
ATCAATAATTTCTTTAGGGTTAATGATGTTTATCTACAGTATTAATGGGGATCATTCACGAGTGTATTTTGGTACAGATACTAGATTA
CAGACATTGTTACTGGGTGTTATTTTAGCTTTTTTATGGCCACCGTTTAAATTGAAAAATGATCCACCTAAAGTTGTAAAATATGTTA
TTGATAGCATAGGTAGTTTATCATTTATAGTACTTATATTATTATTTTTCATTATTAATGATGAGACGAATTGGATATATGATGGTGG
TTTCTATTTAATATCCATATTAACGTTATTTATTATTGCTAGTGTCGTTCATCCATCTACATGGATAGCGAAGATATTTTCAAATCCAG
TGTTAGTATTTATCGGGAAAAGGTCTTATAGTTTATATTTATGGCATTTTGCAGTAATTAGTTTCGTACATAGTTACTATGTAGACGG
ACAGATACCTGTATATGTGTACTTTATAGATATAAGTTTAACAATTATATTTGCAGAGCTATCATATCGCTTTATAGAAACTCCATTT
AGAAAAGAAGGTATTAAAGCTTTAAATTGGCGACCTTCTTATATACCACAATTTATAAGAATGGCAATTGTAGTAACCTTGTTAATT

CCATTTATGTTGATTTTAGTAGGTGCATTCAATAAATATGGTAAAGACATTATTGGAGAAAAAGCGAATAGCTTTGATACCACAATT
GAAGATAATTATTTAATGCGGATAGCACCAATTGATAACATACACATTGATGGCTTAGTAAGTGAGAAGAAAAAGGAATCTTCCGA
CGTATATAATAATATTAAACCTCTTTTAATCGGTGATTCAGTAATGGTTGATATCGGTGAGTCATTTAAGTCATCAGTTCCTAAGTCT
AGAATTGATGGAAAAGTAGGGCGTCAATTGTATCAAACCTTACCTTTAGTTAAAGCGAATTATTCACAATATAAAAAATCATCTGAT
CAAGTCGTATTAGAATTAGGTACAAATGGCGACTTTACTGTCAAACAGCTCGACGATTTACTTAATCAATTTGGAAAAGCCAAGATT
TATTTAGTTAATACACGTGTTCCAAGAATTTATGAGGCAAATGTAAATCGATTATTAGCTGACGCGGCGAAACGAAAGTCCAATGTC
ACATTAATTGATTGGTATAAGCGATCACAAGGACATAGTGAATATTTTGCACCAGACGGTGTACATTTAGAGTACAAAGGAGTCCT
AGCTTTAAAAGATGAAATATTAAAAGCACTTAAAAAGAAATAACATATGTTTAATAAGTCTAGTTAATGTGTAACGTAACATTAGC
TAGATTTTTTTATTCAAAAAAATATTTACAAATATTAGGAAATTTAAGTGTAAAAGAGTTGATAAATGATTATATTGGGACTATAAT
ATAATTAAGGTCAAAGAAAGTCAAAGTCAAAAGTGCAACTTGACCAACTCTACAAATTAGAGGTGAAATTATATGGATATAAATAA
AATGACATATGCTGTTCAAAGTGCTTTACAACAAGCAGTTGAACTGAGTCAGCAACATAAATTACAAAATATAGAAATTGAGGCAA
TTTTAAGCGCTGCCTTAAATGAAAGTGAAAGCTTATATAAAAGTATTTTAGAACGAGCAAATATTGAGGTAGATCAATTAAACAAA
GCTTATGAAGACAAACTAAACACGTATGCATCTGTAGAAGGTGACAATATACAATATGGTCAATATATTAGCCAACAAGCAAACCA
ATTGATAACTAAGGCTGAATCATACATGAAAGAATATGAAGATGAATATATTTCAATGGAGCATATTTTACGTTCGGCAATGGACA
TTGATCAAACAACAAAACATTATATAAATAATAAAGTAGAAGTTATCAAAGAAATTATTAAAAAAGTAAGAGGGGGAAATCACGT
GACATCACAAAATCCAGAAGTTAATTACGAAGCATTAGCTAAATATGGCCGCGACTTAGTAGAAGAAGTTAGACAAGGTAAAATG
GATCCTGTTATAGGAAGAGATGAAGAAATTCGAAATACGATTCGTATTTTAAGTCGTAAAACTAAAAACAACCCTGTGCTCATTGGT
GAACCAGGTGTTGGTAAAACTGCAATTGTTGAAGGATTAGCGCAACGTATAGTTAAGAAAGATGTGCCAGAATCATTATTAGATAA
AACTGTTTTTGAGTTAGATTTAAGCGCATTAGTAGCGGGCGCTAAATATCGTGGTGAATTTGAAGAGAGATTAAAAGCAGTCCTAA
AAGAAGTTAAAGAGTCTGATGGTAGAATTATATTATTTATTGATGAAATCCATATGCTTGTAGGTGCTGGTAAAACAGATGGTGCCA
TGGATGCAGGCAACATGCTAAAACCAATGTTAGCACGAGGAGAGTTACATTGTATTGGTGCAACAACTTTAAATGAATATCGAGAA
TATATTGAAAAAGATTCGGCATTAGAGCGTCGTTTCCAAAAAGTAGCAGTTAGTGAGCCTGATGTTGAAGATACAATTTCAATTTTA
CGTGGTTTAAAAGAACGATATGAAGTGTATCATGGTGTGCGTATTCAAGATAGAGCCTTAGTTGCTGCCGCTGAATTGTCTGATCGT
TACATCACTGATCGTTTTTTACCAGATAAAGCGATTGATTTAGTTGACCAAGCATGTGCAACAATTCGTACGGAAATGGGATCAAAT
CCAACTGAATTGGATCAAGTTAATAGACGTGTCATGCAATTAGAAATTGAAGAAAGCGCACTTAAAAATGAATCTGACAATGCGAG
CAAACAGAGATTACAAGAACTACAAGAAGAGCTTGCCAATGAAAAAGAGAAACAAGCAGCACTTCAATCTCGTGTAGAATCAGAA
AAAGAAAAAATAGCAAATTTACAAGAAAAACGTGCGCAACTAGATGAAAGTAGACAAGCGTTGGAAGATGCACAAACAAATAAC
AATTTAGAAAAAGCTGCTGAACTACAATATGGAACAATTCCTCAATTGGAAAAAGAACTTAGAGAATTAGAGGATAATTTCCAAGA
TGAGCAAGGTGAAGATACAGATCGAATGATTCGTGAAGTTGTAACAGACGAAGAAATTGGCGATATTGTCAGCCAATGGACAGGC
ATACCAGTTTCAAAATTAGTTGAAACAGAACGTGAAAAATTACTTCACTTAAGTGACATCTTGCATAAACGTGTTGTAGGTCAAGAT
AAAGCGGTTGACCTGGTTTCAGATGCAGTAGTTAGAGCAAGAGCAGGTATTAAAGATCCAAACAGACCTATTGGTAGTTTCTTATTC
CTAGGTCCAACTGGAGTAGGTAAAACTGAATTAGCTAAATCATTAGCTGCATCATTATTTGATTCTGAAAAACATATGATTCGTATT
GATATGAGTGAATATATGGAAAAACATGCAGTATCAAGATTGATAGGGGCACCTCCAGGATATATTGGACATGATGAAGGGGGTCA
ATTAACTGAAGCGGTTCGTCGTAATCCATACTCAGTTATCTTATTAGATGAGGTTGAAAAAGCGCATACTGACGTCTTTAATGTATT
ATTGCAAATTTTAGATGAAGGCCGTTTAACTGATTCTAAAGGACGTAGCGTTGATTTTAAAAATACTATTATTATTATGACAAGTAA
TATTGGATCTCAAGTTTTATTAGAAAACGTAAAAGAGACTGGTGAAATTACAGAATCAACAGAAAAAACTGTTATGACAAATTTAA
ATGCATATTTCAAACCAGAAATTTTGAATCGTATGGATGACATCGTATTATTTAAACCTCTATCTATTGATGACATGAGTATGATTGT
AGACAAAATATTAACGCAATTAAATATAAGATTATTAGAACAGCGAATCTCAATTGAAGTTTCTGATGATGCTAAAGCTTGGCTAG
GTCAAGAAGCTTATGAACCTCAATACGGTGCAAGACCATTAAAACGTTTTGTACAACGCCAAATTGAAACACCATTAGCACGTATG
ATGATTAAAGAGGGATTCCCAGAAGGTACAACGATTAAAGTTAATTTAAATTCAGACAATAACTTAACGTTTAATGTTGAAAAAAT
TCATGAATAAAATTGAACCAAGAATGTGATGATTAATCGCATTCTTGGTTTTTTAGATTTGAAATTTATTATAATTGTTCATTGGCAA
TATACTTTTTAAATGATTTAATAAAAACCAATATTTCTGGGCTTTCTTTTTTGCTGTATATATATGTAAATGAAATAGGCGCTTGTAA
AATTTTCGTGTTAATAACCGAAATATTGTAATCACTATCTGTTGTTATATAAATAGGCAGGAATGATATACCTTGATTCATTTCGATT
AATTTAATTGAAGTATGCACATCATTGATAGATAGAAATTGTGCTTTTCCATAAATATTTAAAATATTATTTTTAAGTGATGACCAAT
ATTCTGGATGGTTATCACTTATTATTTTGTATTTTTCAAATAAAGATGCCTCAGTTAGAAGATGATTATTCTCTTTATTGGGAGCAAT
CAATACAATTTTACCTTCGCATACTTTTTCAGAATGAACTTCTCTTAGTTTAGGTTGATTTCTGCTAATCCCGATGTCATACGTATGAT
TATTAATATCTTTTTCAATATTTTCATTTTTGACATGGAGAGAAACATCGATAAACGGATGCTCGTTAAAGAAAGATTTTAAAAATTT
GGGCATAATGAATGTCGCGATATATGAAGACACGACAACATTTAATTTCGATTGAAACATCGTTTTTTTAAGTTGGATATGTTTGAT
GCCACTTTCATATTGTTCAATAAAACTTTGCGCAATTGGAAGAAATGTATGACCATCTTCAGTCAAGATAATTTGATTTTTATAAGTT
TCAAATAGTTTCACATTGAGATGCTGTTCTAAATTTTTAATTTGCTTATGTATAGAAGGTATAGTGAGATTAATTTCTTCACTAGCTA
ATCGATAGTTTAACGTCTTCGCTAAAATGACAAATGTATAGTACCAATCTAAATTCATGAACTTACCTCTTTCAAAAAAGTTAATAA
TTCATTAGATAATATTTAATTTTTATTGCATAAAAATAATATTAATATTATATCAGAGGTTACCGAGGGGGTGAAGTAATATGATTC
GAATTCAAGATAATACAATAAGGGATGGTATGCAACAAAGTAATGTTCGAAAAAGTCTAATTATAAAAAAAGAAGTATTGAAACA
AATTAACAAGTTAAATATAAATTCTGTTGAAGTAGGCATGTGTACAACTATCGAGGATGAATTTAATATTCATCAATTCAGAGACAT
TTTAAGTCCTGAAAAAGAATTAGTAGTATTGACTAGGCTTAATGAAAAAGAAATAAAAAAATAGTCAAATTAAAAATTCATAATTT
AGTGGTAAAAATACTATTGCCAATATCTGACTTGCATATAAAAGAAAAGCTTAATTTTTCAAATAAATATTATATTCAGAAAATCAA
AGACTGCTTGGATATATTAAAGAAAGATAAAAAAGGAGTAGATATTTGTTTTGAAGATGCAACAAGGACTTCTAGAGAAAAATTGA
AGGAATACATGGAAATTATTTCAAAATATCAAGTTAGAACAGTTACCTTTGCGGACACTGTAGGATGTTCGACACCATTAGAATAC
GGAGATATTTTTAATTACTTTGTAAAAAAATATTCTAACATAATTTTTTCTGCTCATTGTCATAACGATCTAGGGTTGGCTACTGCAA
ATACATTAGCTGCAATTTTAAATGGTGCGAAACAAATAGAAACTACATTTTTGGGAATTGGTGAGAGAGCGGCTAATGCTCCTATTG
AGGAAATAATTACTATTTTGACAAAAAAACAAATAAAAAGTACGGAATTCACTTTACCCGACGTATATAAAACTAGTATTAATATTT
CTAAAATTCTCGATTTTCAAATATCAGAAAACAAACCTATAATTGGTGAAAATATATTTAAACATGAATCAGGAATTCATCAAGATG
GTACTAAAAAAAATATAAATATGTATCAATATTTAGTTCCTAGTGATTTAGGATTGAAAAATTCACAAGTTGTTCAGTTTCCAATAA
GTAATATTTCTAGTAAGAAAATCTTGCACAATAAATTTAAATCAATAGTTAACACTGAAGAAATTGATGAAAATATTTCTTTCTATA
AACTTGTGAACAAGTTTTCACCTGAAGTAGCACCTGAAGATACAGTGGATTTACTTCAGATAATAAAAAGGAGGAGTAAAGATGGA
AATTTTAAATGATTCTGTAAAAAGTTTTGAAAAACTTTATTTACAATTACAAGATGGTTTTCCAGTGATTGTTCCTACTGATACTAAT
TATAATTTATGTAGTTTACCTAACAACGATCTTTGTATCGATAAAATATTTGAATATAAAAAGCGATCGAAAGATAAGCCATTATCA
TTATTTATTGATAAGCCAGAGGATTGGAAATTGTATGGAGATAATCAAAATACGGAAATAGTTGATAAACTAGTTGAAATATTTTGG
CCTGGACCATTAAATATTATTTTAAAAAATAAAACAAGCTATAATTATATGCTCAATAATTCAGATAGTATAGCTATAGGATGTGTA
CAAAATAAAACGATGAGAAGATTTATTTCATATATTAATTCACCTATAGCAATTACTTCAGCAAATATATCTGGAACTGCCGATGAT
ATTTTAATAACTGAAAATGAAGCAATTAAGCACATGGGCGAAAATGTAAGATATATGCTAAGAAGTCAAAATAAAACTAACTATAA

AACATCTAGTACGATTATTAAAGTGACAGATAATAAAATTGAATTATTAAGAGAAGGAGATATAAAGTTTGAAGAAATAAAAGAA
AGACTAGGTACAGGTATTATTTATGAATAAGTTAATACTTGGGATTTATTTATACCGAATTTTTTCACGAGCATACTTTTATTTACCG
TTTTTATTAATTTACTTTTTGATTCAAGGTTATTCCATAATACAATTAGAAATATTAATGGCGTCTTATGGCATTGCAGCATTTTTATT
CTCTCTATACAAAGAGAAGTGTTTTAAAATTTGTAACTTAAAAGATTCTAATAAATTAGTTGTTAGTGAAATATTCAAAATCATCGG
TTTATTGTTGTTATTATATCAAAATCAATATTTAATTTTAGTAGTGGCACAAATATTATTAGGGTTAAGTTACTCAATGATGGCGGGT
GTTGATACCGCAATAATTAAAAGAAATATAACAAATGAGAAATACGTACAAAATAAGTCAAATAGCTATATGTTCCTATCATTATT
AATTTCAGGGATTATAGGTAGTTATCTTTATGGAATAAATATTAAATGGCCTATAATAATGACTGGTATATTTTCAATTCTAACAATT
ATAATTATTCGATGCACATTAGTTGAAAATAGGGAATTAAATTTAATAGGAGAAACAAAGGGAAAGATAAAGAAATTTCTACCAGA
AGAGAAGTTTTGGATATTGCATTATTCTTTTTTAAGAGCGTTAATATTAGGATTTTTTATAGGATTTATTCCAATTAATATATATAAT
GATTTAAAACTGAATAATTTACAATTTATTTCAGTATTAACTTGTTACACAGTTATGGGTTTTGTATCTTCACGTTATTTAACTAAAT
ACTTGAATTATAAGTTTGTGTCAGAAATTTGTTTAGTAATATTTTTAATAATATATACATATCAAAGTTTCATAGCAGTTACTATTTC
TATGATATTTTTAGGTATTTCTTCAGGGTTAACTCGTCCACAAACTATAAATAAACTTTCTAGCAGTAGTAACTTAAGAGTGATGCTT
AATTATGCAGAAACGTTATATTTTATTTTTAATATCGCATTTTTACTTATGGGTGGTTACTTATATACAATAGGAACTATTCAATACT
TAATATTATTTATTTCGTTATTAATTTTTATATATTTAATAATAATATTTTATTTTACAAGGAGAGAGCAACATGAAAATAAAAACTG
AATTTAAAGGGAACAATATACCATATGAATACGCAGCAGGTGCAGATGTGAGTGATTCTATTAACGGGAATCCAATTAAGTCATTT
CCATTTGAAGTAATTGAATTACCGGAAGGGACTAAATATCTTGCTTGGTCTTTAATTGACTATGATGCAATTCCTGTGTGTGGCTTTG
CATGGATTCATTGGAGTGTTGCTAATGTAAGTGTTAGTGGCAATTCAATTTCTATAAAAGCAGATTTATCAAGAACAAAGGGTGACT
ATGTACAAGGTAAAAATAGCTTTACTAGTGGGTTATTGGCTGAAGATTTTTCAGAAATAGAAAATCACTATGTAGGACCTACACCAC
CTGATCAAGATCATCAATATGAATTAACAGTTTATGCGTTAGATCATTCTTTAAATTTGAAGAATGGGTTCTACTTGAATGAATTTTT
AAAAGAAGTAAATCAACATAAAATTGATCAAACAAGTATTAACCTTATAGGAAGAAAAATTTAATACTAAATATCTCATCAATATA
AAAATGTTCAATTAAAAGTACAAAGAAACAAAGGTTTTAATTTATATATTAGGTACGGCGTTCGCTATAATGCAAAGAAGTAATTA
AATTTAAGAAATGTAAACTTAGTTATTGTAATGTGAATTTATTTGAAAAAATAGAAAGTATTAACAATTATAGCTTTTACATTAATT
AAAATTTATTTTTAAAAACAAGTAAACAATTTACATACTTATAATTTTTGAAACTTTTCAATTTGTGTTATATTGATTTTGTAAGATA
CTTTAACTCACAAAGGAGAGAGATTATATGAAATTAAAATCATTTATAACTGTAACTTTGGCACTGGGCATGATCGCAACGACTGGC
GCTACTGTGGCAGGTAATGAGGTATCTGCAGCAGAAAAGGACAAACTACCGGCAACTCAAAAAGCTAAAGAAATGCAAAATGTTC
CATATACAATTGCAGTAGATGGCATTATGGCTTTCAATCAATCTTACTTAAATTTACCAAAAGATAGCCAATTATCATATTTAGATTT
AGGAAATAAAGTTAAAGCTTTATTATATGATGAACGCGGTGTAACACCTGAGAAGATTCGAAATGCAAAATCTGCCGTTTACACGA
TTACTTGGAAAGATGGCAGTAAAAAAGAAGTGGATTTAAAGAAAGATAGTTATACAGCAAACTTGTTTGATTCAAATTCAATTAAA
CAAATTGATATTAATGTAAAAACTAAATAAACACTTAAAATTATCAAATTCATCATGAAGCATTTAATTTTACAGTGATGATTATAA
AATAATTGCCTTGATACAAAGATTACTCGTAAATGACATCTTTGTATTAAGGCTTTTTCTAAATTTAAAAGTGATGGGTTAGAGGTC
ATTGAGCTTTAAAATATTCAAAATACAAAACATTAATGGCCAAAAATAAAAGCCGCCTTTATCTGGGCAGCTTCAATAATAAGAAA
GACATATTTCATTTTATACTAAATAGTTATTGTGATGAATCTTTCGGCGGTTTAATTACTGCAGCAAAAATTGCTGTGAAAATCGTGA
ACAATACTGCCATGATAATTGGATTCACTACATTTAAGCTGTCTCCACCTACTAGGCTATTAAGTACAAAGTTAACCATTTGCATTA
ATAATAATGCCCAAAAGAATGTTACGAGGTGTTTCATGTCATTCTACCTCCACTTTAATTATATATATTTTATTTTAAGTGAAAGTTA
GAAATTTGTATAGTAACATCTCATATATTTTGACCATATTATACAGTTTAAATAAATGATTTTATCTGAATGGCTATTCTAATTTAAG
CGCATTAAAACCAATTTCATACTGAAATTTGACGATAATAAAGCATTAAAATTTTATTAACTAGTCAATATTCCTACCTCTGACTTGA
GTTTAAAAAGTAATCTATGTTAAATTAATACCTGGTATTAAAAATTTTATTAAGAAGGTGTTCAACTATGAACGTGGGTATTAAAGG
TTTTGGTGCATATGCACCAGAAAAGATTATTGACAATGCCTATTTTGAGCAATTTTTAGATACATCTGATGAATGGATTTCTAAGAT
GACTGGAATTAAAGAAAGACATTGGGCAGATGACGATCAAGATACTTCAGATTTAGCATATGAAGCAAGTGTAAAAGCAATCGCTG
ACGCTGGTATTCAGCCCGAAGATATAGATATGATAATTGTTGCCACAGCAACTGGAGATATGCCATTTCCAACTGTCGCAAATATGT
TGCAAGAACGTTTAGGGACGGGCAAAGTTGCCTCTATGGATCAACTTGCAGCATGTTCTGGATTTATGTATTCAATGATTACAGCTA
AACAATATGTTCAATCTGGAGATTATCATAATATTTTAGTTGTCGGTGCAGATAAATTATCTAAAATAACAGATTTAACTGACCGTT
CTACTGCAGTTCTATTTGGAGATGGTGCAGGTGCGGTTATCATCGGTGAAGTTTCAGAAGGCAGAGGTATTATAAGTTATGAAATGG
GTTCTGATGGCACTGGTGGTAAACATTTATATTTAGATAAAGATACTGGTAAACTGAAAATGAATGGTCGAGAAGTATTTAAATTTG
CTGTTAGAATTATGGGTGATGCATCAACACGTGTAGTTGAAAAAGCGAATTTAACATCAGATGATATAGATTTATTTATTCCTCATC
AAGCTAATATTAGAATTATGGAATCAGCTAGAGAACGCTTAGGTATTTCAAAAGACAAAATGAGTGTTTCTGTAAATAAATATGGA
AATACTTCAGCTGCGTCAATACCTTTAAGTATCGATCAAGAATTAAAAAATGGTAAACTCAAAGATGATGATACAATTGTTCTTGTC
GGATTCGGTGGCGGCCTAACTTGGGGCGCAATGACAATAAAATGGGGAAAATAGGAGGATAACGAATGAGTCAAAATAAAAGAGT
AGTTATTACAGGTATGGGAGCCCTTTCTCCAATCGGTAATGATGTCAAAACAACATGGGAGAATGCTCTAAAAGGCGTAAATGGTA
TCAATAAAATTACACGTATCGATACTGAACCTTATAGCGTTCACTTAGCAGGAGAACTTAAAAACTTTAATATTGAAGATCATATCG
ACAAAAAAGAAGCGCGTCGTATGGATAGATTTACTCAATATGCAATTGTAGCAGCTAGAGAGGCTGTTAAAGATGCGCAATTAGAT
ATCAATGAAAATACTGCAGATCGAATCGGTGTATGGATTGGTTCTGGTATCGGTGGTATGGAAACATTTGAAATTGCACATAAACA
ATTAATGGATAAAGGCCCAAGACGTGTGAGTCCATTTTTCGTACCAATGTTAATTCCTGATATGGCAACTGGGCAAGTATCAATTGA
CTTAGGTGCAAAAGGACCAAATGGTGCAACAGTTACAGCATGTGCAACAGGTACAAATTCAATCGGAGAAGCATTTAAAATTGTGC
AACGCGGTGATGCAGATGCAATGATTACTGGTGGTACAGAAGCACCAATTACTCATATGGCAATTGCAGGTTTCAGTGCAAGTCGA
GCGCTTTCTACAAATGATGACATTGAAACAGCATGTCGTCCATTCCAAGAAGGTAGAGACGGTTTTGTTATGGGTGAAGGTGCTGGT
ATTTTAGTAATCGAATCTTTAGAATCAGCACAAGCTCGAGGTGCCAATATTTATGCTGAGATAGTTGGCTATGGTACTACAGGTGAT
GCTTATCATATTACAGCGCCAGCTCCAGAAGGTGAAGGCGGTTCTAGAGCAATGCAAGCAGCTATGGATGATGCTGGTATTGAACC
TAAAGATGTACAATACTTAAATGCCCATGGTACAAGTACTCCTGTTGGTGACTTAAATGAAGTTAAAGCTATTAAAAATACATTTGG
TGAAGCAGCTAAACACTTAAAAGTTAGCTCAACAAAATCAATGACTGGTCACTTACTTGGTGCAACAGGTGGAATTGAAGCAATCT
TCTCAGCGCTTTCAATTAAAGACTCTAAAGTCGCACCGACAATTCATGCGGTAACACCAGATCCAGAATGTGATTTGGATATTGTTC
CAAATGAAGCGCAAGACCTTGATATTACTTATGCAATGAGTAATAGCTTAGGATTCGGTGGACATAACGCAGTATTAGTATTCAAG
AAATTTGAAGCATAACTATAAAAATCTTCAGTAACGTTGTTTTAGTTACTGAAGATTTTTTCAGTTTCTTTATACTAAGATGAGCGAC
AGCACAATCGTCATAATAAAATATAAAATATTTATTAATAATAAGGGGATTATCCATGTAGAAACAAAGTAATGCTCTTTTTTTACC
TCTTGTGGGTTGAAAAATGGATCATCAGAGATAGACTTCTTCTTTTTCGAAGATGACATTTGATACTTTAATCTTCTAAAACCATAAC
TTGTCGCATCAAAAATGCCTTCTTGTACAAGTAAAATCAAAAATATGCTAATAAAAATAATTAATGAAACATAAAACAATATATTTA
AATATGTAATGATAGTATGGCTATTAAAAACCCATATAATAAACGTTAACATTGGGGTTATTAGTGCCATTCCAAGCCATTTTTTCA
ACATTTGATCACTCCCACTTATAGAAAACTCTTACGCATAGTTTACATTAAAATGAGACATTGAGGAATGATTTTTTAATTTCTTCAA
CTTTATTGAAATTCTAAAATCAAACATTCTTCATTAGTTTAAAGCAAAAAAACATTGATATATAGTAAATATTGTATATATAATATTA
GTTAAGATTTCAGAAAATTTTGAAGGGAATGGAAATTTAGAAATCGGAATTTGTTAGAGGAGGGGATTAGATGGGAAATATATTT
TCAAACGATTTATTTATATGCTTATTTCTTTATTTATTATTATTACAATTACATTTTTCTTAATGAAATTAATGCCAGGTTCGCCATTT

AACGATGCTAAATTAAATGCTGAACAAAAAGAAATTTTAAATGAAAAATATGGATTAAATGATCCTGTAGCTACGCAGTATTTACA
TTATTTAAAAAATGTTGTTACAGGCGATTTTGGTAATTCATTCCAGTATCATAATCAACCTGTGTGGGATTTGATTAAACCGAGACTA
CTACCTTCTTTTGAAATGGGTCTTACAGCAATGTTCATCGGTGTGATACTGGGACTTATTTTAGGTGTTGCAGCAGCTACTAAACAAA
ATTCTTGGGTTGACTATACAACTACAGTTATTTCAGTTATTGCTGTATCTGTACCATCTTTTGTACTTGCTGTACTTTTACAATATGTA
TTTGCAGTTAAATTAAGATGGTTCCCAGTAGCTGGATGGGAAGGTTTTTCGACCGCGGTATTACCGTCACTTGCATTATCTGCAGCT
GTTTTAGCAACTGTCGCCAGATACATAAGAGCAGAGATGATAGAGGTATTAAGTTCAGACTATATTTTATTAGCGAGAGCTAAAGG
TAATTCGACAATGCGTGTACTTTTTGGACATGCACTTAGAAATGCTTTAATTCCAATTATTACAATTATCGTTCCCATGTTAGCAAGT
ATTTTAACAGGCACTTTAACAATTGAAAATATTTTTGGGGTTCCTGGATTAGGGGATCAATTCGTACGTTCAATTACAACAAATGAT
TTCTCAGTAATCATGGCAATCACACTATTATTTAGCACACTGTTTATCGTTTCTATTTTTATTGTAGATATTTTGTACGGTGTGATAGA
TCCACGAATTCGTGTTCAAGGAGGTAAAAAATAATGGCTGAAAATAAAAACAATTTGTCGATTAACGACGATCATTCTAATGCAGC
TATGACGCATACCTCTGACGCTATCGCATCATCTGATTTTATTATTAGAGATTTAGATTTGAATCAGGAACCTGAAATGCAACGAGA
AAGCAAAAACTTTTGGCAAGATGCTTGGGCTCAGTTAAAACGAAATAAGTTAGCTGTTGTCGGTATGATAGGTTTAATTATCATTGT
AATATTTGCTTTTATCGGTCCAGTTATAAATAAACATGATTATGCTGAACAAAATGTAGAACATAGAAATCTTCCGGCAAAAATACC
TGTATTAGACAAAGTTCCATTTTTACCTTTTGATGGTAAAGATGCAGATGGCAAGGATGCTTATAAAGCAGCAAATGCTAAAGAAA
ATTATTGGTTTGGTACTGATCAGTTGGGTCGAGATTTATGGACAAGAACATGGAAAGGTGCTCAAATTTCATTGTTTATCGGTGTTG
TTGCAGCGATGTTAGATATTTTTATTGGCGTTGTATATGGTGCGATTTCTGGATTCTTCGGTGGACGTGTCGATACGATTATGCAACG
TATACTTGAAGTCATAGCATCTATTCCGAATTTAATTGTCGTAATTTTATTTGTATTAATTTTTGAACCATCCATTTGGACAATTATAT
TGGCTATGTCTATCACAGGCTGGTTAGGCATGAGCAGAGTTGTACGTGGAGAATTTTTAAAATTAAAAAATCAAGAGTTTGTCATGG
CTTCGAAAACATTGGGGGCTTCAAAATTCAAATTGATATTTAAGCATATTTTACCTAATACATTAGGTGCTATCGTGGTTACATCAAT
GTTTACAGTACCTAGTGCTATTTTCTTCGAAGCATTTTTAAGTTTCATTGGTATAGGTGTACCCGCACCTCAAACATCGTTAGGGTCA
TTAGTAAATGATGGGCGCGCAATGTTATTAATTTATCCACATGAATTATTTATACCAGCAATGATTTTAAGTTTATTAATTCTATTCT
TTTACTTATTTAGTGATGGATTACGTGATGCATTTGATCCGAAAATGCGTAAATAAAAGGGGGCATAGCATATGACTGAAAGAATAT
TAGAAGTAAATGATTTGCATGTTTCCTTTGATATTACAGCAGGGGAAGTGCAGGCAGTGAGAGGTGTAGATTTTTATTTAAACAAAG
GGGAAACATTGGCAATTGTTGGTGAATCAGGTTCAGGTAAATCTGTAACAACAAAAGCAATTACAAAATTATTCCAAGGGGACACA
GGAAGAATTAAAAAGGGAGAAATTTTATTTTTAGGGGAAGATTTAGCAAAAAAACCTGAAAATGAGTTAATTAAATTACGTGGCAA
AGATATTTCAATGATCTTTCAAGATCCAATGACATCTTTAAACCCAACGATGCAAATTGGTAAACAAGTCATGGAACCATTAATTAA
GCACAAAAATTATAGTAAAGCACAAGCTAAAAAGCGCGCATTGGAAATACTAAATCTTGTAGGTTTACCAAATGCAGAAAAAAGA
TTTAAAGCATATCCACATCAATTTTCAGGTGGACAAAGGCAAAGAATTGTTATTGCAACCGCATTAGCTTGTGAACCTAAAGTGCTC
ATTGCTGATGAACCAACGACTGCATTAGACGTAACGATGCAGGCACAAATTTTAGATTTAATGAAAGAACTACAACAAAAAATCGA
TACAGCAATTATTTTTATAACGCATGATTTAGGGGTTGTTGCGAATATTGCTGATAGAGTAGCAGTTATGTATGGTGGTCAAATGGT
TGAAACAGGAGATGTTAACGAAATATTTTATGATCCAAAGCATCCATATACATGGGGATTATTATCGTCAATGCCTGATTTATCAAC
AACAAATGACACACCATTACTAGCGATTCCTGGAGCGCCACCTGATTTATTACACCCACCTAAAGGTGATGCATTTGCGAGACGTAG
TCAATATGCATTAGATATTGATTTTAAAGTAGAACCACCGTGGTTTAAAGTTTCACCGACACATTTTGTGAAATCTTGGTTATTAGAC
GCACGTGCACCAAAAGTTGAACTACCCGAGCTGGTAAAACAACGTATGAAACCGATGCCTAATAATTATGAAAAACCACTCAAGGT
AGAAAGGGTGTCGTTCAATGAAAAATGATGAAGTGCTATTATCCATTAAAAATTTAAAGCAATATTTTAACGCAGGAAAGAAAAAC
GAAGTGAGAGCGATTGAAAATATTTCGTTTGATATATACAAAGGGGAAACATTAGGTTTAGTAGGAGAATCGGGGTGTGGTAAATC
TACAACTGGTAAATCAATTATTAAACTTAATGATATTACAAGTGGAGAAATTTTGTATGAGGGTATTGATATACAAAAGATTCGTAA
ACGTAAAGATTTGCTTAAATTTAATAAAAAGATACAGATGATTTTTCAAGACCCATATGCGTCTTTAAATCCTAGGTTAAAAGTAAT
GGATATAGTAGCTGAAGGTATTGATATCCATCATTTAGCAACTGATAAACGTGACCGTAAAAAACGTGTCTATGATTTACTTGAAAC
TGTTGGATTAAGTAAAGAACATGCCAATCGCTATCCTCATGAATTTTCAGGTGGACAACGCCAACGTATTGGAATTGCCCGTGCATT
AGCCGTTGAACCAGAATTTATTATCGCGGACGAACCAATATCGGCATTGGATGTTTCAATCCAAGCTCAAGTAGTTAATTTATTATT
AAAATTACAACGTGAAAGAGGGATTACGTTCCTATTTATAGCTCATGATCTATCAATGGTGAAGTATATTTCAGATCGTATTGCAGT
GATGCATTTTGGGAAAATAGTTGAAATTGGACCGGCAGAAGAAATTTATCAAAATCCATTACACGATTATACTAAGTCTTTATTATC
AGCCATTCCACAACCTGATCCTGAATCAGAACGCAGTCGCAAACGATTTAGTTATATTCATGATGAAGCAAATAATCATTTAAGACA
ATTACATGAAATAAGACCACAGCATTTTGTCTTTAGTACTGAAGAAGAAGCGGCACAACTACGAGAAAATAAATTGGTGACACAAA
ATTAAGGGGAAGGGGGAAATGCAATGACGAGAAAATTTAGAACACTTATTTTAATTTTGATTGCTACAATTGCATTAAGTGGTTGTG
CTAATGATGATGGTATTTATTCAGATAAAGGTCAAGTATTCAGAAAAATTTTGTCATCAGACTTAACATCCCTTGATACATCATTAA
TAACGGATGAAATATCTTCTGAAGTGACTGCGCAAACATTCGAAGGTTTATACACATTAGGAAAAGGTGACAAACCGGTGTTAGGT
GTTGCGAAAGCTTTTCCTGAAAAGAGTAAAGGTGGTAAAACTTTAAAGGTTAAATTAAGAAGCGATGCTAAATGGAGCAATGGTGA
CAAAGTGACTGCGCAAGATTTTGTTTATGCTTGGAGAAAAACAGTTGACCCTAAAACAGGTTCTGAATTTGCATACATTATGGGGGA
CATTAAAAATGCGAGTGATATTAGTACTGGTAAGAAACCTGTAGAGCAATTAGGTATCAAAGCATTAGATGATGAAACATTACAAA
TTGATTTAGAAAAGCCGGTTCCATATATTAATCAATTATTAGCACTTAATACCTTTGCACCTCAAAATGAAAAAGTTGCCAAAAAAT
ATGGTAAAAATTACGGTACGGCAGCTGATAGAGCGGTATACAATGGTCCATTTAAAGTTGATGATTGGAAACAAGAAGATAAAACC
TTACTATCTAAAAATCAGTATTATTGGGATAAAAAGAATGTAAAATTAGATAAAGTGAATTATAAAGTTATTAAAGACTTACAAGC
CGGTGCATCATTGTATGATACTGAATCAGTAGATGACGCAGTTATTACTGCAGATCAAGTAAATAAATATAAAGATAACAAAGGAT
TAAACTTTGTGTTAACGACTGGGACATTTTTTGTAAAAATGAATGAAAAACAATATCCTGATTTTAAAAACAAAAATTTAAGACTGG
CTATCGCACAAGCAATAGATAAAAAAGGATACGTTGATTCAGTGAAAAACAATGGCTCAATTCCTTCCGATACACTAACAGCCAAA
GGAATTGCGAAAGCGCCTAATGGCAAAGATTATGCGAGTACCATGAATTCGCCTTTAAAATATAATCCTAAAGAAGCAAGAGCACA
CTGGGACAAAGCTAAAAAAGAGTTAGGTAAAAATGAAGTGACATTTTCAATGAACACAGAAGATACACCAGATGCAAAAATATCT
GCTGAATATATCAAATCGCAAGTTGAGAAAAATTTACCAGGAGTTACTTTGAAAATTAAGCAATTACCGTTTAAACAAAGAGTATC
ATTAGAACTGAGTAACAATTTTGAAGCATCACTTAGTGGTTGGTCTGCAGATTACCCTGATCCTATGGCTTATTTAGAAACAATGAC
CACAGGTAGCGCACAAAATAATACAGACTGGGGTAATAAAGAATATGATCAATTACTTAAAGTAGCAAGAACCAAATTGGCACTTC
AACCGAACGAACGATATGAAAACTTGAAAAAAGCAGAAGAAATGTTCCTAGGAGATGCACCGGTAGCACCAATTTATCAAAAAGG
TGTTGCACATTTAACAAATCCTCAAGTAAAAGGATTAATTTACCATAAATTTGGTCCAAATAACTCACTTAAACATGTATATATTGA
TAAATCGATAGATAAAGAAACAGGTAAGAAGAAAAAATAATATGCTTTGTAAATTAGGCTGGAGACATATCTCCAGTCTTTTTGTG
TTGGATAAAAACTTTGGGAATAAAAATTTAAAATAAGTCGTTTTTTAAATTACTGAAATTGATTAAATGCATAAATAACTGAATATT
CTAAAAATAAACTTGTAATAATTTTTTCTATGAGTAAACTAAAAAGAAAAAATTAGATTGAAAGTAGGAGGCATATAAATGGGGAA
GCTAATTAAATATATTTCAATACTTATTGTTATCGTTTTAGTGTTGAGTGCTTGCGGAAAAAGCAGTAATAAAGATGAAGGAGTAAA
AGATGCTACTAAAACAGAAACCTCAAAACATAAAGGTGGTACCTTAAATGTAGCATTAACAGCACCTCCAAGTGGTGTTTATTCTTC
GTTATTAAATAGTACACATGCGGATGCTGTAGTTGAGGGATATTTTAACGAAAGCTTATTAGCAACTGATAAAAAAATACGTCCTAA
GGCATATATTGCTTCATGGAAGGACATCGAGCCGGCTAAGAAAATAGAATTTAAAATTAAAAAAGGTATTAAATGGCATGATGGTA

ATGAATTGAAAATTGATGATTGGATTTATTCAATTGAAGTCTTAGCTAACAAGGACTACGAAGGTGCTTATTATCCAAGTGTAGAAA
ATATCCAAGGTGCGAAAGATTATCATGAAGGAAAAACTGATCATATTAGCGGATTGAAGAAAATAGATGACTACACTATGCAGGTT
ACATTTGATAAAAAACAAGAAAATTACTTAACAGGATTTATTACTGGACCTTTATTAAGTAAAAAATATTTATCAGATGTACCAATT
AAAGATTTAGCGAAATCAGATAAAATCCGAAAATATCCTATTGGTATTGGACCGTATAAAGTTAAGAAAATCGTTCCAGGTGAGGC
TGTTCAACTCGTTAAATTTGATGATTATTGGCAAGGTAAGCCTGCACTAGACAAAATCAATTTAAAAGTTATTGATCAAGCTCAAAT
TATTAAGGCAATGGAAAAAGGCGATATTGATGTTGCGAATGATGCTACCGGTGCAATGGCAAAAGATGCTAAGTCATCTAATGCTG
GTCTCAAGGTATTATCTGCGCCAAGCTTAGACTACGGTTTAATAGGATTCGTATCTCATGATTACGATAAAAAAGCTAATAAAACTG
GTAAAGTGAGACCAAAATATGAAGACAAAGAATTACGTAAAGCAATGCTTTATGCAATTGATAGAGAAAAATGGATCAAAGCGTT
TTTCAATGGTTACGCTAGTGAAATCAATAGTTTTGTACCATCTATGCATTGGATAGCAGCCAATCCTAAGGACCTAAATGATTACAA
ATATGATCCTGAAAAAGCTAAAAAAATCTTAGATAAGTTAGGTTATAAAGATAGAGATGGTGACGGATTTAGAGAAGATCCTAAAG
GTAATAAATTTGAGATTAACTTTAAACATTATTCAGGTTCAAATCCTACTTTTGAACCAAGAACTGCTGCGATAAAAGATTTCTGGG
AAAAAGTTGGCTTGAAAACAAATGTGAAGTTAGTAGAATTCGGTAAATATAATGAAGACTTAGCAAATGCATCTAAAGATATGGAA
GTGTACTTCAGATCATGGGCAGGAGGTACAGATCCAGATCCATCAGATTTATACCACACTGATAGACCTCAAAATGAAATGAGAAC
AGTTTTACCAAAATCAGATCAATATTTAGATGATGCATTAGACTTCGAAAAAGTAGGCATTGATGAAAAGAAACGTAAAGATATTT
ATGTTAAATGGCAAAAATATATGAATGATGAGTTACCTGGATTACCAATGTTCCAAGGTAAATCGATAACTATTGTTAACGATAAA
GTACGAAACTTAGACATTGAAATTGGAACTGATCAAAGTTTATATAATTTAACTAAAGAAGCTTAGTAATGTAACCTCGACGATTTT
GTCGAGGTTTACTTATATGGGGGGATGTTATGAATAATGTATTGTTAGAGGTTAAAGATTTAGAAACATCATTAAAAATAAATAATG
AATGGTTAGCAACTGTTGAAAATATTTCTTTTGAATTATCTAAAGGAGAAGTTTTGGGTATAGTAGGGGAATCTGGTTGCGGTAAGT
CCATATTAAGTAAGTCAATTATTAAATTATTACCAGAAAAGATATCTAAACTAAGTAATGGAGAAGTTATATTTGATGGTAAACGA
ATCGATACGCTCAATGAGAAGCAATTGCTAGATATTCGAGGAAATGATATTGCTATGATTTTTCAAGAACCTATGACTGCTTTAAAT
CCTGTATTTACCATAAAAAATCAACTTGTGGAATCTATAAAATCACATAAAAAAAATTTCTAAAAAAGAAGCAAATAACTTAGCAAA
AGATTTACTAAAAAAAGTTGGAATTGCTAGACAAGATGAAATATTAAATAGCTATCCACATCAATTATCTGGTGGTATGAGACAAA
GAGTAATGATTGCAATGGCCATTTCATGTTCTCCTAAATTATTAATTGCTGATGAACCTACAACAGCATTGGATGTCACGATTCAAG
CGCAAATATTAGACTTATTAAAAGAATTGCAAAAGGAAACGCAAATGGCAATTATGATGATTACACATGATTTGAGTGTAGTTGCT
GAGTTTTGCGATAAAGTCTTAGTTATGTATGCAGGTCAAATTGTAGAATTTGGAGGCATAAAAGAAATACTACACAATCCGAAACA
TCCTTATACCCAAAAATTATTATCAACAATTCCAAAACTTAAAGAAGAGCAGAAACGACTTGAAACGATAGAAGGAATTGTGCCAT
CAATCCAAGCATTTCACGTTAATAAGTGCAGATTTGCAAATAGATGTAACAAAAAACTGGATATTTGTAATAATCAATCTCCTAAAA
TGCATGTTTGTGAAGACGTCATTGTACGTTGTCATTTGTACAAAAATGAATATAAGGAGATATAAGAATGGAAAATATTTTAGAAGT
CAACCAAATAAAAAAATACTACAAAATTAAAACTGGATTATTACAAAAAACTCAGTACGTTAAAGCTGTTGATGACGTATCGTTTT
CAATAAAAAAAGGACAAACTTTTGGATTAGTAGGAGAATCGGGTTGTGGTAAGTCAACGTTAGGTAAAGTGATTATCAGGCTTGAA
GATGCAACTTCAGGCTCAATAATTGTTAATGGTGAAGATATAACAAGATTACAAGGTAAAAAACTCAGAAAATCACGACAACAATA
TCAGATGATATTTCAAGATCCGTATGCATCATTGAATCCGATGCAAATGGTTGGAGATATCATTTCAGAACCTATTTTAAATTATAA
AAAATTGCCAAAAGAAGAAATAAAAAAAGAAGTACTATATTTATTAAAATGTGTTGGCCTAAGTGAAGATGCATATTATAAATATG
CACATGAATTTTCAGGTGGACAGAGACAAAGAGTGGGAATTGCAAGAGCATTGGCTTTGCGTCCGAGTTTAATTGTTGCTGATGAG
CCTGTAAGTGCATTAGATGTATCTGTTCAATCTCAAGTACTGAATTTATTAAAAGATTTACAAGAACAATTTAACTTAAGCTATTTAT
TTATCGCACATGATTTAAGTGTAGTAAAACATATAAGTGATGTCATTGGAGTTATGTATTTAGGTCATATAGTTGAAATCGCATCTG
ATAAAGAAATTTATGAAAATCCCAAACATCCATATACAAAAGCGTTGATTTCATCAATACCACAAATTGATAAACATAATAACAAT
AGAATTATATTAAAAGGAGAATTACCTTCGCCAAGTAATCCGCCGCAAGGTTGTCCTTTTCATACAAGATGTCCGATTGCAAAAGAT
AAATGTAAAGAAAATATACCACAATTAAAGGACATTGGTGATGAACATCAAGTTGCTTGTTTTTATGTAAATAAAGTAGGTGATTTA
AATGGTTAAATTAATATTAAAGAGATTAGGTTTAATGATTCCGTTACTAATTTTAATTTCTATTGTTGTATTTTCATTAGCTATCATTC
AACCAGGAGATCCATTTTCAGATTTACAAAACGGAAAAATAAAACAAGAAGCGATAAATGCACAAAGGGAAAAGTTAGGCCTCAA
CGACTCTATATCACATCAATACATTAGATGGGTCAATCATGTTATACATGGTGATTTAGGGGAGTCAATCAAATATAAAAGGCCGGT
AATTGATGTTATTGAGGAAAGAATTCCAAATACAATATTACTCGGTGCTATGTCATTAATTATTACTTATATTATCTCATTTGCTTTA
GGAATAACGTCAGGTAGATATTCTTACAGTTTGACTGATTATACTGTGCAAATATTTAATTATTTGATGTTAGCTATTCCATCTTTTA
TTGCGGGAGTATTTGCAATTTTTATTTTTTCTTTTGAATTACAATGGTTTCCGTTTCAAGGTTCTGTTGATATTAACCTTAAAGAAGGT
ACTTTTGAATATTATATGAGTAAAATTTATCATACATTTTTGCCTGCATTAACTTTAGGATTATTATCTACTGCTGGTTATATTCAATA
TTTACGTAATGATATTATTGAAAATTCTAAAAAAGATTATGTATTGACGGCAAGGTCAAAAGGATTATCTATGAATAAAATTTATAA
TAAACATATATTGAGAAATTCTTTAATACCTATTATTACATTTTTAGGTGCTGATATTGTAAGTATTTTAGGTGGAGCTGTGATTACT
GAGACTATCTTTTCATATAACGGTATCGGTAAATTATTTTTAGAATCGGTAATAGGTCAAGACTATCCATTAATGATGGCATTAACG
TTGTTTTTCTCATTTTTAGGTTTACTGGGTAATTTGATTTCTGATATTACTTATGGATTTATAGATCCAAGAATTAGAAGTAACTAGG
GGGGATTGGAATGCAAAATAAGTCAAAATCGCCTTTTAAAATTGCATTGTCTAGATTTATTCATAATAAAATTGCAATGTTATCGAT
TATTTTTTTATTAATCATAACTATTGTATCAATTATAGCGCCATTAATAGCTCCTTTTCCAGTGAACCAACAAGATTTATTAAATATA
AAAGGTGAAATGACAGCACAAAACATTCTTGGTACAGACTCTGGTGGTAGGGATAACTTTAGTCGTTTGTTATATGCAGGTCGTATT
TCATTATCCATTGGAATTACATCTACAATAGGAATGCTTTTGATTGGAATTACAGTTGGAGTGATTTCTGGTTATTTCGGAGGTATTG
TTGATACATTATTAATGAGAATAACCGAATTTGTTATGTTATTTCCATTTTTAATATTTGCAATTGTATTAAATGCTGCACTTGGAGA
TAAAATTAAAAATCCTTATGGATCTGCCATAATTCTTGTTCTAGTTATTATCGTATTAAGTTGGGGAGGTATTGCAAGACTTGTTCGT
GGTAAAGTACTTCAAGAAAAAGAAAATGAATACTTTTTGGCAGCAAAATCAATTGGTACACCCACATATAAAATTATTTTGAAACA
TCTTTTGCCGAATATATTAAGTGTAGTTATCGTACAAGCAACATTGTTATTTGCCGGTATGATTGTAGTGGAATCAGGATTGAGCTTT
TTAGGATTCGGAATTAGTAAAGCAATACCATCTTGGGGTAATATGTTGAGTGATGCTCAAGAAGGGGATGTTATAAGTGGTAAACC
GTGGATATGGATGCCACCTGCTATAATGATTACATTAACTATATTAAGTATAAACTTTGTAGGGGAAGGGCTTAAAGATGCTTTTAA
TCCTAGAGGTAGACGTTAAATAATAAAAGAGGCACTAGTTAATTCTAGTACCTCTTTATTTTATCTCTTACGTCCTAATCCCATCGCT
TTTTCCATTTTCTTCACTGTTTTAAATGAAACTTTGTGTGCTTTATCTCTACCTTGATCTAAAATATCATCAAGTTTATCTGAGTTATA
GAAACTTTCGTATTTTTCTTGGAATTCTACTAAAAATGCTTTAACTATTTCAGCAAGGTCACCTTTAAATTTACCATAACCTTCCCCTT
CATATTTTGCCTCAATGTCTTTGATAGGCATGTCGGTTAATCCAGCATATATTGAAATTAAATTAGTAATGCCTGGTTTATTGTCGCG
GTCGAATTTAATAATACCATCTGAATCAGTTACAGCGCTTTTAATTTTTTTAGCTGCAACATTCGGCTCGTCTAATAATGAAATGAAG
TTTTTAGTATTATCATCACTCTTACTCATTTTTCTTGTTGGGTCTTGTAAACTCATGACACGTCCACCAACTTTAGGCATACGAATTTC
AGGTTTCACAAGCACATCATTATAGCGACTATTAAATCTATCTACAAGGTTACGAGTCAATTCGATATGCTGCTTTTGGTCATCTCCA
ACTGGAACGATATTAGTATTGTAAAGAACAATATCAGCTGCCATTAAAGGTGGATATGTTAATAGACCAGCAGGTATACCTTCAAC
TGCTTTCTGAGCTTTATCTTTGTATTGCGTCATACGCTCTAATTCTCCAACAGAAGCAATCGTAGTTAACATCCATCCTGCTTGTACG
TGTGCAGGGACTTCAGATTGTATGAACAATGTTGCTTTGTCTGGATCTATACCAGAAGCTAAATAAATCGCTGCTAATTGTCTGGTC
TGTTTACGTAATTTTAAACGATCTTGTGGCATTGTAATTGCATGTTGATCTACGATACAGAAATAACAATCATAGTCATTTTGCACAT

CAACAAATTGTTTTAGTGCGCCAATATAATTTCCAATAGTAGGAATTCCACTAGGTTGGATGCCTGAAAATAATGTCTCCATTTAAA
TGCCTACTTTCTTAGTAATATTTTAATAATTATCATTATAACAGTATTTCTTAAAAATGTAAGATGTGTTAGAATATACGTATAACTT
ACTACTAACAAAGGGATTAATAATCTTTTTTATAAGTGTAGAAACTTATCTGAAAAATTAGGTTTTTCTCATTTAATTTTAATGTTAG
ATAGTTATAATAAATAGTAACGATTAAGAATTTTAGAAAAAAATGTTTTTTCTTGATCTATAACTAAATTTTAAGCAATGAATTTTAT
TCATTCAAATAGGAGAGTGAGATGTATGGTAACATTATTTACTTCACCAAGTTGCACATCTTGCCGTAAAGCGAAAGCATGGTTACA
AGAACATGACATTCCGTATACGGAGCGTAATATTTTTTCTGAACATTTAACAATTGATGAAATTAAGCAAATATTAAAAATGACTGA
AGACGGTACTGATGAAATCATTTCTACACGTTCTAAAACATACCAAAAATTAAATGTTGATATTGATTCACTACCATTACAAGACTT
ATATTCAATCATTCAAGATAATCCTGGCTTATTACGTCGTCCAATTATTTTAGATAATAAACGACTACAAGTTGGTTATAATGAGGA
CGAGATTCGACGTTTCTTACCTAGAAAAGTTCGTACGTTCCAATTACAAGAAGCACAACGTATGGTTGACTAATAACCATTTAACTA
TGTGTCATACTTTACTAAAAGATATGTCAAGCAACACTATTTCTATAGTGTTGCTTTTTTGGTTTTAATTTTACAATAATTGCTTAGTA
TCTTTTAAATCAAATGTGCAATTGTTAATATAGCTCTATATTAGAGTGTTTTGGATGAAACATTTGCTATGATAAGGTAAAATTAAAT
TAAGAAAGTGCTTTAATGCTTTAACTGTATAAATCATTTAAATAGTTTTGCTACAACGAATATGTTATTATAAGTTACCAAAATATTT
TAGAGAAAGAAATCAAACAAATAAATTGTATTTTGTTTGATGCTTTAATACAATGTGGATACTATTCATCGACTGTAAGGAGTGAGA
TGATATGAGAATAGAACGAGTAGATGATACAACTGTAAAATTGTTTATAACATATAGCGATATCGAGGCCCGTGGATTTAGTCGTG
AAGATTTATGGACAAATCGCAAACGTGGCGAAGAATTCTTTTGGTCAATGATGGATGAAATTAACGAAGAAGAAGATTTTGTTGTA
GAAGGTCCATTATGGATTCAAGTACATGCCTTTGAAAAAGGTGTCGAAGTCACAATTTCTAAATCTAAAAATGAAGATATGATGAA
TATGTCTGATGATGATGCAACTGATCAATTTGATGAACAAGTTCAAGAATTGTTAGCTCAAACATTAGAAGGTGAAGATCAATTAG
AAGAATTATTCGAGCAACGAACAAAAGAAAAAGAAGCTCAAGGTTCTAAACGTCAAAAGTCTTCAGCACGTAAAAATACAAGAAC
AATTATTGTGAAATTTAACGATTTAGAAGATGTTATTAATTATGCATATCATAGCAATCCAATAACTACAGAGTTTGAAGATTTGTT
ATATATGGTTGATGGTACTTATTATTATGCTGTACATTTTGATAGTCATGTTGATCAAGAAGTCATTAATGATAGTTACAGTCAATTG
CTTGAATTTGCTTATCCAACAGACAGAACAGAAGTTTATTTAAATGACTATGCTAAAATAATTATGAGTCATAACGTAACAGCTCAA
GTTCGACGTTATTTTCCAGAGACAACTGAATAACGATATTAATTAAGCAACGCACTATGAAAGGAGACTCTTTGGAGTCTCTTTTTT
TATTGATTTCAAAAGCTGAAAAAGTAATAGTAGTTAAACTTACGTGTAATATTTTGAGGTGAAGATATGTTAGTAGCTTTAAATGAA
GAAAAGGAACGTGTGTTAGCAACTACTGCATTGAGAAAGACACAATATTTTTGTCCGGTGTGTGGCAAGCAAGTTATTTTAAAGCG
TGGGCTCAAAGTAATTAGTCATTTTGCACATAAACATTTAGCGGAACAAAAATGTTTTAATAATGAAACGATTAAACATTATAAAA
GTAAATTGATTTTAGCACAGATGATACAGCAACAAGGATTTAAAGTAGAGATAGAGCCATTTTTAAAAGAAATAAAACAAATTCCG
GATATTTTGATTAATAATAAATATGTTATTGAGCTACAGTATTCGCCAATTCCTTATAAACAGATTCTTCAACGAACGGAAGGTTTA
AAGAAAATGGGATATAAAGTAAGTTGGTTATTAAATGATGTTGATTATTGTCATAATAAAGTGAAGTTCAATCATTTTCAAAGTATG
TTTATTAATCCATTCACTCGAAAACTTCATACGTTCAATTTAGAGAAAAAACAAATAATGATGTTTCAACAAATACAATATTTAGGC
GGGCACAAATATGTCGCTGAAAAAAGAAATGCCAAAATTAGTGAGTTGTTTAATGAGGCGCCTTGTGATTATCATGCTGTTTATAAA
TTATCAAAGTTCGCAATTAATCAATATATCAAATATTGTCGCTGGCAAAATTCTGTTTTAGAACCCACTTTAAGTGCAATGTATCAAT
TACAGTTAACTGATCAAGAAGTAGTGCACAATTATGGTTATATTTTTCCAGAGCAAATTTATATTAAAAATCATCCTATTGAGTGGC
AATTACAAGTTGATTTATGGTTAAAGAATGGAAAAAGCAAATTAGTAAATGACAATCTTAATTATTTTAAACTGAAAAAATTTATTG
TTGCTCTAGAAAGTAAAACAGCAATTATAGAAAAACTTATTAACAATTATTTAAATATTTGTTCAGATAAAGGTAATGACGTGCAAA
TTTTGTTCTAAAATGAGAAAATTATATCAATTATATTTTATTAGGAGGTTTACTACATATGAGTCAACAATTATCGAGAGAAGAACA
GGAACGTAAATATCCTGAATATACATGGGACTTAACAACAATTTTCAAAGATGATGAAGCTTTTGAGGCTGCATTTAAAGAAGTTG
AAAATGAGTTAGGCAAAGAAGAACAATTTAAAGGACACATTGGTGATAGTGCTGAGACATTATACAATGCGTTAGAATTAGAAGAT
ACATTAGGTACTAAATTAGAAAAAGTATATGTATACGCGCACCTAAAACAAGACCAAGATACAACGAACGACAAGTATACTGGTAT
GGAGTCAAGAGCACATCAATTAATTATTAAATTTAGCTCGGCATGGAGTTTCTTAGTGCCAGAGATTTTACAAATTGATGAAGATAA
AATTCAATCATTTGTAAATTCATATGATAAATTACAAAAATTCGCATTTGATTTGAAGTTGATTAATGAAAAACGTCCTCATATTTTA
GATGCTGAAACTGAAAAGTTATTAACAGAAGCGCAGGACGCGTTATCAACGCCATCAAATGTATACGGTATGTTTAGCAACGCTGA
TTTAGTATTTGAAGATGCGATTGATAAAGATGGAAATGCACACCCGTTAACACAAGGTACATTTATTAAGTATTTAGAATCAGATGA
TCGCAAACTAAGAGAAAGTGCTTTTAGAAATGTATATAAAGCATATGGTGCTCATAATAATACGCTTGGCGCTACGCTAGCAGGTG
AAGTGAAGAAAAATGTATTTAATGCTCGTACACACAATTACAAAACTGCAAGAGAAAAAGCATTGAGTAATAATCATATTCCAGAA
AATGTATATGACAATCTAGTAAAAACTGTACATAAATATTTACCATTGCTACATAGATATACTGAATTGCGCAAAGAATTGCTAGGT
TTAGATGACTTGAAAATGTATGATTTATATACACCATTAATTAAAGATATTAAGTTTGAAATGCCTTATGAAGAAGCTAAAGAGTGG
ATGTTAAAAGCTTTAGAACCAATGGGTGAAGAATATTTAAATGTAGTTAAAGAAGGCTTAAACAATCGTTGGGTCGATGTCTATGA
GAATAAAGGTAAACGTTCAGGTGGCTATTCATCAGGTGCACATTTAACTAATCCATTTATTCTACTTAACTGGTCTAATACTATTTCA
GACTTATACACATTAGTTCATGAATTTGGGCATTCAGCACATAGTTACTTCAGTAGAAAATTCCAACCGTCAAATTCTAGTGACTAC
ACTATTTTTGTCGCTGAAGTTGCATCAACTTGTAACGAAGCACTTTTAAGTGATTATATGGATAAACATCTTGATGATGAAAAACGC
TTATTATTATTAAACCAAGAATTAGAACGTTTCAGAGCTACATTATTCCGACAAACAATGTTCGCAGAATTTGAGCATAAAATTCAT
GCAATTGAAGAAGCAGGTGAACCATTAACGCCAACTAGAATGAATGAAGAATATGCCAAATTAAATAAATTATACTTCGGTGATTC
TGTAGAAACTGATGAAGATATTAGTAAGGAATGGTCACGTATTCCACACTTCTATATGAATTATTATGTATATCAATACGCAACTGG
TTACAGTGCAGCTCAAAGCTTAAGTCATCAAATTTTAACAGAAGGTAAGCCAGCAGTAGATAGATATATTAATGAATTCTTGAAAA
AAGGTAGCTCAAATTATCCAATTGAGATATTAAAAAATGCTGGTGTAGATATGACAACACCTGAACCAATTGAACAAGCTTGTGAA
GTTTTTGAACAAAAATTGAACGCTTTTGAAAAATTAATGAAAGCTTAGTACTATTGTATTCGCTTACATTATGAACAGATTGTGACA
ATTTAAATTAAGTACTATTAAAAGGTTGAAATGGTAGAGATAGCATGTTATATTATGAACATGAAATTAATCACATAACAAACATA
CCCCTTTGTTTGAAGTGAAAAATTTCTCCCATCCCCTTTGTTTAGCGTCGTGTATTCAGACACGACGTTTTTTTATGTTCTTTTTTAAT
AAGAGGGAAGTGAAGTTGTATAAAAAATGTATTAAATATGCACTTTTTATACATTAACGATTCATATATGTCTATTATGTACCAAATT
TATAATTTGTATAATTTTCGAACTGGTTAAATTCGAAAAATATCACTAATATATTTAGATCGATGTGTAACATTACGTTCTAATAATT
TAATGTTGCAAAATTAAGAGTCAGTAAAAAGACCTCGTTACATTTATGGTGTACGAGGTCTTTAATTTATTTATTTTGATTTGATTTT
AGGCATTTTAGATTTCCAGAAATCGCCATCTGGATATTTAAGTTTTTCAATTTTTTGTTGAATGGCTAACTTCTTTAACTCTTTGTTTA
AAAGTTTTTCTGGCCATTCATAAATAGTAAGTAATTCTTCCATCGTTACAAGTTGTTGTTGCTGTATATAAGTTTCTAATTTAGGAGG
AAGATTCTTTTCGATAGGTTTACCCATCAATTCATTAATTATATAAGTATAGATGTGATATGGGTATAATCCTTCGACTTTTAAACCT
TCTTCATGAACATCTTCACTAAAGAAAACGAGAGAAGGGGCTTGTTCGATTTCCATTTCTCTTGCAATATGCAAATCAATTTTCAAG
CTTTCGGTTAGTTTACTTTTTTGTAAGTCGTCTTTAAATACTTCTAAATCAATACCTGCATTTTGAATACAGTCACAAATCATTGATTC
TGTAATAATATCTCTTTTAGGTATGATTTCATTTTGCATTAAATGTATAAATCGTTCGGCACGTACACGACCTTGTAACTCAGCTGCT
TTATAAGCTAGGGCGATGTTATCAAAGTTGGATGTACTTTGAGCTTGGCATTTCGTTAATACTTTTAACGAAGGATTTAATATATGTC
TGATACGTATATATTGATTATATTCAATTCTTAATTTGGATAAGATTGCTGATAATTTGAAGCAATCGGAGCTAAATGGATCGAAAA
ATGAATAAATTTCGATTTTACTTACAGGTGATAGATTAATATCTTCACGACTCTTATTTTCCATTATTCGTAATTCTCCAGCCATGTTT
ATTCACCTACAATTAATTTTAGGAATTCACCATATGATTAGCAGTTAATCTTAAGCGCTCAAATAAATAATCTCCAACACCTTGTGG

AAACGCAGCGCGATTAATTGCTGTCTGCATATTTTCTAACCATGCATCTCTTTCAAATTCAGTGATTGTAAAATCCATATGTCTTTTT
CTTAGCATAGGATGTCCGTGTTCTTCGGTATAAATGTTTGGACCGCCCAAAAACTGTGTTAAAAATTGTTTTTGTTTACGACTTGTTT
CTGCAAAATCTCCTGGAAACAGGTGATTAAGTCGTTCATCTTTTTCTACAAGGGTGTAAAAATAATCAATCATATCGTATAACGCTT
CTTTACCAATGATGTCATATGGTGTTGTTGTCATTTAATCACCCATTTTCAAAAATTTACTATTACGAACTTAAGTTAATATATAACT
AATATAACATGATTTTAAACATTTGAAAGAAATATGCATATTTGCCAATTTAATTTATATTGTTTGAAAGTGTTTCTTTTTTCTTGAA
AAAACGTTGAACTTTATTTAAAGGTTGATGATGTTCGAGGTTTAGTTCGTTTAATAAAGATTGGAACTTTTGTAAACCTTGATTATAG
TCTTTAACTTCGAACTCTAACTCATAATCCGCAGTATCGAAATACTCACTTTTATCTAAAACGAGTAAATCACCTTTATATTTAGTTT
CTTGGCGATATGTCGTTAATGCACCAAGTATTGATAAAGTTGTATCTTTTACACCAAACTGTTCAACTATAATTTGACGAATGTCATC
TGGAAGATTGTCGTTTGAAATAATCAAGTTCATCTCTGGTTTAATGTCGACGATATAGTTGTATTCTAATAGACCAACCTTTGCTGGT
GTCTTTAAAGTCATTTCATATTGATTGTCTTTAACTCTTATGCGTAGTGCAGAGCGATGTTCCTTTAATTTGAAATCGGGTGTATCAA
TATAGTAATTGACTTGCTTAAAAAGCACACTGTCTTTAAAATATTTCTCTTGCAATTTATTATAGATTGATGCAGTTATCATTTGTTTA
AATTCTATTTCATGATTTGTTGCCATGATATGTATACACCTCGTATCAAATTCAATTTATCTTAACTATATTATGAATGACAAAGTTG
AATTTTAAAAGCAATTTCCTTTATCTATTATCAATGTTAATTTGACCATTAAAAATAGTGTTCGTAAGTGTTTTGTATTATTGAATTGT
GTTAAAATGTTATGGAATAAGAGGAGGATTAAGCATGCGTTTATATATTAATGAAATTAAAATTAAAGATGACATACTTTATTGTTA
TACAGAAGATTCTATTAAAGGATTATCTGAAGTAGGACAAATGCTCGTTGATAGTGATAATTATGCCTTTGCGTATACATTAGATGA
TGGTAAAGCGTATGCTTATCTCATTTTCGTACAAGAAACATGGACGATGTTGCATGAAAACACGACTAAAAAAATTATTATCAATGA
TGAACTAGAATTGACTGAATTCCACCAAGAACTTACTTATATTTTAGACAACATAAAAGGGAATAATAATTATGGTAAGGAATTTGT
TGCAACCGTTGAAGAAACATTCGACATTGAATAAAGCGGGGTGAAGCACTATGAATCAATGGGATCAGTTCTTAACACCTTATAAG
CAAGCGGTTGATGAGTTGAAAGTGAAACTTAAAGGCATGCGCAAACAATATGAAGTTGGTGAACAAGCGTCGCCAATAGAATTTGT
TACTGGTCGTGTTAAACCGATCGCTAGTATTATAGATAAGGCAAACAAACGACAAATACCATTTGATAGGTTAAGAGAAGAAATGT
ACGATATCGCTGGTTTAAGAATGATGTGCCAATTTGTAGAAGATATTGATGTTGTCGTCAATATTTTAAGACAAAGAAAAGATTTTA
AAGTAATTGAAGAACGAGATTATATTCGTAACACTAAAGAAAGTGGTTACCGCTCGTATCATGTCATTATTGAATATCCAATTGAAA
CATTACAAGGCCAAAAAATTTATATTGGCTGAGATTCAGATTCGTACATTAGCAATGAATTTCTGGGCAACGATTGAACATACTTTAC
GATATAAATATGATGGTGCTTATCCGGATGAAATTCAACATCGTTTGGAAAGAGCGGCAGAAGCAGCGTATTTACTTGATGAAGAG
ATGTCTGAAATTAAAGATGAAATTCAGGAAGCTCAAAAATATTACACGCAAAAACGTTCTAAAAAACATGAAAATGATTAACGAG
GTGTTATAAATCATGCGTTATACAATTTTAACTAAAGGTGACTCCAAGTCTAATGCCTTAAAGCATAAAATGATGAACTATATGAAA
GATTTTCGCATGATTGAGGATAGTGAAAATCCTGAAATTGTTATTTCAGTTGGTGGTGATGGTACATTACTACAAGCATTCCATCAG
TATAGCCACATGTTATCAAAAGTGGCATTTGTTGGAGTTCATACAGGTCATTTAGGATTTTATGCGGATTGGTTACCTCATGAAGTTG
AAAAATTAATCATCGAAATTAATAATTCAGAGTTTCAGGTCATTGAATATCCATTGCTTGAAATTATTATGAGATACAACGACAACG
GCTATGAAACAAGGTATTTAGCATTAAATGAAGCAACGATGAAAACTGAAAATGGCTCAACACTTGTTGTGGATGTTAACTTAAGA
GGGAAACACTTTGAGCGATTTAGAGGCGATGGATTATGTGTATCAACACCTTCGGGTTCAACGGCTTATAACAAAGCGCTAGGTGG
CGCACTGATACATCCTTCACTTGAAGCAATGCAAATTACAGAAATTGCCTCAATAAATAATCGTGTGTTTAGAACGGTAGGATCACC
ACTTGTATTACCAAAGCATCATACATGTTTAATATCACCAGTTAATCATGATACCATTAGAATGACGATAGATCATGTTAGTATCAA
ACATAAAAATGTTAATTCAATACAATACCGTGTAGCAAATGAAAAAGTGAGGTTTGCACGTTTTAGACCATTCCCGTTCTGGAAACG
TGTACACGATTCTTTCATATCAAGTGATGAAGAACGATGAAATTTAAGTATCATATATCACAACAAGAAACTGTTAAAGCTTTTTTA
GCACGACATGATTTTTCTAAGAAGACAGTGAGCGCCATTAAAAATAATGGCGCTTTAATTGTTAATGATGAACCAGTCACAGTACGT
AAGCAATTAATGCCAAATGATATATTAGAAATTCATTTACCGCGAGAAATACCGAGTGTTAATTTAATACCTTATGCTCGTAAGCTA
GAAGTATTGTATGAAGATGCTTTTATCATCATAGTTACTAAACCAAACAATCAAAATTGTACGCCTTCGAGAGAACATCCTCATGAA
AGTTTAATCGAACAAGTACTATATCATTGTCAGGAACATGGTGAAAATATTAACCCACATATTGTTACGCGTCTAGATCGTAATACA
ACTGGTATTGTGATATTCGCTAAATATGGACATATCCATCATTTATTTTCTAAAGTAAACTTGAAAAAAATATATACTTGCCTTGTAT
ATGGCAAAACCCATACATCTGGTATTATTGAAGCTAATATTAGACGGTCAAAGGATAGTATTGTAACTAGAGAAGTTGCCTCGGAT
GGTAAATACGCTAAAACATCTTATGAAGTAATAAATCAGAATGATAAATACAGTTTATGCAAAGTTCATTTGCATACGGGACGTAC
ACATCAAATTCGTGTACATTTTCAACATATTGGGCATCCAATTGTGGGAGATTCTTTGTATGATGGTTTTCATGACAAAATTCATGGT
CAAGTACTGCAATGTACGCAAATATATTTTGTTCATCCAATCAATAAGAACAATATTTTATATTACAATTGATTATAAGCAATTACTT
AAATTATTCAATCAACTCTAATTCACACAGGGGGTGTAAGTATGTCAATGAACACAGATGAAAAAGAGCGTGTTCAAGAGGAATTA
TATGATGAGACATTATTAGATCAATACTTAGAAAATGATGATATTGATCAATTTAGAGATGAATTTCTAGCATTACACACATATGAA
CAAAGTGAGTATTTTGAAGATACTACCGATGAAAATAGACAAAAGATTTTTCAATATTTATCACCTGAAGAGGTTGCAAATTTCTTT
GATCAATTAGATATTGATGACGATGAATATGAGTTGCTATTTGATAAAATGAATGCGACATACGCAAGTCACATATTAGAAGAAAT
GTCATACGATAATGCAGTAGATATTTTAAATGAGTTGACTAAACCAAAAGTTGCTAGTCTTTTAACATTGATGAATAAAGATGACGC
GAATGAAATCAAAGCATTACTTCACTATGATGAGGATACGGCCGGCGGTATTATGACGACGGAGTATTTATCACTTAAAGCGCATA
CGCCTGTTAAAGAAGCGTTATTATTGGTCAAAGCGCAAGCGCCAGACGCAGAAACAATATATGTTATATTTGTCGTTGATGATGATG
GTAAATTAGTAGGTGTTTTATCACTAAGAGATTTAATTGTAGCTGAAAATGATGCTTATATTGAAGATATTATGAATGAACGTGTCA
TTAGTGTGAATGTAGCAGACGACCAAGAAGATGTTGCTCAAGTTATGAGAGACTATGATTTCATGGCTGTACCTGTTATAGATTACC
AAGAACATTTGCTTGGTATCATCACGATTGATGATATTTTAGACGTTATGGATGAAGAGGCTAGTGAAGACTACTCTCGTTTAGCCG
GGGTATCAGATATCGATTCGACTAATGATTCAATCATTAAAACAGCATTAAAACGTTTACCATGGTTGATTATTTTAACATTTTTAGG
AATGATTACTGCGACAATTTTAGGGAGATTTGAAAAAACATTAGAAAATGTAGCGCTACTCGCAGCGTTTATTCCTATTATTAGTGG
TATGTCAGGAAATTCAGGTACACAATCTTTAGCCGTTTCAGTTCGTAACATTACGACAGGGGAAATTAATGAGCAAAGTAAATTTAG
AATTGCATTAAGAGAAGCGGGAAGTGGTGTATTATCGGGCGTTGTATGTTCAACAATATTATTTACAATTATTGTTGCAATATATCA
TCAGCCACTTTTAGCATTAATCGTTGCAGGAAGTTTAACTTGTGCGATGACGGTGGGGACGTTTGTAGGTTCGATGATTCCATTATTG
ATGAATAAATTAAATATCGATCCAGCAGTGGCTAGTGGACCATTTATTACAACAATTAATGATATTATTAGTATGTTGATTTATTTTG
GTTTAGCTACATCATTTATGGCTTACTTAATTTAAGGAGGAGTTATGGAGTTTTTATCTTTAGTTATTGTTGTTTTAGCAGCGTTTTTA
ACTCCAATAATTGTCAATCGATTAAATATTAATTTCTTGCCAGTTGTTGTTGCAGAAATTTTGATGGGGATTGTGATTGGAAATTCAT
TTCTAAATATAGTAGAAAGGGATTCAATTCTAAATATTTTATCAACGTTAGGCTTTATCTTTTTAATGTTTTTAAGTGGTTTAGAAAT
TGATTTTAAAGCTTTTAAAAAAGATAAACGCGCACGTCAAGGACAAAATGATGATGAATCCTCAATTCCAGGGCATCTTAATCTAG
CGTTAACTGTATTTGCATTTATTATGATTATTTCGATTCTTTTAGCGTATGTATTTAAATGGCTTGGATTAGTGGATGATGTGTTATTA
ATGGTCATTATCATTTCAACTATTTCCTTAGGCGTAGTTGTTCCAACTTTAAAAGAAATGAATATTATGAGAACAACTATAGGGCAA
TTTATCCTATTAGTAGCAGTACTTGCGGACTTAGTAACTATGATTTTATTAACGGTCTATGGCGCAATCAATGGTCAAGGCGGCAGT
ACAATATGGTTAATAGGTATATTAGTTGTTTTCACAGCAATTTCATATATTTTAGGTGTTCAATTTAAAAGAATGTCATTTTTACAAA
AATTGATGGATGGTACGACGCAAATCGGTATTCGTGCGGTATTTGCATTAATAATATTATTAGTAGCCCTAGCAGAGGGAGTTGGCG
CAGAAAATATATTAGGTGCATTCTTAGCAGGTGTCGTTGTTTCATTATTAAATCCAGATGAAGAAATGGTTGAAAAGTTAGACTCAT
TTGGTTATGGGTTCTTTATTCCTATTTTCTTTATAATGGTTGGTGTAGATTTAAACATACCTTCATTAATTAAAGAACCGAAATTACTA

ATTATCATACCGATTTTAATCGTTGCATTTATCATTTCAAAATTAATTCCAGTCATGTTTATTCGACGTTGGTTTGATATGAAAACAA
CGATTGCATCAGCATTTTTATTAACATCAACATTATCGCTCGTGATAGCTGCAGCCAAAATTTCAGAAAGATTAAATGCTATTTCAG
CTGAAACGTCAGGTATATTAATTTTAAGCGCAGTCATTACATGTGTATTCGTTCCGATTATTTTCAAAAAACTGTTTCCAGTTCCAGA
TGAGTTTAACCGTAAAATTGAAGTTAGTTTAATTGGTAAAAATCAATTAACGATTCCTATAGCGCAAAATTTAACATCTCAGTTATA
TGACGTGACATTATATTATCGCAAAGACTTGAGTGATCGTCGTCAATTGTCAGATGATATCACGATGATAGAAATTGCTGATTATGA
ACAAGATGTTTTAGAACGACTAGGTCTGTTTGACCGAGACATAGTTGTTTGTGCTACGAATGACGATGATATTAACCGAAAAGTTGC
TAAATTAGCCAAAGCACATCAAGTTGAGCGTGTCATTTGCAGACTTGAAAGCACAACGGACGATACAGAGTTAGTTGATTCAGGTA
TTGAAATTTTCAGTAGCTACTTAAGTAATAAAATCTTATTAAAAGGTTTAATTGAAACACCTAACATGTTGAATTTATTAAGTAATGT
TGAAACGTCACTATATGAAATTCAAATGTTAAATTATAAATATGAAAATATTCAATTACGTAATTTCCCATTCGGAGGAGACATCAT
CTTCGTGCGTATTATCCGTAATAATGAGTCGATTGTTCCGCATGGAGATACACAATTGCGATATGGAGATCGCTTAATTGTTACCGG
TGCTAAAGAATACGTTGATGAATTGAAGCAAGAGTTAGAATTTTATTTTTAACAATAATGATATTAAAATGCAGACGTATTTTAGTA
CGACGTAAAATTAATGATTTTAAAATGCTAGTATGTATATGATTTTGATAAATAAATGCTTTTTACGTAAATCAAGTTTGATACAGA
AAGGACTAAATCAAACATTTATCGTTGTAATACGTTTAAATAACTTTATTAAAAAGTCATAATAGTGTTAAAATGTATTGACGAATA
AAAAGTTAGTTAAAACTGGGATTAGATATTCTATCCGTTAAATTAATTATTATAAGGAGTTATCTTACATGTTAAATCTTGAAAACA
AAACATATGTCATCATGGGAATCGCTAATAAGCGTAGTATTGCTTTTGGTGTCGCTAAAGTTTTAGATCAATTAGGTGCTAAATTAG
TATTTACTTACCGTAAAGAACGTAGCCGTAAAGAGCTTGAAAAATTATTAGAACAATTAAATCAACCAGAAGCGCACTTATATCAA
ATTGATGTTCAAAGCGATGAAGAGGTTATTAATGGTTTTGAGCAAATTGGTAAAGATGTTGGCAATATTGATGGTGTATATCATTCA
ATCGCATTTGCTAATATGGAAGACTTACGCGGACGCTTTTCTGAAACTTCACGTGAAGGCTTCTTGTTAGCTCAAGACATTAGTTCTT
ACTCATTAACAATTGTGGCTCATGAAGCTAAAAAATTAATGCCAGAAGGTGGTAGCATTGTTGCAACAACATATTTAGGTGGCGAA
TTCGCAGTTCAAAATTATAATGTGATGGGTGTTGCTAAAGCGAGCTTAGAAGCAAATGTTAAATATTTAGCATTAGACTTAGGTCCT
GATAATATTCGCGTTAATGCAATTTCAGCTGGTCCAATCCGTACATTAAGTGCAAAAGGTGTGGGTGGTTTCAATACAATTCTTAAA
GAAATCGAAGAGCGTGCACCTTTAAAACGTAACGTTGATCAAGTAGAAGTAGGTAAAACAGCGGCTTACTTATTAAGTGACTTATC
AAGTGGCGTTACAGGTGAAAATATTCATGTAGATAGCGGATTCCACGCAATTAAATAATATCATTCAACAGCTTTGTTCACGTTATC
ATATATGTGAGCAAAGCTTTTTTGCTTTTATAATAATCGGACTGATGGAAAATTATTTGATATTTCATCTGACTGATTTTTTTTATGCA
CAAAAAAATCTCCTCAAAAGCTGTAGTTTCAACTTTTGAGGAGATAGTATCTTATATTCTTAAAAGACATCCACAGAATTAGTCCTT
AACATTACTGTTTGCTTTATCAATAATGCGTTGGCGGTATTTGAAAATATTACTAACAACCGTTTTAAGTACAGCATATAATGGCACT
GCAATCAGAACTAATGTAAAGCCACCTAAATCTCCTGCTGCTAAAATAACAACGATAATTGTTAAAGGATGGATACTTAAAGATTT
ACCCATTACATTTGGCGTAATGATATTACCTTCAAGTTGGTGTGCAATTAATGTAATGATACAAACCCATATAAATGTAGTAGGACT
ATCTATAATACCGAGTATTGCTGCAGGTGCAAATGATAACCATGAACCTAAGAAAGGAATTAAGTTTGCGACACCAGCAAATAGAA
CTAATAAAGGAATATATGGTAAGTCAATAATTGAATAACCGATATATAAGAATATACCTAAAATAACACTGACAGTTACTTGACCT
TGAATGTAAGATTTTAATGTAAAGTTTAAATCAGTTAATAAATCTACGAAAAATACTTTACGTTCACCTTTGAAAAATTTAGCAACA
GCTGGGATAAATTTTTCATGGTCTTTTAACATATAAATTAAGAAGAATGGAACCATAATCAATAAGAAGATGGTTGAAATTAATGAT
GTAATGTACTGTAATGAATTAGATAAAATATTAGTAACGCCATCACCCATTGATTTAACCATATTTGTAATTCTACTTGTTACATCTT
CAGGTAATTTATCCATTTGAGCTAGTGCGAATTTAATAATTTGCTCTGCTTCTTTTTGTAGGGCAGGTGTCTGACTAATTAAATTGTT
GATATTTGAAATGATGATTGGTGCAACAAACGCAACAATTAAGCCGATAATAGCAAACAAAGCTAACATGATTGTTGTTATACTAG
CCCATCTTGGAAAACCGACTTTTTCAAGTAAGTTTTGAAAAGGTAGACAGATATAAAATAAAAAACCACTAATTAAAAATGGAAGA
AATACAGAACCGATGATTGTAGCTATTGGAGTAAATACTTCGTGCACTTCCATAAATAGTTTGATGAGAATGAACAGCATAATCAG
AGCGATGCCAGTTCGGAACCAAACCTTGTTTAACATAAACGATGTTCCTCCTATTTTTCTAAACTTAGTTATTATTTAGTATATCCTA
AATAGCAGTGCTTAAAAAGGTTAATATTTTTTAAATATACTTAAATTAACCATTCACTTTCTATTTTAAGATATTTACTTTCAATAGT
TTGTGAGACTTTTCAATGACTCGTAAGTCTGCAATTGGATCAAACAGCACCAGTTTATATGAATAACTTATTTTAATATTAAACGTGT
TTAAATAATGAAATGATCATATCAATATTTATAAGAATATTCTGAAAAATATATTGAAGTGTCTCGTGCTTAATCGTATAATATTTTT
ATGTTTAAACAATAGGGGGAATCTTATGATTGAAAAATTAGTAACCTTTTTAAATGAGGTTGTTTGGAGTAAGCCATTAGTTTATGG
TTTGCTAATTACTGGTGTGCTATTTACATTGCGTATGCGATTTTTTCAAGTTAGACATTTTAAAGAAATGATTCGATTAATGTTTCAA
GGAGAGAAGTCTCCTAATGGTATTTCAAGTTTTCAAGCGATAGCCATGTCTTTAGCAGGCAGGGTTGGTACAGGTAATATTGTCGGT
GTATCTACTGCAATATTTATAGGAGGACCTGGTGCAGTATTTTGGATGTGGATTACTGCGTTTTTAGGTGCAAGTAGTGCTTTTATTG
AATCTACACTTGGTCAAATATTCAAGAGAGTTGAAAATAATGAATACCGTGGTGGACCAGCGTATTATATTGAATATGGTATTGGTG
GTAAATTTGGTAAAATTTACGGAATTATCTTTGCTATTGTTACGATTATCTCAGTAGGTCTATTGCTTCCTGGTGTGCAATCTAACGC
TATAGCAAGTTCTATGCATAATGCGATTCATGTTCCACAATGGTTAATGGGTGCTATTGTTGTAGTTATTTTGGGATTAATTATTTTT
GGTGGTGTACGTAGTATTGCCAATGTTGCAACAGCCGTTGTACCATTTATGGCAATTATTTACATACTGATGGCTGTCATTATCATTT
GTATCAATATACAAGAAGTGCCAGCGTTATTTGCATTAATTTTCAAATCAGCATTTGGATTACAATCTGCTTTTGGTGGTATCGTTGG
CGCAATGATAGAGATTGGTGTTAAACGTGGATTATACTCAAATGAGGCTGGTCAAGGTACAGGTCCACACGCAGCAGCGGCAGCAG
AAGTATCACATCCAAGTAAACAAGGTCTAGTACAAGCATTTTCAGTTTATATTGATACATTATTTGTATGTACTGCAACTGCTCTGAT
TATACTTATTTCTGGTACATATAATGTGACTGATGGTACGGTTAATGCGAATGGCACACCGCATTTAATTAAAGATGGCGGTATTTA
TGTTGAAAATGCAACAGGTAAAGATTATTCAGGTACTGCGATGTATGCACAAGCCGGCATTGATAAAGCGTTCCATGGCAGTGGTT
ATCAATTTGATCCTACTTTCTCTGGCGTAGGTTCGTACTTTATTGCATTTGCTTTATTCTTCTTTGCATTTACTACAATTTTGTCGTACT
ACTACATTACAGAAACAAATGTTGCTTATTTAACGCGTAATCAAAATAATCAAGTTTCATCGATATTTATTAATATTGCTCGTGTGAT
TATTTTGTTCGCTACATTTTACGGTGCAGTTAAAACAGCTGATGTAGCATGGGCATTCGGTGATTTAGGTGTAGGTCTAATGGCTTGG
TTAAATATCATTGCGATTTGGATTTTACATAAGCCTGCCGTAAATGCTTTAAAAGATTATGAAATTCAAAAGAAACGTTTAGGAAAC
GGTTATAATGCAGTTTATCAACCTGATCCGAATAAATTACCTAATGCTGTCTTTTGGTTGAAGACATATCCAGAACGTTTAAAACAA
GCACGTGCCAAAAAGTAATCTACTTTTGTTTATAGTATATGTAGTGATCATTTGATAAAAAAAGAAACAGCATTGATAATTTTAGGT
GCTCAGAAATTTGAATTTTAAAAATATAGTGTCTCTTGGTACAATAACAATACAACTACTAGGGGGCACTTTTTTATGTCAGAATTT
AAAACTGGTAAGATTAATAAACATGTTTTATATAGTAATATTTTAAATAGAGATGTCACGATAAGTATTTATTTACCAGAATCTTAT
AATCAACTTGTTAAATATAATGTCATTCTTTGCTTTGACGGATTAGATTTTTTACGTTTCGGGAGAATACAACGTACATATGAATCGT
TAATCAAAGAAGCGCGTATTGATGATGCGATCATTGTTGGATTCCATTATGAAGACGTTGATAAGCGTAGAGAGGAATTTCATCCAC
AAGGAAGTCGTTCTCATTTAACTATTCAATCAGTCGGTAAAGAAATATTGCCATTTATTGACTCGACGTTTTCTACACTGAAAGTAG
GTAATGCAAGGTTATTAGTAGGGGATAGTTTAGCGGGTAGTATTGCCTTATTAACGGCGTTGACCTATCCAACGATTTTTAGTCGTG
TAGCAATGTTAAGTCCACATTCAGATGACAAAGTATTAGATAAGCTAAATCAATGTGCTAATAAAGAACAATTGACAATTTGGCAT
GTCATTGGTCTAGATGAAAAAGATTTTACTTTACCAACAAATGGTAAGCGTGCCGATTTCTTAACACCGAATAGAGAATTAGCTGAA
CAAATTAAGAAATATAATATAACTTATTATTACGATGAATTTGAAGGTGGTCACCAATGGAAAGATTGGAAACCATTGCTGTCAGA
TATATTATTGTATTTTTTACGTAAAAACACAGATGATCAACATTATGAGTAATTTACATTAGTAGATTTAGTATGAATTGTCTTCATA
TAGTTTGGTCTATAATATAATTTATAAAAGATTTTACTGTTTGATTTAATTTAAATTTGTCGAAATTGCAAAAGATGTATAATGAATT

ATTTTTAATGTAACGGTTTTCAAAGAAATTTGATATAATAGCAATAGGTTAAACAAAGGAGGAATTCAGATGATTTTAGGATTAGCA
TTAATTCCATCAAAGTCATTTCAAGAAGCGGTGGATTCTTACCGTAAAAGATATGATAAACAGTATTCACGAATTAAACCACATGTG
ACAATTAAAGCGCCATTTGAAATTGAAGATGGTGATTTAGATTCTGTCATTGAACAGGTTAGAGCTCGTATTAATGGTATACCAGCA
GTAGAAGTTCATGCTACAAAAGCTTCTAGCTTCAAACCAACGAACAATGTGATTTACTTTAAAGTTGCGAAGACGGACGACTTAGA
AGAATTGTTTAATCGCTTTAATGGAGAAGATTTCTATGGAGAAGCTGAACATGTTTTTGTGCCACACTTTACAATAGCACAAGGACT
ATCTAGCCAAGAATTCGAAGATATTTTTGGTCAAGTAGCATTAGCTGGGGTAGACCATAAAGAAATTATCGATGAATTAACTTTGTT
ACGTTTTGACGATGACGAAGATAAATGGAAAGTTATTGAAACGTTTAAATTAGCTTAAGTAACATAATAGTATTGTTAATCGTAGTA
TGTTTGAATTAATAAGAAAATGGTCATTTTTATTGAATGTAATAAAAATGACCATTTTCTTTATTTTAAAATACGTTTTAACCTTACT
TAGCTTTTTCTCTATTTGCTATAAAGTAGCTTCCATAAAATACAGCTAAGACTAAAAAGATTAATGCCGAGAAATAAAATGTATTGT
TTAAATCGTTGGTAAATTGTGTAATTAATCCGCCAAATAATGGCCCTATCATTGAGCCGAATCCTTGGATACTATTAAAAACACCCC
AAGTTTCTTCTTGTTCATCTGATTTGATAAATCGTGCCATAAAGGTATTCCATGCTGGTAATAAGATGCCATACATTAGACCGATAGC
TAAAGCGATAATCCACACGATGTGAATATTAACAATCATAGATAGAGTAAAAATTAATATCATGTATAAAATAAATCCGCTTAGAA
TAACACCATACATAAAGTTTCTGCTGCGGTTATCTATTAGTTTCGATAAAAATAGCATCGAAACTGCACAGCCGATACCACCAATAA
TGATTGCAACAGTATATTCAATTGTGCTTACGTTAATAACCTTAGTAGCATATGTTGGTAATATAGGAACTAGGGCAGCAATTGCGG
CACCTTGTAAAAGAATACCAGGGAACAACAATAAATGGCGCTTTGTCACATCAACAATTTGTCTCAATTGAGCTTTAACTGGACGA
GTATTATAATTTGTTAACTTTACATCGACAAAATAATATAATATCCATGCAATTAAAACGACTAAAGACATCATGAAGGCAAAGCGT
GTTGGGTGCACTTTGATAAGTAGATTCATAAAAACCATACCTACCAATAGGCCTAACAACCATGAAAAATAAACATAGCCCATTTG
TTTGCCACGTTTATCTTCTTCAACACTGGATAACATAATGACCCAAATAGGACTAACTGCAATACCGAGCATCATAGCACTAAATAT
GATTACAAAAGGTGATGCTGGAAACCAAATAACTAAAAATAAACTTGTAAATGCTAAAATAAATCCAGTCGTTAAAACGATTTTTG
TGCCGAATTTTTTCAGTAAAAATCCTATAACAAAGTTTGTAGATGCATCAGCAATAAAATGTATTGAAAATGCTAGAGACGTTATTG
CTACAGCAATGGATGTAACTGTTGGCAAGAAATTAATATAGCTTAGGATATACATGCCTCTCGCAAATTCCATTAAAAATAAGATA
ATAAGCATTAAAATGAAATTTTTATGATTAGCGTAATTATTTAACGAAGAATCTTGCATATAAAGGAACCTTTCCATAAATCTCTTG
TGGTTGTGATGAATGACCGATTAAATCAAGTAAGTCTCGACATATTGTCTGTGTAGCATACTTAATTTTATCTTGTTCCATTGTACTA
ATCATGTTAGTTAATTGCTCATTACCGTTCGTTAAACTTGCTACAATTTTTATTGCTTCTTCTGGAGTATCAGCGATTTTACCAAAACC
TTTTTCTTCAAAGTAAAGGGCATTTTCAAGCTCTTGACCAGGTGCAGGATTTAGGAAAATCATTGGAATACAACGGGCGAAACCTTC
AGTTATTGTGATACCACCAGGTTTCGTAATCATAAGTTGACTTGATGCCATCCATTCATTCATGTGTTTGGTATAACCTAGAATCAAT
ACATTCTCGTTAGATTTAAACTTAGCTATTAAAGAACGCTTTAGCTCTTTGCTCTTACCACAAATCATAACTACTTGTGCATTTGCAC
TTTTCGCTAATATATCAGTAATCATCGTGTCAAAACCTTTAGATACACCAAATGCACCAGCTGACATTAAAATAGTTTGCTTATCTGG
ATCTAAGTTGTTGTCTATTAACCACTGCTTTTGATTAATAGGCGTTTCAAATTTGTTATCAATAGGAATACCTGTCACTTTAACTGTT
GAAGGATCAATACCTACGTCTATGAAGTCTTGTTTCGTTTCTTTTGTTGCCACATAATATCTTGTTGAATACGGCGTAATCCAGTTTT
TATGTAAGCGATAGTCTGTCATCACTGTAGCAACTGGAATATTAATGTTAAATTGCTCAGTTAGTACCGACATAACTGGTGTAGGAA
ACGTTAATAATATTAAATCTGGCTTTTCTTTTATCAATAAATTAATTAACTTATTAAGTCCATAGTATTTGTAAAAACATTTGTCTAG
TTTATCTGGGCGGCTGTAATAAAACCCTTTGTACATATTTCTAAAATATTTAAAGCTATTGATATACCATTTTTTACAAATAGAAGTC
AAAATTGGATGAGCTTCCATAAATAAATCGTGCTCAATGACGCTTAAATGGTCTAGATTCATATCATTAAGTTGATTAACGATACTC
TGTGTAACTTGCATATGACCGTTACCGAATGAGCCAGTAATAATCAATATCTTTTTATTTTGAGTAACCATTAATAGCCACCCTCCGT
TAGTTTGAAAATTTTATTTAAGTGTAACTTATTTTACGGCATTATAAAAGAAATAAAGACGCAAAGTCGTTACATTTATAGCAATTTT
AATCAATAGATGAATTGATACAAAATAAAACGTTATTTTATAAAGCAATTTATTGTTCTATGTTTTATTTGTATATTTAAAATTATCC
AGTATACAATTATAGCATATTTTTGGAAACAATTATGATATTATACCATGTTACAAGATGGTTTTAATAATTTAAGATGAGCCATAA
TTGTAAAACTAATTCATAATACCGTATGTTTTATTTTTAATAGTAGAAATTAGAAAATGCTGATTAGTAGGATATAACAGTGAAATT
ATAAATTTATTAACATCAACAAAACGTGTATAATAAACATATTGTAGAAAAAGGAGCGGTTCAGTTTGGATGCAAGTACGTTGTTTA
AGAAAGTAAAAGTAAAGCGTGTATTGGGTTCTTTAGAACAACAAATAGATGATATCACTACTGATTCACGTACAGCGAGAGAAGGT
AGCATTTTTGTCGCTTCAGTTGGATATACTGTAGACAGTCATAAGTTCTGTCAAAATGTAGCTGATCAAGGGTGTAAGTTGGTAGTG
GTCAATAAAGAACAATCATTACCAGCTAACGTAACACAAGTGGTTGTGCCGGACACATTAAGAGTAGCTAGTATTCTAGCACACAC
ATTATATGATTATCCGAGTCATCAGTTAGTGACATTTGGTGTAACGGGTACAAATGGTAAAACTTCTATTGCGACGATGATTCATTT
AATTCAAAGAAAGTTACAAAAAAATAGTGCATATTTAGGAACTAATGGTTTCCAAATTAATGAAACAAAGACAAAAGGTGCAAAT
ACGACACCAGAAACAGTTTCTTTAACTAAGAAAATTAAAGAAGCAGTTGATGCAGGCGCTGAATCTATGACATTAGAAGTATCAAG
CCATGGCTTAGTATTAGGACGACTGCGAGGCGTTGAATTTGACGTTGCAATATTTTCAAATTTAACACAAGACCATTTAGATTTTCA
TGGCACAATGGAAGCATACGGACACGCGAAGTCTTTATTGTTTAGTCAATTAGGTGAAGATTTGTCGAAAGAAAAGTATGTCGTGTT
AAACAATGACGATTCATTTTCTGAGTATTTAAGAACAGTGACGCCTTATGAAGTATTTAGTTATGGAATTGATGAGGAAGCCCAATT
TATGGCTAAAAATATTCAAGAATCTTTACAAGGTGTCAGCTTTGATTTTGTAACGCCTTTTGGAACTTACCCAGTAAAATCGCCTTAT
GTTGGTAAGTTTAATATTTCTAATATTATGGCGGCAATGATTGCGGTGTGGAGTAAAGGTACATCTTTAGAAACGATTATTAAAGCT
GTTGAAAATTTAGAACCTGTTGAAGGGCGATTAGAAGTTTTAGATCCTTCGTTACCTATTGATTTAATTATCGATTATGCACATACAG
CTGATGGTATGAACAAATTAATCGATGCAGTACAGCCTTTTGTAAAGCAAAAGTTGATATTTTTAGTTGGTATGGCAGGCGAACGTG
ATTTAACTAAAACGCCTGAAATGGGGCGAGTTGCCTGTCGTGCAGATTATGTCATTTTCACACCGGATAATCCGGCAAATGATGACC
CGAAAATGTTAACGGCAGAATTAGCCAAAGGTGCAACACATCAAAACTATATTGAATTTGATGATCGTGCAGAAGGGATAAAACAT
GCAATTGACATAGCTGAGCCTGGGGATACTGTCGTTTTAGCATCAAAAGGAAGAGAACCATATCAAATCATGCCAGGGCATATTAA
GGTGCCACATCGAGATGATTTAATTGGCCTTGAAGCAGCTTACAAAAAGTTCGGTGGTGGCCCTGTTGATCAATAAAAGATTTATTG
ATGAAGGTAAAACTATTGATGTTTATTTATTCGAAGCATTAAATAACCAGATAATCATTGCTATACCAGATTGGTTTTGGTCATATC
AGATGGCAATGACATTAGATGAAGAAACTTGTTTTGAAGCAATACTCATGCAATTGTTTGTTTTTAAAGAAGAGGAAGAGGCAGAA
TCGATTGCATCACAACTAACAGATTGGATAGAAACATATAAAAAGGAGAAAGACTAATGAACTTAAAGCAAGAAGTTGAGTCTAG
AAAGACTTTTGCGATTATTTCACATCCCGATGCAGGGAAAACAACGTTAACTGAAAAACTATTGTACTTCAGTGGTGCTATTCGTGA
AGCGGGTACAGTTAAAGGGAAGAAGACTGGTAAATTTGCGACAAGTGACTGGATGAAAGTTGAACAAGAGCGTGGTATTTCTGTA
ACTAGTTCAGTAATGCAATTTGATTACGATGATTATAAAATCAATATCTTAGATACACCAGGACATGAAGACTTTTCAGAAGATACG
TATAGAACATTAATGGCAGTTGACAGTGCTGTCATGGTCATAGACTGTGCAAAAGGTATTGAACCACAAACATTGAAGTTATTTAA
AGTTTGTAAAATGCGTGGTATTCCAATCTTTACATTCATTAATAAATTAGACCGAGTAGGTAAAGAACCATTTGAATTATTAGATGA
AATCGAAGAGACATTAAATATTGAAACATACCCTATGAATTGGCCAATTGGTATGGGACAAAGTTTCTTTGGCATCATTGATAGAA
AGTCTAAAACAATTGAACCATTTAGAGATGAAGAAAATATATTACATTTGAATGATGATTTTGAGTTGGAAGAAGATCATGCAATT
ACAAATGATAGTGCTTTTGAACAAGCGATTGAAGAATTAATGTTGGTTGAAGAAGCGGGTGAAGCCTTTGATAATGACGCGCTGTT
GAGTGGAGACTTAACACCTGTATTTTTCGGTTCAGCTTTAGCTAACTTTGGTGTACAAAATTTCTTAAATGCATATGTTGATTTTGCG
CCAATGCCAAATGCGAGACAAACAAAAGAAGACGTTGAAGTAAGCCCGTTTGATGATTCATTTTCAGGATTTATCTTTAAAATTCAA
GCCAACATGGACCCTAAACACCGTGATAGAATTGCCTTTATGCGTGTCGTTAGTGGTGCATTTGAACGTGGTATGGATGTTACTTTG

CAACGTACTAATAAAAAGCAAAAGATCACACGTTCAACGTCATTTATGGCAGACGATAAAGAAACTGTGAATCATGCTGTAGCAGG
CGATATCATTGGACTATATGATACTGGTAATTATCAAATTGGAGATACTTTAGTTGGTGGAAAACAAACCTACAGTTTCCAAGATTT
ACCACAATTTACGCCAGAAATTTTTATGAAAGTTTCTGCTAAAAACGTCATGAAACAGAAGCATTTCCATAAAGGTATTGAACAATT
AGTACAAGAAGGTGCGATTCAATACTATAAAACATTACACACAAACCAAATTATTTTAGGTGCTGTTGGTCAGTTACAATTTGAAGT
TTTCGAACATAGAATGAAAAACGAATATAATGTTGATGTTGTTATGGAGCCAGTAGGCCGTAAAATTGCACGTTGGATTGAAAATG
AAGACCAAATTACAGATAAGATGAACACATCAAGATCGATTTTAGTGAAAGATAGATATGACGATTTAGTATTCTTATTTGAAAAT
GAATTTGCAACAAGATGGTTTGAAGAGAAATTCCTTGAAATTAAATTGTATAGTTTACTTTAACAGCTCAATTGTATAATCGAATTT
GTTACATTAAAAATAATTGTTTCGTTGAAGAAAAATAAATTGTATATTTTAAAAGAAAAAGGTATACTATGATGTATCAAATGAATA
ACCTGTGGCATTTTGTCAGAGGGGAGTAACTTAAGAATCATGACCGTATAAATGATTCGACACTTTATCGTCATTACGAAGATATCT
TCCGGTAAAGTGGGCAATTTAAATTGCTTAGTGAGACCTTTGCTATTTATTTAGCATAGGTCTTTTTGTTTGTACTTAACTTATTTATT
TAAAGGAGTTGTACATGTTAATGGATCCAAGTTTGATCTTACCTTATTTATGGGTACTTGTCGTTTTAGTATTTTTAGAAGGCTTATT
AGCAGCAGATAACGCGATTGTTATGGCTGTAATGGTTAAGCACTTACCACCCGAACAACGTAAAAAAGCTTTGTTTTACGGTTTGTT
AGGTGCATTTGTATTTAGATTTTTAGCATTATTCTTAATTAGTATTATCGCGAACTTTTGGTTTATTCAAGCTGCAGGAGCGGTTTACT
TAATTTATATGTCAATCAAAAATCTGTGGCAGTTCTTTAAACACCCAGAAATTGAAAGTCCTGAAGCTGGAGATGATCATCATTATG
ATGAATCTGGTGAAGAGATTAAAGCAAGTAACAAATCATTCTGGGGAACTGTGTTGAAAATAGAATTTGCAGATATCGCATTTGCC
ATTGATTCTATGCTTGCTGCTTTAGCTATTGCTGTAACACTTCCTAAAGTTGGTATTCACTTTGGTGGTATGGACTTAGGTCAGTTCGT
AGTCATGTTCCTAGGTGGAATGATTGGTGTTATTCTAATGCGTTATGCAGCAACATGGTTTGTAGAGCTATTAAACAAATATCCAGG
ACTTGAAGGTGCAGCCTTCGCGATCGTTGGTTGGGTAGGTGTTAAATTAGTTGTCATGGTATTAGCGCACCCAGACATCGCTGTATT
GCCTGAGCACTTCCCACATGGCGTATTATGGCAATCTATTTTCTGGACAGTACTAATTGGATTAGTAATTATCGGTTGGTTAGGTTCA
GTTGTTAAAAATAAAAAATCGCATAAATAATTGATGTGAAGCGGACAATCTTAATTTAGTTTAAGGTTGTCCTTTTTCATTTAATTGA
GTGATTTATGAAAAATGGATTTTGAAGAATGTGAATCAAAAGATGCGATATAGTATTAAGAAAATGTGCCTTTTATATTTAGCATTT
TTTCAATAGAAATTATATAGATTTTAAAGCAAATTAGGTGTTAATGTGTCATAATGATAAGTGATTTTATTGAATGGAGTGGACATT
AGTGGATATTGGTAAAAAACATGTAATTCCTAAAAGTCAGTACCGACGTAAGCGTCGTGAATTCTTCCACAACGAAGACAGAGAAG
AAAATTTAAATCAACATCAAGATAAACAAAATATAGATAATACAACATCAAAAAAAGCAGATAAGCAAATACATAAAGATTCAAT
TGATAAGCACGAACGTTTTAAAAATAGTTTATCATCGCATTTAGAACAGAGAAACCGTGATGTTAATGAGAATAAAGCTGAAGAAA
GTAAAAGTAATCAGGATAGTAAGTCAGCATATAACAGAGATCATTATTTAACAGACGATGTATCTAAAAAACAAAATTCATTAGAT
TCAGTGGACCAAGATACAGAGAAATCAAAATATTATGAGCAAAATTCTGAAGCGACTTTATCAACTAAATCAACCGATAAAGTAGA
ATCAACTGAAATGAGAAAGCTAAGTTCAGATAAAAACAAAGTTGGTCATGAAGAGCAACATGTACTTTCTAAACCTTCAGAACATG
ATAAAGAGACTAGAATTGATTCTGAGTCTTCAAGAACTGATTCAGACAGCTCGATGCAGACAGAGAAAATAAAAAAAGACAGTTC
AGATGGAAATAAAAGTAGTAATCTGAAATCTGAAGTAATATCAGACAAATCAAATACAGTACCAAAATTGTCGGAATCTGATGATG
AAGTAAATAATCAGAAGCCATTAACTTTACCGGAAGAACAGAAATTGAAAAGACAGCAAAGTCAAAATGAGCAAACAAAAACCTA
TACATATGGTGATAGCGAACAAAATGACAAGTCTAATCATGAAAATGATTTAAGTCATCATACACCATCGATAAGTGATGATAAAG
ATAACGTTATGAGAGAAAATCATATTGTTGACGATAATCCTGATAATGATATCAATACACTATCATTATCAAAAATAGATGACGATC
GAAAACTTGATGAAAAAATTCATGTTGAAGATAAACATAAACAAAATGCAGACTCGTCTGAAACGGTGGGATATCAAAGTCAGTCA
ACTGCATCTCATCGTAGCACTGAAAAAAGAAATATTTCTATTAATGACCATGATAAATTAAACGGTCAAAAAACAAATACAAAGAC
ATCGGCAAATAATAATCAAAAAAAGGCTACATCAAAATTGAACAAAGGGCGCGCTACGAATAATAATTATAGTGACATTTTGAAAA
AGTTTTGGATGATGTATTGGCCTAAATTAGTTATTCTAATGGGTATTATTATTCTAATTGTTATTTTGAATGCCATTTTTAATAATGTG
AACAAAAATGATCGCATGAATGATAATAATGATGCAGATGCTCAAAAATATACGACAACGATGAAAAATGCCAATAACACAGTTA
AATCGGTCGTTACAGTTGAAAATGAAACATCAAAAGATTCATCATTACCTAAAGATAAAGCATCTCAAGACGAAGTGGGATCAGGT
GTTGTATATAAAAAATCTGGAGATACGTTATATATTGTTACGAATGCACACGTTGTCGGTGATAAAGAAAATCAAAAAATAACTTTC
TCGAATAATAAAAGTGTTGTTGGGAAAGTGCTTGGTAAAGATAAATGGTCAGATTTAGCTGTTGTTAAAGCAACTTCTTCAGACAGT
TCAGTGAAAGAGATAGCTATTGGAGATTCAAATAATTTAGTGTTAGGAGAGCCAATATTAGTCGTAGGTAATCCACTTGGTGTAGA
CTTTAAAGGCACTGTGACAGAAGGTATTATTTCAGGTCTGAACAGAAATGTTCCTATTGATTTCGATAAAGATAATAAATATGATAT
GTTGATGAAAGCTTTCCAAATTGATGCATCAGTAAATCCAGGTAACTCGGGTGGTGCTGTCGTCAATAGAGAAGGAAAATTAATAG
GTGTAGTTGCAGCTAAAATTAGTATGCCAAACGTTGAAAATATGTCATTTGCAATACCTGTTAATGAAGTACAAAAGATTGTAAAA
GATTTAGAAACAAAAGGTAAAATTGACTATCCCGATGTAGGTGTTAAAATGAAGAATATTGCCAGTCTAAATAGTTTTGAAAGACA
AGCAGTTAAATTGCCAGGAAAAGTTAAGAACGGTGTTGTTGTAGATCAAGTTGACAACAATGGTTTAGCAGATCAATCTGGTCTGA
AAAAAGGTGATGTAATTACTGAATTAGATGGCAAACTTTTAGAAGATGATTTACGCTTTAGGCAGATTATATTTAGTCATAAAGATG
ACTTGAAATCAATTACAGCGAAGATTTATAGAGATGGTAAAGAGAAAGAAATTAATATTAAACTAAAATAATGTTGAGGTGAACAA
CGTGTCAATTTTTAGCCAGTTTTTAAAAAGATCAAGCCCTCAACAAGGTATTGTATTGTACTATATCGTCGCAATTGTCATTGCATTT
TTATTATTAAACTTACCGTATGTTCATAAACCAGGTGTAGAAGTAAATCCAATTGACACATTATTTGTTGCCGTATCCGGAATTAGTG
TTACTGGATTGTCTCCGATAAGTATTGTCGATACCTATTCTACATTTGGACAATTAATTATCCTCGTGATATTAAATATTGGTGGAAT
TGGCGTCATGGCAATTGGTACGATGTTATGGGTGGTACTAGGTAAACATATTGGAATTAGAGAACGTCAGTTAATTATGTTAGATAA
TAACAAAAACACAATGAGTGGTACCGTCAAATTGATTATTGATATTGTAAAATCAATATTTGTAATCGAACTCGTAGGAGCCATGTT
ATTAGCATTTTACTTTTATCGAGATAATCCAGATTTAAAATATGCAATCATGCAAGGTGTTTTTGTTTCTATTTCTGCCACTACCAAT
GGTGGATTAGATATTACAGGTAAGTCATTAATTCCTTATGCACATGATTATTTTGTACAAGCGATAGTTATATTTTTAATAATTTTAG
GATCAATCGGCTTCCCAGTATTATTAGAAGTTAAAGCTTATATTCAAAATAGGGTTACTAATTTTAGATTTTCATTATTTACTAAAAT
TACGACATCAACATATTTATTCCTATTTATTGTTGGGGTATTAGCCATTCTATTATTTGAACATAACCATGCGTTCAAAGGTTTAAGT
TGGCATCAATCGTTATTCTATTCGCTGTTTCAATCAGCGACTACAAGAAGTGCGGGTCTTCAAACAATTGATGTGACAACACTAAGT
GACCCCACTAATATTATCATGGGTATTTTAATGTTTATAGGATCTTCGCCAAGTTCGGTTGGTGGCGGTATTCGTACAACAACTTTCG
CTATTTTAATTTTGTTTTTAATTAACTTTAGTAATAATGCCGATAAAACATCCATTAAAGTTTACAATAGAGAAGTACACATTATGGA
TATTCAACGTTCATTTGCAGTATTTACAATGGCGACAATTTTAACATTTTTAGGAATGCTAATTATATCAGCTACTGAAAATGGTAAG
CTTACATTTTTACAAGTATTTTTTGAAGTCATGTCTGCATTTGGAACTTGTGGACTATCGCTTGGTGTCACAAGTGATATTAGTGATA
TTTCTAAGGTCGTACTAATGGTACTCATGTTTATAGGACGTGTTGGCTTAATATCATTTATCATTATGATAGCAGGACGTCGAGAACC
AGATAAATTCCATTATCCAAAAGAACGTATTCAAATAGGATAATATAATAGCAATCTAAGTTTAGTTAATATAGATTTTAACTGGAA
CTTAGATTGCTTTTTTAGTTTGTATTTTTAACTTATTTTATAAGACGATTGGTTTTCAAAATGGTAAACTAGTAACAATGAGAGGTGT
AACATGATGGAAAAAAATGAAAACATTAATGTAGAGATTTTAACTACGTCAGATATGCATAGTCATTTCTTAAATGGTGATTATGGT
TCAAATATTTATAGAGCTGGTACTTATGTTAACCAAATAAGAGCACAAAATCATCGCGTCATTTTATTAGATAGTGGCGGAAGTTTA
GCTGGCTCGTTAGCGGCCTATTATTATGCTATTGTTGCACCTTATAAACGACATCCAATGATAAAGTTAATGAACAGAATGCATTAT
GATGCTAGCGGTGTGAGTCCAAGTGAATTCAAGTTTGGTTTATCATTTTTAACTCGTTCAATTGCTTTGGCACGTTTTCCATGGTTAT
CAGCAAATATTGAATACAATGTTACTAAGGAGCCTTATTTTTCAACTCCATATTGTATTAAACATTTTGGTGACTTAAAAATTGCTAT

CGTAGGCGTCACAGCAGATGGTTTAATGGAAAATGAGTATTCTGAAATGGAGCAAGATGTATCTATTGAAAAGACATTAGTGGCAT
CAAAACGTTGGATTAGATATATCCATGAAGTTGAAGAGCCAGATTTTTTGATTGTAATTTATCATGGTGGATTGAATAAAATTAGTA
ATAGTACGAAAAATAAAAAGGCAAGTTCGAATGAAGCTGAAAAATTAATGGAAGAACTCGGTGTTATAGATTTAATGATTACAGCT
CATCAGCATCAAACAATAGTAGGTCAAGATCATGAAACGTATTATGTTCAGGCTGGTCAGGATGCCAAAGAGCTTGTACATCTTTCG
ATTAATTTTAAAAAGAGAACAACAACTTATGATGTTGAAAGCATTGATTCTAAAGTGATTGACTTAAATGAGTATGAAGAGGATCA
AGAATTATTAGATTTAACATTCTATGATAGAAAAGCAGTGGCTTATTGGTCACAGGAAATCATAAGTGATAAAGGTTTGATGTTATC
AGTGAATGGGTTACAAGATTTAGTCTGTCAAACACATCCATTTTCGCAATTATTACATGATGCAATTCAGCTTGCATTTGATAATGAT
ATAACATGTGTCCACGTGCCTATGAACGGAGAGAAGGGGTTGAGTGGACAGATTCGAAATGAAGATTTGTATCATGCATACCCATA
TCCAGATAAGCCAATGGATATGACAATTAGTGGTCAAAATATCAAAGATATATTGGAGTATAGTTATTCACATTTAGATTTTGTTAA
CGAGCAATTAAGCTTAACAATTATTGATGAAACGTTATGTACAATGTGGCAAGGATTCAATTATGAGATTGATATGAATCAAGAAC
CTGGGCAACGAGTAATGTTAGATCAAATTGATTTGACTAAGAGTTATAGAGTTACAATGACTGACTATTGTTATCGTAACTACAAGA
ATTATTTAAAAAATGCTATTATACATGAATCGTACGATGAAACAATGAGTACATTAATTGCAGAGAAGTTAAGAGATCCGAATTAT
CGTATTTCATGTAGTGATAATTTTGTAGTTAAAAACAGGTAAATATTGCTATGTAATAAATTAATTATAAGTTCAACAAGTATAAAA
ACCTTTCTTAGAGAAGATTATCTAGGAAAGGTTTTTAGAATTTTAAAATTGATATAAAAGAACAGTGAAATTCTCATTCATTATATA
TAAGTAAACAAAAAAGCTAAAATGGTATATACCATTTTAGCAAGTAACGGAAACGGAGGGATTCGAACCCTCGCGCCGCTTTCGCG
ACCTACACCCTTAGCAGGGGCGCCTCTTCAGCCAACTTGAGTACGTTTCCAATGGCTCCACAGGTAGGACTCGAACCTACGACCGAT
CGGTTAACAGCCGATAGCTCTACCACTGAGCTACTGTGGATTAATTTGTATGTGTTTGAATATCAAGCACAAATATTATTATAACAA
GATTAAGTTAATTTGCAATAGATACCTTAAAAAAATATGCAGAACATTTACACTATATTTATAAAACATTCCGCATATTTTAAAAAT
AAACAATTTATTTATCTTCAAATAAAGAATAGCGTGTCAATGCTTCAAACACATTCAATGTGGCTTTATTATAATCTATTGGTTGGTC
GGGATTTTTTGATATCATGCGAGATTGTTTATCTACCATGTAATAGTAAAAAATGGAATTGTTATATTGATGCCATAAACTGTGGTAT
GAAACATGAAGAATGATAGATTCATTATTAATAAATGTCACTTTACATTTACGATTTTTTAATTCTTTAATACTTTCGATATAATGCA
TATTTAACCAAATATTTTCATTTTGTCTGTCAGAATGTGTTGGGAAAAAAATAAGTTGGAAATAATGGTGTTAGTAAAATAGGTGGTT
TACTAGAAATGCCAGTAATGCGGTTTGTCTCTGCTTTCTTGCCAAGATAAGTATTACCATAAAATTTGCAAGAGCGTTCAATGATTTT
CTGGACTTTAAAAGGATTTTGAATACGCGTTTTGTCAAACCGAATTATTTCACTACCGTTTCTTTGATCGTCATCATCAAATGCTGGT
CGAATAACCATGTCTCCTTTGCGTATCACATAAATATTTTGAGAATACATAAGCAAAACCTCGCTTTATTAAGTTTTAAACCATTAAT
ATAAAAATACAATTAATGATATAAAGATATTTTATAATAAAATTCTGAGTTGTCAAGTTATTATTTTGGTGAAAATGTACAATAATT
TTTAACTAATAGTCAGAATATTCATTGATTTCAAAATTAGAAAAATGTATACTTATTATAAATTAAAGAAAGGTAGTGGTTTCTATG
AAATACAATACTAATGTTAAACATACAACTTTAGAAGCGTTTGTCACAACTGTCAATGATTTGGGTATTGAATTAATTATCAATGAA
GCACTTCGAGAGGTAAGAAAACGACAGCTCATAGAACTTATAGATGACGCACTCGTCAATAAAGATGAAGCAGCATTTAATCAATA
TACGGCAGAATACAAAAATTTGGAGGCATTTCTCGGTGAATAACATTGATTCGAATTCCATAACGCAATCATAAAAGGACTATTCTT
AACATCTCATACATGTAAGAATAGTCCTTTAATCGTTTATGACATTAATCTAATTAATTCATGTCTATCTATGTCACCGAAATAATGA
TATAAATCATATTCTGATAGTGCTTCTTCTATGTGCTCAAAGTCGTGTAGACAGCCAACTAATGCATTTTCAAGATCAGTGACATCTC
CGACACCAAAGAAATCACCGAAAATTTTTGCATGTTCGATTTACCTCGTTTAACATCAAACTTAATTTGTACAAATCCTTTTTCAAA
TTTTTCCTCGCGTTCAAAGTTATATTTAGGGTTTCTACCATAATTCCATGCCCAAGTTCTATATTTGTCGTTACTTAACTTTTCAATAT
TTTCCCAATCTTCATCCGTTAATTTATATTCTTCTACTTCAGTTTCTCCAAAGATAGTTTTCAAGATGATTTTTTTAAATTCTTCAATTT
CTAAAGGGTCATTTAAAAATTCTTGAATGTTTGCTACTCGTTTACGAACAGATTTAATACCTTTTGATTTAATCTTAGCTGGATTAAC
TTTTAGTGCATTCTGAACTTCGTCTAAATCACTATTTAACATCAACGTACCATGACTAAACATTCTATTTTTAACTTTAACCATAGCA
TTCCCTGAGATTTTCGCTTGTCCAACTTGAATATCGTTACGACCAGTTAATTCAGCATTTACGCCTAAAGATTGTAATGCTTGAACAA
TTGGTTCAGTGAATTTTTGGAAATTGTGGAAACTGTTACCATCATCATCTGTTATAAAACTAAAGTTTAAATTGCCAGTATCATGATA
AACAGCGCCACCACCAGAAATTCTTCTAACTACATCGATGTTATGAGCATCTATATATGTCTGATTTACTTCCTCTATCGTATTTTGA
TTCTTTCCAACAATGATAGATGGTCTATTTATGTAAAATAAAAAGTAACTTTCTTCTGCTGGTAAATTTTTTAAAACATATTCTTCCA
TTGCTAAGTTTAAAGTTGGATCTGTAATATTATTATTACTAATGAATTTCATTACAATCTCTCCCTTATATCTATATATATTCTCTACT
TATTTATGCCTTAACTTTGCTCAAAATGCCAATTATATACTCACAAATCATTCTAAATATCGGCTTAAAGTTACATTTTATTATTTTTG
ATTGTTAATAGTTTATTTGTTAAGTGAATTTTAGTACAATATAATTTAGTTATGATTTAAGGAGGACTTAAAATGACTGTTGCAGAAG
TGGGTAACATTGTTGAGTTTATGGATGGATTAAGAGGTCGTGTTGAAAAAATCAACGATAACTCTGTTATTGTTGACTTAACAATTA
TGGAAAATTTTAATGACCTTGATTTACCGGAAAAAACTGTTATCAATCATAAACGATATAAGATTGTTGAATAAGAAGGTAAGTTAT
AATGAATAAAATCTCGAAGGCTTTAACTTGGTTTATTATAAGTTTCATTATATTTCATCTCATATTATTTATTATGTGGGGCGAACAC
CAAGAATACTGGTATTTATATACAGGTATAATGCTAATTGCTGGTATCAGTTATGTATTTTATCAAAGAGATATTGAATCTAAGCGG
TTGCTTACATCAATTGGTGTTGGTATTATTACGGCAATTATTTTAATTATGCTTCAACTTTTATTCTCACTTATAACTTCTAATTTAAG
TTATAGTTCATTAATTAAAGAATTAGCAAGAACAGGTGTCAATTGGAAGTGGCAAATGTTAGTAACTTTACTTTTTGTCATTCCATGT
CACGAGTTATATATGAGAACTGTTTTACAAAAAGAATTAACACATTTTTCAATACCGAAATGGATAGCTATATTAATAACTGCAATT
TGTTCTAGTTCATTATTTATTTATTTAGATAATTGGTGGATAGTAACATTTATATTTGTTGCACAAGTGATATTATCATTAAGTTATGA
GTATACTCGACGCATAGCAACAACATCCGTTGCGCAAATTGTTGCTATTATATTGTTATTAATTTTTAATGCTTAAATAAGCTTTTCT
TAGACGTCTGTGTTCTAAGAAAAGCTTTTTGCTTTAGAAAAGCAATACGAAGATTCAAGGCATGCTTTATTATAGTGTATACATTTTT
AACGGCAAGCAGTTATTATTGAGGTTATATTTTAGTCAGTTTTTATTACTAAATATTTTATAAAAATAGTATAATATTTTGTGGGCAA
GTTGAATTGATGGTGGCTATCTGAGTAAAGGGGGGTGGTACCTATGGCATTACTTAACACTTTTGAAAGGAAAAGCCTATTGTGATA
TCTATTGCAAACGCATTACATTTAATGTTAAGTTTCGGTATGTTTATCGTCACTTTCATTGGTATAGTAGTAGCAATAATAAATTTAA
GCAATAAAAAAATAACCATCATTTCAAAACTTTGACATAGAATGATGGTTAAAGAAAATCTCATTTAGTCACCGTCTTTTTAACGGGC
TCATTAGGCGACATGTTCCTGCATGTCGTCTTTTTACATTTTTATAGTAACATATCTTTTATTTAATTGTCTATTTTTATTAGATGTGTA
ACAAAATTTTGAAGTATTTTCCGTATATTGCCTGCTAAACATGTCAGATTTGAAAGGAAAATCATACTGTGGTAGAAATCAATTATG
ATTATATGGTAGTACTTATAAATAAGTGATTTCTATTGAATATGTAATGAAGTTCATATATTAATATAACTTTCATTGATATAGAAGT
CGCAATGATTAATTTAAACAATAAAAAACAACCATCATTTCAAAACTTTGACAACGAATGATGGTTAAAGAAAATCTCATTTAGTC
ACCGTCTTTTTAACGGGTTCAATAGGGGACATGTTCCTGCATGTCGTCTATTTATGCACTGCGCAATTTAACAATAATACAATTATAC
TTTGATGTCCATTTAACATAAAGTGGTTGAAATTTGTAGCAAATAAATTAACCTTGACTATTTTCAGAAAATTCTGTATAAATAACG
AAAAAGAGATATGGATTAAATATGAAGAGGTGTTTTTATAGAAGAATAAAATTTTTGGCACTATATTATATTTAACTTTAGCACTTG
GATTATCAACAGCAGCTTATGCATCTACAGAATACGCAGAAGGAGGCACTTGGAGTCACGGTGTCGGCAGTAAGTATGTTTGGTCT
TATTATTATCATGGTCATAAAGGACATGGTGCAACAGCTATTGGAAAATATAGATCATTTAGTGGTTATACAAGAGCTGGTGTAAAA
GCAAAAGCATCAGCTACTAAACATAATTGCTGGGTCAATAGAGCGTATTATAACATTTATTAAATACTAAAAAATCATAGAAAATA
AACAACGCAAGAGGTACAACATGAAATGGTTCAAATTAATACTTGATGTTACAACCTTCATTCTGATTGCTATATTGTTATTTGTTTA
TACATATAAAGAAAACGAAGAAATATTGCCTGATACTAAATACCCTATAGCGGTAACTGACTGGAATAAAAAATATAGTAAAAATG
AGATTTATAAACGTATAAATCAATTCGCTAAAAATGAGAACGTAGCAATCTATAAATCAACTTCAAATTATACAAACAAAAACGTA

GATAAAGATATATATGTATTTAATAAATCAAAAGCAACAACTATCACTCCTTTTAACGCTAAATATAACATTCATTATTTAAGTGAC
GACGAATTATTAAAAAAAGATATCAAAGGAAGTTATTTGTAAAAGACAAAAATTTTGACGTGTCTAAATTCATAAATTTTTTAAAA
GAATATGGTGTTACTGCTGAATCATACAAAATAGATCATATGATGATTGCCGTTGGTGTCATTAAACAAATGAATATAGAAGTTCCG
TTATCCGCACTTTTAATCGTTTATTTTATTTATTATATTTTCGAAAAGAACATTAATTTCAAAGCGTATGCGATTAAGTATTTAAATG
GTTTTACATTAAGAAAAATAATTTTTGAAAATTTTTCAAAAAAATGCACGTATTGGGTAACGTTAATCATAAGTCAAATTCTTTTAA
CTACAAGTGTACTTTGGATATTAAATTACACGGGTAATTTAGATTTATTCATATTAAGATTAGTTCTGCTTTCATGTCTTTTCATTTTA
ACGATTAGTGTTATTAATTTATGGACTTTCTTAATGTTACTAAATTTAAATATTGCTAATATGATTAAAGGTAAGCAGCACTTTAAAA
CAATTCGTTTTATTAATACAGTTTGTAAAAGTATTCTCTTAGTACTAATAGCTAGTGTAATGATAGAAAATACTAGTGTTATTAAAGA
TTTGAATAAAATAAAGGAAACTGAGAAATATTGGAATGTATTAGATGATTATTACACGATTGAATTTGCACCTTATCACGAAACGA
AACAAAGTTTGATTGATAATATGCTGCGATCAGAACAATTAGTAAAGGCTAGTGAAGCAGAAAATAATGCGATTTTATTCAAACCA
AAGGGTGACTCCGTTGACAATGACAACTTTTCGCCTGATGAGGGGAATGTAATATTAGTAAATAATCAGTTCTGGTCGATTTATTAC
AAGCAGTTTCAACCTGATATTCCGATAAAAAATCAAAAAAATAATGTCGAAGTAATTATTCCACAAAAGTTTCATGCAATGCGTAA
TGAAATCAATCAAGCATATCATTCATGGTTTGAATTTGTACAAAATAAAAATAATAAAGAGAATAAGTTATCTATACAGTTTATCAA
CAAAAATGATTATCGAATTTTTTCATTTGATGCACGAGATAGTCGCCATTTGTCATTTATAGAGGCGCCAATCATTGTGAATGTTCAG
GCATCAGATTTATCGAATGATTTTTATTATGCCATGATCAGTCAAGGCGGGTATTTATTCAAAAATTATGACGCGCTAGTAAAAAAT
ATTGAAAATTATCATCTTGATGGGGAAATCAGTGGAATAACCAATTATAAAGATAGCGTGATGGAAATGTATCATGAAAACAATTT
GAAATTAACAGTACTCAACTTTTCACAAATCATTATCGCAATCATTTTAATAATTATTATTTTATTTGATGTGAAATATTATTTTGAA
CAGCATCGAAAATTACTCGTAATCAAAAAGCTATATGGTTATTCAACATTAAGAGCCAATTACCAATACTTATTAATAAATAATATA
GTTGTTGTTTTTATTGGAATATTGACGAATGTAATTTTACATTCTCAGTATATAATGATGATATTTGCAACGATTCTTGTCGTTCAAAT
ATTATTGCAGATTTGCAGCCTATACTATCATGGCCGACGTTTTAATGAAGTTATCAAGGAGTTTTAACTATGAAATTTATCATTGCAA
TATTATTAGGATTAATCGTATCCATTACTATTGCTTTTACAATCATACATCATCCTATACTTGATCGTTTTAATCCTTTCTTAAAAACG
GAGTATAGTTATGCCAAAGTGCCAAAAGGTACGCAACAATATGTTAATATTACGGCTTATAGTGAAAGAGGGGAAAAGCTTGATTA
TAAATTAACATTTAATGGATTTTCACCTAGTAGAACGTATGTCGAAATAAAGCATAAAGGGCAATATGTCATATCGATCACATATGT
TGAAAAAGAGGATATACCAAAAGAGGTAAGACAAGAATGATAGAATTAAAAGATTTAACCATACAAAAAGGTAATATACATATTT
TGAAGAAACTTAATTTGAAATTTCAATGTGGAAAATCATATGCACTTATTGGTAAAAGTGGATGCGGTAAATCTACATTATTAAATA
CTATTGCTGGACTTGAAAAAACGGGGAAACAATATGTCTATTTTAATGGTCAATTAGAACAATTTAAATCTAATTTTTACAGAGATA
AATTAGGATATTTATTTCAAAATTATGGATTAATCGATAATTTGACAGTAAATGAAAATTTAGATATTGGATTAGCATATAAAAAAA
TAAGTAAGAAAGAAAAAGAACAATTAAAGATACGTTATATAGAACAGTTTGGTCTGTCAAACAGTTTAAAAAGAAAAGTTCATACG
CTAAGTGGAGGTGAACAACAACGTGTCGCTTTAATTAGAATGATGTTAAAAGATCCGATTGTTATGTTAGCTGATGAACCAACGGG
TGCGTTAGATCCTAAAACAGGACAGATGATTATTCAATCATTATTTGATTTGGTCGATGAAAATAAAGTGCTGATTTTAGCAACACA
TGATATGGCTATTGCAAATCAATGTGACGAAATAATAGATTTAGAACAGTATAGTAAAGTAGCATCTATGTGATATCCAAAAGATA
AAATTAAAAGCCAATCAATGGGAAATGTTGACTTCAACACTCCGTTTGATTGGCTTTTATAATGTGTTACTAAGTTATGAAAGTAAC
AACGGTAAACTGAAAAAAAGCAATATAATAATATTTAATACGATTAGTAATAAATGTAATTGATTTTTCGTTTTAATATAGCAAAAA
ACAATGACTAAAGATAACAACAATATCATGATTGCACCTGGGAAGAACCATAATGCTGAAGCTGTGTACCAAATCATTGGTAACGC
CATAATAATAAAAGATATAAGACAAGAAAGGATACCAATAATATTATAAGTTCTAGTCAAAATTACACCTCCAATTTTCATTTTAAA
ATGACATGCGTAAAACAATCATTTTAAATACATCATACAATAAAATTAAATTGTGCGGTAGAAGAATTTGAAATTAAATTGAATTG
GCTTGATTAAAAGGTGATTTTATAGAAGTTAATAAAAATGGTTATTTAATAGAAAAACATAATATTAAAGACATGGCTGATAAAAT
ACTAAAGCTTGTTAATAATGATGTTTTAGCAGAGGAGTTTGGTTCGAAAGCGAGAGAAAACATTATAGAAAAATATTCAACGGAAT
CAATATTAGAAAAATGGTTAAATCTTTTCAATAGCTAAATGAAAAAATCCTAGGTATTCATGAGTACCTAGGATTTTTACGCATGAT
ATATACAAAAAAAGAGAGGATAGTCCTCTCAAATAATAGTTAATAAAATTGTAGCCATTTCTTCCAGCTGTAACTACTAATACAAGC
TAAGTTCATTACGATATGTATGATTTCAAATTCATAACCACTTACATATCCTCTTGTAAGTTTAAGTGTGAAGATTGAGCCTAACATA
ACACAAATATAAAAAACATTAACATAAGGCGTTAAAAAGCCGATAATAAGTGCTAAACCTCCAACAACTTCACCAATTGACATCAA
TACTGCGATGGTAGGTGGTAAACCTAAAGAGCCTAAGAAGTGAATTGTTCCATCTAAATCAACAAATTTCAAAGTACCATGCACTA
AGAATGATGTTGCTAACATCCATCTAATTAAAAGCATACCTAATTTCATCATGTCACCTCCAACATTCATATTTTTAAAACATAGTCC
AAATTATATTTGTAAACATGTGGAAAGTAAAGATTTTTTATAAGATTAATTATTAAATATTTATTAAATATTCTATTGTATTGAACAA
TTGTTGTAGAAAGATGTTTAAGTCATCAGTTACTTTTTGTATTTATAAATAAAGATTAGTACAGCATAGTGCAAAGATTATAGGGAA
ATGAATTAACTATTGCATCGCTATTTATGAATCGTAAACACTATTATGAAATTGCGAGAGTAGTATGGATAATTAGAATGATGGTTT
GAGATAAATGAGTATTATTAACCAGTGCGTCATCTTCAAAATTAGAGGCCATTTGCTGTTTATAATAAGAAGATTTGATAATAAACT
ATGATAAGAAAAAAATAATAGCATACCAAATATATCATAAACAAATTAAATGATAAAATAATAATGCAGTTTTCTATTAAAGTTGTTT
GGAGGTAAATATGTGAATAGGAATATCGTTAAATTAGTTGTGTTTATGCTAATCTTAGTTGTAGCAGTAGCGGGTTGTGGTCAAAAA
GATACTGAAGAGAAAACTGAAATGACGACAATAAAAGATGAATTAGGAACTGAAAAAATTAAGAAAAATCCTAAACGTGTTGTTG
TATTAGAATATAGTTTTGCTGATTATTTAGCAGCATTAGATATGAAACCTGTTGGTATTGCAGATGATGGCAGCACTAAAAATATAA
CAAAGTCAGTAAGAGATAAGATTGGGGCATATGAATCGGTTGGATCTAGACCGCAACCGAATATGGAAGTGATAAGTAAATTAAA
ACCGGATTTGATCATTGCAGATGTTAGCAGACATAAGAAAATCAAATCAGAATTGAGCAAAATTGCTCCGACAATCATGTTAGTTA
GCGGTACGGGAGATTATAATGCAAATATTGAAGCATTTAAAACAGTCGCTAAAGCAGTAGGCAAAGAGAAAGAAGGCGAGAAGCG
TCTGGAAAAGCATGATAAAATATTAGCGGAGATTAGAAAGAAAATTGAACAGAGTACGTTAAAATCTGCATTTGCATTCGGTATCT
CAAGAGCAGGTATGTTTATTAATAATGAAGATACATTTATGGGACAATTCTTAATTAAAATGGGTATTCAACCTGAAGTCACAAAA
GACAAAACTACGCATGTTGGTGAACGCAAGGGTGGTCCTTATATATATTTAAATAATGAAGAACTTGCCAATATCAATCCAAAAGT
TATGATTTTAGCCACTGACGGAAAAACGGACAAAAATAGAACGAAATTCATTGATCCTGCAGTTTGGAAATCATTAAAAGCTGTGA
AAGATAACAAAGTTTATGACGTTGACCGAAATAAGTGGTTGAAATCAAGGGGGATTATCGCAAGTGAAAGTATGGCAGAAGATTTA
GAAAAAATTGCAGAAAAAGCAAAATAAAAATACAGCGCTACTCGTAAATCATATAAGAGTGGCGCTGTATTTTTAATTATGTTTAT
TATCTGTCGGATGTGATGATTTACCTGAAAGTTTATTTCGAATAAATTTAATTACATAACCGACAAGGATTGTTTTAACAGTTCTTTT
AATGAATTGGCGCATCGTTACATACCTCATTTCTCTATATCTTACGAACTATATACCCATTCATATATGCTTTTTAAACGTCATTGTC
ACAATTTAATTTTTAGGGAATATAATATAACCATCTTTATCTGCTTTTTTAGTAAAAATGACAAAAATTGCATGTATTATTGAGATGA
TGGTAGGGATACCTGTCCAGAAAAATAATAAGTGAAAAAGACCTTGTCCAAATTTATCAGCATAAAATTTATGAATACCTAAACCT
CCAAGAAATAATGCAACAATAACATAAATGGCTTTATTGACTTTCATTTGTAAATCCTCCTTAACTATAATTCTACTTAAATTCGTTG
TGAAAACCAATATTTCTAACTTTAGAATTTTCAAACTTTCTAAAATTATAAGTATATCTTTTTAAAATAAGCTAGAATTTCTATATAA
TAAATGTTAATAACGTAAAAGGGAATGATGACATAGTGATACGTCAAGCACGTCCAGAGGACCGATTTGATATTGCGAAGTTAGTT
TATATGGTTTGGGATGATATGGAATTAGAATTGGTAAAGCATCTACCTAAAGACATGGTATTAGATGCAATTGAAAAAAGCTGTGTT
GATGCAACATATCGAACTTTTTATCAGCATATTTTAGTTTATGAAGTAGAAAATAAAGTAGCAGGTTGTATTATTAGCTATAGTGGT
GAAAATGAATTGAAATACGAAAAAGCATGGGAACTACTTGACTTGCCAGAAGAAATAAAACAATATGGCACGCCATTACCTGTAA

AAGAAGCTAAAGACGATGAGTATTATATAGAAACAATTGCGACATTTGCAGCATATAGAGGTAGAGGCATCGCGACAAAGTTATTA
ACGTCATTACTTGAATCAAATACACATGTTAAATGGAGTTTGAATTGCGATATTAATAATGAAGCAGCATTAAAGTTATATAAAAAA
GTAGGCTTTATATCTGATGGACAGATTGAATTATACAAGCACATGTATCATCATTTAATTGTTAAATAAAAAAACTCGACAGTTCGA
TGTAAGTCGATTGCCGAGTAGTATCATTTTCTATTAAATGCCTGCAAATAATGCACTAATATAAATACCTAATGCATATAATAAACC
GAAAAATGTATTTGTTTTACCAGCAGCAGCCATTGCTGGCATCATTGTAGGCGGTGTATCATTCTTCTTGAAACGTCTGATAACTTTA
ACAGGCATTGGGAATGATAACAACGCAAGTAAGTAAAATAATGAGCCACCAGGTTTAATAATGATCGTAAGTACAATAAAGGCAT
AAGCGATAAAGTACATGATTGCCATAAATGTTAAAGAAGCATTTTTACCTAATAGAATGGGTAAAGTTTTGCGACCACTTGCTTTAT
CTTTGACACGGTCGCGAATATTGTTAGCCATATTAATTAAACCGATAGTGATTACTATAGGTACACTTAACCAAATTACATAACTTT
GAATATTGCCAGTTTGAATAAAGAATGCAATAACGATAATAAACATACCCATAAATACGCCTGAGAATAATTCACCGAAAGGCGTC
CATGAAATAGGGAAAGGGCCACCTGTATATAGGTAACCAACAGCCATACATACTAATCCAACTGGTAATAACCAAAATGAAGAGTT
AGCAGCTAAAAACAAACCTAATATTGCTGCTAAGATGTAAAATGCAATGGCTAATCGTAGCACAAGCTCTGGGCTCATACCGTTGC
GAACAATGGCACCACCAATGCCTACAGATTCATGATCATCAAGGCCTTTTTTATAATCATAGTATTCATTAAACATATTAGTTGCTG
CTTGAATAAGTAAGCATGCTAGTAACATGGCAATGAATAGGCTGATTTTAATATGATCTTCGCTACCAAGAAAATATATTTTAGATG
CTGCTGTACCAACTAAAACGGGTACTACGGAAGCAGTTAATGTATGAGGACGCATTAAATGCCAATATTTCTTAACTGTAGAATATT
GCTGATATTGATTACTCATTGAATACAAAACCTCTTTAAATCATATTTTTTATAACAAAGATATTTTAATAGAAAGTAGCATAAAGG
TCAATTGACGTGAAAAATTGCAAGATAAGTTTAAGCAATTCTTGTAACACAATTATCCAAAAATGATACAATATAATAATGAGTGA
AAAAAGACGAAGAAAGAAGTGAAATGGATGGCTACGGGCGTATTAGAGGACGATATTGTCAAAGAGATATATGGCAGCTCAAAGG
AATGGGTTTCAGTTGAAGTGAAATTATCACAGTCACTAGACCCGAGCACATTATTTCATCTCACTGACAATGAGGCAGGAGATCGCT
TTTATATGCGTTTGAATGATAATCGAACGTCATATTTTGGCTACAAAGCAATTCAATTATTCAAAAATAATTCTAAAAATAAACAAT
CTATTTTTAAAGACTGGGAAAAATTAAAACATAACATCACATTTATACATCCGCAATCTGAGAAACATCATCTTCGAGTTGTTGGAG
GGTTTCAATTTTCAAGTCATAAATCAGATGATGAATGGCGAGAGTTTGGACTAAATCATTTTGTATTACCTGAAGTTTTAATTTCAAC
TGATAATAATGGGACATTTTTAACTTATACAGTTAAAAGGGAAAGTTTTACTGTTGAGGCATTGAACGATTTAATGGATTTGTTCAA
CAATATATCGGATATAGATGTGGACGAGCAAATTGGGGAAATTACTAGAAATGAAGATATTTATAAAGATGACTGGCGTCAACTTG
TAGTAGAAGCTATAGAATCTATTAATAATGAAGAAAAAAATTGTACTAGCACGTAGACGGTTAATAAAGTTCGATAAAGATATCAGT
ATTCCATATATTCTAAAGCAAGCATATTCTAAAGAAAAAAACAGTTATATATTCTTGTTAGAATCACAAGATTCTATTTTCTTTTCAC
AAACACCTGAACAATTAATAAAGGTCAATAATAAAATACTATCGACTAAAGCTGTAGCAGGTACAATTAAACGTTCACAAGATGAG
GACGAAGATACAAAAAAATGTTGAAGCATTTTTAAAAGATAATAAAAACTTAATCGAACATCGATTTGTTGTTGACAGTATTTTACAT
GATATTAAACCTTATATCACTGAATTACATTATGATAAGACGCCTAAAATTCTAAAAAAATGATCATTTATATCACTTGTACACTGAA
ATAAAGGCGCCACTGAAGGATGATTCGTATATTAGTTTAATTGATCATTTACATCCAACACCTGCTTTAGGTGGCTATCCAAAAGAA
TTTGCGATGGATTTTATTGAACAGAAAGAATTTGGTACACGAGGATTATATGGTGCGCCGGTTGGCTATATAGATATATATGATGAT
TGTGAATTTATTGTTGCAATTCGTTCGATGCTTATTAAGAAAGCACAAGCAACTTTATTTGCTGGGTGTGGCATTGTTAAAGATTCTG
ATCCAGATAGTGAATTGGCAGAAACGAACCTTAAGTTCACACCTATGATGAATGCATTAGGAGTCGATATGAATGGGAAATCATAA
AGCAGCTTTAACGAAGCAAGTTTTTACATTTGCATCTGAGTTATATGCATACGGCGTAAGGGAAGTAGTTATCAGTCCGGGATCACG
CTCAACGCCACTTGCACTTGCATTTGAAGCACATCCAAATATTAAAACATGGATACACCCCGATGAGCGAAGTGCAGCATTTTTTGC
AGTTGGGTTAATTAAAGGCAGTGAAAGACCTGTCGCTATATTATGTACGTCAGGTACAGCAGCAGCGAATTATACGCCTGCAATTG
CTGAAAGCCAAATTAGTAGAATTCCATTAATCGTTTTAACAAGTGACCGTCCGCATGAATTAAGAAGTGTAGGCGCACCACAAGCG
ATTAATCAAGTAAATATGTTTAATAATTATGTAAGTTATGAGTTCGATATGCCTATTGCGGATGATAGTAAAGAGACCATTAATGCA
ATTTATTATCAAATGCAAATTGCTAGTCAATATTTATATGGACCACATAAAGGGCCAATTCATTTTAACTTGCCATTTAGAGATCCGT
TAACACCTGATTTGAATGCAACAGAATTGTTAACTTCTGAGATGAAGATTTTACCGCACTATCAAAAAAGTATAGATGCATCGGCAT
TAAGACACATTTTAAATAAGAAAAAAGGTTTAATTATTGTAGGGGATATGCAGCACCAAGAAGTTGATCAAATACTAACGTATTCA
ACGATATATGATTTGCCTATTTTAGCTGATCCTTTAAGTCATTTAAGAAAATTTGATCATCCGAATGTTATCTGTACATATGATTTGC
TGTTTAGAAGCGGCTTAGACTTAAATGTGGATTTCGTAATTCGTGTTGGGAAACCAGTGATTTCTAAAAAGTTGAATCAATGGTTAA
AGAAAACTGATGCATTTCAAATATTAGTGCAAAACAATGATAAGATTGATGTCTTTCCTATAGCGCCAGATATTTCATATGAGATTT
CTGCAAATGATTTCTTTAGATCATTAATGGAAGACACGACCATCAATCGCGTAAGTTGGTTAGAAAAATGGCAATGCTTAGAGAAA
AAAGGGCGTAAAGAAATTAAATGTTATTTGGAACAAGCTACAGATGAGAGTGCATTCGTTGGTGAATTGATTAAGAAAACATCTGA
AAAAGATGCATTATTTATTAGTAATAGTATGCCTATCAGAGATGTAGATAACTTGTTATTGAATAAAAATATAGATGTCTATGCGAA
TCGTGGTGCGAATGGTATTGATGGTATCGTTTCAACTGCACTGGGTATGGCTGTGCATAAACGAATAACATTATTGATAGGTGATTT
GTCATTTTATCATGATATGAATGGACTATTAATGTCAAAATTAAATAATATTCAGATGAATATTGTATTATTGAACAACGATGGTGG
CGGTATTTTTTCATATTTACCACAAAAAGAAAGTGCAACTGACTATTTTGAACGGTTGTTTGGCACACCGACGGGATTGGATTTCGA
GTATACAGCTAAGTTATATCAATTCGATTTTAAACGTTTTAACAGTGTTTCAGAATTTAAAAATGCCACATTGTTATCTGAAACTTCG
ACGATTTATGAATTGATAACGAATCGCGAAGATAACTTTAAACAGCATCAAATTTTATATCAGAAATTGAGTGAAATGATTCATGAC
ACATTATAAATACTATGAAGCAAACGTTGAGACCAATCAAGTTTTAGTATTACTGCATGGTTTTCTTAGCGACAGTCGTACTTATTAT
AATCACATCGATAAATATACTGATATCTGTCATGTCATCACTATAGACTTACCAGGCCATGGCGAAGATCAGTCTTCAATGGATGAA
ACGTGGAATTTTGATTATATTACGACGTTGTTAGACCGAATTTTAGATAAATATAAAGATAAATCAATAACATTGTTTGGATATTCA
ATGGGTGGGCGTGTTGCATTATATTATGCAATTAATGGTCACATCCCTATATCTAATTTGATATTAGAAAGTACGTCACCAGGTATT
AAAGAAGAAGCAAATCAATTGGAACGCCGTCTTGTTGATGATGCACGTGCTAAAGTATTAGACATAGCAGGTATTGAATTATTTGTT
AATGATTGGGAAAAGTTGCCATTATTTCAATCGCAACTAGAATTACCAGTTGAAATACAACATCAAATAAGACTACAACGATTGTCT
CAATCGCCACAGAAAATGGCCAAAGCATTAAGAGATTATGGTACAGGTCAAATGCCAAACTTATGGCCGCGCCTGAAAGAAATTAA
AGTACCAACATTAATATTAGCTGGAGAATATGATGAAAAATTTGTACAGATTGCGAAAAAAATGGCAAATTTAATTCCTAATAGTA
AATGTAAATTAATTTCTGCTACAGGTCATACAATTCATGTGGAAGATAGTGATGAATTTGATACAATGATATTAGGATTTTTAAAGG
AGGAGCAAAATGACTAACAGACAATGGGAAACACTTAGAGAATATGATGAAATCAAATATGAATTTTACGAAGGGATTGCTAAGG
TAACAATAAATCGCCCTGAAGTACGCAATGCGTTTACACCTAAAACAGTTGCTGAAATGATTGACGCATTTTCACGTGCACGTGATG
ATCAAAACGTTTCAGTTATCGTATTAACTGGTGAAGGTGATTTAGCATTCTGTTCTGGTGGTGACCAGAAGAAACGTGGACATGGTG
GTTATGTAGGTGAAGACCAAATCCCTCGCTTAAATGTATTAGATTTACAGCGTTTAATTCGTATTATTCCAAAACCGGTTATCGCGAT
GGTAAAAGGTTATGCTGTAGGTGGCGGTAATGTACTAAATGTTGTTTGTGACTTAACGATTGCTGCTGATAATGCTATTTTTGGACA
AACTGGTCCTAAAGTAGGTTCATTTGATGCAGGTTATGGTTCAGGATATTTAGCACGTATCGTTGGACATAAGAAAGCACGTGAAAT
CTGGTACTTATGTCGTCAATACAATGCACAAGAAGCTTTAGATATGGGCCTAGTAAATACAGTGGTACCTTTAGATAAAGTTGAAGA
TGAAACTGTGCAATGGTGTAAAGAGATTATGAAACACTCACCAACAGCTTTACGATTCCTTAAAGCAGCTATGAATGCTGACACAG
ATGGTTTAGCTGGTTTACAACAAATGGCTGGGGATGCAACATTGCTTTATTACACAACTGATGAAGCGAAAGAAGGCCGTGATGCG
TTTAAAGAAAAACGTGATCCTGACTTCGATCAATTCCCTAAATTCCCATAAGTTATTTTGAATATGATATTAAGTCACTTGCGTCGTT
TATTAGCGACGATAGGTGGCTTTTTTATTTTATAAGAATTTAAAAGAGAATTTTAATTAATTATCATAAAGATAAGATGGTTTGAGT

AAATTAATAGAGAAAGAATAAATGTTAAAGATTATTATTACAATGAAAAAACGAGCTGGTGAGAAATTGAATTCTCGCCAACCCGC
TTTATGAAGTGAAATCATTTAAATTAGTTATACTAAGCGCTCATAAACGATTGGGCGCGAAGTGCCAATACCTTGTTTGGATGACGT
TTGCATGAGGATATGCTGTGTATCATCATAATCAAGAATATAAGTGATTTCTTCATTATCAATAGATGAAAATTTAATGCGTTGATG
TGCCGTATCTATAAAAAGAATATGATATTGATTGTGACTTGTATCATCGCCATGACGTATACAAATTGATTTTTGTAATTGATGATTA
CTCCAATTACCAATGAATAAATTGATATTGGTTTGTTCTAGATGTGTGAGTTTGGTTGTGTCGTAAACTAAATTTATAAATTGTAGTT
GATACATAGTTTTACCTGCCTTTAATCAATATCTATATTACTTTTTAGTAACCTATCATTGAACCATACCAGTTATAATCACGATTGA
ATGATAAATGTAATAGGCTTGAATCTGCATCTTGGATTGATAATTCTGTATCCCAAGGATTCCAGTAAATAAGTTTTTCTTGGTCATT
AATTTTAGCATTACCAACAACTGCTAGGGCATGTCCTAAATGTGGATCATTAGGGTTTTGAGATACACTTTGTGCAAGAATCATAAT
TCCTACATTATCTTTTGTAAGTTGATCAACTTGTTCATATGATGGTACGCCTTCTTGATAATGAATATCTCTGCCTTGTGATTTACCGT
ATTCAATCATTTGATTAGGGAATGTTGAGCAATTAGGAAGGTCTTGCTCACTTACTTCAGGGTATAATGTACGCATAATATCATGTG
CATTATAAGTGTCTGTATTTTTAGTTGCATTTAATAATGCTGCCATACTAAATCCTGCACACCATGAGTTATCGAATTGTTGTTCTCT
AATTTTGAAGTTTTTAATGTATTTTCATATTGAACTTGATCTTCTTGTATCGCTTCGTTTTCTTCAACTTTAGTAGGTGTTACTGTATT
TTTTAACTCTTGTTTCAATTTTGCTGAAACTGTTTTAGCACTTTCTTTTTCTTTTACATTACCAGGTAGTGGCGTAGCTTTTACTAATCT
AACTTTGCCATCTTCTTCAAAATAAAACCCTTTTTCATCAGTAAGAACAGTGATATTTGAATTTTTATCTTTAATTTGGTCTAAATCTT
TAGCGATGAAGTTTGAAATTTTAACACTGTAATTCATGTCTTCTTTTGATTTGTTTAAATCATCTTTATTTTTAGGGCTAAGTGTTAAA
GTATAAACAATTTTACCGTCTTTTATAACTGGATAATAGTAACTATTATCTTCTTCACCATTAAATTTATAAATTTTAAAAGCTTCGC
CAAGTTCATATTTACCAGTTTTTGCATTACTTTGTTTATCTAATGCTTTAGCATAACCAGCAAATTGTTGTTGTGCTAGATCTTTTACT
TTTTGAGGTACTTTGTCACTCTTAACATTAATTTCTAGCTGTTTAGAGTGTGAATCGGCTTTGGCTTTATTGTTATTAGCAAATGCGCC
TAGTGATAAAATAAGCATTGAAACCATTATGATGCTTATAATATTGAATACTCTAGATTTATATGAACTATTCATATAATACCCTCC
ATTTAAATCAAAATATTTTGCGTTGCTAAGACTTTGCTTCTATTTACTAAATCTAAATTAAGATGAAGTTTATGCAGCGTCAGGGTTG
TCTGAATTATTGTTATCGCCATTGTCTGGATTATCAGGGTTGTTAGGGTTGTCAGGGTTATTTGGTTCATCTGGGTTGTTAGGGTTAT
CAGGATTGTTAGGGTTGTCAGGGTTATTTGGTTCATCTGGGTTGTTAGGGTTATCAGGATTGTTAGGGTTATCAGGATTGTTAGGGTT
ATCAGGATTATCTGGGTTATTAGGTTGGTCATCGTTGGCAAAATGGATATCTTCAATATTTTGTTTTAAGAAGTTGCGTACATTTTCA
TTAATAAATACCGCACCATTAAATTCATTTGGTACACCGCCCCAATGAATTCCGATCACTTCATTTTTTTCATTAAATACAGGTGAAC
CTGAGTTACCACCAGTTGTACTTAAATCATATTGCATAGCTTCGCCTTTGAGGTAAGTGATTTTTCCTTTACTTTCCCACATTGTTGCT
ACAGGTTTATCACCAGGATATCCTGTTACAGTAATATTTTGGTTAACTTGTGTTTCAGCATTATTACTCATTGTTGCTGGTTTAACTAC
TTCACCAATATGTTTGTTTTGCTCATTAGGGGAGAATTTAACTATTGCTAAATCACCTTCGCCTGAATATTTAGTGATTTGTTCAGCA
GTGAAACCACCATTTGGATAATTGTCTTGGTTAATTGCAGAAGGGAATGCTTTTAAAGCATGAGGATCACCGTGCGTAGCATCTACG
ACGTGTTTATTTGTTAAAAGAGTATCTTTACCTACAACTACACCGGAAGCAATAAATGTACCAGTAGGTGCTTCAACTTGAATATAA
GTTACGGGTGCATAATGACCATTCGTTGTATCTGTGATTTGGTGACGATCGTTATTCGGTAATATAACATTTGCGTGTTCACGTTGTT
CTAATGGTTTAAGGTTACCGCCTTTTTTAATCTTAGGTGTTTGCTGTTTGCTTGACTGCGTTTGTTGTGGATGATTGTCCATAGCCTTT
GATGATAACGCGTTTGCTGCTGGAGAACTCACAAGTGTCGCTGTTGTCAAAGTTGCAACGAATAAAGAACTAACTTTTAAAAATTTA
CCTTTCATCTAAAAACCTCCAAAAAATTTATTTACAAGTTAAATATAACACTAAAAATTTTTAAGTCAATAAGAATATATAAATAAA
TTAAAAAGTTTTTGTAAAGATTAGAGTTTACAAAAACATTAAGGGAATTAAAATTGAATTGTATTATACTTTATTCAATTGAGTTAA
ATTGATAAGATCGTCAATTGTTTGCATTTATCTAAAAGAATAAATAAGAAAGGAATTGAGAGCGAATGGAAAGTATTTGTTTATAAT
AATAAATAGTAACTTTTGAATAATAATTGTTTGGCCAAAGGTAGATTAATATGAATGGTGATATATTATTTGAGTGCTTAAGTTTCG
AAGAACAATTATAAGTTAGCATATTAAAACCTTTTGATAGAATCATTTTGAGTGAGGTGTTTAAAGGAGAGGAATATAACTCGGTA
GTATTTACAGTCTCTATAGAGTATTATTTAAAACTTATAGAATATAAAAAACTGAACGTACTATGAATTTAGTTCACGACGTCCAGT
TCAACATATCATTTTTATTTAGTATTTAAATATTTAGCAAGTCTCTTCATACCTTCTTTTAAAACATCCATTTCATAAGCATAGGAAAT
CCTTACAAATCCTTTACCGAATTCTGTAAAGGATGAGCCTGGAACGATTGCTAAATGTGTTGATTCAAGTAAATCGACACAAAATTC
GAAATCGTCATCGGTGATATGTTTAATACTTGGGAAAATATAAAACGCGCCTTCAGGTTGAGCGGTAATCTCAAAACCTAATTTAGT
TAATTCAGATACTAAATAATTTCTTCGTTCTACATAAGCTTCGTTCATATATTTAGGAGCTTCTAATCCTTCGTTAAGTGCTGTTATAC
ATGCTATTTGAGCTGGAACATTGGCACAAATACAATTATAGGCGTGCATAAATGTTAATTTATCAATCAAATATTGAGGCCCTAATA
GAAAACCAATTCTTATTCCGGTTGCTGAGTGTGATTTACTTAAACCACCAATTAATATTAATTGATCACGAATGTCTTCAAATTCAGC
GAAGGATACATGTTTACCACTAAATGTATTTTCAGCATAAATCTCATCGCTAATAATAAATATCGGATATTTTTTTAATACATTTACG
ATATTTAAAACTTCATTTCTTTTTAAAACTACGCCAGTTGGATTAGTCGGATAATTTAACAAGACAGCTTTTGTCTTTGGAGAAATAT
GACTTTCTAATGCATCAGGTGTAATTTTGAATTGTGTTGCTGTTGTATCAATATAAATTGGTTTACCACCTAGTACTTCGATGAGTGG
TATGTAGCCTGCATAAATTGGTCCCGGTATTATAATTTCATCTCCAGGCTCTATGATAGAACGTAACGTTGTGTCTATTGCTTCACTT
GCTCCATTTGTCACAATAATTTCTTCAGGATCATAGGAAAAATGATAACGATTTTTGAAGTATTGACTAATTGCTTCGCGAGTTTCTA
ATAACCCTTTATTGTGAGAGTATGATGTCTTGTCATTGTTAATAGCATCAATATATGCCTTTTTCACAACATCAGGCATCGGGAAGTC
TGGTTGGCCAATAGTTAAATTAACACAATCATCCAAATTATTCATACGATTTGAAAATTGGCGAATACTTGGTGCTCTTAAATATTT
AGAATTAGAATTTAAAGAAAGTTTCATTTGTGACACCTCAAATATAAATCAAATATTGTCTAAAAATTTAGAAATTAATAATATCAT
AACATACTTTTAAACCAAAAAAGGGTAAATTACTTAAGCTTTATTTGAAAAACGAAAAATGTCTAAATACACTGTAGTAACTACTTT
TCAAAATGAATAAAGTGGTTACAGTTAATGTACTTAGACACTATAAAATTATGAATCTTTAAACTGTGATGGTCTTTGTAAATTAAT
TATTGGATTTGTCCATTTACAAACAAAGTTTGTAGATAATACATATACGATGATTACAGATATACTTATTAAATAAAGATAAGTCAT
TAATGATATAGGATTATCGAATGGGTACCATTCAAATCCTCTAACAATGCCAATAATTAAACCATGTAATAAATATACGTATAGCGT
ACGACTACCAATATAAGTATATAATTTTTTCTTTGTTGACATTAAATTTAGAAACGCAGTCATTGCGATTAATATAATTCCATATAAT
ATAAGTCGTTTAAAAGGACTGAATATACTCTGTCCTTCATTTTCAAGTGAAGTATATGGTGAACTTCCCAATAACCAATCTGCATTG
ATAGGATGAATCACGTAAATGATAAAAAACAAAATAAAGGTAATGATAGATACTGGTATTAGTTTTTTATTTTTAAAAATAGCCGT
ATGTTTTTTGGTGAAAATGTAACCTAGGTAAAATATTGGGAAAAATACGATTGTCCTTGAAATGCTCAAGTAGCTATCGATGTTATC
TGAAAAACCTGCTCCAATAGATATAATAATTGAAACTGATAGCACTTTATATGGATTAAATCTTCTAACTATTACTAAAATGACATG
AAAGAAAAATAGCGTGATCAAAAACCATAACGCAAATACTGGGTTAAAAGGATCAAGTTGTAATTCGTCACTTTTACCTGTTAAGA
AATAATATATTGAAAAGAATGCAAAAAATATCATATAAGGTACTATCAAACGTTTTGAAATTTTTTCTAAATAGTATGGTTTATCAA
TATTTTTCGCGAAATAACCAGATATAAACAAAAATGTTGGCATATGGAAACTATAAATAACTAGGTATAATGCTGATAAATATTTAT
CGCCACTAGTGTAGGGTTGTAACATATGTCCAAATACGACTAATAATATTAAAATTGCTCTTGCGTTGTCAAAAAAATAATCTCTCT
CTTTTAATGAAGTCATATTTTATCTCCTTTATGTCCATAAGACTCTTACTAATTTATTATATTTTGTATTAGTTATGCAACAAATTTGT
CTATTGTGTCGAATAGTATTGTAAAAATTATAAAAAATCATATAATATTATAAATAATATGTAGTGGAGTAGAACAACATGTATAAA
CAACTTGAAAAACTTATTACACTGACTAACAATGACTTAAACTTAGTGAATAGAAGATTTGGACAACGCACGGATATCACATCTGA
ACAGCTAGAACTTCTCCGTATTTTATTTAATTACGATCGCTTATCACAGTATGATTTGACGATGAAGATTAGCAGGGAACAATCTAT
AGTTTCAAGGTGGATTAAGAAATTAGTTTTGAAAGGTTACATCACAAGTCAACAATCTAGCGAAGATTTAAGATGTAAAGAATTAA
TTTTGACTGATCAAGCACGTACATTAATTTCACAAATAAATAATGCACGTTGCGAATTGATTGAAGCAAGATGTCAATGTTTATCGG

AAGTCGAATTAGACAATTTAAATCAATTACTTGATAAGTTAAATCAACGACGCATATCGTTGTAACACGACAATAGCAAACACATA
ATTTAGTGTCATTGTGCACACAACAAAAATTGAATCATGAATTACAAGCAAAAGTAGCGGTGATTGTTAAAATTGATGGTAAACAA
TCACCGCTATTTTTGCTTGTGTATGTATAAAAAAGGGATCAAAGGTCATCCCCCATGATTGATAGTGGGGGATGACTTTTGATCCTA
TGTTCATGTTGCTTATTTATATTGTGGGATGTCGAAGTATTTGCCGACTTCGCCAATTTTATCATAGTAGCCTTTGATGATTTTAGCAT
TGATGTTAGCCCAATCTACATCTGTAGCATATTGGTGTGTTCCTGGATGTGCAGGATTCCATCTCATTTTGTAAAGTGTATTTTGACC
AGCTTTTACATATGAGTTGCCGATGAATTTAGCACCACCAACGATTGCTTTTGATACTGTGTCCCAACCAGCTTGTTTAGCATATTTA
ATACCTTCACGTAAAGGATCGTTATCATATGCAGCAATACCAAATACGTTATGGTATTTCGTGTTTGAGTTAGTTACAACTTTGTTGT
TCACTACATCTGCACCTTTCGCTAATTGAGAAGTACCGTTACCTGTTTCTAATAGGGCATGTGAGATAAGATAAACTTCATTAATGC
CATACATTTGAGCAGCTTTGTTAAATGCAGCACCTTGGTTTTCTAATACACCTTTACCTTTTAAGAATTGATTAATTTTATCAATAGA
AATATTTTGTGGTTGGTCTAAGCGTAAGAATTGATATTTTAATGCTGGATCTTGAGCTAAACGCTTCGTATCCATTGCATGCTTAACA
TCATTAAAGTTAGCATCTGTCCACTTACCTGGTACACGTTGTACTTGTGGTTTATATTGTAAACCAGCTTGTATTTGAGCAACTTGGT
TTAATGTCATACCTGTTTGATTATACTTAATTAATTCTTTAGCTAAATCAGTTGATTTAATCCATGCTAATTTACCGTTAGATAATTTA
CCATAGTACCAAGTTTGTCCATTAATGACTTGTTCTTTAACAACTGCGAATGGTTGTTCATTAAATGCTTTTAATGAGTATTTAGCTG
TATCAGAATTTGGTGTTACATAGTAATAACCATTACCATTTTTAATTACATAAGTGTAGTTATAATCTTTGGCAGCTGATGTAGTTGG
TTTCACAGCAGTTGGTGCAGTTAAATCTTTTGCATTTACCCAACCAGTGCGGTTATTAATAGTACCGTATAAATAAACATCTTTGCCT
ACAGATACTTGTTTCGTTGCATTAAATGTACCTTGAGCAATGTTATTGCCTGTTAAAATGACTTGGTTTTTAGTACCCCAAGGAACCA
TTGATAAGCCGTTATTTGATTTATTAACAGTATATTTTTGAGTCGTTTTAACTTCTTTGCCTAAGTTTTGAACATTTAAGTCTTTTACA
TTGAACCAACCTAATGGGATGTTATGGCTTGTATTGTTTAATAATACATACGTTTCATTACCATGAGCACGCTCTTTTGTTACATAGA
ACGTACGGTCTGCATATTTCGCACCGTTTTTCGCTGTTTTTTCATAAACAGAAGCACGAATACCAGTGTTGTTTGGTTTAACTTGAGC
AATCTTGCTAACTGTTTGAGTCGTTTGTGGTTTAGTAACAGTATAAGCTTTTACAGCTGTTTTTGGTTGTGCTACTGCTTTTTTAGGTG
CAGCAGGTACAGCTAAATATGCTTTACTTACCCAACCAGATTTACCATTTACAGTTCCAAATAAATAGATAGATTTATCAATTTGTT
GTTGCTTAGTCGCTTTAAAAGTTTGGTTACCTGTACCAGAAACTGCACCAGCTTCTTGTTTATAAGTGCCCCAAGGTACTGAATATAA
TTTAGTGCCTGGTTTTACTGTATATGTTTGCATTACATTTACAGGTGATTTTGCATTGTTATAAATAACGTCACCTTGTTTAACCCAAC
CAATTAAAGTTGGACTATTGTAATCTTTAACTAAGTAGAATTTGTTTCCACCTAAACTTGCTTCTTTTGTTACAGCAAATGTTTTTTGA
ACTTCTTTCGTTGGCTTACCAGTTTTGTCATAAACTGTAGTGAATAAGCCATTGTTTTTAGCATTAATTTGAGCAACACCGTTTAATG
ATGAAACTGTTAATTTATTATTTGTTGTAGGTGTTGATGGCTTAGGTATTGGTGTAGGCGTAGGTTTAGCAGTATCAACTAAATATGC
TTTACTTACCCAACCAGATTTACCATTCACAGAGCCATATAAATAAATTGATTTATCAATTTGTTGTTGCTTTGAAGCCTTAAATGTT
TGGTTTCCAGAGCCAGACACGCTACCAGCAACTTGTTTAGATGTACCCCAAGGTACTGTATAAAGTTTCGTACCAGATTTGATTGAA
TATGATTGATTTACATTTACAGGTGATTTAGCTGTGTTGTAAACCACATCGCCTTCTTTAACCCAACCAAATTTATTACCAGAATTGT
AATCTTGAACAAGATAGAATTTTTGATTACCTAATGTAGCTGTTTTAGATACAGCAAATGTTTTTTGAACTTCATTAGTTGCTTTACC
AGTTTTGTCGTAAACAGTAGTATATAAACCACTATTTGTTGGTTTGATTTGTGCGACACCATTGTTTGCTGCAACTGTTAATTTACCA
GTTGATGGTTTCGACGGTGTTGTTGGTTTTGATGGTGTAGTAGGGGTAGTTGTAAATTGCGTACCCCATGGCGCCACTTTACCCATTT
TTATTAAATATTTTTCATTAATTAAGTCATATAATTGATCATAACTATAATTATGACTTCTTAAATATCCATGTGGATCGGCATGGTC
CGTACCACCTAAATATTTACTTACAGCGTAGTGAGTCCATACTGTACCATTTCCATCATACTCAGCACTGTCTGGTTTTAAACCATAA
TATTGTAATTGTGTAGCTGCATAGTCAGCATAGTTATTCATTGAACGTGCAAATGAAGCATAGTCGTGTGTGTGTACGATTTCAACA
TTGATGAATCTAGGGTTACCGACTGCACCGACACCCCAAGATAAGTAATCCGTTGGTGCTGTTTCGATTATACGATCCCCATCAACA
AATGCATGTACGAATGCGTTTTGATAGTTATTTTTCATATAACTAATTTCACCATTTATCGTCGAACGATCATTAGCTGTATCATGAA
CTACGATACCTTCAGGACGACCTACGCCGTTACGGTATGCGTATTTAGGGAAGTAAGATGTATAATCTTCTTCAATTTTAGGTGCTTT
TAAGTTATTTTTACGAATGTAATCGTTAATTGAAGAGTTTACTTGTGGTTTATATTTTGGCAAACTCGTTTTTGGTGTTGCAGCAACT
GATCTTGGTTGTGCTGAAGCGCTAAAAGTAGTTACTTTAGGTGTCGCTTCAGTTTTAGCTTTAGGTGCTGCGGCTTTATATTGCGTTT
CAAGAGCTGCAGGTTTAGCAGCTGATTTAATTAATTCTGGATTAATTTGATGTTCTGAATTATCATCTTCATCATCAACTAAACTATA
ACCAGCATTTGTAACATTAGTGTTAGTTTTAGGTGCTGTAGTGCTTGTTGACTTTGCAACAGGCTGCGTATTATTTGTAGTCGCTGAT
TGATTAGCACGAGTGTCACCATTTACTTGTGCAGTATCAACTTTTTGACTTACTTGAGCATTGCCTGTTTTGTTATTTGCTGTTTTTGG
TTGGACAATAGCAGGGTCTTGATATACTTGAGTGCCAGAAATGTTTTGCGTTGGATTTTTTACCTCAGCTTTTGCTTGTTCAGTAGTT
GCTTTAACTTTATTACTATCTAAAACATTTTTATTAGTAGTTTGATCTTGTGTCGTCTCAGCTGCTTGAACTTGATGTGCAGTGACTGC
TGAACCTACAAGCGTTAATGCAACCATTGATGGTAGTTTGTAATTGAATTTTTTCGCCATTCTATTTATTACTCCTAACATTTATTAAT
TATTACTAACATTATAGTACCTGTTTTATATACCTGTGCGTATTTAACCAATTTGTAATATACTCTTAATTTACATTTTCACTGTAAAA
AAATGAAAAAAATTTTGAATCTCAATGGTGACGTGACAAATCAAATAGAATTGATGTCAATTTAAGAATTGGTTTATTACAAATGTT
TCTAAAATAGATACAACTTTTTTTAATTAAGCGAGGTTAACTTTTTTGTCATTTCAATATGCTCGATGCCTTCCTCAAGAAATATATT
ACCTCTCATTTTAAAGTTTAAACTTTCATAAAATGGGATAGCATGACATTGGGCATTCATAGTAGCTACGTAAAAACCTTCATCTTTA
GCTAATGATTCTACAGCTTGCATAAGCATTCTACCCATTCCTTGTCCACGATGTGATTTCATCACAGCTACTCGTTCTATTTTGACAG
TTGTTTCATTAATAGGGCGTATTCGAGCAGTGGCAACTGGCTGTCCATTATCATATCCAATGAGGTGAATAGATTCAGATTCATATT
CATCAATTTCACTTTCCTCAGGGACGCCTTGTTCTTCTACAAACACTTTCTTTCTTATATAGAAGCAATCTTCTAACATCTTTTGATTG
TTTACTTTTGAAAACATAAAATTCCTCCACATCCTAACAAAATCATTTACATTTAATTTACTAATAATAACATAGAATAACGATTGTT
ATTTTCCTCAAAAATAATTAGACATAAAAAAGCTATAAAGATGATTCACAAGAATCATCTTTATAGCTAATTAATTATTAATGTTAT
AGTGCTTCTTTTCTAATAATTGCTGTATATTGCCAAAAGATTCGCATTTGAGCAATATTCCAATTATTCATTCCCATTGTAAATCCTG
AAGGCTTAAATATATTCACATCAGTTACTTCTAATGCTGCTGCAACTAAATATTGTAATGGAATGCTTAGTGATTTCATTGTTTCGGT
AATGCCATTTTCATCATATACCCATGAAAATGGTAACTCTGATTCGAAAACATCAACACTATTGAAAATTTCAGGTAGGGCTAAAAT
ATAACATGCACCATCTACAATAGGATTGTCGATACTATTTTTGTAATAAATTAGGAGTGCTTCATAGTTTTCTCGATGCTCATGATTG
ACGTAAAAAAATTCACTTCTAAAATGAGCCATATCTTGGCTATGAATGATTTGTTGTTCTAACATATTAAACATGCCATTTATATTTG
CAATCTTTTCGTATGTTTTTCTACTCATGTATGATGCTCCTTTCATAAATAGTAAATGTTAATCGTCATATAATATTAATTTACAACAC
CATTTTGGTTATTTGAAGCTTGTGGCGCTTGTTGTGTGCCACCTTGATTTTGATTTAAGTTTTGATCTGTAGCAGGTTGTTGTTGATTG
CTGGAATCACTGTTATTAGTTGAATCATTGTTCTCGTTAGATGTCTTATCTTTATCTGTCGTATCATTTTGTTCTTTTCTCAATAAACT
ACTATCTAAAGGCGTTAACGGTATTAATGAACCATAATGATTAATGACACGTTGATCTAAGAAATCATTTTTATCATTAATAGGTGA
TAATTCTAAGTCTTTACGAAGTAAGTTTGCATATTTTTGAATGCTTTCAACACTTGGATGATAATAGTAAATACCATTTAACATATCA
TCTTTACCTTTTAATTGCGCAGTTTTAATTTCAACATCATTTGTTAAGTACATTTTTGCTAAAGCTTTAATTTCAGAGTTAGTTAAATT
ATGCTTTGCATTTTTGCCTACAATTTGAATCACGTTATCAAGTTTATCAATAGAATCAACTTCCTGTGCTTTTTGGAATAAAATCTTA
ATTAATTCCATTTGACGTTGTCCACGTTTTAAGTCTGAATCATGATGTCTAGTTCTAGCAACTGCTAAAGCCTCATCACCATTTAATT
TTTGGTACCCTTTTTTAATTTTAATCTTACCAGTATCATCTGTGTTAGGTTCATTTAAGTCGTATGGCACATCATAGTATATGCCACCA
AGCTCATTTACAGCCTCGACAAATGCTTTCATATTTACTCTCACATAATAATCAACAGGTACATTCATGGTAGCTTCTACCGAATCCA
TTGCGGCAATTGGACCACCATATGCATGTGCATGGGTAATCTTATCGTAATAGCCAACTTTAGGAATGTAGCTGATAGTATCACGTG

GAATACTAAGCATTCTAATTTGATGTTTTGATTGATTAAAAGTAGTTAAAATCATAGCGTCTGATCTAGAGTGTTCAGCATCCTGTCC
TTTTTTTCTTCTTCCATCGTTATCATCGATACCTAAGAAAAGAATAGAGATAGGTTGTTCTTCAGGATTGACTTTATTATCTCTTAAGT
TGGATTGACGATTAGCATTTTTGCTGTCCTGAGAAGATTCGAATGCATCTTGGGACGTTTTAAAAAGTAACGTAGCGAAGACTATTG
GAACAACAATGAGAACCAATGCTAGAAGGATCAAAAAGTATTTTAAAAATTTATTCATGATTGATGCTCCTATATTAAATTTTTGTT
AAATCATAATCATAATTTTAAAATATAATTAGTTGAACGATATTTATACAACTTCATTACTAATACATTATAGCCTTAAATTGTAGTA
CTTTATAGTAAAAAATACAAATGTTATGTCAAAGTTCCTTGTAATTTGAATGTTTTGTTAAAATCAAAAAGCATTTTTAAAAATATA
ATGCGACTTTAGTATGATGTCTTAAAAAATTTAAAGGCGTAATATAAATTCGAGTGATTTATACAAAGTGATAAGGTATAATTAATT
TTGTTGTATAGATAACATATTAGAAAAACTTATTACTATGTAGCGTAAAGCATAAATGAATGATAAGTTAATTTTTGTAAGTTGTAT
TATTAGGCATCTAGGTTCAAATTTTTAACGATTTAAATATATAAAATGAATACATTGTGAGGCAATTATCAAATGAAATTTAATAAA
GTAAAACTAGTTATACATGCGTGTGTACTATTATTTATCATTATTTCTATAGCGTTAATTTTTCATCGATTACAGACGAAGACACATT
CTATAGACCCAATACATAAGGAAACAAAATTATCAGACAATGAAAAATATTTAGTGGATCGTAATAAGGAAAAGGTTGCGCCGTCT
AAACTAAAAGAGGTATATAATAGCAAGGATCCTAAATATAAGAAAATTGACAAGTATTTACAAAGTTCATTATTTAACGGTTCAGT
AGCTATATATGAAAATGGCAAATTGAAAATGAGTAAAGGTTATGGATATCAAGATTTTGAAAAAGGTATTAAAAACACACCGAATA
CGATGTTTTTAATAGGTTCAGCTCAAAAATTTTCAACAGGGTTACTGTTAAAACAGTTAGAAGAAGAACATAAAATAAATATCAAT
GATCCAGTAAGTAAATACCTTCCATGGTTTAAAACATCTAAGCCTATCCCATTGAAAGATTTAATGTTGCATCAAAGTGGATTATAT
AAATATAAATCCTCAAAAGATTATAAAAATTTAGATCAAGCAGTTAAAGCGATTCAAAAACGTGGTATTGATCCTAAGAAATACAA
AAAGCATATGTATAACGATGGGAATTATTTAGTACTTGCGAAAGTAATTGAAGAAGTTACAGGTAAATCTTATGCTGAGAATTATTA
TACAAAAATAGGAGATCCTTTAAAACTTCAGCACACAGCATTTTATGATGAACAACCTTTTAAAAAATATCTAGCAAAAGGTTATGC
TTATAATAGTACAGGACTTTCATTCTTAAGACCTAATATTTTGGACCAATACTATGGTGCAGGTAATTTATATATGACACCAACAGA
TATGGGTAAATTAATTACTCAAATACAACAATATAAATTATTCAGTCCTAAAATAACCAATCCATTATTACATGAGTTTGGTACGAA
ACAGTATCCAGATGAATATCGATATGGTTTCTATGCTAAGCCAACATTGAATAGACTTAACGGGGGATTCTTTGGACAAGTCTTTAC
TGTTTACTATAATGATAAGTATGTAGTTGTACTTGCATTAAATGTAAAAGGAAACAATGAAGTTCGAATCAAACATATTTATAATGA
TATTTTAAAACAAAATAAACCTTACAATACGAAGGGTGTTATTGTTCAATAATTAATATAGAAGATATAACATGTATATGGCATTAA
GGCATCGACCTTATCTGACCAGTATACGAGTTATATCTTCTTTTTTATAGTGGTAAAAAGTTTAAAGTATAAGGTTGAAGAAGGATG
AGTTTAAAAATATGTGTTAACTGATAAAAGGGGAAATCATTTGGTGAGTTGGCATCAGACTAAAATGAATGAAGACGAATACGTTG
GTCCATGCGTGGTGTGAATGTTTTCTAAATCTAACTGGAAATGATAATTTAATAATGTACACTTTTGATTGTTTAAACATGTACAATT
TTTAAAAACCTAGGGATAATTTTATTGACGTGAACAACATTGAGATTATCTTTAGACATAAATATGGATTTTGAAGTTACTATATTTA
TTAGTTGTGGTTGTTAATGTATTAATTGAATTGATTATAATGATTGTCTTATAAGATATAGAAGGGGACTTAACCATTTAAAAATTGA
AGGAAAATAAAAACCAGCTAACAAAAGTGTTAGCTGGTTAAACTTTACATTATTAAAATGTTGTTTATAAGTGTGAAGAGTGACCG
CCTTGCATAACCCAATATGTTCCGACAACGAAACAAAGTGTAATTACAAGAGCAAAGATAACTTTGAATGTTTGTAAACGTCCATCT
TTACCTTCAGTTAAATGCATGAACATTAATAATTGAAGTCCTGCTTGGACGAATGCAAAGCCAAAGATAATTGTCAACTTCGCGTGG
AATGTTAATGACGTGTATAGTGTTACGTATACTGCTAAAAGCGTTAATACGATAGATGCGATAAATCCTACAGTATGTTTCATTATT
GTACTCATCCGCTATACACCATCCCTATCATATATACGGCAGTAAAGATGAAAACCCAAACAACATCTAAGAAGTGCCAGTATAAA
CTTACTATAAATAATTTTGGCGCATTATATTTGTCTAATCCGCGTCGTTGGATTTGGATTAATAAACAAATGGCCCAAACGATACCTA
GCGATACGTGACAACCATGCGTTCCTAATAGGATAAAGAAACTAGACCAGTAAGAACCAATTGTTGGGTTAACGCCTTCTGATGCA
TAGTGTGCGAATTCATAAATTTCGAATCCAACAAAGACTAAACCTAAAAGTAACGTAATGATCATCCAAAACATCATTAACTTTTGT
TTTTCTTGGCGCATGTAGTAAATAGCAATACCACATGTGTAAGAACTGAATAATAATGCAAACGTCATTATTAAAACAAGAGGCAA
TTCAAATAACTCAGTAGTCATTTTACCTGCATAATCGCCACCATGTTGCAAAGTTAATAGTGTCGCAAATAGGGTACCGAATAACGC
AAATTCGGCTGTAATGAAAATCCAAAAGCCAAGCTTATTTAATTCGCCTTCATGTGTGCGTGAATCAATAGTGTTTGTATCATGACT
CATGACTTACAGCCTCCCTTTCTTTAATACGCGCTTCTCTTAATCTAGCTTCAGTTTCAGCAACTTCAGCAGCAGGGATATGATATCC
GTGATCGATTTGGAAACTGCGATAAATCATAGTACCAAAAATACCGAATAAACAAATTAATGCTGGAATTACAGTTTCGAAAATTA
AGAAGAAACCGCCGATAGTCATAAAGATACCAATCCAGAATCCAACAGGAGTATTGTTTGGCATATGAATGTCTTTGTAATTATGG
TTGTCTAAGTAATGACGACCATGTTCTTTCATATCAACAAATGTGTCGTAGTCATTCCAATCTGGTGTAATGGCAAAGTTGTATTTAG
GTGGAATTGCTGATGCAGTAGTCCACTCTAGAGTACGACCAAGGCCATCCCAGTTATCTCCAGTTGCTTCACGTGGAGATTTGAAGT
GACTGTATACGATACTAACAACAAGGAATAAGAATCCGATTGCCATTAATAATGCACCGATAGTTGAAATTAAGTTTAATAAGAAC
CAACCATCTGATGGCATATAAGTGTATAAACGACGTGGCATACCATCTAATCCAAGAATGAATTGTGGTAAGAAACAAACGTTAAA
TCCGATCATGAAGAACCAGAAGCACCATTTGTTTAATGTTTCGTTTAATTTGTAACCCATCATCTTTGGATACCAGAAGATTAAACC
AGCTAAGCAGGCAAATACAACACCAGTAACCAATGTATAGTGGAAGTGAGCTACTAAGAAGTACGTATTGTGATATTGATAGTCAG
CTGATGCCATTGCTAACATTACACCCGTAACACCACCTAAAAGGAAGTTAGGGATAAATGCTAATGAGAATAGCATTGGTGACTCA
AATGTAATACGTCCTTTATATAATGTTAATAACCAGTTAAACAATTTCACACCAGTTGGAATACCAATCAGCATTGTTGAAATTGAG
AAGAATGAGTTGATTAACGCACCATTACCCATTGTGAAGAAATGGTGAACCCAAACTAAGAAACTAAGGAACGCGATACCGGCAG
TTGCCCATACCATACTTTGATGTCCGAATAAACGCTTACGAGCGAATGTCGGGATAATTTCTGAGTAAATACCAAATGCTGGAAGGA
TAACGATATAAACTTCAGGGTGCCCCCATACCCAGAAGAAGTTAGCCCAAAGCATTGGCATACCGCCATGTGCAACTGTGAAGAAT
GCTGTGTCAAATATTCTATCAGTTGTCATTAATGCTAACGCTACTGTTAAAGGAGGGAAAGCAAGAATAACAATTAATGTAGTAATA
AATGTTGTTACTGTAAACATTGGCATTTGCATAAACTTCATAGTTGGTGTTTTACATCTTAAAATTGTTACAAAGAAGTTGATACCTG
TAGCTAAGGTACCAAGCCCTGAAATTTGTATAGCTATTAAGTAATAGTTAACACCCGGACCAGGACTGAATTCACCTGCTAGTGGCG
CATAGTTTGTCCAACCAGCTGCTGGTGAACCACCAATAATAAATGACAGGTTGAATAAAATCATACCTGCAAAGAATAGCCAGAAA
CTTACGTTGTTTAATACTGGGAATGCAACATCACGTGCTCCAATTTGTAATGGAACAACGATATTCCATAAACCAAAGATAAATGGC
ATTGCCATGAAGATAATCATGATTACACCATGTGTACTAAAAATTTCGTTATAGTGGTTAGATTCTAAAAATTTGTTATCAGGTACT
GTTAATTGCGCACGAATAAGTAACGCATCAATACCACCACGGACGAACATTAATACGGCACAGATTAAATACATAATACCGATTTT
CTTATGGTCTACAGATGTGAACCATTCTTTGTAAAGATATTTCCATAATTTAAAGTAAGTAATTACTGCGATTAAACCAATAACTAA
GAATGGGGCACCAATTTGTGCCATTGTAATCATCCAGTTACCTTTAACTAGTAATTGATCCCATGGAAAATTCATTAATGTCCACCTC
CATGATCATCATTGTCTTGATCTTGCGCATCTTTTGAAATTTTCTTCATTTCTTTCGCATTTTTCGATTCATCTTTCTTGAACTCATTGT
TATATGGTTCGTCATTTCCAAGAATCATCAACTTCATACCATGTCGTTTATAGTTCGCATTTGTAATTTGAGCTTTACGAGCAGGTAT
TAATGGTTTGTCTGATACATCTTTAAACATATTTCTTCACTAGTGAAGTTTGGATCTTTCAATTCGAAATTGAAACGTTTATATGCA
TAGAAGATGTATTCTGGATCGGCTGCTGGATCAACAAACGCCATATGTGTACCATTAAATTCTAAAGCTTTATTAGGTGTGCTTGGT
AATAATTGTTTATCAAATGTATCTTGATCTAACGTTTTCTTACCTTTAACTTCTTTCACCCATTTGTCGTAGTCTTTTTGACTAACGGC
ATTTACTTTAAATGTTTGACGTGAGAATCCTTCACCATTGAAGTTAGAGTTACGACCTCTGAACGTACCAGTTTGAGATGCTTCTAAC
GTCCAATTCATTGTCATGCCAGTCATGGCATATTTTTGACCACCTAATTGTGGAATCCAGAAACTTGTCATTGTATCCATAGCTTGAA
GCTTAAATACAACAGGACGATCTTTAGGGATTGTTAATGTGTTAACAGTCTCTATATGTTCATCTGGATAAGCAAAGAACCATTTGT
ATCCTGCACTTACTGCATATACAACCATTGGATCTTTCTCACTCTTCGGTGGTTTTTCGTAATCGTATAAAGTTTTAACTGTAGGAAT

AGCTAAAGCAGCAACGATTATGATAGGTATTACAAACCATATTGTTTCAATGATGGCATTATGGTGCATCTTACCAGATTCGGCATT
CTTATTATAACTATACTTGTAAATAAAAATGGCGAACATGCCAAGTACAACGAAACAAATAACAAGCATGAAGACGATTGAATAAA
GAATCAAGAACTTCTGACTACTTGCTACTGGCCCTTTTGCGTTGAAAATTTCTATATTTGAACAACCACTAAGTAAAATTAGTGTGCC
AAATAATAGAAGCAAAGACTTAAATTTTGACACTTTTTTGACCTCCTAATACTACAAATGTAGGGCTTAACATTAATTTTAAGTTATT
ACACAATATTTACAAGGGCTTATGGGAAAAAAATTAATAAAATTGTATCAAAAATGTTGATAAATCAAGGTGTGACGTGGGTTCAC
ACATTTGTTAAAATTATGTGTACATTTTGTGACTAATAGCGTTTTTGAAAAAATTGCTTCATATTTAAAAGGAAAGTTGAGAATAGC
TTTCAATTAAAGGTTAATTGAATGAAAATAAAAAAAGTTTGAAATTGTCTAATAAATTGAAAATGATGGCTAGTTGATTGAAAGGA
AGCGCAAACATTTTAATAAGGAATTTGCCAAAAGTAATTTATCGTTAAACAATGAAAAGTGGAAATTTGTTTGTTCAAAATCAAAA
CATTTTTTTAAAAAGAGCAGTTAAGTTTTAATAATTTGATAGGTAGTCCTTTGGTATACAGAAAAAATACAATATTTGGGTTAAAGT
TGCAGATGAAGTTAGAATAACAAATAAAATGGTAGCAAAACATGTTCGTTTGCTACCATTTTATTTGCAGTATAGTGATGAGGGCTA
TTTAACCTCAATATACCTAGTGGAAATTTTAATTGCACCATCTGAATCAGTGACATGATATTTAATTTGATATTTACCAGATTTAGAT
GTATCAATTTGGCCATCGACTTTAATTTTATCGGTTAAATCTCCATCTTCTTTATCAAATGCACTTATGCCGTTTAGAAGGTTATAATC
TTGACCTTTCTTAATAACGATATCATTAGCGCCTTTAATTTGTGGTGTAGTATTCGTCGTTGCATCTGCATTTAAATTTGGTGTTACTA
GTGTAGCAGAAACACCGAGGGCTGATAATGACTGTAATAGTTTATTCATAATGTAACCTCCGAATGATAAGTCATTTCTGAGTTAGC
TTTACTATACCTTAATTTATATTATTTACACATCATACTATAGTCCTAATCTTACCCTATAAAGAATGAAATTTAAATATAAATTTAT
AAATAATGAGGAAATAATTATTATTTTGAAAATCTGCCACAATTGTAAAATTAATAATTTAGGGATAATTTGAAATCAAATATAACT
TTATTCGACAGTTTGAACTTAAATTTTTGTGTTTCAAAACAATAAGAAAAGGGAGGCGAAAATGATAAAGAACCACTAATGATTAA
CTATGTAGTAGTACTTTATCATAGTCACAGCTTCTGTGATGGGGCCCCCACCCCAACTCGCATTGCCTGTAGAATTTCTTTTCGAAAT
TCTCTGTGTTGGGACTCCTCCCCAACTTGCACATTATTGTAAGCTGACTTTTCGTCAGCTTCTTTGTTGGGGCCCATCCCCAACTTGCA
CATTATTGTAAGCTGACTTTTCGTCAGCTTCTTTGTTGGGGCCCCGGACTATAATTGAAAAATGCTTGTTACAAGCTTATTTTCATTC
AATTAACTACTGTCTATATAAAATTGTTGAGCCAAGAACAAAGTGAAATGCTCCCTTCAAAGCCTTCATTTATTCAATGTCTACTTTG
AAGGGAGCATTTGATCGATTAATGTCCCAGATTTTTCTTGTTATTCATTTCAATTGCGAAATAATATTAAATGTGGGTTAAAAGACTT
CATTAAAACTCAGTTATTAGGGAGTGGGACAGAAATGATATTTTCGTAAAATTTATTTCGTCGTCCCACCCCGGCAAGGTTGACTAG
AATTGAAAAAAGCTTGTT

>026-contig_248_RC

ATTGAAAAAAGCTTGTTACAAGCGAATTTTCGTTCAGTCAACTACTGCCATTATAACTTTGTAGAGCATAGAACATTGATTTATGTCT
CAGGCTCTTTACGAATCAATACCTCGACGCATTTTTTCTGCAAGCAAAGTATTATTTAATACCATAGTAATTGTTAATGGACCAACG
CCACCAGGAACTGGTGTAATAGCTCCAGCAATTTCTTTAACCGCATCATAATCAACGTCACCTTTTAATTTGCCATTTTCATCTGGCG
TATTGCCAACATCGATAATTACTGCTCCTTCTTTGACCACATCTTTTGTTACTAAACCAGGCTTACCAACTGCACTGACAATGACATC
AGCATCTTTTAAATATGATGCCATATCTTTTGAACGAGAATGTAAGATTGTTACTGATGCATTTTTTTGAAGTAGTAACTTAGAAACT
GGTTGTCCGACAATATGACTTCGTCCAATTACAACTGCATTTTTACCTTCTAAATCAATATCAGCATGTTTTAATATTTCCATGATGC
CGAGCGGTGTGCAAGGTACAAAAGTTTGTTCATCGATATATAATTTCCCTATATTTATTGGATGAAAACCGTCCACATCTTTTTCAGG
ATTGATTGCTTCTAATATTTTCTGTTCGCTAACTTGTTTTGGTAATGGTACTTGTACCAAAATACCACTTACAGAATCATCATTATTTA
GTCTATTTAGTTCGTTTAATACTTCTTCTTCAGTAGCTGTTTCTTCCAAATGTACGATTTCTGAAATCATACCAATTTTTTCAGCTGCT
TTCTTTTTTGATCTAACATAACTTTGACTAGCGCCATCATTACCAACTAATATAACGGATAATTTAGGTGTAAAACCCTTTTCTTTTA
GCGCTTCAACTTGATCTTGTAACCCCTGTCTGTAGTCTTTGGCAATTTGTTTACCATCTAAAATTTTAGCAACCATAAAAACTTCTCC
TCCTATTGTCAATATCCTAACTTTGTTAGTTTATAAAACTAATTTACATAGCAAAAGACGTAAATTCAAACAAAAAATCAAAATAAA
GTTCGATTTTTGATTGAAAAAGCAGAAATTGCTTGTTATGCTATATCTATAATATACAACTAAAGTAATTCACAATTCAAACTTTTGA
AAGGGTGTACAAAGTGAAAGTAGCAGTCATTATGGGCAGTTCTTCCGATTGGAAAATTATGCAAGAGAGTTGTAACATGTTGGATT
ATTTTGAAATTCCGTACGAAAAACAAGTAGTATCCGCACATCGTACGCCAAAAATGATGGTTCAATTTGCTTCTGAAGCGAGAGAA
AGAGGTATAAACATTATCATTGCAGGCGCTGGCGGTGCGGCACATTTACCAGGTATGGTTGCATCATTAACGACGCTACCAGTTATT
GGAGTGCCGATTGAAACAAAAAGTTTAAAGGGTATAGATTCTTTATTATCAATTGTTCAAATGCCAGGAGGTATTCCGGTTGCAACG
ACTGCAATTGGTGCAGCAGGTGCTAAAAACGCAGGTATACTTGCAGCAAGAATGTTAAGTATTCAAAATCCTTCTTTAGTTGAAAA
ACTAAATCAGTATGAATCTTCGTTAATTCAAAAAGTGGAGGACATGCAAAATGAACTTCAATAAATTAAAGTTTGGTGCGACTATTG
GCATTATTGGTGGTGGTCAGCTTGGAAAGATGATGGCACAATCAGCTCAAAAAATGGGTTATAAAGTGGTTGTATTGGATCCTTCTG
AAGATTGTCCATGTAGATACGTTGCACACGAATTTATACAAGCCAAGTATGACGATGAAAAGGCACTCAATCAATTAGGACAAAAA
TGTGATGTGATTACTTATGAATTTGAAAACATTTCAGCCCAACAATTAAAACTATTATGTGAAAAGTACAATATTCCGCAAGGTTAC
CAAGCTATACAGTTATTACAAGACCGCTTAACTGAAAAAGAAACATTAAAAAGTGCTGGTACCAAAGTTGTCCCGTTCATTTCAGTA
AAAGAATCTACAGATATTGACAAAGCAATTGAAACATTAGGATATCCTTTTATTGTAAAAACTAGATTTGGTGGCTACGATGGCAA
AGGTCAAGTTTTAATTAACAACGAAAAAGACTTACAAGAAGGTTTTAAATTAATTGAAACTAGTGAATGCGTAGCTGAAAAATATT
TGAATATCAAGAAAGAAGTATCTCTTACTGTTACAAGAGGAAACAACAATCAAATCACTTTTTTCCCATTACAAGAAAATGAGCAT
AGAAATCAAATACTTTTCAAAACAATTGTTCCAGCGAGAATAGATAAAACAGCTGAGGCGAAAGAGCAAGTTAATAAAATTATCCA
ATCGATTCATTTCATTGGAACATTTACAGTGGAATTTTTTATAGATAGTAACAACCAATTGTATGTGAACGAGATTGCACCAAGGCC
TCACAATTCCGGACATTATTCAATTGAAGCATGTGATTATTCACAATTTGATACTCATATTTTAGCAGTTACCGGACAATCATTACCA
AATTCAATTGAATTATTAAAGCCTGCAGTCATGATGAACTTACTAGGTAAAGACTTAGATTTATTGGAAAATGAATTTAATGAACAT
CCAGAGTGGCACTTACATATTTATGGTAAGTCTGGGCGTAAAGATAGCAGAAAAATGGGGCATATGACTGTACTAACGAATGATGT
AAACCAAACTGAACAAGATATGTACGCTAAATTTGAGGGGAGTAATTAAAGCATGACATTATTATATGAAGGAAAAGCGAAGCGC
ATTTTCTCAACAAATCAAGAAAATGAATTAAGAGTTGAATATAAAGATGAAGTTACTGCTGGAAACGGGGCTAAGAAAGACACAAT
GGCAGGTAAGGGGCGATTAAATAATCAAATTACTTCTATTATATTTAAATATTTACAAGAAAATGGAATAGAAAGTCACTTTATTAA
ACAATTATCTGAAACAGAACAATTAGTTAAGCCTGTGAAAATAATTCCATTAGAAGTAGTTGTTCGTAATATTGCTAGTGGATCTAT
TACAAAGCGTTTAGGTTTTGAAAATGGTGAAGTTTTTAGAGAACCACTTGTAGAATTTTTCTATAAAAATGATGCGTTAAATGATCC
GTTGATAACGGATGACCATGTTAAATTGCTCAATATAGCATCAGATGAAGATATTGAAATACTAAAATCCAAAGCATTAAAGATTA
ATAATGTGTTGAAACAATTAATGGATGCTATGAATTTAAAATTAGTAGATTTTAAAATCGAATTTGGAAAGACTGAGACTGGTCAA
ATTTTGTTAGCGGATGAAATATCTCCAGATACATGTCGAATTTGGGATAAAGCTACCAATGCAAACTTTGATAAAGATGTATATAGA
AATAACACTGGATCACTGATTGAAACATATCAAATATTTTTAAACAAATTGGAGGATTTAAAATAATGAAAACAATTGAACTACAT
ATCACATTACAACCACAAGTATTAGATACGCAAGGACAAACGCTTACTCGAGCTGTACATGACTTAGGTTATGCACAAGTGAATGA
TATTCGTGTAGGAAAAGTATTATATATGACAGTGGATGAGGTTAGTGATGAAAAGGTACACAACATTATTACAACTCTAAGTGAAA
AATTGTTTGCAAATACAGTGATTGAAGAATATAGCTATAAAGTGTTAGATGATGAAAAGGAGAATGCATAAAATGAAATTTGCGGT
TCTTGTTTTTCCAGGTTCGAATTGTGATAGAGACATGTTTAATGCTGCTATTAAAAGTGGTGTTGAAGCGGAATATGTAGATTATAG

AGAAACATCACTAAGTGGATTTGATGGCGTACTTATTCCTGGTGGATTTTCATTCGGGGATTACTTAAGATCTGGGGCAATGGCTAG
TGTAGCGCCGATTATTTCGGAAGTTAAACGTCTTGCAACTGAAGGTAAGCCAGTATTAGGTGTTTGTAATGGGTTTCAAATTTTAAC
TGAAATAGGCTTATTACCTGGTGCATTATTGCATAACGATTCACATTTATTTATTAGTAGAAATGAAGAGTTAGAAATAGTGAATAA
TCAAACGGCATTTACAAATCTTTATGAGCAAGGTGAAAAAGTTATATATCCTGTAGCTCACGGTGAAGGTCATTATTATTGTACTGA
TGAAATATATCAACAATTAAAAGCTAACAATCAAATTATTCTGAAATATGTGAATAATCCGAACGGTTCATATGATGATATTGCAGG
AATTGTTAACGAAAAAGGCAATGTATGTGGCATGATGCCACATCCTGAAAGAGCTTTAGAAACGTTGTTAGGTACTGATAGTGGTG
TGAAATTATTTGAAGCGATGGTAAAAAGTTGGAGGGAACAACATGTCTAAATTTATCGAACCAAGCGTTGAAGAAATTAAACTTGA
AAAAGTATATCAAGATATGGGATTAAGTGATCAAGAATATGAAAAAGTTTGCGATATTTTAGGCAGACAACCTAACTTTACAGAAA
CAGGTATCTTTTCTGTTATGTGGAGTGAACATTGCTCTTATAAACATTCTAAACCGTTTTTAAAGCAATTTCCTACGTCAGGTGAGCA
TGTGCTTATGGGTCCAGGTGAAGGTGCAGGGGTAGTCGATATAGGTGATAATCAAGCCGTAGTATTTAAAGTAGAGTCTCACAATC
ATCCATCAGCAATTGAACCATATCAAGGGGCTGCTACAGGCGTTGGTGGAATCATTCGTGACATTGTTTCTATTGGGGCTAGACCTA
TTAATTTGTTAAACAGTCTTAGATTTGGAGAATTAGATAATAAACAAAACCAAAGATTACTTAAAGGGGTTGTAAAGGGTATCGGA
GGTTATGGTAACTGCATTGGTATTCCAACAACTGCTGGTGAAATCGAATTTGATGAATGTTATGATGGCAATCCACTTGTTAATGCA
ATGTGTGTTGGTGTTATCAATCACGACATGATTCAAAAAGGCACAGCAAAAGGTGTAGGTAATTCGGTCATTTATGTTGGTTTGAAA
ACTGGTCGAGATGGTATTCATGGTGCTACTTTTGCATCTGAAGAATTGACGGAAGAAAGCGAAAGTAAACGACCTTCTGTACAAAT
CGGTGATCCATTTGTAGGTAAAAAATTAATGGAAGCAACACTTGAAGCAATTACATTTGATGAATTAGTTGGTATTCAAGATATGGG
TGCTGCTGGTTTAACATCTTCATCGTCTGAAATGGCGGCAAAAGGTGGTAGTGGGTTACATTTGAGATTAGAACAAGTGCCAACACG
TGAGCCAGGTATTTCTCCTTATGAAATGATGCTTTCAGAAACTCAAGAACGTATGTTACTAGTTGTTGAAAAAGGTAATGAACAAAA
ATTCTTAGATTTATTTGATAAGCACGAATTGGATAGTGCTGTTATAGGTGAAGTTACAGATACAAATCGTTTTGTTTTAACATATGAT
GATGAAGTTTATGCTGACATTCCAGTTGAACCACTAGCTGATGAAGCACCTGTATATATTTTAGAAGGAGAAGAAAAAGATTATAA
TACTTCTAAAAATGATTATACACACATCGATGTTAAAGATACTTTCTTTAAATTACTTAAGCATCCGACTATAGCATCTAAACACTAT
TTATATGATCAATACGACCAACAAGTTGGTGCCAATACGATAATTAAGCCAGGACTTCAAGCATCGGTAGTACGTGTGGAAGGCAC
AAATAAGGCAATTGCTTCAACAATTGATGGTGAAGCGCGTTATGTATATAACAATCCATATGAAGGTGGAAAGATGGTAGTAGCTG
AAGCTTATCGAAATTTAATTGCCGTGGGTGCAACACCATTAGCAATGACAGATTGTTTAAATTATGGTTCTCCTGAAAAGAAAGAAA
TCTATCAACAGTTGATAGATTCAACGAAAGGTATGGCAGAAGCATGCGACATTCTTAAGACACCAGTAGTTTCTGGAAATGTATCTT
TATATAACGAAACGAAAGGTACTTCTATTTTCCCAACACCAGTTGTTGGAATGGTAGGTTTGATTGAAAATGTAAATTATTTAAATG
ATTTTGAACCTCAAGTTGGAGATAAATTATATTTAATCGGTGATACTAAGGACGACTTTGGTGGTAGTCAACTTGAAAAGTTAATTT
ATGGCAAAGTTAATCATGAATTTGAGTCATTAGATTTGAGTTCAGAAGTTGAAAAAGGTGAATCAATCAAGACCGCTATTCGTGAA
GGACTATTATCACATGTTCAAACAGTTGGTAAAGGTGGCTTACTGATTACTTTAGCTAAACTAAGTGCGCATTACGGTTTAGGATTA
AAGTCTTCAATAGATATAACAAATGCACAATTGTTTAGTGAGACGCAAGGCCGATATGTTGTTTCTGTTAAATCAGGTAAAACTTTA
AATATTGATAATGCAATAGAAATTGGACTTTTAACAGATAGTGATAATTTCAAAGTAACAACACCATATACAGAGATTAGTGAAAA
TGTTTCAGATATTAAACAAATATGGGAAGGGGCAATTGCTCAATGTTTAACTACTCAGGATTAAACGAAGAATGTGGCGTGTTTGGT
ATTTGGAATCATCCTGAAGCAGCGCAACTAACATATATGGGACTTCATAGTTTGCAACATCGTGGTCAAGAAGGTGCAGGTATAGTT
GTTTCTGATCAAAATGAATTAAAAGGCGAGCGAGGATTAGGCTTACTAACTGAAGCGATTAAAGATGATCAAATGGAACGATTAAA
AGGATATCAACATGCAATTGGTCACGTCCGTTATGCTACTTCAGGTAATAAAGGTATTGAAAATATTCAACCGTTTCTGTATCACTTT
TATGATATGAGTGTAGGTATTTGTCATAATGGTAACCTCATTAATGCTAAATCATTGCGTCAGAATTTAGAAAAACAAGGTGCTATC
TTCCATTCGTCTTCTGATACTGAAGTCATTATGCATTTGATACGTCGAAGTAAAGCTCCTACTTTTGAGGAAGCGTTAAAAGAAAGT
TTGCGAAAAGTTAAAGGCGGTTTTACATTTGCGATATTAACTAAAGATGCGTTATATGGCGCAGTAGATCCAAATGCTATCAGACCA
CTTGTTGTAGGTAAAATGAAAGATGGTACATACATCCTTGCAAGTGAAACATGTGCAATAGATGTGTTAGGTGCAGAATTTGTTCAA
GATATTCATGCAGGTGAATATGTCGTGATTAACGATAAAGGTATTACAGTTAAATCTTATACACATCATACGACAACTGCAATTTCT
GCGATGGAATATATTTATTTTGCTAGACCAGACTCAACAATAGCTGGTAAAAATGTCCATGCAGTACGTAAAGCTTCTGGTAAAAA
ATTAGCCCAAGAAAGCCCTGTAAATGCTGATATGGTCATCGGTGTACCCAATTCATCGCTATCAGCTGCGAGTGGTTATGCTGAAGA
AATAGGTTTGCCATATGAAATGGGACTAGTTAAAAATCAATATGTTGCAAGAACATTTATTCAACCAACTCAAGAATTACGTGAGC
AAGGTGTGAGAGTGAAGTTATCTGCGGTAAAAGATATAGTAGATGGGAAAAATATCATTCTTGTTGATGATTCCATTGTTCGCGGTA
CGACAATTCGACGCATTGTGAAAATGTTAAAAGATTCTGGTGCAAATAAAGTACATGTGCGTATAGCATCACCGGAATTTATGTTCC
CAAGTTTTTATGGAATCGATGTTTCAACTACGGCAGAATTAATTTCTGCAAGCAAATCACCTGAAGAAATTAAAGATTATATTGGCG
CTGATTCATTAGCATATCTATCTGTAGATGGGTTAATTGAATCAATTGGTTTAGATTATGACGCGCCATATAGTGGCTTATGTGTAGA
AAGTTTCACTGGAGATTATCCTGCAGGGTTATATGATTATGAAGCAAATTATAAAGCGCATTTAAGTCATCGACAAAAGCAATATAT
TTCTAAAAACAAACACTTTTTTGATAGCGAGGGAAATTTAAATGTCTAAAGCATATGAACAATCTGGTGTAAATATTCATGCTGGTT
ATGAAGCTGTAGAAAGAATGTCTAGTCATGTTAAACGTACGATGCGTAAAGAAGTTATCGGTGGTTTAGGTGGATTCGGTGCTACA
TTTGATTTATCACAATTAAATATGACAGCACCAGTTTTAGTTTCTGGAACAGACGGAGTAGGTACGAAATTAAAACTAGCTATCGAC
TATGGGAAACATGATTCGATAGGTATCGATGCAGTCGCAATGTGTGTTAATGATATTTTAACGACAGGTGCAGAACCATTGTATTTT
TTAGATTACATCGCTACAAATAAAGTTGTTCCTGAAGTTATTGAACAAATTGTTAAAGGTATTAGTGATGCATGCGTTGAAACGAAT
ACTGCACTTATCGGTGGAGAGACTGCTGAAATGGGTGAAATGTATCACGAAGGTGAATATGATGTAGCCGGATTTGCAGTTGGAGC
AGTTGAAAAAGGATGACTATGTAGATGGTTCAGAAGTGAAAGAGGGACAAGTTGTTATAGGGCTTGCGTCAAGTGGCATTCATTCAA
ATGGATATAGTTTAGTGCGCAAATTAATTAATGAATCAGGCATTGATTTGGCATCAAACTTCGATAATCGTCCATTTATAGATGTCTT
TTTAGAACCAACTAAATTATATGTCAAACCTGTACTTGCTTTAAAAAAAGAAGTTTCTATTAAGGCAATGAATCATATTACTGGTGG
AGGCTTTTATGAAAATATTCCACGTGCATTGCCAGCCGGATATGCTGCTAGAATTGATACTACATCATTTCCAACGCCAAAAATATT
TGATTGGTTACAACAACAAGGCAATATAGACACAAATGAAATGTATAACATTTTTAACATGGGTATTGGCTATACGGTTATCGTTGA
TGAAAAAGATGCATCACGCGCTTTGAAGATTTTAGCAGAACAAAATGTGGAAGCTTATCAAATTGGTCATATTGTGAAAAATGAGT
CAACTGCAATTGAATTGTTGGGGGTATAACAATGGTTAAAATTGCGATTTTTGCATCAGGTTCAGGAAGTAACTTTGAAAATATAGT
TGAGCATATTGAATCAGGAAAACTTGAAAATATTGAAGTTACGGCGCTATATACGGATCATCAAAATGCGTTTTGTATAGATAGAG
CAAAAAAGCACGGTATTCCTGTTTATATTAATGAACCAAAACAATTTGATTCAAAAGCAGCGTATGAACAACATTTAGTAACACTAT
TAAATAAAGATAAGGTAGAGTGGATTATTTTAGCTGGCTACATGCGTCTAATAGGTCCAGACTTATTAGCATCATTTGAAGGTAAAA
TATTGAATATACATCCATCTCTATTGCCGAAATATAAGGGGATTGACGCAATAGGCCAAGCATATCATAGTGGCGATACTATTACTG
GTTCGACAGTACATTATGTTGATAGTGGTATGGATACGGGAGAAATTATTGAACAGAGAAAATGTGATATTAGACCGGACGATTCA
AAAGAACAATTAGAAGAGAAAGTAAAAAAATTGGAATATGAATTATATCCAAGTGTTATTGCTAAAATTGTAAAATAAGGAGTGTG
TATGTGATGAAGAAAGCTATTTTGAGCGTATCAAATAAAACAGGTATTGTAGAGTTTGCTAAAGCGTTAACGCAATTAAATTATGA
ATTATATTCAACAGGTGGTACTAAACGTATATTAGATGAAGCAAATGTACCAGTTCGTTCTGTTTCAGACTTAACACATTTTCCAGA
AATAATGGATGGCCGTGTTAAAACATTACATCCGGCTGTTCATGGTGGCATTTTAGCTGATCGAAATAAACCGCAGCATTTAAATGA
ATTATCAGAACAACATATAGATTTAATTGATATGGTAGTAGTTAATTTATATCCATTCCAACAAACTGTTGCAAACCCTGATGTGAC

GATGGACGAAGCAATTGAAAATATTGATATTGGTGGTCCAACAATGTTACGTGCTGCAGCTAAAAACTATAAACATGTAACAACAA
TTGTACATCCGGCAGATTATCATGAAGTATTGACGCGATTAAGAAACGATTCGTTAGATGAGTCATATAGACAATCATTAATGATTA
AAGTTTTTGAGCATACTGCAGAATATGATGAAGCGATTGTACGTTTCTTTAAAGGGGATAAAGAAACTTTAAGATATGGAGAAAAT
CCACAACAATCAGCGTATTTTGTGAGAACTTCGAATGCTAAGCACACGATTGCAGGCGCTAAACAATTACATGGGAAACAATTAAG
CTATAACAATATTAAAGATGCAGATGCTACACTAGCTTTAGTTAAAAAGTTTGATACACCTGCTGCAGTTGCGGTTAAACACATGAA
TCCATGTGGTGTTGGTATCGGTGACACGATTGAACAAGCATTTCAACATGCGTATGAAGCGGATAGTCAATCAATATTTGGTGGAAT
TGTTGCATTAAACCGAGCTGTAACACCTGAGTTAGCAGAGCAATTGCATAGTATCTTTTTGGAAGTCATTATTGCACCAAAATTTAC
AGATGAAGCATTAGATATTTTAAAACAAAAGAAGAATGTAAGATTATTAGAAATTGATATGACTATAGACAGTAACGAAGAAGAG
TTTGTTTCAGTATCTGGCGGATATTTAGTTCAAGATAAAGACAATTATGTCGTGCCAAAAGAAGAAATGAAAGTTGTTACAGAAGTA
GCACCTACTGATGAACAATGGGAAGCAATGTTATTAGGATGGAAAGTTGTACCATCAGTAAAAAGTAATGCAATTATTTTAAGTAA
TAATAAACAAACTGTAGGTATAGGTGCTGGACAAATGAATCGTGTCGGTGCTGCTAAAATTGCGTTAGAGAGAGCTATTGAAATCA
ATGATCATGTAGCGTTAGTATCTGATGGATTTTTCCCTATGGGAGATACAGTTGAACTTGCAGCACAACATGGTATAAAGGCAATTA
TCCAACCGGGTGGTTCGATTAAAGATCAAGATTCAATTGATATGGCTAATAAACATGGTATTGCAATGGTAGTCACAGGCACTCGA
CATTTTAAACACTAATTTTAAAGGGGCGTAACATAAATGAATGTATTAGTAATTGGTGCTGGTGGACGAGAACATGCACTTGCATAT
AAACTTAATCAATCGAATCTAGTTAAACAAGTGTTTGTCATTCCAGGTAATGAGGCAATGACACCTATAGCTGAAGTACACACTGA
AATTTCAGAATCTAATCATCAAGGGATACTAGATTTTGCTAAACAGCAAAATGTTGATTGGGTAGTTATAGGTCCAGAACAGCCGCT
AATTGATGGATTAGCAGACATTTTACGAGCTAATGGTTTCAAAGTGTTTGGTCCAAATAAGCAAGCAGCTCAAATCGAAGGCTCAA
AATTATTTGCTAAAAAGATAATGAAAAAATATAATATTCCAACTGCTGATTATAAAGAAGTTGAGCGAAAAAAGGATGCTTTAACA
TATATTGAAAACTGTGAATTGCCCGTTGTTGTCAAGAAAGATGGGTTAGCTGCTGGGAAAGGCGTTATTATTGCAGATACTATTGAA
GCAGCCAGAAGTGCTATTGAGATTATGTATGGTGATGAAGAAGAAGGTACTGTTGTATTTGAAACGTTTTTAGAAGGTGAAGAGTT
CTCGCTAATGACATTTGTTAATGGTGATTTAGCAGTACCTTTCGACTGTATTGCACAAGATCATAAACGCGCATTTGATCATGATGA
AGGACCAAATACTGGTGGTATGGGGGCTTATTGTCCAGTACCACATATTAGTGACGATGTTTTAAAACTTACAAATGAAACAATTGC
ACAACCCATTGCAAAGGCAATGCTTAATGAAGGTTATCAATTCTTCGGTGTATTATACATTGGTGCTATTTTAACTAAAGATGGTCC
AAAAGTAATAGAATTTAATGCCCGTTTTGGTGATCCTGAAGCTCAAGTATTATTAAGTCGCATGGAAAGTGATTTAATGCAGCATAT
TATTGATTTAGATGAAGGAAAACGTACTGAATTCAAATGGAAAAATGAATCTATTGTAGGGGTCATGTTGGCATCAAAAGGATATC
CTGATGCATATGAAAAAGGGCATAAAGTAAGTGGCTTTGATTTAAATGAAAACTATTTTGTTAGTGGATTAAAGAAGCAAGGTGAT
ACCTTTGTTACTTCAGGTGGTAGAGTTATACTTGCCATCGGAAAAGGTGACAATGTACAAGATGCACAGCGAGACGCATACGAAAA
AGTATCTCAAATACAAAGTGACCATTTATTCTATCGTCATGACATTGCGAATAAAGCACTACAACTTAAATAAGTATATTTAAAAAA
TACTAAGATTAGCTATGAAGAAATCTATAACGATAGTTTTTTTCATAGCTTTTTAGTTGTAGAGTCTAGGACATTGATTTCTGTACC
AAATTTGTGATTATGCATATGTAATACAAAAGAGGCGCCACAACATGTTTGGATGAACAAAATAACATGTTTGTGGCACCTCTTTTG
TTTAGTATGGAATAAATGGTTTTCTTTTTCTATACAATGAATTTCTAATTTAGTATCTATACAATTATGGATAAAATTTAACCTACAC
GACCAAGACGAACATCATCTATGCCCGTGATGGGTAAGGTGATTGAACAATAATATGCCATAGTAATAATGGCAATTAAAACTATA
ATAAAGATTATATCTTTATATGAGAAAGGTACGTTGTAATAGTAAGTACGAGGACCATCTCTAAATCCTTTCGACTCCATCGCAACT
GATAATTGATGTGCCTTTCTAATATTTTGGCTTAATAGAGGTATAATTAAATGCTTAAATCGCTTTAACCCTCTATAATTTGCTGCGT
CTATCATCTGATAGCGCATTTTTAAAGATCTGCGAAGCTGTATTAAAGAACTAATCATTAAAGGTATCATACGAATGGCAGCCATGA
ATGCATAAGCAACTTTTGATTTAACCTTTAAATGTTGCATTAAACTATAAAATATCATGACAACCTGAGATGTAAGTGCGATTAAGA
TACCGAAAAATGAAATAGCAATGGTTCTTAATGATACATGTAAACCACGAACTAAACTTTCTGTTGTAATATGGATAAATCCGAATT
TCAAAATTGTATGGCTACCATTCCCGTATAAAATCATGAACAGGGAAGAGAGTAATGCAAAGCCAATACTTATAGTTATAAAAATT
GCTGTAATTTTAAACTGAGTACCATTAAACATCAATAAGAAAACTAACATTAAGATAGTGATATAAAGCATAAAATCGAAATTATG
CACAAATATAATAAAGAAAAATAGTATAATTCCAAGAAATAGTTTCGTTATAATGTTGACATCATCAACAAATGATTGCCGAACTTT
CCATTGCTCATACATTCGTATCACCATCACAATCTAGTAACGCACCATCTGAAATTTTAAGTCTTCTTGATGGATAACGTTCAATTAT
TTCATCGTCATGTGTAACCATGACAATACTTTGTCCCAAATTAATTCGCTTTTGGAAAAGTTTGATCAACTGGAATGTATTATGGCTA
TCAAGTCCAAATGTCGGTTCATCTAAAAAGATAATATCAGCTTTAGAACTTAGTGCGGTAGCTACGCTAAGGCGTCGTTTTTGACCA
ATAGACAACTCATAAGGATGTTGATCTTTTACATTTTGTAAATCTAAAAGTTTTAAAAGTTGTATCGTTTCATCATCACTTTGATCTT
TAGAAAGGTGATTAAAATGAATGTTAATTTCATCATAAACCGAATTTGTTATAAATTGTAATTCTGGGTTTTGATAAACTAGGTACA
TGTGTTTTGCTGCATGTTTAATTTTTGTTAAACGCTGATTTTCAAAATAAACATCACCTTGATATTTAATCAATTGCATAATTGATTCA
AGCAAGGTTGTTTTACCACTACCATTTGCCCCTGTAATTGTAATCCACTCACCTAGACCAATTTCTAAATCTGAGAATGAGAGCAAT
GTTGATTTACCGCGAATAATACGTCCATTTTTAAATTGTAATAAGTGTGAGTTTGTTGTTGGAAAGTCAACACGACTTGGTGCGAAT
TCCCATGCACGTGGATGCCACACACCATATTCACTGAGTAAATGAACATACTTCTGTAATATGATTTCAGGACATTCATCGGCAATG
ATATTTCCGTTATAATCCATCAAAATGACGCGGTCGACATGATTCCAGATGTGTTTAACTTTATGTTCAACGATTACAACAGTTTGAT
CTTCCCAAAGTTCAATTAGTTTAGTCCATAAATCTTCTGTTGCTTGAACATCTAACATTGCTGTCGGTTCATCTAAAAACAATGTTTT
TGATTGTTGAAGAATGGTTTCAACAATTGCCAATTTCTGTTTCATCCCGCCACTTAAATCTTTGATATACGTTTCAGGGGTAACATTT
AAATTGACCATATTTAAAGCATTGATAATTAACGCATCCATGTCTTCACGTGGTACTTGTCTATTTTCTAAAACGAATGCAAGTTCTT
CGTATACTTTTGGCATACAAAACTGGCTATCAGGGTCTTGGAAAATAACGCCACTTAATGGGTCAACGATTAGTTCATCATATTTCA
TAGGTAATTCAATTAAATTAGGAACAATACCACTTAATACATTCAGAAGTGTACTTTTACCGCAACCAGAAGGACCGAGTAAAAGT
ACTTTTTCTTTGTCTTGAATAGTGATATTTAAATGATCGAAAATTTTACGCTGACCATTTGGATATTTTAATCGTAAATCACTTACTTT
TAACACTCTGAATGCTCCTCTTATAAGTTGTCGTAATCTTCTTTAGCAGCTGGTCTAAATAATTTTGTTACGCCTGTCTTATCTAAAGC
TTTTACTAAAAGGTAAGATAGGACGCCGGCGACTACTGCACCACTAATTAATCTAAATACGATGAATAATGTTAAGTTCCAACCTGC
AACTTCATTTAAATAACCATAGAAATAATCTATAGGGAAAGCCGCGATTGCTGTACAAAAACCTGCTAACATAGCTACCATAACTG
AACGAGATTGATATTTAAAAATTGCAAAGACAAGTTCACACGCTAAACCTTGTATGAAAGCGTAAACGATTGTCGGAATATCGAAA
CGACCCATAATGATAGTTTCGCCGGCACCTGCAGCAAATTCAGCCAGTAAAGCAATACCTGGTTTTGGAATAATTAGATAGCAGAC
AATCGCTGCCATGAACCAAACCCCGTTTGTTAATTGTTCGAGGTGAAGGCCTGTAGCTTGCACACCATTGTAAACAAACCACCATAA
ATTGTAAATAACTGCGAATACTACTGAAATAAGTACGGTTACTAGTATTTCAGATAGCTTTAAACCTTTTGACATTTTTACATCCTCC
TAATAAAAAAACGCACAACCATCCATAGGAAAGTTATGCGTTCACAATATATATTAGTAAAACATATGTATAGTAACACTTTCCTAC
GCTAGTTCAAGCTAGATCAGGTTCAAAGGGTTTGAGGGCAAGCCTCATCTCAGTATAAAACACCCCTAGTGTGTGCGATTTATTTAA
TTAATTATACTGTAAGACGTTTGTAAACTTATGTCAATAGGTTGTCTTCATGAAATTTCGTTTAATTCGATTTAAAATTTATAATATT
AGCATTGGATTTAAATTGAAGATGTAGTAGGAATGTTAGTAATTAAAGATATAAAAATATGTGACATGTAATAAGATTGAGCTGAT
AAATGAAGAGGGATACTTATCAATCATACCTCTTTAACAACAGTGAAGAACCCGTGCATAATGGCTTACGAATTATAGTTTGTAGG
GTAAAAGAGGGATACATGCGCCGAGCACATGCAAAAAAGCCCCTAACAACTAAAAGTTGTAAGGTAAAAGAGGGATACATGCGCC
GAGCACATGCAAAAAAGCCCCTAACAACTAAAAGTTGTAAGGTAAAAGAGGGATACATGCACCGAGCACATGCATAAAATCCCCT
AACAACTAAAAGTTGTAAGGTAAAAGAGGGATACATGCACCGAGCGCAAGCAAAAAAGCCCCTAACAACTAAAGGTTGTAAGGAA

GAAGAGGGATACATGCACCGAGCACATGCATAAAATCCCCTAACAACTAAAAGTTGTAAGGGGATTTAAATTAATTTAGTGTATCT
TGGATATCTTGTTTTGTTTGATTAATATCTTCTGTTTTTTCTTCTTTTTTATCTTTTAATTTTTCTTCAACTTCTTTAGCTTTTTCTGCTGC
TTTTTTATTTTGATTTTCATTAGACATGATTAATTCCTCCCAAATTGGATAATTATTTATATATAAATCTTACCCGGTTGTACTTTCGT
TAAACTTTTCTAAGTCTATAGCACTATTTATTCATTTATCTAAAGACAACAACATTAGATTAATATATAATGATTTTGAGGTGAACAT
AATGTCTTTTCTTAGGAAACACGCCGAAATTATTTTTAGCTATTTAATCGGTATCGTTTCACTCTTCACTGGTCTCATTATTTTAATTA
ACTTGCCATTAATTAAACAATTAAATGGTGGTAAAAAAGTTGATACACATGTTCATAATGTGTGGGAATTTCTGAATGCATTTTTCA
GTGAAATTATTAAAGTAATGAGTCGATTTATAGGTAATTTCCCTATAGTTAGTGCAATTGTGATAATTATATTCGGTATTTTAGTTAT
GTTGATTGGTCATACATTACTTAGAACTATTAAGTATGACTATGATATTTCTATCTTTTTCTTAGTTATCGGTATCATGTACTTTATTA
TTACTCTTATATTAATGACTCAAGTTTATGGATTCTTTGCAGTGATTTTCATTATTCCATTTACAATTCATATAGGATATATCGTCTAT
AAAGATGAATTGAATCAGGAAAATGTAAAAAATCATTTCATGTGGATAATTGTGAGTTATGGTATAAGTTACTTAATTACACAAATT
GCATTGTATGGCAGAATTGATGCTAATGAAATAGAGTCAATTGATATCTTAAGTGTCAATGCTTTCTTTATAATTATGTGGTTACTTG
GTCAAATGGCTATTTGGAATTTCTTGTTCTTGCGCCGAGCTTTACCTTTAACAAAGCAAGAATTAGGTGAAGAGGAGCCAGAATTAT
CAAGAACAAGTAAAGGGAATGTCACGAATCAAACTAAAATTCACTTGAAACAACTCCAAGATAAGACTACAGAATATGCACGTAA
GACAAGAAGAAGTGTCGATTTAGATAAAATTAGAGCTAAAAGAGATAAATTCAAAAAGAAAGTTAATGATATTATCGATATTCAAG
AAGACGATATTCCTGATTGGATGAGAAAACCGAAATGGGTTAAACCAATGTATGTCGAACTATTTTGTGGTGTCGTCATCTTTTTAT
TCACATTTTTAGAATTTAATAATCGTAATGCATTATTTGTATCTGGTGATTGGAAATTATCACAGACACAATATGTTATTGAATGGGT
TACATTATTAATTCTGTTATTCATTATTATCGCATATATCGCTACAACGTTAACTTTCCACTTGAAAGGTAAGTTTTATTATTTACAAC
TATTTATGGGGAGCATTTTATTCTTTAAATTGTTAACGGAATTTATAAATATAATGATTCATGGACTACTACTTTCAGTGTTCATTAC
GCCAACATTACTATTAATGTTATTGGCAATCATCATTTCTTATTCGTTACAATTACGAGAGCGACCATAATTAAAAGCATTATAAAA
GTACTATCTATTAAACATTTTGATGTGTACGCTATAAGTTAGATATATCTCTAACTTATTTAGATACAGGTCAATGAAGTTTATGGAT
AGTACTTTTTTTGTAACTAGATTTGATTGATTCAGGTGATGTGAATTTAGTATTGAAAATTGTATACAAAGTTTTAGTGCAAATAAAA
TAGTTGAAAAGTTATCCATTTGTAAAATCAAGAAAACTAGTAAATAGTTGAAGCGACATATGGAATTTGCGAAACGATATATAGTA
TTTCCTTTGTAGAAATTTAACATATATCATTCGAATTACTAATTTGTTAAAATCAACAGTAAGATTAGAAGTAGATGATATTGAAATT
TGGCAAACAATTTAATCTATATAAAACTACAACGAAACACAGAAAGGAAGTTGTCAGATGAAAATAGCAACTCTGAACAAAGGCA
AAGAAACAAAATATTTTAATGGATATCCTTTAATTGAAGAAGAGGATATCTATTCACAAGATCATTTAAAAGAAGGAGATATTTTTC
AAATTGTGACTGATAAATCACAATATGTTGCAACGGCTTATGTTGGTCGCCAACATAAAGGATTAGGTTGGGTTCTAACATACGATA
AAGCTCAAGAAATCAACACAGCTTTCTTTGTGAAATTGTTTAATACTGCATTAGCAGAACGTGATTATTATTTTAATATAGATGGAA
CAAATGCTTTTAGATTATTTAATGCTGAAGGTGATGGTGTTGGGGGATTAACAATCGACAATTACGATGGTCATTTGTTGATTCAAT
GGTACTCAAAAGGTATTTATAAATTTAAATATGCCATTCTTGAAGCGGTTAGAAAAGTATTTGATTATAAATCTATTTACGAAAAAG
TAAGATTTAAAGACAGCGAATATAGTGGTGGTTTTGTTGAAGGCGATGCACCTGAGTTTCCAATTGTTATCGAAGAAAACTTCACAT
TTTATAATGTAGACCTTGAAGATGGTTTGATGACAGGTATCTTTTTAGATCAAAAAGAAGTGCGCAAGAAATTAAGGGGTCAATAT
GCCAAAGAACGCCATGTTTTAAACTTATTTAGTTATACAGGTGCTTTTTCTGTAATAGCAGCAAGTGAGGCATCTTCAACAACAAGT
GTAGATTTGGCTAATCGTTCTCGTAGTTTAACTGAAGAAAATTTTGGATTAAATGCTATTGATCCTAAATCCCAATATATTTATGTCA
TGGACACTTTTGATTTCTATAAATATGCTGCACGACATGGACATAGTTATGACACGATCGTGATTGATCCACCTAGCTTTGCACGCA
ACAAAAAACGTACATTTTCAGTGCAAAAAGATTATGACAAATTAATTAATGGCGCCTTAAATATTTTATCATCTGAAGGAACATTAT
TGTTATGTACAAATGCAAGTGTATATCCATTAAAGCAATTTAAAAATACTATTAAAAAGACGCTTGAAGAGAGTGGCGTTGATTATG
AATTAACTGAAGTTATGGGATTACCAAAAGATTTTAAAACGCATCCACATTATAAGCCATCTAAATATTTAAAAGCTGTTTTTGTAA
ATATTAGACATTAATTTGATAAAAAGGGGTATAGTAACTTATTGAGAAAAAGAAGGGTGATAATATTATGGGATTCAAAAACAATT
TAACATCAAATTTAACAAATAAAATTGGTAATTCAGTCTTTAAAATAGAAAATGTTGACGGAAAAGGTGCAATGCCAACGACGATT
CAAGAATTGAGAGAAAGACGACAACGTGCTGAAGCAATTGTAAAGAGAAAATCTTTAATGTCATCAACAATGAGCGTTGTTCCAAT
TCCGGGTTTAGATTTTGGTGTTGATTTAAAATTAATGAAAGATATTATCGAAGATGTAAATAAAATATACGGATTAGATCATAAGCA
AGTTAATAGCCTTGGGGATGATGTGAAAGAAAGAATTATGTCTGCAGCAGCAATTCAAGGTAGTCAATTTATTGGTAAAAGAATTT
CAAGTGCATTTTTAAAAATAGTTATTAGAGATGTAGCTAAACGTACTGCTGCAAAACAAACAAAATGGTTTCCTGTTGTAGGACAA
GCTGTGTCTGCATCTATTAGTTACTATTTTATGAATAAAATTGGAAAAGATCACATTCAAAAATGCGAAAATGTTATTAAAAATGTC
ATGTAGGTGCTATAATAGTTTTGCAATTTGCAAATTTTACTGAAACCGGTTTTAAACGAATTGAATTTAAAGCATGGTTTTGGTAAA
GTTAATGTATAAAACTAAGTTAGTATTGTAATAATATTGAAGATTCTAACTATACGAAGGAGAAATGTAATTATGGAACAAAATTC
ATATGTAATCATCGACGAGACTGGTATTCACGCTAGACCAGCAACAATGTTAGTACAAACAGCTTCAAAATTCGATTCTGATATTCA
ATTAGAATATAACGGTAAGAAAGTAAATTTAAAATCAATCATGGGTGTTATGAGCCTTGGTGTTGGTAAAGATGCTGAAATTACAA
TTTATGCTGACGGTAGTGATGAATCTGACGCCATTCAAGCAATCAGTGACGTCTTATCAAAAGAAGGATTGACTAAATAATCATGTC
TAAATTAATTAAAGGTATTGCCGCATCTGATGGTGTCGCAATTGCTAAAGCTTATTTATTAGTTGAGCCAGACTTAACATTCGACAA
AAATGAAAAAGTCACTGATGTTGAAGGAGAAGTTGCAAAGTTCAATAGCGCTATCGAAGCTTCTAAAGTTGAGTTAACTAAAATTA
GAAATAATGCAGAGGTTCAACTAGGTGCTGATAAAGCTGCTATCTTTGATGCACATTTATTAGTTTTAGATGACCCTGAATTAATTC
AACCAATCCAAGATAAGATTAAAAATGAAAACGCTAATGCTGCTACAGCATTAACGGATGTAACAACACAATTTGTTACAATTTTT
GAATCTATGGATAACGAATACATGAAAGAACGTGCGGCTGATATTCGCGACGTTTCTAAACGTGTGTTATCACATATTTTAGGTGTA
GAATTACCGAATCCGAGTATGATTGATGAAAGCGTTGTTATTGTAGGGAATGACTTAACGCCATCTGATACTGCTCAATTAAATAAA
GAATTCGTACAAGGTTTTGCTACAAACATTGGCGGAAGAACAAGTCACTCTGCAATTATGAGTCGTTCTTTAGAAATTCCAGCAATT
GTTGGTACAAAATCAATTACTCAAGAAGTTAAACAAGGCGACATGATTATCGTAGATGGATTAAATGGTGATGTAATCGTTAATCC
AACTGAAGATGAGTTAATCGCTTATCAAGATAAACGTGAGCGTTATTTTGCTGACAAGAAAGAATTACAAAAACTACGTGATGCTG
ATACTGTTACAGTTGATGGTGTTCACGCAGAGCTTGCTGCAAATATTGGTACACCTAATGATTTGCCAGGTGTTATTGAAAATGGTG
CACAAGGTATCGGCTTATATAGAACTGAGTTTTTATATATGGGTCGTGACCAAATGCCTACAGAAGAAGAACAATTTGAAGCTTATA
AAGAAGTATTAGAAGCAATGGGCGGTAAACGTGTTGTTGTACGTACTTTAGATATAGGTGGAGATAAAGAATTATCATACTTAAAC
TTGCCTGAAGAAATTAATCCATTCTTAGGTTACCGTGCGATTCGTTTATGCCTTGCGCAACAAGATATTTTCAGACCACAGCTACGTG
CATTATTACGTGCATCAGTTTATGGTAAGTTAAATATCATGTTCCCAATGGTTGCAACAATTAACGAATTTAGAGAAGCTAAAGCTA
TATTATTAGAAGAAAAAGAAAACCTTAAAAATGAAGGTCATGACATTTCGGATGATATAGAATTAGGAATCATGGTAGAGATACCT
GCAACAGCAGCATTAGCTGATGTCTTTGCTAAAGAAGTAGATTTCTTCAGTATCGGTACAAATGATTTAATTCAATACACATTAGCT
GCTGACCGTATGTCAGAGCGTGTATCATATCTATACCAACCATATAACCCTTCAATCTTACGTTTAGTTAAACAAGTTATTGAAGCG
TCACATAAAGAAGGTAAATGGACAGGTATGTGTGGTGAAATGGCTGGAGATGAAACAGCTATTCCATTATTGCTTGGTTTAGGTTTA
GATGAGTTCTCTATGAGTGCAACGTCTATTCTGAAAGCAAGAAGACAAATTAATGGTTTAAGTAAAAATGAAATGACTGAACTTGC
TAACCGTGCAGTCGACTGTGCAACGCAAGAAGAAGTTATTGAATTAGTTAACAACTACGTAAAATAATTAATTCTTTCGAAAGACA
GAATTATAAATAAACCCGAAGTATTCATTGTGAGTAATTATGAATACTTCGGGTTCTTTTTTATGATTTATGTTAATTTCAATGGAAT
GTTGTTGCTAACTAATTAAACAACTATGTGTCATTAACAAAATGTTGTTCTTTAATTAATGTTAGTTAATTTAAAAATCGATATTTAA

TTTATGATAGCTATAAATGATGAAGTTGATTTAGATGAAAATTAATCTTGGATATTTAATACTTTGTTGATTTCATCAAGATCAACAT
GGTACATTGGATTGCCATTCAACAAAATAAACGGAGTTGAAAAAGCATCAAAATCTATCATTTCGTTTCGATATTGTTGATTGTTGA
TATTTCTCTCTTCAAAATCAATTTGATGCTCATTTAGATAATTTTTTACAAATGTACAAGGTGGACAATCATTCTGCGTATAAACGAT
TATTTCTGACATGTATCATGCTCCTTCGTGCTTTATTCTAATGTAATATACACAATTATACATTGAAATTCAAATTTGTGAACACATT
GTGAACTGACATAAATTATACACAATTTCATAGCGCATAATGTTTTAAAAATACACATTATAGCAATTATAATTAAGTTCATTGTGT
TGTCATCAAAAAAAGAAAAAGGTGATGTTTTTAAATGGATACAGTTGAAATCAGTCGGTTTTTGACAGCTATGACTTTAGCAGTTCA
TATCATTTTTGCAACGATTGGTGTTGGTATGCCTTTAATGTTCGCAATTGCAGAATTTTTAGGTATTCGCAAAAATGATCTTCAATAT
ATAGCTATGGCCAAAAGGTGGGCTAAAGCTTATACAATTACTGTAGCAGTGGGAGTTGTTACAGGTACAATTATAGGACTTCAATT
ATCATTGATTTGGCCTACTTTTATGGAAATGGGTGGACACGTTATTGCACTTCCATTATTTATGGAAACATTTGCGTTCTTCTTTGAA
GCTATTTTCTTAAGTATATATTTATATACTTGGGACCGTTTTAAAAATAAATGGACACATTTCTTAATTAGTATACCAGTAATTTTTG
GTGGCTCTTTCTCAGCATTCTTCATTACTTCAGTGAATTCATTTATGAATACGCCTGCAGGTTTTGAGTTGAAGAATGGAAAGATGGT
CAATGTTCAACCTATAGAAGCGATGTTTAACCCATCGTTTATAGTTCGATCATTTCACGTAATTACTACAGCCGGTATGACGATGGC
ATTTGTTATTGCATCAATAGCAGCTTTTAAATTATTGCGTAATCGTCAACCTAAAGATACTGTCTACCATAAGAAAGCTTTGAAAAT
GTCTATGATAGTTGGATTCTTTTCAACTTTACTTTCTATGTTGGCAGGGGATTTATCTGCAAAATTTTTGCATAAATTCCAACCTGAA
AAATTAGCAGCTTATGAATGGCATTTCGATACATCTTCCCATGCTAAATTATTATTATTTGGTGTGTTAGATGAAAAGACTCAGCAA
GTTAAAGGTGCGATTGAATTACCTGGACTATTGAGTTTCTTAGCAGATAATAGTGTCAAAACTAAAGTGCAAGGGTTAAATGATTTT
CCAAAAAGTTTACATCCGCCTATGATTGTCCATTATTTCTTTGATTTAATGGTAACGATGGGAATTTTATGTTTTGTCATTTCAGGTGT
TTATGTTTTAACTTTAATGTTTAAAAAGCTTAGAAAGTTTTCTACTCATAAATGGATGCTTTACGGAATATTATTAACAGGACCAGCT
TCAATGCTAGCTATAGAATTTGGATGGTTCTTAACAGAGATGGGTAGACAGCCTTGGATTGTTCGTGGTTATATGCGCGTGGCAGAA
GCAGCAACACAAGCAGGCGGAATAACCTTCGTTACAATTTTATTTGGCATATTGTACATCATTTTAATGTATACATGTGCATACGTA
TTAATTCGTATGTTTAAAAATAAACCGGCGTATGAAGATGTAAATCGTTTAGCCAAGAAGCAAGGAGGAGAAATAGAAAAATGATT
TATGCATTTATAGGTATAACAGTGTTATGGTTGTTTTTATTTTGCTATATCATTATTGCTTCTATAGATTTTGGGGCAGGTTTCTTCGC
ATTGCATTCAAAGTTAACTGGTGATGAAAAGAAAATTAATCACTTAATTTCACGTTATTTGAACCCAGTTTGGGAAGTTACGAATGT
ATTCTTTGTTTTCTTCTTCGTAGGATTCGTAGGTTTCTTTCCAGAGTCAATCAAATATTTAGGCACGGTATTGTTAATACCAGGTTCAA
TAGCACTGATTATGATATCATTGAGAAACAGTTTTTATGCATTTGAAAATTATGGTCAAGATACAAAATTAGCATGGATGATCATGT
ATGGGGTAAGTGGATTATTAATTCCAGCTTCATTATCTACTGCTTTAACTATTACAGAAGGTGGCTATATTAATGTTCGAAACAATGT
TATCGATCTAGATTGGGTGCAGTTACTATTAAGCCCATTTGCTTGGTCTGTAGTATTCTTGGCAATTATTTCAGTTTTATATATTTCAT
CAGGATTTTTGACATATTATGCTAAAAAAGCAAATGACGAACCAGCATATAATTTAACAAGACAATGGCACATATTTTTAGGGCCG
CCGATGATTATCATTTGTTTATTCGTATTTCTATCATTACGCATTCAAAATTCTGAACATTTTTATTCAGCTGTTTTTGACTATTGGTG
GATGTTTGCTATAAGTTTCTTATTCTTTGCATTAGCTTCATTGTTAACATTCTTTAAGAAAAAACATGGTTTGGCTTTTGTATTTGTTA
TTTTACAAATGATGTTCGCGTTCTTTGGCTATGGAATTAGTAAATTGCCATACCTTTTATATCCGTTTGTAAAAATTACAGATGCATA
TGTTAATCCAGAAATGGGCTGGACATTAGTGATTGTCTTTATTTTAGGTTTACTTTTATTACTTCCATCGTTAATATTATTATTAAGAT
TATTTGTTTTCGACAAAGAATATGTTGAAGGAAAGAAATCATAAGTTGAGACGATACCCCAACTTTCATTTACTTGCATTACTGCAT
TTAGATAATTTAGTTGGGGTACTCATTTATAATAATTTTAAATTTATATTTCAATGAATTGATGAAAATCATTTAATGGAGGGCGAG
TATGGGTAAAGAATATGTAGTCATCGGTCTAGGTCGTTTTGGAGGTAGTATCGTTCGTGAGTTGAATGCATTAGACATGGATGTAAT
GGCCATCGACCATGATGAAAATAGAGTGAATGAATATAGTGATATCGCTACTCATGCGGTTGTTGCAGACACAACAGATGAAGCAG
TTATGAAAAGTTTAGGTATCCGTAACTTTGATCATGTCATTGTGGCAATTGGTGAAAATATTCAATCAAGTACGTTGACGACTTTAA
TTTTAAAAGAGTTAGGTGTAAAAAAAGTAACTGCTAAAGCACAAAATGATTATCATGCAAAGATTTTAAATAAAATTGGAGCAGAT
ACGGTTGTGCACCCTGAGCGTGATATGGGTAGACGTATTGCGCATAATGTTGCGAGTGCAAGTGTACTTGATTATCTTGAGTTGGCA
GACGAGCATTCTATTGTAGAATTGAAAGCAACTGAAAAGATGGCGGGGCAGTCTATCATTGATTTAGATATAAGAGCACAATATGG
AATTAACATTATTGCAATTAAACGAGGCAAAGAGTTCATCATTTCACCAAATCCAAATATTAATTTAGAAATAGGTGATATTTTAAT
CATGATTGGACATGATAATGATTTAAATCGCTTTGAAAAAAATATTGCGACGAGATAATCGGGAGTGGGACAGAAATGATATTTTC
TCAAAATTTATTTCGTCGTCCCACCCCGGCAAGGTTGACTAGAATTGAAAAAAGCTTGTT

>027-contig_249

AAAAGCTTGTTACAAGCGCATTTCGTTCAGTCAACTACTGCCAATATAACTTTGTAGAGCATTGAACATTGATTTATGTCTCAAGCT
CATCATATATAAAGAAAAAGCTACTAACAATGTGTGGTGAGTTTAATTAAAACTTCACTTCAAAGTTAGTAGCTTTTTGATTATTTAT
TGTTTGATTCTTTTTGTTCGTTTACCTTCATAATGACTGGTAAAATCATTGGTTTTCTAGCTGTTTTTTCAAATAAATAAGGTTGTAAT
GTTTCAATGATAGAAGATTTAATCTGATGCCATTGAATATCTTTATTTTGATTTAACTTACTAATAACATCAGTTTTGATTTTGCGTTG
TGCATCATAAATTAATTGACCTGATTCCCTCATATATACAAATCCTCGAGAAATAATGTCTGGACCAGAAAGTAATTTATTTGTATT
AAAATCAATACTAACAACAACGATAACTAAACCTTCTTCAGATAATAGCTTACGGTCTCTTATTACAACATTACCGATATCACCGAT
ACCACTACCATCAACAAGTACATTACCAGATGGAATGCGACCAGCTTTACGTGCTGAATCGTGTGTTAAAGCTAAGACATCTCCAAT
ATCAAAGATGAAGACATTATCTTCTTCAACGCCGCATTCAACACCAGTCTCACCATGTGCTTTTAACATACGGTATTCACCATGAAT
AGGTAAGAAATATTTCGGCTTGATTAATCGAAGCATTAATTGTTGATCACCTTGAGAACCATGCCCTGAAGTATGGATGTTAGAAAT
CTTGCTATGGATAACATCTGCACCAGCTTTATACAAGGAATTAATAGTTCTGTTAATACTTTTTGTATTACCTGGGATAGGTGATGAA
CTAAATACAACGGTATCTTCAGGTATAATTTTAATTTGCTTATGAGTACCATTAGCAATTCTAGATAATGCTGCCATTGGTTCACCTT
GTGAACCAGTACACAGTATCAATAACTCATGCTTCGGTACGGTATTAATTTTATTAGGTTCAATAAATGTTTCAGGTGGTGCTTTAAT
ATAACCAAGTTCCATACCGATTTTAATATTGTTTTCCATCGAACGACCGAACGTAACAATTTTACGGTTATTTTTGATAGCAGCTTCA
ACTGCTTGTTGAACTCGGTAAATATTAGAAGCGAAGGTAGCAAATATAATACGACCTTTACAATTACGGAAGATCTTATCTACGTTT
TGACCAACTTCACGTTCGCTTAAAGTAAAATCAGGCACAAGTGAATTTGTTGAGTCTGAAAGTAAACATAGAACGCCTTCTTCGCCT
AATTGAGCCATTTTAGCAATGTTTGCTGGTTTGCCTACAGGTGTAAAATCAAATTTAAAGTCACCGGTATGAACTACTTTTCCTTCAG
GTGTATCTACGATGACGCCATAAGTTTCAGGAATACTATGCGTAGTTAAGTAGAAAGAAATCGTAAAGTGCTTAGATTTAATCACAC
TGTCCTCATTGATTTCATTTAGTTTAGCAGTACGTAATAAATGATGTTCTTCAAGTTTATTACGGATTAAACCTAATGCTAAAGGACC
ACCATAAATAGGTATATTAAGTTGTTTTAATAGGAAGGGCACACCGCCTATATGGTCTTCGTGACCATGTGTTATAAATAGGCCAAC
AATTTTATCTTGGTTTTGAACTAGATATGTGTAGTCAGGTATAACATAATCAATCCCTAATAAGTTATCATCAGGGAATTTGATACCT
GCATCGATAATGACAATTTCGTCTTTATACTCAACTGCATAAGTATTTTTACCGATTTCACCTAGACCTCCAAGTGCATATACACCTA
CTTCATTTGGATGTAATTGTTTCATTATTTAGCAATCTCCACATTAAAGTGTTCTGAGTTTTCTTTTTCGTAATCTAAATGTGCGCCCT
CTAATTTAGTGATAAATTCGATATTAAAATTACGATCTTTCAAGTAACGACGTACTTGTTCTTCTGTTTGAGCTTCAACATAAAGTGA
TTGTGTATTTTCACGCACAATTACCTCGTCTCTGTTATGTTGATAAAAAACTTTAAATACTGCCATGTTAAAAATTCCTCCTAAGAAT
GTTTGTTTAATTTATTGTTAACCCTAGTAAAATCGTATTGGAGTATATATCGATAAATTCATTCCAATTATCTCTATAATTTAACTTAA

TAACGATTTGGTTAATAACTAGGTTCATCATGTCGTTCATTTTAAAAAATTAGTGAAATAACACTAAAATTTCAGTTAAATCTTATTT
TACATGATGAATGATAATAAATAAAGCAGTTTATCTCACTAGAAATCTAAGTATTTCGAATAAATGTGCAGTTCTATTTATTAAAAT
AAATGTAGAGATTATAAATTAATGTTAAGAAAGAATTAAAGGATTTTATATGAAACGGTAAAATTTAAATGAGGGGATAAAAAGTT
TGGTAGAAAGGTGAATATATTTAACTGTTGTTTATTCGAAAAAGTTGTACAAGAAAAAGCTTAGGACATCATATTTATCCTAAGCTT
TGATAATTAGTAATAGTGTGTTTTAAACTTCTACTGCATCTGTATGTGGTTGTAATGGGTGGTCTTTGTCAATGTGATCATAGAACAT
TACACCATTTAAATGATCAATTTCATGTTGGAAAACAATTGCTGGATATCCTTTTAGTCGTAATTGTATATCATTACCTTCGATGTCT
TTGGCTTTAATTGTAATTCTATTATGACGGTGAACTAGACCAGCAACATTATCATCGACACTAAGGCAACCTTCACCAGTTGGTAAA
TAAGCTTCTTGAACGCTATGACTTACAATTTTTGGGTTCACAAGCATATAGTCATAAGATTTGCCACTGCCATCATCTGGTATTAAAA
CAGCAATCATACGTTTAGAAATATTAATTTGAGGTGCAGCCAAACCAACGCCTGAACGTAAACCATATCGTTTCGCGATTTCCTCAT
CTTGACTATTTACTAAAAACTCTCTCATGGCGATTAATGTTTCTTTTTCTTCTTTAGTTAATGGTAATTCTAACTCAGCTGCTTTTTGA
CGCAAAGTTGGATGACCATCTCTAATGATGTCTTTCATTGTTAACATATATTGCACCTTCCTTATTTTAATTTGTTTTAGTTGAATGAC
AGTAAAAAGGTTGTTAAGATACTCATGCATTTTTATGTGTAAATATCTACAAAGTTAACCAACTACTGCCAATGTTTATTTTAGATA
GTATATGTAAATTTTCAGATATGCTAATTGCTTAAAAAATGATTAAAGTGTTGGTTTCAAGCAATGATACTTTAGAAATTTATTTATC
ATCTTGACTTTAAAAATTATATTATAAATGACGTAACTGTCAACAGATATACTTAGTAATGAAGATGTGTAATGTAATTGTTTAAAA
TTGATTTCCAAGCAGATTTTATTTATCATTTAATTTAAATAGCAAGTGGAGGTACAAGTAATGAAATTTGGAAAAACAATCGCAGTA
GTATTAGCATCTAGTGTCTTGCTTGCAGGATGTACTACGGATAAAAAAGAAATTAAGGCATATTTAAAGCAAGTGGATAAAATTAA
AGATGATGAAGAACCAATTAAAACTGTTGGTAAGAAAATTGCTGAATTAGATGAGAAAAAGAAAAAATTAACTGAAGATGTCAAT
AGTAAAGATACAGCAGTTCGCGGTAAAGCAGTAAAGGATTTAATTAAAAATGCCGATGATCGTCTAAAGGAATTTGAAAAAGAAG
AAGACGCAATTAAGAAGTCTGAACAAGACTTTAAGAAAGCAAAAAGTCACGTTGATAACATTGATAATGATGTTAAACGTAAAGA
AGTAAAACAATTAGATGATGTATTAAAAGAAAAATATAAGTTACACAGTGATTACGCGAAAGCATATAAAAAGGCTGTAAACTCA
GAGAAAACATTATTTAAATATTTAAATCAAAATGACGCGACACAACAAGGTGTTAACGAAAAATCAAAAGCAATAGAACAGAACT
ATAAAAAGTTAAAAGAAGTATCAGATAAGTATACAAAAGTACTAAATAAGGTTGGTAAAGAAAAGCAAGACGTTGATCAATTTAA
ATAATTAATATAATACAGATGGTAGGAAACAACTAATACAGTTCCTATTATCTGTATCTTTTTTTATTAAAACAGAACTTTTTCAAAT
GGTTTAACAGTCCCATTTATTTGTGGTACAATTAGTAAGGATAAAATGAATTTCTATACAATTATGGGAAAGGTATGGTGAATTGAA
TGGCTCCTAAGTTACAAGCCCAATTCGATGCAGTAAAAGTTTTAAATGATACTCAATCGAAATTTGAAATGGTTCAAATTTTGGATG
AGAATGGTAACGTCGTAAATGAAGACTTAGTACCTGATCTTACGGATGAACAATTAGTGGAATTAATGGAAAGAATGGTATGGACT
CGTATCCTTGATCAACGTTCTATCTCATTAAACAGACAAGGACGTTTAGGTTTCTATGCACCAACTGCTGGTCAAGAAGCATCACAA
TTAGCGTCACAATACGCTTTAGAAAAAGAAGATTACATTTTACCGGGATACAGAGATGTTCCTCAAATTATTTGGCATGGTTTACCA
TTAACTGAAGCTTTCTTATTCTCAAGAGGTCACTTCAAAGGAAATCAATTCCCTGAAGGCGTTAATGCATTAAGCCCACAAATTATT
ATCGGTGCACAATACATTCAAGCTGCTGGTGTTGCATTTGCACTTAAAAAACGTGGTAAAAATGCAGTTGCAATCACTTACACTGGT
GACGGTGGTTCTTCACAAGGTGATTTCTACGAAGGTATTAACTTTGCAGCAGCTTATAAAGCACCTGCAATTTTCGTTATTCAAAAC
AATAACTATGCAATTTCAACACCAAGAAGCAAGCAAACTGCTGCTGAAACATTAGCTCAAAAAGCAATTGCTGTAGGTATTCCTGG
TATCCAAGTTGATGGTATGGATGCGTTAGCTGTATATCAAGCAACTAAAGAAGCACGTGACCGCGCAGTTGCAGGTGAAGGTCCAA
CATTAATTGAAACTATGACATATCGTTATGGTCCTCATACAATGGCTGGTGACGATCCAACTCGTTACAGAACTTCAGACGAAGATG
CTGAATGGGAGAAAAAAGACCCATTAGTACGTTTCCGTAAATTCCTTGAAAACAAAGGTTTATGGAATGAAGACAAAGAAAATGA
AGTTATTGAACGTGCAAAAGCTGATATTAAAGCAGCAATTAAAGAGGCTGATAACACTGAAAAACAAACTGTTACTTCTCTAATGG
AAATTATGTATGAAGATATGCCTCAAAACTTAGCAGAACAATATGAAATTTACAAAGAGAAGGAGTCGAAGTAAGCCATGGCACA
AATGACAATGGTTCAAGCGATTAATGATGCGCTTAAAACTGAACTTAAAAATGACCAAGATGTTTTAATTTTTGGTGAAGACGTTGG
TGTTAACGGCGGTGTTTTCCGTGTTACTGAAGGACTACAAAAAGAATTTGGTGAAGATAGAGTATTCGATACACCTTTAGCTGAATC
AGGTATTGGTGGTTTAGCGATGGGTCTTGCAGTTGAAGGATTCCGTCCGGTTATGGAAGTACAATTCTTAGGTTTCGTATTCGAAGT
ATTTGATGCGATTGCTGGACAAATTGCACGTACTCGTTTCCGTTCAGGCGGTACTAAAACTGCACCTGTAACAATTCGTAGCCCATT
TGGTGGTGGCGTACACACACCTGAATTGCACGCAGATAACTTAGAAGGTATTTTAGCTCAATCTCCAGGTCTAAAGGTTGTTATTCC
TTCAGGCCCATACGATGCGAAAGGTTTATTAATTTCTTCTATTAGAAGTAATGACCCAGTCGTATACTTGGAGCATATGAAATTGTA
TCGTTCATTCCGTGAAGAAGTGCCTGAAGAAGAATATACAATTGACATTGGTAAGGCTAATGTGAAAAAAGAAGGTAATGACATTT
CAATCATCACATACGGTGCAATGGTTCAAGAATCAATGAAAGCTGCAGAAGAACTTGAAAAAGATGGTTATTCTGTTGAAGTAATT
GACTTACGTACTGTCCAACCAATCGATGTTGACACAATTGTAGCTTCAGTTGAAAAAACTGGTCGTGCAGTAGTAGTTCAAGAAGCA
CAACGTCAAGCTGGTGTTGGTGCAGCAGTTGTAGCTGAATTAAGTGAACGTGCAATCCTTTCATTAGAAGCACCTATTGGAAGAGTT
GCAGCAGCAGATACAATTTATCCATTCACTCAAGCTGAAAATGTTTGGTTACCAAACAAAAATGACATCATCGAAAAAGCAAAAGA
AACTTTAGAATTTTAATACATTTTAAAAGTTAACGAAGTTAGCGTATTTTAGTCTCATTGATTAAAATGAAATGTTTAATTTACGAAA
TCTTAGGAGGGCAAAAACGTGGCATTTGAATTTAGATTACCCGATATCGGGGAAGGTATCCACGAAGGTGAAATTGTAAAATGGTT
TGTTAAAGCTGGAGATACTATTGAAGAAGACGATGTTTTAGCTGAGGTACAAAACGATAAATCAGTAGTAGAAATCCCATCACCAG
TATCTGGTACTGTAGAAGAAGTTATGGTAGAAGAAGGTACAGTAGCTGTAGTTGGTGACGTTATTGTTAAAATCGATGCACCTGATG
CAGAAGATATGCAATTTAAAGGTCATGATGATGATTCATCATCTAAAGAAGAACCTGCGAAAGAGGAAGCGCCAGCAGAGCAAGC
ACCCGTAGCTACTCAAACTGAAGAAGTAGATGAAAACAGAACTGTTAAAGCAATGCCTTCAGTACGTAAATACGCACGTGAAAAA
GGTGTTAACATTAAAGCAGTTTCTGGATCTGGTAAAAATGGTCGTATTACAAAAGAAGATGTAGATGCATACTTAAATGGTGGTGC
ACCAACAGCTTCAAATGAATCAGTTGCTTCAGCTACAAGTGAAGAAGTTGCTGAAACTCCTGCAGCACCTGCAGCAGTAACATTAG
AAGGCGACTTCCCAGAAACAACTGAAAAAATCCCTGCTATGCGTAGAGCAATTGCGAAAGCAATGGTTAACTCTAAGCATACTGCA
CCTCATGTAACATTAATGGATGAAATTGATGTTCAAGCATTATGGGATCACCGTAAGAAATTTAAAGAAATCGCAGCTGAACAAGG
TACTAAGTTAACATTCTTACCTTATGTTGTTAAAGCACTTGTTTCTGCATTGAAAAAATACCCAGCACTTAACACTTCATTCAATGAA
GAAGCTGGTGAAATCGTTCATAAACATTACTGGAATATCGGTATTGCAGCAGACACTGATAGAGGATTATTAGTACCTGTTGTTAAA
CATGCTGATCGTAAGTCTATTTTCCAAATTTCAGATGAAATTAATGAATTAGCTGTTAAAGCACGTGATGGTAAATTAACAGCCGAT
GAAATGAAAGGTGCTACATGCACAATCAGTAATATCGGTTCAGCTGGTGGACAATGGTTCACTCCAGTTATCAATCACCCAGAAGT
AGCAATCTTAGGAATTGGCCGTATTGCTCAAAAACCTATCGTTAAAGATGGAGAAATTGTTGCAGCACCAGTATTAGCATTATCATT
AAGCTTTGACCACAGACAAATTGATGGTGCAACTGGCCAAAATGCAATGAATCACATTAAACGTTTATTAAATAATCCAGAATTATT
ATTAATGGAGGGGTAAAACATGGTAGTTGGAGATTTCCCAATTGAAACAGATACTATAGTAATCGGAGCAGGTCCTGGTGGATACG
TTGCAGCAATTCGTGCAGCTCAATTAGGACAAAAAGTAACAATCGTTGAGAAAGGTAATCTTGGTGGTGTTTGCTTAAACGTAGGA
TGTATTCCTTCAAAAGCATTACTACATGCTTCTCACCGTTTTGTTGAAGCACAACATTCTGAAAACTTAGGTGTTATTGCTGAAAGTG
TTTCTTTAAACTTCCAAAAAGTTCAAGAATTCAAATCATCAGTTGTTAATAAATTAACTGGTGGTGTTGAAGGCTTACTTAAAGGTA
ACAAAGTTAACATCGTTAAAGGTGAAGCATATTTCGTAGATAACAATAGCTTACGTGTTATGGACGAAAAGAGCGCACAAACATAC
AACTTTAAAAATGCAATCATTGCAACAGGTTCAAGACCAATTGAAATTCCTAATTTCAAATTCGGTAAACGTGTTATCGACTCAACA
GGTGCTTTAAACTTACAAGAAGTACCAGGTAAATTAGTTGTAGTTGGTGGAGGATACATTGGATCAGAATTAGGTACAGCATTTGCT

AACTTTGGTTCAGAAGTAACCATCCTTGAAGGTGCTAAAGATATCTTAGGTGGCTTCGAAAAACAAATGACACAACCTGTTAAAAA
AGGTATGAAAGAAAAAGGTGTTGAAATCGTTACTGAAGCTATGGCTAAATCAGCTGAAGAAACAGATAACGGAGTTAAAGTTACTT
ATGAAGCTAAAGGCGAAGAGAAAACAATCGAAGCTGATTATGTATTAGTAACTGTAGGTCGTCGTCCAAACACAGACGAATTAGGC
CTAGAAGAATTAGGTGTTAAATTCGCTGACCGTGGATTATTAGAAGTTGATAAACAAAGCCGTACGTCTATCAGCAATATCTATGCA
ATTGGTGATATCGTTCCAGGTTTACCACTTGCTCACAAAGCTAGCTATGAAGCTAAAGTTGCTGCTGAAGCAATTGATGGTCAAGCT
GCTGAAGTTGATTACATTGGTATGCCAGCAGTATGCTTTACTGAACCAGAATTAGCTACAGTTGGTTATTCAGAAGCGCAAGCTAAA
GAAGAAGGTTTAGCAATTAAAGCTTCTAAATTCCCATATGCAGCAAATGGTCGTGCATTATCATTAGATGATACTAACGGATTTGTT
AAACTTATTACACTTAAAGAAGATGATACTTTAATCGGTGCTCAAGTAGTTGGTACTGGTGCATCAGATATTATCTCTGAATTAGGT
TTAGCAATTGAAGCTGGTATGAATGCTGAAGATATCGCATTAACAATCCATGCACATCCAACATTAGGTGAGATGACTATGGAAGC
AGCAGAAAAAGCTATCGGATACCCAATCCATACAATGTAATAACTGATTATCTATAAAGATTCAGTCATTAAAAGCTGTAGCATAT
GCTACGGCTTTTTTGTTTTAGGTAAAGTAATGTAAGGAAATTGATTTGAGATATCGTTAACATGTGACATGCATGTTATACTAGCGA
TGCTAACAAAAGAATTGAAATGGAGGGTTCAACAATGGAATATGAGTATCCAATTGATTTAGACTGGAGTAATGAAGAGATGATTT
CAGTGATAAATTTCTTTAATCATGTAGAGAAGTATTATGAATCCGGCGTGACGGCAGGCGACTTTATGGGTGCGTATAAAAGATTTA
AAGAAATTGTGCCTGCTAAAGCAGAGGAAAAACAAATTTTTAATACTTTCGAAAAAAGTAGTGGCTATAATAGTTACAAAGCAGTT
CAAGATGTAAAAACTCACTCTGAAGAACAAAGAGTAACAGCTAAAAAATAATTCGTTCGAAATTAACACAATTTAATAGGAATTTT
TCTTTAAAACTATTGCTAATAAAGCTATATTTTGATACCTTTATCAAGTGTTAAACAAAATGTTTGATAAAAGTAAACTTAATATAGC
TTTTTTAGGTGGAAAAATAAATGAACATAGGTAATAAAATTAAAAATCTTAGAAGAATTAAAAATTTAACGCAAGAAGAACTTGCT
GAACGTACAGACTTATCGAAAGGCTACATTTCACAAATAGAAAGTGAACATGCCTCACCAAGTATGGAAACTTTCTTAAATATTAT
AGAGGTGTTAGGAACGACGCCAAGTGAATTTTTTAAAGACAGTGAAAATGAAAAAGTATTATACAAGAAGGAAGAACAAGTTATT
TATGATGAGTATGATGAAGGTTATATATTAAATTGGTTAGTTTCAAAGTCAAATGAATATGATATGGAGCCATTAATATTAACTTTA
AAGCCTGGAGCATCATATAAAAATTTTAATCCATCAGAGTCTGATACGTTTATTTATTGTATGTCAGGTCAGATAACACTTAATTTA
GGCAAAGAGATATATCAAGCACAAGAAGAAGACGTTTTGTATTTTAAAGCACGAGATAATCATCGTTTGTCAAATGAATCAAACAA
TGAAACACGAATACTTATTGTAGCGACAGCTTCATATTTATAGGGGGGATCTTATTTGGAACCGTTATTATCATTAAAATCAGTTAG
TAAAAGCTACGATGATCTTAATATCTTAGATGACATAGATATTGATATTGAATCAGGATACTTTTATACATTATTAGGTCCTTCAGGT
TGTGGTAAAACAACAATTTTAAAATTAATTGCAGGGTTTGAATATCCTGACAGTGGTGAAGTGATTTATCAAAACAAACCAATTGGT
AATTTACCACCAAATAAACGTAAAGTGAATACAGTCTTTCAAGATTATGCATTATTTCCACACTTAAACGTCTATGATAATATCGCT
TTTGGTTTGAAATTAAAAAAATTATCAAAAACCGAAATTGATCAAAAAGTAACTGAGGCATTAAAATTAGTAAAACTTTCAGGTTA
TGAAAAAAGAAATATTAATGAAATGAGTGGCGGACAAAAGCAACGTGTTGCAATTGCACGTGCTATCGTAAATGAACCAGAAATA
TTATTGTTAGATGAATCTTTATCCGCATTAGATTTGAAATTGCGTACTGAAATGCAATATGAATTACGAGAATTGCAATCTAGATTA
GGTATTACATTTATATTTGTAACACATGATCAAGAAGAAGCGTTAGCATTAAGTGACTTTCTTTTTGTATTAAAAGATGGGAAAATT
CAACAATTTGGCACACCAACAGATATATATGACGAACCAGTGAATCGATTTGTAGCTGATTTTATTGGAGAATCTAATATTGTTGAA
GGGCGCATGGTTAGAGATTATGTCGTGAATATTTATGGGCAAGATTTCGAATGTGTCGATATGGGTATTCCTGAAAATAAAAAAGT
AGAAGTCGTTATTCGACCAGAAGATATATCATTAATCAAAGCTGAAGAAGGATTATTTAAAGCAACTGTTGATTCTATGTTATTTAG
AGGGGTCCACTATGAAATATGTTGTATAGACAATAAAGGTTATGAATGGGTAATACAAACGACTAAAAAAGCTGAAGAAGGCAGT
GAAGTTGGTCTTTATTTTGATCCTGAAGCCATTCATATTATGGTTCCTGGAGAAACAGAAGAAGAATTTGATAAACGTATTGAAAGC
TATGAGGAAGTAGACAATGCGTAATACTAATAAATTTCTCTTAATCCCGTATTTACTATGGATGGTTATATTTATTATTGTACCAGTT
GTATTACTCATTTATTTTTCATTTTTAGATATCAATGGACATTTTAGTTTCACGAATTATCAACAAATTTTTACTACAAAATATTTGAA
AATGTTTGCATATTCAATTTTATATGCCGCTTTAATAACAATTATTACCTTGACTATCAGTTATCCAGCTGCCTATTATATTACTCGTT
CGAAATTTCAAAATATCTTATTAATGATAATGATTATTCCAACATGGATAAATTTATTGTTAAAGACATATGCTTTTATAGGTTTATT
AAGTCATGATGGCGTGATTAATCAATTTTTCCACTTATTTAATTTACCATCATTCAATTTGTTATTTACAACTGGTGCGTTTTTAGTAG
TGGCAAGTTACATTTATATACCATTTATGATTTTACCTATATTTAATAGCATGAAAGCAATTCCTAATAATTTATTGCAGGCCTCAAG
TGATTTAGGTGCTAGTCCTTTCTATACTTTTAGAAAAGTAATCATGCCGTTAACAAAAGAAGGTGTTATGACTGGAATTCAAGTAAC
CTTTATTCCATCACTTTCATTATTTATGATTACAAGATTAATTGCAGGTAATAAAGTCATAAATATAGGTACGGCAATAGAGGAACA
ATTTTTAACAATTCAAAATTATGGTATGGGATCAACTATAGCTATATTCTTAATTGTATTTATGGCATTCATTTTAATCATTACAAAA
TCATCTAATGGGAGAGGGTGAATCATATGAAATGGTATGGAAAGCTGTATATCGGGATACTTTTAGCGATTTTATACATCCCAATAT
TCTTTTTAATGTTCTATTCATTCAATTCGGCTGGTAATATGATTCACTTTGAACATTTTACATTAGAGCATTATCAATCATTATTTCAA
AATGATCATTTAATGTCGGTCATTTTTAATACGATAGCTGTAGCACTTTTAGCAGCCTCAATTTCTACAGTTATTGGTACATTTGGTG
CCATTGCTATTTATTATTTAAGAAATAAAAAGTTTAAAGTAACTTTACTAACATTGAATAATGTCTTGATGGTATCATCCGACGTTGT
CATAGGTGCATCATTCTTAATTATGTTTACAACGATTGGCCATTTTACTGGTCTTGGTTTAGGATTTTGGACAGTTCTAATATCTCAT
ATTGCATTTTGCATACCTATAGTTGTGATTATCGTCTTACCACAACTGTATGAAATGAATAATAATATGTTAAATGCTGCAAGAGATT
TAGGAGCGACTGAACCACAATTATTAAGCAACATTATTATTCCTAATATTTTACCTTCTATTATAGGAGGTTTCTTTATGGCTTTAAC
TTATTCACTAGACGATTTCACAGTAAGTTTCTTCGTTACTGGTAATGGCTTTAGTGTGTTATCAGTTGAAGTTTATGCTATGGCGAGA
AAAGGAATTAGTATGGAAATTAATGCGATTTCAACATTATTGTTTGCTGTTATTGTATTAGGAATACTAGGATATTATTTGATTCAAT
ACGTGATAAATAAGAAAAAACTAATCAAGCGAGGTGTAAAATAATGAAACGTTTTTTACAACTCATTATAGGTGCATTAGTTGTGG
GTATGCTTTGTCTTACTTTAAGTCATTGGTTTAAATCTAAAGAACAAGTGCATACAAATCAAAAAATTTACGTATACAATTGGGGCG
AATATATTGATCCAGAGTTAATTAAGAAATTTGAAAAAGAAACTGGCATTCAAGTCGTTTATGAAACTTTCGATTCAAATGAAGCG
ATGGAAGCCAAAATTCGCAATGGCGGTACACATTATGATGTTGCTTTTCCTAGTGAATATACAGTTCAAAAATTGAAAAGAGATCAT
TTATTGTTACCAATAGATCATAATAAGGTACCTAATATTAAAAATTTAGATTCAGATTATATGAATATGTCATTTGATAGAGGCAAT
AAATATTCTTTACCTTATTTCTTTGGAACTGTAGGTATTTTATATAATAAAGAAAAGTATCCAAATGAATCATTTGATAGTTGGAAGT
CATTGTATAATCCTAAATTTAAAAACCAAATTTTACTAGTTGACGGTGCTAGAGAGATTATAGGCATGAGTTTGAATAAACTTGGGT
ATAATCTTAATGACCGTAATTCGCACCATTTAAAAGAAGCAGAGCGAGATTTAACCAAACTAGCACCACAAGTAAGAGGTGTCGTA
GGTGATGAAATTACCATGATGCTTCAACAAAATGAAGGTAACATAGCGGTTGTTTGGAGTGGTGTTGCAGCACCTCTAGTGCAAGA
AGGGGATAAATATAATTATGTTATTCCTAAAGAAGGATCGAATTTATGGTTCGACAATATGGTAATTCCAAAAACGGCACAAAATA
AAGAGGGTGCATATAAATTTATGAATTTTTTATTAGATGCTAAAAATAACAAGCAAAATACAGAATTCGTAGGCTATGCAACGCCA
AACAAGGCTGCTCGACAATTGTTGCCTAAAGAGATTAAAGACGACCATCGTTTTTATCCGACTAAGAAAGAGCAAGAACGCCTTGA
AGTTTATAAAGATTTAGGACCTGAAGTTTTAAGTGAATACAATGAAAACTTTTTGAATTTCAAAATGTCATTAAAATAAAATCATAA
ATAGTAGTTATTTACTGTATATTTAGTTATAATAACGGGATAAGTACTATAAGGAGTTGTAACGAATGACTGGAGAACAATTTACTC
AAATTAAACGTCCAGTAAGTAGATTAACTGAAAAAGTTCTAGGTTGGTTATGTTGGGTGATGTTATTAGTGCTTACTGTCATTACGA
TGTTTATAGCACTTGTTTCATTCAGTAATAACACATCGATTGCTAATCTTGAAAATACATTAAACAATAATGCATTTATCCAGCAATT
ATTAGCTGGAAATGGCTATAATACAACACAATTTGTAATATGGTTACAAAATGGTATATGGGCTATTATCGTTTACTTTATTGTTTGT
TTGTTGATTTCATTTTTAGCTCTCATATCTATGAATATAAGGATCTTGTCAGGTTTCTTATTCTTAATATCAGCAATTGTAACGATTCC

TTTAGTTTTACTTATCGTTACTTTAATCATTCCGATATTATTCTTTATTATTGCGATGATGCTATTTATAAGAAAAGATAAAGTTGAAA
TGGTTGCGCCACAATATTATGAAGAGTATAACGGACCGATTTATGATTATCGTGAACCTGTGTATGAGCGCCCCCAACCGAAAGAT
GATTATTATGATGTGCCTAAATATGAAAAAGAATTGGATAAATCAAATACTGTATATGATCAAGAACAGGAAAGAGATAAATATGA
TCAATTTCCTAAACGTGCAGTTGAAAGTGAATATAATCATGATGAGCGCACTGAGGAAGAACCATCAGTATTATCCAGACAGGCTA
AATACAAACAAAAAAGTACTGAAGAACTAGGTATTGAAGATGATGGTTATTATGCAGAACCTGAAGTTGATCCAAAAGAATTGAA
AGCACAACAAAAGCGAGAAAAAGCTGAAATTAAAGCTAAGAAAAAAGAAAAACGTAAAGCTTATAACCAACGAATGAAAGAACG
TAGGAAAAAATCAACCTAGCGCAGTTAGTCAACGTCGAATGAATTTTGAAGAGCGACGTCAAATTTACAACAATGATATTTCTGAAG
AACGCAATTCAAGTGAAGTTAAGGACAAAAAAGAGCAAGAATAAATATTGATGATACCCAGATTAGTAAGCAGAGGTCTTTTGCTT
ATTAATCTGGGTTTTTATACGAGGTTAATTATCGATAACGTTTAATTAAAGTGTTTAGGTGTCATAATTTTAAATGACGATTTCTCAT
TACTATACACCTAAATTATCATCAATCTGAATTCAGATGTTTATTATAAAAATTAGATGAAAAATATGTTAATATACAAGTAATTTA
ATGTGCGTATATCTATAGGCTCGTAGTATACTTATTAAAATATTAAACAAAGAAAGGATTTTAAGATGAATAGAAAACCTGAATTA
ATCATGGCTTGGATTGCAAATAGTATTAGTATTATTTATTTATTAGTTATGGGGCTATCCTATTTTTCTTTAAAAAGTGGTAATGCAT
CACAACGTGAAGAATTAGCGAAGCAATTATCTCAGAACGGTGGCAAGGTTTCTTTAGATATGCTTCAGACAACAATGGGTGCATTA
GCAATTATTTTATTAATTTCAACACTTTATGGTATATTTGCGACAATTTGTATTAAAGGACGTAGAAAATTATCGATTATACTTTTTG
TTATCGCGATAATTGTAAGTTTGATGGCTCTTAATTTAATTGCAATTGTCTTATGGATTATCGTGATGATTATGTTGATTTCTAAAAA
AGAATCAAAAGAAACTACACATAAGGACGATGAGTATATTTATCATTAATATGTTCATAGCAAAAAAAACCATTAAAATGTTAAGT
TGTTAATTATTAGATACAACCAACATTTTAATGGTTTTATTTTTAACTTTGTAGTTCTTGGAATGTTTGAACGATTAAATAGACATTT
AAAATACTTAGAATAATAATAAGTGTCCATGAAATGATATTAACCCATGTTTTATTATAGAAAGGTCCCATCAAGTCTTTATTACTA
GTAGCTAATTGTAATGGTATTAAACAGAATGGCAGAGCAATACTTAAAAATACTTGAGAGAATACAAGTAGTTGCTCAATTTTAGC
TGCATTACCTTTAAAAATGATTAAGCATACGATAACAGGAATGACAGCAAGGGAACGTGTAATTAAACGTCTTAACCAATTTGGTA
TGTGTAATCTTAAAAATCCTTCCATTACAATTTGTCCTGCTAAAGTACCAGTAATCGTTGAATTTTGACCTGACGCTAATAATGCAAC
TGCAAATAATGTACTCATGATTGCACCCATTGTTGCACCTAGTACAGGTTCAGTTTTTAAGGCGTGATATAAATCATAGAAACCACC
TAAATCGTCAGCATTTGAGTTGAAAAATAGTGATGCTCCTAACACTAATAATAAGCAATTGACTACAAATGCGATTGATAACTGAAT
GTTCGAATCTATCGTAGCAAATTTAATCGCTTGCGCCTTTTCTTCATTGTTATGTCTTGAGTATGTTCTAGATTGTACAATTGATGAAT
GTAAGTACAAATTATGAGGCATAATTGTAGCGCCAATAATACCTAATGCAATATAGAGAATGCCGTTATTTGTAATGATTTCACTAT
GTGGTATAAATCCATTTAACACAGCATTCAACTGTGGTGATGAAATATAGACTTCAAATATAAAGATGAATAACACTGTGAAAATT
AATGTACCAACAATAGCTTCAATTTTTCTAAAACCATATTTCATTATAAATAGTAGTAAAAATACATCAAGTACAGTTATTAGTGCA
CCGACGATTAAAGGTATGTTAAATAGGAGATTAAGAGCAATAGCACTACCAATAACCTCAGCAATATCTGTAGCGATAATTGCTAG
TTCTGCAATGATCCAAAAGATTATAGCAATAGGTCTTGATAAATAATGTCGTGTCATTTGAGCTAAGTCCATACCTGTTGCTATTCCT
AATCTCACTGTCATGCTTTGAAGTAACATTGCTGATAAACTTGAAATAAGAATTACGAATAGCAAAGTATAGCCATATTGGGCGCCA
CCTTGCATTGATGTTATCCAGTTTCCGGGATCCATGTAACCAACAGCGACTAATAACCCAGGTCCAAGAAATGATAAAAATTTCTGT
TTATTTGAACTGCGATGATCGAATTTAATTGTATTGTTTATTTCGTCTAAACTTAATTGTTCATTTGTTGAATGTCGTTTATTATTCAT
TTTGACTCACCTCTACTTCTTATGGACAATATAATAACCTAATAAAATGTTTAGGTCAACCTAAATTTATTTTAATTTTTTTAAAGGT
ATGAATTATTATGTTGAACGTTCGATTTAATATTAAGAAAAAAAGAGCACGAAATCATGGATGTGATTACGTGCTCTATTATTTAAA
CAAAGTTATTTATGCAGAATAAAATGGTAAGAACTGATCGAAGGTTTCTTCTAAAAATGCAATAAATGCTTTGTCACTTTTTAATCT
TTTATCTTGTGGTGTGATTGCACGCGCTATAAAGAATTCACCTTTTTTCACATTGATGGCTCTTTGTATCGCTTCTTTTAAATCATCAT
CCGTTAAATCTTTAATAAATGGTTTATCAGGTTTCATATGATCTAAGCAAACACGATAATCATCAGGTAATTGTTGAATAGCTTTAA
ATTTTCTTTCAAAAACTTTTGCACGTGTTGCTTTATCTTTTGCTTCATGCATGATTCCAAACATAACAAACAGTTGATCTTCAAACAT
ACCAATTTGGAAATGAGGTAACATTTTATAGCCTCTTTTGTTTGTTGCAAAAGCAACCCATGTATCTTTAGGAGGATTCACACTTCTT
CTAGCATGCTTTGCTACGTGAGGATAAAATGTTTCACCAGTTTGACTTGTAAAGAAGTCACTAAAATATTCTCCTAATTCATGGAGT
TGTGGTCGTATGTATTCGTTTAAAGCTTCCATTCGTGCGTCTAAGCCTTCTACGTTGAACGCTTTGAAATCTTTAGGTTTAAATGTAT
ATTTTGTCATAGAGTACCTCCTGTATAGTGTTTAAAAATATTGTAGCATATGTTTTATCAATTGCGGCTTAAGACGCTCTCATTAAAA
TTAGTATTTTCATTTTTATCATAAGAAGTTATTTTTATAAAATGTTGATGTATAATAAAGATGTTAGGAGATGATGTTATGGCACTTT
ATGGATTTGCCCAAGGACTTATTCAAGAAGCAGGAATTAGAATTAAACAATTGATGGAGCAAAATTTAACAATTGAAACAAAGTCA
AATCCGAATGACCTTGTTACAAATGTAGATAAAGCAACAGAAGATTTTATTTTTGATACAATTTTAGAAACATATCCCAATCATCAA
GTATTAGGTGAAGAAGGGCATGGTCATGACATCGATACTTCCAAAGGTACGGTATGGGTTGTTGACCCAATAGACGGTACATTGAA
TTTTGTTCATCAACAAGAAAATTTCGCAATTTCAATTGGTATTTATATCGATGGTAAACCTTATGCAGGTTTTGTATATGATGTTATG
GCTGACGTCTTATATCATGCTAAAGTAGGGGAGGGTGCATATCGAGGTAGCCAACCCTTGAAACCATTGAATGATTCTAATCTAAG
ACAAAGCATTATTGGGATCAATCCGAACTGGTTAACTAAACCAATTTTAGGAGAAATCTTTAAAGAAATTGTTAATGATTCTAGAAG
TGCAAGGGCATATGGTAGTGCAGCGCTTGAAATCGTTTCAGTTGCTACAGGTAATTTAGAAGCATATATGACGCCAAGACTTCAACC
ATGGGATTTTGCTGGCGGATTGGTTATTTTATATGAAGTAAATGGACAAGCTTCCAATTTACTAGGAGAACCATTAACAATTAGTGG
TCCAAATTCAATCTTAGTTGGAAATCGTGGTCTCCATCAAGAAATTAGCAATGATTATTTAGAGCCCCACCATGATGCGTTAATACA
ATTACATGAACAACGATTTAAAAGAAAATCAAAATAATTACCAAAAGCGTAATATGTAGTCCTAACAATTAGGTAACATATTACGC
TTTTATACCTTAAATAATAAAAACACGCGCCAATATAAAACCAGTTAAATGTTCATCATGAACATGTGTGGTCTTAAATCAACGCGT
GTTACTGTCGATATAACTTATAGCCAATCGTTTTCTCGATATTTTTTCTTTGTAGTAAATCCAATTCCAAAGGTTGCAACGAGTAATA
TAAATGTTAAAATCATCATTGGTACATTTGATGCACCAACAGCAAAACTAAATAATACTAAGAAAACAACAGCTAATATAGAAAAT
ACCCAAAAGATATTTTTAGATTTTTTTTGTTTCATCCTATCCCCACCTTTTGCTTATCTTTTCTCAATTATATGATATAATAAAAAAGT
TGTAATTAAAAGTGGGATTTTACTTAAGAAAGAAGGAAACTATTTATATGACTAATAAAAGAGAAGATGTCCGCAATATAGCAATT
ATTGCTCACGTTGACCATGGTAAAACAACTTTAGTAGATGAGTTGTTAAAACAATCTGGTATATTCAGAGAAAATGAACATGTCGAT
GAACGTGCAATGGACTCTAACGATATCGAAAGAGAGCGTGGAATTACGATTCTAGCCAAAAATACGGCTGTTGATTATAAAGGTAC
ACGTATTAATATTTTGGATACACCAGGACATGCAGACTTTGGTGGAGAAGTAGAACGTATTATGAAAATGGTTGATGGGGTTGTCTT
AGTAGTAGATGCGTATGAAGGTACAATGCCTCAAACACGTTTTGTACTTAAAAAAGCGCTAGAACAAAACCTGAAACCTGTTGTTG
TTGTTAATAAAATTGATAAACCATCAGCACGTCCAGAGGGTGTTGTAGATGAAGTTTTAGATTTATTTATTGAATTAGAAGCAAACG
ATGAACAATTAGAATTCCCTGTTGTTTATGCTTCAGCAGTAAATGGAACAGCTAGCTTAGATCCTGAAAAACAAGATGATAATTTAC
AATCATTATATGAAACGATTATTGATTATGTACCAGCTCCAATTGATAACAGTGATGAGCCATTACAATTCCAAGTAGCATTGTTGG
ACTACAATGATTATGTTGGACGTATTGGTATTGGTCGTGTATTCAGAGGTAAAATGCGTGTCGGAGATAATGTATCACTAATTAAAT
TAGACGGTACAGTGAAAAACTTCCGTGTAACTAAAATCTTTGGTTACTTTGGATTAAAACGTTTAGAAATTGAAGAAGCACAAGCT
GGAGATTTAATTGCTGTTTCAGGTATGGAAGACATTAATGTTGGTGAAACTGTAACACCACATGACCATCAAGAAGCATTGCCAGTT
CTACGTATTGATGAGCCTACTCTTGAAATGACATTTAAAGTTAACAATTCTCCATTTGCTGGCCGTGAAGGTGACTTTGTAACAGCA
CGTCAAATTCAAGAACGTTTAAATCAACAATTAGAAACAGATGTATCTTTGAAAGTTTCTAACACAGATTCTCCAGATACATGGGTA
GTTGCTGGTCGCGGTGAATTGCATTTATCAATCCTTATTGAAAATATGCGTCGTGAAGGTTATGAATTACAAGTTTCAAAACCACAA

GTAATTATTAAAGAAATAGATGGTGTAATGTGTGAACCATTTGAACGTGTGCAATGTGAAGTGCCACAAGAAAATGCAGGTGCTGT
TATTGAATCATTAGGTGCACGTAAAGGTGAAATGGTTGATATGACTACAACTGATAATGGACTTACACGTTTAATCTTTAATGTACC
GGCTCGTGGTATGATTGGTTATACGACTGAATTTATGTCAATGACAAGAGGTTACGGTATTATTAACCATACATTTGAAGAATTTAG
ACCACGTATTAAAGCACAAATTGGCGGTCGTCGTAATGGTGCATTAATTTCAATGGATCAAGGTTCTGCAAGTACTTATGCCATTTT
GGGACTTGAAGATAGAGGTGTAAACTTCATGGAACCTGGTACTGAAGTTTATGAAGGTATGATTGTTGGTGAACATAATCGTGAAA
ATGATTTAACTGTTAACATCACTAAAACAAAACATCAAACTAACGTACGTTCTGCAACGAAAGACCAAACACAAACAATGAATAGA
CCGCGTATTCTAACATTGGAAGAAGCGTTACAATTCATTAATGATGATGAACTTGTTGAGGTTACACCAGAAAGTATACGTTTAAGA
AAGAAAATTTTAAACAAAAATGTTCGTGAAAAAGAAGCAAAGCGTATCAAACAAATGATGCAAGAAAACGAATAATTTAACATAA
TAAAAGAGAAGCTGGGCGATGAAAGTTGCTTAGCTTCTCTTTTGGCATATAATATTTGAAAAAGTGTTTAAATGCTATTATTTCCTGT
TACTTTCACATTTTCAAATTTCTAACTTGTTTATTCAGATATTAATCGAGCGGATAGTGTTGCGCACGTTGTTTAGGTGTGTCTAATC
GACTTTTACAATCATCACACATAAATGTTCGAATAGGATTGTTTTTAAGTCGTTTAGACTCAGTTGTGCGCTCATCTATAAATACTTT
TGCATCACAGATAATGCATTGCACTTGTCTCAAATTAAATCACCTCAATATGAGTTACTTGGTTGATTCGATATGAATAACCATCTTC
TTGATTGTAAATAAAACTATCTACACCATTATCGCTGTAAAGTCGTTTACCGTCTTTTGCAAATTGGAAAAATAAATAGGGTAATAG
ATCTATCGGTATATCTAAATGATCATGCTCATTTGATAATCGAATAGTAGTTGCAGAGTCATGTGGTTCGGAATGTTTGAAAAATGG
TGTCATATTAATGACAAATGAACCTTCTAACATGGCACGTTTTTTATATTTTATTTCTGAATTTAAAGTAGGCGGATTAGTTTGTCCT
TCTAGGATAGCACGATTCCATTCATGATTATCTTCAAAGTCGATTGGTTTTGAACCATCAAATACACCTTTTTCTAAATCTTCGATGC
TAACTTTTCTATCATCGAAAATCCAAGTCGTACTATCTAATGTTATAGGAAACTTTACGGCTCCTTTAATTTGTATCATTTTCCCACTC
CCTATCAATGTATATAGCATTATTTTAACACAAATTGCTAACGACACATTTTAAATCTACTTGCTTTTTAATATTTAGTAAGATAAAC
TTTTAGTAAGACTTGAGAATTTATATAGAGGGGGAGCGTGTCATGGCGAAACAAGCAACAATGAAAAATGCAGCTTTGAAACAATT
GACTAAAGATGCTGATGAAATCTTGCATCTGATTAAAGTTCAACTAGATAATTTAACATTACCTTCATGCCCATTATATGAAGAAGT
ACTAGATACACAAATGTTTGGACTTCAAAAAGAAGTTGATTTTGCTGTTAAATTAGGTTTAGTTGACCGCGAAGATGGCAAACAAAT
TATGTTACGTCTTGAGAAAGAACTTTCAAAATTACATGAAGCTTTTACACTTGTTTAAGGTTTAATATATAAGCATATTAAAATGGC
GTGAGATATCGACAATTGAGCTGCGCCGATTTTATATTTATAGTTTAATTACTGATTAAAGTGGCTGTTATGTTGTCTTCTTGATGAC
ATGTCAGTCACTTTTATTGGGTTTTAGCAATTCACGATATTAAGTAAACAAATATTCTATAAATTACCTCAAGAGACCTGTTAATATT
TGTGATTATGACTGTGTCATGTATATAATAAGTAACATGGATTTTTAAAATTAAAGTATAAAACGAAATCATTTGAAGTATAAATTG
TAGAATTGGATGACTGAATATATGAAGAATTTTAGAAGTATTTTACGGTATATTGGTAAAACCTCAAAGTTTATTGATTATCCGTTA
TTAGTTACATATATTGTATTGAGTTTAATTGGATTAGTTATGGTATATAGTGCAAGTATGGTTCCAGCAACTAAAGGCACATTGACT
GGTGGTATCGATGTTCCAGGAACGTATTTTTACAACCGACAATTAGCATATGTCATAATGAGTTTTATAATTGTATTTTTTATTGCAT
TTTTAATGAATGTTAAATTACTGAGTAATATTAAAGTGCAAAAAGGTATGATTATAACTATCGTCTCACTATTATTACTGACGTTAGT
AATAGGTAAAGATATTAATGGTTCTAAAAGTTGGATAAACTTAGGATTTATGAACTTACAGGCATCTGAGTTATTAAAAATTGCAAT
CATATTATATATCCCATTTATGATCAGTAAAAAAATGCCTAGAGTATTAAGTAAACCAAAATTAATTTTAAGTCCTATTGTATTAGC
ATTAGGTTGTACGTTTTTAGTTTTCCTACAAAAAGACGTAGGGCAAACATTACTAATATTAATTATTTTAGTTGCGATCATTTTTTAT
TCAGGAATTGGGGTAAACAAAGTCCTAAGATTTGGTATACCAGCAGTGCTAGGATTCTTAGTAGTATTTGTCATTGCATTAATGGCT
GGTTGGTTACCAAGTTATTTAACAGCCAGATTTAGTACGCTAACAGATCCATTCCAATTCGAATCAGGAACTGGATACCATATTTCC
AATTCATTGCTTGCGATAGGTAACGGTGGCGTATTTGGAAAAGGATTAGGAAATAGTGCAATGAAATTGGGCTATTTACCAGAACC
ACATACAGATTTTATTTTTGCAATTATTTGCGAAGAATTAGGTTTAATCGGAGGATTGCTAGTTATTACTTTAGAGTTCTTTATTGTA
TATCGTGCCTTCCAGTTTGCAAATAAAACATCATCATATTTTTATAAACTTGTGTGTGTTGGGATTGCCACATACTTTGGAAGTCAAA
CGTTTGTAAACATTGGCGGTATTTCGGCAACAATTCCATTAACTGGTGTGCCATTGCCATTTATCAGCTTTGGTGGATCATCAATGAT
TAGTTTAAGTATTGCTATGGGATTACTTCTGATTGTAGGTAAACAAATCAAAGTAGACCAGCAACGAAAGAAACAACAACAAAAAG
TTGATATAAGAAGACAATTTAATTAAAAAAATACTAGCCAATATTTAGTACGAGATGACTTCTGTCATTAAGCAATGCTAAACATTG
GCTATTTGTATACTCTTTGTCAAATTGGACTGATGAGTTCAATAACGGAAGTTAGGCAACCAAACAACGCTTAACTTCCTTTTTGTTT
TGATATTACGAGATTACTCATGATTATCAAATTTTATAAACAAAACAACGTATAATTATAACTAAATTTTATGACTAAAAATTACTA
AATGTGGTGGCGAAATTAGAATCATGATGATAAGATAAAGTTATCGCTATTGATAATAAATGTTGATGTTTTGCAATTTTATTTTTA
ATTTTTTAGAGTTGTAGAAATCGAAAATGTTTCACAACAAAAACGTGAACTACCTGTTGTTGCATAGTCATTTTTGAAATTATTATTT
CTGAAGCGGAGGTTTTAATTGTCTCAAGAATGTTAAATTTTGAAAATTTGAAACATAACTATAGCAAAACAGAGCGCTTTAAGATA
AAATTTTATTATCTAATTAGTTAGAAAATTCTAATAGTTTGAGGAGTGTATCGCTATTGAAACAAATAAAAAAGTTACTTGTTGCTA
ACCGTGGAGAAATTGCAATTCGTATATTCAGAGCGGCGGCAGAATTAGACATCAGCACAGTTGCAATTTATTCGAATGAAGACAAA
AGTTCATTACATAGATATAAAGCAGATGAATCCTATTTAGTTGGAAGTGATTTAGGTCCTGCTGAAAGTTATTTAAATATTGAGCGT
ATCATTGATGTAGCAAAACAAGCGAATGTGGATGCGATTCATCCTGGCTATGGATTTTTAAGTGAAAATGAACAATTTGCGCGTCGT
TGTGCAGAAGAAGGAATTAAATTTATTGGTCCTCATTTAGAACATTTAGATATGTTTGGAGATAAAGTTAAAGCTCGTACAACGGCT
ATCAAGGCAGATTTACCAGTTATTCCTGGTACAGACGGTCCAATTAAATCATATGAATTAGCAAAAGAATTTGCAGAAGAAGCTGG
TTTCCCGCTAATGATTAAAGCCACAAGTGGTGGCGGCGGTAAAGGTATGAGAATCGTTCGTGAAGAAAGTGAATTAGAAGATGCTT
TCCATAGAGCAAAATCAGAAGCTGAAAAATCATTTGGTAATAGTGAAGTTTACATAGAAAGATACATTGATAATCCAAAGCATATT
GAAGTACAAGTTATAGGTGACGAACATGGAAATATCGTACACTTATTTGAACGTGATTGTTCAGTACAACGTCGTCATCAAAAAGTT
GTAGAAGTTGCACCATCAGTTGGATTATCACCAACATTACGTCAACGTATTTGTGATGCTGCAATTCAATTGATGGAAAATATTAAA
TATGTCAATGCAGGTACTGTTGAATTTCTAGTATCTGGTGACGAATTCTTCTTTATAGAAGTTAACCCTCGTGTACAAGTTGAGCATA
CAATTACAGAGATGGTAACAGGAATTGATATTGTTAAGACACAAATTTTAGTTGCAGCAGGTGCCGATTTATTTGGTGAAGAAATTA
ATATGCCGCAACAAAAAGATATTACAACATTGGGCTATGCCATCCAATGTCGTATTACAACAGAAGATCCGTTAAATGATTTCATGC
CGGATACTGGAACAATCATTGCTTATCGTTCAAGCGGTGGCTTTGGTGTACGTCTCGATGCTGGAGATGGTTTCCAAGGTGCTGAGA
TATCACCTTATTATGATTCATTACTCGTAAAATTATCTACACACGCGATATCATTTAAACAAGCAGAAGAAAAAATGGTACGTTCAT
TACGAGAAATGCGTATTCGTGGTGTTAAAACTAATATTCCATTCTTAATTAATGTAATGAAGAATAAAAAGTTCACAAGTGGTGATT
ACACAACTAAATTTATTGAAGAAACACCAGAACTTTTCGACATTCAGCCGTCTCTAGATAGAGGTACTAAAACATTAGAATATATA
GGTAATGTAACAATTAATGGTTTCCCAAATGTTGAGAAACGTCCGAAACCAGACTATGAATTAGCATCAATTCCAACTGTATCTTCA
AGTAAAATCGCTTCATTTAGTGGTACGAAACAATTGCTTGATGAAGTAGGTCCAAAAGGTGTAGCTGAATGGGTTAAAAAGCAGGA
TGATGTCTTACTAACAGATACAACCTTTAGAGATGCACACCAATCATTATTAGCTACACGAGTTAGAACTAAGGATATGATTAATAT
CGCATCCAAAACAGCGGACGTATTTAAAGATGGTTTCTCACTTGAAATGTGGGGCGGTGCTACATTTGATGTGGCATATAATTTCTT
GAAGGAAAACCCATGGGAACGACTTGAACGTCTACGTAAAGCTATTCCAAATGTATTATTCCAAATGTTGTTACGTGCTTCAAACGC
AGTTGGTTATAAAAACTATCCTGATAATGTTATTCATAAATTCGTACAAGAAAGTGCTAAAGCAGGCATAGATGTCTTTAGAATTTT
CGATTCTTTAAACTGGGTAGATCAAATGAAAGTTGCCAATGAAGCAGTACAAGAAGCGGGCAAAATCTCTGAAGGTACTATTTGTT
ATACAGGTGACATTTTAAATCCTGAGCGATCAAACATTTATACTTTAGAGTATTATGTCAAACTAGCTAAAGAGTTAGAACGTGAAG
GTTTCCATATTTTAGCGATTAAAGATATGGCAGGCTTATTAAAACCTAAAGCCGCTTACGAATTGATTGGTGAGTTAAAATCAGCTG

TAGATTTACCAATTCATCTTCACACTCATGATACAAGTGGTAATGGTTTATTAACATACAAACAAGCAATAGATGCTGGTGTCGATA
TCATTGATACTGCTGTTGCTTCAATGAGTGGTTTAACAAGTCAGCCAAGCGCCAATTCGTTATATTATGCATTAAATGGCTTCCCACG
CCACCTTAGAACTGATATTGAAGGTATGGAGTCACTTAGTCATTATTGGTCAACTGTACGTACTTATTATTCAGACTTTGAAAGTGAT
ATCAAATCACCGAATACTGAAATTTATCAACATGAAATGCCTGGTGGACAGTATTCGAATTTAAGTCAACAAGCTAAAAGTTTAGG
TTTAGGCGAAAGATTTGATGAAGTCAAAGATATGTATCGCAGAGTGAATTTCTTATTTGGTGATATCGTAAAAGTAACTCCATCGTC
TAAAGTAGTTGGTGATATGGCACTTTATATGGTACAAAATGATCTTGATGAACAATCCGTGATTACAGATGGCTATAAATTAGATTT
CCCAGAATCAGTAGTGTCGTTCTTCAAAGGCGAAATAGGACAACCTGTAAATGGTTTTAATAAAGATTTACAAGCGGTTATTTTAAA
AGGCCAAGAAGCACTAACAGCTCGTCCAGGTGAATATCTAGAGCCAGTTGATTTTGAAAAAGTCCGTGAGTTGCTTGAAGAAGAGC
AACAAGGTCCTGTTACGGAGCAAGATATTATTAGTTATGTATTATATCCAAAGGTATATGAACAATATATTCAAACTAGAAATCAAT
ACGGAAACTTATCGTTACTTGATACGCCTACATTCTTCTTTGGAATGCGTAATGGTGAAACAGTAGAAATCGAAATCGATAAGGGTA
AACGATTAATTATTAAACTAGAAACGATTAGTGAACCAGATGAAAATGGTAATAGAACGATTTACTATGCGATGAATGGTCAAGCG
AGACGTATTTACATTAAAGATGAGAATGTGCATACAAATGCGAACGTTAAGCCAAAAGCAGATAAGAGTAATCCAAGTCATATCGG
TGCGCAAATGCCAGGTTCAGTAACTGAAGTCAAGGTTAGTGTAGGTGAATCTGTGAAAGCTAACCAGCCGTTGCTAATTACTGAAG
CTATGAAAATGGAAACAACAATTCAAGCACCATTTGACGGTGTGATTAAACAAGTAACTGTAAATAATGGTGACACAATAGCGACA
GGCGATTTATTAATCGAAATTGAAAAAGCAACTGACTAAAATGATTAAAATAAAACGAGATTACACAACAATTGACGTGTAATCTC
GTTTTTACATGGCTTAGGTTTAATTTTAAATATTGATATAAAAAACCGTAATTTTAATGGGCGTGCCATTAAAATTACGGTTTGCTTT
TTTGTTATTGCTTGTCACTTCTTACTGATCGTAACATTAGCATGATAAAGTATGTTGTCATACCAAATAAATAAGTGATAAATAATGC
ATGGAAAAGTGCGATTATCAGGTTAACATTTGTCATAATAGATAATGCACCTGTGATAACTTGTAATATAACAAGTATAAATGCAGC
TGTATAACCATAATGAACAGTACGGTTATTTGGATAATTTTTAACAGCGTGAATATAAGTAATCATAATAATCGTAAACACTATAAA
AGCCATGATACGATGCGTGAGTTGAACCCAATCTTGTTCTGAATGTGGTACAAGATCGTGGAATGGCAATGGCCAACCACCATATG
CTAAACTTGCATCCGCATGTCTCACTAGTGCACCAGTATAAACACCACAATAAATGATGATTGCCATTAACCATGTTAAACGTCTTA
ATGGCTTTTTGATATATAATTCGTCAGCTTCATATTTTTGATCTATAGAGAAAATAATCAATGTTATTAAAAATACAGATGAGAAAC
TGATTAATGATATACCAAAGTGCAATGCTAAAACGTAATCGTTTTGTTGCCAAATAACAGCAGCAGCTCCGATTAATGCTTGCAATA
ATAAGAATCCAACACTAATGATTGATAAAGGTTTAATTTCTTTAATATAGCCTATATGTTTCCATGCAGTGATAACTAACCATAAGA
CCATTAATAAAGACAAAGCTGAAACGGCTCTATGACTTAACTCAATAATCGTATCAATAGGAAAGAATTCTGGAATCAACGCACCA
TGACATAGTGGCCAAGAAGAACCACAACCATCAGCTGATCCGGTTTTGGTAACTAAGGCTCCACCAAGTTGTACAAATGTCATCATT
AACGTTGCTACGACACCTAACCATTTTAAATTCTTTTTGCCAAACAATTTATACACCCCATCATAAAAAAACGCATCACTGAGAATT
AGAATTAATAGCCGGATTTGAGATGATACTATAAATTGACCTGATTGTAGGATATGAAAAGATGGGATCCAACGCAGGAAATATGA
TTTAATTTTACAAGTATTGTATGTGGACGACATAAAAAGAACTTAATCGTAATGATTTTTTTATTGGGATAATTATGTAATCCACGTT
TACTCAAATTTGAAGCTTAAATATTAATCTCTTTTAGATGCATGATTCTACTTATTAAAATTTTCTAACATATACATTATAGCAATAA
ATTAAATGTTATTTGTGTCACAAAATTGACATTTTCTCTTGTCGTTAATTTGTCACAATTTAATTTTATTAAACCTAGTCTTTTTATGC
AATCTAAGATATCATAATCTTATATGAACAAATTTAAGGAGGGGGATTATGAGCAAAGAGCATACTTTGTCACAAAATATTAGCAG
AGTTAACTTCAAAGAATTGCAACAGATAATTAAAATGGGACTTGTTCAAGGTAACTTAATTCCGGCTTTTGCGGGCGCATGGCTAGC
AGTTGTAATGACAAATCATTCCTTCTTATCATCAATACCTCAAATTTTATTAATGCTATTTGGATCTACTTTAATTATGGGTGGCGCA
TGTGCGTTAAATAATTATTACGACCAAGATATTGATCGTATTATGCCTAGTAAACAAAATAGACCAACTGTAAATAATAGAATTACA
GATCAAAATTTATTACTATTAAGTTTTGGTATGATGTTAGTTGGAGAAATTTGTTTGTTTTTATTGAATATACCATCAGGCGTACTTG
GTCTTATGGGGATTGTAGGTTATGTGTCTTATTACTCAATATGGTCTAAAAGACATACAACATGGAACACAGTCATTGGGAGTTTTC
CTGGAGCAGTACCACCACTAATTGGATGGGTTGCAATTGAAGGACAAATTAGTTTAACAGCGATTGCGCTGTTTTTAGTTGTATTTT
GTTGGCAACCAATTCATTTTTATGCCTTAGCTATTAAACGTAAAGATGAATATGCACTTGCAAATATTCCAATGTTACCATCAGTTAA
GGGCTTTAAACGTACACGTGTCAGTATGTTTATCTGGTTGATTATTTTATTGCCAGTACCTTTATTACTAATAAATTTAGGTGTCGTA
TTCGTAGTGTTAGCTACATTATTAAATTTAGGATGGATTGCATTAGGTTTAACAACATTTAAGAAAAATTCAGATCAAACAAAATGG
GCAACACAAATGTTTATATATTCACTAAATTATTTAGTGATCTTTTTCGTGTTAGCTGTGATTGTTTCATTACTTACTTTGATCTAGAT
TAATTAAGTTAGGATGAAAAATATGGGCGTTCCAATTTTACCAACGATTAGTACGACATGTATTGTCATTAGTGCAATTTTAATTGC
CATTGGTTGGAGACTTATTTGGAAAAGGGAAATAAATAAACACAAAAATGTTATGTTAGCTGCAGCTGTTTTTGCTTTAACTTTTTC
TTAATCTATGCAAGTAGAACGATTTTTATCGGTAATACAGCATTTGGCGGACCAGCATCAATTAAGAAATATTATACGATTTTCTTA
TTTTTCCATATTAATTTAGCAACAATTGGTGGTATTCTAGGCCTAGTTCAAATTATTACTGCATTTAAAGATAAATATAATGTACACC
GCAAATTCGGGCCATTCGCTTCAGTTATATGGTTCTGTACTGCAATTACAGGTGTAGCAGTTTACTTATTATTATATGTATTATATCC
AGGTGGAGAAACGACATCACTGATTAAAGCAACATTTGGTCATTAATATGCTTATACAAATGATACTAATCTGATAAGGCAATTTGT
TTTATCAGGTTTTTTGATGAAGAATAGTCGGACAGTTGATGGTATTCATTTGATACCTTAACTGTCCATTTTTTTATACATATTTTAAT
CTTAATAATGAAATTCAACCTGAAAACATAAAGATAGGTTATGATATAGAGTGTAGGGAAAATCTTTATGACGTTAAAATTATGCCT
GAAGATGATTTTGTTTTATCGAAACGACACAGTTATGTCAGTGTTGAAAATTTGCTAATTAATGTGTTGTTTTTTACAATAGTATTAA
TACTTTTTAGGTGGTTGATTGATGAAAAAGTTAATTATAAGAGTCGTCGGTGTATTATTTTTAGTTGGATTTTTAATATATCTTTTTTA
TTCACCGAGATTAAAATTTGATGTATTAGAGAACCCGAATAAAGGTAATAAAGTAAATAGATCTGAACAGGTGAATAAATCAAATA
ACCATGCTGAAAATCCAAAGCCTAAAGAAGGTGTTGGTACATGGGTAGGTAAAGATATTAAAGTGCTTACTTCTAAATTTGGACAA
GCAGATCGTGTCTACCCTTTTAGAGATGGTTACAAAAATTATGTGTTTAAAGACAAAAACAGTTATTACATTGTTTCAACTAAACGT
GAAGAGATCGTTTCAGTGTATGCTACAGGTGAGAAAGTCAATGTTAGTCCGTTAAAAATAGGACAACATTCTGCAGAAATTTTTAAT
CATACAAGTATTAATCCAGAACCGTCCTTTAAAGTTGATGGTAAAAAATATGAATTTGAACTTTCAGATGAAGATTTAAAAACACA
AACACTGATTAAATATGGCGACATATATGCACAAGTGTATTCTGATCAACAATCGAAAAAGGTGCTAAGTGTACGATTTTTAACAA
AAGAAATGTTAGCAGATATTGAACCTTATCAATTAAATTCTAATTCTACGTCAGAAGAGCATAATAAGCGTCCAGTTGAGCAAAAT
CCAAATCAATTAATTTCTCTTTATGAAGTAACGAATGAAATGAGAAAATTAAAAGGATTAAAACCATTGAAAATCAATAGCGATTT
AGCACATATTGCATCTAATAACTTATATGAAGCGACCTCTAATGGTTCTGATAGTGTTGAATTTACAGAGGACGCATTAAGAGGGCA
ATTAGATAAAAATCACGTTACTTATAAAACAACTGCTCAAAATGTTGGTTATGCGTTTAATGATGTACCAACATTAATCCATAGTTG
GATGAATTCAGATATACATCGATCTCGTCTATTAAATTCAAAATACGATGAGATGGGTGGAGATGTAATGAGAGATTATTATTCACT
AATTTTCTTAGAAAAATAACAATGAGGGGTATTTATGATTAATGAAGCTTCACTAGCGATATTAGATGATATTGATGAACTAGCTGA
TATGATAGTTGCATCAGATATTTATGCATCATTTGAGCAAGCTAAACAGGCGCTCGAAAACAATGACGAAGCACATTTACTTTATCA
ATCATTTTTAAAATCAAAAGAGAAATATGATGAAGTAATGCGTTTCGGGAAATATCATCCTGATTATAAGAAAGTCATGTTAGAGA
CAAGACAACGCAAAAGAGCATATGAGATGCTTGACGTTGTGATGCATTACAAAGCTAGGGAAATGGCACTTCAACATTTAATTGAT
GAAGTTGTTACGAAAATTGCGTACGCTGTTTCAGAGCATGTCAAAATAGAAACAGGTAATCCATTCTTTCAAACATCACATAGTGGT
TGTGCGACGGGCGGATCCTGTAATTGTTCATTATAAAAAACATCGAGTCAGAAAAAGATGGTTATTGAAACCACTAACTAGCATCT
GACTCGATGTTTTTATTTGTTCGGGATTGTTTGTTTGAATTGTTGTGCTAAATCTGGTCGATCTGTCACAATCGTGTGTGCACCTTTTT
GGTATAAATCATTCATCAGATCTATACTATTTACGCCATAATAGCCTGGAACGATATTCATATCATTTAACCATTTGATAAAACGAG

ATGAAGTCAAATCAATGCCTTTAAAATGAGTAGGCATTTGGAACGTTTGTGCTAATGGTTGGTAGTACCTACCACCTAATAAATGAT
ATTTTAAAAATGCTTCTGTAACTTCCTGTTGGCTAGCACCAATTGCGACGGATCCTTGTGCAATTTTATTAAAACGAACGATTTGTTC
TTTATAAAAACTTGTCACAAGAACGCGGTCAAATGCTTGATTTTCTGCAATTGTATCAAACATAATTTGTGGTGCGATTGAGCCTTC
ATAGGATTCAGGAGCATCTTTTAAGTCTACGTTTATATACATATCAGGATATTGCTTCAGCAACTCATCGAAGGTTAGTATAGCTGT
GTGTGCATGACCACGATATGGTGTTAGTCCATTGATATCTTTGAAGTGATAAGCTGCGTCTAATTTCTTTAATTCTGCTAATGTATGG
GCACTAACTTTTCCAGAGCCGTTCGTCGTTCTATCAACAGTTGCGTCATGAAAAACGATAAGCTGTTGATCTTTTGTGAGTCTCACAT
CTGTTTCAAAGCCATCAACGCCTAATTGTTTAGCATAGTCAAATGCAAGTTGCGTTTGCTCTGGTCTTAAAGCCATACCACCGCGAT
GCGCAAATATATATGGTGCATTGCCTTTGAAAAAAGCAGGGATGGTTTGCTTTTTAGTAATCACTTTATTTTTATTGATCATTAATAG
ACTACTTAAAAATCCAGCACCGACTAGTACCGCATTTAAAATGTTTCTGTTTACTTTTTTCATAGAAAATTCCTCCTCGTTTACTTTTA
TAGCTTAACAAAATCATAGTTCTGACTACAGCGAAATGTGTGGTTAATCATATAGTGATGCTCTTGAGAATTGAATTAATATAATGA
TGTTTAATATAATCTAAATACATTTATCGAGCAATTACATAAGACATAATTAAGGTAGCTTAATATTTTAAAATTAATAAAGACATG
ATATGATTAATGCTGAAAATAAATGGAGTGAGGTTTAAATGAATTTAATCCCAAGAACTAGTATTGTAGTTTATTTAAAACATATGA
AACATGAACGACAAATCCGAAAATATGGACATATCGTTCATTCAAATAGAGATCGTAAATTTGTAATTATGTATGTGAATGAGCAG
GATGTTGATCAAATTGTACATAAACTAATGCAACTTAAATACGTTAGACATATTGATGGCTCACCATATAAATACTTAAAGAAAACT
TACGAAAAAGAGAAACACGAAATATATAATTAATATTATTGAAGTTCTGGTATCCATTTTTGGAGCCTTAACACACTAATTAAATAA
TTATAATATAACGATAAATCATGAAATGCTTGAGGGGGCTGTACATCGATACCTAAAATAGGTATGTCGAATTTTTCAGCACAATCA
TTAATCATTTGAAACTTTTTATTTGAATCTGGATATGGATATTTAATAGCGTTTATCATGATATAAAATGCTTGGAAATGATTGCCAG
TTGTAGGGTTCATAATGATTGCTACAGATGACTTTTTATTTTCAGCTTGTGGTGAATGAAAATAGATAATTTTATCAATACGTTGATG
GTTATATTCAATCATCGTCATGAATTCGAACAAATCTGTTAGACCTTCACCAAGTGTAATAAATGTTTGTTTCATTGGTTTATCATTC
TCCTTTTTCTCAATAAATTATATTAAATAACTAAATGAATTGGAAGTGTGAACAACATGCGCGTCATTGCAGGTAAACATAAAAGTA
AAGCTATAGAAAGTATGGAAGGCCGTAATACGAGACCAACTATGGATAAAGTTAAAGAAGGTATCTTTAATAGTTTATATGATGTG
TCAGGTATAGGTTTAGATTTATTTGCAGGAAGTGGGGCGCTTGGAATAGAAGCACTCTCTCGAGGTATGGATAAGGTAATCTTTGTT
GATCAAAATTTTAAAGCTGTAAAAGTTATTAAATCAAATCTTGCGAATTTGGATTTAGAGGCACAATCTGAAGTTTATAAAAAATAAT
GCAGATAGAGCTTTAAAAGCATTGTCAAAACGGGATATTCAATTTGATGTCATTTTCTTAGATCCACCTTATAATAAAGGTCTCATT
GATAAAGCTTTAAAACTAATTTCAGAGTTTAATTTATTGAAAGAAAATGGTATCATCGTTTGTGAATTTAGCAATCATGAAGAAATA
GACTATCAACCGTTTAATATGATTAAACGTTACCATTATGGGTTGACAGACACATTGTTATTAGAAAAGGGAGAATAGCATGGAAC
ATACAATAGCGGTCATTCCGGGTAGTTTTGACCCCATTACTTATGGTCATTTAGACATTATTGAGAGAAGTACAGATAGATTTGATG
AAATTCATGTCTGTGTTCTTAAAAATAGTAAAAAAGAAGGTACGTTTAGTTTAGAAGAGCGTATGGATTTAATTGAACAATCTGTTA
AACATTTACCTAATGTCAAGGTTCATCAATTTAGTGGTTTACTAGTCGATTATTGTGAACAAGTAGGAGCTAAAACAATCATACGTG
GTTTAAGAGCAGTCAGTGATTTTGAATATGAATTACGCTTAACTTCAATGAATAAAAAGTTGAACAATGAAATTGAAACGTTATATA
TGATGTCTAGTACTAATTATTCATTTATAAGTTCAAGTATTGTTAAAGAAGTTGCAGCTTATCGAGCAGATATTTCTGAATTCGTTCC
ACCTTATGTTGAAAAGGCATTGAAGAAGAAATTTAAGTAATAAAAATAACAGTATTTTAGGTTTATCATGGTTTACAATCCTAAAAT
ACTGTTTTCATTTGTTAACGATATTGCTGTATGACAGGCGTGTTGAAATCTGTTTGTTGTTGCCCGCTTATTGCATTGTATATGTGTGT
TGCTTTGATTTCATTTGTGAAGTAATGTGCATTACTTTTGTTAATATTGGTTATATATTGTCTTTCTGGGAACGCTGTTTTTAAATGCT
TTAAATATTGTCTGCCACGGTCGTTCATCGCTAATACTTTAACTGCGTGAATGTTTCTCGTAACATCTGTAGGTTTAATGTTTAATAA
TACATTCATTAACAGTCTTTGGATATGCGTATATGTATAACGCTTTGTTTTTAGTAATTTTACAAAATGATGAAAATCAGTTGCTTCA
TATATGTTAGATTTCAAACGATTTTCAAAACCTTCAGTAACAGTATAAATATTTTTTAATGAATCTGTAGTCATAGCTATGATTTGAT
ATTTCAAATATGGAAATATTTGATTTAATGTTATATGAGGTGTTACGTACAAGTGTTGAATATCTTTAGGTACCACATGATGCCAAT
GATTATCTTGACTAATGATTGATGTTCTAATAGATGTACCACTTGCAAACTGATGGTGTTGAATTAATGAATCATGATGTTGAGCATT
TTCTCGTTTAATAGAAATTGCATTGATGTTTTTAGCATTTTTAGCAATTGCTTTCAGATAACTAATACCAAGTATGTTGTTAGGACTT
GCTAGTGCTTCATGATGCTCTAATAATTCGCTAATGATACGAGGGTAGCTTTTACCTTCTTTTACTTTTTGTGAAAAGGATTCAGATT
GTTCAATTTCATTAATGCTGTGTGCTAATTGCTTTAATGTTTTGATATCATTATTTTCACTACCAAATGCAATTGTATCGACACTCATA
TAATCTGCGACTTTAACTGCTAGTTCGGCAAAATGATCGCCAGATGATAAACTGGCAGTTGCTGGTAGTTCGATAACTAAATCAGCT
GTTGATAATGCCATTTTTGCACGAGTAAACTTATTATAGATTGCTGGTTCGCCACGCATGACAAAGTTACCACTCATTATTGCAATA
GTAACGTCAGCATTTGTAAGTTTTTTAGATTGATTAATATGATATTGATGCCCATTATGAAAGGGATTATATTCTGTGATTAAGCCAA
CGCTTTTCATTTGCAATTCGTCCTTTCGGTATTCATTATTAATATTGTAACAGAACATGATATGTTAAGAAAAAATCTTGACAACTTG
TTCTTAGAAAGTTAAAATAAATTTTGTGCTTGTTAGAGGTGAAGCCATATGAAATGGTCAATTACGCAATTAAGGAAATATCAAGGT
AAGCCATTTGAATTTGATCAAACGGTGAGTTTTGACAATTTAAAAGAATCATTAGATTTAATTGATTTATCTCCAATTACAATCCAA
GGTCAGTTAACCATTAAGTCAACAGAAGTCGTTGCGGATATTCACATTACTGGAACGTATACAATGCCTTGTGCACGTACTCTTGTA
CCAGTTAAAGTCCCACTAGATGTAACTACTACAGAAGTATTTGATTTAGAAGGGTACAATCAGTATAACGATGATCAAGATGATGT
AGATGAACACTATCACATTATTAAAGATGGTATGGTTAATCTTCAGGATATTGTCGAGGATATAGTTATTATTGAGAAACCAATGAG
AGCTTATTCAGAGCAAAGTGACCAAATGTTGACAGTAGGTAATGGTTGGGAAGTAATCGATGAGGATCAATTAGATGAGCTTGCTA
AACAGCAAGAACAAGATGATTCAGAATCACGACAAGTTGATCCAAGGCTTCAAAAATTACAACAATTATATGATAAAGAGCAATA
AGTAGTTTAATTAATGATATAATGTTGTTAATTAAACTAACATTTAGTATTTTTCGTTTATAATCTAAGGAGGATATATCATGGCAGT
ACCAAAAAGAAGAACTTCTAAAACTAGAAAAAACAAACGTCGTACGCATTTCAAAATTTCAGTACCAGGTATGACTGAATGCCCAA
ACTGTGGCGAATACAAATTATCACACCGTGTATGTAAAAACTGTGGTTCTTACAATGGCGAAGAAGTAGCAGCTAAATAATTTTAGT
TACTCAATATAAAAAGTCCCGCTTAAAATGATTGTTTTAAGTGGGACTTTTTATATTGAGAAAAATAATTGGCGAACGAGGTAACTG
GATACCTCATCCGCCAATTAAAATTTGTTAATTTAATAATTAAATATAAAGACGATTTATTAGTTTTTACGTTTTCTAGGTAATACGA
ATGCAACGATGCTACTTAAAGCTAATAATGCCATTAATGGTAATGTCATATCTTTATTTGATTCTTCACCAGTTTGTGGTAATGATTT
TGCTTTATTTTCTTGTGTATTTTTATTGTTTTGGCTTTGAGTGTGTCCATCATTTGTGTTTTTAATGTTTGCTTTTTGTAATGGAGCACT
ATCTTTTGCTTCGCTAGAACCTGCTGAAGTTTGAACAACATCTTTTGTTGTTTTTGATGAAGCAGTTGTTGGTTTTGCAACATTTTGAG
TCGTAGATACTACCTTAGTTGGAGTTGTACTACTTGATTCTACTTCACCTTTAGTTGGTTTTGTAGCAGGCGTTTTGTCTTTACCTGAC
TCACTAGATGCGTCATTTTCTTTTTCAACACTTGGTAATTGTTTATTGTCATCTTTTTGGCTGTCTTGTTTTTGTGATTCTTTTTCAACA
GGTGATGGTGTTGGTTTGCTAGGCGTAGCTGGAGTAGCTTCCTTCTTAGCTGAGTTATCTTGTTGTTCTTTTTTGTTAGATTTATCGGT
ATTGGCTTTTGTAAATGCTTCTTTATCAACGATTCTGACATGGTATTGTCCATCATAATCAATCGTTTTTACGTGAACTTTAACGATA
GCATCATATAGAGTTTTACCTTCAACATATGGGAAAATAATTGTTCTAGTATTATTTTTAGCATCTTTGCTTATAGTTCTAACACGTT
GACCTTCAACCATGAAATCTTTCCAGTAATCGTCATTAGTAGTTTCCATGACCATATATTTTTTGCCGTTAAGCATACCTGTTTTAAT
AGGGTGTTTAACAAAAGTATCCATCATAGATTCGTTATTCTCAACACTTTCATAAACAACATATTTTGTATCTTGTAAATCAGTCATT
TTTTCATTTGTTGGTTGTACATTTTGGAATTCAGTAATAGCTGATTTCACTTGCTCATCTAAAGCTTTCTTTGTATCCTCTAATTTCTTC
TTGTACTCAGCCTTTAATTTTTCAGGAAGTTTATCTTGAATTTTATTTAATTCATAAACTTGTCTTTCTAGTGTTTTCGCTTTTTTATAT
GGCGCTAATAATTTTTCAGCTTTATAATCTTCTTCAGTTTTGAATTTATCTGCACTGTTATAAATTGGTTGTGCGAATTCCATTAATGT

GTAATCGTATTTTTCTTCTTTGTTATTGAAGTGTGTTGAACTAACAATTTTAACAGCTTTTGTTCCATTTGATACAGAGAAGCGAATG
TAAGCATAATCTTTAACAGTATCGTATGATACTAATTTAATTGGCAACTTTTTGTCACCTTCATAAACTTCAAATTTTCTCCAAAATT
GACCTGATTGTAATCCTAATTCAATTTCTGGTTTTGAATCAGTGAAAATAACTCTAGCAGGTTTAACAGAACTTGCATAATGATAAA
ACTGTTGAGTTCCATCTTTCTTTTTCATTTCAAAATCAATTGGACGAGAGTTTGGTGCGCTATGATCTTTGTCTTTTATTGCAGGGTTT
TTAATCGCTTCTCTAAGTTCCTGATTCAAAATAGGATATGTATTGTTAGTGGCTTTTGCTGCTGGTTTAACTTCTTTTGTTTCAGAAGT
AGGGGCCTCAACTTCTTTATTAGATACTGAGACAGCATTAGCTACTGGTTTAGTTTCTGGAGCTTTTTCAGATGTTGTTGTTGGACTT
GCAACTGCTTCAGTTTTTGGTTGTGCTTCTGTATTTGTACCACCTGTTTCTTCAGCTGCTGCTTGTGCTTCGCCATTTGACATTAATAA
TAAAAGTGTACTAATTGCTACAGATGCAACGCCTAGTGATGACTTTCTAATTGAATAAAATGATTTAAATTCTTTTTGCTGTTTGTTC
ATGTTGTAGAAACAACTCCTAATTGTATATTATCAACTGATAATCATTATCAATTGATTACTTGTATTGTACATTGAAATGGTAGTTA
ATTGCAACGTTATTTTATAAAAAAATAACCTATGTTATAGATTATGAAATATCTATGACATAGGTTAAATAAAATTTAAAATAAAAA
ATATATTAAGTTATGAGTGTGACGATGATTTATTTAGATTCTTTTCTTTTGAAAAGTAATAAAGATAATGAACCTAAAAGGGCAAGT
GTTGCGAAGGCAACTGTGCTAATAAAGTTATCAACTGAAGTTAAACCAGTTTTTGGTAATTCTTTAGCTTTAGATGCTTGTTTAGGCG
TTTCGTTATGTTTTGTAACTTTGTTAGTTTGTTGTGATTTATTATCACTTACAGCTTGATTGTTGCTTTCAGATTTCGCTGTTGCAACAT
CTTTAACAGGTGTTTGAACTTTATTTTGTTCTTGAGCAGTTTGTGCTGTTTTAACTGTATGAGCAGTTTGTGTTTTAGTTTGATCTTTT
GTTGTGTCAGTACTTACAACTTTAGTAGAGTGATTGTCTTCAACTTTGCTTGTTGTAGTTACAGTAGGTTTAACTTTTTCAACTTTAGG
TTGAACTGGTTTAGTTTGCTCAGTAGGTGTTTTAGGTTGAGCTGGTTTTGGTTGAACCGGTTTAACATTGTTTGGTTTTGCTGCGTCA
GCTAATGTAGGAATTGCTTTTTCAAATTCCAAATGCGTAGTATATCTATGATTGTAATTAATTTGTGGCACGACAATATGTACTTTAG
TAGTTAAGCTCTTATATCCAGGTTCAACTGCAACATTGATTGTTCTAGTATCCGCTTTTTTATTATCGTTAACAACAGTTGTTGCTAAT
TCTTGATTGTTTGCATTGTAAAATTTGTATTCTTTCCAGAATGATGCATTGTTTAACACGGTTTGGAAATAATATTTATTATTTTGTTT
AATTACTTTACCAGGGTGTTGCATATAGTCATCCATGTGTGACTTCTCTGAAGAGCCATCTTTTTGCACTTGGAAATTAATTGGTTGT
GATGTTGCTTGAGAAACTTGTGTGCTTTGATTATTAGTTGCGTTCGTAGCTTCTGTTGCCGCATTGACTTGTTGGCTGCCTGCGCCTAT
GTATACAAGGGAACCTAAAATGATAGATGCTGTACCCATTGTAATCTTTTTCATAGCTGATGAACGTTGTTCTGATTGATACTTACTG
TTTAAATAATGTTTTGTCATGTTGTTTTCCTCCTAAGGATACAAATTATGTGTTGTTATATGAGATGATTAACCATTGCAATTGATAA
TGGTTATCAATTATACTAGCACATCTATTTTCGTATAACAATAATAAATTGATTATAAAATAAATATTGACAATGATAATCATTATTA
TTTATGATTTTACTAAAGACTTAAAAGCAATCATAAAAAGGAGGATTATGTTTTGAAAAAATATTTTAAAAGTTTTTAATACAACGAT
TTTAGCGTTAATTATCATCATCGCGACATTCAGTAATTCTGCAAATGCCGCAGATAGCGGTACTTTGAATTATGAGGTTTACAAATA
CAATACCAATGACACGTCAATTGCTAATGACTATTTTAATAAACCGGCAAAGTACATTAAGAAAAATGGTAAATTGTATGTTCAAAT
AACTGTCAACCACAGTCATTGGATTACTGGAATGAGTATCGAAGGACATAAAGAAAATATTATTAGTAAAAACACTGCCAAAGATG
AACGCACTTCTGAATTTGAAGTAAGTAAGTTGAACGGTAAAATAGATGGAAAAATTGACGTTTATATCGATGAAAAAGTAAATGGT
AAGCCATTCAAATATGACCATCATTACAACATTACATATAAATTTAATGGACCAACTGATGTAGCAGGTGCTAATGCACCAGGTAA
AGATGATAAAAATTCTGCTTCAGGTAGTGACAAAGGATCTGATGGAACGACTACTGGTCAAAGTGAATCTAACAGTTCGAATAAAG
ACAAAGTAGAAAATCCACAAACAAATGCTGGTACACCTGCATATATATATGCAATACCAGTTGCATCCTTAGCATTATTAATCGCAA
TCACATTGTTTGTTAGAAAAAAATCTAAAGGCAATGTGGAATAATGAGAAATGTTAAACAAATTGCTACAAAATCTATTATAGCTAT
TATTAGCTTAGGTATACTTACATATACAACAATGATTGGTAGCGTGTTGGCTGATGAGATAAAATATCCATCAGCCAAATTTAATCA
ACCTGAAGCAAAAGATAAAACAGAATTAACTACATCAATTTTTGATGAAAAGATAAAAGAGAATAAAGCGTTAGAGTTACTAATTT
TTAATCAAGAAAATAAAAATGTAACTGAGGAACAACAACTAGTTGACGAAAAGGCGCAATTGATTTCAGATATGACTGGTAAAATT
TACTTGCAAGTAAAGCTAAAAGGTCAAATAGATAAAGAACAACTTGTTTTTCAAAATGACAAAAATGAAGAATTTCCTTTTGTTATA
AAAGATGAAAAGGATGACACAATAGTAAGAATTTTAATTGAACAGCATATGGATAAAATCAATATGCATGTTAAAACGTTGGCTGA
AAAGAAAAATCTAGATAACAAAGAAATGGTGTATTCTATTCATTTTAAAGAGAAAAAAGTACAACATGATGATGCAAAAGAAGTG
CCTTCAAAACATCAAAATCAAGAAAATAATCAAGATCAGCTTAAAAAAGATATTGATGACAAAAAAGATAGTCAAAAATCAGATA
CTAAGGAAAGACGTACTAGCCTTTTTACTGAAAAAGGATTAAATGATATTCCTGTACAAAAAGATAAAGTGCAACAAGACAGTAAT
AAAAAGATTGAAAATGAGCGACCTAAAGCATCAGGTACATTAAAAGTTGAAAATAGCCCTCCAACAATAAAAAAGGTTGAAAATA
ATCACAAAGAGCAACCGAAACATAAAGATGAAAAATCAAAAAAGGAAAAGAAAAAAGTAGTTGAAAAAGAAAAAGCGTTACCAG
CTTTTAATAGAGATGATGATAGCAAGAATAGTAGTCAATTATCTAGTGATATTAAAGAACTTGATGAACCAAATCATAAAAAGCAA
TATATGTTATTTGCAGCTGGCATTGTGTTAGCAACTATTTTACTTATTTCGGCACATTTATACAGCAGAAAGAGAGGTAACCAAGTTT
GAGAATCATAAAGTATTTAACCATTTTAGTGATAAGCGTCGTTATCTTAACCAGCTGTCAATCTTCCAGTTCTCAAGAATCAACTAA
ATCCGGCGAATTCAGAATCGTACCAACAACTGTTGCATTGACAATGACATTGGACAAATTGGATTTACCAATTGTCGGCAAACCCAC
GTCATATAAGACATTGCCTAATCGTTATAAAGATGTACCTGAAATTGGTCAACCAATGGAGCCGAATGTTGAAGCTGTTAAAAAATT
AAAACCAACACATGTTTTGAGTGTGTCAACGATTAAAGATGAAATGCAACCATTTTACAAACAATTAAATATGAAAGGCTACTTTTA
TGATTTTGATAGTTTAAAAGGGATGCAAAAGTCGATTACACAATTAGGTGATCAATTTAATCGTAAAGCACAAGCAAAAGAATTAA
ATGACCATTTAAATTCTGTTAAGCAAAAAATTGAAAATAAAGCAGCTAAACAAAAGAAACATCCCAAAGTATTAATATTAATGGGT
GTACCGGGTAGCTATTTAGTAGCAACTGATAAATCATATATTGGTGATTTAGTTAAAATAGCAGGTGGAGAAAATGTTATTAAAGTG
AAAGATCGTCAATATATTTCGTCTAATACTGAAAATTTGTTGAATATCAATCCAGATATTATTTTAAGATTACCACACGGAATGCCT
GAAGAAGTTAAGAAAATGTTTCAAAAAGAATTTAAACAAAATGATATTTGGAAACATTTTAAAGCTGTGAAAAATAATCATGTTTA
TGACTTAGAGGAAGTGCCATTCGGTATTACAGCAAATGTTGATGCTGATAAGGCAATGACTCAATTATATGATTTATTTTATAAGGA
TAAAAAATAGTGAGTTGATATGATGATAAAAAATAAAAAGAAACTACTATTTTTATGTTTGTTAGTCATTTTAATCGCAACGGCTTA
TATTTCGTTTGTAACCGGTACAATTAAATTGTCATTTAATGACCTATTTACAAAATTTACAACTGGTAGCAATGAAGCAGTGGATTC
AATCATTGATTTGCGATTGCCACGTATATTAATTGCATTAATGGTTGGCGCAATGTTAGCAGTTTCTGGAGCATTATTACAAGCAGC
ACTACAAAATCCTTTGGCAGAGGCGAATATCATTGGCGTTTCTTCAGGTGCACTTATAATGAGAGCACTTTGTATGTTGTTTATTCCA
CAATTGTACTTTTACTTACCATTATTAAGTTTTATTGGAGGTTTAATACCATTTTTAATAATTATATTGTTGCATTCTAAATTTAGATT
CAATGCTGTAAGTATGATATTAGTAGGTGTTGCGTTATTCGTATTATTAAATGGTGTTTTAGAAATTTTAACTCAAAACCCTTTAATG
AAAATTCCTCAAGGCTTAACAATGAAAATATGGAGCGACGTATACATATTAGCAGTATCAGCATTATTGGGATTAATATTAACATTA
CTATTGTCCCCTAAATTGAATTTACTAAATTTAGACGACATACAAGCGCGAAGTATCGGTTTTAATATTGATCGTTACAGATGGTTA
ACAGGTTTATTAGCAGTATTTTTAGCAAGTGCAACTGTTGCGATTGTTGGACAACTAGCCTTTTTAGGTATTATTGTGCCACATGTGG
TTAGAAAGCTAGTTGGGGGCAATTACAGAGTACTTATTCCGTTTTCTACAGTTATTGGTGCATGGCTATTGTTAGTGGCTGATTTATT
AGGACGAGTGATACAGCCTCCTTTAGAAATTCCAGCCAATGCTATTTTAATGATTGTCGGTGGTCCAATGCTAATTTACTTAATTTGT
CAAAGTCAACGAAATCGAATCTAAAACGGTACAACAACTGGTTTAGTGATGACTGAAGAAACGTCGTTAATAAAAGAGGATTTTTA
TGAGAATGAAGCGATTTTTAACTATTGTACAAATTTTATTGGTTGTAATTATTATCATTTTTGGTTACAAAATTGTTCAAACATATAT
TGAAGATAAGCAAGAACGCGCAAATTATGAGAAATTACAACAAAAATTTCAAATGCTGATGAGCAAACATCAAGAACATGTGAGA
CCACAATTTGAATCACTTGAAAAAATAAATAAAGACATTGTTGGATGGATAAAATTATCAGGAACATCATTAAATTATCCAGTACT
ACAAGGTAAGACAAATCACGATTATTTAAATTTAGATTTTGAGCGAGAACATCGACGTAAAGGTAGTATTTTTATGGATTTTAGAA

TGAATTGAAGAATTTAAATCATAATACTATTTTATACGGGCACCATGTCGGTGATAATACGATGTTTGATGTGTTAGAAGATTATTT
AAAGCAATCGTTTTATGAAAAACACAAGATAATTGAATTTGACAATAAATATGGTAAATATCAATTGCAAGTATTTAGTGCATATA
AAACTACTACTAAAGATAATTACATACGTACAGATTTTGAAAATGATCAAGATTATCAACAATTTTTAGATGAGACAAAACGTAAA
TCTGTAATTAATTCAGATGTTAATGTAACGGTAAAAGATAGAATAATGACTTTATCAACGTGCGAAGATGCATATAGTGAAACAAC
GAAAAGAATTGTTGTTGTCGCAAAAAATAATTAAGGTAAGTTAAACAGAAAAGAGGATAATTATGAAATTTATGGCAGAAAATAGG
CTGACGTTAACAAAAGGAACAGCAAAAGATATTATAGAACGATTTTACACGAGACATGGGATTGAAACATTAGAAGGCTTTGATGG
CATGTTTGTTACACAAACTTTAGAACAAGAAGATTTTGATGAAGTGAAAATTTTAACAGTTTGGAAATCAAAGCAAGCTTTTACGGA
TTGGTTAAAATCTGATGTCTTTAAAGCAGCGCATAAACATGTTAGAAGTAAAAATGAAGATGAAAGTAGCCCGATTATCAATAACA
AAGTAATTACATATGATATAGGCTATAGTTACATGAAATAATTGAATTTAAAGAGGTTGCAGTACTTGTTATGACAATTGGTATTGT
TAGCTTTTGTAACGACCATACATGATACCGATGATGGTCGTTTTTTTAATGAACACAAACATGCTAACAACAAATTGCTAAAACATA
GTTTGATTTGAATGTGCTTTGAAAATAATCATCAATTATATCATTTTGATTTATTTTGAAGAAGAAAAGAATTAATAATGATATTTTA
GGGCGTATGATTTATAGTAGTAGTATCGATAAAAGAAATTTATGCAATTTGAAATATTATGAGTGATAAAAGCGATTAATTAATATT
AACTATACAATCAAATGTTTTCATCTTATTAATGATTAATATTTTTATTTTTAAAAATAAAGCGAGGAGCTATCAATGGAACAAATT
ACTTCTGCACAAAATAATAGAATTAAACAAGCGAACAAGCTAAAAAAGAAACGTGAGAGGGATAAAACTGGATTAGCTTTAATTG
AAGGTGTGCATTTAATTGAAGAAGCTTATCAAAGTGGAATTGTAATTACACAATTATTTGCAATTGAACCGGCAAGATTAGATCAGC
AAATTATCGCATACGCGCAAGAAGTTTTTGAAATAAACATGAAAGTTGCTGAATCTTTATCAGGTACAGTGACACCACAAGGGTTTT
TCGCAATCATTGAGAAGCCGCATTATGATATTTCTAAAGCACAACAAGTATTGCTCATCGATCGTGTTCAAGATCCTGGAAATTTAG
GCACATTAATTAGAACTGCGGATGCTGCTGGAATGGATGCTGTAATAATGGAGAAGGGTACGACAGATCCTTATCAAGATAAAGTG
TTGCGAGCGAGTCAAGGTAGTGTTTTCCATTTGCCAGTTATGATACAAGATCTCGATACGTTTATTACTCAATTTAATGGTCCTGTTT
ATGGTACAGCACTTGAAAACGCAGTGGCATACAAAGAAGTTACTTCAAGTGATTCTTTTGCATTACTATTAGGTAATGAGGGAGAA
GGTGTTAATCCTGAATTATTAGCACATACTACAAAAAATTTAATCATACCTATTTATGGTAGTGCTGAAAGTTTAAATGTAGCGATT
GCAGGTAGTATTTTACTTTATCATTTGAAAGGTTGACCGTGTTGAAAGTTTTCCGATATAATTATAATTAATTGTTTAACAGAACATT
TCTACGTGATTGCATACACAATTGAATATAAACCAATAAAAAGGCATGGACATTTATATAAATAATTGTTTTAGGGAGAATAATCGT
GACTGCAAGTTATTCCAATTATTTAAAGTCTTTTCACCTTTTTGGTTACTTAAAGAGATTTAAGTCGGAAAGACAATCCGTTATCAAT
ATTAAACAAGTGTATGCTTAGGCATAAATTTGGGTGGTACCACGGAAATGACTTTCGTCCCTTATTTTTTAAGAGGATGAAAGTCTT
TTTTTAGTTAAACAACAAATATGATAAATATAAAATGAATAGTTCGAATAGGGAGGTCAGTGACATATGTCTGAACAACAAACAAT
GTCAGAGTTAAAACAACAAGCGCTTGTAGATATTAATGAAGCAAATGATGAACGTGCACTGCAAGAAGTTAAAGTGAAATACTTAG
GTAAAAAAGGGTCAGTTAGCGGACTAATGAAATTGATGAAGGATTTGCCGAATGAAGAGAAACCTGCGTTTGGTCAAAAAGTGAA
TGAATTGCGTCAAACAATTCAAAATGAATTAGATGAAAGACAACAGATGTTAGTTAAAGAAAAATTAAATAAGCAATTGGCTGAAG
AAACAATTGATGTATCATTACCAGGTCGTCATATTGAAATCGGTTCAAAGCATCCATTAACACGTACAATAGAAGAAATTGAAGAC
TTATTCTTAGGTTTAGGTTATGAAATTGTGAATGGATATGAAGTTGAACAAGATCATTATAACTTCGAAATGCTGAATTTACCTAAA
TCACACCCTGCACGTGATATGCAAGATAGTTTCTATATTACGGATGAAATTTTATTACGTACGCATACATCACCAGTGCAGGCACGT
ACGATGGAATCACGTCATGGTCAAGGTCCAGTTAAAATTATTTGCCCTGGTAAAGTGTATCGTCGTGACTCTGATGATGCGACACAT
AGTCATCAATTTACACAAATTGAAGGATTAGTTGTTGATAAAAACGTTAAAATGAGTGATTTGAAAGGCACTTTAGAATTGTTAGCT
AAGAAATTATTTGGTGCTGATCGTGAAATTCGTTTACGTCCAAGTTACTTCCCATTCACTGAACCTTCTGTAGAAGTTGATGTGTCAT
GTTTTAAATGTAAAGGAAAAGGTTGTAATGTGTGTAAACACACAGGATGGATTGAAATTTTAGGTGCTGGAATGGTACATCCTAAT
GTATTAGAAATGGCTGGTTTTGATTCTTCAGAGTACTCTGGATTTGCATTTGGTATGGGACCAGACCGTATTGCAATGTTGAAATAT
GGTATAGAAGATATTCGTCATTTCTATACTAATGATGTGAGATTTTTAGATCAATTTAAAGCGGTAGAAGATAGAGGTGACATGTAA
TGTTGATATCAAATGAATGGTTGAAAGAATATGTAACAATCGATGATTCTGTAAGTAATTTGGCAGAACGTATTACGCGCACAGGT
ATTGAAGTGGATGATTTAATTGACTACACAAAAGATATCAAAAATTTAGTTGTCGGCTTCGTTAAGTCAAAAGAGAAACATCCTGAT
GCTGATAAATTAAATGTTTGCCAAGTTGATATCGGAGAAGACGAACCTGTACAAATCGTATGTGGTGCACCGAACGTTGATGCAGG
ACAATATGTCATTGTTGCTAAAGTAGGTGGCAGATTGCCTGGTGGTATTAAAATTAAGCGTGCCAAATTACGCGGTGAACGTTCAGA
AGGTATGATTTGTTCGTTACAAGAAATTGGTATTTCAAGTAACTATGTACCAAAAACGTTTGAATCTGGTATCTATGTATTTAGTGA
AGCCCAAGTTCCAGGAACAGATGCCTTACAAGCTTTATATTTAGATGATCAAGTAATGGAATTTGATTTAACGCCGAATCGTGCAGA
TGCTTTAAGTATGATAGGTACTGCTTATGAAGTTGCAGCATTATATAATACAAAAATGACTAAGCCAGACACAACATCAAATGAGC
TTGAGTTATCTGCAAATGATGAACTGACTGTGACAATAGAAAATGAAGATAAAGTACCATATTATAGTGCACGTGTTGTTCACGAC
GTGACAATTGAACCCTCGCCAATTTGGATGCAAGCACGCTTAATAAAAGCGGGTATACGTCCTATTAATAATGTTGTTGACATTTCA
AATTATGTATTATTAGAATACGGTCAACCATTGCACATGTTTGATCAAGATGCGATTGGTTCACAACAAATTGTTGTTCGTCAAGCT
AATGAAGGCGAAAAAATGACAACATTAGATGATACAGAACGTGAATTATTAACGAGCGATATTGTCATTACTAATGGACAAACTCC
AATTGCATTAGCTGGTGTTATGGGTGGCGATTTTTCAGAAGTTAAAGAACAAACATCAAATATAGTGATTGAAGGTGCTATTTTTGA
TCCAGTTTCAATTCGTCATACATCAAGGCGTTTAAATTTACGCAGTGAATCATCTAGTCGTTTTGAAAAAGGAATAGCTACTGAATT
TGTAGATGAAGCAGTCGACCGTGCATGTTATTTATTACAAACTTATGCAAACGGAAAAGTGCTAAAAGATAGAGTGTCTTCAGGAG
AACTTGGTGCATTTATTACACCAATCGACATCACTGCTGATAAAATTAATCGCACTATTGGATTTGATTTGTCACAAAATGATATTGT
TACTATTTTTAATCAATTAGGGTTTGATACAGAAATAAATGATGATGTTATTACAGTGCAAGTACCATCACGTCGTAAAGATATTAC
AATTAAAGAAGATTTAATTGAAGAAGTTGCACGTATATATGGTTACGACGATATTCCATCAACGTTACCTGTCTTCGAAAAAGTTAC
TAGTGGTCAGCTAACTGATCGCCAATATAAAACTAGAATGGTTAAAGAAGTGTTAGAAGGTGCTGGATTAGACCAAGCTATTACGT
ATTCGTTAGTATCTAAAGAAGATGCTACTGCATTTTCGATGCAACAGCGTCAAACAATTGATTTATTGATGCCAATGAGTGAAGCGC
ATGCGTCATTACGTCAAAGTTTATTACCACATTTAATCGAAGCGGCATCATATAATGTGGCACGCAAAAATAAAGATGTAAAATTAT
TTGAAATCGGCAATGTCTTCTTTGCTAATGGAGAAGGTGAACTACCAGATCAAGTTGAATATTTAAGTGGTATTTTAACTGGAGATT
ATGTAGTCAATCAATGGCAAGGTAAGAAAGAAACGGTTGATTTCTATTTAGCAAAAGGTGTCGTGGATCGAGTATCTGAAAAGTTA
AACCTTGAATTTAGTTATCGTCGTGCCGATATTGATGGATTACATCCAGGTCGTACAGCTGAAATCTTATTAGAAAATAAAGTTGTT
GGTTTTATTGGTGAATTACATCCAACATTAGCAGCTGATAATGATTTAAAACGTACGTATGTTTTTGAGTTGAATTTTGATGCATTAA
TGTCTGTGTCGGTAGGTTACATTAATTACCAGCCAATTCCGAGATTCCCAGGCATGTCTCGTGACATTGCATTAGAAGTAGATCAAA
ATATTCCAGCAGCTGATTTATTATCAACGATTCATGCACATGGTGGCAATATATTAAAAGATACACTTGTCTTTGATGTATATCAGG
GCGAACATTTAGAAAAAGGTAAAAAATCAATTGCAATACGTTTAAATTATTTAGACACAGAAGAAACATTGACAGATGAGCGCGTT
TCAAAAGTACAAGCGGAAATTGAAGCAGCATTAATTGAACAAGGTGCTGTTATTAGATAATGATTTAAACCCCATGTATAAGGATA
TCTGAAGTAGATTGATATCCTTAACATGGGGTTTTATTTTTGGATTCACCTATTAGGTTCCAATTCATTTAAAAAGTCAAAGAGGAAC
AGCGGAATACAGATGATGCTTTGCACAACTGCATATAAAGTCATACTTATGAGCAAAGCAAATTAGTATGACTTACCTAATA

>028-contig_257_RC

GGAACAGCGGAATACAGATGATGCTTCGCATCACTGCATATAAAGTCATACTTATGAGCAAAGCAAATTAGTATGACTTACCTAAT
AGGATCACCAATTAGGTTCCGATTCATAATGATTAAAAATCAAAGAGGCACAGCGGATTACAGTTGATGCTTCGCACAACTGCATA
AAAGCCTCTAATGATTAAAAATCAAAGAGGCTTTAAAATTTTTTGGGCTTTTTCACGATTTTTAAAATGCTTTTTTGAAATGGTATCT
AAACGTGAAAGACCGTATTTTTTTATAATTTTGGCGGCAATTACATCGACTTTAGCACCGGCACCTTTAGGAATCGTCATATTAATAT
TTTTTGATATTTGATCCATATATGTAACAAATGCGTATCGAGAAATTATGCTTGCCACTGCAATGGCTAATGACTTCGATTCTCCTTT
TGTTTCAAATTTTGTTTTCTTTGGAAGTGGTATATCTGATAATGCGTAATGGCTATACACTTCGCGTTTTGCGAACTGATCAATGACG
ATATAGTCTAATTGAGACGAATCAATTTTTTCAAGTACATTTTTGATGGCTTCATTATGAAGAACAGCTTTCATTTTTACTTGAGTCC
AGCCTTTTGCTTGCTGAATATTATATTTTTCATTGTGTAGTGTTAATAGTGAATGTGGGATGAAAGTAACCAATTGCTCAGCAAGTTC
TACAATTTTGGTATCGGTTAATTTTTTTGAGTCATCTACACCCAAAGTTTTTAAAATAGGGACATGCTCTTTGGTAACGAAAGCAGCA
CACACAGTCAACGGACCAAAGTAATCACCACTTCCAGCTTCATCACTACCAATACAGTTAAATTGATCATACATTAAAGTTTGTTCA
AGAAAAGAATTAGCCATATTTTTCTTTTTAGTTTTATTAGTATTCAATTGAGAATGCTGCGGTAGAAGTTCTTCAGACACAGCTTCTG
CATGATTACCCTGAAACATGACTTTACCTGATTGGTAAATATTTACAGTTGTATTTTGATACTTTGCACGTGCTTTCATACCTTGAGG
TAAATTCTCAGTATCAAAAGAAATGCGTGACATTAATGTCGTTATGTCTTTATCCGACAATTTAAAAACGATATTCGCCATTTGTGTA
AATCCTTTCATATAATTTCATTATCATTAGCAAAAATAATCATATCATAAATGATAATAACGTCAGAAACATTTATATATGTTGTAA
AGAATTAATTGCGGATACTTACAAAATACATAGTGGTCATGTATAATGTTTATGAAAATGTTATAGTGACACAATTTAAACATGATA
AAGTATTGAATAGAAAATTCTTTACATTATTTCATTATAAAAAGAACATTAAGTCATCTATAGAGATTGTGTTGTTGTTTTATCATAA
TTGCAATATAACAATATTAGTATCAAATTCAGACAAACTATCCAGTCATATTTTATAACGTGAATTTAGATTTTAAAAAAAGGATCA
GGAGAAATTATAAATGGCACAGTTTAAAAACAAGGTAAATGTATCAATTAATGATCAGCTTTTTACAATTGTTGGGGAAGATAACC
CAGAGCACATACGATATGTAGCACATTTAGTTGATGATAAAATAAAAGAATTAGGGTATAAAGCAGCAGGTTTAGATACTTCAAGA
AAAGCAATACTAACTGCTGTGAATATTATGCATGAAAAAGTACTACTAGAAGAAGAAAATCGACGTTTGAAACAACAAATTCACAA
ATTGCAGCAGCGTGAGCAATAAATGGTCATTGATTTTATCATAATCATTTTCTTTGTGTATTTTGTCATCGTTGGATTCAGACGAGGT
TTTTGGTTATCTATGATACATTTGAGTGCAACGATTGTATCATTGTGGATTGCCAGTCAATTTTACAAATCTATTGTAGAAAGATTAA
TTGTATTTATTCCATATCCTAAAACAACAGCATTTAATACAACTTTTGCGTTTCATTTTAATCATCTACAAAATCGATTTGAAGCGAT
TGTAGCTTTTTTAATGATTACATTGTTTTGTAAGTTCATTTTATATCTAATTATCGTAACTTTTGATAAAATAATAGCGTATCAAAACA
TTCATATTTTCAGTCGTGCAATGGGTATGATAGTTGGTGTGTTTATGACGATAATTGTCTTACACTTTACGTTATATCTATTGGCATTA
TATCCTAACGAAGCATTACAACATCAGCTTAAAATATCTATTGTGAGTCATTCATTGATTTTTCACATCCCGTATTTATCGGCTTTCA
CCATTAATTTATAAATTATCATAAGAGGTCAGGACAAAGGTATATATTAATGATATGTCCTGGTCTTCTTTTTATGGAGTGTTATTAT
GACAAAAAAAGATGTTATCAAACTATTAGAACAAATTGCTACTTATATGGAATTAAAAGGGGAAAATACTTTTAAAATATCAGCGT
ATCGAAAAGCAGCTCAAAGTCTTGAATTAGATGAACGACCATTAGATGAAATATCTGATGTAACGGAGTTAAAAGGCATTGGTAAA
GGTGTTGCAGAAGTAATCAATGATTACCGTGAGACCGGTGAATCTCAGTATTTACAGCAATTACAGGAAGAAGTTCCGGAAGGTCT
TATTCCACTTTTGAAAATTCAAGGACTTGGAAGCAAGAAAATTGCTAAGCTATATAAAGAGTTGAATATTGTTGATAAAGCGTCACT
TCAAGTTGCTTGTGAAAATGGAAAAGTTAGTGAATTAAGCGGATTTGCTAAGAAAACGGAACAAAACATATTAGAAGCTGTGAAAC
AACTTGGTGCTAAGAAAGATAGATATCCAATTGATCAAATGAGAAGACTTAATCAAGAAATCATTGATTATATAGATACATTAAAT
TATATCGATCAATATTCATCTGCAGGAAGCTTCCGTCGTTTTAAAGAAATGAGCAAAGATTTAGATTTCATAATAAGTACCGATAAC
CCAAAAGCAGTGCAGCAGCAATTATTAAATATTCCCAATAAAGTAAAAGAAGTTGCAGTGGGGAACACAAAAGTTTCATTAGAATT
AGCGTATGATGATGAAACGATTGGTGTCGATTTTCGATTAATTGAACCAAGTGCTTTTTATCATACATTGCAGCATTTTACTGGGTCA
AAAGAACATAATATAAGAATTCGACAACTTGCTAAAGCACGTGATGAAAAAGTTAGTGAATATGGAATTGAACAAGCTGATGGTAC
ATTAATTCAATATGATAGTGAAGCCAAGATATATGAACATTTTAATGTGAATTTTATACCACCTGCTATGCGAGAAGATGGTAGCGA
ATTTGATAAAGATCTAAGTAATATCATTACATTAGATGATATTAATGGTGATATTCATATGCATACAACGTATAGTGATGGTGCGTT
TTCTATTCGAGACATGGTAGAAGCAAATATCGCAAAAGGTTATAAATTCATGGTAATTACTGATCATTCACAAAGTTTACGTGTTGC
TAATGGCTTACAAGTGGAAAGACTTTTAAGACAAAATGAAGAAATTAAGGCTTTAGATAAAGAATATAGTGAAATTGATATTTATT
CAGGTACAGAAATGGATATATTACCTGATGGCTCGCTGGATTATGATGATGAAATTTTAGCACAACTTGATTATGTAATTGGAGCTA
TTCATCAAAGCTTTAACCAATCAGAAGAACAAATTATGGAACGATTAGCTAATGCATGTCGCAATCCATACGTGCGACATATAGCG
CATCCAACAGGGCGTATTATAGGTAGAAGAGATGGTTATAAACCGAATATTGAACAATTAATGGCATTAGCTGAAGAAACGAATAC
AGTATTAGAAATTAATGCCAATCCACATCGACTGGATCTGAACGCTGATATCGTTCGTAAATATCCAAATGTGAAATTAACTATTAA
CACTGATGCGCATCATACAAATCATTTAGATTTTATGAATTATGGCGTAGCAACTGCGCAAAAAGGATTTGTAACAAAAGATAGAG
TGATTAACGCATTATCGCGTGAAGCTTTTAAAGACTTTATTGAAAATAATATAAAACTTAAGAAATAGAGGGATTTTATGAGACAA
AAAACATTAGACGTCTTAGAATTCGAAAAAATAAAATCACTCGTTGCCAATGAAACTATTAGTGACTTAGGCTTGGAAAAGGTCAA
TCAAATGATGCCAGCTACTAATTTTGAAACGGTTGTTTTTCAAATGGAAGAAACGGATGAGATTGCTCAAATCTATAATAAGCATCG
TTTACCAAGCTTGAGTGGCTTATCTAAAGTATCAGCATTCATTCATCGCGCTGATATTGGCGGCGTTTTAAATGTATCAGAGCTTAAC
TTGATAAAAAGATTAATTCAAGTACAAAATCAATTCAAGACATTTTATAATCAATTGGTTGAAGAAGATGAAGGTGTTAAATACCC
AATATTAGATGACAAGATGAATCAATTACCTGTGTTAACTGATCTTTTTCAACAAATAAATGAAACATGCGATACGTATGATTTATA
TGATAATGCGAGTTATGAATTGCAAGGGATTAGAAGTAAAATTTCTAGCACGAATCAACGTATTAGACAAAATTTGGACCGTATTG
TTAAAAGCCAAGCAAATCAGAAAAAAATTATCAGATGCTATTGTAACAGTTAGGAATGAAAGAAACGTTATACCTGTCAAAGCTGAA
TATCGACAAGATTTTAATGGGATTGTACATGATCAATCTGCTTCAGGACAAACATTGTATATTGAGCCATCATCAGTTGTTGAAATG
AATAATCAAATTAGTCGATTACGTCATGACGAAGCAATTGAAAAAGAACGCATTTTAACGCAACTAACTGGTTATGTGGCTGCGGA
CAAAGATGCACTACTTGTGGCAGAACAAGTCATGGGTCAGTTAGATTTTTTAATCGCAAAAGCGAGATATAGTAGAAGTATTAAAG
GAACAAAGCCGATATTTAAAGAGGACCGTACTGTATATTTACCTAAAGCATACCATCCATTATTAAATCGTGAGACTGTTGTAGCTA
ATACCATCGAATTTATGGAAGATATTGAAACGGTAATTATTACAGGACCGAATACAGGTGGTAAAACTGTAACATTAAAAACATTA
GGTTTAATTATTGTTATGGCTCAATCAGGATTGTTGATTCCCACACTTGATGGTAGTCAGTTAAGTGTATTTAAAAATGTATATTGCG
ATATCGGAGATGAACAATCAATAGAACAATCATTATCAACTTTTTCATCTCATATGACGAATATAGTTGAAATTTTAAAGCATGCAG
ACAAACATAGTTTAGTTTTATTTGATGAATTAGGTGCAGGTACAGATCCGAGTGAAGGTGCTGCATTAGCAATGAGCATTTTAGATC
ATGTTAGAAAAATTGGTTCTCTAGTAATGGCAACGACGCACTATCCTGAACTTAAAGCATATAGTTATAATCGAGAAGGCGTTATGA
ATGCGAGTGTAGAATTTGATGTAGATACTTTGAGTCCAACGTATAAGTTATTAATGGGTGTGCCGGGTCGTTCAAATGCTTTTGACA
TTTCTAAAAAGTTAGGTCTTAGTTTGAATATTATTAATAAGGCTAAGACGATGATTGGTACTGATGAAAAAGAAATAAATGAAATG
ATTGAATCATTAGAGCGTAATTACAAACGTGTAGAGACACAGAGGTTAGAACTGGACCGTCTTGTAAAAGAAGCGGAGCAAGTGC
ATGATGATTTATCTAAGCAGTATCAACAATTCCAAAATTATGAAAAGTCTCTAATAGAGGAAGCGAAAGAAAAAGCAAATCAGAA
GATTAAAGCTGCAACAAAAGAAGCTGACGATATTATTAAAGACTTAAGACAATTGCGTGAACAAAAAGGTGCAGATGTTAAAGAA
CATGAATTGATTGATAAGAAGAAACGATTAGATGATCATTATGAAGCGAAATCTATAAAGCAAAATGTACAAAAGCAAAAATACG
ATAAAATTGTTGCTGGTGATGAAGTAAAAGTATTATCTTACGGTCAAAAGGGTGAAGTTTTAGAAATTGTCAATGATGAAGAAGCA
ATTGTTCAAATGGGAATTATTAAAATGAAGTTACCTATTGAAGATTTAGAGAAAAAACAAAAAGAAAAAGTTAAGCCAACGAAAA

TGGTTACACGTCAAAATCGTCAAACAATTAAAACTGAACTTGACTTACGAGGCTATCGTTATGAGGATGCTTTAATTGAACTAGATC
AATATTTAGATCAAGCTGTTTTAAGTAATTACGAACAAGTTTATATCATTCATGGTAAAGGTACAGGTGCACTTCAAAAAGGTGTAC
AACAACATTTGAAAAAGCATAAAAGTGTTAGTGACTTTAGAGGTGGTATGCCAAGCGAAGGTGGATTTGGCGTTACCGTTGCAACA
CTAAAATAAATTATAATTTGATAAATTAAATAGCTGCAGTTAAAATAATGTAAAGCAACAAGAATACATTTCAAACATGTTATTTGA
AATAAGCATAAAAATTGAGCAAATAGAAATACATGAAGCATGTTATCTGATATAATTTGAACATCATAATAATAATTAAGGAGGAT
TGGCATTTATGGCAATCGTAAAAGTAACAGATGCAGATTTCGATTCAAAAGTAGAATCTGGTGTACAATTAGTAGATTTTTGGGCAA
CATGGTGTGGTCCATGTAAAATGATCGCTCCGGTATTAGAAGAATTAGCAGCTGACTATGAAGGTAAAGCTGACATTTTAAAATTA
GATGTTGATGAAAATCCATCAACTGCAGCTAAATATGAAGTGATGAGTATTCCAACATTAATCGTCTTTAAAGACGGTCAACCAGTT
GATAAAGTTGTTGGTTTCCAACCAAAAGAAAACTTAGCTGAAGTTTTAGATAAACATTTATAAGTTACAACCAATGACGACTGGGG
CATTTCTTTAATGAATTGCTCCAGTTTTTGTTTGTGTTTTTAATATAAAAAGTTGAATGATAAGTCATCATATTGTTTACGACTTGAGA
ATGGTGGGATTAATAAAATCTATGAACGTTAAATGTTAATCAAGCATGCTGATAGATATGTAGCAGTTGGTTTGATAAAACAATGTT
CAATATTACATGATGTGCATGAAAAGTCATACTCGAAGATGTTGATTATGAGCTAGAATTAGTGGTGATAAATTTGAAGCACTTTTG
TAGCATCATTCATTTTAAAATTAGAAGGGGGGATATTATTGGAAGACTATAAGCAACGAATTAAAAATAAATTAAATGTCGTACCT
ATGGAACCAGGCTGCTATTTAATGAAAGATCGTAATGATCAAGTGATATATGTTGGCAAAGCTAAAAAGCTAAGAAATCGATTGAG
ATCATATTTTACGGGTGCTCATGATGCTAAAACAACGAGACTGGTTGGTGAAATACGTCGCTTTGAGTTTATTGTCACGTCAAGTGA
AACAGAGTCACTTTTACTAGAATTGAATCTGATTAAACAATATCAACCAAGATATAATATATTATTAAAGGATGATAAAAGTTATCC
ATTTATTAAAATTACGAAGGAGAAATATCCTAGACTACTAGTGACGAGAACTGTAAAACAAGGTACTGGCAAATATTTCGGACCGT
ATCCGAATGCATATTCTGCTCAAGAAACTAAAAAGTTATTAGACAGAATATATCCATATCGCAAATGTGATAAGATGCCAGATAAA
TTATGTCTTTATTACCATATTGGACAATGTTTAGGACCATGTGTATATGACGTTGATTTGAGTAAATACGCACAAATGACGAAGGAA
ATTACTGATTTTCTGAATGGGGAAGACAAAACAATTTTAAAAAGTTTAGAAGAGCGAATGTTAACTGCAAGTGAATCACTTGATTTT
GAACGGGCTAAAGAATATAGAGATTTAATTCAACATATTCAAAATCTGACAAACAAACAAAAAATTATGTCATCAGATAAAACGAT
TCGTGATGTCTTTGGTTATAGTGTTGATAAAGGATGGATGTGTATCCAAGTTTTCTTTATACGACAAGGTAATATGATAAAGCGAGA
TACAACGATGATTCCATTACAGCAAACAGAAGAAGAAGAATTTTATACATTTATTGGACAATTTTATAGCTTAAACCAACATATTTT
ACCTAAGGAAGTTCATGTACCACGTAATTTGGATAAAGAAATGATTCAATCTGTTGTGGACACTAAAATCGTTCAACCCGCGCGAG
GTCCCAAAAAAGATATGGTTGACCTAGCTGCACATAACGCTAAAGTATCCTTAAATAATAAATTTGAATTAATATCACGTGATGAGT
CAAGAACGATTAAAGCTATTGAAGAACTTGGAACACAAATGGGAATTCAAACACCAATTAGAATTGAAGCATTCGATAATTCTAAT
ATTCAAGGTGTGGATCCAGTGTCAGCAATGGTTACATTTGTCGACGGTAAACCAGATAAGAAAAATTATAGAAAGTATAAAATCAA
AACGGTTAAAGGTCCAGATGATTACAAATCAATGAGAGAAGTAGTAAGACGACGATATTCTCGCGTTTTAAACGAAGGATTACCAT
TACCTGATTTAATAATAGTAGATGGTGGTAAAGGACATATGAACGGGGTTATTGATGTGCTACAAAACGAATTAGGTCTTGATATCC
CTGTTGCAGGTTTGCAGAAAAATGATAAACACCAAACATCTGAATTATTATATGGCGCTAGTGCAGAAATTGTACCACTGAAGAAA
AATAGCCAGGCATTTTATTTGTTGCATCGTATCCAAGATGAGGTTCACAGATTCGCAATCACATTTCATAGACAAACACGTCAAAAG
ACAGGCTTGAAATCAATACTTGATGATATAGATGGTATCGGTAACAAACGTAAAACATTATTATTGCGTTCATTCGGTTCAATCAAG
AAAATGAAGGAAGCTACACTTGAAGATTTTAAAAATATAGGTATTCCTGAAAACGTCGCAAAGAACCTACATGAACAATTGCATAA
ATAAATTACTGGGCATGAGAATCATTCTCATGCCCCAAAATATATGAAATAATGTTACAATATGGTTAACAAAGTTATTTTTTTGAA
TGTACGTGGAAGCGTTTTCTAAATGAGAAAGTTTATCATCACAAGCAATCTAAAATATCGAATCCAAATGTTACATTGTTCGACATG
CAGTTTGGTGATCGTGCAGTGTGTATTTGTCTTTAAAATGTTTCGCTTAATGATAGTGACAACGCTAGAAACGATATCACTTTAGGG
GGTTAAGCGTACGTTTAATATAAATAGCTCAATAAATTAAATATTCGTTCACAGGGGGGACTCCTTTTGGCTCAATCAAAAAATGAA
TTTTATCTAAGACGTATTCACTCGTTATTAGGTATTATCCCAATAGGTGCATTTTTGGTCGTTCATTTATTAGTGAATCACCAAGCAA
CACAAGGTGCTGAAGCGTTTAATAAGGCATCTAACTTTATGGAATCATTACCATTTCTAATTATTGTAGAATTTTTATTTATATACAT
TCCGTTGTTATATCACGGTTTGTTTGGTATACACATTGCATTTACAGCAAAAGAAAATGTTGGACATTACTCGATTTTTAGAAACTGG
ATGTTCTTTTTCCAAAGAGTGAGTGGTATCTTAACATTTATCTTTATTGGTATCCATTTATGGCAAACACGTTTACAAAAAGCATTTT
ACGGCAAAGAAGTGAATTACGATTTAATGCACGAAACATTGCAACATCTTGGATGGGCAATATTTTATATTATTTGTATTATTGCTG
TTGTGTTCCACTTTGCAAATGGCTTATGGTCATTCTTAGTTACTTGGGGTGGACTTCAATCTCCAAAATCACAACGAGTATTTACATG
GGTTTCATTAATCGTATTCTTAGTTATTTCGTATATTGGTGTTACTGCAATTATTGCCTTTATGTAATACATCGCATTTAACAAATATA
GATTGAAAATTTAGGGGAGTGAAATTTTTATGGCAGAGAAACATCTTATTGTTGTCGGAGGTGGCCTAGCGGGCTTAATGTCAACA
ATTAAAGCGGCAGAAAAAGGTGCACATGTAGATTTGTTCTCAGTTGTACCAGTAAAGCGTTCGCACTCTGTTTGTGCCCAAGGTGGC
ATTAATGGTGCGGTCAATACTAAAGGGGAAGGCGATTCTCCTTGGATTCACTTTGATGATACAGTGTATGGTGGCGACTTCCTTGCA
AACCAACCACCTGTTAAAGCGATGACAGAGGCAGCACCTAAAATTATTCATTTATTAGACCGTATGGGCGTAATGTTCAATAGAAC
AAATGAAGGTCTATTAGATTTTAGACGTTTCGGTGGTACATTACACCACAGAACAGCATATGCAGGGGCAACAACTGGACAACAAT
TATTATATGCATTGGATGAACAAGTTCGTGCATATGAAGTAGATGGATTAGTTACGAAGTATGAAGGATGGGAATTCCTTGGCATA
GTTAAAGGTGACGATGATAGTGCAAGAGGTATCGTTGCACAAAATATGACAACTGCTGAGATTGAAACATTTGGTTCAGATGCAGT
TATTATGGCAACGGGTGGCCCTGGTATTATTTTCGGTAAAACAACAAACTCAATGATTAATACAGGATCAGCGGCTTCCATTGTTTA
CCAACAAGGCGCTATTTATGCTAATGGTGAGTTCATTCAAATTCATCCTACTGCAATCCCTGGTGATGATAAACTGCGACTAATGAG
TGAATCAGCACGTGGTGAAGGTGGACGAATTTGGACATATAAAGATGGTAAGCCTTGGTACTTCTTAGAAGAGAAATATCCTGATT
ATGGTAACTTAGTACCTCGTGATATCGCAACGCGTGAAATTTTCGATGTATGTATTAACCAAAAATTAGGTATAAATGGCGAAAACA
TGGTATATCTTGATTTGTCACATAAAGATCCACATGAGTTAGATGTAAAACTAGGTGGTATCATTGAAATTTATGAAAAATTCACTG
GTGATGACTCACGCAAAGTACCAATGAAGATTTTCCCAGCTGTTCACTATTCAATGGGTGGTCTATATGTAGATTATGATCAAATGA
CAAATATTAAAGGGTTATTTGCAGCTGGAGAATGTGACTTCTCTCAACATGGTGGTAACCGCTTAGGTGCCAATTCATTGTTATCAG
CGATTTATGGTGGTACAGTAGCAGGTCCAAACGCGATTGATTATATTTCAAATATTGATCGATCATATACTGATATGGACGAAAGTA
TTTTTGAAAAGCGTAAAGCTGAAGAGCAAGAACGTTTTGATAAATTATTAGCTATGCGCGGTACAGAAAATGCCTATAAATTACAC
CGTGAACTTGGTGAAATTATGACAGCAAATGTAACTGTTGTTCGTGAAAATGAAAAACTGTTAGAAACAGATAAAAAGATTGTTGA
ATTGATGAAACGTTATGAAGATATTGATATGGAAGATACTCAAACTTGGAGTAACCAAGCGGTATTCTTTACTCGTCAATTATGGAA
CATGTTAGTACTTGCACGTGTTATTACGATTGGTGCATATAACCGTAACGAATCACGCGGTGCCCATTATAAACCAGAATTCCCAGA
GCGTAATGATGAAGAGTGGTTAAAAACGACAATGGCCTCATTCCAAGGCGCATTTGAAAAACCACAGTTTACTTATGATGACGTCG
ATGTGAGTTTAATACCACCTCGTAAACGTGATTACACAAGTAAGTCTAAAGGGGGTAAAAAATAATGACTGAACAATCAGTGAAAA
ACACTCCACAACATGAAACACAATCTAAACCGAAACAAAAAACAGTAAAATTAATTATTAAACGACAAGATACAAGTGATTCTAA
GCCTTATGAAGAAACATTTGAAATTCCATATCGTGAAAATTTAAACGTCATTGCTTGTTTAATGGAAATTAGACGTAACCCAGTTAA
TATTAAAGGTGAAAAAACAACACCTGTTGTCTGGGATATGAACTGCTTAGAAGAAGTATGTGGAGCATGTTCTATGGTTATCAATG
GTCGTGCAAGACAATCTTGTTCTGCGATTGTTGATCAATTAGAACAACCTATTCGTTTAGAGCCAATGAATACTTTCCCAGTTATCCG
TGACTTACAAGTTGATCGTTCTAGAATGTTCGATAACTTAAAACGTATGAAAGCATGGATCCCAATTGATGGAACGTATGATTTAGG
TCCGGGACCACGTATGCCAGAGAAAAAACGTCAAACAGCTTATGAATTATCTAAATGTATGACATGTGGTGTATGTTTAGAGGTTTG

TCCTAATGTTACTGAAAATAATAAATTCGTTGGTGCGCAAGCAATCTCGCAAGTTCGTTTGTTTAATTTGCACCCAACAGGATCTAT
GACTAAAGATGAACGTTTAAATGCATTAATGGGTACTGGTGGCTTACAGCAGTGTGGAAATTCACAAAACTGTGTTAATGCTTGCCC
TAAAGGTATTCCATTAACAACATCCATTGCAGCAATGAACAGAGAAACAACATTCCACATGTTTAAATCATTCTTTGGTTCAGACCA
TGAAGTAGAATAAAAATTAATCCCTTTTGAGTTAAAATGTTATGTTCCTTTCAAAATTAAATTGAATGGAGATACACAATAACTTGG
AAGGGATTTTTTTAATAAGCAGATTACATGACAATATGATAGTGGGCTGATTTAAAATAAATATTATATTATAATGACAAAAATGGT
AAACACAGTGCATAATACTAAAATCCAATGCTTAGTTATGATAAACTAAAAATTAAATTAAAAGTAAGGATGACCGAATATGAATA
AACCAATAGGTGTAATAGACTCTGGTGTCGGAGGTTTGACAGTAGCTAAAGAAATTATGCGTCAGTTGCCAAATGAGACGATTTAT
TACTTAGGTGATATTGGGCGATGCCCATATGGGCCAAGACCAGGAGAACAAGTAAAACAATATACAGTTGAAATCGCTCGTAAATT
AATGGAATTTGATATAAAAATGCTCGTGATTGCTTGTAATACAGCAACTGCTGTAGCTTTAGAATATTTACAAAAGACCTTATCAAT
CCCAGTGATTGGCGTAATTGAACCAGGTGCTAGAACAGCAATAATGACTACTAGAAATCAAAATGTATTAGTACTAGGAACGGAAG
GCACAATTAAATCTGAAGCATATCGTACGCATATTAAACGTATCAATCCACATGTAGAGGTACATGGCGTTGCCTGTCCAGGTTTTG
TGCCACTTGTAGAACAAATGAGATATAGTGATCCAACAATTACAAGCATTGTCATTCATCAAACACTGAAACGTTGGCGTAATAGT
GAGTCTGATACTGTCATTTTAGGATGTACCCACTATCCATTGCTCTATAAACCTATCTATGATTATTTTGGTGGTAAAAAGACAGTGA
TTTCGTCTGGATTAGAAACGGCTCGTGAAGTTAGTGCATTGCTAACATTTAGTAATGAACATGCAAGTTATACTGAAAATCCAGATC
ATCGATTTTTTGCAACAGGTGATCCTACTCACATTACTAACATTATCAAAGAGTGGTTAAATTTATCTGTCAATGTGGAACGTATATC
AGTGAATGACTAGGAGGATTTTTAATGAAAGAGATTGTTATTGCATCGAATAATCAAGGGAAAATAAATGACTTTAAAGTAATATT
TCCAGATTACCACGTAATAGGTATTTCAGAACTAATACCAGATTTTGATGTGGAAGAAACAGGATCAACATTTGAAGAAAATGCTA
TATTAAAATCAGAAGCTGCTGCAAAAGCATTGAATAAAACGGTCATAGCTGATGACAGTGGACTAGAAGTTTTTGCATTAAATGGT
GAGCCAGGTATATACTCTGCACGTTATGCTGGTGAAAATAAAAGCGATGAAGCAAATATTGAAAAATTATTAAATAAGCTTGGTAA
TACAACTGATCGTCGTGCGCAATTTGTTTGTGTCATAAGTATGAGTGGCCCTGATATGGAAACAAAAGTATTTAAAGGTACTGTTTC
AGGTGAAATTGCAGATGGAAAATATGGCGAAAATGGTTTCGGATATGATCCGATATTTTATGTACCGAAATTAGATAAAACCATGG
CTCAACTTTCAAAAGAACAAAAAGGGCAAATTAGTCATAGACGAAATGCGATTAATTTACTTCAAGCTTTTCTTGAAGGTGATAAA
AATGTCTAAATGGATTATTGTGAGTGATAACCATACAGAATCAGGCGTTTTATATCAAATTTATGAAATGCACCCAGATGCAGATGT
ATATTTACATTTAGGAGATTCAGAATTCGCGTATGATGATACGGAACTTAGCTTATTTAATAGAGTAAAAGGCAATTGTGATTTTTA
CCCAGAATTTGAAAATGAAGCGGTCGCAAAATATAATGACGTGAAAGCATTTTATACTCATGGACATTTATATCAAGTCAATCGAA
CAAGAGATTTATTAGCTGAAAAAGGACTTGAATTAGGTTGTTTGTTTGCATTTTATGGACATACACATGTGGCAAAATATGAGTATA
TTAATGGTGTTCATGTTATTAATCCTGGAAGTATATCTCAATCTAGAAGTTCAATGGAAGAAACATATGCTGAAGTTATTATTGATG
ATCAAACTTTACATGGCACCATCAATTTCAAAAATCGACATCACGAAACAATCAGTCATACTACTTTTTAAATAGAAGTAGCTATGG
CTTTTTTTAGTTTATAGACTGATTTAACTAGGAGGTCGTGTCATGAATGTTTGTTTAAAAGTATTTCTGACAATTTTATAAGATAATT
GGAATGAATAACACATTTATGTTATGAAAAGAATTGTAATTTTAGAGAATGTGATTGTTGATGACCCTATATGGTTGAATCATGTTG
ATTATTTGGTTAAAAGTGTATTATTAAAAAGTATAAAATTAAGATTCCAAGAAGTCAGGAAATTTGAAATTTTAGTTAAAATCATGA
TTCGTATACATATTTAAAAGGATTTCTAAAGTCATAAATTATATCAATAATTTGATGGTTAAAATATGGAGTAGCAAAATTCTTGAA
ATTCAGAAATGTAGTGACACAAATATTAATGTTTAGTTAATAAATAGTTAATGTAGTTTTTTGTATCTGTCTATTATTATAAATATAT
ACAAAATAAATATTTGAGGATAGGTGTTATTAATTATGAAAAAGAATTTTATTGGGAAATCAATTTTAAGCATAGCTGCTATTAGTT
TAACGGTATCAACGTTTGCCGGTGAATCTCATGCACAAACTAAAAACGCTGAAACTGTAAAAAAATATAACGAGTATCAAACAAAC
TTTAAAAAACAAGTAAATAAAAAAGTAGTGGACGCACAAAAAGCTGTAAACTTGTTCAAACGTACAAGAACTGTTGCAACACACC
GTAAAGCACAAAGAGCTGTTAATTTAATTCATTTCCAACACAGCTATGAAAAGAAAAAATTACAAAGACAAATCGATCTAGTTTTA
AAATATAATACTTTAAAATAATTGATAGAAGCCATGCTATAGAGTGTGGCTTTTTGCTATATTCAAAGGTATAATTAGTTTATAATA
TTGTGTTCATTTGTATGGTGAGATTATTAGAATAAATCATATAAATAATTACTTAAAAGTGAATGCAATAGTTGTTTATGTTTTGAAC
AAGCGAGGTGGCTATTGTGAAAAGGAAATCGAATGTATTAACAATCATAAAAATTGTAACGGATATAGTGGCAACCGTGTTAATCA
TACGTGCTTTATTAAACAGTCGTAAAGATGCTAAATAAATTGGAAGTTACTTAAAATAAAAAAAACACGCCAATACGTTAGCATTG
TTTTCTAACGTTTGGCGTACAACTATTTAATTCAGTATGTTTTTTAATACCAAGTAATCGAATCGATTTCACCTTTAGCGACTACTTTA
ATATTTTTTCTGTTCTCTTCTAAAGGACGAGTTAAGTCAAAAGTATAGTAATCTCTAGGACCACCATCTTTAATTCTGACAACTGCTT
TCTTCACATCACCTTGGCTTAATTTTTTAACAATAAGGTACAGATCATTAACAGTTTCAGCTTTGTAAGGAGTTAGATTTTTATCAGA
CTCTTTCATTAACTTATCAATACGTTCATCGTCCTTTTTAGCTTGATCTGCTAAATTTTTTGCGATTTCTAAACCTTTCCATTCATAACT
AAAGAAAGCTTTAGCATCATTAGTTTGAGTTGAAAGACCTGCAGCGATTACTGTAGATGCGATAATAGTTTTTGTGATATTTTTTTTC
ATAATAAGTTCTCCCTGTAAAATAAATTTGTACTTACTAATGCGAGTACACTGCTATTTTACAAACGGTTTCATGCATTTACCAGTTA
ACACTTCATTAATTAAAATTAAGTGCAAATTAATAAGGTTTAATGCAATCGAAAAAGAATAATGATGAATTTGATGGTATTATTTTT
ATTTGAGGTATTATTGAATTTGTTGATAGTATAATAATAGTAGTAAACGTCGCTCAACTATGATTGGATAATTTTTATTGTTAAAACG
ATATATATTTCTTTGCATTTTGTTTGCAATGTTGGGTAGACAAACACTCCAAATTGTTATACATATAGCTTTTTTGATAATTAATGTTT
ATAAAACAAAGAAATGATTCATAGTAGAGAAGTAATTAATATGTATAATTTTACTTATAAAGAAATAAGAGATATATTCTAATTAA
GGTTGATATGTTATGATTAATATATAAGTTAAAAATTATACTATTTAATTGAATTATGCATTTTCGAAGTAAGCATAATTCAACACTT
ACATTTTTAAATAGATAAAATAGTGTAAAATCACGTGCCAAGATTACTATAGAGTAGGCATTGAGTATTATTGGCAATCACTTAAGT
GATTGTTATTTTTCGTGGTCAATAATAAAGATATAAATAAATATATTGAGGTCAAACGATGATAATTAAAATTTTAACAATTCTATT
ACTACTTTGTATATTGAGCTATTTGGTTACAAATAGAAAGAAGCCTTTTCTGTTCTTAAAGACACTCTTTATGGGTGTGGTATTTATC
TTTATAGGATATATTTCACTGGCAATATCTGCCGTAATTATTTATGGTATTATTCAATTTATCACAATTGATTTTGGTAGTTTTTTCTT
AATGGGTATTATATTGATCTTGATTTCAAGTATATTCCAATTATTTATAGTTAGATTACTTTTTAGAAAAAAGAATGTCGATTTGACA
GAGGTTGTCGTTTTAGAGCATTTAATTCAATGGTTCTTAGTTTACTTTGCGATCTATCAAGCAGTAAATGAAAAAATGGACATTAAT
GATATTAATATCGACAATTTCCAATCTGTCTTTTTTGACGTGTCTAATTTGAATTTAGTAATTCTACCAACGTTAATCATTAGCTGGG
TCACAATATTTAACTATAGAATGAGAAGTTACAAATAAAATCTATGAAATTATACCATCAGGCACCAACATTCAAATGGTGTCTTTT
TTTGTTGTGTGGTTTTATTTTTGAAATTCGAAAAAGTAGAGGCGTGAATTTTTTGACTAGTGTATCAGTGCTGATGAGTCACAAGATA
GATAACTATATTTGTCTATATTATAAAGTGTTTATAGTTAATTAATAATTAGTTAATTTCAAAAGTTGTATAAATAGGATAACTTAA
TAAATGTAAGATAATAATTTGGAGGATAATTAACATGAAAAATAAATTGATAGCAAAATCTTTATTAACAATAGCGGCAATAGGTA
TTACTACAACTACAATTGCGTCAACAGCAGATGCGAGCGAAGGATACGGTCCAAGAGAAAAGAAACCAGTGAGTATTAATCACAA
TATCGTAGAGTACAATGATGGTACTTTTAAATATCAATCTAGACCAAAATTTAACTCAACACCTAAATATATTAAATTCAAACATGA
CTATAATATTTTAGAATTTAACGATGGTACATTCGAATATGGTGCGCGTCCACAATTTAATAAACCAGCAGCGAAAACTGATGCAAC
TATTAAAAAAGAACAAAAATTGATTCAAGCTCAAAATCTTGTGAGAGAATTTGAAAAAACACATACTGTCAGTGCACATAGAAAAG
CACAAAAGGCAGTCAACTTAGTTTCGTTTGAATACAAAGTTAAGAAAATGATATTGCAAGAGCGAATTGATCAAGTATTAAAGCAA
GGATTAGTGAGATAAAACTTTTATCATTACTGTAGGTTCAAAATAATTTAATCTAATATTAATTTCTATTAATAAAAAGACTATGCTA
TTTAATAGTAGGTTAATGTAACTTTCCTAAAATTGACTTTATAATCATTAAGTATCAACTTTAAAGGAGAGTTTACAATGAAAATTA
AAAAATATATATTAGCAGGCTCATTAGCAGTACTTTTATCGACAACAGCGGTATCAACGTTAAACGGGAACAAAGCAGAAGCAAGT

AGTCAGAAAGATTATTTAATTCAAAGTCAGTTTCATGATAAAAAAATTGCTGAAGAATTGAAATCATTACTTAGACAATCGAACGT
ATATGACTTGGCTGCAGGTAGCTTAAATCGATATTACAAACGTACGATTATGATGAATGAATATAGAGCTAAAGCGGCACTTAAGA
AAAATGATTTCGTATCAATGGCTGATGCTAAAGTTGCATTAGAAAAAATATACAAAGAAATTGATGAAATTATAAATAGATAATAA
ATAAAACAGGTTGAGACAAAAAATGGTCTTAACCTGTTTTCAATTTGCATATGTGATAAATTCTATATCAAAATGCTTATGTATAAT
GAATGACATTTAAAAGTAGGGGAGACAAATATAAATACAATAGTTCCTAGGATTACTCTCAAAATAACTATATCAATTATTTACTTT
GCTCTCCTATTTTTTAAAATATGTACATGTTTAAACAATCAAAAGTGTACAATATTAAATTATCATTTCCAGTTCTAGTGCTATATTG
GTAGTAGTTGACTAAATGAAAATACGCTTGTAACAAGTTTTTTCAATACTTGTGGAGCCACAACAGAGAGAAATAGGATCACCAAT
TCCAACAGACAATGCAAGTTGGCGGGGCCCCAACATAGAGAAATTGGATCACCAATTTCAACAGACAATGCAAGTTGGGGTAGGA
CATCGATAAAAAAATACTTTTTCTTTAGAAATTAGTATTTCNTATGCATGAGTTTTACTCATGTATTCATTTTTTAAGTAGACATTAG
CTACAGCTAATGATAAAGAACCACTACATAATAAATCATTAGTGGTTCTTTATCATTTCTATCTCACTCTTTTACTGGAAGAAAAAGT
TTACGTTTGTAGAACATGCCACAATACCAAAAATAATTAAGAAAAATAAGACGATAAGCATGATGACACTTTTCAAACAACCTCTA
TCAGTTTCTTCCGATTTTCTTTGTTGAACCTTTTTATAATCTTCAAGTAGTTTTGTCGCTTTTTTATTTATATGTTTATTCATGATGTTG
ACTCCTTATAATATATGTTTAATTCATTAAAATAGTTGAAAACATGACTTGAAATAAAGATATAAAGCTAAAGTAAGGATGTTCAAT
TGTTTCAAATTTTATGAAAGTATAACGTTAAATATGTTAATTTGATGATGATATTTGCTTTTTATTTTCCAAATGGAATTTACTTAAAC
TGATGCATTAAAATATTAATGAAGCACTAGAATACATAAATGAATAGTAATGGTGCACAGTATAGAATAATTAAGGCTATATTAAG
TATAAATATCGTTAACTGTAAGCTATCTTTAGTTTTAATATAAACTATTAGGATAATCGACGTAAGAAGAATCATATATATTAATGA
TGAAGAAGTCCATACAAAATCCGCATCATTTGTTGTTAATAATGGGACTATAATTAATCCGAAATTAATCATGCATGCTATATATAC
TATAATGTTATACACAATGTTAATTTTTGTTCACCACCTTATACTTCTATTTTAAAAACTTCTTTATAATGATATATTGTTTAATGTTG
AAATAATTAGATTATCTAATTTTCATTTGCTTTACATGTAAAAGGCTATATATAGTATGCTCTTTATGATTCTAAATGCTTTTTAATAT
TTAATGCTCATCAACATTTGGATTTTGAATATTCAATTCAAAAACTTTATTAGCTACGTCAATTGTAAAATCAGAACCATAGTTGACA
TGAGCTACTTTTAATTTTCCATCTAAATAATAGATTGCGATTGCAACATCGTAAAATTCGTCAATGACAAATAAACTCTTTTCGTTTG
TTACAACCTCATGCTCTCCTGAGTATACAACGTTAATTTCCCAATCATTAAAAACCATTTGTTAACCTCCTTGAACATTTAAATTGAT
TCAACTTAAGTTTAACTTATTCATACAACTTCGTACAATATCTAGATGAACATTAATTGTATTTCTAGAAATCTTTTTCAATTATATGT
ACTAATTATACTTTTAAATTTCTTATTTCAGTATAGTTTTAAAACGATTTTAAAATAATTCTGCAAATATATTAACACATAATGTGTT
CAAAAAGTTTTGAACAATTTCAAAACTTTTATATAAAGGGTTTGACAACATGGAATTCAAATTTCTTATTTTAAAAATTACCTCATAT
AGTGTCATGTTTAGTCTAATTTTTAAGTCAGGAAAGCAAATCAAGTACATAGTTTAAATTAAAAATATCATAAGTTAATTAAACTAA
ATTAAGTATAAAAATTAGCCGAAAAACATCATTTCTGAAGTTATCGGCTAAAGTTATAAATTATATTTATTTGTACATGAACAAATA
ATTTACATTAATTTGTCATTTCTTCTTTTTCCCAATCGATTTTATATCTTTCTGAAGAACGATCTATCCATTTATCTTTAGTATTGGTAC
CTTTCCAATTTGTTGAAGTCCAATGCAATTGGTAGTCATCACGAACTCGTTCGTATATTACATCTATATTTGTTTGTTGTTTGGATGCT
TTTCTATCCATAGTAATAACTGTAGCGAAGTCTGGTGAAAACCCTGAAGATAATAGAGAACTTGCTTTGTTAGGATCAAGGAAGTTA
TCTGCTGCTTTCATAGAACCATTTCTAGTTTTCATGAAAAGTTGATTGCCATATACCGGGTTCCAAGAATCTCTATCATATGGTCCCC
AATTTTGATTCACCATATTGTTAAATATCACTTTCCAGCCTACTTTTTTATCAGTTGGGCTCTCTAAAATTGTTTTGAAATCAGGTTGA
ACATATTTCAGTGTATGACCAATCGAAACATTTGCACCAATAAGGCCGCCAATTTTTCCTGTATCATCACCAGTAACATTACCGTTG
AATCCATAAGTTAAAGTACTCATATACTCTTTTGTATCAATCGAATTTCTTGGATAGCAATCAGATATTTGAGCTACTTCATTATCAG
GTAGTTGCAACTGTACCTTAAAGGCTGAAGGCCAGGCTAAACCACTTTTGTTAGCACCTTCTTCGCTATAAACTCTATATTGACCAG
CAATGGTACCTTTCGTTCTAATAACTAGCAGTTTTTTATTGTGATTTTTATCATCGATAAAACTATAAAATACTTTTTTGTGCATGCCA
TTTTCTTTATCATAAGTGACTAAATCACCTGTTTTTACTGTAGTATTGCTTCCAATATCTGTAGTACCGGTTTTAATATTAATATCAGA
ATCTGCGGCATTAGCGACAGGATTCATTAATATGGAACCTAGCAATAGTGTTGTTGTTACTGAGCTGACTATACGTGTTTTCATTTTC
ATCATCCTTCTATTTTTTTAAACGATTTGAGGAAACAATAATCAATATGTCAATTTAGAATATTGCAGTTGAGACATATCAAATATTT
ATAATCGTTTATATTAGTATATTTAATATAGCAACTGATAAATTACTGAGTGATGATGAGTGATTATTTTAAGAATATGTTTTTAACT
TTTATTTAAAATTTGAAAGGAAGCATTTCAATTTCGAGGGTTAGTCAAAGTTGAATAAATTCTTTATGAAACAAGGAAAAGATATAG
CTAATTTTATTGATTAATTTCTTTAAAACTAATGATTTGTTTGATTTAAAAATGTAATAGATTACAATATAAAAATACAAATATCTTA
GAATTAAATCAATTAATTAACTATTAAATAAAAATTAACTATATATTAACTACTGTAAATTAATAAATAGAAATAGAGAAAAAGGG
TATTAATTATGTTTGGAATTTCGTCGGGAATAATTTTTGCCATTACATAGAAATATCTAATAGATAACGAAAAAGTATCGTATGTATT
TTTAATATAGTGTAAAATATCATATGTAAAATAAAATGTAGATTTTTAGTTAGAGGCATTATAAGAAAATTTTGAGTATAGGTTAGC
TTTTAATTATGAATCTTATTGAAATTTGATTAATAAATATATGATAGGGGATTAAAATGAAACTATTTTATATCGTATTTCTTATTAT
TATATGGCTGAATATATTTTTAGGAAATGAAATTATCCATACACTGACTGTTTTAATAACAACATTGTATATTGTTAATTCAAGAAA
GGGGATTAAAAATGACAGAGTTGAATAATATTATAAACTCTCTTCAATCTTTGTTTGAGTCTGAATCAGGCTATAAAATTTCGAAAA
ATTCAGGAGTTCCATATCAAACAGTACAAGATTTAAGAAATGGGAAAACCAAACTAGAAGATGCTAGATTTAGAACGATTATTAAA
CTTTATAGTTACTATGTCTCATTAAAAGAACATTAATCATGGGACAATAAAGTATTGCTAAAATAATTAAATCATTAAATGAAGGAA
TAGAGAGGCTGGGACATAAATCCCTAAAAAAACAGCAGTAAGATAATTTTCAATTAGAAAATATCTTACTGCTGTTCTCTATTTATA
CAATACTTCGTATTGAATGGCTTCGCT

>029-contig_258_RC

TTATAAAATACATATCAAAAAAGCTGATTTCTATCAAATAATTAATAGAAATCAGCTTTTTTACATTGCCTAAGAACTTAATGTCCC
AAGCCCTCTACAATATTATATTGGTAGTAGTTGACCGAATGAAAATGCCCATGTAACAAGCATTTTTCAATTATAGGCGGGGCCCCA
ACATAGAGAATTTCAAAAAGAAATTCTACAGACAATGCAAGTTGGCGGGGCCCCAACATAGAAGCTGGCGGAAAGTCAGCTTACA
ATAATGTGCAAGTTGGGGAGTGACAACGAAATAAATTTTATAAAATACCATTTTTGTCACATTCCTACTCCCAATTCCATTTAAATA
TATAGAAAATTTCACCTATTATATACATCATACACTTTAAAAATTATCATTTAAATAATCTGATTTGGAATTAAAGTGCATGAAGTAT
AAGTCACCTTCATATACTAATCAAAGAGGACGTCAACAGTTATTTTATTAGGATTTTTAACATAAACATTTGCTAGATCTGAATGTA
ATCTTTTGCTTAAATCAATAGTGTAGTTATTACCGCCACCGGTGATCTTAAGCTTACCTTTATTACGATTTTCGTTATATAATATTTTA
TTTTTTATTAACGCTTCTCGTGCACGGAAATCGATTTCTTTCAATGTTAATACTGGTTTATTGCCTTGGTATATTTTATGCGCACCAAT
AATCGTTTGTAGTTTATCTTTGTATTGCACAAAAAGATTATAAGTTTTATCAGAAGGTTTTGCGGCTGGTGTAACGCCACCTGTAAAT
GTCTCTCTATAAGACCACCATAACTGATCAGTATCTTTGTCTTTTAGTCCAAACACATCTACGTAACGATCTTTTAACTGATTAATAT
TTCCCCAACTTTCAGCGCCCCATAAAGATATATGTGCTGACCATGAATATCTCTTAAGTTCAACATAAATGTTTCCATTATCATATTG
ATATAGCCATTTATTTGAAAATGAAAAATGAGGCTGTGTGTAATATTTAATTAATTCATTGATGTTAGTTTCATCTTGACCAATACTA
TAAGCTTTAGCTTCAGAGTAAAAACTAAATACTGGTTTTTGATTTTGAGGTAATACAGTACCTAATAGTAATAATGTTGTCGTTAAA
ATTATATTTTTCGTGATGTTCTTACTCATTAGAACATCTCCTTTCAGAGGAATCATGATACGAGGAATAAGAAATTTAAATGTGAGC
GAAGTCAATATAGTATTTGCGATTATTTTTATTAACTCCATTATTATTGTTAGTTTGATTTTTCGAGGATAACTTCAATTTTTGCATTT
TGAGGTTTTTTAACATATCTATTTGCATCAGTTGATGGCAACCTTTTACTTAAATCTATTGTGTAGTTATTGTCTGCACCTGTTATTTT

AATTTGTCCTTTATTATAAGAATTATTATATAATTTTTTACTTTTAATTAATGTTTGACGAATACGAAAATCTAATTCTTTTAAAGTTA
AAACAGGCTTATTGCCTTCATAAACTGGAAATCCGCCAGTAAACGTTTCTGCTTTATCTTTATATGTTACATTCAGTTTATAGTGTTT
ATCGTTAGATGTTGCTGCAGGAGTAACACCACCAGTAAACGTTTCTTGAGATAATGCAAAAGAATCAATGGTTTCTTGGTCTTTTAT
GCCAAAAATATCAACGCTTTTATTTCTTAATTGGTTGATATTGCCCCAACTTTCAGGTCCATAAACTTGAATATGACTATACCAAGAA
AACTGTAACAACGTTGCATGAATCGTACCGTTATCTTTTTGCCATAACGTACTGTTTGAGAAGGTTAAATATTTTTGCGAGTAATATT
TAGTTAACTCATTTACGTTAGTTTCGTTTTGATTTATATAATAAGCTTTCGCTTCAGATGAAGAATTGATAGGTGTATTAGGAAATTG
TGTAGATGCTGTACCTAATAGTAACAATGTTGTTGATAAAATAATTTTTTTCGTGATGTTATTGTTCATTCGAATTTCTCCTTTGAGTA
TTGTTGGAAAGTTTAATTATAAAAAGGGGTTAATTAGATAATTGAAATTATCCGCATTTACAAAAGGTAATAGGTTAGTTAGATTTT
TCGAGTATGACTTCAATTTGTGCATTTTTAGGATTTTTAACATAACGGTTTGTGTCAGTTAATTTTAACTTTTTACTTAAATCAATCGT
GTAATTATTTCCATCAGCAGTTATCTTAATTTGACCTTTATTAAATTCTCCGTTATATAACTTTTTATTCTTTATTAATGTTTGACGAA
TACGGAAGTCTAATTCTTTTAGAGTTAATACTGGTTTATTTCCTTTGTAAAATTCATGTCCACCGATGATAGTTTGTTGTTTATCACTA
TATTTTAAAAATAGTCTATAAGGTTTATCAGATGAAGTAGCTGCTGGCGTAACACCACCAGTAAATGTTTCATCATAAGTCCAGTAA
CCTTCAACTGTGTCCTCATCTTTAGTTCCAAATATATCAACGTATTTATTTCTTAACTGATTAATGTTTCCCCAACTCTCGGATCCAAA
CACTTGAATATGACTATACCAAACCCACGTTTGCAATGTTGCATGAATGCTACCATTGGGCTTTTGCCATAACCATTTATTTGATAGT
GATAAATGAGGCTGTGTATAATACTTTATTAACTCATTGATATTAGTCTCGTTTTCACTGATATTATAGGCTTTTGCTTCAGATGAAA
AACTGATTGGTGTTTTAGGAAGTTGTGTTGATGTGGTTCCTAAAAGTAACAATGCAGTTGATAAAACTAATTTATTCATGATGTTCTT
TTTCATATGAAAATCTCCTTTGCGTGAATTACCCAAAGTATATAAGCTATTACACCGATTCGGAATTAAATAAAAGCTAAAACTATG
TTAAATAAACTTAAACAGTTAGTAGTGTTATTTAAGCAAAACTTATCATTTTTAAGTTTTGGACAGAAACAGTACTTAATAAAGTAG
GCGGGAGTTATATATTAAAAACGACACGTTAGAATTATTCTCTAATCAATTGCATTAAATTGTTTGATAATTGAATTTTCTAACTACT
TGAAAAATAGTTATACTTTAAATGTAGTACTTATTTTAATTATTTCCTACTACTTAAATTTAATATTAATAAAATGTTCATTTAATTAT
TGATAAAATATTACAAATTTTAATAGTAGGTTGTGTTTATTTTGTATGCGCTTACAATTTAGGTGTAACTAAAATAAAAGGAGTTGTT
ATTAATGAAAAATTTACGAAACAGAAGTTTTTTAACTTTATTAGACTTTTCACGACAAGAGGTAGAATTCTTATTAACACTCTCCGA
GGATTTAAAACGTGCTAAATATATTGGCACTGAAAAGCCTATGTTAAAAAATAAAAATATTGCACTGTTATTTGAAAAAGATTCTAC
AAGAACGCGATGTGCATTTGAAGTTGCAGCGCATGATCAAGGTGCAAATGTAACTTATTTAGGCCCAACTGGATCACAAATGGGTA
AAAAAGAAACAACTAAAGATACTGCACGTGTGCTTGGTGGAATGTATGATGGCATTGAATACCGTGGTTTTTCACAAAGAACAGTA
GAAACTTTAGCTGAGTATTCAGGCGTACCAGTGTGGAATGGTTTAACTGATGAAGATCATCCTACTCAAGTTCTTGCTGATTTCTTA
ACAGCAAAAGAAGTCTTAAAAAAAGATTATGCAGATATTAACTTTACATATGTTGGAGATGGTCGTAATAACGTTGCAAATGCATT
AATGCAAGGTGCTGCCATTATGGGTATGAACTTCCATTTAGTTTGTCCAAAAGAATTAAATCCAACAGATGAATTATTAAATCGCTG
TAAAAATATTGCCGCTGAAAATGGTGGCAACATATTAATCACAGATAATATTGACCAAGGTGTAAAAGGTTCGGATGTAATTTACA
CTGACGTTTGGGTATCAATGGGTGAACCTGATGAAGTATGGAAAGAACGACTTGAATTATTGAAACCATATCAAGTAAATAAAGAA
ATGATGGATAAAACTGGTAATCCAAATGTTATTTTTGAGCATTGCTTACCATCTTTCCATAATGCTGATACGAAAATTGGTCAACAA
ATTTTTGAAAAATATGGAATTCGAGAAATGGAAGTTACAGATGAAGTATTCGAAAGTAAAGCTTCAGTTGTATTCCAAGAAGCTGA
GAACAGAATGCATACAATCAAAGCAGTGATGATTGCTACATTGGGTGAATTTTAAATGATATAAGGAAGTGAATATGATGGCGAAA
ATAGTAGTAGCATTAGGTGGTAATGCTTTAGGAAAATCACCTCAAGAACAACTCGAGCTTGTTAAAAATACTGCGAAATCATTAGT
AGGATTAATAACAAAAGGACATGAGATTGTTATTAGTCATGGTAATGGACCACAGGTTGGAAGCATTAATTTGGGACTTAACTATG
CTGCAGAACATAACCAAGGTCCGGCATTTCCATTTGCTGAATGTGGCGCAATGAGTCAAGCTTACATCGGCTATCAATTACAAGAA
AGCTTACAAAATGAATTGCATTCTATTGGAATGGATAAACAAGTGGTAACACTAGTGACACAAGTTGAAGTTGATGAAAATGATCC
GGCATTTAACAATCCTTCAAAACCAATTGGGTTATTTTACAACAAAGAAGAAGCTGAACAAATTCAAAAAGAAAAAGGATTTATAT
TTGTTGAAGATGCTGGAAGAGGATATAGACGCGTTGTTCCTTCACCACAACCCATCTCTATTATTGAATTAGAGAGTATTAAAACAC
TTATTAAAAATGATACACTCGTTATTGCTGCTGGCGGTGGAGGTATACCAGTAATTAGAGAGCAACATGATGGTTTTAAAGGTATTG
ATGCAGTTATAGACAAAGATAAAACAAGTGCATTGTTGGGTGCTAATATTCAATGCGATCAATTGATTATTTTAACAGCAATTGATT
ATGTATATATTAATTTTAACACTGAAAACCAACAGCCTTTGAAAACAATAAATGTTGATGAATTAAAACGATATATAGACGAAAAT
CAATTTGCAAAAGGAAGTATGTTACCAAAAATTGAAGCAGCCATATCATTTATTGAAAACAATCCAAAAGGAAGCGTGCTTATAAC
ATCATTAAATGAATTAGATGCTGCCTTAGAGGGTAAAGTAGGTACTGTGATTAAAAAGTAATTGAATTGAAACGCTTTTCAATTACT
ATATGTCAAATGCATGATTTTTATTATTTATGTGCACCCCCTGAAAATAATGCCTCTATTTTGATGCGGGGTGCACTTTCTTAATTTAT
ATTTATAAAATCTTTAAGGTAGAAATGCTAGGTTAAATGTCGAAGGAGATGAAACCGTGGAAAATACAATTAATGAAAGTGAAAAG
AAAAAACGATTTAAATTAAAAATGCCAGGTGCATTTATGATTTTATTCATTTTAACGGTTGTTGCAGTTATAGCAACATGGGTTATTC
CTGCTGGTGCATATTCTAAACTTTCTTACGAACCTTCATCCCAAGAACTAAAGATAGTTAACCCTCATAACCAAGTGAAAAAGGTTC
CGGGTACGCAACAGGAACTAGACAAAATGGGGGTTAAAATTAAGATTGAACAATTTAAATCAGGTGCAATTAATAAGCCGGTATCA
ATTCCGAATACTTATGAAAGATTAAAGCAACATCCAGCTGGACCAGAACAAATAACAAGTAGCATGGTTGAAGGTACGATAGAAG
CGGTCGATATCATGGTATTCATTCTTGTACTAGGGGGACTTATTGGCGTAGTTCAAGCCAGTGGTTCTTTTGAATCGGGATTGTTAGC
TTTAACGAAGAAAACAAAAGGGCATGAATTTATGCTAATTGTGTTTGTATCAATACTAATGATTATCGGCGGGACATTATGTGGTAT
TGAAGAAGAAGCTGTAGCATTTTATCCGATTTTAGTCCCTATATTTATAGCGTTAGGATACGATTCTATCGTTTCAGTTGGTGCCATA
TTCCTTGCCAGCTCTGTCGGTAGTACATTTTCAACTATTAACCCGTTCTCGGTTGTAATTGCCTCTAATGCCGCTGGTACAACTTTTAC
GGATGGCTTGTATTGGAGAATAGGTGCTTGTATTGTCGGTGCGATTTTTGTTATTAGTTATTTATATTGGTACTGTAAAAAAATTAAA
AACGATCCTAAAGCGTCATATTCTTATGAAGACAAAGATGCTTTTGAACAGCAATGGTCTGTATTAAAAGATGACGATAGTGCCCAT
TTTACTTTGCGTAAGAAGATAATCCTTACATTATTTGTACTACCATTTCCAATTATGGTATGGGGAGTTATGACGCAAGGTTGGTGGT
TCCCAGTTATGGCTTCAGCATTTTTAATATTTACAATTATAATAATGTTTATTGCTGGGACAGGTAAATCTGGATTGGGAGAAAAAG
GAACTGTAGATGCATTTGTCAATGGTGCATCAAGTTTAGTAGGTGTATCTTTAATTATTGGTTTAGCTCGAGGTATTAATTTAGTGTT
GAATGAAGGTATGATTTCAGATACAATCTTACACTTTTCATCATCTTTAGTTCAACATATGAGTGGACCATTATTTATCATCGTATTA
CTATTTATTTTCTTCTGTTTAGGTTTTATCGTGCCATCATCTTCTGGCTTAGCGGTATTATCAATGCCTATCTTTGCACCACTAGCTGA
TACAGTAGGTATACCAAGATTCGTCATCGTTACGACATATCAATTTGGTCAATATGCGATGTTATTCTTAGCGCCGACTGGACTTGTT
ATGGCCACACTACAAATGTTAAACATGCGATATTCACATTGGTTCCGATTTGTATGGCCGGTAGTTGCTTTTGTATTGATTTTCGGTG
GCGGAGTACTAATTACGCAAGTACTAATTTATTCATAATTTGAAATGCTATATTATAAAAAATACTAATGGGGTTTTATGCATCTCG
TAGGTTTGTAGAAATACTAAACTAAGCGAGGTGCATTATTATTTTTGATTAAGAAAATAATGACGGTAATGATAACACTAGTAAGTA
ATTGATACAATGCTCTATTTAATAATGATATTTTTAAAATTTGTTTTTAATGTAATGTTAGATCTATGGTATATTATATTTATCGTGGT
AAATATGTATTTGCTGTAAACAGTTTTTGTAACACATGATACATATGGTTGTGAAAATAGCATATATAAAGGATGGCTATAAATGAC
ACATTTGACAAAGGTTTTAGATACACTAACTGGAATATGCGTAGTATTATTATTTAGTAAATATTTTGTGGCGTATGCAAATATGGT
GTTTGATTGGAATTTAAGATGGTATTTGCTAGAAAACATACCACATTTGCCAATTATATTATTTATTCTGATGTTTATTTTCGGAGTA
CCTTCTGAAATGATAAAAGATAGGCAAAGGAAAAATAACGGTGTTTAATTTATCGATATTTAGAGGTGATAAAATTTGTCAACTATT
ACAAAGACTAAAATAAAAATCTTTATTAATTATTTTATTGGTATATTAGCCTTGGCGTTTGCTGCTTATATATTATTTAAATAAAGGT

TAGAGTGAAACGTGTTTATGAACTAGACGTTCTAGTATAGTTACTACAGCTTTAGTCAGAAGGTATCATTGATAAGATCATATTAAA
TCAAAGAGGCATTGATATACACTAAAAAGAGGCAAGATTACCTGCCTCTTTTTAGTTATTAAATATACGTGTTAATTCTTGGTAATA
GTGTTACTGAGACGCATTACGAAGAAATTTATCTTGTTTTTCCTTTTTTAAAAAGAAGTGAAGATATCCTATAAAGACTCTAAGTACT
ATTACAGTGGCTAATAACACTTCGATAAACAAAAGACCTTTCCAAATGTCTGGAAACATAAGTACAGGCAAACTGTTCTTTAAAGC
AGTTGCTGAGATTACTAAAGGGAATGTGAAAGCTGAAAATACGGGCGAAAAAGGTTCTTTTAGTAATTTAGGCAATTGTATAATGA
TATAAAAATAAAATATTTGAGCTAATAGTAAAAATATAATTACGATAAAAGCATTCGCCTTAGGAAAAGCTATAACATATGCTGCA
GCGACTAAAGAAAATGGTGCGCAAATTGTCGATGTGTTCGGTTTGATTGACGTCTGCAATGGAAATGCTTTTAATCGCTTGAAAACT
ATAGGTAAGACAATACAAGTAGCTACAAAGCCATATATTACTGTTAATTGACCTATGAAAAAATATCCGCTAACGGGTGCCGTCAA
TCCTGCGATAGCAATACCAATAAAAAGTACAGTCCACGAAGGATAAACATTTTCAAGTGAAAAATCTTTTAAATATTTTATTGAAAA
AATAATCATATGCGTCATAATTCCCACAAGGCATAAAATCCAAATAGGCGTTATTAAGCTATTGATAAAAGTTATGTTACTAAAAAA
TGTATTTAAATAAGTAGTACCTAAAAAGCCAGACATGAAAAATGTTGTGAACACTGATGAAACTAGAGGACTGTTTAATTGTTCTTT
CACATTATTAAAATATTTAATCATAGTACATAAAAGGTGAATCCAAATCAAGAAAGCAAAGATTCCGCAAACAGCGTTTAAAGTAA
GAGATAAGTCTTTTAATAGATTACCCAGGCCTAATAATCCTAAGACTAGTCCTGACGTTACTAGAGGTGCTTTTTGAAGTCTCATGA
TTTACAACCTTTCTTATGTGATTTTCTTCACTAATTATATCATGATAGCTATGGCCAATTAATAAGAAGAGTGTGTACTATTACGTTA
TTAGAGTATGTATATTGGATTGATAGACACATAATTGACATTTAAATCTCAAAATTAATGATATTTGAGGTATGCTTTGCAACCTAA
ATATTGGAATATGTGGAAAAGTAATTATTTAATTTAAATGATGATTGAAGATTTACAAGGGGGTGTACAAAATGAGAAATCAAATT
CAAAAACTATTAGACAGTGATTTGAGCAGTTTACATATATCGAAACAAACAGGAGTTCCACAAAGCACAATACACAGAATGAGAA
AAAATGAAAGATCATTAGATAATATGTCATTGAAAAACGCTGAACTGCTTTATAAATTTGCCAATAGTATATTTAGCAATGAAAATT
AAGAAGAATAATTAGTGAATTAAATAATTAAAAAACCACGTCTCGATGGACGTGGTTATTTTTTTAGATGTATATAATTATAATTAT
AGGATTTCGGGCCCCTGAAAAGTCCCTAAAATTTTGAATAGTATCATAATCTATAGGAATAATATAATAATAAAAATCTCTACGCAT
GCAAATGGTGAGTGTTAAAAATCTTGGTGTAGTGTTGGTGTAGTTCAGGTGCAGTATAGATGAAATTTGAGCGCAAAATTACTTAAT
TAAAAATGTATAAACAAGTGTCAGACGCGTGTCAATTGTGTGTCAAAAAAATCTATAACGAATAAGCATACTTGTTTTTATTACTGT
ATTTTCGGGGATTATGCGGGGGACGTTTTGGTGACGCATCATACTATATTACTGAAATTCAAAACAAAAAGAGCCCCGTAATCACG
GAACTCTTTTATTTGGTAATGCGTATAAAAATACCTATAAACGTCCTGGGAGGGATTCGAACCCCCGACCGATGGCTTAGAAGGCCA
TTGCTCTATCCAGCTGAGCTACCAGGACACGTTTAACAACACAAGAATTATTATATCTAAATGAACTTAAATTAGCAATACCTTGTA
AATAAAAAATGTTTATATTTTTCACTATTATAGAGCTATTTATCTAAAAAGGTTCAATAAGACTTAAATACGAATTCAGGCAACTTA
ATTGTGTTAAATACAGTTTTGAATGCCTAACTGTATTTCTTTTCTCTTTAAAATACAGTTAAGTACATTATAAGATGTTGTGCGGATA
AACAAACTAATTGCATCAAATTTATTTTAAAATAACAACAACAAAACGTTAAGCGAATAACATTTCGGTGATTTAAAAGCTACGCA
CGTTTTTGTTATCTTCAAATTTAAATTTTAAGGAGTGTTTTCAATGGAAGGTTTATTTAACGCAATTAAAGATACCGTAACTGCAGCA
ATTAATAATGACGGCGCAAAATTAGGCACAAGCATTGTGAGCATCGTTGAAAATGGCGTAGGTTTATTAGGTAAATTATTCGGATTC
TAATATCAATATGTTATGTAAGTAATCAGGATTATTTCAAAGGTGAGGGAGAGATTTAAATGACTGGACTAGCAGAAGCAATCGCA
AATACTGTGCAAGCTGCACAACAACATGATAGTGTGAAATTAGGCACAAGTATCGTAGACATCGTTGCTAACGGTGTGGGTTTACT
AGGTAAATTATTTGGATTCTAATATAATAACTAATATTCTTTAAAATAAACTGGGTGAGCATACTTTAATGTTATGCACTCAGTTTAT
TTTATTTGCAGAAATTTGAGCCTCTGTTAAGATTTAGATACATAGACAATATAGGAGATGGGGAAATTGGGATATAAAAATATTTTG
ATAGACTTTGATGATACAATTGTTGATTTTTATGATGCAGAAGAATGGGCGTTTCACTATATGGCGAATGTTTTTAATCATAAAGCA
ACAAAGGATGATTTTTTAACATTTAAAAAAATCAATCACCAACATTGGGAAGCTTTTCAACAAAATAAATTAACGAAGTCTGAAGT
ATTATCAGAACGATTTGTGAATTACTTCAAACATCATCAAATGGAAGTTGATGGGCATCGTGCAGATGTGTTATTTAGAAATGGATT
AGCAGAAGCTAAAGTTAAATACTTTGATCAAACATTAGAAACAATTGTCGAATTATCGAAAAGACATGATTTATATATTGTTACTAA
TGGTGTAACCGAAACGCAAAAGAGAAGGTTAAATCAGACGCCGTTGCATAAATATATTAAAAAGATATTTATATCTGAGGAAACAG
GATATCAAAAACCTAATCCGGAATTTTTTAATTATGTTTTTAATGATATTGGTGAGGATGAAAGACAGCACTCGATTATAGTTGGAG
ATTCTTTAACATCTGACATTCTAGGTGGAATCAATGCGGGTATAGCTACTTGCTGGTTTAATTTTAGAGGATTTGATCATAATCCAGG
AATTATACCTGATTATGAAATTAATTCATGGAAACAACTAAATGATATTGTACGTTAAGACAAATAATTTGAAAATGGTTTACTTAT
ATTAGCTTAAGAAAGTAATTGTAAAAAAGACACTACGAAAAGATGCATTTTCATGCTCGTAGTGTCGAATATTATTATCTTATGGTT
TCCACAATAAGACATCATGTCCCTCTATATTTTTAGCTTCCACATCTGTAAATCCATGATGTAAAAAGAAATCCTTAGAATCATTTCT
GCCAATGGCTTTAATTGGCATATTGAAACTCTTTGCAAAATCAATCAATTCTGAAGCGTACCCTCTGTTTTGATATTTTGGTAATACT
TCTAACTTCCATAATAATATATAATCTTCAAAATCTGGGAAGTAGATTTCTTCGACATCACCTTTTTTTAGTAATGCCATTCTAGCTC
CTAATTGATCTCCGACAAATATGCCATAAAATGGTGAATCTGAACTTGCATCAATCATTTGACCGTTTAACTCATTGACCATGTATA
AGTCTTTGTTGCCAAACGCTCTAAAGTTTTCGAATAATTCGTCAGTTTTGTAATTAATTTCAAGACGTTTGATTTCACTCATATTTATC
TACCCCTTATTTGTTTACTACTATTATTATACATTAAACCACTTGTGTTTTTCATTGATTTGAAAATGTAAAACAATGAATATTATAA
ATTTTCCATGATTAAATTGATTTTCAAGCCGTAAAATTGTCAATCACCGGTATAGACTTTATACTATAGTTTGTAATCGCGTACTTAA
GGAGAAAAAACATGGACTGTAAAGTAGTTAGTTTAAATGAAAAAGATCAGTTTATACCAAAAATAAAGAGCAGTGACCCTGTAAT
AACAGGATTATTTCAATATGATGCAGCTCAACAAACTAGTTTTGAAAAAAGGATGTCTAAAGAAAATAATGGAAGAGAAGCGGCAT
TAGCGAATGTTATTCGTGAATATATGAGTGATTTAAAGCTTTCTAGTGAACAAGAATTAAACATACAACATTTAGCTAATGGTTCAA
AAGTTGTGATTGGTGGACAACAAGCAGGGCTTTTCGGGGGACCATTGTATACATTCCATAAAATATTTTCAATCATTACTTTATCTA
AGGAATTAACGGATACACATAAGCAACAAGTAGTACCAGTTTTTTGGATTGCAGGAGAAGATCATGATTTCGATGAAGTGAATCAT
ACATTTGTTTATAACGAAAATCATGGGTCGCTGCATAAGGTTAAATATCATACAATGGAGATGCCAGAGACGACTGTCTCTAGATAT
TATCCTGATAAGGCTGAGTTGAAACAAACTTTAAAAACGATGTTCATTCATATGAAAGAAACTGTTCATACACAAGGTCTACTGGA
GATTTGTGACAGAATTATTGACCAATATGACTCGTGGACTGATATGTTTAAAGCACTACTGCATGAAACATTTAAAGCATATGGCGT
TCTATTTATAGATGCGCAGTTTGAGCCGTTAAGAAAAATGGAAGCGCCTATGTTTAAAAAGATTTTGAAAAAACATCAGTTGCTTGA
TGATGCTTTTAGAGCAACACAACAACGCACTCAAAATCAAGGCTTGAATGCGATGATACAAACAGATACAAATGTTCATTTATTCTT
ACATGATGAAAATATGCGTCAATTAGTTTCGTATGATGGTAAGCATTTTAAATTAAATAAAACAGATAAGACATATATAAAGGAAG
AAATTATAAATATTGCGGAAAATCAACCTGAATTATTTTCTAATAATGTAGTGACAAGACCATTAATGGAAGAATGGTTATTTAACA
CGGTGGCATTTGTTGGAGGACCGAGTGAAATTAAGTACTGGGCTGAACTAAAAGATGTATTTGAACTATTTGATGTTGAAATGCCTA
TCGTGATGCCAAGGCTTAGAATTACTTATTTAAATGACCGTATAGAAAAATTACTTTCGAAATACAATATTCCATTAGAAAAAGTGT
TAGTCGATGGTGTTGAAGGAGAAAGAAGTAAGTTTATTAGAGAACAAGCATCACATCAATTTATTGAAAAGGTAGAAGGTATGATT
GAACAACAGCGTCGTCTAAACCAAGACTTATTAGATGAAGTGGCGGGGAATCAAAATAATATTAACCTTGTGAATAAAAATAATGA
AATTCATATACAACAGTATGATTATTTGTTAAAACGTTATCTTTTAAACATTGAAAGAGAAAACGACATCAGTATGAAGCAATTTAG
AGAAATTCAAGAAACACTCCATCCAATGGGAGGATTACAAGAAAGAATATGGAATCCACTTCAAATTTTGAATGATTTTGGGACAG
ATGTGTTCAAGCCCTCCACCTATCCACCACTTTCTTACACTTTTGATCATATTATTATAAAACCTTAATATACCAAGGGTTTAGCCCG
ATTTATCTTAATGATAAATCGGGCATTTTTTTGTTTTTTAAAATAAATTTCACAAATTTTGTATAAATAGTGGTGGATAGTGGGGAGA
TGTGGTAAATTATATATAAGGTGAGGTGATAAAAAATGTTCATGGGAGAATACGATCATCAATTAGATACAAAAGGACGTATGATT

ATACCGTCCAAGTTTCGTTATGACTTAAATGAGCGTTTTATTATCACAAGAGGCCTTGATAAATGTTTATTCGGTTACACTCTAGACG
AATGGCAACAGATTGAAGAGAAAATGAAAACCTTACCTATGACAAAAAAAGACGCACGTAAGTTTATGCGTATGTTCTTCTCTGGT
GCTGTTGAAGTAGAACTTGATAAGCAAGGGCGTATTAACATCCCTCAAAACTTGAGGAAATACGCTAATTTAACTAAAGAATGTAC
AGTAATCGGTGTTTCAAATCGTATTGAGATTTGGGATAGAGAAACTTGGAATGATTTCTATGAAGAATCTGAAGAAAGTTTCGAAG
ATATTGCTGAAGATTTAATAGATTTTGATTTTTAAAATGGAGGAATTGAAGTGTTTCATCATATCAGCGTTATGTTAAACGAAACCA
TTGATTATTTAAATGTAAAAGAAAATGGTGTGTACATTGACTGTACGCTAGGTGGAGCGGGACATGCCCTTTATTTACTAAATCAAT
TAAATGACGACGGAAGATTAATAGCAATCGATCAAGACCAAACTGCAATTGATAATGCTAAAGAGGTATTAAAGGATCATTTGCAT
AAGGTGACTTTTGTTCATAGCAACTTCCGTGAATTAACTCAAATATTAAAAGACTTAAACATTGAAAAAGTAGATGGAATTTATTAC
GACTTGGGTGTTTCAAGTCCCCAACTCGACATTCCAGAACGAGGATTCAGTTATCACCATGACGCAACATTAGACATGCGTATGGAC
CAAACACAAGAACTAACAGCATATGAAATTGTTAACAATTGGTCATATGAAGCGTTAGTGAAGATTTTTTATCGTTATGGCGAGGA
GAAATTTTCAAAACAGATAGCTCGAAGAATCGAAGCACATCGCGAACAACAACCAATAACAACAACATTAGAATTAGTTGACATTA
TAAAAGAAGGTATTCCTGCAAAAGCAAGAAGAAAAGGCGGACATCCTGCAAAACGAGTATTTCAAGCACTACGAATTGCAGTAAA
CGATGAATTGTCAGCTTTTGAAGATTCAATAGAACAAGCGATTGAATTAGTGAAAGTAGACGGCAGGATTTCGGTAATCACTTTCCA
TTCTTTAGAAGATCGTTTATGTAAACAGGTGTTCCAAGAATATGAAAAAGGTCCAGAGGTACCAAGAGGATTACCAGTTATACCAG
AAGCATATACACCTAAGTTAAAGCGTGTTAATCGTAAACCGATTACCGCTACAGAAGAAGATTTAGATGACAATAACAGAGCACGA
AGCGCGAAATTACGTGTAGCTGAAATACTTAAATAAGGAGCAATTTATAATGGCTGTAGAAAAAGTGTACCAACCATATGACGAAC
AAGTTTATAATAGTATACCGAAGCAACAACCACAAACTAAGCCCGAAAAGAAGACTGTTTCGAGAAAAGTGGTTGTACAATTAACT
AAATTTGAAAAAGTTTTATACATAACTTTGATTACTGTAATTGCTATGTTAAGTATTTATATGCTATCTTTAAAAATGGATGCGTATG
ATACGCGAGGAAAGATTGCAGATTTAGATTATAAAATAGATAAACAATCAAGTGAAAACAGTGCTTTACAATCTGAAATCAAAAAG
AATTCTTCTTATGAACGCATATACGAAAAGGCTAAGAAACAGGGGATGAGCCTTGAGAACGATAATGTAAAGGTAGTGCGTAGTAA
TGGCGAAGCAAAAAATTAAAATTAAAAAAAATAAAATAGGGGCAGTCCTACTTGTTGGTTTATTCGGACTGCTCTTTTTTATATTGG
TTTTAAGAATTTCATATATCATGATTACTGGACATTCTAATGGTCAAGACTTAGTCATGAAGGCAAATGAAAAGTATTTAGTTAAGA
ATGCACAACAACCAGAACGAGGAAAGATATATGATCGTAATGGTAAAGTGCTAGCAGAAGATGTAGAAAGATATAAACTTGTTGC
AGTAATAGATAAAAAGGCGAGTGCCAATTCTAAAAAACCTAGGCATGTAGTTGATAAAAAAGAGACTGCAAAGAAATTATCTACA
GTCATTGATATGAAGCCAGAGGAAATTGAAAAGAGACTTAGTCAAAAGAAAGCTTTCCAAATTGAATTTGGACGCAAAGGAACAA
ATTTAACGTATCAGGACAAATTGAAAATAGAGAAAATGAATTTGCCTGGTATTTCTTTATTGCCTGAAACAGAACGCTTTTATCCAA
ATGGCAATTTTGCATCACACTTAATTGGTAGAGCTCAGAAAAATCCGGATACTGGTGAACTTAAAGGTGCACTTGGAGTTGAAAAG
ATTTTTGATAGTTATTTAAGTGGATCTAAAGGATCATTGAGATATATTCATGATATTTGGGGATATATCGCACCAAATACTAAAAAA
GAGAAGCAGCCTAAACGTGGTGATGATGTCCATTTAACAATCGATTCAAATATTCAAGTATTTGTTGAAGAAGCTTTAGATGGCATG
GTTGAAAGATACCAGCCGAAAGATTTATTTGCGGTTGTCATGGATGCCAAAACTGGAGAAATTTTAGCATACAGTCAGCGACCAAC
ATTTAATCCTGAAACTGGTAAAGACTTTGGTAAAAAGTGGGCAAATGACCTTTATCAAAACACATACGAGCCTGGATCAACATTTA
AATCATATGGGTTAGCAGCTGCTATTCAAGAAGGTGCTTTTGATCCTGATAAGAAATATAAATCTGGACATAGAGATATTATGGGTT
CACGTATTTCAGACTGGAATAGAGTCGGTTGGGGTGAAATCCCAATGTCACTCGGATTTACTTATTCATCTAATACATTGATGATGC
ATTTACAAGATTTAGTTGGTGCAGACAAAATGAAATCTTGGTATGAACGATTTGGATTTGGAAAATCAACTAAAGGTATGTTTGATG
GAGAAGCACCTGGTCAAATTGGATGGAGTAATGAGTTACAACAAAAAACGTCATCATTTGGTCAATCGACAACTGTAACACCTGTT
CAAATGTTACAGGCGCAATCAGCGTTCTTTAATGATGGTAATATGTTAAAACCATGGTTTGTGAATAGCGTTGAAAATCCTGTTAGT
AAAAGACAATTTTATAAAGGGCAAAAACAAATCGCAGGCAAACCAATAACAAAAGATACTGCTGAAAAAGTTGAAAAGCAATTGG
ATTTAGTTGTGAATAGTAAGAAGAGTCACGCTGCAAACTATCGTATTGATGGTTATGAGGTCGAAGGTAAGACTGGTACAGCACAA
GTCGCTGCACCTAATGGTGGTGGATACGTTAAAGGTCCAAACCCATATTTTGTAAGTTTTATGGGTGACGCACCGAAGAAAAATCCT
AAAGTTATTGTATACGCTGGTATGAGCTTGGCACAAAAAAATGACCAAGAAGCTTATGAATTAGGTGTTAGTAAAGCGTTTAAACC
AATAATGGAAAATACTTTGAAATATTTAAATGTAGGTAAATCAAAAGATGACACATCTAATGCAGAGTATAGTAAAGTGCCAGATG
TTGAAGGTCAAGACAAACAAAAAGCTATTGATAATGTGAGTGCAAAATCATTAGAACCAGTTACTATCGGTTCTGGCACACAAATA
AAAGCACAATCTATAAAAGCAGGGAATAAAGTCTTACCTCATAGTAAAGTACTGTTATTAACAGACGGAGACTTAACTATGCCTGA
CATGTCAGGATGGACGAAAGAAGATGTCATTGCTTTTGAAAACCTAACAAATATTAAAGTAAATTTAAAAGGTAGCGGTTTTGTGT
CCCACCAATCAATTAGTAAGGGACAAAAACTTACTGAAAAAGATAAAATAGACGTAGAATTTTCATCAGAGAATGTAGACAGCAAT
TCGACGAATAATTCTGATTCAAATTCAGATGATAAGAAGAAATCTGACAGTAAAACTGACAAGGATAAGTCGGACTAACAAGAATG
GCTACATTAAATAAGGGTGTATTGTCACTATTTGTTTTTACTAAAGTAATGCTGTGATAGAAAACAACATCTTCATTTAATCTTTATA
AATAAGTGTTGATAGAGAATTTTAAGGTAGACGAGGTCAGATTAACTTGTTTGTTATAGGCAAACTTTTTATACTGGCCTCTTATGC
CGTAAAAAAGAAAAATTATAAATTTTGATTACAACTTTGTTTATTTAATTGAAAAATTGATAATATATTAGTGGTTAAAAAAGTCTG
GATTAAGAAGAGGAGATAGTTATGATTTTTGTATATGCGTTATTAGCGCTAGTGATTACATTTGTTTTGGTACCTGTTTTAATACCTA
CATTAAAAAGGATGAAATTTGGTCAAAGTATTCGAGAAGAAGGTCCACAAAGCCATATGAAGAAGACTGGTACACCAACGATGGG
TGGACTAACATTTCTATTAAGTATTGTGATAACGTCTTTGGTGGCTATTATATTTGTGGATCAAGCTAATCCAATCATACTGTTATTA
TTTGTGACGATTGGTTTTGGGTTAATTGGTTTTATAGATGATTATATTATTGTTGTTAAAAAGAATAACCAAGGTTTAACAAGTAAAC
AGAAGTTTTTGGCGCAAATTGGTATTGCGATTATTTTCTTTGTTTTAAGTAATGTGTTTCATTTGGTGAATTTTTCTACGAGCATACAT
ATTCCATTTACGAATGTAGCAATCCCACTATCATTTGCATATGTTATTTTCATTGTTTTTTGGCAAGTAGGTTTTTCTAATGCGGTAAA
TTTAACAGATGGTTTAGATGGATTAGCAACTGGACTGTCAATTATCGGATTTACAATGTATGCCATCATGAGCTTTGTGTTAGGAGA
AACGGCAATTGGTATTTTCTGTATCATTATGTTGTTTGCACTTTTAGGATTTTTACCATATAACATTAACCCTGCTAAAGTGTTTATGG
GAGATACAGGTAGCTTAGCTTTAGGTGGTATATTTGCTACGATTTCAATCATGCTTAATCAGGAATTATCATTAATTTTTATAGGTTT
AGTATTCGTAATTGAAACCTTATCTGTTATGTTACAAGTCGCTAGCTTTAAATTGACTGGAAAGCGTATATTTAAAATGAGTCCGATT
CATCATCATTTTGAATTGATAGGATGGAGCGAATGGAAAGTAGTTACAGTATTTTGGGCTGTTGGTCTGATTTCAGGTTTAATCGGT
TTATGGATTGGAGTGCATTAAGATGCTTAATTATACAGGGTTAGAAAATAAAAATGTATTAGTTGTCGGTTTGGCAAAAAGTGGTTA
TGAAGCAGCTAAATTATTAAGTAAATTAGGTGCGAATGTAACTGTCAATGATGGAAAAGACTTATCACAAGATGCTCATGCAAAAG
ATTTAGAATCTATGGGCATTTCTGTTGTAAGTGGAAGTCATCCATTAACGTTGCTTGATAATAATCCAATAATTGTTAAAAATCCTGG
AATACCTTATACAGTATCTATTATTGATGAAGCAGTGAAACGAGGTTTGAAAATTTTAACAGAAGTTGAGTTAAGTTATCTAATCTC
TGAAGCACCAATCATAGCTGTAACGGGTACAAATGGTAAAACGACAGTTACTTCTCTAATTGGAGATATGTTTAAAAAAAGTCGCT
TAACTGGAAGATTATCCGGCAATATTGGTTATGTTGCATCTAAAGTAGCACAAGAAGTAAAGCCTACAGATTATTTAGTTACAGAGT
TGTCGTCATTCCAGTTACTTGGAATCGAAAAGTATAAACCACACATTGCTATAATTACTAACATTTATTCGGCGCATCTAGATTACC
ATGAAAATTTAGAAAACTATCAAAATGCTAAAAAGCAAATATATAAAAATCAAACGGAAGAAGATTATTTGATTTGTAATTATCAT
CAAAGACAAGTGATAGAGTCGGAAGAATTAAAAGCTAAGACATTGTATTTCTCAACTCAACAAGAAGTTGATGGTATTTATATTAA
AGATGGTTTTATCATTTATAAAGGTGTTCGTATTATTAACACTAAAGATCTAGTATTGCCTGGTGAACATAATTTAGAAAATATATTA
GCAGCTGTGCTTGCTTGTATTTTAGCTGGTGTACCTATTAAAGCAATTATTGATAGTTTAACTACATTTTCAGGAATAGAGCATAGAT

TGCAATATGTTGGTACTAATAGAACTAATAAATATTATAATGATTCCAAAGCAACAAACACGCTAGCAACACAGTTTGCCTTAAATT
CATTTAATCAACCAATCATTTGGTTATGTGGTGGTTTGGATCGAGGGAATGAATTTGACGAACTCATTCCTTATATGGAAAATGTTC
GCGCGATGGTTGTATTCGGACAAACGAAAGCTAAGTTTGCTAAACTAGGTAATAGTCAAGGGAAATCGGTCATTGAAGCGAACAAT
GTCGAAGACGCTGTTGATAAAGTACAAGATATTATAGAACCAAATGATGTTGTATTATTGTCACCTGCTTGTGCGAGTTGGGATCAA
TATAGTACTTTTGAAGAGCGTGGAGAGAAATTTATTGAAAGATTCCGTGCCCATTTACCATCTTATTAAAGGGTGTGAGTATTGATG
GATGATAAAACGAAGAACGATCAACAAGAATCAAATGAAGATAAAGATGAATTAGAATTATTTACGAGGAATACATCTAAGAAAA
GACGGCAAAGAAAAAGATCAAAGGCTACACATTTTTCTAATCAAAATAAAGATGATACATCTCAACAAGCTGATTTTGATGAAGAA
ATTTACTTGATAAATAAAGACTTCAAAAAAGAACAAAGCAATGATGAAAATAATGATTCTGCTTCTAGTCGTGCAAATAATAATAA
TATCGATGATTCTACAGACTCTAATATTGAAAATGAGGATTATAGATATAATCAAGAAATTGACGACCAAAATGAATCGAATGGAA
TTGCATTCGACAACGAACAACCTCAATCAGCTCCTAAAGAACAAAATGGCGACTCGAATGATGAGGAAACAGTAACGAAAAAAGA
ACGAAAAAGTAAAGTAACACAATTAAAGCCATTAACACTTGAAGAAAAGCGGAAGTTAAGACGTAAGCGACAAAAACGAATCCAA
TACAGTGTTATTACAATATTAATATTGTTGATTGCTGTTATATTAATTTACATGTTTTCACCACTTAGTAAAATTGCGCATGTAAATA
TAAATGGAAATAACCACGTTAGTACTTCAAAGATAAACAAAGTTTTAGGTGTTAAAAATGATTCAAGGATGTATACGTTTAGTAAA
AAAAATGCTATTAATGATCTCGAAGAGGATCCATTAATCAAAAGTGTTGAGATACACAAGCAATTACCAAACACATTAAACGTAGA
TATCACAGAAAATGAAATTATTGCTTTAGTGAAATATAAAGGTAAATATTTACCTTTATTAGAAAATGGTAAATTGCTTAAAGGTTC
AAATGATGTCAAAATTAATGATGCACCTGTCATGGATGGTTTCAAAGGTACAAAAGAAGATGATATGATTAAGGCGTTATCTGAAA
TGACACCTGAAGTTAGACGATATATTGCCGAAGTGTCATACGCCCCAAGTAAAAACAAACAAAGCAGAATTGAATTGTTTACGACA
GATGGACTTCAAGTAATCGGTGATATTTCGACGATATCTAAGAAAATGAAATATTATCCGCAGATGTCACAATCATTATCAAGGGAT
AGTTCGGGTAAACTAAAAACACGAGGCTATATTGATTTATCAGTCGGTGCTTCATTTATCCCATACCGTGGAAACACGTCTAGTCAA
TCAGAAAGCGATAAAAATGTGACTAAATCATCTCAAGAGGAAAATCAAGCAAAAGAAGAATTACAAAGCGTTTTAAACAAAATTA
ACAAACAATCAAGTAAGAATAATTAAAAAATTTTGATATTGTCTATGTTTATAGTTCACAAGCCATTCAACGTATTGTAAACTAAGG
ATAGTGTATTTTTTTAATAGTAATTTGTCAGGAGGTGCCTATCTATGGAAGAACATTACTACGTAAGTATTGATATTGGATCATCAA
GCGTAAAAACAATAGTAGGCGAGAAATTTCACAATGGTATAAATGTGATAGGTACAGGACAAACCTACACGAGCGGTATAAAAAA
TGGTTTAATTGATGATTTTGATATTGCGCGACAAGCAATCAAAGACACAATTAAAAAGGCATCAATCGCTTCGGGTGTTGATATTAA
AGAAGTTTTCCTGAAATTACCTATCATTGGAACGGAAGTTTATGATGAATCAAATGAAATCGACTTTTATGAGGATACAGAAATCAA
CGGTTCACATATCGAAAAAGTATTAGAAGGTATTAGAGAAAAAAATGATGTGCAAGAAACAGAAGTAATTAATGTGTTCCCGATTC
GTTTTATAGTCGATAAAGAAAATGAGGTTTCAGACCCTAAAGAATTAATTGCCAGACATTCATTAAAGGTTGAAGCAGGCGTAATT
GCTATTCAAAAATCGATTTTAATTAATATGATTAAATGCGTAGAAGCATGTGGTGTTGATGTATTAGATGTTTACTCTGATGCATATA
ACTATGGTTCAATCCTAACAGCTACTGAAAAAGAGTTAGGTGCATGTGTCATTGATATTGGTGAAGACGTTACGCAAGTTGCTTTTT
ATGAACGCGGTGAATTAGTAGATGCTGATTCTATCGAAATGGCAGGGCGTGATATTACAGACGATATTGCACAAGGATTAAACACT
TCTTATGAAACTGCTGAAAAAGTTAAACACCAATATGGTCATGCATTCTATGATTCTGCTTCAGATCAAGATATCTTCACTGTTGAA
CAGGTTGATAGTGATGAAACAGTACAGTATACTCAAAAAGATTTGAGTGACTTTATTGAAGCGCGTGTAGAAGAAATATTCTTCGA
AGTATTTGATGTTTTACAAGATTTAGGATTAACAAAAGTAAATGGTGGGTTTATTGTAACTGGTGGATCTGCAAACTTACTTGGCGT
AAAAGAATTATTATCAGATATGGTAAGTGAAAAAGTTAGAATTCACACGCCATCACAAATGGGAATTAGAAAACCTGAATTTTCTT
CAGCAATTTCTACAATTTCTAGTAGTATCGCTTTTGATGAGTTATTAGATTATGTTACAATTAATTATCATGATAATGAAGAAACTGA
AGAAGATGTTATTGATGTGAAAGACAAAGATAACGAATCTAAATTAGGCGGATTTGATTGGTTTAAACGTAAAACAAACAAAAAA
GATACTCATGAAAATGAAGTAGAGTCAACAGATGAAGAAATTTATCAATCAGAAGATAATCATCAGGAACATAAACAGAATCATG
AACATGTTCAAGACAAAGATAAAGATAAAGAAGAAAGTAAATTCAAAAAACTAATGAAATCTCTATTTGAATGATTATTGGCCAAT
AAAACTAGGAGGAAATTTAAATGTTAGAATTTGAACAAGGATTTAATCATTTAGCGACTTTAAAGGTCATTGGTGTAGGTGGTGGC
GGTAACAACGCCGTAAACCGAATGATTGACCACGGAATGAATAATGTTGAATTTATCGCTATCAACACAGACGGTCAAGCTTTAAA
CTTATCTAAAGCTGAATCTAAAATCCAAATCGGTGAAAAATTAACACGTGGTTTAGGAGCAGGAGCTAATCCTGAAATCGGTAAAA
AAGCTGCAGAGGAATCTCGTGAACAAATTGAAGATGCAATCCAAGGTGCAGACATGGTATTTGTTACTTCTGGTATGGGTGGCGGA
ACTGGTACTGGTGCAGCACCAGTCGTTGCTAAAATTGCAAAAGAAATGGGCGCATTAACTGTTGGTGTTGTAACTCGTCCATTTAGT
TTTGAAGGACGTAAACGTCAAACTCAAGCTGCTGCTGGAGTAGAAGCTATGAAAGCTGCAGTAGATACATTAATCGTTATACCAAA
TGACCGTTTATTAGATATCGTTGACAAATCTACGCCAATGATGGAAGCATTTAAAGAAGCTGATAACGTGTTACGCCAAGGTGTACA
AGGTATCTCAGACTTAATCGCTGTTTCTGGTGAAGTAAACTTAGACTTTGCAGACGTTAAGACAATTATGTCTAACCAAGGTTCTGC
ATTAATGGGTATTGGTGTTTCTTCTGGTGAAAATAGAGCGGTAGAAGCTGCTAAAAAAGCAATCTCTTCTCCATTACTTGAAACATC
TATCGTTGGTGCACAAGGTGTGCTTATGAATATTACTGGTGGCGAGTCATTGTCATTATTTGAAGCACAAGAGGCTGCTGATATTGT
CCAAGATGCTGCAGATGAAGACGTTAATATGATTTTCGGTACAGTTATTAATCCTGAATTACAAGATGAGATTGTTGTAACAGTTAT
TGCAACTGGTTTTGATGACAAACCAACATCACATGGTCGTAAATCTGGTAGCACTGGATTCGGAACAAGCGTAAATACTTCTAGCA
ATGCAACTTCTAAAGATGAATCATTCACTTCAAATTCATCAAATGCACAAGCAACTGATAGTGTAAGTGAAAGAACACATACAACT
AAAGAAGATGATATTCCTAGCTTCATTAGAAATAGAGAAGAAAGACGTTCAAGAAGAACAAGACGTTAATCGGTTAATATATATAC
ACAAATAATTCAACACAAATCATCAGATAACATATCTGATGATTTTTTTACTAATTTTTAGAACATGTAGAAGGACATTTAAGTTTTT
CAAAGTTATTAAAAGTGTTTAAGTATCGTGTCAAAATTAAGTCAAAAATTATTTGCGCAACATTTTAACTTTAAACATAAATGTTAT
ATTATATAATTATTAACTTTGTACAGTTAGACGAAGATAATTTAAATGAAATGATGGTGACGATCGAGTGAATGATAATTTTAAAAA
GCAACCGCATCATTTAATATATGAAGAGTTATTACAACAAGGTATTACTCTAGGTATTACAACTAGAGGCGATGGTTTAAGTGACTA
TCCTAAAAATGCTTTTAATATGGCGAGATATATTGATGATCGCCCATATAATATTACTCAACATCAATTGCAATTAGCTGAAGAAAT
TGCGTTTGATAGAAAAAATTGGGTGTTTCCCATTCAAACACATGAAAATAAAGTCGCTTGTATTACAAAGGATGATATAGGCACAA
ATATAGACACTTTAACTGATGCGCTTCATGGTATTGATGCGATGTACACATATGATAGTAATGTCTTATTAACGATGTGTTATGCAG
ACTGTGTACCAGTATATTTTTATAGTACAAAACATCATTTTATTGCATTGGCGCATGCAGGTTGGCGTGGTACCTATACTGAAATTGT
AAAAGAAGTGCTAAAACATGTGAACTTTGATTTGAAAGACTTACATGTCGTTATTGGACCATCTACATCATCAAGTTATGAAATTAA
TGATGATATTAAAAATAAATTTGAAACATTGCCAATTGATAGTGCCAACTATATTGAAACTAGAGGACGAGATCGTCATGGTATTG
ATTTGAAAAAAGCCAATGCTGCATTATTAAATTATTATGGTGTTCCTAAAGAAAATATTTATACGACAGCGTATGCTACATCTGAAC
ATTTAGAATTATTTTTCTCTTATCGATTAGAAAAAGGTCAAACAGGACGCATGTTAGCATTCATTGGTCAACAGTAAACAAGGAGGA
GATATGCTTGCGTGTGAAAGATAATTTACAACAAATCTCAACACAAATTAATGACAAAAGTGAAAAAAATAATTTTTCAACAAAAC
CAAACGTGATTGCAGTTACAAAATATGTTACAATAGAGCGAGCTAAAGAAGCGTATGAGGCTGGAATAAGACATTTTGGTGAGAAT
AGATTGGAAGGCTTTTTACAAAAGAAAGAAGCATTACCATCAGATGCGGTGATCCATTTTATAGGATCATTACAATCTCGAAAAGTT
AAGGGCGTTATAAACGACGTAGATTATTTCCATGCTTTAGATCGATTGAGCTTAGCCAAAGAAATTAACAAACGTGCAGAACATAA
AATTAAATGTTTCTTGCAAGTGAACGTTTCGGGAGAAGCTTCTAAACATGGTATTGCTTTAGAAGATGTTGATCAGTTTATAGATGA
TCTTAAAAAATATGACAAAATCGAAATTGTAGGTTTAATGACGATGGCACCATTGACAGATGATGAAGCATATATTAGATCGTTATT
TAAACAGTTACGTTTGAAAAAAGAAGAAATACAACGACTCAATTTAGAATATGCGCCTTGTGATGAATTATCAATGGGAATGAGTA

ATGACTATCTTATTGCAGTTGAAGAAGGTGCGACGTTTGTTAGAATTGGGACTAAACTTGTAGGAGAAGAGGAGTGAGCCACTTGG
CTTTAAAAGATTTATTTAGTGGATTTTTTGTAATAGATGATGAAGAGGAAGTAGAAGTACCTGACAAACAACAACAGGTCAATGAA
GCGCCAGCAAAAGAGCAGTCACACAAACAACAAAACAAAACGCAATCAAATCAGTCCCTCAAAAATCTGCATCAAGATATACAA
CAACGTCAGAAGAAAGGAATAACCGTATGTCTAATTATTCAAAAAATAATTCACGTAATGTTGTAACTATGAACAATGCTACACCA
AACAATGCATCACAAGAAAGTTCAAAAATGTGTTTATTCGAACCACGTGTTTTTTCAGATACACAAGATATTGCTGATGAGCTTAAA
AACCGCCGTGCGACACTTGTCAATTTACAACGTATTGATAAAGTATCAGCGAAAAGAATTATTGATTTTTTAAGCGGTACTGTTTAT
GCAATCGGTGGAGATATCCAACGTGTAGGTACTGATATTTTCTTATGTACGCCTGATAATGTGGAAGTAGCTGGAAGCATTACAGAC
CATATTGAAAATATGGAACATTCATTCGACTAAGGAGTTAACATATGGATATAAATGTGCTAGCTACAATATTTAAATTTATCCTTT
TTGTTGTTGAAATTTATTATTTCGGCATGATTATATATTTCTTTACATCTTGGGTACCAAGTATTAGAGAAACTAAGGTAGGTTATTT
TTTAGCGAAAATATATGAACCTTTCTTACAACCATTTAGAAAAGTAATTCCACCTATTGGAATTATCGACATATCATCAATCGCTGC
AATTATCGTTTTAGTATTATTCCAAAAAGGGTTACTCCAAATCTTTAATTGGATTTTAATTCAATTACAATAAATTAAGCGACATAAC
GAGGCAAGGGTACATGATACTTTAGCCTCGTTTTTGATATGTATTTTTCTGAATATAAGGGCAATAGATGGTATTTTATAATTTTTTT
AAGGTAGTGATTAACATAGATATTTATCAACACTTTAGACAGGAAGAATACGAATTAATTGATCAGCTAACGGATAAATGTGATCA
AGCGGAACAGCATTATGCACCAGTATTAACGCATTTTTTAGATCCAAGAGAGCAATATATATTGGAAGTGATTTGTGGCAGTTATGA
AGATTTAAACGTATCTTTTTATGGTGGACCTAATGCTGAAAGAAAAAGAGCAATCATTTCGCCGAACTATTATGAACCTAAAGAAA
GCGACTTTGAATTAACTTTAATGGAAATAGATTATCCTGAAAAATTCGTCACTTTAAAACATCAACATATTTTAGGGACATTAATGT
CTTTAGGTATCGAACGCGAACAAGTTGGAGATATAATTGTGAATGAACGAATTCAATTTGTTTTGACAAGTAGATTGGAATCATTTA
TTATGTTAGAATTACAACGTATTAAAGGCGCATCAGTTAAACTTTATACTATTCCAGTAACAGATATGATACAATCTAATGAGAATT
GGAAAAATGAAAGTGCAACAGTTAGTTCTTTAAGGTTAGATGTTGTTATTAAAGAAATGATACGTAAATCACGTACGATTGCGAAA
CAACTAATCGAAAAAAAAACGTGTTAAAGTGAATCACACTATTGTTGATTCAGCAGATTTTCAATTACAAGCAAATGATTTAATATCC
ATCCAAGGTTTTGGTAGAGCACACATTACTGACTTAGGTGGTAAAACTAAAAAAGATAAAACGCACATTACCTATAGAACATTATT
CAAATAGTAATGATTTAAGGAGGATAACAAATGCCTTTTACACCAAATGAAATTAAGAATAAAGAGTTTTCACGTGTAAAGAATGG
TTTAGAACCTACTGAAGTTGCTAATTTTTTGGAGCAACTAAGCACTGAAATTGAACGTCTTAAAGAAGATAAAAAACAACTTGAAA
AAGTAATCGAAGAGAGAGAGATACTAATATTAAGTCTTATCAAGACGTGCATCAATCTGTAAGTGATGCTTTGATACAAGCTCAAAAA
GCTGGTGAAGAAACTAAGCAAGCTGCAGAGAAACAAGCTGAAGCGATTATAGCTAAGGCAGAAGCGCAAGCTAATCAAATGGTTG
GTGACGCGGTAGAAAAAGCACGCCGTTTAGCATTCCAGACTGAAGATATGAAACGCCAATCAAAAGTATTTAGATCGCGTTTCCGT
ATGTTAGTTGAAGCGCAATTAGACTTATTAAAAAACGAAGATTGGGATTACTTGTTGAATTATGATTTAGACGCTGAACAAGTGACG
CTTGAAAATATTCATCATTTGCATGAAAATGATTTAAAGCCAGATGAAGTTGCAGCAAATGCACAAAATAATGCATCAAATACACC
AGACAATAATCAACAATCCAATGATTCAGAAACAACTAAGAAGTAAGAATTAAATAAAGACAGACGCGTAATATACATTTAACTTT
TCACAGCGAATTAGGTAATGGTGAGAGCCTAGTAAAAGCATGTATGTTATATCACTGGCTTTTTAATATTTAAATAATGTAATGAGA
GAACTCTAAGTTGAGTTAATAAGGGTGGTACCGCGAGCAATCGTCCCTTTTAATTTAACTTAGAGTTTTTTAAATTTTTAAGGAGTG
AAAAAAATGGATTACAAAGAAACGTTATTAATGCCTAAAACAGATTTCCCAATGCGAGGTGGTTTACCAAACAAGGAACCGCAAAT
TCAAGAAAAATGGGATGCAGAAGATCAATACCATAAAGCGTTAGAAAAAAATAAAGGTAACGAAACATTCATTTTACATGATGGC
CCACCATACGCGAATGGTAACTTACATATGGGACATGCCTTGAACAAAATTTTAAAAGACTTTATTGTACGTTATAAAACTATGCAA
GGGTTCTATGCACCATACGTACCAGGTTGGGATACACATGGTTTACCAATTGAACAAGCATTAACGAAAAAAGGTGTTGACCGAAA
GAAAATGTCAACGGCTGAATTCCGTGAGAAATGTAAAGAATTTGCTTTAGAACAAATTGAATTACAGAAAAAAGATTTTAGACGTT
TAGGTGTTCGTGGTGACTTTAATGATCCATATATTACATTAAAACCTGAATACGAAGCTGCACAAATTCGTATTTTTGGAGAAATGG
CAGATAAAGGTTTAATTTATAAAGGTAAAAAGCCAGTTTATTGGTCTCCTTCAAGTGAGTCTTCATTAGCAGAAGCAGAAATTGAAT
ATCACGATAAACGTTCAGCATCAATTTACGTTGCATTTGACGTTAAAGATGACAAAGGTGTCGTTGATGCAGATGCTAAATTTATTA
TCTGGACAACAACGCCATGGACAATTCCATCAAATGTTGCAATTACCGTTCATCCTGAATTAAAATATGGTCAATACAATGTAAATG
GCGAAAAATATATTATTGCAGAAGCCTTATCTGACGCTGTAGCAGAAGCACTGGATTGGGATAAAGCATCAATCAAATTAGAAAAA
GAATACACAGGTAAAGAATTGGAGTATGTTGTAGCACAACATCCATTCTTAGACAGAGAATCGTTAGTGATTAATGGTGATCATGTT
ACTACAGATGCTGGTACAGGTTGTGTACATACAGCACCAGGCCACGGGGAAGATGACTATATTGTTGGTCAAAAATATGAATTACC
AGTAATTAGTCCAATCGATGATAAAGGTGTATTTACTGAAGAAGGCGGCCAATTTGAAGGGATGTTCTATGATAAAGCTAATAAAG
CCGTTACTGATTTATTAACAGAAAAAGGTGCACTATTAAAATTAGACTTTATTACACATAGCTATCCACACGACTGGAGAACAAAA
AAACCTGTAATCTTCCGTGCTACACCACAATGGTTTGCCTCAATCAGTAAAGTAAGACAAGATATTTTAGATGCAATCGAAAATACA
AACTTCAAAGTAAATTGGGGTGAAACACGTATTTACAATATGGTTCGTGACCGTGGCGAATGGGTTATTTCTCGTCAACGTGTGTGG
GGTGTACCGTTACCAGTATTTTATGCTGAAAATGGCGAAATTATCATGACGAAAGAAACAGTGAATCATGTTGCTGATTTATTTGCA
GAACACGGTTCAAATATTTGGTTTGAAAGAGAAGCGAAAGACTTACTACCAGAAGGATTTACACATCCAGGCAGCCCTAACGGTAC
ATTTACTAAAGAAACAGACATTATGGACGTTTGGTTTGATTCTGGTTCATCACACCGTGGCGTGTTGGAAACAAGACCGGAATTAAG
TTTCCCAGCGGATATGTATTTAGAAGGTAGTGACCAATATCGTGGTTGGTTCAACTCTTCTATCACAACTTCAGTTGCTACAAGAGG
AGTATCACCTTATAAATTCTTACTTTCTCATGGTTTTGTTATGGATGGTGAAGGTAAGAAAATGAGTAAATCTTTAGGTAATGTGATT
GTACCTGACCAAGTGGTTAAACAAAAAGGTGCTGATATTGCGAGACTTTGGGTAAGTAGTACGGACTATTTAGCTGATGTTAGAATT
TCTGATGAAATTTTAAAACAAACATCTGATGTTTATCGTAAAATCAGAAATACATTAAGATTTATGTTAGGTAACATTAACGATTTC
AATCCTGACACAGATAGCATTCCTGAATCAGAGTTATTAGAAGTGGATCGTTACTTGCTAAATCGTTTACGTGAATTTACTGCAAGT
ACGATTAACAACTATGAAAACTTTGACTACTTAAATATTTATCAAGAAGTTCAAAACTTTATCAATGTTGAGTTAAGTAATTTCTATT
TGGATTACGGTAAAGATATTTTATATATTGAACAACGTGATTCTCATATCCGTCGTAGTATGCAAACAGTGTTATATCAAATTTTAGT
TGATATGACGAAGTTGTTAGCACCAATCTTAGTGCATACAGCTGAAGAAGTTTGGTCTCATACACCACATGTTAAAGAAGAAAGTG
TTCACTTAGCAGACATGCCTAAAGTTGTAGAAGTAGATCAAGCTTTATTGGATAAATGGCGTACATTTATGAATTTACGTGATGATG
TGAACCGTGCATTAGAAACTGCTCGTAATGAAAAAGTTATTGGTAAATCATTAGAAGCTAAAGTTACGATTGCTAGTAACGATAAA
TTTAATGCATCTGAATTCTTAACTTCATTTGATGCATTACATCAATTATTTATCGTGTCACAAGTTAAAGTTGTAGATAAGTTAGACG
ATCAGGCAACAGCTTATGAACATGGTGATATTGTCATCGAACATGCAGATGGTGAAAAATGTGAAAGATGTTGGAACTATTCAGAG
GATCTTGGTGCTGTTGATGAATTGACGCATCTATGTCCACGATGCCAACAAGTTGTAAAATCACTTGTATAATTGAAATTGTATAAA
GTACTCATACAGATGATATAAATTAAAGCTCTCTTCATAATCATGTTGTAGTTTTTGTTGACATGATGAAGAGAGTTTTTTTGTGAAT
AAAAAAATGACCAAGTTACCGGTCATATATGTAAAAAATGTGCGATTTACTAAAATAAAAATTATTCAGGAATGGTACAAATTCTC
TGAGGCATATAAATGCGTTATAGTTGCTATTCTCAATTATGTTCGCGATAATTTTAAGTAAAAGTAAGCACAGATATTGAATTTGAT
AGGAGTTAATTGAATGTATCATAACAGTAACGCAAACTTTGTAAATGGTATCACTTTAAATGTGAGAGATAAGAATGAATTAAAGC
CATTTTATGAGGACGTATTAGGATTAAATATTATAAATGAGACATTAACATCGATACAATATGAAGTAGGTCAAAATAATCATGTCA
TTACACTTGTTGAATTACAAAATGGACGTGAACCTTTAATGTCCGAAGCGGGACTGTTTCATATCGCAATTAAACTACCTCAAATTA
GTGATTTAGCTAATTTACTAATTCATTTAAGCGAATATGATATTCCAGTTAACGGAGGTATACAGCCTGCTTCGTTATCATTATTTTT
TGAAGACCCAGAAGGAAACGGTTTTAAATTTTATGTTGATAAAGACGAAGCGCAATGGACGAGGCAAAATGATTTAGTAAAAATTG

ATATTAGACCATTAAATGTACCGAGATTAGTGAGTCATGCAACAAAATTGTTATGGTTAGGTATTCCAGATGACGCTATTATAGGTG
CATTGCATATTAAGACAATTCATTTATCAGAGGTAAAAGACTACTACCTCGATTATTTTGGACTAGAGCAATCGGCATATATGGATG
ATTATTCAATATTTTTAGCATCGAATGGCTATTATCAACATTTGGCCATGAATGATTGGGTATCAGCAACGAAACGTGTAGAAAATT
TTGATACGTATGGATTAGCAATTGTTGACTTTCATTATCCTGAAACAACACATTTAAATTTACAAGGTCCGGATGGTATCTATTATCG
CTTTAATCATATCGAAGTTGAAGATTAGTATATACTTTGAATGGACGAACCATATAATGAATCGTTTTTAATGATCTTTTTATACAAG
TTATGAAGGAGGCTGGGACATTAAGTTCTTAGGCAATGTAAAAAGCTGATTTCTATTAATTATTTGATAGAAATCAGCTTTTTTGAT
ATGTATTTTATAATGTACAGCT

>030-contig_240

AAAGTGTCTTATTTTTTAAAAGTATTTCAAAGTAAAATTACATGTTAATACGTAGTATTAATGGCGAGACTCCTGAGGGAGCAGTGC
CAGTCGAAGACCAAGGCTGAGACGGCACCCTAGGAAGGGACCCATCATCAAAAATTCTATTTATAGAATTTTACAGTAATGTGCCA
GATGGGCATAGCGAAGCCATTCAATACGAAGTATTGTATAAATAGAGAACAGCAGTAAGATAATTTTTAATTAGAAAATATCTTAC
TACTGTCTTTTTAGGGATTTATGTTCCAGCCTGCTTTCCTAATTTTTAATGTCATCTTAAATATGATAAATGAATAATTAAGTTCATAT
TTAATGTCAAAACATAGTAGTTTATCAAGTATTGAGTGAGTAACATTAGATTTAATGTAATATGCTTACTTTTTTTATTAGCAGGTGT
AAGCTATAATATAAAGAGTTGTCTCATGGACGATTGATTGGAGGAACGAAAATGCACAAAAAATATTTTATTGGCACTTCCATTTTA
ATAGCAGTATTTGTCGTTATATTTGACCAAGTTACTAAATATATTATAGCTACTACAATGAAAATTGGAGATTCATTTGAAGTGATA
CCGCACTTTTTAAACATAACATCACATCGAAATAATGGTGCTGCATGGGGAATATTGAGTGGAAAAAATGACATTTTTCTTTATTATT
ACCATTATTATATTAATAGCCTTAGTATATTTCTTTATTAAAGATGCTCAATATAATTTGTTTATGCAAGTTGCTATTAGTTTACTTTT
TGCAGGTGCACTTGGAAACTTTATTGATAGAATTTTAACAGGAGAAGTTGTTGACTTTATTGATACAAATATTTTTGGTTATGATTTT
CCAATATTTAATATCGCAGATTCAAGTTTAACAATTGGTGTAATATTAATTATTATTGCCTTATTAAAGGATACTTCCAATAAAAAG
GAGAAGGAGGTTAAGTAATGGAGACTTATGAATTTAACATTACAGATAAAGAACAAACAGGTATGCGTGTAGATAAGTTGCTGCCT
GAATTAAATAGTGATTGGTCTCGTAACCAGATACAAGATTGGATTAAAGCAGGTTTAGTCGTTGCAAACGATAAAGTTGTTAAATCT
AATTATAAAGTGAAACTTAATGATCATATAGTTGTCACTGAAAAAGAAGTGGTTGAAGCTGATATTCTACCTGAAAATTTAAATTTA
GATATTTATTATGAAGATGACGATGTTGCAGTTGTATATAAACCGAAAGGCATGGTAGTTCATCCATCACCAGGGCATTATACCAAT
ACATTAGTTAATGGTTTAATGTATCAAATTAAAAATTTGTCAGGTATTAATGGAGAAATTCGTCCAGGTATTGTTCACCGTATAGAT
ATGGATACTTCTGGTTTATTAATGGTTGCTAAAAATGATATTGCTCATCGTGGGCTTGTAGAACAATTAATGGATAAATCTGTTAAA
AGAAAATATATCGCTTTAGTTCACGGGAATATTCCTCATGATTACGGTACAATCGATGCGCCAATTGGTAGAAACAAAAATGATCGT
CAATCTATGGCTGTTGTTGATGATGGTAAGGAAGCAGTGACACATTTTAACGTACTAGAACATTTTAAAGATTATACGCTTGTTGAA
TGTCAACTTGAAACAGGACGTACGCATCAAATCCGTGTTCACATGAAATATATTGGCTTCCCATTAGTTGGTGATCCAAAGTATGGA
CCGAAAAAAGACATTGGATATTGGTGGTCAAGCTCTACATGCTGGACTTATTGGATTCGAACATCCAGTAACAGGTGAATATATTGA
AAGACATGCTGAATTACCACAAGACTTTGAAGATTTATTAGATACAATTCGAAAAAGAGATGCATAATTGTGTCGTGCTATAATTAC
GATAACGTTATTATGTAAACTATAAGTATTTATCGTTTTCGTTTTTTTAAAATGAAAACACATTATAAAATAGATTAAGGTTAACATT
TTTACTGAGATCGGTTAATTTTTCAATATAGATTGACAAAGCGCCTGATTAGTATTATCCTTATAACAGTTTAAGTACAACATAAAG
AGTCTTTAATTGAGCGTCCAGAGAGACGTCAAAGACATGAAAATATAGATATATACTAAAATGATCATAACACTTTCACATATGCA
AGAATAAGTAGTAATACTACGATTCTTCTATGTAAATAGGCTGATTGTATATACTAAACCTCATGTCGATATTAAAGACATGAGGTT
TTTTGTTTCTAAATTTTGAAAGGGTGAGAATATGTCTGAACGTATCATAATGGATGATGCCGCAATACAACGTACAGTGACGAGAAT
CGCTCACGAAATTTTGGAGTATAACAAAGGTACTGATAATTTAATTCTTTTAGGTATCAAAACAAGAGGTGAATATTTAGCGAATCG
TATACAAGATAAAATTCATCAAATTGAGCAACAACGTATACCTACTGGAACAATTGATATTACATACTTTAGAGATGATATAGAGC
ACATGTCATCACTTACGACAAAAGACGCAATAGACATCGACACAGATATTACAGATAAAGTAGTCATCATTATTGATGATGTGCTG
TATACTGGTCGAACGGTTCGTGCTTCACTTGATGCTATTTTGCTAAATGCTAGACCTATTAAAATTGGTTTAGCTGCTTTGGTTGATC
GAGGACATCGTGAGTTACCAATTCGAGCAGATTTTGTTGGTAAAAATATACCTACTTCTAAAGAGGAAACGGTAAGTGTCTATTTAG
AAGAAATGGATCAAAGAAATGCAGTTATAATTAAATAATACCCCTTTTAATTCAGTACGAGAGACTGAAAAAGGTGGGGGAGTTGA
ATGATAAATAAGTTTTCATATGCTTGTTTATCTAATAATACCCTCATGTTCGACAACTATAAGTCAATTTCTATGGACGAAAATGACT
TGTAGCAACTTGTCACACTTTTTGTCTCTTTACATATACTGATGTAAAGAGATTTTTTTATTATCGGAGGAAATGAATATTATGCAAA
ATGATGAAATGTTTGAACGAACAGTAAAACCAGTACTAGATGTAAATGAAAAACCACAACCAGCGCAATGGGCATTTTTAAGCTTA
CAACATTTATTTGCGATGTTTGGCGCAACAGTACTAGTACCATTCTTAACAGGACTACCAATATCCGCAGCGTTACTAGCTTCGGGA
ATCGGTACATTACTTTATATCTTAATAACGAAGGCGCAAATACCAGCATACTTGGGATCTAGCTTTGCATTTATCACACCAATTATC
ACGGGATTAAGTACGCATAGCTTAGGAGACATGCTTGTAGCATTATTCATGAGTGGTGTTATGTACGTCATCATCGGAATTCTAATC
AAATTAAGTGGGACAGCATGGTTAATGAAATTATTACCACCAGTTGTTGTTGGACCAGTCATCATGGTAATTGGTTTAAGCTTAGCG
CCTACTGCAGTCAATATGGCAATGTATGAAAATCCTGGAGATATGAAAGGTTACAATATCAGTTTCTTAATTGTTGCAATGATAACC
TTGCTTGTAACAATAGTCGTTCAAGGATTTTTTAAAGGATTCCTATCTTTAATACCAGTACTTGTAGGTATTATCGTAGGTTATGTGG
TAGCGATTTTTATGGGGATTGTGAAATTTGATGCAATTATGTCAGCAAAATGGATAGATTTCCCTCATATTTATCTGCCATTTAAAGA
TTATGTACCTTCATTTCACTTAGGACTTGTACTTGTAATGATTCCGATTGTGTTTGTAACAGTAAGTGAACATATTGGGCACCAAATG
GTATTGAATAAAATCGTAGGTAGAAACTTCTTTGAAAAGCCAGGACTTGATAAATCAATCATTGGTGATGGTGTTTCTACAATGTTT
GCCAGTATTATTGGTGGACCACCAAGTACAACATACGGTGAAAATATCGGTGTATTAGCGATTACCAGAATATACAGTATTTACGTC
ATTGGTGGTGCAGCAGTTATAGCAATTGTTTTAGCATTCATTGGTAAGTTCACTGCATTAATTTCTTCTATACCTACACCAGTTATGG
GAGGAGTATCTATATTACTTTTCGGAATTATTGCAGCAAGTGGCTTAAGAATGTTAGTTGAAAGCAAAGTAGATTTTGCGAACAATC
GAAATTTAGTTATAGCTTCTGTAATTTTAGTTGTAGGTATCGGTAATTTAGTATTTAACTTAAAAGAAATTGGTATCAACCTTCAAAT
TGAGGGGATGGCATTAGCTGCACTTTCAGGAATTATTTTGAACTTAATCTTACCTAAAGAGAAAAAACAAAACAATTAAGATTTAC
AAATTAAGGAGGGCGCTTTTATGAATCATTTATTATCAATGGAACATTTATCTACAGATCAAATATACAAACTTATCCAAAAGGCAA
GTCAATTTAAATCTGGTGAACGTCAACTACCAAACTTTGAAGGGAAATATGTCGCAAATTTATTCTTTGAAAATTCTACTCGAACAA
AATGTAGTTTTGAAATGGCAGAACTTAAGCTAGGGTTAAAAACGATTAGCTTTGAAACATCAACATCATCTGTTTCAAAAGGTGAAT
CTTTATATGACACATGTAAAACTTTAGAAAGTATTGGCTGTGATTTATTAGTCATTAGACATCCGTTTAATAACTACTATGAAAAATT
AGCGAATATTAACATCCCAATTGCGAATGCTGGTGATGGTAGTGGACAACATCCAACACAAAGTTTACTTGATTTAATGACGATATA
TGAAGAATATGGATATTTTGAAGGCTTGAATGTATTGATTTGTGGAGACATTAAAAATTCACGTGTCGCACGTAGTAATTACCATAG
TTTAAAAGCATTAGGTGCAAACGTAATGTTTAATAGCCCAAATGCTTGGATTGATGATTCTTTAGAAGCACCTTATGTAAATATAGA
TGATGTTATAGAAACAGTAGATATAGTTATGTTATTAAGAATTCAACATGAAAGACATGGGCTTGCAGAAGAAACTAGATTTGCAG
CAGATGATTATCATCAAAAGCATGGCTTAAATGAAGTGCGCTATAACAAATTACAAGAACATGCTATTGTTATGCATCCGGCACCTG
TGAATAGAGGAGTAGAAATACAAAGCGATTTAGTAGAAGCTTCAAAATCAAGAATTTTTAAGCAAATGGAAAATGGCGTTTACTTA
AGAATGGCAGTCATTGATGAATTATTAAAATAGGTAAGGGGACGAAAATGATGAAATTAATTAAAAACGGTAAAGTATTACAAAA

TGGAGAATTACAACAAGCAGATATTTTAATTGATGGTAAGGTAATTAAACAAATTGCACCTGCAATTGAACCAAGCAATGGTGTTG
ACATCATAGATGCGAAAGGTCACTTTGTGTCACCTGGATTTGTCGATGTTCATGTTCATTTACGTGAACCTGGTGGTGAATATAAAG
AGACAATTGAAACTGGTACTAAAGCTGCTGCTAGAGGCGGATTTACAACTGTATGTCCAATGCCTAACACAAGACCGGTACCAGAT
TCTGTAGAACATTTTGAAGCTTTACAAAAATTAATCGATGACAATGCTCAAGTACGTGTATTACCTTATGCTTCAATTACAACACGT
CAATTAGGTAAAGAATTGGTTGATTTCCCAGCACTAGTAAAAGAAGGTGCCTTTGCGTTTACAGATGACGGTGTAGGAGTACAAAC
TGCAAGCATGATGTATGAAGGCATGATTGAAGCTGCAAAAGTAAACAAAGCCATCGTAGCACACTGTGAAGATAATTCATTAATCT
ATGGTGGTGCAATGCATGAAGGGAAACGCAGTAAAGAGTTAGGTATACCAGGTATTCCAAACATTTGTGAATCTGTTCAAATCGCA
AGAGATGTACTATTAGCTGAAGCAGCAGGTTGTCATTATCATGTATGTCATGTTTCTACTAAAGAAAGTGTTAGAGTCATTCGTGAC
GCTAAACGCGCAGGCATTCATGTTACAGCTGAAGTTACACCACACCATTTATTGTTAACAGAAGATGATATTCCTGGTAATAATGCC
ATTTATAAAATGAATCCACCATTGAGAAGTACTGAAGATAGAGAGGCTTTGTTAGAAGGGTTACTAGACGGTACAATTGACTGTAT
CGCAACAGACCATGCACCACATGCACGTGATGAAAAAGCACAACCAATGGAAAAAGCACCATTCGGAATTGTTGGTAGTGAAACA
GCATTCCCATTATTATATACGCATTTTGTAAAAAATGGTGATTGGACATTACAACAATTAGTAGATTACTTAACAATTAAACCATGT
GAGACATTTAATTTAGAATACGGCACATTAAAAGAAAATGGTTATGCAGATTTAACAATCATTGATTTAGATAGTGAACAAGAAAT
TAAAGGAGAAGATTTCTTATCAAAAGCAGATAATACACCATTTATCGGCTATAAAGTTTATGGAAATCCGATCTTAACAATGGTTGA
AGGCGAAGTTAAATTTGAGGGGGATAAATAATATGCAAAGCAAACGTTATCTAGTGTTAGAAGACGGTTCTTTTTACGAAGGCTAC
CGTTTAGGATCTGATAACTTAACTGTAGGAGAAATTGTATTTAATACAGCTATGACAGGTTATCAAGAAACTATTTCAGATCCATCA
TATACAGGTCAGATCATTACTTTTACGTATCCATTAATCGGTAATTATGGTATCAATAGAGACGATTTTGAATCATTAGTACCTACAT
TAAACGGTATTGTAGTGAAAGAAGCGAGTGCGCATCCAAGTAATTTTAGACAACAAAAGACACTTCATGACGTTTTAGAATTGCAT
CAAATTCCAGGGATTGCAGGTGTTGATACAAGAAGTATTACGCGTAAAATTCGACAACACGGTGTGTTAAAAGCTGGTTTTACTGAT
CGAAAAGAAGATATTGATCAACTTGTCAAACATTTACAACAAGTAGAATTACCTAAAAACGAAGTAGAAATCGTTTCGACTAAAAC
ACCGTATGTTTCGACAGGTAAGGATCTAAGTGTCGTACTTGTAGACTTTGGTAAGAAGCAAAATATTGTTCGAGAATTAAACGTCAG
AGGTTGTAACGTCACAGTTGTACCATATACAACTACTGCCGAAGAAATTTTAGCAATGGCTCCAGATGGCGTTATGCTATCAAACGG
ACCAGGTAATCCTGAAGTTGTAGAATGTGCGATTCCAATGATTCAAGGAATTTTAGGGAAAATTCCGTTCTTTGGTATCTGTCTAGG
ACATCAACTTTTTGCATTATCTCAAGGAGCAAGCTCATTTAAAATGAAGTTTGGTCATCGTGGTGCGAACCATCCAGTTAAAAATTT
AGAGACTGGAAAAGTTGATATTACGAGTCAAAACCATGGATATGCAATAGATATAGATTCGTTAAAAAGTACTGATTTAGAAGTTA
CTCATCTTGCATTAAATGATGGTACTGTAGAAGGTTTAAAACATAAAACATTACCAGCATTTTCTGTTCAATACCATCCTGAAGCAA
ATCCAGGACCGTCAGATTCAAACTATCTATTTGATGATTTTGTAGCAATGATGACTAATTTTAAGGAAAAGGAGCGTCATATCAATG
CCTAAACGTAATGATATCAAAACAATTTTAGTAATAGGGTCTGGGCCAATTATCATAGGTCAAGCAGCTGAATTTGATTATGCTGGA
ACACAAGCATGTCTAGCTTTAAAAGAAGAGGGATATCGAGTTATTCTTGTAAATTCAAATCCAGCGACAATCATGACTGATAAGGA
AATTGCGGATAAAGTATATATCGAACCGTTAACTCATGATTTTATAGCGCGAATTATACGTAAAGAGCAACCTGACGCTTTACTTCC
AACTTTAGGTGGTCAAACAGGTTTAAACATGGCGATTCAACTACACGAAAGTGGTGTGCTTCAAGATAATAACGTCCAATTATTAG
GAACTGAGCTAACATCAATTCAACAAGCAGAAGACCGTGAAATGTTTAGAACATTAATGAATGATTTAAACGTTCCTGTACCAGAG
AGTGACATTGTAAATACAGTAGAGCAAGCCTTTAAATTCAAAGAGCAAGTGGGATACCCGCTAATTGTTAGACCGGCATTTACGAT
GGGTGGTACCGGAGGCGGTATTTGTCATAATGATGAAGAATTACATGAAATCGTCTCAAATGGTCTTCATTATAGTCCAGCAACGCA
ATGTTTATTAGAAAAATCTATCGCAGGTTTTAAAGAAATCGAATACGAAGTAATGCGTGATAAAAACGATAATGCCATCGTTGTAT
GTAACATGGAAAATATTGATCCAGTTGGTATTCATACAGGCGATTCAATTGTTGTGGCTCCTAGCCAAACATTATCAGATGTTGAGT
ATCAAATGTTACGTGATGTTTCATTAAAAGTTATTCGAGCTTTAGGTATCGAAGGTGGTTGTAATGTTCAATTAGCATTAGATCCCCA
TTCATTCGATTATTATATTATAGAAGTAAATCCGCGTGTATCACGTTCATCAGCGTTAGCTTCAAAAGCAACAGGATATCCTATTGC
AAAATTAGCTGCTAAAATCGCGGTTGGTCTAACATTAGATGAAATGTTAAATCCAATTACAGGAACATCTTATGCAGCGTTTGAACC
AACTTTAGACTATGTGATTTCAAAAATACCAAGATTTCCTTTTGATAAATTTGAAAAAGGAGAACGAGAGCTTGGCACACAAATGA
AAGCAACAGGTGAAGTTATGGCCATTGGTCGAACTTACGAAGAATCATTGTTAAAAGCAATTCGATCACTTGAGTATGGTGTGCAT
CACTTAGGATTACCAAATGGTGAAAGCTTCGATCTTGATTATATTAAAGAACGTATTTCACACCAAGATGATGAACGATTATTTTTC
ATCGGCGAAGCAATTAGAAGAGGCACAACATTAGAAGAAATTCATAATATGACTCAGATTGATTACTTCTTCTTACACAAGTTCCA
AAACATTATTGATATTGAGCATCAATTAAAAGAGCATCAAGGTGATTTAGAATATCTTAAATATGCAAAAGATTATGGATTTAGTGA
TAAAACAATAGCGCATCGCTTTAATATGACGGAAGAAGAAGTATATCAATTGCGTATGGAAAATGATATTAAACCTGTTTACAAGA
TGGTTGATACTTGCGCAGCTGAATTTGAATCTTCAACACCATATTATTATGGTACATACGAAACTGAAAATGAATCCATAGTTACTG
ACAAAGAAAAAATCTTAGTATTAGGCTCTGGACCAATTCGAATCGGCCAAGGTGTAGAATTTGACTATGCGACAGTTCACGCCGTTT
GGGCAATTCAAAAAGCAGGGTACGAAGCGATAATTGTGAATAACAATCCAGAAACAGTTTCAACAGACTTCTCAATTTCTGACAAA
TTATACTTTGAACCTTTAACTGAAGAAGATGTGATGAATATCATTAATTTAGAAAAACCTAAAGGTGTCGTTGTACAATTTGGAGGA
CAAACAGCGATTAATTTAGCAGACAAATTGGCTAAACATGGTGTTAAAATACTTGGTACTTCACTAGAAAATCTAAATCGTGCTGA
AGATAGAAAAGAATTTGAAGCACTATTAAGAAAAATTAACGTGCCACAGCCACAAGGAAAAACAGCTACATCACCTGAGGAAGCA
TTAGCGAATGCTGCAGAAATCGGATATCCGGTTGTAGTAAGACCTTCTTATGTATTAGGTGGTCGCGCAATGGAAATTGTAGACAAT
GACAAAGAGTTAGAAAACTATATGACCCAGGCTGTAAAAGCGAGTCCGGAACATCCGGTACTAGTCGATAGATATTTAACTGGTAA
AGAAATTGAAGTTGATGCGATTTGTGATGGAGAAACGGTCATTATTCCAGGAATCATGGAACATATTGAACGTGCTGGTGTGCATA
GTGGTGACTCAATCGCTGTATATCCGCCACAAACTTTGACAGAAGACGAGTTAGCAACACTTGAGGACTATACTATAAAATTAGCT
AAAGGTTTAAACATCATTGGCTTAATCAACATTCAATTCGTTATAGCTCACGATGGTGTGTATGTTTTAGAAGTAAATCCACGTTCTA
GTAGAACGGTACCATTCTTAAGTAAAATTACTGATATTCCAATGGCACAATTAGCTATGCGAGCAATCATTGGGGAAAAACTAACA
GATATGGGTTATCAAGAAGGGGTTCAACCATATGCTGATGGTGTCTTTGTGAAAGCACCGGTATTTAGTTTTAATAAATTGAAAAAT
GTTGATATTACTTTAGGACCTGAAATGAAGTCAACAGGTGAAGTGATGGGGAAAGATACTACATTAGAAAAGGCGTTATTCAAAGG
GTTAACAGGTAGTGGTGTTGAAGTTAAAGATCACGGTACAGTATTAATGACCGTCAGTGACAAAGATAAAGAGGAAGTTGTTAAAT
TGGCACAACGCTTAAATGAAGTTGGCTATAAAATTTTAGCAACGTCTGGAACAGCTAATAAATTAGCTGAGTATGACATACCTGCA
GAAGTAGTAGGCAAAATTGGTGGCGAAAATGATTTATTAACACGTATTCAAAATGGTGATGTTCAAATCGTTATAAATACAATGAC
TAAAGGTAAAGAAGTAGAAAGGGATGGCTTCCAAATTAGACGTACTACAGTTGAAAATGGTATTCCATGTTTGACATCTTTAGATA
CAGCTAATGCCTTAACGAATGTAATTGAAAGTATGACATTTACAATGCGTCAAATGTAAATCAATCAAACTGTATCGGTGGGGCTGT
AATTAACCATTTACTTAAAGAAGTTTATATTACAGCCTCATTATTTTAATGAATTTCTTAATATAAAGGGAGACATATATGATGAAA
GATTTACCAATTATTGCATTAGATTTTGAATCAAAAGAAAAAGTAAATCAATTTTTAGATTTATTTGATGAATCATTATTCGTAAAA
GTAGGTATGGAACTTTTTTATCAAGAAGGTCCTCAATTAATTAATGAGATAAAAGAAAGAGGCCATGATGTATTTTTAGATTTAAAA
CTGCATGATATTCCTAATACAGTTGGTAAGGCGATGGAAGGACTAGCTAAATTGAATGTTGATCTGGTAAATGTTCATGCTGCTGGT
GGCGTAAAAATGATGTCTGAGGCCATTAAAGGATTAAGAAAACATAATCAACATACAAAAATTATTGCAGTAACACAGCTTACGTC
AACAACAGAAGACATGTTACGACACGAACAAAATATACAAACATCGATTGAAGAGGCCGTTTTAAATTATGCCAAGTTAGCAAATG
CAGCTGGTTTAGATGGCGTTGTTTGTTCACCTCTTGAAAGTCGTATGTTGACTGAAAAGTTAGGTACATCATTTTTAAAAGTAACACC

AGGTATTAGACCTAAAGGTGCATCTCAAGATGACCAACACCGTATTACGACACCGGAAGAAGCAAGACAGCTTGGTTCGACGCATA
TTGTAGTCGGTAGACCGATTACACAAAGTGACAATCCAGTCGAAAGTTATCATAAAATTAAAGAAAGTTGGTTAGTATAATGGCTA
AAGAAATTGCAAAATCATTATTAGATATTGAAGCTGTAACATTATCACCAAATGATTTATATACATGGAGTTCAGGTATTAAATCAC
CGATTTACTGTGATAACCGTGTTACGTTAGGTTATCCTTTAGTTCGAGGCGCAATCCGCGATGGTTTAATTAACTTAATTAAAGAAC
ACTTTCCTGAAGTAGAAGTTATTTCTGGTACTGCAACAGCTGGTATTCCACATGCAGCTTTTATTGCTGAAAAATTAAAATTACCAAT
GAATTATGTTCGTTCATCAAATAAGAGTCATGGTAAGCAAAATCAAATCGAAGGTGCTAAAAGTGAAGGTAAAAAAGTAGTTGTGA
TAGAAGATTTAATTTCGACAGGAGGATCTTCAGTCACAGCAGTTGAAGCCTTAAAACAAGCAGGTGCAGAAGTATTAGGTGTTGTA
GCTATCTTTACTTACGGTTTGAAAAAAGCAGATGATACATTTAGCAATATTCAACTACCTTTTTACACTTTAAGTGATTACAATGAAT
TAATTGAAGTAGCTGAAAATGAAGGTAAAATTTCTAGTGAAGATATCCAAACATTAGTTGAATGGAGAGACAACTTAGCATAATAT
AGACACTAGAAGGAGGAATTCAACAAATGAATGACAAAACATCTAATGATTTATATGGGAAGATAAAACATTGTAACGAATTTATC
AATCATTCAAATGATTCCAATCTATCTAGTAGTCACGATGTCGACGAAAGTTCAACGAAGCAAAAACATATAAAAAATAAAACAAC
TATAGATCATAATGATGATTTATTTAAACATGTAAAGGATATATTACGTAAACAAGGACAAATTTAAAAACAAAAAAGCTATAGTG
TTTAATATCAGGGGCTGCTTAACCGCATCCGAATATTACAAACACTATAGCTTTTATTATATAAAAAATTTATTAGCGGATAATTAA
CTTTATTAAAATCCAACCAATTAAGAATACGAGTAATAGGACTAATACTGGAATCACATAATGTAACATAACGTCCCTCCTTTAACT
TAATTTTAATTGTAATCAAATTTGACAATAAGTCAAAACATTAATACCTATGATAAGTATCATTTATTAACATATGTATCATATTTTT
AATCTTGCGTAATTTTTATCGTTAACTATGTGATTTAATCAACAGGCCAATAAGACGTTTGTCATTCGTGCACATAACGGTATGACGC
GCTTTTGTCATTTTAATTATGTTAAGATAATAGTAGTTATATAAGTTCAATCTATAGGAGGCATAGCATGGATATTCCAAAAATCAC
GACATTTTTAATGTTTAATAACCAAGCTGAAGAAGCTGTTAAACTATACACAAGCTTATTTGAAGATAGTGAGATTATAACAATGGC
TAAGTATGGTGAAAATGGACCTGGTGATCCTGGGACTGTACAACACTCAATATTTACATTAAATGGACAAGTATTCATGGCAATTGA
TGCTAATAGTGGCACAGAATTACCAATGAATCCTGCGATTTCATTATTTGTTACAGTAAAAGATACTATTGAAATGGAACGACTATT
TAATGGATTAAAAGATGAAGGTGCCATTTTAATGCCAAAAACGAATATGCCACCATACAGAGAGTTTGCTTGGGTTCAAGATAAGT
TTGGAGTAAGTTTTCAATTAGCATTACCTGAGTAAAAGGATTCGCACAGCGTTGAATGATAAAGAAACACTTTTTCTTATGCATGCG
TTTTACCTATGTATTTCTATTTTAAGTATACATTAGCATTTTAGTTTCACTCATTTTTAAAATCACAAAGATAATTTGTAACTTAAATG
ATGATACGTTATTTAAAAACACGATACTTCGTTTCAATGAACGCATTAAATAATAAATAAACACCTCGCAAAAGAAAGTAGTCCTTT
CTTATGGTGAGGTGTTTATTTGTCGACAACTTGATTATGATTGTTTCATTTTTTGAATAAGTTCATAATCAGGTGTAGCATACAAAGT
TTTTTGATTGTCATATGTTACAAACCCAGGCTTTGCACCTGATGGTTTATGCACATTTTTAATTAACGTGTAATCAACAGGTATTTGT
CCAGAATTACCAGCTTTTGAAAAATATCCTGCTAACATAGCCGCTTCCTTGATTGTCGTATCACTTGGTGCATCATTAAATATAACGA
CATGTGAACCAGGAATATCTTTTGTGTGTAACCATGTGTGAGTTTTTTTAGCTTTTTTATTTGTTAAATAATCATTTTGCTTGTTATTC
TTGCCAACATATATATCGTCGCCATCAGTTGATACATAATGTTGTAATTGAATCTGCGCTTTCTTTTTCTTAGTTTGATTTTTACGCTG
TTTCATAAAGCCTTGTTCTGCTAATTCATCTCTAATTTCATCAATGTCATGGACAGAAATATGATGTAATTGTTGTTCGATTGTTGAA
AAATAATCTATATTGTCTTTCGTCAATTGAATTTGATGTTGTAATTCACGTTCTCTCGTTTTCATACGATTATATTGTTTATAATAATA
TTGAGCATTTGCTGATGGGGATTTTGTAGGATTTAAAGGAATGACAACTTCTTCATTCGTATAATAATTCAATGCCGTCACTTCTTTA
TCGCCTTGCTTAATTCGATATATATTAGCAGTGATCAATTCACCATATAACTGTTCAGTATCTTTATTTTTAGACTGCTCATATTCTTC
AATCAACTTCGCTAATTTATTTTGATATTTGTGCAACTGCTGTTGAACAAATCGAACTAAATCATTCGCACGTTGTTTAACGCGTTCA
CGTTCGCCACGCGCATCATAAAAACGGTCAAGTAAATCATTTAATGAATCGTATGTAACTGTATCATCATTAAATTGATTTAACTTT
ATAAAATAGAAATCCTCTTTACCTGTTTCATGATTTTTATGAAAAATAGGAGTAGGTGGTAACTTGGTTTCTGCCATTACTTCGTCAA
ATGCTTCTGGTAATGTTGATGAAGTCATAAATTGACGACGACTAACGATTTCATTCGTAATTAAAGGGCTAAATCCTTCAAACTGAT
TCAATAATTGTTTAGCAATATTACCTGCGTTAAAATCGATATATTTCAACACCTCTGCACCTGTAATATCATACGGATTTATTTTGTG
CTGAGTAGGTGGTGCTTCATAATTAAATCCTGGCATTACTGTACGATAGTGATTCGTATTTGGTGTTAAGTGTTTAAATCCTTCAATT
ATTTTGCGATTTTCATCTACTAAAATTAAGTTACTATGTTTACCCATAATCTCAAGGATGACAGTGCGGTAAATAGTATCGCCAATTT
CATCTTTACTCTTTATATCGATTTCAATGCGACGATCATTACCAATTTGCTTAATCGATTCGATAATACCACCTTCTAAGTGTTTTCTA
AAAACACGCGCAAACATGGGTGGATTAAATGGATTATCATATTTTTTAGTAGTTAATTGTAATCTTGAAAGGTTTGGATGGATTGAC
AATAACAATTGATGGTTTTGTCTATTTTGACGTACAACCATTAGTATCGTGTCATTATCAGGTTGATTGATTTTGTGAACGCGTCCTG
TTGTTAAAAATTGTAGAGACTCAACCATTTTCTTTGTAAATAAGCCATCATAAGCCATAATTTATCTTCCACCTTCTTCATATCATTC
GTTTCATTTTAACATGATTGTAACTTTTCGGTATAGTTATTGAACCATACATTATGCCATACATTTTACATTATCTAAAAGTTTGCAAT
TCAATTTATTTAATTAAAATTAATCGTGCAATATAACGATATTGTTAGACTTAATAGAAATTATGGCATGCAATTTCAAATATGCTAT
ACAATATAAAGAACAATGTGATATCATATTTAAATAATAGAAGATTAGCTTAGAGAGGTCGTAAGGCATGGATAATGAAAAAGGAT
TGTTAATCGTTTTATCAGGACCATCTGGAGTAGGTAAAGGTACTGTTAGAAAACGAATATTTGAAGATCCAAGTACATCATATAAGT
ATTCTATTTCAATGACAACACGTCAAATGCGTGAAGGTGAAGTTGATGGCGTAGATTACTTTTTTAAAACTAGGGATGCGTTTGAAG
CTTTAATCAAAGATGACCAATTTATAGAATATGCTGAATATGTAGGCAACTATTATGGTACACCAGTTCAATATGTTAAAGATACAA
TGGACGAAGGTCATGATGTATTTTTAGAAATTGAAGTAGAAGGTGCAAAGCAAGTTAGAAAGAAATTTCCAGATGCGCTATTTATT
TTCTTAGCACCTCCAAGTTTAGAACACTTGAGAGAGCGATTAGTAGGTAGAGGAACAGAATCTGATGAGAAAATACAAAGTCGTAT
TAACGAAGCGCGTAAAGAAGTTGAAATGATGAATTTATACGATTACGTTGTAGTTAATGATGAAGTAGAACTTGCGAAGAATAGAA
TTCAATGTATTGTAGAAGCTGAGCACTTAAAAAGAGAGCGCGTAGAAGCTAAGTATAGAAAAATGATTTTGGAGGCTAAAAAATAA
TGTTAAATCCACCATTAAACCAATTAACGTCACAAATTAAATCAAAGTATTTAATTGCAACAACTGCAGCGAAAAGAGCGCGTGAA
ATTGATGAACAACCTGAAACTGAATTATTAAGTGAATATCATTCATTTAAACCAGTTGGTAGAGCGTTAGAAGAAATTGCTGACGGT
AAAATTCGCCCTGTTATTTCAAGTGATTATTATGGTAAAGAATAGTTTCGACCATTAAAAATATGTGAAGCTAGACGTACATCAAAT
ACGTCAAGGCATATCCAAATCAAACGCACCTATGTAGGTGTGTTTTTTTAGTTTATCCAATGATTGATGTTATAATAATACTAAATTT
GTATCTATAAAAAAGTAATGAGCATTTGTGCGCATATGATGATGTAAAGCGTAAATGTCATAGTGATATTTAAGGGAGATTGAATA
TGAAGAAAATATTATTAGCCGTTACAGGTGGCATTGCGGCATATAAAGCGATTGATTTGACAAGTAAGTTAACACAATCTGGGTAT
GAAGTTCGCGTTATGTTAACGAATCACGCACAAAAATTTGTGACACCATTAGCATTTCAAGCAATAAGTCGAAATGCTGTTTATACA
GATACTTTTATAGAAGAAAATCCTTCAGAAATACAGCATATTGCATTAGGTGATTGGGCAGATGCAATCATTGTTGCACCTGCAACG
GCAAATACAATTGCAAAATTGAGTGTAGGTATTGCTGATGATTTGGTGACATCAACGTTGCTAGCAACAGAGACACCGAAATTTATT
GCGCCTGCTATGAATGTGCATATGTATGAAAATAAACGTACGCAGCAAAATATTAATATTTTAAAAGAAGATGGGTATCATTTTATC
GAACCAGGAAGCGGATTTCTAGCATGTGGTTATGTTGCTAAAGGACGTATGGAAGAACCGCTTCAAATCGTTTCTGTTATTGATGCT
CATTTTCAAAATAGTAATCGTTTAGCTAATAGTTCATTTCAAGATAAACGCGCATTGGTTACAGCAGGACCAACTATTGAAGTTATC
GATCCAGTCAGATTTGTATCCAATCGTTCTTCTGGAAAAATGGGCTATGCAATAGCTGAAGCATTGCGAAATCGAGGAGCTATCGTG
ACGTTAGTTGCTGGTCCTACAACACTAGAGGATCCAAAAGATATTGAAGTTATTCATGTTCAAAGTGCTGAAGAAATGTTTGAACAA
GTGACAAGCCGATTTGACGAACAAGATATTGTTGTAAAAGCAGCAGCCGTATCTGACTATACACCAGTTGATGTATTAGAACATAA
GATGAAAAAGCAGGATGGTGATTTATCAGTATCTTTTAAACGTACTAAAGACATTCTTAAATATTTAGGTGAACATAAAACATCACA
GTATTTAATAGGCTTTGCAGCAGAGACTGAAGATATTGAAAATTATGCACAACAAAAATTACGCAAGAAAAATGCAGATGTGATTA

TTTCAAATAATGTTGGGGATATGTCTATCGGATTTAGTTCTGATGATAATGAATTGACAATGCATTTTAAAAATAATGAAAAGGTAA
ATATCAAGAAAGGAAAAAAAGTAGTATTAGCTGCACAAATTTTAGATGAACTAGAAACTAGGTGGCAATAATGATAGCGAAAGTC
ATAGTCGATGTCGCGTCGAAGAGCGTTGACTATAAATTTGATTATATAATTCCTGAACAACTCGAATCTGTCATCCAACCTGGTGTG
CGTGTGATTGTACCTTTTGGACCAAGAACGATTCAAGGTTATGTAATGGAAGTAACAGCAGAACCTGATGCACAACTTGACGTTTCG
AAGTTAAAAAAAATCATAGAAGTGAAAGATATACAACCTGAATTAACATCAGAATTAATAGCTTTAAGTGAGTGGATGGGTTCAAC
TCATGTCATTAAACGTATTTCTATGCTAGAAGTGATGCTTCCGAGTGCTATTAAAGCGAAGTATAAAAAAGCATTTAAGATGAAAGA
TGACAAAGAGGTACCTTCAGCTTTATTACAAAAATTTGATAAGCATGGTTACTATTATTATAAAGATGCGCAAAAAAATAATGATAT
TCAATTGCTTATGAAGTTGTTAAAAGATGATATCGTTGAAGAAAAAACGATTCTCACACAAAATATAACTAAAAAAACCAAGCGTG
CTGTTCGTGTCATTGAAGGGTATCATCCTGATGAAGTATTAGCTAAGTTGGAGAAAGTTATTAAACAATACGATTTGTATGCTTACT
TGTCTGAAGAACAACATAAAACAATATTTTTAACTGATATTGAGGATATGGGCTTTTCAAAATCCAGTTTAGATGGACTTATCAAAA
AAGGTTATGTTGAAAAATATGACGCGGTTGTTGAAAGAGACCCATTTAAAGATCGTGTTTTCGAACAAGAATCAAAACAGCAATTA
ACAGAAGACCAATATAAAGCATATGAAGCGATTAAAGCTAAAATTGTAAGCCAAGAGCAAGAAACATTTTTACTTCATGGTGTGAC
GGGATCAGGTAAAACAGAAGTATATTTACAAACGATAGAAGATGTTTTAAGCCAAGGAAAACAGGCGATGATGTTAGTTCCTGAAA
TCGCTCTAACACCGCAAATGGTTTTACGCTTCAAACGTCGATTTGGTGATGACGTTGCTGTATTACATTCTGGCTTATCTAATGGGGA
ACGTTATGATGAGTGGCAAAAAATTAGGGATGGTCGTGCGAGAGTAAGTGTTGGTGCAAGGTCAAGTGTGTTCGCACCTTTCAAAA
ATTTAGGGTTAATCATCATTGATGAAGAACATGAATCTACATATAAGCAAGAAGATTATCCGAGATATCATGCTAGAGAAATTGCC
CAATGGCGAAGTGAATATCATCACTGCCCAGTCATTTTAGGAAGTGCAACACCATGTCTTGAAAGTTATGCACGAGCTGAAAAAGG
CGTTTATCATTTGCTATCATTACCAAACAGAGTGAACCAACAAGCTTTACCTGAAATTGATATAGTAGATATGCGTGAAGAATTGAG
TGAAGGTAATCGGTCAATGTTTTCAAAAGATTTACGTGAAGCCATACAATTAAGATTAGATCGACAGGAACAAGTTGTTTTATTTTT
AAATCGACGTGGTTATGCATCGTTTATGTTATGTCGGGATTGTGGATATGTACCGCAATGTCCAAACTGTGATATTTCATTAACGTAT
CATAAAACGACAGACTTATTAAAATGTCACTATTGTGGTTACCAAGAGACGCCACCGAATCAATGTCCAAATTGTGAGAGTGAACA
CATTCGACAAGTAGGTACTGGTACTCAGAAAGTTGAAGAACTATTGCAACAAGAATTTGAAGATGCGCGCATAATTAGGATGGATG
TAGATACAACCTCAAAGAAAGGTGCACATGAAAAGTTATTGACTGAATTCGAAAAAGGTAACGGTGATATTTTACTAGGTACTCAG
ATGATTGCGAAAGGATTAGATTATCCAAATATTACTTTAGTTGGTGTGCTGAATGCAGATACAATGTTAAATTTACCTGATTTTCGG
GCGAGCGAACGTACTTATCAACTATTAACGCAAGTGGCTGGTAGAGCTGGTCGTCATGAAAAGGCAGGTCAAGTCATCATTCAAAC
GTATAATCCGGATCATTATTCAATATTGGATGTTCAAAAAAATGATTATTTAACATTTTATCGTCAGGAAATGGAATATCGTAAATT
AGGAAAGTATCCACCGTATTATTATTTGATTAATTTCACAATCTCACATAAAGAAATGAAGAAGGTTATGGAAGCATCGCAGCATGT
TCATAAAATTTTATTACAGCATTTAACAGAAAAAGCGCTTGTACTAGGTCCATCTCCGGCAGCACTTGCGAGAATCAACAATGAATT
TAGATTCCAAATTTTAGTGAAATATAAAAGTGAACCTGGATTATTACAAGCCATTCAGTTTTTAGATGACTATTACCATGAAAAATT
TATAAAAGAAAAATTAGCATTGAAGATTGATATTGATCCACAGATGATGATGTAACATTACTAATTATTAGAAACAAGTTCAGTAT
GTACGAGTATTTGAACCAGTGTTTGAATATTTACTTTAAGTACAGAAAAAGGGCAGAATATACAACTGTTAACCATTTAAATTAGCA
GTTTATATTCTGTCTTTTTATATGGCTTTATAACTTACGTGATTTTGATTTGATTAGGAATTTATTAGTATTTTCATTTACGAATTCTGA
TTTTAATGTAGGTGCCATACTTACTGATGAATAAAAGTACTAATGATTAAACAAACTATTTTTAAATGGCAAATTGAAATAGTGCAA
ATTTTACACTTATAAATTAATGGCTATAAACTATTTTATATCTATCTATGTTTTAACTACTTAAATTATTAGACAAAACAATTTTCTTA
AATTATCAATTAAAGTATTTAAAAATATTGATGGTTAATGTAAAATGTCAATATAAATAAGTTTTTTAAATTTATGTATGTTTATTTG
ATTCAAACAAAATAACTTAAGAGGAGAAAGTTTATGAAAAAGACACTGGGATGTTTACTTTTAATTATGCTTTTAGTCGTAGCAGGT
TGTTCTTTTGGTGGGAATCATAAATTATCATCAAAGAAATCAGAAGAATCAAAACAAGAAACTGTAAAAAAAGAATCGGAAGAAG
AGAAAGATCCAGATTTAGAGAAATATGAAGAAATAGAGAAGAAAATGAAAGGAATTAAAGATGCGCCATCTCTTGATAAGTTGGA
TCCATTAATGACAGAAAAGTCGTTTACGAATAGTAAAGGGATTCAAGGATGGAAAGATTACAAAGAATTAATGGGTAAAGTGGAA
CTTGCAGATTATAGATTTACTAAAGATTCAAAAGGATCTTCAATAAAAGATGTTGATGCATTCTTTAAAGGTAAGAAAGGTATAAA
AAGGAAAGTGATTGAAACACACGATGATGTAAAACAAGTTGATTATTGGTATGTAGATCCAGATGGAAAGAAAATTGGCAATTCAA
ACACACCTGTTTTTTACGCAGAAATTATGACAAAATATAAAGATGGAAAGTTAGTTTATGCATCAGTCGAACCAGGATCTTACGTAA
TACATAAAGATGATGCAATTAAATATGACGATTATTCTAAGTTAAAAAAATTAAGTCAGCTAACTAAACTTGATCATCCAAAACCA
GTTCCATATAGCGTAGCTCAAATCAAATCTTTCGGAGTACCTTTAACAAGCGTTTCATTTATGACACATGGATCAAAGGATACTAAA
GATGAAGTGTTGCCGGCATTGGCCTATTTCACTTTTTCACCAAAAAATTATGAAGACAAGTCTAATCCAGATCCAAAAGTTTTAAAT
TTAGTACATATGGATTTCTTAAATGCATCTAGTGATTTTGGTAACGCACATTTTGTTGTTTTAAGTAAATATATTAAAGAGTATGAAT
CAAACTATGAAACAGCGTCAGATGATTCTTTAAAATAGTATTTACTGTGTGAAAAATAAATAGTGTACTACATTAAATAATCGCAAT
AATAATCCCGATAAACAATCAGCATTACTGCTTATCGTAGAGTTCGTAATAACTATAACTCTATGATTCGCAATTAATAAATGATTG
TCATCGGGATTTATTTTTATCAATTTATAATGTAACATTACCATGTTCGTCAGCTGGTTTGAAAACAGTAATTATCGCACTAATAATT
GCTAATATATGTGGGATGACTGTCCAAAAGAATATTAAGTGTAGGAAACCTTGCATGTTTTTGCCGGCATAAAATTTATGAATACCA
AAACTACCTAAGAACAATGCTAATAAAATATAAATAACTTTGTTTACTTGCATTTCTTTCCCTCCAGTTGAATTGCTTATAATGACAT
TAGCTTCTCTTTTTATTATACCCACTTTTAGTTCAAACATTCTAGTTTAAGCATTCCCAATCATCTAAATTTCAGTTATTCAATCCTTA
CAATAAACTTAGGATTTCACTTCAGTTACATTGTATTATTTTACGTGTGAAATATACGTAATGAATCACATGACATTCTTTAAATTGA
AAAATATACATTCTATGATGTAAGGTCGCATTTTTAATATATTTACGTTATAATGGTTTGATGAATTTTATTGAGGAGTAAGAGCTAT
GGCGATTAAAAAGTTAGTACCAGCATCGCATCCTATTTTAACGAAAAAAGCGCAAGCAGTTATAAAATTTGATGATTCGTTAAAAA
GATTATTACAAGATTTAGAAGATACAATGTATGCACAAGAAGCAGCTGGCTTATGTGCACCTCAAATTAATCAGTCATTGCAAGTG
GCAATCATTGATATGGAAATGGAAGGATTATTACAACTTGTTAATCCGAAAATTATTAGTCAATCAAATGAAACGATAACAGACTT
AGAAGGTTCAATTACATTGCCAGATGTTTACGGCGAAGTGACAAGAAGTAAAATGATAGTTGTCGAAAGTTATGACGTCAATGGGA
ACAAAGTTGAACTAACTGCACATGAAGATGTAGCAAGAATGATTTTGCATATTATAGATCAAATGAACGGTATCCCTTTTACAGAA
CGTGCGGACCGTATTTTAACAGATAAAGAAGTGGAGGCATATTTTATAAATGACTAAAATAATATTTATGGGTACACCAGACTTTTC
AACAACTGTTTTAGAAATGCTTATTGCAGAACATGATGTCATTGCAGTCGTAACGCAACCAGATCGACCTGTTGGACGTAAACGTGT
TATGACACCACCACCAGTTAAAAAAGTTGCAATGAAATATGATTTACCTGTATATCAGCCTGAAAAATTAAGTGGATCAGAAGAAT
TAGAACAATTGCTTCAATTAGATGTAGATTTAATTGTAACTGCTGCTTTTGGACAATTATTACCTGAATCATTGTTGGCATTACCAAA
ACTTGGGGCAATTAATGTACATGCATCATTGTTACCGAAGTATAGAGGTGGTGCACCAATTCATCAGGCAATTATCGATGGTGAACA
AGAAACCGGCATAACAATTATGTATATGGTTAAAAAATTAGATGCGGGTAATATTATTTCGCAACAAGCAATTAAAATAGAAGAAA
ATGATAATGTCGGTACGATGCATGATAAATTAAGTGTATTAGGGGCAGATTTATTAAAAGAAACTTTACCATCTATTATAGAGGGCA
CAAATGAAAGTGTACCTCAAGATGATACGCAAGCAACATTTGCTTCCAATATTCGACGCGAAGATGAGCGAATTAACTGGAATAAA
CCAGGAAGACAAGTGTTTAATCAAATTCGTGGATTATCACCATGGCCAGTTGCTTATACAACTATGGATGACACTAACTTGAAAATA
TACGATGCTGAACTCGTTGAGACTAATAAGATAAACGAGCCTGGAACCATTATAGAAACGACTAAAAAAGCCATTATTGTTGCTAC
AAATGATAATGAAGCTGTTGCAATTAAAGATATGCAATTAGCTGGGAAAAAGAGAATGTTAGCTGCCAATTATTTAAGTGGTGCGC
AAAACACACTAGTAGGGAAGAAACTTATATGATAGAAAACGTGAGAAGTCTTGCTTTTGACACGATTCAAGATATATTAAATGAAG

GTGCGTATAGTAACTTGCGTATTAATGAAGTGTTGTCAGAAAATGAATTAAATGCAATGGATAAAGCCTTATTTACAGAAATTGTCT
ACGGAACCGTTAAAAGAAAATATACGTTAGATTTTTATTTAAAGCCTTTTGTGAAAACAAAGATTAAGGCATGGGTTAGGCAATTAT
TATGGATGAGTATTTATCAATATGTTTATTTAGATAAAGTTCCAAATCATGCCATTATTAATGAAGCAGTTGAAATAGCAAAAGAAC
GCGGTGGCTATCATAATGGTAATGTCGTAAATGGTATTTTACGTACAATGATGCGTAGTGACTTACCTGATTTTAATGAAATTGCAG
ATCCTAAAAAAAGAATGGCAATCGAATATAGTATGCCGAAGTGGATTATAGATCATTGGGCAACACATTATGGTCTCGAAGAAACT
GAAACAATTTTACAGTCATTTTTAGAAACGACATCAACAACTGTGCGTGCCAACCTGACGCGAGCATCATTAGATGATATTATTGAA
AAGTTGCAAGACGAAGGTTATGACGTTGAAAAAGATCATGACTTACCTTATTGTCTCCATATAGGTGGACAACCAATTATTCATTCT
CGTTCATTTAAAGATGGATTCGTTTCAATTCAAGATAAAAGCTCAATGTTTGTTGCACACATTATGAATGTAGACCGACATGATCAC
GTATTAGATGCATGTAGTGCACCTGGCGGTAAAGCTTGTCACATTGCTGAAGTTTTAATGCCAGAAGGGCAAGTTGACGCTTCAGAT
ATACATGATCACAAAATAGACTTAATTAATTTTAATATAAAAAAATTACGATTAACAAATATTAAAGCTTTTCAACATGATGCGACA
AAACCTTATGATAAAACATACGATAAGATACTTGTTGATGCACCATGTAGCGGATTAGGTGTAATGAGACATAAGCCGGAGATTAA
GTATACTCAAAGCAAACAACATATTGAGTCACTAGTTGAATTACAGCTTGAAATATTGGAAAATGTAAAAAACAATGTAAAAATAG
GTGGCGAAATCATCTATTCAACATGTACAATTGAGCAACTAGAAAATGAAAACGTGATTTATACGTTTTTGAAAAATAATAAAAAC
TTCGAATTTGAACCGTTTCAACATCCGATAACTGGAGAGTTGGTCAAAACGTTACAAATCATGCCGCAAGACTTTAATTCAGATGGA
TTCTTTATCACTAAGATAAAAAGAAAGGACAATTAGGAATGATAACTGCTGAAAAGAAAAAGAAGAATAAATTTCTTCCAAATTTT
GACAAGCAATCAATATATTCATTGCGATTTGACGAAATGCAAAACTGGCTCGTTGAACAAGGTCAACAAAAATTTCGAGCGAAACA
GATTTTTGAATGGTTATATCAAAAAAGAGTAGATTCGATTGATGAAATGACGAACTTATCGAAAGACTTACGACAGCTTTTAAAAG
ATAACTTTACTGTTACAACTTTAACAACTGTAGTAAAACAAGAAAGTAAAGACGGTACAATTAAATTCTTATTTGAATTACAAGATG
GCTATACAATTGAAACTGTTTTAATGAGACATGATTATGGAAATTCAGTATGTGTAACGACACAAGTAGGTTGTCGTATCGGATGTA
CGTTTTGTGCTTCTACACTTGGCGGCTTAAAAAGAAACCTTGAAGCTGGCGAAATTGTTTCACAAGTTTTAACAGTTCAAAAAGCCC
TTGATGCTACAGAAGAGCGCGTATCTCAAATTGTCATAATGGGTATCGGTGAACCATTTGAAAATTATGATGAAATGATGGACTTTT
TAAGAATCGTCAATGATGATAATAGTTTAAATATTGGTGCACGTCACATTACAGTATCAACATCAGGTATCATTCCTAGAATATACG
ACTTTGCGGATGAAGATATCCAAATTAATTTTGCTGTAAGCTTACACGCCGCAAAAGATGAAGTGCGATCACGCTTGATGCCAATTA
ACCGTGCATATAATGTTGAGAAGTTAATCGAAGCAATTCAATATTATCAAGAAAAAACAAATCGTCGTGTTACTTTTGAATATGGTC
TGTTTGGTGGTGTGAATGACCAACTAGAACATGCAAGAGAATTAGCACATTTAATAAAAGGCTTAAACTGCCATGTTAACTTAATTC
CTGTCAACCATGTTCCAGAAAGAAATTATGTGAAAACGGCTAAAAATGATATCTTTAAATTTGAAAAAGAATTAAAGAGACTAGGA
ATTAATGCCACAATACGTCGTGAACAAGGTTCGGATATTGACGCAGCTTGTGGTCAATTAAGAGCAAAGGAACGACAAGTAGAAAC
GAGGTAAAGACAAATGCTAGAGGCACAATTTTTTACTGATACTGGACAACATAGAGATAAGAATGAAGATGCGGGTGGTATTTTTT
ATAATCGAACTAATCAACAACTTTTAGTTCTGTGTGATGGTATGGGCGGCCATAAAGCAGGAGAAGTTGCAAGTAAATTTGTTACA
GATGAGTTGAAATCCCGTTTTGAAGCGGAAAATCTTATAGAAGAACATCAAGCTGAAAATTGGTTGCGTAATAATATAAAAGATAT
AAATTTTCAGTTATATCACTATGCACAAGAAAATGCAGAATATAAAGGTATGGGTACAACATGTGTTTGTGCACTTGTTTTTGAAAA
ATCAGTTGTGATAGCAAATGTCGGTGATTCTAGAGCCTATGTTATTAATAGTAGACAAATTGAACAAATTACTAGTGATCACTCATT
TGTTAATCATCTTGTTTTAACGGGTCAAATTACGCCGGAAGAAGCATTTACACATCCACAACGTAATATTATTACGAAGGTGATGGG
CACAGATAAACGTGTGAGTCCAGATTTGTTTATTAAGCGATTAAATTTTTATGATTATTTATTATTAAATTCAGATGGATTAACTGAT
TATGTTAAAGACAATGAAATTAAGCGTTTGTTAGTAAAAGAAGGTACAATAGAAGATCATGGTGATCAATTAATGCAATTGGCATT
AGATAACCATTCGAAAGATAACGTTACTTTCATACTCGCGGCTATTGAAGGTGATAAAGTATGATAGGTAAAATAATAAATGAACG
ATATAAAATTGTAGATAAGCTTGGCGGCGGTGGCATGAGTACCGTTTATCTTGCTGAAGATACGATACTTAACATTAAAGTTGCAAT
TAAGGCGATTTTTATACCACCTAGAGAAAAAGAAGAAACATTAAAACGTTTTGAACGAGAAGTACATAACTCATCACAGCTATCAC
ATCAAAATATAGTAAGTATGATCGATGTTGATGAAGAAGATGACTGTTACTACTTAGTAATGGAATATATTGAAGGTCCGACTTTGT
CTGAGTATATTGAAAGTCATGGGCCATTAAGTGTTGACACGGCGATTAATTTTACGAATCAAATATTGGATGGTATTAAACATGCGC
ATGATATGCGTATTGTACATAGAGATATTAAGCCACAAAATATATTAATTGACAGCAATAAAACGTTGAAAATATTTGATTTTGGAA
TTGCTAAAGCTTTAAGTGAGACGTCTTTAACTCAGACTAATCATGTGTTAGGTACTGTGCAGTACTTTTCGCCAGAACAAGCAAAAG
GTGAGGCAACGGATGAATGTACAGATATTTATTCTATAGGTATTGTGTTATATGAAATGCTTGTTGGTGAACCACCCTTTAATGGAG
AAACTGCAGTTAGCATTGCGATTAAACATATTCAGGATTCTGTGCCAAATGTGACAACAGATGTACGTAAGGATATTCCGCAATCTT
TAAGTAATGTCATTTTACGCGCTACAGAAAAAGACAAAGCGAATCGTTACAAAACAATTCAAGAAATGAAAGATGATTTGAGTAGT
GTTTTACATGAAAATCGAGCGAATGAAGATGTCTATGAACTCGATAAAATGAAAACGATAGCGGTACCTTTGAAAAAAGAAGATCT
AGCAAAGCATATTAGTGAACATAAGTCGAATCAACCTAAACGTGAAACGACGCAAGTACCTATTGTAAATGGGCCTGCTCATCATC
AGCAATTCCAAAAGCCAGAAGGTACGGTTTACGAACCAAAACCTAAAAAGAAATCAACACGAAAGATTGTGCTCTTATCACTAATC
TTTTCGTTGTTAATGATTGCACTTGTTTCTTTTGTGGCAATGGCAATGTTTGGTAATAAATACGAAGAGACACCTGATGTAATCGGGA
AATCTGTAAAAGAAGCAGAGCAAATATTCAATAAAAACAACCTGAAATTGGGTAAAATTTCTAGAAGTTATAGTGATAAATATCCT
GAAAATGAAATTATTAAGACAACTCCTAATACAGGTGAACGTGTTGAACGTGGTGACAGTGTTGATGTTGTTATATCAAAAGGCCC
TGAAAAGGTTAAAATGCCAAATGTCATTGGTTTACCTAAGGAGGAAGCCATGCAGAAATTAAAATCGTTAGGTCTTAAAGATGTTA
CGATTGAAAAAGTATATAATAATCAAGCGCCAAAAGGATACATTGCAAATCAAAGTGTAACCGCAAATACTGAAATCGCTATTCAT
GATTCTAATATTAAACTATATGAATCTTTAGGCATTAAGCAAGTTTATGTAGAAGACTTTGAGCATAAATCCTTTAGCAAAGCTAAA
AAAGCCTTAGAAGAAAAAAGGGTTTAAAGTTGAAAGTAAGGAAGAGTATAGTGACGATATTGATGAGGGTGATGTGATTTCTCAATC
TCCTAAAGGAAAATCAGTAGATGAGGGGTCAACGATTTCATTTGTTGTTTCTAAAGGTAAAAAAAGTGACTCATCAGATGTCAAAA
CGACAACTGAATCGGTAGATGTACCATACACTGGTAAAAATGATAAGTCACAAAAAGTTAAAGTTTATATTAAAGATAAAGATAAT
GACGGTTCAACTGAAAAAGGTAGTTTCGACATTACTAGTGATCAACGTATAGACATTCCTTTAAGAATTGAAAAAGGAAAAACAGC
AAGTTATATTGTTAAAGTTGACGGTAAAACTGTAGCTGAAAAAGAAGTCAGCTATGATGATGTATAAATATAATTGAAGTAAATGT
ACCGAGGTTTCTATTTGGAAGTCTCGGTATTTTTATGTTGAGATTGCGGTAGTTTTAAAATGCTTCATGTCATCATATACAGGTGAGC
ATTGTCTATCAATTCTGTATCACTCTCACAATATTTATATCATTTGAAATGATCATTTATATATTAATTTTCCAGTCTTTGATATAATA
AATGCAGTAAAATTAATCGAGAGGTGCCATTTTGAAGACAGGTCGAATAGTGAAATCAATTAGTGGGGTATATCAAGTAGACGTTA
ATGGCGAACGTTTCAATACAAAACCACGAGGATTATTTAGAAAGAAAAAATTTTCACCGGTAGTTGGTGATATAGTGGAATTTGAA
GTACAAAACATTAACGAAGGCTATATTCATCAAGTGTTTGAGCGGAAAAATGAGTTGAAAAGACCACCTGTAAGTAATATAGATAC
ACTAGTAATTGTAATGAGTGCTGTCGAGCCAAATTTTTCAACGCAATTATTAGATCGATTTTTAGTTATTGCACATTCGTATCAGTTA
AATGCGAGAGTTTTGGTGACTAAAAAAGATAAAACACCAATTGAAAAGCAGTTCGAAATAAATGAGTTGTTGAAAATATATGAAA
ATATTGGCTATGAGACTGAATTTATTGGAAATGATGATGATCGAAAAAAAATTGTAGAAGCTTGGCCAGCTGGACTTATAGTACTTA
GTGGTCAATCAGGTGTCGGTAAGTCCACTTTCTTAAATCATTATCGTCCAGAACTTAATCTTGAGACAAATGATATATCAAAATCAT
TAAATCGAGGAAAGCATACTACAAGACATGTCGAACTATTCGAACGTCAAAACGGTTATATTGCAGACACACCTGGATTCAGTGCT
TTAGATTTTGATCATATAGATAAAGATGAAATAAAAGATTATTTTCTTGAATTAAATCGATATGGTGAAACATGTAAGTTTAGAAAT
TGTAATCATATCAAAGAACCTAATTGTAATGTTAAGCATCAATTAGAGATAGGGAATATTGCGCAATTTAGATACGACCATTATTTA

CAACTATTTAATGAAATTTCAAATAGAAAGGTTAGATATTAAATGACAAAACTATATCCATCATTATTATCTGTTGATTTTTTGGATT
TACAACATGAATTAAAACGACTTGAAGAAGCAGGTGTCGACGGAGTTCATTTTGATGTTATGGATGGTCAATTTGTGCCTAATATAT
CTATTGGTTTACCAATATTAGATGCAGTAAGAAAAGGCACAACATTACCTATAGACGTACATTTGATGATTGAAAATCCAGAAAAG
TATATTGCATCATTTGCAGAACATGGTGCCGATATGATTTCAATTCATGTCGAATCAACGCCTCATATTCATCGTGCTATTCAAATGA
TTAAACATTTAGATAAAAAAGCTGGTGTAGTAATTAATCCTGGTACACCAATATCACAAATTGAACCTATTTTAGACATTGTTGATT
ATGTACTAGTGATGACAGTTAACCCAGGGTTTGGTGGTCAATCATTTATTGATCAATGCGTAGAAAAAATAGCGGGTCTTAATGCTA
TTAAAATGGAACGTCAATTAAACTTTGATATTGAAGTTGATGGAGGCGTAAATACCGATACAGCGAAAGTTTGTGTTGAAAATGGT
GCTACAATGCTAGTAACAGGTTCATTTTTCTTTAAACAAGAGGATTATAAAAAAGTCACACAAGGATTGAAAGGTTGAGTGTATAT
GCATATAAATTTATTATGTTCTGATCGACACTTGCCGCAAGATATTTGGGCCAAAAGTAATGAAGGTAAATGGGGCGGCGTTGATA
GAGGTGCTTTGATTTTATTGAAGCATCAAATTATCCCTTTTTTCTCAGTGGGAGACTTTGATTCAGTCAGTAAAGAAGAACGCCAAC
TTTTAACAGAACAGTTACAAATCAAACCAGTTCAAGCTGAAAAAGCTGATACGGATTTAGCTTTAGCGGTTGATAAAGCTGTTGCAC
TTGGATTTGATAGTATTACAATTTATGGTGCAACAGGCGGACGATTAGATCACTTTTTTGGGGCAATTCAGTTATTATTGAAAAAAG
CATATTATAAACATGATGTTCATATAGAAGTTATCGATCAACAAAATAAAATTGAATTATTGCCTAAAGGTCAACATACAGTTGAAA
AAGATAAGAGTTATCCGTACATTTCATTTATACCGATGACTGATGATGTAGAACTTTCTCTAGCAGGTTTTAAATATAATTTAGCTAG
ACAAATGCTTAATATAGGTTCTACTTTAACTATTTCCAATGAAATTGAGTCTTTGCAAGCGAAAGTAACTGTACATGATGGGTTGAT
TTTGCAAATTAGAAGTACAGATTTAAATTAAGTATATTCAAATTTGTGAGGATTTTTAATAAAGTGCATGAGTTATTTAATAAAGTG
ACGTTTATAGATAGAATTTATAACATAATAATAGATGAATCACGCTGTAATTAATGAAGATTATGGCGTGATTTATTATTGAAAGGA
AAATTGTAGGTCCTAGTACATTGATTTATGTGCCAAAATCGATATTGAACCCATAAGTCCAATTTGACAGAGAACCATTATTAAACG
TATTAACCAAATTATATTGATAGTTCGAATTTCACTATGAACCATTATGTATTGTTTGGTAACAAAAAAAGAACTAAGAGGTTGAGC
TCTTAGTTCTTAAGTAAATGCGTCCTCCACATAAGGTCACATTTTTTATGTGCAATATATTTATTAAACTCTAGTTACTTTACCAGATT
TTAAAGCACGTGCAGAAACCCAAACTTTTTTAGGTTTACCGTCAACTAGGATTCTAACTTTTTGAAGGTTAGCGTTCCATCTACGTTT
AGTAGAGTTTAAAGCGTGTGAACGTCTGTTACCAGTCGAAGCTTTACGACCTGTTACGAAACATTGTTTACCCATATGAGTACCTCC
TTTAATAAATATAAATACACATAACTACTTATATACTTAATTAAGATAGCATAGTTTCATTTGAAAAACAATGAATAATTTTCACAT
AAAAGTCAAAAATACTGAGTTTTGTGATATAATTGTAGACTGTGAAGTTATGTAGTATGATATTTTAGAGAAATAAGTGAATGAAA
CATTTAAAATTTTTATTTAATGATACTACATCTATTAAAAGAAAACGCCTATAGATAATCGTCATGAGTGTAAATTCAATTTACTCAA
AATATAATAGGTGATTTAAGCGTAAGTCATTCTGATAAGTTGTACCATTGATATTTTATAAAAATAATCAATGAAACTTAAAGCGTT
TATGACTACACTAAGTGGTAAAATTTTAAAAGTGATTAGGGAAAACGAATAATAAACTTTGAAATTTAGGAGGGACAAAGATATGA
CATTAGAGATTTCAAATGATTACGGTAAAATTGATATTTCAAACGAAGTGATTGCTTCGGTTGTAGGTGGAAAGGCCGTTGAATGTT
ATGGTATTGTAGGTATGGCATCTAGACAACAAGTTAGAGATGGTATTGCGGAAATACTAGGACATGAAAATTATGCTAAAGGCATC
AAAGTAACTGAAAATAATGGCGTAGTGGATATAGATATGTACATTATTGTAAGTTATGGTGTGAAAATATCTGAAGTTGCCAATAA
TGTACAATCAACAGTGAAATATACTTTGGAAAAATCACTTAATGTATCAGTAAATTCAATCAATATATATGTACAAGGTGTACGTGT
GAATAATACAGGCAAGAAAGCTTAGGAGGACAACTTGAAATGATTAGCAAAATTAATGGTAAATTATTTGCCGATATGATTATACA
AGGGGCACAAAATTTATCTAACAATGCAGATTTGGTAGATTCTTTGAATGTGTATCCAGTGCCAGATGGTGATACAGGAACAAATA
TGAATCTTACTATGACTTCAGGTCGCGAAGAAGTAGAGAATAATTTGTCGAAAAATATCGGCGAATTAGGTAAAACATTCTCGAAA
GGTTTACTAATGGGTGCAAGAGGTAACTCTGGTGTCATCTTGTCACAATTATTCAGAGGATTTTGTAAAAATATTGAAAGTGAATCT
GAAATTAATTCAAAATTGTTAGCTGAAAGTTTTCAAGCTGGTGTTGAAACGGCATATAAAGCTGTTATGAAACCAGTTGAAGGTAC
AATACTTACAGTTGCAAAAGATGCTGCGCAAGCTGCAATAGAAAAAGCAAATAATACTGAAGATTGTATAGAATTAATGGAGTACA
TTATTGTAAAAGCCAATGAATCACTTGAAAACACACCAAACTTATTAGCTGTACTTAAAGAAGTTGGTGTTGTTGATAGTGGCGGTA
AAGGTTTGTTATGCGTTTACGAAGGATTCTTAAAAGCGCTTAAAGGTGAAAAAGTTGAAGCCAAAGTTGCAAAGATAGATAAAGAT
GAATTTGTACATGATGAACATGATTTCCATGGTGTAATTAATACTGAAGATATTATTTATGGCTATTGTACTGAAATGATGGTTCGTT
TTGGAAAGAATAAAAAAGCCTTTGATGAACAAGAATTCAGGCAAGATATGAGTCAATTTGGTGATTCTTTATTAGTCATTAATGATG
AAGAAATTGTGAAAGTTCACGTGCATACCGAATACCCAGGTAAAGTGTTTAATTATGGTCAACAATATGGTGAATTAATTAAACTTA
AGGTTGAAAATATGAGAGAACAGCATCGTGAAGTGATTCGAAAAGAACAGCACACAGCTAAACCGAAAATGGAAACGGTTGAAAC
AGCGATTATTACTATTTCTATGGGTGAAGGTATTTCAGAGATATTTAAATCAATGGGTGCCACACATATCATTAGTGGTGGACAAAC
GATGAATCCTTCTACAGAAGATATCGTTAAAGTCATTGAACAATCAAAATGTAAACGTGCAATTATTTTACCGAATAATAAAAATAT
CTTAATGGCAAGTGAACAAGCAGCGAGTATTGTTGATGCAGAAGCAGTTGTTATTCCAACGAAATCTATTCCTCAAGGTATAAGCG
CACTATTCCAATATGATGTGGACGCAACACTTGAAGAAAATAAAGCGCAAATGGCTGATTCAGTAAATAACGTTAAATCTGGTTCA
TTAACGTACGCTGTTCGTGATACGAAAATTGATGGCGTTGAGATTAAAAAAGACGCGTTTATGGGCTTGATTGAAGATAAGATTGTA
AGCAGCCAAAGTGATCAATTAACAACGGTTACTGAGTTGTTAAATGAGATGTTAGCAGAAGATAGTGAAATATTGACTGTGATTAT
TGGTCAAGATGCAGAGCAAGCAGTTACAGATAACATGATAAACTGGATTGAAGAGCAATATCCAGATGTAGAAGTGGAAGTTCAT
GAAGGTGGACAACCAATTTATCAATATTTCTTTTCAGTAGAATAAAAATTTAAAATAAAAAACTACCAATGATAAATCATCAGTTGG
TAGTTTTTTATTTTGCTATTTTAGTGAGATAGCGGGTTAAAAGTATCATTCTCGAGTTGCTAACAATGTCATGTTCAACTTAGTCATG
ATAAAATAAATAACATACTAAATGATACGTAAAATCAAATAAAACATAGGTGATTTATTTTGGCTAAAGTAAACTTAATAGAAAGT
CCATATTCTCTTTTACAATTAAAAGGTATAGGTCCTAAGAAAATAGAAGTATTGCAACAACTAAATATTCATACAGTGGAAGATCTT
GTTCTTTATTTGCCAACTAGATATGAAGATAATACAGTGATTGATTTGAATCAAGCAGAAGATCAATCTAACGTTACGATAGAAGGA
CAAGTATATACAGCTCCAGTAGTTGCATTTTTTGGAAGAAATAAATCAAAATTAACCGTTCATTTAATGGTAAATAATATTGCTGTC
AAATGTATTTTTTTCAATCAACCGTATTTAAAAAAGAAAATCGAATTAAATCAAACTATAACTGTTAAAGGTAAGTGGAATAGGGTT
AAACAGGAAATTACTGGTAATAGGGTTTTCTTTAATTCACAAGGGACACAAACTCAAGAAAACGCAGATGTTCAATTAGAACCAGT
CTATCGTATTAAGGAAGGTATTAAACAAAAGCAAATACGAGACCAAATTAGACAAGCGTTAAATGATGTGACAATTCATGAATGGT
TAACTGATGAACTAAGAGAAAAAATATAAATTAGAGACCTTGGACTTTACTTTGAACACATTACATCATCCTAAAAGTAAAGAGGAT
TTATTACGTGCTCGTAGAACCTATGCATTTACTGAACTGTTTTTATTCGAATTACGTATGCAATGGCTAAATAGATTAGAAAAGTCAT
CTGACGAAGCAATTGAAATTGATTATGACATAGACCAAGTTAAATCATTTATTGATCGTTTACCTTTTGAACTAACTGAAGCACAGA
AATCCAGTGTTAATGAAATTTTTAGAGATTTAAAAGCACCAATACGTATGCATCGATTACTTCAAGGTGATGTAGGTTCAGGAAAAA
CAGTAGTTGCTGCAATTTGTATGTATGCGTTAAAAACGGCTGGTTATCAATCAGCATTGATGGTACCAACTGAAATTTTAGCAGAGC
AACATGCTGAAAGTTTAATGGCTTTATTTGGAGATTCTATGAACGTTGCATTGTTAACTGGGTCAGTAAAAGGTAAGAAACGAAAG
ATACTTTTAGAACAACTTGAAAATGGTACGATTGATTGTTTAATTGGAACCCATGCTTTGATTCAAGATGATGTGATTTTCCATAATG
TTGGTTTAGTAATTACAGATGAACAACATCGATTTGGTGTGAATCAACGCCAGCTTTTAAGAGAAAAAGGTGCAATGACGAATGTG
TTATTTATGACAGCAACGCCGATACCAAGAACACTAGCAATATCAGTTTTTGGTGAGATGGATGTGTCTTCAATTAAACAATTACCA
AAAGGTCGTAAACCTATCATTACTACTTGGGCAAAGCATGAGCAATACGATAAAGTTTTGATGCAAATGACCTCAGAGTTGAAAAA
AGGTCGTCAAGCATATGTCATTTGCCCGCTTATAGAAAGTTCTGAGCATCTCGAAGATGTTCAAAATGTTGTCGCATTGTACGAGTC
TTTACAACAGTATTATGGTGTTTCCCGTGTAGGGTTATTGCATGGTAAGTTGTCTGCCGATGAAAAAGATGAGGTCATGCAAAAGTT

TAGCAATCATGAGATAGATGTTTTAGTTTCTACTACTGTTGTTGAAGTAGGTGTTAATGTACCGAATGCAACTTTTATGATGATTTAT
GATGCGGATCGCTTTGGATTATCAACTTTACATCAGTTACGCGGACGTGTGGGTAGAAGTGACCAGCAAAGTTACTGTGTTTTAATT
GCATCCCCTAAAACAGAAACAGGAATTGAAAGAATGACAATTATGACACAAACAACGGATGGATTTGAATTGAGTGAACGAGACT
TAGAAATGCGTGGTCCTGGCGATTTCTTTGGTGTTAAACAAAGTGGATTGCCAGATTTCTTAGTTGCCAATTTAGTTGAAGATTATCG
TATGTTAGAAGTTGCTCGTGATGAAGCAGCTGAACTTATTCAATCTGGCGTATTCTTTGAAAATACGTATCAACATTTACGTCATTTT
GTTGAAGAAAATTTATTACATCGTAGTTTTGACTAATTGCCATGCTGATTTGTCAATTTGAGTGCAACACTTCGTTAATTGAGTGATA
TGACACTTGAACTATTTAAATGTAAAGTGGTATTTTAACAATTTATAAATTTTCGACTAAATAATAGCTAAATATTACAGTTATTTGT
TGAGTCGGTTAAATAGAAAGTGTTATGATATGTGAGGAATGTTTAAGACTAGGTACTAAAAAATGAGGGGTGAGACGTTGAAACTA
AAGAAAGATAAACGTAGAGAAGCAATCAGACAACAAATTGATAGCAATCCCTTCATCACAGACCATGAACTAAGCGACTTATTTCA
AGTGAGTATACAAACAATTCGTTTAGATCGCACTTATTTAAACATACCAGAATTAAGGAAGCGTATTAAATTAGTTGCTGAAAAGA
ATTATGACCAAATAAGTTCTATTGAAGAACAAGAATTTATTGGTGATTTGATTCAAGTCAATCCAAATGTTAAAGCGCAATCAATTT
TAGATATTACATCGGATTCTGTTTTTCATAAAACTGGAATTGCGCGTGGTCATGTGCTATTTGCTCAGGCAAATTCGTTATGTGTTGC
GCTAATTAAGCAACCAACAGTTTTAACTCATGAGAGTAGCATTCAATTTATTGAAAAAGTAAAATTAAATGATACGGTAAGAGCAG
AAGCACGAGTTGTAAATCAAACTGCAAAACATTATTACGTCGAAGTAAAGTCATATGTTAAACATACATTAGTTTTCAAAGGAAAT
TTTAAAATGTTTTATGATAAGCGAGGATAAAATTATGGTTAAATTAGCAATTGATATGATGGGTGGCGACAATGCGCCTGATATCGT
ATTAGAAGCCGTACAAAAGGCTGTTGAAGACTTTAAAGATCTAGAAATTATACTTTTCGGTGACGAAAAAAAGTATAATCTGAACC
ATGAACGAATCGAATTTAGACATTGTTCTGAAAAGATTGAAATGGAAGATGAGCCTGTTAGAGCGATTAAACGTAAAAAAGATAGC
TCAATGGTAAAAATGGCTGAAGCTGTGAAATCTGGTGAAGCAGATGGATGTGTGTCAGCAGGTAATACTGGTGCTTTAATGTCAGC
TGGTTTATTCATTGTTGGACGTATTAAAGGTGTAGCTAGACCGGCTTTAGTAGTAACATTGCCAACGATTGATGGAAAAGGTTTTGT
CTTTTTAGACGTTGGTGCAAATGCTGATGCTAAACCTGAACACTTATTACAGTATGCGCAACTAGGGGATATTTATGCTCAAAAAAT
TAGAGGTATTGATAATCCGAAAATCTCATTATTAAATATAGGAACCGAGCCAGCTAAAGGTAATAGTTTAACGAAAAAATCATATG
AGTTATTAAATCATGATCATTCATTGAATTTTGTTGGGAATATTGAAGCGAAGACATTAATGGATGGCGATACAGATGTTGTAGTTA
CCGATGGCTATACTGGGAACATGGTCCTTAAAAATTTAGAAGGTACTGCAAAATCAATCGGTAAAATGTTAAAAGATACGATTATG
AGTAGTACTAAAAATAAATTAGCAGGTGCAATATTGAAGAAAGATTTAGCTGAATTCGCTAAAAAGATGGATTACTCAGAATACGG
TGGTTCCGTATTATTAGGATTGGAAGGTACTGTAGTTAAAGCACACGGTAGTTCAAATGCTAAAGCTTTTTATTCTGCAATTAGACA
AGCGAAAATCGCAGGAGAACAAAATATTGTACAAACAATGAAAGAGACTGTAGGTGAATCAAATGAGTAAAACAGCAATTATTTT
TCCGGGACAAGGTGCCCAAAAAGTTGGTATGGCACAAGATTTGTTTAATAACAATGATCAAGCAACTGAAATTTTAACTTCAGCAG
CAAAGACGTTAGACTTTGATATTTTAGAGACAATGTTTACTGATGAAGAAGGTAAATTGGGTGAAACTGAAAACACGCAACCAGCT
TTATTGACGCATAGTTCGGCATTATTAGCAGCGCTAAAAAATTTGAATCCTGATTTTACTATGGGGCATAGTTTAGGTGAATATTCA
AGTTTAGTTGCAGCTGACGTATTATCATTTGAAGATGCAGTTAAAATTGTTAGAAAACGTGGTCAATTAATGGCGCAAGCATTTCCT
ACTGGTGTAGGAAGCATGGCTGCAGTATTGGGCTTAGATTTTGATAAAGTCGATGAAATTTGTAAGTCATTATCATCTGATGACAAA
ATAATTGAACCAGCAAACATTAATTGCCCAGGTCAAATTGTTGTTTCAGGTCACAAAGCTTTAATTGATGAGCTAGTAGAAAAAGGT
AAATCATTAGGTGCAAAACGTGTCATGCCTTTAGCAGTATCTGGTCCATTCCATTCATCGCTAATGAAAGTGATTGAAGAAGATTTT
TCAAGTTATATTAATCAATTTGAATGGCGTGATGCTAAGTTTCCTGTAGTTCAAAATGTAAATGCGCAAGGTGAAACTGACAAAGAA
GTAATTAAATCTAATATGGTCAAGCAATTATATTCACCAGTACAATTCATTAACTCAACAGAATGGCTAATAGACCAAGGTGTTGAT
CATTTTATTGAAATTGGTCCTGGAAAAGTTTTATCTGGCTTAATTAAAAAAATAAATAGAGATGTTAAGTTAACATCAATTCAAACT
TTAGAAGATGTGAAAGGATGGAATGAAAATGACTAAGAGTGCTTTAGTAACAGGTGCATCAAGAGGAATTGGACGTAGTATTGCGT
TACAATTAGCAGAAGAAGGATATAATGTAGCAGTAAACTATGCAGGCAGCAAAGAGAAAGCTGAAGCAGTAGTCGAAGAAATCAA
AGCTAAAGGTGTTGACAGTTTTGCGATTCAAGCAAATGTTGCCGATGCTGATGAAGTTAAAGCAATGATTAAAGAAGTAGTTAGCC
AATTTGGTTCTTTAGATGTTTTAGTAAATAATGCAGGTATTACTCGCGATAATTTATTAATGCGTATGAAAGAACAAGAGTGGGATG
ATGTTATTGACACAAACTTAAAAGGTGTATTTAACTGTATCCAAAAAGCAACACCACAAATGTTAAGACAACGTAGTGGTGCTATC
ATCAATTTATCAAGTGTTGTTGGAGCAGTAGGTAATCCGGGACAAGCAAACTATGTTGCAACAAAAGCAGGTGTTATTGGTTTAACT
AAATCTGCGGCGCGTGAATTAGCATCTCGTGGTATCACTGTAAATGCAGTTGCACCTGGTTTTATTGTTTCTGATATGACAGATGCTT
TAAGTGATGAGCTTAAAGAACAAATGTTGACTCAAATTCCGTTAGCACGTTTTGGTCAAGACACAGATATTGCTAATACAGTAGCGT
TCTTAGCATCAGACAAAGCAAAATATATTACAGGTCAAACAATCCATGTAAATGGTGGAATGTACATGTAATATATTTGAGCTAAA
GCTCAAGATAAAGACGTAGTAATCAATAAATTGATAACTGATAAGCGGAACGCCTGAGCTAAAGCTAATTGACGCAGTGGTTGACT
GGTCATCCAATGGAGAATTGCCTGACCTAGTCAACTTTGCGGGGGAAATTCTAAGCAACCTAGATAAGGTTCGAGAATTTCTCCCTA
AGAAACACTAATCAATAAATTGATAAGAAGATTAGAGGAACGCTTGAGCTAAAGCTCATGCATAAGAAACACTAATCAATAAATTG
ATAACTGGGATAGAGGTACGCCTGAGCTAAAGCTCATGCATAAGAAACACTAATCAATAAATTGATAAGTGTTTCTAAAATTTCTA
CTTGTTTTTTAGAATTTAAAATGGGAAAATATAATAGTCTATGTATAGGCATTTTTAAAGGAGGTGAATCGACGTGGAAAATTTCGA
TAAAGTAAAAGATATCATCGTTGACCGTTTAGGTGTAGACGCTGATAAAGTAACTGAAGATGCATCTTTCAAAGATGATTTAGGCG
CTGACTCACTTGATATCGCTGAATTAGTAATGGAATTAGAAGACGAGTTTGGTACTGAAATTCCTGATGAAGAAGCTGAAAAAATC
AACACTGTTGGTGATGCTGTTAAATTTATTAACAGTCTTGAAAAATAATAAATCTTACATCTGGGTCGTCAGTATTGTCGACTCAGTT
TTTTTCTTTAATTATCAATAGTTTTAACGTAAAATTAAAGATGATTCAAGAGCAACACATAAAGGAGATAAAATAATGTCTAAACAA
AAGAAAAGTGAGATAGTTAATCGTTTTAGAAAGCGCTTTGATACTAAAATGACAGAGTTAGGCTTTACTTATCAAAATATTGATTTA
TACCAACAAGCATTTTCGCATTCGAGTTTTATTAATGATTTTAATATGAATCGTTTAGACCATAATGAGCGTTTAGAGTTTTTGGGTG
ATGCGGTATTAGAATTGACGGTTTCACGATATTTATTTGATAAACATCCCAACTTGCCAGAAGGGAATTTAACAAAAATGCGTGCCA
CTATTGTATGTGAGCCCTCACTTGTAATATTTGCGAATAAAATTGGATTGAACGAAATGATTTTACTTGGTAAAGGTGAAGAGAAAA
CAGGGGGACGTACAAGACCATCATTAATATCAGATGCATTCGAAGCATTTATTGGGGCATTGTATTTGGATCAAGGACTAGATATA
GTTTGGAAATTTGCTGAGAAAGTCATTTTCCCACATGTAGAACAAAATGAGTTATTAGGCGTGGTAGATTTTAAAACACAATTCCAA
GAATATGTGCACCAGCAAAATAAAGGTGATGTAACCTATAATTTAATAAAAGAAGAGGGACCGGCACATCATCGTCTATTCACTTC
AGAAGTTATTCTGCAAGGGGAAGCAATAGCTGAAGGTAAAGGGAAAACGAAAAAAGAATCAGAACAACGTGCTGCTGAAAGTGCC
TATAAGCAATTAAAACAAATTAAATAGAAATTTATATGATACGACATATAGGTGTTCGATTTAGGATTTAAGGAAGAATTTTTAATA
TTTTATGGAACAACTTCGAAATTTAATATACAAATACTAAAATATCGTATACATGTGTTCTTTAAAATTGTGATAAGGAGTTTAGGA
TGGTTTATTTAAAATCAATAGATGCCATTGGATTTAAGTCTTTTGCAGATCAAACCAATGTTCAATTCGATAAAGGTGTAACTGCAA
TTGTTGGTCCAAATGGAAGCGGTAAAAGTAATATTACAGATGCTATTAAATGGGTGTTGGGTGAACAATCGGCTAAATCATTACGT
GGCTCAAAAATGGAAGATATTATCTTCTCAGGTGCAGAACATCGCAAAGCTCAAAATTATGCTGAAGTACAGTTAAGATTAGATAA
TCATTCTAAAAAGCTCAGTGTTGATGAAAACGAAGTTATTGTAACAAGAAGATTGTATCGAAGTGGTGAAAGTGAGTACTACATAA
ATAATGACCGTGCAAGATTAAAAGATATTGCCGATTTATTTTTAGATTCTGGATTGGGAAAAGAAGCGTATAGCATTATCTCGCAAG
GTAGAGTTGATGAAATACTAAATGCTAAACCAATTGATAGACGTCAAATTATTGAAGAATCGGCTGGTGTACTTAAATATAAAAAA
CGTAAGGCTGAATCATTAAATAAACTTGACCAAACAGAAGATAATTTAACGAGAGTAGAAGACATTTTATATGATTTGGAAGGTCG

CGTAGAACCTCTAAAAGAGGAGGCAGCTATAGCTAAAGAATATAAGACACTTTCACATCAAATGAAACATAGTGACATTGTAGTTA
CAGTGCACGATATTGATCAATATACAAATGACAATAGACAATTAGATCAACGTTTAAATGATTTACAAGGTCAACAAGCAAATAAA
GAAGCTGACAAGCAACGTTTAAGCCAACAAATTCAACAATATAAAGGTAAACGACATCAACTTGATAACGATGTTGAATCGCTTAA
TTATCAATTAGTAAAAGCTACGGAAGCCTTTGAAAAATATACGGGACAATTAAATGTTTTAGAAGAACGTAAGAAAAATCAATCTG
AAACAAATGCACGATATGAAGAAGAACAAGAAAATTTAATGGAGCTTTTAGAAAATATATCAAATGAGATTTCTGAAGCTCAAGAT
ACTTATAAGTCTCTGAAAAGTAAACAAAAAGAACTCAATGCTGTCATTCGTGAACTTGAAGAACAACTATATGTTTCAGACGAAGC
ACATGATGAAAAATTGGAAGAAATTAAAAACGAATACTATACATTAATGTCAGAGCAATCAGATGTTAACAATGATATTCGTTTTT
AAAGCATACTATAGAAGAGAATGAGGCTAAAAAATCAAGACTAGATTCTCGATTAGTTGAAGTTTTGAGCAATTGAAAGATATTC
AGGGTCAAATAAAAACGACAAAAAAAGAATATCAACAGACCAACAAAGAACTTTCTGCTGTAGATAAAGAAATTAAAAATATAGA
AAAATATCTCACTGATACAAAAAAAGCACAAAATGAATACGAAGAGAAATTGTATCAAGCATATCGATATACCGAAAAAATGAAA
ACACGTATTGATAGTTTGGCAACGCAAGAGGAAGAATATACTTATTTTTTCAATGGCGTCAAACATATTTTGAAAGCTAAAAATAAA
GAATTAAAGGGTATTCATGGTGCAGTTGCGGAAATTATTGATGTGCCATCTAAATTAACTCAGGCAATTGAAACAGCATTAGGTGCT
TCATTACAACATGTCATTGTAGATTCAGAAAAAGATGGACGCCAGGCTATTCAATTTTTAAAAGAACGTAATTTAGGTCGTGCGACG
TTTTTACCATTAAATGTTATACAGAGTAGAGTGGTAGCGACTGATATTAAATCTATTGCTAAAGAGGCAAACGGATTTATTAGTATC
GCTTCGGAAGCAGTTAAAGTAGCACCAGAATATCAAAATATTATCGGGAATTTATTAGGTAATACGATTATCGTTGATCATTTAAAG
CATGCAAATGAATTGGCACGTGCGATTAAATATCGAACTCGTATTGTTACTTTGGAAGGTGATATTGTAAATCCTGGTGGTTCTATG
ACTGGTGGTGGCGCTCGTAAGTCCAAAAGTATTCTGTCTCAAAAAGACGAGTTGACAACAATGAGACACCAATTAGAAGATTACTT
GCGTCAAACAGAATCATTTGAACAACAATTTAAAGAGTTGAAGATAAAAAGTGATCAATTAAGTGAACTGTATTTTGAAAAAAGTC
AAAAGCATAATACACTTAAAGAGCAAGTGCATCATTTTGAAATGGAGCTCGATAGATTAACTACACAAGAAACACAAATAAAAAA
TGATCATGAAGAATTCGAATTTGAAAAAAATGATGGTTATACGAGTGACAAAAGTCGACAAACTTTGAGTGAAAAAGAAACTCATT
TAGAAAGTATTAAAGCATCTTTAAAACGACTAGAAGATGAAATTGAACGCTACACAAAACTTTCTAAAGAAGGTAAGGAAAGCGT
AACTAAAACACAACAAACGTTACATCAGAAACAATCTGATCTTGCTGTGGTTAAAGAGCGTATTAAAACACAACAACAGACAATAG
ATCGATTAAATAATCAAAGTCAACAAACTAAACATCAATTAAAAGATGTTAAAGAAAAAATTGCATTCTTTAATTCGGATGAAGTG
ATGGGCGAACAAGCTTTTCAAAATATTAAAGATCAAATTAATGGTCAACAAGAAACGAGAACACGCTTATCTGATGAATTAGATAA
ATTGAAACAACAACGTATTGAGTTGAATGAACAAATCGATGCGCAGGAAGCTACACTACAAGTTTGTCACCAAGATATTTTAGCTA
TCGAAAATCACTACCAAGATATTAAAGCTGAACAATCAAAGTTAGATGTATTAATTCATCATGCGATAGATCATTTAAATGATGAAT
ATCAATTGACTGTTGAACGTGCGAAATCTGAATATACGAGTGATGAATCGATTGACGCATTACGTAAAAAAGTTAAGTTAATGAAG
ATGTCGATTGATGAACTAGGTCCTGTAAACTTAAATGCAATTGAACAATTTGAAGAGTTAAATGAACGTTATACATTTTTAAGTGAA
CAACGTACAGATCTTCGTAAAGCTAAAGAAACATTAGAGCAAATTATAAGTGAAATGGATCAAGAGGTTACTGAAAGATTTAAAGA
AACTTTCCATGCTATTCAAGGACATTTTACAGCTGTGTTCAAACAATTGTTTGGTGGAGGCGATGCAGAATTGCAATTAACTGAAGC
CGATTATTTAACAGCTGGTATTGATATTGTGGTACAACCACCGGGTAAAAAGTTGCAACATTTATCGTTACTGAGTGGTGGTGAGCG
TGCATTAACTGCTATTGCTTTACTATTTGCAATTTTAAAAGTAAGATCTGCACCTTTTGTTATATTAGATGAGGTTGAAGCTGCACTA
GATGAAGCAAATGTTATTAGATACGCAAAATATTTAAATGAGTTATCAGACGAAACACAATTCATTGTTATTACACACCGTAAAGG
AACAATGGAATTTGCGGATAGGTTATACGGTGTAACAATGCAAGAATCAGGTGTTACTAAACTTGTGAGTGTGAATTTAAATACAA
TAGATGATGTGTTGAAGGAGGAGCAATAATGAGCTTTTTAAACGCTTAAAAGATAAGTTTGCAACAAATAAAGAAAATGAAGAAG
TTAAATCCTTAACAGAAGAACAAGGTCAAGACAAATTAGAAGATACACATTCTGAAAGTTCAACGCAGGACGCAAATGATTTAGCA
GAAAATGCTGAAGTGAAAAAGAAGCCACGCAAGTTGAGTGAAGCGGATTTTGATGACGATGGCTTAATATCAATTGAAGATTTTGA
AGAAATTGAAGCTCAAAAAATGGGTGCTAAATTTAAAGCAGGACTCGAAAAATCTCGTCAAAATTTCCAAGAACAATTAAATAATT
TGATAGCGAGATATCGTAAAGTAGATGAAGACTTTTTTGAAGCTTTAGAAGAAATGTTAATCACTGCAGACGTCGGTTTTAATACAG
TGATGACGTTAACTGAAGAATTACGTATGGAAGCACAACGACGTAATATTCAAGATACTGAAGATTTGCGTGAAGTCATTGTTGAA
AAAATCGTAGAGATTTACCATCAAGAAGATGATAATTCAGAAGCTATGAACTTAGAAGATGGTCGTTTAAATGTCATTTTAATGGTT
GGTGTGAATGGTGTTGGTAAAACAACAACAATTGGAAAATTAGCTTACCGATATAAAATGGAAGGTAAAAAAGTAATGTTAGCTGC
GGGCGATACTTTTAGAGCGGGTGCTATTGATCAATTGAAAGTTTGGGGCGAACGTGTTGGTGTAGACGTAATTAGCCAAAGTGAAG
GTTCTGATCCAGCTGCTGTTATGTATGATGCGATTAATGCCGCTAAAAACAAAGGTGTTGATATTTTAATCTGTGATACCGCTGGAC
GTTTACAAAATAAAACAAATCTAATGCAAGAATTAGAAAAAGTTAAGCGTGTAATTAATCGAGCAGTGCCAGATGCGCCTCATGAA
GCATTACTATGTTTAGATGCTACAACTGGTCAGAATGCGTTGTCACAAGCTAGAAACTTTAAAGAAGTAACAAATGTTACAGGTATT
GTATTAACGAAATTAGATGGTACAGCCAAAGGTGGTATCGTATTAGCCATTCGTAATGAATTGCACATTCCAGTTAAATATGTAGGT
TTAGGTGAGCAATTAGATGACTTACAACCATTTAACCCTGAAAGTTATGTCTACGGCTTATTCGCTGATATGATTGAACAAAATGAA
GAAATAACAACAGTTGAAAATGATCAAATTGTAACAGAAGAAAAGGACGATAATCATGGGTCAAAATGATTTAGTTAAAACGTTA
CGAATGAATTATTTGTTTGATTTTTATCAATCCTTATTGACGAATAAACAACGTAATTATTTGGAATTATTTTATCTTGAAGATTATTC
TTTAAGTGAAATCGCAGATACTTTTAATGTGAGTAGACAAGCAGTTTATGATAATATAAGAAGAACTGGCGATTTAGTTGAAGATTA
TGAAAAGAAATTGGAATTATACCAGAAATTTGAGCAACGCCGAGAAATATATGATGAAATGAAACAACATTTAAGTAATCCAGAA
CAAATACAACGTTATATTCAACAATTAGAAGACTTAGAATAGATATAAATTGAGGGAGGACATCGATATGGCATTTGAAGGGTTAT
CAGAACGCTTGCAAGCGACGATGCAAAAAATGCGTGGTAAGGGTAAACTTACTGAAGCTGATATAAAGATAATGATGCGTGAAGT
AAGATTAGCGTTACTTGAGGCTGACGTAAACTTTAAAGTGGTAAAAGAATTTATTAAAACAGTATCAGAACGCGCATTAGGTTCCG
ATGTAATGCAATCATTAACACCAGGGCAACAAGTTATTAAAATAGTTCAAGATGAATTAACGCAGTTGATGGGTGGAGAAAATACG
TCGATTAATATGTCAAATAAACCACCTACTGTTGTTATGATGGTTGGTTTACAAGGTGCTGGTAAAACAACAACTGCAGGTAAATTA
GCATTATTGATGCGTAAAAAATACAACAAAAAACCTATGTTAGTTGCAGCAGATATTTATCGTCCAGCAGCGATAAATCAATTACA
AACAGTAGGGAAACAAATTGATATTCCTGTATACAGTGAAGGAGATCAAGTAAAGCCACAACAAATTGTAACTAATGCATTAAAAC
ATGCTAAAGAAGAACATTTAGACTTTGTAATCATTGATACAGCAGGTCGATTACACATCGATGAAGCATTGATGAACGAATTAAAA
GAAGTAAAAGACATTGCTAAACCAAACGAAATTATGTTAGTTGTCGATTCAATGACGGGTCAAGATGCTGTCAATGTTGCAGAATC
TTTTGACGATCAACTTGATGTCACAGGTGTTACCTTAACTAAATTAGATGGTGATACACGTGGTGGTGCAGCTTTATCTATTCGTTCG
GTGACACAAAAACCAATTAAATTTGTTGGTATGAGTGAAAAGTTAGATGGTTTAGAGCTATTCCATCCTGAACGTATGGCATCACGT
ATTTTAGGTATGGGTGATGTGTTAAGTTTAATTGAAAAAGCGCAACAAGATGTGGATCAAGAAAAAGCAAAAGATTTAGAGAAAA
AGATGCGTGAGTCATCGTTTACTTTAGATGATTTTTTAGAACAACTTGATCAGGTGAAAAATCTAGGACCACTGGATGATATTATGA
AAATGATTCCAGGTATGAATAAAATGAAAGGGCTAGATAAGCTTAATATGAGTGAAAAGCAAATTGATCATATTAAAGCGATTATC
CAGTCAATGACGCCGGCTGAAAGAAACAATCCAGACACATTGAATGTATCACGTAAAAAGCGTATTGCTAAAGGGTCTGGTCGTTC
ATTACAAGAAGTCAATCGTTTGATGAAACAATTTAACGATATGAAGAAAATGATGAAACAATTCACTGGTGGCGGTAAAGGTAAAA
AAGGTAAACGCAATCAAATGCAAAATATGTTAAAAGGTATGAATTTACCGTTTTAATGTTTAATATAACACGCTGATTTTTGTTGCA
ATGAGATGTGCATCCCGAAAGTTATACTAAAGAATATAACTTTATGGGGTGCATATTTTTAGATACACAATAATAATTAAAAACTGA
AATTGCGGATTTGCTAGAACATGAGGTTATTGTTGCTAATATTAATAAGTTATATTGGGGGAACGTGTGCGGTAGAAGTGGTACCAT

TCATGTCCTTACGGGATAAATTAACTAAACACGTATGTGGTGAAGGCTACTATGAATTTTTATAACCTATTAACAGTCTAATTTAGT
AATCACATAGTTATTATTGTAAGAAAAAGGTTCAATAATTTTCCTGGTAAAGAAAAAACTCTTTACAAACATTCACACACCTGTTAA
TATTATTTCTTGTAGAAAATAAAAATTAAAACATGACTTAAAGGAGATTTTATAAATGGCAGTTAAAATTCGTTTAACACGTTTAGG
TTCAAAAAGAAATCCATTCTATCGTATCGTAGTAGCAGATGCTCGTTCTCCACGTGACGGACGTATCATCGAACAAATCGGTACTTA
TAACCCAACGAGCGCTAATGCTCCAGAAATTAAAGTTGACGAAGCGTTAGCTTTAAAATGGTTAAATGATGGTGCGAAACCAACTG
ATACAGTTCACAATATCTTATCAAAAGAAGGTATTATGAAAAAATTTGACGAACAAAAGAAAGCTAAGTAATTTAGCGTAAAATTG
TTCTAACAATAAGAATAACTCGTTTACACTGACAGTTATTACTCAATGATACGTTGGGAATATCACATGTTAGTAATATAGAACGTA
TTGGGTACCATAATGGTGCCCTTTTTCTTTGAATTATTTTCAATTAAAATAGAAGTGTTCAAAGCATAGAGTTGGAGGTAATAGAAT
GAGAGTTGAAGTTGGTCAAATTGTTAACACACATGGTATTAAAGGTGAAATTAAAGTAAAATCCAATTCAGACTTTACAGACGTTC
GTTTTCAACCCGGTCAAGTGCTGACAGTTGTGCATAACAATAACGACCTTGAATATACTGTTAAGTCACATAGAGTGCATAAAGGGC
TTCATATGCTTACATTTGAAGGTATTAATAATATTAATGATATTGAGCATTTAAAAGGGAGTTCTATTTATCAAGAGCGTGATCATG
AAGATATCGTACTTGAGGAAAATGAATTTTATTATTCAGATATTATAGGATGTACAGTTTTTGATGATCAAGAAACACCAATAGGTC
GTGTAATTAATATATTTGAAACAGGTGCGAATGATGTGTGGGTGATTAAAGGATCTAAAGAATATTTGATTCCTTATATTGCTGATG
TTGTAAAAGAAGTGGATGTTGAAAATAAAAAAATTATCATCACGCCAATGGAAGGATTGTTGGATTAATGAAAATTGATTATTTAA
CTTTATTTCCTGAAATGTTTGATGGTGTTTTAAATCATTCAATTATGAAACGTGCCCAAGAAAACAATAAATTACAAATCAATACGG
TTAATTTTAGAGATTATGCAATTAACAAGCACAACCAAGTAGATGATTATCCGTATGGTGGCGGACAAGGTATGGTGTTAAAGCCT
GAACCTGTTTTTAATGCGATGGAAGACTTAGATGTCACAGAACAAACACGCGTTATTTTAATGTGTCCACAAGGCGAGCCATTTTCA
CATCAGAAAGCTGTTGAATTAAGCAAGGCCGACCACATCGTTTTCATATGCGGACATTATGAAGGTTACGATGAACGTATCCGAAC
ACATCTTGTCACAGATGAAATATCAATGGGTGACTATGTTTTAACTGGTGGAGAATTGCCAGCGATGACCATGACTGATGCTATTGT
TAGACTGATTCCAGGTGTTTTAGGTAATGAACAGTCACATCAAGACGATTCATTTTCAGATGGGTTATTAGAGTTTCCGCAATATAC
ACGTCCGCGTGAATTTAAGGGTCTAACAGTTCCAGATGTTTTATTGTCTGGAAATCATGCCAATATTGATGCATGGAGACATGAGCA
AAAGTTGATCCGCACATATAATAAAAGACCTGACTTAATTGAAAAATATCCATTAACTAATGCAGATAAGCAAATATTAGAAAGAT
ATAAAATAGGATTGAAAAAAGGTTAGTATTGTGCTAACATAATCTGAGTGTGGAAACACAAAAATCACTATGATCCGCTGCTATAT
ATTTGTCGAGGCAAGAACATAGGTTGAAGAGGAGAATTTATAATGACAAATCACAAATTAATCGAAGCAGTAACTAAATCACAATT
ACGTACAGACTTACCTAGTTTCCGTCCTGGTGATACTTTACGTGTACACGTACGTATCATTGAGGGTACTCGTGAGCGTATCCAAGT
ATTCGAAGGCGTTGTAATTAAACGTCGTGGCGGTGGCGTTTCTGAAACGTTTACAGTTCGTAAAATTTCATCAGGTGTTGGCGTGGA
ACGTACATTCCCATTACACACACCAAAAATTGAAAAAATCGAAGTTAAACGTCGTGGTAAAGTACGTCGTGCTAAATTATATTACTT
ACGTAGTTTACGTGGTAAAGCTGCTAGAATCCAAGAAATTCGTTAATCAGCATTTAAACAAAGCTATGTATGAGTCAAATTCGACTC
AAACAATAAAGCCATCTAGGTCACTTTTATGGAGTGATATAGATGGCTTTTTTTGCTGTTTAGTTAATGTAAAGTTGATAATGTAATT
GTTTATTCCTTCTCTATTCATAACGTGTTTGCTCAAAATGGTTATATAAACAAAAGGCTGGGACATAAATCAATGTTCTATCTCTACG
AAGTTATATTGGCAGTAGTTGACTGAACGAAAATACGCTTGTAACAAGCTTTTTTCAAT

>031-contig_247

AGAAGCTGGCGGAAAGTCAGCTTACAATAATGTGCAAGTTGGGGTGGGACGACGAAATAAATTTTGCGAAAATATCATTTCTGTCC
CACTCCCATTAAATATATGCGTAGCTACATTATTTTTGTCTTGCCCATCTCGTGAAAATAATACTACATATAATGCCAAATATAGTAA
TTGTTATAAGTAAATAATAGTATGGTGGGGCATAGCTCAATTGAATCTTTGTTATATTTTTAGGTGCTTTAATGCCGGTCATAACACC
ATTTACTTGTTCAACTTTAAGACTTTGATCACCTGATGTCGCTTTCATACCTTGATTATATGCTGTTGGCAAAACAATATACCCAGAT
GAATTTTTATTTTTAGTAATAGTATAACCTTGCTTTGTCTTACTAACTTTGACAGCCTCTAATGAATTTGAAGCGTCTTTAAGCGTGGT
ATAATCTTCGCCGTATATCCCTTTTAAATTTACACGATACTTACCTTTAGGCAATGATAATCTAATCCTATCTGAAGCTTTAATGCGT
ATTGTTACGGGTGTTACAAAGCGTCGATATTTATAAGTGAGTTTATTTCTTTCTTGTGTATATTCATTCACTTTAACATCATGAGCTTT
ATCCGGCGAAAGTAATTCTAAATCCATTTCAAAATACAAATCTTTAAATTGATTAGAAACTGATTTTGGCAACTGTACAGTTAGACC
ACCATTATTTTGTTTAACTTGTAATAAATGTTTTGTAGGAGATTGCCAGGCTGCACTATTTAATTTAATTGTTGAATCTGATAGTAAA
TTTTTATTGGCTTTAAAATGTGTATTAACATCTTTAGTCTTGTTAGAAACAATCCCTTGCAACATTGCTTGTTCTTTATCTAATGGAGA
TTTTAATTCTTTATTGGAAAAGACCTTATTTGTAATATGTGCACTTGGATAATGGATGGTATTTTTAGAATGAATCCAACGAACTTTA
TTGTCTTTGTGTTCAGACTTAATTTTAAATCCATATGGTAAGTTGTCATCATGATTCACTCTAATTCGATCATTAACATTCCAAAGTG
ATAGTAAATTTTGACGATTGCCAAGTAATCTATAAGTGCTGTTTTTATCGATTGGCATATTAATTTGGAGTGTCTTGTCATAATATTT
TAATATGTCTCCATTAAAAATACTAGAATATAATGAAATGCCATTATAATGATATATAAATGGTGAATTTAATGCATAGTCTGACAT
ATAATCAATGCGATTAAATGGACCAGTTGCATTTTGATTTATCTTTTTTATAAGCTGGTTTACATAGTTACTATGGTAATCGTGTTGT
TTCAACGTTGATAATGATTGTTGATAAGGTTTGATTGCCATATTTTTATTGTTATCTAAAATGACGATTTGTTGAATCATAACGATTA
ATACTAATATTGCAACGGTTAATTTTTTATAACGCCATAAACTAAATTTTAAAATAACGGCAAGCACCATTAGCAGGATAATACCTA
CTATAAGTGCAAGTGGGTGTGTCGGTGATAGTAATACATAAAGTAATGCGATGATGCTTACTGGTATTGTTCTGATTAAATAATATT
TCATATTTAATGTTGATAAATGTTGAATAAACAATCCGCAAAGAGCGCTTGATGATAGTGCTAAGATATACACCCAACGCCTTTCTG
GAAATGAAAAACCATTAAAAGCACTGTCGAAATACTGTGATAATGAACCAATAAATAATAACCATGTTACTATTGCGAAAAGTCTA
TAAAAGTAAAAACGATACAGTTTGAATGACAATAATGCAACGATAGTAAGAATTGAAATCGTAATATAAAATCCATCGCTAAAGAA
AAAATAATGGTAATCAAGTGGTGTCAAAAACGGTATATCAACATTGGGATTTTGCTTTCTGTCATTTTCCAAAAACGCAGAAATGCC
AGTGAATAAACCAAATACACTTGATAACACACTCAAAACTGTAGCAGATATGACGCAAATTAATTTTTGTGTTCTAGAGACAATGTC
ATATTTGTAAGTGAAAATGAGTCGATATAAATAGTAGCAACCTATAATAATAGCTTGATAATAACTGAAATAAAAATTGCTAAATA
GTGTTAAGGCTATCGCAACAATGAAAATACCGATTTTGCGTTGTTGAAAATATCTTTCCAAACCAAGAATCGATAATGGTAATAAAT
ATAATAAACTTCCATAAAATGACCAAGTAAAATTAAAGTATATAACGACAGTTGACATGCCGTATAAAATCGTAGCGATCATATTT
GTTGAGCGTTTAAAGTGTAATATTTTAAATAAGTAGAAGGTCACGACAAATGTTATGATAGCTCTTATCATGGCCATAATAAGTTGG
TTTGTCGGCCAAAAATGTATTGTTGTCGGATTAAATATACCAACCGTTTCTCCTATTTTAATGAATAGAAAATTTAGCCACATTAAAG
GTGACAGCGAATAATAATATGATAGTCCTTTCATATAATCGCCACCTAATCCAAACGATGCATCATATAAACTAGAAAAACTACTTA
AATGTTCATACAAATACATTTGAAATGGCATCATTTGACGGAATCCATCTCCAGCCCCACTAAAAACAGTACCATTCACAATATAAT
CATAGATATGAGTAGAAAATAAAATAAGCGTTAATATTACACTAATGAAAGTTATAACAAAGAATTGTTTGACGTTTGAATTTAGC
CACTTTTTTAACACAACATTATCCTCAACTTTCAAATTTAAAATTAAGTTTAACTGAAACTAAAGTTAATGAGGTTCTTGATAGGTAA
AGACGAAGATGACTGTGGAACAGATACCTTATCATAGTTACTTAAACTTTGGATCATTTTCAGTTTATCATTAAACAAATATATTGA
ATAATAAAAATGTCATACTGATAAAGATGAATGTCACTTAATAAGTAACTTAGATTTAACAAATGATGATTTTTAATTGTAGAAAAC
TTGAAATAATCACTTATACCTAAATCTAAAGCATTGTTAAGAAGTGTGACAATGTTAAAATAAATATAGTTGAATTAATGAATTTGT
TCTATAATTAACAGGTTATAGATTTTAATAATGAGAAAAGAATTGACGAAAGTAAGGTGAATTGAATGGTTATTCAATGGTATCCA
GGACATATGGCGAAAGCCAAAAGAGAAGTAAGTGAACAATTAAAAAAAGTAGATGTAGTGTTTGAACTAGTAGATGCAAGAATTC

CATATAGTTCAAGAAACCCTATGATAGATGAAGTTATTAACCAAAAACCACGTGTTGTTATATTAAATAAAAAAGATATGTCTAATT
TAAATGAGATGTCAAAATGGGAACAATTTTTTATTGATAAAGGATACTATCCTGTATCGGTGGATGCTAAGCACGGTAAAAATTTAA
AGAAAGTGGAAGCTGCAGCAATTAAAGCGACTGCTGAAAAATTTGAACGTGAAAAAGCAAAGGGACTTAAGCCAAGAGCGATTAG
AGCGATGATTGTTGGAATTCCAAATGTTGGTAAATCCACATTAATAAATAAACTGGCAAAGCGTAGTATTGCGCAGACTGGTAATA
AACCAGGTGTGACCAAACAACAACAATGGATTAAAGTTGGTAATGCATTACAACTATTAGACACACCAGGGATACTTTGGCCTAAA
TTTGAAGATGAAGAAGTCGGTAAGAAGTTGAGTTTAACAGGCGCGATTAAAGATAGCATTGTGCACTTAGATGAAGTAGCTATATA
TGGATTGAACTTTTTAATTCAACATGATTTAGCGCGATTAAAGTCACATTATAATATTGAAGTTCCTGAAGATGCAGAAATCATAGC
GTGGTTTGATGCGATAGGGAAAAAACGTGGCTTAATTCGACGTGGTAATGAAATTGATTACGAAGCAGTCATTGAACTGATTATTTA
TGATATTCGAAATGCTAAAATAGGAAATTATTGTTTTGATATTTTTAAAGATATGACTGAGGAATTAGCAAATGACGCTAACAATTA
AAGAAGTTAAGCAGTTGATTAATGCGGTTAATACAATAGAAGAATTAGAAAATCATGAATGCTTTTTAGATGAGCGAAAAGGTGTT
CAAAATGCCATAGCTAGGCGCAGAAAAGCGTTAGAAAAAGAACAAGCTTTAAAAGAAAAGTATGTTGAAATGACTTACTTTGAAA
ATGAAATATTAAAAGAGCATCCTAATGCAATTATTTGTGGTATTGATGAAGTTGGAAGAGGACCTTTAGCAGGTCCAGTCGTTGCAT
GCGCAACAATTTTAAATTCAAATCACAATTATTTGGGCCTTGATGACTCGAAAAAAGTACCTGTTACGAAACGTCTAGAATTAAATG
AAGCACTAAAAAATGAAGTTACTGCTTTTGCATATGGTATCGCGACAGCTGAAGAAATAGATGAATTTAATATTTATAAAGCTACTC
AAATCGCCATGCAGCGAGCTATTGATGGATTATCAGTACAACCAACGCATTTATTGATAGACGCGATGACGCTTGATAATGCACTGC
CTCAAGTATCTTTAATCAAGGGTGATGCAAGAAGTGTATCTATTGCAGCGGCAAGTATCATGGCAAAGGTTTTTCGTGATGATTATA
TGACACAGTTATCTAAAGATTATCCTGAATATGGTTTTGAAAAAAACGCGGGTTACGGTACCAAACAACATTTACTAGCAATCGATG
ATATTGGCATTATGAAAGAGCATAGAAAAAGCTTTGAACCTATAAAATCGTTACTGTAAATCGTAAAATTCAATTAAATGATAAAA
AAGTAGCATTTAAGTTTACAATAGCGCTTACATTTTTTATAATTGTAAGGGAACTTAACCTAAGTAACAGGAGGATGGAAGATGAAT
ATCCACGAGTATCAAGGTAAAGAAATATTTCGTTCAATGGGCGTTGCAGTTCCAGAAGGACGAGTAGCATTTACTGCTGAAGAAGC
GGTGGAGAAAGCAAAAGAATTAAATTCTGATGTTTATGTTGTAAAAGCACAAATTCATGCTGGAGGTAGAGGTAAAGCAGGCGGA
GTAAAAATTGCTAAATCTTTATCTGAGGTAGAAACATATGCAAAAGAATTATTAGGGAAAACTTTGGTGACACATCAAACTGGTCC
AGAAGGTAAAGAAATTAAGCGTTTATATATCGAAGAAGGTTGTGCTATTCAAAAAGAATATTACGTTGGATTCGTTATTGATCGTGC
GACTGACCAAGTAACATTGATGGCGTCTGAAGAAGGGGGCACTGAGATTGAAGAAGTTGCTGCGAAGACTCCTGAAAAGATCTTCA
AAGAAACTATCGATCCAGTAATCGGACTTTCACCATTCCAAGCAAGACGAATTGCGTTTAATATTAATATTCCTAAAGAATCTGTTA
ACAAAGCAGCTAAATTCTTATTAGCACTTTATAATGTATTCATTGAAAAAGATTGTTCAATCGTAGAAATCAACCCATTAGTTACAA
CAGCTGATGGTGATGTATTGGCATTAGATGCTAAAATTAATTTTGATGATAATGCATTATTCAGACATAAAGATGTTGTAGAATTAC
GTGATTTAGAAGAAGAAGATCCGAAAGAGATTGAAGCGTCTAAACATGATTTATCATACATTGCATTAGATGGTGACATCGGATGT
ATGGTTAATGGTGCAGGTTTAGCCATGGCAACAATGGATACGATTAATCATTTCGGTGGAAACCCAGCCAATTTCTTAGATGCAGGC
GGAAGCGCTACTAGAGAAAAAGTAACTGAAGCATTTAAAATCATTTTAGGTGATGAAAATGTTAAAGGTATTTTTGTAAACATTTTC
GGTGGCATTATGAAATGTGATGTTATCGCAGAAGGTATCGTTGAAGCTGTAAAAGAAGTAGATTTAACTTTACCACTAGTTGTACGC
TTAGAAGGTACAAATGTTGAGTTAGGTAAAAAAATCTTAAAAGACTCAGGATTAGCAATTGAACCAGCAGCAACAATGGCTGAAG
GTGCACAAAAAATTGTTAAACTAGTCAAAGAAGCATAAGGAAAGGATGGGAGCACTAAGATGAGTGTATTTATAGATAAGAATAC
TAAAGTAATGGTACAAGGTATTACAGGGTCTACTGCCCTTTTCCATACAAAACAAATGCTTGATTATGGTACGAAAATAGTAGCAG
GTGTGACGCCTGGTAAAGGTGGTCAAGTTGTTGAAGGCGTTCCTGTTTTCAACACTGTTGAAGAAGCTAAAAATGAAACCGGGGCA
ACGGTTTCAGTCATTTACGTTCCAGCACCATTTGCTGCAGACTCAATTTTAGAAGCAGCTGATGCAGACTTAGATATGGTTATTTGTA
TCACTGAACATATTCCTGTATTAGACATGGTTAAAGTTAAACGCTACTTACAAGGTAGAAAAACACGTTTAGTTGGTCCAAACTGTC
CAGGTGTGATTACAGCAGATGAATGTAAAATTGGTATTATGCCTGGCTATATTCACAAAAAAGGTCATGTTGGTGTAGTATCTCGTT
CAGGTACATTAACATATGAAGCAGTGCACCAATTGACTGAAGAAGGTATTGGTCAAACTACAGCTGTTGGTATTGGTGGAGACCCA
GTCAACGGAACAAACTTTATTGATGTTTTAAAAGCATTCAATGAAGATGACGAAACGAAAGCAGTTGTTATGATTGGTGAAATCGG
TGGTACGGCTGAAGAAGAAGCAGCTGAATGGATTAAAGCGAATATGACAAAACCAGTTGTAGGCTTTATCGGTGGACAAACAGCA
CCTCCTGGAAAACGTATGGGACATGCTGGTGCAATCATTTCAGGTGGTAAAGGTACTGCTGAAGAGAAAATTAAAACATTAAATAG
TTGTGGTGTGAAAACAGCGGCAACACCTTCAGAAATTGGTTCAACATTAATTGAAGCTGCTAAAGAAGCAGGTATTTATGAAGCAT
TATTAACTGTTAATAAATAAAGTTAAAAGATGATATAAATGGCTATAGCCCATTCCATACTTTATAAAAGTATTGGAAATGGGCTAT
TTGTCGTGCGATGAATTTTAGTACACTTTTCATATTTTGGAAAGACAAGTTAATTATTTTACCTCCTACAATAGCTTTACAAAACAAA
GAAAATTTTAATTAAAATATATAGCGCACTATGTTATAATGTTTGTATATTATTGTAAGGAGTGTTTCATCATGAATAAACAACAAA
GTAAAGTACGCTATTCAATTAGAAAAGTTAGTATTGGAATTTTGTCAATTTCAATAGGTATGTTTTTGGCATTGGGTATGTCGAACA
AAGCATATGCAGATGAAATTGATAAATCTAAAGATTTTACAAGAGGGTATGAGCAAAATGTATTCGCGAAATCAGAGTTAAATGCT
AATAAAAATACGACAAAAGACAAAATAAAAAATGAAGGTGCTGTTAAAACATCGGACACAAGTTTAAAGTTAGACAACAAATCAG
CAATTTCAAACGGAAATGAAATTAATCAAGATATAAAGATTTCAAATACTCCGAAAAACTCAAGCCAAGGTAACAATCTAGTTATT
AATAACAATGAACCTACTAAAGAAATTAAAATTGCAAACTTGGAAGCTCAAAATTCTAATCAGAAGAAAACGAATAAAGTTACTAA
TAATTACTTTGGTTACTACAGTTTTAGAGAAGCTCCAAAAACACAAATCTATACTGTAAAAAAAGGAGACACACTTAGTGCTATAGC
ATTAAAATACAAAACTACAGTTTCAAATATTCAAAATACAAATAATATAGCAAATCCTAATTTAATATTTATTGGTCAAAAATTAAA
AGTGCCAATGACACCATTAGTAGAACCAAAACCAAAAACAGTGTCTTCAAATAATAAAAGTAATAGTAATAGCAGTACATTAAATT
ATTTGAAAACATTAGAGAATAGAGGATGGGATTTCGACGGTAGTTATGGATGGCAATGTTTCGATTTAGTTAATGTATATTGGAATC
ATCTTTATGGTCATGGATTAAAAGGATATGGAGCTAAAGATATACCATATGCAAATAATTTTAATAGTGAAGCTAAAATTTATCACA
ACACACCAACTTTCAAAGCTGAACCTGGGGACTTAGTGGTTTTTAGTGGAAGATTTGGTGGAGGATATGGTCATACAGCTATTGTCT
TAAATGGTGATTATGATGGAAAATTAATGAAGTTCCAAAGTTTAGATCAAAACTGGAATAATGGTGGATGGCGTAAAGCAGAGGTT
GCACATAAAGTTGTTCATAATTATGAAAATGATATGATTTTTATTAGACCATTTAAAAAAGCATAATTTAAATCGAAGGCAGGACAT
TGAAATATGAAATTTTCAACTTTAAGTGAAGAAGAATTTACCAACTACACCAAAAAGCACTTCAAACATTATACACAGTCTATAGA
ATTATATAATTATAGAAATAAAATAAATCATGAAGCACATATTGTGGGAGTGAAGAATGATAAAAATGAAGTTATAGCTGCATGTT
TATTAACAGAGGCACGAATTTTTAAATTCTACAAATATTTCTACTCTCATAGAGGTCCTTTACTTGATTATTTCGATGCTAAATTAGT
TTGTTACTTTTTTAAAGAATTATCTAAATTCATTTATAAAAATAGAGGAGTATTTATTCTTGTTGATCCATATTTAATAGAGAATTTA
AGAGATGCAAATGGTAGGATAATAAAGAATTATAATAATTCAGTGATAGTAAAGATGCTAGGGAAAATTGGGTATCTCCATCAAGG
TTATACAACAGGATATTCAAATAAAAGTCAAATTAGGTGGATTTCTGTATTGGATTTAAAAGATAAAGATGAGAATCAACTTTTAAA
AGAAATGGAATACCAAACTAGAAGAAATATAAAAAAGACTATTGAGATTGGTGTTAAGGTTGAAGATTTATCTATTGAAGAAACAA
ATCGATTTTATAAATTGTTTCAAATGGCTGAAGAAAAACATGGTTTTCATTTCATGAATGAAGATTATTTTAAACGAATGCAAGAAA
TATATAAAGATAAGGCAATGTTAAAGATAGCTTGTATAAATCTTAATGAATATCAAGATAAATTAAAAATACAATTATTGAAAATC
GAAAATGAAATGATGACTGTGAACAGAGCATTAAATGAAAATCCAAATTCTAAAAAAAATAAATCAAAATTAAATCAGTTAAATAT
GCAATTATCTAGTATTAATAATAGAATTAGTAAAACCGAAGAACTAATATTTGAAGATGGACCTGTTTTGGATTTAGCTGCTGCTTT
ATTTATATGTACTGATGATGAAGTTTATTATCTATCAAGTGGATCAAATCCGAAATATAATCAGTATATGGGTGCATATCATCTACA

ATGGCATATGATAAAATATGCAAAATCACATAATATTAATAGGTATAATTTTTATGGAATAACAGGCGTCTTTAGTAATGAGGCGG
ATGATTTTGGTGTTCAACAATTTAAAAAGGGTTTTAATGCACATGTTGAAGAATTAATTGGTGATTTCATCAAACCAGTAAGACCAA
TTCTATATAAATTTGCAAAACTTATTTATAAGGTTTAATTATAAAGTATGTTGGAAATTGAAATTTTAAATTCTTTCCAACATACTTT
TCACTTTTTAAGTCAGTATTTTTACTCCTTAAAATAACATTACATCGACAATGTTTTTGTATTAGAAATGTAAAACAAAAATAGAATA
TGATTTTGTATGATTACAAAAAGGAGTTGATTTATTGATTAAACTATTTTTGCTTAAGTTATACTGGGCACACTTTTCGACTAAACAA
ATTCATCAATTTTTAATGGCATATCCTAATGTAATTAAAGAGGAGGGAAGAAAAAAAGATAGTTATTTATGTGAATGGGTGAATAG
GGAAGAAAATGTTCATTTATTACGTAAATACTATGCTTTTATAAAACTTGATCATAACGATATTATTAAAGAACTGCAGAAATTAAA
AGTAAGTTACATTACATATATGGATTCTGAATACCCAGTGCTATTAAAAGAAATATATCAATTTCCATTACTTCTTTTCTATAAAGGG
AACATCAAATTAATAAATAATATGCATCATTTGGCAGTAGTAGGTGCAAGAGATTCTACAAGTTATACCCAACAGTCTTTAGAATTT
TTATTATCAAATGATAAAAGCAAATATTTAACAATTGTTTCCGGCCTTGCTCAAGGAGCTGATGCAATGGCACATCAAATAGCTTTA
AAATACAATCTCCCTACAATTGCAGTTTTAGCCTTTGGCCATCAAACACATTATCCCAAAAGTACATTAGCATTAAGAAATAAAATA
GAAGAAAAAGGTTTAGTTATATCCGAATATCCACCACATACACCAATTGCTAAATATAGATTTCCTGAGCGCAATAGAATTATCAGC
GGTTTGTCAAAAGGGGTTTTAATTACTGAGGCTAAGGAACAAAGTGGCAGTCACATCACGATAGATTTTGCATTAGAGCAAAATAG
AAATGTTTATGTTTTACCTGGATCTATGTTTAATCCTATGACAAAAGGTAATTTATTACGTATCCAAGAAGGTGCTAAGGTAGTATTA
AACGCTAATGATATATTTGAAGACTACTATATTTAAAACTAAAAATAATGAGTTGCTTCATATTTCTTTTGTTACCCTTTGTAATTTA
TTTTTTAATAAAATCTATAAAAAATAGACAGGGAAAATGATTTGTTTAGATATAAAACGTTGACAAAAGCAAAATTAAGCGTTTATC
ATTTATCTTTAGTAATTAGATTAGCGAGGGGGAAATGACATTGGCAGATAATTTAGTCATTGTTGAATCGCCTGCAAAAGCAAAAAC
CATTGAAAAGTATTTAGGTAAGAAATATAAAGTTATAGCTTCAATGGGACACGTCAGAGACTTACCAAGAAGTCAAATGGGTGTCG
ACACTGAAGATAATTACGAACCAAAATATATAACAATACGCGGAAAAGGTTCTGTTGTAAAAGAATTGAAAAAACATGCAAAAAA
AGCGAAAAACGTCTTTCTCGCAAGTGACCCCGACCGTGAAGGTGAAGCAATTGCTTGGCATTTATCAAAAATTTTAGAGCTTGAAG
ATTCTAAAGAAAATCGCGTTGTTTTCAACGAAATAACTAAAGACGCTGTTAAAGAAAGTTTTAAAAATCCTAGAGAAATTGAAATG
AACTTAGTCGATGCACAACAAGCGCGTCGAATATTAGATAGATTGGTTGGCTATAACATCTCGCCAGTTCTATGGAAAAAAGTAAA
AAAAGGTTTGTCAGCGGGTCGAGTTCAATCTGTTGCACTTCGTTTAGTCATTGACCGTGAAAATGAAATTCGAAACTTTAAACCAGA
AGAATATTGGACTATTGAAGGAGAATTTAGATACAAAAAATCAAAATTCAATGCTAAATTCCTTCATTATAAAAATAAACCTTTTAA
ATTAAAAACGAAAAAAGATGTTGAGAAAATTACAGCTGCACTAGATGGAGATCAATTCGAAATTACAAACGTGACTAAAAAAGAA
AAAACGCGTAATCCAGCAAACCCATTTACAACTTCTACATTACAACAAGAGGCGGCACGTAAATTAAACTTTAAAGCAAGAAAAAC
AATGATGGTCGCACAACAATTATATGAAGGTATAGATTTGAAAAAACAAGGTACGATTGGTTTAATAACATATATGAGAACCGATT
CTACACGTATTTCAGATACTGCCAAAGCTGAAGCAAAACAGTATATAACTGATAAATACGGTGAATCTTACACTTCTAAACGTAAA
GCATCAGGGAAACAAGGTGACCAAGATGCCCATGAGGCTATTAGACCTTCAAGTACTATGCGTACGCCAGATGATATGAAGTCATT
TTTGACGAAAGACCAATACCGATTATACAAATTAATTTGGGAACGATTTGTTGCTAGTCAAATGGCTCCAGCAATACTTGATACAGT
CTCATTAGACATAACACAAGGTGACATTAAATTTAGAGCGAATGGTCAAACAATCAAGTTTAAAGGATTTATGACACTTTATGTAG
AAACTAAAGATGATAGTGATAGCGAAAAGGAAAATAAACTGCCTAAATTAGAGCAAGGTGATAAAGTCACAGCAACTCAAATTGA
ACCAGCTCAACACTATACACAACCACCTCCAAGATATACTGAGGCGAGATTAGTAAAAACACTAGAAGAATTGAAAATTGGGCGAC
CATCAACTTATGCACCGACAATAGATACGATTCAAAAGCGTAACTATGTCAAATTAGAAAGTAAGCGTTTTGTTCCTACTGAGTTGG
GAGAAATAGTTCATGAACAAGTGAAAGAATACTTCCCAGAGATTATTGATGTGGAATTCACAGTGAATATGGAAACGTTACTTGAT
AAGATTGCAGAAGGCGACATTACATGGAGAAAAGTAATCGACGGTTTCTTTAGTAGCTTTAAACAAGATGTTGAACGTGCTGAAGA
AGAGATGGAAAAGATTGAAATCAAAGATGAGCCAGCCGGTGAAGACTGTGAAGTTTGTGGTTCTCCTATGGTTATAAAAATGGGAC
GCTATGGTAAGTTCATGGCTTGCTCAAACTTCCCGGATTGTCGTAATACAAAAGCGATAGTTAAGTCTATTGGTGTTAAATGTCCAA
AATGTAATGATGGTGACGTCGTAGAAAGAAAATCTAAAAAGAATCGTGTCTTTTATGGATGTTCGAAATATCCTGAATGCGACTTTA
TCTCTTGGGATAAGCCGATTGGAAGAGATTGTCCAAAATGTAACCAATATCTTGTTGAAAATAAAAAAGGCAAGACAACACAAGTA
ATATGTTCAAATTGCGATTATAAAGAGGCAGCGCAGAAATAATATTTTTATTTCCTAGATACATTTTAAGGTTGTTAAATAGAATCA
TTAGTGAATCTTATTTTAAAGATAGTAATGGATTAATCTAAATAAGTGCGGATAATATAAACATAACAACATAATTAAAAGACATA
AATGACAATAAAAGGAGTATAGAAATGACTCAAACTGTAAATGTAATAGGTGCTGGTCTTGCCGGTTCAGAAGCGGCATATCAATT
AGCTGAAAGAGGAATTAAAGTTAATCTAATAGAGATGAGACCTGTTAAACAAACACCAGCGCACCATACTGATAAATTTGCGGAAC
TTGTATGTTCCAATTCATTACGAGGAAATGCTTTAACTAATGGTGTGGGTGTTTTAAAAGAAGAAATGAGAAGATTGAATTCTATAA
TTATTGAAGCGGCTGATAAGGCACGAGTTCCAGCTGGTGGTGCATTAGCAGTTGATAGACACGATTTTTCAGGTTATATTACTGAAA
CACTTAAAAATCATGAAAATATCACAGTTATTAATGAAGAAATTAATGCCATTCCAGATGGATACACAATTATCGCAACAGGACCA
CTTACTACAGAAACCCTTGCGCAAGAAATAGTGGACATTACTGGTAAAGATCAACTTTATTTCTATGATGCGGCTGCTCCAATTATT
GAAAAAGAATCTATTGATATGGATAAAGTTTACTTAAAGTCCCGTTATGATAAAGGTGAAGCTGCATATTTAAACTGTCCTATGACT
GAGGATGAATTTAATCGCTTTTATGATGCAGTATTAGAAGCTGAAGTTGCGCCTGTAAATTCATTTGAAAAAGAAAAATATTTCGAG
GGTTGTATGCCTTTTGAAGTAATGGCAGAACGCGGACGCAAGACATTACTATTTGGACCAATGAAACCAGTAGGATTAGAAGATCC
AAAGACTGGGAAACGTCCTTATGCAGTGGTTCAATTAAGACAAGATGATGCTGCTGGCACACTCTACAATATTGTCGGCTTCCAAAC
GCATTTAAAATGGGGAGCTCAAAAAGAAGTTATTAAATTAATTCCAGGTTTAGAAAATGTTGATATTGTTAGATATGGTGTGATGCA
TAGAAATACCTTCATTAACTCACCGGACGTATTAAACGAGAAATATGAATTGATTTCACAACCAAACATACAGTTTGCGGGACAAA
TGACTGGTGTTGAAGGTTATGTAGAAAGCGCAGCTAGCGGCTTAGTTGCAGGTATCAATCTTGCGCATAAAATATTAGGCAAAGGT
GAGGTAGTATTTCCTAGAGAAACGATGATTGGAAGTATGGCTTACTATATTTCTCATGCTAAAAATAATAAGAATTTCCAACCTATG
AATGCTAACTTCGGGTTATTACCATCTTTAGAAACTAGAATTAAAGATAAAAAAGAACGCTATGAAGCACAAGCTAATAGAGCTTT
GGATTACTTAGAAAATTTCAAAAAAACTTTATAAAATAGTTAGAAAGACTAGATATGCTATTCATTCTTAAGTCATCAACGAGTAAG
TAATGACTTTCTAAATGGAAAATACTTATCCTAGTCTTTTTAATTTTGGAATTGTTACGTATTTCTGACAATTTAGAATTCGCATTCA
AAAAATATCTAAATAAATAACACGCAATAAGTTGATTGATGTAACATGTAAGAGAATGTTTTAAATAAACTTTATTTAAAAGGCAA
TGAAATAATAAATGGCAAGGCTATTAATAAAGACTTTTAGTAATTAATTTAAAAAAGAGGTATTCTAATTAACAGGTTTTCCGATTA
GTTACAATTATTTAATTCTCAAAAGATTTAGAATTGATTATCAAATTACTGTAAGCCCTTTGCTGTATATGCTACAATTCTTATTGAT
GGAGGGTAAATGTATTGAATCATATTCAAGATGCGTTTTTAAATACATTGAAAGTTGAACGGAATTTTTCGGAACACACATTGAAAT
CATATCAAGATGACTTAATTCAGTTTAATCAATTTTTAGAACAAGAACATTTAGAGTTGAATACTTTTGAATACAGAGATGCTAGAA
ATTATTTGAGCTATTTATATTCAAATCATTTGAAAAGAACATCTGTTTCTCGTAAAATCTCAACGTTAAGAACTTTCTATGAATATTG
GATGACGCTTGATGAGAACATTATTAATCCATTTGTTCAATTAGTACATCCGAAAAAAGAAAAATATCTTCCGCAATTCTTTTACGA
AGAAGAAATGGAAGCGTTATTCAAAACTGTAGAAGAGGACACTTCAAAAAATTTACGGGATCGAGTTATTCTTGAATTGTTGTATG
CTACAGGCATCCGTGTTTCGGAATTAGTAAATATTAAAAAACAAGATATAGATTTTTACGCGAATGGTGTTACCGTATTAGGAAAAG
GGAGCAAAGAGCGCTTTGTACCGTTTGGTGCTTATTGTAGACAAAGCATCGAAAATTATTTAGAACATTTCAAACCAATTCAGTCAT
GCAATCATGATTTTCTTATTGTAAATATGAAGGGTGAAGCAATCACTGAACGCGGTGTACGATATGTTTTAAATGATATTGTTAAAC
GAACAGCAGGCGTAAGTGAGATTCATCCCCACAAGCTCAGACATACATTTGCAACGCATTTATTGAATCAAGGTGCAGACCTAAGA

ACAGTACAATCGTTATTAGGTCATGTTAATTTGTCAACAACTGGTAAATATACACACGTATCTAACCAACAATTAAGAAAAGTGTAT
CTAAATGCACATCCTCGAGCGAAAAAGGAGAATGAAACATGAGTAATACAACATTACATGCAACAACAATTTATGCTGTAAGACAT
AATGGGAAAGCAGCTATGGCTGGAGATGGGCAAGTAACGCTTGGTCAACAAGTCATCATGAAACAAACGGCAAGAAAAGTGCGAC
GTTTATATGAAGGTAAAGTGTTAGCTGGTTTCGCAGGTAGTGTAGCAGATGCGTTTACGTTATTTGAAAAATTCGAAACAAAATTAC
AACAGTTTAGTGGTAACTTAGAAAGAGCTGCTGTTGAATTGGCACAAGAATGGCGAGGCGATAAACAATTACGTCAATTAGAAGCT
ATGCTAATTGTAATGGATAAAGATGCTATTTTAGTTGTCAGTGGAACTGGCGAAGTTATTGCCCCAGATGATGACCTTATCGCTATT
GGATCAGGAGGCAACTACGCATTAAGCGCAGGACGTGCATTGAAACGCCATGCATCGCATTTGTCTGCTGAAGAAATGGCATATGA
GAGCTTGAAAGTAGCGGCTGATATTTGTGTCTTTACCAACGATAATATTGTTGTTGAAACACTATAATAATCAGAGCACGATAAATA
ATTACGAGCAATTAATTTTAGTTAAAAGACGGAGGAATGAAATTAATGGATACAGCTGGAATAAGATTAACTCCAAAAGAAATCGT
ATCTAAATTAAATGAATACATCGTTGGACAAAATGATGCTAAACGTAAAGTGGCAATTGCCTTACGTAATCGATACAGAAGAAGTT
TATTAGATGAGGAATCAAAGCAAGAAATTTCACCTAAAAATATTTTGATGATTGGACCAACTGGCGTTGGTAAAACTGAAATTGCA
AGAAGAATGGCCAAAGTTGTCGGCGCGCCATTTATAAAAGTAGAAGCTACTAAATTTACTGAGGTAGGTTATGTAGGACGAGATGT
TGAAAGTATGGTTAGAGATCTTGTTGATGTTTCAGTAAGATTAGTCAAGGCACAGAAAAAAATCATTGGTACAAGATGAAGCAACAG
CTAAGGCCAATGAAAAACTTGTTAAGTTATTAGTTCCAAGTATGAAAAAGAAAGCGTCTCAAACGAATAATCCTTTAGAGTCACTTT
TCGGAGGTGCAATTCCAAATTTCGGACAAAATAACGAAGATGAAGAAGAACCACCTACTGAGGAAATTAAAACAAAACGTTCTGA
AATTAAGAGACAGCTAGAAGAAGGCAAACTTGAAAAAGAAAAGGTAAGAATTAAAGTCGAACAAGATCCTGGTGCTTTAGGTATG
CTAGGTACAAATCAAAATCAGCAAATGCAAGAGATGATGAATCAATTAATGCCTAAAAAGAAAGTTGAGCGAGAAGTTGCTGTTG
AGACGGCAAGGAAAATCTTAGCTGATAGTTATGCGGATGAACTAATTGATCAAGAAAGCGCTAACCAAGAAGCGCTTGAATTAGCA
GAACAAATGGGTATCATCTTTATAGATGAAATCGACAAAGTTGCGACGAATAATCATAATAGTGGTCAAGATGTCTCAAGACAAGG
TGTTCAAAGAGATATTTTACCTATACTTGAAGGTAGCGTTATTCAAACCAAATATGGTACTGTGAATACTGAACATATGCTGTTTAT
AGGTGCTGGAGCTTTCCATGTATCTAAGCCGAGTGACTTGATACCAGAATTGCAAGGTCGTTTTCCGATTAGAGTTGAACTTGATAG
TTTATCGGTAGAAGATTTTGTAAGAATTTTGACAGAACCAAAATTGTCATTAATTAAACAATATGAAGCATTGCTTCAAACAGAAGA
AGTTACTGTAAACTTTACCGATGAAGCAATTACTCGCTTAGCTGAGATTGCTTATCAAGTAAATCAAGATACAGACAACATTGGTGC
ACGTCGACTTCATACAATTTTAGAAAAGATGCTAGAAGATTTATCATTCGAAGCACCAAGTATGCCGAATGCAGTTGTAGATATTAC
CCCACAATATGTTGATGATAAATTAAAATCAATTTCAACAAATAAAGATTTAAGTGCATTTATTCTATAAAAATATACAAAAGGAG
AAAAATTCATGAGCTTATTATCTAAAACGAGAGAGTTAAACACGTTACTTCAAAAACACAAAGGTATTGCGGTTGATTTTAAAGAT
GTAGCACAAACGATTAGTAGCGTAACTGTAACAAATGTATTTATTGTATCGCGTCGAGGTAAAATTTTAGGATCGAGTCTAAATGAA
TTATTAAAAAGTCAAAGAATTATTCAAATGTTGGAAGAAAGACATATTCCAAGTGAATATACAGAACGATTAATGGAAGTTAAACA
AACAGAATCAAATATTGATATCGACAATGTATTAACAGTATTCCCACCTGAAAACAGAGAATTATTCATAGATAGTCGTACAACTAT
CTTCCCAATTTTAGGTGGAGGGGAAAGATTAGGTACATTAGTACTTGGTCGAGTACATGATGATTTTAATGAAAATGATTTGGTACT
AGGTGAATATGCTGCTACAGTTATTGGTATGGAAATCTTACGTGAGAAGCATAGTGAAGTAGAAAAAGAAGCGCGCGATAAAGCTG
CTATTACAATGGCAATTAATTCATTATCTTATTCTGAAAAAGAAGCGATTGAACATATCTTTGAAGAACTTGGCGGTACGGAAGGCC
TATTAATCGCATCAAAAGTTGCAGATAGAGTTGGTATTACTAGATCTGTAATTGTAAATGCACTACGTAAATTAGAAAGTGCTGGTG
TAATTGAATCACGTTCTTTAGGAATGAAAGGTACTTTCATTAAAGTTAAAAAAGAAAAATTCTTAGATGAATTAGAAAAAAGTAAA
TAAGTCGATGAGTCTGGGACATAATTCAATGTCCTAGTCGTGACGATGTTATCTTGGCGGTAGTCAATGAAATGACTTTGATGATTA
TGTTGAAGTCATTTCTTTATTTTTGTCTAATTTTCAATCAAATTGATATTTCACATTAACTTGATAGAATTACCTTTAAATTTATGGAA
AAATGCTTGAAAAACATTTGCTTTAGACACGGCATTATGTTATATTTATTTTCGGCTATAAAGCCAATACACACATATCAAACGTGA
TTGTGTAAAGGGTGCAATATTTATATATTGTCTTTACAATAGTTTGCTATGGAGGTAATTAACCAATAGGAGGAATTTATAATGGCA
GTAATTTCAATGAAACAATTACTAGAAGCGGGTGTTCACTTCGGTCACCAAACACGTCGTTGGAACCCAAAAATGAAAAAATATAT
CTTCACTGAGAGAAATGGTATTTATATCATCGACTTACAAAAAACAGTGAAAAAAGTAGACGAGGCATACAACTTCTTGAAACAAG
TTTCAGAAGATGGTGGACAAGTCTTATTCGTAGGAACTAAAAAACAAGCACAAGAATCAGTTAAATCTGAAGCAGAACGTGCTGGT
CAATTCTACATTAACCAAAGATGGTTAGGTGGATTATTAACTAACTATAAAACGATCTCAAAACGAATCAAACGTATTTCTGAAATT
GAAAAAATGGAAGAAGATGGTTTATTCGAAGTATTACCTAAAAAAGAAGTAGTAGAACTTAAAAAAGAATACGACCGTTTAATCA
AATTCTTAGGCGGAATTCGTGATATGAAATCAATGCCTCAAGCATTATTCGTAGTTGACCCACGTAAAGAGCGTAATGCAATTGCTG
AAGCTCGTAAATTAAATATTCCTATCGTAGGTATCGTTGACACTAACTGTGATCCTGACGAAATTGACTACGTTATCCCAGCAAACG
ACGATGCTATCCGTGCGGTTAAATTATTAACTGCTAAAATGGCAGATGCAATCTTAGAAGGTCAACAAGGCGTTTCTAATGAAGAA
GTAGCTGCAGAACAAAACATCGATTTAGATGAAAAAGAAAAATCAGAAGAAACAGAAGCAACTGAAGAATAATCAACTGTTGAAT
CTGACTTAGATATAGTTTAAATGGGTGATAAGATATTAATGCTTATCACCTTTTTTAAAAAGAAAATCGAGGCAAATTACAAATATT
CAATTAGAGTATTGGCAATCTTGCCTATAATAATGCTAAAATCATAATATATAAAATGATAACTTATTGGAGGAATAATGAATGGCA
ACTATTTCAGCAAAACTTGTTAAAGAATTACGTGAAAAAACTGGCGCGGGTATGATGGATTGTAAAAAAGCGCTAACTGAAACTGA
TGGTGACATCGATAAAGCGATTGACTACCTACGTGAAAAAGGTATTGCTAAAGCAGCTAAAAAAGCAGACCGTATTGCGGCTGAAG
GTTTAGTACATGTAGAAACTAAAGGTAACGACGCAGTTATCGTTGAAATCAACTCTGAAACAGACTTTGTTGCTCGTAACGAAGGTT
TCCAAGAGTTAGTTAAAGAAATCGCTAATCAAGTATTAGATACAAAAGCTGAAACTGTTGAAGCTTTAATGGAAACAACTTTACCA
AATGGTAAATCAGTTGATGAAAGAATTAAAGAAGCAATTTCAACAATCGGTGAAAAATTAAGTGTTCGTCGTTTTGCTATCAGAAC
TAAAACTGATAACGATGCTTTCGGCGCTTACTTACACATGGGTGGACGCATTGGTGTATTAACAGTTGTTGAAGGTTCAACTGACGA
AGAAGCAGCAAGAGACGTTGCTATGCATATCGCTGCAATCAACCCTAAATATGTTTCTTCTGAACAAGTTAGCGAAGAAGAAATCA
ACCACGAAAGAGAAGTTTTAAAACAACAAGCATTAAATGAAGGTAAACCAGAAAACATCGTTGAAAAAATGGTGGAAGGACGTTT
ACGTAAATACTTACAAGAAATTTGTGCTGTAGATCAAGACTTCGTTAAAAACCCTGATGTAACAGTTGAAGCTTTCTTAAAAACAAA
AGGTGGAAAACTTGTTGACTTCGTACGCTATGAAGTAGGCGAAGGTATGGAAAAACGCGAAGAAAACTTTGCGGATGAAGTTAAA
GGACAAATGAAATAATCTGTCATAAAGTAAAACAAGGAAGAAGACACCTTTAATGTTGCTTTATTAAAATGTAAATCATTCTAATA
AAACGACAACTGTGTCTTCTTTACTTGTATATGTTACATATATTCACGATAGAGAGGATAAGAAAATGGCTCAAATTTCTAAATATA
AACGTGTAGTTTTGAAACTAAGTGGTGAAGCGTTAGCTGGAGAAAAAGGATTTGGCATAAATCCAGTAATTATTAAAAGTGTTGCT
GAGCAAGTGGCTGAAGTTGCTAAAATGGACTGTGAAATCGCAGTAATCGTTGGTGGCGGAAACATTTGGAGAGGTAAAACAGGTA
GTGACTTAGGTATGGACCGTGGAACTGCTGATTACATGGGTATGCTTGCAACTGTAATGAATGCCTTAGCATTACAAGATAGTTTAG
AACAATTGGATTGTGATACACGAGTATTAACATCTATTGAAATGAAGCAAGTGGCTGAACCTTATATTCGTCGTCGTGCAATTAGAC
ACTTAGAAAAGAAACGCGTAGTTATTTTTGCTGCAGGTATTGGAAACCCATACTTCTCTACAGATACTACAGCGGCATTACGTGCTG
CAGAAGTTGAAGCAGATGTTATTTTAATGGGCAAAAATAATGTAGATGGTGTATATTCTGCAGATCCTAAAGTAAACAAAGATGCG
GTAAAATATGAACATTTAACGCATATTCAAATGCTTCAAGAAGGTTTACAAGTAATGGATTCAACAGCATCCTCATTCTGTATGGAT
AATAACATTCCGTTAACTGTTTTCTCTATTATGGAAGAAGGAAATATTAAACGTGCTGTTATGGGTGAAAAGATAGGTACGTTAATT
ACAAAATAAATTTAGAGGTGTAAAATAATGAGTGACATTATTAATGAAACTAAATCAAGAATGCAAAAATCAATCGAAAGCTTATC
ACGTGAATTAGCTAATATCAGTGCAGGAAGAGCTAATTCAAATTTATTAAACGGCGTAACAGTTGATTACTATGGTGCACCAACAC

CTGTACAACAATTAGCAAGCATCAATGTTCCAGAAGCACGTTTACTTGTTATTTCTCCATACGACAAAACTTCTGTAGCTGACATCG
AAAAAGCGATAATAGCGGCTAACTTAGGTGTCAACCCAACAAGTGATGGTGAAGTGATACGTATTGCAGTACCTGCCTTAACAGAA
GAGCGTAGAAAAGAGCGCGTTAAAGATGTTAAGAAAATTGGTGAAGAAGCTAAAGTATCTGTTCGAAATATTCGTCGTGATATGAA
TGATCAGTTGAAAAAAGATGAAAAAAATGGCGACATTACTGAAGATGAGTTGAGAAGTGGTACTGAAGATGTTCAGAAAGCAACA
GACAATTCAATAAAAGAAATTGATCAAATGATTGCTGATAAAGAAAAAGATATTATGTCAGTATAAAACTAATATACAATGACATA
TTAAAATGCCAGTATTAAACGATAATGTAACATTTAAAATGGGCATGTTTAATTAAATCAAAGATGCATGTGATAATTTAAATTCAC
AATGAGCATAAAAATGGTGTTTAAACAAGTTAATTAAACATATACTTTATAAATAATAGGCATTAGGTATATTGCTATAATAAAGTT
ATGTAATTTTTAACCTCAGTATGTATGTCACATTTCTGGTGTAAACTGTACCGAGTCAGACTTTGGTACAGTTTTTTATTTGCTTATT
CAATGCATTAAATGAGTATGATAAAATGATAATGATTGTTTAGTAACTTATACTATATGACAGAGATGATCAGGCTCGGAGGAAAG
ACCATGTTTAAAAAGCTAATAAATAAAAAGAACACTATAAATAATTATAATGAAGAATTAGACTCGTCTAATATACCTGAACATAT
CGCTATTATTATGGATGGTAATGGGCGATGGGCTAAGAAGCGAAAAATGCCTAGAATTAAAGGTCATTACGAAGGTATGCAAACAA
TAAAAAAAATTACTAGGGTAGCTAGTGATATTGGTGTTAAGTACTTAACTTTATACGCCTTTTCCACTGAAAATTGGTCAAGACCTG
AAAGTGAAGTAAATTATATTATGAATTTGCCTGTCAATTTCTTAAAGACATTCTTACCGGAACTAATTGAAAAAAATGTCAAAGTTG
AAACAATTGGATTTACTGATAAGTTGCCAAAATCAACGATAGAAGCAATTAATAATGCTAAAGAAAAGACAGCTAATAATACCGGC
TTAAAATTAATATTTGCAATTAATTATGGTGGCAGAGCAGAACTTGTTCATAGTATTAAAAATATGTTTGACGAGCTTCATCAACAA
GGTTTAAATAGTGATATCATAGATGAAACATATATAAACAATCATTTAATGACAAAAGACTATCCTGATCCAGAGTTGTTAATTCGT
ACTTCAGGAGAACAAAGAATAAGTAATTTCTTGATTTGGCAAGTTTCGTATAGTGAATTTATCTTTAATCAAAAATTATGGCCTGAC
TTTGACGAAGATGAATTAATTAAATGTATAAAAATTTATCAGTCACGTCAAAGACGCTTTGGCGGATTGAGTGAGGAGTAGTATAG
TATGAAAGTTAGAACGCTGACAGCTATTATTGCCTTAATCGTATTCTTGCCTATCTTGTTAAAAGGCGGCCTTGTGTTAATGATATTT
GCTAATATATTAGCATTGATTGCATTAAAAGAATTGTTGAATATGAATATGATTAAATTTGTTTCAGTTCCTGGTTTAATTAGTGCAG
TTGGTCTTATCATCATTATGTTGCCACAACATGCAGGGCCATGGGTACAAGTAATTCAATTAAAAAGTTTAATTGCAATGAGCTTTA
TTGTATTAAGTTATACTGTCTTATCTAAAAACAGATTTAGTTTTATGGATGCTGCATTTTGCTTAATGTCTGTGGCTTATGTAGGCATT
GGTTTTATGTTCTTTTATGAAACGAGATCAGAAGGATTACATTACATATTATATGCCTTTTTAATTGTTTGGCTTACAGATACAGGGG
CTTACTTGTTTGGTAAAATGATGGGTAAACATAAGCTTTGGCCAGTAATAAGTCCGAATAAAACAATCGAAGGATTCATAGGTGGC
TTGTTCTGTAGTTTGATAGTACCACTTGCAATGTTATATTTTGTAGATTTCAATATGAATGTATGGATATTACTTGGAGTGACATTGA
TTTTAAGTTTATTTGGTCAATTAGGTGATTTAGTGGAATCAGGATTTAAGCGTCATTTCGGCGTTAAAGACTCAGGTCGAATACTACC
TGGACACGGTGGTATTTTAGACCGATTTGACAGCTTTATGTTTGTGTTACCATTATTAAATATTTTATTAATACAATCTTAATGCTGA
GAACAAATCAATAAACGTAAAGAGGAGTTGCTGAGATAATTTAATGAATCTCAGAACTCCTTTTGAAAATTATACGCAATATTAAC
TTTGAAAATTAGACGTTATATTTTGTGATTTGTCAGTATCATATTATAATGACTTATGTTACGTATACAGCAATCATTTTTAAAATAA
AAGAAATTTATAAACAATCGAGGTGTAGCGAGTGAGCTATTTAGTTACAATAATTGCATTTATTATTGTTTTTGGTGTACTAGTAACT
GTTCATGAATATGGCCATATGTTTTTTGCGAAAAGAGCAGGCATTATGTGTCCAGAATTTGCGATCGGTATGGGGCCGAAAATTTTT
AGTTTTAGAAAAAATGAAACACTTTACACTATTAGGTTATTGCCTGTTGGTGGATATGTTCGTATGGCAGGAGATGGCTTAGAAGAG
CCACCAGTCGAGCCCGGTATGAACGTTAAAATTAAACTTAATGAAGAAAATGAAATAACACATATCATATTAGATGATCATCATAA
GTTTCAACAAATTGAAGCGATCGAAGTTAAAAAATGTGATTTTAAGGATGACTTATTCATAGAAGGTATCACTGCTTATGATAATGA
AAGACATCATTTTAAAATTGCTAGAAAGTCTTTCTTTGTTGAAAATGGTAGCTTAGTTCAAATTGCTCCGAGAGACAGACAATTTGC
ACATAAAAAGCCATGGCCGAAATTTTTAACATTATTTGCGGGACCGTTATTTAACTTTATATTAGCTTTAGTCCTATTTATTGGTCTT
GCATATTATCAAGGCACGCCTACGTCTACTGTAGAACAAGTCGCAGATAAGTATCCAGCTCAACAAGCAGGATTACAAAAAGGTGA
TAAGATCGTCCAAATTGGCAAATATAAAATATCTGAATTTGATGATGTTGATAAGGCGTTAGATAAAGTTAAAGATAATAAGACGA
CTGTTAAATTTGAACGTGATGGTAAAACAAAGTCAGTTGAATTAACACCTAAAAAGACTGAAAAAAAACTGACTAAAGTAAGTTCA
GAGACGAAGTATGTTCTCGGATTCCAACCAGCGAGTGAACATACACTTTTTAAACCAATTGTATTCGGATTTAAAAGCTTTTTAATC
GGTAGTACTTATATTTTTACAGCTGTAGTAGGTATGTTGGCTAGTATATTTACGGGCGGATTCTCATTTGATATGTTAAATGGTCCGG
TTGGTATTTATCATAACGTCGACTCAGTTGTTAAAGCGGGTATCATTAGCTTAATTGGTTACACTGCGTTATTAAGTGTAAACTTAGG
TATTATGAATTTAATTCCTATTCCTGCACTAGACGGTGGTCGTATTTTATTTGTTATATATGAAGCGATTTTCAGAAAACCAGTTAAT
AAAAAAGCGGAAACAACGATTATTGCTATTGGTGCCATTTTCATGGTCGTTATAATGATATTAGTAACGTGGAATGATATTCGACGA
TATTTCTTATAATTTAGGAGGATAAATAATTATGAAGCAATCCAAAGTTTTTATACCAACGATGCGTGACGTGCCATCAGAAGCAGA
AGCACAAAGTCATCGTTTATTATTGAAATCGGGTTTGATAAAACAAAGTACAAGTGGGATTTATAGTTATTTACCGCTAGCAACACG
TGTGTTAAATAATATTACTGCAATTGTGCGACAAGAAATGGAACGTATCGATTCTGTTGAAATTTTAATGCCAGCGTTACAACAAGC
TGAATTATGGGAAGAATCAGGACGTTGGGGTGCATATGGCCCAGAATTAATGCGTTTACAAGATAGACATGGAAGACAATTTGCAT
TAGGTCCAACACATGAAGAATTAGTTACATCAATAGTAAGAAATGAATTGAAATCATACAAACAATTACCGATGACATTATTCCAA
ATTCAATCTAAATTCCGTGATGAAAAGAGACCACGTTTTGGTTTACTTCGTGGGCGTGAATTTATTATGAAAGATGCATATTCATTCC
ATGCTGACGAGGCATCATTAGATCAAACGTATCAAGATATGTATCAAGCGTATAGCCGTATTTTTGAGAGAGTTGGCATTAACGCA
AGACCAGTAGTTGCAGATTCAGGTGCTATAGGCGGTAGCCATACACATGAATTTATGGCATTAAGTGCTATCGGTGAGGATACAAT
CGTTTACAGTAAAGAAAGTGATTATGCTGCTAACATCGAAAAAGCAGAAGTCGTTTACGAACCAAATCATAAGCATACTACTGTGC
AACCTTTAGAAAAAATTGAAACACCAAATGTTAAGACTGCGCAAGAATTGGCAGACTTCTTAGGTAGACCAGTAGATGAAATCGTT
AAAACGATGATTTTCAAAGTTGATGGCGAATATATTATGGTTTTAGTGCGTGGCCATCATGAAATTAATGACATTAAATTAAAATCT
TATTTCGGCACAGATAATATTGAATTAGCAACACAAGACGAAATTGTTAATTTAGTTGGTGCAAATCCTGGTTCACTAGGTCCTGTA
ATTGATAAAGAAATCAAAATTTATGCAGATAATTTTGTGCAAGATTTAAATAATTTAGTTGTCGGTGCTAACGAAGATGGTTATCAC
TTAATTAATGTAAATGTAGGTAGAGACTTCAACGTTGATGAATATGGCGATTTCCGTTTTATTTTAGAAGGCGAAAAGTTAAGTGAT
GGTTCAGGCGTTGCACATTTTGCTGAAGGTATTGAAGTTGGTCAAGTATTCAAATTGGGTACTAAGTATTCAGAATCAATGAATGCT
ACATTCTTAGATAACCAAGGAAAAGCTCAACCTTTAATTATGGGTTGTTACGGAATTGGAATTTCTAGAACGCTAAGTGCGATTGTT
GAACAAAATCACGATGATAATGGAATTGTTTGGCCTAAATCAGTTACTCCGTTTGATTTACATTTAATTTCTATTAATCCTAAGAAA
GATGATCAACGAGAACTAGCAGATGCACTATATGCTGAATTTAATACTAAATTTGATGTGTTGTACGATGATCGTCAGGAACGTGCA
GGTGTCAAATTTAATGATGCCGATTTAATTGGTTTACCACTGCGAATTGTTGTTGGTAAACGTGCATCGGAAGGTATTGTAGAAGTT
AAAGAACGTTTAACAGGTGATAGCGAAGAAGTTCACATTGATGACTTAATGACTGTCATTACAAATAAATATGATAACTTAAAATA
ATTAAGATCGAATGAATTATAAGAGTAGGAAAAAGCTGAAAGAAATCTGATGCTTATGTCCTGCTCTTATTATTTTTGATATAATGA
TTATTCGATGAAAAATGACTGAAGACATAGTATAATTAAAGATAAATTTGTTTTAACAATATAATGATTAGCCAAATATAAAGCATT
TAATTTTCTATCATTACTATGCTCACATAATCTAAATATTGTTCGAACACGTAAAAGTAATTTCTATTTAAGGTGGTAATTGTCTTGG
CAATGACAGAGCAACAAAAATTTAAAGTGCTTGCTGATCAAATTAAAATTTCAAATCAATTAGATGCTGAAATTTTAAATTCAGGTG
AACTGACACGTATAGATGTTTCTAACAAAAACAGAACATGGGAATTTCATATTACATTACCACAATTCTTAGCTCATGAAGATTATT
TATTATTTATAAATGCAATAGAGCAAGAGTTTAAAGATATCGCCAACGTTACATGTCGTTTTACGGTAACAAATGGCACGAATCAAG
ATGAACATGCAATTAAATACTTTGGGCACTGTATTGACCAAACAGCTTTATCTCCAAAAGTTAAAGGTCAATTGAAACAGAAAAAG

CTTATTATGTCTGGAAAAGTATTAAAAGTAATGGTATCAAATGACATTGAACGTAATCATTTTGATAAGGCATGTAATGGAAGTCTT
ATCAAAGCGTTTAGAAATTGTGGTTTTGATATCGATAAAATCATATTCGAAACAAATGATAATGATCAAGAACAAAACTTAGCTTCT
TTAGAAGCACATATTCAAGAAGAAGACGAACAAAGTGCACGATTGGCAACAGAGAAACTTGAAAAAATGAAAGCTGAAAAAGCG
AAACAACAAGATAACAACGAAAGTGCTGTCGATAAGTGTCAAATTGGTAAGCCGATTCAAATTGAAAATATTAAACCAATTGAATC
TATTATTGAGGAAGAGTTTAAAGTTGCAATAGAGGGTGTCATTTTTGATATAAACTTAAAAGAACTTAAAAGTGGTCGCCATATCGT
AGAAATTAAAGTGACTGACTATACGGACTCTTTAGTTTTAAAAATGTTTACTCGTAAAAACAAAGATGATTTAGAACATTTTAAAGC
GCTAAGTGTTGGTAAATGGGTTAGGGCTCAAGGTCGTATTGAAGAAGATACATTTATTAGAGATTTAGTTATGATGATGTCTGATAT
TGAAGAGATTAAAAAAGCGACAAAAAAAGATAAGGCTGAAGAAAAGCGTGTAGAATTCCACTTGCATACTGCAATGAGCCAAATG
GATGGTATACCCAATATTGGTGCGTATGTTAAACAGGCAGCAGACTGGGGACATCCAGCCATTGCGGTTACAGACCATAATGTTGT
GCAAGCATTTCCAGATGCTCACGCAGCAGCGGAAAAACATGGCATTAAAATGATATACGGTATGGAAGGTATGTTAGTTGATGATG
GTGTTCCGATTGCATACAAACCACAAGATGTCGTATTAAAAGATGCTACTTATGTTGTGTTCGACGTTGAGACAACTGGTTTATCAA
ATCAGTATGATAAAATCATCGAGCTTGCAGCTGTGAAAGTTCATAACGGTGAAATCATCGATAAGTTTGAAAGGTTTAGTAATCCGC
ATGAACGATTATCGGAAACGATTATCAATTTGACGCATATTACGGATGATATGTTAGTAGATGCCCCTGAGATTGAAGAAGTACTTA
CAGAGTTTAAAGAATGGGTTGGCGATGCGATATTCGTAGCGCATAATGCTTCGTTTGATATGGGCTTCATCGATACGGGATATGAAC
GTCTTGGGTTTGGACCATCAACGAATGGTGTTATCGATACTTTAGAATTATCTCGTACGATTAATACTGAATATGGTAAACATGGTTT
GAATTTCTTGGCTAAAAAATATGGCGTAGAATTAACGCAACATCACCGTGCCATTTATGATACAGAAGCAACAGCTTACATTTTCAT
AAAAATGGTTCAACAAATGAAAGAATTAGGCGTATTAAATCATAACGAAATCAACAAAAAACTCAGTAATGAAGATGCATATAAA
CGTGCAAGACCTAGTCATGTCACATTAATTGTACAAAACCAACAAGGTCTTAAAAATCTATTTAAAATTGTAAGTGCATCATTGGTG
AAGTATTTCTACCGTACACCTCGAATTCCACGTTCATTGTTAGATGAATATCGTGAGGGATTATTGGTAGGTACAGCGTGTGATGAA
GGTGAATTATTTACGGCAGTTATGCAGAAGGACCAGAGTCAAGTTGAAAAAATTGCCAAATATTATGATTTTATTGAAATTCAACCA
CCGGCACTTTATCAAGATTTAATTGATAGAGAGCTTATTAGAGATACTGAAACATTACATGAAATTTATCAACGTTTAATACATGCA
GGTGACACAGCGGGTATACCTGTTATTGCGACAGGAAATGCACACTATTTGTTTGAACATGATGGTATCGCACGTAAAATTTTAATA
GCATCACAACCCGGCAATCCACTTAATCGCTCAACTTTACCGGAAGCACATTTTAGAACTACAGATGAAATGTTAAACGAGTTTCAT
TTTTTAGGTGAAGAAAAAGCGCATGAAATTGTTGTGAAAAATACAAACGAATTAGCAGATCGAATTGAACGTGTTGTTCCTATTAA
AGATGAATTATACACACCGCGTATGGAAGGTGCTAACGAAGAAATTAGAGAACTAAGTTATGCAAATGCGCGTAAACTGTATGGTG
AAGACCTGCCTCAAATCGTAATTGATCGATTAGAAAAAGAATTAAAAAGTATTATCGGTAATGGATTTGCGGTAATTTACTTAATTT
CGCAACGTTTAGTTAAAAAATCATTAGATGATGGATACTTAGTTGGTTCCCGTGGTTCAGTAGGTTCTAGTTTTGTAGCGACAATGA
CTGAGATTACTGAAGTAAACCCGTTACCGCCACACTATATTTGTCCGAACTGTAAAACGAGTGAATTTTTCAATGATGGTTCAGTAG
GATCAGGATTTGATTTACCTGATAAGACGTGTGAAACTTGTGGAGCGCCACTTATTAAAGAAGGACAAGATATTCCGTTTGAAACAT
TTTTAGGATTTAAGGGAGATAAAGTTCCTGATATCGACTTGAACTTTAGTGGTGAATATCAACCGAATGCCCATAACTACACAAAAG
TATTATTTGGTGAGGATAAAGTATTCCGTGCAGGTACAATTGGTACTGTTGCTGAAAAGACTGCTTTTGGTTATGTTAAAGGTTATTT
GAATGATCAAGGTATCCACAAAAGAGGTGCTGAAATAGATCGACTCGTTAAAGGATGTACAGGTGTTAAACGTACAACTGGACAGC
ATCCAGGGGGTATTATTGTAGTACCTGATTACATGGATATTTATGATTTTACGCCGATACAATATCCTGCCGATGATCAAAATTCAG
CATGGATGACGACACATTTTGATTTCCATTCTATTCATGATAATGTATTAAAACTTGATATACTTGGACACGATGATCCAACAATGA
TTCGTATGCTTCAAGATTTATCAGGAATTGATCCAAAAACAATACCTGTAGATGATAAAGAAGTTATGCAGATATTTAGTACACCTG
AAAGTTTGGGTGTTACTGAAGATGAAATTTTATGTAAAACAGGTACGTTTGGGGTTCCAGAATTCGGTACAGGGTTCGTGCGTCAAA
TGTTAGAAGATACAAAGCCAACAACATTTTCTGAATTAGTTCAAATCTCAGGATTATCTCATGGTACAGATGTGTGGTTAGGCAATG
CTCAAGAATTAATTAAAACCGGTATATGTGATTTATCAAGTGTAATTGGTTGTCGTGATGATATCATGGTTTATTTAATGTATGCTGG
TTTAGAACCATCAATGGCTTTTAAAATAATGGAGTCAGTACGTAAAGGTAAAGGTTTAACTGAAGAAATGATTGAAACGATGAAAG
AAAATGAAGTGCCGGATTGGTATTTAGATTCATGTCTTAAAATTAAGTACATGTTCCCTAAAGCCCATGCAGCAGCATACGTTTTAA
TGGCAGTACGTATCGCATATTTCAAAGTACATCATCCACTTTATTACTATGCATCTTACTTTACAATTCGTGCGTCAGATTTTGATTT
AATCACGATGATTAAAGATAAAACAAGCATTCGAAATACTGTAAAAGACATGTATTCTCGCTATATGGATCTAGGTAAAAAAGAAA
AAGACGTATTAACTGTCTTGGAAATTATGAATGAAATGGCACATCGAGGTTATCGCATGCAACCGATTAGTTTAGAAAAGAGTCAG
GCGTTCGAATTTATCATTGAAGGCGATACACTTATTCCGCCGTTCATATCAGTGCCTGGGCTTGGCGAAAACGTTGCGAAACGAATT
GTTGAAGCTCGTGACGATGGACCATTTTTATCAAAAGAAGATTTAAACAAAAAAGCTGGATTATCTCAGAAAATTATTGAGTATTTA
GATGAGTTAGGCTCATTACCGAATTTACCAGATAAAGCTCAACTTTCGATATTCGATATGTAAAATGAAATAATCAAGGTATTTATT
TAATGCGTATGGCGTAGTTAAAGAAATACAAAATTGTTGCTGGACATAAAATTATGCCTGTATTTCTTTTCAACGTCTTACGAGTCT
ATTCGAATGTAATGGTGAAATAAATGAACAAACTTTTACAAGAATCTCTGATTAATAGTGAAGTCATTTGTTTCAAGCATAAACTTA
TGCTATAATTAAGTTGCTTAAAAATTAGTGAACTCAGGCAGAAGAGTGGGAGATTCCCGCTCTTTTCTATTTGCCAAAAAGGGAGGC
CTGTATGAGTAAAATTACAGAACAAGTAGAAGTGATTGTTAAACCAATTATGGAAGACTTGAATTTTGAACTTGTAGACGTTGAAT
ATGTCAAAGAGGGTAGAGATCATTTTCTTAGAATCTCTATTGATAAAGAAGGTGGCGTAGATTTAAATGATTGTACGCTAGCTTCTG
AAAAAATAAGTGAAGCTATGGATGCAAATGATCCTATTCCTGAAATGTATTATTTAGACGTAGCGTCACCTGGTGCAGAACGTCCA
ATTAAAAAAGAACAAGATTTCCAAAATGCAATAACTAAACCTGTATTTGTTTCTTTATATGTACCAATTGAAGGTGAAAAGGAATG
GTTAGGCATTTTACAAGAAGTCAATAATGAAACAATTGTAGTACAAGTTAAAATCAAAGCAAGAACGAAAGATATAGAGATACCG
AGAGACAAAATAGCAAAAGCACGTCACGCAGTTATGATTTAACGTGATGAGGAGGAAAAAACGTGTCAAGTAATGAATTATTATTA
GCTACTGAGTATTTAGAAAAAGAAAAGAAGATTCCTAGAGCAGTATTAATTGATGCTATTGAAGCAGCTTTAATTACTGCATACAA
AAAGAATTATGATAGTGCAAGAAATGTCCGTGTGGAATTAAATATGGATCAAGGTACTTTCAAAGTTATCGCTCGTAAAGATGTTGT
TGAAGAAGTATTTGACGACAGAGATGAAGTGGATTTAAGTACAGCGCTTGTTAAAAACCCTGCATATGAAATTGGTGATATATACG
AAGAAGATGTAACACCTAAAGATTTTGGTCGTGTAGGTGCTCAAGCAGCGAAACAAGCAGTAATGCAACGTCTTCGTGATGCTGAA
CGTGAAATTTTATTTGAAGAATTTATAGACAAAGAAGAAGACATACTTACTGGAATTATTGACCGTGTTGACCATCGTTATGTATAT
GTGAATTTAGGTCGTATCGAAGCTGTTTTATCTGAAGCAGAAAGAAGTCCTAACGAAAAATATATTCCTAACGAACGTATCAAAGT
ATATGTTAACAAAGTGGAACAAACGACAAAAGGTCCTCAAATCTATGTTTCTCGTAGCCATCCAGGTTTATTAAAACGTTTATTTGA
ACAAGAAGTTCCAGAAATTTACGATGGTACTGTAATTGTTAAATCAGTAGCACGTGAAGCTGGCGATCGCTCTAAAATTAGTGTCTT
CTCTGAAAACAATGATATAGATGCTGTTGGTGCATGTGTTGGTGCTAAAGGCGCACGTGTTGAAGCTGTTGTTGAAGAGCTAGGTGG
CGAAAAAATCGACATCGTTCAATGGAATGAAGATCCAAAAGTATTTGTAAAAAATGCTTTAAGCCCTTCTCAAGTTTTAGAAGTTAT
TGTTGATGAAACAAATCAATCTACAGTAGTTGTTGTTCCTGATTATCAATTGTCATTAGCGATTGGTAAAAGAGGACAAAACGCACG
TCTAGCTGCTAAATTAACCGGCTGGAAAATTGATATTAAATCAGAAACAGATGCGCGTGAAGCGGGTATCTATCCAGTAGTTGAAG
CTGAAAAAGTAACTGAAGAAGATGTTGCTTTAGAAGATGCTGACACAACAGAATCAACCGAAGAGGTAAATGATGTTTCAGTTGAA
ACAAATGTAGAGAAAGAATCTGAATAATAGGTTGGAGTGAAGTATCTATGAAAAAGAAAAAAATTCCGATGCGAAAATGTATTCTT
TCAAATGAAATGCATCCCAAAAAAGATATGATTCGTGTTGTTGTTAATAAAGAAGGCGAAATCTTTGCGGATGTTACTGGAAAGAA
ACAAGGCCGTGGCGCATATGTTTCTAAAGATGTTGCTATGGTTGAAAAAGCACAACAAAAAGAAATTTTAGAAAAATATTTTAAAG

CATCTAAAGAGCAATTGGATCCTGTTTACAAAGAAATTATTAGATTAATTTATAGAGAAGAGATCCCAAAATGAGTATAGATCAAA
TATTAAACTTTTTAGGATTAGCAATGAGAGCTGGTAAAGTAAAAACAGGTGAATCAGTCATTGTTAATGAGATTAAAAAAGGAAAT
TTGAAGCTCGTTATTGTTGCAAATGATGCGTCTGATAATACAGCTAAATTAATTACAGATAAATGTAAGAGTTACAAAGTTCCATTC
AGAAAGTTTGGAAATCGAAATGAATTGGGAATAGCACTTGGAAAAGGTGAGCGTGTTAATGTAGGGATTACTGACCCAGGCTTTGC
TAAAAAGTTGCTATCAATGATAGATGAATATCATAAGGAGTGATTATATGAGTAAACAAAGAATTTACGAATATGCGAAAGAATTA
AATCTAAAGAGTAAAGAGATTATAGATGAGTTAAAAAGCATGAATATTGAGGTTTCAAATCATATGCAAGCTTTGGAAGATGACCA
AATTAAAGCATTAGATAAAAAGTTCAAAAAAGAACAAAAGAACGACAATAAACAAAGCACTCAAAATAATCACCAAAAATCAAAC
AATCAAAACCAAAATAAAGGGCAACAAAAAGATAACAAAAAGAATCAACAACAAAATAATAAAGGCAACAAAGGCAATAAAAAG
AATAATAGAAATAATAAGAAAAATAACAAGAATAATAAACCACAAAATCAACCAGCTGCTCCAAAAGAAATACCATCAAAAGTGA
CATATCAAGAAGGTATTACATTAGGCGAATTTGCGGATAAATTAAATGTTGAATCATCAGAAATTATCAAAAAATTATTCTTACTTG
GTATTGTTGCTAATATCAATCAATCATTAAATCAAGAAACAATCGAATTAATTGCCGATGATTATGGCGTTGAGGTTGAAGAAGAA
GTTGTGATTAATGAAGAAGATTTATCAATCTACTTCGAAGACGAAAAAGATGATCCAGAAGCAATTGAGAGACCAGCAGTTGTAAC
AATTATGGGACATGTTGACCATGGTAAAACGACTTTATTAGATTCAATTCGTCATACAAAAGTTACAGCAGGTGAAGCAGGCGGAA
TCACTCAACATATTGGTGCATATCAAATTGAAAACGATGGCAAAAAAATCACTTTCTTAGATACACCGGGACATGCTGCATTTACAA
CGATGCGTGCGCGTGGTGCACAAGTAACAGATATTACAATTTTAGTAGTTGCAGCTGACGATGGTGTTATGCCACAAACAATTGAA
GCAATTAACCATGCTAAAGAAGCAGAAGTACCAATTATTGTTGCAGTAAATAAAATTGATAAACCAACTTCAAACCCTGATCGAGT
TATGCAAGAATTAACTGAATATGGTTTAATTCCTGAAGATTGGGGCGGCGAAACAATTTTCGTTCCACTTTCTGCATTAAGTGGTGA
TGGTATCGACGATTTATTAGAAATGATAGGATTAGTTGCAGAAGTTCAAGAACTTAAAGCAAATCCTAAAAACCGTGCTGTTGGTA
CAGTTATCGAAGCTGAATTAGATAAATCACGTGGTCCTTCTGCATCATTATTAGTACAAAATGGTACATTAAATGTTGGTGATGCGA
TTGTAGTTGGTAATACTTACGGCCGTATCCGTGCAATGGTTAATGACTTAGGTCAAAGAATCAAAACGGCTGGTCCATCAACGCCTG
TTGAAATTACAGGTATTAATGATGTGCCACAAGCTGGGGATCGCTTTGTTGTATTTAGTGATGAAAAACAAGCTCGTCGTATTGGTG
AATCAAGACACGAAGCTAGCATTGTACAACAACGTCAAGAAAGTAAAAATGTTTCATTAGATAACCTGTTTGAACAAATGAAACAA
GGTGAAATGAAAGATTTAAACGTTATTATTAAAGGTGATGTTCAAGGTTCTGTTGAAGCTTTAGCTGCATCATTAATGAAAATTGAT
GTTGAAGGCGTAAATGTTCGTATCATTCATACAGCGGTTGGTGCAATTAATGAGTCAGACGTGACACTTGCTAATGCCTCAAATGGT
ATTATCATTGGTTTCAATGTTCGTCCAGACAGTGGTGCAAAACGTGCTGCAGAAGCTGAAAATGTTGATATGCGTTTACACAGAGTT
ATTTATAATGTTATCGAAGAAATTGAATCAGCGATGAAAGGTTTACTTGATCCAGAATTTGAAGAACAAGTTATCGGACAAGCTGA
AGTTCGTCAAACATTCAAAGTTTCTAAAGTTGGTACTATTGCTGGATGTTATGTTACTGAAGGTAAAATTACGCGAAATGCTGGTGT
ACGTATTATTCGTGATGGTATTGTTCAATATGAAGGCGAATTAGATACACTTAAACGTTTCAAAGATGATGCTAAGGAAGTTGCAAA
AGGTTATGAATGTGGTATTACAATTGAAAACTACAATGACCTTAAAGAAGGCGATGTTATCGAAGCATTTGAAATGGTTGAAATTA
AGCGTTAATTAAATAAATTACAAGCTAAAAGTATAGTTAAGATTGATATGCTCCCTATAAATATTGCACTTTTTAAGTGTCTACTTTA
TAGGGAGCATATTTGATACTAGCTTTTGGTTTTTTATTAGAATAGATTACCTATTAAAAGTTACGTTATATGGACATGATTTTGTATA
AAATTTTGTGGTGGCCTAGAATGATTTTTAATGACAAAATATAATGTCGACTATTATTGGAAAATTTTCTGTTGAAATGCCTATCTTA
CGGCAAACTTTATTTGATTTTATAGGCTTAATTTATTAAAATAACGTGTGAGCTAAAATAATTGTTTAAGCATTGTTACACTAAAAA
ATGCAAATAACAATTGAACTTAAAGATAAAGAGGTGACAAGAATGAGCAGTATGAGAGCAGAGCGTGTTGGTGAACAAATGAAGA
AGGAATTAATGGATATCATCAACAATAAAGTCAAAGATCCTCGAGTTGGTTTTATTACAATTACAGATGTTGTTTTAACAAATGATT
TATCGCAGGCTAAAGTATTTTTAACTGTATTAGGTAACGATAAAGAAGTAGAAAATACATTTAAAGCACTTGATAAAGCAAAAGGC
TTCATTAAGTCTGAATTAGGTTCTAGAATGCGATTACGTATTATGCCGGAATTAATGTATGAATATGATCAATCAATCGAATATGGT
AATAAAATTGAACGAATGATTCAAGATTTACACAAACAAGATAGATAATTTAGTGTTAGGTATCTGGAAAATGTTTGATAATTTCTT
AATATCGGTATATTAACATTAAACAGTTAATACATAGATGTGTAGAAATAGTTAACATTTTCCAGTTTTTTTATGAATAAATTCAGTT
GATACGCTATTAAAATATATTTTAAAAAAAGAAGGTGACTATATGTATAATGGGATATTACCAGTATATAAAGAGCGCGGTTTAAC
AAGTCATGACGTTGTATTCAAATTGCGTAAAATATTAAAAACTAAAAAAATAGGTCACACGGGTACGCTTGATCCCGAAGTTGCAG
GCGTGTTACCGGTATGTATAGGTAATGCAACGAGAGTTAGTGATTATGTTATGGATATGGGCAAAGCTTATGAAGCAACTGTATCG
ATAGGAAGAAGTACAACGACTGAAGATCAAACGGGTGATACATTGGAAACAAAAGGTGTACACTCAGCAGATTTTAATAAGGACG
ATATTGACCGATTGTTAGAAAGTTTTAAAGGTATCATTGAACAAATTCCGCCGATGTACTCATCCGTCAAAGTAAATGGTAAAAAAT
TATATGAATATGCGCGTAATAATGAAACAGTTGAAAGACCAAAGCGTAAAGTTAATATTATAGACATTGGGCGTATATCTGAATTA
GATTTTAAAGAAAATGAGTGTCATTTTAAAATACGCGTCATCTGTGGTAAAGGTACATATATTAGAACGCTAGCAACTGATATTGGT
GTGAAATTAGGCTTTCCGGCACATATGTCGAAATTAACACGAATCGAGTCTGGTGGATTTGTGTTGAAAGATAGCCTTACATTAGAA
CAAATAAAAGAACTTCATGAGCAGGATTCATTGCAAAATAAATTGTTTCCTTTAGAATATGGATTAAAGGGTTTGCCAAGCATTAAA
ATTAAAGATTCGCACATAAAAAAACGTATTTTAAATGGGCAGAAATTTAATAAAAATGAATTTGATAACAAAATTAAAGACCAAAT
TGTATTTATTGATGATGATTCAGAAAAAGTATTAGCAATTTATATGGTACACCCTACAAAAGAATCAGAAATTAAACCTAAAAAAG
TCTTTAATTAAAGGAGATAGAATTTATGAAAGTCATAGAAGTGACACATCCTATACAATCTAAACAGTATATTACAGAGGATGTTGC
AATGGCATTCGGATTTTTCGATGGCATGCATAAAGGTCATGACAAAGTCTTTGATATATTAAACGAAATAGCTGAGGCACGCAGTTT
AAAAAAAGCGGTGATGACATTTGATCCGCATCCATCTGTCGTGTTGAATCCTAAAAGAAAACGAACAACGTATTTAACGCCACTTTC
AGATAAAATCGAAAAAATTAGCCAACATGATATTGATTATTGTATAGTGGTTAATTTTTCATCTAGGTTTGCTAATGTGAGCGTTGA
AGATTTTGTTGAAAATTATATAATTAAAAATAATGTAAAAGAAGTCATTGCTGGTTTCGATTTTACTTTTGGTAAATTTGGAAAAGG
TAATATGACTGTACTTCAAGAATATGATGCGTTTAATACGACAATTGTGAGTAAACAAGAAATTGAAAATGAAAAAATTTCTACAA
CTTCTATTCGTCAAGATTTAATCAATGGTGAGTTGCAAAAAGCGAATGATGCTTTAGGCTATATATATTCTATTAAAGGCACTGTAG
TGCAAGGTGAAAAAAGGGGAAGAACTATTGGCTTCCCAACAGCTAACATTCAACCTAGTGATGATTATTTGTTACCTCGTAAAGGT
GTTTATGCTGTTAGTATTGAAATCGGCACTGAAAATAAATTATATCGAGGGGTAGCTAACATAGGTGTAAAGCCAACATTTCATGAT
CCTAACAAAGCAGAAGTTGTCATCGAAGTGAATATCTTTGACTTTGAGGATAATATTTATGGTGAACGAGTGACCGTGAATTGGCAT
CATTTCTTACGTCCTGAGATTAAATTTGATGGTATCGACCCATTAGTTAAACAAATGAACGATGATAAATCGCGTGCTAAATATTTA
TTAGCAGTTGATTTTGGTGATGAAGTAGCTTATAATATCTAGAGTTGCGTATAGTTATATAAACAATCTATACCACACCTTTTTCTTA
GTAGGTCGAATCTCCAACGCCTAACTCGGATTAAGGAGTATTCAAACATTTTAAGGAGGAAATTGATTATGGCAATTTCACAAGAA
CGTAAAAACGAAATCATTAAAGAATACCGTGTACACGAAACTGATACTGGTTCACCAGAAGTACAAATCGCTGTACTTACTGCAGA
AATCAACGCAGTAAACGAACACTTACGTACACACAAAAAAGACCACCATTCACGTCGTGGGTTATTAAAAATGGTAGGTCGTCGTA
GACATTTATTAAACTACTTACGTAGTAAAGATATTCAACGTTACCGTGAATTAATTAAATCACTTGGTATCCGTCGTTAATCTTAATA
TAACGTCTTTGAGGTTGGGGCATATTTATGTTCCAACCTTAATTTATATTAAAAAAGCTTTTTACAAATATTAACATTTATTATATGT
TAAGCTAATATTGAGTGAATAATAAGGTTACAATGAGATAAAGATGATATAAGTACACCTAGAGTAATAATCAAGATATTAAAAAT
AAAGTATGTTTTTTAAAAAAATATAACTTATATTTATACTGATAAGGGTGGGACGATAAGTCTATTTTGTAAATAATAGATGGATAT
CCCGCTCTCTTTTTTTCCAATTCAATATTTTATAACTAATATTAAAATACGATAATAAATGATATGATATAACTATTAGATTCAAGAG
AGGAGATTTATAATGTCTCAAGAAAAGAAAGTTTTTAAAACTGAATGGGCAGGAAGATCTTTAACGATTGAAACAGGGCAATTAGC

TAAACAAGCAAATGGCGCTGTATTGGTTCGTTATGGAGATACAGTCGTGTTATCGACGGCAACTGCATCAAAAGAACCTCGTGATG
GAGATTTCTTCCCATTAACAGTGAACTATGAAGAAAAAATGTACGCTGCGGGTAAAATTCCTGGTGGATTTAAAAGAGAGAAGGA
CGTCCTGGTGACGATGCAACATTAACTGCGCGATTAATTGATAGACCAATTAGACCTTTATTCCCTAAAGGATATAAGCATGATGTT
CAAATTATGAACATGGTATTAAGTGCAGATCCTGATTGTTCACCACAAATGGCTGCAATGATTGGTTCATCTATGGCGCTTAGTGTG
TCGGATATTCCATTCCAAGGGCCAATCGCCGGTGTAAATGTGGGTTATATTGACGGTAAATATATCATTAACCCAACAGTAGAAGA
AAAAGAAGTTTCTCGCTTAGACCTTGAAGTAGCTGGTCATAAAGATGCAGTAAACATGGTAGAGGCAGGCGCTAGTGAGATTACTG
AACAAGAAATGTTAGAGGCGATCTTCTTTGGTCATGAAGAGATTCAACGTTTAGTTGATTTCCAACAACAAATCGTCGACCACATTC
AACCTGTTAAACAAGAATTTATTCCAGCAGAGCGTGATGAAGCGCTAGTTGAACGTGTAAAATCTTTAACCGAAGAAAAAGGACTT
AAAGAAACAGTTTTAACATTTGATAAACAACAACGAGATGAAAATCTTGATAACTTAAAAGAAGAAATCGTCAATGAATTTATCGA
TGAAGAAGATCCAGAGAATGAATTACTTATTAAAGAAGTTTATGCAATTTTAAATGAATTAGTGAAAGAAGAAGTTAGACGTTTAA
TTGCAGATGAAAAAATTAGACCAGACGGCCGTAAACCTGATGAAATCCGTCCATTAGATTCTGAAGTTGGTATTTTACCTAGAACGC
ATGGTTCAGGTCTATTTACACGTGGTCAGACTCAAGCACTTTCAGTTTTAACATTAGGTGCTTTAGGCGATTATCAATTAATTGATGG
TTTAGGACCTGAAGAAGAAAAAAGATTCATGCATCATTACAACTTCCCGAATTTTTCAGTAGGTGAAACTGGTCCAGTACGTGCGCC
AGGTCGTCGTGAAATTGGACATGGTGCGTTAGGTGAAAGAGCATTAAAATATATTATTCCTGATACTGCTGATTTCCCATATACAAT
TCGTATTGTAAGTGAGGTACTTGAATCAAATGGTTCATCATCTCAAGCGTCAATTTGTGGATCAACATTAGCATTAATGGATGCGGG
CGTACCGATTAAAGCACCAGTTGCTGGTATTGCTATGGGCCTTGTTACACGTGAAGATAGCTATACGATTTTAACTGATATCCAAGG
TATGGAAGATGCATTAGGTGATATGGACTTTAAAGTCGCTGGTACTAAAGAAGGTATTACAGCAATCCAAATGGATATTAAAATTG
ACGGTTTAACGCGTGAAATTATCGAAGAGGCTCTAGAACAAGCGAGACGTGGTCGTTTAGAAATAATGAATCATATGTTACAAACA
ATTGATCAACCACGTACTGAATTAAGTGCTTACGCGCCAAAAGTTGTAACTATGACAATTAAACCAGATAAGATTAGAGATGTTATC
GGACCTGGTGGTAAAAAAATTAACGAAATTATTGATGAAACAGGCGTTAAATTAGATATTGAACAAGATGGTACTATCTTTATTGG
TGCCGTTGATCAAGCTATGATAAATCGCGCTCGTGAAATCATTGAGGAAATTACACGTGAAGCGGAAGTAGGTCAAACTTATCAAG
CCACTGTTAAACGTATTGAAAAATACGGTGCGTTTGTAGGCCTATTCCCGGGTAAAGATGCGTTGCTTCACATTTCACAAATTTCAA
AAAATAGAATTGAAAAAGTGGAAGATGTATTAAAAATCGGTGACACAATTGAAGTTAAGATTACTGAAATTGATAAACAAGGTCG
AGTAAATGCTTCACACAGAGCATTAGAAGAATAATATTTAAAGTCATATGACGACAATGTATCGTCATGTGATTTTTTTATGCCATT
TTTTACGAAGTGACCCGTTTTGAATTTGTTGTATTGAACATTTTAAAACGCTTTATTATTTTGTGTGCAACTGTTAATTATCCTGTATG
TATAGTGATTAATAGTGTACATCAAGTGTTTTTTAACTTATAATGAATAGTGAGTTTATATATGGACGGGTAACAAATTTAGGAGGT
AAGATTTTGAGTTTAATAAAGAAAAAGAATAAAGATATTCGCATTATACCATTAGGCGGTGTTGGCGAAATTGCTAAAAATATGTA
TATCGTTGAAGTAGACGATGAAATGTTTATGTTAGATGCTGGACTTATGTTTCCAGAAGACGAAATGCTAGGTATTGATATTGTTAT
ACCAGACATTTCATACGTACTTGAAAATAAAGATAAATTGAAGGGTATATTCCTTACACACGGACATGAGCACGCGATTGGTGCAG
TGAGTTATGTTTTAGAACAATTAGATGCACCAGTATATGGATCTAAATTGACAATAGCGTTAATTAAAGAAAATATGAAAGCCCGT
AATATTGATAAAAAAGTTCGCTACTATACAGTTAATAATGATTCAATTATGAGATTCAAAAACGTGAATATTAGTTTCTTTAATACG
ACACACAGTATTCCTGATAGTTTAGGTGTTTGTATTCACACTTCATATGGTGCCATTGTGTATACAGGTGAATTTAAGTTTGACCAAA
GTTTACATGGACATTATGCACCAGATATTAAACGTATGGCAGAGATTGGTGAAGAAGGCGTATTTGTCTTAATCAGTGATTCTACTG
AGGCAGAGAAACCTGGATATAATACTCCGGAAAATGTGATTGAACATCATATGTATGATGCTTTTGCAAAAGTGCGAGGTCGCTTG
ATAGTTTCATGTTATGCTTCGAACTTTATACGTATTCAGCAAGTTTTAAATATTGCTAGCAAGCTAAATCGTAAAGTGTCATTTTTAG
GAAGATCACTTGAAAGTTCATTTAATATTGCTCGTAAAATGGGGTATTTCGACATTCCTAAAGATTTGCTAATTCCTATAACAGAAG
TTGATAATTATCCTAAAAATGAAGTGATAATTATAGCTACTGGTATGCAAGGAGAACCTGTAGAAGCCTTAAGTCAAATGGCGCAA
CATAAGCATAAAATTATGAATATCGAAGAAGGCGATTCTGTATTTTTAGCAATTACGGCTTCTGCTAATATGGAAGTTATCATTGCG
AATACATTAAATGAGCTTGTACGTGCTGGCGCACATATTATTCCAAATAACAAGAAGATTCATGCTTCAAGTCATGGTTGCATGGAA
GAATTAAAAATGATGATTAATATTATGAAACCTGAATACTTTATTCCTGTACAAGGTGAATTTAAAATGCAGATAGCACATGCGAA
GCTAGCAGCTGAAGCAGGTGTTGCACCAGAAAAGATTTTCCTTGTGGAAAAAGGAGATGTCATTAATTACAACGGTAAAGATATGA
TATTAAATGAAAAGGTAAATTCAGGAAATATTTTAATAGATGGTATTGGTATTGGGGATGTAGGAAATATCGTGTTGAGAGACCGT
CATCTTTTAGCAGAAGATGGTATCTTTATTGCTGTAGTAACGTTAGATCCTAAAAATAGACGTATAGCTGCGGGACCTGAAATTCAA
TCTCGTGGGTTTGTATATGTACGTGAAAGTGAAGACTTATTACGTGAAGCAGAAGAGAAAGTACGTGAAATAGTAGAGGCTGGTTT
ACAAGAAAAACGCATAGAATGGTCTGAAATTAAGCAAAATATGCGTGATCAAATTAGTAAACTATTATTCGAAAGTACAAAACGTC
GTCCTATGATTATTCCAGTAATTTCTGAAATTTAATCAAAAAGTCATTAACATAAAAGAGGTCAGAACAAGTCACTGAAATATAATG
GTTGTCATGGACAATTTACTTATATTTTATGATGGTCAATTGAAGGGGTAACGATTAATCTGTTATCTTAAGTAAATTGATACATAGA
TGATATTGTTCTAACCTCTTTCATCGTCTGTTTGGACTACATATTCTAAACATCAAATAGGAAATTATATATAATAACGTCGTTTTAA
CTAAGGCAACATAAGGAGGTGCGTCAATTGGCACAAGCAAAAAAGAAATCGACAGCTAAGAAAAAAACAACATCAAAAAAAAGA
ACAAATTCGAGGAAAAAGAAGAATGATAATCCGATACGTTATGTCATAGCTATTTTAGTAGTTGTATTAATGGTGTTGGGTGTTTTC
CAATTAGGAATAATAGGTCGTCTAATTGACAGCTTCTTTAATTATTTATTTGGGTACAGTAGATATTTAACATATATTTTAGTACTCT
TAGCAACTGGTTTTATTACATACTCTAAACGTATTCCTAAAACTAGACGAACGGCTGGTTCGATTGTATTGCAAATTGCATTGCTATT
TGTATCACAGTTAGTTTTTCATTTTAATAGTGGTATCAAAGCTGAAAGAGAACCTGTACTTTCTTATGTGTATCAGTCATACCAACAC
AGTCATTTCCCAAATTTTGGTGGCGGTGTATTAGGCTTTTATTTATTAGAGTTAAGCGTACCTTTAATTTCATTATTTGGTGTATGTAT
TATTACTATTTTATTATTATGCTCAAGTGTTATTTTATTAACAAACCATCAACATCGTGAAGTTGCAAAAGTTGCACTGGAAAATATA
AAAGCTTGGTTTGGTTCATTTAATGAAAAAATGTCGGAAAGAAACCAAGAAAAACAATTGAAGCGTGAAGAAAAAGCAAGACTTA
AAGAAGAACAAAAGGCACGTCAAAATGAACAGCCACAAATAAAAGATGTGAGTGATTTTACGGAAGTGCCTCAAGAAAGAGATAT
TCCAATTTATGGGCATACTGAAAATGAAAGTAAAAGCCAGAGTCAACCAAGTCGAAAAAAACGAGTGTTTGATGCAGAGAATAGTT
CGAATAACATCGTAAATCATCATCAAGCAGATCAGCAAGAACAATTAACAGAACAAACTCATAACAGTGTTGAAAGTGAAAACAC
TATTGAAGAAGCTGGTGAAGTTACGAATGTATCGTATGTTGTTCCACCGTTAACTTTACTTAATCAACCTGCAAAACAAAAAGCAAC
ATCTAAAGCTGAAGTGCAACGTAAAGGACAAGTACTAGAGAATACATTAAAAGATTTTGGGGTAAATGCAAAAGTGACACAAATT
AAAATTGGTCCTGTAGTAACTCAATATGAAATTCAACCAGCTCAAGGGGTTAAAGTGAGTAAAATTGTAAACTTGCATAATGATATT
GCATTAGCTTTAGCAGCAAAAGATGTTAGAATCGAAGCGCCAATACCTGGTCGTTCTGCAGTAGGTATTGAAGTGCCAAATGAGAA
AATTTCATTAGTTTCACTAAAAGAAGTTTTAGATGAAAAATTCCCGTCTAATAATAAACTAGAAGTTGGATTAGGAAGAGATATATC
AGGTGATCCAATTACTGTTCCACTAAATGAAATGCCACACTTATTGGTGGCAGGATCGACGGGTAGTGGTAAATCTGTTTGTATAAA
TGGTATTATTACAAGTATTTTATTAAATGCTAAGCCGCATGAAGTTAAACTTATGTTAATCGATCCGAAAATGGTTGAACTAAATGT
TTATAACGGAATTCCACACTTATTAATTCCGGTTGTTACAAATCCTCATAAAGCTGCTCAAGCTTTAGAAAAAATTGTAGCTGAGAT
GGAAAGACGTTATGATTTATTCCAACATTCATCAACTAGAAACATTAAAGGTTATAACGAATTAATCCGTAAGCAAAATCAAGAAT
TAGATGAGAAGCAACCAGAATTACCTTATATCGTTGTTATTGTAGATGAGCTTGCAGATTTAATGATGGTAGCTGGTAAAGAAGTTG
AAAATGCGATTCAACGTATTACACAAATGGCACGTGCAGCAGGTATACATTTAATTGTAGCGACACAAAGACCTTCTGTGGATGTA
ATTACAGGTATCATTAAAAATAATATTCCATCTAGAATAGCTTTTGCTGTGAGTTCTCAAACAGATTCAAGAACTATTATTGGTACT

GGCGGCGCAGAAAAGTTACTTGGTAAAGGTGACATGTTATACGTTGGAAATGGTGACTCATCACAAACACGTATTCAAGGGGCGTT
TTTAAGTGACCAAGAGGTGCAAGATGTTGTAAATTATGTAGTAGAACAACAACAGGCAAATTATGTAAAAGAAATGGAACCAGAT
GCACCAGTGGATAAATCGGAAATGAAAAGTGAAGATGCTTTATATGATGAAGCGTATTTGTTTGTTGTTGAACAACAAAAGGCAAG
TACATCATTGTTACAACGCCAATTTAGAATTGGTTATAATAGAGCATCTAGGTTGATGGATGATTTAGAACGCAATCAGGTAATCGG
TCCACAAAAAGGAAGCAAGCCTAGACAAGTTTTAATAGATCTTAATAATGACGAGGTGTAAAAAAATGTCAGAAATGAATGCGGT
ATATAACGTTAAACAATACATTTTAAATTTGATTAAGCAAAATAAATTGGAATATGGTGACCAACTTCCAAGTAATTTATCAATTGC
CAGAGAATTAAATGTAAAAACCGACGATGTTTATGAAGCAATTCAGGCATTGATTACTGAACAAGTCATTAAAGATAATTTTGAAG
AGGGCACAAGTGTTAAGTCACTGCCCCCTTTCTTTTATCCATTGAATGAACTTATAAGTATTGGGCAAATGATTAAAAATGCAGGAT
TTGAATGCGGAACTGAATACTTAAATTTTGATGAGCAACCAGCAACTATGTTAGATGCAAATTTGTTGAGCGTTGAAGAAGGATATC
CAGTAACCATTATAGAACGATTACGAACTGCCGATGGAGAACCGGTCGTCTATTGTTTAGATAAAATTGCTAAAAAAGAATTAACA
TGTACAGAGTATCAAATGAGCAATGGATCGATTCTAAGTGCGATAAAAGAACAAAGTAATCATAATATTTGTTATGCTGATACAGA
AATTGAAGCGGTAAATTATGAACCTCGAATATCCGAAGTACTGAATGCTTCGCCACACGAAGGTTTGATTTTATTAAAAATTACGCA
CTATAATGAATCAGATGAACCTATTTTGTATTCATTAAATTATATGAAAAATAGCTTAGTTCAATTTAAAATCACTAGAAAAATATA
GATGTAAGAAGATATAGAGGAGGCTATATATTTGAGTAGACAATCTCAACCAAATATACATATCAAAGTTTCACCAACAACTAAAT
TTAAAACAACTACTATAGTTTTTAAATTTATGGCACCTTTAGAATATGACACAATAACAGCTAGATCATTATTAAGCAAATTATTAG
TTCGAGCAACTAAGAAATGGCCAACCGATAAGTCGTTTAATAATCACTTAGCCGATTTATATGGTGCGTATGTGAATAGTACAATTT
CAAAATTCAAAGATCAGCATGTCATTACATTTTCATTAGAAATTGTTAATGAACGTTATTTAAGAAACGGTGAATCATTATTTAATC
AAGGATTAGATTTATTACAAGAAATCATTTGGAATCCATTAATTGAAAATAAAGCATTCAATGATAATTTTGTTAACCAAGAGAAA
ACATTATTAGCCAAAAAAATAGAAGCAATGGTAGATAATAAAGCACAATATTCGTTTTTAAAATTACTCGACCATATGTTTGAAAAT
GAAGCATATAAATACTTATCTACAGGACAACTAGAACAAATCCCACATATTACTGCTGAAACACTATATCATACATATCAATCAATG
ATTAATAATGATCAATGTTCTGTTTATGTTGTCGGCAATGTAGAACCTGAAAGTGTTGAGAAACAAATACGTGAAAAATTTGCACTT
AAACCATTCGATAAACATCAATTCCAACATTCTACTCATCATTTACACGATGAAGAAGTTGATTATATTGTTGAATATGATGACGTG
GATCAAGCTAAATTAAATATGGGATACCGTTTTCCAACACAATATGGACAAAGTGGATATGCTGCCTTTGTTGTATTTAACATGATG
TTTGGAGGAGATCCTTCATCTGTTTTATTTAATGAAGTGCGAGAAAAGCAAAGTTTAGCGTACTCTATACATTCACAAATTGATGGC
AAAAATGGCTATTTATTTGTTTTGAGTGGGGTTTCAAGTGATAAGTACGAAACTGCAAAAGACACTATTATAAGTGAATTTGAAAAA
ATAAAAGCAGGAGATTTCACTGAAGAAAAATTAGAGTTAGCTAAAAAAGTAATCATTTCTCATCGATATGAATCTGAAGATCGTCC
GAAAAGTATTATAGAGATTATGCATAACCAAATATTATTAGAGCAACCACAAAGCAAAGAAACATTTATAAATGATATACAGAAGG
TAAGTCGCGAAGATATTGTTTCTGTTGCTGAAAAAGCATTTTTAGATACAATCTATGTGTTGACAAAAGGAGGGGATAAATAATGA
AAGAGCGTTATTATGAATTAATAGACGAAAGAGTATTCGAACAAGAATTAGAAAATGGTTTACGATTATTTATTATCCCCAAACCA
GGTTTTCAAAAGACATTTGTCACTTACACTACACAATTTGGTTCATTAGATAATCAATTCAAACCCCTTGGACAAGACCAATTTGTTA
CTGTACCTGATGGAGTTGCTCACTTTTTAGAACATAAATTATTTGAAAAAGAAGAAGAAGACTTATTTACTGCGTTTGCTGAAGATA
ATGCACAAGCAAATGCGTTTACAAGCTTTGATCGTACAAGCTACTTGTTCAGTGCAACTGATAATATTGAAAACAACATTAAACGTT
TACTTACAATGGTTGAAACGCCTTATTTTACAAAAGAAACTGTTGATAAAGAAAAAGGTATTATTGCAGAAGAAATAAAAATGTAT
CAAGAACAACCTGGATATAAATTAATGTTTAATACATTGCGTGCAATGTATCAACAACATCCAATACGCGTTGATATTGCCGGTAGT
GTAGAAAGTATATACGATATTACAAAAGATGATTTGTATCTATGTTATGAAACGTTTTATCATCCATCAAATATGGTTTTATTTGTTG
TTGGCGATGTGGATCCTGAAGCAATATGTCGAATAGTAAAACAACACGAGGATGCTCGTAATAAAGTTAACCAACCCAAAATCGAA
CGAGGACTTGTTGATGAACCGGAGGATGTTAAAGAAGCATTTGTTACTGAATCTATGAAAATTCAATCACCAAGACTAATGCTTGGT
TTTAAAAATAAACCATTACAAGAAGCGCCTCAAAAATATGTACAACGTGATTTAGAAATGTCATTATTCTTTGAGTTAATTTTTGGG
GAAGAAACAGATTTTTATCAGAATTTATTAAACGAAGGACTTATCGATGATACATTTGGTTATCAATTTGTACTAGAGCCGACGTAT
AGTTTTTCAATCGTGACAAGCGCTACTGAAGAACCAGATAAATTAAAAAAATTATTATTAGATGAGTTGCGTGATAAAAAAGGCAA
TTTCCAAGATGCAGAAGCATTTGAACTTTTGAAAAAGCAATTTATAGGTGAATTCATATCAAGTTTAAACTCACCTGAATATATTGC
TAATCAATATACTAAATTGTATTTTGAAGGTGTTAGCGTGTTTGACATGTTAGACATTGTTGAAAATATCACCTTAGATAGTATTAAC
GAAACGTCATCGTTATATTTAAATTTAGATCAGCAAGTCGATAGTCGTTTGGAGATTAAAAAGTAATGAAAGCATTAGTATTAGGTG
GTTCTGGTTCAATTGGTTCTGAGATAGTCAAACAATTATTAACTGATGGATTTGAAGTTTATGTGCAATATTATCGTACTGATATAAA
TGAATTAACTAGCAAATTTAATGATGATAAAGTTCGTTTTATACAAGCGGATTTATCTCAAACAATTGATATTGACAAGACATTTGG
TGACATTAAATCATTAGACTGTTTAATATATGCAAGTGGTCAGTCTTTATATGGTGTTTTACAAGATATGAAAGACCATGATATTGA
TGCATGTTATCAGTTAAATGTCTTGCAATTAATTCGATTATGTAGATATTTCGTTGATGTTTTACGTCAAAGTGTCAATGGAAGAATT
ATTGTAATTTCATCAATTTGGGGTGAGACAGGAGCTAGTATGGAAACTATTTATTCGGCGATGAAAAGTGCACAATTAGGTTTCGTT
AAGGCGCTTAGTCAAGAGCTTGCACTAACATCAGTGACAGTAAATGCTATCGCACCTGGATTTGTAGCCGGTAATATGGCAAGTGA
GTGGCAAGAAGATGAACTTCAAGCAATGATAACTGAATTACCACAACAGCGATTGGTTTTACCGAGTGAGGTTGCTCATACATGCG
CCTATTTATATCACCCAAATGCTAGAAGTGTTACTGGAACTATACAGAAAGTTAATGGTGCTTGGTATATTTAAAATATAGAACTAT
TTTTATGAGCAAATGTTTTTATGATTGATGTTATAATATGAATATTACAATGAATCATAATTTGTTAATTAAACAAGGGGTGAGGTTC
ACCATGACAGTTGCAGAGAAAAAAGAATGGTACCTAGAATACGAAATTGAAATTAATAGACCGGGTCTTTTAGGTGATGTATCTAG
TTTATTAGGTATGTTAGGTATAAGTATTGTTACAATTAATGGTGTTGATCAAGGTAAACGAGGCCTTTTAATTAAAACAGACAATCT
TGAAAAAGTTGAACGTTTTGAGCAAATAGCTCGTGGTATAAATGAAATTGAAATAACAAAGCTTAAAAAACCAGAATTAAGAGACC
GTCTTGCAGTAAGACATGGTAGATATATTGAGCAAGATGCAAAAGATAAGAAAACCTTTCGATTTGAGCGTGAAGATTTAGGCTTG
TTAGTAGACTTTTTAGCTGAATTGTTCAAAGAAGAAGGTCATAAGTTGATTGGCATTAGAGGTATGCCACGAGTTGGTAAAACTGAA
TCAATTGTTGCGGGAAGTGTTTGTGCACATAAGAGATGGTTATTTATTAGTTCTACTTTAATAAAACAAACTGTACGTAGCTCTCTAA
TTAAAGGGGAATATGATGCCAATCATGTATACATTATTGATGGTGCAGTTACTGCCAGAGAATCTAATCCAAAACATCAAGAGCTT
GTTAACGAAGTTATGACGTTACCATCAATCAAAGTCGTTGAACATCCAGATTTATTTGTTGAAACAAGTACTTGTACAATGGAAGAT
TTTGACTATATTATCGAATTGAGAGAAAATGAAAATCAAGAAATACATTACGAAGAAATGAAGAAACAGACAGTCCAAAGTAAGA
ATAATTTAGATTTTGGAGATCCGTTTGGTGGTGGTTTTGGTTTCTTCGAGTAAGTGTAAGGAGGCTATGAATTGAAAACGGTCGGTG
AAGCGCTAAAAGGTAGACGTGAAAGGTTAGGAATGACTTTAACAGAATTAGAGCAACGTACTGGAATTAAACGTGAAATGCTAGT
GCATATTGAAAATAATGAATTCGATCAACTACCGAATAAAAATTATAGCGAAGGATTTATTAGAAAATATGCAAGCGTAGTAAATA
TTGAACCTAACCAATTAATTCAAGCTCATCAAGATGAAATTCCATCGAACCAAGCGGAATGGGACGAAGTAATTACAGTTTTCAAT
AATAATAAAGACTTAGATTATAAGAGTAAATCAAAAGAGCCAATACAATTATTAGTAATCATGGGTATTACAGTTTTAATAACTTTA
TTGTTATGGATCATGTTAGTTTTAATATTTTAACAGAAATAAATTAGTGAGAAATGAGGATGTATAATGAATATTCCGAACCAGATT
ACGGTTTTTAGAGTAGTGTTAATACCAGTTTTTATATTGTTTGCGTTAGTTGATTTTGGATTTGGCAATGTGTCATTTCTAGGAGGAT
ATGAAATAAGGATTGAGTTATTAATCAGTGGTTTTATTTTTATATTGGCTTCCCTTAGCGATTTTGTTGATGGTTATTTAGCTAGAAA
ATGGAATTTAGTTACAAATATGGGGAAATTTTTGGATCCATTAGCGGATAAATTATTAGTTGCAAGTGCTTTAATTGTACTTGTGCA
ACTAGGACTAACAAATTCTGTAGTAGCAATCATTATTATTGCCAGAGAATTTGCCGTAACTGGTTTACGTTTACTACAAATTGAACA

AGGATTCGTAAGTGCAGCTGGTCAATTAGGTAAAATTAAAACAGCAGTTACTATGGTAGCAATTACTTGGTTGTTATTAGGTGATCC
ATTGGCAACATTGATTGGTTTGTCATTAGGACAAATTTTATTATACATTGGCGTTATTTTTACTATCTTATCTGGTATTGAATACTTTT
ATAAAGGTAGAGATGTTTTTAAACAAAAATAAATATTTGTTTATACTAGATTTCATTTTCATATGGAATCTAGTTTTTTTAATCCCAA
TTTTAGAAATTAGCCACGCAATTGTTTATAATGATATATTGTAAAACGATATTTGTTCATTTTTTAGGCAAATGTGTTGTAGCATCTG
ATACATTGAATCTAAAATTGATGTGAATTTTTAAATGGAATACATGAAAAAAAGAATTAAACGATACAAGGGGGATATAAATGTCA
ATTGCCATTATTGCTGTAGGCTCAGAACTATTGCTAGGTCAAATCGCTAATACCAACGGACAATTTCTATCTAAAGTATTTAATGAA
ATTGGACAAAATGTATTAGAACATAAAGTTATTGGAGATAATAAAAAACGTTTAGAATCAAGTGTACGTCATGCGCTAGAAAAATA
TGATACTGTTATTTTAACAGGTGGCTTAGGTCCTACGAAAGATGACTTAACGAAGCATACAGTGGCCCAGATTGTTGGTAAAGATTT
AGTTATTGATGAGCCTTCTTTAAAATATATTGAAAGCTATTTTGAGGAACAAGGACAAGAAATGACACCTAATAATAAACAACAGG
CTTTAGTAATTGAAGGTTCAACTGTATTAACAAATCATCATGGCATGGCTCCAGGAATGATGGTGAATTTTGAAAACAAACAAATTA
TTTTATTACCAGGTCCACCGAAAGAAATGCAACCAATGGTGAAAAATGAATTGTTGTCACATTTTATAAACCATAATCGAATTATAC
ATTCTGAACTATTAAGATTTGCGGGAATAGGTGAATCTAAAGTAGAAACAATATTAATAGATCTTATCGATAAACAGACTAATCCTA
CGATTGCGCCTTTGGCGGGAAGTCATGAAGTATATATTAGATTGACTGCAAATGCCGACTCAAAAGAACAAGCACAATCATTGATT
CAACCTGTTAAACAAGAAATTCTTGATCGTATTGGAGAATATTATTATGGTTCAGATGACACATTAATTGAGCAAGCTGTAATAAAG
AAAATTCATGAACCTTTTGTAATATATGATGGTATTACTAATGGTGCTTTATATCATCGATTGAAAGAAGTGGATTTAAACGATGTT
CTAAAGGGTATGATTAATCACAATGAAAACTTTGTTGATATTAATAAACCTATTGAGCAGCAATTAAAAGATGCAGTGCAATTTGTT
AATAAATTGTTTAATGTGTCATCAGCAATTATTCTATTAGAGTATGATGGTGTAGTCCATATAGGCTATGATAATAACTTAGAATTTA
AAACTGAGCAATTTAAAATGTCTAAATCTAGAAATTTATTAAAGAACAGAAGTCAAAATTATGCGCTCATAAGATTATTAAATTGG
CTTAGAACAACAAATTAATTGTATTATCGATAAAAATATAAGCACGTTTGTTCGTTTTTGCATTTTGATTTTAAGATTTTATACGAAC
AAATATTCGCAAAATACTTGTATTTTATTTTGAATCCTTGTATAGTATTGGTAAGATAATTAAAAGATAGCAATTTCAATTAGGAGG
TCTCGCTTTGGATAACGATCGTCAAAAAGCTTTAGATACAGTAATTAAAAATATGGAGAAATCTTTCGGTAAAGGTGCCGTAATGA
AGTTGGGTGACAATATAGGTCGCCGAGTTTCAACTACATCAACTGGTTCAGTTACATTAGATAATGCGCTAGGTGTAGGTGGCTATC
CTAAAGGACGAATTATTGAAATTTATGGTCCTGAAAGTTCTGGTAAGACAACAGTAGCGCTTCACGCTATTGCTGAAGTACAAAGT
AATGGCGGGGTGGCAGCATTTATCGATGCTGAACATGCTTTAGATCCAGAATATGCTCAAGCATTAGGCGTAGATATCGATAATTTA
TATTTATCGCAACCGGATCATGGTGAACAAGGTCTTGAAATCGCCGAAGCATTTGTTAGAAGTGGTGCAGTTGATATTGTAGTTGTA
GACTCAGTTGCTGCTTTAACACCTAAAGCTGAAATTGAAGGAGAAATGGGAGACACTCACGTTGGTTTACAAGCTCGTTTAATGTCA
CAAGCGTTACGTAAACTTTCAGGTGCTATTTCTAAATCAAATACAACTGCTATTTTCATCAACCAAATTCGTGAAAAAGTTGGTGTT
ATGTTCGGTAATCCAGAGACTACACCAGGTGGACGTGCATTAAAATTCTATAGTTCAGTAAGACTAGAAGTACGCCGTGCAGAACA
GCTTAAACAAGGACAAGAAATTGTAGGTAATAGAACTAAAATTAAAGTCGTTAAAAATAAAGTGGCACCACCATTTAGAGTAGCTG
AAGTTGATATTATGTATGGACAAGGTATTTCTAAAGAGGGTGAACTTATTGATTTAGGTGTTGAAAACGACATCGTTGATAAATCAG
GAGCATGGTATTCTTACAATGGCGAACGAATGGGTCAAGGTAAGGAAAATGTTAAAATGTACTTGAAAGAAAATCCACAAATTAAA
GAAGAAATTGATCGTAAATTGAGAGAAAAATTAGGTATATCTGATGGTGATGTTGAAGAAACAGAAGATGCACCAAAGTCATTATT
TGACGAAGAATAGTACACAAATTTATATCTATAGTTAAACTTAGCAAATATCCTTATAGGATTGATTGAAAGTGATATTCATCTCAT
AAAGCTAGAATAATATCTAACTTTATGGGATACACTACAAATCGAGACTATAAGGTTTTTTATTTTATTTATTATTACATTATCAATA
GTTTTATAATCGAGCTTCAAAACTTTAGAAAATAGTAGAAATAGCATTCAATATAGTGCAAAAGTGCAAATTGATAACTTGACACTT
ATCTCCTATAAACCGTACAATTAATTTGTATGATTTATATATAATTTCATAAAGTCATATTGAATTTCATATAAAGAGCAAACCCTAG
AAAAGGAGGTGTTTGTGTGAATTTATTAAGCCTCCTACTCATTTTGCTGGGGATCATTCTAGGAGTTGTTGGAGGGTATGTTGTTGCC
CGAAATTTGTTGCTTCAAAAGCAATCACAAGCTAGACAAACTGCCGAAGATATTGTAAATCAAGCACATAAAGAAGCTGACAATAT
CAAAAAAGAGAAATTACTTGAGGCAAAAGAAGAAAACCAAATCCTAAGAGAACAAACTGAAGCAGAACTACGAGAAAGACGTAG
CGAACTTCAAAGACAAGAAACCCGACTTCTTCAAAAAGAAGAAAACTTAGAGCGCAAATCTGATCTATTAGATAAAAAAGATGAG
ATTTTAGAGCAAAAAGAATCAAAAATTGAAGAAAAACAACAACAAGTAGATGCAAAAGAGAGTAGTGTTCAAACGTTAATAATGA
AGCATGAACAAGAATTAGAACGCATCTCCGGTCTCACTCAAGAAGAAGCTATTAATGAGCAACTTCAAAGAGTAGAGGAAGAACT
GTCACAAGATATTGCAGTACTTGTTAAAGAAAAAGAAAAAGAAGCTAAAGAAAAAGTTGATAAAACAGCAAAAGAATTATTAGCT
ACAGCAGTACAAAGATTAGCAGCAGATCACACAAGTGAATCAACGGTATCAGTAGTTAACTTACCTAATGATGAGATGAAAGGTCG
AATCATTGGACGAGAAGGACGAAACATCCGCACACTTGAAACTTTAACTGGCATTGATTTAATTATTGATGACACACCAGAAGCGG
TTATATTATCTGGTTTTGATCCAATAAGAAGAGAAATTGCTAGAACAGCACTTGTTAACTTAGTATCTGATGGACGTATTCATCCAG
GTAGAATTGAAGATATGGTCGAAAAAGCTAGAAAAGAAGTAGAGGATATTATTAGAGAAGCAGGTGAACAAGCTACATTTGAAGT
GAACGCACATAATATGCATCCTGACTTAGTAAAAATTGTAGGGCGTTTAAACTATCGTACGAGTTACGGTCAAAATGTACTTAAACA
TTCAATTGAAGTTGCGCATCTTGCTAGTATGTTAGCTGCTGAGCTAGGCGAAGATGAGACATTAGCGAAACGAGCTGGACTTTTACA
TGATGTTGGTAAAGCAATTGATCATGAAGTAGAAGGTAGTCATGTTGAAATCGGTGTAGAATTAGCGAAAAAATATGGTGAAAATG
AAACGGTTATTAATGCAATCCATTCTCATCATGGTGATGTTGAACCTACATCTATTATATCTATCCTTGTTGCTGCTGCAGATGCATT
GTCTGCGGCTCGTCCAGGTGCAAGAAAAGAAACATTAGAGAATTATATTCGTCGATTAGAACGTTTAGAAACGTTATCAGAAAGTT
ATGATGGTGTAGAAAAAGCATTTGCGATTCAGGCAGGTAGAGAAATCCGAGTGATTGTATCTCCTGAAGAAATTGATGATTTAAAA
TCTTATCGATTGGCTAGAGATATTAAAAATCAGATTGAAGATGAATTACAATATCCTGGTCATATCAAGGTGACAGTTGTTCGAGAG
ACTAGAGCAGTAGAATATGCGAAATAATTTTTGTCTCCCTCACAAATTAGTGAGGGAGCTTTTTTAAGTTGTAGTCTCAATCTAATT
AGACAGCACTTTATCGGTAGTAACTATAGTAAACAGTAGTTATTTGAAAGTAAGACGGACCTTATATTAAATAAGAAGTTATTGCTT
TTAATAAAAATGTTTTAGGCTTCATAATTACTATATTTATATTATGTAAACCTTTAAAGATGAGTGCTTTTCTAACCAATAAAAAAGA
AGAGAAGATGTAACACATCTTCTCTTCTGCAATATTAATTAGGATTTATTTCTAAGTTGAGTTATTTTAATTGTAAATCTGTTTTCTTT
AATTCTTTTATAACTTCTGCAGTATCATAACAATTTGTTGCAATTGTTGAATATCTCTCTGCTAAACGATATGCATTAATGTAAAGCT
TTAAACTTTCTTTAGCTATATCCTCTGCATCTTCGAATTTTGATGGGTTAGACATAACCACTAATTCTGCAAATTTTTCTGGATCAATA
TTAATAGACATGTATTTATTTACAACTCCTATTTATTTTGATGTCTTAATACTAACATATTGAAGTTTTCAGACAAAGTAATGTCTCTC
TATAATTGAAGAAAAATAATTCAAAGAATTTAATATTTACTTTAAATATTAGAACGTATGAATTAAACTAGTATTTATGAGAGGATG
AACAAAACATGAGAATAATGTTTATAGGGGATATCGTAGGTAAAATTGGACGAGACGCAATTGAAACGTACATACCTCAACTGAAG
CAAAAGTATAAACCAACAGTTACAATTGTAAATGCTGAAAATGCAGCACATGGTAAAGGTTTGACTGAAAAAATATATAAACAATT
ACTAAGAAATGGTGTAGATTTCATGACTATGGGTAATCACACATATGGTCAACGTGAAATTTATGATTTTATAGATGAAGCAAAAC
GACTAGTAAGACCAGCGAATTTTCCGGATGAAGCGCCGGGAATTGGTATGAGATTTATACAAATTAATGATATTAAACTTGCAGTT
ATTAATCTGCAAGGAAGAGCGTTTATGCCAGATATTGATGATCCTTTTAAAAAGGCAGATCAATTAGTCAAGGAAGCACAAGAACA
AACTCCGTTTATATTTGTTGATTTTCATGCAGAAACAACTTCTGAAAAGTATGCAATGGGATGGCATTTAGATGGTAGAGCTAGCGC
TGTTGTTGGAACGCATACACACATTCAAACAGCAGATGAACGTATTTTACCGAAGGGGACAGGGTATATAACGGATGTTGGTATGA
CAGGTTTTTATGACGGCATTTTAGGAATAAATAAAACAGAGGTAATTGAGCGTTTTATCACTAGTTTGCCACAAAGACATGTTGTTC
CAAATGAAGGTAGAAGTGTATTATCTGGTGTTGTTATTGATTTAGACAAAGAAGGTAAAACGAAGCACATCGAACGTATATTGATA

AATGATGACCATCCATTTTCAACATTTTAAAATTACGTAAGTAAACATTCGAATTGGACCCTATCGTCCATTAGTATGAATTTAATAT
AGTACCACTGTTTACATAGTAAATCGGTGGTTCTTTTTGTTATCATTTAATATGAAATATATCCATAGGAGGCATATAACTATGAAAC
CACAATTATCGTGGAAAGTTGGCGGTCAACAAGGCGAAGGTATTGAATCAACTGGGGAAATCTTCGCTACGGCTATGAATAGAAAA
GGATATTATTTATATGGATATAGACATTTTTCAAGTCGTATCAAAGGTGGACATACGAATAATAAAATTAGAGTTTCTACGACGCCT
GTTCATGCAATTAGTGATGATTTAGATATTTTGATTGCATTTGACCAAGAAACAATTGATGTTAACCATCATGAAATGAGAGAAGAC
AGTACTATTTTAGCTGATGCCAAGGCTAAACCTGTGAAACCAGAAGGATGTCATGCACAGCTTATTGAATTACCTTTTACAGCAACC
GCTAAAGAATTAGGTACAGCATTAATGAAAAACATGGTTGCAATAGGTGCTACTAGCGCATTGATGAATTTGAATACAAATACATT
TGAAGAACTTATTACTAATATGTTTTCTAAAAAAGGTGACAAGGTAGTTGAAGTCAATATCCAAGCATTAAACGAAGGTTATCAATT
AATGCAATCCCGCTTACCTGAAATCGACGGGGACTTTGAATTAGAGTCAACAGATGCACTACCACATCTATATATGATTGGTAACGA
TGCCATTGGATTAGGTGCAATTGCTGCAGGTTCACAATTTATGGCGGCATATCCTATTACACCTGCGTCTGAAGTTATGGAATATAT
GATTGCCAATATATCTAAAGTAAACGGAGCGGTTATTCAAACAGAAGATGAAATTGCTGCTGTAACTATGGCTATTGGTGCAAATT
ATGGTGGAGTCAGAGCGTTTACGGCTAGTGCTGGTCCAGGTTTATCTTTAATGATGGAAGCAATTGGATTATCTGGTATGACTGAAA
CGCCATTAGTCATTATTAATACTCAACGAGGTGGACCTTCTACTGGATTACCTACGAAACAAGAACAGTCAGATTTAATGCAAATGA
TTTATGGTACACATGGTGATATTCCAAAAATAGTTGTAGCACCAACTGATGCAGAAGATGCATTTTATTTAACTATGGAAGCCTTTA
ATTTAGCAGAACAATATCAATGCCCTGTTATAGTTTTAAGTGATTTGCAATTATCTTTAGGTAAACAAACTGTTGAAAAATTAGATT
ATAATCGCATTGAAATTAAACGTGGTGAAATCATTCAATCTGATATTGAACGTGAAGAAGATGATAAAGGTTATTTCAAGCGTTATG
CGTTAACATCCAATGGTGTTTCTCCTAGACCTATCCCCGGTGTTAAAGGAGGTATTCATCATATAACTGGTGTGGAACACAATGAAG
AAGGTAAACCTAGTGAATCTGCGTCAAATAGACAACAACAAATGGAAAAACGAATGCGTAAAATTGAGCAGTTACTAATTGAATC
GCCAGTAGAAGCTAACTTACAACATGAGGATGCAGATATTCTTTATATCGGTTTTATTTCTACAAAAGGTGCAATTCAAGAAGGTAG
TAACCGTTTGAATCAACAAGGCATAAAAGTTAACACTATACAAATTAGACAATTGCATCCATTCCCAACAAGCGTTATTCAAGATGC
AGTTAATAAAGCGAAGAAAGTCGTTGTAGTGGAGCACAATTATCAAGGACAATTGGCTAGTATTATAAAAATGAATGTCAATATTC
ATGATAAGATTGAAAATTATACAAAGTATGATGGGACACCTTTCCTACCACATGAAATCGAAGAAAAAGGCAAAATAATTGCTACT
GAAATAAAGGAGATGGTATAGATGGCGACATTTAAAGATTTTAGAAATAATGTTAAGCCTAACTGGTGCCCTGGATGTGGCGATTT
CTCAGTACAAGCTGCAATTCAAAAAGCAGCCGCAAATATAGGGTTAGAACCTGAAGAAGTAGCTATCATCACCGGTATAGGATGTT
CTGGCCGTCTTTCAGGATATATTAATTCTTATGGCGTTCATTCTATTCACGGACGTGCATTACCTTTAGCTCAAGGTGTAAAAATGGC
GAATAAAGATTTAACTGTTATTGCATCTGGAGGAGATGGTGATGGTTATGCTATAGGTATGGGGCATACAATCCATGCTTTAAGAAG
AAATATGAACATGACGTATATAGTCATGGATAATCAAATTTATGGTTTGACAAAGGGACAAACATCGCCGTCATCAGCAGTAGGAT
TTGTTACTAAAACAACGCCAAAAGGTAATATAGAAAAAAATGTTGCGCCTTTAGAATTAGCATTATCATCTGGTGCCACATTTGTAG
CCCAAGGTTTTTCAAGTGATATTAAAGGATTAACAAAACTAATTGAAGATGCAATTAATCATGATGGATTTTCATTCGTTAATGTCT
TTTCACCATGTGTGACTTATAATAAAATTAACACATACGATTGGTTTAAAGAACATTTAACAAGTGTTGATGACATTGAAAATTATG
ATTCTACAGATAAACAATTAGCGACTAGAACTGTTATTGAACATGAATCTTTAGTAACTGGTATTGTTTATCAAGATAAAGAAACAC
CATCATATGAATCTCAAATTAAAGAGTTAGATGATACACCACTTGCTAAAAGAGATATCAAAATTACTGAAGACACGTTCAATGCA
TTAACTGAACAATTTATTTAATAATTAAAACTGAGTCGTATAAATGTATTTATAACAGATCCATTTATGCTACTCAGTTTTTTTACTA
TTACAAAAATAAAGGAGTTTTTAAAAATGAAAGACACATTAATGAGTATACAAATAATTCCTAAAACACCAAACAATGACAATGTT
ATACCTTACGTAGACGAGGCGATTAAAATAATTGACGAATCTGGTTTGCATTTTAGAGTAGGTCCGTTAGAAACGACAGTACAAGG
AAATATGAATGAATGTTTAATTTTAATACAATCATTAAATGAACGAATGGTAGAACTTGAATGTCCAAGTATTATTAGCCAAGTTAA
GTTTTATCATGTGCCAGATGGCATCACTATTGAAACTTTAACTGGAAAATATGATGAATAACATTAAAAGTGAAGTAAACTGGATTT
GAATTGGCTTGTTAGAGATGACGTATAACTTTAACTGCTTTTGCACTTTATAGTTAAATTTAATATAATTATTAAATGATACGGGCAA
ATAGAAAGGATTTTGTAAAGTGAACGAAGAACAAAGAAAAGCAAGTTCTGTGGATGTTTTAGCTGAGAGAGATAAGAAAGCAGAA
AAAGATTATAGTAAATATTTTGAACATGTTTATCAACCGCCTAATTTAAAAGAAGCGAAAAAAAGAGGTAAACAAGAAGTTCGTTA
TAATAGAGATTTCCAAATTGATGAAAAATATCGCGGTATGGGGAACGAGCGTACATTTTTAATTAAAACATATGGATGTCAAATGA
ATGCACATGACACTGAGGTCATTGCTGGTATACTTAAAGCATTAGGCTATCAAGCAACGACTGATATTAACACTGCAGATGTTATTT
TAATTAATACATGTGCGATTAGAGAAAATGCCGAGAACAAAGTGTTTAGTGAAATAGGTAATTTGAAGCATTTGAAAAAAGAACGA
CCTGATATTTTAATCGGTGTTTGTGGTTGTATGTCACAAGAAGAGTCAGTAGTGAATAAAATTTTAAAATCGTATCAAAATGTAGAT
ATGATATTTGGTACACATAATATTCATCATTTACCAGAAATTTTAGAAGAAGCATACTTATCTAAAGCAATGGTTGTTGAAGTATGG
TCTAAAGAAGGAGACGTTATTGAAAATCTTCCAAAAGTCCGTGAAGGCAACATTAAAGCATGGGTCAATATTATGTATGGTTGTGA
TAAGTTTTGTACATATTGTATTGTTCCATTTACAAGAGGTAAAGAACGAAGCCGTAGACCTGAAGACATTATAGATGAAGTACGTGA
ACTTGCTCGTGAAGGTTACAAAGAAATAACGCTTTTAGGTCAAAATGTAAATTCTTATGGTAAAGATTTACAGGATATAGAATATGA
CTTAGGAGATCTTTTACAAGCAATTTCTAAAATAGCGATTCCAAGAGTTCGTTTCACAACAAGTCATCCTTGGGACTTTACAGATCA
CATGATTGATGTTATTTCAGAGGGTGGTAATATCGTTCCGCATATCCACTTGCCAGTTCAATCTGGAAATAATGCAGTATTAAAAAT
AATGGGTAGAAAATATACACGAGAAAGTTATTTGGATTTAGTAAAACGAATCAAAGATAGAATTCCTAATGTAGCATTAACTACAG
ATATTATTGTAGGGTATCCAAATGAATCAGAGGAACAATTTGAAGAAACTTTAACTCTGTATGATGAAGTTGGTTTTGAACATGCAT
ATACGTACTTGTATTCACAACGTGATGGTACGCCTGCTGCTAAAATGAAAGATAATGTACCTTTAGATGTCAAAAAGGAACGATTGC
AACGTTTGAATAAAAAAGTTGGTCATTATTCACAAATAGCTATGAGTAAGTACGAAGGACAAACTGTAACAGTACTTTGTGAAGGT
AGTAGTAAAAAAGATGATCAGGTTCTTGCTGGCTACACTGATAAAAATAAGCTAGTTAATTTCAAAGCGCCTAAAGAAATGATTGG
TAAACTAGTGGAAGTACGAATAGATGAAGCTAAACAGTATTCATTAAATGGCAGTTTTGTAAAGGAAGTAGAGCCGGAAATGGTGA
TTCAATAAATGTATAATAAAGATGACGTGTTGAACCAAGCGGATAATATTGCAAATAAAATTAAAAATTTGGATACTATCAAAACA
TATCAACAAATTGAAGCACAGATTCATCAGAACCAAACGATAAAGACTAAAATGGATATGTTAAAAAAGCATCAAAAACAAGCAG
TAAACTTTCAAAATTACGGGAAACAAAATGCGCTAGAACAGTCGGAACATACCATTCAGAGTATAGAAGCAGAAATAAATACATTG
CCCATAGTTGAACAGTTTCAAACTTCACAATATGAAGCGAATCAATTATTGAAAATGTTTGTATCAACAATGGAAACACGTTTAAAT
GACCATAATAAAGCCAAGCATAGTGATTAATTACAAACAAAAAGGAGAACAATCATATGAAATCAAGAAAACTGGCTATAACTGC
ACTTTTAATTGCAATAAATGTTGTATTAAGCAGTATTATCATCATTCCTCTAGGACCAGTTAAGGCAGCACCAGTACAGCATTTTGTA
AATGTATTAAGTGCGGTGATAGTAGGTCCTTGGTATGGATTAGCTCAAGCGCTTATATCATCAATTTTAAGAGTTCTTTTTGGTACTG
GTACAGCTTTTGCATTTCCGGGTAGTATGATTGGAGTTTTATTGGCTAGTATGTTTTACATATATCGTAAACATATATTTATGGCCGC
GGTCGGTGAAGTACTTGGAACTGGTGTCATCGGAAGTTTAATTTGTATACCATTAGCATATTTCCTTGGGCTTCAAGACTTCTTCATT
AAACCGTTAATGATTACGTTCATAGTCTCAAGTGCTATCGGATCTATTATAAGTTATTTCTTATTAATTACTCTAAAAAAACGTGGTA
TTCTTCAAAGGTTTATAAAATAATTTTATGATATATGATTTCAAACAGAGTCATTGTTCATTTTAATATTGAACGCCCTTACTTAATT
AAATGTCTTGAATGTTAGAATTTTAATTTAATTCTAAACATGCTATTGACACATTTTATTAGGTTTGAGGCGTTTTCTTATACATTTAA
GATGCTTCGTGATTATATGGTATGAATAGGTTTGTAAAAGTAAGTATGTTAAAATGTATAAGTTAAATAATTAATAATTAAAAAGCT
TACAAACGAATATATTGTAAAGACAAATTTTAAAAAGAGACAAGGGTTGTGAAGAAATAATATGTCTAATGTTACACCAATGATGC
AGCAATATTTAAAAATAAAATCAGAATACCAAGATTGCTTATTATTTTTTAGACTAGGTGATTTCTATGAAATGTTTTATGAAGATG

CCAAGGAGGCATCACGTGTACTTGAAATTACTTTAACTAAAAGAGATGCTAAAAAAGAAAATCCAATTCCGATGTGTGGTGTTCCG
TATCATTCTGCAGATAGTTATATAGATACACTTGTTAATAATGGATATAAAGTAGCTATTTGTGAACAGATGGAAGATCCGAAACAA
ACGAAAGGTATGGTTAGACGTGAGGTAGTAAGAATTGTGACTCCAGGAACTGTGATGGAGCAAGGTGGTGTAGATGATAAACAAA
ATAACTATATTTTAAGTTTTGTTATGAATCAACCTGAAATTGCGCTTAGTTACTGTGATGTTTCTACTGGCGAATTAAAGGTTACACA
TTTTAATGATGAAGCGACTTTATTAAATGAAATTACGACGATAAACCCTAACGAAGTTGTTATCAATGACAATATTTCCGATAATTT
AAAAAGACAAATTAATATGGTGACAGAAACAATAACAGTCAGGGAAACGTTATCATCAGAAATCTATAGTGTGAATCAAACTGAA
CATAAATTAATGTATCAAGCGACACAATTATTGCTAGATTATATTCATCATACACAAAAACGTGATTTATCGCATATCGAGGATGTT
GTTCAATATGCAGCTATAGATTATATGAAAATGGATTTTTATGCTAAGAGAAACCTTGAGTTAACGGAAAGCATTCGATTAAAATCA
AAAAAAGGAACGCTACTTTGGCTAATGGACGAAACGAAAACACCAATGGGAGCACGCCGCTTAAAACAATGGATAGATAGACCAC
TAATAAGTAAAGAACAAATTGAAGCACGATTAGATATCGTTGATGAATTTAGTGCTCATTTCATAGAAAGAGACACCTTAAGAACA
TATCTTAATCAAGTGTATGATATTGAACGTCTTGTTGGGCGTGTTAGTTACGGAAATGTTAATGCGAGAGATTTAATTCAACTTAAA
CATTCCATTTCTGAAATACCGAATATTAAAGCATTACTAAATTCTATGAATCAGAATACTCTTGTACAAGTTAATCAACTAGAACCC
CTTGATGATTTACTTGATATATTAGAACAGAGTTTAGTAGAAGAACCACCAATTTCAGTTAAAGATGGCGGACTATTCAAAGTTGGT
TTTAATACGCAATTAGATGAATATCTTGAAGCTTCAAAAAACGGAAAAACATGGTTAGCAGAATTACAAGCCAAAGAAAGACAAC
GTACAGGAATAAAATCATTGAAAATAAGCTTTAATAAAGTGTTTGGTTATTTTATAGAAATAACACGTGCCAACTTGCAAAATTTTG
AACCAAGTGAATTTGGTTATATGAGGAAGCAAACGTTATCGAATGCTGAACGTTTTATAACTGATGAACTTAAAGAAAAAGAAGAT
ATCATTTTAGGTGCGGAAGACAAAGCCATCGAATTAGAATATCAATTATTTGTTCAGCTACGTGAAGAAGTTAAAAAATATACTGA
ACGTTTACAACAACAAGCTAAAATTATTTCAGAGCTAGATTGTTTACAGAGCTTTGCAGAAATTGCTCAAAAATATAATTACACTAG
GCCTTCATTTAGTGAAAATAAAACATTAGAATTAGTGGAATCTAGGCACCCAGTAGTGGAAAGAGTAATGGATTATAATGACTATG
TGCCTAATAATTGTCGATTAGATAATGAAACATTTATATATTTAATTACAGGTCCGAATATGTCTGGTAAATCGACATATATGAGAC
AAGTTGCCATAATTAGTATAATGGCCCAAATGGGAGCTTATGTCCCTTGTAAAGAGGCAGTGTTACCTATATTTGATCAAATATTCA
CTAGAATAGGTGCGGCAGATGATTTGGTTTCAGGTAAGAGTACGTTTATGGTAGAAATGCTAGAAGCACAAAAGGCATTAACTTAT
GCAACAGAGGATAGTTTGATTATTTTCGATGAAATTGGACGTGGTACTTCAACGTATGACGGTTTAGCTTTAGCGCAGGCAATGATA
GAGTATGTAGCTGAAACATCGCATGCTAAAACGTTATTTTCAACACATTATCATGAATTGACAACATTAGATCAAGCATTACCAAGT
CTAAAAAATGTTCACGTCGCTGCTAATGAATATAAAGGTGAACTTATATTCTTGCATAAAGTCAAAGATGGTGCAGTTGACGATAGT
TATGGTATTCAAGTTGCGAAATTAGCTGATTTACCTGAAAAAGTTATTAGCAGAGCACAAGTGATCCTAAGCGAGTTTGAAGCGTCT
GCTGGTAAAAAATCATCGATATCAAATTTAAAAATGGTCGAAAATGAACCTGAAATTAATCAAGAAAATTTAAACTTAAGTGTTGA
AGAAACAACTGATACTTTATCCCAAAAAGACTTTGAACAAGCATCATTTGATTTGTTTGAAAATGATCAAGAAAGCGAGATTGAAC
TACAAATTAAAAATTTGAATTTATCTAATATGACACCAATTGAGGCATTGGTGAAGTTAAGTGAATTACAAAATCAATTAAAATAG
AGGTGTTGCAAAATGGGGAAAATTAAAGAACTCCAAACCTCATTAGCAAATAAAATCGCAGCAGGTGAAGTAGTTGAAAGACCGA
GTTCTGTTGTGAAAGAACTGTTGGAAAATGCTATAGATGCAGGCGCTACAGAAATAAGCATTGAAGTAGAGGAATCTGGCGTCCAA
TCTATTCGCGTAGTCGATAATGGAAGCGGAATTGAAGCGGAAGACTTAGGATTAGTATTTCATAGACATGCGACTAGTAAATTAGA
TCAAGATGAAGATTTATTTCATATTAGGACATTAGGATTCCGTGGTGAAGCACTAGCCAGTATTTCATCAGTTGCTAAAGTAACATT
GAAGACTTGCACGGATAATGCTAATGGAAATGAAATATATGTAGAAAATGGTGAAATATTAAATCATAAGCCTGCAAAAGCGAAA
AAAGGAACAGATATACTTGTAGAATCATTATTTTATAATACACCAGCACGTTTAAAATATATTAAAAGTTTATACACTGAACTAGGT
AAAATAACAGATATTGTCAACAGAATGGCAATGAGCCATCCGGACATTCGAATAGCACTCATTTCAGATGGCAAAACAATGTTAAG
TACAAATGGTTCAGGACGAACTAATGAAGTGATGGCAGAGATTTATGGGATGAAAGTTGCACGAGATTTAGTACATATATCTGGAG
ATACAAGTGATTATCACATTGAAGGTTTTGTTGCAAAGCCTGAACATTCTAGAAGTAATAAGCACTATATTTCTATTTTTATTAATGG
ACGATACATTAAAAACTTTATGCTAAATAAAGCGATTTTAGAAGGCTATCATACACTCTTAACAATAGGTAGGTTCCCGATTTGTTA
TATTAATATTGAAATGGATCCAATCTTAGTAGACGTAAATGTTCATCCAACAAAACTAGAAGTGCGTTTATCAAAAGAAGAGCAAC
TATACCAATTGATTGTGAGCAAAATACAAGAAGCATTTAAAGACCGTATATTAATTCCTAAAAATAACTTGGATTATGTGCCGAAA
AAAAATAAAGTGTTACATTCATTCGAACAACAAAAAATCGAATTTGAACAAAGACAAAACACAGAGAATAATCAAGAGAAGACGT
TTTCATCTGAAGAAAGTAACAGTAAGCCATTTATGGTAGAAAATCAAAACGATGAAATAGTTATAAGAGAAGATTCATATAATCCA
TTCGTAACGAAAACGTCTGAAAGTTTAATAGCTGATGATGAATCTTCCGGTTATAATAATACACGTGAAAAAGATGAAGACTACTTC
AAAAAGCAACAAGAAATTCTACAAGAAATGGATCAAACATTTGATTCGAATGACGATACATCTGTGCAAAATTATGAGAATAAAGC
GTCTGATGATTATTATGATGTAAACGATATTAAAGGAACAAAAAGTAAAGACCCTAAACGAAGAATTCCATATATGGAAATTGTTG
GCCAAGTACATGGAACGTATATTATTGCTCAAAATGAATTTGGCATGTACATGATTGACCAGCATGCAGCTCAAGAAAGAATAAAA
TATGAATATTTTCGAGATAAAATAGGTGAAGTTACCAATGAAGTACAAGATTTATTAATCCCGTTAACATTTCATTTTTCAAAAGAT
GAACAATTAGTCATTGATCAATATAAAAATGAGCTTCAACAAGTAGGTATCATGTTAGAACATTTTGGTGGTCATGATTATATTGTA
AGTAGCTATCCAGTTTGGTTCCCTAAAGATGAAGTAGAAGAAATTATTAAAGATATGATTGAGCTAATTTTGGAAGAGAAAAAAGT
AGATATCAAAAAATTACGTGAAGATGTAGCAATCATGATGTCATGTAAAAAATCTATTAAAGCGAATCATTATTTACAAAAACATG
AAATGTCTGATTTAATTGATCAATTAAGAGAAGCGGAAGATCCATTTACATGTCCACATGGTCGTCCAATTATCATTAATTTTTCAA
AATACGAATTAGAAAAATTATTTAAGCGTGTGATGTAGAGAGGATGAATCAAGTGAATAACAACATATTGCCTGCCATAAGAAACA
TTAAAGATTTAGAGAAACTGATTAAAACAGATTATAAAATGTGTGTGCTTCTAGATATGCATATAGGACATATAAAAAGTATTATG
GAATTGCTGAAGCAAAATCATATAGAGTGTTTTATTCATATAGATTTGATAAAAGGTTTAAGCCACGATGAATTTGCAAGTGAATTT
ATTATTCAGCAATACAAGCCAAAAGGTATCGTATCGACTAAATCTAAAGTAATAAAAAAAGCTAAATCATTAAATACTTTAACGAT
TTTTAGAGTATTTATTATTGATAGTCAAGCATTGAAACGCAGTATAGATTTGATAAAAAAAGTTGAACCTGATTTTGTTGAAGTACT
TCCAGGTGTTGCGAGTAAAGCGATTCATCATATTCAGAAAGAAACAAACACACAAGTCATTGCAGGTGGCCTAATTAATACAATAG
ATGAAGTCAATGAAGCTGTTAAAAATGGAGCGAAATATGTAACAACTAGTTATGATAAACTTTGGTAAATCATAAGTCTGCAAAAA
TTTCATTTTTCACGAGATTTAGTCATATAACATATTAAAAAAGCCGTTTTCATATGGGATATGTAATAAGGTATTTAAGGTATCATAC
AAAGAATAATTAAATAACATAATTAGCACCTCTAAAACGATTAAAATCATTAAAGTTTTAGAGGTGTTTATTAGTAAAAAATGCAA
CTAGTCATAAAAAATATGGCTGAAGAATGTCAATTAAACATAATGGCGTCATAAATAAATTAGTGCGGATTGATATTTCTAGTTTTC
AAATTAAACTACTTTTATAAAGACTTGAAAAATTAAATTTGACAAAATTTTTACAAAAACCATTGACAACGCTTTCATATCGATAGT
ATCATAACAAATATAATAAAAGTTAATACATAGAATAGAGACGGGAGATTTCTACGAGCCAAACTGCTAGTGTAGGAATCTCTTTG
TCTTTTTGGGAGGACATTTAATATGAATGTATATTTAGCAGAATTCCTAGGAACTGCAATCTTAATCCTTTTTGGTGGTGGCGTTTGT
GCCAATGTCAATTTAAAGAGAAGTGCTGCGAATGGTGCTGATTGGATTGTCATCACAGCTGGATGGGGATTAGCGGTTACAATGGG
TGTGTTTGCTGTCGGTCAATTCTCAGGTGCACATTTAAACCCAGCGGTGTCTTTAGCTCTTGCATTAGACGGAAGTTTTGATTGGTCA
TTAGTTCCTGGTTATATTGTTGCTCAAATGTTAGGTGCAATTGTCGGAGCAACAATTGTATGGTTAATGTACTTGCCACATTGGAAAG
CGACAGAAGAAGCTGGCGCGAAATTAGGTGTTTTCTCTACAGCACCGGCTATTAAGAATTACTTTGCCAACTTTTTAAGTGAGATTA
TCGGAACAATGGCATTAACTTTAGGTATTTTATTTATCGGTGTAAACAAAATTGCCGATGGTTTAAATCCTTTAATTGTCGGAGCATT
AATTGTTGCAATCGGATTAAGTTTAGGCGGTGCTACTGGTTATGCAATCAACCCAGCACGTGATTTAGGTCCGAGAATTGCACATGC

GATTTTACCAATAGCTGGTAAAGGTGGTTCAAATTGGTCATATGCAATCGTTCCTATCTTAGGACCAATTGCCGGTGGTTTATTAGGT
GCAGTGGTATACGCTGTATTTTATAAACATACATTTAATATTGGTTGTGCAATTGCAATTGTTGTAGTTATTATTACTTTGATTTTAG
GTTACATTTTAAATAAATCATCAAAAAAAGGTGATATCGAATCAATTTACTAAAATAAAAAGAAACGTAAATAGCATAATTTAACA
TGTTTGATTCATGGATTATGCTATTTTTTCGCCAAAATTTAACAGATTTTGTACAATGGGTTAGCGATTATTTTTTAATAAAGGAGAT
ACTACTAATGGAAAAATATATTTTATCTATAGACCAAGGAACAACAAGCTCAAGAGCGATTTTATTCAATCAAAAAGGGGAAATTG
CAGGGGTAGCACAACGTGAGTTTAAGCAATATTTTCCACAATCAGGTTGGGTTGAACATGATGCAAATGAAATTTGGACATCTGTGT
TAGCTGTAATGACGGAAGTAATTAATGAAAATGATGTTAGAGCTGATCAAATTGCAGGAATCGGTATTACAAACCAACGTGAAACA
ACGGTTGTTTGGGACAAACATACTGGCCGCCCAATTTATCACGCAATTGTTTGGCAATCACGTCAAACACAATCAATTTGTTCAGAG
TTAAAACAACAAGGATATGAACAAACATTTAGAGATAAGACAGGATTACTTTTAGATCCGTATTTTGCAGGTACAAAAGTTAAATG
GATTCTAGACAATGTTGAAGGTGCACGAGAAAAAGCAGAAAATGGCGATCTATTATTTGGAACGATTGATACTTGGTTAGTATGGA
AATTATCAGGAAAAGCTGCGCATATTACTGATTATTCAAATGCGAGTCGTACATTAATGTTTAATATCCATGATTTAGAATGGGACG
ATGAGTTATTAGAACTACTTACAGTACCTAAAAATATGTTGCCAGAAGTTAAAGCTTCGAGTGAAGTATATGGTAAGACAATTGATT
ACCACTTCTATGGTCAAGAAGTACCAATCGCTGGAGTAGCTGGTGATCAACAAGCAGCATTATTTGGACAAGCTTGCTTCGAACGTG
GTGACGTGAAAAACACATATGGAACTGGTGGCTTCATGTTAATGAATACAGGTGACAAAGCGGTTAAATCTGAAAGTGGTTTATTA
ACAACAATTGCTTATGGTATTGATGGAAAAGTAAATTATGCGCTTGAAGGTTCCATCTTTGTTTCGGGTTCAGCAATCCAATGGTTA
CGTGATGGATTAAGAATGATTAATTCAGCACCACAATCAGAAAGTTATGCGACACGAGTTGACTCTACTGAGGGTGTTTATGTTGTT
CCAGCTTTTGTAGGTTTAGGAACACCATATTGGGATTCTGAAGCACGTGGTGCGATTTTCGGTTTAACACGTGGAACTGAAAAAGAG
CACTTTATCCGTGCAACTTTAGAATCACTATGTTACCAAACTCGTGACGTTATGGAAGCAATGTCAAAAGACTCTGGTATTGATGTC
CAAAGTTTACGTGTCGATGGTGGTGCAGTTAAAAATAACTTTATTATGCAGTTCCAAGCAGACATTGTTAATACTTCTGTTGAAAGA
CCTGAAATTCAAGAAACTACAGCTTTAGGTGCTGCATTTTTAGCAGGTTTAGCAGTTGGATTCTGGGAGAGTAAAGATGATATCGCT
AAAAACTGGAAATTAGAAGAAAAATTCGATCCGAAAATGGATGAAGGCGAAAGAGAAAAATTATATAGAGGTTGGAAAAAAGCT
GTTGAAGCAACACAAGTTTTTAAAACAGAATAAACTTGTAGATTAGACTTTTGTATAAACATTGTGATACAATCAATTTAAGTTAAT
ATTTGAATCGAGAAGCGAGAGATTTGTTCGAACATGTACAATTGAAGGATTGTTTATGTGGGACAAGTCTCTCGTTTTTACATTTTTT
AGGAGGCGTTTTGGAATGGCATTGTCTACTTTTAAGAGAGAACATATTAAAAAGAATTTAAGAAATGATGAATATGATTTAGTAATT
ATTGGTGGCGGTATTACAGGTGCAGGTATTGCACTAGACGCGAGTGAAAGAGGAATGAAAGTTGCATTAGTTGAAATGCAAGACTT
TGCACAAGGAACAAGCTCAAGATCTACAAAATTAGTCCATGGTGGTTTGCGTTACTTAAAACAATTCCAAATTGGAGTAGTTGCCG
AAACTGGTAAAGAACGTGCGATTGTTTATGAAAATGGGCCTCATGTTACGACTCCAGAGTGGATGCTTTTACCAATGCATAAAGGT
GGAACATTTGGTAAATTCTCAACATCAATTGGTTTAGGAATGTATGATCGTTTAGCAGGTGTTAAGAAGTCTGAACGTAAAAAAATG
TTATCTAAAAAAGAAACTTTAGCTAAAGAACCATTAGTTAAAAAAGAAGGTCTAAAAGGCGGCGGTTACTATGTTGAATATCGTAC
TGACGATGCGCGTTTAACTATTGAAGTTATGAAGCGTGCTGCTGAAAAAGGCGCAGAAATTATCAACTATACTAAATCTGAACACTT
CACTTATGATAAAAATCAACAAGTAAATGGTGTTAAAGTTATAGATAAATTAACTAATGAAAATTATACAATTAAGGCTAAAAAAG
TGGTTAATGCAGCAGGTCCATGGGTTGATGATGTTAGAAGTGGTGATTATGCACGCAATAATAAAAAATTACGTTTAACTAAAGGT
GTACATGTTGTTATTGATCAATCAAAATTCCCATTAGGTCAAGCAGTATACTTTGATACTGAAAAAGATGGAAGAATGATTTTTGCA
ATTCCACGTGAAGGAAAAGCGTATGTAGGTACTACAGATACATTCTATGACAATATCAAATCTTCACCATTAACTACACAAGAAGA
CAGAGACTATTTAATCGATGCGATTAATTACATGTTCCCTAGTGTTAATGTTACAGATGAAGATATTGAATCAACATGGGCAGGAAT
TAGACCATTAATTTACGAAGAAGGCAAAGACCCTTCTGAAATCTCTCGTAAGGATGAAATTTGGGAAGGTAAATCAGGTTTATTAA
CTATTGCAGGTGGTAAATTAACAGGCTATCGTCACATGGCTCAAGACATTGTTGATTTAGTATCTAAACGCTTGAAAAAAGACTACG
GTTTAACATTTAGTCCATGTAATACAAAAGGTCTGGCAATTTCAGGTGGCGATGTAGGTGGTAGCAAGAACTTTGATGCGTTTGTAG
AGCAAAAAGTAGATGTAGCTAAAGGATTCGGCATTGATGAAGATGTTGCAAGACGTTTAGCATCTAAATATGGTTCAAATGTTGAT
GAATTGTTCAACATTGCGCAAACATCTCAATACCATGATAGCAAGTTACCATTAGAAATTTATGTAGAACTTGTTTATAGTATTCAA
CAAGAAATGGTATACAAACCTAACGATTTCTTAGTTCGTCGTTCTGGTAAAATGTATTTCAATATTAAAGATGTATTAGATTATAAA
GATGCTGTCATCGATATTATGGTAGATATGCTTGATTACTCTCCAGCTCAAATTGAAGCATATACTGAAGAAGTTGAGCAAGCAATT
AAAGAAGCGCAACATGGAAATAATCAACCAGCAGTTAAAGAATAATTAATTTGTACAATCATAAACTGGTGTCCTGTTTTAAGGGC
ATCAGTTTTTTATACGAGATACATTAGTCAATTGAAACTATGCATCACTAAATGTATGATAATAATAAAAGCGAATATAAAAGCGT
TGTTATTAAAGGTGTGAGATGATGACTGAAAAACAATTTAAATTAACTGTACAAGATAATACGAATATTGAAGTTAAAGTGAATTTT
ACAGATGTAGATTCAAAAGGAATTATTCATATATTTCATGGTATGGCTGAACATATGGAACGTTACGATAAATTAGCACATGCACTT
TCAAAGCATGGCTTCGATGTGATACGTCATAATCATCGAGGACATGGTATTAATATTGATGAATCAACAAGAGGGCATTACGATGA
TATGAAACGAGTTATCGGTGATGCCTTTGAAGTAGCGCAAACAGTGAGAGGCAATGTTGATAAACCATACATTATAATCGGACATT
CAATGGGATCCGTTATAGCTAGATTGTTTGTAGAAACATATCCGCAATATGTTGATGGTCTAATTTTAAGTGGTACTGGTATGTATTC
ATTATGGAAAGGTTTACCAACCGTTAAAGTGTTACAACTGATTACAAAAATTTATGGTGCTGAGAAACGAGTTGAATGGGTTAACC
AGTTAGTATCAAATAGTTTTAATAAAAAAATACGTCCATTACGTACACAAAGTGATTGGATTTCTAGTAATCCAATTGAAGTAGATA
ACTTTATTAAAGATCCATATAGTGGATTTAATGTGTCAAATCAATTATTATATCAAACAGCCTATTATATGCTACATACATCACAATT
AAAAAATATGAAAATGTTAAATCATGCCATGCCTATATTATTAGTTTCAGGATATGACGATCCTTTAGGTGATTATGGTAAAGGGAT
TTTAAAATTGGCGAATATATATTGAAAAGCTGGCATTAAAAATGTTAAAGTGAATCTTTATCATCATAAACGTCATGAAGTGTTATT
TGAAAAAGATCATGACAAAATTTGGGAAGACTTGTTTAAATGGTTGAATCAATTTTATAAAAAATAAAGAAAGTGGAATTAAATAT
GAATAAAAATAAGCCTTTTATTGTAGTAATTGTGGGGCCAACTGCTTCAGGTAAAACAGAGCTTAGCATAGAACTCGCGAAGCGTA
TCAATGGTGAAATCATAAGCGGTGATTCTATGCAAGTTTACAAACATATGAATATTGGAACTGCAAAAGTAACACCTGAAGAAATG
GATGGTATTCCACATCATTTAATTGATATCTTGAATCCTGATGATACATTTTCAGCATATGAATTCAAGCGATTAGCAGAAGATTTA
ATTACTGATATAACGAATAGAGGTAAAGTTCCAATCATAGCAGGTGGAACAGGCTTATATATTCAATCATTAATATATAATTATGAA
TTAGAAGATGAAACAGTTACACCTGCACAATTATCCATAGTTAAACAAAAGTTATCTGCATTAGAACATTTAGATAATCAGCAACTA
CACGATTATTTAGCTCAATTTGATGCGGTTTCTGCAGAAAATATTCACCCTAACAACCGCCAAAGAGTGTTGCGCGCTATTGAATAT
TATTTAAAAACAAAAAAACTTTTGAGTAATCGCAAGAAAGTGCAACAATTTACTGAAAATTATGATACATTATTATTAGGGATTGA
AATGTCGCGTAAAACATTATATTCAAGAATAAATAAACGTGTTGATATTATGTTGGATCACGGATTATTTAGAGAAGTGCAACAACT
TGTTGAACAAGGCTATGAATCTTGCCAAAGTATGCAAGCTATTGGATATAAAGAATTAATACCTGTGATTAACGGACAAATGATTTA
TGAAGATGCTGTCAATGATTTAAAGCAACATTCACGCCAATATGCAAAACGACAAATGACATGGTTCAAGAATAAAATGAGTGTTC
ATTGGTTAGATAAAGAAAATATGTCACTTCAAATGATGTTAGATGAGATTACAACCCAGATTAAGTAAAAGGAGTCCGACAGATGA
TTGCAAACGAAAACATCCAAGACAAAGCACTAGAGAATTTTAAAGCAAACCAAACTGAAGTAACTGTATTCTTTCTAAACGGTTTC
CAAATGAAAGGTGTTATTGAAGAATACGACAAGTATGTCGTAAGCTTAAATTCTCAAGGCAAACAACACTTGATTTACAAACATGC
GATCAGCACTTATACAGTAGAAACTGAAGGTCAAGCATCTACTGAAAGTGAAGAATAAGTTGTAATCATATGGGCACGATTTAATG
ACTCGTGTACATAACGTTTGAAGTATGATAAATAGATATAGAAATAAAGTCATATGTGACATCAATTAAATGATGTTCAAATGACA
AGATACAATAGAGGAATGTTTGTAATAAAAACGCTTCATATAAAGGTCGAGTCAATATATGATACGACTTTATATGAAGCGTTTTAT

TTGCTATGAGCTAGTATATTTTATAATAATTTTTCTATTTCTCTTTCGATTTGAACAGGTTTTTTTGAGGTGCAAATCGTTTAACAAC
GTTACCTTCGCGATCCACTAAAAACTTAGTGAAATTCCATTTGATTTTCTCATTAAAGAATCCGTGTTGTGCCGCAGTCAAATATCTA
AATAAAGGTAATTGATGTTCCCCTTTTACGTCTATTTTTTGATGCATAGGGAAGGTAACACCATAGTTTAATTTACAGTTTTGAGCTG
CTTCTTCGCCTGAACCAGGTTCTTGGCCACCAAATTGATTACAAGGGAAACCTAGAATTACAAACCCTTGATCTTTGTATTTCTCGTA
TAATGATTGCAAACCTTCAAATTGTGAAGTAAAGCCACATTCGCTAGCTGTATTAACAATTAGCATAACGTCACCCTTATATGCATC
TAATTTGTAAGTAACACCTTTATTTGTTTCTACTACAAAATCATAAATTGTCTCCATTGTATCATCCTTTCGATTTACTTAAAATGTAC
CACAAAATCGTGTAATAGTCTTTACTAAAACTCTATGATAGAATACTTTGAGTAGGATTTTATTAAGGAGATGTATAACATGGCTCA
GCAACAAATTCATGATACTAAAAATAAACTAGAAAAAGCTGTCTTAGTCGGTGTACATGCTCAAGATGATAAGCAATTTAATTTTG
AGTCTACAATGGAGGAATTATCATCTTTATCAGAGACTTGCCAACTTGAAGTGTTGGGTCAAATTACTCAAAACAGAGATCGTGTAG
ATCGCAAATATTATGTTGGTAAAGGTAAAATTGAAGAAATTCAAGCATTTATTGAGTTCAAAGATATTGATGTAGTCATCACAAATG
ATGAATTAACGACTGCACAATCCAAATCACTAAATGAAGCTTTAGGTGTAAAAATTATTGATAGAACTCAGTTGATTCTTGAAATAT
TTGCATTAAGAGCAAGAAGTAAAGAAGGTAAATTGCAAGTAGAGCTAGCACAACTTGATTATTTATTACCTAGATTGCAAGGCCAT
GGTAAAAGCCTTTCTCGTTTAGGTGGCGGTATTGGAACTAGAGGCCCTGGTGAAACGAAGTTAGAGATGGATCGCAGACATATTCG
AACTCGTATGAATGAAATTAAACATCAATTGCGGACGGTAGAAGAACATCGCGAAAGATATCGAAATAAAAGAAATCAAAATCAG
GTGTTTCAAGTAGCTTTAGTTGGTTATACAAATGCTGGTAAATCATCATGGTTTAATGTTTTAGCAAATGAAGAGACGTATGAAAAA
GATCAATTATTTGCAACGTTAGATCCTAAAACACGACAAATTCAAATAAATGATGGATTTAATTTAATTATTTCAGATACTGTTGGT
TTTATACAGAAACTACCTACGACGTTAATTGCAGCTTTTAAATCAACTTTAGAAGAGGCTAAAGGTGCAGATTTATTAGTACATGTC
GTAGATAGTAGCCATCCTGAATACCGTACGCAGTATGACACAGTTAATGATTTAATCAAACAATTAGATATGAGTCATATTTCTCAA
ATAGTTATTTTTAATAAAAAGGACTTATGTGATCATGCATCAAATCGTCCAGCAAGTGATTTGCCTAATGTTTTTGTTTCTTCTAAAA
ATGATGGTGATAAATTACTTGTTAAGACGTTATTTATTGATGAAATCAAAAGGCAATTAACTTATTATGATGAGACAATTGCGACGA
ATAATGCAGATCGATTATATTTTCTAAAACAACATACATTAGTGACTGAACTTAAATATGATGAAATTGAAAATGTTTATCGTATAA
AAGGATTTAAAAAATAATAAAAGGACGAAATTCAAATGAAAGATATAAGTAAGATAGTAGCTGACGTCGAATCAACGTTAGCACC
ATATTTTAAAGAAATTGAAGAAACAGCATATATTAATCAAGAAAAAGTATTAAATGCATTTCATCATGTCAAAGCAACCGAAAGTG
ATCTACAAGGATCAACAGGATACGGGTATGATGACTTTGGACGTGATCATTTAGAAGAAATATATGCGCAGGCATTTAAAGCAGAA
GATGCAATTGTTCGTCCGCAAATTATTTCAGGTACGCATGCGATTACTATTGCATTACAAAGTTTATTAAAACATGGTGATGAATTA
ATTTATATAACGGGTAGTCCATATGACACTTTACTTGAAGTCATTGGCGTAAACGGAAATGGTATTGAAAGTTTAATGGAGCACGGC
GTATCGTATAAAGATATTGCACTTAAAGAAGGTAAGATCGATATTGAAAGTGTGTTAGATGGGGTTTCTGAGCGCACCAAAGTAAT
AGCGATTCAACGTTCGAAAGGCTATGATCAAAGACCTTCAATTCCGCTAGATGAAATTGAAAAGGTAATTACTAGGTTGAAAAACG
TGCATCCTAATATTTTAATATTTGTGGATAACTGTTATGGGGAATTTGTTGAAAGACGTGAACCTATAGAATGTGGTGCCGATTTAA
TAGCAGGATCATTAATTAAAAACCCTGGCGGTGGTTTAGCTAAGATTGGTGGATACATTGCTGGTAGAAAAGATTTAATTGAACGA
TGTGGTTATAGATTGACAGCACCTGGTATTGGTAAAGAAGCGGGTGCATCATTAAATGCATTGCTTGAAATGTATCAAGGTTTCTTT
TTAGCACCACACGTTGTCAGTCAGAGTCTTAAAGGTGCATTGTTTACTAGTTTATTTTTAGAAAAAATGAATATGAACACAACGCCG
AAGTACTACGAAAAACGAACTGATTTAATTCAAACAGTTAAATTTGAAACGAAAGAACAAATGATTTCATTTTGTCAAAGTATTCA
ACACGCATCCCCAATTAATGCACATTTTAGTCCAGAACCTAGTTATATGCCTGGTTACGAAGATGATGTTATTATGGCAGCTGGTAC
GTTTATTCAAGGTTCATCGATTGAATTATCTGCAGATGGACCTATTCGTCCTCCTTATGAAGCATATGTTCAAGGAGGATTAACATAT
GAACACGTTAAAATTGCTGTGACAAGAGCTGTTAATCAGTTGAAAGAACAAGGACTTATATAATAGATTAAATTTCACCCTAAATG
ATAATGTGTTTTGGGTGAAATTTTTTGCGGAGATTTATCAGATTATTTTTAAAAATTGAAAAAATATTTTAGCTGTAGATTGTTTTAT
GTCGTTTTAGAAGTCGAAATCGCAGTTATATCAATGTTTTAAGCTTTATGTTAGAAAACCTGACATATTTTTGAAATCCTAAAAAAA
TTATGATAAGTTATTAACAAGTTCAAAAGTAGAGGAGAGGAACAATGATATCGAATGATGCAATCAGACGAAATATGGCTGTCTTC
TCTATGAGTGTAGTAAGTAAGTTAACGGATTTAACACCAAGGCAAATACGTTACTATGAAACACATGAACTCATCAAACCTGAAAG
AACAGAAGGTCAAAAACGTCTGTTCTCACTCAATGATTTGGAAAGATTACTAGAAATTAAATCATTATTAGAAAAAGGATTTAATA
TCAAAGGGATTAAACAAATCATTTATGACTCACAAGAGCATTTAACAACAGATGAACAAGAGATAAGAAAAAAGATGATTGTAGA
TGCCACGCAAAAGCCTATTGGAGAAACTTTGCCAATAAATCGTGGTGATTTATCCCGATTTATTAAATAAAATTTGGAGGATTTTAA
AATGCCAAAACGTACTTTCACTAAAGACGACATTCGTAAATTTGCAGAAGAGGAAAATGTAAGATATTTAAGATTACAATTCACTG
ATATTTTAGGAACAATTAAAAATGTTGAAGTGCCTGTAAGCCAATTAGAAAAAGTACTTGATAACGAAATGATGTTTGACGGTTCTT
CTATCGAAGGTTTCGTACGTATCGAAGAATCAGATATGTACTTACATCCAGATTTAGATACTTGGGTAATCTTCCCATGGACTGCTG
GACAAGGTAAAGTTGCACGTTTAATTTGTGATGTATATAAAACAGATGGAACACCATTTGAAGGGGATCCTCGTGCAAACTTAAAA
CGTGTATTAAAAGAAATGGAAGATTTAGGCTTCACAGACTTTAACCTAGGGCCTGAACCAGAATTCTTCTTGTTTAAGTTGGATGAA
AAAGGGGAACCAACTTTAGAACTTAATGATGATGGTGGATATTTCGATTTAGCACCTACAGATTTAGGTGAAAACTGTCGTCGTGAT
ATTGTTTTAGAATTAGAGGATATGGGCTTCGATATTGAAGCTAGTCACCATGAAGTTGCCCCTGGTCAACATGAAATTGACTTTAAA
TATGCAGATGCTGTTACAGCATGTGATAATATCCAAACATTTAAATTGGTTGTTAAAACAATCGCACGTAAACATAATTTACACGCA
ACATTTATGCCTAAACCATTATTCGGTGTGAATGGTAGCGGTATGCACTTTAACGTTTCATTATTCAAAGGTAAAGAAAATGCATTC
TTTGATCCAAATACTGAAATGGGCTTAACGGAAACTGCATATCAATTTACAGCAGGTGTGCTTAAAAATGCACGCGGATTTACTGCT
GTATGTAACCCGTTAGTAAACTCATATAAACGTTTAGTACCTGGTTATGAAGCACCATGTTATATTGCATGGAGTGGTAAAAACCGT
TCACCATTAATCCGTGTACCATCTTCAAGAGGATTATCTACTCGTATCGAAGTACGTTCAGTAGATCCAGCTGCAAACCCATACATG
GCGTTAGCTGCAATCTTAGAAGCTGGACTAGATGGTATTAAAAATAAATTAAAAGTTCCAGAACCAGTTAACCAAAATATTTACGA
AATGAACCGTGAAGAACGTGAAGCAGTAGGCATTCAAGACTTACCTTCAACACTTTATACTGCATTAAAAGCAATGCGTGAAAATG
AAGTTATTAAAAAAGCTTTAGGAAATCATATCTATAATCAATTTATTAATTCAAAATCAATTGAATGGGATTACTACAGAACTCAAG
TATCTGAATGGGAAAGAGATCAGTACATGAAGCAATATTAATACGCTAAACAATGATGGGAAATCAGTAATATCAAGGGCTAGAG
CGTTTTGCTTTAGCTCTTTTTTGATTTGGAAAGATGGGATTTCACTTGACCACTTTTGTTAAAAAATGGATTTGGTCAAGTTTTGGTCA
AGTAAATGTTTTATGTATTTATTTTTTTAAAGCTGGGGAAGACCATTTTATATTTTAGTTCACATGAATTTTAATATATTTGGAGGTG
ATGAGATGAGGCGATAAAAGAACGTCGCGAAAACGTTGAGTATTACTTGAAAATAGCCATATTCATATTACTTCTCATCGAATTCAT
TTGCAGAACGTTCTAAGGTAAGACCCCGAAAAGAGGGACCTTACTACGCATTATTAAGGGGGTAATACTATATGAAAAGAGAAACA
AAACAAAAAATATCTTTTTGTTTATCGATCGGTATTTTCATTTTAGTTATACTACTATTGATATTCTAAAAAATTAAAAATTTCGCCT
CATCTAATTTATGAATTGCTATGATGAAATATTCAATACAATCAAAAAGTTGATAGAAAACAAAGAGATATCGAGTTATCAAATTA
ATAAAGATACTGGGATAAGTTACGGTAATATTAATGCTATGCGCCGTGGAGAAAGAAGAATAGAAAATTTAAGCTTAAAGAATTCA
AAAATCTTATATGAATATGCGAAAAAGGTATTATAATATAAGCATTGAGTTCAATAGGCGTATAATAACTGCCCAATCTAGTGACTG
AGTGGATGTTACTATAGTATTAAAAGTTTTCTAAAAGTGAAAGAAATCATGTTTAGTGATTTACTTATGTTTTTCGAAATAGTATAAT
AATTTTATATAATATATAGAACTTCAGTATACCTTTTGAGTATATATGGAGTTCTTTCTTTTTTACAAGATGTATATGTATAAATGGA
AATGTTTATTACATTACATAAAAACCTTTAGGTGCTCTTAGATGGATGTTAATCATAAAACAAAGTGATTTTGAAAAATATAGGTTA
TTATAATTTAGAATTGCAAATTATAAATCTTGTCATTGGATATGGGAATTCAAACATATCACTTACAACAATTTGTGGAATGTTAAA

TAACTATAAAGAGGAAAGGAGAAAAAATGATAAGAATGGTTAAAGAAAAAGAATGAATAGCAAAATTAAATTTCTAATAGCTGTG
ACGAGCAAAATAATTCAAGTACTTAATTTTATCAAATCGTTCTTTTAAAAAGGTAAAGAACAAAAGACCTTTACCACATTAAATAGG
GAGGTGGTATTTATGACAAGAGAATTAAGGAAAAAATTAACTCTCTATCTTAACATTGCAACTTTAATATTATTTATTATTAATTTAA
CTAGAAAAAAATAAACTATTCTTTCATTTTGACAAAAATAATGAACCAGTTTAAAGAAATTTATAATACAATAGAAAAATTACTAA
ATGATAAAACAATATCTAATTATAGAATTAATCAAGACACTGGTGTTTCTTATGGTGGTATAAGTGAATTAAGAAGCGGGAAAAGA
AAAGTGAATAATTTAACTTTAGAAACAGCGGAAAAACTCTATAATTACCAAAAACAATTAGAAATAATGATTGAAGATTAAAATAA
GACCATATCTTTTGAAATATCATGGAGTGAGCCTTTTAAATCATCTAATTTTAAAGTATATTAAATATGGATAGCTTAATGTGAATAT
AAATAAAAAATTATCAAATAATATAATATAGAATAGAGGGAGAGAAGAAATTTTATCAATATATAATATAAAAACTCCAATACTAT
TAGGTATTAGTTTATGAATTAACTAGATTGGCAACTAGAAAAAAACAATGTATTGCTAAACGTAGTTACTGCATTAGGATTATTATA
ATTATAAATAGAAAGAGAGTGTGATTAATAATGAATGAGATTGAAACTATTATAAGTGAAATAGAAAAGTTATTAACTAACAATAC
ACCATATAGTATTTCAAAAAACTCAGGTGTACCACGTCAAACAGTTACTGATTTAAAGGTAGGTAAAACTAAAATAAAAGAAGCTA
AATTTAAAACGATAATCAAGTTATATGAATATCAAAGAACATTAGAAAATAAAACAGAATGTTAACAAAAATATTTTGTTAGGTTA
GGTGGACAAGAAATTAAATTTCTAATGATTTCATGGTTGTCTGTCCACGTTTTGTCCACCTCAATAAGATAATCTCTTAGTTTTAGAA
ATTGTACATGGTTAAATAATCAAAAATGCATATTATGAAATTATCATTGCCATTAAATCTTGAATGATCTTTAAGCGATGATATAAA
AGGCTGAACACAGTAAATAAGTTAACTTTAATGCAGTATTCTATACTATACATATTTTGTAATAATGTTTATGCACATAATTTTTCAT
TGACAATTTTTTATATTAAATTGTGTAATTGGCATATATGATGATTTGTGATAATTATATTGTAATATAGCATTTGGTTTTATGATTCT
AAAATTAATAGATTTGAAAAGTATACTTTATTTCATGAAAGTTTTTGGTACATTTATTTGAAAATGTTTTTATTACAAAGTTACATGG
CTAAATGTTTCTTAACAAAATTAATTTTTCGAGTGTAAAGAATCGTGTGGTAAACATAATTTATATAGATTATAAGGAGGTGAAAAT
GGTCGAGGAAGTTAAAACGATAAAATAGTTTGAATGTTTTGATTTCAATTCTGGTAGGACTATTGAATTTGCAATCTAATAAGAATG
TGAGGTTTGATTTAATATGAAGTCATATTCTTTGAATAAAATATTACTTTCTCACCATTAGTAAAATGAAGATGACTATTAAAGTGC
GAAAAACAAATAAAAAAGAGAAAATAGAATTTTTAATAGGGACGTTTATAATTATTCTAGTAATATTAGGGTTCAAAATTATGAAA
TAAATGTATAGTATTTGAGAAGTCTATATGACTCCGGAAAAATTAAAGTCTAAAGATATCTCAATATCTCAAATTTAATAGACTACT
GTAATTAGTAGTTTGAATATTGAAAGACACTAATCTAATAAAAATACATAAAAGGTTAAACTTTAGAAGTGGTTGTAAACTTCAATG
AATACTAAACATTGAAGTTTAGTAAAAGGCGTAAGGTATACAAGACAAATCGTTTATCTTTGACAAAGTAATTGATGATGAAAAGT
CGTTAATATGTAGAAACAAAAATTGAAAATATAAAATTTAACTATTATGAAGTGTTGATAGCCATTAATAAATAGAAAAATAATAA
AATCATTTATAAATAAAAAATACGTTGAGTTCACAGAATTTCGAAAAAAAGCAGAAAACATGGTACAGATATGGAGATGAAAAAA
TGTCAGAATACAAGAAAAAGATAATTGAATTAATTGAAAGTAATTTAACAGGATATGAAATTTCTAAAAAAACTGGAGTTTCTCAA
TATGTACTTTCACAATTAAGACAGGGCAAACGCGAAGTAGATAATCTAACCCTTAATACAACAGAAAAATTATATGAATATGCCAA
TAAAGTTTTGTAATATAAATACTGTATAAATTAATCAAGCTATGTTTATTTTATTTAACTATTAATAAAAATCATATGGGAAATGTAT
ATTATAATAATTAAAATACAAAAATAGTAGATTCCAATTTGTGAAAAAGGAGTATGTCGTTTCATTTAGAATTTATTGCACTAAATT
AGCATCGAATAAGGGAAATTAAGTATTAAGTTTTAAATGATAAAAAAGATTGGAATGGATCGTCTTGAAATGCTCCCTTCAAAGTTT
TCATTTTTTCAATGTCTACTTCGAAGGGGGTATTTCCATTAAATTGTTATAGCTTTTTATATTTGTATAATGAACATATAAGTTTAAGA
AGATGCGAGTGAAGGAAATAAAAAAGCTCAAATGTACCAAATTGTTAATCTTAATAAATCTCTACTTTATAAAGATTGAATGGACA
TTCGAGCATTAATCAGTCAGGAGGGAATTTCACTCCTACAATTTAATAATAATACTTGCTTCACCACTGTACAAGGAGTGATTTGTT
ATGTTCAAAGTGAATTATTCGATTTTAAGTTATTATCCAGAATATAATATCGCAGTAAGTTGGCAACGTTTAAGAGAAGGAAAAACA
ATAAAAAACAAGATTTAATACTGCTGCGTCATGAGGCGCTTGAACATTATTTGATGAATAAGTATAATTTCAACTATGATTATGCAC
ATAAAATTGTATCAAAAAAATACGATTATTCAATTTTTATAAAAAAGAAGGTGGATTAAATGCTTACATTAATAAAATTGAAAGAA
GATGAACAGGTTATAATATATGAATATATACCTGAAGATGATATAAGTAACGGTAAAGGTTCAGTAACTTTTAATAAAAAAGATGC
AGAGGTTATAGATTTCTCATTATCTGAAATAGAAAATGAAGAATATTTTATGTTATATCGTAATAAGTCTTTTTCTGTAGTAAGAGA
CTTTATCGAGAAGCAAGAATTTCCCGAAAATTATAAAATAGCATGGTATTAATGCATCCTTTCTAGACAGATAAAGAGAAAGTGGT
GCTATTTTTATATGCTTTTTTAACTATTTACTGAAATAGCAGAATCACAAAGGTTAACATATTTAAAAGAACTTGGTGAAGATGGCG
AATATAAATATGTTGCCAAAATAGATAGTAAAACGTCTAAACTATGTCATTCGCTCAACGGCAAAGTATTTAAAGTTAAAGATATG
ATACCAGGTGTGAATGCGCCACCTATGCATCCTTGGTGTAGAAGTACCACAGTGCCACATGTTGGCAATTGGTGAGACAAGTTCTTT
AAAGAGCGTGAAGGTAAATATCAAGTAGAAGATGACACAACCAAAGATGAATTACAACAAGCTAAAGTATTAGGAAAGAAAATAT
ATATTACTGATCAAGCAATTGATAAAGTCAGATATGTTGATATTCCAACACATACCAAAGAAGAAAATCAATTTATACAAGAACAA
CATAAAGCCTTGCTTAAAGATGCCAAAGAAAACAATGATAGCAATGAAGTAGCTTATTTATTAAAAGATGGTAAAGTTACAAAAGT
ATATGGTGATCAAGATAGTGTATCTTTTGCACCAGGGGAAAAAGCAACAGAATTGTTATTTAACAGTAAACCGAATTCAATTGTTAT
GTTACATAACCATCCTGGGCAGTCTAGTTTTTCTCTTACAGACTTGTATTTATTTATATTTAATAATTCTATTAAAACACTGACAATTG
TTACAAATAAAGGTCAAACAAAGTACTTAACTAAGACAAAAGAATATTGCAAATCAACTTGTATTGATTGTATTAAAAAATATAAT
AAAAATAAGAATATAAAAAAATTCAATCATAAGGATATTGATATGATTCTAAAGAGATTATATAATAGTGGTAACATAATATATAA
AGTTAGGTGATTAAAATGTATTTTGCACAGTTAGATGGTGAAATAACTAATAAACAGTCACAAGAATTATTGGATAAAGAATACAA
GAAAGCAATAGAATTAGAAAATAAGAATAAAGAACAAAAATTAGAAAAATAGCATCCTTTCTACACAGATAAAGAGAAAGTGGTG
CTATTTTTATACACTTTTTTAACCTTCCAATGTGAAGGTTATTTTTTATTGTCCAAAACGTGCTGATGACATTTTAAAAGCAAGTATG
GAATATCAGTCGACAGACTATAAACGGAGGTATGTCTCATGGAGAAAAATGAAAGTAATATTACTGACGTAACTCAGAACGAAGA
GCAACTAGACAACAGTGATGAACAATCACAACAGAATGAGAAAACATTTTCTCAAGAAGAAGTATCACAATTGATTAAAGAGCGT
ATAGCTAGAGAACGCAAAAAATCAGATGAACGTATTAAAGATGCGGTTCAAGAAGCTGAGAAGTTAGCTAAAATGAAGTAGCTTA
TTTATTAAAAGGTGCTAAAGTTACAAAAGTATATGGCGATCAAGATAGTGTATCTTTTGTGCCAGGGTAACAAGCAACCGAATTATT
ATTTAACAGTAAACCGAATTCAATTGTTATGTTACATAACCATCCTGGACAATCAGGATTTTCATTGAATGGTTTAAAGATGTTTATT
GGAAATAAATCTATCCAAACAACGACTATTGTTACTAACTATAGTGTAGTAAAACATATAAGTAAAACTTCATTATATAATCAGTCT
CAAGTTTATAAAATTATGAAAGATATAAAACAATCTATAACTATGAGAAATAATGAAGCGATAGTTGATAATATTCTAAAGCAACT
TTATAATAAAACATATATAAAACGAAAGGACAAATAGGAGTAATACATTATGTCAATTCAACAATTAGATGGTTACATTTCTATAG
AAGATTTTTTTAAGCATATGGATGAATTATCGAAGAAAGAAGAATTAGAAAAAGAAATTAATCAATCAAAGTCTAAATATCAATAG
CATCCTTTCTACACAGATAAAGAGAAGGTGGTGCTATTTTTATACGCTTTTTTAACTATTTACTGAAAATGCAGAATCACAAAAGTT
AACATATTTAAAAGAACTTGGTGAAGATGGCGAATATAAATATGTTGCCAAAATAGATAGTAAAACATCTAAATTATGTCATTCAC
TCAACGGAAAAATATTTAAAGTTAAAGATATGATACCAGGTGTGAATGCGCCACCTATGCATCCTTAGTGTAGAAGTACCACAGTG
CCACATGTTGGCAGTTGGCGAGACAAGTTCTTTAAAGAGCGTGAAGGTAAATATCAGGTAGAAGTAAAAGAAGCTAAATTACAAG
AAAAAGCTAAAAACCAGATGAAAGAAATGATTGAAAGTTGTAAAATGAAAATAGAAATAAATTGTGAAAAACAAAATGGACATAT
GTTAGGTCATCATCTATATAATGAAAATAAAAAAAGAGCCATTTTAAATAATAAGAAATTGCCTAGCTATACAATACTTTCTATAGA
TTTATTGAATGAATTGTTAAGAGAAAAAATGTCAACAGGCAATCTAATATTAAGTGATGAGCTATTTGATATGAAAGAGATTATTAA
TTTTAATCAAATTATTGGAAAAGTACATATCGATAATGTGTATATTGAAACCAGAAAAGGAAAAGTGCATTATTCGAAGACAGGTG
CTCATATAGTACCTTATATTGATAAGTAGGTGAAAAGTATTGAGAATTGAAGATGCATATCGTAAAGATGTTATTATCACGCTTTTG

AATAATGAAGAATACGAAGGGTTTGTAACTGACTATGAAAATGAATTTGAGAGTGAAACAGGAAATCTTGTTGTAGATATACAGAC
TGATTTTGCTGTTTATTCGTTTGATGAAACTGAGATAAAAAGTATTAGATTATTAAAATAATTTCTAAATAGCATCCTTTCTACACAG
ATAAAGATAAAGTGATGCCATTTTTTGCGCTTTTATAACAACTAAGGATACAAGACACATACCTTCAACAAAATCTTCATCTAAACC
TATCGATTTCCTAAGTTCAATTTTAAACACAAAGTTTATTTGTAAAGATGCACACCTATAAAAATCTTAATCATCGAAAAGTCACAA
ACATCTCCATATTAAAAACTTAAATAGTGCATTTCATTTAAACTAAATATCGATATTCCTACCAAAATAAATTTAGTAATTCAATTGC
TATTACATGACTTTCATAAAATAACTACAACCATCATAATACTTTTCAAATCTCGTCAAATTAAAGTAAATACAGCACAAATTAACA
TTTTTAAATATATAAAATAAAACAAGATACTCAATAATCTGTAGTATCTTGTTTTGGAATAATGTAATTTTATATCATCAACGGCTTC
AAATGAGCAGTTTCATATATAAATAAACATAACTACTCAACTGAACTGGCTTAATTAATAAATGATTTGCCAGACGAAACATAACA
GCATTATTAGATTAATTCTAATAGTTTATTTAAATTTTCTTCGGTTGTCGCCCAGCTGGTTGCGAATCTAACAACACGATGTTGATCA
TCGTATTTTTCCCAAACAGCAAATTTAACTTTTTGTTCTAACTCTGCTATTTTCTCGTTACTTAAAATAAAAAATTGTTGATTGGTTGG
AGAATCAAAGTAAAGACGATAGCCTTTATTTTTAAACCCGTCTTTCATCTTATTTGCCATTTCGATAGCATGTCTGCTTATATTAAAA
TATAAATTGTCCGTAAATAATTCTAAAAATTGTATGCCTGTTAACCGTCCTTTTGCTAAAAGGGCACCGTGGTGTTTGATTCGAGTGG
TAAATTGTTTTGGTTCATTATTTTTTGTGAAAACAATAGCTTCTCCGCAAAGTGCACCAATCTTCGTACCACCTATATAAAATACATC
ACAATATTTAGCGATGTCTTTAATAGTCATATCTGATTGATCACTCATTAATGCATATCCTAAACGTGCACCATCCATAAATAATGG
AAGCTTATATTGCTTACATACTTTACATAACTCTTTCAATTCTGATTTAGAGTATAATGTGCCATATTCTGTAGGATGAGAAATATAT
ACCATTCCTGGGAATACCATATGGTCCTTTTTAAAATCACTTTTAAATGTCTCCATGTAAGTTTCAACATCTGAAGCACTAACTTTTC
CTTCGTTAGATGCTAACGTAATTACTTTATGACCACTAAATTCAATAGCACCGCCTTCATGTACAGCAACATGTCCCGTATCCGCTGA
CAATACACCTTCGTAGCTTTCTAACATTGAATTAATAACAACCTGATTGGTTTGCGTTCCACCTACTAAAAAACGGATAGTAGCATT
TGGACAATCAATTGTATCTTTAATCTTTTCAATTGCCTGAGCTGTGAATTGATCAAAGCCATATCCCGAAGCTTGTACAAGATTTGTA
TCTACTAATCGTTTTAATACTTTTTCATGAGCACCTTCTAAATAATCATTTTCAAATGAGATCACTACATTTCCCCCTAAAACTAATA
TCAGCATTTTAATAAGATAAACTAATTTCAAAACTAGTTCGATATTTAAAATGTATTGTGAATTGTTAAAGTTTGTATCGCATTATCG
CGAAGTTGAATAAATATATTATACATCAAACAATACATTAAGAAAATTAATTAATTTTTAATCATAGCGACTTTTCGTGCTGGAACA
CATATATTAATGATTGAATTTTACTGTTAAAAGTTCTATGATTTAGGAAATAGAGAAAAGAGGGGATTTAAATGGAACAAATTAAA
CTTAAAACTTTTACAGCTGAGACTTTAGAGTTATTAGAAAAAAACATTAATGCTTTTTTAAGTTCTGAAGAAGCTACAAATTTAAAA
TTAGTAAATATTACTATTAAAGAAATAGAAGAAAGAACATTCCCAAATAATGAAGAAGAATTCAATGCAATTTTAACTTTATCTGTG
AATAAATAATATGACGTGAAAGGTGCAAAATGTTTTTTGTGCCTTTCTTTATAGGAGAAAAGACTATGCGATATACATTTTCAAATG
ATTTAGGAACACTTTTTACCATTATTTTAGCCATTGGATTCATCATTAACTTAGTATTGGCTTTTGTTATTATCTTTTTAGAAAGAAAT
AGGCGTACAGCGAGTTCAACTTGGGCATGGCTATTTGTACTTTTTGTCTTACCATTGATTGGTTTTATTCTTTACTTGTTTTTTGGTAG
AACCGTTTCGGCACGCAAATTGAATAAAAACAATGGTAACGTGTTAACGGATTTCGATGGACTTTTAAAACAACAAATAGAAAGCT
TTGATAAAGGTAATTATGGTACTGATAACAAACAAGTTCAAAAACATCATGATTTAGTACGTATGCTTTTGATGGATCAAGATGGTT
TTTTAACTGAAAATAATAAAGTTGATCATTTCATTGATGGAAATGATTTATATGATCAAGTTTTAAAAGATATTAAAAATGCAAAAG
AATATATCCATTTAGAGTACTATACTTTCGCTTTAGATGGTTTAGGTAAAAGAATTTTACATGCTTTAGAAGAAAAATTGAAACAAG
GTCTAGAAGTAAAAATATTATATGATGATGTTGGATCTAAAAATGTTAAGATGGCAAATTTTGATCACTTTAAATCATTAGGTGGCG
AAGTTGAAGCATTTTTTGCTTCTAAATTACCCTTATTAAATTTCCGTATGAATAACAGAAATCATAGAAAAATCATCGTAATCGATG
GTCAACTAGGTTATGTCGGAGGATTTAACATTGGTGATGAATATCTAGGATTAGGTAAACTAGGGTATTGGAGAGATACGCATTTAC
GTATACAAGGGGATGCGGTTGATGCACTGCAGTTGCGATTTATTTTAGACTGGAATTCGCAAGCGCACCGTCCACAATTTGAATATG
ATGTTAAGTATTTCCCTAAAAAGAACGGACCATTGGGCAATTCACCAATTCAAATAGCTGCAAGTGGCCCAGCTAGTGACTGGCAT
CAAATTGAATATGGTTATACAAAAATGATTATGAGCGCTAAGAAGTCGGTATATTTGCAATCGCCTTATTTCATTCCGGATAACTCA
TATATAAATGCCATTAAAATTGCAGCTAAATCAGGTGTAGATGTTCATCTAATGATTCCATGTAAGCCAGATCATCCTTTAGTTTATT
GGGCGACATTTTCAAATGCCTCTGACTTATTATCAAGTGGTGTTAAAATTTATACGTATGAAAATGGATTTATACATTCTAAAATGT
GCTTAATTGATGATGAAATCGTATCAGTGGGCACAGCAAATATGGACTTTAGAAGTTTTGAATTAAATTTTGAAGTAAATGCCTTTG
TATATGATGAAAATCTTGCTAAGGATTTAAGGGTGGCTTATGAACATGATATTACAAAATCAAAACAACTAACCAAAGAATCATAT
GCCAATAGACCGCTGTCTGTTAAATTCAAAGAATCGTTAGCAAAATTAGTTTCGCCAATTTTATAATTTATTTGTAAGGAGTCTCGAT
TATAGAGGCTCTTTTTATTTTGCCCAAAATTAAAATAACGATAAATATATTATGTAAATCTAATAATGACATTTGTCATTTCAAATCT
ATGACAAAGTGTTCTATTTTAAATCTAATAAATTTACTAATATTAAATTAAGAAAAAAATAGAGGTGATTAAATTGATTCAAATATC
TAATATCAACAAGTCATTTAATAAAAGATGTGTTCTAAAAAATATTTCGTTCGATATTGAACAAGGTAAATGTATCGCTTTAATTGG
AAAAAATGGTGCTGGAAAGTCAACGTTAATTGATATATTAATTGGTAATGTTAATGCTAATTCTGGTGAGATATTTGATAAAGACAA
GTTATTACAAAGTGAAAATCGCAGTATAATGTTCCAAAAAACGATGTTTACAGATCAATTAAAAGTTATTGAGATTATCAACTTATA
TCAATCATTTTACGAAAATCCATTACCATTGGAAGAAATAATAGAACTGACGAAATTTGATTCTAGTCAACTGAACCAATTTGTAAA
TAAACTTTCTGGTGGTCAACAACGATTACTCGATTTTGTATTATCTTTAATCGGACAACCACAATTGATCTTATTAGATGAACCAACA
TCGACTATGGATATAGAAATTAGAGAATATTTTTGGTCAATTATTGAAAATTTAAAAGAAGATAATCGAACGATACTCTATACATCG
CACTATATTGAAGAAGTCGAACGTATGTCAGACAAAATTATTCTCATTGAAAATGGAGAAATAATACTTAATGATTCAACGTCACAT
ATTAGAACCAATCAGCAATCTCAGATTACGTTATCCGATGAATATATAAGAAAGTTAAAACTAGATAAAGATGATTTAGTTATTCAA
AAAAATCATAATGGCACTATCAAAATTATTACTTCAAATGTAAATGATACGATTTTATATCTTCAACAACTTCATATTAATTTGGATG
ATATTGAAATACAAAAAGTCTCAATTGTTGATTCATACTTCAACAATAAAAAGCAAAGGGGATCTAATTATGATACTAAGTTACTTG
AAAATCGAATTTAAAGTTATAATGCGTAAAAAAACAACATTAATATTATCTATTTTATTTCCTGTTATATTCTATATATTATTTACTT
CGATATTGGAATTGCCGGAAGATGTTAAACCTAAATTTTATAAAGAGTATATGTATAGTATGACGGTTTATAGTTTGTTAAGTTTTA
GTTTACTAACTTTTCCATTAGATATTATTAATGAAAAACAAAATGAATGGCGCCAAAGATTAATGGTAACACCATTTACTTTTACTA
GTTATTATATTTCAAAAGTAGTGAAAACTATGCTGCAATTTGCAATAGCGATATTAGTTATTTTTATGGTTGGACATTTTTATAAAGG
TGTTGCAATGAGTGCAGTTCAATGGTTAGAGTCAGGAATATTTTTATGGTTAGGTGCGTCTCTATTAATAACTTTTGGCATATTATTT
TCTTTGTTAAATGATATTCAAAAAACAAGTGCTTTAGCTAATATCGTAACAATTGGTTTAGCAGTATTAGGTGGATTGTGGTTTCCGA
TAAACACATTTCCAAATTGGCTTCAACATGTTGCTCATGTTTTACCGAGCTATCATTTGCGTAAACTAGGTGTAGATATTGCTTCAAA
TCATCATATCAATTTAATATCATTTGCTATAATACTCTTGTATGCTTTAGGGAGTATAATAGCAGTATATTGTATTAGTCATTTTAAA
AGGGCGGAATAAAATATGAAATTTTTAAAAGATACTTCAATTGCTGAAATATCGTCTATACTTTATCTGATTTTTCCTATTGCCGGTA
TATTTTTTAATGAAGTATATGGTCCCAAATGGTTGTATATTATATCAGTCATTGTCTTTTCGTTGTCGTATCTTATATTAGTTATAGTA
AATAATAGACTTAATACATTAATGTTTTACATTTTGTTGATTATTCATTATTTTATTATTTGTTATTTTGTTTTCAGTGTACATCCAATG
CTAAGTTTGTTTTTCTTTTATAGTGCTTTTGCCGTTCCATTTACTTTTAAAAATAATGTTAAAAAAACGGCAACTAATCTTTTCATACT
AACAATGATTATATGTACAATAATAACGTACTTATTGTATAACAACTATTTTGTTGCAATGATGGTTTATTATGTCGTTATATCGTTA
ATAATGCTAGATAATTTTAAAAAAATGAAAAACCGTGAATATCAAAAAGAAATAGCAGAAAAAAATAGACATATTAATACATTAA
TTGCTGAACAAGAGCGACATAGAATTGGTCAAGACTTACATGATACGTTAGGGCATGTGTTTGCAAGTTTATCATTAAAATCAGAAT
TAGCTTATAAACTAATAGATGCTGATGTAGAAAAAGTAAAAGCTGAATTATTAGCAATTAATAAATTATCTCGTGAATCATTGAACA

AAGTTCGAGAAATTATTGATGATGTAAAATTACCATCATTTATTGAAGAGATTGATAGTATACGTAAAGTTTTAAAAGATGCTGATA
TTGATTTTACATTTGAAAATAAAGAATTAGCGCAAGTATTAAGTCCTACTAAACAATCTATGTTAGTTATGATTACGCGTGAAGCGA
TAAATAATGTTATTAAACATGCAAATGCTTCAAAAGTTCATGGTAAATTAAAAACTGTAAACAATCATAAATTACTGCTTATGATTG
AAGATGATGGCAAAGGTATCGATAGTGATTGTGAGGTGAAAAGTATTTCACAGCGTGTACAACATTTAAATGGAACTTTAGCAGTC
GACTCAACAAATGGAACTAAAATAATCATTGAAATCTCAACAGGAGGAATAGCATGACATCTTTAATTATTGCAGAAGATCAAAAT
ATGTTACGACAGGCAATGGTTCAATTAATTAAACTACATGGTGATTTTGAAATTTTAGCAGATACTGATAATGGTCTCGATGCAATG
AAACTTATTGAAGAATATAATCCTAACGTTGTTATTTTAGATATAGAAATGCCAGGCATGACTGGACTTGAAGTTTTAGCGGAAATT
AGAAAAAAGCATTTGAATATTAAAGTGATTATTGTAACAACTTTTAAAAGACCGGGATACTTTGAAAAAGCAGTTGTGAATGATGT
GGATGCATATGTTTTAAAAGAACGTTCTATAGAAGAATTGGTGGAAACCATTAATAAAGTAAATAACGGAGAGAAAGAATATAGC
GCCACATTGATGACTTCATTTTTTGTAGATAAAAACCCATTAACGCCCAAAGAACAAATTGTATTAAGGGAAATTGGCAATGGTTTA
AGTAGTAAAGAAATAAGTGAAAAATTATTTTTGACAGATGGAACAGTTAGAAATTATACATCTGTTATAATTGATAAATTATTTGCA
GATAATCGTTTTGATGCTTGGAAAAAGGCAAATGAAAAAGGCTGGATCTAAATACAAACAAAAAAGTATTGAGTGTTTTGATTTAA
GAGACTTGGAAAGTGAGTCAATAATCAAAATCTTACTCAATACTTTTATTTTGGTACTCGCAATTTAGATTTGTTAACTAATTTTTTC
GCTTATTATAAAACGAAACAATCAAGTAAACGATAAAGCCTACAAAGATACCCAATAAAATAGATAGTACTGCCGTCACTATTAAT
GGTAATTTGAAAAATATTTGTAGGAAAATACCAATGATAATTGCGATAATTACTGCAATTAATGTGACTGTATTTTCATTTGAAATG
TTCATTTATTTTCACTCCTTAACAATAACATTATATCATGCTATAGCTTTCCAAAATATTGAAATATGTAGATATGGCTATTGACGAT
ATTTCTTAACTTTTATATGATTAATCGGAATGAAAAAAGAGAAGTAGGTGGCAATATGAAGTCAAATAAATCGCTTGCTATGATTGT
GGTAGCCATCATTATTGTAGGTGTATTAGCATTTCAATTTATGAATCATACGGGTCCTTTCAAAAAGGGGACGAATCATGAAACTGT
ACAAGATTTAAATGGTAAAGATAAAGTACATGTTCAAAGAGTTGTGGATGGTGATACATTTATTGCAAATCAAAATGGTAAAGAAA
TTAAAGTTAGGCTTATAGGGGTTGATACGCCAGAAACGGTGAAACCGAATACGCCTGTACAACCATTTGGCAAAGAAGCATCAAAT
TATAGTAAGAAGACATTAACAAATCAAGATGTTTATTTAGAATATGATAAAGAAAAACAAGATCGCTATGGTAGAACATTGGCGTA
TGTATGGATAAGTAAAGATCGTATGTACAATAAGGAATTAGTGGAAAAGGGACTTGCTAGAGAGAAGTATTTTTCACCAAATGGCA
AATATAGAAATGTATTTATAGAAGCACAAAATAAAGCTAAACAACAGAAATTAAATATTTGGAGTAAATAATTAAATAGGTGATTA
TATATATTAAAGTAAAATATACTTGAAGCATCGATTTATTATTACCATAAATTTATATATAGAAATAATTTAAAGGCATAATAACCA
TAGTGCGTATCGAGCGTAATGGATTATTATGCCTTTGATTTTACTTGAATATAGCTGTGATTTGATCAACCTGTTCTTTATTTAAAGG
GTGTTTAGATAGCTCTATGGCTTCGCTAATAATACGTCGTAACGAAGATTTTGTTGAAACTGTATCAACATATTTATCTCTTTCTTCA
GAACCTTGAATGATATTAACTGTACCATCACTAAAGTAAATAACACCGGTACTTTGGACTTTAAAGTTAAAGTTTTGTTCAATATGA
TCCTTTAATGCTTTCGCATTATTTGCTGCTAATTGATACGGATCATAATCATAAAAATCATAAATAACACGATTTGGTTGAACTGTTT
CTGTAAAAGTATAGATAGACTTTCTTGATGAATTAAACTGATCATGATATTGCTGGCTAATGTAATGACCGACAACTTTTTCAATATT
ACTATTGCTTATTTCTGTATCATGCTCATCAGGTACATCAAAGTGATAAAATGTTTTTTCACCCCAGCTTTTTACATCAACGTTTATTA
AACCGATATCTGAAACAATGATAAAATCAAATGAACGTGCATACTCGAAAAACGGATGTTTAGTAGCTAAATTGCTTGTAACGATA
ATATCGTAATATTTTAATTTACCATTTTCGAGATAGCTATCTAAAATCTTGCGTAATTCTCTTTTAGCATTTTTGTATGCATGGTGGCC
AACATCATTATTAGAACTCAGCATTTGACTTCTTAATTCAGCATTTTCTGCACTTAATGCTTTGTTCTTTTTAATAAGTTGCTTTCTTG
CATAAACTTCGGTATCTATTTTACTATTACTATACCTTTGATTTAAAACTAATATACCAATTAATGCTACAATGATAATGATAAGTAC
AACATAAAAAGACATTTTTTCACCAATCCTTTTTGACTTCTTTAACTTTGTATACAATAATAATTAATAAAGATTAATTGTTATTCAA
TTTCCCACATTTTTATTAGTTGATTTTAGTTCATCATTGTTATAATCAAATTATAAACTGACAGATATTGATGTTCAATGAATATGAC
GTGAAAGATTCGTGAATTCAAGTTTATGTCGAATTTATGTTATAACGGTCATTTAAATGACAGAATTAGGTCACTCATAGTATTTTG
AAGATTGAATTCATTAATTTTAAAATGTATAATGATATTTGTGAAAGCGCTTGCTTAGGAGGTGTATTTGAGAGTGAATGAAATGAA
TGCTAAAGAACAATTAGTGGACAATTTAATGAAAACATCATCGCAATTATTTAAATTTCACGGTGAAGTTGCCATGCAGCTTTTCTT
AAATGATGAATTAAAATTACCTTCTATTGTTGAAATATGCGTAGAACGTAAGCGTTTAAGTGATATTGTGAAAGTTATTCCGCAATC
ATATGCGTTACTATACATAGATAAGCAAGATCAAGCAATAGCTAAAGAAGATTTATCACTTTCAAAAATTGCAAAAGTTTATGTGC
AATATGATGATACAACAATAATGAGTATTTTCGTTTATGATGTAGTAAACGATGAATGGATTTTTAGATTGGATCCGAATATACGTA
TACCTAAGAGTAACATATACTTCCATAGTTTAAATTGGGATGTGGATTATATTAAACCGGAGATCGTTCTAATGTATGATCTAATGC
AACACCATCAGTATCATCATTATTCCAATTATAAACGAGTCATAGATGCATTAAGCTACTATCAATTTTTTATTTTAAAATTTGTAGT
AGGTGAGCAACGTATTAAGGATGCAATCCAGAGAACAATAAATAATTAAGAAAAAGCAATTCATAACGCAGTTGAATACATGTGTT
ACGAATTGCTTTTATATTAGTTTTTATCACACAAGTTTTTTAATGCAACCCCGTGATAGCAAAACTCATATGTAGATAATACAGCTTT
TTCAGCATCATCTACATGAATTCCAAACATCATCGAGATTTCCGAAGCACCTTGGTTAATCATTTTTAAGTTAATTTTTGATTCAGCT
AAGGCATGTGTAATTTTATTTGCAGTACCGATGACTTTATTCATACCTTCGCCGACAATCATTAATATTGCTAAATCATGCTCAATAC
TTAGCTCATCAACATCACATTTTTGACGAATTTCATTTAATACTTTTGTTTCTTTATTTTGAATTTGTTTTGAGCGCATAACGATACTG
ATAGTATCAATACCTGAAGGCATATGATCAAATGAAATATTATTATCCTCTAAGACACCTAATATCTTTCTAGTAAAGCCGACTTGT
CTATTCATTAAATACTTTTTGATATTAATAACGGTAAAATCTTTATCACAACTTATACCGCTAATCACATTTTTCGCATTTATTTCTCT
ATCATGCACTATAAATGTACCTTTATCTTGAGGGCGGTTCGTATTTTTAATCACCACAGGGATGCGATCTTTATAAAGTGGTTGTAAG
GCTTCATCATGGAAAACACTAAAACCAGCATAAGATAATTCACGCATTTCTCGATAAGTGATTTCTTCGATTAATTCAGGATCTTTG
ATGATATTTGGATTAGCTTTATAAATACCAGACACATCGGTGAAATTTTCATAAATTGTAGCTCTAACACCACTTGATATGATGGCG
CCAGTTATATCTGATCCGCCACGTGGAAATGTAACTATATATCCTTCATGAGATACGCCGAAAAATCCTGGGATAATTAGTTTCTCA
TCATAATCTCTTAATTTTTTAATTTCAGAGTAAGCACTATCTAATATTTGTGCTTCTTGTGGGACGTCAGTAACAAAAATACCCGCTT
CCTTCGGTGATATATATTTTGTTGGTATACCTTGACTATTATTATATAAAGCTATCAATTGCGCATTAAAATCTTCACCACAAGAAAG
TAATGCATCTAATAGTCTCTTTGGTTCATTTTTTAATTGATTAATATAATGTTCCAAAGTCACATCTATCGTCCGTAAAATACTTTCAT
CCATTTGCAATTCTTTTACAATATCATCATAACGCTGAATAATTTCTCTTTTTTTATCATGATAATCAAGATGATTAATGACCTTTTCA
TATAATCTGATTAACAAATCAGTTGTTTTAATATCATTATCATGTCTTTTACCTGGAGCAGAAACGATAACAATCTTTCGCTCTGGAT
CAGAATTAACAATATTTAAAACCTTTTTAATTTGAGTAGCATTGGAGACGGAGCTACCACCGAATTTGGAAACTTTCATAGTGCATG
CCAACCTTTCTAAAAAATCAGAAAATTGTATTCAGTTTTGTAAGTAAGAATGATATTATGTATTTATATTTTGAATATTACGAACTTT
ACAAAAATAAAGAATAATTCTATACTATACTATCATCTAGTGTTTTTCAATAATGCATTTGTTTTTCTCAAAAGCACAAACGGAGGT
ACATAAAATGAAAAAATTAAATATAGCATTATTAGGATTAGGTACTGTCGGATCTGGTGTTGTTAAAATCATCGAAGAGAACCGAC
AGCAAATTCAAGATACATTAAATAAAGATATTGTCATAAAGCATATTCTTGTTCGAGATAAATCTAAAAAGAGACCGCTAAATATT
AGCCAATATCATTTAACTGAAGATGTTAATGAAATTTTAAATGATGATTCATTAGATATTATCGTTGAAGTCATGGGAGGAATTGAA
CCAACTGTAGATTGGTTAAGAACAGCACTTAAAAATAAAAAACATGTTATTACCGCAAATAAAGATTTATTAGCAGTACATCTTAA
ACTTTTAGAAGATTTAGCAGAAGAAAATGGTGTAGCTTTAAAGTTTGAAGCGAGTGTAGCAGGTGGTATTCCGATCGTAAATGCCA
TAAATAATGGTTTGAATGCGAATAATATTTCAAAATTTATGGGAATTTTAAATGGTACCTCTAATTTTATTTTATCTAAAATGACTAA
AGAGCAAACGACATTTGAGGAAGCACTTGATGAAGCGAAAAGACTTGGTTTTGCTGAAGCGGATCCAACTGATGATGTAGAAGGG
GTAGATGCAGCGCGTAAAGTTGTCATTACATCATATTTATCATTTAACCAAGTCATTAAATTAAACGACGTTAAACGAAGAGGAATT

AGTGGCGTAACTTTAACTGATATTAATGTAGCCGATCAACTGGGGTATAAAATTAAATTGATTGGTAAGGGAATATATGAAAATGG
CAAAGTTAATGCATCGGTAGAACCAACGTTAATTGATAAAAAGCATCAATTAGCAGCTGTAGAGGATGAATATAACGCGATTTATG
TCATTGGTGATGCTGTTGGTGACACGATGTTTTATGGAAAAGGAGCAGGCAGTTTAGCAACAGGTAGTGCCGTTGTCAGTGATTTAT
TGAATGTAGCATTATTCTTTGAATCAGATTTACACACATTGCCACCACATTTTGAATTAAAGACAGATAAAACACGGGAAATGATGG
ATTCAGATGCAGAAATTAATATTAAAGAAAAATCCAATTTCTTTGTAGTAGTGAATCATGTCAAAGGTTCAATTGAAAATTTTGAAA
ATGAGTTAAAGGCAATATTACCATTTCACCGATCATTAAGAGTTGCAAATTACGATAATCAATCATATGCCGCTGTTATAGTTGGAT
TGGAATCATCACCGGAAGAATTAATCACTAAGCATGGATACGAAGTTGACAAAGTATACCCAGTAGAAGGAGTTTAATTATAATGA
GAAGATGGCAAGGATTAGTAGAAGAGTTTAAAGCACATTTACCAGTAAATGAAAATACACCAAAATTAACATTGAACGAGGGAAA
TACACCACTCATTCATTGTGAAAATATGTCTAAAATACTAGGCATAGATTTATATGTGAAGTATGAAGGTGCCAATCCGACAGGTTC
ATTTAAAGATCGCGGTATGGTAATGGCTGTGACAAAAGCAAAAGAGCAAGGTAAGAAAATTGTAATATGCGCTTCGACTGGAAATA
CATCAGCGTCTGCAGCAGCATATGCAGCGAGAGCAGGTTTAAAAGCTATCGTCGTAATACCAGAAGGTAAAATTGCATTAGGTAAA
TTGTCGCAAGCAGTAATGTATGGTGCAGAAATCGTTTCTATTGAAGGAAACTTTGATGAAGCTTTAGAAATTGTAAAAGAAATTGCA
AAAAGTGGCGAAATCGAGCTTGTAAACTCTGTCAATCCATTTAGAATCGAAGGACAAAAGACAGGCTCATTTGAAATTGTACAACA
ATTAGACGGTGAAGCACCTGATATTTTAGCGATTCCTGTAGGTAATGCAGGTAATATTACTGCATATTGGAAAGGCTTTAAAGAATA
TCATGAAGCTAAAGGATCACAATTGCCGAAAATGTTTGGCTTCCAAGCTGAAGGCGCATCACCAATTGTTCAAAATAAAGTCATTA
AAAATCCTGAAACGATTGCAACTGCTATTCGAATTGGTAATCCTGCTAGTTGGGATAAGGCGACTAATGCTCTTAAAGAATCAAATG
GATTAATAGATAGTGTTACTGATGATGAAATTCTAGAAGCATATCAGTTAATGACAACTAAAGAAGGTGTCTTTAGTGAACCAGCG
AGTAATGCTTCTATTGCAGGTTTAATTAAATTGCATAGACAAGGTAAATTACCTCAAGGTAAAAAAGTAGTTGCTATTTTAACTGGT
AATGGATTAAAAGATCCTGATACTGCTATTTCACTACTAGATAATCCGATAAAGCCATTGCCAAATGATAAAGATAGCATTATCGAT
TATATTAAAGGAGCTTTATAACATGTCGAATGTTTTGGAGTTAACAATTCCTGCATCAACAGCCAACCTTGGAGTTGGCTTTGATTCT
ATAGGTATGGCTTTAGATAAATTTTTGCATCTGTCTGTAAAGGAAACATCAGGGACAAAATGGGAATATATTTTCCATGATGATGCA
TCTAAGCAATTGCCTACTGACGAAACAAACTTTATTTATCATGTAGCACAACAAGTTGCTTCTAAATATAGTGTTGACTTGCCTAATT
TATGTATCGAAATGAGAAGTGATATTCCATTAGCAAGAGGGTTAGGTTCGTCAGCTTCTGCTTTAGTAGGAGCTATATATATCGCAA
ATTATTTTGGTGATATCCAACTGTCTAAACATGAGGTATTACAATTAGCGACTGAAATCGAAGGACATCCTGATAATGTTGCGCCGA
CCATTTATGGTGGTTTAATCGCTGGATATTATAATGATGTCTCGAAAGAAACGTCAGTTGCACATATCGACATACCAGACGTGGATG
TGATTGTAACGATACCAACTTATGAACTAAAAACAGAAGCATCAAGACGTGCTTTACCACAAAAATTAACACATAGTGAAGCGGTT
AAAAGTAGTGCAATTAGTAATACAATGATTTGTGCATTAGCACAGCACAATTATGAATTAGCAGGTAAACTCATGCAACAAGATGG
CTTTCATGAACCGTATCGTCAGCATTTAATTGCTGAATTTGATGAAGTGAAAACAATTGCTATTCAACATAATGCCTATGCAACTGT
AATTAGTGGTGCTGGACCAACTATTTTAATATTTAGTCGTAAAGAAAATAGTGGGGAATTGGTTCGCTCTTTAAATAGTCAGGTAGT
ATCATGCCATTCTGAATTGGTCGATATTAATATCAGTGGTGTTAAAGAACGAATTGTATACCAATAGACGCTTTATATTGTAAAATA
GTATTAAATGCAGAATAGAGAGGAGATTTAATGCGATATGACAAATTATAAAGTTGTCGTTTTAGATATGGATGACACATTGCTAA
ATTCAGATAATGTGATATCAGAAGAAACTGCAAATTATTTAACAGCAATTCAAGATGAAGGTTATTATGTTGTTTTAGCATCTGGTA
GACCTACTGAAGGTATGATTCCAACTGCTAGAGATTTAAAATTACCTGAACATCATAGCTATATTATTAGTTATAACGGTAGTAAAA
CGATTAACATGACTAATGAAGAAGTAGAAGTAAGTAAATCGATTGGTAAGCAAGATTTCGATGAAATTGTAGATTATTGTCGAGAT
AGAGGCTTTTTCGTTCTTACATATCATGATGGTCAAATTATTTACGACAGCGAACATGAGTATATGAATATTGAAGCAGAATTAACA
GGTTTACCGATGAAACGTGTTGATGATATCAAAGCGTATATTCAAGGCGATGTACCCAAGGTCATGGGTGTAGATTATGTAGCGAA
TATTACAGAAGCTAGAATTGATTTGAATGGTGTGTTCAATGATAATGTAGATGCTACGACAAGTAAGCCATTCTTCTTAGAATTTAT
GGCCAAAGACGTTTCAAAAGGTAATGCAATTAAAGCGTTATGTCACAAATTGGGATATTCGGTGGATCAAGTCATTGCTTTTGGTGA
TAGTATGAATGATAAATCAATGTTTGAAGTCGCAGGTCTAGCTATTGCTATGGGGAATGCATCAGATGAACTTAAGCAATATGCAA
ATGAAGTTACGTTGGATCATAATGAAAATGGTATTCCACATGCGCTCAAAAAATTGTTATAAATTTTAAAATAAGCCTTAACACATG
ATATTTGAATAAGATATCTTGTGGTTAAGGCTTTTTATTTTTGTGAAAATGACTTCAGTTATACTATGGAGGATTTGAAATACATATT
TTAGATTAGTAATGATATCAAACGAATAGAGTAAATGTATATTTTTTGAATAAATCAAGTATTAACTAGTCACTGAAGGTAGATAAA
TAAATTTGATCATTCTGAATAATAACGAATGCATTAAAAAATGCAGTGACAATCTAACATTAATTGTTAAAATCACTGCAAGCATCT
TGATTTTATTTAGCTGTTTGTGTCCAGTCATGCTTACCTCTGAAACGTTTTACATGAGCATATATTTGGTCAGCAGTATAATCTAAAT
TTGTATAAACTGTAATACTGAACGCTGATTGGTTTTCAGCAAATGATTCTTCGGAAAATAATTCCTTTAAGTGCATAAATAAATCATT
CATTTGTTTATGGTTTAAACCGGGATATTCCCTTAAAGTTCTCTTTAATAATTGTCCGTTCTTTTCTTCTAATGCTTGAACTACAATAA
CAAATCTTTCATCTTGCTTTAGTACATCATCAAATAAATTTGTTTGTCCAATCATTGAATTTCACCCCTGAAAAGTTATTGTTATCTAT
ATTATACACGATATGCAAAATATCTAAAATAAAGAATGGTAACATGGTAATAATAAAATATAAACAGAATACTAGTTAATACACTT
TATACTAGAAAAAGATATTAAAGTATATCTGCTTTACACCATTAAAAAGCGGCAAATGCTATAGATAATCTAAAGCATCTGCCGTAT
GAGAATTTATTTATTTTTGATTGTCATAATCGTGTGGTTTTAAATTTATTGTTTCTAGCTTTACAAATTTTGTTTTGTGAATGATTTTAT
GAATAAAGTAAATCAACGCTAGAATGATTAAAGGTAAAAAGTTTTTAAAAGCATTTAACCATTGATCTTTTAAAATATATTCAACTG
AGCCACCAAATAGCAAGAATAATAGTGTAGTGATGACAATGATTGGTCCTAATGGATAAAAAGGTGCTTTATATGGTAGGACCTTA
TTAGGGTCTTGACCTTGTTTTTTAATAGCTTGTCGCAATCGTATTTGTGACCAAATGCTTGATCCCCAAACAACTATAATCATTGAAC
CAATAATTTCAAGTAAATTAAAAACGGCATTTGAATTAAAGTTTGCATAAATAATAACAATAACAACGACTGCATAAGTAGTTAAT
AATGCTCTTAAAGGTAACTTAGTTGTCTTGTTTAATTTACTTAAAAATTGGGGTGCTTTTTTGTCTGAACTTAAGGAATACAACATTC
TGCCTGTTGTATAAACACCTGAATTTGCAGCGGATAATAATGAAGTTAAAATAACCGCGTTGATTACTGATGCTGCAAAGGCTATGC
CTACTCTATCGAATACAATTGTAAATGGGCTTTGACTTATTGAACTACTTGCTCTTAATAATGATGGATCTGTGTACGGAATAATTGC
ACCAATTACTGCAATTGATAAGACATAGAATAAAAGAATACGCCAAAATACTTGTTTAATTGCCTTAGGCATAGACTTTTTAGGGTC
ATCTGATTCACCAGCAGTTACTGCTACTACTTCTGTACCACCAACCGAAAATCCGGCGACTAATAATACGCCTAAGAAACCAGAGAT
ACCACCAACAAACGGTGCTTGGCCTTTTGTATAGTTTTCAAATCCATATGTATGACCACCTAAGATACCGAAAATCATTAAAAAGCC
AAAAATAACGAATACGATAATTGTTAACACTTTAATCAATGATAACCAAAACTCAGTTTCTCCAAATGATTTTACAGAAAAAAATGTT
TAATAATAGTAAAATTGTAATAAAGATTAAGCTCCAAGTAATGGGGTGGAAAAATTTAAATGTGTCCCAGAAATAAAGCACATTTG
ACGCTACTATGACATCAACACTTGTAACTAATGACCACAATGCCCAATACAACCATCCCATGGTAAAGCCAAGAGATGAGTCAATA
AAGCGTGTTGAATAAGAGCTGAATGAACCTGATACTGGATAAAATGTTGCCAACTCTCCAATTGATGACATTAAGAAATATAGCAT
GACACCAATAACAAGATAAGCGAGTATAGCGCCTCCAGGACCAGCTTGAGAAATGATATTACCAGTAGCTACAAATAGACCAGTCC
CAATTGCACCACCTATAGCAATCATGGAAATGTGTCTTGAGTTAAGACTACGGTTCATTTTATTATCTTCCATATTTAGTCTCCCATC
TATTTAAATATACCCATTATTGTAAGCTTTTTAAGTGTACTATTCAATAACTATTTAGTACTGTAAAGCGAAAAAATTAAAATTTTCT
GATTTTTTAATCATCTTGAGCATGTTTAATTGTAATTTTGATGGGGTTAAATTATAATATGTATTAAATTATAATTATTATAAATTGTG
GAGGGATGACTATGTCACAACAAGACAAAAAGTTAACTGGTGTTTTTGGGCATCCAGTATCAGACCGAGAAAATAGTATGACAGCA
GGGCCTAGGGGACCTCTTTTAATGCAAGATATTTACTTTTTAGAGCAAATGTCTCAATTTGATAGAGAAGTAATACCAGAACGTCGA
ATGCATGCCAAAGGTTCTGGTGCATTTGGACATTTACTGTAACTAAAGATATAACAAAATATACGAATGCTAAAATATTCTCTGAA

ATAGGTAAGCAAACCGAAATGTTTGCCCGTTTCTCTACTGTAGCAGGAGAACGTGGTGCTGCTGATGCGGAGCGTGACATTCGAGG
ATTTGCGTTAAAGTTCTACACTGAAGAAGGGAACTGGGATTTAGTAGGGAATAACACACCAGTATTCTTCTTTAGAGATCCAAAGTT
ATTTGTTAGTTTAAATCGTGCGGTGAAACGAGATCCTAGAACAAATATGAGAGATGCACAAAATAACTGGGATTTCTGGACGGGTC
TTCCAGAAGCATTGCACCAAGTAACGATCTTAATGTCAGATAGAGGGATTCCTAAAGATTTACGTCATATGCATGGGTTCGGTTCTC
ACACATACTCTATGTATAATGATTCTGGTGAACGTGTTTGGGTTAAATTCCATTTTAGAACGCAACAAGGTATTGAAAACTTAACTG
ATGAAGAAGCTGCTGAAATTATAGCTACAGATCGTGATTCATCTCAACGCGATTTATTCGAAGCCATTGAAAAAGGTGATTATCCAA
AATGGACAATGTATATTCAAGTAATGACTGAGGAACAAGCTAAAAACCATAAAGATAATCCATTTGATTTAACAAAAGTATGGTAT
CACGATGAGTATCCTCTAATTGAAGTTGGAGAGTTTGAATTAAATAGAAATCCAGATAATTACTTTATGGATGTTGAACAAGCTGCG
TTTGCACCAACTAATATTATTCCAGGATTAGATTTTTCTCCAGACAAAATGCTGCAAGGGCGTTTATTCTCATATGGCGATGCGCAA
AGATATCGATTAGGAGTTAATCATTGGCAGATTCCTGTAAACCAACCTAAAGGTGTTGGTATTGAAAATATTTGTCCTTTTAGTAGA
GATGGTCAAATGCGCGTAGTTGACAATAATCAAGGTGGAGGAACACATTATTATCCAAATAACCATGGTAAATTTGATTCTCAACCT
GAATATAAAAAGCCACCATTCCCAACTGATGGATATGGCTATGAATATAATCAACGTCAAGATGATGATAATTATTTTGAACAACC
AGGTAAATTGTTTAGATTACAATCAGAGGACGCTAAAGAAAGAATTTTTACAAATACAGCAAATGCAATGGAAGGCGTAACGGATG
ATGTTAAACGACGTCATATTCGTCATTGTTACAAAGCTGACCCAGAATATGGTAAAGGTGTTGCAAAAGCATTAGGTATTGATATAA
ATTCTATTGATCTTGAAACTGAAAATGATGAAACATACGAAAACTTTGAAAAATAAATTTGATATGTAGTTTCTATATTGCGTAGTT
GAGCAGTTTATGATATCATAATAAATCGTAAAGATTCCTAACAAGAGAGGGTGTTTAACGTGCGCGTAAACGTAACATTAGCATGC
ACAGAATGTGGCGATCGTAACTATATCACTACTAAAAATAAACGTAATAATCCTGAGCGTATTGAAATGAAAAAATATTGCCCAAG
ATTAAACAAATATACGTTACATCGTGAAACTAAGTAATTCTTATCATTCAAATACGACGATTTGAAAATAAAGCGGGCTTACCTATT
ATATTGGGGAGCTCGCTTTTTTATGAAATTTTTGTGAAGAGTGATTAATGGATTGAGTTTCATCGGTAGAACAATATATGATTGTATT
AGTTGTTACTTTATTAAAATTTGAGAATATTTATAGAAGGAAATAGATTACTGATTTTATAAAGTCACTTTGTTAGCGAATGCTTGAA
AGAGTATTTAATATAGTAGAATTTAAAATTTCAAAGCGGAATTTAATAAGTACGAAGTAATTCTGGGTATGTTTTATAAATGTTCGA
TAATACACTTTAATCTTAAATATGATGGTTTAGAAAATGATTTAACAAAGAAATGAAACTTTACTGTTGAATTATGTGAGGATTGTG
TTATTATATAAATCGTAATAATTACGATTTGATAAAAAGTGAGGTAACTATATATGGCTAAGAAATCTAAAATAGCAAAAGAGAGA
AAAAGAGAAGAGTTAGTAAATAAATATTACGAATTACGTAAAGAGTTAAAAGCAAAAGGTGATTACGAAGCGTTAAGAAAATTAC
CAAGAGATTCATCACCTACACGTTTAACTAGAAGATGTAAAGTAACTGGAAGACCTAGAGGTGTATTACGTAAATTTGAAATGTCT
CGTATTGCGTTTAGAGAACATGCGCACAAAGGACAAATTCCAGGTGTTAAAAAATCAAGTTGGTAAAATCAAATTTCGTACTTTAG
CCCATTTACAATATCAATAAAACAATGTACAGTATATACGAATGCTATAAACTGAATGTTTTCTCATATTAATAAGGAAACATTCGG
TTTTTAATTTGCATTTAAAAATAATTAGTTTTTAAAGGGGCTATTTAAAGTGAAAATATTTGATTACGAAGATATTCAATTAATACCT
AATAAATGCATAGTTGAAAGTAGGTCTGAATGTGATACAACTATCCAATTTGGTCCGAAAAAATTCAATCTACCTGTAGTTCCTGCA
AATATGCAAACAGTTATGAATGAGAAATTAGCGAAATGGTTTGCTGAAAATGATTACTTTTATATCATGCATCGTTTTGATGAAGAA
GCAAGAATACCTTTTATAAAACATATGCAAAATTCAGGCTTATTTGCATCTATTTCAGTTGGTGTAAAGAAAGCGGAATTTGATTTT
ATTGAAAAGTTAGCTCAAGAAAAATTAATCCCCGAATATATTACAATAGATATTGCGCATGGTCACTCAGATTCAGTGATAAACAT
GATTAAACATATAAAAAACCATATACCTGATAGTTTTGTTATTGCTGGTAATGTTGGTACGCCAGAAGGTGTTAGAGAATTAGAAAA
TGCTGGTGCTGATGCTACCAAAGTCGGTATAGGTCCTGGTAGAGTTTGTATTACAAAGATTAAAACAGGTTTTGGTACTGGTGGTTG
GCAGTTAGCGGCATTAAACATATGTAGTAAAGCAGCTCGTAAACCTTTGATTGCTGATGGTGGTATAAGAACGCATGGCGACATCG
CTAAATCAATTAGATTTGGTGCATCAATGGTCATGATTGGTTCATTATTTGCGGCACACGAAGAATCACCTGGTGAAACTGTAGAAC
TTGATGGTAAACAGTATAAAGAATATTTTGGTAGTGCATCTGAATTTCAAAAAGGCGAACATAAAAATGTGGAAGGTAAAAAAATG
TTTGTAGAACATAAGGGTTCATTAATGGATACCTTAAAAGAAATGCAACAAGATTTACAAAGCTCAATTTCATATGCCGGTGGAAA
AGACTTGAAATCATTACGTACTGTAGATTATGTTATTGTTAGAAACTCTATTTTCAACGGTGATAGAGATTAATATTTATAGTAGGTG
ATGTAAATTAAAAAATTCATAGTAACTGTTGTTGCGTTTTTATCAATTATTATCATTGCGCCAATAACAGAATTTAAACCATTCATTC
ATTTACAAAATGAAGTAAGACAATATATTGACATTCACATCAATAAAGAAACAATTTCTGCGGAAAATAAATTGGATACGCCGAAG
AAACAACAATTTGCCTTTAACAATATACAAATGAACATGTCAAAATCAGATGTTGAGAAAACATTAAATAAACCAAAAAGAGTGAC
ATTTAATGAATATGGTACGAAGTGGTATACGTATTATGATGACGATTACAATAATTTTATAATGATAAGTTACATTAAAGATAAAGT
TAATGCGTTATATACAAATCAAAATATAATCACTTCAAAATCAAAAATTAAATACAATACACCGAAATCGGTTGTAAGGCAAAGAT
TAGGCGAACCAGAAACAGAGATTGTTAAAGGTAGAGTGCGTTACGAACAAAATAATAAAGAATATGATGTTTTCCATAAAAATCAC
ATTTATACGACGGTATTTTATGATAAGCATCGACGTAATAATGTAACAGCTGTTTTACAAGTAAGTGATGCTATGGAAAATAGATTA
AAAGAACAATATGGAGCACCTTCGAAATCGCTTGCAGATAGTTTTGAACTACAAAATTTTGATTTAGTTAATGCTGAAAGAAAACA
ACATCAATTATCTACATTGAAGTATTCTAAACAGAATTCTGAAACTGCACGTAAGCATAGTAAAGATATGGCCAACAATCATTATTT
TGATCATACAAATTTAAAAGGTCAATCACCATTTGATCGATTGAAAAAAGATGGTATTACATTTAACTCAGCCGGAGAGAATTTAGC
ATATGGTCAAGTTAGTAGTATCTATGCACATCAAGGATTAATGAATTCTATTGGTCACAGAAAAAACATTTTAAATGATACGTTTAA
AATATTAGGTGTTGGTGTTGATTTTAATGATGAAAAACAACCTTTTTGGACAGAAAATTATACTGGTTAATGTTTTTGGCATGAGTTT
AAAGGGTAATGTTTGTCTGTAAGGAAGTAGATAAACATGATGACAACAGATAAACCAAAAGATGCGGATATACTTGAACGCGTCA
AAGATATACTTAATAAAAAAGAAAGAAAAAAATAATAATGGCTCGCTCCTGTAAATTATTACGGGGGCGAGTTTTAATTTGTTTAA
CATTTTGCAGGAATCTGTAATTCAAAATTTATATTAAAGAATGAGATTATCTAACAATTCGCAATTTCTGTTTGAACTGAATTTAGAT
TAGAATAAATAAAACCTCTTGAAAACGTAACTTTTTCAAGAGGTCCAAAATTTATAAAAATGAGGGCTTAACAATTACGATAATAT
ATAACGGTTTAAAATATTACATTTCGCGGTACAAACCAATTACTTTCCCAATTACAGCAACATTGTCGAGGTAAATTGGCTCCATTG
TACTATTTTCAGGTTGTAATCGATAACGATTTTTTTCTTTATAGAAGCGTTTGACAGTTGCTTCATCTTCCTCAGTCATAGCAACAAT
AATGTCTCCATTTTCTGCTATGGTTTGACTGCGAACAATTACTTTGTCTCCGTCTAATATACCAGCCTCAATCATACTGTCGCCTACG
ACGTTTAATATGAATATGTCGCTATTGTGTGTCGATGTTAAGTGTTCAGGTAATGGAAAATATTCTTCAATATTTTCTACTGCGGTAA
TAGGAACACCTGCTGTGACTTTACCAATAACTGGCACATGAATCGTTTCTTCCATATTAATATTTTCATTTGTTTGATCACTTACAAT
TTCTATAGCACGTGGTTTCGTCGGATCTCTTCTTATATAGCCTTTTTCTTCAAGACGTGAAAGGTGACCATGAACAGTTGAACTGGAT
GCTAAGCCAACTGCTTCACCAATTTCGCGAACACTAGGCGGATAACCTTTCATTTGAACAACTTGTTTAATATAGTTATATATTTCGC
TTTGTCGTTTTGTTAATTCTCTCATATATAGGCACTCCCTAAATTAATTTATATACAGTATATCATTAATTCACAACCAAAACAAACA
TTTGTTCGTTAAAATATTGACAGAGAACATAAGTTCTGATAAATTACTAATACAAACAAATGTTCTAGGAGTGGAAATGATGTTTTA
CAATAAATATAAAAACGTATCAACATATATCATCATATTTTTAGTTTCAAGTGCAGCCTTTGCAATATTCTTGTTAAGTGCGAACATT
AGTGCTCACTCGGAACAAGTGTACGAAATGACTGACCATCAAATTAAGAACAATACGATAAATAAAGCATACGAACATAAAGACC
CTACAAACAATAGCGAACAAAGAGATGGGAAAGTGTTCGCTTTAATAAATTGATACATTGTCACAACGTTATTTGCCTATTTTTGC
GAAATAGCGTTTTTTATTACTTTTTTACTGATCTTAAATTTGTTATATTTTGTTAAAGTATTATAATGATTGAATAAACAAATTGAAG
GTAGGTTTTTTAATTGAGTAATTCTGATTTGAATATCGAAAGAATTAACGAGTTAGCTAAAAAGAAAAAAGAAGTAGGATTAACTC
AAGAAGAAGCAAAGGAGCAAACAGCCTTAAGAAAAGCTTATCTTGAGAGTTTTAGAAAAGGGTTTAAACAACAAATTGAAAATAC
TAAAGTAATTGATCCAGAAGGTAATGATGTAACACCTGAAAAAATTAAAGAGATACAACAAAAAAGAGATAATAAAAATTAAATC

ACAAATCTGTAAAGAATTTTCTGACATTATAACTTGAAATAAGTATTTTACTTATCTTTTTATTTTAAAATAAGTTATAATGTATTTG
ATAAAATTGAAGAAGGGAAGATACACAAGATGTTTAATGAAAAAGATCAATTAGCTGTTGATACGCTGCGTGCACTAAGTATCGAC
ACAATCGAAAAAGCGAATTCTGGTCATCCAGGATTACCTATGGGAGCTGCCCCAATGGCTTACACTTTGTGGACACGTCATCTGAAT
TTTAATCCACAATCTAAAGATTACTTCAATAGAGACCGTTTCGTATTATCTGCAGGGCATGGTTCAGCATTATTGTATAGCTTGTTAC
ATGTTTCTGGTAGTTTAGAATTAGAAGAATTAAAGCAATTTAGACAATGGGGTTCTAAAACACCAGGTCATCCTGAATACAGACAT
ACAGATGGTGTAGAAGTTACTACCGGACCACTTGGACAAGGTTTTGCTATGTCAGTAGGATTAGCTTTAGCAGAAGATCACCTAGC
AGGGAAATTTAATAAAGAAGGATATAATGTTGTAGATCATTACACATATGTATTAGCTTCTGACGGTGATTTAATGGAAGGTATTTC
GCATGAAGCAGCTTCATTTGCTGGACATAATAAATTAAGTAAATTAGTTGTTTTATACGATTCAAATGATATTTCATTAGATGGCGA
ATTAAACAAAGCTTTTTCTGAAAACACAAAAGCTCGTTTTGAAGCATATGGTTGGAATTACTTACTAGTTAAAGATGGTAATGATTT
AGAAGAAATTGATAATGCGATTACTACTGCTAAATCTCAAGAAGGACCAACAATTATCGAAGTTAAAACAACAATCGGATTTGGTT
CACCGAATAAAGCAGGAACTAATGGTGTTCATGGGGCACCTTTAGGTGAAGATGAAAGAAAATTAACATTTGAAAATTACGGTTTA
GATCCTGAAAAACGTTTTAATGTTTCAGAAGAAGTATACGAAATTTTCCAAAATACTATGTTAAAACGTGCTAATGAAGATGAATCT
CAATGGAATTCATTATTAGAAAAATATGCAGAAACATATCCTGAATTAGCAGAAGAATTTAAATTAGCAATTAGTGGTAAATTGCC
TAAAAATTATAAGGATGAATTACCACGTTTTGAACTTGGTCATAATGGTGCATCTCGTGCTGATTCTGGTACTGTTATTCAAGCAATC
AGTAAAACTGTTCCTTCATTCTTTGGTGGATCAGCAGACCTTGCTGGTTCAAACAAATCCAATGTAAATGATGCAACTGATTATAGT
TCTGAAACACCTGAAGGTAAAAATGTGTGGTTTGGTGTACGTGAATTTGCTATGGGTGCTGCTGTAAATGGTATGGCTGCACATGGA
GGTTTACATCCATATGGTGCAACATTCTTCGTATTTAGTGATTATTTAAAACCAGCGTTACGTTTATCATCAATTATGGGATTAAATG
CAACGTTCATCTTCACACATGATTCAATTGCAGTAGGTGAAGATGGTCCGACTCATGAACCAATTGAGCAATTAGCTGGATTAAGAG
CCATTCCAAATATGAATGTTATCCGTCCTGCTGATGGTAATGAAACAAGAGTAGCATGGGAAGTTGCCTTAGAATCTGAATCTACAC
CTACTTCATTAGTATTGACACGTCAAAACTTACCGGTATTAGATGTACCAGAAGATGTAGTTGAAGAAGGCGTTCGAAAAGGTGCCT
ATACAGTTTATGGCTCTGAAGAGACACCAGAATTCCTATTATTAGCTTCAGGTTCAGAAGTTAGTCTTGCAGTTGAAGCTGCTAAAG
ATCTTGAAAAACAAGGTAAATCAGTACGTGTTGTTTCAATGCCTAACTGGAATGCATTTGAACAACAATCTGAAGAATATAAAGAA
TCAGTTATTCCATCAAGCGTAACAAAACGTGTTGCGATTGAAATGGCTTCACCGCTTGGATGGCATAAATATGTAGGTACTGCAGGT
AAAGTTATTGCTATTGACGGCTTTGGCGCAAGTGCACCTGGCGATTTAGTAGTTGAAAAATATGGATTTACTAAAGAAAATATCTTA
AACCAAGTTATGAGCTTATAAGAATAATTTATAAAGCGAGTATGTTTAGAAGTCTAGGATGCATAATCTTAGGCTTCTTTTAAAGTG
TTGAAATTTAGAGTATAGCACTTAAACTACATCATAAGTGATAAGTTATGAAAGTATACTATTTCAGATTAATCTTTAAAAGCTCTG
TTATAACAGCATGATTTTTGATATTATTTTTAGTATCGATATTAAAATACTTGAATAAACTAGTTCTTGAAATAATGTGATGAATTTA
GTAAAATTCAGTAGGATAAAGAAAGCGGGTGAAACAATGGCAACTTGGTTAGCAATTATTTTTATAGTAGCTGCATTAATTTTAGGT
TTAATTGGAGGTTTCCTTTTAGCTAGAAAATATATGATGGACTACTTGAAGAAAAACCCACCAATCAACGAAGAAATGCTTCGTATG
ATGATGATGCAAATGGGTCAAAAACCTTCTCAGAAGAAAATTAATCAAATGATGACGATGATGAATAAAAATATGGATCAAAATAT
GAAGAGTGCGAAAAAGTAAATTCGCAATTGATAGAGGCTATTTTCCAGATATGGAAATGGCCTCTTTTTATAATCAAATTAATAAA
AACAAATATGTTTATTAAAATTAAAGTTAACAAAATGACGAATAGACTGAGAAATGCTATAATTCATTTTGTATGATTTACAGAGAG
TTTATTTAACGAGAAGGTGTCTGCGTGCTCTATTTAATATTTTCAATCATTGTAGCTTTATTTATGGGAACTATAGTTATAGTTATTCG
TATGAAAGCTCAAAATTATCCGGTAAATGAGAAAAAAATAGTTTTGCCACCGTTTTTTATGGCGACCGGTGCATTGATGTACGTCGT
TCCATATTTTAGGCTAACAGGATCGGAAATGCTAGAAGCCTTTATAATTGGTTTGCTTTTTTCTACAGTTCTAATTTGGACTTCTCGA
TTTGAAGTCAAAGGTACAGAAATTTATATGAAACGATCTAAAGCATTTCCAGTTATTTTGATTTCATTACTTATCATTCGTACTGTGA
TGAAAATATTCATTAGTAATGAAATAGATCCTGGAGAATTAGGCGGCATGTTCTTTTTATTAGCATTCTGTATGATTGTTCCTTGGAG
AGCAGCAATGCTATATAAATACAAAAAACTAAAGAAAACATTAATCAATTAATTACTTTTAAAACCACTTGTGATCGACTTCTAAAT
CAGTCGATGAGTGGTTTTAATTTTACTTGAAAAGGTGAAATGATTATATTAAAATAAAAGCGAACAAATGTTCTATATGGAGGCAGT
AATGAAAATTATACATACAGCAGACTGGCACTTAGGGAAAATATTAAATGGCAAACAGCTTTTAGAAGATCAAGCGTATATTTTAG
ATATGTTCGTAGAAAAAATGAAAGAAGAAGAACCCGATATCATTGTGATAGCTGGAGATTTATATGACACAACATATCCAAGTAAA
GATGCAATCATGTTATTAGAACAAGCGATTGGAAAGTTAAATTTAGAACTGCGTATACCAATAATTATGATTAGTGGAAATCACGA
TGGTAAAGAGAGGTTAAACTATGGGGCGAGTTGGTTTGAACATAATCAGTTATTTATAAGAACAGATTTTACATCGATTAATTCACC
AATAGAGATAAATGGGGTTAATTTTTATACACTCCCTTATGCTACTGTGAGCGAAATGAAACACTACTTTGAAGATGACACCATTGA
AACACATCAACAGGGAATTACGCGCTGTATTGAAACAATAGCACCGGAAATTGATGAAGATGCCGTCAATATTTTAATTAGTCATC
TGACTGTTCAAGGTGGAAAGACATCTGATTCTGAAAGACCATTAACTATTGGAACGGTTGAATCAGTTCAGAAAGGTGTTTTTGATA
TATTTGATTATGTCATGCTAGGTCACTTGCATCATCCATTTAGTATAGAAGACGACAAAATTAAATATAGTGGCTCCTTATTGCAGTA
TTCATTTTCGGAAGCGGGTCAAGCTAAAGGGTATAGACGTGTAACAATTAATGATGGCATTATTAACGATGTATTTATTCCTCTTAA
GCCACTTAGACAATTGGAAATTATCTCAGGCGAATATAATGATGTTATTAATGAAAAAGTTCATGTGAAAAATAAAGATAATTATTT
ACATTTTAAACTTAAAAATATGTCTCATATTACTGATCCAATGATGAGTTTAAAACAAATTTATCCTAATACTTTAGCGCTGACGAAT
GAAACTTTTAATTACAATGAAGAAAATAATGCTATAGAAATAAGTGGAAAAGATGACATGTCAATTATCGAAATGTTTTATAAACA
TATAACTGATAAAGAATTATCGGATATCCAATCTAAAAAGATAAAAAATATTTTAGAAAACGAATTGAGAAAGGAGGATTAATGAA
TGAAACCATTACATTTAAAGTTGAATAATTTCGGCCCCTTTTTAAAAGAAGAAATTGATTTTTCTAAAATTGATAATAATGAATTGTT
TTTAATAAGTGGTAAGACTGGATCGGGTAAAACAATGATTTTTGATGCAATGACTTATGCCTTGTTTGGTAAAGCATCAACTGAACA
AAGAGAAGAAAATGATTTGAGAAGTCATTTCGCTGATGGTAAACAGCCGATGTCAGTAACATTTGAATTTCAATTAAATCATCGAA
TTTATAAAGTGCATAGACAAGGCCCTTATATCAAAGAAGGTAATACAACAAAAACGAACGCTAAATTTGATGTATTTGAGATGGTG
GATGGCAAGTATGAAATTAGAGAAAGTAAAGTAATTTCAGGTACCCAATTCATTATTGAATTATTAGGAGTAAATGCAGATCAATT
CCGACAATTGTTTATTTTGCCTCAAGGTGAATTCAAACGCTTTTTAATATCAAACAGTCGTGAAAAGCAAGGGATATTAAGAACACT
GTTTGACAGTGAAAAATTTGAAGCTATACGAGAAATATTAAAAGAAGAAGTAAAAAAAGAAAAAGCTCAAATCGAGAATAGATAT
CAACAAATTGACCTTTTATGGCAAGAAATTGAATCATTTGATGATGACAATATAAAAGGCTTATTAGAGGTTGCCACTCAACAGATA
GACAAATTGATTGAAAATATACCACTTTTACAAGCTAGGTCGAAAGAAATACTAGCATCTGTAAATGAAAGTAAAGAAACTGCTAT
TAAAGAATTTGAAATAATAGAAAAGAAAACATTAGAAAATAATATATTAAAAGATAATATTAATCAACTCAACAAAAATAAAATT
GATTTCGTTCAATTGAAAGAACAACAACCTGAAATAGAGGGAATTGAAGCTAAGTTAAAGTTGTTACAAGATATTACAAACCTATT
GAATTATATTGAAAATAGAGAAAAAAATTGAAACTAAAATTGCTAATAGCAAAAAAGATATTTCTAAAACCAATAATAAAATATTGA
ATCTTGATTGTGATAAGCGAAACATAGACAAAGAGAAAAAAAATGTTAGAAGAAAACGGAGATTTAATTGAAAGTAAAATCTCTTTT
ATTGATAAAACTAGAGTATTATTTAACGATATTAATAAGTATCAACAAAGTTATCTCAATATTGAAAGCTTGAGAACTGAGGGTGA
ACAATTAGGTGATGAATTAAATAATCTAATTAAAGGTTTAGAAAAGGTCGAAGATTCAATAGGTAATAACGAAAGTGATTACGAGA
AAATTATCGAACTAAATAATGCGATAACTAACATAAATAATGAAATTAATATAATTAAAGAAAATGAAAAAGCTAAAGCTGAATTA
GATAAACTATTAGGTAGTAAGCAAGAGTTAGAGAATCAAATTAATGAAGAAACAACTATAATGAAGAATCTCGAAATAAAATTAG
ATCACTACGATAAATCAAAATTGGACTTAAATGATAAAGAAAGCTTTATAAGTGAAATTAAATCTGCTGTAAAGATTGGAGATCAA
TGTCCGATATGTGGTAATGAAATTCAGGATTTAGGGCATCATATTGATTTTGACAGTATTGCTAAACGTCAAAATGAAATTAAAGAA

ATTGAAGCAAATATCCACGCAATAAAATCGAATATTGCTGTGCATAATTCTGAAATTAAATTTGTTAATGAAAAAATATCGAATATT
AATATTAAAACGCAAAGTGATTTTTCACTCGAAGTATTGAATAAGCGTCTGCTAGAAAATGAAAATGCATTGAATAATCAAAGAGA
TCTTAATAAATTTATAGAACAAATGAAAGAAGAAAAAGATAATCTAACGTTGCAAATTCATAATAAACAATTGCGACTAAATAAAA
ATGAATCTGAGTTGAAATTATGTCGAGATCTCATCACTGAATTTGAAACACTCTCGAAATATAATAATATCACTAATTTTGAGGTGG
ATTATAAGAAGTATATTCAAGATGTGAACCAACATCAAGAACACTCAAAGGAGATTGAAGATAAGTCAATGCAATTGTCTCAAAGA
AAGTTAATTGAGCAAAATAATCTAAATCACTATGAAAATCAACTAGAAACTTACAATAATGACTTAGAATTGAATGAAAAATCTAT
TGAAATGGAAATGTCGAGGCTGAATTTAACTGACGACAATGATATAAATGAAATAATAGCCTGGAGGGGCGAGCAAGAGGAATTA
GAGCAGAAAAGGGATACTTATAAAAAACGTTATCATGAATTTGAAATGGAAATAGCTAGGTTAGAATCATTAACCAAGGATAAAG
AGTTATTGGACTCTGATAAATTAAAAGATGAATATGAGCAAAAAAAAGAAAAGATGAATACACTGATAGATGAATACTCTGCTGTT
CATTATCAATGTCAAAATAATATTAATAAAACACAATCTATAGTTTCGCATATTAATTACTTAAATCAAGAATTAAAGGATCAACAA
GAAATTTTTCAATTGGCTGAAATTGTCAGTGGTAAGAATAACAAAAATCTTACATTGGAAAACTTTGTCTTAATTTACTATTTAGATC
AAATTATTGCCCAAGCAAATCTGAGATTAGCAACAATGTCAGATAATCGATACCAACTAATTAGGCGAGAAGCGGTTTCTCATGGT
CTTAGTGGCCTAGAAATTGATGTATTTGATTTGCATTCAAATAAGTCTAGACATATTAGCTCGTTATCAGGTGGAGAAACTTTCCAA
TCGTCGCTTGCATTAGCTTTAGGGTTAAGCGAAATTGTACAGCAGCAATCAGGAGGTATTTCACTAGAATCAATATTTATTGATGAA
GGATTCGGTACATTAGATCAAGAAACGCTTGAAACAGCGTTAGACACTTTATTAAATCTTAAATCAACTGGTAGAATGGTTGGGATT
ATTTCACATGTGAGCGAATTGAAAAATAGAATACCTTTAGTTTTAGAAGTGAAATCAGATCAATATCAGAGTTCAACAAGATTCAA
AAGAAATTAAAACATAAAAAATAGAGATGGTACTTTGGAATGTATAGAACAACCTTAGCCCATCTCTTTTTATTTAAAAATTATTTT
TTCTCACGTAATAAATCTCTGATTTCAGTTAATAACACAACATTTTCTTCCACAACTGCTTCTTCTTCGGCTTCTTCTTTCTTCATTAA
TGTATTTGCAATCTTAACAAAGATGAATAAAGCAAACGCGATGATAATAAAGTCGATAACAGATTGGATAAATAAACCGTATTTAA
TACCCCAGAATGACCATTCTTTAGCAAAATCAACTGATCCGAAAATTTTACCAATTAATGGCATGATGATATTTTCTACTAATGAAG
ATATAATCTTGTTGAAAGCTGCACCCATCACAACAGCAATTGCTAAATCTAAGACGTTACCTTTTAAGGCGAACTCTTTGAATTCTTT
TAACATTACACTCAACCTCTCTTTTTTAAATGTAACCTCAATTATACAACAAATAAAAAAATTAGAAAATAGATTATTCAAGAAAAT
TTGATTATCGCAAAGACCTTTGTTATCCTATAAAGGTGCGTGAGTTATTGAATAAAATGTTTAGATAATCTTTTTATTCTACTACATA
AAATTAAATATAGAATTGAAGGGAGTAATTAATTATGAATTCTTCTTCACCAGAGAATCCAAATGGAAAGAAGTATTCACCAGTCTT
CATCTATAGTGCAATTGTTGTTGCTATAGTCGTATTACTTGGTGCATTTTTACCTGAACAATTCAACTATGTTACCAATAATATTAAA
ATGTGGATTACAGAAAAGTTAGGTTGGTATTATCTTATTCTTACTACGATTATCGTGTTCTTCTGTATATTCCTTATTTTTAGTCCTAT
TGGAAAACTTAAACTAGGTAAACCAAATGACAAACCTGAGTTTAATACAATTTCATGGTTTGCTATGTTGTTTAGTGCTGGTATGGG
GATAGGTTTGGTGTTTTATGGTGCAGCTGAACCGATGGCGCACTTTGCTACGCCACCTACAGCAGATCCCAAAACTACTGAAGCTTA
TACTGAAGCTCTACGTTCAACATTTTTCCATTGGGGATTCCATGCTTGGGCTGTTTATGGTGTTGTTGCGTTAGCGTTGGCATATTCG
CAATTCCGTAAAGGTGAACCAGGTTTATTATCTAGAACTTTACGTCCTCTTTTAGGTGATAAAGTAGAAGGTCCTATTGGGATTTTTA
TTGACGTTTTATCTGTATTTGCGACAATCGTTGGGGTAGCCGTTTCGTTAGGTATGGGTGCTCTACAAATTAATGGTGGTTTACATTA
CTTGTTCAATGTTCCAAATAATACGTTTGTACAAGCGATTATCATCATTGTTGTTACTATCTTATTTATAGCAAGTGCATGGTCTGGA
TTAAGTAAAGGTATTCAATACTTAAGTAACTTGAACATTGGTTTAGGTACTATTTTAATGGTAGCTGCTTTAATTGTTGGACCAACTG
TTCTTATTTTAAATATGTTAACTAGCTCTACGGGTAGTTTACTAAACACATTCTTGTTTAATAGTTTTGATACAGCAGCTTTAAATCCT
CAAAAACGTGAATGGATGTCTTCATGGACACTTTATTACTGGGGTTGGTGGTTAAGTTGGAGCCCATTCGTTGGAGTGTTTATTGCA
CGAGTTTCAAAAGGACGTTCAATTAGAGAGTTCATTTCTGGTGTCTTGCTAGTTCCAGCAATTGTTAGTTTTGTTTGGTTTAGTGTCT
TTGGTGTATTAGGCATCGAGACAGGTAAGAAACACAAAGAAATTTTTGATATGACTCCTGAAACACAGCTATTTGGAGTGTTTAATC
ATGTGCCATTTGGCATTGTTTTATCGTTGATTGCATTATTATTAATTGCATCATTCTTTATTACATCTGCTGACTCAGCAACATTTGTA
TTAGGAATGCAAACAACATTTGGTTCATTAAATCCATCTAGTATGGTAAAAGTTGTTTGGGGAATTTCACAGGCCTTAATAGCATTT
GTACTTTTATTAGCTGGTGGCGGTAACGGCGCTGAAGCTTTAAATGCGATTCAAAGTGCTGCAATTATAAGTGCATTCCCATTCTCCT
TTGTCGTCATACTCATGATGGTAAGTTTCTACAAGGATGCGAACCAGGAACGTAAATTCCTAGGTTTAACATTGACTCCGAATAAAC
ATCGCTTACAAGAATATATCAAGAGTCAACAAGAAGATTATGAATCTGACATTCTTGAAAAGCGTCAGTCACGTAGAAATATAGAG
AAAAAAGATAACTAATAAAATTTAGTTAAGTATTTAAATAACAAGTAGTATGTCATTCTAGTAGCTAGAACAGATATACTACTTGTT
TGTTTTTGTGGAAAATTGAGTATATTCAAAAGGATAAGAACGAGAATTTCGTTATTAAACTACGAATTCTCGATTTTTTTTATATTTT
AAGATAGGTTTATTTCTGACAACTTAATAGAAAGGGGTTTGACAAAGCTAAAGTGAAGTACGACGGTATAAAAAATTAACTATACT
TTTATAGAAAATTAACTCAGACTGGGACATAAATCAATATTCTATGCTCTACGAAGTTATATTGGCAGTAGTTGACTGAACGAAAAT

>032-contig_259_RC

AATTGGATTCCCAATTTCTACAGACAATGCAAGTTGGGGTGGGACGACGAAATAAATTTTGCGAAAATATCATTTCTGTCCCACTCC
CATCAAAAGAATGACATTGAAAAGAAATATTAAAGTTTGGTGCCATAATTTTCTTTCTGCTCAATGAAATAATACGTTTAAAAATTG
AATTGAATCATTCGCGAAATTGATAATTATTCTCAATTAATAAAAAATACTTATTACACCTTACCTGTATGATAAGTTTTGCTTATAT
ACTCTGATTAAAAAGTCAAAACCTAAATTAAGCGTTTTCTAAGCATGATGAGTTGTTTATCAATAAATATAATAATACAATTTAATC
AAGGCATATAAATATAAAAATGTATCAAGGGGGATCATTAAATGGCTGCAAATTTTAAAGAGCAATCAAAAAAACATTTTGACTTG
AATGGCCAAAGTTATACTTACTATGATTTAAAAGCTGTAGAAGAGCAAGGTATTACTAAAGTTTCCAATTTACCTTATTCAATTCGT
GTTTTGTTAGAATCTTTACTTCGTCAAGAAGATGATTTTGTAATTACAGACGATCATATTAAAGCTTTAAGTCAGTTTGGAAAAGAT
GGAAATGAAGGCGAGGTACCATTTAAACCTTCTCGTGTTATTTTACAAGATTTCACAGGTGTACCAGCCGTAGTTGATTTAGCTTCTT
TACGTAAAGCAATGGATGACGTTGGGGGAGATATTACTAAAATTAATCCAGAAGTACCGGTGGATTTAGTTATTGACCACTCAGTTC
AAGTGGATAGCTATGCAAATCCAGAAGCTCTTGAACGTAATATGAAATTAGAATTTGAACGTAACTATGAACGTTATCAGTTTTTAA
ATTGGGCAACGAAAGCATTTGATAATTACAATGCAGTTCCTCCTGCAACTGGAATAGTTCACCAAGTTAACTTAGAATATTTAGCAA
GTGTTGTACATGTTCGTGATGTAGATGGTGAAAAAACTGCATTTCCGGATACATTAGTTGGTACTGATTCACATACAACAATGATAA
ATGGTATTGGCGTACTAGGATGGGGTGTTGGTGGTATTGAAGCTGAAGCTGGAATGCTTGGACAACCTTCTTATTTCCCAATTCCAG
AGGTTATTGGTGTACGACTAGTAAATTCATTACCACAAGGCGCAACAGCAACTGATTTAGCGTTAAGAGTAACTCAAGAGCTACGT
AAAAAAGGTGTTGTTGGTAAATTTGTAGAGTTCTTTGGTCCAGGTGTACAACATTTACCACTAGCAGACCGTGCTACAATTGCAAAC
ATGGCACCAGAGTATGGAGCAACTTGCGGATTCTTCCCAGTTGATGATGAATCTCTTAAATATATGAAGTTAACTGGTAGATCAGAC
GAACATATCGCGCTAGTAAAAGAATATTTGAAACAAAACCATATGTTCTTTGATGTTGAGAAAGAAGATCCTAATTATACAGATGTT
ATCGAATTGGATTTATCAACAGTTGAAGCATCGCTTTCAGGACCAAAACGTCCTCAAGATTTAATTTTCTTAAGTGATATGAAATCA
TCATTTGAAAATTCTGTAACAGCTCCAGCAGGCAACCAAGGACACGGTTTAGATAAAAGTGAATTTGATAAGAAAGCTGAAATTAA
CTTTAAAGATGGATCAAAAGCTACAATGAAAACAGGTGATATTGCAATAGCAGCAATTACATCATGTACAAATACATCTAACCCTT
ATGTAATGTTAGGTGCAGGTTTAGTTGCTAAAAAAGCAGTTGAAAAAGGCTTGAAAGTTCCTGAATACGTTAAAACTTCTCTAGCAC
CAGGATCAAAAGTTGTTACCGGATATTTAAGAGATGCTGGCTTACAACCTTATTTAGATGATTTAGGCTTCAACTTGGTTGGTTATG

GATGTACAACTTGTATCGGTAATTCAGGTCCTTTATTACCAGAAATTGAAAAAGCGATTGCTGATGAGGACCTATTAGTGACATCTG
TATTATCTGGTAACCGTAACTTTGAAGGTCGTATCCATCCTCTTGTTAAAGCCAATTACCTAGCTTCACCACAGTTAGTTGTTGCTTA
TGCATTAGCTGGAACGGTTGATATTGATTTACAGAATGAACCTATTGGTAAAGGTAATGACGGTGAAGATGTATATTTGAAAGATAT
TTGGCCATCAATTAAAGAAGTTTCAGATACCGTTGATAGTGTTGTAACACCTGAATTATTTATTGAAGAATATAATAACGTATACAA
TAACAACGAATTATGGAATGAGATTGATGTAACTGATCAACCTCTATATGACTTTGATCCTAATTCAACATACATTCAAAATCCATC
ATTCTTCCAAGGATTATCTAAAGAACCGGGTACGATTGTTCCATTAAATGGTTTACGTGTTATGGGTAAATTCGGTGATTCTGTGAC
AACTGACCACATCTCTCCAGCAGGTGCAATTGGTAAAGATACGCCAGCTGGTAAATATTTACAAGATCATCAAGTGCCTATTCGTGA
ATTTAATTCATATGGTTCAAGACGTGGTAATCACGAAGTAATGGTTCGAGGTACGTTTGCTAATATACGTATTAAAAACCAATTAGC
GCCAGGTACTGAAGGTGGTTTTACAACTTATTGGCCAACAAATGAAGTAATGCCTATCTTTGATGCTGCAATGAAATATAAAGAAG
ATGGTACAGGTTTAGTTGTATTAGCTGGTAACGATTATGGTATGGGTTCATCTCGTGACTGGGCAGCAAAAGGTACAAACTTATTAG
GTGTTAAAACAGTTATTGCACAAAGTTATGAACGTATCCATCGTTCAAATTTAGTTATGATGGGTGTATTACCATTAGAGTTTAAAA
AAGGTGAATCAGCTGATTCTCTTGGTCTAGATGGTACAGAAGAAATTTCTGTTAATATTGATGAAAATGTTCAACCACATGACTACG
TCAAAGTTACTGCTAAGAAGCAAGATGGTGATTTGGTAGAATTTGACGCTATGGTTCGTTTTGACTCACTTGTTGAAATGGATTACT
ATCGTCACGGTGGAATTTTACAAATGGTTTTAAGAAATAAATTAGCGCAATAAAAAATAGATATCACAGTAAAATTTTAATCGGTAT
TTGAGGCACTTTGATAGTGCTCAAAAATACTTCTATAAACTTTATGATATAATAGGTCGGTTCAAAACCTTTGTGTTCTTGGCCGGCC
TCTTCTTGTATTTTATGTTAGAATGAGATATAAAGTTAAACTGAAAGAAGGCTTAAAATGATATATAGTATTACAGAAATAGAAGCG
CGTTATGCTGAAACTGATAAGATGGGTGTAATTTATCACGGGAATTATGCAACTTGGTTTGAAGTTGCGCGGTTGGATTATATATCG
AAGTTAGGTTTTAGTTATGCTGATATGGAAAAACAAGGAATCATTTCACCTGTGACTGACCTCAATGTCAATTATAAAAAGTCTATT
TTTTATCCAGAAAAAGTTAAAGTTAAAACTTGGGTTGAAAAATATTCGAGATTACGTTCAGTGTATAAATATGAGATTTTTAATGAA
AAAGGTGAACTTGCAACTACAGGTTCCACAGAATTGATTTGCATTAAAGAAGATACTTTTAAGCCTATACGGTTGGATCGTTATTTC
CCAGATTGGCATGAAGCTTATAGTAAAGTGCAAACGCTCAATAATGAAGGGAAAACAGTAGAGATAATGGATGATATTGATTCTTT
ATAAGTTACTGTAAATGTTTGGAGAGGCAATATTAACAATTAATCTATAAAAAATAGCCAATACGTTAACATTAATGTAATTAGCGT
GAAGATAAGCTAATATATAGCAAAAACGAACTTGTTCATACAACATCAGATGTGCTAAACCAAAGTACAACTTCCGAGGTTCAGGA
CACATCAGAATGTGTAAACAAGTTCGTTTTATTTTAGTTATTTTGTGGAATAAGAAATTTCATCTTCGTGATCAACTACATTTACAAT
AATGTGGTCATCTTCAAAGTACCACAAATCTTTTTCCGCTACGACAACATTTAAATCGTCATATTGTTGTTCATAGCCAATATCAACA
TCTTCCTTTGGTTCAACTGTAAAAGCAGGACTAAATCCTTGCTTGAGTTGGAATTCGCCACCATATCTTACAAAAAACACGAGCACT
TTATTATTTTCAGGCAACTCAAGTTCATTTTTAAACCAAGTTACTGCTGCATCAGTAAGTTCTATTTGCATAACAATGACCTCCTATA
TTTATTACAGAATGGGAGTGGGCCAACTTGTATTATTTGTGGAATTTCTTATCGAAATTCTCTGTGTTGGAGCCCACCCCAACCTGCT
CATTATTGTAAGCTGAATTTCCATAAGCTTCTATGTTGGGACCCCCGACCAGAATTGAAAAAACCTAGTTATAAGCACATTTTGTTC
GGTTAACTACAGCTAATATAACATTGTGGAGCCTATGACATTGATTTATGTCTCGGTCTCAAATGTTCTTGTAAAAAAACTAAGTAT
AGTTGAATTTTAAGTTAAAAAAATTGTTTTTGCAAAAATAAAATAATCCATCAACTGCAAAAAAATATGCAATGATGGACTAATGTC
AAAAAATTGTTACATCCATTTTATTTTAGGTTCTTCGCCTCTAAAAATCCTTGCGATATTAGAGCGATGTCTAATTATCAATATGATT
GAAACTAAGAAACTAACGACTAATAAAATATAGTCTTGAATGATAAGCGAGCCAATCACACAGCAAATTGCTGCAACGATACTTGC
TAAAGAAACATATTTAAAAATCTTCAATACAATAAAGAAGATAATTGCAAGTATTAGTAAAAGTATCGGATTGACTCCCAAGACGA
CACCTGCACTAGTTGCAACAGCTTTGCCACCTTGGAATTTTAAATAAACAGGATAAACGTGTCCAAGTATAGCGAATAAGCCAACA
ATTAAACCATTTGTAAAAAAAGTACTAATAGGGCCATCTGCGTGAACTGGTAACCATAAAGGGAAGAAAACAGTTATGAACCCTTT
GAAAATATCTAGAAATGTTACCAAGAATCCTGCAGGACGACCTAATACTCTAAAGCTATTAGTAGCGCCAGTATTACCACTACCAA
ATTGTCTAATATCTTTTTTGAAAAATAATTTTCCAATTACGAATCCACTTGGGAAAGCGCCGATAAGATAACTTAGTAGTAACATGA
CGATTATCATCATAAATATTACACATCCTTTAATATCTTAGGACTATTTTATCATATTCGTTTAAATTACGGCTAAAAATTTTAAAAA
CGGGGATTAATATATGGAATTAAGCTATGAAAGTTAATTGATACTTGCATTTTACGCTGATTTATATAAGAATAACTATTGTATAGT
TTTAAAAACGAACGTACGTTTGCAGGAGGCGAAATCATTGGCAATGAATAAACAAAATAATTATTCAGATGATTCAATACAGGTTT
TAGAGGGGTTAGAAGCAGTTCGTAAAAGACCTGGTATGTATATTGGATCAACTGATAAACGGGGATTACATCATCTAGTATATGAA
ATTGTCGATAACTCCGTCGATGAAGTATTGAATGGTTACGGTAACGAAATAGATGTAACAATTAATAAAGATGGTAGTATTTCTATA
GAAGATAATGGACGTGGTATGCCAACAGGTATACATAAATCAGGTAAACCGACAGTCGAAGTTATCTTTACTGTTTTACATGCAGG
AGGTAAATTTGGACAAGGCGGCTATAAAACTTCAGGTGGTCTTCACGGTGTTGGTGCTTCAGTTGTAAATGCATTGAGTGAATGGCT
TGAAGTTGAAATCCATCGAGATGGTAATATATATCATCAAAGTTTTAAAAACGGTGGTTCGCCATCTTCTGGTTTAGTGAAAAAAGG
TAAAACTAAGAAAACAGGTACCAAAGTAACATTTAAACCTGATGACACAATTTTTAAAGCATCTACATCATTTAATTTTGATGTTTT
AAGCGAACGACTACAAGAGTCTGCGTTCTTATTGAAAAATTTAAAAATAACGCTTAATGATTTACGCAGTGGTAAAGAGCGTCAAG
AGCATTACCATTATGAAGAAGGAATCAAAGAGTTTGTTAGTTATGTCAATGAAGGAAAAGAAGTTTTGCATGACGTGGCTACATTTT
CAGGTGAAGCAAATGGTATAGAGGTAGACGTAGCTTTCCAATATAATGATCAATATTCAGAAAGTATTTTAAGTTTTGTAAATAATG
TACGTACTAAAGATGGTGGTACACATGAAGTTGGTTTTAAAACAGCAATGACACGTGTATTTAATGATTATGCACGTCGTATTAATG
AACTTAAAACAAAAGATAAAAACTTAGATGGTAATGATATTCGTGAAGGTTTAACAGCTGTTGTGTCTGTACGTATTCCAGAAGAA
TTATTACAATTTGAAGGACAAACGAAATCTAAATTGGGTACTTCTGAAGCTAGAAGTGCTGTTGATTCAGTTGTTGCAGACAAATTG
CCATTCTATTTAGAAGAAAAAGGACAATTGTCTAAATCACTTGTGAAAAAAGCGATTAAAGCACAACAAGCAAGGGAAGCTGCAC
GTAAAGCTCGTGAAGATGCTCGTTCAGGTAAGAAAAACAAGCGTAAAGACACTTTGCTATCTGGTAAATTAACACCTGCACAAAGT
AAAAACACTGAAAAAAATGAATTGTATTTAGTCGAAGGTGATTCTGCGGGAGGTTCAGCAAAACTTGGACGAGACCGCAAATTCCA
AGCGATATTACCATTACGTGGTAAGGTAATTAATACAGAGAAAGCACGTCTAGAAGATATTTTTAAAAATGAAGAAATTAATACAA
TTATCCACACAATCGGGGCAGGCGTTGGTACTGACTTTAAAATTGAAGATAGTAATTATAATCGTGTAATTATTATGACTGATGCTG
ATACTGATGGTGCGCATATTCAAGTGCTATTGTTAACATTCTTCTTCAAATATATGAAACCGCTTGTTCAAGCAGGTCGTGTATTTAT
TGCTTTACCTCCACTTTATAAATTGGAAAAAGGTAAAGGCAAAACAAAGCGAGTTGAATACGCTTGGACAGACGAAGAGCTTAATA
AATTACAAAAAGAACTTGGTAAAGGCTTCACGTTACAACGTTACAAAGGTTTGGGTGAGATGAACCCTGAACAATTATGGGAAACG
ACGATGAACCCAGAAACACGAACTTTAATTCGTGTACAAGTTGAAGATGAAGTGCGTTCATCTAAACGTGTAACAACATTAATGGG
TGACAAAGTACAACCTAGACGTGAATGGATTGAAAAGCATGTTGAGTTTGGTATGCAAGAGGACCAAAGTATTTTAGATAATTCTG
AAGTACAAGTGCTTGAAAATGATCAATTTGATGAGGAGGAAATCTAGTGAGTGAAATAATTCAAGATTTATCACTTGAAGATGTTTT
AGGTGATCGCTTTGGAAGATATAGTAAATATATTATTCAAGAGCGTGCATTGCCAGATGTTCGTGATGGTTTAAAACCAGTACAACG
TCGTATTTTATATGCAATGTATTCAAGTGGTAATACACACGATAAAAATTTCCGTAAAAGTGCGAAAACAGTCGGTGATGTTATTGG
TCAATATCATCCACATGGAGACTACTCAGTGTACGAAGCAATGGTCCGTTTAAGTCAAGACTGGAAGTTACGACATGTCTTAATAGA
AATGCATGGTAATAATGGTAGTATCGATAATGATCCGCCAGCGGCAATGCGTTACACTGAAGCTAAGTTAAGCTTACTAGCTGAAG
AGTTATTACGTGATATTAATAAAGAGACAGTTTCTTTCATTCCAAACTATGATGATACGACACTCGAACCAATGGTATTGCCATCAA
GATTTCCTAACTTACTAGTGAATGGTTCTACAGGTATATCTGCAGGTTACGCGACAGATATACCACCACATAATTTAGCTGAAGTGA
TTCAAGCAACACTTAAATATATTGATAATCCGGATATTACAGTCAATCAATTAATGAAATATATTAAAGGTCCTGATTTTCCAACTG

GTGGTATTATTCAAGGTATTGATGGTATTAAAAAAGCTTATGAATCAGGTAAAGGTAGAATTATAGTTCGTTCTAAAGTTGAAGAAG
AAACTTTACGCAATGGACGTAAACAGTTAATTATTACTGAAATTCCATATGAAGTGAACAAAAGTAGCTTAGTAAAACGTATCGAT
GAATTACGTGCTGACAAAAAAGTCGATGGTATCGTTGAAGTACGTGATGAAACTGATAGAACTGGTTTACGAATAGCAATTGAATT
GAAAAAAGATGTGAACAGTGAATCAATCAAAAATTATCTTTATAAAAACTCTGATTTACAGATTTCATATAATTTCAACATGGTCGC
TATTAGTGATGGTCGTCCAAAATTGATGGGTATTCGTCAAATTATAGATAGTTATTTGAATCACCAAATTGAGGTTGTTGCAAATAG
AACGAAGTTTGAATTAGATAATGCAGAAAAACGTATGCATATCGTTGAAGGTTTGATTAAAGCGTTGTCAATTTTAGATAAAGTAAT
CGAATTGATTCGTAGCTCTAAAAACAAGCGTGACGCTAAAGAAAACCTTATCGAAGTATACGAGTTCACAGAAGAACAGGCTGAAG
CAATTGTAATGTTACAGTTATATCGTTTAACAAACACTGATATAGTTGCGCTTGAAGGTGAACATAAAGAACTTGAAGCATTAATCA
AACAATTACGTCATATTCTTGATAACCATGATGCATTATTGAATGTCATAAAAGAAGAATTGAATGAAATTAAAAAGAAATTCAAA
TCTGAACGACTGTCTTTAATTGAAGCAGAAATTGAAGAAATTAAAATTGACAAAGAAGTTATGGTGCCTAGTGAAGAAGTTATTTT
AAGTATGACACGTCATGGATATATTAAACGTACTTCTATTCGTAGCTTTAATGCTAGCGGTGTTGAAGATATTGGTTTAAAAGATGG
TGACAGTTTACTTAAACATCAAGAAGTAAATACGCAAGATACCGTACTAGTATTTACAAATAAAGGTCGTTATCTATTTATACCGGT
TCATAAATTAGCAGATATTCGTTGGAAAGAATTGGGACAACATGTATCACAAATAGTTCCTATCGAAGAAGATGAAGTGGTTATTA
ATGTCTTTAATGAAAAGGACTTTAATACAGATGCATTTTATGTTTTTGCGACTCAAAATGGCATGATTAAGAAAAGTACAGTGCCTC
TATTTAAAACAACGCGTTTTAATAAACCTTTAATTGCTACTAAAGTTAAAGAAAATGATGATTTGATTAGTGTTATGCGCTTTGAAA
AAGATCAATTAATTACCGTCATTACTAATAAAGGTATGTCATTAACGTATAATACAAGTGAACTATCAGATACCGGATTAAGGGCA
GCTGGTGTTAAATCAATAAATCTTAAAGCTGAAGATTTCGTTGTTATGACAGAAGGTGTTTCTGAAAATGATACTATATTGATGGCC
ACACAACGCGGCTCGTTAAAACGTATTAGTTTTAAAATCTTACAAGTTGCTAAAAGAGCACAACGTGGAATAACTTTATTAAAAGA
ATTAAAGAAAAATCCACATCGTATTGTAGCTGCACATGTAGTGACAGGTGAACATAGTCAATATACATTATATTCAAAATCAAATG
AAGAACATGGTTTAATTAATGATATTCATAAATCTGAACAATATACAAATGGCTCATTCATTGTAGATACAGATGATTTTGGTGAAG
TAATAGACATGTATATTAGCTAAAAACTATATGCAATCACGAAATTAAATGATAAAATACAGTAATGTTAAATTTTGACTAAATTCA
AGGGATTTATATTAAATGCTGACCAAGTACTTATCGTTAAATTAGCGATATACTTTGACCAAACTAATATAAATCTCTTGTATTGTAT
AAGAAATGATGATGTATCTCTATATTTACTGAAGTTTCAATTCGGTCTAATTTTATAGAAGGTACATGATTCATGTAGAGGATGCGA
GGGGTTTATTTTGAAAGATTTCGATAGTTTAATTCCTGGATGGTTTAAAGAATTTGTCCATGTTGGTACCGATTTAATATGGTCTCAA
TATTTAATTGGTCTATTATTGACAGCTGGATTCTTCTTTACAATTAGTTCTAAATTCGTCCAATTACGAATGTTACCTGAAATGTTTAG
AGCTTTAGTAGAACGTCCAGAAACTTTAGAAGATGGTAAGAAGGGTATTTCGCCATTCCAAGCATTTGCGATTAGTGCTGGTTCGAG
AGTTGGTACTGGTAATATTGCTGGTGTTGCGACTGCGATTGTTTTAGGCGGTCCAGGTGCAGTGTTTTGGATGTGGGTTATTGCATTT
ATAGGTGCAGCGAGTGCATTTATAGAAGCGACTTTGGCTCAGGTTTATAAAGTACATGATAAAGATGGTGGATTCCGTGGTGGTCC
AGCTTACTATATTACTAAAGGTTTAAATCAAAAATGGCTAGGTATCGTATTTGCGATTTTAATTACAATTACATTTGCATTTGTATTT
AACACAGTGCAATCTAATACAATTGCGGAGTCGTTAAATACGCAATATAATATTAGTCCAGTAATCACAGGTATTATTTTAGCAATC
GTAACAGCTATTATTATATTTGGTGGTGTACGTAGTATTGCTACGTTATCTTCGTTAATTGTACCGATTATGGCTATCATTTACATTG
GTATGGTTTTAGTAATATTGCTATTTAATTTAGATCAAATTGTTCCTATGATAGGTACGATTATTAAAAGTGCATTTGGTATCGAACA
AGTAACTGGTGGCGCTGTAGGTGCTGCGGTTCTTCAAGGTATCAAACGTGGTTTATTCTCTAACGAAGCTGGTATGGGTTCTGCGCC
GAATGCAGCGGCAACTGCTGCCGTACCACACCCTGTTAAGCAAGGTTTAATCCAATCATTAGGTGTGTTCTTTGATACAATGTTGGT
TTGTACAGCAACTGCAATCATGATTTTACTATATTCAGGACTGAAATTTGGTGATAACGCACCTCAAGGTGTTGCAGTTACTCAATC
AGCACTTAATGAGCATTTAGGTTCTGCTGGAGGTATTTTCTTAACAATAGCAGTTACACTGTTTGCATTTTCATCTGTGGTAGGTAAT
TACTATTACGGTCAATCTAATATTGAATTTTTATCAACAAACCGTGTAATATTATTTATCTTTAGATGTCTTGTTGTAGTACTTGTCTT
TGTCGGTGCAGTTGTAAAAACAGAAACAGTATGGAATACGGCAGACTTATTTATGGGCTTAATGGCAATTGTAAACATTATTTCCAT
TATAGGACTGTCCAATGTAGCTTTTGCATTGATGAAAGATTATCAAAAGCAGAAAAAAGAAGGCAAGAACCCTGTCTTTAAACCTG
AAAACTTAGAAATTAACTTATTTGGAATTAGTGCTTGGGGCGCTAACAAATATAAGAACTCTGATAAATAAATATTTTTAATGTAAC
GATTCATTATATAAAAATCAAATAAACCCTGAGGTTTTCAAATTAAAGCGAAAATCTCAGGGTTTTAAATTTGTACTCAATAATCAT
CTGTATCATTACACTAGTTAATCTAAGATTTGATTATATATTAATATAGTAAGGTTGAGATTTATTTCCTCCATTGGTGTGCAAAGAC
TATACCATTGATAAGACATATTGTTGGATAGATGAATATAAATTTATTTTAGCTTTGCACAATTTAATAGTACGTTTTAGTTTGTATT
GAGTATTAATTCAGATAATATATAAATAAATAGAATTATTTGTAAATATGGGAGTCAGATTTTTTTATTATAGTTATTCCACTTTGAG
AGGCATCGAAATTAAATTAAGATATATCATATAGGATAGGCGTGTAATAGAACACAAGTATTTAAATTATTATACTTTCAAGTTAGA
CATTTCCGATATATAATCTTAATAATAGAAAAATAGGTGATGATAATGGGAGAATATATTGTTACTAAAACATTGAACAACAATGTC
GTAGTATGTACTAATAATGATCAAGAAGTTATTTTAATCGGTAAAGGTATTGGTTTTAACAAAAAAGAGGGAATGGCGTTAAACGA
CCAAACTATTACAATAGAGAAAATTTATAAATTAGAGAGTGAGCAACAAAAAGCACATTATAAAAGTTTAGTTGAAATCGCTGATG
ATAATGTATTACAAGTAATTATTGATTCGTTGAATTTTATTTCTAATACTGCGATGAATGTTGATTCAAAACAACTTGTAGTTTCATT
AACGGATCATATTATATTTGCTTATAAACGCTTAAAACAAAATCAAGTTATTAGCAATCCATTTGTTATGGAAACTATGCAGTTATA
TAGTGATGCATATCATATTGCTAAACAGGTGATTGATCAGTTAAATGCAGCATTAGATGTACATTTTCCTGAAGATGAGATAGGATT
TATTGCATTACATATTGCATCTAATACAGAAGATTTATCTATGCATGAGATGACCTTGATCAATAATGTTATTAAAAAAGGTATAGA
TATCATTGAATCAGACCTTGTGACAACTGTTGATAAGGAATCATTACAATACCAACGTTTTATAAGGCACGTACAATTTTTAATTCG
CCGATTAAGAAGAAAAGAATATATACATGCACAAGATGATTTTGTGTCTATGATTAAAAATCACTATCCGATTTGCTATAACACAGC
ATATAAAATTTTAACTATGATACAAAAACAATTTGATGTTAATATCAGTGAGTCTGAAATTATATATTTAACATTACACATTCATCAT
TTTGAAGAAAGGATTAATCAATCCTAAATTAAGTGTATTACACGGGCGTTTCGTTACTGTAAAGGGTATTTGTATGGTATCATTGAT
GTTAGTAATTTTACATAAGTGGACTGTTTTTATACAACATCTTATGAATATTTTAAGTGGTGGTGAATCTATAATTGAATGAAAATGA
AAAGAATATAAGAAAGAATTTTTTAAAATTTACCGAATCACGGTATATGAAGTTTGTTGGTGGGAATGATTTAGTCTTCTCATTAAT
AGCGCTAGTATTGTTGGGTATTGTTATTTTTATTTTCGAAAAAGTATCATATGTTTTTGATCCTTTTATCATCGTTTTTAAGACGATAG
CAGCACCTATCATCGTCTCTTTAATTCTATTCTATCTATTTAACCCAATCGTAAATATGATGGAACGTTATAGAATACCAAGAGTTGC
AGGTATTTCTATTATTTATCTAGCTGTAGTAGGTGTTATTACGTTAATTGTTAATTTATTGATACCTATTATTGGTTCGCAAGTAGATA
GTTTAGTTAAAAAATTCACCGCAATATCTAGAAAAATTAATTAATTCTATTGATAAAATAGCAAATAATACGTTTTTCTCTTCGTATTA
TAGTCAAATTAATGATTGGTTAAATTCTTTACCTAAGAAAATACCATCTATGTTAAGTGAATTTACAGATGGCTTTGGGTCTAAAATT
GCAACGTTTGCAGAAACGATTGCTAATATTGGCGTTGTGATTGTCACAACACCATTTGTACTATTCTTTATGCTTAAAGATGGACATC
ACTTCAAAGAATTTTCAACGAATATTATGCCACCGAAATTCCGAAAAGATTTTCATGATCTACTTGAAAAAATGAGTGTTCAAGTTG
GTTCATACATTCAAGGACAAATTATCGTTTCATTCTGTATCGGTATACTGTTGTTTATCGGTTATTCGGTTATCGGGTTGAAATATAG
CTTAGTATTAGCTAGTATTGCGGCAGTTACAAGTGTTGTACCATATTTAGGGCCTACTATAGCGATTTCTCCAGCTATTGTAATAGCT
GCTATAACATCGCCGTGGATGCTCTTAAAATTAGCAGTAGTATGGACTTTAGTACAATTTGTTGAAGGGCACTTCATTTCACCAAAT
ATCATGGGTAAAACACTTAAGATTCATCCACTTACAATCATTTTCATTTTACTGTGTGCAGGCAAATTGCTTGGTATTGTAGGCGTTA
TTTTAGGTATTCCGGGATATGCTATTTTAAAAGTATTAGTTACTCATTTATTCCAATTATTTAAACGTCGATACAATCGTTTCTATGGT
AATGATGTAGGTGAATATGATATTAAAGAAAGTAATAAAATAGTTGAATAAGTAATAAAAAATACCAATGACTATATGTTTGTTGG

TATTTTTTATGGTGCTTATAATGTTGGGCAGTTACATTTATGATTATCAGCACATGTTTAGCTTATAAATTTTTACGAAAGTTTAGCC
GTTTATAAAGCACATGCATAATGAAACGAGTATTTGCCACTTGATTAGTACTTCATTATTATGTCGAAAATAAAAACAAGTGGTATT
TTTAATATATTAAGAAGCACTCATAATCGGCTGTTAATTAATAATATTTTTCATAAGTATTGATTCATCATTTTCTTTATGTTAAATAT
GAATCAGTATAGATAACCATATTGTTCTGTTTGAGATAGAAATATTTTCTATTTACTTCTTAATACAGATATCAATATGACAAAAGTG
TTATATAAATCAAAGGTAAATGATATGCTATTTTTATGGTTTATATGATATATTTTGATTTATAACAGAAATAATTAGAATTGATGTG
AAAAAATGAATCAGGAAGTTAAAAACAAAATATTTTCAATCTTAAAAATTACGTTTGCTACAGCTTTATTTATTTTTGTAGTAATCA
CATTGTATCGGGAGTTATCTGGTATTAACTTTAAAGATACGTTGGTTGAATTTAGTAAGATTAACCGTATGTCCTTAGTTTTACTATT
TATTGGTGGTGGGGCATCGCTTGTTATTCTATCAATGTATGATGTGATTTTATCTAGAGCTTTAAAAATGGATATATCCTTAGGCAAA
GTTTTAAGAGTAAGTTATATCATCAATGCATTGAATGCGATTGTAGGTTTCGGTGGCTTTATTGGTGCAGGCGTTAGAGCTATGGTTT
ATAAAAACTATACGCATGATAAAAAGAAATTAGTTCACTTTATATCCTTAATACTTATTTCAATGTTGACAGGTTTAAGCTTATTATC
ATTGTTAATTGTATTCCATGTTTTCGATGCATCTTTAATTTTAGATAAGATTACATGGGTAAGATGGGTATTATATGTAGTGTCATTTT
TCTTACCATTATTCATTATTTATTCAATGGTTAGACCACCTGATAAAAACAATCGTTTTGTAGGATTGTACTGCACTTTAGTGTCGTG
TGTTGAATGGTTAGCAGCTGCAGTTGTATTATATTTCTGTGGTGTAATTGTTGACGCTCATGTATCATTCATGTCCTTTATTGCAATAT
TTATCATTGCTGCATTATCAGGTTTAGTCAGCTTTATTCCTGGTGGTTTCGGCGCTTTCGATTTAGTTGTATTACTAGGATTTAAAACT
TTAGGTGTCCCTGAGGAAAAAGTATTATTAATGCTACTTCTATATCGTTTTGCGTACTATTTTGTACCGGTAATTATTGCATTAATTTT
ATCATCATTTGAATTTGGTACATCAGCTAAGAAGTACATTGAGGGATCTAAATACTTTATTCCTGCTAAAGATGTTACGTCATTTTTA
ATGTCTTATCAAAAGGATATTATTGCTAAAATTCCATCATTATCATTAGCAATTTTAGTATTCTTTACAAGTATGATCTTTTTTGTAAA
TAACTTAACGATTGTTTACGATGCTTTATATGATGGAAATCACTTAACGTATTATATTCTATTGGCAATTCATACTAGTGCTTGTTTA
TTACTTTTACTGAATGTAGTTGGTATTTATAAGCAAAGTAGACGTGCCATCATCTTTGCTATGATTTCAATTTTATTAATCACAGTGG
CGACATTCTTCACTTACGCTTCATATATTTTAATAACATGGTTAGCTATTATTTTTGTTCTGCTTATTGTAGCTTTCCGTAGAGCACGT
AGGTTGAAACGCCCAGTAAGAATGAGAAATATAGTTGCAATGCTTTTATTCAGTTTATTTATTTTATATGTTAACCATATATTTATTG
CTGGAACGTTATATGCATTAGATATTTATACGATTGAAATGCATACATCTGTATTGCGCTATTACTTCTGGCTTACGATTTTAATCAT
CGCTATCATCATAGGTATGATTGCATGGTTGTTTGATTATCAATTTAGCAAAGTACGCATTTCTTCTAAAATTGAAGATTGCGAGGA
GATTATTAATCAGTATGGCGGTAATTATTTGAGTCACTTGATATATAGTGGTGACAAGCAGTTTTTCACTAATGAAAATAAAACAGC
ATTTTTAATGTATCGTTATAAAGCAAGTTCATTAGTGGTTCTTGGAGATCCGTTAGGTGATGAAAATGCCTTTGATGAATTGTTAGAA
GCATTCTATAATTACGCTGAGTATTTAGGCTATGATGTTATATTCTATCAAGTTACAGATCAACACATGCCTTTATATCATAATTTCG
GTAACCAATTTTTCAAATTAGGTGAAGAAGCAATTATTGATTTAACGCAATTTTCAACTTCAGGTAAAAAACGCCGTGGATTTAGAG
CGACTTTAAACAAATTCGATGAACTTAATATTTCATTCGAAATTATTGAACCACCGTTTTCAACTGAATTTATAAATGAACTTCAACA
TGTAAGTGATTTATGGCTAGATAATCGTCAGGAAATGCATTTCTCTGTGGGTCAATTTAATGAAGAATACTTATCTAAAGCGCCAAT
TGGTGTAATGCGAAATGAAGAAAATGAAGTAATTGCATTTTGTAGTTTAATGCCAACATACTTTAATGATGCCATTTCAGTCGATTT
AATTAGATGGTTGCCAGAGTTAGATTTACCATTAATGGATGGTCTATACTTGCATATGTTACTTTGGAGTAAAGAACAAGGTTATAC
AAAATTTAATATGGGTATGGCAACGTTATCGAACGTTGGTCAATTGCATTATTCATATTTAAGAGAACGACTTGCAGGCCGTGTCTT
TGAACATTTCAACGGTCTATATCGTTTCCAAGGATTACGTCGTTATAAATCTAAATATAATCCGAATTGGGAACCACGCTTTTTAGTT
TATCGTAAAGATAATTCGCTTTGGGAATCACTTTCTAAAGTAATGCGTGTAATACGTCACAAATAATTAAAATCCAAGTGCTAAGAG
GTATACAGTTATGCCTGAAGCGCTTGGATTTTTTGGTTTCTATAAAGAATGATTTATAAAAAACGGTACAGATAAGAATATTTAAGT
TTCCTATCTGTACCGTATTTTAATATTGAAATGAGCTTAAGTTAAGTCGCCTAACTTTTATTATCAGTCCAATCAGTTTCATCAAGTA
TAAATGTTATTGCTTATTTTTGTATTCTTGGCGTATTTTTTGTTCTTCTGCATAGCGCTCTGGATTTTTCTTATAAAAATCTTGGTGATA
GTCTTCGGCTTTGTAAAATTGTGACGCGGGTAATATTTTTGTTGCAATTGCCTTATCAGCATTAATCGTATTTTTAAGCTGCTCGATA
TAAGTCTCAGCGAGTTCTTTTTGATGATCATTAGTGTAGAAAATAGCTGTTTGATATTGAGGACCACGGTCTTGATATTGACCCCCTG
TATCTAATGGGTCAATGACTGAGAAAAATATTTCTAATAACTTATTGTATGAGAATAATGCAACATCATATTGAATTTCAACAGTTT
CTAAATGACCACTCGTACCTGATTTTACTTGTTCGTAAGTAGGATTTTCAATATGTCCGCCCATATATCCAGAAGTTACTTTTTCTAT
GCCGTCAAAGGTGTCAAATGGTTTCGTCATACACCAAAAGCAACCTCCGGCAAAATAAGCTGTATTAATATTCATTTTTGACATCCT
TTCATTAGACCTTAGTACGATTTATTAAGAAATCACTTGCTTTTTGAATTGTTTTTATATAACGTTAATATGTGATTATTATAGTACAC
AAAAATCTATTTTTGAAGATATAAGGTAGGTAAATATGGATAAAGAAACTAATGACAACGAATATAGACGTCAAAGTGAACATCGC
ACTTCGGCGCCTAAGCGAAAAAAGAAGAAGAAAATTAGGAAATTACCTATCATTCTTCTGATTGTTGTAATTTTACTTATCGCATTA
GTTGTATATATTGTGCATAGTTACAATAGCGGTGTAGAATATGCCAAGAAACATGCGAAAGATGTTAAAGTACATCAATTTAATGG
ACCAGTAAAAAATGATGGTAAAATTTCTATTCTTGTACTCGGTGCAGATAAAGCACAAGGTGGACAATCAAGAACAGATTCTATCA
TGGTTGTTCAATATGACTTTATCAATAAAAAGATGAAAATGATGTCTGTCATGCGTGATATTTATGCAGATATTCCAGGATATGGAA
AACACAAAATTAATTCAGCATACGCTTTAGGTGGTCCAGAGCTACTTAGAAAAACACTTGATAAAAATTTAGGAATTAATCCTGAA
TATTATGCAGTAGTTGATTTTACTGGATTTGAGAAAATGATTGATGAATTAATGCCAGAAGGTGTACCAATTAATGTCGAAAAGGAT
ATGTCGAAAAATATTGGTGTATCTTTGAAAAAGGGTAACCATAGGTTGAATGGTAAAGAATTACTTGGTTATGCAAGATTCCGTCAC
GACCCTGAAGGTGACTTCGGACGCGTGCGACGTCAGCAACAAGTGATGCAAACATTGAAAAAAGAAATGGTTAATTTTAGAACAGT
TGTTAAATTACCAAAAGTTGCAGGTATTTTAAGAGGCTATGTGAATACAAACATTCCTGATTCAGGGATTTTCCAAACAGGTTTGAG
TTTTGGTATCCGAGGTGAAAAAGATGTTAAGTCATTGACTGTGCCAATCAAGAACTCATACGAAGATGTCAATACAAATACTGATG
GTAGTGCATTACAGATTAATAAAAACACAAATAAACAAGCTATTAAAGACTTTTTAGATGAAGATTAAAAATAAACAAGGCGATTT
CTATCATACTCAGATAGAAGTCGCCTTGTTCGTTTTCAAAGAATTAATTGGCATGCATCTTATGAAGAATTATTGATCTGACTTTCTT
ACGCCAGCCACACCATAATGGTTTGGTTTCATTTCTTCTATAACAACGTGAATTGCTTGTCTATTTGCCCCCGTTGTTTTTTCTACGGC
GTCAGTTACTTCGCTAACTAAATTTTTTAATTGTTCATCCGAACGACCTTCTAATAATTTTACATTGACGATTGGCATCATCCAAACC
TCCTAATCATTATTAATTTGAGTATATCATGTAATACTTAAAAATTCATTTGCAAAATAACAGAACGTTCGTTCTAAAATAGTTGAA
ATGGAACACGTGTTCGTATATAATGAGATTCAAGTAAGGGAGGAATAACAGTGTATAATTATCATTTATTAGAAGATAGGGATGTT
CTATGTATTGACCAAAAAAGTTTTTTTGCGAGTGTTTCTTGTATTGAAAAGGGGCTAGATCCATTAGAAACAAAGCTAGCTGTTGTT
GCAGATACTAAGCGTCAGGGTTCTGTAATATTGGCTGCGACACCTAAATTAAAAGAATTAGGCATCAAGACAGGGTCGCGATTGTT
TGAAATACCACATAGAAATGATATTTACATTATCAATCCAAGTATGCGTAAATATCTTAATGTTTCAGTTGCTATTTCTAAGATTGCA
TTGCGTTATATTCCACCTGAAGATTTACACCAATATAGTATTGACGAATTTTTTATGGATGTTACTGATAGCTATCATAGATTTAGTT
CTACAGTACATGCATTTTGCGAAAGACTTAAACGTGAAATTTATGAAGAAACAGGCATTTATTGTACTGTGGGCATTGGTTCTAATA
TGTTATTAAGTAAAATTGCTATGGATGTTGAAGCGAAGCATAGTCAAAATGGTATAGCTGAATGGCGATATCAAGATGTACCAACG
AAATTATGGCCAATTCAGCCCTTGCGAGATTTTTGGGGTATTAATCGTCGAACAGAAGCCAAATTGAATAAAAGAGGAATTTTTACT
ATAGGAGATTTAGCGAAATATCCATATAAATTTTTAAAAAAAGAGTTCGGTATTTTAGGTGTTGATATGCATCTACATGCGAATGGG
ATAGATCAGAGTAAAGTACGTGAAAAGCACAAGATCAGCAATCCATCGATATGCAAAAGTCAAATATTAATGAGAGATTATCATTT
TGATGAAGCAAAAGTAGTAATGCAAGAGTTAATTGAAGATGTTGCTAGCAGAGTTCGAGCAAGAAAAAAAGTGGCAAGAACGATA
CATTTTGCCTTTGGCTATAGTGATGAAGGCGGTGTACATAAGCAATATACTTTGAAAGATCCAACAAACTTAGAAAAAGATATTTAT

AAAGTAGTAATGCATTTCGCAGATAAATTATGTAATAAACAAGCACTATATCGTACGCTAAGTATATCTTTGAGTCAATTTATTAAT
GAGGATGAGCGACAGTTAAGTCTGTTTGAAGATGAATACCAACGCAAACGTGACGAATGTCTAGCTAAAACGATAGACCAATTACA
TTTGAAATACGGCAAAGGTATTGTGTCCAAAGCAGTATCGTTTACAGAAGCAGGTACAAAACACGGCAGATTAGGTTTAATGGCTG
GACATAAAATGTAATGACTATACGGTTTAAGTAATATATAACTGTGATTCGTATAAAATAAGTCTCTAAAGATAAATATTTCATATA
TCACAATAGATTTCACAATAATATCTAAGAATACATGGAATTTATCAAAAGAGACTTAATAATTATTGGATATAACAATCAAAATCA
CTCAATGCTTGCATACCGCGTTCTCGGTCAGTAGGGTTTTTGAAACTAATTTTTAAAGCACCGTATATATCTTCGCGTACTTCTAAGA
TTCTTAAGTTGCTTATAGATATGTTATGTAAACTCAGGATATAAGTCACTTTACTTATCATACCTGATTCATCCGGAATGTCTACATA
TAGATCATACGCAGTATTTAGTCCACCTAGTTGTTTAGCGGGTAGTGCGTCGCGATACGATTTAGCTTGGGCAAAAAATGATAACAA
TTTTTCAGAATCATTGCTTTCAATTAGTCTTTCTAAATCTTGAAACTGACTTTTTAGCTGTCGAATCATTTCTAAAATATACGTTTTAT
TACTCAAGGTGATATCTTTCCACATTTGTGCATTACTACTAGCTATACGAGTGATATCACGAAAACCACCAGCTGCAAGTTTATTAA
CTAAATGATGTTCTTGACCGTTCTTTTGACTAACATGAACTAAACTAGATGCAACGATATGAGGTAAATGACTTACGACGCTTGTTA
CGTAGTCGTGTTCTTCAGCAGTAGTTACAATAAATTTAGCAAGAGTAGGTGATAACAGTTCTTTTAACGTGTTTGCTGCTTGCTCATT
TCTTGGCTCATTGTAGACTAAAATATAATAAGCGTTTTCAAATAAGTGCTTTTTAGCATTTAGTACACCAGATTTATGACTACCAGCC
ATTGGATGACCACTGACTAAATGAATATTATGCTTTAATAAATTGCATTCGTGTTGCTGTATCATTGCTTTAGTACTACCAGTATCAG
AAACAATAACACCAGGTTTAGTTGGCATATCTATAAGCTCGCTAAGATATTTATTTGTGATAGCAACAGGTGTTGCATAAATAATTA
CATCGGCTTTTTTAATAGCTTCACTATAATTTAAACATTTTTCATTAATAATGCCGATTGATTTAGCTTTATCTAACTGAGAAGTATCT
GCATCGTATGCAATAATATTAGTATTAGGGTTATGGTATTTTATATTGCTAGCAAGACTTCCACCAATTAATCCAAGCCCAACAAAT
AAAACTGTTGTCATATAAATCACCTTATTTCGAAATTTTCAGAATAATAACATTGTAAATGAGCTGTTGACACAGTGCAATAGTAAA
TAAAAATCGATAATAGCATTAATAGATAAACGGAGATAAATCATCTACAATAAAGAGTATAGTAACACAATGGCAACGGAGGGGT
AAATCAATGGAACCAATATTAGAAATGATTAAAACATTAACAGGTATTAATAGTCCTTCAGGAGACACAGAAGAAGCAATTCAATT
TGTCGAAAAATATGCAAAAGACTTGGGTTATCAAACAACACTAACAAATAAAGGTGCGTTATTAATAACAGTGCCAGGCAAAAATG
ATGAAGTACAACGCTGTATTACTGCTCATGTTGATACTTTAGGTGCAATGGTTAAAGAAATTAAAGAAGATGGTCGCTTAGCAATAG
AATTAATTGGAGGATTCACGTATAACGCGATTGAGGGTGAATATTGCCAAATTAAAACTGATGCTGGTCAAATATATACAGGAACA
ATTTGTCTGCATGAAACAAGTGTTCATGTATATAGAAATAATCATGAAATACCTAGAGATCAAAAGCATATGGAAATAAGAATTGA
TGAAGTAACTACATCAGAAGAAGATACAAAGAGTTTAGGTATTTCAGTAGGTGATTTTGTTAGCTTTGATCCACGTACAGTTATCAC
GTCATCAGGTTTTATTAAATCTCGTCATTTAGATGATAAAGCTAGCGTAGCGATGATACTACAATTACTAAAGAAATTAAAAGAAGA
GCAAATAATATTACCACATACAACGCAATTTTATATTTCTAATAACGAAGAAATAGGTTACGGTGCAAATGCATCAATTGATTCGAA
AATCAAAGAATATATTGCATTAGATATGGGCGCGTTGGGAGACGGTCAAGCATCGGATGAATATACAGTTTCTATTTGTGCCAAAG
ATGCTTCAGGTCCATATCATAAGCAATTGAAATCGCACCTAGTTAATCTTTGCAAAATAAATAACATTCCATATAAAGTAGACATAT
ATCCATATTATGGTTCAGATGCTTCAGCAGCTTTACATGCTGGTGCGGATATCAGACATGGTTTATTTGGCGCTGGCATTGAATCATC
TCATGCAATGGAACGAACACATATTGATTCTATTAAAGCGACAGAGAAATTACTATATGCATATTGCTTATCACCAATTGAGTAAAC
AATTAGTGTTGACAAATGTGAACGACCTATGTAATATAATGAACTATAAAAATAATTAGAATTTTCTAAAGAAATAGTAGCAGATA
TGAAACGTAGCAAATAGAAAGCTAATGGGTGATGGGAATTAGCACGCCATATCTTGTGAATTGGACTTTGGAAAACAATTGAATGA
GTTTTGAAAGTGAACATGAATTATGTTAACTAAGGTGGCACCACGGTAACGCGTCCTTACAGGTATATGCGTTATGTGGTGTCTTTT
TATTTAGACAAAATGTAGTAGTTAATTAAAGGTAGCAACAGAAAGTTAGTGGATGATGTGAACTAACACCGAGATTAATGAAATTG
GGTTTTGTCTGCAACAGAAAAATTATATATAGTAAAGAGTGAACTATGAATATTTCGAATATTCGGTTAATTTAGGTGGTACCACGC
GTCAGCGTCCTTTATATTGATAAGGATGCTGGCGCTTTTTTGAAAGGAGCGTATAGAATGGATATATTTTATAAAAAAATAAAAGCA
AATGTAACGCCCGAAGTTTTAGCACAACTTCATTCCAAGAAGATCATTTTGGAAAGTACAAATCAACAACAAACTAAAGGTCGCTA
TTCAGTTGTTATTTTTGATATTTATGGCACTTTAACTTTAGATAATGATGTATTATCAGTAAGTACTTTAAAAGAATCGTATCAAATC
ACTGAAAGACCGTACCATTATTTAACGACTAAAATAAATGAAGACTACCATAATATTCAAGATGAGCAACTTAAGTCATTACCATTT
ATATCTGGATATGTTGGGACGTGTAGCTTTGATTTAGTAAGACATGAATTTCCTAAATTGCAATCAATACAATTAGAAGATCACAAG
CAGCACGATGTAAGGTTATATATGGTTGAACAAGTTTATGTATTTGACCATTACAAAGATGAGTTATATATCATCGCGACGAATCAA
TTTTCAAATTCAACAAAATCAGATCTTGAGAATCGAGTTATTAAGTCTATCGAAGACTTAACTAAAATCCAACCATTCATGCCTACA
CAAGATTTTGATTTTAAAACTAAAGAAATTCAATCAAACATTTCTGAAGAAAGATTTATCGAAATGATTCAGTATTTCAAAGAGAAA
ATAACAGAAGGGGATATGTTCCAAGTTGTGCCATCAAGAATTTACAAATATGCGCATCATGCTAGTCAGCATTTAAATCAACTTTCG
TTTCAACTGTATCAAAATTTAAAACGACAAAACCCAAGTCCATATATGTATTATCTTAATATCGATCAACCATATATTGTCGGTAGTT
CTCCCGAAAGTTTCGTAAGTGTCAAAGATCAAATTGTAACAACTAATCCTATTGCAGGTACGATTCAACGTGGTGAGACGACACAA
ATAGATAATGAGAATATGAAACAACTACTTAATGATCCAAAAGAATGCAGCGAACATCGTATGCTAGTTGATTTAGGACGTAATGA
TATTCATAGAGTAAGTAAAATCGGTACCTCAAAAATTACTAAATTAATGGTTATTGAAAAATATGAACATGTTATGCATATCGTAAG
TGAAATCACAGGTAAAATAAATCAAAATTTATCACCAATGACAGTTATTGCGAATTTATTACCAACAGGTACCGTTTCAGGTGCACC
AAAATTACGTGCAATTGAAAGAATATATGAACAATATCCACATAAACGAGGCGTTTATAGTGGTGGTGTTGGATACATAAATTGTA
ATCATAACTTAGATTTTGCTTTAGCAATTCGAACGATGATGATAGATGAGCAGTATATCAACGTAGAAGCTGGTTGTGGCGTTGTAT
ATGATTCTATTCCTGAAAAAGAACTGAATGAAACGAAATTGAAAGCTAAAAGCTTATTGGAGGTGAGGCCATGATCTTAGTTGTAG
ATAATTATGATTCCTTTACATATAACCTAGTGGATATTGTTGCTCAACATACTGACGTCATTGTTCAATATCCTGATGATGATAATGT
GCTGAATCAATCGGTGGACGCTGTTATTATATCTCCTGGTCCAGGGCATCCATTAGACGATCAACAGTTAATGAAAATCATATCAAC
CTATCAACACAAACCCATTTTAGGTATTTGCCTAGGTGCTCAGGCACTGACTTGTTATTACGGTGGAGAAGTCATTAAAGGCGACAA
GGTTATGCACGGAAAAGTTGATACACTAAAGGTTATATCGCATCATCAACATCTGTTATATCAAGATATACCAGAACAGTTTTCAAT
TATGAGATATCATTCATTAATAAGTAACCCTGACAATTTTCCAGAAGAATTGAAAATTACTGGACGTACCGAAGATTGTATACAGTC
ATTTGAGCATAAAGAAAGACCGCATTACGGTATTCAGTACCATCCTGAATCATTTGCTACAGACTATGGTGTCAAAATAATTACAAA
TTTTATTAATCTAGTGAAGGAAGGATGAAAACCATGACATTACTAACAAGAATAAAAACTGAAACTATATTACTTGAAAGCGACAT
TAAAGAGCTAATCGATATACTTATTTCTCCTAGTATTGGAACTGATATTAAATATGAATTACTTAGTTCCTATTCGGAGCGAGAAAT
CCAACAACAAGAATTAACATATATTGTACGTAGCTTAATTAATACAATGTATCCACATCAACCATGTTATGAAGGGGCTATGTGTGT
GTGCGGCACAGGTGGTGACAAGTCAAATAGTTTCAACATTTCAACGACTGTTGCTTTTGTTGTAGCTAGTGCTGGCATAAAAGTTAT
AAAACATGGTAATAAAAGTATTACCTCAAATTCAGGTAGTACGGATTTGTTAAATCAAATGAACATACAAACAACAACTGTTGATG
ATACACCTAACCAATTAAATGAAAAAGACCTTGTATTCATTGGTGCAACTGAATCATATCCAATCATGAAGTATATGCAACCAGTTA
GAAAAATGATTGGAAAGCCTACAATATTAAACCTTGTGGGTCCATTAATTAATCCATATCACTTAACGTATCAAATGGTAGGCGTCT
TTGATCCTACAAAGTTAAAGTTAGTTGCTAAAACGATTAAAGATTTAGGCAGAAAACGTGCAATCGTTTTACATGGTGCAAATGGTA
TGGATGAAGCAACACTATCTGGTGATAATTTGATATATGAATTGACTGAAGATGGAGAAATCAAAAATTACACATTAAATGCGACT
GATTATGGTTTGAAATATGCACCGAATAGTGATTTTAAAGGTGGTTCACCTGAAGAAAACTTAGCAATCACACTGAATATTTTAAAT
GGTAATGATCGCTCAAGTCGACGTGATGTGGTTGTCTTAAATGCAGGTTTAAGCCTATATGTTGCAGAGAAAGTGGATACCATCGCA
GAAGGCATAGAACTTGCAACTACATTGATTGATAATGGTGAAGCATTGAAAAAATACCATCAAATGAGAGGTGAATAATATGACGA

TTTTAGCAGAAATTGTTAAATATAAACAGTCACTTTTACAAAATGGCTATTATCAAGACAAACTTAATACCTTAAAAAGTGTGAAGA
TTCAGAATAAAAAATCTTTTATAAACGCAATTGAGAAAGAACCAAGGCTAGCAATTATTGCAGAAATTAAATCGAAGAGTCCTACA
GTTAATGACTTACCTGAACGAGATTTATCGCAACAAATCTCAGATTATGACCAATATGGTGCAAATGCCGTGTCCATTTTAACTGAT
GAAAAGTACTTTGGTGGTAGTTTTGAAAGATTACAAGCATTGACGACAAAAACAACATTACCCGTATTATGCAAAGACTTTATTATA
GACCCGCTTCAAATTGATGTTGCCAAACAAGCTGGTGCATCTATGATTTTATTGATCGTTAACATCTTATCGGATAAACAATTGAAA
GATTTATATAACTACGCTATATCGCAAAATTTAGAAGTGTTAGTTGAAGTACATGATCGCCATGAATTAGAACGTGCCTATAAGGTT
AATGCCAAATTGATTGGTGTAAATAACAGGGACTTAAAACGATTTGTCACAAATGTGGAACATACAAATACTATTTTAGAAAATAA
AAAACCAAATCATTATTATATTTCTGAAAGTGGTATTCACGATGCATCTGATGTAAGAAAAATCTTGCATAGTGGTATCGATGGCTT
ACTAATAGGTGAGGCGCTTATGCGTTGTGACAATCTATCTGAATTTTTACCACAACTGAAAATGCAAAAGGTGAAGTCATGATGAA
ATTGAAATTTTGTGGCTTTACATCAATAAAGGATGTTACAGCGGCCAGTCAATTACCTATTGATGCGATAGGTTTCATCCATTATGA
AAAAAGTAAAAGGCATCAAACAATTACCCAAATAAAAAAGTTAGCGTCTGCTGTTCCAAATCATATCGATAAAGTATGTGTCATGG
TAAATCCTGATTTAACAACAATTGAACACGTATTAAGCAATACGTCAATTAACACAATACAGTTACACGGCACAGAATCTATTGATT
TTATACAGGAAATTAAAAAGAAATATTCAAGCATTAAAATCACTAAAGCTTTAGCTGCAGATGAAAACATAATCCAAAACATAAAT
AAATATAAAGGGTTCGTAGATTTATTTATTATCGACACACCCTCAGTGTCGTATGGTGGTACCGGTCAAACATATGACTGGACTATT
TTGAAGCACATAAAAGACATACCTTATTTGATAGCAGGAGGCATTAACTCTGAAAATATTCAAACAGTTAATCAACTTAAATTATCA
CATCAAGGTTATGATCTTGCATCAGGTATAGAAGTAAATGGGCGAAAAGATATAGAAAAAATGACAGCAATTGTAAATATTGTGAA
AGGAGATAGAGACAATGAATAAACAAATACAAACGGAAGCAGATGAATTAGGTTTCTTTGGTGAATACGGAGGGCAATATGTTCC
AGAAACATTAATGCCAGCAATTATTGAGTTGAAAAAAGCTTATAAAGAGGCAAAAGCAGACCCAGAGTTTCAAAGAGAACTGGAA
TACTATTTATCAGAGTATGTAGGACGCGCGACGCCACTTACATATGCTGCATCATATACTGAAAGCCTAGGTGGCGCTAAAATATAT
TTGAAACGAGAGGATCTAAATCATACAGGCGCCCATAAAATTAATAATGCGTTAGGTCAAGCGTTACTTGCTAAAAGAATGGACAA
GAAGAAGCTTGTTGCTGAAACTGGTGCGGGTCAACATGGTGTAGCTAGTGCTACGGTTGCTGCATTATTTGATATGGAACTTGTTGT
CTTTATGGGAAGTGAAGATATTAAAAGACAACAACTTAATGTATTTAGAATGGAATTACTTGGTGCAAAGGTTGTGGCAGTTGAAG
ATGGTCAAGGGACTTTATCGGATGCAGTTAATAAAGCATTGCAATATTGGGTAAGTCATGTAGATGATACACATTATTTATTAGGTT
CTGCATTAGGTCCAGACCCGTTCCCAACGATTGTTAGAGATTTTCAGAGTGTGATTGGTAAAGAAATAAAATCACAGATATTGAAG
AAAGAAGGTCGACTTCCGGATGCAATTGTAGCATGTATCGGTGGTGGCTCAAATGCAATCGGTACATTTTATCCATTTATTAAAGAT
GATGTTGCATTATACGGTGTTGAAGCCGCAGGTCAAGGCGATGATACTGATAAACATGCACTTGCAATTGGCAAAGGATCACCTGG
CGTATTACATGGTACTAAAATGTATTTAATTCAAGATGAAGATGGGCAAGTGCAACTAGCACATTCTATTTCAGCAGGACTTGATTA
TCCTGGTATTGGACCAGAACATTCTTATTACCACGACATTGGTAGAGTAACTTTTGAAAATGCAAGTGATACACAAGCAATGAATGC
TTTAATCAACTTTACAAAACATGAAGGTATTATACCTGCAATTGAAAGTGCACATGCACTGAGTTATGTTGAAAGACTAGCGCCTAC
GATGTCGAAAGAAGATATTATTGTAGTAACTATTTCTGGACGTGGCGATAAAGATATGGAAACAATTAGACAATATATGGCAGAGC
GAGGTCTTGCAAATGACTAAATTATTTATACCTTATATTATGGGCAATAAAGATTTGATTGAAAATGCAACATTGTTGAGTGAAAAT
GGTGCAGATATAATTGAAATTGGTGTACCTTTCTCTGATCCGGTTGCTGATGGTCCAGTTATCATGGAAGCAGGTCAACAAGCGATT
AAACAAGGCATAACGATAGATTATATTTTCGAGCAATTAGAAAAACACGGTAATCAAATTAAGTGTAACTATGTATTAATGACGTA
TTATAATATTATTTGTCATTATGGAGAACAAGCGTTTTTTGAAAAATGTCGAGATACTGGTGTCTACGGCTTAATTATTCCTGATTTA
CCATATGAATTATCGCAGCGTTTAAAACAACAATTTAGTCACTATGGCGTCAAAATCATATCGTTAGTTGCGATGACTACTGATGAC
AAACGTATAAAAGATATCGTATCCCATGCGGAAGGCTTTATTTATACTGTGACGATGAATGCGACAACAGGGCAAAACGGTGCGTT
TCATCCAGAATTAAAACGAAAAATTGAGTCAATTAAAGCGATAGCCAATGTGCCAGTTGTCGCAGGATTTGGTATAAGAACACCAC
AACATGTTGCAGATATAAAAGAGGTTGCAGATGGCATTGTCATTGGTAGCGAAATCGTTAAGCGATTTAAATCTAACACGCGTGAG
GAAATCATTAAATATTTACAATCTATCCAACAAACATTGAATAATTAAGTTTACTTGATTTAAAAAAATTAGGCGAATACTGTTTGA
AAAAGTGAAAAACGGTGAATTATAAAATTGAATAGAAATGATACAAAAGTAATATGAGCAAACCCAAACGTTCATATTACTTTTTT
TGAAATTGTATTCAAAAATCTAAATATTACTATAAAAGTATACGCAATTAAAGCGTTTATGTTTTAGTTTTAACATTAACTATTGTAT
ACTTATTTAGATTAGATTTATTATTTTTGACATTTGCAGAGGGGAAATAGAAAAACTGCAAATACGGAAATGAAATTAATTAACGAG
AGACAAATAGGAGTAATGATAATGAAGTTTACAAATTTAACAGCTAAAGAGTTTGGTGCCTTTACAGATAGCATGCCATACAGTCA
TTTCACGCAAACTGTTGGCCACTATGAGTTAAAACTTGCTGAAGGTTATGAAACACATTTAGTGGGAATAAAAAACAATAATAACG
AGGTTATTGCAGCTTGCTTACTTACTGCTGTACCTGTTATGAAAGTGTTCAAGTATTTTTATTCAAATCGCGGTCCAGTGATCGATTA
TGAAAATCAAGAACTCGTACACTTTTTCTTTAATGAATTATCAAAATATGTTAAAAAACATCGTTGTCTATACCTACATATCGATCCA
TATTTACCATATCAATACTTGAATCATGATGGCGAGATTACAGGTAATGCTGGTAATGATTGGTTCTTTGATAAAATGAGTAACTTA
GGATTTGAACATACTGGATTCCATAAAGGGTTTGATCCTGTGCTACAAATTCGTTATCACTCAGTGTTAGATTTAAAAGATAAAACA
GCAGATGACATCATTAAAAATATGGATGGACTTAGAAAAAGAAACACGAAAAAAGTTAAAAAGAATGGTGTTAAAGTAAGATATT
TATCTGAAGAAGAACTGCCAATTTTTAGATCATTTATGGAAGATACGTCAGAATCAAAAGCTTTTGCTGATCGTGATGACAAATTTT
ACTACAATCGCTTAAAATACTACAAAGATCGTGTGTTAGTACCTTTAGCGTATATCAATTTTGATGAATATATTAAAGAACTAAATG
AAGAGCGTGATATTTTAAATAAAGATTTAAATAAAGCATTAAAGGATATTGAAAAACGTCCTGAAAACAAAAAAGCGCATAACAA
GCGAGATAACTTACAACAACAACTTGATGCAAATGAGCAAAAGATTGAAGAAGGTAAACGTCTACAAGAAGAACATGGTAATGAA
TTACCTATCTCTGCTGGTTTCTTCTTTATCAATCCATTTGAAGTTGTTTATTATGCTGGTGGTACATCAAATGCATTCCGTCATTTTGC
CGGAAGTTATGCAGTGCAATGGGAAATGATTAATTATGCATTAAATCATGGCATTGACCGTTATAATTTCTATGGTGTTAGTGGTAA
ATTTACAGAAGATGCTGAAGATGCTGGTGTAGTTAAATTCAAAAAAGGTTACAATGCTGAAATTATTGAATATGTTGGTGACTTTAT
TAAACCAATTAATAAACCTGTTTACGCAGCATATACCGCACTTAAAAAAGTTAAAGACAGAATTTTTTAGGAAGGGAATTATCAAA
ACATGAAATTTACAGAGTTAACTGTTACCGAATTTGACAACTTTGTACAAAATCCATCATTGGAAAGTCATTATTTCCAAGTAAAAG
AAAATATAGTTACCCGTGAGAATGATGGCTTTGAAGTAGTTTTATTAGGTATTAAAGACGACAATAACAAAGTAATTGCAGCAAGC
CTTTTCTCTAAAATTCCTACTATGGGAAGTTATGTTTACTATTCGAATCGTGGTCCAGTAATGGATTTTTCAGATTTAGGATTAGTTG
ATTATTATTTAAAAGAGTTAGATAAATATTTACAGCAACATCAATGTTTATATGTTAAATTAGATCCGTATTGGTTATATCATCTATA
TGATAAAGATATCGTGCCATTTGAAGGTCGCGAGAAAAATGATGCCTTAGTAAACTTGTTTAAATCACATGGTTACGAGCATCATGG
CTTTACAACTGAGTATGATACATCGAGCCAAGTACGATGGATGGGCGTATTAAACCTTGAAGGTAAAACACCCGAAACATTGAAAA
AGACATTTGATAGTCAACGTAAACGTAATATTAATAAAGCGATAAACTATGGTGTTAAAGTCAGATTCCTTGAACGTGATGAGTTCA
ATCTTTTCTTAGATTTATATCGTGAAACTGAAGAGCGTGCTGGATTTGTATCAAAAACAGATGATTATTTTTATAACTTTATTGACAC
ATATGGAGATAAAGTATTAGTACCATTAGCATATATTGACCTTGATGAATATGTGTTAAAGTTGCAACAGGAATTGAATGACAAAG
AAAATCGTCGTGATCAAATGATGGCGAAAGAAAACAAATCAGATAAGCAAATGAAGAAAATTGCAGAATTAGATAAGCAAATTGA
TCATGATCAGCATGAATTATTGAATGCAAGTGAATTGAGCAAAACGGACGGCCCAATTCTAAACCTTGCTTCTGGCGTTTATTTTGC
AAATGCATATGAAGTGAATTATTTCTCTGGTGGTTCATCAGAAAAATATAATCAATTTATGGGACCATACATGATGCATTGGTTTAT
GATTAACTATTGCTTCGATAATGGCTATGATCGTTATAATTTCTATGGTTTATCAGGTGATTTTACGGAAAACAGTGAAGATTATGGC
GTATACCGCTTTAAACGTGGATTTAATGTACAAATCGAAGAATTAATAGGGGATTTCTATAAACCAATTCATAAAGTGAAATATTGG

TTGTTCACAACATTGGATAAATTACGTAAAAAATTAAAGAAATAGATGTAAATGATGTTAGGGCTTATAGTTATTGATACTATAGGC
TCTTTTTTATATGTTTTTTAATAAAGCCTCTAATGATATATTAAATGACAAGTTTAACAAGTGATTGCAATAAATATAAATATGAATT
GCACAACCTATTTGTAAATGATTGAGTTGAGTATACTTTTTAATTCATTCAATATGACTCAAAACAAGCCAATATGTATTATATTTTT
GGTGAATAAACCTTAGCATAACAATTTATGTCTGATTATATATGTGTGTGTTTGCAAGTGTGTGATATCAAATGTCATATTTGTCTAA
TTGTAATTTATAAAATGCATGAGCAATGATTGCTGTAACAAATTCTAGCAAAGTATTCAGTTTGTATTTTAGAAATAAAAATGATGA
TAGATGTATGATCGATATAAAAGATTTAATATGCACCAAAACGTGAGTAATTGGTTTAATGAAACATAACATGAATGTAAATTTAG
CTTACTGACATAAACACTACACAAACATACAGTTATATAAGTCCCCTTATGGAATTTCACATTCTAGTTTACATAATATATATTATAG
GAAGTAATGTGTGTGACGCAAAAGGTACCCTACATCATAATCATTATCTAATATCGTCACATAACTTACTTATGCTATAATCATGGT
ATTATATTGTTTGGAGTGATTTGATGAGATTTGTCTTTGATATTGATGGTACGCTTTGTTTTGACGGCCGATTAATTGACCAGACTAT
TATTGATACATTGTTACAATTACAACATGCTGGTCATGAACTTATATTTGCATCAGCACGTCCGATTCGTGATTTGTTGCCAGTTTTA
CCATCAGTATTTCATCAGCACACATTAATTGGCGCAAATGGTGCTATGATTTCACAGCAATCAAAGATTTCTGTTATCAAACCAATT
CATACAGATACATATCACCATATCTTAAAAATAATTCAAAAGTATGAGTTAGATTATATTATTGATGATGATTGGAATTATGCTGCA
CAACTTGACGCTGAGAACGCGATTTTTGAGCGTTTAGATCCACATAAGCTGGCAGTTTGTATTGATGTTGCAAATATCGACACACCA
ATCAAGATCATTTTATAAATATAGACCCGGCACAAATTACAACTATATTAGACGAGCTAAATAAATACCATCAAGAATTGGAAATG
ATTCACCATTCAAATGAGTATAACATTGATATAACAGCGCAAAATATTAATAAATATACTGCATTACAATATATATTTGATGCAGAT
GTTAAATATATAGCATTTGGTAATGACCACAATGATATTGTCATGTTACAACATGCTAGTAGTGGCTATATTATAGGACCATCAGAA
GCATACACACACGCAATATTGAAACTTGATAAAATCAAACACATCAATAATAATACACAAGCTATTTGCAAAGTCTTAAAATCATA
TAAATAAAAGCACCCCTATCAAATGATAATCATTATCAATCGATAGGGGCTATTTTAATAAAATTCGTCCTCGAACATTTCTTCCTCT
TCATCTAATCCAAATAATTCTGCCATTTCTCCATGTTCAATTAACATGTTTAAATATGCATCGCGGAGTTCTTCTTCACTCATATCATT
AATCATTTCTTTAAGACTATCAATCCACATATTTCTGCGTAATTGATAGTCTTCTTCAACTTCGTTTAACATCATTATATGTTTATTTG
CTGCTTCTGGACTAGCTGTAAAGAGTAATGCAATCATATGTTTACATATCACTCGTCTTCCATCAGCATGAGGACAATTACATTTGG
ATTTTCTAGGATGTTCCATATCAATATAACAACGATATACTTTGTTGCCACTGCCCTTTACTTCAGCCTCATGCTGCGTTTCTGAAAA
TGATTTTAAGTTAATGACGCATTCACTTTGATAATAATTAAAGCCTCTTTCTATAGAACGAATACTTGCAATATCAAGTAATCCCATT
AATGATACTCCTTTTTATTATTATTTTTAAATAAAGAAAATAAAATCGATAAGTGTCTAGATTAATATACTTGATTCATCTATATTTT
ATAACAAGTCTAGAATTATCGCATTCTTAAATAGCTAATATGAAAATGCTTGCACTAATTCTTTTGTATAAGGGTGTCTATCAACATT
AAATAATTCCTCTATTGCAAAATCATCGACTATCATGCCATCCTTAAGAACGATAATTCTATTAACTAAGCGTTGTAACACGGATAA
ATCATGAGAAATAACGATAAAATGATTTAAGTTCGTAATCGTTTGCGCTTTTAATATATTGATTACATTTTGTTCAGCTATAACATCT
AAATTTGAAGTTATCTCATCACATATTAAAACGCGAGGCTGTGCTAATAACGAACGCATGACATTAAATCTTTGTAATTGTCCGCCA
CTCACTTCGCTTGGTAATTTAGTCAATAATTGCGCGTTTAACTCAAAAGTAGATAAATGTTGTAATAATAATTGATCCTGAGCAGTA
TTATCAGTTAGGCCTCTGTAATAATATAACGCTTCTTTTAATGAGGTCTCAATCGTCCAATCAGGGTTAAAGCTAGTTAAAGGGTGTT
GGAAAATCGGTAACACAGCATTGTCACTTAAGTAAATTTCTCCTTTAACAGGTTTAAACAAGCCAAGAACCAATGAAGCGAGCGTA
CTTTTACCACAGCCACTTTCGCCTAAAATACCAACATTTTCTCCATCGGGTATAGTAATATTGATATCTTGTAGCACCATCTGCTTTTT
ATTATAACCAAAAGTCACATGTTTTAATTCAATCATAGTAATCCCTCTTTAATTGTGTTCTATATTTAATTAGACGTTCAGTATACGG
ATGCAAATGCTCATACTTGAAATGATTAATATTACCTCGTTCAATGATTTGACCTTCTTTTAAAACATAAATGTACTGACAATATTTC
AATACATGACTTAAGTTATGTGTGATAATAAATAATGTTTGACCATGTTCTAATACAATATGCTGTAATAAATCCATCACTTGATTAC
CGTTCAAAGCATCCAATGATGCAACTGGTTCGTCTGCAATGATTAATTTAGGCTCCAACATGAGAACGCTTGCTATGTATACGCGTT
CAAGTTGGCCCCCAGAAAGTTGGAAACTATATTTATTTAATATATCTTTGCTTTGTAAATTAACCCACGACAAAGCCTTATCAACTTT
GGACAAAGCCTCTTCTTTACTACCTTTATAATGCTTACGATAAATCGCAGTTAACTGTTTACCTAATTTAGTATGGTCGTTAAAACTT
TCTGCATAATTTTGGGAAATATAGCCAATTGTATGACCATAATATTGACTCAATCTACTAACATTTTCTCCATCAAATTGGTATGAAT
CATACGTGCAGGTTAAATCAAATGGTAAATATTCAAGTAAGGCTTTAGCAATCAAACTTTTTCCAGCGCCGCTCTCTCCAATCAAGG
CATTAATCTGTTGACTAAAAATTTTCAAATCAATCCCTTTAATAAGTGATTTCTCACTAGTATTCTTTATTGTTAAATTTTGTATATCA
ATGAGACTCATCATATTCACCCCGTTGTTTCAGCAATCTATCTCTTAGCGCATCTCCGGTTAAATTAAAAATTAAAATAGTTATAGCA
ATGACTGAAGCAGGTGCAATCAACATAATTGGATGAGACGAAATAAAATCACGACCTTGTTGCAACATAGCGCCCCACTCTGGTGT
TGGCGGTTGTGCACCTAACCCAATAAATGATAGTGAACTTATATATAGAATGATTTTACCGAAATCAACGACCATCAAAACGATAA
TAGCCGGTATAATTTTAGGTGTTAAATGACGTATTAATATTGTTCTTGTAGGTACATGAAATAATTGTGCCATTTTTATATAAGGCTT
ATTCATTTCGCTATTAACTATACTTCTAGTCAACCTTGTGTAATTCATCCATTTTATTACTGTAATTGAGATAACTAAATTCCATAAA
GATGGTTGAAAAAAGCTTGCTAAAGCAATCATGATGATAAATTCTGGAATACTTAGACCAACATCAATAAACCTTAACACTAATCG
TTCAATCCACCCTTTTTTATATCCGGCAAATAGACCTAGTGTAACACCTATGACAACGATAGCTATTAATGTTAAAACAGTAACAAA
CAATGTTGAACGTGCACCGATAATAATTCGGGTAAATAAATCTCTCCCATAATCATCAGTTCCTAATAAATGCAACCAACTAATAGG
TTCAAAAGTTTGTGATAAATTGACTTTGGTTGCATTTTCACTACTGACAAAGAATTGCAGTACAATTACCACAAAAATAAATGCAAC
GAATACAAAAAAATATCAGGTTATTTTTTGAAAATATTTTATGCATGACGGTCACTACTTTCTGATATCAATGGTGTATTGGTTTTGTT
TTTTGGATTTCCTAATTGTAAACGCTGCTTCGGATCAAGTAATAACGTTAATAAATCAGCAATCGTATTGATAATAACAACGAAGAA
GCCAATAAATAACACGCATCCTTGAATAACAGGATAATCTCGAGATTTAATACTATCCATTAATAGATAACCAATACCAGGTATATC
AAATAAATTTTCAATCACTACAGTACCACCTATTAGACTGCCAAGTGAAATCCCTAGTAATGGGATAATCGGCAAAATTGTTGGTTT
TAGTAAATCATGAATTAAAATATAACGTTCATTCATACCGCGTAATCTTGATGCTTGTACGATATTACTTTGCAATAACATCAATAA
ATTAGAACGCACTAAACGAATGATGTATGCACACATACCTAAAGATAGCGTGATTACAGGTAATATAAACTGACTTAGTATAACGC
TATCTATATTCATTAAATTTGTGACAATAAATAATAAAATAATACCGATAAAGAACGCTGGTAAACTAATCGATAGTGTTGAGATCA
CTCTAATCACTTTATCCGTCCACTTATGAAATCGTTTGGCTGCTATAATGCCGAGCGGTATAGATATGCATAACGACACTACTAATGT
TGAACAAGATATGAGTAATGTTATGGGTGCATAGTTGAATAATATCTGTGTTACCGGTTCTTTTGATTCAAAACTTTTTCCTAAATTA
AAATGTAATAAATGATTCATCCAATGCCACCACTGTACCAATAAAGAATCATTTAATCCCAATTTATCTTTGGTTGCATTTATTTGTT
CCGTCGACACTTGTGCTACATCAAGATGTAATATTTTATCAACAGGATTGCCTGGTGATAATTTCATTAAAATGAATGTAAGTGTAG
AAATAACAAATAAAACAACTATCATTTGCATCAGTCTATACAACATAGACTTAATTATGAACATAACAGTCCCCCTCCTTGTGTAAG
TTACTAACACTTTCTTTTTACATGAGAATGGCGCATGTATATGCAACTTACATATTAAGAACCAACGTTCATTATAGTATTATCCATA
AAGAAATTGAAGTATATTTAATTTTTTAACAAAATCATTATAAAATATAATATTTTGAATCAAGTCAACCATGTAAAATATAAAAAA
GTCAAAACAAAAACAACTATAGCACTGTATTCCATCTCTTTCGAAATAATTGTTACTGCAGTGTAACTTAAAAGTCGATGATTTTGT
GCATATAGTTGTTGAATATTATTTTTTATCTTTACGGCGAAGTTCAGCGCCCTCATAGCCGTATTTTTCAATTTGCTTTTCTAATTTAC
GCGCTTTTCTTTCTTTACGCCAATTTCTAGTAAAATACCATAATAGAAAACTAATTAATAAACTCATGATCGCTAAAAATGCAGCGT
ATCCTAATAATGGTTGATATTTTATATCTTGAAAATTTGGAATAAAAAATGCAAGCACACCTAATATAACAAATGTAATTACTGCAG
ATACAAACCATTTATTTAAAACTAAGCAACAGAATATTGTTAATAAAATCATTATTAATGTTGTGATCCATAAATAATTAGGTATAT
CGAATAATGTCATATTCATTCTCCTTTTATTTCATTACTTTCCTTGTATACATTTTATTATAAATTTTTAAAAACTTAAACAATAGCAG

TCAGTTTCAAGCAATATTCTATCGGTTCTGTTGCAAAGTTGAATTTATAGTATAATTTTAACAAAAAGGAGTCTTCTGTATGAACTAT
TTCAGATA

>033-contig_261_RC

CTATATAAAAAGAACCGTAGATCTGAAGAGACCTGCGGTTCTTTTTATTAAAATTATATTATAAATTCAACTTTGCAACAGAACCTT
CTATCTACTATTAGAAAAATCATTGTTCCTTGCGACATGGAAATCGTAACATTATCGTTTAGGAGACAAAATTATGTATAATGAATG
TATTATACCAAAGGAGTGATTATATGTCTCAAGGTTTACCTTTAAGAGAAGATGTTCCTGTTTCAGAAACATGGGATTTAGTAGACT
TATTTAAAGATGATCAACAATATTATGAAAGTATTGACGCTCTAGTACAACAAGCAAATCAATTTCATCATACATATGCAACAACAT
TAAATTCAATCGAACAAATTAATACTGCTTTAGCTGAATTAGAAAATATTTTAATTGCCTTAGATCGCTTAAGTAATTATGCAGAAC
TACGTTTAAGTGTAGATACTAGTAATATCGAGGCACAAGTATTGAGCGCTAAATTATCTACTACATACGGTAAAATTGTTAGCCAAT
TATCATTTGTAGAGTCAGAAATACTTGAATTACCAGAAGAAATACTTCAACAATTAGAAGAATCATGTCCATATCAACACTATATTA
AACAGTTAATAAAACAAAAGCCATTCCAATTATCTGCGTCGGTAGAACAAGTATTAGCAACTTTATCACCTACGCTAAACAGTCCTT
ACGATTTATACGGCACGACAAAAATGCTAGATATTACATTCGATTCATTTGAACATGATGGTACAACGTACCCTGTCGACTATGCTA
CGTTTGAAAATGATTATGAAGATAATAAAGATCCTGAGTTTAGACGTAAAAGTTTCAAATCGTTTAGCGATGGGATTCGAAAATATC
AGCATACTACCGCGGCTACATATAATATGCAAGTACAACAAGAAAAAATTGAAGCTGATTTACGTGGATTTGAATCAGTCATCGAT
TATTTATTACATAGTCAAGAAGTAACGCGTGATATGTTTGACCGTCAAATCGATATGATTATGCGTGACTTGGCACCAGTTATGCAG
AAATATGCTAAACTTTTACAACGTATTCACGGATTAGATAACATGCGTTTTGAAGACTTGAAGATTTCTGTAGACCCTGATTATGAA
CCAGAGATTTCAATTGAAGACTCAAAAAATTATATTTTCGGTGCGTTAAGTGTTTTAGGTGATGACTATACAAACATGTTACGTGAA
GCATACGATCAGCGATGGATTGATTTTGCACAAAATAAAGGTAAAGATACAGGCGCATTTTGTGCAAGTCCATACTTTACACATTCA
TATGTGTTTATTTCTTGGACTGGTAAAATGGCTGAAGCATTTGTCTTAGCACATGAATTAGGTCATGCAGGTCATTTTACATTAGCTC
AAAAACATCAACCATATCTTGAATCAGAAGCATCAATGTACTTTGTTGAAGCCCCTTCTACAATGAATGAAATGTTGATGGCCAATT
ATTTATTTAACACAAGTGATAATCCAAGATTTAAGCGTTGGGTTATTGGCTCAATTTTATCTAGAACATATTATCATAATATGGTTAC
CCATTTATTAGAAGCTGCTTATCAACGTGAAGTGTATCACAAAGTAGATCAAGGTGAATCTTTAAATGCGCCGACATTAAATGAAAT
AATGCTAAATGTTTATAAACAATTTTTTGGAGATGCAGTAGACATGACTGAGGGTGCTGAATTAACATGGATGCGTCAACCTCATTA
CTATATGGGATTATATTCGTATACGTATTCTGCTGGCTTAACAATCGGAACTGTCGTTTCTCAAAAGATTAAAAATGAAGGCCAACC
AGCTGTTGATGCTTGGTTAGAAACATTGAAAAAAGGTGGTAGTGTATCACCTGTCGAACTTGCAAACATTGCAGGTGTAGACATTAC
TACAGAACAGCCACTTAAATCTACAATTCAATATATTTCTGATTTAGTCGATGAAGTTGAAAAATTAACAGATGAAATTGAGCAAGC
AAATAACTAAGATGCGAGACAATTTATAGTAGAACAATATAAACCAAGATTTTCACATTACTAATTGTGAGAATCTTGGTTTTTTTG
AGGGTTTATGAATGATAGAAATATATATTTATCGTAAGGGATTTTATAGTAATAACTTTAAGTTATTGTTCGTAATGTGTACCTGTTA
AATAAAAATAAACACTTTCAGCGATGTTAATAATATGATCACCAATACGTTCTAAATGTCTTGCTGCTAAATGAGCTTGTGCAGCGA
CAAATGGATCGTTATCAATAAGATACGTTGCGTTAATAATATGACTATATAAGTCATCGATATCTTCATCACGCTCAATTATTTCTCT
TATTAATACGGTATCTTTCTTTTTAAATGCTTGATCTAAGTCCTTTAACATTAACATAGCTAATTTACCCATTGTCTTTAAACGGGTTA
ACACATAATCATCTGTAATCTTTGTACGCAATCGAATATTGGCAATACTCGATGCATTATCTCCTATTCTTTCTAAATCGGAGGCGAT
TTTTAATGAAGAAATCATCATACGCAAATCACTCGCAATGGGCTGTTGCTTTGTAATTAACATGATAACTCGCTCATTAATATCATA
ATTTAATTGATTGATATGTTTATCGTTTTTAACTGTTTGTCGTGCAAAGCCTCTATCGTCAATACTTAATGATTTTATACCATTTTCAA
TACTCACATAGACATTTGCACCTAACCGACGTAATTCCTTTATTAAATCATCAAGTTGCTCCTGATATCGCTGTCTAATTATTGCCAT
TATATATCAACCAAACCTTCCTGAAATATAATCTTCTGTTTTCTTGTTTGATGGGTTAGAGAAAATTTTATCAGTATCATCATATTCA
TTGACATAACCATTTAAGAAAAATGCAGTTTTATCTGATACACGAGCTGCTTGTTGCATATTATGTGTAACCATAATAATTGTATACT
TTTCTTTTAGTTCTTGAACCAACTCTTCTACTCTTAATGTTGAGATTGGATCTAATGCTGATGTCGGTTCATCCATTAAAATAACTTCA
GGTTCAATTGCTAAACAACGCGCGATACAAACACGTTGTTGTTGCCCACCGGATAAACTATATGCATTTGTGTGCAACCTATCCTTT
AATTCATCCCAAATTGCAGCGCCACGTAATGATTTCTCAACGATTTCATCAAGAACTTTTTTATTTTTAATACCGTGAATCTTTGGAC
CGTAAGTAATATTATCGTATATTGATTTTGGAAATGGATTAGGTTGTTGAAAGACCATGCCCACATTTGTACGTAATTGTTCTTTAGA
ATATTTTTGATCAAAAATGTCTTGATCTCGATATAATATTTTACCAGCTGTTTTCACAGAAGGTACTAACTCAACCATTCGATTCAAA
GTTTTAATATATGTTGATTTGCCACAACCAGATGGACCTATAATGGCAGTAATTTGGTTTTCATAAATATCTAAATTAATATTTTGTA
ATGCATGATTTTCGCCATACCATAAGTCTAAATTTTGTGTTGAATATATAACAGAATGTGAGTTTGTATCATCTGGTGTTTTATGATG
ACTTTGTGAGACATCAAACGTATGACTTTGAGATATTTGTTTAGTTTGTGCAAGTGTTTGCGCCATATTTAAAACTCCCTTTTTAATA
GATAAATGAGATTGATTGTTTAAATTAGAATTTTTTACTAAATTTGTTACGTAAAATAATCGCAACGCCATTCATTAAGATTAAGAT
AACTAGTAAAACGATAATGCCTGCCGATGCAACATTCTGGAATTCTTCTTGAGGCATTTTCGCCCAAGTAAATATTTGGATTGGTAA
TGCTGAAAATTGATCCAATATACTTCTAGGTGTTGCCAATAATATAGTCGGTATACCGATTAGCACAAGTGGCGCTGTTTCTCCCAG
TGCTCTTGAAAGAGACAAAATGAATCCAGTTAAAATACCAGGTAACGCTGCTGGTAAGACAACACGTCTTATCGTTTGCCATTTATT
AGCACCTAAACCATAAGAAGCTTCGCGTACTGAGTTAGGTACAGCTCTAATTGCTTCCTGACTTGAAACAATAATAATTGGTAATAT
CAGTAAGGTCATTGTTAGCGCTGCTGCCAATATACTGTTACCCATTTTCAAGGCTTCAATCCCCGCACCACCAACGAACAAAGTGTA
ACCTAATAACCCAAATACAACTGATGGTACACCAGCTAAATTGGAAATACTGATTTTAACAAACTGAGTAAATTTGTTGTTTTTCGC
ATATTCTTCTAAGTATATAGCTGTACCTATTCCTAGGATGATTGATAATGGAATGATACTTAACATTAACCAAAGTGAACCGATTAA
CGCGCCTTTAACGCCAGCCATAGATGGTGTTGAAGAAGAAAAATTAGTGAAAAATTGTAAATTTAAATGACTTACCCCTTTAATCAA
TGTTTGAGTTAACAACGCAATAAGTACGACAAGTCCTAATAATGTACATGCTAAAAATATGAGTTTGAACACTTTATTTTTAACCGT
TCTGGATGATAAATGTTTTTGGACAAGTTGTTGATCGACGAGTGATTGTCTATTATTATCTGTCGTTTCCATATTAATACTCCTCCCTA
AAACGCTTAGAAATCCACTGAGAAAGTAAATTCATGATTAAGGTAAAGATAAATAGTGTGAACCCTACAGCATAAATACTGTAATA
AATATTTGATCCAAATGTTGCATCACCTGTCGCTATCTCAACAATATATCCAGTCATTGTTTGAATCGAACTTGTTAAACTTAATGAA
GCTGTTGGCGAACTACCTGCCGCTAATGATACAATCATCGTTTCTCCAATTGCTCTTGAAATCGCGAGAACGATTGAAGCTACAATA
CCTGATGTTGCTGCGGGAAGTACGACTTTAGTTGCTACTTCTAATTTAGTTGCTCCAAGTCCATAGGCACCTTCTCGAATTTTATTTG
GTACAGATGCCATTGCATCCTCACTCAAACTTGTGATGAGAGGGACAATCATAATACCGACAACTAAGCCGGGACTTATAGCATTA
AACTCTCCAAGACCTGGTATGAAAGATCTTAATACTGGTGTAACAAAGGTTAATGCAAAGAAACCAAACACAATTGTTGGTATTCC
TGCTAAAATTTCTAATATCGGTTTAATTATGCGTCGTGCACGGTCACTTGCATATTCACTTAAATAAATTGCTGCACCAAGCCCGACT
GGAACTGCAAATATAGTCGCAATAACTGTGATTTTTAAAGTCCCTATTATCAATGCCCAGATACCAAACTTAGGGTCTGAACCGGTA
GGATTCCAAGTAGTAGAAAATAGAAATTCAGTTATTGGAATTCTGGTGAAAAAAGTGATGGTTTCTAAAAGCAATGTGATTAATAT
ACCTAGTGTTGTTAAAATGGAAATCGCTGAAATTGCGGCTAAAATAACTGGTATAATTTTGTCATTATGCTTCCCTTTTTTATTATTA
TTTTTTTCGATTAAAGCTTTAACATTAGTAGATGAAGTCATAATCAAACCTCTTTCTATTACTCCCAAAATAATTAGATTTGTAAAAT
AATGATGAACCACTATTTTAAGTTGATAAGCTTGTAGACTAACTGAATGTCAAAATCAGCGTCTCGATAAATCAAGCTTTGTACTCT
AAATAATGATTCACCATCAAAATCATGTTTCAAGACACATTTGAAATTGCGTCTTATTATTTTTTGTCTTCAGACTTTTTATCATCAG

ATTTCTTGTCGTCTGATTTTTGATTTTTATCAATAAATGCTTTTAAATCATCTAATTGTGATTTGTATGTTTTCTCTGGTGCTGCTACAT
ATCCAGCTTCTTCAGCTGCTTTACCTTTATCTTCTAAGACGAATTTGATAAATTCTGACATTACTTTATTATCTTTCAATGCTTTTTCA
TTTACATAAATGAATAATGGTCTACTTAATGCATAAGAGTTATCTTGAATTGTTTTTTTCGTAGGCTCTGTTGCTTTACCATTTTCATC
TTTGATTTTAACTTCTTTTAATTTATCTTTATTTTGAACGTAGAAGTTATATCCAAAGTATCCGATTCCCTCTTTGTTTTTCGTTACAGA
AGAAACGATAGCATTTGTATCAGCATTTTTTTCTGCTTTAATATCTTCTTTATTCATTACTTCATTTTCAAAGAAGTCATAAGTACCAT
GACTTGAGTTTGGTGATACAGCATTTATTTTTTTATCTGGCCATTTACTATTAACATCTTTCCATGTTTTAGCTTTTCCAGAATAAATT
GCTTTTAATTGCTGTTTGTCTAATTCATCCACAAAATCATTTTCTTTATTTATAGCAACCGTTACACCATCTTGCGCAATTTTGAATTC
TTTGTATTTGATATTCTTATCTTGTAATTTTTGCTTCTCTTCATCTTTAATTGGTCTAGAAGCATCAGCGAAGTCGATATCTCCTGCAA
TGAATTTTTGGAAACCAGCACCTGTACCAGCTTGTCCTGCTGAGATTTTAGCATCCGAGTGATCTTGAGCCCATTTTTCATTTAATTT
CTCCACAATTGGTGCTACTGTTGATGAGCCATCACCTTTAGCTTCCCCTTTTAAATCACTATTACCACTGCCACCATTACCGCCACCA
CAAGCACCTAATAATAGTGTTGCACCTAAAGCTGTAGTACCAACAAATTGCCATTTTTTCATTGAAATATCCTCCCTGTATGAACAA
CAAATAGTATTTGTATTGATTACATCTTTATCTTAACCTTGAATTATTAACCTCATTTTTAGAAACTGTAAATGTTACATAAAAATTT
GTTAAGAAAATGTAAATATTAATTTTTGTTAATCTAAAAAATATTGCTTCATTAGTAAAATTAAAATGCTTCCTGAAGAATTATCGA
AATTTAAATATGAATACAAAAATGGCTGGGACATAAATCCCTAAAAAAACAGCAGTAAGATAATTTTCAATTAGAAAATATCTTAC
TGCTGTTCTCTATTTATACAATACTTCGTATTGAATGGCTTCGCT

>034-contig_264_RC

ATAAAGTTAAACGAGAGCTAGGTTTTGTATTAATGGCACTTAATATAAGGAAAATAGCAGCTCAACGAGCTGTACATTATAAAATA
CATATCAAAAAAGCTAATTTCCATCAAATAATTAATAGAAATCAGCTTTTTTACATTGCCTAAGAACTTAATGTCCCAGCCTATTCGT
TTGAATTATTTAATTTTTTACTTTGAAAGGAATGTTTAAAAAACTACTTCATCTGTCCGACTTAATATTGAATATAAAAAACACGCAT
CCTACGACACGTGTAAAAATGATTATTCTTTTGAGTCAATTCGACTCCAACCTTTTTTAGTTAAAGTGATTTTACCTGTTTCTATATTA
ATAATCTTCTGTTTATATAAGTGACCGATTGCACGTTTGAATGAACCTTTACTCATATTGAATACTTCTTTAATCGCTTCAGGGCTTG
ATTTGTCCCAGAATGGTAATTCACCATCATATTCAACTAGTAAATCAAAGATGACTTGGCCGTCATCGTCTAAACGTTCATGTGCAA
GTGGTAAAAATGAACCATTTAACTCACCTTTATCATTATGCCCGATAATTCTAACTTGAACAGATTCACCTAATCTTGGTTCAGCTTT
ACGTTCTGATTCATGTACGAAAATTTTGTAACCTGATTCGCTTAATAAGAAGCTACCAATTCGTAATACGCGGTAAGGTTTGGCTTC
AATGACTTCGTTTTTTAAATTATCATCGTGTACAGGTGTAAACATATTTTCTACAACAGATTCACTCGCTAAACGTCCATACATATGA
TTCTCACGGTCAATTCGTAATGTAACTAGCAAATGATCACCAGGTTGTGGCCATAGTGATTTCACTTTTGGTAAATCTTCCCATGGTA
CTAACACTTCACGGGGTAAACCAACATCTATACGTGCCCCATCGCGATCCGTTTTAAGTACTTTAGCAAAATCATATTTATCTTTCGT
AATATCAGGCATATTTTGAGTTGCAAATAATTCACCTGAACGGTTTGGATAAATGAAGAAACTATATTCTTCACCTACTTCTAATTC
ATCATCATCGTTCATTTCTGATTGGTTTAACTTTACGTTTTCACCGTTTGGTCCTTTTAAAAGGTAAGTTGAGCCTTGTAACCCTACTA
CTTCAAGGAATTCTATAGAACCTACTATATCTTTGTCTAATGCCATGTTATTCTCCTTCGATTCTTGTATTCTTTATTATTATAACATG
TGTGAGTCATTCTATCATTACAACTTATATGAGGTCGAAACTGTGACGATAATATGATATAATTCTTCCGTTAGAATATCGAATGAA
AAAAAGGAGAAAATGCATGTTACAAGTAACTGATGTGAGTTTACGTTTTGGAGATCGTAAACTATTTGAAGATGTAAATATTAAAT
TTACAGAAGGTAATTGTTATGGATTAATTGGTGCGAATGGTGCAGGTAAATCAACATTCTTAAAAATATTATCTGGTGAATTAGATT
CTCAAACAGGACATGTTTCATTAGGGAAAAATGAACGTCTAGCTGTTTTAAAACAGGACCACTATGCTTATGAAGATGAACGCGTG
CTTGATGTTGTAATTAAAGGTCACGAACGTCTTTATGAGGTTATGAAAGAAAAAGATGAAATCTATATGAAGCCAGATTTCAGTGAT
GAAGATGGTATCCGTGCTGCTGAACTTGAAGGTGAATTTGCAGAAATGAATGGTTGGAATGCTGAAGCTGATGCTGCTAACCTTTTA
TCTGGTTTAGGTATCGATCCAACTTTACACGATAAAAAAATGGCTGAATTAGAAAACAACCAAAAAAATTAAAGTATTATTAGCGCA
AAGTTTATTCGGTGAACCAGACGTACTATTACTGGATGAGCCTACTAACGGTCTCGATATTCCAGCAATCAGTTGGTTAGAAGATTT
CTTAATTAACTTTGATAATACTGTTATCGTAGTATCGCATGACCGTCATTTCTTAAATAATGTATGTACTCATATCGCTGATTTAGAC
TTCGGTAAAATTAAAGTTTATGTTGGTAACTATGATTTTTGGTATCAATCTAGTCAGTTAGCTCAAAAGATGGCTCAAGAACAAAAC
AAGAAAAAAGAAGAAAAAATGAAAGAGTTACAGGACTTTATTGCACGTTTCTCAGCTAACGCTTCTAAATCTAAACAAGCAACAAG
TCGTAAAAAACAACTTGAGAAAATTGAATTAGATGATATTCAACCATCATCAAGAAGATATCCTTTCGTTAAATTCACGCCTGAGCG
TGAGATTGGTAACGACTTATTAATCGTTCAAAATCTTTCTAAAACAATTGACGGCGAAAAAGTATTAGATAATGTATCATTCACAAT
GAATCCAAATGATAAAGCGATTTTAATTGGAGATAGTGAAATTGCAAAAACAACATTACTTAAAATATTAGCTGGCGAAATGGAAC
CAGACGAAGGTTCATTTAAATGGGGTGTTACTACATCATTAAGTTACTTCCCTAAAGATAACTCAGAGTTCTTTGAAGGTGTAAATA
TGAATCTCGTTGATTGGTTAAGACAATATGCTCCTGAAGATGAACAAACAGAAACATTTTTACGTGGTTTCTTAGGTCGTATGTTATT
TAGTGGTGAAGAAGTTAAGAAAAAAGCTAGTGTGCTTTCAGGTGGAGAAAAAGTACGTTGTATGCTAAGTAAAATGATGTTATCAA
GTGCGAATGTACTTTTACTTGACGAACCTACTAACCACTTAGACTTAGAAAGTATTACTGCTGTCAATGATGGTCTTAAATCATTTAA
AGGTTCTATCATCTTTACTTCTTATGACTTCGAATTTATCAACACGATTGCAAACCGTGTTATCGATTTAAATAAACAAGGCGGCGTT
TCAAAAGAAATTCCATATGAAGAATACTTGCAAGAAATCGGCGTTTTAAAATAAACATAATATGTAGAATTTCTTTTCGAAAATCTC
TGTATTGAGGCCCGACCAACTTGCACATTATTGTAAGTTGGCTTACTGTCACCTTCTATATTGGGACTTATTCCGCAACTTGTATTGT
TTGTAGAAATTAGGAATCCAATTTCTCTATGTGAGACGCTGACAAGGATTGAAAAAAGCTAGCTACAAAGTCATTTTCATTCAGTCA
ACTACTGTCAATATAACATTGCAGGGCCTAGGACATTGATTCATGTCCGAGACTATATTCAATTTAATAGACATTAACTGGGCGTCT
CGATGCTAATATTTATAGCATCGAGGTGTCCTTTTTATTTTATTTTTTAGCACATCAGCACAGTGAACACCCCTTCTCTAAATTACAA
ATACAGTTATCAACGCATTAAAAGAGTACATTTATTTAATGTGCATATTGATTATGCTATAAGTATTTTGAATTTTATTTTTCAAAAA
GTCTCATTATCACCACCTTTTAAAATATAAGCAACGCTATAACTTGAATTTTCAGGTTATTCCAAAAAATCTAAATTATCGGTTTACA
TTCATATTTGTCATATGTATAATGAACACATACCAATTTAATAATATTTTGATGAGGCGCATCAATCATGAGTAAAGTTTAGATTACT
GTCTGCTAACAGCTAAATTTGAAAGGGTGCGATGCCGAAGCAATTATAATAGCAGTTATAATTTGTTGGACTTTTTGGTTAAGAGCT
GAGAGTTTGTCATTATTTAAAAATAATGGAGTGCATCACTTGTATATAGATTAAGAGCAAGTTCGCATTCCGAACTTGTTCTTTTTTT
ATTATTGTGTGCCCTTCCTAACAATTAGGAGGATTATATGGTAACAAGAAGTGTGTTGAAATTTGGCGGATCATCCGTCAGTGATTT
TACAAAAATAAAAAGGATCGCTGAAATGTTAAAGGAGCGAGTCAATCAAGATGAACAATTAATTGTCGTTGTAAGTGCTATGGGTA
ACACAACAGATCAATTAATGACGAATGTATCGACCTTGACTAAAGCACCAAAACAACAAGAACTGGCATTATTATTGACAACCGGA
GAGCAACAAACTGTATCTTATTTATCAATGGTATTAAATGATATCGGTGTGAATGCCAAAGCAATGACTGGCTATCAAGCAGGTATT
AAAACCATTGGCCATCATTTAAAAAGTAAAATTGCTCAAATTAATCCTCAAACATTTGAACAAGCCTTTCAAGAAAACGATATTTTA
GTAGTTGCTGGATTTCAAGGCATCAATGAACATCAGGAATTAACAACTTTAGGCAGAGGCGGTTCTGATACGACCGCTGTGGCACTT
GCTGTTAGTAATCAAATACCTTGTGAAATTTATACCGACGTTGATGGTGTGTATGCCACTGACCCAAGACTTTTACCAAAAGCTAAA
CGACTAGACATCGTCTCATATGAAGAAATGATGGAAATGAGCGCTTTAGGTGCTGGTGTACTTGAAACAAGAAGTGTTGAATTAGC
TAAAAACTATAATATCCCTTTATATTTAGGAAAAACTTTATCGAACGTGAAAGGAACATGGATTATGTCAAATGAAGAAATATTAG
AGAAAAAAGCAGTTACTGGTGTGGCTTTGGATAAACATATGATGCATGTAACAATTAGTTATCCCCTACCTGACAATCAGCTACTTA

CCCAACTATTTACGGAACTTGAAGAAGGTGCTGTAAATGTTGATATGATTTCACAAATCGTCAACTTGGATGGACTACAACTATCCT
TCACGATTAAAGATAGTGATTTTCATCAAATTTCTATGATTCTTGAAACATTAAAGAATCAATATGAAGCATTAGCTTATAAAATCA
ATGAGCATTATGTCAAAATTTCATTAATTGGCTCAGGCATGCGTGATATGTCAGGTGTGGCATCAAAAGCATTTTTGACATTAATTG
AAAATAATATACCTTTCTACCAAACAACAACATCTGAAATAAGTATTTCATACGTCATTGATGATTTTAATGGGCAACAAGCGGTAG
AAAAACTATATGACGCATTTAACATTTAATGGTAAAATGATTGTTAAAATATTCTAAAAATTGGAAATTATTATAAAATGGAGTGAC
AAGTTATGACAAAGTTAGCAGTTGTGGGTGCAACAGGATTAGTAGGCACAAAAAATGTTGGAGACATTAAATCGTAAAAATATTCCT
TTCGATGAATTAGTATTATTTTCATCAGCACGTTCTGCAGGGCAAGAAGTTGAATTTCAAGAAAAAACATATACAGTACAAGAATTA
ACTGATGCTCGTGCTAGTGAACATTTCGATTATGTATTAATGAGTGCTGGTGGCGGTACAAGCGAACACTTTGCCCCACTCTTTGAA
AAAGCTGGTGCAATCGTTATAGACAATTCAAGTCAATGGCGTATGGCAGAAGATATTGATTTAATCGTTCCGGAAGTCAATGAACC
TACATTTACAAGAGGTATCATTGCCAATCCAAACTGCTCTACGATTCAATCTGTTGTGCCATTAAAAGTATTGCAAGATGCTTATGG
TTTAAAACGAGTGGCATATACAACATATCAAGCTGTATCAGGTTCAGGGATGAAAGGTAAGAAAGATTTAGCTGAAGGTGTAAATG
GTAAAGCACCAGAAGCATATCCACATCCAATTTATAATAATGTGTTACCGCATATTGATGTGTTTTTAGAAAACGGATATACAAAAG
AAGAACAAAAAAATGATTGATGAGACGAGAAAAAATTTTAAATGCGCCAGACTTAAAAGTAACAGCAACATGCGCACGTGTGCCTGTT
CAAGATAGTCATAGTGTTGAAATTGATGTAACGCTTGACAAAGAAACGACAGCAGAAGATATTAAAGCGTTATTTGATCAAGATGA
CCGCGTTGTTTTAGTAGACAATCCAGAGAACAATGAATATCCAATGGCAATTAATTCCACTAATAAAGATGAAGTGTTTGTTGGCCG
TATACGTAGAGATGATTCATTAGAAAATACTTTCCATGTATGGTGTACATCAGACAATTTATTAAAAGGTGCTGCATTAAATGCTGT
ACAAGTATTGGAACAAGTTATGCGTTTAAAAGGAGCGAATTAAAATGACACATTTATTTGAGGGTGTTGGCGTTGCACTTACAACCC
CTTTTACAAATAACAAAATTAATATTGAAGCTTTGAAAACACACGTTAATTTTTTACTAGAAAATAATGCCCAAGCAATCATCGTTA
ATGGAACTACTGCTGAGAGCCCTACTTTAACAACAGATGAAAAAGAACGCATTCTAAAAACAGTTATTGATCTTGTAGATAAACGT
GTTCCTGTCATAGCAGGAACTGGCACTAATGATACTGAAAAGTCAATCCAAGCTTCAATCCAAGCTAAAGCCTTAGGGGCTGATGC
AATTATGTTAATTACGCCCTACTACAACAAAACGAATCAACGTGGTTTAGTCAAACACTTTGAAGCGATTGCAGATGCTGTGAAATT
ACCAGTCGTGCTGTACAATGTTCCTTCAAGAACGAACATGACAATTGAACCAGAAACTGTAGAAATATTAAGTCAACATCCTTATAT
AGTTGCTTTAAAAGATGCTACGAATGATTTTGAGTATTTAGAAGAAGTGAAAAAGCGAATTGATACAAATTCATTTGCATTATATAG
TGGCAATGATGACAACGTCGTTGAATACTATCAACGTGGCGGTCAAGGGGTTATCTCTGTTATTGCCAATGTCATTCCTAAAGAATT
TCAAGCGTTATACGATGCTCAACAAAGTGGATTAGATATTCAAGATCAATTTAAACCAATCGGCACACTGTTATCAGCCCTATCAGT
TGATATTAACCCAATTCCTATTAAAGCTTTAACAAGTTATTTAGGATTTGGAAATTATGAATTACGTTTACCATTGGTTAGCCTAGAA
GATACAGATACTAAAGTGCTTCGTGAAGCATATGACACATTTAAAGCGGGTGAAAATGAGTGAAAATATTACTAATTGGCTATGGT
GCAATGAATCAACGCGTTGCTAGATTAGCAGAAGAAAAAGGACATGAAATCGTTGGGGTCATTGAAAATACACCGAAAGCAACAA
CGCCATATCAACAATATCAACATATTGCAGATGTTAAAGATGCCGATGTTGCAATAGATTTTTCAAATCCAAATCTGCTTTTCCCTTT
ATTAGATGAAGAGTTTCATTTGCCATTAGTTGTGGCAACAACTGGCGAGAAAGAAAAACTACTTAATAAGTTAGATGAATTGAGTC
AAAATATACCTGTATTTTTCAGCGCGAACATGAGTTATGGCGTTCATGCACTGACTAAAATTTTAGCAGCTGCTGTTCCCCTACTTGA
TGAATTCGACATCGAATTAACTGAGGCACATCATAATAAAAAAGTAGATGCACCAAGTGGTACGTTAGAAAAATTGTATGATGTGA
TCGTATCTTTGAAAGAAAATGTAACACCTGTGTATGATAGACATGAATTAAATGAAAAACGCCAGCCACAAGATATTGGTATACAT
TCTATTCGTGGAGGTACGATTGTCGGTGAACATGAAGTTCTATTTGCTGGCACTGATGAAACGATTCAAATCACGCATCGTGCACAA
TCAAAAGATATTTTTGCGAATGGTGCAATACAAGCAGCAGAACGCTTAGTTAATAAACCAAACGGCTTTTATACGTTTGACAACTTA
TAAACATATTAAAGGAGATTGATTATTTTATGGTACAACATTTAACAGCTGAAGAAATTATTCAATATATAAGTGATGCTAAAAAGT
CTACACCAATAAAAGTATATTTAAATGGTAATTTTGAAGGCATCACATACCCAGAAAGTTTTAAAGTATTTGGTTCAGAACAATCTA
AAGTAATCTTTTGTGAAGCGGATGATTGGAAACCTTTTTACGAAGCATATGGTAGTCAATTCGAAGATATAGAAATTGAAATGGATC
GTCGCAATTCTGCCATTCCATTAAAAGACTTAACAAATACGAATGCACGTATTGAACCAGGTGCATTTATTAGAGAACAAGCCATTA
TTGAAGATGGTGCTGTCGTTATGATGGGCGCAACAATTAATATTGGCGCAGTCGTTGGCGAAGGTACAATGATTGATATGAATGCTA
CTCTCGGTGGTCGTGCTACAACTGGTAAAAATGTACATGTAGGGGCTGGCGCAGTATTGGCAGGTGTGATTGAACCCCCTAGTGCAT
CACCAGTTATAATCGAGGATGATGTATTAATCGGTGCAAATGCAGTTATTTTAGAAGGTGTACGTGTTGGTAAAGGTGCTATTGTTG
CAGCTGGCGCGATTGTGACACAAGATGTACCAGCTGGTGCAGTTGTTGCTGGTACACCTGCAAAAGTGATTAAGCAAGCTTCTGAA
GTACAAGATACTAAAAAAGAGATTGTAGCAGCATTAAGAAAACTGAATGACTAGTGACGTCAACGTATAATAATTTCAAGGTTGAG
ATACGATTATGTCTCAACCTTATTGTTTATCATTGAATAAACTTATGATCACAAATTTCGAAATACGTTACACTATTTATATAGTAAT
TTAACACAAAGGATTGATAATTATGAATGAATTAGAATTTGTTACGAAACATCGCCGTCATTTACATCAACATCCTGAATTAAGCTT
ACATGAATTTGAAACAACTGCTTATATTAAAGCGTTTTTAGATAGTTTAAATATTAAATACGATTGCCCATTGGAAACTGGCGTCAT
TGCATACTTAGAAGGTAATGGCTCACATACGATAGCGTATAGAGCTGATATTGATGCGTTACCTATTTTAGAGGAAAATGATGTGCC
TTATCGCAGTCAATCTGATCATGTGATGCATGCTTGTGGACATGATGGTCATACAACTGCATTAATGCTTTTTGTACAACGTTGCAAA
GACATGCAAGACGCAGGTCAATTACCGCAAAATGTCGTTTTCATTTTCCAACCTGCAGAAGAAACTGGTGGCGGTGCAAATCGATT
AATAAAAGCCGGTGCCTTTGATAAGTATCCAATTGAAGCGGTATTTGGTATTCATGTTAACCCATTTGCTGATGAAGGCATTGCAGT
GATAAGAGATGAAGAAATTACGGCCAGCGCAACAGAGTATCGCTTTTTCTTAACAGGCCTGTCAAGTCATGTTGCTGATAAAGAAC
AAGGTCATTCTTGTGGTGAAGCATTACAACATGTATTAACTCAAATATCACAAATTCAACAATTTCACCTTAACGGTTTGAAACGAA
ATATTGTTCATATTGGTCATTTTAAAGCTGGTGAAGCGATTAACACTGTACCAAGTAATGGTTATTTAGAAGGTACAATTCGTACAT
ATGATATTGATGATTTAACAATCGTTAAAAATCAAATGCACAAGATAGCAGAAAGTGTCAAGCTTCTGTTTAATGTAGAATGTGAA
GTTAAATTTGCAGAAGGTTATCCCCCTACAATCAATAGTCCGAAATTACGTACTCAAATAGAGGACGCCTTAATAAAAGCTGATTTA
AATGTCTATGACAAACCAACGCCATTCTTATTTGGGGAAGATTTTAGTTTTTATGGTCAACAACTAGCTCCAGCTTACTTTGTTTTTA
TAGGAACACGAAATGAAGATAAAGGTTTTGTAACTGGTTTGCACACATCACATTTAAATTTTGATGAAAAAGTGTTAATAAACGTG
GTTAATTTTTACGAAAATTTATTAAATAATTACAAAGAGGTGTAATACATTGACAGCAACATGGTCTGTAAATAAGAAAATATTTTT
ACAAAATGCAATCACAGTCAAAAACAATCAGCCATTAATGGCAGTTGTTAAAAATAATGCATATCACTATGACCTAGAATTTGCTG
TAACTCAGTTTATCCATGCAGGTATAGATACATTTAGCACAACATCACTACGAGAAGCAATTCAAATTAGACAACTTGCTCCAGATG
CAACAATCTTTTTAATGAATGCAGTATACGAATTTGATTTAGTCCGTGAACATCAAATACACATGACTTTGCCGTCGTTGACATATTA
CTATAACCACAAAAATGATTTAGCTGGTATTCATGTTCACTTAGAATTTGAAAATTTATTACATCGGTCTGGATTTAAAGATTTAAAC
GAAATTAAAGAAGTATTGAAGGATCACCATCAAAATCAAAATGCAAAAATGATTATTAGTGGTTTATGGACCCATTTTGGATATGC
TGATGAATTCGATGTGTCAGATTATAATGTTGAACGTTCACAATGGATGGAAATTGTTGAATCACTTTTATCTGAAGGTTATCAGTTC
GACCTAATCCATGCACAAAATAGTGCGAGTTTTTATCGGGAAGGACAAATATTACTACCCCACCATACACATGCGCGTGTAGGTATT
GCGTTATACGGTTCAAGACCATATAGTTCACTGAATCAACATGATATTGTTCAGTCATTGACTGTAAAAGCACATGTTATTCAAGTG
CGCGAAGTGCAAGCTGGTGATTATTGCGGTTATAGCTTTGCCTTTGAAGTGACTAAAAACAATACAAAATTAGCTGTAGTTGATATC
GGTTATGGCGATGGAATTTTAAGAACACGTGCTAAACATGAAGCACTTATCAATGGTAAACGCTACCCGATACGTGCATTAATGAT
GAGCCATATGTTTGTTGAAGTAGATGGCAATGTACATGCACAAGATGAAGTTATTCTTTATAATAATGATATCCGCATCGATGAATA
TACCTTTAAAGGTGTTGGTGCAAATTCTGAACAATTAAGTGCTATGAATCATGATTCTTTAAAAAAAGGAGTACATTTCAAATGACTG

TTAAATATAATCAAAATGGCGAATTAACAATGGATGGTATTAGTTTAAAAACGATTGCACAAAGCTTTGGTACACCTACCATTGTTT
ATGATGAACTACAAATTAGAGAACAGATGCGCCGTTACCATCGCGCATTTAAAGATAGTGGATTAAAATACAATATTTCATACGCC
TCAAAGGCATTTACTTGCATTCAAATGGTCAAACTTGTAGCTGAGGAAGATTTACAGTTAGATGTTGTTTCTGAAGGTGAATTATAT
ACAGCTTTAGAAGCAGGTTTTGAACCGAGTCGCATCCATTTCCATGGTAACAATAAAACGAAACATGAAATTAGGTATGCTTTAGA
AAATAATATCGGTTATTTTGTTATAGATTCATTAGAAGAAATTGAATTAATAGACCGCTATGCTAATGATACGGTTCAAGTTGTATT
ACGAGTTAATCCAGGTGTTGAAGCACATACACACGAATTTATTCAAACTGGGCAAGAAGATAGTAAGTTTGGATTATCAATTCAAT
ATGGCTTAGCTAAAAAAGCAATTGACAAAGTCCAACAATCTAAACACTTAAAATTAAAAGGTGTACATTGTCATATTGGTTCACAG
ATTGAAGGTACAGAAGCATTTATTGAAACTGCTAAAATTGTTTTACGTTGGCTTAAAGAGCAAGGCATTCAAGTTGAATTATTAAAC
CTTGGTGGTGGCTTTGGTATTAAATATGTTGAAGGTGACGAAAGTTTCCCTATCGAAAGTGGTATTAAAGATATTACAGACGCAATA
AAATCCGAAATTAAAGTTCTAGGTATAGATGCACCAGAAATAGGTATTGAACCGGGACGATCAATTGTAGGTGAAGCTGGCGTTAC
TTTATATGAAGTTGGAACCATTAAAGAAATTCCAGAGATTAATAAATATGTTTCAATCGATGGCGGTATGAGTGATCATATCAGAAC
TGCACTTTATGACGCAAAGTATCAAGCATTGCTTGTTAATAGAAATGAAGAAGCAGATGACAGTGTAACTATAGCTGGAAAATTAT
GTGAGTCTGGTGATATCATTATTAAAGACGCTAAATTACCTTCATCAGTCAAACGTGGAGACTATCTTGCTATATTATCAACTGGTG
CATATCATTACTCTATGGCATCCAATTACAATCAAATGCAAAAGCCTTCTGTGTTTTTCTTAAAAGATGGCAAAGCACGTGAAGTTA
TAAAGCGACAATCGTTAAGACAACTCATTATTAATGATACAAAATAAAAATAAACAAAGTAATCCCCGAAGCACAGAAAATTATAC
TGTGACTCGGGGAATTTTTATGCATTATAGATGACTGTAGAAAACATGCTTGGACATATACTCTAAAAAAGGAAGAACAGCACATT
TTTAGTTAGAAAGTATTTATTTCTTTTTGTCATTAATCCTTTATATTTAGTTAGCCCTATTCAACTAATTTAAAGCATCATATACATAC
CACACTTTAAACAAGCATCATGAAATTAGATTTTATGCTTTACTTGCTTTTTCAAATAATATAAAACGATAAGAATTAACAACAAAT
ATAATGTATAAAACAGAATTTCATCAAATCCAAAATCTTTACTTGTAATTAATACATCAATATCTTCTAATTCCATCATCTCTGCTTT
AATATAATTATTAAACTTTGGCAAAGTCATTAAATGTACATTGTAAATATAGTAATAAAAGTCGATAAATATGATGTTAAAAAATAT
GTATGTTTTATCAATATTAGTTTTGAAAAATAGTATCCCAGAAATCATTATCGGAATCACTAATACAATCATCATTGCTGCTAAAAT
ATTAATATTTAATCCAATTGCAGATAATACACCGAAAACTAATAAATTAGCAACTAAACTAAGGATAAGATATTTCATAAAATCAC
CTCGTCGTTGTACACCATAATTATATTATATAAATATAATTTTCTATTGCAAACAATGTTGGTGATATAAAAAAAGAGTTCTCAATTT
GGCTGAGAAACTCATCGCGTTAAGTATTTGTAATAAGCATATAAAAACACCTTACTTCTTGGTAAGGTGTTAGTTAAAATCAATGAT
TCAAATCTAAGAATTATAGTTTAACAACGTTTGCAGCTTGTGGACCGCGGTCGCCTTCAACTACTTCAAACTCAACAGCTTGACCTT
CTTCTAATGATTTGTAACCATCTTGGTTAATTGCTGAAAAATGTACGAATACGTCATTTTCTCCTTCAACTTCGATAAAGCCGAATCC
TTTTTCAGCGTTAAACCATTTAACTGTACCTTGTTTCATAATCTGAAACCTCCAAGACTAAAATTCATTCAATATGCTTATTCGCATA
TTACAAAAAATACATGCAAATTTCACTCAAATATTCTTTGCAATATGGAATAATCATCTGCTTAACTTGTATTATAGTGCAGTTATTA
CCAAATTGCAATACAATAATAAAAAATATTCATTTAATCAATTATTGGTAGTTTATAATATACTTCATTATTTTGTAAAACAATGAA
ACTATAAAATATTTGTAAATCCTCTTCACAATCTTCGCAATAGTTCATGTCACAATTATTATAAAATGCATAAATATTATATTTGCCT
GTGACATAATTATCGAACTGAACTTTACATTGATTAATCAATTGTTGATAGTGCTTAAACATGTCATCAAAATCTTGATATTGATATT
TTTCAACAATGTTTTCTGGCCAGTCACTGAATAACCACCATCCCTCATAATCGGCTCTAATTTTGGTAACTGTCCACATTATTAAAGA
CCCCTTCCATACTTCAAAAATTCTATACTTATTTTTATACCTTTATCCTCTGAAAATCAAGTTTGACGACTTGAAACAAACAAAGTAG
TAACTATAGTAATTATTAGTTTTTTTAAGTAAAACTACGTATAATAACATTATGAGGTGGTAAAAATGAGACATATACATTTACAAG
TATTCGGACGCGTTCAAGGCGTCGGATTTAGATATTTTACACAACGCATTGCAATGAACTATAACATTGTCGGTACTGTTCAAAATG
TAGATGACTATGTAGAGATATATGCACAAGGGGATGACGCAGATATAGAGAGATTTATTCAAGGTGTAATTGAAGGTGCCTCACCA
GCATCAAATGTAACAAGCCATCAACTTGAAGAGTTAGAACTAAATCAAAAATTATCGGATTTTCGATCAATATAAAATGCTAAATA
AAGGAAGATGACAGTGAGATATAATATTTCTCATATATTTGGGGTGTTAGTGGGAATTCCTGTAGCGTTTTTAACAAGCATATTTGG
GATGATTGCACTTGATGTATCTTTTTTAATTGATATGTCTATTGGTATTGTTGGCTTTTTAATGACATACCTACCGATACAAAAACTC
ACTTCACGCAAATATTTAAACGAAATTGGTTTGACTAGAAAAGACTATCGCTATATTCGAAATCAGTTAAATCATACACACCAAAA
ACTTAGAGGTATTTTAAAAACGTATGTCAATATAAGATCAATTAAAGATTTTAGGCAGATTAATGATATATACCAAATTTCACGTTC
TATTTATACGACAGTTAGACAGAGACCTGCATCATTTTATAAAGTTGAAGGCTTTTTTTATTCTCATATTGATAATGCTTTAAATTTG
GTTGATGCATATACACGTCTAGCAAAAATGCCCAAAAAATCAATTAATGAACAGCAAAAGTTAGAACAAACACGAATTACTTTGGA
TGAGGTCAAACGAACATTAATCGCTGATTTAAAGCGTCTCAACGAAGATGATTATGAACGTTTAGATATTGAAATGGAATTAAATA
AGTTACATCAAAAACATCATCAAGATTGATGTAAAATAGCAACTATTGGAGAGATGATTGATGACTGAAAATAAAAGTTTCAAAGA
AAGCCATCCACTAGATGATTTTATAAGCGATAAAGAATTATCGAATACTACTATTCAAAAAGAAAAGTTAACAATTGAACAACAAA
AACAGGTAGACACAATCAGTAAACAAATTAACCCTTTAGACAATGAAGGTTTATTAGCGTTTGGTTCTGATTTACAGAAACAAATGT
CTCAATTTTCACATCAAATGTTGGATGAAGTACAAAGTAAAGATGTTGGTCCTATTGGAGATACTTTGTCAGATCTAATGTCAAAAC
TAAAGTCAGTTAATCCAAATGAGTTAAATACTGATAAACCATCTATGTTAAAAAGAATTTTTAGCAGAGCAAAGTCGTCTATCAATG
AAATCTTTTCAAGAATGCAATCAGTTAGTGCTCAAGTCGATCGCATAACGATTCAACTGCAGAAACATCAAACACATTTAACAAGA
GATATTGAATTATTAGATACGCTATATGATAAAAACAAACAATACTTTGATGACTTATCATTGCATATCATTGCTGCACAGCAAAAA
AAGTTGCAATTAGAAAATGAAAAGCTACCACAATTGCAACAGCAAGCGCAGCAATCCACTAATCAAATGGATATTCAACAAGTTGC
AGATATGCAGCAATTTATAGATAGACTAGATAAACGCATATATGACTTACAGCTTTCAAGACAAATAGCTTTGCAAACTGCGCCAC
AAATTCGTATGATTCAAAATGTTAATCAAGCACTTGCCGAGAAGATACAAAGTTCAATTTTGACAAGTATTCCACTATGGAAAAATC
AAATGGCCATTGCGCTTACATTAATGAGACAGCGTAATGCAGTTGCTGCACAACGAGCTGTCACTGATACAACTAATGATTTATTAA
CAGCAAATGCTGAAATGTTGAAACAAAATGCGATTGAAACTGCAACAGAAAATGAGCGTGGCATTGTTGATCTTGATACATTGAAA
CGTACACAGCGTAACATTATTGAGACAATTGAAGAAACATTAATTATTCAACAACACGGTCGCGAAGAACGACAATTAGCTGAAAA
AGAATTACAACAATTAGAACAAGATTTAAAGTCACATTTAGTGAACATCAAAGGACCGAATAAACAATCATAAATTACGAATTAGA
TATAACAAAAAGGAAGCATCCTACGATTAAATTGGAGATGCTTCCTTTTATATTATTCGTTATTCCTGTTGATATTTAATTGGATCTT
GTTTTACAAATATGCCGACTAGATAGCCTAATATCGTTGCAATAATTGCTACTGGGAACCACTCTAAAGAATACGCTCTTAGAGGCA
ATGATTCTATAAAGTTAATTTTCAGCCAACCTAACTTACTAATAACACTGAAAATCGACAATATAAATACGATAATAACTGGAATTT
GTTGTGAAATGCGTTTTGTCGGTATGAATTTGGCAATTAAAATTAATAATACAACAGTTATTGCTACTGGGTATACAATGCTTAATA
CCGGAATTGACATTGAGATAACAGCATTTAAACCTTGGTTAGCAATAATAAAACTCATTAAAATGAAAACTAATACAAATGCTTTGT
ATGATACTTTAGGTACGATTCTATGGAAATATTCAGAAACTGCAACAATAAGCCCGCATGCTGTAGTTAGACATGCCAGCGCCACA
ATGATGCCCAATAAATATTTTCCGAATGAACCAAATCCTGTTGAAGCCATTGTCGTTAATAAATATGTCCCAATGTTTCGATCTTTGG
ATTTCAATTGATCTAACGTCATGTCACTTACTGGCATATGATTACCAATATAACCTAATGAAATATATATGAAAATTAAAGCTACGG
CTGCAATTAAACCAGCAGTCAAAGTTTGTTTGAATATTTGATTTGTTTTAGTAATGCCTGTTAGTTTTACTGCATTAACAACAATCAT
TGAAAAAGCAATTGCTGCAATGGCATCCATTGTTAAATAGCCTTGTGTAAAGCCTTCAGCAAAACTTGAAAAATTAGAATGATATA
GTGCTTCATTGCCCTTTCCAGCACTATTACCGCTAAAGTCTAAGTATCCTTTAATAATCATCGCTAAAATAGTAATCAATAATAATGG
TGTTAATAATGAACCAATACGATCGATTAACTTAGATGGATTTAAACAAATATACAAAACGACTATGAAGTAGATAATCGTAAATA
TAAATAAAGCGATACTACTATTGCTATGTATAATTGGTGTAATTGTCATTTCAAAAGATGTAGATGCAGTTCTAGGTATTGCGAAAA

GTGGTCCTATAGTCAAATAAATGATGATTAAGAACAATATTGAAAATTTAGGTGAAATTTTATTTAATGCGCCAATATATCCTTCTTT
ATCAAGTGCACCTACAATCACACCTAATAATGGTAAACCAATCCCAGTTAGAACAAACGCTAAAATGGCGGGCCAGAAAAATTGAC
CACTATCCAATCCGAGATTAGGAGGAAAAATTAGATTGCCTGCGCCAAAAAACATAGCGAAGAGCGTAAACCCAATGACCCATGTA
TTTTTATTCATAGAGTTACTCCTTTTTTTAAAACACGAAATAATTTACAAGTAAATATTTTAGCACAAAAAATAATAAAGAGTCTATA
TGAAATATGTTGTACATAGCAGAAACAAAACACATGTGTTGTTTAAATCAAATTATGCCAGAAAATAAAATTCTTCAAATTTACTTT
TTAAATAGTAACTGATGACTTTTATTCACACTTTCATATAAACTCATATTTTAAACTTATTTATAAAGATTTAAGTAGTAATTTTTTTA
ATAATGGAGAGAGATGTCCAGGTAAATGCGCAACACCTTCAACGAAAATAGCATATTGACCATAAATATTATGAATTGTTTGTTCA
ACATCTTCAGTTATTGGATCTTGACTCAAAAACACATTAAATACTTCAATGCCAAATTTACGTGACATTTCTACAGCTTCATACGTAT
CAATAATACCATCTTGACTATAATTAAATGCAGACGGTTCGCCGTCTGAAAATACGATTAAAAATCGTTGATGTTGATTTCGACGCA
TTAATCGTTCACTTGCAACTCTAATAGCAACACCATCACGATTATCATCTTGAGGTTCAAGTGCCATAATACGTGGGCCATCTTTTTC
AAAGGTTGAGTAATCATAGTTAATAATTTCATTAATGATATTTGGTTGTGCATGATCGTCTGAATCAAAGGCATCCTCACTGAATGA
TAAAATTTCATGTTTAATGTTCAATGCTTTTAACGTCTCGTGGAATAACACTACACCTTTCTTCGTTTCAGCCATTTTATCATGCATAC
TTGCTGACGCATCAATTAACAATGTAAACGTCGCATCAAATGATTTACTTAAATCTTGTTTTTTGTAAAACAATTTATATTGATCGTC
GATAAACCAATTAATTAAATCCTTTTGTAATCGTCCTTTTGTTAAATTAAAACGTGCATCTCGTTGTTCTCGTTCAATCGTTTTCTTTA
TAATTTGAATTAAGTCTTTGATTTCATATTGTACGTCTTGTTTCGATTCTTGATATTCTAAAACATATTGTGGCTCAATTTCAGGAATT
TGCCATTTTATTTCTACATTTTTGTTCACACCATCAAGTTGAAATGCTTGGCTTTGACCAACAGCATCGCCTTCTTCACGATTTAGCGT
ATCGTTTGAACCTTTGCCCTTTTTGGTCATCATATCAGTCATATCATCTGTAGCATCGCCTTCACGTGCTTCATCATTACCCAATGTTT
CACTATTTTGTCCTTCGTGAAGTTCCATTTCTAAATAGGCGCCACCTTTTGACTCACTATCTGCAGATTTAGAGTCAGCTTTTTCACTT
CCGTTATCATCATCTTCTGAAGTATCATCTTGCCCATCGACTTGACTTGCATCAGTTCTCTTTAAATCATCAAACTCTGGACTAGCTA
ATGTATTATATAGTGTTTTAGGTAAGTAATAATATTCGTTTAGCATATCTTCTTTTAAAATATCATCTACTTGATACATAATTCTTTGA
GCCAGATACATATTATCTTCAGAATTTTGATTTTGGAAAAAATTAGGTAAATATAAGAACATATTAACTACAATATCATCTAAATCT
GAATGTATAGATGGTATATCAAAGAAATCTTGGCTTAAAAATGCATGTTCTAAATACAAAAATAATAAATCTGTATATTGTGTTTTA
GTACGATACACTTTAATTTGAGATTCCGTATATGATATACGTGTATCTAAGCGAAGATCAATTAATTTAGCAGTACTTGGGCGCTCA
ACTTTAATAGAATTTAATACGCGCATATCTTCTAATAATTTAAAAAGTTGTTGATAAAATTTAGGGTGTTTAAAAGTTTTATCTTGTA
CTACTTCATTTACAATTTGTACATCCATCATATGATAACCGTAAGCAGCTAACATAACATCTGTTTTTAAACCAGCCATTTCGATATG
GCTTTGTCGATGTGACCAAAACCAACTCGTAATCAAAACATTTTGAACAGGATTATAGTAAGGGAATTTTTGGATTTTAACTTGTGT
TTGTTCATTTTTTAATAAGAGTCGAGCTAAGTCTTGCAACATCATTACTTGCTTGGCATCTAACTGTTCGTCGTTAAATTTTATGAAA
CGATCACTCATGTTTTATCCCTCTTTAAAAGTTTAGTTCTACAGCATTATATATTGCTTGTTGTTCACGTTCATCTTCCAATTTATCAA
TAATTGTACGTTTAATTGCACGTTCAACTGGCATTACAGTGATTAAATCACACAAGTCTAATAATGCACGGATACTAGCGGCTTCTT
CAGAAATTTGTCCCTGCTTAGACATAGTACGTAAATCTTCGTTAAACTTAATAATTTGTTCGATTTGTTTATCATCTTGTAATAAACT
TTGCTCTTTAATCACATTTTTTAAAATGTCCCCATCAATATAATCAACGTGAATAACAACAAAGCGATTTTTTAGTGCTTCATTCATT
GGCAAAGTACCAACATAACCTTCATTTATCGCTGCTATAACGTTAAATCCTGGTACAGCTTTGATTACTTCACCAGTGTATGGATTCG
TAATTTGACGACGATAATCTAAGACCCCATTTAATACAGGCAATGTTTCAGGTTTAGCCATATTTATTTCATCAATATATAAAATATG
CCCCTCTTTCATAGCTTTAATAACTGGACCATCTACAAAGACAATTTCTTGTTGACCTTCCGCATTTGTTTTAATTGTTTTAAAGCCTA
ATAAGCTTTCTGTATCTAAATCAACAGAACAATTGACTTGATGCATGGGTGTATCAACAACTTCACTTAATGTTTCTGCCAACTTCGT
TTTCCCTGAACCTGTTGGACCTTTAAGTAAAATATTTTTATTTAAATCAAATAATGCCTTCGCATCATTGAAAACTGTTGAATCTGAA
TTCTTATAATGTTTTAGTGCCATTTTAAAACGTTCTCCTTTAAATAAATTAAAAAACTAAGACATGGAATAAGTTCTCCAAATGCCTT
AGTTTACTATTCTATTCTGATATAAAACGCAATGCTTAGTATGCGAATTGTTTATATTTTACCAAATTTACTAATTGAAAATCTCATA
TTAGGCTTCATTTCAATAATTATGCGTTTAAACTTCTTTGAAATATGTTTTATAGTAACCGCCAACTAATCCAGAATTATCTATTATTT
GATAATATTCTTCAGTTTCGTTGATAACATCATATTGTTTGCCAACCGTTAACATGTCAGAAACTGTAAATTTCTTTGCATCTGTATT
AATGACTTCTACTTTCTTGATTGGTGTACGTTCTTTCCAAGTTTCATGTAACATAAATTTTGTCCTCACTATCGTAAAAAAATTATTTT
TGTAAAGTGAATATAAACGATAATCCACTGTTTTCAGTAATTCTAATTGATTCAATATCATCTTGCTCAAGCGTTTGATATGGTATTT
CCACTTCATCTAAGCGCGATTTCGCTTGATTAAAGAAATCTGCATCTTTCGTTTCAAACTCAACTTGATCGACTGCGCCATATTCCGG
CATTGTCATTTCGGTTTCTACAGGCATTAAATGTATTTCGCCACCTAGACCACCCGTTCCAATTTTGAATACTTGGACATGATAGTCA
GCATTGTCGAAGGGTTGGTATTCTGCAAATACTTCAAGTCCGAATATATTTGTTAAAATTTGACCTGTAATATCTACATGATTCACTT
TAAGAATCACTGGTCCTAAACCTTGCACTTGATGTAACGGATTGACCGCACTCTCAAAAGAAGGCATACCTAAACCAACGCCATAA
TTATTCTCGTTTGAATATATTGAGAAAATATGACCATTGTTATCTTCGAGACTGAAATATTTATTGCCATTTAATTCTTTAACTGTTGT
AAATGGAATATCCTTATTCGATAATATTTCCACATACTCCTCTAAACCTGAGTCGGTTGGTGTACGTAATCCAATACCCACAAAGTG
AGATTCTTCTAATTGCTCACTTGGAACTTGTATAAATTGAAGTCTTGTTCCTGGGCTTATATCTGCATCGCCGAAACGAATTGAGTTT
TCAAGAAGTTCTTCATAATTTAATCCTAATATGTCAACCATGAAATGTTTTGTCTGTTCTATATTTGTTGTACCTAATGTTATACTTCT
AAGTCCACACATTATAGCCACCTCTATCTTTATCTTTACTATTATATTATAACTTATTTTAATCATAAATTATAGAATATCGGTCAGTT
GATTTTGATTATAGATTAATATGTGAAAAATGTATTTCTTTAAAAGAACTATTTCATTTAAATAGGTAACATAATATTGTTATGGTTG
CTATTGAGTCTGTCATTAATGAGTATCTGTATCTAGCAATTGAATATTTCGTTTTTTGTAGTACATAAAAAACATATATATGTCGTGT
TCATAAAGTGAATTTACATCTTGTTCAGATGATTTTAAATTAACTAAAGGCGCTTATAGAATTCATAAATTTCATCGCTTGTTATTAT
CTAAAATGATTTCATTAGCATATTGTAACACGCTATTCTACCTATATTTCTATTGTGAGTCTATTTCAATAGAAATGATATATTTTTTA
AGTAGTCAAATTTAAACTCACATTTTAATACGAAGCATTATAAAAATAAAGTACAGCAGTAAGATATTTTGACTAGAAAATAATCTT
ACTGCTGTTTGTTATTGATTTGATTACGTTTTTAGGAATAGTAAACTATATTGTGTTGTGGATTAAGATTCTAATAATAAGTCTTCTG
GGTTTTCAATTAATTCTTTAATTGTTTTTAAGAATCCAACTGCTTCTTTACCGTCAATAATTCTATGATCATAGCTTAATGCAATATAC
ATCATTGGACGATTTTCGATTGTATCTTGATCAATCGCAATTGGTCTTGTAATAATTGAATGCATGCCTAAGATTGCAGCTTGATTAC
CATTGATAATTGGCGTACTCATCATTGATCCAAAAATACCGCCATTTGTAATCGTAAATGAACCATTAACCATATCATCTAAGCCAA
GTTTTTTCTCTCGTGCTTTAACTGCTAAATTAGCAATTTCTGCTTCGATTTCTGCAAAATTCTTTTTATCACAATCTCTTACAAATGGT
ACTAATAATCCATCATCTGTAGAAACAGCTACACCAATATCATAATATTGTTTCGTAATCATGTCGTCGCCGTCGATTTCTGCATTAA
CTTCTGGATACTTTTTCAAAGCTGCTACAGAAGCTTTAGTAAAGAATGACATAAATCCTAATTTAGTACCATCATGATCTTTCATAAA
TTGTTCTTTCTTACGTTTACGCAATTCCATAACATTTGTCATGTCAACTTCGTTAAATGTTGTTAACATAGCTGTATTATTAGATACCT
CTAATAATTTTTTGGCAGCTGTTTTCTTTCTGCGTGACATTTTTTCACGAATCACTGGTTTTGTAGGATATTGATTGTATTTTTTCTCTT
CTTTTGCAGGTGCTTGTTGTGTTGTTTGTGTTGATGCCGGTGCCTGTTGTTTCTTATCAATATCTTCTTTACGAACCACATCATTTGTTT
TCGGACTTACTTCAGCAAGATTCACACCATTTTCACGAGCATATCGACGCGCAGAAGGCGTAGCATTAATACGTTGCTGATTGTCAT
CATTTGCTTGATTGACTTCAGCTTTATCTACCGAATTATTTGTTGTTTCTTCTTTTTTATTATTTGTTTCTTCATTTTGTTGTGGAGTATT
GTCGTTACTATTTTCTTTAGAAGCATTGCCACTACCTTCGCCGATGACAGCAATTGCTTGTCCAACTTCTACAGTGTCGCCTTCACTT
GCAAGTTGTTCAGATAATACACCTGCTTCTTCAGATACAACTTCGACATTAACTTTATCAGTTTCTAATTCAAGAATAGCTTCACCTT
TTTCTACGCTATCCCCTACGTTTTTCAACCATTCTGCAATGGTACCTTCTGTAATAGATTCTGCTAATTCTGGAACTTTAACCTCTGGC

ATGACTTATTTCCCCCTAGTTATTTTTTAATGCATTTTCTATAATTTTATTTTGAACAAGTTTATGAATTTCTCCATCGCCTTCAGCTG
GAGCAGCCCTTTGAATTCTGCCATGATAACTTAAATCATATTTATCTGCAACTAGCACTTTAACATATGGATAGACATATAACCATG
CACCTTGATTTTTAGGTTCTTCTTGTACCCATGACACTTCTTCAAGGTTTGGCAATTGTGCTAGTAATGCTTCAATCTCTTCCTCTGGG
AATGGATACAATCTTTCAATCGCAACGAGTAATACTGATTCGTCTGGATTTTTAGCTAATGCTTCTTTTAAATCAATGAACATTTTAC
CAGTCGCTAAAATAACTTTTGTAACCTTATCCGATTGATATGATTCTGTCAAAATTGGCTCAAATCCACCAGAAGTAAATTCATCAA
TTGGTTTTGCAACTGTTTTATTTCTCAATAAGCTTTTTGGTGACATAACAACCAATGGTCGCATTTGTTCAGAATCTAAACTAGCCGC
TTGTGCACGCAATAAGTGGAAATAATTACTTGAACTAGATAAGTTGACAACTGTGCAATTATTTTCAGCAGCTAATTGTAAAAACCT
CTCTAATCTTGCTGATGAATGTTCAGGACCTTGACCCTCATATGCATGAGGTAAGAATAATGTTAATCCTGAACGTTCTCCCCATTTT
GAGCGAGAACTGAATAAGAAGTTGTCAAAAATCATTTGTGACATATTTGCAAAATCACCATATTGCGCTTCCCAAATATTGAAGCTT
TTTTTGTTTTCCACATTATAGCCGTATTCAAAACCAACTACTGCTGCTTCTGAAAGCGGAGAATTGTGTATATCAAATGTCGCTTTTT
GATCAGGAACATGATGTAAAGGTGTATATGTTTCACCTGTTTGCTCATCATGTAACACGGCATGCCTATGACTGAATGTACCACGTT
CACTATCTTGACCAGTTAAGCGAATCGGTGTACCATCTTGTAAAATTGTCGCAAATGCAAGTTGTTCTGCTTGTGCCCAATCAACTA
AGCCATCTTCTTTATTAAACGGCTCATGACGCTTCTCAAGAACTTTGTTTAACTTTTTCAAAATGTTAAAGCCATCCGGATATGTTAA
CAATGCATCATTTATTTCTTTCAAATGATCAAACGTAAATGATTGTTCGTCTGCTTGTAACGGTAATGCAAGTTCTGCAGGTTTTTCC
ATATCTGGATTATCCATTTTATCAGCTTTATTAATTTTATCATGAGCTTGTCTTAGTTCCTTTTGGACTTGTTCTATAAATGAATGCAT
TTCATCTTCTGAAATGACACCTTCATTAACAAGCTTTTTACCAAACACATATTCAACAGAGTCATGTTTGCGAATATTCTGATAAGGA
ACTGGATTAGTAATTGATGGTTCATCCATTTCGTTATGTCCGAAACGACGATAACCTACTAAATCAATAACGACGTCTTTATGAAAC
TCTTTTCTAAATTCCATTGCAATATCAATTGCTTCAATAGTAGCTTCAACGTCATCAGCATTGACATGGAATATTGGCACATCATAAC
CTTTGGCCACATCTGTAGAATAAGTTGTTGAACGTGCATCAATTGGTTCTGTAGTAAATCCAATTCTATTGTTAGTAATAATATGCAA
TGAACCACCCGTAGAATAGCCTTTCAAGTTTCCTAAGTTCATTGTTTCGAAGTTAATTCCTTGACCAGGATAAGCAGCATCGCCATG
TATAATAATTGGCATTGCTTTATGATGATCAGTCGTCGGAGCCCCAGCACGTTGTGTATCATCTTGTGCTGCTCTCGTACGCCCCTCA
ACAACAGGTGCAACAATTTCCAAGTGACTTGGATTGTTAGCCAGTGCAATACGCTGCATTGTACCGTATGAATCAGTAGTTTTAATG
CCACCAAGGTGATATTTCACATCACCAGTCCATCCAGCAGTTAACTGCAAGCTACCATCTTCAGGTAAGAATTTCATTGGATCTGTA
TGCATAAATTCTGAAATCATCATTTCGTACGGTTTTTCTAAGACATGCGTTAAAACGTTTAAACGTCCACGGTGAGCCATGCCTATTT
GTATATTTTTAATACCTTCTTTCGCAGCAATCGTAATAGTACGTTGTAACATCGGTACAAGTGCGTCTACCCCTTCAATTGAAAAACG
CTTTGCACCAACGAAGTTTTTATGAAGATATTTTTCAAACCCTTCAACATACGCTAATTGTTTGAATAGTGCCCTTTTTTCGTTATTAT
TTAACGTTACTTTATATGGCGTTTCAATTCTTCTTTTTAACCAACCACGTTCGGTATTGTTATTAATATGTGTATACTCAAATGCAATT
GGTCCTTTGTAACGTTTTTCCATTCTTAAAATTGCTTCATAAGCATTATCATAAATGTCGGCAAAGTGATCTGAAACAATTCCTGCTG
ATATACCTTCCAAAGTCTGTTGATCTAAATCAAAGTCTTCAATCTCTAATTTAGGTACATGTTTCCTTTTTGGAGGATTTACAGGATA
AATATCGGCTTTAAGATGCCCGTATTGGCGAATATTATCAATTAAACGCATGACACGCTTAATTGTGCCGTCGCTATTTTGGCTACTT
GTACTTTTTAAAGCTGGTACAATTGAGTCATCATTCTTAATTGTGCTGAATAAGACTTGTAAATCTTCTGGTACAGATGATGGATCTT
GTAAAAAGTCATCATATAGATCTAACATTAGACCTAAATTCGCACCGAAGTTTACAGGAGCCTCTGAAACTTCTTTTCTTTCGTTAGT
CATTTTACACCCTCCACAAAAATGTTGAAACGCTTACAATGTAATAATATCACTATTTAGTTATTTGTTAAAGTAGATTTACCTATTT
ATCTGCTAAAAAGAAGAATCAACAAAAGCGTTGATTCTTCTAGGTTTATAGATGTATTTTATTGTAAATTTTGGGAGTACAACAGAA
ATGATAAAGAACCACTGTTTATTTGTTTTATAGTTGTTCTTATACATTAGTCACAGCTAATGTGTACTTAAAAAATTGATCCTATGAC
TTTGATTGACGTCTCAGTCATGATTAAAATATGATTTTAAACGTTGTTCCTTTGTTAATTTCACTTTTAATTTTAATCGATCCTCCGTT
TAATTGAATGATTTTTTGAGCAATAGATAATCCAAGTCCATTACCGCCTTGACTTCTTGAACGAGATTTATCCACTCGATAAAAGCG
ATCAAAAATGAAATCTTGATCTTCCTCTGGAATACCAATTCCATGATCTGTAATTTCAATTATTTTTTGCTTATTTTTTAACCTTGTCT
TAACTTTAATTTTCTTATTCTTCACATCATATTTGATTGCATTATCAATAAAGATTAAAAATAATTGTTCGAATTGATGAGGTTTCATT
TTAATTTCTAGATTTTTAGATGTCAGATCCGTATCAAATTGATAATCAGGATGCAATTGTTTTAATGAGTGTATTCGCGAACGAATTT
CATCATTAATATGCACGGTCTGTGCTTCAGAAGAAATGTCATTTACATCTCCTTTAGTCAATTCAAGTAATTCTTCGACTAATTTTAT
GATACGATTCATTTCTTCAATAGAAATATTTAACGATTCTTCTAATACTGCTGGGTCTTTTTTTCCCCATCGCTGAATCAAATTTAAAT
GACCTTGAATAATTTGTAATGGTGTTCGTAATTCATGTGACGCATCTTCAACAAATTGTCTTTGTTGATTAAATGATTCTTCAATTTG
GCTCATCATCTCATTAAACGTATTTGCTAAATTATCTATTTCTTCATAATTTGTATTTAATTGCAATTTATTTTGAAAACCATCTCGTC
GAATCTCAATCATTTTATTTGATAAACTGACAAGCGGTTTAGTAATTTGTGTTGAAAATACATAACTGATTGTGGCAGTTATAATTGT
TGCAATCACTCCAAATGCCAGCGCAATGATATACAATGATTTTACGATGTTATCATAATTTTCTAGTGAATGAATTAACAAGCTATA
CCCTTTGAAATCTTGCGTTGTAATTGGTTCTTTAATAATTAAATATTCAATGCCTTTATAGCGTTTTTTTATTACGCGGTCAAAATAAC
GGTGTTCATAACCTGGTTCAACTCTCACTGTGTTATCATTCGATGTCTCAAATAATTTATTATTATGCTCATCATAAATAATTATCTCT
TGAAAATTACCTAAAGATGCATTCAAGTCTAATGCAGATATATCTTTAACAGGCTTAGAATGAAATAAATTATTAATATCGCTTGAG
CTTCGTTCTGCATCATCAAGCTCACTATTATGCAGTGTATCTTTCAAGAAAAAAATAATAATTAAACAAAACAAAAATATCGTGACA
AACGTAATCATTGTGGTAACAATAATCCAGTTATTGCGCAATTTACGTTTTGTCATCGTATCACATACCCAACGCCACGAACTGTTTC
AATCATTTTGTCACGATCGTATGGTTTTAACTTGTTTCGTAAATATCTTATATAAACATCTACGACATTTGTTTCTACTTCACTATTAT
AACCCCATACATGATTTAAAATTTGTTCCCGTTGCATAACATGGTTTTTATTTTCAGCTAGAAGATATAGTAAATCATACTCTGTTTT
TGTTAATTCAATTTCTGCGCCATTTACCGTCACTTTAAAAGCGTTCTTATCAATTGTAATACCGTTGACATCGATAATATCCTTTTGTG
GCTGACGACGTAAAATTGCACGAATTCTTGCTAAAAGTTCTTCAATATCAAACGGCTTAACTATATAATCGTCTGCACCGTAATCAA
GCCCAGCAACTTTGTCATACGTATCACTTTTCGCTGTAATTATAATGATAGGTGTAGATTGTTGTTGTCTAATTTTGCGACAAATTTC
TAAGCCATTAATTGACGGCAACATTAAATCTAATATGATTAAATCATAGTAATGGCTAAGCGCTTTATCTAAACCGTCTTGTCCATC
ATACTCTGTGTCCACATTGTAATTTTCATGTGTGAGTTCCAATTCAAGAAATCTTGCTAAGTTTTGTTCATCTTCTACTATTAAAATTT
GCGTCATTTGTACACCTCATATTACGACTTTTTCTAATAAGGTAATATATTTTAAATTTTGTATTTTTCTATACAATAGTTGCTTTTTA
AATTAGTTTGTTTACGTTTAAATTTTATCACAAATCAATAACCATGTTTTAAATTTTTCAAGCTACAATCATTAATTATTTATTAACTC
AAATATTTAATAATCAGTTAATGGCATTCTGTTAATTCTGATTGCAGTACTTAAATATAGAATACTGTAACACAAGGCCTTACCGCA
ATATTAATTGTTGTCATATACTGACTTTTCACTATTGTAAAATGAATAGTTATTATCTTTATTTTCACAAAATGACCGTAGTTTAAAC
ATTTCACACATCATTTCAATGTGCGATTTTGAACTTGGAAGTAACATTTCATAATGTATGAATTCAAAAGGAGTATGTAAACAATAT
AATGTTTAAACCTCATCACTTTTAAATATTCTAATTCTAATGAGAAATACAAATTTTTTTATTTTCTAAAATAGCGTAATCATGCGTT
TTATTTACTATTCTTAAAAAATATTCAAAAAAAGTTTTAGTTTCTAATTGACAATGATTCTCACTAATGTATAATAGTAGTTGAAAAT
GATTATCAATACCACATAGAACATCCCCCCCCACAACGTTTCGTTCTTGTTGGATTGGTCATTTTCAAATATTCCCCTTTTATATGCC
CGTAAAAGACAATATACGTTATAACAACGTTTTATAAAAGCAGTAAACCCTTACGACACTTTAGGTTTACTGCTTTTGTTTTTTTTCT
ATTTAATTTATTAATTTATTTCTAAGTAATAACGTTGATAAAATAATGCAAAATACGCCACCAATAATGCCGGCAATAATATCTGTT
GGATAATGTACACCTAGATATACACGTGATATGGAAATCAATAAAATCATAGCTGCACATAACCCTATAAGAATACCTTTTGAATTA
CCTTGATTTAATCGATTTAATAGATAGATACCACTTCCAAAATATGCAGTTGATCCCATAGCATGACCGCTAGGAAAACTAAATCCT
GTTATATCAATTAAACGCAGCAATGTAGGTCTTTCTCTATCGAATATATTTTTTAATGCTGGATTCAAAATTCCAGATAGTGCCATTG

TTAATGCAAAAAATAATGCTTCAATTTTGTGGCGCTTTAACATGAGATATGCCACAAGAAGTAATGAAATACATAACATTGCCCAG
ACTTCACCTACTTTAGTAGCCCCAAGCATGATAGATGTCGTAATAAAGCTCTCTGATGAATATATAAATTCATAAACTTCATTATCA
ATCCATTTCCCTAGTCTTGATTCGTGAAAAAACGCGATAATTCCAAAAACCAATGTAAAAACGATGAGCAAAGAGATACGTTTCCA
TTGACTCATAAAGCATTACCCCCCATTAATTCAATGCGTCTTTAATCATCTTATCAAATAAAGCTTCTTTCGTATAACTTTGTTCATAC
GATTTCATATTATTGATAATTCGAGTTCTTTCCTGTTCCATTTCATTTAGTTCTTGTAATAAAATTTGTGCTGTTAATTGTTCTTCATCA
ATCGTTTTAGCATAACCTTTATCAGCAAAATGATTTGCATTGTCAATTTGGTCGCCTCGGGATTGATCTAAACCTAATGGTACTAATA
ACATTGGTATACGTAATGTTAAGAACTCATAAATCGCATTTGATCCAGCTCTACTTATTACTGTGTCCGTAATTGCTAATAAATCTGT
TAAATCCTCTTTAACAAATTCATATTGTATATATCCTGATTTTTTAACTTGAGCATCTTTTAATCCTTTACCAGTTAAATGTATCACTT
GATATTGTTGTAATAATGCATCTAAGTTTTCGCGAATAATGCTATTTAATTTTTTACTTCCTAAGCTTCCACCCATAACGAGTAAAAC
TTTTTTATTTTCATTAAAGCCTGTTAATTGATAACCATTATGTGCATTACCATTTTTTAAATCTTCTCGAATTGTTGCTCCAATAAAAT
CAGCTTTCTCTTTAGGTAGGTAGTTTAGCGTTTCTTCAAATGTTGTATATATTTTCTTGGCAAATTTAAGTGCTATCTTATTCGCTAAT
CCTGGTGTTAAGTCAGATTCATGAATAATAGTTGGTATATTTAATGATTTGGCTGCAATAACAACAGGCACAGATACAAATCCACCT
TTTGAAAATAATAGATCAGGTTTTTCTTTTTTCAAAACTTTACGAGCATCAAGAATACCTTTCAATACTTTAAATACGTCTTTGGCAT
TTTCTAAAGAAATATATCTTCTTAATTTACCACTCGAAATAGGATAATACTTAATTTCTGGTAGTTGTGATTCAATCATTTCTCTTTCA
ATACCATTTTTAGAACCAATATAAAGCGCTTCATAACCTTGTGATAATGCAGTTGGAATTAAACTTAAATTTACTGATACGTGTCCA
ACTGTTCCCCCTCCGGTAAATGCGATTTTCGTCATTAATATTACCTCTATTCTTTTAAATTTTTATAATATGCATAAAATGGTTCACCT
TTATCATATGGCGGATATTCCATTAATTGTTCACCGACCTTATGAAATCCAAATTTCGCAAATAAACCTTGTGCAGGTTTGTTTAACG
CAAAGGTGTCCGTTAAAATAACTTCTGCACCACGTGCTTTAACTACATCAATAACATAATTGAATAATTCTGTAGCAGCTCCTTTAT
ATTCTTTCGAACCAGTTAATCGATGAATAACAAAGGCGCCTTCTCTATTTACTGGCCAGTCAATGTCATCATACCATTCTGCTTGGTC
TTGGTCGACAACAATAAAGCCATAAATTTTGTCATTTTCCTCTAATACATACAAATAATCTTTAGCAATATCTTCTTCAAAATGTTCT
AAAAGAGGGTACTGATCGTCCCATTGCTCGTTGTCGTGTTCTTTCATTAATTCTTTTGCTTCTTCTACTAGATTTAAGATTTGATCTAA
ATCTGACATTTTACCTAGACGGATCATGTACGTCACTCCTTATATCATTATGTTAAACATGTTTTTCAGTACATGTTAATCTTACACA
CTATTAACATGCTTTAAACCTTTATTATGAATGAAAAATAGTAATTATCACACTATGGGATAAAAATAACCAATAAAAGTATCATAT
ACCAATTAATTGATTGTTAAAACTAGTGAATAATACTATTTAAAAAGTTTATGCTCATAGTATATTAAACCATGAATTGTTAACAGC
GATGATAGAAATTGCGTTATTTACATCATATGTAAATGTCTAATTTATATCTTTTAGAGTTAGACGTCAATAAAAATAATCACGCCG
AGGCTGGGACATACATCCCTAAAAAAAGCAGTAAGATAATTTTCAATTAGAAAATATCTTACTGCTGTTCTCTATTTATACAATACT
TCGTATTGAATGGCT

>035-contig_268_RC

AATTCAAAAACACTATATCGTGACTTAGTTGAAGAAAAGCTGATTTCTATCAAATAATTAATAGAAATCAGCTTTTTTACATTGCCT
AAGAACTTAATGTTCCAGCCTGAGCGTGTCATTAACATAATAATAATACAATTTTAGCAGTGTGTATCTCTTATTTTAAAATATTAAT
CAATTTATCGAGAACATCATCATGTTTATTAGCTTTTTCAACTAATAACTCAGTAAATTTATTATTCGTTTCTTTATTAAATTCACCAG
TTACCTCGAGTTTATTCGCTTGTTGGAAAGCTTTAACTTGATTTTCTAAAGCTTGATCAAATTGCGTTGTTTCATTATCAACTTTATAA
CCTAAAGCTGACAAACCAATTTTAATAGTTTTAATATTTTTATCATCGTCTCCAACTTTAAATGTTTTCGTATTAGGAATGACATTTA
AAGATTGATATTTAGGTGTGTCAATAGTAACGTCTGGTTTAATGCCTTTACCGTGAATATAATGACCATCTGGCGTTAACCATTTCAT
TTCAGTATATTTTAACAATGAACCATCCTTAAACTCTCTTGTAGTTTGTACGACACCTTTGCCGAATGTTTTTGACCCATAAACTTTA
GCTTTATTATAGTCTTTTAGCGCACCAGTAAACACTTCAGAAGCGCTAGCTGAACCTTCATTCACTAAGATGGATATATCCATGTCTT
TCGCTTCTTTTAACGCATCATTAGAAGTTTGAATTGCTTCAGTATCTTTACCTTTTTCTAGTTTAACAACAGTTTTTCCTTTATTGATA
AAAATATTTGCCATTTTAACAGCTTCATCTAGTAGTCCACCTGGATTATTTCTTAAATCTAAAACAATCTTTTTCAAACCATCTTTGT
GAGCTTTTAGAACTGCATCTTTCAATTCACCTGATGTATCATTCTGGAATTTATTAATAGTAATAACTCCAACTTTACCTTTTTTCTTA
TACTCAACACTTTTAACATGAATTTTTTCACGTTTAATCTTAACGTCTTTTTCTTCACTACCTCGTTGAACAGTTAAAGTGACTTCAGT
GTTTTCTTTACCACGAACATCTTTGACAACTTCATCTAATGCTTTTCCTTTAATTGATTTTCCATTTACTTTAGTAATGACATCTTTAG
GACGAATGCCAGCACGTTCTGCTGGAGATCCCTTCATAGGACTAGTAACCATAATTTGATCATTTTTCTTTTGCATTTCTGCACCAAT
ACCTACAAAATCACCTGAAACACCTTCATTAAAGGATTTCGTTTGTTCTTTTGTTAAATATTCAGAATAAGGATCTTTTAATTCTTTG
ACCATGCCATCAATTGCAGCTTTACTTAACTTGTCAGAGTCCTGTTTTTTGTAATAATCACTATTTAAGATTTTATACACATTTTCAAT
TTTATTTAAGTTTGCTTGATCAGTTTTGTTTAAACCACTTATTTTTTGATTTATAAAAATATATGCAACAACTGTGATGACAGCTGTTA
TTAGGATTGTACCAATTAATATTGATATGAATTGCCAACGTTTTAAGTGAACGCGTTTCGATGAATTAGTTTCTTGGTCTTGATTGCT
TGTTGCAATTTCAGCGCGTTCATCATCGGATGAAGATGTGTGTTGCTTATCATCCATTGTATCACTTCCTTATTATGTATTATGTACTT
TTAAATTCTGTGTAGTTAATCATATCAATTTATGCAAGATAGCGCACCATATTTCACTATAATTGATTAATTTTTAGTCACAACTTAG
TTTATTTTAAATTAAAACCAGGATAACGAATTGCATCACCCTGGTTCTAACTATTATTATTAAAAATTATATTAAAATTTTAGCCATT
CCGTATATTCTTCATATAAATCATCAAATACAGACATTGGCAATGTGAAATCACCATGTGTCTCAATATAATCAGACAGTATGTTAA
AATCATCATCGTGTTTTGGGAAAGCAAGATCGTCAAATATCTCTTCTGCTAGACGACCTTTATCGTCGTGTCGACCACGAACTGTCA
TGACAAATTGATAAAACGAATAGTTTTTCATTAGTTCATTGTCACATCAATCACTTTTGTTTCACCTTTAATCACAGCATTTTCATCAT
AAATATTAATTGAAGCTGCTTGATCAGTGTTAGTAATTATAATTGGTGAAATTACAGATTTAGCGTTATTATTAATATATTCAAGGTT
GAATCTTACTAATGGATCTCCGACGTTAACTTCGTCACCACTAGACACTAACACTTCAAATCCTTCACCGTCTAATTGAACTGTGTCT
AAACCGATATGAACTAATAATTCTAATCCGTTATCTGCTTTTAACCCAATCGCATGCTTAGTTGGAAAGACATTGTCAACACGTCCT
GCAATTGGAGACACAACTTCTCCTTCAGTTGGATTAATACCAAAACCTTCGCCCATCATTTTTTGTGCGAATACAGGATCTGGAATA
TCTTCAATTTTCACGAATTCTCCAGTTAATGGTGCATAAATTGCGATATCTTTCTGAACTTCTTTGCCTTTTCCGAATAATTTTTTAAA
CATACTTTCCACTCCTACTTATCAAAATGTGATATTAAATCGCCATAACCCAATTCTTCTAACTTTTCATATGGAATAAATTGAATTG
CAGCGGAATTGATACAGTATCTTAAGCCGCCACTTTCTTTAGGTCCATCATTAAAGACATGTCCTAAATGACTATTTGATTCTTCTGA
ACGCACTTCAGTTCTCAACATACCAAATGATTTGTCGACTAATTCTATAATTTCATCGTCATCAAGCGCTTTGGAAAAGCTAGGCCA
TCCACATTCAGAATGAAACTTTTCTTCAGATGTAAATAAAGGTTTACCAGAAATTTTATCTACATAAATTCCTTTAGCAAAATGATTC
CAATATTCATTCATAAATGGTGGTTCAGTGCCGTTTTCTTGTGTAACAATATATTCTATATCTGTTAGTTCACTTTTATCTTTTTTAAG
CATTTTGATTCCCCCAATGTGATTCTATAAACGCTTTTCTACCTGAACCACGTTGATATTGGTAATAATGTACCGGGTTCTTTTTATA
ATAATCTTGATGGTAGTCTTCAGCTGGATAGAAATTTTTATATGGTTTAATAGGTGTAATCACTGGTTTCTTGAAAATACCTTGTTCA
TTTAATTGTTGCTTTTTAAACTCAGCAGCCTTTTTCTGATGTTCATCATGATAGAAAATGACTGGTTGATAGCTTTCGCCTCTATCGA
AAAATTGCCCTTGATCATCAGTTGGGTCAAATGTTTTGAAATATATGTCTAATATATTTTCAAAGGAAGTAACCTCTGGATCAAACG
TAATTTGTACTGCTTCGACATGGCCGGTTTGATTCGTACATACCTGTTCATAAGTTGGGTTGTCAACATGACCGCCACTATAACCAGA
TACGACTGACTTGATGCCTGGATATGATGTAAATGGTTTAACCATGCACCAGAAACATCCTCCTGCTAATGTTGCATATTCTTTTGTC
ATATGAATTACCTCCTCTATCTATCTAATTATAAATTTAGTCTATAAAGGATTGAAATGGAAGTAATTAACACTGAAAAGATTAAAT

TTACTTCTTAAGGACTACTAGGCCAATCGCACCTTGACCAGTATGTGCAGAAATAACTGGTGTAGTTACATTTATATCGTAATTATTC
ACATGAAAAGCTTCATTAAAAACTTTCTTCAATTTATCAACATATTCAATGACGTTAGCATGTGCGACACCAACGGATTTGATTTCA
TGATCTCCTATAAATTCAGCAATTTCCTTTTTCAAGTATTGGATACTAGAATTTTGAGTTCTCGCATTGTGCACAAGCTCTAAGCGAC
CATCATCTAGTGTACCAATTGGTTTAATTTTCATAAGATTACCAATCAAACCTTTTGTTTTACTAATTCTGCCACCTTTAATTAATTGA
TTCAATTGCCCTATAACTACAAATAATTTAATGTTTTCTCTTAAATGATTTAACTTTTTAACTATTTCAGAAGTTGAGACACCTTCTTT
TACAAGCTCTACTAGGTGTTGTATTTGATACCCTAAACCAAAAGAAATAGATTTTGAATCAATAACAGTTACATTAGCATCTACCAT
TTGACTTGCTTGGTAAGCAGTGTTATATGTACCACTTAATCCTGAAGAAAGATGAATACTTATGATTTCAGAGCCATCTTTTCCTAGT
TCTTCATAAGCAGATATAAATTCACCTATGGCTGGCTGACTTGTCTTTACATCTTCATCATTTTCAATATGATTAATAAATTCTTCTGA
TGTAATATCTACTTGGTCAACGTATGAAGCTCCTTCAATAGTTAAACTTAAAGGAATTACATGAATGTTGTTTGCTTCTAAGTATTCT
TTAGATAAATCGGATGTTGAGTCTGTTACTATAATCTGTTTTGTCATGGTCGTTTTCCCCCTTATTTTTTACGAATTAAATGTAGAAA
GGTATGTGGAATTGTATTTTTCTCATCTAGTTTACCTTCAACTGAAGAGGCAATTTCCCAGTCTTCAAATGTATAAGGTGGAAAGAA
CGTATCACCACGGAATTTACCTTCAATAACAGTAATATACATGTCGTCCACTTTATCAATCATTTCTTCAAATAATGTTTGCCCTCCA
AATATGAAAACATGGCCCGGTAGTTGGTAAATATCTTCAATAGAGTGAATTACATCAACGCCCTCTACGTTGAAACTTGTATCTGAA
GTAAGTACAACATTTCGACGATTCGGTAGTGGTTTACCAATCGATTCAAATGTCTTACGACCCATTACTAAAGTATGACCTGTTGAT
AATTTTTTAACATGCTTCAAATCATTTGGTAGGTGCCAAGGTAATTGATTTTCAAAACCAATTACTCGTTGCAAGTCATGTGCAACTA
GAATGGATAAAGTCATAATTATCCTCCTTCTTCTATCATTTCATTTTTTATTACTAAGTTATCTTTAATTTAACACAATTTTTATCATA
AAGTGTGATAGAAATAATGATTTTGCATAATTTATGAAAACGTTTAACACAAAAAAGTACTTTTTTGCACTTGAAAATACTATGATG
TCATTTTGTTGTCTATATGGTTAGCTAACTATGCAATGACTACACTGCTATTGGAGCTTTTATTGCTGGATGTGATTCATAGCCAACA
ATTTCCAAATCTTCATAATTTATGTCGAAAATAGACTTGTCACTGTTAATTTTTAATGTTGGAGGATTGAAACTTTCACGTGCTAATT
GTGTTTGAATCGCATCAATATGATTTGAATAAATATGTGCATCTCCAAATGTATGCACAAATTCTCCTACTTCAAGTCCACATTCTTT
GGCAATAAGGTGTGTCAATAAAGCGTAGCTTGCGATATTAAATGGCACACCTAAAAAGATATCTGCGCTACGTTGGTATAACTGGC
AACTTAACTTACCATCTTGGACATAAAACTGGAACATGGTATGACAAGGCGGAAGTGCCATTGTATCAATTTCTGTTGGATTCCATG
CAGATACGATGTGTCGTCTTGAATCTGGATTATGCTTAATTTGTTCAATTACTGTTTTAAGTTGATCAAAATGATTACCATCTTTATC
AACCCAATCTCGCCATTGTTTACCATAAACATTTCCTAAATCACCGAATTGCTTCGCAAAAGTATCATCTTCAAGAATACGTTGCTTA
AATTGTTTCATTTGTTCTTTATATTGTTCGTTAAATTCAGGATCACTCAATGCACGATGCCCGAAATCTGTCATATCTGGACCTTTATA
CTCGTCTGATTTGATATAATTTTCAAAAGCCCATTCGTTCCATATATTATTATTATATTTTAATAAGTATTGGATGTTTGTATCTCCTT
TAATGAACCATAATAATTCGGTTGCTACTAATTTAAAAGAAACTTTCTTTGTCGTTAATAGTGGAAATCCTTTAGATAAGTCAAAGC
GAAGTTGATGACCAAATTTTGAAATCGTACCTGTATTTGTGCGATCATTTCGTGTATTTCCTATTTCTAAAACTTCTTCACAAAGACT
GTGATATGCTGCATCAAATGAATTCAACATATGCGATAACACCTCATTTTCATTATTTATAGTATGTATATTTAGTTTGATATAACTT
AACTTTATGTAGCATTTTGTTATCACTCATTTTAGGAATATGATATTAATATCATGAATTCCGTTACTTTATTTATAAAATGCTGATTA
AGTACCTACCCCATCGTAACGTGATATATGTTTCCAATTGGTAATTGTTTACCCAAATCTATAACTTTAATGCTAAAAAATTTTAAAA
AAGAGGTTAACACATGATTTGAATATTATGTTTGATGTCCTATTAAAACAGTTAAATTTCTAGAAAATATAGTTGGTAAAAACGGAC
TTTATTTAACAAATAGAATACAACTATATTCTCTATTTTCAATGACAGACACCATTTTTAATATTATAAAATGTGTTAACCTTTATAT
TTATTTATGTGTACTATTTACAATTTTCGTCAAAGGCATCCTTTAAGTCCATTGCAATGTCATTAATATCTCTACCTTCGATAAATTCT
CTAGGCATAAAATAAACTAAATCTTGACCTTTGAATAAAGCATACGAAGGACTAGATGGTGCTTGCTGAATGAATTCTCGCATTGTA
GCAGTTGCTTCTTTATCTTGCCCAGCAAAAACTGTAACTGTATTTGTAGGTCTATGTTCATTTTGTGTTGCAACTGCTACTGCAGCTG
GTCTTGCTAATCCAGCTGCACAGCCGCATGTAGAGTTAATAACTACAAAAGTAGTGTCATCAGCATTTACTTGGTTCATATACTCCG
ATACTGCTTCGCTCGTTTCTAAACTTGTAAAACCATTTTGAGTTAATTCGCCACGCATTTGTTGCGCAATTTCTTTCATATAAGCATC
ATATGCATTCATATTTAATTCCTCCAATTAAATTGTTCTGTTTGCCATTTGTTTCCATACTGAACCAAGTGCCTCATCTCCGTTTTCAA
TACGAGATATGGCCATTTCAATTTGTAATTTAACTTCAAACGCATTGTCATTAATATGGGCTTTTAGTGCGGGAAGCTGCTCTGCATT
ACCTTCATCAAAGATAAACATAGCAGCACGCCACCTAACGATTTTCTGTGGATCATCTAATAGTAGCACCATTTCTGGTAGTGCCTC
TGGATACCCTAAATCGCTTATGCAATCCCCTGCTGTTCTTCTTACAGCAGGACTTTTATCACGAAGCCCCTTATATAAATACGGTAAA
ATTTCTTTACTTTCAATCATACCTAATAATACAATCGCTTGACGTCTAACCGGTACTTTTTCATCAGATAAAGCTAAATCAAGCAGCG
GTATATCTTCAAAAGTCGGCTTTGGAAAATGGTTTAACATTCGTAATCGAGTCTTCCAATTATCAGTCGCATGATATTCATCCAAAG
AGACATGTCGATAGAAATGATAATTATTAGTTACGTGATTTTCTTCTAAAGCATGCTTTACCAATACAGGTAACTGTGATTCTGGAT
AGGTAGCTAGCACTTCTTCTAGGACACCATCCATTACTTCTTCAATATTTCCATAGCGATTTCCTAAATCTAGCCATTTACGCATAAA
AACAATATTGTCATGTTCTGTTTGCGCTTGAGTCATATAATCAACATATGTTTGTGGTAATTGTTCTCTTAATTCTTGGTCAGCAGAA
GTTAGCTTAATTTGATACGGTATACCCTTAAAGGTTAATAGTTCAGCTTTAATTTCACCAAAATGATTGTCAATTTGAGCTTCATTTA
CAGATTCTAAAACCTGATTCCCATCAGAAAAAACAGCTTTAATATCAGGTAATATGACTTCCCAATCAGCTTTTGGCGCCTTATCAA
CAGCTAAGAAGTTCATGACATGAAAAATAGAAGTGATACCGTCTATAGATAACAACTGATTTATAAATCTTGGTTGTGTTTCTTCTA
CTTTTTTATAAGTATTAGATAACTTGTCTTTTCTTGTAAATGACAAAACAACTTTCATTGTATTTGGACTTGGCGTTGGCTCTATACGT
AAAATTTCCATATACTTTATTCCCCCCGAATACTAGTTGATGAAAAATTTATTCGAATACAGCCTCTACTTTTGGCAATACTGTTTCC
CAATTTGCGTCATTTTCTTTATCTACTGAAATAAAGTCCATAACATGGAAAATTGATTTAACACCTTCAACCTTTAAGATGTCATTAA
TAAATGCTGGCTGTGAATCATCAACTTTAGTATACGTATCTGATGTCATACCTTCTCTGCTTTCACTAAGTGTAATCTTCATTGTGTTG
TGGTTCGGTGTTTCTGATATAGATATAATTTGCATTTGACTTCCTCCTATTGAAGTTCATTTCCTAATTGTTTTATTTCTTTCCCTATTG
AACACTCATTGATACAAAAATGATGGGCCTTCGTTTTCCCCTCTAATTTACGCAGACGAGTTTTGATTGGACATTGATTGCAATAAG
TATTCATTAAATCATCTATTTTCGCGAGTGCATTTTGCTCTGAAAAAGTTAAAATACGCGACACTTCCTTTATTACTTCAAAATATTG
CAACCTTGTAAATAAGTGTTACTGTTTTAAATTTGACATAAAAATATTATAACATTTTTGACCCTAAGATTTGAATGAAACCTTTTAC
AATTCTATACGTAAAATGTATTTATATTCAAATGCTATTGCATCGCAAACGATTTTTCAGTAAAATGAGTTAGTTATTTATTGAGCGG
TTTCTAAACACCCGCATGATCAAAATTTAGGAGGACATTTTAATATGTATAATGAAATTTTAGGACTAGTTACGTTTATTGCAACGTT
CGTACTTATGGTACTTATGTATCGCTTTTTTGGTAAACAAGGTTTAATTGCATGGGTTGCAATTGGCACAATCATTGCCAACATACAA
GTGATTAAAACTGTAGAAATCTTTGGTATTTCAGCAACTTTAGGTAATGTCATGTTTGCTTCTATTTATTTAGCAACAGATATTTTAA
ATGATATTTATGGGCGTAGAGTTGCAAAAAGAGCAGTTTGGTTAGGCTTTTCATCAACATTAATTATGATTATTGTTATGCAATTGTC
ATTACATTTTATTCCTGCACCAGAAGATATGGCACAAAAAGCATTACACGCAATCTTTGATGTTGTGCCACGTATTGCTTTAGGCTC
AATCGTCGCATATATTATTGGTCAACATATTGATGTATTTATCTTTTCACTAATTAAAAAAGTATTTAGTTCTGATAAAACGTTTTTC
ATCCGTGCATATGGTAGTACATTTTTAAGCTCAATAATTGATACAGCTTTATTTGTAGCTATCGCTTTTATCGGAAGTTTACCTGGTA
CAGTTGTATTTGAAATATTTATTACAACTTACGTATTAAAATTAGCTTCAACAGTTTTCAATGTACCATTTGGATATATTGCTAAATC
ATTTTATCGTAAAGGTAAGATTGATAAGTTAGATCAAGGCTATTAACGCACTTACTACACAAAAGAATAAGTTTTAAGGGAGTGGG
ACAGAAATGATATTTTCGCAAAATTTATTTCGTCGTCCCACCCCAACTTGCATTGTCTGTAGAAATTGGGAATCCAATTTCTCT

>036-contig_189

AAAAAGCTTGTTACAAGCGCATTTTTGTTCAGTCAACTACTGCCAATATAACATCGTAGAGCATAGAACATTGATTTATGTCCCAGC
CTGAAGATGATTTTTTTGGAAGTATATTACAGTACTACGTATTAGAAAATCATCTTTGACTCAGCGAGTATTTTAAACACGTGTTACT
TATTTAAGTGATGCGTGTTTTTTAATGGATTACCCAACATATTTTGTATTACAGTAATAGTAACTTTAACTAAGTATTGAATAGAAAG
TAGGTTTATTATGGCGAAAATAAATTTTGATGCTGCGACGAAAGGAAATCCAGGCATAAGTACATGTGCCATTGTAATCAAAGAAG
ATGAGCAGCATTATACATATACACATGAGTTAGGCGAAATGGATAACCACACTGCAGAATGGGCTGCATGTATTTATGCACTAGAA
CATGCACGTGAATTAAATGTTCAAAACGCACTATTATATACAGACTCAAAGCTAATTGCAGATAGCATTGAAGCTGGTTATGTGAA
AAACGCAAATTTCAAACCTTATTTTGATCAAATAGAAATATTTGAAAAAGATTTTGATTTATTATTTGTTAAATGGATACCGAGAGA
ACAAAACAAAGAAGCGAATCAACACGCTCAACAGGCATTGTATAAATTAATTAAAAAGAATAAATAAAAGGAGCACCGACTGATG
TGATGTCAATCGGTGCTCCTCTATTTATCAAAGAAACAAATTATTTTTTATTTTTCTTAGATTTCTTTTTCTTCTTTTTAGCTGCTACCT
TTTTAGAAGTATTTTTTGATGCTGACTTTTTGTCTTTGGATTGGTTATCTTTTTGATTTTTCTTTTTAGCCAATAATAAAGGAACGTCCT
CATCTTTAGATTCAATACTAGTTGTTGTTTCAACATCTTCCTCTTTATCTTTGCGTCGTTTTGCGAATAAGAGTGGTGAATGTTCGTTA
TCTTTCAACACTTTTTCATCTGTGTTTTCATTTGTAACTTCCACATCTTCTTCATCTTCTTTACGACGACGTTTTGGTAAGAAGAACGG
CGTATGTTTAACTTTATCGAGTGACTCGCCATTATTTAGCGAATCTTTTTCTTCAACAGTAACATCTTCTTCATCTTCTTTTCTGCGAC
GTTTCGCAAATAAAAGTGGTAAATGTTTTGTATCGTCTAAAGTCTCTTTTATTGAATCTTTATTATTATCTCTTATTTCTAATTCTTCC
TCTTCATCTTCTTTACGACGACGTTTGGCAATGAAGAACCAGAAACTAGCTAGTAAGCCAGAGATACCCACCACACCAATAGCGTTT
TTAATAACATTACCGAAATTATTACTAAAGTGACGCATATGAAGTGGATCAATGTCATCATCTTCATCAATTTTCTTTTTATGTCCAA
TAGTCGAACTGTTAAATGGATGATTTGCAGTTCCATAACCAATTGTTAAATGGCTATTAGATTCATTTCCAGTACTTGACGATTGTTT
AGCACGATCAGATATTACAATTTGTACTGCTGAAATTCGAGCAATACCATTGTTCAATGCAGCCTCAACTTGCTGTAATGTATGCGC
ATTATTAATATCTCTAATTGTTTCAAGCACAATTTCGTTAATTTGATTCATTGCTGCTTGTTTTTCATCAGTTGTGCCAATTTCACTAT
TTCGAATACTATTTATTTTTTCATTTGAAAAGTCTTTAATTCTACTAATTGCTTCTTGCTTGCGTTTAGCTAGTTCTTCTGCTGTTGGAT
TAGCTGCTTTCATTTGAGCTTTAAATTGTTCCAATTGCTCAGTTATTTCATCGATGCTTTGCGCACGTTGAATCGCTTGAATTGCTTGT
TCTTTTAATTGATTTAACTTAGCAATAGCTTCTTGCTTCTCTTTATCTGTTAGATCAGTACGAGCTTTGATTTCATCAATCATATGTTG
AATTGCATCTTCAATCAATTTAATTGCATTTGATTTTGCTTGTTCAATCGTAAATTGTTCTGGATTAAATTGTTCAATATGCGCTTGTT
CTTGTTGTTGAATTTCTTCAACTGAATGAACATCAGGTGCATTGTTAATATGATCAATCGCTTGTTGTTTAAGCTTATTAACCTCTGC
AATTGCAGCTTCTTTTTGTTCCAGTGTTAATGTCACACTATTATTGATTTCATTAATCTTTTGTTGTGCTGCATTTTCGATTGATTCAAT
TGCCTGTTGTTTGACTACTGACAATTCTTTTTCTACAACTTTTACAGGAACATTAACAATCACTTTTGATCCATCAAGCAATGTAACT
TCAACTTTTGCTGTTAAGCTATCAGAAATCGTTGCAGTTGATGGTGTATCAATAACTTCTGCTGAAAGCTGATCAATTAAGTTTATGT
GTGCAAGAATATCTTGTTCTGTAATGATGTCATCACGATGTACAATGAGTTCACCATTAACTAGGATTTGCTCAGGTGTTGCTTCAA
AAATTTCATTTACAGCAGGTTGTTCATCAACCTCCCATACATGTGTATTTCTAATGTCATCTAAACCTAAGTTTAATCCTCGATTTAA
TTGATCTATAGTTTGAGCATTCTCAACGTTTTGTAGTGCTCGTTTTTCAGCTTCGTCAATTTCTTTGAGCGCTTTTGCTTTTTGCTCAG
GTGTTAAATCTGGATTTGAATTGATTTGATCACGCTTCGCATTAGCTAAGGCTTTTATTGCATTTTTCGCATCTTCTTTAGCTTTCACT
AAATCTTTGATGTCTTGCAATGCTTGTGCAAGTTGTGCTTTGGCTTGTTCAATTTCTTCTTTAGTCATCGCATTGTTAATGCCGTTATG
ACCTTGTTGAAGTATTTGATTAATTCGATCTTTAAGTGCTTGTTTTTCCTTATCTGTTAGATTTGGATTTTGATCGATTTCGTCAATTA
AAGCTTGTACACGTTTATCAATATCTTGTTTCGCATTTTCTTTAGCTTTCACTAAATCTTTGATGTCTTGTAATGCTTGTGCAAGTTGT
GCTTTGGCTTGTTCAATTTCTTCTTTAGTCATCGCATTGTTAATGTCGTTATGACCTTGTTGAAGTATTTGGTTAATTCTATCTTTAAG
CGCTTGTTTTTCCTTATCTGTTAGATTTGGATTTCGATCAATTTCGTCAATTAATGCTTGAACTTGCTTGTCAACATCTTTATTCGCAT
CAATTTTAGCTTTCGGTATTTCATTGGCATGCACTTGTTCAATCGCATGGTTGCCAGCTGTTTGAACTTGAGATACATCCTGATTACT
TGTTGCTTTATTAATGTTGTTGATGATGCTGTTTGCCAATTCTTCTGCTTTATTTTTCGCAATAAGCTTGTCTTGATCCGTCGCATTTG
AAGCTTCGATTTCTTTTAGCTTATTAGCTAAAGCTTGATTAATAGATTGAATTGCCTTTTCTTTAGCATCTTGTAGTCGTTGATCACCA
TTAAGATTATGGATTGCATCATTGACTGCTTGGATTGCGCCATTGATATCATTCACATTTGTGTTATCACTATTTAGCAATGTATTTG
CTAGACGTTTGGCATCATCGAAGTTTGTTTTAGCATTATCGTCAGCGTTTTGGTAATTGACAGTTTGCTCTGCATTTGGAATTTCATT
GTCAACTAAATGTTTCAATGTTTCCATTGCATCATTTAAGTCAATTTGATTATTAACAATATCTGTTACATCTGATACAGTATCGGCA
TTGTTAATTGCTTTATGTGCAAGATCTTGTTGCTGTTGATTTAATCCATTTAACGAATTAACAAACGCATTTGCTTTATCTTTGGCATT
TGCAAGGTTTTGGTCTCCATTTAATGCATTTTGAGCATCGATAATATTTTGTTTCAATTGCTCTGCTTCAGCTTTTGCAATTGCATTAC
CTTGCACTTTATCTAACTCATGTGCTGCATTCGCAATTGCATTATCATAATTTGCTTTCAAATTGTCATCTGCATTGATGTAATTTGTG
CTGCTGATAACACCAAGGTGCTGTTCGTCAATATATTGTTGTACTGCACCCATTGCATTATCTAATTTAGTTGCTCTATTAATTGCTC
GAGATGCTTTATTTAGCGTTTCAGCATTATTAATTTGTTGGATTGCTAATTGTCTTTGAGCATTGTTTAGATGTGTTAATTGATCCAAT
CTTTGTAATGCTTCAGCTTTACGATTATTAAGTCTTTCTTCACCATTTAATGCTTTCTTAGCTGCAGTGACTGCATCATTTAATTTGAT
AACTTGTTCTGCAGTTAAGTTTTGCCCAGACGATTTATCAAGTGCTTGTTTCGCTTTATCAACCGCTTCATCATATACTTGTTTGACTT
GTGTATCTGCATTGACATAGTTCGTGCTACCTTTCGTGCGTCCTTCATTGCCAGTGATTTCTTGTGACAATTGATCCATTGCACGATT
AAGTGCCTTAGCCTCATCTACAATTTGTTGGATTTGATTTAAATCGTTTGATTGATCGATGCGACCTTTAAATCCATCTTGTTGAGCA
TTGTTTAATGCATTCAATTGGTCGATGTCATGTTTACCATTTGTTTTAGCAAGTGCTACACGTTCATCACCATTTAATGCTTGTTTTGC
ATTTAAAATATTTTGAAGTGCTTGATCCACTTGTTGCTTATTCGCATTTTGTTTCAATACATTTTCAGCATCAGCAATCGCTTGTTTAT
AAGCCTTTTGCTTATCTGAATCTGCTTGTGTGTGTAATCTATAGTTTGCTCAACGTTAGCATGTTCATTAACTGCTTGTTGTAATTGATCC
ATTGATTGGTTCAATTGCGTTGCTTGATCTACTAATTCAGCGATTGGTTGAAGTTTCGTCGCTTGATCAATATTTTGTTTAGCAGTTGC
AATTTGATCAGCATTTAAATGTGTTAATTGGTCAATAGTTTGTTTCGCTTGTGTTTTAGCTTCAGCGACTCTCTCATTACCATTTAACT
CATTCACTTTTTCTTGAAGCTTAGTTAATGCTTGTTCTACAGCGTCTTTATTCGCATTTGAACCATTAGTTGGATCTGTAATGCTTTGT
GCAGCTTGTAACGCTTGATCTACTGCTTCTTTTTTATCAGTTGACGCTTCAGTGTAATTTGGTTGAGCCTTATCTGTATTCACTTGATC
AACTTTATTTTTCAATGTTTCCATCGCGTGATCAAGTTCAGTAGCAGTTTGAACATGTTGTGCAACCTCTGTTCTTGTAGGCGCTGCA
TTTATAGCATCAGTTAATGTTTGTTGTTGTGCATGATTTAAGTTTGGCAATGCATTTACAGTTGTTACTGCTTGTTGTTGATCACGAGC
AAGTTTTTGATCACCATGTAGATTATCTTTTGCAGTTGTTACTGCTTGTGTTAATTGTTCTACTTGTGATTTGTCGAGTGTTGGATTAC
CTGTTTGGTTAATTAAATCTTTTGCATTTTGAACTGCTGCTTGGTAAGCATCTTTTTGCGGTTTATCTTCATTGATAAACTTGCTACCC
GCTTCCGTTTGTTGTTGATCTTGAATACTATTTCGTAATGCTTGCATTGCTTGATCAAGCGCTTTTGCTTCAGCTAATTTTTGCGCTAC
TTCGCCACGAGTTGCTGCGTTGTTTATTTGGCTTTCAAGTGCTTGACGTTGCGGATTATTCAAACCATTTAATTGATTTAAATCAGTA
ACCGCATGTTGTTTATCGTCTGCAAGTTTTTGATCACCGTGTAGGTTATCTTTAGCTTGTTTAAAAGCATGTGTCAATTGTTCAACTTG
TGCTTTATCAAGCGTTGGATTACCTGTTTGGTTAATTAAATCTTTTGCATTTTGAACTGCTGCTTGGTAAGCATCTTTTTGCGGTTTAT
CTTCATTAATAAACTTGCTACCAGATTCTGTTTGTTGTTGATCTTGAATGCTATTACGTAAAGCTTCCATTGCTTGGTTAAGTGCTTGT
GCTTCAGTTAATTTTTGTGCTACTTCACCACGAGTTGCTGCATTATTGATTTGATTTTCAAGTGCTTGACGTTGTGGTGTATTCAAGTT
AGACAAGTTATTTAACGTTTCTGTTGCACGTTGCTTATCTTGAGCTAGTTTTTGATCACCATGTAAATTGTTTTTAGCATCAGTCACTG
CCTGTGTCGCTTGATCAATGATTGATTTAGCTAATGTAGGATCAGTTGTTTTGTTAATCAAATCTTTCGCGTGTTGTACTGCTTGCGT
ATATGCATCTTTTTGCGCTTGATCCTCGTTAATAAATTTACTACTTGCTTCAGTTTGTGGTTGATCCTTAATACTTTCTTTTAATGCTTT

CATCGCTTCATTTAATGCTTGCGCTTGTGCAATGATTTCAGCCACTTTATCACGAGTTGTTGCATTATTAATTTGATTTCTAGCGCTT
GTTGTTGAGCTGGTGTTAATTGATCTAAATGATTTAGAGAATTTCCAGCAGTTTGCTTATCTTGAGCTAATCGTTCAACACCATCTAA
TGCATTTTTAGATGATATTACTGCTTGAGTCGCACTTGATACATTACCTTTATTGATAGTTGGATTATTTAATCCTGCAATGATAGAC
TCAGCATTTTGAACTGCTTCATCATAGGCTTGTTTTTTATTCGGTTCTGCATTGACATATGCACTGCTCGCACGTGTTGCATCTTTGTC
AGCAATACTTTGTTGTAATGCATCCATAAGTTGATCTAATGCTTGTACTTCAGTCAAATCTTGCTTAACTGCTGTTCTAGTTGTTTCAC
TATCAATTAACGTATCTTCCATATGTTTTTGTGCATTGTTTAGATGTGCTAATTGGCTAACCGTTTGTTTAGCATGATCTTTATCATTT
TGTAATTTCACATCACCATGTAATGCTGCTTTCGTTGTATTCACAGTTGCCGCTACTTGATTAATCGCATTATTATCTAATGTTGCAGT
TTGTTCATTGATAATATTATTTGCGGCTTGAACAGCTTGATCATAGTTTTGTTGCTCTGGTTGATCTTCGTTGATATATTTGCTATTTG
CTTTAGTTGCTGCGTTATCTTGAATGCTCTGTCTTAAACCATGCATTGCTTGGTTAAGCGTATTCGCATTTTGTTCAATTTGATGAACT
GCAGTTACTAAAGTTGCAGCTGTAACTTGGTCTTTTAATGCTGTTTTTTGAGGATCATTTAAGCTTGTTAAAGCATCAATTGCTTGCG
TCGCATTTGTTTTAGCAACTTGTAAATTATGATCTCCATTTAACGCTTGAAGTGTTGTTTGAACTTTAGTAATAGCTTGTTCTACTTCT
GTTTGGTTCGCATTTTGACCAGTTTGTTTGTTAATCATTGCTGCTGCTTCATTTACCGCTGTATTATAAGCAGTTTGTTTATCAGTATC
AGCATTGATGAAGTTACCACGTTGTTCAACGGCTTCATGATCTGCAATCGCATTAATTAAGTTACCCATCGCAGTATTTAAAGATTCT
GCTGTTTGTTTGTGACCATTAACTGCAGCAATATCAGATGATTGCGTAATTTGTGATTGTAACTGTTGCTTTTGCGCATTATTCAAAT
GATCTAATTGATCAAGTCTGTTGTTTGCTTGTTGTTTAGCTGCTGCAAGGTTTTCATCACCATCTAATGCAACTTTCGTATTATTCACT
TGTGTCGTTGCACCATTAACATCATCTGGATTCATCGTAGGCGATGTAGTTTGATTAATGATATTATTAGCAGCTGTTACAGCTTTGT
TATAGTCATTTTGTCTTTGCGCAGTTGCATCATGATAATCTTCAGACGCTAATGTCGTTTGATTATCCGCAACACTATTTCTTAATAAT
TCCATCGCATGATCTAACGTTTGTGCTTTTTGATTAATCGTAGTGACATCTGTAACAAGTGGCGCTTGTGTAATTTGACTTTCAAATG
ATTGTTTTTGTGCATCATTTAAGTGTGTTAAGTTCGCCAATTGTTGTTTTGCTTGTTGTTTTGCATTTGCCAAATTGTCATTACCATTTA
ATGCTTGTTTCGCTGCGTTCACTTCATTAGTGATGCTTTCAACTTGTGCTTGTACTTCATTAGTACCACTTTGTTTATTCAATAATGCT
TCAGCTTTTGCTACTGCCTGTTTATACGCATTTTGTTTGTCAGGATCTGCATTTAAGTAATTACCGTCCGCTAATATTTGATCTTTATC
ATTAATAGCTTGTGATAATTGATCCATTGCTTGATCTAGGCTCGTTGCTGTTTGTTTAACTTGGTTCACTGTAGCAATTTCAGGCGAT
TGATCGATTTGACCTTTTAATGCTTGTTTTTGAGCATCGTTTAAATGCGTCATTCCACTTACGGCATCTTTAGCATTTTGCTTTTCAGT
GGCTAATTTAGCTTCACCGTTTAAGCTATTTTTAGCATCAGTCACTTGAGTTAATGCACGAGTAATGTCATCTGGGTTAAGCGTTGGA
TTTGTCGTTTGATTGATAATACCTTCCGCTTGAGACACTGCATTTGTATATGCTGTTTGCTTATCGACATCAGCATCGTGGTAGTTCTC
ACTTGCTTTCACAGTATCTTTATTTGCAATACCTTGTTTCAAGTTACCCATTGCTGTATTCACATTTTGTGCATTTTGTTTAGTTTGTTC
AACTGTAGCTAAAGCTTGTGCTTGATTGATTTGGTTTCGTAATGCATCACGTTGTGGTTGATTTAAGTCACGTAACGTATCAAGATTT
GCTAAAGCATCTTGTTTCGCTTGCGCTAATTTTTCATCACCGTTTAATGCATCTTTCGCTTGATTCATAGTTGTAACTGCTTTTGTTAC
TGTATCAGGCGCCAATACAGGTGTTGGTGTGCCATTTGCGATTTGTTGCGCTTGGTTAGCTGCATTGTTATAAGCTTGTTGTTTGTCT
TGATCCGCTTGTGTAAAGTCAACTGATTGTGGTACTTGACTATTCGCTTGAATTTGTTGTTTCAACGTACCCATTGCATTATTTAACG
CATCAGCATTTTGCTTAATAGCATTAACACCTGTAACAAGTTCTGCATGCGACACTTGGTCTTTTAATGCTGTTTTTTGTGGTTGATTT
AAGTTTGGTAACTGATCAATGGCTGTATTTGCATTGTCTTTAGCAACTTGTAAGTTATGGTTACCGTTTAAATCACCCTTAGCTTGAT
TCACTTGTTGTAACGCTTGAGCCACTTGTTGTGGATCCACGTTTGCATTTGGTGTACCACTAATGATTTGTTCAGCATGCGCTACCGC
ATTGTTATATGCATTTTGCTTATCAGTATCAGCATTGACAAAGTTACCATTTGCTTTAGTTTGGTCTTTATCTGCAATACCTTGTTTCA
ATTGTGTCATTGCTTGATTTAAATCTTGTGCTGTTTGCTTAATCGTGTTTACATTTGCAACAGTTTGCGCAGCATCGACTTGTTGTTTT
AATGCATCTTTTTGCGCTTGATTTAAATCATTTGCATTTGTAATTACTTGTTTCGCTGCATCTTTTGCACGTTGTACATTGTCATTACC
ATTCAATGCTTGTTTCGCTTCGTTCACACGTTGCATCGCTTGTTCCACTTCAGTTTGACTTGCATTGCTGCCATTTGCTTTAGATAAAA
TACCTTCTGCATGTGAAACCGCATTAGTATAATCATCTTTCTTAGCTTGATCTGCATCAGTAAAGTTAATGCTATTTAATGTCTCAGT
TTTATCTTGTAATGCATTGTTTAAGTTAGTCATAGCATTATTCAGATTTTGAGCATTTTGCTTAACATTATTAACTGTTGCAATATCTG
GTGCTTGTTCAACTTGAGTTGTTAGCGCTTGTTTTTGAGCATCATTTAAATGATCTAGTGTACCAAGTGTTGTCTTAGCATCTTGTTTG
GCATCAGCTAATTTTTGATTACCGTTAAGTGCTTGATTTGTGTCATTCACTTTAGTTAATGCTTGTTGAATTGTATTTGGATCCATTGT
TGGATTTGTTGTTTGTTTCAACAATTCTTCTGCTTTTGTTACTGCATTATCATATGCTGTTTGCTTGTCAGTATCAGCATCGTGATAGT
TCTCGCTACCTTTAATTTGTGCTTTATTCGCAATACCTTGTTTCAATTGTGTCATCGCGTCATTAAGCGTTGTTGCCGCTTGTTGAATA
GCATTGACGTTTGGTACAAGTGTTGCTTGCGTGATTTGTTTGCTGTATTCATCACGTTGCGCTTGGTTTAAGTTTGGCAATTGATCAA
TAGCATGTTGAACATTTTGTTTCGCCGTTGCTAAGTTTGTATTACCATTTAAATCATTTTTAGCTTGCGTAACTTTATTTAACGCTGCA
GTAATTTCGCTAGGTGTAACGACAACATCAGGCGTACCACTAATTAATGCTTCAGCTTTCGCTACTGCTTGATTATATGCATTTTGCT
TGTCAGAATCTGCATTGACAAAGTTACCATCAGCTTTTGTTTGTTCTTTATCTGCAATGCCTTGTTTTAATTGTGTCATCGCATTGTTT
AACTCTTGCGCCGTTTGTTTAACATTGTTTACACCAGCTACAGTAGTTGCATTTTGTACTTGTTGTTTTAATGCATCTTTCTGTGCTTG
GTTAAGATCATTAGAGTTATTAATTAATGCTGTTGCTTCGTCTTTTGCATGTTGAACGTTGGCATTACCATTTAATGCTTGTTTTGCTG
CATTTACTTGTTGGATTGCTTGTTCAACTTCAGTTTGTGTTGCATTACCACCATTAGCTTTGAAATGATATTTTCAGCATTTGTAACT
GCAGTGTTATATGCTTGTTGTTTTGCTTGATCTGCATCAGTGAAGTTAACTGTTGCTTTCGTAGCATCTTTATCAGCTATCGCTTGTTT
CAAGTTACCCATCGCAGTATTTAAGTTTTGCGCATTTTGCTTAATTTGATTTGCTTCATCAACAGTATGCGCGCCATTAATTTGATTA
GTAACAGCTTGTTTTTGCGCATTATTTAAGTTGTCTAATGAACCTAAAGATTGCGTTGCTTGTTGCTTCGCTGCCTCTAAGTTTTCATT
ACCATTTAAAGCATTTTTAGACGTGCTTACTTGTCCAGCAGCTTGATTGATAACAGTCGGATCTAATGAAGGGTTTGTAGTTTGATCA
ATAATACCTTGTGCAGTTGTGACAGCATTATTGTACGCATCCTTTTTATTCGGACTTGCATCAGTATAATTTTGGTTTTGTTTTGTTGT
CGCATTATCTGCAATACTTTGACGTAATTTGTCCATTGCTGCATCAACATTGTTTGCTTTTTGTTCATTACCTTGTGCTTCTGCAACAG
TAGTCGATTGTTGTACCAATTGTTTTAATGCCTCTTTTTGTGCATTTGTTAAATGGCTTAAACCGTCAATTGCTGTATTTGCGTGTTGT
TTCGCTTTTTCAAGGTTTTGAGTACCATTAAGCGCTGCTTTTGTAGTATTCACTTGATTCATAGCTGCTTCAACTTGATCTTTAGGCAC
GTTCGTACCTGTAGATTTATTTAAAATATTTTCAGCATTACGAACCGCTTCATTGTATGCATTTTTCTTCTCTGGATCTGCATCTGCAA
AGTTTTGACTGCCAAGTGTAGTGTCTTTATCATTCAAGCTATTTTTCAAGTTACCCATCGCTGTATTCAGTTCCTGAGCGGTTTGAAT
GGCTTGCGTTACTTCAGCTACAGTGTGCGCTTGATTGATGTTTCCAGTAACAGCACCTTTTTGTGCATTGTTTAAGTTATCTAACGTA
GATAATGTTTGGTTTGCATTGTTTTTAGCATCTGTTACTTTTCTATCACCATTCAACGCTTGTTTAGTAGTTGTTACAGTTGAAAGTGC
TGCTTCAACTTGTGATTGGTTCGCATTTGTACCATTTGCTTGATTAATAATATTTTCAGCAGCAGTTACCGCATTGTTGTATGCTGTTT
GTTTTTCTGGATCTGCATCTGTAAAGTTAACACTTTGTTTCACTTGATCTTTGTCTTGGATTCCGTTGATTAAACGTTCCATTGCTTGA
TCTAATTGTTCAGCTTTAGTTTTCACTTGATTTACTGTGCTAATATTTGGTGCATTTGTCACTTGTGTATTTAAATCTTGCTTTTGAGC
ATTGTTTAAATGACTTAATCGGTTGATGTTAGCAGTTGTTTCTTGCTTTTTATTTGCTAAGTTTTGCGCACCATTAAGCGCAGTTTTGT
TAGTGTTCACTTGATTAGCTGCTTGTGTTACTGCAGATGGATCAAGTGTCGGCTGACTCGTTGCATTAATGATATTTTCAGCACTTGT
AACTGCTGTATTGTATGCATTTTGTTTATCTGTATCTGCATCAACATATGGTTGACTCGCTTTTACTTCATCTTTATTCGCAATACTAT
TTCGAAGTTGATTCATTGCATTGTCAAGATTTGTTGCATTGTCTTTCACACTTTGTACACCAGTTACTTGTGTCGCGCTATCAATTTGA
CCAGTAATACTTTGTTTTTGTGCATCAGATAAATGTGTCATTTGACGTAATGCATCTTTAGCTTGTTGCTTCGCAGTTGCTAAGTTTGT
ATCACCATTTAAACTTGTTTCAGCATTATTGACACGTTGTGTTGCCGCAGTAACATCTGCTGCATTTGTAATCACATTAGGTGAACCA

TTTACGATATTCTTCGCAGCATTATATGCATCAGTGTAAGCACCTTGTTTATCAGGACTTGCGTTAGTGTAATTACCACCAGCTACAA
TTGTGTCATGATCACCAATTGCTTGTTTTAATTGATCCATCGCATTATTAAGTGATGTAGCACTATTTTTCAGATCAGTTACACCTGA
TACGCGTTGTGCAGCATTCACTTGTTGTTTCAATGCTGTTTTTTGCGGTGTATTCAATTGTGTCAAGTTATCGATAGCTTGTGTAGCTT
CAGTTTTAGCGCGTTGTAAGTTCGCGTCACCGTTCAAAGCATTTTCAGTAGACGTTACACTTTGAATTGCTTGTTCAACAGCTTGCTT
ATCTTTATTCGTACCATGATCTTTATTTAAGTAAGATTCAGCTTGTGTGATGGCATTATCAAATGCTGTTTTCTTAGTTGGATCTGCGT
CAATGTAGTTTTCGCTCGCTTTAGTTTGATCTTTATTTGCGATTGCTGACTCTAATCGTTGCATTGCACCATCTAAGTCTTGTGCTTTC
GTTTTAACACTATTTACACCGTTGACTGTTGTTGCACCTTCGATTTGTGATGTTAAATCTTGTTTTTGTGCATTATTTAAGTGTGTTAA
TGTACCTAAGTTTGCTTTCGCATCTGATTTTGCTTGTCTTACATTTGCATCACCGTTTAAAGCATTCTTAGCAGTCGTTACTTGATTTA
ATGCAGCTTCAACTTGTGCTTTTGTCATATTTTGACCGTTTGCTTTATCTAAAATACCTTGAGCATTCGTAACAGCATTTGTGTAAGC
ATCTTTCTTACCTTGGTCAGCATCTGTAAAGTTCACTTGTTGTTTAACAGTGTTTTGGTCATTAATACCATTTTGAAGTTGACCCATTG
CTGTATTTAATTCATTAGCAGTATTTTGTGCAGCAGTTACGTCTGCTACTGTCGTTGCATTGTCAATTTGACTAGTTGCTGCAGTTGAT
TGCGCGTTATTCAAGTTACTATATGAAGCTAATGCACGTTTTGCTGTTTCTTTCGCTTGAGCAACTTTATTATCACCATTCAAGTCGTT
TTTAGCTTGATTCAATTGGTTAAGCGCTGCTTGAACTTGTGTTTCATTCGCGTTACTGCCATTTGCATTAGTAATTGCTTCTGCTTGTG
TAACTGCAGTATCATACGCTTGTTGTTTTGGTTGATCCGCATCTGTGTAATTGACACTACGTTTCGTATTATCCTTTTCAGCAAGTGC
ATGTTTCAAATTACCCATCGCTGTGTTTAACGATGTTGCTTTATTTTTAGCCGCTGTGACAGCTGCAAGATTTGGTGCTTGATCCACT
TCAGCATTTGCCGCAGTTCGTTGTGCATTGTTCAAGTCTGTCAAACGACCGATATCTGATTTCGCAGTTTGTTTTGCTGCTGCTAATTT
TTCATCACCGTTCAATGCAGACTTCGCACTGTTCACTTGGCTCGCTTTTTGGTTAATTGTATCAGGATTCATTTCAGGGTTATTCGTTG
CACTAATAATACCTTCAGCATTAGTTACCGCATCATTGTATGCATTTTGTAAATCTGCATTCGCGTCTTGATAATCTTCGCTTGATTTA
GTCGCATCTTTAGAAGCAATACTTTGTCTTAATTGATTCATTGCTTGATTTAAAGTACCAGCATTTGCTTGGATGCCTTGAACACCAG
CAACAGTTGTACCACGTTCAATTTGTTCTGTTAAGTTTGCTTTTTGAGCATTAGTTAAATGTGACATTGTCGCTAATTGTTGTTTAGCT
GTGTTCTTCGCTTCATTTAATCGCGCGTCACCATTTAATGCTGTTTTAGCAGTATTCACACGTTGTAATGCTTGTTCAACGGCTACTTT
ATTGTCATTTGAACCAGCTGTTTTATCTAATAACGTTTTAGCTGCTGTTACAGCATCATTGTATGCAGTTTGTTTATCTCTATCTGCTT
CAGTATATTTCTGAGCTGCTTTTGTAGCTGCTTCGTCATTAATACCACGTTGTAAGTTGCTCATTGCAGTATTTAATTCAGATGCCATT
GTAGACACTTGATTTACACCTGCAACTGTTGTCGCACCTTCAATTTGTGATTTCAATGCTGCTTTTTGTGCATTATTAATTGATGTAA
GGTTATTCAATGCATTTTTCGCAGTTGTCTTAGCGTTCGCAACATTTTGATTACCGTTCAATTCGTTTTTAGCACGTTGTACATTTTGT
AACGCAGTTTCGACACCGTCTTTTGCAGTATTTGGACCTTGTGCTTTATTTAAAATTTGTTCAGCTTGCGTCACTGCATTTGTATATGC
ATTACGTTTCGCTTCATCTGCATCAGTGAAGTTAACACCTTGCTTAATCGTATTTTGATCTTGAATACCATTTTTCAAGTTCGTCATAG
CTGTATTTAATGCGTCCGCATTATTTTGCGCTTGTGTTACTTCATTAACATGCGTTGCACCTTCGATTTGGCGTTTCGCTGCATCTTTT
TGTGCATTAGTTAAGTCACTTAAGCCATTCAAATGTTGCTTCGCATTTGTTTGCGCAGTTCTTAAGTTTTCTTGACCGTTTAACGCTTG
TTGTTTAGCTGTCACTTGGTCTTTCGCTTGATTAATTTCTTGCGCAATCATTGATGGACTAGTTGTTTGACCAATGATTGCTTTTGCTG
CAGTGACTGCATTGTTGTAGTCATTTTGTTTGTTTTGACTTGCATCTGTGTAGTTTTGACCTGCTTTAATCGTTGCTTCATTCGCAATG
CTATCTCGTAGACCTTTCATTGCAGTGTTTAATGTTTGTGCATTATCACGAACAGTTTGAACATTTGGCAACGTCGTCGCTTGACCCA
CTTGTTCTTTAAGTTTTGCTTTTTGCGGACCATTTAAAGAAGTTAAACCATCAATTGCTGTGTTAGCATTTTGTTTCGCTACTTCTAAA
TTATGATTACCGTTTAACTGCGTCTTCGCATTCGTTACTTGCGTTGCTGCATTTGTAACATCTGATGGTGTTAACGTTGGTGTTGGTGT
ACCACTAACGATATGTTCTGCAGCTGTCACTTTTTCATCATATGCTTGGCGTTTATTCGCATCAGCATTGACATAATTACCACTAGCT
TTTGTGTCAGCTTTATCAGCAATGGCACGTTTTAAAGCTGTCATCGCAGTATTTAATTCAGTCGCAGTATGTTCAACACCATTTGCTG
CAGATACGCGTCCTGCACTTGTTACTTGTGCTTTCAATGCTTCTTTTTGTTTTGTATTTAAGTCCGAAGCATTTGTAATCGCTGTGTTC
GCAGCCTGTTTCGCACGTTCTAAGTTTTGAATACCGTTTAATGCAGTATTGGCTTGTGTAACAGCTTGCATTGCTCTTTCGACATCTG
CTTTAGGTGTATTTCCTCCAGCTGTTTTATTTAAAATAGTTGCTGCTGCATTTACTGCTTGAGAATAAGCCGTTCGTTTAGCATCATCA
GCATCTTGATAATTTTGACTTTGTAACGTCGTGTCTTTATCACGAATTGATGTTTCTAATTGACCCATAGCACCATCTAATTGTTGCG
CTTTGGCTTTAACTGTATTAACACCTTCAACATTTGTTGCTTGTGTAATTTCATTATCTAACGCATTACGTTGTGCATTATTAATGTGT
GTTAATGTACCTAACGTTTGTTTCGCAGCAGCTTTAGCTTCATTTAATTTCGCATCACCGTTCAACGCCGTCTTCGTACTGTTCACATT
TTGTAATGCTTGTTCAACTGCTGCTTTTTCTACATTTTGACCACTAGCTTTTGTTAAAATTGCTTTTGCTGCATTTACTGCTTGATCAT
AAGCTGATTTCTTACTTGGCTCAGCATCTAGGTATTTCTGAGTTTGTTTTGTTTGTGTCTCATCATTGATACCATTTTGTAAACTGTGC
ATTGCGTTATTTAATTCTGTTGCTTTTGCAGTTTCTTGATTTACACCAGCTACTGTAGTTGCACCATCAATGTTACGCGTTAACGCATC
TTTTTGTGCATTGTTAATTGATGTTAAGTTATTCAAGTTGTTTTTCGCTGTTGTCTTAGCTTGAGCTAAGTTTTGCGCACCATTTAATG
CATGTTCTTTAGTTGTCACTTGTGATGTTGCTTGCGTAATAGTATTTGGTTCCATCGTTGGGTTCGATGTTTGATTAATGATTGCTTTT
GCTGCAGTAACTGCGCTGTCGTACTCGTTACGATTATTTGGACTTGCGTCAGTGTAGTTTTGACCTGCTTTAATCGTTGCTTCATTCG
CAATACTATCTCTTAAGCCTTTCATCGCTGTATTCAACGTTTGAGAACTATTTTTAACAGTTTGAACACCATCTAATGTAGTTGCACT
TTGAACTTGTTCTTTTAATTTTGCTTTTTGTACATTATTCAATTGTGCTAAGCCGTCAATTGTATTGTTAGCATGCTCTTTCGCTACAC
GTAAGTTGTTATCACCATTTAATGCTGTTTTAGCCGCATTGACTTGTGATGCCGCACTATTTACGTCATTGACTGTTAATGTCGCATTT
GGTGTACCATTAATGATATTTTCAGCGTTAGTCACTTTTGAATCATACGCTTGACGTTTTTCTTGATCAGCATTGACATAATTACCGC
TTGCTTTTGTTCTTTCTTTATCCGCAATAGCTGCTTTAAGTGCTGTCATCGCACCGTTCAATTCAGTAGCTGTATGTTGAACGTTATTT
GCATCAGATACACGTTGTGCATTGTTAACTTGTGTTTTTAATGCATCTTTTTGTTTTTGGTTTAAGTCTTGCGCGTTGTTAATTGTGTT
CGTCGCATTTGTTTTAGCTTGAGCTAAGTTTTGTGCACCATTTAACGCTGCTTTTGTTGTATTGACTTGATTTGCCATGTCATTAATCG
CATTAGCATCCATATTTGGATTGTTCGTTGCATTAATAACACCATTTGCATTATTTACAGCAGTATTGTAATTATTTTTGTTGCTATCA
GATGCATCAAGATAATTTTGACTTGTTTTCGTCGTATTATCATTTTGGATACTTGTACGTAATGCACCCATGGCAGTATTTAATGTAT
TACCTTTATCTTTAACACCATTTACACCAACTACATTTTGCGCTTGTTCAACTTGATGCTTCAAGTTGTCTTTTTGTAATTGTGTTAAG
TTAGGTAAACCATTAATCGTATTTGTTGCTGAAGTTTTCGCATTTCTTAAGTTTTCAGCACCATTTAAAGCCGCTTTCGCTCTTGTAAC
TGCATTTAATGCATTATCAACGTCTGCTTTAGATGTGTTACCACCTGTTTGTTTATTTAAAATGCCTTCAGCCGCTGTGACAGCTTGC
GTATATGCATTTCGTTTTGATTCATCTGCATCAAGATAATTTTGATTTCTTAATGTCGCATCTTTATCATTGATTGCACCTTGTAAGCT
ATTCATAGCGCCGTCTAATGTATTGGCATTTGTTTTTACAGTATTAACGCCATCAACAGTCGTCGCTTGATTGATTTGACCTTCTAAC
GCAGTACGTTGTGCATTCGTAATATGGTTTAAAGTACCTAAGTTTTGTCTAGCTGCCGCTTTCGCTTCTGCCAGTTTAGCATCCCCAT
TCAATGCATCTTTCGTACTTGTTACTTGTTGTAATGCACGGTCAACTGCTGCTTTATCTGAATTTGAACCTGTTTGTTTATTTAAAATT
GCTTTCGCAGCAGCTACAGCTTGATCATAAGCAGTTTTCTTACTTTGTTCAGCGTCACGATATTTTTGAGTTTGTTTTGTTGCATTTTC
ATCATCAATACCATGTTGTAATTGACCCATAGCTGTATTAAGTTCATTTGCAGTTTGTTGGATACTTGTTACATTTGCAACACGTTGC
GCACTTGTCACTTGTGCTTTCAACGCATCTTTTTGCGCTTTATTTAAGTTAGTAGCACCATCGATGGCATTTGTTGCTGTTTGTTTCGC
TTGCGTTAAATTATGTGTACCATCTAATGCATTTTTCGTTGATGTCACTTGTGTAGCGATTTGGTTAATTGCATTAGCATCCATATTTG
GATTGCTTGTTGCATTAATGACACCATTAGCACTATCAACAGCATTGTTATAGTTTGTTTTGTTACTTCCTGTAGCATCAAGATAGTT
TTGGCCATTTTTCGTAGCTGTGTTATCTTGAATGCTATTTCTTAACGTACCCATAGCACCATTTAATGTGTTGGCACTTGATTTAACAG
TATCTACACCTGCAACATTTTGTGCTTGTTGCACTTGTTGTTTCAATTTATCTTTTTGAGCAGTTGTTAAATGTTGAAGTCCGTTTATA

GTAGTGTTTGCATTTGATTTAGCTTGCTCTAAATTATGGTTACCATTTAGGGCACCTTTCGCATTAGCAACTTGTGACAACGCGTTTT
CAACGGCAGCTTTATCTAAATTTGATCCACTATTTTTATTTAAAATATTTCTGCGTTCGCAACGGCTTGAGTATAAGCAGTTTTCTT
ATCTGAATCAGCATCATGATAGTTTTCACTTGCTAGTGTATTTGCTTTATCATTAATACCATTTTGCAAGTTAGCCATCGCTGTATTCA
ATTGTGTACCTGTATTAGATACAGCTTCAACACCAGCTACAGTTGTTGCTTGATCAATTTTAGTTGTTAAGTCACGTTTTTGTGCATT
ATTAATTGACGTTAAGTTATTTAATGCATTTTTAGCTGCATTCTTCGCATTTGTAACGTTTTGATCACCATTCAATGCATTTTTAGCAC
TTGAAACATTTTGAATAGCCGCTTCAACATCTTGTTTAGACGTATTTGCACCTTGCGTTTTATTCAGAATTGCTTCAGCTCTGCTTACC
GCATTTGTATACGCATCACGTTTAGCTTTATCTGCATCAGTGAAGTTAACACCTTGTTTAACTGTGTTTTGATCATGGATAGCTTGTT
CCAAGTTGCCCATTTGCGTGTTTAACTCAGTTGCTGCATTTTTAGCAGCTGTTACCTCACTCACATGACCTGCACGGTCAATTTGTGA
TGAGATGGCAGATTTTTGGTTATTTGTTAAGTGTGATAACGTATTTAAGTTTTGCTTAGCAGTGTTTTGTGCATTTCTTAAGTTTTCAG
CACCGTTTAAACCATTTTTAGCATTATTCACTTGTGTTGCAGCTTGGGTAATCGCACTAGCATCCATAGTCGGATTGTTAGTTTGATT
AATGATACCTTTCGCATTTGACACAGCGCTATTATATGTTGATTGGTTATTCGGACTTGCGTCTGTATAGTTTTGACTTGCTTTGACTG
TTGTTTCGTTAGCAATACTATCTCTTAAGCCTTTCATTGCATTGTTCAATGCTTGTCCATTTGTTTGAACAGATTGTACGTCTTCTAAT
CTATTGGCTTGTCCCACTTGTTCTTTTAATTTAGCTTTTTGAGGTGTATTTAATTGTGTTAATGCATCAATAGCAGTATTGGCGTTTTG
TTTTGCTTCACGTAATCTTTCGTCACCATTTAATTCTTGTTTCGCGCTGTTTACTTGATTAGCTGCAGCTGTTACTTCTGAAGGTGTCG
TAACAACCGTTGGCGTACCGCTAATAATATGTTCAGCATTGGTAACTTTAGTTGTGTAAGCATTTTGTTTAGTGCTATCGGCATTAAC
ATATTTACTGCTTGCTAACGTATTCGTCTTATCTGCGATGGCATGTTTTAATGTGCCCATTGCCGTGTTCAGTTCAGTCGCATTGTGCT
GTACATCTTGTGCATTAGATACGCGTTGAGCACCATTAGCTTGTGCTTTTAATGCATCTTTTTGTTTTTGATTTAAATCAGATGCGCC
ATTGATTGCTGTAATCGCTGCTTGTTTCGCATTATTTAAGTTTTGCGTACCATTTAATGCATGTTTCGCACTATTAACATTATTAAGTG
CTTGTTCAACCGCTGTTTTCGCTGTATTCGGTCCAGTTTGTTTATTTAAAATGGTTTCGGCAGCTGATATAGCTTGATTGTAAGCATTA
CGTTTTTGCTCATCAGCATCCAAGAAGTTTTGGCTCGCTAATGTTCCTGACTTATCGTTGATAGCCGTTTGTAAGTTACCCATTGCGC
CATCTAAACTATTTGCACTTTGTTTAACTGATTCAACACCAGCTAAGTTTGTAGCTTGTGAAATTTGATTTGTTAAATCATTGCGTTG
TGCTGTAGTGATATGCGTTAAAGTACCTAAATGTTGTTTCGCTGCGTTTTGAGCTGCAATTAATTTTGCATCACCATTCAATGCATCT
TTCGCAGTATTAACACGTTGCAATGCTGCTTCAACTGCATTTTGCGCAGTGTTTGGACCTGTCGATTTATTTAAAATCGCTTTCGCTG
CAGTAATAGCATTATCATACTCTTGTTGTTTATTTGTATCAGCATCACGATATTTCTCAGATGATTTCACTTGAGATTCGTTGTTAATA
CCATTTTGTAAGTTAGTCATAGCTTGATCTAGATGTTGTGCATTTTGTTTTACAGTATCAACACCACTCACTCTTGTCGCACTAGTAA
TTTGACTAATCAAATTGTTCTTTTGAGCATCTGTAATACTCGTTAATGTGTTTAAGTACGTTTTCGCATTTTGTTTTGCCGTAGCTAAG
TTTTCATCACCGTTAAGTGCCGTTTTAGAACTATTCACTTGCTCTGCTTTTTGTGTAATCGTACTTGGATTCATTTCTGGATTACTATT
TGCATTAATAATCGTTTCAGCAGCAGCTACTGCGTTGTTATATGCTGTTTGCTTATCATTATTAGCATCTACGTAATCTTCACTTGCTT
TAGTCGCATCTTTGTTGGCAATACTTTGTCTTAACGTATTCATGGCTTGATCTAATGTATTGGCATTTGATTGAACCGTATGAACACC
AGCGACAGTAGTACCACTATTAATTTGGTTTGTTAAATTCGTTTTTTGTGCAGTTGTTAAATGCGTCATATTATTTAACTGCTGTTTTG
CTGTTTGCTTCGCTTGATCTAATAAACGCGTACCATCTAAGTTATTTTTAGCAGAATTCACTTGATTCATCGCTTCAGTAACTTGATCT
TTCGTTTTATTTTGACCATTTGATTTATTTAAAATATCTTTCGCAGCTTGTACCGCATTTGTGTATGCTGTTTTCTTACTTGGTGTCGCA
TCTTGATAATTTTGACTATTAAGTGTCGTTTGTTCATCATTGATCGCACCTTGCAAGTTACCCATCGCTGTATTTAAATCTGTACCTTG
TTGTTTAACAGTATTTACGCCAGCAATATTTGATGCAGCATTAATTTTAGATTTAACATCTGCTTTTTGTGCATTATTTAAATGTGGTA
ATGCATCAATTGCTCTTGCAGCATCTGTTTTAGATTGTGCTAATTTTTCATCACCATTTAATGCATTTTTATTAGTAGTAACAGCTGAA
GTTGCTCGATTAACATCATCAACAGACATTGTCGGATTAGCTGTTTGATTAATAATTGTTTCAGCACTTGAAACTGCACTATTATATG
CATTTTGTTTAGCTGTATCTGCATCCGCATAATCTTCTGTACGTTTCACTTGATCTTTATCTGCAACAGCTTGTCTTAAGTTACCCATT
GCACTATTTAAGTTAGTTGCATTTTGCTTAATCGTATTAACTGTCTCGATTTGATGCGCACCATTAATTTGCGATTGTAAGTTTTGAC
GTTGTGCATTATTCAAGTTATTTAATTGACCTAATGCAGTGTTCGCTTCTTGTTTGGCAGCATTTAATTTTGTATTACCATTCAATGCT
TGTTCTTTACTTGTGACATTTGATAAAGCATTGTTAACATCACTTGGTGTTATAACTGGATGTTGTGCATTACCATTAATAATGTCAT
TCGCATGGTTGTATGCATTGTTGTAATCATTTTTCTTATTAGTATCTGCGTTGACATAATTATCACTTTGTACGACTTGGTTATGATTA
GCAACGCCACGTTTTAAACCTGTCATAGCAGTATTTAAGCTTTGAGTCGTTTGTTTAATATCATTTACTGCTTGCACGTTTTGTGCAC
TATTCACTTGTTGTGTTAATGCATTCTTTTGTGCTTGGTTTAAATCACTTGCATGCGTAATCGCATTTGTTGCTTCTGTTTTCGCACGTT
GTAAGTTTTGTTGACCATCTAAAGCATCTTTCGTCGATTTAACAGATGCTGCTTTTTGGTTCACTGTGTTAACATCCATCGTTGGATT
AGTCGTTTCTCCAATGACACCTTTAGCCGCATTAACTGCATTATCATACGCTTGTTTATTAGCTGGTGTTGCGTCAGTGTAATTTTGA
CCTGATTTAATTGTATTATTATCCGCAACACTGTCTTTTAATTTCGTCATCGCAGTATTTAAAGCCGTAATGTTAGACTTAATTGTTTC
AAGCGCAGCATGATTTTGAGCAGCATTAATTTGTTGCGTGAAATTATTTTGTTGTGCTTGGTTTAAGTTAGATGCACCATGTAATGTT
GTTAACGCTGGCTGTCTATCTAATGTTAAACCTTGACGTGCTGTATTTAATTGATCCTTAGCTTGGTTTGCTTCTGCCACTTTATCATT
GATATTTTGGACAGTTGGGTTGCCATTCAACACTTGATTAATTTCAGTTAACTTTTGACGCGCTGCTTGTAATTTTTGGTTGTACGCA
TTTAACGAAGCGGTCGTCATACCTGTTGTATCCGTTGGTTGATTAATGCTTTGTTCTAATTGCGTTTTCGCAGTTTGAAGCGATGCTTT
ATCTGGTGTTAAAGCTTGACGTGCATGATCCAAATCAGATTTAGCTTGATTTGCTGCAGACGTATTTGTATTAATTTGATCTACTGTT
GGTGAACCTGCTAATACTTGATTGATTTGTTGAATCTTATTACGTGCAGCCGTTAACTTCGCATTGTATGCATTTACAGAGTCTTGCG
TCATACCAGTTGTACTTGTTTGCGTATCAATACTTTGTTGTAATTGAGCTTTCGCATTTTCTAAAGGCGCTTTATCGACTGTTAAGCCA
TTACGTGCTTGATCAAGTGCTGATTTAGCGGCATTCGCTGCTGTCACGTTTTGACGAATCGTTGCAACATCTGGATGAGAAGCCAGT
ACGCGATCAATTTCTTGAATTTTAGTTCTAGCTGCTGAAAGTTTGTCATTAAATGCAGCAACAGATGCGCTTGTCATACCAGTCGTAC
TCGTTGGCTGATCAATATCATTTTGCAACTGAGTTTTTGCAGTTTGTAATGGTGCCAAGTCTGGTGTTAATCCAGCAATTGCTTGTTT
TAAGCTATTATATTTTTCTTCTACTTTTGTTTTTTCTGCGGCAATTTGTTGGTCTGTCGCGTCACCATTGTTAATAACATTTTGTGCATT
TGTTGATTCTGTTTGACCTGCACGTTTAGCATTTTCATATGCTTGGATTGATGATTGTGTCATACCATCAGTAGTTACTGATTTATTGA
TTTCTTCATCAAGTTTCGTCTTAGCAGTTCTTAAAGCACTATTATCAGCTAAAGGTACTAATTGATTAATTGCTTGCGTTAATCGCTC
ATTGACACGATTTACATTTGTTAACGCAGATTGTACCTCTTGCACTGTTCTTATTGGCTTCTGAATGATAGCATTAGCGCTATTTTTA
GCACTTGTTAAGTCACTTTGAAGTGCACGAATCGAATTATTGTAAGCAGTAATACTTGCCGGCTTCTTACCAGTCGTTGTACCTGTGC
GATTCAATTGTTGCACTGCTTGCTCTAAGGCATGTGTATCTGCAGTTAAATCATGTTTCGCTTGGTTTAATGCTGTTAATGCGTTATC
AACACGATGTTTTTCATCTGAAATTTGTTGTGCAGTTGCATCGCCATTGTCAATAACACGTTGAGCTGCAGTTATTTCAGTTTCTGCT
TCACGCTTCTTCGCATTATAGTTATCAATACTTTGTTGCGTCATACCAGCAGTTGATGGTACTTGGTTCACAGAACTTTGTAAGTTAT
TTTTAGACGTTACTAATTGGCTATTATCTTCTTTATTTTGAAGTAATGCTTTAGCTTGATCAATCTTAGTTTGTGCTGCACGAACTTTA
GTTAGTGCGTCAGAAACTTGTTGTGGTGTTGCACGCTCATTATTAATCACTTGTTGTGCTTCTGTTTTCGCAGTATTGATTTGTTGTTG
CGCATTATGCATTGCATTATTGTACTGCGTAATTGTACCTGGCTTTTTACCTTCAGTGCTTACTGGATCATCTAAATGATTTTTAGCTG
TGATTAACTCACGTTTATCCGCTTTTGTGAAAATGGACTCTTGTACTTCTTCAGTACTACCATCATTGTAAGTTACTGTCACAGGAAT
CGTCGTTGTGCTACCACCAGCTAAATTAGTAGGCATTGCTGTGCCATTTTTAATCGTTGCAGTACGTTTATTAGCAACTTGAACTGCA
TTGTTAATTTCAGCTGCTGTTACATTTGAACCATAATCTTTCACAATTTCAGTTGTGTTGACAGTATGTGCTGCCGGTGCAGTTAATG
TACTTGGATTACTACTTACTGAGTGACCTGTACCTGCTTTCGGAGTAATTGTGATTGATGAATTTGGTTTTATAGTATTGGCATTGAA

CGTCACTTTACCAGTTTGTGCATCTAACGTTACATAGTCAGGCTTATTCGCAATTGTCCATTGATTATTCTGACCACGAACAACATTA
ATTGTCTTACTATGTTCTGCACCATTACCCACTTTTTCAGTGTAAGCAATATCCATTGCTTGTGTTGGATTAATTAAATGTCCTGATGG
ATTATTAGGCGTGATATCAATATGACCATTTTGCCAAATCTTAGTAGTCGCTTGGTTCGGTTGTGGTGCGACAACTGTGAAATCATC
ACTACGTTGCTCATCACTGATTGTTTCACCACTACCTTGTGTTGCAACAACTTGAATTGTATCAGCAGGATTGAAAGTACCTGCTGCA
ACAGTAATACCATTATTAGTTCCAGCAATACCTGCTACAGTTGCTGCAGATGCTTCTTTCACCCATGGACTTGTATTATTGCGACGTG
TAAATGTTGTCACGACACTGCTGTTACGTTTAATAACTAATTTATCAGCATACGTCGTTACGTTACCGGCATGTGTATTCACTGTTTG
GTTTGCTCCAGGTGCAATTGTAATCGCTCCTGCCGCTGTTTCAGTCACAGTTGGTTTCGCTGGTTGCACATCTTTTACTACAAATTTC
GCTGGTAAAGATGTTGCAAAAGTATGTCCGTTATAGATGACGTCATATTTTGCGTTAACGACTTTAGCCATATTAGGTTTATTCATAG
CTGCCCAGTTTGCATCGTTTGTACCAGTTGCTGCATTATTCCATTTATACGTAAATCCATCTGTTGGTAAACCATTAGCGTTTTGCAT
ATGTGCATATCCTGATGCTTGCGTACCACTTGCTAAAGTGCCACCAACTGTTGTTGTATAAGTAGTTTGAGGGAATTCAAATTGATA
TACGTTCACAGTTACAGGAACTCGTTTAGCAGCTGAAATACCTGGATATGTGACATCGACATTTAAATGTTGAACGCCTGCTTGCTG
GTTATTTGGTTGTTGTCTATTTGCCCATGCTGCTGTAATACCATTCGTATTAGTATTTTGATCAAATGTAATGTAATCAATAGCGTTTG
TACCATGTGTCAAATTTTGACCTTTCACATCACGTGATGGCGCCTTAGCATTAGCAACTGGATAAACTTTGACTGGAACTTCAACATT
ACGCGTACCTTGACCACTAGGTAATGTTACAACCGCAGTTTTATGAGTGTTACCGACTGTATTCTTCCATGTATCTGGACTATCATGC
CATGCAACCGTTGCCCCATGTGGCGGGTTTTGAATAAATCTTTCTACGTGTCCGAAATCAAAACCGTCGCCACCTTTAATAAATACA
GCGCCTTCAGTAGTTGCTTGTAATTGTGGTGTCACTGTTACTGTTGCACTGTCATTTGAATCAACAGATTCATTTCTTGTTACATCTAT
CGCTCGACCATGTTCATCTTGAGTAGTGTACGTCACATTATTCATTGTGATAGAAGACCTTGCCTTAATCACACCATTTGGTAACGCA
TCACTTACCGTTACAACTGCAGTATTACCACTACCATAAGTCGTGTTTGTAATCGTTAATGGCGTATTATCTGCTTTAAATAATTTAA
TAGATGAACTACTTAACACATTATTAATTTTAATTTGTTGGTTAGTAAGACCTGCTTTATAAGTCACAGAGTTGCCATCAATTCGCGG
TGGATCTGGTTTAACTTTTGCTAAAATCTTGATAATTTGCGTTTGGTGATTATCATAAGTAACTCGAATATCCCTTGTTGAAATACCA
ACCACATCTTTACTTGGTCCATATGAATTTGTAAATTGGAAATTCATACGTTGCGCATTTTGTGTCCAAGAATCATTAACTGGTTTAA
CATATTGTTTCGCATCATACACATCAGATACACCTGGGAACACAGCGCCTCTATTCGTCTCAAAGACATGCTTCGGTACTGTACTTA
CTACTTTATATGTTGCTGTTTTCGTAATCGGCGTTGTTTCACCATCAATTAAGATATGTGCAGTTACTGTTATATCTTCACCAATACGT
GTATTATCTTTATTTGGCGCTTGACCACTTACCCAAGTAATCGTCGCATCTGGAATGGCACTACCATTAGATAACTTAAAGTAATCTG
ATGCACTAGAACCATTACCTGCTGGGAAGTCTTGGCCTTGTACAGTGTAATGCGAATGTGCAACGATTTCTGGAATGACATGCTTTA
CAGGTACAGTCACTGTTGATGTTGTTCCATCTTGATAAGTAGCAGTTACTGTAACATTGGCATTATTTCCAGTACGACTGACATTACT
AACTGTTTTACTAGTGATTTCATTCGCACTTGCTCTTGCATAACTTCTATTTGGTACTGTTTCAGTAAACGTTAATGAATTAATAATTG
CTGTTTGATCAGCTTGTGATACTGTCGCATTATTCGAAATATTGGCAATTCTCACAGGATTAGCAGCCGTTGATGAAGTACCAACTC
GATATTTATCACGCAAAGGTTTCACTGTTACATTGAACGAAGTTGTAGCTGTATTGCCGCTTGTATCAGTTGCTAATAAATTGATTGT
CTTATTAGTTGCTGATGTCACATTTGGTGCCGTTGCAGAAACATGTTGATGGTTATTATCAACAGTACCTGTAATTTGTGATGTATTT
GGTACAGTTACAGATTGCACACCAAAGTTATCATTCGCTGTAATAGTATTTGTAAATGTTTCACCTGAGAACACTTGATGATTAGTG
TAATTACCTACTGAAATTGTAGGATTAACTAAGTCACTTGGTACAAAATAAATGTTAATAGCGTCAGTAGTATTTCCTGTATTTTGAT
TTAAATGTTCAACATATTGTTTTGGACCATATGGCGTTAAGTATAACTGTGCTTTATAAACTGCATCACTTGCATTATGTGTAGAATT
ACTTTTTACAACGTGGTCAATTGTAAATGCGCCAGCACGATTAGCTGCCGGTTCGTTTACGTTAACAACAGTTGAGTTACTATGGTTT
GCACGAGTATTTGAATTTGTAACAGGTTGCCCACCATAACCGTTTGAAGGGTTGATGATGTCTAACACTTGAATCACTTGACCATTC
AACGTAAATTGACGTTGACCATCAGTTGTCGCATTTGATTGTGAATAACCAGTCGTTCTAAAGTTAGGTCTTCCGCCAGCTTCTGCTT
GTGTTTTTTCATTACCAGTAATATTATTACGCAATCCAGGCACTGTAGTAGTCGCTTTTAGCATCGTTAAAATATCATTAGTTGTAAT
TTGTGTGCCTAATGTAGCATCATAACGATAAATTAAAGATTTATGGTCATCAGACCATGATAAACCACCATCTGTTGGTCTGTTTGC
AATTTTTGCATTCGTCCATGTCACTGTATAATCTTGCGGGAAATTAACCCAACGCAAGAAATTCATATTAGCTAGGTTTGACGCGAA
TGATTTCGTTAATCGACCTTTATTAATATTTACTGTAATAGTACTTACATTAGCACCATTAGGTGTATTTGTAACTGAAATATCACCC
TCGGCAAGCGTAATTACATCTCTATTTGCATTAATAAAAGCTTGTTTTACTTTTGAGATTTCATCAGTTGTTAAATCATATGTTGACG
TACGATACACACGTTGATCGCCTTCAGGTTGTTTCACATCAACGATTTTTAAACTAATATAGAAATCTTCAGTGTCTTTATGATATGC
ATTTGAAGCAACTATATGATATAACTGCGTGCCTGCATTCATTTGACTAACTTCTTGTGCAGTTGGAATAGTTCTATCAGATGTAATA
TTTGTAAATGTGCCACTTGGAATAGCTTGTCCATTACTTAAAGTAAAATATTGTTTAATATCACCAATACTAAATGATTGTCCTTTAG
CAATCGTTACCGTTGCAGTTTTAGCTGCATTGGCAGTTTGATATTCAGATTTCACAACTGGTTCGTATGTCATCACATTTGTAGCATC
AAGCGTTGTTGATGAGCCATCACGGTAATGTAATGTGACATTACCATTATTATCGACAGTTACTGGATCAGTTGATGCTAAATATCC
TCTTATATTTTGGTTTTTATTCATAAAGGCAGTAATTATGCTTTGCTTTTCATCATTAGATACAGCAGTCGGATTGACTACTACAACTT
TCTCAGTATTTCCTAATACAATCGGATGAGCATCTTCACTAATTTTACCTACATGAATTGACACATGTTTAGATTGACTATCATTTGT
CGTATTATTGACATTGTCTGTCGCTGATACTTTAAATGTAATATCACTGTTAAATGCCTGATTCATAGAGACTCTACCTGTAATAGCT
AATGAGCCGTTTTTGTTGTCGGATTTAGTTAAATTTGATGTCCAACCACTTGGCAATGTCGTAATAGTTGTACTTTTTAATCCAGAAT
GTCTATCTGAAACCCCCATAGTAAAGCTAACTTCATCACCACGATAATATTTGGCATTTATTCCAACAGGATTATTGATTGTTGGTGG
AATGTCATCTGGCAAAATAGTAACAGCATTACTCATATTAGATGCAACTACTTTATTATAATAAACTACTGCTCTAATTTGTTTACCT
ACTGGTGGTATGAATACAACATAATCTTTTGGTTGTATAGTAATTGTGTTATGGACCCCATCAGGTGTACCAGTCGCAAGTATCGTA
TAACCAGATGGTATTTGATCTGCCTCACTACCACCATTCGTAGTTTGATTTGTTGGTGCAACTAAATAAACCAATGCAGATGGATCC
AACGGTATTCCCGAAATATTAACAGGCACTTTTTGTAATGCTGTACCTCTTAATTGCTCAGCTGTCGTTGTAATTGTTGGTGTATGTG
GTTTAACACGAATTTCGAATAATTGTTCTTTACGTCCAAAACGTATCGTACCAAAGTAATCCCCTGGTAATAAATTACCAACAAGTT
CAATGTTACGTTCTCTAGCATTATTAGTTCTATTTGTCGAGTTTTCAATGTGTAATGTACGCGGAATACCTGTAATTTGCCAATCTTTA
TCTGTAAAATTCTCATTACTTGTTAAATTAATACGTAATGGTTGAATAGATGTTACGCTACCACCTAAATTCACGGAAGCATTCCCTT
GGTTACCTGCATATACATAGAAAACTGGATGCCCAGCTTGTTTTGAAAATGGTTTTTCAGCAATATTTTGAGTATCGTCATTTTTAAA
GAATTTAATATTATGTTGGATTTCAGGTAATACCGTTAAGTTAACAGGAACTAAAGTTTCACTATTACCATAACCATCAGGATAGTC
GAAACCAATCATTAATCTTATTGGGTTTCTAGTTCTTGCTGCTTCTTGTAATTTTTGATCAGTAAATAATTGACTTAATTGATATTCAT
TTAATTCTTGAACGCCCCCCGGTCTAAAAGCGTTAGTATTTGCATTAGTTGGGTAAAAACCTAACACTTTATTACTCGGCGTCTTAAC
ATAACTTAAAGGATCATCAACAAAATCATCTATATTTAGGTTTGTACTTTCTAATACCTTTTTCGTTTGTACATTTCCTTGACTCACTT
CTACACGTTTAGTTGTACCTATGCGATATGTACGATTAACCACTTGTCTTAAACCATTAAGACGTTGTTGATGGTCTTGCGTTGATTC
GACGTATAGTTGACTGTACATAAATAATTGATTGTACTCTAAAGCACGCCCACCTGCTGCATAATACAATTTATTAACAGTTGGACC
TTGTGTTTTAAATGAAATTGTATAACTTGCATCACCTTGACCGATGAAAGTATAAACTTTAGGAACACCCGGTGATTCAGCAGGTTT
TTGACCATTAATATATTCAAAATTTTGTCTACCAAATGCTTTAGTAGCAGCTGTTGCTCCAGCTTCACTAAAATAATCACTCGCTCTA
GATAAATCTTCTAAAGAATAAATATGGACAGTTGGCCAGTCATATATTACTTGGCCACTTCTATTTCTTATTTTAAAGTCATGTGAAC
GATGAGGATCATTTACTCCATATTCCCACATTTGTTGAAGTGGTTTATTTGCACCTGCTCCTGCTCCATGACTCCATTGTACATTTGTT
CCATCTGAATTAACTGTTACAAAGTCAGTTCTTCCTACTGGCACTTGGTCTGCTGGTAATGCAAACCAGAAAATCATATTTTGATGTG
GAGCATGTCCTTTATTAAACTTAATAACCCATTGGTACCCGTCTCCAGTTTGTTTCGCTGTCATAAGAGCAATTTCCTTTGAGTCAGA

GGCCTTCAATGTATTCCATGGTGTAGTTGGTAAATTATATGGCGCCCCTTTACTAGAAGCATTGATATATGTACCAACAGGATAGCC
CGAACCATCATTAGCATTTTCAACATAATATGATGAATTTGCATTTTGATAGTCTGATACTGATCTAGGCTCTGCAGCTCTTGATTCA
ACCGAACGCGTTTGAATTCTATTTGAACGTCGGCTAGTCTGTTGACGTTGAGGCATTGTACGAATTAAAGCAACAGCTTGTTCCAAT
TCTTTGTATGCAAGTTGCAAGTCTTTATTGTTTATTGGATCATTACTGCCTAATAACGTATTAATCTTATCAAAAATGCCTTTTACATA
GTTAAATGTGCCAGAATCTGCATTTTCACGGTCAATAAAGCGATAATCATGATAATTTGCATCAAAAAATGCTTGTAATTCATTTCG
GTCATGTTGTACGTTACCATTATCAGATGAATCTGAAGCTGTTACTACATTTGCTTCATGATTTTCTACATGAGCCGCTCTATTGTCG
TTTCCATTTTTATTAGCAGATTGACTTTCGTTTTGTTTATGCTTACTTTGTTCTTTATCTGGTTGTGTTGTCGCAGCCGTTGCCGAATCT
TTCTTTGTATTTACATTTTGAGATGCTGGTTGTTCATCATTAGTAGTAGATGATTGCGCTACTTTTTGATCTACTAAATTAGCTTGACT
AATATTTGGTTGCTCATTTTCATGAGTAGCAGATAATGATTGACCATTTTGTGAATTTTGAGAATTTTGTACTTGAGATTCTCGATTC
TCAGTCTGTTCATTATTACTTTGTTGTTTCTGTTTAACCACGCTTGCTGGTTGATTTGTTTCAGCAGCATGTGCTTGTGATGTATTGAA
TCCTAAAAATACCAATGTCGCAATGACAGTTGAAAATGTACCAACTGTATATTTACGAATACTAAACTTTTGAATTTTATCACGATA
ATTCACAGCTATTGTTCACTCCTTATCTTGTTGTTATGTCTCTGTTGTGTTGTGAATGTATTTACTTTTGATTGCTTGTATATTGTAATG
CTCATTCCATTCTCATTTAAAAATTTAAAACGATGACGATTCAATACAATTTCGCAATCTATCCAAAAAGTATTGAAACACATTACA
ACTATGTGTTGAACAATTTATTTATGCTCCAGAATATAATAACACAAAATATATTGTATGGTAAATTGAATTTGTGACAATTTATAAT
AATTATAAATAAAGTAGCGTAAAAGAGTAATAAAAAACCAATCCCTATGTTCATTTTAAATAATGACACATAAGAATCGGTTTATA
TTTTTGTTTAATATTGTAGCGACTTGTATGATTTCAATTTAATTCTCAATTATCATAATTGAGTGTCGTTTTGTTTAGGCACAAGTAAC
AAAATGATAACGAATGATAATATTCCCATACCTGCATTTAACCATAATGCAATCATTGCACCTGTATAAATGTTTGTCATATTTGAT
ACGATTGCATATACTGCACCACTCAATGCGACGCCAAATGCTCCACCTAATGCAGAAGCCATTTTATAGATACCTGCAGCAACGCCT
ACTTTTTCTAACGGTGCATTTGCAATTGCTGTATCTGTTGATGGTGTAGCATATATCCCTAGTCCTAAACCAAAGAATAAATAACCTA
TAATACAACAAATGACATACAATATTTCTGGCAAGAAAGTTAATGAAATGAGACATTCTCCGACAATAAGAACTCCTGTTCCAATT
AACATTGGTTTCTTGCATCCGAGTGTTTGAAGTAACTTTTCACCAACACGAATCATAATTAGTACCATTACTAAATAAGTGATTGAT
AAACTTCCTGCTTGCAATGAAGAATATCCTAAACCTCTTTGAACAAATGTGTTGGCTACTATTAATGTTCCTGCAACACCATTTAACA
AAAAGTTTGAAGCTGTTGCACCTGTGTAAGCTTTATTTTTAAATAATTTAAAATCGATTAAAGGATTTGTAGCACGCTTTTCAAGAA
CTATAAATAAACTAAAAGATCCAATTGCAATAGCTAATAAAGTAATAAAAAGAAGTGAGGTTACACCTAATTCTGATCCTTTAGTA
ATTAAAATATTTAAACTGAGGAGCATAATGACTAAAAGAACCAGACCTTTAATGTCAAATTTATTTAGAGAAATCGATTTAGATTTA
GTTTCAGGTGTGCCTTTAATAAGAAACAGTGCAATTAATGAAATTATAATTGATAGGATGAAAATCCAACGCCAACCTAAAAGCGT
TGCAACTGCACCTCCAAAAAATGAACAAACACCAGAGCCGCCCCATGAGCCAATTGACCAATAACTTAAAGCGCGTTGTCTATCTT
TCCCAATGTAATATGACTTAATAATAGACAAAGTTGCAGGCATAATACATGCTGCTGAAAGTCCTTGAATTAATCTTCCTATAATAA
GTAATAAAGGAATATTTGAAATAATGATTAATAATGAACCTAATATATTTAAGATAATACCAATGTTCGTGAGTTTAATTCTGCCAT
ATTTATCAGCAAGACCACCTGCTCCTACTACAAACATTCCTGAAAATAAAGCAGTTATACTAACGGCGATATTAACCGTTCCAATAT
CTGTATTGAAACTATCTTCAAGTATTGGTACAACATTAACCAATGATTGTGCAAATAGCCAAAACGTTATTACACTTAAAACAATTC
CTATTAACAACTTATTATTGCCTTCAAATGCCTCTCTTGACGGCTTTTCCATCTCTATTTGCCTCCCTATACTTTTGAAAAACAGACAA
ATTCCATAACAGAAATTTGTCTGTTCGTTAGAATAATTGAATGGTTTTAATTTTAATGTGTAAAACTTTAAATTTATTAGTAGTCCAT
CCATTTATTCTCAATGCTAGTTATCTTACCTTATGCTGCTTTTTGTTTCTTCTTATAGTAATAAACTGGTATACCAAGTGCTGTTATTA
GAATTCCAATGATTGCTAATATAAATTGTGTAAACAGTGTATTAATTAATACAAAAGATCCTGCCAAAATAGCAATTAAAGGTATG
ATCGGATATAACGGTACTTTATATGGACGCTCCATATTTGGTTCACGTTTTCTTAAAATTATTACAGCAACAAATGACATACAATAG
AACAACCAAATAACAAAGATTAACATGTTTGTAATTGTATCAAATGCTCCCATTGACATCATGATGATAGCGATTATAAGTTGTATA
ATTGCGCCAAACCATGGTGCACCAGATTTTGTTAATTTTGCGAATAAGTGGCTAAATGGCAATAATTTTCTTTCAGCCATTGCATATG
GTACGCGCATACCAGTCATAGTATAGCCATTGATCGTACCATAAACAGAAATTAATATACCGATTGTAATAATCTTACCGCCATTTT
CACCAAATAATATTTTTGATGTATCTGAAGCTGCATTTAAATTACCAGCAAGTAGTTCTATTGGCAACGTTAATAAGAATGTAGCGT
TAATTAATAAATACACAGCCATAATACAACCGATACCAACTGAAATCGCTAAAGGTAAATCGCGTTTAGGATTTTTAAGTTCCCCCG
CAACATTTCCTACATGAATCCAACCATCATAAGCAAACATAGTTGCTAATAAACCACTACCAATTGCTGTAAAGAAACCATTTCCTG
AATTACCTGTAGTTGGAATTAATGAAAAAGTGATATCTCCAGATTGAAAAATACCAAAAATTACAATAACGATGATTGGAATCAGT
TTAATTACTAAAGTAACTGATTGTAAAATTCCGCCTGCTTTTGAACCTAGGAAATTTATCAACACAATAGATAACGCAGATGCGATT
GCTATTGGTATTAACGAACCTATAGATAAATGGAATAAATTAATTAGCTGTGTCGCAAATACGATAGACAATGCTGCTACGTTAGCT
GGAAAATAAATAAATGATTGCGCCCAACCTGATAGGAAGCCCCAGAAATCACCGTATGTATATTCTATATACTTCGTTAAGCCACCT
GTTTCAGGGATTGCAGCAGCAAGTTCTGCTGCTGTTAACCCCGCACAAATGGTAATGATGCCGCCTAGGAACCATACAAACAAGGC
CATTCCTGCTGTTCCTGTTACTTCTGTTACGTTTGATATTTTAAAGAATACTCCTGAACCAATAACTGTCCCCATAACAATAGCAAAC
GCTGAGAAGAAACCTATATTTTTTTGTAATTCTTTACCATTTGACATAATTACACCTCATTTTTAAGATTAAATGTTTTAACCTACCA
CACCCTTGCTTGTATCTGCAATATTCAGTCCATGTTCAATGACACCTGAAACTCTAGTTAAGTCAACATTCCCGCCTGAAACTAAAG
CAACAACATTTTTATCTTCAAGCCATTTATTGTTTATTTTTCCACTTAAAATTGCAGCTGTTGGTAATGCGCCTGCACCTTCAGTAATA
ATTTTGGCACGCTGCATTAAATCTTTCATAGCATGTTCAATTTCTTCTTCAGTAACAAGAATAAATTCATCTACTAAATGTTTAACTA
CTTCATATGTTTGTTCACCAGGAACTTTTACATCACAACCATCTGCTATTGTGCTATCCACTCGATGTTCAGTTAAATCTCTCTTATAG
AAAGACTCAGCCATACCATGAACATTCTCAGATTGAACACCGATAATATGAATTGAAGGGTTAAATGATTTTAATGCGGTGGCAAT
ACCTGCAATTAATCCTCCACCGCCAACTGGTACGATGACTGTATTCACATTCCAAATATCATCTAAAATTTCTAAACCAATTGTTCCT
TGGCCTGCCATTACAAACTTATCGTCATATGGATGAACGATTGTCATGCCATTTTCTTTCGCTAATTCTTCCATATAAAGTCTAGTTT
CGTTAAAGTTTTTACCTTTTAAAATGACTTTTGCACCATATCCTTTGGTAGCATTCTGTTTAGCAATTGGTGCTGTTTCTGGCATCACA
ATCGTTGCATCAATACCTAATAGTTTTGCTGTCAAAGCTACACCTTGTGCATGATTTCCGGCTGATGCGCCAATAATACCTTTAGCTT
TTTGTTCATCTGACAAATGATTAATTTTATTGCTAGCACCTCTAAATTTAAATGAACCTGTAAACTGCATATTTTCAAGTTTTAAATA
TACGTTACCTTTAGTGATATTTTGACTTAAATACATAGATTTAATTAGTGGTGTTCGACGAATAAATGGTTTAATGCTTGCTTTAGCT
TCTTCGATATCTCCTAAACTTACAATATGTGCTGTTTGTAATGTAACTGTGTTGGTTGTCATAATAATTACATCCTTCCGTTTTGTAAA
TTTTTAGTCCAAACTATAATGCTATATTTAAAATATTCATTTCACTCAATTTCATTTAAATTATTATCTATTCGATAACATTTAATATT
TCTTTATAATCTAGGTCATGTGATGAAGCTAATCCTTGATTTGTCACTTGTCCTTGGTAAATGTTTACACCAGTACTTAAGGCTTCAT
TATCTTTAATTGCTTGTTCTAAGCCTTTGTCACAAATTTCTAATATATAATCAATATTTCCTTGTGCTAATGCCATTGTTGAAGTTCTT
GGGACTGCTCCTGGTTGATTTGGTACACCATAATGAATCACACCTTCTTCTTCATACACTGGATCAGAAATTGTAGTTGGTCTAATTG
TTTCAATAGTTCCACCTTGGTCAATAGCTATATCGATTAATACTGAACCTTTTTTCATTGATTTAACCATCTCACGAGTAACCAATTTT
GGCGGTTTCGCACCTGGAATTAAAATTGTAGAAATAAATACATCTGCTTTCTTAATTTGTTCTGCTAAATTTTCTGGTGTTGATTTGA
CTACTGTGACATCTTTTTCTGCATACATATCTTCAAGATATTTAATGCGGTCATCGTTTAACTCGATAATGATTACTTTAGCATATAG
TCCCAAGGCAACATTTGCTGCATTTGTTGCTGCTACGCCACCACCGAAAATCACATATGTACTACCAGGTATATCCACATTTTCATGT
ACACCAGTCACTAAAGTACCTTGACCACCATGTTGTGCTTCAGAGTAGTAAGCTCCCATAATTGCTGAGCGTTGACCTGCTATAGCA
CTCATTGGCGCTAATAATTCTGCTTTTCCATTTTTTATAATGGTTTCACCACTAATCGCAGTTACACCAACTTCTTGCATTTTTTCTAC

TATTTCTTTTGAAGATGCTAGATGTAAAAATCCCCAGATAATTTGATTCTTTTTGAAATATTGATATTCGCTTTCATGAGGTTCTTTTA
CTTTGATAACAAGATCAGCTTCCCATGCTTGTTCGTGAGTTACGATCTTAGCGCCTTCTTTTTCATACATATCGTTAGAAAATCCTGA
ACCAATGCCAGCATTTTTTTCAACAATTACTTTATGTCCTGCATCCGTTAACTTACGCACATTTTCGGGTGTGCAAGCTACACGTCCT
TCACCTTGTTTTAATTCTTTGACTACTGCAACTAACATTTGCAACACTCCTCTTTCGTTTGTGAATAATTTCACAATTATTTTATCCTTT
GGTTATGAATATGTAAATACCATTAATTAATTATTCATATTTTATCTATAATTTGTTAATAAATATTTAATATATTATTCTTATTCTTA
AATATTATGTGCAATTATACCACTATATTTATTTCTTATACTCCATTTGATTTATAGTTGTATTAATACATTGACTCAAAAACTAATTA
ATCAAATATGTTTTTTAGATTAATAAAGTTGTAACTGTACTATTTTGTAGTGTATGGTAATTTATTTGGATGAAATATAATTCTTTAT
ACTCTATTGATTAATCAAATATGTATCTATCAAAATTCGGCTTATTTATTCACTCTGACATATCCAAATACGCAAAAAGACTATTTCT
ACCTTGTATCGATAGAAATAGTCTTTTTATACTTAATTGGTTTAATTTTTCATAAAATCTTATACTTTTTAAAAATGGGATGAAATAT
ATTTTCCTGAAACATGTAATTCATGTTCGTTACAAATTGATAAAATATGATTAAGCGTTGTTGCAAATGACATTTTCTCAAAAAGCG
CTTCACTATCAATTGGTACTTGTGTGTGAATTTCAGCAAGTCGCTTCGATAAATATAGTTCATCTAAATTGTCATTTATCTTATTACGT
TGACCAGCTGATAATGCATCAATGTTTCAACCACATTTTCAACGCTTTGATATTGCTGAATTAACTTAATAGCCGTTTTCTCGCCAA
TACCTTTAACACCTGCATATCCATCTGCTGTATCACCCATAAACGCTTTAATATCGATCAGTTGTTGTGGTTCAAGGGCATATTCTTC
GTTAAAACGATGTAATGTATATCTATTATAAATGTTAAAACCTTTTTTAATTAGCCAAACTTCAACATTGTCATTAATACATTGCAGT
AAATCTTTGTCGCCCGTAATAATATAGACATCGTTATCAGTTGAATATTGTTGTGCTAATGTACCTATAACATCATCCGCTTCATAGT
TTTTAACGCCAATATTTACAAAGCCAAATTGCTCTGAAATTTCTTTAACATAATCAAATTGTGGTATCAATTCTTCTGGTGGTGCAGA
ACGATTTTGCTTATAACCGTCAAACATATCATTTCTAAAAGTTGATTGTCCCATATCCCAACATACAGCTACATGTGTAGGGCGTATT
TCATGTATTGCCGAAAAGATATGACGCACAAATCCTTGTATTCCATTTGTAGGTACACCTTGTGAATTGTACATAAATTGTTTATGAA
GACTTGTAGCGTAGAAATGTCTAAATAATAGTGCCATACCATCTACAAGTAATATTTTATTAGGCATTTAAAACGTTCCTTCTTTCTT
AATATAAAATAGTTTTCAGTTGTTGATGTTTATCTTTCAATTGATTGATAAGTGTTGTATCTAAATCAAATGCTAATAAATCGTTTAT
TTGCGTTTGAATATTAGCTTCAAAACTTTCAAATTGCGATTCAGCTTCTACAGCCATTTGCTTAACATATAATTCAAGTTGTTGCCTC
AATAATCCCATTTGTGGTTGTAATAATCCTAACGTACTTTGACAAATTGATTCATGTATATCTCTTTGCCCATTTGGATTCAAAATTTT
ACGTTTTGTTAATTGTTTAGGCAATGCATTTAGCATATCGTTGAAATCGATGTGCAATAATGGTTGCTCTATATTAGCTGATTCAAAG
TTAAACTGAGGATTAATAATGACATGTAAATCTGCTAATTGTTGAACGATTGGTGCAATTTGCTCAGTGAGTTGCTTATTAAAGTAT
TTTTTTATACGTTCTGTAATTAAAGATTGCTCTAAAAACAATCGTTGATGAATTTGATCTAAGTATACTCTCGTAGACACTTTCTTTTC
TTCATTAAAATCACTATTTTGCGTCATTTGAGAATTAAACACTGATTTAACATCGTCAAGTAGTTGTAGTTTTAAACGGGCATTTAAA
TGATAAACTTGTTCTTCAACTTCGTTGTCTGTATGTTGTAACGTCGCATCAATTAATTGATGTTGTAAACGCTGTTTATCTTTATAATC
TGTTAACCTTTGTTGGCGACGTGAAATATCAGCTTTATTTGTTTCAAATTCTGTAATCATCTCTACATAAGAACGATCCATTTGTTGA
AGCTGATGAATCATTTGTTGTTCTAAAATTGATTTAGATTCAACATCAACAAATTGTTGTATGCTTTGTTTTAATTGATCAATGCCCT
TATCTTCAGCTTGCAATGCATTTCGACTTGATACAGCAAAAATGTCTGATTGTAAGTGTACTTGTCTTAATGCATCTGATACATATGT
TTCAACTGCTTCAAGATCATCTTGACTTTCTGCTAAATCAGCAGCATTAATTACCATTTTAAATGCTTGGTTTTCATTCAACTGGTTCA
TATCTTTCATGTGTTCTATAAACGCTTTGTCATTATCAGTAAATGAATGATTAAAATAACTTACATACAATATTAAGTCTGAAGAAGT
TAAAATTTGTTCGGTTTCATTTGTATGCCTTTGGTTATTTGAGTGTAGCCCTAATGAATCAACAATTATTTTACCTTTTAACCAATCAT
GCATTAATGCAATGTGTACTGTTTTAACAAATGTTGCGTATTCATCTTCTGCACTCCACTTTTTCAATTCTTGTTGATTAATGGCATGT
TTTTCACCATTTTCCAACATATTGACATACAAATTATAATGTTTTTCAACTGCATGTACAAAAGCGAGTTGATTTTTATTTAAATGTG
ATTTTAACTTTTCTAAATCTGAATTAATAAAGTCTTCTATAGTTGAAAAAGACATATCTTGGTATTCAACTACTGCATTAATTTCATC
TAATAATTGCGATTGTGATTTTAGCGTTATATAACTCTCGTCTCCATAAGATATTTCTGTAGTAGCTGCTGTAGTAGGATTTGGAGAA
CTGACTAAAATCTGCTCGCCTAATAATGCATTTATCAAACTACTTTTACCAGCACTAAATGTTCCAAATACACCTATTTTTATTAATT
TATTATCTATACGTGTTAATGTTTCGTGGATATCTTGCTTTGTACGCTTGAACAAAGGCACATCCGAAATTATATCAAGCGCTTTTTG
AATATCTATAGACATATTTGTTGTAGCTGTATTTTGATTTAGCTGCTGATTATCACGATTATCCTGAGTATTATCAGTAGCCACTTGA
TATGTTGTCTCTTGTCGACCTATTAATTTATCTAGAGATTCATCTAAATGAATATAGTAGTGACGATAATTCTTAGTCGTCAATGACT
GACGCAGCTCATTTAATTCTGTATAACGTTGATATTCTTTTAAATCATCACTTTCTTCTGTTGGTAATTCATCTGCCTGTACATTTTCT
ATTATTTCTTTAAAAATTGGTGTTGATTGTTGTTCAACATATTTCTTAATGGCTTTAACCACTTCGTCTGAAAATGTAAGTACATAAG
TATTGCTGATTGATGTTTGTGGTTGATATAAATCTTCAATCATTTCCGGCTTAACGTCATAATGCTGATTTAATACTTTATCTGAAGCT
TCTTTTTTATTGATAAAACGCGTTACAAATGACATATCTTCTCGCATTGGTTGACGAATTTGTTGATTAACATGTTCTTGTAACGCAT
CTGTCGCTGTTAATAATCGCTGTTGTTGAATTTGTAGCTTTTTCTTCTTTTTATTAAAAAACCCACCAACATTAAAGTCTTGAGACAT
ACTTTCCAAATAACTTCGTAACATTTCTCTCATATTATGCGGCATAATGTATGCATTTTCTAAAATATTTTTACGCTTATTCTTTAAAA
ATGCCATTAATTCATCTGGATTATTTAAAAGTTGTGCCTCTTCACTAATTGCTTGATGTTGTTGACTATTTAAAAATGCTTGTTCAAA
CTCCGCTTCTTCGATACCTAAATCTTCTAGTACTTCCTGAATTTCAGACTGAATGTAATCTAGCTGAGCTTCGGTAATGTATTCAACC
GTTCTTGATGTATAATCCTCTATTGTCTCTCTATGTTGATCTAATGAAATTAGATAACTTGATAATACTTCAAGTTCATTTTCAGGGTG
ATCAAATTTAGATACATAAAAGGTGCGTTCTAATTTAATGCCCCAATCTGCAATTGATTTTTCAACTCGTGATTTAAACGTAGAGAA
TGACAATTCATCGTCTTGATGCTTGTCAATTTGATTAATGATAAACACAACAGGTATTCCAACATCATTTATATGCTTCATAAACTTA
AAGTTAAGTTCAGATTGAACGTGGTTATAGTCAACCGTATAAAATATCATATTACTTGTATACATATATTGTTCTGTTATTGACTGAT
GTGATGCAACATTTGAATCAACACCTGGTGTATCTTGCAACGTAAACCCATTTTCAAATTTAGCTGATTGAAAATTAATTTCTACAG
ATTCAACGTCGACATTTTGGCGATTCATTTCCCTTACTTCATCATAATTAGATAATTTGGCATACGTTTGATTCGGCAAATTAGCAAT
AATATCGTGATTGTCTGAAACTGACACAATAGCAGTATTACTTGTCGTTGGTACAGGAGAACTTGGTAAGATATCTTGTTCAATTAA
TAAATTTATCAGTGTCGATTTACCTGCAGAAAAATGTCCAACGAACGAACATGTATATTGCTGCAAATATACTTTCTTAATTACTTG
GTTAATTGTATGTAAAAGTGCTTCATTTCGCGACTTTTCAACTTCTTTTTTTAATTTATATAAAAGATCTAATTGTTCTTTATTAATCA
TGTTACTTTCCCCTTTGGTTCGTTCACATAGAAATGCTTGCTTGTTACAACGAATTCATACAATATCGATGTCCATTTAAACATAGAT
CGTTAAATGGCTCGCATTGTATTATGTTTAAATTTTATCATAGCTGCTTGATAAAACATACGAATAATCAATTTTCTTCAAACATTCC
AACTTAAAAGTTCATTTTTCTAAAAATCACAAATCATCGTTAAATTTTTCTAGAAAATAAATATTGTCACAGTTTGTTCACACACGTC
ACAGAATTGACACCGTATAATAGCCAATTGGGAGGGAAATATGAAAAATACATTTAATCCATTACAAAGATTACATTTCTATGCAG
CAATATTTATTGCTCCACTGTTAATCACTTTAACCATTTCGGGCATTGGTTACTTGTTCTTTCCAGAAGTTGAAAATAATATTTATAA
GAATGAGTTTTTTGGTGACAGTGATGTAAAAACGCATCAAACATTAAATGATGCAGTACATCAAGTTGAACAACAATATGAAGGAT
TCTTTGTAAGTAAAGTTAGCATACTTGATGAACCATATAACAAACGAATTACACTGAGTGATATGGTAGGAAATCAACGTTACGTCT
TTCTAGATCATAACAATCAATTTGTTGCAGATCAAAATGCGAAACATACGTATTCTAATGTGATGCGAAGTATACATAGTTCTTTGT
TTACTGAAAATACTATTATTAATTATTTAGTAGAGTTAACCGCATGTTGGACGATATTCATGATTTTATCTGGTACTTATTTACTCATT
AAGAAGCATTTAATTTCTAACAAAAGTAAGGCACTTCGTTGGCAAAAGTGGCACGCAATGATTGGAGTTATCATTGCAATTCCAGT
ATTTGTATTAGTTTTAACTGGATTGCCATGGTCTGGTTTTATGGGCAGTAAAATTGCCGGTATGATGGACACAAACGGTGACCTTGG
TCAAGGTGAATTAGCGATTAATCCACCTAAATCAGATTTGAACGAATTACCTTGGGCTACACGTAAAAATAAACAGCCAGCTTCATC
CGAAAAAGGTTCAAGTGGTCATCATGGTAATGCAGCAATGCCTCAAACCAAATTAGATTATCAAATATCTATTGATAAGGTCGTTG

AACAGGCGCAAAAATCTGGTATTAAAAAGCCGTTTTCAATCGTATATCCAAGTGATAAAAATGGTGCCTTTATTGTATCTAATACTA
GTAATTCAGGTGTTACTGGGCTAGATGTATCACCTTACAAGGAACAAACACTTTATTTCGATCAATATAGCGGTAAAAAGCTAGGTA
CGATTAAATATGATGACTACGGTATTATTGCTAAATGGTTTACATGGGGCATTCCGCTTCACGAAGGTCATTTATTCGGCATTTTAAA
TAAAATCATTAATTTATTTGTATGTATCGCTTTATTAGTAGCCATTGGCATGGGGTTTGTCTCTTGGATAAAGCGTACAAAAAATACT
GCAGTAAAAGTACCACATCGCGTAAAAAAACCAGCATCTATATCACTCATAATATGTTTAATTGTATTAGGATTATTAATGCCATTA
TTTGGATTATCACTTATCCTTGTATTTATAATTGAATTAATATTATATATTAAAGATCGTCGTGCTAAACAATAATGCACTTAAAGTT
TTGAACTGACGAAATTTACAAAATGATTCTCGTCTCTCTAATTACTTAAAACGGGTTCTATAATAAATCATACTGATGGAAAGTTTTT
ACTTTTTATCTGTCAGATTTTTTGAATTTGAAGATAAAAAAGCATCTAAAACGCATAAGAAGATAACTCTACTTCTTCAAAATTACG
TTATAGATGCTTTTTTATTAATTCATATGCATGTCGTGAATTTTCATCATTAAACCGTGTGGGACGTCATCATGTTTAACAATCTCTTG
TTTATAAAATGTCTTCCCATCATCTTTAGAAACTAATTTTACAAAGTCTCCTTTTTTCGGCTTGAAATCATTAGATGGTTCTACTTTGA
TATTATGTTTGACTAATCCATCTTTTTCACTGACAACTTTTTCTGCATTTGTATTACTATTCATATAACCAAAATAAGTCGTTTTTTGA
CTAGAAAAAAATCATTAAACATACAACGGCAATACCAATAACAATCAATGCAATAGATAAACCTAGACCCATTTTTTTACCCATTTAT
AGACTCCTCCATTTTTCAAAAATACCTATTATCATGAGAACTTAATTATTACTAATTTTAGTCACGCATTAGAAACACTTTAGTTTTC
AATAGTTTTACGTTCTGTCTTTTTACCCCAGTATTGATAATAAGTACATTCTATATATCCATTAAATAACTTACGTCGCTTTGTTGCTT
TACGATCTACTAAATATTCAAATTCTTTATTACTTGTTAAAATGTATGTAGATAAAAATGGATGTTGTTTCATTAGTTTACCAATATA
ACGGTACATTTCTTCAACTTCTTCACGATCACCAATACGTTCACCATATGGAGGATTTCCAATTAACGCCACCGGTTCTTCTGTATCA
ATTGTTAATGTATTGACATCTTTTACACTAAATTTAATAATATCAGACAACCCAACTTCTTCAGCGTTACGCTTAGCAATCTCTACCA
TTTCTGGATCGATATCAGAAGCATATACTTCGATTTCTTTATCATAATCAGCCACCTTATCCGCTTCATCACGGTAATCATCATAAAT
ATTTGCTGGCATGATGTTCCATTGCTCTGATACGAACTCGCGATTAAAACCAGGTGCGATATTTTGAGCAATTAAACAAGCTTCTAT
AGCTATTGTACCCGAACCGCAAAATGGATCAATTAAAGGTGTATCACCTTTCCAGTTTGCAAGACGGATTAAACTTGCTGCCAACGT
TTCTTTAATTGGTGCTTCACCTTGTGCTAATCTATATCCACGTCTGTTCAAACCAGAACCTGATGTATCGATAGTCAATAATACATTA
TCTTTTAAAATGGCAACTTCAACGGGGTATTTCGCACCTGATTCATTTAACCAACCTTTTTCGTTATATGCGCGACGTAATCGTTCAA
CAATAGCTTTCTTAGTTATCGCCTGACAATCTGGCACACTATGTAGTGTTGATTTAACGCTTCTACCTTGAACTGGGAAGTTACCCTC
TTTATCAATTATAGATTCCCAAGGGAGCGCTTTGGTTTGTTCGAATAATTCGTCAAACGTTGTTGCGTTAAAACGTCCAACAACAATT
TTGATTCGGTCTGCTGTGCGCAACCATAAATTTGCCTTTACAATTGCACTTGCGTCTCCTTCAAAAAATATACGACCATTTTCAACAT
TTGTTTCATAGCCTAATTCTTGAATTTCCCTAGCAACAACAGCTTCTAATCCCATTGGACAAACTGCAAGTAATTGAAACATATATG
ATTCTCCTTTTATACAGGTATTTTATTCTTAGCTTGTGTTTTTTATACATTTCCAACAAATTTAATCGCTGATACATTAACGCATTCGC
TTATTATTTTAAAACAAGGCAGTGTCATTATATCAAGACAAGGCGTTAATTTTAAGTGTTCTTTTCATGAAAAAAGCTCTCCATCATC
TAGGAGAGCTAAACTAGTAGTGATATTTCTATAAGCCATGTTCTGTTCCATCGTACTCATCACGTGCACTAGTCACACTGGTACTCA
GGTGATAACCATCTGTCTACACCACTTCATTTCGCGAAGTGTGTCTCGTTTATACGTTGAATTCCGTTAAACAAGTGCTCCTACCAAA
TTTGGATTGCTCACTCGAGGGGTTTACCGCGTTCCACCTTTTATATTTCTATAAAAGCTACGTCACTGTGGCACTTTCAAATTACTCT
ATCCATATCGAAAGACTTAGGATATTTCATTGCCGTCAAATTAATGCCTTGATTTATTGTTTCATCAAGCACGAACACTACAATCATC
TCAGACTGTGTGAGCATGGACTTTCCTCTATATAATATAGCGATTACCCAAAATATCACTTTTAAAATTATAACATAGTCATTATTAG
TAAGACAGTTAAACTTTTGTATTTAGTAATTATTTACCAAATACAGCTTTTTCTAAGTTTGAAATACGTTTTAAAATATCTACATTAT
TTGAAGATGTATTTGTTGTTGTATTATTCGAAGAAAAACTTTTATTGTCCTGAGGTCTTGATGTTGCTACACGTAGTCTTAATTCTTCT
AATTCTTTTTTAAGTTTATGATTCTCTTCTGATAATTTTACAACTTCATTATTCATATCGGCCATTTTTTGATAATCAGCAATAATGTC
ATCTAAAAATGCATCTACTTCTTCTCTTCTATAGCCACGAGCCATCGTTTTTTCAAAATCTTTTTCATAAATATCTTTTGCTGATAATT
TCAATGAAACATCTGACATTTTTTCCACCTCATTAGAAACTTTGATCTTCAGACCACTGTAAGTCATTGATGAATGCTGTTAATTCAT
CGAACGTCACAATATCACAAGTATAGTTTGTTTTATCCATAAAATCAACTAACATCTGCTTGAAGAACTTAGGACTTGCCTCTTGTTC
CTCATCATAAATGAGCAATGTTTGATCCGAATGTTCAAGCATAAATTGATCTGCTTGTTTAAATTGAAAAGGACCCTGATACGACGT
ATGAAAAATACTATCAACATAATCTGCATGCTTAATTATGTTGGCATACTTACTTTGATTATGTTCATTCCACTTTTCTGTATGTCCTT
GGAACGGTGTAATTACAGCAAACTTTAACGAATCATATGTTCGTTGTAATTCAATAACAACTTCTGCAGTCCATAATTCTATACCCA
TTTGCCCTTGTATTAACACCCATTCTAATCCTTCATCCAACAGTTGTTCAATTTTATGTTTTATAAATTGTTTTAAATAATGTACTTCA
GGTGCGTCATCTTTAAAAATGTTTAATTCGAATGATTTGTAACCTGTTACATAAACTGTTTTAACCATGAGTACCCTCTTTTGTTACG
CCATCTAATATATTTTGTAAATCATATTGAACACTTTTAAGCTTTTCTATAAATAACTTTCGACTTGTTCGCTTAAAATGACATTCGA
CAGAAAGTTGTTCAATGTTAGCAATCAGTAGCTCAAATTTCCTTGAATTCATATAAGGTACTTCTATAATAAATTCACGATGTAATTT
GATCTCATTGAGCATGCTATCAATATGTTCAGTATATGGCTTTACAGTTTGGTAAAAATCATGATCTTGTTGTTGTGATTTCACATTT
TCAAAATTTTGTTGCATGTTGTTAACTTCATAAATTAGTGATTCGACTACATCATTCATGAGGACGCCTCCTACATTTTTTAATTTATC
ACAATATACTGTATTCGTCATGTTTTAACACTCTATATAATTTGATTTAACTATTTTTTCAAATGTGTTATCTGTTAAATCAAGTAAAT
CTAAAAACTTCCTATATAAATACAAAATTTTATCGTGTATGTTGTTATACGATGAAAATACTTTTAATCTAATAAAATCATTTAAATC
AAATACACCTCTGCTGATTAACAACACATACTTGTACTTGCCTCAAAAATAAAAATTACTGATCATGATTTGACTTTTATAACAAAA
TTCAAAAATATTGTAATGAGTATTCATTTTTATAAGCAAAACTTTTCAATTAATTAAAATTGTGTGTAAAATTCCAACATTAACGGTA
AATGTATTATAATATTATTGTGTTAATGCTGAAATCACGTTAAAATAAGCTTTGGTGTATTGTGTCTTTCGCATTTATACTAATTTAA
GTTTGGTGTTTCACTATAATGTTGTGTATAATAAACAACGTTTACTACCAAACTTGTGTGGTGAAATTTATGAATTATCCAAATGGTA
AACCATATCGTAAAAATAGTGCTATAGACGGAGGGAAAAAGACCGCTGCCTTTAGTAATATTGAGTATGGTGGACGTGGTATGTCA
CTTGAAAAAGATATCGAACATTCAAATACGTTTTATCTTAAAAGCGACATTGCAGTTATTCACAAAAAGCCTACGCCAGTACAAATA
GTTAATGTCAACTATCCTAAGCGGAGTAAAGCTGTGATTAACGAAGCTTATTTTCGTACACCTTCAACAACTGATTACAACGGCGTT
TATCAAGGTTATTATATTGATTTTGAAGCAAAGGAAACTAAAAACAAGACGTCCTTTCCTTTAAATAATATTCATGACCATCAAGTC
GAACATATGAAAAATGCATATCAACAAAAAGGTATTGTGTTTTTAATGATTCGTTTTAAAACGCTAGATGAAGTTTATCTTTTACCC
TATTCAAAATTCGAAGTATTTTGGAAGAGATATAAAGATAATATTAAAAAGTCTATAACAGTTGATGAAATACGAAAAAATGGTTA
CCATATTCCTTATCAGTATCAACCAAGATTAGACTATCTAAAAGCAGTTGATAAGTTGATATTAGATGAAAGTGAGGACCGCGTATG
ACGGAAAACAAAGGATCTTCTCAGCCTAAGAAAAATGGTAATAATGGTGGGAAATCCAACTCAAAAAAGAATAGAAATGTGAAGA
GAACGATTATTAAGATTATTGGCTTCATGATTATTGCATTTTTCGTTGTTCTTTTACTAGGTATCTTATTGTTTGCTTATTATGCTTGG
AAAGCACCTGCTTTTACCGAAGCTAAATTACAAGATCCGATTCCTGCAAAGATATATGACAAGAACGGAGAACTTGTTAAAACATT
AGATAATGGCCAAAGACATGAGCATGTAAATTTAAAAGACGTGCCGAAATCAATGAAAGACGCAGTACTTGCAACTGAAGACAAT
CGTTTCTACGAACATGGCGCACTTGATTATAAACGTTTATTCGGTGCAATTGGTAAGAACTTGACTGGTGGATTTGGTTCTGAAGGT
GCCTCAACATTAACACAACAAGTTGTTAAAGATGCATTTTTATCACAACATAAATCTATTGGACGTAAAGCTCAAGAAGCATACTTA
TCATATCGTTTAGAACAAGAGTATAGTAAAGATGATATCTTCCAAGTATATCTAAACAAAATTTACTATTCTGATGGCGTAACAGGT
ATTAAAGCTGCTGCTAAGTATTACTTTAATAAAGATTTAAAAGATTTAAACTTAGCGGAAGAAGCTTATTTAGCCGGTTTACCTCAG
GTTCCAAACAACTATAATATTTATGATCATCCAAAAGCTGCTGAAGATCGTAAAAACACTGTTTTATACTTAATGCATTATCATAAA
CGCATTACAGATAAACAGTGGGAAGATGCTAAGAAAATCGATTTAAAAGCGAACTTAGTAAATCGTACTCCTGAAGAACGTCAAAA

CATTGATACAAATCAAGATTCTGAGTATAATTCATACGTTAACTTTGTGAAATCTGAATTAATGAATAATAAAGCATTCAAAGATGA
AAATTTAGGTAATGTATTACAAAGTGGTATTAAAATTTATACAAATATGGATAAAGATGTTCAAAAAACATTACAAAATGATGTTG
ATAATGGAAGCTTCTACAAGAATAAAGACCAACAAGTTGGTGCAACGATTCTTGATAGTAAAACTGGTGGTTTAGTTGCTATATCTG
GTGGACGTGATTTCAAAGACGTCGTTAACAGAAACCAAGCAACAGATCCTCACCCTACTGGTTCATCTTTAAAACCTTTCTTAGCGT
ATGGACCTGCCATTGAAAATATGAAATGGGCAACAAACCATGCGATTCAAGATGAATCTTCATATCAAGTTGACGGTTCTACATTTA
GAAACTATGATACGAAGAGTCACGGTACTGTATCTATTTATGATGCTTTACGACAAAGTTTCAATATCCCAGCTTTAAAAGCTTGGC
AATCAGTTAAGCAAAATGCTGGTAATGATGCACCTAAGAAATTCGCTGCCAAACTTGGCTTAAACTACGAAGGCGATATTGGTCCA
TCTGAAGTACTTGGTGGTTCTGCTTCAGAATTCTCACCAACACAATTAGCATCAGCATTTGCTGCAATCGCTAACGGTGGTACTTATA
ACAACGCGCATTCAATTCAAAAAGTAGTTACTCGTGATGGTGAAACAATCGAATACGATCATACTAGCCATAAAGCGATGAGTGAT
TACACTGCATACATGTTAGCTGAGATGCTAAAAGGTACATTTAAACCATATGGTTCTGCATATGGCCATGGTGTATCTGGAGTAAAT
ATGGGTGCTAAGACAGGTACTGGTACTTACGGTGCTGAAACTTATTCACAATATAATTTACCTGATAATGCAGCGAAAGACGTGTG
GATTAACGGCTTTACACCTCAATACACTATGTCAGTGTGGATGGGCTTCAGTAAAGTTAAACAATATGGTGAAAACTCATTTGTGGG
ACATAGCCAACAAGAATATCCACAGTTCTTATATGAAAATGTGATGTCAAAAATTTCATCTAGAGATGGCGAAGACTTTAAACGTC
CTAGCTCAGTAAGTGGTAGTATCCCATCAATCAATGTTTCTGGTAGTCAAGATAACAACACTACAAATCGTAGTACACACGGTGGTA
GTGACACATCAGCAAACAGCAGTGGTACTGCACAATCAAATAACAATACTAGATCTCAACAATCTAGAAACAGCGGTGGATTAACA
GGTATATTCAACTAATCCACTCAACATAAAATCCTCAGTTATACCATATTTATGGTGTAGCTGAGGATTTTTTTAGGTTCTTCATCTT
TTATGGTGGGAAGGTAAAACTTCCTGCTTTTTTTATAAGTATTCATACGCTATTGCTATATTAGTAGAAACCGTAAAATCAGTCATTC
ACATACATTTTGAAACATCTAAATAGTTTTTCATATTCACATCTTTAGCTATGTAAAATTATTGAATTTGAATAAGTGGTTGTTCCCA
TTTTTAAAATTATGATTATATTTTCTCATAGTCATCTTAGACGCTGTTTTAAATTGATATATTTATTAATTCCCTGTGTACGTTAATCA
GTGAAATATCAACATGTTTGCAACATATTTTCACAATCATAGATATAAGAAATCCACCTACGATTCGAAATAGATATCTTAAAGCAT
ATCTCAAGATATCCATTATGATGTAGATGGACAGTTTTTATATTAAGACGTTGTAATGACACGACACCTAACATAAATATAAATATG
TGTGCATGAATTAGTTTTACATATTTTCAACATTAGGAAATGATCAATCTTGTCTATACCTGCTTATTCTTTAACCTTGTTTGATAACT
AGCATTCATTTTAATCAATTCAGTTTTAAAGGTCTTCATTTGTCGATAGCCCATAAAATGATATTTACGTTCAATCATATAATCATAT
CTTTCTTTAAAATTCATTGGCACAACAGGATATTGATTTAAATTATCAAAATCAATATTGCCAGTTATGTCATTAACCTGCTTAAAAT
AATCAATAATTTTACTATGGTATTCATCCAAGTAGGGCTTTGCTTCACTAGAACCTAATGTCTTATTTTTGGCAAATACATCAATCTC
TTGTTCTAAATTATCAAATACCTCTTTTGTAATATTTGTCATGTTATCACGCTTCTTTCAAACTAGCTTTATAACGTTTTTGTCCTTCTC
TACAATCTTCTAATAATGGACAAATATCGCATTTAGGTTTTCGGGCTAAACAGTGGTATCTTCCAAAGAAAATGAGTTGATGATGGC
TCCTATTCCATCTATCTCTAGGTATGACAGAACATAAACGGTCTTCTACCTGTCTCACATTATCTTTCCAACGATTAATACCTAAGCG
TTTAGAAACACGTTCTACATGCGTATCAACAGCTAATGAAGGTTCATCAAATGCTACACTCATGACTACATTAGCTGTTTTACGTCCT
ACACCTGCTAAACTTTCTAATTCCTTATGTGTTTGTGGTATTTCTCCATTAAATTGATCAATCAAAGATTGACAAAGTTTCTTAATATT
CTTAGCTTTGTTACGATACAAACCGATAGAACGAATATCATTCATAAGTTCTTCATCACTGACTGCCAAATAATCTTCAGGCGTTTTG
TATTTTTTAAACAGCTCAGTTGTTACTCTATTTACTAGAACGTCTGTACATTGCGCTGACAATAATACAGCAATAGTTAATTCGAACG
GATTATCATGTTTTAATTCACATTCTGCATCCGGAAACATATTTGCTATAACATCAACCATTTCTAATGCTTTTTTCTTACTTACCATC
AAGGTTCTCCCCATTTAACCAATCAAATTTAGGTACCGTTTTAACTGTGTGCGTCATTTTCGGTTTATTGAATTTTTCTCTTATTTTTC
TAGAATCGTCAATTGTTTTGACATTGTTTTTCTTCCAATTAAGTAAAATACGATCTATATATTTAAAGCTAAGTTTATTCAAACTATT
CGCCTCGTCTAATGCCGCTTGTATAATTGCAGTATCGTGTTTATCAACATCAATCCATTGATTTAACGTTTCTATTTCATATGGAGAT
AACGGCCTTGCAAATGTATCCTCTAAAACTCTAAATAATTGTTTAAATTTTTCTTTACTATTTTGCTCTTTCGTTTCCATACTTTGTTG
CTTCAATATATGACTTAATTTTTCGAAAAAAGGATCTAGATTCATATATTCGGTAAATCTACCTTCTTCATCTTTTTGAACTTGTAATT
CTAGCAATTCACGTTGTATCAAATTTTGAATAACCATTGTAATATCACGTGGTTGCATAGTTGAGCCCTTCTGAAGTAATTCAATTGA
AGGCTGTTTATTTGATGTTTCGGAAGCATAAATCAATTTAAGCAAAATGACTAAATCTTGCTCATCTAAACCTAAGTCACTGTAATG
GTCTAATAATTCTCTTCGTATCACTACAGGTCTTGCTTTTAATTGATATTTATCCATGTTCGTGCCCCCTTTTAAATTTAACCATTACT
TAATAGTATAAATCATTTTCTTTAATTTTCGACTAAAATGCTTAATATATTTAACTAGTAGCAAACAAATTTTAAATAACAGTATTTT
TTAACACTAGATTTTAACAATGAACTTTATTTATAATCAGATGAACTATTTATTTTCCAAATATAGTTACTAAACAAAAGTTTTTTCA
TGATTAATATAAGCCGTTATCAATCTTCATTTTCCAAGCATAAATCGCCCAGCTATTAATATAGGCTGGACGATTCTTTTTATTATCA
TAATATATATTTTAAGTAACGACCCTAAAACTTATGGATATAAACGGTTTAATAATCTTGGGAATGGCGCTGTTTCACGAACGTGTT
CAACACCAGAAATCCATGCTACTGTACGTTCTAAACCTAAACCAAATCCACAGTGTGGCACACTACCATAACGACGTAAGTCTAAG
TAGTAACTATATGCTTCTTCGTCTAATCCATGTTCTTTAACGCGTTGTTCTAACAATTCTAAGTCATCCACACGTTCAGATCCACCAA
TAATTTCACCGTATCCTTCAGGTGCAATTAAGTCTGCACATAATACAGTTTCTTCATTTTCAGGATTTGGTTGCATATAGAAAGGCTT
AATTTTAGTTGGATAATTAGTAATAAACACCGGTAAATCATAATGATTAGCAATGGCTGTTTCATGTGGCGCACCAAAATCTTCGCC
CCATTCAATATCATCAAAGCCTTCTGATTTTAAGAATTCAATTGCATCATCATATGAAATTCTAGGGAATGGTGTCGCAACTTTTTCA
AGTTTTGATGTATCACGCTCTAAAATTTTCAACTCTAGTTTACAATTTTCTAAAACTGATTTTACAACATGTGTTACATATTGTTCTTG
AATTTCTAAACTTTCAGCATGATTTGTGAAAGCCATTTCCCCTTCAATCATCCAGAACTCGATCAAGTGTCTACGTGTTTTTGATTTTT
CAGCTCTGAAAGTTGGACCAAATGAAAATACTTTTCCGTGTGCCATTGCTGCAGCTTCTAAGTATAACTGACCACTTTGAGATAAAA
ACGCATCTTGATCAAAGTATTTAGTATGGAATAATTCACTTGTACCTTCTGGCGCACTTGCTGTCAAAATTGGTGGATCAACCTTTGT
AAATCCATCTTTGTTGAAAAATTCATACGTTGCACGAATAACTTCATTTCTAATTTTCATTACAGCATGTTGTTTTTTAGAACGTAAC
CATAAATGACGGTGATCCATTAAGAATTCTGTACCATGATTTTTAGGTGTAATCGGATAGTCATGCGCTTCTGAAATAACTTCAATT
GATTTCACTTGCATTTCGTATCCTAAGTCAGAACGATTATCTTCTGTAATTGTGCCTGTAACGTATAGAGATGATTCTTGAGTAATTT
CTTTCGCAAGTTTGAATACCTCTTCATCAACTTCTGATTTAACTACTACGCCTTGCATAAAGCCTGTTCCATCACGTAATTGTAAAAA
GGCGATTTTACCACTTGAACGTTTATTTGTTAGCCAAGCACCAATTGTAACGTCTTGGTTTAAATGATCTTTCGCTTGTTTAATCGTT
GTTTTCATAACCATTCTCCTATTTATTTTTTCGTTATACAATACTCATTCATTTTAACAAAATCCGCTTTCAAGTTCTAGAACTAGACT
AAAAGATAACGTGTAAATGGTAATGATTTAAGCACAGATTTGAACATTAATTTATATTAAGACAATACAATCATCAGTTTCAGACA
AAATATATAATAAAGCCTCAATATTATTAGTATGAAGGTTGTTTTTTGTGCTAACATTCAAGTTTAATTATAAAACTCAAATTTTGAC
GACCAAACAAAAATTCTTGTAACAGAACATAGCACATGTCATGTCACAAGAATTTTACTTTGTATTTCACTTTTTCTTTTTTTGAATT
TGTCTTAATAATTTTCCAAACTGTTGAATGTCGCCTTTTTTCTGACGATAATTTTCAAGTGTTTGTTCAAAAAAGTTTTTATAATTACT
GTTTATAAGTCGATCATCAAATGAAACTATTATGCCGCGATCATTTTCACTTCTAATTAATCTTCCAAGTCCTTGTCTAAAACGTGTA
ACTGCATCAGGTAATACATATTCCTTGAAAGTTGAAGTGAATTCAGAATCCATAAGCCAATATTTTGCATTATGCTTGTTCATAAAC
GGTAACTTCGCTATCATCACACATTTAATACCATTTGCTTGAAAATCAAAACCTTCAAAAAATGTTGACGTACCAAGCAGTATGGCC
TTATCAAAATTATTAAACTGTTGTACTATTTTATAATTTTGGTTCTGCTGTTGTGTTAATACAACATAATCTTCAAATTCTGGCAATTC
ATTTAGCATATCTTGTACCATATGCATCATTTTATAACTCGTAAATAAGACTAAACATTTTGATGACGTTATAGTCGTATATTCAATA
ATATAGCTTACAATCGATGCTACATACTCATCTATATTTTTATATTGATAAGATGCTACATCACTCGGTATAAATACACTTGTATTTT
TTGCACTTTGTAACGACGTGTTAACTTCAAATGTATTAAAGTGAACATCTTTGTTGAATAACTGTTTAAAAGCTTCAAACGAATGATT

AAATTTTAAAGTACCAGATATAAAAATGAGTGATTTAAATTTTTCCAATACTTGTTTCGTTAATACATCTTTTACAGCGTAATCTTTC
ACATATAAACGTATTGTTGATTTTTGAGATAAATTTTTAATCGAAATGAAACTAGTATGTCCAGCTTTTAAACTTTGTTCGATATTTT
TAAATTTATCTTTTAAATACAACAACTGTTTCCGTAATGATTTAACTGTTTTATGACTAATGCCATTGAATATTTCTAGCGTTTTATTT
AACTTATCGATAATCGCATGTAAATCCTTCAAAATGTCTTTTGTTTCAAAAGTAAATACATTATGGAAGCGATGAATATCATCATCA
TAAACATCAGAATCATTGATAATCGTAAATATCGTTGAGAACAATTGCTCATTTAACTCATGAATCTCATTCATACTAGCCTTCAAG
CCAAAAATATCAATTGGTGCAATATCTAATTTTTCCAAAATTCGCTGCTTTTCCAGTTGATCAATTGCCTTTAACAATTTTTCATTTTC
GTTTTTACCAATCAAACCAAGCTGATATTTAATATCAGCATAACTCAACTCATTTGTCACTTGATTTAAGGCATAGTCTGGTAAGCG
ATGTGCTTCATCCACTATACAATCATCAAACAATTGATATATTGAATTTTCAACATCAGAATGAATTAAATGTGCATGATTTGTAAT
ACCAATTTGAATGTTCTGTGCATTTCGCTTAATAAAATTATAATAATGAACATCGTGACGTGCCGGTACATATGTTTCAATTTTCTGG
TCAAAATACATCTTTTGACCACCTTTTAAATTTAATTCCTGTATATCTCCGGACGGCGTCTCTGTAATCCAAATCAACAATTGCATTT
TCAAGATATTCACTTCGTAATTACTTGTGTCATCTTTTAAAATTTGACTAATAAGTCCCAATGAAATGTAATCACTTTTACTTTTAATC
AATAGTGCATTAATTTTAAAATTCAACGCTTCATTCATTGCTGGAATATCTTTTTCTAACAATTGACTTTGCAGTAATTTAGTATTGG
TAGAAATCATGACATGCTTCCCAGTTTCAATATTATACATCAAGGCCGCAAGTAAATATGCTAATGATTTACCACTGCCTAGTGATG
CTTCAATCATTGCTTTTTCACTATGCATGAGCTGATCTAATATAGTTTCCGCTAAATATAATTGTTGCGGTCGATATGTTAAGCCAAG
TTGATCTACAGCTTTGCTATATAAAGACTTCAAGCTGCCATTATAATTTGTTGTCGGCTTTTTAAAATCAACTTGCTTACGATAGATA
ATCTGTTCGAACTTTTCGTACGATTTATCCAATGGCTTTGCATCATATTGCCTAACCATCTCAAAGAAAATATCATACAAATCGTATT
TCAACTGTTTACTTAAATAATATAATTGCTTTAAAGTATCTAACGGTAACTTTTCAAATTTTTCAAAAGCTAATATCATCAATTTAGC
AGTAGTAGCGGCATCTTCGTCAGCTCGATGGGCATTTGCTAAGGTAATACCATGTGCCTCTGCTAATTCACTTAATTGATAGCTTTTA
TCTGTAGGAAAAGCTATTTTAAAGATTTCTAGTGTATCTATAACTTTTTTGGGACGATATTGAATATTACAATCTTTAAATGCCTTTT
TAATAAAATTCAAATCAAAATCTACATTATGAGCGACAAAAATGCAATCTTTTATCTTATCGTAGATTTCTTGTGCAACTTGATTAA
AATATGGCGCTTGTTGTAGCATATTTTCTTCAATGGATGTTAACGCTTGAATGAACGGCGGAATCTCTAAATTTGTTCTAATCATAGA
ATGATATGTATCAATAATTTGGTTATTGCGCACAAACGTTATACCAATTTGAATGATATCGTCAAAATCTAATTGGTTGCCTGTTGTT
TCCAAATCCACAACGGCATAGGTTGCCATACCCATAGCTATCTCTCCTTGCTTTAGTGTTAAAAATCTATATCTGCACTAATTAAACG
GTGTGATTCACCCGCTTCATCTCTAACAATTAGATAGCCATCGTAATCTAAATCAATTGCTTGTCCTTTAAACTGTTTATCATTTTCTG
TAAATAGCAACGTTCTATTCCAAATATTAGAAGCTGCAATATATTCTTCACGAATTTCAGAAAAAGGTAACGTTAAAAATTGATTAT
ATCTTTTTTCAATTTCTTGAAGTAATCTCTCTAAAAATTGATATCTATCTAATTTATTTTTATCATGTAATTGTATACTTGTTGCTCTAT
GTCTAATACTTTCATCAAAGTCTTCTAGTTGTTGCGTCAAATTAATACCTATACCACATATTATTGCTTCTATACCATCATTATTAGCA
ACCATTTCAGTTAAGAAACCACACACTTTACCATTATCAATATATATATCATTCGGCCATTTCACTTTGACTTCATCTTGACTAAAAT
GTTGAATCGCATCTCTTATCCCTAATGCAATAAATAAATTAAATTTAGATATCATTGAGAATGCAACGTTAGGTCTTAACACGACAG
ACATCCAAAGTCCTTGCCCTTTTGAAGAACTCCAATGTCTATTAAATCGCCCACGACCTTTCGTTTGTTCATCACTCAAGATAAAAAA
TGAAGATTGATTTCCAACAAGTGACTTTTTCGCAGCAAGTTGTGTAGAATCTATTGAATCGTATACTTCACTAAAATCAAACAAAGC
AGAACTTTTTGTATATTGGTCTATTATACCTTGATACCAAATATCTGGGAGTTGTTGTAATAAATGCCCTTTATGATTTACTGAATCA
ATTTTACATCCCTCTAACTTTAATTGGTCAATCACTTTTTTTACTGCAGTGCGCGATATATTAAGTGATTCCGCAATGCTTTGTCCAGA
TATATAATTCGGTTTATTTTTATAGAGTAATTGAAGTACATCTTGACTATATTTTGACATGATTATCCACCCATTTCAAAATTTCAGTT
TCTTCGTTGCTTACTTTACCTGTTACAATCGCTATCTCAATTTGTCTTAGCACATCTTTTAACCACGGACCACTTTTGGCATTTAAATG
TGCCATAAGTACACCGCCATTAACCATCATGTCTTTTCTATTATGCATAGGTAAACGATGCAATGTTTCATCAATCGTTTGAAGGTTA
ACGATTAATGGTTCATGTCCTTGAATATCATTTGCCTTTAACACGTCTGCTGCAACCATTACATTTTTAATGAGATGCGTATCATAAT
CATAAACAAACATTTTTAATTGTTCTTTTGTAATAATACTTGGTAATGCATTCATAATTTGAATATATTGATTGATATCTTTTACTTGT
CGGTTACTTAGCTTTAAAGGCTTCAATGAGTAATTAATATCAAATTTAACTGACACTATAGCAATCAACAATTCTAAATCAATTGGT
TCAGTTACATTAATTTGATTCATATCAAGTTGTTCGAAATATGGCATATAATTAAATGCTTTCAGCGATTTTAAATGAATAAAACTCT
CTTCAACATTAATACCTCGCATTAATTTAGTTAGTTCAATCACTATACGCTCAATTGATAAAAATTTAATATCTGCCATTTGTGTACG
CATCGCTTCAAATGTTTCCGTTGCAATATCAAATGATAATTGTGACTGGAATCTTAAACATCGAATCATACGTAAAGCATCTTCTTG
GAATCGTTCCTCAGCTATACCTACAGTTCTTATTATTCGATTATTAATATCTTGTTGACCATCAAAATAATCATACAATTTGTATGCT
GTATCCATTGCTATCGCATTCATCGTGAAATCTCTTCGTTGCAAATCTTCGTATAAATCCCGAACAAATGTAACACCACTTGGTCTAC
GGTGATCGACATAATCTTCTTCAGCCCGGAATGTTGTCACTTCATAATTTTCATTATTAAAAACTACATTTATGGTGCCATGTTCTTT
ACCTACAGGTATCGTATGACTAAAGATAGATTCTATTTCATCCGGCGTTGCACTTGTTGTGATATCTATATCATGAATATTTCTTCCC
ATGACATAATCTCTTACAGAGCCACCTACATAATATGCTTCAAAACCATTGTCTTGAATTTGTTCTAATATAGGCCTTGCCTGTTCAA
ATAATGATTTATCCATATTATTACTCGCCTTTACTTTTGTTATGCTCATTTAGCATTTTTTGATAATAATACTCATATTGATCTGTAAT
AAGTTCTGATCCAAAACGTTCAGCAATATCTGCTAGCATGTTTTTCTGAAGTTTGTTGTATAACACCTTATCTTCAAGTAATCGGATA
GCATAGTCACTCGCTGAATCACAATCACCCACATCTACGACAAATCCAGTTTCACCATGTTTAATAACCTCTTTAATTCCACCGGCAT
TTGAACCAATTGGAACGACGCCTGTTTTCATAGCCTCAAGTAAAGTTAGTCCAAAGCTTTCTTTTTCACTTAATAATAATACTAAGTC
AGATAATTGGTAAAATTCACTTACGCAATCTTGTTTCCCTAAAAATAAAACATCCTCTTCTACGTTTAACTCTTTCGTCAATTGACGC
ATTGGCACTAATTCAGGACCATCTCCAAGTAAAATTAATTTACTAGGTATCTTTTCACGTACTTTTGCAAATGTTTCTATAATAGTAT
CTATGCGTTTTACTTGTCTAAAATTCGATACATGTATTAACACTTTTTCATCTGGTGCTATACCAAATTGTGATTTTAGTGCTGTGTTA
TGTTTAGTTGGAAACTCATTTTCACGTACAAAATTATAAATCGGTATAATTTCTTTGTTAGTTTCAATAATTTCATGTGTTTCTTGTGC
TAAAGATTTACTCACACTTGTCACAATATCACTTTTTTCAATGCCAAATTTAATTGCACCTTGGAGTGAATGATCATAGCCCAAAAC
AGTAATATCAGTACCGTGTAGCGTTGTCATAATTTTTATATCTTTACCTGACATCTCACGAGCTAAAATCCCACAAATTGCATGAGGT
ACAGCATAGTGCATATGCAACAAATCAAGATCATATTCTTTAATAACTTCAGCGATTTTAGTACTTAACGTAATATCATACGGTGGA
TACTGAAATACTGCATATTGATTCACTTCAACTTGATGAAAAATCATATTCGGTAATGGTTTTCTTATTCTAAACGGGATATTTGAAG
TGATAAAATGTACTTCGTGACCTCGCTCTGCTAATTTAATTCCTAATTCTGTGGCAATAATTCCAGAACCACCCATGGACGGGTAAC
ATGTTATACCTATCTTCATTCGCTTGCCCATCCTTTCTTTCTATTTCTCTCTATTATTCTCGATGCGTAGATAATTGTTTAAATTTAAGT
TTATAGTAATGTTGAGTTTATAATTTCATATATCTAAAAAACAGGTGTTGTATATATAATCATTTATCTAGTTATATTTACTTTAAAA
ATAATATAATTTCATGCGATGCAATTCATTGGTCTATGTTTTTAATCTTAATCAAATCCAAATAAAGCATATATTTTTATCTTCACTTT
CTTTCGAATCGATTTTTATCTCTTGTATTAAACTTTTCCATAGTTTCATTAAAGCTCTCTGTCATATCTATTCCCATTGAATTCGCTAA
ACATAACAACACAAATAAATTATCACCTAATTCTGCTTTAATCGTATTTGCTTCCTCTGAATCTTTCTTCTTTTTTTCACCATAGGTAT
GATTTATTTCACGTGCAAGTTCGCCCACTTCTTCAGTCAATCTAGCTAAGTTAGCTAATGGTGAAAAATATCCTGTTTTAAATTGTCC
AATATATTCATCAACTTCACGTTGCATTTCTACCATTGATTTCATTTCTACGTTCTCCTTATATTGCATTTCTAATATAGTATATATCA
ATTTGAAGTCTCATGCATGTTTAGTGCAATCTACTGACTCTAGCTAAACTAGATGACAAGTTGTTTATTTTAGAAGTTTAATAATTCA
GTTTATATAAATGTAATGCATTCCTAACTAAATTAAATCAATTGAAATTGGTATTATAACTTTATGATACGTACCACTACAATAAAA
TAATATAGTGAATAATCTACCATTAGAAAAATAAGCACAAAAAAACTAGCAACCACACAAAAATGTGATTAGCTAGTTAATAAGTG
TCTAATTTAAGTTAATTGTTAATCCATAAGATTAATCACTTGAACGCGCAATCAAAATAATACGTACAAGCTCTGCTACAGCGACTG

CAGTTGCTGCAACATAAGTCATTGCTGCTGCAGATAATACTTTACGCGCATGCTTGTATTCTTTTTCATTTACAATGTTCAATGCCGT
AATTTGTTTCATCGCTCTTGAACTCGCATCAAACTCAACTGGTAACGTAACAATTGAGAATAATACCGCTAATGACATTAAACCAGC
ACCAATCCATAAAGCAGTTGAACCAAATGCACTACCTATCGCTGTTAAGATAATACCTAACATGATGATCATATAACTTAATGAACT
CCCTAGGTTTGCAACAGGTACTAATGCTGCTCTGAATCTTAAGAACCAATATCCTTGGTGATCTTGAATGGCATGACCAACTTCGTG
GGCTGCAATTGCAGTTCCAGCAACTGATGGTCTGTCATAGTTTGCAGGAGATAGTGAAACAACTTTCTTTTTAGGATCGTAATGATC
TGTTAAGAATCCTTCACCTTTAACAACTTCGACATCATAAATACCGTTTGCATGTAAAATTTCTAATGCAACTTCACGACCCGTTTTA
CCACTAGTTGATCTAACTTGTGAATATTTCTCATAGTTAGATTTAACTTTGTGTTGTGCCCATAAAGGAAGCACCATTAATATTACGA
AATAAATTATCATAGTAAAAATTGAAGACAATAAACTCACTCTCCTTTATAAATATTTTACTGTCATTTGCCGTTTTTATCAAATCAT
TTACACTTTAATAATTTGTTTAATTCAATATAAAGCAAAAGTCCAAAAACACTTAGACAACATGATAATACACCAATTTGCCACACA
TGTGTAGTTATAAAATCATAATATGGAAATTGAAGGTGAAAATAGTCAATATAATCATTCAAAAACACCCAAATCATCGCTACACT
GATTCCAATCATAGAACGTTTAAACCTAGGATAGAAGTAAATTGCCTGAACAGCCATTATACTGTGGGAAAACATTAATACCAAAC
CATTTACTGTAATATCACCTTGTTCAATAATAAATAATATATTCATTATAACTGCCCAAATCCCATATTTGAATAATGTTACAAATGC
CAGTGCATCGATAATACTATTTTGTTTTTGAATTAATATCAATGAGATAGAAATAACTAAGTATAATATTGCAGTTGGGCTATCTGG
AACAAAAATCTTAAAATGCCACGGCGTATGACTTAATTGTTCACCATACCATATATAACCATAAATCATCCCTAATATATTACAAAT
GAGTAGCAGCATTAACCAAGAACGTTGATAAAGTGTATATTGCCAAAATGTATTAATCGTCATCTGCTAAGTCCTCAAATTGATTAT
GTTTATTTACTAGCTTGAGTGTATTTAAAATTTGCGTTAGTTGATAAAAACGTTGCTTTTCATTCATCTGTAAACTTAAATCAATATT
GTGTAACAAGTAATCTATTAATAACGCATGTTTATGCCGATCTATAGCCATACTATTTAAGTCATGAAGATAAGTTTGATAACTGGG
CGATTCCGTTAGTTGAGCAACTATTAAATCATCTAGCCTTTGCTCACGCTTTGACACGTTTGCGAAGTGAATTTGAATATCAAAAGC
ACAGTTATGATTAGCGATATAATCAAATATTTCATTTGTATTCATTAACTTTATATTACGCTTAGTAAATTGAATTGCAGAAGCGTGA
CTTCCCACTTCTGCAATTTCTAATGTTTCATGATGATTAATTTTTGTATCTACAAAATGAATGTTTGCCAATTTCGCCTCATTCACTTT
TATATAGTTAAGCACCCAAACTGCAATACGCGACTTAAATCGATATTGAAAAAGTAAATATTCAATAAAACTTTCTTTAATTTGATT
GAGTGTCTCTGACATCAAATACCCCATTTTAAGATCGCAATCTTGATAATTCGTCATGCCAATTTTCGTTACTTGGCTCTAGTTCCAA
CAATTGATTTAAAATAATAATTGCTTGTTCCTTTTGACCAATCTCAATTAAATAGAAATAATAATCACTCATAAAATCAATATTTGTT
TTCATCGTTGGATATGCTAATTCAAAGAAATGTTGAGCTTCTTTATCTCGCTCTTCTTGACCATAGGCGAACGCTAAATGCCACATGA
ATGTAGGATCCAAATCTTCTTCATCTACATATGTTAATAATTCAATGATTGCTTCATAATCTTCTTCATTACGATATAAATCGCTTAA
AATCAATAAAGGTTCTTGGTAAGCATTATCAACCTCTAATGCTTGTTTTAACAATAATACACCTTCATTAGCATCGCCGTGTTCTATT
TCCAAACATCCAGTTGTATACATTAACTCTTTATAAAATTGACTTAGTCGTAATCCTTCTTTACCCGTCTCAATGGCATCTGGATAAT
TTTTTTCATTTTCATATAATGATTGTAAATACAAGTAGCCTTGAATATAATCAGGATCTTTAGAAAGTAATGTAGTCATTATTTTAAT
TGCTTCTTGAGTGATGTCATTTTTATCGTAAGAAATGGCTTTTTTGAGATAATCTTCTGAAGTCATTTCATCTTCATTAATTTCATCGT
ATAAGCGAATCGCATCACTATAGTTACCACTTTGTAAACTACAATCTGCCATACGAGAGAATAAGTTTACACCATTAACTTGATATT
CACCAGTTTCTAAAACGGTTTCGTATTCAGAGGTAGCACGTAAATATTGACCATCATAATATAACATTTCAGCCAATGCAAAATGGA
TTATTGGATCATTTGGCTCTAGTTCAAGTGCTTCTTGTAATTTATCAATAGCAACTTCCATCATATTAATTTGTTGATATAAATCTGCT
TCTAACATCAACTTTTCAGGTGATGGTTCAACATAACTTAAATATTCTAACGCTTCGTCAGTTTGATTTTCAGACATTAAACCTTCAA
TAAAATAAATCAGCAATTCACTTTCGTCTGGATATTTTTGATATAACACGCGGAATACTTCCAAACCTTGTGGCATTAATCCAAAAT
TGTAAAGTGTCTCTCCTAGAATAAATAATGCGTCATCGTTGTCAGTAGTTATTGCTTCATTAACACGAGAGTCTAAATTTTCTAGTTT
TTGTAGATTGATATCGTCTATTAATTTATAGATATCTTCCATTTTATATTATCCCTCATTTTCTAAAAGCTTTAGTTTTGGTAAAAATC
CTGGAAATGATACATTTACAGCATCAAATTGTTTGATTTTGACAGGCTCGCTTGAAAGTAGAGAAGCAACTGCAAGCATCATTCCTA
TTCGATGATCAGTTAAACTATCAACTGTTGCATTTGTTTTAAATTCTGACGGATGAATAATCAATCCATCATTAGTTGGTTGTAATTC
AAACCCTAACAAGTTTAACATATCAGCCGTTGTATCAATTCTATTTGTTTCTTTTACTTTTAATTCCTCGGCATCTTTAATTGTACTCG
TGCCAACTGCTTGTGTACAAAGTAATGCTATTACAGGCAGTTCATCAATTGCTTTTGGAACTAATTCTCCTTCGATTGTTATTGGTTG
AAGCATTGGTGTGTATTGAATACGAATAGAAGCAGTAGGTTCAGCACCAGTTGTTTGATTGAAAAGTTGGATATTACCGCCCATTTT
TTCAACAATATCAATAATACCTGAACGTGTTGGATTGATTCCAACATTATGAATTGTTACATCACTTCCTGGTGTGATAAGTGCTGCA
ACAATAAAGAACGCTGCAGATGAAATATCGCCAGGAACATGAAAATCTGCAGGTTTAATGTATCGAATTGCTTCAGGGGTTGTATT
AATTGATAACCCTTCTGCTTCAATTGGAATATTAAAATGTTTGAACATCGTCTCAGTATGATTTCGACTTACATCTAATTCTTTAATG
ATGGTCGGTTCCTTAGAAAACAAACTTGCAAATAAAATGGCACTTTTTACTTGTGCACTTGCAACTCCCATTTGATAATTTATACCTT
TTATGACAGATGGCTTAATAATTAATGGTGTATAATTATCTTCAATACCTTCAATATTCGCATCCATAAGTTTCAATGGTCTCAAGAC
ACGATCCATTGGCCTTTTACCAATTGAAACATCGCCAGACAAAACACTTTCAATACCTAAACCACTTAACAAACCAGCTAATAATCG
TGTTGTCGTACCAGAATTACCTGTATACAATACTTGATGTGGCGTGTTAAAAGATTGATATCCTGGGGAAGTCACAACTAATTTTTC
ATCATCTTCTTTGATTTCTACACCTAACAGTCGGAAAATGTCCATCGTACGACGACAATCTTCGCCAAGTAGTGGCTTATATATAGTA
GATACACCTTCAGCTAGCGACGCCAACATGATTGCACGGTGTGTCATTGACTTATCGCCCGGCACTTCTATTTCGCCCTTTAACGGA
CCTGAAATATCAATGATTTGTTCATTTACCATTTCATTCACCTACTTAAAATATGTTTTTAGTTGTTCACACGCATGTTGCAGTGTTGT
TTGATCAATGTGTTGCACTACAATATCGCCAAAATGTTTAATCAATACCATTTGAACTCCTTGTTTGTCGTTCTTTTTATCACTTAGCA
TGTATTGATATATCGTTTCGAAATCTATGTCGGTTATTGTTTCTAAAGGATAACCTAATTTCGTTAAATAATTAATATAATGTTGGAT
ATCGTGATTAGAATTGAACAATATATTCGCAACAATAAATTGATATATTATGCCTATCATTACGGCATGACCATGAGGTATTTTATG
ATAGTATTCAACAGCATGCCCAAATGTATGACCTAAATTTAAAAATTTACGTACACCTTGTTCTTTTTCATCTGCAACAACAATATCC
AGCTTCGTTTCAATACCTTTAGCAATATATTTATCCATACCATTTAATGACTGTAATATCTCTCTATCTTTAAAGTGCTGTTCGATATC
TTGCGTCGTTGATTCACCATTCAATAACGCATGCTTATAAACTTCTGCATAGCCACTTAATATTTGCTCAAATGGTAACGTCTTTAAA
AAATCTAAATCATAAATCACAGCAGTTGGACGATAAAATGCACCGATAAGGTTTTTACCTTGTTTTGAGTTAATACCCACTTTACCG
CCAACACTAGAATCATGCGCCAAAATCGTTGTAGGAACTTGTATAAAATGGACACCTCTTAATAGTGTTGCTGCTACAAATCCTGCA
AAATCTCCTGTCGCACCACCACCAACAGCTATAATCGCTGTATTACGCGTTACATGATGTGACAAAATGTATTCTAATGTTTCTTGAT
ATTGCTCAAATGTTTTCGTCTTTTCACCAGCTGGAATAATGACTTTATGCACATTTTCATATGATAAAATATCATCAAATTTATCAGC
AAAATATTGATTTACATGCTCGTCAATTAATATAAAACTTTGATCAAACTGATCAATATACGTGCTAATATGGTCAATTGCACCATG
TTCAACAAATATTGGATAATTGTTTGAAGGATAGGTTGTTTGTAATTTCATGATCACACCTCAATTGTTCTCGTTGTTAAAACTCAAC
ATTTAATTGTCTGCGGTCAGCAATTTGTTGTTGAAGTTGTTCAATATGGTTTGATTGAAACTCTTCAAGTAATGCTTTCGCTATTTCA
AATGCAACAACGTGTTCACACACTATACTTGCTGCAGGTACAGCACAACTATCAGAACGTTCAATTGTTGCCTTAAAGTCTTCTTTA
GTATTTATATCAACTGAATTTAATGGTTTATATAACGTAGGAATAGGTTTCATTACACCATTTACAATAATTGGCATACCATTTGACA
TACCACCCTCTAAACCACCTAAATGATTAGATCCACGATAATAACCAATTTCACTATTATATAGAATTTCATCTTGAATCTCACTACC
TGGCTTTTCAGCTGCTTTAAATCCTTCACCAAAGCTTACACCTTTAAAAGCATTTATGCTGACAACACCTTGTGCAATCTTACCATCT
AACTTACGATCATAATGCACATAACTACCAACACCAACAGGCATATTTTCAACTACAACTTGAACGACACCGCCAATTGAATCTCCT
TCATTTTTAGCTTCGTCAATTTTATCTCGCATTGCTTGTGCGATACTGTCATCAATTACACGAACATCATTACGATCAAGATTTGCTTT
AAATGTTTCTGAATCATAAAAATCTTTATCTTTAATTCCACCTATTTCAACAACACGACTGTATATATCGATATCTAACTGTTGTAAT

AACACTTTACATAAGGCACCGACTGCAACTCGAGCTGCTGTTTCTCTAGCAGATGATCGCTCTAGCACATTTCGTAAATCACGATGA
TTATATTTCATACCTCCAACCAAATCTGCATGACCAGGTCTTGGTTTTGTAATAGTACGTTTCATATTTTCACGTTCTTCTTCACTTAT
TGGAGCTGCTCCCATAATTTTTCTCCAATGCGTAAAGTCATCATTGGTTACAACCATAGTAATTGGACTACCTAATGTATAACCATTT
CTAACGCCTGATACTATTTCTACTGTATCTTTCTCAATTTGCATGCGTCGGCCACGACCATAACCCCCTTGACGCTTGAACATTTCTTT
ATTAATATCTTCAACTTTAATTTCTAAATTTGCTGGTATACCTTCGACAATAACTGTTAATTGAGGTCCATGTGATTCTCCTGAAGTT
AGATATCTCATACACACTCGCTCCTTTCATACTTCCGCAATAACATTATAATATGTTGTTAATCTTATCACATTCATTGTGGATTCAT
AAATGTTTGTTTTCAAGCTTAATTAAGAAAATGGATGACATCTCATTTTAAATGTCAGAATTATCTTAATATTAAACGAACTAAAAT
TAATTATAATATATTCGTCTGTAGAAAAGTATCATGAATTTCTAAACAGTTGCATCATATAATCATTAATTAAAAAATGATTAGCCG
TTTTAATATAATCGTATCGCAACTTTAGCAAATAATATACTGACTACATTTCTTAATACAAAAGAACGAACTGTTAATTATTAATAA
AGTTAATCAGTCCGTTTAAAAAAATTATGATATTAAAATATACATATGTTAAGCGCTTTTGTTATACTTTGACTTCAAAAATATAATC
AATTCTTTTACTAATACATAAAAAATATAAATGAGAGTCAGTATAAAAATAATTTTTGTTATAGGATTAAGTGGTTTATCTAAAAAT
ATAGCACCATATAGTAATTCCATTAATTTTAAGAACATTAAAATCCCCAAAATAATCGAAATAATATTCTTCATTTTAACAACCCCTT
CTCTCTGTTTAGTTTCATTTGATAAAGAATATTCCTCTCTTTTTAATACGTGTTACCAAAAAACTATAAAATTTTTCAAGTAATTTACT
ACAACATAAAAAACGGGCCTTGTTATGTATAATCATAATGACTAACCACATAACAAAACCCTTTCTACCTTTATAAATAAATCGTAA
AGGTTTACAGTTTATATTTTATTCATATAACCATGCATCACGTGGTGAAGCATAGCTAGTAATTTCATTTTCATTAAACCATAGATTA
ATTTCACGTTCAGCAGACTCTAATGAATCTGAACCGTGAATGATATTTCTACCAACAGTTAAACCTAAATCACCTCTAATTGATCCT
GGTGAAGCTTCTGAAGGATTGGTGCTGCCAATAATATGTCTAGATACATTAACTGCATCTTCACCTTCAACTACCATTGCGAACACT
GGTGCTGATGTAATAAATGAAATTAAATCATTATAAAACGGTTTGCCTTGGTGTTCACCATAATGTGTTTCAGCAAGTTCCATTGGT
ACTTGCATTAATTTACCACCGACAAGTTTTAGTCCTTTTCTTTCAATTCTTGAAATTACTTCACCAATTAGATTTCTTTGTACTGCATC
TGGTTTAATCATTAAAAATGTACGTTCCACTTTATGTATCCCCCTGTGTTTAATAGGTTTATGTATATTACTATTATATATTAACAGGT
TGGTAAAGCGCTTTCAATACTTTTCATAAATACTACGTGTTTCTTGAACCCATTTTTTTCGTCAAACTTAAAAGTAGTGATTTCGGAT
GTCCATCTGGTAACTCAGAAATCAAATCCAAAGCTTTACTTAAATACTTCGAACTTACTGCTTTAGATTCATTAATACTGTCAGACTT
TCTAATAATTTGAATACATTCTTCAAATTCTTTTCGATCGCTGTCACGTCGTAATTGCTCAATTTTCAATTTGAAGTCTGGGTTTTTAC
GCATTTCTAATAAAATCGGTAACGTAATATGACCATTAAGCAAATCACTTCCGACCGGCTTACCTAATTTCTTTTCGGTACTTGTGAA
GTCTAATACATCATCAATGATTTGGAAGCTCATACCTATATAATGACCAATCATTTTCAATTTTCGTACAGTCTCTTTATCAGATTGA
GATGTAATTGCACCAACTTCAGTTGATAATTGAATTAACAGTGCTGTTTTGCGATTGATACGTCGTAAATAATTGATAATTGTCTGTT
GACTGTTAAATTGGTCTTGAAATTGGAAAAGTTCCCCTCTACAAACATCAACGATAGATTCAGATATCAATTGATGTACACGATTAT
CTTTAACGGCCATTAAGTGTTCAAGTCCTAATGCCAATAAAAAAATTCCCAGTTAAAATAGCCGTTGTCTGATCCCATTTCTTTGATAT
GGTTAACTTGCCTCGACGCTTGTCGCTTTTATCAATAACGTCATCATGAACAAGTGTTGCCATATGAATTAACTCTAATGCGACTGCA
ACATGATACGTTTGTTCAGACGTTTGTTCATCTTTACCAAATTGGCTACTCAGAATCACAAATGCAGGTCGTACACGTTTTCCACCAG
AAGACAATAAATGTAAAGAAGCTTGTTCTAGAACAGAATCTTTACTTTTTATTGCCTTTTCAAGTCGTTGTTCCACTTTCTTAATTTC
ATTGTTCATGTTTAACTTTGCCACGTTAATCACCTTTGGTATTATCTTTTTCTTTATAGCCAAGGTGCATTGCAGCAACGCCCCCTGTA
AAACTACGTACTCTTACATTTATGAAACCCGCTTCTTCAAACATGCGCTTCAACTCTTCTTTTCCAGGAAAATTAAACGTAGATTGCT
GTAACCATTCATATTCTTCTTTTGATTTTGCAAATAATTTTCCAAAAATAGGCATAACAAATTTAAAGTATAATGCATACATTTGTTT
AAAGACTGGCAAAGTTGGTTGGCTCGTTTCAAGACATACCACCATACCACCTGGTTTAAGTACTCTATTCATTTCTTTTAACGCGACT
AAATAGTCTGGCACATTTCTTAATCCAAACCCAATTGTTACATAATCAAAAGAATTGTCTTCAAACGGCAATTCCATTGCATCACCA
TGAACAAGTTTAACATTTTCCATTGAAGCAGTTTTTTCTTTTCCTACTTCTAACATATTCTCACTAAAGTCAATACCAGTAACTTCACC
TGTTGGTCCTACAGCTTTGCTTAATGCGATTGTCCAATCACCAGTACCACAACAAACATCTAATGCTTTCGTCCCTTTTCTAACACCC
ATGTCTTTCATGACGCGTTTTCTCCATACTTTATGCTGCTCAAAACTAATAATATTATTTAATCTATCATATTTTTTTGAAATATTTTG
AAAAACGCGATGTACTTGCTCTTTATTTGCTTTATTGTCAGCCATGCTTAATTACCTCTACTTTTTAAATAACTTTTTTGGATATCGTG
TAAGTAATGCTTTACTTCACTTTGATTATATTTCTTGAAGTATGATGGATAGTAATCAGACATATCTTCAAATAAATAATTATATATT
TCCGACTCATCAATATTGATACCGAAATGAGATAACGTAATATATGGGAAAAGTGTTTCAATTTTTACTATTGCTTGAGAAATTTCA
TAATCATTTAAAGCTTGATGATGTAATGAAGATTTCAATTCATTAATTTCAACAATTGCTTTACTAATTTCATTTTGAAATGATAAAT
CATTGATTTCTGCTAGTAGCGTATAAAAATGTGCACTAATTAAATCTCCAATCAAAATGGAATGTTTAGACAAATGATTATATGTAA
TGTCATCAAGGTGTCTCATTGATGTGTCAATTGTGAGGCATGCCACTTTGGCAACATCTGGAATATCATATGAATCAAGTAACTTAC
CTAACCGATGATTAATATTAATGGATTCATATTCTGATACGCCCTTTAATCTTTCTTCTATTTGTCTTTCCAATTTGCTAACAGTTGTT
TCCATGTCTACACCTCACGATATTATCACTATTCATATTAACATTATATGTAAGAAATTAAAATCTTTTGAAGCATTAAGATTACTTA
TCATTTTTAAATTTCAATTTAAACTAACAGTAATTTATGTAGCTTTTGTAATTCTCATAATAACCTTTACTTCATTTACATTATTCATC
TTTTTAAATAACGTAAAATACTATAGTAGACATGGTTACCTTTTAAGTAAATTCAACGCTAACAATTAATCTCAATATTCATATGTAT
ATTTTGTAGTTGCTATTTTTATCAAAACTATTATGTCATGTAATATTTTGAACGCTTAAACAATGAATGTTCCAAAGATTAAAAACTT
AGAAGAACATTACAATTTATGTACAGGTTGTAAGTATTAAAAGCAAAATAAAAAGCCCTTATTCAGGGCTTTTTAAGTAAAATTATT
TTACAGCATCTTTTAATGCTTTACCAGCTTTGAATGCTGGAACTTTACTTGCTGGGATATCAATTTCTTTACCAGTTTGAGGGTTACG
ACCTTTACGTGCAGCACGTTCACGTACCTCAAAGTTACCGAAACCAATTAATTGTACTTTTTCACCTTTAGCAAGTGAGTTTTGGATT
GATTCGAATACAGCATCTACTGCTGAACCAGCTTCTTTTTTAGTTAAATCAGCTTGCTCTGCAACTGCATTGATTAAATCTGTTTTGT
TCATTAGACATTCACCTCCTGAGGTTTGATAATGATATGTTTATATCATTATGGTAAAACTTTAACACAAGCATTACATAAATCGCA
ATCATTTATGTGGCAAAAACGTTGAATTTAAGCCATTTCTATTCATTTTACGTAATTATTAGCCGTATATGTGTAATAATACACATTT
TATTCAGATTTTTTATCGCGCTCCATTAAATCTTTTACGCATTCTTTTACTGAGATATTTTCAAATAATACTCTATATAATGCATTTGT
AATTGGCATATCCACATTTTTTTCTTTAGCTAAATGATAAACTGATTTAGTTGTATAAATACCTTCAACAACCATATTCATTTCAGAT
AATGCTTGATCCATTGATTCACCTTGTCCAAGTTTATATCCTAATGTGAAATTTCGAGAATGTGTTGATGTGCAAGTAACGATTAAGT
CACCGATACCACCTAAACCTAGAAATGTCATAGGATCGGCACCTAACTTTTCACCTAATCTACTAATTTCCGCTAAACCACGAGTCA
TTAATGCAGCTTTTGCATTATCACCGTAGCCAATTCCAGCTACGATACCACTTGCTACTGCGATGATATTTTTCAATGCACCACCAAG
TTCAACACCAATCAAGTCATCATTCGTGTACACACGTAAATAATCATTCATAAATAAATCTTGCGTTAATTTACTTACATTTTTATCT
TTTGATGAAGCAGCAACTGTAGTTGGTTGCTTGACTACAACTTCTTCCGCATGACTTGGCCCGGACAACACGCCAATACCTGCATTA
TATTCAGGTGAAATAGAATCTTCAATCATTTCTGACACACGTTTAAATGTCCCATTTTCAATACCTTTAGCAACATGTATAAAAGTCT
TTTTAGATGTCAGCTTATCATTAATTTGAGTAGCAACTTCTCGCATTGCTTTAGTAGGTAAAGCCATTAAGTAAATATCTGCAAATTG
AATTGCTTTGGTCATATCTGAAGTAGCGATGATGTTAACATCTAATTTCGCGTATTTTAAATACTTTTTATTTGTATGACATGTATTTA
ATTCATCAACAGCATCTTGATTTTTACCCCACATCAAAACATCATGTCCATTTTCTGCAAGAACATTGGCAAGGGCTGTCCCAAAAC
TTCCCATACCAAAAACGGTAATTTTAGTCATTGTTATCCCCCAATCGTTAATTTCTCTTTCGAGCTATAATATGAATTGGTGTACCTT
CAAAACCAAAAGCGGCACGGATTTGATTCTCTAAATAGCGTTTATAAGAAAAATGCATTAATTCTACATCATTAACAAATACAACA
AATGTCGGTGGTTCTATAGCAACTTGTGTTGCATAAAAGACATTCAAACGTCTACCTTTGTCTGTTGGTGTAGGGTTCATGGAAATT
GCATCAGTAACGACTTCATTTAAAGTTGAACTTTGAACACGTTTTTTATGGTTTTCACTTGCTTCATTAATGTAAGGAATAATGTAC

GTAATCTTGTGCGTTCTTTAGCAGACACAAAAGCAATTTGTGCATAATCTAAAAATTGGAATTCTTTACGTACTTCATCTTCAAATTT
CTTCATCGTTTTACTATCTTTTTCCACAGTATCCCATTTATTTACGACAATCACGACTGCTTTACCTTGTTCATGTGCATATCCTGCAA
CGCGTTTATCTTGTTCAATGATGCCTTGTTCTGCATCAATGACCACTAAAACAACATTTGAACGTTCAATCGCTTTTAAAGCTCTTAG
TACTGAATATTTCTCAGTTGATTCATATACTTTTCCTTTTTTACGCATACCAGCAGTATCGATTAAAACATAATCTTGTCCATCATAAC
TATACTCTGTATCAATAGCGTCTCTCGTTGTCCCTGCAACATTAGAAACGATAACGCGATCTTCACCTAAAATAGCATTTACTAAACT
TGATTTACCTACGTTTGGTCGTCCAATAATGGATAGTCGAATTGTATCTTCATCATAAGGATCTTCTTCCTCTTCACCAAAATGAGAA
ACAACTGCATCTAACAAGTCACCAAGACCTAAACCATGTGATCCTGATATCGGATACGGTTCACCAAATCCTAATGAATAGAAATC
ATACACGTCTGTACGCATTTCCATATTATCTACTTTGTTAACCGCTAATACGACCGGTTTTTAGATTTGTATAAAATTTGAGCGACC
ATTTCATCGCTTTGTGTCAATCCTTCACGCACGTTAACCATAAAAATAATAACATCCGCTTCATCTATGGCGATTTCTGCCTGCGCTC
TAATTTGTGTTTGGAATGGTGCATCACCAATTTCAATACCACCTGTATCAATAATATTGAAATCATGTGTTAACCATTCACCTGAAGA
ATAAATACGATCTCGTGTTACACCTGGCGTGTCTTCCACAATCGAAACACGTTCTCCAACTATTCTATTAAAAATTGTAGATTTACCT
ACATTAGGCCTACCTACAATAGCTACTATAGGTTTAGTCATAAGCTAACTTCCTCTCTTTCTTATATCTTTTTAATAATATCACATGA
GACTGTGTATTCAAAATGAGTTTATTACTGTAATGATAAAAATCAGCACTTAAAACTTTATCTTTTTAAACACTTAGTTAAATTTGTC
CACGTTATCATTCCGAGTATATTTTGTTACATAAAAATACTCCACATGCAATCATAAACATGTGGAGTTGACTATTAAATATTAAATT
ATAGTTTAAGATTTTTAAGTTTATCACCAATCATATCGCCAATTGTTGGATTATCTTCTTCTTCGTTTTCTAAGTACGCCTTAGTCGTA
GAAGGATCACTTTCAACAACATCTTCGTTTGGTAATGTTGCTTTAATAGATAGTGATACTCTTTCATTCTCTTCATCAATACCTAATA
TTTTAACATTTACTTGTTGACCAGGTTCTAACACTTCACCTGGCGTACCAATGTGTTTGTGTGCAATTTCAGAAATATGTACAAGTCC
TTGTACACCTGGTGCAATTTCAACAAATGCACCAAAGTTTGCCAATCTTACTACGACACCTTCAATGACATCATTTTCGTGGAATTG
ACCTTTAATATTTTCGAAAGGTGTTGGTAACGTATCTTTGATTGATAATGAAATACGTTCTGTATCTCTATCAATAGATTTAATTTTA
ACTTTAACATCTTGACCAATTGAAACTACTTCTTCTGGTGTTTGAACATGTTCGTGAGAAAGTTCAGATACATGCACTAAACCATCA
ACACCACCAATGTCTATAAATGCACCAAATTGAGTTAAACGCGCTACTTTACCATCAATAACATCGCCTTCATTTAAAGATTGTAAT
AATTGATCTTTTTTAGCATCGTTTTCTTCTTGTTCAACTGCTTTACGGCTTAAAATGACTCTATTATTTTCAGGATCCAATTCTTCAAC
TTTAATACGAATTGTTTGTCCATCAAACACAGAGAAATCCTCAATGAAGTCTGTTGAAATTAGTGAAGCCGGAACAAAACCTCTTTG
TCCTACATCAACAACCAAACCACCTTTAACAACTTCTGTTACTTTCGCTTCGATGATTTCATTATTATCTAATTTTTCTTGTAAATAAC
TATAAGACTTCTCAGTTTCAAGTTGTCTTCTAGATAAGATGTAAGCTCCAGTTTCATTTTCTTCATCAAACTCAACTTTAGTGACATA
TGCTTCAACTTCGTCGCCCTCTTTTACAACTTCACTTGGGCTATCAATATGATGCGTAGATAGTTGACTAATAGGAATAATCCCATTA
AATTTACCACCGTTGATATGAACAACAACTTGCTTGTCTTCAACTTGTTGTACCTCGCCAGTGACTTTGTCACCTTCTTTAATATCGTT
AATCATTGATTCATTGAATTCTTCAGTCATCTTGTTTGCCTCCTTATACACTACATATTTATAACATCTTATAAACTGACAGAATTGTC
AAGAAATTCTACCTCGATTACATTTTTCGAAATAGTCATATACATAAATATGTCACTTTATCATATTCAAGTCGTCATATCGATAAAT
ATTTATTACAACCATTTTTGTATCAAAGTAATACAGTTTGCTATGTTTATACATATTTTTTGTATAAAAATTAGCATAGCTTACTATTT
TGTTTTATAAGTCTGTCATTTCATTGTTCTCTACAAGATATATTATAGTAAAATTTTATACTTTTATAAAACAAAAAGCCACGTAATT
GAGAAACTTTTACATTTTGTTAGTAAGAAGTGTGTATATTAGGCATCATTCTTTAATCATTATATTTGTTAATCGTAATAATAAGTCA
TTGCTAAATAGCACTTTAATATTGATGTATTTTAAAGCATCTCTTTAATTTTAAAAAAGTAAATTTAAATAACATGCTATTTTAGCTT
TTTTGTTAATACAATTCATAGGTTGTTTATAAATCAGTTGAATTACTAATATGCTTGGTTTAATTACGCACTTTTATTTCTAGTATTAT
CGTTATTCGTTTATAAATTAACAAAAGACCATTTCTATTCGATTAGAATAAAAACAGCCTTTTCCGTTCATCAAGTAATTATAAATTT
TATTAATGTTAGCTTCTTAATTATTTAATTTGACTCACCATCGCTAAAATTTCGTCAGTAACTTCTTCAATCGACTTGCCTGTCGTATC
TAATGTCACTGCATCATCTGCTTTTCTTAATGGTGATATTTCACGGTTCATGTCATATTGATCACGAGCTTCAATATCACGTTTTAAAT
CTTCAAAATTTGATTCGATACCTCTTAATTGATTATCTTTATATCTTCTTTCTGCTCGCTCTTCAACTGATGCAATCATATATACTTTT
AAATCTGCATCTGGTAGCACTACAGTTCCGATATCGCGACCATCCATTACGATACCTTTTTCTGCAGCTAACTCTTTTTGTTTTTTAAC
GGCGAATGAACGTACTGGCTCTTTAGATGCAACGTATGAAACATGTTGCGTCACATCATTATTTCTTAAAAAGTCTGTTACATCTTC
GTTATCTAAAATGACACATTGACCTTTATCTGCTTTATAAGTTAAATCTAATGTTGTTTGGTCAACTAGTTTTGCAAAGTCCTCAGTT
TTGTTTAATTTTAAATATTTGTATGTTAATGCACGATACATTGCTCCTGTATCGACATAAATCATTGATAGTTCACTGGCTACACGTT
TTGCAATTGTACTTTTTCCGGCAGCAGCTGGACCATCTAATGCAATATTAATGGCTTTCATGCGTCATTTCCCTTCCTTGTCGTTTTAT
GAATAATATTTTATCATAATTAGGTAAATGAAATATAGGAGGAAATATATGAAACATCTACTGGTTATTCATACTGGTGGCACCATT
AGTATGTCACAAGACCAATCTAATAAAGTAGTAACAAATGATATCAACCCTATTTCAATGCATAAAGATGTCATAAATCAATATGC
ACAAATAGATGAATTAAATCCTTTTAATGTACCATCACCTCATATGACAATCCAACATGTTAAACAATTAAAGGATATTATTTTAGA
AGCAGTAACAAATAAATATTATGATGGTTTCGTTATCACGCATGGTACCGATACGTTAGAAGAAACTGCCTTTTTACTTGATTTAAT
ATTAGGTATCGAGCAACCTGTTGTTATTACTGGCGCAATGCGCTCGTCTAATGAAATTGGTTCTGACGGATTATATAATTATATTTCC
GCTATTCGCGTTGCCTCTGATGAAAAGGCCCGTCATAAAGGCGTGATGGTTGTATTTAATGATGAAATTCATACGGCGCGTAATGTT
ACCAAAACACATACGTCTAATACAAACACATTTCAAAGTCCAAATCATGGTCCGCTAGGTGTATTGACAAAGGATCGTGTGCAATT
CCATCATATGCCATATCGCCAACAAGCATTGGAAAATGTCAATGACAAACTAAATGTACCATTAGTAAAAGCATATATGGGTATGC
CAGGTGACATTTTTAGTTTTTATAGTCGAGAAGGTATCGATGGTATGGTTTTTGAAGCGTTAGGACAAGGCAACATACCTCCAAGCG
CATTAGAAGGCATTCAACAATTAGTATCTTTAAATATACCTATTGTGCTAGTTTCACGTTCCTTTAATGGTATTGTGAGTCCAACTTA
CGCATACGATGGTGGTGGTTACCAACTCGCACAACAAGGTTTTATTTTTTCTAACGGTTTGAATGGTCCAAAAGCAAGATTAAAATT
ATTAGTTGCGTTAAGCAACAATTTAGATAAAGCTGAAATCAAATCATATTTTGAATTATAATGATTACGTAACATAAAAAGGTCTAT
GTACAATCATTAGTCATCAACCTCATTTGACATATCATGAATGTCAGTCGTTGTATAACTAAGATATTTAGTACATAGACCTTTATTT
TTATGATTCTAACGGCGTTTGTTTCTTAGCTAGCATGCTTTGAGCAATAATGCCACCGTGGAATTTACCATTTTCAATAAAAATGGTA
TTCGCATCATTCCCTGCAGCAATCACACCTGCAATGTAGCAATTTTCGATATTTGTTTCGTATGTCTCTTTATTATACATAGGCGCTGT
TCCAAATTCATTTGTATTAATTTGAATACCTACAGATTTTAAAAATTCATAATCGGGATGATAACCAATCATCGCAAAAACATAATC
ATTGTGTATCGTTTTACTTTCACCATTTACTTCATAAGTCACAGTATCTTCAGTTATTTGGGTAACATTAGCATTAAATTCCATGTCAA
TTTTTTCATGATTTACTAATGCTGTGAAATTTGGAAGTATCCACGGTTTAATTGAAGGCGAATAATCTCCACCACGATATAGAACCG
TCACGTTAGCACCAGCTTTTTCCAACTCCAAAGCAGCATCGATAGCCGAATTCTTACCACCAATAATAACAACATCTTGATCAAAAT
ACGGATGTGCCTCTTTAAAATAATGGAACACTTTAGGTAAATCCGCACCTTCAACCTCTAATGTATTATGCTGACCATAATAGCCTG
TCGCGATTGTTAAAAATCGACATTCATAAACATCTTTCGTCGTAGTAATTGTAAATTTATTATTCATTTTTTTAACAGTTAATACTTCT
TCAAATGCATTTACTTTTAATTGATGATGTTTTACAACTTCTCGGTAATAAACTAGCGCTTGATTACGTCTTGGTTTACTTTCTTCAAC
GATAAACGGTACGTCCCCAATACTTAATTTATCACTTGATGAGAAAAATGTTTGGTGAGTAGGATAATTATATATTGATTCAACGAC
ATTACCCTTTTCAATAATTAAGGTATCAATACCTTTTCTTTTTTGTTCAATAGCCGCACTTAATCCGCATGGCCCTCCACCAATTATGA
TACTTTCAACTTTTTGCATTGTTTCGGTCTCCTTTAATCCCAAACTATTATAACAGTTCAGTGATTGATGCGCTAAGTCTGTGCTTGAA
GATTTATGATATATTTTCGACAATTTGTTTAATATGCATAGAGAATTGTTAGCTACGGAGTTGAATAACACTACAATTTAATATGTTG
AATTATTTTTTATTCTTCCTCGTTAAATTTGTTGATTGTGGTGTTTTATCTTTTTGATGACTATGTAAAAAATTTAATTGATATGCCAA
TATTCGAGACAACACCTTGTTAAATAACAAACTTTGAAAAATGAAAAAACCGCTACATACTTATAAGCGCAATTTAAGTTCTCCAAA

ATATTTAGAGATACTTTTAATATGCACGTTTGTATATAGCAGTTCAGTTACAATTTATATAGTTATATTATGGAATAACGATTTGTTG
ACCGTTTCTAATATTGTTACCACTTAAACCATTGGCACGTCTAATTTTTTCAACATTTTCCGGTGAACCTGAACCGTAGTATTGAATT
GCGATACGGTATAAGTTTCTTGACCATTCACTGTATGTCTTTGGCCACCACCTTGACGTTGTTGTTGCTGTTGTTGATTTTGATTAGC
TTGTTGTTGATTTTGATTATTTTGTGCTTGATTGTTCGCTTGATTAGCGTTGTTTTGATCATTATCAGATTCATCTTTAGTCGCTTTGTC
TTGATCCTCTTTTGATTTATCACTGTCTGTAGATTTTGATTTATCTTTAGAAGCGTCTTTAGATGTGTCTTTGTCTTTACTTTCATCAGC
ATTATTTTTATTTGTATTCGCGATTTTATTTTCTTTTGTACCATTATTATGATTGTTTAATGCCATGCCTCCAAATATCGCTAATGCACC
GATAATTAGTACAGCTGCAATTAATGGTAACAATACTTTGGCCATGCCACCTTTTTTACGTTCTTTATCTCTGTCATGATTGTCATGTT
CATCATGGTTTTGGCTTGCATTATTAGAGGCATGTGGTTTTGAAGCGGCAGAAGCACTTTTACTTGCTGCGCCTCCAGCCAAACCTG
CTGTTCCAGCACCGATCGCTGCACCTTTTTTGCCATTATGATGATCTTTAGACTTATCTTGAGACACTTTATCCTCAGTCGAGTTATTC
GACTTGTCAGAATTACTTTTGTTTTGAGCGTCATTTGAATGTTTCTTAGCTTTAGAAACACCCATTGCACCAGCTGCACCTGCAACAC
CCGCTGTTCCAGCACCAATTGCTGCGCCTTTTTTACCACTATGATGATCTTTAGATTCATCTTTGTCTTGCTTAACAGTTACATCATCA
TGTTTATCTTTTGATGTATTTGCTTGGTTAGCACCTGTTGCAAAATATGGTTTAGGTTGCTGAGATTGTTCAGCTTCACTCTTATCAGA
AACTGTTGAATGCTCAGTGTTATTTTCTGCTTCTTTAATAGTCTCGTGTTTATCATTGTCTTCGATTGGTTCTGGATGTGATTTATCCA
TTGCAAAAGCATTCTTATTATAATATTCCTCTTCATGTTGAGGTGTACTGTCTTGATGGCTAGGTTCTTGACTTTCAGTACTGTGTGAT
GATTCGACTTGACGATCATCTATTGTGCCAGCCTCATTTTGAACATTGTCTTCAGATGTTTGTGATTCATTGTGAACTTGTTTATTATG
ATTCGTTGCTAAATCACGGCGTCTTTTTCTTCTTTGGGCATTTCTTGGCGGAAACTGTTGCTCCGTATTCTCTATTGTATCCTGATGTT
CTAATTCTGATTGGTCTTTTTCAACATCTTCCGTATGGTCTTGATGTGAATTTGTGTCTATCGATTGACGATTTTTTTCAAAGTCATCT
TTAAAATTATTAGACATAGCCATCTTCCTTTCTATTCTATTACAATGATTTTACATAAAATATACCCACATGACATATGTTTATGTAT
ATTTTTCAAAATTATTTCAATCATTTTTCAATTGTGTATACACTAATTATAGCTTGTCAATTTTCTTTTAGTAAAGTAATACTATCTGA
GAAATAAATTTTGCAACTTTTCATCAAATCCAATACTTCTAATTACCTTCTTCTTATTTAAAATTGCGATATCAGTTATATTAGAATC
ATTGTCACAACATCGATCTTGTGCCGGTGGATATTCACCGAAAAATTCTAATAAATACTTCCGTCTACATTGATCCAATTTGCAATA
GCCAATCATGCGAAAGAATCCTAATTGCTTTCGTTTAAATGATTGCTTAAATATCTGTTTCAAGGCGCCGATACTATAGAATGAATG
CAACGTTGTCAAAACGTCTTGTTTATCGGGAGCTAAAAATTCTCCTATTTCGAAATTTTGTACATCTTCTTCTGTTATCATATCTGCA
AATAATAACGTTTCTAAAATATATTTATCGTCCGGTTGGAATAAACTAATTGCCTGACTTAGTTCACCATCGCGACCCGCACGGCCA
ATTTCTTGAATGTAGTTAGAAGGACTTGTTGAAAGATGAAAGTGAATGATTGTGCGAATATCTTTTTTATTAATTCCCATACCAAAA
GCACTCGTTGCGACTATAATCGGAATATCATTATTTAAAAATTGTTGTTGAACTGTGTGTCGCTCTTGATAATTCATATCACCATGAT
AAATACCTGTAAGAAAACCTGAATCATAAATAAGTTGCGCTAAATTCAGACACATCTTTTTCGATGAGACATAAATAATCGTTGGTC
CCGACTGTTGTAGAAACGGCAGCAACCATTCAATTTTATCTTCATCATCATGAAAATTAAGATGCTTAAAGCTTATGTTTGGGCGAT
TCATTGTAGTTTTAATAACATTGAATTGAATCGCTAACATTTCCGTCAAATCATCTTGTAAATGCGGTGGTGCAGTTGCTGTCAATGC
TAAGACAACCGCTTCTTTAAAATGCTTTGTTACTTTTCCTATTAGAGCATAATGTGGTCTGAAATCATATCCCCATTCAGATAGGCAA
TGTGCTTCATCTAGAACAATCATGCCAAAGTCTATCATAGATATTAATTTAAAATTTGACGGTTGCAGGAGAAATTCTGGACTTAGA
AAGATGAAGCGGCTATGTCGTAAACATTTAATATTATGCTTTTTCTCAATTTCATCCATACCAGAGTGAATAGATGTTACATGTTTTT
CTCCATTTATTTTCAACTGCATAACTTGGTCATCCATTAAAGATATTAACGGTGAGATAATTAATGTCGGCTTACCTGATAAATACGT
AGGTATTTGATAACACAAACTCTTTCCACTTCCAGTTGGAAGAATACCTAGAGTGTGCTGTTGAGACATTATACTTTCTATAATTTCC
TGTTGTCCCGGTTTAAAACTCTCGAAGCCAAATTTGTTTCGTAAAATATCATGCAACATTCAGATCACCTCTTTCAAATCCAACGATT
AATACTTTTAATTGAAAATATGATAACGTGTCAAATTGTTCTTTGTAAAATTTTAATCGTTCGCCACGATGTTGTTGATAAAAATTCA
AAAATTGCAGTTGATTTTCTTGTTCAACATAATCATCGTAATTAGACATGTAACCTTTGATTAAGATTTCAAGTACATGATCTTCGAT
AGTATTGATTTTAACTTGTTGTTGAGCCGCTATATCTTCCATCGTGAATTGTTCAAGCAATCTTGTATAAGTAATATAAGTTTGGTTT
AATAATGTAGGTTTCATTATTATTTTTGATAAAATAGTATATTCTCCACTTTCTAATTCAAACATCATTATCACTAAATTATTCATTTC
GTATTCAAACAATTGCTGTTGAGATAGTTTTTCTATTAAACTAACTTGTTGTCTCGTATACATTGGTTCATCATAGCCTTGCAAATAA
TAATGTAAGTAACATGGCCCTTTTAAAGTTATTGCTTTAAACAAGTTATGTAGTTCATTTTCAAAATCAATTTGTAGCTTGTTTTCTTT
AATGTAGTTATAGACAATCTTTACAGTTTCTTGTATCTTATTATTTTGAGAGATTGGTACAAAATGAAAAACATGTTGTTTGGTATTA
GACATGGTTTGCACTAATAGTTGAATTGCTTGAAATGTTTGCCCCATACTTTCAAATGTATATCTAGGATGCAACATGATTTCCATTT
CAGCATTAAATTCAGTTATATTTTCTAAAAATAGCTCAAAAGACGGATATTTTAATAGTGGTAAACTGTGATATAATGACAACTGTT
GTTGACTACAAGCGTCAAAAAAGGTTTGGTGAGATTTCTTACCAACTAAGATATTGTAAATACTTTTATTTGTTTTATAGTTAAATGT
TTGTTGTAATGCTGTTTTTATAATGTGTTGCAAATTTAACCCTCACTCTGTAAAACGGATAATTTGAAAATAAAAATGATGCTGTCTA
AAATTTTAGTCACATTACTATTTTAGCATTTTTATCAAAGGAGGCGAAGACATTGGCAAAATATACAATCGTTGATATGGATACTTG
TATTGCATGTGGTGCATGCGGTGCAGCAGCACCAGATATATATGATTACGACGACGAAGGTATTGCTTTCGTAATCCTTGACGATAA
CCAAGGTACTGCAGAAGTACCTGAGGAATTATATGAAGATATGGAAGATGCAATTGATGGATGCCCTACAGATTCTATTAAAATTG
CAGACGAATCATTTGATGGGGACGCTTTAAAATTTGAATAATTGATTTTTTAGACAAATGTTCTATAGAAAAAGCAGGCAAGCTATC
GTTTATTGATAGTCACCTGCTTTTTATTATTATGTGTATATTATTTTGCTTTAATTTGATTAAATTCTTTTCAAGAAATTCGCAAGCCT
TCTATATAGTAAAATAAATACAATAGAAATGACGATACCTTTAATAATATTGAATGGTATAATTCCTGAAACAATGATTACTTTAAG
ATTATTTGCGATATCAGCTAAGTTAAATATCATACCGTACAAAGGTAATAGAACGAAATAGTTCAAGATACTCAACACGATAGTCA
TAACGATTGTTGCAATGATTAATCCAGTAATCAAAGATTTTGTTGAACGTTTATTTTTATAGATGGCGTAAGCAGTTAATAAGAAAC
TTGCGCCTGCTAAAAAGTTAGCAAATGGTCCAACTGGATCGCCCATACTAAATAAGTAGTTCAATAAATTTTTAACCAGTGCAACTA
CGATACCGGCAACTGGTCCAAACGTAAATGTAGCTAGTAGTGACGGTACATCACTAAAATCTAAAGTTAAGTATGGTGGCAAAAAT
GGTATAGGAAACTTGATAAAAGTTAACACAAACGCAATCGCGCTCAACATACTTATTGTGATAAGACGTTTATTTTGTTGCATATTC
AATTTTCTCCTTCCATTCATCTCGTAAGAATAGGAGGATTATTGCACAACAAAAAACCTCCATCTTCTCCCATCCAGACTATACTGTC
GGCTCTAGAATCTCACTAGATCAGCCACTAATATGATACATACTAGCAGGTCGCAGGCTTAATTTACTGCCGGTTGGGAATTACACC
CGACCCCGAAGATGAATTTTACATTATTAAATTTAATTCCCATTAATTTTATACTATATATCACAAAACCTCAATAACTATGCTTAAA
ATGAAAATTAATTAAGTTGGTGATGTATAATGAATTTGCGATTTGCGAGCATTGGTTGGAATGTTGTTTGTTTGATTAATTTTTGAGA
TGGCTTTTTTGAACCTATCTTTTTATTGTTTTTAGTTTAATCATTCGTGTTAGCTTATTCATGATTAAATACACCTACTTCTATTACATC
CTTCTTACTATAATTAGGTCTTGTTGTATTTATATATTTATTAGAAATTTGAAGAGAAACATTCAAATCATGAGGAATATTCTTTTTCT
TTTTTAGCACTTCAGAAAAATCAATCATCTCGTCTATTGTATTATCTTTAGTAAAATTATCTTTAGTACTAAATAAAGTTGTAACAAC
ATATGGACGTGATTTATCTTTAAGTTGTTTTTTTGAGTTTCTCATACCGTCCCTAAAAGACTTATCATCCTCTTTGATTAAGGGTTGAA
AATACTTTTTATATTCATCTAAAGTATATGGAATTGCAGTCAAATAATAATACTCATTTCTAAAACCACTATTCTGCGTTTTCATAAT
TGCATTTTCAGTATAACCAGTATATTTATATTTACTTTTATATTCTTTTAAAACTTCTGTTAAATTATCCAACTCTTCCTTATGTGCTCG
ATATTCAAAGCCACTCAACACTGTACCAACTAAAGTACTCATATCATCGCCTTTATCCTCACTTCTTAATGAGCTATCACTGTCAATA
ATTGATTTGTCAAATGGAATACTCGCATTAAATACGATATCGTGGTCATCACAATGCACGTATACTTCTACGCCGTCGCCACTACCT
ACAACATTCGTAGCTTTAACTTTTAGACCGAAGTTATCCATAAAAAAATTGTTCGCCACGTTTAGCAATTTTATCTTTATGCTTCTTCG
CAAATTCAATCGCATCTTTTTCTGCAGGTGGTTGGAAGCCTTGACCTACATATTTTGAAGCTTCCATTTCTTCTGGTACTGATTTTGTT

TCTTTATTAGATTCGTTATTGGTAGTTGAACATCCTGATAGTATTATCGTTGCTATTAAGATTAGTTTAATTCTTTTAAACATAGCTCA
TCTCCCATTTATGTGTTTGTTTAGAATATTAAAAGCAATTTTTTAAAATTTTATTCACCTTTACTGTGCACCCCATATTCTATTGGACG
ATTATCATCATAAAAGGCTTTTTAGTATTAATAGTAGGCTTACCTATTTGAATTGTAATTGTAGATTTATCTGGTTTATTTTTTAAAT
TATATAATTTATCACTTAGTTCAATTACATCATCTTCTGTATTGTCATTAGTAAAATTTTTCTTTGTACTAAACAGTGTCGCTACTGCA
TCTGTATCAGCGGCATAATTCACTTCTTTTCTTGCTCGTTCCATTCCTTCTTTGAATTCCTTATCATTTTTTTGAATCAAAGGTTCATAA
TACTTTCGATATTCTTTTAAACTTCTAGAAGAGTATGTAATATAAAAATATTCATTTTTATATCCAACGTTTTGTGTCTTATTAATTGC
TTCTTTAGTAAAACCTGTATATTGATATTTCTTTTCATTTTCTTTGAAAAATTTATATAAGTTATCATACTTTTCTTTTTGCGCTCGATA
TTCAAAGCCACTCAGCACTGTACCCACCATCATACTCATATCATCACCGTTGTCATTACTACGCATTGATCCTTTTTGATGGATGGCA
TCTTTGTACAAGGGCAAACTTGCATTAAATACAATGCCATGATCATCGCAATGCACATAAACTTCTATACCATCATCTTTACCTACA
ACATTGGTAGCCTTTACCTTCAGTCCAAAGTTGTCTTTAAAGAATTGTTCACCTACTTTTTCAAATTCTTTACGATGCTTCTTCGCAAA
TTCAATCGCATCTTTTTCTGCAGGTGGTTGGAAGCCTTGTCCAACATATTTTGAAGCTTCCATTTCTTCTGGTACTGATTTTGTTTCAT
TGTTGGATTCGTTATTGGTAGTTGAACATCCTGATAGCAGTAATGTTGCTATTAAGATTAGTTTAGTTCTTTTAAACATAGCTCATCA
CCCATTTATGTGTTTGTTTAGAATATAGAGAAATTAGCTGATTTCAAGTCATTCGACCTCAATCCTTATAGACTCATTATCACTGTAA
TTAACTCGATTAGTACTAATAGTAGATTTTGCTAGTTGTAAAAATATTTTCGTATTTTCAAAATTAAGGTGTAACTTTTTCGTACTTTC
AGACAAATCTATAACATCATCTAATTTTTTGTCCTTTGAAAAGTTACTACTTCTCGAAAACAATGTTGTATGTACTTCTATTGCAGCT
TTATAGCCTACTCCTTTCCTTGCTTGTTTCATACCTTTTTTAAAATTCAGATTATTTTTCTTTATTAGGGGTTCGTAATATTTCCTATAT
TCTTGGAGCGTCGGTATATTAGCAACTATATAAAAATATTCATTTTCATATCCACTATTTTGTGTCTTGTTAATTGCCTCTTTTGTAAA
TCCTGTATATTGGTATTTCTCTTCATTATCTTTAAAAAAATTTATATAAATTGTCATACTTTTCTTTTTGTGTTCGATATTCAAAGCCACT
CAACACTGTACCAACTAAAGTACTCATATCATCGCCTTTGTCCTCACTTCTTAATGAGCTATCACTCTCAATTATTGATTTATCAAAT
GGAATACTCGCATTAAATACGATATCGTGGTCATCACAATGCACGAATACTTCTACACCGTCGCCACTACCTACAACATTTGTAGCT
TTAACTTTTAGTCCAAAGTTATCCATAAAAAAATTGTTCGCCACGTTTAGCAATTTTATCTTTATGCTTTTTCGCAAATTCAACCGCATC
TTTTTCTGCAGGTGGTTGGAAGCCTTGACCTACATATTTTGAAGCTTCCATTTCTTCTGGCACAGATTTTGTTTCATTGTTGGATTCGT
TATTGGTAGTTGAACATCCTGATAGTAGAATCGTTGCTATTAAGATTAGTTTAGTTCTTTTAAACATAGCTCATCACCCATTTATGTG
TTTGTAAAACTTTTATGTTTGAAAAAGCTACTTATTCTCAATGAAAACAAGTAGCATTTAATAAATTAATTAGTATACAGCTAGTTTT
TCTAATTGTTCTTTAACTTGAATTAAGTTTGACCGTATTAGAGAGGCAGATTGATCCATCGTTTGAATTGCTTGTCCTTCATTTTCGTT
CAAGCCATTACAAACAACTTCAAACTGTTGTGCCATTTGATCAAGACGCGCATGAGCTTGTGTGTTTAAAATAAACATATCGTCATA
ATGTGATGGTAAATTGACATTTCGTCGTTGTACGCAAATGTATAAAACCCTTGTTATACCAAGTTCTTTATTATTTTTAACCTTTTAA
ATTTTCCACAAGTGTTTGCCCTTATTTAAATAATCCGCCCTTTTTTTGCCCTGAAAAAATAACCACGTCAATTAAGACGTGGTTACCC
TAATATAGAAAGAAAGTGTTTGTAAATTTTAACATATTTAAGATTTTTTGATTATCTATCTGTTTAGCTCATAGGATTTTTTTCCTTAG
ATTGAGTATCTATTAATTTAACTGTATGATTTTCCCAATCAACTTCATAAATTGATGTATGAGTTGCTCTATTTTCATCTTTATAATTA
TTACCTATCCAGTGAAGTTGATTCCAAAAGTTTGTATATCTATCCATTTCTCTTTGATAAGTAACAGTAATTTTTGATTTTTTTGCAGC
GTTTTGTTTTCGAGATAGGACACCAATAAATTCTGGATTGAAGTTACCTCTGGATAACACTGGCATTTTGTGATATTCCAAGAAGTTT
TGTCCAGCATTTAAGTTGCTTTGTCTTGAGCCTAAAAACATTTCATTACCATAAGTTGAATGATAACTATCTCTGCCATATGGTCCCC
AACCATTATTCATAATTTTATGTGCTTCAACATCCCAACCAATTTTTTTGAAATTAGTTCTTTTATCTAAGCTAGTTCTATAGCTTTCT
TGTTTATAGTTAATTGTCTCTGAAAAAGATTTTGAACCATTACCTCCACCTGATAAGCCGTTAGAGATATTAATATCTCCACCATAAG
AATAACCTACCGTTTGTTGTACTTGAAATTCTTCATTTTGATTTTTAGGTGCATAATCTACAACGTTTACTGAGTCATTAGAATCTGA
ATTAATTGAAATGTTGTACTTAGAACCCCAATAAAATTGAGAACTAATAGTGTCTTTTGGATTTGGCTTTGTATAGCCAGAATAAAT
GTTTCCAGCAGCTTTGAGTATTAATGTATCTTTATCATAACTTTTATCTTTAATAAAATTAAAAGTTAAAATCTGAGAAATTTTTAAC
TTATCGGAATCTGATGTTGCAGTTGTTTTGTACAAAGTAATTTTATCATCAACCTTTTTCTCACTTACAGGTGTGATATGTTGAGCTG
CATCAACTGTATTGGATAGCAAAAGCAATGCAATTGATGTAACAACTGATGATTTGACTATTTTTTTCATATCAATTATGTCCTTTCA
CTTTAATTTCATGAGTTTTCCAGTTCACTTCATATTTAACTGTGTAATTTCTGTTTACAAATGCGTTGTGTATTCTAGATCCTTCTAAA
TAACTATTGCCATAGTGTGTTGTTCTTCTAGTAGCATGAGTAACATCCATATTTCTGCCATACGTTATTTCAAATTCACTTGTATCTCC
TGAGCCTTTTTCATGAGAAACAGTTGCAATAAATGAAGGATTGAAACCACTGTGTACTAATGGGGGTAATTCATTGTCTGGCACAAA
ATAGTCTCTCGGATTTTGACTATATGGTTTATATCCAACAAATAAATTTGGATCATGTCCAGACATTTTACCTAATGATGTGATAAAT
GAATTAGCTTTTATTCCCCATTGAACACTTTTTGAATTTTGATGTTCTACTTCACTGATATAGTTTTGTTGATTATAACTAATTGTTTTT
GAATAATTAAATGAACCATTACCTCCTGTTGATGGACCACTATTAAAATTACCACCTATGTTATAACCTAATGTTTGACTAACATTTA
CTGAATCTATTTTATTTTTAGGTAGATAATTTATTAAATCTACATTGGGGTCATTTGTTTTGAGACCAATATTGTATTGGAAAGGCCA
CCTCATTGCTTTTATATGATCTGTGTTTTTGTAATTGTAATAAGTAGTCTTTGAATTGATAAAACCTTGCATTTTTAAAATCAAAGCGT
CTTTGTTATACTTTTTATCTTTAACAAAATCAAACTGAATATTTTGTGTGACCCCCCACTTATCGCTACTTGTATCTTCTGTTCTTTTG
ACTACCTCAGCGCCATCACCAATATTCTCAATATTGTTATCAGCTTTAGATTCATGAAACGAAGTAGCAATAGGAGTGATTATTCCT
AACGACAATGTTGCAGCTAATAGTCTTTTTTTGACCATAAAAATCATTTCCTTTCTTTATAAATTTTATTACATTTTTATATTAAACCT
TTTTAACTTTTAATAAAATTAAATATTTATTAAAAGATTTACCGAGTTTAAAATATCTATAAATGTGTTTTTTTACTATTATTTCGAAA
AAAACCCGTTCATTACCAAACAAATTATAAATAATTTATATTTTGATTGATAATAAACATAAAACCACGATATAGTTGACCATAACA
ACTTTTTAATCATAGCACACAATACGAACACAAAATAATTTATATACTAAAAACGTTTGTTTTTTTAAAATTTAATATATTCATGTAA
AAATTAACTGTTTATTAATTTTAAATAATAGGCAAGTACCGAAGTACCTGCCTAAGATTCATCATATACAATTATCAAACTGCACTA
AACTTACCAAAACTGCTTATTCTATTACCTGCCTTGTCTACCTCTCCTGTAGCAATATAACGACGTTGTCCACTATTAGCAATATAAG
TAATCCATCTATACCCATTGATGCAATATGCGCCGTCATATTTGATTGTTGCGTTATTAGGTAATACACCTGTAATTCTTGAATTAGT
TGAATAGCCATCCCTCACGTTATTACCTTTAACATTGGCAACTGTGTAATAACCAGTTTCTTTTTTATACGGTACATTATTTTTATCGG
GTGTATAACCTGCTGGCACTGGCGGATTCTTTTCGTTTTTAACTGGTGTTTTAACCTCACTAGATATCACACCACCGATAGGCTTACC
ATGAATCGCACCGGCTATTAATTTAGAATACAAGTCATAGTTTTTCTTAATCCAATCCATATCATTTTTATTAGTGATAAAACCTAAT
TCAGATAAGCGATAATTTATATTTATTTCTGCTGATACGTTAACATTTAGTAAATCGTTACGAGGTGTTACACCTCTTATTTGTCCTA
AATTATTTTTAATAACATCTTGTATACTTTTATCAATAGTATCTGCATTGAATTGACTTGAGATAATAACATGCCCACCACTTGCGCT
TTCTCCTGCTGCGTCTAAATGTATTTCTAGAACAATGTCATACCCCTGTGATTTAACCCAATATAAGCCATAATCTTTTTTATTACCTA
CATTAACACCGTATGCTGTATCTTGATACATGTCTTGTGATTGACTTGAGCCACCATATAATGCGACTTCATGACCGGCATGTCTTAA
ATACTTAGCGATATTTGGCGTTATATATTTACGTATAAAATCGCGTTCGTTTGTTCCGTTTCCTACTGCTCCAGGATCGTTATAACCA
TGACCGGCTACAAGCATAATTTTTTTAGGTTTAATTACTGCTTGCTTTTTGGCAGTTGCTTGCTTAATAATGCTTTTAGCTTTATCCCC
AACACTTACTTTGTCAGGGAAATTTAATCTAATAAAATACATTGGGTCATCGTAATAATGAACATGTCTTGTAACAGTTTCAGGACC
CCAACCAGGTTGCACAACGCCATTTGTCCAACCTTTACCATTCCAATTTTGGCCAAACGATGTGAAAGTGTTTAAATTTGCGCTCTCA
ATAATTTCAACGTGTCCAGCTCCGCCACCATACTTCGACGGGAAAACGACAATGTCCAACTTTTGCGGTAAAAAGCTATCATAGTTT
TTAATTATTTGCCCGTATTTTTCAATCCTTGCTTTATTATCAAATGGAATATTATAAGCGTATAAACCTTGTAACCTTTCGCCTGTTGC
TATCATAAAAAACATATTTGCGTAATCGTAACACTGAAATCCATAAAACAAATCAGGATTGAACTGCTTCCCTAATGAATTATCAAA

CCATTTTTCTGCTTGGTTTTTTGTTATCAACATTGGTCAACACCTACCCTAAATCATTTGTGTCGTTCATATTCGTAGGTGTCATTACT
TCTTTAATTGGCGCTTGCCCTGTTGCTTTTCTATACTTGTTTTCAGCTTTATATTTCTTTAGCTTTTGATTTGCCCATTTACCTTCTTGA
GATGTTGGATTATCTTTATATGTAGTATATAAAGCAACAACTGTTAAGATAATCGATGAAACACTTTCTTCATCTACTGGTATCGGA
CTTATACCTTTATTCGCTAAAAACTGATTGACTAACGCTAAAATTAATACTATATATCTTGTTATTACTTTTGCATTCATTTGTTTGCT
CCTTTTATCCAAAATAAAAAGACGACTAATAAGCCGTCTATTTGATATTTATATTATGGTGTGTTAATTTATATTTAGAAAAAGGGC
AACATACGCAAACATGTTACCCTAATGAGCCCGTTAAAAAGACGGTGGCTATTTTAGATTAAAGATTAAATTAATAACCATTTAACC
ATCGAAACCAACCAAAGTTAGCGATGGTTATTTTTTTATTGCTTAATTCAATAAGCTTGATTACTAGACCTATCAATGCAATAAGGA
ATAAACCAAACTGCAACATGGTACTAATTGTAATCATTAGGCGTCTCCTTTCTAAAGATTTCAGTAATGCCACCATAGGCACCACCT
CCTTATACTCAGATAGCCACCATCTATCCAACTTGCTCAAAGCATATTATAGCACATATACTTTTTTTACTGCTTGTAAACTTTCAGA
TTATTCTCGGTTCATAATAATCTGAACATTACGTGTTCAAAACTACCATGTTTAACTTGTATATAAATATATATCAATATGCATGTTT
TCAGAGTTAATCGTTTTATTATCTCTATATATTCTTAATAGTGTATTTGAATTCTGTCCTTGAGCACCAAATAAATCGTAATTAACCG
AAGGTTCTGTAGAAGTATGAAACACGATTAATCCCCTCTGAACCTTCCCATCAAAAACATCAGAGTTATATAAATTATATTTTTCCT
GTAAATAACGTCTTGCTTGAAGATCCAACTCCTGAACAGTTACATTTTTCTTATTCGTTTTAACCGTTTCCAAAGGTACTGTATTTTGT
TTACCGTCTAGCCATAAATTGATTGGCACTTTTTTCTCTTCGGTCAATCGATTATTATCATGTAACGTTACACCACCATACATGCAAG
CTGTTTTGTTTGGTGTACCACCCGCACATTGATAACCATAATAAGCACCATATAAGTCTACTTTTTTCCCTTTATATTTATCAACAAT
ATCCTTTGAATCAAAATCTACTAATAAATCGTTATACCATGAATGATTTGTAAAAAAGCCTTTAAACAATATAGTATGCTGTAAAAA
TTGATCGTGACTCTCTTTATTTTCAGTTTTAGCTTTTTCATTGTAATAATAGATTTGTTTAAGATTGCCTAAAGCTGTTCCCTGCAATT
CAGACTTTTTTCGCAAATCTTTTTCATTTATTTCTTCGCTTTTCTCGCTACCATTTACAAGTGGACTTGTTGTCCACGTTAGGGCAATG
AATAAAAGTAGTATAAATGCTGTTTTTTTCATTTTGCTCACCTCTAAAGCATAATTCCATACAAACTTTTTATTATCTAAAATATTTG
GATGATAGATTATTTTTGTCAAAGTAATTATTAGTTATATACAATAATTATAGAATTATCATTATTTACTTATACATACATATATTAC
TATACCTTTTTTATACTTGTCTATAATATAATAAAAAGATTTCATTTTTACTCTATTATACATTTCAGATATCTTACTTACTTGGTATT
GACAACACTTCCCAACTTCACTTGCAGTATGCTTCCTGTCTTTTGAGCTGTGTAATACCATGTACGTACCTCTTTTCAATCAAAATAA
AAAGCCAGTGCTGAAGCACTGACCAAAAACATTATTTACATTTACGACCATACAAATAACATGATAACCATCTTGCCCAACTCATTA
TGTCCACCTCCCTTAAGGTAATAACGCAGTAATTGATGCAGTAATGACTGCAATCATAACAATTGTTACAAGCGCCCATATGGCACC
TACGAGCCATTTATTTTGGGCGAGTGTCTTTTCTTCATTTTTTTGCGCAACATCTACTTGCGTTTGATATCTTTCTTCAATTCTGTTTAA
TATCTTTGTTTGCTCTAAATTCTCATCTACAACTTTATCTTGCTTATCTTTAAGTTCTTTATGAGATTCTCTTAGTTCATTATGATGTTG
CTTATGTTCCTCTCTAAGTTCAAGCACATGATCAGCTGTTTCGTTTGCTAGTATTTCAACATCATCAACACGTTCAACTAATTCAGAA
AGCTCTTTTTTTATTTTCTGAATATCATCCAAAACTACACCTACTTTCTAAGAAAGCTATGAGCATAATGCTCATAACTTAGTATAGT
TACATTATTTCGCTATCAACTGATTTATCAGATGACAAGTCAGTTCTATCCACAACTTCTTTCACAACTTTCACACCGTTTTGATTGCC
TGTTAATTGATATAAAAGATTTAAAGTTTCAGCAATCTTTTTAGCGTTTTCCTCAGATTTAAAATCTTGTGCATAACTTGCTGAATCG
GACGTTGTAAAACTGCCTACAAAATCTTGATACAAAACGCGCTCTGTTCCCTCTTTGTCAATTTGTACTAAAATAAATCTCTCTGTTT
TTTTGATAATTTCATTTGCCATATTAAATGACCTCCTTAAATTTTTGTATAAAAATAGTGCCAAGGATTACTCTTCCTCAGCACTTTTG
CTTTTTTCATTTTGTTCTTGTATATATGCTTTTAACATTGCATTTTCTTGTGTTAGTCTTGTGATTTCTTGCGATAAATAGTGAATTGTA
TATTGTGGATTGGCTTGTAAACCTTGATTGCTATCATTCATTTATTAATTCCTCCAGTTTTTCGATTTTTGATTGCTGGTTTTTAATAAT
AGGTATTAAATGAACCCAAAGTCTATCGTATGCGATACCTTCGATTTCTCCGTTGTCATCATAAATAACAAATTCATTAAATCCTAAT
TCTTCAACCTCTTCCGCTATTAAGCCAGTATGTCGACTAAGTTTAAAAGTATCATCAGATAACTTTTTACCACTTTCCAATTCTTTAG
CCATTATTTCCGATTCATATTTGTCAAACCATGTACGAATTGGAAGCTTTAAAATCTCTTTTGAATGACTGAACTGATCGTCTTCATT
GATGTATTGGTTTTCGATTGAAATTTTATACTTTTTGGCAGAAGTAGCACGCCCAATTGTTCCAGCAGAAGTAATGTGTAAGTTAGC
AGGTGCTGAATAAGTACGCTTATAAATAGAATTAGAAGCGACTCTATCGCCAGCATTATCAGAACCTACCGCCAATAAATCATAAC
TTTGAATGCTAACGTAACTATTTCCATCTCTTCGTTTAACTAAGTTGAATTTGCCCATACCTGATTCAATTGTAGTGTCTCCGCCTGTA
GCATAGTCACCATTAACGACTTGAACCAATCCTTTATTGCTACGTTTAGAAAATCTTAATCCAGCACCGTACTTATAGTTTTCATCTG
AACCAAACATGATATAACCGTCAGTTTCGTATGCACTATCAGCGTTTGATAATGTGAATGCGAATCGGTTTAAACCAGGTTTATTTT
TGGTGTTCGGAGATAAATATATCGGTGCTTCTCTACTTTCAATATTAGCTGAAGCATATGAATCGATAATAATTCGGTTGTAGTCAG
ATGTTAAAGCGACTACACCGCCATAAGAATTGATTGTTATGCCATTCATACCACTATCACTGTAAGTTTTATCCCACCATTGAATAGT
ACCGGATGAACCTCCGTCTTCGCCTTCTCCATCAATATATGTTGAAATACCAAAATGTGACATATAAAGTGAACCGCCTGCGGTATT
ATTTCTAAACCTTAGATGTCCATCTTTAAGACGTGTGAATATATCATCGGTTGATCGTTTGCCTTTCCAAGTTCGTTGTACGATTCCG
CCAAGTTCAATAAAATCATTTTGAACTTGTACATAACGGTTAGCATTTCCGCCTTTAATACCAATTCTATTCACATTGATGTCAAGAC
CTTCATTTGATAAGTTTAGGCTATTAACAATGTCATTTTTACCAACTTTGTTATTAATATTATTTGCGACTACATTAAATTCTCTATTC
GCTGTTATATCAACTTTATCGCCACTAATTTTCACGCCATCTTTATCAATAGTATGTGAAGTAATCGCCCCATTTTCGTCATACCTTA
AATAGATGCCTTTCATAGCATTTACAGTGATGTCTGCTAACACTCTTGATAGTGTTCTTTTAGAAGCATTAAACTCTTCTTTAGTAGT
TCTTAATTTGATTTCCTTACCATTTTGTATAATTTGAGAACCATAGCGAGTCAATGTTTTCCTCTGTGCATCTGTGCTTTCTTTGACCT
TGTTGTCTGTATAAGCATTAGCTTTCTTTTCAGCGTTTCTAGCCTTTAGTTCTGCGTTTTGTTTTGCCTCTTCAAGTTTAGCTTGAGCAT
CTTGTATAGCGCGTTGCTCTTCTTCCGAAATTTTACCATCAGCATACGCTTGCGATTCCTTCTCTTTAAGATCATCTTGAGCATCAATG
TATGATTTTAAAGCTTCTTGCGCTTCTTGATTTGCTTGTTCAATACTTGCTTTAATCTCAGGATTATTGGACAAATCACTTAACTGGTC
ATCAGTATATTGTTTTTGTTCTTCCAATCCGTTTCGATATTCGTTTAACGTAACTTTATCTTTGATTTCACCTTTTAAAGTCGTTCTCTC
AGCTTCAGCAGTATCTAAACGTTCAACAATACCGTCTTTGTCTGTTTTATAGTCCGATGTTTTTACATAGTCACGTAATTGTTCTTTTG
TGGATTCTCTAGCTGCTTCAATAGCTGTTTTAACAACATTAGGTTCTCCGACTAACTGCAAATCTTCATTCACCGTTAAACCAAATTT
TGTTGCTATTATTTCCAACGCTTCTTTATATTTTTCATCAGTGTATTGTGACTGTAATAATTTAAATCTATCTGAAATGGCGATTTTAA
CATCTTCTACATCTGTATAAACATCTTGTAATTTCTTTCTATACTCAAGAAATAAAGCTTGTGTATCTACCAACCGACCAATCGTTGC
AGTTTCGGGTGTCATAGATTCTAAATTATTTTTAATTTGATTATAAACATCAATCACAGCGTCTAAACTTGCTTGTAAGTCCGCTTTC
AAATCATTATCTACTAAGTACTCGCTATTCAGTAATTCTGTAGCTTCTGACAAAAGACTAGCGTGTTGTATAGATAAATTAATAAAA
ATATTGTTTAATTCACTGAATAGCGCTTTCTCTCTTGTTATACCACCTAATTTTTCAACATCATTTGGTGTTGCTTCAATCCATCGACC
ATTCCAATATCTACGCAAGACAGCAACATCAGGGTTACTTGTATCATACCAAAGCATATCATTGACTGGATTTTCTGGCGGTGTATC
ACTTTTGTGTATTTTGCGTTCAAAGTATTCTAATTCACCATCTACAACATCTTTAACTATAGTGTTGATATTGCTAATATTATCGTTTA
ACTTTTGATGTATTATGTTCAATCGCTTGTTAAACTCTTCTCGTAATTCTGATTCTTTGAACTCTTTAGGTTGACCGAATGTATATGTG
CTATTTTCTGAAATTATGTTATATTCTTCGGCAATAACTTCTGCCTCTACATACAATGGCGGGTTAAAATCTCTATGTTTTACTCTGAC
TGTATCACCAATTGATATAATCTCGTGCGGATACGTAACTTCCAAATCAGTAGAAGTAATCTCATATGACATAACTGCCGACTTACG
TTTATTTAACTCTGTTTTGGCTAAAGAACGCAACCGTGTTTCATTCATATTTTGATCATCTGATTGAGGTTCGTATATTCCCCAAATAT
AACGGGTAGGTAAGTTGAATTGACTTTGTGCTTCGTCATCAGTCACAACTAACTCTAAACGCTTTCCTTTGTCATTTTCAGGTCCCAC
AGCAATTAATGCTGTTTTGATTTCTGACATATCAATCTTCCTAGTTAACCCAACCAAATCTTTACCATACTCAATTTCTTTACCTTTGA
ATAAGCTGTTTTTCTTTTTGAGTACCACATATCTACCTTTGACGGTATTAGAACTAAGCTCTATATAAAAATCCAATACCATTTTATA

GGTTGTACATAATTGCTTTAAAACTTCATATCTAGTTTGATAAGAAGTCCATGACGTAGTACGTAAGCCATCGTATTCGGTTTGTTCA
GAAACTTCCCAACCTGTATCGCTCAACACATCTTTCAATGCTTCTGAAGTTGTCTTTTTCTCAAATTTGCCTGGTGCATACGGTTTAG
CTGTTGTTATATCAGCAAGATAAGACGCTATACATTCTATCTCTGTGTAGCCGTCCATCGTATCTTGAACCCAGTTAATAATAAATTC
ACGCCATTGTTTGTTTGAATCCCTTATAATAACACGATGTCGTTCACGGAACTTTTCAGCTCTTTCTGATGATATGAGCAGTTCAAGC
ATTTCTGAATTGTCATTAACATTACGTTTATGAATCGCTCTAACTAAGGAAGGGTCATCAGTAGAAAGGAAATCTATAATCTTGTCG
TTAAAATCTAAAACATGTATCACACTCTCATCTCCTTTCTATAAATATCTATCTTGCCATTTAACCGTCGTATCAAAGACGTTTTCAG
GTTGTATGATTAATTCACTGTACCCAGAATCAACATTGAAATAATTACTTCCAAACGATTTCTCGCTCAACATTGGTTCCTCATTGAT
GACAACACTTTTTGCTTGCATATCTATTTTTACTAAATCACCTTTTTGTATAATGACATCCCTTGCGCCTTTCGGTTTTGGTAGAATCT
CCGTATTGAATGAACCTAATCCATTCATCTCCATCCACTTATAACCATTATACTTCGCACTATAGATAGCTATGATAGAAGCTGGAC
GCTGATAAAACTTACCGCCATCTATCCACTCTTTCTCATCCATATCAATAGGTTTACGTCTATCTGGGTCTTTAATGTGATCAAATTT
CCAAGTTTTAATAGAAAATTTATTACCTACTCTTCTGAGCCGCATATAAACAACGATTCTGTCCAAGTTATACATTATCGGTTTATTC
TGATAGTCGTATATCTTTTTGGGGTCTCCTTTTTGGTTATACAACGTAACAACAATATGTCCTATTTTTCTATCATGATATTTATTTTC
ATAACCAATAGAAGCAAGTAACTTACCATCACTATCATAAATATGTTGTGCTGTTCTTCCGGCACCTTTACCTTTTTGTTCAACAATA
CATTTATAGGTAATTTGAAAATCTGTCATCGCTTTAGGGAGCCCTCGTTTCGTGCCAGCACCAACCCAACCTTTTGCATCAGGAAAA
TTAGTTGCTTTATATCCTTCGCCAAGATTGGATATCACAAAGTCACCGCCGACCTTACCACCTAAATCATTACTTGGAATATCTTCAG
TAATCATCTTAGTCCAACCTTTGAAATCACGAAACTCACTATGATAAACAGGAGGCATGTAATCCTTAACTTCTTTGGTTACCTCATC
ATCACCAACCATAAAATAATCTTCATCATTTTTAGTGATCATAAAGTAACTAGATGGTTTAATTGCTCGGGCTTCAACAATTAAAGG
AGTGTCAGCAGTCCCACTATTTACAACTGAAACTTGGTCTGAAATCGCAGTATTTTTATTTCCTGTTACTGAATATTTGTAAGGGTCT
GTTAGTACTACTTTGATAGTGAACTTAACAGGTATTGTAAATTCTTTGTGCAGCTTTATTGGTCCTTCAAAATAAGCGTTCCAGTACC
AATCTTTAGATTTGAATTGTAATTTAACTTGTTCCTCGTAGTTAAAAAACTTTACTAATTCATTCAAGACGTCATCATATGTTTTAAT
GCCGTTGTGAGATAAATAGTCATTACGTACCACTAAAGGTATATCAAAACTATAAGATTCAAGCCTACGCCCTTTATATATAGCCCC
CGAACGTCCATCTACATTTTCTGTTTTTAAAACATAATTAAAAGAGGGTATTTCAAACCCTCTTTCGACATACAACCAAGGAATTGTT
TTGTTGTTCACTTTAATAGTGTCTATCATTGAATAGCAATTCCTCCTTTTCTAAACTTTACTTTTGTTGATTCTTGCCTTTCTCGCTTTT
CTATAGACGCGTTCACCTTTTTATCAAAAGCGTATTCGTCAATAATCGGCTGATAATCTTTATCTGCAATCACATCGTTTGATTGCGC
TATTTTCAGTAATAAAGCTATTTGTTGTTGCTGTTGTTCAATCATTTTCAATAATAAGCTTGGGTCATCAAACCCATTTACACTAGAC
AATTGACTAGGACGCTTATTTTTACTCGCTTTTCTCCCTCTTACTTCTGCTGCTGCATAATGTAACATCTTCATTGCTTCGTTTTTACGT
GCAGGATCAGTAGGAATAACCCACTCTGGATATCCTTCTTCTCCTAAGTGGTACAATCCGTTGTAGACTTTGCCACCAGTAGCATAT
GCGTAATCACCAGCGCGTTTGAACGCAGCTCTCCATGAGCCCGTTCTTGGTACCCATTTACCCACAATATATCTCATAGCCGATATA
GCTTGATGAGTTGGGTTAAGAGGATTATTGTAACCCGACTTTGCGTACGCTCTAAATGAAGGATCTATCATTTGGAACATACCTCTT
GAAGGTGTACCAGCTCTTGCGTTGCTATCCCAATTATTAACTGCATTAGCTGTATAATTGGATTCACGTCTTGCTACACGCATCATTT
CGTGTGTAATCCAGCTAGCTTTGTATTGACCTCCAAGTATATTTTGAGCTGTTCTAATCGCTCTGCGCGCATTTTCAGAACCACTCCC
TCCAGGTGAATTCTTTCCACCAGTTTTGTCGTTTTTCCGTAACCAAGGAATAGGGTCTGTCGAATACCTATTGGACTCTCCGCCTTGA
TTGACTTGGAAATGTAAATGGCGGTAATTAGTCATAGAACCTGTATTACCTGATTTACCAATTAATTGACCAGCTTTAATTTGTTCAC
CTGTCCTACGCAATTGTTCAGATAAGTGCATGAACCACAAAAATGTTCGACCTTTTTGAACAGTAATTGCTTTACCGCCACCATAGT
TGTCATACCAACTTCTAACACGTCCACCCATTGGCGTACGTATAGGGGTACCGGTCGGCGTATCATAGTCAACACCATGATGAACGC
CTCCGTTAAATGGATAATTGGGGTTAGGAGGTTTTGGCGGTGCTGAATAAGGTTGTAGTATTCTGAAACTATCAAACACAGAACCAT
CTCCCGCTTGGCTCTCTAATCCTTCTTTTATCCAATTAATCGCCTTACTTTTAATCTTATTCCAAGACGCTTTTGTTATATCGCCAACA
ATACCCATACCTTTAGTTAGAGAGCTAAAGTCAACACCAAACGCTTTGAGCACATAATTTAAAAGCTTACCCGGATTATCAATAAAG
TCCATTACATCGCCAACTTTATCGCCAAGCCATTTGGTACCTTTACCTATTTGATCTTTTGTCCAGTTAAATGCCAATGATGCACTAG
ATTTAATATCTTTCCACATAGTAGTACCGAAATGAAATCTCGGAAGCGTTCCGTTTAACATTGAATAAGTTTGTGCACCGTTGTATAC
TTTTGAGCCTTTAGGTAAATAAGCAGTAGTGTCTGTATTAGGTGTGATTACACGTTTACCGTTAGGGAATTCAATCATTTCATTTCTA
AAACCATTTGGACCATTTCCACGTCCTTTATCCCCAACTGTAGCGAATGTATCACGTGCAATCTTACCGTTCTTAACTAATCTTGTAG
TAGTATGTGTGTGCTCTGTACCAGTGTGTAACCTAGGTATTTCATCCATACCTAACTTACCACCGACCCAGTTTAAGCCTTCAATTAA
TTTATTAAGTCCTTTTTTAATAGCATCTACCATACCGCCGATATGATCTTTAATTTTACCAATGATAGATTTTAAACCGTCACGCATG
TTTCCGAAGATATTACGTACTTTATCCCACAAACGACCAGCTATACCTACCGTGTTATCTTTAATAGAGTTCCAGATGTTTGACATCC
AATTTCTTAATTTAGTAAATATATCTTTCGTCGCATTCCATAAACTTGTGAATTTAGACCTTACACCCGTAAATAACGAATGAGCCTT
GCCGACGGTATTGCTTTTGATATTATTCCACGCATTAGATAACCAGTTTTTCATATTAGTGAAAATAGATTTAACAATATTGTATAAG
AAACCAAAAATACTTTTCGTTGCATTCCAAATTGCAGATAATGATTTTGTGAAAATACCTTTGACAACACCCCAGATACCGGATATT
AAACCTTTAAGCAATCCACCAAAGTATCTAACAACACCTAGAATCTTACCTACAAACCACAGTTGTATTAAATTCCAAATTAACTGC
ACAGTGCCTTTTAGTATCATCACAATACCGTCCCAAACACCTCGCCAATTACCAGTGAATAAACTTGAAAAGAACTTAATAAAGCCA
AGTATGATATTTAAAGCACCTTGTATTACTCCTTTTATATTCTCCCAAGTACTGACAATCAAGGCTTTAACCGCCGGCCAAATAAATT
GCATCACTTGCCAAATCGCAAACATGATTGGTTTAATTACAAAATTTAAGATAAATTCAAATATAGCTTTGATAAAATTGCATATAT
TTTGAAGCGCTTGAACAATAGAAATTCCGTTTTCATTAAAGAATCCATTAATTTGACTCCAAATATCTTTAGCGAAATCAACGATTG
CTGAAACCGCTTGTTTAAAGACGTTTTTAACGGAATCAATGAAAGGTTGGATAAATTGAATGAAATTATTAAACGTTTGTTTAACAC
TGTTAATTGCACCATTAACAAAATTTCTGAATGTTTCAGATTTCTTATAAGCTATTGTAAATGCGACTGCTAAACCAGCCAGTACACC
TAACACGATACCAATTGGACCAGTTAATGCTGTGAAGACTGTTCCTAAAATAGGCACTTTAGTTGATAAAAAAACTAATCAATCCGTC
AGCCTTTGCAATACTAGCTAATAATGGAGCTAATACAGTTACTGCGTTGCCAACTGTGCTTATGAATGCACCTAATCCAAAAACTAC
AGGACCAATTGCAGCAGCAATACCACCGAATATAACAATCGACCTTTTAGATCCATCACTTAAACTTGAAAACCAATCAACTGCTA
CAGATAGCTTTTTGATTAATTCTTCCATGACTGGAGCAAACGCACTTTCAATAGAAGCCCATACATCAGCACCTACTAATTTAAGTTT
ATTCATTGCTACTTTAAATCTTTCGGAGCCACTTTCAGAATCTTTAAATGTCTGATTGACCGTTCCTTGCGAATCTTCGATAGTTTTTA
AGAACTCTTGGTAACTAAAGCGACCGCCTTTAATAGCATCTGCTAAATCAGGACCTGCTTTTGCACCAAATGCTTCAATCGCTAAAC
TTGTTGCGCTAGCTATATCCGGTGTCCTTTCAATTTCTGCTAATGTCTTCTTAAATTCTTCTCTTGGGTCTTTACCCGCTTTACCCCAAT
TGGATATAGCTTTTTTCAAACCATTGAAGGCTATTTCAGTATTAACACCTGATTTCTCCCATTGAGAGAATAAAGCGATTGATTCTTT
CATCTCAAAGCCCATAGCCCTCATTGGAGCACCGTATTTAGTAATGCTATCAGCTAATGTATCAACACTTATACCGCTAGCCTGTGC
TGCTTTCGCTACCATATCAAGTACACTTTGATACTCATCAGCTTCAATACCTGCATCACCCATTGCACGCGTAATTAATTGAACGGCT
TGTACGCCGTCAGAACCTGTTATGTGACTAAATTTCAAGAATGACTCTGTGGCACTCTCAAGTTCTTTGCCAGTGAAACCTAACCTT
GTGTTAACTTCCCCTAAAACACCGCCTACAGTCTCAGCGTCTTCTGGAAAGTTGCCATAAACATCTTTAAATGAATTCTGCAACTTCT
TAAGCTCTCCGCCGGTTGCTCCTGTTGCTTGGGTAACTGTATCTAAACCTTTATCAACTTCTGCAAAAGCTTTTCCTGATGCTGCTGC
AATACCTAAAACAGGTGCAGTTACACCAATCATCAAACCTTTACCAATGGATTTTAAACCATCACCCATTTTTGTTAATTTAGGTCCC
ATACTTTCAAAAACTTTACTGGTTTTTCCCCAGCCACTTTCTGCCATTCTTTGAGCTTCAACTTGAGCTTTTTTGAACTCTTCAAACTC
AGTTGTTGTTTTTTCTAGTTCTTTTTCTAAAAAATTCAGCTCATTTGCTTGTTTGTTATATTCTTGTCGTAATTTTTGAGCTTCCGCGCT

GTTTTCGCCCTGTTCTTGAGATACCTTGCCATATTGCTTGGCTAAATCATCAACGTTTTTCTTATAACCTGTGATAGTTCCATCAAGTT
CTTTAATCCTTTGTTTGTAACTATGAGTTGATTTTTCGGTATATTTGAAGTTGTTACCGGTTAACTTTAAGTCAGAATTTAAAGTTTTA
AAGTTTCGTTTGATTTCTGCAAATGATCTATTTAAATTTGCTGCATCTAAATCCAAACCTATAGATAAACCTTTTATTCTTTCTCCCAT
TTTTTACCTCCTTTCTAAAAAAGTTCAAAAAAATAACCCTAACCAAACGGTTAAGGTTAAAACGCATCAATTAAAGCCTCTGCTTTT
TCTTCAGAAATGTTATTGTTTTTATTTTGATATATGGAAAGTACATAATGAAATGGCATTTTTAAAACTTCGTTAGCGTCTTTACCAT
TTTCAATTAAGTCCATCATGAGAGTATCCATATTTTTCAACATTGCTTTATATGTTAAATCTTCAGGCTTTATTTCATGTTCTGGATAA
AATTTCTAGTTTCCTCAGTTTGCTGACCTTGAGTAATGAAAATCACTTGTTCACGAAGTGCATTCATTCCATCAGGTGCATGCATACG
TTCTTTTAGGTCTTTAACTGTGAATTGGTTATCGTAAATTTTTACAACCATATCCATCAATCTGTCAGCGATTTCTCTTGGTTTCATCG
TGCTATTTTCGTCCTCAATATCATCGATTAAATCCATTGCTTCGTATACAATTTCAAATGAAATGAAGTGTGGTGTTAAGTACGTTTG
TAATTTAATTTCATTTGCTTTCGGGTCTTCTACTAATTGAATAATGTTACGTTTTAATTTTGCCATTTTATAATACTCTCCTTATTTTCA
AATAAAATAGAGGGGTTGCCCCCTCTTATGCTTCTACATTTATTGTTATAGTGTCACTCATATTACCAACTGTTGCTTTAACCGTAGC
AATGCCTTGTGCTTCCGCAGTAACTTGACCATCACTATTGATTGATACAATATTCGTTTGATCTGTTGTGTATTTCAATAACTTACTTT
GATTAGATGGCTCTACTACAACATTTAAATCGTATGTGTCGCCAACTTTAAGTGTTTTAATGCTATCTGGTATATTAACCGACTTTAC
CGCAGTTTCCGATGAAGCCGGTTTTGTTACAAAGTTTCTTCGTTACCCTCTGTCACGTTTCCAGTATATTCTTCGCCTAAAATTTTCTT
TAAGAAAGCCTCTTCGCCTTTTTCACCGTCTCCATCATGATTTGTCATGTTAGCTGAATCAAAGATATACTTACGTACTGACTTTTTA
TTATCAACTAAAGGGAAAAGTGCCTCACCTTCAACCTCTTCACTTGAGAAATCCCAATCTTTCTCAGCCGTTTCTCCATCGATTTTAG
GATTTGTAAACATAACTTTAGGTAATAAAACTGTTCTAAATGTACCGTCTCTACGCTCTTGTCTGAACCATACAGCTACGTAATTGTT
TTGTTTACCTTGTTTCTCTTCGTAAACGCCATCTTCATCATAATCTTCATTAAAAACAATTTTGCGAATCTCTTTAGGGAACGCATGC
ATTTGTAATGAGATTTTACCTTCTCCGTCTGTATTCCCTGATTCAATTGGACCGCCATCAGCATAAGCTGTTTTTAGTTCTCCACCAGT
TTCAACACCAATTTTTTGTAATCCTCTTGTTTTTGTAATATCACTATATTTTAATTCCGCGCCTTCTTTCGTTAATTTAGCGAAACCTA
AACCAGTAATGTTAATATACGCCTTTGGCGCACTTGCATGTTTTACTGCCATTTAATTTTCCTCCTTATAAAAAATGCCCTCGTAAAC
GCGAGAGCTTCTATATGTTTTAAATTCTTCTATATATTCCGGTTTTCCATTTGAAACATTTCCCATTTTTAGTTCAGACCATAATAACT
TTTGAATGCGATTAGATATCTTATTTCTTATGATTCTCGCATTATATTCATCATTGTACTTAACAAAAACATCTATTTGGACAATATA
ACTATATGCACACTCATCTCCGTCAGTATAAGTTGTAGGTATTGGGTCGTCGATATCGTCAATAACAATAAAAGGTACATCAGTATC
TTTTACATTAGGGTATTTATTGAACTTAATATTATTGATATTTACGTGCTCTCTAATAATTCTGTCTTGACTAATCACTTCATGAACTT
TGTACAAAATATCAATCACAATTTTTTCAACTCCCTTTTTAGCGTCTCAAAATACTTATTTTGCCCTTGTCTTATTGCTCTATTAACAC
CGCCCATAGCTTTAGGTTTGATAAATTTACCTGTTCCTTTTTGAACGTGTCCATATTCAATTAAATGTACGATTTTATAACGGTCTTTA
GAACCTCGCCAATGAACAGTAATTGTACGTTTTCCGTTTATCCATTCAGGTTTACTAAAACTTACCTCATTAATTAATGCTCCCGTAT
CTTTTGAGGGCTTTAGTTGTTTTTTTACTTCTTCAACAATTACCTTAGCACCAGCTATTAACGCCTTATCTTGAACTTTTACCATCTCT
TTTATGCCAAAACGTTTTTCTAATTCTCTTTCTAATGCTTTATCACCTATCACTTTCACACTCATGAACTATATCCTCCACGAATCATA
ATAAAGTCTTTATTATCCAAATCTGGTGATACTTGCTTTATATTCAAACGATTTTTGAAATATCTTGATTCAATTTCAAGATAATGTT
CTTCACTGGGTAAATAATCACCTTGCGGATCACGAATATACAATTTAATGTCATTTTGGGTTCCGTTTGAGATAGCTTGTTCTAATTC
ACGTAACCAGACACCATCAATACTCGCCCAACAGCTATATAATAATTTTTCTTCTTTTTCTCCAGCTTCTGGACCATTATTTTCAGTA
TACTTATAAAAATGAACACGAGTATTTAAACGTTTAGTTGTAATTCTAGGTTTTTTAAACACTTTCTTCATCTTCTGATACCTCCATTA
GAGATAACGAAAAATCTATTATTTCAGGTCTGTAATTATCGTTGAAGTGTTCTAATAAATCTTGATAAGCATATCTAGCGCGTATAA
GTATCAATTCTTGACCTATTAAATTCTCTAATTCAAAAACTCCGCACTGATTTTTTATACGCTCGTACGACATTTTTAACAACTGCTTT
AAGTACTCATCCTCTGAATTATGGTCAATCTTTTCAAGTGATTTAAATTTGACAAGCAAATCATCAATCGTCATTGTCTTCACCATTC
AATAAGTCGACGATTTCACTTTTAACCATTGAACTAGACGCTTTTTTTTGTAATGATTCGCATAGTTCTAATAATTCTTGTTTTGTCAG
CTTATCTAAAGGTACGATATAAACTTTGTCGTACTTATTTTTGATTTGATTTGTCAACAATTCAACACGAGGATTGTTATACCCTTCA
GCTGGATACAACTCCCCTACTTTGTACTTGTGTTGATTGTGCTCTATGTCTTTAAAAGCTCTAACAACTTTAAATTTCACCATTTTATC
ACCTCATAAAATTTTATAGTGTTTCTTCGGTACCTTCTAAAGCTGGCTTATGTCCTTTTAAATCTAATTTCCAAACAGCAGCAACTTT
ATTATCTTTCGCTTTGCCGTAAGCAAATTGTTTTGCAGTGTATAAATCCATATCATCTAACGCAAGTGTTTCTTTAAATTTCTGAACA
TTAATACCACCAGCTAAATAACCATCATATAAACCTTTAACGTACGTTAAAACCTTACCTGCTTCTTGGACTGTAGACTCGATAACA
TTCAAATTAAATGGTAAAGCAGTAACATATACGCCATTTGCATTTAAATGTGTATACTGTGCTTGAACCTCAAAAGCATCGGACGGA
TTAACAACCATTGTTACATTACCTTTAACCGCTACTGATTTACCTTTCTCGTTAGTTGAGTGGTATTTAAACACTTGCGTCAATTCATT
AACTGTAGCGCGCGGATTAGCAAATGTAAGCGTACCTTGTTCTTCTTTCTCTGGATAAGCACCCTCAGTTACCGATACACCTTTTTGT
ACTTGACGGTTTAAGCCGATTGGTTGGTCTTTACCAGTACCTTTTAAGAACGCAGTTTCAAGCGCCACTGCAAATGCTTCTTCGATTT
GAACACGAACAAATCTTTCAATCCACGCAGGACCAAAATCATTTAAATCTTTTGGTAAAACAACAAACGCTGTCAATTTATTTTGAA
TTGCTGTTTCTTCACTGAACGCAGCATCTAATTGACCTTTAATTTCACCATAGATTTTACCCCAAACGGCTACGCCAGAAGTTTCAGA
TTTTAAGAACTTCAAACGCAAACCAGCGTTTTTAATACCTAAATCAGCTAATAACGGATGATTCGTCGTCAAATCTTCAAAAATTCT
ATCAATTGTTTCTTCTGGCAAAAGTTTTTCTTCTTTATAGTTAACGTTTTTATTGATATCCATGAAGAAACTTCTTTGGTTTGCACTCA
AAGATTGTGCTGATTTAGGTAAACTAGAAACTCTTTCAGCTTCTGCTTTTGCTTGTAATTTAGTTTCTTCAAATAGTTGGTTAATCAT
GTCACCGTACAATTCATTTTGTCTTTCTTGCGGTTCACCGTTGTTTACTGCATTAATAAATTCGTTTTTCGCATTTGCGAATGTTTCCG
ATAAATTTATAGTCATTTTATGACCTCCTATTTTTTGTATTAAAAAAGGAATCTTGAAAATCCATTTGCTGATACTTTACTATCTGCA
ACATCGATTTCTGATTCCTTTTCTTTCATATTTATTTTTTCAATTACTTTATTTGCTATTGCGTCAATATCAATGTTAACCTCTGGCGTT
TTACTTACCAAAGCTGTTACACGATTTAATACATCTTTCGATAACACTTGTGTATTGCTTGCTACAATTTGCATATTGTCGTTTTCAAA
CATTTTACTATCCGCAAAACCTTGTTCAATGGCTTCATCAGCATTTAGCCACGTTTCCCTAGCCATCATTTCTACAAGTTCTTGTTTGT
TTTTACCAGCTCTAACCGCATATGCCGCAGCCATTATTTGACCAACATGTTCTAATGTTTCTGCAGCATGATTTAGATCTTTCGCTTCT
CCTTGCGCAATACTTGAAGGATTGTGAATCATCATTCTAGCAACCGGACTCATTTCGATGTGGTCACCAGCCATTGCGATAAGCGAT
GCCGCACTTGCTGCTATTGCTGTGATACGAACATTCACTTTGCCTTTATGAGCTCTTAAATGTGTATATATTTCACTACCAGCTACTA
GGTTACCACCATTTGAGTTAATTATAATATCAACATCTTCATCACTAAATTCTAGTTGTGTTAAAACATCTTTAGGACAAGTCGAATC
CATACCAAGCATTTCGTAAACCCATTTATCTTCGTTGGAAACGATGACGCCTTTAATCTCCGCTTTCATCTTCATCACCACCTTTCAA
AGTGTTTTCATCTTTTTCTTTTTCATCATTTTCACCACTGTTAGCTTTTTCGTAGTTTTTAGTAATCAGGTATTCGTCTAATTCAGGATT
GTCTGATGATTCTTCACCTAACATAATCCGCACCTCATTCCTTGTAAATGAACCAGAACTTACAAGTTTGTCAATTGCTTCAGCATAT
TGAAGTGGGTCTTTTTTATTCACACCGACAATTTCTATTCTTGTATCTTTCAAATACATACTTTGTGTTATGAGTTTCGCGTTTAATTC
GTTCTGAATCTTTTTTAATAAAGGTGTTAAACAGAACTTCTCAAATACAAGCGTGTTTTTTTCCAAATCAGCTGTTTCTCCGTAAATC
AAACCTGGAGGTATACCAATCATCAACGCAACATTTTTTATTGCATCTCTCATTAGCTCACTCAATTCAGAAAAAGGCATGTTACTA
TTCTTACCACCATTAGATAATTCCTCATAATCAAAACCTTCTATCAAAGGCGCGATTGCTAGTTGATTTTTATTAAAAGTATTGAATA
ATTTATTTGTGAACGCTTGTAATTTTTCTATATTCTTTTCGTCATATGCGCTAGAGGCAGATTTCAAAATCCCTCTTATTTGATAGTTT
TTTAATTGTGCACCTATCATTCTTCCGAATATTTTCCCGTAATCTTCGAATAGACTTTCTACAAAGTGTGTCACTTTATTGTTGTTGTA
CTTTAAATATATGACCTCTTGCATTGTGAAAGTACGTTGATAAGTATAATCTTTAACCGTTACATCTTTGAATATATCATCATACAAA

GCGTACTCTTCTCTGTAAAAGCTATCTGCGATAAGTAATTCTTTGCTGTCACTTACTACGATTAAAACCTCGTTATCATAAATTAGTT
TATATATAACTTGTTGCCAAAAACTATCGCTTGATAAGTCAGTATTTGGTTTTATATTTAACTTGTAGTAAACATCATTCTTTTGAATT
CTATTACCTTCCAATACTTTAAAATGACTTTGAGCGACAGCTCGCGCAACAAATTCAATACAACTATCAATCGCTAAACGTTTCACA
TACGCTTGTTGTGATAGATCTTCTATCATATCTAAATCAAGCATATATGTTATATCTTTCCTAGTTTTAAATATCTTTTCTAGAATACT
CATGTCTCACCTCCTCTATTAGAAATCTATACTCATTAATGCATCAAGCGCTTTAGACATGTCTTGTCTACTATATCGTCTGCTCTAT
ATAATGCGTGAACAAAAGCCATGAACCCATCGGTTTTCTTCTATTTTCATCTTTTTTAATATATTCTTTATTACCATCGGGTTTAACC
TTTACTGCAACATTATTAGTAAACCAACGCATCAAAGGATTGTCTCCATATATTACGTTATGTTTCGCAAACATTGTATCGATACGTG
GTGCAAGTAATCCATGTATTGCTTTTGGATTTCTAAGTACTTCAAGTTTTATGCCAGCATCCTCAAACGCACGTCTTACAATATCAGT
TCTATAATTATCAGCTATGACTTTTTCAAGCCCATATTTTTCTCTAGCCTTTAAAAACCAATCAACTATATATTCAATTTCAATGACAT
CATCATCGACAATGGTCAATAATCCCATTTTTTCCCATTCTTTAATAGGAGGTTCTAATTTGACATCATCCAAAAACCCTTGTCTTAC
AAACGAATGTCCTAACCAAATGTAATCATCGTTTTTTCGGAATAATAGCCCTACACTTGCAAAATCTCGAATGTTTGCAAAGTCTAA
ACCACCAATACACATTTGATTATCTAAATTTGGTATCTCTCTATTAGTCGCTAGTATTTCTTTCCATGGTGCTATTACTTTTTCAAGGT
CAACTTCAGGCAAATTCATTCGCTTAGTCATGAATTCGGGCTTATTTGAACGGTTGAATGGTAAATCGTTATATTCTTCTTCAATCGT
ACTTAGCAGTGTTTTAGCGTATTCTGATAACGGTTTATGTAACATTGGGTTCGCCTTTTCCCACGTCTGTCTGTCATCAACTTCTTTTG
GATCGTCTAACTTACAATAAAAAGCAAACAATCTACTATTTTTAACCTTGCCACTTAATACACTTGCAATTTTGTGCTTCATTGCATC
GATATAACCCTCTCTAACAAAACCATCAGTACTTATATAAAACGTTCTTCTATTTTTCTTTTTACCTAATCCACCACGTTTGACGTTTA
CCATTTCAGGACCAAAGAAATAATGAATTTCATCAAAAATAACACACCCCTCACGTCCACCGTCTTTGGTTTTTGTGTTTGATGTGTT
ATATCGAATAACCGATTTAGTTGCACGGTTTATTATTTTTGCTTTACTAACTTCATAAGGAGCTTTTGGCGTTTTACCCGTCTTATTTC
GTTTGTTATCCATTAAAACGGTTCTGATTTCATCAAACGATGTTTTTGCTTGATCTTCACTATTAGCAACAATGGAGATGTGATATTC
TTTAACTCCGTGTAAGGGCGTAGAAAGAAAATCACTAATAGCACTTATTAGACCGTTTTTCCCGCCTCCACGTCCCATGAAAATAGC
AAATTCTGTAAAGAAAGCTTCATCTGTATTTTTATCTATAAGAAATATATTAGCTATGATAAACCTTTGAAATGGTAATGTTGGAAA
ATACCATTTTTCAATAAATTTGATACAATCCTCGATTTTCTGTTCATCAAAATATACATCATCTCGTGAATATATATGTTTTTGTAGAT
AATTAAAGAGATCAATTCTTTCTTTATTTAAAATTATCTTTCCTTGTTTCCACAAATTTATATATTCATCAACGTATTTATTACTTATC
ATAGGTAATCATCAGATGGCGTTTCTGTGTCTTCTTTCTCTTCGGGCAATAAATCCGATAATTGTTTGATTATTTTTTGATATGCAGC
ATCTCTAGCATTAAATAGTTTGGCTACTGGTCTTTCCCTTTCATATGGTGGCGCCTTTTCAGATTGAGTAAATAAATCATAGTCACCT
TTTTCTTTTATGTCTTCCCACATGTAATCAAGCATTACACGTAGCCTTGCTGCTTGAATAATTAAACCATCAACTACTTTTAATTTATT
GCTAGGTATGTCTTTATATATAATACTTGCAGCCTTTCTTTTTCTTTAAGCACTAAGTTTTCATCAACTATAATCTCCATTTCATCACCTG
CCTTAAAATGGTTATAAGAGGGGGGGTTATACATGGATTTTTAAAATTATCGCGAAGTCGAGCCCCTGCCCGTTCCCCAAGTATTTT
GATCGCTTTTGATTTTTTTGACCCGGGGGTATTTACCATTTTTCGTCTTTCCATTTATTTTCTTTTTTTATAAATCTCTTTTCTTTTTTGT
TGTGACATTTAATACACAGTGTTTCTAAATTGTTTAAGTCATGAGCAAACTCCGGATGATGTTCTAGCGATAATATATGATCTACATC
CAACGACTTACGCTTGCTTTTGTCATATGTCGTTAACTTGCCGTCTCTCTTACATTGTTGACATTCATAATTATCTCTTTCTAGCACTC
TTTTTCTTGTTGTTTGCCATTCTTTAGACTTATAGAATCGTATGCGTTCGTCTTTAGTCATCATAATGTTTCACCTTATATAACTTAAG
TAGTATCAAGACTCATCTATACTTGATGTGTAGTAATGTATTTACTATTAGTTTGAACATGTTCATACCTCATAAATAAAAAGACAC
ATCACATAGTAATGCGCCTCTTGTTCATGCGTCGTATTAGCATTTAATAACTTTAAATATTAATCTGATACTAACATAATAAACTGTT
TTAATGCGGACTTACATAGGGTAAAAGTCCGCTACACATAACCAATATACTTTGCTAACTTATCGATCAGTGCATTCCTTCTACGTA
ATATACTTGTCTTACTTGTACCAAAGTAATGTGCTATATCTTCCCATTCATAACAACCAATAGGACAATCCCAATATCTAAACCTTAA
TAACTCAAGCGTATCCTCATCACTTTCATCTATCAATCTATCTACACCGTTAACTATATTTCTTAATGTATTGTACCTGTTATCACTAA
ACTTCTTTATTGCACATCGTTCAATCGGATTACCCGGCAAATTACTTTTGCCAGCTCCCGCATTATCTGGTTCATGACTTTCAAGTAA
TTCATATTCTCGCATCTTCAACTCTCTTCGATAGTTATCGATGTGCTGAATGTATTCTTCAAGCTTTTTGATATCGTGTTTCTCAATCT
TTATCATTCAATGCAATACCTCCGATAATATAAATTACTTTTTAATATCGTTATTCATTCGCTTTAATTTAATCCTGTATTCTTCTAAC
CCGTTGTATCCTTTAGTTTTAACTACTTCATCAAGTAGATAATCATTCATATATCTGAGTGCTTGTATCTCTCTTGCACGATCACTATT
AATACTGATACAAACTAATAGCAATATAGCAAATACAATAGTCATAGTAATCCACATCACTCACTTACCTCCGCTCGAAAGACGTA
ATCACTCGGCGCCTCTACATCATCATTAGCCGTCATCATAATATATACTTGCTCAGTTACATACTTACCTAACTCATACATCGCTAGT
AAGAATAATAATCTTAGTATTTGCTTAATCATTTTTTATCTACCTTCTTTACTTCGTATAAGACCGGATATAAATTTAAAAAGTGTAT
TCTATATCCAATCGTCTTAACTTTTACTTTATCACCTACTTTTAACCTAGCTTGTATGTCTGCGCTATCAAACTTTCCTTTGAAGAATA
AGTCTGAGTTTTCGATGACTTGTTTATCATCTAATACAATATAGAATTTGTCCTCTTTATCTTGTCTTTTGTTATATTTATCTGTAATTG
TCCCTTGATGTACTTCTTTGTTTTGGTAACTAGCCACTGTATAGATAGGCAATGCGACAACAAGTAGCAATGCGGTTATACCGAATA
ATGACAGTATTCCAACAATAAAGATGTCGAACCCATCCATATTTTTAAGTTTTTTAATCATTTCCCACACTCCCTTATATTTTCAAAC
AACTGACCCACTTTAATAACTGCATCCCTTTTAACTTGTTTCTCGTACTTCTCTTTCGCTTCTTCTTTACTCTCTGCCTCAACAACTGTA
AACCTTTGATTACTCTTAGCTTTAGTTATGTGTGTATGTTTACGTCCTGTTGAATCTTTGAATGTTGTGACTAAGTATTGTGTCATTCC
TCATAGCTCCCTTGAACTTGTTTGAGCTTACTCATAAAAAACATTACTAAAAATGCTATTAAGATATGCGTCTTTTGATGTTTATAAG
CAAATGTAGATATCATAAAGATAGTAGCAAGCATTAACATTTCATATATGTTTGTGTGTATAGTCTTTTTACTCTTAAGAAAAATAA
TTGCTATGCGATAAAAGAGATAAACGCCAAACCCTATTAAAAATATTTCTAACATGTCGCTCACTTCCCCAAAACCTCCTTGACCCG
ATCTAAGATGTCTTTACACTCCGCTACTTCCGAAGCCTTTTGCTCCACGTTCTGAAACACTCTCGAATTCCTCCACTTGCTTTAGTTCA
GGTGTCCATATAGGCACGATAACCAGTTGTGCTAGTTTGTCGCCTTTGTTGATTTGGTAAACTCTTCTTATGCCTCTTCCATCTTGTAC
ATAGTTACCTTTTATATCTAAAATGCTTATTAATCCATCTTCTAATTCAGCGTCTATATCATCATATAAAAAGGGTATTCCATCACGT
TCTTCATCATTCTTAATATTAATCCCTAAATTGCCGTGATATCCGGCGTCTATCTTGCCTGTTTCAATCACTAAATGTGTTTTACTACT
TACACCACTACGGCTAGTTAATAGCCCGACATAGCCCTCTGGTATGCTTACAGCTACATCTGTTTTAATCACTGCCTTTTCTTGTGGC
TCAAGTACGACAGTTTCAGCTGAGAATATGTCATAACCTGCATCCGTCTTATGATTTCGTTCGGGCATTCTAGCATTTTCTGATAATA
GCCTTACTTGTAATGTGTTAGTCATTTTCCTGCTCCTCCTTGATTAAATGAATTGGTTTAACAACAAAGTCTATAAGACTAATAATAG
ATCCGTCAGATAACTTGTAATGCGTGTCTCTAATATCGCCAACAAGTTGTACAATTTCTAAAGTTGAATTTGTTTCTGGATTAAAAAC
CTTGTCTCCTACACTAATGCTCATTTTCCTGTTCCTCCTCATATTTATAGACAACTTGACCTGCCATAATCCCTACTGCTTCATCAAGT
TCAACACCTTCTTTAACTGAATGTTGAATAGCATTTGTCATTCCCTCAAGTATTTCATCAAACGCTTGCGCTTTCTTATACACGTCTTC
AATCTCTTTTAGCAATCCCTCTGTGTCATTGCCGTTATACGCACTAGCACTGATCACTGATTGTTCTATTTGTTCACGGTTATCCATTT
GTGTCATCCTCCGTAAAAATTTTATTGTTTAATTCCATCCCAAATTTAACTCTTTCATCATCGTTGCCGAATTCGTTTATTAAATCTTT
TTCAACGCTCTTGCAATACCTATCCCATGCGCTTGCTTTCTTCTCTAGATCTTTGTTACGTTCTCTTAACTTACCTATATCCCCAATAA
GCTCATCTCGTTGCTTCTTGTACTCATCACGTTGTTTTCTCATCTTCTTCAACCTAGCGTCCATTACACCTAGTTGGACCCCTGTTTCA
TAGTTCATTCTGTTACCTCCAATAAATGTGATGATTCAAATATGTTGCCTTTAACCTCACAGTCATATCTAAGGAAGGATTTTATGTC
TATATACTCAAAGTAATCATTTTCGGAGACTGCGCCCTCAAACATAAAATCTTTTAATTGAATACCATTTACAACATCAATAGATAT
TACTGCTCTATTAATTGTTCCTATTACAGATTCATCGTCTGGCATCTCTAATATTTCATCTTCAAACTCAACTATATCTCCCGCATATA
TTTCGTTGTTGTTTTTGTCTTTAAGTCCTGTACTTTGCATAAGTTCTACATCTTTAAAATCTCTTGCATGTATTAAAGCTTCTGCTTCCG

CGTAGTTTTCATAGTGAACTTCAGTCTCAATGAAGTCGAATCCTACAACATCGTGTATTCTTCCTGTATATTCGTCCCACACTCGATA
TTTCGGCATCATACTACTACCTCCACTTTTTCGACCTCTATGCTTGCAGTTTTAATTCTCATCATTTTCATCTCCTCTAAAATAAAGTT
AGTTGCTTCTGCTCCTCGTATTCCAAACCATGTTGCTTTATATATGTTTCAAGCTCTTTCGATGTATCAAATGTCTTTTTCACGCCTTG
CCAACCTGGTACGATATGCCCGTGAAAGTAATAAGTGCCATTTACTACATGGATATGTGCCACTCGCTCGTTATCCTGATACAGATA
TCTCTTAGAGCCGAAAAATTGGTTTAAGTATTCTTTGCGTGCATTATCTGTCATGATCTACTTCTTAACTTTCACGAATATGTCGTTTT
CCATCAGGTAGCACGCATAACGTCCTCTTGGATGCACTTGTGGCACATTAAACAAATGTGGCTTCTTTCTTCTTAGCTCAGCCTCTTT
ACGTCGTTGCCTAGCCATTTCACGTTCTTTGCTCTCTCGCTCCATGATTTTGGATAACACAATTTCTTTATACTCAGCTAAGCGCATAC
CATAAGGTGCATGTAAGGCTTCTAACAACGCCCAGCCACCTCGTACTCTTTTTGCAACCATTCCTGGAGTTAAACCATTCTTTTTTAT
CAATTCATTTTCATGTTCGGTAAATTTATATGGTTTACCGTTAATCTTTACGATACTCATTTATTCCACCTCTATATATGCGTGTCTTA
TTGTTATGCTGTCATACTCTAGTATTTCGTCCGGATTGTTATATAAGTAATCTGCCAGTGCATCTTTTTCATCATCCACATCATCAAAA
TGCTGATATTCAACTTCGGTAGGTATTCTTATATCAATCGTTGCATTTATATATGCTTGTTGTTGCATTAAATCACTTCATTTCTCTTT
TTCTTTTACGTCTGACTTTCACTAAGTCCTCATATACCATCCATTCTTGACCTGTGTATTTAGGCGCTTTACATATCCACGTTAAATTC
ACATCTCTATACTGATATCTGAATATCTTCGCTTTGATGTTGGCAACTTCAGTCGCCTTACCTTTAACATCTAAAACTTCGACCAGTT
TGCCATCCTTCCACAAAGAGAAATCAGCTATATACGTAATCGGTCTTTGTTTCCCAAATTTAGGTTGTAGTTCGAATTTCGGTTGTAT
TTCGATACGATCATAGTTAGTGCCATTCATATTACTTTCTAAATATTGGTAATATTCACACTCTACTTTGCTATCAAATACAATTCCTT
TGTACTCAACTTTCTTAGCGTTGTATTTACTCATCGTCCACCTCTAAATATCAAATATCGTTGCTTGTAAACCTAGCTCTTGCTCATAT
AGAAGTCCGTGAGCGCCTTTAAATCGTTTTAGGTCACTATCAGTCATAATTTTCTTTTCGTCGCTGAAATGGGCTCCTGTGAGCGAAT
AAACTTCATTCTCATTCTCTTTATACTTGATGACCTTAATATCTTCTGTGCCATCTTCTCGGTATAAGTAATATTTTTCTTTCGGCATTT
TTTAACACTCCTTAATATTCGACGATAGCGGGGCGTGTATGACGTTCTGCAAGTTTTTGGATAAATAGGTCGTACAACCTATTTTCAT
CGCCCTGTGCCTCATCTATGAGTTTCTGAGCGTACATATCTGAACACTCAAGTTTAGTTTTAAAAATTCTTTGGTTACCATGTATCT
CGCTCCCTGAAATCGTCTCCGATTACTCTTACTTTTCTTGCATTGTGTTTCATTCTTGAATTGATACGTTGCCAGTTCATATTTTGATTT
AGTTCTTTATCACTAAAGTTAGTTGTAAAGATGTTGTTTTTACCTACTCTGTTATCAACAATGCTGAAAAGTTTATTTAAAGTGTGCT
CTGTGTTTTCTACACCCATATCATCTAGTACAAGTAAATCAATATCACTTAGCAATCTGACTAGCTCGTCTGTAGTCTCTACTGCATT
TTTGTTGTATGTCGCTTTGATACGATCCATCAACATTGGTATGTGCATAAAAGCAACCGTATGCCCTTTAGCTTTAACTGCTTTTGCG
ATAGCGTATGCTAGGTGGCTTTTACCAGTTCCGTATGAACCTTGCAATATTAATGATTTTGGCTCTTTTGTAGAGAAGCCTTGAACGT
ACTCTATTGCTGTTTGTTTAGCGTGTACTTGTTTTTCATTTTGTGGCTTGTAGTTTTTGACTGTTGCATCTCTTAAAGACGGATTAACG
TTTGATTGATTGAATATGTTGTTTATCTTCCGTTGCTTGTTTCGCTTATATTCCTCATAGATTTCACATTTGCAACCGTCTTTATACTCG
TAACCATTCGGGTGTTTTTTAGTAGGAGCAAACTTATATAAGTCGTATTCACTTCCACATCTCTCACATTTCAATCCTTTTTCGACAT
GAGTAGGTTGATATTTTTTCAAGCTTTCGTTTATCTTTTCGCTGAATAGTGGTTTCATAATATCCCCCTAATCCCAATAACTTTCGTCG
TACTTCATGCGTTCCAATTGATTCGTGCCAGTTGGTTGTATTTTTTGATTGAGGTACCCCTCAAATTTACTGCCAAAAAGTGTTTCTG
GTCTAAGGTATTTATCGCTATCCGTGTTTAACCATTCAGCTGTTTTGATATCAATCACCTTTTTAAAATCCTCCAACCTAAAATCTTG
ATTCCATCTTGCTTTAATAAAATCTTTTGTTTTAGCTGTATTATGTTTAAAATGCTTTCCTGCTTTTTTATTTAAGTATTCGATAATTTC
TTTATAGGGAATGGAAGACACCGTCGGGTTGCCCGACAATATACTTCCTTCATTATTAGTATTGTTATTATTAGTTAAATCATTATTA
GTACTATTATTATTAGTAGTATGCGATTTACCATTAACGGTTTTTCCATTGTTGGTTTTACCGTTAACGGTTTTTCCAACGTTGGAAAA
TCGAATGTGGTGCGGTTGCTCATATACTAAGTACTCATAACCATTTAACCTACCACTTTTATCACGTTTTCTACTACGTTGAATGTAT
CCAATTTCTTCCAGTTCCTTGATTCCACTCTTTAAACCGCTAAGTCCATCAGTTGAATGTTGCTCTAGTTCTGTTTCGTAAATTTGCCA
GTTATCAGGTCGACTTAACAAATAAAGTAGAATACCTTTAGCCTTCCAACTTATATTAGAATCATGTATAAAATCTTTGTGTACTGTG
ACAAAGTTACCTGATTCTTTGTAAACTCTAAATGTTGCCATTTCGTTATCTCCTTTCTGGTATAATTTTGTTATCGCTACTGCGTTAGA
TTGGGGGTGAATAAAATATGGAAAAACCTTATATGTTAACATATGATTTAAACTCACCCGGACAAAAATATGAGGAATTGAGAAAT
GTTATAAAAAAGGAAATTTCTAATGGTCATTGCAATTATTGGAAATCTTCATTTTTATTCCGTTCTTCTTTATCAACTTCAGAAATGA
TAGAAAAGTTGAAACCTTATCTCGATTCTGGAGATAAGCTGTTTGTTACAGAAATAGTCAATAACAAACAAGGGTGGTTAACAAAA
GAACAATGGGATTTTATCAACCATAATATTTTTATTTAGGTTCTTTTATTGAATCTTTTGTTATATCAGGAAAACCTTTAGAATCCTC
AGGGGTAAATTTTTTAATTTTTTTAGCGCTTCTAATCTCTTCCGCCAAGATGACGATTAGGAGTGCTATTTTTATTATTCTTAGTCTAT
TCATTCCTTTTTCTCTCCTTTCAACATTTTGTTTAATCTTCTATCAACTTTTAGCCATGAGTCATGCAAGTGATATTTATCATCAAACG
ACTTAACGCCAATCGCATGTTGCTCGTTGTGATGTTCGCGACATAACGCTAATACATGTTTGTCATAGTGGTTCATTTTGTTTCTGTT
CATGCCTCTACCGACTGCTTCGTAATGTGCTAGGTCAGCGTGAGGCTTTCTGCATATTACACAGTTGCGGTTGATTGTAGCCCAATAT
AATAACGCTTTATCTTCGCTTAACAACTTACTCGTTTCTACACTCATAGGTATTTGATGATGAAACATAAACGCTATAATCAGTTCTA
TTAACTCCCTTGCAACTTTCATAGAACAGTCGCGCAGACTGATTTCTTCATAACCTTTCATAATTTCCAATTCTGTTTGTAATAATTTT
CTAATTGATTCCACCGGTTCTCCCCAGTGAAGTTCTATATCTCTACACATTGCGAATATTTTTTTGCGTTGTTCTATAGATAGTTTTTT
ATTATCCGGAACCTCTACTTCTGCTTTTAGTGGATATCCGTTTTCTAGTAAGTCAATGTGACTTTGTTCAAGTTCAACACCAGTAGCA
ACGACGGAATAAGTGCCGTCATTGTCTTTCTGGTATCTTGTAATGTATTGCATTTAAACCACACCTTAAAACGCTAAATCTTGGTCGT
CATATCCAAATTGGCCACTGCTTTCAAATGGATTGCTTTGTTGAGACATTGATGTTTGTTGTTGTGCCCCGTTATTTTCTTCAGCTTTT
TGCTTATCTGTCTTCGGAATAGGTTTGTTAACAACATCATCGCCCTTTTTGTAAGGTTTAATAAATGAAAAATCCGTAAAATACTTAC
CTTCATCTTCATTGAATTTCCATTTCAATACCAAGTGACAAAACTTACCAATAAGATCATTGGTATCAAAATCTAAGCTAGGAAGAT
TTAACTTAATACCTAATCGAGTAACTAATTCAATCAATTGTTTTTCTTGGAAATCATATTTATACGGCGGTACAAATTGATTATGTTT
ATATTGTTTGCCTTCATCATTTTCAAATACGATTGTGAAATATCTATTTTCTCTATCATTGAATTCAATATTTTTAACTTTCACTGTGA
ATTCTCCAGCTTGAAACCCTGCTGAGCCGTTATAAAACTTTTCTTGATTTGTTTCTTTAGTAAATTGCGCTTGTCCTGTGATTTTCATA
ATTAAATACCGTCCTTTTAATTAATTTTTAGTTTCCATTTCTAATTGCTTCTACTACGTCCGTAATGCTAGGATTTGCAAATTTCTTAT
TGTTAATTGTTATTGAAGGTGAATGTCTAATCTTTGTTTCAAACGTATTAGAAGGTTCAGCGTTTAGAATATATCTAGCTTTCTTTTCT
CCGTTATCATCAAATTCTTCAATCATTGCCCTAGCTAACACATCACTTTGAGAAGTAATAGCTTTTTTAATTTGTTCTTGCGCTTCAAT
AGTGATAGTAGGGTTGATAGTGCTACCTTCATCATCTTTATCTTTGTTGATACCTTCATGACCTGTAATAACAAAGTGGAATTTGTAT
TCTTCTTGAAGTTTTCCTATTAATCTGTACATACTGACAATTCGTTCAGCAACTTCTCCCCAATCATTAAACGTTGGTTTTTTAGACTT
ATTTTTCATCACATCATTCAATGTCATATCTCTAAGTTTTTGAATAGTTTCAATAACTACAACATTGATTTCTTGTCCGTTTTCTCTCA
TCTCCTGTAAAATTTGAGGTAAAAAAATTTACAACATAAACAAAGTGTTGATAGTTCTCGATTTCTACGTCTGATCCTTCGTCAGTAAC
CGTTGTTCCACCTTCGTTAATGTCAATGACGAAAGCGTCTTTATCTCTTGTAGCAAACGTGGTTTTTCCTGAGCCAATTTTTCCGTAT
ACTGCAAATTTATAGAATTTCCTTTTATTTTTCTCAGCGATATTATTTATCTTTAGTTTTTTGAGTATGCTTACTTTTTCTTGTGGTTCT
TGTTTTTCCTCAGTCATGTTCTACCTCCTCATACTCAATTGTTTCTGTCACTGTTTTCTTGATTGCTTTGTGCTTAGACATATCAATAAC
AGTTTTGTCTAGTCCGTCGAATTCTCTTGCGTCTCGCATATCAGTTGAATACTTCACTGTATCGTTCACTTCGGTTGGTCGGTTTGTAA
TAAATAGATTTTCATCTTTATGCTTGATTAGATAAGTTACAGTCTGCTTCATAGCGACCTCCTACCATCTCATGACTAAGTTAATTAG
TCTGTCCTGTTCGTCTGTGTTCTCTTCAATCCATTCATCTATTGCTTGGTTGAATAAGTCTGATGCCATATCTAAGTCATTCTCATCTA
CGACATAAGCATGTTTAATTGGTATGTTGTTCATATCTTTAACTTGTATTGATATGCCCATATGACCTTTTAAAATGAATAGCTTAA

ATCGAATCCGTTAACATGAATATTTTTGCGTATGATATCGCCTATTTCGTAATACATCTTGACTTCCTCCGTTTTTCATTTTATATTTA
ACTTGAAATTTTTCTTAAGTGCTTGATGCTGTTACTTGCTCCAACAAGTAGCAGTTTTTTTATCTTATTATCAAAGATGTTTCGTAAAT
TATGCCTTTTGGTTCGCCTGGCACTACTATTTGGCCGACAAAAAAATATTGATGCGCTCTACTTCTGTATGATTTCTTAAGTTTTAAA
TTGTGTAATACTATGTCACCGGTATGTCTGTCTACATATTCAACAAGATAATTTTTGTTTTGAACCGACATGTAAATATATGGATTGT
TGTACTTCTCTCTGTATTCAGTAATCGTCTTAACTTCATCATCACTTAACACAGCTTGTTCTGCTTTTCTTTCCCATTCCACACTAGGT
TCAACGTGTTCTTCGAACCAAGTCATTTAATCATCCACCTCATATAAGTATTCTTTATAAAATATGAATGTTGCTATACTTGCGAATC
CCGCAATCGACCATGCTGTAGTGAAGTACAGCAATGGCATAAGCACAATCGCTAAGACTGTGAAGCACAATACTGCTATTAAGTAG
CTTTTATATGTGTCGCTCATTTAATATCCTCCTAATACCATTTTTTATGCTTTCTGATCAAATACTCTTCCAATTTAGAAATATTAATC
AGAGTGCCTGTTGGTGAATAATCAATGTATAAATTTTCTACACCTAAATTATCTTTGCGGTAATATTTCAACCAGTTGTATACTGTAC
TTCTACATACTCCAAACAATTGATGGATTTGTGTAGGTGTTGCGTATAACTTTTTCACAAATTTTTCTTCGCCTCTATATGTGTTTTCT
GGTGTTGGTGGTACTATGATTTTTGGCATTTCTATCTTTCCTTTCGTGTATAATGTTGTTATTTGCTAATAGTTTGTTCGGCGAACTTC
AAAAGGCGACGAGCAGATTCAGTAGAATTTCCAGCATCTTTCGGTATGGTTAAAGATTTGTTGTTTAGATAGTCACTCAACGCCCTG
CTACTAATCACAGGTTTTCTAGTGTGCTTCTCAATCTTCCAAACCTTCCACGTCACAACTGCCATTGTGATGAGGAGGGTTGTTTTAT
ACAATTTGTTCACTGTGAATCCTCCTTAAAAAACAAACTTCTAAATCCTGATTTTTCATATCTACCGGGTCTGCCTTTTTCACTCTTTG
CATAATGCTCTATGTTTATGTCGTAACCACCTTCGTAATTTCCGTTTCTAGTTACCCATAAAAATTTAACTACTCGTTTGCTCTTCAGC
TCTCCACCTTTATAAATGACTAATGGAACGCTGTTTTCATCTTTCACTTTGATGACAATTAGATCTTTGTGTCTGATATTTTTGTTGAA
CTTTTTTAAAATCTCCCTCATCTCATGAATTTTTTTCAATATTAATTTCATTACTTTTTGAATGTTCATTTGTTACATCTCCTTTCGTGT
ATAATATTGTTATCTCCTACAGAGAGGAGGTAAGGAATCTATATAAAACCTGTTATCATAGAATCGCGGACAGAACACCGAAAATC
AGAGCCACAAGTGACAGAGTTAACATCAGTAAATAAGGTAAGTGCTCTTTCCAACCCCAAGGATGGTTTTTTAAAGAAGTTTTTATA
TCATTTAAAATCTTAAACATTTGAAATCCTCCTTTTTCGTCACTCTTTAATTGGAGTGGCGTTGATTTTTTTGTCTAACTTTTTCAATG
CTAATTTGTAAATAACTGAAGCATGTTCGGTTTTAAAATGAGATTCAGCAATAATTTTCAATGTTTCTAATTTATTTCTTGCATCACC
GTATGTGGTACTTTCTGATAGAACACCTTCTAAAATTTGTTGAACTCGATAATCTAAAAGTTTTAAGTCTTTATTGATGCATTGTTCG
ACACACTCTTCTTTGGTTAATGTGATTTGTTCCATAGTGTTCTCCTATTAAGATGTTTGTTTTTCTCCTAAAAACTTATTAACAAAGTA
TTGTTGTCCTTTGCCTGTTACTTTTGGTGTACGTGATACTTTACTTGAACCATCTGGATTATTAATTATTCGTTTTTTGATATCCAAGA
TTTTTAGATCCATACTCTTTTGAGTTGGTAAGTTATAACTTTCTCCACTCTTTTTAATGAGATATCCATTATTTCTTAACCATTTGAAT
AATCTGTTTTGTCCTATATCCACACCGTTTTGTTTAAGTATTTTCGCAAGCTCTCCGACGAGTATTGAATTGTCGCTACCAGCTACTG
AGTCAGCGAATAATACTTTTGGTTTGTTAACTTCTACTTGCTGTTGTAAAAGTAAGTTTTGCTCTTTTTCTTTCTTATACTCAGTCAAC
ACTGTAATGATGTAGTCTGGATCTTTTAATGTTTGTTCAATTACATTGTCTGTTGCGTAGATACCGTGTTTGCGAATAGCTGGTAGGA
CATCTGATGTTACCCAGCGTTTGAATTTTCTAGCGTTTTCTCTAATTTTTTCGTTTTTGCTTTGTTTAGAAGCATCGAAGATTAGACTG
TATAATCCTGATTCGTTGATAATGATCATATTTCTGTTTTGACCTGATGCACTAAATTGGTGCGTCAGCTTGTCCTCGCTATCAACAT
GATTTCTGATGGCATTGTCTGTCCTTGCATATCCTAAAATCTCAGCAATATCTTTTCCTACAAAATAAGGTTCGTTTTCAATTTCCACT
GTTCTTACTGGTAGCTCTTTAAAATTAAATGTTTGTAATGCTTGCATTGTTCGTTCCTCCTTTTAAGATATTTGTACAGTTTTCTGTAC
ATTTTGTTCAAAAAAATATCTACCTACTTTTGTTGGTGGGATTTCTAATAATTCACAGATTCGTTTTATTTCCCATTGTGTGAATAAAT
TTTTTCCTTGCAACTTGTGATTAATAGATGTCCTTGAAATAGGGATTGCGTTCGCTAAAGAACTTTGGCTATATCTATACTCTGCCAT
TCTTTCGTACAGCAAACTATAATCGAAATTGTATATCATAAACTCACCTCCCTTCTTGTTCGGTTTTCTGTACAAATCAATTAAAACA
CCTTTGTTTAAATAAGTCAACACATAAAATACATTTTTCTGTACAACATTTGTTAAAAATTATTGATAATCGTCATTGTACGTAGTAT
TATATTCTTAGGAGGTGTTCAGAAATATGAACAGTTTTAAGGATAGATTAAAGCAAATTATGTCTGAACGGAAGATATCTCAATCAG
AGCTATCAAGAAGGACTGGTATTGGCAGAAACTCAATTAGCGATTATTTAAATGGAAAATATGAAGCGAAACAAGACAAAGTCTTT
GAACTAGCAAAGGCTTTAAACGTTAACGAAGCGTGGCTTATGGGTTTTGATATTTCTAAGAATAGAAAAATTGAAAATAACGACAT
CACTTCCATATACAACAAACTCACACCTCCCCGCCAAAAAAACGTACTTAACTATGCAAATGAACAATTGGAAGAACAGAATTCTA
AAGGAGATAACGTTGTAGATATTAATTCATATAAACAGGAGAAAACTCCAGTTAACGTCAATGGTTGCGTCTCTGCTGGTGTAGGA
GAACGTTTACACGATGAAACGCTATTTACTGAAATGGTTAAAGGACCTATCCCCACACACGATTTAGCGTTAAAAGTAAATGGTGA
TTCTATGGAACCTATGTTTAAAGACGGTGAAATTATATTCGTAGAGAAAACTCACAATATAAAGAATGGACAAATTGGTATATTCAT
CATTGAAGAAGAAGCGTACGTTAAGAAAGTCTTTGTTGAAGATGATAGATTGACTCTAGTTTCACTAAATAAAGAATACGATGATC
TACACTTTTACAGGAACGAAAGTGTGAGGTTAATTGGAAAAGTTATTTTATAGTTAAAGGAGAAATGAAAATGAGTAAAAAATTTA
GCGATATTGAAAGAAATGATATTAATGCTAAATGGTTCGATGAAATTGAAAAACTTGAAGAAAAGTTAAACGAAGATTTAACTAAA
AGAGAAAAGAAAAAATAGAAAAAGAAATTACAAAAAATAAGAAATATATAGAAAAATCAGCCTTTTGGCAAAACTTAGCTGATAA
AGCTGAAACTACCGGTAACAAATTACAAAATGTAGGTGGTAAAATGCAAAAAGTTGGCTTAAAAACAACAGCAGCCGTATGGACG
CCGGCGTTATACGGCGCTTATAAAGTTGGTAAATCATTAAAGAATAAACCTAAAGAAGATGATTTGATAAAATTTATAAAAGAATG
TCAAACAGCTTATAAAGATGGAAAAATAACTGAGAATGAAATGAAAGAGCATATTTTAACTTTTACAAATAATTACTATAAATAAT
AGAAAAATTATTTTACAGGAGGCTTTAACATGGATTTTAAAGAAGTTGACATTAACATTGAAGAGTGGGAAATGGTTGAAATCCCC
CTTTATACAGAAGAAGAACTGACTTATAGATTGAAAAACGATTTACCTATAACTAAAAGTGAGTTTGAAGAACAGGGGTCTAAAAA
TGAATTCTTATAAAGAAATTGAACACTTACACATTAACACCGGTGGTAAAGAGTTTACTCAAGAGCAAATAGAAGAAGCTAAAGCT
TTTATAGAAAGTCAAGAATTTAAAGATATGATTCGAGAAGCTAAAGAGTCACGTCAAAGAGTTATGGAGTATAAAATCACTGATAG
AACTAAATTGTGATTAACAGCGCCTGTGTGGCGGTTTGATATAAAATAAAAACGCCTACTATTGTAGACGTTAAAATTTACCACTCT
TAATTGCGTTTGCCCATAAAGATGATTGCTGTTTATTCTTGGCATTTAATTTTCTTTGGTTGTTGAGACCTTTCATTCTTTTGCCATTTT
TTTCACTAAATCTACGATAATAATAATCGACTTCGTTTGAAAATTGTTTAAATTCAATATCGTCTTTAATTTTCATTTTTTTTAAAGTT
TTAAACATACAATCACCACCTAAGGAGGAATTTTATTGAAACATTATCTATATACCGACAAAGAATTTATATATTCTTATTTAAGTC
AACACGGAAAAGGTTTAAATCTATCTTACAGTCAAATGAACAAAAATACAAGTACAGAGAGTGAAAGTAAAAGTACCGCTAACAC
AAAATCTAAAGAAGAATTCGAGGGAAATTCAACTGGAGATTTAGGTTTAGAAATTAATGCAGGAATTGCTAAAGGTTCTTACACTG
ATGGAACTAATTATAAAATTAAAATTAACAAAAACTCTTTGAAAGAAACTTTAAATTTTATCGATTCTAAAAGCGAAGCTCAATCAG
AATTATACAAAATTGAATTGCACGATTATCTTTATGAAATATTTGAAAATACCGCTATGAATAAAGGTGATAAAATTTCCTTATATC
CAGATAAAAAAATAACAATTGCAGAATTGGATGGTAATCATTTTAAATTTTTAGAACAATTAATGAATATTTATATAAAATCTGATA
CATCAAGTTTTATCGGTATAGATAATAATACTCAAGAAGAATTCAAAAGTATAAAAAAAGACTTGCAACCTTTAGAAAAAATGAGT
TCAATTATAGATAAATTAGTTCCTGGGGATTATAAAATTATACTTAATCACGACAATCAATCTTTAATCGGTTCACTTTACGAAGAA
AATTTAAATGTACCATTTAAAGAGCTAAAGTACTTTTATGCTCAAAGTAAATTAAACGTTGTCGGAATAAAAGCTAGCAAAATTGA
ACCTAATAAATTTGAATACGAAAATGTATTTGAAGTTATACAACTAGACGCTTTATTACAAGGACCATTATTAAATGAATTATTATC
AAATGAATCGATTTATTATTTTAAACCTATACTAATTTACAGTAACATTTAATATTTTTCGGGTAGCCCGCCTACCCTTATTATTTTTT
ATAAATTTACAGAACGTACGTTCTTACAGGAGGTATAAACATGTGGATTGAAAAATTTAAAAACAAAAATAACGAAACTAAATACA
GATACTACGAGAAGTACAAAGATCCATACACAGATAAATGGAAACGTGTAAGTATTGTCTTGAACAAAAATACAAAACAATCACA
AAAAGAAGCTATGTTCCGTTTAGAAGAAAAAATAAAAGAAAAACTAAACAACAAGTCGTCAAGCGAATTAAAAACTTTGACTTTTC

ACGCGCTATTAGATGAATGGCTTGAATATCATATAAAAACATCAGGTTCAAAGTTGACTACTCTTAATAATATAAAAATAAGAATTA
AAAACATTAAACGATACTGCTCTGAGAACTTGCTTTTAAACAAACTTGATACAAAATATATGCAGATATTTATTAATAAATTATCAG
ATATCTATTCTCAAAATCAAGTAACCCGTCAACTCGGAGATATGAAAGGAGCTATTAAATATGCAGTTAAATTTTACAATTATCCAA
ACGAATATTTGTTAACTAATGTCAAAATACCTAAAAGAAGAAAAACAATAGAAGATATCGAAAAAGATGAATCTAAAATGTACAA
CTATTTAGAAATGAACCAAGTCCTACAGATACGTGATCATATACTAAATAATAATAAGTTACACAAGCGAAATCGCATTTTAATTGC
CAGTATCTTAGAAGTACAGGCTTTAACTGGTATGCGCATAGGAGAATTACAAGCACTTCAGGAAAAAGATATAGATTTATTAAACA
AAACTATCAATATAACAGGTACAATTCACCGCATTAAATATGAAGAAGGATTCGGATACAAAGATACTACAAAGACTATAAGTTCA
AAAAGAAGTATCAGCATCAATTCTAGAACCGTAGAAATTTTTAAAAAGATAATACTGGAAAACAAAATGTTGAAAAGATGGAATTC
TAGCTATGTTGACAGAGGGTTCATATTCACAACAAAAAAAGGAAATCCTTTATGTAATAATCAAATTGCCAGTGTGCTTAAGAAAA
CTACAAAAGCTTTAAATATGAATAAGAAAGTTACCACGCACACATTTAGACATACACACATAACTTTATTAGTAGAAATGAATGTTT
CTTTAAAAGCAATTATGAAAAGGGTAGGACATGTAGATGAAAAAACAACAATTCGCATATATACTCATGTAACTGAAAGAATGGAT
AGAGAACTAACTCAAAAACTCGAAAACATTCCAAGTTAGCTTAAATCTGCCCTTTTTTTGCCCTTATATTTTTTATAAGCTTTATAAA
ATGCTTGAAAACACTGGCATTAAAGATTTTCTTGGACTAATTATATCATCATAATGTGATGGTTCAAATATCATTTGTACAATCAAA
GGCTTCATGTTCTTAACAATATCATCTAAATGGTTATCTAAAATTGGTGACACTGCTTTTAAATCATTAAGAAAAGGCTCCCATTTGC
CTAAAGTATTATCTAATTCTTCTAATTTAGTTTTAATATAATTACAAGTTACATTAGGAATCAGGGACAAAAATTCTTTCTTTTTTAC
ATTTAACATTTCAATTGCATGTCTTAAATTCTTACGTATTTTGGGAATTGTATTAATCAAATATTTTATTACATCGACAATTTTCGATG
CATATTCATCATATATACCTTGAACATAGTCTGCTATTTTTTTAATACCATCATCGATATGGTCTTTTAATATTTTCATTTTTCTTCCTA
AATAATTAGAAGGTAAAGCTAGACCCTGCACCATATTTTCACCGCTATAATTAATTTGATAATTTCCATCTAAAATTGTTGCATCTTG
TTGCTTCATAATACTTCTAATATCTGCAATTTGCCTACCATAAATATCATTTTGATTTTTTATTTGCTGAATATTCTGTTTTACTACTTT
CAAATGTTTCATCATTTCTTCAGATACTCCATCCTTGAAGTCATGATCAATATTTTTGAAAATTTCTAAAATTTCATTATCTATACTAT
CATACACTTTTTCTATAAAAGATTTTATACCTTTAAACAACTCATTAATTCTTTCTTTTAATGCATCCAATGCAAAATCAGGTAATAA
GTGTTTAACAGCACTAATACTTTCTATTGTTTCATCTGCAACTTCTTCAAGTGCGTTTATTTTACTAATTAAAGTTCTTTCCATTTCTTC
TAATTGAAATAAGTTAATCTTATCCTTAAATCCTTCTGATAATTGTTTCTTTCTATCTGCAAAATTTTTATTTTCATTTTCTGAGATGTT
AAAACTTTCATTTAAAAAGATTACGCATTCTGCTAACATACCACTTGTTTCACCAGTAATCAGTTTACTCAACGCATCAAGATTTTCT
AAATTAAGTTTAATTAAAGTTCCTTTTCCGGAACGTGCAATCGAATCTCCTGTCCAAACATTTATTGGAATTCGCCCATCCATATCTA
ATGTTATGTTAATAGTCTTTTTTACTTTTTTTCCATTTTTAATTTCTGTATCTTTTACCGACTTAATTTTGATTAGTGGTACAGTATCGT
ATGTGTTATCTTTTCTATTTAACTTCCTTTTATAACCTACATGGCTGTCTATTAAAGCATCTAACCTGGGCACACCATCACTAATGTTA
ACGCGTTTTCCTGGCATATCTTTGATGAATGGATCTTGTAACCATGTTAATAAATCGTTGGTACTATTAAAACTAATCATATTATCAA
AGCGTGGTCTAGCAAATTTCTGCCAAGCAGCATAAGGAATCATTGCTGAGTCAGTAGCAACAACTTTTTCATTCGGATGTTTCGCTC
CTTGATATTTTGCTCCTGCACCACCTTCCGAATTACCACCATCCGCCACAATTGTTTTGTTTTTGTAATTATAAGGTTTAGTTTTGTAT
TTTCTTCTAAATTCATTTTCATCAAGAACAGAAGCATCTTTAATTTTTTGTCGATAACTATTTGCGAATTCTTCTACTTGCTTTAAATA
ATCTGTGCTTTCATTATCATTATTCATTAATTTAGCATTTTGGAGCCAATCATCTCCAAAACCCGATGATTTTAATGGATTATTTGGGT
TAATTGCCTCATTAGATGTTCCTTGATAAATTATTGTCTGTTGACCAGTTGGATTCCCCTGATCATTAAGTAACTCGTATGTTTTAATA
TCAGCAGCACCATTCATATTATCATTACTATTATCATTATAATTATCTATTTGTTTGAACCGTTTTCCATTTACTTTAAAATCTTTTTTT
TCATTTATATCTTGATAAACCCAATAACTGCTCAATTCTGCTATGTCCCTATCATTTATTTTATTCACTTTATATTCACTTACCCTTCA
ATTGGATTTATATCGTCATAAAATGGTTTTTTAGTATTTATACTAGATTTTCCTAATTGAATAGTAACTTGAGATTTTTGAGGCATATT
GGGTTTTTCTTTTAATTTATTACTCAATTCAATCACATCATCTACTGTATTGTCTTTAGTAAAGTTTTTCTTAGTTGAAAAAAGTGTAG
AAACTGCATCAGTATTGGCAGTATAATCTAACTCTTTTCTAGCTTGTTGCATACCTTCTTTAAATTCTTTATCATTTTTATGAATCAAC
GGTTCGTAATATTTACGATATTCTTTTAAGTTTCTTGATAAATATGTGATATAAAAGTATTCATTTTGATATCCAACGTTTTGTGTCTT
GTTAATTGCCTCTTTTGTAAAGCCTGTATATTGATATTTCTTTTCATTTTCTTTGAAGAATTTATATAAGTTATCATACTTTTCTTTTTG
CGCTCGATATTCAAAGCCACTCAGCACTGTACCCACCATCATACTCATATCATCACCATTGTCATTACTGCGCATTGATCCTTTTTGA
TGGATGGCATCTTTGTACAAAGGTAGACTTGCATTAAATACAATGCCATGATCTTCACAATGCACATAAACTTCTATACCATCATCT
TTACCTACAACATTGGTAGCCTTTACCTTCAGTCCAAAGTTGTCTTTAAAGAATTGTTCCCCTACTTTTTCAAATTCTTTACGATGCTT
CTTCGCAAATTCAATCGCATCTTTTTCTGCAGGCGGTTGGAAGCCTTGGCCTACATATTTTGAAGCTTCCATTTCTTCTGGTACAGAT
TTTGTTTCTGTGTTTGTGTCTTTATTTGATTCATTTTCCATCGCGGAACATCCCCCTAAAATTAATGTCGTGGTTAAAACTGATCCAAT
GAACTTTTTCATATGACACCTCGAATAATTTAATTAATACAATATATCTATATTTTACCCTATTTTTATAATCAAATAAATAACTTTAT
CAAACTTTATATTTTTGTATAAATTAGTAATATATTTAATAGTTGATATTCGCAAAAACCCTAAGTTCTAATCCACATACAAAATTCC
CCACATCAATCAAAAAACGCATGCTATAAATTAGAGTCTCTAATCAATAACATGCGTTCTGAAATAACTATATTCAATTTTATTCTG
GTTTTGGTAGTTTAATAATAAATGTTGTGCCTTTCCCTAATTCGCTTTTAACATCTATGGAACCACCATGCTCTTCGATAATCATTTTA
CAAATGAACAAACCTAAACCGGTACCTTGTTTACCTCGCGTTCTCGCTGCATCAACTTTATAAAAACGATCAAATACTTGTTGTAAA
TGTTCTGGTGCAATGCCTGTACCTGTATCTTTAATGTATAAAATATCTTCGCTTTCATTTTCATCACAAGTAATTGCAATTTCATCTCC
AGGTTTCGTATAACGTGATGCATTATCAATTAAGTTCGTTAGTACTTGGTCCATGCGATCCATATCATAACTCCAAACACGCTTCTTA
CAATAATTAAAAGTCATATTTAGACCTAAATCATCAGCTTGTTGGCGATACTTAATTTTCATCTTATCTAGTAACGCTGCAATAGGCT
GAACTTCTTTATTTACGGATAACCCTTCAGCATCCATGCGTGCGACATTTAACAATTCATTAACTAAACGATTTAAACGTTTCGATTC
ATCAAGGACAATGGCAAGCGATTCTTTTATTTCATCCGGTTCTGTAACAATACCATCTACAATTGATTCAGTATAACCTTGAAGTAA
TGATATCGGTGTACGTAATTCATGTGATACATTAGCAATGAAATCTTTCTTCATTTGATCTAGATTGTGCTCATTAGTCATATCACGA
ACTGTCACAACAACACCACTTTTACCTCCCTGTTCAATCTTGTCGATATAGCTTGTGGTCACAACAAAGAATCGTGCATTCATTTCTA
AATCGCGCATTTCAGTTTGTTTTGATTTAAAAGTATCTTCTATTTGTCTTAATAAGAAAGCTTTAGCATCTTCATCAATATTGTCCATA
ATATCATTCGCCATCTTATTAGATAAGATAATTTGTCGACTCTCATTAATACCTAGGACACCTTCTACCATAGAGTTAATTAAGCTGT
CTCTAATATTTTTAGATGTGGATAATGCGTCGACATGCTCTTCGATTTCTGTACTCATCTGATTAAATGCCTGCGATAATTGACCAAT
TTCATCTTTCGTTGTGACAGAAGGTTTATAAGAGTAATCCCCTTCAGATACACGTGTAGCTTGGTCTCTTAAACGTCTTAAAGGTTTT
GTAATTCTTGACGATAAGAAAAACGCAAAGACTGTTGTAATTGTTAAGAAAATAACAGCCGTAATTATGGTGATAATCGTAATAGC
ATTATTTGTATCTTCGATTGATTTCAAGTCTTTATATATAAAGACTCCACTATATTTGCTATGACTATTCTTCTGTGCTTTTGTTGGAT
AGCCTAACAAAATATATGTTTGAGATGAGCCCTTTTCTTTAATCGTTACATTTCGAGTAACAGATTTACCTTTATCAAACACATCGTC
AAAATGGTCGTTGTTGACTACTTCATTCAACATTTGCTTTTTAATATTAGAAAGTGAAGCCGTTGATTGACGATGTTTATTATTTATA
ATCATCAACCCACCAGGATTTTCAATTAATGTTTGACTATATTTTATTGCTTCTTCTTTATTATGTGATTGTTCGACCAGTGAACTTAT
ACGTCTAGCATCTTCTCTTATGGCATTTTCGGTTTCTTGTGTGAAATAGTATTGCATAAAGGTAATTAAAGCAATACTTAATAAAATT
AAAACTGTCGTCACTATTAAAATAATAGTTAACCACAGTTTAATTACGACACTATTTAGCCGGCTCATCATTAGATTTAACCTCAAA
TTTATACCCAACGCCCCAGACTGTTTGAATCATATGCGCAGCTTCGCTAGACACACGATTTAACTTTTCTCTAAGTCGTTTAACATGA
GTATCAACTGTTCTTAAATCACCATAGAATTCATAATGCCAAACTTCTTTTAATAATTGTTCACGGTCAAATACTTTATTTGGTGTTTT
AGCTAAATATATTAATAATTCGTACTCTTTAGGAGTCAAATTAACTTCTTGATTATCAGCAAGTACGCGATGTGCATCATTATCTATT

TCTAAATGTTTAAATTCAATCACATCACGTGCGTGAGGTTCGCTTTGTTCTACAGTTGTAGATTGCGTTCTTCTTAGAAGTGCTTTAA
CTCTTAAGACTACTTCTCTTGGTGAAAATGGTTTGACGATATAATCATCTGCACCAGATTCAAAACCTTCAACACGGTTTGTTTCTTC
ACCTTTAGCAGTCAACATAATAATCGGTGTTTGTTTATGTTCACGCAATTTAGTTGCCACCTGGATACCATCCATTTCAGGCAACATT
AAATCTAGTAGTATGCAAGCATAATTATTCTCCATTGCAAGTTCATAAGCCTCTTGGCCATTACTTGCTTCATGGATTTCAAAAGATT
CTCTTTCTAAATACATTTTAAGTAATCTTCTGATTCTATCCTCATCATCTACGATAAGTATTTCGTTCGACATACAGGTCATACCTCCC
ACACATGCTTTTCTTTACAAAAGTATTATATCACAGGAAAGCGCATACAGTTATTAAAAACCTAAAATTTTGTGAAAAAATTCTCAA
ACTTTGTGATAAATACATATTTTATTTAATTATTTCCCATGTTCTGCTAAATGACGCATCACTTTTACTTCATGAGGCGTCAATACAC
GTCCTTCACCAGCATTCAAACCGACAACATTTAAAGGCCCATATTCAATACGAGACAGTTTCGTCACTTGATGACCAAAATGTTCGA
ACATTCTTCTGACTTGGCGATTACGACCTTCTGTAATTGTAATTTCAACCAATGTTGTGTTTTTATCTTTATCTTGTTTCTTAACTTTCA
CTTCAGCCGGTTGCGTCATACCATCTTCTAATTCAATACCTTTTTCTAGCGCTTTCACTTCTTCTCTCATTAAATAACCTTTTAATTTC
GCAACATATTTTTTCTTAATTTGATATCTTGGATGTGTCATTAAATTAGTAAATTCACCATCATTTGTGAGTAATAACAATCCAGAAG
TATCATAGTCTAAACGACCAACAGGATAAATACGTGCTTCTATATCTTTAAAGTAATCTGTAACCACTGTACGTCCTCTATCATCAG
ATACACTTGTTATCACTTGAGTTGGTTTATGGAATAAAATGTAAATTTTGTCTTCTAGTTCTATTTTAATACCTTCAACTTCAATCGTA
TCTGATGGCTTCACTTTTGTTCCTAATTCAGTGACAGTCGTACCATTCACTTTCACTTTTCCTTCAGAAATTAAAGTTTCTGCCTTACG
TCTTGAAGTATAACCGCTATTAGCTATACGTTTTTGTAATCGTTCTAATTCTTTAGTCATTATTTTCTCCTTTTTGATTGACTAGATTA
CTGAAAAAAGCATCCATTTCTTCATCGTCTTCTTCAGTTGTCGGCAAATCTTCTATATTGGAAATACCAAATACATTTAAAAATAAAT
CAGTAGTAATTAACTGTTGGCTACGCTGTTCATTAACCACTTTAGCCTCAACTAGTCCTTTGGCAATCAATGTCTTAACTGCACCATC
TGAATTGATACTACGAATTAATTCAATATCACTTCTTGATAATGGCTGGTTATAAGCAATAATTGATAGTACTTCCATTGCTGCTTGT
GATAATTTCATTTGTGACTTTTGTTCAATTAATTGTTCAATATACGTTGCCGCTTCTTTTTTAGTCGTTAAAACATACGTCGTTCCAAA
TCGTTGTATCATTAATCCATGTGATGAATAATTTTCAATTAATTCAACGAGTTGGTCTTTCGACATATCTAATATTTCTAATAGTTGTT
TTTCATCTAAACCTTCATCGCCAGCTGTAAATAAAAGCGACTCTAATATACCATGATTATCCAAAATGGTAGTTCACTCCTCTAATA
ATGTTAATATCTTCAAAATTACGTTGTTGCTCAATATTAATTATTCCTGCTTTTGACATCTCTAAAATAGCTAAAAAGTGAGTGACTA
CTTGTTCAATTGGCTCAGAAAACGTAAACAGACTAAAGAAGTTAAAATGATCTTTATCTTTCAATCTCGATGTCACTTGTTCTGTAGC
TTGTTGAATGGTAAATGTCTCTTTTCGGATTTCAACAGATTTAGGTGTATTTAACTCAACTCTATTTTTAACTCTTTGATAAGCTACAA
TTAATTCAGTTAAATCAATCGTATGATTTGGATTCCAAGATTCATCTGTTTCCAAATGAGATAAATCTGTCGGTCTTTTTGTAAAATA
AAAATCTCTTTCTTCTTTCATGTCATTTAAAATAGCAGTATATTCTTTATAATTTTGATATTCTATTAAACGCCCAACTAAATCTTCCC
GTGGGTCATCATCAACATCCATATCTGATGTTGATTGTGGTAATAGCATCTTACTTTTAATCATTAAGAGTTCTGACGCTAATACTAG
GTATTCACTTGCAATATTAATTTCAAGCTGTTTCATTGCATGAACGTACTGCATATACTGCTCTGTTAATGCTTGCATAGGAATATCA
TAAATATCTATTTCAAATTTTTGGATAAGATGCAGTAATAAATCTAATGGTCCATTGAAAGCATCTAATTTAACTTCATACATAATAT
CTACCTCGTATTGCGTCATATGTGCATAATTCTCTAAAATATTATAGCATGAAAATAACTTACTTTTAAATTGGGGTGATAACAAAA
TGCAACAGGCACTGATAAATTTCTATTATCAATTTCATACTAAGCAACATTATTTCCTATGTCATGACATTTTAGAAGATGCATGGA
AAGCTGAAAATAACTACAGTAAGCAAGATGCAGTTGTTAGTTTAATCTTGTTTGCAACTGCTTGTTACCATTATCGCCGCAATAATT
TAAAAGGCGCCTATAAGTCTTTTAATAAATCTAAAGAAATAATACAAAATGCTAAAGACAGAGATACGTTATACCTTAATTTAAAT
GATTATCAGTTGTTAATTGAGCAACAGATTGCAAAATTGAATGCAGCAAAGCCTTTTTCATCTGTTATATTACCAATAACTCCAGAC
TTTGAACGCATCATCAAGGCCAACTATCCAGACTATGATTATAATCAGGAAACATCAACAGATCCTTTTATTGTAGACCATCACATG
CGGCGTGATCGTTCAGAAGTGATTGCAGCAAAAGAAGAAGCGATTCAATTAAGAAAACATAGACGTAATTAATCAACCAATTTAAA
AAAGCCACAATAGCACAGACATTTTTAGTCATGTGTATTGTGACTTTTGAGTTATGGTTCACTTTATGCTCTAGGATGAAATTGGTTA
TACATTTTTCTAATTTGAGATTTTGAAACATGTGTATAGAGTTGCGTAGTAGATATGTCAGAGTGACCTAACATCTCTTGCACTGCTC
TTAAATCTGCGCCATTTTCCAATAAATGTGTCGCAAAAGAGTGGCGTAACGTATGTGGCGTTAACGTCTTTTTAATGTTTGCCTTTAC
ACCATTTTGTTTAATCATTTTCCATATTGCTTGTCGTGATAAAGGTTTACCATGCATATTTAAAAATAAGACTTCGGTAACAGTCTTT
TTTAAAAGTTGCGGTCTAATCGTTTCAATATAAGTAGTTAAGTACTCAATGACTGCGTCGCCTAATGGCACAATTCTTTCTTTATCGC
CTTTACCAAATACGCGTACAAACCCCATTATTAAGTTCACGTTCTCTAACTCTAAATGTATCAATTCAGATACACGCATTCCCGTTGC
GTACAGAAGTTCTAACATCGTACGATCACGATAACCATTAATTTTATTTAAATCCGGCGTTTCTAATAAAGCCAACACTTCGTCAAC
ATTTAAAACGTCAGGCAATTTTTTGTCATATTTTGGTGAATCTAATAATACCGTTGGATCTTTCGCCGCATATTTTTCTCTTATAGCAA
ATTGATGAAAACTACGGATTGTTGAAATAAATCGCGCAATAGATTTAGCAGATTGCCCTTGGTCTATTAAATGCCCCAAACACTCTT
GAATTAATTGTCGATCTATAAAATCAATATGCGAGATATGATGTTCAGTCATATAATCTTGATACTTTTTCAAATCACGTCTATAAGC
ACCAATTGTGTTAGAACTTAGTCCTTTTTCAATTTGTATAAAACGCAAATATTCTTCAATAATTGTTTCCATAACCACACCTCTAAAA
AAAGCGAAGGTTAATTTAACCTACGCAATTTACTATCCTTCACCTTTAGCTTGGCACGTTTCACACACACCATGGAAAGTTAAACGA
TGATCTAAAATTTTAAAATTGAACTCATTTTCAACTCGATTTTCAACTTCTGGTAACAAATCTTCATCGATTTCATCTACACGACCAC
ATTCCATACATACTAAATGATGGTGGAAATGTTTTGCGCCTTCTTTTCTTAAATCAAAACGAGCGACGCCATCACCAAAGTTAATTTT
GTCGACAACTTTTAGTTCAGCTAACAACTCTAACGTTCTGTATACTGTCGCCAAGCCAATTTCAGGCGCTTTGTCTTTTACTTTCAGA
TATACGTCTTCAGCACTTAGATGATCTTTTTCATTTTCAATTAGAACTCTAACAGTAGCTTCGCGTTGTGGCGTTAGCTTATATGATG
ATTGTTGTAATTGTTGCTTAACGCGATTTAATCGTTCTTCCAACGATGTCCACTCCCCTACTAATAATTAAAATCATTACAAATTATT
TCAAACTTCACAATTAAAACTAACAGTTTTCTCAATAAAATGCAAGCTTTTCTCATTTGTTATTTAGAATGATTATAATTTAATAATA
GGTGTTGCAATGCAATGATAGTCTTCGCATCTTCTATTTCTTTATTCATTAGCATCGATTTAACATTCTCAATCGGAACTTTAATGACT
TCGACAAATTCATCTTCATCTAAGTGAACTGTACCTTCTTCTAAATTATCCGTAAAATATATTGATAATTGTTCATCGCAAAAACCTG
GTGAACCATACATATCTACAACATGTGTTAACTCTTTGGCAATATATCCTGTTTCTTCTTCTAATTCACGCTTAGCCGCTTCGACTCTA
TCTTCATCATCTTCTAATTTACCAGCTGGAATTTCTAGCAATGGTTTTTCTACTGGTTTACGATACTGTTTCACTAATACGACTTCTTT
TTTAGGTGTCAATGCACAAACAGCAACTGCACCATTATGATAAACTAATTCTCTTGTTGACGTTTCACCGTTTGGTAATGTCACTGTA
TGAATTTCTACATCTACAATTTTGCCATTATAAATAACTGTTCGATCAATTGTTTTTTCATTTAAATCCATTATAATCACGTTCCTTTA
AATTCATATTATATATTGATACACTATGCTTGTTAGCTAAAGTGTATCGAAAGGAGAACAGACATGCAAAAAAATATATTAAAAAG
TGGTATTGCATTATCTGAATTAGGTCTAGGTTGCATGAGTTTAGGTACAGATTTAAAAAAAGCCGAACAAATTATAGATTGTGCTGT
TGAAAATGGTATCACTTATTTTGATACAGCAGACATGTACGATAAAGGTATAAATGAATCAGTTGTTGGTAAGGCACTTCTTAAATA
TCAACAACGCGATGATATTGTCATTGGTACAAAAGTAGGCAATCGTTTAACAAAAGATGGCAGTACAACATGGGATCCGAGTAAAT
CCTATATTAAAGAGGCAGTTAAAGGTTCACTAAAGCGTTTAGGTATCGATCATATCGATTTATATCAACTTCATGGCGGAACCATTG
ATGACCCATTAGACGAAACAATAAGCGCATTTGATGAATTGAAACAAGAAGGAATTATACGTGCTTACGGTATTTCTTCTATTCGCC
CAAATGTAATTGATTATTATTTAAAACATAGTCAAATCGAAACGATAATGTCTCAATTCAATTTGATTGATAATCGTCCAGAATCAT
TATTAGATGCAATCCACACCAATGATGTTAAAGTATTGGCAAGAGGACCTGTGTCTAAAGGATTATTAACTTCAAACAGTGTTAATG
TGCTCGACAATAAATTTAAAGATGGTATTTTTGATTATTCTCATGATGAATTGGGTGAAACAATAGCCTCTATTAAAGAAATTGAAA
GTAATTTATCTGCATTGACATTTAGTTATTTAACATCACATGACGTGCTTGGTTCCATCATTGTAGGTGCAAGTAGCGTCGACCAATT
AAAAGAAAATATTGAAAACTATCATACTAAAGTTAGTTTAGATCAGATTAAAACAGCAAGAGCTCGTGTAAAGGATTTGGAATATA

CCAACCATTTAGTGTAGAAGTCATTTTCAGTAATAAAAACAGCGGCATGAGGCGTTTCATTATAAAAATGCCTTACTGCTGTTGTTT
ATGTACAATTCGCTATAATTTATGATTAAGATTACTCACTTATGATAGAAATTAAAGAGTCGTCCTCACGCATCAGTATTTAGTAATT
TCGCCCTGCGGCAGTGCCTTAAGCAAACTTCTGCCACTTCATCTTTTAATAATTTTATTAAAACATCTTTCTATATTTCACTTCGCATG
TTGATTCATCATTATTAGTTATTATTTGTACACCCAGCACATTTCCTTGCAACACAAGTAGTTTGAATTTTTCACAAGTATAATATAA
TGTACTGTCTGAAATTTGGTCTACAGAAATATCGCCTAAAATATCCAGCACTGTAAATTCTTCAAACACTGATAGTTGTTCCGCATAT
CGTACACAAAGTCTTACCACACTCTCCGATTGACAGTTCATTGCCATCCCACCTATTTATGCTTTATTTTTAAATAATTTAGGAAAAC
ATCGTTCAAAAAATCTAGGCGCAATTTGATACATTTTCAACGCATGGTGCATCCATTTAGGCCGATTAATTTCTAATTGTTTTGTTTT
AATGCCATAAATGATATCTTCTGCAAGCTGGTTAGCATCAAGCATAATTTTCCCCATCTTTTTAGCATACTTCATTGATGGGTCGGCT
TTTTGATGAAAAGGTGTATCAATCGGGCCAACATTAACTGTCATGATATGTAAGTTTGGTGACTCTAGTCTTAAAGCATTCATTAAT
GCATAAAACCCTGCTTTCGATGCCCCATAATGTGCAGCATTTGCTTGAGTGGAAAATGCAGCTTGACTTGAAATACCTACAATATGT
GCGTTAGATGTTAAATATGGTCTCAACACAGTATATAAAACATTAAAACTAATTAAATTAAGCTGATACGTTTCAATCATTTCTGAA
AAACTATGGTCTGAAATAGATTTGAAATAACCTAAACCTGCACTATAAATGAATCCATCGAATGATGTATTGTCTTCAAATTGCAGT
GCCTGTATCGACTTCATATCATTTAAGTCACAAGGAATAACATTTATAGTTTTTCCCAATTCCTGTTCAAAGATTCTAGTTGCTTTAT
CAACATCACGCACCAACAACGTTACATGCACTTTATTTTCTAGTAACTTTCGGACAATCGATAAACCTAAACCACTCGTACCACCAG
TCACTATAAAATGTTGTCCTTTCATCAATTAACCTTCCTTTTCAATTATATAGAATGCAATTTATCAACTTTACATAATTGAGACAAG
TTGATTATCTTTCCTAATATATATACAATAATAAGAAAATATAACATACAAATCAAAAACTAAAGGGATGTGACGTTAATGAAACTC
GTATTTTATGGAGCTGGTAATATGGCACAAGCTATATTTACAGGAATTATTAACTCAAGCAACTTAGATGCCAATGATATATATTTA
ACAAATAAATCTAATGAACAAGCTTTAAAAGCATTCGCTGAAAAACTAGGTGTTAACTATAGTTATGATGATGCGACATTATTAAA
AGATGCAGATTATGTTTTTTTAGGTACCAAACCGCATGACTTTGATGCTCTAGCAACACGCATCAAACCACATATCACAAAAGACAA
TTGCTTCATTTCAATTATGGCAGGTATTCCGATTGATTATATTAAACAACAATTAGAATGCCAAAATCCAGTTGCTAGAATTATGCC
AAACACAAATGCGCAAGTCGGACACTCTGTTACTGGCATTAGTTTTTCAAACAACTTTGACCCTAAATCTAAAGATGAAATTAACGA
TTTAGTTAAAGCATTTGGTTCTGTAATTGAAGTATCAGAAGATCATTTACATCAAGTAACAGCTATCACCGGAAGCGGCCCAGCATT
TTTATATCATGTATTCGAGCAATACGTTAAAGCTGGTACGAAACTTGGTCTAGAAAAAGAACAAGTTGAAGAATCTATACGCAACC
TTATTATAGGTACAAGTAAGATGATTGAACGTTCAGATTTGAGCATGGCTCAATTAAGAAAAAATATTACCTCTAAAGGTGGTACG
ACACAAGCTGGCCTTGATACATTGTCACAATATGATTTAGTATCTATTTTCGAAGATTGTCTAAATGCTGCCGTCGACCGTAGTATTG
AACTTTCTAATGTAGAAGACCAATAAAAACAAACCCGCCAACACATGTATGCATCATCGCATACATAGTTGGACGGGTTATTTTTAT
AAATTATTACTATTTGGCAAGCATTGTTTATTACTTTGTCATTAGACTTTAAAACTATCAAAATCTTTTACAAAATTAAAATTAGGTG
TATCTTCATTTTGTATCAATGTTTGATAAATTTCATTTATATCTTCTGTATTATAGCGATTGCTCAAATGTGTAATCAACGTACGTTTA
ACATTGGCTTCTTTTATCAATGCAAATACGTCTTCAATATGGCTATGATGATAATTGTTGGCTAAATGCTTTTCACCATCTATATAGG
TCGCTTCATGTACCATCACATCAGCATCTCTAGAAATCACACGTTCATTAGAACATGGTTTTGTATCACCAAAAATTGCTACAACTG
GACCCTGTTTGGACTCACCTCTAAAATCTTTTGATTGATAAACTTGACCATTATGTTCAAATGTATCATGAGATTTTACTTCTTGATA
TTTAGGACCTGGTTCAAGACCAATGTTTTTTAACGCTTCAACATTGATTGTACCTGTAGTTTCAGGTGCCATTACTCTATATCCATAT
GATGGAATACCATGATTAAGTAAATGCGCCTCTACAGTAAAACCATCATGATGATATGTCAGATGATCATCGATTTCAATATATGTA
ATTGGATAGTTTAAATGTGACTCTGATAAATTCATAGACATTTCCACATATGCTTTAATTCCTTTTGGTCCAACCAATGTAAGCGGCT
TCTGTTCACCGCCCTGAAAAGAACGACTAGAAAGTAATCCTGGCAAACCAAAAATATGATCGCCATGCATATGAGTAATAAATATA
TGTGTCACTTTTCCTAATTTAATTGCATGATGTAAAATTTGGTGCTGTGTACCTTCACCAACGTCGAAAAGCCATATGGAATTGGAAT
ATGGTTCTAAATTTAAGGCGATTGCTTGTGTATTTCTCTCTTTTGTAGGCAAACCTGCACTCGTTCCAAAAAATGTAACTTCCATATA
TGCCCCTCCTTTTCTTCAATTCATTTTATCATAAAATTTGTATCAATTTAGTTTTAATTTATACTTGAATCAAAATCTGTTAAAAATTC
GTCTTTGCTAGTTAATTTTTCTAACTTTAACGTAGACAAAACTATATAAATTTTGATAATTACGTTATACTTATCATTAATAAGTATC
ACATTAAACATGATACATGAATCGATATTTCATTTAAGACACTGCATACTGTCGAGCATATTGAATGACCTGCTGAATGGATTATCT
TATAATAATAAATCATATATCTAATTAAGAATTGAGGTTTTAATCTTGAGTACTAAAAACAAACACATCCCTTGTTTAATCACAATC
TTTGGTGCAACTGGTGACTTAAGCCATCGTAAGTTGTTTCCATCAATATTCCATCTCTACCAACAAGACAATTTAGATGAACATATTG
CCATCATCGGTATTGGACGTCGTGACATTACTAATGATGATTTCCGTAATCAAGTAAAATCATCAATTCAAAAGCACGTAAAAGATA
CAAACAAAATTGACGCGTTTATGGAACATGTCTTCTATCATAGACATGATGTTAGTAATGAAGAAAGCTATCAAGAATTACTAGATT
TTAGTAATGAATTAGATAGCCAATTTGAATTAAAAGGTAATCGACTATTCTATTTAGCAATGGCACCACAATTCTTTGGCGTTATTTC
TGATTATCTAAAATCTTCTGGTCTTACTGATACAAAAGGATTTAAACGCCTTGTTATCGAAAAACCATTCGGTAGTGATTTAAAATC
AGCCGAAGCATTAAACAATCAAATTCGTAAATCATTTAAAGAAGAAGAAATTTATCGTATTGACCACTATTTAGGAAAAGACATGG
TTCAAAATATCGAGGTATTACGTTTTGCGAATGCGATGTTTGAACCATTATGGAATAACAAATATATTTCAAACATCCAAGTTACAT
CTTCTGAAATACTAGGTGTTGAAGATCGTGGTGGTTATTATGAATCAAGTGGTGCGCTAAAAGATATGGTGCAAAACCACATGTTAC
AAATGGTTGCATTATTGGCTATGGAAGCACCTATTAGTTTAAATAGTGAAGATATCCGTGCCGAGAAAGTAAAAGTACTTAAATCA
CTGCGTCATTTCCAATCTGAAGATGTTAAAAAGAACTTTGTTCGTGGTCAATATGGCGAAGGCTATATCGATGGTAAACAAGTTAAA
GCATACCGTGATGAAGATCGCGTTGCAGATGACTCTAACACACCTACCTTTGTTTCAGGTAAATTAACAATTGATAACTTTAGATGG
GCTGGCGTACCATTCTATATTCGTACTGGTAAACGTATGAAATCTAAAACAATTCAAGTTGTCGTTGAATTTAAAGAAGTACCAATG
AACTTATACTATGAAACTGATAAACTGTTAGATTCAAACCTATTAGTAATCAATATCCAACCTAATGAAGGTGTATCTTTACATCTA
AATGCTAAGAAAAATACACAAGGTATCGAAACAGAACCTGTCCAATTGTCTTACTCAATGAGCGCTCAAGATAAAATGAATACTGT
AGATGCATATGAAAATCTATTATTCGATTGTCTTAAAGGTGATGCCACTAACTTCACGCACTGGGAAGAATTAAAATCAACATGGA
AATTTGTTGATGCAATTCAAGATGAATGGAATATGGTTGATCCAGAATTCCCTAACTATGAATCAGGTACTAATGGTCCATTAGAAA
GTGATTTACTACTTGCTCGTGATGGTAACCATTGGTGGGACGATATTCAATAATTGAATTAAAACGCACATGTTAAACAAAAATAAA
TGAGCGAATGACAAGGATTGATTACGAATCAAGTAAATCAATTGTTCTATCATTCGCTTTTTTTCTAGACTATATTATGAAATTATAT
TTTACAATGCCCAAAACTATTTTAATAATCATTGAACAAATGGGTGTATAATTTATAGAAATAATGTAGAATAAAAATAAATGATTG
AATTAATTGGAGTGAAAGTTTTGGACGTTATCAAGCAAATACAACAGGCAATTGTTTATATTGAAGATCGTTTATTAGAGCCTTTCC
ATTTGCAAGAATTAAGTGATTACGTTGGTCTTTCGCCATACCATCTTGATCAATCATTTAAAATGATTGTCGGCTTATCTCCAGAAGC
TTATGCACGCGCGCGTAAAATGACACTCGCTGCAAATGATGTGATTAATGGTGCTACACGACTTGTAGATATCGCTAAAAAATATCA
CTATGCAAATTCAAATGATTTTGCAAATGATTTTAGTGATTTTCACGGCGTATCACCTATTCAAGCTTCTACTAAAAAAGATGAATTA
CAAATTCAAGAGCGATTATATATCAAATTATCAACTACTGAGAGAGCACCCTATCCATACAGATTAGAAGAGACAGATGATATTTC
ATTGGTTGGATATGCACGATTTATAGACACTAAGTATTTGTCACATCCTTTTAATGTTCCGGATTTTTTAGAAGACTTGCTCATTGAT
GGTAAAATTAAAGAGTTACGACGATATAATGACGTTAGTCCATTTGAACTATTTGTTATTAGTTGTCCTCTTGAAAATGGTTTAGAA
ATATTTGTAGGTGTACCAAGTGAACGTTATCCTGCACACTTAGAAAGCCGATTTTTACCTGGCAAACATTGTGCGAAATTCAATTTA
CAAGGTGAAATTGATTATGCAACTAATGAAGCTTGGTACTATATTGAATCAAGTTTGCAGTTAACATTGCCATATGAACGAAATGAT
TTATATGTTGAAGTGTACCCTCTCGATATTTCATTTAATGACCCATTCACTAAAATTCAGCTTTGGATTCCTGTTAAACAGAGTCCTT
ATGACGAAGTTTAAAAATTAAAAACAAAGAAGCCCCCTAATATATCTATAGGTCTACAAATGGCCTTAGATTCTATTAGGGGGCAT

ATTAATATGTTAATTTAGTTCGATAACACATGCTTCATATGGACGTAACTGTTTTAAATTAACTTTGGCATCATAATTAAATAGCTTT
ACTTCTCCATGGCTTAAATCAAATGGTACAGTTAATTCTGCTTCGTGGTTAGTAAGATTACCTACAATAAGAACTTGCTTTTCATTTA
ATGTTCTCGTGTACGCAAAAACTTGTGAATTTTCAGCATCTACTAAATCAAATTGACCATATACGTATACATCATTAGACTTTCTTAA
TTGAATTAAATCTTTATAAAATTGTAATACTGAATGCTCATCTTCTAATTGTTGTGCAACATTGATAGTTTTATAATTCGGATTCACT
AGGAACCACGGTTCACCATTTGTAAATCCTCCATTTAACGTATCATCCCATTGCATTGGTGTGCGAGAATTATCTCGGTTCTCATCTT
TATATTTCGCAAGTAAAGCGTCTACATCTCCACCTTGAGCTTTCACTATTTGATAGTCATTTTTAACAGCAACATCGTTAAACGTTTC
AATACTTTCAAATGGATAATTCGTCATACCAATTTCTTGACCTTGATAAATGAATGGCGTACCTTGTTGCAAGAAATAAACAGCTGC
ATGACTTGTTGCTGATTCATACCAATACTTGTCATCGTCACCCCACGTCGATACACGTCGTGGTTGGTCGTGATTTTCAATAAACAAC
GCATTCCAACCTTTATTTTCAAGTTGTTTTTGCCATCTATTTAATACAGATTTATACGAATTTACATCAAAGTGAGAATCACCACTAT
TCCACAGTCCCAAATGTTCAAATTGGAATATCATATTAAATTTACCATTTTCTTCCCCGACCCAGTCATCAGCATCATCAGGACTTAC
ACCATTCGCTTCACCAACAGTCATAATGTCATACTTACTTAATGAGCGATCTTTCATCTCTTGTAACCAAGTTTGTATACCTGGCTGA
TTCATATCTACATCAAATGCTGGAGCATATGTTTTATCCTCAGGTACAGGTAAGTCACCCGCTTCAAACGTCTTTTTAATATGCGTAA
TTGCATCTACTCTAAATCCATCAATGCCTTTATCAAACCACCAGTTCATCATTTCAAATACAGCATCTCTAACTTCTGGATTAGCCCA
ATTCAAATCAGGTTGTTTTTTACTGAATAAATGGAAATAATATTGCTCAGTATTAGCATCATATTCCCATGTAGATCCATTAAAGATA
CTTTCCCAGTTGTTAGGTTCAGAGCCATCTGGCTTTGGATCTTGCCAAATATACCAATCGCGCTTTGGATTGTCCTTACTAGACTTAG
ATTCTATAAACCAAGGATGTTCATCAGATGTATGATTTACAACTAAATCTAAAATAAGCTTCATGCCTCTATCATGAACACCTTTTA
ATAAACGATCAAAGTCTTCCATCGTTCCAAATTCATCCATAATCTCTTGGTAGTCACTAATATCATAACCATTGTCATCATTAGGTGA
TTTAAACATTGGACTGAGCCAAATGACATCGATACCTAAATCTTTTAAGTAGTCCAATTTATCAATCATTCCAGGTAAATCCCCAAT
ACCATCGTGATTACTATCATTAAAACTTCTTGGATATACTTGATATGCTACTGCTTCTTTCCACCATTGCTTATTCATTTTAAAACTCC
TTTGCTATCGCTGTGTTGATTTTCTTATTTTTAATTCTATATCTATAATGACGAGTTCAATAACATCCTGTGCTTTATTTTTTAATATAT
TTAAAATTGCTGCACCAGCCTGTTGACCTAACATTCGAGGCTTGATGTCAATACTGGTTTGAGGTGGTGACGCAATTTCGGTTAAAT
AAGAATCATTGAACGTTGCTGTCATTACATCTTTCGGAATTTCAATATTAAGTTCATATAGGACACTTAAAATCGCTAAATGTAACA
CAGCATCTAACGAAATGATTGCCTGTTTAATATTTGGGTCCTTCAAACGCGTATGTAGATTTTGCATGTAATTTAAAATAACTTCTCT
TTCATTACTAGTCTCAATAATTTGATAATTAATTTTATTTTGAGAAGCTATCGTTTCAAATCCTTGAATCCTGTCTTTCGAAACTTCAA
AATCGCCTTTTTCTGTAATAAATATTAATTCATCTACACCTTGTTCAATAACATGTCGTGTCAAATTTTCAGAAGCTAATATATTATC
ATTATCTATATGTGTAAATTGATGATCTATATCCGATGTAGGCTTACCAATCACAATAAATGGCATGCTTTCATCAATTAACATTTGT
TTAATCGGATCATTTTCTTTTGAATAGAGCAGTATAAACGCATCAACCATTCGTTGTTTAATCATTTTATAAACTTCATCCATTAAAT
CATTCATATTATTTGAGACTGTCGTTTGTGTACCATAGCCATGCTGGTTACATGTTTCAGAAATCCCTAGCAATACATTGATGTAGAA
TGGATTCAGTCGAATAGGCTCCTCAGACCCTTTTAACACTAAACCAATTTTATATGTTTGGTTAGTAATTAAAGTTCTAGCAGCAGTA
TTAGGAAAATAATTCAATTCTTCCATAACTTTCTTCACTTTTGAAATTGTCGCTTCGCTAATACGTTCATTTCCTTTTATAACTCTTGA
AACTGTCGAAGGAGAAACACCGGCTTTTAGTGCAACATCTTTAATCGTAACCATTTAATCACCTCCTGTTAATTTCTGCATTGGAAA
ACGCTTCCAAACTCTGTATAATACCAGTTTAGTCACACTTTCTAAAAAAGTCAAAAGATTTGTGCAAACGATTGCATAAAGCGATAA
AAATAAAACCTTCATACTGAAATTCAATCCGAAAATCAATATAAAGGTTTGTATAAATAGGTTCTATAATAAATCGGACTGATGGA
AAAAGTATTTTACTTTTCATCTGTCCGATTTTTTGATTTTGAATATAAAAAAAGCGCAATTATCTCTATAATTAAAAGTGCCTAAACA
AATAACTAAAGGAGATAATGCGCCTATGAATAATGTTATACTAAATACCTTTGATTTTAAAGATAAAAATATTATTTTTGACGGTAA
ACTTGAGAAAATTGAGTATAAAGGGAGAAAGTGTTTCTTTTATTACGCAAAACTAATCTATACACCTGAGGTGTGCCCAAACTGCA
ATTGTAAAAACATTAATGGTAATTTAATCAAAAATGGATCTAAGACTTCTAGAATCACTATGCCTAAAATCTCCGAATATCCAACTT
ATATCATGTTGCGTAAGCAACGTTTTAAATGTAAAACGTGCGAACGATACTTTACAGCCGAAACACCTGAAGTTGATAAATACTGTT
TTATATCAAAAAAACACGATCAGCTGTACTTAATAAAGCAGCAGAAATTCGTTCTGAAAAATCTATAGCGAAATCCTGCTCTGTGTC
AGCTACCACAGTTTCTCGTATCATTGATGAGGCCGCTCAATCGCTTCATCAATCGCCGTATTCTGCACTTCCTGAACATATCATGATG
GATGAATTTAAAAGTTTTAAAAATGTTTCTGGGAAAATGAGTTTTATATACGCTGATGCACAAACGCATCATATTATTGATATTGTT
GAGGATCGTAGATTAAGTGAGTTGAAAAAGTATTTTTATCGTTTTTCACTTAAAGCTAGAAAACGTGTAAAGACTGTTTCCATTGAT
ATGCATGAAGGCTATATGACATTGATTAAAGAGATGTTTCCGAACGCTAAATTAGTTATCGATAGATTCCATATTGTTCAATTATTA
AATCGTGCTTTAAATGGTATTCGAGTCGCTGTTATGAATGAATTAAGAACGAAGAATCAACCTTTATATAATAAGTTTAAAAGATAT
GCGAAATTACTATTAAAACCTGGAGAAGATTTAGAGGCATTTGAATATCGTAAAGTAGCTTTATTTAAGGAATGGAAGACACAAAA
GGGTATCGTCAAGTATTTATTAGATCAAGATGATTCGTTAAATGACGCCTACCAATATATCAATCAGCTACGATTCAAACTTAAACA
TAATGATTATGAAGGCTTTATTCACGAATTAAAACATATGCCATTATCCCAAACGCATTCAGTTGTCCAACGAGCAATTAAGACACT
TAACAAACATGAATACTTTATAAAAAATACATTTGATTATTACAATTTATCAAATGGTCCTTTAGAAGGTATAAATAACAAAATAAA
ATTAATTAAACGTACATCTTTTGGTTATGGAAGTTACAATCATTTGCGCAATCGAATATTATTATGTTCAAAACTTTACGCTCCAAAA
AGTAAAAAGGAAGTTAAGCAATGTTTAGTTGCCTAACTTCCGATATTGAACGCATCATTCCAATTTGACATAGAGCCTATAAATATT
AAAATCGATTGTTTAGTCACTAACTGCAAAATAGTTACCTTGGCCATCTTGAAAATTAAATACACGTTGACCATTCATTTCTACTATA
TCATGCCCAGTTAAACCTAAATCATTTAATTTTGAGTATAATGCATCAAAGTTTTTCTCTTTAAACATTAAAGATGGTGTTCCTAGGT
TCACTTCCGGGCTATACTTTTCAATAAATTCTTTTGCCATAATCGTCAATGACGTTTCAGCATCTTTCGTAGGTGATACTTCAACTGC
AACATAGTCCTCAGCTAACGGTGTTTCACTTACAACAACAAATTCTAAAGTTTCTGTCCAAAATGCTTTCGCTTTGTCAACATCATCA
ACATATAACATAACTTGATTTAACTTTTCCATAAGTTAATACCTCTATTTCTCTATAGTACATGCTATCATAACACAGTAAATATTTT
ATTACTTCACAAAATGCTTAAAAATATGGCGGGATGCTTTTAAGGTCAAAGATAATACTTGTGTAATTTTTTATAGGTTGTAGCTACT
CTATCACACTCTCTTTTATATTTATCAAAAGATATAAAAAAGGATAGTATCTTTCAACTATCCTTTAATCAATATTATTCTTCAATCC
ATTGTGTATGGAATACGCCTTCTTTATCTTTTCTTTCGTACGTGTGAGCACCGAAGTAGTCACGTTGTGCTTGAATTAAGTTTGCAGG
TAAATCAGCTGCACGGTAACTATCATAGTAATTAATACTTGATGAGAAACCAGGTGTTGGTACACCATTTTGAACACCAGTTGCGAC
AACATCACGTAACGCATCTTGATATTCAGTAACGATGTTTTTAAAGTAAGGATCTAGCAATAAGTTTTGTAATCCTGGATTATTATC
GTAAGCATCTTTGATCTTTTGTAAGAATTGTGCACGGATAATGCAACCTTCTCTCCAAATCATAGCTAAATCACCAAGTTTTAAATTC
CATTCATTATCTTCACTTGCTTTACGCATTTGCGCGAAACCTTGTGCATAAGAACAAATTTTACTCATATATAATGCTTTACGAATTT
TTTCTAAAAAGTCTTTCTTGTCACCATCAAATGATGCTTTTGGACCATTTAATTCTTTAGAAGCATTTACGCGCTCTTCTTTGATTGAA
GAGATAAAACGTGCAAATACAGATTCAGTAATGATTGTTAATGGAATACCTAATTCTAATGCGTTAATTGAAGTCCATTTTCCTGTA
CCTTTTTGACCTGCAGTATCAAGAATTTTTTCAACTAATGCTTCTTTATTTTCATCTAATTTCATGAAAATATCACCAGTGATTTCAAT
TAAATAACTTTCTAATTCACCAGCATTCCAGTCTTTGAACGTTTGAGCAATGTCTTCATGAGACATGCCTAATAATTCTTTCATCATA
GCATAACTTTCTGCAATTAATTGCATGTCAGCATATTCGATACCATTGTGTACCATTTTCACATAGTGTCCAGCACCATTAGGTCCAA
TATAAGTAACACATGAAGCACCGTCTTTTGCCTTTGCAGCAATTGCATCAAGAATATCTGCAACTTTGTTATAAGCTTCTTCTTGTCC
ACCCGGCATTAATGACGGACCAGTTAACGCTCCAATTTCACCACCAGAAACGCCCATACCAATAAAGTTGATTGCACTTTGTGCTAA
TGCTTTATTACGTCTGATAGTATCTTGATAGTTTGTATTACCACCATCAATTAAAATATCTCCATCATCTAATAAAGGTAACAAACTA
TCAATCGTTGCATCCGTAGCTTTACCTGCTTGAACCATTAATAAAATTTTACGTGGTTTTTCTAAAGAATTAACAAATTCTTCCAATG

AGTACGTTGGATGAATATTTTTCCCTTTTGATTCTTCAACCATTAAATCAGTTTTTTCACTTGAGCGGTTAAATACAGATACACTATA
TCCGCGTGATTCAATATTCCAAGCTAGGTTTTTACCCATAACGGCTAAACCAATAACTCCAATTTGTTGTGTCATATTACTTACCTCA
CTTGTTGATTTTTCATTAGTATTGTATCACAAAATAGACATACACTACACTAAATCATTTCGATTGTCGCGCAACTATTTTGATTATTT
CTAACACTTGACTTGCAAGCAAGTTCAATGATTTAATCGGCATTCTCTCATTTGTTGTATGGATTTTTTCATAACCCACTCCTAAAAT
GACTGAAGGAATACCAAATGTATTAATAATACTGCCGTCTGAACCGCCACCAGAAATAATTGTATTTGCAGATAACCCTAAATTAC
GAGCACTTTCTTGTGCAATTTTAACAACCGTTTCATTATCATTAATTTTAAAACCTGGATAACTTTGCTCCACTGTAACTACTGCTTTC
CCACCTAATTCTGATGCAGTTGTTTCAAACACATCAGTCATATGTTTGACTTGTGTTTTTATTCTTTCTGGATCGTGAGAACGTGCCTC
TGCTTCTAAAATGACTTCATCTGCAACAATATTCGTAGCTGAACCGCCATGAAACTTACCAATATTGGCAGTAGTTATTTCATCAACT
TGTCCTAATTTCATTCGACTAATTGCTTTCGCCGCAATATTAATAGCACTAACACCCTCTTTTGGCGTACTTGCATGAGCCTTTTTGCC
AATAATTTTAGCTGAAATTAACATTTGCGTCGGTGCACCTACAACCGTAGTACCGACATCAGCACTTGCATCAATAGCATAACCAAA
GTCCGCGTCCAACAACTCTGAATTTAATTCTTTAGCACCAATTAAACCTGATTCTTCTCCAACAGTAATCACAAATTGAATTTGTCCA
TGTGAGATTTGTTGTTCCTTTATCACTTGCAAAACTTCAAGCATAGCTGCTAATCCTGCTTTATCATCTGCACCTAGAATAGTCGTAC
CATCAGAGTATATGTAGCCGTCGTCTTTTACAATTGGCTTTACATTAATTGCGGGTACAACAGTATCCATATGGCTCGTCAAATATA
ATTTAGGTACTTCGCCTTCTTCGATAGTACTTTTCATTGTACACACTAGATTATTGGCACCTAATTTAGGATGTTTAGCCGCTTCATCT
TCTTTAACATCTAACCCTAATGCTATGAATTTTTCTTTTAAAATAGGTTGGATTGTTGATTCATTCCCTGTCTCAGAATCGATTTGTAC
AAGTTCTAAAAACGTATTAAGTAATCTTTGCTCATTAATCATAATGGAACCCCTTTTATCAAAATTTTCAAGTACGCCAAACGGTAA
AAATTGATGAAATAAAATGTTACAGTAATTGACGTTACACAGATTTATCAGGCTTGTAAATTGTGTCATATTATTTTCAATTTAATAT
ATATATTTATTGTAACTCAAACTAAGCTTTGTCAAAAATATATTGATTGATTTTTCAAAGATATCGTATAATGAGGAAAATGACATA
AGCAAACTTACTCATGTTTTTTATAATATTCCTTTATGATGATTGCTAGTTATATCGTCTCAAATTAAAAGTTTTATATCTTATGTCGT
AATTATTAATACAAAGGTTATTCATTTGGAGGCACACAAAATGCAAAATAAAGTTTTAAGAATTATCATTATCGTTATGCTTGTATC
AGTTGTATTAGCATTGTTATTAACGAGTATCATTCCAATTTTATAAACTATATCTCAACTACCTATACAAAATCATACAATTAAAAAT
CCATCCGTTATAAACGCATGTATTAATAAGTTATCGTATTGCAACGATTATTTTCAAACATGGGTCATACGGATGGATTATTTTTTAA
GCTACTTCACTATGCATTTTCAATGAACCAAATTGCGATTTGATTTGTAAATATTCTTCTAATTCATTTAATATTTGAATAATACTTGC
TCTCGAGTTAAGCGCTTTGTGTGTTGTTGGCAATGGAAGTTCATCCAATTTCAAACGCGTCTCATACAAATTGTGTAAACGCATTGCT
GTATAGTCATTACTATTCACATTTAGACCAATTTCTTTCAGCAGTGACGCAACATCATTTAAAAGTGGATCTTTATGACAGATACTTT
CGATGAGCGGTTTCATTCTCATTAACAATTCCACTTGCTCTTCTCGCATATCAAAATAATGATAGTATGAATTTTCGTTTCTAACAAA
ATGATTTTTAACATCTCGGAACGCGATAGACTTCGCCTTTTTAATATTTAAAAGTAACACTTCAAATTCAATCGCAATGGTATCTTCA
TATTTTTCACAAATATAACTATATTTACTAAAAATATCAGCAATTTGTTGCTCAATTTTACATTTGTATTCGTCTAGTTGTTTGTCTAA
ACTTGGCATCATTAAATTCATTGTAAATGCAATGCTTAGTCCAATTAACAGTAATAATGTTTCATTAACAATTAAATGTGCATCAATT
GATTTTGCATTAAAAACATGAAGTAATATAACGCAACTCGTAATGACACCTTCTTGTACTTTTAATACGACAGTTAATGGTATAAAT
AACAATACGATAATACCGAGTACAATTGGACTCTGACCTAATAAACTAAATATTGCTGAACCTAAAAACAATACTAAAAAACATGA
TACTAATCTTGAAATAATCGCTTGTAGCGAATGTACTTTTGTATGTTTAATACATAATACGACTAATATGGCGCTTGAAGCATAATTA
TCTAAACCTAACAGCTTACTAATAATTACACCTAAAGTCATACCCACTGCTGTTTTTATTGTTCTAAATCCAATCTTGTAAGGATTTA
ACTTTAACATGGGTTAGCGCCTCTTATCTTTCTTCACAATATTTATTGAATAATGTTTGTAATTGATTAATTACGTTCATCACATCATG
ACCTTCGATTTGATGTCTTTCAATCATTTCTGTAATCTTTCCATCTTTTACTAATGCAAATGACGGACTTGAAGGCGCATAACCTTCG
AAGTATTCACGCGCTCTTTGTGTCGCTTCTTTATCTTGTCCAGCAAATACTGTCACTAGACGATCAGGTAATACGTCATAATGTAAAG
CATGTGATGCTGCTGGTCTTGCGATACCACCTGCACAACCACATACAGAATTGATCATAACTAGTGTTGTACCATCTTGTTTAAGAA
CTTTGTCAACATCTTCTGCAGTAGTTAATTGCTCATATCCCGCAGATTCAATTTCATTCCTTGCTTGTTCTACAACACCGTTCATGTAT
AAATCGAAATTCATGTCCATAAGTTCAATCACCTATCCCTTTATATTTAAACTATCCTCATTCTACTAATTAATAACATATTGTTCAA
TAAACTAATCTGAATCACACCTATATTTAGACACAATTTTAACAATATACCAAACATTATTGTGCTTAAAATCATGGTAACTAATTT
GTTCACATGTTTTCATTAATATGTTTCAAGTATGATGTCTTATTTTGACTTTACTGCAAAAATTCAATCAACCATGTTGATTATTGTTC
TTTATCTTTTTTGAATATATTGCACATATTTTAGTGCCAAAAAATAATACATCCATCGACAAGAACAAGATAAAACAAGTTGTCGAT
AGATGCATCTATGTTATCACTAATATATATTTGTATTTTCTAAAGTATACTGTTCGATACGCTGTTTAATATGATTCATAAATTTACCT
GTTTGTAAACCATCTAAAATACGATGATCAATTGAAATACATAAATTAACCATGTTACGAATTGCAATCATATCATTAATTACTACT
GGCTTTTTAACGATTGATTCTACTTGTAAAATCGCTGCTTGTGGATGATTTATAATACCCATTGATGATACTGAACCAAATGTACCAG
TATTATTTACCGTAAATGTACCGCCCTGCATATCTTCAGCTGTCAATTGCTTATTACGCGCTTTCATTGCTAAAGTATTAATTTCTCTA
GCTATACCTTTGATTGACTTTTCGTCTGCATGCTTAATCACAGGTACGTATAATTTATTTTCATCAGCAACAGCAATTGAAATATTAA
TGTCTTTATGTAAGACAATTTCATTTCCTTGCCAGCTACTATTTAATAAAGGATATGCTTTTAAAGCATCTGCTACAGCTTTTACAAA
GAAAGCAAAGAACGTTAGATTATATCCTTCTTTATTTTTAAAGCTGTTTTTATAATGATTTCTCGTATTCACAAGATTTGTAGCATCT
ACTTCAATCATCATCCATGCATGTGGAATCTCTGTTACACTATTAACCATATTTTGCGCAATTGCTTTACGCACACCATTTACTGGTA
TTGTGCTGTTTTCACTATTGTCTTCAGATGATTGGTTACTTGATGTATCTACTGATGTTGATTTTGTTTGAACTTGTTTGTCAGATTGA
GCTGTGGTACCACCATTTTCAATAACTGACATTATATCCTTCTTAGTTACACGACCTTCAAATCCACTACCTACAACTTGTGATAAAT
CAATGTCATGCTCTGAAGCGAGTTTAAATACAACAGGTGAAAAGCGACCATTATTACGTGGTTGATTTTGTTTAGCAGTAGATGTCT
GTTCCACTGTTGCACTAGCTTTTTTAGTAGATTTCTGAGTATGCTCATCCACTTTTGCTTGTATCTCTTCAGTTGTTTCATTTGTCTTTT
CATCAGCAGTTTCAATTTTACAGATAATTGTATCAATAGCTACTGTCTGCCCCGCTTCAACTAAAATTTCTGTAATTGTTCCTGATAT
CGTGGAAGGGACTTCAGCTGTCACTTTATCTGTAATAACTTCACATAATGGTTCATATTCATCAATATGATCACCAACAGAAACTAA
CCATTGTTCAATGGTACCTTCATGAACACTCTCACCTAACTTAGGCATTGTTATTTCCATGACTTTCCCTCCCTAGAATTCTGCTAATT
CACGCATTTTATTTAAGATTTTTTCTGGATTCATCATAATTTCATTTTCTAATACAGGTGAAAATGGCATAGATGGTACATCTGGAGC
AGCTAAACGCATGATTGGTGCATCTAAATCGAACAAGCAATGCTCTGCAATAATCGCTGACACTTCTGACATAATACTACCTTCTAA
ATTATCTTCAGTTACAAGTAAAACTTTACCTGTATGTTTAGCACGATCAATAATTGTTTCTTTATCTAATGGATAAACAGTTCGTAAA
TCTACGACTTCAACATTGATACCGTCTGCAGCTAAAATATCAGCGGCTTGTAAGCAATAATTGACCATTAATCCATAACAAAATACT
GTTAAATCTTCACCTTCACGTTTAACATCTGCTTTTCCTAAAGGTACAGTGTAATATTCTTCTGGCACTTCTTCCTTTAAGAAACGAT
AAGCTTTTTTATGCTCAAAGTACAATACTGGGTCATTTGATTCGATAGATGATAATAAAAGCCCTTTAGCATCATACGGTGTGGAAG
GAATAACAATTGTTAAACCTGGTGATGAAGCAAATATACTTTCAATACTTTGTGAATGATATAGTCCTCCGTGAACACCGCCACCAA
ATGGTGCACGAATCGTTAATGGGCATTGCCAATCATTATTTGAACGATAACGCATTTTCGCAGCTTCACTAATAATTTGATTTGTCGC
AGGTAAAATAAAATCTGCAAATTGAATTTCTGCAATTGGTCTTTTACCAACCATAGCTGCGCCAATAGCTGTTCCGACAATATTTGA
CTCAGCTAATGGTGTATCGATAACTCTGTCCTCACCATATTGTTGTTGCAGTCCTTGAGTAGTACCAAATACGCCACCTTTTTTACCA
ACATCTTCACCAAGAATAAATACATCTTTATTTTGTTGTAATGCTAAGTCTTGTGCCTGGCGAATCGCCTCTAAATAAGATAATTTAG
CCATTAGTTAAGACTCCCTTCTTCGTACACAAATGCATAGGCTTCTTCGACACTTGGATATGGCGCGTCTTCAGCAGCCTTTGTCGCT
TTATTGATAATGTCTTTATGTTCTGCTTCTATTTCTGCCAACCAAGCATCATCGATAATGCCAGCTGAAAGCAACTCTTTTTTGAACTT
TTCATTGCAGTCTGCTTTTTTAAGCGCTTCACGCTCTTCTTTCGTACGATATTGATCGTCATCATCTGATGAATGAGCTGTCATACGAC

TTGTTACTGCTTCAATCAAAGTTGAACCTTGACCAGAAATAGCTCGATCTCTTGCTTCTTTCATCGCTTTATACATTGCTAATGGATC
ATTACCATCTACTTGTTCACCATGTATACCGTAACCAAGTGCTCTATCCGATAATTTTTCAGCTGCGTATTGTAATGAATCAGGTACT
GAAATTGCATATTTATTATTTATAATGACACATACAAAAGGAAGTTTGTGTACACCCGCGAAGTTTAAACCTTCATGGAAGTCACCT
TGGTTTGAGCTACCTTCACCAACAGTTGCTGTTGCAATTTTCTTCTTACCATCCATTTTTATAGCTAAAGCAGCACCAACAGCATGGG
GTATTTGAGTTGCTACCGGTGAACTTTGTGACAAAATATTCTTAGCTCTACTACTAAAGTGTGATGGCATTTGTTTTCCACCAGAGTT
AACATCGTCTTTCTTTCCAAACGCTGATAAAAACGTATCATACGCTGAGATACCCATATAAGTAACGAAAGCTAGATCTCTATAATA
AGGCGCTGTAATATCACCTTCTTCTAATGCGTATGCCATCCCAATCTGAGTTGCTTCTTGTCCTTGACCACTTACAACAAATGGAATT
TTACCTGCACGGTTCAATAACCACAGTCTTTCATCTATTTTTCTACCTAAATCCATCCATTTATATATTACTTTTAGGTCTTCTTCGCT
AAGGCCTAATGATTTATAATCAATCATGTTAAATCCTCCTATTTATACGTGAATAGCTCTACTTTCTGCTTTCAATCCTAATTCCATC
AACACTTCAGAGATGGAAGGATGTGCGTGTGTTGTTAGTCCTAATTCTAATGCCGAGCCATTCATGAACTGTAACAGTGATGCCTCA
TTAATCAATTCTGTTACATGTGGACCAATCATATTAATACCCACAATTTCTTCAGTTGATTGATCAATCACCATTTCGCTATACCCTT
CGTTTGCGTCATGGCTATCAATCACTGCTTTACCAATTGCTTTAAATGGTACTTTAAAACTTTTAACTTTCATTCCCTCTGCCTTTGCT
TGTTCAATGTTTAAACCGATAGAAGCAATTTCAGGTTGTGAATAAATACACTTAGGCATCATGTTATAGTTTACTGGGATTGGGTTC
CCCTCAAACATATGATCAACAGCCACAACACCTTCTTTTGATCCAACATGTGCCAATTGTAATTTTCCTATACAATCACCAGCTGCAT
AAATATGTTTATCTTCAGTTTGTTGAAATTCGTTCGTTAAAATATGTCCTGATGTTGAAAGTTTTATTTTAGTGTTGTTTAAACCAATA
TCTGATGTGTTAGGTTTTCTACCAATCGATAGCAACACTTTATCTACTTTAATTATGTCTGAGGAAATTTCAAACGTAACACCATCTT
CGTTAACATTTATATCATTTTCAGAAAGTTTTATTCCCTCATAGAATTTAACACCACGTGCTGACAATGATTTTTTTAATAGTTGTGA
AGCTTGTTTACTTTCAGTTGGTAAAATTCTTTCACCTGCTTCTATAACTGTTACGTCAACACTTAAATCTATCATCAATGATGCAAAT
TCCATTCCGATAACACCACCACCAATAATACCAATACTTGATGGTAACGTCTTTAATGATAATATATCATCGCTAGATAAAATTTTA
TCATGATCAAATGATAAGAATGGCAACTCTGCAGGCGAAGAACCAGTTGCAATTAATACAAATTGGTTGGGTAATAAGTCTGATTC
ACCATCTTCATATTCGACAGAAATTGTGCCACTTTGAGGTGAAAATATAGATGTACCTAGAATACGTCCCGTGCCATTATAAATGTC
AATGTGATTGTGTTGCATTAAATGCTTTACACCTTGATACATTTGATTAATAATGTCTTCTTTTCGTGCCAACATATTTTCAAAATTAA
CATTAGCATCTTTGACATCAACGCCAAACATTGCTGCCTGTTTTACTGTTTGAAATACTTCAGCAGATTTAAGCAGCGATTTAGTAGG
AATACAACCTTTATGGAGACAAGTACCTCCTAATAGTTGTCGTTCTACTATTGCCACTTTTTTACCTAATTGAGACGCACGTATCGCA
GCAACATATCCTGCAGTACCTCCACCGAGAACGACTAAATCATATTGTTTCTCTGACATGTTCTTACTCCTAACTAATGATATATATC
CATTGAAAATTTATTAATACATAGTTTTCATGTCCATTAATTACCTATTTTACATGATTGTCTATTTAGTTTGAATGCACATAAATAA
ATCCATAAATGAGTATTCAACACACTTTGGAAATTATCTACGTCTTTGATTGTGTTGTATCATTTCTCTTGCATTTTCTCGAGTTAAAT
CAGTAACACTTGCACCTGAAATCATTCGTGCAATTTCATCTACTTTATCATCGCCAATTAACTCTTGAACTTGTGTTGTTGTACGATC
ATCTTTTGATGACTTCGAAATTAATAAATGATGGTCGCTCATCGATGCAACTTGTGGCAAGTGAGAAATACAAATAACTTGTATATA
TTCTGCTATATCTCGCATTTTCTCTGCCATTTTTTGTGCGGCTTGTCCAGATACACCTGAGTCAACCTCATCGAATAAAATTGCAGTTT
GACCTCTCGATTTAACAAAAATACTTTTTAACGCTAACATAATTCTAGAAAGTTCTCCACCTGACGCAATTTTATTTAAACTTTTTAA
TGGTTCCCCTTTATTTGGACTGATTAAAAATTCTACAAATTCGATTCCATCAATATTCGGTTCTTCTAATTTTTTAAATGAAATCTCAA
GATTTGCGTCTTTCATTTGTAAGTTTTGAATTTCCGATACAATGTGGTCTCTTAACTCCCTAGCGACTATACGACGCTGCTTTGATAA
CGCTTGTCCAACCTCTATAACTTGATTATACAATGCATTTATTTCTTCTCGTAACTGCGATGTACTTTGTTCATAGTTTTCAATTTTAT
TGATTTCATTATTAAGCTTTTCTTGATATGCGATTAATTCTGAAATATCTTTTCCATATTTACGTTTTAAATTATTCAGCAAATTCATA
CGAGATTCATACTCGTTTAACACTTGTTCGTCGAATTCTGTATTAGCCATTTCATCATATAACTCATGTTTTGCATCTTCTAAAATGTA
ATAAAATTGATCAATATCTTCTTTTAATTTGTCATATTTGTTTGGAACTATATCGTTTATTGTTAACAAATGGTTGCTTAGTTCATACA
AACGATCAGTGATAGCATTTTCATCCGTTAATGTCATATGTGCGTTATTAAGCGCTAAGCTTAATTTTTCAGAGTTTTGAATGCGTTT
AATATCTATTTCAAGTTGCTCTATTTCGCCTTCTTTTAGATGTGCTTCAGACAATTCTTCTAATTGGAATTTCATTAAATCTAAACGCT
GCAGCAATGCTTGGTCTGCTGATTCTAAATCTTCTAACTCTTGCTTTTTGGCTTTATAATTTTGAAAAGTTTGATGATATTTATCCAAC
AAATCTTGATAACGTGATTCTGCGTAATTATCCAATAATGTTAAATGGTATTTTTGTTTCAACAAAGACTGCGTTTCATGTTGGCCAT
GAATATCTAATAATTCTTGCATAACTTTTCGTAAATCTTGTAAAGTAACTGTTTGATTATTAATTTTACAAAGACTTTTACCAGAGCT
GAAAATTTCCCGTTTAACTAATAAAAAATCTTCATCTACATCAATATCCATATTTTTCAATATATGTATAGCATCTTTACTCTCGTCA
ATATCAAATATACCTTCGATGACAGCCTTTTTTTCACCATGTCTTACAAAATCAGATGAAGCTCTCATTCCAATTAATTGTCCAATTG
CATCTATAATAATTGACTTACCTGAACCCGTTTCACCACTTAAAACAGTTAAACCATCAGAAAATTGAATTTCTAACTCTTCAATAAT
AGCAAATTGCTTGATTGATAAGGTTTGTAACATAAACTCATCGCATCCTTATAACAAATTGAAAATTCTTGACTTGATTTCATCACTT
GCCTCTTTGCTTCGACAAATAATTAAACAAGTATCATCACCACAAATTGTGCCTAGTACTTCTTCCCAATTGATTTGGTCTAATATAG
CTCCAATAGATTGTGCATTACCAGGTAATGTTTTTAGAACAAGTAAATTATCAGTACCATCTATATTAACAAAGGAATCCATTAAAT
AACGTCCCAATTTTTCTAAAGGATGGAATTTTCTATCATTTGGTAAACTATAAACATATTGACCTGAAGGTATAGGTACTTTAATAA
GTTGTAGTTCTTTAATATCACGAGAAACAGTTGCTTGAGTGACATTTAAATCATAATCGTTTAATCGTTTAACTAATTCATCTTGTGT
CTCTATCTGTTCATTTGAAATAATTTCTCTAATTTTTATATGCCTAACCGATTTTTTGGGCACAGTAAACACCTCTGATTACGAATATT
TATACATTTATTTTAACACATGCACTGATTTACGACTACTAAACACCTTTACGTAAAAAGGGTAAACATGGTTTATCTATCTTGGTTA
TCTATTTATAAATATTTTTCATATTACGCATAACAATTGCTTAAAATATGTATAAAAATGAATATATGTGTAATAAACTTGCTAATTA
TTAGATTTAATAAGCGTCAATTGTTTGAACATATTTAATTAAAATCACATTGATATCACAGATGCGAATATTGTCGTATAGAAATTG
AAAATTCTATTTTTTAAATGAAAGTCTTCAACATAATTTTAAGTTTCAACATGAGAAAAATCGATTAACAAACAACGTCAGTTGAAT
ATGCCTTTTGAGACATTTCAAACTTTACAATTGTTGCTAATCGATATATTTGCTTTTAGTGATCCCTGCTATAAAATAAATCAACGAT
TTCTAATAAGTGTTTTGTATTGAATTGTTCATCAATTTGCGTTAGTTCATCCACTGCTGCGTCTCTATGATAAGTCAATTTATCTTCTG
CGCCATCTTTCCCTAATAAACTCACGTACGTACTTTTATTATTTTCAAGATCGCTGCCCACTTTTTTACCTAACTTTGCTTCATCACCA
TAGCAGTCTAATAAATCATCTTTAATCTGGAACATCATACCTAAATGATAACTATAACTTTCTAAATGTTCTTTAGTTGCATCATCGA
CATTAGCGATATCTGCTGCACTCATAACCGCAAAAGTTAATAATGCTCCTGTTTTTGTTTTGTGTATCATTTCCAAAGTTTCAAGATC
AATTGGTTGGCCTTCGCTTTGCATATCTAACATTTGACCGCCGACCATTCCAACATGACCACTTGCTATTGACAGCCGTTGTAGAACT
TTTATTTTTACTTCATCAGTTAATCTATCATCACTTGAAATAAGTTCAAATGCTTTAGTTAATAAAGCATCACCTGCTAATATCGCAG
TCCACTCACCATATACTTTATGATTTGTTAATTTCCCTCGTCGATAATCATCATTATCCATCGCTGGTAGGTCATCATGAATAAGTGA
ATATGTATGAATCATTTCTAGTGCAATTGCGCTCTTCACACCTAACTCATACTCGGTATTTAGTGAATCTAAAGTGAGTAATAACAG
AACTGGTCGGATGCGTTTACCTCCAGCATTTAATGAATACAACATGCTTTCTTCTAGCTGAGTATCCATTACTGATTTATTTATCGCA
ACCGATAATTCATTATTGACTTCATCTATTAATTTATTCATCGGTAGATTCGTCATTTTTTACATCCTCAGCTTCTTCTTTTATTAAGTC
ATTCACCTTTTTTTCGGCATTTTTTAAAGTTGTGTCACAAGCTGCTGATAGTTTCATACCACGTTGATATAAATCTAATGATTCCTCTA
AAGATACTGTTTCATTATCTAATTTTTGAACAATTTGCTCTAATTCTTGCATCATTTCTTCAAAACTTTGCGTTTCTTTAGTCATTATT
ACACCTTACTTTCGTAACTTTTGCATCTACTAAGCCATCTTTCATTGTTAACGTCAATTGATCATTTTCTGTTAAATCTTTAGTACTCG
TAATGACTTCGTCTTTTTTATTAACAATTGCATATCCACGCAACATTGTATTAGTTGGACTTAAATTGTTTAAGTTTTCTACTTTATTT
TTCAAATCATTTTTATAACTTAATATCTTAGAATTCAATAATTTAACAAGTTGGTTTGTCAATTGAAGATTATTTTGTTGTTCTTGATT

AACACTACTTAGTAATGCTTTTAAATTATAACGTTGTTGCAACAGCATTAATCGATGTCTCTGTTGTTCAAAAGTTGCCTGAATTTGT
TGTTTCAGTCTCTTTTCTAAATCATCTCGACGTTGTATCTGTTGATCATACAATAAAGTTGGTTGTTTAAACTTGTAATACGATGACA
AATGTTCAACATGTTTACGTTGTTGTTCTAAATGTTTCTTGATGAAACGAGTCAATGTAAACTGATATTGCTGTATTTGTTGCAGCAA
TTCATATTGGTCTGGTGTTGCAATAACAGCAGCTTGAGTTGGAGTCGCAGCTCTGATGTCTGCAGCAAAATCACTTAATGTAAAGTC
TGTTTCATGACCTACTGCTGATATAATCGGTGTCTTACAATTATATATTGCACGGACGACAGCTTCTTCGTTGAAATTCCATAAATCT
TCTATGGATCCACCACCTCGACCTACAATAATGGTATCTACACCTAAACTATCTGCATATTCAATTTTTTCAATAATGTCGTCTTTTG
CTTTTTCACCTTGAACCAAAGTACTAATTTGTATTTGTTCAGCTAATGGAAAACGACTATTTATCGTTGAATGGATATCTCGAATTGC
GGCACCTGTACTCGCTGTTAAAACTGCAATTTTTTTAGGAAACTTAGGTATTGATTTCTTATTCGCTTTATCAAAACAACCTTCTTCA
GTTAATTTTTTCTTTAATGCTTCTAATTTTTGATATAAGTTCCCTATACCATCTAATTGCATTTTATTTACATAAATTTGATAGTTTCCA
CGACGTTCAAAAACAGAAACACGTGCTTCTAATAAGACTTCATCTCCTTCTTTAGGTTCGAAGTTTAATTTAGAAGCACTACCTTTG
AACATCATGGCACTTATAACGCTCTCTTTATCTTTCACATTAAAGTATAAATGACCACTTGAATGCTTTTTGAAATTTGAAAGCTCAC
CTTTAATCAATACAGATTGGAGATGTGGATCTTGATCAAATTTATATTTAATATATTTCGTTAAAGCTGAAACACTTAAATAATCTGA
CATATAACATCACTCAATTTTATTTTTTTATATTACTCAATACACCATTTATAAATTTATAATGATCATCATCACTGAATTGTTTTGTT
AATTCAACTGCTTCATTCATTACGACTTTAGCAGGTGTATCACTGTGTAATATTTCATATGTTGCCATTCTTAAAATAATACGATCCG
TTTTTAATAAACGTGCAATAGTCCAATCTTTTAAATAAGGACTAATTGTCTCGTCTAATACAGGTTCGTGATCTTTAACGCCAGAAAC
TAGCCAATGAATAAATTCAAAGTCTAAATCTGGATTATCGTCTTTAATAAAACTTATCGCTTCATTTATCGTTAAATCACTGTCCTTC
ATTTCTAATTGAAATAAAGTTTGAAAAGCTTGCACTCGGGATTCTTTACGACTCATTTTTAACTCCTTCAAACGTTTGTATTTTTCTTT
ATTTAATTACTGAATTAGGTATGACATTACTTTTCAATAACGATTTGTGTAATGTGAATATTAATTTGCTTAGGTTCTATCGCTGTCA
TATTAGAAATTGAATTAAAAATTGACGTTTGAATTTTGTTTGCAGTTTTTGAAATATTAACACCATGTTTTAATGCACAATATACATC
TATATATATGCCATCTTCTTTACTCTCGATTTTTAAATCACGGCTTAAATTTTTACGACTAACTTTTTCTAAATTTGTTTCTTTTAATTC
AGCAAAATGGCCAGTGATGCCTTCGACTTCCGAAGTAGCTATACTTGCAATAACAGATAGCACTTCTGGCGCTATTTCTACTTTACC
TAATTTTGAATTTGAATAATCAGTTACTTTGACCATGGATTGACCTCCTATTAACCTTCATCATTCATAATGCTATTTTGCTCTAAAA
AGTTTGTATTAAATTTACCGCTTCTAAATATATCGTTATTCAATAATTTAATATGGAATGGAATAGTTGTATCAATACCAAGAACCAC
AAATTCACTTAATGCACGAATGCCAGCCATAATCGCTTCATCTCGTGTCGGTTCATGTATGATTAATTTCGCTACCATCGAATCATAA
TATGGCGGTATCGTATAATTAGTATAACATGCTGACTCTATTCGAACACCATATCCACCTGGTGCAAGATATTGCTCAATTTTACCTG
GTGATGGCATAAAGTTCTTGTAAGGATTTTCAGCATTAATTCTAAATTCAATTGCGTGTCCTGTTAATTTAATATCTTCTTGTTTATAC
GGTAACACGTCACCCATAGCAACCTGTAATTGTAATTTAACTAAATCAATTCCTGTTACCATTTCAGTTACAGGATGTTCTACTTGAA
TACGTGTATTCATTTCCATAAAATAAAATTTATTATCATTTAAATCATATATAAACTCAATTGTTCCCGCATTTTCATAATTTACAGCT
TTCGCTGCACGCACTGCGGCATTTCCCATTTCACGACGTGTTTCATCATCTAAAATTGGGGAAGGTGCTTCTTCCACTAATTTCTGCA
TACGTCTTTGAATTGTACAATCACGTTCTCCTAAATGAATTACATTACCATAGCTGTCCCCAACAATTTGGATTTCAATATGGCGGAA
GTTTTCGATGAATTTCTCCATATAAAGTCCACCATTACCAAATGCAGTTTGAGCTTCTTGTTCTGTCATTCGGAAGCCAGTTTCAAGT
TCTTTTTCATCACGAGCAACACGAATACCTTTTCCGCCACCACCAGCAGTAGCTTTAATGATGACCGGATAGCCAATTTTTTTAGCGA
TTTTCTTAGCTTCTGAGACGTCTTTCATTAAACCATCACTACCAGGAACAACTGGAACATTGGCTTTGATCATTTCTGCCTTAGCAAC
ATCTTTGATACCCATTTTTTGGATAGATTGATAACTTGGTCCAATGAACTTCAATTGGCATGCTTCGCATAATTCTGCAAAGTCAGCA
TTTTCAGCTAAAAAGCCATAACCCGGATGAACGCCATCACAACCTGTAGAAGTTGCAATAGATAAGATGTTCGGAATATTTAAATA
TGAATCTTTAGACAAAGTGGGACCTACGCAATATGCTTCATCAGCAATTTGAGTATGTAGCGCATCTTTATCCCCTTCAGAATAGAT
TGCAACAGTTTGGATGCCTAAATCACGACAAGCGCGAATAATCCTAACTGCGATTTCACCGCGGTTTGCAATTAAAACCTTTTTCAT
TATTTCACCTTAAATAACGGTTGGCCATACTCTACCATTTGTCCGTCTTCTACTAAGATTTCAACAATTTCACCTGAAATTTCTGCTTG
AATTTCATTAAATAGTTTCATTGCCTCTAAAATACACACTGTTGTTTCATTTGAAACAGTGTCCCCAACTTGCACATATGCTTCTTCG
TCTGGAGATGGCGATTTGTAAAATGTACCTACCATAGGTGCATTAATTGTTTTGTGATTATCTGAAGTTGGCTTTGGAGCTTCAGTTT
TATTGCTATCAGTTGATTGTACTTGAGGCATAGGCATTGCCGCAGCTTCAACTGGCATTTGTGAGATTTGTGGCGTGATAATCTCAGT
TTCTTTTTCTTTCTTAAGCGTCACTTTGCCTTTAGTATCTTCAATATTGATTTCCGTTAAAGTTGATTTATCCAGAATTTCAATTAATTC
TTTGATTTCTTTAAAGTTCATTATTACTGACTCCTTCAGTTTGTTTTCATCTACCCGTCTATTTTACTTGAGACAACTCTTCAATTCAA
GCATGTTCATATTGCTGGCGACATTATAAGTCTATCCCAAAGATATAATAAAAACACATTTTAAATTAAAAACACTTGTGTATTTAT
TACTTAACATTGACTCATCTTAACTCTTGATTAAAAATTTTATGCATTCAGTTAAACATATCAATGTTATTTTTATTAGCTATTATGTT
TAATCCTCAACAATATGATATGTAGATATATACCTTTACTCATGTCTATGTTTTTTCACTACCAATTAGACGATTCGTTTTATATTTTA
AAAATTATTTTAAAAATAAAAAGCCTCATATCAGCATATAACCTATAATCATTTTAGGCAATAGCTTTATGAGACTTATTTAGAATA
TCTTACGTTAAATCAGTATAGTGAATACAAGCTATTTGTTAACAAATTAGAGATTATCCTCTTGAAATGTAGCTTCCATCACCAGTGT
TGATAATTAAAACGTCACCTTCGTTTACAAATAAAGGTACATTTAATGTATAACCAGTTTCAACAGTTGCCGATTTAGTGGCACCAG
TTGCAGTATCACCTTTAATACCAGGTTCTGTTTCAGTTACTGTTAATTCAACAGTTTTAGGTAATTCAACACCGATAGTTTCACCTTC
GTATGTTTGAATTTGTACTTCCATACCTTCTTTTAAGTAATTCAATTCTTCTTTTAAGTAATCACTTGAAAGTTCTGTTTGTTCAAAGC
TTTCATTATCCATAAATACATGATTATCTCCGTCAGCATATAAATATTGCATGCGACGATTTTCAATCATTGCTGGTTCAACTTTTTC
ACCAGCTCTAAACGTTTTCTCTTGAATTGCACCAGTTCTTAAATTACGTAATTTTGAACGAACGAATGCTGAACCTTTACCAGGCTTT
ACATGTTGGAAGTCTATAACTTTCCAAATAGCGTTATCAACAGAAATTGTTAAACCTGTTTTAAAATCATTAACCGAAATCATTCAG
TTTCCTCCTCATTTTACACGCTTATGTTAAAACTATAAGGTCTTTTGTGCATTTAGTAAAGACTTGACAACCATTTTCTGTAATTAAA
ATATCATCTTCTATTCTTATACCGCCCAAACCTTCTATATAAACACCAGGTTCTACTGTAACACAGTTGTTAACTTGAAGTTTATCTT
GTATCGTACGAGCCAGCATTGGCCCTTCATGGATTTCTAAACCAATACCATGTCCTAGTGAATGTCCAAATTCTTTTCCATACCCTTT
TGACTCTAAATAGTTTCTTGAAATGGCATCAGCTTCTGCACCAGTCATGCCAGGTCTAATCTCATTAATTGCTTTCATTTGAGATTCA
AGTACTATTTGATATATTTCTTTCAGTTTAGGATCTGGTTCTCCAATAGCAAATGTTCTAGTAATATCTGAACAATAGCCGTTATAAT
ATGCGCCAAAATCTAATGTAATCATGTCGCCTTTTTCAATAATTTTGTCACTTGCAACACCATGTGGTAATGCACCTCTATGACCAGA
TGCTACAATCGTATCGAATGATGGTCCATCTGCTCCTAATTCTAGCATTTTACTTTCTAATATTGCCTTTAATTCTTTTTCAGTCATAC
CTGCTTTTACAACAGTTAAAATATATTCATATGTTTCATCAACAATATTAGCTGCTTTTTGAATTAAAGCAATTTCGTCAGCATCTTT
GACGTCTCTAATTTTATCTACAGTATTAGAAATGCTTATTAATGATATACGACTTTTATTTAATTCAAGGTATGTATCATAACTTACA
TGATGCCCCTCAAAACCTACATTTTCAAAATTTTCTTGGTGTAGCAATTCTTTAATCTCACCAATAATAGTAGATTTACGATTAATAA
TTTCATAATTTGGCGCTTGCTTAGTTGCTTGATCAATATATCTAAAGTCTGTTATCAAATATTGTTTATCTTTAGATATGATAAGTGCT
CCACTGGTACCAGTAAAACCTGATAAATATCTTCTATTGTAATCCGAAAGAATGATAATCGCATCTAAATGTTTTTGTTCTAAAATA
CGATGCACTTGTGTTATTCTGCTCATCTTATTACCTCCTAGTGAAACGTTTGTGCATTTCAACTTTATACATTAAAATAATATCATAAT
GAGGATAAAAAATAATAGATATTGATTTTAGGGAGATAGTAATGAAAAAATTGGTTTCAATTGTTGGCGCAACATTATTGTTAGCTG
GATGTGGATCACAAAATTTAGCACCATTAGAAGAAAAAACAACAGATTTAAGAGAAGATAATCATCAACTCAAACTAGATATTCAA
GAACTTAATCAACAAATTAGTGATTCTAAATCTAAAATTAAAGGGCTTGAAAAGGATAAAGAAAACAGTAAAAAAACTGCATCTAA
TAATACGAAAATTAAATTGATGAATGTTACATCAACATACTACGACAAAGTTGCTAAAGCTTTGAAATCCTATAACGATATTGAGA

AAGATGTAAGTAAAAACAAAGGCGATAAGAATGTTCAATCGAAATTAAATCAAATTTCTAATGATATTCAAAGTGCTCACACTTCA
TACAAAGATGCTATCGATGGTTTATCACTTAGTGATGATGATAAAAAAACGTCTAAAAATATCGATAAATTAAACTCTGATTTGAAT
CATGCATTTGATGATATTAAAAGTGGCTATCAAAATAAAGATAAAAAACAACTTACAAAAGGACAACAAGCGTTGTCAAAATTAAA
CTTAAATGCAAAATCATGATAGGAGTCTTTTAATGCGTAATATAATATTTTATCTTGTACTTATTATTGCTGCGATTGGATTAGTAAT
GAATCTAGATGCCTTTATTTTTTCAATCGTCAGAATGTTAATCAGCTTTGCTGTAATAGCTGGTATTATTTATCTGATTTATTATTTCT
TCATCTTAACTGAAGACCAACGCAAATATCGCAAAGCAATGCGTAAGTATAAAAGAAATCAAAGAAGAAAATAGATAAAAAAACG
GAAGCACTTGTAGGTAAAATAGTCTACGTGCTTCCATTTTTTATTCTAAAAACTACTTTCTAAACATCCATTCATCTGAACGATATTT
TTCAGTTAATTCTTCCACTTCTGCCAATTGAGCTTCTGTTAATTCAAGTGGCTTTAATTCTATATTTAAACCTTTCTTAAAACCTTTCT
CGAAAGCTTCTTCCATTTGACTAATAGTAATGTGTTTATCTGAAATATCATTGATGGCAACTGCTTTTTCAACGAATGCCTCTTTCAT
TTTTAATTTTAATCTTTCATTTTTATAAATAAACATATCAAACAGTTCATCAATATCAATATCTTGTAAAATCGAACCGTGTTGGAGG
ATTACGCCCTTTTGTCTCGTTTGAGCACTCCCAGCAATCTTACGGCCTTCAACAACTAGCTCATACCAACTTGGTGCATCAAAACACA
CTGAACTTCGAGGTTGTTTTAATTTTTGACGCTCTTCAGGCGTTTTAGGTACCGCAAAATAAGTATTAAATCCTAAGTTTTTAAATCC
TTCTAATAATCCTTGTGAAATCACTCTGTACGCTTCTGTAACTGTAGAAGGCATATTCGGATGCGATTCAGGCACAATCACACTGTA
AGTTAACTCTTTATCATGTAGCACCCCACGGCCACCAGTTTGACGCCTTACGAGACCAAAACCTTTCTCTTTAACCTTATCAATATCA
ATTTCTTTTTGTAGCCTTTGGAAATACCCTATTGATAATGTTGCAGGATTCCATGTGTAAAAACGTATAACTGGATCAATTTCACCTC
TAGAGACAAAATTTAATAACGCTTCATCCATTGCCATATTATAATATGGGTCTTTACTTCCTGTATTAATAAAATTCCAAGTTTCAGT
CATATTCAAGAACTCCTATCAGCAAAATGTATAATATTTGATTCGCTAATTAATCAATTTAACTAAATGAATAATAATTGCAATTCTT
TAGTGAAATATTTTGATAATTTGACCTAACAGTCTTATAATTATATTATCGTTTAATTAGGGAGGATGCAAGATGAGTGCTAGTTTGT
ACATCGCAATAATTTTAGTTATAGCAATTATTGCTTATATGATTGTTCAACAAATTCTTAACAAGCGAGCTGTTAAAGAATTAGATC
AAAATGAATTCCATAATGGGATTAGAAAAGCTCAAGTCATCGATGTTAGAGAGAAAGTTGACTATGACTACGGTCACATTAATGGG
TCTCGCAATATTCCTATGACAATGTTCAGGCAACGATTCCAAGGATTAAGAAAAGACCAACCGGTATACTTATGTGATGCCAATGG
GATAGCTAGCTATAGAGCCGCTCGTATTTTGAAAAAAAATGGATATACAGATATCTATATGTTAAAAGGCGGCTATAAAAAATGGA
CTGGAAAAATAAAGTCTAAAAAATAGTTTTGTAAATTTAATATACGATTTAATAAAATCTGAGTGTTAATTGATCATCAATAACAAT
ACTCAGATTTTAATTTTTTAACAAAGTCTGTTACTATATTTCTCTAGCTTCACTAATCATTAAACTTAGTTTCAGCATAATAAAGAAA
GTTCAGCTCATTTTCAATACGATTCAATTACCGCAATCTAAAAAATGAAAAGACAATTTCTATGAAAGAATAATACCAAACCCTAAG
AGTTATTACTTCGGTTTAGTTTTCTTGTTTAAATAGAAATTGTCTTTTTCAATTGATTTTGAAACCATTATCCTTAAATCTTCATACAA
AGTTAGAATAATAATTCTCGGAATATGTGTTTAATACTTTATTTTTCCTGTTTAAGATTTTCAAACTTTAATATTGGTTTACGAGCAG
CTGTAGCTTCGTCTAATCGATCAATCACAGTTGTATGTGGTGCTTCTAGCACTTTATCAGGATCATTTTTAGCTTCTTCAGCAATACT
AATTAATGTATCGATAAAATAATCAAGTGTTTCTTTAGACTCTGTCTCAGTCGGTTCAATCATCATACCTTCTTCAACATTTAATGGG
AAGTATATTGTTGGTGGATGTACACCGAAATCTAATAATCGCTTAGCCATGTCTAAAGTACGTACACCAAATTCTTTTTGACGCACA
CCACTTAACACAAACTCGTGTTTACAATATTGTTTATAAGGTATTTCAAAGTGTTCAGATAAACGTGCTTTAATATAATTCGCATTAA
GAACCGCTGCTTCAGAAACCTCTTTAAGTCCAGTTGCTCCCATAGTTCGAATATACGTATAAGCTCTTAAGTAAATACCAAAGTTAC
CATAAAATGGTTTTACACGTCCGATAGAATTTTTAATGTCATTATCATATTTAAATTTGTCGCCATCTTTAATAACCATTGGCTTTGG
TAAGTAACTTGCTAGTTCTTTTACTACACCGACTGGACCTGAACCAGGACCGCCACCACCATGTGGACCAGTAAATGTTTTATGCAA
GTTTAAATGAACAGCATCAAATCCCATATCTCCTGGGCGAACTTTGTCCATAATAGCGTTTAAATTCGCACCATCATAATATAATAG
ACCACCAGCATTATGGACGATTTCACGGATTTCCATAATATTTTTTTCGAAAATACCTAAAGTGTTTGGATTAGTTAACATAATAGCT
GCTGTATTTTCATTTACAACACGTTTCAAGTCATCAATATCAACTTCGCCACGTTCGTTTGATTTTACAGTAACTGATTTAAATCCTG
CAAATGAAGCTGAGGCTGGATTCGTACCATGCGCAGAATCTGGCACAATGACTTCATCACGATGACCTTCACCATTATTCTCATGGT
AAGCTTTAAATATCATCAATGCAGTCCATTCACCATGTGCGCCAGCAGCTGGTTGTAATGTCACCTCATCCATACCAGTAATTTCTTT
TAATTCTTCTTGCAAACTATAAATAATTTCTAATGAACCTTGAACTTGATCTTCATCTTGTAATGGATGTGATTCACTAAATCCTGGT
ATTCTAGCAACCTTTTCATTAATTTTAGGGTTATACTTCATCGTACATGAACCCAATGGATAAAATCCGTTGTCTACACCGAAATTTT
TATTTGAAAGTTCAGTATAATGACGTACTAAGTCTAGTTCAGCAACTTCAGGAAACTCCGCTTTGTTTTTACGAATAAATTTATCATC
TAACAATGACTCAACAGAATTTGTTTTAATATCACTTTTTGGTAATGAATATGCATATCTGCCTTCACGAGATCTTTCAAAAATTAAT
GGACTTGATTTACTAGTCATTTAACTCACCAGCCTTTTCTACAAATGTATCGATTTCATCTTTTGTTCTTAATTCAGTTACAGCTATTA
ACATGTGATTTTTAAAGTCGTCTGAAACAACACCTAAATCAAAACCACCGATAATATTGTACTTCACTAATTCCTCGTTAACTTGTTG
AATTGGTTTGTCAAATTTGACTACAAACTCATTGAAAGATGTACCATCTAATACTTCAAAACCTTTTTTAATAAATTGTTGTTTAGCA
TAGTTAGCATGTTCTATATTTTGAACTGCAATATCATAGATACCTTGTTTACCAAGTGCTGACATTGCAATTGATGACGCTAAAGCAT
TTAATGCTTGGTTAGAACAAATATTAGATGTCGCTTTATCGCGTCGAATATGTTGTTCACGTGCTTGTAATGTTAATACAAAGCCACG
ATTACCTTCATCATCTTGTGTTTGACCGACTAATCTACCTGGCACTTTACGCATTAACTTTTTCGTCGTTGCAAAATATCCACAATGT
GGCCCACCGAATTGAGCAGGAATTCCGAATGGCTGAGTATCACCTACAACAATATCTGCACCAAATGAACCTGGAGGTGTAAGTAA
TCCCAATGCTAATGGATTTGCATATACGATAAATAATGCTTTTTTATCTTCAATAAAGCTATGAATCTTTTCAAGATCTTCAATTGAA
CCGTAAAAGTTTGGATATTGTACTGCAAAAGCTGCTGTTTCATCATCCACTGCTGCTTCTAATTTTTTCAAATCTGTAACAGTGCCAT
CTAAATCGATTTCCATTACTTCGAATTCCTTACGCGTCTTAGCATAAGTATGAAGTACTTGTAATGCTTGATAATGTAAACCTTTTGA
GACTACAATTTTATTTTTCTTTGTTTGACTAAATGCTAAGATACATGCTTCAGCAAAGCTAGTCATCCCATCATACATAGAAGAATTT
GCTACATCCATATCTGTTAATTCACAAATTAAAGTTTGGAACTCAAAAATGGCTTGTAATTCACCTTGAGAAATTTCCGGTTGATAT
GGCGTATATGCTGTGTAAAATTCTGATCTTGAAATCATAGCATCCACAACTGATGGCGCGTAATGATCATAAACACCAGCACCCAA
AAATGATGTATGCGTTTCTTTAGTGATATTCTTGCTTGCAATGCGATTTAATCTTCTAAGTAACGTTGTTTCCGCTTCGCCTTCAGCAA
TATTTAAATCTCTATTTAATAAAATGTCACTTGGTACATCACCGAATAATTCTCCTATAGATTTTGCACCAATTGTTTGTAACATTTCT
TGCTTGTCTTTTTCAGTTAAAGGTATATAACGATGACTCACAATGCACACCCCTTTTTAATTATTTATCAATTTGATTTTTCTTAACAA
TTTTCGCTTTTAATTGACGCTTACGAACTTGAACAAGTAACTCTCTACCCATTTCAAACTCATCTCTTTTTATCATTGCAAGTGCAATT
GATTTTCCTGATGATGGAGACTGTGTTCCTGAAGTTACTTCTCCAATAATATTTCCATCTAAATCCATAACTTCATAACCAGTTCTTG
CAATTCCTTTTTCAAGTAATTCTAATCCCACTGTTCTTCTTGGTGCACCATTTTCTTTTTGATCTTTTAATACAGATTTACCAATAAAA
TCAGTATCAATTAATGGTTTACTTGCAAAAGCGATACCACCTTCATATGGTGTAATTGATTCAGTTAAATCTTGTCCATGTAATGGCA
ATCCAGCCTCTAATCTTAATGTATCACGAGCGCCTAACCCACATGGCATAACATTATACTCTAATAAACCATCCCAAATTTTTTCAGT
ATCATCGATATTACAATAAATTTCAAACCCATCTTCACCTGTGTAACCTGACTGAGATAAAATGACGTTTGCTCCAAATAATTTGAA
ACCCTGTTTAAATTCAAACATTTTCATTTCAGTTACATCTTCATCAACTAATTGATTAATTAAATCTCTAGCTTTTGGTCCTTGTATTG
CTAATTGACCATATTGGTTTGATACATTTTGTACTTCAACATCAAATTTCTCTTTGTGTTTTAAAATCCAATTAAAATCTTTTTCAGTA
TTAGCAGCATTAACAACTAATAAATAATTGTCGTCAGCTAATTTATATATTACTAAATCATCAATAATACCGCCTTCTTCATTACATA
AAGCAGTATATAATGCTTTTGAAGTAGTTAAATTATCAGTATCATTTGATAATAAATATTGCACAAACTGACTAGCATCTTTACCTGT
TACTTCAATTTCACCCATATGACTAACATCAAACAGGCCAATTTCGTATCGAACAGCATTATGCTCCTCTTTAATACTTGAAAATTGA
ACAGGCATCGCCCATCCTCCGAATTCCACAATTTTTGCACCTCTATCAACATAATTTTGATATAAAGGTGTTTGTTTTAAATCACTTG

ACATTGAAATACCCCCTTAAAATTATAAAAACAGGATAGCAATCGCTACCCTGTTTATAAATCACCAATTCAGGAACGCGTTACAGT
ATTATCCTTTTGCCTGAGAGTTTCGTAATAAATTACTTGCACCTTCGGCGATAGAGTCTTAACGCTCTATTCTCTCTAATACTGATCA
CTCGCTTTTATTAAATTTAGCAATTCATAATATATTTCTGAAATTGATAGCAAATGACTATCAAATTTCTTGAATGCGATTTCATTAT
ATCTTAAATTCCGCGAGCAATACAAGTCATTTAACTGCTTGATTGTCTTATTATTTGCATTAGGTCGATGTGGGTCATCATTGATTCG
ACTATATATAATATCAATATTACAATCTAACCAAATAATGTTTTTTTGATTTTTCAAAAAATTAAATGCCTCTTCACTTTCAATTATA
CCACCACCAGTAGCAATTATATCTGCAGTGTTAATACATTCTTGCAAACATGTGAACTCTAAATTCCTGAAATATTGTTCACCATGTT
TACTAAATATTTCTGGTATTGTTAACTTATACTTCTCTTCGATATATGAATCTATATCAATAAATGATAAATTTTGCTCATCTGCAAC
GTATTTACCAATCGTAGATTTACCGGTACCCATGAAACCAATTAAAATTATTGGTGATTTATCATGATTCATGCGTTTTCCCTTCTTT
CAATAACAAAACAATCTTATAGTTATAGTCAGTTTCAATATTATATAATGTCTTTAATTTTAAACTATATTGACTGATATAAAAAGTG
AAAATGGAAAAATAAATTGCTGTTAGCATCAATAAGAACGGCGTAACATATCCTTTTTCTTTATATAAATCAAAATTAATAAATTAT
TTCACATCCTTTTTAATAGTACTCATTTAAATACTAGTAAGTTATACATAAATAGTTTTAGTCTGCACATTTGTACCGACTTTAACTGT
TATCGTTATTTTAATAATGGATTTATAGTAGATATTTGCAGTAAATGCTGTAACATTATTAATCATTGTTATATTTCCTCTGTCATTTA
CAACTTTAATAATTTTATTATTTATTAATTTGTATTCGATCATTTCTTCACCTTTATGTAAAATGATACGCTGTTGCCTAATTTCAATA
TCGTTTCTATCTACTCCTTTAAAATCCTCTAGAATATCTCTTGAGAAAAATTCGAAATCTACAGTTGTTAATTCCCTACTTTCAATTAG
AAAAGTTTTGCTAAGTCTAATTAAGTCTGGAACAATTAGTAAAGTTATACTTATAACCATCATCGCTACTAACATTTCAATGAGCGA
AAAAGCTTTGACATTAATACTGTATACATGTCTTTTGATAAGAAGTGGTATTTTTTGAAATAGCACAAATTTGTTGGTCACTTTGCTT
AATATCATACTTCCCTATAGTTACCCCCTTCTTAAGTTCTTCTTTATTAATTTTAGATACAGTCGTCAAAATTACTTTAGAAGCATCAA
TTGTTTGTAGTTTATGGTTTATACTCGCTTGCATTTGATTCATCATTGGTATCAATAGTAATGTAATCAATCCAATTAGCAAAAATCC
AGCCATACTATCTATTAAGAATGATCCTTTACACTTATAGCTTTTCATAACGAATTCTTCCTTTTTCAATATGGAATATTATTCTATAA
ATTGAATTGTTATTGTCAATTGTTATACTACCAAATTTATTGATATTCCCTTTTTTATCAAAGGCAATAATATCAACTTTTGCAACATT
AATTATTTTGCCTACTTTTAATTTTAGAAATCGTATTTTATTATTCTCTATTACTTTAATAGTGTCACTGTTTTCATAAAATCTAACATT
GATATATCCTTGATTTGCTATAGCTTGCGACTTAATATAATTCAATTCAGTAATAAAAGAAATGATATTTGCCTCATCATCTATTACT
CTAAGATTGCTTAATCCTTTAGATGTCATTGTCAAAAGTAGAAATATACTGATTAACATCATTACCCACAAGCATCTCAATCATAGTA
AATGCTGACTGCTTTCTAATTTGCAACTGCTTCTCCATTACTAATTGTTATTGTCTCTCCTGATTTACATGTCTTTTGTGCTTCTTTTAT
AAAACCATCTGCAATTAAGTCTTCAATAGACGATGGATTTCTATTATGTTTCAATGCATACGCTTCAATTTGACTATTAACCATTTTT
ACCTGTGCATTACAACCTGTTGATTGTATGTGAGCAGTTTGTTTAGCAATATTTGGAATGATTAAAATTAATAATAAACTGATGATT
AATAACACTAATAGCATCTCTATCAATGTAAACGCTTGAGTTTTCTTAAGAAATTTAAACATATATTAACCTCCATTATTTTATACTT
TGCATCATTTGAAACATTGGTAACATAATTACTAAATAAATTGCGACAATAAATAAACCTAAAATCAAAAATAAAATAGGCTGTAA
AAACTGAGTCTGTTTTATCGCTTTATCTTCTATTTGTTTTACTAATATTTGCGAATATAACTTTAGTTCTACTTCTAGCTTCCCTCTCTT
TTCACCTTGTAGCACAAACTTAATTAATTGAGGCTTAAAGCATTTTAGTTTTTCTAAAATTTGAGGCAAACCATATCCCATTTCTGAA
TAAGTTAATAAGTATTTACCTAGAAACTGTCTAAATGGATCACTACTATGGTTAATATAAACGTCTACTATTGATTGAAGTGTAATA
CCATTTTTATAAAACAACACTAATTCATTAGTTACAAAATAAGTTTTAAATAATTGGAAATAGCCTGATATTAGCGGTAGTTTCATC
ACAAAGTTTATCTTATTGAGCATATTTAAATTGTTATAAATTAATTTCATAATAATAGCCAACATAGATACTATTATGAGCATTACTA
CAATTATAGTAGGTAAGCTGGTAATGAAAAAAGACAATGTTTTTTGAAAAGAGGATAGTTGAATATTCATAGAAGTATATAATTGT
TGAAACTGTGGAATTACTGTGAGGTTTAATATAATAATCATAGCAATAAAAATAGAAACTAGTATTAAGGGGTATTGCAGTGTCTTT
AACAATCGTTGTTCTGACTTTCTATTCACTTTCATATAATTTACGGTTTCTTCTAGAACGTCTATAATATTACCAAATCTTTCTGCCAA
ATATACTTGCATGACGATAGTATCGCTATAACCTATCAGTGATAATATCTGATTGCATGGTGCACCATTTGAAATTTCACTTAGAAT
GGTGGCACCTAATTGCTTATTTTTATATGCCATTTGAAGATTTAAAAATTGAAAACTTTGATACAGAGTGAAACCATATTTCAACAA
ATTACATAAATTTGAAAGTAAGTCGATTTGTTGGGCCTTACTTAATTGTCGCTTCTTAGAATGTAGTTTAAATGTATTTATCCATTGT
AGTTTCACAAATGACACCTGCTTTTGTCATATCATCAAGTTTATCTTCTAAGTTCTTAAATGATGATGGTAATGAATGATTATGGGAA
AAGAAATATCGGAGTTGTTGCTGAGATAGAATTTCACATACTAATTGTCGCTGTTGCTTAATAGTAGTTACAAGTCGTTGGTTTATA
ATTAAGTTAGTTGCCTGTATCAATTCTTGTACAGAAATGCCCATTTCTAATAGCCTTAAAATAGCACCTTTACAATCAGTTGCATGCA
ATGTAGTCAGAACAAGGTGACCACTTAAACTAGCCTGTATAACACACTTGGCAACATCTTTATCTCTGATTTCACCTATTAAAATAA
CATCAGGATCACATCTTAAAATAGCTTTAAACGAATTTACATAGTTTATGCCAGCTTTATCATTCACATTAATTTGGACGATACCAG
GAATTTGCATCTCTACAGGATCCTCTATAGAAATTACATTTAAATTCAAGGCTTTATTCGCGTATGAGACCATTTGATACATTAATGT
ACTCTTTCCTGAACCAGTTGGTCCACTAAACAATAGTAATCCTTGTTTCTTATTCATGAGGTGTTTAAAATCATTGAATTTATAAGTT
GATTTCTGTGGTTGAAAAAAATTGAGGTACAATTCTGATAACACAACTTTCTTGGCCAAGTGACAATGGTAAAGTTGATATTCTCAAA
AAATATATTTTATTGAAAAGGTAACTATATCGACCGCTCTGTGCGACTTGCTGTGTAGAAACATCAAGCCCAGCTTGAAACTTCATA
TAAACTAATAACTTTTGATAAATGCTATTCCCAATTTGTTCATACTGCTCCAAGTTATCATTAATTCTAAATTTAATACTTACTTCATT
TTTAACTGGAATAAAATGTACATCACTCGCTTTCATTTCTATCGCTTTATTAATTATTTCTTGAAATAGAATCTTCAAAAAAACACCT
CCTACATATAATCACGTAGGAGGTGTTTTTATTACTTCAATTTAACCGTGTAAAAATGGATTTAATTGTTCATCATCAACCGTCGTAT
ATGGACCATGTCCAGGGAATAAAGGTAAATCGCCTTCTAATTCAAATATTTTATCTTGAATAGAATCAACTAGCGTTTCATAATCAC
CTTTATATAAATCTGTACGTCCGATTCCATTATTAAATAATGTATCTCCAACAACTGCGAATTCATCGAACACATATGTTAAACTTCC
TGGTGAATGTCCAGGTGTGTGTAACACATTAAACTTAAATCCTTCTATTTCTGTGCTACCTTCGTTTAACTTTTCAGGAGTTACCTTA
CTTGTAATAATTGGTAATCCATATTGCTTAAATTTATCTGCCCCATTTTTAACGGGATCTTTTAGAAAATCAAACTCTGCTTCATGCA
TATAAACCGGGACATCGAATCGATCAACTATATCATCGACTGCTCCGATATGATCAAAGTGTGCATGTGTTAATAAAATAGCTTTTA
ACGGTTTATTTATTTGGTTTAATTTTTTAATAATTTTTTCACTTTCACCTGAAGGGTCAATCAGAATAACAGCTTTGTCATTTTCGATG
AAATACGTATTAGTATCAACTAAGCCTAAAGTTAAGCTTGAAATCCTCATAGGTTTTCACCACTATTTTCTAAATGTTTTTGTACTGA
TGTTAGTTTATCATTCATTTTTCTAGATTTGTCTCGTCGGTCATCAATACGGATATTTGTACAAACTCTACTTAAACCTTTATCAAAAG
GTAATTCATGTATCACTTGCACAACTTCTAATACATCGCTTAATTCACCTTCAATTAGAGTATTCATTGGTGTTAATTGAAAATCAAT
TTTACCCATTGCTTTATATTCTTGAAGTTTTTTCTGAATATCTGCAATATATTTACTAACACTCGGACCTTCCGTTCCAACTGGAATAA
CAACCACATCAACAATAGCCATTATTTTACCCCCTCTTTATCTAATACATAGGTCTTGATTAATCCTGCTGCTCCTGTAATACCTGCA
TCATTACCTAATTTCGCTTGTACAATTTCAGTTTCAAATTGAGCAGGTGCAAATGTTAAATTATGATATTCTGTTTTAATATTTTCAAT
TAAAATAGGTCCTGCAGTAGACATTCCTCCACCTAGAACGATATATTTCGGATTACTTGTAACACTAATAATACTACATAAATATCC
AATATAGTTTGCAACCTTTTCAGTAATGAAAATACAGAATTGGTCACCAGCTTTTGCCGCATCAAAAACAGCTTTTGCTGTAACCTT
ATTTTCTTTAATCAATTCTAATATAGAAGATCTAAACGTCAACTTCGGATAGTAGAAGTTAACTAAGTTAACAACGCCTGTCGCTGA
AGCAACTGTTTCAATACATCCAGAACGACCACAATTACATTTAAATCGTTGATCGAAGTCTGCTCTAAAATGACCTATTTCTGCGCC
AGAGCCATTATGACCATGTACGATTTCACCATTTGAAATAATTCCTCCACCTAGACCTGTACCAAGTGTGATGGCAACAACATCATC
GGCACCTTCACCAGCACCTTTGTGTTTCTCCCCTAAAGCAGCTATGTTAGCATCATTATCTACATACACTGGACAATCAACGAATTGT
TCAAAAATCTCACGTACATTAACTTTTTCTGGCCAATATAAGTTTACTGCTCCATTTACTGTACCTTTTTCAAAGTCAACAGGACCTG
GTACACCAATACCTACGCCAAGTACATTTGAAAAATTATAATTATTTTCATTTACTTTTTCAACAAACGAATCATAAATTCCTTTCAA

AAGTGTATATCCTGTACTATCAGATGTATCAGTGTGAATAGACCATTTATGTAATTGTTCTAATTCAGGTGTGAAAATACCTAATTTA
CAAGTCGTCCCGCCTACATCAGCTGCTAAAATAATTTTTGTCATTTCTGTTCATTCCTTCTCTGATTAATTATCAACGTACATTTCAAA
TATTCTTCCTTAGATAACAGTTCATATTGATACAATGATGAAATCTCCTGTTGAATCATTTCGTACATATCTTCTGGATTTTTAAAAT
ATATTAGAAATCCGTAACTTTTCAATAACTGTTGTACATCATAAAAGTTATTTATTTTTCGACTCATTTGATTTAGTCAACTCTTTTTC
TAAGTTAATATAATCTGTATTTTTAGGGTCTGCATTTAATGCTTTACGCACATATTTTAATGCTTTTTCATCATCATTTAATGAACGAT
TTGCTATCGCTAACTCAAAATTTAATAAACCTGATTTAGGAAACATTCTAAGTCCTCGCTCCCATTCTGTCATACCTTCAGACTTAGA
ATTGATAGTAGCCATAATCATACCACTTAAATAATATGTTTGATCATCGGCATAATTTTTATTTATTGTCTGCTTCACAATATTTTGA
GCATTATCATAATTACCACTAGTCATATCATCTTTGATCAATTTATTATAAATATTATCTTCTTTAATTGTAAAAATTCTAATTTGAAG
TGCAATAAATATAACAAGCATACCAATTAGTAAAATCCAAAAAATATTACGATTCACTTTATAGTAATAGCCAATTAAAGTTATTAA
TAAACCACCAATGAATCCTCCAATATGCGCCACAATATTTATATTTGACATAAACAGAGAAACACCAACTAATATCACTAATGCAAT
TAATAACTGTCCTAACATTTTTTTGTTAAATGTTTTTGAAACATACATCATCGCAAAAATTGATCCAATCAGACCAAATATAGCACC
ACTAGCCCCAACTGAAATTGTAGTCGTATTAAATGATAGTGATACAAAGTTTCCAAACAACCCTGCAATAAAGTATACAGTTAACAT
CCGCCATGAACCAATAATTGCTTCGACTATTTTACCAAAAATAAATAATGAAAGCATATTCATAAGTATATGTTCAAAACTAAAATG
TAAAAACATCGATGTAACAATTCGATACCATTCACCATGTACGACATTAAAATGCACTAACCCGCCAACATCTAATAATTTTACATC
CGAAAAATTATTTAAATATAAAATCATACATAACCATATTAAGACATTTACAAATATTATTGTGTATGTTGCCGGTGAAAATTTCTG
CATATATTTATCTAAAAAGTTATCGGTTAATACTTTGCGTTTATAGAACATATATGTCTTTTTATCATTATCTTCTGAAATAAGTCTAG
CCAAAAATATATTCGGCATATGCTTTATCAAATCTTTTGTGTGTCTTATAACATTGAATTTAATTCTAATTGGCGAAACTTCATTTAA
TTGTTCTTCTGAAAATTCCGATTCTGTAAAATAATAAAATTCAAAACTTTGTGGTTCGAAAGAAATAAAATTTGCTATTTCATCTTTG
TGTTCAAGCACTTTCGCTTTGTCAAAACGTATTTCTTGGGTAGACTTGATATGTTGTTTAAAAATGACTACTTGTTTTTTTCTTTTATG
GGCTAACCAAATTTCTTGGTCATCTTTTTCCCTACTGACAATATCAAAATTCAAGTACCTAATCCAATAATATATTGTTTTCCAAAAT
TGTTTGTCTATGTTCATTTAGTCCTCCACTATGCTGATTGATATATAATCAATTTATCGACTGGTTGATCGAATGATTCCGGTTCAAAT
GATGTTATTTGAAAATCGTATAATAAGCTTATTGTCTTTGTCTGATAATTAGCTAAAAACCTGTCGTAATAGCCACCACCATACCCA
ATTCTATATCCATCGTCTTGAAATCCAACACCAGGAACAACAATTAAATCTAGGTTATTCGTTGTTTCACCTTTTGAAGTTGGATAGT
AAATCCCCTTATTATCGACATCAATATCTTTGAGATTAAATATTTCTTTAAAAGTCATTTGATGATTTAAATAATCCATTTTCGGTAC
AAAAATACGTTTATGATCCATTAAGGCTTGTTCAATAATAGAAAAAGTATCTACTTCATGATTAAAAGAAAGAACTAGCGCAATTG
CGTTTGCTTCTTTGTATTCTTCAGTTGCAAAAAATTGATTTCTTAACCATGTGTCTGCTTTTCGCTTTTCAGCTTTATTAAAATTCTTCA
TTTTATGTAAAATGTATTTTCTAATCTCATTTTTAGTCACATTATCACCCACTTTATTCAATTCTATTATATCGTTAATAACAATACAT
GTCATTATGTTGTCATGATGAATTATGCGACAATTCTAAAATTTATTATTTCAATTGAAAATCTTTTATCATTTCATACGTGTGCATA
ATTAAAAAGGTGAAACCTCTTATTTTGAGATTTCACCTTTTTTATTGTTAATTTAAATTTAATTTTAATGATTATTTTGTTTCACGGTG
TAAAGTTTGTTTGTTTTCACGTGAACAGAATTTCTTCATTTCAACACGTTCTGGATTATTTCTTTTGTTCTTAGTTGTAATGTAGTTTCT
GTCACCACATTCCGTACAAGCTAAAGTTACGTTTACGCGCATGGTTATTCCTCCTTATCATTTCTAAATACGACTTTATTATTATACC
AACATCTATAATATTTGCAAAGTTAAAATTTTACAACCATGCGCTACACAATCGTCAGGTTAAATTTTATTTGTCTTTGTCTTTATTTT
TATCATCTTTACCTAACTGCTTAAAGTAGTAGTTAATTACATCTCTACCTAAGTCTCCACCTGTTAACCATGGTGGTGGTACAGGCTG
ATTTGTATATACAATTGAAAACGCTAATTTTGGATCATCAATTGGCGCGTATCCTATATAAGTAGAGTTAACTCTTGGCTCTCCGTTT
TGGAACACTTCAGCGGTACCCGTTTTACCAGCAGTAGGTACTACTGTATCTTTAAAACTAACATATCCAGTACCATCTTTATCATTAA
ATGCCATTTTGAATCCTTCTTGAATTTGTTTGATTTCCTTTTCAGTATTATTAACCTTGTTCAAGACAGTGCCATTAATTTTCTTCTTGA
GTGGACCAACCTCATCTTTATTAGTTGATTCATGAATCGTTAATCCAATGTGTGGCTGTATTCTATAACCATCATTCGCTATAGTTGA
AACATATTGTGATAATTGTAATGGTGTATAGGTATCATATTGACCAATTGATAAATCTAGATAATTACCTGGATTATTTGTTAATGGT
TCGATTTGACCTCTTGTTTCATTTGGTAAATCTATCCCTGTTTTCACACCTAAGCCTACTTGATTTAATCCTCTTCTAAGCTTTTGGGC
AGGTGAACTTATATCTGAAGGTAAAGCCATACCAGAATAATAAGGGTCTCCCGCTAATTTTAATGCTGTTTTAAACATATATACGTT
TGATGAATGCATCAAAGCTTGCTTATCATTAATAGATACATGCCCGTTTTTATTGAAGTATGATCGTTTTGTCAAACCACCTTGGAAA
TGTAATGGTTCATCGACCATTGTTTCTCCAACTTTGATAGCTTTATTCTGATAACCAGCTAATAATGTTCCACCTTTTACAGAAGATC
CAACCGCAAATTGAGAAGTAAACGTACCAATGTCATAATCAGTCATTTTACCACTCTTATTAATCTGCTTTCCGGCAAGCGCAAGAA
TGTCTCCATTTTTAGGATTTTGTACAACCATCATAGCATTATCCATATCTTTTGCACCTTGGCTGCGAAGCTTCTTAATTTGTTTATCT
AATAATGCTTCTACTTCTTTTTGAAGATCTATATCGATCGTTAATTTCAAATCTTGACCGCGAGCGCCAGGATTTAACACTTCTGAAG
ATGTAACTTTACCAGATTTGTCCGTTGTGTATTTCATTTCTTTCTTCTTACCACGCAATACATCTTCATATTGATATTCTAGGTAAGAT
TTTCCAACACGATCATTGCGTGAATATCCTTTGGATAAGTAATGTTCTGTCAATTCTTTTGGAATACCTTCAGCAGGTGTCGATACAT
CTCCGAATATACCTCTTAAAGTATCGCCATATGGATATTTTCTATCCCAATCCATAGACGTGTTAACACCTGGTAATTTGGAAAGTTG
CTGAGAAACTGCTGCATACTCTTTTTCACTGACATCTTCATTTTTTATCATTTGTGGATCTAAAACTGTTCCTGCATTCATCTCTCGAA
AAATAGCTAAAACTTGTAAATCTTTAGAAGACAATTCATCTAATTGTGATTTTCCGATTTTCGATAACAGTTGTTTATCATATTGATC
TTGTTTAATACTTCCATCTGCTAACATAGCTTGTTCTTTTGTCATCATTGCTTTTGCTTTTTTAGGATGCAACTGAATCCAGAAATCTT
TCTTATCACGTTCTGTAATTTTCTTAGTATCCATCTTGATTAGCTTTGATAACTTTTCAGCCGTATCCAACATTTCCGATTGTGTTGTTT
TTCGACCCCTAGTATATGTAATAGCCATTTTAGAAGCATTATCAACTAAAACTTTCCCATTTCTGTCTAAAATACGACCTCTTGGCAC
AGACTCATTCACTGTAATGTTTTCATCATTTTTTATAATTTGTTTATAATGTGAGCCTTGTGCGATTTGTAAATAACCTAAACGTAGT
ACTAGTACTGCAAAAATAAATACAATCACACCAAATATAAAGTTAATTCTCTTGTTAATTGTATTTTGAACGATTTCATCATTTGATT
TTTCTTTTAGTCTTTTTAACAAAACTACCTACCTCTATTCAAAGTCTTTCACTTTAAATCATATATGAATTTAGAAATTATTTCTATCT
TTTTGACAAAAAAATAACGGTCTCATTTAAGAGACCGAACAAGTAATCATACTTTATTTTGTTGCATTATATAATTCGTCAACTTTTT
CCCAGTTAACTACATTCCAAAATGCGCCAATGTAGTCAGGGCGTTTGTTTTGATATTTTAGGTAATAAGCGTGTTCCCATACGTCTAA
ACCTAAAATAGGTGTTTTACCCTCAGTTAATGGATTATCTTGGTTTGGTGTAGTCACAATTTCTAACTGGCCATTGTTTACGACTAAC
CAAGCCCAACCTGAACCAAAGCGTGCAGCTGCTTTGTCAGCAAATTCTTTTTTAAATTCTTCTAAAGAACCCCATTGTTCTTTAATTT
TTTCTACTACAGTACCTTTTTCTTCTGAGTTTGGTGAAAGTAACTCCCAGAATAATGAATGGTTTAAATGTCCACCGCCATTATTACG
TACAGCAGTTTGGATGTTAGCTGGTACACTGTCTAAATTAGCAACAATTTCTTCAATAGATTTAGATTCTAAATCTGTACCTTCTACT
GCAGCATTTAATTTCGTAACATACGTGTTATGATGTCTGTCATGGTGAATTTCCATAGTTTCTTTGTCAAAATGTGGTTCTAATGCAT
CAAATGCGTATGGTAATTTTGGTAATTCAAAAGCCATAAATAATCATCCTCCTAAAATGTCTGTAAGTAAATAATAACAAGCAGTGA
CTGGTTCAACAAAGAATTTGCTTAAATTCTACTACTTATTATTTTCTCTACTCATTTAATATAACTCAAATCAAAAATAATTAAACAT
TTTGTATATAAAAAGTTAACAGATTTGCCATAAAATCATACGAACGGAGTATGAAATGAACCTTTATCTTCTATAATTTAAAAAATG
AGATTTATGCATACATCTGACCAAATGTGCATAAATCTCATTTCTTTATATTAATCTTGGCAAGACTCACATACACCATAAACTTCAA
GTTTGTGTTTGTGAATATTAACACCAGGTAGTGATAATTTTATCTGATCTATTGGACAATAATCTATTACCTTTGTATCTCCACACTTT
TCACAGATAAAATGATGATGATGATGGTTTGTACAAGCGATTCTAAACTTCATTTCACCATCAAGTTCTGTATTTTCAATAATTCCTA
AATCTTTAAATAAGTGCAGGTTTCTATATATTGTGTCGAATGAAATTCCAGGATAATTTTCATCCATAACTTGTTGTATATACTTTGC
GTTTATATACTTATCTTCTTCGACAAAAATATCTAACATATCTTTACGTTTATCTGTATATTTTAAACCGTTCTCTTTTAAAATTTTAA

TAGCATCATTTGTATTCATTGATATTAGCTCCCTTTTTAAACTTCATTCGCATTTTCTGATAAGCCATTGTAATCATAAGTAAAATAA
CAAGTAGAACTACAATTACACCACCCGGAGAAATGTCCATATAGAAAGCTAGGACTAAGCCTAATATTACTGATAATTCACCTAAA
AATACACTTAGTAATATCAATTGCTTAAAACTTTTTGTTATTCGCATACTTATTGCAATTGGTAACGTGATTAACGCACTTACTAACA
GTATCCCTACAACACGCATTGAGGCAGAAATAACCATCGCTACAATAACAATAAATAAAAATTGAATCCATTTAGGAATGCCAATG
ACTTTACTATATTCCTCATCAAATGACAATATAAATAATTCTTTATAAAACAATGTAATAAACAGAACAACTATGATGGCAATGACA
ATAATCGTTGTTAAATCACTTATATTCACTGCGCTTATTGAGCCAAATAGCAATCCAACAATTTCTTGATTGAACCCATCAGCTAATG
AAATGAAGATTGCACTCAAGGCGATACCAGCACTCATTATAATTGGAATAGCAATTTCTTGGTAAGCAGTGTATGACGTTCTTAATT
TTTCAATTAGAAGCGCACCTACTATTGCGAATAAGATTCCAAACCACATTGGATTAATAAATACTAGTGTTGGCATAATAGTAAGTA
AAAACATACCGAAAGATATACCACCTAAAGTTACATGACTTAGAGCATCAGCTATAAGTGATAGTCGTCTAACAACGATAAAAGCA
CCGATTAGAGGCGCAATAAAACCTATCAAGATACCACTAATTAAAGAGTACCTCATAAAATCAAAATTCAATAATGCATCTATCAA
TTGTGACACGCCTTTCCATTTTAAATAAACTCAAATCTTTATTAATTACAACATTCTCGATTATGCTGATGATCGACAAAACGTACAG
GATGTCCATAAATTTTTGAAATTTCAACTTCATCAAGTGATTTAAACTCATCAGTTGTACCATGGAAATGCAAATGCTTATTTAAACA
TGCTACTTCAGTAGCAGTATCTGCTACAACACCGATATCATGAGTAACTATGATAATGGTGATACCTTCTTGTTTTAATTGATCTAAA
GTATTATAAAATTCACTTACATGTTTTGCATCAATACCATTCGTTGGTTCATCAAGTACTAATACTGCAGGTTCTGAAATCAATGCTC
GAGCAATCATTACACGTTGTTGTTGACCACCTGATAATTCTGCTATATTTTTATGAATTAAATCACTTATATTCAGTCTTTCTAGTACT
TTAATCACTTTTTCATTATCTTTGCTATTAAATGTTTGGAAAAGACGTTTTGTCTTTGTTAATCCGCTTAAAACAACTTCTTTAACACT
TGCTGGGAAACCTGAATTAAAGGCATTTGCTTTTTGTGATACATAGCTTAATTTAATTGATGTTTTCTTATTTTTAAAATCAATACCTT
CAACAAAAATCTCACCACTTTGTAAAGGTAATAACCCTAGAATCAACTTCAATAATGTTGATTTACCAGCACCATTTGGTCCAACAA
TTGCTAAAAATTCACCTTTATTTATTTTAATGTTTATATTTTCTAACACTTTTTTATGATCATAGTAGTAATTGACATTTTTCAATTCA
AAGACTGGTGTCATCGTATTCTCACCTCGCATTCAACTATACAACTCCTAGTAACATATGTAAACAGTAATGTTTACGACTCAAAAT
TAGACAAAATAAAGAGATATGCCCCCTTCAAGTTTTATTTATCGCATTTCTTGAAGAGAGCATTATCATTTTATTGTTGCATAACCTT
ATTTTTTAATTCTGGGTCAAATTGCTGTTGTTTTAACATTTCAATTTCAAGTTTATATGGCGGTTTTTTATTTTTCTTATCTTCACCAAC
ATAAGGTGTTTCTAAGATTTTCGGAATATCTTTAAAACTATCATGATGCACAATGTAATTTAATGCATCAAAACCAATGTAACCGAA
GCCAATATTTTCATGTCGGTCTTTTTGAGCGCCACGGTCATTTTTAGAATCATTGACATGAACAACTTTGATTCTGTCGACTCCAATG
ATTTTATCAAATTCATTTAATACGCCATCAAAGTCCTCTTTAACATTATATCCAGCATCATGCGTATGACATGTATCAAAACATACTG
ATAAACGTTCGTTATTATGAACTCCATCAATAATACGTGCTAACTCTTCAAATGAGCGACCAATCTCTGTACCTTTACCTGCCATCGT
TTCAAGCGCAATACGTACATTATTGTCATTCGTTAAAACTTCATTTAATCCTTCAATAATCTTATTAATTCCGGCATCAACACCAGCT
CCAACATGCGCACCTGGATGTAATACAATATCTTTAGCCCCTATAGCTTGCGTTCTTTCAATTTCTTGTTGCAAGAAATCTACACCAA
GATTAAACGTTTCTGGTTTGGTTGTATTTGCAATATTAATGATGTATGGTGCATGAACAACAATATTAGATAAACCATATTTTTCCAT
CACTTCATGACCTTTAGTTATATTTAAATCTTCAATACTTTTACGGCGCGTGTTTTGAGGTGCACCAGTATAAATCATAAATGTTGTT
TCACCATATTCATGCGCTTCTATAGCAGAACCTTCTAACATCTTTTTACCACTCATTGAAACATGTGATCCTAATAACATAAAACACA
CCTAACCTTTTTTGTTTTGCTTACGTTTTTGTCTATTTTGCTGCTTACTAAATTGCTTACGCTCTTGACGTTTCATTTTTTCAACTTCTTG
TTTAAATTTCTTCTTATAACCTGGTTTAACTTTGTTTTTAATTTTACTTCGAACTTTGTTCTTCACTTGATTAGTTAAATGGTCATCTTT
GCGCATTCTTGCTTGACGCTGATTGTGCGCTTTAACTTCTTTTAACTCACCATCTTTAATATCAACAGTATTGAATACAAAACCGCGA
TCTTCTATTAATGAAATATTGTGTTCTTCATCAGGACTATAAAGCGTAATTGCTACACCTTTATAATTCCCACGACCAGTTCGTCCAA
CTCTATGCGTAAAGAAGTCAATATCATTTGGCACATCAAAATTGATGACATGACTAACACCTTCAATATCAATACCACGAGATGCTA
AATCGCTGGCAATAACGTATTGGAATTCTAAATTACGTATACGTTTCATTTGTTGTTTACGTTCACGTGGCGTTAAACCACCATGAAT
CATACCAACTTTAATACCAGCTTCATTTAGTGAACGTGCTAAATCATTTGCATTATCTCTACTATTACAGAAAATAATACATAAGTAT
GGATTTAGTATATCAATTAAATTTAAAGTTTTTTCAACTTTAGCTGCACCTTTAGTAGGTATTAAAAAGAATTCGATGTTCTTTTTATT
TTGTTTTTTACTGTCGACAGCTACATATTCTGGATGACTTAAATATTTATTTAAAAATGGTTGTAACTGTTGTGGAATTGTAGCACTA
AACACCGCAATATTTGCATTATCTTCCAATCTTGCAGCAATGTAATCTACATCTTCAATTAATCCTAAGTCAATCATAAGATCCGCTT
CATCAATAACTAAATATGATGCTAAGTGCACATGTAAATGTCCCGTTTTAGCTAAGTCATTAATTCTAGTAGGGGTGCCTATAATCA
ATTGTGGTTGTGCATTACAACGTTGTCTATCTTTCTCTATATCTGTACCACCAATAAAAACTTTAACTGAAACACCAGCTTTAAATTG
GCTTAAATGGTTCGCTGCATCGTATAGTTGTTGTGCAAGTTCTCTTGTTGGTGCAACTACGATTGCTTGTGGTTCTTTTATTTCACTAT
CAATTAACTGCATTAATGGTAATAAAAATGCATGAGATTTCCCTGTACCCGTTTGAGATTGACCAATTAAATTTGTTCTCTTTAGTAT
TCTTGGAATAATTCGATTCTGAATTTCAGTTGGTTTTTCAAAATTAAGGTCTTTCACAGCGTCAATTAAACTAGATTCTAGATTAAAT
TGTTCGAATGGATGTTTTGCCATGATTTGGCCTCCTTATATGTTCATCTTTACTATTATAAAGTATAAATGTGCTATTTTAAATAAAA
ATATTAAAACCTCGAAATTTCAAAATTTAGAAATCCCGAGATTTTAGTTTAAATATATTGAAATGGATCTGTATTAATTGTAGATGC
TTCAACATCTATATTTATTTTTTCTGTATTGAACCAATTCATTAGTAACGTTTTTAAACCTTCTTTCATCACATATTCGCTGTAATGAT
TAATATCAATTAAATTCACACCATGAATTTTAGCATCTAAGGCATCATGATGTTTAATATCACCTGTAACAAAGACATCTGCGCCTT
GTTGGACAGCTTGATATTCATATCCAATACCTGAACCACCAATAATTGCAATACGTTTAATTTTCTGATTAGACTCACCAACAAAAC
GGACACTTGGGATATTTAATTTAGATTTAATATCAGCTGCGAAATCTTCCAATGTCATTTGATTATCCACTTCTGCCATAACGCCAAG
TCCATAAAGGGATGTTTGTTTTATCTCAATAAAATCAAATACCGGTGTTTCATATGGATGGTATTGTTTAATTAATTGCTCAGCCCTT
GACTTTTGATATGCATCTATCATAAATTCAATTTTAACTTCATCTACATATTCAATTTTATCAATTTGTCCTATTGTTGGATTAGCTTC
ACCAACTGGTTTGAATTGCCCTCTTCCTTCACTTTCAAAGAAACAATATTCATAATTACCTTCTTGCGCTAATCCATTTTCACTAAGC
TTATCTTTAAATGGTCCAACATTATCCTTAGGTATATATGTTTGAACTTTATAGTATACATCTTGTTGATTATTTATTATTGAAATGTT
CTTCAAACCCATCGCCTTCGCCAACATCATATTGACACCATACGGATTTACATCTAAATTTGTATGCATCGCTATTAAATTAATGTCA
TGTTGAATTAGTTTTCTAATGATCAAACCATAACCATTAGCTTTTAATGATGTTACGCCTTTAAAGATTAGAGGATGATGACTAATA
ATAGTATTATAACCTTTTTCGATTGCTTCATTTACTACTTCCAACGTACAGTCTAATGCTGTTAAAACACCAGTAACTTCAACATCTT
CATCACCTATTAACAATCCTACATTATCCCAAGATTCAGCAGTACTAAATGGCACATGATGATCTAACAATGTCATTAAATCAGCTA
TTTTCATTACTATAACACCCTTTCAATTACAGCAATTTCGTCATTAATTTGAGCTAAACGTTGATGATGTTGTTCAGTATTGAGTTTCG
ATTTAATATGATAAAGTGCTTCTAACTCTCTTTGCCATTTTTTTATAAAATATTCATTTTTGTTGTTCAGCAATTTTGGTCCGAATTTC
AATTCATCAGATGATAGCTCTATTAATTGTGTAGAATATTCTGCTACAACAATTTCATAAATATGGCCTTTTTCTTCCATTATTATTTC
ATCAATTATTTCATAATTCAATTGTTGTAATGTTTGTCTTAAATTTTCAGTTTGGATATTACTTTGTAAAATCAACCTTGGATGTTGAC
TTAACTTATCTTGCCCATCTTTTAAAATTTTAGCAATAAGTGGTCCGCCCATACCACAAATTGTGATATTATCGATTACGTCCTCAGG
ATGAATAACACTTAAGCCATCCCCTAAACGTACATCAATTCTTTCTACTAAATGATTTGCAGCTACATTTTTCACAGCAGCTTGAAA
AGGGCCTTGAATAACTTCTCCTGCAATACCGTAATCGCATAAATGGTTTTGAATTGCATAGATTGGCAAATAAGCATGATCTGAGCC
AATATCCGCGATTGTACCTTGTTTTAAAAATCGACTTACCGTCGTTAATCGGTTATTTAACGAAATCATTTTATTCTCCTTTATGAAA
AAAGAGGTTGAACATTATTCAATGTACCAACCTCACTCAAGTTAAATCTTAATCTAAAAATAAATAAGCGCTTATTCATGTCTTGGT
ATCAATCATTAAAATTGATATAAATTAATCCATAAAGTCTTTCAAACGTTTACTACGACTTGGATGTCTTAATTTTCTAAGTGCTTTT
GCTTCAATTTGTCGAATACGTTCACGTGTAACACCGAAAACTTTACCAACTTCTTCAAGTGTTCTTGTTCTGCCGTCATCAAGACCAA

ATCTTAATCGTAATACATTTTCTTCTCTATCAGTTAATGTATCAAGCACATCTTCTAATTGCTCTTTTAATAATTCATAAGCAGCATGA
TCTGAAGGACTTTGTGCTTCCTGATCCTCAATAAAGTCTCCTAAATGACTATCATCTTCTTCACCAATTGGTGTTTCTAATGAAACAG
GTTCTTGCGCAATTTTTAAAATTTCACGAACTTTTTCTGCTGGTAAATCCATTTCTTCACCAATTTCTTCTGGTGCTGGATCTCGACCT
AAGTCCTGTAATAATTGACGTTGAACACGAATTAATTTATTAATTGTTTCTACCATATGCACAGGGATACGAATCGTACGTGCTTGG
TCAGCAATTGCACGAGTGATTGCTTGTCTAATCCACCATGTTGCATATGTTGAAAACTTAAATCCTTTGTTAAAGTCAAATTTTTCAA
CAGCTTTAATAAGACCCATATTACCTTCTTGGATTAAATCAAGGAATAACATACCACGACCTACGTATCTTTTAGCAATACTTACAA
CTAAACGTAAGTTCGCTTCTGCAAGTCTTGATTTTGCTACTTCATCACCTTGTTCAATACGTTTGGCTAATTCGATTTCTTCTTGTGCA
CTTAATAAGTTAACACGCCCAATTTCTTTAAGGTACATACGAACTGGGTCATTTATTTTAACACCTGGAGGGGCACTAAGATCACTT
GGATTCAGTTTCTCGTCAGTATCTGAACTATCTTTTTCATTAACTAGTGAAATATCATTATCATTTAATTGATCAAAGAAATCATCCA
TTTGATCAGAGTCGATATCAAAATTCTGAAGTTTTTCAGCAATTTCTTCATGACTTAAATGACCCTCTTTTTTACCTTTTTCAATTAAT
TGCTTCTTAACATCTTCTAATGTTAATGTCGGATCAATTGTTTGTTTTTTAATTTTAACTGTGTTATCAGACATGAAACGGCCTCCCGA
TTTTAAATATGAACATTCGAAATTTATTCAATATTGCTATTTTAAACTAAATTCTTAATTAATTCATCCATATTTTTAATTTTATTTTA
CAAATTGGAACTAATCCCAATATTTATTTTTCAATAGTGTTGTTTAAATGCGGTAACTATCATTTACCCTTATACATTTTGACATAAT
CATTATTCGTATTATTCTTTAAAATCACATGCTACATGCGTTCTTTATTCTTAGCAACAATTTGCTGTAAATAGTATTTTTGTAATTCT
ACATCGCCAATCCTTGTAGCTTCCCTTAATTTATGATTCAATGACTCAATTGTTTCTTGTCCTTTTTCATTAATAACATTGACATAATC
ATCAATTTCATTTTCATATGGTTCGTCATTCAAATTATATTGTTCTAAGCTAATTAGTGTTTCTCTCAACTCATTTGAATTAACATACT
GCACAGCATCACTGATATTATATTGATCATTTTCCGCATAAAAATCATGTAAGACTTCGAATACATATTTAAAATGCTGATTTGTGA
AGTTATCCTTATCAACACTTTCATAATAATTTAAAAATGTATCTTTATCTCTCATTAAATGTTTTAAAAATGCTCGCTCCGCTTTTTCT
TGACGGCTCAAATTGTCAAATTGTGCCATACCAATTGGCTCAGGTTCAATATAACCGCCATACTCATCTTCTGGATAATAATTGGCT
GGTGCTTGATTGAATTGTATTTCGTTAGCTAACTGCTCAGGACTAACATTGAAAAATGGCGCAACATCATTTATAGCCTTTTGTTGCA
AAATCGATGACTTCATAAGTGAAATGTCATGACTCAGTTCTTTCAAATAACGTTCATATGAAAGGTCATTATGTGCAATTTCATCTTT
TAATATACTTACTTTATAATGTGCAAATGACTTTTTGTCATTTTTTACAAAAGTAGTAAATGCGTCGTTGCCATACTTACCAATGTAT
TCATCCGGATCCATGCCAGATGGCAATTGTATAACAAATACATTTAGCCCTTGCTGTAACAAATTTTGACCTGTTTTAAGTGTTGCTT
CACTACCCGCAAAATCCCCATCAAACATTAATGTTATATTTGATGTTAACTTTCGTATAAAAGTAATATGTTCATCTGACAACTGTGT
CCCCATTGTTGCAACAACGTTTTTCAAGCCAGCAGTATCAGATTTTATAACATCCATAAAACCTTCTAGTAATACGATTTCATCTAAT
TTTCTAATTGATTTACGCGCTTTATCTAAGTTGTATAACAACTTTCTTTTTTGAAAGATAGGTGTTTCAGGACTATTTAGGTATTTTGG
TTCTTGACCGGTATATGTTCGACCTGAATATCCAACAATTCTTCCTTGCGCATTTTTCAAAGGAAACATAATACGATTTCGAAATCTA
TCGTAATAACTGAAATTTTCTTCGTTACGTGATAATAATCCGGCTTCATATGCTAATTCAATATCGTAACCCTTTTTTTGAAGAAAAT
CATGACAAAAATGTGAGCTATCGGGTGCAAAGCCAATGCCTCGCTCTTTAATAAGCGCATCTGTAAAACCACGTTCTTGTAAGTATG
TTAATGCTTGTTCGCCTTCGACTGTCTTTGTTAAAGCGTAATAATAAAATTCTTGTATTAACTCATGCATTTCAATCATTTGTAAATCA
TCAGAAGCAATTTGAACATTTGAGTTAGATTGTGTTGCCTCAATATCTACAGCAACATTAACTCTATCACCTAATTCTTTAACCGCTT
CAACAAATGATATGTCTTTAATTTCTTGAGTAAATTGAAAAACATTGCCACCTTTTTTACAACCAAAACAATGACAGATTTGTTTATC
TTCAGAAACTGTAAATGAAGGTGTCTTTTCATCATGAAAAGGACACAAACCTATATAATTGCGTCCTCTCTTTTCTAGTTTTACATAT
TCACTTACCAAGTCTAAAATGTCGGTTTTATCTTTTATTTCATTAATGATCGATTGATCTATTCGCAAATTAAATCACCTATTAAGCA
TTAGTTATACTATTATACCTAATTTTTGACAAAAATGAAATCATTTCGATTTATTTTGTTCAATATAATGGATTATATCGTTTGCTGTT
TCTTCGATTGCTTTTTGAGAAACATCAATGACAGGACATCCAATTTCACTTACGATTTCTTCAAAGTAATTCAATTCTTCTTGGATTC
GAGCTTCTGTTGCATAACGAGCTGTGTCACCTAGTCCCAATTGTTTTAATCGCTCTTTTCTAATGCGATTTAATTTTTCTTCACTTATT
TTAAGTGCGATACATTTCTTTGGATCAATATCATATAAGCCATCTGGCGGTGTCACTTCTGGTACAATCGGTACATTCATAACTTTGT
AACTTTTATGCGCTAAATACTGGGATAATGGTGTCTTTGAAGTTCTCGAAATACCAAGTAAAACAATATCAGCTTTAGGTAATCCTT
TAGGATCTTTACCATCATCGTATTTAACTGCAAACTCTATCGCATCAATTTTCTTGAAATATGCATCATCTAATCTATGAACGATACC
TGGCTCATTATAAGGTTTTTCTTCAACCGATGCTGATAATAAATCCATTAATGGCCCCATGATATCGACGGACTTCAATTGGAATTCT
GCTACTTTCTCACTCATATATTGCTTCATTTCAGGTTTAATAAGTGTATAAACAATGATAGCATTTGTATCTTTTGCAACTTGAATCA
CTTCATCAACATCTTCAAAAGATTCAATATATGGATATCTTAATAATTCATTTTTACATTGCTTAGGATTAAATTGTGAAATACCTGC
CCTAGCAACTAACTCTGCCGTTTCACCTATAGAATCTGAAGCTACGATAATTTTAATTTTTTCCATTACTTCTCACCTATTCTTTATAT
AATGCTACTAATAACTTAGCTATTGTTGTTTTGGAAATTCTTCCAATTACTTCATACTTTTGATTATCTTTTTTTCTTACAATTGGAAT
CGAATCAATTTCTTTTTCAATCATTCTATCTGCTGCGTATATGACTAATTCGCTTTCCTCTAAATAAGTGACATTAGGCATACGTGTC
ATATTTACACTAATAGGTACTGTATGAATATCTGCTCCAATCATTGAAGCTCTTAATAAATCTTTTCTTGAACACACACCAACAAAAT
CGTTATCTTCATTAATAATAAATAATGTACTTACATCTTCTAAAAAAAATTGTACAAATAGCGTCATAAACTGTTGTATTCTCTCTTAG
CACAACAGGTTGAGACATATAGTCCTTAACCTCAAATTGTCGAAGTTTTTCATTAAAAAATTTACCTTTTGATTTACCTGAATAATAA
TATCCAACTCGGGGACGCGCTTCTAAAAAACCTGACATTGTTAATATCGCTAAATCTGGTCTGAGCGTTGCTCTTGTTAAATTCAACT
TATCTGCTATTTGTTCACCAGTAATGGGTCCTTGAGTTTTAACAATTTCGATGATTCGTTCTTGTCTTTGACTGAGTTCTATAGGTCTT
CACCCCTTTTTTATTCACTTATTATTATACCGATATTTCTTTCATGTCACAAACACATTAATTTTACTTGCCTTTAAATAATCTATCAA
TTAGAATAAACGTACAGCGAGTTAAGGATAGTGTAAGCTTAACAATAAGATTGGCGCAACGAATCATTTTAAAATAAAAGCGAGTG
ACTACACTAATTTGGGTGGAACCGCGGGTTAAATACGTAACTTATTTTGTATTATATACAATTTAAAATATTAACTCGTCCCATGGA
AGTAAAGCATATTTACTATGCTGTTATTTTCCATGGGGCGTTTTCATGTATGAGGAGAGGTAATTATGGCAAAAGATATGGATACAA
TTGTTTCATTAGCAAAACACAGAGGTTTTGTGTTCCCTGGTAGTGATATTTACGGTGGTTTATCAAACACATGGGATTATGGTCCTTT
AGGTGTTGAATTAAAGAATAATGTTAAAAAAGCTTGGTGGCAAAAATTCATTACACAATCACCGTTTAACGTTGGTATCGACGCTGC
AATCTTAATGAATCCAAAAGTATGGGAAGCTTCAGGACACTTAAACAACTTCAACGACCCAATGATTGATAATAAAGATAGTAAAA
TTCGATATCGCGCTGATAAATTAATTGAAGATTATATGCAAGATGTTAAAGGTGATGAAAACTTCATTGCCGATGGTTTAAGTTTTG
AACAAATGAAAAAAATTATTGACGATGAAGGTATTGTTTGTCCTGTAAGTAAAACTGCTAACTGGACTGAAATTCGCCAATTCAATT
TAATGTTTAAAACATTCCAAGGTGTAACTGAAGATTCTACAAATGAAATTTTCTTACGTCCTGAAACAGCACAAGGTATTTTTGTAA
ACTATAAAAACGTGCAACGTTCAATGCGTAAAAAATTACCATTTGGTATCGGTCAAATTGGTAAATCATTCCGTAATGAAATCACTC
CAGGTAATTTCATTTTCAGAACAAGAGAATTTGAACAAATGGAACTTGAATTCTTCTGTAAACCTGGAGAAGAAATCGAATGGCAA
AATTATTGGAAAACTTTTGCAAGTGACTGGTTAACAAGCTTAAATATGAGCAGTGAAAATATGCGTTTACGTGATCATGATGAAGAT
GAATTATCTCATTACTCAAATGCAACAACTGATATTGAATATAAATTCCCATTTGGTTGGGGTGAGTTATGGGGTATCGCAAGTCGT
ACAGACTTCGACTTACGTAAACATGCTGAACACTCTGGTGAAGATTTCAGATACCATGATCCAGAAACGAACGAAAAATATATTCC
ATATTGTATCGAGCCATCACTTGGTGCAGATCGTGTAACATTAGCTTTCTTATGTGATGCATATGATGAAGAAGGCGTTGAAGGTAG
TAAAGATGCACGTACAGTTTTACACTTCCATCCTGCATTAGCACCATATAAAGCAGCGATTTTACCTTTAAGTAAGAAATTATCTGG
CGAAGCGATTAAGATTTTTGAGCAATTAAGTTCTAAATTCTCAATCGATTTCGATGAATCACAATCTATCGGTAAAAGATACCGTCG
TCAAGATGAAATCGGTACACCTTATTGTGTAACATTCGACTTTGATTCATTAGAAGATAATCAAGTTACAGTACGTGACAGAGATTC
AATGGAACAAGTTCGTATGCCAATCTCAGAGTTAGAAGCTTTCTTAACTGAAAAAACAAAATTCTAATTAAAAAGACTCTCCACCAT

ATGTGGTGGCTATATAATTTATAAAAAGAGATACTGGTTAGTAAAATTGCCAGTATCTCTTTTCTATTCGAAAAAGGTATTCAAATA
AACACATCTATGTGGCTACTTGAATACCTTTTATTATTTTATGTTATTGTTCCAATCTTTTTAATTGGTTGATTAGTTTCTGACTTTTAA
AAAACATACCTGCATATTCACGATATAACATTAAAATGATATCTGACATTTCATCAATAATTTCTTGATGGATATTCAATGAATTCAT
TTTATCTATTGGTAATTTTTGTAATACATCTAATAAATATAGTGTTTTATTCGATAATATAACTGCATGTACATCTTTAGAAGCCTCTT
GCCTTGAAATCGCACCGTCAAACTTAAAACTATAACCTATTAAATCTGCTTGTGTGTCATTACCACTCACAGCACAGCGATTAAATG
ATGCAGTAAAACCAAATCGTTTCATACACTTTAACATAACTACGACTGACATTAACTGTGCAGATGTACCTGATTCTATTTTTTCAAG
AACAAATTGTAATAATTGATAGTTATATGGTGCAATGTCACCTTCATCCATTGAGCGCTCAATAGTTTCAGCTGCCAGAGAGGCATA
ACTGCTTACGTAAAGGTCCATTTGTAATTTATAATGTTGACTAATAACATCTACAGAATTTAACGTTCCCATACCTCGCCACTGATTG
TAAATAAACAAACCATAAACAAACAATTGCGTTTGCGCTTGTAAACCCGTCTTAACTTTTTTAGCACGCCTTGCCATAAGTGGTACT
TTTGCACCATGCTCATTTAAAATCGTGATAATTTTATCAGATTCACCATAATCAACTGCTTTGATGATAATCCCTTTTTGGCGCATTA
ACAATTATCTTCACCACTTTTAAGATTAATCTTGGTCTTCAACATAACCAATTTGGCGAATAAAGTTAACTTTGTTTCGCCAGTCTCT
TTGAACTTTGACCCATAATTCTAAGTAAACTTTAGAGCCTAGAAGCATTTCTATATCACGTCTCGCACGTTTTCCTACTTCTTTTAACT
TTTTACCGCCTTTTCCAATGACAATTCCTTTTTGCGAATCTCTTTCAACATATATAGTTGCTTCGATATGAACACGATCTTCGCTTTCT
TTAACCATACGGTCCACATTAACACCAATCGCATGAGGGATTTCTTCACTTGTAAGATGAAGGATTTTTTCACGAATGATTTCACCC
ACTACAAATTGTTCAGGATGGTCTGAAATTTGATCATCTGGATAATATTTAGGTCCTTCGGGTAAATACGTCTTTAAAACATCAATA
AAATGATCGACATTTAGCCCTTCTAATGCTGAAATAGGTACAATCTCTGTAAAGTCCATATAACTTTGATATTCTTCAATCTTTGGCA
TTAATTCATCTGGATGCACTAAATCTATTTTATTTAATACTAAAAATACTGGTGTCTTAACATTTTTCAACATTTCTATAATATATTCA
TCGCCTCGTCCAATTTCCTCATTGGCATTAACCATAAACATGATTGCATCTATCTCAGATAATGTATTTTTAGCGACTTTCATCATAT
AGTCACCTAATTTGTGTTTAGGTTTATGAATACCTGGCGTATCAATGAATATAATTTGCGCGTCATCTCTTGTCATAACACCTTGAAT
TTTATTTCTAGTTGTTTGAGCTTTATCGGACATGATTGCTATTTTATGGCCGATCACTCTATTAACAAATGTTGACTTTCCTACATTTG
GTCTACCTATAATTGAAACAAATCCTGATTTATGTTCTGTCATTTATTCTAAATCCTTTCCTGAAAATCCAAATGGTAGTAACTCTGC
GACTGTCATCATAACCATATCTCCTTTATGATTTGTCATATACACAGGCATATCATCATCACATAATTCCTTCAAAACTTGACGACAT
GCACCACAAGGTGATGACGGTTTATCTGCATCTACGGTTACAGTTATTGATTCAAAATCACCTGGTCTGTATCCTTGAGAAATTGCC
GATACCAAACTAGCTCGTTCAGCACATATCGATAATGGATAAGAAGCATTTTCTACATTGGTACCATAAAAAGTTCTACCGTCTTTC
GTTTTTAAATAAGCCCCTACTTTAAATTGACTGTATGGCGAATATGATTCTTGTTGTGCTTTTCTAACTTCTTGAAAATAATGAGGTT
GATAACTCATATATGCCTCCCTAAAATAACGCTATAAAATGTGGTAAAAATACTATTAAACCTATAATAAATGCTAATATTGAAACT
ATAAGTACACTAAAAGCCGCAATATCTTTAGCGTATTTAGCTAAATCATGATATTCAACGGTCACTAAATCGACAACATATTCAATA
GCAGTGTTTAAAGCTTCAACAGTGAGAACTAATGCAATAGCAATGAGTATAAATATCCACTCAATCCGATTAATATTTAGTACGAG
ACCAAAGACAATAGCAACAATCATTGCAAACACATGTAAAAGAAATTTATAGTCTTTTTGAATTAAGATTTTCAGCCCATCAAGTGC
ATATTTAAACCTTTTCATAATTAGTCTCGTGTTAATCCATATGCGTTTAATATTGTATCTTGTCGACCAAACATTTCCTTTTCATCCGC
TTCAGTCATATGATCATAACCTAATAGATGCAAAAATCCATGTAATGCTAAAAATCCTAATTCTCGTTCAAAAGAATGTCCGTAATT
GTTTGCTTGTTCTTGCGCTACATCCGTACAGATAATTATATCCCCTAAAACACGTGGTATATCAAGACCACTAAAATCAATCTCTGGC
TCATCTTCTTCTAAAGCAAATGAGATTACATCTGTAACTTTATCTTTATCTCTATATGTTCGATTAATTTCTTGTATTTCTTGTTTATCT
ACAAATGTAACAGAAAGCTCAGCATCGTCTTCTATATGCTCTTCTTTTTTAGCAAATTCTAATAAATCTTCAATTTGTTTATACCAAG
CATCTTTAACTAAGCCTGTGTGATCGCTAAAATCTATCGTAAACATTTAATTCTCTCCTTCATAATGTTCAATGATCTTACTTACCAA
TGGATGTCTTACTACATCGCTCTGATCTAATTTCAATATACTTATACCTTTAACGTTGTGTAACCTACTGACCGCTTCCTTAAGTCCAC
TTTTAACACCTTTAGGTAAATCGATTTGAGTTTGGTCACCAGTAACTACCATTTTTGAGCCAAAACCTAGTCTTGTTAAAAACATTTT
CATTTGCGCATGTGTCGTATTCTGCGCCTCATCAAGAATTACAAATGCATCTTCTAATGTTCGTCCGCGCATATATGCAAGTGGCGCT
ATTTCGATAATGCCTCTTTCAATAAATCGCTCTGTTTGTTCACGCCCAAGAACAGTATATAGACCATCATATAAAGGTCTTAAATATG
GATCTACCTTTTCTTTCAAATCTCCTGGTAAAAATCCAAGTGACTCTCCTGCTTCAACAGCAGGTCTTGTTAATACAATACGTTTAAC
AGCACCTTTACGGAGTTGCTTTGCTGCATAAACTACAGCTAAGAATGTCTTACCTGTACCAGCAGGACCTATACCAAATACTAAATC
ATTATTTTTCATGGCATTAACATATATACGTTGCCCCATCGTTTTCGCACGAATCGTCTTACCAAATGCATCTTTAGTTATCTCTTCAT
CATATAAATCTAACAGATGTTGAATTGTGTTATTATGCGCCATTTTAATAGCTGCTTCAACATCTTTAATTGTAATATTATTACCTAA
ATCAATAACCTTCAGCAAATTGATTAATACTGATTCCGCTTTTTCTACGTTTTCTATTTTTGTACCTTTAACGGCAACTTCTTGTCCTC
TTGCATGGATGACAACATCGAAACTCTCTTCAATTGCTTTTAAATGTTCATCATTATTTCCAATTAAAGCTTGAGATTGGTTCATATC
GTCTATTTGTATAATTCCAGGCATACACGCGCTCCTTTTCATATATCATTTATATTCATTCTATGATGCTTTAATATCTACTTAATTAT
ATCATGCAATTGATTGATTTAAACATCTTTGCATTGACTTACTCATTTTTCTTTAATTTAAAATAATCATTTATACTTTTCACCTTGTC
ATAAAAGCAGATTCAATTTTAGACTAATCAATTTTTTGTTGTAATTTAAATAACTAATATTTCTCGAAAGATTCTCTTTGTTTTTCAAT
TTTTCTTAACGTAAAAAAACAAGCAACTACCATTACGATAGTGCTTGTTTTCAAAAAATTTTATAATTGTTTTGGTTTAGCTAAAATT
TCTGACCATATCATACCATTGATTACTTCATCTTTATCAAATTGGAACGCTGATTTAGTCAAATCTTTTTCGACATTTTGAGAATTAA
GCAACTGCTTTAATTTCAAACGTTTTGTACGTTCAGATAAGTATTTATCCTCAATAATATCTCTAGCACGTTTTTCCATTTTAGCAATT
TGCTTTTCTTTTTCTCTATCAATGTCAGTACGAATCGTTTTAATATCATCTCTAAGTGATTTTTCTAATTGACGTCTAATTTCGTCAGA
ATTATCTTGTCTAGAAGGTCTATGTTTCTGCTCCACCGGTTCTGCTTTAGGCACTGTAATTGGTTTCTCTGTCATCGGTTTTGTTTCTG
GTTGTTTTTTAGGTGCCATAGGTTCTACAACAGGTTTCGATTTAGGCTCTTCCTTTGGAAGTTCATCGAATAAAGGTGGTAACGTATC
ATCATATTTTCGTTTCGATGATTTCTTTTCTTCTTCATTTAATTCTTCACTTATTTCTTTAAACGTTCGCTCAATTTCTTCAAAAAAGCC
ACCTTTTTTTGGTTCATTATCGGTAGATGTTTTTTGAGGTGGCTTTTGATTTTGTCTATCTTTATGACTATTTTCGCGCATAGTAGTAA
TGATAGAAATGATCACTGATATGACAAAAATTAGAATACCGACACTCATTTAATCACCTCTCGACTTAATGTTCAGGTGACTCATCA
TCACTTTGATCAGTTCGTTTATTAATTGCATTTCTCATGCCTGTATCAGCTTCGATATTTTTCAAATTATAATAATCTTTAACACTGAT
ATTACCTGAACGTAATGCTTCAGCCATAGCTAATGGTACTTCAGATTCGGCTTCAACTACTTTAGCATGCATTTCTTGTACACGCGCT
TTCATTTCTTGCTCAGTTGCTACAGCCATAGCTCTACGTTCTTCAGCTTTTGCTTGTGCAATATTTTTGTCTGCTAATGCTTGTTCAGTT
TGTAAGTCTGCACCAATATTTTTACTAATATCAACGTCAGCAATATCAATTGATAAAATTTCAAATGCAGTACCTGAATCTAAACCT
TTGCTTAAAACTGTTTTAGAAATATTATCTGGGTTTTCAAGTACTTCTGTATGATGCTTACTAGAACCAATTGTTGAAACGATACCTT
CACCAACACGTGCGATGATTGTTTCTTCACCAGCACCACCAACAAGTCGAGCAATATTAGCTCTAACTGTGATACGAGCTTTGGCTT
TCACTTCAATACCGTTCATTGCTACACCTGCGATAAATGGTGTTTCAATGACTTTAGGATTAACAGACATTTGAACCGCTTCTAATAC
GTCACGTCCTGCAAGGTCAATTGCAGCAGCACGTTCGAAAGGAAGATCAATGTCAGCACGTTGTGCAGCAATATTAGCGTCAACAA
CTCTGTCAACATTTCCTCCTGCTAGATAATGCGATTCTAATTGGTTTGTTGTTAATGCTAGTCCTGCTTTATGCGCTTTAATTAATGGC
GCTATAACTTTTCTTGGAGATACACGACGTAAACGCATACCAACCAATGTACCTATACCAACATGAACGCCAGCTGCTAACGCTGA
AATCCATAAACCAATGGGTACAAATGAGAATAAAATAAGTAATGCAACTACTATAATAACTGCTATTACGATAAAACTTAAACTAA
ACATGGTATCGCTCCTTTTAATTAATCTACTTCCCTCACAACTACTCTTGTTCCTTCAACTTCAAGGATTTTCACCGTTTTATTGCGTA
AAATAAAGTTGCCATCTGAAACAGCATCAATACGTTCGTTTTCACAAAAAATAATCCCTGCAGGTCGAAGATCTGTAACTGTTTGAG
CAGTCTTTCCTACGAGGTGCGAGCGGTTATCATGAGAATTGTAACCTGACTCAGAATTAGTTGAATCTTTTAAGATAACTTTATCCA

AAAACGGAATCTTTCTGTTGAAAATCTTCACTAATATCACCCATTCTACAATCGTTAAAATCAAGGCAACGATAACATTCGCAAGCA
TAAATAGCAAATTATCACCGAGCGTTGTTATGCTTATAGTTATCAGTATCATGCCAATAATACCAATTACTGCACCAACTACAAATA
ATTCAATTACAACTAATATAACGCCAATTGAGAATATTAAGATAGAATGCATATTGACATTTCCTTGGATTAGAAATCCCAAAAATA
AAATAAGTAATGATAATGTGGCGATAATACCAGCTGCATTGATTTTTTTAGAGTAAAGTTGATATACAAATCCTAAGAATGTCAAAC
AGGTTAATATTAACGTAAAAATAGGTTGAACAATTATATTACTAACTTGTTCAACCCAAGTATCTCCAGCCGTTGATTCCAAGATAG
TTGTCATTTGTAAAAAATTATTATAACTCACAATTTCACCTCGCCCTCACATTAATTATACATGAAATTGAATATACTTTATAGTTAT
AAGAACCTATAGAATATCAATTTGATTATTTATAAACTATATAACTTTTTTTAATTAAAAATTGACAACAAAAAAACATCACATATT
AGTGTAAGACACTAACTTGTGATGTTTGTTTTATATAATTAATATTCGTGTTGAGGGAGTCAACAGAGGTATTAATTATTTGAATTTA
CGTTTACGTGCAGCTTCTGATTTCTTTTTACGTTTTACGCTTGGTTTTTCGTAAAATTCACGTTTACGTACTTCTTGGATTGTTCCACTT
TTAGAAACTGAACGTTTAAATCTACGTAACGCATCTTCAAGTGATTCATTTTTACGTACTACTGTTTTAGACATCTGTATTTCCCTCC
CTCCAAATATCAACGGAACTTTATAGTCAATTGCACAGTAAAACTATGCATTTATAAGTATAATATATCTGTAATTTATGGTCAATT
AGTAAATTGTTTTTTATTTGAAACATATTTCACATCAAAATCACAAAGACTTTTAGATTTTGTTCTAAAAATCTCTTAATAATTTATTT
AATGAGAAGAGTTGCTTATATAGTAAATTGTGAAGCCGTTAAAACAACGTTACAAAACCTATATCTTTAATACGGAACCTTATGGTA
TGAATCAAGGAATACTTAAACTAAAACTTCTCTATCAGATTTATTTGTTGCGAAATCAACAACTTTAATTGCTTGCCCTTCATTTAAT
GGATAATTTGCTTGCGTAATTTTAACTTTTACAATTTGACCTATGAGTGATTCGTCACCTTCAAATTGTACTTTCATATAATTATCTGC
ATATCCAACTAATGTGCCTTCTGTGTCACCCTGTTCCTCAGGAATTACTTCAAGCACATCTTGATCAAATTTAGACGCATATAACTTT
CCGAGTTGATTGCTTAGCGTAATTAACTTATGCACCCGTTCATTTTTAATTTCTTCATCAATTTGGTCATCCATTCTTGCAGCTGGCGT
GCCAATTCTAGGAGAATAAGGGAAAACATGCAGTTCAGAGAACTTATGCTTTACGATAAAATCATATGTTTCTTGGAACTCTGCTTC
AGTTTCACCTGGGAAACCAACAATTACATCACTCGTAACTGCCAAGTCTGGTAAAGCTTTATGCAATTTTGTTAATCGTTCTGAAAA
TCTATCCATTGTATACTTACGTCTCATACGTTTTAATACTGTATCTGAACCAGATTGTAATGGAATATGCAAATGACGCACAACTTTT
GTTGAACGTTCTAAAACGTCAATTACTTCATCTGTAAGTTGACTTGCTTCAATTGAAGAAATTCGAATTCGTTCTAATCCATTAATCG
TTTCAAGATCACGTAATAATTGGGCCAAGTTATAATCTTTTAAATCTTGACCATATCCACCTGTATGAATTCCCGTCAATACAATTTC
CTTATATCCTGAATTCACTAGTTGCGTCGCTTGTTCAACTACTTTTTCCGGATCTCTTGAACGCATTAAGCCACGAGCCCATGGAATA
ATACAGAATGTGCAGAAGTTGTTACAACCTTCTTGAATTTTTAATGACGCACGTGTTCTATCTGTAAAATATGGGACATCTAATTCTT
CATATTTACGATTTTTCATGATATTTCCAACACCATTAATTGGTTGGCGTTCTTTACGGAATTCATCAATGTAACCTAATAGTTTATGT
CTATCTTGTGTACCAACTACTACATCGACACCAGGAATTTCCATAATTTCAGCTGATGAAGTTTGCGCATAACAACCTGTTACACAG
ATTACAGCATCAGGATTTTGTCTTATTGCACGTCTAATTATTTGACGACTTTTTTTATCACCTGTATTCGTTACTGTACAAGTATTAAT
AACAAATACATCAGCATTCGCTTCAAAGTCAACGCGCTCATAGTTTGCTTCTTTAAATAATTGCCAGATTGCTTCAGTTTCATAATGG
TTTACTTTACAACCTAATGTGTGAAACGCAACTGTTGACATAAATATTCACCCCATTAATTCTTTTTCATAACTTATTGCACTTAACG
CATACAATGGCGCAGTTTCTGCCCGTAAAATTCTCGGTCCAAGACCAACAACTGTACTAGTATTACTAAATAATGAAATTTCATTTT
CTGACAAACCACCCTCAGGACCAAAAATCATCAACACTTTATCCTGAGCCTTGAATTGTTGTAAAGTTTGCTTGAAATTGCTTAACT
CACCATCTTTTGCTTCCTCTTCATATGCAATAAGAATATAGTCATAATTATCAATAGTATCACAAATTAATTTTAAATTCGACTCGAA
TTGAATAGATGGAATCACTAAACGATAGCTTTGTTCAGCAGCTTCTTTAATTATTTTTTGCCAACGCTCTATCTTTTTGGCAACTTTCG
CCTCGTTTAATTTAACAATTGAGCGTTCCATGCTCACAGCTATAAATGATGAAGCACCCAATTCAGTAGCTTTTTGTAGCAACCACTC
ATATTTGTCAGCTTTAATTAGTCCACTGCAAATCGTAACATCAACTGGCAATTCTGTATTAATATTTTGTTTTTCTTTTAAATCAACTG
CAATTTTATCACTTGTTATGTCAGCAATTTCACATAAATAAACTGTTTGATCATTAAAAGTTAAAATAATTTTACTACCAACATCATA
TCTCATTACATTTGTTATATGATGAATATCTTCTTTTTTTGTAATAAAAAAACGCTGACTTACATCAGCGTTTTGGTCTATGAAATAA
CGTTGCACATTATTCACTCACTTTCTGGCCAACAAGACAAACCCAACCATTGTCATGTTGTTCTGAAATAATTTTAAAACCTACACGC
TCCATATGTGACTGTATACCTTCATACTTCTCTTTTATAATACCAGAAGTAATAAAATAACCGCCTTCATTTAGAGTATTATAAGCAT
CTTCAATCATTTCATCAATAATATGCGCTAAAATATTTGCTATTACAATATCAAATTTTTCTGTTTCGTCTTTCAATAAGTTACCTGGA
ACAGCTTCAATTAACGTTTCACAATGATTTCTTCTGAAGTTTTCTTTAGCTACACTCACTGCCATTTCATCAATATCCAACGCTTTAAT
ACGTTTTACACCGATTAGATGACTTGCAATACTTAATATACCTGAGCCAGTACCAACATCAATTACTGAATGCTGTGGCAATACATA
TGTTTCTATTGCCTTCAAACACATACTTGTAGTCGGATGATCGCCTGTTCCAAAAGCCATACCTGGGTCGAGCTCAATGCAAAGCTC
TTCATCCGCTTCTTTAGCATATGTTTCCCAACTAGGAACTATTGTGAACTTCTTCGACGCTCGGAATGGATGGAAATAGTTTTTCCAT
TCATTTTCCCAATCCGTCTCTGCAATAATTTGCTCACTGAATTGAATGTTATGTTGATCAAGTTCATCTAAATTTAATAACTCATCTTT
AATTTGCTGTCGCAACTTATCATCATAAGTCATTTCATTAAAATAGGCTTTCAATCTTACTCCCTTATCTGGATAATCCTCTTTTTTCA
AAGCGTAAATTTCACCGTATTTATCTTCTGGTTGGTTAATTAAATCATCTGAATCTTCTATCACGACACCATTTGATCCATGATTTTC
AAGTATATTGGTAGCCAATTCTACTGCTTCATGATTAATAATAATTGAAAGCTCTGTCCAGTTCATACTTTATTCTCCCTTAAAGAAT
CTTTTTGCTCTATCTTTAAAATTCGAAGGTTGTTCATTAATTTCTTCACCATTTAATTGGGCAAATTCTTTCATTAGTTCTTTTTGTCTA
TCTGTTAATTTAGTAGGCGTTACTACTTTAATATCAACATATAAATCTCCGTATCCATAGCCATGAACATTTTTTATACCCTTTTCTTT
TAAGCGGAATTGCTTACCTGTTTGTGTACCAGCAGGGATTGTTAACATAACTTCATTATTTAATGTTGGTATTTTTATTTCATCGCCT
AAAGCTGCTTGTGGGAAGCTAACATTTAATTTGTAATAAATATCATCACCATCACGTTTAAATGTTTCAGATGGTTTAACTCTAAAT
ACTACGTATAAATCACCAGCAGGTCCTCCATTCACGCCTGGAGAGCCTTCACCAGCTAATCTAATTTGTTGTTCATTGTCGACACCTT
CAGGTACTTTCACTTCTAATTTAACTGTTTTATTTTCAGTACCTTTTCCGTGACATGTTGGACAAGCTTCTTCAAATTCTTGACCACTT
CCATTACATTTAGGACAAACTTGTTCAGTACGAACTCTACCTAAAATTGTGTTTTGTTCTACAGCTACATGACCAGCGCCATTACAGT
AACTACAAGTCTTTTTACTTGTTCCAGGCTTTGCACCATCACCATGACATGTTTCGCATGTTACATCTTTACGGATTGAAATTTCTTTT
GTTGTACCAAATACCGCTTCTTCAAATGTTAATGTCATTGTATACTGAAGATCATCACCTTTTTGCGGTGCATTTGGATCTCTTTGTCT
GCCGCCACCGAAGAAAGAGCTAAAGATATCTTCAAAACCGCCGCCACCGAAGCCACTAAAACCGCCAAAGTCAGAGCCATTGAAT
CCTTGTCCACCAAAACCTTGTGGACCATCATGTCCAAATTGATCATAGCTTGCGCGTTTATTATCATCACTTAAAACTTCATAGGCTT
CAGAAATTTCTTTAAACTTTTCATCTGCACCTTCTTCTTTGTTAATATCTGGATGATATTTTTTCGAAAGCTTTCGATACGCTTTTTTG
ATTTCATCTTTTGAAGCATCTTTACTAATGCCTAAAACTTCATAATAGTCTCTTTTGGCCACAGCTATCTCTCCTTTTCTTAATTAACT
CATATAGTTTAACGTAATATGTCATACTATCCAAATAAAAAGCCAAAGCCAATGTTCTATTGACTTTGACTTTTCAGATCATGACAA
CATTCTAATTGTATTGTTTAATTATTTTTTGTCGTCGTCTTTTACTTCTTTAAATTCAGCATCTTCTACAGTACTATCGTTGTTTTGACC
AGCATTTGCACCTTGTGCTTGTTGTTGCTGTTGAGCCGCTTGCTCATATACTTTTGCTGATAATTCTTGAATCACTTTTTCAAGTTCTT
CTTTTTTAGATTTAATATCTTCTATATCTTGACCTTCTAAAGCAGTTTTAAGAGCGTCTTTTTTCTCTTCAGCAGATTTTTTATCTTCTT
CACCAATATTTTCGCCTAAATCAGTTAAAGTTTTTTCAACTTGGAATACTAGACTGTCAGCTTCGTTTCTTAAGTCTACTTCTTCACG
ACGTTTTTTATCTGCTTCAGCGTTAACTTCAGCATCTTTTACCATACGGTCGATTTCTTCGTCTGATAATGAAGAACTTGATTGAATTG
TAATTCTTTGTTCTTTATTTGTACCTAAGTCTTTTGCAGTTACATTTACAATACCGTTTTTATCGATATCAAACGTTACTTCAATTTGA
GGTTTACCACGTTCAGCTGGTGGAATATCAGTCAATTGGAATCTACCAAGTGTTTTATTATCCGCAGCCATTGGACGTTCACCTTGTA
ATACGTGTACATCTACTGATGGTTGATTATCTACTGCTGTTGAATAGATTTGAGATTTAGATGTAGGAATCGTAGTGTTACGTTCAAT
TAACGTATTCATACGTCCACCTAAAATTTCAATACCTAAAGATAGTGGTGTTACGTCTAATAATACTACGTCTTTAACGTCACCTGTG

ATAACGCCACCTTGGATTGCAGCTCCCATTGCCACTACTTCGTCCGGGTTTACACCTTTGTTAGGTTCTTTACCGATTTCTTTTTTGAC
AGCTTCTTGTACTGCTGGAATACGAGTTGATCCACCAACTAAGATAACTTCATCGATATCTGAGTTTGTTAAGCCAGCGTCTTTCATT
GCTTGGCGTGTAGGTTCCATTGTTCTTCTAATTAATGAATCTGATAATTCTTCAAATTTAGAACGAGTTAAGTTTACTTCTAAGTGTA
ATGGACCGTTTTCACCAGCTGAGATAAATGGTAATGAGATTTGAGTTTGTGATACACCTGATAAGTCTTTTTTAGCTTTTTCAGCAGC
ATCTTTCAAACGTTGTAATGCCATTTTATCTTGAGATAAGTCTACGCCATTTTCTTTTTTGAATTCTGCAACTAGGTAGTCAATAATTA
CTTGGTCAAAATCATCACCGCCAAGTTTGTTGTCACCGGCTGTTGATAGTACTTCGAATACACCGTCACCTAATTCTAGGATAGATA
CGTCAAATGTACCGCCACCTAAGTCAAAAACAAGAACTTTTTCATCTTTATCAGTTTTATCTAAACCATATGCTAATGCTGCAGCTGT
TGGTTCATTAATGATACGCTCAACTTCTAAACCAGCAATTTTACCAGCATCTTTAGTTGCTTGACGTTCAGCATCGTTAAAGTATGCA
GGTACTGTAATTACAGCTTTGTCAACTTTCTCACCTAAATAGCTTTCAGCTGTATTTTTTAAGTTTTGTAAAATCATAGCTGAGATTTC
TTGTGGTGTGTATGATTTACCTTCAATATCTACTTTATAATCAGTACCCATATGACGTTTAATAGATTGAACAGTGTTTGGGTTTGTA
ATAGCTTGACGTTTAGCTACTTCACCAACTTGAGTTTCTCCATTTTTGAAAGCTACAACAGATGGTGTTGTACGTGAACCTTCAGGGT
TTTGAATTACTTTTGGCTCATCGCCTTCTAATACTGTTACACATGAATTTGTTGTACCTAAGTCTATACCAATAATTTTACTCATAATA
AAATTCCTCCATTTAATCATTAAATTAATTTAATTTTAAACAATGTCTTTTCGCCAAATTTAAGTTATTGGTTTACTTTGACCATTGAT
GGTCTTAATACTCTATCTTTAAGCTTGTATCCTTTTTGTAGTTCTTGAGTGATTTCGCCAGATTCAAAATCAGGGTTATCATCTTGAAC
TACAGCTTGGTGAATATTTGGATCAAATGCTTCACCTTCAGTTTTAATAACTTCAAGACCATTATCTTTTAGTGCGTTAATCAAACTT
TCATGCATCATTTGTACACCTTTTTGAAGAGATTTAAAAGTCTCATCATCACCTTCAATTTGAAGTGCACGTTCTATATTGTCTATTG
CTGGTAAAATATCTGTTAACACACGTTGTGCTTGATATGTTTTATTTATTTCATTTTCTTTTTGAATTCTACGCTTATAATTTTCAAAC
TCAGCGTAGAGCCTTAAATATTTCTCTTCGTTTTCATCTGCTAATTGTTGAAGTTCATTAATTTTTTGATCTTTTGGATCTATTTCTTCA
ATAACATTCTCGTCAGACGTTTCTTCTATTGCTTCATCTTGTAAATGACCTTTACTTTCTTCAGCTTGTTCAACTGAATCATCAATATT
TTGTTTGACGTTTGTTTCTTCAACTGTTGATTCAGTGTTTTTTTCAACTGATTCGTCTTTATTTGTCATTTTCTGTCCTCCAATACTTTCT
AATCCATCATTACCAAATTCTATTTAATAATTGAATGACATTTTGATAATGCATAGCTGTAGGTCCAATCACAGCGATTTGACCTTTT
AACGTTTCATCAAAATGATATTGACTTGTTACAATTGAAATATCACTTAAGCTGTCATCAATTTCATTACCAATTTTTACATTAATAT
TTGGTGAAGATATATCTTGTAATAATTCTGCAATTCTATTTGATTCTATATATTGTAGAATGGGCTGAATTGAAGATACATTACTTTC
ATTCAATGCATCAATAAGTTTAACCTTTCCACCCATATAAATGCTATTACTTTGATTAGAAATATGATTATTCATCGTATTTAACAAT
TTATTGATAAATATTTCTTCCTGCTCTGATTGAACAAAAGAGACAATATCATCTTGTAAATTCTGATTAAACTCAGTTAGTTTGTTTG
TAACAAAATTTGATATTGTATTTAGTTTGTCATTATTAAACGGTATGTCTGAAGCAAGATGTACATGCTCAACATGACCTGATGAAA
ATACGATAACCATTATAACTAAATTAGGATTAGCACGAATCAAGTGTACATTATTGATAATATCTTGTTTATGATTAGGATGAACAA
CTAAAGTTGTATATTGAGATATATTTGATAATTCATCTGCAAAATATGTCAATGCTGATGATACATCATATTGATTCTCAACTAACAA
TTGATTTAATCGTCTTAATTTATTTGTTTTTTGATGAGATGTTTGTTCAAGTAAACGATTGACATAATACCTAAAACCTAATTGTGAT
GGCGAACGCCCTGAAGAACTATGTGTCTTTTCGATATAGTTTAAATCTTCAAGCTGTTTCATCTCATTTCTAATTGTAGCAGGACTAA
CATTCAAGTTATGTCGCTCAATTAGTGTTTTAGAACCAACGGGTTGTCCAAAATCAACATAATCCTCAACAATTGCGTTTAATATACT
CAATTGCCTATCTGTAATCATGTTTTCACCTCATTAGCACTCACTTATCTCAAGTGCTAATTATAATTTATCAAATTGGTCAAAGTAA
GTCAATGTTAAAGACTCGAAATTTCAATTTTTTTTAATCATTTATTAGGAAAGCTTCAAAAACCTCATTACCTATGACTTTCCCTCGC
TTTGTTAGTGCAATCGCATCGTTCTTTTCTACAATTAATTCCTTCTCTTTTAAATTATTTATTGTTTGACCAAAGACACTTTCAATAGA
TTGGTCAAACTTCTTTTTGAACCTACTACTACTCACACCTTCATTTAAACGCAAACCAAGAAACATTTCTTCTTCCATTCTCTCAGTC
AAAGAAGGTTTATTTGATACTAAAATTGCTTTACTTTCTTTATTTATAGCTTTGATATAATGATTCACTGGATTGATATTCGTATAAC
GCACACCATCTACATAACCACTTGCACCTGCTCCAAATCCATAATATTCCTCATTAAACCAGTAAACCTTATTATGTTCTGATTCATG
GCCATCTAATGCAAAATTAGATATTTCGTATTGATGGAAAGGAGATTGTTCTATCTTAGACATCAGCAACTGATACATGTCAGCACC
TAAATCCTCATTAGGAAGTTTAAGCAACCCTTTTCTATACATATTATAAAATTGGGTTTTAGGTTCAAGTATTAAGCCGTAACTCGAA
ATATGTTGAATATCCATATCTAAAGCTAGATCTAAACTTTGTTCAAAATCTTCAATCGTCTGTTTCGGTAAATGATACATTAAATCTA
AACTGATTGATTTAATACCTGCGTTTTTAGCATTTAACACCGAAGTGTAAATATCTTCAGTATTGTGCGTTCTACCTAAAACAGACAA
TAACTCCGGCTTGAATGTTTGAACGCCCATTGAAATTCTGTTTACTCCATATTTTTCTAATAGTTGGACTTTCTCTTTAGTTAACTCAT
CAGGATTTGCTTCAAATGTATACTCGCCTGTGATTGTAAACGTATCACGTATTGCTTTAAGTAATCTTTCCAACTGATTAATAGAAAG
GGCCGTTGGTGTGCCGCCACCTACATACATGGTCTTTAAGTTCCTATATTTTGCTGTAGACATTTCTGTTATTAGTGCATCTAAGTAC
TCATCTACAGGTTGATTCTGTATAAAATATTTATTGAAATCACAATATGTACATATTCTTACACAAAATGGAATATGTATATATGCAC
TTTGTACCGTCATTTTAATGCCCCGCTTCTCAGACTGTTTATCCAATCGGCTATATCCTGTTGGACTATCTGCAATTATTCTTACTTTT
AACTTTAACGTTTAATCAATGGTTGTCTAATATATCGATTCATTTTGATATTTATTCTTGTTTAGAGGGAGTGAGATAGAAATAATAA
AGAACCACTAATGATTTATTATGTAGTGGCTCTTACACATTAGCCAGATGTAATATGTCCATAAAAATAAAATTACATGAGTAAAAC
TCATGCATAAGGGAGTGGGACAGAAATGATATTTTCGCAAAATTTATTTCGTTGTCCCACCCCAACTTGCATTGCCTGTAGAATTTCT
TTTCGAAATTCTCTATGTTGGGGCCCCACCCCAACTTGCACATTATTGTAAGCTGACTTTTCGTCAGCTTCTATGTTGGGGCCCACAC
CCCAACCTGCATTGCCTGTAGAATTTCTTTTCGAAATTCTCTGTGTTGGGGCCCCATTGTTATAAGCTCATTTTAACTCTGTCGATGA
CTGCCAATATAACATTGTAAAGCCCAGGACGTTAATTTATCCTAGGCTCCCTCTTTTTAGTTATTAAACTTTATTCATTGTAAATTGTT
AATAAATTTTTTAAAAACTTATTCGTCATCCATTTTCAATACAGCCAAGAAAGCATCCTGTGGAATTTCAACATTACCAACTGCTTTC
ATCTTAGCTTTACCTGCTTTTTGTTTTTCAAGTAATTTACGTTTACGGCTTATGTCACCGCCATAACATTTAGCTAAAACGTTTTTACC
CATTGATTTAATATTTGTACGCGCTACAATTTTTTGTCCTATTGCAGCCTGTACAGGTACTTCAAATTGCTGTCTTGGAATTAACGTTT
TAAGTTTTTCAACTAATGCTTTACCACGTTCATATGCAAAATCTCTATGAACTATGAAGCTTAGCGCATCCACTTTATCACCGTTTAA
TAAAATATCCATCTTAACTAAATTACTTTCTTTATTTTCGATGAATTCATAATCAAATGATGCATATCCTTTAGTATTAGATTTAAGTT
GATCGAAGAAATCAAATACAACTTCAGCTAAAGGTAATTCATAAACAATATTTACACGAATATCATCTAAATAGTCCATATTTATAA
ATTGTCCACGTTTACGTTGACATAATTCCATTACTGCACCTACATAGTCATTTGGAACCATCATAGTTGCACGAACATATGGCTCAA
ATATTTTATCAATTTTATCACGATCTGGCATTTGTGCTGGGTTATCAACCGTCACTTCTGAACCGTCCCTTAAAATACATTGATAAAT
TACAGATGGTGCTGTTGCAATTAATTCAATGCCAAATTCTCTTTCAATTCTTTCTTGAATTATTTCCATGTGTAACATACCTAAGAAA
CCAGTTCTATAACCAAAACCTAATGCTTGTGACGATTCAGGCTCAAATTCTAATGATGCATCATTCAATTGTAATTTTTCTAATGCTT
CTCTTAAATCATTATAATTTTTGTTATCTATTGGGAACAGTCCGCAATATACCATTGGATTCATTTTCTTATAACCTTGCAATGGTTCT
GATGCAGGTCTACTAGCTAATGTGATGGTGTCACCAACCCTAGAATCATCAACATTTTTAATACTTGCAATAATATAACCAACATCA
CCAACTGTTAATTCATCAACTGGAAGCTGCTTAGGTGTATTAATTCCAACTTCTGTTACTTCGAACTCTTTACCAGTCGCCATCATTC
GAATTTTATCTCCGGCTTTAACAACACCGTCTACAATTCTTATCGATGAAATTACCCCTCTATATGGATCATACTCAGAATCAAATAT
TAACGCTTTTAGTGGTGCTTCTGGGTCGCCATCTGGAGCTGGCACAACTTCAACTATTTTCTCTAGTATCTCTTCAATTCCAATGTTA
GATTTAGCACTTGCTAAAACAACATCGTCTTGGTCTAAACCTATCATATCTTCAATTTCTTGTTTCACGCGTTCAGGTTCTGCAGCAG
GTAAATCAATTTTGTTAATAACAGGCAATAACTCTAACTCATTATCTAATGCTAAATAAACATTTGCTAATGTTTGTGCTTCGATACC
TTGAGCCGCATCTACTACTAAAATCGCGCCCTCACAAGCTGCCAAAGAACGTGACACTTCATATGTAAAATCGACGTGTCCAGGCGT
ATCGATTAAATGGAATGTATAAGTATTTCCATCTTTAGCTTCGTACTTTAAACGAACTGCGTTTAATTTGATTGTAATACCACGTTCT

CTTTCTAAATCCATTGAATCTAGTAACTGATCTTGCATATCTCTTGTTTCAACTGATTTGGTATTTTCTAAAATTCTATCAGCCAATGT
AGATTTTCCGTGGTCAATATGTGCTATAATCGAGAAATTCCTTATATTCTCTCTTCTTTTTAAGCGTTGCTCATTATCCATTTTATCCT
TCTCACTTTCATAAAATAAACCCGTATTATCTTTCGCATCTTTGATATAATAACGTTTTTAATAGGTAAATGCAAACGCCATAATCTT
GGATGAACTTTGAAATTATAACAATCTTTATTTAAATACATACTTGTTTAAATACATACTTGCGATATGTTAATATGTTAATTACGTA
CTTATAATTATAAAACGTATTGATTGTGTTTCCAACACAATTTATTAAATCTGCAAAATTTTAAGCTAACCCCATCAAATAAATGATT
GCACAACGGTTAGACTTTTGTTAAAATATTTCTTGTTGTAATCAAATAAAATTTTGATAAGATGAACTCACTTTTAGGAGGTGACAG
AAATGGCAAATATCAAATCTGCAATTAAACGTGTAAAAACAACTGAAAAAGCTGAAGCACGCAACATTTCACAAAAGAGTGCAAT
GCGTACAGCAGTTAAAAACGCTAAAACAGCTGTTTCAAATAACGCTGATAATAAAAATGAATTAGTAAGCTTAGCAGTTAAGTTAG
TAGACAAAGCTGCTCAAAGTAATTTAATACATTCAAACAAAGCTGACCGTATTAAATCACAATTAATGACTGCAAATAAATAATCTT
TTTAAATAAAAGTTCAAGCGCATGCTTGAACTTTTATTTTTTATAAAGATAGAATGAATAATTCCAGTATTAACTGTTTATCCATATA
TGATGATTTAAGTTTATAATCAGTTTCCGCACAAGCATCTATAATATTCAATAATTCATCAAGTTGATAATGTCTTACTTGTCCTAAC
GCTAATTTTACTCTGTATGGATGTACGCCTATTGTTTTAGCAATTTGCTGTCCACTATATCCTTTTTGACTCAAAATCTTACATTGATA
AAATAATCGGTAATTACTTGTGATTAGTGCAAGTAATTTAATAGGTTCTTCTTTCATAGTTATTAAATCTTTTACTAAATGAATTGCT
TGTTCTTTCTTTCTTTTCTGAATGTATTCAGTCAGTAAAAATACATTTTGCTCTAAACTTCTATTAATAATTTGGTTAACATCCTGCTT
ATTAATTGTTAGTCTATCGCCTAAAAATAAAATCAACTTTTCTATCTCTTGTGAGACAATATTAAAGTTAATACCTGTCAACTCAATA
AATAAATCTAATGCATCTCTTTTGATATCTTTGAAATTCTCATTTAATTTACTTTGAATCCATTTTTTTATTTCTTCTTCAGACATTTGC
TCTATTTTTTTAAGCCTTGCATGCTTTTTTAGAGTTTTAGTTAACTTTTTTCTTTCATCAAGTTTATTTTGATATATCTCAAAGACAATC
AAATTTTCGCCATCATATTTTTCAATAAATTCTATTAATTGATTTACATTATGAGCCATATCTTTTGGCGCTTTTTCACCTGTAAATAT
ATATGCATTTTTAACCAAAATTGCTTTTTTATCTGAAAAGAAAGGCAATGTTAATGTTTCTTCAACAATTGGTGCAATCTCTGTTTCG
TATAAATTATATTTCACAAAGTTAAAGTCATCTCTATCACTTTTCAAAAATTGTGATATAATTTCTGCACTTTGTTTTTCAACCAATTC
AGGCACATCTCCATAAATAGCTACAATATTGTCGCTCATTCATTACACCCTTTCATTCGTTTGACACATCAATAGATTATATCATGTT
TCTGTATCTATAAACCACTTGCATTTCCATAAGAGTTTGAATCAACTTTTAAATTATCATCTAAGTCAATTGTAACTTGACCGTTTTG
TTGACTATTGTAAATGCGACTGCGAATCCTTTGCAATCGTTTAACAACTTCTATATTAGGAAGATGATACATATTGTTCTTCCCAGAA
GAAATCAAACTTATTTTAGGCTTAATCATCTCTATAAATTCTTTAGAACTACTTGTCTTGCTCCCATGATGACCTACTTTTAAAATAT
CAATCTCCGGCAAGTTATATTTTTTTAGTAGTAAAGATTCATTATTTTTACTAGCATCGCCCATTAATAAAACTTTTTTATTTTGATAT
GTAATCATAGTAATAATCGAATACTCATTTTTATCTCGGCTATTTGGAATAAAACTATCAAAAAATAGAAAACTACTATCTCCAAGT
TTAAAACTACTAACTTGTCTTACATCTATAAGTTTAATGTTGTACTTATGGCTTAATTTCGATAATAACATCAATGTATTACTACTAT
ATCCCTTATTGTATATCACTATATGTTTAATTTTAATATGACTAATAATATATTCCACTTCACCAATATGGTCATTGTGTGGATGTGTT
AAAATTAGATACTCTAATTCATTTATCCCTCTTTCATTTAGCGTTGGTAAAATATGATATTTAGAAATTGAATAACTAGGTTGTTTAG
TATCATCAATCACTTTCCCACCTGTATCAATCAAGACATTTTGGTTCTTACCGCCTTCATATAAAATACTGTCTCCCTGCCCCACATTT
AACATTGTAATTTTGTGATGTGAATTTGTTGGAAACGTTATTAATAATGTCAGAATAATTATAGTCCAAAACGTAACCAATATATAT
TTACGTTTAGCCAATAACCAAAATATGTAATAAACAGAAATTATAAATATTATAAATATCCAATCATTAAAATTGGGAACAGAAAA
ATGTGATTGCTTAATTCTTGTGAATAGGTCTAGTAACCAGTCATGAAAATTAAAACTTAGGTCAACCAAGTAATTTAGCGGCGTTAA
TCCCACAATAAAATGACTTGTAATAAAGAATAAAATAGATAGCGGAAACAATATAATCGAATAGTACGGTACAAAAATCAAATTA
GATAAAAATCCCACCCATTGAAGTTGATGAAAGTTTGGAATGGCAACGATAAATGAAGCTAATTGTGCAATAAACGTAATTATGAA
TAATGATTGTAACTTTGACAATTGCTGTAAAAAAGGAAAAAGTAGCATAATAAAAAATGAAATGATGAATGAAAATTGAAATCCAA
TATCATAAACAACTAGTGGATTTAAAATAAACATAATTATAAATGCAAATGCTAATAGCTGAATACCCTTTATTTTAATTTGCTTAG
TAATAAGCAGTACAAGAGTTGTCATTATTATAGCTCTTACAGCACTAGGTGCATAATTTGTGTATTGAGCAAATAAAGCTAATACAA
TGATTGTAATTCCTTTAATGACAAATAAAGGTAAATTTAATCGTTTTAAAGGTTTGTAAATTAAGAATACAATTGCAGCTATATGCG
AGCCACTAACTGCCAGCAAATGATATATACCTATCTCTTTAACACGTTCCTTAAATTGCTCATTAATTTCTTTTACGTCACCAGTAAT
CAATGCCATAATACGATCCGGAAACTTAATACCCGAATCATAAATTCGATTCATTATAAACTGTTTATGTTTCTCAATTAAATTAGA
CCGGTTCGATTCTAGGCAGCTTTGTACAACTATACTTTGTAATTTAAGAGTTACAAACTTATTGTCATTAACTTTGAATTGTCCTTTA
ACAATACAATTACGACTTTCAATTTTCTTTAAATCAAAATTCTTTTTATTTGTTAAAAAGAAAGGATATATTTCATTTTTTAAACTCA
ACCTACCTTTATATGTGTCACTACCTTGACGTTGAATTTGGATTACTTGAGCACGCTCATTAAACTGAGAATTACGTTCAATATAATT
GATATAATTAAATATTGCTTGTTGTGAATAATGTAAATACCATGAGGAGAAAATGATTAAAAAGAGAGAAATAGGGGCATAAACG
ATTTTATTTTTACGATAAGTAATATACAAAAGTAAAATGAAAAGAAATGTAGAGAGCACTTTGCTAGAATTCCAAAGCACTCCTAC
AATCATTGATAACGCGACATACAGCAAAACATTAACCTTTAGTCAGATATTTAGCAACATATTCTGGTGAAAATGGAATTTTTTTAA
ATTCAATACCAGATTGTTTGAGTAATTTAGTTGCATATTCATGGTTATGATAATCTTCTGCATAGTAGATACGCTTTATACCTGCTTG
AATAATTGACTTTGTACAATTTAGGCATGGAAAATGAGTAACATAGATTGTTGCACCTTCAGTAGATACACCTTGTTTTGCACATTG
TAATAAAGCATTCATTTCTGCATGTATCGTTCTGATACAATGTCCATCTTCAATTAAACATCCTTCATCTATACAATGCACCTCGCCA
GCTACAGAGCCATTATACCCACCAGCAATAATACGATTATCCTTAACAATCGTTGCACCTACAGATAATCTTTGACAAGTTGAACGT
AATGCTAGCAAATGACTTTGTGCCATAAAATATTCTTCCCATTTGATTCTTTCCAAGATGTTCACCTTCCATACTTAAAATTTAGTAA
CATTTTCTAATAATATAAGATTAATCACAAAAAATAAAATTTGCAATTAAAATAATCCATTATGTCGTGAAATAAGATTTCAGTTTA
TCAAAAGTTTTACTTCCAAAACCTTTTACTTTTTTCAAATCGTCAATTTCTTGAAATGCACCTTGTTGGTTGCGATATTCAACAATTGC
ATTAGCTTTAGCTTGCCCTACTCCAGGAACAGACATCAATTCTGATACAGATGCCGTATTTAAATTTACTTTAGTATTATTTGTGTTC
CCATTTTTTACGTGCACACTGTTTACTTCAATTTGTGGTTCAACATTCTTTTGTCCTTTATGAGGTATGAAAATCATTTTTTGATCTGT
TAATTTTTCAGACAAATTAATTCGACTTACATCTGCATCATCCAATAATTGTGCTTTATCAAGTAAATCAACTACTCTATCCTTAGAT
GTCATTTTATAAACATTAGGATGTTTAACAGCACCTTTTACATCGACATATACAGGACCTTTATTTTTGGAATTATCTCCATCTTTGA
CCTGGACATCTTCTAATTTGGACAAACTATTATTTTCACTAGTGCTTTGTTTCAGAGCAGTATCTTTATTTTCAAAATTTCTTGAAGTA
TAATCATCTTGTCTCCAGAATATAAAACCAATTAATACCATAATTAAAACAACAGCACTTATAATATATAACTTCCACTGAGTTAAA
AAATCTTTATAGCGTAATAAAAATTGATACAATAAAACCACTCCTCAACTTTATACACGTCTGAGCGAGTGGTTTTACTTTTATTTTT
TCGCAATGAAAAACAATCTTTCTGCATCTTCATTATGTTCATCTATATTAAAATCAGTAAATGTTTCAACATGTTTAAAACCAACTTG
AGCTAACCAAGACAAATATGTCTTTTCATCAAATGTTCTCTGAAAATGAGACTCATCAAATCTTGAATATGTTTCATCTTCATGTCGA
ATGAAAAATGTCATATCATGATAAACACTTAAAGGTAAATCTCCTTGCACAGCATCCCATGCTAAAAAAATGTCCCCTTTATCATCA
ATATAACTTTGATTATTAAACAAAGTCATCATTTTATAAACAGTATGTACATCAAAAATAAATACACCTGAATCAGTCAGATGATGA
TAAACATTGATGAATGTTTCAATCACTGCTGTTTCATCTTGCAAATAATTTAGAGAGTCACAAAAAATAGTGATGATATCAAATTGT
TGTTGCAAATCAAAAGATGTCATATCTCCTTCAAGCCAATTTACATTTGCTGATTTTTGAGCTGCAACAGTCAACATATCAACACTTA
AATCCATACCAGTAACATTACCTAAAGCTTCTAATTGAACTGTTAAACTACCAGTACCGCATCCAATATCTAAAATATTTGATTCAT
CTTTGCAGTGATTTTTTACAATTTCAAACCATTTTTCATATGGTTGATCTTGAGTCAATTGATCGTACACTAGGCTCATTTCTGCATAT
TGCGACATAATTAATACGCAACCTGACCATATGATTCTAATGGTGCATCTTGATATAACTTTTCAATATTATAATAATTTCTTTCGTC
TTTATGGAAAACATGTACCACAACATCAGCTAAGTCAATTAATATCCAACGCGCTTCATTGTATCCTTCCATACGTTTTACTTCTATA

TTTTGTTCATTGGCTACTTCTTTCACCGCTCTAGCAATCGCTTGAACTTGTCGTTCATTATTTCCGTGCGTTACAACAAAATAATCTGT
CATATCGCTGATACCTTTCATTTCTAAAGAAATCGTATCTTCGCCTTTTTTATTGTCAATTGCATCCACAGCAATTGCTAATAATTCTT
GTGAATTCATTTAATCATCCTTTATTCTTTCGTCACTATAGTTATAATAATTTAAACAGTCAATCGTCTTATTATATACCGTAATATCT
TTCTGTATTAAAAATAGTACTGTGCGTTTAGAAATTTCATAAATTGTCTTATCTAAACTACCTTGATTGTATGCCATATCTCGAATAT
CATCAACTCCTGGGATTGTTCTTCCAGGTTCGATGTAATCTGCAATAAAAATCAGTTTTTCAGTTTTTGTCATTTGTTGACGTCCAGT
AGTATGGTATTTGATAGCCATTAATACTTCCTCATCATTGATACCATATTCATGTTCCATGATTGCTGCACACACAGGGCCATGCAAT
ATTTCACTACCATAACTCAGTAGATCATTACCTAATTCGTATTGTCGAACAATTTGATACATTTTACCTAAATCATCATATTTACAGA
AATCATGTAATACACCTGCTAATTCTACTTTACTAGTGTCTCCATCATAAATTTCTGCCAATTTAATAGCTGTTTCTGCAACTCTTAA
AGAATGATTATAACGTTTCTCTGGCAGTTTCTCTTTTGCAAGCCGTTTTGCTTTTTCAATGTTCATATAATCCTTCCCCCTTAATATAG
TTTTCAACGGATTTAGGAACAAGAACTTGGATAGATTTCCCTTCACTAACTCTTTGTCGAATCATTGTCGAACTTATATCTACCCTAG
GTATCTGAATTGCAATCATAGCATTTTCAACATTTTGACTATTTTTGTCTCGATTTACAACTACAAAAGTAACCATTTCTTTTAAGTAT
TCAATTTGATACCATTTCTCTAGTTGGTTATACTGATCCGTCCCAATAACAAAGTACAACTCACTGTCTTTGTGTTGCTCCTTGAATG
CCTTGATCGTGTCATAGGTATAACTCTGACCACCACGTTTAATTTCATCGTCGCAAATATCTCCAAAACCAAGCTCGTCGATAATCAT
CTGTATCATTGTTAATCTGTGCTGAACATCTATAAAATTATTGTGCTTTTTCAATGGAGACATAAAACTAGGTAAAAAATAAAATTC
ATCTGGCTGTAATTCATGAAATACTTCGCTAGCTACTATCATATGTGCAGTATGGATAGGGTTAAACTGACCGCCGTAAAGTACTAT
CTTTTTCATTATTATGGCAATTCAATTTCTTTATTTTCTTTAGATTCTCTATAAATCACTATCATAGATCCAATCACTTGCACTAATTC
ACTACGCGTAGCTTCGCTTAATGTTTCAGCTAATTCTTTTTTATCATCAAAGTTATTTTGTAGTACATGTACTTTAATCAATTCTCTGT
TTTCTAACGTATCATCTATTTGTTTAATCATATTTTCGTTGATACCGCCTTTTCCAATTTGAAAAATCGGATCAATATTGTGTGCTAAA
CTTCTTAAGTATCTTTTTTGTTTGCCAGTAAGCATATGTTATTCTCCTTTTAATTGTTGTAAAACTGCTGTTTTCATAGAATTAATATC
AGCATCTTTATTAGTCCAAATTTTAAAGCTTTCCGCACCTTGGTAAACAAACATATCTAAGCCATTATAAATATGGTTTCCCTTGCGC
TCTGCTTCCTCTAAAATAGGTGTTTTATACGGTATATAAACAATATCACTCATTAAAGTATTGGGAGAAAGATGCTTTAAATTAATA
ATACTTTCGTTATTTCCAGCCATACCCGCTGGTGTTGTATTAATAACGATATCGAATTCAGCTAAATACTTTTCAGCATCTGCTAATG
AAATTTGGTTTATATTTAAATTCCAAGATTCAAAACGAGCCATCGTTCTATTCGCAACAGTTAATTTGGGCTTTACAAATTTTGCTAA
TTCATAAGCAATACCTTTACTTGCACCACCTGCGCCCAAAATTAAAATGTATGCATTTTCTAAATCTGGATAAACGCTGTGCAATCCT
TTAACATAACCAATACCATCTGTATTATACCCTATCCACTTGTCATCTTTTATCAAAACAGTGTTAACTGCACCTGCATTAATCGCTT
GTTCATCAACATGATCTAAATACGGTATGATACGTTCTTTATGAGGAATTGTGATATTAAAGCCATCTAATTCTTTTTTCGAAATAAT
TTCTTTAATTAAATGAAAATCTTCAATTGGAATATTTAAAGCTTCATAAGTATCATCTAATCCTAAAGAATTAAAATTTGCTCTATGC
ATAACGGGCGACAAGGAATGTGAAATAGGATTTCCGATAACTGCAAATTTCATTTTTTAAATCACCTTATAAAATAGAATTTCTTAA
TACAACATCAACATTTTTAGGAACACGAACGATTACTTTAGCTCCTGGTCCTATAGTTATAAAGCCTAGACCAGAGATCATAACATC
GCGTTTCTCTTTGCCTGTTTCAAGTCTAACAGCCTTTACCTCATTAAGATCAAAATTTTGTGGATTTCCAGGTGGCGTTAATAAATCG
CCAAGTTGATTACGCCATAAATCATTGGCCTTCTCCGTTTTAGTACGATGTATATTCAAGTCATTAGAAAAGAAACAAACTAACGGA
CGTTTACCACCTGATACATAATCTATGCGCGCTAGACCGCCGAAGAATAATGTTTGCGCCTCATTTAATTGATATACGCGTTGTTTTA
TTTCTTTCTTAGGCATAATAATTTTCAATTCTTTTTCACTAACTAAATGCGTCATTTGGTGATCTTGAATAATACCTGGTGTATCATAC
ATAAATGATGTTTCATCTAAAGGAATATCTATCATATCTAAAGTTGTTCCAGGGAATCTTGAAGTTGTTACTACATCTTTTTCACCAA
CACTAGCTTCAATCAGTTTATTAATCAATGTAGATTTCCCAACATTCGTTGTCCCTACAATATACACATCTTCATTTTCTCGAATATTC
GCAATTGATGATAATAAGTCGTCTATGCCCCAGCCTTTTTCAGCTGAAATTAATACGACATCGTCAGCTTCCAAACCATATTTTCTTG
CTGTTCGTTTTAACCATTCTTTAACTCGACGTTTATTAATTTGTTTCGGCAATAAATCCAATTTATTTGCTGCTAAAATGATTTTTTTG
TTTCCGACAATACGTTTAACTGCATTAATAAATGATCCTTCAAAGTCAAATACATCCACGACATTGACGACAATACCCTTTTTATCCG
CAAGTCCTGATAATAATTTTAAAAAGTCTTCACTTTCTAATCCTACATCTTGAACTTCGTTATAATTTTTCAAGCGGAAACAACGTCT
GCAAATCACGTCATCACGAAACATATTATGCTCTGGTACAAAACCAGGTTTATTTTTATCTTCAGATTGAAGTGGCGCACCACAACC
GATACATTTTAAAATGTCAGACAATCAATTTTCCTCCCATGTGATATAACCTTTTTTACTGAAATGACGTAATAATCGTCTTTCAATT
AATCTATTAAACTTAGTAATAAAGCCATCAGTTCGTTTAACTGGAACAACCATAATTGTATATAGACCTCGACGATTACCACCAAAT
ACATCAGTAAGCATTTGGTCACCTATAACAACAGTTTGATCTGGTCTGATATTCATCTTAGTTATTGCTTTATCAAACGCTTTCCCCA
TTGGCTTTCTCGCTTTAAAAATAAAATCGATGTCTAAATGCTGACTAAAACTAGCAACACGAGACTCATTATTATTAGACACGATTG
TAATAGTGATTCCTTTTTCATTAGCTTCCTTAAACCATGCTTTAACACGTTCTGTAGGTTCTTTAACATCCCAACCTACTAGCGTATTA
TCTAAATCTGTAATAATACCTTTAACGCCTTTGTCCACTAACTTGTCTAAATCAATTTGAAATATTGATTGAACATATGAATTCGGCA
TAAAAAACTTGCGAACTAAACCCATTTAACTCACCTTTACCTTTTTATAATTGAGACACTAATGCTTCAACAGTTTGACTTGATGATA
CAGCTGCTTTTTCTAAAAATGCTTCGAAGCTCATTTCCGCTTCTCCATTTGCTAAGTCTGAAACTGCACGAACTACAACAAATGGTAC
ATTAAATTGATAACATGTTTGTGCAATTGCAGTTGCTTCCATTTCAACCGCCATCGCATTTGGAAATGCTTTTTTAATTTTTTGGCGTT
GTTCAACACTACCGATAAAGCTATCACCACTTACAATTAAGCCTACTTTAGCTGTTAATTGTTGTTGTTGTACAACTTGAGATACTTT
TTCTATTAAAGGTTTACTTGATTGAAATGCTACCGGCATCTGTGGTATTTGTCCATATTCATAACCAAATGCTGTTGCATCTGCATCA
TGATATTTTACATCATCACTTATAAGAACGTCACCTACATTTAAACTTTCATCTAAAGCTCCAGCAGAACCTGTATTAATAATGACGT
CCGGTTTAAACTTATTAATTAATAATGTCGTAGAAATTGCAGCATTAACTTTTCCAATGCCACTTTGGGTAATCACTACTTCTCTATC
TTTTAAAATGCCAGTATAAAATTTAACATGTGCAACTGAAATTTCGCTTAATTGTGTTAATTTATTTTTTAATATTGTTACTTCTTCTT
CCATGGCACCAATTATACCAATCATCCTTTGATTCACCTCTTTTAAAAAATCCTAGCATTTGTTATTTTATCACATTTTAATCACTACA
ACGACAAGTATCATGATTTTCATTACTGTATAAAACTTTTGCTTTATTTTTATAAAAAGCTCTTTCAATAACTTGGTATCATAAATAA
TTATTTTTGAACTCATAACAGTGAATTCGATGCTTCTAATTTGTCTATTTTTCAAAAACTAATCATATCAATTACATCAACCAATCGT
GTCAAGAAACTTTATTAAATAACAGGAAAATGATATGTTTAAAATAATAATGATGAAATAAGAGGATTTTACAATGGCTGAAAATA
ATCAAAATAGTCTCGTAACAAAGATAGCTACATACGGCAGTTTTATTGCAATTGCGTCGTTTGTCATTTTATTTATAAGTATATTTTT
AAAGTTTTCATTTAATATCGAGTTTACAGCAACAATTATGAACATATGTCGCTACACTCTAATTCTAGGATTTATACTTATGTCCTTA
CCTGATGTTGTTGATAAAAACATCAAAAAAATTATATTCGATGCATTTATTATCATCGTCTTTATCTTCTTTTTACTATAGTAGTTTTA
TTTTGTTATTACACAGTTAATCATATTTGTTTCGTATCGAATTCCTGTGTGTATTAAAGATAATTCTAATAGAAAACAGCAGTAAGAC
TTTTCTAAGTGTTATCATCCTTACTGCTGTTTTTATTTGTAACAGTATAATGACAGGTACTTTATATTTCACTGTCAAAGATTATTAAT
GATTAACTTAATTAAATTATGATTTGAATAAATAGATATCTAAATGCAATTTGTCACTATTGATAGTTTTATTATCAGCATACATTTT
TAATACCTCATAACCATACTCACCATTTAAATTATAAAAATCATATTCTACATTTTGTTCATCATCATTATGATATTTAACATACCCTT
TAACAATGTCGCCACCTTGTTCGTACAACTTATACTTCTCATTCAAATAATTTCTTAATTGAACATCTAATTCTTGAATGGTAACAAT
TTTCTTTTTCGTTTTTACTGCTGTTAATTCTATTTCTGCTTGATGTCCGTCAATCCATAAATTACAAGGTATATTCTTATAATCATCGTA
TTTATTATTGTCACTTAAAGTAACACCACCATAACTACATTGCGTTTTATTGGTTGCTCCCCCATGACATTCATAGCTATAGCTTCCA
GCAAATATATCGATATCCTTATTTATAAATTTCTTTGAAAGTGCCTCATTTTCAAATTCAACTTTAAAATCCTTTTTCGAAGCAACAT
TTACGAACATGTCATGAAATAACAAATCATGCTTCAACAGTCTATCGTTTGTGTTTTTATTTTCAAGCTGAGTTGGATTGATGAATGA
CATCTTAGCATTACTTAGACGCTTTGAATCAAACTTACTTTTATGATGTAAGTCTGAATATTCGATTGCACTCGCTGAATTTGTAAAA

ATTCCTTCTAACAATAATATTAAAATAAATCCTGTTAAATATTTAACTTCAAACTCCCCCAAAACAATTATTAATTTACATTTGAGAG
CGAGACATAAATGATAAACAGGCTGGGACATAAATCAATGTTCTATGCTCTACGAAGTTATATTGGCAGTAGTTGACTGAACGAAA
ATGCACTTGTAACAAGCTTTTTTCAATTCTAGTCAGGGGCCCCAACACAGAGAATTTCGAAAAGAAATTCTACAGGCAATGCAAGTT
GGGGCGGGGCCCCAACATAGAAGCTGGCGGAAAGTCAGCTTACAATAATGTGCAAGTTGGGTGGGACGACGATAAAGAAATACTT
TTTCTATAGAAATTAGTATTTCTTATACATGAGTTTTACTCATGTATTCCTATTTTTAAGTACACATTAGCTGTGGCTAATGTGTAAGA
ACCACTACATAATAAATCATTAGTGGCTCTTTATCATTTCTGTCCCACTCCCCTGAGAAGTTTAAAATTTTATATGTTGGCTTGTTAT
GTTAAGGGAATTAACATGGTTGTCTTGTTTATATTATGTGATTCAAACATTACTAGTCTTGTAAATCTAATTCGTAAAATGCTAAATC
TAACCATCTATTAAATTTAAAACCTACATTGGTCAGTGTGCCGGCATGCTTAAAAGCAAACTTTTGATGCAACTGAATACTCGCTTT
GTTGGAAGCATCAATGCCTGCAACTAGCGCACGATAACCTTTAGCTTTTGCTTCCACAATTAAATGGTGTAGTAATTGACTAGCAAT
ACCTTTTCCTCTAGCTGAAGCATCGACATAAATAGAATGTTCGATTGTATATAGGTATGCTGGCCAAGGTCTAAATGAACCGAACGT
CGCAAACCCTAAGACACTTCCATTTTCCTCAAATACAAAGATAGGCTCATGCTTACGTTGTTTCGTTTCAAACCATGCGACACGTTC
GTCTATGGTTTGTGGTTCATAAGTATAAACAGCTGTAGTATTGATAATGGCATCATTGTATATCGCTAATATAGCGTTTAAATCCTCT
TTTTTAGCGTATCTAATCATATCAATTCCCCCTTAGTAATTATTAAAAGCGTTTCGTTATTTGAATGCAAATATGTGTAATGAAATCT
AACGTAAAAGTATACATGTAAATTTTATAGTATAAAATGAATTGCTATGAGTCATTTTGAAATTAATGGTATACTATATGAAATGTT
AACAGGCATTGTGAAATGTATAAAAGGAGCCTTAACGTATGAAAAAATGGACAAATCGATTAATGACAATCGCTGGTGTGGTACTT
ATCCTAGTGGCAGCATATTTGTTTGCTAAACCACATATCGATAATTATCTTCACGATAAAGATAAAGATGAAAAGATTGAACAATAT
GATAAAAATGTAAAAGAACAGGCGAGTAAAGATAAAAAGCAGCAAGCTAAACCTCAAATTCCGAAAGATAAATCGAAAGTGGCA
GGCTATATTGAAATTCCAGATGCTGATATTAAAGAACCAGTATATCCAGGACCAGCAACACCTGAACAATTAAATAGAGGTGTAAG
CTTTGCAGAAGAAAATGAATCACTAGATGATCAAAATATTTCAATTGCAGGACACACTTTCATTGACCGTCCGAACTATCAATTTAC
AAATCTTAAAGCAGCCAAAAAAGGTAGTATGGTGTACTTTAAAGTTGGTAATGAAACACGTAAGTATAAAATGACAAGTATAAGAG
ATGTTAAGCCTACAGATGTAGGAGTTCTAGATGAACAAAAAGGTAAAGATAAACAATTAACATTAATTACTTGTGATGATTACAAT
GAAAAGACAGGCGTTTGGGAAAAACGTAAAATCTTTGTAGCTACAGAAGTCAAATAATCTATTACGCTAATGGATGAATATATTGA
GTGGAAAACAGTCTTGATTGCGAGACTGTTTTTTGTTTGGTATGAGGTAGCAATGACGACGTGTCATTGGTGGAGATTGTAAAAATA
CATAATAAAAAGAAGCGGCAATGTATACCGCTCCTTTTTTATACTACATACCGATTTTCAACCATCTCTTTCTACTTAGTAATAAGAC
AATAGTATTAACTATAAATAGAAGAACGAAGAATGATACTATATTTATAATTTCAGTAGGACACATAAATGTTGACTCGTTATTCAA
TATTTTTTCTACGGCACGATACATCGTATTGCTCGCCTCAAATGGAGCAACGATACCAAATATATTTTTATTAATGGCAACTAAGAT
GACTGAACCAATCCAATATACAATGCTGATACCTAAGCTGATTAAAATGTTAGGTGAAACCATACTAATCGTTCCAACAACTAAGA
TATATTGTAAGATAACGAGTGAAAATAAGATTATTAATAGTAAGTAATGTGAGAAATCCGAATATATAATTGAAATAATAGTGATA
CTTAGAATTATGAACACTAAACATTCAAAAAATAACACTGCTACCTTTTTATAGAAGAAGGTAAAGATATTATCGCCAATCAATTTA
TAAAACAGGATATTTTTATTCGAATACTCTTTATTAATAAAATATGCAATAACAAATGAAAATAGTAAGAACCCTAATTGCGTTGCA
ACAGTATATGAACTGAAGAAAAACTGGCTATAGCTTAAACTTTTAACTTTGTCTATACCTATTGGTAAAAAATACCCAAGTAAGAAA
AGGAATGTGAATAGCACAACAAGCGTGTAAATAATTTTATTGGAAATACTTTTTTTAAATTCTAATTTCAAAGTGGACACCTCAATT
ATAAATTAATGTAATCATTTATGACTTCTTCTTTTGATTGGTACTCTTCTATTTGAAGGTCTTTAAAAATAAAGTATTTACCCGGCAA
AGCACTTAAATCGGATAAATTATGTGTAATATTGATAATAGTTTTAGTTTGATGGCTTTGAATAAAATCATTTAAAAATTCATAAATT
TCATTAACTGTTTTCTTGTCTAAAGCGTTTGTAACTTCATCTAATATGATTAAATCATGATCTTCCAATAAGAAACTTAATAATTTAA
GCTTTTGTTTTTGACCATCACTCAAGTTTTTTAGTAGTACGTTAGACGGTATTGCTTCGATGTTTAGTATGTTATAAATCTTATCGAAT
GTTTGTCGATTGTTTTCAAATTTTGATTTTAACAATGATAATAAAAAATCTTTTGTTATATCATTAGGAATATTTGATGAACTTGATA
TTAAGGTTACATTTTGAGAAATGGACTTAGGGATATTTCCACTATTATTTAGTAAGAAATCTTTAGCTAATAAAGATTTTCCTACACC
ATTTTTACCAACAATGTGATTGATCTGACCAAGATAGAAATTTAAATCACAATTGTCAACTAGTTGTTTGTTTTTAACTTTTAAAGAA
TAGTTATTTAGTTTCATGTATACAACTCCTATGTATAAAGGGATTTATTACACCGATATTTAATTGTATTTTTAAAAATTCTTTCACAT
TATGTATAGATGTTATAAATTAGTATATCACACTATATTTTGTCTAAATGATAAATATATCGTTATATTTTTACAATATTCTGAAATTT
ACGTTCGCCTCTGAATGCTATATCCAGTGTAATGTGTTTTGCATATATGAAAGCAATTTCAAAATGTGAATATAGGTTCATTGTGGTA
TGACAAACTTCATTGCTTGTCATGAGATGGATATAATGAAGACGACTAAAAGAGCAATGAGGAGGATAATCATGGTTAAGGCTATT
GCGGTAGATATGGACGGGACGTTTTTAGATTCAAAAAAGACATATGATAAACTTAGATTTGAAGCGATTTTTACTGAACTTAGAAAT
AGAGATATTACATTTATTGCTGCGAGTGGCAATCAATATGCGAAGTTGAAGTCTATTTTCGGGGATAGAGATATGTATTTTATTTCT
GAAAATGGTGCAGTTATTTATAATGGCAATGAGTTATATAATTATAAAAGCTTTAATCGTCAGGTGTTTCAACAGGTTGTCGATTAC
TTAAATATGAAGCAAAGTATTGATCAACTCGTCATCTGTGGTTTGAAAAGCGCGTATATTTTAAAACATACTTCTGAAGCGTTTAAA
GAAGATACGAGATTTTATTATCATCAGTTAAAAGAAATTGACAGTCTACAGCAATTACCTGAGGATGATTATGTCAAAATAGCATTT
AATATTAATCGTGAGACGCATCCGAATGTTGACGAAGAAGTAGCAACGCAATTCAGCAATGATATTAAACTTGTCTCAAGTGGGCA
CGATAGCATTGATATTATTATGCCAAACATGACTAAAGGGCAGGCATTAAAACGATTGTTAGATAAATGGGAAATGTCTCCTTCAG
AGCTCATGGCCTTCGGAGATGCGAATAATGATAAAGATATGTTAGCGTTCGCTAAGCATAGTTATGTGATGGAAAATAGTCATGAT
GAAGAATTGTTCAATATTGCCTCTGCTGTCGCTCCAAGTAATGATAAGCAGGGTGTATTAACGATTATCGAACAAGAAGTGTTGAAA
AAGTAGGTAGTATATCATCAATGAAGCAGCTATCATGATACTAAATACATGTTAAATGAAAACTTTGAATACGAATCATCAAATATT
AATATATCTCAAACAGGTTAGGCAATAATGCCATAGTAAAATAAGCGCACTAAAATCATTTGAATAGATGATAAAATAAAGCGGAT
TTTAATCAAATCGATTTTGATACAGATATACAAAATAAAAAGCAAAGGCACATGGCGCGCCTTTGCTTTTAAATATATATATTAATT
TAAACGTGTTTCACATGTACCAGTGTTAATGACAGATAATGCTGCGTTTAAACCACCTTCAACAAGGTTTTGTACTGCTTCATCAGA
GAAGAAAGCAATATGTGGCGTTACTAAAATTCTTTCATGTTCGATTAACTCTAATAATGTTTTATCGTCAATGTCTTTATTAGTCCAG
TCATTTGTGAAGTATGCAGCTTCATTTTCATAAGTATCAATCGCAGCACCTAACAAAGTACCATCGTTCACTGCAGCGATTAAATCA
GGTGTATTGATGACTGCACCACGTGCTGCGTTAACTAAGATTGCACCTTTTTTCACATGATCAAACATTGCTTTATCGAATAAATGAT
AGCTTTCTTTGTTCGCTGGAACATGTAAAGAAATAATATCGGCATCTTTAATAGCTTCTTTAACACTATCTTTATAAGTTAAAAAGTC
TAAATCTTTATTAGGATAAGCGTCATAAGCTGTAATTGTAGCACCAAATCCTGCATATATTTTAGCTGTAGCAGCACCGATACGACC
CGTACCGATAATTGCAACAGTCATATTTTTAACTGGTTTAGACATGATTTCTGCTTGCCAAGTAAAATCATGTGCTTGTACACGGCGT
TCAATATCTGGGAAGCGACGCACTAATTGTAGGGCGATAGAAACAGAATACTCTGCAATTGTTTCAGGTGAATAACTAGGAACGTT
AGATATCACAATATTGTGTTTTTTAGCTAAATCTAAATCATACATATCAAATCCAGCAGTACGTTGTGCAATTTGTTTAATACCGTAA
GATTCTAATTTAGGATAAACGTCATTTTCTAACTTACCAAATTGCATTGTAGTTACGCCATCGTAATCTTTTAATTGATCGACTGTAG
CACTTGATAATAGCTCTTTAGAAGTAGTTACTTCGACATTATTCTTTTTCCCCCAATTTAATGCCATCTCTTTCTCATAATCACGCGTA
CCAAAGAACATAATTTTTGTCATTATTAAAAACCTCGCTTTTAAAAGATTGAAAAGTAAATGAGTGAAATTAAAGATTATGTACATT
AAAATCACGCCACAATTTAATTGTGAAAAATATCACAAATATATTATAACACTAAATTTCTCAAAATTCAAAAGTGTGTTTTATTGC
AGAAAACCTATAACGTGTGCACAAGTTATAGCGAATTGCAAACGGATTACTTTAGTCTTTTTAAAACATGAAGTATAATTTGTATAG
TAATAAATATAAAAAATTGGAGGCTATGTTCAATGAGCAATATGAATCAAACAATTATGGATGCATTTCATTTCAGACATGCGACTAA
GCAATTCGATCCACAAAAGAAAGTTTCGAAAGAAGATTTTGAAACAATATTAGAGTCAGGTAGATTGTCTCCAAGTTCTCTTGGGTT

AGAACCTTGGAAGTTTGTCGTGATTCAAGATCAAGCGTTACGTGATGAATTAAAAGCGCACAGTTGGGGCGCAGCAAAACAATTAG
ATACAGCGAGCCATTTTGTGCTAATTTTTGCGCGTAAAAATGTAACGTCAAAATCACCGTATGTACAACATATGTTAAGAGATATTA
AAAAATATGAGGCACAAACGATTCCAGCTGTTGAACAAAAATTCGATGCATTCCAAGCAGATTTCCATATTTCTGATAATGATCAA
GCCTTGTATGACTGGTCAAGTAAACAAACGTATATCGCATTAGGCAATATGATGACGACAGCCGCATTGTTAGGTATTGATTCATGT
CCGATGGAAGGTTTTAGTCTGGATACAGTGACAGACATTTTAGCAAATAAAGGTATCTTAGATACTGAGCAATTTGGTTTATCAGTG
ATGGTTGCATTTGGCTACAGACAACAAGATCCACCGAAAAATAAAACACGCCAAGCATATGAAGATGTTATTGAATGGGTTGGACC
AAAAGAATAAATAGAATACAGTATGTCTAAATATATAAAATTAAAAAGTTAGCAATAAAAAAAGCCTGCGATTACATAAATGAATC
GCAGGCTTTTGCGTGAAAAAATTGTATTAATAAAGTATGGATGATTATTTTTCTGGAACAAGGTCAGTATTTGAATGAACTGTGATG
TCAAACCCTTCTGGTGCCGTAAATGTATGTGTTGAGGCGTCGGGTTGATAAATATCAACATGTGTTAATCCATAACTTTGTGCATTGT
TTTGTCTTGCTTGATTGGATTGCCAAGTATTAGCAGCAATATGATGGTGATAATGATTCGTTGACATAAATAGCGCACGTGGAAAAT
CAGACACATGTTGGAATCCCAATTGTTCAATGTAACATTGATATGCTGCGTCTAAATCATGTGTTTTTAAATGTAAGTGTCCAATCAT
GCCTTTTGCTGGCATTCCTTGCCAACCTTCATCAGTACGATGTGTTAATAAGGTTTGGCTATCAACTTCTAAAGTATCCATTTTAACT
TTGCCATTTTGCCATTCCCATGAAGATGAAGGTCTATCGCGATAGACTTCAATACCATTACCTTCGGGGTCATTGAAATATAAAGCT
TCACTTACTAAATGATCACCAGCGCCGATGCCCATATGTTTTTGTGCCACAAAATATAAGAAGTTAGCTAGATCCTCAGTAGTTGGT
AATAAAAATGCTATATGAAAAAGCCCTGCTTCACGTGGGGAAGTCTGACGGCCGTCTTCTAATAAATGTAACGTTAGAGTATGGCC
ACCAGTCCCAACAGATAATACGGTTGTATTATCGTCAGAACTTTTAACGGATAGTCCTAAAATGTTTTTGTAAAATGTTGTCATTAA
GTCTAAGTCTCTTACGTTCAGTTCAATGTTTGTCACTTGTGTTGCTGTTTTATCGTGAAATGCCATTATGCATCGCCTCGTTTTCTATT
TTTCTATAAGTTAGTATAAAAAGTATACCAGAAAAGAAAATGAATTGATAGCATAAAGTTTGAAATGCAAAATAACTAGTCGTTTT
GCAATTTTATATTGATGCGAACAAAAAAGCGATGGTACAGTTGCACCATCGCAAAATTTATTTAACCAAGATATACATCTTGATATG
AATCTTCTTTTTCTAACATATGTTTGGCAAATGAACATGAGGCAATAATTTTTAAATGATTTTCTCGAGCGTGTTCAACAACTGCTTT
AACTAGTTTTTTGCCAACACCTTGACCACCAAGTTCATCAGATACGCCTGTATGATCAATGTTAATTTCATTATTATCCACAAAACGG
TATGTGATTTCAGCTAAAGCATTATTTTCATCATCACCAATATAGAATTTGTTCTCGCCTTGTTTGATTTCAAGGTTACTCATACATAT
CAACTCCTATCATGTTTGATTATAATATTTCCCTATTCTATTTTAACTTAAACGAAGTAAAAGGTGCATGACAGTCATGTGACGACAT
TGCCGCATTTATGTAGTCGTTTTTATTAAGCGCAATTTGAAATAAAGATGAAAACACGTATCTTGACATTAAATCCATTCAGCTATAT
AATTTATCTCGAAATCGAAATAAATTAAAAAGTTGGTGATGATATGGATCGAACGAAACAATCGCTCAATGTCTTTGTCGGAATGA
ATAGGGCGTTAGACACATTAGAGCAAATTACAAAAGAAGACGTAAAGCGATATGGCTTAAATATTACTGAATTTGCAGTGCTCGAG
TTGCTTTATAATAAAGGTCCGCAACCAATTCAACGTATTAGAGACCGCGTATTAATTGCAAGTAGCAGCATTTCATATGTTGTAAGT
CAATTAGAGGACAAAGGTTGGATTACACGTGAAAAGGATAAAGATGATAAACGTGTATATATGGCTTGTTTAACTGAAAAAGGTCA
AAGTCAAATGGCAGATATTTTCCCTAAGCATGCTGAGACATTAACAAAAGCGTTTGATGTGTTAACAAAGGATGAATTAACAATCTT
ACAACAAGCGTTTAAGAAACTAAGTGCACAATCTACAGAAGTGTAAGGCGTGCACTAAAAAATTTACATTAAAATATCTCGATTTCG
AGATAAATACACTGAAAATATAAAGAGGGTATATAAAATGATAAATAATCATGAATTACTAGATATTCACCATGTCACAGCAATGA
CAGATGATGCAGAACGTAATTATAAATTTTTCACAGAAGTATTAGGTATGCGCTTAGTTAAAAAGACAGTCAATCAAGATGATATTT
ATACGTATCATACATTTTTTGCAGATGACGTAGGTTCGGCAGGTACAGACATGACGTTCTTTGATTTTCCAAATATTACAAAAGGGC
AGGCAGGTACAAATTCCATTACAAGACCGTCTTTTAGAGTGCCTAACGATGACGCATTAATGTATTATGAACAGCGCTTTGATGAGT
TTGGTGTTAAACACGAAGGTATTCAAGAATTATTTGGTAAAAAAGTGTTGCCATTTGAAGAAGTCGATGGCCAAGTGTATCAATTAA
TTTCAGATGAGTTAAATGAAGGGGTAGCACCTGGTGTACCTTGGAAGAATGGACCGGTTCCAGTAGATAAAGCGATTTATGGATTA
GGACCCATTGAAATTAAAGTAAGTTATTTTGACGACTTTAAAAATATTTTAGAGACTGTTTACGGTATGACAACTATTGCGCATGAA
GATAATGTCGCATTACTTGAAGTTGGCGAGGGAGGCAATGGTGGCCAGGTAATCTTAATAAAAGATGATAAAGGGCCAGCAGCAC
GTCAAGGTTATGGTGAGGTACATCATGTGTCATTTCGTGTGAAAAATCATGATGCATTAAAAGCATGGGCAACGAAATATAAAGAG
GTAGGTATTAACAACTCAGGCATCGTTAATCGTTTCTATTTTGAAGCATTATATGCACGTGTGGGTCATATTTTAATAGAAATTTCAA
CAGATGGACCAGGATTTATGGAAGATGAACCGTATGAAACATTAGGCGAAGGGTTATCCTTACCACCATTTTTAGAAAATAAAAGA
GAATATATTGAATCGGAAGTTAGACCTTTTAATACGAAACGTCAACATGATTAATTGGAATGAGGAGGATTTGTGATGGAACATAT
TTTTAGAGAAGGACAAAATGGTGCGCCAATACTAATATTATTGCATGGTACAGGTGGTGATGAGTTCGATTTATTACCGTTAGGCGA
AGCATTGAATGAAAATTATCACTTGTTAAGTATTAGAGGACAAGTTTCAGAAAATGGGATGAACCGTTATTTCAAACGTCTTGGTGA
AGGTGTTTATGATGAAGAAGATTTGGCATTTCGTGGACAAGAATTGTTAACGTTCATTAAAGAAGCTGCTGAACGTTATGATTTTGA
TATTGAAAAAGCAGTACTTGTTGGATTTTCAAATGGATCAAATATAGCGATTAACTTAATGTTGCGTTCAGAAGCACCATTTAAAAA
AGCATTGTTATATGCACCGTTATACCCAGTTGAAGTAACGTCAACAAAGGATTTATCAGATGTCAGTGTGTTGCTTTCTATGGGGAA
ACATGATCCAATTGTGCCATTAGCTGCAAGTGAACAAGTCATTAACTTGTTTAATACACGTGGGGCACAAGTCGAAGAAGTTTGGGT
GAAGGGCCATGAAATTACAGAAACTGGATTAACGGCTGGTCAACAAATACTTGGGAAATAACAGTTCTATTAAGAAGCGGACAGA
TGGAAAAGATTTTTACTTTTCATCTGCCCGCTTTTTTGATTTTGAAGTGCTGTACTAAATTTTACAATAGTATAGATATTTTAATCGAT
ATGAGATTTGCCGGTAATACGCTTAATTAAACCTTTATAGAGTACAGGTATGAGTAAGATGAAACCGAACAATCCCATAATAGGGA
ATACTTTTCCAATTAATGAAATGAAACCGATAAATGTACTAATATAAGTGATGACAGCCATTGTAATAATAATGATGAAGTAACGTC
TGCTGAATGGAACGCTGAAACGTGACGCAAATGCATACATTAATCCAACAACAGTATTGTAGATGACAAGTATCATAATGACAGAC
ATAATAATACCAATTGACGGAGACATTTGTGTCGCTAATTTTAATGTAGGTAGATCTACGTGTTTAATTTTATCGAATTGAGAAATT
AAACCTAGATTAATCATCATGAGTAAAAATGTAATGATTAAACCGCCAATCAAGCCCCCGTATAACGTTGAGTCACGATATTTAACT
TTACTACCCATCACTGATAAGAAGCTGAAGGCAGCAGCAATTTGCAAGCTTGCATAGTTAATCGCATCAAACCACCATCCAGGTGA
TAATGATTTCTGCTTATGAATCTGAGCATCATTATTAGCGGCAGTAAAATCAAGATGACTTGTTGTGAAATAGTAGACCGCAATCAT
AATGACAATCGCAATTAAAAATGGGGTAACACCGCCAAGCACAGCAATTAAACGATCGAATTTTAGATACAGTGTTGCTAAAATAA
AGGCGACTAATATGAGTGCGCTCAGCCAATACGGTAAGTTGAAACTTTGATGAATGGTTGACGCACCACCTGCAGTCATAATAATA
GCTAAAGACAACATAAACATTGTTAAAATAATATCAAAACCTCTTGCAATAGAGGGGTATAAGAAATAGTTAATTGAATCAGAATG
ATTTCTGGACTTTAGATGATGACCTGTATGCATGACAACCATTCCACCTAAAGTAATCAATAGTCCTGTTACAATAATGCCTGAAAT
GCTATATGCGCCATGACTTGTGAAAAACTGGAAAATTTCTTGACCAGTAGCAAAGCCGGCACCAACGACAACACCAACAAAGGCAA
ATGCCACAATAATGGACTCTTTTAAGATACGCATGATTTAAAAATGTCCCTTCGTAATTTTAAGTAATATAGAAAATGTAACATACA
TGTTAATGAAAAATATAGTACTAATATAGTATTTTGTTAAATTGGAGTAGAAGCGAGGGTGTCGGTCATTTCATTAATTTATTAGTT
GATTTTGCATTTTTTTGCTGTAAAGTTGTTATAATACAGTTAACAGGAATTAGCATAGATACACCAATCCCCTCACTACTCGCAATAG
TGAGGGGATTTTTTTCGGTGTAGCTAGGTCGCCTATTTATCATCGTGTTTGCGTAGCCAATGCGTAAACACAGTACCACTAAATAAG
TGCACGATACATGCATCAAATGTCGTCTTTAGTCTAAGTAACGATCATGCATTAACATTTTCAAAATATCTATTTGAGCTTGAAGATC
TTTACCAATATTGGTATCACGAATCTTCTTACGTTGTAATTCTTTATCTACGACGCGCTTTATAGAAAGTTCATCGATACCTTCGGAA
AGTATTTTTTCTTTAGCGTTAAATTGTTGGTGTGCAACGAGTTGCATACCGAATGAATTATACAATAGTGTATAGCCTGCAATGCCA
GTTGTTGACTGATAAGCTTTTGAAAAGCCACCATCAATGACAAGCATCTTTCCATCAGCCTTGATAGGATCTTCGCCATTGATTTCTT
TCACTGGTGTGTGACCATTAATAATGCGTCCTTCATCTGGATTTAATCCGAAATCACTGAGCATTTTACGAACCATATTCACATCTTC

ACGAAGATGATAGTACGGATTCTTTTCTTCTTTATGAGAAGCTTTATCTGCAATAAAGTATCGCTCAAACGTAGTCATGGCACGTTT
ACCAAATAGTGACGAATATTTCCCAGTCCATAAATACCAAACTAAATCCGTCGATAAGTCATCAGTATTTTCTTTTTCATCAAATGA
TTTACGGACATGATACTCAAACACATCTAATAATTCTTGGCCGCTGTAAGTATGACCATCAATTTCAAATGATTCCATCTCACCATTT
TCATCAACTGGAATACAACCATGAATGAGTAAATTACCATTATATGGTAAGTAAAGCGAACCTTTACGCATCAAGAAAGACATATG
ACGACGTAATTTTTCAGATTGTTGGAATGACAATAATAGTTTATTCATGACTTCTTCTTCTTCAGGTAGTAATTCTGCTGGATTATTA
CGATTGATAGTTTGGAAACATGTGTCTTTCAACGGGTATGTATTACCATAAACTGTAATTTCATTTGTATCATAATTAACCTTTTCAA
GCACAAGACGTTCTTCCATTTCGAAATTTGGACGACGTTTAATAATTGGTATTTCTAACTTGAATTGAATCATCGCAATAGCTTGATG
AATTTTAGTAATTTGACTTTCTTCACGTTGAGTTAAACGTTCGTGTTTGTCAGGTCTTTTTTTAGGCTTAAAAGCAGGATTATCTGCGT
CATAGTATTTTTCAGCTAAAGTAAGCAGTGGTCTTAAATTAATGCCATAAGCGTCTTCGATAATATCTAAATTATCATAGCGTGCAC
AAATTCGAAGTAAGTTTGCTAAGCATACTTTTGACCCAGCATAGGCTCCAACCCAAAGCACATCATGATTACCCCATTGAATATCTA
GGGAATGATAATTAATCAGTGTATCCATAATTTTATCTGGTTGTGGTCCACGATCATAAATATCACCGACAACGTGTAAATGATCGA
CGACTAAGCGTTGTACGGAATAAGCGAGTCCAATAATTAAATCATCTGCCTGTTTAAGTTCAATTACTTGGTTAACAAGTGTTTCGT
AATATGATTTTTTATTTTGATATTCATTACTTTTATACAGTAGTTCCTCAATAATATAAACGTATTGTTTTGGCAATGCTTTACGCAGT
TTTGAACGCGTATATTTTGAAGAACAATATTTAATCAACTCAATTAAATGTTCGATTGTTGTGATATACCAGACATTAAGTTGACCG
CAACTTTGGAAATCACTTTTAATCAATTTTAATTTGTCTTCTGGATAGTAGACAAGAGCAGTTAAATCATTAAGCTCCTTAGTTGAAA
GTCTCTCTTTGAAAATATCATTGATTTTCGCTCGCACATTCCCAGAACCGTTGCGTAATACGTGTTGGAAAGCTTCGTATTCACCATG
TAAATCGCTGACGAAATGTTCCGTACCTTTAGGTAATTCTAAAATTGATTCTAAATTGATAATTTCAGTTGCAAGTTTTTCTGGAGTA
TCAAAATTTTGGGATAGTAAATCTAAATACTTCTTTTTTAATTCTTTTTCAGTAATTTGAGTCATTACGTGTCACTCCTGTAGTATATT
TCAAACGTTAGTTTATAAAATAAATGCGCTTACAATGATTATCAAGAAAACGTTAACATTTTTCAATAAGTATGCTTGGATTGATAA
AAATAGAGGGCAGGATTTTGGTATTAGGAGTCGTTGCAGTTTAGTTAAGTAAAAGTGGGAAAGTGATATATTTATTTTAAAAATGA
GAGGAGGAATTTGTTATATTCACTAGTTTCCTAAAAAATTACGATATGTAAAATGTGGAATTGCCAAAATTTGGATAAAAAAATCCC
CGCACCATTATTTGGGTGAAGGGATTTGAAAATGATTTTGAATTACGATGGAATATGTGTGTATTTATTCATCTAATACTTTAAGTTA
CTAAATATTTTTCTTCTTGAAAATAAATACTACAAGTATTAAGAAAATAGAAATATAAGCAATATTACCAATAAACAATTCATGAAG
TTCTAATTTCGTCGACTTACTAAAGCCTTTTTCAACTGTTTGAAGCATAATATTCATCATATTAATAGGATTCCACTTTAGCCAGTCT
ATTTTTTCTAAAAGTGCCGTTTGAATAACTGCTAAAATAGAACTTGCAAAATAAAAAACAATACCTACAGCAATGGCTACACCCGTT
GAATTAGTTGCAGATGATAATAGCAACGTTAAGCTTAAAACTAACCAAACGCCAACAAATGTACCTAAACTAACTAATAATAATTG
ATTCAATAAAGATAATTGATTACCGCTACTTTCAAATATATTTAAATCATTAAAGAATAAAGACCCAATAACAATTGAAGCAATAAT
TGTAATAACAAAATAAATTAAAGAAATAATAAATAATGTGATGATTTTGCTAACAATCATAGTTGTTCTTGAATATTCACGATAGAG
TAAATTTTTAATCGTACCGTAATGAAATTCCATTGAAATGATTGTACTTGCTTGAATAATTAATAAAAATGCAAACCATGAAAAACC
ATTATAAGCAGATGTGAAAGATTCCTGTGGCGTAAAAATTTCATTGTATTTTGTAGCGATATAACCTTGAGCAACCATTAGTAGAAT
GAAGACAATAGGTGCGATAAAAGTTGATTTCTTTTTAAATAATTTGAAACATTCTTGTTTAATTAAAGTTCCCATTTTATGCTTGGTC
CTCCCTTTCTATGATTTCTAGTAATACATTTTCTAAATCATTATCTTTAAATTGTTTAAGTTCTTCTTCCGATGTTTCTGTAACAATTTT
ACCTTTGTTAATAATAAGGATAGAGTTTGTGATTTTAACTAACTCACTTAAAATATGACTCGAAATTAAGAAAGTAACACCTTCTTG
CGACTTTTGGACAATCAATTCACGTACATCTCGCACAGCTTTTGGATCTAAGCCGTTCATTGGTTCATCTAAGATAATGAATTGAGGT
TCATTTAAAAATGCTATAGCAATTCCTAATTTTTGTTTCATACCAAGAGAATACGTTTTAGCTTTTTTATGAATGTATTCATCCATATG
AAGTTGTGAGACAATTTTATCGATATCTTGAGTGTTTTTTGATTCATTCAATAACTTCAAGTTTTCATATCCAGACATAAAAGGATAT
ATTCCTGGATTTTCAATCAATGCACCGATATTGCTTTTGCTGTCTTTGCTGTTAATAACATTAAAATTTCCGCTTTGGAAACTAGAGT
AACCTAATATAACTTTCATTAAAGTTGTTTTACCTGCACCATTCGCTCCAACGAGACCAACTATTTGTCCGCGTTTCAGCTTAAATGA
TACATCTTCGAGAATCGTTTTGTTGCCTATCTTCTTTGTTAAATGTTCTATTGTTAAAACACCCATATCACTATCTCCTTTGCTATTAA
ATAATCTGAATCTTAAGATTTTATATATAAAATTAATTTTATCACATAGCAAGTGTTATAAAAATATGGGTTTAAAAAACTTTAATA
ATATAAAATATAAATATGAACACGAGTATTTTTGTAGCGTGCAAATGGTATTGCTACATTTATTAAAATGCGCAATTTGTTATGAGT
AACTTTTTTGGATAGTAGGCACATTAAAGTGTCGAAAACAATTTGTGTGGAGGACAGGGTAAATTCTGTTTATTTAAGATGATAGTT
TGTATTATAGATGGATTTGTGAGATAAGTTTGCGGAATGTATACATACTGAGGGAACTGAACTTTGATGGGAAACTTCATTATATCT
CTTAACGTAAAGGATTTTAGGTGACTTTCAGCTAAAGTATGCCAAATAAACTTAAAAAAGCAGTGAATCATTTCAATAGATGGAAT
GATTTTACTGCTTTTTGTGTATAGAAATTTAAAATTCATGCCTTTATACTAACAACAATATTATTTTACTCTCTTACGTTTGCCAATGT
AACGTATAGCAAAGTAAATGACTGCGATAATGATGACAACATACATAATACGTGAATATGTATGAAGACCAGTCATCAACATACCA
AAGCTATCGCTCAACGTGCGTCCTAGTAAAATTAAACCGAAATTCCAAATTGTTGTACCTATTAATGAAATAACGGTAAATGTCACA
ACATTCATGCGGTTTACACCGGCTGGAATGGTAATCAATACACGTAATACAGGTATGAAACGACAGATAAATACAGCCCATACGCC
ATACTTTTTAAACCAATCATTTGCTCGCTTTAAATCCTTACTTTTCAATTTAATCCACTTACCGTGTCGATCAATAAAACGATATAGA
CGTTCTTCTGAAATCAAACGGCAGATATAATATAAAATTAACAGCCCTATAAACGATGCGATGGTTGCAATAATAAATAAGGTTAA
AATTGATAAATGTGATTTAACAGACATAAGGCCAGCAAATGTCAGAATAATTTCCGATGGTACGATAGGTAAAACATTTTCTAATA
AAATTAATATAAAAATGGCTGCATACCCAAAACGACTAATAAATTCAGTGATAATTTGTTCCATTATTAAAAATAAGCTCCTTTAAT
TTTCAGCAATTGCAGACAAATAGAAACTGGATAAATTCAAATGAATAATGAATGATGTCGTATGCGATGTTGAAACAAGCATTTGT
CTTAAAATGTATTATAAGCAATAAATCAAAAAATTGCTTGCTATAAGTACGAAAAGTCAACAAATGATCATCTAAATAACAGGCGC
TGCACAATGAATGAGCAATGTAGTTTTATGCAATTTAAAATGAATGTCGAATATGTAAGAAAGCCTGCAATATACGTTTTAAAACAC
TAGGATTGCAGTATTTTTAATTAACAAATGATAGGCTACTATGTAACTAAAAGTTCCTTTATATCGTTTTAATAGCAAAAGTTAAGC
GGAATTTAATAAAATTTTTATAGATTCTATTGTAAACGCTTTCCATGTCTGATAATATTATAAAAAATAACTTTGGTACAATTTTGTG
ACAAAGTAGGGGTGGACATGCCAGATGTCCGAAAATTTTAACGTTTCAGGGTAAGTTTTAAAAAATTAATTAGGGAAGTAGGAATA
GTTATGAAGGAAAAACGAACTAACGTAAGGTGGATGTTCGCGCTTGCGTTCTTCTTTATTGGGGTTATTGCGTATATGGATAGAGCA
AACATTTCATATATCGCTAAACAAATGATGGATGATTTAGGGATGACCAAACCACAATTTGGTTTATTGGCATCATTCTTCTCTCTAG
GTTATGCATTAATGCAAGTACCATCGGGGATGTTGGCTGAAAAATTCGGTCCACGTAAGATGATTACAATTGCATTAGTTTGGTGGA
GTGCATTTACAATCTTAACGGGTATGATTAAGAACCACGGTTTAATTTATTTAGTGAGATTCTTATTTGGTGTGGGTGAGGCGCCAA
TGTATCCTTCTAATGCTGTGTTTAACTCATTTTGGTTCTCTAAAAATGAAAAAGGTAGAGCATCAAGTGCATTATTAGCAGGATCAT
ATTTCGGACCTGTATTAGCACCAATAGTTACAATTGCTATTGTTAACGCATTTAACTGGCAAGCAGTATTTTACATTTTTGGTGCAGT
AGGTATTTTAATGGCTGTATTATGGGCGATTATTGCCAAAGACTTACCTGAGCAACATAGAATGGTTAATGAAGCGGAGAAACGTTT
CATTATGGAAAATCGTGATATCGTAGCTACTGAAAAGTCATCACCACCATGGAATAATTTCTTTAAACGTTTTAGCTTCTATGCAATT
GCAATTCAATACTTTGTTGTACAATTTATCATTACATTATTCTTAATTTGGTTACCGACGTATTTAACAGAAGTATTCCACGTTAACTT
TAAAGAAATGAGCATTAGTTCATTACCTTGGTTATTAATGTTCTTCTTAATCTTATCAGCAGGTGCAATTTCTGACCGTGTATTAGGA
TTAGGTCGTTCAAAATTCGTAGCTAGAGGTGTAATTGCAATCGCAGGATTTATTGTGTTTGCAGTTTCAATTATCTTTGCTGTACGTA
CAGGAAATTTATATGTAAGTATTTTCTGGTTATCACTAGGTCTTGGTGGTATCGGTATTTCAATGGGTATGAGTTGGGCTGCAGCAA
CTGACTTAGGACGTAACTTCTCTGGTACAGTATCAGGGTGGATGAACTTATGGGGTAATATAGGTGCATTAATCAGTCCGCTATTAG

CAGGTCTATTCGTAGAACATTTAGGTTGGACAATGACATTCCAATTGTTAATCGTTCCAGCAGTAATCGCTGTGATTATGTGGTTCTA
TGTGAAACCAGATCAACCTTTAATTGTTAGTGATGATAAAGCAATAGAAAAATAATTTAAACAAGCAGTAAGCTTTCACATTGTTGG
GGCTTATTGCTTTTTTACGTTGAAATTGAAACTTTTTAAAACAGATATGGTTTAAGATGAACGGGAAGTTATTAAAATGATATATGT
AAAGAAATAAAGTTTTAAAACATTAGTCAGGTAACGCTTGTAAAAGTACATATAAATTTTAACTAGCCCAAAGGTGGGCGACCAAA
GGTTCAACGATGTTAAATAACATTAGAAATTAATTTTAATTGGACTTTAAAAGTTTTTAAATTTAGATAATTGAGCATAAGGTGTTA
TAATCACATATGTTGCGTAATTAAAATTTATAGCAACAAATTCATTTTAACTATGCTAATAAAAAGATTATGGAAATATTTTGACAA
GGAAAGGAGAAGTCGAAATGACATCTTTTTGACATCACTTATAAAAATCAATCGACTTAACTTAGACTTTTATAAAGGTGTAAGAC
AGGGACTGTTAATGATTATTCCTGCAATAATCGGTTACTTATGTGGTAATTTCCAATTTGGATTATTAGTTGCAACCGGAACACTAGC
CCATATTTATGTTTTTAAAGGTCCGTCGCGATCTAAGCTGCGAACTGTAATAATTTGTAATTTAGCGTTTGCAATATGTATGATGCTT
GGTACGCTAACAGCCAAAACGCCACTCGTTTTTGGAATGACATTATTAATTGTTACGGTTATACCATTTTATATATTTACTGCCTTAA
AAATAGCTGGACCGTCATCGACATTCTTCATTGTGACATTCAGTCTACCCATTAACTTACCTATAGCTCCCGAAGAAGCATTATATA
GAGGCTTTGCGATTTTAGTAGGCGGTATACTTGCCACTATGATGGTGTTAATCACGATCGTATTTTCTAAAAACAAAGCTGAAGAAC
AAGCAATTCAAAATGATTTTAAACTCATATCTAAGTTGTTACACACTTATAATGATAAAGCTGCTTTTTTACAAGTAGCAAAAACAG
CGGTGGATAGTTTTAAAGCATCTGATAAATTATTAATCACTTCTACTTCAAGTAACGATAAATTAAGTAGACGTTTCCAAAAAATTAT
TATTATTACACACATCTGCCCAAGGGATTTATTCTGAACTGTTAGAGTTGAACGCTAAACAAATTCGACCATTGCCAGATGAGTTAA
TTGAAATGATGGATCATATCATTGCACAACTAGATAATAGTGATGAAAATGTAAGATATTGGCGAAAAGAAGTGACAGTAACAGAG
GAATTTCAAAATTTATTCAACCATATATTGAAAATTGATGAAATGGTGCATGCAAATGAAGCGCGTATTGCGTATGAAGCAGACAT
GCGAAAACCTTTATATAGTAAACGCATTTATCAAAATTTAACATTAGACTCTATTGTTTTTAGAAATACATTGAGATATACAGCGAT
TATGATGATAGCGATATTTATTGCGTTAATGTTTGATTTTGAAAAAGCATACTGGATACCGTTATCTGCACATACAATATTACTAGG
AACATCAACTATACATGCAATCGAGAGAGGTATGGCACGAGGTTTAGGTACTATTTTAGGTGTGTTAGTACTTTCAGTCATATTGTT
GTTTTCAATACCAACACCTGTTGCAGTAATTTTAATGGGCATTGCAGCATTGTTTACTGAAGCATTGGTGGGAGCAAATTATGCGAT
TGCAGTAGTTTTTATTACAATACAAGTTATTTTAATGAATGGATTAGCATCACAGAATTTAACAATTAACATTGCGTTTCCAAGAGTT
ATTGACGTTGCAATAGGTATTGTGATTGCAATCATAGGTTTATTTGTCCTTGGACAACGTACCGCATCCGTATTGCTTCCTAATGTAA
TGGCTGAAGTTGTTCGTAAAGAAGCAACGCTCTTCCATTATTTGTTTTCTGAAAACCAATATAAAGATAATGTGTATCAGAAAAACA
CGGCCATGAACCTATCTGTGAAATTAAATAACATGACGCAAGTTTATAATGCAGCTAATGGTGAATTATTTAGTGATAAGACATTGA
TTCAAAATTATTATCCTAGCTTATTCGCATTAGAAGAAATTAGCTTTATGTTAAATAGAGCCATGGCTAATGAAGATAGACTTACAA
TAAATGAACAATTAATGGGCGAGTATTTAGCAACATTTGAAAACATCGCTAAGCATTTAGAATTAAATACAGAATTAGAAATAAAG
ATATTACCAGATTTACCACAGTATAATTATATTCAATCAGCAATGATGAATATTCAACACAATGGTTTTCGTGAGAGAGACAAGAAC
GTTTAAAATAAAATATTTATCAGGTCAGACTAACGTTTGTCTGAAGTTATTATGACGGGAGTCTAAAAAAATTGTGTTTAATATGTTTC
ATTTTATAATTATGGTGGTATATAACATGAATTATAACATATTGGAGGTTAAGGAAATGCCCTTTACAATGAAAGGTTACCCCGAAA
AATGGAAAGAATTTGAAGTAATTAAACGGCAAAAAGCGATAGAAATTGCGAATGCAATGTTAAAAGAAGGATATCTTGAAAAAGA
TGTTGTTCCTATTGCGGCGAAAAAAGCGAAAGATTGGTATCGAGCGTTATCGAAAGAAGAAATCAAAGCATTAGAAGAAGAGAAC
TTCGTACAATTCCATACAGCAACAATGGAAAAAGCAGAGGTGGATAGTGAATATGCAACAACTGATAATGAAGAACATATTCAAG
AACAATCAAATCAAGATAAATCGTACTTTGATAGCGAATCAATTGGCAACCAAAGTGGTGGCGTTCGACAAAGAGAACCGCTTATG
GATGGTGCACATCCAAAAGTTGTGGAATCATTTAAAGACGTATAACATCTCTATCAATTAAGCACTTGTACCTATATAACGGTAACA
GGTGTTTTTTTGCGTTAAATCATTTTAAAATTACTTCCTGTAATTGAAGGTTTTGATACATAATTCGGATTGTTTGAGTTTCTTTGTGC
TGTTTCAGTATCAGCCCAAAGTTCGATAGTGTATTAGTATTATCTTAATAAAATGTTAGGTACAATAAAGATGATTATATATCGGAG
GTTAGTATAAAAATGTATGTAGATCGAAAACCATCACTATATTTAGAGGATTTGCGACATGATTTTAAAAATAGTTTAAGTAAATTT
GAAAATGGTGATGAAGCATTTGATACGTTATTAGGTTTCGTAGAGTTAGATCATATTTATTCGTCAGCACTAAAGGAAATAAGCACT
AAACTGAGTATTTTAGATGACAATTTCAATCACATTTATAAACACAATCCTATACATCATATGGAGCGACGTGTGAAAGAAATGCGT
AGTTTAATAGAAAAGCTTAATCGTAAAGGATTACAGATTAGTGCAGAAACTGCCAAAGAACACATACTGGATATTGCGGGAATTCG
CGTAGTATGTAATTACTTAGATGATATTTATTTGATTGAAGAGATGTTGCTTAAACAAGAAGACGTACAATTGATAAAACGTAAAGA
TTATATTCAGCACCCTAAAGAAAATGGTTACCGCAGTTTACATATCGTTGTATCCATTCCAGTCTTTTTAGCAGAACGCGTTGAGGTA
TTGCCTGTTGAAATTCAAATTAGAACGATAGGTATGGATATGTGGGCAAGTTTAGAACATAAAATACGTTATAAAAACAATGCAGA
GACGGAAAAGTATCGAGATTTACTGAAAGAATGTGCGACAGAGATTACTGAAGTTGAAGATAAATTACAACAAATTCATTCTGAAA
TAACAGAGTAGACGTTTAGTAGGAGTGTTGCTTAATTTGAGTGATTTTGTAAATCAACATGGTACGAAATCATAAAAATAAGACGC
CAAGTATAATAAATTTCTTAAAAATATGGAAATTGTACTTGCTCGTGACGTCGGGTCATCAACTATGGTGATTGTAGGAGGTGGAAT
CTATGTCTAACTATTCGACTGGAGAACTCGCAAAATTATGCAATGTGACAACACGAACGATTCAATATTATGATCGCAAAGGTATTT
TGAAACCACAAGGATTTACAGAAGGAAAGCGTCGTGTGTATACAGAACAACAGCGACAAACATTAGAGTTAATCTTATTGCTTAAA
GATTTAGGTTGTGCGTTAAGCGATATAGATATGTTGCTAAAAGGTGAAGGTACTTTGAAGACACTCAATACCTTACTAACCATGAAA
CAACAAGAAATTAACCAACAAGTCAAACAGCAACAAGCGGTATTAAACAAAATTAAAAATGTTCAATATTACGTAAATGAAGCGT
CGACGTCTCCAATCACACACTTAAAAGACATAGAGCATGTCATGAGTAAATCAGCTGAAATGAAAAGTATTCGTCGTAACATTTGG
ATTAGTGCTGGTATTATAGGAATAATTCAATATTCTAGCATTATGAGTTCAATCTTGATGAAAAACAAATGGCCGTTTTTAATTGCTT
TACCATTTATGAATTGTTACGGCATTGGTGTTACTTTTTATTACCAACAAAAGGTTGCCTATTTATGTCCTAACTGCCAGCATATATT
CTCACCATCTTTGTGGGCAGTTATCAAAGCGAAACATACAGCGACAACACGTCGATTCGAATGTCCGAACTGTCATGAAACGCATT
ATTGCATTGAAGTACCTAAAGCGCATATGAGTACAGAACAATTAGAAATATCCCACATACAACATAACAATTAAATCATTCGTGTG
ATAGTGATCATTCCCTTTAAATTTACATCGAAGAGGGGTGGTCACTATTTTAGTTCGCATAAAGAAAGCGCTTGCAAACACTTAAGA
ACTATATTAACATACTTGTATACAAGTATGTCATGTAATGAAGGAGTGCTTTGATGATGTATGGATATCCAGAGAAATGGTTGGAAG
GTATGACAACTGGAGAAGGTATCGCGGCAGAATTACGCTTAGGTATTGTGAATGGTCACATAGCTGAAGGTACGTTACTCACTGAA
AATCAAATGGCAAAGCAATTTAATGTGAGTCGTTCGCCAATTCGAGATGCATTTAAATTATTGCAACAAAATCAACTCATCCAATTA
GAAAGAATGGGTGCACATGTGTTGCCGTTTGGGGAACAAGAAAAGAAAGAAATGTACGATTTGCGACTGATGCTAGAGTCATTTGC
ATTTTCAAGAGTTAAAAATCAAGAGAGACTACCTATCGTAAAAGAAATGAAGAAACAACTTGAAATGATGAAAGTGGCAGTAAAA
TTTGAAGATGCAGAATCATTTACGAAGCATGACTTTGAATTTCATGAAACATTAATCAAAGCATCTAACCACCAATATTTAAACTCA
TTTTGGAGTCATTTAAAACCAGTAATGATGGCACTCGTTTTAACATCCATGCGACAACGTATGCAACAGAACCCGCAAGATTTTGAA
CGCATACATCATAATCATCAAGTGTTTATTGATGCAGTGGAACAATACGACAGTCAAATTTTGAAGGAAGCGTTTCATTTAAATTTT
GACGATGTTGGTAAAGATATTGAAGGATTTTGGTTGAATTAAAATGAAAGAAAGAAGGATTTATCATGAAATATATGATTGGTGTC
GACATTGGAACGACGAGTACCAAGTCAGTCTTATATGATGAAAATGGAGCTTTTATCATGAAACATCAAATCGGCTATGATTTACAC
ACACCAAACGTTGATGTCTCAGAAGAAAACCCAGATGAATTATTTGATGCGGTATTAATGACAATTAAATACATAATGAGGGAATC
GAAAGTTAATCAAGATGATATTAAATTTGTGTCATTTAGTGCGCAAATGCATAGCTTGATTGCGATGGATCAGCAACATCAAAGATT
AACAAATAATATTACTTGGGCAGATAACCGCGCTGCAAAATATGCAACAGTAATAAATGAAGTGCATGATGGCAATGCGATTTATC
AGCGAACAGGTACGCCTATTCATCCTATGTCGCCTTTAGTGAAAATTTTTTGGATGAAACATGAATGGCAAGATGTATTTCAACGTA

CTGCTAAGTTTGCAGATATTAAAACATACATTTTCTATCATTTATTTGATACATATATCATTGATTATTCAATGGCTTCCGCAACAGG
GATGTTTAATTTAGAAACATTAGATTGGGATGTTGGGGCATTAGAATTGCTTGGTATTTCCAAGGAAATGTTCCCGGAATTAGTGCC
AACAACGTACGTAATGAAAGGCATGAAAGAACGTTATGCAACATTAATGGGGCTTAATGAAGATACACCGTTTGTTATTGGTGCGA
GTGATGGGGTCCTTTCTAATTTGGGTGTCAATAGTGTTGGTAAAGGAGAAGTTGCTGTCACAATCGGTACATCTGGCGCGATTCGTA
CTGTGATAGATAAACCACGTACTGATTACAAAGGTAGAATATTTTGTTATGTCTTAACAGAGGACCACTACGTCATCGGAGGTCCTG
TAAATAATGGTGGTGTCGTATTGAGATGGTTGCGCGACGAGTTGCTAGCGAGTGAAGTCGAAACTGCGAAACGTCTCGGTGTTGAT
CCTTATGATGTCTTAACTCAAATTGCAAAGCGTGTTAAACCAGGTGCAGATGGTTTAATATTCCATCCCTATTTAGCTGGAGAACGT
GCGCCGCTTTGGAACGCAAATGCAAGAGGTTCATTCTTCGGTTTAACTTTATCTCATAAAAAAGAACATATGATTCGCGCTGCATTA
GAAGGTGTTCTTTACAATTTGTATACCGTCTACCTTGCACTCATTGAAGTAATGAATGAAACGCCTAATATGATAAAAGCAACAGGT
GGTTTTGCGAAAAGTGAAGTATGGCGTCAAATGATGTCAGATATATTTGACACAGAGTTAGTGGTTCCTGAAAGTTATGAAAGTTCA
TGCTTAGGTGCCTGCGTGCTTGGACTTAAAGCTGTAGGTGACATTGAAGATTTTTCAATCGTTTCATCGATGGTCGGTGCTACAAAT
AATCATACGCCGTTTGAAGAAAATGTCGCTGTTTACCAAGAGCTCGTATCCATTTTTATCAATTTAAGTCGTTCTTTAACAGAGAATT
ATGAACAAATTGCAGATTTTCAACGCCAACATATGGCTGAAAATAAAACACAATAAATACGTCACTCAAGCATCTTAGATAAAGTT
GTTGTGCCATGACTACTATATATTATCTGGAATACGTGCGCGACGACGGTGTTACATTCTATTGAAAAGAGAAAGAGGGGAATGTC
ATGTTTAACGAAATATGGCCGTTAATCAGTGTTGTTTTAGGTATCGTTATTTTATTAGTATTAATTATTGGGTTTAAATTAAATACAT
TTATTTCATTAATTATCACATCGATGATTACAGCGTTAATGCTAGGTATACCATTGACTAAAATTATGGAGACGATTGAGAAAGGGA
TGGGCAGTACGCTCGGCCACATTGCACTAATATTCGGTTTAGGTGCCATACTTGGGAAATTACTTGCTGATGGTGGTGGTGCAACTC
GAATTGCAGACACGTTGATTCAAAAATTTGGTCAAAAACATGTACAATGGGCAATGCTTGTTGCAGCATTTATCGTCGGTATTGCAT
TGTTCTTTGAAGTAGGTTTAGTCTTACTGATTCCATTAGTATTTACAGTAGCAAAACGCGCAAATGTTTCAGTGTTAAAACTAGGACT
ACCTATGGTAACAGCTTTATCTGTGACACATGGCTTTTTACCACCACATCCAGGACCGGTAGTCATCGCAAAAGAATTAAAGGCAAA
TGTTGGAGATGTATTACTGTACGGTATGATTATTGCCATTCCAGTTACACTCATTGCAGGACCTATATTTAACAAAGTTGCACAAAA
AATGATTCCGTCTGCGTATACACGAGAAGGCGATATTTCAGCGTTAGGTGCACAAAAAGAATTTACGGATCAAGAGATGCCAGGAT
TTGGTATGAGTTTATTAACTGCAACATTACCAGTCATATTAATGTTAGTGTCTACGATAACGCAACTTGTAACTGGGCACGACAAAC
CTACAAATCTATTTGAATCTATCATTTATATGATAGGAACAGCAGGGACAGCCATGCTAATAGCAGTATTATTTGCAATCGTTACGA
TGGGATTAATGAGAAAACGTAAGATGAATCATATTATGGAATCAGTGACGAATGCGATTTATCCAATCGGAATGATGTTATTGATT
ATTGGCGGTGGCGGTACATTTAAACAAGTATTAATTGACGGTGGTGTCGGAAATACAATTGCTAAAATGTTTGAAGGTACAGAGAT
GTCTCCAATTTTACTAGCATGGATTGTTGCAGCTGTGCTACGTATCGCATTAGGTTCGGCTACAGTAGCTGCGATTTCAACTACAGGT
ATTGTCTTACCATTATTACAATCATCAGATGTAAATGTTGCATTAGTTGTACTTGCGATAGGTGCAGGTAGTGTGATTTTGTCTCATG
TAAATGACGCAGGATTTTGGATGTTTAAAGAATATTTTGGTTTAACTGTAAAAGAAACATTCTTAACATGGTCATTATTAGAAACGA
TTATTTCCGTTTCAGGTATCATCTTCATTCTATTCATCAGCTTATTTGTTTAACTAAATATTAAGTAAACGTGTTTTTAGGTAATTGCA
ATATATTGCCTTGAAACACGTTTATTTTTATGCAGAAAATCGTCTGAAATAGTCAGAATCCGAACGATATAAAAAGTATATTGGGGA
TTTTAGTGTAATGAAAGGCGTTTCAAGACATGTTTTTGTGCGTATTGTACAGGCGATAATTATGAAACACTTAGTATATTGTTTTAAA
TTAGATAATGATGAATTTAATTTGAAAAATAAGTATAAAAAATACAAGCCTTGTGTGACAAGGGTTTATGATGACTTGAATACAATT
TATAGGTATATTTCAAATAATAAAATTATCAATTAACATAAAATTAATGACAATCTTAACTTTTCATTAACTCGCTTTTTTGTATTGC
TTTTAAAAACCGAACAATATAGACTTGCATTTATTAAGTTTAAAAAAATTAATGAATTTTGCATTTAAAGGGAGATATTATAGTGAA
AAACAATCTTAGGTACGGCATTAGAAAACATAAATTGGGAGCAGCATCAGTATTCTTAGGAACAATGATCGTTGTTGGGATGGGAC
AAGATAAAGAAGCTGCAGCATCAGAACAAAAGACAACTACAGTAGAAGAAAATGGGAATTCAGCTACTGATAATAAAACAAGTGA
AACACAAACAACTGCTACTAACGTTAATCATATAGAAGAAACTCAATCATATAACGCAACAGTAACAGAACAACCGTCAAACGCA
ACACAAGTAACAACTGAAGAAGCACCAAAAGCAGTACAAGCACCACAAACTGCACAACCAGCAAATGTAGAAACAGTTAAAGAA
GAAGAGAAACCTCAAGTTAAGGAAACGACACAACCTCAAGACAATAGCGGAAATCAAAGACAAGTAGATTTAACACCTAAAAAGG
TTACACAAAATCAAGGGACAGAAACACAAGTTGAAGTGGCACAGCCAAGAACGGCATCAGAAAGTAAGCCACGTGTGACAAGATC
AGCAGATGTAGCGGAAGCTAAGGAAGCTAGTGACGTTTCAGAAGTTAAAGGCACAGATGTTACAAGTAAAGTTACAGTAGAAAGT
GGTTCTATTGAGGCACCTCAAGGAAATAAAGTAGAGCCACATGCTGGTCAACGTGTCGTATTGAAATACAAATTGAAATTCGCAGA
TGGATTAAAAAGAGGAGATTATTTTGATTTTACATTATCAAATAATGTAAATACTTATGGGGTTTCAACAGCTAGAAAGGTACCAGA
GATTAAAAATGGCTCAGTTGTAATGGCTACAGGTGAGATCTTAGGGAATGGTAACATAAGATATACATTTACTAACGAAATTGAAC
ACAAGGTAGAGGTAACAGCTAATTTAGAAATCAACTTATTTATTGACCCTAAAACTGTACAAAGCAATGGAGAACAAAAGATTACT
TCTAAATTAAATGGTGAAGAAACAGAAAAAACAATACCAGTTGTTTATAATCCAGGTGTTAGCAATAGTTATACAAATGTAAATGG
ATCAATTGAAACATTTAATAAAGAATCTAATAAATTTACACATATAGCTTATATTAAGCCAATGAATGGAAACCAGTCAAACACTGT
ATCAGTAACAGGGACGTTGACTGAAGGTAGTAATTTAGCTGGTGGACAACCTACTGTTAAAGTATATGAATATCTAGGGAAAAAAG
ATGAATTGCCACAAAGTGTTTATGCAAATACATCAGATACTAACAAATTCAAAGATGTAACAAAGGAAATGAATGGAAAATTGAGT
GTGCAAGACAATGGTAGTTACTCATTGAATTTAGATAAGTTGGATAAAACGTATGTCATTCATTATACAGGTGAATATTTGCAAGGG
TCAGATCAGGTTAATTTTAGAACTGAATTATATGGGTATCCAGAACGAGCATATAAATCTTACTATGTTTATGGGGGATATCGTTTA
ACTTGGGATAATGGTTTAGTTTTATATAGCAATAAAGCTGACGGCAATGGTAAAAATGGACAAATTATTCAAGATAATGATTTTGAA
TATAAAGAAGATACTGCAAAAGGAACTATGAGCGGGCAGTACGATGCCAAGCAAATTATTGAAACAGAAGAAAATCAAGACAATA
CACCGCTTGACATTGATTACCACACAGCTATAGATGGTGAGGGTGGTTATGTTGATGGGTATATTGAAACAATAGAAGAAACGGAT
TCATCAGCTATTGATATCGATTACCATACTGCTGTGGATAGTGAAGCGGGTCACGTTGGAGGATACACTGAGTCCTCTGAGGAATCA
AATCCAATTGACTTTGAAGAATCGACACATGAAAATTCAAAACATCACGCTGATGTTGTTGAATATGAAGAGGATACAAATCCAGG
TGGTGGCCAAGTAACAACTGAGTCTAACTTAGTTGAATTTGACGAAGAGTCTACAAAAGGTATTGTAACTGGCGCAGTGAGCGACC
ATACAACAATTGAAGATACGAAAGAATATACGACTGAAAGTAATCTGATTGAACTAGTAGATGAACTACCTGAAGAACATGGTCAA
GTACAAGGACCAATCGAGGAAATTACTGAAAACAATCATCATATTTCTCATTCTGGTTTAGGAACTGAAAATGGTCACGGTAATTAT
GGCGTGATTGAAGAAATCGAAGAAAATAGCCACGTTGATATTAAGAGTGAATTAGGTTACGAAGGTGGCCAAAATAGCGGTAACC
AGTCATTCGAGGAAGACACAGAAGAAGACAAACCTAAATATGAACAAGGTGGCAATATCGTAGATATCGATTTCGACAGTGTACCT
CAAATTCATGGTCAAAATAAAGGTGACCAGTCATTCGAAGAAGATACAGAGAAAGACAAGCCTAAATATGAACATGGCGGTAATA
TCATTGATATCGACTTCGACAGTGTGCCACAAATTCATGGATTCAATAAGCATAATGAAATTATTGAAGAAGATACAAACAAAGAT
AAACCTAATTATCAATTCGGTGGACACAATAGTGTTGACTTTGAAGAAGATACACTTCCAAAAGTAAGCGGCCAAAATGAAGGTCA
ACAAACGATTGAAGAAGATACAACGCCGCCAACGCCACCGACACCAGAAGTACCGAGTGAGCCGGAAACACCAATGCCACCGACA
CCAGAAGTACCGAGTGAGCCGGAAACACCAACGCCACCAACACCAGAGGTACCAAGTGAGCCGGAAACACCAACACCACCGACTC
CGGAAGTACCAAGTGAGCCGGAAACACCAACACCACCGACACCAGAAGTGCCGAGTGAGCCAGAAACACCAACACCGCCAACACC
AGAGGTACCAGCTGAACCTGGTAAACCAGTACCACCCGCAAAAGAAGAACCTAAAAAGCCTTCTAAACCAGTGGAACAAGGTAAA
GTAGTAACACCTGTTATTGAAATCAATGAAAAGGTTAAAGCAGTGGCACCAACTAAAAAAGCACAATCTAAGAAATCTGAACTACC
TGAAACAGGTGGAGAAGAATCAACAAACAAAGGTATGTTGTTCGGCGGATTATT

>037-contig_188_RC

CTGTGGCTAATGTGTAAGAACCACTACATAATAAATCATTAGTGGCTCTTTATCATTTCTGTCCCACTCCCTTGAGCCTACAATTTTG
ATTATGTATCACGTTCATAATACTTTCCAATTTGAAATACCCTGTTCGCAATTATTAGTTGAGTTATATCAACCAATACAGTGTAAAC
ATAAAAAATTCCAAACCGTTTAACAGTATATCAGAAAAAATGATAATCATTTCTTTTATTTTTATTAAATTTCATAATGATTCATATAA
TATTCGCATTAAAACATCACAATACTGATACACAACAAAAAGAGCAGTACATTTGTCATGCACCACTCTCAACAAATATATTTTTCA
ATGAAGCAATTACGCGATCTTACAAATACTATAAGTATAGCTGAATCACATATTATATTTATTTTATAATCGAATTTAGAAATTCATC
ACTTATCTCATAAATTGTTGGTCTACTTTTAATTTTAACTAAGTAGTTATCATATTTTTTAATTGTATTATTAATTTTTACTCTTCCTAC
TCCCAATATGTTCTCTAAATCAATTAATGTTAATCTATTTCTTTTATTTCCAAACAATTTATCCATTGAAAGGAGATACAAAAATTCA
TAATCTATCTGTTTATAATGACTAGTTAAATAAAGCGTTAATTTTTCAGTCGCATCCATTTTTGGTATTAACTCATCAAGTATTCTTTC
TTGTCCTGCTATCAATAGCTCAAGCATTGTGTCAATAAATTCCGTCAAATCACCACAATTCAAATGATTTGAAGCAGTCATAAACGC
CTTGTAATATTTCGATTTATTTCTATTTATAACATATGAAAATGTCAAAGCAGTATAATTATCATAATAATCACTCAAGAGCTTGGCA
ATTATAAATCTTCCAACTCTACCATTGCCATCATAAAAAGGATGTATATATTCAAACAGATAATGACTAGCCATGATTTTGAACGGC
TGAGGCGCATCAAAATATTTTAAAAATGTTAGCATTTCACCTATATATTCAACAATTTTGGTTTCAGGTTGTAACCCAACATGTATAT
ATTTATTCGTTGACCCATCATGCACACCGACAAAATTTTTACGAAATAGCTCTCCATCTAACTTATCTTGTTCGTTAATTTCATTTGA
AACTAATTTATCATAAATCGCTCTAATGTCTCTTACATTATCAACTTTAATTTTTTTATTAAGTTCTATCTCTTTATATTGATCCACGA
GGCCTCTGAACTTAAGAAATTCTGATGCTTGGTTATTTAACGCATGTGCAATCTCTTGTTTAGTACTAAATACATTTTCAATTTCATT
AGTACTTTGTAATTCATCGATTAATAAATTATTAAAATATTGTTCTCTAGCTGCATATGGTAAAGAATTAAGTGCTCTATCAATTTTT
CTACTATTAATTGATATTAATTCTTGTTTTTTTGATAGATTTTTAGTCACCATAAAGAATAGAGGATATTCCAAATCATTAACTTTTTT
TCCATTTTCCATAGGTATGATATTAATATTAGTATTGAAAGAAGCTAAAGAATTAAATCTTTTAGTATACTCTTCCTTCATTTTACTTT
CATTGTGTTCATGAAAAATACTTTTTAAAGTTCTGTAACCCATTTGCAGCCTCCTCTACTCATTTAAAATGTATTTTTTGTCCAATAAA
TCCATTTTCATTAGATTAGCTAAATATTATCACTTTTTTTCTTTAAAAACTATATAAATAACTATTTTCTCAATCAATAGAAAAAGAT
TTTTCATCACAACAAAAAGAGCAGTACATTTGTCATGCACCACTCTCCCTTAAAATATATCTAATTATGCTTTATAAATGAATCACCG
GAAATCAAAATATATCTTGCCTAATACTAACAACATTTACAGTGATGCTATTAGTCGAAATGTCTTTTAAAATAACTGAAGTACTTC
TTTGAATGAGAATATACCTGTCAATATTGTAACAAGTACTGCAACGATACCAAAGATGAACATCCAATTTGGGTGTTTATAATCACC
AACAATTGATTTCTTTTTACTTGCAATCAAAATTGCACCTAACGTAATAGGTAAAATCCATCCATTTATCGCGCCTGCTATAATTAAA
AGGCTGATTGGTTTTCCAATAAATAAGAAAATCATTGTTGAAATAACGATAAACACAATCACAATTAAATTACTTCTTTCGTTAAGT
GATTTATGAAGTGTTTTTAAAAATGTTGCGCTTGTGTATGCTGAGCCAATTACTGATGACATAGCTGCAGCAAATAACACAATACCA
AAAATATTCTTTCCAATTGGTCCAATTGCGTGTTCAAAAACTGACGCTGGTGGATTTTCAGAACTTAGTGTCACACCTGTTACAACA
ACTCCTAATACCGCTAGGAATAGTAACGTTCTCATAATACCTGTAGTTAAAATACCAGCAATTGCTGATTGATTTACAAATGGTAAA
TATTGCTTACCTTTAATGCCAGAGTCTAATATACGATGTGCACCTGCAAAGGTAATATAACCACCTACAGTTCCACCAACTAACGTA
ATGATGGGCAAGACTAATTTCATTGGATGTTCTGGCGCAAATGTATGCACAAAAGCATCACCATAAGGTGGATTAGAAACAAACAT
CACATATGCCACAACTAAAATCATCACAATACCAAGAATCATTGAAACAACGTCCATAATTTTTTGGCCACTTTTACTTACAAAGAT
TAATATTGCAAAGATTGCAGTAATAGCTGCGCCCCATTTTACATCTAATCCAAAAATTGCATTTAAACCTAAACCGGCACCAGCAAT
ATTACCAATATTAAAAGCTAGACCACCAAATGCAATCAATATTGAGATAACAGTACCAAGCCCAGGAACAACTTTATTTGATATTTC
TTGACCTCTTAAACCAGTTACAACTAATATGCGCCATATATTAATTTGTGCACCAATGTCAATGATGATAGACAGTAATATGGCAAA
TGCGAAACTTGCAAAAAAATTGTGATGTAAATACTGCTGTTTGCGTTAAAAATGCTGGCCCAATTGCAGAAGTTGCCATCAAAAATAC
AGAACCTAATAATAACCTTTTATGATTTTTTGTGAATTCAAAGTCACTTTCTTTTTTAAGCTTTAAATTTCTCCCCATTTTTTTAGCCC
CCTATAAGGATTGAATATCAATGCCTTCTTTCATTAAAATTTCTCTAATTTTCGAAACAAATAATAATGCATGTTCTCCATCACCATG
CACACAAATTGTATCTGCTTGTAACGTTACTTCCTTATTGTTTTTTGAAATAACTTTATTTTCCTTCACCATCTTTAAAACCTGCTTAA
GTGCTTCGTCAGTATCAGTAATCACAGCATCACTTTCTTTTCTACTAACGAGCTGCCCATCATCTTCGTATCGTCTATCAGCAAACAC
TTCAGAAGCTGTAATTAATCCGACATTCTTTGCTTCTGAAATTAGATATGAATTTGCTAATCCTACTAACACTAGTGATGGATCAAA
GTCATAAACAGCTTGTGCTATAACGTTTGCTATTTCTCTGTCTTTTGCACCCATCTGATACAATGCACCATGCGGTTTAACATGATTA
ATTTTAAGTTGATGAATGCGACAAAACCCTTGTAATGCACCTAATTGATAAATCATCAAATTATAAATCTCGTCGTTAGAGATATCT
ATATTTCGTCTGCCAAAGCCTTTCAAATCAGGTAAACCAGGATGTGCACCTACTGCAACATTATGTGCTTTGGCAAGTTTTACCGTTT
CATTCATTACATTTTCATCACCAGCGTGAAAACCACAAGCAACATTCGCACTTGTAATTAACGGAATAATTTGATGATCACCACCAA
AGGAATAATTTCCAAATGCTTCGCCTAAATCACAATTCAAATCAACTCGCATTATAATTCCACCCCTTTAACAATTTGATGTTTTTCT
AAAAATTTAATATCAACATCTTTTGCATCTCCATCACGATATAGTGGATAATTTAAAACTGCATATAAAAAATCGGCAGTTGTAGAA
AATCCATCTATCACCATTTCATCTAAGGTGACTTTCAACTTATCAATTGCTGAAGCTCTATCATGAGATTTTACAATTACTTTAGCTA
CTAAAGAATCATAATATGGTGAAACTTGATAACCGTGATATAGTAAAGAATCGACTCGCACATTAAAGCCTTGAGGTAAATGTAAC
GCTGTCACTTTACCTGGTGTTGGTTGAAATTTCTTTTCAGGATTTTCGGCATTTATTCTCGCTTCTATCACATGACCATTAAATTGAAT
ATCGCTTTGTGAAAAAGGTAAATGATTATGTTCCAATAAATACAGTTGTGCTGCAACCAAATCACGTTCTGCTCGCATCTCTGTAAC
AGTATGTTCAACTTGTATTCGAGCATTCATTTCAATAAAGTAATGTGCGGTATCAGTTACTAAAAATTCAATCGTACCTGCACTTCTA
TAATTTGCTGCACGTGCAACTTTAACAGCATCGTTACATATTTGTTGTCGTCGTTCTTCAGTTAATGCTGCACAAGGAGATTCTTCGA
TTAATTTTTGATTTTTACGTTGTACAGAACAATCACGTTCCCCTAAATGTACATAATTATCCTGCCCATCTCCCATAACTTGAACTTC
AACATGTTTTGCAACAGGTATAAAAGCCTCAACATAAACACGATCATCATCAAAGTATTTTTTTCCTTCACTTTTAGCTTCTTTAAAT
GCCTTTTCTAAATCTTCAGCTTTCTTTACAATACGTATACCTTTACCACCACCGCCACTGGCAGCTTTGATAACAACTGGATAACCGA
TGTCTTTGGCAAGATTCTCAATTTCAGACACATGATTCACAGCACCATTTGATCCTGGAATCACAGGAACACCTGCATGATGAACTG
TTTGTCTTGCTGTTATTTTATCCCCCATCATTTCCATCGTTTTTTTAGTAGGCCCTATAAACGCTATGCCTTGTTCCTCAACGGTTTGA
GCAAATTTTGTTGATTCTGATAAAAAGCCATATCCTGGGTGAATTGCATTAGCACCAGTGATTTGTGCAGCAGATATGATGCGGTCA
ATATTTAAATAACTATCTAAAGCATTAGCTTCCCCAATACATATAGCTTGATCTGCTAAATGTACATGCAAGCTTTGCTCGTCCCCTT
TTGCATAAACTGCTACAGTTTCAATCCCATATTCTCTGCAAGCTCTTATAATCCTTACAGCAATTTCACCTCTGTTCGCAATTAAACA
ACGAAGCATTTACTTACCCCCTTTACTTAATACGTACCAAAACTTGGTCGTATTCAACATTTGTGCCATGATCAGCTACTATTTCAGT
AATTTCTCCAGCAACATCTGTTGTTACCTCGTTTAATACTTTCATCGCTTCAACATATCCTATAATATCTCCCTTGTTAACTTTGTCAC
CGACATTCACAATTGGTTCAGTTAATTCTTTACTATCTTGTAAAAAGAATGTACCTACCATTGGTGATTTAATGTCATGATAATCATT
TGTCGAAACATCGGAGTTATCATTCGCTTTTGAAGCTGTCAAATCATTATTGTTCATACTTTGATTTGATTGATTACTGTGTGCAGCC
AAATGATTCGAGTCAGTGAAGTCAATTTCTATTTCATCTTCAAAATTTTTATATTTAAATTTCTTAACATCATTTTCCTTCACTAATTT
GATTATTTGTTCGATTTTTTCAATATTCATTTTACAAATCCCCTTTTAAAATTGTTGATAATTTTTTCGAAGTATGTCGCAAGCTAGAT
GTATCAAAAAATTGGAGTCTTTTGATGACTCTTAAGAATTTCATTAAACAGAGACATTTGTTCCCGATTCTTATCTACAGCTTCTTGGA
ATGATATCCATTTAAATTGAATTGTATCTTTTGGTTTCATCTGTGCTAACTTTGGCAGATCAAATTTGCATACAGTTGCAATTTTGGT
ATAACCACCTATCGTTTGTTTATCATTAAGCAGAATAATAGGTTGACCATCATTTGGTACTTGAACACTACCAAGAGCAACCGGTTC

AGAAATGATATCTGCTTGATTAATTGGTGCAACGCTGTCACCTTCCAAACGATAGCCCATACGGTCTGATTGTTCAGTAATTAAATA
TGGATGATTTACAATTTTCGCTCTAGCCTCTTCAGAAAATGCCTCGAATTGAGGTCCTTGAAGAATGTGTATAATATTATTTCTGGC
AATAAATCGTCCTGTAAATGAATAGTCTTTCCAATGTTTTCTTTAAAGTCATTATTTATTTTCACTGTTATTACATCATTAGCTAATAA
CTTTCTACCTTTGAATCCTCCTATACTGCTTCGGGTATGTGTTGCATAACTTTCAGCAATAGAAGGTACGTTGATAGAATGACAAAA
AGTAAGATAACCGCGTGCACCTTTGGTTATAGCACCTATTTTTAAAATGTCACCTTTCTCAGCTAATATGACAGAATTCATTGATATA
GTTTTATTATTTAGCGAAGCATTAATACTACCACCGGTTATAACAAATGTATTTTGCGTATTAAATTGAATGGTAGGACCAATCAAA
GTATATTCAATCGCTGGACCATCATTGTTAATTAATGACTGCGCAACCTTAAAACTAAATTGATCCATGGCACCTGCGCCTGAAAAT
CCAATATGTTCATAACCTATTCTTCCTAGATCTTGTACCGTTGAAAAGAGACCTGGTTGTAAAATCTTAATTGACATTTTCAATCACC
ACCCAGTCATCAACATTAAAGTTGCCATCTGATATATCTCTTTCGATTTGTATAAATTTCTGTTCATCTATTGCATAAAATTGTATCC
ATTCTCCTGCTTCGTACATTGACATTGGTTCACGCTCGCTGCTAAATACTTTTAACGGTGTGCGTCCAATAATTTGCCATCCGCCAGG
AGAATCTGATGGATATAGTCCTGTTTGATTATTCGCAATACCTACAGAACCTGCATGAATTTTTAACCTTGGCTGATTACGTCTAGGT
GTATGTAGTTGTTCATCAAGTCCGCCTAAGTATGGAAATCCTGGCATAAATCCTAGCATATATATTAAATAAGGTTTACTTGTATGTT
TTTCAATAACTTGCTCAACAGTTATTCGATTATGCTTTGCTACTTCTTCAATATCTGGTCCATATGTACCACCATATTGAACAGGTATT
TTAATAATACGATTGGTTTGATTCACAGCATGAACATTTTTTTCATTAAATTTGTTAAGTTCTAAATTTTCAATTAATTTAGAAGATG
TTATAGCTTGTTCATCAAAATATATTAGAACTGCTCGATACGAAGGGACAATATCTTGAATTTCTAATATTTCTTTTTCTCGTATCCA
CCGTACCATTGCTGTGACATTACGATATGTCTCTTCGGATATTTTATTTTCAAAATAAATCATAATTGTCTGCTCGTTAATAAATCTT
ACATCCACTTTAAATCCCCCTTTGTATTGCAATAAACCAGTATTGAATACCTTTTCATTGTATCATTGAGAAGCACAAGTTGTTTAAT
AAGTAATTCAAATCGCATACAATAACAAATGATAATCAATTTGTTTTCTCGGAAATATTTTGCTTTTTTAAAATGGAGTAGTTAATAT
TGTAACTCTTACACTAATTTAGGTTCTGCTATCATTCGGTCTGATGGAAAATTTTTACTTTTCATCTGTCCGATTTTTTGATTTTGAAT
ATAAAAAAGCACGACCGAAGTATCATTAACACACTTCAATCGCGCAATTAAACAATCTATTTGATCATTTATTGGATATTAACAATT
TTTACGTTCATTTCGCCACCATTAGGTAGTGGAACACGAACTTCATCATCTAAACCTTTACCAATTAACGCTTTAGCCATTGGTGATT
CATTTGAAATCTTACCATTAAATGCATCTGATTCAGCTGAACCAACGATTTGATAACTTTCCTCTTCATCACCTGGTAATTCTACAAA
CGTTACTGTTTTACCAATTTTAACAACGTTGTTATCTCCAGTATCTTCAATGATTAATGCATTTCTTAACATATGCTCAATTCTTTGAA
TATCTTGTTCGATGAATCCTTGTTCATCTTTTGCTGCATCATACTCAGAGTTCTCTGATAAGTCACCAAATGAACGTGCAACTTTAAT
TTTCTCTACAACTTCAGGACGCTTAACTGTTTTTAATTCTTCAAGTTCACGCTCTAATTTTTCAAAACCTTCTTGAGTCATTGGATATT
GCTTTTGATTTTCCATATTGTCATCTTCCTTTACTGAATTATACTATTGCTTGCTAACTAAAGACTGAATTTTTGTTGTCATAATATCT
ATTGCAACTTTATTGCTCCCACCTTCAGGAATAATTATATCAGCATATTTCTTAGTCGGTTCAATAAATTGGTCATGCATAGGTCTAA
CAACACTTAAATATTGATTGATAACAGAGTCCATTGAACGCCCACGCTCTTTAGTATCTCGTGTTAAACGGCGTAATATTCTCAAGT
CTGCATCTGTATCAACATATATTTTAACATCCATCATATCACGTAATACCTTATTTTCTAAAGCGAAAATACCTTCTACGATAATAAC
ATCTTTAGGTTTAAAATCAATGGTAATGTCACTTCTTGTATGACTAGCATAATCATATGTCGGTACTTCTACTGCTTTACCATTTTTCA
AGTCTTTAAGATTTTCAATTAATAAATCATTATCGAATGCAAATGGATGGTCATAATTGGTTTCTAGGCGCTCGTCGAAAGTCAAGT
GCTTTTGATCTTTATAATAGTAATCTTGAGCAAGTAAAGCGACACTATGACCTTCTAAGTTTTTCATAATTTCGTTAGTTACAGTTGT
TTTTCCTGAGCCAGATCCACCAGCTATGCCAATGATTGTAGTAGCTTTCATTAGCCAATTTCCTTTCTCATCATGTTGTTTGGATATAT
CGGGCGATCCACTTTAATTTGAACGATTTGTAATGGATGGCGCGCCGCGTCTAAGCTGTTACCTTCTTCATCATAAATTGCTTCTACT
ACTTGTGTAAATGTTTCAATTTCTGGACCAAAGAATTCTATTTCTTGACCTGGTTTAAAGTTATTTCGTTGTTGAATAGTCGCAATTTT
TGTATCTTCATTATAGTCTAATACCAAACCACAAAAATCAAATGGTGATTTTTTAGATTGTTGTTGACCAAACATCTGTTCTTCATAA
CCAGGTGTTCCCTCAAAGAATGCTGGTGCAGTGTCTCTATTTGCACATTTATCTAACTCAATTAACCATTCCGGATTAATCTTAAAGT
TGTCAGGATCTGCCGCATACGCATCAATGACTTTACGATATACTGAGACAACTGTTGCAATATAATGAATTGACTTCATACGTCCTT
CAATTTTTAATGAGTCCACACCAATATCCATCATTTGAGGAATTGATTCGATTAATTTTAAATCTTTAGGACTCATCGCAAACGGTGT
AACTTCACCTTGATTATAAAATACATCAAGTTCACCATTATCATCAACTTCTAATAATTCATAATCCCAACGGCAACTTTGACAGCA
ACCGCCTCTGTTGGAATCCCTTGCAGTCATATGATTACTTAATGTACATCTACCTGAATAGGCGATACACATAGCACCATGAATAAA
TGCTTCGATTTCAATATCTACTTTTTCCTTCATTTCACGCATTTCCATCGCGCCGGTCTCACGTGCTAATACAACACGATCCAATCCTT
CTTCTTTCCAATATTCTACAGCTTTGTAATTAGAAAGTGATTGTTGAGTAGATAAATGAATTTCAAGTTTTGGCGCAACTTCTTTACA
TGTTTCGATAATTAAAGGATCTGCAACAATGATACCTGTCGCACCAGTCTTTTCCAAATTACGCAAATATGATTCTAGACCTTCAAT
ATTCTCATCATGTGCAATAATATTTGTCGTAACATAAATTTTGGCACCGTAACGGTTCGCAAATTCAACACCTTCAGCTATTTCTTCC
ATCGTGAAATTATCAGCATTTGAACGTAATCCATATTCTTGACCACCTAAAAATACGGCATCAGCGCCATAATGTACTGCTATTTTT
AACTTTTCTAAGTTTCCAGCAGGTGCTAATAATTCTGGTTTCTTCATAACTGTTTTAGGAGTTGATTTAATCTCTTCTATTGTCTTCAT
GATTAACCTCCTTAATATACTGTTTGTTTATATAAGAAACCTTCGTCAAATGGTCGATGATCAGGTTGAATTTCTTCAATTGGATCAA
TCAACATAAATTTCTCATCTTCATAGATTTCAGGATCTTCATTGTACAAATCTATCGCTTGACGATACTGTTCCGTTACCACATTAAT
ATATTCTTCCGTTTGTAGAATACCATCGATTTTAAATGAATCTATACCCGCCTCAAAAAATGGTGCTAATTCTTCAATTAAACAAATG
TCGTTTGGTGACATAATGTGCGTACCATTGTAATCTTCGTAAACTGGGTAATTATTTTGTCTTTCTTCATCATAAAGTAATAAAGATT
GTTCATCATTGCGACGTTCAATTTTCATTTGGCGATCTTGGAACGTATAATAATTGCCTAGTAGCATACGCTTTGATTGGAACATACA
AGTCATTCCTTGAACTTGCACCTCAATTTCCACATTTGAATTTTCTTTTATATTAATAATTTCATCCAAATTCAGCTCACGTGCTAAGA
CAGCTCTTGATGCGCCTCTTTTACCCCAGTAATTACATTGAAAATGATTAGTTACTAACGTCTCTGCATTCCAATGAAGTGGTATTGG
ATTTTCTTGCGCCTTCACATACATTACTACTGCTGGATCCCCGAAAATAATTCTGTCAACTCGTATTTCATGTAAAAAATTAATATAA
TCTTCTACAGCATCTAAATGATAATTATGAAATAATCCATTCACTGCCGCATATACTTTTTTATCGTTTTTGTGAGCTAATGCGACAG
CCTCTGTCATTTGTTGTCTATTGAATTCCCCTGGAAGTCTTAAACCAAACTTTTGCTCGCCAATTACAAAAGCATCTGCACCTAAATC
AATAAGTGTTTCCATATGGCTTAATGACTTGGGTGTGACAAGTAATTCTGTCATAGTCATTCTCCTTTAATTGAAATCGCTAATCCAT
CGTCTATATTTAAAAAATTCGTTGTATATCCTGGTTGCTTTATTAACCACTCATTATAATCTTGAACCTTTTTAACCATTTGTCTTACA
TTTCTCGATCTAACAATCCCAATATCCGATACAAAACCGTGATATAAAACATTATCTGTAATTACGAGACCTTGGTGCTTTAAAAGT
GGTGTATATATTTCAAAAAATTTCTTTGATTGCGCTTTTGCTGCATCAATAAATATCATATCATAAACTTTGTCATTTACATTTTCAAA
TTGCTCTAAAGCATTACCTTCAATAATTCGAACCTGGTTTTCAAAATGATAAGTAGCTAAATTTTGTTTAGCATATTGAATCATCGTT
TCATTACGCTCTATCGTTGTGACATGAATGTCATCAGATATAGAAGCGAATTGCATAGAACTATAGCCGATTGCTGTACCAATTTCT
AAAATATTTTTAACATTATTCATACGAATTAATTGCTTAATTAAATCTAATGTTAAACGATCTACAATTGGCACTTCATTTCCCTCGG
CAAATTCACGCAAAACTTCGATTGAACTATTTTGATGTTGATGTAAATCTATTAAATATTTTTTATTTAGGTCATCCATGTTTTAAAC
TTCCTTTATGTAAAATAAGTCAATATGATTATGACAATAAAATAAATCAGCCTTCACAATTGATTATAATTTTGCCAACCAATTAAA
TGACTGATTTCTTGTTAGACGCAAAGCTATTTTATTTATAGAAGCGAATCATTCATATAAAATTTAACTTTAGATATTTTACCATATT
TTCAATAAAATTATAAGCGTTAATTATTTATACATTGCTTGACTTAAAAAAATACTCTTGCCTACCCATCTTAAGGTTAGCAAGAGTA
AAATCTTTTTAATTATTCTTCCATTTCAGTATTTACAACTTCTTCAATCATGTCCCATTCTTCATCAGTTTCGATTGGTACTAACTTAC
CACCGTCACCTGACTCATCTGGTTCATTGATCATTGGTACAAGCTCAATCATATCGTCTTCATCTGATTGAGCACCTTCTTCAGCTAA
GATAACATACTCTTTTTTGAATTCAGGATGATAAAATTCTAAAACTTTTCGGTATAAAACTTCATTTCCCTCTTCATCGAATAAAGTT

AATAATTCTTCTTCGTTATTAATTTCTAGTTGTGAATCATGATTATGTTCAGTCATAGTAAAATCTCCTTTTAATGTAGTGAATCTAAA
TAGCCTTGTAAAATAAATACCGCTGCCATTTTATCAATCACTTGTTTTCTTTTTTGTCTTGAAACATCTGCTTCTAATAATGATCGTTC
AGCAGCCATTGTGCTTAATCTTTCATCCCACATCACAATCTCAATAGAAGGATAAGCTTCTAATAATTTTTCTTTATATGTTAACGAA
GCTTCGCCTCGAAATCCTATTGAATTATTCATGTTTTTAGGTAGTCCTATTACGACTGTACCCACATTATGTTTTTTAATAATGTCTAC
TAATTGGTCAATACCTAATTCATTATTTTCTTCATTGATTCGGAGTGTGTCTAATCCTTGTGCCGTCCAACCCATTATATCACTAATTG
CAATTCCTACCGTTCTACTACCGACATCGAGTCCTAAAATTTTATGTTGTAACATAAATTATTTATTTTGCTCTTTTAAATAGTAAGA
AACAAGTTCTTCCATAATAACATCTCTATCAATATGACGAATTTGATTTCTTGCTTCATTCTGGCGTGGAATATACGCAGGGTCACCT
GATAATAAATAACCTACAATTTGGTTTACGGCATTATATCCTCGTTCATCTAATGTTCGATAAACATTATTTAAAACATCTCTTACAT
CTTGCGTTGGAAGTTCTTCATAGTCGAATTTCATTGTTTTATCAAAGTTTTCCATTTGCGACACTCCTTTAATTACAAATATAACTCAC
TATCATCATACAATATTATGGCTTTAAATTATAGATTTTTAATGTAATCTTTAATAAAGCTTAATGATTTTGAGATATTTTCAGGTTGT
GTACCGCCACCTTGAGCCATATCTGGACGACCGCCACCTTTACCACCAACGATTGGTGCCATTTGTTTGATAAGATCACCGGCTTTA
ACGTTATTTGTTAAAGATTTAGGGACAGTTGCAACCATCGATACTTTATCATCAACATTACTTGCAAGAATGATAATTGTATCTTGTA
GTTTAGATTTAAAATCATCCATTGTCGAGCGAATTGCTTTCGCATTTGGTACATCCACTTCAGTAACCAATACTTTATAGCCATTGAT
TTCTTCAACTTGATCTTCAATATTACCCATTTTAAGTGATGTGATTTCTTTGTCACGTTGCTCTAATTGTTTTAATAATGCTTTTTCTTC
ATCTTGTAATTGTGTTAACTTTTCGACTACTTGATCATCAGATTTCACTTTCATCTGTGATTTCATCGTATTAAATTTCTCTTGAATAT
CTTCTAAATATAAGAAAGCTGCTTTACCTGTTAATGCTTCAATACGACGCACACCAGCTCCTGTACCTGACTCACTTACTATTTTGAA
TAAGCCAATTTCAGAAGTATTGCGGACATGAATACCACCACATAATTCAATTGAAAATGGTGCCATATTTACTACACGCACAACATC
ACCATATTTTTCACCGAATAATGCCATTGCGCCCATTTCTTTAGCTGAAGCAATATCCATTTCTTGAATGTTAACGTCAATACCTTTC
CAAATTTCTTCATTTACTAAGCGTTCAACTTGATCAATTTCATCATTAGTCATTGGACCAAAATGAGAGAAATCAAAACGTAAACGA
TCTGCTTCTACTAGTGAACCAGCTTGGTTAACATGATCACCCAGTACTGATTTCAACGCTGCATGTAATAAATGTGTTGCACTATGGT
TCTTTTGAATGTCACGTCGATCATTTTGGTTCACTTCAGCAGACACTGTAGCGCCAACATTTACTTGGCCAAATTGTACTACTCCTTT
ATGCAAGTTTTGACCATTTGGTGCTTTGGTTACTTCACTAACAGCAATTTCAAAATTGTCATTATAAACAATACCTGTATCCGCAACT
TGTCCACCACTGACTGCATAAAATGGTGTTTCCGTTAACATGAAGTATACTGTTTCACCCGCTTCAACTTGTGAAACTTCTTCACCAT
TATATATCAAGTGTGTTAGTGTTGTTTGAGCTGTCGCAGTATCATAACCAACAAAAGTACTTGCAGATGTAATATTTTTCAATACTTC
ACTTTGAACTTGCATTGATTGAGAATTTTGACGTGCTTGACGTGCACGATCACGTTGTTGTTGCATTTCTGACTCAAATGTTGTCATA
TCAACTTTCAATCCTGCTTGCACTGCTATTTCTTCAGTTAATTCAATTGGGAACCCGTACGTATCATACAATTTAAATGCATCTTTCCC
ATTAATTTCATTTGTTGTCGCTTTAGCTTTTTTAATTAATTCATTTAAAATCGCTAAACCATCTTCTAATGTTTCATGGAATCGTTCTT
CTTCAGACTTTATAACACGCTTAATGAAATCTGCTTTTTCCTTAACATTTGGATAATATGGTTCCATAATGTCTGCAACAATATCAAC
AAGTTTGTACATAAATGGCTCATTGATTCCTAACGTTTGACTAAAACGAACGGCACGACGTAACAATCGACGTAATACATATCCTCT
ACCTTCATTGGCAGGTAATGCACCATCAGAAATTGCAAATGCAATCGTACGAATGTGGTCAGCAATTACTTTAAATGCCACATCTTG
TTCGTTGTTTACTAAATATTGTTTACCTGATACTTTTTCGATTTCATTCATTATAGGCATAAATAAATCTGTTTCATAGTTAGTACGTA
CATTTTGAGAAACTGAGGCCATACGCTCAAGCCCCATGCCGGTATCAATATTTTTATTAGGTAATGGTGTGTAACTATGATCTTTATT
ATGATTGAATTCACTAAATACTAAGTTCCATACTTCAAGATAGCGTTCATTTTCTCCACCTGGATACATTTCTTCTGCCGGATCGTCT
TGTCCATATGCTTCTCCGCGATCATAGAAAATCTCAGTGTTCGGTCCTGAAGGCCCTTCACCAATATCCCAGAAGTTACCTTCAATGC
GAATAATACGACTTTCTTCAAGCCCAATATCTTTATGCCAAATGTTATATGCTTCCATATCTTCCGGATGAATCGTAACGTACAATTT
ATCTGGCTCCATACCCATCCATTTATCACTCGTTAAAAATTCCCAAGCAAATTCAATCGCTTCTTGTTTAAAATAATCACCAATTGAG
AAGTTACCTAACATTTCAAAGAATGTATGGTGACGCGCTGTGAAACCAACATTTTCAATATCATTTGTACGAATAGCTTTTTGAGAG
TTTACAATTCTTGGCTTTTTAGGTGTTTCACGTCCATCAAAATATTTCTTTAATGTTGCTACACCTGAATTAATCCATAATAATGTATC
ATCATCAATTGGCACTAATGGTGCAGAAGGTTCAACCATATGTCCTTTTTCAACAAAGAAATCTAGATATTTTTGTCTAATTTCACTC
GCTTTTAACTTTTTCATCTTTTACACATCCTATTTACTGTTTTTAAATTACCATTCCATAAAAATTGATGACACAGATAGTCGATTTGA
AAAACTAGTATAAATCAATATCATTTTTTATTATTAAAAAATAAAAAACGCCCATCCTCAAAAGGGACGAACGTTATCGCGGTACC
ACCCTAGTTATAAATGCAATTCAACACATTTATCACTTTAAATCGACTATACAGTTGTGCATAAAGTAGCGTTCACTAATGTTTGTTG
TACTTTTCACCAACCAGTACATCTCTGATAAACAAATCATTAACTACTCATCTTTATACGAATTTAATTCTATTTTAGTTACATTTACG
CTTGTTGTCAACGTTCTATAAAGTCATACGGCGTGATTTCTCCCATATTAATCATTGGGTCAATTTTAAACATTGTAGCTTCCGTTAA
TACATTTATATCTGTTTTTGTTGAATCAGACGTAACTTCTCCACTATCATTCGATGACATTGGCGCTTCTACTTGATCATCTATTGTCG
TTTGTGAAGCTCCTGTATCATTAGTTGCTGTGTTTTCCAGCATTTCTTCATCTTCTGAATTAAAATAATTTTTCAACAATGTACATAAT
TGTGTTAAACGCGCTTGACCATTTGTTTTTAATCCAATATCAAATGCTTCTGGATCACCAAGTAGAACTAAACTCGTTTTCGCTCTAG
TTAAACCAGTATATAATATCGGTCTTTGTAACATTCTAAAATACTGTTTAACAATAGGCATGATAACAATAGGAAATTCTGAACCTT
GTGATTTATGGATTGATGTACAATAAGCATGTGTTAATTCCATCATATCTTGTTTCGTAAATGTAATTTCATTACCTTCAAAATCCAC
AACAAGTACATCTTTATTAAGGGCATTTTCTTTCGCCCAAAAAATACCAACAATAACTCCTATGTCACCATTGAATATGTTATCATTT
GGCCTATTAACAAGTTGTAATACTTTGTCACCTTTTCTAAAGACTACATCACCAAACTCAATTTCTCGTGTGTCTTTCTTTTTAGGGTT
TAAAATATCTTGTAAAACTTGATTTAAACGTTTAATACCGGCATTTCCTTTATACATTGGTGCAAGCACTTGAATATCAGCCATAGTA
TACCCTTTATTAACAGCACTAGTAACTACCTTCTCAACAACTGTTGGTATTTGGTTTGCCTGACAGTTAATAAAACTTCTATCATGAA
AACGCTGTGTAATATCAATTTTCTGACCCAATTTCATTCGATGTGCTAATTCTATAATGCTTGAACCATCTTGTTGACGATATACTTC
AGTCAGATTAACTCGTGGTATAGCTTTCGATTCAATTAAATCTTTAAATACTTGACCAGGACCTACAGAAGGCAATTGGTCCTCATC
ACCTACAAATATCAATTGTGCATCTAAAGGAACTGCACTTAAAAATTGGTGAAACAACCAAGTATCTACCATAGACATCTCATCAAT
GATTATGAGTCGTGCGTTTATTTCATTTTCTAATATATCCTCTGGCTTTGTGTCTTGATTCCAACCTATTAAACGATGAATCGTCATTG
CTTCTAATCCAGTTGACTCTTGTAGTCTCTTAGACGCTCTTCCTGTTGGCGCTGCTAATACAACTGGATAATCATCATTGACATAATC
ATCATAATCTAATGATAAGCCATGAATCTCAGCATATAATTCTACAATACCTTTAATTACTGTCGTCTTTCCTGTTCCCGGTCCACCG
GTTAATAGCATCACCTTAGAATTGATAGCCGTTTGCAAAGCTTCTTTTTGTGAAGCTGCATAGTTCACTTGATTCGCATCTTCTATTT
CACCAATATGCATTTGTAAATCTGACTGTTCAATTTCCGTAAGTTTATTTGTATGCGTCTTTATTCTGAATAAGTTTTGAACACTTTTG
ATTTCAGAATAATACAAACTTGGAATTGCAACTTGTTCATTGTCAATAATTAGTCGTTTTTCCTCATTTAAGTATTGCAACATTTCGT
CTAATTTTTCAGGTTCGATGACCTCTTCATCTTGATAATTTAATACATCAACCGTTAAATCTATAACAACATTGATAGGCAAATATGT
ATGTCCCTGTTTAATACATTCTTCTTCTAACGTATAGAGCAACGCAGCTTTTAATCGTTCATTATCGTTATAAGCGATACCAATATTT
CTAGCAAGTTGATCTGCTTTATTAAAACCAATACCTTTAATATCATAAATCAATTGATATGGATTTCGATCTAAAATAGTCAGTGTAT
CGCCGAGATAAAACTGATAAATTGCCATTGAAAGTTTAGGACCAAACCCTAAATCATGTAAACGAATCATTATTTTTTCAGATTCTT
GATTTGCTGAAATTTGTTCTGCAATTTGTTTCTGTTTCTTTTTAGATAATCCCGAAACTTTTTCTAGCACTGAATGGTCATCTAATATA
TCATTTATCGCATTGTCACCTAATGTATTAACAATATTTTGAGCTGTCTTTTTACCTACACCTTTAAACAAATCACTAGATAAATAAC
TTATAATTGCTTCTTTCGTTTGTGGCATTTCTTTTTCAAAAGTCTCTGCTTTTAATTGTTTACCATAACGTGGATGATCAACAACTTGC
CCTTTAAATGTGTAGACATCGCCTTCAACAATATTCGGAAGAAACCCTACAACAGTTGGCATTGTATCAAAGTCTTCATTTGTTTCA
ATAGTATCTACTTTAAGCACTGTATAAAAATTATCACTGTTTTGAAACAATATCGCTTCAACAGTACCTTTGATCATTGAATAATCAA

ATAGTGTAGGGTCTGACATGTTACTCCTCCTCTTTCATTTTAGTGAATGTTTTCAGCGCATGCTGACTTAATAAGTGTTTAGGGTCGA
TAGTCACAGCTTCTTTAAAATGAGTTATTGCTTCATCAATATCTTCATTTTTCATAAATAACGCTAAGCCCAAATTGTATCTTGCATC
AACATGATTTTTATCAATCGTTAATACATGTTTAAGTTGAGTTATGGCTTCATTAAACATTTCTAATTGACATAATACAAGACCATAT
TGAAATTGAACTTCTGCATCTTTGTCTTTATCTAGTTCCGCAGCAGTCATTAAATACGGCAATGCCAGCTTAAATGATTCTAACTGAT
TAAACGCCATACCGATCATATAATTACAATCAACTTGTTCAATCTCTGTTTGTAATGCTTGTTGATATAATTTAATAGCTTCTTGATA
ACGTTGCTGATTATAATATACATTTGCTAGATTATAAAATACGACGCCATTCTTCGGATCTATTGTTAAAGCTTTTTGGAAAAAACGC
TCTGCCTTTTCAATCTCATTCGCATCAGCAAGTACGATACCAGCATTAATATAATTTTCAATAATTGTAGGATCTTCTTCGATATTTC
CGAACAATGCTTGTAACGCTTCTTCTATTTTTCCATTTTGTATGTATTGATAAATTGTTTGTTGATCTATCATTTACGAACCTCATTTC
TCATCAATTATAACATCTTGATAAATTGTATGTCTCGAATCACTTAACAACGAATAAAATATAATCTAATATCATTTTCATTCATGAA
AAAGCGGGAATGGAATAGAAATGCTAAAGAACCATTAACGGTTTATTATGTAATGGTTCTTCCACATTAGCCACCACTATTATGTAC
TTAAAAATAGGAATACATAATTAGATTCATGCATAGGGAGTGGGACAGAAATGATATTTTAACAAAATTAAATTCGTTATCCCCAA
CTGGCATTGCCTGTAGAATTTCTTTACGAAATTCTCTGTGTTGGGGGCCCCATCCCCAACTTGCACATTATTGAAAGCTGACTTTTGG
TCAGCTTCTATGTTGGGGGCCCCGTCCCCAACTTCCATTGCCTGTAGAATTTCTTTACGAAATTCTCTATGTTGGGGCCCCGCCAATA
TAACATTGTAGAGCCTAGGACATTGTGATGTCCCAGACTCTATCATCATGAATTATTCTCATCAAAAACTGTCTTTCGTCATTTTCGA
CGTTGAAACTTCAAATAAGTAATTTATTGTTGCCATTGTTTATACAACATAATTTAATTGACCTTCATTTTTGAACACATCGTCAATT
GTTGCACCACCAAGACACACATCACCTTGATAAAAAACAACTGCTTGTCCAGGTGTGATTGCTCTTACTGGCTCAGCAAAAGTAACA
CGTAGTGCATGGTCGTTTTCACGTTTCACAAAAACTTTCGTATCTTTTTGGCGATATCTAAATTTAGCTGTACATTCAAAACCTTGAT
CTAAGTCATTATCTTCTGGATTTACAAATGAATAGTCTGAAGCAATTAAGTAATCACTGTATAATGCATCGTGATGGAATCCTTGTTC
TACATATAAAACATTATCTTTTAGGTTTTTACCGACAACAAACCAAGGATCGCCATCTCCACCTATACCTAATCCATGTCTTTGTCCT
ATTGTGTAATACATCAAACCACTATGTTTACCCATTTTCTTACCATCAAGTGTTATCATATCACCCGGTTGTGCAGGTAAATATTGTG
ATAAAAATGTTTTAAAGTTTTTTTCGCCGATAAAACAAATGCCTGTAGAATCTTTTTTCTTAGCAGTAACAAGTCCTTGTTCTTCAGC
AATTCGACGCACTTCACTCTTTTCGATGTCGCCAATTGGGAACATCACTTTTGAAAGTTGTTGTTGAGATAATTGATTCAAGAAGTAT
GTTTGATCTTTATTATTATCTACACCACGTAACATTTCAACATGACCATCTTCATGACGATGTATGCGTGCGTAATGTCCTGTTGCTA
CATAATCTGCACCTAAATTCATCGCATGATCTAAAAAGGCTTTAAACTTAATTTCTTTATTACACATAACGTCTGGATTTGGAGTACG
ACCTTTTTTGTATTCATCTAAGAAATACGTAAAGACTTTATCCCAATATTCTTTTTCAAAATTAACAGCGTAATATGGAATGCCAATT
TGATTACACACTTCAATAACATCGTTGTAATCTTCAGTTGCAGTACATACGCCATTTTCGTCAGTGTCATCCCAGTTTTTCATAAATA
TGCCAATGACATCATAACCTTGTTCTTTTAAGACGTGGGCTGTTACAGAACTATCTACACCGCCTGACATACCAACGACAACACGTA
TATCTTTATTTGACAATTATGACTCCTCCTTAAATTTAAAATATATTTTATGAATTTCAGCTACAATTGCATTAATTTCATTTTCAGTA
GTCAATTCGTTAAAACTAAATCGAATCGAATGATTTGATCGCTCCTCATCTTCGAACATTGCATCTAAAACATGCGACGGTTGTGTA
GAGCCTGCTGTACATGCAGATCCAGACGACACATAGATTTGTGCCATATCCAACAATGTTAACATCATTTCAACTTCAACAAACGGA
AAATATAGATTTACAATATGGCCTGTAGCATCCGTCATTGAACCATTTAATTCAAATGGAATCGCTCTTTCTTGTAATTTAACTAAAA
ATTGTTCTTTTAAATTCATTAAATGAATATTGTTATCGTCTCGATTCTTTTCTGCTAATTGTAATGCTTTAGCCATCCCGACAATTTGT
GCAAGATTTTCAGTACCTGCACGGCGTTTCAATTCTTGTTCACCGCCAAGTTGAGGATAATCTAATGTGACATGGTCTTTAACTAATA
ATGCACCGACACCTTTTGGTCCGCCAAACTTATGTGCAGTAATACTCATTGCGTCGATCTCAAATTCGTCAAACTTAACATCAAGAT
GTCCAATTGCTTGAACCGCATCAACATGGAAATATGCATTTGTCTCAGCAATAATATCTTGAATATCATAAATTTGTTGCACTGTGCC
AACTTCATTATTTACAAACATAATAGATACTAAAATCGTCTTATCTGTAATTGTTTCTTCAAGTTGATCTAAATCAATAGCACCTGTA
TCATCAACATCTAGATATGTTACATCAAAACCTTCTCGCTCTAATTGTTCAAAAACATGTAACACAGAATGATGTTCAATCTTCGAT
GTGATAATGTGATTACCCAATTGTTCATTTGCTTTTACTATGCCTTTAATTGCCGTATTATTCGATTCTGTTGCACCACTCGTAAATAT
AATTTCATGTGTATCTGCACCAAGTAATTGTGCAATTTGACGTCTTGACTCATCTAAATATTTACGCGCATCTCTTCCCTTAGCATGT
ATTGATGATGGATTACCATAATGCGAATTGTAAATCATCATCATCGCATCTACTACTTCAGGTTTTACTGGTGTGGTCGCAGCATAAT
CTGCATAAATTTCCATGTTTGGACACTCCTCACAATTTTATCAATGTTCCAATAATAGCACCTTACATACTATTTTTCTACTTTTCTGT
TTAACTTTATTTATAATGTTTTTAATTATATTTTACCATTTTCTACACATGCTTTTCGATAGGCTTTTTTAAGTTTATCGCTTTATTCTT
GTCTTTTTTATAAATTTTAGTATTTGCAGATATTTTTTCATTTGTAAAATGTAACGTACTATTATTTTGGTTATGAGCAATTTAATATT
TATCTGGTTATTCGATTGGTATACTTCTTATATCATAAAAAAGGAAGGACGATATAAAAATGGCGATTAAATATTCAGCATTAAACC
TTGTCCCTATTCGAGAAGGTGACGATGAACGAACAGCAATTAATGATATGGTTAAGCTCGCACAACATTTAGATGAATTATCGTATG
AAAGATATTGGATTGCTGAACACCATAACGCTCCCAACCTAGTAAGTTCAGCAACTGCTTTATTGATTCAACATACGTTAGAACATA
CGAAACACATACGTGTAGGTTCTGGAGGAATCATGTTACCTAACCATGCTCCATTAATCGTTGCGGAACAATTCGGCACGATGGCA
ACATTATTTCCAAATCGTGTCGATTTAGGATTAGGACGTGCACCTGGAACAGATATGATGACCGCAAGTGCATTAAGACGAGATCA
ACATGATGGTGTTTATAAATTTCCAGAAGAAGTTTCATTATTACAACAATATTTCGGCCCAGCTCACCAACAAGCATATGTTCGTGC
TTATCCAGCAGTAGGTAAAAATGTGCCTTTATACATTCTTGGTTCTTCAACAGATTCTGCACATTTAGCTGCTCGCAAAGGGCTTCCA
TATGTGTTCGCTGGACATTTTGCACCTCAACAAATGAAAGAAGCTATCGAAATTTACAAAACGTTATTTGAACCTTCTGATGTATTA
GACGAACCTTATGTTATTGTATGTTTAAATACAATCGTTGCTGAAAATGATGACGAAGCACAATATTTAGCTTCATCTATGGCACAA
GTAATGGTTAGTATCACTCGTGGCAGAATGCAGCCCGTTCAACCGCCAACACATGATCTACAAAATATATTAACGCCGAGAGAATA
TGCGATGGCTATGGAAAGACAGAAAATATCATTAATAGGTTCAGAAAATACTGTTCAACAAAAAATTCAAGATTTTATGGAAACTT
ATGGTGAAGTCAACGAAATTATGGCAATAAGTTATATTTATGATAAAGATATGCAATTAGACTCTTATCGTCGGTTCAAGAATGTTA
TAAATCAGATAAATGAAAAAAACACTTTATAATGTGATAAATAAACTAAGTGAAAGTATGTATCCATAATATTAATAAAAATAAAC
AGTAACAGCATTTTGAATAAAAGATGTCTTTATTGTTCAATCATTTGTTTTAGTAATGATTCAAATTCACTTAAAATTCTAAAGCAAA
TATGAAAGCGCCCCTTCAACTTTACACTGTGTTAGTGTTTATTTGATGGGGCGCTTTCAAAATATTGAAAAGCATATCCAAAATTTA
AAGAAATTTATTTCTCTTTATCTTCATTTTCTTTTTTCTCTTCGTTATTCGATCCTGTATATTCATTTATCTTATCTTTTACATTTTTAAC
TTGTTCATTATCGCTATTTTTAAATTTTTCTACTGCGTCTTTAGCTTTATCCATAAAACTCATATTAATCGCTCCTCTTATATTTGATTA
GTTTAATTGAACTTATTTTTTAAGTTTATCAATTGCATCAGTTATTTTGTTTTTAGCATTTTCAACAACTTCTTTTGCTTTACCAGTCGC
TTTATCTTGCTGACCTTCTTTTTCTAATTCTTTGTTATCAGTAACGTTACCTACTGTTTCTTTAACATTTCCTTTAAATTGATCGAACTT
ACTTTCGTCTGCCATAGTGAAACCTCCTTGGATGTATATATTTATATACCACTAAGGAGGTTCGCTAAACATTATAATATGAAGTTTT
TATGTTATAGTATAGTATTTATACGATTAAATATAAAACATGTATCCGTCTAAATCTTCACTTGTATCTACATATTCCGCTAAATATT
TCAATGTTGTATTATCTAAAACATCTCTCACTGCATCTCTCATGCGAATCCATAGTTGTTTTTGCGCAGGTGGTTCTGATTCAATACTT
TCAACAAATGTAATTGGACCTTCTAACAGTCTTATAATATCCCCTGCTGAGATTTCTTCCGCTGGTACTCTTAATTGGTATCCACCTT
TAGCACCGCGTACACTTCGAATTAACCCCGCATTTCTTAAAGGACCTACAAGCTGTTCTAAATATAAATCACTCAAATTATTTTCTTC
AGCAATTGACTTTAATGATATACATCCTTGCCCCTCTTTTTTAGCAAGAGAAATCATCAATGTAAGTCCATATCTCCCTTTAGTAGAA
ATTTTCATTGTATAACCTCACTTAATTCGAATATTGATATTCCCATTTTAGCATTTTTTGAGTTAAGATAGTATAAGAAAGGTGTGAC
AAATGTGAGTACAGAACCATTAGCATCGAGAATGCGCCCAAAAAATATAGATGAAATCATTTCCCAACAACATTTAGTTGGACCAA
AAGGCATTATCAGAAGAATGGTTGACACAAAAAAATTAACTTCAATGATTTTTTATGGTCCACCTGGTATAGGCAAAACAAGTATT

GCCAAAGCAATTTCGGGCAGTACGCAATATAAATTCAGACAATTGAATGCTGTAACTAACACTAAAAAAGATATGCAACTTGTTGT
TGAAGAAGCTAAAATGTCTGGTCAAGTTATCTTGTTGTTAGATGAAATACATCGACTAGATAAAGCTAAACAAGACTTTTTATTACC
TCATTTAGAAAATGGCAAAATCGTCTTGATTGGTGCTACAACTTCAAATCCTTATCATGCTATCAATCCAGCGATTCGTTCAAGAGC
GCAAATTTTCGAGTTATATCCTTTAAATGACGAAGATGTGCGCCAAGCGTTAACTCGTGCAATAGAAGATGATGAGAATGGTTTGA
AAACATATCAACCCAAAATTGATGAAGATGCCATGACCTACTTTTCTACACAAAGCCAAGGTGATGTTCGTAGTGCGTTAAATGCAT
TGGAATTAGCTGTATTAAGCGCAGATAATGACAAAGACGGTTATCGACATGTTACATTGCAAGATGCTAAAGACTGTTTACAAAAA
GGTGCATTTGTAAGTGATAAGGATGGTGACATGCATTACGATGTTATGAGCGCTTTCCAAAAATCTATCCGTGGTAGCGACGTCAAT
GCCGCTTTACATTATTTAGCACGATTAATTGAAGCTGGAGATTTACCTACAATAGTTCGACGATTACTTGTAATTAGCTATGAGGAT
ATAGGCTTAGCCTCACCTAATGCTGGTCAGAGAACACTTGCTGCTATTGAATCAGCAGAACGTCTAGGTTTACCAGAAGCTAGAATT
CCACTAAGCCAAGCAGTAATCGAACTATGCTTATCACCTAAGTCAAATTCAGCAATGAGTGCCATTGATAGTGCATTGTCCGATATT
AGAAACGGTCATGTGGGTCAAATTCCAAATCATTTAAAAGATGGACATTATAAAGGTGCTAAAGATCTAGGCCGATCTATTGGTTA
CAAATATCCACACCAATATGTTAATGGCTATGTTTCACAGCAATATTTACCTGATAAACTTAAAAACAAAATTTATTATGAACCAAA
AACGACATCTAAAAGTGAACAACAACTCAAAGAAATATATAACAACTTACTTAAACAAAGGCCGTAAAAATAAGATTAAATCGATT
ACCTTTGATATTTTAGCTATAATCAAATCAAAACTGACTAGTACACGAGGCATATTCTACTATGCTTGCGAACTAGTCAGTTTTAGTT
TTATGGTGTGGTTACACTTGCCTTACGTTGAAAATTATGACTTTCAAAATGAATTATTGCCCTTTGTCTTTAATGCGACGAACTGGAA
TATCTTTTAATACGTCATTCACCACATAACTAGCACAAATTAATCCAACAACACTTGGCACAAAGGCATTTGAAGAAGGTGGCATTT
GTCCTTTTCGATTGATAGCATTTTTATCTCCAACTATATCTTTTACATCTTCTCTTATGACAATTGGACTTTCATCTGAAAATACAACT
GGAATCCCTTTACGAATTCCTAGTTTTTTCAATTTTTGACGAATAATTTTGGCCATTGGATCGGTATGTGTTTTAGAGATATCTGAAA
TTGTAAAACGTGTTGGATCTGTTTTATTTGCAGCACCCATACTGGAAATCACTTTAATCCCTCGGTCAAGACACTCTTTCATTAAGTG
TACTTTGTACATTATTGTATCACTTGCATCTACAAAATAATCTATATCGTAGTTATCGAAAATTTCTTCATATGTCTCTTCTGTATAAA
ACATATGTAAGGGCGTGACTTTACAATCTGGATTAATTAATTTAATACGTTCTTCCATCAAAGAAACTTTACTTTGTCCTACCGTTGT
AGTTAAAGCGTGTAATTGTCTGTTTACATTTGTAATATCAACATCATCTTTATCTATTAATATAATATGACCAATATTCGTTCTTGCTA
ATGCTTCAGCAGCAAATGAACCAACACCTCCAACGCCAAGTATGACAACAGTTTGTTGCTTCAATAAATCTAAACCTTGTTGTCCAA
TCGCTAGTTCATTTCTTGAAAATTGATGTTTCATTATTTTACCTCTTTCACTGATTTATACATAAGTACATAGTAACTTAAAATTTTAT
ATTTAGCATTATCGCTTTGATTATTTTCCCAAAATTCAACGAGGAAACATTTATTAAACGCTATAAAACCCAACTAATTCTTTATTAA
AAACTTAAAGAAACGCATAAAAATACGCAAGACAAAGTCTTGCGTATCGATAGAGTCCGTATTGCCGTAGTTATAATAGCTTGATC
ATTCGGCCTGTTATATACAGGTGGGTGCCCTGTTTCTTGTTTTGTACGTCCTTCATATAAGGCGTGTACGCTGCAAGAAAACCCATTG
GGCTCCCTTGATCAAAGAGTGTTAGGCCCAAATTAAAAAGCAAACTTACGAACAACTCAGATGACTATCTTATGATGTTATATTACC
ACATAATTAAAATTAATGAAATTATAACAAACCAAAGTTTATTGATTTTTTAAAATTTAGTGACGAATTCGCAAAGAAAGTTCTTCT
AATTGTTTATCAGAAACTTCACCAGGCGCATTCGTTAATAAACATGTAGCAGATGCTGTTTTAGGGAATGCGATTGTATCTCTCAAG
TTTGTTCTATTAGTCAATAACATGACTAATCGGTCTAATCCTAATGCAATACCGCCATGTGGTGGTGCACCATATTTAAATGCATCTA
GTAAGAAGCCGAACTGTTCTTGTGCTTGTTCTTTAGTAAATCCAAGAACTTCGAACATTTTTTCTTGTAACTCACCATCATGAATTCT
GATTGAACCGCCACCTAATTCATAACCATTTAATACTATGTCATAAGCATTTGCCTCAGCTTCTTCTGGCGCAGTGCCAAGCTTAGCA
ATATCAGCTTCTTTTGGAGATGTAAATGGATGATGTGCTGCAACGTAACGTTTCGCATCTTCATCATATTCTAATAATGGCCAATCTG
TCACCCATAAGAAGTTTAATTTTGTTTCATCGATTAAACCTAATTCTTTAGCTAATTTGACACGTAATGCACCTAAACTTTGTGCAAC
GACATTTGGTTTGTCTGCAACAAACATTACTAAGTCACCAGCTTCAGCACCAGTTAATGTAAGTAATGTTTCAACATTTTCTGTTTCA
AAGAAACGTCCAATTGGACCTGTCAAACCATCTTCCACAACTTTAACCCACGCTAATCCTTTAGCACCATAGATGTTTACAAATTCT
GTTAAAGCATCCATATCTTTACGAGTATATTGTTCAGCTGCACCTTTAGCGACAATTGCTTTAATTTCACCATCATTTTCAACAGTAT
CTTTAAATACTTTAAAGTCCATATCACGTCCTAATTGAGAAACGTCAATTAATTCCATTTCAAAACGTGTATCTGGTTTATCAGAACC
ATAGCGACGCATCGCTTCTTTATATGTCATGCGTGGGAAAGCGCCATTAATTTCAACGCCTTTAACTTCTTTAACAACTTTTTTAAGC
ATTTCTTCACCCATTTGCATCACATCTTCTTGATCTACAAAACTCATTTCAATATCGACTTGTGTGAATTCAGGTTGACGATCTGCAC
GTAAATCTTCGTCACGGAAGCATTTTACGATTTGGTAGTATTTGTCAAATCCACTAATCATCAATAATTGCTTAAATAATTGTGGTGA
TTGTGGTAATGCATAAAATTCACCATCATGAACACGAGATGGTACTAAATAGTCACGTGCACCCTCAGGTGTTGACTTCGTTAGTAC
TGGTGTTTCGATGTCAAAGAACCCTTCATCATCCAAATATTGACGAATAGAACGTGTAATTTGATGTCTCATTTTAAATGTTTGCGCT
AACTCTTGACGACGTAAATCTAAATAACGGTATTTTAATCGAATATTTTCATCAACGTTAACATTTTCTTCATTTATAGAAAATGGTG
GTGTCTCAGATTTATTAATCACTTTAATATTTGTAACTTGTACTTCAACTTGGCCAGTTTTAATTTTAGGATTAACTGTTTCAGGGTCA
CGCTTCGTAACTGTACCTTGAACTTCTACAACATATTCAGAACGTACTGTTTCAGCAATTTTCAATGCCTCTTCTGAAAATGCAGGAT
TAAACACGACTTGTACAATTCCTTCTCTATCTCTTAAATCAACGAAAATCAATCCACCTAGGTCACGACGATTGTTAACCCATCCTTT
TAATGTAATTTCTTGTCCTAAAAATGCTTCAGTAACTAATCCACAATAAGTTGTTCTCTTACTCATATTTTAACGCCCTCTCTACTTCT
TAAAATATTCGACTAATGCGTCTAATTCAATTGTTTCAGATTCACCAGTTGTCATATTTTTAACATCGATTTTATTATTTTCTAATTCT
TGATCACCAATAACGATTGTAAACTTGGCACCTAAACGGTCTGCTTGTTTCATTTGTCCTTTAATTTTACGCTGTAAGTAGTCTTTAT
CTGCTTTAATACCATTATGTCTCAAATGATTTAATAACTTCACAGCATATCGATCTGCTTGATCACCCATTGTAACAATGAATAAATC
TAAGTTTTCTTCAATATCTAATTCGATACCTTCTTCTTCAAGTGCAAGCAATAATCGTTCTATACTTAGCGCAAAACCAATACCTGTT
TCACTTGGACCATCTAGCAATTCTAATAAACCATTATAACGGCCACCACCACAAAGCGTTGTAATGGCACCATCATAGTTAGGATTA
TCCATCATTAATTCAAATGCTGTATGTGTATAATAATCCAATCCACGAACTAAGTTAGGATCTTCAATATATGGAATACCTAAATCA
TCTAAATAAGCTTTTACTTGTTCATAATATGCCTTAGATTCCTCATTTAAGAAATCAGTGATTCTAGGTGCAGTCTTAATCGCTTCTTT
ATCACGGTCAACTTTACAATCCAAAATTCGCATCGGATTTGTATGCAAACGTGATTGACAATCTGAACAAAATTCATGAATTACTGG
TTCAAAGTGTTTCACTAACGCTTCGTTATATTCTTTTCGAGACGCCATATCCCCTACACTATTAATAACAAGCTTTAAATGTTTTAAT
CCAAATGATTGATAAATATGCATAACCATAGCTAATACTTCTGCATCTACGCTAGGATTTTCAGCACCAATAGCTTCTACACCAAAT
TGATTAAATTGACGATAGCGCCCTTTTTGCTTACGTTCATATCTAAACATCGGTCCATTGTAATAAAGTTTAATTGGTTGGTTTGGAT
TACCTTGCATTTTATGTTCAATATATGAACGCACAACTGCAGCTGTTCCCTCAGGTCTTAATGTAATACTTCTATCGCCTTTATCTTTA
AATGTATACATTTCTTTTTGTACGACATCGGTTGAATCACCAACACCTCTTGCAAAAAGATCTGTACTTTCAAAAATTGGTGTTCTTA
TTTCTTTATAATTATAAAATGTCATTAATTCATCTAATTGATTTTCAATGTAACGCCATTTCTTTGAATCTTCAGGTAAAATATCCTGC
GTCCCTCTAGGTATTTTAATCATTTTGACATCTCCCTCGGCAAACTATTTTATTTAATTACCTTTTTAAAACTAAAACTATATACGATC
ACTTAACTTTGCATGATAAATCATAATTTTAAAAGCAAAAGAAAAAGCCCTTGCACAGCCTGAAACTGCACAAGGGACGAATATCC
GTGTTGCCACCCTCATTGGTTTAAAAAACATGCCTAATTGTAATCATCATTACTTTTTATAAGCGTGCATTAAATAAACCCACTCAAA
ACGTTTAACGTACGTTTAACGGCTTTACTTTTCATAAAGCACCTCTTCTTGTGTTCAATGTATATAAACTACTCAGCATAATCTTTCA
GTCTATGGATTATATTCCTACACAATCTTAGTTAGATTGCTACCTATTTCATTTACCATTTCAAGAATACTAACGGTTTTAGTTATAA
AATTCAATATCAATGATAACTACTTTCACATATTTTTGCAAGCCCTACGCAGAAAAATAAATTTTAAGTCCATCAACAATTGCTTGTT
CTAAAATTTGTCTATGTAATTGATCTTTAATCATCGTTTCATCAGTTGGGTTACTAATATAACCTAATTCTAATAAAACAGCAGGAAC
TTTTGTTTGTCTTAACACTTGATAATTTTCTTGTCTTGAACCGCGATTAGAAAGTAAACCTTTCTTCTGAATCGTAGCGTCTAACGTAT

CTGCTAAAGCTCTTTGATTATCATGATACCAATAAACTGTCATTCCATTTGCATTAGATGATTCTAATGCATCATTATGTATACTCAA
ATAGGCATCGCCTTTGATATCACGATTTTCTAGTGAAACATATGTATCGTCTGTTCTTGTCATCTTAACAGTTGCGCCTTCTTTTTCTA
AAGTACGCTGCAATTCTTTTGCTGTTTTCAACGTATAGTCTTTTTCTAAACTTTTATATTTAGTATTGCTTGAAGCACCCTGGTCACTA
CCTCCATGACCAGGATCAAGCACTATTGTTTTACCTTGCAAAGGATTTTTCTCCTTCGTATTATCCGCGACAATATCTAAATTTGTGT
GCCATCCAGCTATCCAACCTTTTTCATTACTGGATGTATCTTCAACTTCAATCCATTTACCTACTTTACCAATCTTTTTAAAATGGTCA
CCTTTCTCAACTTTATATATGACTGGATACGCAGCGTTTGGACCTGTACGTAATTCAGTATTTTCAGTTATCGTGATGTTCCCACTAT
CTTCACTATTGCTATTCAGCAATAAAAATAAAAAGATGATAAATAAGACAAAGGCAATCACTACTATTAGAGTACGTTTATTTTTAA
GACCCTTTTTAGATAACCATGCCTCTATTTTTTTCATTGAATTTTGCCGTCCTGACTTTCATAAATAATAGTGACTGGACCATCATTAT
TTATGCTAACATTCATGTGTGTTCCAAATTCACCTGTTTTCACAGTAAGACCATACGCTCGTAGCGCATCATTAAAATACTCATAAAT
TTTTACCGCTTGATCAGGATTTTTAGAATTTGAGAAACCTGGACGGTTACCTTTTTTTACATCTGCATAGAGAGTAAATTGTGAAACT
GATAGTATTTCACCATTCATTTGTTGGATATTAAAGTTTAATTTATTATTGTCATCTTCAAATAATCTTGCATTAGCAATTTTCTTTGC
AATTACATCTGCATCTTGCTCTGTAGAGTTCTGACCGATACCGACTAATAAACAATATCCTTTTTTGATTTGATTATTTAATGTATCA
TTCGTCACCGATGCTTCTTTAACTCTTTGTACAACTACTTTCATATTTTGCACCTCTAGTTCCAAACTCTTGTTACTGTATAAACATCA
CCAAGTTGTTTGATCTTTTCTACCACACGATAAACATCATTCACGTTTTTCACCATGACACTAATATTTATTATTGCATTTTTATCAAT
ATCTGAACGTCCTGAAACTTTAATTAAATTGCCGGCTGTCGAGCTAACAGCTTGTAGTACTTCATTCAACAAGCCATTTCGGTCATA
CGCAGTTACCTCTAAATCAACCTGATATTTTTGAGTTGCGTCTTTTGATTTTACCCATTCAACATTAATTAGTCGTTCAGTTTCGTTCT
TAATATTTGGGCAATCAGTGCGATGTACTTTAATACCGTGACCTTTGGTGATATAACCTACAATATCATCACCTGGTATAGGATTAC
AACATTTTGACAACTTGATAAGTACATTTTCTAAACCTTCTACATAGACACCACTATCAGTAATGATGTTGTCTTTAATAGGCAATGA
TTTCGTAACTTCTTGTGCTTCATTTAAAGCACGTTGTTTATCTAAAATACGTTGTCTTTCAGTTAATTTATTAACAATCTGTAAGGATG
TCACGCCGCCAAATCCTACAGCTGCGAATAAATCATCTTCATTTGCAAAGTTATATTTTTCATTAACAACCTGAATATTTTTCTCTGT
CAAAATATCTTCGACTCTAAATCCTTGCTCTTTTATTTCAACTTCAACCATCATTCGGCCTTTTTCAATATTAGATGAACGATCTTGTT
TTTTGAAGAAACTTTTAATTTTACCTTTGGCACTAGACGATTTAACAATTTTCAACCAATCACGACTTGGTCCATATGAATGTTTACT
AGTACGTATTTCAACAATATCGCCTGTTTGTAAAATATAGTCAATTGGTACAATTTTGCCATTCACCTTGGCACCAATCATCTTATTA
CCTACTTCACTGTGAATCGCATAAGCAAAATCAATCGGCACAGCACCATATGGCAACTCAATAACATCACTCGCTGGGGTAAATGC
GTATACTTTGTCACTCTGTAAGTCATATTTTAAGGTTTCCATAAATTCTTGAGCGTCAGACGATGTATGATCCGCTTCAGCTAATTCT
TTTAACCAATTTAACTTATTTTGATAAGTTTGATCTTTTTCACTTACTTTTTTACCTTCTTTGTAAGCCCAGTGTGCTGCAACACCATG
CTCAGCAATTTCGTGCATATCAAACGTTCGTATTTGGATTTCGAGCGGGTCTCCATTTGGACCTACTACTGTAGTATGCAATGACTGA
TACAAATTTTGTTTAGGCATTGCAATATAATCTTTAAAACGTCCTGGCATCGGTTTCCATAACGTATGCACCAACCCAAGTATCGCAT
AACAATCATTAATAGAATTGACAATAACACGTATCGCCAACAAATCAAAAATTTGATCAAATTGTTTTTTCTGCTTCATCATTTTCCG
ATAAATACTGTAAATATGTTTAGGTCTACCATTTATATCGCCTTCGATATTCATTCGGTCCATTTCAGTACGTATTCTATCAATAGCC
GTTTCGATATACGCTTCACGTTCACTACGTTTCTTCTTCATTAAATTGACTATTCTAAAATATTGCACATTATCAATATAACGAAGAG
CCGTATCTTCTAGTTCCCATTTAATTGTATTAATACCAAGACGATGTGCTAAAGGTGCATAAATTTCTAATGTTTCTCGAGAAATTCT
AATTTGTTTTTCACGCGGCATGGCTTTCAAGGTACGCATATTATGTAATCTGTCTGCTAATTTCACCAAAATTACGCGTACATCTTTG
GCAATCGCAATAAATAACTTGCGATGATTTTCAGCTTGTTGTTCTTCTTTTGAGCGGTATTTTACTTTTTTAAGCTTCGTCACACCATC
AACAATTCGAGCAACTTCTTCATTGAACATTTCTTTTACATCTTCAAATGTATACGGTGTATCTTCAATTACATCATGCAAAAAACCT
GCGACAATCGTCGGTCCGTCTAATCGCATTTCTGTTAAAATACCTGCAACTTGTATAGGATGCATAATGTATGGTAATCCGTTTTTTC
GGAACTGACCTTTATGTGCTTCATAAGCAATATGATAGCTTTTTAAAACATACTCATATTCATCTGCTGACAAATATGATTTTGCTTT
GTGAAGAACTTCATCTGCACTATATGGATATTCGTTGTTCATTATATGATACACCCCATTCATATTTATTACTTCGCCTTAAACAATG
ATTTAGGTACTCTTGTTGAATAGTATTTGTCCCACACCAATCATACGTCCGTCGACGATAAATATTTATCCTGTCGTCATTAATCGTA
ATATTAATTTTACTTGAGCGAGTTTAATTTGTATACTATTCCTACTTTTAAAACTTTTACAAAAATTCGACCTAAATCTACTGTTTCAT
TTTTTAAATATTAGTTCTATGATACTACAATTTATGAAATAAATAAACGATGTTATTAAGGTATAATGCTCAATCATCTAACATTTTC
AGTAAATAAAAAATCCTAACATCTCATGTTAAGAAAACTTAAACAACTTTTTTAATTAAGTCATTGGTTCTTATACATTTGATGAAG
GATTTCATTTGATAAAATTATATTATTTATTATTCGTCGTATGAGATTAAACTCATAACATCGTAATCTTTAATTTTTTCAATACCATT
TAAATATTTCAATTCAATTATAAATGCAATACCTACTACGATACCGCCTAATTTTTCAACTAATTTTATTGCTGCTTCAATCGTACCA
CCAGTAGCTAATAAATCATCTGTAATTAACACACGTTGACCTGGTTTAATTGCATCTTTGTGCATTGTTAAAACATTTGTACCATATT
CTAGGTCATACTCATAACGAATGACTTCACGAGGTAATTTCCCTTCTTTTCTAACAGGTGCAAAGCCAATCCCCATTGAATAAGCTA
CAGGACAGCCAATGATAAAGCCACGCGCTTCAGGTCCTACAACGATATCAACATCTCTGTCTTTTGCGTATTCTACAATTTTATCTGT
TGCATAGCCATATGCTTCACCATTATCCATAATTGTAGTAATATCCTTGAAACTAACACCTGGTTTCGGCCAATCTTGAACTTCTGAT
ACGTATTGCTTTAAATCCATTAATATTTCCTCCTAAATTGCTCACGACAATTGTGACTTTATCCAATTTTTTATTTCTGAAAAATCTTG
ATATAATAATTGCTTTTCAACATCCATACGTTGTTGTCTTAATTGATATACTTTGCTGGAATCAATCGATCTTTTATCAGGTTGTTGAT
TGATTCGAATTAAACCATCTTCTTGTGTTACAAATTTTAAGTCTAAGAAAACTTTCAACATGAATTTAAGTGTATCTGGTTTCACACT
TAAATGTTGACACAATAACATACCCTCTTTCTGGATATTTGTTTCTTGTTTAGTTATTAATGCTTTATAACACTTTTTAAAAATATCCA
TATTAGGTATACCATCGAAGTAAATCGAATGATTATGTTGCAAAACTATATAAAGTTGAGAAAATTGCAGTTGTTGCAAGGAATTA
GACAAGTCTTCCATTGACGTTGGTAAATCTCTTAATACTACTTTATCAGTTTGTTGTTTAATTTCTTCACCATAATAATATTCATTCGC
ATTTACTTTATCACTTTTAGGATGAATAAGCACGACAATATTTTCATCATTTTCTGTAAAAGGTAAACTTTTTCGCTTACTTCTATAAT
CTAATATTTGCTGTTCATTCATCGCAATATCTTGAATAATTAATTGCGGTGATTGATTACCATTCCATTCGTTGATTTGAACAGATCC
TAATATATTAATTGGCTGTTCATCTTGTAACTCAGGTTCTAAGTGTCCATTTTGCCAAAATAGCGCGGCGATATTACTTTCACCAAGT
GTCAATTTTAGATGATTTTTTTGTTGACCGATCGCCTTAACTGAAGAAACTGATAAATCATCCATTTCAAAAATAGGTCTAGAAAAA
TCTGTTCCGAAGGGTCTTAAACGATTCATATCACGAATATTTTTAATCGTTATATCATTTTCTGTTAATAATACGTCTACTGGCTTTAC
GGGATCTAACGAAGTTGTTTTAGATAATTCTTTCATCCATTTATTTAAACCTTCAGCTAACGATTCTATATTTTCAATATCCATCGTCA
TACCTGCAGCCATATGATGGCCGCCAAATTTAGCGATTAACTCTTGATGTGCTGATAGTATTTCAAACATCGACACTTGATCAATTG
ATCTGGCGGAACCTTTTGCATGATTTTGCTCCCTATCAATATTTAAAATTAATGTTGGCAAAGCAAATGTTTCGACAATTTTCGAAGC
AACAATACCTAAGACACCTTCATGCCAATTTTCTTTTGCTAAAAGTAAAAATAAATCTCCCTTTTTAACTTTCGTTTCTGCCATAGCC
ATTGCTTCTTCTGTGATAGTTGCTACAATATCTTTTCTTTCACGGTTAAAATGTTCAACTTGTTCTGCTAAAAATGCAGCTTCTTCTTC
GTCGTCAGTCATCAACAATTCGCAAGCTAATGATGCGTCATCTAAACGACCTACAGCATTAAGTCTAGGTCCAATAATAAAACCAAT
TGTTTCTTCATCAATATTGTCATTGTATCCCGCTTCTTTTAGCAATGCTTTAACAGAGGTCGGACATTGATCATTTAAGACTTTTAATC
CTTGTTTCACTAATGATCGATTTTCATCAGTTAAGGATACTAAATCCGCAATGGTACCTATCGCAACTAATGCTTTAAAATAATCAG
GTACATTTTCAATCAATGCTTGTGCTAATTTGTATGCAACACCTGCACCACACAATTGTTGGAACGGATAATTAAACGATGGATGCA
TTGGATGTACGATTGCATATGCTTCTGGTAATGTACTACCAATTTCATGATGATCAGTTACAATGACATCAACTCCTAAATCTTGAAC
CATTTTAATTTCATTATGACCTTGTATGCCATTATCAACAGTTATGATTAATGTTATGCCTTCATCATGAGCATTTCTAAATGCTAGTT
CGTTTGGTCCATATCCTTCGGTAAAGCGGTTAGGAATATGCCATCCTACTTGTGCACCTAAAAGTTGTAATGTTGTCACTAAAATTGT

AGTTGAGGTAACACCGTCGGCATCGTAATCACCATAAACTAGGATTTTCTCATCATTCGCTATCGCTCTTTTAATTCTTTCAATAGCC
TTAGTCATATCGCTCAATTGCAATGCATCATGATTGACATCTGTGTCTGAAATGATGGATTCAATTGCTTGTTCATCAATAATCGATT
TACTTTCTAATATTTTTTTTACGATTGGCGTTAACTTTAATTTTGATGTTAATTCATCACTTATGTATTCAGCTGGTTTAGTTAATTTCC
ACTTATACTTCGGTTTAATCATATATATTTACGCCTCATTTCCAAAAGACATTATATCTAAAATTACTTTAAATAAAAAGCACTTATC
TAAATAATCAAGACTTTAATTATACCATTTTTTAAAAATGAATGCGGGAACTTATTTATATATATTTTGTTAAATTTAAATAAAGTAA
AAAGCGGTATGTGAACTCTTTATTGTTTCACATACCGCTTAATTCATTTTAAACTAAAATCTTTTCATCGTTCGATTTCTTTTCTTTAT
ATACAACTAATTTGTGTTTCGGCGATTTTTTCAACTGACGTTTTTTCATTATTCCCCATAGCGGAACGGCAATGAAGATTGAAGAGA
ATACACCAGAAATCAATCCGATAAATAACGCTAAAGTAAAGTTGAATATCGTAGGAGCACCGAAGAATAGTATAGCAACTACTACT
ACAATAACTGTTAATACTGTATTAATTGAACGTGTCATTGTCTGTCTAATTGATCTATTAACGATATCATCAATTTGTTCTGTTGTCGT
AATCACTTTAACCTTTTGTAAGTTTTCACGTACACGGTCAAACGTTACGATTGTATCATTAATTGAATAACCGACAATTGTTAATACA
GCGGCGATAAATGTTAAATCTACTTCAATTCTAAATAAACTGAAAATCGCTACTATAATGAATACATCATGTAATAATGCCAATACA
GATGAAAGACCCATGCGCCATTCAAATCGTAATGATACATAGATGATGATACCTATCGATGCATAGATTAATGCAAGCATTGCATTT
TTTGCTAATTCCTGTCCAATAATTGGTGATACAGTATTAATTTGAGGTGTGTCACCGAATTTCGATTTAATCTTATCACTCAATTTATT
ATCTTGAGCACGCGTTAAATCGTCTTTAAATTGAACAGTTGCTACTTTATTATCTTTACCATTGATTTGAATTTGATCCGCTTTAAGTC
CACTATCTTTTACAACTTGCTCAACCTTTTGTTGAGTAATTGCTTGTTTAGATTGGAAATCTACACGTGTACCACTTGAGAAATCAAT
TCCTAAGTTTAACTTGAAGATATAAAGAATAACTAAACCGACAACTACAATTAAAATACTTACTCCAATTAATGGCTTAGCTAATTT
AACAAAATTCCATTTCTCGAATGAAGTTTTAAGGTCATGAACATCTACACCTTCATTAATATCATGTCGTTTATTCTTTTTAACACCA
AATAACCAAAATTGATTTTTGAATATATTTGATGAAACAAGTAATGATAATAAGAATCTTGATAAGAACACGGCTGTAACAAAGAT
CATTAGAATACCTAATAATAACATTGTCGCGAAACCTTTAACTGAACTTTCACCGAAGAAGAATAATACTGCTGCGGCGATAACTGT
TGTTAAGTTAGAATCAAAAATTGTTAGGAATGAACTTTTGTTTGCTTTAGAAAAGGCTTGCTTTATCGTTCTACCTATTCGAAGTTCA
TCCTTAATACGCTCATACATGATAATATTGGCATCTACAGCCATACCTACACCTAATACCAACGCCGCTAATCCTGGTAAAGTTAGA
ACCCCGGAAATGAAATTAAATGCTACTAACGTTAGATAGATATAAGTTGTCAATGCAATAATCGCTACTAAACCAGGTAATCGGTA
GAATCCAAGCATGAATAAATAAATTAATGCTACACCAATAAACGATGCAAACACAGTTTTATCTAATGCATCTTGACCAAATTGGG
CACCTACTGAGTTTGAATAAATTTCTTTCAAGTCAACTGGTAAAGAACCTGCATTTAACAATTCGGCGATTTGTTTTGCTTTTTTAAC
GCCTTCTTGTCCTTTAAATCCACCCGAGATTTCTACGCTATCAGAATTGATTGGTTGATCAACACTTGCTGCAGAAATAAATTTAGGG
TTTTTCTTTTGTGCTTCTTTTTTATAGCTGTCACCTTTTTTGAAATCTAACCAAACAACCATGACATTATCACGTTTCTTAGAGATTTCT
TCCGTTACTTTTTTAAATTTGTTTTTGTCTTTTACTTTAAAAGTAACTGTAGGCTGGTTTGTTTCCTGTTTAAATTCTTGTTTGGCAGAT
CCCTGTTTAATATCAGAACCGCTTAATTTTACTTTATCTTCTGCATCGCGAATTGTTAAATTAGCTTGAGAAGATAAAATTTTACGTG
CTTCATTCTGGTCTGTTACACCAGCAAGTTGAACTCTAATTCTATTAGGTTCTTCAACTTGAATTTTAGGTTCCGAAACACCTAAAAC
GTTAACACGATTTTCTAATGTTTGCGCTGTTGATTGTAAGGCTTTTTTATCTATTTTGTCGCCTTTATTTAAAGGATCGACTTGATAAA
GCACCTCAAATCCACCTTGCAAATCAAGTCCTAAATTGACATTCTTTATAACACTTTTATAAGTTGCAGCCATTCCGGCAAACAACA
ATACGACTAAAAGCAAGAACGCAATTATTCTACTACTTTTCTTCACATGAACACCTCATTATTTACGTATGTATTTAGAATACTTGAA
TACTATTTTATAACGCAAGTTAAATCTTTCTTACAAAATTTATTAGCCTTATACATATTAACATACTAGAGCAGGGAGTAAAAATAG
TCATAGATATATTTAATTTAAATTTAACAATTATTATGTATCACAATTTCTTTTTTTGGAATAAAAATAAGCTATTCTACATACTACTT
ATAACTTAAAAAGGATGAAACGCTATTCGTTTCATCCTTTTTAATACCACTTCTCGTTATGAAGGGTCAACTTGTTTAATAGCAGGTT
TTTCGAAAGTTAATTCAGTACCATGACCATTAACTGTAATAACAACAGTTGTTTCATCTACTGCTTTAACAGTACCTTTAATACCACC
AATAGTTGTAATTCTTTGACCAGATTGAATGTTATTAATCAACTCACGATGCTGTTTCGCACGTTTTTGTTGTGGTCTGATCATCAAG
AAATACATAACCGCAAAAATTACGACTATATATATTAGTAATGAAAATTGCATTTTATACGCTCCTCTTGCTTAAAAGTTTTTTGGGT
TCTCAACATTTAATCCATATTGCTCGAAGAATTCTTCTTTGAAATCTAAAAGACGATCTTCTCGAATGGCTTGTCTTATATCTTCCATT
AATTTTAGCAGAAAATGTAAATTATGAATAGTAGTAAGACGAATACCAAAAGTTTCCTCTGCCTTGATTAAATGACGTATATACGCT
CTTGAATAGTTTTGACATGTATAACAGTCACAATTCTCATCTAACGGTCTTAAATCATCTGCAAATTTTGCATTTTTAATAACTAAAC
GACCTTGCGATGTCATACAAGTACCATTTCTGGCAATACGTGTCGGTAAGACACAATCAAACATATCCATGCCGCGAATACTACATT
CGATTAACGCATCTGGAGATCCTACACCCATTAAATATCTTGGTTTATCTTTAGGCATAAACTGCTCTGTATGTTCAACCATTTTATA
CATAACCGGTTTAGGTTCACCAACTGACAAACCGCCGATTGCATAACCAGGAAAATCTAATTCTACTAAATCCTTTGCACTTTGTTC
TCTTAAATCTTCATATTCGCCACCTTGTATAATGCCGAACAATGCTTGATCTTCAGGTCTTTGGTGTGCATCTAGACATCTTTTCGCCC
AACGTGTTGTACGTTCAATAGATTTTTTTACATAATCATATTCAGCAGGCATCGGTGGACATTCATCAAATGCCATCATAATATCAG
ATCCTAAATCATTTTGAATTTGCATTGATTTCTCAGGACTCAAAAATAATTTAGACCCATTAGTATGATGTCTAAATTCCACGCCTTC
TTCTGTAATTTTACGTAAATTACTTAAACTAAACACTTGGAAACCGCCTGAATCTGTAAGAATCGGACCATCCCAATTCATGAATTT
ATGTAATCCCCCAGCGTGTTTGATAATATCATTTCCGGGTTGTAACCACAAATGATATGTGTTGCCCAAAATGATTTTTGCTTCAATT
TGTCTTAACTCTTCTGGACTCATTGTTTTAACGGTTGCTTTAGTACCAACTGGCATAAACATAGGTGTTTCAAATGAACCGTGTGGTG
TGTGCACGATACCTAAACGCGCACCTGATTGTTTACAAGTTTTAATGTGTTCGTATGTTACTGCAGGCATATTCAAAATCCTCACATT
CTTTTTATATAATTAACATTGCATCGCCAAAACTAAAGAATCTATATTCTAAATTTACTGCTGTTTTATAAGCATTCAGAACATTTTC
ACGAGTACTAAACGCTGATACTAGCATAACTAATGTTGATTTTGGTAAATGAAAATTAGTAATCTGGCCATCAATTGCTTTAAAATC
AAATCCTGGATAAATAAATATATTAGTCCAGCCACTCGTTTCAACAAATTTATCATGATCGCGTCGAATTGTTTCAAGTGTACGTGTT
GAAGTTGTACCAACTGATATAATGCGATGTCCTTTGGACTTAGTATCATTTAATAAATCAGCTGTTTCTTGTGTCATTTGATAATATT
CACTATGCATTTCGTGGTCATTCACATCATCGACGCTCACCGGTCTAAACGTACCTAACCCAACATGTAATGTAACAAATGCGATAT
TAACGCCTTTATTTTTAATTTCAGTTAATAACTCATCAGTAAAATGTAATCCTGCTGTTGGTGCTGCCGCTGAACCACTTTCTTTAGC
GTAAACTGTTTGATAACGATCTGGATCATCTAAACGTTCTTTGATGTATGGTGGCAGTGGCATTTCCCCTAATTCATCTAATCTTTCT
TGTAAAATACCTTCATAATGTAAACGCATGATGCGTCCACCTTGATCCATTTCTTTTATGCACTCAGCTATAATTTTGCCATTACCAA
AATTCAATTTATTACCAACTTTAATACGCTTAGCTGGTTTCAGTAAGACTTCCCAATCATTACCTTCAATTTGAGTTAACATTAACAT
TTCAACTTTTGCACCAGTTTCTTCTTTTAAACCAAAAAGTCTAGCTGGCATTACTCGCGTATCGTTAAGCACTAATGTATCACCAGGT
CTAAAATACTCAATGATATCTTTGAAATGTAAATGTTTCATTTCACCAGTTTCTCTATCCATGACTAATAAACGACTATGATCACGAT
CTTTTAAAGGCGTTTGAGCAATTAATGATTCTGGTAAGTCATAGTCAAATTCTTCAATATTCACGTTATCCTCTCTCCTCATTCGACTT
TGCAAAATGTTCATAAGCTAATGGTGTTGCTTTTCTGCCACGTGGCGTACGTTCTAAAAAGCCTTTCTGAATAAGAAATGGCTCATA
AACGTCCTCAATTGTAATACGTTCTTCACCAATTGTTACGGCAATCGTATCTAAACCAACAGGTCCGCCATTATACTGCTTAATAATA
CAGTTCATCATTTTATGATCAATGTAATCTAGTCCGTGTTGATCAACTTGAAGTAAACCTAATGCGTGCTTCGTTGTTTCAATGTATA
TTTGTTCATCTTCATTCACTTGCTGGAAGTCTCTTACCCGCTTCAATAGTCGATTTGCTACTCTTGGAGTCCCTCTAGAACGTTTAGCA
AGTTCAATGGCACTTTCTTCATCAATACCTGTGCCTAAAACCTCAGCTGTTCTAATAATGATTTCTTTTAAATCTGATTCATTATAAT
ATTCTAATCTTAAGTGCACACCAAATCGATCCCTTAGTGGACCTGTTAAGCTGCCAGCTCGCGTTGTTGCACCTACCAAAGTGAATG
GAGGTAAGTCGATACGGATACTTCTAGCCTCATCGCCTTTACCAATGATAATATCTAAAAAGAAATCTTCCATTGCAGGGTATAACA
CTTCTTCAACAACACTACTCAGTCTGTGTATTTCATCAATAAACAAAACATCTCCAGGTTGAAGTCCTGATAAAATTGCAGCCAAAT

CACCAGGTCTTTCTAATGAAGGCCCTGATACTGTACGTATATTAACTTCCATTTCATTGGCAATGATATTAGATAATGTTGTCTTACC
TAATCCAGGGGGGCCAAAAAGCAATACATGATCTAATGGTTCATGACGAAGTTTAGCCGCTTTAATAAATACTTCTAAATTACTTTT
TATTGAATTTTGACCAATATATTGTCGTAATCTCGTAGGTCTAAGCGACAATTCGAAATCAGTTTCTTCACTATGCATTGATTGATCA
ACCATACGCTCATTCATGACAACACTTCCCCTATTAATCTATTTAAAATTAAGATACAACTAATTGAAGACCTGCCTTAACAGCTTC
ATCAACTGAGTCATATTTATTTTTATTTAACGTTTTCTCAACTTTTGCAAGCTCTCGTTTAGAATAACCTAATGCTTCTAACGCTAACA
TTGCTTCTTGCACGAATTGATCTTGCACCGTCGAAGTAGCGTCTACTTGTAATAATGAATCGCTATCTTCTTCAGTGATTTTCACTTTA
CCTTTTAAATCTAAGACAATCTGTCTTGCCGTTTTCTTACCAATTCCTGGGAATTTAGTTAAATACGTATCATTTTCATTTTCAATGGC
ACGTTTTACTTCATTAGGCGTACTTGTCGCTAAAATAGCTAAAGCTGATTTCGGACCAATACCAGTAACTTTAATTAAACTCAAGAA
CATATCTTTCTCTTCTTCACTACTAAATCCATACAATAATTGTGCATCTTCACGAACAATTAAAGATGTATGAATTAAAACTTCATGA
TCTAGATGCTTTTGAAAACGATAAGAATTTGGTGTTTGAATTTCATAACCAACACCAGCAGTTTCAACAACTACGTGTGTAGGATAT
AAATGTGTTAACTTACCTTTGACATACGCGTACATTATAGGCACATCCTTACATACTCATACTAATTAATTCTACTTTTGATACATAA
TCTAAATTTCTCAAAGCGCCAATAACATCTTCTACTGAAGTTTCTTTAGATTTAGCATTCAGTGATAATGTTATTGTTGCTTTTTCTTC
CATTGGAATACTTTGATGAATCGTTAATACAGATAGTTCTAACTTTGATATAACATCTAGTACACGTGCCAACATACCCACAATATC
AGTTACATATAAAATTAATGTAAATTCTCGATGGTCAAGCATTTTATCGTCTACTGGAAATATCGTTTCTCTATATTTATAAAAAGCA
CTTCTAGATAGATCAAACTGTTTAACGGCATCATAAATGGACAATGTCGGATCACTTTTTAAGGCATCTTTAATCTTCAATGTTTTAA
CCACGGATTCAGGCAAGACATCTTCTCTAATTAAATAAAACTTTTTATAATCTTTATTGTCCATCATGTTACGCTCCTATTCAACGAA
TTCAAATTCTCCTCCAAGAATTCTAACGATATCACCATTTTTACAACCACGTTCTCTAAGCGCATCATCAATACCCATCGAACGCATT
TGACGAGCAAATCGACGTACTGCTGGATCACTGTTAAAGTCAGTCATTTTAAACATTCTTTCAATAGCATTACCACTTACCACATAA
GCACCATCATCATCTCTTGAAATTGTAAATTTATCTTGTGACGGTGTATGTTTATATAATACTCGGTTAATGCCAACCGACTCCTCTT
CTTCAACTGTGAAGTCAACATCTTTATATTCTTCTAATTTATCTGCTATTGCATATAATAATTGATCAATATTATCACGCGTTATTGTT
GAAACTGGAATAACTGGCACATCTTCGCCAATTTCTTCTTTAAACAAGATTAAATTATCTTGTGATTCAGGTAAATCCATCTTGTTAG
CTACTACGATTTGAGGTCTATCTTCTAAACGTTGCTCGTACGCGGCTAATTCTTGATTAATGACTTTATAATCTTCAATAGGTTCTCT
ACCTTCAGAACCACTCATATCAATCATGTGAACAATAACTTTTGTTCTCTCTACATGTCTTAAAAATTGATGTCCTAATCCAACGCCA
TCAGATGCACCTTCAATTAAACCTGGTAAATCTGCCATAACAAAACTTCGTTGATCAGGTGTTGAAACAACACCTAGATTTGGTTTA
ATCGTTGTAAAATGATATGCCCCAATTTTAGGCTTAGCTTTTGAAACGATAGATAATAAAGTCGATTTACCCACACTAGGGAAACCT
ACTAATCCTACATCAGCTAATAATTTCAATTCTAAAGATACATCTAATTCCTCACCTGGTTCACCTTTTTCACTGAAGTCAGGTGCAG
GGTTTCTAGGTGTTGCAAAACGTGAATTACCTCGGCCACCTCGACCGCCCTTCGCTACTACAGCTCTTTGACCATCTTCAACAAGATC
TGCTAACACTTCGTCTGTTTCAACATTTTTAATAATTGTACCAGGTGGAACTTTTAATACTAAATCTTCCGCATTTTTACCATGCATAT
TGCTACTTTGGCCATTTTCACCTTTGCTTGCTTTAAAATGACGTTGATATCTAAAATCTAATAACGTTCTTAAACCTTCATCCACTTCA
AATACGACTGAAGCACCTTTACCACCGTCACCGCCAGCTGGTCCACCAAATGGTACATATTTTTCTCTTCTGTATGCGGTAATACCAT
TACCACCATCACCGGCTTTAAGAGATATTTTGACTTGATCGACAAACATATATCTCACCTCTTTTACATATAATTTTCATTTTCCCAA
TAATTATAGCATATTAGAGCCCCTTATAGTTTAGCGATTAGGGGCTCTTATGCAGTTGCTTTTATAAATCTTTGCTTTAAAATACTTA
TATTTCCGAGTACCAAATATGATACTTTTTTGCTTTTTATACTGCAACTGTATTCTTTTGTCCCCTTATAGTTTAGCGATTAGGGGCTC
TTATGCAGTTGCTTTTAAGTTTACTTTACTCAAAT

>038-contig_319_RC

CTGTATTCCTTTTAGCCCCTTATATATTGTAAATAATTAGGGGCTCTTATGCAGTTACTTTTATAAATCTTTGCTTTAAAATACTTATA
TTTCTCAGCACCAATTGTGGTACAGTTTATTCTTTAAACGCTGCAACTGTATTCCTTTGTCCCCCTTATAGTTTAGCGATTAGGGGCTC
TTATACAGTTGCACATTTAGTATAACGTCTAAAATTCCCATTTTTTTATTACATAATGAAAATAATAATGAAAAACGTTTTATAAAAA
GTAAAAAACACCAGAAGATTCACTTCTGGTGTTAACTAGACAAAATTATTCAGCTACTGCATATACAGAAACTTGTTTTTTGTCGCG
ACCTTTACGTTCGAATTTAACAACGCCGTCGATTTTAGCGAATAATGTATCATCGCCACCACGACCTACATTTTCACCAGGGTAAAT
TTTAGTACCACGTTGGCGATATAAAATTGAACCACCTGTTACGAATTGACCGTCAGCACGTTTAGCACCTAAGCGTTTTGATTCAGA
GTCACGTCCGTTTTTTGTAGAACTTACCCCTTTTTTAGATGCGAAGAATTGTAAGTTTAATTTTAACATCGGAATGCACCTCACTTAT
AATTTAATCTAATATTCTCATTATATTCTTCTTCAATAGTTTGTAAAGACACAAGCATTGTTTGAAGAATTAGTTGCGCTTCATCGTT
ATTTGTATCAACGCTTCTTATATGAAAATGACCACCATTGTCGTCATAATTGATATCTGGTCTCTCAGATGTCAATCCTATAATCGCA
TTAACACTACCAAACAATACAGCTGAAGCTCCAGCACAAACGATATCATGACCATATTCACCATGGTCAGCATGGCCATCCATAAT
AACGTCTGTTACTTTGCCTTCATCATTAACTGTAATATCAACAGTAATCATAATAATTACGCGTTGATTTTATCGATTGTTAATTTAG
TGTATGGTTGACGATGGCCTTTTTTACGTTTTGAATTTTTACGACGTTTGTATGTGAATACAGTAATTTTTTTACCGCGACCTTGTTTA
TTAACAGTAGCAGTAACTGTTGCACCTTCAACTGTTGGCGCTCCAACTTTAACTGAATCTCCACCTACAAATAATACTTTATCAAATG
TAAAAGTATCTCCTTCGTTTACGTCTAATTTTTCAACGAAGATTTCTTGACCTTCTTCTACTTTGATTTGTTTTCCACCTGTTTCAATA
ATAGCAAACATACTTTGCACCTCCTGTATAATAAGTCACGCCATACATAGGTGACATTTCGTTATTGAGTCAAGCGTTTGCACTCTA
CGTTGTTTCGGTCGCAACTCGAGTCATTTATAAACATAAACTCTTCTCGTTTCTTCCTTCAACGCCTTGATTGACAATCGCTTTCTTTC
AATAACTTCTGCAAATGAGTCAAGCGTTTGCACTCTGTGGGAAAATCGCTTTCTTCATTAAAAATGTTCTTGAACCTATATTTGAGCG
GTTGTATGTAGCTACTTATAACTACTCAACTATTGCATCTTACCATTGACGACGTTTCATGTCAATGTCATTGTTTGTTTTTTTAAGGA
ACTTTATAATCAACGGATAAAGCATAATCAACAGTACAAAATTCATAATTAATGTTGGCAATAATCTAAAGACTACAAAATGAATA
ATATCAAATTGAATGAATCCTAACATACCGTATATTAATGCCACATAGACTTCTAATAATAAGGTGCTGGCTAATATAATAATGAAT
AACATCGAATGATCTTTGTAAAAAATTTTAAAGAATCGATCTATAAGTGCTAAAAACAATATATAGCCAAATAAGTACACTCCATA
AATACTACCAAAGTATACATCAGTCATTACGCCTAAAAATATGCTGAGCAATAATGATACGCCAAAGCCACGATACACTACCATCA
TTAAAATATACATAAATGTAAGGTGTGGTACAAATACAAGTTCAAACTTACCTATGTGCATTGGAATAAGAAGCCCAATTGCAGTA
TCTATATAAAATAGTAAAATACCTATCAAAAAATAATACAGTGTACGCATTATTTATCCCTGCTTTCATCATCAGGAATTGTTTTAGG
ATCTCTTTTTGCAACATAAACATGACTCAAATCTGTTAAGTCTGCACCAGTCTTAACCCTAACTTCTTTAGCTAAGCCGTATTGATCA
TTTTGAACCTTAGTCACTTCTCCTATATATAAATTACTTGGTAGTTGATCAGCTAATCCACTTGTAACGACTTTATCACCTTTTGAGAT
ATTATCTCTATTATTAATGTCACTAATTACAAGTTCTGAGTTCTTTTCATCATAACGATCAATTAAACCAAATATATTTTTAGAACCG
TGTTGTATATTTACAGATAATTTACCCGCACGTGTATTAGTTGAGATTAAATCAACTTGTGAAGAAAATTTATTAACTTTAGTAACTC
TTCCAACAAAACCTTGTGATGTCATCACAGCCATATTTGAAGTTATACCTGATTTAGATCCCTTATCAATTACAATTGTATTCATCCA
CTGATCCGGATTTCTTGCCAAAACCGTAGTAGAAATAGGATCAAATTTTGAAATATCTTTTAAATCAAGCTCTTTTTTTAATTTTTCA
TTTTCCGCTTCTAATTGTTGGTTCTTAGATTCTAACTGGCTAATCTTATTTTTAGATTCTTTAGAATCTCCTTTTTTAAAAAAGTCCCC
AATCGTACCAGCAACAAAATTAACTGGATAACTCACAACTCGTTGTCCAAAAGACACAGAATCACCTATATATTGTTCAGGAGGTG
ATTGAGATTGTGAACGTATGGACAGCCCAATTAATGCAATAAAAACGATAATTGCACATAAAACAACAATTAATTTGTTATTTTTAA
AAAACTTAAGCACCCAGAACACCTCTATTATGTCAAAATATTGTATATCCTTTTCTATTTTATATTACTCCCATTATGAAATAAAGCA

CTAACTACGAATTACTTTGAAAATGAACAACTCCGTAAAAAATTTGTGGCATTTTAAAAACAAAAAACTACTAGCAAACATTCCAG
TCACTAGTAGTTTAAATAATCGATTTAAATTTTAAAATTATTTGTTTGTATCGTTCGAGGATTCACTGTTCGAATTCTGAAGTTGTCTC
CAACGTGCGAATAAGTTCTGGGCCTTTTGTTGTTCATTAGCTTGTTTCTGTTTTGATTGTTCTGCCATTTGAACTACACCAACCTTTTA
AATTTATTCTAGTGACAGGATAACTAAAATATATTTCTTATGCAATAATTTCCTATTGATAAGCATTTTCGGGTTTTAGTTGTAAATT
TTGCCCTAATTCATTTAAGTCTTGTTGCATTTCAAATTCAGTATTGTAAACACGCATTGACTCATCTCCAAATTTATAAAGAATAAAT
TCGTCTCCTCTTTGACCTATAATATATTGATCATTATAAGCCATGCGATTCATTCCAGACACAGCCATAAACTCTTGTTTATCTATCA
TTTTAAATACATTTTTAATTTGGCTTAATGGTACATTCTTTATTAAATCATTTTCTTTTAATGAATATTTCTCACCACTTACATAAACA
TGTTGGGCATCATTAGTGGGTTGCGTAACTTGATTCATTTGATCATTATCGTGATTTACAGGATTCCAATCTTGTTCTTTTAAAAAAG
GTGCGTATTTCAAGGCGATATAAACAATTATCACAATTGCTATTACTGCTATAATGTTTTTAATCATACTAAATATCAATCTCATAGT
TCGCCTCCAATGTCTTCATCTAATTATATATTTTATTATGTATATTTTCCTCAGTCTTCAGCCTTAATCATGATATATCTTAGGACTGA
TGCACGTTCATTTAAAATAGCTTATAATTTTGTTATGATTTAAAAGCGTATGAGGAGGTTTGTAGCATTGAGTATACTAACAATTATT
TTAATAGCACTATTAGTCATCCTCCTTTTTAGAGTTGGACTTTCTATTTTGCGATTTTTAATTTATGTCGGACTTGTTTTATTATGTATT
TACTTAGGATATCAAGGTTTAATATGGTTGCTCGATTTTTTTCAAATAAATAGTGGGTTTTTACCGCACTTTCAATTTAACAATTAAA
TTTATTCTGCATAACAAAACTCCTAATGTACTAGTTTAGAATTTTTTAACTAGCTCCACATTAGGAGTTTTAATTTGTTTTCAAGCAC
GGATACTACTTGATGAACAGATCACTATTACTTACGCCCAAATGCGTTAGATAAAAATCGTATCAGCGCGTAGCTACCACTTAGTAA
AAGGCCCCAAACGATTCCAGTAAAAATTGAGCCAATTGCTACACTTAATAAAGGAAAAGCTTGTTTCGTAACGATCACACCTGCTA
ACGCTGTTACTAAAATACCTAACGTAGATAAAGCTAATGTTATTAATAGATTGCGCTTTGTTACAGTTTCATTTTTAATATTTAACTG
CATGGTATAAACCATTGGCACGAGAACCCCGATAATAATAAATAGAGTCACTATTTTTCCTCCAATGAAAGTACTTCGTAGGTATTA
TACACAAGATACGTCATTAATTCTATCAACTTCAATCATTTTCATCAAAGTAACCCGCTTCTACAAGACTGGTAAATCTATTATCACC
GATTATAATATGATCCAACAAATCTATCCCTAAAATCAAACCACACTCCTTCAACCTCATTGTTGTTATGATATCTTCTTGTGAGGGC
GTTACATCACCGGATGGATGATTATGAACTGCGATAATTGCATTGGCATTTTCTCTCACCGCAATACTAAAAATTTCACGTGGATGT
ACAATCGAACTATTTAATGTACCTTTAAAAACACAGGTTTCTTTAATCACTACATTTTTTGAATTTAACAATAAAATGACAAAATGTT
CTTGTGTTAAATCTTTCATTGTTGGAATCATATAATCAGCAACATCACTTGGTTGCGTTATTTTTATACGATTATTTTCAGCTCTTCTC
CCCATCCTTTCCCCTAGCTCAAATGCTGCTTTTAAAGTAATTGCTTTTTGTAATCCAATCCCTTTAACTTGTATCAAATCGTTAATTGA
AGATTTTTTCAATTCATTCAGATTCGAAGCAGATTTAAGCAGTTCATTACTAATGTCTATGCTCGAGAATCCTTTTCTTCCGGTGTTA
ATTAATATAGCTAATAATTCTGTATTCGAAAGACTTTTTGCACCATGGCTTAACAAACGTTCTCTTGGCATTTCTGAAGTTACCATTT
CTTTAATTTTCAAAAATATACGCCTCCTAAAAATTGATGGATATCATTATAAAAAAGTGAATTGATAAAAAAGGAAATAAATATAA
ATGGAACAAGGGGTAATAGTTTAATCGGCTTAAATATCATGGTAATTAAAGCAACTAAACCAGCAATGACAAATGTAAATAAAATG
ACATAAATAGTGAATTGGAGAGGGAAAAACAAAGAAAGTGCAGATATTAGTAAAACGTCACCATAACCAATATATGCCCGAAATA
AAAAGTAGAATATATGCGTGGTCATACTAATAATGAGAAAAGCTACTGGATAAATCATACTTAACGAGAGAGAAACGATACAATA
AATTATAATTAAACGACAATCTAACATTAAAGAAGTGATATCGGTCATAGTAAAAATAAGCAGAAAAACATATGTAGTTATAAATA
GCGTAGCATTTACGTATGTGAAATCATACTTAATAAAGACGATAGGTATTAAAGCAAAGGTTTCCCCTAAGAAATGTGTTAGGGAA
ATACGCTTTCGACAGTTTCGACATCGCCCTTTTAATAATAAAAAACTAATAATCGGCATTAATTCATACCATTTAAGTGACGAATTA
CAATAATCACATTTCGATCTTCTATGTAAATAATCAAATGACGTTTCTTCTATAGATATAAATTGATATAGAAAACTAAAAATACAA
CTGCAACTATAAGATAACAATACTACCAAATGACAACCTCCTTACGTAAAGTATAGTCACTTATTATCAAAATGTAAATATACACTA
TTTTTCAAGAATTGAACCGCTTTTTCATTTAAATTTTTCAATATTGCTAAGCGTAATTGATGGATACTTTAACAGCCCATTACTGCTC
GGCAAAATTAATAATGGCAAGAAATTGAACCTTATAAACACATACGATTTAGAGCATAAAAAATAACCATGAAGCTCTACCTATTG
ATTAAATAGATTCTTCATGGCTATTTTAGTTTTAGTTTTATAATGCTTCAAAGTCTAATTTTGATTTAACTTCACTTATGAAATACAGA
CTACCGGTAATTACTAATGTATCACCTTGATAATTTTTTATAAATTCAACGTAGTCATCTACTAATTGTATTTCATCATTTTCAATACT
ACCTACAATTTCTTCTTTGCGTAACGCTTTCGGAAAATCAAATTCAGTTGCATAAAACGTATGCGCAATTAAACTTAAATGTTTGACC
ATCTCGTTAATCGGTTTTCCGTTTATTGCTGAGAACAAAATATCTACTTTTTCTTTATCATGGTACTGTTTAATTGTATCAATTAGAGC
ATCTATACTCTCTGAATTATGTGCGCCATCCAAAATGATTAAAGGTTTGTCATGCACCTGCTCAATACGTCCAGTCCAACGAACTGA
TTCAATACCGTCTATCATCTTATTGAAATCTAATTCAATTAATCCTTGTTCATTTAATTCAATAAGAGCTGTTATGGCTAATGCAGCA
TTTTGTTTCTGATGTTCACCTAGCATACTTAAAATGATTGTTTCTAATTCATAATCTTTATAACGGTAAGTAAATTCATCATTTTGCGA
TACAACAACAATTTCTCTATCTAATTCAATTGGCTTCGCATGTTGTTCAATTGCGCGTTCACGAACATATTTTAATGCATCTTCATTTT
TAACAGCATATATCACTGGAACGTTAGGCTTTATAATCGCGCCTTTATCCCTAGCAATATCTAGATAAGTACCACCTAAAATATCTG
TATGGTCTAGACCGATACTAGTTAAGATTGATAAAACCGGTGTAAAGACATTTGTCGAATCGTTCTTTATACCCAATCCAGCCTCAA
CAATGACAAAATCAACAGGATGTATTTCACCAAAATATAAAAACATCATCGCTGTGATTATTTCGAATTCAGTTGCAACACCTAAAT
CTGTTTCACGTTCCATCATTTCACTTACTGGTTTAATACGTGATACTAATTCTACAATAGCGTCATTTGATATTGGCACACCATTTAG
ACTAATTCGTTCATTAAATGTTTCAATAAACGGCGACGTAAATGTACCTACTTCATAACCATTTTCAACTAAAGCTGTTCTAAGGTA
AGCAACTGTAGAGCCTTTACCATTTGTGCCACCTACATGAATACCCTTAATGTTATTTTGAGGATTATTAAATTGTGCTAGCATCCAT
TCCATACGTTTAACACCTGGTTTGATGCCAAATTTAGTTCTTTCGTGTATCCAATACAAGCTCTCTAGGTAATTCATTGTTACTAACT
CCTATGCTTTTAATTGTTCAATTCTTGCCTTCACACCATCATATTTTTCTTGATAATCTTGTTTTTTACGTTTTTCTTCATTTATAACCTT
TTCAGGTGCTTTACTTACAAAGTTTTCATTAGAGAGCTTTTTATCTACTCTATCTAATTCGCTTTGAAGTTTAGCTAATTCTTTTTCCA
AACGGCTGATTTCCTTATCCATATCAATTAGCCCTTCTAATGGTAATACCACTTTACCTGCAATTACAACTGATGTCATTGCTTTCTC
AGGAATTTCCACGTCAGTGCTAATATTTAAGGTACTAGGATTACAGAATTTGATTAAATAATCTTTGTTTTGTGATAAAGTTGTTTCA
ATTTCTTTATCTTTAGCTTGAATTAAAATAGGTATTTCTTTAGACAATGGCGTATTTACTTCTACACGTGATTGTCTTACAGATTTAAT
GATTTCAACAAGTTGTTGCATTGTTTGTTTACTTTCTTCAAAAATCAATGATTCACGCACTTCTGGCCATGAAGCTTTAACAATTGTG
TCACCTTCATGTGGTAAACTTTGCCATATTTTCTCTGTTACAAATGGCATGAATGGATGTAGCATTCTCATAATATTGTCTAAAGTAT
AACTCAATACTGAACGTGTAACTTGTTTTTGTTCTTCATCATTACCATTCATTGGAATTTTACTCATTTCAATGTACCAATCACAGAA
ATCATCCCAAATGAAATTATATAATGCACGTCCAACTTCGCCAAATTCATATTTGTCACTTAGATCAGTAACTGTTGCAATCGTTTCA
TTTAAACGTGTTAGAATCCATTTATCTGCTAATGATAAGTTACCACTTAAATCGATATCTTCAACTTTAAAGTCTTCACCGATATTCA
TTAAACTGAAACGTGCCCCATTCCAGATTTTATTGATAAAGTTCCACACTGACTCAACTTTTTCAGTTGAGTATCTTAAATCATGTCC
TGGAGATGAACCTGTTGCTAAGAAGTAACGCAAGCTATCAGCACCGTATTCGTCAATAACATCCATTGGATCCACACCATTACCTAA
TGATTTACTCATCTTACGCCCGTCTTCAGCACGAACTAAACCGTGTAATAATACATCATTAAATGGACGACGATCTGTAAATTCTAA
GCCTTGGAATATCATGCGTGCTACCCAGAAAAAGATAATATCGTAACCTGTAACTAAGGCATTTGTTGGGTAGTATCGTTTAAAGTC
TTCACTTTCTAAATCAGGCCAACCTAACGTAGAGAAAGGCCATAAAGCACTTGAGAACCACGTATCTAATACATCTTCATCTTGTTG
CCAATTTTCAATATCAGTTGGCGCTTCTTCTCCAACATATATTTCGCCTGTTTCTTTATGATACCAAGCCGGAATTTGATGACCCCAC
CATAATTGTCTTGAAATCGTCCAATCTCTAATATTTTCCATCCATTGGTTAAATGTATGTTCGAAACGTTGCGGATAAAAATCAATAC
GATCATCTGTTTTTTGGTTATCTAATGAACGTTTCGCTAAGTCTTCCATGCGCACAAACCATTGTGTTGATAAATATGGTTCAACAAC
AGCGCCAGATCGTTCTGAATGACCTACAGAATGAACATGATCTTCAATCTTGATAACTAAATCTTGTTCTTTTAAATCTTTAACTAGC

TGTTTACGACAATCAAAACGGTCCATACCTTCGTATTTACCCGCTTTGTCGTTCATTTTACCATTTTCATCCATAACGATAATATTTTC
TAATTGATGTCTTTGACCAATTTCAAAATCATTAGGGTCATGTGCTGGTGTCACTTTCATAGCACCAGAACCGAAGTCTATATCAAC
ATACTCATCTGCTAAAATAGGCAGTTCGCGTCCTACGATTGGTAATATAACAGTTTTACCGATTACATCTTTGTATCGTTCGTCATTA
GGGTTAACAACAATCGCTGTATCACCTAACATCGTTTCTGGTCTTGTTGTTGCAATTTCAATAAAACCTTCACCATCAGCGTAAGGAT
ATTTAAAATGATAAAACGCACCTTGAACATCTTCATGTATTACTTCAATATCAGATAAAGCTGTACGTGCTTTAGGATCCCAATTTAT
AATACGTTCGCCACGATAAATAATTCCTTTATTGTATAAATCAACAAAAACTTTTTTAACTGCTTTACTTAAACCTTCATCTAAAGTA
AAACGTTCTCTACTATAATCTAAACCTAGACCTAATTTAGCCCATTGCGCACGAATAAATGACGCATACTCTTCTTTCCAATCCCATG
CCTGTTCTAAAAACTTTTCACGACCAAGATCATATCTAGTTATTCCTTGTTCATTTAATTTAGCTTCTACCTTTGCCTGTGTCGCAATA
CCAGCATGATCCATACCTGGTAAGTATAACGTATCGTATCCTTGCATACGTTTCATACGTGTAATGATATCTTGTAAAGTCGTATCCC
ATGCATGTCCTAAATGTAATTTACCAGTTACATTTGGTGGCGGGATAACAATTGTATATGTTTCTTTTGATTTATCTTCTGACGGTTT
AAAATAACCATTCTTTACCCATTCTTCATAACGTCCCGCTTCAACTTCACGAGGATCATATTTTGGTTTCATTTCCATTTGCTATACCT
CCTAAAAAAATAAAAATATCCATCCTATATACAAATAGGACGGATATTCCGTGGTACCACCTATATTCAAGAAGGATGATTAATAT
CAAATTCACTCTTTTAACATAATTGGAATAATCATACCAATACTATCATCGTGAAATTTGAAATGCTTCATCTCTTCAAGCACTCTAG
ATTATGATTAACGCTCAAACACGTCTTAGCCTACTATTAATCACGTTCAGCTAAGATACTCTGTGGGCTACCTTCAGTAAAAATCATT
TACATACTCACACCAAATCATATGCTCTCTTTAAAATAATTTGAACTTACTCTTCCCAAATCCTATATTAAACTCTTAACTTATAGTA
TAATGATTGACAAAATAAGTCAATGTATAGGTGGGAATAAAATGAATGAATGCGCATTTGGTACTAAAGATCCAGTCTACTTAGAC
TATCATGATCATGTATGGGGACAACCGCTCTATGATAGCAAGGCATTGTTTAAACTTTTAGCATTAGAATCACAACATGCTGGGCTA
TCTTGGTTAACTATTTTAAAAAAGAAAGAAGCCTATGAAGAAGCATTTTATGATTTCGAACCAGAAAAGGTAGCACAAATGACCGC
TCAAGATATCGACCGCTTAATGACTTTTCCAAATATCGTTCATCATCGTAAAAAATTAGAAGCAATTGTTAATCAAGCTCAAGGGTA
TTTAAAAATTGAACAAGCATATGGTAGTTTTAGTAAATTTTTATGGTCATATGTAAATGGTAAGCCTAAAGATTTGCAGTATGAACA
TGCTTCTGATCGTATCACAGTTGATGATACTGCAACACAACTATCTAAAGATTTAAAACAATACGGGTTTAAATTTTTAGGTCCAGT
AACAGTATTTTCGTTTTTAGAAGCAGCCGGTTTATATGATGCACATTTAAAAGATTGTCCATCAAAGCCTAAACACAATTAATTTGC
TATAGCATCATAAAGTAAACGAGACATCATCTATTTTTAACTAATAGACTGTCTCGTTTTTTGATGTGAAAATTTATTCAAATCATAA
GTTAAAGTTCCAAGTTAATGCACTGCTAATATTAGATATCTTAATCTAAAAATAGATGTACAACCTTTGCTTAAACCTTAATAATGA
ATTCTATCCTAAGCATAAATCAACTTAAATGGATGATAGTTTCTAATCTTCACGCTTATTCGAACGATATTTTAAAAAGTAATTAATT
CCTGGTGCAATAATAAATAGGATGAAAAAGATTCGGAAAATATGATAACTCGTAATCATAGCAACATCGGCACCAGTAGCTAATGC
AACTAAAACTATCTGATTAACCCCTCCTGGTGCTGCACCAAGAAACAATTCATTAATAGGATTATTATCAAAGAAATGTATGACATA
AACCATGATTAGCGCACCAATTATCAACATAATATTTTGAATTGTAATTGCGATTGCTAGTCTACCTTTTAAATCTGACAATAAATGC
GCAATTTGAACTCCAATTCTAATCATATATATTAGTTGTGCCATGTTCAACAACCAATGATCTAGTGTAAATGTTAAACCTGTAGAA
AAATTCCAAACAATTAATACAATGAGTGGTGCTAATAATTGAAATGTTGGAAACTTTATTTTAGACATAATTAGATAAACTATAAAG
ATAGCTATCGCTAAAATAACTATTTGCCTTATGTTTAATACTTGTGATAAAGGCAAGACTTTTGTTAACTTTCCATTCGCATGCATGT
TACCATCATGAAAAAAATATGAAATGAACGGTACTAAAACAACAACAAATATAATTCGTGATGTTTGCGTTAAGCTAACAACTAAC
AAATTAGCACGTTTGTCTTGTTCAGCCATGACCAGCATTTGTGTTAGTGCTCCTGGTATAACACTTAAAATAGCTGTTTCCGTATTAA
TACGTGCAATTTTTTTAAAAATAAATGCCATTACTATTGCAATTAATAATATCGAAATAGATACAACAATAATCGAAAGCCAATTGT
CTTTAATATCCATAACGACATTTTTCGTAAACGTTGATCCGATTTGCACCCCTAATAGTACAATCCCTAATTCACTAAGTAAGAATGG
CCATTTAATATCAAGTTTGAAAACTTTCACACAAATGATTGATGCGATAATAGGACCAAACATAAATGGGAGTAATACGTGCGATG
AATACAATATAATACTAATAAAAAATGATAAAACGAACACAATGAAATTATTTCTATATATCATTGTCATGTTTTCCACTTCTTTCA
ATAAAAAATAAAATGACTAAATTGCTGCTTGAGCTTCACGTTTGTTAAGATAACAATATCCGCTAGCAGTTTTGACTACAAAGCATA
TATGGACTTTCACTATCAAGACGCCCATGCCTGATATACATTTAAAAAGAGCCTGAACAAAGTTCAGGCTCTCAATTTGTCCGTATA
TTTATTTTACAATACGACTTAAAGCCGTATCAAATGCTTGAATCGTTTTTTCAATATCTTCTTTCGTGTGTGCCGTAGATAGGAATGT
ACCTTCAAATTGAGATGGTGGTAAAAACACACCTTCTTTTGCCATTTCTCGATACATTTCTGCAAATAATTTCAAATCACTTTTATTC
GCTTGTTCAAAATTAGTTACAGGTCCTTCATTTAAGAAATAACCAATCATTGAACCTGCTCTATTTACAGTTATTGGTACATTGTGTT
TAGCAAATACACGCTTTAAACCGTCTTCAAGTATATCGCCTAACATATTAAAATACTCATATGTCTCTGGCGTTAATTGGCTTAACGT
TTCATAACCACTTGTCATTGCAAGAGGATTTCCTGATAACGTACCCGCTTGATAAATATTTCCTAATGGTGCTATATGATCCATGATT
TCTTTTTTACCACCAAAAGCACCTACAGGTAGTCCTCCACCGATAACTTTTCCTAAGCAAGTTAAATCTGGTGTCACACCAAAGTAA
CCTTGTGCACAATGATAACCGACTCTGAAACCAGTCATTACTTCATCGAAAATTAGCAATGCGCCGTATTCAGTCGTAATATTTCTT
AATCCCTGTAAAAAACCTTCAATCGGCGGTACGACACCCATATTACCAGCAACAGGTTCTACGATTACACCAGCAATATCGTCTCCA
AATTTTTCGAAAGCGATTTTAAGTGCATCTAAATCATTGTATGGAACTGTAATTGTATTTTTAGCAATACCTTCAGGCACACCAGGA
GAATCCGGCAATCCTAATGTTGCCACCCCAGAACCAGCTTTGATTAATAACGAATCACTATGACCATGATAGCAACCTTCAAATTTC
ACAATTTTATTTCTTCCAGTATAACCACGTGCTAATCTTAAAGTATCCAATGTAGCTTCTGTACCAGATGACACCATACGCACTTTTT
CTATTGAAGGTACTCGGTCAATAACGAGCTGCGCCAATTTATTTTCAAGTAATGTTGATGCACCAAAACTTGTACCTTTATCAATTGC
TTCATGTAAATGACTAATAACTTGAGGGTCTCTATGTCCTAAAATAAGTGGCCCCCAACTTAGTACATAGTCGATATACTCGTTACC
ATCGATATCATAAATTTTTGAACCTTTACCGTGATCCATAAAAATTGCTGGTGTATCTACTGATTTAAATGCGCGTACTGGACTATTT
ACACCACCAGGCATTAAAGTTTCAGCAACCTTCATTGCTTCTTCTGATTTCGTATATCTCATAAAGTAAATTCCTCCTATAGTTTATG
GAAAATCATACATATAAAACCTTATTTATCTAAATAGCGACAAATGTCCTTTGCAAAATACGTAATAATCATATCAGCACCTGCACG
TTTCATTGAAACCATTTGTTCCATAACGACACGTTCTTCATCTATCCAACCATTTTGTGCCGCTGCTTTAGTCATACTATATTCTCCAC
TCACATTATATGCAACAACTGGAACATTCGTATGATTTTTAACATCTCGAACTATGTCTAAATAACTTAGAGCAGGTTTAACAATCA
TCATGTCGCACCCTTCTTTAAGATCACTTTCTAATTCACGAAGTGCTTCCAAACGGTTAGCAGGGTCCATCTGATACGTTTTTCTATC
CCCAAATGATGGCGCTGAATCTGCTGCATCTCTAAAAGGTCCAAAGAAACTTGATGCATACTTGACACCATAACTCATTATAGGAAT
ATTGTAATAGCCGGCTTCATCTAATCCACGACGAATTTCAGCAACAAAACCATCCATCATATTACTTGGCGCAATAATATCAGCACC
AGCTTCCACTTGAGAAATTGCTGTTTTAACAAGTAGTGGCAATGATTTATCATTGTCAACGTCATGTGTATGGTCATCAATCACACC
ACAATGACCATGATCAGTATATTCACATAAACAAGTGTCTGCAACAATTAATAAGTCATCATACATTTTTTTAGCAATACGTGTTGC
CTGTTGAATAACACCATCGTGAATGTATGCACCAGTACCTATATCATCTTTTGAGTTTGGAACACCGAAAAACATAATGGCACGTAT
GCCTAAGTCATAAGCTTCTTTTAATTCACTTTCAAGTAAATTCAAACTGATTTGGTATACACCTGGCAATGACTTAATTTCTTTTTTCA
CATCGTCTTTTTCAACTACAAAAATTGGATATATTAAATCTTCTTTTCTTACATGATTCTCTCTAACCATATCTCTCATTGTCGCTGAT
GATCTCAATCTTCTATGTCTATCAAATTTCATTTTTAGCCCCTACTTTCTAAAATCTTTTCAATTAGTGATTCGAGTGTTTGAATTTCT
GCAATTGTTACTGGTTGTTGATATGATTTAATGGTCCGTGCTGTTTGTTCTCCAATAGCAAAATACGACTTGAATTTTGGTACAAATC
CTTCATTAAAATAATAACGTACTGCCGACGAACTTGAAAATGTTAATGCATCGATTTGTTGATGTTCTATCATTTCTTTAACATCTTG
TATATTTTGTTTGTTAGGCACTGAAGTATATAAATCTATTTTAACAACTTCATTATCTTTAGATAACGCTGCTAATAACAATGGTCTC
GCCAATTCACTCGAAGGCAAAAGTATTTTTTGGTTAGTTTGATTAAATGATTTTAAAAATCCTTCTTGAGAAAAGTCGTTTGGCATA
AAATCAACTCGAATGCCAAGTGATTCACAATATTGCGCTGTCTTACTTCCTATCACAGCAATGTTATCAACATTAATTCCTTTTAGAT

ATTTATAAAAGAATTTCACAGCATTTTTAGATGAAAAAATAAGCCAGTCATAGCGTTGATTTAACAAATGAATATCAAAATTTAGTG
GCTTTATATCAATAAAGGGTTTGTGAATAATTGATACTAAATCACTTTGCATGTCATTTGTTTGTGTCATAACTACAACTGGCTTCAT
ATTTAAACGTCACTCCATTATTTAATGTTGTTCATTTAAGCGTTTTATAATTTCATAAGCACCTTGCTCTTTTAATTTGTTACTCACTG
TTTTGCCTAACTCAACCGGATCTGTTCCGTTCATTGTATATTCAAATCGTTCTTTACCATCTGGGGTCATAATTAAACCTGTAAATTC
GATTTCGTTTTGATCTGAGATTGTAGCATATCCTGCGATTGGCACCTGACAACTACCATCCATTTCTGCTAAAAACGTTCGTTCAGCA
GTCACACATTTTGCAACCTCATCATTATGTACTTTGCTTAATAATGTTAATAGTTCTTCATCGTCACTACGACATTCTATCCCTAAAG
CACCTTGTCCGATTGCAGGTAACAATGTATCTCTATCAAGATAAGATGTTACAATATCATCTGACCAGCCCATTCTTCTTAAACCAG
CTGCAGCTAAAATAATCGCATCATAATCTTCAGTTTGTAACTTTTCTAATCGTGTATCTATATTACCTCTAATCCATTTAATCTCTAAA
TTAGGATACTTAGATAATATTTGTGCACCACGACGTAATGAACTAGTACCAATAATACTGCCTTCTGGCAATTGGGATAGTGGTGTA
TGTGTTTTAGAAATATACGCATCAAAAGGTAATTCTCTATCAGGGATACAACCTAATGTTAAACCTTCCGGAATTACACTTGGTACG
TCTTTAAGCGAGTGTATTGCCATATCGATATTTTTTTCAAAAAGTTCATGTTGTATTTCTTTAACAAATAAGCCTTTGCCTCCGACTTT
AGACAATTGCTTATCTACTATACGATCGCCTTTCGTGACAATTTCTTTAATTTCAATTTCTAGATTTGGCTCGACAGCTTTTAATTTAT
CAATAAATTGCTGGCTTTGTGTTAAAGCTAATTTACTTCTTCTGGAGCCAACGACTAATTTACGCATGTTCAATTCCTCCTAGGAACG
GATTGCTCTAGATTATTTTCTCAATTCACAAAATGTGTTGCAAAAAATAAATTAATCATATTTAAGCAAAATAAAATAATGTTATAG
TATATTAAATACCTTGAATTCAACCATTTGTTGATTCTAAGCAATATATAACTTCCATATAATATTGTAATAATTGAAGAGAGTATTA
CCTTCGGGTCAATGAATATACGTTCACCAACTGAAATTACACCCCACTGTGTACCTAAAATAATACTAAATATGAGAATTATCCACC
CACTTAACGTTGAGTAAAACACAATTGATTCAAGTGTAGCAACGCTACCTATTCTAAAGTATTTTTGATCAAAACGTTTTTCCTTCAA
ATTACGGTATTGCATGATATACAGTAATGCATTGACAAAAGCTAAGGCAAAGAAGACATAACTTAACACAGCTAGACCGATATGGA
CTAACAGTAACTCGTCTACAACAGCAATTTTCTGAACCTTATTAGTATAATGTGTCGGTTGAAATGTATTCATCCCTAAAAGTGTTAA
CCCTATTAAATTCAAGAAAAACACAGAGAAATTCAATACTTTGATAAGGTTAAGAATTAAAGAGATTGAAATAATCAGCCAACTTA
AAGTATAGAATACATCTGAAATAGACCCTAATGGAATATGTCTAGTTTGTATAATAAAAATAGATAAAGAGATTGTTTGTAAAACC
CAAACAATCCCCAATAAATATATGCCTAAACTTCTAATCTTATGACTTTTTTGGACAAAATCATAAAAATAGCAAATGATACTGATT
AAGTATATTAATAATATAATTTCATTGAATCGAATAAACAGGTTTTCTTGCATATTATCACCATATTAATATGCTTATTCAAAACTAA
AGATACGTCGCGCTGAAATTTCCTTGACCTTGCTTTCTTTTTGTTGCTTCGCTTGCTCATGAGGACATTCAGCTTCGATATCAAATAT
ATTTTGAAATAGCTCTAATTTTTCATTACTTTTCTTATCACTACTTAATTCTTTGGCCTGTTTAATAGGATCTTTCAACATTTGATTGAT
GATACTTTTTGTATGTTTAGATATAATTTTACGTTCTCTTTCGCTTAACCCTGGCAATTTACGATCAATACTGTCCATCGTTTCTGCTT
GAATTGTCATAGCTTTTTCACGTAAAGCTCTAATCACTGGAACAACACCCAACATACTAATCCACTCATTGTGTGCATGTATTTCTGC
AGGAATTTGTTCCGAAATTGTTGCAGCCGCTAATTGTCGCTCACGTAAGTTTGCATCAACTAAACCTTTTAAGTCATCAACATCATA
ATTAAAGATGTTTGTGATGGCACTAATACCAGGTTCAATATCTCGAGGAACTGCAATATCAATCAATACTAGTGAATCTTGCTTTCT
ATTTTCTGCAATTCTTTCTATCATTTCATTTGTAATGATATAAGATTGTGCACTCGTTGAACTAATCACAATATCTGCACTTTCAAGTA
AATTTGGTAATGATGATAGTTCATCATATTTCACTTGATGCTTTGCTGCTAATTTCATAGCATTTTCAATTGTTCTATTTACTACTGTA
ATATCAGTAATTCCAGAACCAAGAAGATTTAATAGTGATAATTCACTCATTTCCCCTGCACCAATAATGATAGCTTGCTTACTTTTCA
ATTTGCCAAATACTTTTTTCGCCAACTCGACCGCAGCATAAGACACACTTACAGCATTATCAGCTATATCTGTTTCATTATGTGCTCT
TTTTGCAAAAGTAATTGCCTGTTTAAATAGATGATTAAAAATTGTTCCTGTCGTACCTGTGCTTTGCGCTAAGAAAAATGCATCTCTT
ATTTGACCTAAAATTTGAGTTTCTCCAAGTACGATTGAATCTAAACCAGAAGTGACACGCAATAAATGTTCTACTGCTTCGTCCCCC
ACTTTTACTTCTGACATTGCTTTAATATCATCTACTTCAAATCCAAATGCACGAGCTAGAAATCGTTGAATATAGTAACGACCTGTGT
GAATTTGATCAACAACAGCATATACTTCAGTTCGATTACATGTTGATAATATGACATTTTCTAAAATAGATTTAGTTTCATATAAATC
TTCATGGGCAATTCGTAAGGCATCATCTCTAAAAGCAACTTGCTCTCTTAGTGCAACATCAGCTGTGCGATGATTTATACTAATTGC
AATAAAATGCATTTGAAACGCCCCCATATATACTAACAATACAAATATTATAACATAATTTAAAATCCAATTAAGTACGATGCTTAA
ATAAATTATAATCTTTCTAATTTACTTTTGAGTTTTTGACAAAATGATGAACGTTGAAAAATGTTATCAATTGTGTACTAATTAAAAG
TTTCGTGCTTACATTATTTTTAAAATTTTTGTATAAGTTTTGTCGTACAAAAACTATGAAATATACGGTTCAATTAAATTCCATATTTG
TTGTTGTTTATTATTTTGAATTGATGAATAACTTACAATTGTATCGTCTGGGTCCATATCTAATTGTGTCTTAATATTTTTAATATGCT
TTTGAACTTTACCTTTTGGAATTTTGTCTTCTTTAGTGCATATAACTAAAGTAGGAATATCAAAATGTTTCAAATAATTGTACATTAA
GATATCATCTTGTGTTGGATCATGTCTTAAATCAACTAATTGAATAACTAATTGCAAATTCTCTCTCTTAGTTATATATTCCTCAATC
ATTTTCCCAAATTTTTCACGTTGTGTTTTACTTACTTTAGCATATCCATACCCTGGAACATCCACAAAAATAAGTTGTTCATCTATATT
ATAAAAATTTAACGTTTGCGTTTTGCCGGGTTGCTGTGATGTACGTGCCATATTTTTTCTGCCAATCATACTATTGATAAATGTAGAC
TTACCTACATTAGATCGACCGCTCAGTGCAACTTCAGACAATTCTGTTTCTGGATATTGTTCTTCTTTTACTGCACTAATGATTAATTC
TATATTATTAGGATTAACTTTCATTTTATATCCTCACTTTTTTATATTCTCTTTCAACTCTACCATTATATAATATTTGAACCTTTCGGT
ATACATTATAAAAAAGCTCCGATCAAAGTTAAACTATTTGAGCGTCAACTTTGATTGGAGCTTTTCACTTTTATAACACATCAATGA
TTAAGCTGATGTTTTACTATTATTAATTAAATTGCCTTCTGCGTCGTATAGTTCTGGTTCAGTTTCTTCATTAATTGTTTGTGCTGTAA
TAACTACCTTCGTTACATTTTCGTTAGAAGGCACATCAAACATAATATCGATTAACGATTCTTCTATGATTGAACGTAAACCACGCG
CACCTGTTTTTCTTTCAATTGCTTTTTCACTAATTGCTGATAAAGCTTCTTCAGTGAACTCTAAATCCACATCATCTAATTCCAGCATT
TTAGTATATTGTTTCACAAGTGCATTTTTAGGTTGCGTTAAGATGTTTTTCAACGCAGTTACATCTAATGTTTCTAAATTAGCTACAA
TTGGCACACGTCCGATAAATTCAGGAATCAAACCATAGGCTTGCAAATCTTCTGGGCGAATTTGTGCTAATAATGCTTGTTCGTCAT
ATTTATCAGCTTCATTGCTTGAGAAACCAATAACTTTTTCACCAAGACGGCGCTTAATCACTTCTTCAATACCATCAAAGGCACCAC
CAAGAATAAATAAGATATTTGTTGTATCAATTTGAATCATTTCTTGGTTTGGATGTTTGCGTCCCCCTTGTGGCGGAACACTTGCAGT
CGTACCTTCTAAGATTTTAAGCAATGCTTGTTGAACACCTTCACCTGAAACGTCACGTGTTATAGATGTGTTTTCAGATTTACGTGCA
ATTTTATCAATTTCATCTACATAAATAATACCTTTTTCGGCTTTATCAATGTCAAAGTCAGCTGCTTGAATTAATCTCAACAAGATAT
TTTCAACATCATCGCCTACATAACCAGCTTCAGTTAAACTTGTCGCATCTGCAATTGCAAATGGTACATTCAACGTCTTGGCTAATGT
TTGAGCTAATAATGTTTTACCACTACCTGTTGGCCCAATTAATGCAATGTTACTTTTTTGTAATTCAACATCATCTTCTTTTGGTCCTA
ATTGTTGAATACGCTTATAGTGGTTATAAACAGCTACAGCTAAAGATTTTTTAGCTTTTTCTTGACCAATAACATATTCGTTTAAATG
ATCCATAATTTCTTTAGGAGTAGGTAATTCTGTCATCGCTTCAGAAGTGTTTTGAGCTAATTCTTCTTCGACGATTTCTGAGCATAAT
TCAATACACTCATTACAAATATATACACCACTTCCTGCTACAAGTTTTTTTACTTGATCTTGGTCTTTTCCGCAGAAAGAGCATTTCA
AATTTTCTTCATCTTCATTGAATTTAAACATTCTTTTTACACCCCTATTCGTTAAAGACTATACTAGATTGGATATTACAATGCAACAT
ATTAACATACAAACTTTTTGCTTAAAGAATAGTAGCAGATACATAAGCTATGCATCTGCTACTCATTTTTGTAATTTAATATTTAATG
AAGATTAATCTTCTTTAGTTCCTTCAACGAACTTTGCGTTATCTCTTAATAAATCGATAACTTTTTGGATACGAACATCATTTTTAATG
ATATCAGTATTACCTAAAGTATTTTTGATATCTTCAACTGAGATATTAAATTGTTTACTCATTTTTTCTAATTCTTTATCGATATCTTC
ATCAGTAGCTTCGATTTTTTCAGCTTCAGCGATCGCAGTTAAAGTTAAGTTAGTTTTAACACGTTGTTCTGCATCGTCTTTCATTTGCT
CTCTTAATTGAGTTTCATCTTGACCTGAGATTTGGAAGTACGTTTGTAAATCTAAACCTTGTTGTTGAATTCTTTGTGCAAATTCAGA
CACCATACGATCTAATTCAGTATTAACCATTGCTTCAGGAATATCGATTGTTGTATTATCAGTAGCTTTTGTAATCGCTTCTTCTTTTT
CAACATTTTCAGCATCTGTAGCTTTTTGTTCAGCTAAACGTTTACGTAAGTTTTCTTTGTACTCGTCTACTGTATTTGCTTCTGCATCT

AATTCATTAGCAATTTCATCTGTTAATTCTGGAACTTCTTTAAATTTAATTTCGTTAACTTTTGTTTTGAAAGTTGCTTCTTTACCAGC
TAATTCTTCAGCATGGTATTCTTCTGGGAATGTTACGACAACATCTTTTTCTTCGTCAACTTTCATACCTTCTAATTGCTCTTCGAAAC
CAGGTATGAATGAACCTGAACCGATTTCTAAATCGTAACCTTCAGCTTGTCCACCTTCGAATTCTTCTCCGTCAACTGAACCACTAA
AGTCGATGTTAACTGTGTCGCCATTTTCAACAACACCATCTTCTTTAACTACCATTTCAGCTAAATGTCCTAAGCTGTGGTCAATCGC
TTCTCGTAACTCATCATCAGATAATTCAGTTTCTTGTTTTTCAATTTCAAGACCTTTATAGTCTCCTAATTTAACTTCTGGCTCAACTA
TAACTGTTGCTTCAAAAATGAAATCTTTACCTTTTTCAATTTGAGTAACACTTACTTCTGGTTGTGCAACTGGTTTAATATCAGTTTCG
TCAATTGCTTCACCATAAGCATCTGGTAATAAAATGTCGATAGCATCTTGATATAATGCTTCTACACCAAAGCGTTGTTCAAAAATT
GGACGTGGCACTTTACCTTTACGGAATCCAGGTACGTTAATTTGTTTAACCACTTTTTTGAATGCTTGATCTAACGCTTTGTTTACTTT
TTCTGCAGGAACAGTAACAGTTAATAAACCTTCGTTACCTTCCTTTTTTTCCCAAGTTGCTGTCATGTATATATACCTCCATGATTAA
CTAATTTATTTTTTCAACTTCCCTATTATATCATAGGTCTATTCCCTATACAAACATTGAAATCACAACGTTTATATATTTGTAAATCA
ACTTTTTTCGTCAAAACTATATTAAAATGCTGAGTATTATTTTGCAACAATTTAACTATTATTATTCAAATCTAATTGCTGAATAAAA
TTAAATGCATCGATACTTTGTAATTCACATTTAAGTCCTAACATATTTTTGAAATAACATTCGTATGCATTAATCCATTTATTCGTTTC
AAACAAAGTTTCAATATCAATTGGATAAATCATGATAGAGTGATTATTCATAATATGGTGTGCTTCTTCAGCGATATGTAAAGCACC
ATCATTTAATGTTTCCATAACGTTAGGTATAACTTTTTCTTTCAGTGTTGTATGTTCTAGCCCATTTAAATTAGCTGGTACAAAAGTTA
CATCCATACCATACTTTTCAATTGTCAGTTCTTGTGTACAATTTGCAAACCTTAAATACTCAATCATTAAACTAATGAGGTTATAACT
GTACGTATTAGATTTTAATATATATAATACCGTTTCTTGAAATTGAAAATGACCATTGTCTATTAACTTCAAAATCAAGTGCGTCTGT
TCCCTCATTGATAACGTATCAAAATCAGTTAATGACTGAGTCAATCGTTTTTCATCTTCAATTAATTTTGACTTAGCAAATTCTTTAA
GAGGATGTAATGCCATTCTTGTTTTGTGATCTTTGACTTCATCAATAATTTGATGAATTACTTCTACCGCTTCAAAATATTGTCCCAA
ACCAATCAAACTTTTCACGTAATAAATCATCAATGCATCATATTGTTGTATGCCAGTTTTCAATAAGACAATTGTTTCTTCTCTTAAT
TCAAGAAATGACCCCGTCTCATATAACATACGGCATTTCATCATTGCTATTTCTTCAGTGAGTTCAAATTGACGTTCATATTGCACAA
TATATTCATACATCAGGTCATAATTTTGGTCTTGTTCAGCACGTTTTATATCTTTATATAACTTTTTCGAAGAGTTAGGAAACTGAAT
AATATCTGACATATATGTCACCTCGTTTGATTATAAATCTTGAAGGATTAATGCGCTCCACGTTTCGAAATCATCATATGACTCTATG
TTGCCTTTTTCAACCCATTTTATTCGTAAAGTATCTGTTTCTTTAGCTTCTACATCATTCGTACTTACAGTGATTTTAAATACAACACC
AATATGTACCTTGCCCACTTCATTATTATCGTCATTAATAAAACCGATATATTCCATATTTTGTGAATCTTGCTCACTTAAACCTACTT
CTTCTTCTAATTCTCTCTGTGCATTAACTCTCAATACTTCGTTAATAGATTCTGCTCCTGGAACATCATTCATATGACCGCCTACACCT
ATTGAAGATTGTCCATGCAATCGAGCTTCTCCACCGCCAGATAATCGTTCATACACTAATATCTCGCCATGCTCATTTTCAAGTAAAC
AATAAGAAATGAGTTGTTTATATGATGGATCCTCTTCCATATCGCCGCGTCGCTTAACTTCATATTGACTTAGCGCATCAAAAATATT
TTGACCTTCTGGTTTATTCTTATTTAAAAACCCATTGAAAGTATTCTTTTCATTATTAAAAATAATTTCTCTAGGTACTACAATGATTT
GTTCATCAAATTTAGACATCGTATAAAACTCCTCACTTTAATGTTTGTTCTCTTAAATGCATTTTAGCAAATATGTCATATTTTAGCG
ATTAAATGTGGCATTTTGCTTTTTAAAAAATAAAAAAGAGATGACAATGCCATCTCTTTTTTATAAGTGTTATTATTTTAAAGCATCT
TTAGCTTTAGTTACTAATTGAGCAAATGCTTTTTCGTCAGAAATTGCGATTTCTGATAACATTTTACGGTTAATGTCGATACCAGCTT
TTTTCAAACCGTTCATTAAACGTGAGTAGCTCATTTCATGTTGACGAGCTGCTGCGTTGATACGTGTAATCCATAATTTACGGAAGTC
ACGTTTACGTTGACGACGGTCACGGAAAGCATATTGACCTGATTTCATTACTTGTTGCTTAGCTACTTTGTATAATGTATGTTTTGAA
CCGAAGTAACCTTTAGCTAATTTAATCGTTTTTTTACGACGCGCTCTTGTTACTGTTCCACCTTTAACTCGTGGCATAAAAAAATTCCT
CCTTAGGTAATTCCTACCGATTTCTGTATTTGTTCTTATTTTTTGTATGCTAATAATTGTTTTACACGTTTCATATCGCTCTTAGACACT
AATCTAGCTTTACGTAATTGACGTTTTTGTTTAGTGCTCTTGTTTGCGAATAAGTGAGATGTGAAAGCTCTTGAACGTTTTAATTGAC
CTGAAGCAGTTCTTTTAACACGTTTAGCTGCTCCGCGGTGAGTTTTCATTTTTGGCATAATATAAGTCCTCCTGTAATAGCTTTAATT
ATTTTTCAGCTGTTGGCGCTAACATGATAAACATTTGACGCCCGTCCATTTTAGGTTTTTGTTCAACTGTTGCTATATCTTTGCATTCA
TCTGCATATTTTTCTAGCACACGTTGACCAATTTCCTTATGCGTAATGGCACGCCCTCTGAAACGAATAGATACTTTACATTTATCGC
CTTTAGTTAAGAATTTACGTCCGTTTTTCAACTTAGTTTGGAAATCATGTTCCTCAATTGTTGGACTTAAACGAATTTCTTTAACATTG
ATAATTTTTTGTTTCTTTTTCATTTCTTTTTCTTTTTTCTGTTGTTCGAATTTGAATTTACCGTAATCCATAATTCTTGCAACTGGTGGTT
TCGCATTCGGTGCAACGACCACTAAGTCTAAATCTACACGTTCAGCCATTTCTAAAGCTTCACGCTTTGATTTAACACCAATTTGTTC
ACCATCTTGACCGATTAAACGTAATTCTTTTGCACGAATTTTGTCATTGATTTGAGTTTGATCTTTTGCTATGGTTGACACCTCCAAA
ATTTTTACGAAATTTGCACCAAGCAAAAAGGAAGAGCAGGTATAAAATACCCGCTCTTCCTTATACACAGTTATGTGTAATGTGATT
AACCTGCCAACTGCTTTATGCGTCGCTACAGGTGAGAAGCGGGTGCTTCTACTTGGTTCGTTTCGTATTCAACGTTGTTAATCATATC
AACAATTCACATTTAAGTCAACACTATAACTGTAATTATTTTTATTTTAACCTTTTATTTCATCCATTGACACGTCTTGACGTAAATCT
ACTTGTTCTAATGGAATTTTTTTCGTTTTATATCGAAGCTTATGATAAATAAAGAATGCTAAAAATACTGGGATTCCCATATACGTAA
TTAAGAATCGACTAAAATTAAAATCTCCTGTTTTAATAAAGTCAACATCTTGACCAATAATTACTACAATACATAAAAAGCCAGCAA
ATAATGGTCCGAATGGGAATAATTTAGCAGTATATTTTAATTTAGATTTGTCATAATTTTGTTTATCAAATGCTCTTCTAAATCGATA
ATGACTTACTGCTATACCTACCCAAGCAATAAAACCAGTTAAACCACTTGCTGCAACGATATATTCGTATGCACCTTTTGTAAGGCT
TTGTAATACGAAAATTATCACTACAATGATTGCTGTAACGAGTAACGACATATATGGCACACCGTTTTTATTTGTTTTACCAAATGCT
TCAAACGCTAATTTATCTTTACTCATTGAATATAGCATTCGAGTTGAAGCATACATACCTGAGTTACCTGCTGATAACACAGACGTT
AAAATGACTGCATTCATAAATGATGCTGCAAACGCAAATCCAGCATTTTTAAACACTAATGTGAATGGAGACGTTGCTACATTATCA
CTACCACCCATTAATGCACTACTATCATAAGGAATTAACATACCGATAACAAAAATGGCTAAAATGTAAAATAATAAAATTCTCCA
GAATACTTGTTTAATTGCTTTCGGCACAGCACGTTCAGGATTTTCTGATTCACCAGCCGTAATACCAATTAACTCAGTACCTTGGAAT
GAGAAACCAGCGATTAAGAATACACCTAGAATTGATAATAAACTTCCTCCTAAGTTGCCACCAAGAATAGGACCTTCACCTTTATTA
AATATTTCGAATCCTACAACATGACCACCCATGATTCCGACAATCGTTAATAAACCAATTGCAATGAAAACAATAACTGTAACCACT
TTTATCAATGCCAACCAGTATTCACTTTCACCATAGACGCGAACTGATAACGAATTCAGACTAAAAATTATAACTAAGAACAACGC
ACTCCATGCCCAAGCGGGTATGCCTTGCAATGGTGTCCAATATTGAATGACTTGTGCTGCAATCGTAATATCTGCTGCTACAGTTAC
TACCCAGTTAAACCAATAGTTCCAACCAAGCGCAAACCCTAAAGATGGATCAACAAATCTTGTAGCATATGTACTAAATGAACCTG
ATACTGGCAAATACGTAGCCATTTCGCCAAGTGACGTCATTAAGAAAAATACCATTATTCCGATAATTGCGTATCCTATTAATGCAC
CCAAAGCACCTGCATCATGAATTGCTCCACCAGAAGTTACAAATAAACCTGTACCAATACAACCCCCAATCGCAATCATAGAAATA
TGACGATCTTTAAGTCCCCTTTTGACAACATTGTTACTTTCATTTTGAACTTTTGACATATAAGCTCCCTTTCCATGTTTTAGAGGCCA
AGAAATAAATAACCTTTATTCACTTTCATACCCTTTTTTACAGAACATAGTGATATTAAAAGTAAAAAAGTGGCTACATACCTTTAA
GATATGCAACCACTTTGGCAATTATCTATATAAGGTAGCACATCACATTCTGTGACAGTACAGTTCCTATTCGAAAACTGTCCCAAT
AGATATAATTTATGGTTTATATCTATTTCGGCATCTTTACCTTTCACTTGTTCAACTTAGGTACCATAAATACTTCTGACAAGTTACTA
ATTAAACATGCAACCTCTAACTCAATTTAATATTTTAACTAACTTGTAATATACAGGATTCATCACGCATAATCAACCCTGTAAAAC
TTGATACGCAATAAAAGTTTTACAGCATTTTATTGCGACAACTGTCTATCTATGTTTTTTCAAACGAATTTCATCAACTAGATTCCAG
ATAAATTCATCTTTTTCAACTGTTTCTTGGTCTTGCGATCCATATTGACGCACATTCACTTGATTATTTTCAACTTCCTTATCCCCAAC
TACGATTTGATAAGGTATTTTTTGCATTTGAGCTTCTCTAATTTTATAACCCATTTTTTCATTACGGTCATCAATACTTACACGAACAC
CTTGAGATTTCAATTCATCTTGCAATTGGCGCGCATAATCATAATGTAAATCAACGTTAACTGGAATGATTTGAACTTGTTTTGGCGC

TAACCAAGTTGGGAATGCACCTTTAGTTTCTTCAGTTAAAAATGCTACAAATCGTTCCATTGTTGATACAACACCACGATGAATCAC
AACTGGACGATGATGTTCACCATCTTGACCAATATAAGTTAAATCAAAACGTTCTGGTAATAAGAAATCAAGTTGTGCTGTTGATAA
TGTCTCTTCTTTACCCATCGCTGTTTTAACTTGAACATCTAGTTTCGGACCATAGAATGCCGCTTCACCAATCGCTTCTTCGTACGATA
AGCCAAGCTCATCCGCTGCCTCTTTAAGCATATTTTCAGCTTTATTCCACATGTCATCATCATCAAAGTACTTTTCTTTATCTTCAGGG
TCTCTATAACTTAATCTAAAGCTATAATCCTCGAAACCAAAGTCTTTATACACATCAATAATCATGTTTACAACGCGTTTGAATTCTT
CTTTAATTTGATCAGGTCGAACAAAGATATGTGAATCATTTAAAGTCATACCACGAACACGTTGTAATCCTGATACAGCACCACTTG
CTTCATATCTATGCATCGTTCCTAGCTCAGCGATACGGATAGGTAATTCACGATATGAATGTGGTTTATTCGCATAAATCATCATATG
ATGTGGACAGTTCATTGGACGTAATACCATAGATTCAGTTTCATCTAACTGCATTGGTGGGAACATATCTTCTTGATAGTGATCCCA
GTGACCAGATGTTTTGTATAAATCAACATTAGCAAGTACTGGTGTATAAACGTGGTCATATCCCATGCTAACTTCTTTATCAACAAT
GTAACGTTCAATTTCACGTCTAATTGTTGCACCGTTAGGTAACCATAATGGCAAACCAGCACCAACTAATTGGCTATTTGTGAATAG
TTCTAACTCTTTACCAATTTTACGATGATCACGTTCTTTACGCTCTTCTAACATTTGTAAATGTGCTTTCAATTCTTTTTTATCAAAGA
AAGCAGTACCGTATATACGTTGTAACATTTTGTTGTTACTATCTCCACGCCAGTATGCACCTGCTGTAGATAATAGTTTAAACTCTTT
AATTTTAGCTGTTGATGGAACGTGAACTCCACGACATAAATCAGTAAAATCACCTTGACTATATAATGTTACATTTTCATCTTCAGG
AATCGCGTCGATTAATTCTAATTTGTATTCATCATTGCTGAATAACTCTTTCGCTTCATCTCGTGAAACCACTTTTCGTTCGATTTTCA
TATTTTCGTTAACGATTTGTTTCATTGTTTTTTCAATTTGTTCAAAGTCATCAGATGAGATGTTTTGGTCAATGTCGAAGTCATAGTAG
AATCCACCTTCTATTACAGGACCTACACCAAATTTAACATTACCATATAACCTTTTAATCGCGTGTGCCATTAAATGTGCAGTAGAA
TGACGTAATACCTCTAACGCTTCTTCACTACCTGGTGTCACAATTTCAATTGATCCATCAGTTTCAAGCGGTTTAGTTAAATCTACAA
GTTGCCCGTTAAATTTGCCGGCAACAGCTTTTTTACGTAATCCAGGACTAATTGATTGTGCTATATCTTCAGTAGTAGTACCTTTATC
AAACGCCTTTTTATTACCATCTGGAAATTGAATATTAATTTGTTCCATGTTAAACCCTCCTATTTGCTTTATAAATACTTTTATCTGAC
CTCGTTAAAAAATTGAACAACAATCATCGTTTTAACATTAAACCTATTAACAATAAACAGGGAATTTATGACACCAACAAACATTTA
TGTCTGAAAAAACCAAAAAATCCCGCCCCTATACAAGGGACGAGATCGTCGTGGTACCACCCTAGTTATCTAATACAATTTATGTAT
TAAATATCTCTGCTTAAGATAACGGTCTTGATCCGGGTATTCATTACAAATACCATAAATGAAGTAGTAATCATCTAATTTATTAAC
CATATTCTCATCAAACAATGGCTTTCTGTGTAATATTGATTAGATCATCTTGTCTTCAATCATTTAAACGATTTAGATTTGTATTCATT
ATACACCAATCATTTTAAAATTCAATTGTTTCTGAAATTTTCTCCTGATAAAAAGTATGGTGTTGACAAAGATTTGACACGTTCAATA
ATACGTGCTGCTTTAGTCTTCTCTTCACCATCACGAGTCATCGCTAAATGATGTTCCAATTCACTATAGTCAAAATTAGAACTAAAGA
ATGTTGGTAATTCATGAACCATTCGATAATGTAGCAAAGGTCCAATTACCTCATCTCTCACCCATGGAGTCACTTCTTCAGCCCCAAT
ATCATCAAGCATTAAAATGTTTGCTTCTCTTACGCGATGTAATTTCTTTTCAAAAGAACCATCTTTAAAGCCACCTTTTAATGTTCTA
ATAAATTCCGGTAAATAAATAATTGTCGAACGTACCTTCTTAGATTTGAGCTGATTCGCAATTGCACCTAGAATAAAAGATTTACCT
GTCCCAAATGGACCATAAAGGTAAAGGCCTTTCACTTGTTCCCCATTAGTTATTGCTGTACAAATATCATCTGCTGCCATAGCTACAT
CAAGACGGTCTCGATGATTCATATAAATATCTTTCAATTTGGCATTTAAAGTATCTCGTTGCATATGATGAGATGTAATTAGCTCAGC
TTCAAAGCGTTCTTCGTCGTACTTGATTTTACATGGGCATTGTAAATAGCGTATTTTAATTCGGTTATTATCAACATATAACTCAGGC
ACATGCCCCTTTACGAAATTTGGACAATCAGCAAATTTATGACCGTCATAATGTTTTTGTTGATCTTTATACTCTTGTAACACATTTA
AGTCTTCATCAATCATAGCATTCGTTAATTCAGCTCGATGCGCTTCCAAAAATTGCTTAACATCTGGGTCATTGATTACTTCTTTTTTT
ATCTTTTCTATTCTTTTTTCAAAGTCCTGCGACGTGTTAATTATACTTTTAAATTGCTTCATTATTGACTGTCCTCCTCCCATTTTTTAG
ATAATTTATCTAGAAATGCTTGTCGATCTTGCTCTAATTGTTGATCATCTACGCTATTATCTTTAGCCGAATCTTCTTCACTAGGTTTA
TCTCTATTTTCTAACCATTTAGGTGTTTTTTCTTTTGAAATACGATTACGCTGCCCATAGTATGAACCACGCTTTTGGTAATTTCCGCT
AGAACCCTCATTTTTAGATTGATTAACTTTTTTAGCGTAATTATATGCTTCTTTAGCTGTCTTAATACCTTTTTTCTTCCAATTTGATGC
TATTTCCAAAATATACGCTTTAGGAAGTTTCATATCTTCTTTTAACATGACAAATTGCAACAAAATATTAATGACGCCAAAAGACAT
ATTTTCACGTTCAATTAATTCTTCAACCATTGTCTTTTGCGATATAGTTGGTTCTGATTTAGACCAAGAAGCTAACATATCAATTGGA
CTCGTTTGTTCAAGTAACTCAAACCATTCATCACTTTGTGGCTTTGGATTCACTTCTGAAGATTTACCCGTCGAAGATGATGTAGCAG
GAGATTTCACCTGTAATTTAGGCATTTGATTTTCGTGTTCCATTAAGTAATACGAGCGTGCTTGTTTACGCATTTCTTCAAAGGATAA
CTGTTGTCCACTTGTAATTGAATTTAAAATAACATGCTTCATGCCATCTGCTGTTAAACCATATAAAGTCGCGAGTTGTGTAATTAAA
CGCTTTGCATCTTTGGTAACAATGTCTTGACTAATAAAATGTTTACCTAACATTTGTCTCAACATTTCAAAGTCAAAAGATTCATTTG
ATAAATCGATACCTTGGTACGGTTCATTAATCGGAATATCACTTGTATCGATATCTATTTTTGTAGACGGCACTTTAAAAACATCAGT
AAATTGTCTTGTTACCTGTTTAAATTCACTCAAATCAATTTGTTGATACTCAAAGTATTTCTTCAACTCATGAAATCGACGATGCTCG
ACTTCACTATATAAAAAGATTGACAACATTGGATCATTAAAAAATAAATGTGCTGAAGGCGGTTGAATTAATTGGTAAACAAATTG
TGTTTCTTGTTCATCATGTTTGACAAACGCCTTTAACAATCCAATCGCTTCAAGTAAGTCCATTTGTTGTCTAAACTCTAGTAAATTA
ATTTTAAGTTCATTCATAAAAATATAATGAGAAAGAATCAATGTTTCATTATGACTTTCTTTAACGAATTGAGTCATAAAATGATAT
AAACCCACTGCTTGCGTTCCAATTAGCGGTGTATACAGTCGATTCAATACCTCTAAATGATTCGTATTTAAATCAAAGTGTTGCATA
ACTTTGAATTGATCCTTTGGTCTTAAGCCGAATTCGAAGGCTTGTCGTCCCATTTAAGCATCACTCCGTTTGTTTTCGCTTAAAATCC
CTTGCATCGATGCTAACAATTGATCAACATCTTTAAATTCTTTATAGACTGATGCAAATCTAACATATGAAACTTGATCAACATGCA
TTAACAAGTTCATAACGTGTTCACCTATATCTCGTGAAGACACTTCCGTATGACCTTCATCTCGTAATTGCCATTCAACCTTGTTTGT
TATGTCTTCAAGTTGTTGATATCTAACTGGTCGTTTCTCACAAGAACGCACAAGTCCATTAAGTATTTTTTCTCTTGAAAACTGCTCT
CTTGTGCCATCTTTTTTCACAACTATAAGCTGACTAACTTCGATATGTTCAAATGTAGTGAAACGTGTTCCACAATTTTCACATTCTC
TTCGTCTTCGAATGGCATTTAATTCATCGGCATGCCTTGAATCTACAACTTTAGATTGTGTAGAATTACATTTCGGGCATTTCATTAC
ATCACCCTCTTTATTTTGATTATGCCTAATTATACTATAAATCTAGAGATGAAAAAAGAATCCCTCAATTTAATTCATTTAACCAAAT
AATGAAACAATAAAAAACATTATATCGTTACTTATTAAGTAATTTGCATGACAATATTATTGTATTAAAAAATAAAAAACCTAACTC
CGAAGTCAGAGTTAGGCTATAAATTAATTGTATTAACTTGCACTTACAGTTTCTTTTGATGTCAAAAGTGCTCCAATTTGCTCAGCAA
CATCTACAACTCTATTTGAATAACCCCATTCATTATCATACCAAGCAATAACTTTTACTTTATTCCCTGACATGACCATTGTTGATTTT
GCATCAATAATAGCTGAATTTGGATTAGTATTAAAATCAACAGACACTAGTGGTTGATGTTCGACTTCTATGATACCTTCTAAACCT
GCATTTTCAAAAGCTTGGTTTACTTCTTCTGCAGTTACTTCTTTTTCTAAATCAACAACTAAATCAACGAGCGATACATTCTTTGTTG
GTACACGTAATGCCATGCCGTGTAATTTACCTTCTAATTCTGGTAATACTTCTTTTAAAGCTTTCGCCGCACCAGTAGAAGTAGGAAT
AATGCTTTCATTACATGAGCGTGCACGTCTTAAATCTTTATGTGGATTATCAATATTTTTTTGGTCATTTGTAATAGCGTGAACAGTA
GTCATTAAACCATTAACTATTCCAAACTGATTATTTAAAACTTTTGCAACTGGACCAATGCAATTAGTAGTACATGAAGCATTACTA
AAAATGTCAAATGCTTCTATATCTAATTGGTTATCATTTACGCCTTTAACTACCATTTGAACATGTCCACCTTTTGAAGGACCAGTTA
ACAATACTTTTTTGGCACCTGCTTTAATATGTGCGATGGCTTTATCACCATGATTAAATTTACCAGTTGCATCTATAGCAATATCGAT
ATCTAATTCTTTCCATGGCAAGTTTTCAGGATTGCGATCAGCAACCAATTTAATTTTATGATCACCAACTTGCAATCCATTTTCAATC
GGTTCAACTTTTAGATTATATTTTCCATGTGTTGTATCGTAATTGATTAAATGTGCAATTGTTTCGGGTGGATAACTAGCATTTATCG
CTACTACATTTAAATTTTTATTTTGTAATGCAATACGTAATACCATTCTTCCAATTCTACCCATACCATTAATTGCAATATTCGTTGAC
ATTAAAAGCACCCCTTGATTTTTATGTGTTATACTTTATGGAATTAGTATAGCATATTATATGAAATTTGAAAGCGTTTTCCGTTGAT
TTATTTATCTTTTTTATAAAAAATAGTCATTACTTACATACATCAACGCGTACGTAAAGTAATGACTTATTTATAGTGTAATATTAAT

CTTCTTCTCCGTAATACGGCTTTTCAATATAACCTTCTTCTTCTAACAATCTCTCAAGGTTTTGTTTTAATTCAAGTTTATCCCCTAAA
TTATCGATAACATGATCGGCCATTCGGCTTTTTTTATCAATAGAAATTTGGCTATAGACACGTGCTTTCGCATCTTCTAATGACAGAT
TATTACGTTGCATTAAACGATCCATTTGTATACTTTCAGAAGTGTATACAACCCACACTTCGTCTACTGTATTTTCCAATTCATTTTCA
AATAATAATGGAATATCCATGATTACATTATATCCTTGTTTTAAATATTCTTGCTTTTCTTCTTCCATAATATCTCGCACGATAGGATG
TATGATAGCATTTAATTCTAAGCGTTTTTCTGGATGGTTAAACACTAAATCACCCATATAACGACGATTCATCTCACCATTTTCATCA
ATTGCTTCATCACCAAAGACTTCTCGTACTTGAGCTAAACCTTTACTCCCCTTTTTAACAGCTTCCCTGGCTGCTTTATCAGCATCTAC
TACTTTAAAACCGAATACGGATAAGAGTTCTGATACTGTTGATTTTCCTGAGGCGATTCCACCTGTTAGACCAATAACTTTCGGCAT
AATTTCACTCTTTCTTTATTTTTGACATACTGGACAATAATGACTATTTCTTGTCGCGATGATTTTTGTTTCAATTTGACTTCCACACA
CTTTGCATACCGGCTGCTTATATACATTAAGATGCAATTGCATCTCACCAGTTTTTCCATCAGCATGACGATAATCTGAAATACTTGT
ACCGCCATATTTAATACCTTCTTCTAGTACTTCTCTAACATAATAAAAAACCATTTCTTGTTGTTGGTGTGTTAAGTCTTTTACTTTTT
TATCTGGTAAAACACCTGCACGAAACAACGCTTCACATGCGTAAATATTTCCACAACCTGCGATTACTTTATGATCCAAAATCACTT
GTTTGATTGGTTTATTCTTATTAGACTGTTGATGAATTCGATTTAAATAATACATCAATGCTTCATTTGAAAAAGGTTCAGGCGCTAT
TTCTAAAAATGAAGGATAAGATGCTACAGACGCAACATTTCTAATTTCTCCAAAACGACGTATATCTGAATAAATTAACTTTTTGTC
ATTTGACAACTCAAAAATAACATGCCAATGCTTACGATAATTAGGTATCATAATATCTTCAAGTTCATCTACAATGAAAAAACCACC
CGCCATACCTAAATGACTAATTAATGTACGTTGTTCTCGTTTATTATCTAGCTGAAAAACGATATATTTACTTCTTCGTTCTACATTA
GTAATGGTATAGCCTTCCGATAAAGTTTTAAAAGTATCTAATTCAATTCCTTTTATAATTGTTTCCTTGCCTTGAGCTTTACCTTCGAT
TACTTTATCCGAAAATTTAACGTGTTCAATTTTTTGATTTATAACGTAGGGTTCAATTCCTCTTTTTACATGTTCTACTTCTGGTAATT
CGGGCATACCATTAACCTCACTTTATTTTGCATCATACCAAGTTGCACCATAACTTGAGTCTACTTTTAATGGAACATCTAATTGCAA
TGCATTTTCCATTATTTCTTCTACAAATTCACTAAATGAATCTACTTCTGACTTAGGTACTTCAAAAATTAATTCATCGTGTACTTGTA
ATAATAGTTTAGCTTGATATGTTGTCTCTTTCATTTTTTGTGCAAATTTAACCATTGCCAGTTTAATGATATCTGCAGCACTGCCTTGT
ATTGGCGTATTCATAGCAGTACGTTCAGCAAAGCCGCGTAAATTAAAGTTACGACTCGTAATATCAGGAATATAGCGTCGACGATG
TAGCAATGTTTCCACATAACCTAAAGCTTTGGCATCTTTTACAATATCAGACATATATTGTTTTACACCTGGGAAACTAGCTAAATA
ATCATCAATGAATGCTTTTGCTTTTTTACGAGTAATACCTAAACTTTGACTTAAACCATAATCACTTATCCCATAAACAATTCCAAAG
TTAACCGCTTTTGCTTGACGACGCATTAAACTATCGACTTGATCAGCTTCTACACCAAATACTTTCATAGCAGTTGCTGTATGAATAT
CATCGCCGTTGATAAATGCTTCTTTCATACTCTCATCTTGTGTAATGTGTGCTAATACACGCAATTCAATTTGAGAATAATCTGCTGA
TAATATAACGCTATCTTTTGAAGTTGGTTTAAAGGCTTTTCTTATTTTACGCCCTTCTTCAAGTCTAACCGGAATATTTTGTAAATTAG
GATCTACACTTGATAAACGTCCAGTTTGCGCTAAAGTTTGGTTAAAACGTGTATGGATACGTTGATCATCACTAATTACCTTTTGCAA
TCCTTCAACATAAGTAGACTGTAACTTAGATAATTGACGATATTCTAAAATATAATCAATGATAGGATGTTCACCTTGCAATTGCTC
TAAGACATCTACAGCAGTAGAATATCCTGTTTTCGTCTTTTTAATAACAGGTAATTGTAATGTCTCAAACAATACAACACCTAATTG
CTTAGGAGAATTTATATTAAAATCTTCACCAGCTGCATCATGGATATTTCGAATCAAGACGTCTAATTTTTCTTGAATTTCTTTTTCC
ATTTCTTCTAAATCATGAACATCTGTAAATATACCAATTTCTTCCATTTCACTTAAAATCTTAGCTAGCGGTAGCTCTAAATCAGCTA
AGAGTTCTACCTGATTGTATTCTTCTAATTGTTTATCCATATTTGGTTTCGCAAAGTAAATTGCATCAGTAATAGAAGCAACATATGG
ATTTAAAACATCATCTTCAGGTACCTTAAATTTCTTACCTTTTCCATATATACTCACATCGTCTTTCACAAAACTTTGACCGTACAAT
GAAACAACTGATTGAACATCACTAATCGTACGAGATGGATCAATTATATAACTGGCCAACATAATATCGAAAGAAATATTTTGAAT
ATCAATTCCCAATCTATGTGATGCTACATATGTTTTTTTAGCATCATATACGACTTTTTTCGTATTCGGATTTTCTAACCATGAAACTA
GTTCGGCATAATTATTTATGTCATCCGCATTAATTACAATATGTTTCTCACCTGTAAATAAAGAGAATTTCAAAATATTATTTCGCAA
ATAGTTACCACCGTCTAATTCGAAATGGATGGCCGCTTCTTTCAATAAAGTAAAATCAATATTATCAAAAGACGTTTCAATTTCAAA
TGTCTTTTCTATTGCATCTTCAACGCTTGCTGATTGATCAATGTCAGCCAACAATTGTTTGAATTCTAACTTCTTAAACAATTCGATTT
TTTCTTGTTGTTCATCTTGATGAGTCATTAACGTATCTTCAAGTTTTACTTCAATCGGACTATCTACATTAATCGTTGCTAATTCTTTA
CTCATTAATGCATCTTCTTTGCTATTTTGAAGTTTTTCTTTTAACTTTTTACCTGAAATTTCATCTAAATGTTCATAGACACCTTCTACT
GTGTCAAATTGGTTTAGCAATTTTATTGCTGTTTTCTCTCCAACACCTGCAACACCTGGTATATTATCAGAAGTATCTCCCATTAATC
CTTTCATATCAATAATTTGATTAGGTGTTAACCCGTTGTATTTTTCCGCAATAAAGTCAGGTGTATAATGATCAACATCAGTAACACC
TTTTTTAGTGTAATAAATGGTTACATTATCCGTTGCAAGTTGTGTTAAATCTCGGTCTCCCGTAATAATAATTGTCTGAAAGCCCGCT
TTATCTGCTTCTTTACTTAAAGTTCCGATAATATCATCTGCCTCATAGTTATCTAATTCATAACGTTTAATATGATAAGCATCTAATA
ATTGGCGAATATAAGGAAATTGCTCACTTAGTTCAGGCGGCGTTTTCTGGCGTCCACCTTTATATTCACTATATTTTTCATGTCTGAA
AGTCGTTTTACCTGCATCAAACGCTACTAAAAAATGATTTGGCTTTTCTTCTTTTAAAATCTTCTCTAGTAACATTGCAAAACCATAT
ACTGCATTGGTATGAATGCCTGCTTTGTTTGATAACAAAGGTAATGCATAAAAAGCTCTAAAACTTAAGCTATTACCATCGATTAAT
ACTAATTTATTCACAATTTTAACCTCCAGAACTAATTTATATATATATTTTAGCATATTCGACTGTGATGTCGTAATTAAAGAAACTATC
TCATAGAGTATTTATACACGACCTAATTTCAAATAAAACAAGTGCCTATATAGACTTAGCTAAAAAATAAAAAAGACATAATCATG
ATTGTTCTTGATGAGCACTCCATAAAGTCAGGTTAAAAGTCCAACTTTTGAGATGCACATTATCATTTCATGATTATGTCCACTTTTT
AATATTTTTATGCTGCTATTTTAAATTTTTATAGGTAAGAATAAGTTATGAATCGTCTGAACCAAATGGAATATATTTAACTTTGGTC
ATTTTCGGTAATTCTTCATAATTATTAAACCACTCTTGGTAATTTTTATTGATTGGTGACGGATGATGCATATAATGTTCAAACGGCA
ATGATTCCACTGTATAAACCTCTTGTCGTGGATGATCTTTAATTTGTTGTTTTAATAACTGAACTCTTTTTTCATGTTCATAATGAACA
TAAATAAATGCACTAAGATATATCACAGCTAAAACTAGTGATGCACCTTTAATAAAATTAACATTGATTGACTTATATTTCCGAAAT
TCTTTTAATAAAATAATTAAAATGATTACATGTATCGTATAAACAATCAAAAAATTACCTGGTTCTATTGGAGTAACAATGACTAGT
GTCGACGCCGAAACACATATTGCAATAAGTAAGGAATATAAAGTGATTTGTGTTTTACGATCATTAATAGACAAATAAATACCTAC
AAATATCGAAAACGCAAAGTAATCACATACAATTACGTTCACAAAACCAACCAATCCAATATCTGTATTTTTATTTAATAAAAACTG
ATTGTAAAATAGTAAATAATAAAGCGGTAAAGTGATAAATCCTATCATAATGATACGACGCTTTAATATTGTTAAATGTACATACCG
ATCACTTCTAAGCAGTAGATAAATAATAAGCGCTGCAATCATACTTAAAATTATAATCTGACTGAAAATGACACCATATGGAAGTG
ACGTTGATATCATTTCTGCAAATTTTGAAAAGATACCTTGATTATTTGAAACTTGTTGATATTCTGATCCTTCAAATAAAATTTTGCG
ATAATTTGGATTGGAAAACATAATGATTGTACCTATAGTAGCAATCATAAATGAAAACAATAATTTATAATTAAGTGTTCGATTTGC
AGTAAATTCATATAGAAAAGCTAAAATTAAAATGATACAGTGGAATAATGTCACATTCTCCATAAATAATTGACCGAAGAAGCATA
AAACATAGAATAGTACAGTAACACTTACCGGCTGCTTTTCTTTATATATAATTGCATTAATACAATAATAAATAATAAAGAGTGAAA
TTAGTGTTGATGTCGCATAATTATAAAATCCTGCAAACCAGCCATATGTATCTGCATAAATAGCACTTGGTAAAATTAACATTAAAG
AAAATGCTAACAATAATAGCTTGTCCACGCTTTACAACGTGTAATGTGCATAATCATCCAAATGATGCCCATACTAATGAGACCAT
ATGAAAGCCAACGTAACCAGCTTACATGTACAGCTATAATTTCAAAGATATTTCCGATATAGCGACCATTAAGTGATGCAAATCCTA
CTTTTAAAATATCAGTATTATAATTGCTAAACCATTGTAAATCATCGTGCATGAGTGGTAGTAAGATACCCATAAAAGTATAAAACA
ATAATATCGCAATTAATATCAAAGTTGTCTTGTGTAATTGAATTGTTTTCACTTTGCTAATCCTCAAATCTAGTTAAATTTTCCTCAAC
TTGTAGGTCGAAAAATTAATTCAATATTTTTAATGTATTTCTAATTTTCACCTATGCATGTTTCCTCAATCAAATTAGATAAACAAGG
TATTTAATATTACTTTCAACAATTTATCTAAATCGCCCCTCGTCTTTTTCTATGACGAATGATTACACTTGTCTATTCAAGTGTTTAAA
TTAAGATTTATTTCCGTTCAAATAAAACTTATTACTTTTTAATTAAGCATCTATTTTAAGCGCGTTACTAAAATTTCTTTATTATAATA

AATATATTATAAAATTGCTTGATTCACAACAATCTATTACTTATAACTTACAAATCGACATATGCAAATGACAAAATTGACAACCGA
ATTTATTCCACTTCATTAACTCTAATTTAATACCAATAGAGATTTGAACGAAATAAAAAAGCAGCAACAACTTCGTGAAAAATACAC
GCGCTGTTACCACTTTAATTTTTATTCTTTATAATCTTTTAGAATAACTTTGAACGTTGAGCCTTTGCCAACTTGACTATTCACTTCAA
TATTGCCTTGGTGGGCCTCTACTATATGCTTTGTAATTGATAATCCAAGACCTGTCCCACCAGAATCTCTACTTCGTGCTTTATCTACT
CTATAAAATCTTTCAAAAATACGTTGTTGGTCTTCTAATTTAATACCTATACCAAAATCTTGTACTTCGAAAATGACACGAAAGTCAT
CTCGATACACACGAACGTTAATATCTCCATCTTCATAAGAATAGTTAATTGCATTCGTTAACAAATTCGTGATAACTTGAGCAATTTT
ACTTTCTTGTGCTTTAACAATGACATCTTTTTCAATATCAGTATGAATGGAAATATTTTTTTGATTGGCTTGAGTCATCATATTATCAA
TAATACGCCGCGTTAAATCTGATAAATTCATATAGTCTGTATCTAACTCTGTATGTTGCTCAATATGCGATAAATCTAACAAATCTGT
TACTAAAGATTCTATTCGATTTGATTCTTTTAAAATTATGTTTAAAAACATGTCTAATGATTCCGCATCATTTTTTGCACCATCAATA
AGCGTTTCAGCAAATCCCTTAATTGAAGTAATAGGTGTTTTTAATTCATGTGAAACATTTGCTACAAACTCACGTCTTAGATTTTCAA
GTTGTTTCAGATTTGTTATGTCATGCATCACAACTAAAATCCCTTGCAAACTTTTTTGAGACCTAGTTAAAATCGGAACGCATGAAAT
ATCAAAGTACTTGGCATGGACTTGGTTTATTGCAACTTCCAATTGTTCATAAATAGGTTTTTCAACTTTAAAACTTTCTAAAATTAAT
TGCTCAATTTCAGTATTAACATAGCCGTGATAGCCTACTTGTTCAATATTATGCGAGATGTTGAACTGTTCATAATACGCTTTATTTG
CAACAACGATTTTTCCATTTCGATCTATCATTAAAATAGCACTTGGAATATTTTCAATCGTTGTTTTTAAACGGTTGGATTGAATTTTT
TGCTCATTATTAAGCTTTTGAAGGCGTCGTGCTAAATCATTGGTAGACACAAAAAGCGCTTTAGTTTCTACAACATTACTTTCAGGTA
CACGTATGTGATAATAACCATTTGCCAACAATTGTGTTGCATAAGTAACTTCTTGAATGGGACGGATTAATGTACGCTTAAAACTAC
GGCTTGCAAAATACAGACAAAGGAGTACAACTAAACATGTCAAAATAAGATATTTCCACAACGTCCAATGCATTTCTGTAATATCG
TTATTGTAACCTTTAATCCATACATGATAACCGTTAACCTTCTTATTAAAAATAAAAACGTCCCTTTTTTGAATATAGCTATCACTAT
TTGGGATAGCTTTCAGTTTGCCACTACTAAACACCTTTTTATCATGTTTAGTTATCAATAAATCTATATTTTGCTGTTTTACAATTTCT
TTAACTTTATCAATCTCATTATCTTGGACTAAATAAATATATGATCTTGCATCTGTTGCTAGAGCTTGTTCGTGTTTTTCTGATAAAAC
ATATGTGATGGAAGCGTGAATAATAATGCCTAATGTAACAAAACTGATAATTAATATACTGCTTATCAATAACATTAAGCGGTGGT
GAAACTTCATCATTGTTCTTTAGGTCTTTCCAATTTATAGCCTAAGCCACGCACAGTTTTAATAAGTTGTGGCTTCTTAGGATTATCTT
CTAATTTATCTCTTAAATGACTGATATGTACATCAACAATTCTTGAGTCTCCTGCAAATTCATAATTCCATACCGTATTTAACATATG
CTCTCTCGTAATGACTCTGCCTTGTCTTTCTATCAAATAAAGCAAGAGTTCAAATTCTTTCGGTGTTAGCTCCAATAACTCATTATGC
TTATATACTTCAAAATAATCGGGTCTAATACGTATCGATCCGATGGTAATATCATCGTCAATCTCTTCTACTTCTTTTACAAATTGAG
AACGTCTTAAAATGGCTTTCACACGGGCAACAACTTCTCTAGGTGAAAAAGGCTTAGTCATATAGTCATCGGCACCTAATTCTAAAC
CTAATACCCGATCAAATTCATCATTTTTCGCCGTTAACATTAATATAGGGACTAAATTTTTATTTGTTCTTACAGTCTTACATACGTC
AATGCCATCTTTTTTAGGTAGCATAACATCTAAAATAATTAAATCTGGCTGTTCACTTTCTACCTTTTCTAAAGCCTCATCACCATCA
AATGCGACAACAACTTCATAACCAGCTGTTTCTAAGTTATATTTAAGTAATGTTACGATTGAATGTTCGTCATCTACTACCAACACTT
TTTGCGACATGGTATGCCTCCCTAACTTATAATTATATTTACATTATAACCGAACTATTTATAAAAATAACATCCTACACATTATCTT
TACACATTTTTTACATTACTTTACACATAAATAAAATACTTCTTATATTTCCTTCTATCATTTGCATGACTTACTCTGGGACAACGAA
ATAAACTTTTTGAAAATATCATTTCTATGCCACTACCTATGCATGAGTTTTACTCATTTATTCCTAAGCTTATGTACACAATCGCTTTG
CCTAATGTGTAAGAAACACTATATAATCAATCATTAGTTACTCTTTATTATTTCTATCCTGTTGCCAACTTCAATTCATTTAAAAAGG
CGAACCTAGCAATTAAAGTTGCTAGACTCACCTATTTTTATTTTGCTTATTTTAAATTTTTAATCAATTCATCTGCAAATGCTGATGTA
GAAACTTCTTCAGCACCATCCATTAAACGGGCAAAGTCATAAGTAACAACTTTTGAAGCAATTGTATCTTCAATTGAATCTGTAATC
TTATCTGCCGCTTCTTGCCATCCTAAATGTTCTAACATTAATACAGAACTTAAAATTACTGAAGATGGATTCACTTTATTTAAACCTG
CATATTTTGGAGCTGTACCATGTGTTGCTTCAAAAATAGCATGACCTGTTTCATAATTAATGTTTGCACCTGGCGCAATACCAATACC
ACCAACTTGTGCAGCTAAAGCATCTGAAATATAGTCACCATTCAAGTTCATAGTTGCTACAACATCATGCTCAGCTGGACGAGTTAA
AATTTGTTGTAAGAAAATGTCAGCAATAGAATCTTTAATGATAATCTTGCCTTCTTTTTCAGCTTTTTCTTGAGCAGCATTAGCAGCA
TCTCTGCCTTCTTTTTCAACAATTTCGTCATATTGTTGCCAAGTGAATACTTGATCACCAAATTCAGATAATGCTAAATCGTAACCCC
ACTGCTTAAATGAGCCTTCTGTAAATTTCATAATATTACCTTTATGAACTAAAGTAACTGATTTACGGTTATTATCGATAGCATATTG
TATAGCTGCTCTAACTAATCGCTCAGTTCCTTCTTTAGAAACTGGTTTAATACCAATACCTGAAGTTTCTGGGAATCGAATGTTTGTC
GCACCCATTTCGTTTTGTAAGAAGTCAATTACCTTTTTAACTTCTGTTGTACCTTCTTTAAATTCAATACCAGCATAAATGTCTTCAGT
ATTTTCACGGAAAATAACCATATCAACATCTTGTGGACGTTTAACAGGTGATGGTACTCCTTTAAACCAACGTACCGGTCTTAAACA
AGTAAATAAATCTAATTCTTGGCGTAAAGCCACATTTAATGATCTAATACCACCACCAATTGGTGTTGTTAAAGGTCCTTTAACAGC
AATTAAATATTCTTTAATTGTATCAAGTGTTTCTTGAGGTAACCATTCACCAGTTGTATCAAATGCTTTTTGGCCAGCTAGCACTTCT
TTCCATTCAATGCGTTTTTCGCCATTATAGGCTTTCTCAACAGCAGCATCTATAACTCGGCTTGCTGCCTTCCAAATATCCGGTCCAA
TTCCATCACCGATAATAAATGGGATAATTGGTTCATTAGGTACGTTTAATCCTTCAGTTCCTTGAGTAATTTTTTCTGCAGTCATAGT
TATTTTACCTCCAAATTTTACATCTTCATTTTTAATTTGTATTGATTATTTTCTTTCTTCAAGCGGGATATACTTACGATTCGTTTCGCC
AATATATTTCGCTCTAGGACGCATAATTCTATTATCTTTATATTGTTCTAAAATATGAGCAATCCATCCTGCAGAACGACTTACAGCA
AAGATTGGCGTGAATAAGTCATGAGGTATTTCCATACAGTGATAAACACTCGCACTATAAAAATCAACATTAGGAATTAATCCTTTT
TCTTCTGCCATACGTTTTTCCATTTTCACTGACATTTCAAATAATTCTTCACGACCAGCGTCTTTCGTAATTTGACGGCTCATTTCTCT
TAAATATTTCGCTCTAGGATCACCATCTTTATATACACGATGACCGAAGCCCATTACTTTATCTTTATTAGCAAATTTTTCATCTAAG
TAAGCATCAACATTTTCAATTGACCCAATCTCAGATAACATCGTCATAACTTGTTCGTTTGCACCACCATGTAATGGCCCTTTCAGAG
AACCTACAGCTGCTACAATACCTGAGTACATATCTGACAATGATGATACCGCACAACGTGCTGTAAATGCAGATGCGTTCAACTCAT
GATCAGCGTGTAAAATAAGTGCTTTATTGAAGGCTTCTACTTCTATATCTGTTGGTAATTCCCCACGTAACATATATAGGAAGTTTGC
CGCATAACTTAAGTCAGGATTAGGCTTAAGTGGTTCTTTATCTTGTCTTACTCGAGCAAACGCTGTAACTAATGATGCTACTTTAGCC
TGTATACGCATTGCTCTTTCATAACGATTTTCATCTGATTCATTTTCAGCATCAGGATCGAAATGTGCAATATATGATAATGACGTAC
GTAATGCTGTCATTGGATGCACGTGATCTGTAACATACTCCTCAAAATGTGTATACACACGAGGATTTAATGTCATGTATTGATTTA
ATTTCCCTTTTAGATGAGCAAGCTCTTCTTCGTTTGGCAATCTATAGTTCCATAATAGGAAAATAACTTCTTCAAATTGCGCATTTTC
AGCTAGATCATCAATATCATAGCCGGCATAAGTCAATTGACTTTCAATAATTGAACTTATTTTAGTCTCCGCTGCGATAACCCCTTCT
AAACCTCTTTGTAATTCTGCCATGATAAATTTCCCCTTTACTGTTTCTTTATGAAATGGCTTTCAACTAATATTATAGCTAAACGCATT
TCGTTTGTGAAATATAAAAACTGGCATTATATGTGCAACAATTTTTTTGTAAATCTATGTAAATGTCACAGAATAGCCACAAATAGC
ACCATTAAAGCTTTTTCAAGATGTGAATAATCCATGAATTTTACAATAGAATTAAATTTTCAATTGGTTATTATATATGATTATAAAA
ACCAAAGTATATTAACATGGAATTTTACACATTTTTACCGAAAATAATTGTATATATCTCAATATAAGTGCATAATAAACATATCTA
TATTTAAGGGAGATGTATTATGGCTGAAAAATTACAAAGGGAACTGAGCAATCGCCACATACAATTAATTGCAATTGGCGGTGCAA
TTGGTACAGGCTTATTCTTAGGTGCTGGTCAAACGATTGCATTAACCGGCCCTTCAATTCTATTAACATACATCATTATAGGATTTAT
GTTATTTATGTTTATGCGAGGTTTAGGAGAAATCATTATACAGAATACTGAATTTAAATCTTTCGCAGATGTAACCAATACATATATT
GGGCCTTTTGCAGGATTTGTTACCGGATGGACATACTGGTTCTGTTGGATTATTACAGGTATGGCTGAAGTAACGGCTGTGGCAAAA
TATGTTAGCTTTTGGTTCCCAGAAATTCCAAACTGGATAAGTGCACTATTTTGTGTACTGTTATTAATGTCATTCAACCTACTTAGCG
CAAGACTTTTCGGAGAATTAGAATTTTGGTTCTCTATCATTAAAATAGCGACAATTATTGGTTTAATAGTAGTTGGTTTCGTCATGAT

TCTATTTGCATTTAAAACTCAATTCGGGCATGCCAGTTTCACAAATTTATATGAACACGGCATATTCGCTAAAGGTGCTTCTGGATTC
TTTATGTCTTTCCAAATGGCACTATTCTCATTTGTAGGAATTGAAATGATTGGTGTTACAGCTGGGGAAACAAAAGATCCAGTTAAA
ACAATTCCAAAAGCAATTAACAGTGTACCCATTAGAATTTTAATATTTTACGTTGGGGCGTTAGCGGTTATCATGTCTATTATCCCTT
GGCAGCAAGTTGATCCTGATAACAGTCCATTCGTAAAATTATTCGCATTGATCGGTATTCCGTTTGCTGCGGGCTTGATTAATTTTGT
AGTATTAACCGCTGCTGCTTCATCATGTAATAGTGGTATATTCTCAAATAGCCGTATGCTTTTCGGTTTATCAAGTCAACAACAAGCA
CCTCCGAACTTTTCTAAGACGAATAAATATGGCGTTCCACATGTTGCAATCTTTGCTTCATCAGCATTATTACTTGTGGCAGCATTAC
TAAACTATATTTTCCCAGATGCGACAAAAGTATTTACGTATGTGACTACCATCTCTACAGTGTTATTTTTAGTTGTATGGGGTCTGAT
TATCATTGCATATATCAATTATAGTCGTAAAAACCCAGATCTACATAAAAATGCTACGTACAAACTATTAGGTGGTAAATATATGGG
CTACTTAATATTTGTATTCTTCATTTTTGTGTTCGGGTTATTATTTATTAATGTTGATACAAGACGTGCAATTTATTTTATTCCGATTTG
GTTTATACTTTTAGCATTTATGTACTTAAGATATAAACGTATCGCTGCTAAATCAAATAAATAACAACAAGTTTTACCCCTGTTGAAT
ATACTAAAGTTCAACAGGGGTTTTTATATGCAACTTATAATATTAAATTGCGTACTTGGCTCAATACTTTTACTTTCTCATCTATTTA
ATAATGTATCATTTCAGAAATACATCCATACTTCTATTTTATAATAAATTTCCAAAGTAATATGAGTGAAAGTTTGAAGGTGATAAT
GTACATGTATAAAAGATATAAACATTTATATAGATTGCCATTCATACACTATCATTATCAAATAACCTATTAATTACGTCATAAAAT
ACCAGATGAACCAAAAAACGCCTTTCCATTGTTGATAAATGGAAAGACGTTTTTTTATAAATTATAGTACGTTTGCATATCCTTCAA
AGATTTTACCTTGAGCAGCATCAATCGTAACTAACATGTTATTGCTTATGTTTTTAACAGCTTTTTCTACACCTACAACTGTTGGAAT
ACCTTTTTCTAAACCAACAATTGCACTTGGTGATGTAATACCATTTTCTTCTGTAATTAAGCCTAAAGCTTTTTCTACATAAGGTACA
AACGTTTCATCGATTGAGTTAGTAACGATAACTTTGTCAGATAAATCTTTACCTTCTAAATCTTTAACAGTTTCAGCAACTAACGTAG
TACCAACAACTGATCCACGTCCAATACCTTGACCATTAGCAATTTCGTCACCAACTAGGTGGATTTTCATCATATTAGTAGTTCCAGT
TTCACCAGTTGGTACACCAGCAGTAATAATGATTAAATCACCATTAGATACTCTACCAGTTTCAACAGCTGTTGCAACTGCATTGTTT
AACAATGCATCTGTACTCTTACGTCCTTTTTTAACTACAGGTTGAACTCCCCAAACAATTGAACATTGACGTGCAGTTTCTTCACTTG
GAGTCACCGCAATAATGTCTGAATGTGGACGATATTTAGAGATAGTACGTGCCGTTGAACCACTTTCAGTAGCAGCTACAATTGCTT
TAACATTTAAGTTTAAAGCTGTATGTGCAACCGAAATACCGATAGCATTCACTAATGAAGTTTCAACTAATTTAGTACGATCTGACA
ATAACTTTTTGTAATCTTGGGCTGCTTCAGCTGATACAGCAATATTTCTCATTGTTTTAACAGCTTCTTCAGGATATAAACCAGCAGC
AGTTTCACCAGATAACATTACTGCATCTGTACCATCATAGATTGCGTTGGCAACGTCACTAGCTTCTGCACGTGTAGCACGTGGGTT
ACGTTGCATAGAATCTAACATTTGTGTAGCTGTAATAACTGGTTTACCTAATTTGTTACATTGTCTGATTAAATCTTTTTGAACCATT
GGTACTTTTTCAGGTGGAATTTCAACACCCATGTCACCACGTGCAACCATTAAACCATCAGACACTTCAAGAATTTCCGCAATATTA
TCAATACCTTCTTGATTTTCAATTTTAGGGAATACTGAAATGTTAGCTTTTTGTTCTTCTAAAATTTCACGAATTTCTAAAACATCACT
AGGACGACGTACGAAACTTGCTGCAATGAAGTCAACATTTTCTTTAATACCGAAACGGATATCTTCAGCATCTTTTTCTGTAATACC
AGGTAAACTTACTCTTACGCCAGGTAAGTTAACACCTTTTTTGTTTTTAAGCTCACCAGAGTTTAAAATATCACATTTAACTTCTTTTT
TAGCATGGTCAATATCTTTAACTTGTAATTCAATTAAGCCATCATCAAGTAAAATGTATGAACCTACTTGAACATCGTTAATTAAGTT
TTCATATGTTACTGAGAACTTTTCAGGCGTTCCTTCAACTTCATTCATGCTAACAATAACTTCGTTACCACGTTCAAGTTCAATGATA
CCGTCTTTCATATTATGCGTACGAATTTCTGGACCTTTTGTATCTAATAAAATTGCTACAATTTTGTCTAATCTTTTAGCTACTTTACG
AATTGTATCAATTCTACCTTTATGCTCTTCATGACTACCATGTGAAAAGTTTAATCGTGCAACGTTCATACCAGCATTGATTAATTTC
TCAATCATTTCTTCTGATTCTGAAGCTGGTCCAATTGTACATACAATTTTAGTTTTTCTCATTTTATAATTCCTCCTGAAATCTTATAT
AGATAACTTGTTAGCAAGTTCATATAGACTATAATCAAATTTATGATCTTTACCATCAAAAATTTCATCAAAAGATGTTGCTACAAT
TTTATTGTTCTTAATTCCAACACCCTTAGCTGTTTCACCTTGCATTAATAAGTCTACCGCATATCCACCTAAACGTGATGCTAAAACT
CTATCCGCACCTGTTGGGCTACCACCACGTTGAACGTGACCTAACACAGACACTCTATTATCAACATTGATGTATTGTGATAATTCTT
TTTGACAATCTTGCGCAGTCATACAACCTTCTGCTACAAGAACGATTGAGTGTTTCTTACCACGTTTAATACCTTGTTCAATTTTATC
AGCTATTTCTTTAATATCTGTTTTCACTTCTGGAACTACAATTGTCTCAGCACCAACTGATAATCCAGCCCATAATGCTAGATCTCCA
CAATCACGGCCCATTGCTTCAATGATAAATGTTCGTGCGTGACTTGACGCAGTGTCTCTAATTTTGTCGACTAAGCCAATAATCGTAT
TTAATGCTGTGTCAAATCCAATTGTAAAATCAGTACCATTGATATCATTGTCAATCGTACCAGGAATACCGATAGTTTGAATTTCTTT
GCATTCCTCACTGATGCGTTGTGCACCGCGATAACTACCGTCACCACCAATAACTACAAGGCCCTCAATCCCTCTTTTACGTAAGTTT
TCGATTGCAACTTTACGTACTTCTTGCTCCTTAAACTCTGGACATCTTGCTGAATACAAGAATGTACCTCCACGCTGAATCGTATCCC
CAACTGATCCTAATTCAAGTTTATGAATATCATCATTTAACAATCCTTGGTAACCATGATACACACCATAAACTTCAATTTCATTGTA
AATTGCTGTACGAACAACTGCTCTTACGGCAGCATTCATTCCAGGTGAATCTCCACCACTAGTTAAAACTGCAATTTTCTTCATGAC
GACATACCTTTCTATAACTGATTTATCTTTAACTCTAAATTACCACAAATTGATATGTCATTCCATCAAAACTAATGTATTGAATTGA
TGTAAACGTTTTAAATATTGAATTTTATTTTAACTTAATTAAAAAAAGAGGTTCTCCACCAAATGTGGTGGGT

>039-contig_321

TCCCACCATAAAAGATGAAGAACCAAAAAAGATTAGTAGCCACAATTTTGACTACTAATCTTAGTTAAAATTCAGTATCTAAATATT
AAATTATTGTTTTAATGTACTTCAATTTATGGTAAAACCCAGTATCGATTTAACATAAAAATGCTCAAGTTGATTATTCTATATAAGA
ACCGATATTTCTGAATTTTTCAAAGCGATCATTAGCAATTTCATCACGTGATAATGACTCAAGTGAATCTAACTGTGCAACAAACGC
TGATTTAATAGCTAAAGCTTGCTGTTCAACATCTTTATGTGCACCGCCAAGTGGTTCAGAAATGACATCATCTATAATACCTAATTGC
TTAATATCATGGGCAGTAATTTTCATTGTTTCAGCTGCAATTTTAGCCAAATTACTGTCTTTCCATAATAATGCCGCTGCACCTTCAG
GAGATATAACAGAGTAAGTACTATTCTCTAACATCAATACTTTATTGGCAATACCAATACCTAGAGCACCTCCACTGCCACCTTCAC
CAATGACAATCGCAATAACTGGTACTTTTAGTGAAGCCATCTCAATCAAATTTGTTGCGATAGATTCACTTTGTCCACGTTCTTCCGC
AGCTTTACCAGGATATGCACCTTTTGTATCTATAAATGTAAAGATAGGACGATTGAATTTTTCAGCTTGTTTCATTAAACGTAATGCT
TTTCGATAACCTTCTGGATGCGCCATACCAAAATTTCGATAAATATTATCTTTTGTATCTTTTCCACGTTGTTGTCCAATAACTGTAAC
AGCACGACCATTTAAAAAGCCAATACCACCAATCATTGCTGGATCATCTCTAAAATTACGATCACCATGTAGTTCCATAAACGAATC
AAAGATATATGGAATATAATCTAGGGTCGTAGGTCTTTCTTGCAAACGCGCAATTTGCACACGATCCCATGGTTTTAGATTTGTATA
TATTTTTTTAGTTTCTCGTTCCAATGACGCTTCAAGCATGTCAATTTCTTCTTGTAAATCCACATCATTTTTATCTTGAGATTCTTTTAA
AGATTCAATTTTATTTCGAATTTCAAAAAGTGGTTTTTCAAAATCTAACATTATTTAGTCACCTCTTGATGGATTTTTAGAATTTCAG
ACAATGTTTGACGCATATCATTACGATGTACAACTTTATCCAATTGTCCATGCTCTAATAAAAATTCTGCAGTTTGGAAATCATCTGG
CAATTTTTCGTTTATTGTCTGTTCAATAACTCGACGACCTGCAAAACCTATCAACGCTTTTGGCTCACTTAAATTTATATCACCAACT
GATGCAAAACTTGCAGATACACCACCAGTAGTTGGATGTGTTAAATATGATATATATAATAGTCCAGCGTCAGAATGACGTTTTAAA
GATACACTGGTTTTACCCATTTGCATCAAGGAAATAATACCTTCTTGCATACGTGCACCACCACTTGCAGAGAAAAGAATAAATGGT
AAACGGTTCTCAGTGCAGTAATCAATGATGCGACATATCTTTTCACCGATAACCGATCCCATACTTCCCATTCTAAAACGTGAATCC
ATGACAGCAACGCCAAATTTCATACCATCTAGTTGCGCTGTACCAGTCACAACTGCTTCTTTAAGACCTGTCTTTTGTTGGTCCTTTT
CAATTTTTTCTAAATAACTTGGAAAATCTAATGGATTCGCAGAGGTCATTCCCTTATCGAATTCTGTAAATGATCCTTCATCAGAAAT
TGCTTCTATACGTTTATACGCAGTTAAAGCAATATGATGATCACAATTAAAGCACACATTTAAATTTTCAGCTAATTCTTTTGTGTAC

ATAATTTTCTTACACTTTGGACACTTAGTCATAATACCTGCAGGCACATCATTATTTTTAGAGTCTTGTACTGTAAGATATTTCTTTTT
CTTTGTTCGATTAAAAAAATCTTTAAACATAGGAAAACCTCCAAATAACCTCTGTTCTAACCATTTAGAGCCTTAAACTGAACTTGA
CCAGTTAAAAACGGGTTTAAATTTATTTGATTTATATTCGTTTGACTGTTGAATGTTTATCATAAACTTTCATTTTCATCATTTTAATG
ACTCTTTATTGCTAACTTTTAAGCAATTTAATTAAACGTTAATTATTTTTATTGTAAATCTGTAAGTTTCATTGTTTTATCATACACAT
CTTGCGGATCAACTTCAATCCTAGCTACTCCAGATTCCATTGCCGCTTTAGCAACATTACGAGCAACTGATGGCGCTACACGTTTATC
AAACGGTCCTGGGATACAGTAGTCTTCATTTAATTCAGAACTATCGATTAAATCAGCAATCGCTTCTACAGCTGCCTTTTTCATTTCT
TCATTTATATGTGTAGCTTCAACCTCTAATGCACCTCTAAAAATACCAGGGAAAGCTAATACATTATTAATTTGGTTAGGATAGTCT
GAACGTCCTGTACCAACAACTCGTGCACCTGCCGCTTTGGCATCATCAGGTATTATTTCAGGATTTGGATTAGCCATTGCAAATATA
ATTGGATTATCTGCCATACTCTTAACCATATCTTGTGACAGCGCATTAGCTACAGAAACTCCGATAAATACATCTGCGTCTTTTACGA
CTTCTTCTAAAGACCCTTCAATCTTATCTTTATTTGTCCATTTAGCTACAACATCTTTCGTAGGATTCATACCATATGAACGTCCTTCA
AAAATTGCGCCTCTTGAGTCACACATAACCATATTTCTTACACCATACGCGTATAGTAATTTAACAATGGCTATTCCTGCTGCACCA
GCACCATTTAGTACAACTTTTATTTTAGCAATATCTTTGTTAACAACTCTCAATGCATTTACCAAACCTGCCATTGTTACAATTGCTG
TACCATGTTGATCGTCATGGAATACCGGAATATTAGTTTCTTTTTTCAATCGTTCTTCAATTTCAAAACAACGTGGTGCCGAAATATC
CTCTAAATTAATACCACCATAATTAGGTTCTAACAACTTAACTGTTTTAATGATTTCTTCGGTATCAGTTGTATTTAACGCAATAGGC
ACCCCATTGATACCAGCGAAGCTTTTGAATAATACTGCTTTACCTTCCATTACAGGAATACTTGCTTCAGGTCCAATGTTACCTAAAC
CTAATACCGCTGTTCCATCAGTAATAACTGCAACTGTATTTCCTTTAATTGTGTAATCATATACTTTTCTTTTATCTTCATAAATATCT
TTACACGGTTCAGCAACGCCAGGTGAGTATGCTAAACTTAATTCCTCTTTATTAGTAACTTTTACATTTGGTTTAACTTCTAATTTAC
CTTGATTACGTTTGTGCATTTCCAATGCTTCATCTCTTAATGACATGAAATCAGCCCCTAATTCAATATTTATTTTTAAAAAATAACTT
GGATAAAACGCATTACATTATAAAAGTAAAAATATTGGGTAAATCTGAATGAGTAAGAATTTATGGTTTTGATTATGTAACACAAA
TAGCGATAAACGATAATAAAATAATATTTATAAAGATACATTAAACCATACTATCTAAAGATATACCTTTAATTATTATAATGGATA
GCAAAAACCAATATATCAAAAAGTTATTATTTTTCCGCACGATATATCGACAAAATTCTTTACTCAATTTATGTATACTGCTTTTTGT
GCTAATTATTCTTATGGATTAATCAATAATGTAAAGTGAAACTCATAAAAATAATAAGCATAAAAAACTAATATAAACGCAAACTG
ATGGTTAAAAAATATCTAACCATCAGTTTACTATATCATAATTTATTAGTTGATAAAAGTTATATAAGCCTAATATCACTAGGGTTA
AAGGATTGTATAAAATTATTAAACATACTATCTTTTTGATTAATATAGCCTATAGTAGTCATTTGTTTAATCGTTTCATCATAAAAGG
ATAACATAACATCATTAGCATTCTCTTTCGTAGCTTTAATCATCTCTTCAAACATATCTATTTGTGATTTATTTCTAATTATAATTTGT
TTGGCAAATGCTAATTTTTGTTCTTCAAAAGTGGCTAATGTTTGAATCTCATTTATAATTAGTTGCCGTTGTTGCTTTCTATGGTCAAA
TTTCCCGCTAACTATAAACAAGTCATTATGTGATAACAACTCTTCGTACTTTTTAAACTGATTAGGGAAAATCACACCATCTAAAGTT
TCAATGCCATCATTTAATATGACGAATGCCATATTTTGACCATTTTTAGTTCGAATTTGTTTAACTTTATCAAACTGTACTAATATAG
GTTTATTATTCTGCGCGTTACTCAATTTAAATATCGTTAAATATTGTTTGGCAACAAACTTTTTATCTACTGGGTGTTGCGAAACATA
AAATCCTAAATATTCTTTTTCGTACTGACTAATAAGTGCATCAGGCAATTCTTCTTTATCTTCATACATCTGTTTTGGCGTTAAAATAT
CAAATAAAAAACCATCTTGTTCAATGTTTAAATCGCCATCCAACACTTGATCAATAGCTTGCAACAACGTTGAACGTGTTTTACCAA
AAGCATCAAACGCTCCCACTAAAATCAGTGCTTCAAGTAACTTTCTCGTTTTGACTCTCTTCGGTATACGTCTAGCAAAATCAAAGA
AATCTTTATATTTGCCGTTCTGATAACGTTCATCAACAATCACTTTCACACTTTGATAACCAACACCTTTAATTGTACCAATTGATAA
ATAAATGCCTTCTTGGGAAGGTTTATAAAACCAATGACTTTCGTTAATGTTCGGTGGCAATATAGTGATACCTTGTTTTTTGCTTCT
TCTATCATTTGAGCAGTTTTCTTCTCACTTCCAATAACATTACTTAAAATATTTGCGTAAAAATAATTTGGATAATGGACTTTTAAAA
AGCTCATAATGTATGCAATTTTAGAATAGCTGACAGCATGTGCTCTAGGAAAACCATAATCAGCAAATTTCAGAATCAAATCAAAT
ATTTGCTTACTAATGTCTTCGTGATAACCATTTTGCTTTGCACCTTCTATAAAATGTTGACGCTCACTTTCAAGAACAGCTCTATTTTT
TTTACTCATTGCTCTTCTTAAAATATCCGCTTCACCATAACTGAAGTTTGCAAATGTGCTCGCTATTTGCATAATTTGCTCTTGATAAA
TAATAACACCGTAAGTATTTTTTAATATCGGTTCTAAATGCGGATGTAAATATTGAACTTTGCTTGGATCATGTCTTCTTGTAATGTA
AGTTGGAATTTCTTCCATTGGACCTGGTCTATACAAAGAAGTTACAGCCACAATATCTTCAAAGTGTTCCGGCTTTAATTTTTTTAAT
ACACTTCTTACACCGTCAGACTCTAATTGGAATATGCCAGTCGTATCTCCTTGCGACAACAATTCAAACACTTTTTGATCATCGAACG
GAATCTTTTCGATATCAATATTAATACCTAAATCTTTTTTGACTTGTGTTAAGATTTGATGAATAATCGATAAGTTTCTCAAACCTAG
AAAATCTATTTTTAATAACCCAATACGTTCGGCTTCAGTCATTGTCCATTGCGTTAATAATCCTGTATCCCCTTTCGTTAAAGGGGCA
TATTCATATAATGGATGGTCATTAATAATAATTCCTGCCGCATGTGTAGATGTATGTCTTGGTAAACCTTCTAACTTTTTACAAATAC
TGAACCAGCGTTCATGTCGATGGTTTCGATGTACAAACTTTTTAAAATCGTCAATTTGATATGCTTCATCAAGTGTAATTCCTAATTT
ATGTGGGATTAAACTTGAAATTTCATTTAATGTAACTTCATCAAACCCCATAATTCTTCCAACATCTCTAGCAACTGCTCTTGCAAGC
AGATGACCGAAAGTCACAATTCCAGATACATGTAGCTCGCCATATTTTTCTTGGACGTACTGAATGACCCTTTCTCGGCGTGTATCTT
CAAAGTCAATATCAATATCAGGCATTGTTACACGTTCTGGGTTTAAAAAACGTTCAAATAATAGATTGAATTTAATAGGATCAATCG
TTGTAATTCCCAATAAATAACTGACCAGTGAGCCAGCTGAAGAACCACGACCAGGACCTACCATCACATCATTCGTTTTCGCATAAT
GGATTAAATCACTTACTATTAAGAAATAATCTTCAAAACCCATATTAGTAATAACTTTATACTCATATTTCAATCGCTCTAAATAGAC
GTCATAATTAAGTTCTAATTTTTTCAATTGTGTAACTAAGACACGCCACAAATATTTTTTAGCTGATTCATCATTAGGTGTCTCATAT
TGAGGAAGTAGAGATTGATGATATTTTAATTCTGCATCACACTTTTGAGCTATAACATCAACCTGCGTTAAATATTCTTGGTTAATAT
CTAATTGATTAATTTCCTTTTCAGTTAAAAAATGTGCACCAAAATCTTCTTGATCATGAATTAAGTCTAATTTTGTATTGTCTCTAATA
GCTGCTAATGCAGAAATCGTATCGGCATCTTGACGTGTTTGGTAACAAACATTTTGAATCCAAACATGTTTTCTACCTTGAATCGAA
ATACTAAGGTGGTCCATATATGTGTCATTATGGGTTTCAAACACTTGTATAATATCACGATGTTGATCACCGACTTTTTTAAAAATGA
TAATCATATTGTTAGAAAATCGTTTTAATAATTCAAACGACACATGTTCTAATGCATTCATTTTTATTTCCGATGATAGTTGATACAA
ATCTTTTAATCCATCATTATTTTTAGCTAGAACAACTGTTTCGACTGTATTTAATCCATTTGTCACATATATTGTCATACCAAAAATCG
GTTTAATGTTATTTGCTATACATGCATCATAAAATTTAGGAAAACCATACAATACATTGGTGTCAGTTATGGCAAGTGCATCAACAT
TTTCAGACACAGCAAGTCTTACGGCATCTTCTATTTTTAAGCTTGAATTTAACAAATCATAAGCCGTATGAATATTTAAATATGCCAC
CATGATTGAATGGCTCCTTTCTATTAGTTAAGTTTTGTGCGTAAAGCTGTAGCAAGTTGCTCAAATTCATCCCAGCTGTTCACTGAAA
CTCCTGACGCATTCGGATGACCACCGCCACCAAAATCTTGCGCAATATCATTAATAATCAATTGCCCTTTAGAACGTAATCGACATC
TGATTTCATTACCTTCATCGACTGCAAATACCCATATTTCAATCCTTTGATGTCAGCAATTGTATTAACAAACTGAGATGCTTCATT
TGGCTGAATACCGAATTGCTCCAAAACATCTTCAGTTATTTTAACTTGGCAGAATCCATCATCCATAAGTTCGAAATGTTGTAAAAC
ATAACCTTGAAACGGCAACATTTTTGGGTCCTTCTCCATCATTTTATTTAAAAGCGCATTATGATCAATATCATGCCCAATTAACTTT
CCAGCAATTTCCATAGTATGTTCTGAGGTATTGTTAAAAAGGAATCGCCCAGTATCACCGACGATACCAAGATATAAAACACTCGC
GATATCTTTATTAACAATTGCTTCATCATTAAAATGTGAGATTAAATCGTAAATGATTTCACTTGTAGATGACGCGTTCGTATTAACT
AAATTAATATCACCATACTGATCAACTGCAGGATGATGATCTATTTTAATAAGTTTACGACCTGTACTATAACGTTCATCGTCAATTC
GTGGAGCATTGGCAGTATCACATACAATTACAAGCGCATCTTGATATGTTTTATCATCAATGTTATCTAACTCTCCAATAAAACTTA
ATGATGATTCCGCTTCACCCACTGCAAATACTTGCTTTTGCGGAAATTTCTGCTGAATATAGTATTTTAAACCAAGTTGTGAACCATA
TGCATCAGGATCTGGTCTAACATGTCTGTGTATAATAATTGTATCGTTGTCTTCGATACATTTCATAATTTCATTCAAAGTACTAATC
ATTTTCATACTCCCTTTTTTAGAAAAGTTGCTTAATTTAAGCATTAGTCTATATCAAAATATCTAAATTATAAAAATTGTTACTACCA

TATTAAACTATTTGCCCGTTTTAATTATTTAGATATATATATTTTCATACTATTTAGTTCATAACGATTATAGGACATCAAGCAATATT
ATACTAGCAACAGTTGACCAAATAAAAATGCACAAATAACAAGCTTTATTTAATTCAGGGGCCCCAACACAGAGAATTTCGAAAAG
AAATTCTACAGACCATGCAAGTTGGGGTGGGGCCCCAACACAGAGAAATTGGACCCCCAATTTCTACAGACCATGCAAGTTGGGGT
GGGGCCCCAACACAGAGAATTTCGAAAAGAAATTCTACAGACAATGCAAGTTGGGGTGGGCCACAACACAGAAGCTGACGTAATG
TCAGCTTACTATAATGTGCAAGTTGGGCTCCAACGTTTGTGCGAAATCTATCTTATGCCTATTTTCTCTGCTAAGTTCCTATACTTCGT
CAAACATTTGGCATATCACGAGAGCGCTCGCTACTTTGTCGTTTTGACTATGCATGTTCACTTCTATTTTGGCGAAGTTTCTTCCGAC
GTCTAGTATGCCAAAGCGCACTGTTATATGTGATTCAATAGGTACTGTTTTAATATACACGATATTTAAGTTCTCTATCATGACATTA
CCTTTTTTAAATTTACGCATTTCATATTGTATTGTTTCTTCTATAATACTTACAAATGCCGCTTTACTTACTGTTCCGTAATGATTGATT
AAAAGTGGTGAAACTTCTACTGTAATTCCATCTTGATTCATTGTTATATATTTGGCGATTTGATCGTTAATTGTTTCACCCATCTGAG
GCTGTCTTCCTAAAAGTTGCATAGACTTTAAAACATCTTGTCTATTAATCACACCCACTGTCTTTTTATTACTCGAAACGACAGGAAT
CAATTCAATACCTTCCCAAATCATCATATGCGCACAACTTGCTACTGTACTCATAGCATTTACATAAATAGGATTTCGCGTCATCACT
TTATCTATTTCGTCGTCGTCCTTTGTATTAATCATCTCTCGACTTGTTACAATACCTACTAATTTATAAGACTCATTGACTACCGGAAA
TCTTGTATGGCCAGTTCGATTCGCCATACGCTTATAATCTGCTATTTTCATCGTATCAAACAGCACAGATAAATCATCTAATGGCGTC
ATTATATCTTGAACTATTAAGATATCTTTTCGTATTTTCTGATTAAAAAGTGCTTTGTTGATAATATTTGCAACTAGGAATGTATCAT
AACTTGATGATAGAACAGGTAAATCATGTTCATTCGCAAAATTAATAACTTTATTAGATGGCTTAAATCCACCAGTAATTAATATAG
CCGTACCTCTTTTTAAAGCTTCAATCTGCACATCTTCGCGATTTCCGACAATCAATAATGTCTTTGGACCAATATATTTTAAAATATC
TTTGAGTTCCATTGCTCCAATTGCAAATTTAGATACCATCTTAGTGATACCTTTATTGCCACCTAACACTTGGCCATCAATAATATTG
ACAATTTCATTAAAAGTTAAATGTTCAATTTCATTACGATTACGTTTTTCGATTCGAACCGTACCAACACGATCTATCGTTGCGACCA
TGCCCATTTTATCAGCATCTTTAATTGCACGATATGCTGTCCCTTCAGATACGTTTAAAAATTTAGCGATTTTACGCACCGAAATTTT
AGAGCCTATAGATAACGATTCAATATAATCTAAAATTTGTTCATGTTTTGTCATTCTTTACCTCTTCTTTTCGAACAGTATTAACTAC
ATTATAACTTTATTTTGGATAAAAAGCATTGAAGTGAAATGAAATAATGATCGTTTCACCTATTTTATTTTTTGAAAATATACAACAA
ACACAAAGATCACAAAATCTTTAATTTTAAATGGAAAAATCCATTATTATTTATTAGAATGTAAGTGAGGAGGGATGTACTAATGTA
TAAAAATATATTACTTGGTGTAGACACTCAGTTAAAAAATGAAAAAGCACTAAAAGAAGTGTCTAAATTAGCTGGCGAAGGTACAG
TCGTAACAGTTTTAAACGCAATCAGCGAACAAGATGCTCAAGCATCAATTAAAGCAGGTGTTCATTTAAACAAACTTACTGAAGAA
CGAAGCAAGCGATTGGAAAAAACACGCAAAGCTTTAGAAGATTATGGTATTGATTATGACCAAATAATTGTTCGTGGTAATGCAAA
AGAAGAACTATTAAAACATGCTAATAGCGGTAAATACGAAATTGTTGTTTTAAGTAACCGTAAAGCAGAAGACAAAAAGAAATTTG
TACTTGGAAGTGTCAGCCACAAAGTAGCAAAACGTGCGACTATCCCTGTATTAATCGTTAAATAAAATTTTTATCCAGAATCACAAA
TAATCTTTCAATCATGATGCAGTCTCAAACGACTGAGTAAACACAAGAAATGATTATGACTGTGGTTCTGGATTTTTTATATCGTAG
TAAATTTATAATCAATGTCTAATTGTATAAAACTAAAATTACGAGAGTAGGTCAGAAATGATAAAGAACCACTGATGTCCCCCGTCC
ACGTCGTAACTGAATCAGTAGAATATAAAAACACCCACTAAAAATATGCAGACGATAACTTCCACATAGATTAGCGAGGTGTTTTT
TAGTGTAAAATCTATATTCTATTTAAAACTGAACAGATTCACCTGGTTTTAAAATTTGCACGTCCCCTACATTAACAGCATCTTTAAA
TTGTTGTGGATCTTGTTCGATTAATGGGAATGTATCATAATGAATCGGTACAGAAATTTTTGGTTTAATAAATTCATTAATAGCATAA
CTTGCATCATCAATACCCATCGTAAAATTATCTCCAATTGGTACAAAACATACATCAACTGGATGACGTTTCGCAATAAGTGACATG
TCACTAAACAGACCTGTATCACCAGTATGATAAATTGTTTTTCCTTCAACTTCAAACACGATACCCATTGGCATACCTAAATAAACT
GGAATACCATTTTCATGTGTAAAACTTGAACTATGAAATGCTTGAACAAATTTAACGCTTCCGAAATCAAAGTTTGCTTTACCACCA
ATATTCATACCATGAACATTTTCAACACCGTGATATGAAGAAAGATAGTCAGCCATTTCTGCACTTCCAATTACTGTTGCTCCTGTTT
TCTTTGCTAGTTCCACAACATCACCAAAATGATCAAAATGACCGTGCGTTAAAACGATATAGTCTACCTGCACTGTTTCAATATTCA
AATCACACTTAGGGTTATTTGAAATAAACGGATCTACGATAACCTTTTTGTTGTTCCCTTCTAAATAAATCGTTGATTGACCATGAAA
TGATAACTTCATTTGAGCATCCTCCTATCAATTGCTATATAAATTTAGTACCCTTTTGCCACTTAATTATAACAAATTCTCAAATTTTA
AAAATTGAAAATCTAGTTAATGTATTAGCTCGATTTTGAAATCTAATAATAATTGGCATAAAATGGAAGTAATATTATGTTGAGGAG
TGTTTATAAAATGACAAAAATATCAAAAATAATAGACGAACTGAACAATCAACAAGCTGATGCAGCATGGATTACAACACCGTTGA
ATGTATATTATTTTACTGGATACCGTAGCGAACCCCATGAAAGATTATTTGCATTATTGATTAAGAAAGATGGTAAACAAGTACTAT
TTTGTCCAAAAATGGAAGTCGAAGAAGTCAAAGCATCACCTTTCACAGGTGAAATCGTTGGATATTTAGACACTGAAAACCCTTTCT
CACTTTATCCTCAAACAATCAATAAATTACTAATTGAAAGCGAGCACTTAACAGTAGCACGCCAAAAACAATTAACCTCTGGTTTCA
ATGTCAATTCATTCGGAGATGTTGATTTAACAATCAAACAATTGAGAAATATTAAATCCGAAGATGAAATTAGCAAAATACGTAAA
GCTGCTGAGTTAGCAGATAAGTGTATCGAAATAGGTGTTTCTTATTTAAAAGAAGGTGTGACTGAACGTGAAGTAGTCAACCATATT
GAGCAAACTATCAAACAATATGGCGTCAATGAAATGAGTTTTGATACGATGGTTTTATTTGGAGATCATGCCGCATCACCTCATGGC
ACACCAGGAGATCGCAGATTAAAAAGCAATGAATATGTACTATTTGATTTAGGTGTAATTTATGAGCATTATTGTAGCGATATGACA
CGTACCATTAAATTTGGTGAACCTAGCAAAGAAGCACAAGAAATTTATAATATTGTATTAGAAGCAGAAACATCTGCAATCCAAGC
AATTAAACCTGGAATACCTTTAAAAGATATCGATCATATCGCTAGAAATATTATTTTAGAAAAAGGTTATGGTGAATATTTCCCTCA
TCGCTTAGGTCATGGTTTAGGATTACAAGAACATGAATATCAAGATGTTTCAAGTACTAATTCTAATTTGTTAGAAGCTGGCATGGT
TATTACAATCGAACCAGGTATTTATGTACCAGGTGTTGCAGGTGTAAGAATTGAAGATGACATACTTGTCACTAATGAAGGATATGA
AGTATTAACACATTACGAAAAATAAGGAGTGGGATAAAAATGAAAAGCTTGTTACAAGCGCATTCTCATTCAGTCAAACACTGCCA
ATATAACATTGTAGCGCCTAAGACATAAATTTTTATCCAAGTCTAAATGCAATATGTAACAAACAAGCTAGAAACACATATGCAGG
TATGTTCATCAGTAACATGTAATGAATCAAATCAATATCATTCATGTTCGATGATTTCTTCGCATTGTTTCTAGCTTTAATTTATCATT
ATTTAATTTTAATAACCAAGGAGATGATAACGTCATTCTTTAGTACGCTGTAATCCATTCCCTTTTCATTAAATTCAAATTATAATTG
TAATGCTTCTTCTACAGATTTATATTCCATTTCAAATGCCTCTGCAACGCCTTTATTGGTTACATGACCTTTGTAAGTATTTAAACCTA
ATGATAATGGTTGATTTGATTTAAATGCTTCTCTATACCCTTTATTAGCTAGCATGAGCGCATAAGGTAGCGTAGCATTATTTAAAGC
TAACGTCGAAGTACGCGGTACTGCACCTGGCATATTTGCAACTGCATAATGAACCACACCATGCTTAATATATGTAGGATCATCATG
TGTCGTAATTTTATCAGTTGTTTCAAAAATACCGCCTTGATCAATAGCAATGTCAATAATAACTGACCCATTTTTCATTTGTTTAATC
ATGTCTTCTGTTACAAGTCTTGGCGCTTTAGCACCTGGAATTAAAACTGCACCTATTACTAAATCACTTTGTTTAACATACAACTCAA
TATTCAACGGATTTGACATAATTGTATGTACACGTCCACCGAATAAATCATCTAATTGTTGTAAACGCTTTGGATTAACATCTAAAA
TCGTAACATCTGCACCTAGTCCTAGTGCAATTTTAGCTGCATTTGTTCCTGCTTGACCACCACCGATAATAGTTACTTTACCTTTAGG
TACTCCTGGAACACCACCTAGTAGAATTCCCATACCACCATTGAGTTTTTGTAGGAACTCTGCACCAACTTGAGCTGACATTCTTCCT
GCTACCTCACTCATTGGTGATAACAATGGTAAAGATCGGTCTGGTAACTGCACAGTCTCATATGCAATACTAATTACTTTTCTATCTA
TCAAAGCTTGTGTTAATTTTTCTTCATTTGCTAAATGAAGATAAGTGAATAATACAAGCCCTTCTTTAAAATATGGATATTCAGATTC
AAGTGGTTCTTTAACTTTAATAACCATATCCACATCCCAAACTTTTGCTTGTTCAGCAACAATCTCAGCACCTGCTTCTTTGTAATCT
ACATCTTCAAAGAACGATCCTGAACCCGCATTTGTTTCCACTAAAACAGTATGCCCACTTTCTACTAAAGCGTGCACACCACTTGGT
GATAAACCAACACGATTTTCATTATTTTTAATCTCCCTTGGTATACCAATTTTCATACCATCCACCTCCATAATCATCTTAACGCGAA
CATTTTGAAAGCGCAATCAAAAATCCACAAAATTGTAAAGGTTATTACACTGACTTTTCCGAAAATTGTGGTAAAATATAATTAAGA
AAGAACAAGGAGGCACTTACTATGATTACTTACAAAAATATTTTAATCGCAGTTGACGGTTCACATGAAGCGGAATGGGCATTTAA

CAGAGCAGTTGGTGTTGCTAAACGTAACGATGCGAAGTTAACAATTGTGAATGTAATTGATTCAAGAACGTATTCTTCTTATGAAGT
TTATGATGCTCAATTTACTGAAAAATCTAAGCATTTTGCAGAAGAATTATTAAATGGTTATAAAGAAGTAGCTACTAACGCTGGTGT
TAAAGATGTAGAAACGCGTCTAGAGTTTGGCTCTCCTAAATCTATCATTCCTAAAAAGCTTGCACATGAAATTAATGCAGACTTGAT
TATGAGTGGTACATCAGGCTTAAATGCCGTGGAAAGATTTATTGTTGGTTCTGTATCAGAATCTATCGTTCGTCATGCGCCATGTGA
CGTGTTAGTTGTTCGTACTGAAGAGTTACCAGCAGACTTCCAACCACAAGTTGCAACAACTCAATTACGTGAAAAATATCAAAATTA
AATATAGATGATCATTAGATAACTACTTGATCATTATGAACCAAGTGCAACTTTCTGCACTTGGTTTTTATTGTTTATAAATAAAAC
TCATTTAATAATGTTTTCATAATCTTCTTCGACTACTTAATTCTTTAAGATATTCGTGAAAAGAGACATTACACTAGTTAATTTTCAA
ACAATACAAAAAGCGTCTACCTCCTACATATAATTGTAGCGGAGATAGACGCTTAATATTTATTTAAAAATTATTTTAAACCACCGA
ATGTCATAACATCACGGGCAATCATACTTTCTTCATCTGTTGGAATAACGACAACTTTAACTGGTGAATGAGGATAGTTAATAAATC
CTTCTTTACCACGTAGTAAGTTTTCATTTTTCTTAGGATCCCAGTAAACACCCATAAATTCTAAGCCTTCAAGAACTTTCGCACGAAT
TTCTACTGAGTTTTCACCGATACCTGCTGTAAATACGATAACATCAACACCATGCATTCTCGCAGCATATGATCCAATATATTTGTGA
ATTTTAGAAGCAAATACATCTAAAGCCATTTGTGAACGTGCTTTACCTGATTCAGCTTCTTCTGATAAGTCACGTAAATCACTAGAT
GTACCTGATAATCCTAATAAACCTGATTCTTTGTTTAAGATTTCCAATACTTGTTCAGCAGTTTTACCTGTTTTTTCCATAATAAATGG
AATTAAAGCAGGGTCAATATTACCAGAACGAGTACCCATTGTTACACCAGCAAGTGGTGTGAAGCCCATTGATGTATCAATAGATT
TACCGCCATCGATAGCTGCAATTGATGCTCCATTACCAATGTGACATGAAATAATACGTAAATCTTCAATTGGCTTATCTAACATTTC
TGCCGCTCTTTGTGATACAAATTTATGGCTTGTACCATGGAAACCATACTTACGAATGCCATAATCTTTATAATAATGATATGGCAA
GCTGTATAGATATGCTTTTTCAGGCATTGTTTGATGGAATGCTGTATCAAAAATTGCCACATGAGGGATATTTGGTAATAATTTACG
GAAAGCACGAATACCCATCAAGTTAGCTGGGTTGTGAAGCGGTGCTAATTCGCTTAATTCTTCAATTTCCTTTTCAACCTCATCAGTA
ATAGCTACTGATTCAGGGAATTTTTCACCACCATGTACAACACGGTGACCTGTTCCATCGATATCGTTAATATCATTAATAATATTGT
GCGCTTTAAAAGCATCCAACATGATATCAACTGCCTCAACGTGATCCTTGATATCTTGTACTGTTTTAACTTTTTCCCCGTTGACTTC
AATTGTAAAAATTGAATCCTTCAATCCGATTCTTTCTATTAAACCTTTTGTTACTAATTCCTCTTCAGGCATTCTAATTAATTGAAATT
TTAATGATGAACTACCAGCATTGATAGCCAAGATTAATTTTGACATAAGTAATGATCCTCCATCGTTTGCTTTTTATACTATTTCATT
TCTCATTTTATCATTCATACAGTTTTATGACTATACTATTACAGATTATTTTTTAGGACGATTTGTGTCCATCCACTGATTTAACTCTG
TCATAAATCCTTGAAATTGTGAAGGATTTTTGAAATCAGGAATATTTGCCAATAATACTTCAACTGGCTTTGTTTCACCCGATTTTTT
CTTTTGTAAAATTAATATAGATTTTCGCGCTTTTTCATTTTTAAATAAAGTTGGTGGTAAATTTAAAAATGCTTGCATCTCTGTCTCTG
TTGCAATATATTTTTCAAGCTGTTTTACATGTTCACCTGTAAAAATATTACTTGGTACCACTAGAAAGGCATATCCAGCATCTTTTAA
TACATTTATTGCTTGTTCTATTAATAAATAATGTGAATAACTATGTCCTTCTTCAAAACCTAGCTTAAACTCCTTACTTCTTTCATCAA
TTGGATAATAGCCTACTGGAAAATCACCAATAACGATATCTGCTTCTTCTAATGGTAGTGGCATGATGGCATCTTGAGGATACACAT
CGAAAGGAATTTCTAAGAAGTTTGCTAAATGTACACTAACACGTGATAAAACTGGATCAACTTCAATTAAATGATGCATAACCGCA
ATTTCAGGTAACACTTCTTTTACAGTAGCACTTAAATGACCGGCACCACTTGCAATATCAACAATATGTAATTCTTCTTGGTTGTTCA
TAAAACGCTCAACTAAAAATCCTAGTATCAATCCAATTGAATCTGGTGTAATTTGATGATTTGCTTGTATCTTTTCTTCCTGCATTAA
ACTTAAATATGCAAATTGGAATGCTTTACGTCGATCTTGTAACGTCGATTGTTCTAACAATCCTCTTTCATTGGTATATACTTGTTCC
ATTGCTAGCCCAAGATTTTCAATAAAACTTTGGCCATTTTCATTATTTAATGTTTTAGCTTTTTCATCTAATGTATGAAACAAGCGTTC
CATAATTGTTTGTTGTTCTGCCATATTCGTCCCTCTCCAATAAAAAGTGCCCATAGGTCTTAGCAATCATAGAAAACAATTCTATTTT
AATGCTGTTGACAACGATGGACACGATGTATACATTTTAAGATTTATCTCTTTAATGATTAAATATTTTTGTATGCAGCTAAAGCAGC
ATCAAAATCTGGGAAATCAGTACCTTCACTAACGATTTCTTTATAAACAACTTTATTATCTGCATCTAATACAAATACTGCACGAGC
TAATAAGCGAAGTTCTTCCATAACAACGCCATAGTTTTCACCAAATGATAAGTCACGGTGGTCACTTAATGTAATGACATTGTCTAA
ACCTGCTGAAGCGCACCATCTTTTTTGTGCGAATGGTAAGTCTGCTGAAATTGTAAGCACAATCCCCTCTTCTTTAGAAGCATCAGA
GTTGAATTTGCGAGTCTGCTGATCACAAACACCTGTATCAATTGATGGTACCACACTAATTAATTTCTTTTTACCAGCATAATCTGCT
AATGTTACTTGATTTAAGTCATTATCTAACACTGTAAAATCAGGTGCAAAATCACCTTCATTAATTTGTTGACCTTTTAAGTGGATTG
GTCCACCTTTGAATGTTATTTCAGTCATATACTTGACCTCCTGCATTTTAAAATTCTAACTACATCATACGACAATTTATGGTTTGATT
ACAATTTTCATGCTTTAATCAAAAGCAAATTTAATTTTATACAAATTGTTGAATATAATCAAAAGCATTTTTTAAAATTAAAATAGC
AGTAATAATAATAAATAATACTTTTACATAGCCAACACCTTGTTTGATAGCAAAATGTGCCCCAGTATATGACCCAGCAATCATGCT
TGTAGCCATTATTAAACCTATTACATAATCTACTTGTCCTAATACCATAAATAATACAAGCGCACCTATATTAGAAGCAAAGTTCAA
AACTTTAGCATTTCCTGCTGCACTTAAAAAATCAAAACCAAAGACTAACAATACAAAAAGCATAAATGAACCTGTTCCCCCACCTA
CAAATCCATCATAAAAGCCGATTAATATAAAAAGTGCTGCAAATAGTATGGCTTTCTTAAATGTAAATTGAGTAAACGTACGTGTAT
TGCCCCAATCTTTTTTAAGTAATGTGAATATAAACACCGACGAAAGTGCAATAATGATTAAAGGTTTCAATATTTGTGACGGAACCA
TCGTTGCAATATATGCGCCACATGCAGATGCCAAAAATACAAAACCAAATAATTTGGCAACAACATATAAGTCCACTTTACCGGAC
CTTATAAACTTTATCGTACTAGTTAAAGAACCAAATGAACTTGCCAATTTATTTGTACCTAAAGCCACAGATGGTGGTAGACCGATT
GCTAATAATGCTGGCGTAGAAATTAAACCGCCACCCCCTACAACCGAATCTATAAACGCCGCGATAAAACCAAATAAAATTATGAT
TATAATCATCGTTAAGTTCAAATCCATAGTTTAACATCCCCTTATGTTTAAATTTACTCTAGGATTTTATTTATAAAAAGTCATTTATT
AATTGGTTTGTTTGCTGTTCTTTAATAGTTTTATAATCACTAGTTATTTCAAGTGTTTCAATATTTTCAACAGCACGATTAATCATCTC
TTCAAAATCAAAGACGCTTTCATATTGAACTACCTTATCAAAGTTTGGTTCGGTTACTGGATTTTTAGGGGTGAAAATTGTACAACA
ATCTTCAAATGGTTGAATAGATGTTTCAAATGTACCAATTTCTTTCGATTTAATAATAATTTCTTCTTTATCGTAAGTTAATAAAGGA
CGTAATACAGGAGTAGAAGTTACATTATTAATTGCATACATGCTATGAAGTGTTTGACTGGCTACCTGCCCTAGGTTTTCACCATTTA
CAATAGCTAAAGCCCCTATTTGATGTACTAATTTATCAGCAACACGCATCATCATACGTCTCGTTGAAGTCATTGTATATCTTGGATG
TACAACTTTATTTACCTGTTTTTGCAATTCTGTAAATGGTACAATATGCAATTTAATTGGTCCAACACGTTCAGCTAAAATACGTGTC
AATTCAATAACTTTTTCTTTTGCTTGATCACTTGTAAATGGTGGACTATGGAAATGAATCGCTTCAATTGTTACGCCACGTCTCATCA
CTTCCATCCCAGCAACTGGTGAGTCTATACCGCCTGAAAGCATTAGTAACGTCTTACCACCAGTACCAACTGGTAATCCACCTGAAC
CCGGAACAACTTCTTCATACATATAAATTGCATCTAATCTAACTTCCACTCGAATTTCATGATCTGGACGTTTGACATTCACTGAAAT
ATTGTCGAAGTGCTTCAATACTGCACCACCCAATTCACGCTGTAATTCATACGTATCCATTGGGAAATTTTTATCGGCACGCTTCACA
TCAATTTTAAATGTGCTGTTTTCTTCAAATTGCTGCGCAAATTTAATTGCCGCTGCACTCATTGCCTCTATTGTTTTTTCTACTTTTAAT
ACTGGACTAATAGATTTAATACCGAAAATTTTTGATAATCGATATGTTATTTCATTTATATCTGCATGGTCTTCAAGTTCAATATACA
TACGATCTCGTTTGCCTTTAACGACAAACCCATCAAGTCCTTTTAATGACTTATTTACATTATTTCTTAATTGATTTACAAATTTTTTT
CTATTTGAACCCTTTAATGTTAACTCCCCGTATCTAACAAGCAAGTGATCATACTTCATTATTTTAGCAACTCCTTAATTTCCTCATA
AATGATGATAAATATTTCTTTAAACCTTGCTATATCTTCTTTAGTTGTAGTAGCCCCAAATGATAATCTTATACTACCTTCAATAGAT
TTGTCTGATAATCCCATTGCAGCCAATACTTCATTTAATTTATTACGTTTAGATGAACAAGCACTCGTCGTAGATATCATAATGTCAT
ATTTTGAAAAAGCATTAACTAATACTTCACCTTTTACGCCAGGAAAACTAATATTTAAAACGAATGGTGAACCAGAAGTTGAAGAA
TTAATATAAACTCCATGATATTTATTTAAAAATTGACGGACGTCATTATTTAACACAGTAACAAATGCATTCAATGCTTCAAAGTTTT
CATTAGCTATCTTCATCGCTTTAACCATTGCAATATCATTTGGCAAATTAACTGTTCCACTTCTAACGCCATATTCTTGACCACCACC
ATGGACAGTTGGTTCAACATTTTGAATGTGATTTACAAGTAAGACGCCTTGGCCTTTTAAACCATTAAACTTGTGTCCACTTAAACTA

ATACTATCTATGTTATTGAGATCCATTGAAATTTTGCCGAATGCTTGAACCGCATCTACATGAAAATGTGCCTTAGGATAATTTTTTA
TAACTTTAGCCATTTGTGGAATAGGCTGTATTTGTCCAGTTACATTATTTACATACATACATGTTACTAAACCGACTTTGTCTGACAT
TAATTCTTTGAAGTGTTCTAAGTTAATACTGCCATCTTTCTTTACATCAACATATTTAACTTTAAATCCTTCGTGTGCTTCCAAATATC
TTACAACCTCTAATACGGACGGATGCTCTAACACGGATGTAATTATTTCCTTCGCTGTATCAAATTTACGATAGGCAATACCTTTTAA
AGCAAGATTATTGGATTCAGTTGCACCACTAGTGAATACAACATCATAATTTGTTTTTGAATTAATCATTGCATTAATTTGGGCTTTT
GCTTGTTGTAGTAATTGATTTGCCTGCAAACCAGCTTTATGCGGACTATTCGGATTATAATACATTGATTGATTTACTTTTAAATAAG
TATCTAACACTTCTTCAAATGCTTTCGTCGTTGCCGCATTATCTAGATATATCAAAATAAACACTTCTCTTTCATTAATCATCAATCGT
TAAAATTGTTTTACTCAAGCATTATACTATTATCCGACAAAAAATAAAGGTTATAGCGCAAAATAATCTTATTTCTACTTATTAAATA
AAAATTAGACTATTTCACTCTATTTAAACAGCTGTGTGATTATGCTTTTTTCACTACTTATATTAATTAAAACAACACATTTGATTAC
GTTTATCTTTAAACAATTGCTATTTATATTTATTGTCCCTTTCATTTATCCATATAAAAAAGAAGCTAGACAACGTATCGTTGGCTAA
CTTCAATTATTAAACTCATCAGTCCAATTTGACAGAGTGCTCATATTATTTAAGTATAACTACATACTAGTTTCTATTGCTTAATAAC
TTCTTCTTCAATATGTTTAGTAACACCTGGCTCAACACTTTCAAGAGCTTGCTCTGCAATTTCAATCGCACGCTTATAGCGATTATTTT
TAAATAATCGTTCAGCTTCATTTAAGCTCTTATCAACATTGCTATAGTCCTTACGATATCTATTTCCATATTGAATTAATTTCTCTGCA
TAAACAGCATTAACAAGAACATCATTTGCTTCATCTTCAAATGTATTCATTTGAATCACAATTTTAGACACTTTATCTTTTAACTGTT
TAACGTGTATTGGACGTTCACTAAATTGTTCGTTAACATCACGAACTTCATGATCAATTTCATTTTTCATGATGATAAACCTTTCAGG
AACGCTTGTTAAGTTAGAAGCAAGTAATCGACGATACACTTCTTCTTTCTTCGATTGGACTCGTAGCAGATTGTCTTCTGCTTCTGCT
TCATCTTCACGCAATTGAATCAGATGATTTTGTAGCTTTTCTTGTTTGTCATTAATAACTGTGACATGATCTTCTAAATATTGTAAATT
ATCCTGAACCTCGCTATATCGCACAGCAGATTTAGACATTTCTTTTAAAATATCATCATATACAGAAATTAAACTTTGAATTTCATTT
TCAAATTGACGAACACTTTGAGCATCAGATTCATTTATATAGTAGTTTTCACGTACATATTCAATTTCTGTTTGCAATGTATAATTCA
TGTCTTTAGCTTTGAATAAGTTATCCGTAATGATATCTTTTGTTTCTTCGACATCATTTTTAGCTTTAACTTCATGTTCAATTAAATCA
TACATGTCATCTAACTTATCATTGATATTAGCTAGTTTATCATTAGCTTCTTCTAATTCTAAGCGGCTAATTAATGGTTCAACGAAAC
TAAGCTCTGTTTTTAAGCTTTGTAATGTACTGTCTACTTTTACGTGATCCAGATCATACCCTTCAACTTTAAGATCACGGCAACCATA
TTTTAAATCTTGGAATTGACCAGGTAATTCTTTTTGAGTTTCTCTAATTAATTCTGGTATTTCTTCCATATAAGATCTTAGCTGTTTCA
TTTGTTCATTCAAGGCAGCTATATGGTTGTGCGCTTGTACATAATTACCATCAGCTTTTAGTACTTCATATTGCTCTAACCTTGGCTCA
AACTTTTCAATTTCAGTTTCAAGTAGACTTGCTGCCTCACCAAATTGATGACGATTTGCTAAAACATCACGTTTCATTTCACGATAAT
CAACCTTACATTTGTCATATAATTCATCATTATCTTTGTATAACGCAATAATTTCATTTACATCTTCTAATTGTTGCTGATAGCTTTGT
TCGTAACTATCCATCAACTCATTTGCATCATCAATTTCACTTTGAGATGCGTTGAAACTAAATTTATCTAATAAAGCCTCAGCATTAT
GGATTTTTTCTTCCACAGGAGCTAGATACTTATTTGTACTTTCTACGTTGTCCTTTTTCATTGCATCGTATTTCGTTTTTGTTTCACCTT
TTAAATTCAACTTAGATAATTGTGCAAGGTTTTGATCAAAAGGTAACGTCTCAATTTCATTTTTACGTTCGATTGCTTTTTCAATAAT
TTGTCGTTTATTTGAACGTAAATAGAATAATACACCTACAGCAATCAATATAATCACAATTATTGCCAAAATGATATATAACACCAT
ATGCTTCTCCTCCTAATTTATCATTACTATTATAACGTAATTTTGCTTAAGGATAAATTATAAAATGAATTTATTGCCATAATATTTTC
GAAATTCTATACTATTATAAAATCTATGAAAATAAGAGGTTTTAAATATGACAACAATTAACCCAACAAACTACACATTATTAAAG
AAACAAGCAGCAAGCCTTATTGAAGATGAACATCATATGATTGCTATTTTAAGTAACATGTCTGCCTTATTAAATGATAATCTAGAT
CAAATTAATTGGGTCGGCTTTTACTTATTGGAACAAAATGAACTTATACTTGGACCTTTCCAAGGACACCCCGCTTGTGTCCACATTC
CAATTGGAAAAGGTGTATGTGGTACAGCCGTTTCAGAACGTCGTACACAAGTTGTAGCTGATGTCCATCAATTCGAAGGACATATC
GCTTGTGATGCTAATAGTAAGTCTGAGATTGTCGTTCCAATTTTCAAAGATGATAAAATTATCGGCGTCTTAGATATCGATGCCCCT
ATAACTGATCGATTTGATGACAATGACAAAGAACATCTTGAAGCAATTGTTAAAATTATTGAAAAGCAACTCGCATAAAAGGACAT
CAGCATTTTCAATAAAGTGTTGACAGTTAGCAGGAAAATGTTACAATAATCTTTGTGTGAATTAACGAAAGTAGCAGTTGTATATTA
TTGAGCGCTATGTTGTTCCCAATGCGGACGTGTCACGTAACTGTCGCTATAAGGTGAAGACACATAAAACAATATATCTTAGTAAGC
ATGCAACACTCTTTTTTGTTTATTCATAACAACAAAAAAGAATTAAAGGAGGAGTCTTATTATGGCTCGATTCAGAGGTTCAAACTG
GAAAAAATCTCGTCGTTTAGGTATCTCTTTAAGCGGTACTGGTAAAGAATTAGAAAAACGTCCTTACGCACCAGGACAACATGGTC
CAAACCAACGTAAAAAATTATCAGAATATGGTTTACAATTACGTGAAAAACAAAAATTACGTTACTTATATGGAATGACTGAAAGA
CAATTCCGTAACACATTTGACATCGCTGGTAAAAAATTCGGTGTACACGGTGAAAACTTCATGATCTTATTAGCAAGTCGTTTAGAC
GCTGTTGTTTATTCATTAGGTTTAGCTCGTACTCGTCGTCAAGCACGTCAATTAGTTAACCACGGTCATATCTTAGTAGATGGTAAAC
GTGTTGATATTCCATCTTATTCTGTTAAACCTGGTCAAACAATTTCAGTTCGTGAAAAATCTCAAAAATTAAACATCATCGTTGAATC
AGTTGAAATCAACAATTTCGTACCTGAGTACTTAAACTTTGATGCTGACAGCTTAACTGGTACTTTCGTACGTTTACCAGAACGTAG
CGAATTACCTGCTGAAATTAACGAACAATTAATCGTTGAGTACTACTCAAGATAATACGTTCAATACAAACACCCACAATTGTGGGT
GTTTTTTTTATTAATTTAAAAATGATAGATTCAAAATTATATGGAGCTACCCTATTTTACAATCAACTTAGAATTTACAAGTCTTATT
TGTTTAATTATCTTTAGTATTTCATCATTAGTTTTTAAAATTATCAATTGCGATATACGAATCCTCATCCCTCTATCAAATTACCATTC
ATCGTCTCACTTAATAAATAATTTCGGATTATCTGTAATTAATCCATCTACACCCATTTGTCTTAATTTTTCTCCAGTTTTCAATTTAT
TAACTGTGTATGGCATAACCTGTAATTGATGATGATGTGCTTTATCAACAAATTTTCTGGTCACTAATGCATAATTAGGATTAACATA
ACTAGCAATTTGAGCAATTCTTGAAAAGTTTGGCTTTTTATACCAATAATTACGTTTACTACAAAGCACACCTAATTCATATATACTG
CCCAATGTGTTTAACTTTTCGATGCATTCGATATCAAAAGATTGTATAACTACTTGTGTCGCATCGACTTTCTTTTCTTCCAAAAATG
CCAGCAATTTACATTCTATTTCTGGATATAAATTCGGACTTTTCAATTCAATCAATAGCTTTTTATCATACTTTAAGCATAATGAAAG
TACTTCATCTAACGTGGGTATTCGTTCTCCCTTAAAAGCAACATCTTTATAACTACCAAAATCAAATGATTTTAATTGCGATAATGTG
TAATCAGCAATACGCCCCTTACCATCCGATGTTCTATCAATTGTTTCATCATGTATCACAACAAAATGTTGGTCTTTGGTCAAATGAA
CATCTATTTCTAACATAGCAACATTGAGCCCCATTACCTCTCGATAACCGACCATTGTATTTTCAGGAAAATCACTCGGCAATCCAC
GGTGCGAAACAATTTGTAATTCATCTTTCAGTTTATTCAGAGTCATTGATAAGTCACCTTTCTGTTAAACTTTGATAAACTATTCTAA
AATTAAACTATCACAATTATATTGGAGGTTAAAGCAATTGCATCAACATGACTTTAAAGTCCAAACTTCTTGGCAAGGTGGTCGTAA
CAATGTCGGAAACGTTCAAGGCGACATACTTTCAGAGAATATTTCTATACCTGCTTCCTTAGGTGGTGTTGGTTTAGGAACAAATCC
CGATGAATTGTTAGTATCAGCCGCTTCATCATGTTATATCATCTCATTAGCAGCTACTCTTGAACGTGCAAAGTTCACAGATATTTCA
ATTGAACAACAATCGATTGGAACAGCTTGTTTAAATAACGGAAAATTCAGTATGTCAAAAATTGTGCACCATCCTCAAATTCAAATT
CCAAGTGATCAAATAGCACAATTAGAAAAGCGATTACCAAAATTGATAACAATTGCAGATAATAATTGCATGATTTCAAATGCTGT
AAGAAATAATGTGGACATAAAAATTTATCCCATCATTCAGGCCAAATAAAATCTTTAAGTGATAACGTTTGTTCGACAATATAGCGT
TATCACTTTTTTAGGCTTTTAAATTTTCAGAAAATTTGCTAAAATAGTGAAATCTTATTTATAGGAGGCATATAAATGTATTATCATC
AACCGTTGTTATTAACACCTGGCCCAACCCCTGTACCTGATGCCATTATGAGAGAAATTCAAGCACCTATGGTTGGTCATCGTTCTA
AAGATTTTGAAGACATCGCACAACAAGCATTTCAAGGTCTAAAGCCAATATTTGGGAGTCAAAATGATGTACTTATTTTAACATCTA
GCGGTACAAGCGTCTTGGAGGCTAGTATGTTGAACATTGTAAACCCTGAAGATCACTTCGTTGTCATTGTTTCAGGTGCCTTTGGTA
ACCGATTTAAACAAATTGCACAAACTTATTACAAAAATGTGCATATTTATGACGTAACATGGGGAGAAGCTGTAGATGTCAAAGAT
TTCATCAATTTCCTTTCAACTTTAAATGTTGAAGTGAAAGCAGTATTTAGTCAATATTGCGAAACATCTACGACAGTGCTACACCCTA
TTCACGAGTTAGGAAATGCCATCAATCAATTTAATAGTAATATTTATTTTGTAGTTGACGGCGTAAGTTGCATTGGTGCTGTTGATGT

TGACATTAACAAAGATAAAATTGATGTACTTGTTTCTGGTAGTCAAAAAGCAATTATGTTACCTCCAGGATTAGCTTTTGTAGCTTAT
AGCCACCGTGCAAAAGAACGTTTCAAAGAAGTAACTACGCCAAAATTTTATCTAGACTTAAATAAATACATTTCGTCACAAGCTGA
CAATTCTACACCGTTCACACCAAATGTGTCTTTATTTAGAGGTGTAAATGCATACGTTGAAACCGTAAAAGCAGAAGGTTTCAATCA
CGTAATAGCACGACACTATGCAATTAGAAATGCATTAAGAAGCGCCTTAAAAGCATTAGATTTAACTTTATTAGTCAATGATAAAG
ATGCATCTCCAACGGTTACAGCATTCAAACCTAATACAAATGATGAAGCGAAAATAATCAAAGATGAACTTAAAAATCGGTTTAAA
ATAACAATTGCTGGTGGTCAAGGCCATCTTAAAGGTCAAATTTTAAGAATTGGTCATATGGGGAAAATTAGTCCTTTCGATATTTTA
TCGGTAGTATCTGCTTTAGAAATTATTTTAACTGAACACCGTAAAGTTAACTATATCGGTAAAGGTATATCAAAATATATGGAGGTT
ATTCATGAAGCAATTTAATGTACTCGTTGCAGATCCCATATCAAAAGATGGTATCAAAGCATTATTAGATCACGAACAATTCAATGT
AGATATTCAAACTGGCTTGTCCGAAGAAGCATTAATCAAAATTATACCTTCATACCATGCTTTAATCGTTCGTAGTCAAACTACGGT
TACTGAAAATATCATAAATGCTGCTGATTCTTTAAAAGTAATCGCACGCGCCGGTGTTGGTGTAGATAATATTAATATTAATGCTGC
AACATTAAAAGGTATTTTAGTTATTAATGCCCCAGATGGTAATACGATTTCAGCTACTGAACATTCACTGGCAATGTTATTATCAAT
GGCTCGAAATATTCCGCAAGCACACCAATCACTTACAAATAAAGAATGGAATCGAAATGCATTTAAAGGTACTGAGCTTTATCATA
AAACATTAGGTGTCATTGGTGCTGGTAGAATTGGTTTAGGTGTTGCTAAACGTGCGCAAAGTTTCGGAATGAAAATACTAGCTTTTG
ACCCTTACTTAACGGATGAAAAAGCAAAATCTTTAAGTATTACGAAGGCAACAGTTGATGAGATTGCCCAACATTCTGATTTCGTTA
CATTACATACACCACTAACACCTAAAACAAAAGGCTTAATTAATGCTGACTTTTTTGCCAAAGCAAAACCTAGTTTGCAAATAATCA
ATGTGGCACGTGGTGGTATTATTGATGAAAAGGCGCTAATAAAAGCATTAGACGAAGGACAAATTAGTCGGGCAGCTATCGATGTG
TTTGAACATGAACCTGCAACTGATTCGCCTCTTGTTGCACATGATAAAATTATTGTTACACCTCATTTGGGTGCATCAACAGTCGAA
GCTCAAGAAAAAGTGGCAATTTCTGTTTCAAATGAAATCATCGAAATTTTAATTGATGGTACTGTAACGCATGCAGTGAATGCACCT
AAAATGGACTTAAGCAATATAGATGATACTGTAAAATCATTCATCAATTTAAGCCAAACAGTTGGTGAATTAGCTATTCAATTAATG
TACAATGCACCAAGCTCTATTAAAATTACGTACGGTGGCGACTTAGCCTCTATTGATAGTAGTTTATTAACACGTACAATTATTACTC
ATATTTTAAAAGATGATCTTGGTCCTGAAGTCAATATTATCAATGCTCTAATGTTGTTAAATCAACAACAAGTGACATTAAATATTG
AAAATAATAAAGCAGAGACAGGTTTTAGTAACTACTTAGAGGTAGAACTATCAAACGATAGCGATTCCGTTAAAGTTGGCGCTTCT
GTCTTTACAGGTTTCGGTCCAAGAATTGTTAGAATTAATAATTTTTCTGTAGACTTAAAGCCAAATCAATATCAAATTGTGTCATATC
ATAATGATACTCCAGGTATGGTAGGAAAAACTGGCGCATTGTTAGGTAAATACAATATCAACATTGCATCTATGACTTTAGGTAGA
ACTGAAGCGGGCGGAGATGCGCTAATGATTTTATCCGTTGATCAACCTGTTTCAAACAATATAATTGATGAACTTAAACAAGTTGGT
GAATACAATCAAATTTTCACAACTGAATTGACGGTACAGTCATAAACAATTTATGTAAATTCAAAATGCCAAGAAGACATGGGCTT
GGGACATTAAGTTCTTAGGCAATGTAAAAAAGCTGATTTCTATTAATTATTTGATAGAAATCAGC

>040-contig_241_RC

TACGAAGTATTGTATAAATAGAGAACAGCAGTAAGATATTTTCTAATTGAAAATTATCTTACTGCTGTTTTTTTAGGGATTTATGTCC
CAGCCTGTTGCTTCTTGGCATTTTTTCATTATTAATTTCATTGTAACTCAATATGAAACCTTTGGTTCAATCATTCACTTCAAGTTATG
CTGTCAAACAACAATCAAATTACAAATTATCTAGTACACCTCTAAGTTCACCTAAATGATTAATAACATAGTCGGCATGATGCGCTT
CTAACTCACCTGCAGCATCCTTACCTTTTAAACCTGTTAATGTTCCAATAAACGTTGCACCTATTTTTTGAGCACTTAATAAGTCAGC
TAACGAATCGCCTACTATAAATACGTCATCTTTATTTACAATGTTATCTTGCTTATTGATATAAGATTCATATTTATCGCGATTATTAC
CATATAAAGCTGCGATATAACTAAAAGGATTCGGCTTTCCTAATGGTCGTGCTTGCGGATACATATTCTCTGCTTCTAAAACATCAC
TTGCTGTTGCAATAAAATCAGCTTCAAAATATGGTAACAATCCTAAATTTTCAAATGGCACAACAGTCTCAGTATAAGGACGACCTG
TTGCAATACCTAATTCGAAACCAGCACCTTTTAAATCATTCAGAAGTACCTTAACTTCATCTACTGGTCTCAAAATAATTTCTTGATA
AATATAACCTGTCTTAAAAGTAGTTCGTGCTATTTTCTTTTCAACATCTTCATACAACTTCGATCCTAAATACCATTCTTGATAAACTT
CTTGCGCTAACGTCCATAATGCACCCTTCAAACTAAATAAAGTAGCATCCGAAACATGTAACTCTGTTGTTGCAAACTCTTCCAAAG
CAGCATAAATATTATTTTTACCAACTTTTACATTATCCAAAAATTGTAAAGGTAGTTGTTCATTTAAATTAAAACAGTCTGCTAAGTT
TGTGCTTATATTTTGCAACTTCAATTCGACAGGCTCATCTTGATACATGAATGCCTCAATTTCATCATGTGATAACTTTTTCAAAATA
TCAATTAAATGAATACTAAAAACGATAAATAACATATCCCAATTTGAATTCAGCCCTAGCGATTTTAATTTGTTTAAAATCTTATCTT
TTTGAAAAATTCGATTCCTAATGTCTTGTATATCGTTATCAGTCAAAGTTTCCCAATCTATATGTGAATGAAGACCTAAATAACACTT
ATCCATTAATAATTCATATACCGTTAATGCAGAGACATCGAAACAACGTTCTTCACTTAAAAAAACGCCATCAACATCAAATAAAAT
TTTCTTCACAATCCCCACTCCATTTCTGAAAATTCAGATATAAATCATTCTACTATTTGACTAAAAAAAGCGCAAACCCTATTGAAGT
TGGATTTGCGCTTTAGCTGTTTAAATTTTATAAATGTTTTTCAATTTCATCAGCAACCTGCTGTACGTGTGTACCGACAATAACTTGA
GTTGAATGTTTACCATTAACAGTAACACCAACTGCACCGGCGTTTTTAATCTTCTGTTGATCAATAATAGATGTGTCTTTTAACTCTA
GACGCAACCTTGTTGCACAATTGGTTAAATTAACAATATTCTCTTGACCGCCTAAACCTTCTAATATTTGTATAGCATGTTGATGATA
TTTACTTTGTTTAATATCATTTTCACCAGGAGCAATATTATCTTTTACAACTGTTGGATCAACTAATTCATTTTCACCTCTACCAATCG
TATTCAAGTTAAATACTTGGATTACTACACGGAAAATCACATAGTATAAGATGAAAAATACAACACCTTGAACAAGCAACATCAAT
GGATGATTTGATACTGGATTAATTAGTGATAACACATAATCTATCAAACCTGCACTAAATGAAAATCCAGCTGTCCAATGGAATGTA
GCTGCGATAAATAAAGATAATCCTGTTAATAACGCATGAACAACATATAAGATTGGCGCAACAAACATAAATGCAAACTCAATCGG
CTCTGTAACACCAACGAAAAATGCTGCAACTGAACTCGCTAGGAACCAACCGTAAACTTGTTTTTTCTGAGTCGTTTTAGCTGTATG
ATACATTGCTAACGCAGCCGCTGGAATACCGAACATCATGATTGGGAAGAATCCCGCTTGATAGCGTCCTGTAATACCTTTTATAGC
ATCTTTGCCACTTTGGAATTTACCAATATCATTAATACCAATCGTATCAAACCAGAACACACTATTCAGTGCATGATGTAATCCTGTA
GGAATTAATAATCTATTGGCAACACCATATATGAAAGCTCCAAACGATCCTAAACCGACTATAGATTCACCAAATTTTACAATCCAT
GAATAAAGTAGTGGCCATAAGAATAACAATATGACAACTAAAAATGTACAGTAAAATGCAGTCATAATTGGAACTAGACGTTTACC
ACTAAAAAATGATAATGCTAATGGTAATTCTGTTTCACTAAACTTATTGTATGCATAGGCTGCTATTAAACCTATTACAATACCAAC
AAAGACATTGCCATTATTCATCTTTTCAAAAGCTGAATTTATTTCCGAAGCTTTCATTCCTAATAAAGGCGCTAATTTCATTGGTGAT
AATACAACTGTAACTAAAAAATATCCTAACGTAGCTGCAAGCGCGACTGCACCATCATTTTTCTTTGCCATTCCTATAGCTACACCA
ATTGCAAATAAAATACCTAATTGCTCTAAAATCGTAGTACCTACCGTAGTAAAGAACATTGCGATTTTCGGCGTCGCATGAAGTGCA
TTTAACGTATTACCAATTCCGGCAATAATTGCTGCAGCCGGTAAAATGGCAACTGGTAACATTAACGAACGCCCTAAATTTTGGAAA
AATTTATACATTGAATGTCATCCTTCTTAAAATAAGGTAGAAATATAAAGATTACTAATGTAACTAGAATAACTACTTCGATACTCC
GTTATAGTCACCTAGGCATACTAACCAGCTATATTTCTACCTCAAGTTATTTTATAAACTTTTTACAATTTCATGCAATTCTTGTTGTA
ACTTTGCTGTTCGTGTTTCAATCTCTTTTGTAATGTAATCGATACGCTCGTTTCGTTTTAAATCTTTAGGTAAATCGTTAATATCGATT
GGTTTACCAATATTTATGTATGCTTGTCCTGTTAAAAGACCATGAATCTTAGTAGGACCAACATAAGCAACAGGTAATATTGGTGAC
TTACTTAACATTGCAATTGTTGAAGCACCACGTTTCAATGGTGCACCTTCTTGCGATGTGCGAGAACCTGTTGGGAAGATACCAACT
GTCTTATTATCTTTCAACAAATTGATTGGGCGTTTTAAAGTACTAGGTCCTGGATTTTCACGATCTACAGGAAATGCATTTAAAGACG
TTAAAAATTTACCAATCCATTTATTTTTGAATAATTCTTTTTTAGCCATATAATGAATTTGATTAGGATATAATGCCATACCTAGCAT
AATAACTTCGTTATAACTTTCATGCGTACAAGTTACGACATATTTACTATCCTTAGGAATATTATCTTTACCGATTACGTATAATGAT

TTTGACATTTTAACTAAAATGAAATTCAAAATCTTACTAATCACTGAATACATTGTGCCACCTACTTAACTTATTTGTTTATAACAAC
TAGATTTTATCACTATATTTTTACTAGAACAAGATAGTCTGAAAATACATCTAAAGTTGTATAGTGTATTACTAGCAATAATTATAA
AAAAAAGACATAATTAAAGCATATATAAATCATATAATGGAGGTTAAGTATGTCAGATTTTAATCATACAGATCATTCTACAACAA
ACCATAGCCAAACACCTAGATACAGAAGACCTAAATTTCCATGGTTTAAAACAGTCATCGTTGCATTGATTGCTGGAATTATTGGTG
CACTTCTAGTACTTGGTATAGGCAAAGTATTAAATAGTACAATTTTAAATAAAGATGGTTCAACTGTTCAGACAACAAATAATAAAG
GTGGCAATCAATTAGACGGTCAAAGCAAGAAATTCGGTACCGTTCATGAAATGATAAAATCTGTCTCCCCTACAATTGTTGGAGTTA
TTAACATGCAAAAAGCATCAAGTGTAGACGACTTATTAAAAGGCAAATCATCTAAACCATCTGAAGCTGGAGTAGGTTCAGGTGTT
ATCTATCAAATAAACAACAATTCAGCTTATATCGTTACAAACAATCATGTTATTGATGGCGCAAATGAAATTAGAGTCCAATTACAT
AATAAAAAACAAGTTAAAGCGAAATTAGTTGGTAAAGATGCAGTAACTGATATTGCTGTACTTAAAATTGAAAATACAAAAGGTAT
TAAAGCGATTCAATTTGCCAACTCTTCAAAAGTACAAACTGGCGATAGCGTATTCGCAATGGGTAACCCATTAGGATTACAATTTGC
TAACTCTGTAACATCTGGTATCATTTCAGCAAGCGAACGTACGATTGACGCTGAGACAACTGGTGGCAATACAAAAGTTAGCGTTCT
TCAAACAGATGCTGCTATTAACCCAGGTAACTCAGGTGGCGCATTAGTAGATATTAATGGTAATTTAGTTGGTATTAACTCAATGAA
AATTGCTGCGACACAAGTTGAAGGTATCGGGTTTGCTATTCCAAGTAATGAAGTTAAAGTAACAATTGAACAACTTGTAAAACATG
GTAAAATTGACCGCCCTTCGATTGGTATTGGTTTAATTAATTTGAAAGATATTCCTGAAGAAGAGCGCGAGCAACTTCATACTGATA
GAGAAGACGGTATTTATGTCGCCAAAGCTGATAGTGATATTGATCTTAAAAAAGGTGATATTATTACAGAAATTGATGGCAAGAAA
ATTAAAGATGATGTTGATTTAAGAAGCTATTTATATGAAAATAAAAAACCTGGTGAATCAGTCACTGTTACCGTTATCCGTGATGGT
AAAACAAAAGAAGTTAAAGTGAAATTAAAACAACAAAAAGAACAACCAAAACGTCAAAGCCGATCAGAACGTCAATCACCTGGCC
AAGGCGATAGAGATTTCTTTAGATAATTTACAGACAGAAAAGTACATTAAGAGCCTACTCATTACATTCTTAGGAATGAATATGAGT
AGGCTCTAATTTATTTAGCTATGCAATTATATTTATTGATAGTTAACCATGAAGTATTTTTTCTTACCGCGACGAATAATCGTAAATT
CGCCATCAATTTTATCTTCTGGTGCTAAAGCATAATTAACATCTTGTTGTCTCTCACCATTAATATAAATCGCACCATTGTTAACATC
TTCACGTGCTTGTCGTTTAGAAGGAGAAATGCCTGTTTCAATAAGGACTTCAACGATATTTGTTGTGTCATTTGATAATGTCACTTGA
GGCACATCTTTAAATCCATCTTTTAATTCTTTCGCTGATAATGATTTTAAATCACCACTAAATAATGCTTGTGAAATACGGATTGCAT
CATTTAATGCATCTTCACCATGAATAAATTTAGTTACTTCTTCAGCTAATGTTTTTTGAGCTTCACGTAAATGCGGTGCTTCATTTTTA
GATTGTTCTAAGCGATCAATTTCTTCTTTTCCTAAGAAAGTAAAGTATTTTAAGAATTTAATTACATCTTCGTCTGATTGATTAATCC
AGAATTGATAAAATTCATAAGGACTTGTTTTTTCAGCATCTAACCAAACAGCACCTGACTCAGACTTACCAAATTTCTTACCATCTG
ATTTAGTTACAAGCGGAATAGTTAAACCGTATGCGTCTGTTTGACCATACATACGACGCATTAATTCAATACCACTTGTGATATTAC
CCCATTGATCTGATCCACCTACTTGAATCTTACAATTCAATTCTCTATTCAAATGACCGAAATCAATAGCTTGTAAAATCGTGTATGT
GAATTCTGTATATGAAATACCATGTTCTAAACGACTTTGGATTGAATCTTTACCTAACATGTAATTAACGCCGACGTGTTTACCATAG
TCACGTAAAAAGCTAATTAATGAAATTTGTCCTAACCAGTCTCTATTATTAACAAGCACTGCACCATGGTCTGTTCCAAATTCAAAA
ATATTGTGCATTTGTTTACTAATACCTTCGATATTTTTATCTACTTGTTCTTCTGTTTGTAGCACACGTTCTTCTGATTTACCTGATGGA
TCACCAATCATACCTGTACCACCACCAATTAAAACGATAGGACGATGTCCATGTTCTTGAAAACGTCTTAATGTTAAGAATGGTAGT
AAGTGACCAATATGTAAACTATCTGCCGTTGGATCGGCACCGCAGTATAACGTCACTTGTTCTTTATTTAATAAATCTTCAATACCTT
GTTCATCAGTTTGTTGATAAATAAGACCTCTCCATTTTAAATCTTCAATTAATACATTCGTCATTATTATTTCCTCCTATTTATAATTA
TTTATTGAATACTTGTTAAAATACTTTAAAGTTTTTTGAACGTAAAAAAACCCTTACAACAAATATGTAAGGGCGCGATTGCACGTT
ACCACCAAACTTAAACATAATCATAAGATAATGTTCACTCTATTAATGATACGTTCATTAATAAACGTAGGACATGTTAGTTATAAA
GGTGTATTCATATTATTAAAAACACTAGTTCACAGCGACCACTAGCTCTCTGATGATTTCAAATAATATTACTTGTCCTTTTATCCTA
TTCTTTATGCATTTTCCCTAATTATAATTAACGTTAAAATAAAAGTCAAATTGCTTAAATATGGTATACTATACGTAATTTAGGAGGT
TAAAGATGACGAATCAAGACAACAATCATCAATTGAATCATCGTATATATCATTTTGAAAAGATATATAAAGCTATCAAACATGTC
ATTGTTTTTATATTTATGATTTTCATTGCCATCGTTGCTATCGCTGTGATTGCGATGTCTTTATATTTTCATCATTTAACTAAAACGTC
CGACTCATTATCAGATGATGCTTTAATAAAAAAAGTTCGACAAATACCTGGCGATGAATTATTAGATCATAATAACAAAAATTTATT
ATATGAGTATAACCATTCTCAAAACTCACTCATTATAGGCCCTAAAACATCAAGTCCAAATGTCATTAAAGCATTAACGTCATCTGA
AGACACTTTATTTTATAAACATGATGGCATCTTACCAAAGGCGATTTTAAGAGCAATGATACAAGATATTTTTAATACTGATCAAAG
TTCAGGTGGTAGCACAATTACACAACAACTTGTTAAAAATCAAGTTCTTACCAACGAAAAAACATATAGTAGAAAAGCAAATGAAC
TTCGCCTAGCAATTAGATTAGAACACCTACTCTCAAAAGATGAAATTATATATACATATTTAAATATAGTTCCCTTCGGTAGAGATT
ATAATGGCGCTAATATTTCCGGAATTGCATCCGCTTCATATAGTTTGTTTGGTAATTCACCAAAAGATTTATCAATTGCACAATCTGC
ATACCTTATCGGTTTGTTGCAAAGCCCTTATGGCTATACACCCTACGAAAAAGATGGAACGTTAAAATCGGATAAAGATTTGAAATA
TAGTATTCAAAGACAACATTATGTATTAAAACGTATGTTAATCGAAGATCAAATCACTGAAAAAGAATACAACGACGCATTAAAAT
ATGATATTAAATCACATTTGTTAAATCGAAAAAAGCGTTAATTGATGCTCACTTTTTAAAGTAACCACAACAATGCGTCCAAATATT
AAAAACAGCAGTAAGATTATTTTCAATTAGAAAATTTCTCACTGCTGTTCTTTATTTTAAATACTCTAAAGGAAAGCCATTAAACCA
ATCAATAGTTTATAAATTAATTGCTTTCACATTGTCTACAAAGTTAATCATACATATGCTATATCCAGTTAATTATTTAGATTCTTTTC
TGAATTTAGGAATGAGTAAAGCTAACATACCTAATAACGCATATAAGCCCCACCATGATTGGCTTGAAGTTGTTTCTCCAGTTTTTG
GTAGCATTTTAGATTGACTAAGTTTGTTTTCTTTCGATGGTGTGCCGGCCTTTTCTTTTGTTTTCACTGTTTTGTCCACAGTTTTATGAA
TATCACTCGGCAGATGTTCAGTTGCTTTTTTGTCAGTAACTTTGATAGTACTGATAGGATGATATTTCATTGTATCAATGTTTGTTTCA
GTTCCAGTATTATTAACTTTATTTTTATCGGCAATATGAGCAAGCTGTATGACTTTGTCTTTATTTTTATTAGAGTCTTTATCAGTATC
GACATTCGATGACATACCAACGCTATTATCAACACCCTTATCAGTGTCTTTAGCAATTTGAGTATCCATTTCTTTTAAATCATACTTA
TCGAAGTGATTGTTATCCAACATATCGGATAAATGATCAACATCATGTTGCACATCTTTGTCAACACTACTATCAGTATCTTTAACCA
TGTCAGACTCTGGTTCTATCACATCTGCTTTATCAGACGCATCTTTAGGATTTGTTGCAGTGCTGCTATTTTCAGCTACATCTGTATCA
GCAACCTTATCTTCTTGTCCTGTTTGTACATTTAGCGGTTCACTCGTGTTATTTTGTGATATATCATCATCTTTTGTATTGATATCCTG
ATTTATAATTCTGACATGATATTGACCTTCATAACCAATGTTTGCCACAACGACTTTAACAATCGCATTGTAAACTGCTTTGTCAGGT
ATATATGGGAAAATCAGCGTTCTAGAATTATTTTTAGGATCTTTAGAAACAGTAGTGACACGTTTACCTTCTACAATTAAATCTTTCC
AGTAACTGTCATCCTTTGTTTTCATCACTACATATTTTTGACCATTTAAAGTTGCTGTATAGAATGGATGTTCAACAAAGCCGTCCAT
AACTGACTCACTATTTTCTTCACTTTCAAAAACAACAAAATGCGCTTCTTGTAAATCTGTTAATTGATCATTTGTAGGTGTAACATTT
TCAAATTCCGTCACTGCTGATTTAACTTGATCAGCTAACTCTACTCTAGTTTGATCTAATTTCTTTTTATATTCCGCCTTATATTTTTCT
GGCAATTTCTCTTGTAATTTTTCTAATTCATAAACTTGTCTTTCTAACGTTTTAGCTTTGTGATACGGAGCTAATAATTTTTGTAAATT
GTATGTTTCTTCATCCACATAGTCGTCTGGGTTATTAGTAATAGGCTGTGCAAAGACCATTAGCGTATAATCATAGTCTTCATGGATG
TTCTCACCATATTCAATAGATGACACAATTTTAACTTCTCTCGTACCATTAGATACTGGGAAACGAATATAGGCATAATCTTTATCAG
AATCATATGATACTAATTCGACTGGTAACTTTTTGTCACCTTCATAAACTTCAAATTTCTTCCATGTTGAAGCTGTCTTTAAACCTAA
TTCAATTATTGGTCCTGTTTTTGTAAAAATGACAGTTGCTGGTTCAACAGTACTAGCATAATGATAGAACTGTCTTTCACCTTTATCA
TTTTTCATTTGAAAATCAATTGGTCGCCAATTATCAGCTGTATGTTCTTTATCGATGATAGCCGGGTTTTTAATTGCATCTTGTAGTGA
TTCATCTGCTGGTGGATACTGTTGATTAACATCCGACTGATTACTTGACTCATTCGTTTTATCGTTAGTATTACCATTAGAATTTGTTT
CATGAGCTGGTTGGCTTGACGCTTGATCTGCATTCGTTGACGATTTTGGTTGTGCAGGTTGACTCATATTGGTCGTTGCCTGCGGTTG

ATTATTAGCATTGTTGATCATTGATGTATTTTGATTAGATGTATTCGTGTTTGTTTGATTACTTGCGACTGAACTTGATTGATCATTCG
TTACTACTGCATCATTTGTATCTGATTTTACAAGTGAAGGATCGTTATAAATAGGTTTAGCAAATACTAATTTAGTATAATCATAATT
TGTTTCTTCTCCATCATCAATTTGAGTCGAAGAAACAATTTTCAATTCTTGTGTGCCATCTGAAACTGGGAATCGAATATAGGCATGG
TCTTCTGGTACAGGACTATATGATACAAGTCTCACTGGCAGTTTTTGATTGTTTTCATAGACTTCAAACTTCTTCCATGTTGAAGCAG
TATTGATGTCTAATTCAACTTCTGCTTTCTTTTTAGTGTAATACACATCTGCTGGATCTTTGATGCTGAAAAAGTGATAGTACTGCGT
TTCATTGTTTTTATCTAATAACTGGAAATTGACTTGTTCTCTTGGACCTATATCATGTTCTTTATTTTCTAATGCAGGATCTTTAATTG
CATCTTTAAGTGATTCATCCGCTGCAGGATAGTTTTTCGCAGTGTTTGCTGGTTGCGTAGCAGGTTGTGTTTGATTTGTATCCTTAGG
TGGCTGAGTTGTAGTTGCATTATTATTTTGATTTTCCGAGATTTTATCTGAAGTATTTGTATTTTCTGCTGCTTGTGCTTGATGTTGAG
AAGTAATTAAAAATAGTGTACTGACAATGACCGATGCAACGCCTAGAGTTGATTTTCTAATAGAATAGAAAGACCTTAATTTTGGGT
GATGTTTGTTCATGTTGTTAACAACTCCTATAAGATTATATTTAATTGATAATAATTATCAATATCAATTATTAGTGTTAATTGTAGC
GCATCCCAATTAAATATTGCAACCTATTTTATTAATTTTTTTAATATGGAATATTCATTGCATCGCTTTCCTATTCTTCAAGCCCACTT
TTTATCTTCATATACATTAATTAACCACCTTCAAACATTGTCGTTAGATTCGCCAAATTGAATCTATTTTTAGCACAGCAAAAAACCG
AACTGCTGAAATAATGCATCTCCAACAGTCCGGTCTATTAAACTATTTACTTATCTTTATTAAACAATTGACATGATTTATTAGAATA
ACCCAATTGCATGACCATCATCAGTAACATCCATGTTTAATGCTGCTGGTTTTTTAGGTAAACCAGGCATAGTCATGATTGCACCTGT
CAACGCTACGATAAATCCTGCACCTGTTTTCGCTTCTAATTCACGAATTGTAATTTCAAATCCTGATGGTGCACCTAACAACGTTTGA
TCATCTGAGAATGAATATTGTGTTTTCGCCATACATACTGGGTAATTATCCCAACCATTTTCTTTAAATTGTTTTAATTGTTTTTGCGC
TTTACTGCTAAACGTTACTTTTGAACCGCCATAGATTTCAGTCACAATCTTTTCAATCTTTTGCTCTAATGGTAATTCTAATTCATATA
AAGGTTTAAATGAATTAGGTTGATCAATGACTTCTAATACTTCATTTGCTAAGTCAACGCCACCTTTACCACCTTTTTCCCAAACTTC
AGTTAAGGCAATTCGTACGTTATTTTCTTTAGCCCAAGATTTTACATATTCTACTTCTGCATCGGTATCATGTATAAATGCATTAATT
GCAACAACCGGTTCTACACCGAATTTTTTAATATTATTAACATGACGCTCTAAATTAACAATTCCTGCTTTTACTGCTTCTACATTTTC
TTCTTTTAAATTATCTTTCGCTACACCACCATGCATTTTTAACGCACGAATTGTCGCAACAACAACGACAGCTGCCGGATCAAATCCT
GCTTCACGCGCTTTAATATCCATGAATTTTTCAGCGCCTAAGTCTGAACCAAATCCAGCTTCCGTTACAACGATATCAGCTAAATCA
CGTGCTGTTTCAGTTGCTAAAATTGAGTTACAACCGTGTGCGATATTCGCAAATGGTCCACCATGAACTAATGCAGGTGTCCCTTCA
ATTGATTGTACTAAGTTTGGTTTTATTGCATCTTTTAAAATCATTGCAAGTGCACCTTCCACTTTTAAATCTGCAACTGTAACTGGCTT
GCGATCTCTAGTGTAACCAATAGTAATACGACTAATTTTATCTTTTAAGTCTTTAATACTTCTACTTAAACATAAAATCGCCATAATT
TCAGACGCTACTGTAATATTAAAGCCATCTTCACGTGGTACACCATTTGTAGGTCCACCTAACCCAACGTTTACATGTCTAAGTGCA
CGATCATTCATATCTAATACACGTTTCCACTCAATACGTCTTTGATCGATTCCTAATTCGTTACCTTGGTGAATATGATTATCGATAA
ACGCAGACAATGCATTATTTGCAGTTGTAATCGCATGGAAATCTCCGTTGAAATGTAAGTTGATATCTTCCATAGGTAAGACTTGCG
CATAACCACCACCAGTCGCACCACCTTTGATACCAAATGTTGGTCCTAAAGCAGGCTCTCTTAATGCAACCATAACGTTTTTATTTA
ACTCATGGAATGCATCAGCTAAACCAACTGTAACCGTTGATTTACCTTCACCAGCTGGTGTTGGGCTCATCGCAGTTACTAAAACAA
CTTTCCCTTTGTTTTCTCTTGGCGTAATTTTATTAATGTCGATTTTAGCTTTGTAATGACCATAAGGTTCTAATGCATCCTCTGAAATA
CCTACTGATGCAGCAATATCCTTAATTGGTTGTAGTGTTGATTGATTCGCAATATCTAAATCTGATAAATGAGTCAACTTTTTTCGCC
CCTTTTTGTTTAATTTATGCATTTATAATATGTAAGTTGTAGCTAAGAAATTTTTACTTACATTCATATAATAACGAACTGTTAAAAA
CTTGTCGACTAAATCGGGTTAGCGCTTTCATTTTTGCAATAAATTCACTTATTAAAGTTTTGTATTATAAAACTTTTAGCGTTTGATAC
TATTGATAACTATATTTTTCTATTATTTAAAATAATCATCTTCCAAAGATTATATTAAAAATAAAAAGCAATATAAAGATATGCCATT
ATTGTTATGTCATCATTTTCAAATGCTTTCTACGAAATGATATGACACAAGTTAACAGCTCTTTATATTGCTTATCGTAAAGTAAACG
ATTATTTAAACATTGACATGAAATATGTTGTCACCCTTTATTCTTCGAAAAAAATCTTCAATAACATTCATGTCATTATTCCATTGTA
CTTAAATCCCCAGCATCTAAATTTAATTCCCAAGCTTTTAATACACGTCTCATAATTTTACCTGACCGTGTTTTAGGTAATTTATCTTT
AAATTCGATTTCACGTGGTGCTGCATGTGCCGACAAACCTTCTTTAACAAATATACGAATGTCTTCTTTTAATTCGTCTGTTGGTTCA
TATCCTTTTCTCAGTGCAACAAACGCCTTAATTATTTCACCGCGAACCGGATCTGGTTTACCAATAATTCCTGCTTCGGCAACTGCTT
CGTGTTCAACCAATTTAGACTCAACCTCAAATGGTCCAACTCGTTCACCAGCTGTCATAATTACATCATCAACACGTCCTTGGAACC
AGAAGTAACCATCTTCATCTTTATATGCCGAATCACCAGATACATACCAGTCTCCAATAAAATATGATTTATATTTTTCTGGATTCTT
CCAGATACGATACATCATTGATGGCCAGCCTTTTTTTATAGCAAGGTTGCCCATTCGATTTGGTGGTAATTCATTACCTGCATCATCG
ATAATTGCAGCTTGAATACCAGGTAATGGTTTGCCCATTGAGCCAAGCTTGACGTCCATCGTTGGATAGTTAACAATCATATGTCCA
CCTGTTTCTGTCATCCACCAAGTATCTAACACCGTTAAACCGTATACTTTTTTCGCCCATTTTATAACTTCAGGATTTAAAGGCTCAC
CTACTGATAGAATCGAACGTAACGATGACAAGTCATATTTCTCAACAATATCGTCACCAGCACTCATTAACATTCTTAAAGCTGTTG
GTGCCGTATACCAAATCGTCACTTTAAAATCTTCAATCATACTATACCACTGTTCTGGTGAAAAGCGACCACCAGCTATACAATTTG
TAGCGCCATTTAACCATGGTGCAAAAATACCATAAGATGTTCCTGTAACCCAACCTGGATCTGCTGTACACCAATAAACATCATCTT
CTTGTAAATCTAATACATATTTTCCAGAAATATAGTGCACTAACATTGCTTGTTGAACATGCAATACACCTTTAGGTTGCCCAGTAG
AACCTGATGTATAATGTAAAATCAAACCATCATCCGACTTTAACCATTCAATGTCAAATTCATCGCTAGCAGTTTCCATCAAACTAA
TGAAGTCTATGTAATTGTCTTCTACATCCTCATCTACGACAACAATTTTTTTCAAGTTCGGTAATTTATCTACAGGTACTCGAGGTAA
CAATGCCTTATTAGTAATTAACACTTTAGCTTCACTGTTCTCTAATCTATCCGCAACTGCCTTTTCCATAAATGCTTCAAATAACGGC
CCAACAATTGCACCAATTTTTAAAACACCTAACAACGCAAAATATAGTTCAGGTGTACGCGACATAAATATAAATACTCTGTCACCT
TTGTCAACTTCTGCATGTTCAGACAAAACATTCGCTGCTTTATTAGATAATCGTTGCATATCTTTATAAGTATACGATTCTTTTCTGTA
CTCATCTTTGTAATTTAACGCTATTTTATCCCCTAATCCTTGATCTACATGGCGATCTATACATTCATATGCCATGTTCATTTTTCCAG
TTTCACTCCAAGAAAATGCTTGTTCTACGTCTTTCCAATCAAAAGTATTATATGTTTCTTCATAATCTTTAAGGTTATGTTTACCTTGC
GCTCCTTTATAAACTTCGACTTTCATTTGAAACTCCCCCTTTGTTATGTGAAAACGCTTTCTTTCTCAATATTATACATAACATTTCTA
ATTATTCAAAATTTTTACTTTTATCCTTTTTCTAGTATTAATTATTGTAAATTTTCTATATAATGAATTTAATACAATCACGACGTCGA
TTGAGGAGTTGAAAATATGAATCATTTAAAGACGTATCAATCCGAAGATTATTACATTCATGACAAGCAATTTGTTATTGAAGGTCC
TTTAACATACGAATATTTGAAAGCGCTTACTTTCGATGCGCATTTAACCGCATTTAGAGATGCTGAAGATCAGTATGAAGCTTTGTT
AGAAATTACAACATTACCAGAAGGTAGAATTTATGTTGCTCGCCAAGATCAACTCATTGTGGGTTATGTCACTTTCCACTATCCTGA
TGAAATTGAGCGCTGGTCTACAGGTAACCTTCCATATTTAATCGAATTGGGGGCAATTGAAGTCAGCATCAATTTTAGGCAATTACA
TCTTGCAGAAAAGCTAATACAACTTAGCCTTTCTACACCAGAATTCGAGGATTATATCGTTATAACTACTGAATATTACTGGCATTG
GGATTTAAAAAATTCAAAGTTAGATGTATTTGACTATAAAAAATTAATGCAGCGGTTAATGGCAACTGGTGGACTTGAAATATTCGC
TACAGATGATCCAGAAATAACAAGTCATCCAGCTAATTGTTTAATGGCAAGAATTGGCAAAAATATTACATTAGAACAGCAACAAG
CGTTTGATGATATTCGTTATATGAATCGGTTTTTCTTTTAATTTTTGGAATATCGGAGGGATTATATGCAACAACATTCATCAAAAAC
TGCATATGTTTATTCAGATAAGTTATTACAATATCGATTTCATGACCAACATCCCTTCAATCAAATGCGTTTAAAATTAACAACAGA
GCTACTTTTGAATGCAAATTTATTGTCTCCAGAACAAATAGTACAACCTAGAATTGCAACAGATGACGAATTAATGTTAATTCATAA
ATATGATTACGTCGAAGCAATTAAGCATGCTTCACATGGCATTATCAGTGAAGATGAGGCTAAGAAATATGGATTAAATGATGAAG
AGAATGGTCAATTTAAGCATATGCACCGCCATAGTGCCACAATTGTTGGAGGCGCTTTAACTTTAGCAGATCTTATTATGTCAGGCA
AGTATTAAATGGTTGTCACTTAGGTGGTGGATTGCATCACGCTCAACCTGGTCGAGCTAGTGGTTTTTGTATATACAATGATATTG

CAATTACCGCACAATACTTAGCTAAAGAATACAATCAACGCGTTTTAATCATAGATACCGATGCACATCATGGAGATGGTACACAA
TGGAGTTTCTATGCCGATAACCATGTTACTACTTATTCTATCCATGAAACCGGAAAATTTCTTTTCCCAGGCTCTGGTCACTATACTG
AGCGCGGTGAAGATATCGGCTATGGACACACTGTAAATGTCCCACTTGAACCGTATACAGAAGATGCATCATTTTTGGAGTGTTTTA
AATTAACAGTTGAGCCTGTCGTAAAGAGTTTTAAACCTGATATTATTCTAAGCGTAAATGGTGTCGATATACATTATCGTGATCCAC
TAACTCATCTAAATTGTACGTTACATTCATTATATGAAATTCCATATTTTGTAAAATATTTAGCTGATTCTTATACGAATGGAAAGGT
AATTATGTTTGGTGGCGGAGGCTACAATATTTGGAGAGTCGTACCACGTGCATGGAGTCATGTATTCTTAAGTTTAATTGATCAACC
AATTCAAAGTGGTTATTTACCGTTAGAATGGATTAATAAATGGAAACATTATTCATCTGAATTATTACCTAAAAGATGGGAAGATCG
TTTAAATGATTATACCTATGTCCCCCGCACAAAAGAAATTAGTGAAAAAAATAAAAAATTAGCTTTACATATAGCGAGTTGGTACG
AATCTACTCGTCAATAAGTAACCTAATCTGCAACGATTTTAAAAGATAAATAAAAGGCATTCTTCCAACACCCATATAATTCGGGTC
GTTAGATGAATGCCTAATTTTGTGAATTTATTTTGTAGTTCCTCGGTATTCAATTCTGTGAGGTAAAACTACATTTGGTTCTTCTATCT
TTTCATCGTTCATATATTTTGTTAATAAGCGCATCCCTACTGCACCGATATCATATAATGGTTGAATAACACTAGAAAGTTGTGGTCT
AACCATCTCAACTAAACGTGTATTATTGAAACTAATAATTTGTAATTCCTCTGGAACTTTAATACCAGCATCCATTGCACTATGCATA
ATACCAATAGCTTCTTCGTCACTGATACATAATATAGCATCTGGAAGATTACCTTTCATTTTGGCAAAAGCTTTTACGCCTTCTTTAT
AACTTTCAGCACCAGAACAATTCAATGTATCACCTAATTGAAGACCATTTTTATTTAACACTTCAGTTAAACCTTCTAAAACATCTTC
TTGAGCTTTTTTAGAATGTTCTCCACCTACTAAAGCAAATGATTTAGCGCCTTTTTCAATTAATTCTCCCGTAATTTCTTTCGCAGCTT
CAGTAAAATCAATATTAACTGATGCTATATGTGCATCCTTACCATTTGTTCCTGATACTACTACAGGTACAGATGATTGATTTATCAA
TTCTTTCATTTCTTCAGTAATTGTACCACCAAGGAAAATAATACCATCAACTTGTTTACTTAATAAGTTATTAAAAATTTCTTTTTCTT
TTTCAGGATCGTTATCTGAATTTGAAATAATTGAGTGATATTTATACATTGTTGCAATATCTTCAAGTCCACGAGCAAGTTGTGAATA
ATAGATATTAGATATATCTGGAATGATCACACCTACTGTTGTTGTCTTTTTACTAGCTAAACCTCTAGCAACAGCATTTGGACGATAA
TTCAAACGCTTAATGACTTCGTTAACTTTATTTTTAGTTTCTGCTTTAACATTTTGGTTCCCATTAACAACACGCGACACTGTGGCCAT
AGAGACACGCGCTTCTCTTGCTACATCATATATAGTAACTGTCATAATTTCCTCCTTGTAAACGTTTTATTCATTATAATAACATGTT
TTCATTTCATTTTCATAGTTTACCATATTTTAATTTACAGTAACATATTTTTGTTTGGAAAAATTTAGCGAAAAGCTTGAAATAAAAA
CAAACGCTAAATGATAACGAACGATATATTTTAATTTTATATAAACTTTTTTCATTTTTAATTGTAATGTTTTGAGAAAGCCTTTTCA
AATATCAATTGCATTTGTTGAAAAATGTTCACATTTCAGTAACAAATTGGTGAATCTATTATATTTACAGATAATTCATAGTTATAAT
TTTAATTACTGCTGTCAGTCATATTCTTTAATAATTGTGAAATTGGACAAGTGTCGAAATATTATAACAATCGTTAAGTGTAGTATAG
CCTTTATTTCTATACAAAAAATAATAGCCAATCTAAAAGTAATATACTCTTAGATTGGCTACTAGTTTAAAATTGTCGATTCAAAGA
TAATATTCTACTTCATCATGACATATTAGTTAAGTAGTCTATAGTCTCCTTGGAATATTATTTTAACTTTTTAGCGTTATATAAATCAG
CTAAAGGCTTTAATTCATCATAAAATGCTTGGAATTCATCTAAATCCATTTGTTGACCCGCATCACTAAGTGCAACAGATGGATCTG
GATGCACCTCAGCCATAACACCATCAGCACCTACTGCTAATGCTGCTTTCGCAGTTGGTAACATGATATCTTTACGACCTGTACTAT
GCGTAACATCTACCATGACTGGTAAGTGTGTACCTTGTTTTAAAATTGGTACTGCTGAAATATCTAAAGTGTTACGTGTCGCCTTTTC
ATAAGTTCGGATTCCGCGTTCACATAAAATAATGTTTTGATTACCTTGTGAAGCAATGTATTCAGCTGCATAAACAAACTCTTCGATT
GTAGCAGATAAACCACGTTTTAATAGAATAGGCTTTTTCGTACGCCCAGCTTCTTTTAATAACTCAAAGTTTTGCATATTACGTGCAC
CAATTTGGAATACATCTAAATATTCATCGGCTACTTCAAAATCATTAGGATTTACAATTTCACTAACTACATTTAAATCGTATTTATC
TTTAATCTGTTTAAGTATTTTAAGTCCTTCAACACCTAGGCCTTGGAAGTCATAAGGCGATGTACGTGGTTTAAAAGCACCGCCACG
AATAAACTTTTCACCTTTGGCATGTAAATTTTTAGCTACCGCTTCTACTTGTTCAAATGATTCAACAGAGCATGGTCCAAATACAAAT
GATTTGTTGCCATCTCCAATAATACCCCCATTTTCAAATGTTACAATCGTATCCTCAGGTTTCAACTTACGAGATACGTATAAATGTT
TTTCATTTTCAGATTTTTGTAAATCTGTAGAGGCTTTGAAAATTTCTTTAAATAATTGTTTAATAGTATTATCGTTGAATGGTCCTTTG
TTACTATCGATTAAGTCGTTAAGCATTTCTTTTTCACGTTGTGGATCATAGATACGTGTACCTTGTTTTAATTTTTCTTCCCCAATTTTT
TGTGCTAGTTCACCACGTTTAGATAATAAGTCTAAAATTTGATGATTCAGTGATACAATCTCACTTCTGTATGATTCTAATTTATTAC
TCATCCTTACTCACCTCTACTAATTATTTCAAGTATATTCCACTTTAAAGCAATAAAATATAATGTTAAAAAATATTCTAACAATATC
ATATGTATAGTTCAACCCTAAATTGACAGAAAATAATCAATTTTCTAATTATATGATTCGAGACTGACATTTCGATTTTCTCAGTCAT
TATTGGAAAGTTATATTCTATATTGATATTTATTAGCAAATGTTAATCAATTAAATAAGTGTCATTATTATAGCTTATTTTGAGTCAA
CATTACTGCTTTTAAGAAAAATTTTCGTTTCTATCTTCTATTATAAATCTAAAATTCTCATACATTCAACCTAACGCTTAATAAAAAT
TAACAACCTTTGTCTTTGTATATTAATATAAATCTATAGTGAACATCACTCACCAACACATTCAGCTATATTTAAATTAGTGAACGTC
ATTGTACCTTCTAAGATTCATTGTTTATTTGATTATCGAATATTTTTCCATCTTCAGCTCAAAATGTGCAATATCACTATTGTAATCTA
ACGATGTTACAAACAACATAAATACATGGAATGATCGCGTTGCCTTTCAAAATAAACTGATGATATCAAAAAAGAGGCTGGGACAT
AAATTCCTAAAAAAACAGCAGTAAGATAATTTTCAATTAGAAAATATCTTACTGCTGTTCTCTATTT

>041-contig_187_RC

GCTGATTTCTATCAAATAATTAATAGAAATCAGCTTTTTTACATTGCCTAGGAACTTAATGTCCCAAGCCTTTTATTTTGAAATTCAA
TTTTTAAAAATATAATACATAGCTTACTGCTATGATTGATTATTAAACGATTTTAGTAATCTTAGTCATTGAATGTACGTTTCTCTATT
TTACTATTAGCTTTTTCAACTTTAGCATTTGGCGTTTTTGATGCATTGGATGATTTAGCATTTGTTGTTTTTGAATTCTTATTACTAGTT
TGATTATTCTTTTGAGTTGACTTACTTGAACTACTTTGTTTCTTTGCACCTTGTGATGATTTCTTATTATGTTGCTTTTTCTGACCTGAA
GTTTTATTTGTAGATGCTTTTGATGCATTTCGTTTATTTGAAGGTGTTGTTTTTTTAGATTGTTTACCTGATTTCTTGTCAACAGCTGTA
TTATTAGTTGTTTTTTCATCAGCTTTACGTGTAATAACACCATTTTCAAATTTAGCTTCTTTTTGTTTAACAGTGTCTTTATTATCTATA
TTAACTGTCTCTGTCTCTGATACATCATTCTTTTTAGTAACTAATTGAGGAATTTCTTTCAAGTCATTTTTAGCAACTGGTTTTTCTGC
AAATAACGCTTCAGTTAATTGACTCTCTTTTGAACCTGGTGTTAATTTAGCTTGCTTCTGTTTTGCAGCGTTTGCTAATCTTTCAGCTT
GTGACTCTTCTTTCGATACCTTTAATGCCACTGCTTTTGATTCTTTATTTGATACTTCTGCACTTTTATCTGTTTCTGCTTGTGCTTCTT
TTTTAGCTTCTTGAATCTCTTGTGCCTCTTGTGATGTATCACTCAAATTATTTGCACTTGCTTCTTCTTTTATTGCTGCTTGTTGTGCTT
TTAATGCCACTGGTTTTGATTCTTTATTTGATACTTCCGCACTTTTATCTGTTTCTGCTTGTGCTTTTTTCTTCGCTTCTTGAATCTCTTG
TGCCTCTTGTGATGTATCACTTAAATTATTTGCACTTGCTTCTTCTTTTATCGCTGCTTGTTGTGCTTTTAATGCCGCTTGCTCATTTTT
AGATTTGTTTAAAAATCCTTCAACACGTTCCTTTGTAAAGGCAACCGTTTCTTCAAGTTGCGTTTTTCTTTCTTCAAACTTTTGCGACA
GTTCTTGTTCTTTGACTTTTAAATCATCTGCTTTTTGATAAACTTTATTTTTAAAATACAAACCTAAAGCTGAACCAACAAGCGCGCC
AGTTATAAAACTAACAACAAAATCTTTACGGTTGGGTAATGGTTCATTTTGATAAGTGTGTTTATTTTTTAATGTTCTTTTATTATTTT
GTTGTTGCGTCATTTGTGTATCCTCCTATAACAAATGCACTTAAATAAGTAACAACCTCATTTAAGTGCATTTGTTTAATTATTAATT
ATTTATCTACTCGAGAAGTATAGCTATGATTTGCATCAGTTGCTACATTATTAGCTTTGTAATTTGCACTTCCACGACGGTAGTGTCT
ATTTTGCCATTTGTCTGCAATTTCCATTGCAACATTTGACCATTGAACAACTTGTGAGATTTTATCTTCATTTTGAGAAATATTATGTG
TAATTGAATTTGTTACACGATCTACAGAGCTGTTTAACGTTTGTACTGAGTCACCGATACCTTTAACAGCATCTACAACTGAGTTTAA
ACGATCTACTTTACCTTGGATATCCTCAGTTAAACGGTTTACTTTATGAAGTAAATCTGTTGTTTCACGAGTAATACCTTGAACTTGA
CCTTCTACACCGTCAAGTGTTTTTGCAACATAATCTAAGTTTTTCTTAACAGAATTTAATACAGCTACGATACCGATACATAAAATTA

AGAATGCAATCGCAGCGATAATTCCAGCAATTGGTAAAATCCAATCCATTAAAAACGCCTCCTAATTAACATGTAATAATGTCATTA
ATAATAAATACCCATACTACTCTATTATAAACATATTAAAACGCATTTTTCATGCCTAATTTATCTAAATATGCATTTTGTAATTTTT
GAATATCACCTGCACCCATAAATAAAACAACAGCATTATCAAATTGTTCTAATACATTAATAGAATCTTCATTAATTAACGATGCAC
CTTCAATTTTATCAATTAAATCTTGTATCGTTAATGCGCCAGTATTTTCTCTAATTGATCCAAAAATTTCACATAAGAATACACGATC
TGCTTTACTTAAACTTTCTGCAAATTCATTTAAAAATGCTTGTGTTCTAGAGAAAGTGTGTGGTTGAAATACTGCAACAACTTCTTTA
TGTGGATATTTCTTTCGTGCTGTTTCAATTGTAGCACTAATTTCTCTTGGATGGTGTGCATAATCATCTACAATAACTTGATTTGCAAT
TGTAGTTTCATTGAAACGACGTTTAACACCACCGAACGTTTCTAATGCTTCTTTAATATTTGTAACATCTAGCTTCTCTAAATAACTA
ATCGCAATTACAGCTAATGCATTTAAAACTGTATGGTCACCATATTGTGGAGACAGGAAGTGATCATAAAACTCACCATCCACATAC
ACATCAAAAGCAGTACCTTTATCCGTAATTTGAATATTTTGAGCATAAATGTCATCCGAATCTTTAAATCCATAGTAATAAATTGGA
ACATCTGCTTCAATTTTACGTAGATGTTCATCATCACCCCAAGCAATAATACCTTTTTTAACATTATGTGCCATTTCTTGGAATGCAT
CAAAAACATCATTAATATCTTTAAAATAATCAGGATGATCGAAATCAATATTTGTCATAATTGCGTAATCAGGTTTATAACTTAAAA
AGTGACGTCTATATTCACATGCCTCAAAAGCGAAATAATCACTTTCAGGCAATCCCATACCTGTGCCATCACCAATTAAAAATGAAG
TTTTTTTATCACCATTCATAACATGTGATAATAAACCTGTTGTAGAAGTTTTACCATGTGCACCAGTTACAGCTACTGAAGTATATTG
ATCAATAATCTGTCCTAAAAAATCATTATAACTTACAACATCTAATTTCAATTGATGTGCACGTACTATTTCTTCATGGCTACTCGCG
AATGCATTACCTTGTATAACTACCATATCTTCTTTTATGTTATTAGCATCAAATGGTAATATTTTTATCCCCTTATTTCTAAGAGCAAC
TTCTGTAAATACGTAGTTCTCAATATCCGATCCTTGAACTTCATGTCCTAAATCATGCATGATTTGTGCTAATGAACTCATGCCAGAA
CCTTTAATTCCGACAAAATGATAGTGTGTCATTATATAAAACTCCTTACTCATAATTATTCTTTATTTAAATCTGCTTCCGTAACATA
AACATCCCTTGGTTTTGAACCATTAGCACTCGAAACATAACCGAGTTGCTCTAATTGATCGATAATTCTTGCTGCTCTATTATAGCCA
ATTTGGAAATGTCTTTGGATTAATGATGTTGAAATATGTCCTTCATTAACCATAAATGCACAAACATCATCAAATAATTCATCTTGTG
ATTGTGTTTGTGTTTTTTTCAACAATTCTTTTTCTTCAAATAGATAGTCCGGTTCTCTTTGTTGTTTGATAAAATCAACAACATCATCA
ATTTCGTCATCAGAAACAAATGTACCTTGAACTCTAATCGGTTTATTCATACCGCTACCAAGATATAACATATCGCCATATCCTAAC
AAGCGTTCTGCTCCACCACTGTCTAATATCGTTCTCGAATCTACACTTGATGATACCATAAATGCAATTCTTGTTGGTATGTTGGCTT
TAATTAAACCTGTAATTACATTGACAGATGGTCTTTGCGTAGCTACTAACATATGAATACCACATGCTCTCGCTTTTTGAGCAATTCT
AGCAATAGACTGCTCAACTTCTTGCGGAGCCATCATCATTAAATCAGCCAACTCATCAATTACAATGACAATTTTTGGCATTCTTTCA
TCATATGGTGCTTTTTTGTTAAATGCTGTTATATTACGTACATGGTAATGTGCAAATAACTTATAACGTCGTTCCATTTCTTCTACGGC
CCATTTTAAACTCTGTGTAGCTGCTTTGACATCTGTAATTACCGGTGCAACTAAATGTGGCAAACCATTATAAGGAGCTAATTCAAC
CATTTTTGGATCGATAAGTAATAATCTTAATTCCTCAGGGTGATTTTTATATAGTAAAGACATCAAAATACTATTGATACAAACTGA
TTTCCCTGATCCAGTTGCACCTGCAATTAGTGCGTGTGGCGTTTTAGCAATATCCATAAGTAATGGTTCATTATTAATTCTATACCCC
ATCGCAACTGTTAATTTAGATTCAGCATTTTTAAAACTTGGAGATTCAATAATAGAACGTAAGTTGACTGTCGTTGGATTTTGGTTCG
GAACTTCAATACCAACACGACTAGTTCCTGGAATAGGCGCTTCTATACGAATATCTTTCGCTGCCAATGCCATTTTAATGTCATCTTG
TAATGCCGTAATTCTTGAAACTTTAACACCTTTTTCAACTGATAATTCAAATCTTGTAACACTTGGACCTTCAGTTACATCTTGTACTT
CTGCAGGTACATTAAAGTAAAATAATGCGTCATTCAGTTCTTTCTTTTTATCTGTAATCCAGTCCTCGTCCGACTCAATAACTTGTGG
TTCTTCTAGTAATGAAACACTTGGCAATTTAATATTTGGGCCTTTACGAATCATCGGCTTAGATGTTTGTACAGCTTGATTTGTTGCA
ACATCTTGTTGGTTCGTCCGAGGTCTATTTTCATTAGCTGTATCTTCATTTTGATTTGAAAATGAAGGCTGTAAATCTTTTTGTTGATC
ATCATTATCTTGTTGTCCACTAGTATTGTTTGGATGTGTTGTTTCTTCGCTTTCTTCAGTTATGTCGCTTACTTCTGAAACTGAAGACG
AAGTTGATTGATCAACTTGCTCATTTTCCAATTGATTATCATCTTGGCTTTCTTCAAATATTTCCGTTTGAGTTGAATCAGCAGAAGG
TTTCTGCTCTGAATATTGTTGTGCATTTTGATAATCATTTTCTGTTTCTGCATGACCAATAAGTTGATTGTTCTCAACATTATTTGATG
TCATATTATTTATTTTATATGCATTTGTATTTGACTCTTGTGAATAAGTGCTTGATGATGGTGTGGCTTGACTCGCAGTCTTGCTTTCA
CTCACAGCTTGCTTACTTTGTACAGGCTTCAATTCAGGCACATTGACTTTTGAATGCTTTTTACGATCCATCATACGCTTTTTATCAGA
TGGCGTCATGACAACATTAAATGGTCTTTTACTTAAAGTTGAAACCTTTTCAGTCTGTTTCTTTGGATTTACGTTTTCAATAGTTTTCT
CTTCGACATTTTGTTCACTTTCAGCTGTTTCGTTTTGCACATTTTCTGTAATTTCTGCGTTATGCTTTTCTATTTCATTCATGCTTGAAT
CTTCCACAACATGACCATCATTCACAATATTCGTTTTCAATGAGTTGACACTTAAGTTTGTTTGGTCATCTTTTTTATGACGCGGTGCT
AATTCAATTTCATTATCAACCGTTTTATCGATATTATTTACTTGAGATTCAGTTACTGCGTGCTCTTGCGTTTTAGGAGCGTCTACATG
TATTTCAGACGCGTTTTTATTGGTATTATCTTCGATATTTGAAGTAGTATCCTCATCTACTTCTGTATCATTTAATTCATAAAATTCTG
CATCATCGATTTGACTTCCTGAATATTCTTCTTTATTCGTTGCTCCATCTTCTTCTGAATCAGCAATTAATCTGGATTGATTTTTTAGTT
CATCTTTATCATTTACTTCAACGTTATGTTGTAGTGCTGATTGATGTTGAGACGTTACATTCGAAACCGTAACTTCATCATCTGACAA
TTGTTTTGTTGTCGATACTTGATTCAAACTAACTTCCTCATAATTGTATGGACTTTCATCTACATGAGAGTCATTAGTGTCTTCATTTT
GTATTGACGCTTGTTTATTTTCATCATTTGATACAGTTTCATTCTCTATGCCATTTGTATGAAGCTGACTAGCATCTGTACTATTATCT
GTTAAGTCACTATCATCATTTAATGAACTATCACCAACATAGCGTTCCGCTTGTTTAGCATACATTTCATCAATTGCACGTTGTATTG
CATCTTGCTCTTCGTTTTTATGTTCTTCACGCTTTTGTTGCAACGCTTTTTTAAATCGACGCTTTTGAAGCACTTTACGTTCTCGTTCAC
GTCTAATTTCTTCAACAATTTGTGAAGCATAAATATTTTCAATTTTGATAGTATTATCAACTTTTGAATAATTAGGCATTGATTTTGTT
GTTGATGATACATTTTCATCAGATGATTTAGATGCAGTTCCTTGTTTGACAACACTATCATTTTGTTTTTCTTGTTCTAATTGTTTATTT
TGAGACGTTTGCGTCTTAGCTACATATTTATCATATTTTTGTTTATCTGACTCAACTTTTTCTGAAGGTTTACTTACAGGGATACGACC
ATTTTCTAACTTTTTAGGTTTCATTGTGCCAAAAATAGCTGACGGTACCTCTGAAGTCTTGAAACTTTCTTTGTGATAATCTGGAGTT
GAATCTTTAGCACGATGATTTGTATTATTAGAATACATATTATGTGTTTTTGACTTGTGCGTTTCCTTCTCAATACCATTAATTGCAGA
AACATATGTACCTGGCTTATTCGTATGGTAATGTGAGTGATCTTTATATTTTATACTTTGCTGTGATATTTTAGAATTCCCACGTTGTT
CACTATAATTTTGTTCTTCAGTTGTTTGATTTCTTCTACGGCGATGTCGTTTTTGTGAACGAGAATCGTGGCTTTGTTTGCGATAGTCT
CGATGGTATTGTTCTTTTTCATCTGAAATAATATCTGCAGATTGTTCAACATTTTCATTTTCATAAGCTACGCTCATAGGAAAACGGA
ATTTTCCCCTCGGACGACTATAAATATCATTATTTTGAGGCAGTAATGAGTCATGATCGTTATCTATATTTTGTGATTCTTGACGTCTT
TTTTTCTTTCTATGAATCAAGTCATCATTTGAATCATTATCTTCGCCGAATAATTTATCAAACCAGCTCATATTTTCACTACCTTCCTT
AATTATTCAAAAAATGCTTGTCCAATTTCATAGCTGTCATTTAATACCATAATACCTTTTTCTTCAGGTGCATTAGGTAAATTCAATT
CTTTCATTGAACAAATCATACCGCTTGAGGCAACACCACGTAATTCAGCATCTTTAATTACCATACCGCTAGGCATCACTGCACCTA
CTTTAGCAACAACAACTTTCTGTCCAGCTTCAACGTTAGGCGCGCCACATACAATTTGTAATGTGTCATTTCCAACGTTTACATTTAG
TACACTTAATTTATCTGCATCAGGATGTTTGTCTTTAGTTTCAACGTAGCCAACTACAAATTTCGGTGATAGATCAGCATTTAATTTA
TAATCAAAACCAGCTTCTGAAATACGCTTTTGGAATACATTTACAAGTTCATCAGTTAATTTAATATGACCTTTTTCTTCAATTGTTA
TATCTTTTGAGATTTCAAAAATATTATAACCTACAACATTACCTTCATTAGTAATTTCAACAACATTACCTTTTTTATTGTAGTTTAAT
TCACCTTCAACTGGTTCAATTTGTAAAAATGCGACATCTCCTACATATTTAGGATTGTAAAATAAATTCATTATGACAAAACTCCTTA
TCCAATTATTTCTATTTATCTTTATTAAATTTACGACGATTTGCTTCTAAACGCTGAATCACGTTTGGATCTCTTTTTTGTTTATTATTT
TTACCTAAAATAAATATCGGTTCAAGATGACCCTGTTTATATCCAAAGGATAATGATGTAATTGGAACTAGACCTTTAGTGAAAAAT
TCCATTGTTAAATGTGCCATCACATCATATCCTGTTTTATTGCGTATATCTGCAATAATTAACACATCTTGGTGTGGCACTGCTACGA
GCATTTCGCCTTGACATTGTGCCTCAATTTCATTTAAAAATGCAGTATTTAGTATCCTACTTGCATCATACCCGTCATTTGAGTTAAT

AAAATAAAAAATATTACCTTTTACTTCATCAGTCGTATATGAATTTGACAATTTTCTAACATTAAACAGAGACATTTCTCTTATTTGT
TGTTCAGTTAACTTCAAATCCTCTAACATGCTTTCGTCAATTAGACGATATGATTTCCCTAAATCGACTGCATAATAAACTGCTGTTT
CTGCAGTATGCTCATCATAGATAAAAGGAACACCTTGTTTAGTTTTTTTATCAAAGCTAGTCGCTCTAATGACAGGCATAATTTGACT
AGATGATATACTCTCAAGGGTTTTATCTGCCATTTGTGCAATAGCTTCATCAACGTAATAAACAATTTCATCTACAATTTTTTCTTTTT
TATCTTCATATTTTGCGACTATAGCGTTAAGTTTAATCGTGATACCTTTGTTATTATCTGTTCGATAAATACGCAAAGTTTCTTCTTCA
CGATTAAATTTAAAATCAACGTCTAAATGGCTTAAACGTTCCTTTAATTTATCTCTCATTTGAAAGGTATTCATATGTAACACTCCTA
ATTTCGCACCTTATAATAGTTTACAATAAATCAACACATAGTAACAGAGAAAAACGAGTATTACCCCGTTTTTCTCTAGTCTAAATT
ACACGTATTGAGCTAAAAATGCATCTATCTGTTCAATTGATTTTCGTTCTTTTCCAATATAACTTCCAAGCAGTTCTCCATTTTTATAT
ACTAGAAAACTTGGAATACCCATAATACCATTTTCAATACAAATATCCATAAATTTATCACGGTCTACTGATACGAAGTCAAACATA
GGATATCTCGCTTCTAATTCCGGTAAATCTGGTTCTATCACTCTACAATCTGGACACCAGCCTGCAGTGAATTCAAATACTGTAGCA
CCTTGTTTTAAAGATTCAAATTGTTGTTCTGATTCAAGTTGTTTCATTTTTATGTCTCCTTAATTAATGATTATATATAACTATTTAGA
GATAATAGTTGTTATTACCCGATTATTTATATTGTAATGTTTCTATTTGATTATTATCTAAATTACAAATGGCTTCTGAAAGTAAAGA
TCTAGCTGCAAAATAGTCTCTTATATCAAATACTGAGTCTGTACTATGAATATATCGTGCACATACACCAATAACTGCAGTCGGAAT
ACCAATATTAGCTTTATGAATTTCTCCACCATCTGTTCCACCTGGTGACATATAGTATTGATGTTCAATGTCATGTGCTTCTACTAAC
TTTAATAAATAGTCTCTAAATACAGGCTTTAAAATCATTGTACCGTCTTTTATGCGAATTAACGTCCCTTTACCAAGTTCACCAGATA
ATGGTTGGTTTCCTTTAACGTCATTGGCAGGTGAACAATCAACTACAAATGCAACGTCTGGGTCTATCATCTCTGCAGATGCTTTCGC
ACCTCGTAATCCAACCTCTTCTTGAACATTTGCGCCAACATACAAGTCTACATCTAATTCTATATCTTTTAATAATTCTAGTATTTCA
ATTGCCAAGACACAACCATAACGATTATCCCATGCTTTAGCACTATATCGATGTTCAGATAACTGTGTGAATGGCGTGTGAGGTACA
ATTGTATCTCCTATATCTATTCCGCGCTCACGCACCTCATCTTCATTTTGAGCACCTATATCTAATGTTAAATCTTTAATTTCCGGTGC
ACCTTCACTACCAGTACGAAAATGTTTAGGTATATTAGAAACAACACCGATAATTTTATCGCCATTTCTATTTTTAATTACTAAGCGT
TGTCCTTGCCAAATATCATTTGCAACACCACCTAAATTTGTGAATTGAATCATTCCATTTTTAGTGATATTTGTAATCATAAATCCGA
TTTCATCCATATGTGCTGCAATCATTACACGTTTTGCATTTGGATTTTTAGATTTTTTCACACCAAAAAATCCACCCATACGATTTTCA
ATAAATTCATCTACGTACAGCGCCATTTGCTGAGTCATATAATTTTTTACTTCTTCTTCAAAACCTGGTGCCCCATGAAGCTCAGTTA
AAGTTTGAATTCGTTGTAATGTTACTTTTTTATTTATGTTCATAAAAGTTCTCACTCCTTATAAGTTATTATATCATCTTCAATATGGT
AAACTAATGATATGAATTTGAAATCAAGGGAGCATAAATGATGACTAAACTGAAATATATAATTCCAACAATAATTGCAGTAGCCA
TTGTAATTATTTCTACCATTTCAATCATCCAATATATTAATCGTAAACGCTATAATCCCGTTAAAGTACTTAATGAAGTAAAATCATA
TTTTATGAATGTCAAAGGCTCATATATCGTTTATGAACCATTCGTTCATCCTGAAACTGATAAATACCGTTTAGTTTATCAAGGTGGA
ATTACAACTATTAAAAATGGTCAAAATATTCATTATGATTTTTATGCAGATGCATATACTGGTGAAGTCATTAACATTGTAGAGCGT
TAATACGAATTTTATCCATATTTGATTTGGATCGTAAAACTTAAAAATGAGCCACACCCTAATTAGTAAGATTGGATGTGGCTCAAT
TTTTTATTTTTGAAAATTTGTTTAGTAATACATCTTTATTTTAGTTATTATCAACTAATGTTTCACGTAAAATATATGCATCTATGTTTT
CACCATCATCGCTGTATTTTACAGCAAAGTAGTTTTCATCATGATAAAACAAGAACCAATATTGTTGCTGAATAAAATATGGTATCA
TGCGTTCTTTTTCACGAATCGATTGCATAGGATAATCATCATATGCCGTTACCCATAGAGGATTTTTATGTGCAGTAGTTGGGAATAT
ATCACCCATATGAACTGCTTTATCTCCTTGACTTTCAATCGTAATAATCGTGTGGCCAAAGCTATGACCTCCACTATGTTGCATCTTG
ATACCCGGAACCGGTTCAAAATGTTTTTCGAATAAAATCAACTTGTTACTATAATCGCCTTTATTCTTATCCCAGTAAGTTGATTTAC
TTCTTATATTAGGTGCAATAAACTCATGCCACTCATCTTGTTGCACAACATGAATCGCATTTTCAAAAATTGCATGTCCCGCTTGATC
AGTCAAACCGGCAGCATGATCAAAATGCATATGTGTCATTAGCACATAATCAATATCCTTTGGCGTTAAATTATAATTTGCCAAATC
AGCAATAATATGACTTTCTTCATCTACTCCAAAATTACGTAATTGCTTTTCAGATAATTTACCATTACCAATACCCGCATCTATAATC
AAATTATATTGAGCCGTTTGAATCAAAATTGGATGTGTCGGTAAATTGATTTGATTTCGTTCATTTGCATTGTATTGCTTTGACCACA
ACGGCTTCGGAACAACACCAAACATTGCACCGCCATCCATTTTTGTATTGCCACCATTTAGATAATGAATAGATATATCCCCGATTT
TCATAACATCACCTATTCTTTCTGTATTCGTTATTACAATTAGCATGTGTTTATTACATCATATCACTATTCATAAATGCGAACAAAA
ACGAAACGCCCCACACTAATACACTTCTAACTATTTATAAAGATTAATGCAATCATATTATATGTAAATGCATCATAACCATCGGCC
TGTAATTTTAAATTAATAATTAAAATTTACAAGTAAAATGCAAGCTTTATTCTTTTAAATCAAAGGTATTTAGTATAACATTGTTCAT
AGGCGCATTATCTCCTTTAGTTATTTGTTTAGTCACTTTTAATTATAGAGATAATTGCGCTTTTTTTATATTCAAAATCAAAAAATCG
GACAGATGAAAAGTAAAATACTTTTTTCCATCAGTCCGATTTATTATTGAACCCTAATATGCTTTTTCTATAGAACCATTATAGTACG
AAAAAACACTTATGATTGTATGTGTACATACCCTCATAAGTGCTCTATTTTTGTGAATGGAATTTCGCTTCCATACGATAAATACGTG
ACCCTTTATCCGAAAATTTCTTTTCATATTCTGTTAAAATATTACTGCCATCGTCTTCTTGATGTAAATTTAGATTTATTTTTGTAAAA
TACATTCCAAATTGAGACATACTTTCTAAACTGTAGGCAAATAGTCCTCTGTTATCAGTTTTAAAATGTAAATCTCCTTCATCATTTA
AGATTTGTTGATACAACGCTAAAAACGTATGATACGTTAAACGTCGTTTTGCATGACGATTTTTTGGCCATGGATCTGAAAAGTTCA
AATAAATACGCGAAACTTCGCCGTCTTTAAAATATTCATTTAATTCAATGGCGTCATTACAAATAATCTTTAAATTTGTTAAACCCAT
CTCTTTAACTTTATCCAATACTTTATAAACGATACTTTTCTCACGTTCCATTGAAATATAGTTAATATGAGGATTTTGAGCAGCTAAT
GTTGTAATAAACTGCCCCATACCCGAACCAATTTCAATGTGTATCGGTTGCGTTTTATCAAACCATTCAGTCATTTTCCCTGCATGTT
GACCGTCCATGTCAACCAATTCAGGATGACCTTTTAAATAATCTTCAGCCCATGGTTTGTATCGAACTCTCATATTTTATTCTCCTCTT
AAATAAACATGTTACTATTCATAACTTCATTTAGGAATTTAAGCCAAGTGTTCATATCCTTATATCTTTTTTGCTCTTCATACCATTGA
ACAAGACCTATAGATTGAATTACCGTATACCATTTCATACGTTTATTTAAATTCAAGCTCTCTTGAACACCATATGTTTCAAGCCATT
CAGACCATTGTTGTTGTGGAACATAGTTGTAAAGCAGCATTCCGATATCAATTGCCGGGTCTGCGATCATTGCACCTTCCCAATCGA
CTAAAAATAGTTCATCCCGATCAGATAATAACCAATTATTATGATTCACATCACCATGTACAACAGTGAAAAAACGTGAATCTAAA
CTCGGCATATGCTCTTCTAAATAGGTTAATGATTTTCTCACAATATGATGTGTTAAAACTTCCCTTGATAAAGAGGCATTAATTTTAT
TAAGCATAATCTCAGGAGTAATAGGTTCCATTTCCATACGCTTTAACATACTTAATAAAGGTCTAGAATTGTGTATCTTCTTTAATAA
ATGTGCAACTCTTGTTTGCTTCATTTCGTTTGAAGATAGTTCACGCCCATTTTTCCAATGTTGTGCTGTAACAACCTCGCCTGTTTCTA
TGCGTTTCGTCCATACTAATTTGGGCACAATACCTTCTGCTGATAATGCCGCAATAAATGGATTTGAATTTCGTTTTAAAAACAACTT
TTGTCCATCTTGTTCAGCCATATATGCTTCACCAGATGCACCACCTGCTGAATCAAGTGTCCACCCTAATTGATAAAACTGCTCCAAC
TCGTCCACCTCACTTTCAATTAGAAAATGGCTCTAGAAATAGGTTTTTCAAGAGCCATATATTCTAATTTATAACACCATACTGGTAC
AAATATTATGTCCAGATAATTATTGTAAATCCTAAACTAATGCCTACATTAAACAACTTAAATTAAATCAATATGTCTGTCATTGAC
ACCATACATTCTAAAGTCGCTTACATGACATATAATGTTACCGTGTCTAAAACTACATGTTTTTGAATCTCTGTAGGCGATAAACTCT
AGTTTTCAAAATAATTGCTATCCCATTTTCATGGTTAGCATAAATTTATGAACTGTAACATTTACGTACTTAGTAAAATATGATGCAC
ATCATATTTGTAACTCATAGAAAATTTTATAATTTTTATCATTATATTTCAACTGAAAATGAGAAACAAAATGGCACTTTTTACTAAT
ATGTGTTTTCTAAACAACACTTTTAAGCTTCGTTTTAAATTATAACATAATTCACTTACGAAAGTTGATAAATTTAAGTAATTTAATC
TAAAAATATGATGAAAGAATTTTAAATACTGTGTGACTCTATATACTTTTCAAATCCTTCTTGTAGTTGACGTGTAATTGGGCCAACT
TTACCATCATTAACTGGTTCACCATCTAATTTAATAACAGGTGTAACCTCAGCTGAAGTACTTGAAACAATAACTTCATCTGCGTTTT
TCAAGAAATCTACAGTAAACGTTTCTTCTTTAAATGGGATGTTATAGTCTTCGGCAATTTTTTTAATTACAATTCGTGTAATACCATT
AAGAATATAGTTGTTAATCGGATGTGTATAAATCACACCGTCTTTAATTGCATAAGCATTACTTGAAGATCCTTCAGTTACAGTTTCA

CCTCGATGTTGAATTGCTTCAACTGCATTATATTTCACAGCATATTCTTTTGCTAATACATTTCCTAATAAGTTCAAGCTTTTAATGTC
GCAACGTAACCATCGGATATCTTCAACGGTAACACCATTCACACCATTTTCTAAATGATCATAAGGACGATCATAACTCTTTGTATA
AGCAACAATTGCTGGTTCTACTTCAGGTGTCGGGAAGCTATGATTCCTTTCAGCTACACCACGCGTTGCTTGAATATAAATTGCCCC
AGTTTCAATTTGATTCATATCAACTAATTTACGAGATAGTTCAATTAATTCTTCTACAGAATAATTTAAATCTAAACCAATCTCATTG
GCACTACGTAAAAATCTTTCATAATGTTCTGTTACTGTAAATAACTTACCATTATATACTCGAATGTATTCATAAATACCATCGCCAA
ATACGTATCCTCTGTCGTTGTATGAAACCTTTGCTTCACTTGGACTTACAAACTCACCATTTAAAAAAATTTTTTCCATATATTATTCC
TCCACGCATAATGAATAAATTGCTTCTAAGTAAATACTAGTTGCGTTAAATAACTGTTTTTAGTGATATATTCATTTTTCTGATGCA
TTAAATCTTCAGAATCACTAAACATTGCGCCAAATGCTACACCCTTGTCTAAGTTTCTCGCATAAGTACCGCCACCTATAGTATAAG
GTTCAGTCATATCATTTGTTTGATTTCTATATGCAGTAACTAACTTTTGTACAAAAGGATCATTTTTATCAACATAATGTGGTGGTTG
GACTTTACCTAATTTCACTTCAAAGCCATATTGTTGAATCTCATTTGCAAAACGATCCATAGCTTTTTCAAATTCAAATCCTTCTGGG
TAGCGTAAGTTGATACCGAAAAGACCTGCGTTTTCATTATCATATGTAATAACACCAATGTTAGTTGTCACGTCACCCATGACATCT
GTATGGAATTTCATTCCCATCTTTTCACCAAAATCTGAATTAAATAAGTAGCGATTACTAAATGCTACAAACGCTTGTGCATTATTAT
CAAGATTTAATGATGCTAAGAATTTTAGTAAGTAAAGACCCGCATTCACACCGATAGATGGATCCATACCATGAACCGCTTTACCTT
CAACTGTTAAAACTAGAATGCCACTATCAACAGTACTATCACCTTGTAAATGATTTTGTTCTAAAAAGTACTCAAAGTCTTGAATAA
CATCTGTCATATTTTCTTTAACAAGCACTCTTGCTTCTGCATGATCAGGTACCATGTTGTAACGTTCACCAGATTTAAAAGTTATTAA
TTCATAATCAGGTTCATCTTGATCTTCAGTAAGTTTATTTTGAACTAAATCAAATGTTGTAATGCCTTTTTCACCATGAATACATGGA
AATTCTGCATCTGGTGCAAAACCTAATGTTGGCATTTCTTCTGTTTTAAAATAGCGATCCGTACATTTCCAATCAGATTCTTCATCCG
TACCAATAATCATATGAATACGTTTCTTCCAATCCACATTCATATCTTCTAATATCTTAATTGCATAATAAGCAGCAATTGTTGGACC
TTTGTCATCAAGTGTACCTCTAGCTATGATAGCATCTTCTGTTACAACCGGCTCGAACGGATTACTATCCCATCCATCACCAGCAGG
AACAACGTCAACATGACATAAGATACCTAATACGTCATTTCCTTTACCTGCCTCAATTCTTCCTGCAATATGATCCACATCATGTGTT
GTAAATCCATCTCTATGTGCAATTTCATACATGTAGTCTAATGCCTTACGAGGACCTGGACCAACTGGTGCGTCTTCTGATGCTTTTG
CATCATCTCTCACACTTTCAATTGCTAATAATCCTTTTAAGTCATTAATGATTTGATCTTCGTATTGTTGAACTTTTTCTTTCCACATTC
GAAATCGACTTCCTTTTTTCTATAAGTTAAATTCTATTTTACATGAAAAGATATAAAAACTACAATAAGATGTCAGAAAATAATAAA
AAGGAACAAAACGATGCTATTGATATGACACAAATCATAAATAGCTGCTTTGTTCCTTTTTTAATTTATATATTTAAAATACACATAT
TCAAGAGCTCGAGATATAAGTCAATGTACTAGGCACACAATTTAATATTGACAGTAATTAACCGAACGAAAATGCGCCCCGGGGCC
CCAACATAGAGAATTTCGAAAAGAAATTCTACAGACAATGCAAGTTGGCGGGGCCCCAACATAGAAGCTGGCCAATAGTCAGCTTT
CAATAATGTGAAAGTTGGGGTAAGGGCCCCAACACAGAAGCTGGCCAATAGTCAGCTTTCAATAATGTGCAAGTTGGCGGGGCCCC
AACACAGAAGCTGGCCAATAGTCAGCTTTCAATAATGTGCAAGTTGGGGTAAGGGCCCCAACACAGAGAATTTCAAAAAGATAAA
GAAATACGTTTTCTTTAGATATTAGTATTTTTTATGCATGAGTTTCACGCATGTATTCTTCTTTCTAAATACATATTAGCTATGACTAA
CGATAAAGAACTTGAAACACTAATAAATGTCCTATAGTTTACAATATTATATTGGCAGTAGTTGACTGAATGAAAATACGCTTGTAA
CAAGCTTTTTTCAATTCTAGTCAACCTTGCCGGGGTGGGACGACGAAATAAATTTTGCTAAAATATGATTTCTGTCCCACTCCCTTAT
CATTTCTGTCCTACTCACATCTTATTCTTTATCAGATAATGCATTTTTATTCTTTTTTAAATCTTCTTCAGTGACGATACGTAAATTATT
ATTTGGTGCTGCGCCACCTTCATCATCAAATTTACCTTTTTCAATACTTTCGTCAGTCTTATTGTCATATTCGGTAAATTTTGATTTTT
CTTCTTCGAAAAATGCTTTTGGATTATTTTTTAATCTATTAGCATATTCTTTCGGATTTGTTTTTACTTCTTTAATTGTTTCATTAGCAA
TTGTTCCTAATTGCGTCGCTTTATCCTTAGCATTATCTTTATAGCTTTGAGGATCTTGTTTATATTTATTATATTCTGCTTTCAGCTTGT
CACGACTATCTTTACGTGTAACAAGTACAGCTGCTACAGCGCCACCTATACCTAAAATCGCTTTAAATAAATTACCTTTTGCCATATC
AATCGTCTCCCTTTTATTTATAATTTAATTTGTCAAAATCATTTTCAGTTAATAAACGATATTCTCCTGAATCTAGATTGCTGTCCAAT
TCTAAATCAGCAATTTTGATACGTCTTAAATGTAATACCTCATTTTGAATGCTATGAAACATTCGTTTAACTTGATGATATTTTCCTTC
ATAAATTGTTACGTGTGACGTTTGATTATCAATATAAGTTAAAATTGCAGGCTTAACCTTGCCATCAGACAGTGTTACACCCTCTTTA
AAAGCTTGAATGTCGTCTTCAGTGATAGGATTTGCTGAAATAACTTCATATTTTTTAGAAACATGTTTGTTTGGACTCATTAATTCAT
GATTAAAATCACCATCATTCGTTATCAATAAAAGCCCTTCTGTATCTTTATCAAGACGACCAACCGGAAAAATATTTAGATGTTGGT
ATTCAGGTATTAAATCAATAACGGTTTTTGAATGATGATCTTCAGTTGCCGATATATAACCTTTTGGCTTATTTAACATAATATAGAC
ATTTTCAACGTATTCTATTAATTCTCCACGAACTGTTATCTTATCGTTTTCTGGTTCTATATGTGTTTTTGGTGATTTAATTACTTGTTC
GTTGACATTTACAAGGCCTTTTTTAAGTAACTGTTTGACCTCATTACGTGTACCGACGCCCATATTTGCTAAAAATTTATCTATTCTC
ATCGTAAAAACCTAACTCTACGTCTTAATTTTTCAGGAATTTCACCTAAGAATTCGTCCGCAAGACGCGTTTTAATTGTGATTGTACC
GTAAATTAGAATACCTACTGTAACACCTAAAATAATAATGATTAAGTAACCAAGTTTAGTAGGTTCTAAGAATAGATTTGCAAGGA
AGAATACTAATTCTACACCTAGCATCATAATAAATGAATACAAGAATATTTTTGCAAAATGAATCCAACTATAGCTGAATTTAAACT
TCGCATATTTTTTAAGAATATAGAAATTACATCCAATTGCAAATAATAATGCGATACTAGTACTTAAAATTGCACCAGGTGTATGGA
ATAACATAATTAATGGATAGTTTAACGCTAACTTGATAACTACAGAAGCTAAAATAACATAAACTGTTAATTTCTGTTTATCTATAC
CTTGTAACATTGATGCCGTTACACTTAATAGTGAAATTAGTATTGCTACAGGCGCATAATAGAATAATAAGCGACTACCATCATGGT
TAGGGTCATGACCTAAAACAATTGGATCGTAACCATAGAAAACTGTGAATAATGGTTGTGCCAAGGCCATAATTCCAATACTAGCT
GGAACAGTTATAAACATTAATACACCAATAGATGTTCTAATTTGATGATGCATTTCATGTAAGCGACCTTCTGCAAATGTTTTTGTA
ATATAAGGAATTAAACTCACTGCAAAACCAGCACTTAATGATGTCGGAATCATTACAATTTTATTAGTTGACATATTTAGCATATTA
AAGAATATATCTTGTAACTGTGAAGGTATACCAACTAAAGATAAAGCACCGTTATGTGTAAATTGATCTACTAAGTTAAATAATGG
ATAATTCAAACTTACAATAACGAACGGTATACTATAAGCAATAATTTCTTTATACATCTTGCCATATGACACATCTATATCTGTGTAA
TCAGATTCGACCATACGATCAATATTATGCTTACGCTTTCTCCAGTAATACCAGAGTGTGAATATACCAATAATCGCACCAACTGCT
GCTGCAAAAGTAGCAATACCATTGGCTAATAAAATAGAGCCATCAAAGACATTTAGTACTAAATAACTTCCGATTAATATGAAAAT
CACGCGTGCAATTTGCTCAGTTACTTCTGACACTGCTGTTGGCCCCATAGATTTATAACCTTGGAATATCCCTCTCCATGTCGCTAAT
ACAGGAATAAAGATAACAACCATACTAATGATTCTTATAATCCAAGTAATATCATCGACTGACCAACCGTTTTTATCATGAATGTTT
CTAGCTAATGTTAATTCAGAAATATAAGGTGCTAAGAAATACAGTACCAAGAAACCTAAAACACCGGTAATACTCATTACAATAAA
ACTCGATTTATAAAATTTCTGACTTACTTTATATGCCCCAATAGCATTATATTTCGCAACATATTTCGAAGCTGCTAATGGTACACCT
GCTGTCGCAACTGCAATTGCAATATTATATGGTGCATAAGCGTATGTGAACGGCGCCATATTTTCTTGTCCACCAATTAAATAGTTG
AATGGAATGATAAAAAGTACGCCCAATACCTTGGTAATTAATATACTAATGGTAATTAAAAAGGTTCCACGCACCATTTCTTTACTT
TCACTCATTACGAATCTCCCTATCTCATGTTTATTAAAGTTTTGTAAACTAAAAGCTGTTTCTCTGTAAAATCATTTTTCATTATTATG
AATATATCACAAAACTTTATTTCATTGTCGTATATTCAATGAATTATCATAACAAAATTATCAACACATTGTCATTGAATACTAGATT
TTGATTAGAATATTACGAAATTTCATATAAACATTATACTACTATTTGAGATGAACATCGCATAACAGTAGAAAAATCATTCTTATC
ATACACATACATCTTCATTTTTTATGAAGTTCACATTATAAATATATTCAACATAATTGTCATCTCATAACACAAGAGATATAGCAA
AGTTTAAAAAAGTACTATAAAATAGCAATTGAATGCCCAGTAACAAATTTGGAGGAAGCGTATATGTATCAAACAATTATTATCGG
AGGCGGACCTAGCGGCTTAATGGCGGCAGTAGCTGCAAGCGAACAAAGTAGCAGTGTGTTACTCATTGAAAAAAAGAAAGGTCTA
GGTCGTAAACTCAAAATATCTGGTGGCGGTAGATGTAACGTAACTAATCGATTACCATATGCTGAAATTATTAAGAACATTCCTGGA
AATGGGAAATTTTTATATAGTCCCTTTTCAATTTTTGATAATGAATCCATCATAGATTTTTTTGAGTCTAGGGGTGTTAAATTAAAAG

AAGAAGATCACGGGCGTATGTTTCCAGTTTCCAACAAAGCACAAGACGTGGTTGATACATTAGTGACAACTATCGAACGCCAACAT
GTAACGATTAAAGAAGAAGAAGCTGTTAGTAGAATCGAAGTTAATACAGACCAAACTTTCACTGTACATACTCAAAATAATAGTTA
TGAAAGCCATTCGCTAGTGATTGCTACAGGTGGTACAAGTGTCCCTCAAACTGGTTCAACTGGTGATGGTTATAAGTTCGCACAAGA
TTTAGGTCATACCATTACTGAGTTATTCCCGACCGAAGTTCCAATTACATCAGCTGAACCTTTCATCAAATCCAATCGTCTAAAAGGT
TTAAGTTTAAAAGATGTTGAATTGTCAGTACTTAAGAAAAATGGTAAAAAACGCATCAGTCATCAAATGGATATGTTATTTACTCAT
TTTGGTATCAGTGGTCCAGCTGCATTAAGATGTAGTCAGTTTGTTTATAAAGAACAAAAAAATCAAAAGACACAGCACATTTCTATG
GCAATCGATGCATTTCCTGAATTAAACCATGAACAATTAAAACAACACATCACATCATTATTATCGGACACACCAGATAAAATCATT
AAAAACAGTTTGCATGGTCTAATTGAAGAGCGCTACTTACTGTTCATGCTGGAACAAGCAGGAATCGATGAAAATACCACATCACA
TCACTTATCAAATCAACAATTGAACGACTTAGTAAATATGTTTAAAGGGTTTGTATTTAAGGTGAACGGGACATTACCTATAGATAA
GGCATTTGTCACAGGTGGTGGTGTGTCACTTAAAGAAATTCAACCTAAAACAATGATGTCTAAATTAGTTCCGGGATTATTTTTATG
TGGTGAAGTATTAGATATACATGGTTATACTGGTGGTTATAATATTACAAGTGCACTCGTAACAGGACATGTCGCTGGATTATATGC
CGGACATTACTCACATGCATCAATGGAATAATAGTATAAAATTTGGTTCGATTCTCTTTAGTAGATCAACTTTTTCATTCAAATAAAA
ATGACCTTAATATAACTGAGTCACTAAAAAGTGTCGTTATATTAAGGTCATTTCGTTAATTATGATTCTTTTTCGTTTTTAGTACGTCT
TCTAGCTAACAAAGCCGCACCTGTAATCAGTGCAAATTCTTTCAATGGTAAATCCATTCCTTCAGAACCTGTATTTGGAAGTTCTTTT
TCAACTTTGCGCGATTCATGTGTCTCTTCTTTTTTAATAGGCGTACAAACTTTTGGAGCTGGCTGAATTTCTTTTGGTGATACTTTCGT
CGCTTCAGCTGGTAATTTAATTGCTAAAATTTCATCAACAATGAATTGCGTGTGTTGTTTGATGTCATTTAATGTCGCATCTTCATCA
ATCATTCTATTGCCATCTGCAACATATTGATCAATTAATACTTTTACTTTAGCTAATTGTTCTGGTGTTGCGATCGCTTTGAATTTCGC
ATATGTTTGTTGAGCAATGTTATCAATTCGCAGTAAGCTATTTTCTTTTTCAGTAATTACTGCTTCTATATCGCTTAATGCAACATTAA
ATCGTTTTAAAACTGCATCAACATCAGCATTAGTGCGTGCTGTTTTTAATTCTTCATCCATTTGTAATTTTAAAGCAGTTATAGCTTTT
AATGCATCAGCCTTATTACGATTACTTACTTTTCGATAATTTTGCACTAAAGCAGTGACGCGTGCAAGATCATCATTAATCGTTTTTT
CAGCATCTGGCTTTTTAATAGGATGTACATCTAAATCATGTATTGTTTGTAGATTTAATGATGCTGTTTTATCAACTTGTGCATTGCT
ACGATCTTGATCAATTTGTCCAATAGCAGTGTCATAAATATTTTGTAACTGTGCTAATATACTATTTCTTTCTTCTACCGTTGCTTGAA
TATTCGCTTCAATTGCTTGTTTTTTATCGTTGAATAATGTTGTCAATTGTTCTCGAGCAGACGCCTTTCTGTTAATAACAGGTTCGATT
TCACGAATTTCGTTTTTCTCATCATGCAATAAATATGCCACATCAGCATTAGTCACTGCACTAGCAATTTGTTGTTTAGCTTTAATTA
ACTCTTTTTCAACTTGTGCTATTGCAATATTTTGTTCTTCATCTGTCGCTTCGTTATTTGCTTTAATTAAATTAATTTTATTTGTAGCGA
TATTTTGAATTTGTTGTAATGCTGTTGCTTTAACTGTTGTCGCTGGTTTAATTTTTGAAATAATATTTTGAGCATTTATACTATCTTGA
TTAACTTGGGCAGTCTTATCTGCATGATTGATCTGATCAATAGCCTGATTAAGTGCTTGTTCTACTAAATGTTTAGCAGCTAGTCTTT
CTTCTTCAGTTGATAAATCGCTTTGATCGATTAGTGCATTTTGAGCTTCGGCTTTTACACCAACAGATTGACGCGCTGCTGGTTTAAC
TTGAACTTTAGGTAAAATCACTTTGATGTTGTCGTTGCCATCAGTCTCAGTTCGATCCACTTCTGCATTCGTTTTGTTTTGTGCAATGT
CATTTTTAATTGTATTTACAATTTTATTTAAAGTATCAATAGCAACATCTCTTTCATCTTGAGTAGTATCCAACGTATTTCGGATTGCA
TCGAGTTGATTTTTATTATTTTCTTCAATGCTATCAAGCGCAGCTCGTTTACGTCTTACTTTAGGTTTTATTTGCTCTATTGCCTTGATT
GTTTGATTTCTAACATCAGTAACAGCAGCATCTTGATTTGTATTTTCTATTTCTTGTTGCGCTTGTTTGAGTGTGTCGCTAATTAATTG
ATTCGCTTCATCTAATTCATCAACTGTTGCATGTGGTGTATCTTTTATTGATTCTACTTGATTTTCTGCACTTGCTTTTATTGCTTGTTG
TGCTTCAGGCTTAATTACAATATGAGGTTGTACACCTTTTAGTGTAGCAATGCCATTTGTTTCAACACGTTTCACATCATTATTCGCT
ATTGCTTGATCGATGTTTTGTAATGCACGTTTTTCATTATTCGCTAATTGATTTAAAGCAACTTGTTTTTCTTCATCAGTCGCATGTTC
CGCTTGCTCAATTTCGCGCTTTTTAGCTTCATATTGTTGCTTAACTGCATCTCTAGCAGCTGCTCTAACAATATGGTCAGGCGTCACT
AAAGCAATGCTATCAAGCGCTTGACTTGTTGTATCATCAACTTGTTGATTTGTTCTATTATTCGTAATATCTGTCATGGCTTGATTTAC
AAATTCATTGATTTTATCTAGTGCTACTTGTCTTTCTTCTGCTGTTGCTTCTTTATCCTGATTAATCTTAGCACGTAATTCATTCGCTTT
TTGATTGATTTTTTCACGTGCAGCTGGTTTAACTTTTGTTTCAGGTTGAATAATTTTAATCGCTGATACACCATTTGTTGCTGCTTGAT
TCACCTGACTATTCGTTTGAGCTTGGTCAATAGCTGCAAGTGCTTTTTCTTTTTCTTTTGCTAATGCTTGTGAAGCAACTTCTTTCTCA
TTATCTGTTGAATCAAGACTATTATCAATTTGCTGTTGCTTTTCTTTAACAGCTTTTTCAATATCTGCAATTGCCTTTGGTTTAATTAC
TACTTCAGCTTCAACATTATCTATAGCATTTACCGCTTGATTTGTAGTTGCGTCTACCTGATCATTTGTTTGGTTTTGATTAATTTGAT
TAAATGCTTGATCTTTAAGTTGATTGATTTGATTAACAGCAGCCTGCTTTTCTTCGTCAGTTGCATTAGGTGTTTGTTTAACCGCTTCA
ATACGCTTCGCCACTTCAGCAGTGATTTTATCTCGCGCTGCTTGTTTTTTTACTACGTCAACTTGAACAGCATCGATATTATTCTCTGC
TACTGTCGCAGCTTGGTCTACTTCTGCATTTGTGTTAGCTTGTTTAATACTTTCAATAGCTTGTTGTCTGTCTTGATTTAAAGTATTGA
TCGCAGCATTTTTCTCATCATTCGTTGCATCTGGTGTAGCATTGATTTCAGCTAATTTAGCATTATAATGCTGATTGATTTGTGCTAAT
GCTGCAGGTTTTTTAACAATATTTGGCTGAATCGCATTAATTGCTTTTGTACCTAATTGTTGCGCTTGATCTACTTCCGCATTTGTATC
AGCTTGATTTATATTATTAATTGCCGTTGCTAACTCTTGATCCACTTGATTTAAAGCCACTTGCTTTTCTTCAGTTGTTGCATTTGTGT
TTTGATTAATTTCTTGCTTTTTAGCAGTTGCTAAATCATTTAATACACCTGTAGCAGTTTGTTTCTTCGTTACATGCGGTTGAACTGCG
CCGATTTGATTGACTGCATCATCTCTAATACTATTGACCATCGCAGTAGTAGACGTCACACCAATATCAGTTAATGCTCTATTTTTAA
GTGTATTGACACGATTTATCGCTTCTTGTTTCTCTTCTCGAGTCGCGCCAGGTGTTGCATTGATATTTGTTATCGCCTCTCGCGCTTTA
TCATTTACAACATTGCGAGCATCCGTTTTAACTTGTGTTGCCGGTTGAATCACAACTATATTTTGTGTGCCCTGATCTTTTGCTTGTGC
AACTTCTTGATTCGTATCTGCTGCATTAATATTTTGCTTCGCTGTGGCTACAGCTTGATCAAGTAATTGTTGTGCTGCTTGTTGTTCTT
CGAGCGTCGCATCATTATTATTAAATATCGTATTATGTTGCGTTTCCGCACTTTTATCAATGGCATTTTTTGCATCTGTTTTTACTTTT
GTAGCTGGTGTAATTGCTTGTATTCCTTGTATCGCATTTGTCTTCACTTGTTCCACTTCAGCATTGGTATTAGCGTTTAAAATGTTTGT
GTTTGCTGCAGTTACAGCAGCATTCACTTTGTCAATTGCTGCTTGTCTTTCTTCTTGAGTCGCATCAGGATTAGCATTGATCTCTGCG
ATATGTTGTTGTGCATCATGTGATACAGCATCCCTTGCAGCTTGCTTAACAACAGTTACAGGAGCAATTGGATTAATGGCATTTAGA
CCATCTCCTTTGGCGTTATCAACATCAGCATTTGTTGTTGCTTGATTGATTTGTTCTAAAGCATGGTTGGTTGCTTGAGTTAATTCGTT
CAATGCTGCATTTTTCTCTTCCTGAGTTGCTTCTCTATTACTATTTATTTGTTGTCTTTTCGTTGCTGTTGCTTGGTTAATTGCATCTCT
TGCAGCTTTTTTATGAGTTACTTGAGGAACAACTGCTCCAATAGTATTGATGCCATTATTTTTAGCTGTTTCAACGTCAGCATTTGTA
GTAGCATTCGTAACATTATCAATAGCATCTGTTTCATCCGTAGCTAATTGATTTATTGCATCTTGAATCTCGTCTTCAGTAGCATCTG
GTGTTGCATTGATAATTTCCCTTTGTTTCGTTGCTTTATCACGTATTGCTTTTTTAGCATTTGGTTTAACAACCGGTGTTGCAGTATCC
CCACTTAAGGTCTGTATACCTTGATCTTTGATTCTAGTAACGCCATCATCAGTCGTTTGATCACCAATATCACCAATAATATTGCCTT
TATGTTCCTCGATAACTTGTATTGCTGCTTGTTTTTCTTCATCTGTCAGTTCATCATTTTGATTAACTAAATCTTCCATTTGATCAGCTT
TTTGATTTACTGCATTTTCAGCATCAACACTTCGAATTAAAGTATGTTGCATCTGATTAGTTAATGTATCAATATCTGCTTGAGATAC
TCTTTTATTTAATGGTACATTGTTACGATTTTCAGCTAAAATTGTTTGTGCACGTTTTTTAAGATCATTAAAGATATCTAATGATGCA
AATGTATAATCAGCTTGTTGAATACGTCTGTCAACTTCGGCTTGTAATGCATCTTTATTCATGATGATATCGATAGTATATGGATTTG
TACTTACAGTATGACTTTCTGCAGCTGAATTAATGAAATCTTGTGTATATGTTTTATACGTTAATGTATCATTAAATGTTACTGTTCTT
GGTGTCGGTACATTATTAACACGCAACTTATACTTTAAATCCAATATTTTATCAGGCATTAGTCGTGCAGGTGAATTCGTTGTGCCAC
CACCAGTGCTTTTAATTGTTATCACTCGATTCGCCGCATCATATGTAACATTCATATCATTAACATCAACGCCTGAATTGTTACTTGG
AAAATCTTTAGTCAATGAATTGTTCACATATTCTACCCCTTCAGGTAATTGAATTTTATAAACAAATTCATCTGTGTCTAAAGAAGCA

CCAGAATTACCATTATTTTTTAGTGATGTTGTAACTGTAAATTCTTTATTATTCGTTGCTGTGGGTTCCATTGTTCGTCTTTCAACATA
AACATGTGACCCAGAATGAAGTCCGATAGAGTCAACAAAGCTATAGTATTTGTAACCATCTTTTAGTTGATAAATGCCACGCGCATC
TGTTATTGCGTCATTTTTAGGTACAAATTGAATTTTGAGATTTCTCACATTATCAGGTACTTTAAATAAACGCAAAGTTGGACCGCCT
TCAACAGTCTTTTCAGCAATCGTATCATTAGTATCAGCATTTTTGATAATAACATTTGTTGCGCCTTGACCATTTTTAGTTGGCATTGT
ATTAAATTCAAAGATTAATTCAGAATTTGGATTTACTGTTAAAGATTTTTCAATACCATTAAAATCTCCATGATTACTAGCGTCCGTC
CCGCTTATACGTCCTAATGCAAGTACATTGCCTTGTGCCTGATAGGTTCTGTTGCTCCCAGCCTCAAACATACTTGTTCTAACCATCG
CATGACTTAGCACACCTACTTTACCACCATTGATAAGTGTAAAGCCTGGTAAATTATCAACAACTGTTACTGATGGTACAGAACGGT
TGGTACTTGGTCTAATACTATTGTCATCAAATGACAGCACTTCGTTAGGTGCATTTTGTCCTGCATTATTGGCATTAGGATCAGTTGT
AGGCGTGTATGGCGCTGTAATTGCAACTGGTGCACCACCGTTTCCTGCCGCTGCAGCCCCTGGATCTGCTGGAGTTGCATTAGGATC
TGCCGGTGCCGCACGTCTACTTCTCTTTTTCGGTCTATTAGATGCTGGTTCAGCAATTGCAACTAGCTCTGGTTTATCTGAAGAATGA
CGAACATCTTCTTGAATTTCCTTTAAAGTTAGATGTCCTCCTGAACCATTTTCATTTGTTTTATTTGGTACATTTAATGTGGTATTATT
CGACGCTACATCATTATTATTAGCGTTTGACACTGTCTCAGTTGCTGTTGTATTGCCATCTGTATGATTATTGTTTTGTGCTGAAGGC
GTACTTGATTGCACTGATGTTGGCGTAGCTTGATTTACTATACTACCATTATTATGATTAACCAATGCTTGATTCGTTGCTTGATTGG
TTGTTGCAGATTGATTAGGTGTATTCTGCGCACTATTTGCTAAACCTCTATTATTAGCAACATCTTTATCTTGTGAATTTACAGGTGTT
GCTTGATTAGTATCGCTTTGTACATTATTATCCGTAGTTAAGGCTTGTGCACCATTTGGGTTTGAAAGTAATAAAACTGTTCCGATTA
AAGTAGAGAATATGCCTACTTTATACTTCCTAATACTATATTTATTTTTCTTTAACAAATTCATTTCATTTTCCTCCTGGTTGTCTGTT
CTCTGCTGTGTTGTTAAGAATTATGCTAAAAATATACGCTCCCTCACATTATGTTTATAGCTATATTATTTTGTACAATATTTTAAGTT
CACTCAATTTTATCATCATCATTTTATAAAATAAAGTTTTATTTTATTACAATTTAGTTTCCGTTTTGTAATTAAAATCTTAATAAAAC
ATAACAAGAACGCTTTAGATTGTTTTGTAACAGAAAGTAATTCATACCAAAAAACACCAAGCAACCGAAACGTTACTTGGTGTCAT
TACAAATGTAATATTATTTTAAATTATGTCACTACTTTAAATACTTTTTATTTCCAAACCTTCATCGCCCCATGCGTGCATGCCGCCTT
CGACATTTACGGCATCAATGCCATTTGCCTCTAAATATTCTACAACTTTAGCGCTTCGAACTCCACCAGCACATACAATATAATATAT
TTCATTTTTATTAAATGAATTTAAATTATCCGGAATGGTATCCATTGGAATCAACTTTGCATTAGGAATATATCCCATTGCTGTTTCTT
CGTCAGTACGAACATCAACAATTTGAACTGGTTTAGATTCTAAAAGTTTGTTTTTTAATTCATCTGTAGTAATTGACTTCATTTCAAA
GTCCTCCTTAAAACATTATTTAGCTACAATATTGACTAATTTTTGAGGAACAGCGATGACTTTCATGATGTCTTTACCTTCAATACTC
GCTTTAACATTGTCATTAGATAAGGCAATTTCTTGCATTTCTTCTTTTGATGTATCTTTAGCAATTTTAATTTTAGCTCTCAATTTACC
ATTTACTTGAACAACGATTTCTACTTCATCATCTACAAGTAGTGCTTCGTCATAAGTTGGCCAAGGTTGGTACGTAATAGACTCTTCA
TGTCCTAATTTTGACCATAATTCTTCACCGATATGTGGTGCAATAGGTGCTAACATTTTAACGAAGCCTTCAATGTAAGGTTTATAAA
CTTCATCAACTTTATAACACTCATTAATAAATACCATTAATTGACTAATAGCAGTATTAAATCCTAATGTTTCAAAGTCTTCTGTTAC
CTTTTTAACAGTTTGGTTATAAACTTTATCTAAAGATTTATTATTTGTAGTTACAATTTTTGAACTCAATGTTCCATCTTCATTTACCA
TTAAACGCCATACGCGATCTAAGAATCGACGAGACCCATCTAATCCTTTTTCACTCCATGCAATTGCAGCATCTAAAGGTCCCATAA
ACATTTCGTAAAGACGTAAAGTATCTGCACCATGAGACTGTACTATATCATCAGGATTGATTACATTTCCTTTAGATTTACTCATCTT
CTCATTACCTTCTCCTAAAATCATACCTTGGTTAAATAATTTTTGGAAAGGTTCTTTAGTAGGTACGATACCCAAATCATAAAGGACT
TTATGCCAAAATCTTGCATATAATAAGTGAAGAACCGCATGTTCTACTCCACCGATATATAAATCAACAGGTAACCAATGTTTTAAT
TTTTCAGGATCTGCTAACATATTTTCATTTTTAGGATCGATGTAACGTAAATAATACCAACAACTACCTGCCCATTGTGGCATTGTAT
TTGTTTCACGACGTCCTTTCATACCTGTTTTTTCATCTACAACATTTACAAATGAATCAATATTAGCTAGTGGAGACTCACCAGTCCC
TGATGGCTTGATTTCATCTGTTTCAGGTAACAACAATGGTAGCTCTTCTTCAGGAACAGTTGTCATTGTTCCATCTTCCCAATGAATG
ACAGGAATTGGTTCGCCCCAATAACGCTGACGACTGAATAACCAATCTCTTAATTTGTAATTAACTTTCTTTTCGCCAGCACCTTTTT
GCTCTAATAATTGAATAGCTTTAGTAATTGCCGCTTCATTTTCTAAACCATCAAGTTCACCAGAATTAATATGTTTACCTTCACCAGT
GTATGCTGCTTCTTCAACATTTCCACCTTCGATGACTTCAATGATTGGCAAATCAAACTTTTTAGCAAATTCATAATCTCTGTCATCA
TGCGCTGGTACTGCCATAATTGCTCCAGTACCATATGTTGATAATACATAATCAGCAATCCAAATTTGTACTTTTTCACCAGATAAA
GGATTAATTGCATATGCACCAGTAAACACACCTGATTTATCTTTTGCTAAATCTGTACGTTCTAAATCTGACTTTTTAGAAGCTTCTG
TTTGATAAGCTTTTACTTTTTCTTTATATTCATCAGTTGTAATTGAATTAACTAATGCATGTTCAGGACTTAAGACTAAGAATGATGC
ACCATAGATTGTATCTGGTCTAGTCGTAAATACTTCTACTTTTCCTTCCGCTTCCGTATTATCTACATCAAATGAAACTTTGGCCCCTT
CAGAACGTCCAATCCAATTGCGCTGCATATCTTTTAAAGATTCAGGCCAATCTAAATCATCTAAATCTGCTAATAATTGATCTGCAT
ATTCTGTGATTTTAAGTACCCATTGTTTCATCGGCTTACGATAAACTGGATGTCCACCACGTTCAGAGACACCATCAATCACTTCTTC
GTTAGATAAAACAGTGCCTAATGCTGGACACCAGTTAACTGCAACTTCATCAACGTATGCTAAACCTTTGTTATATAACTGTATGAA
AATCCACTGTGTCCATTTATAGTATTCTGGATCTGTTGTATTAACTTCACGATCCCAATCATAACTGAACCCTAATTCTTTAATTTGTC
GTTTAAAAGTTTGGATATTTTTCTTTGTAAATTCACGTGGGTCGTTGCCAGTGTCTAAAGCATATTGCTCTGCTGGTAATCCGAATGC
ATCCCACCCCATCGGATGTAATACATTATATCCTTGCATTCTTTTATATCTTGAAATGATATCTGTTGCTGTATAGCCCTCAGGATGT
CCAACATGTAAACCAGCACCTGATGGATATGGAAACATGTCTAAAGCATAAAATTTCTTTTGACCTAAGTTATCATTTGTTTTAAAT
GTTTTATTTTCGTCCCAATAGTCTTGCCATTTCTTTTCAATTTGATTGTGGTTGTAATTCAACACATTTCCTCCTATTCAATAAAAAAG
ACACCTCTAACTATTCTATTTACATAGGGACGACATTTGCCGCGTTACCACCCTATTTCACAAAAATTGTGCACTCATTTTAATAGTA
AGAGATGTCATTCATTCAATTTAAAATTAATACGATTTACATTTAGTCCTTAACACTAGCTCTCAGCATCACTAGCTTTCTGTAGAAA
AGCTCCAAACGAAATTAAATTATACAAATTAATATTATGTTATAACGACATTTATTTCAAGTCTATAACGTTAATTCTATTTACTCAT
TATGCATCTATTTTTTTAGGTTGCGTATTATTCTCCTTGAAAACCATTAATAATATTAATGCAAATACAAGTAGTAGCATCATACCGA
TAAACATCATGCGCATATTAAACGCATCAACTAATACACCACCAAGAAATGGACCAAATGCTTTTCCTACTGTAGCAGCTGAATTCA
CAAAACCTTGGTACTGTCCTTGCTTACCATCTGGCGCTAACTGATTGGCTATAGTTGGAACTGCTGGCCATACAAACATTTCTCCAA
AAGTTAAAATAATCATACCGACAACAAATATTGTAAAGTTTTCGGCAAAACTCGTGACAAAGAACGACAACATAAAAATGATGATG
CCAACAAACATTTGCTTCTTTAAGTTTCCTTTTAACAGATAGAGAATCGGTTTAATTAATGGTTGTGCTACTAAAATCATTATTCCGT
TAATTGTCCATAAAACACTATATTGTGCCATTGAAATATTAATAGATTGTGTAAATGATGCGATTGTAGACTCCCATTGAATATACG
CAACCCAACAAATTGCAAACATTGCACAAATTAGTACTAATGAAATAAATCTTGCTTTATTCTTTTTACCAGTAATATCTAAATGAG
TTGGATATTTAACTTTCGCATTAATTTCAATATTAAATTGCGTTACCGCGACAAGCGCAAACACAACATACATAATAAGATTGGCTA
AAAAGATATAGTTAAAGCTAAATTCTGCGACAAAGCCGCCCATTGCAGCACCGACAGCCACACCAATATTTTGCGCTAAGTATATC
GCATTAAACGTTTGTCTTCCGCCATTTGGCCACACTGCTCCAGCCATAGCGTATATCGCAGGAATAATCATTCCGCCACCAAACCCT
AACATTACAAGCCATACAGCATACCAAGGCCACCCGTGAAAGAAATTAAGTAGCGTTGTACTACAAAGACAAGTGAAAGTTCCAAT
TAAAATCGTCTTGTATCCACCTAATTTATCAAATAGTGAACCACCTAATAAGTTTCCAATAACCATGCCAAATGAATTTATCATTAG
CACTAAACCAGCAACAGTTAAACTTTTTCCAAGTTCTTGTTTCATATAAATTGTATTTAAAGGCCACAAAAAAACTGGAACCAGTAAT
ATTTAACGCCATGCCAATTACTAGCCACCAGACTGATTTAGGTATATTCATATTTCATAGTTCCTCTCTCTTTTTTGTCAAACTATAAT
AAACACTATAACATTAAAATATGTTAAAATCTGTACTTAGAACTCTTTTTTTATAGAAAGGAAAATGACATATGGGCAATCATTTCC
AATACGCTTTTGAAAACAACCGTTATCACACATGGAATTACCATTTAAAAAATAAATTTGGACAAAAAATATTTAAAGTTGCATTGG
ATGGCGGGTTTGACTGTCCTAACCGCGATGGCACTGTAGCACATGGTGGATGTACATTTTGTTCTGCTGCAGGTAGCGGAGACTTTG

CAGGTAATCGTGCAGATTCAATCGCAGTACAATTTAAAGAAATTAAAGAAAAGATGCATGAGAAATGGCACGAAGGAAAATATAT
TGCTTATTTTCAGGCATTTACAAATACACATGCACCGGTTGAAGTATTAAAAGAAAAATTCGAACCTGTACTTAAAGAACCGGGTGT
TGTGGGATTATCTATTGGTACGCGTCCTGACTGTCTACCAGACGATGTTGTCGAATATTTAGCAGATTTGAATCAACGAACATACTT
ATGGGTTGAATTAGGACTACAAACAATCCATCAGTCAACATCTGATTTAATCAATCGTGCCCATGATATGAAAACTTATTATGATGG
TGTGGCAAAATTACGTAAGTATAATATCAATGTATGTACACACATCATTAATGGCTTACCTGGCGAAGACTATGACATGATGATGGC
TACTGCCAAAGAAGTTGCACAAATGGATGTACAAGGTATTAAAATTCATTTACTTCATTTGTTAAAAGGTACACCGATGGTAAAACA
ATACGATAAAGGTTTATTAACTTTTATGACTCAAGAAGAATACACAAACCTAGTTGTGGATCAATTAGAAGTGATTCCCCCTGAAAT
GATCGTTCACCGAATTACCGGTGATGGTCCAATAGATATCATGGTCGGTCCAATGTGGAGTGTTAATAAATGGGAAGTATTAAATG
GCATCGATGCTGAATTAGCACGTAGAAATTCTTATCAAGGCTTGCGTTACAAGTCTAAGGTGAAGCAATGAAATTAGAACGTATAC
TCCCTTTTTCAAAAACACTTATTAAACAACATATAACACCAGAAAGTATTGTTGTAGACGCAACTTGCGGTAACGGCAATGACACTT
TATTTTTAGCCGAACAAGTACCAGAAGGACATGTTTATGGTTTCGACATTCAAGATTTAGCTTTGGAAAATACACGTGATAAAGTTA
AGGATTTCAATCATGTTTCTTTAATAAAAGATGGACATGAAAATATTGAACATCATATAAATGATACACATAAAGGTCATATTGATG
CAGCCATCTTTAACCTAGGTTATTTGCCTAAAGGTGATAAATCTATCGTGACAAAGCCTGACACGACAATCCAAGCTATTAATTCAT
TGCTATCATTAATGTCAATTGAAGGTATTATTGTACTTGTTATATATCATGGTCATAGCGAAGGACAAATTGAGAAGCATGCATTGC
TTGATTACTTGAGTACTTTGGATCAAAAGCATGCGCAAGTTTTGCAATATCAATTTTTAAACCAACGTAATCATGCTCCATTCATTTG
TGCCATAGAAAAAAATTTCTTAAGCATTTGCTGCGCTATACTATTTATAATTTAGGATTGAGTAGTCAACATTTGAAAAATTAATACC
AACAACAAAAAGAGGTTTTCAACTTAATGAAAACCTCTTTTTATGCTATTAAACTTACACAGCAATAATTGCGTTTAAACTATTTTG
CATTGCTGTTGCTCTACTTGCAATCGCATCACTGATGAAATTCAACAACTCTACTTTCTCTTGTTGTAACTTTTCATTGAAATGAATA
ATAACTGTTCTTTCATCGTCAACAGGACGCTCTTTGTAAATCCATTCTAATTCAACTAAATTATTATACGTTCTCGTACGCTTATACG
GTTTAACTTCAACAAATCTGTCCATTTCTTTAAGCGTCATAGAACCTTTTTGCCATAAAGTTAGTAAAATTAAAATTTCTTCTCTAGA
CATTTTGTATTCGCTTTCAATCTCGCTGAAAATTGAGTTAATGTCACCCAAAAGTGTTTCTAATTGCAAAATCCCAAATACAGTGTCG
TTATTTACTTTTTTCATGCTAAACATCTCCCAATTAATAATCACATATTTAGCTACCATACAAGTATTAACAAACATCCCAACAATCC
CAAAACTTGTATGTGCTAACTTATGCATAAAACTACAAGTGTAAATAAACTTGCTTTCTATTCAATTGTCAAAGTTGAATAAAATTA
AATAAGTATAAAAACCAATAATCAATAGAGTTAATATTTAGAAATAAGATAATAGTACATAAAACAAATCACTCTATTACATATTA
ATTTTATAATTTATATACAAAGAAAATATATCAATATTACCATTAAATTGCAACAATTCTACATATATTTCTTCAAAAAAGATACGC
ATTGAATTTTTATTACATATAACTCTCATGAAAATTGATTAAACTAAATTAATTAGTTAGCCTTAAATTTAACAAATTAAATCAAACC
GAATGAGGCTAAATTGGTTATTTTTCAAGATATGATTTGTATAAATCAAAACATTTCTTACAAATCAAATGTGTATAAAATGTGATA
TACATTCTCAACCTTAAAAAAGTTAAAAAAAAGATGAGCATCTACTGTCTCATCTTTCAGTTCTTTTTTACGGGTCTGTTTTCTAATT
TGAGCACAATCTTCGATTTCTTTATCTAAA

>042-contig_273_RC

AAGTATTGTATAAATAGAGAACAGCAGTAAGATATTTTCTAATTGAAAATTATCTTACTGCTGTTTTTTAGGGATTTATGTCCCAACC
TTTTTAGAATATTAAATTTCTACAATTTCGTCATCTTCAACAATAAAGCCCATTGTATTGACGCTGTTATTTAAGAAAGTCAGAATAT
AACGCATTACTTCATCACGTTCTGGCTCATTGTGAACCTCGTGGTAAAAACCTTGCCAAGCTTTAAAATATAATTCAGGTGTTTGATA
TTTTTCTTTAAACTCATCAATTGCCCTAGTATCAACAATTAAATCCTTCGTTCCATACATTAATAGCGTTGGCATTGGTTGAATGTCA
TGAATATGAGCCATCGTATCTTTCATCGTCTCATTAATTGTATTATACCAATGATACGTTGCTTTTTTTAACATTAAACCATCGTTAAC
TGTTTCTTCAACAATTTCTAAATTACGTGTTAAATCTTTTGGTTCTACACCAACATTAAAACGTGTGTCTTTTGAAATTTTACCTATAT
TTGAAACAAGTTTATCTTTACGATTTTTTCCATTCTTTTGAAGTTCTAGCATAGGAGAAATTAACATCATCCCCTCGATTGGCAATTC
TACTTTTTCAAGTAAATTTAATAAAATCAAACCGCCAAGTCCTACCCCTAATACATAAGTAGGAATTTTATATTCATTAGCTATCTTT
AACCAGTCTAGCAAACTTTCGTGATACGTTTGAAAGTTTTCAATTTGTCCTTTATTAGCTCTTGAAGTTTGACCTTGACCAGGCAAAT
CTCCCATAATCACATGATAGCCATTTCTTCTTAACATCGTAATAACATATGCATATCTTCCCGTATGTTCTAATATATTATGAGCAAT
AACAACGACGCCTTTCGCATCATTTTCAGCTTCCCACTTCCACATTATTATACTGCCCCTTTTTCATTAATCTTCAATAACATAATTAT
AGCAAATTCACTATGTAGATTTCTATTTATAGTATTATTGTTGTCCATATTATTATATATAAATGAAATCAACATCAATAATAGTGTA
ATTATACATAATTATTTTTGATTGTTTTTGATGAAAACGCTTTCTCGAATATTTTTTTCATGCTAAACTTATTGTAAACACAAGGGTTT
GGAGGAGTAGCAATGGCACTATTAAAGAATTTTTTTATCGGATTATCTAATAATAGTTTTTTAAACAACGCAGCAAAAAAAGTGGG
CCCACGTTTGGGCGCCAATAAAGTCGTTGCCGGAAATACAATTCCAGAGTTAATTAATACAATCGAATACTTAAATGACAAGAATA
TCGCTGTTACGGTAGACAATTTAGGGGAATTTGTCGGTACAGTTGAAGAAAGTAATCATGCTAAAGAACAAATTTTAACAATTATG
GACGCGCTTCATCAACATGGCGTAAAGGCACATATGTCTGTTAAATTGAGTCAGTTAGGTGCAGAATTCGACTTAGAATTAGCTTAC
CAAAATTTAAGAGAGATTTTACTTAAAGCAAATACTTACAACAATATGCATATAAATATTGATACTGAAAAATATGCTAGCCTGCA
ACAAATTGTTCAAGTTTTAGATCGCTTAAAAGGCGAATTTAGAAATGTTGGTACTGTAATTCAAGCATATTTATACGATAGCCACGA
ATTAGTTGATAAGTACCAAGATTTACGATTACGTTTGGTTAAAGGTGCATATAAAGAAAACGAATCAATTGCATTTCAATCTAAGGA
AGACGTAGATGCAAATTACATCAAAATAATTGAACAACGTTTGTTAAACGCACGCAATTTCACTTCAATTGCAACACATGACCATCG
CATCATTAATCATGTAAAACAATTTATGAAAGAAAATCACATTGAAAAAGATCGTATGGAATTCCAAATGCTCTATGGTTTTAGATC
AGAGTTAGCAGAAGAAATCGCAAATGAAGGCTATAATTTCACTATTTATGTACCTTATGGCGATGATTGGTTTGCGTATTTTATGAG
AAGATTAGCAGAACGCCCACAAAACCTATCTCTTGCTGTAAAAGAATTTGTGAAACCTGCTGGCTTAAAACGTGTTGGCATAATTGC
AGCTTTAGGAGCTACAGTTATGTTAGGTTTAAGTACAATTAAAAAATTATGCCGTAAATAGAGCAAGACATAAACAATAATTTAGG
AGTCTGGAACAATAATCAATGTTCTAGGCTCCTAAATGTTATATTGGCAGTTCTGAAACATTTATAAATCTTTGATTATGAACTCACA
ACAGTGCTCTATGCTTTTATAGATTTTAATAAATTAGCCATTTCAATTGCACTTACTGCTGCTTCAGCACCTTTATTGCCAGCTTTCGT
ACCTGCTCTTTCCACAGCTTGTTCAATACTTTCAGTCGTTAAAATACCAAATATGACTGGTACATTAGTTTGATCATTCACTTTAGAA
ACACCTTTCGCGACTTCATTACAAACATAATCATAATGAGACGTAGCACCGCGAATTACGCATCCTAATGTAATTACTGCATCATAA
TTTCCTGATGAGGCTAATTTTTTAGCTACTAAAGGAATTTCAAACGCACCTGGCACAAATGCTACATCAATATTGTCTTCATTAACAT
CATGTCGAATCAAAGTATCTTTTGCACCTTCAAGTAATCTTCCAGTGATAAAATCATTAAATCGACTAACTACGATTGCAACTTTCA
AATCTTTTCCAATTAATTTACCTTCAAAATTCATGTTAAAGTCCTCCTATATTAAATGACCCATTTTTATTTTTTTCGTTTCCATATAA
TCATGATTATGTACCGTTTCTGGTACGATAACTTCAATTCTTTCTGCAATATCAATGCCATATTGTTTTAATCCCTCAAATTTACTTGG
ATTATTACTTAATAAATTGATATGTTCGATGTTAAAATATTTTAAAATCTGTGCAGCAATATGATAATCTCGTAAATCTTCATCAAAA
CCTAATGCTAAATTTGCAGTTACTGTATCATATCCTTGCTCAATTAATTCATATGCGCGTAATTTGTTTAACAATCCTATGCCACGAC
CTTCTTGAGGTAGATAAATAATCATGCCACCATGTTCATTGATATACTTCATAGACGATTCAAGTTGAGCACCACAATCACAACGTT
GACTATGGAAAATATCGCCTGTAAGGCACGCAGAATGTAAGCGTACATTTTCATGTTGTCGAATTGCACCTTTTGTCAGTACAACTA
TCTCTTCATCTGTGTATGTCGCTTTAAAACCATACATATCAAATGTTCCGAAATCTGTAGGCATTTTCACTTTTGCCTTAAATTCAATT
TCTGGTTCTAATTTTTTACGATATTCAATTAAATCATCAATCGTAATCATCTTTAATTGATGTTTTTCTTTAAACTTTTGTAAATCTTGT

CCTTTCGCCATCGTGCCGTCATCATTCATAATCTCACAAATGACACCAGCGGGCTTGGCACCAGTAAGTTTAGCTAAATCAACAGCC
GCTTCTGTGTGTCCATTTCTAGCTAATACGCCTTTATCTTGTGCTACTAATGGAAATAAATGACCAGGACGATTAAAATCTTTAGCTT
CACTACTAGGATCAATGAGCTTTTTGGCAGTCAATGTACGTTCATAAGCACTAATTCCTGTTGTTGTATCTACATGATCAATACTCAC
TGTAAATTGCGTACCAAAGATGTCGGAGTTATCATCAACCATTTGTACCAAATCCAAACGTTGTGCAATATCTTTAGACACTGGTGC
GCATATTAATCCCCTTGCTTCTTTCGCCATAAAATTAATGGTATTATCGTTCATCCATTCAGTGACCGCTACTAAATCACCTTCATTTT
CACGATTCTCATCATCTACTACAATAATTGTTTCTCCATTTTTTAAAGCCATTAAAGCACTGTCAATATTATCGAATTGCATGCTACC
CCTCCTAAAAACCAAATGCTCTTAATTTATCTACAGATAATTGGTCTTTATCTTTATTTAAAATATTTTCAACATATTTAAACAAAAC
GTCTGTTTCTAAATGTACTTTATCTCCTAATTTTTTGGATGATAAAATCGTTGAACGCCTCGTTTCTGGAATAAGATGAATGTCAAAA
CTGTTATCATGCTTATCAAATACCGTTAGACTTACACCATCCACAGTAATAGACCCTTGCTTAACTAACTGATTATTAATATGTTGGC
TACATTGAATCGTAATAATTTTGGCATTGGCTGTTTCATTTATTTTTGAAACTGTTCCTAGTTCATCTACATGACCGAGGACAAAATG
TCCACCAAACCTACCGTTACCACCCATGGCACGCTCTAAATTTACTTCTGATTGTCGCTTAACATCTGCTAAATAGGTTTTATTTTCA
GTGCCTTTAATTACTTGAACAGTAAAAGATGTCTGATTAAAATCAATCACTGTTAAACATGCACCATTAACACTGATGGAATCACCA
ATATGCATATCTGCCGTAATCTTATGTGCTTCGATTTCAATCGTCCTGACTGATTGACGAATTTGAACACTTTTAACGACACCTATTT
CTTCAACGATGCCAGTAAACATGCATCATCACTTCTTTCGTAAAGTTAATTTAACATTTTGATTTAATAACTCGGAATGAACAATTTC
AAATTGGTTCGCATCTGGTATCTCAATCACATCATTTGTTTGATAAAATTGATAATTTCCAGATCCGCCAATTAATTTCGGGGCATAA
TAGAGAATAAATTCATCTATATAATTAGATTGGAGAAATTCTGAAGTAGTGGTTGGACCTGCCTCGACTAGCAAAGTTCCAACTCCT
CTTTTATATAAATTGTGAAGAATTGTTGTTAAATCGCAAGACTTCAAGTAAATAATTTCAATATGTGTTTGATTGGTTGTTAAATTTG
GATTTTCAGTATATATCCAAATTGGTGTTGATTCATCTTGATAAATTTGCTGATTAAAATGAATATTCCCAGACTTAGACAATATTAC
TTTTATAGGGTTTTTTCCATCTTGAATACGTGTAGTATATTGTGGATCATCTAATTCAACTGTACGTCTTCCAGTTAACACTGCGTCGT
GTCGATTTCTTAACTTATAGACATCTTGTTTAACCTCTTTGTTAGTAATCCATTGACTTTGTCCATTATCATTCGCTTGTTTACCATCT
AAACTTGCAGATACTTTCACTGTAATTTGTGGCAGTTGCTTTGCTTTTGCTTTAAAAAAGGCTTGGTATAATTGTGATGCCCGTTCAT
CATCAACGCATTCAACCTCAATACCGTGAGCCCGTAACGTCTCATCACCATGTGTGTCTAACGAATTGTCTTTTGTTGCGTATACTAC
TTTTGCTATCTTACAATCAATTATTTTGTTAACACAGGGTGGTGTTGAACCAAAATGACTACATGGCTCTAACGTAATATAAATCGTC
GCACCTTCAGCATTTTGTTGTGCCATATCAAGTGCTTGAACTTCCGCATGCTTGTCACCTTTTCTCAAGTGTGCACCAATACCAACAA
TCCTACCTTCTTTAACTACAACAGCGCCAACGGGTGGATTAACACCTGTTTGACCTTGTACCATATTTGCAAGTTGAATCGCATAATC
CATAAATTGACTCAAATGATCACCTCTATAAACAAAAATCCTCACATCATGAATTAAGATGCAAGGAGAAAAATTTATCGTTAAAT
AAGCCTATTTGTACACATTTTTACAAATACGCTACATTATCTTTGTCGATAATTAACATTCTTTCTCCCATCCAGACTTTAACTGTCGG
CTCTAGAATCTCACTAGATCAGCCACTAATATGAAACATATTAGCAGGTCGCAGGCTTTATTTACTGCCGGTTGGGAATTTCACCCT
GCCCCGAAAGAATTATATATGAAATTGTTATAGATTATTTGAGTACGTAGTATGTCAACTACATTTAAAATGATACTATATGTTTTCT
GAAAAAACAATTAATGACGGTTTTAATTTAATATAATCTGAGTACTATAGGCATCTCATTGATATGATTCTTACTAACAGACATTAA
AATCAAACCTTCAATTCGTCTCTATAGAGCGTTCTCTTTATTATCTTCTAGTTACAAATTATTGATTGTCACTGCGCTGTTGTTGCTCA
TTCGATTCTAAAGCATCATATAATTGAGATACTGTATGCGCAACTTGTTCTACTATCATTTTCACACCATTTCGTAGTTTATTAACAC
CGTTTGTCATTTGACCTATCGCAATCATATTTGTTAATGTTCCAAACCTTGGACTAATAACTTGATTGGTTTCCGGTATGATTTGTATG
CCTCCCATTGGGTGTGCTTGTACAATTTGTCTATTTTCAAGATTTTTAATTAATTGATCATCTTGATCCAATTCATTTAAATGACTTTT
TGCACCTGTCGCGTTAATGACAACATTATATATGTCTACTGATTCTTGGTTTTTGTATGAAAAATAATACAACTTGCCATCATGTTTC
ACATCTTCTAAATCTTTTTTCAAAATTAAAGACTTATTTTCGATTAATTCAATAATTAGTTCAGCAGTTCTTGGAGGCATTGGATTTG
AATTTAATTGAATCATCTTTGAGTATTTTTGATTAAATTGATGTTGGTCTTCAATACTTAAGCTATTCCATATCCAATTTAAATTCTCT
TTCAAATGTTCAATCATACTTTGGAAAATGCCCATTTCTGTTGGACGCGCTAAATCATACTTCAAATCTGCAATATGATTTCCTGTAC
GTCTATGTACTAATTTTTTAAAATCAATGTCATATTCAGCACATTCTTTTAAAAATAAAGAAACTAAAGTATCAAGCGGTGCATTGC
CGAAATGATGTTTTTTAATGTCATTTAATTTGTCTTTAGTTAAGTACTTGAATGTCACGTCTATCATTGTACCTCTTACACTTGGTAAA
TGAGCAGAACGACTCGTCATAGTAATTGGTAATTTCGGATGATGAGCAGCAACATAACGGACAACATCTAAACTGGCAAGGCCTGT
ACCAATAATCGCAATATCGTCCAGTTCATTTACTTCGTCTAACGTATTATATGTTGGATAAGGCGTAGCGATATATCCTTTTTTACCC
TTTAAGTTATATGGATCATGGTAGGCAAATGTACCACATGTTAAAAATACATAATCGTACGCTTGCCATGATTGTTCTGAATTTGTA
GTACATATGTAATAAGTTAAATTCGTTTCATCGATATTAGAATTTGTATAAATCTCTTGAACTTTATTATAATTAGTTGATATATTTG
GATATTTTTTCGTGAACATAGATAAATAAGATTTCATATAATGTCCGAATACAAATCTAGGTAAATATGCAGGTTCATCAAAATTAA
AATCAGTTTGTTGTTTATACCACTTCCAAAATTCAGTCTCATCATCTAAATTTAAACTCATCTTTTTCGAAGGCATATTAATTAGCAG
CTCAGAACTATCATTTTGAAATGGTACGCCCTGTCCCATATTTACTTTATCATCGTATAAATCTATATCTAATTGATTAAACTTCGGG
TGCTTAACTAACTCTCTCAATACACTTACACCAGCAGTTCCCATGCCTATTATTGCTACACGCATATATCATCCATCCTTTTGTAATTC
AAAATATTATATTTAAACTATTACCTATAATTATAGCAATTTGGTATTTTTAATAAAATTTATTAAGGCAAGCTATTATTTCACTTTC
ACAACCATTAAACCATTTAAAACTTTTCATTTAATATCAACCTTAGTGTTTGTGTTAAAATATTTTTAACGAGGTGAATTAAATTGAA
ATTTATAGCAAAGATATTAATCTCATTAGTAATTCTATTTATGATTTTTAGTCGTAAAAACAAAGCTTCTTAATCAATACCCTTGATT
ACAAAATAAATTCAACTAAGGTGATTGGCCATACACTCAAGATATGGCTAATCACCTTTTTCAATGCAAAACAAAGAGTATATTTTG
CAAAATCAATATCTATAATTTATCTTTAATACCCTTTTAATACTAAAACCAAATTTAAATTACATAGTCATATCGTGTAATATACCAA
AGGAACATAGACATTCATCTATATAGATGATAATCTATTTAACGTCTAGGAGGTATGTTATGACGTATAAAGAACTAGCAACATTTT
TAAAAGTTTTATCAGATTCAAGCAGATTAGAAATACTAGATTTACTTTCTTGTGGAGAGTTATGCGCTTGTGATTTGTTAGCACATTT
TCAATTCTCTCAACCTACACTTAGCTATCATATGAAAGCATTAGTAAAATCCAACTTAGTTACGACACGAAAAATCGGAAATAAACA
TTTATACCAGCTCAATCATAATATTTTTGAGTCCGTAATTAATAACTTGTCTAAGGTTCATACCTCTAATCAACGATGTATTTGTCAT
AACCTTAAGACTGGTGAATGCTAATGATGACAACTTTAGCGACACTCATTTTTCTAGTAACTTTATTATTTGTATTATGGCAACCTAA
AGGCCTAGATATTGGCATTACCGCATTAACTGGTGCCTTTATTGCTGTTATTACTGGTGTTGTAAGTTTTTCCGATGTTTTAGAAGTA
ACAGGTATTGTTTGGAATGCTACTTTGACTTTTGTCTCAGTCATTCTTATTTCATTAATATTAGATAAAGTTGGATTATTCGAATGGTC
AGCTATTCACATGCTTCATGCTTCAAAAGGCAATGGTTTAAAAATGTTCGTTTATATCATATTATTGGGTGCCCTTGTTGCTGCATTT
TTCGCAAATGATGGCGCAGCGTTAATCTTAACGCCTATTGTATTAGCAATGGTTAAAAATATTGGATTTAATAAGCGCGCAATATTA
CCATTTATTATTGCAAGTGGTTTTATAGCTGACACAACTTCTTTACCTTTAATCGTGAGCAATCTAGTGAATATTATATCTGCTGATT
ATTTTCATGTAGGATTCGTTCGATATTTTAGTAGAATGATTATACCTAATTTATTCTCACTTTTAGCAAGTATTATAGTATTGTGGTTA
TATTTTAGAAAAGCGATACCTAAAACGTTTGATGATAATAATATAAAGCATCCTAAAGATGCCATTAATGATTTAAAGCTATTTAAA
ATTTCATGGATTGTTCTAGTTATATTACTTTTCGGCTATCTAATCAGTGAATTTACTAAAATTCCGGTATCAATTTTCACTGGAATCAT
TGCTTTTATTTTTCTAATGTTGGCTCGTAAATCAAATGCTGTAAATATTAAGCAAGTCATTAAGGGCGCACCTTGGAATATAGTATTA
TTTTCAATTGGTATGTATATCGTCGTATTCGGCTTAAGAAATGCTGGCATTACTTTAATATTGGCTAAAATATTAGAATATATTTCCA
ATTACGGTCTATTTAGCACTATTTTGGGAATGGGCTTCATTTCAGCGTTTTTATCATCAATAATGAATAATATGCCTACAGTTTTAAT
AGATGCGATTGCTATTGGTCAATCAAATGTCCATGGCATGTTAAAAGAAGGCCTAATTTATGCGAATGTTATCGGTTCTGATTTAGG
TCCAAAAATTACACCGATAGGCTCTTTAGCTACATTACTGTGGTTACACGTCTTAACACAAAAAGATGTTAAGATTTCTTGGGGCAC

ATACTTTAAAACTGGTATCATCATTACAATTCCAGTACTATTTATAACCCTCATAGGGTTGTATCTAACACTTATCATATTTTAATTA
ATTAGGAGATTGTTATGACTAAAAAAACAATTTATTTATATGTACAGGCAACTCATGTCGAAGTCAAATGGCTGAAGGTTGGGCTA
AACAAATCTTAGCAGATGATTGGAATGTATATTCTGCTGGTATCGAAACACACGGTGTTAATCCCAAAGCGATAGAAGCTATGAAA
GAAGTAGGCATTGATATATCAAATCATACATCAGATTTAATCGATAATAATATTATTAAAAATTCAAATTTAGTTGTTACATTATGT
AGTGATGCAGACGTAAATTGCCCTTCTTTACCAACAAATGTTAAGAAAGAACATTGGGGATTTGATGATCCTGCAGGCAAGCCTTG
GTCAGAATTCCAACGTGTAAGAGATGAAATTAAAATCGCAATTGAAAATTTCAAATCACGATGAGAGTGGGACAGAAATGATAAA
AAAACACTAATGATTTATTATGTAGTGGTTCTTTATCATTAGCTATAACTAATGTGTACTTAAAAATAGGAATACATGAGTAAAACT
CATGCATAAGAAATCCTAATTTCTAAAGAAAAAGTATTTCTTTATCGATGTCCTGCCCCACTTGCATTGGTGGGATTATCAATCAAT
GTGTTTTCTCTAATGCAAAAAGACGCTTTCTATCAAAACAAGATAGAAAGCGTCTTTTTAGGGTTCTTCATCTTTTATGGTGGG

>043-contig_278

CACATTTGGTGGAGAACCCTTTTTAGTATGTCCGAGTCATAAAAACAACTCGTAGCTTATCAAATTACTTATTCAAATGTTTACTGTC
ATCTTTATACACAAAGTATTTGAAGTATTTTCCTTCAGTCTTCATGTTCTTATAAAAGTCAGCGATAATTGTTGCATTACTTTCTGCCC
ACTTAATATCTGTAGCATATTGATGTTCTCCTGGATTTTTTGGATTCCATCTCATACTATACAATGTATTTTGATCTGTGCTTGATAAG
AAGTGCTTATGAATGAAATCAGCACCGCCTGAAATAGCTTTTTCAGGTGTATCCCAACCATGCTTTTTAGCATATTCTGCACCTGTTT
TAATTGGGTCTTTATCAAGGGCTCCTACTCCATAGAAATTGTAGTATTTTTTGCCATCAATTTCGACTCCATTAGCTAATTCACTTTTA
ACTGCGCCAGTTTCTAATAATGCATGTGAAATTAAATAAACTTCGTTAACGTGCTTATCTTTAGCAGCTTTTAAGAAATCATCCGTAT
GTTTCAATAACGTTGGTCTATCTACTAACATACGTTTAATTCTATTTTTATCAATCCCTTGATACTTTGATAAATCTAAAAATTGATAT
TTTTGCTTTTCATTATCGATAAAAGTACCGCTATCCATTGCACTTTTAATTTCAGTTGCAGATGCATCTCTCCATGCATCATTCTTTTT
ATTTGATACCTGTTGACTCGTATAATTATTTATTTGTTTCTTTGCTGCATCGTTTAATGTAACATTTAACTTTTCAATCTTAATGTCGG
ATTTAACATGTTTGAAAAATATCTGATCGGATATCATTGAGAAAAATAAAAATGAGACAACAGCAAATATGACAACAAGTCCTATT
ATTCCAAAAATAGAACCTTTCTTGTGTTTATTCATATCCACACCTCTTAGGTCATTGTTGTTATACATTCAGTTTGACTACGATAATA
AAGTTTAACATTATTAAAACCGTTTAACAATTTTATCTATGCATTGTTACATATTCATAAATAAACAGTAATGTAAATGAAACGTTTT
AAACCATTTATCAACATTACTTTACGTTGATCTACGAATCATAAATGTTTTATCTTTTTGTATAACCTTTGAAAACCTTTATATCAATA
ATCTTATTATTCACGTATTAAAAAACAAAAAATTCTTTTGTTCGTTTACTTAGTTTCTTTTTTATAATCCATCATAATAAAACTTGCTG
CATTTGATCTATTTTTATAATAGTACTGATTTAAATCAATCGTGCCTTCAATTTGACCATCACGATACATCATTTCATTAGTGAAGTT
ATTTATCATCACGAAATCAGCGCGATCTCTTATAACATCTAAATCGTCGTTTCGTGCAAAAAAATGTTCTAAATTTAAATGTGCGTA
ATCCATTGTTACCTCCCGATTACAAATTTTGTTCTATCTACTATTTAGCAAAGTAATAATACATCATATCACATTAAATTAATTACTT
ATGTCTTATATTTAACACTTAATTTAGCGATTACGCAAATGAATGATTTTTCTAAATATTTTATTCTAACTTTATCCGGAAATATTTGT
TATACGTATAACTTGGAGAATGACTACTTGAAATTTAATGACGTATACAACAAACACCACAAAATCATACACCATCTTTTAAAAAA
ATATAATATTAGCTATAATTATGATGAGTATTATCAACTACTCTTGATAAAAATGTGGCAATTGAGTCAGATATATAAACCCTCAAG
CAAGCAATCTTTATCCTCTTTTTTATTCACTCGATTACATTATTACCTTATCGATTTATTCAGACAACAAAATCAATTAAAAGATGTC
ATTTTATGTGAGAATAATTCACCAACATTAACTGAACAACCAACATACTTTAATGAACATGACCTTCGATTACAAGATATCTTCAAG
CTTTTAAATCATAGAGAAAGACTATGGCTCAAACTATACCTTGAAGGATACAAGCAATTTGAAATTGCTGAAATCATGTCATTATCG
CTTTCAACGATTAAATTAATTAAGATGTCCGTTAAGCGTAAATGCCAACATAATTTTAATTAGTTTATTGTGAAATTTCGGTCGATGT
GTTAATGATGTTGAAACACCCTAACATCTTCATATAAATTTATTTTAACCATTAAATTAACAACTTATTATACGAGGAGTACTATATT
TGCAAGACAATTCTACTAAATATCTACTTTATATCCAAACTGCTACTTCAAACCATCTCGAAACAAATTGTGTCTTTTTACATTGCGA
TTATATTCTTAAAGTTCCAATTAACAAACTCGTTTCATATTATGCGAAATTGCATTTGTCATCACAAAGTGTGCTAATTGAGACTGCA
AAAAACATACTAAATATTAATAAACTGGTTCCTATTTACATCAACCCAAAAACTATACTTTTTCCGTTAAAACATAAACGTGCACCG
ATACAAATTTATATCAATGCACATTATATTGTTGGTATGACTGCCATAGAGAATTCAACATTAATACATTTTCAAGAAGGCATTCAG
CTAGAAGTTGATGAGCCATTTTCTCTAGTTTCAAAAAAATGTCATGAAAGTTTAGCTTTGAAGCACTTCATTGAAAATACTATTTCG
AATTAAATTTAATTTCTAAAAGTTTCTTAGTAAAGTATGATGTCACAATTTTTATAAAATATATGCTAACAATCATTAATAAAATCGC
TCCAATAATTGTTAAAATAACAACAGTTGTTGATAAATCTACCTTAGGACTAACGAAAAATTGTACGCCAGATAGACTTGTAATTAG
CCCAAATAAAAACAAGCCTAATAAAGCAACATAAGCAAACATCATCACTACTAATGTAACAAACATACTATAACTTTTTGTTTCTTT
ATTTCGAAATACATTTTGTCTTAAGTAAGCATTAGCAAAGGTAACAGGCTTATTTAAGTTAACGCTGTCATCATCAGATATAGAAGA
TAATCGTTGATCTAATGCTTGTTTCTCCTTCTTGTTCAAAAACCATAATTGCTTTTAACTTTTTTATCAAATGATGCTTTATTCATAA
TTAAGGATTTCTCTCCTTTATAATATTGACGTAATTTTAAACATTAGCGTCAATTGAAAGTGTATTTTATATATTTTACAGAAATATA
TGTATCAAATCTACAAAAATCCATATTACAATTTCATTTATGGTGATTTGACGCTAAAATACAGTCTGCTAATTTTTCAAACTAATGC
TTTTTAGGTAGGTACAATGATAAATTTTAATCGTAAATATGTTTCATATATTATGAATCTAGCCTTAATCATATTGCCTTCAAAATAA
TTTTAAAAAATGAAAAGAAAGGATTTAGCATGCAAAAATTCAAAGACTTTTTTTACGATGATTTATCGGTTACACGAGGAAATTATT
TTTTAACTTTAATGGCAGCATTTTTTATTACTATCATTTTATTTATCGGCATAGTTGTCAGTGAAGTACATTTACTTTATAGCATGCTA
ATTGTATTAGTAGGTTTAATTCTATTGAGGCTATTCAAAATCAATTTAATCTCTTTTAAAAAATTAACATTGTCTCAAGTTATTTATAT
TATAGGCGGTGCACTATTAATTTATGGGTTAGATAATCTTTATTTATATTTCATGACGTACCGGCAAATGAACAACAATTAGAGCA
AGAAATACGAAATACACCATTCTATATTTCTATTTCACTGTTACCATCATCCCCGCTATTGTGGAAGAAATTGTTTTTCGCGGTATG
ATAATAAGGGTTATCTTCAGAAAACACTTGTTTTTAGGGTTAATTGTGTCTAGTTTAGTTTTTGCATCATTACACGAATCTGACACTT
GGATTGGTTATTTACCTTACTTATATTCTGGTTTGATTTTTGGTCTAATTTATATAAAAACAAAACGATTAGAAGTGGTAATATTCAT
GCACTTCTTAAATAACTTGTTAGCTCTGCTCTTTATAATATGGGGATAATTTTGAGATAACCTTTCATATTATTTTCAAATTATCTATC
TGATATTTATTTTATTGAACTTGAGTCACCACTTAAACTCAAAAATACGTGTCATCAAGTGCAAAATAATTTAGAACTAATACAAAC
ATAAAAACTTTTGCTTTACGCTTAAAATCAAAAGTCACATACCTAGATATAAGTACAACTTGTATATTTTGTACTCTAATAGGTATGT
GGCTTAAATAATATTTAATTCTTGAGTTGCATATAGTAATTGTTTTTATTCATTTTCTAAAATATGCGAGACTAATTTCCTTTTCTACT
CCTGTTGAATCAATTTAGACTTTTTTATAACTCAAGCTTTTACTTAGCTGAATTTTGACTTCTTCTTCATTAATAACTATTATGGAAAA
CCCCCATAAGCACAATGCCTATGAGGGTTTAAATATATTTATATATTATAGAATAGAAAGACCTGAAGATTGAATATCTTTCGCAAA
GCCTTTAACTGTATCTACTGATAATTCGTTAATATCGCGACCTAAGTTTGTATTCACTTTTTTCACAACATCTGCAGGGCATGTAATA
ATATCTGCACCAATTTCATCAGCTTGAATCACATTGAATAATTCGCGGCAACTTGCCCATAATAATTTAACGCCGTCTTTACTATGCG
TAACTTTTACAGCCTCTTTCATTAATGGTAATGGATCTACGCCTGTATCTGCAATACGTCCTGCAAATACTGAAACATATGTTGGCAC
ACCTTCAGTTACTGCTTCAGTTATTTCTTTAACTTGTTCAATTGTGTAAACAGCCGTAACGTTTAATCTCACATTGTCAGCTGAAAGC
TTTTTAATTAAAGGAATCGTTGATTCACCTTTTGTATTTACAATAGGAATTTTAACAAATACATTTTCGCCATATTGTTTTAGAATTGC
TGCTTCTTTTTCCATAGTTTCTAAATCGTCTGCAAATACTTCAAATGAAATTGAAGCATCTGGAATTTCTTTCACAGCTTCTTCAGCA
AAAGCTTTGTAATCTGTTACGCCCCGCTTTCGCCATTAAACTAGGATTTGTTGTAAAACCATCCACTTGTTTGTTTTTATAAGCTGCTTT
CATTTCTTCAATATCTGCACCGTCCGCAAATACTTCTACATTTAGTTTAGCCATATAATATAGCCTCCTTGATTCTTATTAAAATTTTA

ACAACATCTGCATGTCTTTTTCTTACAACCATTTGTAAAAAATGATTTTTATTTCTTTGTTATATAAAACATTTATATCACGCTTTTAT
AATATATAGCTAATTTTTTGCCTATATTTTTTAAAAATGACTTTAGCAAACATTGTGTCTACAAGTTAATAAAACTATTGTTTTATGA
TTGTTTATGATACGATATGTAAATGATAACCATCCAGGAGGTTTAGCATGTCTCGTTCAAAAAAATACTTTTACTTATCTAGCTTAAT
GATTATTTTAAGCTTTTTCTTTAATACAAATAACGTTTTCCTAAGTGGACTTTTTAATTCTTTTATTAAATTAATACTTTTCTGCAGTG
TTATTAACTCAATTGTACTAATTTTGTCTATAATTTTTGCAGATCGTTCAATTAAATCACTAAAGCCTGATGCAGATTGGATTAGAAT
TGCGAGTAAAAGTTTGCCTTGGATTATTCTAATTGTTATTTTAGTACATATCTTTTCAATTGTTCGTACATTCGGTTTTATTTAAAAAA
GATAAAATGTCAATGTAGCGTTAATACAAAACAATACAATGTATCATGCTATAATGAGTAAAACAATTTGATGACGTTGTTGCGTAT
AAAAAATATTAGATTTTCGAAATCATAACTATGCATCTAATCGCTATAGTTATACAACAAGATATAACATATAATGAGGTTTGATAA
TGCATCGACAATTTTTGTCGTCGTGTTGCCAAAACCTCTTTTTTAAATTCAAACTACTTCTTTTCGAGGTGAACCAAATGCAATATGT
ATATATTTTTATCGGTGGTGCTTTAGGCGCTTTATTACGTTACCTCATTTCTTTTCTGAATACTGACGGAGGTTTTCCAATCGGAACAC
TGATAGCCAATTTGACTGGTGCCTTTGTAATGGGATTGCTAACAGCCTTAACAATTGCATTTTTTTCAAACCATCCGACCCTAAAAA
AAGCTATTACGACTGGTTTTCTTGGTGCTTTAACGACTTTTTCAACATTTCAATTAGAATTAATACATATGTTTGATCATCAACAATT
TATAACTTTACTACTATATACTGTAACAAGTTATGTCTTTGGTATTTTGTTATGTTACGTCGGTATAAAACTAGGTGGTGGTTTATCA
TGATATCAATCATTTTAGTCATGATTGGCGGCGGTTTCGGCGCAATTGCTAGAAGTGCCATTACTGATTATTTTAATCATAAATTTAC
TTCAAAGTTACCTATCGCAACATTGATAGTAAATCTAGTTGGTAGTTTTTTAATTGGATTAACTATAGGCTTATCAATTTCAATCTCA
TGGTTCCCTGCGTTCTTTGTTACCGGTTTTTTAGGTGGCTTAACAACTTTCTCAACGTTAGCTAAAGAACTTACACTAATGATGACGC
CAAAATTTAATATTAACCTTTTTCTCAATTATTCACTTTTACAATTCATCATTGGATTTATAGCTTGTTATATTGGCTATCATATTTAA
AAATAAAATGCTTCATTCAGCAAATAGGTAAATTACGACACCTTCCTGAACGAAGCATTTTTTAATTTTCATGCAAATTTTTTAACAC
CATATAATGCCTACCAAAATAAAACAATCTTTGTTGCCGTTTAAATAATGTGAATGTCAATACATTCTCCAAACTAGTCGAAAATAA
AGGGAGTGGGACAGAAATGATATTTTCTCAAAATTTATTTCGTCGTCCCACCCCGGCAAGGTTGACTAGAATTGAAAAAAGCTTGTT

>044-contig_322_RC

AAAAAGCTTGTTACAAGCGCATTTTCGTTCAGTCAACTACTGCCAATATAACTTCGTAGAGCATAGAACATTGATTTATGTCCCAGC
CTGTGACAATCCATCTAACCTTTTCATCAGTTGAGCGATTTTAATCTATCCTTCAAAAGTTTTTGGATCAGGTCCAATTCTCTTATCTT
GATTTAAACCATCAATTCGCGTCATTTGTTCATCTGATAATTCGAAATCAAATATTTGGAAGTTTTCAGAGATTCTGTTTGGTGTTAC
CGATTTAGGGATTATAACCACACCATGCTGCACATTCCATCTTAAAACAACTTGGGCAGGTGACTTTCCTAATTCTTGAGCAATGTC
TTTAATTGTCTCATCATTTAAAATTTGTGCATTCATCAATGGTGACCAAGATTCCATCACGATATGTTGTGCTGCCAAATATAATTTC
AATTTATGTTGCGTTAAATATGGATGATATTCAACTTGATTAATTACAGGTTTAATTGACACTTGTGCCAACAAAGCTTCCAAATGTT
CAGGTTCAAAATTGCTGACACCTATATTTTTAACTTTATTATTTTTATATAAATCTTCCATACCTTTCCATGTATCAACCATTACGGCT
TCGTTCGTACCTGGCCAATGTACTAGATACAAATCTAAGTATTTTAAACCTAATCTAGATAAACTAGCTTCGTAAGCAGCTGCTACA
TTTTCACGACCGAAATCCTCAAAATATAATTTTGAAGTAATAAATAAGTCTTCTCTAGCAATACCAGTTGACTCCAATCCGGCACGA
ATGCCAGCACCTACTTGTTCTTCATTCCCATAAACTTTTGCGGTATCAATACTACGATATCCTTGTTCAATGGCATACTTAACACTTT
CCATGCAATTTTCATCATTTTCCACACGAAATGTCCCTAAACCAATTTGTGGCATCGTGTTTCCATTATAAAATGTTTTAACCTCCAT
AAATATCGCCTCACCTTTTTGATGTATTATACCCTGTTATCATAACAAATCTGAGTTGAATACATGAGAAAAAACACTTAGAGAAAT
CAACCACTAAAATTCTAGTAATATCTCTCAAACATTAATCAAAATGTAAAAGCAATTCTGTTTAAATTATGACAAACTAAAAAAGCC
GAAGTCACAACATATAGTCATCACTTCAGCCTAACATTTAGTTGAATGATTCAATTTTATCCATCATTTGTTGTAAGTCTTCCACGTT
GTATTGAATACGACCATGGAATACAAATTTGTTAAAGAACTCGTCTAATTGTTCAGCACCGACAAGCACTTTGACAGCACTATTTTG
ATTATAATTTGAAATCGTTACATCGCCTTCATTTTTAGGATTAAAGTATAAAATTGAAGTTGGCGTATATTTGGCACCTAATTCTTTT
TGTAAGTCTTCAGCCAATTGTTTAATCGCCTCAATTTGATCTGAATAATTTACAAAAGATAATGAACGTTTGTCATCATTTTGATCCA
TCACAATAGTTTGTGGTCTAGATTTATCTAAATCCAATGTATCAAATACTTGTTCCATTGGTGGTAAATCTTTAAATTGACCGCCACT
AATACCATTATAAACATGACCTTTTAACAATTGAGAATCAATAATATAAAGACCAGTTCTTGTTAATACTAAATGACTAATTCGTTC
AATATTATTAAAGCCATCCTTTGGTAAAAAGATATTTGCCATAATGTGCATATCTTCTGGTCGAATTCGTTTTTCTTTAACTAATCTTT
CACGAATACCAATTAATCTCATGTCCGTTACATATTCACTATGATTTTTCGAGAACAATTTTAATGCGTCAATCTCACGATCTTTTGT
ACTAACCATGTGATTATAATCTTCTTGTTGTTTTGTAATTGTCTTTTTATTTTGAATACGCTCTTTCTCTAAAGCTTCTTCATGAGACTT
TTTAATGTTTTGTTCTTGTTGTTCATACTTTTCTTCTGTTTGTCGCTTAACTTTTTTCTTACTACCTAAGGCAACTAAGAAAAGGACAA
AAAAGATTAATGCAATGACTACTGCAATAATGAGTCCAATGACTACCGGTGAAGATAAATCCATCACAACAACGCTCCTTTTTAAT
ATATGAATAACTTTAATTATAATAGAAAAGCTAAAGATTTTCGATACATATTATCATTTATATATCGAAAATCTTTTATTTAGCTATA
TTCAATTCATCTTATTATTTTACTGCGTCTTTTAATTCTTCCACTTTGTCTAATTTTTCCCATGGGAATAAGACATCTGTACGTCCAAA
ATGACCATAAGCAGCAGTTTGTTTGTAAATTGGTTGTTTCAAATCAAGCATTTTAATAATACCAGCAGGTCTTAGGTCAAAGTGTTTT
CTAACTGCTTCAACAAGTTGCCCTTCAGAAACTTTACCTGTTCCAAATGTATCAATTGCAATTGACACTGGTTCTGCAACACCAATCG
CATATGCCAATTGTACTTCACATTGATCTGCTAAACCTGCTGCAACAATATTTTTAGCCACATAACGTGCAGCGTATGCAGCTGAAC
GGTCTACTTTTGTAGGATCCTTACCACTGAAGCATCCGCCACCATGACGTGCATAGCCACCGTACGTATCAACAATGATTTTACGTC
CTGTTAATCCTGCATCACCTTGAGGTCCACCGATTACAAAGCGTCCTGTAGGATTGATGTAGAATTTAGTTTGTTCATTAATCAAGTT
TTCTGGAACAGTTGGATAAATGACATGTGCTTTAATGTCTTCTTGAATTTGTTCAAGTGTCACATCCTCAGCATGTTGTGTTGATACG
ACAATCGTATCAATACGTACTGGGTTATCATTTTCATCATATTCAACAGTGACCTGAACTTTACCGTCTGGTCGTAAATAATTTAACG
TACCATCTTTACGCACATCTGATAAACGTTTTGCCAATTGATGTGATAAATAAATTGCTAGAGGCATATACGTCTCTGTTTCATTCGT
TGCGTAACCAAACATTAAACCTTGGTCACCTGCACCTGTTGCTTCAATTTCTTCTTCGCTATCTTTATCACGATACTCTAATGCATTAT
CCACGCCTTGTGCAATGTCAGGTGATTGTTCATCAATCGCAGTTAAAATTGCCATTGTTTCATAATCATAACCATATTTTGCTCTTGT
GTATCCAATTTCTTTAATTGTTTCTCTAACAACTTTCGGAATATCAACATATGTTGTTGTAGAAATTTCGCCGGCGATCAATGCCATA
CCTGTTGTAACAGTTGTTTCACAAGCTACACGTGCATTTGGGTCGTCTTTTAAAATAGCATCTAATATTGCATCTGACACTTGGTCAG
CGATTTTATCTGGGTGTCCTTCTGTAACAGACTCTGAAGTAAATAATCGTTTGTTATTTAACATAGTTTGCTCCTTTAAATTTATATTA
CGAAAATTCTCTCTCTGTGAGCTAAATAAAAAAGACCTTCTAACTATTAATATAGAGAGAAGGCCTAATACGTCCATTCGCTCTTAT
CGTTCAGACCTATTTGTCTGCAAACGGTTTGGCACCTTTCTTTTATAAAAAAAGAGGTTGCTGGGTTTCATTGGGTCCATGTCCCTCC
ACCACTCAGGATAAGAGAATCCGTTAAAAATAATAGTACCTAATTAATGAATTAATGTCAATTTTTCACAAATAAATTTACAGTAAA
ATATTGTAGATTAATTATGTTAATGTGTTATACTAATTAAATGTAAAGGCTTACATTTAAATTATCGCTTTGGAGGGATTTAGGATGT
CAGTAGACACATACAATGAAACAACTAAAATTGACAAATTACTGAAAAAACCAACGTCACATTTTCAACTTTCGACGACACAACTT
TATAATAAAATCTTAGACAATAACGAAGGGGTATTAACAGAACTTGGTGCTGTTAATGCAAGTACTGGAAAATATACTGGTCGTTC
GCCTAAAGACAAATTTTTTGTCTCTGAACCTTCATATAGAGATAACATTGATTGGGGAGAAATTAATCAACCTATCGATGAAGAAAC
TTTCTTGAAGTTATACCATAAAGTACTAGACTATTTAGATAAAAAAGATGAACTATACGTATTTAAAGGCTACGCTGGTAGCGATAA
AGATACAATGTTAAAACTTACAGTCATCAATGAATTAGCATGGCATAATTTATTTGCTAAAAATATGTTTATTAGACCTGAATCAAA

AGAAGAAGCTACAAAGATTAAACCTAACTTCACTATCGTTTCTGCACCACATTTTAAAGCAGATCCAGAAGTTGATGGTACTAAATC
TGAAACCTTTGTCATTATTTCATTTAAACACAAAGTCATTTTAATCGGCGGTACTGAATACGCTGGTGAAATGAAAAAAGGTATCTT
CTCTGTAATGAATTATCTCTTACCGATGCAAGATATTATGAGCATGCATTGCTCAGCAAACGTTGGTGAAAAAGGCGATGTTGCATT
ATTCTTTGGTCTATCTGGCACTGGTAAAACAACCTTATCGGCTGACCCACACCGTAAACTAATCGGTGATGATGAACACGGATGGAA
TAAAAACGGGGTCTTTAATATCGAAGGTGGCTGCTATGCAAAAGCAATTAATCTTTCCAAAGAAAAAGAACCACAGATTTTTGACG
CAATCAAATATGGTGCAATTTTAGAGAACACTGTAGTTGCAGAAGATGGTTCAGTGGACTTTGAAGACAATCGTTATACAGAAAAC
ACGCGTGCCGCTTATCCAATTAATCACATTGACAATATTGTAGTACCATCAAAAGCAGCACATCCAAATACAATTATTTTCTTAACT
GCGGATGCATTTGGTGTTATTCCACCGATTTCAAAGTTAAATAAAGACCAAGCAATGTATCATTTCTTGAGTGGTTTCACTTCTAAAT
TAGCTGGTACAGAGCGTGGTGTGACAGAACCTGAACCATCATTCTCAACATGTTTCGGTGCACCGTTCTTCCCGTTACACCCTACTG
TTTACGCTGATTTATTAGGTGAACTTATCGATTTACATGATGTTGATGTTTATCTTGTTAATACTGGATGGACTGGCGGAAAATATGG
TGTAGGACGTAGAATCAGCTTACATTACACACGTCAAATGGTAAACCAAGCGATTTCTGGCAAATTGAAAAATGCAGAATATACAA
AAGATAGTACGTTTGGTTTAAGCATTCCTGTAGAAATTGAAGATGTACCGAAAACAATTTTAAATCCAATTAATGCTTGGAGCGACA
AAGAGAAATATAAAGCACAAGCAGAAGATTTAATTCAACGTTTTGAAAAGAACTTCGAAAAATTTGGTGAAAAAGTTGAACATATT
GCTGAAAAAGGTAGCTTCAACAAATAAATTTGAATACTAATTAAAGAGCCAACCGTTGTTATAGTCTAACAATGGTTGGCTCCTCTT
ATTTTATGTGCTAAAAATTTATAGGCAATTTTATTACAACAATGTACATTTAAGGTGACCTTCATGCCAAAATCGCATCACTCATTTA
ATGGAAGCAGCACGTCTTCATATAAAGTACCGATCCCTAATTCAACGCATGTAGTACCACATCTTCAAAGCTTGATAGTTCCCATGC
GCACACCACGTTTCATACTAGCTATGCGACTCAACTTGGTTCATAAACTCTTTAATATAAGTCAATGTTTCAACCATCGCTGGTGGTC
TTGGCACATGTCCTTCTGCCATTTGATAAAATGTTTCATGCGTGGCACCTTTTAACTCTAGTTGGTCCGCTAAATAATACGCATGATG
AATACCAACTTGCTGGTCTTTCCCTCCATGTACAATTAATATTGGCGGACTGTTTTCATTAATGTTTGGAATCGCTTGGCGTGCCTCA
TATGCCGCTCGATCTTTTTTCGGATGACCAATCATTCTTCGTAGCATGCCTCTTAAATCGACACGTTCTTCATACATTAAATCAATAT
CTGAGACACCACCCCAGATTGTATAACTTGTTACTGGTAAGTCTTGAAATGTCAACAATCCTTGTAAACCACCTCGCGAAAAACCAA
CCATGTGGATAAATGCATGTGGATATTTATCATGTAGCAACCTTAATAATTGCGTCACATCATTTAAATCGCCACGGTAAAATTCGT
CTTTACCTTCACTCCCATTGTTACCTCGGTAGTATGGCCCAATCACTAAAGTTTGACTATCTGAAAATTGCATTAATCTACCTGCGCG
CACACGTCCTACTTGACCTTTGCCACCTCGCAAATATACTACAATGCGATTTACTTCATGATGTGGTGTCATCATTAAAGCTTTTACT
TGTAAGTCATCTGACAAATATGTAATTTCTTCGAATTGATGCGTAAAATATTCAATTGGCATTCGTTTACGTTTGATAAAACCCAAGT
GATTGCACCCTCTCTACGCATTTTAAAATGGTACTATCTTGCAGTAAGAAACTCCGTTGTGCGAGTTCAATATCATTGATACAGTTAA
ACAACACTGGCCCTGCTGTTTCTAAATAATCGTTCTTGCTTACCAATGATTCAACTTCGATAAAATATACATCTTTTACAAAATCAGT
TTGATCATGTGTTTCAATGGTATATTGTGCTATGTAATAAATATTTTTAACTTTGGCGCCTGTTTCTTCATATAATTCACGTGTAACTG
CTTCAGCACTACTTTCCCCGCGTTCCCTTTTACCACCAGGAAATTCAATCCCCCGTAAATTATGTTTGGTAAAAAGCAATTGATTTTT
AAACGTTGGAATAGCTAGCACATGATTGCCATCTGCTATCTCATTATCCTTTTTAAATGTCAAATTAACTTGACGATTATCTTTATCC
CTAAACTTCACGCGCATCACATCCCTACATTGTATGTTAATATAATAGTTAATTACTATCGTTGGAGGCATTAATTATGAAAAAGAT
ATTCTTGGCGATGATTCATTTTTATCAACGTTTCATTTCGCCACTCACTCCACCAACTTGTCGTTTTTATCCAACATGTTCAGAGTACA
CTAGAGAAGCGATTCAATACCACGGTGCTTTCAAAGGCCTTTATTTAGGTATCCGTCGTATTTTAAAATGTCATCCGCTTCATAAAG
GCGGCTTTGACCCTGTTCCGTTAAAAAAAGACAAGTCAGCAAGCAAGCATTCACATAAACATAACCATTAATATGGTTGTAATTGA
GTTATATCCACTAAAGGGGGGCGAAATTCGAGTCGCCCCTCTTTTAATATGCCTGAATGCGCCACCACATCTTGTTCAAAATAATAA
CCTGCTGGTGTAACATCTCCTGGATAATCACCTTTACGAGCAAGCATCGCTGTAAAATAGCGGCTTAAACCATATTCGTACATGCCG
CCAATAACCACTTTTGCACCATGACTTTTCAAAGTATCAATTGCCGTTTGCACTTTATCAATGCCACCTAGACGAAATGGTTTTAATA
CAACAACTTTCACATTGTATAATTCTATCAAATTAATTATGTCCGACAATGATGTCGCCTTTTCATCTAGAGCAATCGGAGGTATTGT
TCCATCCACTACTTCATCAAGCATGGAGATATCTTTAAATGGCTCTTCGATATAAAGAACCTGTTCACGCGCTAATAACTGTAACTG
TGTGAAATCTTGACGATTCAAGGACTCATTTGCATCTATAACCAATTGAAAGTGAAAGTCTAATTCTCGTAACACTCTAATTTGATC
CATGATTTGAGGTGTCCATTTTAATTTAATTCTAGTCGGCTTTGTTGCTTTCAATGACTCTAGTTGTTTATTTGATAAGCCGCTCGCTG
TCGCTCCATATGCTACTGAAAATGAAGGCAGTACATGAAACATTTGATACAATGCCATGACAATAGTTGCCCTTGCAGAAGGCGTA
TTTTCCAATGAATCTACTAATTTTAGTGCTGCTTCATACGTTTCAAATGATTTATTTCTATTATCTTCGAACCATTGCTCAATTACATG
TTTCACTGAAGCAATTGTTTCATGATCATACCAATCTGTTTGAAAAGCGTTACACTCCCCGAAATATGCATTTCCCTTATCATCAATC
AATTCGATAAACAAACAATCACGATGCATTAATGTGACTTTCGGTGTTACAATTTGTGCCTTAAATGGCTCACTATATTTATAAAAA
TGCAAAGCTGTCAACTTCATCAAATCATCCTCTATACAACTTATTTCTCTGTAATTTACCCGTTGATGTATAAGGTAAAGTATCAACC
TTTTCAAAGTGTTTCGGTACTTTATATTTCGCTAAATGTTTTGATAAATATGCAATCAATTGTGCCTTTGAAATGTCACTTTCACTGAC
AAAATATAATTTAGGCACTTGGCCCCAAGTATCATCAGGATGCCCTACACATACTGCGTCACTGATACCTGGAAATTGCTTCGCTAC
CGTTTCAATTTGATATGGATAAATATTTTCACCGCCACTAATAATTAAATCTTTACGTCGGTCATAAATCATGACATAACCTTCATAA
TCTATTTCAGCAATGTCACCCGTATTAAAATAACCATTTTCAAACGTATCCGTTAAATCTGTTGGATACAAATATCCATTCATCACAT
TGGCGCCTTTAATCATTAATTCTCCATGACCTTCTTTATTAGGATTTTTAACTTTTACATCAACATTGGCACTTGGCATACCAACAGT
GTCAGGACGTGCATGCAACATTTCCGGTGTTGCTGTTAAAAATTGCGAACATGTCTCAGTCATACCAAATGAATTATAAATTGGCAG
GTTATATTGTAATGCCGACTCTATCATAGTGGCAGATAATTTAGCACCGCCGAGTAATATTTTTTGTAAATTATAAGGTTCATGTAAA
CCTTGTTGCATAAGCCAATTTAAAGTTTGTGGCACAAGCGAAATGTGCGTGATTCGTTCATTTTTAATCATCGTTAAAATTTGTTCGG
CATTGAATTTATCAACAATGCGCACAGTAAAACCTTCAATAACAGCTCTTAAAAGTACACTGAGACCCGAAATATGATAAATCGGC
AAGACAGATAGCCAATTAGTGTCACGATCAAATCCCAAGCTCTCTTTACATCCGATTGCACTGGCATAATGATTACGAAACGTTTGT
GGCACCGCTTTTTGAGGGCCCGTTGTCCCTGATGTAAACATAATTGATGCAATGTCATCTAAATTAAATGATGTATTTAATATGTTGG
ACGGCGACTCTTTCGGCACCACAGTTTCATTCGATGTATCATATTGGATACCCATTGTGTTGTCCAACAAACCGTTCGTTGTAATATC
CCTTCCAGCGAATTCAATATCATCCAGCGATACAATTTGAAACCCTCGCAATTCCAGTGGCAAGGTACAAAAAATCAATTGTACATC
GATTGACCTCATCTGATTCGTCATCTCATTAGGTGTCAACCTTGTATTAATCATCGCAATTTCAATATTTGCCAACCAACATGCATGT
ATTAAAATGATCGATTGAATCGAATTATCTATGTATAGCCCGACACGAGATTGTTGATAAGCCTTGAATCTTTTAGCCAATAGACTC
GCTTCACAGTATAAATTTTGATAAGTATAAGATTCTTGACCGTCTGTTATCGCAATATGATGTCCATTTTGTTGTGCTTGTTTATATA
ACCAAAAGTCCATGCGTTATTCCTCCAAAATCATTTACATTATAATTATAACGATTTTATGACATTCTAGCAGTGGTTATGTTTAAAA
ATATAAAAAAGTAGACGAATTGATTCATTGATATGATTGTTATAATGCTCAATACATATCGTTATATCATTCGTCTACTATTATCAGT
TATTTTTATTTAATTTTAGTGTCATTCTGTCATTTTGATGTGGTGATTTACCCATTGTTGCCACATCATCTGCAATGTCAATTGGTATA
CGATTCATGTCTTGTAATGCACTTAAATGGAATACTTCATCATCTAAATTTTCAATGAGATATACATAATATGTTACCTTGTCCTTTTT
ATATTTTAACGTTTTCCAAAAGTCCGGCTTGCAATTCAATACATTATCCGGAATATATTCAATAAATAAGTAACGTTTGCTGCCTACT
TTGTCTACGAAATATTTTACAGTGCCTTTTTCTATACCTCTTATATGTGCATAGTCTGCTGAAAAGTAAATACTACCTATTGTTTCATT
ATGTTGTTGTATTTCAAATCGTTGGCCTACTATTTTATTATTTGTGCCACAACTACTTAAAAAAATCAGTAGATATAGCATTAAACAT
ATTTTCATCCCCTTGAATTTTAAAAACTTTTTCAAAAGCAACACCTCTAAAAATAAATACAATATATTATATCAAATAGAAATTATTA
TTTTGTTAAATTATGTTGTCATGTTCGTGTCATTTTGAGTCGTAATACAACTTTACAATTATTAAATTAACACTTCTACAATCCAAAA

ATATTTTTAAAGTTTTGAAAAAAATTAAAGTATTTCCACTACTAATTTGGTAAAATATGATTATGTGATATAGTGAAAGCGTTTTACA
CTTAATAACTCCCTCTTAAATGCATCCAGGTTTTATGTAGTAAATCATGAAAATAACATATAAATATAGAGGAGATTTACCTTTGAA
TACAGAGAACAACAAGAATCAAAACCAATCTGTTAAAAATTCTGAAAGACGCGGCATGTTAAAAGGATGCGGCGGTTGCCTTATTT
CTTTTATTTTATTAATAATCTTATTATCAGCCTGTTCAATGATGTTTAGTAATAATGACAATTCCACTAGTAATCAATCATCAAAAAC
GCAATTAACTCAAAAAGATGAAAATAAAAATGAAGATAAGCCTGAGGAAAAATCAGAAACAGCAACAGATGAGGATTTACAATCA
ACCAAAGAAGTACCCGCAAATGAAAATACTGAAAATAATCAACATGAAATTGATGAAATAACAACAAAAGATCAATCAGACGATG
ATATTAACACACCAAACGTTGCAGAAGATAAATCACAAGACGACTTGAAAGATGATTTAAAAGAAAAGCAACAATCAAGTAACCA
TCATCAATCCACGCAACCTAAGACCTCACCATCAACTGAAACAAACACGCAACAATCATTTGCTAATTGTAAACAACTTAGACAAG
TATATCCGAATGGTGTCACTGCCGATCATCCAGCATATCGACCACATTTAGATAGAGATAAAGATAAACGTGCATGTGAACCTGAT
AAATATTAAACAACAAGCGAATTGAATTCAAATTGTATTTAGCTTTATGCACTAATCACATAGTAAATAATGAGGGAGATTTTTTTG
GCATGAGCAATCAATTCAAAAGCGAAGAAGAGCGAAGACAATGGGAACAATTCCAAGCTTTCCAAAATCAACAAAACCAACAGCA
CCAGCAATACGGACAAAAGAAATCTAAAAAAAGATGGTTCTGGGGCTGTGGTGGTTGTCTATTATTATTTATTTTAATTATCATCGG
TATTTCAGCTTGTACAGCTGGTATTACAGGTAACCTTGGCGGAAATAGTTCTAAAGAAACGAACAAAACCCATAAAATCGGTGAAA
CTGTTAAAAATGGCGACCTTGAAGTCACTGTAAATTCAGTGGAAACTATGAAATCTGTAGGACCATCTCTTGCACCAACAAACGCTA
AAGGTATATTTGTCGTTGCTGATGTGACGATTAAAAACAAAGGTAAAGAAGCGTTAACAATTGATAGTTCAATGTTTAAGCTAAAA
TCCGGTGATAAAACATTTGAAGCAGATAATACAGGTTCAATGTCTGCTAATCAAAGTGACAACGGTAGTATAGAAAATTCATTTTTC
TTACAGCGTATAAATCCTGATAGCACTGCACAAGGTAAAATTGTTTTCGATGTGTCAGAAAACATAGCCAACGCAAAAGATAAAAA
ATTAGAAGTTATTTCTAGTTTATTTAGCGTCAAGAAGATTACATTTGATTTATCCGATGCTAAAAAAACATCAAAAGCTAAAAAAGA
CAAGCAAGATACAGAAGTAGCTGTTGCGAGTTCAAATAGCGATAATGTAAATTATGAAGCTTCGGCTACTACACCTGCTACAACTT
CTAGTGCGGATACTGATTCTGAAGATAGCGAAAAGTCTAGTAAAGATGAGGATAAACAGAATGCGTCTAAAAGTGATAAAGATAG
TGTAGAAAAAAGTGAATCTAATGAGGAAACTGCTCCTGTAGAGCCCATGCCCCAGAGCAAACCTACCACTAGTGAAGCACCACCTA
GTCAAAATAATCACAACGAAAATAACATGTATGATGCTTCAACAGAATAAATTTTCAGGGTAGCTCAGCTACCCTTCTTTTTCGGTA
AAAATAAATATACATAGCAATATAAGGAGATTAAAAAATGAAATTCAAAGCTATCGTTGCAATCACATTATCATTTTCACTATTAAC
TGCCTGCGGTGCTAATCAACATAAAGAAAATAATAGTAAATCAAATGACACTACTAAAAAGACGCAAGAAACTGACAACACTACA
CAGTCAAATACAGAAAAGCAAATGACACCACAAGAAGCCGAAGATATCGTTCGAAACGATTACAAAGCAAGAGGTGCTAACGAAA
ATCAAACATTAAATTATAAAACAAATCTTGAACGGAGTAATGAACATGAATATTATGTTGAACATCTAGTCCGCGATGCAGTTGGC
ACACCTTTAAAACGTTGCGCTATTGTTAATCGACACAATGGTACGATTATTAATATTTTTGATGATATGTCAGAAAAAGATAAAGAA
GAATTTGAAGCATTTAAAAAGAGAAGCCCTAAATACAACCCAGGTATGAGTGATCATGATAAAACAGATGGTGAGTCAGAAGACA
TTCAACATCATGACAATGATAATAACAAAGCCATTCAAAATGATACACCAGATCAAAAAGCCGTTGATAAAAACGATAAAAATGCT
GTTAATAAAGAAGAAAAACACGATAACCGTGTAAATAAATCAGCAGAAACTAAAGTTAAATAATGGCATACCAAAATTTTAAATCT
AATATGGAAGGTATTATTAATTTGAAGAAATTATATATTTTTTTAATAGCGATTAGTTTACGCATGTTAACTAACTCTGAAGAAGCTT
ATGCTGATGTAGGTGTTCAAAATTTAAGGAATTATTATGGGAGCTATGAATTTACAGATTTGAAAAATCAAAGTGATAAAAACAAT
CCTCAAAGTCATCAATTAGAATATGAAGTTGAAGGTAAAGCACTTTATTCACAACTTTCAAGTGAACATGATGTTAAAAGACTTAAA
TCAAAAAAAGTTGATATTTTTGGAATTCCCTATACTGGTATCACACAAAACGTTGAATATATATATGGTGGAATAACTTTATCTAAT
GATTACTTAGATAATGCTAGAAATATTCCGATAAATCTTTGGATAAATGGTCAACACAAAACAATTTCTACTGATAGAGTATCCACA
AATAAAAAACAAGTAACCGCTCAAGAAATTGATGTTAAATTACGAAGATATCTTCAGGAAGAATATAATATATATGGTCATAATAA
AACAAATAAAGGGCGAGAATATGGAAACGAATCTAAGTTTAACTCTGGATTTGATAAAGGAAATGTAATTTTTCATTTAAACAATG
GTACTCAATTTTCGTATGATCTTTTCTATACTGGTCATGGTCAACCTGAAGACTTTCTTAAAGTTTATAATGATAACAAAACAATTGA
TTCAGAAAATTTTCATTTAGATGTAGATCTCTTTCTTTCTAAAACCATTTAAAATACAATACAACTTAGGAGCCGCACATGAAACTAT
TCTTTATATTTAACTTCATTCTAAATACTATTATGTCTCAAACAGACTATTCTGATCCAACGTCAAACCAACTAAACAAAACAAGTCA
ATATGAAGGTTTAATGTATAATATGAAGGCGCTCTATGAATCAGATCCTGTATCTTTTGAAAAGATAAAATCATATGATCAATTTTT
ATCTTTTGACTTGATTTATTTTTATCAAACTTTACAATATAACAGAATTAAAACCGAATTAACCAATGATCTTTCAAATTATTATAAA
GATAAAGACGTAGATTTATTTGGAGTAAAGTACTTTGTGAATTGTTATTTCTCAGATAATAAAGACATAAAAGAAAATTCACCAAGT
AACGATAGGGTTTGTATGTATGGTGGTGTCACAGAGCATGAAGGTAATAGAACTAAACTGTTTGGTTATACGATAGTAGTTTCTGTA
TATATCGACAATAGAAATAGTCGCTCATTTACCTTATCAACAAATAAAGATCATATTACAGCTCAAGAATTAGATTATAACGTTAGA
AATCAGTTAATTAAAGATGAACAATTATATGAATTTGAAAGTTCTCGTTTTGAAACAGGCTATATTAAATTTATTGAAAACGGTAAA
GCTGTATTTTGGTATGATCTAATGCCACCCCCAAATTTTACGCAGAGTAAATATCTAATGATTTATAAAGATAATAAAACTTTGTTAT
CTTCCAAAACTGCAATTGAAGTTCATTTAACAAAGAAAAAATAACAATGATTATTTAAATATAAACTTAAATTAAAAATAATGAAT
CACTACAACGAACTCTACATTCCACCTTAAAAAGCGATTTACGATAGCCTGTAAATCGCTTTTTATACAACTTAAACGGATAATATT
TATCAAATATGTATTCCTAAATTATACAGCCTTAATCCAGCAGCTACTTTCGAAACTTCCAACTTAATTGATATAAGATTTAATAGTT
TGTTTCGTTCTTTTTCAGATAAACCAGAATTTAAATTGATATTATTGACTTCATAAAAATTATAGACTAATGCCTCTATTTGCTTTTTA
GGCATAAGTAAGTCGACTGAAAACTGATTTACGTCGCTTTCATAAATCATTTCATGTAAATTCTTTAGACTATTATCGTTGTTATCTT
TATATTTGTATAAATAACGACCGAGTTCACGAGCTATTGCAAATCTTGTATTATTAATCGAGTGATTATTATTGATATAAATCGTTCT
TCCACTTAAATAACCCGAAAAATTACCCTCTATTTTAATATATTTAACTTTTAAATTAAGTTGAAATAATAGCTTGTCTATGTCAATA
GCAAAATGTTCAGAAGTAATAAAAAGTTGATCCATTTTGTCCTTTATAAATGGCTGAAATAATCGAACTATTTTTGGTTCTAAAATA
TCTTCATAATGAACTTTCTCAATAACTTTCAATTGATTCCCCACCCTAACTAAAAAATTAACCTACATCATTTAATAAATTTATAACT
ATAGGTGCAAACATTATTAAAATAAAACCCCTTTACTACTATATGTAACGAAGGGACATGAATTCAAAATAAAATACCTTTTTTATA
AATTTATTATAATATTTTTCATTATGCTGTACAACCGAATCTAATCCAACAATCCCGAAAAGAAGAACAAAGGTGGTTCTGCTTCAT
AATCATTTTTGTCTAAGTGTTCTTTTTCAATTAATTCATCCACTGTTAATCGGTCTTTAGCATCTTTCTCTTTCAATAATCGCCCTATTT
TTTCTTCCTTATATCCTATGCTACTATTAAACTGATTTCCTTCATCTCCTGTATTTATATATGCCTGAAATTTATATTGTTTATCATTAT
TTATATATTCTTTAATAAAGTAAGCATTCACCGGGCTTTTTTGAAACTTTGTTATTTTCATAGACTTATAGTGATTCAGGTTATGCTTA
AAATACAAATCGATTCGCTTTTCTTGTGTCTCAATATAATGTTCTTTTTTACTTTTGTGAAACGCGTATGCAATAACAATTAGTACTA
TTAATAATACAACAACTACCACAAATACATATCTCTTTTTCATTTTAACCCCCAAAGTTTAGTATTTATTTCTTATAGATTTTAGATTT
AAAAAAACAATTCACTTTGACATGCTATCTTTTTTCTATGTTCCGTTTACGCCCCTCATGAAACGTTGTTTCAAAATACTTTTAAAAA
TTATTTGCTTGACATAATTATAAATACATAAAATAACGCTGTTATATATGGCGTTTATAAAATGTAGGAAAATGGCGAAATGAAAAT
GCTGTTTTTCCTAACGTCAAAGGTGTTAAATCATACTCGTGTATAATTTCATTTCTTGGCTTACCATTATCATATAATCTAACCATTTA
TAACTTAAACACTGAACTAAATGTTCTTCTTTCTTAAATTAACAAAATCTACTCTCATAGAATTTGTCCAATTAGATGATATAATAGT
AGAGTCGCCTATCTCTCAGGCGTCAATTTAGACGCAGAGAGGAGGTGTATAAGGTGATGCTTATTTTCGTTCACATCATAGCACCAG
TCATCAGTGGCTGTGCCATTGCGTTTTTTTCTTATTGGCTAAGTAGACGCAATACAAAATAGGTGACATATAGCCGCACCAATAAAA
ATCCCCTCACTACCGCAAATAGTGAGGGGATTGGTGTATAAGTAAATACTTATTTTCGTTATCTCAATTATACTGCTAATTTTTCTTT
TTGTAAAATATGCAAGGTTTTAAAGAGAAAAATCAAGAACTAAAAAAGGCTCTATGCCAAATTGGACTGATGAGTTCAATATCGGA

AGTTAGGCAACTAAACATTGCGATATATAGCAACTTGGGAAATGGAAGAAATAGAATACTTACATTTAGAGGATGAATACAAAGA
AGATATACTTTATTTTGAAAAGAGGATGATAAAGTAATAGCAGTTACAAATATTTTATGGACTTGCTCTGGTTTCAATGATATTTGA
TCAACTCAATAATGAGTCGCCTAGCTTTATTAAAAAACTCCTTTAAAGGGTTGTTTAATTTTAAATGTAGGCGATTTTATTAATGCTT
GAAGAAAGAGAATCTATCCTCTTAAAACTGAATTCGTCAAATTAAAGTACAAGATATTTGAGAAACTATAGAGTACCAGTCTAAGT
AAAATAATTCCCCAAAAAGCTGATTCATTTTAATAGACTGGTCATCTGTAAAGAAAGTTGTTCCTTCGATTTTACGACATTTTTTATA
TAGTTATACTAAGTCATTTATTTTGAAACAAAATAATCATAAATATGATTTAAATGTTTATCTAAATTTTTAATTATCGATTTCACAT
CATCTATTGAAATATTTACATATGGATAATGAATACTTTTTTGTAGTATTCCTAACTTTTTCGTTGCATCTTTTCTAGCTTTACTAATA
CTTCTCGAAAATTCATTTTTATTATTTTTTTCTAACTTTTCTAAGATGCTATTAAGAAATCCTTCTAAATTTTTATCCTTTAATTCCATT
TCAGCTTCGCTCATGTTCATAGCCTGTAAAGCATAATATTTAAATGAGTATTCTAAAAACATTCTAACAGCTATTTTGATTAAAACAC
TGTATTCTCCATTAATATTTTCTTTACTTAAAAAGTAAATTAATTTATTTAATTCAACCGCATCAAAACATTCTATTTTCTCATAATAT
TTATTATAAAATTTATTCGAAAGACATTTTTCGCTTCTTACTTCTCTATTCTTCTCTTTAATCTCGATACTTATAGTTCTCGAATCAATT
AATATACTTTTCTTATTATTCTTCCATAAAAATATATTTAAGCTATATTTTCCTGGTATATTAGGTGAAACTATCTTATAATCTTCTAT
ATTTAATTCGTCACTCTTAAATTCAATATATTTATCTCCCTCAATTTTAGTTTTTATATTTTCCATTAGATTAACATTGTCTTTTTCGGA
AATAATTAAAGATTCTCTATTAACCTCTATCTTTATTGTTTCTTTCTCGTTTTCCACATCTACCTTTTGACTTTTAGAATTATCAATTTG
CTGGTTAAATTCCTTTATTTCTTCCTTTTTCACCTTATTATTAGATACTTCTTTTTCTATTTTATTAATTGTTTTAGTCACCGAATCATT
AACATTACTTAAATCATTATTCTGTTCATAATTATCTATTAAAGGAATAATAAAATCTTCAATTTCTCTTGCTCTATTTAATAACCTTG
ATACTGCTATTTTCTTTTCTCTAGCTTCTTTTTCAACAGTATCTATTAAATAATTTATTATTAATAATTTATTTTTAGGAATAGCATTTC
CGCTATCTAATTCTTCTTTATTTAAACCCAACCTTTTTTTTACAGCAATATTGTTAAATAATCTATCCAAGGTGGTTAACGGAATTTTT
TTAGTGACATCAGTGCTTAAATTTTTGGTTAAAGTATCTGCAATTATAAAACCAATATTAACCTTTTTTTTCCTTCTTTTTGAGAATTC
TTGCTTTTCCTTTGAATTCCATTGTTTCACACCTTTACCTTGATTCTCTCCGGAGTGCATTATTTCCATTATATAGTAAGCACGCTCTTT
AGTGGTTAATAAACATTTAATTTCATCAATTTTATTATGCTTTTCATCTTTTTTTAATTTCAAAACTTTCTTCATTAATTTCTCGTCGAT
ATCTTTTAATATATTAGGATTATTAATAATTTTAATACAACTAATTCTTCTATTTCCCTCGTACACAATATATTTATCTATTTTGCTAT
CAAAAACTAAAACCGGTAAATTAGCCCCTATAAGTCCATTTAAATAAATATCTTCTAATAAATTATACATTTGACTCGAGCCAACTT
TAGATATCAGTTTGTCTAAGGTTTCTCTTTGATTTAATGTAACTTCATGTCTAGGATTTTCAGTATATAAAAGTATATTTTCAACATTA
ATATTTTGAATAGTTTCCATATATAGCTCCTTTTGACTTAATTGCTTTTGAATAAAACATATACTCAATAGCTGTCTTTTTGTTTTGTA
ATGAATAATTCAAATTAATTTCTTTAAAGACTAAATTATCATACATTTTCTTAATTTCATCACATTTATCATATGTGATTATCCATTTT
CTATTTTTTAACTTTCTTTGAATATAGTTAGCTAAATTGTGATGATCTTCATGTAAATAATAATTTGTATATAATCCAGACCCCTTTTT
ATAATATGGTGGGTCAAAAAATGTAAATACACTTCTCCTTTTCATTAATACTTTATCTATAAATTCCAATGCATCCATATTATAGACT
TCTATGTTCTCTTTTAAGTTTGATATTTGAACTATTTCTTTATTAAACTTTCTTTATTGAACCTACAATCTATTAGATAATTCCCATTT
TGTTTAAAACCCCCAATAGGACCAGCCTTATCTATAATTCCAGATCTATTAGTTCTATTTAAAAAAAATGTTGAAAACCCTATTTCTA
AAATACTATAATCTGTTAAGTTTTTCTTATATTTTTTTGAATATGCCATGTATCAATATCTATTGTAGTAGATTTAATGAGATTTATA
AAATCTTCAGTATAATATAATATACTATACCAAAAGCAATATATTGTGTAATCAAAATCATTTATTATAATTTTTTTTACTGCCTCAC
TTAATAACAACTCTATTGATAATGCTGAACCTCCAGCAAATACTTCACAGTAGGTATTACATCCACTATTTCTACAAGACCTTTTAC
ACTTTTTAATAATTTATACTTTCCACCAGGATATCTAAGCGGTGAAGGATTCATGAAATCACCTTTTCTGTTTATTTTTTAATATTTTT
AATTTAAATGAACAACTTTTGAAGAAATCCTTGTTTACGATTTTTTAGGCTTTCAATCTTTTTTTCAGATTGTTCAATACGTCGATTTA
TAATAAGTATAAATTGACTAATTTTATCCTGTTCAAACTTTACAGGTAATGGAAATGGGATTAATTTCAATTCTTTTACAGGCACTAA
ATTGGTTATTGCCGACCCAGGATTTGCCTCTAGGATTTTTCTTTGCATTTGATTGCTCCTCATAAATATCAAAAAGAATTCACTTCTA
ATTTTATGGTTAGTTCTAAATCTAATAAGCGCTTGGTTTATAATTCCCTTTGTAAAATTCTGAGGAATGAGTGATAATCTTCCAATAG
TACCTGAGCAGCTCATTATTATATCATTTGGTTGAACCGAAAATGATAGCATTTCATTATATTTATTTTCGTCTATATAATACCTGAA
GTTACTTATATCATAGATTGCATTCCTTTGTTCATAAACTGCATAACCGCTTTCTACAAAGATATCTTTCTTTAATGCTCCTCCAAAA
GGCCCCCTTTTTATACCTTCAGTTTTATCCTTCAAAACTTTTTGAAGCATTACATTTTCCCAATCTGGATAATCATTTCCATTCTCATC
CTTGAATCGCAATTCCTGTGAGAAGATTTTCTGCATATAGCCTTTTTTCTGTTGTTTAAGTAATTCAAGTTTTTGTTCTTCTAATTCAA
TTTGTCGGTCGAGTTTACTAAATAAGTCACCGATTTTTTGTTGCTCTTTAATTGATGGAAACTTCACATTTATTTTTCTTAATATATCT
TGACTAACATTAAATCTTGTACTACCACCAGCCGCTGAACCAATTTCATATCTTTTTTTAGGTATATTCAAAGCTTCTTTGAGAAATA
TAGGATTATAGTCCGATACCTTTTTCCCTCTAATTATAAACCCACCATACAATGCATAATTTTTATCTAAATACACATTGCATAAGCC
AACATCTTCTCGAGTTTCTGAACTTCTTAAAAACACTAAATCTCCAAATTCAACTTTATTATTTTTTTCGACATTTTCAGGTACAGAA
ACTTTACCAATTATACTTTCATAAGTAATGAAATTGTTGTTTAATATATCTAAAACATTTATAAATTTTCTACCCATTCCATACTGTTC
TTTTTTAGCATTTATTCCATTATTAAACTCACAAAACTCCCCTAACTTCTTTTCTTCCCATTCGCCTTCAAACCCTGGGAATCTCAACT
CTGGCAAATTTTTCTTTTGTGTATTACTCATCTTTCAACACCCCAAGTTCTTTCAGGTATGCATTGATTTCTTGTTCAACTTGTGCGAT
TTCTTTGTCGATATTTTTCAAATCTTGTTGGACTTGATCTAAATCAATTGGCGCTTCTTCTTCGAATGTATCGACATACCTCGGTATGT
TTAGGTTGTAATCGTTATCGGCGATCTCTTGTAATGTCGCGCTGTAGCTATATTTATCAATCGTTTCTTTACGCTTATATGTGTTAATA
ATGCGTTCGACTTGGGCATCGCTTAAA

>045-contig_58

ATGTGGAGAATTTTGATGTTGGTGAAAATCAATCATTGATTGGCTTTATTTGAGACATTCTTTTCTAATTTATTATAAATTTCCAGCT
ATAAACTTGTTTAATACTTACTGATTAAAATACTCACCAACAATCTTTCGATAAAAACACTTTTTCTCTAATTTTTATAATTCAAAAC
ACTAAAACGATGTTAAAAAATAATTCTATTTTTTAGTTAATAGATAGTTAATGCATTTTTGATATTTAGTTAATTATTCTTTTTAAAA
AATATTATTATATTTACATTGTAAACGTTTACAATATAAAAAAGGAGCAATTAAAATGAATACAAAATTTTTAGGTAAAACATTAGT
AGCAAGTGCTTTAGTATTAACAACATTGGGAACAGGATTACATTCTTCATATTTAGGATTAGATACAAACAAAGTTGTTAAAACAGC
AAAAGCAGAAGAAAATATGACAGATGGTCAGTTGTGGAAAAAAGTTAAAGATTCATTAATTGATTCAGATATTATTAGTGGTAACC
GAGATGAAGAAATAAAAGTAACTTATTCACTTAAAGATGGTAATTCGCCTAGTGTGACTGCACCTGGAAATGATAGCGGCGATAAT
TTTAATCAAAAAATTGATTTTGGTAGTTTAAAACAAGTTGATATAAGTAAACAAAATATTGGTGAAGAAGATTTTAACAAAAGATT
AGATGCGAATAAAACTTGGAACACTTTTACTCAAAAATTAAAGGATGCTGGTTTATTGAAAAATGGTCAAAAAGTAACCATTCAAA
CTACTAATCCAACAACATCTTCGACAAACAGTATTACCGGCACAGTAGGTGAAACATTGAATGATAAAAATGCTGATCCATTAGAA
AAACGCTTAATCAACAAAATCACAATTGAATAATCGCTAAACTCAAATTTCCTTAATTCATAAAACCTTAATAACTAACCCTTTCTT
ACAAACCACCTAGCCCATAATAAATTAAAACGTCATTTTTTAATTCATACAGCGGATAAAATAAAAAACCATTCCGCATTAACTTTG
GCAAGTTGGAATGGTTGAAAAATTAATTTAGAAGCCACTAGACGGCTTACTAGGACGACATGTTAGCATATGTCGTCTTATTTATAT
TCATATAATAACATACAATTCAATAAAATGTCTAATCGATAAAACGCAATTGTATTTAAATGTAGAGGGAAATTTATGTTCCTCTTC
TCTTTTCTCTTATTCGGCTACTTAATTAAAAAGTAACTCATACAAATTTTATTTATCATTTTCAGCACTAAGATTAATCCTTAATCGTT
CTGCCACTCCATTTATTAAATCCTTTGAAAAGTAAAACTGCTAATTACAACGTGATAATAATATAGGTTACCATTATCATCATCTATA

GTATATTTCCTTCAATATATATACATAACGCGTGGCATATGTGATTGGTAAATAGAACCACGAATGATCTCCAAGCACTTCTAATCC
AAAATAGACATTAAATATAAATATTAAAACACCGGCAACAATAGCTAATATATCTTTAATAAAATGATAAATTTAGAATTAATAAC
AAATTCATTTTTACATAAGGACTAAACTTTTCAAAAGTTTTTAGATATTGAAGCTATTACCGAAGAAGAATGTAACACTTAAAAAAT
TATTAGGTTTATATAATACAATTTTATAACTACTTGTACTGAAAATAATATAAGGATTTAAGATCGTAAAATCTAAACTAAGGAGAT
GTTTAAATGCATTTGAAACTGAACGATGAAGATTTAAATTATTTAGTAAATATAAGTAAAGAAATACTTGAACAATACAATATTAA
ATTAGACGAAACTTGTAAGGGAGATATTAACATAACATCATGTAATTCTGAATACTTTTTCACTTTAAGCTATTTTTTAAAGCCTGGA
AAAGCAACTATAAATTTCAGAGAATGCCGTTACAACTATAGCTTGTTACGTTTAAATCTTAATGACGGTTTTCATAAAAACTCTAAT
AATGAGAAAATAAGGGGAAATAGAATTAATATATTTTCTGAAAATGAATTTATACAAAAAGCTGATGGCGCAACCCATATGAAAGC
ATATCCTTTACCTTATAATATATTTGACGATAGCTCTGATTTTGTAAAACAATTATTTACTTTGCTTGAATATACAAAAACTAATCAT
AATGATAATATTAAAATTGAAACTAATCTATTCATGAGTTGGTGATACTATGCATGACAACACAATTAAATCTATTAGCAAAGACTA
TTTTGATTACTTACAAACAGAATCAAAGTTTATCCCATTAGAAAATGAAACTATAGAATTTTATTCACCTATCGTCGATTATTTTGGT
GATTCTATTTCTGTAAATATATCTTTTTCAGGGGATCGATATAAACTAACTGATCATGGTGAAACTTTGTGGAACATGGAACAATTT
GGTTTAGATCTAACTAATCATAAAAAACAAAAAAAGTATCAACTTCTCAATAATATTATACAAAATTACGGTCTAGTCATTGAAAAT
AAGCACATAAACTTTTATACAAATAGAATAGACTTATCTCAAGCCATTCATGATTATGTTTTGGCTATTTCCGAGATTAGTTATTTGG
GAATATTAAAGAAAGAAAATATTAAGTCATTATTTAAAGACGAAGTGATGCAATATTTTCTATCAAACAGAAAGATTTATCCTAAT
ATATTTCCCGAATTTAAGGTAGAGGGGAAATCTAAGTTAATTCATAGTTTCGAAGCAGTATTTCCAGGAGAAGCTACAGAGTATGTT
AAAACAATTAAACGAATCGATAAAAACAATGCAAAAAATGTCCTTTTCGATTGGAATGATGTTGAAGTACATAGAAATAAAAATTT
TGATTCTAATGCTAGACTTAATATCATTACAGAACAACAAGGCGATATTAACGAAGCAGTATCAACAATGTTGTCTCAATATAATGT
AGAGGTATTTTCATTTGAAAACAAAAAACAACTTGAAGAAAAGTTTAGTAACGTATAAATATCATGCCACATAGTGATACTTGTGA
TAATACTTTTAAATCCACATTACAAATCACTTAAACTTACTCAATATGATAAATTTTCTGCTACCATTCTTATGATGAACTTTCACCG
TCATACAATTTTAGATGCCATATATACAAAAAGAACCACGCCGATTATGACGTGGTTCCATTTTTTTATTATTTTTTATATGATGAAA
GTTTTAAATAAATCTTCTGTCTCTAGTTTAGCACCCTGATATTTATCAAAACATTCTATGTTTCACTTACTAAAAATCATCATGTTTGT
AGATAAAAAATTAAATGCTATGATTTAGCCGAATTAAAAACATATATACTTATTTACTATGAACTAAAAGTTTATCCTAAAAATAAA
AAACCATGATTTCGAAGTCTTCATAAACTGAAGCTTCTACTCTCATGGCAATTTATACTATGATATTCAATTATAATGCCTAGTCTCA
TAGTAAACGGCTACTACATTCGCATGTACTTCGTTTTACTGTCCACGCATGTTCGTTGACTTATTTATATTATAATATAAATATTTTGT
TTTTCAATACTTAATTTTAAAAATCCCCTATGAAAATGTTGATGAGCTGCATCTTTAAATCTTATTATACATTGTATGAAATTATATT
GCGAGGTACTAAATTGATAATATACACTTTTGGTTGTGTATGCATCTCACCTATCTTTTTTTGCTTTTTTCAAATAAGAAAGTATTAA
AAATTAATGCCTATACAACGTTAAAGGAGGAACAGACATGGCTAAACTCGATTTAAATAGTCTTGACGACGAGCACGTAAAATTAT
TAATAAATGAATTAAAATATCCAGAAACTCATATCGATGTAAATGAATTAAAAACAATAGTTGCTAGTCGAATAAATGAAAGGCAA
GAAATAATAAGTTTTAAGTTAGGAATAAAGTACTTATTAACAATAAAAAGAGGGAACATAGAAAAAGATAGGTTTTCAATTTCAAT
CATTTTTAAAGATACCTATCACACCCTAGTTAGAATAGATATTAACGGTGGTACTCACGATAATCCAGATGGAACATTCGCTCCGAA
AAGTCATATTCACATATATAATGATAAGTATGATAAAAAGGATAGGTTTGCTTATGAAATTAACTTGAAAGATTTCCCCGACATCTA
TAACTTGTATAATGTGTATATGTCGTTTTTAGAGTATAATAATATAAAAGACCTTGAATAAAGGAGGGATAATATGACTACATTTGA
TGCTAAAAAATTAAAAAAAGAATATCTTGATTGGTATAATCAGACCTTAGAGTTTTCTAATTTATCAAACAATGTAGTAAGAATAGA
TACTCCTTTTAAAGATAATTCTTTAGATAATTTAATAATTTACGCTTTATACGATCAGTCCAGAGACATGATTACACTGACAGATGAC
GGCTATACTATATTTGATTTAGAAAATAATGGTATTTTTTTAAATAAATCAAAAAAACATAAAAAGATTTTTGAAGAGCACCTTTCA
GCTTACGGTATTAAATATAACGATAAAACTCACGAAATTTTTGTTCAAACTAACTTTAAAAATTTTAATAAATCGAAACATAATTTA
TTACAGTGCCTTATATTTGTTAATGATATGTACTTACTTTCTAATCCTAAGTCACAGAACATATTTACAGAAGATGTTGCAAACAAAT
TGGATGAACATAACATTTATTACGGAAGAGATTTACCTATTATAGGAAGCAGTGGTGTTGTTCATAATTTCGACTTTTTTATTAGCGC
TAAGAAAAATCAAAAAGAAAAATTTATCAATGCTATTTCTAACCCTAATAATTCTATGATTATTAAGTCGAAAATAACGGATGCTAT
GCAAGCAAAAAAAATAAAAAGACACAGGCAAAATGAGTTTATTTTTATTTTAAATGACTCAAAAAAAGAAATAAATGAACATAAT
AAAAACCTTCTTCATGAAAACTATATTAGTACTATAGATTATAGCGAATTAGACGAAAAGATAGGTTTATTGATTTAATATTTCTGG
ACGTGATAATATCAATTTTTTTATTAAATGAGAGAAATTTGACAATTTCAAAACAGATTAAGCTAGCAAATTGCATAACCTTATTGA
TAATGCAGAATCGTCTACAAATAGTTAAACAAATTATTTGAGAATAATTATTGTTAAACTATATGGGTAGGCGATTTTATTTTGACA
AGAGACAGAAGAACATATATTAAAGACTTTAAATTGCAAATGGTTAAACTTTATGAAAATGGTAAGTCTAGATACGAAATTATACG
CGAATACGATTTAACACTCTCAATCTTCTCGAACTGAATAAATAATGGGTCATTCAATCATCAAGACAACTAAATATGTGATAAAAA
ATGGTCAAATCATTTTGCAAATTTAAATAGACACTCTCAATAAATTGCAGAGTTCAAAATTAAAAATGTCAACATTATAGTAAACTA
ACATAGTCCCCTGTTCATATTCTTCAATATTTAAATCTTATCGAACAAATAAAAGCAGATAATTTTTTAATTTGAACGTTTTATTTTA
GAACAAAAAAGAGCAAGGTTAACGAGTCAGAAAATCTTTTTCTTGATACGTATATGACACTTGCTCTTTTAAGAATCTGAATATTTC
ACGCTATTTCCATTAAATTAGCGGAGAATGAATGATATCTTTATACTAATAAATTTAACTTTTTAACGTATAAAATTAATGCATTGTA
CTAAAGTTTCAATTATTTTATAATTGTATGAATGAATCGTATACACTTAGTTAGTCAAATCTAAGAATTTAGATATTGTTTAATTTTA
AAACTATCGATTCACATATAAATAAAAGGAGATAACAGCATGAGGAGATTACTCAGTCTATTACTAGCTAGTACAATGATTTTAGTC
GCATGTGGGAATGCTAACAATGAAAATAAGAAAAAGGAAGACGAAAAAAAAATCAGAAGTAAAAAAAGAAGCTAAGAAAAATAAT
GATAAACCAAAGAACGAAAAGAAGAATCAAGATATAAATAAAAACAATAATGAGCAAGTTCAAAATGTCAATAATAAACAACCAG
TCTTAAATAATAATAGTAATCATAACAATCAACAACAAAGCAATAAAAACAATTACACAGTTATAGAAAACGGGAATACAGTTACA
GAAATTTATAATGGACAATCACACACAACTACAAATAACCCTATTAGAGAAGAATATGTAGAGGGAGAAACAGACACGATATATA
AAAGAGAGTATAAGCATTTTTATACACCTGAAGAAGCTCAAAGAGCACAAGAAAATAGCGACCAAATTTTAAGAGAAATGGGTCT
CGAACCAGAAAGATATGAATAAAATCAAACTAAATAATTTCGGGTAGCCCCCCTACCCTTATTATTTTTTTGCTAAGAAGTAATAAT
TATATTTCTAATATGATTAGGTTCGATGTTAATGGTAGTGATCATGCAAATCCACCAAATAATGAAAGAATACCTACGCCACATATA
CACATATATACTGAAGAATATAATAATGGTGGTATTGCGATTCCATTAAAAGATATTGAAGATTTAGAATTAACAGATGAAATTATA
GAATCATTAGACTTCTTTATGAAGTATACTAATATAAAACACGATAATGTTATAATAGAACAAAGATTACTGTAAAAGAAATGGAG
GGATACTTAGTGAAGACAATCGAAGAAAGAATGAACGAATATTTCAACTGGTTAAAGCAAAATTATATCTTCAAAGAATTAGATAG
TTCTACAGAAATTACTACTCCCTTTAAGAACCATTTGAATGATTTTATTAGAATATATGCTGATACACTACCTAATAATGAAATTTGT
CTATCAGATGACGGATTGACGATTAATGAATTAGAAATGTTGGGTATTGATATAAACACAAAAACGAGAACTAAACTTATACAAAA
TATTTTAAATCAATTTAATTTAAAATTAGTTGATAAAGAAATTACAGCGGATGTTAAAAATGAGAGTTTTGCTCAGTCTAAACATAA
TTTAATTCAAGGCATATTGAAAATTTATGACCTTACTTTAACTACTAAATCTAACGTTACAAACATATTCTATGAAGAAGTATTTGAA
TTCTTATATGACCAAGAAATAAGAGGTTTAGCACAAGTTTCTGTTTCTGGAGAATCAGGTTTAAAATATTCTATTGATTATATTGTTT
CTGAAACAAAATCACAACCAGAAAAACTAGTTAACTTTACAAACAACTTAGATTTCAACAAAATAACTAATGAAGCTTATATTTAC
CGTGATATCAAACCAAACAGACCATCCAGAAACAAATTAGAACCTATCATGTTGATAATTGTAAATGATGTAGATCATCCGATAAA
TGAAAGAGCACAAACTGTAGCAGAACATGAAAACCTTGAGATATTAAAATGGTCTAATAAATCAAAAATCATTGAAACTTTAACCT
CATAAATTAGCAGTAGTCCCTTATTTATATTCTTTAATTTCCAAACCATATCGTGCAAATAGAAAAGCAGATAACTTTTTTATTTGCA

CGTTTTATTTTAGAACAAAAAAGAACAAGGTCAACGAGTCAGAAAAACTTTTTCTTGATACGTATTTGACACTTGCTCTTTTAAGAA
AACTAACAATTCATTGTATTTCCGTTAAATTAACGGAGAATTCGGATTACCTCGATAGATTTACCTCTAGAGTGTTAAAAAATTGTA
CATTCTTAAACAATCATAAATGTACGTTATTAAATTATCATTTCCACTGGAGAATCATGTCGACTAAAAGATTATAAAATTAAAAAT
ACATATAAAAGCACGTCATTTTCAAACCGCCATAAAACAGCATTTTTAAACCGCTATTGACATAAAACATATTTAGAATAAATCATT
TAGAAAAATTAAATAATTTAACCGGCAATAAACTAACTATTTACATGAATCTATAAAACACTTAGAACAAAAGTTCGAAAAACGCA
CTTTATGATTGTATAGTGATATCAAATATACTTATCAGTGAGTATTAAGAAATACTTCCATTTTAATACTCTTAGAATCAACAACTTT
ATTATCTCCGTAAATATTTAAAAACTTATATGGAACAAAAGGTACTAGTTCTTTTTTAGGAAATAAGTCAAACCAAAAACTTGTATT
GTTCTTTTCAGTAAATTTTATATATCCAGATTCAAATGCAGAACCATCAAACTCGTATAGCTTTTTTTCTTTAGTGAGCTAGTGTCTTG
CTTTGTAATCTAGTTCCTGAATAGTAACCATATTCTTATTTGTAGTTATTGTAAATCCAAGTGATTGTCTATTGTCGATTGTTACCTGT
ACAGTAATTAATTTATCTCTTTCATTTTCTGAACTATTAAATGTAAGACCACCATACATACAACAACCTCCAAAATTTTGGTTTATAT
CCGGTTCAGATTTAGGTATTATACATGTATAAAAATATGGAACGCCAAAAATGTCTACTTTTTTATCTTTATAATTGTTAGCTAATTC
TGTATTTTCTAATTCAGTTTTAACCTCATTATAACTCTTATACTCAATTGGAAAAATTAAATCATGAGATAAAAACTGTCTAGAATTA
ATAACTCCTCTTCCTTCAACAGGTGGAGACGTATAAAGATTCATTACATTACCCATAGTTCCCTTATTATTTTTATAATCACTTACTTT
ATTTAGTTCGTCTAATTTAGGATCGGGTTGAGCATTCACATAATTCATATTATGAAAAACTATTTCTAGAATCAAAATAATAATTAC
AGTAGATAATTTCTTCATAAAACCTCCTCTAACTTATTCAGTCTATTTGACAATGAACCTTATTATAAATTCACGCTTATAGATAGTT
CAGGTTGTAGTGTGTCGATAGCATTGTTTTAAAACTAAAAATTCACTAGCAGAATAAATTAAAATTCTATTTATCATTATTACTAATT
ACAACAAATAATTTTGAATCCGTATTCAAGATAATATATAAATTTCAAATTTTACTACAATTCAAATCATTTTCCTCTCATATAATGG
TTTAATTTCATGATTCAGTTGATAATCATCAATCACTTAATCTTTATATAAAAATACATCAATATGATAATTAGATGAGCTAACTGTT
CTATTATCACTATAAAATTGAAAAAACTCTGCTCCCACTGAACCTTTTACGTTATATAAATCATAATAAAATGATTGATCTTGATGAT
TATGAGAATGAAAGAAAATGCATCCTTTTTGTATGTTACCGGTATCTTTATTGTATATTTTATATAAATTTTCTAATTTAAATCGAAC
TTTAGTGTCTAATTCTTGTACTGTTACTTTAGCCTTTTTGGTTTTTATAACAAAACCTTCTTGTTGGACACCATCTTTAAATACATTAA
CGCCTATAACTTTCTCTTCATCTAATTGATTTCCATCATGTATCGTAACTCCTCCGTATAAGCATGATGTTTTTTCTTCAGTTAAGCCT
ACACATTTATTTCCAAAATACAGTCCATAAATATCTATATTTTTTCCTTTAAATTGATTTGCTAAATCTGATGAGTTAAACTCAACTTT
CAAACTAGAAGTTTTAAGTACGGATATATCAATATTTTTTAATATTATAGTATTATTCAGTAGTTGATCTGTACTAATTTTATTTGAC
TCGTCTAATTGCCACGTTATATCAGTATAATAGCTTGTTAAATTAAATAACTTACTACTATCTAGATCAGATTTTTTCTTTAAATCTTT
TTTGTCTACTTCAGCATTAACATAATTATTATTAATAAGACATAATAAAGTTATTATAATTGCAGCTATGTAGAACAATCTCATAAGC
TTTTTAATATTTTTTATAATATTCTCTCCCTCAATTATTTTTTGGTTAAATGAACTTCTACATTAATAGATTTACTCTCAACTGTTTTAT
TATCATTATAAATTAGTAAATATTTAGTCGGATAAAATTTTTTACCAGATTCAGGCATCAAATCATACCAAAAAGAATGACCACTTC
CTTCGATAAACTTTATATAGCCAGTCTCATAAGGCGAACTGTTAAATTCATATAAATTTTTATGCTTAAGTAAATAGTTTCTAACTTT
ATAATCTATTTCTTGTGCTGTTATGTTTTTCTTATTAGTAGGTATATCAAAAGATAATGAATTTCTCTCGTTTTCATAAACTTTAATTA
AGATATTATGAGAGTTATCAGTTGAATTATTTTTATCTATTTGATTTCCATCATGCTCGGTCACACCGCCATACATACACGTTTTTTCA
ATTAATCTACCATCATTTAATTCCATATTATCCGATGAAAAATAGCATTGATTATAATAGTTTGTCCCAAACAAATCTACGTTTTTAT
TTTTATATTTATCCGAAAGGCTTTTATTATTAAATTCTGTTTTTAAAATTTTAGAACCATTTATTTTAAATAATAAATCATGTTGTAAA
AACTTTTCTTGGGATTTAATGTTTGTTTCTGATACGTGTTTATCATCATACAAATACCTCATATTATCCATTAGACCAGTGAATTCACT
CGCTTTATTCAATTGTTCTGGTTTAGGATTGCCATTTAACATAAATAGTAAGCTGCAAGACTTAACACATATGAAGATAAAAGCAAA
TAACTTCATTACAACAACTCCATATTTTAATTTTTTATAAATTTGATGTATTACAGGGAATATTTAACGTACACATTAGCCCTAGAGA
CTTTAAAATTTAAATAGTTAATACATATATTAATTTCGTATAAATTCGCATTTTAGTGTTAAAACTAATTATCATTAGTTACTATCTA
CATATGATATTTCGACATCAAGATGAAATTTTTCAGATTCTATTATTTTATTATCTTCATAAATTTTCAAAAAACTTACAGGCAGTCC
ATCTCCTGTATAAAACAAATCATATGAAAATGATTTTTCATTATTTAAATGAAATAAAACTTTCCCATTATTAAAACCTGAATAAAA
TTTAGATTTATATCCATATTCTTTGCCTTTACCAGTGTTATTATGACCATATATATTGTATTCTTCTTGAAGATATCTCCTTAATTTAA
CATCAATTTCTTGAGCTGTTACTAGTTTTTTATTAGTTGCTATTTTGTCAGTAGAAATTGTTTTATGTTTGCCATTAACCCAAAGATTA
ATAGGGATTTTTCTAGCAGAATTTAAGTATTGTCCTGATAAAGTGGCCCCTCCAAACATGTATTTAGATTTACAAGGACCATTATAA
TCAATGCCAAAAATATCCAGTTTCTTTCCTTTAAATTTACTTATTTCGTCCCAATTATTAGATTCTGAGATCAAATCATTGGTACCTGT
TGAAAATTCGAGTTGATTTGCAATAGGTAGATTTTTATCTGTGACGCCTTTTAAATCTATATAATCATGTTTTGTATAGAAATTTCTT
AAGTTACCTACACCAATATCACCTTGAGCATACGTAAGATCTTTAATGTTAAAAAGTAATATAAAAACTAATATAAAACTATATTTA
AATTTTTTCATGGCATTTCCTTTACTAAGTTATGATTGAAATACTTCAACTTTCGTCCTTATAAGATATTTCTACATCTAAATGGAATT
TTTCAGTTTCGACAGTTTTGTTGTCATTATATATTTTTAAGAAACTTTCAGCTTGTCCTGTTCCAGTATCAAATAAGTCATAAGAAAA
TGATGAACCATCATTCAAATGAAATAATATTTTTCCTGCATTAAATCCAGAACTAAATTTTGACTTATTACCATAATTTCTTCCTTTA
TTTGTATCATTAAAGCCATAAATATTATATTCTTCTTGTAGATATCTTCTTAATTTAGTATCAATTTCTTGAGCTGTTACTAACTTTTT
ATTAGTTGATACTTTGTCAGTAGATATAGTTTGATGTTCTCCATTAACCCAAAGATTAATAGGAATACGTCTAGATTTCTCTAAATAA
TCACCTGCTAATGTAACTCCACCGTATATATATTTGTTTTTCAAACAATATCCACTATAACTTAGACCATATACATCTACAGCATGAT
TTTTGAATTTCTTTACATCATCAACGTTCTTAAATTCAGCTGTTATTGTCGAATTATCCATAGAAAAAACTAATTGATGCGAAAGATG
ATTATTTTCAGGATTAATACTTTGGTGGTCTTCAATTGGATAGCTACCATAATAGTTCCTAAGATTCAAAACTCCGACATCAGCGGTT
GCGATATGATTTTGCGAATAGCAAAACAATAAAACAACAATGATAAGTATTCTTTTCATATTTTATTTCTCCTATATTTAAACTTCTC
TATCTTTATTATCTTTTATTGTAAAAATAACCAATTCTTCTTAAATCATCATGATTCTATCCCACTTATATATTATTACTCGTTAATCT
GGTTTTGTTATCTATACTAAAATAGAAAGCTTTTTATAGTAATAAAGTTAGATTTGAATCACTATTCGTTCTTCATCATTTACTAAGC
TACTTTGTAAAAATATGTTAATATCTATAGTTCTTTATAGGATTGTAAGTTGATATCCATCAACTTTCAGATTGTTATGTAAATAAAT
AAACATCAATATGATAGTCTGATGAATCTATTGTTTTATTATCATTATAAATTTGCAAATATTGATCTGGTAAATTTCCTTTAATATA
AAATAAATCATAATAAAATGATTCTTTATGCTCCGAATGAGAATGAAATTTAATATAACCTTTTTGTACATCACTAGTCATTCTGTCA
TACAATTTATATGCATTATTTAATTTTGTTCTTACTTTAATATCAAGTTCTTGTGCAGTAACTTTCTTTTTATCTGTAGTAACGATAAA
TGTATTAACATTTACATTATCCTTATACACAGCTACTCCTATATTTTTAGGCTCGCTTAATTTATTATTTTCATGAGGTGTTACCCCAC
CATATGTACAGGCAGTATCCACTTGATGCTCACCATGACAATGTGCTTTATAGTAAACACCATAAATATCAACAGTCTTTCCTAGAA
ATTCTTTAGAAATCGCTGATGAGCTAAATTCCACTTTTAAATCTTTAAATTCAGCAGATATTCCATCTAACCAATTAATACTTTTAAA
TAATAAATCGAAGTCTAAGAATTTTTCTGTAGTTGATACAATTGATTTTACTGTCGTAAATCGATTATTTATATAATCATCATTAATA
TTATGTAAAGCAATAGGGTCTACACTTGACTTCTTACACAAACTCTCTATTTTAGGATCTTCTTCATTTGCATATGCATTGTTTACACT
ACATATTGCAATTAAATTTAAAATTAATAATAATACATTTAACATTACTTTACTATTTTTAATAATATCCCTCCTTCACAAAAATTTTT
TCTTAATAAACTGAAAAATTCTGTTTTTAATCTTTAAATATTCACTAATATTATTAGATTACGATTTTAAACTTAACAATCCTACTTAC
TATTGAACAAACAAATTTTAAATTAAGTGACTCAGATAATAGACAAAAAACCTTAAAAATAGCGCTTAAAATGGGTAAAACCATTT
TAAGCGCTATAACGGAGAGTGAGGGATTCGAACCCTCGAGACGCTTGTGGCGCCTACACACTTTCCAGGCGTGCTCCTTCGGCCAA
CTCGGACAACTCTCCTCAACAATAAATATGTAATAAAAAAACAGAAGCGATATTTCACTTCTGTTTGTATGACTCCTACGGGACTCG
AACCC

>046-contig_53_RC

GGGCGGCTGAAGGGGATCGAACCCTCGAATGTCGGAACCACAATCCGATGTGTTAACCACTTCACCACAACCGCCATGAATTAAGA
ATGGTTCAGGACAGAGTCGAACTGCCGACACATGGAGCTTCAATCCATTGCTCTACCAACTGAGCTACTGAACCGTAATGGTAATA
ATAATGGCGGTCTCGACGGGAATCGAACCCGCGATCTCCTGCGTGACAGGCAGGCGTGTTAACCGCTACACTACGAGACCTATTAA
ATTAAAAACTATGTATTGCGGGAGGCGGATTTGAACCACCGACCTTCGGGTTATGAGCCCGACGAGCTACCGAACTGCTCCATCCC
GCGCTAATATTATTTTGAATTACCTAATTAATATACCATAATCAAAAACCTAAAGTCAATAACTTTTTGAATTTAATTTAAATGTTAT
CTCTTAAATAATTACTTAAATATCGTAGCAACATGTTCTCTGTTGAACACAAATATTAGTATATTCATTTTTGTAGTGTACGTCAACG
ACATTTTCAAAGTTTTTTGTGTAAAAAAACGCTTCTTATTCCCTTTTATCATATAAGTGTCTAATAGTTGTCATAAATAGTGTTAAAG
CATTTAAAAAGGTATAGGAGTTATAAAGTTTACAACGCCTATACCTTCTGAAAAAGAAATTATAAAAGCTTGTTACACCGCATATTC
TTTCAGTCAGCGACTACCAATATAACATTGTAGCCCTAAGACATTGCTTGACGCCTCAATTACAACAATTTTTCAAAATCAGCAGCT
ACCTACTGACACAACATAACACAACCCATACACTAACTATCGTGTCATGTAATCTTGCATCCGATCTTGCAACGCTGTAAATGTTTC
GAAGCCATCCTCTTCTAAGAAGTGCCCTCCATCTTCCACGATTCGCAAGTTCCCCTCTAATGCATTCATTAAACGCTGGGTTTCTTTA
TAAGAAACGTATTTGTCATTTTTAGAACTCAATCCGTAAAAATTGTCAACTTTCTTTTTAATATTATCGTAATCAATGGTTACATTAC
TTAAATCAATATCTAAATCTATATTTTCTGCATCTTCTTTAAAGCCCGCTATACTAAAAAAGCCTTCAATCGGCTGATCAATCATTTC
AATATATTTTAAAGCTGTGATTGAACCTAAACCATGTGTTACAAAATATGTATCCTTTTTGCGTACATTAATTTGTTTCGTCATAGCT
TCAATCCACTGATCCACTGTCTTCGCTTCAGGGGATTCAAAATTAAATAATGTTACGTCATATCCTTCTAAAGTTAAGTTATGCTCCA
ACCACTGATACCAATGATTTCTACTATTTCCATGCATAGAATGTACAATAATTACATCTGTCATCTCATTCTCTCCTTTCAACTTACTA
CTTCTTTTCTATTTTTAAAAAAATGACTGATTACCTATAATTGTAAAATAAAAACACCTTAATTAGAAATGTTATATCGCAAAGTGAC
ATTTCTAATTAAAGTGTATTGTCATCATTTCAATATCATTCAAAAACAGCTAAACCTTTGTCTCTGCTTCAATTTCACAAAAATAATT
CCCGCTGAAAGTATCTATATTTACACACTACTTCTACCATCATATAACTTAAAATGACTATATTTCATCAAACATTATCTAAAGGCGT
CGCACCTACACCACACCATCCAACAATTAACTTACAACTCTGCGATTACTTCTTCAGCAGCAACTTTACCTTGCGTAATACAATCAG
GTAGTCCAACCGCTTCAAAAGATGCACCAGTTACTCTAAGTCGTGGATATGTTTGTTTAATATGTGCTTGAATCTGTCTAATTTGTTG
AATATGACCGACATGGTACTGTGGCATACTTTTCGGCAAACGATTGACAATTGTAAATTCAGGATCACCTTTAAATGTCATCATTTG
ACTTAAATCTCTACGTACAATCGATACTAATTCATTATCTGTATGATCATCAACCACAGTATCACCTGGTTTACCTACATACGCACGA
ATCAAAACCTTACCTTCTGGTGTAGTAAATGGCCATTTTTTCGATGTCCAAGTACATGCGGTAATGTCTGTATCACTTGTTCTCGCAA
TCACGAAGCCAGTACCATCATAAGTATTTTCAATGTCTTTTTCATCAAATGCCAATACAACAGTTGCAACAGTCGTACTATCCATCGT
TTTAAAGTAATCAAATGCTGGATCTTGCCCGAACCAATTCAAAAAGACTTGATGTGGTGTTGTCACTAATATTCCATCGAATACATC
TTCTTGTTGATTACTGTAGACAATTTTATATTGCTTTTGAGATGTAATAATATCATCCACTGACGTATTGTAGCGTATTGTCACACCTT
TATTTTTAACATCTTGTTCTAATGCTTCAATAAATGAGCTTAAACCATGCTTAAATTGTTTGAATTGTCCTTTTGGTGCGCCAGGATA
TAATTGTCTTTGTTTCAGACGCTTATTTTTCTCATCCTTCATACCTTTTATCAGACTTCCGAATGCCTCTTCTTTTTCTTTAAAATTAGG
AAACGTACTCATCAAACTTAATTTATCAATATCGGTACCATAAATACCACCCATTAAAGGCTCTATTAAATTCTCAAGTACCTCATT
ACCTAATCTTGCTCTGAAAAATGCACCAACAGAAATGTCACCATTCTGCATTTGAGTAGGTTTTTTTAATAAATCAAACCCTGCTCTT
AACTTTCCAAATGGTGAAATTAATTTAGTTGTCACAAACGGTTTGATATCTGTCGGAATTCCCATAATTGATCCACCTGGAATAGGA
TACAATTTGTTTTTCGCAAAAATATAAGATTGTCCAGTCGTATTTGTAAAAATATCTTGTTCTAATCCAATATCTTTCGCTAATTCTGT
CATGATTGTTTTTCTACCTAAATAAGATTCAGGCCCTAGTTCAATCATATAACCATCTTTACGATACGATTGAATCTTTCCCCCCGGA
CGATTCGATGCTTCAAAGATTGTTATGTCAATATTAGGATCCTGCTGTTTTAAAAAATATGCACTTGATAAACCTGTGATCCCCGCTC
CTATTATAGCCACTGATTTAGTCACAACGTTTCACGCTTCTTTCATAATTAAAATATGGACTTTATTTCATCAACAATTGCACCGATA
AATAATGGATGTGTATTCGGCATTTCCGGTCGATAATAATTCGCACCAATATCATCGCAAACAACTTTACATTCATAATCATTGTCAT
AAAGCACCTCTAAATGCTCACATACAAAACCTACTGGTGTATATATAAAGTTTTTATACTGATGTTTTTCATATAAATCACGTGTTAA
ATCTTGTACATCTGGCCCTAACCAAGGTGTACCTGTATTACCTTCAGATTGCCAACCAATCGCGATATGTTCAATATTAGATTGTTCT
TTAATTAAAAGTGCAGTATGTTCTAGTTCTTGTGGATATGGATCATTATTCTTTTCGATTAAACCTTTTGGCAAACTATGTGCCGAAA
CAACTAATACCGTGTCTTTATGTTCCTCTTCCGGTATTTGAGCTAATGTTTCGTTGACTTTATTCGTCCAATATTCAATAAATTTAGGT
TGTTCATAATAATGTTTCACATGTGTAAGTTGAATACCATATTTTGCAGCTTCTTCATCAGCACGTTTGTCATATGATCCTACTGAAA
ATGAAGAATAATGTGGTGCTAGTACTACAGTAATTGCTTCAGTAATGCCATCATTGTGCATTTGTTCAACCGCATCTTCGATAAATG
GTGAAATGTGTTTTAATCCTAAGTATAGTTTAAATTCAACATCTGCATATGCTTTATTTAATGCTGAAACTAGCGCATCAGCTTGGTC
ATCTGTTGTACCTGCTAATGGTGATAAACCACCTATAAATTCATATCTATCTTTCAAATCTTGAAGTTCTTCTTCAGATGGACGTTTA
CCATGTCTAATATCTGTATAATATGGCTCTATGTCACTTTCTTTATAAGGTGTGCCATAAGCCATAACTAATAATCCCATTTTTTTAGT
CATTGATAATACCTTCCTTTAAATGAATTATCTTTCATGTGCTTCAATGTAATACTATGATTATCTTTGTGTATATGTGTGTACGAATT
CGCTTACTTTACGTAACGTCTCTGGTTGCACTTCTGGGAAAACACCGTGTCCTAAATTAAAGATGTGTTTACCGTTCTCCATACCTTG
ATCTAATATTGGTTTCAATCTCTCTTCAATGACATTCCATGGTGCTAATAAAATTGATGGATCTAAATTCCCTTGTAATGTTTTAGTA
ACGCCTAATTGTTGAGCCTGATTAATAGACGTTCTCCAATCTAGGCCTAATACATCAATCGGTAAATCATTCCATTCATTGATTAAAT
GACTGGCACCTACACCGAATAAAATTACCGGCACATCATGTTTTTCTTTAACCTCACTGATTAATCGAATCATATGTGGTTTAATGTA
ACGTCTGTAATCCTCGACATTTAATGCACCTACCCATGAATCGAAAATTTGAATCAATTCGGCGCCTGCTTCGACTTGAGCTGTTAC
ATATTTAACAGATACATCAACTAAATGATTCATTAAAGCAAACCATGTTGCTTCATCTCTATACATCATCGCTTTTGTAAAATTGTAA
TTTTTCGATGGTCCGCCTTCAATCATATATGACGCTAATGTAAATGGTGCCCCAGTAAATCCTATTAGCGGCACATTTAACTTTTCTT
CTGTTAAAAGTTTAATTGTATCTAATACATATGGTACATCTCGTTCGGGGTCTATTTGAGAAAGTTTCTCAACATCTTGAATTGTTTT
GATTGGATTATGAATCACTGGACCAATACCCGATTTAATTTCTACATCGACACCAATTGGCTTTAATGGTGTCATAATATCTTTGTAT
AAAATTGCTGCATCTGTATGATAATTATCAACTGGTAAATGTGTTACATAAGCGCACAACTCCGGCTGATGTGTAATATCGAATAGT
GAATATTTTTCTTTCAATTTTCGATATTCTGGTTGCGAACGGCCAGCTTGTCGCATAAACCAAACAGGTGTATGTGATGTTTCTTCAC
CTTTGATCATTTTTAAAATTGTATTGTTTTTATTATGCACCTTAAAGGCCTCCTAAATTAAAATCATTCTTATCTATATTATCATATCG
CTCATTCGTTCGTATTTTCAATAAATAAATGTCATAAAACTGACATTTAATCATAGAACTATTTATTGTAAATTTAAATTCTAAAGTC
CATTATTTTGTATCATTACTTCTAAATATCTCGCAAGATTCATTATAGTAATTTTAATCAATTATTAATAGTGGTAATGACTAGTTTAT
CATCGTATAATAAATAAAAACATAAGGGGGACCTTTCATATGAAGAAACTATATACATCTTATGGCACTTATGGATTTTTACATCAG
ATAAAAATCAATAACCCGACCCATCAACTATTCCAATTTTCAGCATCAGATACTTCAGTTATTTTTGAAGAAACTGATGGTGAGACT
GTTTTAAAATCACCTTCAATATATGAAGTTATTAAAGAAATTGGTGAATTCAGTGAACATCATTTCTATTGTGCAATCTTCATTCCAT
CAACAGAAGATCATGCATATCAACTTGAAAAGAAACTGATTAGTGTAGACGATAATTTCAGAAACTTTGGTGGCTTTAAAAGCTAT
CGTTTGTTAAGACCTGCTAAAGGTACAACATACAAAATTTATTTCGGATTTGCTGATCGACATGCATACGAAGACTTTAAGCAATCT
GATGCCTTTAATGACCATTTTTCAAAAGACGCATTAAGTCATTACTTTGGTTCAAGCGGACAACATTCAAGTTATTTTGAAAGATAT
CTATACCCAATAAAAGAATAGATTCGAAGAAAACTTCCATTGGTTATGCTGTTAACGTATAGATGCTAATGTAATCTGAACGCCGTA
TCATTTGGCGTTCAACATTACATGCACCTTTACAACATACATGCCAATGGAAGTTTTTTAGTCTCGTAATAATGTTTCCTGATATTTC

AACTTTTTAATAATACTTCTAATCGTTAATAAACCTACAATATAGAAAATGAGTACAACATAGAATAACGTCATATGTTTTATAATA
AATGTCACTGCAAACACAGTACAAATAACAAACATTAATCTATATAAAAACTGTTCATACCCTTTGATTACCTTCTCTTCAGGTACA
GGCCACACTTGAGGCCATAAGCCATATGCTTGCTGTGAATAAAATTGTGCCATTTGTAACAATATAATATATACAAATAAACACCCA
ATAATTGCTGTCACTAATGGATATGATAACCAAACCATTAATAAAACTGCAATAATTACTAACCTAAAGATAATATTAAATGCGTCT
CTCCCTCTTATAAAGCTTCTAATAAATAAGAATAAATACATCGCATTAGAGTTAAATTTACTACCCTTTGGAACTGGTAAAAGTATA
TCTAGATAACTTCTTCTGACTGCAGATTCTTTCAAATGTTTTACATCGGTGAACATATTAACAAATTTATAATAATTCATATGATGTC
GATGTTCGATTGCAATCATTTTCTCCCAAGGATACAAAAAGCCTGGTTTATATTTTTTAACTAAAAATTCTATTAACACAGGCAAAG
CAACCATCACAAATGCGATGTACCATTTTGGAGCTAATAGTAAGTAATATGTTAGAGCAAAGGTGATGAATGATATTAAATTAACTT
GCCATGTTTTAAGTCCCGATTGATACCATTGCCATCTTAAGCGTAAACCAACATATGGAAAAATTAATGCACTGACTCCAAAACAAA
TATAAAATGCCACATTATGTTGATTAATATTGTAAAACAACGGGAACATTACAATAACAATAATGAGTTGGATTAATATGCGCGCA
AAGTAACTATATAAAATCGCATGACGCATAAATTGAGACATGTGTTTTTCAAATGGTAATAAAAAGATTTTATCCGCTTCTTTTAAC
AGTGGTCGCATTGGAAAAATAGATGTCAACGCAACAATCACTGCTGCTATTAATGAAAAATTGATATTCGTTGGAATATGTTTTAAC
CATTCACCATATCCAAAAATAAATGCACCCAGCAAAATAAGTAAAAAGACCATGAAATGACCATTAAATATAAACTTATTATATAATA
ATTTTTCTCTTTACGAAGGGCATGTAATCTTTTATTAAATAATGTGGTCGCTTGGTTATGCATGTACATCTCCACCTTGCGTCACATG
AATATATATATCGTCTAATGTTTGTTTATGTAAGCCAGTTTGTTGTCTCAATGCTTCTAAATCTCCAAATGCAACGACTTCACCTTCG
TCTAGTATGATAAAACGATCACAATAACGTTCAGCTGTTGCTAAAATATGTGTACTCATTAGAACGGTTCTACCTTCGTTTTTCTTTT
CAACCATTAAATCTAACATGGATTGAATTCCTAATGGATCTAGGCCAAGGAATGGTTCGTCTATAATATACAATTCGGGATTAACGA
TAAACGCACAAATAATCATGACTTTTTGTTTCATCCCCTTAGAAAAATGACTCGGAAAAACTTTCAATTCATTTTCTAAACGGAATG
TCTTTAATAATGGCATTGCTCGATTCATCGCTTCATCACGATCAATATCATATGCCATTGCTGTCATCTCAATGTGTTCCTCTAATGTG
AGTTCTTCATAAATAACCGGTGATTCCGGAATATAAGATAACTTTCTTCTATAAGCCTCTATGTCATCATTAATGTTGATATCTGAAA
TTGATAGAGATCCTTCCATAGGTGTAAGCAATCCTAGCATATGTTTAATCGTTGTACTCTTACCAGCGCCATTAAGGCCAATAAGTC
CAACGATTTCGCCTTTGTTTAATTCAAAATTTATATCTTTAATTACAGGGCGTTTTCCATATCCACCTGTAAGCTGTTCTACTTTAACT
GTCATAAGGCACCTCCATGACTTATATTGTACCAAAAATTATAAAATGCTCATATTAAATACACATGTCCTAATATCGAATTTTTAG
CGACAATGTTATAATGAATGGTAATACAAGTTGAAAAGGAGTGTAGTCATCATGTCAGAAACAATTTTCGGCAAAATTTTAACTGG
AGAAATTCCTAGCTTTAAAGTATATGAAGACGATTATGTCTATGCCTTTTTAGATATATCACAAGTTACTAAAGGACATACGTTATT
AATTCCTAAAAAAGCTTCTGCTAATATCTTTGAAACTGATGAAGAAACAATGAAACATATCGGTGCAGCATTACCTAAAGTAGCAA
ATGCTATTAAGCGTGCATTTAATCCTGATGGTTTAAACATTATTCAAAATAATGGTGAGTTTGCAGATCAATCTGTATTTCATATTCA
TTTCCACTTAATTCCTCGATATGAAAATGATATTGATGGATTTGGTTATAAGTGGGAAACACATGAAGACATTTTAGATAACGATGC
AAAACAACAAATTGCTGAACAAATTCAAGCACAATTTTAAATGTATGCTTAATCTAAGCTCGAACGGGTATAATATGATTAATATTA
TAACAATTGCGTTTGAAGTGATAACATCAAGGTTAGCAATTTTAAACAAAATGAGTTATCAAGATAACAGATGTTAAAAGTGAGGA
GAATATAAATGAAAGCATCACGCATTCTATTCGGTATCGGTGTTGGCGTAGCAGCTGGTTTTGTAGTTGCACTTCAAGGACGAGACG
ACAAAAGTGTCAAGAACAACACGATCGATCGTACTGCCCCTACTGGTTCAAAATCAGAACTACAACGTGAATTTGAAACGATTAAA
CAAAGTTTTAATGACATTTTAAACTATGGTGTTCAAATTAAAAACGAAAGTGCGGAATTTGGTAGTTCAATTGGTGGTGAAATTAAG
TCATTACTTGGAAACTTCAAATCTGACATTAATCCTAATATTGAACGTTTACAGTCACACATCGAAAATTTACAAAATCGTGGCGAG
GATATTGGAAACGAAATTTCTAAGTAGCACGTTACGTTCTCGATCACAACTATTATTATTAGTAACAGCATATTTATTTTTTTAATT
AAATGCCAAATAAACGAGATGACATTAGAAATTAGATATTTCTTGTCATCTCTTTTTTATAACTCAAATGAACTTATGTTTACAAATT
ACAGGAATACATCGTTTGTAGTGATTTTCGCTTAAATCATATTTATGAATTGATTGAAAACATTGCTTAGGATTCATTGTGTTATCCT
TGCACTTTGATTACGCTTTACTTAAATCATTATCGACAAACAACATACTTATATTTTCATTGAGCCGAACCTTTTATGCACATTACAT
ATACCTTACTTGCACAAATTATTAATCTGGTATTTATTATAATTACATATCACTATATTTTTAGCATTTGTATAACTTAGTTGGTCAAA
AGATGCTTTTGCATATGCCCTGCCATATCCATTTTTTATATTATTCAAGTTTTCAAATACTCATGTCACACGCGCCTTTTTCATAAGTG
ATGCTTTATTAGCAAGAAGATGTGTTCGCAGAAATTTGTTCTGCATTCTACTTCTACGCTAGTCAATCAGCCAATTTTACCAAACTCC
ACTTTCGCGTTTCAAATCAAACAATACTTCGCCCCTTTCTTCTTATATAACAATTCTTCTAACATGATATGTTACTATTGAATTACTGA
ACCTGAGTTAGTTATAATCTAACTTATATTGAAAAGAGATGAGGCGTAAGATATGTTTTTATGTAAAAGACAAATTGATATCAATGC
ACGATTTGGTTTGCCTAGAATTGCATTTATGAGTGCAGTTGCAACCATCATTATGTTTTTAGTTAGTTATGAAGCAATGTATTTTTTA
TCTAATACGCCATTATCAGATAGACATTTTCTCATCTTTTTATTACTTGTATTTATGACGTATCCATTACATAAAAGTATACATTTGTT
ATTTTTCTTACCATATAGAAAATCGTTTAAAGTTCATAAGTTGACTAAAAGAAAATGGCTTATATTCTATAATACCTACGTCAATCA
ACCTGTACACAAATTTTATTTTTGCATTAACTTAATATTGCCGTTAATTATCTTATCTGCAATGTTCGTTTATCTAACAATTTTATTCC
CGCAATATGGACATTATTTTATGTTCTTATTGGCATTGAATTTCGGTATTTCCATTACAGATTTATTATATTTAAAAATAATTATATTT
TCTAATTATGGACAATATATATAGAAGAACATAGTACAGGTATTAATATTTTGAAAAAAATTAAAAATCCATATCATTTATAACAAAAT
AATTATAGCAAGGTGTTATTATTTGTTTTTAGGCTATGTAATAGCTTACAATCAAATGTATATAGACCTTGTTTTTTTATTTTCATCAA
TTTCTACCCCTAAACCTAATGCTCTAGTCTGATGTCATGGGTTATTGATTGGTGATAATATAAAACTATGTTATATTCACGATGATTA
ACTTACAAAGGAGTTTCAACTATGAAGATGATAAACAAATTAATCGTTCCGGTAACAGCTAGTGCTTTATTATTAGGCGCTTGTGGC
GCTAGTGCCACAGACTCTAAAGAAAATACATTAATTTCTTCTAAAGCTGGAGACGTAACAGTTGCAGATACAATGAAAAAAATCGG
TAAAGATCAAATTGCAAATGCATCATTTACTGAAATGTTAAATAAAATTTTAGCTGATAAATATAAAAATAAAGTTAATGATAAGA
AGATTGACGAACAAATTGAAAAAATGCAAAAGCAATACGGCGGTAAAGATAAATTTGAAAAGGCCCTTCAACAGCAAGGTTTAAC
AGCCGATAAATATAAAGAAAATTTACGTACTGCTGCTTATCATAAAGAATTACTATCAGATAAAATTAAAATCTCTGATTCTGAAAT
TAAAGAAGACAGCAAGAAAGCTTCACATATTTTAATTAAAGTTAAATCTAAGAAAAGCGACAAAGAAGGCTTAGATGATAAAGAA
GCGAAACAAAAAGCTGAAGAAATTCAAAAAGAAGTTTCAAAAGATCCAAGTAAATTTGGTGAAATCGCTAAAAAAGAATCAATGG
ATACTGGTTCAGCTAAAAAAGATGGCGAATTAGGTTATGTTCTTAAAGGACAAACTGATAAAGATTTTGAAAAAGCACTATTTAAG
CTTAAAGATGGTGAAGTATCAGAGGTTGTTAAATCAAGCTTTGGATATCATATTATTAAAGCTGATAAACCAACAGACTTTAACAGT
GAAAAACAAAGCCTGAAAGAAAAATTAGTCGATCAGAAAGTACAAAAAAATCCAAAATTATTAACTGATGCATACAAAGATCTAT
TAAAAGAATACGATGTTGACTTTAAAGATCGTGATATTAAATCAGTTGTCGAAGATAAAATCTTAAACCCTGAAAAACTTAAACAA
GGTGGCGCACAAGGCGGACAATCCGGCATGAGCCAATAACACAAAACCGAGCGACCGTGGTTCAAAAATCATACCACGGCCGCTC
GGTTTTTTCGCATTAAAAATCGGACAGATGAGCTCATGTTTCAGTATACTCATCTGTCCGATATCTTTTAATTCTTAATCGAGTGATT
CAGGATTGTAGAATCTACGATTTTCAAGACCAAATATTTTATCTGTAAACTGACCCTTGTCAGTTTTTTTATATGCCTTTTCAAACAT
ATTCATTCTAGCATCGATATTATCGATATAGCATAAAATTTCTGCTTCTTTTAAGTATGGCAGTTTTGGCGAACCATACTCTAACTTA
CCATGATGAGATAAAATCATATGTCTTAACAACATGATTTCTTCTCCTTCAATGTTCAATTCACGAGCTGCTTCAACTACTTCATCAC
TCGCAATCGAGATGTGTCCTAATAAGTTACCTTCGACTGTATACGACGTCGCAACAGGACCACTCAATTCTCTAACTTTACCAATAT
CATGCAAAATAATACCACTATATAACAAACTTTTGTTTAACAATGGATAAATGTCACAAATTGATTTTGCAATACGTAACATCGTTA
ATACATGATAGCTTAAGCCACTCGCAAAGTTATGATGATGAGAACTAGCAGCTGGATATGTGTAAAATCGTTCTTGATATTTTTTCA
ATAAATGACGTGTGATACGTTGTAAATTAGCATTTTCAATATCTAGCAAATAATGAGAAATCTCTTCTTGTATTTCTGCCGGTGATAA

AGGTGCACCATCTACAAATTGTTCTGTTTTTAATTGATCTTCAGTTGTCGCTAGTCTAATTTGGTTGACTTTCATCTGTTTATTTCCGC
GATAGTTTATGATGTCACCTTTAACATGTACAATTTCTTCAGGCTTGATTGTTGCCATATCATTTTTTGTAGCCGTCCAAAATTTCGCT
TCAATTTCACCACTTTTATCTTGCAAATGTAATGTCATATAATCTTTACCTTGTGCTGTTACACCCTGTGTAGCTTTATGCACTAAGAA
AAAGTGATCAACTGAATCTCCGGGATTTAGATTCTCTATATTTCTCATCGTTTCCCGCCTTCCTCTATTTTGTTTAATGTAATCACTTC
TTTTGATGGAACAATATTATCTTTTACACATGTAAAGTATAGTACTTGATAGTGTTCTGATAATGATCGTAAATAATTCAACATTTTT
TCAGTACGCTTTTTATCAAAATGAACAAATGCATCATCAACAATTAATGGGAACGGATAATATGGTCTTAGTACCTTAATTAAACTG
ATACGTAAAGCTACATAAAGTAATTCTTTTGTAGATTGACTTAGTTCAACAGGATCATATAATTGACCATTAACATGTTTAACCGTA
ATTGAATCTTCATTATAGTTAATCATCGTATATCTGCCATCTGTTAAATGCTTCAATATTTCTACCGCTTCATTAATAACTTGAGGCA
AACGTTTATCTTTAATTTGTTTAATGTGTTCATCAACTAAACTTTGTAAATAACTTAAACTTGCCCAATCTTTTGCGATATCATTAAGT
TGATTTTTAAGACTGTGATATTCATGTCTTAAATTAGCAAGCGTTGTATCAGTTTCCATGTGATTGATTTGTGCACTTAAATCACTGA
CTTGTGCTTGCATTTCAAGATATTGCTCATTATATTCGTCAACTTGAGTAGCCAATAAATGATCTTCTTCTTCAAGTTGTGCAGTTGTT
TTTTCACTTAAACTAGAACTTAATTCATAAGAATAGTTTTGGTTCTCAAGATATTTAGTTAAATCATTAAAACGACTCAAATTACTAG
TATAAGTTTGGTAATCTTCATGATGTTGGTAAAAATCTTCTTCAGTACCAACATTGATAAAATCGAATAGTGCTGTAATTTCTTTATT
ATTTTCTTCTAATTGAGCATTTAAATGATTTAATTCATTTGTAACAAGTTTGGTATTTTCAGCATTAATACGCCATTTTTCATTCGTGT
CTTCAGCTGATTTCAACCATTGTTGCACATCGTGGAATAAAGATAATTTGTTGAAATAAACAAATTGTGATTTTGTAACAGCTTCAG
CATGATTGTAGAATGTATCTAATTCTTGAACCAATTGCTGGCGTTGTTGATTTAAATCACTGATATGTTGATCTAATGCTTTAATATT
CGCCATTGTAGAAATACTATCAACAATTAAATCATTTGAAATTTTAGATGATAAGTATAATTCATCCTTAACGTTTTCAACTGTCGAT
TGTAATTCATCATGACGCCCTTTCGCATCATTTAAACGACCTTCAATATACTGACGTTTCTCTTCTAAAATATCTTTATTTTTCAATGC
CTGTTGCCAGTGATCACGAATGCGATATTGCTCATCAAGATCAAAATCTAAGTCATAGTTTTCATCTAAAATGGCTAGTTGTGCTTTA
ATTTCTTCGATTTCATCTGTGATGGCCTCGCTATAATCTACTTCTTTTGATTTAGACATGATGATACCGATAACAAATACTAAAGTTA
ATACTGCGAAAATAATACCAAACAGCATGTTGTTTGAAATAAATGAGAAGGCAGTTAAACCAATACCTACTAATGTTAAAAGAATA
AACGTTGTTCGTAACAATTTTTGACGTTTTTGTTTTTCTTGTTGCTCAATTTCGAAACGTTCTTTCAATTTGCTATACAAGTTCTCTTTT
TCGTTTAATTCAATGACTTGTTGTGAGTATTCTTTTTTCTTTTCAAAAGTCTCTTCAGGAACTATTTTTTCTTCAACAGAATCTAGTTC
GCTATGAACCGCATTATCTTCGATTTTATTTTCTTCTAAACTACGTTCTAATTGTTTAATGTATGCAGCTTGTTCTTGTTTATTCTTGAT
TTGCTCACTGACATAACTTTTCATTGCCTCTGAACTATCTACGTCATGATGCGTTTCAGACCAACCAATATTTGCTTGCAATTCATCTT
TATCACGTTGTTTATTCGCAATATCCTTTTCGATTGCAGTAAGTTCAAATTCTTTATTTTTAATTTCATTTTCTTGTTGATTCAAACTAA
TAAAGGCGTCAATATCAGATTGCTTAACTGGCTCTAATTGAGTCGCTTCTTCTTTAAGTTGAGCTAAACGCTCATTTCTTAAACCAAT
ATCTCTTTCTAACGATTGCTTATGCGCTCGTGCTTTTTCGTAACGATCCACACCTTTTTCTGGGAATGTGATTGGCTCAATATTTAACT
GTTGTTCTAGAGACTTCCATTCTTGTGAATGATCATGTAAAGCAACCTCTTTTTGTTTTTCTTCATGCATTTTTGATAATTGATTTAAA
TTATGCTTTAAATTCTCTAATCGACGTGATGATTTATCACGATCATCTACTAAGCGATGATATGTTTCTAGTTTAGCTTCTTCTTCACG
AATTTGACTTTCTAGTTGTTTTAATTGCTCAATTTGTTGATTAATGATCGGATTTTTACCTGATTTTTTATATAATTCATCTTTTTTACG
ATTAATCACTTCGCGCATTGACGTGAATTCAGTTGATCCTAAAGCCCCTGCTTGTAATAAATAATCTTGCAATTGTTTTTCATTTAGA
TTTCTATGAATGTCTTGAAGCCCTAGTACATCAAATGAAAAGATACCTTGATATGTCTTTTTAGAAATATAATTAAGTTTCTTTTGTA
ACCAAGCATCATCACGCACAGCACCATTAGGTAAATATACTTTCACATCACCTTGAGCACTGCCTTTAATTCGTTCAACTTCAATCTC
TAAGCCATCATCAAGAATAAGTACTAATTTACCACCGTATTGGTTACCTAGACGTGGTTCTAGTCTTGGCTCTTTAGACTTTTTAGTT
GGAAAACCAAATAATATCGAATGGATGAATGCTTGAATCGTCGATTTACCCGCTTCATTTTCACCAAAAATTTCAGTGAAGTTTTTA
TTAAATTCAATTTTACGTTGAACAAATTGACCGTAACCATAAATTTCAAGTGATTTAATTATCATTTTGTTCACCTCTCATTTCAGCTT
TTAATATTTCTTCAGCACGATTAACTAATGCTGTATGGTCGAATGTTCCATAATCGTCTAGGAACTTTGATGCCCTTGGATTTAAATA
TAAATCTGACATCGCTTTATCAAAAACAGTTTGATCGACTAATAATTCCGCTGAAAATTCATTAACTAAAGGTGACTCATCATTTTGT
GCATATTGTATTTTTAACTCATCAATATATACAAATTGATTTTCGTTTTCTTCATAATCTGTAATCATTTCTTCAACTTGTAATAAATC
TTGAGGTGAAATTAATGTCTCACTATTAATAACAAGCGTTAAACGATAAAAGGCTTTTCCTTCTTCTCTCACTTGTTCTTTAAAGTTT
TGAATGACCTCGTATAAACCTTGCTTAGATGTCTTATCCGTTTCAATAGTTGCTTCTTCAAATCTAATATACTGTGTAGGATAAAATT
TAGTCTTTAATTTTAAGTGGTCACCCTCGATTAATAAGCAACCTTTTTCACCTTGCTCATTAAAATGTCTACCTTGAATATTACCTGA
ATAGTTAATTACAGGCATATCACTTAATTGTTGACGTTCATGTATATGACCTAAAGCCCAATAATGATACAATTTACTGTTTAAATCT
TCTAAAATGAACTCGGTATATCTTTCGTTAACTGAAGATTTACTATACGTACCATGCAAGACACCAATATGTATGCCTTTTTGGCCTT
GACTTGATGGATATTCATCAATCTTGTTCTCATAACTTGCTCTATTTTCATAACTAAATCCGTGAATATAAATTGTTTCACCAGATTT
AGTAATTGCTTCATACGTCTCAACTTTATTTGAAAATACAGAAACATTATCTGGCCAGTTTGATGAAATCTTTGATGATAATGGGTC
GTGATTACCATGACAAACATAAACAAAGATTTGTTCATTTTGTAAACGTTCAAATTGCTGTTTTAAGAAAATTTCAGCACGTAATGT
TCTGTTTTCACTATCAAATAAATCACCCGCGATAATTACAAAATCAACATCTTGTTGTAATGCAATATCTACAATATTTTTAAAACTT
TCATAAGCACTTTTTTGAACATCCTCAAAAATTTTAGGGCTTATATGACTCTTAGATTTGAAAGGACTATCTAAATGTAAATCCGAA
CAATGAATAAATTTAACCATACATCCATATCTCCTTAATTAAAGGTTCTAACATCAATTTTGCAAAATCCAACTATACAAAGCATTC
TAATCACTATAATCATACTTATAGGTGTAGTGATTCATTAGCTGATTTATTAAAACCAGTCGCCATTTTTATTTCGTTAAAAAACTAT
TATCTACACAATAATAAAGTGCTTTAGTCAAATCGTGATGTTTTATTTATTAACAAGTTTATTATTTCTTCATTTTACCATAATACGCT
TCATATCGTCGATGAACATATGAAATTGAGACACATTTATAACAGTTTAAAATTTTATTTGTAGCGTACGTTGATATTTTTCAAAAAC
TTCAGTCGAAAATGCAATTTCCGTAATTGTTAACACCGCTAGTGAACCAACTGACACATTAAAGATTTACATGATTACTCTACTAGA
TTTGCTCGGCAGTAATGACCAATTAGAAACTAAATGTCAAAACTATCATTGCACTTAATACGCCAAACATTTTCAGGCCGTTCTTAA
TGAGTCATTAAAAGTGAAACGCTTTTCCATCAGTCCGATACATGACCGAACTAATAGTATTATGTTGAAAACTTTACACTTCAAAAT
GCAAAAAGGAAGTTAGGACACGTTTACTAGCCTAACTTCCGTTATCAAGCTCATCCGCCCGTTTTGGCATAGAGCTAGCCATGTTTG
ATTGCCCAGCTTCCGTCGTCCGTTCTGAGCTAAAGCTAGGACATGTTTAGTAGCCTAAATTCCGATACTGAACGCATCTGTCCAATTT
GACAGAGAGCCTCTAACTTCCGTTATCAAGCTCATCTGCCCGTTTTGGCATAGAGCTAGCCATGTTTGATTGCCCAGCTTCCGTCGTC
CATTCTGAGCTAAAGCTAGGACATGTTTAGTAGCCTAAATTCCGATAGTGAACGCATCTGCCCAATTTGACAGAGAGCCTCTAACTT
CCGTTATCAAAACTAAAAAAACTACCCACAAAGTCTCGGACTCTGTCGGCAGTCTATTCCTCTATTTAAAGGATTGAAAAATATTAT
TTTAATATTAGTCAGCGTAAATTTCGTCTAATGGTTTAACGATAATTTGGTTGATTTCTTGGAATACTTGACTCATTTTTTGTTCAGCA
TTCATTAATGCAGAGATGTTTTCATCTTTTTCAATTGCTTGCGCTTGTTCTTGCGCTTTTTGTAAATCTTCTTCAGCAATTTCTTCACCT
TGCATTTGTTTTTGTTGGAAGTTAATTTGAGTTTCACGGAACTCGTCGAATAACTTTTTAGATTCTTCGTTAGCTTTTACATTAGCGAA
TGCTTCTTTGATTGCTTTGTATTCTTCGCTTTCTCTTAAAGCTTGTTCTAATTGATTTGCATAATCATATAAATTTACTGCCATGGTTAT
AGCACTCCTTTGTTGTGATGTTTATTAAAACGTTCGATATTACTTTACCACATCTCGCTTTTTTAGACAAAGATTGCAACCAAACCTT
GGAAAAATCCAATAATACCACCTAAAATGAAACCTAATGACATAATCAACTTTAGTTCTTTGTTAGCAATTTCTATAATTAATTTTTC
AATATAATCTAAGTCAAACGTATTAATTTGTTCTTCAATTAATCCTCGTAAATCCACTTTTTTCATAATTGTAGATAAGTGAATCGAT
AATTTTTCAATGATTAAATTTGCTAATTTGCTAGAGAGTTGACCTTCTAAATAATCAACGAATTGAGGCATTAACGTAACCACTGGC
TTATTCGCTTGTTTGCTTGCATATGTTGTTACATACACTGATAGATTTTCAGCGATTTCATTAAACTGCGATGCATCTAACAGTTCATT

CAATGGTTTATCTTTAAAAGTTTGATATTCATTGGTAATTAACGATGTCACAATTGTTCTTGCTTTAGGATGAGATGTTAAACGTATA
AGTTCTTGTTGAATGCGATCTGCAATGCTCTCTTTTGTCATAAACATTTGCAACATACCAATTAACTTACCTTTCTCATTGAAAAAAG
TATCCAACATATCATTAATATCTTGTGTGCCTTTTGCAGATGATAAATAATTCCTTGCACGATCACATAATAAGTCTGTTGCGTTATC
TACATGCTGATCTAAAAATGTTACAAGTTGATTTGGCAATAAAGATGCTATTGTTTGGTTTTGATGCTTTGTATAATAATTATTCAAT
TGTGATTCAATATATTGATTTCCATTAGTTTGTAATACTTGTTCTAAATCAATATCGATTTGAGAAGTAATTTGTTTTATTGACAATTG
ATCTTTCGTCAACTTTTGTAACTGCTGTTGAATCATAGATTCTATTGCTTGCTGTGATTGCTCGCTTTTTAATTTTTCATTAATTAAAG
TTTCTGTAAGCAAATGCTCTTCAATCACTTGGCCAATTTTAGTTGCAATTTCTTCGCGTCTTTTCGGTATTAAACCTGGTGTAAATGG
GACTCTAAATTTAAATATATAGTATGGTTTAAAGGGGTGAAACAGCATTCTAATTGCAATTACATTAGTAATGCCCCCAATTATCGC
CCCTACTACAATCATAAATATGATAATAAATAGTGCATTCATAATTTTGACTCCTTTGTGACAACATAGTTATTCATTATACCACTAT
TTTTATTTTTTAATTCATTAATCATTTCACTTTCTGCTCAATTTATTATTAGTTTACATACAAAAAGCGATGCCTCACCTCTCGAAACA
GGTGTGTTACATCGCTTTTATTATTTATTAAAAGTATATAGGCCTAGATATGTACTAATTCTTCTTTAGCATTTCTGTTAAAGTATGTT
TCAGCTTCTCTCATTTTAGAAGTACCGAAAATTGGTTGGTTATCTGGATTTAATACACGATAAATATTGCTTACTTTGTTACTTTGTA
AAATGTACCCATTACGAGTCTCAATGTTATTCCAATAAATATCACTTGTTGGTGCATGGTTTGGATATGCACAGTGATTTCTTGAAAT
TGTTTGAACGATTTCTAATGGATCACAGCCAAATGTACTGTTTAATACTTCAGAGTCTCTGAATAATGCACAAATTGAAATACAAGT
TGTCCAGTTTGGTAATACTCTCTCTTTTTCGATTTGTACTAAAGTCTTTTTAGAAAGTCCAATTGTTTGCGCCATAGTATCTTGCGTAT
AACCAGCCTCTATACGAACCATTTTAAATTTTGTTTGAATTAAATCTGTAAAACTCTGTCTATCCATTCTGTTATCTACCTTTCTGTTT
GGGGAATTTTATCCGGACACAAGAAATTGCAATAATACACATTTCTTGAAACACAGATTACATCTTAATATATTTTTAATAAAATGA
AAAGAGTCAATTTCACATTTGTATTAAATTTTGATCAAGTCAACAAGAGTTAAGTTTACTTTATAATGAAGCGAAAGTAAATGAAGT
AAAATTTTATGCATAATCACTAGATTTGATAAAACTTACACTTTTATAATGATATCAAATTTACAAAGAAAACTGTATAATAAACCC
TATCGCTATAAAAAATTCATCAAAATCAGATTAAAAATTGTCTATTTTTCGACAAATATTTAAAAGCTCATTCTAAATCAACTTATTT
TCCATTGCATAAATTGCTGCTTGTGTACGATCGCTAACTTGTAATTTACTAAATATATGACTGACATGTGTTTTAATTGTTTTTTCAGA
TACAAATAAAGTTTCTGCAATCTCTTTATTTGTTTTACCTTTAACCATTTCACGTAACACTTCAATTTCTCTCTTTGACAACTTATTCG
TGTAGTGTGGTTTTTGGCTAACTGTTTCGAATACATCTTGTGCCTTAGGATGTATCATTTTTTCACCGTTCATAACTCGTCTAATAGTT
TCAATTAATTGCTGAGGCTCAACGTCTTTCATTTCATAACCATCAGCACCTTTATTAATTGCTGAAATTACATGTTCATCATCAACAT
AACTTGTTAATACCAAAACTTTAATATCCGGATAATGTGCCTTAATATATTCCGTAATTTCAATACCATTCATGCCAGGCATCACTAA
ATCTAATAGCACAATATCAGGGTGCTCATGCTCTTTTAAATATTCTAAAAATGTTTCTCCATCTGCAAAGTCTTGTAAAACTTCTATG
TTTTCAATCGTGGATAATAAAAATCGCAATCCTTGTCGCACAATATAATGGTCATCTACTAATATTACTTTGTTCATGGGTTATCTCC
TTAAATCAAGCTATTTTATAGGAATTGTGAATTGTATTTGTGTACCCTTTGTTGGCTGAGAATGAAAGGTCACTTTACCTCTTAATAA
TTTAACTCTTTGTTTTATGTTATTAATACCGTGTGATGAAGCTATCTGAACATTATCGATCTCAAATCCTTGACCATAATCAATCACG
TCAATATATAGTATATCGTTCATTTGTTTTAATGTAAGATCCATTTTATTCGTATCAGCATGTTTCTTAACATTATTAATACACTCTTG
TAATGCTCTGTATATGTTTTCTTCGATTTCATTAGATAAATCGATTAAACCTTCTACATTTACATTTAATTGTATATGCATTAATTTAC
TATATGCTGTCAAAGCATGAATTAAACCTTGCTCAAGTCCAACTGGCTTAAGTTGCCAAATCAATGCACGCATTTCATTAACGGCAT
TTTGACTCGTTTCCTCAATCGTCTTGAATGCTTGTTTAGCGATGGATTCGTTTGACATGCCATACGCAGCATGTGCTGTTAGTTTTAC
AGAAAATAACATTTGATTTACTGAATCATGTAAATCTCTAGCTAAACGATTACGTTCATTAATTTTTGCCGCTTCTTTTTCACGGTCT
GTTAAATAAATACGTTTGATGGCTGACCCTAATTGAAATGCGACAGACTCTAGCAACTCTAAATCTTCATCGCTATATATTTCAGTA
TTTGGAGACGCTACATTTAAAATGCCGAATTGTTCTTGACCCGATTTAAGTGGTACCGTTGCATGATGTGTAATATTGTCATTTTGGC
TAGGAAATGCTTTAGAGGCTAAGTTAATACGAGAACAATTGACGATATTCGACGCTTTCATTAGCCTACGTTGATTAAATGCTTTCA
CACACCAACAAGACCCATCTTTAATATAGTGACAGTGGTCTGCTGTCAAAGATTGTGGTAGAGCCACATGTGATACAAGTTCATGTT
CACCTACGCTATTGATGAAAAATATCCAGCCTGTCGTGAAATTACTGCCCTCAATTAAATATTTTAACGCACCTTGGGTCATGCTAT
ACATTTCAGTTTCTTCGTTTAAAAATTCGGCAATCTCTTTTAATAAAGCTAGTCGCGTCCTTTGTTCCATCAAATCGCTCCAATTCATT
TTTACGTGTATTAACTATTATACATTGAGTTATTATATTTTTAAATCTTAGACGTAAACATGATAAAATGGCCTTGATTACTCAATAG
TTATATTTCGGAGAACTGATTTGTGATATGATATTAAAGACTATAGGAGGATTTTATGAAATTTAAAATACCAGAAAACTTTAATGA
CTTAAGTTTACGAGATATTTTCCAACAACTTAAGGTACCTAAAAAAGATTTACATCATTTAAATATGTCTAAAGATATTACTATTAAT
GATAAACCTGCGCGATTAATGGATAAAGTGCATACTGGCGACGATGTATTTGTTCCAACCATCGATGAAAAAAGTAATTATGTTCCA
AGTTATCGTTATGCACAAATTAAATACGAAGACGATGATATGGCAATCGTAATGAAACCTAAAGGTGTTAAGACTCACCCTAATGA
TTTAAAAGAAAGCAATACTTTAATGAATCATGTGATTTACACTATTGATAGTGACTATGTCGAACCAATTCATCGACTGGACCAGGA
AACAGTAGGATTATTAATTGTTGCTAAAAATCCTTTAATGAAAAAAATTCTTGATCGCATGTTAGAAGACAATGATATTACGCGGAT
ATACAAAGCAAATGTTAAGGCACTTTTACCTTTAAAACCACAAACGATTGATATGCCAATTGGTAAAGATAAATTCCATTCGAATAA
ACGACGTGTGTCTCCTACTGGACAGCGTGCAATTACACACATTTTAACTTCAAAAATGATAAAAGAAGCTGTGTGCCAACTTGAAAT
CAAGTTGGATACTGGACGTACTCATCAAATTCGTGTGCATTTAGCTGAAATTGGTCACCCTGTTATTGGTGATCCTTTATATGGTGAT
TCAACGTTAAGACAATTAGAACTTGAAAGTTACAAAATAGAGTTTGTGCATCCCTTGACTAAGGAAGTCATTTCCGTTTCTTTGGAT
GACTAATTTGATTAGTTTTGCATGATATTTAAACATGCAATACCGCATTGTAACTAAATCAAGTATCAACTTAAATCGGATAGATGG
AAAATTATTAATTTTTTCAGATGTTCGGTTTTTTTTTTACGATGCTTAGGATTTTATATTTTGATATTTTAGTAATTATTCATTTTATAA
CATCCTTGGATAATGACTTGTAGTCTTTTTCAACTGCGTTACGTGTATCTATGGACAATACATGACATCATAAGATTTTTATCACAGG
TTGTTTGGCCAATACATGTACAACAATTCATCATATAAAAAATAGGTTCTATAATAAAACGGACTCCATGAAAAGTTTTTCCTTTTC
ATGGCTCTATATCAAATCAGACTTATAAGTTCAATTTGACATAGACAAAAAATAAAGCTTGAACACTACATCTACCTGTAATGCCCA
AACTTTATTTTATATTAATATTTAATTAATGAGGATCTACCATATCTTCTGGTTTAATCCATGCTTCAAATTGTTCTTCTGTAACATAT
CCAGTTTGAATTGCAGATTCTTTTAAAGTTAAACCTTCTTTATGGGCTTTCTTAGCAATTTGAGCTGCTTTTTCATAACCAATATGTGG
ATTTAATGCAGTAACTAACATTAATGATTGATTTAAATAATTATCAATATTCTCTTCGATTGGTTCAATGCCCACTGCACAATTGTTA
TTAAATGTTTCCATACCATCAGCTAAAAGGTAAATTGATTGTAGTGTATTATGCATAATAACTGGTTTATAAACATTCAATTCAAAG
TTACCTTGTGAACTTGCGAAGCCAACAACTGTATCATTACCCATTACTTGGACTGCAACCATTGTTAACATTTCACATTGTGTAGGAT
TAACTTTACCAGGCATAATTGATGAACCTGGTTCATTTTCAGGGATGGAAATTTCTGCCAAACCAGCTCGTGGCCCTGAAGCCAACC
ATCTCACATCATTAGCAATTTTCATTAAGTCTCCTGCTAGCGCCTTCAATGTTCCATGTAGTTGTACTACTTCATCATGTGCTGTTAAT
GCATGGAATTTATTTTCAGAAGATACAAATGGATAACCCGTATTTTCTGAAATATAATGTGCCACTTTATCACCAAATTCAGGATGC
GCATTAATACCAGTACCAACAGCCGTACCACCGATGGCAAGATTTAAAATGTGCTTCTTAGATTCAGATAACATTATTTCGCAACGG
TCAAGCATATAACGCCAGCCACTAATCTCTTGTCCTAGTTTGATCGGCGTTGCATCTTGTAAATGTGTACGACCAATTTTAATAATTG
AATCAAATTTGTCTTCTTTTTCTTTCAAAGTATTTCTTAAAAGTTTTAATGCAGGTTCTAATTTTGTTTCAACCTCTTGATATAATGCA
ACGTGCATAGCAGTTGGGAATGTATCATTCGAACTTTGTGATTTATTTACATCATCATTTGGGTGGATACTTTCATCAATTTGATGAT
CTTTTAAATACATATTAGCAACATAACTTACTACTTCGTTCACATTCATATTACTTTGTGTACCGCTTCCTGTTTGCCATACAACTAGT
GGGAAGTGTTCATCTAATTCACCTGATAAAATTTGATCACATGCGTATACAATGGCATCTTTCTTTGCCTCGCTTAATTTTCCTAAAT
CAAAATTAGCTAATGCTGCTGCACGCTTTAGTTGTGCAAAACCATAAACTACTTCGATTGGCATACGCTCTTTACCAACTGGGAAAT

TACGTTTACTTCTTTCTGTTTGAGCACCCCAATATTTATCTGCAGGTACTTCTATTTCTCCAAAAGTATCATGTTCAATTCTTACTGAC
ATTCAATTTCTCCCCTTATCACTGTTTATTTAACTGTAGTATATCATTAAATAATTTAATTGAGCAATTTATGATTAAAACGTTTTCAT
AATTTGAAATAAAAATACACTAATCGCACGTGTTCACCCTTTATTACAGTGATACGGTCATACGATTAGTGTGTTATCTATCATTATT
TAGTTATTGTTGAACTAAGTTTAATTACGATACTTTGTTTTAGTAGCTTCAACCGTAGCAATAGCTGTAAGTATATATAATACAGCAC
TAACAATTGTCGTATATGGATTTAGAGCAACAAGCGTACCTAATACTCCTGTTAAACTCGCATAAAACCCATCAAGTGTATAATACA
ATATTCCTGTTATTAATAAACAACAAATTACACTTATTACAATACCTGATTGATTACTTTTAATGAATGTTTGCGCATTAACATCATC
GATTAATCCTTTTGATAAATTGAGTTGTAATTTTATTACTTTGAAAATAACAGGTAAATATAATGCCCCAATTGCCAATGGAAAAGC
TTTAATTGATATTAAACTTATAATAACTGTTGCTATCAATAATTGAATCCAGTATTTTCCTAACATAAATATATAAATCTCCTCTAAT
TTCATTCTTCAATAGCATATCATAATCTTGGCATATTAAGAAACGCGGTTTAATGATTTCATTAAAAATATTACTGATAGATGACTTC
TTTCAATTATGTCTGGAGTAATTAATTATCAATTCCGTTTAAATGGTGTTTTAATATTTAAAATTGAATTTTTGATATATTACTATGTC
TGGTACACAAATCAATGTTTTATGCTTTACAAAGTTATATTGGCAGTAGTTGACTGCGGGCCACAAACTCAGAAGCTGGCGGAAAGT
CAGCTTACAATAACGTGCAAGTTGGCGGGGCCACAACATAGTGGAATTGGATTCCCAATTTCTACAGACATTGCAAGTTGGCGAGG
CCACAACATAGAAAAATTGGATCCTCAATTTCTACAAACATTGCAAATTAGGGAAACGGGCCACAAACTCAGAAGTTGGTGGAAAG
TCAGCTTAAAATAACATGCAAGTTGGCGGGGCCACAACATAGAAAAATTCGATTCCCAATTTCTACAGACGATGCAAGTTGGCGGG
GCCACAACATAGAAAAATTGGATCCTCAATTTCTACAAACAATGTAAGTTGGGGAAACAGCCCCAACACTGAAACTGGCAGAAAGT
CAGCTTAAAATAACATGCAAGTTGGCGGGGCCCCAACATAGAAAAATTGGATCCTCAATTTCTACAGACGATGCAAGTTGGGGAAA
CGGGCCACAAACACAAAAGCTGGCGGAAAGTCAGCTTCTATGAATATAATAAAAAAGCTAGGTAACAAAATGCTACCTAACTTCAT
ATTCAAGATTATCAATCCTATTTGATATAGGTTCTATACTATTCATTGTTTACATGATAAATAGCTGAATATTACACAATTATAATAC
TTTACTGACTGTCTTCTTCAGAATTCTTTTCTTGATCATTTTGATCAGAAATTTGTTCCATTTCTTTACCTAATTCTTTTAAATCTTCAA
AATCCGTTACCATACTGTTTTCTTCTTCATGATAATTTAATTTTGGATCTTTGTCTTTAGACATAATCAATACCTCACAGATTTTTAAT
TAAGCAAAGCGTGACGTAAAGTAAGCTTTAACATCTTCAGGTAAACCTGCAGCCGCTTCTTTATCAAGAATAATATTTACCATTCTA
TGTGCTTTTAAATCGGCTGGTTCGAAGCTTGTTTTACCATTTTCTTGATATAATTTTTCAACTACATCTCGTTTATTAGCACCTGTCAC
TACTAAGAAAATTTCTCTTGCTTCCATTAGTCCTTGACGAATACTAACATTTAACTTACCTTGCTCATCGATAGAAACAACTTGTAAT
GTTAATTTCCCTTTATTTTCTTTAGTTTTAATCTTATCAGCGATTAATTCGATTGCATCTTTTTCATAAGCAATTGGATAAACTTGACC
TGCTGGTACACCTAACCCTTCGAAATATGATTTTTTATCGTCATAATCTAAAATATTTATTTGGCTAAAATCAACAGCATGTTTTTCA
ACATTTTTCTTTAATTCATCTAGAACTGGCGCTTGATCTGTATCTAAATGAAAACCTGCAATTGTAGTAGGATTATTGTTAAATTGCT
TTCTAATAATATCAGCAGCATATTCTGCTACAAGTTGACTATTGTCAAAGACTTTAAAGTTCATTGCCATGATTTCTACACTCCTCTT
AGTTTGCATTTTACATAAAAACGGCGATATTAATAATTATTATACCCTAACTTTCAATATATCAAACCATTTAAGTTTAACATGCTTA
TACTCTAAATATAGCACTTAAGCATCATTTTATAATGAAAATAGACGAATTTTAATTCAATCCTGGAAAATCTTGTTGACGTAACGC
TTCATAAATTAACAACGCAGCAGTATTTGATAAATTTAATGAACGAATATGTTCACTCATAGGAATTCTTAATGCTGTGTCTTGATAT
TTCTCTTTCACCCAGTCTGGTAATCCTGTCGTCTCTTTTCCAAAAATGAAGTAAAAATCTTTGTCATGATTTGAAAAATCAAAATCAC
TATAAGTCTTTTTACCAAATTTTGTTAATAAGTAATACTCGCCATTTGTGGCTTCAAAAAATGCTTCAATACTATCATGATACGTAAT
ATTCACAAATTCCCAATAATCTAAACCGGCTCTTTTTAACATTTTATCATCAGTTCTAAATCCAAGAGGTTTAATTAAATGTAAATGT
GTGTTTGTACCTGCACACGTACGTGCAATGTTACCAGTATTAGCTGGGATTTCTGGTTGATATAAAACGATATGATTTGTCATATTAC
TATTCTCTCCTTGTGTCTAATCCTTTTATCATTTCATTCTGAACTTCTGCATCCTCTTGATCATAATTAGCATTGATAAAATCTCTTGCT
TCTTCCCCAAGAATTTGACCAATGGCCCAATAAGCAGTTGCTCGAATCATCGGTCTTTCATCTGTTATTGCAACTTTTTTCAATTCTG
GAATTGCATCCACTTCATTAAAATGCGCCAATGCTAAAATAGCATTTCGTTGTATCGGTTTTTTACCACGCCAAGCACCTGCAAGGT
GACCATATGTTTGTTTGAATTCTTTATTAGACATACGTAGTAAAGGTACTAATCTTGGCTTTAAAATTTCTGGTTCCAAAATAATGTC
ATCTTGTTCGGTATTAATACCTCTATTTTTCGGACAAACTTGTTGACACGTATCGCAACCATATAATCTATTCCCAATTTTATAACGA
TATTGGTCAGGCATATAGCCTTTTGTTTGCGTTAAAAAACTAATGCATTTCTGACTATTTAATTGGCCATTTCCAACTAATGCACTTG
TTGGACAACGATCAACACAAATTGTACAATCACCACAGCTATCTAATAATGGATCATCAGGTTCAAAAGGTATACTGACTAACATTT
CACCGAGGTATGTCCATGTTCCTAGTTTAGGATTGATGACAAAGCCATTACGACCAACAAATCCTAAACCTGCACGTTCTGCTACTG
CCCTATCTGATAATACACCCGTATCTACCATAGATTTTATTTCAACATCTGGAACTTTAGATTCAATAAATGAAGCTAACATGTCTAA
TCGTTTACGCATAATTGTATGATAATCTTGACCCCACGATGCTCTAGCAAATAAGCCTCTGCGATCACCTCTAACACTCTTAGGTGCA
CCTTTCAGTTTGTTAGGATAACCAACTGCAATTGCTATGATTGACCTTGCTGTTGGTAAGGATAATTTAGGCTCCGTTCGTAAAGCAA
TATCAGATTCTTCAAATCCTGAGGCATAACCATTTGCATGATATGCTTCTAGCTTTTGCTTCAATTCATCAAAGGGATCGGCAGTTGT
AAATCCAATACTGTCGATACCAATTGTATATGCATAGTCAATGATATCTTGCTTTAACTGCTTTGTATCCAATCGTATGCCCCCCATA
AAATAATACTATGTTATTTTAACACAACCATTATTCATATTGAGGTTTCATTTCACATAATTTTAATAATAAAAAGCTTGAATATATG
ATATAACTTTGTGGTGCGTATCATATATTCAAGCTTCGTTAGGTTGTTATAATACTCTTGCTAAGAAATTTTGTGTTCTTTCATGTTGC
GGTTGTTCAAATATTTCGACTGGTGTGCCTGACTCTACGACAACGCCATCTGCCATAAATATGACTTTGTCACTTACATCTTTGGCAA
ATCCCATTTCATGTGTCACAACCACCATGGTCATACCTTCTTTGGCTAGGTCTTTCATTACTTTTAATACATCACCAACTACCTCAGG
ATCTAATGCTGAAGTTGGTTCATCGAATAAAATAACATCTGGATGCATTGCTAAAGCTCTTGCAATTGCTACCCTTTGCTTTTGACCA
CCTGATAATTGATTCGGATATACATCTGCTTTTTCTTTTAATCCCACTTTATCTAATAACGACAATGCTTCCTTATGTAATTCATCGTT
ATTATCTTTCTTTAATAATTTAGGAGCTAAAATAATATTATCGACAACTTTTTTATGTGGAAATAGGTTGAAGTTTTGAAATACCATA
CCCATTTTTTGACGTAGTTTATCTACTTGTGTCCCTTTTTCCGTTAAGTCATTGCCTTCAAAAATCACTTGACCTTTAGTGGGTACTTC
TAATAAATTCATACATCTTAACAATGTACTTTTACCACTACCAGATGGACCTATTATTGCTACTACTTCCCCTTGATTGATTTCAAGA
TTGATATCTTTTAAAACTTCATTATCTCCAAAAACTTTATTAAGATTGTTTATTTTAATCACTGGCATTCAATCTCCCTTCAATCATGT
TCATAATGCGTGTAAGTACAAATGTTAAGACAAAGTATAATGCTGCTGCCACTATTAATGGTGTGAATGGGTCAAATGAAATACCTT
GAACCACTTGTGCATTAAACATAATTTCGCCAACTCCAATTGTTGACACAATAGATGATTCTTTAATTAAAGTGACAAATTCATTAC
CTAAAGCTGGTAAAATATTTTTAATTGCTTGTGGCATAATTACACTTTTCATCGTTTGTCTATAATTCAAACCTAGACTACGTGCGGC
TTCCATTTGGCCTTTATCAACAGCATTTATACCTGCACGAATAATTTCAGCAATGTAAGCTGAGGAATTAATAACTAATGCAATTGTT
CCACAAACAAGAGCTGAAATGTCTAGTCCTAATGCAGCAGTTATACCAAAGAATACGATAAATACTTGAACTAACATTGGTGTTCC
TCTTAATATTTCGATATAGATAGAAGCAATCCATGAAATAATTTTTATTTTACTTAATTTCATTAACGCAACGAATGCACCTAAAATA
GAACCTAATGCAACACCGATAAGTGAAATTAATATTGTAATCTTAATTCCTTTCAAGAAAAAACTACCATACTTAGAAATAAAACC
ACTGTCATCATTCATCGCATTTGCAGCATTAGTCATATATTTATCGATTAATCCTTTATCTTTAACCTCCTTAATCGTTTTATTAATTT
GTGACAATAATTTTGGTGAATCTTTTGGCACTGCTATCACTGTATCTTTTTCTTCTTCATTAAATTTCACATTGGAAATTCCTAATTTA
GGATTTTGTTTTAAATATGCTTCTGCTACAGGTTTTTCAACTACAGCACCTTCAACCTTTCCACTTTTAAGTGCTAATATAACGTCTGG
TAAACGGCTTAATGAAGTAATAGATGCATTTTCAATTTCGGTTTGAGCGATTTTTTCTTGTTCAGTTCCCTTTTGTGCCCCTACTTTTT
TATTATTAAAGTCTTTGATATCTTTATATTCATTAACTTTATCTTTCTTTACAAGCATGATATTTTTAGTCATCATATATGAATCTGAA
AAATCAACTTGCTTCTTACGTTCAGGCGTTGAAGTCATTCCGGAAATAATAATATCAATTTTTCCAGTTTTAAGAGCTCCTAACAAAC
TATCAAATGACATATTGACGATTTTTAATTTTAAATTATTATCTTTCGCAATTTTTTTAGCTAAATCAATATCTACACCTGCATACTCA

GTCTTACCATTAACTGTATGCTCAAATTCCATTGGTGCATAATCTGCAGAAAGACCCACTCTAAGTTCACCGCGTTCTTTAATCTTCT
CCCATGTTTGATCTTGTTCAGCATGTGCTGTGGGATTGATATACACCATAGCACTGCTAATTAACAACCCTAATACTAATATAAACCT
AATTAAACACTTCATTTATACAGCCCTCCCTAATTAATATTCATAATTATACATATTAATACATTTTGATGCAATAGTGTTTTATAAA
TGTATTTTTATTAATATAAAGAAAAGCTTCTTTTTAAAAGACTGTACATAAAAAAATCCGCTATTATACGTGAACAAATCTCACATTT
TAAAATCAATCAAAAAAGCGCTACTATCAATATCTTATTGACAGTAACGCCTATATTTATCATATATTAAAACTTATTCTTGTCATCA
AGCTATAAATTATTGTTTTGGAACTTCAAATTTATAAATATCATTAGAATATATAGGAATGAATTTTTTACACAATTCATGAACAATA
AATGCCGATACAAATGGTATGACAAAATAACCAAGTGATAATAAAATAATATTCATTGTTGGATCACCTGACATTCGGTTAAATGC
ATTTATTGGGCCAACTAATCCTGTATATCCAAATCCAGCAGACAATGGTGTTCCTTTAACCTGAAGAACGTAAGCAATGATTCCAGT
AATTATTCCATTTATAGTCAATGGTATCGAAATAATTAAATTTTTCAAGTAAACTGGGATCATCATTTTTGCTGCTCCTATGAGTAAA
ACTGCATTTACACCAATAGAATTGACACGCAATGAGCCAAATAAGAAAGTAACACAGGCAGCCACTATACCTAGGTTTGCTGCTCC
ACTTCCTAGTCCGTTTAAACTAATCGCAGTTGCAATCGCTACTAACGATATTGGTGTTACCATTAATAATGAAAATGCCACACTAAT
AAGTATAGACATTAACAACGGATTTAAGTCTGTAAAAGAATGAATTACATTTCCAATTGCTTGAGTAATTTTTCGAATGTAAGGTAA
TGTGATTAGACCGATACCCCCACTAACGATAGGTACTAAAACTGGTAATATAATTAATTCAAAAGATCCAAGTTTGTTTTGTAATAC
CATATATATAAGACATGCAATAATAACAACCAAACTCGTATTTATAATGTCACCTATACCTTTTAACATAAAACTATTATTGCTATAT
ACAACAGCACCTGAACCAATCATAGCTGATGTACCTACTATAGCAGCACCTGCGCCATTAAATTTAAATTGATGAGCAGCTAAAAC
CCCAATAATAAATGCCATAAATGATTGAATTAGTATCACTAACTGATACGTTAATTCTAAAATTTCATTACCACTTTTAAATATTTTT
AATACTTCACCTAATAAAGCATTCGGAACAAGTGCAATAACAACACCAGCACCAATAGAATTTAAAATCTTACTGAAAAACTGTTT
ATTATCAGCATTCTTTGCGTTACTCATAAACGACCTCCAATTTGAACATTAAACTCATCATATTATAGAAAATTAACTTTAACAATGA
TTAGTTGGCTAATTTAATCTAAATATAACAATTCACAAAATGAATAAAGTAAATTTAAACAATCTACTCACTAATCAATGTCATGCT
AATTTTAAAATACTACTAATTACATCCTGTGACTAATAATGTAATAAATTGTAAATATTAAAATAGCCATATTACTATTAAGATAGA
TAAATTGAATCGACTTGGTATAAAGCAGAATAAAATAGCACTTTTACAATTATTTTATTAGAAAAGTTAAAACAAATACATAACCCC
AGATAATATAATCATTATCATTAATGTAATACCAAAAAAACAAAAAAAGAAGTTAAACAATGTATAGTTGCTTAACTTCTAATATTG
AACTCATCATTACAATTTGACATAGAGCCTATTAAAGCGTGTACCATTTGAGTCCACTTTTATTTGTATTGTATAGAGAGAAATAAA
AAGAAACCTTGTTTTACAAGGTTTCTAGTACGTTATGTTATGTAAATAACAGTTAATTATACCGGTGGTCGGGGTCGAACCGACACT
CCACAAGTGGAACGGGATTTTGAGTCCCGCGCGTCTGCCAATTCCGCCACACCGGCTTAATGGTAAACAAAAAACTTCCCTTTGGAA
GCAATTATGGAGCGGAAGATAGGATTTACACCTATACCTCGTTCCGGGAAGGAACGTGTTCTAAAAGTTGAACTACTCCCGCAAAT
ATTAAATTATGGAGCGGAAGATAGGATTTACACCTATACCTCATTCCAGGAAGGAATGTATTCTAAGAGTTGAACTACTCCCGCATT
ATTATTAAATTATGGAGCGGAAGATAGGATTTGCACCTATACCTCGTTCCGGGAAGGAACGTGTTCTAAAAGTTGAACTACTCCCGC
ATAAACCTGGAGGCGGCAACCGGATTTGAACCGGTGATAAAGGTTTTGCAGACCTCTGCCTTACCACTTGGCTATGCCGCCAATAAC
TGGGCTAGCTGGATTCGAACCAA

>047-contig_203

ATGAATATGATGTAGATTCAAGGATCGAACCCACACCAAAGGTTTTGGAGACCTCTATTCTACCGTTGAACTATGCCCCTATTAAAA
ATAATAAATGGAGGGGGGCAGATTCGAACTGCCGAACCCGAAGGAGCGGATTTACAGTCCGCCGCGTTTAGCCACTTCGCTACCCC
TCCATAAATGGTGCCGGCCAGAGGACTTGAACCCCCAACCTACTGATTACAAGTCAGTTGCTCTACCAATTGAGCTAGGCCGGCTAA
GAAATGGTTCAGGACAGAGTCGAACTGCCGACACATGGAGCTTCAATCCATTGCTCTACCAACTGAGCTACTGAACCATAATAAAA
ATGTAATGATGGCGGTCTCGACGGGAATCGAACCCGCGATCTCCTGCGTGACAGGCAGGCGTGTTAACCGCTACACTACGAGACCT
ATAAAATATTGCGGGAGGCGGATTTGAACCACCGACCTTCGGGTTATGAGCCCGACGAGCTACCGAACTGCTCCATCCCGCGATAA
TAAAAAATAATGGCGGAGGAAGAGGGATTCGAACCCCCGCGGCCCGTTAAGGCCCTGTCGGTTTTCAAGACCGATC

>048-contig_124_RC

ATTCCTCCAAAATTATATGGACCTTGCAGGACTCGAACCTGCGACCGAACGGTTATGAGCCGTTAGCTCTAACCAACTGAGCTAAAG
GTCCTAAATATAATTTTACAACTAATAAATAGTGGCGGTGGAGGGGATCGAACCCCCGACCTCACGGGTATGAACCGTACGCTCTA
GCCAGCTGAGCTACACCGCCTTATATAGTTTGTAAATAATATGGTGGAGACTAGCGGGATCGAACCGCTGACCTCCTGCGTGCAAA
GCAGGCGCTCTCCCAGCTGAGCTAAGCCCCCATAATAATTACAGTATATCGGGAAGACAGGATTCGAACCTGCGACCCCTTGGTCC
CAAACCAAGTGCTCTACCAAGCTGAGCTACTTCCCGTATAATTAACGCGCCCGATAGGAGTCGAACCCATAACCTCTTGATCCGTAG
TCAAACGCTCTATCCAATTGAGCTACGGGCGCATATGTTTTTATTGAAAATGGTGCCGAGGACCGGAATCGAACCGGTACGGTGATC
ACTCACCGCAGGATTTTAAGTCCTGTGCGTCTGCCAGTTCCGCCACCCCGGCACTATAAAAATGGAGCAGAAGACGGGATTCGAAC
CCGCGACCCCAACCTTGGCAAGGTTGTATTCTACCGCTGAACTACTTCTGCATATGCGGGTGAAGGGAGTCGAACCCCCACGCCGTA
AGGCGCTAGATCCTAAGTCTAGTGCGTCTGCCAATTCCGCCACACCCGCAAATGGTGAGCCATAGAGGATTTGAACCTCTGACCCTC
TGATTAAAAGTCAGATGCTCTACCAACTGAGCTAATGGCTCTTCCATGGTGCCGGCCAGAGGACTTGAA

>049-contig_251_RC

ATGATGTTTGATTAGCTCATAAATACTAAATAATGTTTGTAACTAATAGTTACGTTTTTGGAATTAACGTTGACATATTGTCATTCAG
TTTTCAATGTTCGTTTGTCACTTACAAGATTTAATTATAACTCGTAAGTTTTTGTAAGTCAAGAATTATTTTTAATTATTTTATTTCTA
AAAAATAAATTTCCTAACTCAATTAATTCAACAAATATTATTTTATTATGTATTTGTTCGAATTTCAATAGTCAAATTTACACTTTATT
AATATTTTAAATCAACAACTTAAAACATTTGTTTTTTAAATATTAGTGCCGTTTGACGACTTTTATATAATATCAAGTTATTCTAGTA
GAGTCAACATAAAACAACACTTATTTTTGTTTAAAAATTACTGTTGAAGGGATTATTTTCTATTCGTAAGTACTATATGTATTTAAAG
TGTAATTCCTTACGCTTTTTACTTCAGTTTTACAAACAAACTTATACTCACTTTATGGATAGTTATTTAGTAGACCTTCGTTTGAATAT
TTTTATGTTTATTTATAAAGGATACTAGTTATTAGTTAATACCACTTCCGTAGTTAGAATATTCTTTTCTTCAATTTAAATATTTTTCA
GAATAAGCATAAAAAAAGAAGTTAAACAACTTTCATGTCGTTTAACTTCTTAAATATTACACTTACTTATATGAAGTAAGCTAACGCT
TTTTTAATTTGTCATACTACCAAAGTTAAATTATTTATCTTGGCATTCTTTACAAACTCCATAAATTTCCATTCGATGATGTGTTACGT
CAAAGTCAGTCATATGCTGAGCTAATCTTTCAATTTCATTTAACTGTGGATATTGAAAATCAACAATCTTACCACATTGTTCACATAT
AATATGATAATGATTATGTGTATTAAAGTCGAATCGACTTGATGAGTCTCCATATGTTAATTCTTTTACAATTCCAATATCTTTAAAC
ACTCTTAAGTTATTATATATTGTCGCAACACTTATATTTGGAAAATCAGGTGAAAGTGCTTGATAAATTTCATCAGCTGTTGGATGA
GTATGTGAAGAAATTAAATAACGTAATATTGCTTGTCTTTGAGGTGTAATTCTTACGCCTGCTTGTCGCAATGATGCAATTGATTCTT
CTAGTTCATGTTCAATTGATTCTATTTCAACACTCATCTATATCACCATCTTTCTTATATAATAATAATTATTACTTACTATAAATATA
ACTGTAGTATTGCGCGCGTGTCAATATTTACATGATGATTTCATTTAATAGCCTTTTTAGTATCCACTTCATTCTCAATTAGACCATT

CTTATTGAGAAAATTAACTAGATTGTTTTTAAAAATATCTAATAAGTCATACTTTGCTTCATAATCATTACCAGTTATATGTGCTGTT
ATGGTTACATTTTCCAATTCATATAATTCATAATTAGGTTTCAAAGGTTCATTTTCAAACACATCTAAATATGCATGTCGAATAACTT
TACTTTTTAATACTTCTATTAAGACCGCTTCTTTAACTATGCTACCTCGTCCTATATTTATAAAAAGTGCTTCATCTTTCATTAATTCA
AAATGTTTTTTCTTTAGTAAATGAATCGTTTCTTGCGTTTCTGGTAAAGCATTTATAATAATGTCAGCATTTGGTAATGTGCTTTCAA
GTGATTCGATAGTATATATCTCGTCAAACTCATCTTTGTTCTGACCTGACTTGCTCAGACCAATTAAATTCATATTAAAAGCCTTTGC
TAACTTCGCAGTTCTAGTAGCAATTGCACCTGTACCTAAAAATAAAACTGTTTGTCCTGATAGGCGTTTACCAGTTATTTTCGAATCA
TATATATGTTGTCGTTGGTTATCATATGATAGTTTCATCTTTTTATAATCATCTAAAATGAAAGCTAAAATGTATTCAGATAATTGTT
TAGCTTGAACACCTTTTCCATTAGTTAAAAGTATGCCGTGATCTGCAATATAATCTAACGGTAATGTATTTACACCCGTTGCAAACC
ATGCAATCCATTTTAAATTCGGGCAACGTTGTAAAAATGCCTCATTGATACCGCCATCATAACCAACTAATATATCTAAATCAGCCA
AGTCACTCTCAGGTATTTCTGATGCTTTTTTATAAAATTTAAAATCTAAATCTGAAAAGCGTTGTTGTAATTCAGTTTCAACTTCACG
CATACGATTCAACCCAACAACTTTCATATATGTCACCCCAAAATGTTTTTAAGTTCTTCTATTTGTGTTTTAACCTTAACCTTCTCGAT
AACATCTAATACTTTACCTTGTTCATCTATTATAAAAGTCGTTCTTACAATGCCCATACTTTCTTTGCCAAATGATTTTTTCAACTGAT
ATACGCCAGTTTCTTTAGCTAATTTAAAATCTTCATCTACTAATAAATCGAAATTCAATCCGTGTTTCTCAATAAAATTTTGGTGTTTT
TTCTTTGAATCACCGCTTATACCATATACTGCAACATCTAAATCATTGAACATTTCTAAATTGTCTCTAAAGTCACAAGCTTCTGTGG
TACAAGTAGGTGTATTATCTCTAGGATAAAAATATATAATCGCCTTTTTACCTTTTAATGTATCATTTGTAATGACAGTTCCGTCTTG
ATTTTCTAATTTAAATATTGGAAATTGTTCTCCTTTTTGCAACATATAATCACCTTTTTCTTCATTATAATAATTTTATGATACGATAT
TAGATGAAAATATTAAATTAAAAGAGGTTGATATAAATGAATTTTAGTGAAAGTGAACGTTTACAACAACTTTCAAACGAATATAT
TCTAGGCGGTGTCAATTCCCCTTCTCGTTCTTATAAAGCTGTAGGAGGCGGTGCACCTGTTGTTATGAAAGAAGGACACGGTGCATA
TTTATATGATGTCGATGGCAATAAATTTATTGATTACCTTCAAGCATACGGTCCAATTATTACGGGGCATGCACATCCTCATATTACT
AAAGCAATTCAAGAACAAGCAGCCAAAGGTGTTTTATTTGGTACACCGACTGAACTAGAAATTGAATTCAGCAAAAAATTACGTGA
TGCAATTCCATCTCTTGAGAAAATTCGCTTTGTAAATTCTGGAACAGAAGCAGTCATGACAACAATTCGTGTTGCACGTGCATATAC
TAAAAGAAATAAAATTATAAAATTTGCTGGATCTTATCATGGCCATTCTGATTTAGTATTGGTTGCAGCAGGTAGTGGCCCATCTCA
GCTCGGTTCTCCAGACTCAGCTGGTGTTCCAGAAAGCGTCGCACGTGAAGTCATTACTGTACCTTTCAATGATATTAACGCATATAA
AGAAGCAATTGAATTTTGGGGTGATGAAATTGCCGCAGTATTAATAGAACCAATTGTTGGTAACTTTGGAATGGTAATGCCTCAACC
TGGATTTTTAGAAGAGGTTAATGAAATTTCGCATAACAATGGGACACTAGTGATTTATGATGAAGTAATTACTGCATTCCGTTTCCA
TTACGGTGCCGCTCAAGATTTATTAGGTGTTATCCCTGATTTAACTGCATTTGGTAAAATTGTTGGCGGTGGTTTACCAATTGGAGGC
TATGGTGGACGTCAAGATATTATGGAACAAGTAGCACCTCTAGGACCTGCATATCAAGCTGGTACAATGGCTGGTAACCCGTTATCT
ATGAAAGCAGGTATTGCATTACTCGAAGTACTAGAACAAGACGGTGTTTATGAAAAATTAGACAGCTTAGGCCAACAACTAGAAGA
AGGTTTACTTAAATTAATCGAAAAACATAATATCACAGCTACAATTAATCGTATTTATGGATCTTTAACATTGTACTTTACAGATGA
AAAAGTCACACATTATGATCAAGTTGAACATTCTGACGGCGAAGCGTTCGGTAAATTTTTCAAATTAATGTTAAATCAAGGTATCAA
TTTAGCACCTTCTAAGTTTGAAGCTTGGTTCTTAACAACTGAACATACAGAAGAAGATATTCAACAAACTTTAAAAGCTGCAGACTA
TGCTTTTAGTCAAATGAAATAACTTTTAAGGAAGTATATTTTTAATTGAATTAAAATAAAACAAATGCTCACATAGTAGATTCATTTT
ATATCGTTAACTATTAACGTTTAACTTGATATGTCACTATGTGAGTTTTTATTTTACAAATTTATACTCTTTTAATCTCTGACTAAAGT
TTTACATCGCTATCAGTATTATGTATGATTTATTTTAACAACTATAAATAAGCTTGAATTTTGTAACTAGTAAGTGTATAACAGTAAT
GAATACGAATATTAGATATTCAATAAACTTAAAAGGGGCGTCATTATTTTTGAGACTAGGAGCTCGGATTTTTAAAACTGGTATAGC
CATTATTTTAGCTATGTCTATCGCTTCTTTACTACCGGATGATGTTGGTCTGAAAGCCTTAGCTGGTGTCAGTGCTGTTGTTGCAATG
CAACCTAGTATTTATCGTTCATTTAAAACAGTTTCTGATCAAGCATTGGGTAACATTATAGGTGCCATACTATCTGTTACAATGGTAA
CAATTTTTAGTGATAATTTTATCATTATGGGTGTTACCGTCATAGTACTTATTGCTATTTTATTTAAATTTAATTTAGCACATGTTGCT
ACTTTGGCGAGTGTAACAGCATTAATCATTATGGGACAACATACCGGTTCTTTCTATATTACCGCCTTTTACAGGTTTGTACTTGTAA
TGATTGGTGTCATAAGTTCATCTCTAGTTAACTTTGTGTTTCTACCACCTAAATTTGAAACAAAAATTTACTATAATTCATTAAATAT
ATCCTCAGATATTTTTATGTGGTTTAAATTAGTATTAAATGATACGACGGAGTTTAATAATATTAAACAAGATAGTCATAATTTAAA
ACAACGTGTCGAAAAGTTAGAGAAAATTTATGATTACTATAGCGAAGAACGTCCTATTACAAAAAAACATATTCACCAACAGAATA
GAAAGAAAATACTTTTCAGAGAAGTAGTTCAGACGACTAGACAAGCTTATGAAGTATTAAACAAATTGTCGCGTTATCAAAACGAC
TTATATTTACTTAATAACAATTTCCTATTACAAATCAAATTAGATTTAGATTCATTAACTGCTTTTCACGAGCAAATTTTAGCTAGCC
TTTCTAAAAAAGCACGTTATAATGTGACACATGTTGATTATGAGTTAGATAATCCGCAGAAAAAAGATTTATTGTCTACATTTCAGC
ATGAGTTAATAAATCATCCTTATCAAACGGAATATTCGTTTGCCAATGTTATGCAAATTGTTGCAGCCATCGAAGAGTATCGACACC
ATTTAGAGCATTTAGACCGCATAAGAATTAGCTTTTTCACATATCATAGATCTGATGCAGACATTGAAATTGTTGAAGAAGACTTTG
ATTTATAACATTAAAACAATTCCTGTAATTAATACCACAATGAGATAAAGTAATATTGAATCTCGAAATTAAATTTAAAAAAGCAGT
AAGATTATTTTCAATTAAGAAAATCTCTTACTGCTTTTTCTATGTTATGACCCCACCATCCTGTTACGAATTTTCTCTTCAAGATTGTA
CTGAAGTCAAACAACTAATACTTTATAAGTTTTGAATGCTATATAAATGCTCGTAAGCACCTTGTTTTGCAATCAATTCACGATGCGT
ACCTGTTTCAACAATATGTCCATTTTCAATTACGACAATTTTGTCAGCATGTGTAATAGTGGACAAGCGATGCGCTACGATAAGTGT
CGTTCGATCTTTACTCAACACATCTAATGCTTCTTGAATAATGGATTCACTTTCTAAATCAAGTGCACTTGTTGCTTCATCCAAGATA
AGAATTGGCGGATTATTTAAAAATATTCTAGCAATCGATAATCTTTGTTTTTGACCACCTGATAATTTAACACCTCGTTCACCTACTT
CAGTGTCATATCCCTGTGGCAAGTTCATAATAAAGTCATGTGCATTAGCCATTTTCGCCGCTTCAACTACTTCTTCATCTGTTGCTGT
TGGACGACCAAGTAAAATATTTTCCTTAACTGTATCAGAGAATAAAATATTATCCTGTTGCACCAATCCTATTTGATTTCTTAAACTT
CCCGTTAAAAAATCTTTAATATTGTGACCATCTATTAAAATTTGCCCAGAAGTTACATCGTAAAATCTCGGTATTAAGTTAATTAATG
TTGATTTACCACCACCACTCATACCTACGAAAGCAACTGTTTCTCCTTTTTCAATACTCAAATTAATATCTTTTAAAATTGGATCTTC
GTTATCGTTATATTGAAAACTAACATGATCAATATCAATACGACCTTGTTTAATTTCAATAGGTTGAGCACCAACACCATTTTTAATG
TCATAATCTTCGTCAATTAATTGGAATACACGGTCCATTGAAGCAAAACTTTGCGTTAAAGTTGTAAATGATGCGACTAAACGACGT
AAAGGCCCGAACAATAACTCTAAGTATCCAACAAATGCTGCAAGTGTACCTACTGTGATTGATCCAGAAATAGCAAGATATGCACC
AACACCGATGACAATAATTGGTCCAATATCTGTAACTGTATTAATTGCGGCAAAGGAATAGGCATTCCATCTTGTATGTTTCAACGC
ACGTGTTAAGAAATTAGTATTCTTTTTATCAAAGTTTTTCGCTTCATTGTCTTCAATCGCAAAACTTTTAACGACTGAAATACCTTGA
ACACGTTCATGCAAGAATCCTTGAACCTCAGCTAATGCTTGAGATCTTTCACGTGTCAATTTTCTTAATCTTCCAAAGAAAACGTAC
ACCGTTAAAATGTAAAATGGGAAGATAAACAGTGCTGCTAAAGTCAATTTCACATCTAAAAAGAACATTATGGATAGTGCAATAAT
AATTGTTATACAATCTAACCAAATATTCATTAACCCGGTTAAAATGAAATCTTTTGTTTGTTCAACATCATTAATCACTCTAGATATT
ACTTGACCTACTTGATTATTAGCATAAAATCTCGCACTTAAAGCTTGTAAATGGTTATATAACTTTTTACGTATATCATACAATATTT
TATTACTTGTCCATTGCGCCAAATATTGACGTATAAATTCAATTGGTGGTCTCACTATTACAAAAATAAATAATGCGATACCAATGG
CAATAGTTAAATGATGAACTTTTTCATCAGTCGTTAATGCGTGGTTATTAATCACGCCATCTATTGCATATTTAATTAAAAGTGGTAT
AAGCATTGGTATACCAAACTTAATTATTCCAACAATAATCGTTGCAAAAATACGATATTTGTATGGCTTAACAAATTGCAAATATCG
TTTAATCATACAATCCCCCCTAATCTATTGCCCTATCCTATTCATAAGCATAAAAATGAATAGAGGTTGGATACATAATTTGTAGAT
GTAAATTCTTCTTACAATTTACATTTTTAAAATTAAGATATCTCAACCTCTGTCATATTCATGTGATTAAATACGTTGTCTTATTCGTA

TGTTATATTCTATTCAATTTAATCTATGGATACTGTGTCCCCACACGACAGCAAAAGTTATCATACTTCTTTACATCACTAAGTCAAT
ATAAATGATTTAATCAGTATTTACACTTTATTTGCTTAATACTGTCTAATTTTTTTGTAACGTTCTTTCCAAACTTTGATAAAATCTGG
CGCGAATGGGCCCTTCTTCTGTTCTATCCATTGTTGAAGAATGTCCACGTTGCGTCTTAAAATAATATCAATATCATGCGGATAATTC
ATTTGATTCATATGTTGCTCATATTCATCTTCATCTAATAAATGATACTTTCCGTTTGGATATACTTTAATATCTAAATCATAGTCTAT
ATATTTTAATGCCTCTTCATCACAAACAAATGGTGATGACAAATTGCAATAGTAATAAATTCCATCTTCTCTAAACATGCAGATAAC
ATTAAACCAATATTCTGAGTGAAAGTAAACAATTGCCGGTTCACGTGTTATCCAAGTTCTTCCGTCACTTTCAGTTACTAACGTATGA
TCATTGCCACCAATGACAACATGATCAGTACCCTTTAATATTGTTGTTTCAGACCAAACGCGATGAATCTTACCATCATGTTTATAAC
TCTGAATTTTAATGTTTTCCCCTTCTTTAGGTATGGATTCTCTGACCATACTCCACACCACCTTCTGTTAATTTAACCATTATAAATTA
TAGCATATTTCAGAAATAGTATTATATAAATACATATTTTTACGAAATAAGATTTTACTACTTAATAATTAAACTAGGTAATATTGCT
AAGTACTACAACAGAGATTTACATGTCCCATTTAAAGTATATAAAATCATCACTTTTATAAATCAACACTTTAACTTTTTGACATTGT
TATCCTATGAGATTTAAAGATATCATTTATACATTTTTAAAATTAATGTCACTATGTTTTCCGATAATATTACCAATCATCGAATGTT
ACCCATTTATAAATTGATAAATCTTTGACATAGGTACAGGGAATGTATATTGGTCTCGATCACTTAAATTAAACCAAATCATGTCAT
CTGGTAATGTTTCAATGTTAATTGCTCCTGAAACGGCGTATACTTTAATCTTCCATGTTAAATGAGTAAATTGATGCTTCAACTCAAA
AATAGGTGTTTCTACTGGTTGAATGTCATGACCGATTTTTTCAGTCATTTTACGTCTAGCATGCTCACTTTCAAACATAGGAAATTGC
CACATACCATGCAATAATTTTTCGCTACGCTTTTGCAACAGATATTGACCTTGATTATTTCTAATTAAAAAGACGGATTGCTCAATTA
CTTTTTTACTTACATTTTTAGATTTAACAGGTAACTTTTCAAATGTACCTTTATCAAATGCCTCACAGTTTTCTTGAACTGGACAAAAT
AAGCATAATGGATTTTTTGGTGTACAAATTAACGCCCCTAATTCCATCATAGCTTGATTAAACGTTCCAGCTTCTGTAGTAACATACG
GTAATAATTCTTGTTCGTACGATTTCCTCGTCGATTGTAATTTAATATCTCGATAGTCATCATTCAATCTAGACCATACTCGAAAAAC
ATTTCCGTCTACAGTTGCTAGTGGTACATTATATGCAATGCTCATTACTGCAGCTTGTGTGTATGGGCCAACACCTTTTAACGCTTTA
AATTGATCAGGATCTTTGGGAACTAAGCCTTCATATTTATCATGAACTTCTTTAATCGCCGTATGAAAATTTCGAGCTCTACTATAAT
ATCCTAAGCCTTCCCAATACTTTAACACTTCATCTTCCGAAGCTTGACTCAAAACTTCCACAGTTGGAAATCGTTCAACAAAACGAT
GATAATAGTCAATAACTGTTTTAACTTGTGTCTGTTGTAACATGACCTCACTTAACCAAATATAGTACGGATTGGTCGTTTGTCGCCA
TGGCATTTCTCTTTGATTTTCATCAAACCAGTGTATCAAATTTTCTTTAAAACTAGACTGCTGATACATTTATAAAACCCTTTCCTCAC
CAAAATTAATTGTCTTTACTCATAATGTTTTTATTGTACATTAAAATCATGATTAGTATGTAAGTTAATTTAGTTATTTGCGAAATTG
GATTATAATAGTATATATAATATTATGAAATGAGTGAACTGATATGGACACTGCAACACATATCGCAATTGGGGTGGGCCTTACATC
ACTTGCAACTCAAGATCCAGCAATGGCTTCTACGTTTGGTGCAACAGCTACAACCCTTATCGTTGGTTCATTAATTCCTGATGGGGA
TACTGTTCTTAAATTAAAGGACAATGCAACATATATTTCGCATCATAGAGGTATCACGCATTCCATCCCTTTCACAATACTATGGCC
AATTTTAATTACATTTTTAATATTCACGTTCTTTAGTGGAACCAACCCATTTCATGTATGGATGTGGGCTCAGCTCGCAGTATTTTTA
CATGTCTTTGTAGATATATTCAATTCTTATGGTACACAAGCGCTTAGACCTATCACAAACAAATGGATTCAATTAAGTGTGATTAAC
ACATTTGACCCTATTATTTTCACAGTTCTTTGTATTGGTATTGTATTATGGGTTATAGGCTTGCATCCATTTGCAGTCTTCTTTCCTAT
AATCGCTTTACTAATCATTTATTACATGATTCGTTTTAAAATGAGAGCCGTAATTAAGCAACAAGCTTTAAAAGCAATTCAACAAGA
GCATCACCCTGTTAAAGTATTTGTTGCGCCAACAATAAAATTTATGGAATGGCGTGTCGCGATACAAACTGATGCACATGACTATGT
TGGAAAAGCATATGGTAGGAATGTGGTGTTTAGTGATAAAGTGGAACGTCAAACATTATCAACAGACTCCATTTTATGGAAAGTCA
AAGGTAATAAAGATATACGTACTTTTTTAAACTTTTCATCAATCTATCGTTGGCAAACAACAACGTTAGCAGATGGTTCTACTGAAA
TTCGTTTGATTGATTTGCGTTATTTAAAAAATGATCATTATTCATTTGTGGCAATTGCACATGTAACAAACGATAATGTCATAGACCA
CTCTTATATTGGCTGGGTATTTACAGAAGATAAGTTACAACGTAAACTGTATGCTAAATAATTTCAAGTTATTATTCACTAAAGTTA
ATCTATAAAAAATGAACAACCGGGCAGAATGAAAATCAAAACGATTTTTACTCTGTCCGGTTTTTTAATGTAAAACTATGAATGCTT
TTACAAAATCTAAAATTTATATTGTTGCTAACAAACTACCTTTAATGACTCGAAATATCAAAATCAGTATAGGAAAACAATATCTAG
ATGATATTCTAATTGTTTCTGATTCTCACAGATTAATTTACACAACAGGTCAGCTAAACATCATGAAGAAGTATCCGCCTCGTCTGTA
CTATCATTTGAAACATCCTGTTGATTATCAGTTTGTGTCACTTTAGATGTTTTATTATAAATTGCGTGTGTCGTATACCTTGCTAATAC
GAAATTTACTGCAGCTATTAAACATAAGAATAAAATAAAGTAAAAATGATATGGAATGTTTTCCATACTTGATATACCAAATATGAT
AGATTGTGCTATACCATTAACAATGTAATACATTGGATTTAGCATTAGGATGTGATTGATAAATACATGATTTGGATTTGGTATGAA
AATAATTGGTAACAATAAGAAACACAATACACAAACCCCATAAAATATGATATTTATTTTTTCAGTTAACAGTCGAATAAGACCAA
AAGTAACGGATATTAATCCTACAAAAATAGTTGCCATCACAATAAAATAGAATAGCGCTATATATGATGTTTCGAAGTTTACTGGTT
TAACCAATGCACTAATCATCGTCAAAATGACTAGCATAATAAAACTTAAAATAGACATAATAACTACTGGCGTCGCTGCGGAAATA
TTAAATAACTTAGTAATTAAAAAGTCATTCATATAATGATGATAAGAGATATAAATTGTGAACATCACTACCGCAAAAACAATCAA
TACAATGATTCTAAATAACCATCTCACCTCTTTAACATCGGTCAAATGACTAAAGGAAAATAGCGTTTCACTTGCAATAATAAGTAT
TAAACATGCAGCTAATACGATTAGAAAACTTTTCCAACTTTGTTTTAAACGTTCTGTTACATACCTTTTAGTATGCGGTAAGTTTTTA
AAATATAAAATAATATTATCTAGCATCTTTACACCTACAATTCAATATAAATCCATTTGTCTTCTTTCAAATTGGCAATACAATATCC
ATTCCCAACTTTAGTAATCCAAATGTTATTGTTAATATTAAACTTATCTTTTAATTCTTTTTTATCTACAATTGGATAAACAAAACCGT
ATAACTGATTATTATCATTGAAAAGTAATTGTATCGGTTGTTGTGTAACGGATGCAACGAAACGGTTATCATCATCAGGAACTAAAG
TTTTTATAACTGAACTATGCTTTTTATGTAATTGACTATTAAAGTATGCATAGTAATTACTATAATTTGATTTCATATATGGTGCTAAT
TTTTTAAAACTATGCGTTTGATATAAACCTGCCGGGTTAAAATATTCTAATTTATCTTTACTAACATAGTAAGTCTTATTATCGGCGG
TAACACGATAATTTTTACTATTTTCGCCAGTAATTGTAACTACACTATATTTTGGAATTGTGACTTGTTTATCCCCTTGCATATCCACA
CTGCCATTAAAAGCAATTCCATAAGCTAACTTTTCGCCATATGGATCTTTATTTTCATTTTGCATCGTAGCACGATTCGTAAAATCTG
TTATCGAAATAATTCCTATATTATTGAAAATGAGCAACATCATCAATGCTAAGCCTAAAATAGTACCTGAGGCTAAAGTCCAAAAA
CGCACTAAAAACTTAGGCGGTTTTGCATGATTATAGCGCTCAACACGTTTGAAATTATAGGTCATCTCTGGTATTCTTGTACGATTCT
TTTTCCAATCTAAATCAAAGTTTTCAATTTCTTCTTTTGAGTTTAGACTTAATCGATCACGTTCATGTTCTTTAAAAGATGGAATAACT
TGTTTAAGTGACCCTTCCATTCTTAATTGACCATGTGAAAACCAAGCTATGTAGTTACTCACTTGTGAAATTTTATCAATATCATCAC
CAATTGACACAATCGTTAAATTATTTTCAATATAATCATTTGTTAATTCAATCGCACGTTCCATAAATTGTGATGTTAAATAGTCAAT
AACATGATTTAAAATAATAATATTTGATTTTGATGAGCGTGCAATACTTAATAGTAATTGAGCGTATGCCGCTTTCGATATATGGTT
AACCGGCTTCGTTTTATAATCGCCTAAATGTGCATATTGAATAATCTGTTCAGCTTTATGATCATTAATTTCATATGGAAATAGTTGA
ACTAACTGCGCTGTATAAGCTTCAACAGTTTGATGAATTAACGATTGATCTTCAATATATCCGTAGAACAAATCTTCAGTACAAACT
ACTTTACCTTTATCAGGTTTAATTGCACCTGCCAACAATTGACCTACCAACGCTTTGGAAGATTCAGGTTCACCAATTATACCTAATG
CTTCTCCTTGATAAATATGTAAACTAATATTGTTTAAATCGATATCTTCAGCATCATATCCAAAAGGTAAATACCATTTCTTATTCTG
TTTATTCCTATAGTAGTGTGTTACTTTTAGTAACTTTAAAACAATTGAACTTCCCATCTATTTTCATCCTTCTATAATTGTAATAACGC
TATAGGTAAGCCTTCTTCGGGTTCGTTGCTATTAAATCTAAACCCCCACGCAAAAATACCTTTTAATCTTTCTACTTTAAAATATTGA
TTACTGCCATCAGTCAGTTTATAAATTTTACCAATTTCTATCTTTTTTCTGTCTACTAAGTAACTTTCGGCAACAATTACTTTACTTTG
ATAAACGTCATATTCATTTAAAATACCATTCATCTCAGCTTTTCTCATTTTCTCTTTATAAAGTTGTATTTCGTGTCTTAATTCTGGCT
CAGACATCTCACTCAATTTCTTCTGTTCCATCGGCAATACCACTTTCTTCTAATTTAGCTTTAATTTTATCATATTTATATCCTTTTCTC
ATAAGGCCTTCGATAGTTTTTGAAATTAATTTCTGTTGCGTGTACTTCTTTCGATTTTTATTATAAATTTTTTCTAAATCTCGTTGTAA

TAAATCGTCTAAAACCGCTTCATCTTGTGTAAAATCCATTTCATTCAATACAGCATGAATGGTTTCCATTTCAAACCCTTTTTGAATT
AAAGATTGCATTACTTTTTCTTTAACCTTATTTTGTGGCCCTTTTTTTGTTTTAGATATTTTTTCAGCAATTTGGATAATATCATCAAGT
TCCTGTTGTTCTCTATAAAGTTCTGTAAACATTTCAATGATATTTGGTTCTATACCAAGTTGATATAGTTTTTGTTGATAAATTTTAGG
TCCTTTATCTGTCGTGCGAATCATTGTATTTTTTAAACTTTCCGCATAATCTTGATGGTCGATTAACTTTTCGCGATAACAATATTCAA
TCACTTCAGAAATCGCTTGCTCTGATATCTCTTCTTTTTGTAAATATTGTATAACTTCTTTTTCAGTTCTCTTTTTATATGATAAATATT
GGATTGCTTTGTTTAAACCTATGCGATAATGATCATACTTTTGAATCTCTGCCATGTCAGCAGCTTCAAGTTGTTGCCCTTTTTTTAAA
TTAAATTTGACTAATGTATCGATATCTATACCCATTTCAAATTGTTCGTCTAAAAAAAGATTAAAACGTTCTTTGTTTTTCTTTTGAAC
TTCTATTTTAGTAATCTTCGGCACTGGATCACCCCGTTACTTTCTCAAGTTTTAATACAATACTCTTTTATTATTCAATAAGCCACTTC
CTATAGCAAATGTTTAACTTTAAATATTTTTCGATGCTAACAAAAAATCACACTATCATCTTTTAAAATGAAAGTGTGATTACAAGC
AAATCTGTAAAATTTATAAAGCAGAAACAATTCAACTTTATCATTATGACATTTCAATTAAACCTTCTACATTATAGTTCCAAGCATC
TTACACATGAATGCAAGTATTTAACGATTTAATTGTGACATAGCCTGTTGATATTGTGTTTCATTGATATAATTTTGTTGCTTCATTTT
TTCTAAGTTCGTGCTTACACGTTGCGTAAAATTCTCTGACATATTATTGATATTATATACGCTAGGTGCATTGACTTTACTAGCTAAA
ATAGCGCTTTGTAAAACTGTTATGTGAGACATTGTTGTACTATTTTTATTCACGGTTGTTCCAAAGTAATGGTTTGCTGCGCCCTCAA
GCGTATATTGATTATCCCCAAAGTAAATATTATTTAAATAAAAGCTTAAAATTTCGTTCTTATTATATTGTTTTTCAACTCGATGAGC
TACAAATAATTCTTTTACTTTTCTAGTAAATGAACGATCATTATCATAAAAATAATTTTTGACAACTTGTTGTGTAATGGTACTACCA
CCTTGCACATCTCTGTCGCTAATCGTTGAAAATAAAGCTCTAGTTGTACCTTTCAAATCGAATCCATGATGATTGTAGAATCGTTCAT
CTTCCATTGAAATAAAGGCACCTTTAACATACTCTGGCATGTTATCAGCTGACACAAAACTACTTTTATTTTCAATTTTTCTTAGTTC
ATCCACATTATCGCGTGTAGATAAAAAATACATGATACCAATAAACAATGCGATAATGATTAGAATGGTTAATAATATTTTTAATAG
TATTCGTTTACTTTTTTTCTTTTTCGGCGGTTTGCCAACTGGTTGATAATACGTATTATAGTGAGGTTCGTGTTTCATATGCTCAAAAT
GTTCATTTGAGTTTGAGTACTTATCGCTTCTTTTCATGCGTTTGCTCCTTCTTTTAAAACTCACTTAGTATATACCTTGAGTTTACCAG
TACTATCACAAATAGGCTACACTTTTTGGGAAAATCAGTCCAAGGGCTTACAATCGTATACGCCATCATACTTACTTTTTTGTTTTTT
GAAAAAATTATAGATAAATCATTGCAATTTTAAATATTAATCATGTCAAATATTGTTATATTTTATAAAAATAAAAGACCATCCCTA
TTAAATGCCAATAGAGACGACCTTTTATTTGTTATTCATTTATTAAAACTAAAATCCATATTTCATTTCAAACGAAAATATATAAATT
TTAACAATCGATAACCACAATACTTCTATTGTAATTGTTTAACGATTTCTCGATTAAAATCATCTAAATCGTCTGGTACTCGACTTGT
TACAATATTGTTGTCTACAACTACTGACTCATCAACTACATGTGCGCCTGCATTTGATAAATCTTTGCGCACATTTAATACTGCTGTT
AACGTACGACCTTTTAAATCGTCTGTATCTATTAGTATTTGTGGCCCATGACAAATGGCAAATGTTGGTACATCATTTTTAGTAAAGT
ATTTAGCAAATGTGCCATATCGACCTTCTGTATCTCCACGTAAATGATCTGGTGAAAATCCTCCAGGAATTAATAATGCATCATAAT
CTTCTGGTTTAGCTTCTGCAATGCCTACATCGACAGTAACTTTTTCACCGTGTTTACCAACAACTTCACTATTTGCAGTATCTCCAATC
ACTACAGTATTAAAGCCTGCATTCTCTAATGCCTCTTTAGGGCTTGAATATTCTATATCTTCAAATTCGTTTGCTAGAATAATTGCTA
CTTTTTTAGTCATTGAAAATCACCTTTCTATATATCATTGATATAATTACTATAGACAAGTAAATCAGTGATTAAACATACAAGATAT
AAAAAATATTAAGCGACTATCGCGATATCTAACTCTAACACATCTTATGCGGCATTTACTTAGATACTAATTTAACCTTTTCTTCAAG
CTGATCTAACAATCCAATCCATTCATCTATATCTTCAACACGTACTTCATCAGGATTTACATGATCGATATCCTCAATAAACTTATTT
AAACGCGCTTTTATCTGTTCGATTGTTTGCTGTTCATTCATAAAAAGTTAACTCCTTTTATTTTGTTTTCTTTTTCATTATTATCCTAAC
AGAAATTGCGTTAAAGCGATATAATCTTAGCTATATTTATGACATTCAAATTATTTTGACTTTTAAAAATCCCCTTTTCAATTAACTA
AAATTAAGAGATAATTTGTTACGAGTGATAATACGAAGTGGTATCATACCGATATGAACCAAATAGAAAGAAGGAAGTTTAAGACG
ATGAATAGCGTCAAATTGAAGCAACCTGTTAGCATTTACAATGATCCATGGGAATCATATAACGATGTTAAAGAACATGGCCAATT
AACTTTAAGTAACATCGAATTTACAACTACAAATCTTTGTAATATGCGTTGTAGCCACTGTGCAGTTGGTTATACTTTACAAACTGTC
GACCCCGAGCCTTTAGATATGGACTTAATTTATCGTAGACTTGATGAAATTCCAAATCTGCGAACGATGTCAATTACAGGTGGCGAA
CCAATGTTTTCTAAAAAGTCTATTAGAAATGTTGTTAAACCTCTATTAAAGTATGCACATCATCGAGGTATATATACACAAATGAAT
TCAAACCTAACATTGCCTCAAGATCGTTATTTAGATATTGCTGAATATATCGATGTTATGCATATCTCACATAACTGGGGAACAACT
GATGAATTCGCAAATGTTGGCTTTGGCGCAATGAAGAAGCAACCACCGTTAAAAGCTAAGTTAAAATTATATGAACAAATGATTTC
GAATGCACGTACATTATCAGAACAAGGAATGTTTGTATCTGCGGAAACAATGCTCAATCAAAGTACGCTACCACATTTACGAAAAA
TACATCAAGAAGTCGTTCATGATATGAAATGTAGCAGACACGAGATTCACCCTATGTATCCAGCTGACTTTGCAAGTCAATTAAATG
TGTTAACTCTAGCGGAAATGAAAAAGACAATTCATGATATATTGGATTTCAGAGATGAAGATATTTGGATGTTATTTGGTACTTTGC
CTGTGTTTCCATGCTTAAAAGATGATGAAGATCAAAAGTTACTATCACGTTTAAGAACTGCTAAAAATGTAACGACTAGAAATGAC
CCGGATGGCCGTAGTCGTTTAAATGTCAATGTATTTACAGGTAATGTAATCGTAACTGATTTCGGAGATGAAACAGGTACAATTTCG
AATATACAAAAAGATAAATTAACAGATGTATTTGATAAATGGTTATCCTCTGATCTTGCTAAATCATTAAATTGTCATTGTTCCGAG
TTTAGTTGTTTAGGACCAAATGTTCTTGTTAAAAATATGTACTATCCGAATATGGATTTTAAAGATAATGAGCGTCATATGCACAAA
CAACCACAAATTATACAATTTTAAAAACGCTTAATTATGCGGAGAAGCACTTTATCGATAAGTAGTCTCCGCATATTTTAATGCTAT
TATAAAATAAAAAAACAATTAATTGCTGGCAGTACTCTACTTTAATAATAAAGGTCATTTAATAGGACTAATAGTCTATAATAAAAG
GGGTAAATTTTAACTAAAAGCATAAACGTGCATAATCAAAAAGACAGATTGTAGGTGGAATATTCGAACATAACAGTTCAATTCAT
CCTTAACAATCTGTCTTTATATTTTTAGTCTCTAATATGTTGCACTTGAGCTAAATATTCAATTGTTTGTTTACTTTCAATGCGACGTG
CTTTTCTTCGTTCAACACGTTGTGGTGCTGTATCATAAAACCATTTTTCAACATCATCTTCTGGATATACACCAGGTACATGTTTAGG
TTTGCCTTCATCATCTAACGCAACAAATGTTAAGTAACTTAATGCAGCTAAATCATGCTTGTTATTAAATACATCGTCAATTCTAATT
TGAACGACCACTTCCATTGAACTAGTCCCAGCGTATGAAACCATCGCTACGTATTGTAATATGTCCCCTGTTTTAATCGGCTTTAAGA
AATCTACTGAGTCTGTAGATGCGGTAACTACTTGCGCACCAGCATGTTTCATAGCTGTGATTGCTGCAATTTCATCAATATTAGCCAT
CAATGTACCACCAAACATTGTATGATGGTGATTCGTATCTTGAGGGAAAACTTGTCTATTTTTATAACATTTTGATTCTGACATAGAT
TTCATTGGTCTGTCCTGATTTGTCATTATTTTATCTCCCAATCTATACTTTAATCACGTATTTGACCTTTAAATACATGTTTCATTTCCT
ACTCAGCACCTCAAAATAACATTTATTAAAATGTTGATGCCCAATTTCCATTTTCAAATACTAGTTCTTTTGAACCATCTTCGAATAT
GCCATAAATAGTCAAATCACTACTACCAATCATGAAATCGACATGTACATTTGAATCATTTAATCCGCTTGCAATCTTTTCCTCAACA
GTCATTTCCGTTCCACCTTGAATATTAAAGGCGTAAGCAGATCCAATCGCTAAATGACATGCTGCATTTTCATCAAATAAAGTATTG
TAAAAAATGGTATTACGATTTGATATCGGTGAATCATCAGGTACTAATGCTACTTCACCTAATCTTCTTGAACCTTCATCAGTATTGA
TTAAATCTTTCAATACCGTTTCACCTTTTTCAGCTGAAAAATCAATAATCTCTCCATCTTTAAACATCAATTTAAATTGATCGATAAT
TGTGCCATTATAACTTAAAGGCAATTTATTAGTAACATATCCATCTACTCTATTTCGATCTGGTGCTGTAAATACTTCTTCAGTTGGT
ATATTAGCAATAAAAGCTTGTTCCTTGCCGTTAACATAACTTGTAGCATCTTCCCAGATATGATTTTTCGCTAAACCTACCGTTAAAT
CAGTACCTTCAGATACATAATGCAACGCATGGTAATTTTTTTGTTGTAACTTTTGAGCGTAAACACTTAAATTAGCGATGTGTTGACG
CCAATTTTCAACTGGATCGTTACCATCAATTCGCACAATATCGAATACCTCGTCAATAAACTTAATATATGCTTCTTCTACAGATAGT
TCAGGATACACTCTCTTCGCCCAAGCTTTAGATGGAAAAGCAGCTACGACCCATGGAAATTGATTTTTTTGTACACTTTCCATATATC
CTTTAAATGCGCGTGCGTTTTGTTGTTGAAATGCTTGTAATTTTTGGCTATCGATACCATCCATTAAATCAGGATCTTCACTAATAAG
TGCTAAATTAGCAGCGCCACGTTTAACGTAATCCATTCTCGCTTCAACATCATATGATTTTAACTCATGATTAGCAAAATGTTCCACA
GATTCATTTTCAAATTTTAAGCGTTTTAAGGTTGGATCGCTATATACAACACGTACATCTGAAGCGCCACAATGATATGCTTCTTCTA

CAATTAAATGCGTTAATTCTAATGTTTCTACTGATGATCTAATAAATACTGGCTGTTTTGGTTGAACATTCATACCAACCTTAACTAA
TAGTTCAGCGTATTGTTGTAACTTTTCTTTGTAATTAGTCAAAATTCCCACTCCTAGTCGTGTCGCAATTGTCCTAAAGCGTCAGCAA
TAGCTGTCACTTCACTTGGCGACAATCTTTCTCTTGATTGAACAAAATCATATATATCTGTTAATTCTTCTTCATTTGCATTTTTATAT
TTTTCAGGATCTAATAATCCTTGATTAACAATATTTAACTTCTCTCTAATAGCTAATACTTTCTCTTCATTTGTCATAGCCACAATGTT
CACCTCTAATAATAGTTATGTTATCAAAGTATCACAATTTAATAAACTTTAATAGAATTTTATATAATCTGCTTTCTTATTGTTTAAA
GTCGCTATAAAATGGGGATATAACTACATAGTTTGGAGGAATCAAAATGGTAGATGTAGCATTTGTCTGTCTTGGCAATATATGTCG
TTCTCCAATGGCAGAAGCAATCATGCGACAAAGACTTAAAGACAGAAATATTCATGATATTAAAGTACATTCAAGAGGTACTGGTA
GCTGGAATTTAGGAGAGCCGCCTCATGAAGGTACACAAAAAATTCTCAACAAACACAATATTCCATTTGATGGCATGATTAGTGAA
TTATTCGAAGCGACAGATGATTTTGATTACATTGTGGCTATGGATCAAAGTAACGTTGATAATATTAAATCTATCAATCCTAATCTTA
AGGGACAATTGTTCAAACTGTTAGAATTTAGTAATATGGAAGAGAGTGATGTACCAGATCCATACTACACGAATAATTTTGAAGGT
GTATACGACATGGTATTATCATCTTGTGATAATTTAATAGACTACATCGTAAAAGATGCAAATTTGAAAGAGGGGTAGTTTTTTATG
GAAAATAAATTTGTTCCTGGTATTTTAATTGGTGCCGTAATTGGTGGTGCAATTAGTTTAGCTGATAAATCTACACGTCAAGCTTTAG
TTCAATCAGTTAAAGATGCAAAAAAATGGTAACCGCACTCGTAAGCCTTCTAAAGTCAGCAAGATTAAAGACGAAGTTTTATACTGG
AAAGATGTTGTTGAAGAAATTCGTCGTAATAATCCTGAATTAGAACGTTCATTAAAGGATGCGAAAGAAACATTTGTTAATAGAAA
AAATCAACGCTAAGATGATTTTGTTTTTATAAGGCAATACTTAACAATTTGGTGTACGCCTTTCATAGTTATCATTATTAAATGATAA
TACAATGATTGGCCAAACTGCATTGTTTCAGTATTGCCTTTGTTTTAATTTTAAATCAAAATAGCCTATGAAAGATTTAAATCAATTA
ATTTCTATAATATTATCATTTTTAAAGCATATCATTGTTTAGTTTTTTTATAATTGGATAAATACTAATAGTTACTTTATAAAACATTA
CATAGAGAAAGGTTAAGGAGTGCACATGTCGAAAAAGGATCACTCTTCTTCAAAATACCTTAATTCTGTTAAGGAAGCGCAAGAGG
AGTCAAAAAAGAAAAATAAAAGTAATCCCAAAATTGATGTTGATCGTACATATATTGAACCTCAACAATTCCAATCTAAGAAACCT
AAAAAAGATGATCAGGTTTTCTTCTTATCAAGATTAAATAAACCTGCAAAATATAAGAAAGACTCTAATTTCTTATCATATCTCATC
TATCGCATAGGAAAAGATGATGCCTCAGGACTAGCAGCACAAATGACTTACCATTTCGTACTTGCTATGTTCCCTATGTTGCTTTTCC
TATTAACATTATTACCATTTTTCAATATTAAGCAGAGTCAAATTACTAATATGTTAAGCAATGCACCCGCTGAAACATCTACTCTAAT
TAAGAGTGTAATTGGTGATATAACTCAAAACTCCAGTGGTGGCTTATTATCTATCGGTTTGATTTTAGCAATTTGGTCAGCTTCAAAT
GGAATGACTGCAATTATGAATTCTTTCAATGTTGCTTACGATGTAGAAGATAGCCGTAATGGAATCGTATTAAAACTACTAAGTGTT
GTCTTCACTGTAGTTATGGGCGTTGTGTTTGTAGTTGCTCTAGCATTACCAACGCTTGGTTCTGTAATTAGTCATTTCCTATTCGGTCC
ACTTGGATTTGACGAACAAGTGAAATGGATTTTTAACCTTATTAGAATTGTGTTACCAATCATTATTATATTTATCGTATTTATCGTG
TTATATTCGGTTGCACCTAACGTTAAAACGAAGCTTAAGTCAGTATTACCAGGTGCAGTATTTACTTCAATTATTTGGTTAGCTGGTT
CATTTGGTTTTGGTTGGTATATTTCAAATTTTGGTAACTATTCTAAAACATATGGCAGTATCGCGGGTATCATCATTTTGTTACTATG
GTTATATATCACAAGTTTTATTATAATTGTCGGTGCTGAAATCAATGCAATCATTCATCAGCGTAGTGTAATTAAAGGTAAAACACC
TGAAGAAGCAGCATTAGAACATGATGACAATAATAAAAATCATTATAATGAGAACACTAGGTACGAATATGATGAGGATAATAAT
GCAACACATCAACGTACGTATCATGTTGATGAACATCCTAGTGATACCTATCCAGAAGATGATAAAAATATAAAAGACAAAATCGT
TGATAAGCTTAAAAAAGACTAAACAACAACAAAACAGAGGTTTTCGCTAATGATTGCGAAGACCTCTGTTTTTTGTGTCTATTTGAT
GTTATATTGTGATTGGCGTAAGTAACTTTTCTTAATTCGATATGAACTATTGAATTAAATTATGTTGGAATGCATAGATGACAGCTTG
TGTTCTATCTTGCACTTCTAACTTACTTAAAATGTTACTCACATGTGTCTTAACCGTTTTAATAGTAATATGCGATGCACTAGCAATTT
CTTGATTTGAGTAACCTTTCGCAATCAATAATAATATTTCCATTTCTCGTTCTGTAAGCATTTCATATAACTCTGCGCGCTTTTTCATA
CGGTTACGCATTTTCACTAAAACTTCCGGTTCAAAAACAGATTCTCCTCTAGAAGTTTTACGAACTGCATCGGCGATATCTTTTGCAC
TTGTTGTTTTTAAAATGTAACTATCGACACCTGCATCTAATGCACGATATACCTCTTTATCTTCAATAAAACTAGTTAATATTAATAC
TTTAATTTGCGGTAAATCTTTTTTAATCTGAGTCGTCGCTTCTACACCATCCATGTCATCCATAAGTAAATCCATTAAAATTAAATCT
GGCTTCAACTCATGGGCTTTGGCAATTGCTTCTTTACCAGAAGCGCCTTCACCAACTACTTCAATATCACTTTGCGTTGATAGATAAC
TTGAAATTCCTATACGTACCATTTCATGATCATCCACAAACAATACTTTAATCGTCATACGAATCCTCCTTATTTAAAGGTGCTTTCA
CCTCGATACGTGTACCTGAATCTGGCAATGATACAATATGGAACGTTGCACCAATTTCCAAAGCTCTTTCACGCATATTTTTAAGTCC
ATAACTTTGTTCTAATTTTTCATCAACATTAAAACCTTTACCATTATCTTGAATTCTCAACAATAAATAATCGTCTTTATTAAACAATT
CTACTGTCACTTTTGTACCGTTTGAATGACGCAATGTATTCGAAATTGCTTCCTGTGTAATTCTGAACAAATGATCTTCAATACCTTT
AGGCACTTTAAAATCTTGTATTTCATGCACAACTTTCATTGGCACTTTTTTTTGTAAATCAATAACTAAATCTTTAATACCCTCACCTA
AAGATTTGTCTTTTAAACCAAGCGGTCTTAAATGTAACAGCAAAGCACGCATTTCTAACTGCGAATCTTGAACCATTTTCTCTAAAA
TAGGAATTTGTTGGTCTAATGGTGGTTCTAACTTCGTTTCTTTGATAGCAGATAGCATCATACTTGCCGCAAAAAGTTGCTGACTAAC
AGAATCGTGAAGTTCTCGTGCTAGTCTTTGACGTTCATCTTCAATAATCTTTTTAACTTTCACATCATTAATATTATAATTTTCATTGG
TTAAGTTTTGAGTTTTAAGTCGCAACTTATGCAATTCTTGATTTAAAGGTACGAGTGTATGGTATAAATCTAACGTTTCACTATATAT
TTCTATATTTTGATCATTAATGCCAACTGTTTCGCCTTCCATTGAACGCTCAATTTGCGTCTTAATCCAATCATTTTGCTGATTGATTT
TGTAAGCGAGTACCGAACCAACAATAATACACAATAATATGATGATGAGATTTAAAAATAAAAAGACTGGTATTCCGAATATTTGT
GTATAAAACATACCTTGAAAATAGATGATATTTACAAAAACTTTATCGATGAACAGAAATGCAGCTAGCATGCTATATACTAAGAT
GAGCATTGAACCAATTGTTCTAATGTAGTGGTTCATCGATAAATCACCTCTACGTCTCCGATAAACGTTGATACGTAGATATTAACT
GTATAGTTATCCGGTTTCATCATTTCTTCAATATGAATATTGTTATTTTCAACTTTATATGATTTTTCATTCACGTAAGTACTTCCATA
AAAAGCAGCTACATGTAAATTAATATTGTAATTAACCGGCAATATAACCTGCACTTTACCTAAAATGTGTCTAACAACAATGGTATT
ATTTTCCTTAATATTTGCAGCTTTTGTTAAGTCAATATGTAGGTCGCCAATTCCATGTTGAATTTGTACATCTTCCCACTTATATACAT
AAACTGGTGTACGTTGCTCACCAAACCACTTTTGTTTAATAAAAGATGGTGAAGTCATAACCTCATCCGTCGCAACTACTTTTTTTGG
TTTAAACTTGTGTATTAAATAACGCACAATAAGTAGTAATAAAAAGACGAACAAGATGATGATTGTATACTTATTAGACAATAATG
TGAATGCAATTAATAGCGCGCCTATCCAAAACGCCAAAAGGCCACGTATTTTATGAAAATAAAGATATCCTACATAAACTAATACA
CATCCCAATAATAGAACGAGTAAAAAGCCAATTTTTTCAAAAAATATGTAGTAAAAATTGGCAATAATCATTAATGCAGTAAAAAT
GATCAACATTTGCGTTGATATATATTTGTGTGTCATGTTTCGCCGCCTTTCTATGCCACAGCGTTCATATAATTTATTAGATACGATG
TTTTTTCTGTTGCTATTTTTTCAGTTGAAGTTTGTTGAATTTGAGTTTCAATTTGTGCATAATATGCTTCAACTTTATTCAACTCTGATT
TAATATTTTCAATTTCTTGATTATTGATTTCTCTTGCACATTGAATTTGAAATGCTTCAGCAATATCAATATATTTATTTTCTAATGTT
AGCATTAGACTGTCAATTAACTTGCTAAGTTGTTCTTTTTTAGTAATTAAATAACGCATATAATCATCTAAATTTTTCATTTTTTCATC
TAGCATTAAAAGGTAGTGCTTGATATTATTTCTTACTGTTGCTCTCAAAAACTGTTCAATATAACGTTCAACATAGTTCATAACTATC
ACCTTTTATAATAAGTTTTAAAATACCAAATGCGCTATGTATCTTCGTTGATGTCGATGATATGTTTGCTTAAAAACTACTTTATTAC
AACATATTTTTGAAATGACGCATTGATTGTGTTCTTAATTATTATAAAACAGTTCCGAAACCAATATGAATAGGCTCCGGAACTAAA
TGATGTTCATACTTTAGTCTTAGTATATGTTGAACTATTCTTCTTCAATTTTTGTCGTTAAAATCGGACCATCCTTAGTCACGATAACC
GTATGCTCAATTTGAGCAACAAAACTTTTATCGCTCGTTTCAAAAGCCCATTCATTTTTACCTTCTGTAACAAATGATGCATTTGATG
AGATAAACGGTTCAATAGCTAATACCATACCTTCAGTTAATAATGTTTTGTCTTTTGGATCAAAGTAATTAAGTACATGTGCTGGTG
CTTCATGTAATGATAAACCAACACCATGACCTGTTAAGTTTTTAATGACTTTCAAATCATTTTGTCTAGCTGTATTATGCACCGCTTT
ACCAATGTTACTTAACTTAGTACCCGGTTTTACTTTTGCAATTGCATTCTCAAATGCCATCGTTGCTACGTCACATACTTTTTGTTTCA

TTGGATCATCTGATTCTCCAACGACAAATGAAATACCTGTATCTGCATAATAGCCATTCTTCAAAGCCGATACATCAATATTTACTA
AATCTCCTTCACGAATGACACGCTTACTTGGAATCCCATGTGCCACCTCTTCATTGACACTAATACACGTTTGACCAGGAAAATTTTC
ATCATGAATTGGCGCAGAAATAGCACCGTATTCTTCAAATAACTCTTTCGCAATATTATCAAGCTCTTTCGTAGTGATACCTGGTTTG
GTTGCAGCTTGCATTGTATTGCGCACTTTAGCGCATATGTATCCAATTTCTTTTAACGCTTGTAATTCTTCTTCTGTTTTTACAATCAT
TTTATCCCGTTCCTTTTTTATATAATTATAACTTATTATAGCAAAAATTCTTTGATATACTAAAGATGTTACTTTAGTATTTTGGAAAG
TGAGTGAGACATAGTGTGATAAAGAAAAAATATTTCTTTATCGTTGTACCCCAACTTGCATTGCCTGTAGAAATTGTAGATTCAATT
TCTCTATGTTGGGGCCCCGCCAACCTGCACATTATCGTAAGCTGACTTTTCGTCAGCTTCTGTGTTGGGGCCCCGGACTATAATTGAA
AAATGCTTGTTACAAGTGCATTTTATTTCAGTCAACTACTAACAATATAACATTGTGGAGCCCAGAACTTTGATTAATGTATATGAA
AATCAAAGTAATGCTTAAGTATGATTATTTCAAATATTTTACATACATGAACTTTTCCAATGTACGATACTATTATTATAAAGCGCTC
GCTAAGATTTTACGATGATTAGAGGTTAAAAAATGAACGATCAATGGTATAAACATTTAATTGGTGCCAGAACAATTAAAACTGGT
ATTGCCATTTTTTTAACAGCTGTCTTTTGTATGGCACTAGATTTAACACCCATCTATGCCATTTTAACAGCTGTAGTCACAATTGAAC
CAACTGCCAAGGCATCACTTATTAAAGGTTATCGTAGATTACCTGCTACGGTAATTGGTGCAGGCTTTGCAGTATTATTTACATATTT
ATTCGGCGATCAATCACCTTTTACATATGCATTGAGTGCAACGTTCACGATTTTATTCTGTACAAAACTCAAATTGCAAGTTGGTACG
AATGTCGCTGTACTAACATCATTAGCTATGATTCCAGGTATTCATGATGCCTACATATTCAACTTTTTGTCTCGAACATTGACAGCAA
TTATTGGACTCGTGACATCAGGTTTAATTAACTTCATGGTGTTTCCACCTAAGTATTATGGTCAGGTCGAAGAAAAATTAAGTAAGA
CAGATGCCTTGATGTATAAATTATTTTACAATCGCTGCCAAGAACTTATCTTATCAAGACTGCAATCTGATAAAAGCGAAAAATCAT
ATAAAAATATTTTCAATTTAAATAATCAAGTTGAAACGTTAATAAGTTATCAACGTGATGAATTAAGCTATCATAAGAAAAAAGAA
TGTGATTGGAAATTACTCAATCAATTAACTAAACGTGCATATACGAATCGTTTGTTTATCACTCATTTATCAAATATCATTTACTTAC
CTAAAAATACTCGTGTTAACTTTTCAGGTGACGAAAAAATGGCATTGCTAAAAATTAGTAGTAGCATCAAAGATATATTTTATGATG
GTTCTTTTAAGAGAGAAGATGATTCTGTAGAAACTTTACGTTCGACAATAAAAGCACTTGAAATTAGTGGTGAAAATCAAATTAAA
AGTCATATACTATACGAAGTTCTAATGATTTATCGACTGCTAGACAGCAGATATGCTTAATTTAAGTACCTTATACTAATTGGCACC
ATGGGAGTGGGACAGAAATGATATTTTCGTAAAATTTATTTCGTCGTCCCACCCCAACTCGCATTGCCTGTAGAATTTCTTTT

>050-contig_246_RC

GCCCCTGACTAGAATTGAAAAAAGCTTGTTACAAGCGCATTTTCGTTCAGTCAACTACTGCCAATATAACTTCGTAGAGCATAGAAC
ATTGATTTATGTCCCCGCCTCTTTTTATCGTATATAGCATGTCCCTTTTTAAAATTACAAGCATAAATATTCAGACTCAAATATAGCC
ACACCCCTAAACTACGGAGGGATGTGGCTGTCTTTTTTGATTATAATTATGTATCAGATTTAAATCATTAATGTAAAGAGGCTAATT
CATTAATAAATTTAGTAAAATCTGATTTAGCAACATTTATCCTTAATGAAATACCAGATTCTGTTGCCATGTTTGAATGCCTTAAACC
AGAATCAAAATCAATATAAATACAAAGCAGTTCTTGATTAAAATCAATAATTATAAAATGTAAATCAGGTTCTATAAAATATATTTC
[truncated: 774,760 more chars]
