# Supplementary material for: Genome sequencing and molecular characterisation of Staphylococcus aureus ST772-MRSA-V, “Bengal Bay Clone”
Source: BMC Res Notes. 2013 Dec 20;6:548. doi: 10.1186/1756-0500-6-548 (PMC3878137; doi:10.1186/1756-0500-6-548)
Supplement: Additional file 3 — Annotated draft genome sequence for isolate 07–17048. [file 1756-0500-6-548-S3.pdf]

> **dnaA**
Function: protein coding sequence; chromosomal replication initiator protein (core genome, constant)
Best match: dnaA_D89066.1[456:1817] (completely identical)
Position: 001-contig_254_RC: 9187 … 10549; Length: 1362 bp
Sequence:
ATGTCGGAAAAAGAAATTTGGGAAAAAGTGCTTGAAATTGCTCAAGAAAAATTATCAGC
TGTAAGTTACTCAACTTTCCTAAAAGATACTGAGCTTTACACGATTAAAGATGGTGAAGC
TATCGTATTATCGAGTATTCCTTTTAATGCAAATTGGTTAAATCAACAATATGCTGAAATT
ATCCAAGCAATCTTATTTGATGTTGTAGGCTATGAAGTTAAACCTCACTTTATTACTACTG
AAGAATTAGCAAATTATAGTAATAATGAAACTGCTACTCCAAAAGAAACAACAAAACCT
TCTACTGAAACAACTGAGGATAATCATGTGCTTGGTAGAGAGCAATTCAATGCCCATAAC
ACATTTGACACTTTTGTAATCGGACCTGGTAACCGCTTTCCACATGCAGCAAGTTTAGCTG
TAGCCGAAGCACCAGCCAAAGCGTACAATCCATTATTTATCTATGGAGGTGTTGGTTTAG
GAAAAACCCATTTAATGCATGCCATTGGTCATCATGTTTTAGATAATAATCCAGATGCCA
AAGTGATTTACACATCAAGTGAAAAATTCACAAATGAATTTATTAAATCAATTCGTGATA
ACGAAGGTGAAGCTTTCAGAGAAAGATATCGTAATATCGACGTCTTATTAATCGATGATA
TTCAGTTCATACAAAACAAGGTACAAACACAAGAAGAATTTTTCTATACTTTTAATGAAT
TGCATCAGAATAACAAGCAAATAGTTATTTCGAGTGATCGACCACCAAAGGAAATTGCAC
AATTAGAAGACCGATTACGTTCACGCTTTGAATGGGGGCTAATTGTTGATATTACGCCAC
CAGATTATGAAACTCGAATGGCAATTTTGCAGAAGAAAATTGAAGAAGAAAAATTAGAT
ATTCCACCAGAAGCTTTAAATTATATAGCAAATCAAATTCAATCTAATATTCGTGAATTA
GAAGGTGCATTAACACGTTTACTTGCATATTCACAATTATTAGGAAAACCAATTACAACT
GAATTAACTGCTGAAGCTTTAAAAGATATCATTCAAGCACCAAAATCTAAAAAGATTACC
ATCCAAGATATTCAAAAAATTGTAGGCCAGTACTATAATGTTAGAATTGAAGATTTCAGT
GCAAAAAAACGTACAAAGTCAATTGCATATCCGCGTCAAATAGCTATGTACTTGTCTAGA
GAGCTTACAGATTTCTCATTACCTAAAATTGGTGAAGAATTTGGTGGGCGTGATCATACG
ACCGTCATTCATGCTCATGAAAAAATATCTAAAGATTTAAAAGAAGATCCTATTTTTAAA
CAAGAAGTAGAGAATCTTGAAAAAGAAATAAGAAATGTATAA

> **dnaN**
Function: protein coding sequence; DNA polymerase III subunit beta (core genome, constant)
Best match: dnaN_CC001-ST772_118_AJGE01000004.1[31202:32335:r] (completely identical)
Position: 001-contig_254_RC: 10826 … 11960; Length: 1134 bp
Sequence:
ATGATGGAATTCACTATTAAAAGAGATTATTTTATTACACAATTAAATGACACATTAAAA
GCTATTTCACCAAGAACAACATTACCTATATTAACTGGTATCAAAATCGATGCGAAAGAA
CATGAAGTTATATTAACTGGTTCAGACTCTGAAATTTCAATAGAAATCACTATTCCTAAA
ACTGTAGATGGCGAAGATATTGTCAATATTTCAGAAACAGGCTCAGTAGTACTTCCTGGA
CGATTCTTTGTTGATATTATAAAAAAATTACCTGGTAAAGATGTTAAATTATCTACAAATG
AACAATTCCAGACATTAATTACATCAGGTCATTCTGAATTTAATTTAAGTGGCTTAGATCC
AGATCAATATCCTTTATTACCTCAAGTTTCTAGAGATGACGCAATTCAATTGTCGGTAAAA
GTGCTTAAAAACGTGATTGCACAAACAAATTTTGCAGTGTCCACCTCAGAAACACGCCCA
GTACTAACTGGTGTGAACTGGCTTATACAAGAAAATGAATTAATATGCACAGCGACTGAC
TCACACCGCTTGGCTGTAAGAAAGTTGCAGTTAGAAGATGTTTCTGAAAACAAAAATGTC
ATCATTCCAGGTAAGGCTTTAGCTGAATTAAATAAAATTATGTCTGACAATGAAGAAGAC
ATTGATATCTTCTTTGCTTCAAACCAAGTTTTATTTAAAGTTGGAAATGTGAACTTTATTTC
TCGATTATTAGAAGGACATTATCCTGATACAACACGTTTATTCCCTGAAAACTATGAAATT
AAATTAAGTATAGACAATGGGGAGTTTTATCATGCGATTGATCGTGCCTCTTTATTAGCAC
GTGAAGGTGGTAATAACGTTATTAAATTAAGTACAGGTGATGACGTTGTTGAATTATCTT
CTACATCACCAGAAATTGGTACTGTAAAAGAAGAAGTTGATGCAAACGATGTTGAAGGT
GGTAGCCTGAAAATTTCATTCAACTCTAAATATATGATGGATGCTTTAAAAGCAATCGAT
AATGATGAGGTTGAAGTTGAATTCTTCGGTACAATGAAACCATTTATTCTAAAACCAAAA
GGTGACGACTCGGTAACGCAATTAATTTTACCAATCAGAACTTACTAA

> **recF**

Function: protein coding sequence; DNA replication and repair protein F (core genome, constant)
Best match: recF_CC001_MW2-USA400_BA000033.2[3912:5024] (completely identical)
Position: 001-contig_254_RC: 12582 … 13695; Length: 1113 bp
Sequence:
ATGAAGTTAAATACACTCCAATTAGAAAATTATCGTAACTATGATGAGGTTACGTTGAAA
TGTCATCCTGACGTGAATATCCTCATTGGAGAAAATGCACAAGGAAAGACAAATTTACTT
GAATCAATTTATACCTTAGCTTTAGCAAAAAGTCATAGAACGAGTAATGATAAGGAACTC
ATACGTTTTAATGCTGATTATGCTAAAATAGAAGGTGAGCTTAGTTATAGACACGGCACG
ATGCCATTAACAATGTTTATAACTAAAAAAGGTAAACAAGTCAAAGTGAATCACTTAGAG
CAAAGTCGTCTAACTCAATATATTGGACACCTCAATGTGGTTCTATTTGCGCCAGAAGATT
TGAATATTGTAAAAGGCTCTCCTCAAATAAGACGACGCTTTATAGATATGGAGTTGGGCC
AAATTTCTGCTGTTTACTTAAATGATTTAGCTCAATACCAACGTATTTTAAAGCAAAAGAA
TAATTACTTAAAGCAGTTACAATTAGGCCAAAAAAAGGACTTAACAATGTTGGAAGTATT
AAATCAGCAGTTTGCTGAATATGCAATGAAAGTAACTGATAAACGTGCACATTTTATTCA
AGAGCTAGAGTCGTTAGCTAAACCGATTCATGCTGGTATCACAAATGATAAAGAAGCGTT
GTCGCTGAATTATTTACCTAGTCTTAAATTTGATTATGCTCAAAATGAAGCGGCACGACTT
GAAGAAATTATGTCTATTCTTAGCGATAATATGCAAAGAGAAAAAGAACGAGGCATTAG
CTTATTCGGACCACATCGAGATGATATAAGTTTTGATGTGAATGGCATGGATGCTCAAAC
ATATGGTTCTCAAGGACAGCAACGTACAACGGCTTTGTCCATTAAATTAGCTGAAATTGA
GTTAATGAATATCGAAGTTGGGGAATATCCCATCTTATTATTAGACGATGTACTCAGTGA
ATTAGATGATTCGCGTCAAACGCATTTATTAAGTACGATTCAGCATAAAGTACAAACATT
TGTCACTACGACATCTGTAGATGGTATTGATCATGAAATCATGAATAACGCTAAATTGTA
TCGTATTAATCAAGGTGAAATTATAAAGTAA

## > **yaaA**

Function: protein coding sequence; RNA-binding S4 domain protein (core genome, constant)
Best match: yaaA_CC001_MW2-USA400_BA000033.2[3670:3915] (completely identical)
Position: 001-contig_254_RC: 12340 … 12586; Length: 246 bp
Sequence:
GTGATTATTTTGGTTCAAGAAGTTGTAGTAGAAGGAGACATTAATTTAGGTCAATTTCTA
AAAACAGAAGGGATTATTGAATCTGGTGGTCAAGCAAAATGGTTCTTGCAAGACGTTGA
AGTATTAATTAATGGAGTGCGTGAAACACGTCGCGGTAAAAAGTTAGAACATCAAGATC
GTATAGATATCCCAGAATTACCTGAAGATGCTGGTTCTTTCTTAATCATTCATCAAGGTGA
ACAATGA

## > **gyrB**

Function: protein coding sequence; DNA topoisomerase type II subunit B (core genome, constant)
Best match: gyrB_CC001-ST772_118_AJGE01000004.1[27523:29457:r] (completely identical)
Position: 001-contig_254_RC: 13704 … 15639; Length: 1935 bp
Sequence:
ATGGTGACTGCATTGTCAGATGTAAACAACACGGATAATTATGGTGCTGGGCAAATACAA
GTATTAGAAGGTTTAGAAGCAGTACGTAAAAGACCAGGTATGTATATAGGATCGACTTCA
GAGAGAGGTTTGCACCATTTAGTGTGGGAAATTGTCGATAATAGTATCGATGAAGCATTA
GCTGGTTATGCAAATCAAATTGAAGTTGTTATTGAAAAAGATAACTGGATTAAAGTAACG
GATAACGGACGTGGTATCCCAGTTGATATTCAAGAAAAAATGGGACGTCCAGCTGTCGA
AGTTATTTTAACTGTTTTACATGCTGGTGGTAAATTCGGCGGTGGCGGATACAAAGTATCT
GGTGGTTTACATGGTGTTGGTTCATCAGTTGTAAACGCATTGTCACAAGACTTAGAAGTA
TATGTACACAGAAATGAGACTATATATCATCAAGCATATAAAAAAGGTGTACCTCAATTT
GACTTAAAAGAAGTTGGCACAACTGATAAGACAGGTACTGTCATTCGTTTTAAAGCAGAT
GGAGAAATCTTCACAGAGACAACTGTATACAACTATGAAACATTACAGCAGCGTATTAG
AGAGCTTGCTTTCTTAAACAAAGGAATTCAAATCACATTAAGAGATGAACGTGATGAAGA
AAACGTTAGAGAAGACTCCTATCACTATGAGGGCGGTATTAAATCGTACGTTGAGTTATT
GAACGAAAATAAAGAACCTATTCATGATGAGCCAATTTATATTCATCAATCTAAAGATGA
TATTGAAGTAGAAATTGCGATTCAATATAACTCAGGATATGCCACAAATCTTTTAACTTA
CGCAAATAACATTCATACGTATGAAGGTGGTACGCATGAAGACGGATTCAAACGTGCATT

AACGCGTGTCTTAAATAGTTATGGTTTAAGTAGCAAGATTATGAAAGAAGAAAAAGATA
GACTTTCTGGTGAAGATACACGTGAAGGTATGACAGCAATTATATCTATCAAACATGGTG
ATCCTCAATTCGAAGGTCAAACGAAGACAAAATTAGGTAATTCTGAAGTGCGTCAAGTTG
TAGATAAATTATTCTCAGAGCACTTTGAACGATTTTTATATGAAAATCCACAAGTCGCAC
GTACAGTGGTTGAAAAAGGTATTATGGCGGCACGTGCACGTGTTGCTGCGAAAAAAGCG
CGTGAAGTAACACGTCGTAAATCAGCGTTAGATGTAGCAAGTCTTCCAGGTAAATTAGCC
GATTGCTCTAGTAAAAGTCCTGAAGAATGTGAGATTTTCTTAGTCGAAGGGGACTCTGCC
GGGGGGTCTACAAAATCTGGTCGTGACTCTAGAACGCAGGCGATTTTACCATTACGAGGT
AAGATATTAAATGTTGAAAAAGCACGATTAGATAGAATTTTGAATAACAATGAAATTCGT
CAAATGATCACAGCATTTGGTACAGGAATCGGTGGCGACTTTGATCTAGCGAAAGCAAG
ATATCACAAAATCGTCATTATGACTGATGCCGATGTGGATGGAGCGCATATTAGAACATT
GTTATTAACATTCTTCTATCGATTTATGAGACCGTTAATTGAAGCAGGCTATGTGTATATT
GCACAGCCACCGTTGTATAAACTGACACAAGGTAAACAAAAGTATTATGTATACAATGAT
AGGGAACTTGATAAACTTAAATCTGAATTGAATCCAACACCAAAATGGTCTATTGCACGA
TACAAAGGTCTTGGAGAAATGAATGCAGATCAATTATGGGAAACAACAATGAACCCTGA
GCACCGCGCTCTTTTACAAGTAAAACTTGAAGATGCGATTGAAGCGGACCAAACATTTGA
AATGTTAATGGGTGACGTTGTAGAAAACCGTAGACAATTTATAGAAGATAATGCAGTTTA
TGCAAACTTAGACTTCTAA

> **gyrA**

Function: protein coding sequence; DNA topoisomerase type II subunit A (core genome, constant)
Best match: gyrA_CC005_04-02981_CP001844.2[7005:9674] (completely identical)
Position: 001-contig_254_RC: 15675 … 18345; Length: 2670 bp
Sequence:
ATGGCTGAATTACCTCAATCAAGAATAAATGAACGAAATATTACCAGTGAAATGCGTGA
ATCATTTTTAGATTATGCGATGAGTGTTATCGTTGCTCGTGCATTGCCAGATGTTCGTGAC
GGTTTAAAACCAGTACATCGTCGTATACTATATGGATTAAATGAACAAGGTATGACACCG
GATAAATCATATAAAAAATCAGCACGTATCGTTGGTGACGTAATGGGTAAATATCACCCT
CATGGTGACTTATCTATTTATGAAGCAATGGTACGTATGGCTCAAGATTTCAGTTATCGTT
ATCCGCTTGTTGATGGCCAAGGTAACTTTGGTTCAATGGATGGAGATGGCGCAGCAGCAA
TGCGTTATACTGAAGCGCGTATGACTAAAATCACACTTGAACTGTTACGTGATATTAATA
AAGATACAATAGATTTTATCGATAACTATGATGGTAATGAAAGAGAGCCGTCAGTCTTAC
CTGCTCGATTCCCTAACTTGTTAGCCAATGGAGCATCAGGTATAGCGGTAGGTATGGCAA
CGAATATTCCACCACATAACTTAACAGAATTAATCAATGGTGTACTTAGCTTAAGTAAGA
ACCCTGATATTTCAATTGCTGAGTTAATGGAGGATATTGAAGGTCCTGATTTCCCAACTGC
TGGACTTATTTTAGGTAAGAGTGGTATTAGACGTGCATATGAAACAGGTCGTGGTTCAAT
TCAAATGCGTTCTCGTGCAGTTATTGAAGAACGTGGAGGCGGACGTCAACGTATTGTTGT
CACTGAAATTCCTTTCCAAGTGAATAAGGCTCGTATGATTGAAAAAATTGCAGAGCTCGT
TCGTGACAAGAAAATTGACGGTATCACTGATTTACGTGATGAAACAAGTTTACGTACTGG
TGTGCGTGTCGTTATTGATGTGCGTAAGGATGCAAATGCTAGTGTCATTTTAAATAACTTA
TACAAACAAACACCTCTTCAAACATCATTTGGTGTGAATATGATTGCACTTGTAAATGGT
AGACCGAAGCTTATTAATTTAAAAGAAGCGTTGGTACATTATTTAGAGCATCAAAAGACA
GTTGTTAGAAGACGTACGCAATACAACTTACGTAAAGCTAAAGATCGTGCCCACATTTTA
GAAGGATTACGTATCGCACTTGACCATATCGATGAAATTATTTCAACGATTCGTGAGTCA
GATACAGATAAAGTTGCAATGGAAAGCTTGCAACAACGCTTCAAACTTTCTGAAAAACA
AGCTCAAGCTATTTTAGACATGCGTTTAAGACGTCTAACAGGTTTAGAGAGAGACAAAAT
TGAAGCTGAATATAATGAGTTATTAAATTATATTAGTGAATTAGAAACAATCTTAGCTGA
TGAAGAAGTATTACTACAATTAGTTAGAGATGAATTAACAGAAATTCGAGATCGTTTCGG
TGATGATCGTCGTACTGAAATCCAATTAGGTGGATTTGAAGATTTAGAAGATGAAGATCT
CATTCCAGAAGAACAAATTGTAATTACACTAAGCCATAATAACTACATTAAACGTTTGCC
GGTATCTACATATCGTGCTCAAAACCGTGGTGGTCGTGGTGTTCAAGGTATGAATACATT
GGAAGAAGATTTTGTCAGTCAATTGGTAACTTTAAGTACACATGACCATGTATTGTTCTTT
ACTAACAAAGGTCGTGTATACAAACTTAAAGGTTATGAAGTGCCTGAGTTATCAAGACAG
TCTAAAGGTATTCCTGTAGTGAATGCTATTGAACTTGAAAATGATGAAGTCATTAGTACA
ATGATTGCTGTTAAAGACCTTGAAAGTGAAGACAACTTCTTAGTGTTTGCAACTAAACGT
GGTGTCGTTAAACGTTCAGCATTAAGTAACTTCTCAAGAATAAATAGAAATGGTAAGATT

GCGATTTCGTTCAGAGAAGATGATGAGTTAATTGCAGTTCGCTTAACAAGTGGTCAAGAA
GATATCTTGATTGGTACATCACATGCATCATTAATTCGATTCCCTGAATCAACATTACGTC
CTTTAGGCCGTACAGCAACGGGTGTGAAAGGTATTACACTTCGTGAAGGTGACGAAGTTG
TAGGGCTTGATGTAGCTCATGCAAACAGTGTTGATGAAGTATTAGTAGTTACTGAAAATG
GTTATGGTAAACGTACGCCAGTTAATGACTATCGTTTATCAAATCGTGGTGGTAAAGGTA
TTAAAACAGCTACGATTACTGAGCGTAATGGTAATGTTGTATGTATCACTACAGTAACTG
GTGAAGAAGATTTAATGATTGTTACTAATGCAGGTGTCATTATTCGACTAGATGTTGCAG
ATATTTCTCAAAATGGTCGTGCAGCACAAGGTGTTCGCTTAATTCGCTTAGGTGATGATCA
ATTTGTTTCAACGGTTGCTAAAGTAAAAGAAGATGCAGAAGATGAAACGAATGAAGATG
AGCAATCTACTTCAACTGTATCTGAAGATGGTACTGAACAACAACGTGAAGCGGTTGTAA
ATGATGAAACACCAGGAAATGCAATTCATACTGAAGTGATTGATTCAGAAGAAAATGAT
GAAGATGGACGTATTGAAGTAAGACAAGATTTCATGGATCGTGTTGAAGAAGATATACA
ACAATCATCAGATGAAGATGAAGAATAA

## > **txbi_gyrA**

Function:  bidirectional rho-independent terminator of gyrA (core genome, constant)
Best match: txbi_gyrA_CC398_SO385-pig_AM990992.19665:9724
Position: 001-contig_254_RC: 18335 … 18398; Length: 63 bp
Sequence:
TGAAGAATAATAAAAAAATAAGACTTCCCTATATGTAGGGGAGTCTTATTTTTATGCTAG
AAA

## > **yjeF**

Function: protein coding sequence; putative carbohydrate kinase (core genome, variable)
Best match: yjeF_CC005_N315_BA000018.3[9762:10592:r]RC (completely identical)
Position: 001-contig_254_RC: 18432 … 19263; Length: 831 bp
Sequence:
TTAACTTTCTAATTGTTTCATTGCGTAAGGTATTTCATTGATAAGTCTTGATGGTGGTACC
ACATACATATCTTTTGCAAGGTTTTCGCCAATAAAACTATGTGTATATGTGGCACTCATAA
CCGCTTCTTTTAAGTTATCAAATTGACCGACAAAACTTGTAATCATACCAGCAAGTGTATC
GCCCATACCACCAGTCGCCATTGCTGGGCTACCGATTGTTAATTTAAAGTCTTCATCTTTA
AAGAAAATTTCAGTACCATGTTTTTTAAGTACAACAGTTGCACCTAAACGATCAACTGCT
TCACGATTACGCTCATATGTCTGTTCCTCAATAGGAATACCACTTAATCGTTCCCATTCTT
TGAGGTGTGGAGTAAAGATCACACGACATGTAGGTAATTGCGGTTTCAGTTTACTAAAGA
TTGTAATCGCATCGCCGTCTACGATTAAATTTTGATGCGGTTGTATATTTTGTAGTAGGAA
TGTAATGGCATTATTTCCTTTGAAATCAACGCCAAGACCTGGACCAATTAGTATACTGTCA
GTCATTTCAATCATTTTCGTCAACATTTTCGTATCATTAATATCAATAACCATCGCTTCTGG
GCAACGAGAATGTAATGCTGAATGATTTGTTGGATGTGTAGCTACAGTGATTAAACCACT
ACCGCTAAATACACATGCACGAGCCGCTAACATAATGGCACCACCTAAGTTAGCAGATCC
ACCAATTAATAAAATTTTGCCATAATCACCTTTATGTGAATCTTCTTTACGCTTAGGAATG
TTAATAGAATTTAACGTTTCCATAGTGATATAACCTCCCAT

## > **hutH**

Function: protein coding sequence; histidine ammonia lyase (core genome, constant)
Best match: hutH_CC001-ST772_118_AJGE01000004.1[22077:23591:r] (completely identical)
Position: 001-contig_254_RC: 19570 … 21085; Length: 1515 bp
Sequence:
ATGACTTTATATTTAGATGGTGAAACACTAACAATTGAGGATATTAAATCATTTTTACAAC
AACAATCAAAGATTGAAATTATTGATGATGCGTTAGAACGTGTCAAAAAAAGTAGAGCG
GTAGTTGAACGTATTATTGAAAATGAGGAAACGGTTTACGGTATCACTACAGGTTTTGGG
TTATTTAGTGATGTACGTATAGACCCGACGCAATATAATGAATTACAAGTGAATCTGATA
CGCTCACATGCCTGTGGACTAGGTGAGCCATTTTCAAAAGAAGTAGCATTAGTCATGATG
ATTTTACGATTGAATACATTATTAAAAGGTCATTCAGGTGCCACTTTAGAATTAGTGAGA
CAATTACAATTTTTTATAAATGAACGTATTATACCGATAATCCCACAACAAGGCTCTCTCG
GTGCATCAGGAGATTTAGCGCCATTATCACATTTAGCATTAGCATTAATTGGTGAAGGGA

AAGTATTGTACAGAGGGGAAGAAAAGGATAGTGACGATGTATTAAGAGAATTAAATAGA
CAACCTTTGAACCTTCAGGCTAAAGAGGGTTTAGCATTGATTAATGGTACGCAAGCTATG
ACAGCTCAAGGTGTCATTAGTTATATAGAAGCAGAAGATTTAGGTTACCAATCTGAATGG
ATTGCTGCATTAACGCATCAGTCTCTTAATGGCATTATAGATGCATATCGACATGATGTGC
ACTCTGTTCGTAATTTTCAAGAACAGATTAATGTGGCAGCGCGTATGCGTGATTGGTTAG
AAGGATCAACATTAACGACGCGACAAGCAGAAATACGTGTACAAGATGCATATACGTTG
CGTTGTATACCACAAATCCATGGCGCGAGTTTTCAAGTATTCAATTATGTTAAACAGCAA
TTAGAATTTGAAATGAATGCGGCTAATGATAATCCACTTATATTTGAGGAAGCAAATGAA
ACGTTTGTTATTTCAGGTGGTAACTTCCATGGACAACCTATTGCTTTTGCATTAGATCATC
TTAAATTAGGTGTAAGTGAATTAGCAAACGTATCGGAACGTCGTCTAGAGCGACTAGTAA
ATCCTCAATTAAATGGTGATTTACCAGCATTTCTTAGTCCAGAGCCAGGATTGCAAAGTG
GCGCGATGATTATGCAATATGCTGCTGCAAGTCTCGTTTCTGAAAATAAAACTTTAGCGC
ATCCAGCGAGTGTTGATTCTATCACTTCATCTGCGAACCAAGAAGATCACGTATCTATGG
GAACTACAGCTGCTAGACATGGTTATCAAATTATTGAAAATGCAAGACGTGTGTTGGCAA
TCGAATGTGTTATTGCATTACAAGCAGCAGAGTTGAAAGGTGTCGAAGGATTATCACCAA
AAACACGTCGTAAATATGAGGAGTTTCGAAGTATCGTGCCATCCATTACACATGACCGTC
AATTTTATAAAGATATTGAAGCGGTTGCACAGTATTTAAAGCAATCAATTTATCAAACGA
CTGCATGTCACTAA

## > tbox01

Function: noncoding RNA T-box leader element (core genome, constant)
Best match: tbox01_CC001-ST772_118_AJGE01000004.1[21788:21999:r] (completely identical)
Position: 001-contig_254_RC: 21162 … 21374; Length: 212 bp
Sequence:
CATTGTTCGTAGGACAAGTAATATATAGTGTTCGATATCAGAGAGCTTGTGGTTAGTGTG
AACAAGAATCAACATATATATGAATCTACCTACTTAATTTAAAAGAACAATCGGTGATAA
CCGTTATTTTAGTGAAGTGCAATTTAGGTTTAGTTTATCTTTATAACTTAAATTGTTAAAT
AGGGTGGCAACGCGTAGACCACGTCCCTTGT

## > serS

Function: protein coding sequence; seryl-tRNA synthetase (core genome, constant)
Best match: serS_CC005_Mu50-VRSA_BA000017.4[12793:14079] (completely identical)
Position: 001-contig_254_RC: 21463 … 22750; Length: 1287 bp
Sequence:
ATGTTAGACATTAGATTATTCAGAAATGAGCCTGACACAGTTAAGAGCAAAATTGAATTA
CGTGGAGATGATCCAAAAGTTGTAGATGAAATTTTAGAATTGGATGAGCAACGACGTAA
ATTAATTAGTGCAACAGAAGAAATGAAAGCACGTCGTAATAAAGTAAGCGAAGAAATCG
CATTAAAAAAACGTAATAAAGAAAATGCTGATGATGTGATTGCTGAAATGCGCACATTA
GGTGACGATATTAAAGAAAAAGATAGTCAATTAAATGAAATTGATAATAAAATGACAGG
TATCCTTTGTCGTATTCCAAATTTAATAAGTGATGATGTACCTCAAGGTGAATCTGATGAA
GATAACGTTGAAGTTAAAAAGTGGGGTACACCACGTGAGTTTTCATTTGAACCAAAAGCA
CATTGGGATATTGTAGAAGAATTGAAAATGGCTGATTTTGATCGTGCAGCAAAAGTTTCA
GGTGCGCGTTTTGTATATTTAACAAATGAAGGTGCGCAATTAGAGCGTGCTTTAATGAAC
TATATGATTACAAAACATACAACACAACATGGTTATACAGAAATGATGGTACCACAGCTT
GTGAACGCAGATACAATGTATGGTACAGGTCAATTACCTAAATTTGAAGAAGATTTATTT
AAAGTAGAAAAAGAAGGATTATATACAATTCCAACTGCTGAAGTACCATTAACGAATTTC
TACCGTAATGAAATTATTCAACCAGGTGTACTTCCTGAAAAATTCACTGGTCAATCTGCAT
GTTTCCGTAGTGAAGCAGGATCAGCAGGTAGAGATACAAGAGGATTAATTCGTTTACATC
AATTCGATAAAGTGGAAATGGTACGTTTTGAACAACCTGAAGATTCATGGAATGCTTTAG
AAGAAATGACAACAAATGCAGAAGCAATTCTAGAAGAGTTAGGTTTACCATACCGTCGT
GTTATTTTATGTACAGGTGATATTGGATTTAGTGCAAGCAAAACATATGATATAGAAGTT
TGGTTACCAAGCTACAATGATTATAAAGAAATTAGTTCATGCTCAAACTGTACGGATTTC
CAAGCGCGTCGTGCTAACATCCGCTTCAAGCGTGACAAAGCAGCTAAACCAGAATTAGC
ACATACATTAAATGGTAGTGGTTTAGCAGTTGGACGTACATTTGCTGCTATTGTTGAAAAT

TACCAAAATGAAGATGGAACAGTAACAATTCCAGAAGCATTAGTACCATTTATGGGTGGT
AAAACACAAATTTCAAAACCAGTTAAATAA

> **tnpIS431-06**
Function: protein coding sequence; transposase for IS431 (mobile element)
Best match: tnpIS431-06_circ_S-epidermidis_GQ900381.1[43799:43807] (completely identical)
Position: 001-contig_254_RC: 21994 … 22003; Length: 9 bp
Sequence:
ATGAACTAT

> **azlC**
Function: protein coding sequence; branched chain amino acid transporter (core genome, variable)
Best match: azlC_CC005_Mu50-VRSA_BA000017.4[14730:15425] (completely identical)
Position: 001-contig_254_RC: 23400 … 24096; Length: 696 bp
Sequence:
ATGGCAACACATTTAAGTTTTAGACAAGGCGTACAAGAGTGTATCCCAACATTATTGGGT
TATGCCGGTGTTGGTATTTCATTTGGTATTGTGGCTTCGTCTCAAAACTTTAGTATTTTAGA
AATTATCTTGTTATGTCTTGTTATATATGCCGGTGCTGCGCAATTTATTATGTGCGCGTTGT
TTATAGCAGGTACACCGATATCAGCGATTGTACTAACTGTATTTATCGTAAATTCAAGAA
TGTTCCTTTTAAGTATGTCGCTTGCACCAAACTTCAAGACATATGGGTTTTGGAACCGTGT
TGGATTAGGTTCATTAGTAACTGACGAAACGTTTGGCGTCGCCATTACACCTTATTTAAAA
GGAGAAGCTATCAATGATCGTTGGATGCATGGTCTTAATATCACAGCATATTTATTTTGG
GCAATTTCATGTGTAGCTGGGGCTTTATTTGGCGAATATATCTCAAATCCGCAAACGCTA
GGGTTAGATTTTGCTATCACGGCTATGTTTATCTTTTTGGCCATTGCGCAATTTGAATCAA
TTACTAAATCGCGATTAAGAATTTACATAGTACTCATTATTGCCGTCATAGTAATGATGTT
ATCGCTAAGTATGTTTATGCCTTCATATCTAGCAATATTAATTGCAGCCACAATTTCAGCA
GCGTTAGGAGTGATGATGGAACGATGA

> **azlD**
Function: protein coding sequence; branched chain amino acid transporter (core genome, constant)
Best match: azlD_CC005_Mu50-VRSA_BA000017.4[15422:15751] (completely identical)
Position: 001-contig_254_RC: 24092 … 24422; Length: 330 bp
Sequence:
ATGATAACTCATATGAACATGTTAATACTTATTTTATTGTGTGGTATCGTAACGCTATTAA
TTCGAATTATACCTTTTATCATGATTTCAAAAGTGCAATTGCCTGATGTTGTGGTTCGATG
GCTATCATTTATCCCAATCACACTATTTACGGCACTTGTCATTGACAGCATTATTCAACAG
ACGCCTCATGGTGAGGGGTATACATTAAACATCCCTTACATTATCGCGCTCATTCCGACG
GTTATTTTATCTATAATCACGCGTAGTTTAACTATTACAATTATTAGTGGGATTGTTATCA
TGGCAACATTACGATTTTTCTTTTAA

> **sam-L1**
Function: noncoding RNA SAM riboswitch locus 1 (core genome, constant)
Best match: sam-L1_CC001_MW2-USA400_BA000033.2[15949:16047] (completely identical)
Position: 001-contig_254_RC: 24626 … 24725; Length: 99 bp
Sequence:
TTCTTATTGTGAGAAGTTGAGGGACTTGGCCCTGTGATACTTCAGCAACCGACTTTATAGC
ACGGTGCTAAAACCAACGAGTTACTCGAATGATAAGTA

> **metX**
Function: protein coding sequence; homoserine O-acetyltransferase (core genome, constant)
Best match: metX_CC005_Mu50-VRSA_BA000017.4[16115:17083] (completely identical)
Position: 001-contig_254_RC: 24784 … 25753; Length: 969 bp
Sequence:
ATGACAAATTACACAGTAGATACTTTAAATCTAGGGAAATTTATTACAGAATCTGGGGAA
GTCATAGATAACTTGCGTTTGAGATATGAGCATGTTGGTTATCATGGACAACCATTAGTT

GTAGTTTGTCATGCATTAACTGGCAATCATTTAACATATGGAACAGATGATTATCCGGGTT
GGTGGCGAGAAATTATTGATGGGGGATATATACCCATTCACGATTATCAATTTTTAACAT
TTGATGTTATTGGTAGTCCTTTCGGTTCAAGTTCACCTTTAAACGACCCTCATTTTCCTAAA
AAATTAACATTAAGAGATATAGTCAGAGCGAATGAACGAGGTATACAAGCCCTTGGTTAT
GATAAGATTAATATTTTAATAGGGGGAAGTCTTGGAGGTATGCAAGCAATGGAACTACTT
TACAATCAACAGTTTGAAGTAGATAAAGCCATTATCCTTGCTGCAACAAGTCGAACATCA
TCTTATAGTAGAGCTTTCAATGAAATTGCAAGGCAAGCCATTCATCTTGGTGGTAAGGAA
GGTCTAAGTATTGCACGCCAATTAGGTTTTTTGACATATCGATCATCAAAAAGTTATGATG
AACGTTTCACGCCGGATGAAGTAGTCGCATACCAACAACATCAAGGTAATAAATTTAAAG
AACATTTTGATTTGAATTGTTATCTGACACTGCTAGATGTATTGGATAGTCACAACATTGA
CCGAGGTCGCACAGACGTAACGCATATTTTTAAAAATTTAGAAACGAAAGTCTTAACGAT
GGGGTTCATAGATGATTTGCTATATCCGGACGATCAAGTTCGTGCATTAGGTGAACGTTTT
AAATATCATCGTCATTTCTTCGTGCCTGATAATGTTGGACATGATGGATTCCTACTAAACT
TTAGTACCTGGGCACCTAACTTATATCATTTCTTAAATTTAAAGCATTTTAAGCGTAAGTA
A

## > Q6GD77

Function: protein coding sequence; putative membrane protein (core genome, constant)
Best match: Q6GD77_CC001-ST772_118_AJGE01000004.1[16178:17164:r] (completely identical)
Position: 001-contig_254_RC: 25997 … 26984; Length: 987 bp
Sequence:
TTGTTAATTTCCATTAAAGAGGTTAAAATAATAGCTATAGTTAAAAATATGGGAGTGGCT
TATGTGTTTTCAAAAATACAACCTAAAGCAACAATAATTGCAACGATTACGTTGGTATTT
GTCGCTTTAGCTTTATATCTAGTGCCTGGTTTGGGACTAATATTTGCATTATTTGCAACCA
TACCAGGTATCGTTTTATGGAATAAATCAATACAATCTTTCGGGGTTAGTGCACTTATTAC
AGTAATTATAACAACTGTTTTAGGTAATACTTTCGTTTTAAGTGCCATCATATTAGTCTTA
ATTGCAAGTTTAATTATTGGTCAATTGCTCAAAGAAAGAACGTCTAAAGAAAGAATGTTA
TACGTAACAACAGTAGCGATGAGCTTAATTTCATTAATCGCTTTTATGTTACTACAAACAT
TCGGAAGGATTCCACCATCAGCGAGCATAGTAAAACCTTTCAAGCAAACATTACATGAAG
CGATTACGATGAGCGGTGCCGATGCGAATATGACCCAAATATTAGAAGAAGGGTTTAGA
CAAGCGACCGTTCAATTACCAGGTTTCATCATTATCATTACATTTTTAATTGTCTTAATTA
ACTTAATCGTTACATTTCCGGTTTTACGAAAATTTAAAATCGCTACACCTGTATTTAAGCC
ACTTTTCGCGTGGCAAATGAGCGGTATTTTATTATGGATATACATTATTGTTATCATATGT
TTATTATTTACAGGTCAACCGAGTGTGTTCCAGAGCATTCTTTTAAACTTCCAACTTGTGT
TATCATTAGTAATGTATATTCAAGGTTTAAGTGTTATTCATTTCTTTGGTAAAGCGAAAGG
TTTGCCGAATGCAGTAACGATTTTACTATTGATTATCGGTACAATACTGACACCTACGACA
CATATTGTAGGACTACTTGGTGTTATCGATTTAAGTTTGAATTTGAAGCGAATCATGAAA
AATAATTCTAAAAAGTGA

## > gdpP

Function: protein coding sequence; cyclic di-AMP phosphodiesterase (core genome, constant)
Best match: gdpP_CC001-ST772_118_AJGE01000004.1[14196:16163:r] (completely identical)
Position: 001-contig_254_RC: 26998 … 28966; Length: 1968 bp
Sequence:
ATGAATCGGCAGTCCACTAAGAAAGCTTTACTAATACCATTTGTCATCATGATCATCACA
GCAATTGTTTTAATGGGTGTATGGTTTATCTTTAATAGTCTTATAGCATTAATTGCATCTAT
CGTTCTTGTCGTGATGATTATTGTTAGCATCATTTTATTCAGACAAGCTTTAATGAAAATG
GATAGTTATGTAGATGGTTTGAGTGCTCAAATTTCAACAACAAATAATAAAGCAATCAAA
CATTTACCAATTGGTATCATTGTTTTAGATGAAAATGATCACATCGAATGGGTTAACCAAT
TTATGACAAATCATATGGAAGCAAATGTCATTTCTGAATCTGTAAATGAAGTATTTCCAA
ACATTTTAAAGCAATTAGATAGAGTGAAATCCGTTGAAATAGAATATAATCAGTATCATT
TCCAAGTACGTTATTCTGAGAATGATCATTGCCTCTATTTCTTTGATATAACTGAACAAGT
ACAAACAAATGAACTATATGAAAATTCTAAACCAATCATTGCGACATTATTTTTAGATAA
CTACGATGAGATTACTCAAAATATGAATGATACGCAGCGTTCGGAAATCAATTCAATGGT
AACGCGTGTTATTAGTCGATGGGCAACTGAGTATAATATATTCTTTAAAAGGTATAGTTC

CGATCAATTCGTAGCCTATTTAAATCAAAAAATATTAGCTGACTTAGAAGAATCTAAATT
TGATATCTTGAGTCAATTACGTGAAAAAAGTGTTGGTTATCGTGCCCAATTAACATTAAG
TATTGGTGTTGGTGAAGGTACTGAAAATTTAATCGATTTAGGTGAATTATCACAATCAGG
CCTAGACTTAGCATTAGGGCGCGGTGGCGACCAAGTTGCAATTAAAAGTATTAATGGTAA
TGTGCGTTTCTATGGCGGTAAGACTGACCCGATGGAGAAACGTACTCGTGTAAGAGCACG
AGTGATCTCACATGCGTTAAAAGATATCCTTGCAGAGGGTGACAAAGTCATTATCATGGG
ACATAAACGTCCTGACTTAGATGCAATTGGTGCAGCAATCGGTGTGTCTAGATTTGCAAT
GATGAATAATTTAGAAGCATACATCGTATTAAATGAGACTGACATTGATCCAACATTACG
ACGCGTGATGAACGAAATTGATAAAAAGCCAGAGTTAAGAGAGCGATTTATAACATCAG
ATGATGCTTGGGATATGATGACATCTAAGACAACCGTAGTGATTGTTGATACGCATAAAC
CGGAACTGGTTTTAGATGAAAATGTCTTAAATAAAGCAAACCGTAAAGTTGTTATCGATC
ATCATAGACGTGGTGAAAGCTTCATCTCTAATCCATTGTTGATATATATGGAACCATACG
CAAGTTCAACAGCTGAATTGGTAACAGAGTTACTGGAATATCAACCAACAGAACAACGTT
TAACACGTCTTGAATCAACAGTGATGTATGCAGGTATTATTGTAGATACAAGAAACTTTA
CATTACGAACAGGATCAAGAACATTCGATGCAGCGAGTTATTTACGTGCACATGGTGCAG
ATACGATTTTAACGCAACATTTCTTAAAAGATGATGTGGATACTTACATTAATCGATCTGA
ATTAATTCGAACTGTAAAAGTTGAAGATAATGGCATAGCCATTGCGCATGGTTCAGACGA
TAAAATTTATCATCCAGTAACAGTTGCACAAGCAGCAGATGAACTGTTAAGTTTAGAAGG
TATTGAAGCATCATATGTTGTTGCGAGACGTGAAGATAATCTGATTGGTATATCTGCGCG
TTCACTCGGTTCAGTAAATGTTCAGTTAACAATGGAAGCACTTGGTGGCGGTGGACATTT
AACCAATGCTGCAACACAACTTAAAGGTGTGACAGTCGAAGAGGCGATAGCACAATTAC
AACAAGCAATTACAGAACAATTAAGTAGGAGTGAAGATGCATGA

## > rplI

Function: protein coding sequence; 50S ribosomal protein L9 (core genome, constant)
Best match: rplI_CC005_Mu50-VRSA_BA000017.4[20293:20739] (completely identical)
Position: 001-contig_254_RC: 28962 … 29409; Length: 447 bp
Sequence:
ATGAAAGTAATTTTTACACAAGATGTTAAAGGTAAAGGTAAAAAAGGTGAAGTTAAAGA
AGTACCAGTAGGTTATGCAAATAACTTCTTATTGAAAAAGAATTATGCTGTAGAAGCAAC
ACCAGGTAACCTTAAACAATTAGAGTTACAGAAAAAACGTGCAAAACAAGAACGCCAAC
AAGAAATTGAAGATGCTAAAGCATTAAAAGAAACGTTATCAAACATTGAAGTTGAAGTA
TCAGCAAAAACTGGTGAAGGTGGTAAATTGTTTGGATCAGTAAGTACAAAACAAATTGCC
GAAGCACTAAAAGCACAACATGATATTAAAATTGATAAACGTAAAATGGATTTACCAAA
TGGAATTCATTCCCTAGGATATACGAATGTACCTGTTAAATTAGATAAAGAAGTTGAAGG
TACAATTCGCGTACACACAGTTGAACAATAA

## > dnaC

Function: protein coding sequence; replicative DNA helicase (core genome, constant)
Best match: dnaC_CC005_Mu50-VRSA_BA000017.4[20771:22171]
Position: 001-contig_254_RC: 29440 … 30841; Length: 1401 bp
Sequence:
ATGGATAGAATGTATGAGCAAAATCAAATGCCACATAACAATGAAGCTGAACAGTCTGT
CTTAGGTTCAATTATTATAGATCCAGAATTGATTAATACTACTCAGGAAGTTTTGCTTCCT
GAGTCGTTTTATAGGGGTGCCCATCAACATATTTTCCGTGCAATGATGCACTTAAATGAA
GATAATAAAGAAATTGATGTTGTAACATTGATGGATCAATTATCGACGGAAGGTACGTTG
AATGAAGCGGGTGGCCCGCAATATCTTGCAGAGTTATCTACAAATGTACCAACGACGCGA
AATGTTCAGTATTATACTGATATCGTTTCTAAGCATGCATTAAAACGTAGATTGATTCAAA
CTGCAGATAGTATTGCCAATGATGGATATAATGATGAACTTGAACTAGATGCGATTTTAA
GTGATGCAGAACGTCGAATTTTAGAGCTGTCATCTTCTCGTGAAAGCGATGGCTTTAAAG
ACATTCGAGACGTCTTAGGACAAGTGTATGAAACAGCTGAAGAGCTTGATCAAAATAGT
GGTCAAACACCAGGTATACCTACAGGGTATCGAGATTTAGACCAAATGACAGCAGGGTT
CAACCGAAATGATTTAATTATCCTTGCAGCGCGTCCATCTGTAGGTAAGACTGCGTTCGC
ACTTAATATTGCACAAAAAGTTGCAACGCATGAAGATATGTATACAGTTGGTATTTTCTC
ACTAGAGATGGGTGCTGATCAGTTAGCCACACGTATGATTTGTAGTTCTGGTAATGTTGA

CTCAAACCGCTTAAGAACGGGTACTATGACTGAGGAAGATTGGAGTCGTTTTACTATAGC
GGTAGGTAAATTATCACGTACGAAGATTTTTATTGATGATACACCGGGTATTCGAATTAA
TGATTTACGTTCTAAATGTCGTCGATTAAAGCAAGAACATGGCTTAGACATGATTGTGAT
TGACTACTTACAGTTGATTCAAGGTAGTGGTTCACGTGCGTCCGATAACAGACAACAGGA
AGTTTCTGAAATCTCTCGTACATTAAAAGCATTAGCCCGTGAATTAGAATGTCCAGTTATC
GCATTAAGTCAGTTATCTCGTGGTGTTGAACAACGACAAGATAAACGTCCAATGATGAGT
GATATTCGTGAATCTGGTTCGATTGAGCAAGATGCTGATATCGTTGCGTTCTTATACCGTG
ATGATTACTATAACCGTGGTGGCGATGAAGATGATGACGATGATGGTGGTTTCGAGCCAC
AAACGAATGATGAAAACGGTGAAATTGAAATTATCATTGCTAAGCAACGTAACGGTCCA
ACAGGCACAGTTAAGTTACACTTTATGAAACAATATAATAAATTTACCGATATCGATTAT
GCACATGCAGATATGATGTAA

> **purA**

Function: protein coding sequence; adenylosuccinate synthetase (core genome, constant)
Best match: purA_CC005_Mu50-VRSA_BA000017.4[22449:23732]
Position: 001-contig_254_RC: 31119 … 32403; Length: 1284 bp
Sequence:
ATGTCATCAATCGTAGTAGTTGGGACACAATGGGGAGACGAAGGAAAAGGAAAAATAAC
GGATTTCTTGGCAGAACAGTCAGATGTTATCGCGCGTTTTTCAGGTGGTAATAATGCAGG
CCATACCATTCAATTTGGCGGAGAAACATATAAATTACATTTAGTACCATCTGGTATCTTT
TACAAAGACAAATTAGCGGTAATCGGTAACGGTGTCGTTGTTGATCCAGTTGCACTATTG
AAAGAATTAGACGGATTAAATGAACGTGGCATTCCTACAAGTAATTTACGTATATCTAAT
CGTGCACAAGTGATTTTACCATATCACTTAGCACAAGATGAATATGAAGAACGTTTACGC
GGTGACAATAAGATTGGTACAACTAAAAAAGGTATCGGTCCAGCATATGTAGACAAAGT
TCAACGTATCGGTATTCGTATGGCAGATTTACTTGAAAAAGAAACATTCGAAAGATTATT
AAAATCAAACATTGAATATAAACAAGCATATTTCAAAGGTATGTTTAACGAAACATGTCC
ATCATTTGATGATATCTTTGAAGAATACTATGCTGCAGGTCAACGTTTAAAAGAATTTGTA
ACAGACACATCAAAAATCTTAGACGATGCATTTGTAGCAGATGAAAAGGTACTTTTCGAA
GGTGCGCAAGGTGTAATGTTAGATATCGACCATGGTACATACCCATTCGTTACATCAAGT
AATCCAATTGCAGGTAACGTTACTGTTGGTACAGGTGTAGGTCCTACATTCGTTTCAAAG
GTAATTGGTGTATGTAAAGCTTATACATCACGTGTTGGTGATGGTCCATTCCCTACTGAAT
TATTCGATGAAGATGGACATCATATTAGAGAGGTTGGTCGTGAATACGGTACAACAACAG
GACGTCCACGTCGTGTAGGTTGGTTTGATTCAGTTGTATTACGTCACTCTCGTCGTGTAAG
TGGTATTACAGATTTATCTATTAACTCAATCGATGTTTTAACAGGCCTAGACACAGTGAA
AATCTGTACAGCTTATGAATTAGACGGTAAAGAAATTACTGAGTACCCAGCAAACTTAGA
TCAATTAAAACGTTGTAAACCAATCTTTGAAGAGTTACCAGGTTGGACAGAAGACGTAAC
AAGTGTGCGTACTTTAGAAGAATTACCTGAAAATGCACGTAAATATTTAGAGCGTATTTC
AGAATTATGTAATGTACAAATTTCTATCTTCTCAGTTGGTCCAGATAGAGAACAAACAAA
CCTATTAAAAGAATTGTGGTAG

> **tx_purA**

Function:  rho-independent terminator of purA
Best match: tx_purA_CC005_Mu50-VRSA_BA000017.4[23758:23813] (completely identical)
Position: 001-contig_254_RC: 32428 … 32484; Length: 56 bp
Sequence:
ATTATAAATACATGAGCCTTCTATCTTTATTGGTAGGAGGCTTTTGCTTTGTTTGC

> **tRNAglu**

Function: tRNA tRNA glutamate
Best match: tRNAglu_CC001_MW2-USA400_BA000033.2[24156:24230] (completely identical)
Position: 001-contig_254_RC: 32831 … 32906; Length: 75 bp
Sequence:
GGCTCCTTGGTCAAGCGGTTAAGACACCGCCCTTTCACGGCGGTAACACGGGTTCGAGTC
CCGTAGGAGTCACCA

> **tRNAasp**
Function: tRNA tRNA asparagine
Best match: tRNAasp_CC001_MW2-USA400_BA000033.2[24238:24313] (completely identical)
Position: 001-contig_254_RC: 32913 … 32989; Length: 76 bp
Sequence:
GGTCTCGTAGTGTAGCGGTTAACACGCCTGCCTGTCACGCAGGAGATCGCGGGTTCGATT
CCCGTCGAGACCGTAC

> **tx_tRNAasp**
Function:  :  rho-independent terminator
Best match: tx_tRNAasp_1_CC001_MW2-USA400_BA000033.2[24302:24353] (completely identical)
Position: 001-contig_254_RC: 32977 … 33029; Length: 52 bp
Sequence:
TCGAGACCGTACAAATGCCTATCCAAGAGGATAGGCATTTTTTTGCGTTTAA

> **vicR**
Function: protein coding sequence; two-component sensor/response regulator (core genome, constant)
Best match: vicR_CC001_MW2-USA400_BA000033.2[24930:25631]
Position: 001-contig_254_RC: 33598 … 34300; Length: 702 bp
Sequence:
ATGGCTAGAAAAGTTGTTGTAGTTGATGATGAAAAACCGATTGCTGATATTTTAGAATTT
AACTTAAAAAAAGAAGGATACGATGTGTACTGTGCATACGATGGTAATGATGCAGTCGA
CTTAATTTATGAAGAAGAACCAGACATCGTATTATTAGATATCATGTTACCTGGTCGTGAT
GGTATGGAAGTATGTCGTGAAGTGCGCAAAAAATACGAAATGCCAATTATAATGCTTACT
GCTAAAGATTCAGAAATTGATAAAGTGCTTGGTTTAGAACTAGGTGCAGATGACTATGTA
ACGAAACCGTTTAGTACGCGTGAATTAATCGCACGTGTGAAAGCGAACTTACGTCGTCAT
TACTCACAACCAGCACAAGACACTGGAAATGTAACGAATGAAATCACAATTAAAGATAT
TGTGATTTATCCAGACGCATATTCTATTAAAAAACGTGGCGAAGATATTGAATTAACACA
TCGTGAATTTGAATTGTTCCATTATTTATCAAAACATATGGGACAAGTAATGACACGTGA
ACATTTATTACAAACAGTATGGGGCTATGATTACTTTGGCGATGTACGTACGGTCGATGT
AACGATTCGTCGTTTACGTGAAAAGATTGAAGATGATCCGTCACACCCTGAATATATTGT
GACGCGTAGAGGCGTTGGATATTTCCTCCAACAACATGAGTAG

> **vicK**
Function: protein coding sequence; two-component sensor/histidine kinase subunit (core genome, constant)
Best match: vicK_CC001-ST772_118_AJGE01000004.1[7023:8849:r] (completely identical)
Position: 001-contig_254_RC: 34312 … 36139; Length: 1827 bp
Sequence:
ATGAAGTGGCTAAAACAACTACAATCCCTTCATACTAAACTTGTAATTGTTTATGTATTAC
TGATTATCATTGGTATGCAAATTATCGGGCTGTATTTTACAAATAACCTTGAAAAAGAGC
TGCTTGATAATTTTAAGAAGAATATTACGCAGTACGCGAAACAATTAGAAATTAGTATTG
AAAAAGTATATGACGAAAAGGGCTCCGTAAATGCACAAAAAGATATTCAAAATTTATTA
AGTGAGTATGCCAACCGTCAAGAAATTGGAGAAATTCGTTTTATAGATAAAGACCAAATT
ATTATTGCGACGACGAAGCAGTCTAACCGGAGTCTAATCAATCAAAAAGCGAATGATAG
TTCTGTCCAAAAAGCACTATCACTAGGACAATCAAACGATCATTTAATTTTAAAAGATTA
TGGCGGTGGTAAGGACCGTGTCTGGGTATATAATATCCCAGTTAAAGTCGATAAAAAGGT
AATTGGTAATATTTATATCGAATCAAAAATTAATGACGTTTATAACCAATTAAATAATAT
AAATCAAATATTCATTGTTGGTACAGCTATTTCATTATTAATCACAGTCATCCTAGGATTC
TTTATAGCGCGAACGATTACCAAACCAATCACCGATATGCGTAACCAGACGGTTGAAATG
TCCAGAGGTAACTATACGCAACGTGTGAAGATTTATGGTAATGATGAAATTGGCGAATTA
GCTTTAGCATTTAATAACTTGTCTAAACGTGTACAAGAAGCGCAGGCTAATACTGAAAGT
GAGAAACGTAGACTGGACTCAGTTATCACCCATATGAGTGATGGTATTATTGCAACAGAC
CGCCGTGGACGTATTCGTATCGTCAATGATATGGCACTTAAGATGCTTGGTATGGCGAAA

GAAGACATCATTGGATATTACATGTTAAGTGTATTAAGTCTTGAAGATGAATTTAAACTT
GAAGAAATTCAAGAGAATAATGATAGTTTCTTATTAGATTTAAATGAAGAAGAAGGTCTA
ATCGCACGTGTTAACTTTAGTACGATTGTGCAGGAAACAGGATTTGTAACTGGTTATATC
GCTGTGTTACATGACGTTACTGAACAACAACAAGTTGAACGTGAACGTCGTGAATTTGTT
GCCAATGTATCACATGAGTTACGTACACCTTTAACTTCTATGAATAGTTACATTGAAGCAC
TTGAAGAAGGTGCATGGAAAGATGAGGAACTTGCGCCACAATTTTTATCTGTTACCCGTG
AAGAAACAGAACGAATGATTCGACTGGTCAATGACTTGCTACAGTTATCTAAAATGGATA
ATGAGTCTGATCAAATCAACAAAGAAATTATCGACTTTAACATGTTCATTAATAAAATTA
TTAATCGACATGAAATGTCTGCGAAAGATACAACATTTATTCGAGATATTCCGAAAAAGA
CGATTTTCACAGAATTTGATCCTGATAAAATGACGCAAGTATTTGATAATGTCATTACAA
ATGCGATGAAATATTCTAGAGGCGATAAACGTGTCGAGTTCCACGTGAAACAAAATCCAC
TTTATAATCGAATGACGATTCGTATTAAAGATAATGGCATCGGTATTCCTATCAATAAAG
TCGATAAGATATTCGACCGATTCTATCGTGTAGATAAGGCGCGTACGCGTAAAATGGGTG
GTACTGGATTAGGACTAGCCATTTCGAAAGAGATTGTGGAAGCGCACAATGGTCGTATTT
GGGCAAACAGTGTAGAAGGTCAAGGTACATCTATCTTTATCACACTTCCATGTGAAGTCA
TTGAAGACGGTGATTGGGATGAATAA

> **yycH**

Function: protein coding sequence; putative protein (core genome, variable)
Best match: yycH_CC001-ST772_118_AJGE01000004.1[5696:7030:r] (completely identical)
Position: 001-contig_254_RC: 36131 … 37466; Length: 1335 bp
Sequence:
ATGAATAATAAGGAGCATATTAAATCTGTCATTTTAGCGCTACTTGTCTTGATGAGTGTCG
TATTGACATATATGGTATGGAACTTTTCTCCTGATATTGCAAATGTCGACAATACAGATAG
TAAGAAGAGTGAAACGAAACCTTTAACGACACCTATGACAGCCAAAATGGATACAACTA
TTACGCCATTTCAGATTATTCATTCGAAAAATGATCATCCAGAAGGTACGATCGCGACGG
TTTCTAATGTGAATAAGCTGACGAAACCTTTAAAAAATAAAGAAGTGAAGTCCGTGGAAC
ATGTTCGTCGTGATCATAACTTGATGATTCCTGATTTGAGCAGTGATTTTACATTATTCGA
TTTTACGTATGATTTACCGTTATCAACATATCTTGGTCAAGTACTGAACATGAATGCGAAA
GTACCAAATCATTTCAATTTCAATCGTTTGGTCATAGATCATGATGCTGATGATAATATCG
TGCTTTATGCTATAAGCAAAGATCGCCACGATTACGTAAAATTAACAACTACAACGAAAA
ATGATCATTTTTTAGATGCATTAGCAGCAGTGAAAAAAGATATGCAACCATACACAGATA
TCATCACAAACAAAGATACAATTGATCGTACGACGCATGTTTTTGCACCAAGTAAACCTG
AAAAGTTAAAAACATATCGCATGGTATTTAACACGATTAGTGTTGAGAAAATGAATGCTA
TACTATTTGACGATTCAACCATCGTTCGTAGTTCAAAGAGTGGTGTTACTACTTATAACAA
TAATACAGGTGTCGCAAACTATAACGATAAAAATGAAAAATACCATTATAAAAACCTGTC
CGAAGATGAAGCAAGTTCCAGCAAAATGGAAGAAACGATTCCAGGAACCTTTGATTTTAT
TAATGGTCATGGTGGTTTCTTAAACGAAGACTTCAGATTGTTTAGTACGAATAATCAGTC
AGGCGAGTTAACATATCAACGTTTCCTTAACGGTTATCCAACGTTTAATAAAGAAGGCTC
TAATCAAATTCAAGTCACTTGGGGTGAAAAAGGCGTCTTTGACTATCGTCGTTCGTTATTG
CGCACCGACGTTGTGTTAAATAGTGAGGATAATAAAACGTTGCCGAAATTAGAGTCTGTA
CGTTCAAGCTTAGCGAACAATAGTGATATTAATTTTGAAAAAGTAACAAACATCGCTATC
GGTTACGAAATGCAGGATAACTCAGATCATAATCACATTGAAGTGCAGATTAACAGTGA
ACTCGTACCGCGTTGGTATGTAGAATATGATGGCGAATGGTATGTTTATAACGATGGGAG
GCTTGAATAA

> **yycI**

Function: protein coding sequence; putative protein (core genome, variable)
Best match: yycI_CC001-ST772_118_AJGE01000004.1[4907:5695:r] (completely identical)
Position: 001-contig_254_RC: 37466 … 38255; Length: 789 bp
Sequence:
ATGAACTGGAAACTGACAAAGACACTTTTCATTTTCGTGTTTATTCTTGTCAACATCGTGT
TAGTATCGATTTATGTTAATAAAGTCAATCGCTCACACATTAATGAAGTCGAGAGTAACA
ATGAAGTGAATTTTCAACAAGAAGAAATTAGGGTGCCAGCCAGTATTTTAAATAAATCTG
TTAAAGGCATACAATTAGAACAAATTACTGGACGATCAAAAGACTTTAGTTCAAAAGCTA

AAGGTGATTCTGATTTATCCACATCAGATAGTGGAAAATTATTAAATGCGAACATCAGCC
AATCGGTAAAAGTTAGTGATAATAACTTAAAAGATTTAAAAGATTACGTTAACAAACGCG
TATTTAAAGGTGCTGAATATCAATTAAGCGAGATTAGTTCAGATTCTGTAAAATATGAAC
AAACGTATGATGATTTTCCAATTTTAAATAACAGCAAAGCGATGTTGAACTTTAAAATAG
AAGATAACAAAGCAACTAGTTATAAACAGTCAATGATGGATGACATTAAGCCGACTGAT
GGTGCAGATAAGAAGCATCAAGTTATTAGTGTGAGAAAAGCAATCGAGGCATTATATTAT
AATCGTTACTTGAAAAAAGGTGATGAAGTCATTAATGCTAGACTCGGTTACTACTCAGTC
GTGAATGAAACGAATGTTCAATTGTTACAACCAAACTGGGAAATTAAAGTGAAGCATGA
CGGTAAGGACAAAACGAATACTTACTATGTCGAAGCGACAAATAATAACCCTAAAATTA
TTAATCATTAA

> **yycJ**

Function: protein coding sequence; metallo-beta-lactamase family protein (core genome, variable)
Best match: yycJ_CC001-ST772_118_AJGE01000004.1[3711:4511:r] (completely identical)
Position: 001-contig_254_RC: 38650 … 39451; Length: 801 bp
Sequence:
ATGAGCCGCTTGATACGCATGAGTGTATTAGCAAGTGGTAGTACAGGTAACGCCACTTTT
GTAGAAAATGAAAAAGGTAGTCTATTAGTTGATGTTGGTTTGACTGGCAAGAAAATGGA
AGAATTGTTTAGTCAAATTGACCGTAATATTCAAGATTTAAATGGTATTTTAGTAACCCAT
GAACATATTGATCATATTAAAGGATTAGGTGTTTTGGCGCGTAAATATCAATTGCCAATTT
ATGCGAATGAAAAGACTTGGCAGGCAATTGAAAAGAAAGATAGTCGCATCCCTATGGAT
CAGAAATTCATTTTTAATCCTTATGAAACGAAATCTATTGCAGGTTTCGATGTTGAATCGT
TTAACGTGTCACATGATGCAATAGATCCGCAATTTTATATTTTCCATAATAACTATAAGAA
GTTTACGATTTTAACGGATACGGGTTACGTGTCTGATCGTATGAAAGGTATGATACGTGG
CAGCGATGCGTTTATTTTTGAGAGTAATCATGACGTCGATATGTTGAGAATGTGTCGTTAT
CCATGGAAGACGAAACAACGTATTTTAGGCGATATGGGTCATGTATCTAATGAGGATGCG
GGTCATGCAATGACAGACGTGATTACAGGTAACACGAAACGTATTTACTTATCACATTTA
TCACAAGATAATAATATGAAAGATTTGGCGCGTATGAGTGTTGGTCAAGTATTGAACGAA
CACGATATTGATACGGAAAAAGAAGTATTGCTATGTGATACGGATAAAGCTATTCCAACG
CCAATATATATACAATATAA

> **sasH**

Function: protein coding sequence; cell-wall attached adenosine synthase A (core genome, variable)
Best match: sasH_CC001-ST772_118_AJGE01000004.1[1166:3484:r] (completely identical)
Position: 001-contig_254_RC: 39677 … 41996; Length: 2319 bp
Sequence:
ATGAAAGCTTTATTACTTAAAACAAGTGTATGGCTCGTTTTGCTTTTTAGTGTAATGGGAT
TATACCATGTCTCGAACGCGGCTGAGCAGCATACACCAATGAAAGCACATGCAGTAACA
ACGATAGACCAAGCAGCAACAGATAAGCAACTAGTAACGCCAACAAAGGAAGCGGCTCA
TTATTATGGTGAAGAAGCGGCAACCAACGTATCAGCATCAGCGCAGGGAACAGCTGATG
ATACAAACAACAAAGTAACATCCAACGAACCATCTAATAAACCATCTACAGCAGTTTCAA
CAACAGTAAACGAAACGCGCGATGTAGATACACAACAAGCCTCAACACAAAAACCAACT
CACACAGCAACATTCACATTATCAAATGCTAAAACAGCATCACTTTCACCACGAATGTTT
GCTGCCAATGTACCACAAACAACAACACATAAAATATTACATACAAATGATATCCATGGC
CGACTAGCCGAAGAAAAAGGGCGTGTCATCGGTATGGCTAAATTAAAAACAGTAAAAGA
ACAAGAAAAGCCTGATTTAATGTTAGATGCAGGAGACGCCTTCCAAGGTTTACCACTTTC
AAACCAGTCTAAAGGTGAAGAAATGGCTAAAGCAATGAATGCAGTAGGTTATGATGCTA
TGGCAGTCGGTAACCACGAATTTGACTTTGGATACGACCAGTTGAAAAAGTTAGAGGGTA
TGTTAGACTTCCCGATGCTAAGCACTAACGTTTATAAAGATGGGAAACGCGCGTTTAAAC
CATCAACGATTGTAACAAAAAATGGTATTCGTTTTGGAATTATTGGCGTAACGACACCAG
AAACAAAGACGAAAACAAGACCTGAAGGCATTAAAGGTGTTGAATTTAGAGATCCATTA
CAAAGTGTGACAGCAGAAATGATGCGTATTTATAAAGACGTAGATACATTTGTTGTTATA
TCACATTTAGGGATTGATCCTTCAACGCAAGAAACATGGCGTGGTGATTACTTAGTGAAA
CAATTAAGTCAAAATCCACAATTGAAGAAACGTATTACAGTTATTGATGGTCATTCACAT
ACCGTACTTCAAAATGGTCAAATTTATAACAATGATGCATTAGCACAAACAGGTACAGCA

CTTGCGAATATCGGTAAGATTACATTTAATTACCGCAATGGAGAGGTATCGAATATTAAG
CCGTCATTGATTAATGTTAAAGACGTTGAAAATGTGACACCGAACAAAGCATTAGCTGAA
CAAATTAATCAAGCTGATCAAACATTTAGAGCACAAACTGCAGAGGTAATTATTCCAAAC
AATACCATTGATTTCAAAGGAGAAAGAGATGACGTTAGAACGCGTGAAACAAATTTAGG
AAACGCGATTGCAGATGCTATGGAAGCGTATGGCGTTAAGAATTTCTCTAAAAAGACTGA
CTTTGCCGTGACAAATGGTGGAGGTATTCGTGCCTCTATCGCAAAAGGTAAGGTGACACG
CTATGATTTAATCTCAGTATTACCATTTGGAAATACGATTGCGCAAATTGATGTAAAAGG
TTCAGACGTCTGGACAGCTTTTGAACATAGTTTAGGCGCACCAACAACACAAAAAGATGG
TAAGACAGTATTAACAGCGAATGGTGGTTTACTACATATCTCTGATTCAATCCGTGTTTAC
TATGATATGAATAAACCATCTGGCAAACGAATTAACGCTATTCAAATTTTAAATAAAGAG
ACAGGTAAGTTTGAAAATATTGATTTAAAACGTGTATATCACGTAACGATGAATGACTTC
ACAGCATCAGGTGGCGACGGATATAGTATGTTCGGTGGCCCTAGAGAAGAAGGTATTTCA
TTAGATCAAGTACTAGCAAGTTATTTAAAAACAGCTAACTTAGCTAAGTATGATACGACA
GAACCACAACGTATGTTATTAGGTAAACCAGCAGTAAGTGAACAACCAGCTAAAGGACA
ACAAGGTAGCAAAGGTAGTGAGTCTGGTAAAGATGCACAACCAATTGGTGACGACAAAG
TGATGGATCCAGCGAAACAAACAGCTTCAGGTAAAGTTGTGTTGTTACCAGCGTATAGAG
GAACTGTTAGTAGTGGTAGAGAAGGTTCTGATCGCGCATTGGAAGGAACTGCTGTATCAA
GTAAGAGTGGGAAACAATTGGCTAACAAGTCAGCGCCTAAAGGTAGCGCACATGAGAAA
CAGTTACCAAAAACTGGAACTGATCAAAGTTCAAGCCCAGCAGCGATGTTTGTATTAGTA
GCAGGTATAGGTTTAATTGCGACTGTACGACGTAGAAAAGCTAGCTAA

> **tx_sasH**
Function:  rho independent terminator of sasH
Best match: tx_sasH_CC001-ST772_118_AJGE01000004.1[1099:1164:r] (completely identical)
Position: 001-contig_254_RC: 41997 … 42063; Length: 66 bp
Sequence:
ATATATTGAAAACAATACTACTGTATTTCTTAAATAAGAGGTACGGTAGTGTTTTTTTATG
AAAAA

> **orfX**
Function: protein coding sequence; 23S rRNA methyltransferase (core genome, constant)
Best match: orfX_CC001-ST772_118_AJGE01000004.1[319:798:r] (completely identical)
Position: 001-contig_254_RC: 42363 … 42843; Length: 480 bp
Sequence:
ATGAAAATCACCATTTTAGCTGTAGGGAAACTAAAAGAGAAATACTGGAAGCAAGCCAT
AGCAGAATATGAAAAACGTTTAGGCCCATACACCAAGATAGACATCATAGAAGTTCCAG
ACGAAAAAGCACCAGAAAATATGAGCGACAAAGAAATCGAGCAAGTAAAAGAAAAAGA
AGGCCAACGAATACTAGCCAAAATCAAACCACAATCCACAGTCATTACATTAGAAATAC
AAGGAAAGATGCTATCTTCCGAAGGATTGGCCCAAGAATTGAACCAACGCATGACCCAA
GGGCAAAGCGACTTTGTATTCGTTATTGGCGGATCAAACGGCCTGCACAAGGACGTCTTA
CAACGCAGTAACTACGCACTATCATTCAGCAAAATGACATTCCCACATCAAATGATGCGG
GTTGTGTTAATTGAACAAGTGTATAGAGCATTTAAGATTATGCGAGGAGAAGCGTATCAC
AAATAA

> **attSCCx**
Function:  attachment site of SCC/right part (SCCmec)
Best match: attSCCx-Mu50_part_AY751822_AY751822.1[1:19:r]
Position: 001-contig_254_RC: 42794 … 42813; Length: 19 bp
Sequence:
AGTGTATAGAGCATTTAAG

> **attSCCy**
Function:  attachment site of SCC/left part (SCCmec)
Best match: attSCCy-HU25_CC001-ST772_118_AJGE01000004.1[306:337:r] (completely identical)
Position: 001-contig_254_RC: 42824 … 42856; Length: 32 bp

Sequence:
AGAAGCGTATCACAAATAAAACTAAAAAATAG

> **sccterm**
Function:  terminus of SCC towards orfX (SCCmec)
Best match: sccterm11_ref_CC001-ST772_118_AJGE01000004.1[1:318:r] (completely identical)
Position: 001-contig_254_RC: 42843 … 43161; Length: 318 bp
Sequence:
AACTAAAAAATAGATTGTGTATAATATAAAAGGAAGGGATTTATATTAAAATTTTGAATT
CAAAAATTATTGAAAGGGAAGCTACCTTAGAAATTGAATCTATGGCCACTAATACATTGA
AAATAAACCCAGACATTAATTCTTACTATACAGAAATGTCTTTCGATGGAGAATTGGAAG
TGTATGATCCTGAAAATTTGAATAAAAAATTTCGTTGGAAAAATACAAGTTCAAGTTAAA
GGAAAAGAAGTAGCTAAAAGAGGAGGTAAGATTATTCGTCGAAGTAATGGGTTCTGTTG
CAAAGTAAAAAAATATAGC

> **tnpIS431-04**
Function: protein coding sequence; insertion sequence IS257e (mobile element)
Best match: tnpIS431-04_CC005_CIG1165_AHVE01000014.1[1655:2329]RC
Position: 001-contig_254_RC: 43191 … 43221; Length: 30 bp
Sequence:
TTAACTTGCTAGCATGATGCTAATTTCGTG

> **tnpIS431-06**
Function: protein coding sequence; transposase for IS431 (mobile element)
Best match: tnpIS431-06_circ_S-epidermidis_GQ900381.1[43799:43807]RC (completely identical)
Position: 002-contig_329_RC: 1 … 10; Length: 9 bp
Sequence:
ATAGTTCAT

> **mvaS-SCC**
Function: protein coding sequence; truncated 3-hydroxy-3-methylglutaryl CoA synthase (SCCmec)
Best match: mvaS-SCC_trunc-ref_CC001_MW2-USA400_BA000033.2[37179:37531]
Position: 002-contig_329_RC: 82 … 435; Length: 353 bp
Sequence:
ATGTTGGTAACATTTATACAGGATCATTATACTTAAGTTTAATTTCGTTATTACAGAACCA
CACATTCCAACCAGAAGAGAAAGTATGTCTATTTAGTTATGGTTCAGGAGCAGTAGGAGA
AATCTTTAGTGGTTCAATCGTTAAAGGATATGACAAAGCATTAGATAAAGAGAAACACTT
AAATATGCTAGAATCTAGAGAGCAATTATCAGTCGAAGAATACGAAACATTCTTTAACAG
ATTTGATAATCAAGAATTTGATTTCGAACGTGAATTGACACAAGATCCATATTCAAAAGT
ATACTTATACAGTATAGAAGACCATATCAGAACATATAAGATAGAGAAATAA

> **Q5HJW6**
Function: protein coding sequence; putative protein (SCCmec)
Best match: Q5HJW6_CC001_MW2-USA400_BA000033.2[37629:37859]
Position: 002-contig_329_RC: 532 … 562; Length: 30 bp
Sequence:
ATGCTTATTTTTTTATCTTACGATTTTGTA

> **Q5HJW6**
Function: protein coding sequence; putative protein (SCCmec)
Best match: Q5HJW6_part_M06/0171_LCL_10048.1[1:176]
Position: 003-contig_276: 2 … 258; Length: 256 bp
Sequence:

CTGGAGCCATTAGTCTCTCGCACATACTATTCCCTCAGGCGTCAGCACTTACAAAATCGGT
TGTAATTTTCATTTTTATACGCATTCTTACTGAGATTATACTAATAAGAGGAATAGTAAAA
GCAATTCTAAGTAAAATTGCAGATAAGAGGTTTGTTAAAAGCAGTTCTCAGTAAAATTAC
AGATAAGAGGTACGTTAAAAGCAGTTCTAAGTAAAATTGCAGATAAGAGGTTTGTTAAA
AGCAGTTCTAAGTAA

> **dru**
Function: SCC direct repeat units (SCCmec)
Best match: dru-45394F_CC008-ST239_T0131-ST239_CP002643.1[37657:38014]
Position: 003-contig_276: 87 … 525; Length: 438 bp
Sequence:
TCTTACTGAGATTATACTAATAAGAGGAATAGTAAAAGCAATTCTAAGTAAAATTGCAGA
TAAGAGGTTTGTTAAAAGCAGTTCTCAGTAAAATTACAGATAAGAGGTACGTTAAAAGCA
GTTCTAAGTAAAATTGCAGATAAGAGGTTTGTTAAAAGCAGTTCTAAGTAAAATTGCAGA
TAAGAGGTACGTTAAAAGCAATTCCATGCAAAATTGCTGATAAGGGGTAAGTTAAAAGC
AGTTCTCAGTAAAATTGCAGATAAGAGGTACGTTAAAAGCAGTTCTAGGCAAAATTGCAG
ATAAGAGGTGCGTTAAAAGCAGTTCTCAGTAAAATTGCTGATAAGGGGTAAGTTAAAAG
CAATCCTAAGTAAAATTGCAGATAAGAGGTAAGTTAAAAGCAATCCTAAGTAAAATTGC
AGATAAGGGGTACAGAAAAAC

> **ugpQ**
Function: protein coding sequence; glycerophosphoryl diester phosphodiesterase (SCCmec)
Best match: ugpQ_CC001_MW2-USA400_BA000033.2[38288:39031] (completely identical)
Position: 003-contig_276: 726 … 1470; Length: 744 bp
Sequence:
ATGCTAACTGTATATGGACATAGAGGATTACCTAGTAAAGCTCCGGAAAATACAATTGCA
TCATTTAAAGCTGCTTCAGAAGTAGAAGGTATAAACTGGTTGGAGTTAGATGTTGCAATT
ACAAAAGATGAACAACTGATTATCATTCATGATGATTATTTAGAACGGACTACAAATATG
TCCGGGGAAATAACTGAATTGAATTATGATGAAATTAAAGATGCTTCTGCAGGATCTTGG
TTTGGTGAAAAATTCAAAGATGAACATTTGCCAACTTTCGATGATGTAGTAAAAATAGCA
AATGAATATAATATGAATTTAAATGTAGAATTAAAAGGTATTACTGGACCGAATGGACTA
GCACTTTCTAAAAGTATGGTTAAGCAAGTGGAAGAACAATTAACAAACTTAAATCAGAAT
CAAGAAGTGCTCATTTCAAGCTTTAATGTTGTGCTTGTTAAACTTGCAGAAGAAATCATG
CCACAATATAACAGAGCAGTTATATTCCATACAACTTCGTTTCGTGAAGACTGGAGAACA
CTTTTAGATTACTGTAATGCTAAAATAGTAAACACTGAAGATGCCAAACTTACTAAAGCA
AAAGTAAAAATGGTAAAAGAAGCGGGTTATGAATTGAACGTATGGACTGTAAACAAACC
AGCACGTGCAAACCAACTTGCTAATTGGGGAGTTGATGGTATCTTTACAGACAATGCAGA
TAAAATGGTGCATTTGTCTCAATAG

> **ydeM**
Function: protein coding sequence; putative dehydratase (SCCmec)
Best match: ydeM_CC001_MW2-USA400_BA000033.2[39128:39556] (completely identical)
Position: 003-contig_276: 1566 … 1995; Length: 429 bp
Sequence:
ATGAAATACGATGATTTTATAGTAGGAGAAACATTCAAAACAAAAAGCCTTCATATTACA
GAAGAAGAAATTATCCAATTTGCAACAACTTTTGATCCTCAATATATGCATATAGATAAA
GAAAAAGCAGAACAAAGTAGATTTAAAGGTATCATTGCATCTGGCATGCATACACTTTCA
ATATCATTTAAATTATGGGTAGAAGAAGGTAAATACGGAGAAGAAGTTGTAGCAGGAAC
ACAAATGAATAACGTTAAATTTATTAAACCTGTATACCCAGGTAATACATTGTACGTTAT
CGCTGAAATTACAAATAAGAAATCCATAAAAAAAGAAAATGGACTCGTTACAGTGTCAC
TTTCAACATACAATGAAAATGAAGAAATTGTATTTAAGGGAGAAGTAACAGCACTTATTA
ATAATTCATAA

> **txbi_mecA**
Function: bidirectional rho-independent terminator of mecA (SCCmec)

Best match: txbi_mecA_CC001_MW2-USA400_BA000033.2[39547:39611:r]RC (completely identical)
Position: 003-contig_276: 1985 … 2050; Length: 65 bp
Sequence:
TAATTCATAATAAAACAGTGAAGCAACCATCGTTACGGATTGCTTCACTGTTTTGTTATTC
ATCT

> **mecA**
Function: protein coding sequence; penicillin binding protein 2a (SCCmec)
Best match: mecA_CC398_SO385-pig_AM990992.1[47524:49530:r]RC (completely identical)
Position: 003-contig_276: 2040 … 4047; Length: 2007 bp
Sequence:
TTATTCATCTATATCGTATTTTTTATTACCGTTCTCATATAGCTCATCATACACTTTACCTG
AGATTTTGGCATTGTAGCTAGCCATTCCTTTATCTTGTACATCTTTAACATTAATAGCCAT
CATCATGTTTGGATTATCTTTATCATATGATATAAACCACCCAATTTGTCTGCCAGTTTCTC
CTTGTTTCATTTTGAGTTCTGCAGTACCGGATTTGCCAATTAAGTTTGCATAAGATCTATA
AATATCTTCTTTATGTGTTTTATTTACGACTTGTTGCATACCATCAGTTAATAGATTGATAT
TTTCTTTGGAAATAATATTTTTCTTCCAAACTTTGTTTTTCGTGTCTTTTAATAAGTGAGGT
GCGTTAATATTGCCATTATTTTCTAATGCGCTATAGATTGAAAGGATCTGTACTGGGTTAA
TCAGTATTTCACCTTGTCCGTAACCTGAATCAGCTAATAATATTTCATTATCTAAATTTTTG
TTTGAAATTTGAGCATTATAAAATGGATAATCACTTGGTATATCTTCACCAACACCTAGTT
TTTTCATGCCTTTTTCAAATTTCTTACTGCCTAATTCGAGTGCTACTCTAGCAAAGAAAAT
GTTATCTGATGATTCTATTGCTTGTTTTAAGTCGATATTACCATTTACCACTTCATATCTTG
TAACGTTGTAACCACCCCAAGATTTATCTTTTTGCCAACCTTTACCATCGATTTTATAACTT
GTTTTATCGTCTAATGTTTTGTTATTTAACCCAATCATTGCTGTTAATATTTTTTGAGTTGA
ACCTGGTGAAGTTGTAATCTGGAACTTGTTGAGCAGAGGTTCTTTTTTATCTTCGGTTAAT
TTATTATATTCTTCGTTACTCATGCCATACATAAATGGATAGACGTCATATGAAGGTGTGC
TTACAAGTGCTAATAATTCACCTGTTTGAGGGTGGATAGCAGTACCTGAGCCATAATCAT
TTTTCATGTTGTTATAAATACTCTTTTGAACTTTAGCATCAATAGTTAGTTGAATATCTTTG
CCATCTTTTTTCTTTTTCTCTATTAATGTATGTGCGATTGTATTGCTATTATCGTCAACGAT
TGTGACACGATAGCCATCTTCATGTTGGAGCTTTTTATCGTAAAGTTTTTCGAGTCCCTTTT
TACCAATAACTGCATCATCTTTATAGCCTTTATATTCTTTTTGTTTTAATTCTTCAGAGTTA
ATGGGACCAACATAACCTAATAGATGTGAAGTCGCTTTTCCTAGAGGATAGTTACGACTT
TCTGTTTCATTAGTTGTAAGATGAAATTTTTTTGCGAAATCTCTTAAATATTCATCCATTTT
TTTAACGGTTTTAAGTGGAACGAAGGTATCATCTTGTACCCAATTTTGATCCATTTGTTGT
TTGATATAGTCTTCAGAAATACTTAGTTCTTTAGCGATTGCTTTATAATCTTTTTTAGATAC
ATTCTTTGGAACGATGCCTATCTCATATGCTGTTCCTGTATTGGCCAATTCCACATTGTTTC
GGTCTAAAATTTTACCACGTTCTGATTTTAAATTTTCAATATGTATGCTTTGGTCTTTCTGC
ATTCCTGGAATAATGACGCTATGATCCCAATCTAACTTCCACATACCATCTTCTTTAACAA
AATTAAATTGAACGTTGCGATCAATGTTACCGTAGTTTGTTTTAATTTTATATTGAGCATC
TACTCGTTTTTTATTTTTAGATACTTTTTTTATTTTACGATCCTGAATGTTTATATCTTTAAC
GCCTAAACTATTATATATTTTTATCGGACGTTCAGTCATTTCTACTTCACCATTATCGCTTT
TAGAAATATAACTGCTATCTTTATAAACTTGTTTGAAATTTTTATCTTCAATTGCATCAAT
AGTATTATTAATTTCTTTATCTTTTGAAGCATAAAAATATATACCAAACCCGACAACTACA
ACTATTAAAATAAGTGGAACAATTTTTATCTTTTTCAT

> **mecR**
Function: protein coding sequence; signal transducer protein MecR1 (SCCmec)
Best match: mecR_part_CC008_NCTC8325_X52593.1[1:40:r]
Position: 003-contig_276: 4146 … 4186; Length: 40 bp
Sequence:
GTGTTATCATCTTTTTTAATGTTAAGTATAATCAGTTCAT

> **tnpIS431-06**
Function: protein coding sequence; transposase for IS431 (mobile element)

Best match: tnpIS431-06_circ_FM207105.1[6418:7045]
Position: 004-contig_253_RC: 30 … 60; Length: 30 bp
Sequence:
ATAAAAAGAACCGCAGGTCTCTTCAGATCT

## > Q4LAG7

Function: protein coding sequence; putative protein (SCCmec)
Best match: Q4LAG7_CC001-ST772_118_AJGE01000059.1[137:565:r]RC (completely identical)
Position: 004-contig_253_RC: 166 … 595; Length: 429 bp
Sequence:
CTAATTTTTTCTCTTTGTAAATAAATGCAAAGAGATACCATAAGGATCTCTTACATAACCA
TAACCTTCAGTATAGAATTCTGGACTAAATGTTTTCAATACCTCACTGCCTTTTTCTATTA
ACTGGTCATATACATGTTTAGTTTCTTCTACTTGGTCAAAAGTGAGACAAAGAGATATATT
ATTACCTTGTGTTATGGGCAAACCTTCAGTGTCATCTGCGATCATAATTTTTATATCTCCA
AATTGAAGTACACATTGATCAATTTTATTTAAATCATTTTCGTCAATATTAAGTTGCTTAT
CTATAGGTCTATCTTTAATACGTTGAATATACAGTGTTTTAGCGCCAAACAGCTCTTCATA
CAACTTTTTTAAACCCTCTGCATTTTGAGTGATTAAAAAATATGGACTTACTTGAAATTTC
AT

## > Q3T2N0-var1

Function: protein coding sequence; transcriptional regulator (SCCmec)
Best match: Q3T2N0-var1_CC398_SO385-pig_AM990992.1[51021:51950] (completely identical)
Position: 004-contig_253_RC: 675 … 1605; Length: 930 bp
Sequence:
ATGAAAAAATCTGTTAGATTATATAATATGATTGAATATTGTAATGAAAATAGGAACTTC
AAATTAAATGATTTAATGTCAGAATTTAATATTTCTCGTAGTACCGCTTTAAGGGATATAA
AAGAAATTGAAGCATTAGGAGTACCTTTATATAGTAATCCAGGGAAAAATGGTGGTTATA
CGATCATAGGTAATCGAGACCAAACGAAAATAGCAATCTCAGATGAAGAGTTGAAAGCT
TTAGTATTTACACTTTCGAGTATTTCAAATGTGAGTAATCTACCTTTTCAAACAGAATATC
AAGAAATATTAAAAAAATTATATAATAACTCGAATAAAAAAGAGTTAATAAACCAATAT
AATGATCTATTTCAATATTTTAATGAAGATAAGTATCAGTTCAAAAGTTATAAGTTATTTA
ATGAAATCATTAGATTGATAATTGAGAATAAGTCTTTTGAAACCTGTTATTCACAAAACT
ATATTAAAGAACAATATAAAGGTATTGGCATTATGTATAAAAATCATCAATGGTATTTTG
TTGTAGCTAATATAGAATCAAAGTTAGTGAATCTATTAAATATTTCGAAAATAAAAGAAC
TATATGAAATGGGAGAGACTCAAGAGTGTAATGATATAACTATGCAGAATTTTCAACAGT
TCATGGTTAAAAATGAAACAGCTATTGATATTCTTATTAGAAGCAATGTTATGGGATTGA
ATATCTTGAAAGGCTACCTGTGGAGTGACTATATGATTGAAAATATTGACGAAGAGACAT
ATTTATTTAAATCAAAAGTGAACGCGAAAGATATAGATTTTATAGCTAAGTTAATTGTCA
CAGGTGGTGTCAATGTAAAAGTAGAGACCCCTAATAGTTTGAAAAATGCTGTTAAAGTTG
AATTAACTAAAATAATAAACATGTATTAA

## > Q4LAG4

Function: protein coding sequence; putative protein in SCCmecV (SCCmec)
Best match: Q4LAG4_CC001-ST772_118_AJGE01000059.1[1737:3725] (completely identical)
Position: 004-contig_253_RC: 1766 … 3755; Length: 1989 bp
Sequence:
GTGAATAAAAATATTACAGCTCTAAAATCTACTGAAAATACTACATATATGCAGTATACG
GTTCATATCGATGATCAAATTATAAATAATATAAAAGAGTGTACCAAAAAGTTTAAAATT
TTGCCTATGGAAGATAAAATTCCATTATCACCGTTGTTACAACCAGAATATGCAGGAGAG
GTACAAGATTTTATTAGTACATATGAGCAGTTTATGATTAATTTTGGTAAAGTAATATTGG
ATAGTCAAGGCATAAAAAATACAGTTTGAAAGTGAATCATTAAGTAGTATTCAACGAGGC
ATTCAAGAACATTGTTACTTAAATGAGCGAACAAATGACATTGATGTGACTAAAGAATGG
TATTTATGCAAATTTTCAAATCAAACATTAGAAGAGGATAAAAATCAATTGTATAATGCT
TTAAAGCAACTCATGAATGATTCTAAAAATAAAAAGCAAGCTTTTCAAGAGGTGTTTAAG
GTTCATATTGATATTTATCTCTACAATAAAGAAAAATCTGAATATAAGTATCAAGTATCTA

GTTATTTCAATCTTGTAAAAGAAAATCCTAAAATAACTTATAAAAAAAGACATTTACAAG
AGAAACAAGGTGTCAAGGGTACAACTTTCACGAACAAATTAAAATTATTGAACAAAATG
TATGGTGTAGATGTTGCAAAATATCAGCCATTTTATAATTCTAATAATCCTGAATATGAAA
GAGGTCAATTTGGTGAAAGATACATTTCTCAAAGATCTAACTATGAATTTAATAGACTTC
AATATCAAATTATAGATATGTTATCGAAAATACTTGATAAGCATCCATTACCAAAGTCAG
ATAATAATTATAAACATATTCCAACTATTGAAAAAGCAATATTAAGTGGGGATTCTCATA
GTTTTTATGAGTATTTTGAAGATATAATGAAAGAAATTATAAGTATGGAAAATTCTTCTTT
AAAGGAAAAATTATTGACTGATTTTACATATCAATCTCAATGTAGATGGTACTCTGAAAG
TGAAAAATTAAAATTGCAACTTGAAAGTTTTATGCATAAAGTTTTGGAGAGCAATTATTA
TGAGGGTAACAAATTATATAGAATGTTGTCTCATGCAATTGAAGAAACAATCAATGAGGC
TGATGAAGATAAGGTTCATTTCAATTATTTTAAAGATTATTTTTTGACAGACGGTGGGGTG
AAGAATTGGGAGCAGATTAGCGAGAAAATTACTGAATTCAATGGTAAAGTTATTAACGA
TATCCAAAATGAATATAATAAGATTCAATTTAATAACGCAAATCGAAATCAGAAACTAAA
CTTTAATTATTTATACCATTGCTTTGAGTTTAGTAATAACTTAGTAAAAGCAAGGGTAAAT
GGAAATAGAGGTTACATTTTATATTTTGTAGATAAATATAAAATAAAAATGCCCTTTTTG
GATCAATTATTAATCAATTATTTGAATCAAGAAATAGGTCAGGAAAGTATTAATTATAAT
ATGCAAACATTGTTTGAAAAAGAAAGATATGATAGAAGTAGTACCATTGAAAAATTAGT
AGCAACAAGCAAATTTAAGTATGAAAAAGATGATTCAGATTTATTCAAACAACTTTTCAA
TGATGTTGAAAATTCAATAGACAGATTAGGTATTTACTTACTAAATAATGGTATAAATTC
GAATGATGAAAATGCAAGATATTATAGATCGTTTTTAAAGGAACTTAGTAGAATAAAAA
GTAAATTAACGCCATTTTCTCTTGAAATAAGTAAGTCTAGCGGAAGAGAGCAACATTATC
CTGATGATGCTATTGATGATAAAGATGAGAGAAGAAAAAATAAAGAAGAAACATATCAT
GCTTTTGATGATAAAAGCGATATTGACTCCAAATTAAAGAATAAAATAAATGTTTCTATC
GATAATTTATTGTCAGTTAAAATTAAGAGCAATTTGTATGATATTAGTCCAAATACAAAA
AAACAATAA

## > Q4LAG3

Function: protein coding sequence; putative protein in SCCmec III &V (SCCmec)
Best match: Q4LAG3-PM1_CC398_SO385-pig_AM990992.1[54295:55404] (completely identical)
Position: 004-contig_253_RC: 3949 … 5059; Length: 1110 bp
Sequence:
ATGTATCAAGCAAATATACGTGATTTAATTACAAAATTGCCTCAAAGCAATAAAACAGAA
CACTTTTTAATGAACAAATTTTCAAATCAAGATAAAGTTCAGCAGCTACAAAGACAAATT
AGCCAACAGCTAGATCAACAATATAATGAGCTTTTGGCTAATGAAAAAGCTAAGCTAGA
CCAATACGTGGAAGTACACCATAATTTAGAACCATTAAAGAAAGAGATTGAATCAGAAT
CTATTAACCTTGATACCGATAAATTACCTGATATCAAAGCGACAATGCTTGAAAAGGCTA
AGAACGATGAACATTTTGATAAAATCGAACAGCTATTTGATAGATTAGATCAGTCATTAA
ATGGTACGAATCGATTATATACGCAATTATCGTTGATTGGCACACGAACACATCGGATCA
CAACGAAAAGATTTAATGTTCAAGGCTTGCCTAAATTAGTCCAACAAATGATTTTACCTT
CGCAATTTAAAAAGGTTTATACAATAGATTTTAAATCATTCGAACCATCAGTTGCTGCGT
ATATGACACAAGACGAACAACTGATTGACTACTTGAATCATGAAGAAGGGTTATACGAT
GCATTACTGAGAGACTTATCTTTGTCAAAAGAGAAGCGCGTGAGTGTGAAACGTGCATTT
ATAGGGTCATTTCTTTTTGGCGGTCGTTATAGTAGCTCTAAATTCAAAATCAATCAAGAGG
TTAGTGAAATTAACTGGCTACAAGTAATGAGCAAATTCAAGAAGGTCATTGAATTTAAGG
AGCAAGTCGAAAAATATAAAACAATGCCTACGCCTTACGGCATTGAACATGATATGAGC
GCATTTCAAGGTAGTAGTATTATGGCAATTTATGTACAAACGGTAGCAAGCTATATTTTC
AAGCACATTTTGTTGGAAGTGTACAAAGCACAGTGCGAAAAAAAAACGTTCAAGATTAT
AGTGCCGATACACGATGCGATTATGATTGAATGTAATGATAAGGGGATTGCACAAAATGT
AGCACAGCTCATGAAAGATACAGCTAATCAACTGTTTAATGGTGAATTTGCACATGTGAC
AGTGGAAGCTTTAGGAGGTATAGACAATGAATAA

## > Q3T2M7-var1

Function: protein coding sequence; putative protein (SCCmec)
Best match: Q3T2M7-var1_CC398_SO385-pig_AM990992.1[55397:55765] (completely identical)
Position: 004-contig_253_RC: 5051 … 5420; Length: 369 bp

Sequence:
ATGAATAATGATAGAGGAAAAAGTCTTCAAATTCCCCAAAGTACATTGTTAAAAGAAGG
ATCTATATACGTTGCTACGTTACATTCTGTGTACGAGAAGAACTTCTCAGGTGATATTAAA
CATCAGTTTACGTATGAAGTAGAACTTAACCAAGAAACGCACTATGTAAATCGCAATATT
ACCGTAAAATCTATGAGTCACCAATTATCAATTGCTGATTGGATTAAACGTCACAGTAAC
TATAACGTAAACCACATTAATTATGATCCATACATTGATCGAAAACATTTGGTCCTTGTAG
GGCAATATAACGGAAATTATTATATTCAAGATGTAGCACCATTAGATGAATTTGGAGGGG
TATTGTAA

> **ccrAA**

Function: protein coding sequence; cassette chromosome recombinase AA (SCCmec)
Best match: ccrAA-TSGH17_CC398_SO385-pig_AM990992.1[55765:57381] (completely identical)
Position: 004-contig_253_RC: 5419 … 7036; Length: 1617 bp
Sequence:
ATGAATCATATTTTAGAAATGTTAATAAAATTATTAAAAGTGGGTATGGAGGCAATCGAC
CGTAAAGGTCTGATTGCCATCCTAACAAGTAGTATTGGAAATGATGAAATGGATGATTCT
GAACAAGCTGTAATGGTGTATAACGAGCTTATCGATAAGCTACAGCTTAACATTCCTAAA
GATGTCGACTATAGACCTAACATATACAGTTATTTTGGTATTCAAAAAAAGCCAAATGAC
ACAATATTAGTGGAAATGATGATATGTATTTTTCATATCAAGCGTTTTGATTCAGAGTTGT
TTGTTTTCAAAGATAAAGGTTGGCAAAAGGTAAGTGAAGATGAATTGCAAGGGTTGATAT
CTAAAATGATACAAGTGTTGCTAGTTGATTATAAACCTTCACTAAGTACTTTGAAAAACG
TAGTAGATGGCATACAGAAATCAACGGACATAGAAAAACTTGTTGAGCATAGACAGTAC
ATTGGTTGTGGACGAAATATGTTCAATCTAAAGACCTTTAAAGTGGTTGATAATGACCTT
GAAATATTCCCTAAAACACGCTTAGATTTGGAATTAGATATAAATGACACGATTACGGAC
AAGATACCCCCGAATTTCAAACAATATATGTTAGAGTTGGCGAATTATGACCATGATTTA
CAATATTTCCTTTTCCAACATATGGCAGTGTTATTGACGGCAGATACTAAACTACGTCGTG
GACTTTTTTTGTATGGAACTGCAAAAAATGGGAAATCGGTCTATATTAAATTAGTTAAGT
CATTCTTTTATAGTAATGATATCGTATCTAAAACACTTAATGAACTTGGCGGGCGTTTCGA
TAAGGAAAGTCTAATTGGTAAACGAATTATGGCAAGTGATGAAGTGGGGAAAGCTAATA
TTGATGAAGCAACTGTGAATGATTTCAAAAAATTACTATCTGTTGAACCAATTCATGCTG
ACCGTAAAGGAAGAACGCAAGTAGAAGTTACTTTAGATTTAAAACTCATTTTTAATACGA
ATGCTGTACTCAATTTTCCATCATCACATGCAAAAGCATTAGAGCGTAGAATTGCTGTTAT
TCCATGTGAATATTATGTTGAAAAAGCTGACCCTGACTTAATTGAAAAGTTACAGGATGA
AAAGAAAGAAATCTTTCTTTACTTGATGTATGTGTATAAGCAAATTGTAAAAAATGATAT
CGAGTACCTCCAAAATGATCGTGTTACTGAAATTTCTCATGATTGGTTAAATTTTGGATAT
GAATTTGTTTCTAGTAAATCAGCAAATATTGCACATCAGAAAGCGTGTATTAATTTACTCA
GAAAACTTATAGAAATCAAACCAGGGTCACGTATCAAAGTGTCTAGGCTAAATGAGGTT
ATTAGAGATGAAATTAAAGTAAGCTCTCAAGTTATTAATGATTTGGTTCAAGCTAACTTT
AATGTACAAAGTAGACTAAATAATGGTTATAAGTATTGGGTCGATTTAGGATGGAAAGA
AACTGATAAAAAAGATGACATGATTTCATTCGATAAAAATGAGAATGTAACAGATGATG
AATTCTTATACGAAGATGATTTGAACTTAGGTTGGGAGGACTTTGACGATGAATAA

> **ccrC**

Function: protein coding sequence; cassette chromosome recombinase C (SCCmec)
Best match: ccrC-TSGH17_CC001-ST772_118_AJGE01000059.1[7230:8909] (completely identical)
Position: 004-contig_253_RC: 7260 … 8940; Length: 1680 bp
Sequence:
ATGAAGGGTAAAATTGCACTTTATTCACGTGTTAGTACGTCTGAGCAGTCAGAACATGGT
TATTCTGAAAAGGAGCAGGAACAACTACTCATCAAAGAAGTTATGAAAAATTTCCCAGGT
TATGACTATGAGACATATACTGACTCAGGCATTTCAGGTAAAAATATTGAAGGTCGTCCG
GCAATGAAACGTCTATTACAAGATGTTAAGGATAATAAAATCGAAATGGTATTAAGTTGG
AAATTGAATCGTATTTCTCGCTCAATGAGAGACGTGTTTAATATTATTCATGAATTCAAAG
AACATGACGTAGGGTATAAATCGATTTCTGAGAATATTGATACATCCAATGCTTCTGGAG
AAGTACTCGTTACAATGTTTGGACTAATAGGATCTATAGAACGCCAGACTTTGATTTCCA
ATGTGAAACTTTCTATGAATGCTAAGGCAAGGAGCGGAGAGGCAATCACCGGTCGTGTTT

TAGGCTACAAATTATCACTTAATCCATTGACACAGAAAAATGATTTAGTTATTGATGAAA
ATGAAGCTCATATTGTACGGGAAATCTTTGATTTATATTTGAATCACAATAAAGGATTTA
AAGCAATCACGACAATTCTAAATCAAAAAGGATATCGTACCATTAATCAAAAACCATTTT
CAGTGTTTGGCGTGAAATACATTTTGAATAATCCAGTCTATAAAGGCTATGTCAGATTCA
ATAATCATCAAAATTGGGCTGTTCAGCGAAGAAGTGGTAAAAGTGATAAAAATGATGTG
ATATTGGTCAAAGGTAAGCATGAAGCCATTATAAGTGAAGATGTATTTGATCAAGTTCAT
GAGAAACTAGCTTCTAAAAGTTTTAAACCGGGTCGACCTATTGGTGGAGATTTCTACTTA
CGTGGCCTTATTAAATGCCCAGAATGCGGAAATAATATGGTATGTCGACGGACGTATTAT
AAAACGAAAAAGTCCAAAGAACGCACAATCAAACGCTATTACATTTGTTCATTATTCAAT
CGCTCAGGAAGTTCTGCCTGTCATAGTAATGCGATTAATGCTGAAGTCGTCGAACGCGTA
ATCAATGTTCATTTGAATCGTATTCTTTCACAACCTAATGTTATTAAGCAGATTGCGTCAA
GTGTGATAGAAGAACTGAAACAAAAGCATAGTAAACAAACAGAAATAAAATATGATATT
AATAGTCTAGAAAAACAAAAAGCAAAAGTTAAAACACAACAAGAACGATTATTGGAATT
GTTCTTAGATGATGAAATGGATAGCGAAATGTTAAAAGCTAAACAAAGTGAAATGAATC
AACAGTTAGAAGTATTAGACCAACAAATTAAAGAAGCAAAACAAGCAAATCAATCACAG
GATGATATACCCAATTTTGATAAGTTAAAAGCACGACTCATTTTGATGATAACACGATTC
AGTGTGTACTTAAGAAAGGCTACACCCGAAGCTAAAAATCAACTTATGAAAATGTTAATT
GATTCAATTGAAATTACGAAAGATAAACAAGTGAAACTCGTAAGGTATAAAATTGACGA
AAGTCTTATCCCTCAATCTTTGAAAAAAGATTGGGGGTCTTTTTTTATACCTAAATTTAAC
TTTGTGATAAATGTCACAAAGAAAAATAGGATTGAAAATTTATCACTTTTACCACTTTTTT
AG

## > Q4LAF9

Function: protein coding sequence; putative protein (SCCmec)
Best match: Q4LAF9_CC398_SO385-pig_AM990992.1[59374:59712] (completely identical)
Position: 004-contig_253_RC: 9028 … 9367; Length: 339 bp
Sequence:
ATGACGCTAAGCAAACAACTTAAAACGTATATCACTGAACGATTTAAATTAAATTATCAA
GAAACTTGGGCTTGTGAAACCATAGATGCGGTGGCTGAAGATGTATTACCTGAAAAATAT
ATTAAAAATAGTCCACTTGAACATAAAATTTTAAATACTTTTACCTATTACAATGATGAAT
TACATGAAATCAGCATTTACCCTTTTTTATGTTATCTAGATAAGGAATTAGTAGCAATAGG
TTATTTAGATAATTTTGATTTAGACTTTATATTTTTAAATGACACTCATCAAGTTATTATTG
ATGAACGCTACTTGTTACAAAAAGGGGGCCAGTAA

## > Q7A206

Function: protein coding sequence; putative protein (SCCmec)
Best match: Q7A206_trunc_AY894416_TSGH17_AY894416.1[5033:5119]
Position: 004-contig_253_RC: 9372 … 9402; Length: 30 bp
Sequence:
AATTGGATCAAGGTCGCTCAACTATCTGTC

## > Q7A207

Function: protein coding sequence; putative protein (SCCmec)
Best match: Q7A207_CC001-ST772_118_AJGE01000059.1[9430:9741] (completely identical)
Position: 004-contig_253_RC: 9460 … 9772; Length: 312 bp
Sequence:
ATGAACATCAATCGATACATCACAAGAGGAATTAATGAAAGTATTCCACTAGACCTTCAA
ATCTTACTTTGGCACATGGTAGAAGAAAAAGATAACCAGCCTCATACCGATTACCTACAC
ATTTTCAAACTACAAGAAGATGATAATATGTTGTCAATTAAACATGAACAAGAACAGCCC
GCATACAAGTTAGAATATCACTATACAAACTATGAAAAAAATCAAAATGCATTACCTAAG
AAAGTCTACGTCATTCGTGAAGATGGCGTAGACGCTTTTTATTATGTGATGCTTTTACCAG
AAGAATACTAA

## > Q9KX75-v1-TYH1

Function: protein coding sequence; putative protein (SCCmec)

Best match: Q9KX75-v1-TYH1_CC001-ST772_118_AJGE01000059.1[9759:10262] (completely identical)
Position: 004-contig_253_RC: 9789 … 10293; Length: 504 bp
Sequence:
ATGAATACAATCAAAAATACGATATACACAGAAGCTATATTTAGCAAGGATGAAAAACA
CCGCTATTTACTCAAGAAAACATGGGATGAAAAGAAACCTGCTTGTACAGTGATAACGAT
GTACCCTCATTTAGATGGTGTATTATCACTCGATCTCACAACTGTTCTTATTCTTAACCAA
TTAGCGAATTCTGAACAATATGGTTCTGTATATCTTGTAAATCTATTCTCTAATATTAAAA
CACCAGAGAACCTTAAACATATTAAAGAACCTTATGATGAGCACACAGATATACACTTAA
TGAAAGCGATAAGTGAAAGTGACACAGTGATTTTAGCCTATGGTGTTTATGCAAAGCGTC
CCGTTGTTGTCGAACGTGTTGAGCAAGTGATGGAAATGTTAAAACCTCATAAAAAGAAAG
TAAAAAGGCTCATAAATCCAGCAACGAATGATATTATGCATCCGCTTAATCCTAAAGCGC
GTCAAAAATGGACATTGAAATAA

## > A9UFT0
Function: protein coding sequence; putative protein (SCCmec)
Best match: A9UFT0_CC001-ST772_118_AJGE01000059.1[10276:10497] (completely identical)
Position: 004-contig_253_RC: 10306 … 10528; Length: 222 bp
Sequence:
ATGAACCATGAAACTAAACAATCAGATTGGCATACGGTTGCTAATTGTTTAGAATCGCAA
AATTATACATCGATCGTAAAAGGATTAGTACATCATTTCACAGCGATTGAAGATGAAGAA
ATACTTGATAAAATCTATGATGATTTTATGAATGATGACTCTATAACAACGGTACTTAAC
AATGATTTACAGATAATTATTAATCATTACCTATCAAAATGA

## > tx_hsdR_WIS_Shae
Function: bidirectional rho-independent terminator of hsdR_WIS in S.haemolyticus (SCCmec)
Best match: tx_hsdR_WIS_Shae_85-2082_AB037671.1[46796:46857]RC
Position: 004-contig_253_RC: 10571 … 10601; Length: 30 bp
Sequence:
CGGTGAGGGATGCTCTTTTAGTTTATTTTT

## > hsdRx
Function: protein coding sequence; type I restriction-modification system endonuclease
Best match: hsdR_WIS_CC001-ST772_118_AJGE01000059.1[10571:13690:r]RC (completely identical)
Position: 004-contig_253_RC: 10601 … 13721; Length: 3120 bp
Sequence:
TTATTGGTTACTCGCCATAAGCTCTGCTTGTTCTACGACTTGCTCTACAGCCATTTTTTGTA
AATCTGGTGGATAGCCATATTTTTTAAGCAATCTTCTAACAGCTACGCGCATTTTAGCTTT
TGCGCTATCACGTTTAGACCAATCAACACCCATGTTTTCTTTCACTGTTTTAGTTAGCTCAT
GAGCAATCGCACGTAGTTCTTTATCTCCCATGGCTTCTTTTGCTGTTTCATGTGAAGCTAA
AGCATCGTAAAACGCAATCTCATCTGAATTCAGGCCTAATTCTTTTCCTCGTTGTTGTTCT
TGTTTGATATCTTTAGCGAGTTGAATAAGTTCTTCAATCACTTTAGATGTTTCAATGGAAC
GACTATTATATTTATTAATCGAGTTTCCTAACATTTCAGAGAAACGCTTAGATACTGTCGC
GTTCGTTTTCATTAATGATTTGACTTGCCCTTTGAGTAATCGATTTAATAATTCTACTGCAA
CATTTTTTTGTTTCAATCCTTCCACGTCTTTTAAGAAATCATCTGATAGGATTGATAAATC
GGGTTGTTCAAGACCTAGCGTTTGATAAACATCAATGACATCTTCAGTCACAACAGATTG
TGACACAAGTTGATTAATCTCTGCTTCAACTTCTGCAGGTGTTTTACGTATTTTTCCTTCTT
TTGGCGGTTGTAACAATTTAACAAGTCCTGCTTTAACCGCTTTAAAGAAGGCAATTTCATC
ATTGAGTTCTTGGGCTGTTGGTTCAGTCGCACAAAGAGCAAAGGCTTTACCTAACTCTGT
GACCGTTTTAATAAAACGTTGACGTTCATCTTCACCTAAACCAATCACATAATCCATCGTA
TTCGATATTGTATAATAACGTTCCAATTTTTTATTTGAATTAAATTTAGAATAATCAAGAT
TATATAACATATCTTGAATTACATCATACTTCAATAACATCAATTCAACCGCTTTATCTGT
ATCTATCGCTGTTTGAGCTTGATCAGATTCTGTATACTCTTTAAGTGCTTCTTTCAAACTTT
CGGCAATACCCACATAATCGACAATCAATCCACCCGGTTTATCTTTAAACACTCGATTAA

CTCGGGCAATTGCTTGCATCAAATTATGGCCTTTCATTGGTTTATCAATATACATCGTATG
CATAGAAGGAACATCAAACCCTGTCAGCCACATATCTCGAACAATCACGAGTTGTAATTC
ATCATTCACATCTTTCATACGTTTTTCTAATAAATTACGACGTTTTTAGGACCAATATGTC
TTTGGAAAGAAGCTGGGTCACTAGAGGAGCCCGTCATTACCACTTTAATGACCCCTTTAT
CATCATCATCTGAATGCCATTCTGGTTTTAGACGAATGATTTCATCATATAAATCAACAGC
AATTCGACGACTCATCGTTACAATCATTCCTTTGCCTTTCATCGCTTGTTGACGTGTTTCAA
AGTGTTGGATGATATCTTTGGCTAGGGCTTCGATACGAGGTTTTGCACCTGCTAAGGCTTC
AATTCGTGACCATTTTGATTTTAAACGCTGTTTTACATCCTCTTCTTGGTCTTCTGTAATGT
CATTATATGCTTCATCTAAATCTAAATTTTGGGGTAGATTTAATGGAATTACACGACTTTC
ATAGTAAATTTTAACTGTACTTCCATCAGCTACAGCTTGTGTCATGTCATAAACATCGATA
TAGTTTCCGAAAACCATTTGTGTATTTTTATCCGTTGAAGCTACGGGTGTGCCCGTAAATC
CGACGAATGTTGCATTCGGTAAAGCATCTCTTAAATATTTAGCATAACCATATTTAATGCC
TTCACCTTTATCATTATACTTTGCATTAAAGCCATATTGTGTACGATGAGCCTCATCTGCC
ATAACAATCACATTTTTACGTTCTGTTAGGGCAGCCATGGTCGTTTCATTTTGTTCAGGTT
CAAATTTTTGCATTGTTGTAAATACAATACCACCCGACTCAACAGATAATAACGATTTTA
ATTCTTTACGTGTTTCAGCTTGTTTTGGCGTTTGTCTTAATAATCCTTTACCAGAACGTCCT
TTTGATTTAACAAATGTACTGTATAGTTGGTTATCTAAATCATTACGATCTGTAACAACGA
CTAAGGTAGGATTATTCAGCATTTGAATTAATTTTCCAGAGAAAAAGACCATGGTTAAAC
TTTTACCAGACCCTTGGGTATGCCAAATAACGCCGCCTTTACCATCACCCGGCCCAGATG
AAGCTAATAAAGCTCTATCAACAGCTTTATTAACAGCATAGTATTGATGATATGCTGCTA
GAATTTTACTGATATGCCCTTTACCATCATCTTGGAATAATACAAAATATCGAATTAAATC
AAGTAGAGTATTAGGATTTAACATCCCATGAATCAGTACGTCTAAGCTAGCTAAACTTGA
CGAAGATTCTGTTTCTCCATCTTTAGAACGCCAAGTCATAAAACGATCATAGTTCGCAGTT
AGTGAACTGGCTTTAGTATTAATACCATCACTTGTAACAAGCACTTCATTAAATGTAAAT
AATTGTGGAATACGCATCTTATACGTTTCTAATTGATGATAACCATCTTCGACGCCTACGG
TTTCATTGGTTGAATTTTTAAGTTCGATCACAACGATAGGCAAGCCATTGATAAAGAGGA
CAATATCGGGACGTTTTGTATAGTCTCCGTTAATGACAGTGAATTGATTGACTGCTAAAA
AATCATTGTTTTGTGGATGTTCAAAATCAACGATTTTAACAATTTCTACTACCGATTGTCC
TTCGTCATCATAGTCTTCGATTTCAATACCATTGATCAAATTTTCATGAAAGGTAAGGTTA
TTTTCTAAAAGATTGGGCGACTTCTCTAAAGTTAGTTCATGTATGGCTTTTTCGATAAAAC
GATGATGAATATCTGAATTAATCTTTCTTAATGATTTTTCTAATCGTTCATGAAGGACAAC
ATCTTTATCACTTTTACGTTCTGGCGCTAGACCTGTCATACTAATCTCATTACCTTTTTTGT
AGTCATAGCCCAGTGATTGTAGCCATTCTAATGCGACTTGTTCTAAATCATCTTCACTAAA
TTGAAAGCTCAT

> **hsdS_CC22**
Function: protein coding sequence; type I restriction-modification system site-specificity determinate (genomic island)
Best match: hsdS_CC22_CC093_JKD6159-ST93_CP002114.2[64670:65902]RC
Position: 004-contig_253_RC: 13704 … 13734; Length: 30 bp
Sequence:
CTAAATTGAAAGCTCATCTTCATTCACCTC

> **hsdM2-scc**
Function: protein coding sequence; type I restriction-modification system DNA methylase (SCCmec)
Best match: hsdM2-WIS_CC001-ST772_118_AJGE01000059.1[14821:16335:r]RC (completely identical)
Position: 004-contig_253_RC: 14851 … 16366; Length: 1515 bp
Sequence:
TTAAACTCCATAGCCTAATCCCTCCAATGATTTGCGGATTTGGTTTTCAAGTTCTTTTGATT
TCGCAAATTGTTCGCTTAATTCAGAAGTAATGCGTTCCATTTTTTGCTCAAACGGTTCTTT
ATCTTCTTCAACATCAGTTAAACCTACATAACGTCCTGGCGTTAAAATATATTCATTATTC
TTAATTTCTTCAAGGTTAGCTACTTTACAAAAACCAGCTATATCTTCATAGGATTTGTCGT
TTGTACCTTTCCACGCATGATACGTTTGTGCTACTTTTTGAATATCTTCATCTGAAAATTCT
TTTAATGTTCTAGATACCATATGACCGATTTCACGAGCATCAATAAATAAAATTTCATTTT

TACGTTCTTTTTTACCATTTTGACCTTTATTATTACTAATGAACCAAAGACATACAGGTAT
TTGTGTTGAATAAAAGAGTTGACCTGGTAAGGTAACAATACATTCCACTAAATCTTGTTC
GATAAGATTTTTTCGGATTTCTAATTCATCTTTTCCACTTGTAGACATTGAACCGTTGGCT
AATACAAATCCTGCTGTACCATTAGGTGCTAATTTTGAAATCATATGTTCAATCCATGCAT
AGTTGGCATTACCTTTTGGCGGAATACCAAATTGCCAACGGTAATCATCAAGTAATCGTT
CTTGACCCCAATCACTTGCGTTAAAAGGAGGATTGGCTAATATGTAATCTGCTTTTAATCC
TTTATGTAAATCGTTATGGAATGTATCAGCATTTCGTTCACCTAAGTCATTATCAATACCA
CGAATCGCTAAGTTCATTTTAGCTAATTTCCAAGTTGTAGGATTAGATTCTTGCCCATAAA
TCGCAATATCGTCTAATCGACCTTGATGTCTTTCAACGAAGCGTTCACTTTGTACAAACAT
CCCACCTGAACCACAGCATGGATCGTAGATACGACCTTTATAAGGTTCAATCATCTCAAC
CAATAATTTTACAATTGATGAGGGGGTATAGAACTCTCCGGCATTTTTCCCTTCAGCGCTT
GCAAACTTAGCAATAAAATACTCATACACTCGGCCTAATACATCTTGCTTACGACTTTCA
GTATCGCCTACCTTAAAAGTAAATAAATCAATAATATCGCCTAATTTTTCTTTATCTAACG
CAGGGCGCGCATATTCTTTAGGTAACACACCTTTTAATGATTCATTTTCTTTTTCAATCGC
AATCATGGCTTTATCAATAATTTGTCCAATTTCTGGTTTTTTCGCATTATCATTAATATATT
GCCATCTTGCTTCTTTTGGCACCCAGAAAATATTTTCTGCTAAATATTCATCTTGATCCTCT
TCATCAGCATAAGGATCTTGCTTCAATTCTTCATACTTTTCTTCAAAAGAATCTGATACAT
ATTTTAAAAAGATTAAACCTAATGCTACGTTCTTATATTCAGCAGCATCCATACTTCCTCT
TAATTTATCGGCAGCTTGCCATAATTTTTCTTCAAATCCGATTGTCGCCAT

> **dus**
Function: protein coding sequence; tRNA-dihydrouridine synthase (core genome, constant)
Best match: dus_part_CC005_MR1_ACZQ01000311.1[1:278:r]
Position: 005-contig_59: 43 … 73; Length: 30 bp
Sequence:
TGAAGCCCAAATGGACGAAGACGTTAAAAT

> **entCM14**
Function: protein coding sequence; enterotoxin-like protein (genomic island)
Best match: entCM14_CC001-ST772_AJGE01000059.1[26448:27227]RC (completely identical)
Position: 005-contig_59: 265 … 1045; Length: 780 bp
Sequence:
CTACTTTTTAGTTAAGTGCACTTCTATTTCCGTTTTGGCTGATTCAACTGTTTCATTACCTC
GATATATCATCAAATATTTAGACTGAGTAAAGCCAGGGTCTGGCATCATATCATACCAAT
AATATCGATTACCATTTTCTATAAATTTAATGTATCCCGTTTCATATGAAGTGCCACCTAA
ATGGTATAAATTGATTTTAGAAATTAATTTATTTCTCACTTTATAATCTAACTCTTGAATA
GTTACAGTCTCTTTATCAATATTGATATCAAAAGAAAGAGTAACACTGTCATTCTCGTAA
ACTTTTACAACAATAGGCTGAATTCTATTATTGGTATTGTTCTCATTTAATGTTATACCAC
CATACATACAAGTTTTTTTACTTCCAGCTCCTTGATTACTATCACACTGTATATTTTCATTT
GAAAAATAGCATTGATCAAAATAATTGACGCCAAAAATATCGACTTCTTTCCCATCAAAT
GAACTAGCCATTGTACTGTCTTTTAACTCAGTTTTAACATAGTCATAATGACCAGGGTTAT
TTATTGGAACAATGAAGTCATGACTCAAAAATTTGTCACTTAGCTTTATTTTTTTAGCATT
AACTGGATCGCTATTATATAAATACCATATATTATGCCAAGAACCCGTATATTGACTAGA
TTTGTTTAATTCACTTATATTTGGATCATTTTGTGTCTCTGCTCTAGCTTCCAACAAAGAAT
AGCTACTGTAACCAAATAACAAAGTGATTAGTATAAATACTTTTCTCAT

> **tx_entCM**
Function:  rho independent terminator of entCM_bov (genomic island)
Best match: tx_entCM_CC001-ST772_AJGE01000059.1[27283:27330]RC (completely identical)
Position: 005-contig_59: 162 … 210; Length: 48 bp
Sequence:
AAGTAATTAAAAAAAGCAACTTTTCCAAGTCGCTTTAATTCATTAAAA

> **Q6GKL6**
Function: protein coding sequence; putative protein (genomic island)

Best match: Q6GKL6_CC001-ST772_118_AJGE01000059.1[25912:26070]RC (completely identical)
Position: 005-contig_59: 1422 … 1581; Length: 159 bp
Sequence:
TTATTTAAAAGCGATTTTACGAATAAGTTCATTTGTAATTTCTTTCTCATTATTAATTGCAT
GAAGAATTTGTATTTGCAGTTCTCGTGTAACATACATACCTTCAAGTGAAAGGTTTTTAGC
GATTTGTTTATTAATCTTTGAATCAATAGTAATCAT

## > tnpIS232

Function: protein coding sequence; transposase for IS232
Best match: tnpIS232_trunc_15584_EU272081.1[7731:7833:r]
Position: 005-contig_59: 1819 … 1922; Length: 103 bp
Sequence:
AAATTATTTTTAGATATAAATGCGAATTTTGAAGTTGCTACTCTTTAAGGTTTACTAAAAT
AAAAGTTTATATAAAGAAACTTGAACATGAGAACAAATAAAA

## > mcrC

Function: protein coding sequence; type IV 5-methylcytosine-specific restriction enzyme subunit C (genomic island)
Best match: mcrC_trunc_15584_EU272081.1[7531:7640]RC
Position: 005-contig_59: 2012 … 2122; Length: 110 bp
Sequence:
TTATTTTAGTAAAAATATATCATTAACTATCCCATTCAACTGTTTTTTAATAATAGTAAAA
TCACAGTTCAAATCTAATGTCTTAATAATGATTTGACTACCACTCATAT

## > Q9KX84-M10

Function: protein coding sequence; putative protein (SCCmec)
Best match: Q9KX84-M10_trunc_CC012_21266_AFTT01000014.1[137215:137286:r]
Position: 005-contig_59: 3779 … 3851; Length: 72 bp
Sequence:
ATGAACAGCAATTGGAGACGTGGTTGCAAGCAATAATTGGAAATATGGATACGAAAGAT
GTAAAAGATGTGA

## > Q8VUY2-GI

Function: protein coding sequence; putative protein (genomic island)
Best match: Q8VUY2-GI_CC001-ST772_118_AJGE01000054.1[314:1069:r]RC (completely identical)
Position: 006-contig_274: 241 … 997; Length: 756 bp
Sequence:
TTATAAAATGAGTTGAACTATAGCAAAAACGATTAAAATACTGATAATCCATTTTTGTAT
TATGTTAGGGACTTTTTTACTTAATTTTAACCCTATTGGAGCAAATATAATACTCCCTATT
ATAAGGAATAAGGCGTCATATAAAGGGATATAACCTTGAATAAGTTTGATGACAAAAGC
ACCAATTGAAGATATAAAAGCAATTACTATACTATTAGCGACTACAGTATTCATTGGTAA
TTTGAATAAAACCAATAATATAGGAATAATAATGAAGGCACCACCTGCACCTACTATACC
TGAAATAATACCAATGAAAAGGCCAATGATAACTAATAAATATTTATTAAATGAAGACTT
TTCGGAACTAGGTTTCACTTTAATAAACATTAATGTTAATGCAAGTAAAGCAATAATGAT
ATATACCGTATTTACAAATGTAGCATCAAATAAATTTGCTAGAAATGCACCTAACATACT
CCCTATAATCATGCCGCCACCCATATAAAGAACTAATTGTGGCGAGAACTCTGTTTTTTTT
CGAGCTTTTAATGAGCCACTTAATGTACTGAAAAAGACTTGGCTAGAAGTAAGACCTGAT
GCGATATATGCGCTATATGCAGGGGCTCCGAATAATGGTGGTAATAATAAAATAGCTGGA
TAAATAATGATAGCACCACCTACGCCTACTAGACCAGATATGAACCCGCCGAATACCCCA
ATGAGTAACATGATAACTATATTAACAATATCCAT

## > Q9XBC0-GI

Function: protein coding sequence; putative protein (genomic island)

Best match: Q9XBC0-GI_CC001_MW2-USA400_BA000033.2[64924:65184:r]RC (completely identical)
Position: 006-contig_274: 996 … 1257; Length: 261 bp
Sequence:
TTACTTACTTTTCACCAATAAGTTTACAGCTTCATTAATTAACTCTTGGGAGCTTTCTTCAT
CATCCGCAGCTGCTTTTACACATTCTATTAAATTCTCACTAATAATGATACCCATCAAGCG
TTGGAGTGAACTCTTTGATGCACTTATTTGTGTAATGACATCTTTACAGTCTTTTCCTTCCT
CCATCATTTTAATAATTCCATTTAGTTGCCCTTGTATTCTATTAATACGATTAATCATTTTT
TTATCATAATTCAT

## > Q9XB85

Function: protein coding sequence; putative protein (genomic island)
Best match: Q9XB85_CC001-ST772_118_AJGE01000054.1[1466:2533] (completely identical)
Position: 006-contig_274: 1393 … 2461; Length: 1068 bp
Sequence:
ATGAAACAATACGGAGAAAAGTTTATCGATGAATTTAGTAAAGCAGAATTGGAAAAACT
AGCCAAGCAAGGGCAATTAATTGATGTTAGAACAGAAGAGGAGTATGCATTAGGACATA
TCAATGGTTCCATACTTCATCCTGTTGATGAGATTGAGTCATTCAATAAAGAAAAAAATA
AAACCTATTATGTAATCTGTAGAAGTGGTAACAGAAGTGCTAATGCTAGTAAATATTTAG
CTAAACAAGGTTATAACGTTATAAATCTTGATGGTGGTTATAAAGCTTATGAAGAAGAAA
ACGATAGTTATGATACACAAGAAGAATATAAAAGTATAGAAATTAAAGCAGATCGTAAA
CAATTTAACTATCGTGGTCTTCAATGTCCAGGGCCAATTGTAAAAATTAGTCAAGAAATG
AAGAATATTGAAGTAGGTGACCAAATTGAAGTCAAAGTCACAGACCCTGGATTCCCTAGT
GACATTAAAAGTTGGGTGAAACAAACAAGGCATACTTTAGTTAAGCTTGATGAAAATAA
CAATGGAATTAATGCGATTATTCAAAAAGAAAAAGCAAAAGATTTAGATATAAATTATTC
TGCTAAAGGTACTACAATTGTATTATTTAGTGGAGAATTAGACAAAGCTGTAGCAGCATT
GATTATTGCAAATGGCGCTAGAGCTGCTGGAAAAGATGTAACTATCTTCTTTACTTTTTGG
GGGCTTAATGCATTAAAAAAAGTGCAAACAGTTAATGTTAAAAAGCAAGGTATTGCAAA
AATGTTTGATTTAATGTTGCCCAAAAAGAATATACGAATGCCTCTTTCCAAAATGAATAT
GTTTGGTTTAGGAAATATGATGATGCGCTACGTAATGAAAAAGAAAAATGTTGATTCATT
ACCAACACTTATCAATCAAGCTATTGAGCAAAATATCAAATTAATCGCTTGTACGATGAG
TATGGATGTCATGGGTATTCAGAAAGAAGAACTTAGAGATGAAGTTGAGTACGGTGGTGT
AGGCACTTATATTGGTGCTACTGAAAATGCGAATCATAATTTATTTATCTAA

## > Q2G1R6

Function: protein coding sequence; putative hydroxyacylglutathione hydrolase (?) (genomic island)
Best match: Q2G1R6_CC001-ST772_118_AJGE01000054.1[2564:3898] (completely identical)
Position: 006-contig_274: 2491 … 3826; Length: 1335 bp
Sequence:
ATGTTTTTTAAACAGTTTTACGATAATCATTTATCTCAAGCATCATATTTAGTGGGTTGTC
AACGTACAGGAGAGGCAATAATAATAGACCCTGTTCGTGATTTATCGAAATATATAGAAG
TTGCAGATTCTGAAGGTTTAACAATTACACAAGCTACAGAAACACATATTCATGCTGATT
TTGCTTCAGGAATTCGTGATGTGGCTAAACGTTTAAATGCAAATATATATGTGTCTGGCG
AAGGTGAAGATGCATTAGGGTATAAAAATATGCCATCAAAAACACAATTTGTTAAACAT
GGAGATATCATTCAAGTAGGCAATGTTAAATTAGAAGTTCTGCATACTCCAGGGCACACG
CCTGAAAGTATTAGCTTTTTACTCACTGATTTAGGCGGTGGTTCAAGTGTTCCGATGGGAT
TATTTAGTGGTGACTTTATTTTTGTTGGTGATATAGGTAGACCTGATTTATTAGAAAAATC
TGTTCAAATAAAGGGTTCTACAGAAATTAGCGCGAAACAAATGTATGAGTCCGTTCAAAA
TATTAAAAATTTACCAGACTATGTTCAAATCTGGCCGGGTCATGGTGCTGGAAGCCCTTG
TGGTAAAGCATTAGGTGCCATACCTATATCTACAATAGGTTATGAGAAAATTAATAACTG
GGCATTTAATGAAATTGATGAGACTAAATTTATTGAATCATTAACATCAAATCAACCAGC
ACCACCGCATCATTTTGCACAAATGAAACAAGTTAATCAGTTTGGTATGAATTTATATCA
ATCATATGATGTTTATCCTAGTTTAGATAATAAGAGAGTAGCATTTGATCTTCGTAGCAAA
GAGGCCTTTCACGGTGGCCACACAAAAGGAACAATCAATATACCATACAACAAAAACTT
TATTAATCAAATTGGTTGGTACTTAGATTTTGAAAAAGATATAGATTTAATTGGAGATAA

ATCTACTGTTGAGAAAGCGAAACACACTTTACAATTAATTGGGTTTGATAAGGTAGCAGG
CTATCGTTTGCCAAAATCAGGCATTTCAACCCAGTCCGTTCATAGCGCTGATATGACAGG
TAAAGAAGAACATGTATTAGACGTACGTAATGATGAAGAGTGGAATAATGGACACTTAG
ATCAAGCAGTTAATATTCCACATGGTAAATTATTAAATGAAAATATTCCTTTTAATAAAG
AGGATAAAATATATGTACATTGTCAGTCAGGTGTTAGAAGTTCAATTGCAGTGGGTATAT
TGGAAAGCAAAGGTTTTGAAAATGTGGTGAATATTAGAGAAGGCTATCAAGATTTTCCAG
AATCATTAAAATAA

## > Q2G1R5-GI

Function: protein coding sequence; putative sulfide-quinone reductase (genomic island)
Best match: Q2G1R5-GI_CC001-ST772_118_AJGE01000054.1[3917:5110] (completely identical)
Position: 006-contig_274: 3844 … 5038; Length: 1194 bp
Sequence:
ATGAATAAGCATTATCAAATTGTTATTATTGGTGGCGGTACAGCAGGTGTTACCGTAGCA
TCAAGACTATTAAGAAAAAATCAAAACTTAAAAGAGAAAATAGCAATTATAGACCCAGC
AGACCATCATTACTATCAACCATTATGGACGTTGGTTGGTGCAGGGGTATCTAGTTTGAA
AAGTTCTCGTAAAGATATGGAAAGTGTTATACCTGAAGGTGCTCACTGGATAAAACAGGC
TGTTTCAAGTTTTCAACCTGAAAATAATAGCGTTATTTTAGGAGATAATACAGTCGTTTAT
TATGATTTTTTAGTAGTAGCTCCAGGATTACAGATTAATTGGTCTTCAATTAAAGGACTAA
AAGAAAATATAGGTAAAAATGGTGTTTGCTCTAACTATTCACCTGACTATGTTAACGAAA
CTTGGAACCAAATTTCTAATTTTAAACAAGGAAATGCCATTTTTACGCATCCAAACACCC
CTATAAAGTGTGGAGGTGCGCCTATGAAAATTATGTATTTAGCTGAAGATTATTTTAGGA
AACATAAAATCCGTTCTAACGCTAATGTGATATATGCAACGCCAAAAGATGCTTTATTTG
ACGTAGGAAAATATAATAAAGAATTAGAGAGGATTGTTGAAGAAAGAAATATAACAGTC
AATTATAATTATAACCTTGTTGAAATCGACGGTGACAAAAAAGTGGCTACATTCGAACAT
ATCAAAACATACGATAGAAAAACAATAAGTTATGATATGTTACATGTAACACCACCTATG
GGTCCCTTAGATGTAGTAAAAGAAAGTACACTTTCAGATAGTGAGGGTTGGGTAGATGTT
AACCCAACCACATTACAGCATAAAAGCTACTCTAATGTATTTGCACTTGGTGATGCTTCA
AATGTACCTACTTCAAAAACAGGCGCAGCTATTCGTAAGCAAGCACCTATCGTCGCTAAT
AATTTATTGCAAGTGATGAATAATCAAATGTTAACGCATCATTATGATGGTTATACTTCAT
GTCCTATTGTTACTGGATATAATAGGTTAATACTTGCAGAGTTTGATTATAATAAAAATAC
TAAAGAAACAATGCCGTTTAATCAGGCCAAAGAACGTAGAAGTATGTATATATTTAAGA
AAGATTTATTACCTAAAATGTATTGGTACGGCATGCTAAAAGGATTAATATAA

## > txbi_Q5HJT6

Function:  rho independent terminator of Q5HJT6
Best match: txbi_Q5HJT6_ST425_LGA251-cow_FR821779.1[78806:78878:r]RC (completely identical)
Position: 006-contig_274: 5216 … 5289; Length: 73 bp
Sequence:
TTATGAAAATAGAAACAGCAGTAAGATGTTTTCTAATTGAAAATCATCTCACTGCTGTTTT
TTTAAAGGTTTA

## > Q5HJT6

Function: protein coding sequence; putative protein (genomic island)
Best match: Q5HJT6_CC001-ST772_118_AJGE01000054.1[5378:5479:r]RC (completely identical)
Position: 006-contig_274: 5305 … 5407; Length: 102 bp
Sequence:
TTATTTAATAATAATTAATGGTATTTGAGCAAGTTTAGCGACTTTATGACTGACATTACCA
ATTTCCATTTCTTGCCAGATATTCAAACCACGTGTACTCAA

## > dus

Function: protein coding sequence; tRNA-dihydrouridine synthase (core genome, constant)
Best match: dus_CC005_21318_AFNQ01000029.1[56403:57389]RC
Position: 006-contig_274: 5701 … 6688; Length: 987 bp

Sequence:
CTATAATTCTATTTTAACGTCTTCGTCCATTTGGGCTTCAAATTCATCGAGTAGTGCTCGT
GCTTCTGCAATTGATTGTGTGTTCATCAATTGATGGCGAAGTTCGCTAGCGCCTCTTATGC
CACGCACATAGATTTTAAAGAATCTACGCAAGCTCTTGAATTGTCGTATCTCATCTTTCTC
ATATTTGTTAAACAATGATAGATGCAATCTCAACAGATCTAATAGTTCTTTGCTTGTGTGT
TCACGTGGTTCTTTTTCAAAAGCGAATGGATTGTGGAAAATGCCTCTACCAATCATGACG
CCATCAATGCCATATTTTTCTGCCAGTTCAAGTCCTGTTTTTCTATCGGGAATATCACCGTT
AATTGTTAACAATGTATTTGGTGCAATTTCGTCACGTAAATTTTTAATAGCTTCGATTAAT
TCCCAATGTGCATCTACTTTACTCATTTCTTTACGTGTACGAAGATGAATAGATAAATTGG
CAATGTCTTGTTCGAAGACGTGCTTCAACCAATCTTTCCATTCATCGATTTCATAGTAGCC
AAGGCGTGTTTTAACACTTACCGGAAGCCCACCTGCTTTAGTCGCTTGAATAATTTCGGCA
GCAACGTCAGGTCTTAAGATTAAGCCGGAACCCTTACCCTTTTTAGCAACATTTGCTACA
GGACATCCCATATTTAAGTCTATGCCTTTAAAGCCCATTTTAGCTAGTTGAATACTCGTTT
CACGGAACTGTTCTGGCTTATCTCCCCATATATGAGCGACCATCGGCTGTTCATCTTCGCT
AAAAGTTAAGCGTCCACGCACACTATGTATGCCTTCAGGGTGGCAAAAGCTTTCAGTATT
TGTAAATTCAGTGAAAAACACATCCGGTCTAGCTGCTTCACTTACAACGTGTCGAAAGAC
GATATCTGTAACGTCTTCCATTGGCGCCAAAATAAAAAATGGACGTGGTAATTCACTCCA
AAAATTTTCTTTCAT

## > tnpIS431-06
Function: protein coding sequence; transposase for IS431 (mobile element)
Best match: tnpIS431-06_circ_S-epidermidis_GQ900381.1[43799:43807]RC (completely identical)
Position: 006-contig_274: 6875 … 6884; Length: 9 bp
Sequence:
ATAGTTCAT

## > A6TXM6
Function: protein coding sequence; putative protein (core genome, variable)
Best match: A6TXM6_CC001-ST772_118_AJGE01000049.1[1164:1367] (completely identical)
Position: 006-contig_274: 6996 … 7200; Length: 204 bp
Sequence:
ATGGAAGATTTAAAGCATTCTTTAAAAAGTTTAGGTTGGTGGGATTTATTTTTTGCGATAC
CTATGTTTCTACTATTCGCATACCTTCCAAACTATAATTTTATAACGATATTTCTTAACATT
GTTATCATTATTTTCTTTTCCATAGGTTTGATTTTAACTACGCATATAATTATAGATAAAAT
TAAGAGCAACACGAAATGA

## > A6QD71
Function: protein coding sequence; putative protein (core genome, variable)
Best match: A6QD71_CC001-ST772_118_AJGE01000049.1[1416:1712] (completely identical)
Position: 006-contig_274: 7248 … 7545; Length: 297 bp
Sequence:
ATGGCGACTAAGAAAGATGTACATGATTTATTTTTAAATCATGTGAATTCAAATGCCGTT
AAAACTAGAAAGATGATGGGAGAATATATTATTTATTATGATGGTGTTGTTATAGGTGGT
TTGTATGATAATAGATTATTGGTCAAGGCGACTAAAAGTGCACGTCATCAATTTCAAGAT
AATACATTAGTATCGCCGTATCCTGGTGCTAAAGAGATGATATTAATTCCAGATTTTGAC
GAAGCAACAAATCTCACTGATTTATTTAAGACCATAAAAAATGATTTGAAAAACTAA

## > Q6GKK6
Function: protein coding sequence; putative protein (genomic island)
Best match: Q6GKK6_CC008_NCTC8325_CP000253.1[49481:50092] (completely identical)
Position: 006-contig_274: 7743 … 8355; Length: 612 bp
Sequence:
GTGAACGATATGTTAATTAGTCTTGTAATCCCAGTTTTTGCTTTGTTAGTTATTGGTGGTAT
TATTTGGATGATTATAGAAGGTATAGTACATATTTCAAAAAAGAATAAAGCAATTGATAA
CTTTTTTAACCAAGTTAATAAAGTAAGTGAGACATATAAATTCGCTACTACTTTTTTATTT

CTAATCTTGGCTACGGCTGGTATTTCTCAATTTTATCTATATTATATAGTATCAGCGTTTCT
TTTTTGGCTTGTCATACTTACCTTTGGCATTGCAGGTATCATTTTTTTAATGCCATATGGAT
TATGTTTTCTACCGTTTTATAAGCAAAAAAAGAAAAAACAGACATTTAAAAAATACATGG
TTTACACTACGATTGGTTTGTCAATTTGTCTAGGCTTATCTCTAGTTTTGGTTCACACTACG
AAAATTTATATGGACGAAGGTGGCGTAAGATACTATTACGGTAGTTTTGTAATGAAACAA
GCGGGCGGTTATGCTTATTTAGCTTTAGCGGTACTTTCAACGTTGTTAATTGTTGCGAAAA
AAGCTACAAATAAAAATAAAGAAATCGAAACCGTCGACAATACAAATATAACGGAAAGA
TAA

> **Q7A890**

Function: protein coding sequence; putative protein (genomic island)
Best match: Q7A890_CC001_MSSA476_BX571857.1[76016:79168]
Position: 006-contig_274: 8621 … 11774; Length: 3153 bp
Sequence:
ATGAGTCAATTGCTAAATGATACGTTATCGGCTTGGTTGTTAATTGAATCTTTAAGTGCAG
GAGAAGTAAATTTTACAGCGGAAGATATACTCTCAGCTGAACATTTTAAAAATGGTACAA
AGCAAGCACAGCTTCAAAGTTTTGATGAATATTTTGAAATATGGAACGATGAACGTTTTA
TTGTGTCAGAAGAAAAATCAGAGACTGGGGAACTTATATTTAAATTTTATAGACATTGCT
TCCGCTATAATGAAATTAATTTGAAAATTCAAGATATTTTTGAAAATCACTCTGATATTCA
TAATCCAAATGGGACACACTGTTATGGTTACACATTTAACACAGATAAACACGGCAAAGT
GATAGTTGATTCTATACATATTCCGATGATTATGAGTGCATTAAAAGAAATTGAAAAGAA
CAAAAATGCCAATATAGAAGAAAAATTTAATGATTCTGTTGAAAAATTTGTTCAAAAAGT
AAAAGAAATTTTAGCAGATGAACCAATTAATGAATTTAAATTGAAGAAGATGGACAAAG
CTTATGATGAGTACTTTTCTGTATTAAATTCAAAGAAAGATGGATTATTTGGACATTATGT
AGCAATAGAATATGTGAAAGATAGTGATTTACCACAGCCGGAATTTAACAGTTTCTTCAT
AAGTGATATTGAGAAAGCAAGAAAATCTCCCAACCAAACTTTAATTGATTACATTGAAGG
TGTAGAAGAAAGTCAGCGCATAGAAGTAGATGAAAATAAAGAAATGTTTGACAAATTTT
TACATCCTTCACGTTTGCCTGATGGACGATGGCCATCACAGACTGAGTTTAGATTGTCTTT
AATGCAACAACTTGCTGTAAACCAAATTACGAGTGGTAATGAAAGAATAAGTTCAGTTAA
TGGGCCACCAGGGACAGGTAAGACTACTTTATTAAAAGATATATTTGCTCATCTAGTAGT
TGAAAGAGGTAAAGAGTTAGCTAAACTAAATAATCCTAAAGATGCATTTGTCAAAACAA
AAATTCATGAAACGGATGATAAATACGTATACTTACTAAAGGAATCTATTGCCAAATATA
AGATGGTAGTCGCATCTAGTAATAATGGAGCTGTTGAAAATATATCTAAAGATTTACCGA
AAATTGAAGAAATTATAAGAAATCCCGAAAAATGTAAATTCCCTAAATATGAACAGAAT
TATGCAAATTTAGCACATGAATTAAAAGATTTTGCTGAAATAGCTGAAGGTTTGATTGGT
GAAAGTGCCTGGGGCTTATTTTCTGGAGTATTTGGAAAAAGCAGTAACATTAATAAAGTA
TTGGAGCACATGCTAAAAAAAGATGGGAATGATATTGGCTTTGCTAAATTACTACAAAAT
GAGAATAATCGTATGAGTTATAACGAGTTAATGAGTGAATGGCAATCACATCAACGTGCA
TTTTTAGAAGAGTTGAGGCATGTTGAAATGTTAAAAGAAGAATCTATTAGAGCATATGAT
GTTTATAAAAATTGTGAGTCTTTCTCTAAGATTGAACAGGTTATTAATAGTGAAAAAAACA
AGTATTGAAGAACAGGTATATCATTTAGATAATGAAACGTTACGAGACAATAAAGAAAT
AGAAGATTTGGATAATCGAATTAATTATATTGTTAAGCAAATAGAAACTTTAAATGAGTT
AATTAAATCCATCAAAGAAAGCAACAAAGGTTTTATTAACAAACTGAAAGCGATGTTTAA
TTCAGAAGAAGATGAAAGCTATAAAGATCATAATAAAGAGAAGCAACAATTATTAACAC
AACAGTTAGAGTTAGAGAAATGTAAAAAAAACAAACATGAAGACCTTGTTAGCAAACTA
AAAGAAAAAGAGAAATTAATTAAACAATTAACTAAAGTACAGTTGCAATTAGACGAGTT
AAATTCACAGTTACAAGAGTTAGAAGCATATCGTATTGAGTCAAAAATTACAATTCCAGA
AAAAGATTTTTGGAGTGACAACAATTATGATGAGCGCCAAGTTACTAATCTGTGGACGAG
TGACGAACTTCAATACAGACGTGCCATGCTCTTTTTAAGAGCAATGATATTGCATAAATT
ATTATTGATTGCTAATAATACAACTATTTATTATGCGATTAATGATTTTAAAGATAGAAGG
AAATTAATTGATGCAAATCCAGATAAAGTACACAACGCATGGAATGTGATGCATTTAATA
TTTCCAGTAGTTAGTACGACGTTTGCAAGTTTTAAATCTATGTATGGGGGCATACCAAAA
GATTTCATAGACTACTTATTTATTGATGAAGCAGGACAAGCAATACCTCAAGCAGCTGTG
GGAGCATTATATCGTTCAAAAAAAGTTGTAGCTGTAGGTGATCCGATTCAAATAGAACCG
GTTGTGACTTTAGAAAGTCATTTAATTGATAACATTCGTAAAAATTATCATGTTCCGGAAT
ATCTAGTTTCTAAAGAAGCTTCTGTGCAGTCTGTTGCAGACAACGCCAATCAATATGGTTT

TTGGAAATCTGATGCTACTGATAGTAATCAAAAAACCTGGATAGGCATACCTTTATGGGT
GCACAGACGATGTTTAAAACCTATGTTCACGATAGCTAACCAAATCGCTTATAATAATAA
AATGGTGTTGCCAAGTAATATTACAAAAGTAGGTAAAACAGGTTGGTATGACGTTAAAG
GAAACGCAGTTCAAAAACAATTTGTGAAAGAGCATGGTGAAAAAGTAGTGGGATTATTA
GCTGATGATTGGATTGAAGCAATTAAGGAAGGTAAAAATGAACCGAGCTCATTTGTAATA
TCGCCTTTTTCAGCAGTACAGCAACAGATTAAACGTATGTTAAAGCAACAACTACCGACT
AGAATTGATATTGAACGTACAAAAATTAATCAATGGGTCGATAAATCCATTGGTACTGTT
CATACTTTTCAAGGTAAAGAGGCTCAGAAGGTGTATTTTGTAATAGGTACTGATAATACC
CAAGATGGTGCTGTGAACTGGTCATGCGAAAAACCAAACTTGTTAAACGTTGCAGTGACA
AGAGCTAAGAAAGAGTTTATGTAATTGGCGACATGCAAAGAATACAGATGAAACCATT
TTATGAGACGATTTTTAAAGAAAGAAATGTAAAATAA

## > **Q2YUT2**

Function: protein coding sequence; putative protein (core genome, constant)
Best match: Q2YUT2_CC001-ST772_118_AJGE01000049.1[5998:6480] (completely identical)
Position: 006-contig_274: 11830 … 12313; Length: 483 bp
Sequence:
ATGGAGAAATTTAACAACTGGATATTAAATGCAATAAGTGGATCTCAAACAGACAAGAA
TGGAACAACTAAAGAATTAAAAGGGGCAAAATTTATCATTTTATATGCATATTCAATGCT
CGTTTTGCTTGCGTTAGTAATTTCTAACATATTCATTCACATTTTGGAGCCTAAACTATCA
ATCACCACTCAAATCATCATCGTTTTGATTTTAATTGAAGCACTAATTGGACTGCGTTTCT
TGAAAGCGTACGATGTTAAGCGTGGCAAAGATAAAGAAAATAAGAAAAATAGTAAGGAT
TTCGTTAAACTAAAATCAATTTTAGTAGCAATTTTATTTACATCATTGGCGCTGACAGCAG
GTACTGTAGCTGATATATACGGTTTCACTGACTTAGGAAATACTAGAAGTGATTTAATCG
TTTGGAGCATAGGTGGTATTATATTTGGCCTCGTATGTTACACAATGGAAGATAAAAGAT
AA

## > **plc**

Function: protein coding sequence; phosphatidylinositol-specific phospholipase C (core genome, constant)
Best match: plc_CC001-ST772_118_AJGE01000049.1[6685:7671] (completely identical)
Position: 006-contig_274: 12517 … 13504; Length: 987 bp
Sequence:
ATGAAAAAGTGTATTAAGACTTTGTTTTTAAGTATCATTTTAGTAGTGATGAGTGGTTGGT
ATCATTCAGCACATGCGTCAGATTCGTTGAGTAAAAGTCCAGAAAATTGGATGAGTAAAC
TTGATGATGGAAAACATTTAACTGAGATTAATATACCGGGTTCACATGATAGTGGCTCAT
TCACTTTAAAGGATCCATTAAAATCAGTTTGGGCAAAGACTCAAGATAAAGATTACCTTA
CCCAAATGAAGTCGGGAGTCAGGTTTTTTGATATTAGAGGTAGAGCAAGTGCTGATAATA
TGATTTCAGTTCATCACGGCATGGTTTATTTGCATCATGAATTAGGAAAATTTCTCGATGA
TGCTAAATATTACTTGAGTGCTTATCCAAACGAAACAATTGTGATGTCTATGAAAAAGGA
CTACGATAGCGATTCTAAAGTTACGAAGACATTTGAAGAAATTTTTAGAGAATATTATTA
TAATAACCCGCAATATCAGAATCTTTTTTACACAGGAAGTAATGCGAATCCTACTTTAAA
AGAAACGAAAGGTAAAATTGTCCTATTCAATAGAATGGGGGGTACGTACATAAAAAGTG
GTTATGGTGCTGACACGTCAGGTATTCAATGGGCAGACAATGCGACATTTGAAACGAAAA
TTAATAATGGTAGCTTAAATTTAAAAGTACAAGATGAGTATAAAGATTACTATGATAAAA
AAGTTGAAGCTGTTAAAAATTTATTGGCTAAAGCTAAAACGGATAGTAACAAAGACAAT
GTATATGTGAATTTCTTGAGTGTAGCGTCTGGAGGCAGCGCATTTAATAGTACTTATAACT
ATGCATCACATATAAATCCTGAAATTGCAAAAACGATTAAAGCAAATGGGAAAGCTAGA
ACGGGTTGGCTGATTGTTGACTATGCAGGATATACGTGGCCTGGATATGATGATATCGTA
AGTGAAATTATAGATAGTAATAAATAA

## > **lpl-SAOUHSC_00054**

Function: protein coding sequence; tandem lipoprotein-like (genomic island)
Best match: lpl-SAOUHSC_00054_CC008_COL_CP000046.1[91050:91823]
Position: 006-contig_274: 13724 … 14495; Length: 771 bp

Sequence:
ATGATGAAACGATTAAACAAATTAGTGTTAGGCATTATTTTCTGTTTTTAGTCATTAGTA
TCACTGCTGGTTGTGGCATAGGTAAAGAAGCGGAAATAAAGAAAAGCTTTGAAAAAACA
TTGAGTATGTACCCTATTAAAAATCTAGAGGATTTATACGATAAGGAAGGCTATCGTGAT
GATCAGTTTGATAAAAATGATAAAGGTACATGGATTATAAATTCTGAAATGGTTATTCAA
CCTAATAATGAAGATATGGTAGCTAAAGGCATGGTTCTATATATGAATAGAAATACCAAA
ACAACAAATGGTTACTACTATGTCGATGTGACTAAGGACGAGGATGAAGGAAAACCGCA
CGACAATGAAAAAAGATATCCGGTTAAAATGGTCGATAATAAAATCATTCCAACAAAAG
AAATTAAAGATGAAAAAATAAAAAAAGAAATCGAAAACTTTAAGTTCTTTGTTCAATATG
GCGACTTTAAAGATTTGTCGAAGTATAAAGATGGAGACATTTCATATAATCCAGAGGTGC
CGAGTTATTCAGCCAAATATCAATTAACTAATGATGATTATAATGTAAAACAATTACGTA
AAAGATATGATATACCAACAAATAACGCGCCGAAGTTGTTGTTGAAAGGTACAGGGAAT
TTAAAAGGTTCATCAGTTGGATATAAAGATATTGAATTTACGTTTGTAGAGAAAAAAGAA
GAAAACATTTACTTTAGTGATGGGTTAATTTTTAAACCAAGTGAGGATAAATAA

## > lplTerminator-2

Function: protein coding sequence; (genomic island)
Best match: lplTerminator-MW2-2_CC001_MSSA476_BX571857.1[83756:85132]
Position: 006-contig_274: 16558 … 16708; Length: 150 bp
Sequence:
GAACCACATAATGAAATAAAAGACAAACTAAATAAAATTACGGACTTATCTAAGAAATA
TAATTCGTATTTACAGCAAATCGAAAAAAGTATTAATGAAATAGTTGCTAAGGATCAACA
ATTGGCAGGGCAAATAGGTGATTTAATATGA

## > Q8NYT6

Function: protein coding sequence; HTH-type transcript regulator (core genome, variable)
Best match: Q8NYT6_CC001-ST772_118_AJGE01000049.1[13632:15869] (completely identical)
Position: 006-contig_274: 17600 … 19838; Length: 2238 bp
Sequence:
ATGCAACGTGATTATTTAATTCGAGTAGAAACTGAGAGTATGTCAGATTTCAAAAGGCTC
AATGGTTTAATGATTGGTTTTGTTATTAAAGGTGAGGCGCATATTTATGATGAAAATAAT
ATGACGCAATACAACAGTGGCGACATTTTCATCATTAACCACCGCGACTTGTATCGATTT
CAACTTCAACAAGAGGGCATCATATGTTATATCCAATTCCAAATGAAATATTTAGCAGAC
AAGTTTGATGATGCGCATTGTCTATATTTTCACTTAACAGATGCGACCACAACCAAGAAT
ATACATCAACTGAGAAATATAATGGCAAGACTGGTTTCAACACACATTCGACATAATGAG
TTGTCTAAATTGACTGAGCAACAACTTGTGATTCAGTTGCTTATGCATATGATTCATTATG
TCCCGCGTACATATCATTCGAACCAAAGTATCTTAAATGATGATAAAGTGAATCAAGTAT
GCGACTATATCGAGTTACATTTTCATGAAGATTTAAGCCTTTCAGAATTAAGCGAATACG
TTGGGTGGTCAGAGAGCCATCTGTCTAAAAAGTTTACAGAATCGCTAGGTGTAGGATTCC
AACATTTCTTAAATACGACGCGAATTGAGCATGCGAAACTCGATTTAACATACACAGATG
AAACGATTACTGATATTGCATTGCAAAATGGCTTTTCAAGTGCAGCGAGCTTTGCGAGAA
CATTTAAACACTTTACGCATCAAACGCCTAAACAATATCGAGGTGATCGTCCAGCAATCA
CTGAAAACCAGCAATCGTTACAACATAATTATCACGACCGTGAATTGATATTACTTTTAA
ATGACTACATTGAAGAAATGAATCATTTCATTGAAGATATTGAAAAGGTGAACTATAAAG
AGATTGCCTTTAAACCAACTAATCAACAACTAAATCAATTTAATCATATTATTCAAGTGG
GCTATTTGAGGAATTTGCTCAATACACAGTATCAATCACAGTTACTTACATGCCATCATGA
TTTTCATGTCAATGAAGTATTAGCATATGATGTGATGCCATATATTATGAAAAAGCTCAAT
GCGCCATTCACGTATGATGCAGAGATTTCGAATATATTTTATGATATCGATTTGTGTTTAG
ACTTTTTATTAGATCATAACTTTAGTTTGACAATGCATTTGGATCAGTATGACTCACGTGA
TTATATCGATGCATTCAAAGTATTTATCCATCACGTTGCCCTGCATGTCAGTCATAGAAAA
GATTTGAAGTTCAACTTGTATGTGACGACATTGCACAATTCTTTGATTGAAATGATTGATT
ATTTTAAAGCATTATTCCCCAATGGTGGCTTGTACGTTCACTTAGATCAAGCTACGGAAA
GACATCTACCATTGTTGAAACGACTTGAGCCACACATCGACCATTTTGTATTTGATGCCAA
TTCAAATGACGCTGTTGATTTTAATAAAATGAATGATGATGAATTTAAAACCGCAAGTCA
AATGATTATTAATAAAACGAATTACCTTATCGACTTAATGCATCGCCATAACCTAAAGCG

TCCACTCATTTTACTCAATTGGAATACATTGACGGGTGATACATTTATTACAAACGGCGA
ATATTTTAGAGGTGGTATCATCATTGAGCAATTATTAAAATTAAGTTCTAAAGTAGAGGG
TATCGGGTATTGGTTGAATTATGATTTGCACGTCAGTCATTGTAGAAATGAACGGGATTA
TATGAATTCTATTGAACTGTTTCATCAATATAATGGAAAACGTCCGGTCTATTTCACGGCA
TTGCTATTTAATAAATTAACAAGTAATATTTTATATTCTGATGATACATGTATTGTCACGG
GAACTGATTCAAATTTTCAAATATTGTTATATGATGCAAAGCATTTTAATCCGTACTTAGC
GTTGGACAATCAAATGAATATGCGTGCAACGGAAATGATCCATTTGAACATTAATGCCTT
GGAAGAAGGTATGTATAAGATTAAACATTTTACCTTAGATAAAGAAAATGGTGCGTTATT
TAATCTTTGGCGCAAACATCATACGATACATGGCATGGACAAGGACTCTATAGATTACGT
TAATCGAATGAGTTTTCCGAAATTAGAAGTATATGATATAGATATCACGGACACACTGGC
ATTAAACATTAAAATGATTACGAATGGGATTCACTTAATTGAAGTAAAACGTTACCCAAG
TTCATAA

> **Q8NYT5**

Function: protein coding sequence; aminoacylase (core genome, constant)
Best match: Q8NYT5_CC001_MSSA476_BX571857.1[88413:89591] (completely identical)
Position: 006-contig_274: 19988 … 21167; Length: 1179 bp
Sequence:
ATGAATCAACAATTAATTGAAACTTTAAAATCTAAAGAAGGCAAAATGATTGAGATCAG
ACGTTATTTACATCAGCATCCAGAATTATCTTTTCATGAAGATGAAACGGCGAAATACAT
CGCTGAATTTTACAAAGGTAAAGATGTGGAAGTAGAAACGAATATCGGACCACGTGGAA
TTAAAGTAACGATTGATTCAGGGAAACCTGGTAAAACATTAGCAATCCGTGCAGACTTTG
ATGCATTACCTATTACTGAAGATACAGGATTATCTTTTGCATCACAAAATAAAGGTGTTAT
GCACGCATGTGGTCACGATGCACATACAGCATACATGCTTGTATTAGCAGAGACGCTTGC
TGAAATGAAAGATAGTTTTACAGGAAAAGTCGTTGTGATACATCAACCAGCTGAAGAAG
TACCACCAGGTGGTGCTAAAGCAATGATTGAAAATGGTGTATTAGACGGTGTTGATCATG
TATTAGGTGTACACGTCATGAGCACAATGAAAACAGGTAATGTGTATTACAGACCTGGTT
ATGTTCAAACAGGACGCGCATTTTTCAAATTGAAAGTTCAAGGTAAAGGTGGTCATGGTT
CATCACCCCATATGGCCAATGATGCCATTGTTGCAGGTAGCTACTTCGTCACAGCGTTAC
AAACAGTTGTATCTAGACGACTAAGTCCATTTGAAACCGGTGTTGTCACAATCGGTTCAT
TTGACGGTAAAGGTCAATTCAATGTCATTAAAGATGTTGTTGAAATTGAAGGTGATGTAC
GTGGATTAACAGATGCTACAAAAGCAACAATTGAAACAGAAATTAAACGTTTATCAAAA
GGATTAGAAGCATTGTATGGTGTAACTTGCACATTAGAATATAACGATGATTATCCTGCA
TTATATAATGATCCAGAGTTTACTGAGTACGTGGCTAAGACGTTAAAAGAAGCAAACCTT
GATTTTGGTGTCGAAATATGTGAACCACAACCACCTTCAGAAGACTTTGCATACTATGCT
AAAGAACGTCCAAGTGCCTTTATTTATACAGGTGCAGCTGTGGAAGATGGTGAAATTTAC
CCACATCATCATCCTAAATTTAACATTTCAGAAAAATCATTACTTATTTCGGCAGAAGCTG
TAGGGACAGTTGTTTTAGATTACCTTAAAGGAGATAACTAA

> **norC**

Function: protein coding sequence; multidrug efflux pump (core genome, constant)
Best match: norC_CC001-ST772_118_AJGE01000049.1[17200:18588] (completely identical)
Position: 006-contig_274: 21168 … 22557; Length: 1389 bp
Sequence:
ATGAATGAAACGTATCGCGGGGGCAACAAGTTAATCTTAGGTATTGTATTAGGTGTTATT
ACATTTTGGTTGTTTGCACAATCACTTGTAAATGTTGTACCAAATTTACAACAAAGTTTTG
GTGCAGACATGGGAACAATTAGTATTGCGGTAAGTCTAACCGCACTATTTTCAGGCATGT
TTGTTGTTGGAGCAGGCGGCCTAGCAGATAAAATTGGGCGCGTGAAAATGACGAATATC
GGTTTATTGTTAAGTATTATTGGTTCAGCATTAATTATTATTACGAATTTACCGGCATTATT
AATTTTAGGTCGTATTATACAAGGCGTATCAGCAGCGTGTATTATGCCTTCCACATTGGCC
ATTATGAAAACTTATTATGAGGGTGCTGAACGTCAGCGCGCCTTAAGCTACTGGTCTATC
GGTTCTTGGGGTGGAAGTGGTATCTGTTCACTCTTCGGTGGGGCAGTTGCGACAACTATG
GGTTGGAGATGGATTTTCATCTTCTCAATTATCGTTGCCGTACTTTCAATGTTACTCATCA
AAGGGACGCCTGAAACGAAATCAGAAGTTACCAATACACATAAATTTGACGTTGCAGGG
CTAATTGTTCTAGTAGTTATGTTGCTAAGTTTAAACGTTGTCATTACTAAAGGTGCAGCAC

TTGGTTACACATCATTATGGTTCTTTGGTTTAATTGCAATCGTAATTGTAGCATTCTTTATT
TTCTTAAAAGTTGAGAAAAAAGTAGATAATCCGCTTATTGATTTTAAATTATTTGAAAAT
AAACCATATACAGGTGCAACGATTTCGAACTTCTTATTAAACGGTGTTGCAGGTACATTA
ATTGTAGCGAATACATTCGTGCAACAAGGTTTAGGTTATACAGCATTGCAGGCAGGATAC
TTATCAATTACTTATTTAATCATGGTGTTATTGATGATTCGAGTTGGTGAAAAATTATTAC
AAAAAATGGGTTCTAAGCGACCAATGTTATTAGGTACATTCATTGTGGTCATTGGTATTG
CACTTATTTCATTAGTATTCTTACCAGGCATATTTTATGTTATCAGTTGTGTCGTAGGATAT
TTATGTTTCGGACTAGGCTTAGGTATTTATGCAACACCTTCTACAGATACAGCTATTTCAA
ATGCACCGTTAGATAAAGTTGGCGTTGCTTCAGGTATTTATAAAATGGCTTCATCACTTGG
TGGTGCATTCGGTGTCGCAATTAGTGGTGCAGTATATGCCGGTGCAGTTGCTGCAACGAG
CATTCATACAGGTGCGATGATTGCACTTTGGGTTAACGTATTAATGGGAATCATGGCATTT
ATCGCAATTTTATTTGCGATTCCTAATGATGATAAACGTGTCAAAGATGCGAAATAA

## > txbi_universal1

Function: bidirectional rho independent terminator
Best match: txbi_universal1_CC022_HE681097.1[2619148:2619217:r] (completely identical)
Position: 007-contig_191: 14 … 84; Length: 70 bp
Sequence:
TGTATAAATATAGAACAGCAGTAAGATATCTTCTAATTGAAAATTATCTTACTGCTGTTTT
TTAGGGATT

## > Q1Y1J7

Function: protein coding sequence; putative Na+/Pi-cotransporter protein (core genome, constant)
Best match: Q1Y1J7_CC009_21334_AGTW01000007.1[298140:299801:r]RC (completely identical)
Position: 007-contig_191: 159 … 1821; Length: 1662 bp
Sequence:
TTAAATTTCAGTTGTTGCAATTTCTTCATCTGTAGGTACATCATCGTTAAGGCCAACAAGT
GCTTCAGAAACATTTCGTGAATGATAACCGATACGTTCAAGAACACCAATCATATCGATA
TATAGTAATCCGCCTTTTGTTGTACATTCACCACGATTAAGGCGTTTAATATGACCTTTGC
GTAGTTTATGTTCAATATTAAATGATTCTCTACTACGTTCTACAATTTCATCTTTTTTCGTT
TTGTCATAAACATCTAACATGTCGATGGCTTTATCAAATGACTCAGCAACATGGTTGAAT
AATTTATCCATACCGCGTTGTGCATCTTCTGTAATGCGAATATCTTCATCATGTTGGCGTT
TTAATTGAGCGACATACTCTTCTGTTAGCTCTGCTACTTTTAAAATAGAGCGATTGACATC
AAACATAACTGCTAAACGCTCAACGTCAGCCTTCGTTATGGCTTTTGTAGAAATTCTAACT
AAATAATTTCGAATGCTATCATTGATTGTTTCAACAGCTTGGTGCTTTTGTTCAAGCTTTTT
GATCAATTTTTTATCGTCTTTTGTAATTTCGCGAATGTCTTCAAACATTGATAAGACAATC
TGACCAACATTTTGTAATTCTTTTTGAGTTTCTTGTAATGCAACACCAGGTGCGTGATAAA
CAAGATCTTTGTTTAAGTGCTGAGGTTTATAGTCATCAGCAATATCTTTACCTGGGACAAG
CTTTGTAACAATCCATGCTAAACCTGCTACAAATGGTAATTGAATCAAAGTATTTGTTATG
TTGAAGATACCATGTGATACTGCAATCGTCATCGCTGGTTTTAAGTGCCATACATCTTGTA
ACAAACTAATCAAATGAATCACAACTGGCAAGAAAATTGTGAAGATAATTACCCCGATT
AAGTTAAAGATGACGTGTACAAGCGCCGCACGTTTTGCAGCGATTGAGCCGGCTAAACTA
GCTAAGATAGCTGTAATCGTTGTACCAATATTATCGCCTAGTAACACAGGGATTGCTGCG
TTTAAGCTAATTAAATCTTGTTGATAAAATTCTTGTAAAATACCAATCGTCGCACTTGAAC
TTTGAACTAGTGCTGTTAACCCTGCGCCGACAATGACAGCAAGTATTGGATTTGTAGACA
TATCAAGCATTAATTGCTTAAATCCATCTAATGATGCTAAAGGTTTAACGGCATCACCCAT
AAATTCTAGACCGAAGAATAGAGAACCGAAACCGAATAGTATGCGGCCAATGTTATTGA
TTTTAGAGCGTTTAAAGAAAAAGATTAAAAATGCACCTAATGCTAAAATTGGCATTGCAT
ATTCGCCTAAATCTATACCGATAATAAATGCGGTTACCGTTGTTCCGATATTGGCACCCAT
TATCACTCCAATAGCTTGTTTTAACGTCATAAATCCAGCTGTTACCAGTCCGATTGTGATA
ACGGTCGTACCTGAACTACTTTGTATTAAAATAGTTACAACGATACCTGCAATAACACCT
AATACTGGATTTGATGTAAATTTGTTTAAAATATCTCGTAGCCTGTCTCCTGCTGATGCTT
GAAGCCCGTCTCCCATGATTTTTAAGCCGTAAAGGAAAATACCTAAACCACCTAAAAAGG
AGAAAATGACTTCTGTAACCGACAT

> **Q2YUS5**
Function: protein coding sequence; 67 kDa myosin-crossreactive streptococcal antigen homologue (core genome, constant)
Best match: Q2YUS5_CC001-ST772_118_AJGE01000014.1[33670:35445]RC (completely identical)
Position: 007-contig_191: 2144 … 3920; Length: 1776 bp
Sequence:
TTATAACAATTTGTGTTCTTTTAATAATGTCTCAATGTACGTACCTTTTACCTTTTTAAGGA
ATCCAGCTAATGCGAGTTTTTGTATTTTCGAATCTTTAGTAATCTCGCGCAAATCTTGATG
GTCATTCAGTTCGTATACGGCATCCATTAAGACGCGAAGATCAAATGTACTATTGATGAC
CTCTGGAATACCACGATCTATATTTAGTAATTGATAAACAGCTTCCATGGCAGTACGAAC
CGAATATTCTGTTGTAAATACAGTGTCTCGCTCTGTTTCTGCAAAGTTACCAATAAATGCT
AAATTTTGTGATTGATGCGGAACGACTAAAGGTCTGTCGCCGATAGCACGCGTCATGAAA
TAAGAGGTAATATATGGCATATAAACAGGAATCGTATTAGATGCATGTTTTGCTAAGTCT
TCAATTTTGTCAGTTGGTACACCTAAGTGATATAGCCATTCTTGACATATTTCATTACCAC
TACATTCTGTGATTGGCTTTTTAATATAATCGCCGTTTACATCTGAATATAAAGCATAAAT
CCACGTAGATATTTCATTTTTAGGTTGGTCTTTAAACTGTTGCTGACGATTGATTGTAAAA
CTGATTTGCCATGCAGAATCATTGATTGTAATAATTCCGCCAGTAACCGTTTTGCCTGCAA
GAGGGTCACGTTTACAAATGCTTTCTATTGTATCGATAATATCTTTATTGTTTGTTGTCGAT
GTTGCAGAAACAAACCAACTCTTTTGAGGAATATTTTGGCAAAACTTATCAGGATTACCA
AATTCAGGACTTTGTCGCGCTAAATTTTTCCATAGTGTCCAACTACCACCTAATTCGTCAG
TTGGTGGTGCTGGTGTATCATTATCACCATACGTAGAGCTTTCCGTAATACTACCGTTTGT
CACAAAGACAAGATCATCTACAGTCAGTTTAATTGACTCTGCTTTGCCATGTCGGTGAATT
AATATTTCTCGGGCAATTTTTTGACTTGTCGTAACATCTACTTTAATATCTTCTACTTTTAC
ATCGTATTCAAATTGAACCCCATGCGATTTTAAATATTCAACCATAGGTAATACTAAAGA
TTCATATTGATTATATTTAGTGAATTTTAAAGCTGAAAAGTCTGCGAGACCACCAATATG
ATGAACGAATCGCATTAGATAGCGACGCATTTCCATAGCAGAATGCCACGGTTCAAATGC
AAACATCGTTTTCCAGTAAATACAAAAGTTTGAATTAAAGAAGTCATCGGAAAATACATC
TGTTATTTTGACATCATCTAAATCTTCTTCATTCGTTAAGCATAAATCTAAAATTTCTTTAA
TCGCTTTTTTGGTCAAAGTGAAGTCTCCATCGGTAACTAAACGTTGACCCTGTTTTTCAAT
AACTCGACAGCGAGAATAGTTAGGGTCTTCTTTGTTTAGCCAATAGAACTCATCTAATAC
AGACGCGTTATCGATTTCTAATGAAGGGATAGATCTGAATAAGTCCCACAAACATTCAAA
GTGGTTCTCCATTTCACGACCACCTCGGACAACATAGCCTTTTAAAGGCATATTTTCACCA
TCAAGACTACCACCTGCTTTAGGTAACTCTTCTAAAATATGAATCTTCGAACCTTCCATTT
GACCATCCCTAATTAAGAAACAAGCTGCCGCAAGTGAAGCTAACCCAGACCCGATTAAA
TAAGCAGATTTGTTTTCTACATTTTCAGGTTTTTTAGGGCGCGCAAATGCTTCATAATTTC
CATAACTGTAATACAT

> **A5INY0**
Function: protein coding sequence; putative protein (core genome, constant)
Best match: A5INY0_CC009_21334_AGTW01000007.1[302251:302724] (completely identical)
Position: 007-contig_191: 4270 … 4744; Length: 474 bp
Sequence:
ATGTCTAAAATCAGGTCTTTTACAATATTAAGTCTACTTATTTACTTAGCTATGATGTGCT
ATACAGTAGTGACCTATTCAAAATTACCAACCAAAGTACCTATTCATTATAATTTAGCAG
GGGATGCTGATAACTTTGCTGATAAATGGGTACTGCTTTTGATAAATAGCGCATTTATAGT
GATTTGGCTTATATTTTTCATTGCAGGTAGATACTATGAACGATTTGCCAAATGGTCACAT
TATAATCATACACCACGTGAAATTCGAGCGATTAAATTATTTTTAAGTACGTTAAATTTAG
AGATTATGAGCTATATGTCTATCTTCACAGTATTAGAAATTTGGCAAATACAACACCATC
ATCAATTAAATTTACTATGGTTTAATATGATATTTATCATTATCATTGGTTTGACGCTTGTC
ATATTTTGTTTACTTCCTACAATTCACAAAATGAGAGATTCTCAATAA

> **lctP-L1**
Function: protein coding sequence; L-lactate permease (core genome, variable)
Best match: lctP-locus1_CC009_21334_AGTW01000007.1[302986:304578] (completely identical)
Position: 007-contig_191: 5005 … 6598; Length: 1593 bp

Sequence:
ATGACACTACTTACTGTAAATCCATTCGATAATGTTGGATTATCAGCCTTAGTTGCAGCAG
TACCTATTATTTTATTTTTATTATGCTTAACCGTTTTTAAAATGAAAGGCATTTATGCAGCA
TTGACAACTTTGGTTGTTACATTGATTGTGGCTTTATTTGTATTTGAATTACCAGCGCGTGT
ATCAGCAGGTGCGATTACAGAAGGCGTTGTTGCCGGTATTTTCCCAATAGGATATATCGT
TTTAATGGCAGTTTGGTTATATAAAGTTTCTATTAAAACAGGACAATTTTCTATTATTCAA
GATAGTATTGCAAGTATTTCAGTGGACCAAAGAATCCAACTATTATTAATTGGATTTTGTT
TCAACGCATTTTTAGAAGGTGCAGCAGGATTTGGTGTGCCAATTGCGATTTGTGCAGTATT
ATTAATTCAACTTGGATTTGAACCATTAAAAGCAGCGATGTTATGTTTAATTGCTAATGGT
GCGGCGGGTGCCTTTGGTGCAATTGGTTTACCAGTTAGTATTATTGATACGTTTAACTTAA
GTGGAGGCGTTACAACATTAGATGTTGCGAGATACTCAGCATTAACACTTCCAATTTTAA
ACTTTATTATTCCATTTGTTTTAGTATTCATTGTAGATGGTATGAAAGGTATTAAAGAAAT
TTTACCTGTCATTTTAACAGTGAGTGGTACATATACTGGATTACAATTATTATTAACAATA
TTCCATGGTCCAGAACTAGCAGACATTATTCCATCACTAGCAACAATGGTGGTGTTAGCA
TTTGTTTGTCGTAAATTTAAACCGAAAAACATTTTCAGATTGGAAGCGTCTGAACATAAA
ATTCAAAAACGAACGCCTAAAGAAATTGTCTTTGCTTGGAGTCCGTTCGTAATTTTAACTG
CCTTTGTATTAGTATGGAGTGCACCATTCTTCAAAAAATTATTCCAACCTGGAGGTGCACT
TGAAAGTTTAGTAATAAAATTGCCAATTCCAAATACTGTGAGTGATTTATCGCCTAAAGG
AATTGCGTTGCGTCTCGATTTAATTGGTGCAACTGGGACAGCGATTTTATTAACAGTAATT
ATTACAATTTTAATTACGAAGTTAAAATGGAAAAGTGCAGGTGCTTTATTGGTCGAAGCA
ATTAAAGAATTATGGTTACCGATCCTTACAATTTCAGCTATCCTAGCTATTGCTAAAGTTA
TGACATACGGTGGTTTGACTGTAGCAATTGGACAAGGTATTGCTAAAGCGGGAGCAATTT
TCCCATTATTCTCTCCAGTATTAGGTTGGATTGGTGTGTTTATGACGGGTTCAGTTGTAAA
TAACAATACTTTATTCGCACCTATTCAAGCGACAGTGGCACAACAAATTTCAACAAGCGG
TTCATTACTTGTGGCAGCTAACACTGCAGGTGGTGTAGCAGCGAAACTTATTTCACCACA
ATCAATTGCCATTGCGACTGCAGCTGTTAAAAAAGTTGGTGAAGAATCTGCATTATTAAA
AATGACGCTAAAATACAGTATTATATTTGTTGCTTTTATTTGTGTTTGGACGTTTATACTA
ACGTTAATATTCTAA

## > txbi_lctP

Function:  bidirectional rho-independent terminator of lctP (core genome, constant)
Best match: txbi_lctP_CC001_MW2-USA400_BA000033.2[99244:99315] (completely identical)
Position: 007-contig_191: 6641 … 6713; Length: 72 bp
Sequence:
CTAATTATTCATGAATCGAACTAGTACGAAATGCATTGAGCATCTTGTCTAGTTCGATTTT
TTAATGTCTAA

## > txbi_proteinA

Function:  bidirectional rho-independent terminator of protein A (core genome, constant)
Best match: txbi_proteinA_CC705_RF122-cow_AJ938182.165518:65579
Position: 007-contig_191: 6772 … 6838; Length: 66 bp
Sequence:
AAGTAAGTAAAATAAAAAATGCACTGAGCAACAAAAGATGTTGCTAGTGCATTTAGATG
ATTCTTA

## > spa

Function: protein coding sequence; immunoglobin G binding protein A chv
Best match: spa_CC001-ST772_118_AJGE01000014.1[29545:30663]RC
Position: 007-contig_191: 6926 … 8417; Length: 1491 bp
Sequence:
TTATAGTTCGCGACGACGTCCAGCTAATAACGCTGCACCTAACGCTAATGATAATCCACC
AAATACAGTTGTACCGATGAATGGATTTTCTTCACCAGTTTCTGGTAATGCTTGAGCTTTG
TTAGCATCTGCATGGTTTGCTGGTTGCTTCTTATCAACAACAAGTTCTTGACCAGGTTTGA
TCATGTTTTTATCAGCTAATTTGTTATCTGCAGCAATTTTGTCAGCAGTAGTGCCGTTTGCT
TTTGCAATGTCATTTACTGTATCACCAGGTTTAACGACATGTACTCCGTTGCCGTCTTCTTT

ACCAGGTTTTTTGTTGTCTTCTTTGCCAGGCTTGTTGCCATCTTCTTTACCAGGTTTTTTGTT
GTCTTCTTTACCAGGCTTGTTGCCGTCTTCTTTGCCAGGCTTGTTGTTGTCTTCTTTACCAG
GTTTGTTGCCGTCTTCTTTACCAGGTTTTTTGTTGTCTTCCTCTTTTGGTGCTTGAGCATCGT
TTAGCTTTTTAGCTTCTGCTAAAATTTCTTTGCTCACTGAAGGATCGTCTTTAAGGCTTTGG
ATGAAGCCGTTACGTTGTTCTTCAGTTAAGTTAGGTAAATGTAAAATTTCATAGAAAGCA
TTTTGTTGTTCTTTGTTGAAATTGTTGTCAGCTTTTGGTGCTTGTGCATCATTTAGCTTTTTA
GCTTCTGCTAAAAGGTTAGCGCTTTGGCTTGGGTCATCTTTTAAGCTTTGGATGAAACCAT
TGCGTTGTTCTTCGTTTAAGTTAGGTAAATGTAAGATTTCATAGAAAGCATTTTGTTGTTC
TTTGTTGAATTTGTTATCCGCTTTCGGTGCTTGAGATTCATTTAACTTTTTAGCTTCTGACA
ATAGGTTAGCACTTTGACTTGGGTCATCTTTTAAGCTTTGGATGAAACCATTGCGTTGTTC
TTCGTTCAAGTTAGGCATGTTCAAGATTTCATAGAAAGCATTTTGTTGTTCTTTGTTGAAA
TTGTTGTCAGCTTTCGGTGCTTGAGATTCGTTTAATTTTTTAGCTTCACCTAAAACGTTAGT
GCTTTGGCTTGGATCGTCTTTAAGACTTTGAATGAAACCATTGCGTTGCGCTTCGTTTAAG
TTAGGCATGTTCAAAATTTCATAGAAGGCGCTTTGTTGATCTTTGTTGAAGTTATTTTGTT
GCGCATCAGCTTTTGGAGCTTGAGAGTCATTAAGTTTTTTAGCTTCACCTAAAACGTTAGC
ACTTTGGCTTGGATCATCTTTAAGGCTTTGGATAAAACCATTGCGTTGATCAGCATTTAAG
TTAGGCATATTTAAGACTTGATAAAAAGCATTTTGTTGAGCTTCATCGTGTTGCGCAGCAT
TTGCAGCAGGTGTTACGCCACCAGATATAAGTAATGTACCTAAAGTTACAGATGCAATAC
CTACACCTAGTTTACGAATTGAATAAATGTTTTTCTTTTTCAAATTAATACCCCCTGTATGT
ATTTGTAAAGTCATCAT

## > **tx_sarS**
Function:  rho independent terminator of sarS (core genome, constant)
Best match: tx_sarS_CC001_MW2-USA400_BA000033.2[101369:101431:r]RC (completely identical)
Position: 007-contig_191: 8742 … 8805; Length: 63 bp
Sequence:
TTAAAAAATAGAAAACACAAGTGCACTTTAGAATACAGCACACTTGCGTAATAGAGATA
TTAT

## > **sarS**
Function: protein coding sequence; HTH-type transcript regulator (core genome, constant)
Best match: sarS_CC001_MW2-USA400_BA000033.2[101428:102180:r]RC (completely identical)
Position: 007-contig_191: 8801 … 9554; Length: 753 bp
Sequence:
TTATTCAAAAACAAGATGTAAATGATCTTTATCTGCTAATAATTGATTCACTTGAGCTAAT
AATTGTTCAGCATGGTCTTGCTGCGCGTCATCCATATGAATTAAAATTTTTCTTTCATCTTC
AGTTGAGCGTTCTTTTATTAGATAGCCTTGCTTTTTTAAATTATTGAGAGCTCTAACAGTTT
GAGGGTATTTATGGTGGATTGTTTCAATTAAATCTTTAAGAAGAACGATGTTTTTATTTTG
AGAAGTGATAATAGCTAGAATTGTGAATTCTACAAAACTTAATGTTAGATGTTTTTTGAT
AATATTCTTGAAATACATTGTATACATCATCAAGTTTAGAAATTCTTTACTATCTTTTGGT
ATCATCTGTGATTCACTTTGATCTGCAAGGTTAAATTGTTTAATGATTTGATCAAACAATG
TAACACGTTCTGCAATTTTCTCTCGTTGTTCTTCAGATATTGAAATGTAAGTATTACGCTC
ATCAATTTTACTTCGAACTTTACTAATATATGAATGTTTCACAAGTACTTTTATATGCTGT
ACTAAATCCGATTGTTTATAACATAAATCTGAAACAATCTTCTTAAATGGAAGTGTGTTTT
CTTGCTGATGAAATAAATAAGTCAGTAATATAAATTCTTTTATAGTCATATCGACTTCAGG
CTTGACTTTTTTCTTAAAACGAAACATATATGCTTCAATGATTATAAAATCTCTAATTTTG
TCATGGTTATTATATTTCAT

## > **sirC**
Function: protein coding sequence; siderophore 'staphylobactin' ABC transporter/permease (core genome, constant)
Best match: sirC_CC005_ED98-hen_CP001781.1[88085:89083:r]RC
Position: 007-contig_191: 9922 … 10921; Length: 999 bp
Sequence:

TTATAACTTTTTCACGGTTAATAATAAGTATATGAAGAATGGGGCACCAAAAGCAGCAAT
AAATACACCTGCTGGCACTTCTTTAGGCAAGAATAAGGTACGCCCAATTAAGTCTGCAAT
AACAATTGATATGGCACCAATCATTGCTGACATTAGTAACTTTTTAGCATAACTTCCGCGA
ACGATTGTTTTCGCGATATGTGGTGCGATTAAACCGACAAACCCAATGTTACCTACTAAA
CTGATTGCCATAGATACGAGTATAGTAGAAGTGATTAATTGGATTAGTTTCATACGTTGT
ACATGTAAGCCTAAGCCAATCGCTACAGGGTCATCAAGTATAGATATTTTCATTTTTGGTA
TAACAAGAAATAACAACGGCACAACAGCTAAAATAACCATACCCAAAATGATTGTATCT
TTAAACGTAGCACCGTAAAGACTTCCGACTAGCCATGTATAAGCTTTGGCAGCAGATAAT
TGCTTCGTTGTAATGAGTAATCCTTGGACAAGCGCAATAAACAACGTTTGCATCGAAATA
CCGATGATTATGAGTGTTGTCGGGCGTATTTGTCCTTTCGTTTGAAACACTAATAGTATCA
TCATTGCAACTGCGCCACCTAATACTGCAAATAGTGAAAGTAAATGTATCGTTAAATGGC
TGAAAAATGCAATAAAGACAACAGCACTTAAGCTAGCACCACCTGTGATACCGATAATA
TCAGGTGAGGCAATTGGATTTTTTAATACATTTTGCAACATTAAACCACTCATTCCTAGTG
CGGCACCTGCTAAAATCGCAAGTGTAATGCGAGGTAAGCGTAATACTTCTAAAGTGAATT
GATCCATACTGTCATTTGGATTTATAAAGTACATCAGTACGCGTTGTAATGGTATAAAGCT
TGAACCAATCATCATACTTACCACTGAAACGATGGCTAAAAAGATTAACGCGAAGATGA
GATGGTAATTGTCTTTTTTATTAATCTTTTCGGTCAT

## > **sirB**

Function: protein coding sequence; siderophore 'staphylobactin' ABC transporter/permease (core genome, constant)
Best match: sirB_CC005_Mu50-VRSA_BA000017.4[126499:127494:r]RC (completely identical)
Position: 007-contig_191: 10917 … 11913; Length: 996 bp
Sequence:
TCATAAGCGTTGACGTCCTTTCTTCATAATATAGATTAAGACAATAGCGCCAATGACAGC
GGTAACGACACCGATAGGCAACTCTAGTGGCTTAATTATTATACGAGCAACAATGTCTGA
AATGATCATTAGGATTGCTCCAGCTAATGCAGTAAAAGGAATTAAATACTTATAGTTTGG
TGGTAATAATCGTTTGCTAATATTCGGTACGATAAGACCCACAAAGACGATTGATCCAGC
TACGGCTACCGAAATACCGGCTAACATACTGATGAGCATAATAATCATCCATTTGATTAA
TTTTATGTTTTGACCGAGGCCGGTTGCAATGTCGTCACTTGTCATCAAGATGTTGATGTGT
GCAGCCATGCTAAATGCAATTAAAATAAGTATCAATACAAGCGGAATAATCCATGGGAT
ATCCCAAATATTACGTAATGAAACGGAGCCACTTAACCAAAATAATAGGCCTTGTAAGTC
TGTTTCGTTCATAATAAGTATGCCTTGAGTAAAGGCTGTAAATAGCATCGCAATCGCAGC
ACCTGCCAAAATGACACGGTGAGGTGAGAATAGTGTTTGTCTAAACATACCTAGCGCAAC
AACTAATATAGTAACAACAATGGCACCTAAAAATGCAATAACTACAATCATTTTAAAAGA
TTGAATTTGGATAAATGTAATACTAAAAATGACAAAAAATACTGCGCCTGCATTGACACC
GAAAAGCCCTGGTGAGGCTATTGGGTTTCGTGTAAGTGCTTGCATCAACAAACCTGAGAC
AGCAAGGGCAGCACCAGTCAATAACGCAATGATTGTTCTCGACGCCCGTGCACCAGTGAC
AACATCATGTAAATCGTTTTCACTATCAAAGTTGAATAACGCCTGTATCACCGTACCTGGT
GACACAAGCGTATTTCCAATCATTAAACTTAAGATAGCTACTATTGCAAGACATAAACCA
GCAATAACGATTTGGTATTTTGGTTTAAGTAGCAT

## > **sirA**

Function: protein coding sequence; siderophore 'staphylobactin' ABC transporter/substrate-binding protein (core genome, variable)
Best match: sirA_sabac-114_AC025949.4[1:518]RC (completely identical)
Position: 007-contig_191: 11928 … 12446; Length: 518 bp
Sequence:
TTATTTTGATTGTTTTTCAATATTTAACTTTTCATATAAATCGTCAATAAGTTTTAATGAAG
ATTTATATCCGCCAGCTAAGTTCCAAGTGATTTCATCTAAATCATCAGATACTTGGTTGTT
TTTAACTGCGTCTAAATTTTTCCACTCTTTACTTGAAGTCCATTCGCTTTCAGTCTTTTTAA
CTAATGCAGCATCTTTCGCATTTGGATCTGATTTTACTACAAAAATATGATCAGCGTTCAT
TAATGGAATGCTTTCTTTAGATGTAAGTTGGATAATATCTTTACCATTATCAACTTGTTTTT
GTAAGTCTTTATTACGTTTGAATCCTAAATCATTTAAGATTTCACCAGCATATCCACCAGC
ATAAATTCTTGTATGATCAGCACGGAAGTTAACAACTGAAGCTTTCAATGGCCATGCATC

TTTATACTTTGCTTTTGCATCTTTTTGGAATGCAGCTACTTTATCATCGTACTTTTTAAGTA
AATCTTCAGCTTCTTTTTCTTTCCCTA

> **sbnA**
Function: protein coding sequence; siderophore biosynthesis protein A (core genome, constant)
Best match: sbnA_CC001-ST772_118_AJGE01000014.1[23830:24810:r] (completely identical)
Position: 007-contig_191: 13151 … 14132; Length: 981 bp
Sequence:
TTGATTGAAAAAAGTCAAGCATGTCACGATTCATTGTTAGATTCTGTAGGGCAAACACCT
ATGGTTCAACTTCATCAACTATTTCCGAAACATGAAGTGTTTGCAAAGTTAGAGTATATG
AATCCTGGAGGCAGCATGAAAGATCGACCTGCCAAGTACATCATTGAACATGGTATTAAA
CATGGTTTAATCACTGAGAATACACATTTAATTGAAAGTACTTCTGGTAATTTAGGCATTG
CGTTGGCAATGATATCTAAAATCAAGGGATTAAAACTCACGTGTGTTGTTGATCCTAAAA
TATCACCAACAAATTTGAAAATTATTAAAAGTTATGGTGCCAATGTAGAAATGGTTGAAG
AACCTGATGCACATGGGGGTTATTTAATGACTCGTATTGCAAAGGTGCAAGAAATGTTAG
CCACTATTGACGATGCATATTGGATTAATCAATATGCGAATGAGTTAAATTGGCAATCCC
ATTATCATGGTGCAGGCACAGAGATTGTTGAAACAATTAAGCAACCTATAGATTATTTTG
TCGCGCCAGTCAGCACGACAGGTAGCATTATGGGTATGAGTAGAAAAATAAAAGAAGTG
CATCCAAACGCACAAATTGTTGCTGTTGATGCGAAAGGGTCAGTCATTTTTGGTGACAAA
CCTATTAATAGAGAATTACCTGGTATCGGTGCTAGTCGTGTACCCGAAATATTGAATAGA
TCAGAAATTAATCAAGTGATCCATGTAGATGATTATCAATCTGCTTTGGGCTGTCGAAAA
CTGATTGATTATGAAGGCATATTTGCCGGAGGTTCAACAGGCTCGATTATTGCAGCGATT
GAGCAGTTGATAACGTCAATTGAAGAAGGTGCAACAATTGTCACGATTTTACCAGATCGA
GGCGATCGTTACTTAGATTTAGTTTATTCAGATACATGGTTAGAAAAAATGAAATCAAGA
CAAGGAGTTAAATCAGAATGA

> **sbnB**
Function: protein coding sequence; siderophore biosynthesis protein B (core genome, constant)
Best match: sbnB_CC001-ST772_118_AJGE01000014.1[22823:23833:r] (completely identical)
Position: 007-contig_191: 14128 … 15139; Length: 1011 bp
Sequence:
ATGAATAGAGAGATGTTGTATTTAAATAGATCAGATATTGAACAAGCGGGAGGTAATCAT
TCACAAGTTTATGTGGACGCATTAACAGAAGCATTAACAGCCCATGCGCACAATGATTTT
GTACAACCGCTTAAGCCGTATTTAAGACAGGATCCTGAAAATGGACACATCGCAGATCGA
ATTATTGCAATGCCAAGTCATATCGGTGGTGAACACGCAATTTCAGGTATTAAGTGGATA
GGTAGTAAGCACGACAATCCATCGAAACGTAATATGGAGCGTGCAAGTGGTGTCATTATT
TTGAATGATCCAGAAACGAATTATCCAATTGCAGTTATGGAAGCAAGTTTAATTAGTAGT
ATGCGTACTGCAGCAGTTTCAGTGATTGCAGCTAAGCATTTGGCTAAAAAAGGATTTAAA
GACTTAACAATCATTGGATGCGGGCTAATCGGAGACAAGCAATTACAAAGTATGTTAGA
GCAATTCGATCATATTGAACGCGTGTTTGTTTACGATCAATTCTCTGAAGCATGTGCACGC
TTTGTTGATAGATGGCAACAACAGCGTCCGGAAATTAATTTTATTGCGACAGAAAATGCT
AAAGAAGCAGTATCAAATGGTGAAGTAGTCATTACATGTACCGTAACGGATCAACCATA
CATTGAATATGATTGGTTACAAAAGGGTGCATTTATTAGCAACATTTCTATCATGGATGTG
CATAAAGAAGTCTTTATTAAAGCTGACAAAGTCGTAGTAGATGACTGGTCACAATGTAAT
CGAGAAAAGAAAACTATTAACCAATTGGTGTTAGAAGGTAAATTCAGCAAAGAAGCACT
TCATGCTGAACTAGGACAACTTGTGACAGGTGACATACCAGGACGTGAAGACGATGATG
AAATCATATTACTTAATCCGATGGGTATGGCTATCGAAGATATTTCAAGTGCTTACTTTAT
TTATCAACAGGCACAACAACAAAATATTGGGACAACATTGAACCTATATTAA

> **sbnC**
Function: protein coding sequence; siderophore biosynthesis protein C (core genome, variable)
Best match: sbnC_CC001-ST772_118_AJGE01000014.1[21048:22802:r] (completely identical)
Position: 007-contig_191: 15159 … 16914; Length: 1755 bp
Sequence:

TTGCAGAATCATACAGCAGTCAATACAGCACAAACGATAATATTAAGAGATTTAGTTGAT
GCATTATTATTTGAAGATATAGCCGGTATTGTATCGAATAGTGAGATTACTAAAGAAAAT
GGACAAACCATTTTGATATACAAACGTGAAACACAGCAAATAAAGATACCTGTTTATTTT
AGTGCTTTAAATATGTTTCGTTATGAAAGTTCACAACCAATTACGATAGAGGGAAGGCTG
TCTAAGCAACCTTTAACGGCAGCTGAATTTTGGCAAACAATTGCTAATATGAATTGTGAT
TTAAGTCATGAATGGGAAGTGGCTCGCGTTGAAGAAGGACTGACTACTGCTGCCACACAG
CTTGCTAAACAATTATCAGAATTAGATTTAGCGTCACATCCTTTTGTGATGTCAGAGCAGT
TTGCAAGTTTAAAAGATCGTCCATTTCATCCATTAGCTAAAGAAAAAAGAGGATTAAGAG
AAGTGGATTATCAAGTGTATCAAGCTGAATTAAATCAATCATTTCCTTTAATGGTTGCAGC
AGTTAAAAAGACACATATGATTCATGGCGATACTGCAAATATCGATGAATTAGAAAATTT
GACAGCACCTATAAAAGAACAAGCGACAGACATGTTAAATGATCAAGGGTTATCAATAG
ATGACTATGTACTATTTCCAGTACATCCTTGGCAATATCAGCATATTCTGCCGAACGTCTT
TGCGAAAGAGATTAGTGAAAAGTTGGTTGTACTATTACCGTTAAAATTTGGAGATTATCT
GTCGTCTTCAAGTATGCGTTCATTAATTGATATTGGCGCACCGTATAACCATGTCAAAGTA
CCATTTGCAATGCAGTCATTAGGCGCATTAAGGCTAACGCCTACGCGTTACATGAAAAAC
GGAGAACAAGCAGAACAATTATTACGTCAGCTTATAGAAAAAGATGAAGCACTAGCTAA
GTATGTCATGGTTTGTGATGAAACAGCTTGGTGGTCATATATGGGTCAAGATAATGATAT
TTTCAAAGATCAATTAGGTCATCTAACTGTTCAGCTAAGAAAATATCCCGAAGTGCTAGC
CAAAAATGATACGCAACAGCTAGTGTCAATGGCAGCACTCGCGGCAAATGATCGCACTTT
ATATCAAATGATTTGTGGAAAAGATAATATTTCTAAAAATGATGTCATGACGTTATTTGA
AGATATCGCACAAGTCTTTTTAAAGGTAACACTATCATTTATGCAATACGGCGCATTACC
AGAGTTGCATGGTCAAAATATATTGTTGTCATTTGAAGATGGACGTGTACAAAAATGCGT
GTTACGTGATCATGATACTGTCAGAATTTATAAACCATGGCTAACAGCACATCAGCTTTC
ATTGCCGAAGTATGTCGTCAGAGAAGATACACCTAATACGCTAATTAATGAAGATTTGGA
AACATTCTTTGCTTATTTTCAAACATTAGCTGTATCGGTAAATCTATATGCCATTATTGAT
GCAATTCAAGATTTATTTGGTGTAAGTGAGCATGAACTTATGTCGTTGTTAAAACAAATTT
TAAAAAATGAAGTGGCAACTATTTCCTGGGTTATAACTGATCAGCTAGCTATCAGACACA
TTTTATTTGATAAACAGACGTGGCCATTCAAACAAATTTTATTACCATTGCTATATCAACG
TGATAGTGGTGGAGGTAGTATGCCTTCAGGTTTAACTACCGTACCAAATCCAATGGTGAC
ATATGATTAA

> **sbnD**

Function: protein coding sequence; siderophore biosynthesis protein C (core genome, constant)
Best match: sbnD_CC001-ST772_118_AJGE01000014.1[19799:21055:r] (completely identical)
Position: 007-contig_191: 16906 … 18163; Length: 1257 bp
Sequence:
ATGATTAATCAGTCTATATGGCGCAGTAACTTTCGCATTTTATGGCTCAGTCAGTTTATAG
CGATTGCTGGACTGACAGTACTTGTGCCATTATTGCCAATTTATATGGCATCACTACAAAA
TCTATCAGTCGTAGAAATACAGTTGTGGAGTGGTATAGCGATTGCTGCTCCAGCTGTAAC
GACGATGATAGCTTCGCCGATATGGGGGAAGCTAGGTGATAAGATCAGCCGAAAATGGA
TGGTGTTAAGAGCGTTACTTGGTTTGGCGGTATGCTTATTTTTAATGGCATTGTGTACGAC
ACCATTACAGTTTGTACTTGTGAGGTTATTGCAGGGACTATTTGGTGGTGTTGTTGATGCA
TCAAGTGCGTTTGCGAGTGCAGAGGCGCCAGCTGAAGATCGTGGAAAGGTATTAGGAAG
ACTGCAAAGTTCAGTCAGCGCAGGGTCTCTTGTGGGGCCATTAATTGGCGGTGTTACAGC
TTCGATATTAGGTTTTAGTGCGTTACTGATGAGTATTGCCGTTATTACTTTTATTGTCTGTA
TTTTCGGTGCATTAAAATTGATTGAAACGACACATATGCCAAAATCACAAACACCAAATA
TTAATAAAGGTATTCGCCGTTCATTTCAATGTCTATTATGCACACAACAAACATGTCGATT
TATTATCGTTGGCGTTTTAGCAAACTTTGCTATGTATGGCATGCTAACTGCATTATCACCA
CTTGCTTCATCAGTGAATCATACAGCGATAGATGACCGTAGTGTGATTGGATTTTTACAGT
CCGCATTTTGGACGGCTTCGATATTAAGCGCGCCTTTATGGGGACGCTTTAATGATAAATC
ATATGTTAAATCAGTATATATATTTGCCACGATTGCATGTGGTTGTAGTGCGATACTGCAA
GGTTTAGCGACGAATATAGAGTTTTTAATGGCTGCAAGAATACTTCAAGGATTAACATAT
AGTGCATTGATTCAAAGTGTCATGTTTGTTGTCGTGAATGCGTGTCATCAACAACTTAAAG
GCACATTTGTTGGAACGACGAACAGTATGTTAGTTGTTGGTCAAATTATTGGCAGTCTTA
GTGGCGCTGCCATTACAAGTTATACTACACCAGCTACTACGTTTATTGTTATGGGCGTAGT

ATTTGCAGTAAGTAGTTTATTTTTAATTTGTTCAACCATCACTAATCAAATCAACGATCAC
ACATTAATGAAATTATGGGAGTTGAAACAAAAAAGTGCAAAATAA

## > sbnE

Function: protein coding sequence; siderophore biosynthesis protein D (core genome, variable)
Best match: sbnE_CC001-ST772_118_AJGE01000014.1[18073:19809:r] (completely identical)
Position: 007-contig_191: 18152 … 19889; Length: 1737 bp
Sequence:
GTGCAAAATAAAGAATTAATACAACATGCAGCGTATGCGGCTATCGAACGCATTTTAAAT
GAATATTTTAGAGAAGAAAATTTATATCAAGTACCACCTCAAAATCATCAATGGTCTATA
CAATTATCAGAGCTCGAAACTTTAACGGGTGAATTTCGCTATTGGTCTGCGATGGGGCAT
CATATGTATCATCCAGAGGTATGGCTTATCGATGGAAAAAGTAAAAAAATAACAACTTAT
AAAGAAGCAATTGCGCGTATTTTGCAACATATGGCTCAAATTGCAGATAATCAAACGGCA
GTGCAACAACATATGGCGCAAATTATGTCTGACATCGATAATAGCATTCATCGCACGGCG
CGTTATTTGCAAAGTAACACAATAGACTACACAGAAGATCGCTATATCGTTTCAGAACAT
TCTTTATACTTAGGTCATCCATTTCATCCGACTCCTAAGAGTGCAAGTGGGTTTTCAAAAG
CAGATTTAGAGAAATATGCACCCGAATGTCATACATCATTCCAATTGCATTATTTAGCTGT
GCATAAAGATGTTCTGCTCACGCGCTATGTAGAAGGTAAAGAAGATCAGGTTGAGAAAG
TGTTGTATCAATTAGCAGATATAGACATATCAGAGATACCCAAAGATTTTATTTTATTACC
AATACATCCTTATCAAATCAATGTGTTGCGACAGCATCCACAGTATATGCAATATAGTGA
ACAAGGTTTAATAAAAGACCTTGGCATTTCCGGTGATTCAGTGTACCCGACGTCTTCGGTT
AGAACTGTATTTTCAAAAGCATTAAACATTTATTTAAAATTACCGATACACGTTAAAATT
ACTAATTTTATACGTACGAATGATCTTGAACAGATTGAACGGACAATCGATGCCGCGCAA
GTTATTGCATCAGTCAAAGATGAGGTTGAAACACCCCATTTTAAATTGATGTTTGAAGAA
GGATATCGTGCATTGTTACCGAATCCGTTAGGGCAAACAGTTGAACCTGAAATGGATTTA
TTAACAAATAGTGCCATGATTGTTCGTGAAGGGATACCGAATTACCATGCTGATAAAGAT
ATTCATGTATTGGCGTCATTATTTGAAACGATGCCTGATTCACCGACCTCTAAGTTATCAC
AAGTGATTGAGCAAAGTGGTTTAGCACCAGAAGCATGGCTTGAATGTTATTTGGATCGTA
CATTATTGCCGATATTAAAGCTGTTTAGTAACACAGGCATTAGTCTAGAAGCACATGTAC
AAAATACATTAATTGAATTAAAAGATGGCATACCCGACGTGTGCTATGTCAGAGATTTAG
AAGGCATTTGTCTATCTAGAACGATTGCTACTGAAAAACAGCTTGTGCCAAATGTTGTGG
CAGCATCAAGCCCTGTTGTATATGCACATGATGAAGCATGGCATCGTCTTAAATATTACG
TTGTAGTAAATCACTTAGGACATTTAGTATCAACTATTGGTAAAGCGACTAGAAATGAAG
TTGTGTTATGGCAAATTGTAGCGCATCGTCTTATGACATGGAAAAAAGAATACGCGAATA
ACGCAGTATTTGTTGACTGTGTAGAAGATTTATATCAAACGCCGACCATTGCGGCTAAAG
CGAATTTGATGAGTAAATTGAATGATTGTGGTGCAAACCCCTATTTATACACATATACCAA
ATCCAATTTGTCATAACAAGGAGGTATCGTATTGTGAATCAAACAATTCTTAA

## > sbnF

Function: protein coding sequence; siderophore biosynthesis protein F (core genome, constant)
Best match: sbnF_CC001-ST772_118_AJGE01000014.1[16314:18053:r] (completely identical)
Position: 007-contig_191: 19908 … 21648; Length: 1740 bp
Sequence:
ATGCACCAACTGGTATCATCACTTATTTATGAGAATATTGTTGTGTATAAAGCGTCATATC
AAGACGGTGTCGGTCATTTTACAATAGAAGGACATGATTCAGAGTATCGTTTTACTGCTG
AAAAGACACATAGCTTTGATCGTATACGTATCACATCACCAATTGAGCGTGTCGTAGGAG
ATGAGGCAGATACAACAACAGACTATACACAATTATTGAGAGAGGCTGTATTTACATTTC
CTAAAAATGATGAAAAGCTAGAACAATTTATTGTTGAGTTATTACAGACAGAATTAAAAG
ATACACAAAGTATGCAGTATCGAGAATCAAACCCACCAGCAACACCTGAGACATTTAAC
GACTATGAATTTTATGCGATGGAAGGGCATCAGTATCATCCAAGTTACAAATCACGTTTA
GGATTTACGTTGAGTGATAATTTGAAATTTGGTCCTGATTTTGTACCAAACATTAAACTGC
AGTGGTTAGCTATCGACAAAGGTAAAGTAGAAACGACGGTATCAAGAAATGTTGTAGTT
AACGAAATGTTACGTCAACAAGTTGGCGATAAGACTTATGAACATTTTGTACAGCAAATT
GAAGCGTCTGGCAAACATGTAAATGATGTTGAGATGATACCTGTACACCCATGGCAGTTT
GAACATGTCATCCAAGTTGATTTGGCTGAAGAAAGGCTTAATGGCACAGTACTATGGTTA

GGGGAAAGTGATGAGCTATATCACCCTCAACAATCGATTCGTACGATGTCGCCAATAGAC
ACGACAAAATATTATTTAAAGGTACCAATAAGTATAACGAACACTTCAACGAAACGAGT
GTTGGCGCCTCATACAATTGAAAATGCAGCGCAAATTACGGATTGGTTAAAGCAGATACA
GCAACAAGATACGTATTTAAAAGATGAATTAAAGACAGCTTTTCTAGGGGAAGTCTTAGG
ACAGTCTTATTTAAATACACAACTTTCGCCTTATAAACAAACTCAAGTTTATGGTGCGTTA
GGTGTTATATGGCGTGAAAATATATATCACATGTTAATCGATGAAGAGGATGCGATACCA
TTTAATGCACTTTATGCAAGTGATAAGGATGGTGTACCATTCATTAAAAATTGGATTAAA
CAATATGGTTCTGAAGCTTGGACAAAGCAATTTTTAGCTGTAGCGATTCGTCCAATGATTC
ATATGCTTTATTATCACGGTATTGCCTTTGAATCGCATGCACAAAATATGATGCTCATTCA
TGAAAATGGTTGGCCTACACGTATTGCCTTAAAAGATTTCCATGATGGTGTTCGTTTTAAG
CGTGAGCATTTAAGTGAAGCTGCTTCACACCTGACATTAAAGCCAATGCCAGAAGCACAT
AAAAAAGTGAATAGTAATTCATTTATTGAAACAGATGACGAACGTTTAGTACGCGACTTT
TTACACGATGCATTTTTCTTTATTAATATCGCCGAAATCATCTTATTTATTGAAAAGCAAT
ATGGTATCGATGAGCAGCGACAATGGCAATGGGTTAAAGGCATCATCGAGGCGTATCAA
GAAGCATTTCCAGAGTTGAATAACTATCAACATTTCGATTTGTTTGAACCTACGATTCAAG
TTGAAAAGTTAACGACACGTCGATTATTAAGTGACTCCGAGTTAAGAATTCATCATGTTA
CAAATCCATTAGGTGTAGGAGGTATCAATGATGCAACAACTATCTCTGAAACATAG

> **sbnG**

Function: protein coding sequence; siderophore biosynthesis protein G (core genome, constant)
Best match: sbnG_CC001-ST772_118_AJGE01000014.1[15563:16339:r] (completely identical)
Position: 007-contig_191: 21622 … 22399; Length: 777 bp
Sequence:
ATGCAACAACTATCTCTGAAACATAGATTAAACAATGGTGATTCAGTTTATGGCATTTTTA
ATTCTATACCGGACCCATTGATGATCGAGGTTATCGCAGCAAGCGGGTATGACTTTGTTG
TGATTGATACAGAACACGTGGCGATTAATGATGAGACACTAGCGCATTTAATTCGTGCAG
CTGAAGCAGCGCATATTATACCAATTGTACGTGTCACTGCAGTGATAGATAGAGATATCA
TTAAAGTGTTGGATATGGGTGCGAGAGGTATTATTGTGCCACACGTTAAAGATCGTGAGA
CAGTTGAGCATATTGTGAAATTAAGTCGTTATTACCCGCAAGGATTAAGAAGTTTGAATG
GTGGTCGCATGGCAAGATTTGGACGTACACCATTACTTGATGCAATGGAGATGGCTAATG
AGCATATTATGGTGATTGCCATGATAGAAGATGTTGAAGGGGTTATGGCCATTGACGATA
TAGCTCAAGTCGAAGGTTTAGACATGATAGTCGAAGGTGCCGCAGATTTATCGCAGTCAC
TTGGCATACCATGGCAAACGCGTGATGATCAAGTAACATCACATGTTCAACATATTTTTG
AAGTTGTGAATGCACATGGTAAACATTTTTGTGCATTACCACGTGAAGATGAAGATATTG
CAAAATGGCAGGCACAAGGTGTACAAACATTTATTTTAGGTGATGATCGCGGAAAAATAT
ATCGCCATTTAAGTGCATCTCTAGCGACGTCTAAACAGAAAGGGGATGAAGGCTAA

> **sbnH**

Function: protein coding sequence; siderophore biosynthesis protein H (core genome, variable)
Best match: sbnH_CC001-ST772_118_AJGE01000014.1[14361:15563:r] (completely identical)
Position: 007-contig_191: 22398 … 23601; Length: 1203 bp
Sequence:
ATGCGTATAGTTCAACCTGTTATTGAACAATTAAAAGCACAATCTCATCCAGTTTGTCATT
ATATCTATGATTTAGTCGGACTGGAACATCATTTGCAACATATTACATCGTCATTGCCGAG
TAATTGTCAAATGTACTATGCAATGAAAGCAAATAGTGAACGAAAAATCCTAGATACAAT
TAGTCAGTATGTTGAAGGATTCGAAGTTGCATCTCAAGGTGAAATAGCAAAAGGTCTTGC
TTTTAAACCAGCAAATCATATTATTTTTGGTGGCCCTGGTAAGACAGACGAGGAACTAAG
ATATGCAGTAAGTGAAGGTGTTCAGCGTATTCATGTTGAAAGTATGCATGAATTACAACG
GCTAAATGCCATCTTAGAACATGAAGATAAGACACAACACATTTTATTGCGTGTTAATTT
AGCAGGACCATTTCCCAATGCAACGTTGCATATGGCAGGACGCCCAACACAATTTGGTAT
TTCTGAAGACGAAGTTGATGATGTCATTGAAGCTGCGCTCGCAATGCCAAAGATTCATCT
AGATGGATTTCATTTTCATTCTATTTCTAACAATTTAGACTCGAATTTACATGTCGATGTA
GTGAAACTTTATTTTAAAAAAGCAAAGGCATGGTCTGAAAAACATCGATTTCCACTCAAA
CATATCAATCTTGGTGGTGGCATAGGCGTTAACTATGCAGATTTAACTAACCAATTTGAA
TGGGATAATTTTGTAGAAAATTTTAAAACACTTATCGTTGAGCAAGAAATGGAAGATGTG

ACATTGAACTTTGAATGTGGGCGCTTTATTGTGGCACATATTGGTTACTATGTGACAGAA
GTGCTAGATATTAAGAAAGTGCATGGTGCTTGGTATGCCATTTTAAGAGGAGGTACGCAA
CAATTTAGACTGCCGGTATCTTGGCAGCATAACCATCCTTTTGAAATTTATCGTTATAAGG
ACAATCCATATTCATTTGAAAAAGTTTCAATTTCGAGACAGGACACAACGTTAGTCGGTC
AATTATGTACACCGAAAGATGTCTTTGCTAGAGAAGTGCAGATAGACTCAATCAGTACAG
GCGACGTTATTGTTTTCAAATATGCAGGTGCATACGGATGGTCTATTTCACATCACGATTT
CTTAAGCCATCCACATCCTGAATTTATTTATTTAACGCAAACAAAGGAGGATGAATAA

> **sbnI**

Function: protein coding sequence; siderophore biosynthesis protein I (core genome, constant)
Best match: sbnI_CC093_JKD6159-ST93_CP002114.2[122755:123519] (completely identical)
Position: 007-contig_191: 23604 … 24369; Length: 765 bp
Sequence:
TTGAATCATATTCATGAACATTTAAAATTGGTACCAGTAGATAAGATTGATCTTCACGAA
ACATTCGAACCTTTAAGATTGGAAAAGACGAAAAGTAGTATTGAAGCAGATGATTTTATA
CGTCATCCTATTTTAGTGACAGCGATGCAACATGGTAGATATATGGTTATAGATGGTGTG
CATCGGTATACAAGTTTGAAAGCGTTAGGATGTAAGAAAGTTCCAGTGCAAGAAATCCAT
GAAACACAATATTCAATTAGTACATGGCAACATAAAGTTCCATTTGGTGTGTGGTGGGAA
ACGTTACAACAAGAACATCGCTTGCCATGGACTACTGAGACAAGACAAGAAGCGCCATT
TATTACGATGTGTCATGGTGATACAGAACAATATTTGTATACAAAAGATTTAGGCGAAGC
ACATTTTCAAGTATGGGAAAAGGTTGTCGCAAGTTATAGTGGTTGTTGTTCTGTAGAGAG
AATTGCACAAGGTACATATCCTTGTCTTTCTCAACAAGATGTACTCATGAAGTATCAGCC
ATTGAGTTATAAGGAAATTGAAGCGGTTGTTCATAAAGGGGAAACTGTGCCAGCAGGTGT
GACACGCTTTAATATTTCAGGACGATGTCTTAATCTTCAAGTACCACTGGCATTACTTAAA
CAAGATGATGATGTTGAACAACTGCGCAATTGGAAGCAGTTTTTAGCAGATAAGTTTGCC
AATATGAGATGCTATACTGAAAAAGTATACTTGGTGGAGCAATAG

> **Q5HJP3-var1**

Function: protein coding sequence; putative protein (core genome, variable)
Best match: Q5HJP3-var1_CC001_MW2-USA400_BA000033.2[117648:118274]
Position: 007-contig_191: 25021 … 25648; Length: 627 bp
Sequence:
ATGGAAAAAAATGTAGAAAAATCATTCATAAAGATAGGTTTATATTTTCAAATAGCTTAT
ATAGTACTCATGGCTATAACTTTATGTGGGTTTGTAATTTGCTATGGACTAATTTTCGGCC
TTTTCTATTTATTATCAGGTAGCAAAGTTGATTATTTAATAGTAACAATAGTTATATCGGC
AATAATTTCTATATTTGTAATTATACTTTCAATCGTACCTGTCATCGTATTGGCATCTGACT
TATTTAAAGAAAGGATTTCAAAAGGTGTCATATTAATTGTATTGGCTATTATTGCTTTAGT
ATTATGCAACTTTGTATCTGCAATACTCTGGTTTGTTTCAGCCATATCTATTTTAGGTAGA
AAAAAATTAGTAGCTGCAGCAGATACTACCACTATTCAAAAAAGTAAAGGGAACGCAAA
TCAAGCATCACATAAAGACACGTGTAAAAAGGAACTTGATAGTCAAGACATGATGGAAC
ATCCTGAGGTTAAAAATCCCACGACTAAAAACCTTGAAGGATTTAACGAAGAAATACAT
AAAGATGAAGCTACAACTAAAGTTGTCAGTGATAACACGGAACCGCCGATTGAATCAAA
AGACCATGTCTCGAAAAAAGATTGA

> **butA**

Function: protein coding sequence; acetoin(diacetyl) reductase
Best match: butA_CC005_Mu50-VRSA_BA000017.4[140984:141760] (completely identical)
Position: 007-contig_191: 25859 … 26636; Length: 777 bp
Sequence:
ATGACAAACAACAAAGTAGCATTAGTAACTGGCGGAGCACAAGGGATTGGTTTTAAAAT
TGCAGAACGTTTAGTGGAAGATGGTTTCAAAGTAGCAGTTGTTGATTTCAATGAAGAAGG
GGCAAAAGCAGCTGCACTTAAATTATCAAGTGATGGTACAAAAGCTATTGCTATCAAAGC
AGATGTATCAAACCGTGATGATGTATTTAACGCAGTAAGACAAACTGCCGCGCAATTTGG
CGATTTCCATGTCATGGTTAACAATGCCGGCCTTGGACCAACAACACCAATCGATACAAT
TACTGAAGAACAGTTTAAAACAGTATATGGCGTGAACGTTGCAGGTGTGCTATGGGGTAT

TCAAGCCGCACATGAACAATTTAAAAAATTCAATCATGGCGGTAAAATTATCAATGCAAC
ATCTCAAGCAGGCGTTGAGGGTAACCCAGGCTTGTCTTTATATTGCAGTACAAAATTCGC
AGTGCGAGGTTTAACACAAGTAGCCGCACAAGATTTAGCGTCTGAAGGTATTACTGTGAA
TGCATTCGCACCTGGTATCGTTCAAACACCAATGATGGAAAGTATCGCAGTGGCAACAGC
CGAAGAAGCAGGTAAACCTGAAGCATGGGGTTGGGAACAATTTACAAGTCAGATTGCTT
TGGGCAGAGTTTCTCAACCAGAAGATGTTTCAAATGTAGTGAGCTTCTTAGCTGGTAAAG
ACTCTGATTACATTACTGGACAAACAATTATTGTAGATGGTGGTATGAGATTCCGTTAA

## > capX_epi

Function: protein coding sequence; UDP-glucose 4-epimerase (core genome, variable)
Best match: capX_epi_CC001_MW2-USA400_BA000033.2[119607:120578] (completely identical)
Position: 007-contig_191: 26980 … 27952; Length: 972 bp
Sequence:
TTGGAAAGAGTTTTGATAACTGGTGGGGCTGGTTTTATTGGGTCGCATTTAGTAGATGATT
TACAACAAGATTATGATGTTTATGTTCTAGATAACTATAGAACAGGTAAACGAGAAAATA
TTAAAAGTTTGGCTGACGATCATGTGTTTGAATTAGATATTCGTGAATATGATGCAGTTGA
ACAAATCATGAAGACATATCAATTTGATTATGTTATTCATTTAGCAGCATTAGTTAGTGTT
GCTGAGTCGGTTGAGAAACCTATCTTATCTCAAGAAATAAACGTCGTAGCAACATTAAGA
TTGTTAGAAATCATTAAAAAATATAATAGTCATATAAAACGTTTTATCTTTGCTTCGTCAG
CAGCTGTTTATGGTGATCTTCCTGATTTGCCTAAAAGTGATCAATCATTAATCTTACCATT
ATCACCATATGCAATAGATAAATATTACGGCGAACGGACGACATTAAATTATTGTTCGTT
ATATAACATACCAACAGCGGTTGTTAAATTTTTTAATGTATTTGGGCCAAGACAGGATCC
TAAGTCACAATATTCAGGTGTGATTTCAAAGATGTTCGATTCATTTGAGCATAACAAGCC
ATTTACATTTTTTGGTGACGGACTGCAAACTAGAGATTTTGTATATGTATATGATGTTGTT
CAATCTGTACGCTTAATTATGGAACACAAAGATGCAATTGGACACGGTTATAACATTGGT
ACAGGCACTTTTACTAATTTATTAGAGGTTTATCGTATTATTGGTGAATTATATGGAAAAT
CAGTCGAGCATGAATTTAAAGAAGCACGAAAAGGAGATATTAAGCATTCTTATGCAGAT
ATTTCTAACTTAAAGGCATTAGGATTTGTTCCTAAATATACAGTAGAAACAGGTTTAAAG
GATTACTTTAATTTTGAGGTAGATAATATTGAAGAAGTTACAGCTAAAGAAGTGGAAATG
TCGTGA

## > capX_5M

Function: protein coding sequence; capsular polysaccharide biosynthesis glycosyltransferase
Best match: capX_5M_CC001_MW2-USA400_BA000033.2[120541:121233] (completely identical)
Position: 007-contig_191: 27914 … 28607; Length: 693 bp
Sequence:
TTGAAGAAGTTACAGCTAAAGAAGTGGAAATGTCGTGAAAATGACATTGAAGCTGTCCA
TAATAATAAGGGTTATGCCTATCAAAGAAAATTAGACAAACTAGAAGAAGTGAGAAAAA
GCTATTACCCAATTAAACGTGCGATTGACTTAATTTTAAGCATTGTTTTATTATTTTTAACT
TTACCGATTATGGTTATATTCGCCATTGCTATCGTCATAGATTCGCCAGGAAACCCTATTT
ATAGTCAGGTTAGAGTTGGGAAGATGGGTAAATTAATTAAAATATACAAATTACGTTCGA
TGTGCAAAAACGCAGAGAAAAACGGTGCGCAATGGGCTGATAAAGATGATGATCGTATA
ACAAATGTCGGGAAGTTTATTCGTAAAACACGCATTGATGAATTACCACAACTAATTAAT
GTTGTTAAAGGGGAAATGAGTTTTATTGGACCACGCCCGGAACGTCCGGAATTTGTAGAA
TTATTTAGTTCAGAAGTGATAGGTTTCGAGCAAAGATGTCTTGTTACACCAGGGTTAACA
GGACTTGCGCAAATTCAAGGTGGATATGACTTAACACCGCAACAAAAACTGAAATATGA
CATGAAATATATACATAAAGGTAGTTTAATGATGGAACTATATATATCAATTAGAACATT
GATGGTTGTTATTACAGGGGAAGGCTCAAGGTAG

## > capX_cps2H

Function: protein coding sequence; O-antigen polymerase
Best match: capX_cps2H_CC001_MSSA476_BX571857.1[121296:122534]
Position: 007-contig_191: 29963 … 31202; Length: 1239 bp
Sequence:

ATGGAAAATCAACACAATAGTAAATTACTAACATTGTTACTTATCGGTTTAGCGGTTTTTA
TTCAGCAATCTTCGGTTATTGCCGGTGTGAATGTTTCTATAGCTGACTTTATCACATTACT
AATATTAGTTTATTTACTGTTTTTCGCTAACCATTTATTAAAGGCAAATCATTTTTTACAGT
TTTTCATTATTTTGTATACATATCGTATGATTATTACGCTTTGTTTGCTATTTTTTGATGATT
TGATATTTATTACGGTTAAGGAAGTCCTTGCATCTACAGTTAAATATGCATTTGTAGTCAT
TTATTTCTATTTAGGGATGATCATCTTTAAGTTAGGTAATAGCAAAAAAGTGATCGTTACC
TCTTATATTATAAGCAGTGTGACTATAGGTCTATTTTGTATTATAGCTGGTTTGAACAAGT
CCCCTTTACTAATGAAATTGTTATATTTTGATGAAATACGTTCAAAAGGATTAATGAATGA
CCCTAACTATTTCGCGATGACACAGATTATTACATTGGTACTTGCTTACAAGTATATTCAT
AATTACATATTCAAGGTCCTTGCATGTGGTATTTTGCTATGGTCTTTAACTACAACGGGGT
CTAAGACTGCGTTTATCATATTAATCGTCTTAGCCATTTATTTCTTTATTAAAAAGTTATTT
AGTAGAAATGCGGTAAGTGTTGTGAGTATGTTAGTGATTATGCTGATATTACTTTGTTTTA
CCTTTTATAATATCAACTACTATTTATTCCAATTAAGCGACCTTGATGCCTTACCGTCATT
AGATCGAATGGCGTCTATTTTTGAAGAGGGGTTTGCATCATTAAATGATAGTGGATCTGA
GCGAAGTGTTGTATGGATAAATGCCATTTCAGTAATTAAATATACATTAGGTTTTGGTGTT
GGCTTAGTGGATTATGTACATATTGGCTCGCAAATTAATGGTATTTTACTTGTTGCCCATA
ATACATATTTGCAAATTTTTGCGGAATGGGGCATTTTATTCGGTGCGTTATTTATCATATT
TATGCTTTATTTACTGTTTGAATTATTTAGATTTAACATTTCTGGGAAAAATGTAACAGCA
ATTGTTGTAATGTTGACGATGCTGATTTACTTTTTAACAGTATCATTTAATAACTCAAGAT
ATGTCGCTTTTATTTTAGGAATTATCGTCTTTATTGTTCAATATGAAAAGATGGAAAGGGA
TCGTAATGAAGAGTGA

> **capX_wzy**

Function: protein coding sequence; lipopolysaccharide flipase (wzy)
Best match: capX_wzy_CC005_Mu50-VRSA_BA000017.4[146316:147746]
Position: 007-contig_191: 31191 … 32622; Length: 1431 bp
Sequence:
ATGAAGAGTGATTCACTAAAAGAAAATATTATTTATCAAGGGCTATACCAATTGATTAGA
ACGATGACACCACTGATTACAATACCCATTATTTCACGTGCATTTGGTCCCAGTGGTGTGG
GTATTGTTTCATTTTCTTTCAATATCGTGCAATACTTTTTGATGATTGCAAGTGTTGGCGTT
CAGTTATATTTTAATAGAGTTATCGCGAAGTCCGTTAACGACAAACGGCAATTGTCACAG
CAGTTTTGGGATATCTTTGTCAGTAAATTATTTTTAGCGTTAACAGTTTTTGCGATGTATAT
GGTCGTAATTACTATATTTATTGATGATTACTATCTTATTTTCCTACTACAAGGAATCTAT
ATTATAGGTGCAGCACTCGATATTTCATGGTTTTATGCTGGAACTGAAAAGTTTAAAATTC
CTAGCCTCAGTAATATTGTTGCGTCTGGTATTGTATTAAGTGTAGTTGTTATCTTTGTCAA
AGATCAATCAGATTTATCATTGTATGTATTTACTATTGCTATTGTGACGGTATTAAACCAA
TTACCTTTGTTTATCTATTTAAAACGATACATTAGCTTTGTTTCGGTTAATTGGATACACGT
CTGGCAATTGTTTCGTTCGTCATTAGCATACTTATTACCAAATGGACAGCTCAACTTATAT
ACTAGTATTTCTTGCGTTGTTCTTGGTTTAGTAGGCACATACCAACAAGTTGGTATCTTTT
CTAACGCATTTAATATTTTAACGGTCGCAATCATAATGATTAATACATTTGATCTTGTAAT
GATTCCGCGTATTACCAAAATGTCTATCCAGCAATCACATAGTTTAACTAAAACGTTAGC
TAATAATATGAATATTCAATTGATATTAACAATACCTATGGTCTTTGGTTTAATTGCAATT
ATGCCATCATTTTATTTATGGTTCTTTGGTGAGGAATTCGCATCAACTGTCCCATTGATGA
CCATTTTAGCGATACTTGTATTAATCATTCCTTTAAATATGTTGATAAGCAGGCAATATTT
ATTAATAGTGAATAAAATAAGGTTATATAATGCGTCAATTACTATTGGTGCGGTAATGAA
TTTAGTATTATGTCTTGTTTTGATATATTTTTATGGAATTTACGGTGCTGCTATTGCGCGTT
TAATTACAGAGTTTTTCTTGCTCATTTGGCGATTTATTGATATTACTAAAATCAATGTGAA
GTTGAATATTGTAAGTACGATTCAATGTGTCATTGCTGCCGTTATGATGTTTATTGTGCTT
GGTGTGGTCAATCATTATTTGCCCCCTACAATGTACGCTACGCTGCTATTAATTGCGATTG
GTATAGTAGTTTATCTTTTATTAATGATGACTATGAAAAATCAATACGTATGGCAAATATT
GAGGCATCTTCGACATAAAACAATTTAA

> **sodA-L1**

Function: protein coding sequence; superoxide dismutase/locus 1 (core genome, constant)
Best match: sodA-L1_CC001_MW2-USA400_BA000033.2[125516:126115] (completely identical)

Position: 007-contig_191: 32888 … 33488; Length: 600 bp
Sequence:
ATGGCATTTAAATTACCAAATTTACCATATGCATATGATGCATTGGAACCATATATAGAT
CAAAGAACAATGGAGTTTCATCACGACAAACATCACAATACGTACGTGACGAAATTAAA
CGCAACAGTTGAAGGAACAGAGTTAGAGCATCAATCATTAGCGGATATGATTGCTAACTT
AGACAAGGTACCGGAAGCGATGAGGATGTCAGTCCGTAATAATGGCGGTGGTCATTTTA
ACCATTCATTATTCTGGGAAATACTATCACCTAATTCTGAAGAAAAAGGTGGCGTAATAG
ATGACATCAAAGCGCAGTGGGGCACTTTAGATGAATTTAAAAATGAATTTGCAAATAAA
GCAACAACATTATTTGGATCAGGTTGGACTTGGTTAGTTGTTAATGATGGCAAATTAGAA
ATTGTGACAACGCCAAACCAAGATAATCCATTAACAGAAGGCAAAACACCAATCTTACT
ATTTGATGTTTGGGAGCATGCCTACTATCTGAAATATCAAAATAAACGTCCAGACTATAT
GACTGCATTTTGGAATATTGTTAACTGGAAAAAAGTTGATGAATTATACCAAGCAGCAAA
ATAA

> **sasD**
Function: protein coding sequence; Staphylococcus aureus surface protein D (core genome, variable)
Best match: sasD_CC001-ST772_118_AJGE01000014.1[3380:4105:r] (completely identical)
Position: 007-contig_191: 33856 … 34582; Length: 726 bp
Sequence:
ATGAAAAAATTAGCAACAGTAGGTTCTTTAATTGTAACAAGCACTTTAGTATTCTCAAGT
ATGCCTTTTCAAAATGCGCATGCCGACACAACTTCAATGAATGTGTCGAATAAACAAAGC
CAAAATGTACAAAATCATCGTCCTTATGGCGGAGTAGTACCACAAGGAATGACGCAAGC
ACAATATACTGAATTAGAGAAAGCTTTACCCCAATTAAGCGCTGACAGTAATATGCAAGA
CTATAATATGAAATTGTATGATGCGACGCAAAATATTGCTGATAAATACAATGTGATAAT
TACAACTAATGTAGGGGTATTTAAACCACATGCTGTTAGAGATATGAATGGCCATGCGTT
ACCTTTAACAAAAGATGGCAATTTTTATCAAACGAATGTAGATGCAAATGGTATTAATCA
TGGTGGTAGTGAAATGGTGCAAAATAAAACAGGTCATATGAGTCAACAAGGCCATATGA
ATCAGAACACACACATGAACCAACAGCCACACATGCAACAAGGTCATATGCAATCATCA
AACCATCAAATGATGAGTCCAAAAGCAAATATGCATTCATCAAATCATCAAATGAACCA
AAGTAACAAAAAAGTTTTACCAGCTGCTGGTGAAAGTATGACATCAAGTATTCTTACTGC
AAGTATTGCCGCACTACTATTAGTATCTGGGTTATTCTTAGCATTTAGACGACGTTCAACA
AATAAATAA

> **Q5HJN3**
Function: protein coding sequence; transcript regulator/GntR family (core genome, constant)
Best match: Q5HJN3_CC001_MW2-USA400_BA000033.2[127399:128154:r]RC
Position: 007-contig_191: 34772 … 35528; Length: 756 bp
Sequence:
TTAATAATCCGATTGTCTTATACGTGTCAGTGTTAATTCAGAGATTTCCTGTGGAATATAC
CACTTATTAATCATAATTGGATAAGGTGTTTGTGCGTACAATGTTTCAATAATCAGCCAAC
AATGTGTATCACCATCAAACACGTGACTATGATTTTTGAAGTGGGGCGCTTTGGTAATAG
ACATTTTTAAATCTGATTGATATGCATTGTTATAAATCGTTTGCTCAACGAACGTCTTCAT
GTCGTCTTCGTTTTGTGTATTCACTTTAAATGTGTCAATGACATTTAACGGTATAAAGGTA
AAACAAAATGCATCAGCTTGCTTAGAATGATTGTCCTTTTTTTGATAATAGCGTTCCATTG
CAATGACGGCAGAAGGATGGTTTGCAAACAAATGATTTGTATATTCACTTTCTAAATCAA
CACGATAATTAATTGATGACATAGATACGCGAGCTAGCAATATTTGATCAAGTGGATGCT
TAAATTGATCCATACTTGAAGCGTGTTGGGCATTTGTTTGTGGAATAACAAAGTGTCCCTT
CCCTCTTGTACTCTCTACGATGCCATCTTCGGCTAACAATTTTATAGCTTGGCGCAAAGTC
ATGCGACTGACATCAAAGCGCGCACAAAGTTCCTTTTCAGTAGGTAATGCATGGCCACTC
GGATATTTTCCTATTTGAATTTCTTTATATAACGTATTATAAATCGTTAAAAATTTTGGTTG
TGTTTGCGTCACGTAGACAACCTCCAT

> **deoD-L1**
Function: protein coding sequence; purine nucleoside phosphorylase locus 1 (core genome, constant)
Best match: deoD-L1_CC005_Mu50-VRSA_BA000017.4[150887:151594]

Position: 007-contig_191: 35763 … 36471; Length: 708 bp
Sequence:
ATGAAATCAACACCACACATTAAACCAATGAATGACGTCGAAATTGCAGAAACGGTTCT
ATTGCCAGGAGATCCGTTAAGAGCTAAGTTCATTGCAGAAACTTATTTGGATGATGTGGA
ACAGTTCAATACAGTGCGAAACATGTTTGGTTTTACCGGAACATATAAAGGTAAAAAAGT
TTCTGTCATGGGTTCCGGTATGGGTATGCCATCTATTGGCATTTACTCTTATGAATTAATT
CATACATTTGGTTGTAAAAAATTAATTCGCGTTGGCTCTTGTGGCGCGATGCAAGAAAAC
ATTGATTTATATGATGTGATTATTGCACAAGGTGCCTCTACTGATTCAAATTACGTTCAAC
AATATCAATTACCAGGTCATTTTGCGCCAATTGCTTCTTATCAATTATTAGAAAAAGCAGT
TGAAACAGCACGTGACAAAGGTGTACGTCATCATGTAGGTAATGTGTTATCAAGTGATAT
TTTCTATAACGCGGATACAACAGCGAGTGAACGTTGGATGCGTATGGGTATTTTAGGTGT
AGAAATGGAATCAGCTGCATTATACATGAATGCAATTTACGCTGGTGTCGAAGCATTAGG
TGTGTTCACAGTGAGCGATCATTTAATTCATGAAACGTCAACAACACCTGAGGAAAGGGA
ACGTGCATTTACAGATATGATTGAAATTGCACTGTCATTGGTGTAG

> **tet38**
Function: protein coding sequence; membrane transport protein (core genome, variable)
Best match: tet38_CC008_A9765_ACSN01000018.1[60782:62134]
Position: 007-contig_191: 36477 … 37830; Length: 1353 bp
Sequence:
ATGAATGTTGAATATTCTAAAATAAAGAAAGCAGTACCTATTTTATTATTCTTATTTGTAT
TCAGTTTGGTTATAGACAACTCATTTAAATTGATTTCTGTAGCCATTGCTGATGACTTAAA
CATATCTGTAACGACAGTAAGTTGGCAAGCGACATTAGCCGGTTTAGTAATTGGTATTGG
CGCTGTAGTATACGCTTCATTATCTGATGCCATTAGTATACGCACACTATTTATTTATGGC
GTGATATTAATCATTATCGGATCAATTATTGGTTACATTTTCCAACATCAATTCCCATTAC
TTTTAGTTGGACGTATTATTCAAACTGCCGGTTTAGCTGCTGCAGAGACATTATATGTGAT
ATATGTTGCAAAGTATCTTTCTAAAGAGGACCAGAAGACTTACCTTGGCTTAAGTACGAG
CAGTTATTCCTTGTCATTAGTTATCGGTACATTATCAGGTGGATTTATTTCTACGTATTTAC
ACTGGACAAATATGTTTTTAATTGCATTAATCGTAGTATTTACGTTGCCATTCCTATTTAA
ATTATTACCAAAAGAAAATAATACGAATAAAGCTCATTTAGATTTTGTTGGCTTAATTCTA
GTGGCAACTATTGCTACAACAGTCATGCTGTTTATTACGAACTTTAATTGGTTATATATGA
TTGGTGCCTTAATTGCGATTATCGTTTTTGCGCTATATATTAAAAATGCGCAACGTCCATT
AGTAAATAAATCATTTTTCCAAAATAAACGTTATGCTTCATTTTTATTTATAGTATTTGTA
ATGTATGCTATCCAATTGAGTTATATTTTTACGTTCCCATTCATAATGGAGCAAATTTATC
ATCTGCAACTAGACACAACATCACTGTTATTAGTACCGGGTTATATAGTAGCAGTCATTG
TTGGTGCACTAAGTGGTAAAATCGGCGAATATCTGAATTCAAAACAAGCGATTATCACAG
CAATTATTTTAATAGCACTGAGCTTGATTTTACCTGCATTTGCAGTAGGTAATCACATTTC
AATCTTCGTCATTTCTATGATATTCTTTGCAGGTAGCTTTGCTTTAATGTATGCACCTTTAC
TTAACGAAGCCATTAAAACAATAGATCTTAATATGACAGGTGTGGCTATTGGTTTTTATA
ATTTAATTATTAATGTGGCGGTATCTGTAGGTATTGCGATTGCTGCGGCTCTAATCGATTT
TAAAGCATTAAATTTCCCAGGCAATGATGCATTAAGTTCACATTTCGGTATTATTTTAATT
ATTTTAGGTTTAATGAGTATTGTCGGATTAGTTTTATTCGTCAGCTTAAATCGTTGGACAC
AATCTGAAAAATAA

> **deoC-L1**
Function: protein coding sequence; deoxyribose-phosphate aldolase locus 1 (core genome, constant)
Best match: deoC-L1_CC001_MW2-USA400_BA000033.2[130537:131199]
Position: 007-contig_191: 37910 … 38573; Length: 663 bp
Sequence:
ATGAAATTTGAGAAATATATAGATCACACTTTATTGAAGCCTGAGTCAACACGTACGCAA
ATCGATCAAATCATCGATGAAGCGAAAGCATACAATTTTAAATCTGTATGTGTGAATCCA
ACGCATGTTAAATATGCAGCAGAGCGACTAGCTGATTCAGAGGTGCTCGTTTGTACGGTA
ATAGGATTCCCATTAGGTGCGTCGACAACTGCAACGAAAGCATTTGAAACAGAAGATGC
AATTCAAAATGGTGCAGATGAAATTGACATGGTCATCAACATCGGCGCATTAAAAGATG
GACGTTTTGATGATGTACAACAAGACATTGAAGCAGTGGTGAAAGCTGCGAAAGGTCAC

ACAGTAAAAGTGATTATTGAGACGGTATTGTTGGACCATGACGAAATCGTAAAAGCGAG
TGAATTAACAAAAGTGGCTGGTGCGGACTTCGTTAAAACTTCAACAGGTTTTGCAGGTGG
CGGTGCGACTGCAGAAGACGTTAAATTAATGAAAGATACAGTAGGTGCTGATATAGAAG
TAAAAGCATCAGGTGGCGTACGTAATTTAGAAGATTTCAATAAAATGGTTGAAGCAGGTG
CGACACGTATTGGTGCGAGCGCAGGCGTTCAAATTATGCAAGGTTTAGAAGCAGATTCAG
ATTACTAA

> **deoB**

Function: protein coding sequence; phosphopentomutase (core genome, constant)
Best match: deoB_CC005_Mu50-VRSA_BA000017.4[153724:154902] (completely identical)
Position: 008-contig_256: 46 … 1225; Length: 1179 bp
Sequence:
ATGACAAGACCATTTAATCGTGTACATTTAATCGTAATGGATTCAGTAGGTATTGGTGAA
GCGCCAGACGCAGCTGATTTTAAAGATGAAGGTTCACATACTTTAAGACATACCTTAGAA
GGTTTCGATCAAACTTTACCAAACCTTGAAAAGTTAGGTCTAGGGAACATCGATAAATTA
CCAGTAGTAAATGCAGTTGAACAACCAGAAGCATACTATACTAAATTGAGTGAAGCTTCA
GTTGGTAAAGATACAATGACTGGTCACTGGGAAATTATGGGATTAAATATTATGCAACCT
TTTAAAGTATACCCTAATGGATTCCCTGAAGAGTTAATTCAACAAATTGAAGAAATGACA
GGTCGTAAAGTTGTTGCTAACAAACCGGCATCGGGTACGCAAATTATCGATGAGTGGGGC
GAGCACCAAATGAAAACTGGTGACTTAATTGTTTATACAAGTGCAGACCCAGTATTGCAA
ATTGCTGCACATGAAGACATTATCCCATTAGAAGAGTTATATGATATTTGTGAAAAGGTT
CGTGAGTTGACAAAAGACCCTAAATATTTAATTGGTCGTATTATCGCACGTCCATATGTTG
GTGAACCAGGAAACTTTACACGTACATCTAATCGACATGACTATGCGTTAAAACCATTTG
GTAAAACTGTCTTAGATCATTTGAAAGACGGTGGTTATGATGTTATTGCCATCGGTAAAA
TTAATGACATTTATGATGGTGAAGGTGTAACAGAAGCGGTTCGTACGAAGAGTAACATGG
ACGGTATGGATCAATTGATGAAAATTGTTAAGAAAGATTTCACAGGTATTAGCTTCTTAA
ACTTAGTAGACTTTGATGCATTATACGGTCATCGTCGTGATAAACCAGGTTATGCACAAG
CAATTAAAGATTTCGATGATCGCTTGCCAGAACTGTTTAGCAACTTAAAAGAAGACGATT
TAGTAATTATTACAGCAGACCATGGTAATGACCCGACAGCGCCAGGTACGGACCATACG
AGAGAATATATCCCAGTAATTATGTACAGTCCGAAATTTAAAGGTGGTCATGCACTAGAA
AGTGATACTACATTCAGTTCTATCGGTGCAACTATAGCAGATAATTTCAACGTAACATTA
CCAGAGTTCGGTAAAAGTTATTTAAAGGAATTGAAATAG

> **phnE2**

Function: protein coding sequence; phosphonate ABC transporter/permease protein 2 (core genome, variable)
Best match: phnE2_CC188_21340_AGTX01000018.1[42261:43076:r]RC
Position: 008-contig_256: 1355 … 2171; Length: 816 bp
Sequence:
TTAAACAATACGTTTTCGGATTGAACCGGAAATTAAATCTACAATTGCGACCATTAGTAC
TAAACCGATTAATATAATACCTACACGGTCCCAAGAACGTGTTTGAATGGCAAATATGAG
TGGTGTCCCGATACCACCAGCCCCAATTAGCCCCAGTATAGAAGCTGAACGTAAGTTTAG
TTCAAAGCGATAAAGTATGAGAGATAGAAAGGCAGGCATAATTTGTGGTATGACTGCAA
ATACGAGTGTTTTAATCTTATTTGCACCACTGGCCTTTAATGATTCTATAGCACTGAAATC
TAGACCTTCAATATCTTCAGCTAAAAGTTTCCCAAGCATACCTACAGAATGGATACCTAA
AGCTAATACACCTGAAAATGAACCTGGGCCAACAGCTTTGATAAATATAAGTGCCATTAC
AATTTCTGGGAAGACACGTATAACACTTAAAATAAATTTGCTAACACCTGAAACCGGGCG
TAACTTTACCATATTATTTGCACCTAGAAATGCTAATGGAATACAGATAATTGCGGCGAT
GAAAGTACCTACAACGGCTATCGCAAAGGTTTCAAGTAAACCACGTAATAAGTCTTCGCC
ATCTGGTATATAGATATAGCTGATGTCAGGATGGAATAATCCGCTGAATATGGATTTTAA
GATTTCTAATGATTTACTTTTAAGTTCTAAACTTGGTACACCTGCAAATGCCCAGATGATA
ATAGCTAAGACGACAATTGCAATAAGCCATCTTTTAATCAATTTTCGTTTGTGTGCTTTTG
TGTGAACATTATATTTTGCTATTTGCTGTGTCAT

> **phnE1**

Function: protein coding sequence; phosphonate ABC transporter/permease protein 1 (core genome, variable)
Best match: phnE1_CC188_21340_AGTX01000018.1[43073:43873:r]RC (completely identical)
Position: 008-contig_256: 2167 … 2968; Length: 801 bp
Sequence:
TCATGCGAGATGTGCCCTCACTTTCGTACTGATGTAATCAATGACGACGACGATAACTAA
AGTAAATAAAATAATCGTTGCTGTTTTTGGATATTGAAATAAACCAAGTGTTTGATCATA
AAACAATCCAATACCGCCAGCGCCGACTAATCCAAGAACAGCTGAAGCACGTATATTTAC
TTCAAATGCATATAATACGTATGACATAAATGACGATATGGCTTGTGGTACAACACCGAA
AACAATCCATTTTATTTTATTAGCACCAACAGCCGTCATTGCTTCCATTGGACCTGGATCT
ATCGTTTCCAATGATTCATATAATAATTTTCCAATAATACAGATAGTTAAAATAAACAAT
GCTAATATCCCTGGAATTTGACCGATTCCAAATACCGCCACAAAGATTGCTGCTAATAGC
AAATCCGGTATAGTACGAACTATATTTAAAATAAAACGCGAGGGTATTGAAATCCACTTT
TGATGAACGATATTGCTAGCACATAATAACGCAATTGGTATTGAAACGATGCTACCTAAT
ACTGTACTTACGATAGCCATTCGAATGGTATCTAACATTGGCGTTGTAATTTGTTGTAAAT
ACTCGAAATCAGGTGGAATCATTTGTTTGAATAGATCACCTATTTGAGGTATTCCTATCAT
TAAATCTCCAAAATTAAACCCCGTATAAATGAAGCTCCAAATGATAAGCACGATGATTAA
CATGAAGGTAAAACTCGTTTTTAAAGAAACCTTTTTCTTTAAAAGGGAGTCATACTTTGTA
GGTGTTTCTAAAGGCAT

> **phnC**
Function: protein coding sequence; phosphonate ABC transporter/ATP-binding protein (core genome, variable)
Best match: phnC_CC005_Mu50-VRSA_BA000017.4[156645:157418:r]RC
Position: 008-contig_256: 2969 … 3743; Length: 774 bp
Sequence:
TTAGTTCACTCCTAGCTTTTCATCTTCTTTAATTGTACGTCCATATATTTCACTAAATACGT
CATCTGTTGCTTCAGATGCAGGACCATCATAGACAACTTCACCATCACGTAAACCAATGA
TGCGTGTGCCATATTCTTTTGCCAAGTCAACAAAATGTAAATTAATTAAAATTGTAATGCC
TAATTCTTGGTTGATTTTTCTTAAATCATCCATAACCTGTTTCGTTGTTAATGGGTCTAATG
AAGCAACTGGTTCATCTGCAAGAATAATTTCGGATTCTTGGCATAGCGCACGTGCAATAG
ATATACGTTGTTGTTGGCCACCTGATAATTCATCAGAGCGTTGATTATATTTATCTAAGAT
GTTGACCCGTTCTAGTGCATCCATTGCTTTAATTTTGTCTTCTTTTGGAAATAAACCTAATA
CCATTTTCCAAGTAGGGTGATAACCTACACGTCCACTTAGTACATTTCGTAATACACTTGA
CCGTTTAACTAAATTAAAATGTTGGAAAATCATACCTATATTTCGGCGCATTTCTAATAAT
GCTTTACCATGGGCTTTAGTGATTGATTTACCTTGGATGAAAATTTCACCTGACGTGATAT
TATGCAAACGATTTACAGATCTTAATAACGTGGATTTCCCAGCACCAGATAGTCCGACAA
TAACTGCAAATTCACCTTTTTCAATATTTAAGTTAATATTTTTCAAGCCTACATGACCGTT
AGGATAGACTTTACTGACGTTTTTAAATTCGATTTGACTCAT

> **phnB**
Function: protein coding sequence; phosphonate ABC transporter/substrate-binding protein (core genome, variable)
Best match: phnB_CC007_TCH959-USA300_AASB02000033.1[3918:4874:r]RC
Position: 008-contig_256: 3956 … 4913; Length: 957 bp
Sequence:
TTATTTCATATCTTTAACTAATTTTTCGTACTCTCTTACAATGTCGAAATTTGAATCTTTCG
TTTCTGTGTATCCTTCATGTGAATAAACTTCGCTAATAATTTTGTGACCTTCTTTTGATTTA
GCAATGTCTATAAAAGCTTTTTTCAATTTTTCTTGAAAATCTTTATCCATATCTGGTCTTAC
AGAAATTGTGTCATTCGGAATAGCTTGTGTTAATTTTAAAATTCGTGTGTCTTTAAATACA
TTTGGTTGGTCTTTTTTCACAGTATTACGTGCATCGTTAAATACAGCTGCAGCATCTACAT
CTCCATTTAATAATGAGATAACTGCTTGGTCATGACCTTTAACATTCACAATTTTCATATC
TTTAGTTGCATTAATACCTGCTTCGTTTTTTAACATCGCAAGTGGGAATGTATATCCAGCA
GTTGATGTTACATCTTGTAAGGCAATTTTCTTACCTTTTAAATCTTTCAAGCTTTTAATTTT
TGAGTCTTTTTTAACAAGAATTTCTGATTTATAGCTATCTACAAGTTCTTTACTTGCTGAAC

CATCTTCTTTTACACCGAAACGTTGTGCTTGTAATAATAAATCAGCTGCTTTTTGATCATG
TGCTAATGTGTATGCCGTTGGTGGTAAGAAACCAACATCAACTTTTTAGACTTCATAGCT
TCAACAATTGTATTGTAGTTAGTTGATACAGACACTTTAACTGGAATCCCTAATTCTTTAG
ATAGTAATTTTTCTAATGGTTTTGCTTTAGCTTCTAATGTTCCAGCATTTTGCGAAGGTAC
AAATTGAACGGTTAATTCTTTAGGTTTGTATCCTCCTGATTTAGAATCCGAATCATTACTA
GCGTTCTTTTGATTATCTAAAGAACTTGAATTTCCACATGCTGCTGCAAAAACAATGACTG
CTAACATTAATACAAATAAACACTTAAAATTTTTCAT

## > Q5HJM4

Function: protein coding sequence; putative exported protein (core genome, variable)
Best match: Q5HJM4_CC001-ST772_118_AJGE01000016.1[101146:102690:r] (completely identical)
Position: 008-contig_256: 5142 … 6687; Length: 1545 bp
Sequence:
ATGAAAAAAATATATAAGTCATTAACTGTCTCTGCAATTGTTGCAACGGTATCATTAAGT
GCTTTACCGCAATCTTTAGCTATAACGCATGAATCGCAACCTACAAAGCAACAGCGAACG
GTATTATTCGATCGTTCTCATGGTCAAACAGCTGGTGCTGCAGATTGGGTTAGTGATGGTG
CATTTTCAGATTATGCGGATTCAATACAAAAACAAGGTTATGACGTTAAAGCTATTGATG
GTCATTCGAACATAACAGAAGCAAGTTTGAAAATTTCCAAAATATTTGTAATTCCTGAGG
CTAACATTCCTTTCAAAGAATCAGAACAGGCAGCAATTGTTAACTATGTGAAACAAGGTG
GCAATGTTGTCTTTATTTCAGATCATTACAATGCTGACCGAAATTTAAATCGTATTGATTC
ATCAGAGGCAATGAATGGTTATCGACGTGGAGCGTATGAAGATATGTCGAAAGGTATGA
ATGCAGAAGAAAAAAGTTCTACTACAATGCAAGGTGTGAAAAGTTCAGATTGGTTATCTA
CAAACTTTGGCGTACGTTTTCGATATAATGCACTAGGTGATTTAAATACGAGCAATATTGT
TTCTTCAAAAGAAAGTTTCGGTATTACTGAAGGTGTGAAATCTGTCTCTATGCATGCCGG
ATCGACATTAGCAATTACTAATCCAGAGAAAGCAAAAGGTATTGTGTATACACCAGAAC
AATTGCCAGCGAAAAGTAAATGGTCACATGCTGTAGATCAAGGTATTTATAATGGGGGCG
GTAAAGCAGAAGGCCCCTATGTAGCAATTTCTAAAGTTGGAAAAGGTAAAGCAGCATTT
ATCGGTGATTCATCACTTGTGGAAGATAGTTCGCCCAAATATGTAAGAGAAGATAATGGA
GAAAAGAAGAAAACATATGATGGTTTTAAAGAACAAGACAACGGTAAGCTATTAAATAA
TATAACGGCTTGGATGTCTAAAGATAATGATGGGAAATCACTTAAGGCGAGTGGCCTAAC
ATTAGATACAAAGACTAAGTTGCTTGATTTTGAACGACCAGAGCGTTCAACTGAGCCTGA
AAAAGAGCCATGGTCACAACCGCCGAGTGGTTATAAATGGTATGATCCAACAACATTTAA
AGCAGGTAGTTATGGCAGCGAAAAAGGCGCAGATCCTCAGCCAAACACACCAGATGATC
ATACACCACCAAATCAGAACGAAAAAGTAACATTTGATATCCCGCAAAATGTTTCTGTAA
ATGAGCCATTTGAAATGACAATACATTTAAAAGGATTTGAAGCAAATCAAACACTTGAAA
ATCTTAGAGTTGGTATTTACAAAGAAGGCGGACGTCAAATCGGACAATTTTCAAGTAAAG
ATAACGATTATAACCCACCAGGTTACAGTACTTTGCCAACAGTTAAAGCAGATGAAAACG
GAAATGTCACAATTAAGGTCAATGCTAAAGTACTTGAAAGTATGGAAGGTTCAAAGATTC
GTTTAAAACTCGGTGACAAAACCTTGATTACAACAGACTTCAAATAA

## > cpdB

Function: protein coding sequence; putative 5' nucleotidase (core genome, constant)
Best match: cpdB_ST425_LGA251-cow_FR821779.1[139788:141323]
Position: 008-contig_256: 6737 … 8273; Length: 1536 bp
Sequence:
ATGTCAAACATAGCATTTTATGTCGTGAGTGACGTACATGGTTATATTTTCCCAACAGATT
TTACGAGTAGAAATCAATATCAACCTATGGGATTGTTACTAGCGAATCATGTTATAGAAC
AAGACAGAAGGCAGTATGACCAAAGTTTTAAAATAGATAATGGTGATTTTTTGCAAGGGT
CACCATTTTGTAATTACTTAATCGCGCATAGCGGCAGTAGCCAGCCTTTAGTTGATTTTTA
TAATCGAATGGCATTCAACTTTGGTACGCTTGGTAATCATGAATTTAATTATGGATTACCA
TACTTAAAAGACACTTTACGCAGACTCAATTATCCAGTTTTGTGCGCTAATATTTATGAAA
ATGATAGTACATTGACTGATAACGGTGTGCAGTATTTTCAGGTTGGAGATCAAACGGTTG
GTGTGATAGGTTTAACGACACAATTTATTCCCCATTGGGAACAACCAGAGCATATTCAGT
CACTTACGTTTCATAGTGCTTTTGAAACACTTCAACAACACTTACCTGAAATGAAGCGAC

ATGCAGATATCATTGTGGTTTGTTACCATGGTGGATTTGAAAAGGATTTAGAAAGTGGTA
TGCCGACCGAAGTGTTAACGGGTGAAAATGAAGGATATGCCATGTTAGAAGCATTTTCTA
AAGATATAGATATCTTTATTACGGGTCACCAACATCGACAAATTGCTGAAAGATTTAAGC
AAACGGCTGTGATTCAACCTGGTACGAGAGGTACAACTGTAGGCAAAGTTGTCTTGAGTA
CTGATGAATATGAAAATGTATCCGTTGAATCATGTGAATTACTTCCTGTTATAGATGATTC
CACATTTACTATTGATGAAGATGACCAACATTTACGAAAGCAGTTAGAGGACTGGTTAGA
TTACGAAATTACTACATTGCCATATGATATGACGATTAATCATGCATTTGAGGCACGTGT
GGCACCGCATCCTTTTACTAATTTTATGAATTACGCTTTATTAGAAAAAAGTGGTGCAGAT
GTTGCCTGTACAGCTTTGTTTGATTCTGCTAGTGGTTTCAAGCAAGTCGTGACGATGCGAG
ATGTTATTAACAATTACCCATTTCCAAATACATTTAAAGTTTTAGCTGTAAGTGGTGCCAA
ACTTAAAGAAGCCATTGAACGATCAGCAGAATATTTTGACGTGAAAAATGATGAAGTAA
GTGTGAGCGCAGACTTCCTTGAACCCAAACCCCAACACTTTAATTATGATATATATGGTG
GCGTAAGTTATAACATTCATGTTGGAAGGCCAAAGGGACAACGTGTGAGCAATATGATG
ATCCAAGGGCACGCAGTTGATTTAAAGCAGACATATACAATTTGTGTAAATAATTATCGT
GCAGTTGGCGGTGGTCAGTATGATATGTATATCGACGCGCCAGTTGTAAAAGATATTCAA
GTTGAAGGCGCACAATTACTTATTGATTTTTTATCAAATAATAATTTGATGCGCATCCCGC
AAGTTGTTGATTTTAAAGTTGAAAAGTGA

> **A5IP16**

Function: protein coding sequence; putative protein (genomic island)
Best match: A5IP16_CC005_N315_BA000018.3[162124:162891]
Position: 008-contig_256: 8429 … 9197; Length: 768 bp
Sequence:
TTGGAAACAATTAAACAAGAAGTAAGAGAATCTATTGTTAAAGGAATTATCGCGGGATA
TAAGGACTATGTTAATGAGAGAAATGAAGTCAAGAAACGTATGGTGATTAGTGATGCCT
ATGCCTTTACTAAATCTAATCATATTGAAAGTCAAGTTGCTAAGCATTTGGAAAATTTTGT
TAAGTATATTAAAGAAAATGCTGGACCGTCATGGAAGTATTTAAAGTTTATATTTAATAT
GGATGAAGAGAAAAATAATATCATGTTTATATTGAAAAATGAAGATTACTTTGATGAAAA
AAATGTAAGCGTAGGGAAGAGTTTAGTTGCCGATAAAAATCAAAAAAGTAAAAACTATC
TAGAGGGACTTATGGCTAAGAATAGGGACATTAATTTTGATACTGTTGAAGAAGATTTTA
AAATTAGGCATCAAATGACTGCAGATAGTATATTATTTAATATAAAAGAAGGCAGTAATA
GAGATATCAATTCTTATTTTTTAATTATTACATATCGTATTGATAAAGAATCAAAGCAATT
AGCTGCAATCAAACAATGGTTACCAAATCCAGAAACAAATTCTGCAATTATGGTTGATGA
TTTGATAGAGTTGATAGAAAAAGTAATAGTAGAAAGAGAAGATTATCATATTGACGAAG
AAGAGTTAGAAGTACTTAAAAACGATGGAGAGTTAGAACTCATAGATGTGGAACATGCA
TTTGGTATCAGTATCGATGAAGGTAACGATATGATTGAAAGTGAAAGGTGA

> **Q1Y4B9**

Function: protein coding sequence; putative DNA-binding protein (core genome, variable)
Best match: Q1Y4B9_CC005_N315_BA000018.3[162897:164072]
Position: 008-contig_256: 9202 … 10378; Length: 1176 bp
Sequence:
ATGTTTATTGGCAAGAATTTAGAATACGTGAGAAAGCTTAATGCTCTAAGTAGAAAAGAA
TTATCAGAAAAAATAAATGTGAGTGAACAAGCAATTTGGCAATATGAAACAAAAAATAT
GATGCCAGAAATTAGTAAAATTTATGATATGACATCTATTTTTAATGTTAAATCTAGCTAC
TTTATTAGCGAGCAGCCCGAAGAGCTCCTAATAAATTCTGTCGATAAACATAGTATAGCT
TTTAGAGCAAAAAATTATAAAGTATCTACGAAACTATTAAATAAGCAATATTATCAAGCA
ATGTATTTATCTAATTTAACTAACTACTTATTTTCCTTTGTAAAGATACCGGATAATATTAT
TTTATCGTTAATAAATAATTTAGATGACTTGTTGAATGGCAATTTAGAATCACTAAATAAA
AAAGAAAGCATAAAAGAAACTGCTAAAGTTGTGAGGGCTAAAATTCTTCAAGACGAAAG
CAATGAAGCACTATTATTTATGCTTGAAAAAGCGGGTATAGTTATATACGAAAAGCGAAT
TAATGATAGCATAGACGCATATAGTTTTTGGAGTAAAGATCTGACACCTTTTATTATACTG
GGTACAAATAAGGGGGTAGCAGTTAGAAGGAATTTTGATCTTGCACATGAGCTGGGACA
TCTCGTTCTACATCGTCATATTCAATTCGACTTATTATCTCCTGAAGAATATAAGACAATT
GAACATGAGGCAGATATTTTCGCCTCTGAATTTTTACTACCTGAAGAAGCGTTTAAAAAA

GACTTTGATCAAATGACCAAAAAATCAAATCCTGACTATTTAGCTGTATTGAAAGAAAAA
TGGTATGTTTCTATACAAGCAATTGCAATGAGAGCGTATTATCTAGGCCTAATGTCTAGTA
CTCAGTACAGATATTTTTGGGCATCATTAAATAAAAAAGGCTATAAAAGTAAAGAGCCTT
TAGACGACGTGATTGAAATGTCACGTCCTGTCAAAATGAATAGTTTATTAAAGTTGTATTT
TGATAGAAATTTACTGACACCACAAAAATTATTGAATTATTTAAAAGTCGATGAAACATT
TTTGAATCATCTAGCAGGTATTAATTTAAAACTCTTTAAGGATTATGTTAATGAAAATAGA
GAATATAATATAACGAATCTATATAAATAA

> **txbi_Q1Y4B9**

Function: bidirectional rho independent terminator of Q1Y4B9 (core genome, constant)
Best match: txbi_Q1Y4B9_CC005_Mu50-VRSA_BA000017.4[164035:164097] (completely identical)
Position: 008-contig_256: 10379 … 10442; Length: 63 bp
Sequence:
GCACCAATATTTAAAACTCGAGCTGAAAAGATCATCATTTCAGTTCGAGTTTTTTTGTGTA
AC

> **adhE**

Function: protein coding sequence; alcohol-acetaldehyde dehydrogenase (core genome, constant)
Best match: adhE_CC001_MW2-USA400_BA000033.2[142665:145274]
Position: 008-contig_256: 10762 … 13372; Length: 2610 bp
Sequence:
ATGTTAACTATACCTGAAAAAGAAAATCGTGGATCGAAAGAACAAGAAGTGGCAATTAT
GATTGATGCTCTAGCTGACAAAGGGAAAAAAGCATTAGAAGCATTATCTAAAAAGTCAC
AAGAAGAAATTGATCATATTGTTCATCAAATGAGCTTAGCAGCTGTTGATCAACATATGG
TGCTAGCAAAATTAGCACATGAAGAAACTGGAAGAGGTATATACGAAGATAAAGCGATT
AAAAATTTATACGCTTCTGAATATATATGGAATTCAATAAAAGACAATAAGACAGTAGGG
ATTATTGGTGAAGATAAAGAAAAAGGATTAACGTATGTAGCGGAACCAATTGGTGTTATT
TGTGGTGTTACGCCAACAACAAATCCTACGTCGACAACTATTTTTAAAGCGATGATTGCA
ATTAAGACAGGAAATCCAATCATTTTTGCATTCCATCCAAGTGCACAAGAATCGTCGAAG
CGTGCAGCAGAAGTTGTATTAGAAGCGGCAATGAAGGCAGGTGCACCTAAAGATATTAT
TCAGTGGATTGAAGTGCCTTCTATCGAAGCAACAAAACAATTAATGAATCACAAAGGTAT
TGCATTAGTTCTAGCAACAGGTGGTTCGGGCATGGTTAAGTCTGCATATTCAACTGGCAA
ACCGGCATTAGGTGTGGGACCAGGTAACGTGCCGTCTTACATTGAAAAAACAGCACACAT
TAAACGTGCAGTAAATGATATCATTGGTTCAAAAACATTTGATAATGGTATGATTTGTGC
TTCTGAACAAGTTGTAGTCATTGATAAAGAAATTTATAAAGACGTTACTAATGAATTTAA
AGCACATCAAGCATATTTTGTTAAAAAAGATGAATTACAACGCTTAGAAAATGCAATTAT
GAATGAACAAAAAACAGGTATTAAGCCTGATATTGTCGGTAAATCTGCAGTTGAAATAGC
TGAATTAGCAGGTATACCTGTCCCCGAAAATACAAAACTTATCATAGCCGAAATTAGCGG
TGTAGGTTCAGACTATCCGTTATCTCGTGAAAAATTATCTCCAGTATTAGCCTTAGTAAAA
GCCCAATCTACAAAACAAGCATTTCAAATTTGTGAAGACACACTACATTTTGGTGGATTA
GGACACACAGCCGTTATCCATACAGAAGATGAAACATTACAAAAAGATTTTGGACTAAG
AATGAAAGCTTGTCGTGTACTTGTAAATACACCATCAGCGGTTGGAGGTATTGGTGATAT
GTATAACGAATTGATTCCGTCTTTAACATTAGGTTGTGGTTCCTACGGTAGAAACTCAATT
TCACATAATGTTAGTGCGACAGATTTATTAAACATTAAAACGATTGCTAAACGACGTAAT
AATACTCAAATTTTCAAGGTGCCTGCTCAAATTTATTTTGAAGAAAATGCAATCATGAGT
CTAACAACAATGGACAAGATTGAAAAAGTGATGATTGTCTGTGACCCTGGTATGGTAGAA
TTCGGTTATACAAAAACAGTTGAGAATGTATTAAGACAAAGAACGGAACAGCCTCAAAT
TAAAATATTTAGCGAAGTCGAACCGAACCCATCAACTAATACAGTATATAAAGGTCTGGA
AATGATGGTTGATTTCCAACCGGATACAATCATTGCACTTGGTGGTGGTTCAGCGATGGA
TGCTGCAAAAGCAATGTGGATGTTCTTTGAACACCCTGAGACATCATTCTTCGGTGCTAA
ACAAAAGTTCCTAGACATCGGTAAACGTACTTATAAAATAGGCATGCCTGAAAATGCGAC
GTTCATTTGTATCCCTACGACATCAGGTACAGGTTCAGAAGTAACACCATTTGCAGTTATC
ACAGATAGTGAAACAAATGTAAAATATCCGTTGGCTGATTTTGCTTTAACACCTGACGTT
GCAATTATTGACCCTCAATTTGTGATGAGTGTGCCAAAAAGCGTTACAGCAGATACAGGA

ATGGATGTACTAACGCATGCAATGGAATCATATGTATCTGTAATGGCTTCAGACTATACA
AGAGGTTTGAGTCTACAAGCGATTAAATTGACGTTCGAATATTTAAAATCATCTGTTGAA
AAGGGTGATAAAGTTTCAAGAGAGAAAATGCATAACGCATCAACTTTGGCTGGTATGGC
ATTTGCAAATGCATTCTTAGGTATTGCACACTCAATTGCACATAAAATTGGTGGCGAATA
TGGTATTCCGCATGGTAGAGCGAATGCGATATTACTACCGCATATTATCCGTTATAATGCC
AAAGACCCGCAAAAACATGCATTATTCCCTAAATATGAGTTCTTCAGAGCAGATACAGAT
TATGCAGATATTGCCAAATTCTTAGGATTAAAAGGGAATACGACAGAAGCACTCGTAGA
ATCATTAGCTAAAGCTGTCTACGAATTAGGTCAATCAGTCGGAATTGAAATGAATTTGAA
ATCACAAGGTGTGTCTGAAGAAGAATTAAATGAGTCAATTGATAGAATGGCAGAGCTCG
CATTTGAAGATCAATGTACAACTGCTAATCCTAAAGAAGCACTAATCAGTGAAATCAAAG
ATATCATTCAAACATCATATGATTATAAGCAATAA

## > tx_adhE

Function:  (core genome, constant)
Best match: tx_adhE_CC001_MW2-USA400_BA000033.2[145327:145383] (completely identical)
Position: 008-contig_256: 13424 … 13481; Length: 57 bp
Sequence:
CCACATAAACTTTAAGTCGATAATCATTATACGGTTATCGGCTTTTATTTATTGCCA

## > capA-L1

Function: protein coding sequence; capsular polysaccharide biosynthesis protein A/locus 1 (core genome, constant)
Best match: capA-L1_CC001_MSSA476_BX571857.1[144318:144986] (completely identical)
Position: 008-contig_256: 13716 … 14385; Length: 669 bp
Sequence:
ATGGAAAGTACATTAGAATTAACAAAAATTAAAGAAGTATTACAAAAAAACTTGAAGAT
TTTAATTATTTTACCGCTATTATTTTTAATTATTAGCGCTATTGTTACATTTTTCGTCTTATC
ACCTAAATATCAAGCTAATACTCAAATCTTAGTGAATCAAACTAAGGGTGACAATCCTCA
GTTTATGGCACAAGAGGTTCAAAGTAATATTCAACTTGTAAATACGTATAAAGAAATTGT
TAAAAGTCCTAGAATTTTAGATGAGGTGTCAAAGGACTTAAATGATAAGTATTCACCATC
TAAATTGTCGAGTATGTTGACAATTACAAACCAAGAAAATACGCAACTTATCAACATCCA
AGTTAAAAGTGGTCATAAACAAGATTCGGAAAAAATTGCGAATAGCTTCGCTAAAGTTAC
AAGTAAACAAATTCCGAAGATTATGAGTGTGGATAACGTATCAATTTTATCTAAAGCAGA
CGGTACAGCAGTTAAAGTCGCACCAAAAACTGTAGTGAATCTAATCGGTGCATTCTTTTT
AGGATTAGTTGTCGCGCTTATATATATCTTCTTCAAAGTAATTTTCGATAAGCGAATTAAA
GATGAAGAAGATGTAGAGAAAGAATTAGGATTGCCTGTATTGGGTTCAATTCAAAAATTT
AATTAA

## > capB-L1

Function: protein coding sequence; capsular polysaccharide biosynthesis protein B/locus 1 (core genome, constant)
Best match: capB-L1_CC005_Mu50-VRSA_BA000017.4[168054:168740] (completely identical)
Position: 008-contig_256: 14400 … 15087; Length: 687 bp
Sequence:
ATGTCAAAAAAGGAAAATACGACAACAACACTATTTGTATATGAAAAACCAAAATCAAC
AATTAGTGAAAAGTTTCGAGGTATACGTTCAAACATCATGTTTTCAAAAGCAAATGGTGA
AGTAAAGCGCTTATTGGTTACTTCTGAAAAGCCTGGTGCAGGTAAAAGTACAGTTGTATC
GAATGTAGCGATTACTTATGCACAAGCAGGCTATAAGACATTAGTTATTGATGGCGATAT
GCGTAAGCCAACACAAAACTATATTTTTAATGAGCAAAATAATAATGGACTATCAAGCTT
AATCATTGGTCGAACGACTATGTCAGAAGCAATTACGTCGACAGAAATTGAAAATTTAGA
TTTGCTAACAGCTGGCCCTGTACCTCCAAATCCATCTGAGTTAATTGGGTCTGAAAGGTTC
AAAGAATTAGTTGATCTGTTTAATAAACGTTACGACATTATTATTGTCGATACACCGCCA
GTTAATACTGTGACTGATGCACAACTATATGCGCGTGCTATTAAAGATAGTCTGTTAGTA
ATTGATAGTGAAAAAAATGATAAAAATGAAGTTAAAAAAGCAAAAGCACTTATGGAAAA

AGCAGGCAGTAACATTCTAGGTGTCATTTTGAACAAGACAAAGGTCGATAAATCTTCTAG
TTATTATCACTATTATGGAGATGAATAA

> **capC-L1**

Function: protein coding sequence; capsular polysaccharide biosynthesis protein C/locus 1 (core genome, constant)
Best match: capC-L1_CC001_MW2-USA400_BA000033.2[146991:147755]
Position: 008-contig_256: 15089 … 15854; Length: 765 bp
Sequence:
ATGATTGATATTCATAACCATATATTGCCTAATATCGATGACGGTCCGACAAATGAAACA
GAGATGCTGGATCTTTTAAAACAAGCGACAACACAAGGTGTTACAGAAATCATTGTAACA
TCACATCACTTACATCCTCGATATACCACACCTATAGAAAAAGTGAAATCATGTTTAAAC
CATATTGAAAGCTTAGAGGAAGTACAAGCACTAAATCTAAAGTTTTATTATGGTCAGGAA
ATAAGAATTACCGATCAAATTCTTAATGATATTGATCGAAAAGTTATTACCGGTATTAAT
GATTCACGCTATTTACTAATAGAATTTCCATCAAATGAAGTTCCACACTATACTGATCAAT
TATTTTTCGAATTACAGAGTAAAGGCTTTGTACCGATTATTGCACATCCAGAGCGGAATA
AAGCAATAAGTCAAAACCTTGACATACTATACGATTTAATTAACAAAGGTGCTTTAAGTC
AAGTGACAACGGCGTCATTAGCGGGTATTTCCGGTAAAAAAATTAGAAAATTAGCAATTC
AAATGATTGAAAACAATCTGACACATTTCATCGGTTCAGATGCGCATAACACAGAAATCA
GACCGTTCTTAATGAAAGACTTATTTAATGATAAGAAATTACGTGATTATTATGAAGATA
TGAACGGATTTATTAGTAATGCGAAGTTAGTTGTTGATGATAAAAAAATTCCTAAACGAA
TGCCACAACAAGATTATAAACAGAAAAGATGGTTTGGGTTATAA

> **capD**

Function: protein coding sequence; (core genome, constant)
Best match: capD_CC001_MW2-USA400_BA000033.2[147775:149598]
Position: 008-contig_256: 15873 … 17697; Length: 1824 bp
Sequence:
ATGGCACATTTATCTGTGAAATTGCGGCTTTTAATACTAGTATTAATCGATTCACTGATAG
TGACATTTTCAGTATTCGTAAGTTATTACATTTTAGAACCGTATTTCAAAACATATTCTGT
CAAATTATTAATATTGGCAGCTATATCACTATTCATATCGCATCATATTTCAGCATTTATT
TTTAATATGTATCATCGAGCGTGGGAATATGCCAGTGTGAGTGAATTGATTTTAATTGTTA
AAGCTGTGACGACATCTATCGTTATTACGATGGTGGTCGTGACAATTGTTACAGGCAATA
GACCGTTTTTTAGATTGTATTTAATTACTTGGATGATGCACTTGATTTTAATAGGTGGCTC
AAGGTTATTTTGGCGTATTTATCGGAAATACCTTGGAGGTAAGTCATTTAATAAGAAGCC
AACTTTAGTTGTTGGTGCTGGTCAAGCAGGTTCAATGCTGATTAGACAAATGTTGAAAAG
TGACGAAATGAAACTTGAACCGGTATTAGCAGTCGATGATGACGAACATAAACGCAATA
TCACAATTACTGAGGGTGTAAAAGTCCAAGGTAAAATTGCGGATATTCCAGAACTAGTGA
GGAAATATAAGATTAAAAAAATCATCATTGCAATTCCAACTATTGGTCAAGAGCGTTTGA
AAGAAATTAATAATATTTGCCATATGGATGGCGTTGAGTTATTGAAAATGCCAAATATAG
AAGACGTCATGTCTGGTGAGTTAGAAGTGAACCAACTTAAAAAAGTTGAAGTAGAAGAT
TTACTAGGCAGAGATCCTGTTGAATTAGATATGGATATGATATCAAATGAATTGACGAAT
AAAACTATTTTAGTTACGGGTGCAGGTGGTTCAATAGGATCAGAAATTTGTAGACAAGTT
TGTAATTTCTATCCAGAACGTATTATTCTACTTGGCCATGGTGAAAACAGTATTTATTTAA
TCAATCGTGAATTGCGAAATCGCTTCGGAAAAAAATGTTGATATCGTTCCTATTATAGCGG
ATGTGCAAAATAGAGCGCGTATGTTTGAAATTATGGAAACGTATAAACCATACGCAGTTT
ATCATGCAGCAGCACACAAGCACGTGCCGTTAATGGAAGACAACCCTGAAGAAGCAGTA
CGTAATAATATTTTAGGTACGAAAAATACTGCTGAAGCTGCTAAAAATGCAGAGGTAAA
GAAATTCGTTATGATTTCTACGGATAAAGCCGTTAATCCGCCTAATGTCATGGGCGCTTCA
AAGCGAATTGCAGAAATGATTATTCAAAGTTTAAATGATGAAACGCATCGAACAAATTTT
GTTGCAGTGAGATTTGGTAATGTACTTGGATCGAGAGGATCTGTGATTCCACTTTTCAAA
AGTCAAATTGAAGAAGGTGGGCCAGTTACTGTGACACATCCTGAAATGACACGTTACTTT
ATGACAATTCCTGAAGCTTCTAGACTAGTTTTGCAGGCAGGGGCATTAGCAGAAGGTGGC
GAAGTATTTGTGCTAGATATGGGAGAACCAGTGAAAATTGTTGATTTGGCACGTAATTTA
ATTAAGCTAAGTGGTAAAAAAGAGGACGACATACGCATTACTTATACAGGGATTAGACC

CGGCGAAAAAATGTTTGAAGAGCTTATGAATAAAGATGAAGTTCATCCTGAACAAGTATT
TGAAAAAATTTATCGTGGCAAAGTACAACATATGAAATGTAATGAAGTTGAAGCCATTAT
TCAAGACATCGTCAATGACTTTAGTAAAGAAAAAATTATTAACTATGCCAATGGCAAAAA
GGGAGATAATTATGTTCGATGA

> **capE**

Function: protein coding sequence; (core genome, constant)
Best match: capE_CC001_MW2-USA400_BA000033.2[149588:150616]
Position: 008-contig_256: 17686 … 18715; Length: 1029 bp
Sequence:
ATGTTCGATGACAAAATTTTATTAATTACTGGGGGCACAGGATCATTCGGTAATGCTGTT
ATGAAACGGTTTTTAGATTCTAATATTAAAGAAATTCGTATTTTTTCACGCGATGAGAAA
AAACAAGATGACATTCGAAAAAAATATAATAATTCAAAATTAAAGTTCTACATTGGTGAT
GTGCGTGATAGTCAAAGTGTAGAAACAGCAATGCGAGATGTTGATTACGTATTCCATGCA
GCAGCTTTAAAACAAGTGCCGTCATGTGAATTCTTTCCAGTTGAGGCAGTGAAGACAAAT
ATTATTGGTACAGAAAATGTCTTACAAAGTGCTATTCATCAAAATGTTAAAAAAGTCATA
TGTTTATCTACAGATAAGGCAGCGTATCCTATTAATGCTATGGGTATTTCAAAAGCAATG
ATGGAAAAAGTATTCGTAGCCAAATCAAGAAATATTCGTAGTGAACAAACGCTTATTTGT
GGTACAAGATACGGTAATGTGATGGCTTCAAGAGGATCAGTAATACCTTTGTTTATCGAC
AAAATCAAAGCTGGAGAACCTTTAACGATTACAGATCCTGATATGACAAGATTTTTAATG
AGCTTAGAAGATGCGGTAGAACTAGTTGTTCATGCATTTAAGCATGCAGAGACAGGAGAT
ATTATGGTTCAAAAAGCACCAAGCTCAACGGTAGGGGATCTTGCGACCGCATTATTAGAA
TTGTTTGAAGCTGATAATGCAATTGAAATCATTGGTACGCGACATGGAGAGAAAAAAGC
AGAAACATTGTTGACGAGAGAAGAATACGCACAATGTGAAGATATGGGTGATTATTTTA
GAGTGCCGGCAGACTCCAGAGATTTAAATTATAGTAATTATGTTGAACCCGGTAACGAAA
AGATTACGCAATCTTATGAATATAACTCCGATAATACACATATTTTAACGGTGGAAGAGA
TAAAAGAAAAACTTTTAACACTAGAATATGTTAGAAACGAATTGAATGATTATAAAGCTT
CAATGAGATAG

> **capF**

Function: protein coding sequence; (core genome, constant)
Best match: capF_CC001_MW2-USA400_BA000033.2[150629:151738]
Position: 008-contig_256: 18727 … 19837; Length: 1110 bp
Sequence:
TTGAATATTGTAATTACAGGAGCAAAAGGTTTTGTAGGAAAAAACTTGAAAGCAGATTTA
ACATCAACGACAGATCATCATATTTTCGAAGTACATCGACAAACTAAAGAGGAAGAATT
AGAGTCAGCATTGTTGAAAGCAGACTTTGTCGTGCATTTAGCGGGTGTTAATCGACCTGA
ACATGACAAAGAATTCAGCTTAGGAAACGTGAGTTATTTAGATCATGTACTTGATATATT
AACTAGAAATACGAAAAAGCCAGCGATATTATTATTGTCTTCAATACAAGCAACACAAG
ATAATCCTTATGGTGAGAGTAAGTTGCAAGGGGAACAGCTATTAAGAGAGTATGCCGAA
GAGTATGGCAATACGGTTTATATTTATCGCTGGCCAAATTTATTCGGCAAGTGGTGTAAG
CCGAATTATAACTCAGTGATAGCAACATTTTGTTACAAAATTGCACGTAACGAAGAGATT
CAAGTTAATGATCGGAATGTTGAACTAACGCTAAACTACGTGGATGATATCGTCGCTGAA
ATAAAGCGTGCTATTGAAGGAACTCCAACGATTGAAAATGGTGTACCTACAGTACCAAAC
GTATTTAAAGTGACATTGGGAGAAATTGTAGATTTATTATACAAGTTCAAACAGTCACGT
CTCGATCGAACATTGCCGAAATTAGATAACTTGTTTGAAAAAGATTTGTATAGTACGTAT
TTAAGCTATCTACCTAGTACAGACTTTAGTTATCCCTTACTTATGAATGTGGATGATAGGG
GTTCTTTTACAGAATTTATAAAAACACCGGATCGTGGTCAAGTTTCTGTAAATATTTCTAA
GCCAGGTATTACTAAAGGTAATCACTGGCATCATACTAAAAACGAAAAATTTCTAGTCGT
ATCAGGTAAAGGGGTAATTCGTTTTAGACATGTTAATGATGATGAAATCATTGAGTATTA
CGTTTCTGGCGACAAATTAGAAGTTGTAGACATACCAGTAGGATACACACATAATATTGA
AAATTTAGGTGACACAGATATGGTAACTATTATGTGGGTGAATGAAATGTTTGATCCAAA
TCAGCCAGATACGTATTTCTTGGAGGTATAG

> **capG**

Function: protein coding sequence; (core genome, variable)
Best match: capG_CC001_MW2-USA400_BA000033.2[151742:152866]
Position: 008-contig_256: 19840 … 20965; Length: 1125 bp
Sequence:
ATGGAAAAACTAAAATTAATGACAATAGTTGGTACAAGGCCTGAAATCATTCGTTTATCA
TCAACGATTAAAGCATGTGATCAATATTTTAATCAGATATTAGTACACACTGGTCAAAAT
TATGATTATACATTGAATCAAATTTTCTTTGATGATTTGGAATTGAGACAACCGGACCACT
ACTTAGAGGCAGTTGGAAGTAACCTTGGAGAAACGATGGGGAATATTATTGCGAAGACA
TATGACGTTTTATTACGCGAACAACCAGATGCACTTTTAATTCTTGGTGATACAAATAGTT
GTTTAGCAGCAGTATCTGCTAAACGATTAAAGATTCCTGTGTTCCACATGGAAGCGGGTA
ATAGATGCTTTGATCAGAATGTACCTGAAGAAATCAATCGTAAAATTGTTGACCATGTCA
GTGATGTGAATCTACCTTATACGGAACATAGCAGACGTTATTTATTAGATGAAGGCTTCA
ATAAAGCGAATATCTTTGTGACAGGATCACCGATGACAGAAGTGATAGAAGCGCATCGA
GATAAAATTAATCACAGTGACGTTTTAAATAAACTAGGATTAGAACCGCAACAATACATT
TTAGTATCTGCGCATAGAGAAGAGAATATCGATAATGAAAAGAATTTTAAATCATTAATG
AATGCGATAAATGATATTGCCAAAAAGTATAAAATGCCTGTGATTTATTCAACGCATCCA
AGAAGTTGGAAGAAAATTGAAGAAAGTAAATTTGAATTTGATCCATTAGTTAAACAGTTA
AAGCCATTTGGTTTCTTTGATTATAATGCATTGCAAAAAGATGCATTTGTTGTGCTATCAG
ATAGTGGAACATTGTCAGAAGAGTCGTCTATTTTGAAGTTCCCTGGTGTCCTTATTCGAAC
TTCCACAGAAAGACCGGAAGTACTAGATAAAGGTACGGTTATTGTAGGTGGTATTACCTA
TAACAATCTAATCCAATCCGTTGAACTAGCAAGAGAGATGCAAAACAATAACGAACCGA
TGATTGATGCTATTGATTATAAAGACACTAACGTTTCGACAAAGGTAGTTAAAATTATTC
AAAGCTATAAAGATATTATCAATCGAAATACTTGGAGGAAATGA

## > **capH**

Function: protein coding sequence; O-acetyl transferase (genomic island)
Best match: capH5_CC001-ST772_118_AJGE01000016.1[86222:86848:r] (completely identical)
Position: 008-contig_256: 20967 … 21594; Length: 627 bp
Sequence:
ATGAGGATAGCGATTGAAAAGATAATTGGTTTGCTGAAAAACCAGTCCTCTAAAGAATCG
AATGTTAAGATTCATCGCTTGGCGTATATTACAAACTCAAAATTTGATGGCAATAACTAT
ATAGATAGATGGTGTAAAATCAGGAATTCTCACATTGGTGAATACAGTTATATTGGATTT
GGTAGTGATTTTAATAATGTAGAAGTAGGAAGATATTGTTCGATATCTTCGGATGTAAAA
ATTGGGTTAGGAAAACATCCTACACACTTTTTTAGCTCATCACCGATTTTTTATTCTAATA
ATAATCCATTTAACATAAAGCAAAAGTTTATAGACTTTAATGACCAACCAAGCCGTACAA
CAATTAAAAATGATGTGTGGATTGGTGCAAATGTAATTATTATGGATGGTTTAACAATAA
ATACTGGTGCAGTCATAGCAGCCGGCTCAGTTGTTACTAAAAATGTAGGAGCATATGAGG
TTGTTGGTGGGGTTCCTGCAAAAGTGATTAAGAAGCGATTTGACAATAAAACAATTGAAA
AACTTTTGGAAAGCAAGTGGTGGGAGAAAACGCCTGATAAACTAAAAGGATTTTCGGTT
GAATATTTAAATAAAAAGGATACTTAA

## > **capI**

Function: protein coding sequence; capsular polysaccharide biosynthesis protein Cap5I (genomic island)
Best match: capI5_CC001-ST772_118_AJGE01000016.1[85108:86217:r] (completely identical)
Position: 008-contig_256: 21598 … 22708; Length: 1110 bp
Sequence:
ATGAGAATTTTAAATATTGTATCGAGTAATATTGTTCAAGACCCAAGGGTACTTAAACAA
ATGGAAACAATTAAAGACGTTACGGATGATTATAAAATTGTTGGAATGAATAATTCACAA
GCTACTAATAAGCGATTGGAAAATTTAGATTGTAATTATCGTTTGTTAGGTAGCAAGGTA
GATCCAAAAAATATTCTTTCTAAATTAATTAAGCGTATAAGATTTGCAACAGGTGTTATCC
GAGAAATTAAAGCTTATAAACCTGACGTGATTCATGCAAATGATTTCGACGTATTATTAA
TGGTCTATTTAAGCAATTATAAAAAAGCTAATATTGTTTATGATGCGCATGAAATATATG
CGAAAAATGCCTTTATTAATAAAGTTCCACTTATTTCAAAGTTTGTAGAAAGTATAGAAA
AACACATAGTAAAACATCGTGTTAATGCCTTCGTAACAGTAAGTCATGCAGCAAAAGAAT

ATTATCAATCTAAAGGATATAAGAAGGAAGCGAATGTTATTACGAATGCACCTATTTTAA
ATGATAGCAGAGAATTTAAAGAAATCGAAAACTTTAAAGAAATTGTATATCAAGGTCAA
ATTGTAATGGACAGAGGATATGAAGAGTTTATTATTGCTTCATCAGCTTTTAAACAAAAT
GCTCCTTCATTCATAATTCGAGGGTTTGGTCCGCATGAAGAAGTGATAAAAGAACTGATT
AGTTATAACTCGGAAAATATTAGGTTGGATAAACCAGTTGAAGTAAAAGAATTGGTTGAT
AAGTTAGCAGAAAGTAATGTTGGTGTTATCTTGACGAAACCTGTATCTATTAATTTTGAAT
ATACAGTATCTAATAAAATTTTTGAATGTATACATGCTGGTTTACCAGTAATTTTATCTCC
TGTCAAAGAGCATATTTATCTCAATGAAAAATATAAATTTGGCATTGTTTTAAAGGAAGT
TACGCCGTTAGAAATTGAAAAGGCGGTTAGAAAATTAAGAGATAATCACGATTTGTTTAA
TCATTTACGTCAAAATGCAATTAAGGCGTCTAAAATTTTGAATTGGCAAATAGAAAGTGA
ACGATTAGTAGAATTATATAAATTTTAA

## > capJ

Function: protein coding sequence; O-antigen polymerase (genomic island)
Best match: capJ5_CC009_21334_AGTW01000044.1[118940:120106:r] (completely identical)
Position: 008-contig_256: 22721 … 23888; Length: 1167 bp
Sequence:
ATGAAATTTTTTGTACTTTGTGCAATTATCAGCATGAACATATTTATAGTAATCTCTACAT
TTACTAAAGAAGTATTAGGGTTCCCTATAGAGCCGGTGTATTACTCTACCATGGTTGGTAT
AGCATTAATTACTACGGTGTTTGCTATTTATAAGATAATTGTCACGCAAGAAATTCCGCG
AGGGTTAATATTATTAATTGCTATATGTTTGCTTTATCTAGCTTTTTATTATTTTTCACCAG
ATAAGGAAGAGAAACTAGCTAAAAATAATATTCTATTCTTTTTAACATGGGCAGTTCCAG
CGGCAATTAGTGGTATTTATATTAAATATATAAACAAGGCTACGGTAGAAAGATTTTTTA
AATTAGTATTTTTCATATTTTCTATTTCATTTATTTTTGTAATTTTAATACCAAAACTTACA
GGTGAGATACCTAGCTATATCAATTTTGGACTTATGAACTATCAAAACGCTTCGTACCTTT
CAGCATTTACTGCCGGATTAGGCATTTATTTCATTATGAAAGGTTCAGTGAAACATAAGT
GGATATATGTTCTATTTACAATAATTGATATCCCTATTGTGTTTATACCAGGAGGGCGTGG
AGGTGCTATTTTTATTAATTCTTTACGGCTTATTTGCATTTATACTTATTACGTTTAAAAGAG
GAATACCTATTGCAGTAAAAAGCATTATGTATATTTTTGCATTAAGCATATCTAGTGTATT
GATTTACTTTCTTTTTACAAAAGGTTCGAATACTAGAACATTTTCATATCTACAAGGTGGA
ACACTTAATTTAGAAGGTACTTCTGGAAGAGGACCGATTTATGAAAAAGGTATTTACTTT
ATTCAACAAAGTCCGTTATTAGGCTATGGGCCATTTAACTATTATAAACTAATCGGAAAT
ATACCACATAACATCATTATTGAGTTGATTCTATCATTTGGCTTATTAGGGTTTTTTATCAT
AATGATTTGCATTTTGCTACTAGTTTATAAAATGATTAGGAACTATGATCCAAACACTATA
GATTTACTCGTTATGTTTATAGCAATCTATCCAATCACATTATTAATGTTTAGTTCAAATT
ATTTAGTTGTAAGTGAATTTTGGTTTGTGTTGTTCTATTTTATTACAAAAGGACGGCGTCA
TCATGGTTAA

## > tnpIS431-06

Function: protein coding sequence; transposase for IS431 (mobile element)
Best match: tnpIS431-06_circ_S-epidermidis_GQ900381.1[43799:43807] (completely identical)
Position: 008-contig_256: 23180 … 23189; Length: 9 bp
Sequence:
ATGAACTAT

## > capK

Function: protein coding sequence; capsular polysaccharide biosynthesis protein Cap5K (genomic island)
Best match: capK5_CC005_Mu50-VRSA_BA000017.4[177534:178739] (completely identical)
Position: 008-contig_256: 23880 … 25086; Length: 1206 bp
Sequence:
ATGGTTAAGAAAGTTTTTATTATGGATAGCGTAAAGACAATAATTGGTACGTTGCTTATA
GCTTTAGGATTACAATTTTTAGCTTATCCAATTATTAATCAACGAGTAGGTAATGAAGCGT
TTGGTTCTATTTTAACGATTTATACAATAATAACAATCACGAGTGTTGTATTAGGCAATAC
GCTTAACAATATACGATTAATTAATATGAATCTATACAAATCCAATCATTACTACTGGAA

ATTTGTGTCGATACTTTTAATTTCAATTCTGATTGAGAGTATAGCTTTAATTATTGTATTTC
TTTACTTTTTTAATTTGAACACCATCGATATTATCTTTTTAATTCTACTTAATATTTTAATG
TGTTTAAGGATTTATCTGAATGTATTTTTTAGGATGACTTTAAAATATAATCAGATTTTGT
ATATTGCTCTTATTCAATTTTTAGGTTTGCTGATAGGACTATTTCTATATTATTTAATCCAA
AACTGGATTGTTTGTTTTATTACCAGTGAATTGTTTGCAACGATATATACATTGGTTAAAT
TACGGGGATTAACTATAGGCGAGTATCAAAGTGAAGATAATAATGTGGTCAAAGATTAT
GTGATGCTACTGAGTACAAATAGCCTTAATAATTTGAATCTCTACTTAGATAGATTAATCT
TATTACCAATTATAGGTGGAACAGCTGTAACTATATCATTTCTTTCAACATTTATTGGGAA
AATGTTAGCTACATTTCTGTATCCGATTAATAATGTAGTACTTTCATATATTTCTGTAAAT
GAAAGCGACAATATAAAGAAGCAATATTTGAAAACTAATCTATTTGCTATAGCTGCACTA
TGTTTAGTCATGATTATATGTTATCCAATTACATTAATTATTGTCTCTTTACTGTATAACAT
TGATTCAAGTTTATATTCGAAGTTTATTATTTTAGGTAATATAGGTGTTTTATTCAATGCA
GTGAGTATTATGATCCAAACTTTAAATACAAAACACGCATCAATAACATTACAAGCGAAT
TATATGACGCTTCACACGATTACATTTATATTCATAACTATTTTAATGACAATTGCGTTTG
GTCTAAATGGATTCTTTTGGACAACGCTGTTCAGCAACATTATTAAGTATGTGATTTTAAA
TATTATAGGTTTAAAGTCTAAATTCATTAATAAAAAGGACGTCGATTAG

## > capL

Function: protein coding sequence; capsular polysaccharide synthesis enzyme (core genome, variable)
Best match: capL_CC001-ST772_118_AJGE01000016.1[81524:82729:r] (completely identical)
Position: 008-contig_256: 25086 … 26292; Length: 1206 bp
Sequence:
ATGAGTGAAAAAAAGATTTTGATTTTATGTCAGTATTTTTATCCGGAATATGTATCTTCTG
CGACGTTACCAACTCAATTGGCGGAAGATTTAATTGCGAATCACATTAATGTCGATGTCA
TGTGTGGATGGCCATATGAATATAGTAATCATAAACAGGTTTCTAAAACCGAGATGCATC
GTGGTATTCGCATTCGACGTCTCAAGTATTCGAGGTTTAATAACAAAAGTAAGGTTGGAA
GGATCATCAATTTCTTTAGTTTATTTTCAAAATTCGTGATTAATATACCTAAAATGTTGAA
ATATGATCAGATTCTTGTTTACTCTAATCCACCAATCTTGCCATTAATACCAGACGTTTTA
CACAGACTGCTTAAGAAAAAATATTCTTTTGTGGTGTATGATATAGCACCTGATAATGCG
ATTAAGACAGGTGCAACTCGTCCAGGTAGCATGATTGATAAGCTGATGCGTTACATTAAT
AGACATGTCTACAAGAATGCTGAAAATGTCATTGTCCTTGGTACGGAAATGAAAAACTAC
TTACTAAATCATCAAATTTCTAAAAATGCTGACAATATCCATGTGATTCCTAACTGGTATG
ACATGCGTCAATTACAAGACAATCGTATCTATAATGACACATTTAAAGCTTACCGTGAGC
AATACGACAAAATTTTATTGTATAGCGGTAATATGGGGCAGTTACAGGATATGGAGACAC
TTATCTCATTTTTAAAATTAAATAAGGATCAGCCTCAAACGTTAACAATACTTTGTGGTCA
TGGTAAGAAATTTGCAGATGTCAAAACGGCAATAGAAGACCATCGTATTGAAAATGTTA
AAATGTTTGAGTTTTTAACAGGTACAGACTATGCTGACGTATTAAAAATTGCGGATGTAT
GTATTGCATCGCTGATTAAAGAAGGCGTCGGTTTAGGCGTGCCGAGCAAGAATTATGGCT
ACCTTGCAGCTAAGAAACCGTTGGTACTCATCATGGATAAGCAATCTGATATCGTTCAAC
ATGTTGAACAATATGATGCGGGTATCCAAATTGATAATGGCGATGCACATGCCATTTATA
ACTTCATCAACACTCACTCGAGTAAGGAATTGCACGAGATGGGTGAGCGCGCACATCAAC
TGTTTAAAGATAAATATACGAGAGAAATTAATACTATGAAGTATTACAATCTGTTGAAGT
GA

## > capM

Function: protein coding sequence; capsular polysaccharide synthesis enzyme (core genome, variable)
Best match: capM_CC005_Mu50-VRSA_BA000017.4[179956:180513] (completely identical)
Position: 008-contig_256: 26302 … 26860; Length: 558 bp
Sequence:
ATGAAGCGATTATTCGATGTAGTGAGTTCAATATATGGTTTAGTAGTTTTAAGTCCGATTC
TGTTAATTACAGCATTACTAATTAAAATGGAATCACCTGGACCAGCCATTTTCAAACAAA
AAAGACCGACGATTAATAATGAATTGTTTAATATTTATAAGTTTAGATCAATGAAAATAG
ACACACCTAATGTTGCAACTGATTTAATGGATTCAACATCGTATATAACAAAGACAGGGA
AGGTCATTCGTAAGACCTCTATTGATGAATTGCCACAATTATTGAATGTTTTAAAAGGAG
AAATGTCAATTGTAGGTCCTAGACCAGCGCTTTATAATCAATACGAATTAATCGAAAAAC

GTACAAAAGCGAACGTGCATACGATTAGACCAGGTGTGACAGGACTAGCTCAAGTGATG
GGGAGAGATGATATTACTGATGATCAAAAAGTAGCGTATGATCATTATTACTTAACACAT
CAATCTATGATGCTTGATATGTATATCATATATAAAACAATTAAAAATATCGTTACTTCAG
AAGGTGTGCATCACTAA

> **capN**
Function: protein coding sequence; capsular polysaccharide synthesis enzyme (core genome, variable)
Best match: capN_CC001_MSSA476_BX571857.1[157643:158530]
Position: 008-contig_256: 27717 … 27747; Length: 30 bp
Sequence:
GATGAAACGACAACCAAAGATAAGGCATAA

> **capO**
Function: protein coding sequence; capsular polysaccharide synthesis enzyme (core genome, constant)
Best match: capO_CC001-ST772_118_AJGE01000016.1[78753:80015:r] (completely identical)
Position: 008-contig_256: 27800 … 29063; Length: 1263 bp
Sequence:
ATGAAGTTAACAGTAGTTGGCTTAGGTTATATTGGTTTACCAACATCAATTATGTTTGCAA
AACATGGCGTCGATGTGCTTGGTGTTGATATTAATCAGCAAACGATTGATAAGTTACAAA
GTGGTCAAATTAGTATTGAAGAACCTGGATTACAAGAGGTTTATGAAGAGGTACTGTCAT
CGGGAAAATTGAAGGTATCTACAACGCCAGAAGCATCTGATGTTTTTATCATTGCCGTTC
CGACGCCGAATAATGATGATCAGTACCGGTCATGTGACATTTCGCTAGTTATGCGTGCAT
TAGATAGTATTTTATCATTTTTAGAAAAAGGAAATACTATTATTGTAGAGTCGACAATTGC
GCCTAAAACGATGGATGATTTTGTAAAACCAGTCATCGAAAATTTAGGATTTACAATAGG
TGAAGATATTTATTTAGTGCATTGTCCAGAACGTGTACTGCCAGGAAAAATTTTAGAAGA
ATTAGTTCATAACAATCGTATCATTGGCGGTGTGACTAAAGCTTGTATTGAAGCGGGTAA
ATGTGTCTATCGCACATTCGTTCAGGGAGAAATGATTGAAACAGATGCACGTACTGCTGA
AATGAGTAAGCTAATGGAAAACACATATAGAGACGTGAACATTGCTTTAGCTAATGAATT
AACAAAAATTTGCAACAACTTAAATATTAATGTATTAGATGTGATTGAAATGGCAAACAA
ACATCCGCGTGTTAATATCCATCAACCTGGTCCAGGTGTAGGCGGTCATTGTTTAGCTGTT
GATCCGTACTTTATTATTGCTAAAGACCCTGAAAATGCAAAGTTAATTCAAACTGGACGT
GAAATTAATAATTCAATGCCGGCCTATGTTGTTGATACAACGAAGCAAATCATCAAAGCG
TTGAGCGGGAATAAAGTCACAGTCTTTGGTTTAACTTATAAAGGTGATGTTGATGATATA
AGAGAATCGCCAGCATTTGATATTTATGAGCTATTAAATCAAGAACCAGACATAGAAGTA
TGTGCTTATGATCCACATGTTGAATTAGATTTTGTGGAACATGATATGTCACATGCTGTCA
AAGACGCATCGCTAGTATTGATTTTAAGTGACCACTCAGAATTTAAAAATTTATCGGACA
GTCATTTTGATAAAATGAAGCATAAAGTGATTTTTGATACAAAAAATGTTGTGAAATCAT
CATTTGAAGATGTATCGTATTATAATTATGGCAATATATTTAATTTTATCGACAAATAA

> **capP**
Function: protein coding sequence; capsular polysaccharide synthesis enzyme (core genome, constant)
Best match: capP_CC001-ST772_118_AJGE01000016.1[77531:78706:r] (completely identical)
Position: 008-contig_256: 29109 … 30285; Length: 1176 bp
Sequence:
ATGTGTTTGAACTTCAGAGAGGATAATGTTATGAAAAAAATTATGGTTATTTTCGGTACG
AGACCCGAAGCAATAAAAATGGCACCATTAGTAAAAGAAATTGATCATAATGGGAACTT
TGAAGCGAACATTGTGATTACAGCACAACATAGAGATATGTTAGATAGTGTGTTAAGTAT
ATTTGATATTCAAGCTGATCATGATTTAAATATTATGCAAGATCAACAAACGTTAGCGGG
CCTTACGGCGAATGCGCTTGCTAAACTTGATAGCATCATTAATGAGGAACAACCGGATAT
GATTTTAGTACATGGTGATACTACAACGACTTTTGTAGGAAGTTTGGCAGCATTTTATCAT
CAAATTCCGGTCGGACATGTAGAAGCTGGACTTCGAACACATCAGAAATACTCACCATTT
CCTGAAGAGTTAAATCGAGTCATGGTAAGTAATATTGCTGAATTGAATTTTGCGCCAACA
GTAATTGCAGCTAAAAATTTACTTTTTGAAAACAAAGACAAAGAGCGTATCTTTATTACT
GGAAATACAGTTATTGACGCATTGTCAACAACAGTTCAAAATGATTTTGTTTCAACGATT
ATTAATAAACATAAAGGCAAGAAAGTTATTTTACTAACAGCGCATCGTCGTGAAAATATT

GGGGAACCGATGCATCAGATTTTTAAAGCAGTAAGAGATTTGGCAGATGAATATAAAGA
TGTTGTCTTCATTTATCCAATGCATCGTAATCCAAAGGTAAGAGCGATTGCCGAAAAATA
TTTATCTGGGAGAAATCGGATTGAATTAATTGAGCCATTAGATGCGATTGAGTTCCATAA
TTTTACAAATCAATCGTACCTCGTGCTGACAGATTCTGGTGGTATTCAAGAGGAGGCTCCT
ACATTTGGAAAACCTGTGTTGGTATTAAGGAATCATACAGAGCGTCCCGAAGGCGTTGAG
GCGGGAACATCGAGAGTAATTGGCACAGATTATGACAATATTGTTCGAAATGTGAAACA
ATTGATTGAGGATGATGAAGCGTATCAACGTATGAGTCAAGCGAATAATCCATATGGTGA
TGGACAAGCATCACGACGTATTTGTGAAGCAATAGAATATTATTTTGGATTGCGCACAGA
CAAGCCGGATGAATTCGTACCTTTACGTCACAAATAA

> **isdI**

Function: protein coding sequence; heme-degrading monooxygenase (core genome, variable)
Best match: isdI_CC008_NCTC8325_CP000253.1[136125:136451:r]RC (completely identical)
Position: 008-contig_256: 30349 … 30676; Length: 327 bp
Sequence:
TTATTTTTGATAGTGGTAGCCAATATCATATTTGAATACTTTATTTGATAATATTGGACTTT
GCTGTCCATCGTCATCACTTTTTAAACGTACATTTTTATGAGCTTCTTTAAATACATCGGA
ATTCAACCAATTATTAAAGCTATCTTCAGATTCCCAAATAGTTAAGATTTTAACTTCGTCT
GTATCCTCGGTATTTAATGTTTTAGTGACAAACATTTGTTGGAAGCCTTCAATAGTTTCAA
TACCTTGTCTATTGTAAAAACGTTCAATCGTTTCTTCCGCACTGCCTTTTTGTAATTGTAAT
CTATTTTCTGCCATAAACAT

> **aldA1**

Function: protein coding sequence; aldehyde dehydrogenase/locus 1 (core genome, constant)
Best match: aldA1_CC001_MSSA476_BX571857.1[162276:163763]
Position: 008-contig_256: 31492 … 32980; Length: 1488 bp
Sequence:
ATGGCAGTAAACGTTCGAGATTATATAGCAGAGAATTATGGTTTATTTATCAATGGGGAA
TTTGTTAAAGGTAGCAGTGACGAAACAATCGAAGTGACTAATCCAGCAACTGGAGAAAC
ACTATCACATATTACAAGAGCAAAAGATAAAGATGTCGATCATGCAGTCAAAGTGGCGC
AAGAGGCATTTGAATCATGGTCATTAACTTCTAAATCAGAACGTGCACAAATGTTGCGTG
ATATTGGTGATAAATTAATGGCACAAAAAGATAAAATTGCAATGATTGAAACATTAAATA
ATGGTAAACCGATTCGTGAGACAACAGCAATTGATATTCCATTTGCTGCAAGACATTTCC
ATTATTTCGCAAGTGTTATTGAAACAGAAGAAGGTACAGTAAATGATATCGATAAAGACA
CAATGAGTATCGTACGACATGAGCCGATTGGCGTCGTAGGTGCTGTTGTTGCTTGGAACT
TCCCAATGCTATTAGCTGCATGGAAGATTGCGCCAGCCATTGCTGCAGGTAATACAATTG
TGATTCAACCTTCGTCTTCAACACCATTAAGTTTATTGGAAGTTGCTAAAATTTTCCAAGA
GGTATTACCTAAAGGTGTTGTCAATATACTAACGGGTAAAGGTTCAGAATCAGGTAATGC
AATTTTCAATCATGATGGTGTAGATAAATTATCATTTACGGGCTCAACTGATGTAGGTTAT
CAAGTTGCCGAAGCTGCAGCAAAACATCTAGTACCCGCTACATTAGAGCTTGGTGGTAAA
AGCGCCAATATCATATTAGATGATGCTAATTTAGACCTTGCAGTTGAAGGTATTCAGTTA
GGTATTTTATTCAACCAAGGTGAAGTATGTAGTGCAGGTTCTCGATTATTAGTTCATGAAA
AAATTTATGATCAATTGGTGCCACGTTTACAAGAGGCATTTTCAAATATTAAAGTTGGAG
ATCCACAAGATGAAGCTACACAAATGGGTAGTCAAACTGGTAAGGATCAATTAGATAAA
ATTCAATCATATATTGATGCAGCAAAAGAATCAGATGCACAAATTTTAGCAGGCGGTCAT
CGCTTAACTGAAAATGGATTAGATAAAGGGTTCTTCTTTGAGCCGACATTAATTGCTGTG
CCAGACAATCATCACAAATTAGCACAAGAAGAAATATTTGGACCAGTGTTAACAGTTATT
AAAGTGAAGGACGATAAAGAAGCAATTGATATAGCTAATGATTCTGAGTATGGTTTAGC
AGGCGGTGTATTTTCTCAAAATATCACACGTGCATTAAATATTGCTAAAGCTGTACGTAC
AGGACGTATTTGGATTAACACTTACAACCAAGTACCAGAAGGCGCACCATTTGGTGGTTA
TAAAAAATCAGGTATCGGTCGAGAAACTTATAAAGGTGCGTTAAGTAACTATCAACAAGT
TAAAAATATTTATATTGATACAAGCAATGCTTTAAAAGGTTTGTACTAG

> **tauB**

Function: protein coding sequence; taurine ABC transporter/ATP-binding protein (core genome, variable)
Best match: tauB_CC001-ST772_118_AJGE01000016.1[71192:71932:r] (completely identical)
Position: 008-contig_256: 35883 … 36624; Length: 741 bp
Sequence:
ATGATTAAAATTCAACAATTACAACATCACTTTGGATCACATAAAGTAATTCATAACTTT
AATTTGGACATTAGCAAGGGAGAAATAGTCACTTTCATAGGGAAAAGTGGTTGCGGAAA
GTCTACTTTACTCAATATTATCGGTGGATTTATTCATCCATCGTCTGGTCGTGTCATTATTG
ATAACGAAATTAAACAACAGCCATCTCCAGATTGTTTAATGTTATTTCAACATCATAATTT
GCTGCCATGGAAAACGATTAATGACAACATTAGGATTGGATTTCAACAGAAAATTAGTGA
TGAAGAGATTAACGCACAGCTTAAATTAGTTGATTTAGAAGACAGGGGAAAGCATTTTCC
CGAGCAACTGTCCGGGGGTATGAAACAACGTGTGGCACTATGTCGAGCGCATGTGCATA
AGCCTAACGTTATATTGATGGATGAGCCATTAGGTGCATTAGATGCATTTACACGTTATA
AACTTCAGGATCAACTAGTGCAACTAAAACATAAAACGCAATCAACTATTATTTTAGTGA
CGCATGACATTGATGAAGCTATTTATCTTTCTGACCGCATTGTTCTGTTAGGTGAAGGGTG
CAATATTATTTCTCAATATGAAATTACAGCATCACATCCACGCAGTCGTAATGATAGCCA
CCTACTTAAGATTCGTAATGAAATTATGGAAACATTTGCATTGAATCATCATCAAGTTGA
ACCTGAATATTATTTATAA

> **tauA**
Function: protein coding sequence; taurine ABC transporter/substrate-binding protein (core genome, variable)
Best match: tauA_CC001_MW2-USA400_BA000033.2[168952:169926] (completely identical)
Position: 008-contig_256: 36637 … 37612; Length: 975 bp
Sequence:
ATGAAAAGGTTAAGCATAATCGTCATCATTGGAATCTTTATAATTACAGGATGTGATTGG
CAAAGGACGTCTAAAGAACCGTCTAAAAATGCCCAAAATCAGCAAGTGATTAAAATTGG
ATATTTGCCGATTACACATTCAGCTAATTTGATGATGACTAAAAAATTATTATCACAATAC
AATCATCCGAAATATAAACTAGAATTAGTTAAATTCAATAATTGGCCAGATTTAATGGAC
GCATTAAACAGTGGTCGTATTGATGGTGCATCAACTTTAATAGAGCTAGCGATGAAATCA
AAACAGAAGGGCTCAAATATAAAGGCTGTGGCATTGGGCCATCATGAAGGCAATGTCAT
TATGGGACAAAAAGGTATGCACTTAAATGAATTTAATAATAATGGCGATGATTACCATTT
TGGTATACCACATCGTTATTCAACACATTATCTTTTACTTGAGGAATTACGTAAACAATTA
AAGATTAAACCGGGGCATTTTAGCTATCATGAAATGTCGCCAGCAGAAATGCCAGCCGCA
TTGAGTGAACACAGAATTACAGGGTATTCTGTAGCCGAACCATTCGGTGCACTGGGTGAA
AAGTTAGGCAAAGGTAAGACTTTGAAACATGGTGATGACGTTATACCTGATGCGTATTGC
TGTGTGCTAGTACTGAGAGGGGAATTGCTTGATCAACACAAGGATGTAGCGCAAGCATTT
GTACAAGATTATAAAAAGTCTGGCTTTAAAATGAATGATCGCAAGCAAAGTGTAGACATT
ATGACGCATCATTTTAAACAAAGTCGTGACGTTTTAACACAGTCAGCGGCATGGACATCC
TATGGTGATTTAACAATTAAGCCATCCGGCTATCAAGAAATTACGACATTGGTAAAACAA
CATCATTTGTTTAATCCACCTGCATATGATGACTTTGTTGAACCGTCATTGTATAAGGAGG
CATCGCGTTCATGA

> **DUF2294**
Function: protein coding sequence; putative protein (genomic island)
Best match: DUF2294_CC001_MW2-USA400_BA000033.2[171945:172304] (completely identical)
Position: 008-contig_256: 39631 … 39991; Length: 360 bp
Sequence:
GTGAATACTATAGATACGCATACTAAAGAACAACAATTCTCGAATCTAGTAAGATCTTAT
CGTAAAGAATACGTGGGTAAAGGACCCAATAGTATTCGAGTGTCGTTTAAAGATAATTGG
GCGATTGCACATATGACAGGTGTTTTGAGTAAAGTTGAGAGTTTTTACCTAAACGACAAA
CGCAATGAATCGATGCTCCATTATACACGCACAGAGAAGATTAAACAGATGTATAAAGA
AATAGATGTAAATGAGATGGAAAGTCTTGTAGGCGCTAAGTTTGTAAAATTATTTACAGA
TATTGATTTGAATGATGATGAAGTCATTTCAATATTTGTTTTCGATAAGTCAATAGAATAA

> **fdh**

Function: protein coding sequence; NAD-dependent formate dehydrogenase (genomic island)
Best match: fdh_CC001-ST772_118_AJGE01000016.1[66627:67652:r] (completely identical)
Position: 008-contig_256: 40163 … 41189; Length: 1026 bp
Sequence:
ATGAAAATCGTAGCGTTATTTCCAGAAGCAGTAGAAGGTCAAGAAAATCAATTACTTAAT
ACTAAAAAAGCATTAGGATTAAAAACATTTTTAGAGGAAAGAGGACATGAGTTCATTAT
ATTAGCAGATAATGGTGAAGACTTAGATAAACATTTACCAGATATGGATGTGATTATTAG
TGCGCCATTTTATCCTGCATATATGACTCGTGAACGTATTGAAAAAGCACCGAACTTGAA
ATTAGCAATTACAGCAGGTGTAGGATCTGACCATGTAGATTTAGCGGCAGCAAGTGAACA
CAATATTGGTGTCGTTGAAGTTACAGGAAGTAATACAGTTAGTGTGGCAGAACATGCGGT
TATGGATTTATTAATACTTCTTAGAAACTATGAAGAAGGTCATCGTCAATCAGTAGAAGG
TGAATGGAACTTGTCTCAAGTAGGTAATCATGCGCATGAATTACAACACAAAACAATTGG
TATTTTTGGATTTGGTCGAATTGGACAACTTGTTGCTGAAAGATTAGCGCCATTTAATGTA
ACATTACAACACTATGATCCAATCAATCAACAAGACCATAAATTGTCTGAATTTGTAAGC
TTTGATGAACTTGTTTCAACAAGTGATGCGATTACAATTCATGCACCATTAACACCAGAA
ACTGATAACTTATTTGATAAAGATGTTTTAAGTCGTATGAAAAAACACAGTTATTTAGTG
AATACTGCACGTGGTAAAATTGTAAATCGCGATGCGTTAGTTGAAGCGTTAGCATCCGAG
CATTTACAAGGATATGCTGGTGATGTTTGGTATCCACAACCTGCACCTGCTGATCATCCAT
GGAGAACAATGCCTAGAAATGCTATGACGGTTCACTATTCAGGTATGACTTTAGAAGCAC
AAAAACGTATTGAAGATGGAGTTAAAGATATTTTAGAGCGTTTCTTCAATCATGAACCTT
TCCAAGATAAAGATATTATTGTTGCAAGTGGTCGTATTGCTAGTAAAAGTTATACAGCTA
AATAG

> **lmrP**

Function: protein coding sequence; integral membrane transport protein (core genome, variable)
Best match: lmrP_CC008_NCTC8325_CP000253.1[147348:148598] (completely identical)
Position: 008-contig_256: 41574 … 42825; Length: 1251 bp
Sequence:
ATGAAACGCTTAAGTACGACTTTGAAAGTACGATTGATTAGCAATTTTTTACAGCTAATT
ATTACGACAGCATTTATACCGTTTATAGCACTATATTTAACAGATATGTTAAGTCAATCAA
TTGTCGGTATATATCTTGTTGGTTTAGTGGTTCTAAAATTTCCATTGTCCATTATATCTGGT
TACCTTATTGAGATATTTCCGAAAAAGTTGCTAGTACTTATTTATCAAGCGACGATGGTGA
TAATGCTTGTGTTCATGGGCGTATTTGGGTCACATCAATTGTGGCAAATTATTGGTTTTTG
TGTTGCATATGCCATATTTACAATCGTTTGGGGATTACAATTTCCAGTTATGGACACATTA
ATTATGGATGCAATTACCGAAGACGTGGAACATTATATTTACAAGATTAGCTATTGGATG
ACAAACTTATCGGTAGCTATTGGGGCATTGTTAGGTGGCTTGATGTATGGCTACAGTATG
TTACTACTTTTCTTAATAGCAGCTTGTATATTTTTAATTGTACTCTTTATTTTATATATTTGG
TTACCTCAAGACCGAAATCAAGTAAAGCAAAGTGATGACAAGAGGCATGCAAGTCGTTA
TCAAAAATTACAAATAATGAATATATTTCGCAGTTATAAATTAGTTTTGAAAGACCGTAA
TTATATGTTATTGATTTCGGGGTTCAGTATCATCATGATGGGTGAATTTTCAATCTCCTCA
TATATTGCTATTAGACTAAAGGATCAGTTTGAAACAATAAGTATAGGTTCATATGATATT
ACAGGTGCTAAGATGTTAGCAATCTTGCTAATGATTAATACGGTCGTCGTCATTTTACTCA
CGTATTCAATCTCGAAAGTTGTATTGAAAATAGATTTTAAAAAAGCTTTAATCACTGGTTT
GCTGATTTATATTGTTGGCTATAGTGGTCTAACCTATCTTAATCAGTTTGGCTTATTAGTTG
TTTTTATGATAATTGCGACTGTAGGTGAAATTATTTATTCGCCTATAGTTTCAGAACAACG
CTTTAAAATTATTCCTAAAGCTAAAAGAGGAACATATAGTGCAGTTAATGCATTAGGTAT
TCATTTTTCAGAAACACTAGCTAGGTTAGGGATTGTGTTGGGTGTTTTCTTAACGTCATTA
CAAATGGGACTGTATATGTTTATCGTTTTAACAATTGGTGCTAGCATGCTTGTTGCTGGTG
TATTTGGGGGACAAAAACAAGTGAATACAAATTGA

> **ausA**

Function: protein coding sequence; non-ribosomal peptide synthetase (core genome, variable)
Best match: ausA_CC001-ST772_118_AJGE01000016.1[57369:64544:r] (completely identical)
Position: 008-contig_256: 43271 … 50447; Length: 7176 bp

Sequence:
ATGATTATGGGTAATTTGAGATTTCAACAGGAATATTTTCGTATATACAAAAATAATACA
GAATCAACGACACACCGTAATGCGTATTGGGTTAAACTCGCTAAAAATGTTGAAGCTACT
AAAATGATGTATGCATTATCGACAATTGTGCAACAACATGCATCTATAAGACATTTTTTTG
ATGTTACTACCGATGACAATTTAACAATGATACTTCATGAATTTCTGCCTTTTATTGAGAT
AAAACAAGTTCCATCTTCTTCCGCAAACTATGATTTAGAAGCTTTTTTTAAGCAAGAATTA
AGTACTTACCATTTTAATGATTCACCTTTATTCAAAGTTAAATTGTTTCAGTTCGCTGATG
CTGCATATATACTATTAGATTTTCATGTGTCCATTTTCGATGATAGTCAAATTGATATTTTT
CTTGATGATTTATGCAATGCATATCGTGGCAATACTGTTATTAACAATACTCGACAGCATG
CACATATAAATAGAAATGATGATAAAGACAATCAAGATGCATCGCATATAGCATTAGAC
TCAAACTATTTTCGGTTAGAGAATAACTCTGACATCCATATTGATAGTTATTTTCCAATTA
AGCATCCATTTGAACAAGCTTTATATCAAACGTATTTGATTGATGATATGACATCAATAG
ATATGGCATCGTTGGCTGTTAGTGTGTATTTAGCTAATCATATAATGAGTCAACAACATG
ATGTCACATTAGGTATACATGTACCATCACATTTACCAAATGATTTACACGGAAATATTGT
GCCGTTAACGTTAACAATCGATGCAAAAGATGTATGTCAACGTTTTACAACAGATTTTAA
TAAATGTGTGTTGCAAAATATGTCGCAATTACAGTGCGCGAAGTCTTCGCTTTCACTAGA
GACTATTTTTCATTGTTATCATCATATGATGTCTTGTTGTAATGATGTTATTGAGGATGTAC
ATCAAATACATGATGCACATACATCTTTAGCGGATATTGAAATTTTTCCACATCAACACG
GGTTCAAAATTATATATAACAGTGCAGCATATGATTTGCTCTCAATCGAGACGCTGAGTG
ACTTAGTTCGAAATATTTATTTGCAAATTACTGAAGAAAATGGAAATAAACGAACAACTG
TAGATGAACTTAATTTGATGACAGAACGTGATATTCAATTATATGACGATATCAATTTAA
GTTTGCCTGAGATAGATGATGCGCAAACAGTTGTTACCTTATTTGAGCAACAAGTTGAAG
CAACGCCGAATCATGTCGCTGTGCAATTTGACGGAGTGTTTATAACATATCAAACATTGA
ATGCACGCGCGAATGATTTAGCACACCGTTTGAGAAACCAGTATGGTGTTGAACCTAATG
ATCGTGTCGCTGTCATAGCTGAAAAAAGTATTGAGATGATAATAGCGATGATAGGTGTGT
TGAAAGCTGGTGGGGCTTACGTGCCAATTGATCCGAACTATCCAAGTGATCGTCAGGAGT
ACATTTTAAAAGATGCAACGCCTAAAGTTGTAATAACGTACCAAGCTTTATATGAAAATG
GTAAACAAAATATTAATCACATTGATTTGAATAAGATAGCGTGGAAAAATATTGATAATC
TTTCTAAATGTAACACGTTAGAAGATCATGCTTATGTTATTTACACGTCGGGGACAACTG
GTAACCCTAAAGGGACATTAATTCCGCACCGAGGTATTGTTCGCTTGGTCCATCAAAATC
ATTATGTACCATTAAATGAAGAGACGACGATTTTGTTATCAGGAACTATAGCCTTTGATG
CTGCAACATTTGAAATATATGGTGCATTGCTCAATGGTGGAAAGTTGATTGTTGCTAAAA
AAGAACAATTATTAAATCCAATAGCGTTAGAACAATTAATCAATGAAAATGACGTTAATA
CTATGTGGTTAACCTCCTCATTATTTAATCAGATTGCTAGTGAACGAATAGAAGTATTGGT
ACCGTTAAAGTATTTATTAATTGGTGGAGAAGTATTGAATGCTAAGTGGGTGGATTTGCT
TAATCAAAAACCGAAGCATCCTCAAATTATTAATGGTTATGGACCAACTGAAAATACAAC
ATTTACAACGACTTATAATATACCTAACAAAGTTCCAAATTGTATTCCTATTGGTAAACCG
ATTCTTGGTACTCATGTTTATATCATGCAAGGCGAGCGTCGATGTGGCGTTGGTATTCCTG
GAGAATTATGTACAAGTGGCTTTGGGTTAGCTGCAGGTTATTTAAATCAGCCAGAATTGA
CAGCAGATAAATTTATCAAAGATTCAAATATAAATCAGCTGATGTATAGAAGTGGTGATA
TCGTTCGTTTGTTACCCGATGGCAACATAGATTATTTATATCGAAAGGACAAACAAGTTA
AGATTCGAGGGTTTAGGATTGAGTTGTCAGAGGTTGAGCATGCGCTCGAGCGTATACAAG
GTATTAATAAAGCAGTTGTTATTGTTCAAAATCATGATCAAGATCAGTATATCGTTGCTTA
TTATGAAGCGATGCATACATTATCACATAATAAGATTAAATCACAATTACGTATGACCTT
ACCGGAGTACATGATACCAGTTAATTTCATGCATATTGAGCAAATTCCTATTACTATTAAT
GGGAAATTAGATAAGAAGGCATTGCCTATCATGGACTATGTCGATACGGATGCCTATGTA
GCACCGAGTACAGATACCGAACACTTGCTATGCCAAATTTTTGCAGATATTTTACATGTG
AATCAAGTAGGTATTCATGATAATTTCTTTGAATTAGGTGGCCATTCATTAAAAGCAACG
TTAGTGGTGAATCGGATAGAGGCATCTACTGGGAAACGATTACAAATTGGTGATTTATTA
CAAAAGCCAACTGTATTTGAACTAGCACAAGCGATTGCTAAGGTTCAAGAACAAAACTAT
GAAGTGATTCCAGAAACTATAGTTAAAGATGATTATGTGCTGAGCTCTGCACAAAAGCGT
ATGTATTTATTATGGAAATCAAACCATAAAGATACGGTGTATAACGTACCTTTTTTATGGC
GGTTATCATCAGAACTTAATGTAGCTCAATTGCGACAAGCAGTGCAGCGTTTGATAGCGC
GACATGAGATTTTACGAACACAATATATTGTTGTAGATGATGAGGTTCGACAACGTATTG
TGGCAGATGTTGCAGTTGACTTTGAAGAAGTTAACACGCATTTTACGGATGAACAAGAAA
TCATGCGCCAATTTGTAGCACCTTTTAATTTGGAAAAGCCAAGTCAAATTAGAGTGAGAT

ACATTAGAAGTCCCTTACATGCATACCTCTTTATAGATACACATCATATCATTAATGACGG
TATGAGTAATATACAATTAATGAATGATCTTAACGCACTTTATCAACATAAATTATTGTTA
CCACTTAAATTGCAATATAAAGACTATAGTGAGTGGATGTCGCATCGTGATATGACGAAA
CATAGACAATATTGGTTATCTCAATTCAAAGATGAAGTACCTATTTTAAGCTTACCGACA
GACTATGTTAGACCAAATATTAAAACGACAAATGGAGCAATGATGTCATTTACAATGAAT
CAACAAATGAGACAGCTACTTCAAAAGTATGTAGAAAAGCATCAAATTACTGATTTTATG
TTCTTTATGAGTGTGGTCATGACGTTGTTAAGCAGATATGCTCGAAAAGATGATGTTGTTG
TCGGTAGTGTGATGAGTGCGCGTATGCATAAAGGCACGGAGCAAATGCTAGGCATGTTTG
CTAATACGTTGGTATATAGAGGGCAACCGTCACCTGATAAAATGTGGACACAGTTTTTAC
AAGAGGTTAAGGAAATGAGTTTGGGTGCATACGAGCATCAAGAATACCCATTCGAATGTT
TAGTAAATGACTTAGATCAATCACATGATGCCTCACGGAATCCATTATTTGATGTCATGTT
AGTACTACAAAACAATGAAACGAATCATGCTCATTTTGGGCATAGTAAATTAACACACAT
TCAACCCAAATCAGTGACGGCGAAATTTGATTTATCTTTCATCATTGAAGAAGATCGCGA
TGACTATACAATCAATATCGAGTATAATACCGATTTATATCACTCAGAAACAGTTCGTCA
CATGGGTAATCAATGTATGATTATGATTGATTATATTTTGAAGCATCAAGATACACTACA
AATTTGTGATATACCAAACGGTACGGAGGAACTTCTAAATTGGGTCAATACGCATGTTAA
CGATCGAATGCTTAATGTCCCGGGAAATAAATCTATCATAAGTTACTTTAATGAAGCTGT
CTCACGACAAGGTAATCATGTTGCGCTAGTCATGAATGATTTGACAATGACGTATGAAAC
ATTACGCAACTATGTGGATGCCATTGCGCACATGCTCCTATCAAATGGTGTGGGCAATGG
TCAACGGGTTGCCTTGTTTACAGAACGTAGTTTTGAAATGATTGCGGCGATGTTGGCGAC
AGTTAAAGTAGGTGCATCTTATATACCTATCGATATTGATTTTCCGAATAAACGACAAGG
TGCAATTTTGGAGGATGCTAAAGTAACTGCAGTCATGTCTTACGGCGTTGAAATTGAAAC
GACATTACCAGTCATTCAATTGGAAAATGCTAAAGGCTTTGTTGAATCAAAGGAAAATGA
ACAATATGATGATTTACATGGCAATCAACTTGAAAACACAGCGATGTTAGATAATGAGAT
GTATGCTATTTACACATCTGGTACGACCGGGATGCCTAAAGGGGTTGCCATACGACAACG
AAATTTGTTGAATTTAGTGCATGCATGGTCAACTGAATTGCAATTAGGCGACAATGAAGT
ATTTTTGCAACATGCAAATATTGTTTTTGATGCATCAGTTATGGAGATTTATTGTTGTTTGT
TAAATGGTCATACGCTTGTGATTCCAGATAGAGAGGAACGTGTTAATCCAGAACAGTTAC
AACAACTCATTAATAAGCATCGTGTGACGGTTGCGTCGATTCCGTTACAGATGTGTAGTG
TTATGGAAGACTTTTATATTGAAAAGTTGATTACAGGCGGGGCAACTAGTACGGCATCCT
TTGTTAAATATATTGAGAAGCATTGTGGCACGTATTTCAATGCCTATGGACCATCTGAGTC
AACAGTCATCACATCGTATTGGTCACATCATTGTGGTGATTTGATACCTGAGACGATTCCA
ATTGGCAAACCCTTATCTAACATCCAAGTGTATATTATGTCAGATGGTTTGTTATGCGGTA
TTGGTATGCCAGGCGAGTTGTGTATTGCAGGTGATAGTTTAGCGATAGGATATATTAATC
GTCCAGAATTAATGGCTGATAAATGGCAAAATAATCCATTTGGTAAAGGAAAGTTGTATC
ATAGTGGTGATTTAGCACGTTATACATCTGATGGTCAAATTGAATTTTTAGGAAGAATAG
ATAAACAAGTGAAAGTTAACGGGTACCGTATTGAACTTGATGAAATTGAAAATGCAATAT
TAGCTATTCGTGGTATATCTGATTGTGTTGTAACAGTAAGTCACTTTGATACGCATGATAT
ATTGAATGCTTATTATGTTGGAGAGCAACAAGTGGAGCAGGATTTGAAGCAATATTTAAA
TGATCAGCTGCCTAAGTATATGATTCCTAAGACTATAACGCATATCGATTGTATGCCATTA
ACCACGAATGACAAGGTGGATACTACGCGTTTGCCAAATCCATCACCTATACAACAGTCT
AATAAAGTGTATAGCGAACCCTCTAATGAAATTGAGCAGACATTTGTTGATGTATTTGGA
GAGGTATTGAAACAAAATGATGTCGGTGTTGACGATGATTTCTTTGAACTTGGTGGTAAC
TCATTAGAGGCGATGTTAGTTGTCTCGCATTTAAAACGATTTGGCCATCATATTTCAATGC
AGACATTATACCAATATAAAACCGTGCGACAGATTGTTAATTATATGTACCAAAATCAAC
AATCATTAGTTGCATTACCGGATAATCTTTCGGAATTACAAAAGATTGTTATGTCTCGTTA
TAACTTGGGTATTTTAGAGGATAGTCTAAGTCATCGACCTCTAGGAAATACACTATTGAC
TGGCGCGACAGGTTTTTTAGGTGCTTATCTGATTGAAGCACTACAAGGATACAGTCATCG
CATTTATTGTTTCATACGTGCTGATAATGAGGAAATAGCATGGTATAAGTTGATGACGAA
TTTAAATGATTATTTTTCAGAAGAGACGGTTGAAATGATGTTATCAAACATTGAAGTCATT
GTTGGTGATTTCGAGTGTATGGATGATGTTGTTTTACCAGAAAACATGGATACGATTATTC
ATGCAGGTGCTCGTACAGATCACTTTGGTGATGATGATGAATTTGAAAAAGTAAATGTTC
AAGGTACTGTTGATGTCATACGTTTGGCACAACAACATCATGCAAGGTTAATATATGTGT
CTACGATAAGTGTGGGAACTTATTTTGATATAGACACAGAAGATGTGACATTTTCAGAAG
CGGATGTCTATAAAGGGCAACTACTAACATCACCATATACACGGAGCAAATTTTATAGTG
AATTAAAAGTATTAGAAGCTGTAAATAATGGCTTAGATGGTCGGATTGTACGTGTTGGTA

ATTTGACGAGTCCTTACAATGGAAGATGGCATATGAGAAATATAAAGACTAACCGTTTTT
CAATGGTAATGAATGATTTGTTACAACTGGATTGTATCGGGGTTAGCATGGCTGAAATGC
CTGTAGATTTTTCTTTTGTGGATACGACTGCAAGACAAATTGTCGCATTAGCACAGGTCAA
CACACCACAAATCATTTACCATGTGCTATCACCTAATAAAATGCCGGTGAAATCTTTGTTA
GAATGCGTTAAGCGCAAAGAAATTGAACTCGTCAGCGATGAATCATTTAATGAAATTTTA
CAGAAACAAGACATGTACGAAACGATTGGATTAACTAGTGTTGACCGTGAACAACAACT
AGCAATGATAGATACAACATTAACATTAAAAATAATGAATCACATCAGTGAAAAATGGC
CAACGATAACTAACAATTGGCTGTATCATTGGGCACAATATATCAAAACAATATTCAATA
AGTAA

## > **ausB**

Function: protein coding sequence; holo-acyl-carrier-protein synthase (core genome, constant)
Best match: ausB_CC001-ST772_118_AJGE01000016.1[56712:57356:r] (completely identical)
Position: 008-contig_256: 50459 … 51104; Length: 645 bp
Sequence:
ATGACAGTATTTGTAATGCAATTACAGAGTAACTTGAAAAGTATTGAAGAATTAATATCA
CAAAGTCGTTGGTCATATAAAAAACCGCGTACAGTCAACTATAGATACAATCAAGATAA
ACTCATGCACAGATTGGGAGATATTTTAGTGCAATATGGAATTCAACATGACACAGGTTT
ATTACCACATGAATGGCATTATCACATTTCGCCACGAGGTAAGGCAGATATTGTTCAACA
CAATCGTGATGGACAGCCCATCTATGTGAGCTTATCATATAGTTATCCTTATATCGTGTGT
GTTGTCGATAAAGAACCAGTTGGTATTGATATCGAAAAGATATCACAACGTTTAGACTGG
CGTACGTTAGTGACGTGTTTCTCTACAAACGAAGCACATCAAATATGTAGTTTAAATGAT
TTTTATCAAATATGGACACAAAAAGAAAGTTTTACAAAATTGATTGGTGAAGGTTTAATC
AAAGGATTGGATATTTATGATATGACACAATCACACTTTTATCAATCACGTGAAGTGAAG
TTCAAACAATTTATTTTTGATCAGTTTATGGTACAGGTATGTTTCTTAGGAGAGGCACCCT
GGGGTTATAAAAAAGTGTCTGTATTTCAGTTATTGAGTAGTTAA

## > **argB**

Function: protein coding sequence; acetylglutamate kinase
Best match: argB_CC001_MW2-USA400_BA000033.2[184495:185259:r]RC
Position: 008-contig_256: 52178 … 52943; Length: 765 bp
Sequence:
TTATGAATTAAGGATTGTTGTGCCAACAAAATCATTATTGTAAATAGATTCAATGATATTT
GGCTTGTTTCCTGATGCAATGATAACTTTAGGACAGCCATTTTCAATCGCATTTTTGGCAT
CTAGCACTTTGGGAATCATACCTCCATAAATATCACCATGTTCAATATATTGATGAATATC
GACTAATGGCAATTGAGGTATAACCATATCATTGATGAGTACACCTGCAATATTACTTAA
TACATAAATAGGCGCTTTTAATGATGATGCAATAAAATAGGCAAGCGTGTCAGCATTAAT
ATTGTAAAATTCTCCATCATGGTTATTGAAACCAATCGAATTGATGATAGGTACAAATTT
AGTACATATATACTGTAAAGCATCCTTATTTAAAGCGGTCGGGACACCGACATATCCATA
TTGTTGATCAAAACGTTTAATTTCAAATAATTGAGCATCCAAACCACATAAGCCTATCGC
AGAACATTGGTGCTGGTTAAATTGAGCTACTAATGCAGTGTTAACGTCTGCAATGAGCGT
GTGTTTAGTAATGGTCATGGTTGCTTTATCAGTCACTCTTAGGCCATTAACAAAGTGTGGC
TCGATTTGCTGGTTTGATAATGCATCATTAATAAATGGGCCACCGCCATGAACGATAATA
GGGTAGATGTTGTTTAATCGTAAATGCTTAATATTGTTAATAATTGATGGATGCATGTCAC
TAAGTGTACTGCCACCAATTTTAATGACAATAAATTTCAT

## > **argJ**

Function: protein coding sequence; arginine biosynthesis bifunct protein
Best match: argJ_CC001_MW2-USA400_BA000033.2[185275:186516:r]RC (completely identical)
Position: 008-contig_256: 52958 … 54200; Length: 1242 bp
Sequence:
TTATGTTCGATATGATGCGTTGATACGCACATAATCATAGGATAAATCACAACCGTATGC
AGTCGCTGCAGCGTTACCTAAACCAAGCTGAACGTCAATTGTGACATTTTCATGAGTTAA
TGTATTCGACATAGCTTGCTCATCAAATAGTACAGCCATACCTTTATCAACGACAGGTATT
TGGTTCAGTTGAACATATGTGCAGTTAGGATCAATTTCACATCCGCTGTAGCCAATAGCT

GTAATGATTCGACCAAAATTGGCATCTTCGCCAAAAATAGCTGATTTTACTAGATTTGAA
CTTACGATAGTTTTACCGATTTTTCTTGCATCTGATATTGATTTAGCGCCTGACACATTGA
CGCTGATTAACTTTGTTGCGCCTTCGCCATCTCTGGCTATAGCTTTAGCTAAAAATGTACA
GACAAAATTGAATGCATCAACAAATGTTTCCCATTGTGGATGGTCTTGACTAAGTATTTG
GTGTTCAACTTGGTGATTTGCCATGACTAATACCATGTCATTTGTACTTGTATCGCCATCA
ACAGTAATCATATTAAATGTATGGTCAGTCGAAGATTTTAATAATTGATGAAGTGTATTC
GATTCAATCGATGCATCGGTTGTTATAAAAGCAAGCATGGTAGCCATATTTGGGTGAATC
ATACCTGAACCTTTGGTGCTACCACCAATTGTAACGGTTTTACCATCGATTTTTAGTGATA
CAGCGATATGTTTTGTACAGGTATCAGTTGTTAAAATTGCCTCGTTAAACGCACCTGGCGT
TGCAAAATTAGCATCCTTAATATGTTCGGTCCCAGTCTTAATTTTATCCATAGGCAAATAT
TCACCAATGACCCCAGTTGAAGCAACAGCAACATGCTCAGATGGTATTTGAAGTTGTTGA
GCAACCCATGTTTGTGTTTGTCGTGCATCATCTATGCCTTGTTGACCGGTACAAGAATTTG
CATTAGCTGAATTAACAACAAGTGCTTGTAATTTTCCTTTAGACTTTTGTAAAGTGTCTTC
AGTGACAATAAGTGGTGCAGCTTTAAACTGATTTAAAGTATATACGGCAGCTGCACTTGC
CAAAGACGATGAGTAAATCCACCCAAAGTCTTTTTTGTTAGCGCGTAAACCGATGTGCAT
ACCACCAGCCGTGAAGCCTTGAGGTGTACTGATATCGCCATGTTTAATAATTGAAAAGTT
ATATTGTTGTGATGTCGTTTCTTGATGTTTCAT

> **argC**
Function: protein coding sequence; N-acetyl-gamma-glutamyl-phosphate reductase
Best match: argC_CC001_MW2-USA400_BA000033.2[186528:187562:r]RC
Position: 008-contig_256: 54211 … 55246; Length: 1035 bp
Sequence:
TTATGGATAAACTGGTGATTGATTTAGGCCAGTCGTCACTTCAAAATCATATAATATATTT
AAATTTTGAATGGCTTGCCCACTTGCGCCTTTGACAAGGTTATCAATCACTGATACTAAAA
TTGCTGTTTGCGTTGTTTCATCTACATAGATGCCGATATCGCAGTAGTTACTACCGAGTAC
TTCTTTTGTGGTTGGAAAAGTCCCAATATCTCTAATTCTGACAAATGGCTGATTAGCATAA
TAAGAGGTCATTAATTTATGTAATGATTCAGTCGTATATTCAGATGATAATTTGACATATA
TTGTTGATAAAATACCTCGTGTCATTGGTACGAGATGTGGTGTAAATATGACTGATACAT
CTTGACCCGCAATGATAGATAAATATTGCTCGATTTCCGGTTTGTGTTTATGGTTTCCGAT
TGCATAAGCGCTTAGATTTTCATTCATTTCTGAAAAATGAACACGTTGTGATAATGAACG
ACCAGCACCTGACACGCCGGTCTTAGCATCAATAATAATAGATGACAAATCTACTATTTT
TTCGCTAATAAGTGGATGTAATGCTAATAATGTTGCTGTAGGGAAACAGCCAGGATTAGA
AATGAGCTTCGTTCCATTGTTATCAAACGATTGCCATTCTGAAATGCTGTAAATAGCATGA
TTCAAATCATCTTGTGCTGCAGCAGTTTCTTTGTAATATGCTTCATATATTTCACGATTCTT
AATTCTAAATGCGCCAGATAAATCGATAACATGAATACCTTTTTCTACTAAGGGAGGGAT
ACATGTTTTACTTACGGGTGCTGGTGTCGCAAAGAAAATCACATCACAGTCATTATTGTCC
ACTGTAAGTGCTTCGAAATGTTGCATAATATGTTGTAAATGTGGAAATGTTAATTTCAAC
GGTTCATCTACTTTTGAATGTGAGTAGATGTGTGCAATCGTTACATGAGGATGTGTTTGTA
ACAATCGAATTAATTCAATTGCGCCATAACCGCTACCGCCAACGATACCTACTTTAATCA
TCAT

> **argD1**
Function:
Best match: argD1_CC001_MW2-USA400_BA000033.2[187598:188782:r]RC
Position: 008-contig_256: 55281 … 56466; Length: 1185 bp
Sequence:
TTAATTTTTAAAAACGTCTTGAAAAGCTGCAACAATTTGATGGATTTCCTCTTTATCAATG
ACTAGAGGTGGTGACAATCGAATGATAGTACGATGCGTGTCTTTGCACAAGATTCCACGT
TGAATCAGTTGATCCACAAAAGGTGCAGCATCTGTGTTAAGCTCTATGCCTATAAATAAA
CCACGACCTCTAATTTCTTTAATACTAGGATGTTTAAGTTGTAGCAACGCTTTTAATAAAA
ATGAACCTAAGCGTTCTGATCGTTCAACCAGTTGTTCATCTTTAAGTACATCAAGCGCTGC
CGTCGATATTGCAATGGCTAAAGGGTTACCACCAAATGTCGAACCATGTGTACCTGGTGT
TAGAACACGCATGACATCATTATTTGCAAGTACAGCAGATACAGGGTATAAGCCGCCACC
CAATGCCTTACCTAAAATATAAATGTCTGGAACAACTTGCTCCCATTCCATCGCAAACCA

TTTCCCAGTTCTACCAAGACCAACTTGAATTTCATCTGCAATCAATAATATTTGATATTTA
TCACATAGATGACGCACAGCTTGAATATATCCTTTCGGTGGTATATTAACGCCACCTTCAC
CTTGAATTGGTTCCAAAATAATTGCTGCTGTATTTGGTGAAATAGCTTGTGTTAATTGTTC
AATGTCTCCAAAATCTACAGTTGTAGTGCCTTGAAGTAGGGGGTGAAATCCTGCTTTATA
TGCGTCGTGGTTAGATAGTGATAATGAGCCAAGTGTACGACCGTGAAAATTGTTATTCAT
AGCGATGATTTCAACTTGTCCGTCAGTAATGCCTTTAACTTCAGAGCCCCATTTTCTAGCA
ATTTTAATGGCTGCTTCAACAGCTTCAGTACCAGAGTTAAGGGGGAGTACTTTGTCTTTCT
TAGCAAGATGACAAATTTTTTCTTCCCATTTCCCGAGATTGTCACTATAAAGGACACGTGA
AATGATAGACAACTTTGAAGCTTGTTCTGTCATCGCTTTAACAATTGTTGGATGACAATGG
CCTTGGTTTGCAACTGAAAAACCCGAAATGCAATCTATATATTGTTTGCCATCAGTATCCC
AAACTTTGACACCTTTACCTTTAGAAATGACAAGCTTAAGTGGTGCATAATTATTAGAGC
TATAATAATCAGTTAATTCAATGATTGAATTCAT

## > **rocD1**

Function: protein coding sequence; ornithine aminotransferase 1 (core genome, variable)
Best match: rocD1_CC001-ST772_118_AJGE01000016.1[51350:52534]RC (completely identical)
Position: 008-contig_256: 55281 … 56466; Length: 1185 bp
Sequence:
TTAATTTTTAAAAACGTCTTGAAAAGCTGCAACAATTTGATGGATTTCCTCTTTATCAATG
ACTAGAGGTGGTGACAATCGAATGATAGTACGATGCGTGTCTTTGCACAAGATTCCACGT
TGAATCAGTTGATCCACAAAAGGTGCAGCATCTGTGTTAAGCTCTATGCCTATAAATAAA
CCACGACCTCTAATTTCTTTAATACTAGGATGTTTAAGTTGTAGCAACGCTTTTAATAAAA
ATGAACCTAAGCGTTCTGATCGTTCAACCAGTTGTTCATCTTTAAGTACATCAAGCGCTGC
CGTCGATATTGCAATGGCTAAAGGGTTACCACCAAATGTCGAACCATGTGTACCTGGTGT
TAGAACACGCATGACATCATTATTTGCAAGTACAGCAGATACAGGGTATAAGCCGCCACC
CAATGCCTTACCTAAAATATAAATGTCTGGAACAACTTGCTCCCATTCCATCGCAAACCA
TTTCCCAGTTCTACCAAGACCAACTTGAATTTCATCTGCAATCAATAATATTTGATATTTA
TCACATAGATGACGCACAGCTTGAATATATCCTTTCGGTGGTATATTAACGCCACCTTCAC
CTTGAATTGGTTCCAAAATAATTGCTGCTGTATTTGGTGAAATAGCTTGTGTTAATTGTTC
AATGTCTCCAAAATCTACAGTTGTAGTGCCTTGAAGTAGGGGGTGAAATCCTGCTTTATA
TGCGTCGTGGTTAGATAGTGATAATGAGCCAAGTGTACGACCGTGAAAATTGTTATTCAT
AGCGATGATTTCAACTTGTCCGTCAGTAATGCCTTTAACTTCAGAGCCCCATTTTCTAGCA
ATTTTAATGGCTGCTTCAACAGCTTCAGTACCAGAGTTAAGGGGGAGTACTTTGTCTTTCT
TAGCAAGATGACAAATTTTTTCTTCCCATTTCCCGAGATTGTCACTATAAAGGACACGTGA
AATGATAGACAACTTTGAAGCTTGTTCTGTCATCGCTTTAACAATTGTTGGATGACAATGG
CCTTGGTTTGCAACTGAAAAACCCGAAATGCAATCTATATATTGTTTGCCATCAGTATCCC
AAACTTTGACACCTTTACCTTTAGAAATGACAAGCTTAAGTGGTGCATAATTATTAGAGC
TATAATAATCAGTTAATTCAATGATTGAATTCAT

## > **brnQ1**

Function: protein coding sequence; branched-chain amino acid transport system II carrier protein
Best match: brnQ1_CC008_NCTC8325_CP000253.1[162494:163849:r]RC (completely identical)
Position: 008-contig_256: 56718 … 58074; Length: 1356 bp
Sequence:
TTATGCTTGTGGAATTTTACGATGCTTAATTTTATAAATAATGAAGCCGATAATGAAACCA
ATCAAACTGAGAACAACCCAGCCCATACCAATGTCTGATAATGGTAAATATTTTTGGCTG
AAATTAATCAAAGTTTGTGAGAATGATGTGCTTGAAATGAACTCTGGACTAGCTTTTAAT
CCATCTACTAATGCAGCAATCATTGTAAAGAAAATGGTACATTGATAAATAAGTTTTGAA
TGATGGAATTTGCTACTAAATAATGTTAGTACAATCAAGGCAATTGCTAATGGATATAAG
AACATTAACACTGGGACTGAGTACATAATAATCTTAGTTAAACCAACATTCGCGAATAAG
AACGAAATAAAGCTTACAACTGTTGCAATCGCTAGGTAATTCATTTTAGGGAAAAGGTGT
TCGAATGTTTCTGAAAATGCCGTAATCAAACCGATGGCTGTTTTTAAACAAGCAACCATA
ACGATAAGTGACAACAGGACGATACCGTAGTTACCTAAGTAGTATTGAGTAATTTGCGCT
AAGGCAATACCACCATTTTCACTAAGTTTGAAATGACCAATACTTAATGTACCCATGATT
GCTAGTAGGGTATAAATGATCCCCATCATAATGATACTGATAGTACCAGACTTAATTGTT

TCTTTAGCGATATCAGTTGGATTTTCGATACCTAACTTTTTAATCGTTGCAACAATGATAA
TACCAAATGCCAATGACGCTAGCGCATCTAAGGTATTGTATCCATCTAAAAAGCCGTTAA
ATAAGGCATGTGATTGATATTGTTTACTAATAGGTGCATCAGATATGCCACCTAATGGAT
GGATAAAAGCAAATAATAAAATAATTGCTAATAATACTAAGAATACCGGATTTAAAAAT
TTACCGATATATTCTAAAATTCTTGATGGCTTTCTCGCAAAAAACCATGCAATCACAAAG
AAGACGAAGCTAAAAATAAATAAATATAAAGTGATTTGCTTTGGTGATAAAAATGGCGA
AAATGCAATTTCAAATGATGTCGTTGCCAGTCTAGGTAAGGCGAAAAATGGTCCGATAAC
TAGATATAAGGCAATCGTGAAAATGTAAGCATATGTTTTATTAACACGCGATGCAATTTC
AAATAAACCAGATGTCTTTGAAATGCCAATAGCAATGATACCTAGAAATGGTAAGCCAAT
TGCTGTAATTAAAAATCCTAAGTTAGCGATAAAAACGTTAGAACCAGCAGCTTGACCCAA
GTGTATTGGGAAGATAAGATTGCCGGCACCAAAGAATAAACCAAATAACATAGAACCTA
TAAACATGTTTTCTTTAAATGTTAGTTTCTTCTTCAT

> **Q5HJI6**

Function: protein coding sequence; isochorismatase (core genome, variable)
Best match: Q5HJI6_CC005_Mu50-VRSA_BA000017.4212238:212735:rRC
Position: 008-contig_256: 58350 … 58848; Length: 498 bp
Sequence:
TTAGTTCAATATATTTAGTAATTCGCTTGTGGTTACTGTTTGCCCCATTATAGGGAATACA
TTATCTATTGGAAATTGATGTAGCGTTTCGTTTTGTGCACTCATCATATCTGTAACAAAAA
ACTGATTGTAGTTTAATTGATAGGCATCTCGCGCTGTCGTATCTACGCCAATATGCGTTGC
GACACCACCAAGAACAATCGTATCAATTCCTCGACGTCGCAATTGTAAGTCCAAATCTGT
TCCTACAAATGCACTAAAATGTCGTTTGTCTATGACAAAATCGTCATCTCTCTTGTCTAAT
AAATGATGGAAACGACTGTAGTCGTCGCCTTCTTTTGGTGGTAATGAGATCATTGCATTTG
GTTGCAATACATCTTTACCATCATAGAAATTCACGCGAACAAAAGCGATAAAGCCATTGT
TTTTTCTAAAAACATCTATTAATTTATTAGCGTTTTGAACGACATTTTCAGCTGTATGTGG
CGCATAATCCAT

> **ipdC**

Function: protein coding sequence; indole-3-pyruvate decarboxylase (core genome, constant)
Best match: ipdC_CC001_MW2-USA400_BA000033.2[191290:192930:r]RC
Position: 008-contig_256: 58974 … 60615; Length: 1641 bp
Sequence:
TTATTTATTTTGTTGTGAAAAAGCTTTAGCGATATCGATGAGTTTCTTCGGTGCGTCTTCG
ACAGACATTTTGACTTCGACAAAATGCATCACATCGGGATGACCATTAATTGCATTAAAC
GTGTCTTGTAAATCTTTTGATGATTCAACGTCATGAATTTCAACATTTTTACCACCAAATA
CAGCTGGTAAAGCTTTATAATCCCACATGTGAATTTCATTATAAGGTTCATACATGCCGTG
AATAAGTCGTTCTACCGTATAGCCGTCATTATTAATCACAAATAATACCGGTTTAATATGC
TGTCTAATCATAGTTGAAATAGCTTGAACAGTTAGTTGCAATGAGCCATCACCAATTAAT
AATAAGTTACGACGATCTTTGTCTGCTAATTGTGAACCTAATGTTGCAGGTAATGTATAGC
CGATAGAACCCCATAACGGTTGCCCTATAAAAGTATTGTTTTTGTATAATGCTAAATCATA
AGCACCAAAGAATGATGTACCTTGATCAGCAATAATGACATCATTTGGTTTTAAGAAATT
TTGCATCATTTTAAAATAAGTTTGTTGTGTTAATGGTTCTGTGCCAACTGTATAATCGGGT
GATGTTGGACGATGATACGCAGGGAACGTTGCGTTATTCGTATGTGAAATATTGGATAAC
TGTTTTAACAATGATGGTAGAGATATTTCATCATTTGTAACATCGTCAATTTTGATATTGT
GATGATTTAACATAACGACATCATCGATATTGAATTGGTATGAAAAACCTGCTGTTGCTG
AATCTGTTAATTTGGCTCCAATATTTAAAATTAAATCGCTGTTGTCCACATAATCTCGTAT
TTTATCTTCGGCAATTTTCCCATCGTAAATACCCATATAATATGGATTTTCCTCATTAAAA
GCACCTTTTCCTAATGAAAGTTGTGCTACTGGTATCTGTGTTTGATTTACAAAATCTTCTA
ATTCTTGATGAAGGTGAAAACTGTTAATTTCATGTCCAGTAATGATGATAGGCTGCTTCG
ATTGATGCAATTTAGATGTTAATAACTCTATATATGTTGATGCATCCGTATCTTTGGCTGC
CGTTACTTCAAATGGTGTCGGTATCTCAATTTCAGAGATTGCGACATCGATTGGTAAATGT
AAATGAACTGGGCGTCTTTCGGCGATTGCTGTATTAATTAAACGTGGTATTTCGGTTGTTG
CATTTTCAGGTGTGATATAACCTTGTGCAACGGTTATATGTGCAAACATTTTTCGGTAGTC
GTCAAATGTACCTTCACCAAGTGAGTGATGTACATATTTACCGGCATGTTCAACAGCACG

TGTCGGCGCACCTGTAATCGCAATGACAGGTATGCGTTCAGCATATGAACCTGCGATACC
GTTGACGGCACTTAATTCGCCAACACCAAATGTAGTAACTAATGCAGCGAGTCCATTAAG
ACGGGCATAACCGTCCGCTGCGTAACTTGCGTTTAATTCATTTGTATTTCCTACCCAATCT
ACATTGGGATTGCTGATAATATCGTCTAGAAAAGCGAGATTAAAATCACCAGGAACACC
AAAAATTTTATCGACGCCTGCTCGATGAATAGCGTCAATTAAGTAAGCTCCAATGCGTTG
TTTCAT

> **glcA**

Function: protein coding sequence; glucose-specific PTS transporter protein/IIABC component (core genome, constant)
Best match: glcA_CC005_Mu50-VRSA_BA000017.4[214774:216819:r]RC
Position: 008-contig_256: 60887 … 62933; Length: 2046 bp
Sequence:
TTATTTAGCTTCAAATAATTGATCGCCAAATGAAATGTTGCCATGTTCACCTTGTTTAAAA
TCAAGGTTTGTAATGTTTCCTTGTGTCACGATAATAGGCGTAATATCACTCTTTGCATGAT
TGCGGATGTAGTCTAAATCAAAGTTGATTAATAAATCACCTTGTTTAACTTCTTGACCTTC
CTCAACATGTAAAGTAAAGCCTTCTCCGTTTAATTTAACAGTGTCTAAACCGATGTGGATT
AATAGTTCTAAACCACTATCTGATACAAGACCAATTGCATGTTTTGTTGGGAAAATCATTT
GTACTTTACCGTTGAATGGTGCACGAACTTCACCTTGTGAAGGTTTGATAGCGATACCGTC
ACCCATCATTTTTTCGCTGAACACTTGATCAGGCACTTCTGATAAAGGTGTTACTTCACCA
GTTAATGGTGCATGAACGATATGGCTCAATTCGCTTGTTGCAGATTTATCTTCTGCAACAA
CAACAGTTTCGTCTTTATCGTCTTCCATAGTAGTAGGGTTTTCTACTACTTGACCATTCATA
ATCTGTTGCATTTCATGTTTGATTTGGTCAGATTTAGGACCAAAAATTGCTTGCATATTAT
TGCCGACTTCTAATACACCAGATGCGCCTAAATCTTTCAAACCAGGAACATCAACTTTAG
ATTTGTCGTTAACTTCAACACGTAGACGTGTGATACAAGCGTCTAAATGTTTAATGTTTGC
TTTGCCACCCATAGCTTCTAATACTGCATATGGTAATTCAGTTGCTGAAGCAGTAGCCGCT
TGTGATTGTTTATCTTCACGACCTGGTGTTTTGTATTTTAATTTTACAATTAAGAATCGGA
ATACGAAGTAGTAAATAACTGCGTATACAAGACCTACAGGAATGACTAACCACCATTGTG
TCTTATTAGGTAGTATACCGAGTAAGAAGTAGTCGATGAAACCACCTGAGAATGTATAAC
CTAGATGAAGATCTAATAAGTACAATGTTAAGAATGATAAACCATCAAGTACTGCGTGAA
TAAAGAATAATAATGGTGCTACAAATAAGAATGAGAATTCTAATGGTTCTGTAATACCAG
TTAAGAATGATGTTAAAGCAGCAGAACCCATTAAACCTGCTACCACTTTCTTATTTTCAGG
TTTAGCTGTGTGATAAATTGCTAAAGCTGCTGCAGGTAAACCGAACATCATAACAGGGAA
TTCACCTTGCATGAATTTACCAGCTGTCAAATGTGCGCCTTCACGAATTTGTTCGATAAAG
ATACGTTGGTCACCGTGAATAATTTCACCAGCTGCATTTTTCCATGAACCAAACTCGAACC
AGAACGGTGCGTGGAAAATGTGATGTAGACCGAATGGAATTAATAAACGCTTGATGAAA
CCAAATAAGAATACGGCAACACCAGTATTTGAATCTAATAATCCTGTACTGAATGCATTT
AATCCTGATTGAATCGTTGGCCAAATTAATGCCATTGGGAATGCTAAAATAAATGATGTT
GTAGCCATCATAATAGGTACGAAACGCTTACCAGCAAAGAAACCTAAATAAGATGGTAA
GTTAATGTTATAGAACTTGTTATAACACCAAGCTGCCAGGGCCCCGATAATAATACCGCC
GAACACACCTGTTTGTAATGTTGGAATACCTAAAATGCTAGCGTAACCACTCGCTGGATC
AGTAACATTTTTAGGTGTAACTTGTAAAAAGTCGCCCATTGTTTTGTTCATGATTATGTAA
CCGACGAATGCTGCGATAGCTGCTACGCCATCACCGCCAGCTAATCCGATTGCGACACCT
AATGCGAAAATCATAGGTAAGTTTTCAAAAATAATACTACCGGCAGCTGTCATTAATTTA
GCGACATTTTGAACGCCACCATTTTGTATAAACGGCAAGTAGTGTTGTAATGCTTCGCCTT
GTATAGCTGTACCGATAGCTAATAACAGACCCGCTGCTGGTAAAATTGCAACAGGTAACA
TTAGCGCTTTACCAATACGTTGCAATTGACCGAAAAGTTTCTTCCTCAC

> **rsaK**

Function: noncoding RNA ncRNA of Staphylococcus aureus K (core genome, constant)
Best match: rsaK_CC001_MW2-USA400_BA000033.2[195307:195518:r]RC (completely identical)
Position: 008-contig_256: 62991 … 63203; Length: 212 bp
Sequence:
AATAGACTGTGTTGGTAACCATCACAGCTTAATTTAACTCATGCCTAATCTTACTTAGTAA
CACGTTGGTGTATGTAATTAATGTAAAAGAAGCAATTGACTAAGTAGTATATAAAACTGA

TACGTTAGTTTATCTAGCTTACCATCACATCTTATTGAATCTTATTTGTTTGCGACTCCTAT
TTTAGCATGGTGAAGTATGCGTTTTCAAT

> **murQ**

Function: protein coding sequence; N-acetylmuramic acid 6-phosphate etherase (core genome, constant)
Best match: murQ_CC001_MW2-USA400_BA000033.2196885:197784
Position: 008-contig_256: 64569 … 65469; Length: 900 bp
Sequence:
GTGATGGAAAATAGTACGACCGAAGCACGTAATGAAGCGACGATGCATCTTGATGAAAT
GACTGTGGAAGAGGCTTTAATTACGATGAATAAAGAAGATCAGCAAGTCCCGTTAGCAG
TTCGAAAGGCAATACCACAATTGACAAAAGTAATTAAAAAAACAATTGCACAGTATAAA
AAGGGTGGACGATTGATCTATATCGGTGCAGGTACAAGTGGAAGATTGGGTGTCTTAGAT
GCAGCGGAGTGTGTACCTACATTCAATACTGACCCTCATGAAATTATAGGTATTATTGCT
GGTGGACAACATGCTATGACGATGGCTGTAGAAGGTGCGGAAGATCACAAAAAATTAGC
GGAAGAAGATTTGAAAAATATAGATTTAACATCAAAAGATGTCGTTATAGGAATTGCCGC
GAGTGGCAAAACGCCATATGTTATAGGCGGTTTAACATTTGCTAACACAATCGGTGCTAC
AACAGTATCTATTTCATGCAATGAACATGCAGTTATAAGTGAAATTGCGCAGTATCCAGT
AGAAGTTAAAGTTGGTCCAGAAGTATTAACTGGTTCAACGCGTTTAAAGTCTGGTACAGC
ACAAAAATTAATTTTAAATATGATTTCAACCATCACAATGGTTGGTGTCGGAAAAGTTTA
TGATAACCTCATGATTGATGTTAAAGCAACCAATCAAAAACTGATCGACCGTTCAGTGCG
TATTATTCAAGAAATATGTGCTATCACATATGATGAAGCAATGGCGTTATATCAGGTATC
TGAGCATGATGTGAAAGTTGCGACAGTTATGGGTATGTGTGGCATTTCTAAGGAAGAAGC
AACAAGACGGTTATTAAACAATGGTGACATTGTTAAACGAGCAATCAGAGATAGACAAC
CTTAG

> **A6QDH7**

Function: protein coding sequence; putative phosphosugar-binding transcript regulator/RpiR family (core genome, constant)
Best match: A6QDH7_CC001_MW2-USA400_BA000033.2[199250:200128] (completely identical)
Position: 008-contig_256: 66934 … 67813; Length: 879 bp
Sequence:
ATGATGACAAATATTTTATATCGCATTGATAAGCAGTTGAGTGATTTTACGAAGACAGAA
AAGATAATCGCTGATTACATTTTAAAGAATCCACATAAAATCATTGATATGACTGTGAAT
GATTTGGCAGATGTTACGAATGTTAGTACAGCATCAATTGTTAGATTTAGTCGGAAAATG
ACACATCAAGGTTTTCAAGAGCTAAAGATTGCGATATCTCGATACTTACCCGAAGATATT
GCAACCAATCCACATTTAGAATTGATTGAAAATGAATCTGTAGAAACTTTGAAAAATAAA
ATGATTGCTAGAGCAACGAATACGATGCGATTTGTAGCTACTAATATTATGGATGCGCAA
ATTGATGCAATTTGTGATGTGTTGAAAAATGCCAGGACAATATTTTTATTTGGATTTGGCG
CATCGAGTTTGACTATTGGTGATCTTTTTCAAAAGTTATCTCGTATTGGCTTAAATGTCAG
GTTATTACATGAAACGCATTTACTTGTGTCAACATTTGCGACGCATGATGATAGAGATTG
CATGATTTTTGTGACGAATCAAGGTAGTCATAGTGAATTGCAGTCAATTGCACAGGTGGC
CACACATTACAGTATTCCCATCATAACTATATCTAGTACAGCTAATAATCCAGTGGCTCA
AATTGCAGACTATGCATTGATTTATGGCAGAACTGATGAAAATGAAATGCGTATGGCGGC
TACAACGTCACTATTTGCACAGTTATTCACGGTAGATATATTGTACTATCGATTTGTAGCA
TTAAATTATCATGCGATTCTAGATTGTATAACCCAATCGAAAATGGCACTTGATAATTAC
AGGAAGCATCTTGCGACGATAGATTTTAAACATTAG

> **A5IP63**

Function: protein coding sequence; putative protein (core genome, constant)
Best match: A5IP63_ref_CC001_MW2-USA400_BA000033.2[200322:200603:r]RC
Position: 008-contig_256: 68125 … 68275; Length: 150 bp
Sequence:

TAGGTAGTAGTACATAAAGAGTATGGCTGCGAACATGCCGAATATTGAAGTGAAGTTACC
TACAGTGACTGCCATGTAATGATTTAAAGTTAAATAGTAACTTAGACTAATAATAAAAAA
TAAAATGATTTGATAGGTAATTAACACCAT

## > **hsdR**

Function: protein coding sequence; type I restriction-modification system endonuclease (core genome, constant)
Best match: hsdR_CC001_MW2-USA400_BA000033.2[200816:203605]
Position: 008-contig_256: 68487 … 71277; Length: 2790 bp
Sequence:
ATGGCATACCAAAGTGAATACGCATTAGAAAATGAAATGATGAATCAACTTGAACAATT
GGGTTACGAAAGAGTAACAATACGTGATAATAAGCAATTGCTTGATAATTTTAGAACGAT
TTTAAATGAGCGTCATGCGGACAAATTAGAAGGCAATCCCTTAACAGATAAAGAATTTCA
ACGTCTGTTAACGATGATTGATGGGAAAAGTATTTTCGAGAGTGCCCGTATTTTACGTGA
TAAATTACCACTTAGACGTGATGATGAGTCTGAGGTTTATTTGTCGTTTTTAGATACGAAA
AGTTGGTGTAAAAATAAGTTTCAAGTGACGAATCAAGTATCTGTCGAGGATACATATAAA
GCACGTTATGATGTAACGATATTAATCAACGGACTACCCCTTGTCCAAGTTGAATTGAAA
CGTCGAGGTATTGATATTAATGAGGCGTTTAACCAAGTAAAACGTTACCGCAAACAAAAT
TACACAGGCTTATTCCGCTACATACAAATGTTTATCATTAGTAATGGTGTTGAAACGCGAT
ACTTTTCTAATAATGATAGCGAACTATTGAAGAGTCACATGTTTTATTGGAGTGATAAAC
AGAATAACCGAATCAATACATTACAATCGTTTGCTGAGTCGTTTATGAGACCTTGTCAATT
AGCTAAGATGATATCGCGCTATATGATTATTAATGAAACAGATAGAATACTGATGGCAAT
GCGTCCGTATCAAGTGTATGCGGTAGAAGCACTTATTCAACAAGCGACTGAGACAGGGA
ATAATGGATATGTATGGCATACAACTGGAAGTGGTAAGACATTGACTTCTTTTAAAGCGA
GTCAGATTTTATCACAGCAAGATGACATTAAGAAAGTTATCTTTTTGGTTGACCGTAAAG
ACTTGGATAGTCAAACAGAAGAGGAATTTAATAAATTTGCTAAGGGTGCTGTAGACAAA
ACTTTTAATACCTCGCAACTGGTACGCCAACTAAATGATAAAAGTTTGCCACTTATTGTAA
CGACGATACAAAAAATGGCTAAAGCGATTCAAGGTAATGCCCATTTATTAGAACAGTATA
AAACGAATAAAGTTGTATTTATTATTGATGAGTGTCATCGCAGTCAATTTGGTGACATGC
ATCGTCTAGTTAAACAACATTTCAAAAATGCCCAATACTTTGGATTCACTGGTACGCCAC
GTTTTCCAGAAAATAGTAGTCAAGATGGTAGAACAACTGCAGATATTTTCGGTAGATGCT
TACATACGTATTTAATTAGAGATGCCATTCATGATGGTAATGTACTTGGTTTCTCAGTTGA
CTATATTAATACTTTTAAAAATAAAGCTTTAAAAGCAGAAGATAACAGCATGGTTGAAGC
AATTGATACGGAAGAAGTATGGTTAGCGGATAAACGTGTGGAATTAGTAACACGACATA
TCATCAATAATCATGATAAATATACACGTAATCGTCAATATTCAAGTATATTTACAGTCCA
AAGTATTCACGCGCTTATTAAATATTATGAGACATTTAAGCGACTTAACAAAAAGTTGGA
ACAACCGTTAACGATAGCTGGTATATTTACGTTTAAACCTAATGAAGATGATCGTGATGG
TGAAGTGCCATATCATTCACGTGAAAAATTAGAGATAATGATTAGCGATTATAATAAAAA
GTTCGAGACGAATTTTTCAACAGACACAACTAATGAGTATTTTAATCATATTTCAAAAAA
CGTTAAAAAGGGCGTTAAAGATAGTAAAATTGATATCTTAATCGTTGTTAATATGTTCTTA
ACTGGTTTTGATAGTAAAGTACTGAACACTTTATATGTTGATAAGAATTTAATGTATCATG
ATTTAATTCAAGCGTATTCACGTACAAATAGGGTTGAAAAAGAATCAAAGCCATTTGGTA
AAATTGTAAACTATCGTGACTTGAAAAAAGAGACAGACGATGCACTGAGAGTATTCTCAC
AAACAAATGATACGGATACAATTTTAATGCGCAGTTATGAAGAGTATAAAAAAGAATTT
ATGGACGCTTATCGTGAGCTTAAAATGATTGTGCCGACACCACACATGGTTGATGACATT
CAAGATGAAGAAGAGCTAAAGCGCTTTGTTGAAGCTTATCGTTTATTAGCTAAAATAATA
TTACGTTTAAAAGCATTTGACGAGTTTGAGTTTACAATTGATGAAATTGGAATGGATGAA
CAAGAGAATGAAGACTATAAAAGTAAATATTTAGCTGTGTACGATCAAGTAAAAAGAGC
GACGGCTGAGAAAAATAAAGTATCCATTTTAAATGATATTGATTTCGAAATAGAAATGAT
GCGTAATGATACGATTAATGTGAATTATATTATGAATATATTGAGACAAATTGATCTTGA
AGACAAAGCGGAACAACGTCGTAACCAAGAACAAATTAGACGCATTTTAGATCATGCAG
ATGATCCGACATTGAGGTTAAAACGAGATCTAATTAGAGAATTCATCGACAATGTTGTAC
CTTCTTTAAATAAGGATGATGATATCGATCAAGAATATGTTAATTTCGAAAGTATTAAAA
AAGAAGCGGAGTTCAAAGGATTTGCTGGAGAGAGATCTATCGATGAACAAGCCCTAAAA
ACAATTTCAAATGACTACCAGTATAGTGGTGTTGTAAACCCACATCACCTTAAAAAAATG

ATTGGTGATTTGCCATTGAAAGAAAAGCGTAAAGCAAGAAAAGCCATTGAATCTTTCGTG
GCAGAAACAACTGAAAAATACGGTGTGTAA

## > **hsdRx**

Function: protein coding sequence; type I restriction-modification system endonuclease
Best match: hsdR_part_DQ413432.1[1:432]
Position: 008-contig_256: 69888 … 70320; Length: 432 bp
Sequence:
TGGTTAGCGGATAAACGTGTGGAATTAGTAACACGACATATCATCAATAATCATGATAAA
TATACACGTAATCGTCAATATTCAAGTATATTTACAGTCCAAAGTATTCACGCGCTTATTA
AATATTATGAGACATTTAAGCGACTTAACAAAAAGTTGGAACAACCGTTAACGATAGCTG
GTATATTTACGTTTAAACCTAATGAAGATGATCGTGATGGTGAAGTGCCATATCATTCAC
GTGAAAAATTAGAGATAATGATTAGCGATTATAATAAAAAGTTCGAGACGAATTTTTCAA
CAGACACAACTAATGAGTATTTTAATCATATTTCAAAAAACGTTAAAAAGGGCGTTAAAG
ATAGTAAAATTGATATCTTAATCGTTGTTAATATGTTCTTAACTGGTTTTGATAGTAAAGT
ACTGAACACT

## > **Q5HJH7**

Function: protein coding sequence; putative protein (genomic island)
Best match: Q5HJH7_CC080_11819-97_CP003194.1[219535:220377] (completely identical)
Position: 008-contig_256: 71481 … 72324; Length: 843 bp
Sequence:
ATGGATAATAGAAATATGATTAATCGTGTTTTTAGTCAAAAGATATTACATCAAATTGCA
ATCAAAAATAAAAGTGATGTTGTTGATGAGGCATATGATTTTTATATACAGGGACCTAAA
AATATCAATGTAATACAGAAGATGAAATCTTTATATAATTATCTTAAAAAGTCTTATCGT
AACGAATATTTTTACAAAAACACAATACTTAATAAACTCCTTCTAGGACGACATTCTATT
AATACAACTACTGCACTTTCTGAGATGCCCATAGGGAAAAGTATTGCTGATTTTATATTGT
TAAACGGCAAAGGCGTTGTCTATGAGATTAAAACAGAATTAGATAAGCTGGATAGATTA
GATAATCAAATTAATGATTATTATGAAGTGTTTAATTATGTAGTAGTTATTACAAATGACA
AACATCTGAATAAAGTTATGGCTAGATACAAAGATACAACAGTTGGAATTTTAGTGTTAA
CAACTAGAAATACACTGAGTGAAGTTCAAAAACCTAAAGAAAACAATAGTCTCTTAAAC
ACAAAAGCGATGTATAACTTTTTACGAAAAGAAGAAAGAAAAAGAGTTATTGCACAAAA
TCATATGGATGTGCCAACTTATAATGATTTCACAGAGTATGATGTGTTATTTGACGTGTTT
AAAGAAATACCAATGACGAAACTGCATAACAATATGATTTCTGAGTTGAAAAAAAGAGG
CAACATGAAAGAATACAAAGATGAATTTTTAGCAGCGCCGGCTGAAATTAAGTTCTTGTT
ATATTTCGCAAAAATGACAAAGAAAGATAAAAATAAACTATATCATTTTCTTAAGGAGGA
TTAA

## > **Q9RL82**

Function: protein coding sequence; putative protein (genomic island)
Best match: Q9RL82_CC012_21266_AFTT01000027.1[229333:230256:r] (completely identical)
Position: 008-contig_256: 72325 … 73249; Length: 924 bp
Sequence:
ATGTATTATCCTTATTTGCGTGGGAAACAAAATGAACTATTTGCAATTAGAGAATTGTTA
GAGAAAGGTTTGATTGGTGATTGTATTCAACCTATAATTGAACCGATTAAGTATACAACC
ACGTTTAAAAATGTTTTGAAATATTGTGGTGAAAAAGAATTTCGTATAAATTTAGTAGTA
AATTCGAAGTTAACTGAAGAAGAGATTAGTAACGAAACTGTTGAAGACTTAAGCGAAAT
AATAACAAAAAACAAAAGTGTTATTCAAAAAGCTTACTTGGGTCCTTCTGATGAAGGCGA
TGATAGGTTGAAACAGCAATTTTCAAGTAATAGTTTAGCTATTTTAACAAGTGTAGATGA
CTGGGAAATGTTTGGAGATAAAAATAAACTTGAAATGGTTTTTGTACCAGATGATAGACA
CATTAAACGTAAATTGCGTAATATTCCAAACAAAGGCATCATTATGGATCCTTTTAATAA
ACTAAGTCGTAATGTTGATTATTTAGATAATGATGACGAGTTTTATAGCGACGATCACCTT
TATTATAAGGAAGATGGATACGTAGCATTTTCAGACTATTCTGTTATAGGTGGAGAATAT
GTAGACGGTGGCTTTTCGCCATTAGCTATTGCGATACATATTGTCTATTTTGATGAGGCTA
ATGAGTTAAGAGTTAAACATTTTGTCTCTGATTCTAATAACGATAGATTAAATCCAGCTA

AAAAGTTTTTTGAGGCTGTAGATAAATTAGTAACTTGGTCTAAAAACTTAGATAATAAAA
ATAGATCTTATGCAATCGGACAATTTGAAGAATTAAATGAAAATAATAAGTATCCAGGAT
TAGGTTTAATTAAAAGGTTATCTATCATGCATCATCTAGAAATTATGAATAGATACTTGG
AGTCTCAAAATGAAAATATGTGA

## > A6QDI2

Function: protein coding sequence; putative protein (genomic island)
Best match: A6QDI2_CC080_11819-97_CP003194.1[221289:222401] (completely identical)
Position: 008-contig_256: 73235 … 74348; Length: 1113 bp
Sequence:
ATGAAAATATGTGAAAAATGTTTTAATAATACTGAAATCGTAGAAATCATTGTAAATGAT
AATAGCAAATTTGACAATTGTGATATTGATAACGATCATCTTGGTGTTAAAATATTTGATA
CGACTCGACACATAGATAAATTAGAGCAGATTAGAGATTATTTAAGACCAGCGTTAGAAT
TATATGATATTAGTATAAATTTACCAGATACTTTTAGCCAAAAAGAAGGTAAAAAAATTG
AAACAGCATTAAAAGATGATTGGAGTATATTTAATGTTGAAGAAGCTCAAATAAGTTGTA
TTTTAAATGAACTTTTTAAAGATGATGAAAATATAGATAGACGGGTGTTGGAAGGTTTAG
TAAGCGCTAAAATCATTAAAGATAAAAAATATACAAATGAAAATCTAATTGTAGCAAAT
AATGACTGGGATGGATTTTGTGAGAGCTTGAAGTATAAAAATAGATTTCATAATAATATG
ATCAATTTAGAGAATTTAGCGTTCTTTCTCGATATCACTACTAATTACTATAGTATTGAGG
AATTTAGAGAAAGATTTGAACCATTATATAGATCTAGAATAGTTAAGTTCATTACAGAGA
AAAGTGAACTAATGTCACCACCAAAGGGATTTGCAACGGCAGGAAGATTGAATTCTAAA
TGGATAAGCGTATTATACCTTAGTACAAAAGAACAGGTCAGTATAGAAGAAGTTAAACCT
AAGCATAATGACATTATTTATATAGGTAAGGTTAAGTTACAAAAAAACGTTACAAAAGA
GAAATTGAAAATTGCGAATTTAACTAATTTAACTAGTAATGCAATCAAAGCTGGCGATGA
TGGATTTAGAAAGTATTTTGTAAATCACCAAACATTAAAAAAGATTCATAAAGGAATAAC
AAATCCAAGCGATGAACAAGGAATAGATTATTTGCCATTTCAATATTTAGCTGATTATAT
TCGAAGCCTTAAATATGACGGGATAATGTATGAGAGCATATTGCAAGATGGTACTTTTAA
CTTTGTGTTTTTTGATCAAAATTTATTTGAATGTGTTAATTATGAAAGAAAAAGAGTAAGT
GATGTTAAATATACATTGACTAAGCTAAGTTGA

## > tx_oppF2

Function:  rho independent terminator
Best match: tx_oppF2_CC008_TCH1516_CP000730.1[234728:234786:r]RC (completely identical)
Position: 008-contig_256: 74373 … 74432; Length: 59 bp
Sequence:
ATAAAATATAAATAATCCCCCGCACTTCACATAGTGGCGGGGGATTTATTCTGGCGTTA

## > oppF2

Function: protein coding sequence; putative oligopeptide ABC transporter/ATP-binding protein (core genome, constant)
Best match: oppF2_CC001-ST772_118_AJGE01000016.1[31611:33203]RC (completely identical)
Position: 008-contig_256: 74612 … 76205; Length: 1593 bp
Sequence:
TTATTTAGCAATAACTGCTACTTCTGAAATAAGTTGCTTTGCATAGTCTGATTGCGGATGT
TTGATAATATCTTCTGTGTTATTCAGTTCAACGATTTCGCCATTTTTCATAACTGCTACGCG
ATCACATATTTCATTGATAACACCCATGTCATGTGTGATGAATAAATAAGTGATGCCGAA
GTCTAACTGTAATTGTTTTAATAACTCGATGATATCTTTTTGAATTGAAACGTCTAAAGCG
GACACTGCCTCGTCGCAAACAATCACTTTAGGTTCTACAGCAAGTGCTCTCGCGATACTT
ACACGCTGACGTTGCCCACCAGATAATTCGTGTGGATAGCGATATAAGAAACTTTGATCT
AGGCCAACTTTTTCTAACAACGATACGACAGTTTTAATAATGTCATCATTATCTTTGACTT
TCCCATGAATGATTAGTGGTCGTTTAATCACATCAATGACTTTAAATCTTGGATTAATAGA
TGCGAATGGATCTTGAAAAATCATTTGTATCTCTTGTCGTAAAGATTTCAATTCATCATCT
TTAAATAAACTTAATGGTAATTCGTTATACCAAATAAAGCCTTCTGACACTTCCTTTAGAC
CGACGACCGTCTTAGCTAATGTCGATTTCCCTGACCCTGATTCACCGACAATGCCTAATGT
TTCGCCTTTTCTAATACCCAAGTTAATATCATTAACTGCTCGGTATAGGCTGCCACTCGGT

GATGTGTAATCCACGCTCACGCGATCGAATTTTAATAAAATATCATTGTTTAACGGTCTTG
GCGGACGCGTTTGATGAATATCAGGAATCGCATCTATTAAGCGTTTTGTATAGGTATGTT
GTGGCGATTTAAAAATACTTTCAACCGTGCCACTTTCAACGACACTTCCATCTTTCATTAC
AATCACATCGTCGCAAAATTGATACACAGCGCCTAAATCGTGAGTGATAAAAATAATAG
ATGTTTCTGTGTACTCATAAAGGGACTTCATTAACTGCAGTAATTGATTTTGTGTACTGGC
ATCTAATGCCGTTGTTGGTTCATCTGCGATTAAAATTTGTGGCTTTAAAATCAATGCCATT
GCTATCATGACACGTTGACGCATACCACCAGAAAGTTCATGTGGATAAGCATCAAATTGT
CGAGTTGCATGTTTTATACCTACTTTTTCTAAAATGTCTATTGTCATCGACTTTGCTTCAGA
TTTAGATACACGTTTATGTTGAAATAGTACTTCTGTAATTTGTTTGCCAATCGTTAATCTTG
GATTCAACGAAGAGAGTGGATCTTGAAAAATCATTGAAATATCCTTACCTCGAATTTGTT
GTAACGCTGAAGTTGATAAATTATTTAACGATTGCCCATTAAAAATAATTTCTCCTGTTAA
TGTGTGATCTGGATAATCTGGTAGTAGCCCTAAAATAGATTTAGCAGTAATACTTTTTCCT
GATCCTGATTCACCAACAATACCTAGGATATGTTTTTTTCGTAATTCGAAAGAGACATTTT
TTACCGCTTGAACTGTAGTTTCATCATAATTGAATTGTACATTCAGACTGTTGACTTCTAA
TAAATTTGACAT

## > oppB4

Function: protein coding sequence; putative oligopeptide ABC transporter/permease protein (core genome, constant)
Best match: oppB4_CC001_MW2-USA400_BA000033.2[213096:214403]
Position: 008-contig_256: 76332 … 77640; Length: 1308 bp
Sequence:
TTGTATCACAAGGCATTTTACAAGTTTGTATTATCGGTATTAAGCTTGCCAATATTTTTAT
ATGCAGTGATAAAGTTTTTCTTTTCTGCTAAGAGAAAAAATTTTTACGCTAATAATTCTGA
AATTTCAGAAATTGAACGAGCGTTACATCAAAAATATAAATATTTATCGCAGCAAAAGTC
ATCCACACAAATACATAAAGAAGCATTAAAAATATTCAAGGCACAAAGTTCTAATACGA
GTTCAAAGAATATTGAACAAGCACATTTTTCAACATACTTTGAAAATTTATTATTTCATAA
GTTCATCATGATCAAAGTAATATTGGCCTTGCCGATGTTCATCTTATTGACTATTTATTTA
CAGCCATTAGTTAGATATATTTTTGAGAGAATTGTCATGGCTGTGATTGTCATCATTGGTG
TTATTGTCAGTGTGTTTACCATTCTGTATTTTTCACCGCTTGATGCGGCTTATAGCATACTG
GGACAAAATGCAACAAAGGCACAGATACATCAATTCAATGTATTACATCATCTTAACGAA
CCTTATTTTATTCAATTGTGGGATACCATCAAGGGTGTTTTTACCTTTGACTTAGGTACGA
CTTACAAAGGGAATGAGGTTGTGACTAAAGCAGTTGGCGAAAGAATTCCAATTACAATA
ATTGTCGCAGTATTAGCGCTAATTGTGGCATTAATTATTGCAATACCAATTGGTATTATCA
GTGCGATGAAGCGAAATAGTTGGCTTGATATCACGTTAATGATAATTGTATTAATTGGTTT
ATCTATTCCAAGTTTCTGGCAAGGGCTATTATTCATTTTAGCGTTCTCATTGAAATTGGAT
ATTTTGCCACCATCTTATATGCCAGAACATCCAATATCGTTGATTTTACCTGTACTTGTCA
TTGGAACAAGTATTGCTGCTTCTATCACGCGTATGACAAGGTCTTCTGTACTTGAAGTAAT
GCGCAGCGATTATGTTTTAACTGCTTATGCAAAAGGATTATCGACGACACAAGTTGTTAT
TAAACATATTTTGAAAAATGCCATTATTCCAATTGTAACGTTAGTTGGTCTTCTAGTGGCA
GAGTTACTAGGCGGTTCAGCAGTGACGGAACAAGTATTTAACATTAATGGTATCGGGCGT
TATATCGTCCAAAAACAACTAATACCTGATATCCCAGCAGTCATGGGTGGGGTCGTATAT
ATATCAATTGTAATATCTTTAGCAAACTTAATTATTGATATATTTTATGCTTTAATCGATCC
AAAATTACGTAGTGAAATTAACGAAAGGAAGTGA

## > Q8NYL1

Function: protein coding sequence; putative oligopeptide ABC transporter/membrane protein (core genome, variable)
Best match: Q8NYL1_CC001_MW2-USA400_BA000033.2[214409:215572] (completely identical)
Position: 008-contig_256: 77645 … 78809; Length: 1164 bp
Sequence:
ATGGTAAAACTTACAACAAAGATAGCTTCCTTAAAACTATTCGCAAGTTATGCTATAGCA
ACTTATATTTTAGTTATATTAACGAGTGCATTAAATCTTTTTAAAGGTTATGTGGCCGATA
CGTTCTATATTGCTGAAACATTGCTAATCGTTTTAACCATCATTTTAATTATCATTTTAACA
ACGGAACAAACATGGAAGCATCATGACTTATGGCGACGTATCGTCGAAGTGTTGTTATTG

TTGATGACATTAACAGGCAACGTATTTACATTATTAATGTTTGTAAGTATTAGACGTTACC
AACGTACATCGCAAATACATAGTTATAACGGGTGGAATCGTTTATACGAAAAACTACTA
GACATCGTATTGCGATTATCGGGTTACTTATTTTAGTCTACATGCTGACATTATCAATTGT
GTCACAATTTACATTTGATACGACATTGGCTACTAAAAATCAGTTCAATGCACTGTTACAT
GGACCGAGTCTAGCCTATCCGTTTGGTACTGATGATTTCGGTAGAGACTTATTTACACGCG
TAGTTGTAGGAACGAAGCTGACATTTTCAATTTCAATTATTTCAGTAGTTATTGCAGTTAT
TTTTGGTGTGTTACTAGGCACTATCGCAGGTTATTTTAATCATATTGATAATTTAATAATG
CGAATTTTAGATGTAGTGTTTGCAATTCCATCATTATTGTTAGCGGTGGCAATTATTGCAT
CATTTGGAGCAAGTATTCCAAATTTAATTATTGCTTTAAGTATCGGTAATATACCATCATT
TGCACGGACAATGCGTGCCAGTGTTTTAGAAATTAAACGCATGGAATATGTAGATGCAGC
ACGTATCACTGGTGAAAACACTTGGAATATCATATGGCGTTATATTTTACCGAATGCGAT
TGCGCCTATGATTGTACGTTTTTCATTAAATATAGGTGTGGTTGTATTAACAACAAGTAGT
TTAAGTTTCCTAGGACTTGGTGTTGCACCTGATGTAGCTGAATGGGGCAACATTTTACGTA
CCGGTAGTAACTACTTGGAAACGCACAGTAATTTAGCTATTGTACCTGGTGTTTGTATTAT
GTTCGTCGTTTTAGCATTTAATTTTATAGGTGATGCAGTGCGTGATGCACTAGATCCAAGA
ATTCATTAA

> **rlp**

Function: protein coding sequence; RGD-containing lipoprotein (core genome, constant)
Best match: rlp_CC005_Mu50-VRSA_BA000017.4[237518:239293]
Position: 008-contig_256: 78825 … 80601; Length: 1776 bp
Sequence:
GTGAAGAAAATCATTAGTATCGCAATTATAGTTTTAGCGTTGGTATTAAGTGGTTGTGGT
GTCCCTACGAAATCAGAAGTGGCTCAAAAGTCATCGAAAGTTGAAGTGAAAGGCGAGCG
ACCAACAATACATTTCCTAGGACAAGCAAGTTATGAAAATGATATGAATATCGTTAAAGA
TCAATTGGAAAATGCAGGATTTAACGTGAAGATGAATATCCAACCAGATTATGGTAGCTA
TCGTACACAACGTCAAGCCGGCAATTATGATATCCAAATTGATGACTGGATGACAGTGTT
TGGTGACCCGAACTATGCTATGACGGCATTATTTAGTTCTACAGGATCAAATAGTTTATTG
AAAGATAAACATGTAGACCAGTTGTTAAATAAAGCTTCTACTCAAAATGAAGCAGATGTT
AAACAAACATATAAGCAAATTGAAGATGAAGTTGTATTTGATAAAGGGTATATGGCGCCT
TTATATGGATCAAAAAAGAATTTAGTATATGACAATAAAGTGTTAGATAAAAATAGTGTT
GGATTGCCAAATTCACGTGCATTAATATGGCAACAATTTGATTACAACAATAGTAGAGAA
CGAGATACGCGGCCACTTGTGATGACACAACAAGATGGTGAAATTCCTACATTGGATCCA
ATACGTTCAATTGCGCCGTCAGTATATTCAATTAATATGAATATGTACACAAGGTTATTAT
TATTAGATGAAAATGATCACTTAACAACGAAAGGTTCGTTAAGTCGTGATTATGCTGTGA
ATAAGGACAATAAAGCATTTTATTTCTTGTTAAGAGATGATGATTATTTTGTGAAAGTGGT
CAATGGACAAGCACGTAATACTGGAGAGCGTGTATCGGCTGAAGATGTTAAGTTTTCTTT
AGATAGAGCACGTGATAAAAAGTCTGTGCCTAACAATAATACTTACAATATGCACAAAC
ATATAAATGACATCAAGATATTAAAAGATGAGGACATCGATCAGTTGCGTAAAGAGAAA
GACAAGGACGATAAATCAATCTATGATAAGTTGATTAAAGCTTATAACGTCAAATCGTTA
ACGACAGATGGTCAAAAAGTAAATAATAAAGACGGTATTTATCAAATTGTTAAAATTACG
ACAGATCAATCGATGCCTCGAGAGGTAAATTACTTAACACACTCTTCGGCAGGCATTTTA
TCTAAAAAATTTGTTAATCAAGTAAATCAAGAATATCCAAAAGGATATGGGGATAGCAGT
ACAATTCCTGCAAATTCAGATGGGAAAAATGCGCTGTATGCAAGTGGCGCATACATTATG
ACACAGAAAAATGCATATCAAGCAACGTTTCAACGTAATCCAGGATTCAACGAAACAGA
AAAAGGTAGTTATGGACCAGCTAAAATTAAAAATATTACATTGAAGTTTAATGGTGACCC
GAATAATGCATTGTCAGAACTTAGAAATCATTCAATTGATATGTTGGCAGATGTGAATCA
AAAACATTTTGATTTAATTAAGTCGGATAAAAATTTAAGCATTATTCGCAAAAATGGACG
CAAGTCAGTCTTTTTAATGCTAAATATTAAAAAAGGTATATTTAAGACGCATCCAAACTT
GAGACAAGCAGTAGTTAATGCGATAGATCAGGATCAATTTATTAAGTTTTATCGTGGCGA
TAAATTTAAAATTGCATCACCGATTACACCACTTGTCGATACTGGTAACGAGCAACGTCA
AGATATAGAAAAAGTAGAAAAAGCCATTAATCAATAA

> **ggt**

Function: protein coding sequence; gamma-glutamyltransferase (core genome, constant)

Best match: ggt_CC005_Mu50-VRSA_BA000017.4[239331:241337]
Position: 008-contig_256: 80638 … 82645; Length: 2007 bp
Sequence:
ATGGTCATTAACTTAAATGACAAACAGACAAAAACATCTAAAGAAGGGTTAATTTCCGTA
TCACATCCTCTTGCGGCTAAAATTGGTAAGGATGTATTAGATCAAGGTGGCAACGCCATG
GATGCAGTGATTGCAATTCAACTGGCATTGAATGTGGTAGAACCATTTGCATCAGGTATT
GGTGGTGGCGGGTATTTGCTATATTATGAGCAAAGTACTGGCAGTATAACTGCTTTTGAT
GCGCGTGAGACAGCCCCTGCACATGTAGACAAACAATTTTATCTAGATGATTCAGGCGAA
TATAAATCATTTTTTGATATGACTACACATGGTAAAACTGTCGCTGTGCCAGCAATTCCAA
AGCTGTTTGATTATATTCACAAGCGTTATGCTAAGTTGTCATTGGAAGATTTAATTAATCC
TGCAATTGAACTAGCTATTGAAGGTCATGCAGCCAATTGGGCTACTGAAAAATATTCGCG
CCAGCAACACGCACGATTGACAAAGTATCATGAAACGGCACAAGTATTTACGCATGAAA
ATCAATATTGGCGTGAAGGTGATTGGATTGTACAACCCGAATTAGGTAAGACATTTCAAA
TATTAAGAGAACAAGGGTTTAATGCATTTTATAAAGGTGACATTGCGAAACAATTAGTCA
ATGTTGTTAAAGCATGTGGTGGGACAATCACTTTAGAGGATCTAGCCAATTATGACATTC
AGATTAAAGCGCCAATCAGTGCGACATTTAAAGACTATGACATTTATTCAATGGGACCAT
CTAGTTCTGGCGGTATCACGGTCATTCAAATATTGAAGTTATTAGAACATGTCGATTTACC
ATCTATGGGTCCAAGATCTGTCGATTACTTGCATCATTTGATACAAGCGATGCATTTAGCA
TATAGTGATCGCGCGCAATACTTGGCGGATGATAATTTTCATGAGGTGCCTGTACAGTCA
TTAATTGATGACGATTATTTAAAAGCACGCAGTACGCTCATTGATAGCAATAAAGCAAAT
ATTGATATAGAGCATGGTGTTGTGTCTGATTGCATTAGTCATACAGATGTTGAAGAAAAT
CATACCGAAACAACTCATTTTTGTGTGATTGATAAGGAAGGTAATATTGCTTCATTTACGA
CATCAATCGGTATGATTTACGGATCGGGTATTACGATTCCAGGGTACGGCGTGTTATTGA
ATACCACAATGGATGGTTTTGATGTAGTAGATGGTGGTATTAACGAAATTGCACCATATA
AACGACCACTAAGTAACATGGCTCCAACGATTGTGATGCATCACGGGAAGCCGATATTAA
CAGTAGGTGCACCTGGTGCCATAAGTATCATTGCTAGTGTTGCGCAAACATTAATCAATG
TATTAGTGTTTGGCATGGATATTCAGCAGGCTATAGATGAACCTAGAATTTATAGTAGCC
ATCCTAATCGCATTGAATGGGAGCCTCAATTTTCACAATCTACAATATTAGCATTGATTGC
ACGTGGACATGCAATGGAACATAAACCAGATGCCTATATTGGAGATGTACATGGGTTACA
GGTTGACTTGAATACACGTGACGCGTCGGGAGGTGCTGATGATACGAGGGAAGGCACAG
TGATAGGTGGCGATGTATTATCAATTAGAAAACAACCATTACCTAGTCCGAAAATATACG
ATAATGACACCCATCGAGTATATTTCAATGATATGCAGTTGCCATTATATGCTGAACAAG
TGCGGTGGATGCATGACAAATATTGGGTTGATGAAAGTGTTATTAGAATCATTTTCCCTG
AAGTTAGTGTGCATATTGAAGATTTAAGAAGTTATGAAATCGCCGGAAAAAATTATATAG
ATATTGCCTGGTTGGCACGGAAGAAAGGTTATCAAGTTACCTTAAAGGATGACAGTTTAT
ACTTAACTGATGAAACATATCATTCAGTGAAAGCAAACACAAATGCATACTATAGATATG
ATCGAGATAGTATCACAAGATAA

## > **acpD**

Function: protein coding sequence; FMN-dependent NADH-azoreductase (core genome, variable)
Best match: acpD_CC001_MW2-USA400_BA000033.2[220684:221310:r]RC
Position: 008-contig_256: 84381 … 84531; Length: 150 bp
Sequence:
TGGAATATAAGTTTCAAATAAATCAATATGTTTCACTTCATCATCAGGATGTTGTTGTTGA
TATGTTTCGATAAATGCTTTACCAGCCGCCATAGAATTTGATACCAGTTCATTAAAAGGGT
GTGCTGTAATATATAATACTTTTGCCAT

## > **msmX**

Function: protein coding sequence; maltose ABC transporter/ATP-binding protein (core genome, constant)
Best match: msmX_CC030_MRSA252_BX571856.1[238058:239155]
Position: 008-contig_256: 85701 … 86799; Length: 1098 bp
Sequence:
ATGGCAGAACTAAAGTTAGAGCATATTAAAAAGACGTATGATAACAACAATACTGTAGT
GAAAGATTTTAATCTACATATTACTGACAAAGAATTCATTGTATTTGTTGGACCATCGGG

ATGTGGTAAATCAACAACATTACGAATGGTTGCTGGACTAGAGTCTATCACATCTGGAGA
TTTTTATATTGATGGGGAACGCATGAACGATGTTGAACCAAAGAATAGAGATATTGCGAT
GGTATTTCAAAACTATGCATTATATCCACATATGACTGTTTTTGAAAATATGGCATTTGGG
CTAAAGCTACGTAAAGTAAATAAAAAAGAGATTGAACAAAAAGTTAATGAAGCAGCTGA
AATATTAGGATTAACTGAGTATCTTGGTCGTAAACCAAAAGCGTTATCTGGCGGACAGCG
TCAACGTGTTGCTTTGGGCAGAGCTATTGTTAGGGATGCGAAAGTCTTTTTAATGGATGA
ACCATTATCGAATCTTGATGCGAAGCTTCGAGTACAAATGCGCACAGAAATATTGAAATT
ACATAAGCGACTTAATACTACGACAATTTATGTTACACATGATCAAACTGAAGCATTGAC
GATGGCTAGTCGAATTGTTGTTTTGAAAGATGGCGACATTATGCAAGTCGGCACACCTAG
AGAAATATATGATGCCCCTAATTGCATATTTGTGGCGCAATTTATCGGCTCACCAGCAAT
GAATATGTTGAATGCTACAGTTGAAATGGACGGATTGAAGGTAGGAACACACCATTTTAA
ATTACATAATAAAAAATTTGAAAAGTTAAAAGCTGCTGGCTACTTAGACAAGGAAATTAT
TTTAGGTATTCGAGCTGAAGACATTCATGAAGAACCAATATTTATTCAAACTTCTCCAGA
GACACAATTTGAATCTGAAGTAGTTGTATCCGAACTGTTAGGTTCAGAAATTATGGTACA
TAGCACATTCCAAGGAATGGAATTGATTTCTAAATTAGATTCAAGAACACAAGTGATGGC
GAACGACAAGATTACACTAGCATTTGATATGAATAAGTGTCACTTTTTTGATGAAAAAAC
AGGAAATCGTATCGTCTAA

## > STAR

Function:  Staphylococcus aureus repeat element
Best match: STAR_CC001_MW2-USA400_BA000033.22371925:2372149:r
Position: 008-contig_256: 90261 … 90370; Length: 109 bp
Sequence:
GGGAGTGGGACAGAAATGATATTTTCGCAAAATTTATTTCGTCGTCCCACCCCAACTTGC
ATTGTCTGTAGAAATTGGGAATCCAATTTCTCTATGTTGGGGCCCCGCC

## > Q2FZB6

Function: protein coding sequence; putative protein (genomic island)
Best match: Q2FZB6_CC001_MSSA476_BX571857.1[1167133:1167311]RC
Position: 008-contig_256: 90348 … 90378; Length: 30 bp
Sequence:
TTCTCTATGTTGGGGCCCCGCCAACTTGCA

## > repeat_nySAgamma

Function:  repeat element
Best match: repeat_nySAgamma_CC001_MSSA476_BX571857.1[2756252:2756375]
Position: 008-contig_256: 90357 … 90387; Length: 30 bp
Sequence:
TTGGGGCCCCGCCAACTTGCACATTATTGT

## > mviM

Function: protein coding sequence; NADH-dependent dehydrogenase (core genome, variable)
Best match: mviM_CC001-ST772_118_AJGE01000016.1[15157:16197:r] (completely identical)
Position: 008-contig_256: 91618 … 92659; Length: 1041 bp
Sequence:
ATGACAAAATTAAAAGTTGGTGTGATAGGTGTTGGTGGTATTGCACAAGACCGTCATATT
CCAGCATTGCTGAAACTCAAAGACACAGTCTCATTAGTTGCAGTACAAGATATTAATACA
GTGCAGATGATTGATGTTGCGAAGCGCTTTAATATACCTCATGCAGTTGAGACACCTAGC
GAGCTGTTTAAACTTGTTGATGCGGTGGTCATTTGTACACCCAATAAATTCCATGCTGATC
TTTCTATAGAAGCATTGAACCATGGTGTCCATGTTTTATGCGAAAAGCCAATGGCAATGA
CGACGGAAGAGTGTGACCGCATGATTGAAGCGGCTAATAAAAATCACAAATTACTAACT
GTCGCTTATCATTATCGCCACACAGATGTGGCAATTACTGCTAAAAAAGCAATTGAATCA
GGTGTGGTTGGTAAACCTTTAGTAGCACGTGTACAAGCGATGCGTAGGCGTAAAGTACCT
GGGTGGGGCGTTTTTACCAATAAAGCGTTGCAAGGTGGCGGTAGTTTAATCGATTATGGT
TGCCACTTGTTAGACTTATCTTTGTGGCTATTAGGCAAAGATATGGTGCCGCATGAAGTGC

TAGGAAAAACATATAATCAATTGAGTAAACAACCGAATCAAATTAATGATTGGGGAACA
TTTGATCATACTAAATTTGATGTCGATGATCATGTTACTAGTTATATGACATTTGCCAATC
GAGCAAGCATGCAGTTTGAATGTTCGTGGTCTGCAAATATAAAGGAAGATAAGGTACAC
GTTAGTTTATCAGGCGAAGATGGCGGTATCAATTTATTTCCATTTGAAATATATGAGCCCC
GCTTTGGAACTATTTTTGAAAACAAAGCTAATGTTGAGCATAACGAAGACATTGCTGGTG
AGAGACAGGCGCGTAACTTTGTCAATGCGTGTTTAGGGATAGAAGAGATTGTGGTGAAA
CCGGAAGAAGCACGCAATGTAAATGCCCTTATAGAAGCGATTTATCGTAGCGATCTTGAT
AACAAGAGCATACAACTTTAA

## > iolE

Function: protein coding sequence; sugar phosphate isomerase/epimerase (core genome, variable)
Best match: iolE_CC005_Mu50-VRSA_BA000017.4[251172:252140] (completely identical)
Position: 008-contig_256: 92713 … 93682; Length: 969 bp
Sequence:
ATGAAAATAGGTGTATTTTCAGTATTATTTTACGATAAAAATTTTGAAGATATGTTAGATT
ATGTCGCAGAATCTGGATTGGATATGATTGAAGTTGGAACAGGTGGTAACCCAGGAGAT
AAATTTTGTAAGTTAGATGAGTTGTTAGAAAATGAAGACAAGCGACAAGCATTTATGAAG
TCAATCACAGACAGAGGCTTACAAATAAGTGGTTTCAGTTGTCATAACAATCCAATTTCT
CCAGATCCGATAGAAGCGAAAGAAGCCGATGAAACGTTACGTAAAACAATCCGTTTAGC
AAATCTATTAGACGTGCCAGTTGTTAATACATTTTCTGGCATTGCAGGGTCAGATGATACC
GCTAAAAAGCCTAATTGGCCTGTTACACCTTGGCCAACAGCCTACTCTGAAATTTATGATT
ATCAGTGGAATGAAAAGTTGATACCATATTGGCAAGATTTAGCTGAGTTTGCAAAAGAGC
AAGATGTAAAAATTGCCATAGAGTTACATGCAGGATTTTTAGTTCATACACCATATACGA
TGTTAAAGTTACGTGAGGCTACAAATGAATATATCGGTGCTAACTTAGATCCTAGTCATTT
ATGGTGGCAAGGTATTGATCCAATTGCTGCGATTCGCATATTAGGCCAAGCAAATGCAAT
TCATCACTTCCATGCTAAAGATACGTATATTAATCAAGAAAATGTAAATATGTATGGTCT
AACTGATATGCAACCATATGGTAACGTTGCGACAAGAGCATGGACATTCCGTACAGTTGG
TTATGGACATAGTCCATATGTATGGGCAGATATCATAAGTCAACTTATTATTAATGGATAT
GATTATGTATTAAGTATTGAACATGAAGATCCTATTATGTCAGTAGAAGAAGGTTTCCAA
AAAGCTTGTCAAACTTTGAAATCTGTTAATATTTACGACAAGCCAGCAGACATGTGGTGG
GCATAA

## > Q7A1X1

Function: protein coding sequence; putative protein (core genome, constant)
Best match: Q7A1X1_CC001_MW2-USA400_BA000033.2[230572:231066:r]RC (completely identical)
Position: 008-contig_256: 94042 … 94537; Length: 495 bp
Sequence:
TTAAATATTCATTAATTTCTCTTCTTGTTTAATACGTACATATAAGAAATACGCATACGGT
ACTAATAAAATAGTTGTATATGTTGCGTGTGTTAATAATAATACACCGATTAATTCAGGA
ATGATGTTTAAGAAGTAATTTGGGTGTTTTGTAATTTTATATAATCCAGATTTAATAATAG
GATGGTTAGGTAAAATGAATAATTTTAATGTCCAAATACCACCTAAAGTTTTAATAACCA
TAAATAACATGATATAAGCAAAGATTAATATAACTAAGCCAATACCATTTGCAAAGCTAA
ATGTATCTTTATTAATAAATGCCTCTACACCAGCCAATACATAAATTAAAACGTGTGTTAT
TGCTAAAAACTTCGAATTTTTAACGCCATATTCAACTGCACCGTCTGCTTTTAATTGTTTT
GAGTGATTAATAGATATCTTTAAGCTGACAAGTCTGATACAGAAAAAGATAAGTAATATA
GATAGAATCAT

## > uhpT

Function: protein coding sequence; putative hexose phosphate transporter (core genome, constant)
Best match: uhpT_part_AJ566817.1[1:1373]
Position: 008-contig_256: 94769 … 96142; Length: 1373 bp
Sequence:
ATGAACTTTTTTGATATCCATAAGATTCCGAACAAAGGCATTCCATTATCGGTACAACGT
AAATTATGGCTTAGAAACTTCATGCAAGCTTTCTTTGTAGTGTTCTTTGTTTATATGGCTAT

GTATTTAATTCGAAACAACTTTAAGGCGGCCCAACCGTTTTTAAAAGAGGAAATTGGATT
ATCTACATTAGAACTTGGTTATATCGGATTAGCATTTAGTATCACGTACGGTTTAGGGAA
AACATTACTTGGATATTTTGTCGATGGACGTAACACAAAACGTATTATCTCGTTCTTACTT
ATCTTATCTGCGATTACAGTTTTAATTATGGGATTTGTTTTAAGTTACTTTGGTTCTGTAAT
GGGATTATTAATTGTACTTTGGGGACTTAACGGGGTGTTCCAATCAGTTGGTGGACCTGC
AAGTTATTCAACGATTTCAAGATGGGCGCCAAGAACGAAACGTGGCCGATACTTAGGATT
TTGGAATACATCACATAATATCGGTGGTGCCATTGCAGGTGGTGTTGCACTTTGGGGTGC
TAATGTATTCTTCCATGGAAATGTTATAGGGATGTTCATTTTCCCATCGGTGATTGCATTA
CTTATTGGTATCGCAACATTATTTATCGGAAAAGATGATCCGGAAGAATTAGGATGGAAT
CGTGCTGAAGAAATTTGGGAAGAGCCGGTCGATAAAGAAAATATTGATTCTCAAGGTAT
GACGAAATGGGAGATCTTTAAAAAATATATCCTGGGAAATCCTGTTATATGGATTCTATG
TGTTTCAAACGTCTTTGTATACATTGTACGAATCGGTATTGATAACTGGGCACCGTTATAT
GTGTCAGAGCATTTACACTTTAGTAAAGGCGATGCAGTTAATACGATATTCTACTTTGAA
ATTGGTGCATTAGTTGCAAGTTTATTATGGGGCTACGTATCAGACTTATTAAAAGGTCGTC
GTGCAATTGTAGCTATTGGCTGTATGTTTATGATTACATTTGTTGTCTTATTCTACACAAAT
GCTACAAGTGTCATGATGGTTAACATTTCATTGTTTGCATTAGGTGCGTTAATCTTTGGTC
CGCAATTATTAATTGGTGTATCATTGACTGGTTTTGTTCCTAAAAATGCCATCAGTGTAGC
AAACGGAATGACAGGTTCATTCGCGTATCTATTCGGTGACTCAATGGCGAAAGTTGGTTT
GGCGGCTATTGCTGATCCAACACGTAACGGTTTAAACATCTTTGGATATACATTAAGTGG
ATGGACAGATGTTTTCATCGTCTTCTATGTTGCATTATTCCTAGGCATGATTCTATTAGGA
ATCGTTGCTTTCTATGAAGAAAAGAAAATTAGAAGTTTAAA

> **rsaG**
Function: noncoding RNA ncRNA of Staphylococcus aureus G (core genome, constant)
Best match: rsaG_CC001_MW2-USA400_BA000033.2[232733:232926] (completely identical)
Position: 008-contig_256: 96203 … 96397; Length: 194 bp
Sequence:
CCCCGACGGCATGTGCGTGAAGAGATGAAAGATACTGCTTCTACCCTTGCAAATATATCA
TCTCTATGTCTCGGGGCAGATCATAATTCCCTGTTATGAAGTATCCTTATTTGCCCGACTT
AGGGTGACTCAATGAATTTACTCCTTACAATAAAGACATATAGCGGTGTCAATATTGTAG
GGAGTATTGTTTT

> **txbi_rsaG**
Function:  bidirectional rho-independent terminator of rsaG (core genome, variable)
Best match: txbi_rsaG_CC001_MW2-USA400_BA000033.2[232932:233001] (completely identical)
Position: 008-contig_256: 96402 … 96472; Length: 70 bp
Sequence:
TAAACTCTCTAAAAAGCGGACTGAAAGAAAAGTGAAAACTTCTCTATCAGTCCGCTTTTT
CATAGAACAA

> **yesN**
Function: protein coding sequence; two-component sensor/response regulator (core genome, variable)
Best match: yesN_CC001-ST772_118_AJGE01000016.1[10550:11308]RC (completely identical)
Position: 008-contig_256: 96507 … 97266; Length: 759 bp
Sequence:
CTATTTTGCTTGCTTACAATAATCACTTGGCGACATTTGTAAATATTTTTTAAAATGATAG
CTAAACATTTTATACTCTGAAAAGCCTACTTTGTCTGCAATTTCATAGTGTTTGTAATGTC
GATCTAACAATTGCAGAGATTGTAAAATACGATAGCGATTTAAATAATCGACAATTGTAA
TACCAACATGATCTTTAAATGTTCGCATCGCATACGATTCACTAACATCGATATGTTGAAT
TAAATCTGAAACAGTCACTTTCGTTTGATAAGATTGCTTAATTTGATCAACAATCTGATTT
ACATAATAATCATCGTATTCTACTTTTAATAGTGGTTGGAAGGCATCATGACAAGATGCT
AAGCTACGGCCGTTCTGTGATTGTTGCTCTAATAAGGTACGGACAAGTCTTCCTAAAATA
ACTTCTAATTGTGCATGGTCTACTGGTTTTAATAAATAATCAAGAACATGATGTTGAATAC
CGGCTTTCATATATTCAAAGTCATCGTAACTCGATAATATGATGACATTACAATCTAGATG
CGCAATATCATTGAGTAAATCGACGCCATTCTTACGTGGCATACGAATATCAGTAATTAC

TAATTCTGGCTGATGTTGTTGAATTAGTGATAATGCTTCAACACCATCTTTAGCAGTGTAT
ATTGTATTGAAATGATAGTCTCCCCACGGAATGATTTGCTTTAAACCTTCTCGAATAATAC
GCTCATCATCACAAATAACTACCTTAAACAT

> **yesM**
Function: protein coding sequence; two-component sensor/histidine kinase subunit (core genome, variable)
Best match: yesM_CC001_MSSA476_BX571857.1[232488:234044:r]RC
Position: 008-contig_256: 97258 … 98815; Length: 1557 bp
Sequence:
TTAAACATCTACATTCCCCCTTGAAAGTGGTATTTTATAACAAATTAACGTACCTTGATTA
CGCTTTGAAAAAATATGGAGTCGTGCATGTGAACCATATTGAATCATTGCTTTATTATGTA
AATGATTTAATCCCAAATGCTTAGTATCAAATACATCATTATTAAGAGATTGGCGTACAT
ATTGCAGGCGAGATGACGACATCCCAATACCATTGTCGCAAACTAAAACATGTAAATTCT
GACGTGCCAATGTCAGGCGTATAGTAATGTCCAATGACTCAGTATCTCTACCATGTTTAAT
AGCATTTTCTATGAGTGGCTGAAGCATCATTTTACCAATTGTCTGGTGACGCGCTTCTTCA
GAACTTTCGATATGGAGCTTAATCATGTCATCAAAACGGATGTTTTGTATCGCAACATACT
GTTCAATGTAGTTCAACTCTTCGTTTAATTCCACTGTATGTGAGTTTGTACGTAATGAGTA
ACGTAACATTTGCGATAATTGTTGGACCACAGTTTGTGCTAATTTAGGAGATAACGTAAT
TAAATATTGTATTGTTTGCATCGTATTGAATAGGAAATGAGGTTGGAATTGGCGCTCTATT
TCCTTTAACTGAATATCACGCAAGCGACGTTCTGTATGCTCGATAGAATGGATTAGTTGCT
CATTTGATTCAAATAAATCGTAAATATAATTATTAATTTCTTCTAGTTCACTGTTGTTTTTT
AAAGGCGTATATGTACCTAGATGACGATTTTTGGCATAGTAAATTTTTTGAATAATCGTTT
CGATATCTTTTGTTTGTCGTTTAGCCATATTATCTGCGCTAATGAAACCAAATATTACTAG
TAAAACAAGAACTACGGCCATAACAATTAACAACGTGATACCATCTTCAATGTTTTCATG
TATATCTTTATAAATAATGAGACGATGGTCAGCATGGTTTAATTTTACAGATTCATTCATA
AATCCGAATTGTTGCGGTCTATACTTTTCACCTATAGTAAAACGGTCATCGTTGGCGTATA
AAATATTGTCATATTGATCAACGATAAGTGCGAATTGTCGGCTATCTTTCTTAATTTCACT
TAAACGTGGGGTGTTAGCCATATAAATTTTAAGCATATATGTACTATTATTGAATTTAAGC
TGATGCGTTGAAAATAAATACATATTTTTAGTGTTTAAATGTTCATAATTATTGGTTATAA
ACTGATTTGGTCCAGATAATTCATAATAAAGTGTTGCGGGCTGTTGGTGTATTAATTTTAA
TAATTCACGTTTTGTAGCGGTAACATCATGATGATTTGTTAAATCGAGCTCTTGAAACGAA
TTATTATGCTGTGTAATAAATGTCTGAATCTGCTTTTCAGTGTGATGTAAAGATGACTGAC
TTTCATCAACATGTTGATGAATCGTACGATGCTCAATCCAAATATAGATGGCATAGAAGC
TTACTAGTCCAATAATAATGACTAAAAATACTGGAAAAATAGTAGACGCAAATAACGAT
CGTCTCAATTGATGTCTATAAGGTTTGTATGCCGTCAT

> **Q8NYJ7**
Function: protein coding sequence; iron(III) transport system substrate-binding protein (core genome, variable)
Best match: Q8NYJ7_ref_CC001_MW2-USA400_BA000033.2[235341:236309:r]RC (completely identical)
Position: 008-contig_256: 98811 … 99780; Length: 969 bp
Sequence:
TCATTGAATCATCTCCAAAAATTTATGATGTGGAATGTCCGGTAATTTAGATTTCGGTATT
AAAGGTATGTTCTTAAGATTTTCGATAGACTGATCGCTTTGTTCACTAACATCCTTTCGAA
TTGACTTGGCATCGAACTCTGCAACTAATCGTTGTTGTACTGAGCGGCTTGTTAAATATTG
CACTAACTTTTTACGCTTAGGATGAGGGTGTGCATTTTTAACTAAAGCAATACCATCAAC
ATTTAACATTGTTCCTTCAATTGGATAAACGATTGATACAGGATAACCTTTGTTTTTCCAT
GTGCGTGCATCTTGTTCGTAGCTTAGACCTGCGTAATATTTACCTTTTGCAACATCTTCAA
TGACTTTAGACGTCTTTGACAGTTGCATCGCATGGTTTTGGAATTGATGCACATCACTTAC
TCGATGATGCATGCTATAAATAGCGCGCATATGTTGATAGCCTGTCGTTGTTGTATTTGGA
TTTGAGTACGCAATTTTACCTTTAAGTATAGGTTGTAATAAATCTTGATAACCTCGAATCT
TAATATCTCCTTGTAAATCTGAATTCACTACTATAACTGTTGGCATTAATAGAAAACTAGT
AACATATTTATTGTTCGAGCGATAATCCTCTAATTGCTGTGTTACAGATGTATCTTGATAG

GGAACAAAATCTTCTGGATGATCAATTGTTTCTGATAACACACCACCCATAAAGACGTCA
CCACGCTCCGAAAAATCTTCGTTATGCAAGTTTGAAAGCAGTACTTGAGTAGATCCATGT
TTGATTTCAATTTTGACATGCTCTTGTTTTTCAAATTCATTTAAAATTGGACGAATCAAGTT
TGATTGATACGGAGAATAAACTGTTAATACATTTTTATCTGATTCAGAGTGACGCGTATTA
GCGCATGCTGATAAAAAAATGAGAAATAATAGCAAGATATAAATTTTTGATTTCAT

> **pflB**

Function: protein coding sequence; formate acetyltransferase (core genome, constant)
Best match: pflB_CC001_MW2-USA400_BA000033.2[236897:239146]
Position: 008-contig_256: 100367 … 102617; Length: 2250 bp
Sequence:
ATGTTAGAAACAAATAAAAATCATGCAACAGCTTGGCAAGGATTTAAAAATGGAAGATG
GAACAGACACGTAGATGTAAGAGAGTTTATCCAATTAAACTACACTCTTTATGAAGGTAA
TGATTCATTTTTAGCAGGACCAACAGAAGCAACTTCTAAACTTTGGGAACAAGTAATGCA
GTTATCGAAAGAAGAACGTGAACGTGGCGGCATGTGGGATATGGACACGAAAGTAGCTT
CAACAATCACATCTCATGATGCTGGTTATTTAGACAAAGATTTAGAAACAATTGTAGGTG
TACAAACTGAAAAGCCATTCAAACGTTCAATGCAACCATTCGGTGGTATTCGTATGGCGA
AAGCAGCTTGTGAAGCTTACGGTTACGAATTAGACGAAGAAACTGAAAAAATCTTTACA
GATTATCGTAAAACACATAACCAAGGTGTATTCGATGCATATTCTAGAGAAATGTTGAAC
TGCCGTAAAGCAGGTGTAATCACTGGTTTACCTGATGCATACGGACGTGGACGTATTATC
GGTGACTATCGTCGTGTAGCTTTATATGGTGTAGATTTCTTAATGGAAGAAAAAATGCAC
GACTTCAACACGATGTCTACAGAAATGTCAGAAGATGTAATTCGTTTACGTGAAGAATTA
TCAGAACAATATCGTGCATTAAAAGAATTAAAAGAACTTGGACAAAAATATGGTTTCGAT
TTAAGCCGTCCAGCAGAAAACTTCAAAGAAGCAGTTCAATGGTTATACTTAGCATACCTT
GCTGCAATTAAAGAACAAAACGGTGCAGCAATGAGTTTAGGTCGTACATCAACATTCTTA
GATATCTATGCTGAACGTGACCTTAAAGCAGGCGTTATTACTGAAAGCGAAGTTCAAGAA
ATTATTGACCACTTCATCATGAAATTACGTATTGTTAAATTTGCTCGTACACCTGATTACA
ATGAATTATTCTCTGGAGACCCAACTTGGGTAACTGAATCTATCGGTGGTGTAGGTATTG
ACGGACGTCCACTTGTTACGAAAAACTCATTCCGTTTCTTACACTCATTAGATAACTTAGG
TCCAGCACCAGAACCAAACTTAACAGTATTATGGTCAGTACGTTTACCTGACAACTTCAA
AACATACTGTGCAAAAATGAGTATTAAAACAAGTTCTATCCAATATGAAAATGATGACAT
TATGCGTGAAAGCTATGGCGATGACTATGGTATCGCATGTTGTGTATCAGCGATGACAAT
TGGTAAACAAATGCAATTCTTCGGTGCACGTGCGAACTTAGCTAAAACATTACTTTACGC
TATCAATGGTGGTAAAGATGAAAAATCTGGTGCACAAGTTGGTCCAAACTTCGAAGGTAT
TAACAGCGAAGTATTAGAATATGACGAAGTATTCAAGAAATTTGATCAAATGATGGATTG
GCTAGCAGGTGTTTACATTAACTCATTAAATGTTATTCACTACATGCACGATAAATACAG
CTATGAACGTATTGAAATGGCATTACATGATACAGAAATTGTACGTACAATGGCAACAGG
TATCGCTGGTTTATCAGTAGCAGCTGACTCATTATCTGCAATTAAATATGCACAAGTTAAA
CCAATTCGTAACGAAGAAGGTCTTGTAGTAGACTTTGAAATCGAAGGCGACTTCCCTAAA
TACGGTAACAATGACGACCGTGTAGATGATATCGCAGTTGATTTAGTAGAACGCTTCATG
ACTAAATTACGTAGTCATAAAACATATCGTGATTCAGAACATACAATGAGTGTATTAACA
ATTACTTCAAACGTTGTATACGGTAAGAAAACTGGTAACACACCAGACGGACGTAAAGCT
GGCGAACCATTTGCACCAGGTGCAAACCCAATGCATGGCCGTGACCAAAAAGGTGCATT
ATCTTCATTAAGTTCTGTAGCTAAGATCCCTTACGATTGCTGTAAAGATGGTATTTCAAAT
ACATTCAGTATCGTACCAAAATCATTAGGTAAAGAACCAGAAGATCAAAACCGTAACTTA
ACTAGTATGTTAGATGGTTACGCAATGCAATGTGGTCACCACTTAAATATTAACGTATTTA
ACCGTGAAACATTAATAGATGCAATGGAACATCCAGAAGAATATCCACAGTTAACAATC
CGTGTATCTGGTTACGCTGTTAACTTCATTAAATTAACACGTGAACAACAATTAGATGTA
ATTTCTCGTACATTCCATGAAAGTATGTAA

> **pflA**

Function: protein coding sequence; pyruvate formate-lyase-activating enzyme (core genome, constant)
Best match: pflA_CC001_MW2-USA400_BA000033.2[239169:239924]
Position: 008-contig_256: 102639 … 103395; Length: 756 bp
Sequence:

ATGCTTAAGGGACACTTACATTCTGTCGAAAGTTTAGGTACTGTCGATGGACCGGGATTA
AGATATATATTATTTACACAAGGATGCTTACTTAGATGCTTGTATTGCCATAATCCAGATA
CTTGGAAAATTAGTGAGCCATCAAGAGAAGTCACAGTTGATGAAATGGTGAATGAAATA
TTACCATACAAACCATACTTTGATGCATCGGGTGGCGGTGTAACAGTCAGTGGTGGCGAA
CCATTGTTACAAATGCCATTCTTAGAAAAATTATTTGCAGAATTAAAAGAAAATGGTGTG
CACACTTGCTTAGACACATCGGCTGGATGTGCTAATGATACAAAAGCATTTCAAAGGCAT
TTTGAAGAATTACAAAAACATACAGACTTGATATTATTAGATATAAAACATATTGATAAT
GACAAACATATTAGATTGACAGGAAAGCCTAATACACACATCCTTAACTTCGCGCGCAAA
CTGTCAGATATGAAACAACCTGTATGGATTCGACATGTCCTTGTGCCTGGTTATTCTGATG
ATAAAGACGATTTAATTAAACTAGGGGAATTTATTAATTCTCTTGATAACGTCGAAAAGT
TTGAAATTCTGCCATATCATCAGTTAGGTGTTCATAAGTGGAAAACATTGGGCATTGCAT
ATGAATTAGAAGATGTCGAAGCGCCCGATGATGAAGCTGTTAAAGCAGCCTACCGTTATG
TTAACTTCAAAGGGAAAATTCCCGTTGAATTATAA

## > glpQ1

Function: protein coding sequence; glycerophosphoryl diester phosphodiesterase (core genome, variable)
Best match: glpQ1_AB158555.1[1:1764]
Position: 008-contig_256: 103730 … 105494; Length: 1764 bp
Sequence:
ATGAAGAGAATTAGTAAAGATATATGGGCAGTATTTAAATTACTGTATCAAAATAAAGG
GCGTTTTAGCATTAATGCCTTACTATTGCAGTTAATCATGATTTTTATTAGTAGTACATAC
TTAATTTTACTATTTAATATGATGTTAAAAGTAGCTGGGCAAAGCCAACTTACGATTAAC
AATTGGACGGAAATCATAAGTCATCCTGCCAGTGTGATACTTCTTATTATATTCATATTAA
GTGTTGCTTTTCTGATTTATGTAGAGTTTTCATTGTTAGTTTATATGGTTTATGCCGGCTTT
GATCGACAGATTATTACATTTAAATCCATTTTTAAAAATGCCTTTGTAAATGTGCGTAAAC
TCATAGGTGTACCAGTTATTTTCTTTGTCATTTATTTAATGTTAATGATACCCATTGCCAAC
CTAGGACTAAGTTCAGTATTAACAAAAAATATTTACATACCTAAATTTTTAACGGAAGAA
CTTATGAAAACGACGAAAGGTATAATCATTTACGGTACCTTTATGATTGCTGTATTTATAT
TAAACTTTAAATTAATATTTACCTTACCGTTAACGATTTTAAACCGTCAGTCGTTATTTAA
AAATATGAGACTAAGTTGGCAAATTACGAAGCGAAATAAGTTTCGACTTGTTATAGAAAT
AGTTATATTGGAACTCATCATTGGTGCGATTTTAACATTAATTATTTCAGGAGCAACATAT
CTTGCTATTTGTGTAGATGAAGAAGGAGATAAGTTTTTAGTCTCATCAATTTTATTTGTTG
TATTGAAAAGCGCATTGTTCTTCTATTATTTATTTACGAAATTATCATTAATCAGTGTGTT
AGTACTGCACTTAAAACAAGAGAATGTATTAGACCAACCGGGCTTAGAATTTAAATACCC
AAAACCGAAACGGAAGTCTAGGTTCTTTATAATTTCAATGGTGCTTGCAGTGACATGTTTT
ATCGGTTATAACATGTACTTACTTTACAATAATACTATCAATACAAATATCTCCATTATTG
GCCATCGTGGTTTCGAAGATAAAGGCGTTGAAAATTCTATTCCGTCATTGAAAGCTGCTG
CAAAAGCGAATGTCGAATACGTTGAGTTAGATACAATTATGACGAAAGATAAACAATTT
GTTGTTAGTCATGATAACAATTTGAAACGTTTAACAGGTGTTAATAAAAACATTTCTGAA
TCTAATTTCAAAGATGTCGTCGGTTTGAAAATGCGTCAAAATGGACATGAAGCAAAACTT
GTATCCTTAGACGAATTTATTGAAACGGCTAAACAATCAAATGTGAAGCTATTAGTAGAG
TTAAAGCCACATGGTAAAGAACCAGCAAATTATACACAACGTGTTATTGATATTTTGAAA
AAGCATGGTGTTGAACATCAATATCGTGTGATGTCATTGGATTATGATGTGATGACTAAG
TTGAAAAAAGAAGCGCCATATCTCAAGTGTGGTTATATCATTCCGTTGCAGTTTGGTCATT
TTAAAGAAACATCATTAGATTTCTTTGTCATCGAAGATTTTTCTTATTCGCCAAGACTTGT
TAATCAAGCGCACTTGGAAAATAAAGAAGTCTATACTTGGACTATTAACGGCGAAGAAG
ATTTAACGAAATACTTACAAACCAATGTTGATGGTATTATCACAGATGACCCAGCATTAG
CTGATCAGATTAAAGAAGAAAAGAAAGACGAAACATACTTCGATCGTTCTATAAGAATTT
TGTTTGAATAA

## > Q2YV49

Function: protein coding sequence; putative protein (core genome, variable)
Best match: Q2YV49_CC001-ST772_118_AJGE01000016.1[1814:2158]RC (completely identical)
Position: 008-contig_256: 105657 … 106002; Length: 345 bp

Sequence:
TTAATTGTTTTGTGAATGCAAAGGGTTAGAAATTGAATCGTAAATACTTTCTAATCTATGT
TTCGCTTTAGTCATTTGATCCAAATTTTTAGTGCGTATAACGGATTTTGCAATATAATGTG
CAGCTAAAATATCGCGTTTTTGATAAGCGTCTAAATTTAGGTACGATAATTTATTTAAGTC
AGTGTTTGCTATTAATTCATGTAATTGATCTACAAGCGCTTGATGTTGATACGTATGTGAT
GTAGTTTCAGATTTGCTTGCTAATTTAATACCAGTCGTATCAAGGAGCGCCGCTTTAATAC
CAGCAACTAAATATGTTTTGATTTTCATTTGTGTTGTCAT

> **coa**

Function: protein coding sequence; staph coagulase chv
Best match: coa-SV06_ref_AB158552.1[2462:4450] (completely identical)
Position: 008-contig_256: 106191 … 108180; Length: 1989 bp
Sequence:
ATGAAAAAGCAAATAATTTCGCTAGGCGCATTAGCAGTTGCATCTAGCTTATTTACATGG
GATAACAAAGCAGATGCGATAGTAACAAAGGATTATAGTGGGAAATCACAAGTTAATGC
TGGGAGTAAAAATGGGACACCGATTTCTAATGGTTACTTTTGGGGGAAAATCGATAGTCT
AGAATCACAATTTTCTAAAGCATTAGCAATAATTGAAGAGTATCAATATGGTGAGAAAGA
ATATAAAGATGCAAAAGATAAGTTTATGGATAGAATTTTGTCAGAAGATCAATATCTTTT
GGAGAAAAAGAAAGCTCTGTATGAGAAATATAAAGAGTGGTATAAAAAGCACAAAGAG
ATAAACCCAACTTATCCAAAAATGCAAACATTCCATGAATTTAGTGTATATAATCTAACT
ATGGAAGAATACAATGAGATTTCTAAATCTTTGAAAGATGCGGAAGAAGAGTTTCGTAA
AAATGTAAGAGAAGTTCAAGAGAAAGAAGTCAAATTAAAAACATATAGCGAAGATGAA
GAAGAAAAGGCAACTAAAGAAGTATACGATCTTGTAGCCGAAGTTGATACTATATATGT
ATCTTATTTTGGACATGATAAACATGGTCAAAGTGCTAAAGAATTGCGTGCGAAATTAGA
TTTAATAATTGGAGAGGAAAAAAACGCCTTTCGTATTACTAATGAACGTATAAAAAATGA
AATGATTTATGACTTAAATTCTATTATTGACGATTTCTTTATGGAAACTAACCAAAATAGA
CCGGAAAATATAACAAAATTTGATTGGGATAAGCATGATTATAGAAATAAACCAGAAAA
CAAAGCAAATTTTGAGGCTCTAGTTAAAGAAACTAAAGGTGCGGTCGATAAGGCAGATG
ATTCTTGGAAAACTAAAACTGTCAAAAATTACGGTGAATCTGAAACAAAATCGCCAGTAG
TAAAAGAAGAGAAGAAAGTTGAAGAACCTCAAGCACCTAAAGTTGATAACCAACAAGAA
GTTAAAACTACTGCTGGTAAAGCTGAAGAAACAACACAACCAGTGGCACAGCCATTAGT
AAAAATTCCACAAGGAACAATCTATGGTGAAACTGTAAAAGGTCCAGACTATCCAACTAT
GGAAAATAAAACGTTACAAGGTGTAATTGTTCAAGGTCCAGATTTCCCAACAATGGAACA
AAGCGGCCCATCATTAAGCAATAATTATACAAACCCACCGTTAACGAACCCTATTTTAGA
AGGTCTTGAAGGTAGCTCATCTAAACTTGAAATAAAACCACAAGGTACTGAATCAACGTT
AAAAGGTACTCAAGGAGAATCAAGTGATATTGAAGTTAAACCTCAAGCATCTGAAACAA
CAGAAGCATCACATTATCCAGCGAGACCTCAATTTAACAAAACACCTAAATATGTTAAAT
ATAGAGATGCTGGTACAGGTATCCGTGAATACAACGATGGAACATTTGGATATGAAGCG
AGACCAAGATTCAATAAGCCATCAGAAACAAACGCATACAACGTAACGACAAATCAAGA
TGGCACAGTAACATATGGCGCTCGTCCAACACAAAACAAGGCAAGCAAAACAAACGCAT
ATAACGTAACAACACATGCAAACGGTCAAGTATCATACGGAGCTCGTCCGACATACAAG
AAGCCAAGCAAAACAAATGCATACAACGTAACAACACATGCAAATGGTCAAGTATCATA
TGGCGCTCGCCCGACACAAAACAAGCCAAGCAAAACAAATGCATATAACGTAACAACAC
ATGCAAATGGTCAAGTATCATACGGAGCTCGCCCGACATACAAGAAGCCAAGCGAAACA
AATGCATACAACGTAACAACACATGCAAATGGTCAAGTATCATATGGCGCTCGCCCGACA
CAAAAAAAGCCAAGCGAAACAAACGCATATAACGTAACAACACATGCAGATGGTACTGC
GACATATGGGCCTAGAGTAACAAAATAA

> **tx_coa**

Function:  rho-independent terminator
Best match: tx_coa_CC001_MW2-USA400_BA000033.2[244890:244937] (completely identical)
Position: 008-contig_256: 108447 … 108495; Length: 48 bp
Sequence:
CAAGTCTAGTTCGAACACACACTGATGTGAGTGGTTTTCTTTATTTTA

> **fadA**
Function: protein coding sequence; 3-ketoacyl-CoA transferase (core genome, variable)
Best match: fadA_CC005_Mu50-VRSA_BA000017.4[267199:268383:r]RC (completely identical)
Position: 008-contig_256: 108766 … 109951; Length: 1185 bp
Sequence:
CTAACGCACATACTCAAATATAGCAGCTGCACCCATGCCGACACCAATACACATCGTAAC
CATGCCGTAACGGCTATCGGGACGTCTACCCATTTCATTAAGTAAACGCGCGGTTAACAT
TGCCCCTGTAGCACCTAATGGATGACCTAAAGCAATAGCGCCACCATTCACATTCGTACG
TGATATATCTAGACCTACTTCTTTAATAGATGCAATCGTTTGAGAAGCAAATGCTTCGTTC
AATTCGATCAAATCAATGTCTTCAACAGATAGATTGCTGAGTGACAATACTTCAGGAATC
GCATATGCAGGCCCAATACCCATAATTTTCGGGTCAACGCCTACTGCCTTATAACCAACG
AATCGTGCAATAGGTGTCACGCCTAGTTCTTTCACTTTATCTCCAGACATTAAAACTACAA
ATCCTGCACCATCAGAAAGTGGGGCAGATGTTCCCGCAGTCACAGTGCCGTCAGCTTTAA
ATACTGTACGTAATTTGGCTAATGCCTCCATCGTGGTGTCAGGGCGTATAAATTCATCTTG
GTCAAAGATATTTGTGTGTACTTTTGGTCCTGCGTTTGTATATTCAACTGAGTTTACCCGT
ATTGGAATAATTTCATCTTTGAACCGACCATCACGTTGTGCGTCATAGGCACGTTGATGAC
TTCTGACAGCATAAGCATCTTGATCTTCGCGTGATACGTCAAATTGGGATGCTACATTTTC
AGCAGTTAAACCCATAGGATATGACGCACCTATATCATCATATTGTAAGGTTGGATTGTT
TGTGGGCTCGTTGCCACCCATTGGTACGGCACTCATCAATTCAACGCCACCAGCTACAAG
TATATCTCCTTGACCAGCCATAATTTGATTGGCTGCAATCGCGATGGTTTGTAATCCTGAT
GAGCAGTAGCGATTCACTGTTTGACCCGGTACCGTGTCAGATAATCCCGCACGCAATGCA
ATCGTTCGTGCAATGTTTTGTCCTTGTAATCCTTCTGGAAAAGCCGTACCAACAATGACAT
CTTCAATCATATTCTTATTGAATTTTCCGTCAATACGTTTCAATACGCCTTGTAATACTTTG
GCTGCGACATCATCAGGTCTTTCGTGGAATAATGCGCCTTGCTTTGCTTTCGCTGCGGCTG
AACGCCCATAAGCTACAATGTATGCTTCTTGCAT

> **fadB**
Function: protein coding sequence; NAD binding 3-hydroxyacyl-CoA dehydrogenase (core genome, constant)
Best match: fadB_CC005_Mu50-VRSA_BA000017.4[268413:270674:r]RC
Position: 008-contig_256: 109980 … 112242; Length: 2262 bp
Sequence:
TTAATTACGTAATGGCTTACCAGTTTTTAACATATGTGCAATTCTTTCATATGATTTTTTAG
ATTTTAGTAAGTCAATAAAGCCAATTTTCTCCAACGATTGAATGTAACGTTGATTTATAAA
TGTATTTCTTGGTAAATCACCACCCGCTAAAATTGTGGCGATATTTAAGGCGATATGATA
ATCATGGTCGCTAATAAAATGCCCCCGTCTTTGCGCATCTAATTGTCCTTGGATCAATGCT
TTGAAGTCTTCACCTAAAGCGATATATTGATGTCTAGGATTCGGAATATAGTTTGTTTCTG
CTTCATATTTCGCACGTTTGAGCGCAACTTCGACACGTTGTGCTGTATTGAAAATAATCGT
ATCTGTATCACGTAAATAACCATAACGACGTGCCTCAAAGGCATTTGTAGAGACTTTCGC
AAATGCGATATTCGTCAGTACTTTTGTCATGGAAGCTTGTTTGTCATCAAACTTATGCGAT
GTGCGTAATATGCGATCAGCCATTTCTGCAAGGCCACCGCCACTCGGTAATAAGCCAACG
CCTGCTTCAACAAGACCTATATATGTTTCACTTGCAGCGACAACAATAGGTGAGTAAAGT
ACAAGCTCACAGCCACCGCCTAAGGCACGACCTTGAACAGCTGTGACTACTGGTTTCAAA
CTATACTTCAAACGATTAAAGCTATAATGTAATTTATCAATTGATTGTGCAACGACATCAT
CTACAAGACCGTCTTCATGCGCCTTTTTCATTAAGAAAAGGTTAGCACCCACACTGAAAT
TATTACCATCTGCATAAATGACCATACTTGTGTAATGGTCATTTTCCAGTAAATCAATGGC
ATCAACTAACGCATCGTTGAATTCATCGGTAATGACATTATTTTTACTTTGTAATTTCAGT
AACAGTTGATCATCATGAGTTACGGAAAGTTTGGCATCACCTTTATCCCAAAGTTCATCTT
TTACGAAGTGAGAAACAGGTGTTGCATATTCGATGGTCTCATCTTGTTTATAAAAGCCAC
CATCTAAATCACTAATCCATTGTGGTAAGTCTCCAATTTCGTCTTCCATACGTGTTTTAAC
ACGTTCATATCCCATTGCATCCCATAATTGGAATGGACCAAGTTTCCAGTTGAACCCCCA
GACAAGCGCACGGTCTATGTCTCGGAAATCATCGGTAGCTTTAGGTACATTGATAGCAGA
GTAATAGAAATTATTACGTAATGTCTCCCATAAAAATAGTCCCGCTTCGTCTTGCGCATTG
AATATGACATCAAGGTTATGCACTAAGTCTTTATTAAATTCATTTAAAATTGGTAATTGTG
GTTGCGATACAGGTACATAATCTTGTTTTTCAACATCGTAAACAAGTCGAGCTTTAGTTTC

TTTATCCTTTTTGTAAAATCCTTGTTTCGTTTTACGTCCGAGTGCGCCATTGTCAAACAACG
TATTTACAATTTTGACATCATGAAAATAAGGCGTTTCTTCAGGTACTTGTTGCATGCCTTT
AATTACAGACACTGCAATATCTAAACCGACTAGGTCAGATAGGGCATATGTACCTGTTTT
AGGACGACCAATCGCTTGCCCAGTTAAAGCATCCACATCTACAATGCTTAACTTGTGTTG
CTCGGCGCGATACATAATATCATTCATTGTTTGCGTGCCGACTCTATTTGCGACAAAGCCA
GGCACATCATTGACGACAATGACACCTTTACCTAACACATTTTGCGCGAAATTTTTTACAT
CTAATATGATAGATTCCTTCGTGTGTGACGTAGGTATTAACTCCACTAATTTCATAATACG
TGGTGGGTTAAAGAAATGTAGACCAAAGAATCGTTCTTGATCCTTCTCGTTAAATGTTTG
AGCAATCGCATTAATTGGAATACCTGATGTATTTGTAGCGAATAAAGCATCTTCTTTAGC
ATGTTGTAGAACTTGTTGCCAAACAGCATGCTTAATTTCAATATCTTCTTTAACTGCTTCG
ATATATAAATCAGCATCATCATTTACCAAGTCATCATCAAAATTACCATATGTTAAATGA
CTCGCTAGATTTAAGTCGAATAGTAGCGGCCGTTTCTTATCTGTAATTTTATCGTAAGATT
TTTTCGCAATGAGATTTGGATCGTTTTTGTCCACTACAATATCTAATAGTTTTACTTTAAGT
CCAGCATTCACAAAGAGTGCTGCCAGTTGAGCGCCCATCGTGCCTGCGCCAAGAACGGTT
ACTTTATTAATTGTCAT

## > fadD

Function: protein coding sequence; putative acyl-CoA dehydrogenase (core genome, constant)
Best match: fadD_CC001_MSSA476_BX571857.1[247587:248798:r]RC (completely identical)
Position: 008-contig_256: 112428 … 113640; Length: 1212 bp
Sequence:
TTATACGAAAGCAGAATCTCCAGTCAAAGCGCGTCCAATTACTAAGGCATTAATTTCATG
TGTACCTTCGTACGTGTAAATCGCTTCTGCATCAGAGAAGAAACGTGCAATATCATAATC
GTCAGCTAGTATGCCATTACCACCTGTAATACCACGGCCCATAGCTACTGTCTCACGCAA
ACGTAAGGCATTCATCATCTTCGCCGTTGAAGTTGCAACCTCGTCATATTCACCATGTGCT
TGCATATTAGCTAATTGAGCACATGTTGCCATTGCTTGAGCTAAATTACCTTGCATCATTG
CTAGCTTTTCTTGTATTAACTGATATTTACTAATTGGTTTGCCGAATTGCTTACGCTCAGTG
ACATAATCTAATGTGGCACGTAAAGCGCCAGCCATACCACCTGTAGCCATATAAGCAACG
CCTGCTCTCGTTGAATAAAGAATTTTGGCAATATCTTTAAAGCTTGTTATGTTTTGTAAGC
GATCCGCTTCATCTACTTTGACATTAGTTAATTTAATTAGGGCGTTAGGAACAATGCGAA
GTGCGATTTTATTATCAATGACTTCAATATCGACGCCATCTTGTTCTGGTCTGACTACAAA
GCAATGGGGTTTGCCAGTTTCTTTATTTACTGCGAATACTGGAATGACATCAGATACATGT
GCACCACCAATCCATTTCTTTTCACCATTGATAACCCAAGTATCGCCCTGGCGTTCAGCGA
CTGTTTCAAGACCTCCCGCAACGTCCGAACCGTGTTCTGGTTCAGTTAAAGCAAAGCATG
TACGCAGTTCATGTGACTGTAATTTAGGTACATATTTCGCAATTTGTTCTTTGCTACCTCC
GAAATAGAAAGTGTTATGCCCTAAACCTTGGTGAACACCGAGTAGGGTAGCTAAGGAAA
TATCAAATCGCGCGAGTAGGTAAGACATGAAAAACTGAAATAGTTGACTAGGCATTTTGG
CGTTTGGACGATCCTTGTAAAGTAATGGATTGTTAAAATAATTTAATTCTCCCAGATCTTT
AAAATAGTCCTCGGGTACAGTAGCGTCTATCCAATGTTGATTAATATTTTCACGGTACTTA
CTTTCTAGCAATGAATCTACTTGTTGTAAAAATTCGACTTCACCGTCTGTTAAACCTTTAG
CAATACTAAGTACATCTTCAGGAAATAATGTTTTTAAGACCGTTTCTTTTTCAAATGTCAT

## > fadE

Function: protein coding sequence; putative acyl-CoA synthetase (core genome, constant)
Best match: fadE_CC001_MW2-USA400_BA000033.2[250210:251715:r]RC (completely identical)
Position: 008-contig_256: 113751 … 115257; Length: 1506 bp
Sequence:
TTAAAGTGTTTGTGAGACTTTGTCATTCATCATTTGTCGAATCGCAAGTTTATCTGGTTTCT
GCGTACTGTTTAACGGCATATGTGTCACTGGTACATACATTCTTGGGACTTTATAACCTGC
TAAACGACTTCGCATATGTTGATCTAAAATTTCAGCGTAATGAGGTTCATCTTCGCGAAGT
ATAATGGCTGCAGCAATTGATTCACCATATTTTGGATGATCATAGCCAACGACCACACAC
CGGTCTACTAGTGGATGCTCAGCTAAAGCATTTTCGACTTCGGATGGTAAGACATTTTCGC
CACCAGTTATGATTAATTCTTTTTTGCGGTCAATAATAAATATATCGCCATCGTCGTCCAT
CTTCGCTAAGTCACCAGTTAATAAATAACGACCATGAAATGCTTTGGCAGTCTCTGCTGG
TTTATTCCAATATCCTGGCGTGACATTTTTAGCCTTAATTGCAAGTTCGCCAATCTCACCA

GTAGGTACTTCCTCACCGTTATCATCAAGGATACGTGCATCAACGAACATGACTGCTTTA
CCAATACTCATTGGCTTACGTTTTGAATTTTCCGGTGTATTAACAAGTACAAGAGGTGCTT
CAGTTAAACCATAGCCGTTAATAATGTTTATGCCATATTGTTTAAAAGCTGCTTGGATACT
TGGTAATGGTTGTGAACCACCTTGGATGATATAATCCATAGCTTTAAAATTTTCAGGATTA
AAATTACTAGCACGTAGCGTACTATAATACATTGTCGGAATCATGATAATAAATGTAGGG
TGATATTGTGCAATCATGTCATTCAATTCTTCGCCGTTAAAGTAACGTTGAAGAATAAGTG
TGCCACCTGACATCAATACTGGTAATACAGTATCGTTAAACCCTAAAACATGGAACATTG
GTGTTGATACAATCGTAATATAGTTTGAATTGAACTTATACGTCAGCTCTAAGTTTGCACC
GTTATGAACAAATGATTCATATGAGAACATCACACCTTTAGGTGATCCGGTTGTACCACT
TGTATAAATTAATGCTGCAAGATCTTGTGGTTCGACAGGTGTTGCTTGAAAAGGTTGGTG
ATAATCTGGATTTACGATTTCATCATATTGCGCTACATCAATATCCATATGCAATAAGTTT
TGGTCAATATCGGTGAGTGAACTTAAATGTTTTTCAGCATAGAAGAGCAGTTTTAATTGT
GCATCTTCCACAATGGCTGCAATTTCTTTTGGGTTAAGCCGCCAATTCAATGGTAAAAAA
ACCGCACCTGTTTTAAAACAAGCAAACAATAAATCTAATATTGCAATATCATTTGGCGCA
AAAATACCGATAACATCGCCTTTTTTAACACCTTGAGATGTTAAATAATGTGCCATATTAT
CAGCGCGTGCATTGAGTTGTTGGTATGTCCAAGATGTTTGTTTTGCGTGATCAATAACGGC
AGGCTTGTCATCATCGAAGTCTGAACGCGTTTTTATCCAATCGAAATTCAT

## > **fadX**

Function: protein coding sequence; putative acetyl-CoA/acetoacetyl-CoA transferase (core genome, constant)
Best match: fadX_CC001_MSSA476_BX571857.1[250441:252003:r]RC (completely identical)
Position: 008-contig_256: 115282 … 116845; Length: 1563 bp
Sequence:
TCATACTTTATGAATTGATTGTTTAAGTTGTCCCCATTTTTCTTTGTAAATGCTGGTATCAA
TTAATTTTAAATGATCAGCAATAATTGGTTTAAAAGCCATTTGATTCAAAATATCTTTATG
CAAATCAAGACCTGGTGCAATTTCAATTAGTTTCAAGCCTTGATTGGTGAGTTCGAATACT
GCACGCTCAGTAACAAAATAGACTTCTTGCTCGAGTGATTGTGAATATTGTGCATTAAAG
TCGATATGGCTCACATCTGATACAAATTTCTGGTTTTGTCCTTCAGTTTCAATGTTTAATCG
TTGATTATGGCATGAGACATGACTGCCAGCTACAAAAGTACCTGAAAAGATAATTTTATT
TACAGATTGCGTAATGTCTATAAAGCCACCACATCCATTTAGTCGGTCATTGAAGTAAGA
CACGTTGACATTGCCGTATTGATCAACCTCAGCAAAGCTAAGATAGGCAACTGATACACC
ATTGTTATAAATAAAATCCCATGCTCGATCATGAGGCATGCGCACATCTGCATTGTAATTC
ATACCAAAATGTTCACGACTCCCAACGAATCCACCGAAAATGCCAACATCTAAAATCGGT
TGCACATCATGTTCAACACATTCTTCGTGCAATAAATTAGAGAGTTCATTATTGATGCCAT
AACCGATGCTAATTGTATCGCCATAAGTTAAAAACTGAGCAGCACGTCGGAGAATCAATT
TGCGACTATTAAAAGGTAATGCGGGTTCAGGTATTCCATCAATTCGTTCTTCTCCAGACAA
GGCTGGTAAATAATGACTCTGAATTACTTGGCGGTGATTCTTTTCATCTTCTGTGACGTAT
ACATAATCGACAAGATTTCCTGGGATAACAACTTCATTCGGTTTTAGTTGATATCCGTCAA
CTAAAGCTTTAACTTGTACAATAACTTTCCCATGATTGGCTTTCGCGTTTAATGCGACATG
ATAACACTCGCTCAAGTACGCTTCTTGAGTTAAATAAATGTTACCTTGTTGATCTGCGTAT
GTTCCTCTCAGTAGTGCCACATCAACGCTAGGGAATGTGTAATGTAAGTATGTTTCATCGT
TGATGGTTACTAATGAAACTAAATCATCAGTTGTTCGTGTATTTACTTTACCGCCACCGTA
TCTAGGATCAACAGCTGTGTTTAATCCGATTTTAGTAATAACTCCAGGTAATAATTGATTA
CTCTGACGATAATGAGTTGCAATGATACCTTGTGGTAAAAAATAAGCTTCAATGTCATTA
TTTTTCATTGCTTGTGCCGTTTTGGAAGAAGCCGTTAAAATGCTCATAATGACACGTTTAA
TCATGCGACGTTCTATAAAATCATCTAAATCCGGTGCGGCACCTAAACTATGGATATCAT
TCGCTAATATAAACGTTAAATCTTTGGGCGTATGATATGTGTCATGTTGCGCTAACACAGC
ACGTAGAACTTCGGCGGGTAAGTTGGCTACAGCTAATGCTGGTAAACCAATCACATCACC
ATCTTTAATGATATGTTGTAAGTCGTGCCATGTGATTTGTTTCAA

## > **Q5HJE2**

Function: protein coding sequence; putative protein (core genome, variable)
Best match: Q5HJE2_CC001-ST772_118_AJGE01000065.1[68669:69811:r] (completely identical)
Position: 008-contig_256: 117300 … 118443; Length: 1143 bp

Sequence:
ATGTCATCAGCAAGTACACAATCAACTAAGACGTCCGACATACATAATGAATCTATCAAT
AAACAAATGGAAGCCAAAGCGCATGAAACAGCGCAAAATGCAGATTTAAAAACCGAAG
CAAGAAGTTTATTTGATAATGCAACCAAATCAATCGGTAGACTAGCCGGCAATGATGAAA
GCTTAAATCTTAATTTAAAAGATATGTTTTCTGAAGTATTTAAGCCGCATACTAAAAACG
AAGCAGATGAAATATTTATAGCGGGTACTGCTAAAACTACGCCAGCAATTTGTGACATAT
CAGAAGAATGGGGGAAGCCATGGCTCTTTTCTCGAGTATTCATCGCTTTCACAGTAACAT
TTATTGGATTATGGGTCATGGCAGCGATTTTTAATAACACTAACGCGATTCCGGGTCTCAT
TTTTATAGGGGCTTTAACAGTACCATTATCGGGTTTGTTCTTCTTTTATGAATCAAATGCG
TTTAAAAATATTAGCATTTTTGAAGTTATTATCATGTTCTTTATTGGCGGCGTATTTTCATT
ACTAAGTACGATGGTATTATATAGATTTGTCGTTTTTAGTGATCAATTCGAAAGGTTTGGT
TCTTTAACATTTTTCGATGCATTTTTAGTAGGATTAGTTGAAGAAACTGGAAAAGCACTCA
TTATTGTTTATTTCGTCAATAAATTGAAAACAAATAAGATTTTGAATGGATTATTAATCGG
TGCTGCTATTGGTGCAGGGTTCGCAGTTTTTGAATCAGCAGGTTATATTTTGAATTTCGCT
TTAGGGGAAAATGTCCCATTATTAGATATTGTCTTCACACGTGCGTGGACTGCGATTGGT
GGTCATTTAGTTTGGTCAGCGATTGTTGGTGCTGCAATAGTTATTGCGAAAGAACAGCAT
GGCTTTGAATTCAAAGATATTTTTGATAAACGCTTTTTAATATTCTTTTTATCAGCCGTTGG
TTTACATGGCATTTGGGATACATCTTTAACTGTACTTGGCAGTGATACGTTGAAAATATTT
ATTTTAATCGTTATTGTGTGGATACTTGTATTCATTTTAATGGGGGCAGGTTTAAAACAAG
TGAATTTACTGCAGAAAGAATTTAAAGAACAACAGAAAAAAGTAGGCGAATAA

> **hmp**
Function: protein coding sequence; FAD-binding oxidoreductase (core genome, variable)
Best match: hmp_CC001-ST772_118_AJGE01000065.1[64825:65970]RC (completely identical)
Position: 008-contig_256: 121141 … 122287; Length: 1146 bp
Sequence:
TTATACTGCAACGCTTAGTCTTGGAATAAATGTTTCGTAGTGTACGCGATCCATATCGTAA
TTTAAAGATTTAAGTGCTTCGATCATAGATTGTAAGAATTTTGTACCACCACAGATATAA
ATTTCAGGTTTATTTGCTAAAAATACTTGTAATTCTTCAGCGCCAATATAGCCTTGTTTAT
CTTTTAAGTGAGTATATAACTTAGCGTTGTCATGATGGCTTGCGATACTGTTGAAGTTGTC
TTTGAAAGGTAAATGTTGTTCATTTTCAGCAACTTGAACCATCTGTGTATCTAAACCTTTG
GCAGAGGCAGCTTCATACATAGCTACTAAAGGTGTAACACCAATACCTGAACCTAAGAA
AAGTTGTGGTTCAGTCGTATTCTCTAATACGAATCCACCTACAGGTGCAACTAAATTAATC
ATATCGCCTTCTTTAATCTCATCGTGTAAAATTGTTGAAACTTCGCCTTCATGTTCTGTTGT
GACATCACGTTTAACGCCAAAAGTTAAATGGTTTTTATCACCTGATACGATAGAATAGTG
ACGTTTAGCTCTATATGGAAGTTTATCACTAGAAACATCAACTGTGATGTATTGGCCTGGT
ATAAATTCACTAAAGTCATATTCTTCAGTTTCAACTGTAAATGATTTAATGTCTTCAGATT
CTTGTTTAATATTGGTAATTTTGAATGGTTTAAAACCAATCCACATCATTTGATCATAAAT
TTCTTTTTCAATTTGGATGAACACGTCCGCAATAACGCCATATGCTTTCGCCCAAGCTTGA
ATGACAGGGTCATTTTCTTCTAATCCTGTCACATCTTGAATGGCTTTTAATAAATTTTCCCC
CACAATTGGATAATGTTCAGCATAAACTTGTAGTGCGCAGTGTTTATATGCGACTGGCAT
AATGACTGGTTTAATAACACTTAAATTATCGATATTAACCGCTGCAGCCATTACAGCTTGT
GCTAATGCTGAAGATTGCATGCCTCGTTTTTGGTTCGTTTGATTAAACATGTTTAAAAGTT
CAGGATGCGCTTTAAACATTTTTGGATAAAAGATTGACGTAATTTCTGTCCCTTTCTCTTT
AAGTAAAGGCACCGTTTGTTTGATAATGTCTTTCTCTTGTTCTGTAATCAT

> **lctE**
Function: protein coding sequence; L-lactate dehydrogenase 1 (core genome, constant)
Best match: lctE_CC001-ST772_118_AJGE01000065.1[63299:64252:r] (completely identical)
Position: 008-contig_256: 122859 … 123813; Length: 954 bp
Sequence:
ATGAACAAATTTAAAGGGAACAAAGTTGTATTAATAGGTAATGGTGCAGTAGGTTCAAG
CTACGCATTTTCATTAGTGAACCAAAGCATTGTTGATGAATTAGTCATCATTGATTTAGAC
ACTGAAAAAGTTCGAGGAGATGTTATGGATTTAAAACATGCTACACCATATTCTCCAACA
ACAGTTCGTGTGAAAGCTGGCGAATACAGTGATTGTCATGATGCGGATCTAGTTGTCATC

TGTGCTGGTGCTGCACAAAAACCTGGAGAAACACGTTTAGATTTAGTATCTAAAAACTTG
AAAATATTCAAATCAATTGTTGGTGAAGTAATGGCATCAAAATTTGATGGTATTTTCTTGG
TAGCTACAAATCCTGTTGATATTTTAGCGTATGCAACATGGAAATTCTCTGGTTTACCTAA
AGAACGTGTTATAGGTTCTGGTACAATTTTAGACTCTGCACGCTTTAGATTATTGTTAAGC
GAAGCGTTCGATGTTGCGCCACGTAGCGTCGATGCTCAAATTATTGGTGAACATGGTGAC
ACTGAATTACCAGTATGGTCACACGCTAATATTGCGGGTCAACCTTTGAAGACATTACTT
GAACAACGTCCTGAGGGCAAAGCGCAAATTGAACAAATTTTTGTTCAAACACGTGATGCA
GCATATGACATTATTCAAGCTAAAGGTGCCACTTATTATGGTGTTGCAATGGGATTAGCT
AGAATTACTGAAGCGATTTTCAGAAATGAAGATGCCGTATTGACTGTATCAGCATTATTA
GAAGGCGAATATGATGAAGAAGATGTTTATATTGGTGTTCCAGCAGTCATCAATAGAAAC
GGTATTCGCAACGTCGTAGAAATCCCATTAAACGACGAAGAACAAAGCAAGTTCGCACA
TTCAGCTAAAACATTAAAAGATATTATGGCTGAAGCAGAAGAACTTAAATAA

## > ptsIIBC

Function: protein coding sequence; putative PTS transport system/IIBC component (core genome, variable)
Best match: ptsIIBC_CC001-ST772_118_AJGE01000065.1[61449:62978]RC (completely identical)
Position: 008-contig_256: 124133 … 125663; Length: 1530 bp
Sequence:
TTAATCCCCGAGCAATTCTTCAATCTCATTTTTGATAACTGTAACGTGAGGCCCATAAATT
ACTTGCACACCAGTGCCTTGCTGGATTACACCTTTGGCACCAGTACTTTCGAGTAATACTT
TATCGACTTTGTCATTTTGATGAAGTGTGACGCGTAGTCTCGTTGCACAACAGTCAACGAT
TTCAATGTTATCCTTGCCTCCCAAACCAGCAACAATAGTTTGTGCTCTTTCAGTAGCCTCA
ACTTGTTGTGCTGCAGCTTTATCTTCTCGACCAGGTGTTTTGAAATTAAATTTCGTAATTA
AGAATCTGAAAACGATGTAATACAAACAGAACCACACAATTCCAATAGGTATGACGTAT
AGGTAGTTTGTTTTACTATTACCTTGTAGCACACCAAAAAGTAAGAAATCGATAAAGCCT
CCACTGAAGGTTTGACCAATTGTAATGTTGAAAATGTCTGCCATCATAAATGCTAATCCA
TCAAAGAAGGCATGGATTACATAAAGAATAGGTGCGACAAACAAGAAACTAAACTCTAA
AGGTTCGGTAATACCTGTTAAAAATGAAGTGAGTGCAGCGGATAACATTAAACCGCCGA
CAACTTTTTTATGTTCAGGTTTAGCTGTGTGATAAATTGCAAGTGCGGCACCACATAAGCC
GAACATCATCGTAATAAAACGGCCTGACATAAAGCGTGACACACCTGAATAATACTTCGT
CACATCTGGATCACCAAGTTGAGCAAAGAAGATGTTCTGCGTACCTTGAACTAAGTGCCC
TTTGACTTCTAAAGTACCACCAAGTGCCGTCTGCCAAAACGGTAAGTAAAAAATATGGTG
TAAACCGAGTGGACCTAACAATCTTAAGATGAAGCCATAAACAAAAGTACCGATGGCAC
CTGTTTTCGTTACAAATCCACCAACATGATAAATGCCGGCTTGTATGCTTGGCCAAATGA
AAAACATCAATACACCTAAAAAGATTGCGGCAAATGCTGTGACAATAGGGACAAATCTA
GAGCCACCAAAGAAACCTAAATACGGTGGTAATACCACTTTGTGATATTTGTTGTGAAGT
ATTGCGGTCATAATACCTGTGATAATCCCGCCAAAAACACCGGTTTCAACCGTTTGTATA
CCGAGCACCATGCCTTGTCCATTTTGTGCAAGCTGATCTTTTGCCAATGTGCCCGTGATAG
TTAATAAGCCATTCATAGTTACGTTCATAATTAAGAAACCGAGCAGCGCAGCTAAACCTG
CAGTACCTTTATCGCTTCTAGATAATCCGATTGCGACACCAATGGCAAAGATGACCGGTA
AATTTGGAAAACAATACTACCTGCAGCTGACATTAATGTAAAAATATTTTGTAATAAGG
TAATATCTAAAATAGGGTATGCTTTAACGGTGTTTGGATTACTTAATGCACCACCGATACC
CAACAATAGACCTGCAGCTGGTAAGATTGCGATAGGTAGCATAAAGGACTTGCCGAACT
GCTGTGCTTTTTCAAATAAAGATTTCAT

## > rihA

Function: protein coding sequence; inosine-uridine preferring nucleoside hydrolase (core genome, variable)
Best match: rihA_CC001-ST772_118_AJGE01000065.1[60152:61087:r] (completely identical)
Position: 008-contig_256: 126024 … 126960; Length: 936 bp
Sequence:
ATGAAAAGAAAGATTATTATGGATTGTGATCCAGGACACGATGATGCAATAGCATTAATT
TTAGCGGGGGCAATTGACAGTCCACTAGAGATATTAGCTGTAACAACAGTCGCAGGTAAT
CAATCAGTTGACAAGAATACGACAAACGCCTTGAACGTATTGGATATTATGGGACGCCAA

GATATAGCAGTAGCGAAAGGTGCGGATAGGCCGTTAATTAAACCAGCTGCCTTTGCTTCT
GAAATACATGGGGAATCTGGATTAGATGGTCCGAAACTACCGTCGACACCATCACGTCAA
GCAGTTGCAATGCCAGCATCAGATGTGATTATAAACAAAGTGATGACGAGTGATACACCT
GTAACAATTGTAGCGACAGGTCCTCTTACGAATGTAGCAACGGCATTGATTCGTGAGCCA
AGAATCGCTGAGCATATTGAATCTATTACTTTGATGGGTGGTGGTACATTTGGAAATTGG
ACGCCTACAGCAGAATTCAATATTTGGGTAGATGCTGAAGCAGCGAAGCGTGTTTTTGAA
AGTGGGATTACTATAAATGTGTTTGGTCTAGATGTAACACATCAAGTTTTAGCCGACAAT
CACGTGATTGAACGCTTTGAAAGTATCAATAATCCTGTTGCACAGTTCGTCGTAGAATTAT
TGCAATTCTTTAAGAAGACATACAAGACTCACTTTAATATGGATGGTGGTCCAATACATG
ATGCTTGTACAATTTTGTATTTGTTACAACCAGAATTGTTTACAATGGTACCCGTTAATAT
CGACATTGAACATCAAAGTCCACTAACTTATGGCACTATGGCTGTCGATTTAAATCATGTT
ACAGGTAAGCCTGTCAATGCTTATTTTGCTACAGCAGTTGATGTTGAAGAAGTGTGGAAC
TTGATAGATTATAAGTTACGTACTTACGAATAA

> **bglG**

Function: protein coding sequence; transcript antiterminator/PTS regulator (core genome, variable)
Best match: bglG_CC001-ST772_118_AJGE01000065.1[57718:59814:r] (completely identical)
Position: 008-contig_256: 127297 … 129394; Length: 2097 bp
Sequence:
ATGAACGGGGATAATCAGCAAATACTCAGAGAAATTGTATTGAATCCTACTATTCATGGT
AAAGAACTTGAATCGATATTTGGTTTGTCTCGTAGACAACTAGGATATCGCATTCAAAAA
ATCAATTTGTGGCTTGAACAAGAGGGTTATCCAAAACTTGAAAGAACAAGCCAAGGAAA
TTTTATTGTAAGTTCTGAAATCATGACGTTATTCAAGCGAGATGTATCGGAGCAGCAAAT
GTTAAACGGCAACAATGTCATTTTTAGCATAGAAACACGTCGTTATTATTTAATGCTCATG
CTTTTTAGTAAGGAAAATGCAATGTCTCTAAACCATTTTTCAATTGATTTACAAGTCAGTA
AAAATACTGTCATTCACGATATAAATCATGTGAAAGAGCAATTGGAAGATCATGGTTTGT
CACTTAAGTATTCTCGAAAGCATGGTTATGAAATTGTTGGTGATGAATTTGAAGTTCGCC
GTTTCTTCATTAAGTTGATTGATCAAAGGTTGAATCATGATATTACTAAAAGTGAAGTTTT
AAAGGCGCTCAACTTAACATTCGAAGATATCGCATATCAAAAAGACAAGATCAAACAGG
TAGAACAATTTTTGAAGAGTCGCTTTATAGACAAATCACTTAGTTCATTGCCTTATGTCCT
TTGTGTGATTCGTAGACGAATTCAAAGTGGTCATGTGATGAATCCATTAAATATTAATTAT
CAGTATTTGAGGGATACGAAAGAATATCAAGCAACGGAGATTATGACGCAACATGAGCC
GGATTTGCCAGAAGCGGAAAAGTTATATTTGACATTACACTTACTTTCAACAAGTGTGCA
ATGGACTGATTTGCAGGAATCAGATAACATATCGAATTTAACGATGGCCATCGCTCAAAT
GATTCACCATTTTGAACAAATCACTTTTATTAACATTGAAGATAAGGAGAAATTATCACA
GCAACTCTTGTTACATTTAACGCCTGCTTTTTATAGGATTAAATATAACTTAACGGATCGT
GATGAATTAATAAATCCTTTACAAGGAAATTATCAATCCTTATTTCATATGGTGAAACAA
TCATGTCAATCGTTAACTGAATATTTCGGAAAATCGTTGCCTGATAATGAAATAGCATATT
TAACCATGTTGTTCGGAGGTAGTTTGAGACGTCAAGATGAAAACTTCGATGGCAAGATAA
AAGCTATTATCGTGTGTACACAAGGCACGTCAGTATCACAAATGATGTTATACGAGTTGC
GAAACTTATTTCCAGAAATTATTTTCTTAGATGCGATTTCACTTAGAACATTTGAAAATTA
CACATTAGATTATGACATCGTCTTTTCACCAATGTTTGTCCTAACACATAAAAAATTATTT
ATCACAAAAGTAGCTTTATCTGAAAATGAGCAACGAAAGTTACGTAAAGATGTGATGAA
GTACATTAATAAGGAATCGGCTGACATTGATAAGGAAATAAACAAGTTAATGGCATTAAT
TGAACGCACAACGACAGTTAATGACATTACAGAACTACGTGATGGTTTAGAAGATTTTAT
TGCCAATTATAATTCAATTTCAACCATTAATGGATCGATTGTCACACAAAATAAGACATT
AGATTTAGCTGACTTGATACCGGCAAGGCACGTGAAAAGAATGCATCATGTTGAAAATAT
TGATGAAGCTATTGCTAAAGCAAGTGATGTGTTAGTTGCTAATCATTTTATTGATATTAAA
TATATTCATGAGATGCAACAGGTATTTGATGATTCGTATATGGTTATCATGCAAAATATTG
CTATTCCACATGCATACTCTGAAAAGCATGTACATAAAACAGCGATGAGTATGTTGATAT
TACAAGAACCAATATACATGTCAGATGGCACAGCAATCCATATTATTGTACCTATTGCTG
CTGTTGATAAAGTGACACACTTAAGAGCGTTACTACAATTGAGAGATGTGGCGCAAGACA
ATGACGCAATTAAGCGCATCATACAAAGTCGCAAAAATTCTGATGTAAATGAGATTTTAA
AAAATTATTCAAATAAAGAAGCGAGGGAAAATGGATGGGACAGCAATTAG

## > Q5HJD2

Function: protein coding sequence; phosphotransferase system sugar-specific component IIA (core genome, variable)
Best match: Q5HJD2_CC008_NCTC8325_CP000253.1[234857:235324] (completely identical)
Position: 008-contig_256: 129378 … 129846; Length: 468 bp
Sequence:
ATGGGACAGCAATTAGTGCATAAAGAAAATATAATGCTCAATTTGTCGGCAACTGATAAA
GAATCCGTATTGTCACAAATGTCAGATGTGTTATTTCAAAATGGGTTCGTGAAGTCAACG
TTTAAAGATGCAGTCATCGACAGAGAAAAAGAATTTGCTACTGGTTTACCAACGCATCTA
TGTTCGGTCGCTATACCGCATACAGATGTCGAACATATTAACCATAGAACGATAGGTGTG
GCTGTTCTAGAAAAAGAAGTGCCGTTTATTGAAATGGGAACACTTGATCAACAGACAGA
AGTGAAAATCGTTTTTATGTTAGCAATGGATAAAGTAGATGATCAACTTAAGCTGTTACA
ACAGTTGATGCAAATTTTTCAAAGTGAAGAAAAATTGGAGCAGATTCTACGAACGAAAG
ATGAAACAATTTTAGCAACACTAATCAATGATTATTTGGAATATAACTAA

## > Q5HJD1

Function: protein coding sequence; phosphotransferasesystem sugar-specific IIB component (core genome, constant)
Best match: Q5HJD1_CC001-ST772_118_AJGE01000065.1[56965:57243:r] (completely identical)
Position: 008-contig_256: 129868 … 130147; Length: 279 bp
Sequence:
ATGAAACAAGTATTAGTAGCGTGTGGTGCAGGTATTGCAACGTCAACAGTAGTAAATAAT
GCAATTGAAGAAATGGCAAAGGAACACAATATTAAAGTAGATATTAAACAAATCAAAAT
TACAGAAGTTGGACCTTATGAAGACACTGCAGATTTATTAGTTACAACTGCAATGACAAA
AAAAGAATATAAATTCCCAGTTATCAACGCACGTAATTTTTTAACTGGTATTGGTATTGA
AGAAACAAAACAACAAATCTTAACAGAGTTACAAAAATAA

## > gatC1

Function: protein coding sequence; PTS system/sorbitol-specific IIC component (core genome, constant)
Best match: gatC1_CC001-ST772_118_AJGE01000065.1[55479:56738:r] (completely identical)
Position: 008-contig_256: 130373 … 131633; Length: 1260 bp
Sequence:
ATGAGTTACTTCACTGATTTTGTAAGGGGATTTTTAGATTTAGGTGCAACTGTTATTTTAC
CGGTTGTCATATTCTTGCTTGGCCTATTCTTTAGGCAGAAAATTGGAGCGGCATTTAGGTC
TGGTTTAACAATAGGTGTGGCTTTTGTAGGGATTTTCTTAGTCATCGATTTATTAGTTAAA
AATTTAGGGCCAGCAGCACAAGCGATGGTTAAAAATTTAGGCGTCAGTCTGAATGTGATT
GATGTAGGTTGGCCAGCAACATCATCTATCGCTTGGGCATCATCTGTCGCAGCATTTATTA
TTCCACTCGGAATCATAGTTAACGTTGTATTGCTAGTAACTAAAGTGACAAAGACGATGA
ATGTAGATATTTGGAATTTTTGGCATTATACGTTTACAGCAGCAATGGTTTATGCCGTATC
AGGCAGTATTTGGCAAGCGTTATTAGCAGCAGTTATTTTCCAAGTTATCTGTTTGAAAGTA
GCAGATTGGACAGCACCGATGATGAGTGAGTTCTTTGATTTACCAGGTGTATCGATTGCT
ACAGGAAGTACAATTTCTTATGCACCAGGTATTTACTTAGTTAAATTGTTACAAAAAGTA
CCCGGTCTGAATAAGTTAGATGCTGATCCTGAAACAATTCAAAAACGTTTTGGCGCATTT
GGAGAGTCTATCTTTGTCGGCTTAATTTTAGGTTTAGGTATTGGTGTGTTAGCAGGTTACA
AACCTGGAGACATCATTAATTTAGGAATGTCAATGGCTGCAGTAATGGTATTAATGCCTA
GAATGGTAAAAATCTTAATGGAAGGTTTAATGCCAGTTTCAGAGTCTGCAAGAACATGGC
TAAATAAACGTTTTGGCGAACGTGAAATTTATATTGGTTTGGATGCAGCAGTAGCATTAG
GTCATCCAGCGGTTATTTCGACAGCATTAATTTTAGTACCTATCACTGTTTTATTAGCCGT
TATTTTACCAGGAAACCAAGTACTACCTTTTGGTGACTTAGCAACGATACCATTTGTTGTC
GCGTTTATTGTTGGTGCAGCAAGAGGAAACATTATTCATTCTGTCATTGTGGGTACGATTA
TGATTGCAATTTCACTATATATTGCAACAGACGTAGCACCTATTTTCACAGATATGGCGA
AAGGTACGAATGTACAAATGCCAAAAGGTTCATCTGAAATTTCAAGTATTGATCAAGGTG
GTAATATCGTTAACTATCTTATCTTTAAACTATTTAGTCTATTCAATTAA

> **gutB**
Function: protein coding sequence; L-iditol 2-dehydrogenase (core genome, variable)
Best match: gutB_CC001-ST772_118_AJGE01000065.1[54406:55461:r] (completely identical)
Position: 008-contig_256: 131650 … 132706; Length: 1056 bp
Sequence:
GTGAAAGCTTTAGTAAAAACAAGAGAAGGACATGGCAACTTAGAACTTCTTGATAAAGA
AGTTGCAACACCGCTAGATGATAAAGTAAAGATTAAAGTACATTATGCAGGAATTTGTGG
CACAGATATTCATACTTATGAAGGTCATTATAAAGTTAATTTTCCAGTGACATTAGGTCAT
GAATTTTCTGGTGAAATCGTTGAAGTTGGAGCAGACGTTAAAGATTTTAAAGTTGGTGAC
CGTGTCACTTCTGAAACGACATTCTATGTTTGTAATGAGTGTGAATACTGTAAATCAAAA
GACTATAATTTATGCAACCATCGAAAAGGTATTGGAACACAAGTTGATGGCGCATTTACT
AATTATGTCATTGCACGTGAAGAAAGTTTGCATCATATTCCAGATGAAGTATCGTATCAG
TCTGCAGCTATGACAGAACCATTAGCATGTGCACATCATGGCGTTTCTAAGATTCAAGTC
AATTCAGGCGATGTAGCAGTTGTAATGGGACCTGGGCCAATCGGATTACTTGTAGCACAA
GTGTTAAAAAGTAAAGGCGCAACTGTTGTGGTAACTGGATTGGACAATGACAAAGTCAG
ATTAGATAAAGCAGAAGCATTGCACATGGATTATGTAGTCAATTTACAACAAACAGACTT
AAAAACGTATATCAATGGAATTACAGACGGTTACGGTGCAGATGTTGTTGTTGAATGTTC
AGGTGCAGTTCCAGCAGCACGACAAGGTTTGGATATTTTACGCAAAAAAGGTTTCTACAG
TCAAATAGGTATTTTTAAGGATGCTGAAATTCCATTTGATATGGAAAAAGTGATTCAAAA
AGAAATAACAGTTGTTGGTAGTAGAAGTCAAAAGCCAGCAGATTGGGAACCTTCATTGC
AACTTATGGCGGATGGTTTAGTAAATGCTGAAGCTTTGGTGACAAAAAATATATGATATTT
CGAAATGGGACGAGGCGTATCAACATTTAAAATCCGGCGAAGGTATTAAAGCATTACTTA
AGCCGCTCGATTTAGATGAAAATGAAGGAGAGAATTAA

> **Q5HJC7**
Function: protein coding sequence; putative protein (core genome, variable)
Best match: Q5HJC7_CC001_MW2-USA400_BA000033.2[269164:269310] (completely identical)
Position: 008-contig_256: 132707 … 132854; Length: 147 bp
Sequence:
ATGGTAGAATCAATGCTAACTTTTATGCTTGGGCCATTAAGACAAATCACTGATTTTTATA
TGGAACATTTACTCGTAAGTAATTCCATTGTCATTGCAGGTTATTTTGCGACAGGTATTTT
TAAAAAGAAAAAAGTTGTGAATTAA

> **Q5HJC6**
Function: protein coding sequence; putative sugar-phosphate dehydrogenase (core genome, variable)
Best match: Q5HJC6_CC001-ST772_118_AJGE01000065.1[53191:54234:r] (completely identical)
Position: 008-contig_256: 132877 … 133921; Length: 1044 bp
Sequence:
GTGAAAGCATTGAAATTATATGGCGTGGAAGATTTACGGTATGAGGATAACGAAAAGCC
AGTCATTGAAAGTGCGAATGACGTTATTATTAAAGTACGAGCGACTGGCATATGTGGTTC
AGACACGTCACGATACAAAAAAATGGGGCCATACATTAAAGGTATGCCATTTGGTCATG
AATTTTCAGGTGTAGTAGATGCCATTGGAAGTGATGTTACGCATGTTAATGTGGGCGACA
AAGTGACAGGTTGCCCAGCAATACCTTGTTATCAATGCGAGTATTGTTTAAAAGGTGAAT
ATGCACGATGTGAAAAGTTATTCGTCATTGGCTCATATGAACCTGGATCGTTCGCGGAAT
ATGTCAAATTGCCAGCGCAAAATGTTTTAAAGGTTCCAGACAATGTTGATTACATTGAAG
CAGCAATGGTTGAGCCATCAGCCGTTGTTGCGCATGGGTTTTATAAATCGAATATACAAC
CTGGTATGACTGTTGCAGTAATGGGGTGTGGCAGTATAGGTTTGTTAGCTATTCAATGGG
CACGAATATTTGGTGCTGCACATATCATCGCTATAGATATAGATGCGCATAAACTAGATA
TTGCAACATCATTGGGCGCACATCAAACAATCAATTCAAAAGAAGAAAATCTTGAGAAA
TTCATCGAAAATCATTACGCCAATCAAATCGATTTAGCTATAGAATCATCAGGTGCTAAA
GTTACGATTGGTCAAATATTGACGCTACCTAAAAAAGGTGGCGAGGTGGTATTACTCGGA
ATACCATATGATGATATTGAGATTGATCGCGTTCATTTTGAAAAAATTCTGCGTAACGAG
TTGACAGTATGTGGCTCTTGGAACTGTTTGTCCAGTAATTTTCCGGGCAAAGAGTGGACG
GCAACCTTACATTATATGAAGACGAAAGATATTAATGTAAAGCCTATTATTTCTCATTTTT

TACCGTTAGAAAAAGGCCCGGAGACATTTGATAAATTAGTTAACAAGAAAGAACGATTT
GATAAAGTCATGTTTACGATTTATTAG

## > tarI1

Function: protein coding sequence; 2-C-methyl-D-erythritol 4-phosphate cytidylyltransferase 1 (core genome, variable)
Best match: tarI1_CC001-ST772_118_AJGE01000065.1[51947:52663:r] (completely identical)
Position: 008-contig_256: 134448 … 135165; Length: 717 bp
Sequence:
ATGATTTATGCAGGTATTTTAGCAGGAGGTATTGGTTCGAGAATGGGGAACGTGCCATTA
CCAAAACAATTTTTAGATATTGATAATAAACCGATTTTAATCCATACAATTGAGAAGTTC
ATTTTAGTGAGTGAATTTAATGAGATTATTATCGCAACGCCAGCACAGTGGATTTCCCAT
ACACAGGATATTTTAAAAAAATATAACATTACAGATCAACGTGTCAAAGTAGTTGCAGGT
GGTACGGATCGAAACGAAACAATTATGAACATTATCGACCATATTCGCAATGTAAATGGA
ATTAATAATGATGATGTGATTGTAACTCATGATGCCGTAAGACCATTTTTAACTCAACGTA
TTATTAAAGAGAACATTGAAGTAGCAGCAAAATATGGTGCAGTAGATACAGTCATTGAA
GCAATTGATACGATTGTAATGTCTAAAGATAAACAGAACATACACAGTATCCCTGTAAGG
AATGAAATGTATCAAGGCCAAACACCACAATCATTTAATATTAAATTATTACAAGATAGT
TATCGCGCCTTAAGTAGTGAACAAAAAGAAATCTTATCAGATGCATGTAAAATCATTGTC
GAATCTGGATATGCAGTTAAATTGGTACGTGGAGAACTATACAACATTAAAGTGACAACA
CCGTATGATTTAAAAGTAGCAAATGCCATTATTCAAGGTGATATTGCCGATGATTAA

## > tarJ1

Function: protein coding sequence; CDP-ribitol synthetase (core genome, variable)
Best match: tarJ1_CC001-ST772_118_AJGE01000065.1[50929:51954:r] (completely identical)
Position: 008-contig_256: 135157 … 136183; Length: 1026 bp
Sequence:
ATGATTAATCAAGTATATCAACTCGTTGCACCGAGACAGTTCGACGTCACATATAATAAT
GTTGATATTTATGGTAATCATGTCATCGTAAGACCTTTATACTTGTCTATTTGTGCAGCTG
ATCAAAGGTATTACACAGGTCGAAGAGATGAAAATGTACTGCGCAAAAAATTGCCAATG
TCATTAGTTCATGAAGCTGTTGGTGAAGTTGTATTCGATAGTAAAGGCGTATTTGAAAAA
GGTACGAAAGTAGTAATGGTGCCGAATACACCTACAGAGCAACATCATATTATTGCGGA
GAATTACTTAGCCTCTAGTTATTTTAGATCTAGTGGTTATGATGGTTTTATGCAAGACTAC
GTTGTGATGGCACATGATCGTATCGTTCCGCTGCCTAATGCCATTGATTTGAGTACGATTT
CATACACAGAGTTAGTGTCAGTAAGTTATCATGCTATACAACGATTTGAACGTAAATCTA
TACCTTTGAAAACCAGCTTTGGTATTTGGGGTGATGGTAACTTAAGTTATATTACTGCTAT
TTTGCTACGTAAGTTGTACCCAGAAGCTAAAATTTATGTATTTGGTAAGACAGACTATAA
ATTAAGTCATTTTTCATTTGTAGATGACATCTTTACAGTAAATCAAATACCAGATGATCTT
AAAATTAATCATGCATTTGAATGTGTTGGAGGTAAAGGAAGTCAAGTTGCACTTCAACAA
ATAGTTGAACATATTTCACCAGAAGGCAGTATTGCTTTGTTAGGCGTAAGTGAATTACCC
GTGGAAGTGAATACACGATTAGTACTTGAAAAAGGATTAACATTGATTGGTAGTAGTCGA
AGCGGCTCTAAAGATTTTGAGCAAGTTGTTGATTTATATCGTAAGTACCCAGACATTGTTG
AAAAGTTAGCATTATTAAAAGGACATGAAATTAATGTATGTACGATGCAAGATATCGTCC
AAGCGTTTGAAATGGATTTATCGACATCTTGGGGAAAAACAGTATTGAAATGGACGATTT
AA

## > tarL1

Function: protein coding sequence; glycosyl/glycerophosphate transferase (core genome, variable)
Best match: tarL1_CC001-ST772_118_AJGE01000065.1[49213:50907:r] (completely identical)
Position: 008-contig_256: 136204 … 137899; Length: 1695 bp
Sequence:
ATGACAAAAACGAAACAAGCAATACATATTGATAACATATACTGGGAACGTGTTCAGTT
ATATATTGAAGGACATAGTGAAGGTGTCGATTTAACATCAGGACAATTTGTTCTGAGGAA
TTTAACCGAAACAAAAACATTAGAAGCAAATGAAATGAAAATAGACGGTAATACATTTA
TATGTAGATTCAACGTCGCAATATTAGACGATGGGTATTATTTACCAATGGATAAATATTT

ATTTGTTTATCATGACCAGTTAGAGTATATTGGACAACTTAATCCAAATATTATTGATCAA
GCTTATGCGGCATTAAATGAAGAGCAAATTGAAGAATACAATGAGCTGACTACACAAAA
TGGAAAAGTGAACTATTTATTAGCGTATGATGCTAAAGTTTTCCGTAAAGGTGGCGTATC
ACAACATACGGTCTATACCATTACTCCGGAAATAGCAAGTGACGTTAACGAATTTGTATT
TGATATTGAAATCACCTTACCTCAAGAGAAATCAGGGGTCATTGCGACAAGCGCACACTG
GCTTCATAAACAAGGACATAAAGCTTCATTTGAAAGTAGAAGTTTCTTATTTAAAGCTAT
TTTTAATATTACAAAGTTACTACATATTAAAAGAAGCAAAACAATATTATTCACATCAGA
TTCGCGTCCGAATTTATCAGGGAATTTCAAGTATGTATATGATGAGTTACTTCGCCAAAA
AGTAGATTTTGATTATGATATTAAAACGGTATTTAAGGCGAATATTACGGATAGACGTAA
ATGGAGAGACAAGTTTAGATTGCCATATTTACTTGGTAAGGCAGATTATATTTTTGTTGAT
GATTTCCATCCATTAATTTATACGGTTCGCTTTAGACCATCACAAGAAATTATTCAAGTGT
GGCATGCCGTTGGTGCTTTTAAAACAGTTGGCTTTAGTCGTACAGGAAAAAAAGGTGGTC
CGTTTATCGATTCATTAAACCATCGTAGTTACACGAAAGCATATGTTTCATCAGAAACCG
ATATTCCATTTTATGCTGAAGCATTTGGAATTAGAGAAGAAAATGTTGTACCAACAGGTG
TACCACGTACTGATGTACTATTTGATGAAGCTTATGCAACACAAATTAAACAAGAGATGG
AAGATGAATTGCCAATTATAAAAGGTAAGAAAGTTATTCTATTCGCACCGACATTTAGAG
GTAATGGTCACGGTACGGCACATTATCCATTTTTTAAAATTGATTTTGAACGTTTAGCAAG
ATACTGCGAGAAGCATAATGCAGTTGTGTTATTCAAAATGCATCCGTTCGTAAAAAATAG
ACTTAATATTTCACGTGAACATAGACAATACTTTATCGATGTGTCAGATCATCGTGAAGTT
AACGATATTCTCTTTGTTACAGACTTGTTGATTAGTGATTATTCATCTTTAATATATGAAT
ATGCAGTATTTAAAAAGCCGATGATTTTCTATGCATTTGACTTAGAAGATTACATTACGAC
GCGTGATTTCTATGAACCATATGAATCATTTGTTCCAGGTAAAATTGTACAATCCTTTGAT
GCATTAATGGATGCTTTGGACAATGAAGATTATGAGGTTGAAAAAGTTGTGCCATTCTTA
GATAAACATTTTAAATATCAAGATGGTCGCTCAAGTGAACGTTTAGTCAAAGATTTGTTT
AGACGCTAA

## > tarL2

Function: protein coding sequence; glycosyl/glycerophosphate transferase (core genome, constant)
Best match: tarL2_part_CC009_21334_AGTW01000002.1[1:1438:r]
Position: 008-contig_256: 137633 … 137663; Length: 30 bp
Sequence:
CTTTAATATATGAATATGCAGTATTTAAAA

## > tarF

Function: protein coding sequence; CDP-glycerol-poly(Glycerophosphate) glycerophosphotransferase (core genome, variable)
Best match: tarF_CC001-ST772_118_AJGE01000065.1[47616:48785:r] (completely identical)
Position: 008-contig_256: 138326 … 139496; Length: 1170 bp
Sequence:
ATGATTAAAAATACAATTAAAAAATTGATAGAACATAGTATATATACGACTTTTAAATTA
CTATCAAAATTGCCAAACAAGAATCTAATTTATTTTGAAAGCTTTCATGGTAAACAATAC
AGCGACAACCCCAAAGCATTATATGAATACTTAACTGAACATAGTGATGCCCAATTAATA
TGGGGTGTGAAAAAAGGATATGAACACATATTCCAACAGCACAATGTACCATATGTTACA
AAGTTTTCAATGAAATGGTTTTTAGCGATGCCAAGAGCGAAAGCATGGATGATTAACACA
CGTACACCAGATTGGTTATATAAATCACCGCGAACGACGTACTTACAAACATGGCATGGC
ACGCCATTGAAAAAGATTGGTTTGGATATTAGTAACGTTAAAATGCTAGGAACAAATACT
CAAAATTACCAAGATGGTTTTAAAAAAGAAAGCCAACGTTGGGATTATCTAGTGTCACCT
AATCCATATTCGACATCGATATTTCAACATGCATTTCATGTTAGTCGAGATAAGATTTTGG
AAACAGGTTATCCAAGAAATGATAAATTATCACATAAACGCAATGATACTGAATATATTA
ACGGTATTAAGACAAAATTAAATATTCCATTAGATAAAAAAGTGATTATGTACGCGCCAA
CTTGGCGTGACGATGAAGCGATTCGAGAAGGTTCATATCAATTTAATGTTAACTTTGATA
TAGAAGCTTTGCGTCAAGCGCTGGATGATGATTATGTTATTTTATTACGCATGCATTATTT
AGTTGTGACACGTATTGATGAACATGATGATTTTGTGAAAGACGTTTCAGATTATGAAGA
TATTTCGGATTTATACTTAATCAGCGATGCGTTAGTTACCGACTACTCATCTGTCATGTTC
GACTTCGGTGTATTAAAGCGTCCGCAAATTTTCTATGCGTATGACTTAGATAAATATGGC

GATGAGCTTAGAGGTTTTTACATGGATTATAAAAAAGAGTTGCCAGGTCCAATTGTTGAA
AATCAAACAGCACTCATTGATGCATTAAAACATATCGATGAGACTGCAAATGAGTATATT
GAAGCACGAACGGTATTTTATCAAAAATTCTGTTCATTAGAAGATGGACACGCGTCACAA
CGAATTTGCCAAACGATTTTTAAGTGA

## > tarI2

Function: protein coding sequence; 2-C-methyl-D-erythritol 4-phosphate cytidylyltransferase 2 (core genome, variable)
Best match: tarI2_CC001-ST772_118_AJGE01000065.1[46624:47340:r] (completely identical)
Position: 008-contig_256: 139771 … 140488; Length: 717 bp
Sequence:
ATGAAATACGCTGGTATTCTAGCTGGAGGTATAGGCTCAAGAATGGGTAACGTACCTTTA
CCTAAACAATTTTTAGATTTAGACAACAAACCGATTTTAATCCATACATTAGAAAAATTT
ATTTTAATTAATGATTTTGAAAAAATTATTATCGCGACGCCACAACAATGGATGACGCAT
ACGAAAGATACACTTAGAAAATTCAAAATTTCTGATGAAAGAATTGAAGTCATTCAAGGT
GGTAGCGATCGTAACGATACAATTATGAATATCGTTAAACATATTGAATCAACAAATGGT
ATTAACGATGACGATGTTATTGTGACACATGATGCAGTTAGACCATTTTTAACGCATCGT
ATTATTAAAGAAAATATTCAAGCTGCTTTAGAGTACGGTGCAGTAGATACAGTGATTGAT
GCTATAGATACGATTGTTACATCTAAAGATAATCAAACGATTGATGCAATTCCAGTGCGT
AATGAAATGTACCAAGGTCAAACACCTCAATCGTTTAATATTAATTTATTAAAAGAAAGC
TATGCACAGTTGAGTGATGAGCAAAAGAGTATTTTATCTGATGCTTGTAAGATTATTGTA
GAAACAAACAAACCGGTTCGACTTGTAAAAGGTGAGTTATATAACATTAAAGTAACAAC
ACCTTACGATTTAAAAGTAGCGAATGCTATTATTCGAGGTGGTATTGCCGATGATTAA

## > tarJ2

Function: protein coding sequence; CDP-ribitol synthetase (core genome, variable)
Best match: tarJ2_CC001_MSSA476_BX571857.1[276235:277260] (completely identical)
Position: 008-contig_256: 140480 … 141506; Length: 1026 bp
Sequence:
ATGATTAATCAAGTATATCAATTAGTTGCACCTAGACAATTTGAAGTTACGTATAACAAC
GTAGATATTTACAGTGACTATGTCATTGTACGTCCTTTATATATGTCAATTTGTGCTGCCG
ATCAAAGATATTATACTGGTAGCCGTGATGAGAATGTCTTATCTCAGAAATTGCCAATGT
CTTTAATTCATGAAGGTGTTGGTGAGGTCGTATTTGACAGTAAAGGTGTGTTTAATAAAG
GTACAAAAGTAGTTATGGTACCGAATACGCCGACAGAAAAAGACGATGTCATTGCTGAA
AACTATTTAAAATCGAGCTACTTCAGATCAAGTGGACATGATGGGTTTATGCAAGATTTT
GTGTTGCTAAATCATGATAGAGCTGTACCACTACCTGATGATATTGATTTAAGTATTATTT
CATATACAGAGCTTGTAACAGTAAGTTTGCATGCTATTCGTCGTTTTGAAAAGAAATCTAT
TTCAAATAAAAATACATTTGGTATTTGGGGTGATGGTAACTTAGGTTACATTACAGCCATT
TTATTACGTAAATTATATCCAGAGTCTAAAATATATGTCTTTGGTAAAACAGATTATAAAT
TGAGTCACTTCTCATTTGTTGATGATGTCTTCTTTATTAATAAAATACCTGAAGGCTTAAC
ATTTGATCATGCATTTGAGTGTGTGGGTGGTCGCGGTAGTCAATCAGCCATAAATCAAAT
GATCGATTACATTTCACCAGAAGGAAGCATTGCACTGTTAGGTGTAAGTGAGTTCCCAGT
AGAAGTTAATACACGTCTAGTATTGGAAAAAGGACTAACGTTGATTGGTAGTAGTCGAAG
TGGTTCAAAAGATTTCCAAGATGTTGTAGACTTATACATTCAATACCCAGATATTGTAGAT
AAATTAGCGTTGTTAAAAGGTCAAGAATTTGAAATTGCAACAATTAATGATCTTACAGAA
GCTTTTGAAGCAGACCTGTCTACATCTTGGGGTAAAACAGTATTAAAATGGATTATGTAA

## > Q5HJB8

Function: protein coding sequence; glycosyl transferase/group 2 family protein (core genome, variable)
Best match: Q5HJB8_CC001-ST772_118_AJGE01000065.1[42138:43863:r] (completely identical)
Position: 008-contig_256: 143248 … 144974; Length: 1726 bp
Sequence:
ATGATGAAATTTTCAGTAATAGTTCCAACATACAATTCAGAAAAGTATATAACAGAATTA
CTTAATAGCCTTGCGAAACAAGATTTTCCGAAAACTGAATTTGAAGTGGTTGTAGTTGAT

GACTGTTCAACAGATCAAACGTTACAAATAGTTGAAAAGTATCGTAATAAATTGAACTTG
AAAGTAAGTCAACTCGAAACAAATTCTGGTGGTCCAGGTAAACCTAGAAATGTGGCGTTA
AAACAAGCAGAAGGTGAATTTGTATTATTTGTGGACTCCGATGACTATATAAACAAAGAG
ACTTTAAAGGATGCAGCAGCATTTATTGATGAACATCACTCAGATGTCTTGTTGATTAAA
ATGAAAGGTGTTAATGGTCGTGGTGTACCACAATCTATGTTTAAAGAAACAGCACCTGAA
GTTACTTTGTTAAATTCAAGAATTATCTATACTTTAAGCCCAACTAAAATCTATAGAACAG
CATTACTAAAAGATAATGACATTTATTTTCCAGAAGAATTAAAGAGTGCAGAAGATCAAT
TATTTACAATGAAAGCATATTTGAATGCAAATCGAATCAGTGTGTTAAGTGATAAAGCGT
ATTATTATGCTACAAAGCGTGAAGGTGAACATATGAGTAGTGCGTATGTTTCACCTGAAG
ACTTTTACGAAGTCATGAGATTGATTGCTGTAGAAATATTAAATGCAGATTTAGAAGAAG
CTCATAAAGATCAAATCTTAGCAGAATTTTTAAATCGTCATTTTAGTTTTTCTCGTACGAA
TGGCTTCTCACTTAAAGTTAAACTAGAAGATCAACCGCAATGGATTAATGCTCTAGGAGA
CTTTATACAAGCAGTTCCAGAACGTGTAGATGCATTGGTGATGAGTAAATTACGACCATT
GTTGCACTACGCGAGAGCGAAAGATATAGACAACTATAGAACTGTAGAAGAAAGTTACC
GTCAAGGTCAATACTACCGTTTTGATATTGTAGATGGTAAATTAAACATTCAATTCAATG
AAGGCGAACCATACTTTGAAGGCATTGATATCGCTAAGCCAAAAGTGAAAATGACAGCA
TTTAAATTTGATAATCATAAAATTGTTACAGAGCTAACGTTAAATGAATTTATGATTGGCG
AAGGACATTATGATGTCAGACTTAAATTACATTCACGAAACAAGAAGCACACAATGTATG
TACCTTTAAGTGTCAATGCGAATAAACAATATCGTTTTAACATTATGTTAGAAGATATTAA
AGCGTATTTACCTAAAGAAAAAATTTGGGATGTTTTCTTAGAAGTCCAAATAGGTACGGA
AGTATTTGAAGTGCGTGTTGGTAATCAACGTAATAAATATGCATATACTGCAGAAACAAG
TGCATTAATTCATTTGAATAATGATTTTTATAGATTAACACCGTATTTCACAAAAGACTTT
AATAACATTTCGTTATACTTTACAGCTATTACATTAACGGATTCAATCTCAATGAAGTTAA
AAGGTAAAAACAAAATCATTTTAACTGGTCTGGATCGTGGTTATGTATTTGAAGAAGGTA
TGGCTAGTGTCGTGCTAAAAGACGACATGATTATGGGAATGTTAAGCCAAACGTCAGAA
AACGAAGTGGAAATCTTACTTAGTAAAGATATTAAGAAGCGAGACTTCAAAAATATTGTT
AAGTTAAACACTGCACATATGACTTACTCGCTAAAATAAATAA

## > scdA

Function: protein coding sequence; cell wall metabolism protein (genomic island)
Best match: scdA_CC001-ST772_118_AJGE01000065.1[41324:41998:r] (completely identical)
Position: 008-contig_256: 145113 … 145788; Length: 675 bp
Sequence:
ATGATAAATAAAAATGACATAGTAGCAGATGTAGTAACAGATTATCCGAAAGCAGCAGA
TATTTTTAGAAGCGTAGGTATAGACTTTTGTTGCGGTGGACAAGTAAGTATAGAAGCAGC
ATCCTTAGAAAAGAAATATGTAGATTTGAACGAATTATTACAGCGTCTCAATGACGTTGA
ACAAACGAATACACCAGGTTCGCTTAACCCTAAATTTTTAAATGTTTCGTCACTTATTCAA
TATATTCAAGCAGCATATCATGAACCTCTTAGAGAAGAATTTAAAAATTTAACACCTTAT
GTGACGAAATTATCGAAAGTACATGGACCTAACCATCCATATTTAGTCGAGTTAAAAGAA
ACATATGATACATTTAAAAATGGCATGTTAGAGCATATGCAAAAAGAAGATGATGTTGAT
TTTCCAAAACTAATTAAATATGAACAAGGTGAAGTAGTAGACGATATTAATACAGTGATT
GATGATTTAGTATCTGATCACATTGCAACAGGACAATTGTTAGTGAAAATGAGCGATTTA
ACATCTAGCTATGAACCACCGATAGAGGCATGTGGTACGTGGCGACTCGTTTATCAGAGA
TTAAAAGCACTTGAAGTGTTAACACATGAGCATGTTCATTTAGAGAATCATGTTTTATTTA
AAAAAGTATCATAA

## > lytS

Function: protein coding sequence; sensor histidine kinase/subunit S (core genome, constant)
Best match: lytS_CC001-ST772_118_AJGE01000065.1[39325:41079:r] (completely identical)
Position: 008-contig_256: 146032 … 147787; Length: 1755 bp
Sequence:
GTGCTATCGCTAACAATGTTATTACTTGAGCGTGTAGGTTTAATTATTATTTTGGCCTATG
TGTTGATGAATATTCCATATTTTAAAAACTTAATGAATCGTCGACGTACATGGAAAGCAC
GTTGGCAATTATGTATTATTTTCAGTTTGTTTGCCTTAATGTCTAATTTAACTGGTATCGTC
ATCGATCATCAACATAGTTTGTCAGGAAGTGTGTACTTCCGTTTAGATGATGATGTATCTT

TAGCTAACACACGTGTATTAACGATAGGTGTCGCAGGATTAGTTGGTGGTCCTTTTGTAG
GTCTATTTGTTGGCGTTATTTCAGGTATTTTCAGAGTGTATATGGGTGGGGCGGATGCACA
AGTTTATCTTATCTCATCTATATTTATCGGTATAATTGCTGGTTATTTTGGCTTACAAGCTC
AAAGACGCAAGCGTTACCCGAGTATTGCGAAAAGTGCCATGATTGGAATTGTTATGGAA
ATGATTCAAATGTTGAGCATTTTAACATTTTCCCACGACAAAGCATATGCGGTTGACCTCA
TATCATTAATTGCACTACCAATGATTATTGTTAATAGCGTTGGTACGGCGATTTTTATGTC
TATTATCATTTCAACATTAAAGCAAGAAGAGCAAATGAAGGCTGTTCAAACACATGACGT
ACTGCAATTGATGAACCAGACATTGCCGTATTTTAAAGAAGGATTGAATAGAGAATCGGC
ACAGCAAATTGCGATGATTATTAAAAATTTAATGAAAGTATCTGCCGTAGCAATTACAAG
CAAAAATGAAATCTTATCGCATGTAGGTGCAGGTAGTGATCATCACATACCAACAAATGA
AATATTAACAAGTCTGTCTAAAGATGTATTGAAATCAGGAAAGTTGAAAGAAGTTCATAC
TAAAGAAGAGATTGGTTGTAGTCATCCGAATTGCCCGCTTAGAGCAGCTATCGTGATACC
ACTTGAGATGCATGGTTCTATCGTCGGTACATTGAAGATGTATTTTACAAACCCTAATGAT
TTAACTTTTGTGGAACGTCAACTTGCAGAAGGATTGGCAAATATTTTTAGTAGCCAAATT
GAACTTGGTGAAGCCGAAACGCAAAGTAAGTTATTGAAAGATGCTGAGATTAAGTCATT
ACAGGCACAAGTGAGTCCACATTTTTTCTTCAATTCAATTAACACGATTTCAGCTTTAGTT
AGAATAAATAGCGAAAAGGCACGAGAGTTACTATTAGAATTGAGTTATTTTTTCAGAGCG
AATTTACAAGGCTCAAAGCAACATACGATTACTTTAGATAAAGAGTTAAGTCAAGTGCGT
GCATACTTATCACTCGAACAAGCACGTTATCCAGGAAGATTTAATATCAATATTAATGTT
GAAGACAAATATCGCGATGTGCTTGTACCACCATTTTTAATTCAAATTTTAGTTGAAAATG
CCATCAAACATGCGTTTACGAATCGAAAGCAAGGTAACGATATTGACGTGTCAGTGATTA
AAGAAACTGCAACACATGTACGTATTATTGTACAAGATAATGGTCAGGGTATTTCTAAAG
ATAAAATGCATTTGTTGGGAGAAACATCTGTAGAATCAGAATCTGGAACTGGTAGTGCTT
TAGAAAATTTAAACTTACGCCTAAAAGGATTATTTGGAAAATCCGCAGCATTACAATTTG
AATCGACATCGAGCGGTACCACTTTTTGGTGTGTACTTCCTTATGAAAGACAAGAGGAGG
AATAA

> **lytR**

Function: protein coding sequence; sensor histidine kinase/subunit R (core genome, constant)
Best match: lytR_CC001-ST772_118_AJGE01000065.1[38582:39322:r] (completely identical)
Position: 008-contig_256: 147789 … 148530; Length: 741 bp
Sequence:
ATGAAAGCATTAATCATAGATGATGAGCCATTAGCACGTAATGAATTAACATATTTATTA
AATGAAATTGGTGGTTTTGAAGAAATTAATGAGGCAGAAAATGTAAAAGAAACATTGGA
AGCACTACTGATCAATCAATATGACATTATATTTTTAGATGTCAATTTAATGGATGAAAAT
GGGATCGAATTAGGAGCTAAGATTCAAAAGATGAAAGAGCCACCTGCGATTATTTTTGCA
ACTGCACATGACCAATACGCAGTACAGGCATTTGAATTAAATGCGACAGACTATATTTTG
AAACCGTTTGGTCAAAAACGTATTGAACAAGCAGTCAATAAAGTGCGTGCGACTAAAGC
CAAAGATGATAATAACGCAAGTGCAATTGCGAATGATATGTCGGCGAATTTTGATCAAAG
TTTACCTGTTGAAATTGACGATAAAATTCACATGTTAAAGCAACAAAATATTATTGGGAT
TGGCACACATAATGGTATTACAACCATACATACAACGAATCATAAATACGAAACAACAG
AGCCATTGAATCGTTATGAAAAACGATTGAATCCCACTTATTTTATACGTATTCATCGTTC
ATATATTATTAACACTAAACACATTAAAGAAGTGCAACAATGGTTTAACTACACTTATAT
GGTAATATTGACAAATGGTGTCAAGATGCAAGTTGGACGTTCATTTATGAAAGATTTTAA
AGCGTCGATAGGATTACTTTAA

> **lrgA**

Function: protein coding sequence; holin-like protein (core genome, constant)
Best match: lrgA_CC005_Mu50-VRSA_BA000017.4[307072:307515] (completely identical)
Position: 008-contig_256: 148642 … 149086; Length: 444 bp
Sequence:
ATGGTCGTGAAACAACAAAAAGACGCATCAAAACCAGCACACTTTTTTCACCAAGTCATT
GTAATTGCTTTAGTACTCTTTGTATCGAAAATAATTGAATCATTTATGCCAATTCCTATGC
CTGCATCAGTAATCGGTTTAGTATTATTATTTGTATTATTATGTACTGGTGCTGTTAAGTTA
GGCGAAGTCGAAAAAGTAGGAACGACACTAACAAATAACATTGGCTTACTCTTCGTACC

AGCCGGTATCTCAGTTGTTAACTCTTTAGGTGTCATTAGCCAAGCACCATTTTTAATCATT
GGACTAATAATCGTCTCAACAATACTATTACTTATTTGTACTGGCTATGTCACACAAATTA
TTATGAAAGTTACTTCGAGATCTAAAGGTGACAAAGTCACAAAAAAGATCAAAATAGAG
GAGGCACAAGCTCATGATTAA

> **lrgB**

Function: protein coding sequence; antiholin-like protein (core genome, constant)
Best match: lrgB_CC001-ST772_118_AJGE01000065.1[37332:38033:r] (completely identical)
Position: 008-contig_256: 149078 … 149780; Length: 702 bp
Sequence:
ATGATTAACCACTTAGCACTAAACACACCTTACTTCGGAATACTGTTATCCGTTATACCAT
TTTTCTTAGCGACCATATTATTTGAAAAAACTAATCGTTTCTTCTTATTCGCACCGCTATTT
GTCAGTATGGTATTTGGTGTGGCCTTCCTCTATTTAACAGGCATTCCGTATAAGACTTACA
AAATAGGTGGAGACATTATTTATTTCTTCTTAGAACCGGCAACAATCTGTTTTGCGATTCC
GTTATATAAAAAGCGTGAAGTGCTTGTTAAACATTGGCATCGTATCATCGGAGGTATTGG
CATCGGTACAGTTGTAGCGTTATTAATTATTTTAACTTTTGCGAAGTTAGCACAATTTGCC
AATGATGTTATTTTATCAATGTTACCTCAAGCAGCAACTACAGCGATTGCGTTACCAGTAT
CAGCTGGTATCGGTGGTATAAAAGAATTAACATCATTAGCAGTTATTTTAAATGGTGTCA
TTATTTATGCCCTAGGTAATAAATTCTTGAAGCTTTTCCGAATTACTAACCCTATTGCCCG
AGGATTAGCACTTGGAACAAGTGGTCACACATTAGGTGTAGCACCAGCCAAAGAATTAG
GACCTGTAGAAGAATCAATGGCAAGTATAGCTTTAGTGTTAGTTGGTGTAGTTGTTGTAG
CAGTTGTGCCTGTCTTTGTAGCAATATTCTTCTAA

> **Q6GK48**

Function: protein coding sequence; putative nitric oxide reductase (genomic island)
Best match: Q6GK48_CC022_21310_AFNP01000014.1[33698:35989:r]
Position: 008-contig_256: 150119 … 152411; Length: 2292 bp
Sequence:
ATGAGTATGCAAAGTTCATATAAACGTTTAGTTAAAGTGCTACTAACGATTTTAGTGATT
GTGTTTTCAATTTTACTAGTAGGTGGTTGGTATATTTTTAAAAATGAAGCACCTCGTCCAA
CAAAGATAGTAGATCAACAAGGTCATACGCTTGTTACAAAAGACGAGCTGATCAGTGGG
CAAGCGATATATGAGAAATATGGGTTAACAGATTATGGCTCCTACCTAGGCAATGGTTCT
TACTTAGGCCCAGATTATACAGCAGAGGCATTACATCATTACCTGATTGGTATGCGTAAA
TATTATGCACAAGATATTTATCATAAGCAACTTCATAAATTAAATCATGCAGAGTTAGCC
GTAGTTAAAGATAAAGTGATGAAAGAAATACGGATTAATCGTTATTCTGAAAAAGAAGA
TCAACTTGTATTAACGCCAGGACAAGTATACGGTCTAAAATATTTGCAAGATTATTATAA
AGAAGAGTTTGTTAATAACCCTAAACAAGTAGGGTTAAATGAAAATATGATTAAACAGTT
TCAAAATGATGATTATATGGTCGCAGGAAATAAAGTTGAACATTTAAGTCAATTCTTTTTC
TGGGGTGCTTGGCTATCGTCAACAGATAGACCTGGAAAAACATTTTCATATACAAATAAT
TGGCCATACGATGTGGATGCAGGAAATACGTTGCCTAGTGCAGGGATTTTATGGACTGCT
ATATCTGTAACATTACTGATTGCAGGCTTGGCATCCATTATATATATTCAAAAGCGTTATC
AATTTGACATGAAGCCGACGTATGAGTCGGAACGGGAACTACCAAAGAGTGAAGTGGAT
CGTAAAATCACTGATAGTCAACGAAAAGTTGGGAAATATTTAGTCGTTGTCATGTTACTA
TTTTTGGTTCAAATATTATTAGGCGAATTACTGGCACATTATTATGTAGAGAATAAATTTT
TTGGTATAGAAATACAAAGGCTCTTTCCATTTAATATTGCGAAGACATGGCATGTGCAAT
TAGTTATTTTCTGGGTTGCTACGACTTGGTTAGCAGCAGGAATATACATTGTACCTAAAGT
ATTGGGCAAAGAACCAAAGAAACAAGGCGTATTAGTAGATATATTGTTTTGGGCCTTAAT
CATAGTTGTGATGGGCAGCATGATTGGAGAATGGGGTCATATTTTAGGTTGGATTGATTC
TCATTGGTGGCTATTCGGGCACTTTGGTTGGGAATATATAGAATTAGGTAAATTTTGGCA
AATATTATTTATAGTAGGTATGATCTTATGGGTTATCATTCTTTGTCGAGGTTTTTTACCAG
CTATTCGCAACAAAGTACAAGTGAATCATGATAAACGTCACTTATTGACGCTATTGTTTGT
TGGAGCAATTGCAATTCCGTTATTTTATCTTGCATCATTATTTATAATGCCGAATTCACAT
GTGACGTTTGCCGATTACTGGCGATGGTGGATTGTTCACTTATGGGTTGAAGGTATATTTG
AAGCATTTGCCGTAGTGTTGATTGGTTTCTTAATGGTTAATATGAAATTAACTACCATTGA
AACGACGATTCGTGCATTATATTTTCAATTAATCTTATTACTAGGTACAGGTATTGTTGGT

ATGGGTCATCATTACTATTGGCAAGGTGATCATTCCATATGGCTAGCACTGGGTTCTTGTT
TTTCAGCATTAGAAGTCGTGCCGCTTTGTTTATTAATTTGGGAAGCATACACACACTATCG
CTTATATAAGAATAGTGACATCGCTTTTCCTTACAAAGGTACATTTATTTTTCTTGCTTCA
ACAGGATTGTGGAATGCAATTGGTGCTGGAGCATTAGGCTTTTTAATTAACACACCAGCA
GTTAACTACTTTGAACATGGTACGCAATGGACTGCAGCACATGCCCATGGTTCTATGGCT
GGCGTGTATGGTATGTTCTCAATTGCAATTGCGTTGTATGTTTTTAAAAATATTACTAAAA
AAGAATTTTGGACACCGAAAGTTGAAAAATGGATAAAAATATCATGTTGGGCTTTAAATA
TTGGCTTGGCAGGTATGGTTTTCGTTACATTAATGCCAGTAGGTTATATCCAACTTAAAGA
TGCATTAGAACATGGTTATTGGCATTCAAGACTAACGTCATTTTATGAACAACCACTTGTA
AAAGCAATCATGTGGGGGAGAATGCCTTGGGACATTATATTCACAGTAGGTGTCATTATA
TTAGTAGTTATTTTTATAAGGGGATACAGACACTTAAAACCAAAATACAGAGTATAG

## > **Q5HJB2**

Function: protein coding sequence; putative transcript regulator/GntR family (core genome, variable)
Best match: Q5HJB2_CC025_21193_AFEG01000009.1[165880:166584]RC
Position: 008-contig_256: 152636 … 153341; Length: 705 bp
Sequence:
CTACTTTTTACTTGGAATATAAAACTGTGCATGACGATAATGAAATACGATGTCAGACGA
ATCAAAAGGTTTGCCAGTCATTGTATAAAACGTTTGGTGATAACGTAAACATGGTTCGCC
TGCAGATAATTGTAGTAATGTCGCTTCACTTGAAGTGAGTTTATCTACATTAAAGAAAAT
ATCTGAAAAACCAATACGAAGTTTCATGTTTGATTCTAAATAATCGAAGATAGAGCCCTT
AGCAATATCATCATTTAAATATTTCACGATTTCTTTATGATAATAAGAATATTCGATACAT
AAAACATCATCGTCCACGAATCTTAATCGCTCTAAATAGTAGACGGTATCATCTGCATTT
AATTGGAGTTCATCTTGTACAGATTTAGGTGGCGTTGCCATCTCCTTAAAAACAAGTACCT
TACTTGTCATTCGATGTTCACCTAAACTTTTAGAGAAACCATTAGTCTTAAAGACGTTGAT
ACGATTGGCATCTGCAATATTTCTCACATAAATACCACTGCCTTGTGCTTGATAGATCAAA
CCATCTTGTTCCAATAAGCCTAATGCTTTAATGATAGTACTCTTACTTACTTGATAACGTT
CTTTTAATTGCGTCACGCTTGGCAATTTATCACCGGGTTTGAAATTAGATTGATGTATAAA
CGCATTAAGTTGCTTAGCAATATGTTCATACTTTAACAA

## > **Q1YAD5**

Function: protein coding sequence; PTS system/IIA component (core genome, variable)
Best match: Q1YAD5_CC001-ST772_118_AJGE01000065.1[32831:33622:r] (completely identical)
Position: 008-contig_256: 153489 … 154281; Length: 792 bp
Sequence:
ATGAGCAATAAATATAAAGAACAAGCCCAAGACATTCTTACAGCTGTAGGTGGTGTCGA
AAACATTGTTGATGCAACGTATGATACGAAGTGCATTACAATTCATATGCAACATACAAT
TCCTTCTACAGCAAATGAAGTGAAACAAATAGTTGATGTGACATCTGTAGCAGAAAATGA
TGCGCAGTTAGTTATAAAATTAAATGGAAATGTTGATGAAGTGTATCAGCAATTACAGCG
ATTAATTAAGAATGCTAATGTCGAAGAGAGTGAGAATACTGACAATATTAATAGCCAAG
ATACAAGTTATACACCTCAAGTAAAAGTAACAACACCAATTTTAGTGAAAGCACCAATCG
CTGGTCGTCGTATTTTACTTAAAGAAGTAAGAGATTCAATTTTTAGAGAGAAAATGGTAG
GCGAAGGCTTGGCAATCAAAGCTCATGAAGAATCCAAAGTAATCGCACCGTTCAATGGTT
TAGTATCTATGATTGTACCAACTAAGCATGCAGTTGGTATTCAATCAGAAGACGGTGTGG
ACATAGTCATTCATATTGGCGTGAATACAGTTGACTTGGAAGGTAAAGGGTTCAAGTGCT
TTGTAAAGCAAAATGATCGTGTTGAAGCAGGGCAAACGTTGTTACAATTCGACCAGCAAT
ATATACAACAACAAGGCTACAATGCTGACGTTATTGTCGTTATTAGCAACTCTGCCGATTT
AGGAAAAGTAGAACTGACAATGAATGAAATCATTACGACTGAAGATGTTATTTTTAAAAT
ATTTAAAAACTAG

## > **bglA**

Function: protein coding sequence; putative 6-phospho-beta-glucosidase (core genome, variable)
Best match: bglA_CC001-ST772_118_AJGE01000065.1[31379:32815:r] (completely identical)
Position: 008-contig_256: 154296 … 155733; Length: 1437 bp
Sequence:

ATGACAAAATTACCGCAAAATTTCATGTGGGGTGGCGCACTTGCCGCAAATCAATTTGAA
GGTGGATATGATAAAGGTGGTAAAGGGTTAAGTGTAATTGATGTTATGACGAGTGGTGCA
CATGGCAAAGCACGTCAGATTACAGAATTTATAGATCCCAATCACTATTATCCAAATCAT
GAAGGTATTGATTTTTATCATCGTTATAAGGAAGATATTGCCTTGTTTAAAGAAATGGGA
TTGAAATGTTTACGTACGTCGATTGCGTGGACACGTATCTTTCCGAATGGGGATGAAGAT
GTGCCAAACGAAGAAGGACTCGCCTTTTATGATCGTATCTTTGATGAATTAATTGCACAA
GGTATTGAACCTGTTGTGACGTTATCACATTTTGAGATGCCACTTCATTTAGCGAAACATT
ATGGTGGATTTAGAAATAGAGAAGTTGTCGATTATTTTGTGCATTTTGCGCGTGTTGTATT
TGAAAGATATAAAGATAAAGTTACATATTGGATGACGTTTAATGAAATTAATAATCAGAT
GGACACATCAAATCCTATCTTTTTATGGACGAATTCTGGGGTAGCATTGACAGAAAATGA
TAATCCTGAAGAAGTCTTGTATCAAGTAGCACATCATGAACTTTTAGCCAGTGCTTTAGC
AGTTCGTCTTGGTAAAGAGATTAATCCGAAGTTTAAGATTGGAACAATGATTTCACATGT
ACCCATTTATCCATATTCGTGTCATCCGAAAGATATGATGGAAGCACAAATTGCGAATCG
CTTACGTTTCTTTTTCCCGGATGTCCAAGTGAGAGGTTATTATCCAAGCTATGCTAAAAAA
ATGTTGGCACGAAAAGGATATGATGTTGGATGGCAAGAAGGGGACGACAGTATTTTACA
GCAGGGCACGGTTGATTATATTGGCTTTAGTTATTACATGTCTACGGCTGTAAAACATGAT
GTTGATACTACAGTTGAAAACAACATCGTCAACGGTGGTTTGAATCATTCTGTGGAGAAT
CCGCATATCGCAACGAGTGATTGGGGTTGGGCGATTGATCCAGATGGCTTAAGATATACA
TTGAATGTGTTATATGATCGTTATCAGTTACCACTTTTTATTGTGGAAAATGGTTTTGGTG
CAGTTGATGAAGTGGTAGATGGACATATTCATGATGATTATCGCATTGAATATTTAAAAG
CACATATTACAGCAGCGATAGAAGCAGTTGATCAAGATGGTGTAGATTTAATCGGTTATA
CACCGTGGGGAATCATTGATATTGTTTCATTTACAACCGGTGAAATGAAGAAACGCTATG
GTTTAATATATGTTGATCGAGATAATGATGGTCATGGCACGATGGAACGCTTGAAAAAAG
ATTCGTTCTATTGGTATCAACAAGTGATAGCATCAAATGGAGATAAATTATAA

## > **Q5HJA9**

Function: protein coding sequence; putative methyltransferase (genomic island)
Best match: Q5HJA9_CC001-ST772_118_AJGE01000065.1[30213:30974]RC (completely identical)
Position: 008-contig_256: 156248 … 157010; Length: 762 bp
Sequence:
TTAGTGCTCTTTTTTAGCGACAAAAGTAATATAATTCATATCTTTACGCAATTTAGTCATC
GTTTTAAACATTTTACAAAACATTGGTCGATTTTCTTTTTTTAAAGCATTGTTGATAATCTT
TATAGTTCCAACAATACCTTCGTCATAAATTAAACCTTTTGGTGTCATTAAACTCATTGGA
CCAGTATGATAATGCACATGATTAAAACCAGCTTGATTATATAAATCTAACCAGCCAAGT
TTCGTCTGCGGTGAGACATTGACATTAATTGCTGCAGATAATGATTTAACTACATGTGTGG
CATGTGATTCATTAACGATGACAATATCATGTGTTAACAAGATACCCCCAGGCTTTAAGA
CTCGGTAGTACTCGCGTAATGCTTTTTCCTTTATGGCGATGGGTAACATTGTTAACATTGC
TTCATTTAAAACGATATCGAATTGATTGTCATCAAAGGGCAATTTAACAGCATTCGCTTGT
TGAACTTGAATATATGATTCAAGACCTGCTGCTGAAATATTTTCCTGTGCTTTTTCTAATG
CTTTCTTATTTATATCAACGCCTTGAATGTGACAGCCATATGTATGAGCTAGATAAATAGA
TGTTGTGCACATATTACATGCCACTTCTAACACTTTTTTATCTTGTGAAAATGCCCCTTGTT
GTATTAACCAATCTGTTGCTGCTTTACCACCGGGGCGTAGACGAGTTTTTCCTAATTTAGC
TAAAAATGTATGACCAGCTTCTTTGGACAT

## > **rbsK**

Function: protein coding sequence; ribokinase (core genome, variable)
Best match: rbsK_CC001-ST772_118_AJGE01000065.1[29048:29962]RC (completely identical)
Position: 008-contig_256: 157260 … 158175; Length: 915 bp
Sequence:
TTACACTTGATTTACTTCTTCTAGTAGAGGAATAGATGCTTGCGCGCCGTGTTTTTGTACA
GTGAGTGAGCTCGCTTTATTACCAAAATCAATAGCATCTGCTAAGTTATCTTGCGACTTGT
TTAAGCGACTGACAAATGCACCAATAAATGTGTCGCCTGCAGCAGTTGTATCAATCGCAT
TTACTTTATAAGCTTCGATGTGTTGGCTTTGATTTTTAGTAGCAAAATATGTACCTTGCTTA
CCTAGCGTAATCAAAACAGTCTTAATGCCTAAAGATAAAAAGTAATTGGCATTGTCTTTC
ATAGATTGTTCATTAGTTACTTTAATCCCAGATAACAATTCGGCTTCTGTTTCGTTTGGCA

CAATAATATCGATTAATGATAATAATTCATTAGGTAATGCTTTCGCTGGTGCAGGATTTAA
TACTGTCGTCACACCATGTGCCTTGGCAATTTCAAATGCAGATATAATAGCCGGGATGGG
TACTTCTAATTGTGCAACGACAAAGTCTGCATTGATTATAGCGTCTTTTGCGTTAATAACA
TCTTCAGGTGTCATCGTCATATTCGCACCACCATAAACATAGATGGTATTTTGTCCTTCTG
CATTCACAGTGATAAAGGCTTGGCCCGTTTTTGCTTCAGTTGATTTGATAATATATGATGT
ATCAATATGAGCTGCTTTAAAATCTTCTAAGATGAAATCAGCAACGCCATCAGTGCCAAT
TTTAGTAATAAATGTTGTGTCTGCTTGCATGCGTGCAGTGGCAATAGCCTGGTTGGCACCT
TTACCTCCGCCGAATGCTTTTTGTGCTTCTTCAACATGTAATGTTTCGCCTGGTTGTGCATA
TCTTTCAACTGTTAAAAATTGATCGACATTCGTTGAACCTAAAATAACAACTTTGTTGGTC
AT

> **rbsD**

Function: protein coding sequence; D-ribose pyranase (core genome, constant)
Best match: rbsD_CC001-ST772_118_AJGE01000065.1[28616:29020]RC (completely identical)
Position: 008-contig_256: 158202 … 158607; Length: 405 bp
Sequence:
TTAAAAAGTAACATTCGATTCTAATGCAATATTAGAGTAGGGCGTTGTTTCACCAGTACG
AATATTACCTTTATTTAATGGGTGAGCTAAGTTACTTTTCATTTCTTCGTGAGGAATGAAA
ATGATTTCGATTTCCGATGAAATCAATTGTTTAATTTGTTGCAATTGTGTAGGGTTATGTT
CTTTTATTTCTTCTGCTAAGTATATTTTTTGTATTTCCATTTCTTCTAACACTGTAGCTAAG
ACATCAATAAAGCGTGGTAAGTTTTTAGTTACAGCTAGGTCGATACGACGATGATCATTT
GGAATTGGCATGCCAGCGTCATTAATCGTTAATAGATCAAAATGACCAATTGTCGCGATT
GCTTTTGAAATATGTTCATTTAAAACAGCTGTTTTTTTCAT

> **rbsU**

Function: protein coding sequence; ribose uptake protein (core genome, constant)
Best match: rbsU_CC001-ST772_118_AJGE01000065.1[27720:28601]RC (completely identical)
Position: 008-contig_256: 158621 … 159503; Length: 882 bp
Sequence:
TTATTTTATAAATACTGTAACAGAAGCGGCTACTAAAATGAGTACTAAGCCGATGATTGT
AATAACCATTTCTTTTGACGTTTTATGTTGTTTTAAGAAATAAATACCAGTTAATGTAGCA
AGCACAACGGATGTTTGAGAAAGAATAAATCCAGTTGCTAAACCATTCATATTAGGTTGT
GCTGAAATAAGATATGTTAAGGCACCAAATGCAAAGAAGAAACCTGAAATAATTTGTAA
CCACGTAATTTTATTACGGAATGGATTCTCTGCTTTCATATTCATAAAGCCATAAATGACT
GCAACAATTACCATACCCATTGCTTGAGGTAAAAAGGCAGTTAGGCCATCAATAGAAGTT
GCTTGCGGTGCAGCTGAATATAACCAGTATCCAAATTCACCAATTAACAGAAGTACCACT
GCACGACGTAAATTTTTGGCGTTACTTGCTTCTTTGCGTTCACTCCAAACTGTCATACGCG
CACCAATTAGAATAACGACTAAAGCTGTAAATCCAATGATTTTATGACCAATGCCTGGCC
AATTTCCTAATGCAAAGACACCCCATAAAGATGCGCCTAATAATTGGAATGCTGTTGTGA
CTGGCATGGCACGAGATGAGCCGACTAATTCGAACGCTTTAAATGTAATGATTTGTCCGA
ATCCCCATCCTGCACCTGATAATAAGGCGAATAGCAAATTGGTTCCAGTAGGGAAGCCAC
TTGATGTGACTACGGCTAATATAATAGCGAAGATTAACGTACCTACAGTAGCACCGATAA
TTTGATGTACAGGTTTACCACCAAACTTTGAAGCGACTGTTGGGAAGAAGCCCCAGCCAA
TTAAGGGGCCTAACCCGATAAGTAATGCAACAATGCTCAT

> **rbsR**

Function: protein coding sequence; ribose operon transcript repressor (core genome, variable)
Best match: rbsR_CC001_ATCC51811-ST1_ADVP01000041.1[145517:146515]RC (completely identical)
Position: 008-contig_256: 159734 … 160733; Length: 999 bp
Sequence:
TTAGTTTGAAAGATGATAGCCAGTTGTTGCACGAATTTTTAAAGTCGTTGGTAATTCAATC
ATATCAATGGATTTATCTAAGTGCTGTAATCGTTGAAGTAATAAGGTTAAAGATGTTTTGC
CAATATCAGTTATAGGTTGTGCCACAGTAGTTAAAGGTGGCGAGACGTACGCTGCATAAT
CAATGTCGTCATAACCTATTAATGAGATATCTTTCGGAATACTGATGCCATGTTCAATTAG

TCCTCGTAAAATGCCAATAGCGAGTTCATCGTTAATAGCGAAGATTGCAGTGGCAGATTG
AACCATGATGTCATCAACAATGGTTAGCCCACCGCGCTTAGATAATTCAGTATGGACGAT
TTGTGGTTCTGGCAATTGATTCGCGCGCAAAGTATCAACAAATCCAGCGACACGAGTCGA
CATATTCGCCATCATGTCATATGGTGCAACAATCATCATATTGTTGTGACCGAGTTCTATT
AAATGTTGTGCTGCAAGTTGTCCGCCTTGATATTCATTTGTCCGAACAAAATCTGTATAGC
CTTGATGGTCATTTTGATCCAGTACGACATAAGGTACATGATGTTTCTTTAGATAGTTATT
TAGGGCGTCCGGGGATGAAATGTATTGTGCGATAATTAATCCGTCAATACCTCGATCAAT
TAAATGTTTAATATTGTCATACAAATCAGTTGCTGTAGATGTTAAAAAGCATAAATCAAC
ATCAGATGGTTTATGGTCATGAATACTTTGCATCAGTGCTGAGAAAAACGGATTTGTTAA
GCTAGGCAAAATGACGCCAATAGTTTGAATTTTACTGCCGCGCAATTGTTTTGCATGTTTA
TTAGGGGCATAGCCTAAACGTTCTGAAACAGCATGTACGTTTTTTATCGTTGTTGCGGAA
AAACGACTATCATTATGATTTAAAATATGTGACACAGTTGTAACTGATACACCAGCTTCT
CTAGCAACATCTTTAATTGACACTTTTTTCAT

## > **lytM**

Function: protein coding sequence; lysostaphin (core genome, variable)
Best match: lytM_CC001_MW2-USA400_BA000033.2298898:299848 [4_50ANF10END]
Position: 008-contig_256: 164560 … 165511; Length: 951 bp
Sequence:
ATGAAAAAATTAACAGCAGCAGCGATTGCAACGATGGGCTTCGCTACATTTACAATGGCG
CATCAAGCAGATGCAGCAGAAACGACAAACACCCAACAAGCACATACACAAATGTCAAC
ACAATCACAAGACGTATCTTATGGTACTTATTATACAATTGATTCTAATGGGGATTATCAT
CACACACCTGATGGTAACTGGAATCAAGCAATGTTTGATAATAAAGAATATAGCTATACA
TTCGTAGATGCTCAAGGACATACGCATTATTTTTATAACTGTTATCCAAAAAATGCAAAT
GCCAATGGAAGCGGCCAAACATATGTGAATCCAGCAACAGCAGGAGATAACAATGACTA
CACAGCGAGTCAAAGCCAACAGCATATTAATCAATATGGCTATCAATCAAATGTAGGTCC
AGACGCGAGCTATTATTCACATAGTAACAACAACCAAGCGTATAACAGCCATGATGGTA
ATGGAAAGGTCAATTCCCCTAATGGCACGTCTAATCAAAATGGCGGATCAGCAAGTAAA
GCGACAGCTAGTGGTCATGCGAAAGACGCAAGCTGGTTAACAAGTCGTAAACAACTACA
ACCATATGGACAATATCACGGTGGTGGTGCGCATTACGGTGTCGACTATGCAATGCCTGA
AAATTCACCAGTTTACTCATTAACTGATGGTACAGTAGTACAAGCAGGTTGGAGTAACTA
TGGTGGTGGTAATCAAGTAACGATTAAAGAAGCGAACAGTAATAACTACCAATGGTATA
TGCATAATAATCGTTTAACTGTTTCAGCTGGTGATAAAGTCAAAGCTGGTGACCAAATTG
CATATTCAGGTAGTACGGGTAATTCAACAGCGCCTCACGTACACTTCCAACGTATGTCTG
GTGGCATCGGTAATCAATATGCAGTAGACCCAATGTCATACTTGCAAAGTAGATAA

## > **ssaA**

Function: protein coding sequence; staph secretory antigen A (core genome, constant)
Best match: ssaA_CC001-ST772_118_AJGE01000065.1[16080:16982]RC (completely identical)
Position: 008-contig_256: 170240 … 171143; Length: 903 bp
Sequence:
TTAATGGATGTAATTATATGATGAAACTTCTGAAGCAGAGATGGTTCTTGATGAAACGAT
ATATTCACCGATCCAATTCATTTCTGAAATTAGAATGCTTCCATCAATATTAACTTTTTCA
ACGTAGGCTACATGACCAAATGGACCGTTTACTGTTTGTAAAATTGAACCTCGTGTTGGG
TGTCTATCTACTTTGAAGCCATTGCTTGAAGCTTGGCCTGCCCAGTTTTTAGCATCTCCCC
AAAATGTACTAATCGTGTGTCCATCTTTGGCACGTTTATCAAAGACATACCATGTACATTG
TCCAGCAGTATATAAGTTGTTCTTACTTGTGATAAGAGGCTGATCAATGATTTTACCGTTA
CCTAATGCTAAAGGTTTACCGTCAGCAGTCTTTGCTTTGTCATTAAATTCGGCGATTTGTA
ATTCGTCATACAATTCGTCTAAATCGATGCCTGTAATAAGCCCTTTGTTATCTTTTGAAAA
AGCGTTATTTAATTCATCGTCATTGTCTTCGACATTCGGTATTGCTGGTGTCAAAGGATTG
CTTGGTGACGTTTGAGGCGGTGTGTGTGAATCAATTGCGTCATTAATGTGCGTATACTGAC
CACTTAATGAAGAATGGTACTGATTGTTGTTAAGATCACGTTGATTTGCGTGTTGATGATT
GTCGTTTGTACGTGACTGGTTTTGATGATTGTTGTTTGGCGTGTTGTTTTTGTCATATGTAT
AAGTATACGCGCCGGTGTCTTTATTCACTTTGAACTGTGCGTTTGGGTGTGCTTTCTTTGCT

TCTTCTAATGTTTTGCTATCATTCGTATATGCTTGAGCCGAGTTAGGCGACATACTAAATA
AAGTAAGAGTTGTCATCGTCAGTAAAATTGTTTTCTTCATAATAACCAT

> **esxA**

Function: protein coding sequence; virulence factor esxA (core genome, constant)
Best match: esxA_CC001_MW2-USA400_BA000033.2[305719:306012] (completely identical)
Position: 008-contig_256: 171381 … 171675; Length: 294 bp
Sequence:
ATGGCAATGATTAAGATGAGTCCAGAGGAAATCAGAGCAAAATCGCAATCTTACGGGCA
AGGTTCAGACCAAATCCGTCAAATTTTATCTGATTTAACACGTGCACAAGGTGAAATTGC
AGCGAACTGGGAAGGTCAAGCTTTCAGCCGTTTCGAAGAGCAATTCCAACAACTTAGTCC
TAAAGTAGAAAAATTTGCACAATTATTAGAAGAAATTAAACAACAATTGAATAGCACTG
CTGATGCCGTTCAAGAACAAGACCAACAACTTTCTAATAATTTCGGTTTGCAATAA

> **esaA**

Function: protein coding sequence; protein esaA (core genome, constant)
Best match: esaA_CC705_RF122-cow_AJ938182.1[273193:276222]
Position: 008-contig_256: 171757 … 174787; Length: 3030 bp
Sequence:
ATGAAAAAGAAAAATTGGATTTATGCATTAATTGTCACTTTAATTATTATAATTGCCATAG
TTAGTATGATATTTTTTGTTCAAACAAAATATGGAGATCAATCAGAAAAAGGATCCCAAA
GTGTAAGTAATAAAAATAATAAAATACATATCGCAATTGTTAACGAGGATCAACCAACG
ACATATAACGGTAAAAAAGTTGAGCTGGGTCAAGCATTTATTAAAAGGTTAGCAAATGA
GAAAAACTATAAATTTGAAACAGTAACAAGAAACGTTGCTGAGTCTGGTTTGAAAAATG
GTGGATACCAAGTCATGATTGTTATCCCAGAAAACTTTTCAAAATTGGCAATGCAATTAG
ACGCTAAAACACCATCGAAAATATCGCTACAGTATAAAACAGCTGTAGGACAAAAAGAA
GAAGTAGCTAAAAACACAGAAAAAGTTGTAAGTAATGTACTTAACGACTTTAACAAAAA
CTTAGTCGAAATTTATTTAACAAGCATCATTGATAATTTACATAATGCACAAAAAAATGT
TGGCGCTATTATGACGCGTGAACATGGTGTGAATAGTAAATTCTCGAATTACTTATTAAA
TCCAATTAACGACTTCCCGGAATTATTTACAGATACGCTTGTAAATTCAATTTCTGCAAAC
AAAGACATTACAAAATGGTTCCAAACATACAATAAATCATTATTGAGTGCGAATTCAGAT
ACGTTCAGGGTGAACACAGATTATAATGTTTCGACTTTAATTGAAAAACAAAATTCATTA
TTTGACGAGCACAATACAGCGATGGATAAAATGTTACAAGATTATAAATCGCAAAAAGA
TAGTGTGGAACTTGATAACTATATCAATGCATTAAAACAGATGGACAGCCAAATTGATCA
ACAATCAAGTATGCAAGATACAGGTAAAGAAGAATATAAACAAACTGTTAAAGAAAACT
TAGATAAATTAAGAGAAATCATTCAATCACAAGAGTCACCATTTTCAAAAGGTATGATTG
AAGACTATCGTAAGCAATTAACAGAATCACTGCAAGATGAGCTTGCAAATAACAAAGAC
TTACAAGATGCGCTAAACAGTATTAAAATGAACAATGCTCAATTTGCTGAAAATCTAGAA
AAACAGCTACATGATGATATCGTTAAAGAACCAGATAAAGATACAACATTTATCTATAAC
ATGTCTCAACAAGACTTTATAGCTGCAGGTTTAAATGAGGATGAAGCTAATAAATACGAA
GCAATTGTCAAAGAAGCAAAACGTTATAAAAACGAATATAATTTGAAAAAACCGTTAGC
AGAACACATTAATTTAACAGATTACGATAACCAAGTTGCGCAAGACACAAGTAGTTTGAT
TAATGATGGTGTCAAAGTGCAACGTACTGAAACGATTAAAAGTAATGATATTAATCAATT
AACTGTTGCAACAGATCCTCATTTTAATTTTGAAGGCGACATTAAAATTAATGGTAAAAA
ATATGACATTAAGGATCAAAGTGTTCAACTCGATACATCTAACAAGGAATATAAAGTTGA
AGTCAATGGCGTTGCTAAATTGAAAAAGGATGCTGAGAAAGATTTCTTAAAAGATAAAA
CAATGCATTTACAATTGTTATTTGGACAAGCAAATCGTCAAGAAGAACCAAATGATAAGA
AAGCAACGAGTGTTGTGGACGTAACATTGAATCATAACTTTGATGATCGCTTATCGAAAG
ATGCATTAAGCCAGCAATTGAGTGCGTTATCTAGGTTTGATGCACATTATCAAATGTACA
CAGATACAAAAGGCAGAGAAGATAAACCATTCGATAACAAACGTTTAATTGATATGATG
GTTGACCAAGTTATCAATGACATGGAAAGTTTCAAAGACGATAAAGTAGCTGTGTTACAT
CAAATTGATTCAATGGAAGAAAACTCAGACAAACTGATTGATGACATTTTAAATAACAAA
AAGAATACAACAAAAAATAAAGAAGATATTTCTAAGCTGATTGATCAGTTAGAAAACGT
TAAAAAGACATTTGCTGAAGAGCCACAAGAACCAAAAATTGATAAAGGCAAAAATGATG
AATTTAATACGATGTCTTCAAATTTAGATAAAGAAATTAGTAGAATTTCTGAGAAAAGTA

CGCAATTGCTATCAGATACACAAGAATCAAAAACAATTGCAGATTCAGTTAGTGGACAAT
TAAATCAATTAGATAATAATGTGAATAAACTACATGCGACAGGTCGAGCATTAGGCGTAA
GAGCGAATGATTTGAACCGTCAAATGGCTAAAAACGATAAAGATAATGAGTTGTTCGCTA
AAGAGTTTAAAAAAGTATTACAAAATTCTAAAGATGGCGACAGACAAAACCAAGCATTA
AAAGCATTTATGAGTAATCCGGTTCAAAAGAAAAACTTAGAAAATGTTTTAGCTAATAAT
GGTAATACAGACGTGATTTCACCGACATTATTCGTATTATTGATGTATTTACTATCAATGA
TTACAGCATATATTTTCTATAGTTATGAACGTGCCAAAGGACAAATGAATTTCATTAAAG
ATGATTATAGTAGTAAAAACCATCTTTGGAATAATGTCATTACGTCAGGTGTTATTGGTAC
AACTGGTTTGGTAGAAGGATTAATTGTCGGTTTAATTGCAATGAATAAGTTCCATGTATTA
GCTGGCTATAGAGCGAAATTCATCTTAATGGTGATTTTAACTATGATGGTCTTCGTACTTA
TTAATACGTATTTACTAAGACAGGTAAAATCTATCGGTATGTTCTTAATGATTGCTGCATT
GGGTCTATACTTTGTAGCTATGAATAATTTGAAAGCGGCTGGACAAGGTGTGACTAATAA
AATTTCACCATTGTCTTATATCGATAACATGTTCTTCAATTATTTAAATGCAGAGCATCCT
ATAGGCTTGGCGCTAGTAATATTAACAGTACTTGTGATTATTGGCTTTGTACTGAACATGT
TTATAAAACACTTTAAGAAAGAGAGATTAATCTAA

> **essA**
Function: protein coding sequence; protein essA (core genome, constant)
Best match: essA_CC001_MW2-USA400_BA000033.2[309124:309582] (completely identical)
Position: 008-contig_256: 174786 … 175245; Length: 459 bp
Sequence:
ATGTTGATGAATAGCGTGATTGCTTTAACTTTTTTAACAGCATCTAGCAATAATGGCGGAC
TTAATATTGACGTGCAACAAGAAGAGGAAAAGCGAATCAATAATGATTTAAATCAATAT
GATACAACGCTATTTAATAAAGACAGCAAAGCGGTTAATGATGCGATTGCTAAGCAGAA
AAAAGAACGACAACAACAAATAAAAAATGATATGTTTCAAAATCAAGCGAGTCACTCGA
CTCGCTTGAATGAAACTAAAAAAGTGTTATTTCCAAATCTAACTTAGAAAAGACTTCGG
AGAGTGATAAAAGCCCCTATATTCAAAACAAGCAGGAGAAAAAAATATTCCCGTACATT
TTGATGTCTGTAGGGGCTTTTTTGACTTTAGGATTTGTCATTTTTTCAATTCATAAAGGGA
GACGAACGAAAAATGAATCAGCACGTAAAAGTAACATTTGA

> **esaB**
Function: protein coding sequence; protein esaB (core genome, constant)
Best match: esaB_CC005_Mu50-VRSA_BA000017.4[330865:331107] (completely identical)
Position: 008-contig_256: 175216 … 175459; Length: 243 bp
Sequence:
ATGAATCAGCACGTAAAAGTAACATTTGATTTTACTAATTATAATTACGGCACATATGAC
TTAGCAGTACCAGCATATTTACCGATAAAAAATTTAATAGCTTTAGTATTGGATAGTTTGG
ACATTTCAATATTTGATGTCAATACACAAATTAAAGTGATGACGAAAGGTCAATTACTTG
TTGAAAATGATCGACTCATTGATTATCAAATCGCTGATGGAGATATTTTGAAGTTACTATA
G

> **essB**
Function: protein coding sequence; protein essB (core genome, constant)
Best match: essB_CC001_MW2-USA400_BA000033.2[309809:311143]
Position: 008-contig_256: 175471 … 176806; Length: 1335 bp
Sequence:
ATGGTTAAAAATCATAACCCTAAAAATGAAATGCAAGATATGTTAACGCCTTTAGATGCT
GAAGAAGCAGCTAAAACAAAATTACGCTTAGATATGAGAGAGATTCCTAAGTCTTCAATT
AAACCAGAACATTTTCATTTAATGTACTTATTAGAACAACATTCTCCATATTTTATAGATG
CTGAATTAACTGAACTACGTGATAGTTTCCAAATACATTATGACATTAATGACAATCATA
CACCTTTTGATAATATTAAATCATTTACTAAAAATGAAAAATTACGTTACTTACTCAATAT
CAAAAATTTAGAAGAAGTAAATCGTACACGCTACACATTTGTGTTGGCACCAGATGAATT
ATTTTTCACAAGAGATGGATTACCAATTGCTAAAACAAGAGGTTTACAAAATGTTGTTGA
CCCATTACCGGTGTCAGAAGCTGAATTTTTAACAAGATATAAAGCACTGGTTATCTGTGC
ATTCAATGAGAAACAATCATTTGATGCTTTAGTTGAAGGAAACTTAGAATTACATAAAGG

AACGCCATTTGAAACTAAAGTTATTGAAGCGGCAACGTTAGATTTACTAACGGCATTTTT
AGATGAACAGTATCAGAAACAAGAACAAGATTATAGTCAAAATTATGCATATGTACGCA
AAGTAGGGCATACCGTTTTTAAATGGGTTGCTATCGGTATGACAACGTTAAGTGTTTTATT
AATTGCATTCTTAGCCTTTTTATATTTTTCAGTAATGAAGCATAATGAGCGCATTGAAAAA
GGATACCAAGCATTTGTAAAGGATGATTATACGCAAGTACTAAATACGTATGATGATTTA
GATGGTAAAAAATTAGATAAAGAGGCACTTTACATTTATGCCAAAAGTTATATCCAAACA
AATAAACAAGGTTTAGAAAAAGATAAGAAAGAAAATTTACTTAATAACGTGACACCAAA
TTCAAATAAAGACTACTTATTATATTGGATGGAATTAGGACAAGGACATCTTGATGAAGC
GATTAATATTGCCACTTATTTAGATGATAACGATATTACAAAGTTAGCGTTGATTAATAA
GTTAAATGAGATTAAAAATAACGGAGATTTATCGAATGATAAACGTTCTGAAGAAACGA
AAAAGTATAACGATAAATTGCAAGATATTTTAGACAAAGAAAAACAAGTTAAAGATGAA
AAAGCGAAATCTGAAGAAGAGAAAGCAAAAGCGAAAGATGAGAAATTAAAGCAACAAG
AAGAGAACGAAAAGAAACAAAAAGAACAAGCACAAAAAGATAAAGAAAAACGCCAAG
AAGCTGAAAGAAAAAAATAG

> **essC**
Function: protein coding sequence; protein essC (core genome, variable)
Best match: essC_CC001_MW2-USA400_BA000033.2[311165:315604]
Position: 008-contig_256: 176827 … 176857; Length: 30 bp
Sequence:
ATGCATAAATTGATTATAAAATATAACAAA

> **orfHyp-MRSA252**
Function: protein coding sequence; putative protein (genomic island)
Best match: orfHyp-MRSA252_ref_CC030_MRSA252_BX571856.1[333582:334646]
Position: 008-contig_256: 180211 … 181276; Length: 1065 bp
Sequence:
ATGGAAGAATATGAAGAAATGATGTCTAAGGTGACACGTGAAGGTTTAGCATTAGGGGT
ATACATCATTCTTTCAGGATCAAGATCAAGTGCAATAAAATCAGCTATATTTACTAACATT
AAAACAAGAGTTGCACTATACCTCTTTGAAAATAATGAATTAACAAATATTATTGGTTCT
TATAAAAAAGGTGTGAAAGATGTTAAAGGTAGAGCTGCAATAAATGATGATAACTTCAC
ACAGTTCCAAATTGCGCAACCATTTGAATTAGCAGAAGGACAAACATATAACGAGCGCA
TTAAAAATGAAGTAGCACAAATGAAAGAATTCTATGTAGGAGATTATCCAAAACATATCC
CAATGATGCCAGATAAGGTATTAATGGATGATATTCAAGAAACATATGATTTGGAAAAA
ATCATTCATGAAGAACACAAATTACCATTAGGATTAGACTTTGAGGATGTGGAACTAGTT
AGCTTAGATCTGACAAGTTCTTCGATTGTAACAGCAATTAAACCAACTGAGATGGAAAAA
ATGAATGATGTTATAATGTCTAGTTTATCAGTATATAGTAAAAACCAATTTGTTATCTTGG
TTGATGCAGAAGATAATATGAGCCAGTATGCAGAAGATGTAACCTCTTATTATTCAGCAC
CATCAGATTTGAGCAACATTAGATTAGGATTTAAGCAAGAAATAGAAGCAAGGAAAAAT
GGCGAGAAAAGTATAGAGGAATGCAAGATTGTATTTATAAATAATATTAAACGATTCAAT
CAATTAACTGGAATGACAGAAGATGAAATTCGTGTGTTATTTAATGAAGGTCAAAAAGTT
AATATTATCATTATTGCAAGTGGACTATACTCAGATACAATTGGTGCATTTGATAGAGAA
AGTAAAATGATGGTAAGAACTATAAATCAAGCTTTGATTAGTCATAAGATATCGGAACAA
GAATTTATAAGAGTTAAAGATCGATTTGGTGAACCAGAATTAAAAGTGGGAGAAATGTA
TTACATTAATAATCAAGAATATCAAAAAATAAAACTAATGGAGGGATAG

> **Q6GK23**
Function: protein coding sequence; putative protein (genomic island)
Best match: Q6GK23-MRSA252_ref_CC030_MRSA252_BX571856.1[334649:334915]
Position: 008-contig_256: 181395 … 181545; Length: 150 bp
Sequence:
TTTAAAGAATATGTAAATATGTTTGAAGATTATCAATCAGCTCTATCTAATTATGAAAAC
ATAATTGAACAAGATACGACGGCTATGGATACAACGGTAACAGAAATTGTTGAAAACGA
TAGAGAAATAGCAGGACAAATTAATAAATAA

> **Q6GK22**

Function: protein coding sequence; putative protein (genomic island)
Best match: Q6GK22-MRSA252_ref_CC030_MRSA252_BX571856.1[334938:335333]
Position: 008-contig_256: 181567 … 181963; Length: 396 bp
Sequence:
ATGAGTAATAAGGGAGAAATAAGAAGGCAAATTGCTAATAAAGAAAGAGAAAAAGCAA
GTAAAGAAGCACAACTAACAGATTTAAAAGAGGATTTAAGAAGGTTGAAAGACGCTTCG
AAAAAATTGGATACAGCAGGAGAAGATTTTAACAAAGGACAGAGTAGTTATAACAAAGT
AGAAATTAGTACTTCAGATTGGAAAGGAGAAAGAAGGACTAAAAGTGACAGCAAAAAG
AAAGATGTCGATTCTGAATTAAAAAAAGTTGAACAAGACTTTGATGATGCGAAGAAGGC
TATTAAAAAAGATATTCAGGATAAAGAAGGAGAAATCAAGGGAGTCGAAGGAGAAATC
AGTACTATAAATGCAGCTATAGATGCACTTAAATCAAAATTATAA

> **Q6GK21**

Function: protein coding sequence; putative protein (genomic island)
Best match: Q6GK21-MRSA252_ref_CC030_MRSA252_BX571856.1[335355:337025]
Position: 008-contig_256: 181984 … 182134; Length: 150 bp
Sequence:
ATGGGGTACAAAGTTGATATGTCTGAAGTGCACAATATGCAAAAGTCTATTGATTCAAGC
TTAAGTGCAATAAATACAAAGGTTAGCACGCTTAGCAGTAGCATAAACAACCTTATAAAT
ACTGAAGGATTCGAAGGTCAGACAGCAAGT

> **Q6GK19**

Function: protein coding sequence; putative protein (genomic island)
Best match: Q6GK19-MRSA252_ref_CC030_MRSA252_BX571856.1[337685:338140]
Position: 008-contig_256: 184314 … 184770; Length: 456 bp
Sequence:
ATGGAAGTAGGAATGTTAATTATGCCGTTTTGTGTGAAGATTACATTTGAAATTGATAAA
AATGATAATTGGTTAAATGCATTAGATACAGTAAATGAATTTTTTATGGATAAAGATATA
TATCCTACTGGACCTATAATTTTCCAAAGAGAATTAGCAGGTTTGGGTGAATATAAATAT
ACAGCATATATAGCTCTTAATGATGAACTCCAAGATATACCTGAATTGAACATTAAATAT
ATTGATTGTTTAGAGGTAGGACCTACGTTATCAACTAAGTGTTTTGAAGAAGAGGAATTT
GAAAGAGCCTATAAAGAAATTGAATATGTAGCTAACGAAAATAATATTAAGATTCTAAA
TCAACCATATTATCATGTAATGGTGGATTATTTTGGTGGTACGGCTTTTGAAATTTATGCT
CAAGTAGATTTGGATGAGAGTGAAGTATATGGATAA

> **Q6GK17-var1**

Function: protein coding sequence; putative protein (genomic island)
Best match: Q6GK17-var1_CC012_21266_AFTT01000027.1[118923:119510:r] (completely identical)
Position: 008-contig_256: 185245 … 185833; Length: 588 bp
Sequence:
ATGATAATTTTTATTTTAATAACAATATTTGCTATTTATTATATAGCTATGATTGCTAGTTT
GTTTAAAAGTGAAGGTTTTTCAATAATAGGTTTAATATTGGATATAGTTATTTTGACAACG
TTAATTTTTTATTATTTTGTAGGTGCGCGTTTTGTTGATCATGATTTAAGCAACTTTTTAGC
ATTTATGCATTTGGGTTCATTTGTGTATATTTACTTTGCAATTAAGTGTTTATGGGTGAAA
CCTAAGGTAGTAAATTATTTGATTGCAAAAGAATTGGATGAATCTAAAGAGGTCATTGAA
GAGCAGGAATTAGATTTACAGACATCAAAGATAAGAGGTATCTACTTTTTCATTATTGCA
GTAGTGATGTTAATTATTGCTAAATTAAGGATGCAACCAGAATTACAAGTAGATGCGTTA
TCAATGAATCCAGTTTTTATTTTTATAGGAGTTATTATCATCTTAATTTGGCTAGTACTGG
ATATATATCGTAAGAAAAAATACGGTATATTCTTATTCAAGACGATAGTGCCATTAGTTG
TTACGACTTGGATTATTATAGCTACTATTGTACTTTCGTAA

> **Q9L3N6**

Function: protein coding sequence; putative protein (genomic island)

Best match: Q9L3N6_CC030_MRSA252_BX571856.1[339379:339597] (completely identical)
Position: 008-contig_256: 186013 … 186232; Length: 219 bp
Sequence:
ATGGAAAACCAAAAACAAGGCAATGGCTTAAAAATTGCAACATGGGTATTTATTGTATTA
ACAGTAGTTACACCGCTATTTGGTATTGGAAGTATTGTTTGTAGTATTAATTACAAAAAT
ACGATGCAGAAAAAGGTTCGAAGTTATTGAAAATTGCAATTATCGTAACAATAATTGTTT
TTGTTTTAAATTTATTAGCATATTTAGGTTTAAGATAA

## > Q9L3N5-locus1
Function: protein coding sequence; putative protein (genomic island)
Best match: Q9L3N5-locus1_CC030_MRSA252_BX571856.1[339729:340229]
Position: 008-contig_256: 186363 … 186393; Length: 30 bp
Sequence:
ATGACTTTCGAAGAGAAGTTAAGTCAAATG

## > Q9L3N5-locus10
Function: protein coding sequence; putative protein (genomic island)
Best match: Q9L3N5-locus10_CC008_NCTC8325_CP000253.1[293073:293573]
Position: 009-contig_245_RC: 57 … 207; Length: 150 bp
Sequence:
GATTGGGCGAATTCAGAGTTTGGACAAATGGGAAGAGAACATTATTACATGTATAAAAA
ATTTGGAATTTGGCCTGAAAAAGAATATGCCATAAATTGGGTAAAAAAAATAAAAGATT
ATGTTAAAGAGCAAGATGAAGCTGAACTATAG

## > Q9L3N5-locus4
Function: protein coding sequence; putative protein (genomic island)
Best match: Q9L3N5-locus4_ref_CC001_MW2-USA400_BA000033.2[325134:325622]
Position: 009-contig_245_RC: 217 … 1217; Length: 1000 bp
Sequence:
ATGACTTTCGAAGAAAAGCTAAGTCAAATGTACAATGAAATTGCAGATGAAATCAGTGG
AATGATACCAGTTGAATGGGAAAATATATATACAATTGCCTATGTAACTGATCAAGGTGG
AGAGGTCATTTTTAATTATACTAAACCAGGTAGCGATGAATTGAATTATTACACGGATAT
CCCTAGAGAGTATAATGTCTCTGAAAAAGTATTTTATGATTTGTGGACGGATTTATATAG
ATTGTTTAAGAAGTTAAGAGAAACTTTTAAAGAAGAAGGGCTTGAACCATGGACATCAA
GTGAATTTGACTTTACAAGCGAAGGTAAATTAAAAGTTTCATTTGATTATATTGATTGGAT
AAATACAGAGTTTGATCAATTAGGCCGTGAAAACTATTATATGTATAAAAAGTTTGGTGT
TTTACCAGAAATGGAATACGAAATGGAAGAAGTTAAAGAAATCGAGCAATATATTAAAG
AGCAAGATGAAGCTGAACTATAGGGGCGATAACATGACTTTCGAAGAAAAGCTAAGTCA
AATGTACAATGAAATTGCAAATGAAATCAGTGGAATGATACCAGTAGAATGGGAAAAAG
TATATACAATTGCCTACGTAGATGATGAAGGTGGAGAGGTTGTTTTTAATTATACTAAAC
CAGGAAGTGAAGATTTGAATTATTATTCAGATATTCCTAAAGATTGCAATGTCTCAAAAG
ATATTTTTAAGAATTCATGGTTTAAAGTTTATCGAATGTTTGATGAGTTAAGAGAAACTTT
TAAAAAAGAAGATTTAGAACCGTGGACATCATGTGAATTTGACTTTACAAGAAAGGGAA
ATTTAAAAGTATCATTTGATTATATAGATTGGATTAAATTAGGTTTTGGCCCATCAGGAAA
GGAAAACTACTATATGTACAAAAAAATTGGTATTTTACCAGATATGGAATATGAAATGGA
AGAAATTCGAGAAGTAGAGAAGTATGTTAAAGACCAAGAGTAG

## > Q1Y4S0
Function: protein coding sequence; putative protein (core genome, variable)
Best match: Q1Y4S0_CC001-ST772_118_AJGE01000018.1[109069:109443:r] (completely identical)
Position: 009-contig_245_RC: 1839 … 2214; Length: 375 bp
Sequence:
ATGAAAAGAATATTGGTAGTATTTTTAATGTTAGCAATTATATTGGCAGGTTGTTCTAATA
AAGGTGAAAAGTATCAAAAAGATATTGATAAAGTGTACAAAGAACAGAATCAAATGAAT
AAAATTGCCTCGAAAGTACAAAACACTATTAAAACAGACATTAAACAAGAAGACAGTAA

TACACATGTTTATAAAGATGGTAAAGTCATTGTTATTGGTATTCAATTATATAAAGATCGT
GAAAAAATGTATTATTTCGCATATGAAATAAAAGATGGTAAGGCAGAAATCAATAGAGA
AATAGACCCAATTAAGTATATGAAAGACCATAAAGCAGATTATGAAGATGAAAATGTAG
AAGTGGAAAAAGATTAA

> **Q2G173**

Function: protein coding sequence; putative membrane protein (core genome, variable)
Best match: Q2G173_CC001_MW2-USA400_BA000033.2[326771:327169]
Position: 009-contig_245_RC: 2363 … 2762; Length: 399 bp
Sequence:
ATGGAAAAATCGATCAAAATAATGACAATAATAGGAATTGTGATTCAGGGTTTAGCAAC
GGTATTTAGTTTACTATTGATGGTTTTAGCAGCATCAGGTGTAATGACTACAGATGTGTCA
ACAACAGTTAATGGTGAGGTTGACCCAGTTGATGCAGAAACAGCAGCAGCAATTTTCACT
GTATTATTCTTATCCCTATTCATATTTGGAATCATTTCAATTATTTTAGGTGCAATCGGTAT
GTTTAAAGCATCTAAAAACAAAAAAATGAGTGGTATATTGTTGATTATTGGAGCTGTAAT
AAGTGGTAACATAATTACATTTGCTTTATGGTTAGTCAGTGGTATTAAACTCCTTACTAAT
AACAAGCCTAAAGATGAAATAAGCGACTTATCATAA

> **nirC-L1**

Function: protein coding sequence; formate/nitrite transporter/locus 1 (core genome, variable)
Best match: nirC-L1_CC005_Mu50-VRSA_BA000017.4[347433:348257:r]RC
Position: 009-contig_245_RC: 2976 … 3801; Length: 825 bp
Sequence:
TTAATTATTTTTATAAAATTGATGATGATCATTCAAGTAAGCATAGAATAAACCTATAATG
AGTCCGCCTCCAATATAGTTACCGATAAAAGCCGCAGCGATATTCGAAATAGCTGGTATG
AAGTGCAATGTATCAACTTGATAAATTAAACCACCCATAAATAAGCAACTGTTGTAAACG
ACATGTTCATAACCCATAAAGGCGAATATGGTAACACCGAACATCATGACAAACATTTTT
GCGAGTACATCGTCAATTTGCATGGCAATAACTAATGAAATATTGATAAAGAAATTGGCG
AATATCGCTTTCATTAATATACTTACAAAACCAGTAGACAACGTTTTATGCTCTATAACTG
CTGATAACTGATTTAACATATCTGGCGTCATTACATTTGAAAAACGCATGAAACTAAATA
AAATAGCAGCACCTAAAATATTTCCTGCAAAGCACAATAAAAATATTTTCAATACTCTAG
TCGGTTTAATTACTTTATAATACAGGCCTACAGTAAAGTACATGAAGTTACTGGTTAGTA
GTTCGGAGTTTGTAAATAAAATGAGTACTAACGCAAAACTGAATGTAATGGCGCTGGCCA
TATTCACAATGCCTGGCGGTAAATCTGGTTCGTGTGTTGCTTTAACTGATAATACGAAGAC
CGTAATAATCCCGATAATAAATCCTGCCATCATAGCGCGTAATAAATAACGTTTTAAATA
AACGCTTTGTAATATATCTTTCGTTCTTATCGTTTCGACTACGTTATTTACCCAGTCGTCCC
CATAAAAAATCTTATCCCATTTAATATGTTTCTCCTTCAC

> **brnQ2**

Function: protein coding sequence; branched-chain amino acid transport system II carrier protein (core genome, variable)
Best match: brnQ2_CC008_NCTC8325_CP000253.1[297408:298715:r]RC
Position: 009-contig_245_RC: 4039 … 5347; Length: 1308 bp
Sequence:
TTAAGTTGTCGCTTGTTTCGGACGGCGTATAACTACATCGATGATGAAACCTATGATTGCC
AAAAGAATGAATGGTACAAGCCAAGCTAAATCGATATCTGCTAAAGGTAACATCATAAA
CCATTTCAAAATAACACCGTGTAATAAGTTGAAACTATTTAGTATTTGTAAAATTGAAAT
AATCAATGTAATAACAGTTGCGAGTCGATAGGCCCAACTGAATCTGAATGTGCTAAACAT
GTTAGCAAATGATATCAGTACAAGTGCAATAGACACGGGATATATTAATGTCAACAATGG
TACAGCAATTTTTAAAATCATTTCTAAACCAAGTGTTGTAAATAAGAACCCTATGATAGA
GAAAATAAGTGCGAATATTTTATAAGAAAACTTAGGTACGTGTTTCTTAGTAAATGTGGC
GCAAGCATTGACGAGTCCTATACATGTTGTTAAACATGCAAGTATCACTGTCATACCAAA
TACTATGTTGCCAAATGAACCAAATACGCGTAATGAATTGTACGTCAATATATCAGTACC
ATCTTTAAAGTTTCCTGGAGCTGTTGACGCACCTACATATGCCAACGCAAAGTAAATCAT
ACCAAGTAATATAGCTGCAATAAATCCTGAAAAGCAGACATACTTTAATATTTTCATGCG

ATCTGTGAGGCCTTTAAACTTATAGCCATTGACAATGACTACGGAAAAAGCTAACGCAGC
AACAAGATCCATTGTAAAATAGCCTTCCAAACTTCCTGAAATGAAAGGATGTGTTATATA
TTTATCCTTAGGTGCACTTAGTGCAGATTCAGGGTTGAAAATGACAGCAATACTTAATAG
AGCGACCATTAATAGTAATAACGGTGTTAATAATTTACCTAAATTATCAACGATTTTCGAT
GGATTTAAACTAATCCAGTAAACGATGGCAAAAAAGACTGCTGCGAATATAATTAAAGT
CCATTGGTTGTGCACAGGTAAAATGTGTCTTGTACCAATTTCGTACGCGACATTTGCAGCA
CGTGGAATACCGTAAAATGCTCCGATAGACATGTAAATCACGACAGCAAAAATAAACCC
GAACCATGGATGTATACGATTGCCTACACTTTCAACACCTTCATCATAAAATGCAACAAC
AATAACAGTAATAAAGGGGAGTAATATGCCTGTAAGGGCAAAGCCTAACATACCAATCC
ACATATTTTGACCTGCTGTATGGCCAAGCATGGGCGGAATATTAAATTTCCGGCTCCAA
AAAATAGTGAAAATAACATGAGGCCCGAAATAATAACTTGTTTTTTCAA

## > Q99WS0

Function: protein coding sequence; acid phosphatase precursor (core genome, variable)
Best match: Q99WS0_CC001_MW2-USA400_BA000033.2[330373:331263]
Position: 009-contig_245_RC: 5930 … 6821; Length: 891 bp
Sequence:
ATGAATAAAATTTCAAAGTATATTGCAATAGCATCATTATCGGTAGCGGTTACAGTTTCG
GCACCACAAACGACAAATTCTACAGCGTTTGCCAAAAGTTCTGCTGAAGTTCAACAAACA
CAACAAGCTTCTATACCAGCATCACAAAAGGCGAATCTTGGTAATCAAAATATTATGGCA
GTGGCTTGGTATCAAAATTCAGCTGAAGCAAAAGCATTATATTTACAAGGTTATAACAGT
GCAAAGACTCAGTTAGATAAAGAGATTAAAAAGAATAAAGGTAAACATAAGTTAGCTAT
TGCTTTGGATTTAGATGAAACAGTTTTAGATAATTCTCCATATCAAGGCTATGCATCAATA
CATAATAAACCTTTCCCAGAAGGTTGGCATGAATGGGTACAAGCTGCTAAAGCTAAACCT
GTCTATGGCGCAAAAGAATTCTTGAAATATGCTGACAAAAAAGGTGTCGATATCTACTAT
ATTTCTGATAGAGATAAAGAAAAAGATTTAAAGGCAACACAAAAGAACTTAAAACAACA
AGGTATCCCTCAAGCTAAGAAGAGTCATATTTTACTAAAAGGTAAAGATGATAAGAGTA
AAGAATCACGCAGACAAATGGTTCAAAAGGATCATAAACTTGTCATGCTATTTGGAGATA
ATTTATTAGACTTTACAGATCCAAAAGAAGCTACAGCTGAATCTCGTGAAGCATTAATTG
AAAAACATAAAGACGATTTCGGTAAGAAATATATCATTTTCCCTAACCCAATGTATGGTA
GTTGGGAAGCTACAATTTACAACAATAACTATAAAGCAAGTGACAAAGCAAAAGATAAA
TTACGTAAAAATGCTATTAAGCAATTCGATCCTAAAACAGGCGAAGTTAAATAA

## > Q2G168

Function: protein coding sequence; ABC transporter/permease protein (core genome, constant)
Best match: Q2G168_CC005_Mu50-VRSA_BA000017.4[351524:352573]
Position: 009-contig_245_RC: 7070 … 8120; Length: 1050 bp
Sequence:
ATGTTTTTAGCTTGGAATGAAATACGGCGCAACAAATTGAAGTTTGGACTAATTATTGGT
GTGTTAACGATGATTAGTTACTTGCTATTTTTATTATCTGGATTGGCGAATGGTCTTATCA
ATATGAATAAAGAAGGCATTGATAAGTGGCAAGCAGATGCCATTGTTCTAAATAAAGAT
GCCAATCAAACTGTGCAACAATCTGTTTTTAACAAGAAAGATATTGAAAATAAATACAAG
AAGCAAGCTACTTTGAAGCAAACAGGGGAAATTGTGTCTAATGGCCATCAAAAAGACAA
TGTTTTAGTGTTCGGTGTTGAAAAGTCATCATTTTTAGTTCCGAGTTTAATAGAAGGGCAT
AAAGCGACTAAAGATAATGAAGTGTTAGCTGATGAAACACTTAAAAATAAAGGATTTAA
AATTGGCGACACATTATCACTATCTCAATCAGATGAAAAATTGCATATCGTAGGTTTTAC
AGAAAGTGCAAAATATAATGCGTCACCAGTCATTTTCACGAATGACGCTACCATTGCCAA
GATCAATCCTAGATTGACTGGAGATAAAATTAATGCAGTTGTTGTACGTGATTCAAATTG
GAAAGACAAAAAATTAAACCAAGAGCTTGAAGCGGTAAGTATTAATGACTTTATTGAAA
ATTTACCAGGTTATAAACCGCAGAACTTAACATTAAACTTTATGATTTCATTCTTATTTGT
CATTTCAGCTACAGTTATAGGCATTTTCTTATATGTCATGACATTACAAAAAACAAGTTTA
TTTGGCATATTAAAGGCTCAAGGATTTACGAATGGCTATTTAGCGAACGTAGTTATTTCGC
AGACGCTCATATTAGCATTATTTGGTACGGCATTTGGCTTACTGTTAACAGGCGTTACAGG
TGCATTTTTACCTGATGCAGTACCTGTCAAATTCGATGTACTAACATTGCTCGTATTTGCA

ATTGTGTTAATGATTGTCTCTGTATTAGGAAGTTTATTCTCCATTTTAACAATTAGAAAAA
TAGATCCGTTAAAGGCGATTGGGTAG

> **A5IPH6**
Function: protein coding sequence; ABC transporter related protein (core genome, constant)
Best match: A5IPH6_CC001_MW2-USA400_BA000033.2[332575:333252] (completely identical)
Position: 009-contig_245_RC: 8132 … 8810; Length: 678 bp
Sequence:
ATGTTGAAATTTGAAAATGTAACAAAGTCATTTAAAGATGGGAATCGTAACATTGAAGCG
GTTAAAGATACAAATTTTGAGATAAATAAAGGTGATATTATAGCATTGGTTGGACCTTCT
GGCTCTGGTAAAAGTACATTTCTAACTATGGCAGGTGCTTTACAAACGCCGACATCTGGG
CACATTTTAATCAATAACCAAGATATTACGACAATGAAGCAAAAAGCATTGGCAAAAGTT
AGAATGTCTGAAATAGGTTTTATTTTACAAGCTACAAACCTTGTGCCATTTTTAACGGTAA
AGCAACAATTTACATTATTGAAAAAGAAAAATAAGAATGTTATGTCTAATGAAGACTATC
AGCAACTTATGTCACAATTAGGGCTAACTTCATTGCTTAATAAGTTACCTTCAGAAATTTC
AGGTGGTCAGAAACAACGTGTGGCGATAGCTAAAGCGTTATATACGAATCCGTCGATTAT
TTTAGCGGATGAACCTACCGCGGCGTTAGATACTGAAAATGCGATTGAAGTCATTAAAAT
TCTACGTGATCAAGCCAAACAAAGAAAGAAAGCATGTATTATTGTTACACATGATGAACG
ACTTAAAGCATATTGTGATCGTTCATATCATATGAAAGATGGCGTCCTTAATCTTGAAAAT
GAAACAGTAGAATAG

> **pfoR**
Function: protein coding sequence; putative perfringolysin O regulator protein (core genome, constant)
Best match: pfoR_CC001_MW2-USA400_BA000033.2[333460:334491]
Position: 009-contig_245_RC: 9017 … 10049; Length: 1032 bp
Sequence:
ATGGATTTATTGATAGGTACTTTATTTTTATTTTTGGTCTTAGTGATTTTTACATTATTTAC
ATATAAAGCACCTAATGGTATGCGTGCCATGGGAGCATTAGCTAATGCAGCAATCGCAAC
ATTTTTAGTAGAAGCATTTAATAAATATGTTGGTGGCGAAGTATTCGGTATTAAATTTTTA
GAAGAGCTAGGAGACGCTGCGGGAGGTCTAGGTGGTGTCGCTGCCGCTGGATTAACAGC
ATTAGCTATCGGTGTGTCACCAGTATATGCATTAGTTATAGCAGCCGCGTGCGGTGGTAT
GGATTTATTACCAGGTTTCTTTGCGGGTTATATGATTGGATATGTGATGAAATATACAGAG
AAATATGTGCCGGATGGTGTCGACTTAATTGGATCGATTGTCATCTTAGCGCCATTAGCTC
GTCTTATTGCAGTATTATTAACGCCAGTAGTGAATAGTACATTGATTCGAATTGGTGATAT
TATCCAAAGTAGTACGAATACGAATCCAATTATCATGGGTATCATTTTAGGTGGTATTATT
ACGGTTGTCGGCACAGCGCCATTGAGTTCAATGGCATTGACAGCATTATTAGGTTTAACG
GGTGTACCTATGGCTATTGGTGCCATGGCAGCATTTAGTTCGGCATTTATGAATGGGACG
CTATTCCATCGCTTAAAATTAGGTGATCGTAAGTCTACGATTGCAGTAAGTATTGAACCTT
TATCACAAGCAGATATTGTATCAGCCAATCCAATTCCAATCTATATTACAAATTTCTTTGG
TGGTGCGATTGCTGGTTTAATTATTGCTATGTCAGGTTTAATTAACGATGCGACAGGTACA
GCTACACCGATTGCAGGATTTTTAGTTATGTTTGGATTTAATCATCCGACGACAATTGTGA
TTTATGGTGTAGTAATGGCGATTGTAGGTGCGCTTGCAGGTTATCTTGGTTCAATTGTATT
TAAAAAATATCCAATTGTTACTAAGCAAGACATGATTAATCGAGGTGCAGTAGACGCATA
G

> **Q5HJ57**
Function: protein coding sequence; PfkB family carbohydrate kinase (core genome, constant)
Best match: Q5HJ57_CC001_MW2-USA400_BA000033.2[334834:335952]
Position: 009-contig_245_RC: 10391 … 11510; Length: 1119 bp
Sequence:
ATGAGCGATTCTGAGAAAGAAATTTTAAAAAGAATTAAAGATAATCCGTTTATTTCACAA
CGTGAACTTGCTGAGGCAATTGGATTATCTAGACCCAGCGTAGCAAACATTATTTCAGGA
TTAATACAAAAGGAATATGTTATGGGAAAGGCATATGTTTTAAATGAAGATTATCCTATT
GTTTGTATTGGCGCAGCGAATGTAGATCGTAAGTTTTATGTGCATAAAAATTTAGTTGCA

GAAACATCAAATCCTGTAACGTCAACACGCTCTATTGGTGGCGTAGCAAGAAATATTGCT
GAGAACTTAGGTAGGCTTGGCGAAACGGTCGCTTTTTTATCTGCTAGTGGACAAGATAGT
GAATGGGAAATGATTAAACGATTGTCCACACCATTTATGAATTTGGATCATGTTCAACAA
TTTGAAAATGCGAGTACAGGTTCATATACAGCTTTAATTAGTAAAGAAGGCGACATGACA
TATGGCTTAGCAGATATGGAAGTATTTGACTACATTACGCCTGAATTTTTAATTAAGCGTT
CACACTTATTGAAAAAGGCTAAGTGCATTATTGTCGATTTGAATTTAGGCAAAGAGGCAT
TAAACTTCTTATGTGCCTATACCACGAAACATCAAATCAAATTAGTTATCACCACGGTTTC
TTCCCCAAAAATGAAAAATATGCCTGATTCATTACATGCTATTGATTGGATTATCACGAAT
AAAGATGAAACAGAAACATACTTAAATTTAAAAATAGAATCTACTGATGATATAAAAAT
AGCTGCTAAACGCTGGAATGATTTAGGTGTTAAAAATGTTATTGTGACAAATGGCGTGAA
AGAACTCATTTATCGAAGTGGTGAGGAAGAAATCATCAAGTCAGTTATGCCATCAAATAG
TGTGAAAGATGTTACAGGTGCAGGTGATTCATTCTGTGCTGCAGTAGTGTATAGCTGGTT
AAATGGGATGTCTACTGAAGATATATTAATTGCTGGTATGGTTAACGCAAAGAAAACGAT
AGAAACGAAATATACAGTTAGGCAAAACCTAGATCAACAGCAACTTTATCACGATATGG
AGGATTATAAAAATGGCAAATTTACAAAAGTATATTGA

> **psuG**
Function: protein coding sequence; pseudouridine-5'-phosphate glycosidase (core genome, variable)
Best match: psuG_CC001-ST772_118_AJGE01000018.1[98875:99798:r] (completely identical)
Position: 009-contig_245_RC: 11484 … 12408; Length: 924 bp
Sequence:
ATGGCAAATTTACAAAAGTATATTGAGTATTCTCGAGAAGTTCAGCAAGCACGGGAGAA
CAATCAACCGATTGTAGCATTAGAATCAACAATTATTTCGCATGGTATGCCGTACCCACA
AAATGTTGAAATGGCAACAACAGTAGAGCAAATTATCAGGAATAATGGTGCCATTCCAG
CAACCATAGCCATTATAGATGGCAAAATTAAAATTGGTTTAGAAAGCGAAGATTTAGAA
ATACTGGCAACTAGTAAAGACGTTGCTAAAGTATCTAGAAGGGATTTAGCAGAAGTTGTT
GCGATGAAGTGTATTGGTGCTACTACTGTAGCGACGACGATGATATGTGCTGCAATGGCT
GGTATTCAATTTTTTGTTACAGGAGGTATTGGGGGCGTCCATAAAGGTGCAGAACATACG
ATGGACATTTCAGCAGACTTAGAAGAACTGTCTAAAACAAATGTCACTGTTATCTGTGCA
GGTGCCAAATCAATTTTAGACTTACCTAAGACGATGGAGTATTTAGAAACAAAAGGCGTT
CCAGTTATTGGATATCAAACGAATGAATTGCCAGCATTCTTCACTCGCGAAAGCGGTGTT
AAGTTAACAAGTTCGGTTGAAACGCCAGAACGACTTGCTGACATTCATTTAACAAAACAG
CAGTTAAATCTTGAAGGCGGCATTGTTGTTGCTAATCCAATTCCATATGAGCATGCCTTAT
CAAAAGCATATATTGAGGCAATCATAAATGAAGCTGTTGTTGAAGCGGAAAATCAAGGT
ATTAAAGGTAAGGACGCCACACCGTTCTTGTTAGGGAAAATTGTAGAAAAAACGAATGG
TAAAAGTTTAGCAGCAAATATAAAACTTGTTGAAAACAATGCGGCGTTGGGTGCTAAAAT
TGCTGTCGCTGTTAATAAATTATTGTAG

> **nupC2**
Function: protein coding sequence; nucleoside permease C/locus 2 (core genome, variable)
Best match: nupC2_CC015_CIGC93_AHVD01000006.1[17555:18775]
Position: 009-contig_245_RC: 12418 … 13639; Length: 1221 bp
Sequence:
ATGAATATTTTATTCGCTATCACAGGGATAGCATTTGCACTATTTGTTGCGTTTTTATTCA
GTTTTGATCGTAAAAACATAGACTTCAAAAAGACGTTAATAATGATATTTATTCAAGTGT
TGATTGTGTTATTTATGATGAACACAACGATTGGTTTAACAATCTTAACTGCATTAGGCTC
ATTTTTTGAAGGGTTAATAAATGTTAGTAAAGCAGGTATAAACTTTGTTTTTGGAGATATA
CAAAATAAAAATGGCTTTACGTTCTTTTTAAATGTGTTGCTGCCATTAGTGTTCATTTCTG
TATTAATAGGCATTTTTAATTATATTAAGGTATTACCATTTATTATCAAATATGTAGGTAT
CGCTATTAATAAAATAACGAGAATGGGGCGCTTAGAAAGTTACTTTGCTATTTCAACAGC
AATGTTTGGACAACCAGAAGTATATTTAACAATAAAAGATATTATTCCAAGATTATCTAG
AGCGAAATTATATACAATTGCGACGTCTGGTATGAGTGCTGTTAGTATGGCAATGTTGGG
TTCATATATGCAGATGATTGAGCCCAAGTTCGTAGTTACAGCTGTAATGTTAAATATTTTT
AGTGCGCTTATCATCGCCAGTGTAATCAATCCCTATAAATCTGATGATAGTGATGTTGAA
ATTGATAACTTAACTAAATCAACGGAAACGAAATCAGTGAATGGAAAAACAGGAAAACC

TAAGAAAGTTGCCTTTTTCCAAATGATTGGTGATAGTGCGATGGATGGGTTTAAAATCGC
TGTTGTAGTAGCCGTGATGTTGTTAGCGTTTATTTCATTAATGGAAGCAATCAATATCATT
TTTGGTAGTGTTGGTTTGAATTTTAAACAGTTAATTGGTTATTTGTTTGCACCTATCGCATT
CTTAATGGGGATTCCTTGGAGTGAAGCTGTTCCAGCTGGCTCTTTAATGGCTACAAAATTA
ATTACAAATGAGTTTGTAGCAATGCTAGATTTTAAAAATGTTTTGGGTGACGTATCAGCT
AGAACACAAGGTATTATCTCAGTTTACTTAGTGAGTTTTGCTAATTTCGGTACTGTTGGTA
TCATCGTTGGTTCAATTAAAGGAATTAGTGATAAGCAAGGAGAAAAAGTTGCATCCTTTG
CAATGAGGTTGCTACTTGGTTCAACTCTAGCTTCAATCATTTCAGGATCAATTATTGGCTT
AGTATTGTAA

> **sss**
Function: protein coding sequence; sodium solute symporter (core genome, variable)
Best match: sss_CC001-ST772_118_AJGE01000018.1[96007:97539]RC (completely identical)
Position: 009-contig_245_RC: 13743 … 15276; Length: 1533 bp
Sequence:
CTACTTTTTCGTAGCCGTTTTTGAAATGTATGTTGATGGTTTATCTTTTTCAAAAATTGTTA
ATCCCGTTATATCTTTTTTATGTTTTGAAGGGACAATGAAGCTAAGTATATAAGCAAAGA
CAAAAGCAACTGTAAATGAAATGGTAGATACATAGAAAGGTGAGTTACCTTTGCCAACA
CCATTATAGATATAAGCAAAGATGATACCCAATATTAATCCACAAATAACACCGAATGTA
TTCGTACGTTTAGTGAAAATACCAACTGCAAATACACCAGCTAATGGAACACCGAATAAT
CCAGTTACAAACAAGAATAAATCCCATAAGTCATTTGAATTAGAAGCAATTAAGTATAGT
GACATTCCAAAACCGAAAATACCTGCAATGATGATAATCAAACGTGCAAAGTTAACTTCG
TGTCGCTCGCTACCTTTTCCGAAGAAGCGTTGCTTAATGTCGATTGAAATACAAGCAGAT
ATAGAATTTAAACTAGATGAAATGGTAGACTGTGCAGCGGCGAAAATGGCTGCAATAAG
TAATCCTGCTACAAATGGTGGCATCTCAGTCAAAATGAAATATGGCACTACAGATGATGT
ATTGAAGCCTTTTGGTAAAACAGCTTCATGTGTATAAAATGAATACAGCATTGTACCCAT
ACCATAAAATAAGGGTGCTGAAATTAAAGCTAGGATACCATTTGTCCATAACGATTTATT
TGTTTCTTTTAAACTATCAGAAGCTTGATAACGCTGCACGACGTCTTGACTCGCTGTGTAT
TGATACAAGTTGTTGAAAATATTTCCTAGGAAAATAATTGGAATGGCAGCTGCCGCAGTG
TTTAGCTTCCAATTATCTGCACTAATTAATTTTTTGTGCTCAATCGCATCTGCAAAGACAG
TGCCAAAACCGCCTTTAATGTTCATAACACCTAGAATAATAATAACTAAAGCGCCGCCTA
ATAAAATGACGCCTTGAATGAAATCACTCCAAACCACACCTTCGAATCCACCTAAAAATG
TATATAAAATACATAGTAAACCAACGAGTGATGCAACGATATAAGGGTTCATGTCTGATA
CAGATGTGATTGCTAATGTTGGTAAGTAGATAACGATTGCAACACGCCCTAAATGGTAAA
CGACAAATAATAATGAGCCAATAACACGTATGCTAGGGCCGAATCTAGCTTCTAAATATT
CATATGCAGATGTTACCTTTAACTTTTTAAAGAATGGAACATAGAAATAAATGAGTAATG
GAATAATTGCTACGATAGCGATGTTACCTGCAATATATGACCAATCTGTTAAAAATGCTT
TCTCTGGTGTAGACATAAACGTAATTGCACTTAATGTAGTAGCATAAATGGAAAAACCAA
CTACCCAAGATGGCAAGCGACCACTTGCGGTAAAGAAACTATTCGTACTTTGACTCGCGC
GCTTGGTAAAATAAACGCCAATGAGTAACATAGCTAGTAGATAAATGATAACGGCAACC
CAGTTTAGTGTGCCAAATCCAACTTCTTTCAT

> **nanA**
Function: protein coding sequence; N-acetylneuraminate lyase (core genome, variable)
Best match: nanA_CC001_MW2-USA400_BA000033.2[339759:340640:r]RC
Position: 009-contig_245_RC: 15315 … 16197; Length: 882 bp
Sequence:
CTATAAATCGTATTTTGCAATGAGTTGATCTAATGTTTGTCGATGTGCTTCGTTAAAAGGT
TTGAAAGGTCTTTTCGGTAATCCTGCATCAATGCCACGATGACGTAATATTTCTTTCAATG
TTGGATAAATCCCCATTGATAACACTGTTTCGATAATGTCGTTTGAATCATGTTGTAGTTG
GTAAGCTTCTTGAATTTGACCTTGTCTTGCTAAGTCGAAGATTTTTCTAGCGCGGCGACCA
TTAACGTTATATGTAGAACCAATTGCACCATCTACGCCAGAAATCGTAGCTTGAACTAAC
ATTTCATCAAAGCCAGATAAGATTAATTTGTCTGGGAATGCTTTTCTAATACGTTCGAGTA
GGAAGAAGTTTGGCGCTGTATATTTAACACCAACAATTTTTTCATGATTAAATAGCTCGCT
GAATTGTTCAATAGAAATATTCACACCTGTTAAATCTGGTATTGCATAAATAATCATATTG

TTCTGAGTTGCTTCGATAATATCGAAATAGTAATCTCTAATTTCTTCAAAAGTAAATGGAT
AGTAGAATGGTGTTACGGCGGAAAGTGCATCATAACCGAGTTCTGTGGCATATTTTCCAA
GTTCAATGGCTTCATTTAAATCTAACGAACCTACTTGAGCAATCAATTTCACTTTATCCCC
AACTGCCTCTTTGGCAACTTTGAAAACTTGCTTCTTCTGCTCTGTATTTAATAAAAAGTTTT
CGCCTGAGCTACCATTTACATAAAGACCGTCTAATTCTTCAGTTTCAATGGCATTTTGAGC
AATTTGTTTGAGTCCTTGTTCATTTACTTGACCATTTTCATCAAAAGGAACGAGTAACGCT
GCATATAAACCTTTTAAATCTTTGTTCAT

> **rok**

Function: protein coding sequence; putative protein/repressor/ORF/kinase-family (core genome, variable)
Best match: rok_CC001_MW2-USA400_BA000033.2[340798:341658] (completely identical)
Position: 009-contig_245_RC: 16354 … 17215; Length: 861 bp
Sequence:
GTGTATTACATCGCAATCGATATTGGAGGCACTCAAATTAAATCGGCAGTTATTGATAAG
CAATTGAATATGTTTGACTATCAACAAATATCAACGCCGGACAACAAAAGTGAGCTTATT
ACTGACAAAGTATATGAGATTGTAACAGGATATATGAAGCAATATCAGTTGATCCAACCT
GTCATAGGTATTTCATCAGCAGGCGTTGTTGATGAACAAAAAGGCGAAATTGTATACGCA
GGGCCAACCATTCCGAATTATAAAGGTACTAATTTTAAGCGATTATTAAAATCACTGTCT
CCTTATGTCAAAGTAAAAAATGATGTAAACGCTGCATTACTAGGCGAATTGAAATTACAT
CAATATCAAGCAGAACGGATCTTTTGTATGACGCTTGGTACAGGCATTGGGGGTGCGTAC
AAGAATAATCAAGGTCATATTGATAATGGTGAGCTTCATAAGGCAAATGAAGTTGGGTAT
TTATTGTATCGTCCAACTGAAAATACAACGTTTGAGCAACGTGCTGCAACGAGTGCATTG
AAAAAGCGCATGATTGCCGGAGGATTTACGAGAAGCACACATGTGCCAGTATTGTTTGAA
GCAGCTGAAGAAGGTGATGATATTGCAAAACAAATATTGAATGAGTGGGCAGAAGATGT
AGCAGAAGGGATTGCCCAAATACAGGTCATGTATGATCCAGGGCTTATATTAATTGGGGG
CGGTATATCTGAACAAGGAGATAATCTCATTAAATATATCGAGCCGAAAGTTGCACACTA
TTTACCAAAAGACTATGTTTATGCACCAATACAAACGACTAAGAGTAAAAATGATGCAGC
ATTATATGGCTGTTTGCAATGA

> **nanE**

Function: protein coding sequence; N-acetylmannosamine-6-phosphate 2-epimerase (core genome, constant)
Best match: nanE_CC008_NCTC8325_CP000253.1[311800:312468]
Position: 009-contig_245_RC: 18431 … 19100; Length: 669 bp
Sequence:
ATGTTACCACATGGATTAATAGTATCTTGTCAGGCACTACCAGATGAACCATTGCATTCAT
CTTTTATTATGTCGAAAATGGCATTAGCTGCGTATGAAGGTGGTGCTGTTGGTATTCGCGC
AAATACTAAGGAAGACATTTTAGCAATTAAAGAAACGGTAGATTTACCAGTTATTGGCAT
TGTGAAACGTGACTATGATCACTCAGATGTTTTCATTACTGCAACGTCAAAAGAAGTTGA
TGAACTGATAGAAAGCCAATGTGAAGTCATTGCATTGGATGCAACGTTACAGCAACGTCC
GAAAGAAACGTTAGACGAATTAGTATCATATATTAGAACACATGCACCGAACGTTGAAA
TCATGGCTGATATCGCGACCGTTGAAGAAGCTAAAAATGCCGCACGACTTGGCTTTGATT
ATATTGGCACGACGTTACATGGCTATACTAGTTATACGCAAGGACAATTACTTTATCAGA
ATGACTTCCAATTTCTAAAAGATGTACTACAAAGTGTTGATGCAAAAGTTATTGCGGAAG
GTAATGTCATTACACCGGATATGTATAAACGTGTGATGGACTTAGGCGTTCATTGTTCAGT
CGTTGGTGGTGCGATAACACGACCAAAAGAAATTACGAAACGTTTTGTTCAAATTATGGA
AGATTAA

> **A6TYA3**

Function: protein coding sequence; putative membrane protein (core genome, constant)
Best match: A6TYA3_CC008_NCTC8325_CP000253.1[312595:313908:r]RC
Position: 009-contig_245_RC: 19226 … 20540; Length: 1314 bp
Sequence:

TTATCCAAAAATAAGTAAAGCGACGGGGATGGTGATTAATAGCGACAACGCCACGCGTA
AAAACCAAATGATGATGAGTTTCCAGACAGGTATTTTAATTTCAGTTGCTAGTATACATG
GCACTAATGCTGAGAAAAAGATAATGGCTGATACGCTTACTACACCGACGACAAATTTAG
TACTCATTGCAGCTTTAGTTACTAACAAAGATGGTAGAAACATTTCTACAATAGAAATCG
CTGACGCTTTTGCAAGTACAGCCTGATCAGCAATTGGGAAGATATAAATAAATGGATAGA
ATATATAGCCAAGCCAATCAATGAATGGTGTATAGTTCGCTACAATCAGTCCTAAAAAAC
CAATCGATAATATAGAAGGTAAAATACCAACAGTCATTTCTAAACCGTCTTTCAAATTGT
CCCAAACGTTCTTCACGAGAGATGGTGTTAATGCATTTTGTATCATCGCCTCTGCATATGC
AGTTTTCAGTCTGCTTCCTTCAATAGCAACTTCTTGTTCTCCTTCTTGTCCGTTATAATATT
CTGTTGATTCATTGCTGATTGGCGGTAGCCATGCAGTAATTGCAGTCACGACAAATGTGA
TGACTAAAGTTATCCAAAAGTATAAATTCCAATGCGGCATTAATCCTAAAGTTTTAGCAA
CGATAATCATAAAAGTTGCTGAAACTGTTGAAAAGCCAGTCGCAATAATCGTGGCTTCTC
GTTTGTTGTACATCCCTTGCTTATAGACACGATTAGTAATCAATAATCCTAAGGAATAACT
GCCGACAAACGAAGCCACTGCATCGACAGCGGATTTTCCTGGTGTTTTAAAAATAGGTCT
CATAATAGGCTCCATATAAACACCGACAAATTCTAATAAGCCATAGCCCACTAATAAAGA
AAGCGCAATTGCACCTACTGGAATTAAGATACTTAATGGCATCATTAATTTTTCAAACAA
AAACGGACCATAGTTAGCTTTAAATAGTATTGATGGACCGATTTTAAATACATACATTAT
ACCAATCATTGCACCTGCAACTTTAAATAATGTAATGACCAAGTTTGTGATTGAAGTCAT
AAAAGTACGTCTCACTATTGGTAACGCTGTACCAATTAAAATCATAATCAGTGCAACATA
GGGCATAAGTGGACCTATGATTGAGCGAATGGCTAGATGAACATGATCGACGAAAATAG
TGTTGTTACCATTAATCGTAAAAGGAATAAAGAAACATAGTATGCCCACTAAACTATAGA
CAAAAAAACGCCATGCACTTGGTTGTTGTGCATTAGAATGATATTGATTCAT

> **lip2**

Function: protein coding sequence; lipase 2 (core genome, constant)
Best match: lip2_CC001-ST772_118_AJGE01000018.1[88253:90325:r] (completely identical)
Position: 009-contig_245_RC: 20957 … 23030; Length: 2073 bp
Sequence:
ATGTTAAGAGGACAAGAAGAAAGAAAGTATAGTATTAGAAAGTATTCAATAGGCGTGGT
GTCAGTGTTAGCGGCTACAATGTTTGTTGTGTCATCACATGAAGCACAAGCCTCGGAAAA
AACACCAACTAATGCAGCGGCACAAAAAGAAACACTAAATCAACCGGGAGAACAAGGG
AATGCGATAACGTCACATCAAATGCAGTCAGGAAAGCAATTAGACGATATGCATAAAGA
GAATGGTAAAAGTGGAACAGTGACAGAAGGTAAAGATACGCTTCAATCATCGAAGCATC
AATCAACACAAAATAGTAAAACAATCAGAACGCAAAATGATAATCAAGTAAAGCAAGAT
TCTGAACGACAAGGTTCTAAACAGTCACACCAAAACAATGCGACTAACAAAACTGAACG
TCAAAATGATCAGGTTCAAAATACCCATCATGCTGAACGTAATGGATCACAATCGACAAC
GTCACAATCGAATGATGTTGATAAATCACAACCATCCATTCCGGCACAAAAGGTAATACC
CAATCATGATAAAGCAGCACCAACTTCAACTACAACCCCGTCTAATGATAAAACTGCACC
TAAATCAACAAAAGCACAAGATGCAACCACGGACAAACATCCAAATCAACAAGATACAC
ATCAACCCGCGCATCAAATCATAGATGCAAAGCAAGATGATACTGTTCGCCAAAGTGAA
CAGAAACCACAAGTTGGCGATTTAAGTAAACATATCGATGGTCAAAATTCCCCAGAGAA
ACCGACAGATAAAAATACTGATAATAAACAACTAATCAAAGATGCGCTTCAAGCGCCTA
AAACACGTTCGACTACAAATGCAGCAGCAGATGCTAAAAAGGTTCGACCACTTAAAGCG
AATCAAGTACAACCACTTAACAAATATCCAGTTGTTTTTGTACATGGATTTTTAGGATTAG
TAGGCGATAATGCACCTGCTTTATATCCAAATTATTGGGGTGGAAATAAATTTAAAGTTA
TCGAAGAATTGAGAAATCAAGGCTATAATGTACATCAAGCAAGTGTTAGTGCATTTGGTA
GCAACTATGATCGTGCTGTAGAACTTTATTATTACATCAAAGGTGGTCGCGTAGATTATG
GTGCAGCACATGCAGCTAAATACGGACATGAGCGCTATGGTAAGACATATAAAGGAATC
ATGCCTAATTGGGAACCTGGTAAAAAGGTACATCTTGTAGGGCATAGTATGGGTGGTCAA
ACAATTCGTTTAATGGAAGAGTTTTTAAGAAATGGTAACAAAGAAGAAATTGCCTATCAT
AAAGCGCATGGTGGAGAAATATCACCATTATTCACTGGTGGTCATAACAATATGGTTGCA
TCAATCACAACATTAGCAACACCACATAATGGTTCACAAGCAGCTGATAAGTTTGGAAAT
ACAGAAGCTGTTAGAAAAATCATGTTCGCTTTAAATCGATTTATGGGTAACAAGTATTCG
AATATCGATTTAGGATTAACGCAATGGGGCTTTAAACAATTACCAAATGAGAGTTACATT
GACTATATAAAACGCGTTAGTAAAAGCAAAATTTGGACATCAGACGACAATGCTGCCTAT
GATTTAACGTTAGATGGCTCTGCAAAATTGAACAACATGACAAGTATGAATCCTAATATT

ACGTATACGACTTATACAGGTGTATCATCTCATACTGGTCCATTAGGTTATGAAAATCCTG
ATTTAGGTACATTTTTCTTAATGGATACAACGAGTAGAATTATTGGTCATGATGCAAGAG
AAGAATGGCGTAAAAATGATGGTGTCGTACCAGTAATTTCGTCATTACATCCGTCCAATC
AACCATTTGTTAATGTTACGAATGATGAACCTGCCACACGAAGAGGTATCTGGCAAGTTA
AACCAATCATACAAGGATGGGATCATGTCGATTTTATCGGTGTAGATTTCCTAGATTTCA
AGCGTAAAGGTGCAGAACTCGCCAACTTCTATACAGGTATTATAAATGACTTGTTGCGTG
TTGAAGCGACTGAAAGTAAAGGAACACAATTGAAAGCAAGTTAA

## > attB_phiSa5

Function:  attachment site in bacterial chromosome for int1-phiL54a (mobile element)
Best match: attB_phiSa5_empty_ST425_LGA251-cow_FR821779.1[342202:342299:r]RC (completely identical)
Position: 009-contig_245_RC: 22831 … 22929; Length: 98 bp
Sequence:
TGAACCTGCCACACGAAGAGGTATCTGGCAAGTTAAACCAATCATACAAGGATGGGATC
ATGTCGATTTTATCGGTGTAGATTTCCTAGATTTCAAGC

## > A5IPQ2

Function: protein coding sequence; esterase/lipase-like protein (core genome, variable)
Best match: A5IPQ2_CC001-ST772_118_AJGE01000018.1[87184:88011]RC (completely identical)
Position: 009-contig_245_RC: 23271 … 24099; Length: 828 bp
Sequence:
TTACATCATTGTTATAGCGTTTAAGAAATCAACAACTTTACGATAAATAGTGATTGCTTCG
TCATTAGGTCTACGATCAAAATCATGCTCGTTTTTATTCACGCGTTCAAATGTTGAATGTG
GAACATGATTCATGATATGTTCGCTTTCCTCAACGGGAACATCATAATCGCCATTACAAT
GCGTAATGAACACCGGGGGAAGTGTTTTAAGTTCATATGGTGCAATATTATATTTTGAAT
CAGTATAATCAGCAATGTTAATCATATTTATCCATTTACCTGTGCCACGTGCATAAACGTA
GATTAAAAAACGTTGTGCGATTTGATCTTGAACAACCGGTGTTGGTGAAGTGAGTTGTGC
AATCATTGTTTCGTTTACGCTTTGAGCTATTTTTGCGTAATAACTATTAGTTGTTTTAAAAG
GTTCAGTGTTGATGCGACTATAACCATAAAAATCAATAACACCATCAATATCTCTGTCTC
GTGCAATTAATAGACTTAAATATGCACCTGATGATCTGCCAAAGGTAAAAATAGGGCAAT
TAGAATATTGTGATTGAATCGCATCGAATGATGCGTAGACATCCTCAATAATGCAATCGA
GACTTACTTCTGGTAATAAACGATAACTTAGTTGAATTAAATCGTAATGTTCCGTAAGGA
TATCGATATACTGTGGGGATAAATCGTTAGCTTTACCGAACATTAATCCACCACCGTGGA
TGTAGACAATAGCGCCTTTTGTTGGTTGATTTTTTGCTTTAATAATTGTGTAAGGTAATGC
AAATGCATCTTTAGTAATTACTTTATCTTTAATTTCAGTCAC

## > Q6GJZ4

Function: protein coding sequence; putative NADH-flavin oxidoreductase (core genome, variable)
Best match: Q6GJZ4_CC001_MW2-USA400_BA000033.2[348620:349819:r]RC
Position: 009-contig_245_RC: 24171 … 25371; Length: 1200 bp
Sequence:
TTAAGACGATGAGTCATGATAATTCTGTTCCAATTGACGTAAAGCGTCACGGGTATGCTT
CTTTAGACCTTCCCCATAATCCATCATTTTAACAATATCTTTAAAAGCAGCATGTGGAATG
GCTAAATCTTCTAAATCTGCCATAGAAAATTCAAGATTGATATCATGTGGTCGCTGTTCAG
CAAGTTTATGCACAAAGTCAGGTTCTGTGACAAAAGGCGAAGACATGCCGACCATATCTG
CATGTTGTAAAGCATCTAAAGCAGACTCTGGAGAATTAATCCCGCCACTTGCAATTAAAG
GGATACGACCTGCTAAATGTTCATAGACAATTTGGTTAACTGGTCGACCGAAATGATCAC
CTGGTGTACGAGACGTATTTTGATAAATATGTCGACCCCAGCTAGCGATTGCTAAGTATT
GGATGTTTGAAACGTCCATGACCCAATCGATTAATTGGTTGAACTCGTCAATGGTATATC
CTAAATCACTGCCTCTGGTTTCTTCTGGCGTTGCTCGAAATCCTAAAATAAAATTGTCAGG
TGCTTCTTTATCAATCACTTCTTGTACCGCACGCATAACTTCTAAACATAATCTTGCACGA
TTTTTTAATGAGTCGGCACCGTAATGGTCTGTACGTTTATTCGAAAAAGTTGAGAAAAAT
GTTTGAATCAGCAAACGTTGTGCAATCGAAATTTCCACACCATCAAAACCTGCTTTAATC
GCGCGTAATGTAGCATCGCGATACTGCTGAATGATGCTATTGATTTTCTCATGAGACATG

GCGATAACATCGTGTTCAATCGGTGAATGCAAAGTCATAGGACTTGGTCCATACACCTTT
CCAAAATTTAAAATGGCTTGATTTGAAAAACGACCAGCATGCGCTAGCTGGATAATAGCG
AGGCTACCATGTTGTTTCATCGTAGATGCCATGTTAGTTAATCCAGGGATACAAGCATCA
TGATCAATATTAAAGCCATATTCAAACAATTGACCATAAGGTTCAATGTAAGCAGCGCCG
GTGACTTGCATTCCAGCTGAATTAGAGCGACGTGCAGCATAAGCCAAGTCTGCTTTTGTA
ATATAGCCTTCTTTTGTTGATGTGTTTACGGTCATTGGTGATAATACAAAGCGATTCGAAA
TTTTGATGCCATTAGGTAAGTGGATTGATTGTAAAAGTGGTTTGTATCGGTACAT

## > lplA1

Function: protein coding sequence; lipoate-protein ligase A/locus 1 (core genome, variable)
Best match: lplA1_CC008-ST239_16K-ST239_BABZ01000038.1[6384:7406]
Position: 009-contig_245_RC: 28752 … 29775; Length: 1023 bp
Sequence:
ATGTACTTAATAGAACCGATTAGGAATGGAGAATATATTACTGATGGTGCGATTGCACTC
GCTATGCAAGTTTATGTTAATCAGCATATCTTTTTAGATGAAGATATTTTATTCCCTTATTA
TTGTGATCCAAAAGTGGAAATTGGACGTTTTCAAAATACTGCTATAGAAGTGAATCAAGA
TTATATAGATAAACACAGTATTCAAGTAGTTCGCCGAGATACTGGTGGTGGCGCTGTGTA
TGTTGATAAAGGTGCCGTTAATATGTGTTGTATTTTAGAACAAGACACTTCAATTTATGGT
GATTTTCAACGATTTTATCAGCCAGCTATAAAGGCGTTGCATACATTAGGTGCAACAGAT
GTGATACAAAGCGGTAGAAATGATTTAACATTGAATGGTAAAAAAGTGTCAGGCGCCGC
AATGACATTAATGAACAATCGTATTTATGGCGGTTATTCGCTATTACTTGATGTTAATTAT
GAAGCAATGGATAAAGTGTTAAAGCCTAATCGCAAAAAGATTGCATCGAAAGGGATTAA
ATCTGTGCGTGCACGTGTTGGTCATCTTAGAGAAGCACTGGATGAAAAGTATCGTGATAT
AACAATTGAAGAATTTAAAAATTTAATGGTGACGCAGATTTTGGGAATCGATGACATTAA
AGAGGCAAAACGATACGAATTATCTGATGCAGATTGGGAAGCGATTGAAGAATTGGCGA
GTAAAAAGTATAAAAATTGGGATTGGAATTATGGGAAGTCACCTAAATATGAATATAATC
GAAGTGAAAGATTATCATCAGGTACGGTAGACATAACAATTTCTGTTGAACAAAATCGTA
TCGCAGATTGTCGTATTTATGGGGATTTCTTTGGACAAGGTGATATAAAAGATGTGGAAG
AAGCATTACAAGGAACAAAAATGACAAGAGAAGATTTAACGCACCAGTTAAAGCAATTA
GACATCGTTTATTATTTTGGCAATGTTACGGTAGAATCATTGGTTGAGATGATTTTAAGTT
AA

## > Q5HIW6

Function: protein coding sequence; putative NAD-dependend oxidoreductase (genomic island)
Best match: Q5HIW6_CC001-ST772_118_AJGE01000018.1[80167:81192:r] (completely identical)
Position: 009-contig_245_RC: 30090 … 31116; Length: 1026 bp
Sequence:
ATGAATAATAAAGTATTAGTAACCGGTGGTACAGGGTTTGTTGGCATGCGAATTATTTCA
CGATTATTAGAACAAGGTTATGAGGTACAAACAACGATACGTGATTTAAGTAAAGCTGAT
AAAGTAATTAAAACAATGCAAGACAATGGCATTTCCACAGAGCGATTAATGTTTGTCGAA
GCGGATTTATCACAAGATGAACATTGGGATGAAGCAATGAAAGATTGTAAATATGTCTTG
AGTGTAGCATCTCCGGTGTTTTTCGGTAAAACAGACGATGCAGAAGTGATGGCGAAGCCT
GCCATTGAAGGCATACAACGTATTTTAAGAGCTGCAGAACATGCTGGCGTGAAGCGTGTG
GTGATGACTGCAAACTTTGGTGCAGTTGGTTTTAGCAATAAAGATAAAAATTCAATCACA
AATGAAAGTCATTGGACAAATGAAGATGAACCAGGCTTATCAGTATATGAAAAATCAAA
ATTGTTAGCTGAAAAGGCAGCGTGGGATTTTGTTGAGAATGAAAATACAACAGTAGAATT
TGCCACAATCAATCCAGTTGCAATTTTTGGGCCATCATTAGATGCACACGTTTCAGGAAG
CTTTCATTTATTAGAAAATTTATTGAATGGTTCAATGAAACGTGTACCGCAAATTCCATTG
AATGTTGTTGATGTGAGAGACGTAGCTGAACTACACATTTTGGCAATGACAAATGAACAG
GCTAATGGCAAGCGATTTATTGCTACAGCTGATGGACAAATTAATTTGTTGGAAATTGCA
AAATTAATTAAAGAAAAGAGACCTGAAATAGCTCAAAAAGTTTCTACTAAAAAATTACC
AGACTTTGTTTTGAGTCTAGGTGCTAAATTTAATCATCAAGCTAAAGAAGGTAAACTTTTA
TTAGATATGAATCGAAATGTAAGTAACGAACGAGCAAAAATACTTCTTGGTTGGGAACCG
ATTGCGACACAAAAAGAAGCAATTTTAGCAGCTGTCGATAGTATGGCTAAGTATCATTTA
ATATAA

> **break**
Function:  integration site of chromosomal replacement
Best match: break_ST239_2_CC001_MW2-USA400_BA000033.2[355545:355593] (completely identical)
Position: 009-contig_245_RC: 31096 … 31145; Length: 49 bp
Sequence:
CTAAGTATCATTTAATATAATACAAAACACCGTCCATAAGTAAGTGGCT

> **ulaA**
Function: protein coding sequence; putative sugar-specific permease/SgaT/UlaA (core genome, constant)
Best match: ulaA_CC001_ATCC51811-ST1_ADVP01000041.1[83583:84929]RC
Position: 009-contig_245_RC: 31208 … 32555; Length: 1347 bp
Sequence:
CTATTGCTGTGGCTTTTGACGGCCTTTTAATAAAATTGCTGTCGCACCTACGATAATAATA
AATAGAATCGCACCAAATAATCCCATATATTTTACTGCGTTACCGAACACGATACCGACA
GTTAAAAAGTCTGTATCTGAGAATGTTGTTGCAGCACCACCTAATTCGCCTAAAAATGGC
AAGAATAATAATGGTAAAAACGTGATTAGGATACCATTTAGAGCGGCGCCAGCAACAGC
ACCTTTAATACCGCCTCTTGCATTACCGAATACAGCAGCCGTTGCACCTAAGAAGAAGTG
TGCAACTACGCCAGGTAAAATGACGACGCCACCAAATAAGAATAAGATAAACATACCGA
TGACACCTGTAATAAAGCTGACAAAGAATCCAATTAATACTGCATTTTGTGCATAAGGGA
ACACAATAGGGCAGTCTAATGCAGGTTTAGAATTTGGTACAAGCTTTTCAGAAATTCCTT
TAAATGCTGGGACGATTTCAGCTAAGATTAAACGAACGCCCGTTAAAATAATAAATACAC
CAGCAGCAAATGTCACACCTTGAATTAAAGAAAAGACAATAAAGTTTTGACCATCACTAA
TAGATTCGTGTACATAACTAACGCCCGCAAATAAGCATGCGATGAAGTAAAGTAATGCCA
TCGTAATCGAGATACTAATTGTACTTTCTCGTAAGAAACTTAAGCCTTTTGGAAATTTAAT
CTCTTCCGTTGATTTAGACTTACCTTTGAATAATTGACCTACAGCACCTGCGGCAAAGTAA
CTGATTGAGCCAAAATGACCTAACGCTACTTGGTCATTCCCTGTAATTTTTCGCATTGTAG
GTTGGAGTAATGCAGGTAAGACTGCCATGATTAATCCTAATACGAGTGCGCCGATAACAA
TCGTTAGCCAGCCTTTAATATGACTGACTGTTAAAATGATTGCTAAAAACGCAGCCATGT
AAAATGTATGATGACCTGTTAAAAAGATATATTTTAAATTAGTGAAGCGGGCAATTAAAA
TATTAACAATCATGCCACAGACCATGATGAGTGCAGCTGTTGTTCCAAAATCTTTTAAGG
CTAGTGAGACGATAGCTTCGTTGTTAGGTACGATACCTTGCACACCAAATGCGTGTTGGA
ATATTTTGCCGAATGGTTCAAGAGATCGAACGACGACATCAGCACCTGCACTTAAAATTA
AGAAGCCTAATATCGTTTTAATGGTTCCTGAAGTGATCGTTGCGGCAGGTTTTTTCTGAAC
GATTAAACCTATAAAGGCAATCAGTGCAACAAGAATGGCTGGTTGACTTAAAATATCGAC
TATAAAATTAAGGATTGCTTGCAT

> **A8YZF7**
Function: protein coding sequence; transcript antiterminator/BglG family (core genome, variable)
Best match: A8YZF7_CC001-ST772_118_AJGE01000018.1[76024:77979]RC (completely identical)
Position: 009-contig_245_RC: 33303 … 35259; Length: 1956 bp
Sequence:
TTAAGCCTCCTTTGTCATAGTTAAAGCAATGTGTTGTTTAATTTTAAAAATATTCCCATCT
AAGAAATCTTGTCGATATAAGTCGTTGCTTAAGCATTCGCTTAACTGTCCCAATGCCTTTA
AATGTGCATTGGGGTGGTCCGTTGCTAATGTAATTACAAGGTGAACGGGATCGTTAGCTT
TACTACCAAAGATAATCCCTTCAGTGAAATATGTTAGTGCGAAACCTACACCATTCTGTA
CATAATCAGTACCAGCGTGAATAAGTGCAATATGTGGACTAATGACCATATATGACCCGA
ATTGTTCAAATTGTTTTAAAATTGCAGCTGTATAATTTGAATAGACAATGCCATCATTGAT
TAAAGGTTGCACAGCCACTGCAATTGCGGATTCAATTGATAATGGTTGTTTATTTATAATG
ATGCGATGTTCAGGCAATAAATCTGCGAGTGACTTGCCATCAGTTGCCATTTTCATGACTC
GTTGTTCTCTTGAGTCATTGATAATTTGATTCAATTTTTGACGAGATTGTTGATTGATAAA
TGGATCGACATGAATAACTGGTACAGCTGATATTTCACAAGGTACTGTTGAAATGACATA
ATCAATGTTATCTTGCAATAATCGACTTTCTTCCAATTGATAAATGGAATAGGCATCCCAA

ATGTGAAACTCAGGATACAGGTGATTTAGTTTTGATTTTAAAAGTTGCGACGTGCCTATA
CCAGAACCACATAGTAAGACAACCTTAATCATTGATTGTTTATGTGTTGCAACACGCTCT
ATACTTGATGCGAAGTGAATTGTAATGTATGTTAATTCATCTTCGTTGAAGCGAATAGCA
GCATCTTGTTCAATTGGGCTAATATGCTTGCTAACGGCTTCAATGATTTGAGGATAGCGAC
GCATAACTTCTTGCCTCAAAGGATTAGGTTGTAGCATATCATATTTAATACGATGTATAGC
TGGTTTGATATGTGTGATCAGACTGGTATGTAACTTGTTGTCTTTTGACATATCAATGCCT
AATTCTTGGCTAACACAAGTGATTAATTCATGTATATTTTGCGATAAATCATGGTATTCAA
AGGCAATTGAAGACGCTGTATGTTCGGTCATTTTAGAGCCTAGTAAATGTAACGTGATAA
AGATAATTTCAGACTCTGGAAATGTGACATTACAACTACGTTCTAAGTTTTCTATCATTTT
TGAAGCAATAGCATACTGATTAGTATGTCGCCATTTATCAATTTCATTGATAGGTATATCG
AACGAAAAATTTTCATTTAGACGCTGAATGGCAATGAGTATATGATAGATTAAGCCATCG
ATAGCCGACTGAACTAAATGATAATTTTCACTATTTAATGTCTTAATAATGGCACGGCGA
ACCAATGCGATTGATTCTGAATTAAAGATATCCGCCTCTATAAAAGGTGCAGCTTGTTTC
ATATATTGATGTATAAAGTGTGCATACGCTTTACGATAGTGATCTTCCTCACCAATAATAT
TGAATCCTTTATTGTGGACATAATTTAACTTTAAATGGTATTGATCTAGTTGGGCTTGAAT
CATTTTAATATCATCTGCAATTGTCCGACGCGAAACATTAGCATCTTGCGCAAGTTGCTTT
GTTGAAACAGGATCGGTTGTTTCGAATAACTTTAAAGCGATATGTGTGAGTCGTTCATCTT
TTGAAAAATGAATTTGATTTGTTAGATTGTGCTCTAATTCATTTAATAGTGTCGCGTGAGC
TGTTGTTACTTTGATGCCTGCAGCTTTATTACGGCTGACTTGGTAATGATAAGTTTCAGCA
TATTGCTCAATATATGCTATATCATATTGAATGGTACGAGGTGATACACCAAGTTGATTA
GCAATGGTATTGATTGGAATAAACGTTTGCTCATGAATTAAAAGATACAAAATTTCGATT
TGTCTATAACTTAACAA

> **mepR**

Function: protein coding sequence; MATE regulatory protein (core genome, constant)
Best match: mepR_CC001_MW2-USA400_BA000033.2[359919:360338] (completely identical)
Position: 009-contig_245_RC: 35470 … 35890; Length: 420 bp
Sequence:
ATGGAATTCACTTATTCGTATTTATTTAGAATGATTAGTCATGAGATGAAACAAAAGGCT
GATCAAAAGTTAGAGCAATTTGATATTACAAATGAGCAAGGTCATACGTTAGGTTATCTT
TATGCACATCAACAAGATGGACTGACACAAAATGATATTGCTAAAGCATTACAACGAAC
AGGTCCAACTGTCAGTAATTTATTAAGGAACCTTGAACGTAAAAAGCTGATCTATCGCTA
TGTCGATGCACAAGATACGAGAAGAAAGAATATAGGGCTGACTACCTCTGGGATTAAAC
TCGTAGAAGCATTCACTTCGATATTTGATGAAATGGAACAAACACTCGTATCGCAGTTAT
CTGAAGAAGAAAATGAACAAATGAAAGCAAACTTAACTAAAATGTTATCTAGTTTACAA
TAA

> **mepA**

Function: protein coding sequence; staph virulence regulator A (core genome, constant)
Best match: mepA_CC001_MW2-USA400_BA000033.2[360445:361800]
Position: 009-contig_245_RC: 35996 … 37352; Length: 1356 bp
Sequence:
ATGAAAGACGAACAATTATATTATTTTGAGAAATCGCCAGTATTTAAAGCGATGATGCAT
TTCTCATTGCCAATGATGATAGGGACTTTATTAAGCGTTATTTATGGCATATTAAATATTT
ACTTTATAGGATTTTTAGAAGATAGCCACATGATTTCTGCTATCTCTCTAACACTGCCAGT
ATTTGCTATCTTAATGGGGTTAGGTAATTTATTTGGCGTTGGTGCAGGAACTTATATTTCA
CGTTTATTAGGTGCGAAAGACTATAGTAAGAGTAAATTTGTAAGTAGTTTCTCTATTTATG
GTGGTATTGCACTAGGACTTATCGTGATTTTAGTTACTTTACCATTCAGTGATCAAATCGC
AGCAATTTTAGGGGCGAGAGGTGAAACGTTAGCTTTAACAAGTAATTATTTGAAAGTAAT
GTTTTTAAGTGCACCTTTTGTAATTTTGTTCTTCATATTAGAACAATTTGCACGTGCAATTG
GGGCACCAATGGTTTCTATGATTGGTATGTTAGCTAGTGTAGGCTTAAATATTATTTTAGA
TCCAATTTTAATTTTTGGTTTTGATTTAAACGTTGTTGGTGCAGCTTTGGGTACTGCAATCA
GTAATGTTGCTGCTGCTCTGTTCTTTATCATTTATTTTATGAAAAATAGTGACGTTGTGTCA
GTTAATATTAAACTTGCGAAACCTAATAAAGAAATGCTTTCTGAAATCTTTAAAATAGGT
ATTCCTGCATTTTTAATGAGTATCTTAATGGGATTCACAGGATTAGTTTTAAATTTATTTTT

AGCACATTATGGAAACTTCGCGATTGCAAGTTATGGTATCTCATTTAGACTTGTGCAATTT
CCAGAACTTATTATCATGGGATTATGTGAAGGTGTTGTACCACTAATTGCATATAACTTTA
TGGCAAATAAAGGTCGTATGAAAGACGTTATCAAAGCAGTTATCATGTCTATCGGTGTTA
TCTTTGTTGTATGTATGATTGCTGTATTTACAATTGGACATCATATGGTTGGACTATTTACT
ACTGATCAAGCCATTGTTGAGATGGCGACATTTATTTTGAAAGTAACAATGACATCATTA
TTATTAAATGGTATAGGTTTCTTGTTTACTGGTATGCTTCAAGCAACTGGTCAAGGTCGTG
GAGCTACAATTATGGCTATTTTACAAGGTGCGATTATCATTCCAGTATTATTTATTATGAA
TGCTTTGTTTGGACTAACAGGTGTCATTTGGTCATTATTAATTGCTGAGTCACTTTGTGCA
TTTGCTGCAATGTTAATTGTCTATTTATTACGTAATCGTTTGACAGTTGATACATCTGAATT
AATAGAAGGTTAA

> **mepB**

Function: protein coding sequence; putative protein (core genome, variable)
Best match: mepB_CC022_HE681097.1[355767:356197]
Position: 009-contig_245_RC: 37455 … 37886; Length: 431 bp
Sequence:
ATGTATAAATCTAAAATACTGTTGAAATATATTTTTAGTGAAGAATCAGAAGTTAAAGAC
TTAACTGAAGAAAAATATAATCAAGATTACGAAGCATTAACATTTAGCTTTAAAGAGGAA
ACATATCAAAGTAGGTTAGCTAAGAAAACACCGACTAAAACAGGGTATTTTGTTACATGT
TGGACAAAAGACGAGAACAATTGCAATCAACCATACTCAAAAGAGGCATTTGCAGATTA
CTTAATGATTATAGTTATTGATGAAGAATTAAGCGGTTATTTCTTATTTCCTAGGGAATTA
TTGGTAGAAAAAGGCATCTTAACTACATTTGAACATAAAGGTAAGATAGCTTTTAGAGTT
TATCCTAAGTGGTGTAATCAATTGAATAAAACAGCAGGGCAAACACAAAAGTGGCAATG
TAAATATTTTTT

> **txbi_glpT**

Function:  bidirectional rho-independent terminator of glpT and mepB (core genome, constant)
Best match: txbi_glpT_CC001_MW2-USA400_BA000033.2362340:362428 [4_50ANF10END]
Position: 009-contig_245_RC: 37888 … 37977; Length: 89 bp
Sequence:
AATACTAATAAAAAGTCATATGGTTGAACTTATAAGAGATGAATATATCATCTTTCGATA
AGATAACCCTATGACTTTATTTTTTATCA

> **glpT**

Function: protein coding sequence; glycerol-3-phosphate transporter (core genome, constant)
Best match: glpT_AJ563809.1[1:1359]RC
Position: 009-contig_245_RC: 37978 … 39337; Length: 1359 bp
Sequence:
TTAATGATGAACGGTTTCTTGTCCTACTTTATTCCAAGTGAGGATAAAGCTCAACATTGCA
AACACACTGATTGCTGTTAATAAAATAAAACCGACATCCCATCCGAATTTATCAACTACA
GCACCTAAGACGATGTTGGCCATTACAGCACCAAACAGATAACCAAATAATCCTGTTAAT
CCAGCTGCTGTGCCAGCTGCTTTTTTAGGTACATAATCTAATGCTTGTAAACCAATTAACA
TAACTGGTCCATATATTAAGAAACCAATGGCAATTAATGAGACATTGTCTAACCAAGCAT
TGCCTGGAGGATTTAACCAATAAATTAATACAAATACTGTGACACCTAACATAAAGAAGA
AACCTGCAGGTCCACGACGACCTTTGAATAATTTATCAGAAATGTAACCACATAATAATG
TACCAGGAATTCCAGCCCATTCGTATAAGAAGTATGCCCAACCTGATGCTTTTAAGTCGA
AATGTTTTTCTTCACTTAAGTAGACTGGCGCCCAATCAAGTACACCATAACGCACGAAAT
AAACAAATATATTTGCAAAGGCAATTGCCCATACCCATTTATTGTTCAGTACATATTTAAA
TAAAATTTCTTTTGTAGTTAATTCTGTTTCTAATGTTTTCTTATCGCTTGTAGCAAAGTCAT
TTTTATAAATTTCGATTGGAGGTAAACCTTGAGATTGAGGTGTGTCTCTAATCAATACGTA
TGAAATTGCGGCAATGATAAGTGCTAAGAGTGCAGGGTAAATGAATACACCTTCGAAAC
CTTTTAAATAACCAAAGTTGATAAATGCTGTTGTTGTAATACCCCAAGCAGCAATAGGTG
CCATAATACCTCCACCAACATTATGCGCAACGTTCCAAAGGGCAGTCTTACTTCCGCGTTC
ACTTACACTAAACCAGTGAACGAGAACACGGCCTGAAGGTGGCCAGCCCATACCTTGAA
ACCATCCATTTAAGAATAATAGGACAAACATAATACCGATACCTGATGTAAAGAACGGT

ACAAATCCCATTAACAAATTGACGATAGCAGTGAGTGCTAATCCAAGAACTAAGAATATC
CGAGCATTGCTCCGATCACTTACAGTACCCATAAAGAACTTACTAAATCCATATGCGATG
GAAACAGCAGAAAGTGCAAAACCTAGTTCCGCTTTTGTAAAACCTTGCTCTTGCAATGCC
GGCATCGCTAATGAAAAGTTTTTACGTAATAAATAGTACCCAGCGTAACCGATGAAAATA
CCAAGAAATACTTGGAGACGTAATCGTTTATAGGTATCATCTATCTGATTTTCTGGCAAA
GGCTTAATATGCTTTGCAGGTTTAAGAAAATTCAT

## > **yeiH**

Function: protein coding sequence; UPF0324 membrane protein (core genome, variable)
Best match: yeiH_CC001_MW2-USA400_BA000033.2[366741:367736:r]RC
Position: 009-contig_245_RC: 42289 … 43285; Length: 996 bp
Sequence:
TTAACTATATAGCCAATGAACGACGATAAAGGCAAGTGATGACAAGCATATTGAGGTAA
TAATGATTGTCATAAGCGGTTTAAGTGCGCGATTTTTAAGATCTTTAAATGCCACATTTAA
CCCTAAAGCAACCATGGCCATTAATAAGCAAATTGTTGATACAGTATTTAAAATATTTAG
CAATGCTGACGGAATAGTTACATATGTATTCACTAAGGCCATAATGACAAATCCAATTAA
AAAGTATGGAATGCTTATTCGACCCTTGCTAGATGATTCTGATGAACGGAAACGCATAAT
TAAAATAAGTACGATGGTTAATGGAATCAGTAAGAATACTCTACCAAGTTTGCCCAGAAG
TGCAATTTTAAGTGCATCACTACCACCAAAGCCACCAGCTAAGACAACGTGTGCAATTTC
ATGAAGACTAACACCAGACCAAGCGCCATAAACATTTGTCGTCATTGAAAAGATAGCGT
AGATAGCTGTATATATAAGTGAGAATATCGTACCAATCAATGCGATGATACCGATACTAA
TAGCTGTATCCTTTTCACGTGATTTGAATATTGGAGCTACTGCAGCAATAGCGGCAGCAC
CACAAACGCCTGTACCTACACCTAGTAATAATGCGATGTTTTTGTCACCATGTAACAGTTT
GTTGACAAAGAGCATCATTACAATACTAAAAATAACGACACCTACATCGATGGCTAATAG
TCTACTACCTTGACCGATAATATCGAATATATTGAGTTTAAGTCCATATAGGATGATTGCA
AATCTTAATAAATATTTAGATGAAAACGTAATACCTGAGCTATATTGTTCAGGATATCCTC
TAAAGTGACGATATAGAATAGCGATTAATATCGCGATAGTTAATGCGCCAACCTTATCTA
AGATTGGCAATTTAGCTGCTAAAAAGCTAAATAATGCGACTATAAATGTTAATGATAGTC
CAATCATAAAATGCTTATTTTTCAATGATGCCAT

## > **STAR**

Function:  Staphylococcus aureus repeat element
Best match: STAR_CC121_21269_AFTU01000042.1[72955:73177]RC
Position: 009-contig_245_RC: 43434 … 43464; Length: 30 bp
Sequence:
GGCGGGGCCCCAACAAAGAAGCTGACGAAA

## > **repeat_nySAgamma**

Function:  repeat element
Best match: repeat_nySAgamma_CC705_RF122-cow_AJ938182.1[1695238:1695347:r]RC
Position: 009-contig_245_RC: 43461 … 43491; Length: 30 bp
Sequence:
AAAAGTCAGCTTACAATAATGTGCAAGTTG

## > **Q2FZB6**

Function: protein coding sequence; putative protein (genomic island)
Best match: Q2FZB6_CC008_COL_CP000046.1[1182242:1182414]
Position: 009-contig_245_RC: 43462 … 43492; Length: 30 bp
Sequence:
AAAGTCAGCTTACAATAATGTGCAAGTTGG

## > **furbox-fep**

Function:  binding site of ferric uptake regulator next to fep
Best match: furbox-fep_CC001_MW2-USA400_BA000033.2[368688:368722:r]RC (completely identical)

Position: 009-contig_245_RC: 44405 … 44440; Length: 35 bp
Sequence:
ATAATGATTTTGATAATTATTATCAACTAGATAAT

> **fepA**

Function: protein coding sequence; lipoprotein (core genome, variable)
Best match: fepA_CC001_MW2-USA400_BA000033.2[368853:369707]
Position: 009-contig_245_RC: 44570 … 45425; Length: 855 bp
Sequence:
ATGAAAAAGTTAACAACGCTATTATTAGCATCAACGTTATTAATTGCTGCATGTGGGAAC
GACGATAGTAAGAAGGATGATTCAAAGACATTGAAAAAAGATGATGGTGTTAAAGCAGA
ATTAAAACAAGCAACAAAAGCATATGATAAATATACTGATGAACAGTTAAATGAATTTTT
AAAAGGTACAGAAAAATTTGTTAAAGCGATTGAAAATAATGATATGGCCCAAGCAAAAG
CGTTATATCCAAAAGTTCGTATGTATTATGAACGCTCTGAACCAGTTGCAGAAGCATTTG
GAGATTTAGATCCTAAAATTGATGCACGTCTTGCAGATATGAAAGAAGAGAAAAAGGAA
AAAGAATGGTCAGGATATCATAAGATTGAAAAAGCATTATACGAAGATAAGAAAATTGA
TGATGTGACTAAAAAAGATGCACAACAATTATTGAAAGATGCAAAAGAATTGCATGCCA
AAGCTGATACATTAGATATCACACCAAAATTAATGTTACAAGGTTCTGTTGACCTATTAA
ATGAAGTTGCAACTTCTAAAATCACAGGTGAAGAAGAAATTTATTCACATACAGATTTAT
ATGATTTTAAAGCGAACGTTGAAGGCGCACAAAAAATTTATGACTTATTTAAACCTATTT
TAGAGAAAAAAGATAAAAAATTAAGTGATGATATTCAAATGAACTTCGATAAAGTGAAT
CAATTATTGGATAAATATAAAGATAACAACGGCGGTTATGAGTCATTTGAAAAAGTATCG
AAGAAAGACCGTAAAGCATTTGCGGATGCTGTTAATGCATTAGGAGAGCCACTAAGTAA
AATGGCTGTGATTACTGAATGA

> **fepB**

Function: protein coding sequence; iron-dependent Dyp-type peroxidase (core genome, variable)
Best match: fepB_CC001-ST772_118_AJGE01000018.1[64632:65861:r] (completely identical)
Position: 009-contig_245_RC: 45421 … 46651; Length: 1230 bp
Sequence:
ATGACAAATTATGAACAAGTTAACGATAGTACGCAATTTTCAAGACGTACATTTTTGAAA
ATGTTAGGTATTGGCGGTGCCGGTGTTGCAATTGGCGCAAGTGGTGTTGGTAGCATGTGG
TCTTTCAAATCAATGTTCAATACACCAGAAGATCCGGAAAAAGATGCGTATGAATTTTAT
GGTAAAGTGCAACCAGGCATTACCACACCCACGCAAAAAACATGCAATTTCGTTGCGTTA
GATTTGAAGTCAAAAGATAGAGATGCAATTAAGGCAATGTTTAAAAAGTGGACGGTTAT
GGCTGATCGTATGATGGATGGTGATACAGTTGGCAAGCCGAGTAACAATCCTTTAATGCC
ACCAGTAGATACCGGTGAATCGATAGGATTAGGTGCAAGCAAGTTAACGATTACCTTTGG
GATTAGTAAGTCTTTGATGAAGAAAATTGGGTTATCTAGTAAAATTCCCGATGCCTTTAA
AGATTTACCGCATTTTCCGAATGATCAGTTAATAGACGATTACAGCGATGGTGATATTAT
GATTCAAGCATGCTCAAATGATTCGCAAGTATCCTTTCATGCGGTTCATAATTTAGTTCGT
CCATTTCGAGATATTGTTAAGGTACGTTGGGCGCAATCTGGTTTTATCTCTGCTAAAGGTA
AGGAAACACCTAGAAATTTAATGGCATTTAAAGATGGAACAATTAATCCTAGAAAAAGT
AATCAACTTAAAGATTATGTGTTTATTGATGACGGATGGGCGAAACATGGAACTTATTGT
GTTGTCAGACGTATTCAAATACACATTGAAACGTGGGATCGTACTGCACTGGAAGAACAA
GAGGCTACATTTGGTCGGAAACGACATAGTGGTGCGCCGTTAACAGGTGGGAAAGAGTT
TGATGAAATTGACTTAAAAGCGAAAGATAGTCATGGCGAGTATATTATTGATAAAGATGC
CCATACGAGACTAGCGAAAGAAGCAAATACGTCAATTTTACGTAGAGCCTTTAATTATGT
TGATGGTACGGATGACCGCACAGGTAACTTCGAAACAGGCTTGTTGTTCATTGCTTTTCA
AAAAGCGACAAAACAATTTATCGATATACAAAATAATTTAGGTAGTAATGATAAATTAA
ATGAATATATTACACATAGAGGTTCTGCTTCATTTTTAGTATTACCAGGTGTTAGTAAGGG
AGGATACCTTGGTGAAACATTATTTGACTAA

> **fepC**

Function: protein coding sequence; high-affinity iron transporter (core genome, variable)
Best match: fepC_CC005_04-02981_CP001844.2[390150:391862]

Position: 009-contig_245_RC: 46631 … 48344; Length: 1713 bp
Sequence:
GTGAAACATTATTTGACTAAATTTGTAGCAATGCTAATAACTGCTGCTATGGTGTGTAGCT
TTGGGTTACTGAAAAGTCAGGCAGCAGAACAACAAAGTATTAGTGATGTATATAGTGTGA
TAACGGATGCGAAATCTGCACTTTCTAATAATTCGATATCGAATGACAATAAGCAGAAAG
CAATTGAGCAAGTGGTAAGTGCAGTTAAGAAATTATCGCTTGAAGATAATAGTGAAAGT
AATGCTGTCAAATCAGATGTGAGAAAGCTTGAAGATGCAAAAGCGAATGATAATCAAAA
AGATACACTTTCGCAATTAACGAAGTCATTAATTGCTTATGAAGAGAAATTGGCTAGTAA
AGATGCGGGTTCTAAAATTAAACTATTGCAACAGCAAGTCGATGCCAAAGATGCAGTAAT
GACAAAAGCGATTAAAGATAAAAATAAAGCAGAGCTAGAATCGCTGAACAATAGTTTGA
ATCAGATTTGGACAAGTAATGAAACGGTAATTCGCAATTATGACGCAAATCAATATGGAC
AAATTGAAGTCGCATTACTGCAACTTAGAATCGCAATTCATAAGTCGCCATTAGATACAG
CGAAAGTGTCACATGCTTGGACAACTTTTAAATCAAATATTGATCATGTCGATAAGAAAA
GTGATACGTCTGCAAATGATCAATACCGTGTATCACAATTAAATGATGAGTTAGAGAAGG
CGATTAAAGCTATCGACGACAATCAATTGTCGGATGCTGATGCTGCGCTTACACATTTTAT
AGAAATTTGGCCGTATGTTGAAGGTCAAATTCAAACTAAAGACGGTGCTTTGTATACGAA
AATTGAAGATAAAATACCATATTATCAAAGTGTATTAGACGAACATAATAAAGCACATGT
GAAAGATGGTTTAGTAGATTTAAATAACCAAATTAAAGAGGTTGTTGGCCATAGTTATAG
CTTCGTCGATGTGATGATTATCTTTTTACGTGAAGGGCTAGAAGTGTTGTTAATTGTAATG
ACATTGACTACCATGACGCGTAATGTAAAAGATAAGAAAGGGACTGCAAGTGTGATTGG
TGGTGCAATTGCCGGACTTGTACTGAGTATTATCTTAGCAATTACGTTTGTAGAAACTTTA
GGGAATAGTGGCATTCTTCGTGAAAGTATGGAAGCGGGATTAGGTATCGTTGCGGTCATA
TTAATGTTTATCGTTGGTGTTTGGATGCACAAACGTTCAAATGCAAAACGTTGGAATGAC
ATGATTAAAAATATGTATGCTAATGCGATTAGTAATGGTAATTTGGTATTGTTAGCGACG
ATTGGTTTAATATCTGTGTTGCGTGAAGGTGTTGAGGTTATCATTTTCTATATGGGGATGA
TAGGTGAGCTAGCGACAAAAGATTTTATTATTGGTATTGCTTTAGCTATCGTTATATTAAT
TATCTTTGCGTTATTATTTAGATTTATCGTCAGATTGATACCTATATTCTATATATTTAGAG
TATTGTCGATCTTTATTTTTATTATGGGATTCAAAATGCTTGGCGTAAGTATTCAAAAATT
ACAATTATTAGGTGCGATGCCGAGACATGTTATCGAAGGATTCCCAACGATTAACTGGTT
AGGATTCTATCCAAGTTATGAACCATTGATAGCACAAGCTGCCTATATTATGGTAGTTGCT
ATCTTAATCTTTAAATTTAAAAAATAA

> **tatC**

Function: protein coding sequence; twin-arginine translocation protein C (core genome, variable)
Best match: tatC_CC188_21340_AGTX01000031.1[74906:75607:r]RC
Position: 009-contig_245_RC: 48435 … 49137; Length: 702 bp
Sequence:
TTAATGTGTTGGCGGTTTGCCTCGGTATGTAAATTTAACGATGAACGTACTGAACTCAAA
GAGCAATATGAGTGGCAATGTGAGTAATATATTTAATGTTAAATCGGGTGGTGCAATGAT
ACTTGCTAATACAAAGCAAGCGAAATAAATATATTTTCGATAATGCTTCAATGATGTGGT
ATCTATAAGACCGAATTTTGCAAGTCCTATGAATAATATTGGCAATTGGAATAAAATGCC
AAAAGTGAAAAGCCAACGGATAAGTTCAACTAAATACGCTTTAAAGCCAATGACAGGCG
AAATGTTTAAAGTTAATGATAATTTTAACGCGAATTGAATGATCATTGGAAAGCTAACAT
AAAATGCAAAAGCGACGCCAGCACAGAATAATAACACGCTGAAAAAACTATATTTATAA
ATAAATTGGCGCTCATTATTATGCAAACCAGGCGCAACAAACGCCCACAATTGATAAAAC
ATAACCGGTGAAATGAGACAAAACGCGATGAAAAATATAATCATCACGTATATTTGGAT
CATTTCTGTGAATGAAAATGCATGTAAGGACACATGTGCACGGGTAATATACGTTATGAA
TGGTGTCATCCACCAAAATGATGAAACATATACGACGATGACCGTAATGACGAACGACA
ATAAAATTTTTACTAACCGATGGCGTAGTTCGCTAAAGTGAACCAT

> **tatA**

Function: protein coding sequence; twin-arginine translocation protein A (core genome, constant)
Best match: tatA_CC001_MW2-USA400_BA000033.2[373436:373651:r]RC (completely identical)
Position: 009-contig_245_RC: 49153 … 49369; Length: 216 bp
Sequence:

CTATTGCTCTCGCTGTTGCTTCGATTCCTTACTGGGTGTATCGTGAGACTCTTTATCTAAAT
CTTCTGTTGCAGATTTAAATTCTTTTAAAGTAGAACCGATAGCACGACCAAATTGTGGTA
ATTTTTTCGGACCAAAAATAATTAAAGCGATAATGCTAATGACGACAAGACTTGTTGGAC
CTGTGATGCCTAAAATAAAAGTGTTAGTTATCAT

## > ltrA

Function: protein coding sequence; low temperature requirement A (core genome, constant)
Best match: ltrA_CC001_MW2-USA400_BA000033.2[377187:378320:r]RC (completely identical)
Position: 009-contig_245_RC: 52884 … 54018; Length: 1134 bp
Sequence:
TTATTCATGTTTATTACGTAGACGTTGATTTTTAAAATAAATCAGCATGATGCAGAAAGTC
GCAACGGATACCGAGATAATCATTACTTGGTCATGCCCCTTAAAGATAAGGCTGATGATA
TATGATACTACAAGTATGCCTAGTGAAGAAGAAATATATTTTGCGTTTGTCAGTTCATTAT
GGAAATAAGGCGTGATTAACCATAATCCAATATAGAATATTAAAACACTGATATACATCA
TATTAATTTCAAACAAGTCATTTAGTTTATTGTTATTACTAAAAACAATTGCAGCATTAAT
CACACCTAAAGCGATATTGATTAATAGATGCGTATACGATAAACGGAAACCGATAGATG
ACAATTTATGATTAATGTAATTTTCAGTAATGATCCAATATACACCGAAAAGACTAATTA
AAATCATAAATTGGAATATATAAATGTAACTAAAATGATCAATGCTAAATGATGACGAA
GCTAAACCAACCAGTACCTCGCCAAAGATAATAATTGTTAGTAACGAAAAACGTTCTACT
AAATGCATCATATTAACAGGTGATAATACAAGATATTTCTGAAATGGAATAAGTCCTGTC
GCTGCAATGAATACGCCTAAAAATCCAGGGATGTAATGGATACTTTGTGGTAGTACTAAT
GATAGAAATGATAAAAATGAAATCACAAAGGCTACGCTCGCAAAAGCTTGACATGTACG
CTTATCGCCATAATCTAACCCTGTACGTATATGTAATAAATACTGTAATCCGATACTTAAA
TACATAATTGCCACGCATAAGAAGAATGGGAAGAATGTCTTTTCAAAGTCCGGATATAGG
CTGTTAGATAGGAAGACCATGATGAACATATTAAACATCATAAACGAGACGTCTTTGAAT
GTAACTTGACCAAATCGATTTGTAAAAAATGTTTGATGAGACCACATTAACCATAAGAAC
AAACTCATGACGATGTATTTGAAAAATAAATCAGCTGAAATGGAACCGTTTTGTGTTGTT
AAAATCACATGTGCAATTTTTTGAATGGCATAGACGAAAATTAAATCAAAGAACAACTCA
TGGAATCCTGCACGCTTTTCAGCTAAATGTTTTGGTGTTAATGCATTAACCAT

## > thlA/yqiI

Function: protein coding sequence; acetyl-CoA acetyltransferase (core genome, constant)
Best match: thlA_CC001-ST772_118_AJGE01000018.1[55545:56726:r] (completely identical)
Position: 009-contig_245_RC: 54556 … 55738; Length: 1182 bp
Sequence:
ATGACGAGAGTCGTATTAGCAGCAGCATACAGGACACCTATTGGCGTTTTTGGAGGTGCG
TTTAAAGACGTGCCAGCCTATGATTTAGGTGCGACTTTAATAGAACATATTATTAAAGAG
ACGGGTTTGAATCCAAGTGAGATTGATGAAGTTATCATCGGTAACGTACTACAAGCAGGA
CAAGGACAAAATCCAGCACGAATTGCTGCTATGAAAGGTGGCTTGCCAGAAACAGTACC
TGCATTTACAGTGAATAAAGTATGTGGTTCTGGGTTAAAGTCGATTCAATTAGCATATCA
ATCTATTGTGACTGGTGAAAATGACATCGTGCTAGCTGGCGGTATGGAGAATATGTCTCA
GTCACCAATGCTTGTCAACAACAGTCGCTTCGGTTTTAAAATGGGACATCAATCAATGGT
TGATAGCATGGTATATGATGGTTTAACAGATGTATTTAATCAATATCATATGGGTATTACT
GCTGAAAATTTAGTGGAGCAATATGGTATTTCAAGAGAAGAACAAGATACATTTGCTGTA
AACTCACAACAAAAAGCAGTACGTGCACAGCAAAATGGTGAATTTGATAGTGAAATAGT
TCCAGTATCGATTCCTCAACGTAAAGGTGAACCAATCTTAGTCACTAAGGATGAAGGTGT
ACGTGAAAATGTATCAGTCGAAAAATTAAGTCGCTTAAGACCAGCTTTCAAAAAAGACG
GGACAGTTACAGCAGGTAATGCATCAGGAATCAATGATGGTGCTGCGATGATGTTAGTCA
TGTCAGAAGACAAAGCTAAAGAATTAAATATCGAACCATTGGCAGTGCTTGATGGCTTTG
GAAGTCATGGCGTAGATCCTTCTATTATGGGTATTGCACCAGTTGGCGCTGTAGAAAAGG
CTTTGAAACGTAGTAAAAAAGAATTAAGCGATATTGATGTATTTGAATTAAATGAAGCAT
TTGCAGCACAATCATTAGCTGTTGATCGTGAATTAAAATTACCTCCTGAAAAGGTGAATG
TTAAAGGTGGCGCTATTGCATTAGGACACCCTATTGGTGCATCTGGTGCTAGAGTTTTAGT
GACATTATTGCATCAACTGAATGATGAAGTTGAAACTGGTTTAACATCATTGTGTATTGGT
GGCGGTCAAGCTATCGCTGCAGTTGTATCAAAGTATAAATAA

## > Q5HIT9

Function: protein coding sequence; cyclase family protein (core genome, constant)
Best match: Q5HIT9_CC001_MW2-USA400_BA000033.2[380123:380875:r]RC (completely identical)
Position: 009-contig_245_RC: 55820 … 56573; Length: 753 bp
Sequence:
TTAGTCATTTGATGGTTTAATTGCAAATGCTCTAACAGGGAACCCAGGTGCATCTTTTGGT
TTAGGGCTGATAGCGTAAATGATGGCGCCACGAGTTGGTAATTGATCTAAATTAGTTAAT
AACTCGACTTGGTATTTATCCTGACCAAGAATATAACGTTCGCCAACTAAATCACCATTTT
TTACAACGTCCACAGATGCATCGGTATCGAATGTTTCATGACCAACAGCTTCAACACGAC
GTTCTTCAATTAAGTACTTCAAAGCATCTAATCCCCAACCCGGTGCATGTTGTTGTCCGTT
CGCATCTTTGTTTTCAAACTTTTCAATATTAGGCCAACGTTTTGACCAATCGGTACGAAGT
GCAACAAAAGTGCCAGGTTCAATAGTACCATGCTCTTTTTCCCATGCTTCTATATGCGCAC
GTGTTACAATGAAATCATTGTTGTTCGCTACTTCTGTTGAAAAGTCTAATACAATTAACGG
CAATACCAATTCTTTTAAATCAATGTCTTCTAAATAACGTTTATTCTCGACAAAGTGAATT
GGTGCATCAATGTGAGTACCATATTGCGTTACAATATTCCAACGTTGCACATAGAAACCA
TGATCTTTAACCGTGAATAAAGTTGAAACTTCGCCTTTTTCAAACTCACTAAAACGTGGTA
TTTCCGGATCAAATGTATGCGTTAAATCAACCCAAGTTGCTTGTTTTAAAGTATTTAATTG
TTGCCATAAAGGATATTGTGTCAT

## > metE

Function: protein coding sequence; 5-methyltetrahydropteroyltriglutamate--homocysteine methyltransferase (core genome, constant)
Best match: metE_CC030_MRSA252_BX571856.1[397296:399524:r]RC
Position: 009-contig_245_RC: 56615 … 58844; Length: 2229 bp
Sequence:
TTATTCTTGGCGTTTAGCTTTAACAGCATTCACAAGCACAGTCAATGCATCTTTAACTTCT
TCTTCTTTTCGCGTTTTCAAACCACAGTCAGGGTTTACCCAGAATAATGAGCGGTCGATTT
GTTGTAGTGAACGATTGATTGCTGTAGTAATTTCTTCTTTTGTTGGAATACGTGGACTATG
AATATCATACACACCTAGACCAATACCTAAATCATAATTAATATCTTCAAAGTCTTTAATT
AAATCACCATGGCTACGAGATGTTTCAATTGAAATAACATCAGCATCTAAGTCATGAATA
GCATGAATGATTTGACCGAATTGAGAATAACACATATGTGTATGGATTTGAGTTTCATCA
CGAACTGAAGACGTTGCAAGTTTAAATGATAAAACAGCATCTTTAAGATATTGTTCGTGA
TATTCAGAGCGTAATGGTAAGCCTTCACGTAATGCAGGTTCGTCAACTTGGATAACTTTG
ATTCCTGCAGCTTCAAGTGCTAATACTTCTTCGTTGATTGCTAAAGCAATTTGATCTTGAA
CGACTTTACGTGGTAAATCAACACGTTCAAATGACCAGTTTAGAATTGTTACAGGTCCAG
TTAACATACCTTTAACTGGTTTATCTGTTAAGCTTTGTGCATAAACTGTTTCATCAACAGT
TAAAGGCGCTGTCCATTTTACATCACCATAAATGATTGGTGGTTTTACGGCACGTGAACC
ATATGATTGCACCCAACCGAATTTAGTTACTAAGAAACCTTGTAATTTTTCTCCGAAGAAT
TCAACCATGTCATTACGTTCAAATTCACCGTGAACTAATACATCTAAGCCAATGTCTTCTT
GAATTTTAATCCATCGAGCAATTTCATTTTTTAAGAATGTTTCATATGCTTCGTCTGTAAT
GCGTTTGTTCTTCCAATCTGCACGGTATTTTCGAACTTCTCGGCTTTGTGGGAATGATCCA
ATAGTTGTTGTTGGTAAATCCGGTAAGTTCAAACGTTTTTGTTGTTGTTCAATACGTTGCG
CGAATGGTGATTGTCTTGAAGTACGCACGCTTTCGAAATCATAATCTAAGTTTTTGAATGA
TTGATTTTGGAAACGCTCATAACGTGCTTTTAATTTATCATATTTAACACTATCGTTTTGAT
TAAATAGGCGACGCAATGCATCTAATTCGTCTAATTTTTCAGTTGCAAAGCTTAAGCCTTC
GCCAACACTTGTATCTAATGTTTCATCATCTAAAGATACTGGAACATGTAATAATGAAGA
TGATGGTTGAATGACAAGTTCATTAGTGTGTGCTAACAATTTATCGATTAAGACTTTTTTA
GCTTCAATGTCACTTGCCCACACATTACGACCATCAATAATTCCAGCGTATAATGTTTTTG
ATTTATCAAAATCTCCAGCTTCAATTTGTTTAAGGTTATAGCCATTATCATGGACAAAGTC
TAAACCTAAACCACCAACAGGTAAAGAACTTAAGAATTTAAGATGTGCACGTTCAAAGT
ATGTTTGAATGACTAATTTTTTAGCAACACCAGCTTTTTCGAAATAGTCATAAGCTTCACG
TGTAATATTTTCATAGCTTTCGCTGTCGTCTGTAACTAAGATTGGCTCATCAACTTGAATG
TACTCAGCACCTGCATCAATTAATGATTCAAACACTTCTTTATAAAGTGGTAATAACGTTT

TAACTTTTTCTTCAAAAGTTTGGTGACCGCCTTTTGATAATTTAACAAAAGTAATCGGACC
AACAATGACAGGGTGAGCGTTAACGTTTAAAGATTGGGCATATTTAAAGCGATCTAATAA
TACATTGCGACTCACTTTAGGCTCAACATTGTCCCATTCAGGTACGATGTAATGATAGTTA
GTGTTAAACCATTTTATAAGTGCACTTGCAACATGGTCTTTATTACCGCGAGCAATATCAA
ATAATAAATCATCATCAATAGTTCTTCCTTGGAAACGTTCAGGGATGATGTTGAATAATA
ATGACGTATCTAATATATGGTCATATAAAGAGAAATCACCAACTGGGATGCTATCTAAGT
GATAGTACTTTTGTAATAATAAATTTTCTTTATGTAGATCAGTTAATGTTTGATCTAATTCT
TCTTTAGAAATCTTCTTTGCCCAATAACTTTCGATGGCTTTTTTCCATTCTCTTTTTCTACCT
AATCTTGGGAATCCTAAGTTTGATGTTTTAATTGTTGTCAT

> **metH**

Function: protein coding sequence; methylenetetrahydrofolate reductase (core genome, constant)
Best match: metH_CC008_NCTC8325_CP000253.1[352150:353991:r]RC
Position: 009-contig_245_RC: 58840 … 60682; Length: 1842 bp
Sequence:
TCATAATATTGCCTCCTTGTGAGCAGTAATAGATTTTGAGTATGCTGCAAGTTCTAATGAA
TCTTCGACATTTTGAAACGGTGTGATAATGTATAAACCATTAAAATATTCATGAACAGTA
TCGATTAAATCCTTTGAAAGCTTAAGACTTAGTTCTCGTGTTTTGGCTTTATCATCTTTAAC
TGCTTCAAATTGTTGTAAAATTTCATCTGACATCTTGATTCCTGGCACTTCATTATGCAAA
AAGAGTGCGTTTTTGTAACTTGCGATAGGCATAATGCCTATGAAAAATGGTTTGTTCAAG
TGCTTAGTGGCATGGTAAATTTCAATGATTTTCTCTTTGCTGTACACGGGTTGTGTTATAA
AATAAGACATTCCGCTTTCTATCTTTTTCTCTAATCTTTTGACGGCACCATCTAATTTACGA
ACATTAGGGTTAAAGGCGCCAGCGATGTTGAAGTGTGTACGTTTCTTCAGCGCATCACCG
TCAGTGTTAATACCTTGATTAAATCTTAGAGCGAGTTCAGTTAATCCTTTAGAATTAACAT
CATAGACATTGGTTGCACCTGGTAAGTGACCAACTTTTGAAGGATCACCAGTTATGGCTA
ATATTTCGTTAACGCCAATGAGCGATAATCCAAGTAAATGGGACTGCAAGCCGATTAAGT
TTCGGTCTCGACATGTAATATGTACGAGTGGTTCAATATTGTAATATTGCTTAATTAAGCT
AGCAGCAGCAATATTGCTAATTCTGACAGTTGCCAATGAATTATCTGCGAGTGTTACCGC
ATCTACATTAGCTTTATCAAGTTTAGCGATATTTTCAAAAAATCTATCCGTGTCTAAATGT
TTCGGTGTATCCAATTCGATAATAACGGTTGGACGTTCTTGAACCTTAGATGTTAATGATT
GTCTAACTTTATTTTGAGATGGATTGAAAAGTGCTTTCGTTGGTATCGGAATCACTTTTTT
GTCATTAACAGGTTTAAGTGTCTGAATAGATTCTTTAATAAATTTGATGTGCTCTGGCGTT
GTACCACAGCAACCACCAATTAAACGAACACCTTCGCGAATTAGATTTTGAGCAACTTGA
CCGAAATATTGTGCATTGTCACTATACTTAAATTCACTATTTTCAATATCTAATAAGCTGG
CATTTGGATAACAAGATAAGAATGCGTGCTCTGGTAATTCAATATGTGTGAAAGACTCTT
GCATATGGTGCGGGCCATGATGACAATTGAGTCCCACGATGTTTGCACCACATTGAACGA
GTTGTTTTAATCCTTCATTGATTGCCTGACCATTAACTAAGTAATTTGTGTTTGAAGCGGT
TAATTGAGCAATGATTGGAATGTCGTATTTCTTTCTCGTTCGTGAAATGACATTTGTTAAC
TCTTCTAGGTCGTAATACGTTTCGAAAAGTAGCGCGTCAACGCCTTCTTCAATTAAGGTGT
CTATTTGAATTTCAGTATGATAAAGAATAGTTTGTAAGCTGATATCCTCTTGTTTGATACC
TCTAAACCCACCAACTGTGCCTAATATATACGTATCTTTATTTGCTGCTTTTTTTGCGATGC
GAACGGCGGCTTGATGTATTGCTTTAACTTTATCTTCAAGACCGAATCGTTTTAACTTTTC
AAAATTTGCACCATAAGTATTGGTTTGAATGACATCAGCACCGGCTTCAATATATGAACG
ATGGATGCGTTCAACTTTATCTGGATGGCTAAGATTATATGCTTCTGGACAGGTGTCTAAT
CCTTCAGAGTATAAAATGGTTCCTATAGCGCCATCAGCTACTAAAACATTATCTTTCAATT
GTGTGAGGAATTGACTCAT

> **A5IPT9**

Function: protein coding sequence; putative cystathionine beta-lyase (core genome, variable)
Best match: A5IPT9_CC007_TCH959-USA300_AASB02000040.1[61053:62213:r]RC (completely identical)
Position: 009-contig_245_RC: 60650 … 61811; Length: 1161 bp
Sequence:
TTATCTTTCAATTGTGTGAGGAATTGACTCATTGAATGCCTCCTTTAATGCGTATTTGATG
TCTGCAATGAGTTCATCAGGATCTTCGAGACCAACACTTAATCGGAATAGACCGAAAGTG

ATACCACGTTCTTGCCTCACTTCTTCAGGTAGTGTTGCGTGAGACATTGTTGCTGGGTGCG
AAAGTATTGTTTCCACACCACCTAGACTCACTGAAACGAGTGGTAATGTCAGTGCATCGA
CAAATTGTTGTGCTTTAGACTCATCAGCTAAACGTAAGCCAATAACGGCACCGCCATTTTT
AGCTTGTTCTAAATGAGATGCAGTAAGTCCCGGATAATAAACTTCTGAAATTTCATCTTGC
TTTATTAAAAATGACACGATTTTTTGCGCGTTTTCGACAGATTGTTTAAATCTGATTGGAA
ATGTTTTTAAATGTTTAGCAAGTGTCCAGCTATCTTGAGCAGATAACATATTGCCTGTACC
ATTTTGTATTAAATAAAGAGCTTCACTAATTGCCTCATTATTAGTAATGACAGCACCAGCA
ATTAAATCGCTATGTCCACTTAAAAATTTTGTAGCACTATGAATGACAATATCAGCGCCA
AGTAATAAAGGTGATTGACCTAACGGTGTCATGAATGTATTGTCCACAGCTACCAGTAGT
TCATGCTTTTCGGCTATTTTAGAAACAGCTTTGATATCAGTAATTTTAAAACAGGGATTCG
ATGGTGTTTCGATATAAATTAATTTTGTGTTTGATTGAATGGCACCCTCGATTTGTTCGAG
CTTGGTAGTATCTACGGTTGTAAATTCAATATTAAATCGATTCAAAATTTGCTCAGTGAGG
CGAAAAGTACCGCCATATACATCATCGGGTAAGATGACATGATCACCAGATTTGAAAGTC
AAAAGTACTGCTGAAATCGCAGCAATACCTGATGCAAAAGCAAAAGCGAATTTCCCCTGT
TCTAATCGTGCTAACTTCTCTTCTAAAAGTTCACGGTTAGGGTTGCCACTTCGTGCATAAT
CATATTTAACATCGCCACCAAGACTTGTTTGATGGAATGTTGAAGAATCATAGAGTGGTG
GGTTAGCTGAATGATATTCCACACCTCTACGCCAATCGAATATCACTTCTGTCTCTTTTGA
AAGTGTCAT

> **A5IPU0**

Function: protein coding sequence; putative cystathionine gamma-synthase/cysteine synthase (core genome, variable)
Best match: A5IPU0_CC001_MW2-USA400_BA000033.2[386110:387213:r]RC
Position: 009-contig_245_RC: 61807 … 62911; Length: 1104 bp
Sequence:
TCATACAATCTCTCCAATCTGAGCTTTATCTAATGCTTGGATGATATCGCGTTCGATGTCT
TCATAATTTTCAACACCTAGTGATAAGCGGATTAAATACTCATCAATGCCACGTTTATCTT
TTTCAGCATCTGGCATATCAACATGTGTTTGGGTGTAAGGGAAGGTCACTAATGTTTCAGT
ACCTCCTAAACTTTCTGCAAAAATGCAAATGTCTAAATTTTTTAATAATTTAGCGACGCTA
TAGGCCTTGTTAAGTCTTAAACTAAGCATGCCAGTTTGCCCGCTATATAGTACTTCGTCAA
TTGCTTGAAGTGACTGACATTTTTTAGCAAGTTTTCTAGCGTTTGATTGCGCACGCTCAAT
GCGTAAATGCAAAGTTTTAAGTCCACGTAACAACAAATAACTATCTATTGGTGAAAGTGT
TGCGCCAGTCATGTTGTGAAAATCAAACAACTGTTGCGCGAGTGATTCACCTTTGACGGT
TACGACACCTGCTAGTACATCATTATGTCCACCAATATATTTCGTAGCTGAATGTAAGACT
ATATCAGCACCTTCTGCTAATGGTGTTGAAAGATAAGGTGTTAAAAATGTATTGTCGATA
ATTGACAATAAGCCTTTAGCTTTACAAAGTTGATAGTACGGCTTTACATCAATAGCAATC
ATTTGTGGGTTAGATATTGGTTCAATGAATAATGCAACTGTTTTATCAGTGATTTCTTTTTC
AACTTGTTCATAATCTGTAAAATCAACGTACTTAAATTTGATATTGTATTGTTGCTCATAA
AATTCAAATAATCTAAATGTGCCGCCGTATAAATCGAATGAAACTAAAATTTCATCGTGT
GGTTTAAATAAATTACATATTAATTGAATGGCTGACATTCCACTTGATGTAGCGAATGAT
GCAATACCATGTTCGAGTTTGGCAAAACAGGTTTCAAATGTTGAGCGTGTAGGATTTTTA
GTACGTGTATAATCAAAACCTGTCGATTGTCCTAGTTTTGGATGCTTGTAGGCAGTAGATA
AATGGATTGGATTCGCTATAGCACCGGTTGAATCATCGGTTAATGTGATTTGGGCTAACT
GTGTATCCTTCAT

> **tbox02**

Function: noncoding RNA T-box leader element (core genome, constant)
Best match: tbox02_CC001_MW2-USA400_BA000033.2[387279:387648:r]RC (completely identical)
Position: 009-contig_245_RC: 62976 … 63346; Length: 370 bp
Sequence:
AAAAAGGACGAAAGCTTATGTTTCGCGGTACCACCTTTATTTGTTATTCCATCGCTGAAAT
AACCTTATTCAGTACGCATTAAAAGTAAATATGCTTACTGAACAATTATCACAATTAAAG
TCAGTAAGTAAGGATATAGTAATGTGCTATCCCATACTTATTAACAAAAAATCGTGCGTA
AAGAATCCAGTACGCCATTTAACATCAATGTTAATACTGTATCGCTATAACGGGCGAACC

CGTAGACACCTCATATTGGCATCAACACTCCAAGGCCATTTTCAAACACGCTTTCAAAAT
CTTCTCTCAGCTACTAAAGACTCTCTGTATAAGCAGGGTGTGTTTTACTTTCCTCTTTATTG
TGTTTAC

> **spo0J**

Function: protein coding sequence; chromosome partitioning protein (core genome, variable)
Best match: spo0J_CC001_MW2-USA400_BA000033.2[387877:388722] (completely identical)
Position: 009-contig_245_RC: 63574 … 64420; Length: 846 bp
Sequence:
GTGAGTGAATTGTCAAAAAGTGAAGATCAACGTATTACTAAAACAAAAGATGAACAAAT
TAAGCAAATAGATATATCGGATATCAAACCGAATCCGTATCAGCCCCGAAAAACTTTCGA
TGAAAATCATTTAAATGATTTGGCAGATTCAATTAAGCAATATGGAATTTTGCAACCAAT
TGTGCTTAGAAAAACAGTTCAAGGTTATTACATTGTAGTTGGTGAAAGAAGGTTTAGAGC
TTCGAAAATTGCTGGTCTAAAATACGTATCAGCGATTATCAAAGATTTAACAGATGAAGA
TATGATGGAACTGGCGGTCATCGAAAATTTACAACGAGAAGACTTAAATGCGATTGAAG
AAGCTGAAAGTTATCAACGTTTGATGACAGATTTGAAAATTACACAACAAGAAGTAGCG
AAGCGATTGAGTAAGTCGCGCCCGTATATAGCGAATATGTTGAGGTTATTACATTTGCCG
AAAAAGATTGCTGACATGGTAAAAGATGGGCGACTGACAAGTGCACATGGACGAACGTT
ATTGGCAATTAAAGATGAACAACAAATGCTTAGGTTAGCGAAACGGGTTGTTAAAGAAA
AGTGGAGTGTTAGATATTTAGAAAACCATGTTAATGAATTAAAAAATGTTTCGTCAAAGT
CGGAAACAGACAAAGTAGATATAACTAAGCCTAAATTTATAAAGCAACAAGAACGACAG
TTGCGAGAACAGTATGGTACCAAAGTAGATATATCAATAAAAAAATCGGTTGGTAAAAT
CTCATTTGAGTTTGATTCACAAGAAGATTTTCTAAGAATAATTGAACAATTAAATCGTAG
GTATGGTAAATAG

> **engD**

Function: protein coding sequence; GTP-dependent nucleic acid-binding protein EngD (core genome, constant)
Best match: engD_CC001_MW2-USA400_BA000033.2[390005:391102]
Position: 009-contig_245_RC: 65702 … 66800; Length: 1098 bp
Sequence:
ATGGCTTTAACAGCAGGTATCGTTGGATTGCCAAACGTTGGTAAATCAACATTATTTAAT
GCAATAACAAAAGCAGGTGCTTTAGCAGCGAACTATCCATTCGCTACGATTGATCCTAAT
GTAGGGATAGTAGAAGTGCCAGATGCTAGATTACTTAAATTAGAAGAAATGGTTCAACCT
AAAAAGACATTGCCGACTACATTTGAATTTACAGATATCGCTGGTATTGTGAAAGGTGCT
TCAAAGGGAGAAGGGTTAGGTAATAAATTCTTATCACATATTAGAGAAGTAGATGCGATT
TGTCAGGTCGTTCGTGCATTTGATGATGATAACGTAACTCATGTTGCTGGTCGAGTAGACC
CTATTGATGATATTGAAGTTATTAATATGGAATTAGTACTAGCGGACTTAGAATCTGTTGA
GAAACGTTTGCCTAGAATTGAAAAATTAGCACGTCAAAAAGATAAGACTGCTGAAATGG
AAGTACGTATTTTAACAACTATTAAAGAAGCTTTAGAAAATGGTAAACCCGCTCGTAGTA
TTGACTTTAATGAAGAAGATCAAAAATGGGTGAATCAAGCGCAATTACTGACTTCTAAAA
AAATGCTTTATATCGCTAATGTTGGTGAAGATGAAATTGGTGATGATGATAATGATAAAG
TAAAAGCGATTCGTGAATATGCAGCGCAAGAAGACTCTGAAGTGATTGTTATTAGTGCAA
AAATTGAAGAAGAAATTGCTACATTAGATGATGAAGATAAAGAAATGTTCTTAGAAGAT
TTAGGTATCGAAGAACCAGGATTAGATCGATTAATTAGAACAACTTATGAATTATTAGGA
TTATCAACATATTTTACTGCTGGTGTGCAAGAAGTACGTGCTTGGACATTTAAACAAGGT
ATGACTGCACCTCAATGTGCTGGTATCATTCATACTGATTTTGAACGTGGATTTATCCGTG
CCGAAGTAACAAGTTATGACGACTATGTAAAATATGGTGGCGAAAGTGGCGCTAAAGAA
GCGGGCAGACAACGATTAGAAGGTAAAGAATATATTATGCAAGATGGCGATATCGTTCA
TTTCAGATTTAATGTATAA

> **rpsF**

Function: protein coding sequence; 30S ribosomal protein S6 (core genome, constant)
Best match: rpsF_CC008-ST72_21259_AFTS01000010.1[98755:99033:r] (completely identical)
Position: 009-contig_245_RC: 67338 … 67617; Length: 279 bp

Sequence:
ATGTACATCGTACGCCCAAACATTGAGGAAGATGCTAAAAAAGCGTTAGTTGAACGTTTC
AACGGCATCTTAGCTACTGAAGGTGCAGAAGTTTTAGAAGCAAAAGACTGGGGTAAACG
TCGCCTAGCTTATGAAATCAATGATTTCAAAGATGGCTTCTACAACATCGTACGTGTTAA
ATCTGATAACAACAAAGCTACTGACGAATTCCAGCGTCTAGCTAAAATCAGTGACGATAT
CATTCGTTACATGGTTATTCGTGAAGACGAAGACAAGTAA

> **ssb**
Function: protein coding sequence; single-stranded DNA-binding protein/chromosomal (core genome, constant)
Best match: ssb_CC008-ST72_21259_AFTS01000010.1[98231:98734:r] (completely identical)
Position: 009-contig_245_RC: 67637 … 68141; Length: 504 bp
Sequence:
ATGCTAAATAGAGTTGTATTAGTAGGTCGTTTAACGAAAGATCCGGAATACAGAACCACT
CCCTCAGGTGTGAGTGTAGCGACATTCACTCTTGCAGTAAATCGTACGTTCACGAATGCT
CAAGGGGAGCGCGAAGCAGATTTTATTAACTGTGTTGTTTTTAGAAGACAAGCAGATAAT
GTAAATAACTATTTATCTAAAGGTAGTTTAGCTGGTGTAGATGGTCGCTTACAATCCCGTA
ATTATGAAAATCAAGAAGGTCGTCGTGTGTTTGTTACTGAAGTTGTATGTGATAGCGTTC
AATTCCTTGAACCTAAAAATGCGCAACAAAATGGTGGCCAACGTCAACAAAATGAATTCC
AAGATTACGGTCAAGGATTCGGTGGTCAACAATCAGGACAAAACAATTCGTACAATAATT
CATCAAACACGAAACAATCTGATAATCCATTTGCAAATGCAAACGGACCGATTGATATAA
GTGATGATGACTTACCATTCTAA

> **rpsR**
Function: protein coding sequence; 30S ribosomal protein S18 (core genome, constant)
Best match: rpsR_CC001_MW2-USA400_BA000033.2[392495:392737] (completely identical)
Position: 009-contig_245_RC: 68192 … 68435; Length: 243 bp
Sequence:
ATGGCAGGTGGACCAAGAAGAGGCGGACGTCGTCGTAAAAAAGTATGCTATTTCACAGC
AAATGGTATTACACATATCGACTACAAAGACACTGAATTATTAAAACGTTTTATCTCAGA
ACGCGGTAAAATTTTACCACGTCGTGTAACTGGTACTTCAGCTAAATATCAACGTATGTT
GACTACAGCTATCAAACGTTCTCGTCATATGGCATTATTACCATATGTTAAAGAAGAACA
ATAA

> **txbi_rpsR**
Function:  bidirectional rho-independent terminator of rpsR
Best match: txbi_rpsR_CC001_MW2-USA400_BA000033.2[392742:392795] (completely identical)
Position: 009-contig_245_RC: 68439 … 68493; Length: 54 bp
Sequence:
TAATTTATTGTCAAACCCCGTAGGCATAGGCTTACGGGGCTTTTTGTGTTTTGG

> **txbi_Q1XZL6**
Function:  bidirectional rho-independent terminator of Q1XZL6 (genomic island)
Best match: txbi_Q1XZL6_CC001_MW2-USA400_BA000033.2[392889:392950] (completely identical)
Position: 009-contig_245_RC: 68586 … 68648; Length: 62 bp
Sequence:
AGATGCATAAAAAAAGAACTACGCATTTTAAATAAAATGCATAGCTCTTCTTTTTCTTGC
AT

> **Q1XZL6**
Function: protein coding sequence; Abi-like protein (genomic island)
Best match: Q1XZL6_CC005_A6300_ACKF01000002.1[51616:52554:r]RC
Position: 009-contig_245_RC: 68700 … 69639; Length: 939 bp
Sequence:

TTATACTTTTGGTCTGTTGAAGTCAATAATTTTATCTAAAGCTATAAAAAATCTTTTGATA
GCTAATGCATTATTATAATAGCTTTCGTTTCTTTTATATCGCTTTTGAAGTTGGTCCAAATC
GTGATATCTTGCTTGGATAATTGCATTACTACAAACTTGATTATGTAATTCTAGCGTAGCG
AAAGTATCTATGAAATTTTTTATTCCGAACATGTTTCTAGATATGCCTATATTATTCCCTTT
TTCAAATAAATATTGAGGAGATCTACTGTCATAATTTAGATTTGCTATGATGGGTTGGTTA
TGAGCTGATTTGTTTCTTATATTTTTAACTAAAGGCATTAAAATATTAGCAACTCTCAATT
CTTCGTCATTGTACTTCTTGTAATAGAAGTTGAGAAACGAAACGAATTGACCTAGTTGCA
TGAATTCAATGCAAACTCATGCGGGTGGATTTTGATAGTATTTATTCAACTTCTCGGGTAG
TTGTCCTCGTTTATTCATATGCTTGAATATTTCGTTTTTATTTTTGATTTTGGTTTCCATAAC
TTCTTCTGGTGTTCTTGAATTTGTGTCAAAATTTGAATTGCTATATGATTTATCAATACATA
AGAACTCATCTATTATTTTATAACCATCTTCTTGGTTATTTTCTGTTATTAGTTTTAAGACT
AGACACTTTAAACTATGTTCAATATCTAAAGTTAAATGCAACATTGTGTATCTTAATTTCA
TATCTATAGTTGCTAAATCTGATAAATAAGCAAATTCTATGAAATAGCCGCCATTCTTTTT
TTCGAAATTTTTTCGGAAATAAGCTAGTTTGAAGAAGTAATTATTTTTTCTAAGAATTTCA
TTTGCTTTTTCGGTGTCAATAATATTAAAAAATATATTCATCTGTTTTAATTTCGCTATTTG
CTCATCAAAATTGAGCAT

> **int5-setC**

Function: protein coding sequence; (mobile element)
Best match: int5-setC-COL_trunc_CC008_NCTC8325_CP000253.1[362987:363688:r]RC
Position: 009-contig_245_RC: 69677 … 70379; Length: 702 bp
Sequence:
TTAACTCCCCAATCGTTCAAATTTATTCATCATATCTTTCGCCATCTGATTAGTAACATGT
GTGTATATCTCTAGATTTTTTTTATAATCTGAATGACCTACATGCTCTTGCATTGCTTTTAA
GTTAATTCCTAATTGAGCAAGTGTAGATATATGCGAATGATGTAATGTATGCGTCGTTAT
AGGTTTCTTAATAGAACTAATATCAGCGCCCCCCTTTAATAATGTGGCTAATTTTGTTCGA
GTCGATAGGGCTACCAGCCGTATTTGTGAATATGTACTCTCTATCAATAAACTTATCATTC
CAAGCATAAGTGTTCTTAGTAAGTCGATGCTTTGGGTAGTGAGCCCTATGGCCTTATAGCT
ATTACTTCTTTCAGTTGTCTCCTTTACTCCGAATGCTCCCGTCTTTTTTCAGTTATCCAATT
AATTGTACCATCGATATCTAGCGTTTTATCTTCATAGTTTATATTTCTCTCTTTATTGCAA
GTAGCTCACCGATACGCATGTCATTAGCAATTTGAAACTGTACCATAGCTTTTACCATTTT
ATAATTACGTTTTGTCGTTGGATATTTTTATACTTAATTACATAGTCGAAACAATCCAGTA
ACTCCTTATCTTCATTATCTTCTAAAGTGTTATTACGTTTAGCTAGTAACGCATCACTATA
GGGATATCTATTTTATCTATTACACATAT

> **setC**

Function: protein coding sequence; staph enterotoxin-like toxin X (genomic island)
Best match: setC_CC001-ST772_118_AJGE01000018.1[40087:40698:r] (completely identical)
Position: 009-contig_245_RC: 70584 … 71196; Length: 612 bp
Sequence:
ATGTTCAAAAAACATCATTCTAAAAATTCAATTGTATTAAAATCTATTCTATCGCTAGGTA
TCATCTATGGGGGAACATTTGGAATATATCCAAAAGGAGACGCGTCAACACAAAATTCCT
CAAGTGTACAAGATAAACAATTCCAAAAAGTTGAAGAAGTACCAAATAATTCAGAAAAA
GCTTTGGTTAAAAAACTTTACGATAGATACAGCCAAAATACAATAAACGGAAAATCTAAT
AAATCTAGGAATTGGGTTTATTCAGAGAGACCTTTAAATGAAAATCAAGTTCGCATACAT
TTAGAAGGTACATACACAGTTGCTGGCAGAGTGTATACACCTAAGAGGAACATTACTCTT
AATAAAGAAGTTGTCACTTTAAAAGAATTGGATCATATCATAAGATTTGCTCATATTTCTT
ATGGCTTATATATGGGAGAACATTTGCCTAAAGGTAACATCGTCATAAATACAAAAGATG
GCGGTAAATATACATTAGAGTCGCATAAAGAGCTACAAAAAGATAGGGAAAATGTAGAA
ATTAATACTGATGATATAAAAAATGTAACTTTCGAACTTGTGAAAAGTGTTAATGACATT
GAACAAGTTTGA

> **tx_setC**

Function:  rho-independent terminator of setC (genomic island)
Best match: tx_setC_CC001-ST772_118_AJGE01000018.1[39995:40056:r] (completely identical)

Position: 009-contig_245_RC: 71226 … 71288; Length: 62 bp
Sequence:
TATCGCTAATACTTTGAAAGTTAGGTATCCGAAGATGCCTAACTTTCTTTGTTATGATTAG
C

> **Q2G106**
Function: protein coding sequence; putative exported protein (core genome, variable)
Best match: Q2G106_CC080_11819-97_CP003194.1[407424:407792]
Position: 009-contig_245_RC: 71564 … 71933; Length: 369 bp
Sequence:
TTGAAAACGATTTTAAAAACAATAACATATCTTGCACTTACTATCATTGGCGCTTATGCTG
CTTTATTCATTTTAAAAACAATAGACTCTCATGGTATAACAGATCAATTTAACCCATTAGT
AAAGGAAGATGATTCTTATGTTAAAACGACAGAGGTGTCTACTAGAATGGATGATCAACT
CCGAAGTTATACTCAAAGTGCTTTTAATAAAGAAGGGAAAGAAACGCAATTAATGTATAC
TGCTACATTTGATGTTAAACCGCATAGATACTTGAAAATTACACATAAAGGTCATCATGT
AGAAACTTTTGAAGAAGTTGAAAAGGAAGAAGTACCTAAAAAAGCATTAGACAAACTGA
GTCGATAA

> **Q1Y9M1**
Function: protein coding sequence; putative protein (core genome, constant)
Best match: Q1Y9M1_CC001_MW2-USA400_BA000033.2[396429:397001]
Position: 009-contig_245_RC: 72113 … 72686; Length: 573 bp
Sequence:
ATGAAATTAAAATCATTAGCAGTGTTATCAATGTCAGCGGTGGTGCTTACTGCATGTGGC
AATGATACTCCAAAAGATGAAACAAAATCAACAGAGTCAAATACTAATCAAGACACTAA
TACAACAAAAGATGTTATTTCTTTAAAAGATGTTAAAACAAGCCCAGAAGATGCTGTGAA
AAAAGCTGAAGAAACTTACAAAGGCCAAAAGTTGAAAGGAATTTCATTTGAAAATTCTA
ATGGTGAATGGGCTTATAAAGTGACGCAACAAAAATCTGGTGAAGAGTCAGAAGTACTT
GTTGCTGATAAAAATAAAAAAGTGATTAATAAAAAGACTGAAAAAGAAGATACAGTGAA
TGAAAATGATAACTTTAAATATAGCGATGCTATAGATTACAAAAAAGCCATTAAAGAAG
GACAAAAAGAATTTGATGGTGATATTAAAGAATGGTCACTTGAAAAAGATGATGGCAAA
CTTGTTTACAATATCGATTTGAAAAAAGGTAATAAAAAACAAGAAGTTACTGTTGATGCT
AAGAACGGTAAAGTATTAAAGAGTGAGCAAGATCACTAA

> **Q5HIS2**
Function: protein coding sequence; putative protein (core genome, constant)
Best match: Q5HIS2_CC008_NCTC8325_CP000253.1[366133:366396:r]RC (completely identical)
Position: 009-contig_245_RC: 72823 … 73087; Length: 264 bp
Sequence:
CTATTTATCCATGCGAATATCGACTTCTTCTAAATGTTTCTGATATTCTTTAACCTTACTTT
CTAAAAACATTTCATATGGTGCATCAAAGAAGTCAGCTAAATGCATGGCATCTTTAAATT
TAGGTTCATGGTGATGATTCTCCCATTCCCAAATTTGATGTGCTTCATATTTAGTACCATA
TTTCTCATTTAATTGCTGTGCTAATTCGTCAATTTCTAAATTATGTTTAGTTCGTAAGTTAT
ATAAAATATGCATATTCAT

> **Q7A7J4**
Function: protein coding sequence; putative protein (core genome, constant)
Best match: Q7A7J4_CC001_MW2-USA400_BA000033.2[398373:398954]
Position: 009-contig_245_RC: 74060 … 74642; Length: 582 bp
Sequence:
ATGACGATTTATTTAGTTAGACATGGCGAATCAAAATCGAATTATGATAATAAACATTTT
AGATCTTATTTTTGTGGACAATTAGATGTGCCGTTAACGGATACTGGCACAAAAAGTGCG
GACGATTTATGTGATTATTTTAAAGAGAAACAGATTAAACATGTATATGTTTCAGACTTAT
TAAGAACACAGCAAACGTTTGAACATATTTTTCCATATGACATTGCATCAACGACTACGC
CTCTATTAAGAGAACGTTCACTTGGCGTATTTGAGGGTGAATATAAAGATGAAATCAGTG

CGAATCCGAAATATGAAAAATATTTCAATGATTCAAACTTTAAAGACTTTCGTCATAGTTT
TTCACAAAAAGCGCCTGAAGGAGAAAGTTATGAAGATGTATATCAACGCGTAGAACATT
TTATGAATCATGTTGTCAATGAAGATACACAAAAAGATGATATTGTCATTGTTGCACATC
AAGTTGTCATTCGTTGTTTAATGGTTTATTTTAATAACGTTTCACGGGAAGAAGCTGTGGA
TTTAAAAGTTGAAAATTGCAAACCATATATCATTGAATAG

> **A5IPV8**

Function: protein coding sequence; putative lipoprotein (core genome, constant)
Best match: A5IPV8_CC008_NCTC8325_CP000253.1[368658:369284:r]RC (completely identical)
Position: 009-contig_245_RC: 75346 … 75973; Length: 627 bp
Sequence:
TTATTTATCGATAACATCACTCTTGATACCTTTAGATTTTAAGAAATCTTTAATTTTATCTT
GTTGCTTTTTATTAACATCACCGGCATATTTTGTTGGCACGTCGACAACATTGATTTTATTT
TGCGGTTGATAGCTAAGCTTTTCAATATCTTCATCAACATTGGCGATTGTACTATTTAAAG
CTTTGAAGTAATTCATCATTAATTCAACGGGTTTCTTATATTCTTTAGGAATATTGTTTTCA
GTGACAAATTTCTTGAAATGCAAATCGTTTTTAACAGCTAAGTTAGATAAGTGGCTAAGT
GTTTCTGCTTGTTTTTCAGTCACTTTTGTTTGACTGTCAATTTGTTTATCTAGTTTATGTTGC
ATAATATATTTGTTATCAAGTATATCGCTATTTACAGACAAATACTTTTCTATAGCTTGCT
TCATCTCTGCATCACTAATATCACTATTTTTCTTATCTGAGTTAAAGATATCTTTTGTTTCT
AATTTTTTAGCGCTTTTAGGTGCATGGATGCCAGTACTTGTATGATGATCTTCGTTATCAG
ATTGATCGGACGCGCAACCTGTAAGAATTAATGTCGATGCTAAAAATGTACTTAGTAGTA
ATCTCTTTTTCAT

> **ahpF**

Function: protein coding sequence; alkyl hydroperoxide reductase subunit F (core genome, constant)
Best match: ahpF_CC001_MSSA476_BX571857.1[400727:402250:r]RC
Position: 009-contig_245_RC: 76417 … 77941; Length: 1524 bp
Sequence:
TTAGTTTCTGATAATATAGTCAAAGGCATTTAATGCTGCATTTGCACCAGCGCCCATTGAA
ATAATAATTTGTTTGTTCTTCTGATCTGTGACATCGCCAGCAGCAAATATTCCAGGTACGT
CAGTATTATTGTTACGATCAATAACAATTTCACCACGTTCGTTTAATTCAACAGCATCTTT
TAACCATGATGTGTTTGGAAGTAAACCAATTTGAACAAAGATACCATCTAAGTTAAGTAG
ATGTTCTTCGCCGGTGCTCATGTCTTCGTAACGTATACCTGTAACATGGTCTTCTCCGACA
ACTTCAGTAGTTTTGGCATTTGTTTTGATATCAACATTTGATAAAGAACGTAAACGATCTT
GTAACACGTTGTCTGCTTTTAATTCGCTAGCGAATTCGAATAATGTAACATGATTAACGAT
ACCAGCAAGGTCAATTGCTGCTTCAACCCCAGAGTTACCGCCACCGATAACTGCTACGTC
TTTATTTTCAAATAGAGGTCCGTCACAGTGAGGGCAGAATGCAACACCTTTATTAATCAA
TTGCTCTTCACCTGGAATGTTTAGCTTACGCCAACCTGCACCAGTAGCAATAATGACTGTT
TTACTTTCTAAGACAGCACCGTTTTCTAACGTAACTTTAATTGCTTCGTCAGTCTTTTCGAT
ATCTGTAGCACGTATACCTGTCATTGCATCAATGTCATATTGATCAATGTGCGCTGCTAAG
TTAGAAGAAAATTCAGAACCAGTTGTTTCTTTAACAGTAATGAAGTTCTCAATACCAGCA
GTATCATTAACTTGGCCACCGATACGATCAGCAACTATACCAGTACGTAAACCTTTACGT
GCTGTGTAAATCGCTGCACTACCACTAGCAGGACCACCACCAACGATTAAGACATCATAA
GGTTCTTTATTTTCAAACTCAGATGCATCTGCCGTACTGCCTAGTTTCGAAAGAATATCTT
GGATTGTCATACGACCATTGCCAAATTCTTCGCCGTTTAAAAAGACAGCAGGGACTGCCA
TGATGTTTTCAGATTCTTCACGGAACACTGCACCATCAATCATAGAATGCGTGATGTTAG
GGTTGATCACACTCATTAAGTTAAGTGCTTGAACGACATCAGGACATTTTTGACACGTTA
AACTAATGAATGTTTCAAAATGGAATGAACCTTCTAAGTTTTTAATTTGGTCAATAATTGA
CTGTTTTTCTTTAGGTGCACGACCACTAACCTGTAAAATTGCTAAAACAAGTGAGTTAAA
CTCGTGACCTAATGGAATTCCTGCAAATGTTACACCTGTTTCTTCGCCAGGACGGTTGACT
GAGAAACTTGGTGTACGTTTTAAAGACTTTTCAGAAAGTGAAAGTCTAGGTGACATATCA
GAAATTTCTGTTAATAATTCTTTGAGTTCCTTAGATTTTTCATCTGAACCAAGGCTGGCAA
CGAATTCAACGTTGCCCTCCATTAGTTCTAATAATTGTTTAAGTTGTTGTTTTAAATCAGC
ATTAAGCAT

> **ahpC**
Function: protein coding sequence; alkyl hydroperoxide reductase subunit C (core genome, constant)
Best match: ahpC_CC001_MW2-USA400_BA000033.2[403621:404190:r]RC (completely identical)
Position: 009-contig_245_RC: 77956 … 78526; Length: 570 bp
Sequence:
TTAGATTTTACCTACTAAATCTAAACCAGGTTGCAATGTTTTAGCGCCTTCTTCCCATTTA
GCTGGGCATACTTCGCCAGGGTTTTTACGAACATATTGAGCTGCTTTGATTTTGTGAGCTA
ATGTACTAGCGTCACGGCCAATTCCGTCAGCGTTAATTTCAGATGCTTGTACAACACCGTC
TGGGTCGATAATGAATGTACCACGTTGAGCTAAACCAGTAGCTTCATCTAATACATCAAA
ATTACGAGTGATTGTTTGTGATGGGTCACCAATCATAGTGTAAGTGATTTTGCTAATTGCA
TCTGAATGGTCATGCCATGCTTTGTGTACGAAGTGAGTATCAGTTGATACTGAGAATACA
TTTACGCCTAATTTTTGTAATTCTTCATATTGGTTTTGTAAGTCTTCTAATTCAGTTGGACA
AACGAATGAGAAGTCAGCAGGATAGAAGCATACTACGCTCCAAGAACCTTTTAAATCTTC
TTGTGTAACTTCTTTAAATTGATCTTTTTTTGGATCGAAAGCTTGCGCTGTAAATGGTAAG
ATTTCTTTGTTAATTAATGACAT

> **nfrA**
Function: protein coding sequence; NADPH-dependent oxidoreductase (core genome, constant)
Best match: nfrA_CC008_NCTC8325_CP000253.1[372329:373084] (completely identical)
Position: 009-contig_245_RC: 79017 … 79773; Length: 756 bp
Sequence:
GTGTCAGAACATGTATATAATCTTGTGAAAAAGCATCATTCTGTTAGAAAATTTAAGAAT
AAACCTTTAAGTGAAGACGTTGTTAAGAAATTGGTAGAAGCTGGACAAAGCGCTTCGAC
GTCAAGTTTCCTGCAAGCATACTCAATTATTGGTATCGACGATGAGAAGATTAAAGAAAA
TTTACGAGAAGTTTCTGGACAACCTTATGTTGTAGAAAATGGCTATTTATTCGTCTTTGTT
ATTGATTATTATCGTCATCATTTAGTTGATCAACATGCTGAAACTGATATGGAAAATGCAT
ATGGTTCAACGGAAGGTTTGCTAGTAGGTGCAATCGATGCAGCATTAGTTGCCGAAAATA
TTGCGGTAACTGCTGAAGATATGGGGTATGGCATTGTCTTTTTAGGATCATTAAGAAATG
ATGTTGAACGCGTTCGAGAAATTTTAGACTTACCTGACTATGTCTTCCCGGTATTTGGTAT
GGCAGTAGGGGAACCCGCAGATGACGAAAATGGTGCAGCCAAGCCACGCTTACCATTTG
ACCATGTCTTCCATCATAATAAGTATCATGCTGATAAGGAAACACAGTATGCACAAATGG
CAGATTACGACCAGACAATCAGCGAGTACTATGATCAACGTACAAACGGGAATCGCAAA
GAAACATGGTCGCAGCAAATTGAGATGTTCCTAGGAAACAAAGCAAGATTAGATATGTT
AGAACAATTGCAAAAATCAGGCTTAATACAGCGATAG

> **A6TYN5**
Function: protein coding sequence; putative transporter (core genome, constant)
Best match: A6TYN5_CC008_NCTC8325_CP000253.1[373165:374553:r]RC (completely identical)
Position: 009-contig_245_RC: 79853 … 81242; Length: 1389 bp
Sequence:
TTAGTGTGAAGTTAATGCAGCATTATCATTTGAATCGAAAGTATCTTTATCCCAATGTTTA
GTTAACTTGGCGGTACCTGTACCAGCTAGCATTGAATCGTTCACGTTTAATGCTGTTCTAC
CCATGTCAATCAATGGTTCAACGGAGATGAGCACGCCGGCTAAAGCGACTGGCAAGTTTA
ACGTTGACAACACCAATATGGATGCAAATGTAGCCCCGCCACCGACGCCAGCAACGCCG
AATGAACTAATAATCACGACAGCGATTAACGTTACAATAAATTGTAAATCAATTTCTACA
TTAGCGACGGGTGCGACCATAATTGCAAGCATGGCAGGGTAAATGCCTGCACAACCATTT
TGTCCAATCGACAATCCAAATGTCGCAGCGAAATTGGCAATACCTTCTGGCACGCCTAGA
CGTCTTGTTTGTGTTTGTACATTCAATGGTAAGGCACCCGCGCTTGAGCGTGATGTGAATG
CAAAGATTAATACTTCCAAAGTCTTTTTAACATAGCGAATTGGGCTAATACCTAACAGGC
TTAAAATAATTAAGTGAATGATATACATCGTAATTAATGCAGCGTACGATGCGATTAAGA
ATTTTCCTAAAGTCCAAATGGCGCCAAAGTCACTTGTCGATAATGTGTTGGCCATAATTGC
TAATACACCGTATGGCGTTAAACGTAAGACGAACGTCACAATCGCCATTACTAGTGAATA
GATAGCGTCAATCGCACGCTTAAGCAATTCACCATGATCAGGTTGTTTGCGTGCTACGCG
TAAATAAGCAAATCCTATAAACGAAGCAAATATCACGACAGCAATCGTGGAAGTTGCAC
GTTGTCCAGTGAAATCTAAGAATGGATTTTTAGGCAATAATTCCAAAATTTGTTGTGGTA

ACGTATGTGCTGTTAAATCTTTCGCTTGTTTAGCAATTTCGCTTCCACGTGCTTGTTCAGCG
TTACCAAGGTTAATTGTTGATGCATCTAAACCAAACACCAAGGCATACACAACACCAACA
ATCGCAGCAATGGTGACAGTGCCAATTAAAAAGATAAAAATGAGACTACCAATTTTAGC
AAACTTTTCTCCGATTTGAATTTTAGTGAATGCAGCTACAATAGAAATGAAAATTAAAGG
CATAACAATCATTTGCAACAATGCAACGTAACCTTGTCCGACAATGTTGAACCAGTCACT
TGTTGATGTAATAACATTCGAATGTGTGCCATAAATAAGATGCAATAACACACCGAATAC
TATACCAATCCCTAAAGCTGTAAACACACGTTTCGCAAAAGATATATGTTTGCGAGCCAT
CATGTGCAATATTACGATGAAAATCACCAATACAATAATATTAATCAGTGTAAGAAAAGC
ATTCAT

> **yflT**
Function: protein coding sequence; heat induced stress protein (core genome, variable)
Best match: yflT_CC008_NCTC8325_CP000253.1[377409:377816:r]RC (completely identical)
Position: 009-contig_245_RC: 84097 … 84505; Length: 408 bp
Sequence:
CTAAGCTTTGGGACCTTTAGATGCTTCAGCAAAATGTGTAATATCAATCTCTTCATAAGCT
GAATTATTTTCATGCACTTCTTGATGTGATGATTTGTCACGAACCGCTACAACTAACATTT
TATCGTCTAAAATAAGTTGTTTATATTTTTCTAATTCATCAGGCGCTAAGTTGTAGCGTGA
TAAAACTGCATGTTCACCATCTTCTCCTGTTAACAGTTTAGTCATTCTATCACTAAATGTT
CCACTTGTTGAGATAAGGGAGATTTCAGAGTCGTGTAAGTCATTTAGGTGTAATTTACTTT
TACTAATAATTGTTAGCTCTGATTCTAAATAACCTTCAGATTTCTTTTGATTGATTACGTTG
TATAATTCGCCAGTGTCATTTACTACAGTAATATCTGCCAT

> **RF00167**
Function: noncoding RNA purine riboswitch (core genome, constant)
Best match: RF00167_CC001_MW2-USA400_BA000033.2[410546:410648] (completely identical)
Position: 009-contig_245_RC: 84684 … 84787; Length: 103 bp
Sequence:
GTTAAATAATTTACATAAACTCATATAATCTAAAGAATATGGCTTTAGAAGTTTCTACCAT
GTTGCCTTGAACGACATGACTATGAGTAACAACACAATACTA

> **xpt**
Function: protein coding sequence; xanthine phosphoribosyltransferase (core genome, constant)
Best match: xpt_CC001_MW2-USA400_BA000033.2[410879:411457]
Position: 009-contig_245_RC: 85017 … 85596; Length: 579 bp
Sequence:
GTGGAGTTACTAGGACAAAAAGTAAAGGAAGACGGCGTTGTCATTGATGAGAAGATTTT
AAAAGTCGATGGATTTTTAAATCATCAAATTGATGCAAAGTTAATGAATGAAGTTGGTCG
CACTTTTTACGAGCAATTTAAAGATAAAGGGATTACTAAAATCTTAACCATTGAAGCTTC
CGGTATCGCACCTGCAATCATGGCTGCACTGCATTTTGATGTGCCATGTTTATTTGCGAAA
AAAGCAAAACCTAGCACTTTGACGGATGGTTATTATGAAACATCTATTCATTCATTTACTA
AAAATAAAACAAGTACGGTCATTGTTTCAAAAGAGTTTTTATCAGAAGAAGATACTGTAC
TTATCATCGATGACTTTGTAGCAAATGGTGATGCTTCATTAGGATTATACGATATCGCACA
GCAAGCGAATGCTAAGACAGCTGGTATTGGTATTGTTGTTGAAAAGAGTTTCCAAAATGG
GCATCAACGTTTAGAAGAAGCAGGTTTAACAGTTTCTTCTCTCTGCAAGGTTGCTTCACTA
GAAGGAAACAAAGTGACATTGGTGGGAGAAGAATAA

> **pbuX**
Function: protein coding sequence; xanthine permease (core genome, constant)
Best match: pbuX_CC007_TCH959-USA300_AASB02000040.1[87381:88649]
Position: 009-contig_245_RC: 85595 … 86864; Length: 1269 bp
Sequence:
ATGAAAAATTTAATCCTAAGTGTTCAACATCTTTTAGCTATGTACGCAGGTGCTATCTTAG
TTCCAATCATTGTTGGTACAAGTTTGAAGTTTACACCTGAACAAATCGCTTACTTAGTTAC
AGTAGATATATTTATGTGTGGGGTTGCCACATTTTTACAAGCCAATAAAGTAACAGGAAC

AGGATTACCAATCGTTCTTGGATGTACATTCACGGCTGTTGCGCCCATGATTTTAATTGGT
CAAACGAAAGGAATAGATGTACTTTATGGTTCGCTATTTTTATCAGGGATATTAGTTATTA
TCATCGCGCCTTTCTTTTCACATCTTGTAAAATTCTTCCCACCAGTAGTAACGGGTAGTGT
TGTTACTATCATTGGTATCAATTTAATGCCAGTAGCAATGAATTACTTAGCTGGAGGTCAA
GGTGCAAAGGACTATGGAGATGTTAAGAACATTTTGTTAGGTTTAATGACATTAATCATT
ATTCTTGTTTTACAAAGATTCACAACTGGATTTATTAAGAGTATTGCCATATTAATTGGAC
TCGTTTTAGGAACGATAGGTGCTGGCTTACTTGGGATGGTCGATATTAATCAAGTCAATC
ATGCCGGTTGGTTAGGCATCCCAGTGCCGTTTAGATTCTCAGGATTTAGCTTTGATGTGAC
ATCGACGTTAGTGTTCTTTATTGTAGCTATCGTTAGTTTAATTGAGTCGACAGGTGTCTAT
CATGCGTTAAGTGAAATTACCGGTAAGAAGTTAGAAAGAAAAGATTTTCGTAAAGGTTAT
ACTGCGGAAGGTCTAGCGATAGTGTTAGGTTCTATATTCAATTCATTTCCGTATACAGCCT
ATTCGCAAAATGTAGGACTTGTTTCTTTATCCGGCGCTAAGAAAAACAATGTTATATACG
GCATGGTCGTGTTATTACTTATATGTGGTTGTATACCTAAGCTTGGCGCATTAGCAAATAT
CATACCGCTACCTGTGTTAGGCGGTGCGATGATAGCTATGTTTGGCATGGTAATGGCATA
TGGTGTTAGTATATTAGGACATATCGATTTTAAAAATCAAAACAATTTATTAATTATCGCT
GTATCAGTAGGATTAGGTACTGGTATAAGCGCTGTACCACAAGCATTTAAAGGTTTAGGT
GAACAATTTGCATGGTTGACTCAAAACGGAATTGTTTTAGGCGCAATCTCTGCAATTATTC
TTAATTTCTTTTTAATGGAATAAAGTATAAACAAACGGAAGAAAATGTGAAATAA

> **guaB**

Function: protein coding sequence; inosine-5'-monophosphate dehydrogenase (core genome, constant)
Best match: guaB_CC009_21334_AGTW01000029.1[87100:88566]
Position: 009-contig_245_RC: 86901 … 88368; Length: 1467 bp
Sequence:
ATGTGGGAAAGTAAATTTGCAAAAGAATCATTAACGTTTGATGATGTGTTATTAATTCCA
GCACAATCTGATATTTTACCGAAAGACGTTGATTTAAGCGTACAATTATCAGACAAAGTT
AAATTAAATATTCCAGTTATTTCTGCTGGTATGGATACTGTAACTGAATCTAAAATGGCG
ATTGCTATGGCTCGTCAAGGTGGTTTAGGTGTTATTCATAAAAATATGGGCGTTGAAGAA
CAAGCGGACGAAGTTCAAAAAGTAAAACGCTCAGAAAATGGTGTCATTTCAAACCCATTT
TTCTTAACGCCAGAAGAAAGCGTTTATGAAGCAGAAGCATTAATGGGTAAATACCGTATT
TCAGGTGTACCAATTGTTGATAATAAAGAAGATCGCAACTTAGTAGGTATTTTAACAAAC
CGTGACTTACGTTTTATTGAAGACTTCTCGATTAAAATTGTAGATGTAATGACGCAAGAA
AATTTAATTACAGCTCCAGTGAATACAACACTTGAAGAAGCAGAAAAAATTCTCCAAAA
ACATAAGATTGAAAAGTTACCATTAGTTAAAGACGGACGTCTAGAAGGTCTTATTACTAT
TAAAGATATTGAAAAAGTTATCGAATTCCCTAATGCAGCAAAAGATGAACATGGTCGTCT
ACTTGTAGCCGCAGCAATTGGTATTTCAAAAGATACTGATATTCGTGCTCAAAAATTAGT
CGAAGCAGGTGTGGATGTCTTAGTTATCGATACAGCACATGGTCACTCTAAAGGCGTTAT
CGATCAAGTGAAACATATTAAGAAGACTTACCCAGAAATCACATTAGTAGCTGGTAACGT
AGCAACTGCAGAAGCAACAAAAGATTTATTTGAAGCGGGTGCAGATATTGTTAAAGTTG
GTATTGGCCCAGGTTCAATTTGTACGACGCGTGTTGTAGCAGGTGTTGGTGTACCACAAA
TTACAGCAATTTATGATTGTGCAACTGAAGCACGCAAACATGGTAAAGCTATCATTGCTG
ATGGCGGTATTAAATTCTCAGGAGATATCATTAAAGCATTAGCTGCTGGTGGACATGCGG
TTATGTTAGGTAGCTTATTAGCAGGTACTGAAGAAAGTCCAGGTGCTACAGAAATTTTCC
AAGGTAGACAATACAAAGTATATCGCGGTATGGGTTCTTTAGGTGCGATGGAAAAAGGTT
CAAACGACCGTTACTTCCAAGAAGACAAAGCGCCTAAGAAATTTGTACCTGAAGGTATCG
AAGGACGTACAGCATATAAAGGTGCTTTACAAGATACAATTTACCAATTAATGGGCGGA
GTGCGTGCTGGTATGGGTTATACTGGTTCACACGATTTAAGAGAATTACGCGAAGAAGCA
CAATTCACACGTATGGGTCCTGCTGGTTTAGCAGAAAGCCACCCACATAATATTCAAATT
ACGAAAGAATCACCGAACTACTCATTCTAA

> **guaA**

Function: protein coding sequence; GMP synthase glutamine-hydrolyzing (core genome, variable)
Best match: guaA_CC001-ST772_118_AJGE01000018.1[21349:22890:r] (completely identical)
Position: 009-contig_245_RC: 88392 … 89934; Length: 1542 bp
Sequence:

ATGGAAATGGCAAAAGAACAAGAGTTAATCCTTGTCTTAGACTTTGGTAGCCAATACAAC
CAATTAATTACACGCCGAATTCGTGAAATGGGCGTTTATAGTGAATTACACGATCATGAA
ATTTCAATTGAAGAAATTAAGAAAATGAATCCAAAAGGTATTATCTTATCAGGTGGTCCA
AATTCAGTTTATGAAGAAGGTTCATTTACAATTGATCCGGAAATATATAATTTAGGAATT
CCAGTACTTGGTATTTGTTACGGCATGCAATTAACTACTAAATTATTAGGTGGTAAAGTTG
AACGTGCCAATGAACGTGAATACGGTAAAGCAATCATTAATGCGAAGTCAGATGAGTTA
TTCGCTGGCTTACCAGCAGAACAAACTGTTTGGATGAGTCATTCTGATAAAGTTATTGAA
ATTCCAGAAGGCTTTGAAGTTATCGCTGATAGCCCAAGTACAGACTATGCAGCAATCGAA
GATAAGAAACGTCGCATTTATGGTGTTCAATTCCATCCAGAAGTACGTCATACAGAATAT
GGTAATGATTTATTAAATAATTTTGTCCGTCGTGTTTGTGATTGTAAAGGTCAATGGACAA
TGGAAAACTTTATCGAAATCGAAATTGAAAAGATTCGTCAACGAGTAGGAGACCGTCGT
GTATTATGTGCGATGAGTGGCGGCGTAGATTCATCTGTTGTTGCTGTATTATTGCATAAAG
CAATTGGGGATCAACTAACATGTATCTTTGTAGACCATGGCTTACTTCGTAAAGGTGAAG
GCGACATGGTTATGGAACAATTCGGTGAAGGGTTCAACATGAATATTATTCGTGTTAATG
CGAAAGATCGTTTTATGAATAAATTAAAAGGTGTTTCAGATCCTGAACAAAAACGTAAAA
TCATTGGTAATGAATTTGTATATGTATTTGATGATGAAGCATCAAAACTGAAAGGTGTAG
ACTTCCTTGCGCAAGGAACACTTTATACAGACGTTATCGAATCAGGTACCAAGACAGCAC
AAACAATCAAATCACATCACAATGTTGGTGGATTACCAGAAGACATGGAATTCGAATTAA
TCGAACCAATCAATACATTGTTTAAAGATGAGGTACGTAAATTAGGTATTGAATTAGGTA
TTCCAGAACATTTAGTATGGAGACAACCATTCCCAGGACCTGGTCTTGGTATTCGTGTACT
TGGAGAAATTACTGAAGATAAACTAGAAATCGTTAGAGAATCAGACGCAATTTTACGCC
AAGTGATTAGAGAAGAAGGTCTTGAAAGAGAAATTTGGCAATACTTCACAGTGTTACCA
AACATTCAATCAGTAGGTGTTATGGGAGACTACCGTACGTATGATCACACAGTAGGTATC
CGTGCAGTAACATCTATCGACGGCATGACAAGTGATTTTGCACGCATCGATTGGGAAGTC
TTACAAAAGATTTCAAGTCGTATCGTAAACGAAGTAGACCACGTTAACCGCGTAGTCTAT
GACATTACATCAAAACCACCAAGCACAATTGAGTGGGAATAA

> **attB_nySa2**
Function: attachment site for int8-tetM (mobile element)
Best match: attB_nySa2_trunc_CC001_MW2-USA400_BA000033.2[415744:415804:r]RC
Position: 009-contig_245_RC: 89882 … 89943; Length: 61 bp
Sequence:
CGTAGTCTATGACATTACATCAAAACCACCAAGCACAATTGAGTGGGAATAATTATATAT
A

> **tx_guaA_int7**
Function: rho independent terminator of guaA
Best match: tx_guaA_int7_CC001_ATCC51811-ST1_ADVP01000041.1[24733:24786:r] (completely identical)
Position: 009-contig_245_RC: 89938 … 89992; Length: 54 bp
Sequence:
ATATAGCAAATGATAGGCTGGAGTTACCGTTACTATGCGGTTTCCAGCCTTTTT

> **Q8NY68**
Function: protein coding sequence; putative protein (genomic island)
Best match: Q8NY68_ref_CC001_MW2-USA400_BA000033.2[416307:417413] (completely identical)
Position: 009-contig_245_RC: 90445 … 91552; Length: 1107 bp
Sequence:
ATGGATAAATCACTTGAAATTAACTTAAAAGAATTAGAAGGCATAATGTATAAAGAAAA
AGTAATCGACGTTAAAGAGTATGATAATTTAAAAGTAACTTTTGAGACACTAAACGATGC
TCCTAAAAGTGAAATACCCACTCCCTTTACAATAGAACAAATGTTATATAAATTAGAAAC
TAAAAATTTATATTTTCCTGAAATTAAGTCAAAAGAAAAATGTGTGGAGCTTTTACATAG
TGTTGGCTATTATAATTTGAAGCATTTTATGTATAATGAATTTGGTAAAGATGAGCCGAA
AAATTTTGATGAAGTATATATACTTTATAAATTTGATAGATATTTATTGAAACAATTATTT

AGTTTAGTAAATATGTTGGAAACTCATATAAGAAATATTATTTTAGAAGTATATATGTTA
GAAATTGAGTATAATAACAAGCCTTCGACACTGTTCTATTTAGATAAAGATTTATATTTTG
AGAAAGTTAATGATGAGTATAAATATTCTGCCAAAAAGCTAAAAGAGTTCAATAGGTTAC
AAAATGTTTTTTGGAGAGCTATAGAAAAAAAGAAAAGTAACGATAACGTTAAACATAAT
ATTAAAAAATACAATATTATACCAGCTTGGGTTTTATTCCAAAATTTTAGTTTTGGAGATT
TGTCTACTTTTTATAGAACTACATTACCAACTTATAGAAATAAAGTAAGTAAGCGAATTG
AAAATTTAATAGAAAAAAATACTGGTATAAGCATAAAACTTCCAGAAAAGTTATTGTGTG
CATGGTTGAATAGTATCAGGTTTTTGAGAAATAGAATAGCTCACACAGATATAATTTATG
GAATTAATTTTACAAACACCTGTGCAAAACATCATAGTGATGAGGAAATGTATGTAAATA
TAGAAAAGTATAAATATCAGCAAAGGTTAGTTACATTTTTATTAGCTATGAAGAAAATAT
TTATGAGTATGCCAGAAAATAATATTATTGAATGGAATGAGACTCTAACTAAGATAGAGA
ATAAATGTTCAGAACATAACTTTATTAAACTTTCTAGGTTAGGAGTTATTGAAAATAACCT
TTCTTACTTTAAAATAACTAAATAA

> **Q6GC78**
Function: protein coding sequence; putative protein (genomic island)
Best match: Q6GC78_CC009_21334_AGTW01000029.1[92374:92766:r]RC (completely identical)
Position: 009-contig_245_RC: 92176 … 92569; Length: 393 bp
Sequence:
TTAAAAATCTATACTATTTATATTGAAATTTTTATTTATGGTTATGCCATAACTACTTTGTA
TTTCCGGTACTAAACTTTGCAATATTGGGTAATAGTTATTAGCGAAACCAATAATAATGG
AATTAATCACATTGATACTAAGTTCTTTATCTTCAGGTAATGCATTTTCTATTATTTTGATT
AAGTCTATATATTTTTATTTCATCAGAATTTTTATGTGCTAAATACTTCATAAAATCAGTTT
GCACTTTTTCGAAAACGCTATATCTTAAGTCCATTTGTACAAATATTAAGCTGACAGTTTT
AGTTTCGAATTGACTTTGACCCAAAACTGAAGCAAGTTCTTCTATTGATTGATATAGCAAT
CTGATATCGCCGCTATCTTCAAGCAT

> **Q6GC73**
Function: protein coding sequence; putative protein (genomic island)
Best match: Q6GC73_CC009_21334_AGTW01000029.1[93820:94242:r]RC (completely identical)
Position: 009-contig_245_RC: 93621 … 94044; Length: 423 bp
Sequence:
TTAGTTAATATATTTAATTGCTAACTTATTATTAGGGTCTCTTTCAAGAATTTTATTTGCGA
CCAATTGAATATTCTCATGTGAAATCATATCAAAGTCTGGTTCTCCAAGGATTTCTAAAAA
TTCGTATAATAAATTAGCATTATTAAATTGCGAAATCTCTAACGATTGCCAATAATGATA
GTATGCTAAATCATAGGCATTATCTATGTAATTGAAATGAAAGTGATAGATGCTTGCGAC
CATTTCATGATATTCAATTGTATTTATTTGATTCGCAAGATTTGATAAATATGATACTAAT
TTTTCATCTGAAATTTCGTTCGACATAAAGTATGTTAGAATATTCTCCATATCTTTATTCTC
TAACATATTTTTTAATTCAGTTGGTAATAAATTTAATAGAATGTTAACGCTTTTCAT

> **Q8NY63**
Function: protein coding sequence; putative protein (genomic island)
Best match: Q8NY63_ref_CC001_MW2-USA400_BA000033.2[419858:420190] (completely identical)
Position: 009-contig_245_RC: 97533 … 97866; Length: 333 bp
Sequence:
GTGGCAACACTAAAAGATGTAAAGACTGGTAAAAAGGAAATTTTGCCATCAACTGCAAA
AGATAAAAATGGTAAAAATGTTACGTTAATTTATTTTGAAAAAGACGGTAAATTAGGTTT
TTATGTGCAGAAAACACAAAAAGAACGTGGAGTAGGGAAATGCGTTTCTGGTATAGCGG
GTGGCGCAGTGACAGGAGGCACTACTTTAGGTCTTGCAGGTGCAGGAGTAGGAACAGTT
ACTATTCCAGTAATTGGGACAGTTAGTGGAGGCGTAGTTGGAGCTGTTGGTGGTGCTGTC
GGCGGTGGTCTAACCGGTGGAGCCACATTCTGCTAA

> **Q2YVN4**
Function: protein coding sequence; putative protein (core genome, constant)

Best match: Q2YVN4_CC008-ST72_21259_AFTS01000010.1[65761:66347]RC
Position: 009-contig_245_RC: 98465 … 99052; Length: 587 bp
Sequence:
TTACTTAAGCTTGTAAAATGCGGAAATAAGTTTTCCTTTTTTAGTTATTTTTTCTTGGTATA
TTTTAAATCCACAATTATTGCAATAAAATTCTTTCAAATTCTTATTATCATACCAATCAAC
ACAAACATGTCTTAGTGCAGTACGATCATAAATTTCAGAAGCAATAGATAAGGCATAGTC
CATTACAATTTCTCCTGGTATATGCTTTTTGTGTTTATCGCTCCTACCTAATTTAGTTATTA
ATAATGCTTGAAATTCTCCATTCTTTTTAGCATCAGACGAGATTTTTCTTTTTTCGTTTTTC
CACTAACGTCTTTTTCAAGCTTTATATTTACAATTTTTAAGAAGAAGTATCCCATTATTTC
AAGGTCTGATTTATCTATAATTAAATACGTACGTAAGGAATTTTGATCGTTCATTGTTATT
GCTTTATGTTTTAGGGAATGTGCTATTGAAGGGTTTAAAGATGTTTGAAATGTCTTGGTTT
CTTGACTTATATAGTTATGAAAGAGGGCGTTGTCCTTTATAGCATTTCTGAGATTAGAAAT
GCTATAAATAAAGTGATTAAGATTTTTTGTTTTCAT

## > Q5HIQ1

Function: protein coding sequence; putative membrane protein (core genome, variable)
Best match: Q5HIQ1_CC008_NCTC8325_CP000253.1[386599:386958:r]RC (completely identical)
Position: 009-contig_245_RC: 99708 … 100068; Length: 360 bp
Sequence:
TTACCAAAGCATTGATATGAGTGCCAATACTGGCAATGTGCCTTGTTTGAAAAAGATACT
AATATTGCTTGAAAGGCCACCATAAATAGCAACGCCAATGATATACACTAAAATAGCTGC
GCATATTTCTTTTGGATTACTGCTGATAAACAAACCGTATATTAGCAAAACTCCGATTAAA
CCGTTATATACGCCTTGGTTCTTCAAAAGTAGGTTAATATTTTTGTCTTTCAATTTATCGAC
GCTTATATTAAATGTCTCGCTAGTCTTTTTGGAAGTTGTAGCAATCGTTTCAAGGTACATA
ATATAGAAAAACTCTAATGCCACAAATATGATTAAAATTGTTGAGATGATATTCAC

## > Q2G0Y0

Function: protein coding sequence; putative protein (core genome, variable)
Best match: Q2G0Y0_CC001-ST772_118_AJGE01000018.1[10351:11196]RC (completely identical)
Position: 009-contig_245_RC: 100086 … 100932; Length: 846 bp
Sequence:
TTAAATATTTTCTTGTAAAAATGATTGCAGTGTCTGTGGTTGATCATTGACTAATTGTTGG
AAATCATTGGATTCTTGGTCTAATAGTCCTCTTGCTCCTGCGTCGTACATTGATGCCAATA
ATGCACCAAAGCCTTTAGGTTCATCGTACATTTCTGCAAATGTCTCTAATGAAACGGGCTC
ATATTTAATTTCTGTGCCTGATGCCTCAGATAAAATTGCAGCAAGTTCTTTCATATCATAA
CTGTAGCCTGATAATAAGTAGCGTTTGCCCCAAGTATCTGGATTTTTAATAATAGCAATG
ACACCTCTAGCAATATCATTTCTAGTAATATAATTAATACGACCATCGCCAGCTGGATAA
ATCAGTTTATGCATATTCATCAATTCTGGTAAATATGGTTTAAGTGGATCCATGTACATTG
CCATTCTTACATACGTATAGTCAATGCCACTTGTTGCCAATAGACGTGCTGCATAACCAA
AATAAGGACTCATATGGAATGGATTATTATGCTGATCTGCGTAATAACCTATGAAAATGA
TATGAGCAACGCCGCTCTGCTTTGCCGCATATACTAAATTTTCCACTTCAGGAATACGTTT
GAATGATGGATGGATAATACTTGGAATAAACACAACGGTATCCATTCCTTTAAATGCTTC
TACCATGCTTTCTTGATTAAAATAATCTAATTGTCGAACAGGAACTTTTCCGCGCCAATCT
TCTGGAACTTTCTCAACATTTCTAACACCAATGTGAAAATGATCTATGTGATTTGCAATGG
CTTGATTTGTAATATGTGTGCCTAAATGACCTGTAGCACCTGTTAACATAATATTCAT

## > ssl01

Function: protein coding sequence; staph superantigene like protein locus 1 (genomic island)
Best match: ssl01-COL_CC001-ST772_118_AJGE01000018.1[9199:9879:r] (completely identical)
Position: 009-contig_245_RC: 101403 … 102084; Length: 681 bp
Sequence:
ATGAAATTTAAAGCGATAGCAAAAGCAAGTTTAGCATTGGGAATGTTAGCAACAGGTGT
AATTACATCGAATGTACAATCAGTACAAGCGAAAGCAGAAGTTAAACAACAAAGTGAAT
CAGAGTTAAAACACTATTATAATAAACCAATTTTAGAGCGTAAAAATGTGACTGGATTTA
AATATACTGATGAGGGTAAACACTATTTAGAAGTCACAGTAGGGCAACAGCATTCTCGAA

TCACTTTACTTGGATCTGATAAAGATAAATTTAAAGACGGAGAAAACTCAAATATAGATG
TGTTTATCCTTAGAGAAGGTGACAGTAGACAAGCAACAAATTACTCAATTGGTGGCGTTA
CAAAATCAAATAGTGTGCAGTATATTGATTATATCAATACGCCAATTTTAGAAATCAAGA
AAGATAATGAAGATGTACTTAAAGATTTTTACTACATTTCAAAAGAAGACATCTCATTAA
AAGAACTTGATTATAGATTAAGAGAACGTGCGATTAAACAACACGGCTTGTATTCAAATG
GTCTTAAACAAGGTCAAATTACAATTACAATGAATGATGGCACAACACATACAATCGATT
TAAGTCAAAAACTTGAAAAAGAACGTATGGGTGAGTCAATCGACGGCACTAAGATTAAT
AAAATTCTAGTAGAAATCAAATAA

> **ssl02**
Function: protein coding sequence; staph superantigene like protein locus 2 (genomic island)
Best match: ssl02_CC001-ST772_118_AJGE01000018.1[8218:8913:r] (completely identical)
Position: 009-contig_245_RC: 102369 … 103065; Length: 696 bp
Sequence:
ATGAAAATGAAATCAATTGCAAAAATAAGTTTGTTATTAGGAATATTAGCAACAGGTGTA
AACACTACAACGGAAAAACCAGTTCATGCCGAAAAGAAACCTATTGTAATAAGTGAAAA
TAGCAAAAAATTAAAAGCTTATTATAATCAACCTAGTATTGAATATAAAAATGTGACAGG
TTATATCAGTTTCATTCAACCAAGTATTAAATTTATGAATATCATAGATGGTAATTCTGTT
AATAATATTGCTTTAATTGGCAAAGATAAGCAACATTATCATACGGGTGTACATCGTAAT
CTTAATATATTTTACGTTAATGAGGATAAGAGATTTGAAGGTGCAAAGTACTCTATTGGG
GGTATCACGAGTGCAAACGATAAAGCTGTCGACCTAATAGCAGAAGCAAGAGTTATTAA
AGAAGATCATACTGGTGAATATGATTATGACTTTTTCCCATTTAAAATAGATAAAGAAGC
GATGTCATTGAAAGAGATTGATTTTAAATTAAGAAAATACCTTATTGATAATTATGGTCTT
TACGGTGAAATGAGTACAGGAAAAATTACAGTCAAAAAGAAATACTATGGAAAGTATAC
ATTTGAATTGGATAAAAAGTTACAAGAAGACCGTATGTCCGATGTTATCAATGTCACAGA
TATTGATAGAATTGAAATCAAAGTTATAAAAGCATAA

> **ssl03**
Function: protein coding sequence; staph superantigene like protein locus 3 (genomic island)
Best match: ssl03_CC001-ST772_118_AJGE01000018.1[6818:7927:r] (completely identical)
Position: 009-contig_245_RC: 103355 … 104465; Length: 1110 bp
Sequence:
ATGAAAATGAGAACAATTGCTAAAACCAGTTTAGCACTAGGGCTTTTAACAACAGGCGC
AATTACAGTAACGACGCAATCGGTCAAAGCAGAAAAAGTACAATCAACTATAGTTGACA
AAGTACCAACGCTTAAAGCAGAGCGATCAGCAATGATAAACATAACAACAGGTGCAAAT
ACAGGGGCAACGCAAGCAGCTAACACAAGACAAGAACGCATGCCTAAACTCGAAAAGG
CACCAAATACTAATGAGGAAAAAACTTCAATTTCCAAAATAGAAAAAATATCACAACCT
AAACAAGAAGCGCAGAAATCGCTTAATATATCCACAACGCCAGCGCCTAAACAAGAACA
TTCACAAACGACAACCGAATCCACAACGCAGCAAACTAAAGTGACAACACCTCCATCAA
CAAACACGCAGCAAACTAAAGTGACAACACCTCCATCAACAAACACGCCACAACCAATG
CAATCTACTAAATCAGACACACCACAATCTCCAACCATAAAACAAGCACAAACAGATAT
GACTCCTAAATATGAAGATTTAAGAGCGTATTACACGAAACCGAGTTTTGAATTTGAAAA
GCAGTTTGGATTTATGCTCAAACCATGGACGACGGTTAGGTTTATGAATGTTATTCCAAAT
AGGTTCATCTATAAAATAGCTTTAGTTGGAAAAGATGAGAAAAAATATAAAGATGGACC
TTACGATAATATCGATGTATTTATCGTTTTAGAAGACAATAAATATCAATTAAAAAAATA
TTCTGTCGGTGGCATCACGAAGACTAATAGTAAAAAAGTTGATCACAAAGCAGAATTAA
GCGTTACTAAAAAAGATAATCAAGGTATGATTTCACATGATGTTTCAGAATACATGATTA
CTAAGGAAGAGATTTCCTTGAAAGAGCTTGATTTTAAATTGAGAAAGCAACTCATTGAAA
AACATAATCTTTACGGTAACATGGGTTCAGGAACAATCGTTATTAAAATGAAAAACGGTG
GGAAGTATACGTTTGAATTACACAAAAAACTGCAAGAGCATCGTATGGCAGATGTCATA
GAAGGTACAAACATTGATAAAATTGAAGTGAATATAAAATAA

> **ssl04**
Function: protein coding sequence; staph superantigene-like protein locus 4 (genomic island)

Best match: ssl04-USA300_delta_CC001-ST772_118_AJGE01000018.1[5551:6453:r] (completely identical)
Position: 009-contig_245_RC: 104829 … 105732; Length: 903 bp
Sequence:
ATGAAAATAACAACGATTGCTAAAACAAGTTTAGCACTAGGCCTTTTAACAACAGGTGTA
ATCACAACGACAACGCAAGCAGCAAAAGCGTCAACACCATCTTCCACTAAAGTGGAAGC
ACCACAATCAACACCGCCCTCAACTAAAGTAGAAGCACCGCAATCAAAACCAAACGCGA
CAACACCATCTTCCACTAAAGTGGAAGCACCGCAACAAACAGCAAATGCGACAACACCT
CCATCAACAAACACGCCACAACCAATGCAATCTACTAAATCAGACACACCACAATCGCC
AACCACAAAACAAGTACCAACAGAAATAAATCCTAAATTTAAAGATTTAAGAGCGTATT
ATACGAAACCAAGTTTAGAATTTAAAAATGAAATTGGTATTATTTTAAAAAAATGGACGA
CAATAAGATTTATGAATATTGTTCCAGATTATTTCATATATAAAATTGCTTTAGTTGGTAA
AGACGATAAAAAATATGGTGAAGGAGTACATAGGAATGTCGATGTATTTGTCGTTTTAGA
AGAAAATAATTACAATCTGGAAAAATATTCTGTCGGTGGTATCACAAAGAGTAATAGTAA
AAAAGTTGATCACAAAGCAGGAGTAAGAATTACTAAGGAAGATAATAAAGGTATAATCT
CTCATGATGTTTCAGAATTCAAGATTACTAAAGAGCAGATTTCCTTGAAAGAACTTGATTT
TAAATTGAGAAAACAACTTATTGAAAAAAATAATCTGTACGGTAACGTTGGTTCAGGTAA
AATTGTTATTAAAATGAAAAACGGTGGAAAGTACACGTTTGAATTGCACAAAAAATTACA
AGAAAATCGCATGGCAGATGTCATAGATGGCACTAATATTGATAACATTGAAGTGAATAT
AAAATAA

> **ssl05**

Function: protein coding sequence; staph superantigene like protein locus 5 (genomic island)
Best match: ssl05_CC001-ST772_118_AJGE01000018.1[4483:5187:r] (completely identical)
Position: 009-contig_245_RC: 106095 … 106800; Length: 705 bp
Sequence:
ATGAAAATGACAGCAATTGCGAAAGCAAGTTTAGCACTAGGTATTTTAGCAACAGGAAC
AATAACATCAACGCATCAAACTGTAAATGCGAGTGAACATGAAGCAAAATATGAAAATG
TGACAAAAGATATTTTTGACTTAAGAGATTACTATAGTGGCGCAAGTAAGGAACTTAAAA
ATGTTACTGGTTATCGTTATAGCAAAGGTGGCAAGCATTACCTTATCTTTGATAAAAATA
GAAAATTCACAAGAGTACAGATATTTGGTAAAGATATTGAAAGATTTAAAGCACGCAAA
AATCCGGGATTAGACATATTTGTTGTTAAAGAAGCGGAAAACCGTAATGGCACAGTGTTT
TCATATGGTGGTGTCACTAAGAAAAATCAAGACGCTTATTATGATTATATAAACGCACCA
AGATTTCAAATCAAGAGAGATGAAGGTGACGGTATTGCTACGTACGGTAGAGTACACTA
CATTTATAAAGAAGAGATTTCACTTAAAGAACTCGACTTTAAATTGAGACAGTATTTAAT
TCAAAATTTTGATCTGTATAAAAAGTTTCCTAAAGATAGTAAGATAAAAGTGATAATGAA
AGATGGCGGCTATTATACGTTTGAACTTAATAAAAAATTACAAACAAATCGCATGAGTGA
TGTCATTGACGGTAGAAATATTGAAAAAATAGAAGCCAACATTAGATAA

> **ssl06**

Function: protein coding sequence; staph superantigene like protein locus 6 (genomic island)
Best match: ssl06-MW2_CC049_21262_AHJW01000044.1[53855:54553]
Position: 009-contig_245_RC: 107230 … 107260; Length: 30 bp
Sequence:
ATGAAATTAAAAACGTTAGCTAAAGCAACA

> **ssl07**

Function: protein coding sequence; staph superantigene like protein locus 7 (genomic island)
Best match: ssl07_CC001-ST772_118_AJGE01000018.1[3357:4052:r] (completely identical)
Position: 009-contig_245_RC: 107230 … 107926; Length: 696 bp
Sequence:
ATGAAATTAAAAACGTTAGCTAAAGCAACATTGGCATTAGGCTTATTAACTACTGGTGTG
ATTACATCAGAAGGCCAAGCAGTTCAAGCAAAAGAAAAGCAAGAGAGAGTACAACATTT
ATATGATATTAAAGACTTACATCGATACTACTCATCAGAAAGTTTTGAATTCAGTAATATT
AGAGGTAAGGTTGAAAATTATAACGGTTCTAACGTTGTACGCTTTAACCAAGATGGTCAA

AATCACCAATTATTCTTATTAGGAGAAGATAAAGCTAAATATAAACAAGGACTTCAAGGT
CAAGATGTCTTTGTGGTAAAAGAATTAATTGATCCAAACGGTAGACTATCTACTGTTGGT
GGTGTAACGAAGAAAAATAACCAATCTTCTGAAACTAATACACCTTTAGTAGTTAATAAA
GTGTATGGAGGAGATTTACATGCATCAATTGACTCATTTTCAATTAATAAAGAAGAAGTT
TCACTGAAAGAACTTGATTTCAAAATTAGAAAGCAATTAGTTGAAAAATATGGTTTATAT
AAAGGTACGACTAAATACGGTAAGATCACTATCAATTTGAAAGACGAGAAAAAGGAAGT
AATTGATTTAGGTGATAAACTGCAATTCGAGCGCATGGGTGATGTGTTGAATAGTAAGGA
TATTCAAAATATAGCAGTGACTATTAATCAAATTTAA

> **ssl08**
Function: protein coding sequence; staph superantigene like protein locus 8 (genomic island)
Best match: ssl08_CC005_Mu50-VRSA_BA000017.4[472430:473128] (completely identical)
Position: 009-contig_245_RC: 108264 … 108963; Length: 699 bp
Sequence:
ATGAAATTTACAGCGATAGCTAAAGCGATATTTGTATTAGGAATATTAACAACAAGTGTA
ATGATAACAGAAAATCAATCGGTTAATGCAAAAGGAAAGTATGAAAAAATGAACCGTTT
ATATGATACAAACAAGTTACATCAATACTATTCAGGACCTAGTTATGAGTTAACAAATGT
TAGTGGCCAAAGTCAAGGTTATTATGACTCTAACGTTTTGCTTTTTAACCAACAAAATCAA
AAGTTCCAAGTATTTTTATTGGGAAAAGATGAAAATAAATACAAAGAAAAAACACATGG
TTTAGATGTCTTTGCGGTACCAGAATTAGTAGATTTAGATGGAAGAATATTTAGTGTTAGT
GGTGTAACGAAGAAAAACGTAAAATCAATATTTGAGTCTCTAAGAACGCCGAACTTACTA
GTTAAAAAAATAGACGATAAAGACGGTTTTTCTATTGATGAATTTTTCTTTATTCAAAAGG
AAGAAGTGTCATTGAAGGAACTTGATTTTAAAATAAGAAAACTGTTGATTAAAAAATACA
AACTGTATGAAGGGTCAGCTGATAAAGGTAGAATTGTTATTAATATGAAAGATGAAAAT
AAGTATGAAATTGATTTAAGTGATAAATTAGATTTCGAGCGTATGGCAGATGTCATTAAT
AGTGAACAAATTAAGAACATCGAAGTGAATTTGAAATAA

> **ssl09**
Function: protein coding sequence; staph superantigene like protein locus 9 (genomic island)
Best match: ssl09-var1_CC005_Mu50-VRSA_BA000017.4[473505:474203] (completely identical)
Position: 009-contig_245_RC: 109339 … 110038; Length: 699 bp
Sequence:
ATGAAATTGACTGCATTAGCAAAAGCAACATTAGCATTAGGAATATTAACTACAGGTGTG
TTTACAGCAGAAAGTAAAGCTGTTCACGCGAAAGTAGAACTTGATGAGACACAACGCAA
ATATTATATCAATATGCTACATCAATACTATTCTGAAGAAAGTTTTGAATCAACAAACATT
AGCGTTAAAAGTGAAGATTACTATGGTTCTAACGTTTTAAACTTTAACCAACGAAATAAA
ACTTTCAAAGTGTTTCTGCTTGGTGACGATAAAAATAAATATAAAGAAAAAACACATGGC
CTTGATGTCTTTGCAGTACCTGAATTAATAGATATAAAAGGTGGCATATATAGCGTTGGC
GGTATAACAAAGAAAAATGTGAGATCAGTGTTTGGATTTGTAAGTAATCCAAGTCTACAA
GTTAAAAAAGTTGATGCTAAACATGGCTTTTCGATAAATGAGTTGTTCTTTATTCAAAAG
GAAGAAGTATCGTTGAAGGAACTGGATTTTAAAATAAGAAAAATGTTAGTCGAAAAATA
TAGATTGTATAAAGGCGCGTCAGATAAAGGTAGAATCGTTATTAATATGAAAGACGAAA
AGAAATATGTAATTGATTTAAGTGAAAAATTAAGTTTTGATCGTATGTTTGATGTAATGG
ATAGTAAGCAAATTAAAAATATTGAAGTGAATTTGAATTAG

> **ssl10**
Function: protein coding sequence; staph superantigene like protein locus 10 (genomic island)
Best match: ssl10_CC001-ST772_118_AJGE01000018.1[196:879:r] (completely identical)
Position: 009-contig_245_RC: 110403 … 111087; Length: 684 bp
Sequence:
ATGAAATTAACAGCGATAGCTAAAGCTGCATTAGCTTTAGGAATTTTAACAACAGGAACT
TTAACAACAGAAGTTCATTCAGGTCATGCAAAACAAAATCAAAAGTCAGTAAATAAACA
TGACAAGGAAGCATTATACCGATACTACACTGGAAAGACTATGGAAATGAAAAATATTA
GTGCTTTGAAACATGGTAAAAACAACTTACGTTTTAAGTTTAGAGGTATTAAGATTCAAG
TTTTACTGCCTGGAAATGATAAAAGTAAATTTCAACAGCGTAGTTATGAGGGGTTAGATG

TTTTCTTTGTTCAAGAAAAAAGAGATAAGCACGATATATTTTATACTGTTGGTGGTGTAAT
ACAGAATAATAAAACATCTGGAGTTGTCAGTGCACCAATATTAAATGTTACAAAGGAAA
AAGGTGAAGATGCTTTTGTGAAAGGGTACCCTTATTATATTAAAAAAGAAAAAATAACAT
TAAAAGAGTTAGATTTTAAATTGAGAAAGCATCTAATCGAAAAATATGGACTTTATAAAA
CAATCTCAAAAGATGGTAGGGTTAAAATTAGCTTGAAAGATGGCAGTTTTTATAACCTTG
ATTTAAGATCTAAATTAAAATTTAAATATATGGGGGAAGTCATAGAAAGCAAACAAATTA
AAGATATTGAAGTTAACTTAAAGTAA

## > hsdM-Lx

Function: protein coding sequence; type I restriction-modification system DNA methylase unknown locus (genomic island)
Best match: hsdM_AB057421.1[3606:5162:r]
Position: 010-contig_255: 241 … 271; Length: 30 bp
Sequence:
AAAGAACTTGGGGTGTTGAAAGATGAGTAA

## > hsdS-etD

Function: protein coding sequence; type I restriction-modification system site-specificity determinate (mobile element)/(genomic island)
Best match: hsdS-etd_trunc_CC080_11819-97_CP003194.1[450654:451318]
Position: 010-contig_255: 263 … 928; Length: 665 bp
Sequence:
ATGAGTAATACACAAAAGAAAAATGTGCCAAAATTGAGATTCCCAGGGTTTGAAGGCGA
ATGGGAAGAGAAGCAATTTGCTGATTTTACTAAAATAAATCAAGGATTACAGATTGCTAT
TAATGAACGTAAAACTGAATATTCTCCAGAGTTGTATTTTTATATAACAAATGAATTTTTA
AGACCAAATAGTCAAACTAAATATTTTATCGAAAATCCCCCTCAATCAGTAATTGCAAAT
AAAGAAGATATTTTAATGACTAGAACAGGTAATACTGGAAAAGTAGTAACTAATGTATTT
GGAGCGTTTCATAATAATTTTTTTAAAATTAAATTTGATAAAAATCTGTATGATAGATTGT
TTTTAGTAGAGGTTTTAAATTCATCTAAGATACAAAATAAAATACTATCTTTAGCAGGATC
TTCGACGATACCAGATTTAAACCATAGTGATTTTTATAGTATTAGTTCTTCTTATCCGCTG
CTTAGAGAACAGCAAAAAATAGGTAAATTCTTCAGCAAACTCGACCGACAAATTGAATT
AGAAGAACAAAAGCTTGAATTACTTCAACAACAGAAAAAAGGCTATATGCAGAAAATCT
TCTCACAGGAATTGCGATTTAAGGACGAGAATGGAAATGATTATCCGGATTGGGAGAAA
AAGAA

## > ssl11

Function: protein coding sequence; staph superantigene like protein locus 11 (genomic island)
Best match: ssl11-CC80_CC001-ST772_118_AJGE01000070.1[2117:2815] (completely identical)
Position: 010-contig_255: 1861 … 2560; Length: 699 bp
Sequence:
ATGAAATTAAAAAATATTGCTAAAGCAAGTTTAGCACTAGGGATTTTAACAACAGAGATG
ATTACAACTACTGCTCAGCCAGTAAAAGCAGACGAAGCTAGTAGCAGATTATCAGTTACT
TCAAAAGATACACAAAATTTAAAGACGTATTATACTGGAGCAAGTTTTGATCTTAGAGGA
TTAAGCGGATATAAAGAAGGAGATAAAGTAATATTTCCTCAAAATGGTCAACAAATTGAT
GTTACATTAACAGGTAAAGAGGGAGACGTGGTTCAATCTAATGACGACGTTACAAATGTT
GATGTGTTTGCTGTTCCTGAAGGTACAGGTCGTTCTGCCATAATGACTACAATTGGTGGG
ATTACTACACCAAACACTCAAAGTTATAAAGACACTACAAATAATGTGAACTTAAGAGTA
TCAAAGAGTACAAGTCCCAATACCATATCTGTGTCTGTAAAAAATTATGATATTTTTAAA
GAAAAAATTTCATTAAAAGAACTTGATTTCAAATTAAGAAAGCATTTAATTGATAATCAT
GATCTTTATAAAACAGAACCTAAAGACAGTAAAATTAGAATTAATATGAAAGATGGCGG
CTACTATACGTTTGAATTAAATAAAAAGTTACAAACTCATCGTATGGGTGACGTAATTGA
TGGTACTAAAATAAAAGAAATTAATGTTGAATTAAACTAA

## > tnpIS431-06

Function: protein coding sequence; transposase for IS431 (mobile element)

Best match: tnpIS431-06_circ_S-epidermidis_GQ900381.1[43799:43807]RC (completely identical)
Position: 010-contig_255: 2984 … 2993; Length: 9 bp
Sequence:
ATAGTTCAT

> **Q6GC50**
Function:
Best match: Q6GC50-COL_CC008_NCTC8325_CP000253.1[403207:404715]
Position: 010-contig_255: 4112 … 4142; Length: 30 bp
Sequence:
GAACGTAAGGCACCAAAATTAGAAAAATAA

> **txbi_Q6GC50**
Function:  rho independent terminator
Best match: txbi_Q6GC50_CC001_MW2-USA400_BA000033.2[445740:445826]
Position: 010-contig_255: 4198 … 4228; Length: 30 bp
Sequence:
TCAACGTCATTGAGCTGTTTTTTGTGCTTT

> **Q2G0X2**
Function: protein coding sequence; putative protein (genomic island)
Best match: Q2G0X2_CC005_A5937_ACKC01000031.1[393792:394100:r]RC
Position: 010-contig_255: 4248 … 4557; Length: 309 bp
Sequence:
TTATTTTACGTGTTCATATTTTGAAACATCAAAGCCGTCTTGCTTAGCTTTGTTGATAATAT
CTTTGATTGAATGTAGTCCTTTATCGGCGAAGTATGATCTTAAGTTGTCTTTTGTAGCTTG
GTCAGCATTCTTATCTAATAACACATCGATATAACTTAACTCGTGTTCTAAGAAGTTTGCG
TCATCATGTAGTACGAGTCCATTTTGAGAATACACTTTCGCATCTGCTTGATTACCATATC
CAACAACGCCAGTTGCTAAAACACCTACCATTGCCGTAGCTACTAAAACCTTTTTAAATTT
CAT

> **lpl**
Function: protein coding sequence; tandem lipoprotein-like (genomic island)
Best match: lpl1_CC008_Newman_AP009351.1[453191:453994]
Position: 010-contig_255: 4893 … 4923; Length: 30 bp
Sequence:
ATGAAGTATAAAACAGAGAGACGTGAAGCG

> **lpl**
Function: protein coding sequence; tandem lipoprotein-like (genomic island)
Best match: lpl13_CC001_MW2-USA400_BA000033.2[447351:448121], lpl-SAB0394_ref_CC705_RF122-cow_AJ938182.1[429813:430607], lpl-SACOL0482_CC008_TCH1516_CP000730.1[467620:468411] MANUAL OVERRIDE
Position: 011-contig_288_RC: 228 … 1081; Length: 853 bp
Sequence:
TATTTAAGTTATAAGCCAGCGAAAAAATAGTGTTGATAATTAAATATTAGGGTGTGAAAT
GATGAGATATTTAAAAAGAGTTGTACTGTACATAATTGTTATGGTTTTGAGTGTTTTTATA
ATAGGTTGTGATAAATCAAGCGATACTTCAGAAAAGCCAAAAGAAGATTCAAAAGAAGC
ACAAATTAAAAAGAGTTTTGCAAAAACGTTAGACATGTATCCTACTGAAAATCTAGAAGA
CTTTTATGACAAAGAGGGATATCGAGATGGAGAATTTAAAAAAGGTGACAAAGGGACTT
GGCTAATTAGATCTGAAATAGTTAAACAGCCAAAGGGCAAAGTGATGAAAACAAGAGGT
ATGCAATTATATATTAATAGAAATACCAAAACAGCCAAAGGTTTCTTTGTTTTGAAAGAA
ATAAGTGAAAATAATAATCGTGTAAATAAAGATAAGGAGGAAAAATACGAAGTGAAAAT
GGTAGGAAATAAAATTATTCCTACTGAACAAATTAATGATGAGAAAATAAAAAAAGAAA
TTGAAAACTTCAAGTTTTTTGTGCAATATGGAAACTTTAAAAATTTCGAAAAATACAACA

ATGGTGAGTTTTCATATAATCCTGAAGCACCAATCTATTCGGCTAAATATCAATTACACA
ACGACGATTACAATGTAAGGCAACTACGTAAAAGATATGACATTTCAACAAAAGAAACA
CCGAAGTTACTTTTGAAAGGTGGAGGAGATTTAAAAAATTCCTCAGTTGGTCAAAACGAT
ATTGAATTTACTTTTGTTGAAAGAAAAGGTGAGAATATTTATTTTAACGATAGTGTTGAAT
TCATACCAAGTAAGTAA

> **lpl4**

Function: protein coding sequence; tandem lipoprotein-like (genomic island)
Best match: lpl4_CC005_Mu50-VRSA_BA000017.4[484932:485717]RC, lpl10_CC005_JH9_CP000703.1[494856:495617]RC, lpl-SAB0392_ref_CC705_RF122-cow_AJ938182.1[428201:428986]RC, lpl2_CC005_N315_BA000018.3[457923:458717]RC
Position: 012-contig_282_RC: 1000 … 2763; Length: 1763 bp
Sequence:
GTACAATACTACTCTTTTTGAATCCCTCATTTTTTCACTTCTCTCTATCATGTTAATGTTTG
TTGAGTACTATCAACCTTTGTGTTATTTTTAGTCACTTGGAATTAAGTTTAATCCATCTGA
AAAATATATATTTTCTTTTTTATTTTCTATAAATATAAATTCAATTTCCTTATATCCAACTG
AAGACCCTTTCAAATCCCCCGAACCTTTCAACAACAATTTAGGCGCCTTTTCTGTTGGAAT
ATTATATCTTTTTCGTAATTCTTTAACATTATAATCATCATTTTTCAGTTTATATTTCGCTG
AATAAATAGGAGCTTCAGAATTATAGGAAATGTCACCATTTTCATAATTCTCTATTCCTTT
AAAACTGCCATATTGCACAAAAAACTTAAAGTTTTCAATTTCCTTTTTCAACTTTTCATCT
TTTATCTCCTCAGTTGGAATAATTTTATTGTTTATCATTTTCACAGGATACTTTTTTTCTGTT
TCTCTTTCATCAAGGCTTTTTCTTGAATCTATTTCTTCTATTGAAAAATAACCGGTTGTGGT
CCTAGTATTTCTATTCATGTATAATATCATGCCTCTTGAAGTTAAACTTTTACCTTTTGGTT
GAATTATCATTTCAGATCTAACAACCCACGTCCCTTTGTCTCCCTTTTTAAATTCTCCATCT
CGATATCCTTCTTTGTCATATAAGTCCTCGAGATTCTTAATTGGGTACATATCTAACGTTTT
CGCAAAGCTCTTTTTGATTTGTGTTTCTTTTGAATCTTCAGGAGTGTCAAATACACCACTG
CAACCTGACACTAATAGAATTAAGACTGACGTACCTATACACAACGCGTATTGCCTAAAA
TTATTCATTGTTCCGTATCCCCCCTTTTTTCATCATAGTTAATAGTCCTCACTTGGGGTAAA
CTCCAAACTATCAGTAAAAAATACATTCTCTTCTTGATTCTCAACGAAGGTAAACTCAAT
GTCCTTATGTCCGATTGATGAACCTTTTAAATCTCCAGTACCTTTCAACAACAATTTAGGA
CCTTTTTTGGTTTGAATGTCATACATTTTACGTAATTGTTTTACATTATAATCATCATTACT
TAATTGAAATTTAGCAGAATAAGTAGGTGCTTCTGAGTTATATGAATAATCACCATTTTG
ATAATTCTTTAATCCTTTAAAATCACCATATTGTGAGAAAAACTTAAAGTTTTCAATTTCT
TTTTTTACACCTTTATCATTAATTGGATCAATAGGTACTATTTTATTATTCACCATCTTTAC
TGGATAACGTTTTTCTTTATCCTCTGTCTTTTTTAAAGTATCATTACTTGTTTCATCTACAA
TAAAATATCCTTTTGCGGTTCTTGTATTTCTATTTAAAAATAGATACATCCCTCTTGATTTC
ATAATTTCACCTTTTGGCTGTTTAACCATTACTGAACTGATAACCCATGTCCCTTTGTCAC
CTTTTTTAAATTCGCCATCTCGATATCCTTCTTTGTCATATAAGTCCTCGAGATTCTTAATT
GGGTACATATCTAATGTTTTCGCAAAGCTCTTTTTGATTTGTATTTCTTTTGAATCTTCTTT
TGTTTCATTACCTTTGCCACAACCTGCTACAATAACTATTAAAATCAAGATACTTATGTAC
AATGCAAAACTTTTTAAATTTCCCATCATTTCGCGTCTCTCTGTTTTAGAATTATTTCTACA
TCTAATAAACTTTAGCTATCTTCATCAGACGGCCCAAAATT

> **lplTerminator-RF122**

Function: protein coding sequence; (genomic island)
Best match: lplTerminator-RF122_ref_CC705_RF122-cow_AJ938182.1[432435:433760]
Position: 013-contig_317_RC: 1836 … 1866; Length: 30 bp
Sequence:
CTTGCATCTCAAATAGGTGGATTACTATGA

> **Q5HIM7**

Function: protein coding sequence; putative protein (core genome, variable)
Best match: Q5HIM7_CC001-ST772_118_AJGE01000070.1[20119:20313:r]RC (completely identical)
Position: 013-contig_317_RC: 2864 … 3059; Length: 195 bp

Sequence:
TTACAAACCATAAAGCGCTGCAGTTAAGTATTGAATGGCATAGCCAATTGAAATGAAAAT
AGCAATCGTTATAATAGCACTACCAATGGTATATAACGTATGTTTCGTAGATAATCCTTCA
GTCTTTGAACCAAAAACACTGAGTAGAATAAATGCAATGCCAATGACATATAAGGCTAA
AATGAAAAATAACAT

## > **cobW**

Function: protein coding sequence; cobalamin biosynthesis protein (core genome, constant)
Best match: cobW_CC001_MW2-USA400_BA000033.2[453431:454633]
Position: 013-contig_317_RC: 3245 … 4448; Length: 1203 bp
Sequence:
ATGGCTAAAATTCCAGTTACGGTATTAAGTGGTTATTTAGGCTCGGGGAAGACAACGTTG
TTAAATCATATTTTACAAAATCGAGAAGGTCGACGTATCGCGGTAATTGTAAATGATATG
AGTGAAGTCAATATCGATAAAGATCTTGTCGCAGATGGTGGGGGACTATCGCGTACAGAT
GAAAAATTAGTCGAACTTTCTAATGGTTGTATCTGTTGTACACTTAGAGACGATTTATTAA
AAGAAGTTGAGCGTTTAGTGAAAAAAGGTGGCATCGATCAAATTGTTATTGAGTCAACAG
GGATTTCAGAGCCAGTACCTGTTGCACAAACTTTCTCATATATTGATGATGAACTTGGCAT
TGATCTTACAGCGATTTGCCGTTTAGATACAATGGTTACAGTTGTGGATGCTAACCGCTTC
GTACATGACATCAACTCAGAAGATTTATTGATGGATCGTGATCAAAGCGTTGATGAAACA
GATGAGCGTTCGATTGCTGATTTATTAATTGACCAAGTTGAATTTTGTGATGTATTGATTA
TTAATAAAATTGATTTAATTAGTGAAGAAGAACTAGCGAAGTTAGAAAAAGTGTTAAGTG
CATTGCAACCGACTGCTAAAATTATTAAGACAACAAATTCTGAAGTAGATTTAAAAGAAG
TCTTAAATACGCAGCGTTTTGATTTTGAAAAAGCGAGCGAGTCAGCAGGATGGATCAAAG
AACTTGAGTCTGGTGGTCATGCATCGCATACGCCTGAAACAGAAGAATATGGCATATCAT
CATTTGTATATAAACGTCGTCTACCTTTCCATGCTAAAAGGTTCAATGATTGGTTAGAAAG
CATGCCAAATAATGTCGTTCGATCAAAAGGTATCGTATGGTTAGCACAATACAATCATGT
AGCATGTTTATTATCTCAAGCAGGGTCATCTTGCAATATTCATCCAGTTACATATTGGGTG
GCTAGTATGTCTGAAGCGCAACAAACACAAATATTAGCAGAACGTCAAGATGTCGCAGC
TGAATGGGATCCAGAATATGGCGATCGTCATACACAATTTGTCATTATTGGTACAGAATT
AGATGAAGAAAAATTAACAAAAGAACTCGACGCATGCTTAGTCAATGCGCAAGAAATTG
ATGCAGATTGGCAACAATTTGAAGATCCATATCAATGGCAAATTAGACCAGCACGATAA

## > **psmA4**

Function: protein coding sequence; phenol-soluble modulin alpha 4 (core genome, constant)
Best match: psmA4_CC001_MW2-USA400_BA000033.2[455290:455352:r]RC (completely identical)
Position: 013-contig_317_RC: 5104 … 5167; Length: 63 bp
Sequence:
TTATTTTGCGAAAATGTCGATAATTGCTTTGATGATTTTAATGATAGTACCTACAATAGCC
AT

## > **psmA3**

Function: protein coding sequence; phenol-soluble modulin alpha 3 (core genome, constant)
Best match: psmA3_CC001_MW2-USA400_BA000033.2[455416:455484:r]RC (completely identical)
Position: 013-contig_317_RC: 5230 … 5299; Length: 69 bp
Sequence:
TTAGTTGTTACCTAAAAATTTACCAAGTAAATCTTTAAAGAATTTGAATAATTTTGCTACG
AATTCCAT

## > **psmA2**

Function: protein coding sequence; phenol-soluble modulin alpha 2 (core genome, constant)
Best match: psmA2_CC001_MW2-USA400_BA000033.2[455536:455601:r]RC (completely identical)
Position: 013-contig_317_RC: 5350 … 5416; Length: 66 bp

Sequence:
TTACTTACCAGTGAATTTCTCAATTAATCCTTTAATGAATTTAATGATTCCTGCAATGATA
CCCAT

> **psmA1**
Function: protein coding sequence; phenol-soluble modulin alpha 1 (core genome, constant)
Best match: psmA1_CC001_MW2-USA400_BA000033.2[455633:455698:r]RC (completely identical)
Position: 013-contig_317_RC: 5447 … 5513; Length: 66 bp
Sequence:
TTATTTACCAGTGAATTGTTCGATTAAGCTTTTGATAACTTTAATGATGCCAGCGATGATA
CCCAT

> **ndhF**
Function: protein coding sequence; NADH dehydrogenase subunit 5 (core genome, variable)
Best match: ndhF_CC001_MW2-USA400_BA000033.2[456218:457702]
Position: 013-contig_317_RC: 6033 … 7518; Length: 1485 bp
Sequence:
GTGTTAAGTTTTCAATTGCTATTTTCACTGTTTGTTATTGCGCTTATCATTGCATTGATAAG
TGGCTTGTTGTTTTTAGCACCAGTTATGCCAATGAGATATATTAAATTACATTTATACATA
CTAGTCATGCCAGTATTATTTGCAGTCATTGGCTTTTTCGGTATTCATGGTCAACATGTCTT
AGGTCCATTTAAAATAGATCGTTTATCTTGGTTATTAGCTGGCTTTGTAATGGCGCTTGGT
TTTATTATTCAAAAGTTTTCAATGCGATATTTACTAGGTGATCATCATTATAGACATTACT
TTCCATTGTTCACTGCGATTACGTCGTTTGCATCTTTAGCATGGATGTCTGAAGACTTAAG
ACTGATGGCACTCTGCTGGGGTATGACATTATTATGTTTAACATTGCTGATGAACGTTAAT
CGTTTTTGGAAAGTGCCACGTGAGTCTGCGAAATTATCAAGTATGACATTTTTATGTGGTT
GGCTTGCATTCGTTGGAGCAATTGTAACTATTTATATTGCGACTGGCGAGTGGCGCGTGC
CTCAACATATGTCTAATTCGACATGGTCATTGTTGACGAATGTACTACTTGTATTAGCTGT
CATGATACCGGCAGCACAATTTCCTTTTCATCGATGGTTGATTGAATCTGTAACGGCACCA
ACGCCAGTATCGGCAATTATGCATGCAGGAATTGTGAATGCAGGTGGTGTTATTCTAACT
CGTTTTGCGCCGATATTTGATAATGGATTTGCGTTATCATTATTACTTATCTTTTCTAGTAT
TTCTGTATTGTTAGGATCGGGTATTAGCTTAGTTCAAGTTGACTATAAACGCCAATTAGTG
GGCTCTACGATGAGTCAAATGGGCTTTATGTTAGTTCAATGTGCATTGGGTGTATATTCAG
CAGCGATTATTCATTTAATATTGCACGGTATTTTTAAAGCAACATTATTTTTACAATCAGG
TTCTATCGTGAAGCGATTCAATATTCCAAAACAAGCATCTGCTAAAGACGCTTATGGCTG
GATTGTCATGGGACGTGTATTAGCTATTATCGTGGCATTTCTGTTTTGGATGAGTAGTGAC
AGAAGTGCATATGAAGTGTTAAGTGCACTCATTCTAGCTTGGTCATTACTTGTATCTTGGA
ATCAAATGGTAGCCTTTAGTAAAGGACGCATGGCACGTCTTGTTGGTATGATTTTGATTGC
AATTGTGACATTTATCTACATCATCACACATAATTATTTTTACGATGTATTGCGAAATATA
ACAACACATGCGACAACACCGCCTACAGTGAGTGTCATCATTAGTGTTGTCATTTTAATCT
TTGGTAGTTTATTAAGTATTTGGGTGGCGCGTCATCGATACTCTAAGGGTTTTGCGGTATT
GTACGTGTGGTTAGTTAATCTAGGTGAAGCACGCTCGAAAGCGATAGAAAGTCATCCGAA
TTATTTGAAGAAGTATTTATAG

> **Q5HIM4**
Function: protein coding sequence; putative protein (core genome, variable)
Best match: Q5HIM4_CC001-ST772_118_AJGE01000070.1[24785:27248] (completely identical)
Position: 013-contig_317_RC: 7530 … 9994; Length: 2464 bp
Sequence:
ATGACAACACAGTTAAATATCAATTCAGTCATTGAAAATGCGAAACGTGTTATTACACCA
TTATCACCGATTTCGATTTTTGCAGCACGAAATCCATGGGAAGGATTAGAAGCGGATACG
TTTGAAGATGTTGCAAAATGGTTACGTGACGTTCGAGATGTGGATATTTTCCCAAATAAA
GCATTAATAGAAAGCGCTGTGGCACGTGGTGAATTAGATGAAAGTGTCTTTAATCAACTT
GTTACTGATATGTTACTTGAACATTACTACAATATCCCGCAACACTACATCAATCTTTATA
TTGATAACATTAAAACATTAAAAGACGTACCTGCATCATATATGGATCATTCAAATGTTG

ATGTTGTTGCTGATCTACTATTAGAAAAATCAAAACGTGATATGGCTGAATCATATCATC
ACTATGATGTACGTCCGATGAGTGATGCAATAATAGATGAACAAGGTGAGCCACTTAGCG
AACAAGTGAATCGTCAAATGATTAAATGGACGAAACTTTATATCGATCAATTTCTATCGA
GTTGGACAATGCCGAAGCGTGAGCAAAGTTTTTACCATGCATGGTTGCATTTAGCGCAAC
ATGACCATAGTTTTACTAAAGCACAGCGCCAAGTGATTAAAGGCTTACCCAATGATCCTG
AAATGACGATAGAGTCAGTATTAACTCATTTTTCAATAGATCAGGAAGACTACCAAGCTT
ATGTTGAAGGACATCTTTTGGCGTTACCGGGTTGGGCAGGTATGTTATATTACCGTTCACA
ACAGCATCACTTTGAACAACATTTGTTAACGGATTATTTGGCAATTCGGTTAGTTGTCGAA
CAATTGCTAGTTGGTGATGAGTTTAAGTCAGTCACTAAAGATTGTGAAAGTAGATCGGAA
AATTGGTTTAAGCAAACTGTTGCATCATGGTGTTACTACAGTGATATGCCTAGCGATGTAT
TACTACAACATGACGTCCATGAAATTCAAACATTTATTCATTTTGCAGCAATTATGAATAA
AAATGTATTTAAAAATTTATGGCTAATTGCCTGGGAAATGACATACGAATCTCAGTTAAA
ACAAAAAATTAAAGCAGGTCATGAAAGTGTGGCGGGCGCATTAGATGTAAACCAAGTAA
ATGTCTCAGAAAATGATAACGTTAATCAGCCACATTCAGTATTGTTAAATGACACACAAG
CAGTTGATGAAAATAATAGCGAGCTAAATCAGGTGGGCACATCAACGAAAGCGCAAATT
GCATTTTGTATAGATGTTCGTTCAGAACCATTTCGTAGACATATCGAAGCAGCAGGGCCC
TTTGAAACGATTGGTATTGCAGGCTTCTTTGGATTACCTATTCAAAAAGATGCCGTCGACG
AACAATTCAAACATGATTCATTACCTGTCATGGTAACGCCGGCATATCGCATTAAAGAAT
TTGCAGACCGCTACGATATGAATGTTTATCGACAACAGCAACAGACAATGTCATCGATGT
TTTACACATTTAAATTGATGAAAAATAATGTTATGCCTAGTCTGTTATTGCCTGAATTAAG
TGGGCCATTTTTAAGCTTGAGTACCATTGTCAATTCGATTATGCCTAGAAAAAGTCGCGC
GTCTTTACAAAAAATAAAACAAAAATGGTTGAAAAAGCCTGAAACAAAGTTAACGATTG
ATCGTGAGTTTGACCGAACATCAGATTTACCTGTTGGATTTACTGAGCAAGAGCAAATTG
ATTTCGCGTTACAAGCGTTGAAATTGATGGATTTAACCGAAGCATTTGCGCCGTTCGTTGT
GTTAGCAGGTCATGCTAGTCATTCTCACAATAATCCACATCATGCATCACTTGAATGTGG
GGCTTGTGGTGGCGCATCAAGCGGTTTTAATGCTAAGTTATTAGCGATGATATGTAATCG
TCCAAATGTCAGACAAGGATTAAAACAAGCAGGCGTGTATATTCCAGAGACAACTGTTTT
TGCGGCAGCAGAACATCATACATCTACTGATACGTTGGCATGGGTATATGTGCCAGACAC
ATTATCAGCTTTAGCTCTAGATGCATATGAATCATTGAATGACGCGATGCCGATGATTTCT
GAACAATCGAATCGCGAACGTTTGGACAAACTGCCAACGATTGGTCGTGTGAATCATCCA
GTGGAAGAAGCGCAGCGGTTTGCGAGTGATTGGAGTGAGGTACGTCCAGAATGGGGCTT
GGCTAAAAATGCATCATTTATAATTGGACGACGCCAATTAACAAAAGGTATTGATTTAGA
AGGGCGGACATTTTTACACAATTATGATTGGCGTAAAGATAAAGATGGCACATTATTAAA
TACCATCATTTCTGGTCCAGCGCTTGTGGCACAATGGATTAATTTACAATATTATGCGTCG
ACAGTTGCGCCGCATTTTTACGGAAGTGGGAATAAAGCGACACAAACCGTCACGTCAG

> **ybcL**
Function: protein coding sequence; putative protein (core genome, constant)
Best match: ybcL_ST425_LGA251-cow_FR821779.1[448524:448886] (completely identical)
Position: 013-contig_317_RC: 10397 … 10760; Length: 363 bp
Sequence:
ATGAAAAGAACGAAAGGTGAAATCGAAGCTGAAATCAGTAAAGCCATTACGCAATGGGA
AAAAGATTTCCTTGGCAGAGGTTCCTTGTCGGTTAAATCAGACATTTTAAGAGATATGGT
GATTATTAGTTTACAAGGTATCTTAACGCCAGCAGAATATCGTGTGTGTAGTACGAATGA
AGGATTACTAAATATTAAACGAACACGTTCTGAATTAGTTGAATCCGGTGAGCAAGATTT
GAATGATATCATTCTTAAAATTACAGGTATCAAAGTGATGAGCTTCCACAGTGATTTAAG
TACAGTTACAGGTGAACGTATTATCGTATTCAAACTTGAGGATAATTTGGAAAAGCATAT
TTAA

> **Q5HIM2**
Function: protein coding sequence; putative protein (core genome, constant)
Best match: Q5HIM2_CC001_MW2-USA400_BA000033.2[461173:461520:r]RC (completely identical)
Position: 013-contig_317_RC: 10988 … 11336; Length: 348 bp
Sequence:

TTACATAAAGAGTATATGTGTGACGTAGGCATATAATCGATAAAGTATTCCTAAAAAATT
AGGTATAATGACCATTGTTGCAATAAGTTTTTCGGCCTTGAAACCGATAACGCATAGTAT
AATAGGAATAAAATATAATACAAATATCCAGTAAATCAAATTTGAAAACGATGGATGAA
TAGGGGTAAAGAATCTAACTAATGTAATGATTAACGCTATATAGACAAAAATAACTCTAT
TGATACGCCTTACCCCCTCTGTATAATAAATATAGATCGTACTAAATTGAAAATAGCAAA
ATATTGTTATTTCAATTATACAATGTTTTATTTGCAATATACATAACAA

## > Q5HIM1

Function: protein coding sequence; lipid phosphate phosphohydrolase 2 family protein (core genome, constant)
Best match: Q5HIM1_CC001-ST772_118_AJGE01000028.1[1476:2150] (completely identical)
Position: 013-contig_317_RC: 11442 … 12117; Length: 675 bp
Sequence:
ATGATAGATAAAAAATTAACATCACCGAAAATGACAGTGCCTTTATTTTTAATCGCGCTG
ATTGTATTTATAGGTATGTTTTACAGTGTAGTGACAAATCAAGAATGGCTTAAAAATATA
GATATGGGATCATTAACATGGTTTACAGATTATTTCGGTGAGCCACAACGTCAGTATGTT
AACAATTTATTTAATTACTATATGACGTTTAGTGCGGAAATTGGAGATGTCAAAGGTGTC
GTGTTGATTTCCATTATCGTCACAATCGTACTGTTTATTAAACAGAGGCATTTAGCGGTTT
GGTTTGTGACATATTTGGTTTCAGGCGTCATCATGAACAAATTAATTAAAGATACTGTTTT
ACGTCCAAGACCATATAATCATTTAGCCGTTGATACAGGCTTTTCATTTCCAAGTGGACAT
TCCAACGCCAGCACATTATTATATTTCGCCTTAATGATCATAATTATTTCACTTGCTGCTA
AGACAATAACAAAAGTGTTGAGTGCGCTAGTTATGGGAATATTATGGCTTAGCATATTAT
TTTGTCGCCTTTATTTTCATGCGCATTACTTTTCAGATGTCATTGGCGGCACGTCACTAGC
AATCATTTGGGTAGCGTTATTCTTAATGGTATACCCATACTTTATTAATCATCGACGACAA
CGCGTTTAG

## > Q5HIM0

Function: protein coding sequence; putative esterase (core genome, variable)
Best match: Q5HIM0_CC001-ST772_118_AJGE01000028.1[2187:2921] (completely identical)
Position: 013-contig_317_RC: 12153 … 12888; Length: 735 bp
Sequence:
ATGAGAATTAAAACACCGAGTCCATCGTATTTAAAAGGCACAAATGGACATGCGATATTA
TTATTACATTCATTTACAGGTACAAATCGGGATGTGAAGCATCTTGCAGCTGAGTTAAAT
GACCAAGGATTTAGTTGTTATGCACCGAATTATCCAGGTCATGGTTTATTGTTGAAAGATT
TCATGACATATAATGTAGATGATTGGTGGGAAGAAGTTGAGAAAGCTTACCAATTTTTAG
TCAATGAAGGTTATGAATCTATCAGTGCAACGGGTGTGTCTTTAGGTGGATTAATGACAT
TAAAATTGGCACAACACTATCCTTTGAAACGTATCGCTGTCATGTCAGCACCAAAGGAAA
AGAGTGATGATGGTTTAATAGAACATTTAGTTTATTATAGTCAACGCATGTCGGATATTTT
AAATTTAGATCAGCAAGCATCGAGTGCGCAATTAGCAGCAATTGATGATTATGAAGGTGA
AATTACGAAGTTTCAACATTTTATTGATGATATCATGACAAATTTAAATGTTATTAAAATG
CCAGCTAATATATTATTTGGTGGTAAAGATGCGCCATCCTATGAAACAAGTGCACACTTT
ATTTATGAACATTTAGGATCAGTAGACAAAGAATTAAATGGTCTGAAGGATTCGCATCAT
TTAATGACGCATGGAGAAGGCAGAGATATTTTAGAAGAAAATGTTATTCGCTTTTTCAAT
GCTTTAACATAA

## > Q5HIL8

Function: protein coding sequence; transporter (core genome, variable)
Best match: Q5HIL8_CC001_MW2-USA400_BA000033.2[463528:464865]
Position: 013-contig_317_RC: 13343 … 14681; Length: 1338 bp
Sequence:
ATGAAAAGACAACAATCACAATGGAAGTCATCAACTGGATTTATTTTAGCTAGTGCGGGT
TCTGCAATCGGTCTTGGTGCCATGTGGAAATTCCCATATATGGCAGGGATTTATGGCGGC
GGTGCCTTTCTAGCTATGTTCTTAATATTCACCATTTTTGTTGGGTTGCCATTACTCATTAT
GGAATTCACTGTTGGGAAAATGGGACGGACATATACAACACAAATATATAGTAAATTAA
CTGGTAAAAAATGGCTCAATATCATTGGCTGGAACGGTAATTTGGCAGTGTTTATTTTATT

TGGCTTCTATAGTGTTATCGGTGGTTGGATTGTCATTTACATCGGACAAGTATTATGGCAA
TTAGTTATATTTCAACGCATCAATCATCTCCAAGAAATGAATTTTGAAGCGGTAATATCA
AATCCTTGGTTAACCGTTCTAGGGCAAGGTATATTCATATTCGCTACGATGATTATTGTCA
TGTTAGGTGTTGAAAAAGGATTAGAAAGGCATCGAAAGTTATGATGCCATTGCTGTTTG
TCTTTTTAATCGTCATTGTGATTAAGTCTTTAACATTAGATGGCGCCTTAGAAGGTGTGAA
ATTTATTTTACAACCAAGAGTATCAGAGATTACTGCTGATGGCATCTTGTTTGCGCTAGGT
CAATCATTCTTTACGTTATCATTAGGAACTACAGGTATGATTACTTATGCGAGTTATGCCT
CTAAAGACATGACGATTAAGTCATCAGCTATTTCTATCGTTGTTATGAATATCTTTGTATC
TGTATTGGCAGGTCTAGCTATATTTCCGGCTTTACATAGTTTTGGCTATGAACCACAAGAA
GGGCCTGGATTATTATTTAAAGTACTGCCAATGGTCTTTAGTCAAATGCATCTAGGCACAT
TATTCTATTTGGGATTCTTAGTGCTGTTCTTATTTGCGGCTTTAACGTCATCTATTTCTTTA
TTAGAATTAAATGTTTCTAACTTCACGAAGAATGACAATACAAAACGTAAAAAAGTCGCA
GTGATCGGTAGTATTTTAGTATTTATCATTAGTATTCCAGCAACCTTATCTTTTGGTATCTT
AAAAGATGTAAGATTCGGTGCGGGAACGATTTTTGATAATATGGATTTCACCGTTTCGAA
TGTATTGATGCCATTAGGCGCATTAGGTACTACGCTTGTCGTAGGACAATTATTAGATAA
AAAATTATTACAACAATATTTTGGTAAAGATCGATTTAGATTATTCAGTGGTTGGTATTAC
TTAATTAAGTATGCGATGCCTGTCGTTATTATTTTAGTCTTTATCGTGCAATTATTTAGTTA
A

> **cysM**

Function: protein coding sequence; cysteine synthase (core genome, variable)
Best match: cysM_CC008_21209_AGRP01000021.125427:26341:r
Position: 013-contig_317_RC: 14045 … 15811; Length: 1766 bp
Sequence:
ATGATTACTTATGCGAGTTATGCCTCTAAAGACATGACGATTAAGTCATCAGCTATTTCTA
TCGTTGTTATGAATATCTTTGTATCTGTATTGGCAGGTCTAGCTATATTTCCGGCTTTACAT
AGTTTTGGCTATGAACCACAAGAAGGGCCTGGATTATTATTTAAAGTACTGCCAATGGTC
TTTAGTCAAATGCATCTAGGCACATTATTCTATTTGGGATTCTTAGTGCTGTTCTTATTTGC
GGCTTTAACGTCATCTATTTCTTTATTAGAATTAAATGTTTCTAACTTCACGAAGAATGAC
AATACAAAACGTAAAAAAGTCGCAGTGATCGGTAGTATTTTAGTATTTATCATTAGTATT
CCAGCAACCTTATCTTTTGGTATCTTAAAAGATGTAAGATTCGGTGCGGGAACGATTTTTG
ATAATATGGATTTCACCGTTTCGAATGTATTGATGCCATTAGGCGCATTAGGTACTACGCT
TGTCGTAGGACAATTATTAGATAAAAAATTATTACAACAATATTTTGGTAAAGATCGATT
TAGATTATTCAGTGGTTGGTATTACTTAATTAAGTATGCGATGCCTGTCGTTATTATTTTA
GTCTTTATCGTGCAATTATTTAGTTAATATATAGAAGCATCTGACACACAAGTATTTGTGT
TGCTCAGGTGTTTTTTGTTTGGAATTGAAAATTTAGTACTGGTTCTGAATTAATCGAAACT
TAGATAATTGCAAAATAACAAAATGTAGTTTATAATTCTTATCTGAAAAGTAAGGATTAA
AATTGAGACATGAAGAAAGGTTTCATTCATTTGATCGGCATGTCAGATGAGGGTGCATCT
ATGATTACTTATGATTTAATTGGCAATACGCCATTAGTACTGTTAGAACATTATAGTGATG
ATAAAGTTAAAATTTATGCCAAGCTTGAACAATGGAATCCTGGAGGCAGTGTTAAAGACA
GACTCGGGAAATATTTAGTAGAGATGGCAATTCAAGAAGGGCGTGTGCGTGCAGGTCAA
ACTATTGTTGAAGCGACTGCTGGCAATACAGGCATAGGGTTAGCTATTGCAGCAAATAGA
CATCATTTGAAATGTAAGATCTTTGCGCCGTATGGTTTTTCAGAAGAAAAGATTAATATTA
TGATAGCGCTTGGTGCAGATGTTTCAAGAACGAGTCAGTCTGAAGGTATGCATGGGGCAC
AATTAGCTGCACGTTCCTATGCTGAAAAATATGGTGCCATTTATATGAATCAATTTGAATC
CGAACATAATCCGGATACATATTTTCATACATTGGGGCCCGAATTGACTTCAGCATTACA
GCAAATTGATTATTTTGTGGCTGGTATTGGCTCAGGCGGTACATTTACAGGTACCGCACGT
TATTTAAAGCAACATCACGTGCAATGTTATGCCGTTGAGCCAGAAGGGTCCGTGTTAAAT
GGAGGGCCAGCTCATGCACATGACACTGAAGGTATCGGTTCTGAGAAATGGCCGATATTT
CTAGAGAGACGTCTTGTAGATGGTATATTTACGATTAAAGATCAAGATGCCTTTCGAAAT
GTCAAAAGTTTGGCTATAAATGAAGGGTTGTTAGTAGGCAGTTCTTCAGGTGCAGCATTA
CAAGGTGCATTGAATTTAAAAGCGCAATTATCTGAAGGTACGATTGTTGTCGTATTTCCA
GATGGTAGTGATCGCTATATGTCTAAGCAAATATTTAATTATGAGGAGAATAATAATGAA
CAAGAAAACTAA

> **metB**

Function: protein coding sequence; cystathionine gamma-synthase (core genome, variable)
Best match: metB_CC008-ST72_21259_AFTS01000010.1[24276:25418:r]
Position: 013-contig_317_RC: 15794 … 16937; Length: 1143 bp
Sequence:
ATGAACAAGAAAACTAAATTAATTCATGGTGGGCACACAACAGACGATTATACAGGTGC
CGTTACAACACCAATTTATCAAACAAGTACATATTTACAAGATGATATTGGTGATTTACG
TCAAGGATATGAATATTCTCGTACTGCGAATCCAACAAGAAGTTCTGTAGAAAGCGTTAT
TGCGACATTAGAAAATGGCAAACATGGCTTTGCATTTAGTTCAGGTGTTGCAGCAATCAG
TGCAGTTGTTATGCTGTTGGACAAAGGAGATCATATTATTTTAAATTCAGATGTATACGGC
GGTACTTATCGCGCATTGACAAAAGTATTTACACGATTTGGCATTGAAGTGGATTTTGTA
GATACAACGCATACAGATTCAATTGTACAAGCGATACGCCCAACAACAAAGATGTTGTTT
ATTGAAACACCTTCTAATCCATTATTACGTGTTACTGACATTAAAAAGTCTGCTGAAATTG
CGAAAGAACACGGTTTGATTTCAGTTGTTGATAACACATTTATGACACCTTATTATCAGA
ATCCATTAGATTTAGGTATCGATATTGTCTTACATTCTGCAACGAAATATTTAGGTGGACA
TAGTGATGTCGTTGCTGGTTTAGTTGCAACATCGGATGACAAGCTTGCAGAACGTTTAGC
ATTTATTTCAAATTCAACAGGTGGCATTTTAGGACCTCAAGATAGCTATTTACTTGTGAGG
GGTATTAAAACATTAGGTTTACGTATGGAACAAATTAATCGCAGTGTTATTGAAATTATT
AAAATGTTACAAGCACATCCAGCTGTGCAACAAGTGTTCCATCCAAGTATTGAAAGTCAT
TTAAATCATGATGTCCATATGGCTCAAGCGGATGGCCATACAGGTGTGATTGCATTTGAA
GTGAAAAATACAGAAAGTGCCAAACAATTGATTAAAGCAACATCGTATTACACATTAGCT
GAAAGTTTAGGTGCAGTGGAAAGTTTAATTTCAGTACCTGCATTGATGACACATGCATCC
ATTCCAGCAGATATTCGAGCTAAAGAAGGTATTACAGACGGACTTGTAAGAATTTCTGTA
GGTATTGAAGATACTGAAGATTTAGTCGATGATTTAAAACAAGCACTAGATACGTTATAA

> **metN1**

Function: protein coding sequence; methionine import ATP-binding protein 1 (core genome, variable)
Best match: metN1_CC005_CF-Marseille_CABA01000058.1[22942:23967:r]
Position: 013-contig_317_RC: 17230 … 18256; Length: 1026 bp
Sequence:
ATGATTGAGTTTCGACAAGTTAGTAAATCATTTCATAAGAAAAGGCAAACAATAGATGCT
TTGAAGGACATATCATTTACGGTCAATCGCAATGATATTTTTGGTGTGATTGGATATAGTG
GTGCAGGAAAAAGTACGTTGGTAAGACTCGTGAATCATCTTGAAGCTGCCTCGAATGGAC
AAGTGATTGTAGATGGACATGATATTACGAATTATAGCGAAAAAGGCATGCGAGAAATT
AAGAAAGATATCGGTATGATATTTCAGCATTTCAATTTATTAAATTCAGCTACCGTATTTA
AAAATGTAGCAATGCCACTCATTTTAAGTAAGAAAAGCAAAACAGAAATTAAGCAACGA
GTAACAGAAATGCTTGAATTTGTAGGATTGAGTGATAAAAAAGACCAATTTCCTGATGAA
TTATCTGGTGGGCAGAAGCAAAGGGTGGCTATTGCAAGAGCGCTTGTTACTAATCCGAAA
ATACTCCTATGCGATGAAGCAACAAGCGCATTGGATCCAGCAACGACTGCTTCGATATTG
ACGTTATTAAAGAATGTCAATCAAACCTTTGGCATTACAATTATGATGATTACACATGAA
ATGCGCGTTATTAAAGACATTTGTAATCGTGTTGCTGTAATGGAAAAGGGGCAAGTGGTT
GAAACAGGAACTGTTAAAGAGGTGTTTAGTCATCCTAAAACGACGATTGCTCAAAATTTT
GTGTCTACAGTTATACAGACTGAGCCAAGTACATCATTGATTCGTCGATTGAATGACGAA
CAAGTTGACGATTTTAAAGATTATAAAATCTTCGTCGAGGAAACTCAGGTGACACAACCG
ATTATAAATGACTTGATTCAAATTTGTGGCAGAGAGGTTAAAATTTTATTTTCATCTATGT
CAGAAATACAAGGTAACACCGTATGTTATATGTGGCTTCGATTTAATATAGATCAACAAT
TTGATGACACGGCAATAAATCAATATTTCAAAGAGAAAAATATTCAATTTGAGGAGGTGC
ATTAA

> **metI**

Function: protein coding sequence; D-methionine transport system permease (core genome, variable)
Best match: metI_CC008_COL_CP000046.1[506065:506724]
Position: 013-contig_317_RC: 18889 … 18919; Length: 30 bp
Sequence:
GTTCTAGCTAGATTCATACGTAGACATTAA

> **metQ**

Function: protein coding sequence; D-methionine transport system substrate-binding protein (core genome, constant)
Best match: metQ_CC001_MSSA476_BX571857.1[467806:468648]
Position: 013-contig_317_RC: 18955 … 19798; Length: 843 bp
Sequence:
ATGAAAAGATTGATTGGGTTAGTTATCGTAGCACTTGTATTATTAGCAGCGTGTGGTAGT
AACAATGATAAAAAAGTAACAATTGGTGTCGCATCAAATGACACTAAGGCTTGGGAGAA
GGTTAAAGAATTAGCTAAAAAAGATGATATTGATGTGGAGATTAAGCACTTCTCCGATTA
CAATTTACCGAATAAAGCATTAAACGATGGTGATATTGATATGAATGCATTCCAACATTT
TGCATTTTTAGATCAGTATAAGAAGGCACATAAAGGAACAAAGATTTCAGCATTGAGTAC
AACAGTTTTAGCACCGTTGGGCATTTACTCAGATAAAATTAAAGATATCAAAAAGGTTAA
AGATGGTGCTAAAGTTGTCATTCCAAATGATGTATCAAACCAAGCACGTGCACTTAAACT
ATTAGAAGCAGCTGGTTTAATAAAACTGAAAAAAGATTTCGGATTAGCAGGTACAGTGA
AAGATATAACGTCAAATCCAAAACATTTAAAAATTACTGCAGTAGATGCACAACAAACT
GCACGTGCTTTATCTGATGTCGATATTGCAGTTATTAATAACGGTGTAGCAACTAAAGCG
GGGAAAGACCCTAAAAATGATCCGATATTTTTAGAAAAATCAAATTCAGATGCTGTAAAG
CCATATATTAATATTGTTGCAGTTAATGACAAAGACTTGGATAACAAAACATATGCTAAA
ATCGTAGAATTGTATCATTCAAAAGAAGCTCAAAAAGCGTTGCAGGAAGATGTCAAAGA
TGGAGAGAAACCTGTTAATTTATCTAAAGATGAGATTAAGGCAATAGAAACGTCATTAGC
AAAATAA

> **aaa**

Function: protein coding sequence; N-acetylmuramoyl-L-alanine amidase (core genome, variable)
Best match: aaa_CC008_A5948_ACKD01000044.1[32597:33601]
Position: 013-contig_317_RC: 20129 … 21137; Length: 1008 bp
Sequence:
GTGCAAAAAAAAGTAATTGCAGCTATTATTGGGACAAGCGCGATTAGCGCTGTTGCGGCA
ACTCAAGCAAATGCGGCTACAACTCACACAGTAAAACCGGGTGAATCAGTGTGGGCAAT
TTCAAATAAGTATGGGATTTCGATTGCTAAATTAAAGTCATTAAACAATTTAACATCTAAT
CTAATTTTCCCAAACCAAGTACTAAAAGTATCTGGCTCAAGTAATTCTACGAGTAATAGT
AGCCGTCCATCAACGAACTCAGGTGGCGGATCATACTACACAGTACAAGCAGGCGACTC
ATTATCATTAATCGCATCAAAATATGGTACAACTTACCAAAATATTATGCGTCTTAATGGT
TTAAATAATTTCTTTATTTATCCAGGTCAAAAATTAAAAGTATCAGGTACTGCTAGCTCAA
GTAACGCTGCGAGCAATAGTAGCCGTCCGTCAACGAACTCAGGTGGCGGATCATACTATA
CAGTACAAGCAGGTGACTCATTGTCATTAATCGCATCAAAATATGGTACAACTTATCAAA
AAATTATGAGCTTAAATGGCTTAAATAATTTCTTTATATATCCGGGTCAAAAATTAAAAG
TAACTGGTAATGCAACATCTTCAAACTCAGGATCTGCAACAACGACAAATAGAGGTTACA
ATACACCAGTATTTAGTCACCAAAACTTATATACATGGGGTCAATGTACATATCATGTATT
TAATCGTCGTGCTGAAATTGGTAAAGGTATTAGTACTTATTGGTGGAATGCTAATAACTG
GGATAACGCAGCGGCAGCAGATGGTTACACTATCGACAATAGACCTACTGTAGGTTCTAT
CGCTCAAACAGATGTAGGTTACTATGGTCATGTTATGTTTGTAGAACGTGTAAATAACGA
TGGTAGTATTTTAGTTTCAGAAATGAACTATTCAGCTGCACCAGGTATTTTAACTTACAGA
ACGGTACCAGCTTACCAAGTAAATAATTATAGATATATTCACTAA

> **tnpIS431-06**

Function: protein coding sequence; transposase for IS431 (mobile element)
Best match: tnpIS431-06_circ_S-epidermidis_GQ900381.1[43799:43807] (completely identical)
Position: 013-contig_317_RC: 21053 … 21062; Length: 9 bp
Sequence:
ATGAACTAT

> **gltC**

Function: protein coding sequence; transcription activator of glutamate synthase operon

Best match: gltC_CC001_MW2-USA400_BA000033.2[474976:475860:r]RC
Position: 013-contig_317_RC: 24790 … 25675; Length: 885 bp
Sequence:
TTAATGGAATGTAGACGTTTTAGTCATTAATTGCTGAATGAGTGTTAATAAGATACCAAT
ATCACTCTTCGTATAAGGTTCTTTCGTAATAGCACATATCGTTCTTTTTAATTCAGTATGAT
CTAATTTTATATCTATCCATGATTTAGATTCTGGTAAATGTATATTTTGTGATGAAATGAT
GTAACCTTCTTTTTGACGAAGGAGATACTGCGCAAGTGGTTGGCTACTGATTGTGTATAC
ATCTGATTTAGTAATCTTGCGCAATTGTTTTTTTACAGTTTCGGCAAATGGTGCCAAGCAA
TAAATATGACTATGCTCAAACTGAATTAATGGTGGGTGTGTCGCCATCGTAATTGGATCG
TCTGAAGGCGCATATAAATGATAGTGCTCTTCGAATAAAGGTAGCATATGTAATTGTTTG
TGTTTACGTATTTCTGGTGTAAGTTCCGTGAAACCAATGTCTATATTCCCATTTAATACGC
TATTTATAATTGTGTCATGTTCTAATAAGCTCGGTATGACATGTGTATCATTTTGTAAATG
AAACGTTTGGATAAGTGGTAGTAACATGTGGGATACGTCACTCTCATCATAGCCAATGTA
GATACTTTTATTTTTAGTTAATCCATGGCTTTGAAATTGTTCAATCGTGCTATCTAAATGTT
CAATAATACGCAGAGCTTCATTAAATAATAATTTCCCTTCAGATGTGAGCGTAATATTGC
GTCCTTGCTTTTTAAATAAAGACACATTAAGTTCTTGTTCTAATAATGTAATTTGACGGCT
TATCGCTGATTGAGCAATGTTTAGTTCAAGTGCTGTTTCAGAGATATGTTCTCTTTTAGCG
ACCTCGATAAAATATCTTAATTGTTTAATTTCCAT

## > gltB

Function: protein coding sequence; glutamine 2-oxoglutarate amidotransferase
Best match: gltB_CC001_MW2-USA400_BA000033.2[476042:480541]
Position: 013-contig_317_RC: 25855 … 30355; Length: 4500 bp
Sequence:
ATGCACAATGAGAAATTAATTAAAGGCTTATATGACTATCGTGAGGAACATGATGCGTGT
GGTATTGGTTTTTATGCGAATATGGATAATAAAAGGTCTCACGACATCATTGATAAATCG
CTTGAAATGTTGCGACGCTTAGATCATAGGGGCGGGGTCGGCGCAGATGGCATCACTGGT
GATGGCGCAGGTATTATGACTGAAATACCTTTTGCATTTTTCAAACAACATGTAACGGAC
TTTGATATCCCAGGTGAAGGTGAATATGCCGTGGGGTTATTTTTTTCCAAAGAACGCGTTT
TAGGTTCTGAACATGAAGCAGTTTTTAAAAAAAATTTTGAAGGCGTAGGGTTATCAATTC
TTGGTTATCGTAATGTACCAGTTAATAAAGATGCCATTGCTAAACATGTAGCAGATACGA
TGCCAGTCATTCAACAAGTGTTTATTGATATTAGGGACATTGAAGATGTTGAAAAGCATT
TGTTTTTAGCGAGAAAACAATTAGAGTTCTATTCGACTCAGTGCGATTTAGAATTGTATTT
TACGAGCTTATCACGCAAAACAATTGTATATAAAGGTTGGTTACGATCAGACCAAATTAA
AAAACTATATACAGATTTATCGGATGATTTATATCAATCAAAGCTAGGGTTAGTGCATTC
GAGATTTAGTACGAATACATTCCCGAGTTGGAAAAGGGCACATCCTAACCGTATGTTAAT
GCATAATGGTGAGATTAACACGATTAAAGGTAATGTAAACTGGATGCGAGCACGCCAAC
ATAAATTAATCGAAACATTATTTGGCGAGGATCAACATAAAGTGTTTCAAATTGTCGATG
AGGATGGTAGTGACTCTGCTATTGTAGATAATGCGCTAGAGTTCTTATCGTTAGCCATGG
AGCCAGAAAAGGCAGCGATGCTACTCATACCTGAACCTTGGTTATATAATGAAGCGAATG
ATGCAAATGTACGTGCGTTTTATGAATTTTATAGTTATTTAATGGAACCGTGGGATGGTCC
TACAATGATTTCGTTCTGTAACGGTGACAAACTTGGCGCGCTTACAGATAGAAATGGATT
ACGTCCAGGTCGTTATACGATTACTAAAGATAACTTTATTGTCTTTTCATCTGAAGTGGGT
GTTGTGGACGTACCTGAAAGTAATGTTGCTTTTAAAGGTCAATTGAATCCTGGAAAGTTA
TTGCTTGTTGATTTTAAACAGAATAAAGTCATTGAAAATAATGATTTAAAAGGTGCGATT
GCTGGAGAATTACCATATAAAGCGTGGATTGATAACCATAAAGTTGACTTTGATTTTGAA
AATATACAATATCAAGATTCGCAATGGAAAGATGAGACGTTATTTAAATTACAACGTCAG
TTTGCATACACGAAAGAAGAGATTCATAAATATATTCAGGAACTTGTAGAAGGTAAGAA
GGATCCTATCGGTGCAATGGGATATGATGCGCCAATTGCAGTGTTGAACGAGCGACCAGA
ATCACTATTTAATTACTTTAAACAGCTGTTTGCACAAGTTACGAATCCACCAATTGATGCG
TATCGTGAAAAAATCGTAACGAGTGAACTTTCTTATTTAGGTGGCGAAGGTAACTTACTA
GCACCTGACGAAACGGTTTTAGATCGTATTCAATTGAAAAGGCCGGTATTGAATGAATCA
CACTTAGCAGCGATTGATCAGGAACATTTTAAATTAACTTATTTATCAACGGTATATGAA
GGGGATTTGGAAGATGCGTTAGAAGCATTAGGACGAGAAGCAGTGGATGCTGTAAAGCA
AGGCGCTCAAATTCTAGTGTTAGATGATAGTGGATTAGTTGATAGCAATGGCTTTGCGAT
GCCGATGTTACTCGCAATAAGTCATGTGCATCAATTACTTATTAAAGCAGATTTACGTATG

TCTACAAGTTTAGTCGCTAAATCTGGTGAGACACGAGAAGTGCATCATGTTGCTTGTTTAC
TCGCATATGGCGCGAATGCAATTGTGCCATACCTAGCGCAACGTACAGTTGAACAACTGA
CATTGACAGAAGGGTTACAAGGCACCGTTGTCGATAATGTTAAGACATATACGGATGTAT
TGTCCGAAGGTGTCATTAAAGTAATGGCTAAGATGGGAATTTCGACAGTGCAAAGTTATC
AAGGTGCACAAATATTTGAAGCGATTGGCTTGTCTCATGATGTGATTGATCGTTATTTTAC
TGGGACACAGTCTAAGTTATCGGGTATTTCGATTGATCAAATTGATGCTGAAAATAAAGC
ACGTCAACAAAGTGATGATAATTATCTTGCATCAGGTAGTACATTCCAATGGAGACAACA
AGGTCAACATCATGCTTTTAATCCGGAATCTATTTTCTTATTGCAGCACGCATGTAAAGAA
AATGACTATGCGCAATTTAAAGCATACTCTGAAGCGGTGAACAAAAATAGAACAGATCA
CATTAGACATTTACTTGAATTTAAAGCATGTACACCGATTGACATCGACCAAGTTGAACC
GGTAAGTGACATTGTCAAACGCTTTAATACAGGGGCGATGAGTTATGGATCGATTTCAGC
GGAAGCACATGAAACGTTAGCACAAGCCATGAACCAATTAGGTGGAAAGAGTAATAGTG
GTGAAGGTGGCGAAGATGCAAAACGTTATGAAGTACAAGTTGATGGAAGCAACAAAGTA
AGTGCGATTAAACAAGTTGCTTCTGGGCGTTTTGGTGTAACTAGTGATTATTTACAACATG
CCAAAGAAATTCAAATTAAAGTTGCGCAAGGTGCAAAGCCTGGTGAAGGTGGTCAATTA
CCTGGTACTAAGGTATATCCGTGGATTGCGAAGACAAGAGGGTCAACGCCAGGTATCGGT
CTGATTTCACCACCGCCACATCATGATATTTATTCAATAGAAGATTTAGCGCAACTGATAC
ATGATTTGAAAAATGCGAATAAAGATGCGGATATCGCGGTAAAATTAGTTTCGAAAACA
GGTGTTGGTACCATTGCATCTGGGGTGGCAAAAGCATTTGCAGATAAAATTGTCATCAGT
GGTTACGATGGTGGTACAGGGGCTTCACCTAAAACGAGTATTCAGCATGCCGGTGTTCCT
TGGGAGATTGGTTTAGCAGAAACACATCAAACATTAAAACTAAATGACTTAAGAAGTCGT
GTTAAGTTAGAAACAGACGGTAAGTTATTAACTGGTAAAGATGTAGCGTACGCATGTGCG
CTTGGAGCGGAAGAATTTGGATTTGCAACTGCACCATTAGTGGTGTTGGGCTGTATTATG
ATGCGTGTATGCCATAAAGATACATGTCCAGTAGGAGTTGCAACTCAAAACAAAGATTTA
CGTGCTTTATATAGAGGTAAAGCACATCATGTTGTTAATTTTATGCATTTTATTGCACAAG
AATTAAGAGAAATTTTAGCATCTTTAGGTTTGAAACGTGTAGAAGACTTAGTTGGAAGAA
CTGATTTATTACAACGATCATCAACATTAAAAGCGAATAGCAAAGCGGCTAGTATTGATG
TTGAAAAACTGTTATGTCCTTTCGATGGGCCAAACACAAAAGAAATTCAACAAAATCATA
ATCTTGAGCATGGATTTGATTTAACAAATTTATATGAAGTAACGAAGCCATATATTGCTG
AAGGGCGTCGCTATACAGGTAGCTTTACAGTAAATAATGAACAACGTGATGTAGGGGTTA
TTACAGGTAGTGAGATTTCGAAACAATATGGAGAAGCAGGACTTCCTGAAAATACAATTA
ATGTTTATACGAATGGTCATGCTGGTCAAAGTCTTGCAGCATATGCACCGAAAGGCTTAA
TGATTCATCATACTGGAGATGCGAATGACTATGTTGGTAAAGGATTATCTGGTGGTACGG
TCATTGTCAAAGCACCTTTTGAAGAACGACAAAATGAAATTATTGCTGGTAACGTCTCAT
TCTATGGTGCGACAGGTGGTAAGGCATTTATTAACGGTAGTGCAGGAGAAAGATTCTGTA
TTAGAAATAGTGGTGTAGATGTTGTCGTTGAAGGTATCGGCGACCATGGATTAGAGTATA
TGACTGGTGGACATGTCATTAATTTAGGTGATGTAGGTAAGAACTTCGGTCAAGGTATGA
GTGGTGGTATTGCTTACGTTATCCCGTCTGATGTAGAAGCTTTTGTTGAAAATAATCAACT
AGATACGCTTTCGTTTACAAAGATTAAACACCAAGAAGAAAAAGCATTCATTAAGCAAAT
GCTGGAAGAACATGTGTCACACACGAATAGTACGAGAGCGATTCATGTGTTAAAACATTT
TGATCGCATTGAAGATGTCGTCGTTAAAGTTATTCCTAAAGATTATCAATTAATGATGCA
AAAAATTCATTTGCACAAATCATTACATGACAATGAAGATGAAGCGATGTTAGCTGCATT
TTACGATGACAGTAAAACAATCGACGCTAAACATAAACCAGCCGTTGTGTATTAA

## > gltD

Function: protein coding sequence; glutamine 2-oxoglutarate amidotransferase
Best match: gltD_CC030_MRSA252_BX571856.1[503158:504621]
Position: 013-contig_317_RC: 30372 … 31836; Length: 1464 bp
Sequence:
ATGGGTGAATTTAAAGGATTTATGAAGTATGACAAACAGTACTTAGGTGAATTATCACTG
GTAGACCGTTTGAAGCATCATAAAGCATATCAACAACGATTTACTAAAGAAGATGCCTCT
ATCCAAGGTGCGCGATGTATGGATTGTGGAACGCCGTTTTGTCAAACCGGACAACAGTAT
GGTAGGGAAACAATAGGTTGTCCAATTGGAAACTACATTCCTGAATGGAACGACTTAGTG
TATCATCAAGATTTTAAAACTGCTTATGAACGCTTAAGCGAAACAAATAACTTTCCTGAC
TTTACAGGGCGTGTATGTCCTGCACCATGCGAAAGTGCTTGTGTGATGAAGATTAATAGA
GAATCGATTGCGATTAAAGGTATTGAACGCACGATTATTGATGAAGCTTTTGAAAATGGT

TGGGTTGAGCCGAAAATTCCAAGTCATCGTAGAAATGAAAAAGTTGCAATCATTGGTAGT
GGTCCTGCAGGATTGACTGCCGCTGAAGAACTGAATTTATTAGGGTACCAAGTGACAATT
TATGAGCGTGCTAGAGAATCAGGCGGTTTATTAATGTATGGTATTCCAAATATGAAGCTT
GATAAATATGTCGTTCGTCGTCGTATTAAATTAATGGAAGAAGCGGGCATAACTTTCATT
AACGGCGTTGAAGTAGGTGTTGATATTGATAAAGAAACTTTAGAATCTGAGTATGACGCT
ATTATATTATGTACTGGGGCACAAAAAGGTAGGGATTTACCTTTGGAAGGTCGCATGGGT
GAAGGTATACATTTTGCTATGGACTATCTGACTGAACAAACACAACTTTTAAATGGTGAA
ATTGATGATATCACTATTACAGCAAAGGATAAAAATGTCATCATTATTGGTGCTGGTGAT
ACAGGAGCAGACTGTGTAGCAACGGCATTAAGAGAAAACTGTAAATCGATTGTTCAATTT
AATAAATATACGAAATTGCCAGAAGCAATTACATTTACAGAAAATGCATCATGGCCTTTA
GCAATGCCGGTGTTTAAAATGGACTATGCGCACCAAGAGTACGAAGCTAAGTTTGGTAAG
GAACCACGTGCATATGGTGTTCAAACAATGCGTTACGATGTTGACGATAAAGGACACATA
CGTGGTTTGTATACTCAAATTTTAGAGCAAGGCGAAAATGGTATGGTCATGAAAGAAGGA
CCTGAAAGATTTTGGCCTGCTGACCTTGTATTATTATCAATCGGCTTCGAAGGTACAGAAC
CAACTGTACCGAATGCTTTTAACATTAAAACGGATAGAAATCGAATCGTGGCGGATGATA
CAAACTATCAAACTAATAATGAAAAAGTATTTGCTGCTGGAGATGCTAGACGTGGTCAAA
GTTTAGTTGTATGGGCGATTAAAGAAGGTAGAGGCGTAGCGAAAGCAGTAGATCAGTAT
TTAGCGAGTAAAGTTTGTGTATAA

## > tx_universal3

Function: bidirectional rho-independent terminator
Best match: tx_universal3_CC001_MW2-USA400_BA000033.2[1397772:1397835:r]RC
Position: 014-contig_260_RC: 31 … 61; Length: 30 bp
Sequence:
TTAATAGAAATCAGCTTTTTACATTGCCT

## > tnpIS1272

Function: protein coding sequence; transposase of insertion element IS1272
Best match: tnpIS1272_15653_EU272084.1[3547:5193]
Position: 014-contig_260_RC: 33 … 63; Length: 30 bp
Sequence:
AATAGAAATCAGCTTTTTACATTGCCTAA

## > treP

Function: protein coding sequence; PTS system/trehalose-specific IIBC component (core genome, constant)
Best match: treP_CC008_NCTC8325_CP000253.1[440144:441571]
Position: 014-contig_260_RC: 491 … 1919; Length: 1428 bp
Sequence:
ATGGCTGTAAAAAGAGAAGATGTAAAAGCCATCGTAACCGCTATTGGGGGAAAAGAAAA
TCTTGAAGCTGCAACGCATTGTGTAACACGATTACGTTTAGTGCTGAAGGATGAAAGTAA
AGTTGATAAAGACGCATTAAGTAATAACGCGTTGGTCAAGGGGCAGTTTAAAGCAGACC
ATCAATATCAAATTGTCATTGGTCCAGGAACAGTCGATGAAGTGTATAAGCAGTTTATTG
ATGAAACAGGTGCTCAAGAAGCTTCGAAAGATGAAGCGAAACAAGCAGCTGCGCAAAAA
GGGAATCCAGTACAACGTTTGATCAAATTATTGGGGGATATTTTTATACCAATATTACCTG
CGATTGTGACAGCTGGTTTGTTAATGGGAATCAATAATTTACTAACAATGAAAGGTTTAT
TTGGTCCGAAAGCACTTATTGAGATGTATCCACAAATTGCTGATATTTCAAACATCATTAA
TGTGATTGCGAGTACGGCATTTATTTTCTTACCAGCATTAATTGGTTGGAGTAGTATGCGT
GTATTTGGTGGTAGTCCGATTCTAGGCATAGTCTTAGGTTTGATTTTAATGCATCCGCAAT
TAGTATCTCAGTATGATTTGGCAAAAGGGAATATTCCGACATGGAACTTATTTGGCTTAG
AGATTAAGCAGTTGAATTACCAAGGTCAAGTGTTGCCAGTTTTAATTGCAGCTTATGTTCT
AGCTAAAATTGAAAAAGGATTAAATAAAGTCGTTCACGATTCGATAAAAATGTTGGTCGT
TGGCCCCGTAGCGCTTTTAGTTACTGGATTTTTAGCATTTATTATCATTGGACCAGTTGCA
TTATTGATTGGTACAGGAATTACATCTGGTGTTACATTTATATTCCAACATGCAGGATGGC
TTGGCGGAGCAATATATGGATTGTTATATGCACCACTTGTAATTACAGGACTACACCATA

TGTTTTTAGCAGTAGATTTCCAATTGATGGGTAGCAGCTTAGGTGGTACGTATTTATGGCC
AATCGTTGCGATTTCCAATATTTGTCAGGGCTCTGCAGCATTTGGAGCATGGTTTGTCTAT
AAACGTCGTAAAATGGTTAAAGAAGAAGGCTTGGCATTAACATCTTGTATTTCTGGTATG
TTAGGTGTTACTGAACCAGCTATGTTTGGTGTGAACTTACCTCTGAAATATCCATTTATCG
CTGCGATATCAACGTCTTGTGTATTGGGGGCAATCGTTGGTATGAATAACGTGCTTGGAA
AAGTTGGTGTTGGTGGCGTGCCAGCATTCATTTCAATTCAAAAAGAATTTTGGCCAGTAT
ATCTTATTGTGACAGCTATTGCTATTGTTGTACCATGTATACTAACAATTGTGATGTCTCA
TTTTAGTAAACAAAAAGCGAAAGAAATTGTTGAAGATTAA

> **treC**

Function: protein coding sequence; alpha,alpha-phosphotrehalase (core genome, constant)
Best match: treC_CC001_MW2-USA400_BA000033.2[484344:485984]
Position: 014-contig_260_RC: 1982 … 3623; Length: 1641 bp
Sequence:
GTGTCGAAAGAAATAGATTGGAGAAAATCCGTTGTATATCAAATTTATCCTAAGTCGTTT
AATGATACGACGGGGAATGGTATAGGAGATATCAATGGAATTATAGAAAAATTGGATTA
TATCAAGTTATTGGGTGTTGATTATATTTGGTTAACACCAGTGTATGAATCACCGATGAAT
GATAATGGCTATGATATCAGCAATTATTTAGAAATCAATGAAGACTTTGGAACGATGGAT
GATTTTGAAAAGTTAATCAAAGTTGCGCATCAAAAAGACTTGAAAGTGATGTTAGATATT
GTCATTAATCATACGTCGACGGAGCATGAATGGTTTAAAGAAGCCCGTAAATCTAAAGAT
AACCCTTATAGAGATTATTACTTTTTCAGATCATCTGAAGACGGGCCGCCAACAAATTGG
CATTCTAAATTCGGTGGTAATGCATGGAAGTATGATTCTGAGACAGATGAATATTATTTA
CATTTATTTGATGTCAGTCAAGCTGATTTAAATTGGGATAATCCGGAAGTACGTCAATCGT
TATATCGCATAGTCAATCATTGGATAGACTTCGGCGTTGATGGTTTTCGATTTGATGTCAT
TAACTTAATTTCTAAAGGTGAATTTAAGGACTCTGACAAAATAGGTAAAGAATTTTATAC
GGATGGTTCTAGAGTGCATGAGTTTCTGCATGAATTAAATCGTCAAACGTTTGGTAACAC
TGACATGATGACTGTAGGAGAAATGTCTTCGACGACGATTGAAAATTGTATTAAGTATAC
ACAACCAGAACGCCAAGAATTGAATAGTGTTTTTAATTTTCATCATCTAAAGGTTGATTAT
GTTGATGGTGAAAAGTGGACAAATGCGAAGCTTGATTTCCATAAGTTAAAGAAAATTCTG
ATGCAATGGCAACGAGGTATTTATGACGGTGGTGGATGGAACGCGATTTTCTGGTGTAAT
CATGATCAGCCACGGGTAGTGTCTAGATTTGGTGATGATACGTCGGAAGAGATGAGGATA
CAAAGTGCTAAAATGTTAGCTATCGCACTGCATATGTTGCAAGGGACGCCATATATTTAC
CAAGGGGAAGAAATTGGTATGACGGACCCACATTTTACATCAATAGCACAATATCGCGAT
GTTGAATCGATTAATGCCTACCATCAGTTGTTAAGTGAAGGGCATGCTGAAGCGGATGTG
TTAGCGATTTTAGGACAGAAGTCACGAGACAATTCGAGAACGCCTATGCAATGGAGTGAT
GATGTTAATGCTGGATTTACAGCTGGTAAGCCTTGGATTGATATTTCGGAAAATTATCATC
AGGTCAACGTTAGACAAGCACTTCAGAATAAAGAGTCTATTTTCTATACGTATCAAAAAT
TAATACAATTAAGACATACGCATGATATTATTACGTATGGAGACATTGTGCCACGTTTTAT
GGATCATGATCATTTATTTGTTTATGAACGTCATTATAAGAATCAACAATGGCTAGTAATT
GCGAATTTCTCAGCATCGGCTGTTGATTTGCCAGAAGGATTGGCTAGAGAAGGTCGTGTT
GTGATTCAAACAGGCACAGTGGAAAATAATACGATAAGCGGGTTTGGTGCAATTGTAATC
GAAACAAACGCGTAA

> **treR**

Function: protein coding sequence; trehalose operon repressor (core genome, constant)
Best match: treR_CC030_MRSA252_BX571856.1[508595:509323]
Position: 014-contig_260_RC: 3647 … 4376; Length: 729 bp
Sequence:
ATGGCGAAACAAAAAAAGTTTATGAAGATTTATGAGGCGTTGAAAGAAGATATATTAAA
TGGGCAGATTCAATATGGTGAACAAATTCCATCTGAACATGATTTGGTGCAATTGTACCA
GTCATCTCGAGAGACCGTGCGTAAGGCATTAGATTTGTTGGCATTAGACGGCATGATTCA
AAAGATTCATGGTAAAGGGTCACTTGTCATTTATCAGGAGGTTACAGAGTTTCCATTTTCT
GAACTTGTTAGTTTTAAAGAAATGCAAGAAGAAATGGGCGTCGCATATTTAACTGAAGTT
GTTGTGAATGAGGTTGTTGAAGCGCATGAAGTTCCAGAAGTTCAACATGCTTTAAACATC
AATTCTAGTGAATCACTCATTCATATTGTTAGAACTCGTCGGCTTAACCAACATGTGAAG

ATTGTTGATGAAGATTATTTTCTAAAGTCGATTGTTTCAGATATAGGTAATGATGTTGCGA
GTGATTCTATTTATGATTATTTGGAAAAGGTATTAAATCTTAATATTAGTTATTCAAGTAA
GTCTATTACTTTTGAACCGTTTGATGAACAAGCATATCAATTGTTTGGTGATGTATCGGTG
GCTTATTCAGCAACAGTTCGAAGTATTGTGTATTTAGAAAATACAATGCCGTTTCAATATA
ATATTTCAAAACATCTTGCAAATGAATTTAAATTTAATGACTTCTCAAGACGTCGTACAA
AGTAA

> **ffs**

Function: noncoding RNA 4.5S-RNA (core genome, constant)
Best match: ffs_CC001_MW2-USA400_BA000033.2[486799:487068] (completely identical)
Position: 014-contig_260_RC: 4437 … 4707; Length: 270 bp
Sequence:
CCGTGCTAGGTGGGGAGGTAGCGGTTCCCTGTACTCGAAATCCGCTTTATGCGAGGCTTA
ATTCCTTTGTTGAGGCCGTATTTTTGCGAAGTCTGCCCAAAGCACGTAGTGTTTGAAGATT
TCGGTCCTATGCAATATGAACCCATGAACCATGTCAGGTCCTGACGGAAGCAGCATTAAG
TGGATCATCATATGTGCCGTAGGGTAGCCGAGATTTAGCTAACGACTTTGGTTACGTTCGT
GAATTACGTTCGATGCTTAGGTGCACGG

> **dnaX**

Function: protein coding sequence; DNA polymerase III gamma and tau subunits (core genome, constant)
Best match: dnaX_AC025951.19[17480:19177]
Position: 014-contig_260_RC: 5607 … 7305; Length: 1698 bp
Sequence:
TTGAATTATCAAGCCTTATATCGTATGTACAGACCCCAAAGTTTCGACGATGTCGTCGGA
CAAGAACATGTCACGAAGACATTGCGCAATGCGATTTCGAAAGAAAAACAGTCGCATGC
TTATATTTTTAGTGGTCCGAGAGGTACGGGGAAAACGAGTATTGCCAAAGTGTTTGCTAA
AGCAATCAACTGTCTAAATAGCACTGATGGAGAACCTTGTAATGAATGTCATATTTGTAA
AGGCATTACGCAGGGGACTAATTCAGATGTGATAGAAATTGATGCTGCTAGTAATAATGG
CGTTGATGAAATAAGAAATATTAGAGACAAAGTTAAATATGCACCAAGTGAATCGAAAT
ATAAAGTTTATATTATAGATGAGGTGCACATGCTAACAACAGGTGCTTTTAATGCCCTTTT
AAAGACGTTAGAAGAACCTCCAGCACACGCTATTTTTATATTGGCAACGACAGAACCACA
TAAAATCCCTCCAACAATCATTTCTAGGGCACAACGTTTTGATTTTAAAGCAATTAGCCTA
GATCAAATTGTTGAACGTTTAAAATTTGTAGCAGATGCACAACAAATTGAATGTGAAGAT
GAAGCCTTGGCATTTATCGCTAAAGCGTCTGAAGGGGGTATGCGTGATGCATTAAGTATT
ATGGATCAGGCTATTGCATTTGGTGATGGTACGTTAACATTGCAAGATGCGTTGAATGTC
ACAGGTAGCGTACATGATGAAGCGTTGGATCACTTGTTTGATGATATTGTACAAGGTGAC
GTACAAGCATCTTTTAAAAAATACCATCAGTTTATAACAGAAGGTAAAGAAGTGAATCGC
CTAATAAATGATATGATTTATTTTGTCAGAGATACGATTATGAATAAAACATCTGAGAAA
GATACTGAGTATCGAGCACTGATGAACTTAGAATTAGATATGTTATATCAAATGATTGAT
CTTATTAATGATACATTAGTGTCGATTCGTTTTAGTGTGAATCAAAACGTTCATTTTGAAG
TGTTGTTAGTAAAATTAGCTGAGCAGATTAAGGGTCAACCACAAGTGATTGCGAATGTAG
CTGAACCAGCACAAATTGCTTCATCGCCAAACACAGATGTATTGTTGCAACGTATGGAAC
AGTTAGAGCAAGAACTAAAAACACTAAAAGCACAAGGAGTGAGTGTCGCTCCTGTTCAA
AAATCTTCGAAAAAGCCTGCGAGAGGCATACAAAAATCTAAAAATGCATTTTCAATGCA
ACAAATTGCAAAAGTGCTAGATAAAGCGAATAAGGCAGATATCAAATTGTTGAAAGATC
ATTGGCAAGAAGTGATTGATCATGCCAAAAATAATGATAAAAAATCACTCGTTAGTTTAT
TGCAAAATTCGGAACCTGTGGCGGCAAGTGAAGATCACGTACTTGTGAAATTTGAGGAA
GAGATCCATTGTGAAATCGTCAATAAAGACGACGAGAAACGTAGTAGTATAGAAAGTGT
TGTATGTAATATCGTTAATAAAAACGTTAAAGTTGTTGGTGTACCATCAGATCAATGGCA
AAGAGTTCGAACGGAATATTTACAAAATCGTAAAAACGAAGGCGATGATATGCCAAAGC
AACAAGCACAACAAACAGATATTGCTCAAAAAGCAAAAGATCTTTTCGGTGAAGAAACT
GTACATGTGATAGATGAAGAGTGA

> **yaaK**

Function: protein coding sequence; DNA binding protein (core genome, constant)
Best match: yaaK_CC005_Mu50-VRSA_BA000017.4[528695:529012] (completely identical)
Position: 014-contig_260_RC: 7394 … 7712; Length: 318 bp
Sequence:
ATGCGCGGTGGCGGAAACATGCAACAAATGATGAAACAAATGCAAAAAATGCAAAAGA
AAATGGCTCAAGAACAAGAAAAACTTAAAGAAGAGCGTATTGTAGGAACAGCTGGCGGT
GGCATGGTTGCAGTTACTGTAACTGGTCATAAAGAAGTTGTCGACGTTGAAATCAAAGAA
GAAGCTGTAGACCCAGACGATATTGAAATGCTACAAGACTTAGTGTTAGCAGCTACTAAT
GAAGCGATGAATAAAGCTGATGAGCTTACTCAAGAACGTTTAGGTAAACATACTCAAGG
CTTAAACATCCCTGGAATGTGA

## > recR
Function: protein coding sequence; recombination protein R (core genome, constant)
Best match: recR_CC001_MW2-USA400_BA000033.2[490084:490680] (completely identical)
Position: 014-contig_260_RC: 7718 … 8315; Length: 597 bp
Sequence:
ATGCATTATCCAGAACCTATATCAAAACTTATTGATAGCTTTATGAAATTGCCAGGCATTG
GTCCAAAGACAGCCCAACGTCTGGCTTTTCATACCTTAGATATGAAAGAAGACGATGTTG
TTCAGTTTGCCAAAGCATTAGTAGATGTTAAGAGAGAATTAACATATTGTAGCGTATGTG
GTCACATTACTGAAAATGATCCATGTTATATTTGTGAAGATAAGCAAAGAGATCGTTCAG
TTATTTGTGTTGTGGAAGATGACAAAGATGTCATAGCTATGGAAAAAATGAGAGAATACA
AAGGTTTATATCACGTTTTACATGGGTCTATTTCGCCTATGGATGGCATTGGACCAGAAG
ATATTAATATTCCTTCATTGATTGAACGCTTGAAAAACGATGAAGTTAGCGAATTAATCTT
AGCTATGAACCCGAACTTAGAGGGGGAATCTACAGCCATGTATATTTCTAGATTAGTTAA
GCCTATAGGTATCAAAGTGACGAGATTAGCACAAGGGTTATCGGTAGGTGGCGATTTAGA
GTATGCTGACGAAGTAACATTATCTAAAGCAATCGCAGGTAGAACAGAAATGTAA

## > txbi_Q1Y6R4
Function:  bidirectional rho-independet terminator (core genome, constant)
Best match: txbi_Q1Y6R4_CC005_Mu50-VRSA_BA000017.4[536170:536241] (completely identical)
Position: 015-contig_316_RC: 230 … 302; Length: 72 bp
Sequence:
ATTATAAAAAGTGAACAGCAGTAAGATAGTTTTTAATCATAAATCATCTTACTGCTGTTTT
TAGATTTTATG

## > A5IQ33
Function: protein coding sequence; putative Orn/Lys/Arg decarboxylase (core genome, constant)
Best match: A5IQ33_CC001_MW2-USA400_BA000033.2[497404:498741]
Position: 015-contig_316_RC: 466 … 1804; Length: 1338 bp
Sequence:
ATGAAGCAACCTATTTTAAATAAATTAGAAAGTTTAAATCAAGAAGAAGCGATTTCTTTG
CATGTTCCGGGTCATAAAAAATATGACTATTGGTCATTTATCTCAATTATCAATGACAATGG
ATAAAACTGAAATACCTGGATTAGATGATTTACATCATCCTGAAGAAGTCATTTTGGAGA
GTATGAAGCAGGTGGAGAAGCATTCAGATTATGATGCTTATTTCTTAGTGAATGGCACCA
CTTCAGGAATATTATCTGTCATCCAGTCTTTTTCACAGAAAAAAGGCGATATCTTAATGGC
AAGAAATGTACATAAATCTGTATTACATGCGCTCGATATTAGCCAACAAGAAGGGCATTT
TATTGAAACGCATCAAAGTCCGTTAACGAATCATTATAATAAAGTTAATTTAAGCCGTTT
GAATAATGACGGTCACAAACTTGCTGTGTTGACTTATCCTAACTATTACGGTGAAACATTT
AATGTAGAAGAGGTTATCAAATCTTTGCACCAATTAAATATTCCTGTACTCATTGACGAA
GCACACGGCGCGCACTTTGGATTGCAAGGATTTCCAGATTCTACATTAAATTATCAAGCT
GACTATGTTGTTCAATCTTTTCATAAAACGTTACCAGCTTTAACGATGGGCTCGGTACTTT
ATATTCATAAAAATGCACCTTATAGAGAAACTATTATAGAATATCTAAGCTACTTCCAAA
CATCTAGTCCTTCGTATTTGATTATGGCTAGTTTAGAGTCAGCTGCCGAGTTCTATAAAAC
ATATGATAGTACCGTGTTTTTTGATAAGAGAGCGCAATTAATCGAATGTTTGGAGAAGAA

GGGTTTTGAAATGCTTCAAGTTGATGATCCGTTGAAGTTGCTGATAAAATATGAAGGTTTT
ACAGGTCATGATATTCAAAATTGGTTTATGAATGCACATATCTATTTAGAATTAGCGGAC
GACTATCAAGCATTAGCGATATTGCCGTTATGGCATCATGATGATACGTATTTATTTGATT
CGCTTTTACGTAAAATTGAAGATATGATTTTACCGAAAAAATCAGTTTCTAAAGTTAAAC
AAACACAACTTTTAACAACTGAAGGTAACTATAAACCAAAACGCTTTGAATATGTTACTT
GGTGTGATTTGAAAAAGGCAAAAGGTAAAGTTCTGGCGCGACATATTGTCCCGTATCCGC
CAGGGATTCCTATTATTTTCAAAGGAGAAACAATAACTGAAAATATGATAGAATTGGTAA
ATGAATATCTGGAAACTGGAATGATAGTTGAAGGAATTAAAAATAATAAAATTTTAGTTG
AGGATGAATAA

> **tmk**
Function: protein coding sequence; thymidylate kinase (core genome, constant)
Best match: tmk_CC001_MW2-USA400_BA000033.2[498743:499360]
Position: 015-contig_316_RC: 1805 … 2423; Length: 618 bp
Sequence:
ATGTCAGCTTTTATAACTTTTGAGGGCCCAGAAGGCTCTGGAAAAACAACTGTAATTAAT
GAAGTTTACCATAGATTAGTAAAAGATTATGATGTCATTATGACAAGAGAACCGGGCGGC
GTTCCTACTGGTGAAGAAATACGTAAAATTGTATTAGAAGGCAATGATATGGACATTAGA
ACTGAAGCAATGTTATTTGCTGCATCTAGAAGAGAACATCTTGTATTAAAGGTCATACCA
GCTTTAAAAGAAGGTAAGGTTGTGTTGTGTGATCGCTATATCGATAGTTCATTAGCTTATC
AAGGTTATGCTAGAGGGATTGGCGTTGAAGAAGTAAGAGCATTAAACGAATTTGCAATA
AATGGATTATATCCAGACTTGACGATTTATTTGAATGTTAGTGCTGAAGTAGGCCGCGAA
CGTATTATTAAAAATTCAAGAGATCAAAATAGATTAGATCAAGAAGATTTAAAGTTTCAC
GAAAAAGTAATTGAAGGTTACCAAGAAATCATTCATAATGAATCACAACGGTTCAAAAG
CGTTAATGCAGATCAACCTCTTGAAAATGTTGTTGAAGACACGTATCAAACTATCATCAA
ATATTTAGAAAAGATATGA

> **tnpIS431-06**
Function: protein coding sequence; transposase for IS431 (mobile element)
Best match: tnpIS431-06_circ_S-epidermidis_GQ900381.1[43799:43807]RC (completely identical)
Position: 015-contig_316_RC: 2088 … 2097; Length: 9 bp
Sequence:
ATAGTTCAT

> **holB**
Function: protein coding sequence; DNA-directed DNA polymerase III delta' subunit
Best match: holB_CC001_MW2-USA400_BA000033.2[499931:500857]
Position: 015-contig_316_RC: 2993 … 3920; Length: 927 bp
Sequence:
ATGGATGAACAGCAACAATTGACGAATGCATATCATTCAAATAAATTATCGCATGCCTAT
TTATTTGAAGGTGATGATGCACAAACGATGAAACAAGTTGCGATTAATTTTGCAAAGCTG
ATTTTATGTCAAACAGATAGTCAATGTGAAACAAAGGTTAGTACATATAATCATCCAGAC
TTTATGTATATATCAACAACTGAGAATGCAATTAAGAAAGAACAAGTTGAACAACTTGTG
CGTCATATGAATCAACTTCCTATAGAAAGCACAAATAAAGTGTACATCATTGAAGACTTT
GAAAAGTTAACTGTTCAAGGGGAAAACAGTATCTTGAAATTTCTTGAAGAACCACCGGAC
AATACGATTGCTATTTTATTGTCTACAAAACCTGAGCAAATTTTAGACACAATCCATTCAA
GGTGTCAGCATGTGTATTTCAAGCCTATTGATAAAGAAAAGTTTATAAATAGATTAGTTG
AACAAAACATGTCTAAGCCAGTAGCTGAAATGATTAGTACTTATACTACGCAAATAGATA
ATGCAATAGCTTTAAATGAAGAATTTGATTTATTAGCATTAAGGAAATCAGTTATACGTT
GGTGTGAATTGTTGCTTACTAACAAGCCAATGGCACTTATAGGTATTATTGATTTATTGAA
ACAGGCTAAAAATAAAAAACTGCAATCTTTAACTATTGCAGCTGTGAATGGTTTCTTCGA
AGATATCATACATACAAAGGTAAATGTAGAGGATAAACAAATATATAGTGATTTAAAAA
ATGATATTGATCAATATGCGCAAAAGTTGTCGTTTAATCAATTAATTTTGATGTTTGATCA
ACTGACGGAAGCACATAAGAAATTGAATCAAAATGTAAATCCAACGCTTGTATTTGAACA
AATCGTAATTAAGGGTGTGAGTTAG

> **metS**
Function: protein coding sequence; methionyl-tRNA synthetase (core genome, constant)
Best match: metS_CC001-ST772_118_AJGE01000045.1[7369:9342] (completely identical)
Position: 015-contig_316_RC: 7453 … 9427; Length: 1974 bp
Sequence:
ATGGCTAAAGAAACATTTTATATAACAACCCCAATATACTATCCTAGTGGGAATTTACAT
ATAGGACATGCATATTCTACAGTGGCTGGAGATGTTATTGCAAGATATAAGAGAATGCAA
GGATATGATGTTCGTTATTTGACTGGAACGGATGAACACGGTCAAAAAATTCAAGAAAA
AGCTCAAAAAGCTGGTAAGACAGAAATTGAATATTTGGATGAGATGATTGCTGGAATTA
AACAATTGTGGGCTAAGCTTGAAATTTCAAATGATGATTTTATCAGAACAACTGAAGAAC
GTCATAAACATGTCGTTGAGCAAGTGTTTGAACGTTTATTAAAGCAAGGTGATATCTATTT
AGGTGAATATGAAGGTTGGTATTCTGTTCCGGATGAAACATACTATACAGAGTCACAATT
AGTAGACCCACAATACGAAAACGGTAAAATTATTGGTGGCAAAAGTCCAGATTCTGGAC
ACGAAGTTGAACTAGTTAAAGAAGAAAGTTATTTCTTTAATATTAGTAAATATACAGACC
GTCTATTAGAATTCTATGACCAAAATCCAGATTTTATACAACCACCATCAAGAAAAAATG
AAATGATTAACAACTTCATTAAACCAGGACTTGCTGATTTGGCTGTTTCTCGTACATCATT
TAACTGGGGTGTCCATGTTCCGTCTAATCCAAAACATGTTGTTTATGTTTGGATTGATGCG
TTAGTTAACTATATTTCAGCATTAGGCTATTTATCAGATGATGAGTCACTATTTAACAAAT
ACTGGCCAGCAGATATTCATTTAATGGCTAAGGAAATTGTGCGATTCCACTCAATTATTTG
GCCTATTTTATTGATGGCATTAGACTTACCGTTACCTAAAAAAGTCTTCGCACATGGTTGG
ATTTTGATGAAAGATGGAAAAATGAGTAAATCTAAAGGTAATGTCGTAGACCCTAATATT
TTAATTGATCGCTATGGTTTAGATGCTACACGTTATTATCTAATGCGTGAATTACCATTTG
GTTCAGATGGCGTATTTACACCTGAAGCATTTGTTGAGCGTACAAATTTCGATCTAGCAA
ATGACTTAGGTAACTTAGTAAACCGTACGATTTCTATGGTTAATAAGTACTTTGATGGCG
AATTACCAGCGTATCAAGGTCCACTTCATGAATTAGATGAAGAAATGGAAGCTATGGCTT
TAGAAACAGTGAAAAGCTACACTGAAAGCATGGAAAGTTTGCAATTTTCTGTGGCATTAT
CTACGGTATGGAAGTTTATTAGTAGAACTAATAAGTATATTGACGAAACAACGCCTTGGG
TATTAGCTAAGGACGATAGCCAAAAAGATATGTTAGGCAATGTAATGGCTCACTTAGTTG
AAAATATTCGTTATGCAGCTGTATTATTACGTCCATTCTTAACACATGCGCCGAAAGAGA
TTTTTGAACAATTGAACATTAACAATCCTCAATTTATGGAATTTAGTAGTTTAGCGCAATA
TGGTGTGCTTACTGAGACAATTATGGTTACTGGGCAACCTAAACCTATTTTCCCAAGATTG
GATAGCGAAGCGGAAATTGCATATATCAAAGAATCAATGCAACCGCCTGCTACTGAAGA
GGAAAAAGAAGAGATTCCTAGCAAACCTCAAATTGATATTAAAGACTTTGATAAAGTTG
AAATTAAGGCAGCAACGATTATTGATGCTGAACATGTTAAGAAGTCAGATAAGCTTTTAA
AAATTCAAGTAGACTTAGATTCTGAACAAAGACAAATTGTATCAGGAATTGCCAAATTCT
ATACACCAGATGATATTATTGGTAAAAAAGTAGCAGTTGTTACTAACCTGAAACCAGCTA
AATTAATGGGACAAAAATCTGAAGGTATGATATTATCTGCTGAAAAAGATGGTGTATTAA
CCTTAGTAAGTTTACCAAGTGCAATTCCAAATGGTGCAGTGATTAAATAA

> **tatP**
Function: protein coding sequence; deoxyribonuclease (core genome, variable)
Best match: tatP_CC001-ST772_118_AJGE01000045.1[9373:10146] (completely identical)
Position: 015-contig_316_RC: 9457 … 10231; Length: 774 bp
Sequence:
ATGTTAATCGATACACATGTCCATTTAAATGATGAGCAATACGATGATGATTTGAGTGAA
GTGATTACACGTGCTAGAGAAGCAGGTGTTGATCGTATGTTTGTAGTTGGTTTTAACAAA
TCGACAATTGAACGCGCGATGAAATTAATCGATGAGTATGATTTTTTATATGGCATTATC
GGTTGGCATCCAGTTGACGCAATTGATTTTACAGAAGAACACTTGGAATGGATTGAATCT
TTAGCTCAGCATCCAAAAGTGATTGGTATTGGTGAAATGGGATTAGATTATCACTGGGAT
AAATCTCCTGCAGATGTTCAAAAGGAAGTTTTTAGAAAGCAAATTGCTTTAGCTAAGCGT
TTGAAGTTACCAATTATCATTCATAACCGTGAAGCAACACAAGACTGTATCGATATCTTA
TTGGAGGAGCATGCTGAAGAGGTAGGCGGGATTATGCATAGCTTTAGTGGTTCTCCAGAA
ATTGCAGATATTGTAACTAATAAGCTGAATTTTTATATTTCATTAGGTGGACCTGTGACAT
TTAAAAATGCTAAACAGCCTAAAGAAGTTGCTAAGCATGTGTCAATGGAGCGTTTGCTAG

TTGAAACCGATGCACCGTATCTTTCGCCACATCCGTATAGAGGGAAGCGAAATGAACCGG
CGAGAGTAACTTTAGTAGCTGAACAAATTGCTGAATTAAAAGGCTTATCTTATGAAGAAG
TGTGCGAGCAAACAACTAAAAATGCAGAGAAATTGTTTAATTTAAATTCATAA

> **rnmV**

Function: protein coding sequence; ribonuclease M5 (core genome, variable)
Best match: rnmV_CC007_TCH959-USA300_AASB02000182.1[10255:10791]
Position: 015-contig_316_RC: 10397 … 10934; Length: 537 bp
Sequence:
ATGAAAATCAATGAGTTTATAGTTGTAGAAGGACGAGATGATACTGAGCGTGTTAAACG
AGCTGTTGAATGTGATACGATTGAAACGAATGGTAGTGCCATCAACGAACAAACTTTAGA
AGTAATTAGAAATGCTCAACAAAGTCGAGGCGTTATTGTATTAACAGATCCAGATTTCCC
AGGAGATAAAATTAGAAGTACAATTACTGAACATGTCAAAGGTGTTAAACATGCGTATAT
TGATAGAGAAAAAGCTAAAAATAAAAAAGGGGAAATTGGTGTTGAACATGCCGACTTAA
TTGATATTAAAGAAGCGTTAATGCATGTTAGTTCACCCTTTGATGAAGCTTATGAATCAAT
TGATAAATCTGTGCTTATAGAGTTGGGGTTAATTGTTGGGAAAGATGCAAGGCGCCGTAG
AGAAATTTTAAGTAGAAAATTGCGAATCGGCCATTCCAATGGTAAGCAGTTATTGAAAAA
GTTAAATGCATTTGGTTATACCGAAGCGGATGTAAGGCAAGCTTTAGAAGATGAATGA

> **ksgA**

Function: protein coding sequence; ribosomal RNA small subunit methyltransferase A (core genome, constant)
Best match: ksgA_CC001_MW2-USA400_BA000033.2[507882:508775]
Position: 015-contig_316_RC: 10944 … 11838; Length: 894 bp
Sequence:
ATGTTGGATAATAAAGATATTGCAACACCATCAAGAACGCGAGCGTTGTTAGATAAATAT
GGCTTTAATTTTAAAAAAAGTTTAGGACAGAACTTTTTGATAGATGTGAATATCATTAAT
AATATCATTGATGCAAGTGATATTGATGCACAAACTGGGGTGATTGAAATTGGTCCAGGT
ATGGGGTCGTTGACAGAACAATTGGCCAGACATGCTAAAAGAGTATTGGCATTTGAAATT
GATCAACGTTTAATACCTGTATTAAATGATACACTATCACCTTATGATAATGTGACGGTG
ATTAATGAAGATATTTTAAAAGCGAATATTAAAGAAGCTGTTGAAAATCATTTACAAGAT
TGTGAAAAAAATAATGGTTGTTGCAAACCTGCCGTACTATATTACGACGCCAATTTTATTA
AATTTAATGCAACAAGATATACCAATTGATGGCTACGTGGTGATGATGCAAAAAGAAGT
GGGCGAACGCTTAAATGCTGAAGTAGGTTCAAAAGCATATGGTTCGTTATCAATTGTCGT
ACAATACTATACAGAGACTAGTAAAGTATTAACGGTACCTAAATCTGTATTTATGCCACC
ACCTAATGTTGATTCAATAGTTGTAAAACTGATGCAGAGAACTGAACCGTTAGTAACAGT
AGATAACGAGGAAGCATTCTTTAAGTTAGCAAAAGCAGCATTTGCACAAAGAAGAAAGA
CAATTAACAATAACTATCAAAATTATTTTAAAGATGGTAAACAACACAAAGAAGTGATTT
TACAATGGTTGGAACAAGCAGGTATTGATCCAAGACGTCGCGGTGAAACGCTATCTATTC
AAGATTTTGCTAAATTGTATGAAGAAAAGAAAAAATTCCCTCAATTAGAAAATTAA

> **veg**

Function: protein coding sequence; Veg protein (core genome, constant)
Best match: veg_CC001_MW2-USA400_BA000033.2[508875:509138] (completely identical)
Position: 015-contig_316_RC: 11937 … 12201; Length: 264 bp
Sequence:
ATGCCAAAATCAATTTTGGACATCAAAAATTCTATTGATTGTCATGTAGGAAATCGTATT
GTACTGAAAGCCAATGGAGGCCGTAAGAAAACAATAAAACGTTCTGGAATTTTAAAAGA
AACATATCCGTCAGTTTTCATTGTTGAGTTAGATCAAGACAAACACAACTTTGAGAGAGT
ATCTTATACATACACTGATGTGTTAACTGAAAATGTTCAAGTTTCATTTGAAGAGGATAAT
CATCACGAATCAATTGCACACTAA

> **ispE**

Function: protein coding sequence; 4-diphosphocytidyl-2-C-methyl-D-erythritol kinase (core genome, constant)

Best match: ispE_CC001_MW2-USA400_BA000033.2[509448:510296] (completely identical)
Position: 015-contig_316_RC: 12510 … 13359; Length: 849 bp
Sequence:
ATGATATATGAAACGGCACCAGCCAAAATTAATTTTACGCTCGATACACTTTTTAAAAGA
AATGATGGCTATCATGAGATTGAAATGATAATGACAACAGTTGATTTAAATGATCGTTTA
ACTTTTCATAAAAGAAAAGATCGAAAGATAGTTGTTGAGATTGAACATAATTATGTGCCT
TCTAATCATAAAAATCTCGCATATCGTGCAGCGCAACTATTTATTGAGCAATATCAACTA
AAGCAAGGTGTAACAATTTCTATCGATAAAGAAATACCTGTTTCTGCTGGCTTAGCTGGA
GGTTCGGCTGATGCAGCAGCAACGTTAAGAGGATTGAATCGACTTTTTGATATAGGGGCG
AGTTTGGAAGAATTGGCTCTACTAGGCAGTAAAATCGGGACAGATATTCCGTTTTGTATT
TATAATAAAACTGCACTATGTACTGGAAGAGGAGAGAAAATCGAGTTTTTAAATAAACC
ACCTTCAGCTTGGGTGATTCTTGCTAAACCAAACTTAGGCATATCATCACCAGATATATTT
AAGTTGATCAATTTAGATAAGCGTTACGACGTACATACGAAAATGTGTTATGAGGCCTTA
GAAAATCGAGATTATCAACAATTATGTCAAAGTTTGTCTAATCGATTAGAGCCAATTTCT
GTTTCAAAACACCCACAAATCGATAAATTAAAAAATAATATGTTGAAAAGTGGTGCAGAT
GGTGCGTTAATGAGTGGAAGCGGACCGACTGTGTATGGGCTAGCACGAAAAGAAAGCCA
AGCAAAAAATATTTATAATGCAGTTAACGGTTGTTGTAATGAAGTGTACTTAGTTAGACT
ATTAGGATAG

> **purR**

Function: protein coding sequence; purine operon repressor (core genome, constant)
Best match: purR_CC001_MW2-USA400_BA000033.2[510310:511134] (completely identical)
Position: 015-contig_316_RC: 13372 … 14197; Length: 825 bp
Sequence:
ATGAGATATAAACGAAGCGAGAGAATTGTTTTTATGACGCAATATTTGATGAACCATCCG
AATAAATTGATTCCATTAACTTTTTTTGTGAAAAAATTTAAACAGGCGAAGTCTTCAATAA
GTGAAGATGTCCAAATTATAAAAAATACATTCCAAAAAGAAAAGTTAGGTACAGTAATT
ACTACTGCTGGCGCAAGTGGTGGTGTTACGTATAAACCAATGATGAGTAAAGAAGAGGC
GACTGAAGTTGTTAATGAGGTCATTACTCTATTAGAAGAGAAAGAACGTTTGTTACCTGG
CGGGTATTTATTTTTATCAGATTTGGTAGGTAATCCATCGCTACTAAACAAAGTTGGTAAG
TTAATTGCCAGTATTTACATGGAAGAAAAATTAGATGCTGTTGTTACCATTGCGACAAAA
GGTATTTCATTGGCAAATGCGGTTGCTAATATTTTAAATTTACCAGTAGTAGTGATTAGAA
AAGACAACAAGGTGACTGAAGGTTCTACAGTTTCAATTAATTACGTTTCAGGATCTTCAA
GAAAAATAGAGACAATGGTACTTTCGAAGAGAACTTTAGCAGAAAATTCAAATGTTTTAG
TTGTCGATGATTTTATGAGGGCTGGTGGCTCTATTAATGGTGTTATGAATTTAATGAATGA
GTTTAAAGCCCATGTAAAAGGGGTATCAGTACTTGTAGAATCAAAAGAAGTTAAACAAA
GATTGATTGAAGATTATACTTCCTTAGTGAAATTATCTGATGTAGATGAATATAATCAAG
AGTTTAACGTAGAACCTGGCAACAGTTTATCTAAGTTTTCATAA

> **yabJ**

Function: protein coding sequence; translation initiation inhibitor (core genome, constant)
Best match: yabJ_CC001_MW2-USA400_BA000033.2[511151:511531] (completely identical)
Position: 015-contig_316_RC: 14213 … 14594; Length: 381 bp
Sequence:
ATGAAAATCATTAACACAACAAGATTACCGGAAGCACTTGGACCATATTCGCATGCAACA
GTTGTGAATGGTATGGTTTATACTTCTGGTCAGATTCCATTGAATGTTGATGGGGAAATCG
TAAGCGCTGATGTTCAAGCACAGACAAAACAAGTTTTAGAAAATTTAAAGGTTGTTTTGG
AAGAAGCAGGATCTGATTTGAATTCTGTTGCGAAAGCGACCATTTTCATTAAAGATATGA
ATGATTTCCAAAAAAATAAATGAAGTGTATGGTCAATATTTTAATGAACACAAGCCAGCGC
GTAGTTGTGTAGAGGTTGCGCGTTTGCCAAAAGATGTGAAAGTAGAAATTGAATTAGTAA
GTAAAATTAAGGAATTATAA

> **spoVG**

Function: protein coding sequence; septation protein spoVG (core genome, constant)
Best match: spoVG_CC001_MW2-USA400_BA000033.2[511603:511905] (completely identical)

Position: 015-contig_316_RC: 14665 … 14968; Length: 303 bp
Sequence:
ATGAAAGTGACAGATGTAAGACTTAGAAAAATACAAACAGATGGACGAATGAAAGCACT
CGTTTCCATTACATTAGATGAAGCTTTCGTAATTCATGATTTACGTGTAATTGAAGGAAAC
TCTGGCTTGTTCGTTGCAATGCCAAGTAAACGTACACCAGATGGTGAATTCCGCGACATC
GCGCATCCTATTAATTCAGATATGAGACAAGAAATTCAAGATGCAGTGATGAAAGTATAT
GATGAAACAGATGAAGTAGTACCAGATAAAAACGCTACATCAGAAGATTCAGAAGAAGC
TTAA

> **glmU**
Function: protein coding sequence; UDP-N-acetylglucosamine pyrophosphorylase (core genome, constant)
Best match: glmU_CC001_MW2-USA400_BA000033.2[512249:513601] (completely identical)
Position: 015-contig_316_RC: 15311 … 16664; Length: 1353 bp
Sequence:
ATGCGAAGACACGCGATAATTTTGGCAGCAGGTAAAGGCACAAGAATGAAATCTAAAAA
GTATAAAGTGCTACACGAGGTTGCTGGGAAACCTATGGTCGAACATGTATTGGAAAGTGT
GAAAGGCTCTGGTGTCGATCAAGTTGTAACCATCGTAGGACATGGTGCTGAAAGTGTAAA
AGGACATTTAGGCGAGCGTTCTTTATACAGTTTTCAAGAGGAACAACTCGGTACTGCGCA
TGCAGTGCAAATGGCGAAATCACACTTAGAAGACAAGGAAGGTACGACGATCGTTGTAT
GTGGTGACACACCGCTCATCACAAAGGAAACATTAGAAACATTGATTGCGCATCATGAG
GATGCTAATGCTCAAGCAACTGTATTATCTGCATCGATTCAACAACCATATGGATACGGA
AGAATCGTTCGAAATGCGTCAGGTCGTTTAGAACGCATAGTTGAAGAGAAAGATGCAAC
GCAAGCTGAAAAGGATATTAATGAAATTAGTTCAGGTATTTTTGCGTTTAATAATAAAAC
GTTGTTTGAAAAATTAACACAAGTGAAAAATGATAATGCGCAAGGTGAATATTACCTCCC
TGATGTATTGTCGTTAATTTTAAATGATGGCGGCATCGTAGAAGTCTATCGTACCAATGAT
GTTGAAGAAATCATGGGTGTAAATGATCGTGTAATGCTTAGTCAGGCTGAGAAGGCGATG
CAACGTCGTACGAATCATTATCACATGCTAAATGGTGTGACAATCATCGATCCTGACAGC
ACTTATATTGGTCCAGACGTTACAATTGGTAGTGATACAGTCATTGAACCAGGCGTACGA
ATTAATGGTCGTACAGAAATTGGCGAAGATGTTGTTATTGGTCAGTACTCTGAAATTAAC
AATAGTACGATTGAAAATGGTGCATGTATTCAACAGTCTGTTGTTAATGATGCTAGCGTA
GGAGCGAATACTAAGGTCGGACCGTTTGCGCAATTGAGACCAGGCGCGCAATTAGGTGC
AGATGTTAAGGTTGGAAATTTTGTAGAAATTAAAAAAGCAGATCTTAAAGATGGTGCCAA
GGTTTCACATTTAAGTTATATTGGCGATGCTGTAATTGGTGAACGTACTAATATTGGTTGC
GGAACGATTACAGTTAACTATGATGGTGAAAATAAATTTAAAACTATCGTCGGCAAAGAT
TCATTTGTAGGTTGCAATGTTAATTTAGTAGCACCTGTAACAATTGGTGATGATGTATTGG
TGGCAGCTGGTTCCACAATCACAGATGACGTACCAAATGACAGTTTAGCTGTGGCAAGAG
CAAGACAAACAACAAAAGAAGGATATAGGAAATAA

> **prs**
Function: protein coding sequence; ribose-phosphate pyrophosphokinase (core genome, constant)
Best match: prs_CC005_Mu50-VRSA_BA000017.4[552813:553778]
Position: 015-contig_316_RC: 16810 … 17776; Length: 966 bp
Sequence:
ATGTTAAATAATGAATATAAGAATTCGTCATTAAAGATTTTTTCATTGAAAGGAAACGAA
GCATTAGCGCAAGAAGTTGCTGACCAAGTAGGAATTGAACTAGGTAAATGTTCAGTTAAA
CGTTTTAGTGATGGAGAAATTCAAATTAATATCGAAGAGAGTATTCGTGGTTGTGACGTA
TTTATTATTCAACCAACATCATATCCTGTGAATCTACATTTAATGGAATTATTAATTATGA
TTGACGCTTGTAAACGTGCTTCTGCAGCAACAATCAATATTGTAGTGCCATATTATGGATA
TGCAAGACAAGATAGAAAAGCCCGTAGCCGTGAGCCAATCACAGCTAAATTAGTTGCAA
ACTTAATCGAAACAGCTGGCGCAACTCGTATGATTGCGTTAGACTTACATGCACCACAAA
TTCAAGGATTCTTTGATATTCCAATTGACCACTTAATGGGTGTGCCAATTCTTGCTAAACA
TTTCAAAGATGATCCGAATATTAACCCAGAAGAATGTGTCGTTGTTTCACCAGACCATGG
CGGTGTTACACGTGCACGTAAATTAGCTGACATTTTAAAAACTCCAATTGCAATTATAGA
TAAACGTCGTCCTAGACCAAATGTTGCTGAAGTGATGAACATTGTTGGTGAGATTGAAGG

ACGTACGGCAATTATTATTGACGATATTATTGATACAGCAGGTACAATCACTTTAGCTGC
ACAAGCATTAAAAGATAAAGGTGCTAAAGAAGTATATGCTTGTTGTACACACCCTGTTTT
ATCAGGACCGGCTAAAGAACGTATCGAAAATTCTGCTATAAAAGAATTAATCGTAACAA
ACTCAATTCATTTAGATGAAGATCGCAAACCATCTAACACTAAAGAATTATCTGTTGCTG
GTTTAATCGCACAAGCTATCATTCGTGTATACGAAAGAGAATCAGTTAGCGTATTATTTG
ACTAA

## > **rplY**

Function: protein coding sequence; 50S ribosomal protein L25 (core genome, constant)
Best match: rplY_CC001_MW2-USA400_BA000033.2[514863:515516]
Position: 015-contig_316_RC: 17925 … 18579; Length: 654 bp
Sequence:
ATGGCTTCATTAAAGTCAATCATCCGTCAAGGTAAGCAAACACGTTCAGATCTTAAACAA
TTAAGAAAATCTGGTAAAGTACCAGCAGTAGTATACGGTTACGGTACTAAAAACGTGTCA
GTTAAAGTTGATGAAGTAGAATTCATCAAAGTTATCCGTGAAGTAGGTCGTAACGGTGTT
ATCGAATTAGGCGTTGGTTCTAAAACTATCAAAGTTATGGTTGCAGACTACCAATTCGAT
CCACTTAAAAACCAAATTACTCACATTGACTTCTTAGCAATCAATATGAGTGAAGAACGT
ACTGTTGAAGTACCAGTTCAATTAGTTGGTGAAGCAGTAGGCGCTAAAGAAGGCGGCGT
AGTTGAACAACCATTATTCAACTTAGAAGTAACTGCTACTCCAGACAATATTCCAGAAGC
AATCGAAGTAGACATTACTGAATTAAACATTAACGACAGCTTAACTGTTGCTGATGTTAA
AGTAACTGGCGACTTCAAAATCGAAAATGATTCAGCTGAATCAGTAGTAACAGTAGTTGC
TCCAACTGAAGAACCAACTGAAGAAGAAATCGAAGCTATGGAAGGCGAACAACAAACTG
AAGAACCAGAAGTTGTTGGCGAAAGCAAAGAAGACGAAGAAAAAACTGAAGAGTAA

## > **pth**

Function: protein coding sequence; peptidyl-tRNA hydrolase (core genome, constant)
Best match: pth_CC001_MW2-USA400_BA000033.2[515827:516399]
Position: 015-contig_316_RC: 18889 … 19462; Length: 573 bp
Sequence:
ATGAAATGTATTGTAGGTCTAGGTAATATAGGTAAACGTTTTGAACTTACAAGACATAAT
ATCGGCTTTGAAGTCGTTGATTATATTTTAGAGAAAAATAATTTTTCATTAGATAAACAAA
AGTTTAAAGGTGCATATACAATTGAACGAATGAACGGCGATAAAGTGTTATTTATCGAAC
CAATGACAATGATGAATTTGTCAGGTGAAGCAGTTGCACCGATTATGGATTATTACAATG
TTAATCCAGAAGATTTAATTGTCTTATATGATGATTTAGATTTAGAACAAGGACAAGTTC
GCTTAAGACAAAAAGGAAGTGCGGGCGGTCACAATGGTATGAAATCAATTATTAAAATG
CTTGGTACAAACCAATTTAAACGTATTCGTATTGGTGTGGGAAGACCAACGAATGGTATG
ACGGTACCTGATTATGTTTTACAACGCTTTTCAAATGATGAAATGGTAACGATGGAAAAA
GTTATCGAACACGCAGCACGCGCAATTGAAAAGTTTGTTGAAACATCACGATTTGACCAT
GTTATGAATGAATTTAATGGTGAAGTGAAATAA

## > **mfd**

Function: protein coding sequence; transcription-repair-coupling factor (core genome, constant)
Best match: mfd_CC001_MSSA476_BX571857.1[515030:518536]
Position: 015-contig_316_RC: 19461 … 22968; Length: 3507 bp
Sequence:
ATGACAATATTGACAACGCTTATAAAAGAAGATAATCATTTTCAAGACCTTAATCAGGTA
TTTGGACAAGCAAACACACTAGTAACTGGTCTTTCCCCGTCAGCTAAAGTGACGATGATT
GCTGAAAAATATGCACAAAGTAATCAACAGTTATTATTAATTACCAATAATTTATACCAA
GCAGATAAATTAGAAACAGATTTACTTCAATTTATAGATGCTGAAGAATTGTATAAGTAT
CCTGTGCAAGATATTATGACCGAAGAGTTTTCAACACAAAGCCCTCAACTGATGAGTGAA
CGTATTAGAACTTTAACTGCGTTAGCTCAAGGTAAGAAAGGGTTATTTATCGTTCCTTTAA
ATGGTTTGAAAAAGTGGTTAACTCCTGTTGAAATGTGGCAAAATCACCAAATGACATTAC
GTGTTGGTGAGGGTATCGATGTGGACCAATTTCTTAACAAATTAGTTAATATGGGGTACA
AACGGGAATCCGTGGTATCGCATATTGGTGAATTCTCATTGCGAGGAGGTATTATCGATA
TCTTTCCGCTAATTGGGGAACCAATCAGAATTGAGCTATTTGATACCGAAATTGATTCTAT

TCGGGATTTTGATGTTGAAACGCAGCGTTCCAAAGATAATATTGAAGAAGTCGATATCAC
AACTGCAAGTGATTATATCATTACTGAAGAAGTAATCAGCCATCTTAAAGAAGAGTTAAA
AACTGCATATGAAAATACAAGACCCAAAATAGATAAATCAGTGCGCAATGATCTGAAAG
AAACGTATGAAAGCTTTAAATTATTCGAAAGTACATACTTTGATCATCAAATACTACGTC
GTTTAGTAGCGTTTATGTATGAAACACCTTCGACAATTATTGATTATTTCCAAAAAGATGC
AATCATTGCAGTTGATGAATTTAATCGTATTAAAGAAACTGAAGAAAGTTTAACAGTAGA
GTCTGATTCGTTTATTAGCAATATTATTGAAAGTGGTAATGGATTTATAGGACAAAGTTTT
ATAAAATATGATGATTTTGAAACATTGATTGAAGGCTATCCTGTCACTTATTTTTCATTAT
TCGCTACAACAATGCCGATAAAACTAAATCATATTATTAAATTTTCATGTAAACCTGTCCA
ACAATTTTATGGGCAATATGACATTATGCGTTCTGAATTTCAACGATATGTTAATCAAAAC
TATCATATCGTGGTTTTGGTCGAAACCGAAACTAAAGTTGAACGTATGCAAGCGATGTTA
AGTGAAATGCATATTCCATCAATAACAAAATTGCATCGCTCAATGTCATCGGGGCAAGCA
GTGATTATTGAAGGCAGTTTATCTGAAGGATTTGAACTACCTGATATGGGATTAGTTGTC
ATTACTGAGCGTGAGCTTTTTAAATCAAAACAGAAAAAGCAACGAAAACGTACGAAAGC
TATCTCAAATGCTGAAAAAATTAAGTCTTACCAAGATTTAAATGTGGGAGATTATATTGT
TCATGTGCATCATGGTGTTGGTAGATATTTAGGTGTTGAGACGCTCGAAGTGGGGCAAAC
GCATCGTGATTATATTAAATTGCAATATAAAGGTACGGATCAACTATTTGTTCCAGTAGA
TCAAATGGATCAAGTTCAAAAATATGTAGCTTCGGAAGATAAGACGCCAAAATTAAATA
AACTCGGTGGCAGTGAATGGAAAAAAACAAAAGCTAAAGTTCAACAAAGTGTTGAAGAT
ATTGCTGAAGAGTTGATTGATTTATATAAAGAAAGAGAAATGGCAGAAGGTTATCAATAT
GGGGAAGACACAGCTGAGCAAACAACATTTGAATTAGATTTTCCATATGAACTTACGCCT
GACCAAGCTAAATCTATCGATGAAATTAAAGATGACATGCAAAAATCGCGTCCAATGGA
TCGCTTGCTATGTGGTGATGTTGGTTATGGTAAAACTGAAGTTGCAGTGAGAGCAGCATT
CAAAGCTGTAATGGAAGGAAAGCAGGTTGCATTTTTAGTTCCTACAACTATTTTAGCTCA
GCAACATTATGAGACGTTAATTGAGCGTATGCAAGATTTTCCTGTTGAAATTCAATTAAT
GAGTCGTTTTAGAACGCCTAAAGAGATAAAACAAACTAAGGAAGGACTTAAAACTGGAT
TTGTTGACATAGTTGTTGGTACACACAAATTACTTAGTAAAGATATACAGTATAAAGATT
TAGGGCTGTTGATTGTAGATGAAGAACAACGATTTGGTGTACGCCATAAAGAGCGTATTA
AAACATTAAAACATAATGTAGATGTACTAACATTGACTGCAACCCCAATACCTAGAACAT
TGCATATGAGTATGCTAGGTGTGCGCGATTTGTCAGTGATTGAAACGCCGCCAGAAAATC
GTTTCCCAGTTCAAACATATGTATTAGAACAGAACATGAGTTTTATCAAAGAAGCTTTAG
AAAGAGAACTATCCCGTGATGGCCAAGTGTTTTATCTTTATAATAAAGTGCAATCCATTT
ATGAAAAACGAGAACAACTCCAGATGTTAATGCCAGATGCTAACATTGCAGTTGCTCATG
GACAAATGACAGAGCGCGATTTAGAAGAAACGATGTTAAGTTTTATCAATAATGAATATG
ATATTTTAGTAACGACGACGATTATTGAAACAGGTGTCGATGTCCCAAATGCAAATACTT
TGATCATTGAAGATGCAGATCGCTTTGGATTGAGTCAGTTGTATCAATTAAGAGGTCGTG
TTGGTCGTTCAAGTCGTATTGGTTATGCATACTTCTTACATCCAGCAAATAAGGTACTAAC
TGAGACTGCAGAAGATCGATTACAAGCGATTAAAGAATTTACGGAGTTAGGCTCAGGATT
TAAGATTGCGATGCGTGATTTGAACATTCGTGGTGCTGGTAATTTGTTAGGTAAACAACA
GCACGGCTTTATTGATACAGTTGGATTTGATTTGTACAGTCAAATGTTAGAAGAAGCTGT
AAATGAAAAACGTGGTATTAAGGAACCAGAATCTGAGGTGCCAGAAGTCGAAGTTGATT
TAAACTTGGATGCATATTTGCCGACAGAATATATTGCAAATGAACAAGCTAAAATTGAAA
TTTATAAAAAGCTACGAAAAACTGAAACATTTGATCAAATTATCGACATTAAAGATGAAT
TAATTGATCGTTTTAATGATTATCCTGTTGAAGTAGCACGTTTGCTTGATATAGTGGAAAT
AAAAGTACACGCATTACATTCAGGTATCACGTTGATTAAAGATAAAGGGAAAATAATTG
ATATTCATTTATCTGTAAAAGCCACTGAAAATATTGATGGCGAAGTGCTGTTCAAAGCAA
CACAACCTTTAGGTAGAACAATGAAGGTTGGTGTTCAAAATAATGCAATGACAATTACTT
TAACGAAACAAAATCAATGGCTTGATAGTTTGAAGTTTTAGTTAAGTGCATTGAAGAAA
GTATGAGAATCAGTGATGAAGCATAA

> **ltrB**

Function: protein coding sequence; similar to low temperature requirement protein B (core genome, constant)
Best match: ltrB_CC001_MW2-USA400_BA000033.2[519895:521421] (completely identical)
Position: 015-contig_316_RC: 22957 … 24484; Length: 1527 bp
Sequence:

ATGAAGCATAAAGAAGCATTTAATGGCGTTGTCGTGTTAACTGCTGCATTAATTGTCATT
AAAATTCTGAGTGCTGTATATCGAATTCCATATCAAAATATATTAGGTGATACAGGTTTGT
ATGCATATCAACAAGTGTATCCAATTGTAGCATTAGGAATGATATTATCGATGAATGCCA
TTCCTAGTGCAATTACACAAAATATAGGGAAGTATCATAGTGACGAAGCATATGCAAAA
GCGGTCGCTTATATACAATTAGTTGGTATATTATTATTTATTGCTATTTTTGTGTTTGCGAA
CAATATTGCACATATGATGGGTGATAGCCATTTAACACCAATGATTCAAGCAGCAAGTTT
AAGCTTTATATTTATAGGTATGCTTGGCGTGTTAAGAGGTTATTATCAATCTGCAAATAAT
ATGACAGTTCCGGCTATTTCCCAGGTTATAGAACAAGTTATACGAGTAGGTATTATCATT
GTTACTATTGTTATTTTTGTAGACAGAGGTTGGACGATATATGAAGCGGGAACAATTGCT
ATTTTAGCATCAACGATAGGTTTTTTAGGTTCTTCAATTTATTTAGTAGCGCACCGACCTT
TTAAGTTTAAAATGGTAAATAACACTGCAAAGATTGTTTGGAAACAGTTCGCACTTTCGG
TTTTGATTTTCGCTATCAGTCAATTAATCGTAATTTTATGGCAAGTGATTGATAGTGTTAC
TATTATTAAGTCACTTCAAGCGATACGCGTGCCATTCGATGTTGCCATAACTGAAAAAGG
AGTCTATGACCGTGGTGCATCATTTATTCAGATGGGATTGATTGTAACTACAACATTTAGT
TTTGCGCTCATTCCTCTGTTAAGTGACGCAATCAAAATGAATAATCAGGTACTTATGAATC
GTTATGCAAATGCGTCATTAAAGATTACGATTTTAATAAGTACAGCAGCGGGAATAGGAT
TAATTAATTTATTGCCTTTAATGAACGGTGTGTTTTTTAAGACGAATGATTTAACCTTAAC
GTTAAGTGTTTATATGATTACGGTCATTTGTGTATCGTTAATTATGATGGATATGGCTTTA
TTACAAGCGCAACATGCTGTGAGACCTATTTTTGTTGGTATGACGGCAGGATTGGTTATTA
AATTTATACTTAATATCATTTTGATTCGTTTAAGTGGCATTATTGGTGCGAGCATTAGTAC
TGTTGTATCATTAATTATATTCGGTACGATTATCCATATTGCTGTCACGAGAAAATACCAC
TTACATGCGATGAGACGATTTTTTATCAATGTTGTTTTAGGTATGGTATTTATGTCGATTG
TTGTTCAATGCGTGTTAAACATAGTGACAACACACGGTAGATTCACTGGACTCATTGAAT
TATTATGTGCAGCAGTATTAGGTATCATTGCATTGTTTTTCTATATTTTTAGATTTAATGTT
TTGACATATAAAGAGTTAACTTATTTACCATTTGGTTCAAAGTTGTATCAAATTAAGAAA
GGAAGACGTTGA

> **mazG**

Function: protein coding sequence; tetrapyrrole methylase (core genome, variable)
Best match: mazG_CC001-ST772_118_AJGE01000044.1[12202:13395:r] (completely identical)
Position: 015-contig_316_RC: 24483 … 25677; Length: 1194 bp
Sequence:
ATGGCACATACCATTACGATTGTTGGCTTAGGAAACTATGGCATTGATGATTTGCCGCTA
GGGATATATAAATTTTTAAAGACACAAGATAAAGTTTATGCAAGAACGTTAGATCATCCA
GTTATAGAATCATTGCAAGATGAATTAACATTTCAGAGTTTTGACCATGTTTATGAAACA
CATGACCAATTTGAAGATGTCTATAATGATATTGTGGCGCAATTGGTTGAAGCTGCTAAT
GAAAAAGATATTGTCTATGCGGTTCCGGGTCATCCTAGAGTTGCTGAGACAACTACAGTG
AAATTACTGGCTTTAGCAAAGGACAATACTGATATAGATGTGAAAGTTTTAGGTGGTAAA
AGCTTTATTGATGATGTGTTTGAAGCAGTTAATGTAGATCCAAATGATGGCTTCACACTGT
TAGATGCGACATCATTACAAGAAGTAACACTTAATGTTAGAACGCATACATTGATTACGC
AAGTTTATAGTGCAATGGTTGCTGCTAATTTGAAAATCACTTTAATGGAACGATATCCTG
ATGATTACCCTGTTCAAATTGTCACTGGTGCACGAAGCGATGGTGCGGATAACGTTGTGA
CATGCCCATTATATGAATTGGATCATGATGAAAATGCATTCAATAATTTGACGAGTGTAT
TCGTACCAAAAATCATAACATCGACATATTTGTATCATGACTTTGATTTTGCAACGGAAGT
GATTGATACTTTAGTTGATGAAGATAAAGGTTGTCCATGGGATAAAGTGCAAACGCATGA
AACGCTTAAGCGTTATTTACTTGAAGAAACATTTGAATTGTTCGAAGCTATTGACAATGA
AGATGATTGGCATATGATTGAAGAACTAGGAGATATTTTATTACAAGTGTTATTGCATAC
TAGTATTGGTAAAAAAGAAGGGTATATCGACATTAAAGAAGTGATTACAAGTCTTAATGC
TAAAATGATTCGTAGACACCCACACATATTTGGTGATGCCAATGCTGAAACTATCGATGA
CTTAAAAGAAATTTGGTCTAAGGCGAAAGATGCTGAAGGTAAACAGCCAAGAGTTAAAT
TTGAAAAAGTATTTGCAGAGCATTTTTTAAATTTATATGAGAAGACGAAGGATAAGTCAT
TTGATGAGGCCGCGTTAAAGCAGTGGCTAGAAAAAGGGGAGAGTAATACATGA

> **divIC**

Function: protein coding sequence; cell-division protein (core genome, constant)

Best match: divIC_CC008-ST72_TCH130-ST72_ACHD01000274.1[26209:26601] (completely identical)
Position: 015-contig_316_RC: 25954 … 26347; Length: 393 bp
Sequence:
ATGAAAAATAAAGTAGAACATATAGAAAATCAGTACACGTCGCAAGAGAACAAGAAAA
AACAACGTCAAAAAATGAAAATGCGTGTTGTTCGTAGGCGTATTACAGTATTTGCGGGCG
TATTACTTGCGATAATTGTTGTTTTATCAATCTTGCTTGTTGTCCAAAAACATCGCAATGA
TATCGATGCACAGGAGCGAAAAGCGAAAGAAGCACAGTTTCAAAAGCAACAAAATGAA
GAAATTGCGTTAAAAGAAAAGTTGAATAATCTGAATGACAAAGATTATATTGAAAAAAT
TGCGCGTGATGATTATTACTTAAGCAACAAAGGTGAAGTGATTTTTAGGTTGCCAGAAGA
CAAAGATTCGTCTAGCTCAAAATCTTCGAAAAAATAA

> **tilS**
Function: protein coding sequence; tRNA(Ile)-lysidine synthase (core genome, constant)
Best match: tilS_CC001_MW2-USA400_BA000033.2[523970:525265]
Position: 015-contig_316_RC: 27032 … 28328; Length: 1296 bp
Sequence:
ATGCAGTTAAATAGTAATGGTTGGCATGTTGATGACCATATTGTTGTCGCTGTTTCTACAG
GTATTGATAGTATGTGTTTATTGTATCAACTACTAAATGATTATAAAGATAGTTATAGAAA
ACTAACATGCTTACATGTCAATCATGGCGTTAGGTCAGCTTCAATCGAGGAAGCCAGATT
TTTAGAAGTATACTGCGAACGTCATCACATCGATTTACATATCAAAAAGTTAGATTTGTC
GCATAGTCTCGACCGAAATAACAGCATTCAGAATGAAGCTCGAATTAAACGTTACGAATG
GTTTGATGAAATGATGAATGTATTAGAAGCGGATGTATTGCTAACGGCGCATCATTTGGA
CGATCAATTAGAAACTATTATGTATCGTATTTTTAATGGGAAATCGACGCGTAATAAACT
AGGATTTGATGAGTTATCGAAGCGAAAAGGTTATCAGATTTATCGACCACTTTTAGCTGT
CTCTAAAAAAGAAATAAAACAATTCCAAGAGAGATATCATATTCCATATTTTGAAGATGA
ATCTAATAAAGATAACAAATATGTTAGAAATGATATTCGTAATAGAATTATTCCAGCTAT
TGATGAAAATAATCAACTTAAAGTATCGCATTTATTAAAATTAAAACAATGGCATGATGA
ACAATATGATATTTTGCAATATTCAGCTAAACAATTTATTCAAGAATTTGTGAAGTTTGAT
GAACAGTCAAAATATTTAGAGGTTTCTAGACAAGCTTTTAATAACTTACCAAACTCATTA
AAGATGGTTGTGTTGGATTGCCTATTATCAAAGTATTATGAGTTGTTTAATATTAGTGCTA
AAACATACGAAGAGTGGTTTAAACAATTTAGTAGTAAGAAAGCACAATTCAGTATTAATC
TCACGGATAAATGGATAATTCAAATTGCATATGGTAAATTAATAATAATGGCTAAAAATA
ATGGCGATACATATTTTAGAGTTCAAACTATTGAAAAGCCAGGTAATTATATTTTTAACA
AATATCGATTAGAGATACATTCTAATTTACCAAAATGTTTATTTCCGCTTACAGTGAGAAC
ACGACAAAGTGGCGATACATTTAAACTGAATGGGCGCGATGGTTATAAGAAAGTGAATC
GCCTGTTTATAGATTGTAAAGTGCAACAGTGGGTTCGGGATCAAATGCCAATCGTATTGG
ATAAACAACAGCGCATTATTGCGGTAGGAGATTTATATCAACAACAAACAATAAAACAA
TGGATTATAATTAGTAAAAATGGAGATGAATAG

> **hpt**
Function: protein coding sequence; hypoxanthine-guanine phosphoribosyltransferase (core genome, constant)
Best match: hpt_CC001_MW2-USA400_BA000033.2[525270:525809] (completely identical)
Position: 015-contig_316_RC: 28332 … 28872; Length: 540 bp
Sequence:
ATGCATAATGATTTGAAAGAAGTATTGTTAACTGAAGAAGATATTCAAAATATCTGTAAG
GAATTGGGAGCACAATTAACAAAGGATTATCAAGGTAAACCATTAGTATGCGTGGGTATC
TTAAAAGGCTCAGCAATGTTTATGTCAGATTTAATTAAACGAATTGATACCCATTTATCAA
TTGATTTCATGGATGTTTCTAGTTATCACGGAGGCACTGAGTCAACTGGTGAAGTTCAAAT
CATTAAAGATTTAGGTTCTTCTATTGAAAATAAAGACGTATTAATTATTGAAGATATCTTA
GAGACTGGTACTACACTTAAGTCAATTACTGAATTATTACAATCTAGAAAAGTTAATTCA
TTAGAAATAGTTACTTTATTAGATAAACCAAACCGTCGTAAAGCGGACATTGAAGCTAAG
TATGTAGGTAAAAAAATACCAGATGAATTTGTTGTTGGTTACGGTTTAGATTATCGTGAA
TTATACCGAAACTTACCATATATCGGTACGTTAAAACCTGAAGTGTATTCAAATTAA

## > **ftsH**

Function: protein coding sequence; cell-division protein H (core genome, constant)
Best match: ftsH_CC001_MW2-USA400_BA000033.2[526066:528159] (completely identical)
Position: 015-contig_316_RC: 29128 … 31222; Length: 2094 bp
Sequence:
ATGCAGAAAGCTTTTCGCAATGTGCTAGTTATCGTAATAATAGGCGTTATTATTTTTGGTC
TATTTTCATATTTAAACGGTAATGGAAATATGCCGAAACAGCTTACATATAATCAATTTAC
TGAGAAGTTGGAAAAAGGTGACCTTAAAACTTTAGAAATCCAACCACAACAAAATGTCT
ATATGGTAAGTGGTAAAACGAAAAATGATGAAGACTATTCATCAACTATTTTATATAACA
ACGAAAAAGAATTACAAAAAATTACTGATGCTGCTAAAAAGCAAAACGGTGTAAAATTA
ACGATTAAAGAAGAAGAAAAACAAAGTGTCTTTGTGAGTATACTTTCAACATTAATTCCA
GTTGTAGTCATAGCGTTATTATTTATTTTCTTCCTAAGCCAAGCACAAGGTGGCGGTAGTG
GCGGTCGTATGATGAACTTTGGTAAATCTAAAGCAAAAATGTACGATAATAATAAACGTC
GTGTTCGTTTCTCTGATGTAGCAGGGGCAGATGAAGAAAAACAAGAATTAATTGAAATTG
TTGATTTCTTGAAAGATAATAAAAAATTCAAAGAAATGGGATCTAGGATTCCTAAAGGTG
TCTTACTTGTTGGACCTCCAGGTACTGGTAAAACATTACTTGCTAGAGCGGTTGCAGGTG
AAGCTGGCGCACCATTCTTCTCTATTAGTGGTTCAGACTTTGTAGAGATGTTTGTTGGTGT
TGGTGCGAGCCGTGTTCGTGACTTATTCGATAATGCTAAGAAAAACGCGCCTTGTATCAT
CTTTATCGATGAGATTGATGCTGTTGGTCGTCAACGTGGTGCAGGTGTTGGTGGCGGTCAT
GATGAACGTGAACAAACCCTAAACCAATTATTAGTTGAAATGGATGGTTTCGGTGAAAAT
GAAGGTATCATTATGATAGCTGCTACAAACCGTCCTGATATCCTTGACCCAGCCTTATTAC
GTCCAGGTCGTTTTGATAGACAAATTCAAGTTGGTCGTCCAGATGTGAAAGGCCGTGAAG
CAATTCTTCATGTTCATGCTAAAAACAAACCACTTGATGAAACGGTTGATTTAAAAGCAA
TTTCACAACGTACACCTGGTTTCTCAGGTGCTGATTTAGAGAACTTATTAAATGAAGCATC
TTTAATTGCTGTACGTGAAGGTAAAAAGAAAATTGACATGAGAGATATCGAAGAGGCAA
CGGATAGAGTTATAGCCGGACCTGCTAAGAAATCTCGAGTTATTTCTAAGAAAGAACGTA
ATATTGTTGCTCATCACGAAGCTGGTCATACAATTATCGGTATGGTACTTGATGAGGCAG
AAGTAGTGCATAAAGTTACTATTGTTCCACGTGGACAAGCAGGTGGTTATGCAATGATGC
TACCTAAACAAGATCGTTTCTTAATGACTGAACAAGAGTTATTAGATAAAATCTGTGGTT
TACTTGGTGGACGTGTATCAGAAGATATTAACTTTAACGAAGTATCAACAGGTGCTTCAA
ATGACTTCGAACGTGCAACACAAATCGCACGCTCAATGGTTACGCAATATGGTATGAGTA
AAAAATTAGGACCATTACAGTTCGGTCATAGCAATGGTCAAGTATTCTTAGGTAAAGATA
TGCAAGGTGAGCCTAATTATTCAAGCCAAATCGCATATGAAATTGATAAAGAAGTTCAAC
GAATCGTTAAAGAACAATACGAACGTTGTAAACAAATTTTATTAGAGCACAAAGAACAA
TTAATTTTAATTGCTGAAACATTATTAACAGAAGAAACATTAGTTGCTGAACAAATTCAA
TCATTATTCTACGAAGGTAAATTACCTGAAATTGATTATGATGCAGCTAAAGTTGTTAAA
GATGAAGATTCTGAATTTAATGATGGTAAATTCGGTAAATCTTATGAAGAGATTCGTAAA
GAGCAATTAGAAGATGGACAACGTGACGAAAGTGAAGATCGTAAAGAAGAAAAAGATA
TTGCTGAGGATAAAAAAGAAGCTGATAAATCTGATGAAAAAGATGAACCAGCACATCGA
CAAGCCCCAAATATCGAAAAACCTTACGATCCAAATCACCCAGACAATAAATAA

## > **hslO**

Function: protein coding sequence; 33 kDa chaperonin (core genome, constant)
Best match: hslO_CC005_A9299_ACKH01000011.1[31361:32242]
Position: 015-contig_316_RC: 31450 … 32332; Length: 882 bp
Sequence:
ATGACACACGATTATATTGTTAAAGCATTAGCATTTGATGGGGAGATTAGGGCTTATGCT
GCTTTGACAACTGAAACTGTTCAAGAAGCACAAACGAGACATTATACATGGCCGACAGC
ATCTGCTGCAATGGGAAGAACAATGACAGCAACAGCTATGATGGGCGCAATGTTGAAAG
GTGATCAAAAATTAACTGTCACTGTAGATGGCCAAGGACCTATTGGACGAATTATTGCCG
ATGCAAATGCTAAAGGCGAGGTGCGTGCTTATGTAGACCATCCACAAACTCATTTTCCAT
TAAATGAGCAAGGTAAACTTGATGTAAGACGAGCAGTAGGGACAAATGGATCTATTATG
GTTGTTAAAGACGTTGGAATGAAAGACTATTTCTCTGGAGCAAGTCCAATTGTTTCAGGA
GAACTTGGTGAAGATTTTACTTATTATTATGCTACAAGTGAACAAACACCTTCATCGGTA

GGTCTTGGTGTATTGGTAAATCCTGATAATACGATTAAAGCAGCAGGAGGATTTATCATT
CAAGTTATGCCAGGTGCCAAAGATGAAACAATTTCAAAATTAGAAAAAGCAATTAGTGA
AATGACACCAGTTTCTAAATTAATTGAACAAGGATTAACGCCAGAAGGATTACTAAACGA
AATCTTAGGTGAAGACCATGTGCAAATTTTAGAGAAAATACCTGTTCAATTTGAATGTAA
TTGTAGTCATGAGAAATTTTTAAATGCTATTAAAGGATTGGGCGAGGCTGAGATTCAAAA
TATGATTAAAGAAGATCATGGTGCTGAAGCAGTATGTCATTTCTGTGGAAATAAATATAA
ATATACTGAAGAAGAATTAAACGTGTTGCTAGAAAGTTTAGCGTAA

## > **cysK**

Function: protein coding sequence; cysteine synthase (core genome, constant)
Best match: cysK_CC001_MW2-USA400_BA000033.2[529448:530380] (completely identical)
Position: 015-contig_316_RC: 32510 … 33443; Length: 933 bp
Sequence:
ATGGCACAAAAACCAGTAGATAATATTACTCAAATTATTGGCGGTACACCGGTAGTCAAA
TTGAGAAATGTAGTAGATGACAATGCAGCAGATGTTTATGTAAAATTGGAATATCAAAAT
CCAGGTGGTTCTGTAAAGGATAGAATTGCTTTAGCAATGATTGAAAAAGCAGAGCGAGA
AGGCAAAATTAAACCTGGCGATACAATTGTAGAACCAACAAGTGGTAATACAGGTATCG
GTTTAGCATTTGTATGTGCTGCTAAAGGATATAAAGCAGTATTTACTATGCCCGAAACAA
TGAGCCAAGAGCGTCGTAATTTATTAAAAGCATACGGTGCGGAATTAGTTTTAACGCCTG
GATCAGAAGCGATGAAAGGTGCAATTAAAAAAGCTAAAGAATTGAAAGAAGAACATGGT
TACTTCGAGCCACAACAATTTGAAAACCCTGCGAACCCTGAAGTTCATGAGTTAACTACA
GGTCCTGAGTTATTACAACAATTTGAAGGGAAAACTATCGATGCGTTCCTAGCTGGTGTT
GGTACTGGTGGTACGTTATCTGGTGTAGGTAAAGTTCTGAAAAAAGAATATCCTAACATC
GAAATTGTTGCTATAGAGCCTGAGGCTTCTCCAGTATTGAGCGGTGGTGAGCCAGGTCCA
CATAAATTACAAGGTTTAGGTGCTGGATTTATTCCAGGCACTTTGAATACAGAAATCTAT
GACAGTATTATTAAAGTAGGAAATGATACAGCGATGGAAATGTCTCGTCGAGTTGCTAAA
GAGGAAGGTATTTTAGCAGGTATTTCATCAGGTGCTGCGATTTATGCTGCCATTCAAAAA
GCAAAAGAATTAGGAAAAGGTAAAACAGTAGTAACAGTATTGCCGAGTAATGGTGAACG
CTACTTATCAACACCTTTATATTCATTCGATGACTAA

## > **folP**

Function: protein coding sequence; dihydropteroate synthase (core genome, constant)
Best match: folP_CC009_21334_AGTW01000006.1[3929:4732:r] (completely identical)
Position: 015-contig_316_RC: 33658 … 34462; Length: 804 bp
Sequence:
ATGACTAAAACAAAAATTATGGGCATATTAAACGTCACACCAGATTCATTCTCAGATGGT
GGAAAATTTAATAATGTTGAATCAGCTATAAATAGAGTGAAAGCCATGATAGATGAAGG
TGCTGACATTATAGATGTTGGAGGTGTTTCAACGAGACCCGGTCATGAAATGGTTTCATT
AGAAGAAGAGATGAACAGAGTATTACCTGTTGTTGAAGCTATTGTCGGTTTTGATGTAAA
AATTTCAGTCGATACATTTCGAAGTGAGGTTGCTGAAGCATGTTTAAAATTAGGCGTTGA
TATGATTAATGATCAATGGGCGGGTCTGTATGATCATCGTATGTTCCAAATTGTAGCTAA
ATATGACGCGGAAATTATTTTAATGCATAATGGAAATGGTAATCGTGATGAACCGGTTGT
CGAAGAAATGTTAACATCTTTGTTAGCACAAGCACATCAAGCTAAAATAGCTGGTATACC
TTCAAATAAAATTTGGCTAGATCCAGGTATAGGTTTCGCTAAAACTAGAAATGAAGAAGC
CGAAGTTATGGCAAGACTGGATGAACTTGTTGCAACAGAATATCCAGTTTTATTAGCGAC
AAGCCGGAAACGTTTCACTAAAGAGATGATGGGTTATGATACAACACCGGTTGAAAGAG
ATGAAGTAACTGCAGCTACGACTGCATATGGTATTATGAAAGGCGTTAGAGCAGTACGCG
TTCATAATGTCGAGTTGAATGCTAAATTAGCTAAAGGTATAGATTTTTTAAAGGAGAATG
AAAATGCAAGACACAATCTTTCTTAA

## > **folB**

Function: protein coding sequence; dihydroneopterin aldolase (core genome, constant)
Best match: folB_CC001_MW2-USA400_BA000033.2[531377:531742] (completely identical)
Position: 015-contig_316_RC: 34439 … 34805; Length: 366 bp
Sequence:

ATGCAAGACACAATCTTTCTTAAAGGTATGCGCTTTTATGGATATCATGGTGCTTTATCAG
CTGAAAATGAAATAGGGCAAATTTTCAAAGTGGATGTAACTTTGAAAGTAGACTTAGCTG
AAGCTGGGCGTACTGATAATGTTATTGATACAGTTCATTATGGTGAAGTGTTCGAAGAGG
TTAAATCAATTATGGAAGGTAAGGCCGTTAATTTACTTGAGCATCTAGCTGAACGTATTG
CAAATCGTATAAATTCACAATATAATCGTGTAATGGAAACGAAAGTGAGAATCACTAAA
GAAAACCCACCGATTCCGGGTCATTATGATGGAGTAGGTATCGAAATAGTGAGGGAGAA
TAAATGA

## > **folK**

Function: protein coding sequence; 2-amino-4-hydroxy-6-hydroxymethyldihydropteridin e pyrophosphokinase (core genome, constant)
Best match: folK_CC008_NCTC8325_CP000253.1[489065:489541] (completely identical)
Position: 015-contig_316_RC: 34801 … 35278; Length: 477 bp
Sequence:
ATGATTCAAGCATACTTAGGATTAGGTAGTAATATTGGTGATAGAGAAAGCCAGTTAAAC
GATGCTATAAAGATTTTGAATGAATATGATGGTATTAACGTATCTAATATTTCTCCGATTT
ATGAAACAGCACCAGTTGGGTATACTGAGCAACCTAACTTTTTAAATTTGTGTGTTGAAA
TTCAAACAACACTCACAGTATTACAACTGTTGGAATGTTGTTTGAAGACAGAAGAATGTT
TACACCGTATTAGAAAGGAACGATGGGGTCCTAGAACTTTAGATGTGGATATTTTGTTGT
ATGGAGAAGAAATGATAGATTTACCAAAACTGTCGGTGCCACATCCGAGAATGAATGAA
CGTGCATTTGTTTTAATCCCATTAAATGATATAGCAGCAAATGTCGTAGAACCACGTTCG
AAATTGAAAGTGAAAGATTTAGTTTTTGTCGATGACAGTGTAAAGAGATATAAATAA

## > **lysS**

Function: protein coding sequence; lysyl-tRNA synthetase (core genome, constant)
Best match: lysS_leader_CC001_MW2-USA400_BA000033.2[532609:532701] (completely identical)
Position: 015-contig_316_RC: 35671 … 35764; Length: 93 bp
Sequence:
ATGGTTAATGATAAAGTATTAGAAACATCGAAAGAGATGTATGTTGAGCAAAAATGTCTG
ATATTTTATAAAACTTTAAAGGAAAATGTTTGA

## > **rrf**

Function: rRNA 5S ribosomal RNA
Best match: rrf_part_CC005_21172_AFEF01000015.1[3008:3119]
Position: 016-contig_250: 0 … 30; Length: 30 bp
Sequence:
CTTACGTTCCGCTAGAGTAGAACGTTGCCA

## > **txbi_universal1**

Function:  bidirectional rho independent terminator
Best match: txbi_universal1_CC001_MW2-USA400_BA000033.2546526:546597 [4_50ANF10END]
Position: 016-contig_250: 444 … 516; Length: 72 bp
Sequence:
TGTATAAATAGAGAAGAGCAGTAAGACGGTATCTAATTGAAAATGATCTTACTGTTCTTT
TATATACTTTAT

## > **Q1Y6R4**

Function: protein coding sequence; transcript regulator/GntR family (core genome, variable)
Best match: Q1Y6R4_CC001-ST772_118_AJGE01000068.1[799:2181:r]RC (completely identical)
Position: 016-contig_250: 595 … 1978; Length: 1383 bp
Sequence:
TTACTTTATAATTAATGATTTTATTAGAGCGTCTACATGCGGTTTTAAAGCATCATCGTCT
ATACCGCCAAAGCCTAATATAAATTTAGGGGTTTTCTTATAGTCTTGATCATCATCAAAAT
TATAAACTTGTAATTTTAACTTTACTTTGTTTGCTCTATCAAGACACTCTTGTAATGTTAAT

CCATTTTTTACTGTAATTGTAAAATGCATACCCGTTTCAGCACCTTGAATATCAAGCTGCT
CTTTGTAAGGTTTCAATCTTTTTAAAATATAGGTTAGTTTTCTACGATAAATTCGTCTCATT
TTATTTAAATGCCTTTCAAAACCACCGGAAGATATAAACGTTGCAATAAGGTTTTGCATA
TGAACAGGTACAGTGTTGCCTTCAATGTGATTTTGAGAATGATATTTTTTCATTATAGAAT
AGGGTAACACCATATATGCAACTCGACAGCTAGGAAAAATAGACTTTGAAAATGTACTG
ATATAAATCACTTTTTCTCCTCTTGAATATAGACCTTGAATTGCTGGAATGGGTTTGCCGA
AATATCTAAACTCGGAATCATAATCATCTTCTATAATAAATCGTTCTTCTTTTTCTTGAGC
CCATTGTATTAATTGAGTTCGCTTTTTTAAGTCCATCACATATCCAGTTGGAAATTGATGG
GAAGGCGTTATATATACTATATTTTTTTGTGATTTAATAACTTCATCTACGTTTATTCCATT
ATCTTCAACTTCAATTTGTTCATATTCAACTTGTTTTTTATCTAAAATATTTTTGATTGGTG
GATAACTAGGTTTTTCGATAATAAATGTTGAAGTATAAAGTAAATCGACTAATTGATTTA
CTAATTGTTCGGTAGATGAGCCAATTATAATTTGATTAGGATCACAAATTACACCACGAT
TAGTAAATAAATAAAATGCCAGTTGAAATCTTAAATGTAATTCTCCTTGAAAATGTCCTCT
ACGTAATTGATTTAAATGATTTGTATCATAAAGATCTTTGGAATACTTTCTGAAAAGTTCT
ATAGGGAAATGTTTCGTATCTATTTCATCCAAATTAAAAGCATAATCATAAGCTTCATCAC
TCGCTTTTGGTTTATATGAATCATCATCAAAAAGAGAGGGGATAGGTTGATTGTTTAAAA
TTGTTAAAGATTCAATTTCGGACACAAAATATCCAGAGCGAGGTCTTGAATAAATGTAAC
CTTCGTCTAATAGAAGTTGATATGCATGCTCTACGGTTGTTTGGCTGATAGACAAATGTTT
GCTTAATTGTCTTTTAGAATAAAATTTATCGCCTTCTTTAAATTGACCTTCAATTATTTGTT
TTTTTAATTTTTCATAAAGTTGATGGTATAAAGTGTTTTTCAA

> **pdxS**

Function: protein coding sequence; pyridoxal synthetase (core genome, variable)
Best match: pdxS_CC001-ST772_118_AJGE01000068.1[2285:3172] (completely identical)
Position: 016-contig_250: 2081 … 2969; Length: 888 bp
Sequence:
ATGAGTAAAATTATTGGATCAGACAGAGTCAAAAGAGGTATGGCTGAAATGCAAAAAGG
CGGCGTTATTATGGATGTCGTTAATGCTGAGCAAGCAAGAATTGCAGAAGAAGCTGGCGC
GGTAGCAGTTATGGCATTAGAACGAGTACCTTCTGATATTAGAGCTGCTGGTGGCGTTGC
ACGTATGGCAAACCCTAAAATTGTAGAAGAAGTAATGAATGCTGTTTCTATTCCAGTCAT
GGCTAAAGCACGTATTGGTCATATCACTGAAGCAAGAGTATTAGAGGCGATGGGTGTTGA
CTATATTGATGAATCAGAAGTGTTAACACCAGCAGATGAGGAATATCACTTAAGAAAAG
ATCAATTTACAGTACCATTTGTATGTGGATGTCGTAATTTAGGTGAAGCTGCGCGTAGAA
TTGGTGAAGGTGCTGCTATGTTACGTACTAAAGGTGAACCAGGTACAGGTAATATTGTTG
AAGCTGTAAGACATATGAGACAAGTTAATTCAGAAGTTAGTCGTTTGACTGTAATGAATG
ATGATGAGATTATGACTTTTGCGAAAGATATCGGTGCGCCTTATGAAATTTTAAAACAAA
TTAAAGACAATGGTCGTTTACCGGTAGTTAACTTTGCAGCTGGTGGCGTTGCGACTCCTCA
AGATGCTGCTTTAATGATGGAATTAGGTGCCGACGGTGTATTCGTTGGATCAGGTATTTTT
AAATCAGAAGATCCAGAAAAATTTGCTAAAGCAATTGTTCAAGCAACAACACATTACCA
AGACTATGAGCTAATTGGAAGATTAGCAAGTGAACTTGGCACTGCTATGAAAGGTTTAGA
TATCAATCAATTATCATTAGAAGAACGTATGCAAGAGCGTGGTTGGTAA

> **pdxT**

Function: protein coding sequence; amidotransferase involved in pyridoxal biosynthesis (core genome, variable)
Best match: pdxT_CC001-ST772_118_AJGE01000068.1[3176:3736] (completely identical)
Position: 016-contig_250: 2972 … 3533; Length: 561 bp
Sequence:
ATGAAAATAGGTGTATTAGCATTACAAGGTGCAGTACGTGAACATATTAGACATATTGAA
TTAAGTGGTCATGAAGGTATTGCAGTTAAAAAAGTTGAACAATTAGAAGAAATCGAGGG
CTTAATATTACCTGGTGGCGAGTCTACAACGTTACGTCGATTAATGAATTTATATGGATTT
AAAGAGGCTTTACAAAATTCAACTTTACCTATGTTTGGTACATGCGCAGGATTAATAGTT
CTAGCGCAAGATATAGTTGGTGAAGAAGGATACCTTAACAAGTTGAATATTACTGTACAA
CGAAACTCATTCGGTAGACAAGTTGACAGCTTTGAAACAGAATTAGATATTAAATGTATC
GCTACAGATATTGAAGGTGTCTTTATAAGAGCGCCACATATTGAAAAAGTAGGCCAAGGC

GTAGATATCCTATGTAAGGTTAATGAGAAAATTGTAGCCGTCCAGCAAGGTAAATATTTA
GGCGTATCATTCCATCCTGAATTAACAGATGACTATAGAGTAACTGATTACTTTATTAATC
ATATTGTAAAAAAAGCATAG

## > nupC1

Function: protein coding sequence; nucleoside permease C/locus 1 (core genome, constant)
Best match: nupC1_CC001_MW2-USA400_BA000033.2[549820:551034:r]RC (completely identical)
Position: 016-contig_250: 3738 … 4953; Length: 1215 bp
Sequence:
TTAAATAAAGAATCCAGCGATTGCAGCTGAAATGAAAGATACTAGTGTTGCACCGAATA
ATAGTTTCAAACCAAAGCGGGCAACTGTATCTCCTTTTTGTCATTAAGTGATTTAATCGC
ACCTGAAATAATACCAATAGAGCTAAAGTTAGCAAATGATACTAAGAATACAGATGTAA
CACCTTTTGCGTGTTCAGATAAATCACTAAGTTTACCAAGTGCTTGCATTGCTACAAATTC
GTTAGATAATAGTTTTGTCGCCATAACTGAACCGGCTTGAACTGCATCTTGCCATGGCAC
ACCGACTAAGAATGCAAATGGTGCAAAGACAAAACCAATTAATGTTTGGAAATCCCAAG
AAATAGCGCCACCTGAAACTGTACTAAAGATATTGCTTACAATTCCATTTAATAGAGCGA
TAATAGCAATGTATCCGATTAACATTGCGCCTACAATGACAGCTACTTTAAATCCATCTA
AAATATATTCTCCTAGCATTTCGAAGAATGATTGTTGTCTTTCTTCAGTTTCTTCAACTAAT
AATTTGTCATCTTCTTCATTAACTTTATAAGGGTTAATAATTGAAGCGATGATGAAACCAC
CAAATAAGTTTAAGACAACAGCCGTTACAACATATTTAGGTTCAATTAAGGTAAAGTATG
CACCGATAATTGAAGCAGAAACAGTCGACATTGCTGAAGCTGTTAATGTGTATAAACGTT
GCTTAGGTATGTATGGTAATTGTTTTTTAATTGAAATAAATACTTCAGATTGTCCCAAAAT
TGCTGCAGCAACTGCATTGTATGATTCTAAACGTCCCATACCATTAATTTTAGAAATTAAG
AATCCTAAAACATTAATGATTAAAGGTAAAATCTTTGTGTATTGAAGGATACCGATAATC
GCTGAAATAAATACGATAGGTAATAATACACTGAAGAAGAATGGTGGTTGCTTAGGATC
GATATATTGAATACCACCGAATACAAAGTTAACACCATCTGCTGCTTTTAATAATAAGTA
GTTAAAACCGTTTGAAATACCACCAATAACCTTGATTCCCATTGTAGTTTTAAGCAAGAT
AAATGCAAAGATAAGCTGAATTGCAAGTAAAATTCCTACATATTTCCAGCGAATATTTTT
CCTGTCTGAGCTAAATAGAAACGCAAGTGCTAAAAAGAAGATAATTCCGATAATCCCAAT
TAGAATATGCAT

## > ctsR

Function: protein coding sequence; transcript regulator (core genome, constant)
Best match: ctsR_CC001_MW2-USA400_BA000033.2[551192:551653] (completely identical)
Position: 016-contig_250: 5110 … 5572; Length: 462 bp
Sequence:
ATGCACAATATGTCTGACATCATAGAACAATACATCAAACGTTTATTTGAAGAGTCGAAT
GAAGATGTCGTTGAAATTCAGAGAGCGAATATCGCACAGCGTTTTGATTGTGTACCATCA
CAATTAAATTATGTAATCAAAACACGATTCACTAATGAACATGGTTATGAAATCGAAAGT
AAACGTGGTGGTGGTGGTTACATCCGAATCACTAAAATTGAAAATAAAGATGCAACAGG
TTATATTAATCATTTGCTTCAGCTGATTGGACCTTCTATTTCTCAACAACAAGCTTATTATA
TTATTGATGGGCTTTTAGATAAAATGTTAATAAATGAACGTGAAGCTAAAATGATTCAAG
CAGTTATTGATAGAGAAACGCTATCAATGGATATGGTTTCTAGAGATATTATTAGAGCAA
ATATTTTAAAACGTTTGTTACCAGTTATAAATTATTACTAA

## > clpC

Function: protein coding sequence; chaperone-like protein C (core genome, variable)
Best match: clpC_CC001_MSSA476_BX571857.1[551880:554336]
Position: 016-contig_250: 7167 … 7197; Length: 30 bp
Sequence:
ATGTTATTTGGTAGATTAACTGAGCGTGCA

## > radA

Function: protein coding sequence; DNA repair protein A (core genome, constant)

Best match: radA_CC008-ST72_21259_AFTS01000028.1[45190:46554:r] (completely identical)
Position: 016-contig_250: 10107 … 11472; Length: 1365 bp
Sequence:
TTGGCCAAGAAAAAAGTGATTTTTGAATGTATGGCTTGTGGTTATCAATCTCCTAAATGG
ATGGGGAAATGTCCTAATTGTGGCGCTTGGAATCAAATGGAGGAAATTGTTGAAAAAGC
AGCCAATCCTAAACATGGAGTTAAAACCAAGGAATTAGCAGGTAAAGTACAAAAATTAA
ATAGTATTAAACATGAAACAACGCCGAGAGTGTTAACAGATTCAGCAGAATTCAACCGT
GTATTAGGTGGAGGTATTGTGAGCGGATCGTTAGTACTTATTGGTGGGGATCCAGGTATT
GGTAAGTCAACGTTACTTTTACAAATTTGTGCATCGTTATCTCAAAAGAAAAAAGTACTA
TATATTACTGGAGAAGAATCGCTTAGTCAGACTAAATTACGTGCAGAGCGATTAGATGAA
GATTCAAGTGAATTGCAAGTATTAGCTGAAACAGATCTTGAAGTTATTTATCAAACAGTA
AAAGAAGAACAACCTGATTTATTAGTAGTGGATTCGATTCAAACAATATATCATCCTGAA
ATCAGCTCTGCGCCAGGTTCTGTTTCACAAGTTCGTGAAAGTACACAAAGTTTAATGAAT
ATTGCTAAACAAATGAACATTGCAACTTTTATAGTGGGTCATGTAACGAAAGAAGGTCAA
ATTGCTGGCCCAAGATTGCTAGAACACATGGTTGATACTGTGCTTTATTTTGAAGGCGAT
GAACACCACGCATATCGAATTTTGCGAGCTGTTAAAAACCGTTTTGGTTCAACGAATGAA
ATGGGAATCTTCGAAATGAAGCAAAGTGGATTAAAAGGTGTAAATAATCCATCTGAAAT
GTTTTTAGAAGAACGTTCAACAAATGTTCCAGGTTCAACAATTGTTGCAACCATGGAGGG
AACCAGACCACTTTTAATAGAAGTTCAAGCGCTGGTAACTCCAACGACTTTTAACAATCC
GAGACGAATGGCAACAGGGATTGATCATAATCGATTAAGTTTGTTGATGGCTGTTTTGGA
AAAGAAAGAAAATTATCTATTACAACAACAAGATGCTTATATCAAAGTAGCTGGCGGTGT
AAAGTTAACGGAGCCAGCAGTTGATTTAAGTGTAATTGTAGCAACTGCATCTAGCTTTAA
AGATAAAGCTGTCGACGGATTAGATTGCTATATTGGAGAAGTTGGTTTAACGGGCGAGGT
ACGTCGTGTATCTCGGATAGAACAACGCGTGCAAGAGGCTGCAAAACTAGGTTTCAAAC
GTGTAATTATTCCTAAAAATAATATAGGCGGATGGACATATCCTGAAGGTATACAAGTAA
TAGGTGTAACTACTGTACATGAAGCATTGTCATTTGCTCTTCATTCATAA

## > gluS
Function: protein coding sequence; glutamyl-tRNA synthetase (core genome, constant)
Best match: gluS_CC001_MW2-USA400_BA000033.2[559208:560662]
Position: 016-contig_250: 13126 … 14581; Length: 1455 bp
Sequence:
ATGAGCGATCGTATAAGAGTAAGATATGCACCAAGTCCAACTGGTTATCTTCATATTGGT
AATGCAAGAACAGCATTATTCAATTACTTGTATGCTAAACATTACAACGGAGATTTTGTG
ATTCGAATTGAAGATACTGATAAAAAACGTAATTTAGAAGATGGAGAAACATCACAATTT
GATAATCTTAAATGGTTAGGATTAGATTGGGATGAGTCTGTAGATAAAGACAATGGCTAC
GGACCATATCGTCAATCTGAACGTCAACATATCTACCAACCATTAATAGATCAGTTACTA
GCAGAAGATAAAGCATATAAATGCTATATGACAGAAGAAGAATTAGAAGCTGAACGTGA
AGCGCAAATCGCTCGTGGTGAAATGCCTCGCTATGGTGGTCAACATGCGCATTTGACTGA
AGAACAACGTCAACAATTTGAAGCAGAAGGACGCCAACCATCAATTCGTTTCCGAGTACC
TCAAAACCAAACGTATTCATTTGATGATATGGTAAAAGGAAATATTTCATTTGATTCAAA
TGGTATTGGTGACTGGGTTATCGTAAAAAAAGATGGCATTCCAACGTACAATTTTGCAGT
AGCTATAGATGATCATTACATGCAAATTTCAGATGTAATTCGTGGTGATGATCATATTTCA
AACACGCCTAAACAAATTATGATTTATGAAGCATTTGGCTGGGAGCCACCTCGTTTTGGT
CATATGTCATTAATTGTTAATGAAGAACGTAAAAAGTTAAGTAAACGTGATGGGCAAATT
TTACAATTTATTGAGCAATATCGTGACTTAGGTTATTTACCTGAAGCGTTATTTAATTTTA
TTGCGTTATTAGGTTGGTCTCCTGAAGGTGAAGAAGAAATCTTTTCTAAAGAAGAATTTA
TCAAAATCTTTGATGAAAAGCGTTTGTCAAAATCACCAGCATTTTTCGATAAGCAAAAAT
TAGCATGGGTTAATAACCAATATATGAAACAAAAAGATACTGAAACAGTATTCCAATTAG
CATTACCTCATTTAATTAAAGCAAATTTGATTCCTGAGGTGCCGTCAGAAGAGGATTTATC
TTGGGGACGCAAATTAATTGCGCTTTATCAAAAAGAAATGGGTTATGCCGGTGAAATTGT
ACCTTTATCAGAAATGTTCTTTAAAGAAATGCCAGCTCTTGGTGAAGAAGAACAACAAGT
GATTAATGGAGAGCAAGTACCAGAGTTAATGACGCACTTATTCAGTAAATTAGAAGCACT
TGAACCATTTGAAGCGGCTGAAATTAAAAAGACAATTAAAGAAGTTCAAAAAGAAACAG
GAATAAAAGGCAAGCAATTATTTATGCCTATTCGTGTTGCTGTAACAGGCCAAATGCATG

GTCCTGAATTACCAAATACAATTGAAGTACTTGGTAAAGAAAAAGTGCTAAACCGTTTAA
AACAATATAAGTAA

> **tbox03**
Function: noncoding RNA T-box leader element (core genome, constant)
Best match: tbox03_CC001_MW2-USA400_BA000033.2[560767:560992] (completely identical)
Position: 016-contig_250: 14685 … 14911; Length: 226 bp
Sequence:
ATAAAACGAAGGATTAGTAATTAAATTTATACGATGCAGAGAGTGTACGGTTGCTGTGAG
TACAACGTAGAAATTAATGAATGCACCTTCGTAAAATGAATTAAATATATAATGAGAGTG
ATGAGCATTAAGTTGACTTAGTTTCCTTGATAATTTGGAAGCGCCCGCAATATTATTAATG
TTATTCGCTAAATTCAGAGTGGAACCGTGCGGAAGCGCCTCTAAC

> **cysE**
Function: protein coding sequence; serine acetyltransferase (core genome, constant)
Best match: cysE_CC001_MW2-USA400_BA000033.2[561091:561732]
Position: 016-contig_250: 15009 … 15651; Length: 642 bp
Sequence:
TTGTTAAAAAGAATGAGAGACGATATAAAAATGGTATTTGAGCAGGATCCAGCGGCACG
TTCAACATTAGAAGTCATTACAACGTATGCAGGTTTACATGCAGTTTGGAGTCATTTGATT
GCACATAAGTTATACAACCAAAAAAAATATGTTGCAGCACGCGCGATATCTCAAATTTCA
AGATTTTTCACAGGTATAGAAATCCATCCAGGTGCTAAAATTGGAAAGCGTCTATTTATA
GATCATGGTATGGGCGTTGTAATAGGAGAAACATGTACAATTGGTGATAATGTGACAATC
TATCAAGGCGTGACACTTGGTGGGACAGGGAAAGAAAGAGGGAAAAGACACCCAGATAT
AGGAGACAATGTTTTAATAGCAGCGGGTGCGAAAGTTTTAGGAAATATTAAAATAAATTC
AAATGTAAATATTGGTGCAAATTCAGTTGTTTTACAATCAGTTCCAAGCTATTCAACGGTT
GTTGGTATACCAGGACATATTGTTAAGCAAGATGGTGTTCGAGTTGGAAAAACATTTGAT
CATCGCCATCTACCTGATCCAATTTATGAACAAATTAAGCATTTAGAACGACAACTTGAA
AAGACTAGGAATGGAGAGATTCAAGATGATTACATTATATAA

> **cysS**
Function: protein coding sequence; cysteinyl-tRNA synthetase (core genome, constant)
Best match: cysS_CC001_MW2-USA400_BA000033.2[561716:563116]
Position: 016-contig_250: 15634 … 17035; Length: 1401 bp
Sequence:
ATGATTACATTATATAATACGCTTACACGTCAAAAAGAAGTGTTCAAGCCTATAGAACCA
GGAAAAGTAAAAATGTATGTATGTGGTCCTACTGTATATAACTACATTCATATTGGTAAC
GCAAGACCAGCAATTAATTATGACGTAGTGAGACGTTACTTTGAATACCAAGGATATAAT
GTAGAATATGTATCAAATTTTACAGACGTAGATGATAAATTAATTAAACGTTCTCAAGAA
TTAAATCAGTCTGTTCCCGAAATTGCAGAAAAATATATCGCAGCTTTTCATGAAGATGTT
GGTGCGTTAAATGTTAGAAAAGCGACTTCAAATCCAAGGGTAATGGACCATATGGATGA
CATTATTCAATTTATTAAAGAGTTGGTGGATCAAGGTTATGCATATGAAAGTGGTGGCGA
TGTTTACTTTAGAACACGTAAATTTGAAGGTTATGGTAAATTAAGTCATCAATCCATAGAT
GACTTAAAAGTGGGTGCTCGTATAGATGCAGGAGAGCATAAAGAAGATGCACTTGATTTT
ACATTGTGGAAAAAAGCGAAGCCTGGCGAGATTAGTTGGGATAGCCCATTTGGTGAAGG
TAGACCAGGATGGCATATAGAATGTTCTGTAATGGCATTTCATGAGCTAGGACCTACAAT
TGATATACATGCGGGTGGTTCAGATTTACAATTTCCACATCATGAAAATGAAATAGCACA
ATCAGAAGCACATAATCATGCGCCATTTGCTAATTATTGGATGCATAATGGTTTCATTAAT
ATTGATAATGAAAAAATGAGTAAATCACTAGGCAACTTTATTTTAGTTCACGATATTATT
AAAGAAGTTGATCCAGATGTACTAAGATTCTTTATGATTAGCGTACATTATAGAAGCCCA
ATTAACTATAATCTAGAATTGGTAGAATCAGCACGTAGTGGACTAGAGCGTATTCGCAAT
AGTTATCAATTAATTGAAGAGCGCGCACAAATTGCTACTAATATTGAAAATCAACAGACA
TATATTGATCAAATTGATGCGATTTTAAATCGTTTTGAAACAGTTATGAATGATGATTTTA
ATACAGCTAATGCAATTACAGCTTGGTATGATTTAGCAAAACTTGCGAATAAATATGTAC
TAGAGAACACAACATCAACAGAAGTAATTGATAAATTTAAAGCAGTTTATCAAATTTTCA

GCGATGTTTTAGGTGTACCGTTAAAATCTAAAAATGCAGATGAATTATTGGATGAAGATG
TTGAAAAATTAATCGAAGAGCGTAATGAAGCAAGGAAAAACAAAGATTTTGCACGAGCA
GATGAAATTCGAGACATGCTGAAATCACAAAACATTATATTAGAAGACACACCTCAAGG
GGTTAGATTTAAACGTGGATAA

> **mrnC**
Function: protein coding sequence; minimal RNase III (core genome, constant)
Best match: mrnC_CC001_MW2-USA400_BA000033.2[563109:563513] (completely identical)
Position: 016-contig_250: 17027 … 17432; Length: 405 bp
Sequence:
GTGGATAATCAACAAGATAATCACATTAAATTATTGAATCCATTGACCTTAGCATATATG
GGAGACGCAGTCTTAGATCAATATGTACGTACCTATATCGTTTTAAAGCTTAAAAGTAAG
CCTAATAAACTACATCAAATGTCTAAAAAATATGTATCTGCCAAAAGTCAGGCGCAAACG
TTAGAATATTTAATGGAGCAAGAATGGTTTACAGACGAAGAAATGGATATTTTGAAGCGA
GGGCGTAACGCGAAAAGTCATACTAAAGCTAAAAACACTGATGTTCAAACATATCGTAA
AAGTTCAGCGATAGAAGCAGTGATAGGTTTTCTTTATTTAGAAAAAAGAGAAGAACGATT
AGAGGCATTATTAAATAAAATAATAACAATAGTAAACGAAAGGTAG

> **yacO**
Function: protein coding sequence; tRNA/rRNA methyltransferase (core genome, constant)
Best match: yacO_CC001_MW2-USA400_BA000033.2[563521:564267]
Position: 016-contig_250: 17439 … 18186; Length: 747 bp
Sequence:
GTGGAAGATACGGTTATTGTTGGTAGGCATGCTGTTAGAGAAGCGATTATTACTGGGCAT
CCGATAAATAAGATATTGATTCAAGAAGGTATTAAAAAGCAACAAATTAATGAAATTTTA
AAAAATGCAAAAGATCAAAAAATCATTGTTCAAACTGTACCAAAATCTAAATTAGATTTT
TTAGCAAATGCACCACATCAGGGTGTTGCAGCGCTTATTGCACCATATGAATATGCTGAC
TTCGATCAATTTTTAAAACAGCAAAAAGAAAAAGAAGGTTTATCGACAGTACTTATATTA
GACGGCTTAGAAGACCCACATAACTTGGGATCAATTTTAAGAACAGCCGATGCAACGGG
AGTTGATGGTGTTATTATTCCTAAACGTCGTTCAGTTACACTAACGCAAACAGTTGCAAA
AGCCTCAACAGGTGCAATTGAACATGTACCAGTTATTCGAGTGACAAATTTAGCTAAAAC
TATCGATGAACTAAAAGATAATGGCTTTTGGGTAGCTGGCACTGAAGCTAATAATGCAAC
AGATTATAGAAATCTAGAAGCGGACATGTCATTGGCTATTGTAATTGGTAGCGAAGGACA
GGGTATGAGTCGCCTAGTAAGTGATAAATGCGATTTTTATATTAAGATTCCAATGGTTGG
ACATGTAAACAGTTTGAATGCTTCGGTTGCAGCAAGTTTAATGATGTACGAAGTATTTCG
AAAAAGACATGATGTTGGAGAAATATAA

> **yacP**
Function: protein coding sequence; putative protein (core genome, constant)
Best match: yacP_CC001_MW2-USA400_BA000033.2[564267:564791] (completely identical)
Position: 016-contig_250: 18185 … 18710; Length: 525 bp
Sequence:
ATGAAAGAACGTTACTTAATCATTGATGGATACAATATGATAGGACAATCACCAACGCTA
AGCGCCATTGCAAAAGAGAATTTAGAAGAAGCTAGAATGCAATTAATAGATGCAATTGC
AAATTATAATGCAGTTATTTCAGATGAAATTATTTGTGTTTTCGATGCTTATGACCAATCG
GGTGTTGAAAGAGAATACATGTATCATGGCGTTAAAACGATTTTTACCAAGGAAAAAGA
AACAGCTGATAGTTTCATAGAACGTTATGTTTATGAACTTTATGACAAGCATACTAAGCA
TATTACAGTTGTAACAAGTGATATGAGTGAGCAACATGCTATCTTTGGATCAGGTGCATA
TAGAATATCATCTCGCGAAATGTGGAGAGATTTAAAAGAAAATGAAATTGATGTGAGTA
AATCATTAGATGATATAAGTGAAAACAAGCCAAGAACTCGAATTCCGTTATCTTCTGAAA
TCCTTGCAGAATTTGAAAAAAATACGAAGAGGACATCATAAGAAATGA

> **sigH**
Function: protein coding sequence; alternative sigma subunit of RNA polymerase (core genome, variable)

Best match: sigH_CC001-ST772_118_AJGE01000068.1[19075:19563] (completely identical)
Position: 016-contig_250: 18871 … 19360; Length: 489 bp
Sequence:
ATGGATCTAAAACCATTAATTATTCGACGCATCAAAACATTTGGATTTAATCATTATGATT
TAGAAGACTTATATCAAGAAATACTTATACGGATGTATAGGTCGGTCCAAACATTTGATT
TTAGTGGAGAGCAGCCTTTCACAAATTATGTTCAATGTTTAATTACGTCTGTAAAGTATGA
TTATTTGAGAAAATATTTAGCTACAAATAAAAGAATGGATAATTTGATTAATGAATATAG
AGTTACGTATCCATGTGCAATAAAGCGTTATGATGTTGAAAACAATTATTTGAATCAATT
AGCAATTAAAGAGTTGATTCGTCAGTTTAAGTATTTGAGTGCATTTGAAAAAGATGTCAT
GTATTTAATGTGTGAACAATATAAGCCGAGAGAAATTGCTCAACTGATGCATGTAAAAGA
GAAAGTGATTTATAATGCCATACAACGATGTAAAAATAAAATAAAACGTTATTTCAAAAT
GATTTGA

> **L10**
Function: noncoding RNA ribosomal protein L leader RNA (core genome, constant)
Best match: L10_leader_CC001_MW2-USA400_BA000033.2[568089:568235]
Position: 016-contig_250: 22007 … 22154; Length: 147 bp
Sequence:
CTTATTGTGATTATTTTACCTAAGACAGTAGGAGTTATTTATAACTTAAAACTTATCCTGC
CGAGGCTAAAATTGACTTGAACGTGATGATCTATGATCTTTCAAGCACTTTTTGCCGTGGG
TAGAAAGTGCTTTTTTTATTAATTT

> **rpmG**
Function: protein coding sequence; 50S ribosomal protein L33 (core genome, constant)
Best match: rpmG_CC001_MW2-USA400_BA000033.2[565556:565699] (completely identical)
Position: 016-contig_250: 19474 … 19618; Length: 144 bp
Sequence:
GTGAGAAAAATACCTTTAAATTGTGAAGCTTGTGGCAATAGAAATTATAATGTTCCTAAG
CAAGAAGGCTCGGCAACAAGATTAACCTTAAAGAAATATTGTCCAAAATGTAACGCGCA
CACAATTCATAAAGAATCGAAATAA

> **secE**
Function: protein coding sequence; preprotein translocase subunit (core genome, constant)
Best match: secE_CC001_MW2-USA400_BA000033.2[565755:565937] (completely identical)
Position: 016-contig_250: 19673 … 19856; Length: 183 bp
Sequence:
ATGGCTAAAAAAGAAAGTTTCTTTAAAGGCGTTAAGTCTGAAATGGAAAAAACAAGTTG
GCCGACGAAAGAAGAGCTATTTAAATATACTGTAATTGTAGTTTCTACTGTTATATTCTTC
TTAGTCTTTTTCTATGCCTTAGATTTAGGAATTACAGCATTGAAAAATTTATTATTTGGTTA
G

> **nusG**
Function: protein coding sequence; transcription antitermination protein (core genome, constant)
Best match: nusG_CC001_MW2-USA400_BA000033.2[565950:566498] (completely identical)
Position: 016-contig_250: 19868 … 20417; Length: 549 bp
Sequence:
ATGTCTGAAGAAGTTGGCGCAAAGCGTTGGTATGCAGTGCATACATATTCTGGATATGAA
AATAAAGTTAAAAAGAATTTAGAAAAAAGAGTAGAATCTATGAATATGACTGAACAAAT
CTTTAGAGTAGTCATACCGGAAGAAGAAGAAACTCAAGTAAAAGATGGCAAAGCTAAAA
CGACTGTTAAAAAAACATTCCCTGGATATGTTTTAGTGGAATTAATCATGACAGATGAAT
CATGGTATGTGGTAAGAAATACACCAGGTGTTACTGGTTTTGTAGGTTCTGCAGGTGCAG
GGTCTAAGCCAAATCCATTGTTACCAGAAGAAGTTCGCTTCATCTTAAAACAAATGGGTC
TTAAAGAAAAGACTATCGATGTTGAACTCGAAGTTGGCGAGCAAGTTCGTATTAAATCAG
GTCCATTTGCGAATCAAGTTGGTGAAGTTCAAGAAATTGAAACAGATAAGTTTAAGCTAA

CAGTATTAGTAGATATGTTTGGCCGAGAAACACCAGTAGAAGTTGAATTCGATCAAATAG
AAAAGCTTTAA

## > **rplJ**
Function: protein coding sequence; 50S ribosomal protein L10 (core genome, constant)
Best match: rplJ_CC001_MSSA476_BX571857.1[566904:567404] (completely identical)
Position: 016-contig_250: 22191 … 22692; Length: 501 bp
Sequence:
ATGTCTGCTATCATTGAAGCTAAAAAACAACTAGTTGATGAAATTGCTGAGGTACTATCA
AATTCAGTTTCAACAGTAATCGTTGACTACCGTGGATTAACAGTAGCTGAAGTTACTGAC
TTACGTTCACAATTACGTGAAGCTGGTGTTGAGTATAAAGTATACAAAAACACTATGGTA
CGTCGTGCAGCTGAAAAAGCTGGTATCGAAGGCTTAGATGAATTCTTAACAGGTCCTACT
GCTATTGCAACTTCAAGTGAAGATGCTGTAGCTGCAGCGAAAGTAATTTCTGGATTTGCT
AAAGATCATGAAGCATTAGAAATTAAATCAGGCGTTATGGAAGGCAATGTTATTACAGC
AGAAGAAGTTAAAACTGTTGGTTCATTACCTTCACACGATGGTCTTGTATCTATGCTTTTA
TCAGTATTACAAGCTCCTGTACGCAACTTCGCTTATGCGGTTAAAGCTATTGGAGAACAA
AAAGAAGAAAGCGCTGAATAA

## > **rplL**
Function: protein coding sequence; 50S ribosomal protein L7/L12 (core genome, constant)
Best match: rplL_CC001_MW2-USA400_BA000033.2[568816:569184] (completely identical)
Position: 016-contig_250: 22734 … 23103; Length: 369 bp
Sequence:
ATGGCTAATCATGAACAAATCATTGAAGCGATTAAAGAAATGTCAGTATTAGAATTAAAC
GACTTAGTAAAAGCAATTGAAGAAGAATTTGGTGTAACTGCAGCTGCTCCAGTAGCAGTA
GCAGGTGCAGCTGGTGGCGCTGACGCTGCAGCAGAAAAAACTGAATTTGACGTTGAGTT
AACTTCAGCTGGTTCATCTAAAATCAAAGTTGTTAAAGCTGTTAAAGAAGCAACTGGTTT
AGGATTAAAAGATGCTAAAGAATTAGTAGACGGAGCTCCTAAAGTAATCAAAGAAGCTT
TACCTAAAGAAGAAGCTGAAAAACTTAAAGAACAATTAGAAGAAGTTGGAGCTACTGTA
GAATTAAAATAA

## > **Q5HID4**
Function: protein coding sequence; 16S rRNA methyltransferase C (core genome, constant)
Best match: Q5HID4_CC001_MW2-USA400_BA000033.2[569359:569967] (completely identical)
Position: 016-contig_250: 23277 … 23886; Length: 609 bp
Sequence:
ATGAGTCATTATTACGATGAAGATCCAAGTGTAATTAGCAATGAACAACGTATTCAATAT
CAATTAAACCATCATAAAATTGATTTAATAACTGATAATGGAGTGTTTTCGAAAGATAAA
GTAGATTATGGTTCAGATGTTCTTGTTCAAACTTTTTTAAAAGCGCATCCACCTGGTCCAA
GTAAGCGAATTGCCGATGTTGGTTGTGGTTACGGACCAATTGGTTTGATGATTGCTAAAG
TATCACCACATCATTCAATTACAATGCTAGATGTTAATCACAGAGCGCTAGCCTTAGTTG
AAAAAAACAAAAAAAATAAATGGTATTGATAATGCGATCGTAAAGGAAAGTGATGCTTTG
TCTGCTGTGGAAGACAAAAGTTTTGATTTTATTTTAACCAATCCACCAATAAGAGCAGGG
AAAGAAACCGTGCATCGTATATTCGAGCAAGCATTACATAGATTAGACTCGAACGGTGA
ACTATTCGTTGTAATTCAGAAGAAGCAAGGTATGCCATCTGCAAAGAAAAGAATGAATG
AACTTTTTGGAAATGTAGAAGTGGTAAATAAAGATAAAGGATATTACATTCTGAGAAGTA
TAAAAGCTTGA

## > **rpoB**
Function: protein coding sequence; DNA-directed RNA polymerase beta subunit (core genome, variable)
Best match: rpoB_CC001-ST772_118_AJGE01000068.1[24304:27855] (completely identical)
Position: 016-contig_250: 24100 … 27652; Length: 3552 bp
Sequence:

TTGGCAGGTCAAGTTGTCCAATATGGAAGACATCGTAAACGTAGAAACTACGCGAGAATT
TCAGAAGTATTAGAATTACCAAACTTAATAGAAATTCAAACTAAATCTTACGAGTGGTTC
CTAAGAGAAGGTTTAATCGAAATGTTTAGAGACATTTCTCCAATTGAAGATTTTACTGGT
AATTTGTCATTAGAGTTTGTGGATTACCGTTTAGGAGAACCAAAATATGATTTAGAAGAA
TCTAAAAACCGTGACGCTACTTATGCTGCACCTCTTCGTGTAAAAGTGCGTCTAATCATTA
AAGAAACAGGAGAAGTTAAAGAACAAGAAGTCTTTATGGGTGATTTCCCATTAATGACT
GATACAGGTACGTTCGTTATCAATGGTGCAGAACGTGTAATCGTATCTCAATTAGTTCGTT
CACCATCCGTTTATTTCAATGAAAAAATCGACAAAAATGGTCGTGAAAACTATGATGCAA
CAATTATTCCAAACCGTGGTGCATGGTTAGAATATGAAACAGATGCTAAAGATGTTGTAT
ACGTACGTATTGATAGAACACGTAAACTACCATTAACAGTATTGTTACGTGCATTAGGTT
TCTCAAGTGACCAAGAAATTGTTGACCTTTTAGGTGACAATGAATATTTACGTAATACTTT
AGAGAAAGACGGCACTGAAAACACTGAACAAGCGTTATTAGAAATCTATGAACGTTTAC
GTCCAGGTGAACCACCAACTGTTGAAAATGCTAAAAGTCTATTGTATTCACGTTTCTTTGA
TCCAAAACGCTATGACTTAGCAAGCGTGGGTCGTTATAAAACAAACAAAAAAATTACATTT
AAAACATCGTTTATTTAATCAAAAATTAGCTGAGCCAATTGTAAATACTGAAACTGGTGA
AATTGTAGTTGAAGAAGGTACAGTGCTTGATCGTCGTAAAATCGACGAAATCATGGATGT
ACTTGAATCAAATGCAAACAGCGAAGTGTTTGAATTGCATGGTAGCGTTATAGACGAGCC
AGTAGAAATTCAATCAATTAAAGTATATGTTCCTAACGATGATGAAGGTCGTACGACAAC
TGTAATTGGTAATGCTTTCCCTGACTCAGAAGTTAAATGCATTACACCAGCAGATATCATT
GCTTCAATGAGTTACTTCTTTAACTTATTAAGCGGTATTGGATATACAGATGATATTGACC
ATTTAGGTAACCGTCGTTTACGTTCTGTAGGTGAATTACTACAAAACCAATTCCGTATCGG
TTTATCAAGAATGGAAAGAGTTGTACGTGAAAGAATGTCAATTCAAGATACTGAGTCTAT
CACACCTCAACAATTAATTAATATTCGACCTGTTATTGCATCTATTAAAGAATTCTTTGGT
AGCTCTCAATTATCACAATTCATGGACCAAGCAAATCCATTAGCTGAGTTAACGCATAAA
CGTCGTCTATCAGCATTAGGACCTGGTGGTTTAACACGTGAACGTGCTCAAATGGAAGTA
CGTGACGTTCACTACTCTCACTATGGCCGTATGTGTCCAATTGAAACACCTGAGGGACCA
AACATTGGATTGATTAACTCATTATCAAGTTATGCACGTGTAAATGAATTCGGCTTTATTG
AAACACCATATCGTAAAGTTGATTTAGATACACATGCTATCACTGATCAAATTGACTATTT
AACAGCTGACGAAGAAGATAGCTATGTTGTAGCACAAGCAAACTCTAAATTAGATGAAA
ATGGTCGTTTCATGGATGATGAAGTTGTATGTCGTTTCCGTGGTAACAACACAGTTATGGC
TAAAGAAAAAATGGATTATATGGATGTATCGCCGAAGCAAGTTGTTTCAGCAGCGACAG
CATGTATTCCATTCTTAGAAAATGATGACTCAAACCGTGCATTGATGGGTGCGAACATGC
AACGTCAAGCAGTGCCTTTGATGAATCCAGAAGCACCATTTGTTGGAACAGGTATGGAAC
ACGTTGCAGCACGTGATTCTGGTGCAGCTATTACAGCTAAGCACAGAGGTCGTGTTGAAC
ATGTTGAATCTAATGAAATTCTTGTACGTCGTCTAGTTGAAGAGAACGGCGTTGAGCATG
AAGGTGAATTAGATCGCTATCCATTAGCTAAATTTAAACGTTCAAACTCAGGTACATGTT
ACAACCAACGTCCAATCGTTGCAGTTGGAGATGTTGTTGAGTATAACGAGATTTTAGCAG
ATGGACCATCTATGGAATTAGGAGAAATGGCATTAGGTAGAAACGTAGTAGTTGGTTTCA
TGACTTGGGACGGTTACAACTATGAGGATGCCGTTATCATGAGTGAAAGACTTGTGAAAG
ATGACGTGTATACTTCTATTCATATTGAAGAGTATGAATCAGAAGCACGTGATACTAAGT
TAGGACCTGAAGAAATCACAAGAGATATTCCTAATGTTTCTGAAAGTGCACTTAAGAACT
TAGACGATCGTGGTATCGTTTATATTGGTGCAGAAGTAAAAGATGGAGATATTTTAGTTG
GTAAAGTAACGCCTAAAGGTGTAACTGAGTTAACTGCCGAAGAAAGATTGTTACATGCA
ATCTTTGGTGAAAAAGCACGTGAAGTTAGAGATACTTCATTACGTGTACCTCACGGCGCT
GGCGGTATCGTTCTTGATGTAAAAGTATTCAATCGCGAAGAAGGCGACGATACATTATCA
CCTGGTGTAAACCAATTAGTACGTGTATACATCGTTCAAAAACGTAAAATTCATGTTGGT
GATAAGATGTGTGGTCGACATGGTAACAAAGGTGTCATTTCTAAGATTGTTCCTGAAGAA
GATATGCCTTACTTACCAGACGGACGTCCGATCGATATCATGTTAAACCCTCTTGGTGTAC
CATCTCGTATGAACATCGGACAAGTATTAGAGCTACACTTAGGTATGGCTGCTAAAAATC
TTGGTATTCACGTTGCATCACCAGTATTTGACGGTGCAAACGATGACGATGTATGGTCAA
CAATTGAAGAAGCTGGTATGGCTCGTGATGGTAAAACTGTACTTTATGATGGACGTACAG
GTGAACCATTCGATAACCGTATTTCAGTAGGTGTAATGTACATGTTGAAACTTGCGCACA
TGGTTGATGATAAATTACATGCGCGTTCAACAGGACCATATTCACTTGTTACACAACAAC
CACTTGGCGGTAAAGCGCAATTCGGTGGACAACGTTTTGGTGAGATGGAGGTATGGGCAC
TTGAAGCATATGGTGCTGCATACACATTACAAGAAATCTTAACTTACAAATCCGATGATA
CAGTAGGACGTGTGAAAACATACGAGGCTATTGTTAAAGGTGAAAACATCTCTAGACCA

AGTGTTCCAGAATCATTCCGAGTATTGATGAAAGAATTACAAAGTTTAGGTTTAGATGTA
AAAGTTATGGATGAGCAAGATAATGAAATCGAAATGACAGACGTTGATGACGATGATGT
TGTAGAACGCAAAGTAGATTTACAACAAAATGATGCTCCTGAAACACAAAAAGAAGTTA
CTGATTAA

## > **rpoC**

Function: protein coding sequence; DNA-directed RNA polymerase beta' subunit (core genome, constant)
Best match: rpoC_CC008_NCTC8325_CP000253.1[525848:529471] (completely identical)
Position: 016-contig_250: 27788 … 31412; Length: 3624 bp
Sequence:
TTGATTGATGTAAATAATTTCCATTATATGAAAATAGGATTGGCTTCACCTGAAAAAATC
CGTTCTTGGTCTTTTGGTGAAGTTAAAAAACCTGAAACAATCAACTACCGTACATTAAAA
CCTGAAAAAGATGGTCTATTCTGTGAAAGAATTTTCGGACCTACAAAAGACTGGGAATGT
AGTTGTGGTAAATACAAACGTGTTCGCTACAAAGGCATGGTCTGTGACAGATGTGGAGTT
GAAGTAACTAAATCTAAAGTACGTCGTGAAAGAATGGGTCACATTGAACTTGCTGCTCCA
GTTTCTCACATTTGGTATTTCAAAGGTATACCAAGTCGTATGGGATTATTACTTGACATGT
CACCAAGAGCATTAGAAGAAGTTATTTACTTTGCTTCTTATGTTGTTGTAGATCCAGGTCC
AACTGGTTTAGAAAAGAAAACTTTATTATCTGAAGCTGAATTCAGAGATTATTATGATAA
ATACCCAGGTCAATTCGTTGCAAAAATGGGTGCAGAAGGTATTAAAGATTTACTTGAAGA
GATTGATCTTGACGAAGAACTTAAATTGTTACGCGATGAGTTGGAATCAGCTACTGGTCA
AAGACTTACTCGTGCAATTAAACGTTTAGAAGTTGTTGAATCATTCCGTAATTCAGGTAA
CAAACCTTCATGGATGATTTTAGATGTACTTCCAATCATCCCACCAGAAATTCGTCCAATG
GTTCAATTAGATGGTGGACGATTTGCAACAAGTGACTTAAACGACTTATACCGTCGTGTA
ATTAATCGAAATAATCGTTTGAAACGTTTATTAGATTTAGGTGCACCTGGTATCATCGTTC
AAAACGAAAAACGTATGTTACAAGAAGCCGTTGACGCTTTAATTGATAATGGTCGTCGTG
GTCGTCCAGTTACTGGCCCAGGTAACCGTCCATTAAAATCTTTATCTCATATGTTAAAAGG
TAAACAAGGTCGTTTCCGTCAAAACTTACTTGGTAAACGTGTTGACTATTCAGGACGTTC
AGTTATTGCAGTAGGTCCAAGCTTGAAAATGTACCAATGTGGTTTACCAAAAGAAATGGC
ACTTGAACTATTTAAACCATTCGTAATGAAAGAATTAGTTCAACGTGAAATTGCAACTAA
CATTAAAAATGCGAAGAGTAAAATCGAACGTATGGATGATGAAGTTTGGGACGTATTGG
AAGAAGTAATTAGAGAACATCCTGTATTACTTAACCGTGCACCAACACTTCATAGACTTG
GTATTCAAGCATTTGAACCAACTTTAGTTGAAGGTCGTGCGATTCGTCTACATCCACTTGT
AACAACAGCTTATAACGCTGACTTTGACGGTGACCAAATGGCGGTTCACGTTCCTTTATC
AAAAGAGGCACAAGCTGAAGCAAGAATGTTGATGTTAGCAGCACAAAACATCTTGAACC
CTAAAGATGGTAAACCTGTAGTTACACCATCACAAGATATGGTACTTGGTAACTATTACC
TTACTTTAGAAAGAAAAGATGCAGTAAATACAGGCGCAATCTTTAATAATACAAATGAA
GTATTAAAAGCATATGCAAATGGCTTTGTACATTTACACACTAGAATTGGTGTACATGCA
AGTTCGTTCAATAATCCAACATTTACTGAAGAACAAAACAAAAAGATTCTTGCTACGTCA
GTAGGTAAAATTATATTCAATGAAATCATTCCAGATTCATTTGCTTATATTAATGAACCTA
CGCAAGAAAACTTAGAAAGAAAGACACCAAACAGATATTTCATCGATCCTACAACTTTA
GGTGAAGGTGGATTAAAAGAATACTTTGAAAATGAAGAATTAATTGAACCTTTCAACAA
AAAATTCTTAGGTAATATTATTGCAGAAGTATTCAACAGATTTAGCATCACTGATACATC
AATGATGTTAGACCGTATGAAAGACTTAGGATTCAAATTCTCATCTAAAGCTGGTATTAC
AGTAGGTGTTGCTGATATCGTAGTATTACCTGATAAGCAACAAATACTTGATGAGCATGA
AAAATTAGTCGACAGAATTACAAAACAATTCAACCGTGGTTTAATCACTGAAGAAGAAA
GATATAATGCAGTTGTTGAAATTTGGACAGATGCAAAAGATCAAATTCAAGGTGAATTGA
TGCAATCACTTGATAAAACTAACCCAATCTTCATGATGAGTGATTCAGGTGCCCGTGGTA
ACGCATCTAACTTTACACAGTTAGCAGGTATGCGTGGATTGATGGCCGCACCATCTGGTA
AGATTATCGAATTACCAATCACATCTTCATTCCGTGAAGGTTTAACAGTACTTGAATACTT
CATCTCAACTCACGGTGCACGTAAAGGTCTTGCCGATACAGCACTTAAAACAGCTGACTC
AGGATATCTTACTCGTCGTCTTGTTGACGTGGCACAAGATGTTATTGTTCGTGAAGAAGA
CTGTGGTACTGATAGAGGTTTATTAGTTTCTGATATTAAAGAAGGTACAGAAATGATTGA
ACCATTTATCGAACGTATTGAAGGTCGTTATTCTAAAGAAACAATTCGTCATCCTGAAAC
TGATGAAATAATCATTCGTCCTGATGAATTAATTACACCTGAAATTGCTAAGAAAATTAC
AGATGCTGGTATTGAACAAATGTATATTCGCTCAGCATTTACTTGTAACGCACGACATGG

TGTTTGTGAAAAATGTTACGGTAAAAACCTTGCTACTGGTGAAAAAGTTGAAGTTGGTGA
AGCAGTTGGTACAATTGCAGCCCAATCTATCGGTGAACCAGGTACACAGCTTACAATGCG
TACATTCCATACAGGTGGGGTAGCAGGTAGCGATATCACACAAGGTCTTCCTCGTATTCA
AGAGATTTTCGAAGCACGTAACCCTAAAGGTCAAGCGGTAATTACGGAAATCGAAGGTG
TCGTAGAAGATATTAAATTAGCAAAAGATAGACAACAAGAAATTGTTGTTAAAGGTGCT
AATGAAACAAGATCATACCTTGCTTCAGGTACTTCAAGAATTATTGTAGAAATCGGTCAA
CCAGTTCAACGTGGTGAAGTATTAACTGAAGGTTCTATTGAACCTAAGAATTACTTATCT
GTTGCTGGATTAAACGCGACTGAAAGCTACTTATTAAAAGAAGTACAAAAAGTTTACCGT
ATGCAAGGTGTAGAAATCGACGATAAACACGTTGAGGTTATGGTTCGACAAATGTTACGT
AAAGTTAGAATTATCGAAGCAGGTGATACGAAGTTATTACCAGGTTCATTAGTTGATATT
CATAACTTTACAGATGCAAATAGAGAAGCATTTAAACACCGTAAGCGTCCTGCAACAGCT
AAACCAGTATTACTTGGTATTACTAAAGCATCACTTGAAACAGAAAGTTTCTTATCTGCA
GCATCATTCCAAGAAACAACAAGAGTTCTTACAGATGCAGCAATTAAAGGTAAGCGTGA
TGACTTATTAGGTCTTAAAGAAAACGTAATTATTGGTAAGTTAATTCCAGCTGGTACTGGT
ATGAGACGTTATAGCGACGTAAAATACGAAAAAACAGCTAAACCAGTTGCAGAAGTTGA
ATCTCAAACTGAAGTAACGGAATAA

## > rplGAe

Function: protein coding sequence; 50S ribosomal protein L7Ae (core genome, constant)
Best match: rplGAe_CC001_MW2-USA400_BA000033.2[577630:577884] (completely identical)
Position: 016-contig_250: 31548 … 31803; Length: 255 bp
Sequence:
TTGTCTAAGGAAAAAGTTGCACGCTTTAACAAACAACATTTTGTAGTTGGTCTTAAAGAA
ACGCTTAAAGCGTTAAAGAAAGATCAAGTTACATCTTTGATTATTGCTGAAGACGTTGAA
GTATATTTAATGACTCGCGTGTTAAGCCAAATCAATCAGAAAAATATACCTGTATCTTTTT
TCAAAAGCAAACATGCTTTGGGTAAACATGTAGGTATTAACGTCAATGCGACAATAGTAG
CATTGATTAAATGA

## > rpsL

Function: protein coding sequence; 30S ribosomal protein S12 (core genome, constant)
Best match: rpsL_CC005_Mu50-VRSA_BA000017.4[611666:612079] (completely identical)
Position: 016-contig_250: 31900 … 32314; Length: 414 bp
Sequence:
ATGCCAACTATTAACCAATTAGTACGTAAACCAAGACAAAGCAAAATCAAAAAATCAGA
TTCTCCAGCTTTAAATAAAGGTTTCAACAGTAAAAAGAAAAAATTTACTGACTTAAACTC
ACCACAAAAACGTGGTGTATGTACTCGTGTAGGTACAATGACACCTAAAAAACCTAACTC
AGCGTTACGTAAATATGCACGTGTGCGTTTATCAAACAACATCGAAATTAACGCATACAT
CCCTGGTATCGGACATAACTTACAAGAACACAGTGTTGTACTTGTACGTGGTGGACGTGT
AAAAGACTTACCAGGTGTGCGTTACCATATTGTACGTGGAGCACTTGATACTTCAGGTGT
TGACGGACGTAGACAAGGTCGTTCATTATACGGAACTAAGAAACCTAAAAACTAA

## > rpsG

Function: protein coding sequence; 30S ribosomal protein S7 (core genome, constant)
Best match: rpsG_CC001_MW2-USA400_BA000033.2[578461:578931] (completely identical)
Position: 016-contig_250: 32379 … 32850; Length: 471 bp
Sequence:
ATGCCTCGTAAAGGATCAGTACCTAAAAGAGACGTATTACCAGATCCAATTCATAACTCT
AAGTTAGTAACTAAATTAATTAACAAAATTATGTTAGATGGTAAACGTGGAACAGCACAA
AGAATTCTTTATTCAGCATTCGACCTAGTTGAACAACGCAGTGGTCGTGATGCATTAGAA
GTATTCGAAGAAGCAATCAACAACATTATGCCAGTATTAGAAGTTAAAGCTCGTCGTGTA
GGTGGTTCTAACTATCAAGTACCAGTAGAAGTTCGTCCAGAGCGTCGTACTACTTTAGGT
TTACGTTGGTTAGTTAACTATGCACGTCTTCGTGGTGAAAAAACGATGGAAGATCGTTTA
GCTAACGAAATTTTAGATGCAGCAAATAATACAGGTGGTGCCGTTAAGAAACGTGAGGA
CACTCACAAAATGGCTGAAGCAAACAAAGCATTTGCTCACTACCGTTGGTAA

> **efg**
Function: protein coding sequence; translation elongation factor G (core genome, constant)
Best match: efg_CC001-ST772_118_AJGE01000068.1[33176:35257] (completely identical)
Position: 016-contig_250: 32972 … 35054; Length: 2082 bp
Sequence:
ATGGCTAGAGAATTTTCATTAGAAAAAACTCGTAATATCGGTATCATGGCTCACATTGAT
GCTGGTAAAACGACTACGACTGAACGTATTCTTTATTACACTGGCCGTATCCACAAAATT
GGTGAAACACACGAAGGTGCTTCACAAATGGACTGGATGGAGCAAGAACAAGACCGTGG
TATTACTATCACATCTGCTGCAACAACAGCAGCTTGGGAAGGTCACCGTGTAAACATTAT
CGATACACCTGGACACGTAGACTTCACTGTAGAAGTTGAACGTTCATTACGTGTACTTGA
CGGAGCAGTTACAGTACTTGATGCACAATCAGGTGTTGAACCTCAAACTGAAACAGTTTG
GCGTCAGGCTACAACTTATGGTGTTCCACGTATCGTATTTGTAAACAAAATGGACAAATT
AGGTGCTAACTTCGAATACTCTGTAAGTACATTACATGATCGTTTACAAGCTAACGCTGCT
CCAATCCAATTACCAATTGGTGCGGAAGACGAATTCGAAGCAATCATTGACTTAGTTGAA
ATGAAATGTTTCAAATATACAAATGATTTAGGTACTGAAATTGAAGAAATTGAAATTCCT
GAAGACCACTTAGATAGAGCTGAAGAAGCTCGTGCTAGCTTAATCGAAGCAGTTGCAGA
AACTAGCGACGAATTAATGGAAAAATATCTTGGTGACGAAGAAATTTCAGTTTCTGAATT
AAAAGAAGCTATCCGCCAAGCTACTACTAACGTAGAATTCTACCCAGTACTTTGTGGTAC
AGCTTTCAAAAACAAAGGTGTTCAATTAATGCTTGACGCTGTAATTGATTACTTACCTTCA
CCACTAGACGTTAAACCAATTATTGGTCACCGTGCTAGCAACCCTGAAGAAGAAGTAATC
GCGAAAGCAGACGATTCAGCTGAATTCGCTGCATTAGCGTTCAAAGTTATGACTGACCCT
TATGTTGGTAAATTGACATTCTTCCGTGTGTATTCAGGTACAATGACATCTGGTTCATACG
TTAAGAACTCTACTAAAGGTAAACGTGAACGTGTAGGTCGTTTATTACAAATGCACGCTA
ACTCACGTCAAGAAATCGATACTGTATACTCTGGAGATATCGCTGCTGCGGTAGGTCTTA
AAGATACAGGTACTGGTGATACTTTATGTGGTGAGAAAAATGACATTATCTTGGAATCAA
TGGAATTCCCAGAGCCAGTTATTCACTTATCAGTAGAGCCAAAATCTAAAGCTGACCAAG
ATAAAATGACTCAAGCTTTAGTTAAATTACAAGAAGAAGACCCAACATTCCATGCACACA
CTGACGAAGAAACTGGACAAGTTATCATCGGTGGTATGGGTGAGCTTCACTTAGACATCT
TAGTAGACCGTATGAAGAAAGAATTCAACGTTGAATGTAACGTAGGTGCTCCAATGGTTT
CATATCGTGAAACATTCAAATCATCTGCACAAGTTCAAGGTAAATTCTCTCGTCAATCTG
GTGGTCGTGGTCAATACGGTGATGTTCACATTGAATTCACACCAAACGAAACAGGCGCAG
GTTTCGAATTCGAAAACGCTATCGTTGGTGGTGTAGTTCCTCGTGAATACATTCCATCAGT
AGAAGCTGGTCTTAAAGATGCTATGGAAAATGGTGTCTTAGCAGGTTATCCTTTAATTGA
TGTTAAAGCTAAATTATATGATGGTTCATACCATGATGTCGATTCATCTGAAATGGCCTTC
AAAATTGCTGCATCATTAGCACTTAAAGAAGCTGCTAAAAAATGTGATCCTGTAATCTTA
GAACCAATGATGAAAGTAACTATTGAAATGCCTGAAGAGTACATGGGTGATATCATGGG
TGACGTAACATCTCGTCGTGGACGTGTTGATGGTATGGAACCTCGTGGTAATGCACAAGT
TGTTAATGCTTATGTACCACTTTCAGAAATGTTCGGTTATGCAACATCATTACGTTCAAAC
ACTCAAGGTCGCGGTACTTACACTATGTACTTCGATCACTATGCTGAAGTTCCAAAATCA
ATCGCTGAAGATATTATCAAGAAAAATAAAGGTGAATAA

> **tuf**
Function: protein coding sequence; translation elongation factor Tu (core genome, constant)
Best match: tuf_CC001-ST772_118_AJGE01000068.1[35474:36658] (completely identical)
Position: 016-contig_250: 35270 … 36455; Length: 1185 bp
Sequence:
ATGGCAAAAGAAAAATTCGATCGTTCTAAAGAACATGCCAATATCGGTACTATCGGTCAC
GTTGACCATGGTAAAACAACATTAACAGCAGCAATCGCTACTGTATTAGCAAAAAATGGT
GACTCAGTTGCACAATCATATGACATGATTGACAACGCTCCAGAAGAAAAAGAACGTGG
TATCACAATCAATACTTCTCACATTGAGTACCAAACTGACAAACGTCACTACGCTCACGT
TGACTGCCCAGGACACGCTGACTACGTTAAAAACATGATCACTGGTGCTGCTCAAATGGA
CGGCGGTATCTTAGTAGTATCTGCTGCTGACGGTCCAATGCCACAAACTCGTGAACACAT
TCTTTTATCACGTAACGTTGGTGTACCAGCATTAGTAGTATTCTTAAACAAAGTTGACATG
GTTGACGATGAAGAATTATTAGAATTAGTAGAAATGGAAGTTCGTGACTTATTAAGCGAA
TATGACTTCCCAGGTGACGATGTACCTGTAATCGCTGGTTCAGCATTAAAAGCTTTAGAA

GGCGATGCTCAATACGAAGAAAAAATCTTAGAATTAATGGAAGCTGTAGATACTTACATT
CCAACTCCAGAACGTGATTCTGACAAACCATTCATGATGCCAGTTGAGGACGTATTCTCA
ATCACTGGTCGTGGTACTGTTGCTACAGGCCGTGTTGAACGTGGTCAAATCAAAGTTGGT
GAAGAAGTTGAAATCATCGGTTTACATGACACATCTAAAACAACTGTTACAGGTGTTGAA
ATGTTCCGTAAATTATTAGACTACGCTGAAGCTGGTGACAACATTGGTGCATTATTACGT
GGTGTTGCTCGTGAAGACGTACAACGTGGTCAAGTATTAGCTGCTCCTGGTTCAATTACA
CCACATACTGAATTCAAAGCAGAAGTATACGTATTATCAAAAGACGAAGGCGGACGTCA
CACTCCATTCTTCTCAAACTATCGTCCACAATTCTATTTCCGTACTACTGACGTAACTGGT
GTTGTTCACTTACCAGAAGGTACTGAAATGGTAATGCCTGGTGATAACGTTGAAATGACA
GTAGAATTAATCGCTCCAATCGCGATTGAAGACGGTACTCGTTTCTCAATCCGTGAAGGT
GGACGTACTGTAGGATCAGGCGTTGTTACTGAAATCATTAAATAA

> **txbi_1000-1**

Function:  bidirectional rho-independent terminator
Best match: txbi_1000-1_CC001_MW2-USA400_BA000033.2582708:582783 [4_50ANF10END]
Position: 016-contig_250: 36626 … 36702; Length: 76 bp
Sequence:
GACATTGTAAAATGCTCCCTTCAAAGTTTTCATTTTTTCAATGTCTACTTTGAAGAGAGCA
TTTCATTAGTTTATG

> **kbl**

Function: protein coding sequence; 2-amino-3-ketobutyrate coenzyme A ligase (core genome, constant)
Best match: kbl_CC008_NCTC8325_CP000253.1[536142:537329] (completely identical)
Position: 016-contig_250: 38082 … 39270; Length: 1188 bp
Sequence:
GTGGTTCAATCATTACATGAGTTTTTAGAGGAAAATATAAATTATCTAAAAGAAAATGGT
TTGTATAATGAAATAGATACAATTGAAGGTGCAAACGGACCAGAAATCAAAATCAATGG
GAAATCATACATTAACTTATCTTCAAATAATTATTTAGGACTAGCAACAAATGAAGATTT
GAAATCAGCTGCAAAAGCAGCTATTGATACACATGGTGTAGGTGCAGGCGCTGTTCGTAC
AATCAATGGTACATTAGATTTACACGACGAATTAGAAGAAACACTAGCAAAATTTAAAG
GAACAGAAGCTGCAATAGCTTATCAATCAGGATTTAATTGTAATATGGCTGCTATTTCAG
CTGTCATGAATAAAAATGATGCTATTTTATCAGATGAGCTTAATCATGCATCAATTATTGA
TGGATGTCGCTTATCTAAAGCTAAAATTATTCGAGTTAACCATTCAGACATGGATGATTTA
CGTGCGAAAGCAAAAGAAGCAGTTGAATCAGGTCAATACAATAAAGTGATGTATATCAC
TGATGGCGTTTTTAGTATGGATGGTGATGTGGCTAAATTACCTGAAATTGTAGAAATTGC
AGAAGAATTTGGTTTATTAACTTATGTTGACGACGCTCATGGTTCAGGTGTTATGGGTAA
AGGCGCTGGTACGGTTAAACATTTTGGTTTACAAGATAAAATCGATTTCCAAATAGGTAC
GCTTTCTAAAGCAATTGGTGTCGTTGGCGGTTATGTAGCAGGTACAAAAGAGTTAATAGA
TTGGTTAAAAGCACAATCACGACCATTCTTATTCTCTACATCATTAGCACCTGGGGATACC
AAAGCAATAACTGAAGCAGTTAAAAAGTTAATGGATTCAACTGAATTACATGATAAATTA
TGGAACAATGCACAATATTTAAAAAATGGATTGTCAAAATTAGGATATGATACAGGTGA
GTCAGAAACTCCAATTACACCAGTAATTATTGGTGATGAAAAAACAACTCAAGAATTTAG
TAAGCGTTTAAAAGACGAAGGTGTCTATGTGAAATCTATCGTTTTCCCAACAGTACCAAG
AGGTACAGGACGTGTAAGAAATATGCCTACAGCTGCACATACAAAAGACATGTTAGATG
AAGCAATTGCGGCTTATGAAAAAGTAGGAAAAGAAATGAAGTTGATTTAA

> **hchA**

Function: protein coding sequence; chaperone Hsp31 and glyoxalase 3 (core genome, variable)
Best match: hchA_CC005_Mu50-VRSA_BA000017.4[619305:620183] (completely identical)
Position: 016-contig_250: 39535 … 40414; Length: 879

ATGTCACAAGATGTAAATGAATTAAGTAAGCAACCAACGCCAGATAAAGCAGAAGATAA
CGCATTTTTCCCATCACCATATTCCCTTAGTCAATATACAGCACCTAAAACAGATTTTGAT
GGTGTTGAACACAAAGGTGCCTATAAAGATGGTAAATGGAAAGTATTGATGATTGCTGCT

GAAGAGAGATATGTATTATTGGAAAATGGAAAAATGTTCTCTACGGGTAATCATCCTGTT
GAAATGTTATTACCTTTACATCATTTAATGGAAGCAGGTTTTGACGTTGATGTTGCGACAT
TATCTGGTTATCCAGTTAAATTAGAATTATGGGCTATGCCAACTGAAGACGAGGCAGTTA
TAAGTACTTATAATAAATTGAAAGAAAAATTAAAACAGCCAAAAAAATTAGCAGATGTG
ATTAAAAATGAATTAGGACCTGATTCAGACTATTTATCTGTCTTTATCCCAGGCGGACATG
CTGCAGTTGTTGGTATTTCTGAAAGTGAGGACGTTCAACAAACATTAGATTGGGCATTAG
ACAATGACCGCTTTATAGTTACATTATGTCATGGACCAGCAGCACTACTTTCAGCAGGGC
TTAACAGAGAAAAATCTCCATTAGAAGGATACTCTGTTTGTGTCTTCCCTGACTCATTAGA
TGAAGGTGCAAATATTGAAATAGGTTATTTACCTGGACGCTTGAAATGGTTAGTTGCTGA
TTTATTAACTAAACAAGGATTAAAAGTAGTTAACGACGATATGACAGGAAGAACGTTAA
AAGATCGTAAATTATTAACAGGTGACAGTCCTTTAGCTTCAAATGAGTTAGGAAAATTAG
CAGTTAATGAAATGTTAAATGCAATACAAAATAAATAA

> **araB**

Function: protein coding sequence; ribulokinase (core genome, constant)
Best match: araB_CC001_MW2-USA400_BA000033.2[586655:588292]
Position: 016-contig_250: 40571 … 42209; Length: 1638

ATGTCTTATAGCATTGGAATTGATTATGGAACAGCTTCAGGCCGTGTGTTTTTAATTAATA
CAACTAACGGTCAAGTAGTATCAAAATTTGTGAAACCATATACACATGGTGTCATTGAGA
GTGAATTAAATGGTTTGAAAATACCACATACATATGCACTTCAAAATAGTAATGATTATT
TAGAAATTATGGAAGAAGGAATATCATATATAGTACGTGAATCAAAAATAGATCCAGTC
AATATAGTAGGTATTGGTATAGACTTTACTTCATCTACTATTATTTTTACTGACGAAAACC
TTAACCCGGTACATAATTTAAAACAATTTAAAAACAATCCACATGCGTATGTGAAACTTT
GGAAACATCATGGTGCATATAAAGAAGCAGAGAAATTATATCAAACTGCTATTGAAAAT
AATAATAAGTGGTTAGGCCATTATGGATATAATGTTAGTAGTGAATGGATGATTCCCAAA
ATAATGGAAGTCATGAATCGAGCACCAGAAATTATGGAAAAAACGGCTTATATTATGGA
AGCGGGCGATTGGATTGTAAATAAATTAACTAATAAAAATGTACGCTCGAATTGTGGATT
AGGTTTCAAAGCATTTTGGGAAGAAGAAACAGGGTTTCATTATGATTTATTTGATAAAAT
AGACCCCAAATTATCAAAAGTAATTCAAGATAAAGTATCTGCACCGGTTGTTAATATTGG
TGAAGTAGTAGGGAAACTGGACGATAAAATGGCACAGAAATTAGGATTATCAAAAGAGA
CTATGGTAAGTCCTTTTATTATTGATGCCCATGCTAGTTTATTAGGTATTGGGTCTGAAAA
AGATAAAGAAATGACTATGGTGATGGGAACAAGCACATGCCATCTTATGTTAAATGAAA
AGCAACATCAAGTGCCAGGTATATCAGGTTCTGTAAAAGGAGCAATTATTCCAGAATTAT
TTGCTTATGAAGCGGGGCAATCAGCAGTAGGTGATTTGTTTGAGTATGTCGCTAAGCAAG
CACCAAAGTCATATGTAGATGAAGCAGCAAATAGAAATATGACTGTATTTGAATTAATGA
ATGAAAAGATAAAACATCAAATGCCAGGTGAAAGTGGGCTCATTGCTCTTGATTGGCATA
ATGGAAATCGAAGTGTATTAAGTGATAGCAATTTAACAGGTTGTATCTTTGGATTAACTTT
ACAAACTAAGCATGAGGATATTTATAGAGCATATTTAGAAGCTACAGCATTTGGTACTAA
GATGATTATGCAACAGTATCAAGATTGGCATATGGAAGTAGAAAAGGTATTTGCATGTGG
CGGTATACCTAAAAAGAATGCTGTTATGATGGATATCTATGCGAATGTACTGAATAAAAA
ACTAATTGTTATGGATAGTGAGTATGCACCAGCAATAGGCGCAGCAATATTAGGTGCAGT
CAGTGGTGGCGCACATAATTCAATTAATGATGCAGTTGATGCTATGAAAGAGCCAATTTT
ATACGAAATTAATCCAGAAGCGGAAAAAGTACAAAGGTATGAAACATTATTTAAAGCTT
ATAAGGCTTTACATGATATCCATGGTTATAAAAAAGCTAATATAATGAAAGATATCCAGA
GTTTAAGAGTTGAGGGATAA

> **ilvE**

Function: protein coding sequence; branched-chain-amino-acid aminotransferase (core genome, variable)
Best match: ilvE_CC001_MW2-USA400_BA000033.2[589806:590882] (completely identical)
Position: 016-contig_250: 43723 … 44800; Length: 1077

ATGTCACAAGCAGTTAAAGTTGAACGACGAGAAACATTAAAACAAAAACCAAATACATC
TCAACTAGGTTTTGGTAAATATTTTACTGATTATATGTTGAGTTATGATTATGATGCAGAT

AAAGGATGGCATGATTTGAAGATAGTACCTTATGGTCCTATTGAAATTTCACCTGCTGCA
CAAGGTGTTCATTATGGTCAATCGGTATTCGAAGGATTAAAAGCATATAAAAGAGATGGG
GAAGTTGCACTTTTCCGTCCTGAAGAAAATTTTAAGCGTCTTAATAACTCGTTAGCACGAT
TAGAAATGCCTCAAGTAGACGAAGCAGAATTGTTAGAGGGGCTAAAACAATTAGTTGAT
ATAGAAAGAGATTGGATTCCTGAAGGGGAAGGTCAATCATTATATATTCGTCCATTTGTT
TTTGCAACAGAAGGGGCACTTGGCGTTGGTGCATCACATCAGTATAAATTATTAATTATTT
TATCTCCTTCAGGTGCATATTATGGTGGTGAGACTTTAAAACCAACTAAAATCTATGTAG
AAGATGAATATGTGCGTGCTGTTCGTGGCGGTGTAGGCTTTGCAAAAGTTGCAGGTAACT
ATGCGGCAAGTTTATTAGCACAAACAAATGCAAATAAATTAGGTTATGACCAAGTATTAT
GGCTTGATGGTGTTGAACAGAAATATATCGAAGAAGTTGGTAGCATGAATATTTTCTTCG
TTGAAAATGGAAAAGTAATTACACCAGAGTTGAATGGCAGTATTTTACCTGGTATTACAC
GTAAATCTATTATCGAATTAGCTAAAAACTTAGGATATGAAGTCGAAGAGCGCCGCGTTT
CAATCGATGAATTATTCGAATCATATGATAAAGGTGAGTTAACAGAAGTATTTGGTAGTG
GTACTGCAGCAGTTATTTCACCTGTGGGTACATTGAGATACGAAGATCGTGAAATCGTTA
TTAATAATAATGAGACTGGTGAAATTACTCAAAAATTATACGACGTCTATACTGGTATTC
AAAATGGTACTTTAGAAGATAAAAATGGTTGGAGAGTCGTTGTACCAAAATATTAA

> **tadA**

Function: protein coding sequence; nucleoside deaminase (core genome, constant)
Best match: tadA_CC001_MW2-USA400_BA000033.2[593271:593741] (completely identical)
Position: 016-contig_250: 47188 … 47659; Length: 471 bp
Sequence:
ATGACAAATGATATATATTTTATGACATTAGCGATTGAAGAAGCTAAAAAAGCAGCTCAA
CTAGGCGAAGTACCTATAGGTGCTATCATCACTAAAGATGATGAAGTTATCGCTAGAGCA
CATAATTTAAGAGAAACACTACAACAACCAACGGCGCATGCTGAACATATTGCAATTGA
ACGTGCAGCCAAAGTGTTAGGTAGTTGGCGTTTAGAAGGTTGCACATTATATGTAACCTT
AGAACCATGTGTCATGTGCGCAGGAACAATTGTAATGAGTCGCATTCCAAGAGTCGTCTA
TGGCGCAGATGATCCTAAAGGTGGTTGTAGTGGCAGTTTAATGAATTTATTGCAACAATC
TAATTTTAATCATCGTGCAATTGTTGATAAAGGTGTACTTAAAGAAGCATGTAGCACATT
ATTAACAACATTTTTTAAAAACTTAAGAGCCAATAAGAAATCCACCAATTAG

> **A5IQB3**

Function: protein coding sequence; putative hydrolase/haloacid dehalogenase-like family (core genome, constant)
Best match: A5IQB3_CC001-ST772_118_AJGE01000068.1[48009:48878] (completely identical)
Position: 016-contig_250: 47805 … 48675; Length: 870 bp
Sequence:
ATGATAAAACTAATAGCCACTGATATGGATGGCACGCTACTTAATGCAGCACATGAAATT
TCTCAACCTAATATTGATGCGATTAAATACGCTCAAGAACAAGGGATAACGGTTGTTATC
GCGACAGGTCGAGCATTTTATGAAGCACAAGCACCAGTTGCTGACACAGATTTAACAGTA
CCATATATTTGTTTGAATGGTGCTGAAGTACGTGATGAAACTTTCAATGTAATGAGCACTT
CACACCTTAATAAATCGTTAGTACACAAAATTACAAATGTTTTAAAAGATGCAGGTATTT
ATTATCAAGTATACACGAGTCGTGCGATTTATACTGAAGATCCACAAAGAGATTTAGACA
TTTACATAGATATTGCTGAGCGTGCAGGTCAACATGCAAACGTTGAGCGTATTAAAAATG
GTATTCAAAGACGCATAGATAATGGTACGTTGAAAGTTGTTGATAATTATGATGCTATTG
AAAACATACCTGGTGAATTAATTATGAAAATATTAGCATTTGATGGAAATTTAGAAAAAA
TTGACAAAGCTAGTAAAATTTTAGCTGAATCTCCGAATTTAGCTATATCATCATCTTCGAG
AGGAAATATAGAAATAACGCATTCAGATGCACAAAAAGGTATTGCGCTAGAAACAATTG
CCGAAAGATTAGGGATTGAAATGAAAGAAGTCATGGCAATAGGTGACAATTTAAATGAC
TTATCAATGTTAGAGAAAGTTGGCTATCCAGTTGCGATGGAAAATGGTGCAGAAGAAGTT
AAAAAAATAGCGAAATATGTCACAGATACGAATGAAAATAGTGGTGTTGGAAAAGCTAT
TATGAAATTATTACGTGAACAACAAGTTTAA

> **azo1**

Function: protein coding sequence; FMN-dependent NADPH-azoreductase (core genome, variable)

Best match: azo1_CC001-ST772_118_AJGE01000068.1[48899:49465] (completely identical)
Position: 016-contig_250: 48695 … 49262; Length: 567 bp
Sequence:
ATGAAAGGATTAATTATTATTGGCAGTGCACAAGTGAATTCACATACAAGTGCACTAGCA
AGATACTTAACTGAGCATTTTAAAACACATGATATTGAAGCGGAAATATTCGATTTAGCA
GAAAAGCCGTTAAATCAATTAGATTTTTCAGGAACAACACCGTCTATTGATGAAATCAAA
CAAAATATGAAAGATTTAAAAGAGAAAGCAATGGCGGCGGACTTTTTAATATTAGGAAC
GCCAAACTATCATGGTTCATATTCTGGAATATTGAAAAATGCATTAGATCATCTAAATAT
GGATTATTTTAAAATGAAACCTGTAGGCTTAATAGGAAATAGTGGTGGTATTGTTAGTTC
AGAGCCATTGTCACATTTAAGAGTAATCGTCAGAAGTTTACTAGGCATTGCTGTACCAAC
TCAAATAGCAACACATGATTCTGATTTTGCTAAAAATGAAGATGGTTCATATTACTTAAA
TGATAGTGAATTCCAATTACGAGCAAGATTATTTGTCGATCAAATTGTATCTTTTGTGAAT
AATAGTCCATATGAACATTTAAAATAA

> **tx_azo1_1**
Function: rho independent terminator
Best match: tx_azo1_1_CC001_MW2-USA400_BA000033.2[595361:595442] (completely identical)
Position: 016-contig_250: 49278 … 49360; Length: 82 bp
Sequence:
AATATACATTAAAAATAGGTATGTAGCTTAAGGTATTTCATTGAGAATGCTTTTAAGTCA
CATGCCTATTTTTTGTTCTAAT

> **tx_azo1_2**
Function: rho independent terminator
Best match: tx_azo1_2_CC001_MW2-USA400_BA000033.2[595725:595769] (completely identical)
Position: 016-contig_250: 49642 … 49687; Length: 45 bp
Sequence:
GCAAAATATTATATTTATCAAATTTTGATATTTTAAAGGAGTATT

> **sdrC**
Function: protein coding sequence; unidentified bone sialoprotein-binding protein, probably sdrC chv
Best match: sdrC_CC012_21266_AFTT01000023.1[32762:35593:r]
Position: 016-contig_250: 49692 … 51688; Length: 1996 bp
Sequence:
ATGAATAATAAAAAGACAGCAACAAATAGAAAAGGCATGATACCAAATCGATTAAACAA
ATTTTCGATAAGAAAGTATTCTGTAGGTACTGCTTCAATTTTAGTAGGGACAACATTGATT
TTTGGGTTAAGTGGTCATGAAGCTAAAGCGGCAGAACATACGAATGGAGAATTAAATCA
ATCAAAAAATGAAACGACAGCCCCAAGTGAGAATAAAACAACTGAAAAAGTTGATAGTC
GTCAACTAAAAGACAATACGCAAACTGCAACTGCAGATCAGCCTAAAGTGACAATGAGT
GATAGTGCAACAGTTAAAGAAACTAGTAGTAACATGCAATCACCACAAAACGCTACAGC
TAGTCAATCTACTACACAAACTAGCAATGTAACAACAAATGATAAATCATCAACTACATA
TAGTAATGAAACTGATAAAAGTAATTTAACACAAGCAAAAAACGTTTCAACTACACCTAA
AACAACGACTATTAAACCAAGAACATTAAACCGTATGGCTGTGAACACAGTAGCAGCAC
CACAACAAGGTACAAATGTTAATGATAAAGTTCATTTTTCAAATATTGATATTGCTATTGA
TAAAGGACATGTAAATTCAACAACTGGCAAAACTGAATTTTGGGCGACTTCTAGTGATGT
TTTAAAATTAAAAGCGAATTACAAAATCGATGATTCTGTTAAAGAGGGCGATACATTTAC
ATTTAAATATGGACAGTATTTCCGTCCGGGTTCTGTAAGATTACCTTCACAAACTCAAAAT
TTATATAATGCCCAAGGTAATATTATTGCAAAAGGTATTTATGATAGTACAACAAACACA
ACAACATATACTTTTACGAACTATGTGGATCAATATACAAATGTTAGCGGTAGCTTTGAA
CAAGTTGCATTTGCGAAACGTGAAAATGCAACAACTGATAAAACAGCTTATAAAATGGA
AGTAACTTTAGGTAATGATACATATAGCGAAGAAGTCATCGTCGATTATGGTAATAAAAA
AGCACAACCGCTTATTTCAAGTACAAACTATATCAATAATGAAGATTTATCACGAAATAT
GACTGTTTATGTAAATCAACCTAAAAATACATATACAAAAGAAACATTAGTAACAAATTT
AACTGGTTATAAATTTAATCCAGATGCTAAAAACTTTAAAATTTATGAAGTAACTAACCA
AAATCAGTTTGTGGATAGTTTCACCCCAGATACTTCAAAACTTACAGATGTCACTGATAA

ATTTAAAATTACATACAGTAATGATAATAAGACGGCGACAGTTGATTTATTGAATGGTCA
ATCTAGTAGTGATAAACAGTACATTATTCAACAAGTTGCTTATCCAGATAATAGTTCAAC
AGATAATGGGAAAATTGATTATACTTTAGAAACACAAAATGGAAAAAGTAGTTGGTCAA
ACAGTTATTCAAATGTGAATGGTTCATCAACTGCAAATGGCGACCAAAAGAAATATAATC
TAGGTGACTATGTATGGGAAGATACAAATAAAGATGGTAAACAAGATGCCAATGAAAAA
GGGATTAAAGGTGTTTATGTCATTCTTAAAGATAGTAACGGTAAAGAATTAGATCGTACG
ACAACAGATGAAAATGGTAAATATCAGTTCACTGGTTTAAGCAATGGAACTTATAGTGTA
GAGTTTTCAACACCAGCCGGTTATACACCGACAACTGCAAATGCAGGAACTGATGATGCT
GTTGATTCAGATGGTTTAACAACAACAGGTGTCATTAAAGACGCTGACAACATGACATTA
GATAGTGGATTCTATAAAACACCAAAATATAGTTTAGGTGATTATGTTTGGTACGACAGT
AATAAAGATGGTAAACAAGATTCGACTGAAAAAGGAATTAAAGGTGTTAAAGTTACTTT
GCAAAACGAAAAAGGCGAAGTAATTGGTACAACTGAAACAGATGAAAATGGTAAATATC
GTTTCGATAATTTAGATAGCGGTA

> **sdrD?**
Function: protein coding sequence; unidentified bone sialoprotein-binding protein, probably sdrD
Best match:  [MANUAL OVERRIDE/BLAST]
Position: 017-contig_194_RC: 1 … 756; Length: 756 bp
Sequence:
ATTTCACACTTGATAACGGATACTTCGAAGAAGATACATCAGATAGCGACTCAGATTCAG
ATAGCGATTCAGATTCAGATAGCGACTCAGACTCAGACAGCGACTCAGATTCAGACAGC
GACTCAGACTCGGATAGCGATTCAGATTCAGACAGTGATTCAGATTCAGACAGCGATTCG
GATTCAGACAGCGACTCAGACTCGGATAGCGACTCAGACTCAGATAGCGACTCAGATTCG
GATAGCGACTCAGACTCAGACAGCGACTCAGATTCAGACAGCGACTCAGACACAGATAG
CGACTCAGACTCAGACAGCGACTCAGACAGTGATTCAGACTCAGATAGCGATTCAGATTC
AGACAGCGACTCAGACTCAGATAGTGACTCAGATTCAGATAGTGACTCAGACTCAGACA
GTGATTCAGACTCAGACAGTGATTCAGATTCAGATAGCGACTCAGACTCAGATAGCGACT
CAGATTCAGACAGCGATTCAGACTCAGATAGTGACTCAGATTCGGACAGCGATTCAGACT
CAGATAGCGACTCAGATTCAGACAGTGATTCAGACTCAGATGCAGGTAAGCACACACCT
GTTAAACCAATGAGTACTACTAAAGACCATCACAATAAAGCAAAAGCATTACCAGAAAC
AGGTAATGAAAATAGCGGCTCAAATAACGCAACGTTATTTGGCGGATTATTCGCAGCATT
AGGATCATTATTGTTATTCGGTCGTCGTAAAAAACAAAATA

> **tx_sdrD**
Function:  rho-independent terminator (genomic island)
Best match: tx_sdrD_CC001_MW2-USA400_BA000033.2[603049:603097]#[1_PERFECT MATCH]#tx_sdrD#[ter:rho-independent terminator]#[gi:21.5894:21.5475] (completely identical)
Position: 017-contig_194_RC: 757 … 806; Length: 49 bp
Sequence:
ATAATACAGCTTATACCAGGTCCGTGAGGCCTGGTTTTTATTTCAAAA

> **sdrE/bbp?**
Function: protein coding sequence; unidentified bone sialoprotein-binding protein, probably sdrE/bbp
Best match:  [MANUAL OVERRIDE/BLAST]
Position: 017-contig_194_RC: 807 … 3656; Length: 2850 bp
Sequence:
TTTATGAAATGTATCTTATGTTTGAGATTAAATAGTGCAATTTTTTAAAAAATATAACATA
AATCAACATAAATTTAATTCATCTTTACATTAAAAGTACAAACGAATAAGAAAAGCAATT
AAACACAGTATCAAACCATTTAAAACAATTGATATAAAAGACAAACGAATAATTAAAAA
TTACTAGAATTAATTTATTCAAACCAAAGTGTAAAATAACAGTCTTAGATAAATAAATTT
ATTTAAAGTATTGTGCTTTATCTAAAAATGTATTACGATGGGAATACAAATTTTTATATGT
AAAAATATTAATTTTTTGCAAATATTGATATTTTAAAGGAGATTTATATGATTAACAGGG
ATAATAAAAAGGCAATAACAAAAAAGGGTATGATTTCAAATCGCTTAAACAAATTTTCG
ATTAGAAAGTATACTGTAGGAACTGCATCGATTTTAGTAGGTACGACATTGATTTTGGTC
TAGGGAACCAAGAAGCTAAAGCTGCTGAAAACACTAGTACAGAAAATGCAAAACAAGAT

GATGCAACGACTAGTGATAATAAAGAAGTAGTGTCGGAAACTGAAAATAATTCGACAAC
AGAAAATAATTCAACAAATCCAATTAAGAAAGAAACAAATACTGATTCACAACCAGAAG
CTAAAAAAGAATCAACTTCATCAAGTACTCAAAAACAGCAAAATAACGTTACAGCTACA
ACTGAAACTAAGCCTCAAAACATTGAAAAAGAAAATGTTAAACCTTCAACTGATAAAAC
TGCGACAGAAGATACATCTGTTATTTTAGAAGAGAAGAAAGCACCAAATAATACAAATA
ACGATGTAACTACAAAACCATCTACAAGTGAAATTCAAACAAAACCAACTACACCTCAA
GAATCTACAAATATTGAAAATTCACAATCGCAACCAACGCCTTCAAAAGTAGACAATCAA
GTTACAGATGCAACTAATCCAAAACAAACGGTAAATGTATCAAAAGAAGATCTTAAAAA
TGATCCTGAAAAATTAAAAGAATTAGTTAGAAGTGAAAATAACACTAATCATTCAACTAA
ACCAGTTGCTACAGCACCAACAAGTGTTGCACCAAAACGATTAAATGCGAAAATGCGTTT
TGCAGTTGCACAACCAGCAGCAGTTGCGTCAAATAATGTAAATGATTTAATTACAGTGAC
GAAACAGACGATCAAAGTTGGCGATGGTAAAGATAATGTGGCAGCAGCGCATGACGGTA
AAGATATTGAATATGATACAGAGTTTACAATTGACAATAAAGTCAAAAAAGGCGATACA
ATGACGATTAATTATGATAAGAATGTAATTCCTTCGGATTTAACAGATAAAAATGATCCT
ATCGATATTACTGATCCATCAGGAGAGGTTATTGCCAAAGGAACATTTGATAAAGCGACT
AAGCAAATCACATATACATTTACAGATTATGTAGATAAATATGAAGATATAAAAGCACGT
TTAACTTTATACTCATATATTGATAAGCAAGCAGTACCTAATGAAACTAGTTTGAATTTAA
CGTTTGCAACAGCAGGTAAAGAAACTAGCCAAAACGTTTCTGTTGATTATCAAGACCCAA
TGGTTCATGGTGATTCAAACATTCAATCTATCTTTACAAAGTTAGATGAAAACAAACAAA
CTATTGAACAACAAATTTATGTTAATCCTTTGAAAAAAACAGCAACTAACACTAAAGTTG
ATATAGCTGGTAGTCAAGTAGATGATTATGGAAATATTAAACTAGGAAATGGTAGTACCA
TTATTGACCAAAATACAGAAATAAAAGTTTATAAAGTTAACCCTAATCAACAATTGCCTC
AAAGTAATAGAATCTATGATTTTAGTCAATACGAAGATGTAACAAGTCAATTTGATAATA
AAAAATCATTTAGTAATAATGTAGCAACATTGGATTTTGGTGATATTAATTCAGCCTATAT
TATCAAAGTTGTTAGTAAATATACACCTACATCAGATGGCGAACTAGATATTGCTCAAGG
TACTAGTATGAGAACAACTGATAAATATGGTTATTATAATTATGCAGGATATTCAAACTT
CATCGTAACTTCTAATGACTCTGGCGGTGGTGACGGTACTGTTAAACCTGAAGAAAAGTT
ATACAAAATTGGTGACTATGTATGGGAAGACGTTGATAAAGACGGTGTCCAAGGTACAG
ATTCGAAAGAAAAGCCAATGGCAAACGTTTTAGTTACATTAACTTACCCAGACGGTACTA
CAAAATCAGTAAGAACAGATGCTAACGGTCATTATGAATTCGGTGGTTTGAAAGACGGA
GAAACTTATACAGTTAAATTCGAAACGCCAGCTGGATATCTTCCAACAAAAGTAAATGGA
ACAACTGATGGTGAAAAAGACTCAAATGGTAGTTCTGTAACTGTTAAAATTAATGGTAAA
GATGATATGTCTTTAGACACTGGTTTTTATAAAGAACCTAAATATAATCTTGGTGACTATG
TATGGGAAGATACAAATAAAGATGGTATCCAAGATGCTAATGAACCTGGTATCAAAGAT
GTTAAGGTTACATTAAAAGATAGTACTGGAAAAGTTATTGGTACAACTACTACTGATGCC
TCGGGTAAATATAAATTTACAGATTTAGATAATGGTAACTATACAGTAGAATTTGAAACA
CCAGCAGGTTACACGCCAACGGTTAAAAATACTACAGCTGATGATAAAGATTCTAATGGT
TTAACAACAACAGGTGTCATTAAAGATGCAGATAATATGACATTAGACAGTGGTTTCTAT
AAAACACCAAAATACAGTTTAGGTGATTATGTTTGGTAC

> **bbp**

Function: protein coding sequence; unidentified bone sialoprotein-binding protein, probably sdrE/bbp chv
Best match: bbp_CC005_A9299_ACKH01000033.1[20944:24243:r]
Position: 018-contig_252: 1 … 171; Length: 171 bp
Sequence:
ACACCTGTTAAACCAATGAGTACTACTAAAGACCATCACAATAAAGCAAAAGCATTACC
AGAAACAGGTAGTGAAAATAACGGCTCAAATAACGCAACGTTATTTGGTGGATTATTTGC
AGCATTAGGTTCATTATTGTTATTCGGTCGTCGCAAAAAACAAAACAAATAA

> **tx_bbp**

Function:  rho independent terminator
Best match: tx_bbp_CC001_MW2-USA400_BA000033.2[606868:606916] (completely identical)
Position: 018-contig_252: 167 … 216; Length: 49 bp
Sequence:

ATAATACAATATGACCCAGGTCCTTGTGGCCTGGTTTTTTATAATTAC

## > **Q2FJ76**

Function: protein coding sequence; putative poly(glycerol-phosphate) alpha-glucosyltransferase (core genome, variable)
Best match: Q2FJ76_CC001-ST772_118_AJGE01000030.1[342:1814] (completely identical)
Position: 018-contig_252: 289 … 1762; Length: 1473 bp
Sequence:
ATGAATTACATTTTAGGAACAATTTTAGAAAGTAAAATTACAGGTGTAGAAAAAGCGCA
AATAAATAGATTGAAGTTGTTCAAACAACACGGCATATCTTCAAAATGTGTATATGTTAA
ATGGAATCCTTATTCATACACATATGCGAAGCAACATCAGATTGAAAATGATGTATTTAC
AATGTATGACTATTTTCAAAAAGCAATCAATTATAAAAAGACAAAGCAAGTTAACTGGAT
ACAGTATTGGGAAAAGTCATGTAGGTACACATTGAAATTTGTGGAAAATTCAAATGATGT
CAGAATATATGATGAAGAGCAATTTATAATGTATGCTCATTTTTTAGATAAACAGTATCA
TCATTTAAACTATGTGAATTATTTTGATCATAAAAGAAGAAAAGTAAAACGCGAATTGTA
TGATGGAAGAGGCTTTTTAAGTTGTTCTCGAATTTTAGGTGAAGGACAACGGATTGTACT
CGAAAATTACTATACACCTAATGGGGAAATCGTCATCCAAAAATATTTCGACGATATAAA
AGGGAAAAACACGCTCACAAAGGTTATCTTAAATGAAGATCAGCATCAACAATTTTTTGA
TACAGAAGATGAATTAGTTCAATATTTTCTCCATCAATTATGTAAAAATAATGATCAAAT
CATATTAGATCGTCCTCATGAATTAGGAAATGTTATAGCGGGATTAAATCAAAGTATTCC
AGTTGTTGTTGTGCTCCATAGTACACATTTATTCGGTACCGGTAATGGTATAAAAAGTTTT
TATAAAACAGTATTTAATAATTTAACACGTTATAAAGCGATTGTTGTATCAACAGAAAAG
CAATGCCAAGATATTTCACAATATATTGAAAATAAAATACCAGTTATCAATATTCCGGTT
GGCTACGTGGCAAATTTAAAGTATCAATTTGACATCAATCAAAAGGAGAAAAATCATATC
ATATCAATTGCTCGCCTCGTTGAAAATAAACAAATTAAACATCAAATTGAAGTGATCAAG
CAATTAGTAACAAAACATCCCAATATTCAATTGAATATTTATGGACATGGAAATGGTTTG
TCAGAATATCGACAACTTGTAGAAGATTATCATTTATCGGAACATGTTAAATTTCATGGTT
TTAAGACGCATATTAATGAAGAGATTGCTAAAGCAGAACTGATGTTATCGACAAGTAAA
ATGGAAGGTTTTGGCTTAGCAATTTTAGAGTCGCTTTCAGTAGGTACACCAGTGATCAGTT
ATGATGTAGATTATGGTCCATCAGAACTGATTCAAGATGGATTTAATGGCTATTTAGTAC
CTCAAGGTGACATCAATCAAATGGTTGAAAAGGTCGACCAATTACTAAATAATACTCAAA
AATTGCAACAGTTTTCAATTAATAGCATAGAATCTGCACAACAGTACAATGCAACTACTA
TCAGTACAAAGTGGCAAAATATTTTAAACTAA

## > **Q5HIB0**

Function: protein coding sequence; putative poly(glycerol-phosphate) alpha-glucosyltransferase (core genome, variable)
Best match: Q5HIB0_CC001-ST772_118_AJGE01000030.1[1939:3429:r]RC (completely identical)
Position: 018-contig_252: 1886 … 3377; Length: 1491 bp
Sequence:
TTATATAAGACGAACAAATTGGTCCCATTGTTTAATTAACGACGCTTTACTATATTGTTGT
GCTTTTGCCAAACTACCTTTTGACAGTCGTTGCTGTACTTCAGGATGATCAATCACATATT
TTACTTTATCAAATAGGGCATCTTCATCATTTTTAGTAATTAAATAACCATTGAAATCTGA
AGTAATCAGTTCGTTAGGTCCATATTTAATATCATAACTAATAACTGGAACACCATGTGCT
AAAGATTCAAGTAGCGCTAAAGAGAAACCTTCCATGTTACTTGTTATTAAACTCAAATAG
GCATCGCTATATTCTTGGTCTAGATTGCTTAAAAAGCCGCGTAAGTAAACATGATTTTCCA
ATCCATATTTTTGTATCAATTCATTTAATTTTTTACTTTCAGAACCAAAACCATACATATG
AAGCTCTATTTTTGGGACATACGATACTAAGCGTTTAATTAATTCAATTTGTTGATGTAAT
TGTTTTTCAGGTGAATAACGAGCAACGGAAATTAATTTAACACTGCGCTGATCTAATGTTT
GGACTGGTGTATCAATTGTTTCACTATAGCCGACAGGAATATTAACAACTGGAATAGTAT
GGTTAATACGTTTTTCAACATCTAATTTTTGCTGCTCAGTAGAAACGATAATTGCACGATA
TCGAGATAAATTTTCAAACATCGCTTTATATACATTTTTAAATGGCGATGAATCTAATGCA
TCAATATTTTTAATGTGTGTACTGTGAAGCACAGCTACTACTGGGATTGACTCAGGCGTTA
AGTTGAAAATAGGTGCTGTGTACACATTACGATCACTGAAAAATAAATCCCCATGTTGAT
ATAGTTGTTTAATGAAAAATGCGCCTAATTCCGTTTCATTATTAAAGAAATATTGTTTGTT

AGCATAGTAAACAATAATTTTTTGTACTTCTGGTTTGCCATCCTTGTAAGAAAAATACTTT
TCTAATTTTGTGTCACCTTCTGGATTATAGAAAAATTCACATAATGTTTGTTGTTTATCAA
CAAGAATCCTACTACAACTTAAAAAGCCACGCACATCATAAAAATCACGTTTTACTTTTC
GTCTTTGACTATCAAAATGATTCACATAATCTAATATACGATATTTAGGATCTTGAAAATG
GGCATACATTAAGAAACGCTCTTGATCATATATTCTAAAGTCATGACTATTTTCAACATGT
TTTAAAGTATAATGACATTCATCAGTCCAATACGACAACCAGTCAAATGGTTCATTGCGT
TCTAAATATGTTGCTTCTTGGAAGAAATCATACATATTAATATAGTCAGAACTAGTAATAT
AATTTTGGGCATTTCTATATAAATATCTATTCCATGACAGAAATACACATTGCGCTGGTCT
TCCCATTTCTTTAAATAAATTTAAACGATTAATAATTGCTTTCTCTATCCCAGTTAAATTA
ACACCTAAACTATTACCTACAAAATAATTCAT

> **folE2**
Function: protein coding sequence; GTP cyclohydrolase (core genome, variable)
Best match: folE2_CC001-ST772_118_AJGE01000030.1[3711:4589:r]RC (completely identical)
Position: 018-contig_252: 3658 … 4537; Length: 879 bp
Sequence:
TTATTTGCGATACTTTAATTTAGCGAAAGCATCATGTTGATGGATAGACTCTTCATTACGA
CATTCGATATCGAAACCGTCTAACCAATCAAATTCAACTAAGTCCGCGGCAATTAAACGA
ATTAAGTCTTCGACAAAACGTGGATTTTCATATGCACGCTCTGTCACACGTTTTTCATCAG
GACGTTTTAAAATAGGGTATAGAATTGAACTTGCATTAGCTTCCATTGCATCTAAAATTTT
ATTTTTATAGTCATCAACTATGTCTTGATCTTTATTAATATATGTTTTAACAGTGACAACA
CCACGTTGGTTGTGCGCTGAATACTCACTTATTTCTTTTGAACAAGGGCATAGCGTTGTGA
CAGTTGCTTCAATAGTAAGTTCTTTACGTGTAACTTTATCACCGTCAATTGCTAATCCATA
AGTGACATCGGCATTGCCAACTGCTTTAATATTTGTGGTTGGACTATAGCGATCAAAGAA
CCATTTCCCAGAAACATCAACGCCTGCCGCATTTTGTTTCATATTCGTTTGTAAAGTGCGT
AACACCTGATAAAGTGTATTAAATTCAAGTTCAATACCATTATCATAGTGCTTTTCAACAC
TTTCAATTATACGGCTCATATTAATACCTTTTTCGTCTTTTGTTAAACTTGTTGAAAAACTA
AATGTGCCAGCTGTTTGATACTGGTCAACAAGTACAGGGTACACTAAGTTTTTAATACCA
ACTTCTTCTATTTCAAATAAAAAATCTTTATGTGTACTTTGTAAATCTGTCATTTCGTTCTT
AGTAGTAGGTTTCGTGCCTTCAATAGGATCTACGGAACCAAAGTGTTTCCAACGACCTTC
TCGTGTCGATAAATCAAATTCAGTCAT

> **nagB**
Function: protein coding sequence; glucosamine-6-phosphate deaminase (core genome, constant)
Best match: nagB_CC001-ST772_118_AJGE01000030.1[5919:6677] (completely identical)
Position: 018-contig_252: 5866 … 6625; Length: 759 bp
Sequence:
ATGAAAGTATTAAACTTAGGATCGAAAAAACAAGCATCATTCTATGTTGCATGTGAGTTA
TATAAAGAGATGGCATTTAATCAGCACTGTAAACTTGGTTTAGCAACTGGTGGTACAATG
ACAGATTTGTATGAACAACTTGTTAAGTTATTAAATAAAAATCAGTTAAACGTAGACAAT
GTATCCACGTTTAATTTAGACGAATATGTAGGTTTAACTGCATCACATCCGCAAAGTTATC
ACTATTATATGGATGACATGCTTTTCAAACAATATCCTTATTTTAATAGAAAGAACATTCA
TATTCCAAATGGAGATGCCGATGATATGAATGCGGAAGCGTCAAAATATAATGACGTTTT
AGAACAACAAGGTCAACGTGATATTCAAATTTTAGGTATTGGAGAAAATGGTCATATTGG
ATTTAATGAACCTGGTACGCCGTTTGATAGCGTTACTCATATCGTTGATTTGACTGAAAGT
ACTATTAAGGCTAATAGTCGATATTTTGAAAACGAAGATGATGTTCCAAAGCAAGCCATT
TCGATGGGACTTGCTAATATTCTTCAAGCCAAACGTATCATTTTACTCGCATTTGGTGAAA
AGAAACGTGCTGCTATTACACATTTATTAAATCAGGAAATTTCTGTTGATGTTCCAGCCAC
ATTACTTCACAAACACCCGAATGTTGAGATATATTTAGACGACGAAGCTTGCCCGAAAAA
TGTTGCGAAAATTCATGTCGATGAAATGGATTGA

> **hxlA**
Function: protein coding sequence; 3-hexulose-6-phosphate synthase (core genome, variable)
Best match: hxlA_CC001-ST772_118_AJGE01000030.1[6754:7386] (completely identical)
Position: 018-contig_252: 6701 … 7334; Length: 633 bp

Sequence:
GTGGAATTACAATTAGCAATTGATTTATTAAACAAAGAAGACGCGGCTGAGTTAGCAAAT
AAAGTAAAAGATTATGTAGATATCGTAGAAATCGGTACGCCAATCATTTACAACGAAGGT
TTACCAGCAGTTAAACATATGGCAGACAACATTAGTAATGTAAAAGTATTAGCAGACATG
AAAATTATGGATGCAGCTGATTATGAAGTTAGCCAAGCAATTAAATTTGGCGCGGATGTA
ATTACAATACTAGGTGTTGCAGAAGATGCATCAATTAAAGCAGCTATTGAAGAAGCTCAT
AAAAATAATAAACAATTACTAGTTGATATGATTGCTGTTCAAGATTTAGAAAAACGTGCA
AAAGAACTAGATGAAATGGGTGCGGATTATATTGCAGTACACACTGGTTATGATTTACAA
GCAGAAGGGCAATCACCATTAGAAAGTTTAAGAACCGTTAAATCTGTTATTAAAAATTCT
AAAGTTGCAGTAGCAGGTGGAATTAAACCAGATACAATTAAAGATATTGTCGCTGAAAG
TCCTGATCTTGTTATTGTTGGTGGCGGAATCGCAAATGCAGATGATCCAGTAGAAGCTGC
AAAACAATGTCGCGCTGCAATCGAAGGTAAGTAA

## > sis

Function: protein coding sequence; sugar isomerase (core genome, variable)
Best match: sis_CC005_Mu50-VRSA_BA000017.4[647833:648381] (completely identical)
Position: 018-contig_252: 7335 … 7884; Length: 549 bp
Sequence:
ATGGCTAAATTTAGTGACTATCAATTAATTCTAGATGAATTAAAGATGACTTTGTCACATG
TTGAAGCGGATGAGTTTTCAACTTTTGCATCCAAAATACTACATGCTGAACATATATTTGT
AGCTGGCAAAGGACGTTCAGGATTCGTGGCGAATAGTTTTGCAATGCGCTTAAATCAGCT
CGGCAAACAGGCACATGTTGTTGGAGAATCAACGACACCTGCGATTAAGTCGAATGATGT
ATTTGTAATTATCTCTGGTTCAGGTTCCACGGAACATTTAAGATTATTAGCAGACAAAGC
AAAATCAGTAGGTGCTGACATCGTATTAATTACTACAAATAAAGATTCTGCAATAGGCAA
TCTAGCTGGGACGAACATCGTTTTGCCTGCAGGTACAAAATATGATGAACAAGGCTCGGC
ACAACCATTAGGAAGTTTGTTTGAACAAGCATCTCAATTATTTTTAGATAGTGTTGTAATG
GGATTGATGACTGAAATGAATGTTACGGAACAAACGATGCAACAAAATCATGCTAATTTA
GAATAA

## > Q2FJ68

Function: protein coding sequence; putative haloacid dehalogenase-like hydrolase (core genome, variable)
Best match: Q2FJ68_CC008_COL_CP000046.1[651653:652300]
Position: 018-contig_252: 7996 … 8644; Length: 648 bp
Sequence:
TTGAAGTTTGACAATTATATTTTTGATTTTGATGGTACGTTGGCAGACACGAAAAAATGTG
GTGAAGTAGCAACACAAAGTGCATTTAAAGCATGTGGCTTAACGGAACCATCATCTAAA
GAAATAACGCATTATATGGGAATACCTATTGAAGAATCATTTTTAAAATTAGCAGACCGA
CCATTAGATGAAGTAGCATTAGCAAAGTTAATCGATACATTTAGACATACATATCAATCT
ATTGAAAAGGACTATATTTATGAATTTGCGGGTATAACTGAAGCCATTACAAGTTTGTAT
AACCAAGGGAAAAAACTTTTCGTGGTGTCTAGTAAGAAGAGTGATGTATTAGAAAGAAA
TTTATCGGCTATTGGATTAAATCACTTGATTACCGAAGCTGTTGGATCCGATCAAGTAAGT
GCATATAAACCAAATCCTGAAGGCATACACACAATTGTGCAACGCTACAATTTAAATAGC
CAACAAACGGTGTATATTGGTGATTCAACGTTTGATGTTGAGATGGCACAACGTGCTGGT
ATGCAATCTGCAGCTGTCACTTGGGGTGCACATGATGCAAGGTCATTACTTCATTCAAAT
CCGGATTTTATTATTAATGATCCATCAGAAATTAATACCGTATTATAA

## > proP

Function: protein coding sequence; putative proline/betaine symporter (core genome, variable)
Best match: proP_part_CC005_CF-Marseille_CABA01000111.1[9182:9701]
Position: 018-contig_252: 9635 … 9665; Length: 30 bp
Sequence:
TTGGGACATTATCAGGTTACATAGCTGCTT

## > vraA

Function: protein coding sequence; long chain fatty acid CoA ligase (core genome, constant)
Best match: vraA_CC001-ST772_118_AJGE01000030.1[11142:12518] (completely identical)
Position: 018-contig_252: 11089 … 12466; Length: 1377 bp
Sequence:
ATGAACGTAATTTTAGAACAGTTGAAAACACATACTCAAAATAAACCTAATGACATAGCA
TTACATATAGATGATGAAACAATTACATATAGTCAACTAAATGCCCGCATCACTAGCGCA
GTTGAATCTTTGCAGAAATATTCACTTAACCCTGTCGTTGCTATTAATATGAAATCATCGG
TTCAAAGTATTATTTGTTATTTAGCTTTGCATCGTGTACATAAAGTGCCTATGATGATAGA
AGGTAAATGGCAAAGTACTATACATCGTCAATTGATTGAAAAATATGGTATTAAAGATGT
AATTGAAGATACAGGTCTCATGCAGAGTATAGACTCACCGATGTTTATTGATGCAACGCA
ATTACAGCACTACCCCAATTTATTACATATTGGTTTTACTTCAGGGACAACTGGACTGCCA
AAAGCATATTATCGTGATGAAGATTCATGGTTGGCTTCTTTTGAAGTTAATGAAATGTTGA
TGTTAAAAAATGAAAATGCAATAGCAGCCCCTGGACCACTATCGCACTCGTTAACATTAT
ATGCGTTATTGTTTGCTTTAAGTTCCGGTCGTACTTTTATAGGACAGACCACTTTTCATCCT
GAAAAGTTACTTAATCAATGTCGTAAAATATCATCATACAAAGTTGCTATGTTTCTTGTTC
CAACGATGATTAAATCATTATTGTTAGTTTACAACAATGAACATACAATCCAATCATTTTT
TAGCAGTGGAGATAAGCTGCATTCTTCTATTTTTAAAAAGATAAAAAATCAAGCAAATGA
CATAAATTTGATTGAATTTTTTGGTACATCGGAAACCAGTTTTATCAGCTATAACTTGAAT
CAGCAAGCACCAGTTGAATCAGTAGGTGTGCTATTTCCAAATGTGGAATTGAAAACAACG
AATCACGATCACAATGGTATAGGAACTATTTGTATAAAAAGTAATATGATGTTTAGTGGC
TATGTAAGTGAACAATGTATAAATAATGATGAATGGTTTGTTACTAATGATAATGGCTAT
GTAAAAGAGCAGTATTTATATTTAACGGGACGTCAACAGGATATGTTAATTATTGGTGGT
CAAAATATATATCCAGCACATGTTGAACGCCTTTTAACGCAATCTTCGAGCATTGATGAA
GCAATTATCATCGGTATTCCAAATGAGCGTTTTGGTCAAATAGGCGTATTGCTTTATTCTG
GTGATGTGACACTTACACATAAAAATGTAAAACAATTTTTAAAAAAGAAAGTGAAACGC
TATGAAATTCCATCGATGATTCATCATGTAGAAAAGATGTATTACACTGCAAGTGGTAAA
ATTGCTAGAGAAAAAATGATGTCGATGTATTTGAGAGGTGAATTATAA

> **vraB**
Function: protein coding sequence; acetyl-CoA c-acetyltransferase (core genome, constant)
Best match: vraB_CC008_NCTC8325_CP000253.1[567450:568589] (completely identical)
Position: 018-contig_252: 12467 … 13607; Length: 1140 bp
Sequence:
ATGAATCAAGCAGTCATAGTTGCAGCTAAACGAACTGCATTTGGGAAATATGGTGGCACT
TTAAAACATTTAGAGCCAGAACAATTGCTTAAACCTTTATTCCAACATTTTAAAGAGAAG
TATCCAGAGGTAATATCTAAAATAGATGATGTAGTTTTAGGTAATGTTGTTGGGAATGGT
GGCAATATTGCAAGAAAAGCATTGCTTGAAGCGGGGCTTAAAGATTCAATACCTGGCGTC
ACAATCGATCGGCAATGTGGGTCTGGACTTGAAAGTGTTCAATATGCATGTCGCATGATC
CAAGCCGGAGCTGGCAAGGTATATATTGCAGGTGGTGTTGAAAGTACAAGTCGAGCACC
TTGGAAAATCAAACGACCGCATTCTGTGTACGAAACAGCATTACCTGAGTTTTATGAGCG
TGCATCATTTGCACCTGAAATGAGCGACCCATCAATGATTCAAGGTGCTGAAAATGTGGC
CAAGATGTATGATGTTTCAAGAGAATTACAAGATGAATTTGCTTATCGAAGTCATCAATT
GACAGCGGAAAATGTAAAGAATGGAAATATTTCTCAGGAAATATTACCTATAACCGTTAA
AGGAGAAATATTCAACACTGATGAAAGTCTAAAATCACATATTCCGAAAGATAACTTTGG
CCGATTTAAGCCCGTGATCAAAGGTGGGACCGTTACCGCTGCGAATAGTTGTATGAAAAA
TGATGGTGCAGTTTTATTGCTTATTATGGAAAAAGATATGGCATACGAATTAGGTTTCGA
GCATGGTTTATTATTTAAAGATGGTGTTACGGTAGGTGTTGATTCTAATTTTCCTGGCATT
GGTCCAGTACCAGCCATTTCCAACTTACTAAAAAGAAATCAATTAACGATAGAAAATATT
GAAGTCATTGAAATTAACGAAGCGTTCAGTGCACAGGTAGTTGCCTGCCAACAAGCTTTA
AATATTTCAAATACGCAATTAAATATATGGGGTGGTGCATTAGCATCAGGTCATCCATAC
GGTGCAAGCGGTGCCCAATTAGTGACTCGATTATTTTATATGTTTGACAAAGAGACTATG
ATTGCATCTATGGGGATAGGGGGAGGTCTAGGAAATGCAGCATTATTTACTCGATTCTAA

> **vraC**
Function: protein coding sequence; putative protein (core genome, constant)

Best match: vraC_ref_CC001_MW2-USA400_BA000033.2[620281:620646] (completely identical)
Position: 018-contig_252: 13581 … 13947; Length: 366 bp
Sequence:
ATGCAGCATTATTTACTCGATTCTAACCAGCGATTAAATGTGTCATTTTCTAAGGATAGTG
TGGCTGCATATTATCAGTGTTTTAACCAACCTTATAGAAAAGAAGTACCACCATTAATGT
GTGCGTCATTATGGCCAAAATTTGATTTATTTAAAAAATATGCAAATAGCGAACTGATTTT
AACAAAATCAGCAATTAATCAAACTCAAAAGATAGAAGTAGACACAATATATGTAGGGC
ATTTAGAAGATATTGAATGCCGACAGACTCGCAATATCACACGTTATACAATGGCTTTAA
CATTAACTAAAAATGATCAACATGTCATAACGGTTACACAAACTTTTATTAAGGCGATGA
AGTAG

## > ssr128
Function: noncoding RNA small stable RNA 128 (core genome, constant)
Best match: ssr128_CC001_MW2-USA400_BA000033.2[620913:621305:r]RC (completely identical)
Position: 018-contig_252: 14213 … 14606; Length: 393 bp
Sequence:
AAATAACCGATATTAGCTGCATGAACGCATATTAATTAGGAGATGAAAGGACAGCTAAT
ATCAGTTATGTATTGTTATTATTATTGGGAACAGAGATGAATATAGGTTACGTTTCTTTCT
TTGCACGGGGATGCATTAATCTAAAATAATAATAACAACTATATCAATGTTTAATAAATT
CTGGATTATTGGAACGATTAGTCAATTTAACTAACTTTCATATGATCTATATCGTCTTGTA
ATAAAGAGAGCAATTTGAATATTTCAGTATCACTAAATGAATCGTCACATTTAATTGAAA
CATGCTGAAACGTTTTGGTTATAATTTCATAAACTGGTGCGCCTTCATGGTGATACTGTCG
ATAAATAATCATAACCTATATTACCTCCTTT

## > thiD1
Function: protein coding sequence; phosphomethylpyrimidine kinase (core genome, constant)
Best match: thiD1_CC005_Mu50-VRSA_BA000017.4[655591:656421:r]RC (completely identical)
Position: 018-contig_252: 15093 … 15924; Length: 831 bp
Sequence:
TTAAACCTCTGTTACTTCAACATCGATATGTTCAATACGGTTGTATGCACCGTGATCCACA
GGACCAACAAAATCATTCATTTTCCAACCGTTTTTAATAGCAGAAGCGACGAAAGCTTTC
GCGCTAATCACAGCTTCTTTCGGTGACTTACCGTTAGCTAAATATGCAGTTGTTGCCGCAG
CAAATGTACAACCAGCACCATGGTTATAACTTTGTTGGAACATGTCTGTTGTTAGTTGATA
AAATGTTTGACCATCATAGTATAAGTCATACGATTTATCTTGATCTAAAGCTTTGCCACCT
TTAATGATGACATGCTGTGCGCCTTTATCAAAGATAATTGTTGCAGCCTTTTTCATATCTT
CAATTGAATTTAATTTACCTAATCCTGATAATTGACCCGCTTCAAATAAGTTTGGTGTCAC
TACCGTTGCTTTAGGTAGTAAATATTTAATCATCGCCTCAGTATTTCCAGGATTAAGCACT
TCATCTTCGCCTTTACAAACCATGACAGGATCTACTACAAAATATTGTGCATTAGATGCCT
CATATACTTCTCCAGCACGTTTGATTATCTCCTCAGTACCTAACATACCTGTTTTAATAGC
ATCAGGTCCGATTGATAAAGCCGTTTCAAGTTGTTTTTCAAATACATCCATTGGTAATGGT
GTAACATCGTGTGACCATGTATCTTTATCCATAGTAACGATGGCAGTTAAAGCGACCATG
CCATACGTATCTAATTCTTGGAACGTTTTCAAATCTGCTTGCATACCTGCGCCAGCACTTG
TGTCAGAACCGGCAATTGTTAAAACTTTCTTTAAAGCCAT

## > ung
Function: protein coding sequence; uracil-DNA glycosylase (core genome, constant)
Best match: ung_CC001_MSSA476_BX571857.1[621498:622154]
Position: 018-contig_252: 16107 … 16764; Length: 657 bp
Sequence:
ATGGAATGGTCGCAAATTTTTCATGACATAACAACGAAACATGACTTTAAAGCTATGTAT
GATTTTTTAGAAAAAGAATATTCGACTGCAATCGTATACCCTGATAGGGAAAATATATAT
CAAGCGTTTGATTTAACACCGTTTGAAAATATCAAAGTTGTTATATTAGGACAAGACCCG
TATCATGGTCCAAACCAAGCACATGGATTAGCATTTTCAGTGCAACCTAACGCAAAATTC
CCTCCATCTTTACGTAATATGTATAAAGAATTAGCAGATGATATTGGATGCGTTAGACAA
ACACCGCATTTACAAGATTGGGCAAGAGAAGGCGTCTTGTTATTGAATACAGTTTTAACC

GTAAGACAGGGTGAAGCAAATTCTCATCGTGATATTGGTTGGGAAACATTTACTGATGAA
ATTATTAAAGCAGTGTCTGATTATAAAGAACATGTTGTCTTTATTTTGTGGGGGAAACCTG
CACAGCAAAAAAATAAAGCTTATCGATACATCTAAACATTGTATTATAAAATCAGTGCATC
CTAGTCCACTGTCTGCATATAGAGGATTCTTTGGATCAAAACCGTATTCCAAAGCGAATG
CCTATTTAGAGTCAGTAGGAAAATCACCAATTAATTGGTGTGAAAGTGAGGCGTAG

> **rsaA**
Function: noncoding RNA ncRNA of Staphylococcus aureus A (core genome, constant)
Best match: rsaA_CC001_MW2-USA400_BA000033.2[627561:627699] (completely identical)
Position: 018-contig_252: 20860 … 20999; Length: 139 bp
Sequence:
AGTTAACCATTACAAAAATTGTATAGAGTAGCGACTGTATAATTTCTATTGAGGTTAACG
TTTATATGTAGTGATAGTAGTTAAAGTTCTCCCAAGGAAGACTACTCGGGTACACTTTGCT
ATGAGCAAAGTGTACTTT

> **pta**
Function: protein coding sequence; phosphotransacetylase (core genome, constant)
Best match: pta_CC001-ST772_118_AJGE01000030.1[22141:23127] (completely identical)
Position: 018-contig_252: 22088 … 23075; Length: 987 bp
Sequence:
ATGGCTGATTTATTAAATGTATTAAAAGACAAACTTTCTGGTAAAAACGTTAAAATCGTA
TTACCTGAAGGAGAGGACGAACGTGTTCTAACAGCTGCAACACAATTACAAGCAACAGA
TTATGTTACACCAATCGTGTTAGGTGATGAGACTAAGGTTCAATCTTTAGCGCAAAAACT
TGATCTTGATATTTCTAATATTGAATTAATTAATCCTGCGACAAGTGAATTGAAAGCTGAA
TTAGTTCAATCATTTGTTGAACGACGTAAAGGTAAAGCGACTGAAGAACAAGCACAAGA
ATTATTAAACAATGTGAACTACTTCGGTACAATGCTTGTTTATGCTGGTAAAGCAGATGG
TTTAGTTAGTGGTGCAGCACATTCAACAGGAGACACTGTGCGTCCAGCTTTACAAATCAT
CAAAACGAAACCAGGTGTATCAAGAACATCAGGTATCTTCTTTATGATTAAAGGTGATGA
ACAATACATCTTTGGCGATTGTGCAATCAATCCAGAACTTGATTCACAAGGACTTGCAGA
AATTGCAGTAGAAAGTGCAAAATCAGCATTAAGCTTTGGCATGGATCCAAAAGTTGCAAT
GTTAAGCTTTTCAACAAAAGGGTCTGCTAAATCAGACGACGTGACAAAAGTTCAAGAAG
CTGTCAAATTAGCACAACAAAAAGCTGAAGAAGAAAAATTAGAAGCAATCATTGATGGC
GAATTCCAATTTGATGCTGCGATTGTACCAGGTGTTGCTGAGAAAAAAGCGCCAGGTGCT
AAATTACAAGGTGATGCAAATGTCTTTGTATTCCCAAGTTTAGAAGCTGGTAATATTGGTT
ACAAAATTGCACAACGTTTAGGTGGATATGATGCAGTTGGTCCAGTATTACAAGGTTTAA
ATTCTCCAGTAAATGACTTATCACGTGGCTGCTCAATTGAAGATGTATACAATCTTTCAAT
TATCACAGCAGCGCAAGCCTTACAATAA

> **lipL**
Function: protein coding sequence; octanoyl-GcvHb protein N-octanoyltransferase
Best match: lipL_CC001_MW2-USA400_BA000033.2[629778:630614] (completely identical)
Position: 018-contig_252: 23077 … 23914; Length: 837 bp
Sequence:
ATGGATTTAGCGAGTAAATATTTTAATGGCGTCAACTGGCGATATATCGATCATTCTTCTG
GATTAGAACCTATGCAATCTTTCGCATTCGATGATACATTTTGCGAAAGTGTGGGCAAAG
ATATATCAGATAATGTTGTGCGTACTTGGATTCATCAACATACTGTTATTCTTGGTATTCA
TGATTCAAGATTGCCGTTTTTAAAAGATGGCATTGATTATTTAACGAATGAGATTGGTTAT
AATGCCATTGTTAGAAATTCTGGTGGCTTAGGTGTCGTTCTAGATCAAGGTGTATTAAATA
TATCGCTGATGTTCAAAGGACAAACAGAAACAACGATTGATGAAGCGTTTACTGTGATGT
ACCTCTTAATTAGCAAAATGTTCGAAAATGAGAATGTTGATATTGATACGATGGAAATTG
AACATTCTTATTGCCCAGGAAAATTTGACTTAAGTATCGATGGTAAGAAATTTGCAGGCA
TATCGCAACGAAGAGTTAGAGGCGGTATTGCTGTACAAATTTATCTTTGTGTTGAAGGCT
CTGGTTCAGAACGTGCATTGATGATGCAAACATTTTATGAACATGCTTTAAAAGGTGAAG
TGACTAAATTTAAATATCCTGAAATTGAACCATCTTGTATGGCCTCATTAGAGACATTGCT
TAACAAAACGATTACTGTTCAAGATGTAATGTTTTTACTATTATATGCAATCAAAGATCTT

GGCGGTGTATTAAATATGACGCCAATTACTCAAGAAGAATGGCAAAGATACGATACGTA
TTTTGATAAAATGATTGAAAGAAACAAGAAAATGATAGATCAAATGCAATAG

> **mvaK1**
Function: protein coding sequence; mevalonate kinase 1 (core genome, constant)
Best match: mvaK1_CC001_MW2-USA400_BA000033.2[631201:632121] (completely identical)
Position: 018-contig_252: 24500 … 25421; Length: 921 bp
Sequence:
ATGACAAGAAAAGGATATGGGGAATCGACAGGTAAGATTATTTTAATAGGAGAACATGC
TGTTACATTTGGAGAGCCTGCTATTGCAGTACCGTTTAACGCAGGTAAAATCAAAGTTTT
AATAGAAGCCTTAGAGAGCGGGAACTATTCGTCTATTAAAAGCGATGTTTACGATGGTAT
GTTATATGATGCGCCTGACCATCTTAAGTCTTTGGTGAACCGTTTTGTAGAATTAAATAAT
ATTACAGAGCCGCTAGCAGTAACGATCCAAACGAATTTACCACCATCACGTGGATTAGGA
TCGAGTGCAGCTGTCGCGGTTGCTTTTGTTCGTGCAAGTTATGATTTTTTAGGGAAATCAT
TAACGAAAGAAGAACTCATTGAAAAGGCTAATTGGGCAGAGCAAATTGCACATGGTAAA
CCAAGTGGTATTGATACGCAAACGATTGTATCAGGCAAACCAGTTTGGTTCCAAAAAGGT
CATGCTGAAACGTTGAAAACGTTAAGTTTAGACGGCTATATGGTTGTTATAGATACTGGT
GTGAAAGGTTCAACAAGACAAGCAGTAGAAGATGTTCATAAACTTTGTGAGGACCCTCA
GTACATGTCACATGTAAAACATATCGGTAAGTTAGTTTTACGTGCGAGTGATGTGATTGA
ACATCATAACTTTGAAGCCTTAGCGGATATTTTTAATGAATGTCATGCGGATTTAAAGGC
GTTGACAGTTAGTCATGATAAAATAGAACAATTAATGAAAATTGGTAAAGAAAATGGTG
CGATTGCTGGAAAACTTACTGGCGCTGGTCGTGGTGGAAGTATGTTATTGCTTGCCAAAG
ATTTACCAACAGCGAAAAATATTGTAAAAGCTGTAGAAAAAGCTGGTGCAGCACATACTT
GGATTGAGAATTTAGGAGGTTAA

> **mvaD**
Function: protein coding sequence; mevalonate diphosphate decarboxylase (core genome, constant)
Best match: mvaD_CC001_MW2-USA400_BA000033.2[632126:633109] (completely identical)
Position: 018-contig_252: 25425 … 26409; Length: 984 bp
Sequence:
TTGATTAAAAGTGGCAAAGCACGTGCACATACGAATATTGCACTTATAAAATATTGGGGT
AAAAAAGATGAAGCACTAATCATTCCAATGAATAATAGCATATCTGTTACATTAGAAAAA
TTTTACACTGAAACGAAAGTCACTTTTAACGACCAGTTAACACAGGATCAATTTTGGTTG
AATGGTGAAAAGGTTAGTGGCAAAGAATTAGAGAAAATTTCAAAATATATGGATATTGT
CAGAAATAGAGCTGGCATCGATTGGTATGCAGAAATTGAAAGCGACAATTTTGTACCAAC
AGCAGCAGGGTTGGCTTCATCGGCAAGCGCATATGCAGCTTTAGCAGCAGCTTGTAATCA
AGCGCTAGACATGCAGCTGTCAGATAAGGATTTATCGAGATTGGCGCGAATTGGTTCGGG
TTCTGCGTCGCGTAGTATTTATGGTGGATTTGCAGAATGGGAAAAAGGGTATAGTGATGA
GACGTCATATGCCGTTCCACTTGAATCGAATCATTTTGAAGATGACCTTGCCATGATATTT
GTTGTGATTAATCAACATTCTAAAAAGGTACCTAGTCGATATGGTATGTCATTGACACGA
AACACATCAAGGTTTTATCAATATTGGTTAGATCATATTGATGAAGATTTAGCTGAAGCA
AAAGCAGCGATTCAAGACAAAGATTTTAAACGCCTTGGTGAAGTAATTGAAGAAAATGG
TTTGCGTATGCATGCCACGAATCTAGGATCAACACCGCCGTTCACATATCTTGTGCAAGA
AAGTTATGATGTCATGGCGCTTGTTCACGAATGCCGAGAAGCGGGGTATCCGTGTTATTT
TACAATGGATGCGGGACCTAATGTGAAAATACTTGTAGAAAAGAAAAACAAGCAACAGA
TTATAGATAAATTATTAACACAGTTTGATAATAACCAAATTATTGATAGTGACATTATTGC
CACAGGAATTGAAATAATTGAGTAA

> **mvaK2**
Function: protein coding sequence; phosphomevalonate kinase 2 (core genome, constant)
Best match: mvaK2_CC008_NCTC8325_CP000253.1[581406:582482] (completely identical)
Position: 018-contig_252: 26421 … 27498; Length: 1077 bp
Sequence:
ATGATTCAGGTCAAAGCACCCGGAAAACTTTATATTGCTGGAGAATATGCTGTAACAGAA
CCAGGATATAAATCTGTACTTATTGCGTTAGATCGTTTTGTAACTGCTACTATTGAAGAAG

CAGACCAATATAAAGGTACCATTCATTCAAAAGCATTACATCATAACCCAGTTACATTTA
GTAGAGATGAAGATAGTATTGTCATTTCAGATCCACATGCAGCAAAACAATTAAATTATG
TGGTCACAGCTATTGAAATATTTGAACAATACGCGAAAAGTTGCGATATAGCGATGAAGC
ATTTTCATCTGACTATTGATAGTAATTTAGATGATTCAAATGGTCATAAATATGGATTAGG
TTCAAGTGCAGCAGTACTTGTGTCAGTTATAAAAGTATTAAATGAATTTTATGATATGAA
GTTATCTAATTTATACATTTATAAACTAGCAGTGATTGCAAATATGAAGTTACAAAGTTTA
AGTTCATGCGGAGATATTGCTGTGAGTGTATATAGTGGATGGCTAGCGTATAGTACTTTT
GATCATGAATGGGTTAAGCATCAAATTGAAGATACTACGGTTGAAGAAGTTTTAATCAAA
AACTGGCCTGGATTGCACATCGAACCATTACAAGCACCTGAAAATATGGAAGTACTTATC
GGTTGGACTGGCTCACCGGCGTCATCACCACACTTTGTTAGCGAAGTGAAACGTTTGAAA
TCAGATCCTTCATTTTACGGTGACTTCTTAGAAGATTCACATCGTTGTGTTGAAAAACTTA
TTCATGCTTTTAAAACAAATAACATTAAAGGTGTGCAAAAGATGGTGCGTCAGAATCGTA
CAATTATTCAACGTATGGATAAAGAAGCTACAGTTGATATAGAAACTGAAAAGCTAAAA
TATTTGTGTGATATTGCTGAAAAGTATCACGGCGCATCTAAAACATCAGGCGCTGGTGGT
GGAGACTGTGGTATTACAATTATCAATAAAGATGTAGATAAAGAAAAAATTTATGATGA
ATGGACAAAACATGGTATTAAACCATTAAAATTTAATATTTATCATGGGCAATAA

> **ykgC**
Function: protein coding sequence; FAD-dependent pyridine nucleotide-disulphide oxidoreductase (core genome, variable)
Best match: ykgC_CC001-ST772_118_AJGE01000030.1[28120:29442:r]RC (completely identical)
Position: 018-contig_252: 28067 … 29390; Length: 1323 bp
Sequence:
CTAGAAATTAAATAAATCATTAAATGATTCGGCCATCGTAGGATGCGTATAAATATTATC
TCGTAATACGGTATATGGAATGTTTTGATCAATCGCAAGTTTAATTATATTAATTAATTCT
TCAGATTGCTTACCATATAATGTAGCACCTAAAATCATATTATTTTCATTATTAATGACTA
CTTTAAATAAACCTCTTGGATCATTGTTAATTTTGTGACGAGGTATAGCACTTACTAAAAG
TTGATGTTCAGTGTAATCATAATGTTGAGCGGCAGCTTCTTTACCAGTTAATCCAACACGT
GATAATGGTGGATCTATAAATACTGTATAAGGCACGCTGCCTCTATTGTCAGTCGTACGT
GACTGATTACCATATAACGCTGATTTGATAATTCGATAATCATCTAAAGATATATACGTA
AATTGAAGTCCGCCTTTAACATCACCTGCAGCATAAATATGCGGCACAGTTGTTTGAAGA
TGAGCATTGACTTTAATTTCGCCTCTGTCGCCTAATTCGATATCAGTATTTTCTAAAGCTA
AATCCGTATTCGGTTTGCGACCGATAGCCAAAAGTACTGCATCAGCCTCAAAGTTACCAA
CGTTAGTATGGACTGTTGTATGATGATTGTCAGATGACAATTCAGTCGTTTCAACATTTGT
ATACAATGCAATGCCTTTATTTTCTAAGTCAGTAATAGCATGTGCAACGACATCTTGATCT
TCACGTGGCATAAATGATTCACCACGTTCTAATACTGTAACCTTACTACCTAAATTCGCAA
ACATTGAAGCAAATTCTAAGGCGATATAACCGCCACCTACAATAACGAGGTGCTTAGGTT
GATAGCTAATGTTTAATAAACCTGTCGAATCGAAGACGTGTTTAGCTTGATCAAGGCCTT
TAATGTTAGGAATGACAGAGGTAGCACCGGTATTAATAATGATATGAGGTGCAGTAATAC
TATCGACGATATCGTCATGTTGATCTAATAAATTCACTTCAGTATTAGATTTAAACTGCGC
TTTAAAATCCAGTACATCAATGTTGTTATCGTCTGCTAATAAGTGGTAATTTTTATTGTTT
AGCGCATTGACAACATCGTTTTTACGGTTATAACTTGCTTCAAAAGATTTGCCTTCTAATC
CATCATGTACAAGTGTCTTCGAAGGTATACATCCTATATTTATACAAGTGCCTCCATACAT
TTTCGAAGATTGTTCGATAACTGCGACGTGTTGACCTGTTGATGCAGCGTATTTCGCTAAA
GTTTTACCAGCTTTCCCAAATCCTATTACAATTAAATCATATGTTTTCAT

> **Q5HI81**
Function: protein coding sequence; transcript regulator (core genome, variable)
Best match: Q5HI81_CC008_NCTC8325_CP000253.1[584361:584801:r]RC (completely identical)
Position: 018-contig_252: 29376 … 29817; Length: 441 bp
Sequence:
TCATATGTTTTCATGACATAAATCCTCCTTTTGAATGTCTTCAATGACATCTTTGATTGTTT
TTCCATTATAAAAATTAATAATGATATTCTGTTCGTCTTGCTGATAATGTGACATGGTAGT
TGCAATATTACGAGCAATTTGACAGTGACTGCCTTCGTCGCCAGTAAATAGACGTGTGTG
GTGTTCTTTCTCTAAGACAAAATGTTTATATAATGTTGCTAGAGAGACATCAGCACTTTGA

TCATTTGCTAAATAACCGCCATCTTTACCTCGTATTGTGTCAATCATTTTTAAATCGACAA
GTTGAGTCGTCACGCGTCGTAATTGAACAGGATTTAAACAAGTTAATTCTGCTAATGAAC
TACTATTGAATTTTTCTGAATGATGCTTAGTTAAAAAAGCTAATACATGCACGGCAATGTT
AAATTCTAAATTCAA

> **Q5HI79**

Function: protein coding sequence; putative protein chv
Best match: Q5HI79-CC25_CC025_21305_AFNO01000011.1[183738:185144:r]
Position: 018-contig_252: 30441 … 31848; Length: 1407 bp
Sequence:
ATGAGTATTGACATGTATTTAGACAGATCTCGAAACCAAGCTTCAAGTGTGGGGAATTTG
AGTCAAACAATGAATTCAAATTATGATGCGTTGGAAAAAGCAATTACTCAATTTATTAAT
GATGATGCGCTTAAAGGGAAAGCGTATACGTCAGCTAAGCAATTTTTTAGTACGGTGTTA
ATTCCATTATCAACAAGTATGAAAACATTGAGTGATTTAACGAAGCAAGCTTGCGATAAT
TTTGTGTCACGTTATACGAGTGAGGTTGATAGCATATCTTTAAAAGAATCAGAGCTTGAA
GAAGATATCAGATCATTAAGTCAACAAATTACGCGATATGAAAATTTGAATAACAATTTG
AAAAAGCATGCTTCCGATAATCAGCAAGCCATTTCATCGAACCAACAAATAATACGAAC
ATTAGGTCAACAAAAACATGAATTAGAAGAGAAGCTACGCAAATTGCGTGAGTTTAATC
AAAAATCACCAGAAATATTTAAAGAAGTTGAAGAATTTCAAAAAATTGTCCAACAAGGA
CTTACCCAAGCGCAGAATTTTTGGAACTTTTCAACAAATCAATTTAATATACCTTCAGGTA
AAGAACTTGATTGGGCTAAAGCAAGTCATGAAAAATATTTGAAAGTTGCTATGGGGAAA
ATTGAACATAAAGCAGAGAAAGAAACTTTAAATAAAGCAGACTTTGCTGTTATAAAGGC
ATATGCCAAAGAGCATCCAGAAGACGATATCCCGAAAAGTATACTGAAATATATAAATG
ACAATAAAGACAGTATTAAAAGAGATATAGGATTAGATATCACTTCAACACTTTTAGAGC
AAGGCGGTATAAATGCAAGTAAATTCGGTGTATTTATCAATACAGCAGGTGGAGTGAAA
GGCCCAGCAGGTCCAAATTCATTTGTGGAAGTCAAACGTACATCAGGTAATGTGTTTATA
GAAAATGGTAGTAAATTTGCAAAAGGCGGAAAATACCTAGGTAAAGGTGTTGCTGGTGT
AGGATTTGGTATAGGTATGTATGATGACCTTGCAAATGATGATAAAACATTTGGAGAGGC
ATTATCGCATAATGGTATGACGCTTGCAGCTGGATCTGCAATGGGGACTATTGTGTCTGT
AGGTATAACTATGCTGGCAAGTAATCCAGTAGGATGGGCGGTTTTAGGAGGTTTTGCTGC
AGCAACAGGTTTTGCAATAGCGGCTGATTGGGCATATGAAAATAATTTGTGGGGATTAAA
AGATGAAACAGACTGGGTAGGACATAAAATAGATGACGGTATCGATGTTGTAAAAAAAT
CTACAAAAAAGGCTGTTGATAGTGTAGAAAAAGCTGTTGGTGAAGCTACAAAAAGTATA
TCTAATCATATAAATCCTATGAAATGGAGCTGGTAA

> **DUF443-var1**

Function: protein coding sequence; putative protein (genomic island)
Best match: DUF443-var1_CC001_MW2-USA400_BA000033.2[642305:642946] (completely identical)
Position: 018-contig_252: 32602 … 33244; Length: 642 bp
Sequence:
GTGGAGACATTGCTTTGCGAATCTAAAGTTATTAATAAAAACCCTAAATATCGAATAATC
AAATACGATAGTGAATATTTAATGATTGATTTGGCAAGTAATTGGATTGTCTTCTTCTTTC
CATTTATTAACTGGCTCATACCGAAAACATATGTCAAAATCACTAAGAATGATTATGAAA
AATTAAATATTGTCAAACCAGTTAAAAATAAATCGATAGGATGGACCATATTCGCGGGTA
TTGTGTTACTTGGTGGTACTGTAAGAAGAAATACTTATTTATTTGATTTTCAATTAGAAGA
ACTAATTGTTTGGAGCAGCTGTTTCATTGGGTTTTTAGAGATTATATTTTTTTATTGTTATC
TAAATAAGAAATTAACATTAAATATTTATAATGAAAGTAAAAATAATGAACTTAAATTAA
GATTATTACCCTCCTTTAAAAATATTTGTTTCACAATTTTTTATTACCTATTTACTGGTTTC
ATGTCTTATGGGGCATTTTACTTGTTGGTATTTGAAAATGTGCAAAATTTAATCTTATATG
TTTCTTGGCTTTTCATGACTATGCTATTTATGTTTATGAATATGCATTCAATTATAGATAAA
AAAGTACATATATTCTTAAAGTCTAATAAATAG

> **DUF443-var10**

Function: protein coding sequence; putative protein (genomic island)

Best match: DUF443-var10_ref_CC008_COL_CP000046.1[679951:680580] (completely identical)
Position: 018-contig_252: 33290 … 33920; Length: 630 bp
Sequence:
GTGCTTTGTGAATCTAGACAAATTTATAAAAATCCTAAATATCGAGTTATTAGATATAAT
AATGAATATTTCATGGTCGATTTAGTAAGTACTTGGATTACTTATTTTTTCCCTATGATTA
ATTGGTTTTTGCCCAAAAAATACGCAAAAATTAGCGAAAATGAATTTGAAAGGTTAAATA
TAGTCGAGCCTGTTAAAAATAATGTTTTTTGGCCGGTTGCAGGAAGTTCAGTTCTATTTGG
AATTATATTGAGAAAGTACGGTAACTTCTTTAATGTTCAGTTTGAAAAACAACTAGCAAT
CACTGTATTTTTTATCATGTTAATAGGGATGTTAATTTTTTATTTTTATCTAAATAAAAAAT
TAACATTAAAAATTTTTAATACCAACGTGGTTAATAAGAATAGAGTTGTATTAATACCGA
CTTTCAAACAAGGATTGTTAATAGTTTTTGCGTACTTTTTTTTAGGAAGCGCTTCAATATTT
ACTTTAACCATTCTTTTGACGACAGAGTCACAAAATATAATAATATTTTTAACTTGGGTTA
TTATCACGATGTTTTTCTTTTTAGTGAATATGGCTTCGATAGGTAATAAAAATGTTCATGT
TATTTTAAGAATAAACGAGTAG

> **DUF443-var7**
Function: protein coding sequence; putative protein (genomic island)
Best match: DUF443-var7_CC001_MW2-USA400_BA000033.2[644075:644704] (completely identical)
Position: 018-contig_252: 34372 … 35002; Length: 630 bp
Sequence:
ATGTTGCTTTGCGATGTCAGAGTCATTTATAAAAATCCGAAATACAAAGTCATTCAACAT
AACGGTGAATACTTATTAGTCGATTTAGTAAGCACTTGGTTCGTGTACTTTTTTCCTTTCAT
TAATTGGTTCATTCCAAAAAAGTACGCGATAATTAGCGAAGAAGAATTTGAAAATTTAAA
TGTTGTTAAACCAAATAAAAATAATGTTTTCTGGTCAGTTATAGGAAGTTCGGTTTTGTTT
GGAGTTACTTTAAGGAAATACATACATGTTTTTGATGTTCAATTAGATAAGCTAGTTGTAA
TGATATTGTGTGCTCTCGCTTTAATTTGTGTTATAGTTTTTTATTTTAACTTAAATAGAAAG
CTTAAGTTAAAAGTGTTTGATACAAATATTGAAAAAAAATAAGAGAGTTATATTAATACCA
ACGTTTAAACTTGGCTGTTTTTTAGTTTTCGGATATATTTTCGCTGGAAGTTTTTCAATATT
TTCATTAATTGCCCTTATGACAATCGAACCTCAAAATATAATAATATTTATTTATTGGATT
ATGATGACAATGCTTTTCTTTTTGTTAAATATGACTTCGATAGGTAATGAAAAAGTTCGCG
TTATAATGAAAAAATAATTGA

> **DUF443-var4**
Function: protein coding sequence; putative protein (genomic island)
Best match: DUF443-var4_CC001_MW2-USA400_BA000033.2[644819:645469]
Position: 018-contig_252: 35116 … 35767; Length: 651 bp
Sequence:
GTGGAGACATTGCTTTGTGAAACTGAAAATATTAATAAGAATCCCAAATATAGAATTATC
AAATACAAAGATGAATATTTGATGATTGATTTAGTAAGTACATGGTTAGCACTCTTTTTCC
CAATGATTAATTGGCTGATTCCAAAAAAGTACGTCAAAATCAGCGAAAAAGATTTTGAAA
CTTTAAACATTGTGAAGACAACTAAAATCAATTCTTTTTGGCCAGTGGCAGGAAGTACGG
TCTTATTTGGTGTTATGTTAAGAAGGTATTCCCATTTATTTATCGTTAAATATGAATATAG
TATAGTAATTTTAATTTGTTGCATCATAATACTAGGTATTTTTCTGTTTTTTTTATATTTAA
ATCAAAAGTTAAAGTTACAAATCTATAATGAAAACAAAAATAAAAGCAATAAGATAATC
ATATTTCCCACTTTAAAGAGTCTTTGTTTATCAATAGTTTTATATATTTATTTAGGTGGTGG
TTCATTCTTTACTATTTATATGTTATTGACGATTGAAGTTCAAAATATAATATTATTTATAA
CTTTGTTTGTAATATTTTTTGTTTTTCTTATTTTTAAATATGTGCTCACTATATGACAATAAA
GTTCATGTATTATTTAAATCAAATGGAATTGAAAAGTTTTAA

> **DUF443-var5**
Function: protein coding sequence; putative protein (genomic island)
Best match: DUF443-var5_CC001_MW2-USA400_BA000033.2[646376:647002]
Position: 018-contig_252: 36673 … 37299; Length: 626 bp
Sequence:

ATGTTGCTATGCGAATCTAAAATCATCAATAAAAACCCAAAATATAGAATTATTAAATAT
AATGATGAATACTTAATGGTCGATATAATAAGCACTTGGATTAGTTTATTTTTCCTTTTAT
TAATTGGTTCATCCCAAAAGAATACGTCAAAATTAGTAGAGAAGAGTTTGAAAACTTAAA
TATTGTTAAACCTGCTAAAAAGAATGTTTTTTGGCCAGTTGCAGGTAGCTCTGCTTTGCTG
GGAGTTGCATTAAGAAAATATACACATTTACTTGACATTCAACTTGATAAAAAATTAGTT
ATTGCCATATGTTGCATCACATTTATAGGGATTTTAATATTTTATGTACGCCTAATTAAAA
AATCATCTTTAAATATTTATAATACTAAAAATAAAAGGTCAAAAATTTTTTTAATACCTAC
ACTAAAAAATGTTTGTTTCACATTATTTGGATATATTTTATTTGGCGGATTGACTATGCTA
TTCCTAGATGCACTATTATCAATGAGTTATCAAAACATAATAGTATATTTTGTTTGGATTG
CAGTTATAATGGGTTTTTTTCTAGTTAATATAGCTTTAATTATAGATAAAAACATTCATGT
CATACTTAAAAACCAATAG

> **Q5HI67**

Function: protein coding sequence; oxidoreductase (core genome, variable)
Best match: Q5HI67_CC001_MW2-USA400_BA000033.2[647272:648210] (completely identical)
Position: 018-contig_252: 37568 … 38507; Length: 939 bp
Sequence:
ATGAAGCAATTTGTAAACTTAGGTAAATCTGATGTTGAAGTGTTTCCAATCGCACTTGGG
ACGAACGCAGTAGGTGGGCATAATTTATATCCGAACTTAGATGAAGAACAAGGAAAAGA
TGTTGTTCGTCAAGCCATTAATCATGGTATTAATTTATTAGATACGGCATATATTTATGGG
CCAGAACGATCAGAAGAATTGGTTGGAGAAGTTGTTAAAGAATATCCGCGAGAGCAAAT
TAAAATTGCTACGAAAGGGTCTCATGAATTTGATGAAAATCAAGAAGTACATCAGAACA
ATCAACCGGAATATTTAAAACAACAAGTTGAGAATAGTTTGAAACGTCTACAAACTGATT
ATATCGATTTATATTATATTCATTTTCCGGATAACAACACTCCGAAAGATCAAGCAGTTGC
AGCATTACAAGAGCTTAAGGAACAAGGGAAGATTAAAGCAATTGGTGTATCAAATTTCA
CATTAGATCAACTTAAAGAAGCAAATAAAGATGGTTACGTTGATGTTGTACAGTTAGAAT
ATAATTTATTGCATCGCGAAAATGAGGCAGTATTACAATATTGTGTTGATCACCAAATCA
CATTTATTCCATATTTCCCATTAGCATCCGGTATTTTAGCTGGAAAATATGATGAGAACAC
TAAATTTAGTGACCATCGTACTACACGTCGTGATTTTAAACCAGGTGTATTTGAAGAAAA
TGTGCGTCGCGTAAAAGCTTTGGAAAGCATAGCTGCAGCACATCAAACTTCAATTGCGAA
CATTGTATTAGCATTTTATTTAACGAGACCAGCTATCGATGTGATTATTCCTGGTGCAAAA
CGTGCAGAACAAGTCGTTGAAAATATTAAAGCTGCAGATATCGTTTTATCAGATGATGAG
ATTCAATATATCGATGAACTGTTTCCGATTGAAGACTAA

> **Q5HI66**

Function: protein coding sequence; NADPH-dependent FMN reductase (core genome, variable)
Best match: Q5HI66_CC001_MW2-USA400_BA000033.2[648421:648957] (completely identical)
Position: 018-contig_252: 38717 … 39254; Length: 537 bp
Sequence:
ATGATTACTGTTTTGTTTGGTGGGAGTAGACCAAACGGTAATACTGCACAATTAACAAAA
TTCGCTTTGCAAGATTTAGAGTATCAATGGATTGACGTGACACAACATCAGTTTAAACCG
ATACGTGACGTGAGACATACAGCAGAGACTATTACTTCATATGACGATGACTATTTGCCG
ATTCTAGATAAAATATTGGCTAGTGATACAATTATTTTTGCATCACCAGTGTATTGGTATA
GCATTTCAGCACCATTGAAAGCATTTATCGAACATTGGTCAGAAACATTACAAGATAAAC
GATATCCTAATTTTAAGGCACAAATGGCCGAAAAGGATTTTAGAGTTATTTTAGTTGGTG
GAGATTGTCCAAAAATAAAAGCGAAGCCAGCAATTACGCAAATGAAATATAGTTTAGAC
TTTTTAGGTGCCACTTTAAATGGTTATATTATTGGAACTGCTGAAAAGCCTGGTGACATCA
TGAAAGACAACTATGCCTTAGCACGTGCAACTGAGTGGAATAGTATATTGCAATAA

> **Q8NXU4**

Function: protein coding sequence; GNAT family acetyltransferase (core genome, constant)
Best match: Q8NXU4_CC005_Mu50-VRSA_BA000017.4[676945:677421] (completely identical)
Position: 018-contig_252: 39422 … 39899; Length: 477 bp
Sequence:

ATGTTCAAGGTAAGACAAGCAACTGAAAAAGATGTTGTTCAAATTAGAGATGTCGCAACT
AAAGCTTGGTTTAATACATACTTAAATATATACGCTGCGACAACAGTTAATCACTTGTTA
GAAGCTTCATATAATGAACATCATTTAAAGAAAAGACTTCAAGAACAATTATTCTTAGTC
GTTGAAGAAGGTAATGACATCGTTGGCTTTGCTAACTTTATTTACGGTGAAGAATTATATT
TATCAGCTCATTATGTTAAACCAGAATCGCAACATACAGGTTATGGTACAGCATTGTTAA
ATGAAGGATTATCACGTTTTGAAGATAAATTTGAAGGTGTTTACTTAGAAGTAGATAATA
AAAATGAAGAAGCAGTAGCTTACTATAAAGAGCAAGGTTTTACAATCTTACGCTCTTATC
AGCCAGAAATGTATGGCGAAAAGTTAGACTTAGCACTTATGTACAAAGCATTTTAA

## > Q5HI65

Function: protein coding sequence; phosphohydrolase (core genome, constant)
Best match: Q5HI65_CC001_MW2-USA400_BA000033.2649642:650937
Position: 018-contig_252: 39938 … 41234; Length: 1296 bp
Sequence:
ATGACTAACGCATATGTAGGTTTAAAATTAGTAGAAGAAAAAGTTTTTAAAGACCCGATA
CATCGATATATTCATGTTGAAGATCAATTGATATGGGATTTAATTAAAACTAAGGAATTC
CAAAGGTTACGTCGAATTAGACAACTAGGAACACTGTACCTATCTTTTCACACAGCAGAA
CATAGTCGCTTTGGACATTCTTTAGGTGTGTATGAAATAGTTAGACGATTAATTGATGAGT
CATTTATTGGTCATGATGCATGGGACAATAAAGATAGACCGTTGGCATTATGTGCTGCAT
TATTACATGATTTAGGACATGGTCCATTTTCACATAGTTTTGAAAAAATATTTAATACAGA
CCATGAAGCATACACACAAGCGATTATTACTGGAGATACTGAGGTGAATGCTGTATTACG
TAAAGTGTCGCCTGAGTTTCCAAGAGAAGTTGCGGAAGTAATTAATAAAACGCATCATAA
TAAATTGGTCATTTCGATGATTTCGTCACAAATCGATGCGGATAGAATGGATTATTTACA
ACGTGATGCGTATTTCACAGGTGTATCATATGGTGCTTTTGATATGGAACGTATTTTAAGA
TTAATGCGACCTTCTAAAGATGAAGTACTAATTAAAGAAAGTGGTATGCATGCAGTTGAA
AACTTTATTATGAGTCGTTATCAAATGTATTGGCAAATTTATTTCCACCCAGTTAGTCGTG
GTGGAGAAGTGCTGCTTAATAATTGTTTGAAACGCGCAAAACAGCTTTATAATGAAGGCT
ATGAATTTAAGTTGCATCCACATGATTTTATTCCATTTTTTGAAGAGACAGTTACGATTGA
ACAATATGTTGAACTCGATGAAGCGGTAGTTACGTATTATTTGGAAAAATGGACAAAAGA
AGATGATGCCATTTTAAGTGATTTAGCAAGTCGATTTATTAATCGAGACTTATTTAAATAT
ATTCCATTTGATGGCTCAATTATTACAATATCAGAACTGCAAGAACTGTTTGAAGCAGGT
GGTATTAATCCAGATTATTATTTTGTGAGTGAAGCATTTTCGGATTTGCCATATGACTATG
ATCGACCTGGGTCAAATCGCAAACCGATTCATTTATTAAGACAAGATGGTACGATTAGAG
AAATAAGCAATCAATCTTTAGTCATTCATAGTATTACAGGCATTAATCGCCAAGACTATA
AATTATATTATCCTAGAGAAATGGTTGCAAAGATTAAAGATAAGACAATTAGAGAAGCT
ATTGAAAATTTGATTAATGAGCTTAATTAA

## > adhA

Function: protein coding sequence; alcohol dehydrogenase I (core genome, constant)
Best match: adhA_CC001_MW2-USA400_BA000033.2[651985:652995]
Position: 018-contig_252: 42282 … 43293; Length: 1011 bp
Sequence:
ATGAGAGCAGCAGTTGTAACGAAAGATCACAAAGTAAGTATTGAGGACAAAAAGTTAAG
AGCTTTAAAACCTGGTGAAGCGTTGGTACAAACGGAATATTGTGGCGTTTGTCATACCGA
TTTACATGTTAAGAATGCTGATTTTGGTGATGTTACAGGCGTTACTTTAGGTCATGAAGGT
ATTGGTAAAGTCATCGAAGTTGCGGAAGATGTAAAATCATTAAAAATTGGAGACCGTGTG
TCTATCGCTTGGATGTTCGAAAGCTGTGGAAGATGTGAATATTGTACAACAGGTCGTGAA
ACACTTTGCCGTAGTGTGAAAAATGCTGGTTATACAGTAGATGGTGCAATGGCTGAACAA
GTTATTGTTACTGCAGACTATGCTGTGAAAGTACCTGAAAAATTAGATCCAGCAGCAGCG
TCTTCTATTACATGCGCAGGTGTGACAACTTATAAAGCTGTAAAAGTAAGTAATGTAAAA
CCTGGACAATGGTTAGGTGTTTTTGGTATAGGTGGTTTAGGTAACCTAGCTTTACAATATG
CTAAAAACGTTATGGGGGCTAAAATTGTTGCCTTCGACATCAATGATGATAAATTAACAT
TCGCGAAAGAATTAGGTGCAGATGCTATTATTAATTCTAAAGATGTTGATCCAGTTGCAG
AAGTTATGAAATTAACTGATAACAAAGGATTAGATGCAACAGTGGTAACTTCAGTTGCTA
AGACGCCATTTAACCAAGCGGTTGATGTTGTAAAAGCTGGTGCAAGAGTTGTTGCCGTTG

GTTTACCTGTTGATAAAATGAACTTAGATATCCCAAGATTAGTGCTTGACGGTATTGAAG
TAGTAGGTTCACTTGTTGGTACAAGACAAGACTTACGTGAAGCGTTTGAATTTGCTGCTG
AAAATAAAGTAACACCTAAAGTTCAATTAAGAAAATTAGAAGAAATTAATGATATTTTTG
AAGAAATGGAAAATGGTACTATAACTGGTAGAATGGTTATTAAATTTTAA

> **argS**

Function: protein coding sequence; arginyl-tRNA synthetase (core genome, constant)
Best match: argS_CC008_NCTC8325_CP000253.1[601966:603627]
Position: 018-contig_252: 43981 … 45643; Length: 1662 bp
Sequence:
ATGAATATTATTGATCAAGTGAAACAAACATTAGTAGAAGAAATTGCAGCAAGTATTAAC
AAAGCAGGATTAGCAGATGAGATTCCTGATATTAAAATTGAAGTTCCTAAAGATACAAA
AAATGGAGATTATGCTACTAATATTGCGATGGTACTGACTAAGATTGCAAAGCGTAATCC
TCGTGAAATTGCTCAAGCGATTGTTGATAACTTAGATACTGAAAAAGCACATGTAAAACA
AATTGACATTGCTGGTCCAGGATTCATTAATTTTTACTTAGATAATCAGTATTTAACAGCA
ATTATTCCTGAAGCAATTGAAAAAGGTGATCAATTTGGACATGTAAATGAATCAAAAGGG
CAAAATGTATTGCTTGAGTATGTTTCAGCTAACCCTACAGGAGATTTACATATTGGTCATG
CTAGAAATGCAGCAGTTGGTGATGCTTTAGCTAATATTTTAACTGCAGCTGGCTATAATGT
AACACGTGAATATTATATTAATGATGCTGGTAATCAAATTACTAACTTAGCGCGTTCGATT
GAAACACGTTTCTTTGAAGCTTTAGGTGACAATAGTTATTCAATGCCAGAAGATGGCTAT
AATGGTAAAGATATTATTGAAATAGGTAAAGATTTAGCAGAGAAACATCCTGAAATTAA
AGATTATTCTGAAGAAGCACGTTTGAAAGAATTTAGAAAATTAGGCGTAGAATACGAAA
TGGCTAAACTGAAAAATGATTTAGCAGAGTTCAATACGCATTTTGATAATTGGTTTAGTG
AAACATCTTTATATGAAAAAGGCGAAATTCTTGAAGTTTTAGCAAAAATGAAAGAATTAG
GTTATACGTATGAAGCTGATGGCGCTACATGGTTACGTACAACTGATTTTAAAGACGACA
AAGACAGAGTATTAATTAAAAATGACGGTACATATACGTATTTCTTACCAGATATTGCGT
ACCACTTCGATAAAGTTAAACGTGGTAATGACATTTTAATCGATTTATTTGGTGCTGATCA
TCATGGTTATATTAATCGTTTGAAAGCATCTCTTGAAACGTTTGGTGTAGATAGTAATCGT
TTAGAAATTCAAATCATGCAAATGGTTCGTTTAATGGAAAATGGTAAAGAAGTGAAGATG
AGTAAACGTACTGGTAATGCGATTACATTAAGAGAAATTATGGACGAAGTTGGCGTTGAC
GCTGCACGTTATTTCTTAACTATGCGTAGTCCTGATAGTCACTTTGATTTTGATATGGAAT
TAGCGAAAGAGCAATCTCAAGACAATCCAGTTTACTATGCTCAATATGCACATGCGCGTA
TTTGTTCAATTTTAAAACAAGCGAAAGAGCAAGGAATTGAGGTAGCTGCTGCGAATGATT
TTACAACGATTACAAATGAAAAAGCGATTGAATTGTTGAAAAAAGTAGCTGATTTCGAAC
CTACAATTGAAAGTGCAGCTGAGCATAGATCAGCACATAGAATTACTAATTATATTCAAG
ATTTAGCTTCTCATTTCCATAAATTCTATAATGCTGAAAAAGTGTTAACAGATGATATTGA
AAAAACAAAAGCACATGTTGCTATGATTGAAGCGGTCAGAATTACATTGAAAAATGCATT
GGCAATGGTCGGTGTAAGCGCACCTGAATCAATGTAA

> **tx_universal2**

Function:  rho indendent terminator
Best match: tx_universal2_CC001_MW2-USA400_BA000033.2[655556:655596]
Position: 018-contig_252: 45852 … 45882; Length: 30 bp
Sequence:
CTGAACGAAAATTCGCTTGTAACAAGCTTT

> **A5IQG3**

Function: protein coding sequence; similar to iron-binding transport protein (core genome, constant)
Best match: A5IQG3_CC008_NCTC8325_CP000253.1[605030:605917] (completely identical)
Position: 019-contig_265: 1036 … 1924; Length: 888 bp
Sequence:
GTGAAGAAATCGTTAATTGCTTTTATTTTGATTTTTATGCTTGTCCTGAGTGGCTGTGGTAT
GAAAGATAATGATAAACAAGGTAGCAATGATAATGGCTCGTCTAAATCGCCGTACCATA
GAATTGTTTCGTTAATGCCTAGTAATACTGAAATTTTATATGAATTAGGATTAGGTAAATA
CATAGTTGGTGTTTCAACGGTTGATGATTATCCAAAAGATGTGAAAAAGGGTAAGAAACA

ATTTGATGCTTTGAATCTAAATAAAGAGGAACTTTTAAAGGCAAAGCCAGATCTAATTCT
TGCGCATGAGTCGCAAAAGGCAACTGCTAATAAAGTATTGTCATCATTAGAGAAACAAG
GCATCAAAGTAGTGTATGTTAAAGATGCACAATCAATTGATGAAACTTACAACACATTTA
AGCAAATTGGGAAATTAACGCATCATGATAAGCAGGCTGAACAACTTGTTGAGGAAACT
AAAGATAATATCGATAAAGTCATAGATTCAATTCCTGCTCATCATAAAAAATCAAAAGTA
TTTATTGAGGTTTCATCAAAGCCTGAAATATATACAGCAGGGAAGCATACATTTTTTAAT
GATATGTTAGAAAAATTAGAAGCCCAAAATGTGTATAGTGACATTAATGGTTGGAACCCT
GTAACGAAGGAAAGTATTATTAAAAAGAACCCAGATATATTAATTTCGACGGAAGCTAA
GACAAGATCAGATTATATGGATATCATCAAAAAAAGAGGTGGATTCAATAAAATTAATG
CTGTCAAGAATACACGTATTGAAGTTGTAAATGGTGATGAAGTATCAAGACCAGGTCCAC
GTATTGATGAAGGATTAAAAGAATTAAGAGATGCAATTTATAGAAAATAA

## > tx_universal3

Function:  bidirectional rho-independent terminator
Best match: tx_universal3_CC001_MW2-USA400_BA000033.2[662739:662803] (completely identical)
Position: 019-contig_265: 7091 … 7156; Length: 65 bp
Sequence:
AGGCAATGTGAAAAAGCTGATTACTATTCATTATTTGATAGAAATCAGCTTTTTTTGAAAT
GTAT

## > sarA

Function: protein coding sequence; staph accessory regulator A (core genome, constant)
Best match: sarA_CC001_MW2-USA400_BA000033.2[662954:663328:r]RC (completely identical)
Position: 019-contig_265: 7307 … 7682; Length: 375 bp
Sequence:
TTATAGTTCAATTTCGTTGTTTGCTTCAGTGATTCGTTTATTTACTCGACTCAATAATGATT
CGATTTTTTTACGTTGTTGTGCATTAACAAGAATTAATACAGTTCTTTCATCATGCTCATTA
CGTTTTTTATCGAAGTAATCTTCTTGAGATAAAATTTTAACTGCTTTAACAACTTGTGGTT
GTTTGTAGTTTAAATGATTAATAATATCTTTAAGATAGTATTCTTTCTCTTTGTTTTCGCTG
ATGTATGTCAATACAGCGAATTCTTCAAAGCTAATTGAAAATTCCTTTTTAATTAAACTTT
TTAATTTGTCAGCATAAGTGACCATTGATAACAACTCAAAGCAATCATTGATTTTTGTAAT
TGCCAT

## > A6QES9

Function: protein coding sequence; putative protein (core genome, variable)
Best match: A6QES9_CC008_NCTC8325_CP000253.1[612385:612534:r]RC (completely identical)
Position: 019-contig_265: 8391 … 8541; Length: 150 bp
Sequence:
CTATGAAATATTAGCACGAATAACATAATTTTTGCTATTAATTTGGTGTAAATTATTAAAA
AATAACATTAATTTAGGTCTTAAGAATGAGTTGACTATCGAATTTGCTAAATTAAAATTTG
TTGTGTCAAAGCATACTACCCAAATCAA

## > Q2YSV8

Function: protein coding sequence; putative protein (core genome, variable)
Best match: Q2YSV8_CC001-ST772_118_AJGE01000030.1[54626:55555:r]RC (completely identical)
Position: 019-contig_265: 8629 … 9559; Length: 930 bp
Sequence:
TTAAAATAATCTTAAAAGTATAATGCCTGTCAAAATACATAATAGACCAATAGTTTTTCT
GGATGTCATTGCTATTTTAGGTGAACCAAATAATCCAAAGTGATCTATCAATATGCCCATT
AGAATCTGGCCAAACATCCCAATAAGTGTTGTTAATGCTGCACCCATATGAGGCATTAAG
ATAATGTTAGCTGTTACAAAAGCCATGCCAAGTATACCGCCAGTAAAATAGATAGGCTTT
AATTTACCGAATTTTAAATGACTTGTTTTTAGTTTTAAAGAACGATTAAAAATAGCGGTTA
AAATCAATAGCGCTATTGACCCAATTGTAAATGATACTAATGATGCAAAGGCTGGTGAAT

GAGTATGACTTGCTAAAGCACTATTAATTGTCGTTTGAATAGGTGGAAAGAAACCAAAAA
TAAATCCTAAGAGAAGCCAAAACAGTAAATACTTTTGATCAGTTAGTAATAAATTATTCT
TGTTAAATTGATTCATTATGACGATGCCGATAATGAGTAACAATACTCCAATTGCTTTAAT
TAAATTAAAATCATGAATTGTAGCGCCAAATAAGCCCAATGTATCAATAATGACACCCAT
AATAATTTGACCCGCAACTGTTGCAATTACAGTTAATGTTGCACCTAATTTTGGCAATAAC
AATAAATTGCCAGTTAAAAAGCTAACCCCAAGCAAACCACCGACTACCCATGTGTAGTTA
AATGATTGATTATTGTAAAAGTGAATAGTAAATACTTCTGGATTGATAATGATATTTAAA
ATAATTAAACAAATTGTTCCAACTGAAAATGAAATGAATGAAGTATAGAAAGGCGATTT
AGTATACAGTGATAGTCTTGAATTGACAGATGTTTGGATAGGAATAAGCATTCCAACTAG
GACACCAATGATATAGAAAAGAAGCAT

## > A6QET1

Function: protein coding sequence; putative protein (core genome, variable)
Best match: A6QET1_CC001_MW2-USA400_BA000033.2[665400:665624:r]RC
Position: 019-contig_265: 9753 … 9978; Length: 225 bp
Sequence:
CTATTTCTTATCTTCAAATAAAACAGTTGTTGTTTTATCAATAATTTGAGCAGAATGTACT
TTGGTGATAGTACCACTATTTGACTTTATAATATTTAAATCTACTAATGCATTCATACTAT
CACGTGCAACTTGCTCGGTCACTACGTTGTTTAATTGTGATAATTGCAGTTTTACAGGCTT
GTCTAGTGAAGATTTAAAACTTAATTCAAGTGTTTTAGTCAT

## > Q7A1N3-var1

Function: protein coding sequence; putative protein (core genome, variable)
Best match: Q7A1N3-var1_CC008_COL_CP000046.1[702663:702866:r]RC (completely identical)
Position: 019-contig_265: 9994 … 10198; Length: 204 bp
Sequence:
TTAAATTGATAAAGATTTGATGAGTTCGATATTGCTATATGTTTCTCCAGTAAGTCGCTCA
ATAAGTTTGCTGAATGTTTTAATTTGGTCGTTTGATGCATCAGGGTTAATGTTAGCGAATC
GACGCTTAAATTCTGTTTGTTTGCCGTTAGCGTCTACTTTAGTAAATGATAATACAATAGT
GATGTGGTTTATTTTACTCAT

## > A5IQH4

Function: protein coding sequence; putative phage integrase family protein ?? (core genome, constant)
Best match: A5IQH4_CC001_MW2-USA400_BA000033.2[666003:666563] (completely identical)
Position: 019-contig_265: 10356 … 10917; Length: 561 bp
Sequence:
ATGAATAAAGTAGAAGCGATTAAATTTAATGATGATATTGTTAAAATGTATGAAGCGCTC
AAGATAAAATCTGAACGTGACTATTTATTCTTTAAGTTAGCTATACATAGTGGATTGAAA
GTATCAGAATTATTAACAATTACAGTCTCTCAAGTTAAGAGACTAATTGAAAAGTGTACG
TTATCAGAAATGTGTAAAGCACATTTTCATTCGTTGATTAAAATTAGGTTACCAGAAACA
TTATCGAAAGAACTACTTCAATATATAGAGGACAGGAGTCTTTCGAATGAAGACGTTCTT
TTTCAATCACTACGAACAAATCAAGTATTATCTAGACAGCAAGCATATCGAATAATTCAC
CAAGCATCAATTGAAGCTGGTATAGATAATGTAGGACTAACGACATTGCGTAAGACATTT
GCATATCATGCTTATCAAAAAGGTATACCTATACCAGTCATTCAAAAGTATTTAGGGCAT
CAATCTGCTATTGAAACACTAAATTTTATCGGTTTAGAAAATGAGTGTGAACATAGTATTT
ATATTTCATTACAATTATAG

## > mrpA

Function: protein coding sequence; Na+/H+ antiporter/MnhA component/putative (core genome, constant)
Best match: mrpA_CC001_MW2-USA400_BA000033.2[666582:668984]
Position: 019-contig_265: 10935 … 13338; Length: 2403 bp
Sequence:
ATGAGTTTGGTTTATTTATTAATTGCTATACTTGTGATTATGGCGATGATACTTCTAATGTC
TAAACGTAGAGCATTGGCTAAATATGCCGGGTACATAGCGTTGGTTGCACCTGTAATTTC

ATCTATCTATTTTTTGATTCAAATACCATCAGTAGCTAAACTGCAATATCTTTCTACCTCTA
TTCCATGGATTAAGACATTAGATATTAATTTAGATTTACGTTTAGATGGTTTAAGTTTAAT
GTTTTCTCTTATTATTTCACTTATTGGAATTGCAGTATTCTTCTATGCAACTCAATATTTAT
CCTCTCGAAAAGACAATTTACCAAGGTTTTATTTTTATTTAACGTTATTTATGTTCAGTAT
GATTGGTATTGTATTATCAGACAATACGATATTGATGTACATTTTTTGGGAATTAACGAGT
GTATCATCATTTTTATTGATTTCATATTGGTATAACAACGGAGATAGTCAATTTGGTGCGA
TGCAATCATTTATGATTACAGTATTTGGTGGTTTGGCATTATTAGTTGGTTTTATCATGTTG
TATATTATGACAGGAACGAATAACATCACAGAGATATTAGGACAAGCAGATTATATTAA
GAATCATGGATTGTTTATCCCTATGATTTTTATGTTTTTATTAGGTGCATTTACAAAATCA
GCACAATTTCCATTTCATATTTGGCTACCTAGAGCAATGGCTGCACCTACACCTGTAAGTG
CTTATTTACATTCAGCCACGATGGTAAAAGCTGGTATCTTTTTATTACTTCGATTTACACC
ATTATTAGGTCTTAGCAATATGTACGTATATATCGTTACGTTTGTTGGTTTAATAACAATG
TTATTTGGTTCAATTACAGCTTTAAAACAATGGGATTTAAAAGGTATCCTAGCGTACTCTA
CAATCAGTCAACTTGGGATGATTATGGCTATGGTGGGTATAGGTGGCGGATATGCTCAAC
ACCAACAAGACGCAATAGCATCTATTTATGTATTTGTATTATTTGGTGCGCTATTTCATCT
AATGAATCATGCCATCTTTAAATGTGCGCTTTTCATGGGAGTAGGTATTTTAGATCATGAA
GCAGGTTCAAGGGATATACGAATTTTAAGTGGAATGCGTCAACTATTTCCTAAAATGAAT
CTAGTCATGACGATAGCGGCTCTATCTATGGCTGGAGTACCATTTTTAAATGGATTTTTAA
GTAAAGAAATGTTTTTAGATGCATTAACACAAACTGGACAATTATCCCAATTTAGTTTGA
TTTCAATGATAGCTATCGTGTTTGTTGGTGTTATTGCGAGTGTTTTTACATTCACATATGCA
CTATACATGGTAAAAGAAGTATTTTGGACAAAATATGATTCTAAGGTTTTTACTAAAAAA
AATATCCACGAACCATGGTTGTTTAGTTTACCATCTCTTATATTAATGGTGCTAGTACCTG
TAATCTTTTTTGTACCAAATATATTTGGGAAGGGGATTATCGTTCTAGCATTAAGAGCTGT
ATCAGGTGGTAATCATCAAATTGATCAATTGGCACCACATGTTTCGCAATGGCATGGATT
TAACATACCGCTTCTTTTAACCATCATCATTATTTTATTGGGTAGTGTACTAGCAATCAAA
GTAGATTGGAAAAAAGTGTTCACAGGTAAAATTAGACAGATTTCAGTTTCAAAAAGCTAT
GAGATGGTATATCGACATTTTGAAAAGTTTGCTACGAAGCGATTTAAACGTGTTATGCAA
GATCGTTTAAACCAATACATTATTATGACCTTAGGCATATTTATGATTATCATTGGATATG
GTTATATTCGAATTGGACTTCCTAAAGTACATCAGTTACATGTTTCTGAATTTGGGGCATT
AGAAATTATATTAGCAATCGTAACTGTCACAATTGGTATTTCTTTAATTTTTATACGTCAA
CGACTGACAATGGTCATTTTAAATGGAGTCATCGGATTTGTTGTGACCTTATTCTTTATAG
CAATGAAAGCCCCTGATCTAGCATTGACTCAGCTAGTAGTTGAAACAATAACGACGATAC
TATTTATTGTCAGTTTTTCAAGATTACCAAACGTGCCAAGATCTAACGCTAACAAAAAAA
GAGAAATAATTAAAATTTCTGTATCACTCTTGATGGCACTTATTGTTGTATCATTAATTTT
TATTACACAACAAACAGATGGTTTATCATCAATATCAGACTTTTATTTAAAAGCTGACAA
ACTAACAGGTGGTAAAAATATTGTAAATGCGATACTTGGTGACTTTAGAGCATTAGATAC
ATTATTTGAAGGATTAGTGTTAATTATTACTGGGCTAGGTATTTACACATTATTAAATTAT
CAAGATCGGAGGGGACAAGATGAAAGAGAATGA

## > mrpB

Function: protein coding sequence; monovalent cation/proton antiporter protein B (core genome, constant)
Best match: mrpB_CC001_MW2-USA400_BA000033.2[668971:669396] (completely identical)
Position: 019-contig_265: 13324 … 13750; Length: 426 bp
Sequence:
ATGAAAGAGAATGATGTCGTGTTAAGAACGGTCACGAAACTTGTTGTATTTATTTTATTG
ACTTTCGGATTCTATGTCTTCTTCGCAGGTCATAATAATCCTGGTGGTGGGTTTATTGGTG
GTTTAATATTTAGTTCAGCGTTTATTTTAATGTTTCTGGCTTTTAATGTTGAAGAGGTTTTA
GAAAGTTTACCGATTGATTTTAGAATTTTAATGATTATTGGAGCATTGGTATCATCTATTA
CTGCGATAATACCTATGTTTTTTGGAAAACCATTTTTGTCTCAATATGAAACAACTTGGAT
ACTTCCAATTTTAGGACAAATTCATGTAAGTACAATAACACTTTTTGAATTAGGTATTTTA
TTCTCAGTTGTTGGTGTTATTGTCACAGTGATGTTGTCGCTTAGCGGAGGTCGATCATGA

## > mrpC

Function: protein coding sequence; monovalent cation/proton antiporter protein C (core genome, constant)
Best match: mrpC_CC001_MW2-USA400_BA000033.2[669393:669737] (completely identical)
Position: 019-contig_265: 13746 … 14091; Length: 345 bp
Sequence:
ATGAATTTAATATTATTACTAGTTATAGGATTTTTAGTGTTTATAGGAACATATATGATTT
TATCAATCAATTTAATTCGTATTGTAATCGGAATTTCAATATATACTCATGCTGGTAATCT
CATTATTATGAGTATGGGAACGTATGGTTCTAGTAGATCAGAACCACTAATAACTGGTGG
AAACCAATTGTTTGTTGATCCCTTGTTACAAGCTATTGTACTAACTGCAATAGTTATAGGG
TTTGGGATGACTGCGTTTTTACTTGTACTTGTTTATAGAACTTATAAAGTAACAAAAGAAG
ATGAAATTGAAGGCCTAAGGGGGGAAGATGATGCTAAGTAA

## > mrpD

Function: protein coding sequence; monovalent cation/proton antiporter protein D (core genome, constant)
Best match: mrpD_CC001_MW2-USA400_BA000033.2[669727:671223] (completely identical)
Position: 019-contig_265: 14080 … 15577; Length: 1497 bp
Sequence:
ATGCTAAGTAACTTATTGATTTTACCAATGTTATTACCATTCCTTTGTGCCTTAATCCTTGT
ATTTTTAAAAAATAATGATCGTATTTCTAAATATTTATACTTAGGTACAATGACTATCACC
ACAATTATTTCATTAATGCTATTAATTTATGTTCAGCGTCACCGTCCAATTACGCTAGACT
TTGGAGGATGGTCAGCGCCCTTTGGTATACAGTTTTTAGGAGATTCTTTAAGTTTAATTAT
GGTTACAACCGCTTCGTTTGTGATTACTTTAATTATGGCATACGGATTTGGGCGTGGCGAA
CATAAAGCAAATCGTTATCACTTGCCATCGTTCATATTATTTTTAAGTGTTGGCGTGATAG
GCTCTTTTCTAACATCAGATTTATTTAATTTATACGTCATGTTTGAAATTATGTTACTAGCG
TCATTTGTACTCATTACACTTGGACAATCTGTAGAACAATTACGTGCTGCAATTATTTATG
TTGTCTTGAATATTATTGGTTCATGGCTATTCTTATTAGGTATAGGTTTACTTTATAAAACA
GTAGGTACATTAAACTTTTCACATATTGCAATGCGTTTGAATGACATGGGAGATAATCGC
ACTGTTACAATGATTTCATTAATCTTCTTAGTCGCATTTAGTGCGAAAGCAGCGCTGGTCC
TTTTTATGTGGCTACCCAAAGCCTACGCTGTGTTAAATACTGAGCTTGCAGCATTATTTGC
AGCGTTAATGACCAAAGTAGGGGCCTATGCATTAATTCGATTCTTCACTTTACTATTTGAT
CAACATAATGATCTCATACATCCATTGCTAGCAACTATGGCTGCTATAACTATGGTCATCG
GCGCTATAGGTGTCATTGCTTATAAAGATATTAAAAAGATTGCAGCTTACCAAGTCATAA
TCTCAATAGGATTTATCATTTTAGGTTTAGGAACAAACACGTTTGCAGGTATTAATGGTGC
AATATTTTATTTGGTAAATGACATTGTTGTAAAAACATTGCTATTTTTTATTATTGGTAGTT
TAGTTTACATTACAGGCTATCGACAATATCAATATTTGAATGGCTTAGCTAAAAAAGAAC
CTTTATTTGGAGTTGCGTTTATTATAATGATTTTTGCTATTGGCGGCGTGCCTCCATTTAGT
GGCTTTCCGGGGAAAGTACTTATTTTCCAAGGTGCATTGCAAAATGGCAATTATATTGGA
CTAGCGTTAATGATTATTACTAGTCTAATTGCAATGTACAGTTTATTTAGGATACTTTTTT
ATATGTATTTTGGAGATAAAGATGGGGAGGAAGTTAATTTTAAGAAAATCCCGCTATATC
GAAAAAGAATTTTAAGTATTTTAGTAGTTGTGGTTATCGCAATCGGAATTGCTGCACCTGT
TGTGTTAAATGTTACAAGTGATGCAACTGAGTTGAACACGAGTGATCAATTATATCAAAA
ACTTGTAAATCCGCATTTGAAAGGAGAGGACTAA

## > mrpE

Function: protein coding sequence; monovalent cation/proton antiporter protein E (core genome, constant)
Best match: mrpE_CC001_MW2-USA400_BA000033.2[671224:671706] (completely identical)
Position: 019-contig_265: 15577 … 16060; Length: 483 bp
Sequence:
ATGAATCAAATAGTTTTAAATATTATCATTGCATTCTTATGGGTATTATTTCAAGATGAAG
ATCATTTTAAATTCTCGACTTTCTTTTCTGGATATCTAATTGGTTTAATTGTCATTTATATA
TTACACAGGTTTTTCAGCGATGATTTTTATGTTAGAAAAATATGGGTAGCTATTAAATTTT
TAGGTGTTTATTTATATCAATTAATAACATCTAGCATTAGCACGATTAATTATATTCTTTTT
AAAACAAAAGATATGAACCCTGGATTACTTTCATATGAAACAAGACTAACAAGTGATTG

GTCAATAACATTTTTAACAATTTTAATTATTATAACTCCAGGGTCTACAGTAATACGAATT
TCTCAAGACTCTAAAAAGTTTTTTATTCATAGTATCGACGTGTCAGAAAAAGAAAAAGAT
AGTTTGTTAAGAAGTATTAAGCATTATGAAGACTTAATATTGGAGGTGTCGCGATGA

## > mrpF

Function: protein coding sequence; monovalent cation/proton antiporter protein F (core genome, constant)
Best match: mrpF_CC001_MW2-USA400_BA000033.2[671703:672005] (completely identical)
Position: 019-contig_265: 16056 … 16359; Length: 303 bp
Sequence:
ATGATACAAACAATAACACATATTATGATTATTAGTTCACTCATTATTTTTGGAATTGCAT
TAATCATCTGTTTATTTAGATTAATCAAGGGACCTACAACAGCAGATCGTGTCGTTACATT
TGATACAACAAGTGCTGTCGTAATGTCAATTGTGGGTGTGTTAAGTGTACTTATGGGCAC
CGTTTCTTTCTTAGATTCAATCATGCTCATTGCCATTATATCTTTTGTAAGTTCTGTTTCAA
TATCACGCTTTATTGGTGGGGGGCATGTGTTTAATGGAAATAACAAAAGAAATCTTTAG

## > mrpG

Function: protein coding sequence; monovalent cation/proton antiporter protein G (core genome, constant)
Best match: mrpG_CC001_MW2-USA400_BA000033.2[671980:672417] (completely identical)
Position: 019-contig_265: 16333 … 16771; Length: 438 bp
Sequence:
ATGGAAATAACAAAAGAAATCTTTAGTCTTATTGCTGCTGTGATGTTGTTGTTAGGTAGTT
TTATTGCTCTTATTAGTGCAATAGGTATCGTGAAATTCCAAGATGTTTTCTTAAGAAGTCA
CGCTGCGACAAAAAGTTCAACTTTATCCGTGTTATTAACTTTAATCGGTGTATTAATTTAT
TTTATTGTGAATACAGGATTTTTCAGTGTGCGTTTATTACTGTCACTTGTTTTTATTAATTT
AACTTCACCAGTCGGCATGCACTTAGTCGCTCGCGCTGCTTATCGCAACGGCGCTTATATG
TATCGAAAAAATGATGCTCACACACATGCATCAATATTATTAAGTTCAAATGAACAAAAC
TCTACAGAAGCATTACAATTACGTGCTGAAAAACGAGAAGAGCATCGTAAGAAATGGTA
TCAAAACGATTGA

## > Q5HI38

Function:
Best match: Q5HI38_L1_CC001_MW2-USA400_BA000033.2[672757:674799] (completely identical)
Position: 019-contig_265: 17110 … 19153; Length: 2043 bp
Sequence:
TTGGAAATATTTGAAACAATTCTTATATTTATAGCTGTTGTGATACTAAGTTCGTTTGTCC
ATACTTTCATACCTAAAGTACCCCTAGCATTTATACAAATTTTCTTGGGCATGTTACTATT
TATTACCCCAATCCCTGTTCAATTTAATTTTGATTCTGAATTGTTTATGGTAACAATGATTG
CGCCTTTGTTATTTGTAGAAGGTGTTAATGTTTCTAGAGTCCATTTAAGGAAATATATTAA
GCCAGTGATGATGATGGCATTAGGATTAGTCATTACTACTGTGATAGGTGTAGGTTTATTT
ATTCATTGGATTTGGCCAGATTTACCTATTGGAGCAGCATTTGCAATTGCTGCCATTCTTT
GTCCTACTGATGCAGTAGCAGTGCAAGCAATCACTAAAGGAAAGGTCTTGCCAAAAGGA
GCAATGACAATTCTTGAAGGTGAGTCATTATTGAATGATGCTGCTGGTATTATTTCATTTA
AAATAGCTGTTGGAGTATTAGTTACAGGTGCTTTTTCACTTGTTGATGCTGTTCAGTTGTT
TTTAATTGCATCAATTGGTGGCGCAGTGGTTGGTTTACTTATAGGTATGGCATTAGTAAGG
TTCCGATTAACATTGATGCGTCGAGGATATGAAAACATTAATATGTTTACAATTATTCAAT
TGTTAACACCATTTGTTACGTATTTAATTGCTGAATTGTTTCACGCATCAGGAATCATTGC
AGCAGTAGTTGCAGGACTTGTACATGGTTTCGAACGTGACAGAATTATGCAAGTACGTAC
ACAACTGCAAATGAGTTACAATCATACATGGAATATACTAGGTTATGTTTTAAATGGCTT
TGTTTTTTTCAATATTAGGATTTTTAGTACCTGAAGTTATTATTAAAATTATCAAAACAGAA
CCGCACAATTTAATCTTTTTAATAGGCATCACTATTGTTGTTGCTTTAGCTGTCTATCTATT
TAGATTTGTTTGGGTTTATGTCTTATATCCTTATTTTTATTTAGCCATCAGTCCATTCCAAA
AAATGATGACTAAAAATGATGATGATAATCCAACGACTGAGAAACCACCAAAGCGAAGT

TTATACGCTTTAATTATGACGTTATGTGGTGTGCATGGAACAATTTCTTTAGCAATCGCAT
TAACGTTACCGTATTTTTAGCAGGGCATCATGCTTTTACGTATAGAAACGACTTATTATT
TATTGCATCTGGTATGGTTATTATTAGTTTGGTAGTTGCGCAAGTATTATTGCCATTATTA
ACGAAACCTGCACCTAAAACAGTAATTGGCAATATGTCGTTTAAAGTTGCTAGAATTTAT
ATATTAGAACAAGTTATTGATTATCTAAATCAAAAATCTACTTTCGAAACAAGTTTTAAAT
ATGGTAACGTGATTAAAGAATATCATGATAAATTAGCATTTTTAAAAACTGTAGAGAAAG
ATGATGAAAACTCTAAAGAATTAGAACGTCTACAAAAAATTGCTTTTAATGTAGAAACAA
AAACATTAGAGTCTTTAGTAGATGAAGGACAAATAACGAATAGTGTACTTGAAAACTATA
TGCGTTATGCTGAAAGAACACAGGTATATAGACAAGCATCATTAATAAGAAGAATGATT
GTATTATTACGAGGTGCTTTATTAAAACGAAGAGTACAAACGAGAGTGAACTCCGCATCT
TCACTTAGTGTTACGGATAACTTAATGGAATTAAATAAAATTAATAAATTAGTCCATTAT
AATGTGGTTAGTCGTTTGTCTAAGGAAACAACAAAAGATAATACACTTGAAGTTGGAATG
GTTTGTGACGGTTATTTAATGCGAATTGAAAACTTAACACCATCAAATTTCTTCAACTCAG
CAAGTGAAGATACGATTACTAAAATTAAATTAAATGCATTGAGAGAACAACGTCGCATTT
TACGTGAGTTGATTGATACAGATGAAGTATCAGAAGGTACAGCGTTAAAACTAAGAGAA
GCCATCAATTACGATGAAATGGTTATTGTAGATAGTATGACGTAG

> **txbi_Q5HI38**
Function:  rho independent terminator
Best match: txbi_Q5HI38_L1_CC001_MW2-USA400_BA000033.2[674803:674870:r]RC (completely identical)
Position: 019-contig_265: 19156 … 19224; Length: 68 bp
Sequence:
CTAATTATGCTAAAAGGGATTGATGAAAAACTGAAGGGCTTTTCATCAATCCCTTTTATTT
TATGGGA

> **tx_rsaC**
Function:  rho-independent terminator of rsaC
Best match: tx_rsaC_CC001_MW2-USA400_BA000033.2[674987:675048:r]RC (completely identical)
Position: 019-contig_265: 19340 … 19402; Length: 62 bp
Sequence:
TATTCACAAAAAATAGCCACACTCATATGACATCGGATGAGTGTGGCTTAAGGATCTATG
GG

> **rsaC**
Function: noncoding RNA ncRNA of Staphylococcus aureus C
Best match: rsaC_ref_GQ403617.1[1:533]RC
Position: 019-contig_265: 19351 … 19884; Length: 533 bp
Sequence:
AATAGCCACACTCATATGACATCGGATGAGTGTGGCTTAAGGATCTATGGGGGGAGGAA
ACCATAGATGTTTACTTTGATAGGCCAGATTAAATATCAAAGTATGCGATTATTTATAGCT
TGATGCAAAAGTGGTATGCCTATTAAAAGTTACTGCACATAGCTTTTAATATTCCGTTCAA
AGGAAAGGGGCATACAATTGAACAATCTGTAATAGTACTTTTAACCAGCTATGCTAAAAG
TCTAGTAGGGAGAACAGTTGTCCAATCACATAAGAACCTCTAACTTCGTTAGTACGATTA
AGAAAAGCTTTTTAGTTAGTATGTAATACAATTTATTGACGCGCGTGAATCTCTTTTATAA
GAGTGTGTAGGGAATGGCGTTGTATAAATTGTATTAGAAGAACTTCTAACGCATCTCTGT
GGTTAAAAGAGATGAAGGGAACGACAGTTTAATTAAAACTGCATAAGAACTTCTAGCTTT
TCTCTCTCGTTCAAAGAGAAGCAGCTGTTCGCAGTTTAATCAAAACCACAT

> **rsaC**
Function: noncoding RNA ncRNA of Staphylococcus aureus C
Best match: rsaC-CC8_CC001_MW2-USA400_BA000033.2[674998:675725:r]RC
Position: 020-contig_263 RC: 37 … 67; Length: 30 bp
Sequence:

AGTGACAAATGTTTACAGTTTAATTAAAAC

> **tx_orphan01**

Function: rho-independent terminator
Best match: tx_orphan01_CC001_MW2-USA400_BA000033.2[676003:676063] (completely identical)
Position: 020-contig_263 RC: 211 … 272; Length: 61 bp
Sequence:
TATGTTGATGTGTGGCCTAAAATATTGGAGATACCAATATTTTAGGTTGCATCAACATCA
A

> **mntC**

Function: protein coding sequence; manganese(II) ABC transporter/substrate-binding subunit (core genome, constant)
Best match: mntC_CC001_MW2-USA400_BA000033.2[676093:677022:r]RC (completely identical)
Position: 020-contig_263 RC: 301 … 1231; Length: 930 bp
Sequence:
TTATTTCATGCTTCCGTGTACAGTTTCAATATTTGATTTCATCATTTTGTAGTAAGAGTCAC
CTTTAGTGCCTTCTTTACCGATTGAATCTGTGTACACTTCACCAAAGATATCTTTCTTCGTT
TCTTCAGATAAACTTTCCATTGCTTTCTTATCAACACTTGTTTCTACTAATAAGTGTTTTAA
TTTGTGCTTTTTAACAAACTCAATAGCTTGTCTCATTTGTTCAGGTGTACCTTGTTTTTCAG
TGTTAATTTCCCAAATATAACCTGGTGTAATACCGTATTGTTTTGAGAAGTACTTGAAGGC
ACCTTCACTTGTAATCATGGCACGTTGTTCTTTTGGAATGTCATTAAATTTGTCTTTACTGT
CATTATTTAATTTTTCCAATTGAGCAATGTATTTGTTACCTTGCTTTTCATAATCTGCTTTA
TGTTTTTTGTCGTTATCGATAAATGTTTGTTGAATTGTTTTTACGTATTTAATACCGTTATC
TAAACTTAACCATGCGTGTGGATCTTGTTTATCTTTGTTGCCTTCTTCACCGTTTAAATAGA
TAGGTTTAACATCTTTTGATACTGCGATAACTTTTTTATCTTTTAATGATTTACCAGCCTGT
TCTAAGGCTTTTTCAAACCAACCGTTACCAGTCTCTAAATTTAATCCGTTGTATAAAATAA
CGTCAGCGTCAGTTAACTTTTTAATATCTTTAGGTTTAACTTCATATTCATGAGGATCTTG
ACCAACAGGTACAATACTATGAATATCGACGTTGTCTCCACCAACATTTTTAGCCATATC
ATATAAAATTGAATTCGTCGTTACTACTTTTAATTTGCCATTTGACTTATCACTGCTTTGTT
TACCACCAGTACCACATGCAGCAACTAGAAGTAATAAGGCTAATAATAAAGGTACTAATT
TTTTCAT

> **mntB**

Function: protein coding sequence; manganese(II) ABC transporter/permease subunit (core genome, constant)
Best match: mntB_CC001_MW2-USA400_BA000033.2[677019:677855:r]RC (completely identical)
Position: 020-contig_263 RC: 1227 … 2064; Length: 837 bp
Sequence:
TCATGTTAAACTTCCTCGTTTCTTTCTATTCGTAAATTTTGTGAAAAATAATGTGATGATAT
AAATTACAAACGTACAAAGTACGATTGTCGCACCACTAGGAATGTTGTAAATATAGCTGT
AATAAAGTCCGACAATTGAACTTATGACACTTATTAAACTTGCTATAATCATCATTGAGT
ATAGTTTTTTACTAATTAAAAATGCTGTAGATGCAGGTGTAATTAATAATGCAACTACAA
GAATAATACCTACCGTTTGAATACTTGCTACTGTTACTAATGAGAGTAACAACATCACAA
AGTAATGTAATAACGTCGTATTTAGACCACTCATTCTACTAAACGTTGGATCGAATGTAG
AAATCATTAATGGACGATAGAAAATAATGATTAGAATAAGGACGATTGAACCAATCACA
ATAGTTGTTAAAAATGCACTATTTGTGATTGCCAGTAAATTACCAAACAGAATATGGTAC
AAATCTGTCGTAGTGTTTATTAAGCTAATAATAATAATCCCCGAAGCTAAGAAAGCGGTA
AAACTAATTCCAATAGCGGCGTCAGGTTTCGTTTTACTACTAGATGTGATATAACCGATA
AAAATACTTGCGATCATACCAGTTATAAGTGCGCCTACAAACATTGGAATACCAAATAAG
AATGATAGGGCAACACCAGGTAATACTGCGTGACTCATTGCATCTCCCATTAATGAAAGA
CCACGTAATACAATTAAACTACCAACTGTACCACAAACTATCCCTACAATAATTGAAGTT
ATCAATGCTCGATTCAAGAATTGATATGTAAATAAATGTTCGACAAACTCTAACAT

> **mntA**
Function: protein coding sequence; manganese(II) ABC transporter/ATP-binding subunit (core genome, constant)
Best match: mntA_CC001_MW2-USA400_BA000033.2[677849:678592:r]RC
Position: 020-contig_263 RC: 2057 … 2801; Length: 744 bp
Sequence:
CTAACATGTTATATTGCTCCTTTGACTAGGGTCACTACAGTCAGTGCTACTCATAAATGTT
TCGTTTAAGCGAGTGACACTCATAGCCTCTTCACTATCACCAAAGTATCGTAATGTTTGAT
TTAATAGAATAATGCGATCAAAGTATTGCTTTGCTTTTGATAGATCATGGTGGATGATAA
GAATAAGTTTTCCTTGTTGTTTTAAGTTCTCGATTTTTGTCATGATTAATTTTTCGCTACTA
AAATCAATTCCGACAAACGGCTCATCTAGAAAATAAACTTCACTTTCGGACATCAATGTT
CTTGCTACTAGCACACGTTGTAATTGTCCACCACTTAATTCTGAAATTTGTCGATGACGTA
AAGATTCTAATTCTAAATCGCTTAATAACTGTTTGAGTTTATCCCTTGCTGATTTATTAGG
TCGTCTAAACCATCCAATTTCTTTGTAGCAACCTGATAAAATCACTTGTTCCACACTTATA
GGAAAATCTAAATCAATATGTGCTTTTTGTGGAATATATGTAATATGTTGCAGTTGTTGTT
GTATAGGTTTGTTATATAACAATTTAGTACCGGTAGCATTAAATTCACCAATTAAAGACTT
GATAAGGGAAGATTTACCAGCACCATTCGGGCCCATGATACCAATTATTTCGCCGCGTAC
TGGTATCGATAAGGAAATGTTTTTAAGTACATGCTTATTACCTAAAAACAGATTTAAATCT
TTTGTTTCTAACAA

> **mntR**
Function: protein coding sequence; manganese-dependent transcript repressor (core genome, constant)
Best match: mntR_CC001_MW2-USA400_BA000033.2[678714:679358]
Position: 020-contig_263 RC: 2922 … 3567; Length: 645 bp
Sequence:
ATGTTAACTGAAGAAAAAGAGGACTATTTAAAGGCAATCCTTACGAATAATGGCGATAA
AAACTTTGTGACAAATAAAATCTTATCTCAATTTTTAAATATTAAGCCTCCATCTGTAAGT
GAAATGGTAGGACGTCTTGAAAAAGCAGGCTATGTTGAAACAAAACCATACAAAGGTGT
TAGATTAACAGAGGATGGTTTAACGCATACGCTTGATATCATTAAGAGACATCGACTATT
AGAATTATTTTTAATAGAAATATTGAAATATAATTGGGAAGAAGTACATCAAGAAGCAG
AAATTTTAGAACATCGAATTTCAGATTTATTTGTTGAAAGGCTGGATAGCTTGTTAAATTT
CCCAGAAACTTGCCCGCACGGCGGTGTGATTCCTAGAAATAATGAATATAAAGAGAAAT
ATATAACAACGATATTGAATTATGAACCTGGCGATATCGTTACAATCAAACGTGTGAGAG
ATAAGACCGATTTGCTAATATATTTGTCTAGTAAAGATATTTCTATTGGTAATGAAGTGGA
AATTGTATCGAAAGATGAAATGAATAAAGTAATTATCATTAAACGTAATGATAATGTAAT
TATTGTCAGTTACGAAAATGCAATGAACATGTTTGCTGAAAAATAA

> **tarA**
Function: protein coding sequence; N-acetylmannosamine transferase (core genome, constant)
Best match: tarA_CC001_MSSA476_BX571857.1[678925:679689]
Position: 020-contig_263 RC: 4634 … 5399; Length: 765 bp
Sequence:
ATGACTGTTGAAGAAAGATCCAATACAGCCAAAGTTGACATTTTAGGGGTCGATTTTGAT
AATACAACAATGTTGCAAATGGTTGAAAATATTAAAACCTTTTTTGCAAATCAATCAACG
AATAATCTTTTTATAGTAACAGCCAACCCTGAAATAGTGAATTACGCGACGACACATCAA
GCGTATTTAGAGTTAATAAATCAAGCGAGCTATATTGTTGCTGATGGGACAGGAGTAGTC
AAAGCTTCGCATCGTTTAAAGCAACCTCTAGCGCATCGTATACCTGGTATTGAGTTGATG
GATGAATGTTTGAAAATTGCTCATGTAAATCATCAAAAAGTATTTTTGCTAGGGGCAACT
AATGAAGTTGTAGAAGCGGCACAATATGCATTGCAACAAAGATATCCAAACATATCGTTT
GCACATCATCACGGTTATATTGATTTAGAAGATGAGACAGTAGTAAAACGAATTAAACTG
TTTAAACCTGATTACATATTTGTAGGTATGGGATTCCCTAAACAAGAAGAATGGATTATG
ACACATGAAAACCAATTTGAATCTACAGTGATGATGGGCGTAGGTGGTTCTCTTGAAGTA
TTTGCTGGGGCTAAAAAGAGAGCGCCTTATATCTTTAGAAAATTAAACATTGAATGGATA
TATAGAGCATTAATAGATTGGAAACGTATTGGTAGATTAAAGAGTATTCCTATATTTATG
TATAAAATAGCCAAAGCAAAAAGAAAAATAAAAAAGGCGAAATAA

> **tarH**

Function: protein coding sequence; teichoic acid ABC transporter ATP-binding protein (core genome, constant)
Best match: tarH_CC001_MW2-USA400_BA000033.2[681251:682045:r]RC (completely identical)
Position: 020-contig_263 RC: 5459 … 6254; Length: 795 bp
Sequence:
TTATTTAATAACGAAGCGGGACTCATCGAGTTTGTTTCTAAATTCTTTTTGTTCGGCTTTG
GATTTCTTTTTAAAATCGTTAAGGAAAGCTTCATATTTAGGTAATACATCATCAAGTTCAC
CGTAATCTTTTAACTTTCCGCCTTCAATCCAAGCAATCTTAGTACAAAATTGTCTCACTTG
TCCTAAGTTATGACTAACGAAAAAGATGGTTTTGTTTTGCTCTTTAAACTCGTAAATTTTA
TCTAAACATTTTTGTGCAAAAGTTTGGTCACCTACAGATAAAGCTTCGTCAATGACTAAG
ATATCTGGATTAACTGTGATATTAATTGAAAAACCAAGTTTTGCACGCATACCACTTGAA
TACTTTTTAACTGGTTGATAAATAAACTCACCAAGTTCACTAAATTCAATAATCTTAGGTG
TCATCGCTTTAATTTCTTTTCGCTTAAAGCCCATACATAACATTTTAAATTCGATATTTTCA
ATCCCTGTAAGTTGTCCACTCAAGCCAGCACTAATTGCGATAACGCTGACTTCACCATTAC
GATCCACTTTGCCAACAGTAGGCGACAAAGAACCGCCAATGATATTGCTCAACGTTGATT
TGCCGGAACCATTGATGCCAACAAGCCCTATGACGTCGCCTTCATATGCTTTTAAACTAAT
GTCATCTAAAGCGAAAAATGTTTTGTTTTTATGTTTGGGAATGAGCGCATCTTTCATACGT
TCTTTATTTGTACGATAAATACGATATTCTTTTGTTACATTTTTAATGTTTACCGAAACGTT
CAT

> **tarG**

Function: protein coding sequence; teichoic acid ABC transporter permease protein (core genome, constant)
Best match: tarG_CC005_Mu50-VRSA_BA000017.4[711266:712099] (completely identical)
Position: 020-contig_263 RC: 6579 … 7413; Length: 834 bp
Sequence:
TTGAAAGTGTGGTTTAATGGAATGTCAGCAATAGGAACAGTTTTTAAAGAACATGTAAAG
AACTTTTATTTAATTCAAAGACTGGCTCAGTTTCAAGTTAAAATTATCAATCATAGTAACT
ATTTAGGTGTGGCTTGGGAATTAATTAACCCTGTTATGCAAATTATGGTTTACTGGATGGT
TTTTGGATTAGGAATAAGAAGTAATGCACCAATTCATGGTGTACCTTTTGTTTATTGGTTA
TTGGTTGGTATCAGTATGTGGTTCTTCATCAACCAAGGTATTTTAGAAGGTACTAAAGCA
ATTACACAAAAGTTTAATCAAGTATCGAAAATGAACTTCCCGTTATCGATAATACCGACA
TATATTGTGACAAGTAGATTTTATGGACATTTAGGCTTACTTTTACTTGTGATAATTGCAT
GTATGTTTACTGGTATTTATCCATCAATACATATCATTCAATTATTGATATATGTACCGTTT
TGTTTTTTCTTAACTGCCTCGGTGACGTTATTAACATCAACACTCGGTGTGTTAGTTAGAG
ATACACAAATGTTAATGCAAGCAATATTAAGAATATTATTTTACTTTTCACCAATTTTGTG
GCTACCAAAGAACCATGGTATCAGTGGTTTAATTCATGAAATGATGAAATATAATCCAGT
TTACTTTATTGCTGAATCATACCGTGCAGCAATTTTATATCACGAATGGTATTTCATGGAT
CATTGGAAATTAATGTTATACAATTTCGGTATTGTTGCCATTTTCTTTGCAATTGGTGCGT
ACTTACACATGAAATATAGAGATCAATTTGCAGACTTCTTGTAA

> **tarB**

Function: protein coding sequence; teichoic acid biosynthesis protein B (core genome, variable)
Best match: tarB_CC001-ST772_118_AJGE01000015.1[7433:8536] (completely identical)
Position: 020-contig_263 RC: 7511 … 8615; Length: 1104 bp
Sequence:
ATGAACGTTTTAATAAAGAAATTTTATCATTTGGTAGTTCGAATACTTTCTAAAATGATTA
CGCCTCAAGTGATTGATAAACCGCATATCGTATTTATGATGACTTTTCCAGAAGATATTAA
GCCTATCATCAAAGCATTAAATAATTCGTCGTATCAGAAAACTGTTTTAACAACACCAAA
ACAAGCGCCTTATTTATCTGAACTTAGCGACGATGTTGATGTGATAGAAATGACTAATCG
AACATTGGTAAAACAAATTAAGGCTTTGAAAAGCGCGCAGATGATTATTATCGATAATTA
TTACCTATTACTAGGTGGATATAATAAGACTTCTAATCAACACATTGTTCAAACGTGGCAT
GCAAGTGGTGCATTAAAAAACTTTGGCTTAACAGATCATCAAGTCGATGTGTCTGACAAG

GCAATGGTTCAGCAGTACCGTAAAGTTTATCAAGCGACGGATTTTTACTTAGTGGGTTGT
GAACAAATGTCACAATGTTTTAAACAGTCTTTAGGTGCAACAGAAGAGCAAATGCTGTAT
TTTGGGCTTCCGAGAATTAATAAATATTACACAGCTGATAGAGCAACGGTTAAGGCAGAG
TTAAAGGATAAATATGGAATTACAAATAAGTTGGTATTATATGTACCAACATATAGAGAA
GATAAAGCAGATAATAGGGCTATTGATAAAGCTTATTTTGAAAAATGTTTACCAGGATAT
ACACTGATTAATAAATTACATCCATCAATTGAAGATTCAGACATTGATGACGTATCTTCA
ATCGACACGTCTACATTAATGCTAATGTCAGATATAATTATTAGCGACTATAGTTCGCTGC
CAATAGAAGCTAGCTTGTTAGATATTCCAACTATATTTTATGTGTATGATGAAGGAACAT
ATGATAAAGTGAGAGGCCTGAATCAATTTTACAAAGCAATACCGGATAGCTACAAAGTG
TATACTGAAGAAGATTTAATAATGACGATACAAGAAAAAGAACATCTATTAAGTCCGTTA
TTTAAAGATTGGCATAAGTATAATACTGATAAAAGTTTACATCAGCTCACAGAATATATA
GATAAGATGGTGACAAAATGA

> **tarX**
Function: protein coding sequence; teichoic acid biosynthesis protein X (core genome, constant)
Best match: tarX_CC001-ST772_118_AJGE01000015.1[8533:9594] (completely identical)
Position: 020-contig_263 RC: 8611 … 9673; Length: 1062 bp
Sequence:
ATGAGGCTTACGATAATCATACCTACATGTAATAATGAGGCAACAATTCGACAATTGTTA
ATATCTATTGAGAGTAAAGAACACTATAGAATCCTTTGTATTGATGGTGGTTCTACTGATC
AAACAATTCCTATGATTGAACGGTTACAAAGAGAACTCAAGCATATTTCATTAATACAAT
TACAAAATGCTTCGATAGCTACGTGTATTAATAAAGGTTTGATGGATATCAAAATGACAG
ATCCACATGATAGTGACGCATTTATGGTCATAAATCCAACATCAATCGTATTGCCAGGTA
AATTAGATAGGTTAACTGCAGCTTTCAAAAATAATGATAATATTGATATGGTAATAGGGC
AGCGAGCTTACAATTACCATGGTGAATGGAAATTGAAAAGTGCTGATGAGTTTATTAAAG
ATAATCGAATCGTTACATTAACGGAACAACCAGATTTGTTATCAATGATGTCTTTTGACG
GAAAGTTATTCAGTGCTAAATTTGTTGAATTACAGTGTGACGAAACTTTAGCTAACACAT
ACAATCACGCAATACTTGTCAAGGCGATGCAAAAAGCTACGGATATACATTTAGTTTCAC
AGATGATTGTCGGAGATAACGATATAGATACACATGCTACAAGTAACGATGAAGATTTTA
ATAGATATATCATAGAAATTATGAAAATAAGACAACGAGTCATGGAAATGTTACTATTAC
CTGAACAAAGGCTATTATATAGTGATATGGTTGATCGTATTTTATTCAATAATTCATTAAA
ATATTATATGAATGAACACCCAGCAGTAACGCACACGACAATTCAACTCGTAAAAGACTA
TATTATGTCTATGCAGCATTCTGATTATGTATCGCAAAACATGTTTGACATTATAAATACA
GTTGAATTTATTGGTGAGAATTGGGATAGAGAAATATACGAATTGTGGCGACAAACATTA
ATTCAAGTGGGCATTAATAGGCCGACTTATAAAAGATTCTTGATACAACTTAAAGGGAGA
AAGTTTGCACATCGAACAAAATCAATGTTAAAACGATAA

> **tarD**
Function: protein coding sequence; glycerol-3-phosphate cytidylyltransferase (core genome, constant)
Best match: tarD_CC001_MW2-USA400_BA000033.2[685527:685925] (completely identical)
Position: 020-contig_263 RC: 9735 … 10134; Length: 399 bp
Sequence:
ATGAAACGTGTAATAACATATGGCACATATGACTTACTTCACTATGGTCATATCGAATTG
CTTCGTCGTGCAAGAGAGATGGGCGATTATTTAATAGTAGCATTATCAACAGATGAATTT
AATCAAATTAAACATAAAAAATCTTATTATGATTATGAACAACGAAAAATGATGCTTGAA
TCAATACGCTATGTCGATTTAGTCATTCCAGAAAAGGGCTGGGGACAAAAAGAAGACGA
TGTCGAAAAATTTGATGTAGATGTTTTTGTTATGGGACATGACTGGGAAGGTGAATTCGA
CTTCTTAAAGGATAAATGTGAAGTCATTTATTTAAAACGTACAGAAGGCATTTCGACGAC
TAAAATCAAACAAGAATTATATGGTAAAGATGCTAAATAA

> **txbi_tarD**
Function:  bidirectional rho-independent terminator of tarD and pbpD (core genome, constant)
Best match: txbi_tarD_CC001_MW2-USA400_BA000033.2[685975:686043] (completely identical)
Position: 020-contig_263 RC: 10183 … 10252; Length: 69 bp
Sequence:

AAAATAAATATAAAACGGACAAGTTTCGCAGCTTTATAATGTGCAACTTGTCCGTTTTTA
GTATGTTTT

## > pbpD

Function: protein coding sequence; penicillin binding protein D (core genome, constant)
Best match: pbpD_CC001-ST772_118_AJGE01000015.1[10172:11467:r]RC (completely identical)
Position: 020-contig_263 RC: 10250 … 11546; Length: 1296 bp
Sequence:
TTATTTTCTTTTTCTAAATAAACGATTGATTATCATATGAACAATTAGTGCTAATCCAGCG
ACAAGGCATGCACCACCAATGATAGTGAATAATGGATGTTCTGCCCACATACTTTTAGCA
ACAGTATTTGCCTTTTGAATAATTGGCTGATGAACTTCTACAGTTGGAGGTCCATAATCTT
TATTAATAAATTCTCTTGGATAGTCCGCGTGTACTTTACCATCTTCGACTACAAGTTTATA
ATCTTTTTTACTAAAATCACTTGGTAAAACATCGTAAAGATCGTTTTCAACATAATATTTC
TTACCATTTATCCTTTGCTCACCTTTAGACAATATTTTTACATATTTATACTGATCAAATGA
GCGTTCCATTAATGCATTCCCCATCATATTACGTTGCTTCTCGCCACCAAGGTTTTTATAG
TCTCCCGCACCCATGATAACTTGATTAATTCTAAATTTACCTCGTTTGGTAGTAATCGTAT
GGTTGTAATTTGCTGTATCACTTGATCCAGTTTTTAAACCATCTGTACCAGTCAAACTCAT
TTTTGCACCTTCCAATGAAAAGTTGAATGTGTAATACGTAACTGCATGCGTTGTTGGTGCT
AACTGCTTTGTAAAGTCTAATATTTTAGGTGTCTCTTTAATCACGTGTAAATCTAAAATGG
CGTAGTCTCTAGCAGTCGTTACAGTACGTTCTTGGTCTTTATACTTTGTTGGTGCAAATGT
ACGTAATCTTGAATTTTCAGCACCCGTTGGATTGACGAAATGTGTATTTTTCATTCCGATA
GCTTTAGCTTTGTTATTCATTAAATCAACGAAATCGCTGGTGTTTTTTGAAACCTTCTTAG
CTAAAATTAATGCCGCGGCATTACTAGAATTAGATACTGTAATTTGTAATAGGTCTGCGA
TTGTCCATACTTGTCCAGGATATAGTTTCGTATTACTCAACTCAGGTAGTGTAGACATAAT
ATATTCTTTGTTCGTCATTGTGACAGTGTCATCAAGTGAAAGCTGCCCCTTATTTACAGCT
TCCAATGTTAAGTACATTGTCATTAATTTAGTCATAGACGCTGGATTCCACTTAGTATCGA
TATTGTATTGATACAGTAATTGTCCAGTTTGACTTACATTAACAGCACTCGTCGGTTCGTA
TGCAGCTGACAAACCTGCATAACCATATTGATTTGCTGCTTGTACAGGGGTTACGTCACT
GTTAGCAGCTTGTGCATATGGTGTCATAATACTTAATGTTAAACATAAAATGATGATAAT
AGATATTAAATTTTTCAT

## > abcA

Function: protein coding sequence; ABC transporter ATP-binding protein (core genome, constant)
Best match: abcA_CC398_SO385-pig_AM990992.1[755418:757145]
Position: 020-contig_263 RC: 11966 … 13694; Length: 1728 bp
Sequence:
ATGAAACGAGAAAATCCATTGTTTTTCTTATTTAAAAAACTATCATGGCCAGTGGGTCTTA
TCGTTGCAGCTATCACTATTTCATCACTAGGGAGCTTAAGTGGACTATTAGTGCCACTGTT
TACTGGTCGAATTGTAGATAAATTTTCCGTGAGCCATATCAATTGGAATCTAATCGCATTA
TTTGGTGGTATCTTTGTTATCAATGCTTTATTAAGCGGATTAGGTTTATATTTATTAAGTAA
AATTGGTGAAAAAATTATTTATGCGATACGCTCAGTTTTATGGGAGCATATCATACAATT
AAAAATGCCATTCTTTGACAAAAATGAAAGTGGTCAATTAATGAGTCGATTAACTGACGA
TACGAAAGTGATAAATGAATTTATTTCACAAAAGCTACCTAACTTATTACCATCAATCGTT
ACATTAGTTGGGTCACTAATCATGTTATTTATTTTAGATTGGAAAATGACATTATTAACAT
TTATAACGATACCGATATTCGTTTTAATTATGATTCCTCTAGGTCGTATTATGCAAAAGAT
ATCGACAAGTACACAATCTGAAATTGCAAACTTCAGTGGTTTGTTAGGGCGTGTCCTAAC
TGAAATGCGTCTTGTTAAAATATCAAATACAGAGCGTCTTGAATTAGATAATGCACATAA
AAATTTGAATGAAATATATAAATTAGGTTTAAAACAGGCTAAAATTGCGGCAGTTGTACA
ACCAATTTCAGGTATAGTTATGTTGCTAACAATTGCAATTATTTTAGGTTTTGGTGCATTA
GAAATTGCGACTGGTGCAATCACTGCAGGTACATTAATTGCAATGATATTTTATGTTATTC
AGTTATCTATGCCTTTAATCAATCTTTCAACGTTAGTTACAGATTATAAAAAGGCAGTCGG
TGCAAGTAGTAGAATATACGAAATCATGCAAGAACCTATTGAACCGACAGAAGCTCTTG
AAGATTCTGAAAATGTATTAATTGATGACGGTGTATTGTCATTTGAACATGTAGACTTTAA
ATATGATGTGAAGAAAATATTAGATGATGTGTCGTTCCAAATCCCACAAGGTCAAGTGAG
TGCTTTTGTAGGTCCTTCTGGGTCTGGTAAAAGTACGATATTTAATCTGATAGAACGTATG

TATGAAATTGAGTCAGGTGATATTAAATATGGCCTTGAAAGTGTCTATAATATCCCGTTAT
CTAAGTGGCGACGCAAAATTGGATATGTTATGCAATCAAATTCGATGATGAGTGGTACAA
TTAGAGACAATATTTTATACGGAATTAATCGTCATGTTTCAGATGAAGAACTTATTAATTA
TGCTAAATTAGCGAACTGTCATGATTTTATCATGCAATTTGATGAAGGATATGACACGCTT
GTAGGTGAACGAGGATTGAAACTGTCTGGCGGACAACGTCAACGTATTGATATTGCTAGA
AGTTTTGTTAAAAATCCTGATATTTTGTTACTTGATGAAGCAACAGCTAATCTCGATAGTG
AAAGTGAATTGAAAATTCAAGAAGCTTTAGAAACATTGATGGAAGGTAGAACAACGATT
GTCATTGCGCATCGTTTGTCTACAATTAAAAAAGCCGGTCAAATTATATTCTTAGACAAA
GGACAGGTAACAGGTAAAGGTACGCATTCAGAACTGATGGCATCACATGCGAAGTATAA
AAACTTTGTAGTGTCTCAAAAATTAACAGATTAA

> **nupG**
Function: protein coding sequence; purine nucleoside transporter (core genome, constant)
Best match: nupG_CC005_CGS03_ABWY01000024.1[17092:18321:r]
Position: 020-contig_263 RC: 14050 … 15280; Length: 1230 bp
Sequence:
ATGTTTTTATTAATCAACATTATTGGTCTAATTGTATTTCTTGGTATTGCGGTATTATTTTC
AAGAGATCGCAAAAATATCCAATGGCAATCAATTGGGATCTTAGTTGTTTTAAACCTGTT
TTTAGCATGGTTCTTTATTTATTTTGATTGGGGTCAAAAAGCAGTAAGAGGAGCAGCCAA
TGGTATCGCTTGGGTAGTTCAGTCAGCGCATGCTGGTACAGGTTTTGCATTTGCAAGTTTG
ACAAATGTTAAAATGATGGATATGGCTGTTGCAGCCTTATTCCCAATATTATTAATAGTGC
CATTATTTGATATCTTAATGTACTTTAATATTTTACCGAAAATTATTGGAGGTATTGGTTG
GTTACTAGCTAAAGTAACAAGACAACCTAAATTCGAGTCATTCTTTGGGATAGAAATGAT
GTTCTTAGGAAATACTGAAGCATTAGCCGTATCAAGTGAGCAACTAAAACGTATGAATGA
AATGCGTGTATTAACAATCGCAATGATGTCAATGAGCTCTGTATCCGGAGCTATTGTAGG
TGCGTATGTACAAATGGTACCAGGAGAACTGGTACTAACGGCAATTCCACTAAATATCGT
TAACGCGATTATTGTGTCATGCTTGTTGAATCCAGTAAGTGTTGAAGAGAAAGAAGATAT
TATTTACAGTCTTAAAAACAATGAAGTTGAACGTCAACCATTCTTCTCATTCCTTGGAGAT
TCTGTATTAGCAGCAGGTAAATTAGTATTAATCATCATCGCATTTGTTATTAGTTTTGTAG
CGTTAGCTGATCTATTTGATCGTTTTATCAATTTGATTACAGGATTGATAGCAGGATGGAT
AGGCATTAAAGGTAGTTTCGGTTTAAACCAAATTTTAGGTGTGTTTATGTATCCATTTGCG
CTATTACTCGGTTTACCTTATGATGAAGCGTGGTTGGTAGCACAACAAATGGCTAAGAAA
ATTGTTACAAATGAATTTGTTGTTATGGGTGAAATTTCTAAAGATATTGCATCTTATACAC
CACACCATCGTGCGGTTATTACAACATTCTTAATTTCATTTGCAAACTTCTCAACGATTGG
TATGATTATCGGTACATTGAAAGGCATTGTTGATAAAAAGACATCAGACTTTGTATCTAA
ATATGTACCTATGATGCTATTATCAGGTATCCTAGTTTCATTATTAACAGCAGCTTTCGTT
GGTTTATTTGCATGGTAA

> **rsaD**
Function: noncoding RNA ncRNA of Staphylococcus aureus D (core genome, constant)
Best match: rsaD_CC001_MW2-USA400_BA000033.2[691221:691397:r]RC (completely identical)
Position: 020-contig_263 RC: 15429 … 15606; Length: 177 bp
Sequence:
AAAAACCAAGTGCACATGGTAATACACTTGGCTTTTATGGGAAATGAATATTATTGTACA
TATGACAGTAAGGACTAGGTACAGTCATAGTACTTCGAGCAAAATTTGTTTTGTTATTATA
AACAACACAAAGGAGATAACTTCTCTATTGAAGAAGTTAAAAACATTATAGCAGAC

> **yxkD**
Function: protein coding sequence; putative membrane protein (core genome, constant)
Best match: yxkD_CC001_MSSA476_BX571857.1[690095:690928]
Position: 020-contig_263 RC: 15804 … 16638; Length: 834 bp
Sequence:
GTGAATAAAACGGTTAAAGATTTAATACTAGTTGTCTTAGGTTCATTTATCTTTGCTGCAG
GTGTAAATGCATTTATTATTTCTGGTAACTTAGGTGAAGGCGGGGTTACAGGTTTAGCAA
TTATTTTATATTATGCGTTTCATATTTCACCAGCCATCACTAACTTCTTGGTCAACGCAGTA

TTGATTGCCATTGGTTATAAATTTTTGAGTAAGAGAAGTATGTACTTAACTATTCTTGTAA
CAATTCTTATTTCAATATTTTTGAGTTTAACAGAATCATGGCAAGTAGAAACTGGAAACA
GCATTGTGAATGCCATTTTTGGTGGTGTAAGCGTTGGACTAGGAATCGGAGTAATTATCC
TTGCAGGCGGTACAACAGCAGGTACAACAATTTTGGCAAGAATTGCAACGAAATACCTC
GATGTAAGCACGCCATATGCTTTGCTTTTCTTCGATATGATCGTTGTTGCAATTTCACTTA
CAGTTATTCCACTTGATAAAGTATTAGTAACAGTAATATCACTTTATATAGGAACAAAAG
TGATGGAATATGTCATAGAAGGTTTAAACACTAAAAAAGCTATGACGATTATTTCAACTA
ATCCCGACAAACTTGCCAAAGCAATAGACGAGCAAATTGGAAGAGGTTTAACCATTTTAA
ACGGACATGGCTATTATACGCGTGAAGAAAAAGATGTCTTATACGTTGTTATTTCTAAAA
CACAAGTTTCAAAAGCAAAGCGATTAATTAAACAAATCGATAAAGATGCATTCCTCGTAA
TTCATGATGTAAGAGATGTCTATGGTAATGGCTTTCTTGCAGATGAATAA

## > **fhuC**

Function: protein coding sequence; iron-hydroxamate uptake/ATP-binding protein
Best match: fhuC_CC005_Mu50-VRSA_BA000017.4[721606:722403] (completely identical)
Position: 020-contig_263 RC: 16919 … 17717; Length: 798 bp
Sequence:
ATGAATCGTTTGCATGGACAACAAGTTAAAATTGGTTACGGGGATAACACGATTATAAAT
AAATTAGATGTTGAAATACCAGATGGCAAAGTGACGTCAATCATTGGTCCTAACGGCTGC
GGGAAATCTACTTTGCTAAAGGCATTGTCACGTTTATTGGCAGTTAAAGAAGGCGAAGTA
TTTTTAGATGGTGAAAATATTCATACACAATCTACGAAAGAGATTGCAAAAAAAATAGCC
ATTTTACCTCAATCACCTGAAGTAGCAGATGGCTTAACTGTTGGGGAATTAGTTTCATATG
GTCGTTTTCCACATCAAAAAGGATTTGGTAGATTAACTGCTGAGGATAAGAAAGAAATTG
ATTGGGCAATGGAAGTTACAGGAACTGATACATTCCGACACCGTTCAATCAATGATTTAA
GTGGTGGTCAAAGACAACGTGTTTGGATTGCAATGGCATTAGCACAAAGAACTGATATTA
TCTTTTTAGACGAACCAACAACATATTTAGATATCTGTCATCAATTAGAAATACTAGAATT
AGTTCAGAAGCTAAATCAGGAACAAGGTTGTACAATTGTCATGGTTCTTCATGATATCAA
CCAAGCGATTCGTTTCTCAGATCATCTTATTGCGATGAAAGAAGGGGATATCATCGCTAC
AGGTTCAACAGAAGACGTATTAACACAGGAAATATTAGAAAAAGTTTTTAATATTGATGT
TGTTTTAAGTAAAGATCCTAAAACTGGAAAACCTTTACTGGTAACTTATGACTTATGTCGC
AGAGCTTATTCTTAA

## > **fhuB**

Function: protein coding sequence; iron-hydroxamate uptake/permease
Best match: fhuB_CC005_Mu50-VRSA_BA000017.4[722439:723443]
Position: 020-contig_263 RC: 17752 … 18757; Length: 1005 bp
Sequence:
ATGACAAATAGAGAGAACCCAACGCCATTGAAGTTTTTATCCTATATTATAGGTTTAAGT
ATGATACTACTAATCACACTATTTATTTCTACATTAATAGGTGACGCCAAAATTCAAGCCT
CTACAATTATAGAGGCTATTTTTAATTATAATCCTAGCAATCAACAGCAAAACATCATCA
ATGAGATTAGGATTCCCAGAAATATAGCAGCAGTAATTGTAGGTATGGCGCTTGCAGTTT
CTGGTGCGATTATACAAGGTGTTACTCGTAATGGTCTTGCTGATCCGGCGCTCATTGGTTT
AAATTCAGGTGCTTCATTTGCTTTAGCATTAACATATGCAGTTTTACCAAACACTTCATTT
TTAATATTGATGTTTGTTGGATTTTTAGGTGCTATTCTAGGAGGTGCTATTGTATTAATGA
TAGGCCGATCTAGACGTGATGGATTTAATCCGATGCGTATTATTTTAGCGGGTGCAGCAG
TAAGTGCTATGTTAACAGCGCTAAGTCAAGGTATTGCATTAGCTTTTAGACTAAATCAAA
CAGTAACATTTTGGACTGCTGGAGGCGTTTCAGGCACAACATGGTCACACCTTAAGTGGG
CAATTCCATTAATTGGTATTGCGTTATTCATTATATTAACAATTAGTAAACAACTTACCAT
TTTAAATCTTGGTGAATCATTAGCTAAAGGTTTAGGTCAAAATGTAACAATGATCAGAGG
CATATGTTTAATTATTGCTATGATTCTAGCAGGTATTGCAGTTGCTATCGCTGGACAAGTT
GCATTTGTAGGTTTGATGGTACCTCATATAGCAAGATTTTTAATTGGAACTGATTATGCTA
AAATTCTACCATTAACAGCCTTGTTAGGTGGGATACTCGTGCTTGTTGCCGATGTGATAGC
ACGATATTTAGGAGAAGCGCCTGTTGGTGCAATCATTTCATTTATCGGTGTTCCTTACTTT
TTATATTTAGTTAAAAAAGGAGGACGCTCAATATGA

> **fhuG**
Function: protein coding sequence; iron-hydroxamate uptake/permease
Best match: fhuG_CC001_MW2-USA400_BA000033.2[694546:695562] (completely identical)
Position: 020-contig_263 RC: 18753 … 19770; Length: 1017 bp
Sequence:
ATGATTAGTTCAAATAATAAACGCAGACAATGGATTGCACTGGCTGTTTTTAGCATTCTA
CTATTTCTAGGTTGTACTTGGAGTATTACCTCAGGTGAATACAACATACCTGTTGAAAGAT
TTTTCAAAACTTTAATTGGACAAGGTGATGCCATTGATGAGTTAATCTTATTAGATTTCAG
ATTACCTCGGATGATGATTACTATTTTGGCTGGCGCAGCGCTTAGTATTAGTGGTGCAATA
GTGCAAAGTGTCACGAAAAATCCAATAGCTGAACCCGGTATATTAGGTATTAACGCAGGT
GGCGGATTTGCAATCGCATTATTTATTGCAATTGGTAAAATTAATGCTGACAACTTTGTTT
ATGTACTGCCGTTAATAAGTATACTAGGTGGTATCGCCACTGCATTGATTATTTTTATTTT
CAGTTTTAATAAAAATGAAGGTGTTACACCTGCGAGTATGGTATTAATAGGTGTAGGTTT
ACAAACAGCATTATATGGTGGCTCAATTACAATTATGTCAAAATTTGATGATAAGCAATC
TGATTTCATCGCTGCTTGGTTTGCAGGTAATATTTGGGGTGACGAATGGCCATTTGTCATT
GCATTTTTACCGTGGGTGTTGATTATTATTCCTTACTTACTATTTAAATCGAATACACTAA
ATATTATTCATACGGGTGATAATATTGCACGAGGTCTAGGTGTAAGGTTAAGCAGAGAAC
GTTTAATATTATTCTTTATCGCAGTGATGTTATCATCTGCTGCTGTAGCAGTAGCAGGTTC
GATTTCGTTTATCGGATTAATGGGTCCGCATATTGCCAAACGTATCGTTGGACCACGTCAC
CAGTTGTTTTTACCAATTGCCATTTTAGTAGGGGCATGTTTACTTGTTATAGCTGATACAA
TTGGCAAAATTGTATTACAACCAGGTGGGGTTCCAGCAGGTATTGTCGTAGCAATTATTG
GTGCACCGTATTTCTTATATTTAATGTACAAAACGAAAAATGTATAG

> **dakK**
Function: protein coding sequence; putative dihydroxyacetone kinase/K subunit (kinase) (core genome, constant)
Best match: dakK_CC001_MW2-USA400_BA000033.2[695796:696764] (completely identical)
Position: 020-contig_263 RC: 20003 … 20972; Length: 969 bp
Sequence:
ATGATGAAAAAGTTAATCAATAAAAAAGAAACATTTTTAACTGATATGCTTGAAGGATTG
TTAATTGCGCACCCAGAGTTAGATCTGATTGCTAATACAGTTATTGTAAAAAAAGCTAAG
AAAGAACATGGTGTAGCAATAGTCTCTGGAGGTGGAAGCGGACATGAACCTGCGCATGC
CGGTTTTGTTGCAGAAGGTATGCTAGATGCAGCGGTTTGTGGCGAAGTATTTACATCACC
TACACCTGATAAAATATTAGAAGCTATTAAAGCAGTAGATACTGGTGATGGTGTATTACT
AGTTGTAAAAAACTATGCAGGTGACGTGATGAATTTCGAAATGGCACAAGAGCTTGCAG
AAATGGAAGGTATAAATGTTCAAACTGTTATTGTTCGTGACGACATTGCTGTGACAAACG
AAGTACAACGTCGTGGTGTTGCAGGAACAGTGTTTGTTCATAAGCTTGCCGGTTATCTTGC
TGAAAAAGGTTATTCATTAACAGAGATAAAATCGCGTGTAGAAGCGTTGTTACCTGAAAT
TAAAAGTATTGGTATGGCAATTGAGCCACCGCTTGTTCCAACTACTGGAAAATATGGCTT
TGATATTGAAGACGACAAAATGGAAATCGGTATTGGTATACATGGTGAAAAAGGTATTC
ATAGGGAAGAAGTAAAGGATATTGATCATATTGTTGGAACATTGTTAGACGAATTGTATA
AAGAAGTTACTGCCAATGATGTCATATTAATGGTAAATGGTATGGGTGGTACGCCGTTAT
CTGAATTAAATATCGTAACTAAATATATTCAACAAAATTTAGCTGCAAGAACGGTTAATG
TTGCTAAATGGTTTGTTGGTGATTATATGACATCTTTAGACATGCAAGGTTTTTCTATAAC
TATCGTGCCTAATAAACCAGAATATTTGGAAGCATTTTTAGCACCAACAACAAGTCAATA
CTTTAAATAA

> **dakL**
Function: protein coding sequence; putative dihydroxyacetone kinase/L subunit (core genome, constant)
Best match: dakL_CC001_MW2-USA400_BA000033.2[696806:697390]
Position: 020-contig_263 RC: 21013 … 21598; Length: 585 bp
Sequence:
ATGAAAGTGAATGATATGAAAGCACGTTTATTAAATTTAGAAGAAACGTTTAAAAAACAT
GAATCTGAATTAACTGAATTAGATCGAGCAATTGGTGATGGTGACCACGGGGTTAACATG

GTTCGTGGGTTTAGTAGTCTTAAAGACAAACTTGATGATAGCTCAATGCAATCATTGTTCA
AATCAACTGGTATGGCATTGATGTCAAATGTTGGGGGTGCATCTGGACCACTGTATGGCT
TTAGCTTTGTTAAAATGTCTGCAGTCACCAAAGATGATATGGATAATCAAGATTTCATTAC
ACTAATTCAGGCATTTGCCGAAGCGGTTGAATCACGTGGTAAAGTTACTTTAAATGAAAA
GACAATGTATGATGTAATAGCGCGAGCAGCAGAGAAGCTTAAAAATGGTGAAACTTTAA
CATTCAATGATTTACAGCAATTAGCAGATAATACAAAAGATATGGTAGCAACGAAAGGT
AGAGCTGCATATTTTGGAGAAGAATCAAAAGGTTATATTGATCCAGGTGCTCAAAGTATG
GTTTATATTTTAAACGCTTTGATTGGAGATGAAGATAATGCCTAA

## > dakP

Function: protein coding sequence; putative dihydroxyacetone kinase/phosphotransfer subunit (core genome, constant)
Best match: dakP_CC001_MW2-USA400_BA000033.2697383:697745 [4_50ANF10END]
Position: 020-contig_263 RC: 21590 … 21953; Length: 363 bp
Sequence:
ATGCCTAAAATTATACTTGTTAGCCACAGTAAAGAAATTGCAAGTGGTACAAAATCTTTG
TTAAAGCAAATGGCAGGTGACGTTGATATTATACCAATCGGGGGATTACCAGATGGTTCA
ATTGGAACTTCATTTGATATCATCCAAGAAGTTTTGACTAAATTAGAGGATGATGCATTGT
GTTTTTACGATATTGGATCTTCAGAAATGAATGTAGATATGGCAATTGAAATGTATGATG
GTAATCATCGTGTGTTAAAAGTTGATGCACCAATTGTTGAAGGCAGTTTTATCGCAGCAG
TAAAGCTATCAATCGGCGGTTCAATTGATGATGCATTAGCAGAAATGAAACAATCATTTT
AG

## > graX

Function: protein coding sequence; cofactor of GraRS (core genome, constant)
Best match: graX_CC001-ST772_118_AJGE01000015.1[26790:27713] (completely identical)
Position: 020-contig_263 RC: 26868 … 27792; Length: 924 bp
Sequence:
ATGAAACCTAAAGTTTTATTAGCAGGTGGAACAGGATATATTGGTAAGTATTTAAGTGAA
GTGATTGAAAATGATGCTGAACTTTTTACTATATCAAAATATCCAGACAATAAAAAAACA
GATGATGTTGAAATGACTTGGATTCAGTGTGATATATTTCATTACGAACAGGTTGTTGCA
GCAATGAATCAAATAGATATTGCTGTATTCTTTATCGACCCAACAAAGAATTCTGCCAAA
ATAACACAATCATCAGCAAGAGATTTAACATTAATCGCAGCAGATAATTTTGGTCGAGCA
GCGGCTATTAATCAAGTAAAAAAAGTAATCTACATACCTGGGAGTCGTTATGATAATGAA
ACAATTGAACGCCTAGGTGCATATGGTACTACTGTAGAAACAACAAATTTAGTTTTTAAA
CGTTCTTTAGTTAATGTAGAATTACAAGTTTCAAAGTATGATGATGTTAGATCAACGATG
AAGGTAGTTTTACCAAAGGGATGGACATTAAAGAACGTTGTAAACCATTTTATTGCATGG
ATGGGTTACACTAAAGGAACTTTTGTGAAAACAGAAAAATCACATGATCAATTTAAGATA
TATATTAAGAATAAGGTGCGACCGCTCGCAGTATTTAAAATAGAAGAAACAGCTGACGG
AATAATAACTTTAATTTTATTGAGTGGAAGTTTAGTGAAAAAATACACAGTTAATCAAGG
GAAGTTAGAATTTAGATTAATCAAAGAGTCGGCAGTCGTTTATATACATCTATACGATTA
TATCCCTCGATTATTTTGGCCGATTTATTACTTTATACAAGCACCAATGCAAAAAATGATG
ATTCATGGCTTTGAAGTTGACTGCCGGATTAAAGATTTTCAAAGTCGATTAAAATCAGGA
GAAAATATGAAATATACTAAATGA

## > graR

Function: protein coding sequence; response regulator of two-component sensor system (core genome, variable)
Best match: graR_CC001-ST772_118_AJGE01000015.1[27729:28403] (completely identical)
Position: 020-contig_263 RC: 27807 … 28482; Length: 675 bp
Sequence:
ATGCAAATACTACTAGTAGAAGATGACAATACTTTGTTTCAAGAATTGAAAAAAGAATTA
GAACAATGGGATTTTAATGTTGCTGGTATTGAAGATTTCGGCAAAGTAATGGATACATTT
GAAAGTTTTAATCCTGAAATTGTTATATTGGATGTTCAATTACCTAAATATGATGGGTTTT
ATTGGTGCAGAAAAATGAGAGAAGTTTCCAACGTACCAATATTATTTTTATCATCTCGTG

ATAATCCAATGGATCAAGTGATGAGTATGGAACTTGGCGCAGATGATTATATGCAAAAAC
CGTTCTATACCAATGTATTAATTGCTAAATTACAAGCGATTTATCGTCGTGTCTATGAGTT
TACAGCTGAAGAAAAACGTACATTGACTTGGCAAGATGCTGTCGTTGATCTATCAAAAGA
TAGTATACAAAAAGGTGACGATACGATTTTTCTATCCAAAACAGAAATGATTATATTAGA
AATTCTTATTACCAAAAAAAATCAAATCGTTTCGAGAGATACAATTATCACTGCATTATG
GGATGATGAAGCATTTGTTAGTGATAATACGTTAACAGTAAATGTGAATCGTTTACGAAA
AAAATTATCTGAAATAAGTATGGATAGTGCAATCGAAACAAAAGTAGGAAAAGGATATA
TGGCTCATGAATAA

> **graS**

Function: protein coding sequence; sensor histidine kinase (core genome, variable)
Best match: graS_CC005_Mu50-VRSA_BA000017.4[733161:734201] (completely identical)
Position: 020-contig_263 RC: 28474 … 29515; Length: 1041 bp
Sequence:
ATGAATAATTTGAAATGGGTAGCTTATTTTTTGAAATCTCGCATGAACTGGATATTTTGGA
TATTGTTTTTAAACTTGCTTATGTTAGGCATTAGTCTAATCGATTATGATTTTCCAATAGAC
AGTTTATTTTATATTGTTTCTTTGAATTTAAGTTTAACAATGATTTTTCTTATATTGACATA
TTTTAAAGAAGTAAAATTATATAAGCATTTTGACAAAGATAAAGAAATAGAAGAAATTA
AACATAAAGATTTAGCGGAAACGCCATTTCAACGTCATACAGTTGATTATTTATATCGTC
AAATCTCAGCGCACAAAGAAAAGGTTGTTGAGCAACAGTTACAATTGAACATGCATGAA
CAAACCATTACAGAATTTGTGCACGACATAAAAACACCTGTGACAGCCATGAAATTATTA
ATTGATCAAGAAAAAAATCAAGAAAGAAAACAGGCATTACTATATGAATGGTCTCGTAT
AAACTCGATGCTGGATACACAGCTGTATATTACTAGATTAGAATCTCAACGCAAAGATAT
GTATTTTGATTACGTGTCACTTAAACGCATGGTCATTGATGAAATACAATTAACAAGACA
TATTAGTCAGGTTAAAGGTATTGGTTTTGATGTTGACTTTAAAGTGGATGATTATGTTTAT
ACAGATACAAAATGGTGTCGTATGATTATTAGACAGATTTTGTCAAACGCATTGAAATAT
AGTGAGAATTTTAATATTGAAATTGGGACAGAATTAAATGATCAACATGTTTCGTTATAT
ATTAAAGACTATGGCAGAGGTATTAGTAAAAAAGATATGCCGCGAATATTTGAACGAGG
ATTTACGTCAACGGCTAACAGAAATGAAACGACGTCTTCAGGTATGGGTCTATATTTAGT
AAATAGTGTAAAGGATCAATTAGGTATTCACCTGCAAGTCACGTCGACTGTTGGTAAGGG
GACAACTGTCAGATTGATTTTCCCATTACAAAATGAAATTGTTGAACGCATGTCGGAAGT
GACAAATTTGTCATTTTAA

> **vraF**

Function: protein coding sequence; ABC transporter/ATP-binding protein (core genome, variable)
Best match: vraF_CC133_ED133-shep_CP001996.1[721175:721936]
Position: 020-contig_263 RC: 29658 … 30420; Length: 762 bp
Sequence:
GTGGCAATTTTAGAAGTAAAACAATTAACAAAAATATATGGAACTAAAAAAATGGCACA
AGAAGTGTTGCGAGATATCAATATGTCTATTGAAGAAGGCGAGTTTATTGCTATTATGGG
TCCCTCTGGATCTGGGAAAACGACATTATTAAATGTTTTAAGTTCAATTGATTATATTTCA
CAAGGTTCTATTACATTAAAAGGAAAAAAATTAGAAAAGCTTTCAAACAAGGAATTATCT
GATATACGCAAGCATGATATTGGTTTTATTTTTCAAGAGTATAATTTACTGCATACATTGA
CTGTTAAAGAAAACATAATGTTACCACTAACGGTTCAGAAGTTAGATAAAGAACATATGT
TAAATCGTTATGAAAAAGTAGCAGAAGCATTAAATATATTGGATATTAGTGATAAATACC
CTTCTGAATTGTCTGGTGGACAAAGACAACGAACATCTGCTGCAAGAGCGTTTATTACAT
TACCTTCTATTATATTTGCTGACGAACCAACAGGTGCACTGGATTCTAAAAGTACTCAAG
ATTTATTAAAACGATTAACAAGAATGAATGAAGCATTTAAGTCTACAATTATTATGGTAA
CGCATGATCCTGTTGCAGCAAGTTATGCCAATAGAGTAGTGATGCTAAAAGATGGTCAAA
TTTTCACTGAATTATACCAAGGGGATGACGATAAACATACCTTTTTCAAAGAAATAATAC
GTGTACAAAGTGTTTTAGGTGGCGTTAATTATGACCTTTAA

> **vraG**

Function: protein coding sequence; ABC transporter/permease (core genome, variable)
Best match: vraG_CC005_ED98-hen_CP001781.1[670281:672170]

Position: 020-contig_263 RC: 30409 … 32299; Length: 1890 bp
Sequence:
ATGACCTTTAACGAGATAATATTTAAAAATTTCCGTCAAAATTTATCACATTATGCCATCT
ATCTTTTTTCATTAATTACGAGTGTAGTATTGTATTTTAGCTTTGTAGCATTAAAATACGC
GCATAAACTAAACATGACAGAGTCATATCCAATTATAAAGGAAGGCTCACAAGTCGGAA
GCTACTTTCTATTTTTCATCATAATTGCATTTTTGTTATATGCCAATGTGTTATTTATTAAA
CGACGAAGTTATGAGCTTGCATTATATCAAACATTAGGTTTATCTAAATTCAACATTATTT
ATATACTAATGCTCGAACAATTACTAATATTTATAATTACGGCAATATTAGGTATTATTAT
TGGTATTTTTGGTTCGAAACTGTTATTAATGATTGTCTTTACATTATTAGGAATTAAAGAA
AAGGTTCCAATTATTTTTAGTTTGAGGGCGGTATTTGAAACATTAATGTTAATCGGTGTCG
CTTATTTTTTAACATCTGCTCAAAATTTTATATTAGTGTTCAAACAATCTATTTCACAGATG
TCAAAGAATAACCAGGTTAAAGAAACAAATCATAATAAAATTACATTTGAAGAGGTTGTT
TTAGGCATCTTAGGTATAGTATTGATTACCACAGGATACTATCTATCTTTGAACATTGTTC
AATATTATGATTCTATCGGTACACTTATGTTTATTTTATTGTCAACTGTGATTGGGGCATA
CTTATTTTTTAAAAGCTCTGTTTCTCTAGTTTTTAAAATGGTGAAGAAGTTTAGAAAAGAT
GTTATAAGTGTAAATGATGTCATGTTCTCATCATCTATTATGTATCGTATTAAGAAAAATG
CTTTTTCACTTACGGTCATGGCAATCATTTCAGCGATTACTGTTTCAGTTCTTTGCTTTGCT
GCTATAAGTAGAGCGTCCTTATCAAGTGAAATAAAATATACTGCACCACACGACGTTACA
ATTAAAGACCAACAAAAAGCTAATCAGTTAGCAAGTGAATTAAACAATCAAAAAATTCC
TCATTTTTATAATTATAAAGAAGTAATTCATACGAAATTGTATAAAGATAATTTATTTGAT
GTAAAAGCGAAAGAACCATACAATGTAACAATTACTAGTGATAAATACATCCCTAATACT
GATTTGAAACGTGGGCAAGCTGATTTATTTGTAGCGGAAGGTTCTATCAAAGATTTAGTG
AAACATAAGAAGCATGGTAAAGCAATTATAGGAACGAAAAAACATCATGTTAATATTAA
GTTACGTAAAGATATTAATAAAATCTATTTTATGACAGATGTTGATTTAGGTGGACCAAC
GTTTGTCTTAAATGACAAAGACTATCAAGAAATAAGAAAGTATACAAAGGCAAAGCATA
TCGTCTCTCAATTTGGATTCGATTTGAAACATAAAAAAGATGCTTTAGCATTAGAAAAAG
CGAAAAATAAAGTTGATAAATCTATTGAAACAAGAAGTGAAGCGATAAGCTCAATATCA
AGTTTAACCGGAATATTATTATTTGTAACATCATTTTTAGGTATTACATTCTTGATTGCTGT
ATGTTGCATTATATACATAAAGCAAATAGATGAAACCGAAGATGAGTTAGAGAATTATA
GTATTTTGAGAAAGCTTGGATTTACACAAAAAGATATGGCAAGGGGACTAAAGTTTAAA
ATTATGTTTAATTTTGGGTTACCTTTAGTTATTGCACTATCACATGCATATTTTACATCATT
AGCATATATGAAATTAATGGGTACAACGAATCAAATACCGGTTTTCATAGTAATGGGATT
ATACATTTGTATGTATGCTGTTTTTGCAGTGACGGCTTATAATCATTCCAAGCGAACAATT
AGACATTCCATATAA

> **pitA**

Function: protein coding sequence; phosphate transporter (core genome, variable)
Best match: pitA_CC001-ST772_118_AJGE01000015.1[33578:34585] (completely identical)
Position: 020-contig_263 RC: 33656 … 34664; Length: 1008 bp
Sequence:
ATGTCATATATAATCATCGTCACTATAGCTGTAGTTATTTTCTCGCTGATATTTGACTTTAT
CAATGGATTCCATGATACAGCCAATGCAGTAGCTACTGCTGTATCTACTAGAGCGTTAAC
GCCTAAAACGGCAATTTTAATGGCAGCAGTGATGAACTTTATAGGTGCTTTAACATTTAC
GGGCGTTGCAGGCACCATTACTAAAGACATTGTCGATCCATTTAAATTGGAAAATGGATT
AGTTGTTGTGTTAGCTGCAATACTTGCGGCTATTATTTGGAATTTAGCTACTTGGTTTTAC
GGAATTCCAAGTTCGTCTTCACATGCACTTATAGGTTCAATTGCGGGTGCAGCAATCGCA
TCTGAAGGCTCATTTGGAGTGTTACATTACCAAGGTTTCACAAAAATTATTATTGTATTAA
TCGTTTCACCGATTATCGCATTTTGTGTTGGTTTCTTGATGTATTCAATTTTTAAAGTTATC
TTTAAAAATGCAAATTTAACAAGAGCGAATCGTAACTTTAGATTTTTCCAAATTTTCACAG
CAGCGTTACAATCATTCTCTCACGGTACGAATGATGCGCAAAAGTCAATGGGTATTATTA
CGTTGGCATTAATTGTTGCTAATGTACAGAATGATGGCAGCGTTGAACCACAGTTATGGG
TAAAATTTGCCTGTGCGACAGCAATGGGGCTTGGTACTGCAATTGGTGGCTGGAAAATTA
TCAAAACTGTAGGTGGTAATATTATGAAAATACGTCCAGCAAATGGTGCTGCGGCCGATT
TATCATCTGCATTAACAATTTTTGTTGCATCATCGCTACATTTCCCATTATCAACAACTCA
CGTTGTGTCATCATCAATCTTAGGTGTTGGTGCTTCTAACCGAGCTAAAGGTGTAAAATG

GAGCACTGCGCAACGAATGATCATTACATGGGTGATTACATTACCTATTTCAGCATTGTT
AGCAGGTTTACTATTCTATATACTTAACTTATTTTTCTAA

## > ssaA5

Function: protein coding sequence; staph secretory antigen A5 (core genome, constant)
Best match: ssaA5_CC005_Mu50-VRSA_BA000017.4[739936:740733:r]RC (completely identical)
Position: 020-contig_263 RC: 35252 … 36050; Length: 798 bp
Sequence:
TTAATGGATGAATGCATAGCTAGAAACTTCTGAAGCTGGAATTGTACGGTAGTTCATATT
GTATGGACCATATGTGTAATTCATTTCAGAAATCAAGATACTACCATCACCATTGACACG
TTCAACATAAGCAACATGACCATATGGACCAGGTGTGCTTTGCATAATTGAACCAACTGA
TGGTGTGTTGTTTACTTGGTAACCATCATTAGCTGCGTTACCAGCCCAATACTTAGCGTCT
GACCAATATGTGCTAATTGGACTACCAGCTTGAGCACGACGGTCAAATACGTACCATGTA
CATTGACCAGCAGTGTATAAATTTTGGTGATTAAAAGATGATGCATTGCCATTGCTACCT
GTTGTAGCTGTTGGTGTTGTACCACCTGATCCACCATTAGGAATTTGTAATGTTTGGTTAG
GCATAATTAAATAACCACGTAAGTTATTGGCTGCCATTAATTGATCAACTGAAACACCAT
ATCTGCTAGCAATGATATTTAATGATTCACCAGCTTGTACAGTATGAGATGATGCTGAAC
CAGCTTGTGGAGAAGTGTTTGACGTATTTTGTGCATCACTTCCACCTACTGAGATAACTTG
ACCAGGGAATACCAAGTTGTTATCTAATTGGTTATTTTGTTTAATACTCTCTACTGAAGTG
TTGTATTTTTGAGCAATACTCCATAATGATTCACCAGATTGTACTGTATGTTGTGTAGAAG
CTTGTGCATCATGATGCGTTAAAAATGCAGCTGCACCAGATGTTGCTGTTATTGCAAATG
CTAATTTTTTCAA

## > rbf

Function: protein coding sequence; regulator of biofilm formation (core genome, variable)
Best match: rbf_CC005_CF-Marseille_CABA01000037.1[29963:32114]
Position: 020-contig_263 RC: 37751 … 39902; Length: 2151 bp
Sequence:
ATGGCAAAATCATGCTTGCATATACTTACTAATAATGAATATGCGACAACGCGTTGCCAA
GATGGCATAGTCTTATTTTGGCCAATTGACGGGGAAATCGAACTACAAAAATTTCGTAAA
AGTAAAATAATTGAAGATGATATATATATTATTAATCATCTGGATGTATTTAGTATTAAG
AATAATAAAAAAACGATCATGTTGTATTTGAGTAGCGATTGGTTTGCGGAATTAGGCTTT
ACTTTCTTTAATTACCACTATACAGCAAAGTTGATTAAATCATCCTATAATTTGAAATGTC
TACTATTAAAATTGACATATCGATACCTTGATAATCAGCCTCTTAATGACGCTGATATTAG
AAAATTACAGGATATTATTAAAATCATTGCAAAAGAAGCAAGTATGGATAAAAAGATTG
CACAAAATCAATATCGATATGCCTATTATGGTGATTTGCGTGACGAGCTCGAATATATTT
ATCAAAATGTAAATCAACGATTGACATTAAAAAGTGTCGCTGATAAATTATTTGTCTCAA
AGTCAAATTTGTCATCACAATTCCACTTACTTATGGGCATGGGTTTTAAAAAATATATTGA
TACTTTGAAAATTGGTAAATCGATTGAAATTCTACTTACTACTGATAGTACTATTAGCAAC
ATAAGTGAACATTTAGGTTTTAGTAGTAGCTCCACTTACTCTAAAATGTTTAAAAGTTATA
TGGATATAACACCGAATGAATATCGTAATTTATCAAAATATAATAAATGTTTAATGCTAA
AGCCAGAACCACTAGTAGGCAAAATGGTACAAGAAGTAAAAGAAATCATATTGAATTAT
ATTGAACATTATAAAAACCACCTAACTGATGTTATACATATTGATGAAGACAAATTTGAA
ACACCTAAATTGTTTCAAACGGTTATTCAAATAAATACTTATACAGAAATGAAATTAGTT
TTCTTAGAAGGAATCTTTAAAACCTTATTGAATAAGAACAGTCAAGTTGTCTTTTTCATCA
TGCCATCGATTCTAAAAAGTAAAAATACCATGTCCGAAGAAGAAAAATTCACAATCATTA
AAACAATAATTGAAAGTGATCTAAAGATAGCATTTAATATAAATGATATTGAAACAACTT
ATTTTGTTGAAGAAGCTTTTATGAGTGTTTTCAGACAAATATCTCCAAACGAATTAAGTAA
TCATAATAATTACGAAGTGCATTTTGTTTTTGATTTATCATTGATGGAAATTAGAACAATT
TATCGAATGATATTAAAATTACATAACATCATGTTGAATGTGAAATTAGGATTGAACATT
ACCTGTTTATTTGAAAAACCTTCAGTTTTTAAATCATTAGTATCACAAATAAAGCGACTTA
AATTCGATTCGTTAATAATAGATAATGCAAATTTAAGTAGCCCTTATTTGATGGGGGAAA
GTGATGAGTTACTATTGAAAAATATTTTGCATTTTAAAAATTTAAAACAAGTAATTAATG
AATTGGATATTGAACAAGAAAAGCTTATTTTTCTAAATGTTGAAAATCATAAACTGCTTA
ATAATAAAGAACGAGATTTAAGTAATAGTGCTCCATTAATTTATAAGACATTAAGTGCGC

TGTATCACAACTTTGATGGCTTTGGATTAAACATTTTTGATAATCATCATACATTTAATGC
GATGCATCTATATGATAAAAATGGATTTAAAACAACACTAGGTCTTATATTGGAAAAATT
TATCGAATATGTCTCGAAACCAAAATACGAAAACAGTTATTATTCTATTTTTGATATAGA
GAATTATTATTGCCTGGTTATTTATGATTGGCGAGTGATTGAGAGCGAGACAATTATGAG
CAATTTTGAGGATAGTCAAGTTTATATAAATTTCAAAAATAATGTTTTAAACGATAAATA
TCTAATTGTAATAGAAACATTGGACGAAAATAGTGGCAACATTAATCATTTGATTTCTAA
AGAATTAAGAGATAAATATGAATGGAACCCTAGTTTACTATCTAAAATTGACAACTACCT
TAAACCAGCAATCGAGATTAAAGAGCATAATTTTAGTGATAATTCCTTGAATATTAACGT
TACTTTTAATGCGTTATACATAATTAAAATAGGAAAAAAATAA

> **sarX**
Function: protein coding sequence; HTH-type transcript regulator sarX (core genome, constant)
Best match: sarX_CC001_MW2-USA400_BA000033.2[715773:716198] (completely identical)
Position: 020-contig_263 RC: 39981 … 40407; Length: 426 bp
Sequence:
ATGGCAAAAAAATTTAAGATAATAATGACAGAAGCATTGTCTTTATATATTTGGGGGTGC
AACATTTTGAATACTGAGAAATTAGAAACATTGCTTGGCTTCTATAAACAATATAAAGCA
TTATCTGAATATATTGATAAAAAATATAAGTTGTCGCTAAATGATTTAGCAGTCTTAGATT
TAACGATGAAGCATTGCAAAGATGAAAAAGTACTTATGCAATCATTTTTAAAAACTGCAA
TGGATGAGCTAGATTTAAGTAGGACAAAATTATTAGTTTCTATAAGAAGACTAATTGAAA
AAGAAAGACTTAGTAAAGTTAGATCATCTAAAGATGAGCGTAAAATTTATATTTATTTAA
ATAATGATGATATATCTAAATTTAATGCTTTATTTGAAGATGTAGAACAATTTTTAAATAT
TTAA

> **A5IQM5**
Function: protein coding sequence; putative protein (core genome, constant)
Best match: A5IQM5_CC008_NCTC8325_CP000253.1[666418:667062] (completely identical)
Position: 020-contig_263 RC: 42138 … 42783; Length: 645 bp
Sequence:
GTGAAAGTAAAGTATATAGATAAACGTCACTGGCGTCGCCTAATTGATAGGGAATACAC
AGAGGTAAAAGTTAATAATAATAGGTTTAAGGGTATTATAGGCTTAGTCACGATGAAAA
AGGTTCGTGATCCTTTAGAGGTGACGGTAGTTGGACAAAATATCATTGTCGCAGATGACA
ATTATAAATGGTTGCAAATACTACCTGAAAAGAAACGTTATAGTATAACTGTAATGTTTG
ATAATAAAGGCAATCCATTAGAATATTATTTTGATATAAATATCAAAAATATAACGCAAA
AAGGTAATGCGCGTACAGTAGATTTATGTTTAGATGTTTTAGCATTACCAAGTGGTGAAT
ATGAGTTGGTAGATGAAGATGATTTAATGTTTGCATTAGAAAGTGAGCAAATTACAAAAA
AGCAATTTCATGAAGCATATATGATTGCACATCAAATTATGGCAGAGTTAGAAAATGATT
TTAAAGGATTCCAAAAGAAAATCATGTACTGCTTTAATAAAATTAATGCAAAGGCTCAAA
AAAATCATCAAAAGCCACAAAATAAAACTAATATTGAAAAAAGCAAACAAATAAAGCCT
AAGCAATATAATCAAACTAAAAATCACCAACAACAAAAGAAAAACTAA

> **A5IQM6**
Function: protein coding sequence; transcript regulator/LysR family (core genome, constant)
Best match: A5IQM6_CC001_MW2-USA400_BA000033.2[718690:719556]
Position: 020-contig_263 RC: 42898 … 43765; Length: 867 bp
Sequence:
ATGAAGATTGAAGACTATCGTTTACTAATAACATTAGACGAAACGAAAACGTTACGTAAA
GCGGCTGAAATTTTATATATATCTCAACCTGCTGTTACACAAAGACTAAAAGCTATTGAA
AATGCTTTTGGAGTAGATATTTTTATCAGAACAAAAAAACAATTGATTACAACAACTGAA
GGAACAATGATTATTGAGCATGCCCGTGACATGTTGAAAAGAGAGCGATTATTTTTTGAC
AAAATGCAGGCACATATTGGTGAAGTGAATGGAACAATATCAATCGGGTGTTCTTCTTTG
ATTGGACAAACCTTACTTCCTGAAGTTTTGAGCCTATATAATGCCCAATTTCCTAATGTTG
AAATACAAGTGCAAGTTGGTTCAACTGAACAAATTAAAGCAAATCATAGAGATTATCATG
TTATGATAACTCGTGGAAATAAGGTAATGAATTTAGCTAACACACATTTATTTAATGATG
ATCATTATTTTATTTTTCCAAAAAATAGAAGAGATGATGTTACAAAGTTACCATTTATAGA

GTTTCAAGCTGATCCGATTTATATAAATCAAATAAAACAATGGTATAACGATAATTTAGA
ACAAGATTACCATGCAACTATTACAGTGGATCAAGTAGCAACTTGCAAAGAAATGTTGAT
TAGTGGTGTAGGTGTTACAATCTTGCCAGAAATTATGATGAAAAATATCAGCAAAGAACA
ATTTGAGTTTGAAAAAGTAGAAATTGATAATGAACCGCTGATTCGTTCGACATTTATGAG
TTATGATCCGAGCATGTTGCAATTGCCACAAGTTGATTCTTTTGTAAATCTCATGGCGAGC
TTTGTTGAACAACCAAAGGCGTAG

> **A8Z196**

Function: protein coding sequence; putative sugar efflux transporter (core genome, constant)
Best match: A8Z196_CC001_MW2-USA400_BA000033.2[719965:721185]
Position: 021-contig_267_RC: 116 … 1337; Length: 1221 bp
Sequence:
ATGGATATGTTTGCAGCGTTATTACAAATAAAGAATTATAAACTCTTTGTTGCTAATATGT
TTCTACTAGGTATGGGTATTGCGGTTACGGTCCCATATCTTGTTCTTTTTGCAACTAAAGA
TTTAGGTATGACAACAAATCAGTATGGATTACTTCTAGCATCTGCAGCGATTAGCCAGTTT
ACAGTAAATTCAATTATTGCTAGATTTTCGGATACGCATCACTTTAATAGAAAAATTATTA
TTATTCTCGCATTATTAATGGGTGCGCTTGGTTTTTCAATATACTTTTTTGTAGATACAATC
TGGTTATTCATATTACTATATGCGATTTTCCAAGGATTATTTGCACCAGCAATGCCCCAAC
TTTACGCATCTGCTAGAGAATCTATCAATGTTTCAAGCTCTAAAGATAGAGCTCAATTTGC
CAACACAGTATTACGTTCAATGTTCTCATTGGGCTTTTTATTTGGTCCATTTATTGGTGCCC
AATTAATCGGATTAAAAGGCTATGCTGGATTGTTTGGTGGAACAATAAGTATCATTTTATT
TACTTTAGTACTTCAAGTGTTTTTCTATAAGGATTTAAACATTAAACACCCTATTAGTACG
CAACAACATGTTGAAAAAATTGCTCCTAATATGTTTAAAGACAAAACGCTTTTATTACCA
TTTATTGCATTTATTTTATTACACATTGGACAATGGATGTATACGATGAATATGCCTTTAT
TTGTTACTGATTATTTAAAAGAAAATGAGCAACATGTCGGTTATTTAGCTAGTTTATGTGC
TGGTTTAGAAGTGCCATTTATGATCATTCTTGGCGTTTTATCATCTAGATTACAGACTCGA
ACATTGTTGATTTATGGAGCGATTTTTGGTGGTTTATTCTACTTCAGCATTGGGGTATTTA
AAAACTTCTATATGATGTTAGCAGGACAGGTGTTTTTAGCTATTTTCTTAGCGGTTCTTTT
AGGAATTGGTATTAGTTATTTCCAAGATATCTTACCAGATTTTCCAGGATACGCCTCAACA
CTATTTTCTAATGCAATGGTTATTGGACAGTTAGGCGGTAACCTATTAGGTGGTGCTATGA
GTCACTGGGTAGGTTTGGAAAATGTATTTTTTGTATCAGCAGCATCAATCATGTTAGGTAT
GATACTTATATTCTTTACTAAAAATCAAAAAATTACAAAAGAGGATGTGATATCAACATG
A

> **Q2YSN2**

Function: protein coding sequence; putative membrane protein (core genome, constant)
Best match: Q2YSN2_CC005_JH1_CP000736.1[767471:767958]
Position: 021-contig_267_RC: 1333 … 1822; Length: 489 bp
Sequence:
ATGACAATTATTTTATGGCTACTTATCATCGCTGCCTTCATGTTAGCATTTGTTGGGTTGAT
TAAGCCGATTATTCCTTCTGTTTTAGTATTATGGGTTGGCTTTCTAATCTATCAATTTGGCT
TTCATAATCAGCATTTATCATGGGTGTTTTATGTATCTATGACATTGCTAACAATACTAAT
TTTATGTGCGGACTTTTTAGCTAATAAATATTTTGTGAATCGCTTCGGTGGTTCTAAGTTT
GGAGAGTATGCAGCTTTAATTGGTGTGGTTATTGGATGTTTTGTTTTACCGCCATTTGGAA
TTATTATTATACCTTTTATTTTGGTATTCATAGTTGAATTAATACAAGGCTATTCATTTGAA
AGAGCAGTTAAAGTAAGTATAGGTTCAATCGTAGCATTTTTAACAAGTAGTATAGCTCAA
GCAATCATTATGTTTATAATGATTGTATGGTTCTTTATAGATGCTTTATTGATTAATTAA

> **Q2YSQ6**

Function: protein coding sequence; putative uncharacterized protein (core genome, constant)
Best match: Q2YSQ6_CC001_MW2-USA400_BA000033.2[721769:722458:r]RC
Position: 021-contig_267_RC: 1920 … 2610; Length: 690 bp
Sequence:
TTATAAAAATGTATGAGAATGTTTTTTCGAAATATTTCTTTCAATGCGTAATCCAATAGGT
ATAACTATAAATGAAAATATAATAAATACCCAATTGCTTACCTGAATATATGGTCCAAAG

GCAAGTAATGATTGTGGAATAAAGAATACATAGAAAAATCCTACAATTAGTAGAATGAT
TGCTAAATAGGTGAATATCCATATCCAATTTGAATTGTTAAAACATTGCAGTTGTATTGTT
TTAAAACTAAACCATATAAATAAAAAGACAATAAAGAATCCGACGACTACTGAAAACGG
GAATGAAACAAAATATAAATTACTTCCATTTTTTTCCATGAAAAATCCTAAAAATCCTTTG
AGAAAACTAACAATCCCAATTAATAGAACGATGTGTTGATAGATATATTTAAAAATATTT
TTAAATGTTTCATTAGGCATCGCTTTTAGTTCTTTTATTGCATGTGCTTTTGGGTCGTGATT
GAAAAAATCTAAGGCTAATAAACCATGTTGTTCTGCGCTTAATAATTGTTTGAGTATACG
GTTAATAATTAACTCTGTATCATGAGGGTTGACGCGAAAGTCAGAGCGCATATAAGTCAT
ATAATTCTCGAAGATTTCTCTATCAGTATTGCTTAATCTTAATGATTTAACATTATTTTCTT
TTGTTAATTGCGCAGTACTTTTCAT

> **A5IQN0**

Function: protein coding sequence; putative acetyltransferase (core genome, constant)
Best match: A5IQN0_CC001_MW2-USA400_BA000033.2[722585:723028]
Position: 021-contig_267_RC: 2736 … 3180; Length: 444 bp
Sequence:
ATGAGGACACTTAATAAAGATGAACATAATTATATCAAGCAAATAGCTAATATACATGA
GACATTATTGTCGCAAGTAGAATCCAACTATAAATGTACTAAACTGAGTATTGCTCTTAG
GTACGAGATGATATGTTCAAGATTAGAACATACAAATGATAAAATTTATATATATGAAAA
TGAAGGTCAATTAATAGCGTTTATTTGGGGACATTTTAGTAATGAAAAAAGTATGGTTAA
CATTGAACTGCTATATGTTGAACCACAATTTCGCAAACTAGGAATAGCTACGCAACTGAA
GATTGCGCTTGAAAAATGGGCAAAAACTATGAATGCAAAGCAAATAAGCAGTACAATTC
ATAAAAATAATTTGCCAATGATATCTTTGAATAAAGATTTAGGTTATCAAGTGAGTCATG
TGAAAATGTATAAAGATATTGATTAG

> **A8YZU8**

Function: protein coding sequence; putative lipoprotein (core genome, variable)
Best match: A8YZU8_CC007_USA300_TCH959_AASB02000218.1[115805:116200] (completely identical)
Position: 021-contig_267_RC: 3246 … 3642; Length: 396 bp
Sequence:
ATGAAGAAATTAATCATCAGTATTATGGCGGTCATGCTATTTTTAACAGGTTGTGGTAAA
AGTCAAGAGAAAGCCACTCTGGAAAAGGATATCGATAATTTACAAAAAGAAAATAAAGA
ATTAAAAGACAAAAAAGAAAAGCTTCAACAAGAAAAAGAAAAATTAGCAGATAAGCAA
AAAGACCTTGAAAAAGAAGTGAAAGATTTAAAACCATCAAAAGAAGATAACAAGGATG
ATAAAAAAGACGAAGACAAAAAATAAAGACAAAGATAAAGATAAAGAGGCATCACAAGA
TAAGCAATCAAAAGATCAAACTAAGTCATCGGATAAAGATAATCACAAAAAGCCTACAT
CAGCAGATAAAGATCAAAAAGCTAATGACAAACACCAATCATAA

> **rimL**

Function: protein coding sequence; acetyltransferase/GNAT family (core genome, variable)
Best match: rimL_CC001-ST772_118_AJGE01000051.1[4090:4632] (completely identical)
Position: 021-contig_267_RC: 4158 … 4701; Length: 543 bp
Sequence:
ATGATTTATTGTGAAACAGAGCGTTTAATATTAAGAGACTGGCATGAAGATGATCTGTTA
CCTTTTTAAAAAATGAATGCGAATTATGACGTACGTAAATATTTTCCAAGTTTATTGAGTT
ATCGTCGTTCAGAATTAGATATGAGAACTATGGATGCGGTTATTAAAGATTATGGCATTG
GATTATTTGCTGTAGAAGATAAAGAGTCACATCAATGGATAGGCTTTATAGGTTTGAATT
ATATTCCAGAAACAAGCGATTATCCATTTAAAGAATTACCGCTTTATGAAATAGGTTGGC
GCTTGTTGCCAGAATTTTGGGGAAAAGGATTAGCAACTGAAGGCGCAAAGGCAACTTTG
AAGTTAGCAGAAGAACATCAAATATACGATGTCTATAGTTTTACAGCAGAAGCAAATAA
AGCTTCACAACGTGTAATGGAAAAAATTGGCATGACAGTGTATGATCATTTCGAATTACC
CAATCTAAGTAAGTATCATTTATTAAAAAGGCAAGTACGCTATTACATTAATCTTCGAAA
GTGA

> **uppP**

Function: protein coding sequence; undecaprenyl-diphosphatase (core genome, constant)
Best match: uppP_CC008_NCTC8325_CP000253.1[674809:675684:r]RC (completely identical)
Position: 021-contig_267_RC: 6680 … 7556; Length: 876 bp
Sequence:
TTAAATACCTTTACCAATGCCAAATCCGAAGTAAAGTATAGCAATAAAGATTACTAATAC
AATTCTGTAAATGGCAAATGGAATTAGTTTGATTTTGTTAATTAGATGCAAGAATGTTTTG
ATTGCAATTAGTCCAACAGTAAATGCAGCTAAAAAGCCTAAAATATAAAAAGGTATATC
AGCAATCTGAATATCTTGATAATGTTTTAATAAAGATAAACCACTAGCTGCTAACATAAT
TGGAACAGCCATAATAAATGTAAAGTCCGATGCTGCTTTATGATTTAATTTCATTAATACC
CCAGTTGAAATTGTTGAGCCTGAACGGCTGAAACCAGGCCACATAGCTACTGCTTGAGAA
ATACCAATTACAAATGCTTGGAAATAACTGATTTGATCTACTGTTTGTGGGTTTTTAACTT
TAGCTGAGTATTTATCAGCAATAATCATATAGATAGCACCTACGAATAAGCCAATCATAA
CAGTTGGCACACTAAATAAATGTTCTTCGATGAAATCATCAAATAGTAAGCCTAAAATAC
CTGCTGGCACCATACCCACTAATACATGTAATAAATTTAAACGTCTTGGCTTTGAACGTCT
TTGTTGATCGTTATCTCCTTCAACATGTTTGTGTTTACCAATATGTAAAATCTCTAAGAAG
CGTTCGCGGAACACCCATGCTGCTGCAAAGACGGATCCTAATTGGATGACGATTTTAAAT
GTAAATGCTGACTGAGAACCTAAAAATTCAGATGATTTTAACCACATATCATCAACTAGG
ATCATATGTCCAGTAGAGGAAACAGGTGCAAATTCTGTTAATCCTTCGACGACCCCTAAG
ATAATACCTTTTATTAATTCAATGATAAACAT

> **graA**

Function: protein coding sequence; ATP binding cassette transporter
Best match: graA_CC001_MW2-USA400_BA000033.2[727623:729254]
Position: 021-contig_267_RC: 7774 … 9406; Length: 1632 bp
Sequence:
GTGAAAAAATTAACAACAATACTGTTTCAATATAAAATTTTTCCGGTACTCATGTTCTTGG
TCAGTACTGGTCTCGGCATACTCGTTATAACGCAAAATATTTTAATAGCAGATTTTTTAGC
TAAAATTATAAGACATCAATTTCAAGGTTTATGGATTGTATTATTTATTTTATTAGGTGTT
TTACTTTTAAGAGCAACTGTGCAATTTCTAAATCAATGGTTAGGTGATACATTAGCATTTA
AAGTTAAGCATATGCTTAGACAGCGGGTTATTTATAAAAATAATGGTCATCCAATCGGTG
AACAAATGACTATACTCACAGAAAACATTGATGGTCTAGCACCTTTTTATAAGAGTTATT
TGCCTCAAGTGTTCAAATCAATGATGGTTCCGCTCATCATAATCATTGCAATGTTTTTCAT
CCATTTCAATACCGCATTAATTATGTTAATAACTGCACCATTTATTCCTTTGTTTTATATTA
TTTTCGGTTTGAAAACGCGAGATGAGTCAAAAGATCAAATGACTTATTTGAATCAATTTA
GTCAACGGTTTTTAAATATTGCTAAAGGTTTAGTGACGTTAAAGCTATTTAATCGTACAGA
GCAAACAGAGAAGCATATTTACGACGATAGTACTCAGTTTAGAACTTTAACAATGCGCAT
TTTACGCAGTGCTTTTTTATCGGGATTAATGCTCGAATTTATAAGTATGTTAGGTATTGGA
TTGGTTGCATTGGAAGCAACGCTAAGCTTAGTAGTATTTCATAATATTGATTTTAAAACTG
CGGCAATTGCGATTATTTTAGCGCCTGAATTTTATAATGCAATTAAGGACTTAGGGCAAG
CGTTCCATACTGGAAAACAAAGTGAAGGTGCCAGTGACGTTGTGTTTGAGTTTTTAGAAC
AACCGAACTATAATAATGAATTTCTATTAAAGTATGAGGAAAACCAAAAGCCATTTATTC
AGTTAACAGACATATCATTTCGATATGATGATTCTGATAGATTGGTATTAAATGATTTAAA
TTTGGAAATATTTAAAGGTGATCAAATTGCACTTGTAGGTCCAAGCGGGGCAGGTAAATC
CACTTTGACACATCTTATTGCAGGTGTTTATCAGCCAACAATAGGTACTATAAGTACAAA
CCAGCGTGATTTAAATATAGGAATACTTAGTCAACAGCCATATATTTTCAGTGCTTCTATA
AAAGAGAATATTACGATGTTTAAAGATATAGAAAATAATACTATTGAAGAAGTGCTAGA
CGAAGTAGGTTTATTAGACAAAGTGCAATCTTTCACAAAAGGCATTAACACAATAATAGG
TGAAGGAGGCGAAATGTTATCTGGTGGACAGATGAGACGCATAGAACTTTGCCGTCTTTT
AGTTATGAAGCCAGATCTCGTTATATTTGATGAGCCTGCAACTGGTTTAGATATTCAAAC
AGAACACATGATTCAGAACGTTCTGTTTCAACATTTTAAAGATACAACGATGATTGTCAT
TGCACATAGAGATAATACAATTCGCCATTTACAACGACGCTTGTATATAGAAAATGGAAG
ACTGATTGCTGATGATCGCAATATTTCAGTAAATATAACAGAAAATGGTGATGACTTATG
A

> **A6TZG3**

Function: protein coding sequence; ABC transporter ATP-binding protein/MsbA family (core genome, constant)
Best match: A6TZG3_CC001_MW2-USA400_BA000033.2[729251:730924]
Position: 021-contig_267_RC: 9402 … 11076; Length: 1674 bp
Sequence:
ATGAAAACACGACTAAAATTTCAAGTAGATAAGGATTTATTGTTAGCTATAGTTGTTGGT
GTTTGTGGAAGTTTAGTTGCGCTCGCCATGTTTTTCTTAAGTGGTTATATGGTGACACAAA
GTGCACTTGGTGCGCCACTATACGCTCTGATGATTTTAGTCGTAACAGTAAAATTGTTTGG
GTTTTTAAGAGCTATTACTCGATACGTAGAGCGCCTTATTTCTCATAAAGCTACATTTACA
ATGCTACGTGATATTCGGGTACAGTTTTTCGGTAAATTAGTAAATGTCATTCCTAATGTTT
ACCGTAAACTGAGTTCTAGTGATTTAATTTCACGTATGATTAGTCGTGTTGAGGCATTACA
AAATATATATTTACGTGTTTATTATCCACCAGTCGTCATCGGTTTGACAGCGCTAGTTACA
GTCATAGTTTTGGCGTTCATTTCAATCGGCCATGCGCTATTGATTATGGTTAGCATGTTGT
TTACTTTACTCATTGTTCCTTGGTTAAGCTCAAAAAAAGCACGTACTTTAAAGAAACATGC
AGCTAATGAACAGGCCCGATTTTTAAATCATTTTTATGATTATAAAGCTGGTATGGATGA
ACTACGTCGATTTAATCAAATTAATCATTATCGAGATAATTTGATGGCTAAATTAAATCAT
TTTGATAAATTACAACTTAAAGAGCAACGCTTTTTAACGATTTATGATTTTATATTAAATA
TTATTGCTATGCTTTCGATTTTTGGTAGTTTAGTTCTAGGATTAATTCAAATTAATGCAGG
CCAACTAAATATTATTTATATGACGAGTATAGTTTTAATGGTCTTAACTTTATTTGAACAA
GCTGTACCAATGACAAATGTCGCGTATTATAAAGCGGATACTGACCAAGCATTGCACGAT
ATTAATGAAGTGATATCTGTACCTTCTACTAATGGAAAAAAACGTCTTAATGATAAGTAT
GATGCAACGAACATTTATGAAGTTAAGGATGCTAGTTTTAAGTATTGGAATCAGCAAACG
TATGTGTTGTCGGATATTAATTTTAATGTTAATAGAGGCGAAAAGATTGCGATTGTGGGT
CCTTCTGGTTCAGGAAAAAGTACATTACTACAAATTATGGCAGGGTTATATCAATTAGAT
AGTGGCTCTGTTCGTTTCGAAAATATGGATATGTTTGAAATAGATGACAAAGATAAGTTT
GAATCGTTAAATGTCTTGCTACAATCTCAACAATTATTTGATGGTACAATACGTCAAAATT
TATTTACCGATGAAAAAGATGAAGCGGTGCAAGCAATATTTAAGCAATTAGATTTAGAAC
ATTTGGCACTAGAACGTCAAATTGACTTAGATGGTCATACATTATCTGGCGGAGAAATTC
AGCGTTTAGCGATTACGAGGATGTTATTAAAAGATACTGCATCAACATGGATTTTAGATG
AACCAACAACTGCATTAGATAAACAAAATAGTTTAAAAGTTATGGATTTAATTGAAGCAC
ATGCAGAAACATTAATTGTTGCTACACACGATTTAACTTTATTGTCACGTTTTGAGACCAT
CATTGTGATGATAAATGGTAAAATAGTTGAAAAGGGAAACTATCAACAATTACTCGCTAA
TCAAGGTGCTTTATGGAATATGATTCAATATAATGCATAA

> **mgrA**

Function: protein coding sequence; regulator of autolytic activity (core genome, constant)
Best match: mgrA_CC001_MW2-USA400_BA000033.2[731051:731494:r]RC (completely identical)
Position: 021-contig_267_RC: 11202 … 11646; Length: 444 bp
Sequence:
TTATTTTTCCTTTGTTTCATCAAATGCATGAATGACTTTACCTAATAAGCGATTAAGTTCTT
TAACTTCATCTTGAGATAAAGAAGAAGCTGAAGCGACTTTGTCAGATGCATTACTTAATT
CTGGTCTAATAGTTTCACTTTTGTCAGTCAAGTGAATAAATACTTCACGTTGATCGACTTC
GGAACGTTCACGCTTAATTAAGTCTACTTGTTCCATTCGTTTTAATAATGGTGATACTGTA
CCAGTATCGAGTGCTAATTCAGTTACGACTTTCTTGACGTTTACAGGAGATTCATCCCATA
AAATTGTTAAGACAAGAAATTGTGGGTATGTTAGATTGTACTTCTTAAAAACTTTGTTAG
AGTAGTAGCGATTAACTTGTCTTTGAGCATTGTACAAACTAAAGCATAGCTGTTCTTTTAA
ATTATGTTGATCAGACAT

> **cobW2**

Function: protein coding sequence; cobalamin (vitamin B12) biosynthesis protein
Best match: cobW2_CC007_TCH959-USA300_AASB02000218.1[124431:125357] (completely identical)
Position: 021-contig_267_RC: 11872 … 12799; Length: 927 bp
Sequence:

ATGAAAAATAATAAAGATGAAAAAATAAGAATATCCATAATTAACGGATTTTTGGGTAG
TGGTAAAACCACGTTACTGACACATTATATTAGTGAATTATTAAAAAATGATGAGAAAAT
TAAAATCATCATGAATGAATTCGGTACTTTTGATATTGATAGCAATAGTATTTCAAATGA
AATTGAAGTCCATTCATTGATTAATGGTTGTGTTTGTTGCGATCTTAAACAAGAACTTGTC
TATGAACTAAAAGCCATTGCTTTAAAAGGGGACGTTAATCATGTCATCATAGAAGCGACA
GGCATTGCGCATCCTTTGGAATTACTAGTTGCATGTCAAGATCCGCAAATCGTTAATTTCT
TTGAAAAGCCGATTATTTATGGTGTATTAGATGCGACTCGATTTTTAGAACGTCATCAATA
TACCGAAAATACAGTTTCGCTGATGGAAGATCAGTTGAAACTAAGTGACATGATTATTAT
TAATAAAATTGATCTTATAACTGATGACAGTCTTGAGAAAATTGATAAGCAATTAGGTAT
GATTTGTGCAAGTATTCCAACTTATAAAACAACCTATGGAAAAGTTTCGTTGGAAGAATT
GGACTTAACTGTTAAAGACAGAGAGATATCGTCTCATCATCACCATCATCATGGGATTAA
AAGTATGACTTACACGTTTACAGGTCCGATTGATCGTCAATTGTTTTATCAATTTATAATG
AAATTACCGGAATCTGTTCTACGTTTGAAAGGTTATGTGTCATTTAGAGATCAACCAAAT
GCAATTTATGAATTTCAATATGCATATGGTTTACCAGACTATGGAATAATTGGCATGCAA
TTACCATTAACGATTGTTATTATTGGTGAAACTTTAGATACAAATCACATACGTAATCAAT
TGGATATGCTACAATTTACGTAA

> **phrB**

Function: protein coding sequence; deoxyribodipyrimidine photo-lyase
Best match: phrB_CC001_MSSA476_BX571857.1[734330:735703]
Position: 021-contig_267_RC: 16053 … 16203; Length: 150 bp
Sequence:
ATGGCAATTGCTGTGTTATTAAATCGAATGTTTCGAATGGAACACAATCCATTATTTGAAT
ATATTTATCAACAAAAAGAAGACATTGATGCATGTTATTTTATCATTCCGGAAGAGGACA
TGTCTTCAGCTTCTGATTTGAAAGCACAG

> **Q2G0A5**

Function: protein coding sequence; putative membrane protein (core genome, variable)
Best match: Q2G0A5_CC001_MSSA476_BX571857.1[735858:736142:r]RC
Position: 021-contig_267_RC: 17581 … 17866; Length: 285 bp
Sequence:
TTATCTTAGTATGTCCGTAAATAAAGTGAGGTATAGTACGACATACTCTAAAAACGTAGT
GAGATAAATATATTTCAATCTAACTTTTATGTTTTGAGGCACTTGCCATTTAGGATATTGT
CGTTCGTAATACGACACTTGTTGTATAAATACACCTAGTCCAAATGGCAGCATCATGAGT
AAGATACTTCTTAAATAACTTAAACCAATATCATACCATATGTGTCCAATAATCAATTGA
AAGCCAATGATAGATACTATTAAAACGATTATATTTATTGTCAC

> **norA**

Function: protein coding sequence; multidrug efflux pump (core genome, variable)
Best match: norA_CC001-ST772_118_AJGE01000051.1[19228:20394] (completely identical)
Position: 021-contig_267_RC: 19296 … 20463; Length: 1167 bp
Sequence:
ATGAATAAACAGATTTTTGTCTTATATTTTAATATTTTCTTGATTTTTTTAGGTATCGGTTT
AGTAATACCAGTCTTACCTGTTTATTTAAAAGATTTGGGATTAACTGGTAGTGATTTAGGA
TTACTAGTTGCTGCTTTTGCGTTATCTCAAATGATTATATCGCCGTTTGGTGGTACGCTAG
CTGACAAATTAGGGAAGAAATTAATTATATGTATAGGATTAATTTTGTTTTCAGTGTCAG
AATTTATGTTTGCAGTTGGCCACAATTTTTCGGTATTGATGTTATCGAGAGTGATTGGTGG
TATGAGTGCTGGTATGGTAATGCCTGGTGTGACAGGTTTAATAGCTGACATTTCACCAAG
CCATCAAAAAGCAAAAAACTTTGGCTACATGTCAGCGATTATCAATTCTGGATTCATTTT
AGGACCAGGGATTGGTGGATTTATGGCAGAAGTTTCACATCGTATGCCATTTTACTTTGC
AGGAGCATTAGGTATTCTAGCATTTATAATGTCAATTGTATTGATTCACGATCCGAAAAA
GTCTACGACAAGTGGTTTCCAAAAGTTAGAGCCACAATTGCTAACGAAAATTAACTGGAA
AGTGTTTATTACACCAGTTATTTTAACACTTGTATTATCGTTTGGTTTATCTGCATTTGAAA
CATTGTATTCACTATACACAGCTGACAAGGTAAATTATTCACCTAAAGATATTTCGATTGC
TATTACGGGTGGCGGTATATTTGGGGCACTTTTCCAAATCTATTTCTTCGATAAATTTATG

AAGTATTTCTCAGAGTTAACATTTATAGCTTGGTCATTATTATATTCAGTTGTTGTCTTAAT
ATTATTAGTTTTTGCTAATGGCTATTGGTCAATAATGTTAATCAGTTTTGTTGTCTTCATAG
GTTTCGATATGATACGACCAGCCATTACAAATTATTTTTCTAATATTGCTGGAGAAAGGC
AAGGCTTTGCAGGCGGATTGAACTCGACATTCACTAGTATGGGTAATTTCATAGGTCCTTT
AATCGCAGGTGCGTTATTTGATGTACACATTGAAGCACCAATTTATATGGCTATAGGTGTT
TCATTAGCAGGTGTTGTTATTGTTTTAATTGAAAAGCAACATAGAGCAAAATTGAAAGAA
CAAAATATGTAG

## > **ybaK**

Function: protein coding sequence; transcript regulator (core genome, variable)
Best match: ybaK_CC080_11819-97_CP003194.1[786508:786990] (completely identical)
Position: 021-contig_267_RC: 21381 … 21864; Length: 483 bp
Sequence:
ATGGCTAAAAATAAGAAAACGAACGCGATGCGTATGCTTGATCGTGCAAAAATTAAATA
CGAAGTTCATAGCTTTGAGGTACCAGAAGAACATTTATCTGGTCAAGAAGTCGCAGAACT
CATACAAGCAAATGTTAAAACAGTATTTAAAACGCTTGTTCTAGAAAATACAAAACATGA
ACATTTTGTATTTGTTATCCCAGTAAGTGAAACTTTAGATATGAAAAAGGCAGCTGCTTTG
GTTGGAGAGAAGAAATTGCAGCTTATGCCTTTAGATAATTTGAAAAATGTAACGGGGTAC
ATTCGTGGTGGGTGTTCGCCTGTTGGTATGAAAACATTGTTTCCAACAGTCGTTGACAAAT
CGTGTGAAAATTATAGTCATATCAGTGTGAGTGGTGGGCTTCGAACAATGCAAATCACAA
TAGCTGTTGAGGATTTGATTACAATAACTAAAGGCAAAATTGGAGCAGTTATCCATGAAT
GA

## > **fruR**

Function: protein coding sequence; transcript regulator of fructose operon (core genome, constant)
Best match: fruR_CC001_MW2-USA400_BA000033.2[741964:742725]
Position: 021-contig_267_RC: 22116 … 22878; Length: 762 bp
Sequence:
ATGATAATTACAGAAAAAAGACACGAGTTAATTTTAGAAGAACTTTCGCACAAAGATTTT
TTGACTTTACAAGAATTAATAGATCGAACTGGGTGCAGTGCTTCAACAATACGAAGAGAT
TTATCTAAACTACAACAATTAGGGAAATTGCAACGTGTGCATGGTGGTGCAATGTTAAAA
GAAAATCGTATGGTTGAGGCGAATTTAACTGAAAAATTAGCAACGAATCTTGATGAAAA
GAAAATGATTGCTAAAATAGCAGCTAATCAAATCAACGATAATGAATGCTTATTTATCGA
TGCTGGTTCATCTACATTGGAGCTAATTAAATATATTCAAGCGAAAGATATCATTGTGGT
AACCAATGGTTTAACACATGTAGAAGCTTTACTTAAAAAAGGTATTAAAACAATTATGCT
AGGTGGTCAAGTTAAAGAAAATACACTTGCTACGATTGGTTCTAGTGCCATGGAGATATT
AAGACGATATTGTTTCGATAAAGCTTTTATCGGGATGAATGGATTAGATATTGAACTTGG
ATTAACTACTCCCGATGAGCAAGAGGCATTAGTTAAACAAACAGCAATGTCATTAGCCAA
TCAATCATTTGTACTTATTGATCATTCTAAGTTTAATAAAGTATATTTTGCTCGTGTACCTT
TGCTAGAAAGTACGACAATCATCACATCTGAAAAAGCATTAAATCAAGAATCGTTAAAA
GAATACCAACAAAAGTATCACTTTATAGGAGGGACTTTATGA

## > **fruB**

Function: protein coding sequence; fructose 1-phosphate kinase
Best match: fruB_CC001_MW2-USA400_BA000033.2[742722:743642]
Position: 021-contig_267_RC: 22874 … 23795; Length: 921 bp
Sequence:
ATGATTTATACAGTGACTTTCAATCCTTCAATTGACTATGTCATTTTTACGAATGATTTTA
AAATTGATGGTTTGAACAGAGCAACAGCAACATATAAATTCGCTGGGGGAAAGGTATT
AATGTCTCGCGCGTCTTAAAGACATTGGATGTTGAGTCAACTGCCTTGGGATTTGCAGGT
GGATTTCCTGGAGAATTCATTATAGATACATTAAATAACAGTGCAATTCAATCGAATTTT
ATTGAAGTTGATGAAGATACACGTATTAATGTGAAATTAAAAACAGGACAAGAAACAGA
AATCAATGCACCGGGTCCTCATATAACGTCAACACAATTTGAACAACTGTTACAACAAAT
TAAAAATACAACAAGCAAAGATATAGTTATTGTTGCTGGAAGTGTACCAAGTAGTATTCC
AAGCGATGCGTATGCGCAAATTGCACAAATTACAGCACAGACAGGTGCTAAATTAGTAG

TCGACGCTGAAAAAGAATTGGCTGAAAGCGTTTTACCATATCACCCACTATTTATTAAAC
CTAATAAAGATGAATTAGAAGTGATGTTTAATACAACAGTGAACTCAGACGCAGATGTTA
TTAAATATGGTCGTTTGTTAGTTGATAAAGGTGCGCAATCTGTTATTGTCTCGCTTGGCGG
TGATGGTGCTATTTATATTGATAAAGAAATCAGTATTAAAGCAGTTAATCCACAAGGGAA
AGTGGTTAATACAGTTGGCTCTGGTGATAGTACAGTTGCAGGCATGGTGGCTGGAATTGC
TTCAGGTTTAACGATTGAAAAAGCATTCCAACAAGCAGTCGCATGCGGTACTGCCACGGC
ATTTGATGAGGACTTAGCAACACGGGACGCTATAGAAAAAATAAAATCACAAGTTACGA
TTAGCGTACTTGATGGGGAGTGA

## > fruA

Function: protein coding sequence; fructose specific permease
Best match: fruA_CC001_MW2-USA400_BA000033.2[743648:745606]
Position: 021-contig_267_RC: 23800 … 25759; Length: 1959 bp
Sequence:
ATGAGAGTAACAGAGTTATTAACAAAAGATACGATAGCAATGGATTTAATGGCAAATGA
CAAAAATGGTGTTATTGATGAGTTAGTAAATCAATTAGACAAAGCAGGTAAATTAAGTGA
TGTCGCGTCATTTAAGGAAGCGATTCACAATCGAGAATCACAAAGTACAACTGGTATCGG
CGAAGGTATTGCCATTCCACATGCCAAAGTGGCCGCAGTTAAGTCACCAGCTATTGCGTT
TGGTAAATCTAAAGCAGGCGTAGATTATCAAAGTTTGGATATGCAACCAGCACACTTATT
CTTTATGATTGCAGCGCCAGAAGGTGGCGCCCAAACACATCTAGATGCTTTAGCTAAGTT
GTCTGGTATTTTAATGGATGAAAATGTACGTGAGAAATTATTACATGCTTCATCACCTGA
AGAAGTACTAGCGATCATAGATGAGGCTGATGATGAAGTGACAAAAGAAGAAGAGGCA
GAAGCTGAAGCACAACAAGTTGCAACTGCAGAACAATCATCTAAACAATCTAATGAGCC
ATATGTGTTAGCAGTAACTGCTTGTCCAACAGGTATTGCACACACATATATGGCACGTGA
TGCATTGAAAAAGCAAGCGGATAAAATGGGTATTAAAATTAAAGTTGAAACGAACGGTT
CAAGCGGCATTAAAAACCATTTAACTGAACAAGATATTGAAAATGCAACAGGTATCATTG
TTGCTGCTGATGTTCATGTTGAGACGGATCGCTTCGATGGTAAAAATGTCGTAGAAGTAC
CAGTAGCAGATGGTATTAAACGCCCAGAAGAATTAATTAATAAAGCATTAGATACAAGT
CGTAAACCTTTTGTTGCCCGTGATGGTCAAAGAAAAGGTAACTCAAATGACAGTCAAGAA
AAATTAAGCCCAGGTAAAGCTTTCTATAAACACTTAATGAACGGTGTTTCTAACATGTTG
CCACTTGTAATATCTGGTGGTATTTTAATGGCAATTGTATTTTTATTTGGAGCAAATTCAT
TTAATCCAAAAAGCTCAGAGTACAATGCGTTTGCAGAGCAGCTTTGGAACATAGGTAGTA
AAAGTGCATTCGCGTTAATCATTCCAATTTTATCTGGATTCATTGCACGTAGTATTGCGGA
TAAACCTGGTTTCGCTTCAGGTCTTGTAGGTGGTATGTTAGCAATTTCAGGTGGTTCAGGA
TTTATTGGTGGTATTATTGCAGGTTTCTTAGCAGGTTACTTAACACAAGGTGTTAAAGCGA
TGACACGTAAGTTACCACAAGCATTAGAGGGATTAAAACCAACATTAATTTACCCACTAT
TGACAGTAACAGCTACAGGCTTATTGATGATTTATGCCTTTAATCCACCAGCATCTTGGTT
AAATCATTTGTTATTAGATGGATTAAACAATTTATCAGGTTCTAATATTGTATTATTAGGT
TTAGTTATTGGCGCTATGATGGCGATTGATATGGGCGGTCCATTCAACAAAGCGGCATAT
GTTTTTGCAACAGGTGCGTTGATTGAAGGTAATGCAGCACCAATTACAGCTGCAATGATT
GGTGGTATGATTCCACCGTTAGCAATTGCGACAGCGATGTTAATTTTCAGACGTAAATTT
ACAAAAGAACAACGTGGTTCAATTATCCCTAACTATGTGATGGGTATGTCCTTTATTACA
GAAGGTGCGATTCCATTTGCAGCTGCCGATCCATTACGTGTTATTCCTTCAATGATGATTG
GTTCAGGTATAGGTGGCGCAATTGCTTTAGGCTTAGGTTCACGAATTACTGCGCCACATG
GTGGTATTATTGTAATTGTTGGTACGGATGGTGCACACTTACTTCAAACTCTTATTGCACT
TCTAGTTGGTACATTAGTTTCAGCATTAATTTACGGTTTAATCAAACCCAAGTTAACTGAA
ACAGAAATCGAAGCTTCAAAATCAATGGACGAGTAG

## > nagA

Function: protein coding sequence; N-acetylglucosamine-6-phosphate deacetylase
Best match: nagA_CC001_MW2-USA400_BA000033.2[745914:747095]
Position: 021-contig_267_RC: 26066 … 27248; Length: 1182 bp
Sequence:
GTGTCAGAATTAATTATATATAACGGCAAAGTTTATACTGAAGATGGCAAAATCGATAAT
GGTTACATTCATGTGAAAGATGGACAGATTGTTGCAATTGGAGAAGGGGATGATAAAGC

AGTAATTGATAATGATACGACAAATAAAATTCAAGTGATTGATGCTAAAGGTCATCATGT
ATTACCAGGTTTTATTGATATACATATTCATGGTGGTTATGGTCAAGATGCAATGGATGG
GTCATACGATGGCTTAAAATATCTATCCGAAAATTTGTTATCTGAAGGGACGACATCATA
CTTGGCCACTACAATGACGCAATCTACTGATAAAATAGATAAAGCACTTACAAATATTGC
TAAATATGAAGCGGAGCAAGATGTTCACAATGCAGCGGAAATTGTAGGTATACATTTAG
AAGGACCATTTATATCTGAAAATAAAGTTGGTGCTCAACATCCGCAATACGTTGTACGCC
CATTTATCGATAAAATTAAACATTTTCAAGAGACTGCTAACAGATTAATAAAGATTATGA
CGTTTGCACCTGAAGTTGAAGGTGCAAAAGAAGCGCTTGAAACGTATAAAGATGACATT
ATTTTTTCAATTGGTCATACAGTGGCAACATACGAAGAAGCAGTCGAAGCTGTTGAGCGA
GGAGCTAAACATGTCACGCATTTATATAATGCAGCGACGCCATTCCAGCATAGAGAACCA
GGTGTTTTTGGAGCAGCATGGTTGAATGATGCTCTACATACCGAAATGATTGTTGATGGC
ACACATTCTCATCCGGCATCGGTTGCTATTGCTTACCGTATGAAAGGTAATGAACGTTTTT
ATTTAATTACCGATGCAATGCGTGCAAAAGGTATGCCTGAAGGAGAATATGATTTGGGTG
GACAAAAAGTAACTGTTCAATCGCAACAAGCACGTCTTGCAAATGGTGCGCTTGCTGGTA
GTATTTTAAAAATGAATCATGGGTTACGTAACTTAATATCATTTACAGGTGATACATTAG
ATCATTTATGGCGAGTAACAAGTTTAAATCAAGCCATTGCATTAGGTATCGATGATAGAA
AAGGTAGTATTAAAGTAAATAAGGATGCAGATCTTGTTATTCTAGATGATGATATGAATG
TAAAATCTACAATAAAACAAGGTAAGGTTCACACATTTAGCTAA

## > **corC**

Function: protein coding sequence; similar to magnesium and cobalt efflux protein (core genome, constant)
Best match: corC_CC001_MSSA476_BX571857.1[745744:747093]
Position: 021-contig_267_RC: 27468 … 28818; Length: 1350 bp
Sequence:
TTGGAAACTTCGACCATAATTAGTTTGATTATATTTATTCTATTAATTGCATTAACCACTG
TATTTGTTGGTTCAGAATTTGCATTAGTAAAAATTAGAGCAACAAGAATTGAACAGCTAG
CAGATGAAGGAAATAAACCTGCTAAAATAGTAAAAAAGATGATTGCTAATCTAGATTATT
ATCTTTCTGCTTGTCAGTTAGGTATAACAGTAACATCTTTAGGGTTAGGTTGGCTTGGTGA
ACCAACGTTTGAAAAGCTATTACACCCAATATTTGAAGCAATCAATTTACCAACTGCATT
AACGACGACGATTTCGTTTGCAGTGTCATTTATAATCGTTACGTATTTGCATGTAGTACTT
GGTGAATTAGCGCCTAAATCTATAGCGATTCAACATACTGAAAAGCTTGCTTTAGTATAT
GCAAGACCATTGTTCTATTTCGGTAACATTATGAAACCATTGATTTGGCTGATGAATGGTT
CTGCACGTGTTATTATTAGAATGTTTGGTGTAAATCCTGATGCCCAAACTGATGCAATGTC
AGAAGAAGAAATCAAAATTATTATTAACAATAGTTATAATGGTGGAGAAATCAACCAAA
CTGAATTGGCATATATGCAAAATATCTTTTCATTCGATGAAAGACATGCAAAAGATATAA
TGGTACCTAGAACTCAAATGATTACACTAAATGAACCTTTTAATGTAGACGAATTACTAG
AAACAATAAAAGAACATCAATTTACGCGTTATCCAATTACTGATGATGGTGATAAAGACC
ACATTAAAGGATTTATTAACGTCAAAGAATTTTTAACTGAATACGCTTCTGGAAAAACGA
TTAAAATAGCAAACTATATACATGAGTTGCCAATGATTTCAGAGACAACACGTATCAGTG
ATGCATTAATTAGAATGCAACGTGAACATGTACATATGAGTCTTATTATAGATGAATATG
GTGGAACGGCAGGTATTTTAACGATGGAAGATATTTTAGAAGAAATCGTTGGGGAAATTC
GTGATGAATTTGATGATGATGAAGTGAATGATATCGTTAAAATTGATAATAAGACATTCC
AAGTAAATGGTAGAGTACTATTGGATGATTTAACTGAAGAGTTCGGTATAGAATTTGATG
ACTCTGAGGATATTGATACGATAGGTGGATGGTTACAATCTCGTAATACCAATTTACAAA
AAGATGATTACGTGGATACAACTTATGATCGCTGGGTTGTTTCAGAAATCGATAACCACC
AAATTATTTGGGTGATATTAAACTATGAATTTAATGAAGCGAGACCTACTATCGGACAGT
CTGATGAAGATGAAAAATCAGAATAG

## > **txbi_saeS**

Function:  bidirectional rho-independent terminator of saeS
Best match: txbi_saeS_CC001_MW2-USA400_BA000033.2[750836:750889:r]RC (completely identical)
Position: 021-contig_267_RC: 30988 … 31042; Length: 54 bp
Sequence:

AAATAAACAAAAAAAGAAGCCCTCATTAATGGGAGCTTCTTTTTAGTCTTTGCA

> **saeS**

Function: protein coding sequence; histidine protein kinase/sae locus (core genome, constant)
Best match: saeS_CC001-ST772_118_AJGE01000051.1[30997:32052:r]RC (completely identical)
Position: 021-contig_267_RC: 31065 … 32121; Length: 1056 bp
Sequence:
TTATGACGTAATGTCTAATTTGTGTAATGTTACAGTCATCGTAGTTCCTACATCTATATCA
CTGCTTACACTGATTTTTGCGTTATTTTGTTGCGCGAGTTCATTAGCTATATATAAGCCTA
ATCCAGAACCACCCGTTTTTGTATTACGAGAGTTTTCTACTCTGAATGTACGTTCGAATAT
ACGTTCTTGTAGTTCTGGTATAATGCCAATACCTTCATCGCTAATAGCAATGTCGATAGTA
TCTTGATCTTTGTTTTCACTAATATTAATATCAATGCGACTACCAACATTTGAAAATTTTA
GCGCATTATCAAGTAAGTTTGTTAAAATACGCTCAAGTGGCGTTCGATATTGATAAAATG
CATCAATTTCGCTACAGAAATTCACTTCTAATGTGCGGTTTTCATGTTTGATACGTTGCTC
ATATGGTTGCAATATTGATACAAGTAATTGGTCTAGTTGTATTAATTCTGGGGGATATGTT
TTACCTGTATTTAAAGTGATAATATGAGTCATATCATCAAATAATGTTGATAATCTGTTTG
CTTGTTTAATTAATATGTCGTATGACTCTTTAATCTCATGATCCTTAGTGATTATACCATCA
CGTAGTCCTTCAGAATATGAAATAATGCTTGCTAAAGGTGTTTTTAAATCATGGGCTAAG
TTTTGAATCAGTTCTGTTTTTTCTTGTTGTTCGGATTTAATTTGATTCATTTGTTGCGTAATT
TCAGAAGCCATTTTATTAAAAGATTGATTTAATTCATAAATTTCTTTTGGTGAATTAAACG
TTTTATCATTGCTTGCGTAATTTCCGTTAGCAAATTGCTTAGTTTTTATATTAAACTGCTTA
ATTTTTTGTATAAGTGGATTAATAAAAATACTACATATTAATAAGGTTAAACAGCTTGTA
ATTATTGTCGTTAAGGTCAAAGTTAGTGTCATATGGCCGTTAAACCACATTAAAATATAT
GCAATTGCTAAAATAGTTGAAGTTAATAGTATACTCGATACGACGCCAATAATGATTTGA
CTTCTAATTGATAACACCAT

> **saeR**

Function: protein coding sequence; response regulator/sae locus (core genome, constant)
Best match: saeR_CC001_MW2-USA400_BA000033.2[751968:752654:r]RC (completely identical)
Position: 021-contig_267_RC: 32120 … 32807; Length: 687 bp
Sequence:
TTATCGGCTCCTTTCAAATTTATATCCTAATCCCCATACAGTTGTGATGGTATATGTTGTA
AAGCTCTCTTTTTCTAATTTTTCTCTAATACGGTGTATATGGACATTCACGGTATTAGCATC
TTCGTAATAGTCATATCCCCAAACTTTTTCAAGTAATTCTGATTTAGAAATAACTTCATTT
TCTCTAGAAGCTAAATACCACAATAACTCAAATTCCTTAATACGCATAGGGACTTCGTGA
CCATTTACAGTCACAACTTTACTTAAGTTAATAAGTGTTAATTCATCAAACGACAGTTGTT
CAACTGGTTGATGATGGTATTTCTTCATTCTTGTAAGTAAATTATTAATACGTAAAACGAG
TTCCCTTGGACTAAATGGTTTTTTGACATAGTCATCTGCACCTAAAGTTAAGGCGTAAATG
GTATCATGTTCTTGTGTTTTGGCAGTTAAATAGATAAAGGGGATATCTAATTTTTGCCTTT
TCATTTCTTTGACAATGTCGTAACCATTAACTTCTGGCATCATGATATCAAGTACCATGAT
ATCAATATCATTTGATAGTAAAGAAATTGCTTCTTTACCGCTAGTTGTCGTTGTTACTTTG
TAACCTTCATATTCAAAATAGGTTTGACAAATGTCTACAATGTCTTGTTCATCATCCACGA
TCAGTAAGTGGGTCAT

> **saeQ**

Function: protein coding sequence; putative protein (core genome, constant)
Best match: saeQ_CC001_MW2-USA400_BA000033.2[752629:753102:r]RC (completely identical)
Position: 021-contig_267_RC: 32781 … 33255; Length: 474 bp
Sequence:
TCATCCACGATCAGTAAGTGGGTCATCTATTTTTTCACCTCTGTTCTTACGACCTCTAAAG
TAATTAATGATTTCTTTAAGTGAAATCTGTTTTAACAATGAGTGACTCATTAGTAAAAGGA
TAAAGAAAGTTAATTGAAGAGGATACGTAAATATCATATCTGCTAAGATATAATTTATCA
TAACAAAGGCTCCAAAGAAACTAGCAGCATATGCAAAAACTCCAAAAATTAAACCTAAT
CCAATTGCAATCTCTCCGAGTGGGACAACAATATCAAATAATGACGTCGTATGTGCAACT
ATATTTGCGAAAAACCACTTATACCACTCTGGTGAATCAGTATTGTTAGCGATGACTGGT

ACTAAACCTTTCAGCGTAAATCCGCCCGTTAATTTTTCGTAGCCTTGCATTAACATAACAA
TACCTGAACCCACACGAATGATAAATGTAACGAGTAGCAATAATTTATTCAT

> **saeP**
Function: protein coding sequence; putative lipoprotein (core genome, variable)
Best match: saeP_CC001_MW2-USA400_BA000033.2[753445:753885:r]RC (completely identical)
Position: 021-contig_267_RC: 33596 … 34037; Length: 441 bp
Sequence:
TTATTTTAATTTAGCGCCGCCGAAGATGACGTGAGCTTTTTTACAATAGATTGTTACTTCA
TCATATTTGCTTAAATCTACATTTTTAAGATCAAATGTTTGTTTTTCTTTATCGTAGTCAAC
CATTGCGATTTCTTTACCGTTTTTAATGTCGCCATTTTTTGTTAGGTAGACGTATAAATCTG
GACCTTTTGATGATTTGTAGTTAGTAAGCATTAATTTACCATTTTTAATCTCAGCTTTACCT
TCAACAGTTTCACCGTTTTTAGAACTGAATGTACCTGTTAGGTGTTTTGTTTTATCAGTTTT
AACATTGCTATCTTCTGACTTTGTTTTTTGTTCAGTTTTGTTACCTTGATCTTGTGAATTAG
AATTACCACAAGCGCCTAAAGTTAATACAGAGGCAACAGCACCAACTGCTAAAAAATAT
TTTGTATTCAT

> **queE**
Function: protein coding sequence; 7-cyano-7-deazaguanosine (PreQ) biosynthesis protein E
Best match: queE_CC008_132-ST8_ACOT01000023.1[78584:79297:r]RC
Position: 021-contig_267_RC: 34999 … 35713; Length: 714 bp
Sequence:
TTATACACCTTTTTTATTGCTCCAAAGTAATGTATGAAGTTGTGGTAACACATAAACGTGA
TTCATATCATTACTTTGCATAACTAAATCCACCAACTGCTCGTAGCGTTCTAACAACTTTT
CGGTATGATTATCTACGCTGTCTGATAAATATGGGTTACCAACTTGTAAATAGAAGGGAA
TATCTGGATAACGGTGGTGTATCATTTTGGCAAAATCATAATCTTTATCGTCGAATACAAC
TACTTTTAAGTTTAATGAGGAAGGTACGCATTGTGTAATCACTTCATCTAACTTTTTTAAA
TCAGGTGTCATAGTTGAACTTGGTGGTTTTGGACTAATCGTTAAATCATCAATTTGTGTCA
TCCAAGGTTGGAATTTACTGCCTTGTGTCTCCAGTGCGCTGAAAATACCTTTATCTTGAAA
TAAGTCAACTAACTCTTGGATACCTTTAATTAATGCTGGGTTACCACCAGAAATTGTAAC
GTGATTAAATAAATCGCCACCAATTCGTTTTAATTCATCATAAATTTCTTCAGCGGTCATG
AGTTTTATATCGCCTTTAGCACTACCATCCCAAGTAAATGCAGAATCACACCAGCTACAG
CGATAATCACATCCAGCTGTTCTCACAAACATCGTTTTTCTACCGATTACTCTTCCTTCAC
CCTGAATGGTTGGGCCGAATATTTCGAGTACAGGAATTTTAGCCAT

> **queD**
Function: protein coding sequence; queuosine biosynthesis protein D
Best match: queD_CC030_MRSA252_BX571856.1[798825:799244:r]RC
Position: 021-contig_267_RC: 35716 … 36136; Length: 420 bp
Sequence:
TTACACCTGTTCCTTTGGTCTAAATACGACATAACTTGTTGGTGTTTCTCTTACAAATACTT
GAATACATTTTGGTTGGTGTTCGAGCGATGCCAAATTTTCTTTAACAATTTGATAAATTGT
TTCCGCTACGATTTCAGTTGAAGGGATTTTGTTTTTAAAAGCAGGTAAGTTATTTAACAGT
TGATGGTCAAATTTACCGTGTATCATCTTTTTTAAATGGCTAAAGTTCACTAAAAAGCCAG
TGTCATCTAGTTTATCACCGACAATTGTTAAATTAACAAAGTAAGTATGACCATGGACAT
TTTGACAAATACCTGCTTCTTCACACGGAATGTGATGTGCAGCTGAAAAATTAAAATCTTT
ATTTAATTCGAATTGATATGGATGCGTTGTACTAGGATAGATTTGTTGTAACAT

> **queC**
Function: protein coding sequence; 7-cyano-7-deazaguanine synthase
Best match: queC_CC001_MW2-USA400_BA000033.2[755986:756654:r]RC (completely identical)
Position: 021-contig_267_RC: 36137 … 36806; Length: 669 bp
Sequence:
TTAAAGCGCTCCTTTACTTTCAAGATATTGATTTAGTCCACGTTGACGTAAATGACAAGCT
GGACATTCACCACAGCCATCCCCAATGATACCGTTATAGCATGTTAATGTTTTTGTACGAA

TATAATCTAAAACTTCGAGTTCATCACTTAATTTCCACGTTTCTGCTTTGTTTAACCACATT
AAAGGAGTATGAATGACAAAATCTTTGTCCATAGCTAGGCTTAATGTTACGTTCATTGAT
TTTATAAAACTATCGCGACAGTCTGGGTAGCCTGAAAAGTCTGTTTCACATACGCCTGTA
ATAATATGCTTAGCCCCAATTTGATAAGCTAGAGCGCCTGCAAACGACAAGAAAAGTAA
ATTTCTAGCTGGAACAAATGTATTAGGTATACCATCTTCATTATTAGTAATTTCCATATCA
TGTTGTGTTAATGCGTTTGGAGTAAGTTGTGATAATAATGACATATCTAAAACGTGATGTT
TCATTCCTTGATCTTGTGCAATTTGTTTTGCGACTTCAATTTCAGTATCATGTCTTTGGCCA
TAATTAAACGTTACGAGTTCAACTTCTTTGAAATGTTTTTTTGCATAAAAGAGACATGTTG
TACTGTCTTGACCACCACTAAAGACAACGATGGCTTTTTCATTATTTAATACACTTTCCAT

> **PreQ1_riboswitch**
Function: noncoding RNA PreQ1 riboswitch (core genome, constant)
Best match: PreQ1_riboswitch_CC001_MW2-USA400_BA000033.2[756824:756870:r]RC (completely identical)
Position: 021-contig_267_RC: 36975 … 37022; Length: 47 bp
Sequence:
CAATGTCTAGTTTTTTATAGAGGGTTTCAGCTAGGAACCTCTGATAT

> **pabA**
Function: protein coding sequence; putative para-aminobenzoate synthase/glutamine amidotransferase/component II (core genome, constant)
Best match: pabA_CC005_Mu50-VRSA_BA000017.4[785621:786214]
Position: 021-contig_267_RC: 37156 … 37750; Length: 594 bp
Sequence:
ATGATTCTAGTCATAGATAATAATGATTCATTTACATATAATTTAATAGACTATATTAAGA
CTCAAACGAAACTAACAGTTCAAGTTGTTGGTATTGATAATCTGCTGATAGAAGACGTCA
TTAATATGAAGCCAAAAGCAATTGTTATTTCACCTGGGCCGGGTAATCCGGATGATTATC
CTATCTTGAATGAAGTGTTAGAACAATTTTATCAGCGTGTACCTATACTAGGTGTATGTTT
AGGATTTCAATGTATCGTGTCTTATTTTGGTGGAAATATCATTCACGGCTATCATCCTGTA
CACGGACATACTACACAGTTACGCCATACCAATGAAGGTATTTTTCAAGGACTGCCTCAA
AATTTCAATGTAATGCGTTATCATTCATTAATTGCTGACGGAGCGACTTTTCCAAATTGCT
TAAAGATTACAGCAAAAAACGATGAAGCGATTATTATGGCATTTGAGCATATTAGATTTC
CGGTTTTTGGTGTGCAATATCATCCTGAATCTATTTTGAGTGAATACGGTTATCGACAAGT
TGAATTATTTTTATCGAAGGTAGGTGATTACTGTGAGAATAGAATATAA

> **pabB**
Function: protein coding sequence; anthranilate/para-aminobenzoate synthase component I (core genome, constant)
Best match: pabB_CC008_COL_CP000046.1[794399:795550]
Position: 021-contig_267_RC: 37733 … 38885; Length: 1152 bp
Sequence:
GTGAGAATAGAATATAATTATCGCTACTATTTAACTGAAAATGAATATAAGCAATACCAT
ATTCAATTAAAGGGATTTATAAAGAAGTATGTTGCTACTAAGTTGGCTGATGTGGGAGAA
GTGATACACTTTGCACAAGCGCAGCAACGACAAGGTAGATATGTCTCGTTATATTTAAGT
TACGAAGCGGCAAAGTATTTTAATCATGTTATGTGTACACATTCATTAGCTAAAGATGAT
ATTTATGCAGTAGCTTATAGTTTTGAAAAAGCGGAAAGCATAAATTCAACATATGAACAT
CAAACTTCTTATGTATCAAAGCATCATTTTTCATTTGTTGAATCTTCTGAGGTTATGATGA
CTAATATTAACCGTGTCCAACAAGCAATTGTTGAAGGCGAAACGTATCAAGTGAACTATA
CGGCGCGCTTAACAGATAACATTTATTATCCTATTAGTACTTTATATGAACGATTAACTCA
ATTTAGTAATGGTAATTATACTGCGTTATTACAAACAGATGAAATCCAAGTAGCGTCTAT
CTCACCAGAATTATTTTTTCAAAAAGGACAATTTAACAATGTCGATAACGTTATCATAAG
CAAACCGATGAAAGGGACAATGCCTAGAGGTAAAACGGAAGCTGAAGATCAACAGTATT
ATAAAACATTGCAAACTTCTTCGAAAGATCGTGCAGAAAATGTCATGATTGTTGATTTAC
TAAGAAACGATATAGGGAGAATATCACAGAGTGGCTCAATTAAGGTGTATAAACTATTTT
TTATTGAGGCATATAAAACTGTATTTCAAATGACTTCGATGGTAAGTGGAACTTTAAAAA

ATAATACAGACTTAACTCAAATTTTAACATCGTTATTTCCTTGTGGTTCGATTACAGGTGC
ACCGAAACTGAATACAATGAAATATATTAAACAATTAGAAAGTTCACCTCGTGGTATATA
CTGCGGAGCAATTGGACTATTACTTCCAACTGAAGATGATAAAATGATTTTTAATATTCC
GATTCGCACTATTGAGTATAAATATGGACAAGCGATTTATGGAGTCGGAGCAGGTATTAC
AATTGATTCTAAGCCAAAAGATGAAGTGAATGAATTTTACGCAAAAACCAAGATTTTGGA
GATGTTATAA

## > **pabC**

Function: protein coding sequence; 4-amino-4-deoxychorismate lyase (core genome, constant)
Best match: pabC_CC005_Mu50-VRSA_BA000017.4[787349:787957] (completely identical)
Position: 021-contig_267_RC: 38884 … 39493; Length: 609 bp
Sequence:
ATGCAATTATTTGAAACAATGAAAATTGATAATGGACATATCCCTAGACTTACTTATCAT
ACTAATCGCATAAAATGTTCTTCTGAGCGATTAAACTTTAAATTTGATGAACATGCATGG
CGAAATGAATTAAACGATGTAACAACAAAGTATCACAGTGGTCAATATAGACTTAAAAT
CGTATTAAATGCTGAAAGCAAATTTGAAACGATAGTGTCACCTTTACCTGAGAAAAGTAG
TTTTACAGCAAAATTTCAAGTGTTGCCCAAAGTAGTTAATCCAACTTTTATAAAAAATAA
AACGACAGAACGAAAGCATTTAGCACACAATCATGAAACAGATTTAATATTGCTAACTTC
AGAGGACGGCAAGGTCCTTGAATTTGATATTGGCAACATTGTCATTGAAGAGGATGGAA
AATGGTACACACCAAGTTATAAAGATGATTTCTTAAAAGGATGCATGCGTGATTATTTAA
TAGATAGTGACAAACTTGTTGAAAAAGACTTTAATAAAAACGAATTGATTTATAAATATC
ATAACAATGAGATACGTTTATTTTTGATAAATAGTTTACGAGAGGTTGCCGATGTCCACCT
TTGCCTTTAA

## > **tnpIS431-06**

Function: protein coding sequence; transposase for IS431 (mobile element)
Best match: tnpIS431-06_circ_S-epidermidis_GQ900381.1[43799:43807] (completely identical)
Position: 021-contig_267_RC: 39783 … 39792; Length: 9 bp
Sequence:
ATGAACTAT

## > **ltaS**

Function: protein coding sequence; glycerol phosphate lipoteichoic acid synthase (core genome, constant)
Best match: ltaS_CC008_NCTC8325_CP000253.1[710153:712093] (completely identical)
Position: 021-contig_267_RC: 42024 … 43965; Length: 1941 bp
Sequence:
ATGAGTTCACAAAAAAAGAAAATTAGTCTTTTTGCGTTCTTCTTATTAACCGTAATAACGA
TTACCTTGAAGACGTATTTTTCTTATTATGTTGATTTTTCTTTAGGTGTTAAAGGTTTAGTA
CAAAACTTAATATTATTGATGAATCCTTATAGTTTAGTAGCACTGGTTTTAAGTGTGTTCC
TATTCTTTAAAGGCAAAAAAGCATTTTGGTTCATGTTCATAGGCGGCTTCTTATTGACGTT
CCTATTATATGCCAATGTTGTGTACTTTAGATTCTTCTCTGATTTTTTAACGTTTAGTACTT
TAAACCAAGTAGGTAACGTAGAATCTATGGGTGGTGCGGTTAGTGCATCATTCAAATGGT
ATGACTTTGTTTATTTCATTGATACGTTAGTTTACTTATTCATTTTAATATTTAAAACAAAA
TGGTTAGACACAAAAGCATTTAGTAAGAAATTTGTTCCTGTCGTAATGGCGGCTTCAGTA
GCATTATTCTTCTTAAACTTAGCTTTTGCTGAAACTGACAGACCAGAATTATTAACACGTA
CATTTGACCATAAATATTTAGTGAAATATTTAGGACCTTATAACTTTACAGTATACGATGG
TGTTAAAACTATCGAAAATAATCAACAAAAAGCGCTAGCATCTGAAGATGACTTAACAA
AAGTATTAAATTATACGAAACAACGTCAAACAGAGCCTAACCCAGAATATTATGGGGTG
GCAAAGAAGAAAAATATTATTAAGATTCATTTAGAAAGTTTCCAAACCTTCTTAATTAAT
AAAAAGGTTAATGGTAAAGAAGTAACACCGTTTTTAAACAAATTATCAAGTGGGAAAGA
GCAATTCACATACTTCCCTAACTTTTTCCATCAAACAGGTCAAGGTAAAACATCTGACTCT
GAATTTACAATGGATAACAGTTTATACGGTTTACCGCAAGGTTCTGCCTTTTCATTAAAAG
GAGATAATACGTATCAGTCATTACCAGCAATTTTAGATCAAAAGCAAGGCTACAAATCTG
ATGTCATGCACGGTGACTATAAAACATTCTGGAACAGAGACCAAGTATATAAACACTTTG

GTATCGATAAATTCTATGATGCAACATACTATGACATGTCAGATAAAAACGTTGTAAACT
TAGGCTTGAAAGACAAAATTTTCTTTAAAGATTCTGCTAATTATCAAGCTAAGATGAAAT
CACCATTCTATTCTCATTTAATTACATTGACTAACCACTATCCATTCACATTAGATGAAAA
GGATGCAACTATTGAGAAATCAAACACAGGTGATGCAACAGTTGATGGTTATATTCAAAC
AGCACGTTATTTAGACGAAGCATTAGAAGAATATATTAATGACTTGAAGAAAAAAGGAT
TATATGACAATTCAGTGATTATGATTTATGGTGACCACTATGGTATCTCTGAAAACCATAA
CAATGCCATGGAAAAACTATTAGGTGAAAAAATCACACCGGCTAAATTTACAGATTTAAA
CAGAACTGGTTTCTGGATTAAAATCCCTGGTAAATCTGGTGGTATCAATAATGAATATGC
TGGTCAAGTCGATGTAATGCCAACAATTTTACATTTGGCTGGTATAGATACGAAGAACTA
TTTAATGTTCGGTACTGATTTATTCTCTAAAGGTCATAATCAAGTAGTTCCATTCAGAAAT
GGTGACTTTATAACAAAAGATTATAAATATGTTAATGGTAAGATTTATTCTAATAAAAAT
AATGAACTCATAACTACTCAACCAGCTGATTTCGAAAAGAATAAAAAGCAAGTTGAAAA
GGATCTCGAAATGAGTGACAACGTGCTTAATGGTGATTTGTTTAGATTCTACAAAAATCC
AGACTTCAAAAAGGTAAATCCTTCGAAGTATAAATATGAAACAGGACCTAAAGCAAACT
CTAAAAAATAA

## > **recQ1**

Function: protein coding sequence; ATP-dependent DNA helicase (core genome, variable)
Best match: recQ1_CC008_NCTC8325_CP000253.1[714259:716040] (completely identical)
Position: 021-contig_267_RC: 46130 … 47912; Length: 1782 bp
Sequence:
ATGATGCAACAAACATTATCGCATTACTTTGGGTATGAAACGTTTCGACCAGGACAAGAA
GAAATTATTAGCAAAGTATTAGACCATCGTAATGTGCTTGGTGTCTTACCAACTGGTGGA
GGTAAGTCTATATGCTATCAAGTACCAGGTTTATTGTTAGGTGGTACAACAATTGTAATA
AGTCCACTAATATCATTAATGAAAGATCAAGTGGATCAATTAAAAGCGATGGGAATTCAA
GCTGCTTTTTTAAATAGTAGTTTGACTCAAAAAGAGCAACAACGTATTGAAAAAGCATTA
TCAAATGGAGAAATTCAATTTTTGTATGTTGCACCAGAACGATTTGAAAACCGATATTTTT
TAAATATGCTTCAGCGTATAAAGATTCACTTAGTCGCGTTTGATGAAGCGCATTGTATTTC
TAAATGGGGTCATGATTTCAGGCCGAGTTACCAAAATGTTATTTCAAAAGTATTTACGTT
ACCTCAAGATTTTACAATAATAGCGTTGACAGCAACTGCCACGGTTGAAGTACAGCAAGA
TATTAGAGAAAAGTTAAATATCGCTCAAACTGATCAAATTAAAACGAGTACTAAGCGTAG
AAACTTAATTTTTAAAGTAAATCCTACTTATCAACGTCAAAAATTTATATTGGATTATATT
AAAACACACGATGAAGATGCAGGTATTATTTATTGTTCTACACGTAAGCAAGTTGAAGAG
CTTCAAGAAGCCTTAGAAAGTCAGAAAATTGAAAGTGTTATATATCATGCAGGTTTGAGC
AATAAAGAAAGAGAAGAAGCGCAGAATGATTTCTTATTTGATCGTGTTAAAGTAGTCGTT
GCTACAAATGCTTTTGGTATGGGTATTGATAAATCCAATGTACGCTTTGTTATTCATTATA
ATATGCCTGGAGATTTAGAATCTTATTATCAAGAAGCGGGTCGTGCAGGTCGTGACGGGT
TGAAAAGTGAATGTATTTTGTTATTTAGCGAACGCGATATCAATTTACACGAGTATTTTAT
AACAGTCTCTCAAGCTGATGATGACTATAAAGATAAAATGGGCGAAAAGTTAACTAAAA
TGATTCAATATACAAAAACAAAAAAATGTCTAGAAGCAACAATTGTCCATTATTTTGAAC
CGAATGAAAAATTAGAAGAATGTGAACAATGTAGTAATTGTGTTCAACAAGATAAATCA
TATAATATGACACAAGAAGCTAAGATGATTATTAGTTGCATCGCTCGTATGAAACAACAA
GAGAGTTATAGTGTTATCATTCAAGTGTTAAGAGGAGAGTCAACAGATTATATTAAGTAT
AAAGGTTATGACCAAATTTCAACCCATGGTTTAATGAAAGGTTACACAACATCAGAATTA
AGTCACTTAATAGATGAATTAAGATTCAAAGGGTTCTTAAATGAAAATGACGAAATATTA
ATGTGTGATACTTCAATTAAAAAATTACTCAGTAATGAAGTAGAAGTATTCACAACACCA
TTTAAGCAAAAAGCGACTGAAAAAGTATTTATAAATACGGTTGAAGGGGTTGACCGAGT
ATTATTCAGTCAGTTGGTAGAAGTTCGTAAAAAGTTAAGTGACAAATTAACGATAGCACC
TGTAAGTATATTTTCTGATTACACGTTGGAGGAATTTGCTAAACGTAAGCCTGCTTCGAAA
CAAGATATGATTAATATTGATGGCGTAGGTAGTTACAAATTAAAACATTATTGTCCAGCA
TTTTTAGAAACGATTCAAAATTATAAAGCCAAAGTATAG

## > **opuBA**

Function: protein coding sequence; osmoprotectant ABC transporter/ATP-binding protein (core genome, constant)

Best match: opuBA_CC008_NCTC8325_CP000253.1[716262:717239] (completely identical)
Position: 021-contig_267_RC: 48133 … 49111; Length: 978 bp
Sequence:
GTGATTAAGTTTAAAAATGTAACTAAGCGTTATGGCAAACATGTTGCTGTCGATAACATT
AGTTTCAATATTAATGAGGGTGAATTTTTTGTGCTAATTGGACCTTCAGGTTGTGGAAAAA
CTACGACATTAAAAATGATTAATCGACTCATTCACTTAAGTGAAGGTTATATTTATTTTAA
AGATAAACCAATAAGTGATTATCCAGTATACGAAATGCGTTGGGATATTGGATACGTATT
GCAGCAGATTGCATTATTCCCACATATGACAATCAAAGAAAATATTGCACAAGTGCCACA
AATGAAAAAGTGGAAAGAAAAAGATATAGATAAAAGAGTAGATGAATTACTTGAAATGG
TTGGATTAGAACCTGAAAAATATAAAAACAGAAAACCTGATGAATTGTCAGGGGGGCAA
CGACAACGTGTAGGAGTTATACGTGCGTTAGCAGCTGATCCACCAGTTATTTTAATGGAT
GAACCGTTTAGTGCATTAGACCCAATCAGCCGAGAAAAACTTCAAGATGATTTAATTGAA
TTACAAACTAAAATTAAGAAGACAATCATATTTGTTACACATGATATTCAAGAGGCGATG
AAACTTGGTGATAAGATTTGTCTTTTGAATGAAGGGCATATTGAACAAATTGACACACCA
GAAGGATTTAAAAATAATCCTCAAAGTGAATTTGTTAAACAATTTATGGGTAGTCATTTA
GAAGATGATGCGCCATGTGTTGAAGAGAACGCAATTATCCGTGACTTGGATATTATGAAA
CCAATCGATGAGGTTACATCTATGAGCGCTTATCCAATTGTTTATGACAATCAACCAATTG
AAGTATTGTATCAACTTTTATCAGAGAGCGAGCGTGTCATTGTCATGCAAGAAGATAGCG
TAGGTCAATATGTTATTGATAGGAAAGATATCTTCAAATATTTGTCCCAGAAAAAGGAGG
TAGCTCAACATGACTAA

> **opuBB**

Function: protein coding sequence; osmotically activated L-carnitine/choline ABC transporter/permease protein (core genome, variable)
Best match: opuBB_CC001_MW2-USA400_BA000033.2[768952:770466]
Position: 021-contig_267_RC: 49103 … 50618; Length: 1515 bp
Sequence:
ATGACTAACTTTTTCGACATATTGAGTGAACGTAAGGGGCAACTCTTTTCGACAATGATA
GAACATATTCAAATATCATTTATCGCATTATTGATTGCAACTGCTATTGCGGTACCATTAG
GTATTTTATTAACGAAGACTAAAACGATATCTGAAATCGTAATGAATATTGCGGCAATTC
TTCAAACCATACCATCGTTGGCATTATTAGGTTTAATGATTCCTTTATTTGGTATCGGTCG
TGTGCCAGCAATTATTGCACTTGTAGTGTATGCGTTGTTACCAATTTTAAGGAATACGTAT
ACTGGAATTAAAGAAGTTGATCCATCACTCATTGAAGCGGCTAAAGGTATAGGTATGAAA
CCATTTAGACGTTTAACTAAAGTCGAACTTCCGATAGCAATGCCTGTTATAATGGCTGGT
GTAAGAACGGCTATGGTATTAATTATAGGTACAGCAACACTAGCAGCATTAATTGGTGCA
GGCGGACTAGGAGATTTAATTTTATTAGGTATAGACCGTAACAATGCATCGTTGATATTA
TTAGGTGCAATTCCAGCAGCCTTATTGGCAATTATATTTGATTTAATTTTAAGATTTATGG
CTAAATTATCTTATAAAAAGTTATTGATGACGTTAGGTGTTATAGTGATGATTATTATACT
GGCTATCGCTATTCCTATGTTTGCACAAAAAGGTGATAAAATTACGTTAGCTGGAAAGCT
TGGCTCCGAGCCTTCGATTATTACAAATATGTATAAAATTTTAATAGAAGAAGAGACCAA
AAATACTGTAGAAGTGAAAGATGGTATGGGGAAAACAGCATTTTTATTTAATGCTTTAAA
ATCTGATGATATAGATGGGTATTTAGAGTTTACTGGAACAGTTTTAGGTGAATTAACAAA
AGAACCATTGAAGTCAAAAGAAGAGAAAAAAGTTTATGAACAAGCTAAGCAAAGTCTTG
AAAAGAAATATCAAATGACTATGTTAAAACCAATGAAGTATAACAATACGTATGCTTTAG
CTGTAAAACGTGATTTTGCTAAACAACATAATATACGTACAATTGGTGATTTAAATAAGG
TTAAAGATCAACTTAAACCAGGATTTACATTGGAATTTAATGATCGTCCAGATGGTTACA
AAGCTGTTCAAAAGGCTTATAATTTAAATTTAGATAACATACGTACAATGGAACCTAAGT
TGAGATATCAAGCGATCAATAAAGGTAATATTAATTTAATAGATGCATATTCAACTGACG
CTGAATTAAAACAATATGATATGGTTGTGTTAAAAGATGATAAGCACGTATTTCCACCAT
ATCAAGGAGCACCATTATTTAAAGAAAGCTTTTTAAAGAAACATCCAGAAATTAAGAAA
CCGTTAAACAAACTAGAAAACAAAATATCTGATGAAGATATGCAAATGATGAACTATAA
AGTAACAGTTAAAAATGAAGACCCATATACAGTTGCGAAAGATTATTTAAAAGCAAAAG
GGTTAATCAAATAA

> **hisC2**

Function: protein coding sequence; histidinol-phosphate aminotransferase locus 2 (core genome, variable)
Best match: hisC2_CC007_TCH959-USA300_AASB02000218.1[163415:164473] (completely identical)
Position: 021-contig_267_RC: 50857 … 51916; Length: 1059 bp
Sequence:
ATGAAAGAACAACTTAATCAACTATCAGCATATCAGCCTGGTTTATCTCCAAGGGCATTG
AAAGAAAAGTATGGCATTGAAGGAGATTTATATAAACTTGCATCAAATGAAAATTTGTAT
GGACCATCGCCTAAAGTTAAAGAAGCGATATCAGCACACTTAGATGAGTTATATTATTAT
CCTGAAACAGGATCACCGACATTAAAAGCGGCGATTAGTAAACATTTAAATGTAGATCA
ATCACGCATTTTATTTGGTGCGGGATTAGATGAAGTTATATTAATGATTTCTAGAGCTGTA
TTAACGCCAGGGGATACTATTGTTACAAGTGAAGCGACATTCGGTCAATATTATCACAAT
GCGATTGTTGAATCAGCTAATGTGATACAAGTACCTTTAAAAGGTGGTGGCTTCGATTTA
GAAGGTATTTTAAAAGAAGTTAATGAAGATACGTCATTGGTATGGTTATGTAATCCAAAT
AATCCTACAGGTACATATTTTAATCATGAGAGCTTAGATTCGTTTTTATCTCAAGTACCTC
CACATGTACCAGTAATTATAGATGAAGCTTATTTTGAATTTGTGACAGCAGAGGACTACC
CGGATACACTTGCTTTGCAACAAAAATATGACAATGCTTTCTTATTACGTACATTTTCAAA
GGCGTATGGATTAGCGGGTTTACGTGTGGGATATGTGGTAGCAAGTGAACATGCGATTGA
AAAATGGAACATCATTAGACCACCATTTAATGTGACACGTATATCTGAATACGCAGCAGT
TGCAGCACTTGAAGATCAACAATATTTAAAAGAGGTAACACATAAAAATAGTGTTGAAC
GCGAAAGATTTTATCAATTACCTCAAAGTGAGTATTTCTTGCCAAGTCAAACGAATTTTAT
ATTTGTAAAAACAAAGCGGGTAAATGAACTTTATGAAGCACTTTTAAATGTAGGGTGTAT
TACGCGACCATTTCCAACTGGTGTTAGAATTACAATTGGTTTTAAAGAACAAAATGATAA
AATGTTAGAAGTTTTATCAAATTTTAAATACGAATAG

> **tx_universal2**
Function:  rho indendent terminator
Best match: tx_universal2_CC001_MW2-USA400_BA000033.2[1067981:1068021:r]RC
Position: 021-contig_267_RC: 51987 … 52017; Length: 30 bp
Sequence:
TAGAATTGAAAAAAGCTTGTTACAAGCGCA

> **tx_universal2**
Function: rho indendent terminator
Best match: tx_universal2_CC007_TCH959-USA300_AASB02000023.1[4549:4580:r]RC (completely identical)
Position: 022-contig_262: 0 … 32; Length: 32 bp
Sequence:
AAAAAGCTTGTTACAAGCGCATTTTCGTTCAG

> **STAR**
Function:  Staphylococcus aureus repeat element
Best match: STAR_CC008_NCTC8325_CP000253.1[721055:721280:r]RC (completely identical)
Position: 022-contig_262: 875 … 1101; Length: 226 bp
Sequence:
TGCGGGGCCCCAACATAGAGAAATTGGATTCCCAATTTCTACAGACAATGCAAGTTGGGG
TGGGCCCCAACATAAAGAAATACTTTTTCTTTAGAAATTAGTATTTCTTATGCATGAGTGT
AACTCATGCATTCATATTTTTAAGTACACATTAGCTGTGACTAATGATAAAGAATCGCTAC
ATAATCAATCATTAGTCGTTCTTTATCATTTCCGTCCCGCTCTC

> **dgkA1**
Function: protein coding sequence; lipid kinase (core genome, constant)
Best match: dgkA1_CC012_21266_AFTT01000025.1[69867:70784]RC (completely identical)
Position: 022-contig_262: 1381 … 2299; Length: 918 bp
Sequence:

TTACAAATCATTGACAGTAAGTAACTGAATGGCATTTGGTATAACCTCAATATCAATAGG
TGTTTCTAATGAAATTTCGCCATCAATATCAACTTTCATTGCTGGATCTGTTGTAAGTGAA
ATCTTTTTACCAGGTATATGCTCAATACCTTGAGTAATTTCATTCCAATTCATGCTATCAC
GCTTTTTAAAAATATCATTTAAAATACTGAAACTTTGTTCATTAAAAATGAAAGTGTTCAG
TTCACCATCTTGAGGAGACAAATCAGTCAATGGTATACGACTACCACCAATGAATGGACC
ATTTGCTGTTAGTATCATGGTCGTTTCGCCAGAATATGTCTTATCATCTATTGATAATTGA
TAATTAAATTGTGTTGGATTTAGCAGTGTTTTGACAGTTGATCCAATATAACTCAATTTAC
CAAATATATCTTTTGAACCATCTTGTACGTTTTCAGCGTTTTGAACAATGAGACCTAAGCC
AACAAAGTTGAGTGCATATTGATTATTTATTTTAATTACATCGTATGTACCAACTTGTGCA
GAAATCATTTGTTCACTAGCTTGTTTATGATTAGGTGCTATATTTAGCGTTTTTGTAAAAT
CATTAAAAGTACCGCCTGGTAAAATGCCAATAGGGAGTTGAAGGTCATGTGACATAACA
CCATTTATAAGTTCGTTAACCGTGCCATCACCGCCAAGAATAAATAATATATCTACATCTT
TTGCATAGTTTTTAGTTTTGATTTCTTGGCAATATTTAATAATGTCACCTTCGTTTTCACTC
AATTGAATAGAAAGATGCTTACAAATTGAACTTAATGCTGTTGTAACTTCCCCAATACCTT
GATTAATATTTTTTAATCCACTGTGTTCATGGTAAAAGAGGACACCATGTGTATATTTATT
TTCCAT

> **queF**

Function: protein coding sequence; NADPH-dependent 7-cyano-7-deazaguanine reductase (core genome, variable)
Best match: queF_CC001-ST772_118_AJGE01000051.1[56341:56841:r]RC (completely identical)
Position: 022-contig_262: 4413 … 4914; Length: 501 bp
Sequence:
TTAACGATTATCTATTTTTTCGGGATATAAATCATGATTCATCAAACGATGCTCAGCCATT
TTTTCATATTTAGAATTTGGACGTCCATAGTTTGTATAAGGATCAATAGAAATTCCACCAC
GTGGTGTGAACTTGCCCCAGACTTCAATATAATGTGGGTCCATAAGCTCTATCAAATCATT
CATAATAATATTCATACAATCTTCGTGAAAATCACCGTGATTTCTGAAACTAAATAAGTA
TAATTTCAAAGATTTTGATTCAACCATTTTAACATTTGGAATATATGAAATATAGATAGTT
GCAAAATCTGGTTGCCCAGTAATTGGACATAATGATGTAAATTCTGGACAGTTGAATTTT
ACGAAATAGTCACGACCTTGATGCTTATTATCAAACGATTCTAATACATCAGGACGGTAG
TCGAAATTGTAAGTATTGTCTTGATTTCCTAATAAAGTTATATCTTGTAATTCATCTTGTTG
ACGGCCATGTGCCAT

> **Q5HHU3**

Function: protein coding sequence; putative integral membrane protein (core genome, constant)
Best match: Q5HHU3_CC001_MW2-USA400_BA000033.2[776834:777700:r]RC
Position: 022-contig_262: 4933 … 5800; Length: 867 bp
Sequence:
TTATTTTTTTATTATTTTGGCGTCTCGGCGTGCTTTTTCAAACATGTAATAACTTGCACCGA
TAATAACGACGTAACCTAATGTTGCATAGAAATCTGGAGATTCTCCGAATAGAATAAATC
CAAGTATTGCTGTGAAAATTATAGATGCATACGTAAAAATAGAAATATCTTTTGCGGCTG
CAAAACTATATGCTAAAGTAACACCAATTTGACCCACAGCGGCAGCTAAGCCAGCCCCTA
ATAGATAAAGTATTTGCATCTGACTCATTGGTTCATAAGTATATGCAGTGAAAGGTATTA
AAACGATGACAGAAAATAAGGAGAAGTAAAATACTATAGTATATGGTGCTTCTCTTGTAC
TAAGTGCTCGAACACATGTATATGCTGATGCTGCAAAAATACCTGAGAATAAGCCAGCTA
ATGATGGAATCATAGATGATGAAAATTCAGGTTTCACTATTAAAAGCATACCTAAAATAG
CAATTATCATTGCTGTTATTTGATACTTCCTTACTTTTTCATGTAAGAAAACAATGCTTAAT
AAAATCGTCCAGAAAGGATTGAGTTTCATTAATGAATCGGCATCACTAAGTACCATATGA
TCAATGGCATAAATATTTAACAATACACCAATAAGTCCAAGTGTTGATCGTGTTATTAAT
AAGGGTTGACTTGAAAGTCTGCCAAACATTGGCTGATGGTATTTATATATAAAAAATAAT
GGAATAAACATTGCTACTAAGTTTCGTGCTAATGATTTTTGAAAAACAGGAAGGTCACCT
GCAAGTCTGAAAAACACTGACATAAAACTGAAACCAATAGCCGAAATTAAAATGGCAAT
GATACCTTTTACTTTAGGATTCAA

> **rnaIV**

Function: noncoding RNA regulatory RNA IV (core genome, variable)
Best match: rnaIV_CC008_NCTC8325_CP000253.1[726391:726620] (completely identical)
Position: 022-contig_262: 6210 … 6440; Length: 230 bp
Sequence:
TAATATAGTGTTGGTGAAACGAAAAAGACACAATATCTTGTGTTTTGTATGCAAATGCTT
TATTTATGAAGAAATTACATTTAAAAGTAATTTAACACAGAAATTTAATAGTTATTATCA
ATTAATAGTCATATTTTTAGAAAATGTACTGAGCAAATGGAAGATATCCAATGATGTAAA
CACTACATATAGTGATTTTTATACATTCAACCCATATAAGCTACTATTTT

> **nrdI**
Function: protein coding sequence; stimulator of ribonucleotide reduction
Best match: nrdI_CC001_MW2-USA400_BA000033.2[778501:778899] (completely identical)
Position: 022-contig_262: 6600 … 6999; Length: 399 bp
Sequence:
ATGAAAATAATATATTTTTCATTTACTGGAAATGTCCGTCGTTTTATTAAGAGAACAGAAC
TTGAAAATACGCTTGAGATTACAGCAGAAAATTGTATGGAACCAGTTCATGAACCGTTTA
TTATCGTTACTGGCACTATTGGATTTGGAGAAGTACCAGAACCCGTTCAATCTTTTTTAGA
AGTTAATCATCAATACATCAGAGGTGTGGCAGCTAGCGGTAATCGAAATTGGGGACTAA
ATTTCGCAAAAGCGGGTCGCACGATATCAGAAGAGTATAATGTCCCTTTATTAATGAAGT
TTGAGTTACATGGAAAAAACAAAGACGTTATTGAATTTAAGAACAAGGTGGGTAATTTTA
ATGAAAACCATGGAAGAGAAAAAGTACAATCATATTGA

> **nrdE**
Function: protein coding sequence; ribonucleotide-diphosphate reductase alpha chain
Best match: nrdE_CC001_MW2-USA400_BA000033.2[778862:780967]
Position: 022-contig_262: 6961 … 9067; Length: 2106 bp
Sequence:
ATGAAAACCATGGAAGAGAAAAAGTACAATCATATTGAATTAAATAATGAGGTCACTAA
ACGAAGAGAAGATGGATTCTTTAGTTTAGAAAAAGACCAAGAAGCTTTAGTAGCTTATTT
AGAAGAAGTAAAAGACAAAACAATCTTCTTCGATACTGAAATCGAGCGTTTACGTTATTT
AGTAGACAACGATTTTTATTTCAATGTGTTTGATATTTATAGTGAAGCGGATCTAATTGAA
ATCACTGATTATGCAAAATCAATCCCGTTTAATTTTGCAAGTTATATGTCAGCTAGTAAAT
TTTTCAAAGATTACGCTTTGAAAACAAATGATAAAAGTCAATACTTAGAAGACTATAATC
AACACGTTGCCATTGTTGCTTTATACCTAGCAAATGGTAATAAAGCACAAGCTAAACAAT
TTATTTCTGCTATGGTTGAACAAAGATATCAACCAGCGACACCAACATTTTTAAACGCAG
GCCGTGCGCGTCGTGGTGAGCTAGTGTCATGTTTCTTATTAGAAGTGGATGACAGCTTAA
ATTCAATTAACTTTATTGATTCAACTGCAAAACAATTAAGTAAAATTGGGGGCGGCGTTG
CAATTAACTTATCTAAATTGCGTGCACGTGGTGAAGCAATTAAAGGAATTAAAGGCGTAG
CGAAAGGCGTTTTACCTATTGCTAAGTCACTTGAAGGTGGCTTTAGCTATGCAGATCAAC
TTGGTCAACGCCCTGGTGCTGGTGCTGTGTACTTAAATATCTTCCATTATGATGTAGAAGA
ATTTTTAGATACTAAAAAAGTAAATGCGGATGAAGATTTACGTTTATCTACAATATCAAC
TGGTTTAATTGTTCCATCTAAATTCTTCGATTTAGCTAAAGAAGGTAAGGACTTTTATATG
TTTGCACCTCATACAGTTAAAGAAGAATATGGTGTGACATTAGACGATATCGATTTAGAA
AAATATTATGATGACATGGTTGCAAACCCAAATGTTGAGAAAAAGAAAAAGAATGCGCG
TGAAATGTTGAATTTAATTGCGCAAACACAATTACAATCAGGTTATCCATATTTAATGTTT
AAAGATAATGCTAACAGAGTGCATCCGAATTCAAACATTGGACAAATTAAAATGAGTAA
CTTATGTACGGAAATTTTCCAACTACAAGAAACTTCAATTATTAATGACTATGGTATTGAA
GACGAAATTAAACGTGATATTTCTTGTAACTTGGGCTCATTAAATATTGTTAATGTAATGG
AAAGCGGAAAATTCAGAGATTCAGTTCACTCTGGTATGGACGCATTAACTGTTGTGAGTG
ATGTAGCAAATATTCAAAATGCACCAGGAGTTAGAAAAGCTAACAGTGAATTACATTCA
GTTGGTCTTGGTGTGATGAATTTACACGGTTACCTAGCAAAAAATAAAATTGGTTATGAG
TCAGAAGAAGCAAAAGATTTTGCAAATATCTTCTTTATGATGATGAATTTCTACTCAATCG
AACGTTCAATGGAAATCGCTAAAGAGCGTGGTATCAAATATCAAGACTTTGAAAAGTCTG
ATTATGCTAATGGCAAATATTTCGAGTTCTATACAACTCAAGAATTTGAACCTCAATTCGA
AAAAGTACGTGAATTATTCGATGGTATGGCTATTCCTACTTCTGAGGATTGGAAGAAACT

ACAACAAGATGTTGAACAATATGGTTTATATCATGCATATAGATTAGCAATTGCTCCAAC
ACAAAGTATTTCTTATGTTCAAAATGCAACAAGTTCTGTAATGCCAATCGTTGACCAAATT
GAACGTCGTACTTATGGTAATGCGGAAACATTTTACCCTATGCCATTCTTATCACCACAAA
CAATGTGGTACTACAAATCAGCATTCAATACTGATCAGATGAAATTAATCGATTTAATTG
CGACAATTCAAACGCATATTGACCAAGGTATCTCAACGATCCTTTATGTTAATTCTGAAAT
TTCTACACGTGAGTTAGCAAGATTATATGTATATGCGCACTATAAAGGATTAAAATCACT
TTACTATACTAGAAATAAATTATTAAGTGTAGAAGAATGTACAAGTTGTTCTATCTAA

> **nrdF**

Function: protein coding sequence; ribonucleotide-diphosphate reductase beta chain
Best match: nrdF_CC001_MW2-USA400_BA000033.2[781085:782056] (completely identical)
Position: 022-contig_262: 9184 … 10156; Length: 972 bp
Sequence:
ATGATAGCTGTTAATTGGAACACACAAGAAGATATGACGAATATGTTTTGGAGACAAAAT
ATATCTCAAATGTGGGTTGAAACAGAATTTAAAGTATCAAAAGACATTGCAAGTTGGAAG
ACTTTATCTGAAGCTGAACAAGACACATTTAAAAAAGCATTAGCTGGTTTAACAGGCTTA
GATACACATCAAGCAGATGATGGCATGCCTTTAGTTATGCTACATACGACTGACTTAAGG
AAAAAAGCAGTTTATTCATTTATGGCGATGATGGAGCAAATACACGCGAAAAGCTATTCA
CATATTTTCACAACACTATTACCATCTAGTGAAACAAACTACCTATTAGATGAATGGGTTT
TAGAGGAACCCCATTTAAAATATAAATCTGATAAAATTGTTGCTAATTATCACAAACTTT
GGGGTAAAGAAGCTTCGATATACGACCAATATATGGCCAGAGTTACGAGTGTATTTTTAG
AAACATTCTTATTCTTCTCAGGTTTCTATTATCCACTATATCTTGCTGGTCAAGGGAAAAT
GACGACATCAGGTGAAATCATTCGTAAAATTCTTTTAGATGAATCTATTCATGGTGTATTT
ACCGGTTTAGATGCACAGCATTTACGAAATGAACTATCTGAAAGTGAGAAACAAAAAGC
AGATCAAGAAATGTATAAATTGCTAAATGACTTGTATTTAAATGAAGAGTCATACACAAA
AATGTTATACGATGATCTTGGAATCACTGAAGATGTGCTAAACTATGTTAAATATAATGG
AAACAAAGCACTTTCAAACTTAGGCTTTGAACCTTATTTTGAGGAACGTGAATTTAACCC
AATCATTGAGAATGCCTTAGATACAACAACTAAAAACCATGACTTCTTCTCAGTAAAAGG
TGATGGTTATGTATTAGCATTAAACGTAGAAGCATTACAAGATGATGACTTTGTATTTGA
CAACAAATAA

> **tnpIS431-06**

Function: protein coding sequence; transposase for IS431 (mobile element)
Best match: tnpIS431-06_circ_S-epidermidis_GQ900381.1[43799:43807] (completely identical)
Position: 022-contig_262: 9815 … 9824; Length: 9 bp
Sequence:
ATGAACTAT

> **sstA**

Function: protein coding sequence; iron compound ABC transporter/permease protein (core genome, variable)
Best match: sstA_CC008_COL_CP000046.1[819637:820608] (completely identical)
Position: 022-contig_262: 10530 … 11502; Length: 972 bp
Sequence:
ATGAAGTATTTATTAAAGGGAAATATTTTGCTTCTATTACTAATATTGTTGACAATTATTT
CGTTGTTCATAGGTGTGAGTGAACTATCAATTAAAGATTTACTACATTTAACTGAGTCACA
GCGGAATATTTTATTCTCAAGCCGAATACCAAGGACGATGAGTATTTTAATTGCTGGAAG
TTCGTTGGCTTTAGCAGGCTTGATAATGCAACAAATGATGCAAAATAAGTTTGTTAGTCC
GACTACAGCTGGAACGATGGAATGGGCTAAACTAGGTATTTTAATTGCTTTATTGTTCTTT
CCAACCGGTCATATTTTATTAAAACTAGTATTTGCTGTTATTTGCAGTATTTGCGGTACGT
TTTTATTTGTTAAAATCATTGATTTTATAAAAGTGAAAGATGTCATTTTTGTACCGCTTTTA
GGAATTATGATGGGTGGGATTGTTGCAAGTTTCACAACCTTCATCTCATTGCGCACGAAT
GCTGTTCAAAGCATTGGTAACTGGCTTAACGGGAACTTTGCCATTATCACAAGTGGACGC
TATGAAATTTTATATTTAAGTATTCCTCTTTTAGCATTGACATATCTTTTTGCTAATCATTT
CACGATTGTAGGAATGGGTAAAGACTTTACTAATAATTTAGGTTTGAGTTACGAAAAATT

AATTAACATCGCATTGTTTATTACTGCAACTATTACAGCATTGGTAGTGGTGACTGTTGGA
ACATTACCGTTCTTAGGACTAGTAATACCAAATATTATTTCAATTTATCGAGGTGATCATT
TGAAAAATGCTATCCCTCATACGATGATGTTAGGTGCCATCTTTGTATTATTTTCTGATAT
AGTTGGCAGAATTGTTGTTTATCCATATGAAATAAATATTGGTTTAACAATAGGTGTATTT
GGAACAATCATTTTCCTTATCTTGCTTATGAAAGGTAGGAAAAATTATGCGCAACAATAA

> **sstB**
Function: protein coding sequence; iron compound ABC transporter/permease (core genome, variable)
Best match: sstB_CC005_N315_BA000018.3[787998:788954] (completely identical)
Position: 022-contig_262: 11488 … 12445; Length: 957 bp
Sequence:
ATGCGCAACAATAATAAAAAAATAATGCTTTTAATTGCAGTAACGTTATTAATTAGTATG
CTGTACTTATTTGTAGGTATTGATTTTGAAATATTTGAATATCAATTTTCAAGTCGTTTAA
GAAAGTTCATATTAATTATTTTAGTAGGTGCTGCCATTGCAACTTCAGTGGTGATTTTTCA
AGCGATTACAAATAACCGTCTATTGACACCATCAATAATGGGGTTAGATGCAGTTTATTT
ATTTATCAAAGTATTGCCAGTCTTTTTATTTGGAATTCAATCGGTATGGGTTACTAATGTA
TATTTGAACTTTATATTAACACTTATAACGATGGTGTTATTCGCACTAATCCTATTCCAAG
GTATCTTTAAAATTGGACATTTTTCAATTTATTTTATCTTACTTATTGGTGTCCTTTTAGGA
ACATTTTTTAGAAGCATAACAGGTTTTATTCAACTGATTATGGATCCTGAGTCATTTTTAG
CAATACAAAGTAGTATGTTTGCTAATTTTAATGCTTCTAATTCGAATTTAGTTACTTTCTC
AGCAGTGCTATTAGTAATCTTATTAGTCATTACAATTTTACTATTGCCTTATTTAGATGTAT
TGCTTTTAGGTCGTGCTGAAGCAATTAATCTTGGGATATCGTATGAAAAATTAACGCGAA
TTCTACTTGTAATAGTCTCAGTTTTAGTTTCTGTGTCAACTGCATTAGTAGGACCAATTAC
ATTTTTAGGTTTATTAACTGTAAATCTAGCGCATGAACTAATGAAGACGTATGAACATAA
GTATATTTTAATTGCGACAATTTGCTTGAGTTGGATTAGTTTATTTAGTGCGCAATGGGTA
GTTGAAAATGTGTTTGAAGCTACGACAGAAATGAGTATACTTATTGATTTGATTGGTGGA
AGTTATTTCATTTATCTATTAGTTAGAAGGAGAAATGCGCAATGA

> **sstC**
Function: protein coding sequence; ABC transporter ATP-binding protein (core genome, variable)
Best match: sstC_CC001_MW2-USA400_BA000033.2[784675:785436] (completely identical)
Position: 022-contig_262: 12441 … 13203; Length: 762 bp
Sequence:
ATGATTCAAGTTGAAAATTTAACTAAAACTATAAATAATCAAATGATATTGGAAGATATT
AGCATAGATATCGAAAAAGGTAAATTGACTTCTTTAATTGGACCTAATGGTGCGGGTAAG
AGTACTTTACTTTCAGCGATATGTAGGTTAATTCGTTTTGATAACGGTGAAGTGAAAATA
GATGGACAGCTCATGTCTGATTATAAAAATAATGACTTGTCGAAAAAAATATCTATATTA
AAACAAACAAACCATACTGAAATGAATATTACGGTAGAGCAGTTGGTAAACTTTGGACG
ATTCCCTTATTCTAAAGGTCGTTTGACGAAAGAGGATCATGATATTGTCAATGATGCGCT
AGATTTGTTGCAACTACAAGATATCAGAAATCGTAATATTAAGTCATTATCTGGTGGACA
ACGTCAGCGTGCATACATTGCAATGACAATAGCACAAGATACTGAATATATTTTGCTAGA
TGAACCATTAAATAATTTAGATATGAAGCATGCTGTTCAAATTATGCAAACGTTAAAAAT
GTTAGCGCATAAAATGAATAAAGCGATTGTCATTGTGTTACATGATATTAACTTTGCGTCC
TGTTATTCAGATCAGATTGTAGCATTGAAAAACGGACAACTAGTTAAGTCAGATTTGAAA
GATAATGTCATTCAAAGTAGTGTTTTAAGTGATTTATATGACATGAATATTCAAATTGAAC
ATATAAGAAATCAAAGGATTTGTTTATATTTTAAGGATTGA

> **sstD**
Function: protein coding sequence; ferrichrome ABC transporter lipoprotein (core genome, variable)
Best match: sstD_CC001_MW2-USA400_BA000033.2[785555:786583] (completely identical)
Position: 022-contig_262: 13321 … 14350; Length: 1029 bp
Sequence:
ATGAAGAAAACAGTCTTATATTTAGTATTAGCAGTAATGTTTTTATTAGCGGCATGCGGT
AACAATTCTGATAAAGAACAATCAAAATCAGAAACTAAAGGTTCTAAAGATACAGTAAA
AATTGAAAATAACTATAAAATGCGTGGCGAGAAAAAAGATGGTAGTGACGCTAAAAAAG

TTAAAGAAACTGTTGAAGTACCAAAAAATCCTAAAAATGCAGTTGTGTTAGACTATGGCG
CATTAGATGTAATGAAAGAAATGGGCTTATCAGATAAAGTAAAAGCATTACCTAAAGGG
GAAGGCGGTAAGTCATTACCGAATTTCTTAGAATCATTTAAAGATGATAAATATACAAAC
GTTGGTAATTTAAAAGAAGTGAATTTTGATAAAATTGCTGCGACGAAACCCGAAGTAATC
TTTATCTCTGGACGTACAGCTAATCAAAAGAATTTAGATGAATTCAAAAAAGCTGCACCT
AAAGCGAAAATTGTTTATGTTGGTGCAGATGAAAAGAACTTAATTGGTTCAATGAAACAA
AACACTGAAAATATCGGTAAAATTTACGATAAAGAAGATAAAGCTAAAGAATTAAATAA
AGATTTAGATAACAAAATTGCTTCAATGAAAGATAAAACGAAAAACTTCAATAAAACTG
TTATGTATTTACTAGTTAACGAAGGTGAATTATCAACATTTGGACCTAAAGGTCGTTTTGG
TGGATTAGTTTACGATACATTAGGATTCAATGCAGTTGATAAAAAAGTAAGTAATAGCAA
TCATGGACAAAATGTTTCTAACGAATATGTTAATAAAGAAAATCCAGATGTTATTTTAGC
GATGGATAGAGGTCAAGCGATAAGTGGTAAATCAACTGCGAAACAAGCATTAAATAATC
CTGTATTAAAAAATGTTAAAGCAATTAAAGAAGACAAAGTATATAATTTAGATCCTAAAT
TATGGTACTTTGCAGCTGGATCAACTACAACTACAATTAAACAAATTGAGGAACTTGATA
AAGTTGTAAAATAA

> **murB**

Function: protein coding sequence; UDP-N-acetylenolpyruvylglucosamine reductase (core genome, constant)
Best match: murB_CC001_MW2-USA400_BA000033.2[787232:788155:r]RC (completely identical)
Position: 022-contig_262: 14998 … 15922; Length: 924 bp
Sequence:
TTACGATTCCTTTGGATGTTCACCAATAATGCGAACTTCACGATTTAATTCAATGCCAAAT
TTTTCTTTGACGGTCTTTTGTACATAATGAATAAGGTTTTCATAATCTGTAGCAGTTCCATT
GTCTACATTTACCATAAAACCAGCGTGTTTAGTTGAAACTTCAACGCCGCCAATACGGTG
ACCTTGCAAATTAGAATCTTGTATCAATTTACCTGCAAAATGACCAGGCGGTCTTTGGAA
TACACTACCACATGAAGGATACTCTAAAGGTTGTTTAGATTCTCTACGTTCTGTTAAATCA
TCCATTTTAGCTTGTATTTCAGTCATTTTACCAGGAGCTAAAGTAAATGCAGCTTCTAATA
CAACTAAGTGTTCTTTTTGAATAATGCTATTACGATAATCTAACTCTAATTCTTTTGTTGTA
AGTTTAATTAACGAGCCTTGTTCGTTTACGCAAAGCGCATAGTCTATACAATCTTTAACTT
CGCCACCATAAGCGCCAGCATTCATATACACTGCACCACCGATTGAACCTGGAATACCAC
ATGCAAATTCAAGGCCAGTAAGTGCGTAATCACGAGCAACACGTGAGACATCAATAATT
GCAGCGCCGCTACCGGCTATTATCGCATCATCAGATACTTCGATATGATCTAGTGATAAT
AAACTAATTACAATGCCACGAATACCACCTTCACGGATAATAATATTTGAGCCATTTCCT
AAATATGTAACAGGAATCTCATTTTGATAGGCATATTTAACAACTGCTTGTACTTCTTCAT
TTTTAGTAGGGGTAATATAAAAGTCGGCATTACCACCTGTTTTAGTATAAGTGTATCGTTT
TAAAGGTTCATCAACTTTAATTTTTTCATTTGGGATAAGTTGTTGTAAAGCTTGATAGATG
TCTTTATTTATCAC

> **pepT**

Function: protein coding sequence; peptidase T (core genome, variable)
Best match: pepT_CC001_MW2-USA400_BA000033.2[792003:793229:r]RC
Position: 022-contig_262: 19774 … 21001; Length: 1227 bp
Sequence:
TTAGTGATTTTCAGCGATATCTTCTACAATTCCAATGATTACTTGTACTGCTTTTTCCATAA
CATCAATGGATGCATATTCATATGGGCCGTGGAAGTTACCGCAACCTGTAAAGATGTTTG
GAGTTGGTAACCCCATAAATGACAATTGTGAACCATCTGTACCACCACGAATAGGTTCAG
TGTTTGCTGGAATATCTAATTTGGCAAAGACACGTTTAGGTATATCAATAATATGAGGCA
ATGGTAATATTTTTTTCTGCCATATTGAAATATTGATCCGATATATCAACTTTAACTGGATA
ATTTTCAAAATGGGAATTGATATCGTCACGTATTTCTAAAATACGTTTCTTACGCAATTCG
AATTGTTTTTTATCATGATCACGAATAATGTATTGCAAAGTTGCTTTTTCAACAGTTCCTTC
AAAGTTCATTAAGTGATAAAAGCCTTCGTATCCTTCTGTTCGCTCCGGAACTTCACTATCA
GGTAGCAAACTATCGAATTGTTCACCTAAACGTATTGCGTTTACCATTGCATTTTTAGCTG
AACCAGGATGAACATTTACACCGTGGCATGTAATAACCGCTTCAGCAGCGTTAAAGCTTT
CATATTGTAATTCTCCATATTGACTACCATCCATAGTATAAGCAAAATCAGCATTGAAGC

GGTCAACATCAAATTTATGTGGACCACGACCGATTTCTTCGTCTGGTGTAAATCCAATGC
GAATGGTACCATGTTTAATTTCTGGATGTTCTTGTAAATAACAAATAGCTTCCATAATTTC
CACAATACCCGCTTTATCGTCTGCACCTAGTAACGATGTACCATCAGTTACCATTAATGTG
TGACCAACTAAACTGTTAAGTTCTGGAAATACTTTAGGATCTAAGACACGTTTAGTATTG
CCTAGTTTGTATGGCTTACCATCATAGTTTTCAATAATTTGCGGTTTAACATTTGAAGCAT
TGAAATCAGGTGATGTATCAACATGCGCCAAAAATCCAACTGTTGGGACGTCGACATCGA
TGTTACTTTCTAATGTAGCAAATAAGTAGCCATTTTCATCTAAATCAGTTGGCAATCCTAA
TTGTTGTAATTCTTTTTCTAATAAATGTAACAAATCCCATTGCTTTTCAGTTGAAGGTGTTG
TTGTAGATTTTGGATCAGATTGCGTATCAATTGTCGTATATCTTGTTAATCTATCTATCAAT
TGGTTCTTCAT

## > gdpS

Function: protein coding sequence; membrane bound diguanylate cyclase GGDEF domain-containing protein (core genome, constant)
Best match: gdpS_CC001-ST772_118_AJGE01000051.1[74411:75481:r]RC (completely identical)
Position: 022-contig_262: 22483 … 23554; Length: 1071 bp
Sequence:
TTATAAATTGATAATAGGGTTAAACATTACTTTGTTTCGCCCTTGATTTTTGGCTACATGC
ACCATATCGTCTGCATCTTTAAACACTTTACGCTGTGATTTTGGATCGTCATCTGTTAAAT
AACCAACACCGATAGACACTGACAATTTAATAACTTCTTTGTTTGGTAAATGGAATGATG
ATTTTTCAACACCCGAACGAATATTTTCAGCCAATTTAACACTTTGATCAAGTGAATAATT
GTGAATGACAACTGAGAACTCTTCGCCACCATTTCTAAAAATTTTAAATTGATTCGGCAC
ATAGTTTTTAAGTAATTGAGACATTTGTTTTAATACAGCATCACCTGATTTGTGTGAGTAG
GTATCATTGACATCTTTAAATCCATCGATATCGATTAATAATAATGCGATACTTTGATGTT
CTTTTTCAGCTTTTCGTGAAATTTCATTTAAATGTCTATCAAATTCTTTTACATTACCTAAG
CCTGTTAAGTAATCATATTTATCTTCGTTTTCATAACGATTTACGAGTGAGAAGAAATGCC
AAATATCGACAAATGTTATCGCTGAAGCTAAAGTGATAATTAATGAAATTGGTATTAAAA
TGATAACTTCCGATAGTGTGTAAATAGGACTCACTAACGCGACACCAAATAAAATGATTA
TTGTAACAACATTAAGTATTAATAATGATAGCACATCATTTTGTTTTAAAAATGGTCCAAT
AGCACTTGTTACTGCAGCAATAACAATCAACGTAACACCGTACATAATCGAGTTGTTAAA
TACTACAATTTCAACAATTGCTACAATTACTGTGGCAGATAATGTATAGACCATATTTGTA
AATCTACCTAAAAACAATAAAGGAACGAATGTTAAGTGAATTAAATAATCTTCACGATAA
GGGATAGGGTAGACAGATAATAATAATGATACGATTGTCATTAAAACAGTGACATAAGC
CTTAGAAAAAACCATACGTTTGTTTTCTGAATACTGTAAGCGATGGAATAAATAGATTCC
AGCGACTATAACAGATATATTGTATATAAATGCTTCGAACAT

## > tarO

Function: protein coding sequence; N-acetylglucosamine-1-phosphate transferase (core genome, variable)
Best match: tarO_CC008_NCTC8325_CP000253.1[744387:745442] (completely identical)
Position: 022-contig_262: 23871 … 24927; Length: 1056 bp
Sequence:
ATGGTTACATTATTACTAGTTGCAGTAACAATGATTGTCAGTTTGACGATAACACCAATTG
TTATTGCAATATCGAAAAGATTAAATTTAGTTGATAAACCAAATTTTAGAAAAGTACACA
CTAAACCTATTTCAGTTATGGGTGGTACAGTGATTCTCTTTTCATTTTTAATAGGTATTTGG
ATTGGTCATCCTATTGAAACAGAAATCAAACCACTTATTATTGGTGCGATTATTATGTACG
TACTTGGGCTTGTAGATGATATCTACGATTTGAAACCGTATATAAAATTGGCTGGTCAAA
TTGCCGCTGCCTTAGTAGTTGCTTTTTATGGTGTGACTATTGATTTTATTTCGTTGCCAATG
GGTACAACGATTCATTTTGGATTTCTTAGTATTCCAATTACTGTGATTTGGATTGTTGCTAT
TACAAATGCAATTAACTTAATTGATGGACTCGATGGTTTGGCGTCGGGTGTTTCTGCAATC
GGACTCATTACAATAGGGTTCATTGCAATTTTACAAGCTAATATTTTCATAACGATGATTT
GTTGTGTTTTATTAGGCTCTTTAATTGGGTTTTTATTTTACAATTTCCATCCTGCCAAAATA
TTTTTAGGTGATAGTGGGGCTTTAATGATTGGATTTATCATCGGATTCCTTTCTTTACTCGG
ATTCAAAAATATTACAATTATTGCATTGTTCTTCCCAATTGTTATCTTAGCAGTTCCATTCA
TTGATACTTTGTTCGCAATGATTCGACGTGTGAAAAAAGGGCAGCATATAATGCAAGCTG

ATAAATCGCATTTGCATCATAAACTATTAGCTTTAGGCTACACACATAGACAAACAGTAT
TATTAATCTATTCAATCTCTATTTTATTTAGTCTTTCGAGCATTATTTTGTATGTATCGCCA
CCATTAGGTGTTGTATTAATGTTTGTATTAATCATATTTAGTATTGAATTAATTGTTGAATT
TACAGGATTAATAGATAACAACTACCGACCAATATTAAATTTAATTAGTCGTAAGTCATC
TCATAAAGAGGAATAG

## > **comFA**

Function: protein coding sequence; type II DNA/RNA helicase (core genome, constant)
Best match: comFA_CC008_A5948_ACKD01000058.1[123307:124389]
Position: 022-contig_262: 27111 … 28194; Length: 1083 bp
Sequence:
ATGGATAATGTAACAAGATATAAAATAACAGAGAGTTCGCAAAGTTCATCACAAGCATA
TTATCATCTCTCATTTGAATTGTCGGAACAGCAGTCTTATGCCTCAGAACATATTGTTCGA
GCCATTAGAAAGAGACAAACGATTTTGTTATATGCCGTAACAGGTGCAGGTAAGACAGA
AATGATGTTTCAAGGCATTCAATATGCAAGAATACAGGGAGATAATATAGCTATTGTGTC
ACCACGTGTAGATGTTGTTGTAGAAATTAGTAAACGTATTAAAGACGCATTTCTTAATGA
AGATATAGACATACTACACCAGCAATCAAGACAACAATTTGAAGGGCATTTTGTTGTATG
CACAGTGCATCAACTTTACCGATTCAAACAGCACTTTGATACTATTTTTATTGATGAAGTC
GATGCCTTTCCTTTATCAATGGATAAAAATCTACAACAAGCATTGAAGTCATCTTCTAAA
GTTGAACATGCAACAATTTATATGACAGCAACACCACCGAAACAACTTCTGTCAGAGATT
CCCCACGAAAATATAATTAAATTGCCAGCTCGCTTTCATAAAAAATCACTTCCAGTTCCTA
AATATCGTTATTTCAAACTTAATAATAAGAAGATTCAGAAAATGTTATACCGAATTTTAC
AAGATCAAATTAATAATCAACGTTATACACTGGTGTTTTTTAACAATATAGAAACAATGA
TTAAAACATTTTCGGTTTATAAGCAGAAAATTACTAAATTAACATACGTCCATAGCGAGG
ATGTTTTTCGCTTTGAAAAAGTTGAACAATTAAGGAATGGACATTTCGATGTCATTTTTAC
TACGACAATATTAGAACGTGGATTTACAATGGCAAATTTGGATGTTGTTGTTATCGATGC
ACATCAATATACTCAAGAGGCTTTAATACAAATTGCTGGACGTGTTGGACGAAAATTAGA
ATGTCCTACTGGAAAAGTATTGTTTTTTCATGAAGGGGTAAGTATGAATATGATTCAAGC
TAAAAAAGAGATTCAAAGGATGAACAAATTAGCATTAAAAAGAGGTTGGATTGATGAAT
AA

## > **comFC**

Function: protein coding sequence; type II DNA/RNA helicase (core genome, constant)
Best match: comFC_CC001_MW2-USA400_BA000033.2[800414:801091] (completely identical)
Position: 022-contig_262: 28183 … 28861; Length: 678 bp
Sequence:
TTGATGAATAATTGTTTGAGTTGTGGTGCTAAGTTATATGAAAATATAACCATTTATAATT
TGTTCAAGAAACCTAATAGATTATGTGACAGATGCAAAGAGAATTGGGACAATATTAAA
CTTGATATTAAAGCAAGGCGATGTTCAAGGTGCTTAAAACACTTAAATCAAGATGAAGCG
TATTGTTTAGACTGCAAGTTTCTATCGGCACACTTTAATTTAATGGAACAATTATATTGTC
AATTTCAATATGACGGTTTAATGAAAGAGATGATACATCAGTATAAATTTTTGAAAGACT
ATTATTTATGTGAATTATTGGCACATTTGATTGAAATACCACAAACATCTTATGACTATAT
TGTGCCAATTCCTTCTTCGCCGGCACATGATTTATCTAGAACATTTAACCCGGTAGAAGCA
GTACTAAAAGCTAAAGGGATTCGCTTTGATAAGATTTTAAAGATGTCAAATAGACCAAAA
CAGTCTCATTTAACTAAGAAAGAGCGTCTGGCAGATGAAAATCCATTTATTATTGATACG
GAATTAGATTTAAATGGCAAGGAAATATTACTCGTTGACGATATTTATACAACTGGATTA
ACAATTCATCGTGCAGGGTGTAAATTATATGCTAAAAATATCAGAAAATTCAAAGTGTTT
GCGTTTGCACGATAG

## > **yfiA**

Function: protein coding sequence; sigma-54 modulation protein (core genome, constant)
Best match: yfiA_CC001_MW2-USA400_BA000033.2[801152:801724] (completely identical)
Position: 022-contig_262: 28921 … 29494; Length: 573 bp
Sequence:

ATGATTAGATTTGAAATTCATGGAGATAACCTCACTATCACAGATGCTATTCGCAACTAT
ATTGAGGAAAAAATTGGTAAGTTGGAACGTTATTTTAATGACGTACCAAATGCAGTGGCG
CATGTTAAAGTTAAAACTTATTCAAATTCAGCTACTAAAATTGAAGTAACAATTCCATTG
AAAAATGTTACGTTAAGAGCTGAAGAGCGAAACGATGATTTATACGCAGGTATTGATTTA
ATTAATAATAAACTTGAAAGACAAGTTCGAAAATATAAAACACGTATTAATCGTAAGAG
CCGTGATCGAGGAGATCAAGAAGTGTTTGTTGCCGAATTACAAGAAATGCAAGAAACAC
AAGTTGATAATGACGCTTACGATGATAACGAGATAGAAATTATTCGTTCAAAAGAATTCA
GCTTAAAACCAATGGATTCAGAAGAAGCGGTATTACAAATGAATCTATTAGGTCATGACT
TCTTTGTATTCACAGACAGAGAAACTGATGGAACAAGTATCGTTTACCGCCGTAAAGACG
GTAAATATGGCTTGATTCAAACTAGTGAACAATAA

> **secA1**

Function: protein coding sequence; preprotein translocase subunit secA1 (core genome, variable)
Best match: secA1_CC001-ST772_118_AJGE01000051.1[81835:84366] (completely identical)
Position: 022-contig_262: 29907 … 32439; Length: 2532 bp
Sequence:
ATGGGATTTTTATCAAAAATTCTTGATGGCAATAATAAAGAAATTAAACAGTTAGGTAAA
CTTGCTGATAAAGTAATCGCTTTAGAAGAAAAAACGGCAATTTTAACTGATGAAGAAATT
CGTAATAAAACGAAACAATTCCAAACAGAATTAGCTGACATTGATAATGTCAAAAAGCA
AAATGATTATTTAGATAAAATTTTACCAGAAGCATATGCACTTGTTAGAGAAGGCTCTAA
ACGTGTATTCAATATGACACCATATAAAGTTCAAATTATGGGTGGTATTGCAATTCATAA
AGGTGATATCGCTGAGATGAGAACAGGTGAAGGTAAAACATTAACAGCGACAATGCCAA
CATACTTAAATGCATTAGCTGGTAGAGGTGTTCACGTTATTACAGTCAATGAATACTTATC
AAGTGTTCAAAGTGAAGAAATGGCTGAGTTATATAACTTCTTAGGTTTGACTGTCGGATT
AAACTTAAACAGTAAGACGACAGAAGAAAAACGTGAAGCATACGCACAAGACATTACTT
ACAGTACTAATAATGAGCTAGGTTTTGATTACTTACGAGATAACATGGTGAATTATTCTG
AAGATAGAGTAATGCGTCCATTACATTTTGCAATCATTGATGAGGTTGACTCAATTTTAAT
CGACGAGGCACGTACGCCATTAATTATTTCTGGTGAAGCTGAAAAGTCAACATCACTTTA
TACACAAGCAAATGTTTTTGCGAAAATGTTAAAACAGGACGAAGATTATAAATACGATG
AAAAAACGAAAGCCGTACATTTAACAGAACAAGGTGCGGATAAAGCTGAACGTATGTTC
AAAGTTGAAAACTTATATGATGTACAAAATGTTGATGTTATTAGTCATATCAACACAGCT
TTACGTGCGCACGTTACATTACAACGTGACGTAGACTATATGGTTGTTGATGGCGAAGTA
CTAATTGTCGATCAATTTACAGGACGTACAATGCCAGGCCGTCGTTTCTCAGAAGGTTTA
CACCAAGCTATTGAAGCGAAGGAAGGCGTTCAAATTCAAAATGAATCTAAAACTATGGC
GTCTATTACATTCCAAAACTATTTCAGAATGTACAATAAACTTGCGGGTATGACAGGTAC
AGCTAAAACTGAAGAAGAAGAATTTAGAAATATTTATAACATGACAGTAACTCAAATTCC
GACAAATAAACCTGTGCAACGTAACGATAAGTCTGATTTAATTTACATTAGCCAAAAAGG
TAAATTTGATGCAGTAGTAGAAGATGTTGTTGAAAAACACAAGGCAGGGCAACCAGTGC
TATTAGGTACTGTTGCAGTTGAGACTTCTGAATATATTTCAAATTTACTTAAAAAACGTGG
TATCCGTCATGATGTGTTAAATGCGAAAAATCATGAACGTGAAGCTGAAATTGTTGCAGG
CGCTGGACAAAAAGGTGCCGTTACTATTGCCACTAACATGGCTGGTCGTGGTACAGATAT
CAAATTAGGTGAAGGCGTAGAGGAATTAGGCGGTTTAGCAGTAATAGGTACAGAACGAC
ATGAATCTCGTCGTATTGATGACCAGTTACGTGGTCGTTCTGGACGTCAAGGTGATAAAG
GGGATAGTCGCTTCTATTTATCATTACAAGATGAATTAATGATTCGTTTTGGTTCTGAACG
TTTACAGAAAATGATGAGCCGACTAGGTTTAGATGACTCTACACCAATTGAATCAAAAAT
GGTATCAAGAGCTGTAGAATCAGCACAAAAACGTGTAGAAGGTAATAACTTCGACGCGC
GTAAACGTATCTTAGAATACGATGAAGTATTACGTAAACAACGTGAAATTATCTATAACG
AAAGAAATAGTATTATTGATGAAGAAGACAGCTCTCAAGTTGTAGATGCAATGCTACGTT
CAACGTTACAACGTAGTATCAATTACTATATTAATACAGCAGATGACGAGCCTGAATATC
AACCATTCATCGACTACATTAATGACATCTTCTTACAAGAAGGTGACATTACAGAGGATG
ATATCAAAGGTAAAGATGCTGAAGATATTTTCGAAGTCGTTTGGGCTAAGATTGAAGCAG
CATATCAAAGTCAAAAAGATATCTTAGAAGAACAAATGAATGAGTTTGAGCGTATGATTT
TACTTCGTTCTATTGATAGCCATTGGACTGATCATATCGACACAATGGATCAATTACGTCA
AGGTATTCACTTACGTTCTTATGCACAACAAAATCCATTACGTGACTATCAAAATGAAGG
TCATGAATTATTTGATATCATGATGCAAAATATTGAAGAAGATACTTGTAAATTCATTTTA
AAATCTGTAGTACAAGTTGAAGATAATATTGAACGTGAAAAAACAACAGAGTTTGGTGA

AGCGAAGCACGTTTCAGCTGAAGATGGTAAAGAAAAAGTGAAACCGAAACCAATCGTTA
AAGGCGATCAAGTTGGTCGTAACGATGATTGTCCATGTGGTAGTGGTAAAAAATTCAAAA
ATTGCCATGGAAAATAA

> **prfB**

Function: protein coding sequence; peptide chain release factor 2/programmed frameshift (core genome, constant)
Best match: prfB_CC001_MW2-USA400_BA000033.2[804984:806094]
Position: 022-contig_262: 32753 … 33864; Length: 1111 bp
Sequence:
ATGGAATTATCAGAAATCAAACGAAATATAGATAAGTATAATCAAGATTTAACACAAATT
AGGGGGTCTCTTTGACTTAGAGAACAAAGAAACTAATATTCAAGAATATGAAGAAATGA
TGGCAGAACCTAATTTTTGGGATAACCAAACGAAAGCGCAAGATATTATAGATAAAAAT
AATGCGTTAAAAGCAATAGTTAATGGTTATAAAACACTACAAGCAGAAGTAGATGACAT
GGATGCTACTTGGGATTTATTACAAGAAGAATTTGATGAAGAAATGAAAGAAGACTTAG
AGCAAGAGGTCATTAATTTTAAGGCTAAAGTGGATGAATACGAATTGCAATTATTATTAG
ATGGGCCTCACGATGCCAATAACGCAATTCTAGAGTTACATCCTGGTGCAGGTGGCACGG
AGTCTCAAGATTGGGCTAATATGCTATTTAGAATGTATCAACGTTATTGTGAGAAGAAAG
GCTTTAAAGTTGAAACTGTTGATTATCTACCTGGGGATGAAGCAGGGATTAAAAGTGTAA
CATTGCTCATCAAAGGGCATAATGCTTATGGTTATTTAAAAGCTGAAAAAGGTGTACACC
GACTAGTACGAATTTCTCCATTTGATTCATCAGGACGTCGTCATACATCATTTGCATCATG
TGACGTGATTCCAGATTTTAATAATGATGAAATAGAGATTGAAATCAATCCGGATGATAT
TACAGTTGATACATTTAGAGCTTCTGGTGCAGGTGGTCAGCATATTAACAAAACTGAATC
AGCAATACGAATTACCCACCACCCCTCAGGTATAGTTGTTAATAACCAAAATGAACGTTC
TCAAATTAAAAACCGTGAAGCAGCTATGAAAATGTTAAAGTCTAAATTATATCAATTAAA
ATTGGAAGAGCAGGCACGTGAAATGGCTGAAATTCGTGGCGAACAAAAAGAAATTGGCT
GGGGAAGCCAAATTAGATCATATGTTTTCCATCCATACTCAATGGTGAAAGATCATCGTA
CGAACGAAGAAACAGGTAAGGTTGATGCAGTGATGGATGGAGACATTGGACCATTTATC
GAATCATATTTAAGACAGACAATGTCGCACGATTAA

> **lysM**

Function: protein coding sequence; LysM domain protein (core genome, constant)
Best match: lysM_CC001-ST772_118_AJGE01000051.1[86200:87039] (completely identical)
Position: 022-contig_262: 34272 … 35112; Length: 840 bp
Sequence:
ATGAAAAAATCTCTTACAGTGACGGTTTCGTCAGTGTTAGCTTTTTTAGCTTTAAATAATG
CAGCACATGCACAACAACATGGCACACAAGTAAAAACACCTGTTCAACATAATTATGTAT
CAAATGTTCAAGCACAAACGCAATCACCGACAACTTATACAGTAGTTGCTGGCGATTCAT
TATATAAGATTGCTTTAGAGCATCACTTAACGTTGAATCAATTATATTCATACAATCCTGG
TGTAACACCTTTAATTTTTCCTGGTGACGTGATTTCACTTGTGCCTCAAAATAAAGTGAAA
CAAACTAAAGCGGTTAAATCACCAGTAAGAAAAGCAAGCCAAGCTAAAAAGGTAGTAAA
ACAACCTGTACAACAAGCATCTAAAAAAGTAGTAGTTAAGCAAGCACCTAAGCAAGCAG
TAACTAAGACAGTTAATGTAGCATACAAACCTGCTCAAGTACAAAAATCAGTACCAACTG
TACCTGTTGCACATAACTACAATAAATCAGTTGCTAACAGAGGAAACTTATATGCTTATG
GAAACTGCACATATTATGCTTTCGATCGTCGTGCACAATTAGGTAGAAGTATAGGAAGTT
TATGGGGCAATGCAAATAACTGGAATTACGCAGCAAAAGTTGCAGGATTTAAAGTAGAT
AAAACACCAGAAGTTGGCGCTATTTTCCAAACAGCTGCTGGCCCATATGGACATGTTGGT
GTTGTTGAATCTGTAAACCCTAATGGAACAATTACTGTTTCTGAAATGAACTATGCTGGAT
TTAATGTTAAATCTTCAAGAACAATTTTAAATCCAGGAAAATATAATTACATCCACTAA

> **uvrB**

Function: protein coding sequence; ultraviolet response system protein B (core genome, constant)
Best match: uvrB_CC008_Newman_AP009351.1[814429:816420]
Position: 022-contig_262: 36431 … 38423; Length: 1992 bp
Sequence:

GTGACAATGGTTGAACATTATCCTTTTAAAATACATTCTGATTTTGAGCCTCAAGGTGACC
AACCGCAAGCAATTAAAGAAATCGTGGAAGGTATTAAAGCGGGGAAAAGACATCAAACT
TTATTAGGTGCTACTGGCACAGGGAAAACATTTACGATGAGTAATGTTATTAAAGAAGTT
GGGAAACCAACGTTAATTATCGCGCATAACAAAACATTAGCAGGACAATTATATAGTGA
GTTTAAAGAATTTTTTCCTGAAAACAGGGTGGAATACTTTGTAAGTTACTATGATTATTAT
CAACCAGAGGCATACGTACCGTCTACTGACACTTTTATTGAAAAAGATGCCTCAATCAAT
GATGAAATTGATCAACTACGACATTCTGCTACAAGTGCATTATTTGAACGCGATGATGTA
ATTATTATTGCTAGTGTAAGTTGTATATATGGTTTAGGTAATCCTGAAGAATATAAAGATT
TAGTAGTAAGTGTTCGAGTTGGTATGGAAATGGATAGAAGTGAATTACTTAGAAAACTTG
TAGATGTGCAATATACACGAAATGACATCGATTTCCAACGAGGAACGTTTCGAGTGCGTG
GTGATGTAGTGGAAATATTCCCAGCCTCTAAAGAAGAACTTTGTATAAGGGTTGAGTTTT
TCGGCGATGAGATTGACCGTATCCGAGAAGTTAACTACCTAACAGGTGAAGTGTTGAAAG
AAAGAGAACATTTTGCGATATTCCCAGCTTCTCACTTCGTAACACGTGAAGAAAAGTTGA
AAGTTGCGATTGAACGTATTGAAAAAGAATTGGAAGAACGATTGAAAGAATTACGAGAT
GAGAATAAATTACTAGAAGCGCAAAGGTTAGAACAGCGTACCAACTATGATTTAGAAAT
GATGCGAGAGATGGGATTCTGTTCAGGAATTGAAAACTATTCCGTACATTTAACTTTGCG
ACCACTGGGTTCGACACCATATACTTTATTGGATTACTTTGGCGATGATTGGTTAGTAATG
ATTGATGAATCACATGTGACATTACCGCAAGTTCGAGGCATGTATAACGGAGACAGAGC
GCGTAAACAAGTTTTGGTGGATCATGGGTTTAGATTACCGAGTGCATTAGATAACCGTCC
ACTTAAATTTGAAGAATTTGAAGAAAAGACAAAACAACTTGTGTATGTATCTGCAACGCC
TGGACCATACGAAATTGAACATACGGATAAGATGGTTGAACAAATTATTCGTCCTACTGG
TTTACTGGATCCTAAGATTGAGGTTAGACCTACTGAAAATCAAATTGACGATTTATTAAG
TGAAATTCAAACAAGAGTTGAGCGTAATGAACGCGTACTTGTTACAACGCTCACTAAAAA
GATGAGTGAAGATTTAACCACATACATGAAAGAAGCGGGTATTAAAGTTAATTATCTGCA
TTCAGAAATCAAGACATTAGAACGAATCGAAATAATTAGAGACTTACGAATGGGTACAT
ATGATGTTATCGTAGGTATTAATTTATTAAGAGAGGGTATTGATATACCAGAAGTTTCTCT
AGTTGTCATATTAGATGCAGATAAAGAAGGGTTTTTACGTTCTAACCGCTCATTAATTCAA
ACAATAGGTAGAGCTGCGCGTAACGATAAAGGTGAAGTCATTATGTATGCCGATAAAAT
GACTGATTCGATGAAGTATGCAATTGATGAGACACAACGTCGTCGAGAAATACAGATGA
AACATAATGAAAAACATGGTATTACACCTAAAACAATTAATAAAAAAATACATGATTTA
ATTAGTGCTACTGTTGAAAATGACGAAAATAATGACAAAGCACAAACTGTGATACCTAA
GAAGATGACGAAAAAAGAACGTCAAAAGACAATCGACAATATAGAAAAAGAAATGAAA
CAAGCAGCGAAAGATTTAGATTTCGAGAAAGCTACAGAATTAAGAGATATGTTATTTGAA
TTAAAAGCAGAAGGGTGA

> **uvrA**

Function: protein coding sequence; ultraviolet response system protein A (core genome, constant)
Best match: uvrA_CC001_MW2-USA400_BA000033.2[810661:813507]
Position: 022-contig_262: 38430 … 41277; Length: 2847 bp
Sequence:
ATGAAAGAACCATCCATAGTAGTAAAAGGTGCTCGTGCGCATAACTTGAAAGATATTGAT
ATCGAACTACCTAAAAATAAATTAATTGTTATGACAGGTTTATCTGGGTCAGGTAAATCG
TCATTAGCATTCGATACTATATATGCTGAAGGACAACGACGTTATGTTGAATCATTAAGT
GCCTATGCGCGTCAATTTTTAGGCCAAATGGACAAACCAGATGTTGATACAATTGAAGGA
TTATCGCCAGCAATTTCAATAGATCAAAAAACAACAAGTAAAAATCCAAGATCAACTGTA
GCAACAGTAACAGAAATATATGATTATATACGTTTGTTATATGCACGTGTTGGTAAACCT
TACTGTCCAAATCACAATATAGAAATTGAATCGCAAACAGTACAACAAATGGTTGACCGC
ATTATGGAATTAGAGGCACGTACAAAGATTCAATTATTAGCACCTGTCATCGCTCATCGT
AAAGGTAGTCATGAAAAGCTAATCGAAGATATTGGTAAAAAAGGTTATGTACGTTTAAG
AATCGATGGCGAAATTGTTGATGTAAATGATGTACCTACTTTAGATAAGAACAAGAATCA
TACAATAGAAGTTGTTGTAGACCGATTAGTTGTTAAAGATGGAATTGAAACACGACTAGC
TGACTCTATAGAAACTGCGTTAGAGCTTTCAGAAGGACAATTAACAGTTGATGTCATTGA
CGGGGAAGACCTTAAGTTTTCAGAAAGCCATGCTTGTCCTATATGTGGATTTTCAATCGG
AGAGTTAGAACCAAGAATGTTTAGCTTTAACAGTCCTTTTGGTGCTTGTCCGACATGTGAT
GGCTTAGGCCAAAAGTTAACAGTCGATGTAGACTTGGTTGTTCCCGACAAAGATAAGACG
CTAAACGAAGGTGCAATAGAACCTTGGATACCGACGAGTTCTGATTTTTATCCAACATTG

TTAAAACGTGTTTGTGAAGTTTATAAAATCAATATGGATAAACCTTTTAAAAAGTTAACA
GAACGTCAACGTGATATATTATTGTATGGTTCGGGTGACAAAGAAATTGAATTTACATTT
ACACAACGTCAAGGTGGTACTAGAAAACGAACAATGGTTTTCGAGGGTGTAGTTCCTAAT
ATAAGTAGACGATTCCATGAATCTCCTTCAGAATATACACGTGAAATGATGAGTAAATAT
ATGACCGAACTACCTTGTGAAACTTGTCATGGAAAGCGATTGAGTCGTGAAGCTTTATCT
GTTTATGTAGGTGGTTTAAATATTGGTGAAGTAGTCGAATACTCAATCAGTCAAGCGCTG
AACTATTATAAAAACATTGATTTGTCAGAACAAGATCAAGCGATTGCAAATCAAATATTG
AAAGAAATTATTTCCCGACTCACTTTTTTAAATAATGTGGGACTTGAATATTTAACGCTAA
ACAGAGCTTCAGGTACACTTTCAGGTGGTGAAGCACAACGTATTCGATTGGCAACGCAAA
TTGGGTCGCGTTTGACTGGTGTCTTATATGTATTAGATGAGCCATCAATTGGACTGCATCA
AAGAGATAATGATCGATTAATTAATACACTTAAAGAAATGAGAGATTTAGGAAATACTTT
AATTGTAGTTGAACACGATGATGATACAATGCGTGCGGCTGATTACTTAGTGGACATAGG
GCCTGGTGCTGGTGAACATGGAGGGCAGATTGTGTCTAGTGGGACACCTCAAAAGGTAAT
GAAAGATAAAAAATCATTAACAGGACAATACTTGAGTGGTAAGAAACGTATTGAAGTAC
CTGAATATCGCAGACCGGCTTCAGATCGTAAAATTTCTATACGTGGAGCTAGAAGCAACA
ATCTTAAAGGGGTTGATGTGGACATACCACTATCAATCATGACGGTTGTTACAGGTGTAT
CAGGTTCTGGTAAAAGCTCATTAGTAAATGAAGTATTATACAAATCATTAGCTCAAAAAA
TTAATAAATCTAAAGTAAAGCCAGGATTGTACGATAAGATTGAAGGTATTGATCAACTTG
ATAAAATTATTGATATTGATCAATCACCGATAGGTAGAACGCCACGCTCTAATCCAGCAA
CATATACTGGTGTGTTTGATGATATACGTGATGTGTTTGCGCAAACAAATGAAGCTAAAA
TTCGAGGATATCAAAAAGGGCGTTTTAGTTTTAATGTAAAAGGTGGACGCTGTGAAGCTT
GTAAAGGTGACGGTATTATTAAAATTGAAATGCATTTTTTACCTGATGTTTATGTTCCTTG
TGAAGTGTGTGATGGTAAACGATATAATCGTGAGACACTAGAGGTTACTTACAAAGGTAA
AAATATTGCTGACATTTTAGAAATGACTGTTGAAGAAGCAACACAATTTTTTGAAAATAT
TCCTAAGATTAAGCGCAAGTTACAAACACTAGTTGATGTTGGTCTTGGATACGTCACATT
AGGTCAACAAGCTACAACGTTATCAGGTGGTGAGGCTCAACGTGTGAAACTTGCATCTGA
ACTTCATAAACGTTCAACTGGTAAATCTATTTATATCCTAGATGAACCGACAACAGGGTT
ACATGTTGACGATATTAGTAGATTATTAAAAGTATTAAACCGATTAGTTGAAAATGGTGA
TACTGTTGTAATTATTGAACATAACCTAGATGTTATCAAAACAGCAGACTATATTATAGA
CTTAGGTCCTGAAGGTGGTAGTGGCGGTGGTACTATTGTTGCGACTGGCACACCCGAAGA
TATTGCTCAGACAAAGTCATCATATACAGGAAAGTATTTAAAAGAAGTACTTGAACGAGA
TAAACAAAATACTGAAGATAAATAA

> **repeat_nySAgamma**

Function:  repeat element
Best match: repeat_nySAgamma_CC001_MW2-USA400_BA000033.2[819195:819303]
Position: 022-contig_262: 41622 … 41652; Length: 30 bp
Sequence:
TTCTTTTCGAAATTCTCTGTGTTGGGGCCC

> **hprK**

Function: protein coding sequence; HPr kinase/phosphorylase (core genome, constant)
Best match: hprK_CC001_MW2-USA400_BA000033.2[814114:815046] (completely identical)
Position: 022-contig_262: 41896 … 42829; Length: 933 bp
Sequence:
ATGTTAACGACAGAAAAACTAGTTGAAACATTAAAGTTAGATTTAATCGCTGGTGAAGAA
GGACTATCGAAGCCAATTAAAAATGCTGATATATCAAGACCGGGCTTAGAGATGGCAGG
TTATTTTTCACATTATGCGTCAGATAGAATACAACTATTAGGAACAACGGAACTATCGTTT
TACAATTTATTACCAGATAAGGATCGCGCAGGTCGTATGCGTAAACTATGCAGACCAGAA
ACGCCTGCAATTATTGTGACGCGTGGATTGCAGCCACCAGAAGAATTAGTTGAAGCTGCA
AAAGAATTAAATACCCCACTTATAGTTGCTAAAGATGCGACTACAAGTTTAATGAGTCGC
TTAACAACGTTTTTAGAGCATGCACTTGCAAAGACGACATCTTTACATGGTGTTTTAGTAG
ATGTTTACGGTGTTGGTGTACTAATTACCGGTGATTCAGGAATAGGTAAAAGTGAGACTG
CGTTGGAATTAGTTAAACGTGGGCATAGATTAGTAGCAGATGATAATGTAGAAATACGTC
AAATTAATAAAGATGAACTAATAGGGAAACCACCAAAGTTAATAGAACATCTATTAGAA

ATACGTGGACTAGGTATTATCAATGTTATGACTTTATTTGGCGCGGGTTCAATATTAACTG
AAAAACGAATTAGATTAAATATTAATTTGGAAAACTGGAACAAGCAAAAGTTATATGAC
CGCGTAGGTCTTAATGAAGAGACGCTAAGTATTTTAGATACTGAAATCACTAAAAAAACA
ATACCTGTAAGACCTGGTAGAAATGTTGCGGTAATTATTGAGGTCGCTGCAATGAACTAT
CGATTAAATATCATGGGCATTAACACTGCCGAAGAATTTAGTGAAAGATTAAATGAAGA
AATTATCAAGAACAGTCATAAGAGTGAGGAGTAG

> **lgt**

Function: protein coding sequence; prolipoprotein diacylglyceryl transferase (core genome, variable)
Best match: lgt_CC001-ST772_118_AJGE01000051.1[94762:95601] (completely identical)
Position: 022-contig_262: 42834 … 43674; Length: 840 bp
Sequence:
ATGGGTATTGTATTTAACTATATAGATCCTGTGGCATTTAACTTAGGACCACTGAGTGTAC
GATGGTATGGAATTATCATTGCTGTCGGAATATTACTTGGTTACTTTGTTGCACAACGTGC
ACTAGTTAAAGCAGGATTACATAAAGATACTTTAGTAGATATTATTTTTTATAGTGCACTA
TTTGGATTTATCGCGGCACGAATCTATTTTGTGATTTTCCAATGGCCATATTACGTGGAAA
ATCCAAGTGAAATTATTAAAATATGGCATGGTGGAATAGCAATACATGGTGGTTTAATAG
GTGGCTTTATTGCTGGTGTTATTGTATGTAAAGTGAAAAATTTAAACCCATTTCAAATTGG
TGATATCGTTGCGCCAAGTATAATTTTAGCGCAAGGAATTGGACGCTGGGGTAACTTTAT
GAATCACGAGGCACATGGTGGACCTGTGTCACGCGCTTTTTTAGAACAATTACATTTGCC
TAATTTTATAATAGAAAATATGTATATTAACGGCCAATATTATCATCCAACATTCTTATAT
GAATCCATTTGGGATGTCGCTGGATTTATTATTTTAGTTAATATTCGTAAACATTTAAAAT
TAGGAGAAACATTCTTTTTATATTTAACTTGGTATTCAATTGGTCGATTCTTTATAGAAGG
ATTACGTACAGATAGCTTAATGCTCACAAGTAATATTAGAGTTGCACAATTAGTATCAAT
TCTTTTAATTTTAATAAGTATAAGTTTAATTGTATATAGAAGGATTAAGTATAATCCACCG
TTGTATAGCAAAGTTGGGGCGCTTCCATGGCCAACAAAAAAAGTGAAGTAG

> **trxB**

Function: protein coding sequence; thioredoxin reductase (core genome, variable)
Best match: trxB_AJ223781.2[1265:2200]
Position: 022-contig_262: 45682 … 46618; Length: 936 bp
Sequence:
ATGACTGAAATAGATTTTGATATAGCAATTATCGGTGCAGGTCCAGCTGGTATGACTGCT
GCAGTATACGCATCACGTGCTAATTTAAAAACAGTTATGATTGAAAGAGGTATTCCAGGC
GGTCAAATGGCTAATACAGAAGAAGTAGAGAACTTCCCTGGTTTCGAAATGATTACAGGT
CCAGATTTATCTACAAAAATGTTTGAACACGCTAAAAAGTTTGGTGCAGTTTATCAATAT
GGAGATATTAAATCTGTAGAAGATAAAGGCGAATATAAAGTGATTAACTTTGGTAATAA
AGAATTAACAGCGAAAGCGGTCATTATTGCTACAGGTGCAGAATACAAGAAAATTGGTG
TTCCGGGTGAACAAGAACTTGGTGGACGCGGTGTAAGTTATTGTGCAGTATGTGATGGTG
CATTCTTTAAAAATAAACGCCTATTCGTTATCGGTGGTGGTGACTCAGCAGTAGAAGAGG
GAACATTCTTAACTAAATTTGCTGACAAAGTAACAATCGTTCACCGTCGTGATGAGTTAC
GTGCACAACGTATTTTACAAGATAGAGCATTCAAAAATGATAAAATCGACTTTATTTGGA
GTCATACTTTGAAATCAATTAATGAAAAAGACGGCAAAGTGGGTTCTGTGACATTAACGT
CTACAAAAGATGGTTCAGAAGAAACACACGAGGCTGATGGTGTATTCATCTATATTGGTA
TGAAACCATTAACAGCGCCATTTAAAGACTTAGGTATTACAAATGATGTTGGTTATATTG
TAACAAAAGATGATATGACAACATCAGTACCAGGTATTTTTGCAGCAGGAGATGTTCGCG
ACAAAGGTTTACGCCAAATTGTCACTGCTACTGGTGATGGCAGTATTGCAGCGCAAAGTG
CAGCGGAATATATTGAACATTTAAACGATCAAGCTTAA

> **tx_universal2**

Function:  rho indendent terminator
Best match: tx_universal2_CC001_MW2-USA400_BA000033.2[819367:819407:r]RC (completely identical)
Position: 023-contig_244_RC: 20 … 61; Length: 41 bp
Sequence:

TAGAGTTGAAAAAAGCTTGTTGCAAGCGCGTTTTCATTCAG

## > Q2YSG2

Function: protein coding sequence; P-loop ATPase protein (core genome, variable)
Best match: Q2YSG2_CC008_NCTC8325_CP000253.1[768883:769794]
Position: 023-contig_244_RC: 251 … 1163; Length: 912 bp
Sequence:
ATGGATAATAATGAAAAAGAAAAAAGTAAAAGTGAACTATTAGTTGTAACAGGTTTATC
TGGCGCAGGTAAATCTTTGGTTATTCAATGTTTAGAAGACATGGGATATTTTTGTGTAGAT
AATCTACCACCAGTGTTATTGCCTAAATTTGTAGAGTTGATGGAACAAGGAAATCCATCC
TTAAGAAAAGTGGCAATTGCAATTGATTTAAGAGGTAAGGAACTATTTAATTCATTAGTT
GCAGTAGTGGATAAAGTTAAAAGTGAAAGTGACGTCATCATTGATGTTATGTTTTTAGAA
GCAAGTACTGAAAAATTAATTTCAAGATATAAGGAAACGCGTCGTGCACATCCTTTGATG
GAACAAGGTAAAAGATCCTTAATCAATGCAATTAATGATGAGCGAGAGCATTTGTCTCAA
ATTAGAAGTATAGCTAATTTTGTTATAGATACTACAAAGTTATCACCTAAAGAATTAAAA
GAACGCATTCGTCGATACTATGAAGATGAAGAGTTTGAAACTTTTACAATTAATGTCACA
AGTTTCGGTTTTAAACATGGGATTCAGATGGATGCAGATTTAGTATTTGATGTACGATTTT
TACCAAATCCATATTATGTAGTAGATTTAAGACCTTTAACAGGATTAGATAAAGACGTTT
ATAATTATGTTATGAAATGGAAAGAGACGGAGATTTTCTTTGAAAAATTAACTGATTTGT
TAGATTTTATGATACCCGGGTATAAAAAAGAAGGGAAATCTCAATTAGTAATTGCCATCG
GTTGTACGGGTGGACAACATCGATCTGTAGCATTAGCAGAACGACTAGGTAATTATCTAA
ATGAAGTATTTGAATATAATGTTTATGTGCATCATAGGGACGCACATATTGAAAGTGGCG
AGAAAAAATGA

## > tRNAarg1

Function: tRNA tRNA-Arg (core genome, constant)
Best match: tRNAarg1_CC001_MW2-USA400_BA000033.2[822868:822942:r]RC (completely identical)
Position: 023-contig_244_RC: 3521 … 3596; Length: 75 bp
Sequence:
CAGCCTCCTTGAAGGGAATTGAACCCCTATCTTAAGAACCGGAATCTTACGTGTTATCCA
TTACACCACAAGGAG

## > clpP

Function: protein coding sequence; ATP-dependent chaperone protease/proteolytic subunit
Best match: clpP_CC001_MW2-USA400_BA000033.2[823129:823716] (completely identical)
Position: 023-contig_244_RC: 3782 … 4370; Length: 588 bp
Sequence:
ATGAATTTAATTCCTACAGTTATTGAAACAACAAACCGCGGTGAACGTGCATATGATATA
TACTCACGTTTATTAAAAGACCGTATTATTATGTTAGGTTCACAAATTGATGACAACGTAG
CAAATTCAATCGTATCACAGTTATTATTCTTACAAGCGCAAGACTCAGAGAAAGATATTT
ATTTATACATTAATTCACCAGGTGGAAGTGTAACAGCTGGTTTTGCGATTTATGATACAAT
TCAACACATTAAACCTGATGTTCAAACAATTTGTATCGGTATGGCTGCATCAATGGGATC
ATTCTTATTAGCAGCTGGTGCAAAAGGTAAACGTTTCGCGTTACCAAATGCAGAAGTAAT
GATTCACCAACCATTAGGTGGTGCTCAAGGACAAGCAACTGAAATCGAAATTGCTGCAA
ATCACATTTTAAAAACACGTGAAAAATTAAACCGCATTTTATCAGAGCGTACTGGTCAAA
GTATTGAAAAAATACAAAAAGACACAGATCGTGATAACTTCTTAACTGCAGAAGAAGCT
AAAGAATATGGCTTAATTGATGAAGTGATGGTACCTGAAACAAAATAA

## > txbi_clpP_Q2G035

Function:  bidirectional rho-independent terminator of clpP and Q2G035
Best match: txbi_clpP_Q2G035_CC001_MW2-USA400_BA000033.2[823827:823898] (completely identical)
Position: 023-contig_244_RC: 4480 … 4552; Length: 72 bp
Sequence:

TGAACCGTAACTATATGAAATGTTCCCTTCAAAGTAGACATTGAAAGGAACATTTCAATC
CTTTGTTTGTAA

## > Q2G035
Function:
Best match: Q2G035_CC005_Mu50-VRSA_BA000017.4[852653:853555:r]RC
Position: 023-contig_244_RC: 4612 … 5515; Length: 903 bp
Sequence:
TTATTCTTTGATTAAATCTTCAAGTGCCATTTTTAAATTACTATATTTAAATTGGAATCCCA
ATGCTTGAATTTTATTAGGTAATACTTTTTGAGTATCCAATACTACTGTTGACATTTGACC
AAGTATGAGACGCATTGCAAGACTTGGTGCCCAAGTTTCATGGGGCTTATGCATAGCTCT
TGCTAAAGTGTAGCCAAATAAATTTTGACGCTCAGGTATAGGTGCAGTTAAATTAAACGG
ACCACTAGCTGACTCGTTATTTATTAAAAATAAAATAGCTTGAATTAAATCATTGATATG
AATCCATGAATACCATTGTTGACCAGAACCTAATTTACCACCAATGTAATATTTGTATGGT
AGTTTCATTGTTTGTAACGCACCGCCTTCATTCGATAAAATCATACCGAAACGACCGATG
ACAACTCGCGTACCTAATTGTTCAAATTGTTGTGCGAAACGTTCCCATTGATACACAATAT
CTGATAAGAAATCAAATGGTAAAGTTTTATAAACTTCTGTGTAACTCATAAATAAATCAG
GAGGATAGTAACCAGTGGCACTAGCATTAAATAAAACTTTAGGTGCTTTATTACGAGATT
TAAACAATTCATATAAAGCTTGCGTAGATTGAACTCTACTTAGCATTAGCGTTTGTTTATA
TTCCGGTGTCCATCGTTTATTCAGTGTAGCACCTGCTAAGTTGATGACCACATCGATATTT
TGAGGAACTTTGTGTTCCCACCCAGATTTAGCCCAGTTGACATATGAAATTTTCTTATCAT
TTGAAATTTGGTCGTGTCGCGTTAATATCGTGATATGTGAATCTGATTTTTTAATTTCATTA
ACTAATTGAGATCCAACCATACCAGTCCCACCAGTAATTAAGTATTGTTTCAT

## > yfcH
Function: protein coding sequence; cell-division inhibitor (core genome, variable)
Best match: yfcH_CC001-ST772_118_AJGE01000036.1[29661:30563]RC (completely identical)
Position: 023-contig_244_RC: 4612 … 5515; Length: 903 bp
Sequence:
TTATTCTTTGATTAAATCTTCAAGTGCCATTTTTAAATTACTATATTTAAATTGGAATCCCA
ATGCTTGAATTTTATTAGGTAATACTTTTTGAGTATCCAATACTACTGTTGACATTTGACC
AAGTATGAGACGCATTGCAAGACTTGGTGCCCAAGTTTCATGGGGCTTATGCATAGCTCT
TGCTAAAGTGTAGCCAAATAAATTTTGACGCTCAGGTATAGGTGCAGTTAAATTAAACGG
ACCACTAGCTGACTCGTTATTTATTAAAAATAAAATAGCTTGAATTAAATCATTGATATG
AATCCATGAATACCATTGTTGACCAGAACCTAATTTACCACCAATGTAATATTTGTATGGT
AGTTTCATTGTTTGTAACGCACCGCCTTCATTCGATAAAATCATACCGAAACGACCGATG
ACAACTCGCGTACCTAATTGTTCAAATTGTTGTGCGAAACGTTCCCATTGATACACAATAT
CTGATAAGAAATCAAATGGTAAAGTTTTATAAACTTCTGTGTAACTCATAAATAAATCAG
GAGGATAGTAACCAGTGGCACTAGCATTAAATAAAACTTTAGGTGCTTTATTACGAGATT
TAAACAATTCATATAAAGCTTGCGTAGATTGAACTCTACTTAGCATTAGCGTTTGTTTATA
TTCCGGTGTCCATCGTTTATTCAGTGTAGCACCTGCTAAGTTGATGACCACATCGATATTT
TGAGGAACTTTGTGTTCCCACCCAGATTTAGCCCAGTTGACATATGAAATTTTCTTATCAT
TTGAAATTTGGTCGTGTCGCGTTAATATCGTGATATGTGAATCTGATTTTTTAATTTCATTA
ACTAATTGAGATCCAACCATACCAGTCCCACCAGTAATTAAGTATTGTTTCAT

## > rsaH
Function: noncoding RNA ncRNA of Staphylococcus aureus H (core genome, constant)
Best match: rsaH_CC001_MW2-USA400_BA000033.2[825010:825133] (completely identical)
Position: 023-contig_244_RC: 5663 … 5787; Length: 124 bp
Sequence:
GTACCTTCGATAACGAATAAACATCTCTTAAAAGTATGTGTAAAACGCTGCATGATACAA
ACGAAGGTAAAAATTTGACTCCCTTTAGTAGTGGACCCGTACGTTAATCGTGCGGGTCGT
TTTT

## > Q5HHP8

Function: protein coding sequence; putative exported protein (core genome, constant)
Best match: Q5HHP8_CC005_Mu50-VRSA_BA000017.4[854258:854887] (completely identical)
Position: 023-contig_244_RC: 6217 … 6847; Length: 630 bp
Sequence:
ATGTCGAATCAAAATTACGACTACAATAAAAATGAAGATGGAAGTAAGAAGAAAATGAG
TACAACAGCGAAAGTAGTTAGCATTGCGACGGTATTGCTATTACTCGGAGGATTAGTATT
TGCAATTTTTGCATATGTAGATCATTCGAATAAAGCTAAAGAACGTATGTTGAACGAACA
AAAGCAGGAACAAAAAGAAAAGCGTCAAAAAGAAAATGCAGAAAAAGAGAGAAAGAA
AAAGCAACAAGAGGAAAAAGAGCAGAATGAGCTAGATTCACAAGCAAACCAATATCAG
CAATTGCCACAGCAGAATCAATATCAATATGTGCCACCTCAGCAACAAGCACCTACAAAG
CAACGTCCTGCTAAAGAAGAGAATGATGATAAAGCATCAAAGGATGAGTCGAAAGATAA
GGATGACAATGCATCTCAAGATAAATCAGATGATAATCAGAAGAAAACTGATGATAATA
AACAACCAGCTCAGCCTAAACCACAGCCGCAACAACCAACACCAAAGCCAAATAATAAT
CAACAAAATAATCAATCAAATCAGCAAGCAAAACCACAAGCACCACAACAAAATAGCCA
ATCAACAACAAATAAACAAAATAATGCTAATGATAAGTAG

> **repeat_nySAgamma**

Function:  repeat element
Best match: repeat_nySAgamma_CC001_MW2-USA400_BA000033.21142439:1142558:r [4_50ANF10END]
Position: 023-contig_244_RC: 7098 … 7163; Length: 65 bp
Sequence:
TTGGGGCCCCGCCAACTTGCACATTATTGTAAGCTGACTTTCCGCCAGCTTCTGTGTTGGG
GCCC

> **gapR**

Function: protein coding sequence; regulator of glyceraldehyde 3-phosphate dehydrogenase (core genome, constant)
Best match: gapR_CC001_MW2-USA400_BA000033.2[826835:827848] (completely identical)
Position: 023-contig_244_RC: 7544 … 8558; Length: 1014 bp
Sequence:
GTGAAAGACTTATTGCAAGCACAGCAAAAGCTTATACCGGATCTCATAGATAAAATGTAT
AAACGTTTTTCTATTCTTACTACTATCTCAAAAAATCAGCCTGTCGGACGTCGAAGTTTAA
GCGAACATATGGATATGACTGAACGTGTACTGCGTTCTGAAACAGATATGCTTAAGAAAC
AAGATTTGATAAAAGTTAAGCCTACCGGAATGGAAATTACAGCTGAAGGTGAGCAACTG
ATTTCGCAATTGAAAGGTTACTTTGATATCTATGCAGATGATAATCGTCTGTCAGAAGGT
ATTAAGAATAAATTTCAAATTAAGGAAGTTCATGTTGTTCCTGGTGATGCTGATAATAGT
CAATCTGTTAAAACAGAATTAGGTAGACAAGCAGGTCAATTACTTGAAGGCATATTACAA
GAAGACGCGATAGTTGCTGTAACTGGCGGATCCACGATGGCATGTGTTAGTGAAGCAATT
CATTTATTACCATATAATGTATTCTTCGTACCAGCCAGAGGTGGACTAGGCGAAAATGTT
GTCTTTCAGGCAAACACAATTGCAGCCAGTATGGCACAACAAGCTGGCGGTTATTATACG
ACGATGTATGTACCTGATAATGTCAGTGAAACAACATATAACACATTGTTGTTAGAGCCA
TCAGTCATAAACACTTTAGACAAAATTAAACAAGCAAACGTTATATTACACGGCATTGGT
GATGCGCTGAAGATGGCGCATCGACGTCAATCACCTGAAAAGGTCATTGAACAACTTCAA
CATCATCAAGCTGTCGGAGAGGCATTTGGTTATTATTTTGATACACAAGGTCAAATTGTCC
ATAAGGTTAAAACAATTGGACTTCAATTAGAAGACCTTGAATCAAAAGACTTTATTTTTG
CAGTTGCAGGAGGCAAATCGAAAGGTGAAGCAATTAAAGCATACTTGACGATTGCACCC
AAGAATACAGTGTTAATCACTGATGAAGCCGCAGCAAAGATAATACTTGAATAA

> **gapA**

Function: protein coding sequence; extracellular glyceraldehyde 3-phosphate dehydrogenase/locus 1 (core genome, constant)
Best match: gapA_CC001_MW2-USA400_BA000033.2[827901:828911] (completely identical)
Position: 023-contig_244_RC: 8610 … 9621; Length: 1011 bp
Sequence:

ATGGCAGTAAAAGTAGCAATTAATGGTTTTGGTAGAATTGGTCGTTTAGCATTCAGAAGA
ATTCAAGAAGTAGAAGGTCTTGAAGTTGTAGCAGTAAACGACTTAACAGATGACGACAT
GTTAGCGCATTTATTAAAATATGACACTATGCAAGGTCGTTTCACAGGTGAAGTAGAGGT
AGTTGATGGTGGTTTCCGCGTAAATGGTAAAGAAGTTAAATCATTCAGTGAACCAGATGC
AAGCAAATTACCTTGGAAAGACTTAAATATCGATGTAGTGTTAGAATGTACTGGTTTCTA
CACTGATAAAGATAAAGCACAAGCTCATATTGAAGCAGGCGCTAAAAAAGTATTAATCT
CAGCACCAGCTACTGGTGACTTAAAAACAATCGTATTCAACACTAACCACCAAGAGTTAG
ACGGTTCTGAAACAGTTGTTTCAGGTGCTTCATGTACTACAAACTCATTAGCACCAGTTGC
TAAAGTTTTAAACGATGACTTTGGTTTAGTTGAAGGTTTAATGACTACAATTCACGCTTAC
ACAGGTGATCAAAATACACAAGACGCACCTCACAGAAAAGGTGACAAACGTCGTGCTCG
TGCAGCGGCAGAAAACATCATCCCTAACTCAACAGGTGCTGCTAAAGCTATCGGTAAAGT
TATTCCTGAAATCGATGGTAAATTAGATGGTGGTGCACAACGTGTTCCTGTAGCTACAGG
TTCATTAACTGAATTAACAGTAGTATTAGAAAAACAAGACGTAACAGTTGAACAAGTTAA
CGAAGCTATGAAAAATGCTTCAAACGAATCATTCGGTTACACTGAAGACGAAATCGTTTC
TTCAGACGTTGTAGGTATGACTTACGGTTCATTATTCGACGCTACACAAACTCGTGTAATG
TCAGTTGGCGACCGTCAATTAGTTAAAGTTGCAGCTTGGTATGATAACGAAATGTCATAT
ACTGCACAATTAGTTCGTACATTAGCATACTTAGCTGAACTTTCTAAATAA

> **pgk**

Function: protein coding sequence; phosphoglycerate kinase (core genome, constant)
Best match: pgk_AJ133520.1[2995:4185]
Position: 023-contig_244_RC: 9759 … 10950; Length: 1191 bp
Sequence:
ATGGCTAAAAAAATTGTTTCTGATTTAGATCTTAAAGGTAAAACAGTCCTAGTACGTGCT
GATTTTAACGTACCTTTAAAAGACGGTGAAATTACTAATGACAACCGTATCGTTCAAGCT
TTACCTACAATTCAATACATCATCGAACAAGGTGGTAAAATCGTACTATTTTCACATTTAG
GTAAAGTGAAAGAAGAAAGTGATAAAGCAAAATTAACTTTACGTCCAGTTGCTGAAGAC
TTATCTAAGAAATTAGATAAAGAAGTTGTTTTCGTACCAGAAACACGCGGCGAAAAACTT
GAAGCTGCTATTAAAGACCTTAAAGAAGGCGACGTATTATTAGTTGAAAATACACGTTAT
GAAGATTTAGACGGTAAAAAAGAATCTAAAAATGATCCAGAATTAGGTAAATACTGGGC
ATCTTTAGGTGATGTGTTTGTAAATGATGCTTTTGGTACTGCGCATCGTGAGCATGCATCT
AATGTTGGTATTTCTACACATTTAGAAACTGCAGCTGGATTCTTAATGGATAAAGAAATT
AAGTTTATTGGTGGCGTAGTTAACGATCCACATAAACCAGTTGTTGCTATTTTAGGTGGA
GCAAAAGTATCTGACAAAATTAATGTCATCAAAAACTTAGTTAACATAGCTGATAAAATT
ATCATCGGCGGAGGTATGGCTTATACTTTCTTAAAAGCGCAAGGTAAAGAAATTGGTATT
TCATTATTAGAAGAAGATAAAATCGACTTCGCAAAAGATTTATTAGAAAAACATGGTGAT
AAAATTGTATTACCAGTAGACACTAAAGTTGCTAAAGAATTTTCTAATGATGCCAAAATT
ACTGTAGTACCATCTGATTCAATTCCAGCAGACCAAGAAGGTATGGATATTGGACCAAAC
ACTGTAAAATTATTTGCAGATGAATTAGAAGGTGCGCACACTGTTGTATGGAATGGACCT
ATGGGTGTATTCGAGTTCAGTAACTTTGCACAAGGTACAATTGGTGTATGTAAAGCAATT
GCAAACCTTAAAGATGCAATTACGATTATCGGTGGCGGTGATTCAGCTGCAGCAGCAATC
TCTTTAGGTTTTGAAAATGACTTCACTCATATTTCAACTGGTGGCGGTGCGTCATTAGAGT
ACCTAGAAGGTAAAGAATTGCCTGGTATCAAAGCAATCAATAATAAATAA

> **tpi**

Function: protein coding sequence; triosephosphate isomerase (core genome, constant)
Best match: tpi_CC001-ST772_118_AJGE01000036.1[23343:24104:r] (completely identical)
Position: 023-contig_244_RC: 11071 … 11833; Length: 762 bp
Sequence:
ATGAGAACACCAATTATAGCTGGTAACTGGAAAATGAACAAAACAGTACAAGAAGCAAA
AGACTTCGTCAATGCATTGCCAACATTACCAGATTCAAAAGAAGTAGAATCAGTAATTTG
TGCACCAGCAATTCAATTAGATGCATTAACTACTGCAGTTAAAGAAGGAAAAGCACAAG
GTTTAGAAATCGGTGCTCAAAATACGTATTTCGAAGATAATGGTGCGTTCACAGGTGAAA
CGTCTCCAGTTGCATTAGCAGATTTAGGCGTTAAATACGTTGTTATCGGTCATTCTGAACG
TCGTGAATTATTCCACGAAACAGATGAAGAAATTAACAAAAAAGCGCACGCTATTTTCAA

ACATGGAATGACTCCAATTATTTGTGTTGGTGAAACAGACGAAGAGCGTGAAAGTGGTA
AAGCTAACGATGTTGTAGGTGAGCAAGTTAAGAAAGCTGTTGCAGGTTTATCTGAAGATC
AACTTAAATCAGTTGTAATTGCTTATGAGCCAATCTGGGCAATCGGAACTGGTAAATCAT
CAACATCTGAAGATGCAAATGAAATGTGTGCATTTGTACGTCAAACTATTGCTGACTTAT
CAAGCAAAGAAGTATCAGAAGCAACTCGTATTCAATATGGTGGTAGTGTTAAACCTAACA
ACATTAAAGAATACATGGCACAAACTGATATTGATGGGGCATTAGTAGGTGGCGCATCAC
TTAAAGTTGAAGATTTCGTACAATTGTTAGAAGGTGCAAAATAA

> **gpmI**

Function: protein coding sequence; 2,3-bisphosphoglycerate-independent phosphoglycerate mutase (core genome, constant)
Best match: gpmI_CC001_MW2-USA400_BA000033.2[831126:832643] (completely identical)
Position: 023-contig_244_RC: 11835 … 13353; Length: 1518 bp
Sequence:
ATGGCTAAGAAACCAACTGCGTTAATTATTTTAGATGGTTTTGCGAACCGCGAAAGCGAA
CATGGTAATGCGGTAAAATTAGCAAACAAGCCTAATTTTGATCGTTATTACAACAAATAT
CCAACGACTCAAATCGAAGCGAGTGGCTTAGATGTTGGACTACCTGAAGGACAAATGGG
TAACTCAGAAGTTGGTCATATGAATATCGGTGCAGGACGTATCGTTTATCAAAGTTTAAC
TCGAATCAATAAATCAATTGAAGACGGTGATTTCTTTGAAAATGATGTTTTAAATAATGC
AATTGCACACGTGAATTCACATGATTCAGCGTTACACATCTTTGGTTTATTGTCTGACGGT
GGTGTGCACAGTCATTACAAACATTTATTTGCTTTGTTAGAACTTGCTAAAAAACAAGGT
GTTGAAAAAGTTTACGTACACGCATTTTTAGATGGTCGTGACGTAGATCAAAAATCCGCT
TTGAAATACATCGAAGAGACTGAAGCTAAATTCAATGAATTAGGCATTGGTCAATTTGCA
TCTGTGTCTGGTCGTTATTATGCAATGGACCGTGACAAACGTTGGGAACGTGAAGAAAAA
GCTTACAATGCTATTCGTAATTTTGATGCCCCAACTTATGCAACTGCCAAAGAAGGTGTA
GAAGCAAGCTATAATGAGGGCTTAACTGACGAATTCGTAGTACCATTCATCGTTGAGAAT
CAAAATGACGGTGTTAATGATGGAGATGCAGTGATCTTCTATAATTTCCGACCTGATAGA
GCAGCACAATTATCGGAAATTTTTGCGAACAGAGCATTCGAAGGCTTTAAAGTTGAACAA
GTTAAAGACTTATTCTATGCAACATTCACTAAGTATAACGACAATATCGATGCGGCTATC
GTTTTCGAAAAAGTTGATTTAAATAATACAATTGGTGAAATTGCACAAAATAACAATTTA
ACACAATTACGTATTGCAGAAACTGAAAAATATCCTCACGTTACTTACTTTATGAGTGGT
GGACGTAACGAGGAATTTAAAGGTGAACGCCGTCGTTTAATTGATTCACCTAAAGTTGCA
ACGTATGACTTGAAACCAGAAATGAGTGCTTATGAAGTTAAAGATGCATTATTAGAAGAG
TTAAATAAAGGTGACTTGGACTTAATTATTTTAAACTTTGCTAACCCTGATATGGTTGGAC
ATAGTGGTATGCTTGAGCCGACAATCAAAGCAATCGAAGCGGTTGATGAATGTTTAGGTG
AAGTCGTTGATAAGATTTTAGACATGGACGGTTATGCAATTATTACTGCTGACCATGGTA
ACTCTGATCAAGTATTGACGGATGATGATCAACCAATGACTACGCATACAACGAACCCAG
TACCAGTGATTGTAACAAAAGAAGGCGTTACACTTCGAGAAACTGGTCGCTTAGGTGACT
TAGCACCTACATTATTAGATTTATTAAATGTAGAACAACCTGAAGACATGACAGGTGAAT
CTTTAATTAAACACTAA

> **eno**

Function: protein coding sequence; enolase (core genome, constant)
Best match: eno_CC001_MW2-USA400_BA000033.2[832773:834077] (completely identical)
Position: 023-contig_244_RC: 13482 … 14787; Length: 1305 bp
Sequence:
ATGCCAATTATTACAGATGTTTACGCTCGCGAAGTCTTAGACTCTCGTGGTAACCCAACTG
TTGAAGTAGAAGTATTAACTGAAAGTGGCGCATTTGGTCGTGCATTAGTACCATCAGGTG
CTTCAACTGGTGAACACGAAGCTGTTGAATTACGTGATGGAGACAAATCACGTTATTTAG
GTAAAGGTGTTACTAAAGCAGTTGAAAACGTTAATGAAATCATCGCACCAGAAATTATTG
AAGGTGAATTTTCAGTATTAGATCAAGTATCTATTGATAAAATGATGATCGCATTAGACG
GTACTCCAAACAAAGGTAAATTAGGTGCAAATGCTATTTTAGGTGTATCTATCGCAGTAG
CACGTGCAGCAGCTGACTTATTAGGTCAACCACTTTACAAATATTTAGGTGGATTTAATG
GTAAGCAGTTACCAGTACCAATGATGAACATCGTTAATGGTGGTTCTCACTCAGATGCTC
CAATTGCATTCCAAGAATTCATGATTTTACCTGTAGGTGCTACAACGTTCAAAGAATCATT

ACGTTGGGGTACTGAAATTTTCCACAACTTAAAATCAATTTTAAGCAAACGTGGTTTAGA
AACTGCAGTAGGTGACGAAGGTGGTTTCGCTCCTAAATTTGAAGGTACTGAAGATGCTGT
TGAAACAATTATCCAAGCAATCGAAGCAGCTGGTTACAAACCAGGTGAAGAAGTATTCTT
AGGATTTGACTGTGCATCATCAGAATTCTATGAAAATGGTGTATATGACTACAGTAAGTT
CGAAGGCGAACACGGTGCAAAACGTACAGCTGCAGAACAAGTTGACTACTTAGAACAAT
TAGTAGACAAATATCCTATCATTACAATTGAAGACGGTATGGACGAAAACGACTGGGAT
GGTTGGAAACAACTTACAGAACGTATCGGTGACCGTGTACAATTAGTAGGTGACGATTTA
TTCGTAACAAACACTGAAATTTTAGCAAAAGGTATTGAAAACGGAATTGGTAACTCAATC
TTAATTAAAGTTAACCAAATCGGTACATTAACTGAAACATTTGATGCAATCGAAATGGCT
CAAAAAGCTGGTTACACAGCAGTAGTTTCTCACCGTTCAGGTGAAACAGAAGATACAAC
AATTGCTGATATTGCTGTTGCTACAAACGCTGGTCAAATTAAAACTGGTTCATTATCACGT
ACTGACCGTATTGCTAAATACAATCAATTATTACGTATCGAAGATGAATTATTTGAAACT
GCTAAATATGACGGTATCAAATCATTCTATAACTTAGATAAATAA

## > Q2YSE7
Function: protein coding sequence; putative membrane protein (core genome, constant)
Best match: Q2YSE7_CC008_NCTC8325_CP000253.1[783701:784159] (completely identical)
Position: 023-contig_244_RC: 15126 … 15585; Length: 459 bp
Sequence:
ATGGCTGATAGAACGAATAAAGAAATTAAAACAGGACGCTTTATTGCAACTGCATCAATC
GTATTCTCAATATTATTGATTATTCATTACTTTGTTTCGTTGGATAATGCGACTGCCAAAG
CATTACTTAATTTAACGAATCAAAACACTTCAGATAAAGCGATTGATTACATTTTAAACA
GCTTTAGATTCACTGGTATTATGTATATTTTGGCTTATCTAGCAGGCTTCATCACTTTTTGG
AATCGACATACTTATGTGTGGTGGTTTATGTTTGCAGTTTATGTATCAAATAGTTTGTTTA
CGTTGATTAATTTATCAATCACAATTCAAGCAATAAAAGCTGCACACGGTGCGTACTTAA
CATTGCCAATTTTAATTGTTATTATAGGTTCGGTTGCATTAGCGATTTATATGCTTGTTGTT
TCTATCAAACGTAAAAGTACATTTAATCGCTAG

## > secG
Function: protein coding sequence; preprotein translocase/SecG subunit (core genome, constant)
Best match: secG_CC001_MW2-USA400_BA000033.2[834941:835174] (completely identical)
Position: 023-contig_244_RC: 15651 … 15885; Length: 234 bp
Sequence:
ATGCATACATTTTTAATCGTATTATTAATCATTGATTGTATTGCATTAATAACTGTTGTACT
ACTCCAAGAAGGTAAAAGCAGTGGACTTTCAGGTGCCATCAGTGGTGGTGCTGAGCAGTT
ATTCGGTAAACAAAAACAACGTGGCGTCGATTTATTCTTAAATAGATTAACAATTATTTT
ATCAATATTATTTTTTGTACTTATGATTTGCATAAGTTATCTTGGTATGTAA

## > est
Function: protein coding sequence; putative carboxylesterase (core genome, constant)
Best match: est_CC001_MW2-USA400_BA000033.2[835290:836030]
Position: 023-contig_244_RC: 16000 … 16741; Length: 741 bp
Sequence:
ATGCAGATAAAATTACCAAAACCTTTCTTTTTTGAGGAAGGTAAACGTGCCGTGTTATTA
CTACATGGTTTTACAGGCAATTCGTCTGATGTTCGTCAATTAGGTCGATTTTTACAAAAGA
AAGGTTATACATCATATGCACCGCAATATGAAGGCCATGCAGCACCGCCAGAAGAGATA
CTTCAATCAAGTCCTTTTGTTTGGTTTAAAGATGCCTTAGATGGCTATGACTACCTTGTTG
AACAAGGTTATGATGAAATCGTTGTTGCTGGCTTGTCATTAGGTGGCGATTTTGCATTAAA
ATTAAGCTTAAATAGAGATGTAAAGGGTATTGTAACGATGTGTGCACCTATGGGTGGCAA
AACTGAAGGTGCCATTTATGAAGGCTTTTTAGAATATGCACGCAATTTTAAAAAGTATGA
GGGTAAAGATCAAGAAACGATTGATAACGAAATGTATCATTTCAAACCAACTGAAACTTT
AAAAGAATTAAGTGAAGCATTAGATACGATTAAAGATCAAGTTGATGAGGTGTTTGACCC
AATTTTAGTGATTCAAGCAGAAAACGACAATATGATTGATCCACAATCCGCAAATTATAT
ATATGAACATGTAGACTCTGACGATAAAAATATCAAGTGGTACAGTGAATCTGGACATGT

TATTACGATTGATAAAGAAAAAGAACAAGTATTTGAAGATATTTATCAATTTTTAGAGTC
ATTAGACTGGTCAGAATAA

> **rnr**
Function: protein coding sequence; ribonuclease R (core genome, constant)
Best match: rnr_AC074317.5[11831:14203:r]
Position: 023-contig_244_RC: 19117 … 19147; Length: 30 bp
Sequence:
AAAAAGAAAGCACGTCGTAAGAAAAAATAA

> **ssrP**
Function: protein coding sequence; tmRNA and ssrA-binding protein (core genome, constant)
Best match: ssrP_CC001-ST772_118_AJGE01000036.1[15543:16007:r] (completely identical)
Position: 023-contig_244_RC: 19168 … 19633; Length: 465 bp
Sequence:
ATGGCTAAGAAGAAATCACCAGGTACATTAGCGGAAAATCGTAAAGCTAGACATGATTA
TAATATTGAAGACACGATTGAAGCAGGAATTGTATTGCAAGGCACAGAAATAAAATCAA
TTCGCCGAGGTAGTGCTAACCTTAAAGATAGTTATGCGCAAGTTAAAAACGGTGAAATGT
ATTTGAATAATATGCATATAGCACCATACGAAGAAGGGAATCGTTTTAATCACGATCCTC
TTCGTTCTCGAAAATTATTATTGCACAAGCGTGAAATCATTAAATTGGGTGATCAAACAC
GTGAGATTGGTTATTCGATTGTGCCGTTAAAGCTTTATTTGAAGCATGGACATTGTAAAGT
ATTACTTGGTGTTGCACGAGGTAAGAAAAAATATGATAAACGTCAAGCTTTGAAAGAAA
AAGCAGTCAAACGAGATGTTGCGCGCGATATGAAAGCCCGTTATTAA

> **ssrA**
Function: noncoding RNA tmRNA/regulatory RNA (core genome, constant)
Best match: ssrA_CC001-ST772_118_AJGE01000036.1[15093:15452:r] (completely identical)
Position: 023-contig_244_RC: 19723 … 20083; Length: 360 bp
Sequence:
GGGGACGTTCATGGATTCGACAGGGGTCCCCCGAGCTCATTAAGCGTGTCGGAGGGTTGT
CTTCGTCATCAACACACACAGTTTATAATAACTGGCAAATCAAACAATAATTTCGCAGTA
GCTGCCTAATCGCACTCTGCATCGCCTAACAGCATTTCCTATGTGCTGTTAACGCGATTCA
ACCTTAATAGGATATGCTAAACACTGCCGTTTGAAGTCTGTTTAGAAGAAACTTAATCAA
GCTAGCATCATGTTGGTTGTTTATCACTTTTCATGATGCGAAACTTTTCGATAAACTACAC
ACGTAGAAAGATGTGTATCAGGACCTCTGGACGCGGGTTCAAATCCCGCCGTCTCCAT

> **attB_nySa3**
Function:  attachment site of gemomic island nySa3 carrying entL/entC in MW2 (mobile element)
Best match: attB_nySa3_empty_CC025_21305_AFNO01000021.1[16544:16637]RC (completely identical)
Position: 023-contig_244_RC: 20028 … 20122; Length: 94 bp
Sequence:
TAGAAAGATGTGTATCAGGACCTCTGGACGCGGGTTCAAATCCCGCCGTCTCCATTATAT
AGTCTGCAACCTTAGTGGTTGTAGGCTTTTTGTT

> **tx_ssrA**
Function:  rho independent terminator of ssrA (tmRNA)
Best match: tx_ssrA_int4_CC001-ST772_118_AJGE01000036.1[15047:15101:r] (completely identical)
Position: 023-contig_244_RC: 20074 … 20129; Length: 55 bp
Sequence:
CGTCTCCATTATATAGTCTGCAACCTTAGTGGTTGTAGGCTTTTTGTTTTTGAGT

> **int4-nySa3**

Function: protein coding sequence; bacteriophage integrase (mobile element)
Best match: int4-nySa3_CC001-ST772_118_AJGE01000036.1[13874:14980]RC (completely identical)
Position: 023-contig_244_RC: 20195 … 21302; Length: 1107 bp
Sequence:
TTATAAGTATTTAACTATTTCTTTCTGTGTACTAGGATATAAATGACCATATCGGTTATAT
ACTTCAGTCGTATCGCTGTGACCTAAACGCTGAGCTATAATCATTACACTGGCCCCTAGAT
TAACTAACATAGATGCATGACTATGTCTTAACTCGTGTATAACGATTCTAGGGAAATTTTG
TCCATTTGGTAATTGCTCATCCAACACTTTTAATGCGTTAGTAAACCAACGATCAATGGTA
GACTCGCTGTAAGCTTTATAGAATGTACCAAATAATACATAATCATCTTTAAATATGTTAT
TCTCTTTATACCAATTTAAATAACCTTTGATGTCATCCATCATGTGAGTAGGCAAATATAT
ATCGCGTATGGCTGATTTCGTTTTAGGGGCTGTCACTTCACCGTGATAGTCCGTTTTGTTT
ATATGGATAAAATCATCATCAAAGTTAATATCGCGCCATGTGAGCGCTCTGATTTCGCCTT
TGCGTGCTCCAGAGTAGAACAATAGTTTAAAGAATAACTTTTGTTGTTGTGTCACTAGCG
CACCATAAAATTGATTGAATTGTTCTAATGTCCAATAATTCAATCGTTTCTGTGATTCTAT
TTCAAAATTCCCTACAAGAGATGCAACATTTTGTTTTAACTCATGAAACTTCATTGCATGA
TTCAGTAATGATACTAAATATACATGCATCTTCTTTAAGTAGTCTCCAGAATGCCCCTCTT
TTAACTTCTTATTCTGAAATTTCATCACATCTTGTGTAGTCATTTTAAATACATCCATAGAT
TTAAAATAAGGTAGCAAGTGGTTATTTGTATGTGTTTTTAATGCTTTTACACTTGATGTCT
TTCGACGTGCTGAATACCAGTCTATATATTCTTCAACGAGCTTGTCAAAAGGTAATCTATT
AAGGTGTCCGACTCCCTCTAATTCGTCCATCATTTCGTTACATTTCTTAACTGCGTCTTTAC
GTTGTTTAAACCCCTTACGTGTTATGTACTTACGAGTATTGCTCTTGTCATAGTAAGTGAT
ACGGAAATAATAAGTACCGCGTTTAGTGTCTTTATATATGTTATGGGATAGGTTTAAGTTA
TGTGTCAT

## > str-nySa3
Function: protein coding sequence; putative protein (mobile element)
Best match: str-nySa3_CC001-ST772_118_AJGE01000036.1[12488:12706:r] (completely identical)
Position: 023-contig_244_RC: 22469 … 22688; Length: 219 bp
Sequence:
ATGACAAAACTAGCTTATCCAATGTTATACATTACTAGAAAAGAAAAAGGTGACACACA
AAAGAAAGTTGCTAGCAAACTTGGTATTAGTCCACAACGTTACCAGTTAAAGGAAAGTG
GTAAAGCAATATTTAATTTAAATGAGTGTCAGATTCTTTCAGAAATGTATGATATGCCAA
TTGATGAGTTATTTTCATCAAAAATTAAAGTAGGAGAATAG

## > Q5HHJ1
Function: protein coding sequence; putative protein (mobile element)
Best match: Q5HHJ1-COL_ref_CC008_COL_CP000046.1[908684:909004]
Position: 023-contig_244_RC: 23050 … 23200; Length: 150 bp
Sequence:
ATGAATTGGGAAATTAAAGATTTAATGTGTGACATTGAAGTGATAAAACAAAAAATTAAT
GATGTAGCTACCAAACATGCTTGGTTTGTTGAAGATAGATTTGTAAAAAATGAATTAGAA
ACAAAACGGGAACATATTAATTTTTCTGCT

## > Q5HHJ1-RN3984
Function: protein coding sequence; putative protein (mobile element)
Best match: Q5HHJ1-RN3984_CC001-ST772_118_AJGE01000036.1[11808:12125:r] (completely identical)
Position: 023-contig_244_RC: 23050 … 23368; Length: 318 bp
Sequence:
ATGAATTGGGAAATTAAAGATTTAATGTGTGACATTGAAGTGATAAAACAAAAAATTAAT
GATGTAGCTACCAAACATGCTTGGTTTGTTGAAGATAGATTTGTAAAAAATGAATTAGAA
ACAAAACGGGAACATATTAATTTTTCTGCTAGCTATTTAGAACATCGTATACAAAATGAA
CATACAGTTGAGTTATTACATGTGTACTTAAAAGAATTCGGTGAACTTATACAAAAATTT

CATGAAATAGAAAAAGCATCATCTGAGAACTTTGGCGAGGTATCAGATGACGCACAAAA
ATTAAAAATCACAGAGTAA

> **O54473-nySa1**

Function: protein coding sequence; putative protein (mobile element)
Best match: O54473-nySa1_CC001-ST772_118_AJGE01000036.1[10875:11744:r] (completely identical)
Position: 023-contig_244_RC: 23431 … 24301; Length: 870 bp
Sequence:
ATGAATGAAATTAAATTAGAATATGACACACATGTTTCAGTGGTACATTATGAAAGTTTA
GACTCACGTTCATTTAATAGCTTTTCAAAAATTAATTGGAGTAAGTTGGTTAATAAACTGT
CTGTACCTATAGAAGCAAATTATAAGTATGCACGTGGTGTTGCTGTTTACGGTGATATTA
AAAACGGTGCAAATGATCATGGTGAAATTATCAAAAAGCATCGCAATGACGTTAATGTC
GTATACAGAGATGTGATTGTACTTGATTACGATGAAATAAATGATTTAAAGCAATTACAT
GAAGCAATCAGCTCAGCTTTAAGCAATGTTGCATGGTTTTGGCACACAAGTTACTCGCAC
AGAACTGAACAAGCTAGAATACGCCTGTATATCCCTCTAAATGAGCGAATAAGTGCAGAT
GATTATCGTAATTATACAAAAGTATTAGCGAATAAAATTGGCCACAAAGTTGATGAAGGT
TCATATCAGCCAAGTAGATGTTTTGCGTTACCAGTTATTCAAAAAGGACACATATTTATTA
AACGAGTGAATGACTGTCCGATTATGAATGTTGATATGCTCGAGCAGTGGTCAAAGGGGT
ATAAACAATCAAATGATAGTCCTAATATCAAAGGGTACACACGACGTGATAGTGCGTATT
GGCGAGATATAGCTTTTGGTGTAAGTGAGGGAGAGCGCAATTCAACATTGGCTTCAATTA
CAGGTTATCTTTTGCGTAGGTATGTAGATCCAAACTTAGTTTATGGGTTAGTGAGTGCGTG
GGCAAGTGTATGCAAACCACCTATTAATCAAAGTGAAGTAAACAATACTTTTAAAAGTAT
TTTGAAAAAAGATAGTAAATACAGTTAG

> **vapE**

Function: protein coding sequence; bacteriophage virulence associated protein (mobile element)
Best match: vapE_CC001-ST772_118_AJGE01000036.1[9401:10825:r] (completely identical)
Position: 023-contig_244_RC: 24350 … 25775; Length: 1425 bp
Sequence:
ATGATTGATAGCAGAACCGGTGTTTTAAATGCTAATGATTGGAAAAGTCAATTAAGGCGT
TCTGCTACTACACAAGCATTGAAAAAAACGACTACAAATGCTGAAATCATATTGTGTAAT
GATGAGAGTTTAAAAGGGCTAGTACAATATGACGCTTTTGAAAAAGTAACCAAACTGAA
ACGTCTACCGTATTGGAGGTCAAAAGGGGATGCGAATTATTATTGGGCTGATATAGATAC
CACACATGTGATTTCACATATTGATAGATTGTATAATGTGCAGTTTAGCCGTGATCTTATT
GATACTGTGATTGAAAAGGAAGCTTATCAAAATAGATTCCACCCTATTAAATCGATGATT
GAATCTAAATCATGGGACGGAATCAAAAGAATTGAAACGCTCTTCATTGATTATTTAGGT
GCTGAAGATAACCATTACAATAGAGAAGTTACAAAAAAATGGATGATGGGTGCAGTTGC
TAGAATCTATCAGCCAGGTATTAAATATGATTCCATGATTATTTTATATGGTGGTCAAGGT
GTTGGGAAATCTACGGCAGTGAGTAAATTGGGAGGTCATTGGTATAACCAAAGTATTAAA
ACGTTTAAAGGTGATGAGGTCTATAAGAAATTGCAGGGTTCTTGGATATGTGAAATTGAA
GAACTATCGGCATTTCAAAAGTCTACTATTGAAGATATTAAGGGTTTTATAAGTGCCATT
GTAGATATTTATAGAGCTTCGTATGGTAAACGCACAGAGCGTCATCCTAGACAGTGTGTG
TTTGTAGGGACAACCAATAACTATGAGTTTTTAAAAGACCAAACAGGCAATCGTCGTTTT
TTCCCTATTACGACAGATAAAAATAAAGCAACTAAAAGCCCATTTGACGATCTAACACCA
GATGTTGTGCAACAAATGTTTGCCGAAGCTAAAGTATATTTTGATGAGAATCCGACGGAT
AAAGCATTGTTGCTAGATAAAGAAGCGAGTGAAATGGCTTTAAAAGTCCAAGAAGCTCA
TTCTGAAAAAGATGCTTTAGTTGGAGAAATAGAAGAATTTCTTGAACGTCCTATTCCGTC
AGACTACTGGTATAGAACGTTAGAAGAAAAAAGAGTGTCTGCGCATGGTGTTATAGACC
AAGACTATATTAAATTATATGGTGATGGTAAATTGATTGAATTACCGAATACAAAACCAG
GTGCTTATGTATGGCGTGACAAGGTATGTAGCATGGAAATTTGGAAAGTGATGATGAAAC
GAGATGACCAACCACAACAACACCATTTAAGAAAAATTGATAAAGCGTTAAGAAATACA
AATTATTGTGACATTGTGAAAAAGCAAACGCGATATGGTGAAGGTATTGGTAAGCAATAT
GGCTTTAGTGTAGATTTAGCTTCTTATTATCAGAAACTTAAAGTTTAG

> **Q5HHI6**
Function: protein coding sequence; putative protein (mobile element)
Best match: Q5HHI6_CC001-ST772_118_AJGE01000036.1[7814:8455:r] (completely identical)
Position: 023-contig_244_RC: 26720 … 27362; Length: 642 bp
Sequence:
ATGAACATAGAAACTGTAGTAAATGAATTTGAAACACAAGCAGGCACGTTACTAAGGTA
CTACACTGGATTATTAGAACATAGTAAAGTACAACCGTATTGCTTTAAGTTATACAATGA
TCCGTTTGATATGGTTTATGTGATGATGAACGGGAAGTTATTCGGTCATGTATATATTAAA
GATTGTAAAGTAAGGCAATCATTTGAATTAGCGTCACCTAAGCACACTGAGGGGCTTATA
AGAAGTATAGAAGGTCATTATGTAGGTTATGAATTACATGACGGTAAACAGCTTTCTATT
AGTGATATGATGGCCAGTCAATTATTTGAAGATGAGTATTTTATGTATGGATTACAAACA
TATGCAGAATCAAATAATAGTGATGTGTTTGAGTACCTAGAAAATGGATTTGATACCGAT
ACACTTGAGGGCATTCAATCGAGTAATACTGATGTGATATCGAATATTGAAATGTTGTAT
CAGATAGCTACGGGAATCAATGAACCAGCACCAGAGTTAGTTGAGGGGTTGAGATTAGT
AACTGAGTTTGTACAAGATGAGAAGGCTGCACAAGAGGATTACACGGGGTTAGAACGTA
AATTGAATGATCTAAAAGCGTCTTATTATAGTATAAGTAAATAA

> **Q5HHI1**
Function: protein coding sequence; putative protein (mobile element)
Best match: Q5HHI1_CC001-ST772_118_AJGE01000036.1[5047:5388:r] (completely identical)
Position: 023-contig_244_RC: 29787 … 30129; Length: 342 bp
Sequence:
ATGGATAAAAAGCAAATAAAAGGCTTCGTGTGTGATTATCATAAGCGAACTAGAAGTGA
TGTATTAATAGATGATGATATAAATACTGATGAATTCTTTTCAATAGGTGATGAAAATTCT
AATGAATGGATGACAGACGATAATGTTGATGATCATATTATAAAGAATCACTTAGAAATG
ATTGTTGACCGAGTAGCTAATGATAAAGAGTTTTATATTTTCGATTCTTTAATACAAGGAC
GTAGTTATCAAGATATTAGTAGTGTCTTAGATTGTTCTGAACAATCTGTAAGATTATGGTA
TGAAACCTTATTAGATAAAATTGTGGAGGTGATAGAATGA

> **terminaseS-phiPT1028**
Function: protein coding sequence; bacteriophage terminase small subunit (mobile element)
Best match: terminaseS-phiPT1028_CC030_MRSA252_BX571856.1[420468:421037] (completely identical)
Position: 023-contig_244_RC: 30125 … 30695; Length: 570 bp
Sequence:
ATGAGTGAGTTAACGGCAAAGCAAGCGCGTTTTGTGAATGAGTATATAAGAACACTTAAT
GTGACACAAAGTGCCATAAAAGCAGGTTATAGCGCAAATAGTGCACATGTGACAGGGTG
TAGGTTATTAAAGAAGCCACACATCAAGCAATATATACAAGAACAAAAAGATAAGATTA
TAGATGAGAATGTATTAACTGCAAAAGAGTTACTACATGTGCTTACGAATGCGGCAGTCG
GTGACGAAACAGAAACGAAAGAAGTTGTGGTCAAGCGTGGGGAATATAAAGAGAATCCA
CAAAGTGGCAAAGTACAGTTAGTCTATAATGAACATGTTGAACTGATAGAGGTGCCAATT
AAGCCAAGTGATCGTTTAAAAGCTCGTGATATGTTGGGTAAATACCATAAGTTATTTACA
GATAAGCATGATATCAACGGGGATGTTCCTATATTCATTAACATTGGTGAATGGGACGGA
GACGATGAGGAATTAGATAAAACTGTAAAAGATGTATCTAACGCTAATCCTAACCATACT
GTGATTGTGGATGATATACCGTTAGAGGATTGA

> **Q6GJT2**
Function: protein coding sequence; putative bacteriophagal protein (mobile element)
Best match: Q6GJT2_CC005_Mu50-VRSA_BA000017.4[878889:879857] (completely identical)
Position: 023-contig_244_RC: 30971 … 31940; Length: 969 bp
Sequence:
ATGAAGCCATTTGAAAGTCATAATAAACAATTGAAAATTCTAAGAAGAAGAGGAATGGA
AGTACCGAGTAGTGCTAAAAGAGATTTAGAAAATGAAAATTATTATAATATCATAAATGG
TTATAAAGATTTATTTTTAGAACTAGATGTTAATGGTAATTTTTTGGTTCCTGATAAATAT
AAGCAAGGTACTCATTTTAAAGAAGTCTTTTCTTTATACAAACTAGATAGAAAATTTAGG

AATGTTTTATTAGAGTATTTGTTAGTATTTGAAACTCATATTAAATCAAGAATTTCATATT
ATTTTAGCGAAAAATATAGAGAACCACATTCATATTTATACTTTAAAAATTATTCATCTGA
CACAAGTAAGACAGATAGCATCGTGAAAATGGTTGCTACATTTAGCTCGGTTATGAGTAA
TAGAAAAAATAAACCATTAAAACATTATATTAATACTCATAATGGAGTGCCACTATGGAT
ATTGGTGAATTATTTAACTTTAGGTAATGTTTCAAAAATGTATTCCAATTTGGATGATGAT
CTTCGATTGGAAGTTGCTAAAGACTATAAAAGGAAATTGGAAAGAGATTATAAAACACG
TGTTCAAATAACTCCATCAGATGTAGACAGTATACTACAACAAGCACATATGTTTCGTAA
CGTGTGTGCGCATGAAGAAAGATTGTATGATTATAAAATAGACAGGGCTAAAAGTAGAG
CTAATATATTCGCCAATTATAACAAAATATACGATAAAGAATACGTTCCTACAATGAATG
GTAGTTATGTATTCGATTTGTTGATTTCACTATGTCTATTTTTGAATAAACATGATTACATA
AAATTGATGAAAAATATGGATAAACTAATAAGTAATTATTCACATTCTTTCTATACAATT
ACTATAGATGACCTATATACAAAAATGAATTTTCCAGATCAAACAAAAATACTGGATATG
TTATAA

> **ear**

Function: protein coding sequence; enterotoxin-linked ampicillin resistance protein (mobile element)
Best match: ear-MNDon_CC001-ST772_118_AJGE01000036.1[2485:3039]RC (completely identical)
Position: 023-contig_244_RC: 32136 … 32691; Length: 555 bp
Sequence:
CTAGTTTATAGTTATTTTATCTATGTCTTTCTTGTATAACATATAATTCTTATGCTCAGTAA
AATCTTGTCCAACTATTCCAGAGATTTTAGGATCTTTTTGGTCTTTACTATGAATGGTTACT
TTATCACCATCTTTAATGATTTTCTGTCCTTTTAATTTGTTAGTTAAATTCTTCCATGTATT
ATTAGCATCAATTCTTTCGTTGAAATTACTAATATTTACTTTTTTGATATTAATAGTAGTTA
AACTTTCAATATCTATATTTTGTTTCGAAAAATCTCCATTAGAGTTCCCATTTGCTGAAAT
TTTATTTTCGCTACCATTTTTCAATTTATAATTAACATCTATTGTTTCATTTTCATTTCCATC
AATTATATTAGCCTCTTTCAAAGCATCTCTTACATTTTTCCACAACTGTCCGTCTGTTGTTT
CAGCAGCTTTTGCAACGTTATTAATACCATTATAATTTGAAGAAGAATGAAAACCTGAAC
CTACTGTTGTTAAAACTAATGCACTTGCTATCAATGTTTTTGTTAATAGTTTTTTATTCAT

> **entC**

Function: protein coding sequence; enterotoxin C (mobile element)
Best match: entC-MW2_CC001-ST772_118_AJGE01000036.1[901:1701:r] (completely identical)
Position: 023-contig_244_RC: 33474 … 34275; Length: 801 bp
Sequence:
ATGAATAAGAGTCGATTTATTTCATGCGTAATTTTGATATTCGCACTTATACTAGTTCTTTT
TACACCCAACGTATTAGCAGAGAGCCAACCAGACCCTACGCCAGATGAGTTGCACAAAT
CAAGTGAGTTTACTGGTACGATGGGTAATATGAAATATTTATATGATGATCATTATGTATC
AGCAACTAAAGTTATGTCTGTAGATAAATTTTTGGCACATGATTTAATTTATAACATTAGT
GATAAAAAACTAAAAAATTATGACAAAGTGAAAACAGAGTTATTAAATGAAGATTTAGC
AAAGAAGTACAAAGATGAAGTAGTTGATGTGTATGGATCAAATTACTATGTAAACTGCTA
TTTTTCATCCAAAGATAATGTAGGTAAAGTTACAGGTGGTAAAACTTGTATGTATGGAGG
AATAACAAAACATGAAGGAAACCACTTTGATAATGGGAACTTACAAAATGTACTTATAA
GAGTTTATGAAAATAAAAGAAACACAATTTCTTTTGAAGTGCAAACTGATAAGAAAAGT
GTAACAGCTCAAGAACTAGACATAAAAGCTAGGAATTTTTTAATTAATAAAAAAAATTTG
TATGAGTTTAACAGTTCACCATATGAAACAGGATATATAAAATTTATTGAAAATAACGGC
AATACTTTTTGGTATGATATGATGCCTGCACCAGGCGATAAGTTTGACCAATCTAAATATT
TAATGATGTACAACGACAATAAAACGGTTGATTCTAAAAGTGTGAAGATAGAAGTCCAC
CTTACAACAAAGAATGGATAA

> **entL**

Function: protein coding sequence; enterotoxin L (mobile element)
Best match: entL_part_CC001-ST772_118_AJGE01000036.1[28:734]RC
Position: 023-contig_244_RC: 34441 … 35148; Length: 707 bp
Sequence:

TCATCTTTTTGAAATTTCGACATCTAGATGAAATTGTGTTGAACCAACAGTTTTATTATCT
TTGTAAATTTTTAAGAAGCTTTCTGGAAGACCGTATCCTGTGTAAAATAAATCATACGAG
AAATTAGAACCATCATTCATATGAAATACTACACTCCCCTTATCAAAACCGCTATAAAAT
TTTGAAGAAGTGCCGTATTCTTTACCTTTACCAGTATCATTGTGTCCATAAATATTAAATT
CATCTTGCAAGTATTTTCTTAGTTTGACATCTATTTCTTGTGCGGTAACCATTTTTTTATCT
GTAGAAACACTTTTAGTTGTAACTGTTTGATGCTTGCCATTGATAATTAAATTTATAGGTA
TTATTCTTGGCGAATCTAATTTATTGTTTTCTGCTTTAGTAACACCACCATATATAGTACG
AGAGTTAGAACCATATTTATAGCTTATACCAAAAACATCGACATTTTTCCCTTTAGATCAC
TAGTTATATATTCATTGTCAAATTCAGCATACAATGTATCATTTTTATACGAGTATTCTAA
GCGGTGTGATTCTGGTGAATTTTTGTCTTTAACATTCTTTAAATTCACATATTCATATTTAG
TATAAAAATTTCTTAGGTTTCCTGTACCTACATCGCCGTTAGATAAGACTGTATGATTAGA
AGAAAAAATAAATAAAGTAATAACAATTACAAATAA

## > dfrG

Function: protein coding sequence; dihydrofolate reductase (mobile element)
Best match: dfrG_CC008_930918-3_ABFA01000044.1[6452:6949:r] (completely identical)
Position: 023-contig_244_RC: 35419 … 35917; Length: 498 bp
Sequence:
ATGAAAGTTTCTTTGATTGCTGCGATGGATAAGAATAGAGTGATTGGCAAAGAGAATGAC
ATTCCTTGGAGGATTCCCAAGGACTGGGAATATGTTAAAAATACTACAAAGGGACATCCG
ATAATATTAGGTAGGAAGAACCTTGAATCAATCGGAAGAGCCTTACCTGACAGAAGAAA
TATTATTCTGACGAGAGATAAGGGGTTTACCTTTAATGGTTGTGAAATTGTTCATTCAATA
GAAGATGTTTTTGAGTTATGTAAAAACGAAGAAGAAATTTTTATTTTCGGAGGAGAACAG
ATTTATAATTTGTTTTTCCCTTATGTTGAGAAAATGTACATCACAAAAATACATCATGAAT
TCGAAGGAGATACTTTTTTTCCAGAAGTGAATTATGAGGAATGGAATGAGGTATTTGCCC
AAAAAGGGATAAAGAATGATAAAAATCCGTATAACTACTATTTTCATGTATATGAAAGA
AAAAACTTATTGAGTTAA

## > attB_nySa3

Function: attachment site of gemomic island nySa3 carrying entL/entC in MW2 (mobile element)
Best match: attB_nySa3_occupied_CC001_MW2-USA400_BA000033.2[853791:853848:r]RC (completely identical)
Position: 024-contig_320: 318 … 376; Length: 58 bp
Sequence:
AACTCCCGCCGTCTCCATATTTGTAGCCTACAACCTTTGTGGATGTGGGCTTTTTTAT

## > tx_ssrA

Function:  rho independent terminator of ssrA (tmRNA)
Best match: tx_ssrA_CC001_MW2-USA400_BA000033.2[853801:853855]
Position: 024-contig_320: 328 … 358; Length: 30 bp
Sequence:
GTCTCCATATTTGTAGCCTACAACCTTTGT

## > Q6GIK8

Function: protein coding sequence; putative membrane protein (genomic island)
Best match: Q6GIK8_ref_CC030_MRSA252_BX571856.1[884556:885170]
Position: 024-contig_320: 850 … 1465; Length: 615 bp
Sequence:
ATGAATGACTTGAGTTTATCTTCATTTTTGAAACGCAGTAACAAATTTATGCAATTCAATT
GTTTTATTTGTTTGATTCTAATAATAGTATTTTACATAATTGGTATGAATATACAGGACTTT
AGTGATTTTCCTAGTAAAGATTTAAATCATAAAGTGACTTATAATTTTAATGGGTTTTTGG
AGATATTTGTTAATAATGCTTTTATAGTTCCTTTGGTGTCACTTATATTATCGATAATACCA
ATACCATATTTGTATTTTATTCCTACAATTTCTACAATTTATTCATTGTCGGTTATTATTGG
GGTTACATTTTCATATAAATTAAACGAAGGGATAGCTATTTTTATTGGTATTTTGCCTCAT
GGTATTTTAGAAATATACTTAACAAGCATTGAACTATCAATGTTATTCTTATTAAATGCGT

ATATTAGAAAAAATTCAATGAATTTATTTAGAAAAAGAAAAGAGACATTGCCAAAATTTT
TTGTTTTATTAAAATCAATAGTAAAGTGTTACCTGCTAATATTTTTACCTGTGGCATTTTTA
TGCGCTTTAATTGAAATTACTGTTACACCAACTGTTTACAAATTTTTAACTAATATAATAT
AG

## > Q1YB79

Function: protein coding sequence; putative lipoprotein (core genome, variable)
Best match: Q1YB79_CC001-ST772_118_AJGE01000024.1[1795:2523:r]RC (completely identical)
Position: 024-contig_320: 1749 … 2478; Length: 729 bp
Sequence:
TTATACTTCATCTAAACCACTGTGGTCGTCATCTTTTTGCTTTTCTTTTTCTTTCTCTCGTTC
TTGTTCTTTTTTGTACTCTTCTTCAAATTCTTTTTCTTTCTTTTCTACTTCTTCTCTTGTTTCC
GCTCTATGAGAAAAATCTTCGGTTTTAAGTTTACTAAATTTGAATGATTTAGAATCAACTG
TTTTATCTTCTGAGTATTTATGGACATTTAAATTAATATTTCCATCACCTCTTAACTCATAG
ATGAACATGGCTTGTGCAGTTTTGTCTTTTTTAATTTGATCTTGGTTATGTTCTGTCCAATC
TTTATATTTTTTATCACTTAAAAGATAACCATCTCTTAATTTATTTACTGTATTTTTATCAT
CTTGAGTAATATTAATATAGTCATGAGAAATAGAAGATGGATTTAAATCTTTATCGTCTTT
TTTAGCAGTAATTTCCATTTTAAAAGCGATATATTTCTTTTTCTCATCTTTTTCATTGATGA
TAAACGGTTCTTTTATTTTAGCTTCAAATTTGTCACTAACAATAGTATCGCCTTTAATTTTT
ATATCCATATTTTTTTTGCTTTTAAATTCTTTAAGTTCTTCATTTAATTCTTCATTGTCATTT
TCTTTCTTTTTGTGACTAGTGCTCTCTTTTTTTGCACTATCTTGATGATGTCCACAAGCACC
TAAGATAAGTGTACTTGCTAATAATATCCCCATTACTTTTTTCAT

## > Q5HHN0

Function: protein coding sequence; putative membrane protein (core genome, constant)
Best match: Q5HHN0_CC001-ST772_118_AJGE01000024.1[3121:3642] (completely identical)
Position: 024-contig_320: 3075 … 3597; Length: 522 bp
Sequence:
ATGATTAACATTATTTCAGCTATAGGATCTATTGGAACATTTATTATGGCTTTATTTTATTT
TGTATCAGTTTCAGTTCAACTTTATCAAATGAAAATTAGCTTTCTGCCAGCTTTAGGTTTT
AACCAAATTTTATTAGAAAGGGAGGAGGATCAACTTAATATAATGAATTCGGCAACAGA
AGAGCATCATCATAAAGATTATATTAAACTATATAATTTAGGTGGCGGTGCTGCTAAAAA
AATTGCAATAGAGGTTTTATTGGGTAATGATAAAGTCATTCAGAAAAAATACGTGAATAT
TTTACCTAGTAAAGAAGGGGACATGTTACCAATTAATAAAAATGTGTACGAAGAATTAGA
AAGAACGATTGAGAACAATGGTTATGAAGCTGATTTGAATGTACGTATGACTTATTATCA
TAATGTAAGTCGCAAACAACAGGAAGTTATATTAAAAGGTCAAATCGACCGTTTTAATAC
TTATAATAATAAAGAAATTTATGATTTGCAGTTTATCTAA

## > Q1XZI1

Function: protein coding sequence; putative acetyltransferase/GNAT family (core genome, constant)
Best match: Q1XZI1_CC005_Mu50-VRSA_BA000017.4[886369:886899] (completely identical)
Position: 024-contig_320: 3735 … 4266; Length: 531 bp
Sequence:
ATGCAAATTAGACAAATACATCAACATGACTTTGCTCAAGTTGACCAGTTAATTAGAACG
GCATTTGAAAATAGTGAACATGGTTATGGTAATGAATCAGAGCTAGTAGACCAAATTCGT
CTAAGTGATACGTATGACAATAACTTAGAAATAGTAGCTGTTCTTCAAAACGAAGTTGTA
GGGCACGGTTTACTAAGTGAAGTTTATCTTGATAACGGAGCACAACGGGAAATTGGATTA
GTGTTAGCACCTGTATCTGTTGATATTCATCATCAAAATAAAGGTATTGGGAAGCGATTG
ATTCAAGCATTAGAACGAGAAGCAATATTAAAAGGATATAATTTTATCAGTGTATTAGGA
TGGCCGACGTATTATGCCAATCTAGGATATCAACGCGCAAGTATGTACGACATTTATCCA
CCATATGATGGTATACCAGACGAAGCGTTTTTAATTAAAGAATTAAAAGTGAACAGTTTA
GCGGGAAAAACAGGTACCATAAATTACACATCTGCTTTTGAAAAAATATGA

## > clfA

Function: protein coding sequence; clumping factor A chv

Best match: clfA-Mu50_CC005_04-02981_CP001844.2[845954:848671]
Position: 024-contig_320: 4528 … 7408; Length: 2880 bp
Sequence:
ATGAATATGAAGAAAAAAGAAAAACACGCAATTCGGAAAAAATCGATTGGCGTGGCTTC
AGTGCTTGTAGGTACGTTAATCGGTTTTGGACTACTCAGCAGTAAAGAAGCAGATGCAAG
TGAAAATAGTGTTACGCAATCTGATAGCGCAAGTAACGAAAGCAAAAGTAATGATTCAA
GTAGCATTAATGCTGCACCTAAAACAGACAACACAAACGTGAGTGATACTAAAACAACG
TCAAACACTAATAATGGCGAAACGAGTGTGGCGCAAAATCCAGCACAACAGGAAACGAC
ACAATCATCATCAACAAATGCAACTGCGGAAGAAACGCCGGTAACTGGTGAAGCTACTA
CTACGACAACGAATCAAGCTAATACACCGGCAACAACTCAATCAAGCAATACAAATGCG
GAGGAATTAGTGAATCAAACAAGTAATGAAACGACTTCTAATGATACTAATACAGTATCA
TCTGTAAATTCACCTCAAAATTCTACAAATGCGGAAAAAGTTTCAACAACGCAAGATACT
TCAACTGAAGCAACACCTTCAAACAATGAATCAGCTCCACAGAGTACAGATGCAAGTAA
TAAAGATGTAGTTAATCAAGCGGTTAATACAAGTGCGCCTAGAATGAGAGCATTTAGTTT
AGCGGCAGTAGCTGCAGATGCACCGGCAGCTGGCACAGATATTACGAATCAGTTGACGA
ATGTGACAGTTGGTATTGACTCTGGTACGACTGTGTATCCGCACCAAGCAGGTTATGTCA
AACTGAATTATGGTTTTTCAGTGCCTAATTCTGCTGTTAAAGGTGACACATTCAAAATAAC
TGTACCTAAAGAATTAAACTTAAATGGTGTAACTTCAACTGCTAAAGTGCCACCAATTAT
GGCTGGAGATCAAGTATTGGCAAATGGTGTAATCGATAGTGATGGTAATGTTATTTATAC
ATTTACAGACTATGTAAATACTAAAGATGATGTAAAAGCAACTTTGACCATGCCCGCTTA
TATTGACCCTGAAAATGTTACAAAGACAGGTAATGTGACATTGGCTACTGGCATAGGAAG
CAATACTGCTAGTAAGACAGTTTTAATCGACTATGAGAAATATGGACAATTCCATAATTT
ATCAATTAAAGGTACGATTGATCAAATCGACAAAACAAACAATACGTATCGTCAAACGA
TTTATGTCAATCCAAGTGGAGACAATGTTGTATTACCAGTGTTAACTGGTAATCTAATTCC
TAAGAGTAATAGTAATGCTTTAATAGATGCCAACAATACTAATATTAAAGTTTATAAAGT
GGATAATGCTGATGATTTGTCACATAGTTATTATGTGAATCCAAATGATTTTGAAGATGTA
ACAGATCAGGTCAGAATTTCATATCCTAATGATAATCAATATAAAGTAGAGTTTCCAACA
GACGATGATCAAATTACAACACCGTATATTGTAGTTGTTAATGGTCATATTGATCCGAAT
AGCAAAGGTGATTTAGCTTTACGTTCAACTTTATATGGATATGACTCAAGGTTTGTATGGA
GATCTATGTCATGGGACAACGAAGTAGCATTTAATAACGGATCAGGTTCTGGTGACGGTA
TCGATAAACCAGTTGTTCCTGAACAACCTGATGAGCCTGGTGAAATTGAACCAATTCCAG
AGGATTCAGATTCTGACCCAGGTTCAGATTCTGGCAGCGATTCTAATTCAGATAGCGGTT
CAGATTCGGGTAGTGATTCTACATCAGATAGTGGTTCAGATTCAGCGAGTGATTCAGATT
CAGCAAGTGATTCAGACTCAGCGAGTGATTCAGATTCAGCAAGTGATTCAGATTCAGCAA
GTGATTCAGACTCAGCAAGTGATTCAGATTCAGCAAGCGATTCAGATTCAGCGAGTGATT
CCGACTCAGCGAGCGATTCAGACTCAGGTAGTGACTCAGATTCCGATAGCGATTCCGACT
CAGATAGCGACTCAGATTCAGACAGCGATTCTGACTCAGACAGTGACTCAGATTCCGATA
GCGATTCCGACTCAGACAGTGACTCAGATTCCGATAGCGATTCCGACTCAGACAGTGACT
CAGATTCAGATAGCGATTCAGATTCCGACAGTGATTCCGACTCAGATAGCGATTCCGACT
CAGATAGCGACTCAGATTCAGACAGCGATTCAGATTCAGACAGCGATTCAGATTCAGATA
GCGATTCAGATTCCGACAGTGACTCGGATTCAGATAGCGATTCAGATTCCGACAGTGACT
CAGATTCCGACAGTGACTCAGACTCAGACAGTGATTCGGATTCAGATAATGATTCGGATT
CAGATAGTGATTCCGACTCCGACAGTGACTCGGATTCAGATAGCGATTCCGACTCAGACA
GTGACTCGGATTCAGACAGCGATTCAGACTCAGATAGCGACTCAGAATCAGACAGCGAC
TCAGATTCAGATAGCGACTCAGACTCGGATAGCGATTCGGACTCAGATAGCGATTCAAAC
TCAGACAGCGATTCAGATTCAGACAGCGACTCAGACAGTGACTCAGATTCAGATAGTGAC
TCGGATTCAGATAGCGATTCAGATTCCGACAGCGACTCAGATTCAGATTCAGCGAGTGAT
TCCGACTCAGAAAGTGATTCAAATAGCGATTCCGAGTCAGGTTCTAACAATAATGTAGTG
CCGCCTAATTCACCTAAAAATGGTACAAATGCTTCTAATAAAAATGAGGCTAAAGATAGT
AAAGAACCATTACCAGATACAGGTTCTGAAGATGAAGCGAATACGTCACTAATTTGGGG
ATTATTAGCATCATTAGGTTCATTACTACTTTTCAGAAGAAAAAAAGAAAATAAAGATAA
GAAATAA

> **vwb**

Function: protein coding sequence; van Willebrand factor binding protein chv
Best match: vwb-21266_ref_CC012_21266_AFTT01000017.1[724348:725869:r]

Position: 024-contig_320: 7628 … 9158; Length: 1530 bp
Sequence:
TTGAAAAATAAATTGCTAGTTTTATCATTGGGAGCATTATGTGTATCACAAATTTGGGAA
AGTAATCGTGCGTGTGCAGTGGTTTCTGGGGAGAAGAATCTATATGTATCTGAGTCATTG
AAACTAAATGATAACAAAAGTAAAAGCTTATCATTAGAAAAGTATAAAGAGAGTCTTAG
AAGTATAATGTGTACAAAAGAAATCAACAAAAATGATGGATATGATGAGCCTGAATATA
AAGAAGCACTGAATACTTACAGAAAAAAATTATTTGCTGAATTAGATGCACTTAATAAAT
TTTTAGATGAAGAGAGAAAAATAGCCAGTTACATTAAAAAAAATATGGAAGTGCCAAGT
AATATTTTAGGATTAACACACGGTAGGTATACAGCAATATATAATGCAATTAAAGAAAAT
AAAAATGAATTCGAAAAAAATGTAGAAAATATTGAAAGTAAGCATCTGGATTTAAAAAG
GTTTGATGAAGACAAAGATTATGAAGCAAGAGTTAAATTAAATGAATTAGAAAATAAAG
TTTTAATGTTAGGTCAAGCTTTTCCTGATAAAGTAGATGCTAGAGAAAGTCTTTATAATAA
ATTGGATATGATTGTTGGTTTAAGTAATGATGAAATTGAAGAAAGACATCCTCAAAATGA
AAGGTTATTAAAAGAACGAGTTGAAGATTTAGAAACAATTATCGATGAATTCTTTAATGA
CATAGGTGAAAATAGACCGGAGAATATATCGCCATTAACTAACAATGAATATAAAAATA
AGGAAATGATTACAAAGCTAAAATCTGACACTGAAGCAGCTAAAAATGACGAAAACAAG
AGATGTAATCGAAGTAAAAGAAGTTTAGATTCTCAAAATTATAAATCTGCTTCACAAGAA
GTAACTGCAGAGCAAAAAGCAGAATATGAAAAAAGAGCTGAAGAAAGAAAAGCGAGAT
TTTTGGATAATCAAAAAATTAAGAAAACACCTGTAGTGTCATTAGAATATGATTTTGAGC
ATAAACAACGTATTGACAACGAAAACGACAAGAAACTTGTGGTTTCTGCACCAACAAAG
AAACCAACATCACCGACTACATATACTGAAACAACGACACAGGTACCAATGCCTACAGTT
GAGCGTCAAACTCAGCAACAAATTATTTATAATGCACCAAAACAATTGGCTGGATTAAAT
GGTGAAAGTCATGATTTCACAACAACGCATCAATCACCAACAACTTCAAATCACACGCAT
AATAATGTTGTTGAATTTGAAGAAACGTCTGCTTTACCTGGTAGAAAATCAGGATCACTG
GTTGGTATAAGTCAAATTGATTCTTCTCATCTAACTGAACGTGAGAAGCGTGTAATTAAG
CGTGAACACGTTAGAGAAGCTCAAAAGTTAGTTGATAATTATAAAGATACACATAGTTAT
AAAGACCGATTAAATGCACAACAAAAAGTAAATACTTTAAGTGAAAGTCATCAAAAACG
TTTTAATAAACAAATCAATAAAGTATATAATGGCAAATAA

## > **sspECM**

Function:
Best match: sspECM_CC008-ST72_21259_AFTS01000012.1[77685:78707:r] (completely identical)
Position: 024-contig_320: 9508 … 10531; Length: 1023 bp
Sequence:
ATGAAAAAGAAATTATTAGTTTTAACTATGAGCACGCTATTTGCTACACAACTTATCAATT
CAAATCACGCTAAAGCATCAGTGACAGATAGTGTTGACAAAAAATTTGTAGTTCCAGAAT
CAGGAATTAATAAAATTATTCCAGCTTACGATGAATTTAAGAATTCGCCAAAAGTAAATG
TTAGTAATTTAACTGACAATAAAAACTTTGTAGTTTCTGAAGACAAATTGAATAAGATTG
TAGATTCATCGGCAGCTAGTAAAATTGTAGATAAAAACTTTGCCGTACCAGAATCAAAGT
TAGGAAACATTGTACCAGAGTACAAAGAAATCAATAATCGCGTGAATGTAGCAACAAAC
AATCCAGCTTCACAACAAGTTGATAAGCATTTTGTTGCTAAAGGCCCAGAAGTAAATAGA
TTTATTACGCAAAACAAAGTAAACCACCACTTCATTACTACGCAAACCCACTACAAGAAA
GTTATTACTTCATACAAATCAACACATGTACATAAACATGTAAATCATGCAAAGGATTCT
ATTAATAAACACTTTATTGTTAAACCATCAGAATCGCCTAGATATACACATCCATCTCAAT
CTTTAATTATCAAGCATCATTTTGCAGTTCCTGGATATCACGCGCATAAATTTGTAACACC
TGGGCATGCTAGCATTAAAATTAATCACTTTTGTGTTGTGCCACAAATAAATAGTTTCAAG
GTAATTCCACCATATGGTCACAATTCACATCGTATGCATGTACCAAGTTTCCAAAATAAC
ACAACAGCAACACATCAAAATGCTAAAGTAAATAAAGCATATGACTATAAATACTTCTAT
TCTTATAAAGTAGTTAAAGGTGTGAAGAAATATTTCTCATTTTCACAATCAAATGGTTATA
AAATTGGGAAACCATCATTAAATATCAAAAATGTAAATTATCAATATGCTGTTCCAAGTT
ATAGCCCTACACACTACGTTCCTGAATTTAAGGGTAGCTTACCAGCACCACGAGTATAA

## > **vwb2**

Function: protein coding sequence; truncated "van Willebrand factor" binding protein chv
Best match: vwb2-Mu50_CC001_MW2-USA400_BA000033.2[863297:863818]

Position: 024-contig_320: 10865 … 11387; Length: 522 bp
Sequence:
ATGAAAAGGAAAGTATTAGTACTAACAATGGGTGTAATTTGTGCAACTCAATTATGGCAT
TCTAATCAAGCAAACGCATTAGTGACAGAGAGTGTTGAAACTAATTTTGTTGTAAAAGAT
TCGGATAATAAAAATATTTTACAACCCCATACTGAAATTACTACTGAGGAGAAATTTTCA
ATAGTAGAAAAGAGTCCATTAAATAAATTAAAATCACTATCTAATGACAACTACATAGAA
TATGATTTACATACTAATCAAACAGGCATTAAAAAAGGTTGGTTTTATGGTTATAGTGAA
ATTGACTCATCACATTTCACAGACCGTGATAAACGTGTTATTAGACGTGATCATGTTAAA
GAAGCACAAAGCTTAATTAACGATTATAAAGATACACAAAGCTATGAAGATCTCGCTAA
GGCAACTGCAAAAGTAAGTACACTTAGTCAGTCTCACCAAAATTATTTAAATAAACAAAT
TGATAAAGTGAATAATAAGATAGAGAAAACTGAAAAACGCTAA

> **tx_vwb2**
Function:  rho indepenednet terminator of vwb2
Best match: tx_vwb2_CC001_MW2-USA400_BA000033.2[863934:863988] (completely identical)
Position: 024-contig_320: 11502 … 11557; Length: 55 bp
Sequence:
TGTCTCGATATGATAGTCTGCAACGATTCATGTTGTAGGCTTTTTAATTTTACAA

> **nuc1**
Function: protein coding sequence; thermostable extracellular nuclease (core genome, variable)
Best match: nuc1_CC001-ST772_118_AJGE01000037.1[4954:5640] (completely identical)
Position: 024-contig_320: 11727 … 12414; Length: 687 bp
Sequence:
ATGACAGAATACTTATTAAGTGCTGGCATATGTATGGCAATTGTTTCAATATTACTTATAG
GGATGGCTATCAGTAATGTTTCGAAAGGGCAATACGCAAAGAGGTTTTTCTTTTTCGCTA
CTAGTTGCTTAGTGTTAACTTTAGTTGTAGTTTCAAGTCTAAGTAGCTCAGCAAATGCATC
ACAAACAGATAACGGCGTAAATAGAAGTGGTTCTGAACATCCAACAGTATATAGTGCAA
CTTCAACTAAAAAATTACATAAAGAACCTGCGACATTAATTAAAGCGATTGATGGTGATA
CGGTTAAATTAATGTACAAAGGTCAACCAATGACATTTAGACTATTATTGGTTGATACAC
CTGAAACAAAGCATCCTAAAAAAGGTGTAGAGAAATATGGTCCTGAAGCAAGTGCATTT
ACGAAAAAAATGGTAGAAAATGCAAAGAAAATTGAAGTCGAGTTTGACAAAGGCCAAA
GAACTGATAAATATGGACGTGGCTTAGCGTATATTTATGCTGATGGAAAAATGGTAAACG
AAGCTTTAGTTCGTCAAGGCTTGGCTAAAGTTGCTTATGTTTATAAACCTAACAATACACA
TGAACAACTTTTAAGAAAAAGTGAAGCACAAGCGAAAAAAGAGAAATTAAATATTTGGA
GCGAAGACAACGCTGATTCAGGTCAATAA

> **tx_nuc1**
Function:  rho-independent terminator of nuc1
Best match: tx_nuc1_CC001_MW2-USA400_BA000033.2[864845:864896] (completely identical)
Position: 024-contig_320: 12413 … 12465; Length: 52 bp
Sequence:
ATGCTCATTGTAAAAGTGTCACTGCTGCTAGTGGCACTTTTATAATTTTTGG

> **csp**
Function:  csp_thermoswitch
Best match: csp_thermoswitch_CC001-ST772_118_AJGE01000037.1[5892:6239] (completely identical)
Position: 024-contig_320: 12665 … 13013; Length: 348 bp
Sequence:
AAGAGCGTGAAGAAAAATGTGAGTTATTTATATAGAATATTCTCCTTTTCATTTATGAATT
TGTTACAAAATATTTAGTGCAAAAGCACGACGGAGGTATTCAATATGAATAACGGTACAG
TTAAATGGTTTAATGCAGAAAAAGGTTTTGGTTTCATCGAAAGAGAAGATGGTAGCGACG
TATTCGTACATTTCTCAGCAATCGCTGAAGATGGATACAAATCATTAGAAGAAGGCCAAA

AAGTTGAATTCGACATCGTTGAAGGCGACCGTGGCGAGCAAGCTGCAAACGTAGTTAAA
ATGTAATTTTAACTTATTCAAACAGCCCTTACTATAGGGCTGTTTTTT

## > cspC

Function: protein coding sequence; cold shock protein C (core genome, constant)
Best match: cspC_CC001-ST772_118_AJGE01000037.1[5997:6197] (completely identical)
Position: 024-contig_320: 12770 … 12971; Length: 201 bp
Sequence:
ATGAATAACGGTACAGTTAAATGGTTTAATGCAGAAAAAGGTTTTGGTTTCATCGAAAGA
GAAGATGGTAGCGACGTATTCGTACATTTCTCAGCAATCGCTGAAGATGGATACAAATCA
TTAGAAGAAGGCCAAAAAGTTGAATTCGACATCGTTGAAGGCGACCGTGGCGAGCAAGC
TGCAAACGTAGTTAAAATGTAA

## > Q1Y1Z8

Function: protein coding sequence; putative protein (core genome, constant)
Best match: Q1Y1Z8_CC005_Mu50-VRSA_BA000017.4[895999:896217:r]RC (completely identical)
Position: 024-contig_320: 13466 … 13685; Length: 219 bp
Sequence:
TTAATTTACAGCGTCAAATATACTTATTTCTAATGCTTTGGGGTCTACTGAAACAAGTAAA
GAATGATCGATGTTACTAATATTGCCATTCTCCAAATTTATTTCTGTGAGTATTTGGAAGC
TACCATTAGGCAATGGTTTAACAATAGACAATTGCTTTTCCGCTTGTTGTATTAAAAAAGG
TTTTGTAGATTGATTATTAATATGCCATTCACTCAT

## > Q2G007

Function: protein coding sequence; putative protein (core genome, constant)
Best match: Q2G007_CC005_Mu50-VRSA_BA000017.4[896279:896563:r]RC (completely identical)
Position: 024-contig_320: 13746 … 14031; Length: 285 bp
Sequence:
TCAACCAAAATTTTGTTCAAATTCAATAAATGTCTTGTTTAAAATAGAAATATTGTAAATG
TTATCGTCCAAAATTTCACCAGTTAAGTATTTGTTTTGAATTAAAATTTGGCAGTTAGTTA
AGAAGTCTTGATAATCACGATCGCAAAAATAGTTTTCACGTGCATCTTTAGCATCGCCAA
AAAAGTTAGCGACTGTTTCTGTTTCTCCATTATTCGAACGTTCAATATATAATTTGTAAAA
TTTAGCTATTGTATACTTTTGTTCTTTAGTTAGTTCATTCAA

## > Q1YB69

Function: protein coding sequence; putative exported protein (core genome, constant)
Best match: Q1YB69_CC001-ST772_118_AJGE01000037.1[7345:7914:r]RC (completely identical)
Position: 024-contig_320: 14118 … 14688; Length: 570 bp
Sequence:
TTAATAAGCTTTAACAAGCCTTAGTTTGTATGGATCTATAAAATTATCTTTAATTGCATAG
GGTGAAATAATATGTAGTCCATAACTTTTAACTGATTTTTCACTTACACCAAATTTATAAG
CTTGGTAGATAATTTTAGTACAATACGTAAATTTTTTGCTGTTCAAATTTAATGTAACTAG
ATAACGATGATTTGTATTCTCATAGTTTTTCTTAACCCAGTCAGCCGCTTTTTTACCTGCAC
CAGGATAGCTGCAACGATAAACTTTCATCCAATCATTTTTGCCACTTGCATAATTATATTT
AAAAGATTCGAAGGATTGTGTAGTTGGTTTGTCGCCAGGCCCCTCAATTTGTAAAATCGT
TTTATCATCAATCGCGATACTACAATGACCAAAAAATCCCCACATGACAGGGCCTTTTGT
AACAATAATATCACCAGGTTGTAATTGGAATTTGTCATCTTGAATTTCTGAATACTTATTA
TCTGCAATTGTTTTTGGTGAGTTTATAGGGGATACGACAACGAATAATATAAGTAAAATT
ATCGTTCGTTTAATATAGTTCAC

## > gpmA1

Function: protein coding sequence; putative phosphoglycerate mutase
Best match: gpmA1_CC001_MW2-USA400_BA000033.2[868781:869365]

Position: 024-contig_320: 16351 … 16936; Length: 585 bp
Sequence:
TTGTTGGCTAAAACGTTATATTTAATGCGCCACGGACAAACTTTGTTTAATTTTAAGGGAC
TAATTCAGGGATTTGGAGATTCGCCGCTAACAGAACTTGGAATTGCTCAAGCTCAAAAGG
CACGTAGTTATTATGAAACTAAGGGGATAAACTTCGATTTATATGCATCATCAACGCAAG
AACGCGCAAGTGACACACTTGAAAATGTTGCACCTAACCAATCGTATCAACGTTTTAAGG
GACTGAAGGAATGGCATTTTGGATTATTTGAAGGTGAGTCAGTCTATCTATTTGATAATCT
ATACAAGCCTGAAGACATATTCGGAGATCGAATTGTTCCTTTCAAAGGAGAGGCAAGGC
AACAAGTTGAAGATCGCATTGTGAAAACTTTACATGACATTATGTCTCAAACAAAGAATA
ATGCATTAGTCGTGAGTCATGGAACAATAATGGGAGTATTTTTAAGATATTGCCTTAAAC
TAGATGAAGCATTAAAGCATAATATCGGTAATTGTAATATCCTGAAATTTGAATATGACA
ATGGAACATTTAAATTTGTTGAGTTAATTGATCCAAATTTATAA

> **osmC**

Function: protein coding sequence; osmotically inducible protein
Best match: osmC_CC008_NCTC8325_CP000253.1[806980:807402:r]RC (completely identical)
Position: 024-contig_320: 18673 … 19096; Length: 423 bp
Sequence:
CTAATCTACAACATTTACATTTAAATCGACATTAATATTTCCTTGAGTCGCTTTTGAATAT
GGACAAAATTCATGAGCCATTTGTAAATATTTTTCAGCTTCTTCTTGAGATATAACATTTT
TAATTGTCGCATCAATTGAAACACTTAATTTAGGACTTTCTGAGTCTGAATCATCTTCTAG
TCTCACTGTTAGTGTTACTTCTGGATGAGCATCACGCACTTTGTTTTGCTTTAAAATTAGG
TCGAAAGCACCGTTGAAGCAAGATGCATAACCTGCTGCAAATAATTGTTCGGGGTTAGTA
GCTTTACCATCTGCTTGAGCAGGCGGAACGATATCAATATCTAATGCTCGATCATCAGTA
TAAACATGTCCTTTACGTCCGCCAACATTCGTCGCTTTAGTTTCATAATGTATTGCCAT

> **aroD**

Function: protein coding sequence; 3-dehydroquinate dehydratase type I (core genome, constant)
Best match: aroD_CC001_MW2-USA400_BA000033.2[871673:872389]
Position: 024-contig_320: 19243 … 19960; Length: 717 bp
Sequence:
ATGACACATGTGGAAGTAGTAGCGACTATCGCGCCACAATTATCTATCGAAGAAACTTTA
ATTCAAAAAATTAATCATCGTATTGATGCAATAGACGTATTAGAATTACGAATTGATCAA
ATTGAAAATGTCACAGTTGATCAAGTGGCAGAAATGATTACAAAGCTGAAGGTTATGCA
AGATTCATTCAAATTATTAGTTACGTATCGTACAAAGTTACAAGGTGGCTATGGGCAATT
TACAAATGACTCGTATCTTAATTTAATATCAGACTTAGCAAATATCAATGGCATAGATAT
GATTGATATAGAATGGCAAGCAGATATTGACATTGAAAAACATCAACGAATCATTACAC
ATTTGCAACAGTATAATAAAGAGGTGGTTATATCACATCATAATTTCGAAAGTACGCCTC
CATTAGATGAATTGCAATTTATATTTTTTAAAATGCAAAAATTCAACCCAGAATACGTTA
AATTAGCAGTAATGCCACATAATAAAAATGATGTGTTAAATTTATTGCAGGCAATGTCTA
CATTTTCAGATACTATGGACTGCAAAGTTGTTGGTATTTCAATGTCTAAACTTGGACTAAT
AAGTAGAACGGCTCAAGGCGTTTTTGGTGGTGCATTGACTTATGGTTGTATCGGAGAACC
ACAAGCTCCAGGACAGATTGATGTTACTGATTTAAAAGCACAAGTGACTTTATACTAA

> **arsC1**

Function: protein coding sequence; arsenate reductase
Best match: arsC1_CC008_NCTC8325_CP000253.1[809502:809858] (completely identical)
Position: 024-contig_320: 21195 … 21552; Length: 357 bp
Sequence:
ATGATTAAATTTTACCAATATAAGAATTGTACAACTTGTAAAAAGGCAGCAAAGTTTTTA
GATGAATATGGCGTAAGTTATGAACCAATTGATATCGTTCAACATACACCTACAATAAAT
GAATTTAAAACAATAATTGCAAATACAGGCGTAGAAATTAATAAATTGTTTAATACACAC
GGCGCGAAATATCGTGAGCTTGATTTGAAAAATAAATTACAAACTTTATCAGATGATGAA
AAGTTAGAGTTGTTATCATCTGATGGTATGTTAGTAAAGCGTCCTCTAGCAGTAATGGGC
GATAAGATAACATTAGGATTTAAAGAAGATCAATATAAAGAGACTTGGTTAGCGTAA

> **gcvH**
Function: protein coding sequence; glycine cleavage system protein H (core genome, constant)
Best match: gcvH_CC001_MW2-USA400_BA000033.2[874139:874519] (completely identical)
Position: 024-contig_320: 21709 … 22090; Length: 381 bp
Sequence:
TTGGCAGTACCAAATGAATTGAAATATTCAAAAGAGCATGAATGGGTTAAAGTTGAAGG
TAATGTAGCAACAATTGGAATCACAGAATACGCACAAAGCGAGTTAGGTGATATTGTTTT
CGTTGAATTACCAGAAACAGATGATGAAATTAATGAAGGGGATACGTTTGGTAGCGTAG
AATCAGTTAAAACTGTATCAGAATTATATGCACCAATCTCTGGTAAAGTAGTTGAAGTCA
ACGAAGAACTAGAAGATAGTCCCGAATTTGTAAATGAATCTCCATACGAAAAAGCATGG
ATGGTAAAAGTAGAAATTAGTGATGAAAGTCAGATTGAAGCTTTATTAACAGCTGAAAA
ATATTCAGAAATGATTGGTGAATAA

> **ywqG**
Function: protein coding sequence; putative protein (core genome, constant)
Best match: ywqG_CC001-ST772_118_AJGE01000037.1[15495:16373] (completely identical)
Position: 024-contig_320: 22268 … 23147; Length: 879 bp
Sequence:
ATGCTAAACATTCAAGACGTTAGTCATCTTTCTAAAAAGGAGCAAAAAGCATATAACCGT
TTCGTAGAATCTGTAGAAAACGGTAATTTACCAGTACTACCATGTATTGAAATGGATCTA
AAAGAGATGAAAGAAGAAACATTAAACCAGAGTAAGATTGGTGGAATGCCATTTTTAAA
ATCTTTTAAAGATATACCATTAGATGAAAATAATGTACCAATGGTATTGTTAGCACAGAT
TAATTTGGATAATCTTCCAGAACAACAAGAATTATTTCCTGTAAATGAAGGGATATTGCA
GTTTTGGATTAGTTCAGAAGATCAAATGTATGGTATGACTGAAAATTTAAAGGGAAACAA
TATAAACTCAAGGCTTGTTTATATAAAAGAGCCAATTACAGATTTATCACTCGAAAATAT
TCAAGCGCATTTGAAGTCATTAGATGCTGATAATGAGGATATCCCGTTCAGTGGAGCATT
TTCTATAAAATTTAGATTGTCGAAACAAACTATTACATGTACTGATTATAAGTACGATGA
GGACGTGCTTGCATTGTGGAATAAAGTCAATCCATCCTTCGCGCTAAAATCAATGTTTGG
TGGTTATGATGAATTGATGGAACCTGTGTGTAACACATTTACTGCTAAGGAACCATTTAA
TCAACTTGGTGGTTATCCATATTTTGACCAAATAGATCCAAGAACGAACGATCAAGAACT
GAAAATGTATGATAGAGTCTTACTGCAAATTGATTCTACAAGAGATGGTAATTCTTCGAT
TATATGGGGTGATTTAGGTATTGCCAATATCTTAGTAAAATCTACTGACCTTGAGGCTATG
AAGTTTGATGATTACATGTATTCATGGGATTGCAGCTAA

> **tx_universal2**
Function:  rho indendent terminator
Best match: tx_universal2_CC001_MW2-USA400_BA000033.2[1067981:1068021:r]RC (completely identical)
Position: 025-contig_192_RC: 9 … 50; Length: 41 bp
Sequence:
TAGAATTGAAAAAAGCTTGTTACAAGCGCATTTTCGTTCAG

> **repeat_nySAgamma**
Function:  repeat element
Best match: repeat_nySAgamma_CC001_MSSA476_BX571857.1[2756252:2756375]
Position: 025-contig_192_RC: 209 … 239; Length: 30 bp
Sequence:
TTGGGGCCCCGCCAACTTGCACATTATTGT

> **Q2FZB6**
Function: protein coding sequence; putative protein (genomic island)
Best match: Q2FZB6_CC008_COL_CP000046.1[1182242:1182414]RC
Position: 025-contig_192_RC: 220 … 250; Length: 30 bp
Sequence:

CCAACTTGCACATTATTGTAAGCTGACTTT

> **yusF**
Function: protein coding sequence; small primase-like protein (toprim domain) (core genome, constant)
Best match: yusF_CC001_MW2-USA400_BA000033.2[876317:876703]
Position: 025-contig_192_RC: 529 … 916; Length: 387 bp
Sequence:
ATGGCTATTGTAAATAAAGTGATAATTGTTGAAGGAAAATCTGATAAAAAAAGGGTGCA
ACAGGTTATTGCAGAACCAGTCAATATTATTTGTACTCATGGAACAATGAGTATAGATAA
GCTTGATGATATGATAGAATCACTGTATGATAAACAAGTTTTTGTATTAGCCGATTCTGAT
GACGAAGGAGATCGAATTAGAAATTGGTTTAAACGTTATTTGAGTGAAAGTGAACATATA
TTTATTGATAAAACTTACTGTCAAGTTTCGAATTGCCCCAAACAATATTTGGCGCATGTAC
TTTCAAAACATGGCTTTACTTGTAAGAAAGAAACACCTCTTTTACCGAATATAAATAATG
AAAGGTTAGTTTTAGTAAATGAATAA

> **sam-L2**
Function: noncoding RNA SAM riboswitch locus 2 (core genome, constant)
Best match: sam-L2_CC001_MW2-USA400_BA000033.2[877050:877153] (completely identical)
Position: 025-contig_192_RC: 1262 … 1366; Length: 104 bp
Sequence:
CTCTTATCGAGAGTGGTGGAGGGATGTGCCCTACGAAGCCCGGCAACCGTCTTATATAGA
AATGGTGCCAATTCACATAAAGTTTTAACTTTTGAAGATGAGAG

> **metN2**
Function: protein coding sequence; methionine import ATP-binding protein 2 (core genome, constant)
Best match: metN2_CC001_MW2-USA400_BA000033.2[877242:878267]
Position: 025-contig_192_RC: 1454 … 2480; Length: 1026 bp
Sequence:
GTGATTGAATTAAAAGAAGTTGTTAAAGAATATCGGACTAAAAATAAAGAAGTCCTTGCT
GTAGATCACGTTAATTTATCGATTCGAGCAGGATCGATTTATGGCGTCATTGGTTTTTCTG
GAGCAGGAAAAAGTACTTTGATTCGAATGTTTAATCATTTAGAAGCGCCTACATCAGGTG
AAGTTATTATAGATGGAGACCATATAGGTCAATTGTCCAAAAATGGATTAAGAGCAAAA
AGACAAAAAGTAAGTATGATCTTCCAACATTTTAATTTGTTATGGTCAAGGACTGTGTTA
AAAAATATTATGTTTCCGCTTGAAATTGCAGGTGTCCCTAGAAGGAGAGCTAAGCAAAAA
GCATTAGAACTTGTCGAACTCGTCGGTTTAAAAGGTAGAGAAAAGGCTTATCCATCAGAG
TTATCAGGTGGACAAAAGCAACGTGTTGGGATTGCACGAGCGTTAGCTAATGATCCAACG
GTCTTGCTTTGTGATGAGGCAACAAGTGCACTTGATCCGCAAACAACAGATGAAATTTTA
GATCTACTACTAAAAATTAGAGAACAACAAAATTTAACAATTGTACTAATTACGCATGAA
ATGCATGTCATTCGTCGTATTTGTGATGAAGTTGCAGTTATGGAAAGTGGTAAAGTGATA
GAACAAGGACCGGTGACACAGGTTTTTGAAAATCCGCAACACACTGTGACAAAGCGATT
TGTGAAAGACGATTTAAATGATGATTTCGAAACATCTTTAACAGAATTAGAGCCATTAGA
AAAAGATGCTTATATCGTTAGATTAGTTTTCGCTGGTTCAACAACAACCGAGCCTATTGTA
TCGAGTCTATCAACTGCCTATGATATTAAAATTAATATTTTAGAAGCAAATATTAAAAAT
ACAAAAAATGGAACAGTCGGCTTTTTAGTTCTGCATATTCCATATATTTCAAGTGTAGATT
TTGGAAAATTCGAAAAAGAGTTAATTGAGCGACAAGTTAAAATGGAGGTGTTAAGACAT
GGGTAA

> **A6QFC1**
Function: protein coding sequence; ABC transporter/permease protein (core genome, variable)
Best match: A6QFC1_CC001-ST772_118_AJGE01000009.1[2226:2921] (completely identical)
Position: 025-contig_192_RC: 2472 … 3168; Length: 696 bp
Sequence:
ATGGGTAAATCATTTAGTGAAATTATAAATGAAATGATTACAATGCCTAATATTCAGTGG
CCAGAAGTTTGGACTGCAATAGTCGAAACACTATACATGACAGTCGTCTCAACTATATTT

GCATTTATACTCGGTCTTATTTTAGGTGTGTTATTATTCTTGTCTGCTAAAGGTAAGTCTAT
CGGTGCAAGGTTATTTTATTCTATCGTTTCTTTCATTGTTAACTTATTTAGAGCGATACCAT
TTATTATTTTAATTTTATTATTAATTCCATTTACAAGTTTGATACTTGGAACGATAAGTGGT
CCGACAGGTGCGTTACCAGCCTTGATTATTGGCGCAGCACCGTTTTATGCAAGGCTCGTA
GAAATTGCTTTTAAAGAAATTGATAAAGGTGTCATCGAAGCGGCCTGGTCAATGGGTGCC
AATACTTGGACAGTAATTCGTAAAGTCCTCTTACCTGAAGCCATGCCAGCGCTAGTGTCT
GGCATTACAGTTACAGCAATCGCTTTAGTTGGTTCAACAGCAGTTGCAGGTGTAATTGGT
GCCGGTGGTTTAGGAAATTTAGCATACTTAACAGGTTTCACTCGAAATCAAAATGATGTC
ATTTTAGTATCAACTGTTTTTATTTTAATTATTGTATTTATAATCCAATTCATTGGGGATTG
GCTTACAAATAAACTTGATAAACGATAA

> **Q2FII0**
Function: protein coding sequence; putative lipoprotein (core genome, constant)
Best match: Q2FII0_CC001-ST772_118_AJGE01000009.1[2938:3759] (completely identical)
Position: 025-contig_192_RC: 3184 … 4006; Length: 822 bp
Sequence:
ATGAAAAAATTATTTGGTCTTATTTTAGTATTAACATTTGCAGTTGTATTAGCAGCTTGCG
GTAATGGAAACAAAAGTGGCAGTGACGATAAGAAAATAACAGTAGGTGCTTCACCAGCA
CCACATGCTGAAATTTTAGAAAAAGCAAAACCATTATTAGAGAAAAAAGGTTATGAACT
AGATATTAAAACAATTAACGATTACACTACGCCTAATAAATTACTAGATAAAGGTGAAAT
TGACGCAAACTATTTCCAACATACACCATATTTAAACACAGAGAAAAAGGATAAAGGTT
ACAAAATCGTAAGTGCCGGTGATGTTCACTTAGAACCTATGGCTGTATACTCTAAAAAGT
ATAAAAGTTTAAAAGAATTACCAAAAGGTGCGACAGTTTATGTGTCTAATAATCCAGCTG
AACAAGGACGCTTCTTAAAATTCTTCGTTGATGCAGGTTTAATTAAGATCAAAAAAGGCG
TAAAAATTGAAGATGCTAAGTTTAGTGATATTACAGAGAATAAAAAAGATATTAAGTTTA
ATAATAAACAATCAGCAGAATTCTTACCTAAAATTTATCAAAATGAAGACGCTGATGCTG
TTATCATTAATTCAAACTTTGCAATCGAACAAAAACTAAATCCTAAAAAAGATTCTATTG
CTGTAGAAAGTGCGAAAGATAATCCTTATGCAAACTTAATTGCTGTTAAAGAAGGACATC
AAGATGATAAGAAAATCAAAGCATTAATTGAAGTATTACAATCTAAAGATATTCAAGACT
TCATTAATGAAAAATACAACGGTGCAGTTATTCCTGCTAAATAA

> **attB_nySa1_var1**
Function:  attachment site in bacterial chromosome of int1-SaPI1 (mobile element)
Best match: attB_nySa1_var1_empty_CC005_Mu50-VRSA_BA000017.4[909898:909994:r]RC (completely identical)
Position: 025-contig_192_RC: 3949 … 4046; Length: 97 bp
Sequence:
GATATTCAAGACTTCATTAATGAAAAATACAACGGTGCAGTTATTCCTGCTAAATAAATC
ATATTAATGGCTCAGTAACGTGATGTTGCTGGGCTTT

> **tx_Q2FII0**
Function:
Best match: tx_Q2FII0_var1_CC001_MW2-USA400_BA000033.2[879792:879846] (completely identical)
Position: 025-contig_192_RC: 4003 … 4058; Length: 55 bp
Sequence:
TAAATCATATTAATGGCTCAGTAACGTGATGTTGCTGGGCTTTTTAATTTAAACA

> **csbD-L1**
Function: protein coding sequence; stress response protein/locus 1 (core genome, constant)
Best match: csbD-L1_CC001_MW2-USA400_BA000033.2[880148:880342] (completely identical)
Position: 025-contig_192_RC: 4359 … 4554; Length: 195 bp
Sequence:
ATGGCAGACGAAAGTAAATTTGAACAAGCAAAAGGTAATGTTAAAGAAACAGTAGGTAA
TGTTACTGATAATAAAAATTTAGAAAACGAAGGTAAAGAAGATAAAGCTTCTGGTAAAG

CGAAAGAATTCGTTGAAAATGCAAAAGAAAAAGCAACTGATTTTATTGATAAAGTAAAA
GGTAACAAAGGCGAGTAA

> **Q2YWN7**

Function: protein coding sequence; putative membrane protein (core genome, constant)
Best match: Q2YWN7_CC001_MSSA476_BX571857.1[864544:865395:r]RC
Position: 025-contig_192_RC: 4787 … 5639; Length: 852 bp
Sequence:
TTATTCATTTTCAGCGGTAATTCGACCTAAAGTCAAACTTACAATAAAACCGATGATAAA
TACTACTAATGAAACGAACCACATCACGATATTAGTTGGTAAACCTGGAAATACTGCAAA
GAGGGAGCCAACAACAAAACCAATGATTAATGCAAAAGTCATTAGTTTATGATGTGTTAG
GAAATACTGGATAATTTTGCTTGAAATAATGAATCCAGCAAGCACCCCAAATCCGACTGC
AAGTAATATAGGAAGACCGGTAAAGTTAAGTTTAACAACTTCAGATATTGCTAGCATGAC
CGTACCATAGACGCCAAATACTAATAACATAAATGACCCTGAAATACCTGGGAGTAACAT
AGCACTAGATGCACACATACCTGCAATAAAATATTTAATAATAAGACTAGTTGATAGAGT
AAGTGTTTCTCCAGCATGTTTATCACCATTATTCATTAATGTAATAACAATTAAGATAGCG
ATACCAGCTATAACCATCATGTAATGTTTAGTTGTAAATGACGTTTTATAGTTAGAAATTT
TCAATAAATATGGAACGATACCAATGATTAATCCACCAAAGAAAAACATAGTTGGAATA
TGGTGTTGGCTTAATAAATAATTAAAAAGATTACTTAGTGATCCCATTGCCAGTAACATTC
CAATTATAATGGGGATTAAAAATGTAAAACTTGGCCAAAAACGTCGTGAGAATATGCCG
CTAATTGAAGCGATAAATTGATTGTAAATACCTAACAATAATGCGATAGTCCCACCGCTA
ACACCAGGTACCAAGTCACTCGTTCCCATAGCAAAACCTTTTAGAATATTAATCCATTTA
AATTGTTGCAT

> **sufC**

Function: protein coding sequence; FeS assembly ATPase SufC (core genome, constant)
Best match: sufC_CC001_MW2-USA400_BA000033.2[881754:882515] (completely identical)
Position: 025-contig_192_RC: 5966 … 6728; Length: 762 bp
Sequence:
ATGGCATCAACATTAGAAATCAAAGACCTACATGTGTCTATTGAGGATAAAGAAATCTTA
AAAGGTGTTAACTTGACAATTAACACTGATGAAATACATGCGATTATGGGACCAAACGG
GACAGGTAAATCAACTTTATCATCTGCAATTATGGGACACCCAAGCTATGAAGTAACTAA
AGGAGAAGTACTTTTAGACGGTGTAAATATTTTAGAATTAGAAGTTGATGAAAGAGCAA
AAGCAGGATTATTCTTGGCAATGCAATATCCATCAGAAATTACAGGTGTTACAAATGCTG
ATTTCATGCGTTCAGCAATCAATGCGAAACGTGAAGAAGGACAAGAAATCAACTTAATG
CAATTTATTAAGAAATTAGATAAAAACATGGATTTTCTAGACATAGATAAAGACATGGCA
CAACGTTATTTAAATGAAGGTTTCTCAGGTGGAGAGAAGAAACGTAACGAAATCTTACAA
TTAATGATGTTAGAACCTAAGTTTGCAATCTTAGATGAAATCGATTCAGGGTTAGACATC
GATGCATTAAAAGTTGTATCTAAAGGTATTAACCAAATGCGTGGGGAAAACTTTGGTGCA
TTAATGATTACACACTATCAACGATTATTAAATTACATTACTCCTGATAAAGTACATGTAA
TGTATGCTGGTAAAGTCGTTAAATCTGGTGGTCCAGAATTAGCAAAACGTCTTGAAGAAG
AAGGATATGAATGGGTTAAAGAAGAGTTCGGTTCAGCTGAATAA

> **sufD**

Function: protein coding sequence; putative FeS assembly protein SufD (core genome, constant)
Best match: sufD_CC001_MW2-USA400_BA000033.2[882613:883920]
Position: 025-contig_192_RC: 6826 … 8134; Length: 1308 bp
Sequence:
ATGACAACTGATATTTTGAACATTTCTGAAGAACAACTTGTTGATTATTCTAAAGCCCACA
ATGAACCTTCTTGGATGACAGAATTACGTAAAAAAGCTTTGAAATTAACAGAAACTTTAG
AAATGCCAAAACCTGATAAAACAAAATTAAGAAAATGGGATTTTGATTCTTTTAAACAAC
ACGATGTAAAAGGTGATGTTTATCAATCTTTATCACAATTACCTGAGTCAGTAAGAGAAA
TTATTGACGTAGATCATTCTAAAAACTTAGTAATTCAACATAATAATACGATTGCGTACA
CACAAGTTGATGATAATGCATCTAAAGATGGCGTTATCGTTGAAGGTTTAGCAGACGCTC
TTATGAACCATAGTGATTTAGTACAAAAGTACTTTATGAAAGATGCAGTAACAGTAGATG

AACATCGTATCACAGCGCTACACACGGCATTAGTTAATGGTGGCGTATTTGTTTATGTTCC
TAAAAATGTAGTTGTAGAACATCCAGTACAATACGTTGTGTTGCACGACGACAAAAATGC
AAGCTTTTATAACCATGTTATCATCGTTACTGAAGAAAGCGCCGAAGTCACATATGTTGA
AAATTACTTATCAAATGCATCTGGTGAAGGAAATCAATTAAATATTATTTCTGAAGTGAT
TGCTGGTGCAAATTCAAATATCACATATGGCTCAGTGGACTATATGGATAAAGGCTTTAC
AGGTCATATCATTCGACGTGGTATTACTGAAGCGGATGCCTCAATTAATTGGGCACTAGG
TTTAATGAATGAGGGTAGCCAAATTATTGATAATACAACAAATTTATTTGGTGATCGTTC
AACAAGTTCACTTAAATCAGTAGTTGTAGGTACAGGCGAACAAAAAATTAATCTAACATC
TAAAATCGTACAATATGGTAAAGAAACAGATGGTTATATCCTTAAACATGGTGTTATGAA
AGAACATGCATCGTCTGTATTTAATGGTATCGGCTACATTAAGCATGGTGGAACTAAATC
AATTGCTAATCAGGAATCACGTGTATTAATGTTATCTGAACATGCTCGTGGTGACGCGAA
TCCTATTTTATTAATTGATGAAGATGATGTACAAGCTGGTCATGCTGCATCAGTAGGTCGT
GTTGATCCAGATCAACTTTACTATTTAATGAGTCGTGGTATTTCTCAAAGAGAAGCGGAA
CGTCTTGTTATACATGGTTTCTTAGATCCAGTAGTACGTGAATTACCTATCGAAGACGTTA
AACGTCAATTGAGAGAAGTAATTGAACGCAAAGTTTCTAAATAA

> **sufS**

Function: protein coding sequence; cysteine desulfurase (core genome, constant)
Best match: sufS_CC007_TCH959-USA300_AASB02000223.1[8002:9243]
Position: 025-contig_192_RC: 8248 … 9490; Length: 1242 bp
Sequence:
GTGGCCGAACACTCATTTGACGTTAATGAAGTAATCAAGGATTTTCCGATATTAGATCAA
AAAGTCAATGGCAAACGTTTAGCATATCTTGATTCAACAGCGACAAGTCAAACGCCTGTG
CAAGTGTTAAATGTTTTAGAGGATTACTACAAGCGTTATAATTCAAACGTTCATCGTGGT
GTTCATACATTAGGATCATTGGCAACTGATGGTTATGAAAATGCCCGTGAAACTGTTCGT
CGTTTTATTAATGCGAAGTATTTTGAAGAAATCATTTTCACACGCGGAACAACTGCGTCG
ATTAACCTTGTAGCACATAGCTATGGTGATGCAAATGTTGAAGAGGGCGATGAAATTGTT
GTCACTGAAATGGAACATCATGCCAATATTGTTCCTTGGCAACAGTTAGCAAAGCGTAAA
AATGCGACATTGAAATTTATACCAATGACAGCTGACGGTGAATTAAACATCGAAGATATT
AAGCAAACGATTAATGATAAAACAAAGATCGTTGCTATTGCACATATTTCTAATGTACTC
GGTACAATTAATGATGTTAAAACCATTGCAGAAATAGCTCATCAACATGGTGCAATTATC
AGTGTTGATGGGGCGCAAGCAGCACCACATATGAAACTTGATATGCAAGAAATGAATGC
TGATTTTTATAGTTTTAGTGGTCATAAAATGCTTGGACCAACAGGTATTGGCGTATTATTT
GGTAAACGTGAGTTACTACAAAAAATGGAACCGATTGAGTTCGGTGGCGACATGATTGAT
TTTGTAAGTAAGTATGATGCAACATGGGCTGATTTACCTACTAAATTTGAGGCGGGTACT
CCATTAATTGCACAAGCAATTGGGCTTGCAGAAGCTATTCGCTATTTAGAACGCATAGGT
TTTGATGCAATTCATAAATATGAACAAGAATTAACGATATATGCTTATGAGCAAATGTCT
GCAATTGAAGGAATTGAAATTTATGGCCCGCCAAAGGATCGTCGTGCAGGTGTAATAACG
TTTAATTTACAAGATGTACATCCACACGATGTTGCTACAGCCGTAGATACAGAAGGTGTA
GCGGTTAGAGCTGGGCATCATTGTGCGCAACCGTTAATGAAATGGTTAAATGTGTCTTCA
ACAGCTAGAGCGAGTTTTTATATATACAACACGAAAGAAGACATTGATCAGTTAATAAAT
GCCTTGAAACAAACGAAGGAGTTTTTCTCTTATGAATTTTAA

> **sufN**

Function: protein coding sequence; SUF system FeS assembly protein/NifU family (core genome, constant)
Best match: sufN_CC001_MW2-USA400_BA000033.2[885266:885730] (completely identical)
Position: 025-contig_192_RC: 9479 … 9944; Length: 465 bp
Sequence:
ATGAATTTTAATAATCTAGATCAATTATATAGATCTGTCATTATGGATCATTATAAAAATC
CTAGAAATAAAGGTGTATTAGATAACGGGTCTATGACAGTAGATATGAATAACCCGACAT
GCGGTGACCGTATACGACTAACATTTGATATAGAAGACGGCATTATAAAAGATGCTAAGT
TTGAAGGTGAAGGTTGTTCGATTTCAATGGCAAGTGCATCGATGATGACACAAGCTGTTA
AAGGTCATTCACTTGGAGAAGCAATGCAAATGAGCCAAGAATTTACGAAAATGATGCTT
GGTGAAGACTATGTGATTACAGAAGAAATGGGAGATATTGAAGCATTGCAAGGTGTATC

TCAATTCCCAGCTCGTATTAAATGTGCCACATTAGCTTGGAAAGCATTGGAAAAAGGTAC
TGTTGCTAAAGAAGGTAAAGCAGAAGGTACGACTGAAGAAGAATAG

> **sufB**
Function: protein coding sequence; iron-regulated ABC transporter membrane component (core genome, constant)
Best match: sufB_CC001_MW2-USA400_BA000033.2[885881:887278]
Position: 025-contig_192_RC: 10094 … 11492; Length: 1398 bp
Sequence:
ATGGCTAAAAAAGCACCTGATGTTGGGGATTATAAATATGGATTCCACGACGATGATGTG
TCCATTTTCAGATCAGAACGTGGTTTAACTGAGAATATCGTTAGAGAAATTTCTAACATG
AAAAATGAGCCGGAATGGATGTTAGATTTCCGTCTTAAATCATTAAAATTGTTTTATAAA
ATGCCAATGCCTCAATGGGGTGGCGACTTATCAGAATTGAATTTCGATGACATTACTTAC
TATGTAAAGCCTTCAGAACAAGCTGAACGTTCATGGGATGAAGTGCCAGAAGAAATTAA
AAGAACTTTCGATAAATTAGGAATTCCTGAAGCTGAACAAAAATATTTAGCTGGTGTTTC
TGCTCAATATGAATCTGAAGTTGTTTACCATAATATGGAAAAAGAACTTGAAGAAAAAGG
TATTATCTTTAAAGATACAGATAGTGCTTTACAAGAAAATGAAGAATTATTCAAAAAATA
CTTTGCTTCTGTAGTACCTGCAGCAGATAACAAATTTGCGGCGTTAAACTCAGCAGTATG
GTCAGGTGGTTCGTTCATTTATGTACCTAAAAATATCAAACTAGATACGCCACTACAAGC
TTATTTCCGTATTAACTCTGAGAACATGGGTCAATTTGAACGTACATTAATCATTGCTGAT
GAAGGTGCTTCTGTACATTACGTAGAAGGTTGTACTGCACCAGTTTATACAACTAGTTCTT
TACACTCTGCTGTTGTGGAAATCATTGTGCATAAAGATGCGCACGTTCGTTATACTACGAT
TCAAAACTGGGCGAACAATGTATACAATTTAGTTACAAAACGTACTTTTGTTTATGAAAA
CGGAAATATGGAATGGGTAGATGGTAACTTAGGTTCTAAGTTAACGATGAAATATCCAAA
CTGTGTTCTTTTAGGTGAAGGTGCAAAAGGTAGTACATTATCTATTGCATTTGCTGGTAAA
GGACAAGTTCAAGATGCCGGTGCTAAAATGATTCATAAAGCACCAAATACATCTTCAACG
ATTGTTTCTAAATCTATTTCTAAAAATGGTGGTAAAGTTATTTATCGTGGTATTGTACATT
TTGGACGTAAAGCAAAAGGCGCTCGTTCAAATATTGAGTGTGATACATTAATCTTAGATA
ACGAATCAACATCAGATACAATTCCATATAACGAAGTATTCAACGATCAAATATCATTAG
AACATGAAGCAAAGGTTTCAAAAGTTTCTGAAGAACAATTATTCTATCTAATGAGTCGTG
GTATTTCTGAAGAAGAAGCGACAGAAATGATTGTTATGGGATTCATCGAACCATTTACAA
AAGAACTTCCAATGGAATACGCGGTCGAAATGAACCGTTTAATCAAGTTCGAAATGGAA
GGTAGTATTGGATAG

> **attB_phiSa1**
Function: attachment site for int6-phiETA (mobile element)
Best match: attB_phiSa1_empty_CC045-agrI_21200_AFTN01000029.1[429654:429823]RC
Position: 025-contig_192_RC: 11426 … 11596; Length: 170 bp
Sequence:
CCAATGGAATACGCGGTCGAAATGAACCGTTTAATCAAGTTCGAAATGGAAGGTAGTATT
GGATAGCTTTAAACCGCGATGTTAAGCTATTCTTGACTTCCGGAAATGGCTATTGATACC
ATTTTGATACTGAATATAACAAAAAGCCACATTACTGTGGCTTTTTTGTT

> **txbi_sufB**
Function: bidirectional rho independent terminator of sufB and Q2YWM5
Best match: txbi_sufB_AB046707.1[489:534]
Position: 025-contig_192_RC: 11556 … 11586; Length: 30 bp
Sequence:
TGAATATAACAAAAAGCCACATTACTGTGG

> **Q2YWM5**
Function: protein coding sequence; putative membrane protein (core genome, constant)
Best match: Q2YWM5_CC001-ST772_118_AJGE01000009.1[11576:11890:r]RC (completely identical)
Position: 025-contig_192_RC: 11822 … 12137; Length: 315 bp

Sequence:
TTAAAATAAGAAAACTATTAATAATATTAAGTTCACTACAGATGTTGCTAATGGACCGTA
AGTTTTAAAGACATCTTTACTTTTATAACCAACAATCGCATCTAAAAATTGAACTAAAATC
ATTGCAATGGATATAGTTATCAAAAATATAGCACTATGAATGACTAAAGAAAAAATAGCT
AATAAAAATAAAGGTAAGCTTCGACTAAGTGCATAATATGCATTTATATTATGGCTAGAT
GAACATGCTTGAATTGAATAACCTAAACTTACACTGGCACTGATTATTGTAAATATTGCT
AAAACAAAATACAT

> **hl**

Function: protein coding sequence; putative membrane protein (core genome, constant)
Best match: hl_CC001-ST772_118_AJGE01000009.1[12255:13295] (completely identical)
Position: 025-contig_192_RC: 12501 … 13542; Length: 1041 bp
Sequence:
GTGATCATTGCCATAATTATATTGATATTTATTTCGTTTTTCTTTTCAGGAAGCGAGACGG
CATTAACGGCTGCCAATAAAACAAAATTTAAAACTGAAGCTGACAAAGGTGATAAAAAA
GCAAAAGGCATTGTAAAGTTACTTGAAAAACCAAGTGAGTTTATTACAACGATTCTAATT
GGGAATAATGTCGCGAATATTTTATTACCAACACTTGTTACAATTATGGCTTTACGTTGGG
GGATTAGCGTTGGTATTGCATCAGCTGTTTTAACAGTTGTTATCATTTTGATTTCCGAAGT
GATTCCCAAGTCTGTCGCTGCAACATTTCCAGATAAAATAACAAGGCTTGTATATCCAGT
TATTAATATTTGTGTCATTGTGTTTCGCCCTATCACATTACTTTTAAATAAGTTGACGGAC
AGTATTAATCGAAGTTTATCTAAGGGCCAACCTCAAGAACATCAATTTTCAAAAGAAGAA
TTTAAAACAATGTTAGCAATTGCTGGACATGAAGGTGCTTTAAATGAAATTGAGACGAGT
AGGTTGGAAGGTGTCATTAATTTTGAAAATTTAAAAGTAAAAGATGTAGATACAACACCT
AGAATTAATGTGACGGCATTTGCTTCAAATGCGACATACGAAGAAGTTTATGAAACGGTT
ATGAATAAGCCATACACTAGATATCCAGTGTACGAGGGAGATATTGATAACATTATTGGA
GTGTTTCATTCTAAATATCTGTTGGCTTGGAGTAATAAAAAAGAAAATCAAATTACAAAC
TATTCAGCTAAGCCATTATTTGTGAATGAACACAATAAAGCTGAATGGGTATTACGTAAG
ATGACTATTTCTAGAAAACATTTAGCAATTGTGTTGGACGAATTTGGTGGTACCGAAGCG
ATAGTGTCACATGAAGACTTAATTGAAGAATTATTAGGTATGGAAATTGAAGATGAGATG
GATAAAAAGGAAAAAGAAAAACTTTCTCAACAGCAAATTCAATTTCAACAACGGAAAAA
TCGCAACGTATCTATATAA

> **npd**

Function: protein coding sequence; putative 2-nitropropane dioxygenase (core genome, constant)
Best match: npd_CC001_MSSA476_BX571857.1[873310:874377]
Position: 025-contig_192_RC: 13555 … 14623; Length: 1068 bp
Sequence:
ATGTGGAATAAGAATCGACTTACTCAAATGTTAAGTATTGAATATCCAATTATACAAGCA
GGTATGGCAGGAAGTACGACACCGAAATTAGTTGCATCAGTAAGTAACAGTGGTGGATT
AGGCACAATAGGCGCAGGTTACTTTAATACGCAGCAATTAGAAGATGAAATAGATTATGT
ACGCCAATTAACGTCAAATTCTTTTGGTGTAAATGTCTTTGTACCAAGTCAACAATCATAT
ACCAGTAGTCAAATTGAAAATATGAATGCATGGTTAAAACCTTATCGACGCGCATTACAT
TTAGAAGAGCCGGTTGTAAAAATTACCGAAGAACAACAATTTAAGTGTCATATTGATACG
ATAATTAAAAAGCAAGTGCCTGTATGTTGTTTTACTTTTGGAATTCCAAGCGAACAGATT
ATAAGCAGGTTGAAAGCAGCGAATGTCAAACTTATAGGTACAGCAACAAGTGTTGATGA
AGCTATTGCGAATGAAAAAGCGGGTATGGATGCTATCGTTGCTCAAGGTAGTGAAGCAG
GTGGACATCGTGGTTCATTTTTAAAACCTAAAAATCAATTACCTATGGTTGGAACAATAT
CTTTAGTGCCACAAATTGTAGATGTCGTTTCAATTCCGGTCATTGCCGCTGGTGGAATTAT
GGACGGTAGAGGAGTTTTGGCAAGTATTGTCTTAGGTGCAGAAGGGGTACAAATGGGCA
CTGCATTTTTAACATCACAAGATAGTAATGCATCAGAACTACTACGAGACGCAATTATAA
ATAGTAAAGAAACAGATACAGTCATTACAAAAGCGTTTAGTGGAAAGCTTGCACGCGGT
ATCAACAATAGGTTTATCGAAGAAATGTCCCAATACGAAGGCGATATCCCAGATTATCCA
ATACAAAATGAGCTAACAAGTAGCATAAGAAAAGCCGCAGCAAACATCGGCGACAAAG
AGTTAACACATATGTGGAGTGGACAAAGCCCGCGACTAGCAACAACGCATCCCGCCAAC
ACCATCATGTCCAATATAATCAATCAAATTAATCAAATCATGCAATATAAATAA

## > **Q2FZX5**

Function: protein coding sequence; putative 5'-nucleotidase (core genome, constant)
Best match: Q2FZX5_CC001_MSSA476_BX571857.1[876268:877587]
Position: 025-contig_192_RC: 16594 … 17914; Length: 1320 bp
Sequence:
TTGAGGCTTACAATTTATCATACGAACGATATTCATAGTCATTTACATGAATACGAACGC
ATTAAAGCATATATGGCAGAACAACGGCCACGACTTAATCATCCTTCTTTATATGTTGATC
TAGGTGATCATGTAGATTTATCCGCACCTATAACTGAAGCAACTTTAGGTAAAAAGAATG
TGGCATTACTAAATGAAGCAAAATGTGATGTTGCAACAATCGGTAATAATGAAGGGATG
ACCATTTCACACGAAGCTTTAAATCACCTTTACGACGAAGCAAAATTTATAGTGACATGT
AGCAATGTTATAGATGAATCAGGTCATTTACCAAATAATATCGTTTCTTCTTATATTAAGG
ATATAGACGGTGTGAAAATACTATTCGTTGCAGCGACAGCACCTTTTACCCCATTTTATCG
TGCACTAAATTGGATTGTTACCGATCCACTTGAATCTATAAAAGAAGAAATTGAACTTCA
ACGAGGTAAATTTGATGTATTAATCGTGCTAAGTCATTGTGGCATTTTCTTCGATGAAACA
TTATGCCAAGAATTGCCTGAAATTGATGTCATTTTTGGTAGTCATACGCATCATTATTTTG
AACATGGTGAAATCAATAATGGTGTACTGATGGCGGCAGCTGGAAAGTATGGTAATTATC
TTGGAGAGGTTAATTTAACTTTTGAGGCACATAAAGTAGTACATAAAACTGCAAAGATTA
TTCCTTTAGAAACATTACCTGAAGTTGAAACTTCATTTGAAGAAGAAGGAAAAACGTTAA
TGTCCAATTCAGTAATTCAACATCCAGTAGTGCTTAAGCGTAGTATGAATCACATAACTG
AAGCTGCATACTTATTAGCTCAAAGTGTTTGTGAGTATACACATGCACAATGTGCCATCA
TCAATGCTGGCTTACTCGTTAAAGATATTGTAAAAGATGAAGTGACAGAATATGACATTC
ATCAAATGTTACCGCATCCGATTAATATGGTAAGGGTTAGACTTTCTGGTGTGAAATTAA
AAGAGATTATAGCTAAAAGTAATAAACAAGAATATATGTATGAACATGCACAAGGTTTG
GGTTTCAGAGGGAATATATTTGGAGGATATATTCTTTATAATTTAGGGTACATTCATTCTA
CAGGGCGTTACTATCTGAATGGAGAAGAAATCGAAGACGACAAAGAATATGTATTAGGT
ACGATAGACATGTATACGTTCGGTCGTTATTTCCCAACACTGAAAGAATTACCAAAAGAG
TATTTAATGCCAGAGTTTTTAAGAGATATATTTAAAGAAAAATTATTGGAATATTAA

## > **lipA**

Function: protein coding sequence; lipoyl synthase (core genome, constant)
Best match: lipA_CC001_MW2-USA400_BA000033.2[893702:894619]
Position: 025-contig_192_RC: 17997 … 18915; Length: 918 bp
Sequence:
ATGGCGACAAAAAACGAGGAAATATTACGTAAACCGGATTGGTTGAAAATAAAATTAAA
TACCAACGAAAACTATACAGGACTTAAGAAGATGATGAGGGAAAAAAATCTTAATACTG
TATGTGAAGAAGCTAAATGTCCTAATATACATGAATGTTGGGGTGCACGTCGTACAGCGA
CATTTATGATTTTAGGTGCCGTATGTACAAGAGCTTGTCGTTTTTGTGCGGTTAAGACAGG
TTTACCTAATGAACTTGATTTAAATGAGCCTGAACGTGTAGCTGAATCAGTTGAATTAAT
GAACTTGAAACACGTTGTTATCACTGCTGTTGCGCGTGATGATTTAAGAGATGCTGGTTC
AAATGTTTATGCTGAGACAGTACGTAAAGTTAGAGAAAGAAATCCATTTACAACGATTGA
AATTTTACCATCAGATATGGGCGGGGACTATGATGCGTTAGAAACATTAATGGCTTCAAG
ACCTGACATTTTAAACCATAATATTGAAACTGTTCGTCGATTAACACCGAGAGTTCGTGC
GCGTGCGACTTACGACAGAACATTAGAGTTTTTACGTCGTTCAAAAGAATTACAACCGGA
TATCCCAACTAAATCAAGTATTATGGTTGGATTAGGTGAAACTATAGAAGAAATTTATGA
AACGATGGATGATTTACGTGCGAATGATGTAGATATTTTAACGATTGGTCAATATTTACA
ACCTTCACGTAAACATTTAAAGGTTCAAAAATATTACACGCCTTTAGAGTTTGGTAAATT
AAGAAAAGTGGCAATGGATAAAGGGTTTAAACATTGCCAAGCTGGACCTTTAGTACGTA
GTTCTTATCATGCGGATGAGCAAGTAAATGAAGCTGCTAAAGAAAAGCAACGCCAAGGT
GAGGCACAGTTAAATAGTTAA

## > **nagD**

Function: protein coding sequence; nagD-like protein (core genome, variable)
Best match: nagD_CC005_Mu50-VRSA_BA000017.4[972381:973160] (completely identical)
Position: 025-contig_192_RC: 20304 … 21084; Length: 780 bp

Sequence:
ATGAAACAGTATAAAGCGTATTTAATCGATTTAGATGGCACAATGTATATGGGAACAGAT
GAGATTGATGGAGCAAAACAATTCATCGATTATTTAAATGTAAAAGGCATTCCTCATTTA
TACGTAACTAATAATTCAACAAAAACACCTGAGCAAGTAACTGAAAAATTACGTGAAAT
GCACATTGATGCTAAACCAGAAGAGGTTGTAACGTCAGCGTTAGCCACTGCTGATTATAT
TTCAGAACAATCACCAGGAGCATCAGTATATATGTTAGGTGGGAGTGGTTTAAATACTGC
GTTAACCGAAGCGGGACTTGTCATTAAAAATGACGAGCATGTTGATTATGTAGTTATTGG
ACTTGACGAACAAGTTACATATGAAAAGCTTGCGATTGCAACGTTAGGTGTAAGAAATGG
TGCAACATTTATTTCTACAAATCCTGATGTATCAATTCCTAAAGAGCGTGGTTTATTACCT
GGTAATGGTGCTATTACAAGTGTTGTAAGTGTATCGACAGGTGTATCGCCACAATTTATT
GGTAAACCAGAACCGATTATTATGGTTAAAGCATTAGAAATTTTAGGATTAGATAAATCC
GAAGTTGCTATGGTAGGCGATTTGTACGATACCGATATTATGTCTGGTATTAACGTAGGT
ATGGATACGATTCATGTACAAACAGGTGTATCTACGTTAGAAGATGTGCAAAATAAAAAT
GTGCCACCAACGTATTCTTTTAAAGATTTAAATGAAGCAATAGCTGAATTAGAAAAATAG

> **gyaR**
Function: protein coding sequence; glyoxylate reductase (core genome, variable)
Best match: gyaR_CC001-ST772_118_AJGE01000009.1[20870:21829] (completely identical)
Position: 025-contig_192_RC: 21116 … 22076; Length: 960 bp
Sequence:
TTGGTAAAAATAGTTGTTTCGAGGAAAATCCCAGATAAATTTTATCAACAATTAAGTAAA
CTTGGTGACGTTGTTATGTGGCAAAAATCATTAGTGCCTATGCCTAAAGATCAATTTGTGA
CAGCACTTCGTGACGCAGATGCTTGTTTTATTACATTAAGTGAACAGATCGATGCAGAAA
TTTTAGCGCAATCACCAAATTTAAAAGTAATTGCGAATATGGCTGTAGGATATGACAACA
TCGATGTTGAAAGTGCAACAGCGAATAACGTGGTTGTCACGAATACACCAAATGTACTTA
CTGAAACAACTGCAGAATTAGGATTTACATTAATGCTTGCTATAGCACGCCGTATTGTAG
AAGCTGAAAAATATGTAGAAGCAGATGCATGGCAAAGCTGGGGTCCTTATTTATTGTCAG
GTAAAGATGTCTTCAATTCAACTATTGGAATATATGGTATGGGAGATATTGGTAAAGCTT
TTGCAAGAAGGTTGCAAGGGTTTAATACTAATATTCTTTATCATAATCGATCAAGACATA
AAGATGCAGAGGCGGACTTTAATGCAACATATGTTTCTTTTGAAACGTTGTTAGCAGAAA
GTGATTTTATCATCTGTACAGCGCCACTTACAAAAGAAACACATCATAAATTTAATGCTG
AAGCATTTGAACAAATGAAAAATGATGCAATTTTTATTAATATCGGTAGAGGACAAATTG
TAGATGAAACAGCATTAATCGATGCACTAGACAATAAAGAAATTTTAGCATGTGGTTTAG
ATGTATTAGCAAATGAACCGATTGATCATACACATCCATTAATGGGACGTGATAATGTTC
TGATTACACCACACATTGGTAGCGCATCAGTAACAACACGGGACAATATGATTCAATTAT
GTATTAATAATATAGAAGCGGTTATGACAAATCAGGTACCACATACTCCAGTAAATTGA

> **dltX**
Function: protein coding sequence; putative protein located in teichoic acid biosynthesis gene cluster (core genome, constant)
Best match: dltX_CC001_MW2-USA400_BA000033.2[898192:898344] (completely identical)
Position: 025-contig_192_RC: 22487 … 22640; Length: 153 bp
Sequence:
ATGAAATCTAAAAGTAAACAGCCACCTAATAAATATGTTGAAGCATTCAAACCATATTTA
TTAACACTATTGTATTTGGCAATATTTATTACTTTATATTTAATTTATGGCAGTGGCGACA
CACACAATAACTTCATTTATAATGAGTTCTAA

> **dltA**
Function: protein coding sequence; D-alanine-D-alanyl carrier protein ligase (core genome, constant)
Best match: dltA_CC001_MW2-USA400_BA000033.2[898360:899817]
Position: 025-contig_192_RC: 22655 … 24113; Length: 1458 bp
Sequence:
ATGACAGATATTATTAACAAGCTGCAAGCGTTTGCGGATGCAAATCCACAAAGCATTGCT
GTTAGACACACAACTGATGAATTAACTTATCAACAGTTAATGGATGAGTCTAGTAAATTA
GCACATCGATTACAAGGTAGTAAGAAACCGATGATTTTATTCGGTCACATGTCACCATAT

ATGATTGTTGGGATGATTGGTGCCATTAAAGCAGGATGTGGATATGTACCTGTAGACACT
TCAATTCCTGAAGACCGTATTAAAATGATTATTAACAAGGTTCAACCAGAGTTTGTATTTA
ATACGACTGATGAATCATTTGAAAGTTTAGAAGGCGAAGTATTTACAATAGAAGATATTA
AAACATCTCAAGACCCAGTAATTTTTGATAGTCAGATTAAAGATAACGACACAGTATACA
CAATCTTTACATCTGGTTCTACTGGGGAACCTAAAGGTGTTCAAATTGAATATGCAAGTTT
AGTTCAATTTACTGAGTGGATGTTAGAACTTAATAAATCAGGAAATGAACAACAATGGCT
TAACCAAGCGCCATTTTCATTTGATTTATCTGTAATGGCTATTTATCCATGTTTAGCATCA
GGCGGTACATTAAATCTTGTAGATAAAAACATGATTAATAAACCTAAATTATTAAATGAA
ATGCTAACAGCAACACCGATTAACATTTGGGTATCAACACCATCATTTATGGAAATGTGT
TTATTATTACCAACGCTTAATGAAGAACAATATGGTAGTCTTAATGAATTCTTCTTCTGTG
GTGAAATTCTACCTCACAGAGCAGCAAAAGCGTTAGTAAACCGTTTCCCAAGTGCGACGA
TTTACAACACATATGGTCCAACTGAAGCTACGGTAGCAGTTACAAGTATTCAAATTACAC
AAGAAATCTTAGATCAATATCCGACATTACCTGTTGGCGTTGAAAGACCAGGCGCAAGAT
TATCTACTACAGATGAAGGTGAACTTGTTATCGAAGGTCAAAGTGTAAGTTTAGGATACT
TAAAAAATGACCAAAAAACAGCTGAAGTATTTAATTTCGATGACGGTATTCGTACATATC
ACACTGGTGATAAAGCGAAGTTTGAAAATGGTCAATGGTTCATTCAAGGTCGTATTGATT
TCCAAATCAAATTGAATGGCTACAGAATGGAATTAGAAGAAATTGAAACACAATTACGC
CAGTCTGAGTTCGTAAAAGAAGCGATTGTTGTACCTGTATATAAAAATGATAAAGTTATT
CATTTAATTGGTGCAATTGTGCCAACGACTGAAGTTACGGACAATGCAGAAATGACTAAA
AATATTAAAAATGACTTGAAATCACGCTTACCAGAGTATATGATTCCTAGAAAGTTTGAA
TGGATGGAACAATTGCCATTGACTTCAAATGGTAAAATTGACAGAAAGAAAATTGCAGA
GGTAATTAACGGATGA

> **dltB**

Function: protein coding sequence; hypothethecal membrane transporter (core genome, constant)
Best match: dltB_CC001_MW2-USA400_BA000033.2[899814:901028] (completely identical)
Position: 025-contig_192_RC: 24109 … 25324; Length: 1215 bp
Sequence:
ATGATTCCATATGGTGATTTTACATTCTTCTTAATTGCTTTAATTGCATTATTACCAGTCAT
TATACTTGGATTTTTAGGTAAGCGAAGTTACATTTATAATGGCGTAGTTACAGCATTTATG
ATTGTGTTAATCTTTTCTTCTGATAAACATAATCTGTTTGACCAAAAGTATTTAAGTGTTC
AATTAATTAGTTTTATTATTTACGTCGTATGGCAAGTTTTATTGATAATGTTTTATTATCAT
TCAAAACCAAAAAATAATTCATTTTCAAAATTTGTAACTGTAATGGTTTTATCAATATTGC
CATTAGCACTTGTGAAAGTGTTACAAAGTACATGGTTAGGTGGACATCAGATTCACTTCC
ATGAAAGTAAATTAATTGAATTTGTTGGTTTCTTAGGAATTTCTTATGTTACATTCAAAAG
TGTGCAGTTAATTATGGAAATTCGTGATGGTTCTATCAAAGAAATTAAAGTATGGAAATT
AATTCAATTTATTTCATTCTTCCCAACGATTTCATCTGGACCAATCGATCGTTACAAACGT
TTCGTTAAAGACGATAAAAAAGTACCAACAGGCAATGAATATCGTGAATTAGTATTAAA
AGCAATTCACATGATTATGCTTGGTTTCTTGTATAAATATATTGTTGCTTACTTTATTAACA
CATATGCAATCATGCCGTTACAATTAGACTTACATGGCTTTGTCAATTTGTGGTTATATAT
GTACGCATACAGCTTATATTTATTCTTTGACTTTGCAGGTTATAGTTTATTTGCGATAGCA
TTTAGTTATTTATTCGGTATTAAAACACCACCAAACTTCGATAAACCTTTCAAAGCGAAA
AATATTAAAGATTTCTGGAATAGATGGCATATGACATTATCATTCTGGTTCAGAGATTGT
ATTTACATGAGATCTTTATTCTACATGTCTCGTAAAAAATTATTGAAGAGTCAATTTGCAA
TGTCTAACGTGGCATTCTTAATCAACTTCTTCATAATGGGAATTTGGCATGGTATCGAAGT
GTATTACATTGTTTATGGTTTATACCATGCAGCATTGTTTATAGGTTATGGCTATTATGAA
CGTTGGCGTAAGAAACATCCGCCACGTTGGCAAAATGGTTTCACAACAGCACTTAGCATT
GTGATTACATTCCACTTTGTAACATTTGGCTTTTTAATCTTCTCAGGTAAACTTATATAA

> **dltC**

Function: protein coding sequence; D-alanine--poly(phosphoribitol) ligase subunit 2 (core genome, constant)
Best match: dltC_CC001_MW2-USA400_BA000033.2[901046:901282] (completely identical)
Position: 025-contig_192_RC: 25341 … 25578; Length: 237 bp
Sequence:

ATGGAATTTAGAGAACAAGTATTAAATTTATTAGCAGAAGTAGCAGAAAATGATATTGTA
AAAGAAAATCCAGACGTAGAAATTTTTGAAGAAGGTATTATTGATTCTTTCCAAACAGTT
GGATTATTATTAGAGATTCAAAATAAACTTGATATCGAAGTATCTATTATGGACTTTGATA
GAGATGAGTGGGCAACACCAAATAAAATCGTTGAAGCATTAGAAGAGTTACGATGA

## > dltD

Function: protein coding sequence; putative exoprotein (core genome, constant)
Best match: dltD_CC001_MW2-USA400_BA000033.2[901279:902454] (completely identical)
Position: 025-contig_192_RC: 25574 … 26750; Length: 1176 bp
Sequence:
ATGAAATTAAAACCTTTTTTACCCATTTTAATTAGTGGAGCGGTATTCATTGTCTTTCTATT
ATTACCTGCTAGTTGGTTTACAGGATTAGTAAATGAAAAGACTGTAGAAGATAATAGAAC
TTCATTGACAGATCAAGTACTAAAAGGCACACTCATTCAAGATAAGTTATACGAATCAAA
CAAGTATTATCCTATATACGGCTCTAGTGAATTAGGTAAAGATGACCCATTTAATCCTGC
AATTGCATTAAATAAGCATAACGCCAACAAAAAAGCATTCTTATTAGGTGCTGGTGGTTC
TACAGACTTAATTAACGCAGTTGAACTTGCATCACAGTATGATAAATTAAAAGGTAAGAA
ATTAACATTTATTATTTCACCACAATGGTTTACAAACCATGGTTTAACGAATCAAAACTTT
GATGCTCGTATGTCTCAAACTCAAATTAATCAAATGTTCCAGCAGAAAAACATGTCTACT
GAATTAAAACGTCGTTATGCACAACGTTTATTACAGTTTCCACATGTACACAATAAAGAA
TACTTGAAATCTTATGCTAAAAACCCTAAAGAAACTAAAGATAGTTATATTTCTGGTTTTA
AAGAGAATCAATTGATTAAAATAGAAGCGATTAAATCATTGTTTGCAATGGATAAATCTC
CATTAGAACATGTTAAACCTGCTACAAAACCAGACGCTTCTTGGGATGAGATGAAACAAA
AAGCAGTTGAAATTGGTAAAGCTGATACTACATCGAATAAATTTGGTATTAGAGATCAAT
ACTGGAAATTAATTCAAGAAAGTAAGCGTAAAGTTAGACGTGACTACGAATTCAATGTTA
ATTCTCCAGAATTCCAAGATTTAGAATTACTTGTAAAAACAATGCGTGCTGCTGGTGCAG
ATGTTCAATATGTAAGTATTCCATCAAACGGTGTATGGTATGACCACATTGGTATCGATA
AAGAACGTCGTCAAGCAGTTTATAAAAAAATCCATTCTACTGTTGTAGATAATGGTGGTA
AAATTTACGATATGACTGATAAAGATTATGAAAAATATGTTATCAGTGATGCCGTACACA
TCGGTTGGAAAGGTTGGGTTTATATGGATGAGCAAATTGCGAAACATATGAAAGGTGAA
CCACAACCTGAAGTAGATAAACCTAAAAATTAA

## > tnpIS431-06

Function: protein coding sequence; transposase for IS431 (mobile element)
Best match: tnpIS431-06_circ_S-epidermidis_GQ900381.1[43799:43807] (completely identical)
Position: 025-contig_192_RC: 27569 … 27578; Length: 9 bp
Sequence:
ATGAACTAT

## > ampA

Function: protein coding sequence; cytosolic leucine aminopeptidase (core genome, constant)
Best match: ampA_CC001_MW2-USA400_BA000033.2[907224:908699] (completely identical)
Position: 025-contig_192_RC: 31519 … 32995; Length: 1476 bp
Sequence:
ATGAATTTTAAATTAAATAACACACTAAGCAACGAAATAAATACATTGATTATTGGTATA
CCAGAACATTTAAATCAGTTAGAGCGCATTAGTTTTAATCATATCGATATTACAGAATCA
CTTGAAAGACTAAAACATCAACATATTATTGGTAGTAAAGTTGGGAAGATTTATACAACT
GCATTTGATGTACAAGATCAAACATATCGTTTAATTACAGTTGGTTTAGGAAACTTAAAG
ACACGTAGTTATCAAGATATGTTGAAAATATGGGGACATCTTTTCCAATACATAAAGTCA
GAACACATTGAAGATACGTATTTACTTATGGATTCATTTATTTCAAAATATGATCAGTTAT
CAGATGTATTAATGGCATGCGGTATTCAAAGTGAGCGTGCAACATATGAATTCGATCATT
ATAAATCAAGTAAGAAGGCACCGTTTAAGACGAATTTAAACCTTATTAGTGAATCATTAA
TTGAATTAGATTTTATTCATGAGGGTATCAGTATTGGCCAATCCATTAATTTGGCAAGAGA
CTTTAGTAATATGCCACCGAATGTATTAACACCACAAACATTTGCAGAAGATATTGTTAA
TCATTTTAAAAATACAAAGGTCAAAGTAGATGTTAAAGATTATGACACTTTAGTTTCTGA
AGGATTCGGACTTTTACAAGCAGTAGGTAAAGGTAGTAAGCATAAACCGAGATTAGTAA

CCATCACATATAATGGCAAAGACAAAGATGAAGCACCAATTGCCTTAGTTGGTAAAGGT
ATAACGTATGATTCTGGTGGTTATAGTATTAAAACGAAGAATGGCATGGCTACAATGAAG
TTTGACATGTGTGGCGCTGCGAATGTCGTTGGTATCATTGAAGCGGCTAGTCGTTTACAAC
TGCCTGTAAATATTGTCGGAGTGCTTGCGTGTGCTGAAAATATGATAAATGAAGCATCAA
TGAAGCCAGATGATGTATTTACAGCATTAAGTGGTGAAACTGTAGAAGTAATGAATACAG
ACGCTGAAGGTAGATTAGTCCTTGCAGATGCTGTGTTTTATGCAAATCAATATCAGCCTA
GTGTGATTATGGACTTTGCTACATTAACGGGTGCAGCAATTGTTGCACTAGGCGATGATA
AAGCTGCTGCATTTGAATCGAATAGTAAAGTGATATTAAACGATATATTACAAATAAGTT
CTGAAGTCGATGAAATGGTATTTGAATTACCGATTACTGCAACCGAACGTGCAAGTATTA
AACACAGTGATATCGCTGATTTAGTTAACCATACGAATGGACAAGGTAAAGCGCTATTTG
CGGCAAGTTTTGTAACACATTTTAGTGGTCAAACACCTCACATTCATTTCGATATTGCAGG
TCCAGCAACGACTAATAAAGCTTCATATAATGGTCCAAAAGGGCCAACAGGATTTATGAT
TCCGACGATAGTACAATGGTTAAAACAACAATAA

## > nhaC3

Function: protein coding sequence; Na+/H+ antiporter (core genome, variable)
Best match: nhaC3_CC001_MW2-USA400_BA000033.2[909110:910426] (completely identical)
Position: 025-contig_192_RC: 33405 … 34722; Length: 1317 bp
Sequence:
ATGATAAATGCAGTAGTAATAGCAGTAATTTTAATGATTGTGCTATGTTTATGTCGATTAA
ACGTAGTTATAAGCTTATTTATCAGTGCGCTAGTTGGTGGCTTAATTTCAGGCATGAGCAT
TGAAAAAGTTATAAATGTATTTGGGAAAAATATAGTCGATGGTGCTGAGGTAGCATTAAG
CTATGCTTTATTAGGTGGATTTGCAGCATTAATTTCATACAGTGGTATCACAGACTATTTA
GTAGGAAAAATTATAAATGCAATTCACGCTGAAAATAGTCGATGGTCAAGAGTTAAAGT
CAAAGTGACAATAATCATTGCATTATTAGCTATGAGTATCATGAGTCAAAACTTAATTCC
TGTACATATTGCATTCATTCCAATTGTCATCCCACCATTGTTAAGTCTGTTTAATGACTTA
AAAATAGATAGACGTTTAATCGGTTTGATTATCGGTTTTGGTTTATGTTTCCCGTATGTGT
TATTACCATATGGATTCGGTCAAATTTTCCAGCAAATTATTCAAAGTGGCTTTGCAAAGGC
GAATCACCCAATTGAGTTTAATATGATTTGGAAAGCAATGCTTATTCCTTCAATGGGGTAT
ATTGTTGGCTTACTTATCGGTTTATATGTATATCGTAAACCACGTGAATATGAAACACGTA
AAATTTCAGATAGTGACAATGTTACAGAGTTAAAACCATATATCTTAATAGTAACAATTG
TAGCAATACTAGCTACATTTTTAGTACAAACATTTACAGATTCAATGATTTTTGGTGCACT
GGCAGGGGTACTCGTATTCTTTATTTCACGTGCATATAATTGGTATGAATTAGATGCTAAG
TTTGTTGAAGGTATTAAAATTATGGCTTATATTGGTGTAGTTATTTTAACAGCAAATGGAT
TTGCTGGTGTAATGAATGCTACTGGTGATATAGATGAATTAGTTAAAACTTTAACAAGTA
TTACTGGTGATAATAAATTATTTAGCATTATCATGATGTATGTGATAGGTTTAATTGTCAC
TTTAGGTATTGGATCATCATTTGCAACAATTCCTATTATCGCATCATTATTCATTCCTTTTG
GAGCGTCAATTGGACTAGATACAATGGCATTAATCGCATTGATTGGAACAGCGAGTGCAT
TAGGTGACTCAGGTTCACCTGCAAGTGATTCAACATTAGGACCAACTGCGGGATTAAATG
TTGATGGCCAACATGATCATATACGCGATACATGTGTACCAAACTTCTTGTTTTATAATAT
TCCTTTAATGATTTTCGGTACTATTGCTGCTATGGTACTATAA

## > mnhG

Function: protein coding sequence; Na(+)/H(+) antiporter/subunit G
Best match: mnhG_CC008_COL_CP000046.1[951804:952160:r]RC (completely identical)
Position: 025-contig_192_RC: 36573 … 36930; Length: 357 bp
Sequence:
CTACAATTTTGTGTCTTTTAAGTCTTCCGAAATTTCATCGACTTTAGTCTTTTTAGTATAAG
GCGTTTTAATATTATATGCTGCTTTCATAATCATATGACTTGAAAGAGGACCTGTAATTAA
TACAAAGATAATCGCAACGATTAATTGCATATTTACAAAACCTTGAGTAGCAATAAAATA
TAAAAACGTACCAAATAGTAATGACATTGCACCTAATGTTGATGCTTTTCCGGCAGCATG
TGCACGTGAATATACATCTTCAAGTCTCAATAATCCTATAGCTGCTAGGGCGCTAATTAA
AGCACCGATGATAACAAAGATAAGTGCAAGACTAATCAGTATGATTTTGATCAT

## > mnhF

Function: protein coding sequence; Na(+)/H(+) antiporter/subunit F
Best match: mnhF_CC001_MW2-USA400_BA000033.2[912612:912905:r]RC (completely identical)
Position: 025-contig_192_RC: 36907 … 37201; Length: 294 bp
Sequence:
CTAATCAGTATGATTTTGATCATGTTCAATCACCTTACCTTTGTCCATAAATTTAGAGAAT
ACTGCAGTACCTAAAAAAGCTAATATACCAATCATCATAATAACGACAATCATGTATTTA
ATATTTAATAAAATACTGAATAATGCTATAACTGCCATTAATTGAAGACCAATCGCATCT
AATGCGACAACACGATCGGCAAGTGATGGGCCTAGCACAACGCGAATGAGCATAGCTAA
CATAGAAATGACAACTATGATTAATGCAATAACGATAATAACATTATGATTCAT

> **mnhE**
Function: protein coding sequence; Na(+)/H(+) antiporter/subunit E
Best match: mnhE_AB015981.1[5474:5953]RC
Position: 025-contig_192_RC: 37200 … 37680; Length: 480 bp
Sequence:
TTATATTTCGCCCACCTCTCTTACAATTTTCTCTAATGATGTTTTAATACTTTCTACTTCTTG
CTCTTTAGTTGAAAAATCTATGGCATGAATATAAATTTTTGTACGATCGTCACTTACACCA
AGCACTACAGTACCAGGTGTTAATGTAATTAAATTAGACAGCAAGACAATTTGCCAATCT
TTTTTTAAATCTGTGTGATAAACAAAGAATCCTGGTTCATTTTTAATCGAAGGTTTAATAA
TAATTTTCAAAACATCAAAATTAGCTTTAATCAGTTCGATTAAGAAAATAATAACTAATTT
AATAATACGATATAGCGTGATGACATAAAATCTACCTGGTAACACCCTGTGTAAGAGGTA
AACAAGAACTAGGCCAAAGATGAAACCTAACACAAAGTTATTTGTTGTGTAACTATTTGT
CACAAACAACCAAAACACTGCGATAATAAAGTTTAATACTAATTGTACAGCCAT

> **mnhD**
Function: protein coding sequence; Na(+)/H(+) antiporter/subunit D
Best match: mnhD_AB015981.13976:5472RC
Position: 025-contig_192_RC: 37681 … 39178; Length: 1497 bp
Sequence:
TTATTTACCTCCTAATACAGCTTTAACATAGGTTGATGGATTGTAGAATGTTTCTGCACCA
GCTTTTACCATTGGATATAAGTAATCTGCTGACAATCCATATAAAACAGTTATCACAACT
GCAACGATTGCAATCGTAGTTAAATAATTGACGTCGACTTTGTTATTAAGATCATATCCTT
TTGGTTGACCGAAAAAGCCTTGTAGGAATATGCGAATGACAGAATATAATACGACTAAA
CTTGATAATAAGACAATGACACCACTTAAATAAAATCCTCTTTCAAATGTTGATTGGACA
ATGAAAAATTTTCCATAAAAGCCACTGAGTGGGGGAATGCCAGCTAAACTTAATGCTGCG
ATAAAGAATGACCAACCAAGTACAGGATATCGTTTAATTAAGCCACCAAATTGTCTTAAA
TCAGCAGTGCCTGTAATTTTAATCATAATTCCGATAAGCAAGAATAATGCAAGTTTTACT
AACATGTCGTGCAATGTATAGTAAATAGCCCCAATCATACCTGACTCTGTCATCATTGCA
ACGCCGACTAAGATCACACCTACAGCAATCATGACATTGTATAGGATGATTTTTTTAATG
TTGGCATATGCAACAGCACCGACACAACCAAAGATGATCGTTAATAATGCTAAGAATAA
AATGACATAATGTGAAAAGCTTACATTATCACTAAAGAATAGGCTCAATGTTCTAGCGAT
TGCATAAACACCAACTTTTGTTAACAAAGCACCAAAGAATGCAATGATTGGAATTGGTGG
TGCATAGTATGCACTAGGTAACCAAACAAACATTGGGAATACGCCAGCTTTTGTAGCAAA
AACAAAGATAAATAGTATGAAAACGATATTGACTAAGCCACTGTCATGCGCTGAAAGGT
TAGCTAATTTATTGCTTATATCTGCTAGATTCAATGTTCCTACTACTGAATATAAAATCGC
TACACCCATTACGAAGAAGGATGACGATACAACGTTAACAAGAACATATTTGATTGTTTC
TTGTAGTTGAATTTTTGTAGAACCAATTACTAATAAGAAATAAGATGACATTAAAAATAC
TTCGAAAAATACGAATAGGTTGAAAATGTCACCAGTTGTGAATGCACCAATGATACCTAT
TAACATAAATAGTACTGAAAAATAATAATAATATCTTTCACGTTCAATACCAATTGTTTG
GTATGAATATAAAATCACAATAGCTGTAATAATAATACTAGTAATTATTAGTAGGGCACT
GAATATGTCTAATACAAAGACAATACTGTATGGTGCTTTCCATGAACCTAGCTCTACGCG
TATTGGTCCATGTTTAACAACATTTGCTAAATTGATAATTGCCACGACCAAGGTTAATAAT
GTACCGCCTAGTGCGACATAACGCTTTATAATAGGACGCTTTCCAATAAAGACAAGTAAT
ATGGCTGTAATTACTGGAATAACTAGCGTTAACACAAGCATATTACTTTCAATCAT

## > **mnhC**

Function: protein coding sequence; Na(+)/H(+) antiporter/subunit C
Best match: mnhC_CC001_MW2-USA400_BA000033.2[914875:915216:r]RC (completely identical)
Position: 025-contig_192_RC: 39170 … 39512; Length: 342 bp
Sequence:
TCAATCATCTTCTGGAACTCCTTTCATACTCTCAACGTTATCTGTGCCTAATTCTTTATATG
TTCTAAATGCTAATACTAAGAAAAAGGCTGTTGTCGCAAAGGCGATAACGATTGCTGTTA
AAATAAGTGCTTGCGGGATAGGATCAACATAGCTTTTTACGTTCGCTTCATAAATTGGAA
CAGTACCATGTTTAAGTCCGCCCATAGTTATTAAAAATAAATTTGCTGCATGTGTTAATAG
TGTAGTTCCCATAACAATTCGTATCAGACTTTTAGACAAAACGAGATAGACACTAATTGC
TGTGAGAATACCACTAACAAAAATCATAATAATTTCCAC

## > **mnhB**

Function: protein coding sequence; Na(+)/H(+) antiporter/subunit B
Best match: mnhB_AB015981.1[3214:3642]RC
Position: 025-contig_192_RC: 39511 … 39940; Length: 429 bp
Sequence:
CTATTCGTTCTCTCCAATCGAAATAATAATTGTCATGACAGTACCAACTACTGCACATAAA
ACACCGAAATCAAAGAATACTGCTGTTGTCATATGAACAGGTTCTAATATAAATAACGGT
ATATCAAATGTGACATGCGTAAAGAAATTTTTGCCTAAAAACCAACTTGCGATAGGCGTC
GCAATACAAAAAACTAATCCGATACCTATCAAGATTTTAAAATCTAATGGGAAAATTTTA
CGCATTGTTTCTATATCAAATGCAATCGTAATGATAACAAGTGAACTTGCGAATAATAAT
CCGCCGACGAATCCGCCACCAGGTGTATAATGTCCTGCTAAGAAAAGTGAAAAACCAAA
GACCATTACCATGAAAAAGATAATAACTGCAGCAAATTGCAAAATTAGATCATTTTGTTG
TCTATTCAT

## > **mnhA**

Function: protein coding sequence; Na(+)/H(+) antiporter/subunit A
Best match: mnhA_CC001_MW2-USA400_BA000033.2[915637:918042:r]RC
Position: 025-contig_192_RC: 39932 … 42338; Length: 2406 bp
Sequence:
CTATTCATGATTTTTCACCTCGTTACCTTGCGTTTGACGCTTTTTACGTAATTTAATCATTG
TATATACAGCTAATCCTGCGATACCAAGCACAGATGACTCGAATAAAGTATCCATACCAC
GGAAATCAACAAGTATGACGTTTACCATGTTTTTACCGTGAGCTAAATCATAAACGTGCT
CTTGATAAAACTTAGATATCGATTCAAAATGTCTATTTCCGTATGCAATTAAACCGATAAT
AATGACGGACAAACCAACACCACCAGCAATTAAAGCATTAGTAAGCTGGAATGAGCGCT
TTTCATTATAACGATTTAAATTTGGTAAGTGGTAGAAGCATAATAAGAACAATGCTGTTG
AAATAGATTCAACGACAAACTGTGTCAATGCTAAGTCGGGTGCTTTAAAGAATATAAACA
ATACAGACACAGCATATCCAACTGCACTTAACATAATGATGCTAAATAATCTTGATTTAG
CGAAAAGAATTAAAAAGGCAGCACTTAATAATAAAATTACGATACAAACTTCGAAAATT
CTAATCGGACTAACGTCTTTAAAATTAATGTTGAAAGGTACTGAGAATATAGTGACAAAT
GTTAATAAAATTAATGCACCAAAAATGATAACTAAATTATTACGTGAATAATCGGTAACA
TAGCTATTCGTCATCTTTTCAGAGTAGTTTGGAATAACATTTGCACTTCTGTTGTACCAAT
AATTGAATGTTAGTTTACCAGGTTGTCGTTGCAACAATTTCACCCAATAACTAAATGTCAC
AATTAGTAAGATACCTAAAATATAAATCACTAATGTTGATAAAAAGGCAGGCGTTAATCC
ATGGAACATATGGAATTCAACATCATCAATTACCGTATGATTAATCGAAGATGTAGCTGG
TTCAATAATCGAATTAGTTAAAATGCCAGGGAATAAACCGAATACAATTACTAATGTAGC
TAAAATAGCTGGTGATAAAAGCATTAATATTGATACTTCGTGTGCTTTTTTAGGTAATTGT
TCAGGTTTATATTGTCCGAAAAATATATGCATTATAAATTTAATTGAATATACAAATGTGA
AGACACTGCCCACTATACCAATGATTGGGAATAGATAGCCTAATGTATCAACACTGAATA
AATTTGCTTGGCTTGCTGTAAATGTTGTTTCTAAAAATGATTCTTTTGATAAGAAACCATT
GAACGGTGGTACACCAGCCATACTTAATGCTGTAATAACAGTGATTGTAAATGAAATAGG
CATAATTGTTAGTAAGCCACCTAATTTCTTAACATCACGTGTACCAGTAGAATGATCCACT
GCACCTGTAATCATAAATAGGGCACCTTTAAATGTTGCATGGTTGATTAAATGGAATATT
GCAGCCGTAAATGCAGCAGCATATATTTTGCTATCATCGCCTTGATAGTGATAACTAATG

GCACCGATTCCAAGCATCGCCATAATCATACCTAATTGGGATACTGTTGAAAATGCCAGT
ATACCTTTCAAGTCTTGTTGTTTTGTTGCGTTTAGCGAAGCCCAGAATAACGTAATTAAAC
CAACGAGTGTGACAGTCCATACCCAACCTTGCGATGCTGCGAAGATTGGTGTCATTCGAG
CGATTAAATATAACCCTGCTTTAACCATTGTTGCTGAATGAAGATAAGCACTGACTGGTG
TAGGTGCTTCCATTGCATCTGGTAGCCAAATATAAAATGGAAACTGAGCAGATTTTGTAA
AAGCACCAATCATGATTAAAATCATCGCAAAAATGAAGAATGGGCTATTTTGAATTTCAG
AAGCATGTTGAATCATGTACTGAATGCTAAATGATTGTGTTGGTATAGCGAGTAAGATGA
TACCACCTAATAATGATAGACCACCAAATACTGTGATTATGAGCGATTTTTGAGCACCAT
ATATAGATGCTTGTCGTTCGCGCCAGAATGAAATAAGTAAAAAACTAGAAAATGACGTTA
GCTCCCAGAATAAATATAGAATAATAACATTATCTGAAAGTACGACACCTAACATTGCAC
CCATAAATAGTAATAAATAACAATAAAAATTCCCTAGTTGTTCTGACTTACTTAAGTAGC
CGATTGAATATAATACTACTAAACTGCCGATTCCTGAAATAAGCAAACTAAAGAGTAAAC
CTAAGCCATCAAGATATAAATCAAAGTTCATACCAAAATGAGGCATCCAATTTAAGGTTT
TCATTACAGTATTACCTGACATCGTCGTTTTAATTAATGTAAGCATATAAATAAATATGAC
GATAGGGACAGGTAATACGAACCATCCTAAATGTATACGTTTAAAAAATCTATACAGGAT
AGGAATAATGAGTGCGAATATTAACGGTAATATCACCGCAATATGTAACAAACTCAC

> **kapB**

Function: protein coding sequence; kinase associated protein B (core genome, constant)
Best match: kapB_CC001_MW2-USA400_BA000033.2[918173:918556:r]RC (completely identical)
Position: 025-contig_192_RC: 42468 … 42852; Length: 384 bp
Sequence:
TTATTTAAAATTTTCTTTGTATTGAAGTGAATAATCTTCTTTTAAGCGTGCTAAACTAGCT
AAAGACATTTCAGCATGTTTTGTTTGCTGAGCTTTAAGTTTAGTTTCTAAATCTGTAATTG
CTTGTTGAAGTGAATCTTCATAGCGCAATACATCAACATTGAAGTCGCGTAATTGTGAAC
GTTTCGTATAGCGTTTTTCAAAATGGCTTAATGCTTTGCGTTCATGGAAAAATACACCTTC
AGTTTCAGTAGGGTTATGTAAATCACCTTGTTTCGGGTGTTTGATAACTTGTTCAACTTTA
ACAAGGACATCGTCTCCATTTTCTTCAACAATCGTGACACCATAGCTACCTGTTTTGTGTG
AAAATCGATATAGCTTCAT

> **prsA1**

Function: protein coding sequence; peptidyl-prolyl cis-trans isomerase (core genome, constant)
Best match: prsA1_CC001_MW2-USA400_BA000033.2[918620:919213]
Position: 025-contig_192_RC: 42915 … 43509; Length: 594 bp
Sequence:
ATGGCTAACTATCCACAGTTAAACAAAGAAGTACAACAAGGTGAAATTAAAGTGGTTAT
GCACACAAATAAAGGTGACATGACATTCAAATTATTTCCAAATATTGCACCAAAAACAGT
TGAAAATTTTGTGATACATGCAAAAAATGGTTATTATGATGGAATAACATTCCACCGTGT
CATTAATGACTTCATGATTCAAGGTGGCGATCCAACAGCTACTGGTATGGGTGGCGAAAG
TATTTATGGCGGTGCTTTTGAAGATGAATTTTCATTAAATGCATTTAACTTATATGGCGCA
TTATCAATGGCTAACTCAGGACCTAATACTAATGGTTCACAATTTTTCATTGTTCAAATGA
AAGAAGTACCTCAAAATATGTTAAGTCAACTTGCAGATGGTGGTTGGCCACAACCAATCG
TTGATGCATATGGCGAGAAGGGTGGTACACCATGGTTAGATCAAAAACATACAGTATTCG
GTCAAATCATTGATGGTGAAACTACATTAGAAGATATTGCAAATACAAAAGTGGGACCA
CAAGATAAACCACTTCATGATGTTGTAATTGAATCTATTGATGTTGAAGAATAA

> **namA**

Function: protein coding sequence; NADH-flavin oxidoreductase (core genome, constant)
Best match: namA_CC001_MW2-USA400_BA000033.2[920367:921494]
Position: 025-contig_192_RC: 44663 … 45791; Length: 1128 bp
Sequence:
ATGAAAAGTAAATACGAACCATTGTTTGATAAAGTAGAATTACCAAATGGAGTAGAGTT
GAGAAATCGATTTGTGTTAGCCCCTTTAACACATATTTCTTCAAATGATGATGGTACTATT
TCAGATGTAGAACTTCCTTATATTGAAAAGCGTTCACAAGATGTTGGTATTACAATTAAT
GCTGCGAGTAATGTGAGTGATGTCGGAAAAGCATTTTCAGGACAGCCATCAATCGCGCAT

GACAGTAATATTGAAGGACTAAAACGATTAGCTACAGCAATGAAGAAAAACGGTGCCAA
AGCACTCGTACAAATACATCATGGCGGTGCACAAGCATTGCCTGAATTAACACCTGGTGG
AGACGTCGTAGCACCAAGTCCAATTTCTTTAAAAAGTTTCGGTCAGAAACAAGAACATAG
TGCTAGAGAAATGACGAATGAAGAGATTGAACAAGCAATCAAGGATTTTGGTGAAGCAA
CGCGACGTGCAATTGAAGCAGGATTTGATGGTGTTGAAATACATGGCGCGAATCATTACT
TAATTCATCAATTTGTATCACCATACTATAATAGAAGAAATGATGTATGGGCAAATCAAT
ATAAATTCCCGGTTGCTGTGATTGAAGAAGTGCTTAAAGCGAAAGAAGTGTATGGCAATA
AAGACTTTATTGTTGGATATAGATTGTCTCCGGAAGAAGCGGAGTCTCCAGGAATCACAA
TGGAAATTACAGAGGAACTCGTTAATAAAATTAGCCATATGCCAATCGACTATATTCATG
TTTCAATGATGGATACGCATGCAACGACACGTGAAGGTAAATACGCTGGACAAGAAAGA
CTGCCTTTAATTCACAAATGGATAAATGGTCGTATGCCACTTATCGGTATTGGTTCAATTT
TCACAGCTGACGAAGCTTTAGATGCAGTTGAAAATGTTGGTGTTGACTTAGTAGCCATTG
GTAGAGAGCTACTATTGGATTATCAATTTGTTGAAAAAATTAAAGATGGACGGGAAGATG
AAATTATTAATTACTTTGATCCAGAGAGAGAAGATAATCATCACTTAACTCCTAATTTATG
GCATCAATTTAATGAAGGATTCTATCCATTACCACGTAAAGATAAATAA

> **rocD**
Function: protein coding sequence; ornithine aminotransferase
Best match: rocD_CC001_MW2-USA400_BA000033.2[921802:922992]
Position: 025-contig_192_RC: 46098 … 47289; Length: 1191 bp
Sequence:
ATGACTAAATCTGAAAAAATTATTGAGTTAACAAATCATTACGGAGCACATAATTATTTA
CCATTGCCAATTGTCATTTCAGAAGCTGAAGGGGTATGGGTTAAAGATCCTGAAGGCAAT
AAATATATGGATATGTTATCTGCATATTCCGCTGTTAACCAAGGTCATAGACATCCGAAA
ATTATTCAAGCATTAAAAGATCAAGCTGATAAAGTGACTCTAGTTTCACGTGCTTTTCATA
GTGATAACTTAGGTGAATGGTACGAAAAAATTTGTAAACTGGCAGGTAAAGATAAAGCT
TTACCAATGAATACAGGTGCTGAAGCAGTAGAAACAGCTTTGAAAGCAGCACGACGCTG
GGCATACGATGTTAAAGGAATTGAGCCAAATAAAGCAGAAATCATTGCATTTAATGGTA
ACTTCCATGGTCGAACAATGGCGCCAGTTTCATTATCTTCAGAAGCAGAATACCAACGTG
GTTATGGTCCGTTATTAGATGGATTTAGAAAAGTTGATTTTGGAGATGTAGATGCATTGA
AAGCTGCAATTAATGAAAATACTGCAGCAGTTTTAGTAGAACCAATTCAAGGTGAAGCG
GGTATAAATATACCGCCAGAAGGATATTTGAAAGCAATTAGAGAATTATGTGATGAACAT
AATGTCTTATTTATTGCTGACGAAATCCAAGCAGGATTAGGTCGTTCGGGTAAATTATTTG
CTACGGATTGGGATAATGTAAAACCTGATGTCTATATTTTAGGTAAAGCACTAGGTGGTG
GTGTCTTCCCAATTTCTGTTGTATTAGCAGATAAAGAAGTATTAGATGTCTTTACACCTGG
CTCACATGGTTCAACATTTGGTGGTAATCCACTTGCTTGTGCTGCATCAATTGCTGCATTA
GATGTTATCGTTGATGAGGATTTACCAGGGCGCTCTTTAGAATTAGGAGATTATTTTAAA
GAACAATTAAAGCAAATTGATCATCCATCAATTAAAGAAGTCCGTGGACGTGGTTTGTTT
ATAGGTGTGGAACTTAATGAAAGTGCTAGACCATATTGTGAAGCTTTGAAAGAAGAAGG
TTTATTATGTAAAGAAACGCATGATACTGTCATTCGTTTTGCACCACCATTAATTATTACT
AAAGAAGAATTGGACCTTGCACTTGAAAAAATAAGACATGTATTTCAATAA

> **rocD2**
Function: protein coding sequence; ornithine aminotransferase 2 (core genome, constant)
Best match: rocD2_CC001-ST772_118_AJGE01000009.1[45852:47042] (completely identical)
Position: 025-contig_192_RC: 46098 … 47289; Length: 1191 bp
Sequence:
ATGACTAAATCTGAAAAAATTATTGAGTTAACAAATCATTACGGAGCACATAATTATTTA
CCATTGCCAATTGTCATTTCAGAAGCTGAAGGGGTATGGGTTAAAGATCCTGAAGGCAAT
AAATATATGGATATGTTATCTGCATATTCCGCTGTTAACCAAGGTCATAGACATCCGAAA
ATTATTCAAGCATTAAAAGATCAAGCTGATAAAGTGACTCTAGTTTCACGTGCTTTTCATA
GTGATAACTTAGGTGAATGGTACGAAAAAATTTGTAAACTGGCAGGTAAAGATAAAGCT
TTACCAATGAATACAGGTGCTGAAGCAGTAGAAACAGCTTTGAAAGCAGCACGACGCTG
GGCATACGATGTTAAAGGAATTGAGCCAAATAAAGCAGAAATCATTGCATTTAATGGTA
ACTTCCATGGTCGAACAATGGCGCCAGTTTCATTATCTTCAGAAGCAGAATACCAACGTG

GTTATGGTCCGTTATTAGATGGATTTAGAAAAGTTGATTTTGGAGATGTAGATGCATTGA
AAGCTGCAATTAATGAAAATACTGCAGCAGTTTTAGTAGAACCAATTCAAGGTGAAGCG
GGTATAAATATACCGCCAGAAGGATATTTGAAAGCAATTAGAGAATTATGTGATGAACAT
AATGTCTTATTTATTGCTGACGAAATCCAAGCAGGATTAGGTCGTTCGGGTAAATTATTTG
CTACGGATTGGGATAATGTAAAACCTGATGTCTATATTTTAGGTAAAGCACTAGGTGGTG
GTGTCTTCCCAATTTCTGTTGTATTAGCAGATAAAGAAGTATTAGATGTCTTTACACCTGG
CTCACATGGTTCAACATTTGGTGGTAATCCACTTGCTTGTGCTGCATCAATTGCTGCATTA
GATGTTATCGTTGATGAGGATTTACCAGGGCGCTCTTTAGAATTAGGAGATTATTTTAAA
GAACAATTAAAGCAAATTGATCATCCATCAATTAAAGAAGTCCGTGGACGTGGTTTGTTT
ATAGGTGTGGAACTTAATGAAAGTGCTAGACCATATTGTGAAGCTTTGAAAGAAGAAGG
TTTATTATGTAAAGAAACGCATGATACTGTCATTCGTTTTGCACCACCATTAATTATTACT
AAAGAAGAATTGGACCTTGCACTTGAAAAAATAAGACATGTATTTCAATAA

## > gluD

Function: protein coding sequence; NAD-specific glutamate dehydrogenase (core genome, constant)
Best match: gluD_CC001_MW2-USA400_BA000033.2[923101:924345]
Position: 025-contig_192_RC: 47397 … 48642; Length: 1245 bp
Sequence:
ATGACTGAGAACAATAATTTAGTAACTTCTACTCAAGGAATTATTAAAGAAGCATTGCAT
AAATTAGGATTTGACGAAGGAATGTACGATTTAATTAAAGAACCTTTAAGAATGTTACAA
GTGCGTATCCCTGTACGAATGGATGATGGCACTGTTAAAACATTCACAGGTTACCGTGCG
CAACATAATGATGCTGTTGGACCAACAAAAGGGGGCGTGCGTTTCCACCCAGATGTTGAT
GAAGAAGAAGTAAAAGCATTATCAATGTGGATGACTTTGAAATGTGGCATTGTAAACTTA
CCATACGGTGGTGGTAAGGGTGGTATCGTTTGTGATCCACGTCAAATGAGCATTCATGAA
GTTGAACGTTTATCACGCGGATATGTAAGAGCAATTTCACAATTCGTAGGTCCGAACAAA
GATATTCCAGCACCAGATGTATTTACAAACTCACAAATTATGGCTTGGATGATGGATGAA
TATAGTGCATTAGATAAATTTAATTCACCAGGTTTCATCACAGGTAAACCAATTGTATTGG
GTGGTTCTCATGGACGCGACAGATCAACTGCACTAGGTGTAGTTATTGCAATTGAACAAG
CTGCAAAACGTCGTAATATGCAAATTGAAGGTGCCAAGGTTGTTATTCAAGGTTTCGGTA
ATGCCGGAAGTTTCTTAGCTAAATTCTTATATGATTTAGGTGCAAAAATTGTAGGTATCTC
TGATGCTTACGGTGCATTACACGATCCAAATGGCTTAGATATAGATTATTTATTAGACCGT
CGTGATAGTTTTGGTACGGTAACAAATTTATTTGAAGAAACAATCTCAAATAAAGAATTG
TTTGAATTAGATTGTGACATTTTAGTACCAGCGGCTATTTCAAACCAAATTACAGAAGAC
AATGCACATGATATTAAAGCTAGTATCGTTGTTGAAGCTGCTAATGGACCTACAACACCA
GAAGCAACACGTATTTTAACTGAACGTGGTATATTATTAGTTCCAGACGTATTAGCAAGT
GCTGGTGGTGTAACGGTTTCTTACTTCGAATGGGTACAAAATAATCAAGGTTATTATTGGT
CTGAAGAAGAAGTTAATGAAAAGCTACGTGAAAAATTAGAAGCGGCATTTGATACGATT
TACGAATTGTCTCAAAACCGAAAAATAGATATGAGACTTGCAGCATATATCATAGGTATT
AAACGTACAGCAGAAGCAGCTAGATATCGTGGTTGGGCATAA

## > txbi_universal1

Function:  bidirectional rho independent terminator
Best match: txbi_universal1_CC001_MW2-USA400_BA000033.2[924625:924696:r]RC
Position: 025-contig_192_RC: 49024 … 49054; Length: 30 bp
Sequence:
AATATCTTACTGCTGTTTTTTATAGTAATA

## > glpQ2

Function: protein coding sequence; glycerophosphodiester phosphodiesterase
Best match: glpQ2_CC001_MW2-USA400_BA000033.2[924735:925664:r]RC
Position: 025-contig_192_RC: 49872 … 50022; Length: 150 bp
Sequence:
CGTTAAATTTGTATGCCATTGAATAGCCTGAGGTTTATTTGCAATTTGATTTGTTTGTTCA
GCGCCAGCAGTAGGTACTGATAAAAATCCCATAGTAAAAACAGCAGAAGCAGCCATAAA
TTTAGTGAAGCTTTTCGAAGAGTTAGTCAT

> **argH**
Function: protein coding sequence; argininosuccinate lyase
Best match: argH_CC001_MW2-USA400_BA000033.2[925906:927285:r]RC
Position: 025-contig_192_RC: 50263 … 51643; Length: 1380 bp
Sequence:
TTATTGTGATAGTAATTGTTTAGCAACATCAAGTTGTTGTTTGACCGATGATTGACCTGTT
GAACCGTAACTTTGACGTCGTTTTAAACAATTTTCAGGCTGCAAATAATCGTAAATATCG
GCATCAATACTAGAATGATGTTGTTGATATGTTGCTAAAGGAACATCTAATAAATAATGA
CCTTGTTGTATACATTCTAAGACAATTTTTCCTACAATTTCATGTGCAGTTCTAAATGGAA
TATTTTTAGTTACTAAATAATCTGCTAGTTCCGTTGCATTTGAAAAATCTTCTTTAACAGTT
TGATTGAGTCGTTCTTTATTAATTGTCATCGTTTGAATCATACCTTCGAAAATACGTAAAG
AACCTTTAATTGTATGGACAGCATCGAATAAACCTTCTTTATCTTCCTGCATATCTTTGTT
ATATGCTAGAGGTAATCCTTTTAAAGTCATAAGCATGCTCATTAAATGACCAGTCGTTCG
ACCAACTTTACCTCTAATTAATTCTGCCATATCAGGATTTTTCTTTTGTGGCATAATAGAT
GAGCCAGTTGAAAATGCATCTGATAATGTAATGAATTTAGCTTCGTCTGTGGACCAGAAA
ATAATTTCCTCTGCAAAGCGTGATAAGTGAACCATCGTTAAAGAAATATTATGCAATATT
TCAATAATATAGTCTCTGTCACTAACAGCATCTAGGCTATTCTCATAGAGACTGCCAAAG
TTCAACAATGCTGTTGTCTCGTGTCTATCGATAGGGTATGTGGTACCACTTAAGGCTGCTG
CACCTAAAGGATTAATATCGATTCGTTTTAAACTATCTTCAAATCGTTGTTGGTCTCGTTG
TAACATCCAAAAATAAGTCATAATATGATGTGCAAATGAAATTGGCTGTGCACGCTGTAA
ATGAGTATAACCAGGCATAATTGTATCAACATTATTGGAAGCGATGTCTACAATTACACT
TTGTAACGACTTAATTAATGCGATGATATCTTGCACTTGTTTCTTAGTGTACAAGTGCATG
TCTGTTGCAACTTGATCGTTTCTACTGCGTCCAGTATGCAACTTACCACCAGCATCACCAA
TACGTTTAATTAATTCATGTTCAATATTTAAATGAATATCTTCTAATGATGCACTAAATTG
AATTTGATCTTGATGATAATCATGTTGAATAGATTTTAGTCCTTGTATAATTTGTTCGCTGT
CTTGTTGACTAATAATTCCTTGATTCGCAAGCATAGTTGCATGTGCAATGCTGCCTTCGAT
ATCTTGATCTATGAGCGTTTGATCAAAAGTAATGGATGCGTTAAAGTCGTCAACCCACTC
TTCAGGTTGTACTTCAAATCTACCGCCCCAAGCTTTATTGCTCAT

> **argG**
Function: protein coding sequence; argininosuccinate synthase
Best match: argG_CC001_MSSA476_BX571857.1[911244:912449:r]RC
Position: 025-contig_192_RC: 51632 … 52838; Length: 1206 bp
Sequence:
TTATTGCTCATTGCTATAGCCTCCATGTAGCATCGCATTTACTTGAGTAGGTAAACCATAG
ATATCGATAAAGCCAACAGCAGCGTCTTGATTAAATGCATCTTCTTTTGTATAAGTTGCTA
ATTTTTCATCATATAATGTGTAAGGTGATTTTCTACCATTTACGATGGCATTACCTTTGAA
TAATTTAATTCTGACATCACCACTTACGTATTGCTGAGTACTATCAATAAATAATTTTAAG
CTATCAGTTAAAGGTGAGAACCAAAGTCCATTGTATAGTTGTTCAGCAAATTGCTTCTCG
ATGATTGGTTTAAAGTGTGCGACATCTTTCGTTAACGTAATCGTTTCTAATGCTTTATGCG
CTTTTAAAATAACTTCTGCAGCAGGTGCCTCATAAATTTCTCTTGATTTGATACCTACAAG
TCTATTTTCTACATGGTCAATTCTTCCGATACCATGCTTACCAGCTAATGCATTCAACGTT
AAAATTAAATCGTCTAATTCATATGTTTTGCCATCAATTTGAACTGGGATGCCTTTATCAA
ACGTTAAAATGATTTCATCAGCAGTATCTGGTGTTTCTTCTAAAGCATTTGTTAGATCGAA
CGCATCCTCTGGTGGCGCAGCATAAGGATCTTCTAAAATACCACATTCATTCGCTCTGCCC
CATAGATTTTGATCGATAGAATAAGGTGAATCATGGTTGATTGATACAGGGATATTATGT
TTAATTGCATAATCGATTTCTTCTTCACGACTCCATGCCCACTCACGTACAGGTGCGAATG
CTTTCAATGATGGGTTTAATGCTTTAATGGCAACTTCGAAACGTACTTGGTCATTCCCTTT
ACCAGTACAACCATGTGCAATACCTACTGAATTTGTTTTCTCAGCAATCTCTACTAATTTT
TTAGCGATTAATGGTCTTGATAAAGCTGAAACTAATGGATATGCATTTTCATACATTAAAT
TTCCTTTGATTGCATAACTTACATACTCATCACTAAATTCTTTTGTTGCATCAATAATATGA
CATTCAACTGCTCCCATATCTAAAGCTTTTTTATAAACGATGTCTAAATCTTTACCTTCAC
CAACATCTAGGCAACAAGCTACAACTTCGTATCCTTTGTCGATAAGCCATTGAACGGCCA
CACTTGTATCTAGTCCTCCTGAATATGCTAAAACAATTTTCTCTTTCAT

> **pgi**
Function: protein coding sequence; glucose-6-phosphate isomerase (core genome, constant)
Best match: pgi_CC005_Mu50-VRSA_BA000017.4[1005150:1006481] (completely identical)
Position: 025-contig_192_RC: 53188 … 54520; Length: 1332 bp
Sequence:
ATGACTCATATTCAATTAGATTTTAGTAAAACGTTAGAATTTTTCGGTGAACACGAATTAA
AACAACAACAAGAAATTGTTAAATCAATTCACAAAACAATTCATGAAGGTACTGGTGCA
GGTAGTGACTTCTTAGGCTGGGTTGATTTACCAGTTGATTACGACAAAGAAGAATTTTCA
AGAATTGTTGAAGCATCAAAACGCATTAAAGAAAATTCTGATGTTTTAGTAGTCATCGGT
ATTGGTGGTTCTTACTTAGGTGCACGTGCAGCAATCGAAATGTTAACGTCATCATTTAGA
AACAGCAATGAATACCCTGAAATTGTATTTGTTGGTAATCACTTATCATCAACATATACG
AAAGAGTTAGTTGATTATTTAGCAGACAAAGATTTCTCTGTAAACGTTATTTCTAAATCTG
GTACAACTACAGAACCAGCAGTTGCATTTAGATTGTTCAAACAATTAGTTGAAGAAAGAT
ACGGTAAAGAAGAAGCACAAAAACGTATATTTGCAACAACGGATAAAGAAAAAGGTGCT
TTAAAACAGTTGGCTACAAACGAAGGTTATGAAACGTTTATCGTACCTGATGATGTAGGT
GGAAGATATTCTGTTTTAACAGCAGTAGGATTATTACCAATTGCAACAGCTGGAATTAAC
ATCGAAGCTATGATGATTGGTGCTGCAAAAGCACGTGAAGAATTATCTTCAGATAAATTA
GAAGACAACATTGCATACCAATATGCGACAATTCGAAACATTTTATATGCAAAAGGTTAT
ACAACAGAAATGTTGATTAACTATGAACCATCTATGCAATACTTTAATGAATGGTGGAAA
CAATTATTTGGTGAATCAGAAGGTAAAGACTTCAAAGGTATCTATCCTTCAAGTGCCAAC
TACACAACTGATTTACATTCTTTAGGTCAATATGTACAAGAAGGCCGTCGTTTCTTATTCG
AAACAGTGGTAAAAGTAAATCATCCTAAATATGATATTACTATTGAAAAAGATAGTGATG
ATCTAGACGGATTAAATTATTTGGCTGGTAAAACAATCGACGAAGTTAACACAAAAGCAT
TCGAAGGTACATTATTAGCGCATACTGATGGTGGTGTTCCTAACATGGTAGTGAACATTC
CACAATTAGATGAAGAAACTTTCGGTTATGTCGTATACTTCTTCGAACTTGCTTGTGCAAT
GAGTGGATACCAATTAGGTGTAAATCCATTTAACCAACCTGGTGTAGAAGCATATAAACA
AAACATGTTCGCATTATTAGGTAAACCTGGTTTTGAAGACTTGAAAAAAGAATTAGAAGA
GCGTTTATAA

> **spsA**
Function: protein coding sequence; signal peptidase IA (core genome, variable)
Best match: spsA_CC001-ST772_118_AJGE01000009.1[55178:55702] (completely identical)
Position: 025-contig_192_RC: 55424 … 55949; Length: 525 bp
Sequence:
GTGAAAAAAGTTGTAAAATATTTGATTTCATTGATACTTGCTATTATCATTGTACTGTTCG
TACAAACTTTTGTAATAGTTGGTCATGTCATTCCGAATAATGATATGTCACCAAGCCTTAA
CAAAGGGGATCGTGTTATTGTAAATAAAATTAAAGTTACATTTAATCAATTGAATAATGG
TGATATCATTACATATAGGCGTGGTAACGAGATATATACTAGTCGAATTATTGCCAAACC
TGGTCAATCAATGGCGTTTCGTCAGGGACAATTATACCGTGATGACCGACCGGTTGACGC
ATCTTATGCCAAGAACAGAAAAATTAAAGATTTTAGTTTGCGCAATTTTAAAGAATTAGA
TGGAGATATTATACCGCCTAACAATTTTGTTGTGCTAAATGATCATGATAACAATCAGCA
TGATTCAAGACAATTTGGTTTAATTGATAAAAAGGATATTATTGGTAATATAAGTTTGAG
ATATTATCCTTTTTCAAAATGGACGATTCAGTTCAAATCTTAA

> **spsB**
Function: protein coding sequence; signal peptidase IB (core genome, constant)
Best match: spsB_CC001-ST772_118_AJGE01000009.1[55718:56293] (completely identical)
Position: 025-contig_192_RC: 55964 … 56540; Length: 576 bp
Sequence:
TTGAAAAAAGAATTATTGGAATGGATTATTTCAATTGCAGTCGCTTTTGTCATTTTATTTA
TAGTAGGTAAATTTATTGTTACACCATATACGATTAAAGGTGAATCAATGGATCCAACTT
TGAAAGATGGCGAGCGAGTAGCTGTAAACATTATTGGATATAAAACAGGTGGTTTGGAA
AAAGGTAATGTAGTTGTCTTCCATGCAAACAAAAATGATGACTATGTTAAACGTGTCATC
GGTGTTCCTGGCGATAAAGTAGAATATAAAAATGATACATTATATGTCAATGGTAAAAAA

CAAGATGAACCATATTTAAACTACAATTTAAAACATAAACAAGGTGATTACATTACTGGG
ACTTTCCAAGTTAAAGATTTACCGAATGCGAATCCTAAATCAAATGTCATTCCAAAAGGT
AAATATTTAGTGCTTGGAGATAATCGTGAAGTAAGTAAAGATAGCCGTGCGTTTGGCCTC
ATTGATGAAGACCAAATTGTTGGTAAAGTTTCATTTAGATTCTGGCCATTTAGTGAATTTA
AACATAATTTCAATCCTGAAAATACTAAAAATTAA

> **rexB**

Function: protein coding sequence; ATP-dependent nuclease subunit B (core genome, variable)
Best match: rexB_CC001-ST772_118_AJGE01000009.1[56453:59929] (completely identical)
Position: 025-contig_192_RC: 56699 … 60176; Length: 3477 bp
Sequence:
ATGACATTACATGCTTATTTAGGTAGAGCGGGAACAGGTAAGTCTACGAAAATGTTGACC
GAAATAAAACAAAAAATGAAAGCAGATCCGCTTGGAGATCCAATCATTTTAATTGCGCC
AACTCAAAGTACATTTCAATTAGAACAAGCCTTTGTCAATGATCCGGAATTAAATGGTAG
TTTAAGAACAGAAGTGTTGCATTTTGAACGATTAAGTCATCGTATTTTCCAAGAAGTTGGT
AGTTATAGCGAACAAAAGTTATCTAAAGCTGCAACGGAAATGATGATTTATAACATTGTT
CAAGAACAACAAAAGTATTTAAAACTTTATCAATCACAAGCAAAATATTATGGGTTTAGT
GAAAAATTAACAGAACAAATTCAAGATTTTAAAAAATATGCAGTAACGCCTGAACATTTA
GAACACTTTATTGCTGATAAAAATATGCAAACTCGAACTAAAAATAAGTTAGAGGATATT
GCTTTAATATACCGTGAGTTCGAACAACGCATTCAAAACGAGTTTATTACGGGTGAGGAT
TCATTACAATATTTTATTGATTGTATGCCGAAATCAGAGTGGCTAAAACGTGCTGATATAT
ATATTGATGGTTTTCACAACTTTTCAACGATTGAGTATTTAATAATCAAAGGATTAATTAA
ATATTCGAAGAGTGTCACAATTATATTGACGACAGATGGTAACCACGATCAATTTAGTTT
ATTTAGAAAACCATCGGAAGTGTTACGACATATTGAAGAAATAGCAAATGAACTCAATAT
TTCTATTGAACGTCAATATTTCAAGCAATTATATCGCTTCAATAATCAAGATTTAAAGCAT
CTTGAACAAGAATTTGATGCACTTCAAATCAATCGAGTGGCATGTCAAGGTCATATCAAT
ATTTTAGAATCTGCGACTATGAGAGAGGAAATAAATGAAATTGCGCGACGTATCATCGTT
GATATTCGTGATAAGCAATTACGATATCAAGATATTGCAATTTTATATCGTGACGAGTCTT
ATGCTTATTTATTTGATTCCATATTACCGCTTTATAATATTCCTTATAACATTGATACAAAG
CGTTCGATGACACATCATCCGGTCATGGAAATGATTCGTTCATTGATTGAAGTTATTCAAT
CTAATTGGCAAGTGAATCCAATGCTACGCTTATTGAAGACTGATGTGTTAACGGCATCAT
ATCTAAAAAGTGCATACTTAGTTGATTTACTTGAAAATTTTGTACTTGAACGTGGTATATA
CGGTAAACGTTGGTTAGATGATGAGCTATTTAATGTCGAACATTTTAGCAAAATGGGGCG
TAAAGCGCATAAACTGACCGAAGATGAACGTAACACATTTGAACAAGTCGTTAAGTTAA
AGAAAGATGTCATTGATAAAATTTTACATTTTGAAAAGCAAATGTCACAAGCGGAAACTG
TAAAAGATTTTGCAACTGCTTTTTATGAAAGTATGGAATATTTCGAACTGCCAAATCAATT
GATGACAGAGCGAGATGAACTTGATTTAAATGGTAATCATGAAAAGGCGGAGGAAATTG
ATCAAATATGGAATGGCTTAATTCAAATCCTTGATGACTTAGTTCTAGTATTTGGAGATGA
ACCAATGTCGATGGAACGTTTCTTAGAAGTATTTGATATTGGTTTAGAACAATTAGAATTT
GTTATGATTCCGCAAACATTGGACCAAGTAAGTATTGGTACGATGGATTTGGCTAAAGTC
GATAATAAGCAACATGTTTACTTAGTAGGTATGAATGACGGCACCATGCCACAACCAGTA
ACTGCATCAAGTTTAATTACTGATGAAGAAAAGAAATATTTTGAACAACAAGCAAATGTA
GAGTTGAGTCCTACATCAGATATTTTACAGATGGATGAAGCATTTGTTTGCTATGTTGCTA
TGACTAGAGCTAAGGGAGATGTTACATTTTCTTACAGTCTAATGGGATCAAGTGGTGATG
ATAAGGAGATCAGCCCATTTTTTAAATCAAATTCAATCATTGTTCAACCAATTGGAAATTA
CTAACATTCCTCAATACCATGAAGTTAACCCATTGTCACTAATGCAACATGCTAAGCAAA
CCAAAATTACATTATTTGAAGCATTGCGTGCTTGGTTAGATGATGAAATTGTGGCTGATA
GTTGGTTAGATGCTTATCAAGTAATTAGAGATAGCGATCATTTAAATCAAGGTTTAGATT
ATTTAATGTCAGCATTAACGTTTGACAATGAAACTGTAAAATTAGGTGAAACGTTGTCTA
AAGATTTATATGGTAAGGAAATCAATGCCAGTGTATCTCGATTTGAAGGTTATCAACAAT
GCCCATTTAAACACTATGCGTCACATGGTCTGAAACTAAATGAACGAACGAAGTATGAAC
TTCAAAACTTTGATTTAGGTGATATTTTCCATTCTGTTTTAAAATATATATCTGAACGTATT
AATGGCGATTTTAAACAATTAGACCTGAAAAAAATAAGACAATTAACGAATGAAGCATT
GGAAGAAATTTTACCTAAAGTTCAGTTTAATTTATTAAATTCTTCAGCTTACTATCGTTAT
TTATCAAGACGCATTGGCGCTATTGTAGAAACAACACTAAGCGCATTAAAATATCAAGGC
ACGTATTCAAAGTTTATGCCAAAACATTTTGAGACAAGTTTTAGAAGGAAACCAAGAACA

AATGACGAATTAATTGCACAAACATTAACGACAACTCAAGGTATTCCAATTAATATTAGA
GGGCAAATTGATCGTATCGATACGTATACAAAGAATGATACAAGTTTGTTAATATCATT
GACTATAAATCCTCTGAAGGTAGTGCGACACTTGATTTAACGAAAGTATATTATGGTATG
CAAATGCAAATGATGACATACATGGATATCGTTTTACAAAATAAACAACGCCTTGGATTA
ACAGATATTGTGAAACCAGGTGGATTATTATACTTCCATGTACATGAACCTAGAATTAAA
TTTAAATCGTGGTCTGATATTGATGAAGATAAACTAGAACAAGATTTAATTAAAAAGTTT
AAGCTGAGTGGTTTAGTGAATGCAGACCAAACTGTTATTGATGCATTGGATATTCGTTTA
GAACCTAAATTCACTTCAGATATTGTACCAGTTGGTTTGAATAAAGATGGCTCTTTGAGTA
AACGAGGCAGCCAAGTGGCAGATGAAGCAACAATTTATAAATTCATTCAGCATAACAAA
GAGAATTTTATAGAAACAGCTTCAAATATTATGGATGGACATACTGAAGTTGCACCATTA
AAGTACAAACAAAAATTACCATGTGCTTTTTGTAGTTATCAATCGGTATGTCATGTAGAT
GGCATGATTGATAGTAAGCGATATCGAACTGTAGATGAAACAATAAATCCAATTGAAGC
AATTCAAAATATTAACATTAATGATGAATTTGGGGGTGAGCAATAG

## > rexA

Function: protein coding sequence; ATP-dependent nuclease subunit A (core genome, variable)
Best match: rexA_CC001-ST772_118_AJGE01000009.1[59930:63583] (completely identical)
Position: 025-contig_192_RC: 60176 … 63830; Length: 3654 bp
Sequence:
ATGACAATTCCAGAGAAACCACAAGGCGTGATTTGGACTGACGCGCAATGGCAAAGTAT
TTACGCAACTGGACAAGATGTACTTGTTGCAGCCGCGGCAGGTTCAGGTAAAACAGCTGT
ACTAGTTGAGCGTATTATCCAAAAGATTTTACGTGATGGCATTGATGTCGATCGACTTTTA
GTCGTAACGTTTACAAACTTAAGCGCACGTGAAATGAAGCATCGTGTAGACCAACGTATT
CAAGAGGCATCGATTGCTGATCCTGCAAATGCACACTTGAAAAACCAACGCATCAAAATT
CATCAAGCACAAATATCTACACTTCATAGTTTTTGCTTGAAATTAATTCAACAGCATTATG
ATGTATTAAATATTGACCCGAACTTTAGAACAAGCAGTGAAGCTGAAAATATTTTATTAT
TAGAACAAACGATAGATGAAGTTATAGAACAACATTACGATATCCTTGATCCTGCTTTTA
TTGAATTAACAGAGCAATTGTCTTCAGATAGAAGTGATGATCAGTTTCGAATGATTATTA
AACAATTGTATTTCTTTAGCGTTGCAAATCCAAATCCTACAAATTGGTTGGATCAATTGGT
GACACCATACGAAGAAGAAGCACAACAAGCGCAACTTATTCAACTACTAACAGACTTAT
CTAAAGTATTTATCACAGCTGCTTATGATGCTTTAAATAAGGCGTATGATTTGTTTAGTAT
GATGGATAGCGTCGATAAACATTTAGCTGTTATAGAAGATGAACGACGTTTAATGGGGCG
TGTTTTAGAAGGTGGCTTTATTGATATACCTTATTTAACTGGTCACGAATTTGGCGCGCGT
TTGCCTAATGTAACAGCGAAAATTAAAGAAGCAAATGAAATGATGGTCGATGCCTTAGA
AGATGCTAAACTTCAGTATAAAAAATATAAATCATTAATTGATAAAGTGAAGAGTGATTA
CTTTTCAAGAGAAGCTGATGATTTGAAAGCTGATATGCAACAATTGGCGCCACGAGTAAA
GTACCTTGCGCGTATTGTGAAAGATGTTATGTCAGAATTCAATCGAAAAAAGCGTAGCAA
AAATATTTTGGATTTTTCTGATTATGAACATTTTGCATTACAAATTTTAACTAATGAGGAT
GGTTCGCCTTCAGAAATTGCCGAATCATACCGTCAACACTTCCAAGAAATATTGGTCGAT
GAGTATCAAGATACGAACCGAGTTCAAGAGAAAATACTATCTTGCATCAAAACGGGTGA
TGAACATAATGGTAATTTATTTATGGTTGGAGATGTTAAGCAATCCATTTATAAATTTAGA
CAAGCTGATCCAAGTTTATTTATTGAAAAGTATCAACGCTTTACTATAGATGGAGATGGC
ACTGGACGTCGAATTGATTTGTCGCAAAACTTCCGTTCTCGAAAAGAAGTACTGTCAACG
ACTAACTATATATTCAAACATATGATGGATGAACAAGTCGGTGAAGTAAAATATGATGAA
GCGGCACAGTTGTATTATGGTGCACCATATGATGAATCGGACCATCCAGTAAACTTAAAA
GTCCTTGTTGAAGCGGATCAAGAACATAGTGATTTAACTGGTAGTGAACAAGAAGCGCAT
TTTATAGTAGAACAAGTTAAAGATATCTTAGAACATCAAAAAGTTTATGATATGAAAACA
GGAAGCTATAGAAGTGCGACATACAAGGATATCGTTATTCTAGAACGCAGCTTTGGACAA
GCTCGCAATTTACAACAAGCCTTTAAAAATGAAGATATTCCATTCCATGTGAATAGTCGT
GAAGGTTACTTTGAACAAACAGAAGTCCGCTTAGTATTATCATTTTTAAGAGCGATAGAT
AATCCATTACAAGATATTTATTTAGTTGGGTTAATGCGCTCCGTTATATATCAGTTCAAAG
AAGACGAATTAGTTCAAATTAGAATATTGAGTCCAAATGATGACTACTTCTATCAATCGA
TTGTAAATTACATTAATGACGAAGCAGCAGATGCAATTTTAGTTGATAAATTAAAAATGT
TTTTATCAGATATTCAAAGTTACCAACAATATAGTAAAGATCATCCGGTGTATCAGTTAAT
TGATAAATTTTATAATGATCATTATGTTATTCAATACTTTAGTGGACTTATTGGTGGACGT
GGACGACGTGCAAATCTTTATGGTTTATTTAATAAAGCTATCGAGTTTGAGAATTCAAGTT

TTAGAGGTTTATATCAATTTATTCGTTTTATCGATGAATTGATTGAAAGAGGCAAAGATTT
TGGTGAGGAAAATGTAGTTGGTCCAAACGATAATGTCGTTAGAATGATGACAATTCATAG
TAGTAAAGGTCTAGAGTTTCCATTTGTCATTTATTCTGGATTGTCAAAAGATTTTAATAAA
CGTGATTTGAAACAACCAGTTATTTTAAATCAGCAATTTGGTCTCGGAATTGATTATTTTG
ATGTGGATAAAGAAATGGCATTTCCATCTTTAGCTTCGGTTGCATATAGAGCTGTTGCCG
AAAAAGAACTTGTGTCAGAAGAAATGCGATTAGTCTATGTAGCATTAACAAGAGCGAAA
GAACAACTTTATTTAATTGGTAGAGTGAAAAATGATAAATCATTACTAGAACTAGAGCAA
TTGTCTATTTCTGGTGAGCACATTGCTGTCAATGAACGATTAACTTCACCAAATCCGTTCC
ATCTTATTTATAGTATTTTATCTAAACATCAATCTGCGTCAATTCCAGATGATTTAAAATT
TGAAAAAGATATAGCACAAATTGAAGATAGTAGTCGTCCGAATGTAAATATTTCAATTGT
GTACTTTGAAGATGTGTCTACAGAAACCATTTTAGATAATGATGAATATCGTTCGGTTAAT
CAATTAGAAACTATGCAAAATGGTAATGAAGATGTTAAAGCACAAATTAAACACCAACT
TGATTATCGATATCCATATGTAAATGATACTAAAAAGCCCTCAAAACAATCTGTTTCTGA
ATTGAAAAGACAATATGAAACAGAAGAAAGTGGCACAAGTTACGAACGAGTAAGGCAAT
ATCGTATCGGTTTTTCAACGTATGAACGACCTAAATTTCTATGTGAACAAGGTAAACGAA
AAGCGAATGAAATTGGTACGTTAATGCATACAGTGATGCAACATTTACCATTCAAAAAAG
AGCGCATATCTGAAGTTGAGTTACATCAGTATATCGATGGATTAATCGATAAACATATTA
TCGAAGCAGATGCGAAAAAAGATATCCGTATGGATGAAATAATGACATTTATCAATAGT
GAGTTATATTCGATTATTGCTGAAGCAGAGCAAGTTTATCGTGAATTACCGTTTGTAGTTA
ACCAAGCATTAGTTGACCAATTGCCACAAGGAGACGAAGACGTCTCAATTATTCAAGGTA
TGATTGACTTGATCTTTGTTAAAGACGGTGTGCATTATTTTGTAGACTATAAAACCGATGC
ATTTAATCGTCGTCGTGGGATGACAGATGAAGAAATTGGTACACAATTAAAAAATAAATA
TAAGATACAGATGAAATATTATCAAAATACGCTTCAAACGATTCTTAATAAAGAAGTTAA
AGGTTATTTATACTTCTTCAAATTTGGTACATTGCAACTGTAG

> **Q5HHB6**

Function: protein coding sequence; putative fumarylacetoacetase (core genome, variable)
Best match: Q5HHB6_CC001_MW2-USA400_BA000033.2[939638:940540] (completely identical)
Position: 025-contig_192_RC: 63995 … 64898; Length: 903 bp
Sequence:
ATGAAATTCTTATCATTCAAGTATAATGACAAAACTTCATATGGCGTTAAAGTAAAACGC
GAAGATGCTGTATGGGATTTAACACAAGTATTTGCTGACTTTGCAGAAGGAGATTTCCAT
CCTAAAACATTGTTAGCTGGTTTACAACAAAATCATACTTTAGATTTTCAAGAACAAGTA
CGTAAAGCAGTTGTAGCAGCAGAAGATAGCGGCAAAGCTGAAGACTATAAAATTTCATT
TAATGACATTGAATTCTTACCACCAGTAACACCTCCGAATAATGTGATTGCTTTTGGTAGA
AATTACAAAGATCATGCGAACGAATTAAATCATGAAGTAGAAAAATTATATGTATTTACA
AAAGCAGCGTCATCTTTAACAGGAGATAATGCAACAATTCCAAATCATAAAGATATTACT
GATCAATTAGATTATGAAGGTGAATTAGGTATTGTTATTGGTAAGTCTGGTGAAAAGATT
CCAAAAGCATTAGCTTTAGATTATGTTTACGGCTATACAATTATTAACGATATCACTGATC
GCAAAGCACAAAGTGAACAAGATCAAGCATTTTTATCAAAAAGTTTAACTGGCGGTTGCC
CAATGGGTCCTTATATCGTTACTAAAGACGAACTACCATTACCTGAAAATGTAAATATTG
TTACAAAAGTTAACAATGAAATTAGACAAGATGGTAACACTGGCGAAATGATTCTTAAA
ATTGATGAATTAATAGAAGAAATTTCAAAATATGTTGCACTACATCCGGGAGATATTATT
GCAACTGGTACACCAGCAGGCGTTGGTGCAGGTATGCAACCACCTAAATTTTTACAACCA
GGTGATGAAGTTAAAGTGACTATTGATAATATTGGAACGCTGACAACTTATATCGCTAAA
TAA

> **UPF0344**

Function: protein coding sequence; unknown protein fold UPF0344 (core genome, variable)
Best match: UPF0344_CC001-ST772_118_AJGE01000009.1[64979:65368] (completely identical)
Position: 025-contig_192_RC: 65225 … 65615; Length: 390 bp
Sequence:
ATGTTACATTTACATATATTAAGTTGGGTATTAGCGATTATTTTATTTATCGCTACATACTT
AAACATTTCAAAAAATCAAGGCGGATCACCATTTTTCAAACCGTTGCACATGATTTTACG
CTTATTTATGCTGTTGACGTTAATTTCAGGATTTTGGATATTAATTCAGTCATTTATGAATG

GCGGGGCAAATCATATGTTGCTTACATTGAAAATGCTGTGTGGTGTTGCAGTAGTTGGAT
TGATGGAAGTGTCGATTGCTAAAAGAAAGAGACATGAACAAAGTCACACAATGTTTTGG
ATAACAATGGCATTAATTATCATCACAATGGTATTAGGTGTCATTCTACCGTTAGGGCCTA
TATCAAAATTATTCGGTATTGGCTAA

## > cdr

Function: protein coding sequence; coenzyme A disulfide reductase (core genome, variable)
Best match: cdr_CC001-ST772_118_AJGE01000009.1[65540:66856:r]RC (completely identical)
Position: 025-contig_192_RC: 65786 … 67103; Length: 1317 bp
Sequence:
TTATTTAGCTTTGTAACCAATCATATTGATTAAATCTTTAGGGTGGCTATATGGTGGTGCA
TAAGCCACTTCAAACTCAGTTAACTCATCTACAGTTAGCTGGTTCATCATTGCCATCGATA
GTACATCAATACGTTTATCTGCACCTTCTTTTCCTACTGCAGCTGCTCTTAAAATCTGACG
GTTTGAAGTGTCATAATATACCCTTAAGTGTAAAGGGGAATTTCCTGGGTAATAATTCGC
GTGTGCACCTTGAGTGACTTCTACCATTTTATAGTCAAATTGCTTTAGTTCATTTGGTTTAA
CGCCGACACTCGCAAATGTATAATCAAAGAACTTCACAATATTGTTGCCTAAGAAGCCTT
TGAATTCAATAGTGTCATTTCCAGCAATTTGTTCGGCAACAATACTTGCTGCACGGTGAGC
GCCCCAAGCTAAAGGAACACTAGCCGGTAGATCGACATGTCGATAATGTGATGTTGCAAT
ATCGCCTATTGCATAAATGTTTGGAACATTTGTTTCAAATTTATCGTTTACCGGTATGAAA
CCTTTTCGATCAAGTTTGATATTTGAACTTTCGATAAATTTTGAATTGGGGTGAGTACCGA
CACCTTCAATAATCATATCGTAATGTTCAACTTTTCCTGATTTAAATGTAATTTCATTTCCA
TTGATAGCATCAATTTCCTCATTTAAACGGTATGGAATCTCCCGCTTATCTAATTCATCAA
GTATAGGTTGATTCATGTCAGCATCCATTAATTTATTTATCTTATCAGATCGATGAATTAA
AGTAGGGTGTAAACCACGTTCGTAAAGATTTTCAAGAACTTCTAATGAAACATACCCTGC
ACCTACAACTAATACTTTATCAACTTGATTTGCTTTGATGAATTGATCGATAGCATCAGTG
TCTTCTAAATTTCTAAGTGTAAATGTAATATCACTTTCAAAGCCAAGGCTATTTGCACTTG
CACCAGGGCTTAAAATGAGTTTATCGTAAGATTCTTCAAATTGTTCGTTGGTCTTTCTATT
TAATACAGTTACAGTTTGTCTTTCATCATTGATTGCAATAACTTCATGATAAGTTTTTACT
GTAATTTGCTTTCTATCATAAAATTTTTCAGGTGTATACGCTAAAGCATATTTTCTATCTTC
AACAACTTCGCCAATGACATAAGGCAATGCACAATTAGCAAAGCTCATATCACGATCTTT
TTCAAAAATAATAATGTCACTTTCTTTATCTAAACGTCGAATTTGGCTGGCACATGTTGCA
CCGCCAGCGACTGCTCCGACTACGACTATTTTGGGCAT

## > paaD

Function: protein coding sequence; putative protein (core genome, constant)
Best match: paaD_CC005_Mu50-VRSA_BA000017.4[1020055:1020363] (completely identical)
Position: 025-contig_192_RC: 68092 … 68401; Length: 309 bp
Sequence:
ATGGAAGAGGCATTGAAAGATAGTATCTTAGGTGCATTAGAAATGGTAATTGACCCTGAA
TTAGGAATTGATATCGTTAATTTAGGTTTAGTATACAAAGTGAATGTTGATGATGAAGGC
GTATGTACAGTTGATATGACTTTAACATCAATGGGATGTCCAATGGGACCTCAAATTATT
GATCAAGTTAAAACAGTATTAGCAGAGATTCCTGAAATTCAGGATACTGAAGTGAATATC
GTATGGAGTCCACCTTGGACAAAAGATATGATGTCACGTTACGCTAAGATTGCACTTGGT
GTGAGCTAA

## > Q5HHB1

Function: protein coding sequence; putative O-acetyltransferase (core genome, constant)
Best match: Q5HHB1_CC001_MW2-USA400_BA000033.2[944592:946406]
Position: 025-contig_192_RC: 68948 … 70763; Length: 1815 bp
Sequence:
ATGAACAAAACAAAGGGTTTTACAAAGTATAAGAAAATGAGATATATGCCAGGGCTCGA
TGGTTTGAGGGCAATCGCTGTTCTAGGAATTATTATTTACCACTTAAACAAGCAATGGTTG
ACAGGTGGCTTTTTAGGTGTGGATACATTTTTTGTGATCTCTGGTTATTTAATTACAAGCT
TATTACTCAAAGAGTATGATGACACAGGTATCATTAAATTGAAAAGCTTTTGGATACGTC
GTTTAAAACGTTTATTACCAGCAGTCATAGTTTTATTAATGGTTGTAGGGACAGCAACCTT

ATTATTAAAATCAGATAATATCATTAGGGTTAAACATGATATTATTGCTGCGATATTTTAT
GTATCAAACTGGTGGTATATAGCAAAAGATGTTAATTATTTTGAGCAATTTTCATTTATGC
CATTAAAGCATTTATGGTCTTTAGCAATTGAAGAACAGTTTTACATATTTTTCCCAGTTAT
TTTGGTTACATTATTGTTAACAATTAAAAAGCGATACAAAATAGGATTTATTTTTTTGGGA
GTATCAATAATTTCTTTAGGGTTAATGATGTTTATCTACAGTATTAATGGGGATCATTCAC
GAGTGTATTTTGGTACAGATACTAGATTACAGACATTGTTACTGGGTGTTATTTTAGCTTT
TTTATGGCCACCGTTTAAATTGAAAAATGATCCACCTAAAGTTGTAAAATATGTTATTGAT
AGCATAGGTAGTTTATCATTTATAGTACTTATATTATTATTTTTCATTATTAATGATGAGA
CGAATTGGATATATGATGGTGGTTTCTATTTAATATCCATATTAACGTTATTTATTATTGCT
AGTGTCGTTCATCCATCTACATGGATAGCGAAGATATTTTCAAATCCAGTGTTAGTATTTA
TCGGGAAAAGGTCTTATAGTTTATATTTATGGCATTTTGCAGTAATTAGTTTCGTACATAG
TTACTATGTAGACGGACAGATACCTGTATATGTGTACTTTATAGATATAAGTTTAACAATT
ATATTTGCAGAGCTATCATATCGCTTTATAGAAACTCCATTTAGAAAAGAAGGTATTAAA
GCTTTAAATTGGCGACCTTCTTATATACCACAATTTATAAGAATGGCAATTGTAGTAACCT
TGTTAATTCCATTTATGTTGATTTTAGTAGGTGCATTCAATAAATATGGTAAAGACATTAT
TGGAGAAAAAGCGAATAGCTTTGATACCACAATTGAAGATAATTATTTAATGCGGATAGC
ACCAATTGATAACATACACATTGATGGCTTAGTAAGTGAGAAGAAAAAGGAATCTTCCG
ACGTATATAATAATATTAAACCTCTTTTAATCGGTGATTCAGTAATGGTTGATATCGGTGA
GTCATTTAAGTCATCAGTTCCTAAGTCTAGAATTGATGGAAAAGTAGGGCGTCAATTGTA
TCAAACCTTACCTTTAGTTAAAGCGAATTATTCACAATATAAAAAATCATCTGATCAAGT
CGTATTAGAATTAGGTACAAATGGCGACTTTACTGTCAAACAGCTCGACGATTTACTTAA
TCAATTTGGAAAAGCCAAGATTTATTTAGTTAATACACGTGTTCCAAGAATTTATGAGGC
AAATGTAAATCGATTATTAGCTGACGCGGCGAAACGAAAGTCCAATGTCACATTAATTGA
TTGGTATAAGCGATCACAAGGACATAGTGAATATTTTGCACCAGACGGTGTACATTTAGA
GTACAAAGGAGTCCTAGCTTTAAAAGATGAAATATTAAAAGCACTTAAAAAGAAATAA

> **clpB**

Function: protein coding sequence; chaperone-like protein B (core genome, variable)
Best match: clpB_CC001-ST772_118_AJGE01000009.1[70719:73328] (completely identical)
Position: 025-contig_192_RC: 70965 … 73575; Length: 2610 bp
Sequence:
ATGGATATAAATAAAATGACATATGCTGTTCAAAGTGCTTTACAACAAGCAGTTGAACTG
AGTCAGCAACATAAATTACAAAATATAGAAATTGAGGCAATTTTAAGCGCTGCCTTAAAT
GAAAGTGAAAGCTTATATAAAAGTATTTTAGAACGAGCAAATATTGAGGTAGATCAATTA
AACAAAGCTTATGAAGACAAACTAAACACGTATGCATCTGTAGAAGGTGACAATATACA
ATATGGTCAATATATTAGCCAACAAGCAAACCAATTGATAACTAAGGCTGAATCATACAT
GAAAGAATATGAAGATGAATATATTTCAATGGAGCATATTTTACGTTCGGCAATGGACAT
TGATCAAACAACAAAACATTATATAAATAATAAAGTAGAAGTTATCAAAGAAATTATTA
AAAAAGTAAGAGGGGGAAATCACGTGACATCACAAAATCCAGAAGTTAATTACGAAGCA
TTAGCTAAATATGGCCGCGACTTAGTAGAAGAAGTTAGACAAGGTAAAATGGATCCTGTT
ATAGGAAGAGATGAAGAAATTCGAAATACGATTCGTATTTTAAGTCGTAAAACTAAAAA
CAACCCTGTGCTCATTGGTGAACCAGGTGTTGGTAAAACTGCAATTGTTGAAGGATTAGC
GCAACGTATAGTTAAGAAAGATGTGCCAGAATCATTATTAGATAAAACTGTTTTTGAGTT
AGATTTAAGCGCATTAGTAGCGGGCGCTAAATATCGTGGTGAATTTGAAGAGAGATTAAA
AGCAGTCCTAAAAGAAGTTAAAGAGTCTGATGGTAGAATTATATTATTTATTGATGAAAT
CCATATGCTTGTAGGTGCTGGTAAAACAGATGGTGCCATGGATGCAGGCAACATGCTAAA
ACCAATGTTAGCACGAGGAGAGTTACATTGTATTGGTGCAACAACTTTAAATGAATATCG
AGAATATATTGAAAAAGATTCGGCATTAGAGCGTCGTTTCCAAAAAGTAGCAGTTAGTGA
GCCTGATGTTGAAGATACAATTTCAATTTTACGTGGTTTAAAAGAACGATATGAAGTGTA
TCATGGTGTGCGTATTCAAGATAGAGCCTTAGTTGCTGCCGCTGAATTGTCTGATCGTTAC
ATCACTGATCGTTTTTTACCAGATAAAGCGATTGATTTAGTTGACCAAGCATGTGCAACA
ATTCGTACGGAAATGGGATCAAATCCAACTGAATTGGATCAAGTTAATAGACGTGTCATG
CAATTAGAAATTGAAGAAAGCGCACTTAAAAATGAATCTGACAATGCGAGCAAACAGAG
ATTACAAGAACTACAAGAAGAGCTTGCCAATGAAAAAGAGAAACAAGCAGCACTTCAAT
CTCGTGTAGAATCAGAAAAAGAAAAAATAGCAAATTTACAAGAAAAACGTGCGCAACTA
GATGAAAGTAGACAAGCGTTGGAAGATGCACAAACAAATAACAATTTAGAAAAAGCTGC

TGAACTACAATATGGAACAATTCCTCAATTGGAAAAAGAACTTAGAGAATTAGAGGATA
ATTTCCAAGATGAGCAAGGTGAAGATACAGATCGAATGATTCGTGAAGTTGTAACAGAC
GAAGAAATTGGCGATATTGTCAGCCAATGGACAGGCATACCAGTTTCAAAATTAGTTGAA
ACAGAACGTGAAAAATTACTTCACTTAAGTGACATCTTGCATAAACGTGTTGTAGGTCAA
GATAAAGCGGTTGACCTGGTTTCAGATGCAGTAGTTAGAGCAAGAGCAGGTATTAAAGA
TCCAAACAGACCTATTGGTAGTTTCTTATTCCTAGGTCCAACTGGAGTAGGTAAAACTGA
ATTAGCTAAATCATTAGCTGCATCATTATTTGATTCTGAAAAACATATGATTCGTATTGAT
ATGAGTGAATATATGGAAAAACATGCAGTATCAAGATTGATAGGGGCACCTCCAGGATA
TATTGGACATGATGAAGGGGGTCAATTAACTGAAGCGGTTCGTCGTAATCCATACTCAGT
TATCTTATTAGATGAGGTTGAAAAAGCGCATACTGACGTCTTTAATGTATTATTGCAAATT
TTAGATGAAGGCCGTTTAACTGATTCTAAAGGACGTAGCGTTGATTTTAAAAATACTATT
ATTATTATGACAAGTAATATTGGATCTCAAGTTTTATTAGAAAACGTAAAAGAGACTGGT
GAAATTACAGAATCAACAGAAAAAACTGTTATGACAAATTTAAATGCATATTTCAAACCA
GAAATTTTGAATCGTATGGATGACATCGTATTATTTAAACCTCTATCTATTGATGACATGA
GTATGATTGTAGACAAAATATTAACGCAATTAAATATAAGATTATTAGAACAGCGAATCT
CAATTGAAGTTTCTGATGATGCTAAAGCTTGGCTAGGTCAAGAAGCTTATGAACCTCAAT
ACGGTGCAAGACCATTAAAACGTTTTGTACAACGCCAAATTGAAACACCATTAGCACGTA
TGATGATTAAAGAGGGATTCCCAGAAGGTACAACGATTAAAGTTAATTTAAATTCAGACA
ATAACTTAACGTTTAATGTTGAAAAAATTCATGAATAA

> **Q5HHA9**

Function: protein coding sequence; transcript regulator/LysR family (core genome, variable)
Best match: Q5HHA9_CC001-ST772_118_AJGE01000009.1[73387:74256:r]RC (completely identical)
Position: 025-contig_192_RC: 73633 … 74503; Length: 870 bp
Sequence:
TTATAATTGTTCATTGGCAATATACTTTTTAAATGATTTAATAAAAACCAATATTTCTGGG
CTTTCTTTTTTGCTGTATATATATGTAAATGAAATAGGCGCTTGTAAAATTTTCGTGTTAAT
AACCGAAATATTGTAATCACTATCTGTTGTTATATAAATAGGCAGGAATGATATACCTTG
ATTCATTTCGATTAATTTAATTGAAGTATGCACATCATTGATAGATAGAAATTGTGCTTTT
CCATAAATATTTAAAATATTATTTTTAAGTGATGACCAATATTCTGGATGGTTATCACTTA
TTATTTTGTATTTTTCAAATAAAGATGCCTCAGTTAGAAGATGATTATTCTCTTTATTGGG
AGCAATCAATACAATTTTACCTTCGCATACTTTTTCAGAATGAACTTCTCTTAGTTTAGGT
TGATTTCTGCTAATCCCGATGTCATACGTATGATTATTAATATCTTTTTCAATATTTTCATT
TTTGACATGGAGAGAAACATCGATAAACGGATGCTCGTTAAAGAAAGATTTTAAAAATTT
GGGCATAATGAATGTCGCGATATATGAAGACACGACAACATTTAATTTCGATTGAAACAT
CGTTTTTTTAAGTTGGATATGTTTGATGCCACTTTCATATTGTTCAATAAAACTTTGCGCA
ATTGGAAGAAATGTATGACCATCTTCAGTCAAGATAATTTGATTTTTATAAGTTTCAAATA
GTTTCACATTGAGATGCTGTTCTAAATTTTTAATTTGCTTATGTATAGAAGGTATAGTGAG
ATTAATTTCTTCACTAGCTAATCGATAGTTTAACGTCTTCGCTAAAATGACAAATGTATAG
TACCAATCTAAATTCAT

> **leuA2**

Function: protein coding sequence; 2-isopropylmalate synthase/locus 2 (genomic island)
Best match: leuA2_CC001-ST772_118_AJGE01000009.1[74366:75510] (completely identical)
Position: 025-contig_192_RC: 74612 … 75757; Length: 1145 bp
Sequence:
ATGATTCGAATTCAAGATAATACAATAAGGGATGGTATGCAACAAAGTAATGTTCGAAA
AAGTCTAATTATAAAAAAAGAAGTATTGAAACAAATTAACAAGTTAAATATAAATTCTGT
TGAAGTAGGCATGTGTACAACTATCGAGGATGAATTTAATATTCATCAATTCAGAGACAT
TTTAAGTCCTGAAAAAGAATTAGTAGTATTGACTAGGCTTAATGAAAAAGAAATAAAAA
AATAGTCAAATTAAAAATTCATAATTTAGTGGTAAAAATACTATTGCCAATATCTGACTT
GCATATAAAAGAAAAGCTTAATTTTTCAAATAAATATTATATTCAGAAAATCAAAGACTG
CTTGGATATATTAAAGAAAGATAAAAAAGGAGTAGATATTTGTTTTGAAGATGCAACAA
GGACTTCTAGAGAAAAATTGAAGGAATACATGGAAATTATTTCAAAATATCAAGTTAGA

ACAGTTACCTTTGCGGACACTGTAGGATGTTCGACACCATTAGAATACGGAGATATTTTT
AATTACTTTGTAAAAAAATATTCTAACATAATTTTTTCTGCTCATTGTCATAACGATCTAG
GGTTGGCTACTGCAAATACATTAGCTGCAATTTTAAATGGTGCGAAACAAATAGAAACTA
CATTTTTGGGAATTGGTGAGAGAGCGGCTAATGCTCCTATTGAGGAAATAATTACTATTTT
GACAAAAAAACAAATAAAAAGTACGGAATTCACTTTACCCGACGTATATAAAACTAGTA
TTAATATTTCTAAAATTCTCGATTTTCAAATATCAGAAAACAAACCTATAATTGGTGAAA
ATATATTTAAACATGAATCAGGAATTCATCAAGATGGTACTAAAAAAAATATAAATATGT
ATCAATATTTAGTTCCTAGTGATTTAGGATTGAAAAATTCACAAGTTGTTCAGTTTCCAAT
AAGTAATATTTCTAGTAAGAAAATCTTGCACAATAAATTTAAATCAATAGTTAACACTGA
AGAAATTGATGAAAATATTTCTTTCTATAAACTTGTGAACAAGTTTTCACCTGAAGTAGC
ACCTGAAGATACAGTGGATTTACTTCAGATAATAAAAAGGAGGAGTAAAGATGGAAATT
TTAAATGA

## > Q5HHA7

Function: protein coding sequence; Sua5/YciO/YrdC/YwlC family protein (genomic island)
Best match: Q5HHA7_CC001_MW2-USA400_BA000033.2[951385:952026] (completely identical)
Position: 025-contig_192_RC: 75740 … 76382; Length: 642 bp
Sequence:
ATGGAAATTTTAAATGATTCTGTAAAAAGTTTTGAAAAACTTTATTTACAATTACAAGAT
GGTTTTCCAGTGATTGTTCCTACTGATACTAATTATAATTTATGTAGTTTACCTAACAACG
ATCTTTGTATCGATAAAATATTTGAATATAAAAAGCGATCGAAAGATAAGCCATTATCAT
TATTTATTGATAAGCCAGAGGATTGGAAATTGTATGGAGATAATCAAAATACGGAAATAG
TTGATAAACTAGTTGAAATATTTTGGCCTGGACCATTAAATATTATTTTAAAAAATAAAA
CAAGCTATAATTATATGCTCAATAATTCAGATAGTATAGCTATAGGATGTGTACAAAATA
AAACGATGAGAAGATTTATTTCATATATTAATTCACCTATAGCAATTACTTCAGCAAATAT
ATCTGGAACTGCCGATGATATTTTAATAACTGAAAATGAAGCAATTAAGCACATGGGCGA
AAATGTAAGATATATGCTAAGAAGTCAAAATAAAACTAACTATAAAACATCTAGTACGA
TTATTAAAGTGACAGATAATAAAATTGAATTATTAAGAGAAGGAGATATAAAGTTTGAA
GAAATAAAAGAAAGACTAGGTACAGGTATTATTTATGAATAA

## > Q5HHA6

Function: protein coding sequence; putative protein (genomic island)
Best match: Q5HHA6_CC001_MSSA476_BX571857.1[935988:937106] (completely identical)
Position: 025-contig_192_RC: 76374 … 77493; Length: 1119 bp
Sequence:
ATGAATAAGTTAATACTTGGGATTTATTTATACCGAATTTTTTCACGAGCATACTTTTATT
TACCGTTTTTATTAATTTACTTTTTGATTCAAGGTTATTCCATAATACAATTAGAAATATTA
ATGGCGTCTTATGGCATTGCAGCATTTTTATTCTCTCTATACAAAGAGAAGTGTTTTAAAA
TTTGTAACTTAAAAGATTCTAATAAATTAGTTGTTAGTGAAATATTCAAAATCATCGGTTT
ATTGTTGTTATTATATCAAAATCAATATTTAATTTTAGTAGTGGCACAAATATTATTAGGG
TTAAGTTACTCAATGATGGCGGGTGTTGATACCGCAATAATTAAAAGAAATATAACAAAT
GAGAAATACGTACAAAATAAGTCAAATAGCTATATGTTCCTATCATTATTAATTTCAGGG
ATTATAGGTAGTTATCTTTATGGAATAAATATTAAATGGCCTATAATAATGACTGGTATAT
TTTCAATTCTAACAATTATAATTATTCGATGCACATTAGTTGAAAATAGGGAATTAAATTT
AATAGGAGAAACAAAGGGAAAGATAAAGAAATTTCTACCAGAAGAGAAGTTTTGGATAT
TGCATTATTCTTTTTTAAGAGCGTTAATATTAGGATTTTTTATAGGATTTATTCCAATTAAT
ATATATAATGATTTAAAACTGAATAATTTACAATTTATTTCAGTATTAACTTGTTACACAG
TTATGGGTTTTGTATCTTCACGTTATTTAACTAAATACTTGAATTATAAGTTTGTGTCAGA
AATTTGTTTAGTAATATTTTTAATAATATATACATATCAAAGTTTCATAGCAGTTACTATT
TCTATGATATTTTTAGGTATTTCTTCAGGGTTAACTCGTCCACAAACTATAAATAAACTTT
CTAGCAGTAGTAACTTAAGAGTGATGCTTAATTATGCAGAAACGTTATATTTTATTTTTAA
TATCGCATTTTTTACTTATGGGTGGTTACTTATATACAATAGGAACTATTCAATACTTAATA
TTATTTATTTCGTTATTAATTTTTATATATTTAATAATAATATTTTATTTTACAAGGAGAGA
GCAACATGAAAATAAAAACTGA

> **Q5HHA5**
Function: protein coding sequence; putative protein (core genome, variable)
Best match: Q5HHA5_CC001-ST772_118_AJGE01000009.1[77230:77745] (completely identical)
Position: 025-contig_192_RC: 77476 … 77992; Length: 516 bp
Sequence:
ATGAAAATAAAAACTGAATTTAAAGGGAACAATATACCATATGAATACGCAGCAGGTGC
AGATGTGAGTGATTCTATTAACGGGAATCCAATTAAGTCATTTCCATTTGAAGTAATTGA
ATTACCGGAAGGGACTAAATATCTTGCTTGGTCTTTAATTGACTATGATGCAATTCCTGTG
TGTGGCTTTGCATGGATTCATTGGAGTGTTGCTAATGTAAGTGTTAGTGGCAATTCAATTT
CTATAAAAGCAGATTTATCAAGAACAAAGGGTGACTATGTACAAGGTAAAAATAGCTTT
ACTAGTGGGTTATTGGCTGAAGATTTTTCAGAAATAGAAAATCACTATGTAGGACCTACA
CCACCTGATCAAGATCATCAATATGAATTAACAGTTTATGCGTTAGATCATTCTTTAAATT
TGAAGAATGGGTTCTACTTGAATGAATTTTTAAAAGAAGTAAATCAACATAAAATTGATC
AAACAAGTATTAACCTTATAGGAAGAAAAATTTAA

> **eap-L1**
Function: protein coding sequence; putative exported protein/locus 1 (core genome, constant)
Best match: eap-L1_CC001-ST772_118_AJGE01000009.1[78056:78490] (completely identical)
Position: 025-contig_192_RC: 78302 … 78737; Length: 435 bp
Sequence:
ATGAAATTAAAATCATTTATAACTGTAACTTTGGCACTGGGCATGATCGCAACGACTGGC
GCTACTGTGGCAGGTAATGAGGTATCTGCAGCAGAAAAGGACAAACTACCGGCAACTCA
AAAAGCTAAAGAAATGCAAAATGTTCCATATACAATTGCAGTAGATGGCATTATGGCTTT
CAATCAATCTTACTTAAATTTACCAAAAGATAGCCAATTATCATATTTAGATTTAGGAAAT
AAAGTTAAAGCTTTATTATATGATGAACGCGGTGTAACACCTGAGAAGATTCGAAATGCA
AAATCTGCCGTTTACACGATTACTTGGAAAGATGGCAGTAAAAAAGAAGTGGATTTAAA
GAAAGATAGTTATACAGCAAACTTGTTTGATTCAAATTCAATTAAACAAATTGATATTAA
TGTAAAAACTAAATAA

> **A6U088**
Function: protein coding sequence; putative protein (core genome, constant)
Best match: A6U088_CC001_MW2-USA400_BA000033.2[954638:954823:r]RC (completely identical)
Position: 025-contig_192_RC: 78993 … 79179; Length: 186 bp
Sequence:
TTATTGTGATGAATCTTTCGGCGGTTTAATTACTGCAGCAAAAATTGCTGTGAAAATCGTG
AACAATACTGCCATGATAATTGGATTCACTACATTTAAGCTGTCTCCACCTACTAGGCTAT
TAAGTACAAAGTTAACCATTTGCATTAATAATAATGCCCAAAAGAATGTTACGAGGTGTT
TCAT

> **fabH**
Function: protein coding sequence; beta-ketoacyl-ACP synthase III (core genome, constant)
Best match: fabH_ST425_LGA251-cow_FR821779.1[986175:987116] (completely identical)
Position: 025-contig_192_RC: 79473 … 80415; Length: 942 bp
Sequence:
ATGAACGTGGGTATTAAAGGTTTTGGTGCATATGCACCAGAAAAGATTATTGACAATGCC
TATTTTGAGCAATTTTTAGATACATCTGATGAATGGATTTCTAAGATGACTGGAATTAAAG
AAAGACATTGGGCAGATGACGATCAAGATACTTCAGATTTAGCATATGAAGCAAGTGTA
AAAGCAATCGCTGACGCTGGTATTCAGCCCGAAGATATAGATATGATAATTGTTGCCACA
GCAACTGGAGATATGCCATTTCCAACTGTCGCAAATATGTTGCAAGAACGTTTAGGGACG
GGCAAAGTTGCCTCTATGGATCAACTTGCAGCATGTTCTGGATTTATGTATTCAATGATTA
CAGCTAAACAATATGTTCAATCTGGAGATTATCATAATATTTTAGTTGTCGGTGCAGATA
AATTATCTAAAATAACAGATTTAACTGACCGTTCTACTGCAGTTCTATTTGGAGATGGTGC
AGGTGCGGTTATCATCGGTGAAGTTTCAGAAGGCAGAGGTATTATAAGTTATGAAATGGG
TTCTGATGGCACTGGTGGTAAACATTTATATTTAGATAAAGATACTGGTAAACTGAAAAT

GAATGGTCGAGAAGTATTTAAATTTGCTGTTAGAATTATGGGTGATGCATCAACACGTGT
AGTTGAAAAAGCGAATTTAACATCAGATGATATAGATTTATTTATTCCTCATCAAGCTAA
TATTAGAATTATGGAATCAGCTAGAGAACGCTTAGGTATTTCAAAAGACAAAATGAGTGT
TTCTGTAAATAAATATGGAAATACTTCAGCTGCGTCAATACCTTTAAGTATCGATCAAGA
ATTAAAAAATGGTAAACTCAAAGATGATGATACAATTGTTCTTGTCGGATTCGGTGGCGG
CCTAACTTGGGGCGCAATGACAATAAAATGGGGAAAATAG

## > fabF

Function: protein coding sequence; 3-oxoacyl-acyl-carrier-protein synthase 2 (core genome, constant)
Best match: fabF_CC001_MW2-USA400_BA000033.2[956071:957315]
Position: 025-contig_192_RC: 80426 … 81671; Length: 1245 bp
Sequence:
ATGAGTCAAAATAAAAGAGTAGTTATTACAGGTATGGGAGCCCTTTCTCCAATCGGTAAT
GATGTCAAAACAACATGGGAGAATGCTCTAAAAGGCGTAAATGGTATCAATAAAATTAC
ACGTATCGATACTGAACCTTATAGCGTTCACTTAGCAGGAGAACTTAAAAACTTTAATAT
TGAAGATCATATCGACAAAAAAGAAGCGCGTCGTATGGATAGATTTACTCAATATGCAAT
TGTAGCAGCTAGAGAGGCTGTTAAAGATGCGCAATTAGATATCAATGAAAATACTGCAG
ATCGAATCGGTGTATGGATTGGTTCTGGTATCGGTGGTATGGAAACATTTGAAATTGCAC
ATAAACAATTAATGGATAAAGGCCCAAGACGTGTGAGTCCATTTTTCGTACCAATGTTAA
TTCCTGATATGGCAACTGGGCAAGTATCAATTGACTTAGGTGCAAAAGGACCAAATGGTG
CAACAGTTACAGCATGTGCAACAGGTACAAATTCAATCGGAGAAGCATTTAAAATTGTGC
AACGCGGTGATGCAGATGCAATGATTACTGGTGGTACAGAAGCACCAATTACTCATATGG
CAATTGCAGGTTTCAGTGCAAGTCGAGCGCTTTCTACAAATGATGACATTGAAACAGCAT
GTCGTCCATTCCAAGAAGGTAGAGACGGTTTTGTTATGGGTGAAGGTGCTGGTATTTTAG
TAATCGAATCTTTAGAATCAGCACAAGCTCGAGGTGCCAATATTTATGCTGAGATAGTTG
GCTATGGTACTACAGGTGATGCTTATCATATTACAGCGCCAGCTCCAGAAGGTGAAGGCG
GTTCTAGAGCAATGCAAGCAGCTATGGATGATGCTGGTATTGAACCTAAAGATGTACAAT
ACTTAAATGCCCATGGTACAAGTACTCCTGTTGGTGACTTAAATGAAGTTAAAGCTATTA
AAAATACATTTGGTGAAGCAGCTAAACACTTAAAAGTTAGCTCAACAAAATCAATGACTG
GTCACTTACTTGGTGCAACAGGTGGAATTGAAGCAATCTTCTCAGCGCTTTCAATTAAAG
ACTCTAAAGTCGCACCGACAATTCATGCGGTAACACCAGATCCAGAATGTGATTTGGATA
TTGTTCCAAATGAAGCGCAAGACCTTGATATTACTTATGCAATGAGTAATAGCTTAGGAT
TCGGTGGACATAACGCAGTATTAGTATTCAAGAAATTTGAAGCATAA

## > Q2YWV0

Function: protein coding sequence; putative protein (core genome, constant)
Best match: Q2YWV0_CC001_MW2-USA400_BA000033.2[957370:957741:r]RC
Position: 025-contig_192_RC: 81725 … 82097; Length: 372 bp
Sequence:
TTATACTAAGATGAGCGACAGCACAATCGTCATAATAAAATATAAAATATTTATTAATAA
TAAGGGGATTATCCATGTAGAAACAAAGTAATGCTCTTTTTTTACCTCTTGTGGGTTGAAA
AATGGATCATCAGAGATAGACTTCTTCTTTTTCGAAGATGACATTTGATACTTTAATCTTC
TAAAACCATAACTTGTCGCATCAAAAATGCCTTCTTGTACAAGTAAAATCAAAAATATGC
TAATAAAAATAATTAATGAAACATAAAACAATATATTTAAATATGTAATGATAGTATGGC
TATTAAAAACCCATATAATAAACGTTAACATTGGGGTTATTAGTGCCATTCCAAGCCATTT
TTTCAACAT

## > oppB1

Function: protein coding sequence; oligopeptide transport system permease protein (core genome, constant)
Best match: oppB1_CC005_Mu50-VRSA_BA000017.4[1034300:1035226] (completely identical)
Position: 025-contig_192_RC: 82339 … 83266; Length: 927 bp
Sequence:
ATGGGGAAATATATTTTCAAACGATTTATTTATATGCTTATTTCTTTATTTATTATTATTAC
AATTACATTTTTCTTAATGAAATTAATGCCAGGTTCGCCATTTAACGATGCTAAATTAAAT

GCTGAACAAAAAGAAATTTTAAATGAAAAATATGGATTAAATGATCCTGTAGCTACGCA
GTATTTACATTATTTAAAAAATGTTGTTACAGGCGATTTTGGTAATTCATTCCAGTATCAT
AATCAACCTGTGTGGGATTTGATTAAACCGAGACTACTACCTTCTTTTGAAATGGGTCTTA
CAGCAATGTTCATCGGTGTGATACTGGGACTTATTTTAGGTGTTGCAGCAGCTACTAAAC
AAAATTCTTGGGTTGACTATACAACTACAGTTATTTCAGTTATTGCTGTATCTGTACCATC
TTTTGTACTTGCTGTACTTTTACAATATGTATTTGCAGTTAAATTAAGATGGTTCCCAGTA
GCTGGATGGGAAGGTTTTTCGACCGCGGTATTACCGTCACTTGCATTATCTGCAGCTGTTT
TAGCAACTGTCGCCAGATACATAAGAGCAGAGATGATAGAGGTATTAAGTTCAGACTAT
ATTTTATTAGCGAGAGCTAAAGGTAATTCGACAATGCGTGTACTTTTTGGACATGCACTTA
GAAATGCTTTAATTCCAATTATTACAATTATCGTTCCCATGTTAGCAAGTATTTTAACAGG
CACTTTAACAATTGAAAATATTTTTGGGGTTCCTGGATTAGGGGATCAATTCGTACGTTCA
ATTACAACAAATGATTTCTCAGTAATCATGGCAATCACACTATTATTTAGCACACTGTTTA
TCGTTTCTATTTTTATTGTAGATATTTTGTACGGTGTGATAGATCCACGAATTCGTGTTCAA
GGAGGTAAAAAATAA

> **oppC**

Function: protein coding sequence; (core genome, constant)
Best match: oppC_CC001_MW2-USA400_BA000033.2[958910:959980]
Position: 025-contig_192_RC: 83265 … 84336; Length: 1071 bp
Sequence:
ATGGCTGAAAATAAAAACAATTTGTCGATTAACGACGATCATTCTAATGCAGCTATGACG
CATACCTCTGACGCTATCGCATCATCTGATTTTATTATTAGAGATTTAGATTTGAATCAGG
AACCTGAAATGCAACGAGAAAGCAAAAACTTTTGGCAAGATGCTTGGGCTCAGTTAAAA
CGAAATAAGTTAGCTGTTGTCGGTATGATAGGTTTAATTATCATTGTAATATTTGCTTTTA
TCGGTCCAGTTATAAATAAACATGATTATGCTGAACAAAATGTAGAACATAGAAATCTTC
CGGCAAAAATACCTGTATTAGACAAAGTTCCATTTTTACCTTTTGATGGTAAAGATGCAG
ATGGCAAGGATGCTTATAAAGCAGCAAATGCTAAAGAAAATTATTGGTTTGGTACTGATC
AGTTGGGTCGAGATTTATGGACAAGAACATGGAAAGGTGCTCAAATTTCATTGTTTATCG
GTGTTGTTGCAGCGATGTTAGATATTTTTATTGGCGTTGTATATGGTGCGATTTCTGGATT
CTTCGGTGGACGTGTCGATACGATTATGCAACGTATACTTGAAGTCATAGCATCTATTCCG
AATTTAATTGTCGTAATTTTATTTGTATTAATTTTTGAACCATCCATTTGGACAATTATATT
GGCTATGTCTATCACAGGCTGGTTAGGCATGAGCAGAGTTGTACGTGGAGAATTTTTAAA
ATTAAAAAATCAAGAGTTTGTCATGGCTTCGAAAACATTGGGGGCTTCAAAATTCAAATT
GATATTTAAGCATATTTTACCTAATACATTAGGTGCTATCGTGGTTACATCAATGTTTACA
GTACCTAGTGCTATTTTCTTCGAAGCATTTTTAAGTTTCATTGGTATAGGTGTACCCGCAC
CTCAAACATCGTTAGGGTCATTAGTAAATGATGGGCGCGCAATGTTATTAATTTATCCAC
ATGAATTATTTATACCAGCAATGATTTTAAGTTTATTAATTCTATTCTTTTACTTATTTAGT
GATGGATTACGTGATGCATTTGATCCGAAAATGCGTAAATAA

> **oppD**

Function: protein coding sequence; (core genome, constant)
Best match: oppD_CC398_SO385-pig_AM990992.1[1032752:1033834]
Position: 025-contig_192_RC: 84351 … 85434; Length: 1083 bp
Sequence:
ATGACTGAAAGAATATTAGAAGTAAATGATTTGCATGTTTCCTTTGATATTACAGCAGGG
GAAGTGCAGGCAGTGAGAGGTGTAGATTTTTATTTAAACAAAGGGGAAACATTGGCAAT
TGTTGGTGAATCAGGTTCAGGTAAATCTGTAACAACAAAAGCAATTACAAAATTATTCCA
AGGGGACACAGGAAGAATTAAAAAGGGAGAAATTTTATTTTTAGGGGAAGATTTAGCAA
AAAAACCTGAAAATGAGTTAATTAAATTACGTGGCAAAGATATTTCAATGATCTTTCAAG
ATCCAATGACATCTTTAAACCCAACGATGCAAATTGGTAAACAAGTCATGGAACCATTAA
TTAAGCACAAAAATTATAGTAAAGCACAAGCTAAAAAGCGCGCATTGGAAATACTAAAT
CTTGTAGGTTTACCAAATGCAGAAAAAAGATTTAAAGCATATCCACATCAATTTTCAGGT
GGACAAAGGCAAAGAATTGTTATTGCAACCGCATTAGCTTGTGAACCTAAAGTGCTCATT
GCTGATGAACCAACGACTGCATTAGACGTAACGATGCAGGCACAAATTTTAGATTTAATG
AAAGAACTACAACAAAAAATCGATACAGCAATTATTTTTATAACGCATGATTTAGGGGTT

GTTGCGAATATTGCTGATAGAGTAGCAGTTATGTATGGTGGTCAAATGGTTGAAACAGGA
GATGTTAACGAAATATTTTATGATCCAAAGCATCCATATACATGGGGATTATTATCGTCA
ATGCCTGATTTATCAACAACAAATGACACACCATTACTAGCGATTCCTGGAGCGCCACCT
GATTTATTACACCCACCTAAAGGTGATGCATTTGCGAGACGTAGTCAATATGCATTAGAT
ATTGATTTTAAAGTAGAACCACCGTGGTTTAAAGTTTCACCGACACATTTTGTGAAATCTT
GGTTATTAGACGCACGTGCACCAAAAGTTGAACTACCCGAGCTGGTAAAACAACGTATG
AAACCGATGCCTAATAATTATGAAAAACCACTCAAGGTAGAAAGGGTGTCGTTCAATGA
AAAATGA

> **oppF1**

Function: protein coding sequence; oligopeptide ABC transporter/ATP-binding protein/putative (core genome, constant)
Best match: oppF1_CC001_MW2-USA400_BA000033.2[961068:962009] (completely identical)
Position: 025-contig_192_RC: 85423 … 86365; Length: 942 bp
Sequence:
ATGAAAAATGATGAAGTGCTATTATCCATTAAAAATTTAAAGCAATATTTTAACGCAGGA
AAGAAAAACGAAGTGAGAGCGATTGAAAATATTTCGTTTGATATATACAAAGGGGAAAC
ATTAGGTTTAGTAGGAGAATCGGGGTGTGGTAAATCTACAACTGGTAAATCAATTATTAA
ACTTAATGATATTACAAGTGGAGAAATTTTGTATGAGGGTATTGATATACAAAAGATTCG
TAAACGTAAAGATTTGCTTAAATTTAATAAAAAGATACAGATGATTTTTCAAGACCCATA
TGCGTCTTTAAATCCTAGGTTAAAAGTAATGGATATAGTAGCTGAAGGTATTGATATCCA
TCATTTAGCAACTGATAAACGTGACCGTAAAAAACGTGTCTATGATTTACTTGAAACTGT
TGGATTAAGTAAAGAACATGCCAATCGCTATCCTCATGAATTTTCAGGTGGACAACGCCA
ACGTATTGGAATTGCCCGTGCATTAGCCGTTGAACCAGAATTTATTATCGCGGACGAACC
AATATCGGCATTGGATGTTTCAATCCAAGCTCAAGTAGTTAATTTATTATTAAAATTACAA
CGTGAAAGAGGGATTACGTTCCTATTTATAGCTCATGATCTATCAATGGTGAAGTATATTT
CAGATCGTATTGCAGTGATGCATTTTGGGAAAATAGTTGAAATTGGACCGGCAGAAGAA
ATTTATCAAAATCCATTACACGATTATACTAAGTCTTTATTATCAGCCATTCCACAACCTG
ATCCTGAATCAGAACGCAGTCGCAAACGATTTAGTTATATTCATGATGAAGCAAATAATC
ATTTAAGACAATTACATGAAATAAGACCACAGCATTTTGTCTTTAGTACTGAAGAAGAAG
CGGCACAACTACGAGAAAATAAATTGGTGACACAAAATTAA

> **oppA1**

Function: protein coding sequence; transport system extracellular binding lipoprotein (core genome, constant)
Best match: oppA1_CC001_MSSA476_BX571857.1[945997:947652]
Position: 025-contig_192_RC: 86383 … 88039; Length: 1656 bp
Sequence:
ATGACGAGAAAATTTAGAACACTTATTTTAATTTTGATTGCTACAATTGCATTAAGTGGTT
GTGCTAATGATGATGGTATTTATTCAGATAAAGGTCAAGTATTCAGAAAAATTTTGTCAT
CAGACTTAACATCCCTTGATACATCATTAATAACGGATGAAATATCTTCTGAAGTGACTG
CGCAAACATTCGAAGGTTTATACACATTAGGAAAAGGTGACAAACCGGTGTTAGGTGTTG
CGAAAGCTTTTCCTGAAAAGAGTAAAGGTGGTAAAACTTTAAAGGTTAAATTAAGAAGC
GATGCTAAATGGAGCAATGGTGACAAAGTGACTGCGCAAGATTTTGTTTATGCTTGGAGA
AAAACAGTTGACCCTAAAACAGGTTCTGAATTTGCATACATTATGGGGGACATTAAAAAT
GCGAGTGATATTAGTACTGGTAAGAAACCTGTAGAGCAATTAGGTATCAAAGCATTAGAT
GATGAAACATTACAAATTGATTTAGAAAAGCCGGTTCCATATATTAATCAATTATTAGCA
CTTAATACCTTTGCACCTCAAAATGAAAAAGTTGCCAAAAAATATGGTAAAAATTACGGT
ACGGCAGCTGATAGAGCGGTATACAATGGTCCATTTAAAGTTGATGATTGGAAACAAGA
AGATAAAACCTTACTATCTAAAAATCAGTATTATTGGGATAAAAAGAATGTAAAATTAGA
TAAAGTGAATTATAAAGTTATTAAAGACTTACAAGCCGGTGCATCATTGTATGATACTGA
ATCAGTAGATGACGCAGTTATTACTGCAGATCAAGTAAATAAATATAAAGATAACAAAG
GATTAAACTTTGTGTTAACGACTGGGACATTTTTTGTAAAAATGAATGAAAAACAATATC
CTGATTTTAAAAACAAAAATTTAAGACTGGCTATCGCACAAGCAATAGATAAAAAAGGA
TACGTTGATTCAGTGAAAAACAATGGCTCAATTCCTTCCGATACACTAACAGCCAAAGGA

ATTGCGAAAGCGCCTAATGGCAAAGATTATGCGAGTACCATGAATTCGCCTTTAAAATAT
AATCCTAAAGAAGCAAGAGCACACTGGGACAAAGCTAAAAAAGAGTTAGGTAAAAATG
AAGTGACATTTTCAATGAACACAGAAGATACACCAGATGCAAAAATATCTGCTGAATATA
TCAAATCGCAAGTTGAGAAAAATTTACCAGGAGTTACTTTGAAAATTAAGCAATTACCGT
TTAAACAAAGAGTATCATTAGAACTGAGTAACAATTTTGAAGCATCACTTAGTGGTTGGT
CTGCAGATTACCCTGATCCTATGGCTTATTTAGAAACAATGACCACAGGTAGCGCACAAA
ATAATACAGACTGGGGTAATAAAGAATATGATCAATTACTTAAAGTAGCAAGAACCAAA
TTGGCACTTCAACCGAACGAACGATATGAAAACTTGAAAAAAGCAGAAGAAATGTTCCT
AGGAGATGCACCGGTAGCACCAATTTATCAAAAAGGTGTTGCACATTTAACAAATCCTCA
AGTAAAAGGATTAATTTACCATAAATTTGGTCCAAATAACTCACTTAAACATGTATATAT
TGATAAATCGATAGATAAAGAAACAGGTAAGAAGAAAAAATAA

> **oppA2**

Function: protein coding sequence; transport system extracellular binding lipoprotein (genomic island)
Best match: oppA2_CC005_Mu50-VRSA_BA000017.4[1040211:1041926]
Position: 025-contig_192_RC: 88250 … 89966; Length: 1716 bp
Sequence:
ATGGGGAAGCTAATTAAATATATTTCAATACTTATTGTTATCGTTTTAGTGTTGAGTGCTT
GCGGAAAAAGCAGTAATAAAGATGAAGGAGTAAAAGATGCTACTAAAACAGAAACCTC
AAAACATAAAGGTGGTACCTTAAATGTAGCATTAACAGCACCTCCAAGTGGTGTTTATTC
TTCGTTATTAAATAGTACACATGCGGATGCTGTAGTTGAGGGATATTTTAACGAAAGCTT
ATTAGCAACTGATAAAAAAATACGTCCTAAGGCATATATTGCTTCATGGAAGGACATCGA
GCCGGCTAAGAAAATAGAATTTAAAATTAAAAAAGGTATTAAATGGCATGATGGTAATG
AATTGAAAATTGATGATTGGATTTATTCAATTGAAGTCTTAGCTAACAAGGACTACGAAG
GTGCTTATTATCCAAGTGTAGAAAATATCCAAGGTGCGAAAGATTATCATGAAGGAAAA
ACTGATCATATTAGCGGATTGAAGAAAATAGATGACTACACTATGCAGGTTACATTTGAT
AAAAAACAAGAAAATTACTTAACAGGATTTATTACTGGACCTTTATTAAGTAAAAAATAT
TTATCAGATGTACCAATTAAAGATTTAGCGAAATCAGATAAAATCCGAAAATATCCTATT
GGTATTGGACCGTATAAAGTTAAGAAAATCGTTCCAGGTGAGGCTGTTCAACTCGTTAAA
TTTGATGATTATTGGCAAGGTAAGCCTGCACTAGACAAAATCAATTTAAAAGTTATTGAT
CAAGCTCAAATTATTAAGGCAATGGAAAAAGGCGATATTGATGTTGCGAATGATGCTACC
GGTGCAATGGCAAAAGATGCTAAGTCATCTAATGCTGGTCTCAAGGTATTATCTGCGCCA
AGCTTAGACTACGGTTTAATAGGATTCGTATCTCATGATTACGATAAAAAAGCTAATAAA
ACTGGTAAAGTGAGACCAAAATATGAAGACAAAGAATTACGTAAAGCAATGCTTTATGC
AATTGATAGAGAAAAATGGATCAAAGCGTTTTTCAATGGTTACGCTAGTGAAATCAATAG
TTTTGTACCATCTATGCATTGGATAGCAGCCAATCCTAAGGACCTAAATGATTACAAATA
TGATCCTGAAAAAGCTAAAAAAATCTTAGATAAGTTAGGTTATAAAGATAGAGATGGTG
ACGGATTTAGAGAAGATCCTAAAGGTAATAAATTTGAGATTAACTTTAAACATTATTCAG
GTTCAAATCCTACTTTTGAACCAAGAACTGCTGCGATAAAAGATTTCTGGGAAAAAGTTG
GCTTGAAAACAAATGTGAAGTTAGTAGAATTCGGTAAATATAATGAAGACTTAGCAAAT
GCATCTAAAGATATGGAAGTGTACTTCAGATCATGGGCAGGAGGTACAGATCCAGATCC
ATCAGATTTATACCACACTGATAGACCTCAAAATGAAATGAGAACAGTTTTACCAAAATC
AGATCAATATTTAGATGATGCATTAGACTTCGAAAAAGTAGGCATTGATGAAAAGAAAC
GTAAAGATATTTATGTTAAATGGCAAAAATATATGAATGATGAGTTACCTGGATTACCAA
TGTTCCAAGGTAAATCGATAACTATTGTTAACGATAAAGTACGAAACTTAGACATTGAAA
TTGGAACTGATCAAAGTTTATATAATTTAACTAAAGAAGCTTAG

> **appD**

Function: protein coding sequence; (core genome, constant)
Best match: appD_CC005_Mu50-VRSA_BA000017.4[1041977:1042963]
Position: 025-contig_192_RC: 90016 … 91003; Length: 987 bp
Sequence:
ATGAATAATGTATTGTTAGAGGTTAAAGATTTAGAAACATCATTAAAAATAAATAATGAA
TGGTTAGCAACTGTTGAAAATATTTCTTTTGAATTATCTAAAGGAGAAGTTTTGGGTATAG
TAGGGGAATCTGGTTGCGGTAAGTCCATATTAAGTAAGTCAATTATTAAATTATTACCAG

AAAAGATATCTAAACTAAGTAATGGAGAAGTTATATTTGATGGTAAACGAATCGATACGC
TCAATGAGAAGCAATTGCTAGATATTCGAGGAAATGATATTGCTATGATTTTTCAAGAAC
CTATGACTGCTTTAAATCCTGTATTTACCATAAAAAATCAACTTGTGGAATCTATAAAATC
ACATAAAAAAATTTCTAAAAAAGAAGCAAATAACTTAGCAAAAGATTTACTAAAAAAAG
TTGGAATTGCTAGACAAGATGAAATATTAAATAGCTATCCACATCAATTATCTGGTGGTA
TGAGACAAAGAGTAATGATTGCAATGGCCATTTCATGTTCTCCTAAATTATTAATTGCTGA
TGAACCTACAACAGCATTGGATGTCACGATTCAAGCGCAAATATTAGACTTATTAAAAGA
ATTGCAAAAGGAAACGCAAATGGCAATTATGATGATTACACATGATTTGAGTGTAGTTGC
TGAGTTTTGCGATAAAGTCTTAGTTATGTATGCAGGTCAAATTGTAGAATTTGGAGGCAT
AAAAGAAATACTACACAATCCGAAACATCCTTATACCCAAAAATTATTATCAACAATTCC
AAAACTTAAAGAAGAGCAGAAACGACTTGAAACGATAGAAGGAATTGTGCCATCAATCC
AAGCATTTCACGTTAATAAGTGCAGATTTGCAAATAGATGTAACAAAAAACTGGATATTT
GTAATAATCAATCTCCTAAAATGCATGTTTGTGAAGACGTCATTGTACGTTGTCATTTGTA
CAAAAATGAATATAAGGAGATATAA

## > appF

Function: protein coding sequence; (genomic island)
Best match: appF_CC001_MSSA476_BX571857.1[950619:951599] (completely identical)
Position: 025-contig_192_RC: 91005 … 91986; Length: 981 bp
Sequence:
ATGGAAAATATTTTAGAAGTCAACCAAATAAAAAAATACTACAAAATTAAAACTGGATT
ATTACAAAAAACTCAGTACGTTAAAGCTGTTGATGACGTATCGTTTTCAATAAAAAAAGG
ACAAACTTTTGGATTAGTAGGAGAATCGGGTTGTGGTAAGTCAACGTTAGGTAAAGTGAT
TATCAGGCTTGAAGATGCAACTTCAGGCTCAATAATTGTTAATGGTGAAGATATAACAAG
ATTACAAGGTAAAAAACTCAGAAAATCACGACAACAATATCAGATGATATTTCAAGATC
CGTATGCATCATTGAATCCGATGCAAATGGTTGGAGATATCATTTCAGAACCTATTTTAA
ATTATAAAAAATTGCCAAAAGAAGAAATAAAAAAAGAAGTACTATATTTATTAAAATGT
GTTGGCCTAAGTGAAGATGCATATTATAAATATGCACATGAATTTTCAGGTGGACAGAGA
CAAAGAGTGGGAATTGCAAGAGCATTGGCTTTGCGTCCGAGTTTAATTGTTGCTGATGAG
CCTGTAAGTGCATTAGATGTATCTGTTCAATCTCAAGTACTGAATTTATTAAAAGATTTAC
AAGAACAATTTAACTTAAGCTATTTATTTATCGCACATGATTTAAGTGTAGTAAAACATAT
AAGTGATGTCATTGGAGTTATGTATTTAGGTCATATAGTTGAAATCGCATCTGATAAAGA
AATTTATGAAAATCCCAAACATCCATATACAAAAGCGTTGATTTCATCAATACCACAAAT
TGATAAACATAATAACAATAGAATTATATTAAAAGGAGAATTACCTTCGCCAAGTAATCC
GCCGCAAGGTTGTCCTTTTCATACAAGATGTCCGATTGCAAAAGATAAATGTAAAGAAAA
TATACCACAATTAAAGGACATTGGTGATGAACATCAAGTTGCTTGTTTTTATGTAAATAA
AGTAGGTGATTTAAATGGTTAA

## > oppB3

Function: protein coding sequence; putative oligopeptide transport system permease protein (genomic island)
Best match: oppB3_CC001_MW2-USA400_BA000033.2[967623:968585] (completely identical)
Position: 025-contig_192_RC: 91978 … 92941; Length: 963 bp
Sequence:
ATGGTTAAATTAATATTAAAGAGATTAGGTTTAATGATTCCGTTACTAATTTTAATTTCTA
TTGTTGTATTTTCATTAGCTATCATTCAACCAGGAGATCCATTTTCAGATTTACAAAACGG
AAAAATAAAACAAGAAGCGATAAATGCACAAAGGGAAAAGTTAGGCCTCAACGACTCTA
TATCACATCAATACATTAGATGGGTCAATCATGTTATACATGGTGATTTAGGGGAGTCAA
TCAAATATAAAAGGCCGGTAATTGATGTTATTGAGGAAAGAATTCCAAATACAATATTAC
TCGGTGCTATGTCATTAATTATTACTTATATTATCTCATTTGCTTTAGGAATAACGTCAGG
TAGATATTCTTACAGTTTGACTGATTATACTGTGCAAATATTTAATTATTTGATGTTAGCT
ATTCCATCTTTTATTGCGGGAGTATTTGCAATTTTTATTTTTTCTTTTGAATTACAATGGTT
TCCGTTTCAAGGTTCTGTTGATATTAACCTTAAAGAAGGTACTTTTGAATATTATATGAGT
AAAATTTATCATACATTTTTGCCTGCATTAACTTTAGGATTATTATCTACTGCTGGTTATAT
TCAATATTTACGTAATGATATTATTGAAAATTCTAAAAAAGATTATGTATTGACGGCAAG

GTCAAAAGGATTATCTATGAATAAAATTTATAATAAACATATATTGAGAAATTCTTTAAT
ACCTATTATTACATTTTTAGGTGCTGATATTGTAAGTATTTTAGGTGGAGCTGTGATTACT
GAGACTATCTTTTCATATAACGGTATCGGTAAATTATTTTTAGAATCGGTAATAGGTCAAG
ACTATCCATTAATGATGGCATTAACGTTGTTTTTCTCATTTTTAGGTTTACTGGGTAATTTG
ATTTCTGATATTACTTATGGATTTATAGATCCAAGAATTAGAAGTAACTAG

## > appC

Function: protein coding sequence; oligopeptide ABC transporter permease (genomic island)
Best match: appC_CC005_Mu50-VRSA_BA000017.4[1044913:1045794]
Position: 025-contig_192_RC: 92952 … 93834; Length: 882 bp
Sequence:
ATGCAAAATAAGTCAAAATCGCCTTTTAAAATTGCATTGTCTAGATTTATTCATAATAAA
ATTGCAATGTTATCGATTATTTTTTTATTAATCATAACTATTGTATCAATTATAGCGCCATT
AATAGCTCCTTTTCCAGTGAACCAACAAGATTTATTAAATATAAAAGGTGAAATGACAGC
ACAAAACATTCTTGGTACAGACTCTGGTGGTAGGGATAACTTTAGTCGTTTGTTATATGCA
GGTCGTATTTCATTATCCATTGGAATTACATCTACAATAGGAATGCTTTTGATTGGAATTA
CAGTTGGAGTGATTTCTGGTTATTTCGGAGGTATTGTTGATACATTATTAATGAGAATAAC
CGAATTTGTTATGTTATTTCCATTTTTAATATTTGCAATTGTATTAAATGCTGCACTTGGAG
ATAAAATTAAAAATCCTTATGGATCTGCCATAATTCTTGTTCTAGTTATTATCGTATTAAG
TTGGGGAGGTATTGCAAGACTTGTTCGTGGTAAAGTACTTCAAGAAAAAGAAAATGAAT
ACTTTTTGGCAGCAAAATCAATTGGTACACCCACATATAAAATTATTTTGAAACATCTTTT
GCCGAATATATTAAGTGTAGTTATCGTACAAGCAACATTGTTATTTGCCGGTATGATTGTA
GTGGAATCAGGATTGAGCTTTTTAGGATTCGGAATTAGTAAAGCAATACCATCTTGGGGT
AATATGTTGAGTGATGCTCAAGAAGGGGATGTTATAAGTGGTAAACCGTGGATATGGATG
CCACCTGCTATAATGATTACATTAACTATATTAAGTATAAACTTTGTAGGGGAAGGGCTT
AAAGATGCTTTTAATCCTAGAGGTAGACGTTAA

## > trpS

Function: protein coding sequence; tryptophanyl-tRNA synthetase (core genome, constant)
Best match: trpS_CC005_Mu50-VRSA_BA000017.4[1045836:1046825:r]RC
Position: 025-contig_192_RC: 93875 … 94865; Length: 990 bp
Sequence:
TTATCTCTTACGTCCTAATCCCATCGCTTTTTCCATTTTCTTCACTGTTTTAAATGAAACTT
TGTGTGCTTTATCTCTACCTTGATCTAAAATATCATCAAGTTTATCTGAGTTATAGAAACT
TTCGTATTTTTCTTGGAATTCTACTAAAAATGCTTTAACTATTTCAGCAAGGTCACCTTTA
AATTTACCATAACCTTCCCCTTCATATTTTGCCTCAATGTCTTTGATAGGCATGTCGGTTA
ATCCAGCATATATTGAAATTAAATTAGTAATGCCTGGTTTATTGTCGCGGTCGAATTTAAT
AATACCATCTGAATCAGTTACAGCGCTTTTAATTTTTTTAGCTGCAACATTCGGCTCGTCT
AATAATGAAATGAAGTTTTTAGTATTATCATCACTCTTACTCATTTTTCTTGTTGGGTCTTG
TAAACTCATGACACGTCCACCAACTTTAGGCATACGAATTTCAGGTTTCACAAGCACATC
ATTATAGCGACTATTAAATCTATCTACAAGGTTACGAGTCAATTCGATATGCTGCTTTTGG
TCATCTCCAACTGGAACGATATTAGTATTGTAAAGAACAATATCAGCTGCCATTAAAGGT
GGATATGTTAATAGACCAGCAGGTATACCTTCAACTGCTTTCTGAGCTTTATCTTTGTATT
GCGTCATACGCTCTAATTCTCCAACAGAAGCAATCGTAGTTAACATCCATCCTGCTTGTAC
GTGTGCAGGGACTTCAGATTGTATGAACAATGTTGCTTTGTCTGGATCTATACCAGAAGC
TAAATAAATCGCTGCTAATTGTCTGGTCTGTTTACGTAATTTTAAACGATCTTGTGGCATT
GTAATTGCATGTTGATCTACGATACAGAAATAACAATCATAGTCATTTTGCACATCAACA
AATTGTTTTAGTGCGCCAATATAATTTCCAATAGTAGGAATTCCACTAGGTTGGATGCCTG
AAAATAATGTCTCCAT

## > spxA

Function: protein coding sequence; transcript regulator (core genome, constant)
Best match: spxA_CC001_MW2-USA400_BA000033.2[970804:971199] (completely identical)
Position: 025-contig_192_RC: 95159 … 95555; Length: 396 bp
Sequence:

ATGGTAACATTATTTACTTCACCAAGTTGCACATCTTGCCGTAAAGCGAAAGCATGGTTA
CAAGAACATGACATTCCGTATACGGAGCGTAATATTTTTTCTGAACATTTAACAATTGAT
GAAATTAAGCAAATATTAAAAATGACTGAAGACGGTACTGATGAAATCATTTCTACACGT
TCTAAAACATACCAAAAATTAAATGTTGATATTGATTCACTACCATTACAAGACTTATATT
CAATCATTCAAGATAATCCTGGCTTATTACGTCGTCCAATTATTTTAGATAATAAACGACT
ACAAGTTGGTTATAATGAGGACGAGATTCGACGTTTCTTACCTAGAAAAGTTCGTACGTT
CCAATTACAAGAAGCACAACGTATGGTTGACTAA

## > **mecA2**

Function:
Best match: mecA2_CC001_MW2-USA400_BA000033.2[971570:972289] (completely identical)
Position: 025-contig_192_RC: 95925 … 96645; Length: 720 bp
Sequence:
ATGAGAATAGAACGAGTAGATGATACAACTGTAAAATTGTTTATAACATATAGCGATATC
GAGGCCCGTGGATTTAGTCGTGAAGATTTATGGACAAATCGCAAACGTGGCGAAGAATTC
TTTTGGTCAATGATGGATGAAATTAACGAAGAAGAAGATTTTGTTGTAGAAGGTCCATTA
TGGATTCAAGTACATGCCTTTGAAAAAGGTGTCGAAGTCACAATTTCTAAATCTAAAAAT
GAAGATATGATGAATATGTCTGATGATGATGCAACTGATCAATTTGATGAACAAGTTCAA
GAATTGTTAGCTCAAACATTAGAAGGTGAAGATCAATTAGAAGAATTATTCGAGCAACG
AACAAAAGAAAAAGAAGCTCAAGGTTCTAAACGTCAAAAGTCTTCAGCACGTAAAAATA
CAAGAACAATTATTGTGAAATTTAACGATTTAGAAGATGTTATTAATTATGCATATCATA
GCAATCCAATAACTACAGAGTTTGAAGATTTGTTATATATGGTTGATGGTACTTATTATTA
TGCTGTACATTTTGATAGTCATGTTGATCAAGAAGTCATTAATGATAGTTACAGTCAATTG
CTTGAATTTGCTTATCCAACAGACAGAACAGAAGTTTATTTAAATGACTATGCTAAAATA
ATTATGAGTCATAACGTAACAGCTCAAGTTCGACGTTATTTTCCAGAGACAACTGAATAA

## > **trfB**

Function: protein coding sequence; putative competence protein (core genome, variable)
Best match: trfB_CC001-ST772_118_AJGE01000009.1[96519:97505] (completely identical)
Position: 025-contig_192_RC: 96765 … 97752; Length: 987 bp
Sequence:
ATGTTAGTAGCTTTAAATGAAGAAAAGGAACGTGTGTTAGCAACTACTGCATTGAGAAAG
ACACAATATTTTTGTCCGGTGTGTGGCAAGCAAGTTATTTTAAAGCGTGGGCTCAAAGTA
ATTAGTCATTTTGCACATAAACATTTAGCGGAACAAAAATGTTTTAATAATGAAACGATT
AAACATTATAAAAGTAAATTGATTTTAGCACAGATGATACAGCAACAAGGATTTAAAGTA
GAGATAGAGCCATTTTTAAAAGAAATAAAACAAATTCCGGATATTTTGATTAATAATAAA
TATGTTATTGAGCTACAGTATTCGCCAATTCCTTATAAACAGATTCTTCAACGAACGGAA
GGTTTAAAGAAAATGGGATATAAAGTAAGTTGGTTATTAAATGATGTTGATTATTGTCAT
AATAAAGTGAAGTTCAATCATTTTCAAAGTATGTTTATTAATCCATTCACTCGAAAACTTC
ATACGTTCAATTTAGAGAAAAAACAAATAATGATGTTTCAACAAATACAATATTTAGGCG
GGCACAAATATGTCGCTGAAAAAAGAAATGCCAAAATTAGTGAGTTGTTTAATGAGGCG
CCTTGTGATTATCATGCTGTTTATAAATTATCAAAGTTCGCAATTAATCAATATATCAAAT
ATTGTCGCTGGCAAAATTCTGTTTTAGAACCCACTTTAAGTGCAATGTATCAATTACAGTT
AACTGATCAAGAAGTAGTGCACAATTATGGTTATATTTTTCCAGAGCAAATTTATATTAA
AAATCATCCTATTGAGTGGCAATTACAAGTTGATTTATGGTTAAAGAATGGAAAAAGCAA
ATTAGTAAATGACAATCTTAATTATTTTAAACTGAAAAAATTTATTGTTGCTCTAGAAAGT
AAAACAGCAATTATAGAAAAACTTATTAACAATTATTTAAATATTTGTTCAGATAAAGGT
AATGACGTGCAAATTTTGTTCTAA

## > **pepF1**

Function: protein coding sequence; oligoendopeptidase F (core genome, constant)
Best match: pepF1_CC005_JH1_CP000736.1[1052488:1054296]
Position: 025-contig_192_RC: 97799 … 99608; Length: 1809 bp
Sequence:

ATGAGTCAACAATTATCGAGAGAAGAACAGGAACGTAAATATCCTGAATATACATGGGA
CTTAACAACAATTTTCAAAGATGATGAAGCTTTTGAGGCTGCATTTAAAGAAGTTGAAAA
TGAGTTAGGCAAAGAAGAACAATTTAAAGGACACATTGGTGATAGTGCTGAGACATTAT
ACAATGCGTTAGAATTAGAAGATACATTAGGTACTAAATTAGAAAAAGTATATGTATACG
CGCACCTAAAACAAGACCAAGATACAACGAACGACAAGTATACTGGTATGGAGTCAAGA
GCACATCAATTAATTATTAAATTTAGCTCGGCATGGAGTTTCTTAGTGCCAGAGATTTTAC
AAATTGATGAAGATAAAATTCAATCATTTGTAAATTCATATGATAAATTACAAAAATTCG
CATTTGATTTGAAGTTGATTAATGAAAAACGTCCTCATATTTTAGATGCTGAAACTGAAA
AGTTATTAACAGAAGCGCAGGACGCGTTATCAACGCCATCAAATGTATACGGTATGTTTA
GCAACGCTGATTTAGTATTTGAAGATGCGATTGATAAAGATGGAAATGCACACCCGTTAA
CACAAGGTACATTTATTAAGTATTTAGAATCAGATGATCGCAAACTAAGAGAAAGTGCTT
TTAGAAATGTATATAAAGCATATGGTGCTCATAATAATACGCTTGGCGCTACGCTAGCAG
GTGAAGTGAAGAAAAATGTATTTAATGCTCGTACACACAATTACAAAACTGCAAGAGAA
AAAGCATTGAGTAATAATCATATTCCAGAAAATGTATATGACAATCTAGTAAAAACTGTA
CATAAATATTTACCATTGCTACATAGATATACTGAATTGCGCAAAGAATTGCTAGGTTTA
GATGACTTGAAAATGTATGATTTATATACACCATTAATTAAAGATATTAAGTTTGAAATG
CCTTATGAAGAAGCTAAAGAGTGGATGTTAAAAGCTTTAGAACCAATGGGTGAAGAATA
TTTAAATGTAGTTAAAGAAGGCTTAAACAATCGTTGGGTCGATGTCTATGAGAATAAAGG
TAAACGTTCAGGTGGCTATTCATCAGGTGCACATTTAACTAATCCATTTATTCTACTTAAC
TGGTCTAATACTATTTCAGACTTATACACATTAGTTCATGAATTTGGGCATTCAGCACATA
GTTACTTCAGTAGAAAATTCCAACCGTCAAATTCTAGTGACTACACTATTTTTGTCGCTGA
AGTTGCATCAACTTGTAACGAAGCACTTTTAAGTGATTATATGGATAAACATCTTGATGA
TGAAAAACGCTTATTATTATTAAACCAAGAATTAGAACGTTTCAGAGCTACATTATTCCG
ACAAACAATGTTCGCAGAATTTGAGCATAAAATTCATGCAATTGAAGAAGCAGGTGAAC
CATTAACGCCAACTAGAATGAATGAAGAATATGCCAAATTAAATAAATTATACTTCGGTG
ATTCTGTAGAAACTGATGAAGATATTAGTAAGGAATGGTCACGTATTCCACACTTCTATA
TGAATTATTATGTATATCAATACGCAACTGGTTACAGTGCAGCTCAAAGCTTAAGTCATC
AAATTTTAACAGAAGGTAAGCCAGCAGTAGATAGATATATTAATGAATTCTTGAAAAAA
GGTAGCTCAAATTATCCAATTGAGATATTAAAAAATGCTGGTGTAGATATGACAACACCT
GAACCAATTGAACAAGCTTGTGAAGTTTTTGAACAAAAATTGAACGCTTTTGAAAAATTA
ATGAAAGCTTAG

> **rsaE**
Function: noncoding RNA ncRNA of Staphylococcus aureus E (core genome, constant)
Best match: rsaE_CC001_MW2-USA400_BA000033.2[975352:975451] (completely identical)
Position: 025-contig_192_RC: 99707 … 99807; Length: 100 bp
Sequence:
ATGAAATTAATCACATAACAAACATACCCCTTTGTTTGAAGTGAAAAATTTCTCCCATCCC
CTTTGTTTAGCGTCGTGTATTCAGACACGACGTTTTTTT

> **rsaF**
Function: noncoding RNA ncRNA of Staphylococcus aureus F (core genome, constant)
Best match: rsaF_CC008_COL_CP000046.1[1015068:1015171] (completely identical)
Position: 025-contig_192_RC: 99785 … 99889; Length: 104 bp
Sequence:
TATTCAGACACGACGTTTTTTTATGTTCTTTTTTAATAAGAGGGAAGTGAAGTTGTATAAA
AATGTATTAAATATGCACTTTTTATACATTAACGATTCATATA

> **ltaB**
Function: protein coding sequence; dithiol-disulfide isomerase (core genome, constant)
Best match: ltaB_CC001_MW2-USA400_BA000033.2[975712:976518:r]RC (completely identical)
Position: 025-contig_192_RC: 100067 … 100874; Length: 807 bp
Sequence:
TTATTTTGATTTGATTTTAGGCATTTTAGATTTCCAGAAATCGCCATCTGGATATTTAAGTT
TTTCAATTTTTTGTTGAATGGCTAACTTCTTTAACTCTTTGTTTAAAAGTTTTTCTGGCCAT

TCATAAATAGTAAGTAATTCTTCCATCGTTACAAGTTGTTGTTGCTGTATATAAGTTTCTA
ATTTAGGAGGAAGATTCTTTTCGATAGGTTTACCCATCAATTCATTAATTATATAAGTATA
GATGTGATATGGGTATAATCCTTCGACTTTTAAACCTTCTTCATGAACATCTTCACTAAAG
AAAACGAGAGAAGGGGCTTGTTCGATTTCCATTTCTCTTGCAATATGCAAATCAATTTTCA
AGCTTTCGGTTAGTTTACTTTTTTGTAAGTCGTCTTTAAATACTTCTAAATCAATACCTGCA
TTTTGAATACAGTCACAAATCATTGATTCTGTAATAATATCTCTTTTAGGTATGATTTCATT
TTGCATTAAATGTATAAATCGTTCGGCACGTACACGACCTTGTAACTCAGCTGCTTTATAA
GCTAGGGCGATGTTATCAAAGTTGGATGTACTTTGAGCTTGGCATTTCGTTAATACTTTTA
ACGAAGGATTTAATATATGTCTGATACGTATATATTGATTATATTCAATTCTTAATTTGGA
TAAGATTGCTGATAATTTGAAGCAATCGGAGCTAAATGGATCGAAAAATGAATAAATTTC
GATTTTACTTACAGGTGATAGATTAATATCTTCACGACTCTTATTTTCCATTATTCGTAATT
CTCCAGCCAT

> **relA1**

Function: protein coding sequence; GTP pyrophosphokinase (core genome, constant)
Best match: relA1_CC005_N315_BA000018.3[978184:978819] (completely identical)
Position: 025-contig_192_RC: 102508 … 103144; Length: 636 bp
Sequence:
ATGAATCAATGGGATCAGTTCTTAACACCTTATAAGCAAGCGGTTGATGAGTTGAAAGTG
AAACTTAAAGGCATGCGCAAACAATATGAAGTTGGTGAACAAGCGTCGCCAATAGAATT
TGTTACTGGTCGTGTTAAACCGATCGCTAGTATTATAGATAAGGCAAACAAACGACAAAT
ACCATTTGATAGGTTAAGAGAAGAAATGTACGATATCGCTGGTTTAAGAATGATGTGCCA
ATTTGTAGAAGATATTGATGTTGTCGTCAATATTTTAAGACAAAGAAAAGATTTTAAAGT
AATTGAAGAACGAGATTATATTCGTAACACTAAAGAAAGTGGTTACCGCTCGTATCATGT
CATTATTGAATATCCAATTGAAACATTACAAGGCCAAAAATTTATATTGGCTGAGATTCA
GATTCGTACATTAGCAATGAATTTCTGGGCAACGATTGAACATACTTTACGATATAAATA
TGATGGTGCTTATCCGGATGAAATTCAACATCGTTTGGAAAGAGCGGCAGAAGCAGCGTA
TTTACTTGATGAAGAGATGTCTGAAATTAAAGATGAAATTCAGGAAGCTCAAAAATATTA
CACGCAAAAACGTTCTAAAAAACATGAAAATGATTAA

> **ppnK**

Function: protein coding sequence; putative inorganic polyphosphate/ATP-NAD kinase (core genome, constant)
Best match: ppnK_CC005_Mu50-VRSA_BA000017.4[1055120:1055929] (completely identical)
Position: 025-contig_192_RC: 103160 … 103970; Length: 810 bp
Sequence:
ATGCGTTATACAATTTTAACTAAAGGTGACTCCAAGTCTAATGCCTTAAAGCATAAAATG
ATGAACTATATGAAAGATTTTCGCATGATTGAGGATAGTGAAAATCCTGAAATTGTTATT
TCAGTTGGTGGTGATGGTACATTACTACAAGCATTCCATCAGTATAGCCACATGTTATCA
AAAGTGGCATTTGTTGGAGTTCATACAGGTCATTTAGGATTTTATGCGGATTGGTTACCTC
ATGAAGTTGAAAAATTAATCATCGAAATTAATAATTCAGAGTTTCAGGTCATTGAATATC
CATTGCTTGAAATTATTATGAGATACAACGACAACGGCTATGAAACAAGGTATTTAGCAT
TAAATGAAGCAACGATGAAAACTGAAAATGGCTCAACACTTGTTGTGGATGTTAACTTAA
GAGGGAAACACTTTGAGCGATTTAGAGGCGATGGATTATGTGTATCAACACCTTCGGGTT
CAACGGCTTATAACAAAGCGCTAGGTGGCGCACTGATACATCCTTCACTTGAAGCAATGC
AAATTACAGAAATTGCCTCAATAAATAATCGTGTGTTTAGAACGGTAGGATCACCACTTG
TATTACCAAAGCATCATACATGTTTAATATCACCAGTTAATCATGATACCATTAGAATGA
CGATAGATCATGTTAGTATCAAACATAAAAATGTTAATTCAATACAATACCGTGTAGCAA
ATGAAAAAGTGAGGTTTGCACGTTTTAGACCATTCCCGTTCTGGAAACGTGTACACGATT
CTTTCATATCAAGTGATGAAGAACGATGA

> **rluA1**

Function: protein coding sequence; RNA pseudouridine synthase (core genome, constant)
Best match: rluA1_CC005_04-02981_CP001844.2[1014383:1015237]
Position: 025-contig_192_RC: 103966 … 104821; Length: 855 bp

Sequence:
ATGAAATTTAAGTATCATATATCACAACAAGAAACTGTTAAAGCTTTTTTAGCACGACAT
GATTTTTCTAAGAAGACAGTGAGCGCCATTAAAAATAATGGCGCTTTAATTGTTAATGAT
GAACCAGTCACAGTACGTAAGCAATTAATGCCAAATGATATATTAGAAATTCATTTACCG
CGAGAAATACCGAGTGTTAATTTAATACCTTATGCTCGTAAGCTAGAAGTATTGTATGAA
GATGCTTTTATCATCATAGTTACTAAACCAAACAATCAAAATTGTACGCCTTCGAGAGAA
CATCCTCATGAAAGTTTAATCGAACAAGTACTATATCATTGTCAGGAACATGGTGAAAAT
ATTAACCCACATATTGTTACGCGTCTAGATCGTAATACAACTGGTATTGTGATATTCGCTA
AATATGGACATATCCATCATTTATTTCTAAAGTAAACTTGAAAAAAATATATACTTGCCT
TGTATATGGCAAAACCCATACATCTGGTATTATTGAAGCTAATATTAGACGGTCAAAGGA
TAGTATTGTAACTAGAGAAGTTGCCTCGGATGGTAAATACGCTAAAACATCTTATGAAGT
AATAAATCAGAATGATAAATACAGTTTATGCAAAGTTCATTTGCATACGGGACGTACACA
TCAAATTCGTGTACATTTTCAACATATTGGGCATCCAATTGTGGGAGATTCTTTGTATGAT
GGTTTTCATGACAAAATTCATGGTCAAGTACTGCAATGTACGCAAATATATTTTGTTCATC
CAATCAATAAGAACAATATTTATATTACAATTGATTATAAGCAATTACTTAAATTATTCAA
TCAACTCTAA

## > **mgtE**

Function: protein coding sequence; magnesium transporter E (core genome, variable)
Best match: mgtE_CC005_Mu50-VRSA_BA000017.4[1056801:1058186] (completely identical)
Position: 025-contig_192_RC: 104841 … 106227; Length: 1386 bp
Sequence:
ATGTCAATGAACACAGATGAAAAAGAGCGTGTTCAAGAGGAATTATATGATGAGACATT
ATTAGATCAATACTTAGAAAATGATGATATTGATCAATTTAGAGATGAATTTCTAGCATT
ACACACATATGAACAAAGTGAGTATTTTGAAGATACTACCGATGAAAATAGACAAAAGA
TTTTTCAATATTTATCACCTGAAGAGGTTGCAAATTTCTTTGATCAATTAGATATTGATGA
CGATGAATATGAGTTGCTATTTGATAAAATGAATGCGACATACGCAAGTCACATATTAGA
AGAAATGTCATACGATAATGCAGTAGATATTTTAAATGAGTTGACTAAACCAAAAGTTGC
TAGTCTTTTAACATTGATGAATAAAGATGACGCGAATGAAATCAAAGCATTACTTCACTA
TGATGAGGATACGGCCGGCGGTATTATGACGACGGAGTATTTATCACTTAAAGCGCATAC
GCCTGTTAAAGAAGCGTTATTATTGGTCAAAGCGCAAGCGCCAGACGCAGAAACAATAT
ATGTTATATTTGTCGTTGATGATGATGGTAAATTAGTAGGTGTTTTATCACTAAGAGATTT
AATTGTAGCTGAAAATGATGCTTATATTGAAGATATTATGAATGAACGTGTCATTAGTGT
GAATGTAGCAGACGACCAAGAAGATGTTGCTCAAGTTATGAGAGACTATGATTTCATGGC
TGTACCTGTTATAGATTACCAAGAACATTTGCTTGGTATCATCACGATTGATGATATTTTA
GACGTTATGGATGAAGAGGCTAGTGAAGACTACTCTCGTTTAGCCGGGGTATCAGATATC
GATTCGACTAATGATTCAATCATTAAAACAGCATTAAAACGTTTACCATGGTTGATTATTT
TAACATTTTTAGGAATGATTACTGCGACAATTTTAGGGAGATTTGAAAAAACATTAGAAA
ATGTAGCGCTACTCGCAGCGTTTATTCCTATTATTAGTGGTATGTCAGGAAATTCAGGTAC
ACAATCTTTAGCCGTTTCAGTTCGTAACATTACGACAGGGGAAATTAATGAGCAAAGTAA
ATTTAGAATTGCATTAAGAGAAGCGGGAAGTGGTGTATTATCGGGCGTTGTATGTTCAAC
AATATTATTTACAATTATTGTTGCAATATATCATCAGCCACTTTTAGCATTAATCGTTGCA
GGAAGTTTAACTTGTGCGATGACGGTGGGGACGTTTGTAGGTTCGATGATTCCATTATTG
ATGAATAAATTAAATATCGATCCAGCAGTGGCTAGTGGACCATTTATTACAACAATTAAT
GATATTATTAGTATGTTGATTTATTTTGGTTTAGCTACATCATTTATGGCTTACTTAATTTA
A

## > **Q8NXC6**

Function: protein coding sequence; monovalent cation-H+ antiporter (core genome, variable)
Best match: Q8NXC6_CC005_Mu50-VRSA_BA000017.4[1058196:1060040] (completely identical)
Position: 025-contig_192_RC: 106236 … 108081; Length: 1845 bp
Sequence:
ATGGAGTTTTTATCTTTAGTTATTGTTGTTTTAGCAGCGTTTTTAACTCCAATAATTGTCAA
TCGATTAAATATTAATTTCTTGCCAGTTGTTGTTGCAGAAATTTTGATGGGGATTGTGATT
GGAAATTCATTTCTAAATATAGTAGAAAGGGATTCAATTCTAAATATTTTATCAACGTTA

GGCTTTATCTTTTTAATGTTTTTAAGTGGTTTAGAAATTGATTTTAAAGCTTTTAAAAAAG
ATAAACGCGCACGTCAAGGACAAAATGATGATGAATCCTCAATTCCAGGGCATCTTAATC
TAGCGTTAACTGTATTTGCATTTATTATGATTATTTCGATTCTTTTAGCGTATGTATTTAAA
TGGCTTGGATTAGTGGATGATGTGTTATTAATGGTCATTATCATTTCAACTATTTCCTTAG
GCGTAGTTGTTCCAACTTTAAAAGAAATGAATATTATGAGAACAACTATAGGGCAATTTA
TCCTATTAGTAGCAGTACTTGCGGACTTAGTAACTATGATTTTATTAACGGTCTATGGCGC
AATCAATGGTCAAGGCGGCAGTACAATATGGTTAATAGGTATATTAGTTGTTTTCACAGC
AATTTCATATATTTTAGGTGTTCAATTTAAAAGAATGTCATTTTTACAAAAATTGATGGAT
GGTACGACGCAAATCGGTATTCGTGCGGTATTTGCATTAATAATATTATTAGTAGCCCTA
GCAGAGGGAGTTGGCGCAGAAAATATATTAGGTGCATTCTTAGCAGGTGTCGTTGTTTCA
TTATTAAATCCAGATGAAGAAATGGTTGAAAAGTTAGACTCATTTGGTTATGGGTTCTTTA
TTCCTATTTTCTTTATAATGGTTGGTGTAGATTTAAACATACCTTCATTAATTAAAGAACC
GAAATTACTAATTATCATACCGATTTTAATCGTTGCATTTATCATTTCAAAATTAATTCCA
GTCATGTTTATTCGACGTTGGTTTGATATGAAAACAACGATTGCATCAGCATTTTTATTAA
CATCAACATTATCGCTCGTGATAGCTGCAGCCAAAATTTCAGAAAGATTAAATGCTATTT
CAGCTGAAACGTCAGGTATATTAATTTTAAGCGCAGTCATTACATGTGTATTCGTTCCGAT
TATTTTCAAAAAACTGTTTCCAGTTCCAGATGAGTTTAACCGTAAAATTGAAGTTAGTTTA
ATTGGTAAAAATCAATTAACGATTCCTATAGCGCAAAATTTAACATCTCAGTTATATGAC
GTGACATTATATTATCGCAAAGACTTGAGTGATCGTCGTCAATTGTCAGATGATATCACG
ATGATAGAAATTGCTGATTATGAACAAGATGTTTTAGAACGACTAGGTCTGTTTGACCGA
GACATAGTTGTTTGTGCTACGAATGACGATGATATTAACCGAAAAGTTGCTAAATTAGCC
AAAGCACATCAAGTTGAGCGTGTCATTTGCAGACTTGAAAGCACAACGGACGATACAGA
GTTAGTTGATTCAGGTATTGAAATTTTCAGTAGCTACTTAAGTAATAAAATCTTATTAAAA
GGTTTAATTGAAACACCTAACATGTTGAATTTATTAAGTAATGTTGAAACGTCACTATATG
AAATTCAAATGTTAAATTATAAATATGAAAATATTCAATTACGTAATTTCCCATTCGGAG
GAGACATCATCTTCGTGCGTATTATCCGTAATAATGAGTCGATTGTTCCGCATGGAGATA
CACAATTGCGATATGGAGATCGCTTAATTGTTACCGGTGCTAAAGAATACGTTGATGAAT
TGAAGCAAGAGTTAGAATTTTATTTTTAA

> **fabI**

Function: protein coding sequence; trans-2-enoyl-ACP reductase (core genome, constant)
Best match: fabI_CC005_Mu50-VRSA_BA000017.4[1060318:1061088] (completely identical)
Position: 025-contig_192_RC: 108358 … 109129; Length: 771 bp
Sequence:
ATGTTAAATCTTGAAAACAAAACATATGTCATCATGGGAATCGCTAATAAGCGTAGTATT
GCTTTTGGTGTCGCTAAAGTTTTAGATCAATTAGGTGCTAAATTAGTATTTACTTACCGTA
AAGAACGTAGCCGTAAAGAGCTTGAAAAATTATTAGAACAATTAAATCAACCAGAAGCG
CACTTATATCAAATTGATGTTCAAAGCGATGAAGAGGTTATTAATGGTTTTGAGCAAATT
GGTAAAGATGTTGGCAATATTGATGGTGTATATCATTCAATCGCATTTGCTAATATGGAA
GACTTACGCGGACGCTTTTCTGAAACTTCACGTGAAGGCTTCTTGTTAGCTCAAGACATTA
GTTCTTACTCATTAACAATTGTGGCTCATGAAGCTAAAAAATTAATGCCAGAAGGTGGTA
GCATTGTTGCAACAACATATTTAGGTGGCGAATTCGCAGTTCAAAATTATAATGTGATGG
GTGTTGCTAAAGCGAGCTTAGAAGCAAATGTTAAATATTTAGCATTAGACTTAGGTCCTG
ATAATATTCGCGTTAATGCAATTTCAGCTGGTCCAATCCGTACATTAAGTGCAAAAGGTG
TGGGTGGTTTCAATACAATTCTTAAAGAAATCGAAGAGCGTGCACCTTTAAAACGTAACG
TTGATCAAGTAGAAGTAGGTAAAACAGCGGCTTACTTATTAAGTGACTTATCAAGTGGCG
TTACAGGTGAAAATATTCATGTAGATAGCGGATTCCACGCAATTAAATAA

> **yrbD**

Function: protein coding sequence; alanine or glycine carrier protein (core genome, constant)
Best match: yrbD_CC005_Mu50-VRSA_BA000017.4[1062712:1064280] (completely identical)
Position: 025-contig_192_RC: 110752 … 112321; Length: 1569 bp
Sequence:
ATGATTGAAAAATTAGTAACCTTTTTAAATGAGGTTGTTTGGAGTAAGCCATTAGTTTATG
GTTTGCTAATTACTGGTGTGCTATTTACATTGCGTATGCGATTTTTTCAAGTTAGACATTTT

AAAGAAATGATTCGATTAATGTTTCAAGGAGAGAAGTCTCCTAATGGTATTTCAAGTTTT
CAAGCGATAGCCATGTCTTTAGCAGGCAGGGTTGGTACAGGTAATATTGTCGGTGTATCT
ACTGCAATATTTATAGGAGGACCTGGTGCAGTATTTTGGATGTGGATTACTGCGTTTTTAG
GTGCAAGTAGTGCTTTTATTGAATCTACACTTGGTCAAATATTCAAGAGAGTTGAAAATA
ATGAATACCGTGGTGGACCAGCGTATTATATTGAATATGGTATTGGTGGTAAATTTGGTA
AAATTTACGGAATTATCTTTGCTATTGTTACGATTATCTCAGTAGGTCTATTGCTTCCTGGT
GTGCAATCTAACGCTATAGCAAGTTCTATGCATAATGCGATTCATGTTCCACAATGGTTA
ATGGGTGCTATTGTTGTAGTTATTTTGGGATTAATTATTTTTGGTGGTGTACGTAGTATTG
CCAATGTTGCAACAGCCGTTGTACCATTTATGGCAATTATTTACATACTGATGGCTGTCAT
TATCATTTGTATCAATATACAAGAAGTGCCAGCGTTATTTGCATTAATTTTCAAATCAGCA
TTTGGATTACAATCTGCTTTTGGTGGTATCGTTGGCGCAATGATAGAGATTGGTGTTAAAC
GTGGATTATACTCAAATGAGGCTGGTCAAGGTACAGGTCCACACGCAGCAGCGGCAGCA
GAAGTATCACATCCAAGTAAACAAGGTCTAGTACAAGCATTTTCAGTTTATATTGATACA
TTATTTGTATGTACTGCAACTGCTCTGATTATACTTATTTCTGGTACATATAATGTGACTG
ATGGTACGGTTAATGCGAATGGCACACCGCATTTAATTAAAGATGGCGGTATTTATGTTG
AAAATGCAACAGGTAAAGATTATTCAGGTACTGCGATGTATGCACAAGCCGGCATTGATA
AAGCGTTCCATGGCAGTGGTTATCAATTTGATCCTACTTTCTCTGGCGTAGGTTCGTACTT
TATTGCATTTGCTTTATTCTTCTTTGCATTTACTACAATTTTGTCGTACTACTACATTACAG
AAACAAATGTTGCTTATTTAACGCGTAATCAAAATAATCAAGTTTCATCGATATTTATTAA
TATTGCTCGTGTGATTATTTTGTTCGCTACATTTTACGGTGCAGTTAAAACAGCTGATGTA
GCATGGGCATTCGGTGATTTAGGTGTAGGTCTAATGGCTTGGTTAAATATCATTGCGATTT
GGATTTTACATAAGCCTGCCGTAAATGCTTTAAAAGATTATGAAATTCAAAAGAAACGTT
TAGGAAACGGTTATAATGCAGTTTATCAACCTGATCCGAATAAATTACCTAATGCTGTCTT
TTGGTTGAAGACATATCCAGAACGTTTAAAACAAGCACGTGCCAAAAAGTAA

## > Q2FI63
Function: protein coding sequence; putative esterase (core genome, variable)
Best match: Q2FI63_CC001_MW2-USA400_BA000033.2[988108:988866] (completely identical)
Position: 025-contig_192_RC: 112465 … 113224; Length: 759 bp
Sequence:
ATGTCAGAATTTAAAACTGGTAAGATTAATAAACATGTTTTATATAGTAATATTTTAAATA
GAGATGTCACGATAAGTATTTATTTACCAGAATCTTATAATCAACTTGTTAAATATAATGT
CATTCTTTGCTTTGACGGATTAGATTTTTTACGTTTCGGGAGAATACAACGTACATATGAA
TCGTTAATCAAAGAAGCGCGTATTGATGATGCGATCATTGTTGGATTCCATTATGAAGAC
GTTGATAAGCGTAGAGAGGAATTTCATCCACAAGGAAGTCGTTCTCATTTAACTATTCAA
TCAGTCGGTAAAGAAATATTGCCATTTATTGACTCGACGTTTTCTACACTGAAAGTAGGT
AATGCAAGGTTATTAGTAGGGGATAGTTTAGCGGGTAGTATTGCCTTATTAACGGCGTTG
ACCTATCCAACGATTTTTAGTCGTGTAGCAATGTTAAGTCCACATTCAGATGACAAAGTA
TTAGATAAGCTAAATCAATGTGCTAATAAAGAACAATTGACAATTTGGCATGTCATTGGT
CTAGATGAAAAAGATTTTACTTTACCAACAAATGGTAAGCGTGCCGATTTCTTAACACCG
AATAGAGAATTAGCTGAACAAATTAAGAAATATAATATAACTTATTATTACGATGAATTT
GAAGGTGGTCACCAATGGAAAGATTGGAAACCATTGCTGTCAGATATATTATTGTATTTT
TTACGTAAAAACACAGATGATCAACATTATGAGTAA

## > ltaA
Function: protein coding sequence; glycolipid permease (core genome, variable)
Best match: ltaA_CC005_Mu50-VRSA_BA000017.4[1065999:1067189:r]RC (completely identical)
Position: 025-contig_192_RC: 114039 … 115230; Length: 1191 bp
Sequence:
TTACTTAGCTTTTTCTCTATTTGCTATAAAGTAGCTTCCATAAAATACAGCTAAGACTAAA
AAGATTAATGCCGAGAAATAAAATGTATTGTTTAAATCGTTGGTAAATTGTGTAATTAAT
CCGCCAAATAATGGCCCTATCATTGAGCCGAATCCTTGGATACTATTAAAAACACCCCAA
GTTTCTTCTTGTTCATCTGATTTGATAAATCGTGCCATAAAGGTATTCCATGCTGGTAATA
AGATGCCATACATTAGACCGATAGCTAAAGCGATAATCCACACGATGTGAATATTAACAA
TCATAGATAGAGTAAAAATTAATATCATGTATAAAATAAATCCGCTTAGAATAACACCAT

ACATAAAGTTTCTGCTGCGGTTATCTATTAGTTTCGATAAAAATAGCATCGAAACTGCAC
AGCCGATACCACCAATAATGATTGCAACAGTATATTCAATTGTGCTTACGTTAATAACCTT
AGTAGCATATGTTGGTAATATAGGAACTAGGGCAGCAATTGCGGCACCTTGTAAAAGAAT
ACCAGGGAACAACAATAAATGGCGCTTTGTCACATCAACAATTTGTCTCAATTGAGCTTT
AACTGGACGAGTATTATAATTTGTTAACTTTACATCGACAAAATAATATAATATCCATGC
AATTAAAACGACTAAAGACATCATGAAGGCAAAGCGTGTTGGGTGCACTTTGATAAGTA
GATTCATAAAAACCATACCTACCAATAGGCCTAACAACCATGAAAAATAAACATAGCCC
ATTTGTTTGCCACGTTTATCTTCTTCAACACTGGATAACATAATGACCCAAATAGGACTAA
CTGCAATACCGAGCATCATAGCACTAAATATGATTACAAAAGGTGATGCTGGAAACCAA
ATAACTAAAAATAAACTTGTAAATGCTAAAATAAATCCAGTCGTTAAAACGATTTTTGTG
CCGAATTTTTTCAGTAAAAATCCTATAACAAAGTTTGTAGATGCATCAGCAATAAAATGT
ATTGAAAATGCTAGAGACGTTATTGCTACAGCAATGGATGTAACTGTTGGCAAGAAATTA
ATATAGCTTAGGATATACATGCCTCTCGCAAATTCCATTAAAAATAAGATAATAAGCATT
AAAATGAAATTTTTATGATTAGCGTAATTATTTAACGAAGAATCTTGCAT

> **ugtP**

Function: protein coding sequence; processive diacylglycerol glucosyltransferase (core genome, constant)
Best match: ugtP_CC005_Mu50-VRSA_BA000017.4[1067167:1068342:r]RC (completely identical)
Position: 025-contig_192_RC: 115207 … 116383; Length: 1176 bp
Sequence:
TTATTTAACGAAGAATCTTGCATATAAAGGAACCTTTCCATAAATCTCTTGTGGTTGTGAT
GAATGACCGATTAAATCAAGTAAGTCTCGACATATTGTCTGTGTAGCATACTTAATTTTAT
CTTGTTCCATTGTACTAATCATGTTAGTTAATTGCTCATTACCGTTCGTTAAACTTGCTACA
ATTTTTATTGCTTCTTCTGGAGTATCAGCGATTTTACCAAAACCTTTTTCTTCAAAGTAAA
GGGCATTTTCAAGCTCTTGACCAGGTGCAGGATTTAGGAAAATCATTGGAATACAACGGG
CGAAACCTTCAGTTATTGTGATACCACCAGGTTTCGTAATCATAAGTTGACTTGATGCCAT
CCATTCATTCATGTGTTTGGTATAACCTAGAATCAATACATTCTCGTTAGATTTAAACTTA
GCTATTAAAGAACGCTTTAGCTCTTTGCTCTTACCACAAATCATAACTACTTGTGCATTTG
CACTTTTCGCTAATATATCAGTAATCATCGTGTCAAAACCTTTAGATACACCAAATGCACC
AGCTGACATTAAAATAGTTTGCTTATCTGGATCTAAGTTGTTGTCTATTAACCACTGCTTT
TGATTAATAGGCGTTTCAAATTTGTTATCAATAGGAATACCTGTCACTTTAACTGTTGAAG
GATCAATACCTACGTCTATGAAGTCTTGTTTCGTTTCTTTTGTTGCCACATAATATCTTGTT
GAATACGGCGTAATCCAGTTTTTATGTAAGCGATAGTCTGTCATCACTGTAGCAACTGGA
ATATTAATGTTAAATTGCTCAGTTAGTACCGACATAACTGGTGTAGGAAACGTTAATAAT
ATTAAATCTGGCTTTTCTTTTATCAATAAATTAATTAACTTATTAAGTCCATAGTATTTGTA
AAAACATTTGTCTAGTTTATCTGGGCGGCTGTAATAAAACCCTTTGTACATATTTCTAAAA
TATTTAAAGCTATTGATATACCATTTTTTACAAATAGAAGTCAAAATTGGATGAGCTTCCA
TAAATAAATCGTGCTCAATGACGCTTAAATGGTCTAGATTCATATCATTAAGTTGATTAAC
GATACTCTGTGTAACTTGCATATGACCGTTACCGAATGAGCCAGTAATAATCAATATCTTT
TTATTTTGAGTAACCAT

> **murE**

Function: protein coding sequence; UDP-N-acetylmuramoylalanyl-D-glutamate--2,6-diaminopimelate ligase (core genome, constant)
Best match: murE_CC005_Mu50-VRSA_BA000017.4[1068775:1070259] (completely identical)
Position: 025-contig_192_RC: 116815 … 118300; Length: 1485 bp
Sequence:
TTGGATGCAAGTACGTTGTTTAAGAAAGTAAAAGTAAAGCGTGTATTGGGTTCTTTAGAA
CAACAAATAGATGATATCACTACTGATTCACGTACAGCGAGAGAAGGTAGCATTTTTGTC
GCTTCAGTTGGATATACTGTAGACAGTCATAAGTTCTGTCAAAATGTAGCTGATCAAGGG
TGTAAGTTGGTAGTGGTCAATAAAGAACAATCATTACCAGCTAACGTAACACAAGTGGTT
GTGCCGGACACATTAAGAGTAGCTAGTATTCTAGCACACACATTATATGATTATCCGAGT
CATCAGTTAGTGACATTTGGTGTAACGGGTACAAATGGTAAAACTTCTATTGCGACGATG
ATTCATTTAATTCAAAGAAAGTTACAAAAAAATAGTGCATATTTAGGAACTAATGGTTTC

CAAATTAATGAAACAAAGACAAAAGGTGCAAATACGACACCAGAAACAGTTTCTTTAAC
TAAGAAAATTAAAGAAGCAGTTGATGCAGGCGCTGAATCTATGACATTAGAAGTATCAA
GCCATGGCTTAGTATTAGGACGACTGCGAGGCGTTGAATTTGACGTTGCAATATTTTCAA
ATTTAACACAAGACCATTTAGATTTTCATGGCACAATGGAAGCATACGGACACGCGAAGT
CTTTATTGTTTAGTCAATTAGGTGAAGATTTGTCGAAAGAAAAGTATGTCGTGTTAAACA
ATGACGATTCATTTTCTGAGTATTTAAGAACAGTGACGCCTTATGAAGTATTTAGTTATGG
AATTGATGAGGAAGCCCAATTTATGGCTAAAAATATTCAAGAATCTTTACAAGGTGTCAG
CTTTGATTTTGTAACGCCTTTTGGAACTTACCCAGTAAAATCGCCTTATGTTGGTAAGTTT
AATATTTCTAATATTATGGCGGCAATGATTGCGGTGTGGAGTAAAGGTACATCTTTAGAA
ACGATTATTAAAGCTGTTGAAAATTTAGAACCTGTTGAAGGGCGATTAGAAGTTTTAGAT
CCTTCGTTACCTATTGATTTAATTATCGATTATGCACATACAGCTGATGGTATGAACAAAT
TAATCGATGCAGTACAGCCTTTTGTAAAGCAAAAGTTGATATTTTTAGTTGGTATGGCAG
GCGAACGTGATTTAACTAAAACGCCTGAAATGGGGCGAGTTGCCTGTCGTGCAGATTATG
TCATTTTCACACCGGATAATCCGGCAAATGATGACCCGAAAATGTTAACGGCAGAATTAG
CCAAAGGTGCAACACATCAAAACTATATTGAATTTGATGATCGTGCAGAAGGGATAAAA
CATGCAATTGACATAGCTGAGCCTGGGGATACTGTCGTTTTAGCATCAAAAGGAAGAGAA
CCATATCAAATCATGCCAGGGCATATTAAGGTGCCACATCGAGATGATTTAATTGGCCTT
GAAGCAGCTTACAAAAAGTTCGGTGGTGGCCCTGTTGATCAATAA

## > **prfC**

Function: protein coding sequence; peptide chain release factor 3 (core genome, constant)
Best match: prfC_CC005_N315_BA000018.3[994216:995778] (completely identical)
Position: 025-contig_192_RC: 118540 … 120103; Length: 1563 bp
Sequence:
ATGAACTTAAAGCAAGAAGTTGAGTCTAGAAAGACTTTTGCGATTATTTCACATCCCGAT
GCAGGGAAAACAACGTTAACTGAAAAACTATTGTACTTCAGTGGTGCTATTCGTGAAGCG
GGTACAGTTAAAGGGAAGAAGACTGGTAAATTTGCGACAAGTGACTGGATGAAAGTTGA
ACAAGAGCGTGGTATTTCTGTAACTAGTTCAGTAATGCAATTTGATTACGATGATTATAA
AATCAATATCTTAGATACACCAGGACATGAAGACTTTTCAGAAGATACGTATAGAACATT
AATGGCAGTTGACAGTGCTGTCATGGTCATAGACTGTGCAAAAGGTATTGAACCACAAAC
ATTGAAGTTATTTAAAGTTTGTAAAATGCGTGGTATTCCAATCTTTACATTCATTAATAAA
TTAGACCGAGTAGGTAAAGAACCATTTGAATTATTAGATGAAATCGAAGAGACATTAAAT
ATTGAAACATACCCTATGAATTGGCCAATTGGTATGGGACAAAGTTTCTTTGGCATCATT
GATAGAAAGTCTAAAACAATTGAACCATTTAGAGATGAAGAAAATATATTACATTTGAAT
GATGATTTTGAGTTGGAAGAAGATCATGCAATTACAAATGATAGTGCTTTTGAACAAGCG
ATTGAAGAATTAATGTTGGTTGAAGAAGCGGGTGAAGCCTTTGATAATGACGCGCTGTTG
AGTGGAGACTTAACACCTGTATTTTTCGGTTCAGCTTTAGCTAACTTTGGTGTACAAAATT
TCTTAAATGCATATGTTGATTTTGCGCCAATGCCAAATGCGAGACAAACAAAAGAAGACG
TTGAAGTAAGCCCGTTTGATGATTCATTTTCAGGATTTATCTTTAAAATTCAAGCCAACAT
GGACCCTAAACACCGTGATAGAATTGCCTTTATGCGTGTCGTTAGTGGTGCATTTGAACG
TGGTATGGATGTTACTTTGCAACGTACTAATAAAAAGCAAAAGATCACACGTTCAACGTC
ATTTATGGCAGACGATAAAGAAACTGTGAATCATGCTGTAGCAGGCGATATCATTGGACT
ATATGATACTGGTAATTATCAAATTGGAGATACTTTAGTTGGTGGAAAACAAACCTACAG
TTTCCAAGATTTACCACAATTTACGCCAGAAATTTTTATGAAAGTTTCTGCTAAAAACGTC
ATGAAACAGAAGCATTTCCATAAAGGTATTGAACAATTAGTACAAGAAGGTGCGATTCA
ATACTATAAAACATTACACACAAACCAAATTATTTTAGGTGCTGTTGGTCAGTTACAATTT
GAAGTTTTCGAACATAGAATGAAAAACGAATATAATGTTGATGTTGTTATGGAGCCAGTA
GGCCGTAAAATTGCACGTTGGATTGAAAATGAAGACCAAATTACAGATAAGATGAACAC
ATCAAGATCGATTTTAGTGAAAGATAGATATGACGATTTAGTATTCTTATTTGAAAATGA
ATTTGCAACAAGATGGTTTGAAGAGAAATTCCTTGAAATTAAATTGTATAGTTTACTTTAA

## > **terC**

Function: protein coding sequence; putative integral membrane protein/TerC family (core genome, constant)

Best match: terC_leader_CC001_MW2-USA400_BA000033.2[995865:995993] (completely identical)
Position: 025-contig_192_RC: 120222 … 120351; Length: 129 bp
Sequence:
CATTTTGTCAGAGGGGAGTAACTTAAGAATCATGACCGTATAAATGATTCGACACTTTAT
CGTCATTACGAAGATATCTTCCGGTAAAGTGGGCAATTTAAATTGCTTAGTGAGACCTTT
GCTATTTAT

## > attB_phiSa4
Function:  attachment site in bacterial chromosome for int1-phiSa4 (mobile element)
Best match: attB_phiSa4_occupied_CC001_MSSA476_BX571857.1[980957:981023:r]RC (completely identical)
Position: 025-contig_192_RC: 121345 … 121412; Length: 67 bp
Sequence:
TAGCATTTTTTCAATAGAAATTATATAGATTTTAAAGCAAATTAGGTGTTAATGTGTCATA
ATGATA

## > htrA
Function: protein coding sequence; serine protease (core genome, constant)
Best match: htrA_CC005_Mu50-VRSA_BA000017.4[1073446:1075755] (completely identical)
Position: 025-contig_192_RC: 121441 … 123751; Length: 2310 bp
Sequence:
GTGGATATTGGTAAAAAACATGTAATTCCTAAAAGTCAGTACCGACGTAAGCGTCGTGAA
TTCTTCCACAACGAAGACAGAGAAGAAAATTTAAATCAACATCAAGATAAACAAAATAT
AGATAATACAACATCAAAAAAAGCAGATAAGCAAATACATAAAGATTCAATTGATAAGC
ACGAACGTTTTAAAAATAGTTTATCATCGCATTTAGAACAGAGAAACCGTGATGTTAATG
AGAATAAAGCTGAAGAAAGTAAAAGTAATCAGGATAGTAAGTCAGCATATAACAGAGAT
CATTATTTAACAGACGATGTATCTAAAAAACAAAATTCATTAGATTCAGTGGACCAAGAT
ACAGAGAAATCAAAATATTATGAGCAAAATTCTGAAGCGACTTTATCAACTAAATCAACC
GATAAAGTAGAATCAACTGAAATGAGAAAGCTAAGTTCAGATAAAAACAAAGTTGGTCA
TGAAGAGCAACATGTACTTTCTAAACCTTCAGAACATGATAAAGAGACTAGAATTGATTC
TGAGTCTTCAAGAACTGATTCAGACAGCTCGATGCAGACAGAGAAAATAAAAAAAGACA
GTTCAGATGGAAATAAAAGTAGTAATCTGAAATCTGAAGTAATATCAGACAAATCAAAT
ACAGTACCAAAATTGTCGGAATCTGATGATGAAGTAAATAATCAGAAGCCATTAACTTTA
CCGGAAGAACAGAAATTGAAAAGACAGCAAAGTCAAAATGAGCAAACAAAAACCTATA
CATATGGTGATAGCGAACAAAATGACAAGTCTAATCATGAAAATGATTTAAGTCATCATA
CACCATCGATAAGTGATGATAAAGATAACGTTATGAGAGAAAATCATATTGTTGACGATA
ATCCTGATAATGATATCAATACACTATCATTATCAAAAATAGATGACGATCGAAAACTTG
ATGAAAAAATTCATGTTGAAGATAAACATAAACAAAATGCAGACTCGTCTGAAACGGTG
GGATATCAAAGTCAGTCAACTGCATCTCATCGTAGCACTGAAAAAAGAAATATTTCTATT
AATGACCATGATAAATTAAACGGTCAAAAAACAAATACAAAGACATCGGCAAATAATAA
TCAAAAAAAGGCTACATCAAAATTGAACAAAGGGCGCGCTACGAATAATAATTATAGTG
ACATTTTGAAAAAGTTTTGGATGATGTATTGGCCTAAATTAGTTATTCTAATGGGTATTAT
TATTCTAATTGTTATTTTGAATGCCATTTTTAATAATGTGAACAAAAATGATCGCATGAAT
GATAATAATGATGCAGATGCTCAAAAATATACGACAACGATGAAAAATGCCAATAACAC
AGTTAAATCGGTCGTTACAGTTGAAAATGAAACATCAAAAGATTCATCATTACCTAAAGA
TAAAGCATCTCAAGACGAAGTGGGATCAGGTGTTGTATATAAAAAATCTGGAGATACGTT
ATATATTGTTACGAATGCACACGTTGTCGGTGATAAAGAAAATCAAAAAATAACTTTCTC
GAATAATAAAAGTGTTGTTGGGAAAGTGCTTGGTAAAGATAAATGGTCAGATTTAGCTGT
TGTTAAAGCAACTTCTTCAGACAGTTCAGTGAAAGAGATAGCTATTGGAGATTCAAATAA
TTTAGTGTTAGGAGAGCCAATATTAGTCGTAGGTAATCCACTTGGTGTAGACTTTAAAGG
CACTGTGACAGAAGGTATTATTTCAGGTCTGAACAGAAATGTTCCTATTGATTTCGATAA
AGATAATAAATATGATATGTTGATGAAAGCTTTCCAAATTGATGCATCAGTAAATCCAGG
TAACTCGGGTGGTGCTGTCGTCAATAGAGAAGGAAAATTAATAGGTGTAGTTGCAGCTAA
AATTAGTATGCCAAACGTTGAAAATATGTCATTTGCAATACCTGTTAATGAAGTACAAAA

GATTGTAAAAGATTTAGAAACAAAAGGTAAAATTGACTATCCCGATGTAGGTGTTAAAAT
GAAGAATATTGCCAGTCTAAATAGTTTTGAAAGACAAGCAGTTAAATTGCCAGGAAAAG
TTAAGAACGGTGTTGTTGTAGATCAAGTTGACAACAATGGTTTAGCAGATCAATCTGGTC
TGAAAAAAGGTGATGTAATTACTGAATTAGATGGCAAACTTTTAGAAGATGATTTACGCT
TTAGGCAGATTATATTTAGTCATAAAGATGACTTGAAATCAATTACAGCGAAGATTTATA
GAGATGGTAAAGAGAAAGAAATTAATATTAAACTAAAATAA

> **A6U0J2**

Function: protein coding sequence; putative Na+-transporting ATP synthase (core genome, constant)
Best match: A6U0J2_CC005_Mu50-VRSA_BA000017.4[1075772:1077130] (completely identical)
Position: 025-contig_192_RC: 123767 … 125126; Length: 1359 bp
Sequence:
GTGTCAATTTTTAGCCAGTTTTTAAAAAGATCAAGCCCTCAACAAGGTATTGTATTGTACT
ATATCGTCGCAATTGTCATTGCATTTTTATTATTAAACTTACCGTATGTTCATAAACCAGG
TGTAGAAGTAAATCCAATTGACACATTATTTGTTGCCGTATCCGGAATTAGTGTTACTGGA
TTGTCTCCGATAAGTATTGTCGATACCTATTCTACATTTGGACAATTAATTATCCTCGTGA
TATTAAATATTGGTGGAATTGGCGTCATGGCAATTGGTACGATGTTATGGGTGGTACTAG
GTAAACATATTGGAATTAGAGAACGTCAGTTAATTATGTTAGATAATAACAAAAACACAA
TGAGTGGTACCGTCAAATTGATTATTGATATTGTAAAATCAATATTTGTAATCGAACTCGT
AGGAGCCATGTTATTAGCATTTTACTTTTATCGAGATAATCCAGATTTAAAATATGCAATC
ATGCAAGGTGTTTTTGTTTCTATTTCTGCCACTACCAATGGTGGATTAGATATTACAGGTA
AGTCATTAATTCCTTATGCACATGATTATTTTGTACAAGCGATAGTTATATTTTTAATAAT
TTTAGGATCAATCGGCTTCCCAGTATTATTAGAAGTTAAAGCTTATATTCAAAATAGGGTT
ACTAATTTTAGATTTTCATTATTTACTAAAATTACGACATCAACATATTTATTCCTATTTAT
TGTTGGGGTATTAGCCATTCTATTATTTGAACATAACCATGCGTTCAAAGGTTTAAGTTGG
CATCAATCGTTATTCTATTCGCTGTTTCAATCAGCGACTACAAGAAGTGCGGGTCTTCAAA
CAATTGATGTGACAACACTAAGTGACCCCACTAATATTATCATGGGTATTTTAATGTTTAT
AGGATCTTCGCCAAGTTCGGTTGGTGGCGGTATTCGTACAACAACTTTCGCTATTTTAATT
TTGTTTTTAATTAACTTTAGTAATAATGCCGATAAAACATCCATTAAAGTTTACAATAGAG
AAGTACACATTATGGATATTCAACGTTCATTTGCAGTATTTACAATGGCGACAATTTTAAC
ATTTTTAGGAATGCTAATTATATCAGCTACTGAAAATGGTAAGCTTACATTTTTACAAGTA
TTTTTTGAAGTCATGTCTGCATTTGGAACTTGTGGACTATCGCTTGGTGTCACAAGTGATA
TTAGTGATATTTCTAAGGTCGTACTAATGGTACTCATGTTTATAGGACGTGTTGGCTTAAT
ATCATTTATCATTATGATAGCAGGACGTCGAGAACCAGATAAATTCCATTATCCAAAAGA
ACGTATTCAAATAGGATAA

> **Q1XY52**

Function: protein coding sequence; putative 5'-nucleotidase (core genome, constant)
Best match: Q1XY52_CC005_N315_BA000018.3[1000937:1002451] (completely identical)
Position: 025-contig_192_RC: 125261 … 126776; Length: 1515 bp
Sequence:
ATGATGGAAAAAAATGAAAACATTAATGTAGAGATTTTAACTACGTCAGATATGCATAGT
CATTTCTTAAATGGTGATTATGGTTCAAATATTTATAGAGCTGGTACTTATGTTAACCAAA
TAAGAGCACAAAATCATCGCGTCATTTTATTAGATAGTGGCGGAAGTTTAGCTGGCTCGT
TAGCGGCCTATTATTATGCTATTGTTGCACCTTATAAACGACATCCAATGATAAAGTTAAT
GAACAGAATGCATTATGATGCTAGCGGTGTGAGTCCAAGTGAATTCAAGTTTGGTTTATC
ATTTTTAACTCGTTCAATTGCTTTGGCACGTTTTCCATGGTTATCAGCAAATATTGAATAC
AATGTTACTAAGGAGCCTTATTTTTCAACTCCATATTGTATTAAACATTTTGGTGACTTAA
AAATTGCTATCGTAGGCGTCACAGCAGATGGTTTAATGGAAAATGAGTATTCTGAAATGG
AGCAAGATGTATCTATTGAAAAGACATTAGTGGCATCAAAACGTTGGATTAGATATATCC
ATGAAGTTGAAGAGCCAGATTTTTTGATTGTAATTTATCATGGTGGATTGAATAAAATTA
GTAATAGTACGAAAAATAAAAAGGCAAGTTCGAATGAAGCTGAAAAATTAATGGAAGAA
CTCGGTGTTATAGATTTAATGATTACAGCTCATCAGCATCAAACAATAGTAGGTCAAGAT
CATGAAACGTATTATGTTCAGGCTGGTCAGGATGCCAAAGAGCTTGTACATCTTTCGATT
AATTTTAAAAAGAGAACAACAACTTATGATGTTGAAAGCATTGATTCTAAAGTGATTGAC

TTAAATGAGTATGAAGAGGATCAAGAATTATTAGATTTAACATTCTATGATAGAAAAGCA
GTGGCTTATTGGTCACAGGAAATCATAAGTGATAAAGGTTTGATGTTATCAGTGAATGGG
TTACAAGATTTAGTCTGTCAAACACATCCATTTTCGCAATTATTACATGATGCAATTCAGC
TTGCATTTGATAATGATATAACATGTGTCCACGTGCCTATGAACGGAGAGAAGGGGTTGA
GTGGACAGATTCGAAATGAAGATTTGTATCATGCATACCCATATCCAGATAAGCCAATGG
ATATGACAATTAGTGGTCAAAATATCAAAGATATATTGGAGTATAGTTATTCACATTTAG
ATTTTGTTAACGAGCAATTAAGCTTAACAATTATTGATGAAACGTTATGTACAATGTGGC
AAGGATTCAATTATGAGATTGATATGAATCAAGAACCTGGGCAACGAGTAATGTTAGATC
AAATTGATTTGACTAAGAGTTATAGAGTTACAATGACTGACTATTGTTATCGTAACTACA
AGAATTATTTAAAAAATGCTATTATACATGAATCGTACGATGAAACAATGAGTACATTAA
TTGCAGAGAAGTTAAGAGATCCGAATTATCGTATTTCATGTAGTGATAATTTTGTAGTTAA
AAACAGGTAA

> **tRNAser2**
Function: tRNA tRNA serine (core genome, constant)
Best match: tRNAser2_CC001_MW2-USA400_BA000033.2[1002595:1002686:r]RC (completely identical)
Position: 025-contig_192_RC: 126952 … 127044; Length: 92 bp
Sequence:
TAACGGAAACGGAGGGATTCGAACCCTCGCGCCGCTTTCGCGACCTACACCCTTAGCAGG
GGCGCCTCTTCAGCCAACTTGAGTACGTTTCC

> **tRNAasn**
Function: tRNA tRNA asparagine
Best match: tRNAasn_CC001_MW2-USA400_BA000033.2[1002689:1002763:r]RC (completely identical)
Position: 025-contig_192_RC: 127046 … 127121; Length: 75 bp
Sequence:
TGGCTCCACAGGTAGGACTCGAACCTACGACCGATCGGTTAACAGCCGATAGCTCTACCA
CTGAGCTACTGTGGA

> **comK**
Function: protein coding sequence; putative competence transcription factor (core genome, constant)
Best match: comK_CC005_Mu50-VRSA_BA000017.4[1079266:1079835:r]RC (completely identical)
Position: 025-contig_192_RC: 127262 … 127832; Length: 570 bp
Sequence:
TTATTTATCTTCAAATAAAGAATAGCGTGTCAATGCTTCAAACACATTCAATGTGGCTTTA
TTATAATCTATTGGTTGGTCGGGATTTTTTGATATCATGCGAGATTGTTTATCTACCATGT
AATAGTAAAAAATGGAATTGTTATATTGATGCCATAAACTGTGGTATGAAACATGAAGAA
TGATAGATTCATTATTAATAAATGTCACTTTACATTTACGATTTTTTAATTCTTTAATACTT
TCGATATAATGCATATTTAACCAAATATTTTCATTTTGTCTGTCAGAATGTGTTGGAAAA
AATAAGTTGGAAATAATGGTGTTAGTAAAATAGGTGGTTTACTAGAAATGCCAGTAATGC
GGTTTGTCTCTGCTTTCTTGCCAAGATAAGTATTACCATAAAATTTGCAAGAGCGTTCAAT
GATTTTCTGGACTTTAAAAGGATTTTGAATACGCGTTTTGTCAAACCGAATTATTTCACTA
CCGTTTCTTTGATCGTCATCATCAAATGCTGGTCGAATAACCATGTCTCCTTTGCGTATCA
CATAAATATTTTGAGAATACAT

> **rli28**
Function: noncoding RNA Listeria sRNA rli28 homolog, locus 1 (core genome, variable)
Best match: rli28_L1_CC005_N315_BA000018.3[1006154:1006337] (completely identical)
Position: 025-contig_192_RC: 130478 … 130662; Length: 184 bp
Sequence:
ATAAAAATAGTATAATATTTTGTGGGCAAGTTGAATTGATGGTGGCTATCTGAGTAAAGG
GGGGTGGTACCTATGGCATTACTTAACACTTTTGAAAGGAAAAGCCTATTGTGATATCTA

TTGCAAACGCATTACATTTAATGTTAAGTTTCGGTATGTTTATCGTCACTTTCATTGGTAT
AGT

> **txpA-var1**
Function: protein coding sequence; toxin involved in plasmid maintenance (core genome, constant)
Best match: txpA-var1_CC001_MW2-USA400_BA000033.2[1006172:1006279] (completely identical)
Position: 025-contig_192_RC: 130588 … 130696; Length: 108 bp
Sequence:
GTGATATCTATTGCAAACGCATTACATTTAATGTTAAGTTTCGGTATGTTTATCGTCACTT
TCATTGGTATAGTAGTAGCAATAATAAATTTAAGCAATAAAAAATAA

> **Q7A194**
Function: protein coding sequence; similar to bacteriocin (core genome, variable)
Best match: Q7A194_CC001_MW2-USA400_BA000033.2[1006908:1007201] (completely identical)
Position: 025-contig_192_RC: 131324 … 131618; Length: 294 bp
Sequence:
GTGTTTTTATAGAAGAATAAAATTTTTGGCACTATATTATATTTAACTTTAGCACTTGGAT
TATCAACAGCAGCTTATGCATCTACAGAATACGCAGAAGGAGGCACTTGGAGTCACGGT
GTCGGCAGTAAGTATGTTTGGTCTTATTATTATCATGGTCATAAAGGACATGGTGCAACA
GCTATTGGAAAATATAGATCATTTAGTGGTTATACAAGAGCTGGTGTAAAAGCAAAAGCA
TCAGCTACTAAACATAATTGCTGGGTCAATAGAGCGTATTATAACATTTATTAA

> **yujE**
Function: protein coding sequence; putative membrane protein (core genome, variable)
Best match: yujE_CC005_N315_BA000018.3[1007337:1009301] (completely identical)
Position: 025-contig_192_RC: 131661 … 133626; Length: 1965 bp
Sequence:
ATGAAATGGTTCAAATTAATACTTGATGTTACAACCTTCATTCTGATTGCTATATTGTTAT
TTGTTTATACATATAAAGAAAACGAAGAAATATTGCCTGATACTAAATACCCTATAGCGG
TAACTGACTGGAATAAAAAATATAGTAAAAATGAGATTTATAAACGTATAAATCAATTCG
CTAAAAATGAGAACGTAGCAATCTATAAATCAACTTCAAATTATACAAACAAAAACGTA
GATAAAGATATATATGTATTTAATAAATCAAAAGCAACAACTATCACTCCTTTTAACGCT
AAATATAACATTCATTATTTAAGTGACGACGAATTATTAAAAAAAGATATCAAAGGAAGT
TATTTTGTAAAAGACAAAAATTTTGACGTGTCTAAATTCATAAATTTTTTAAAAGAATATG
GTGTTACTGCTGAATCATACAAAATAGATCATATGATGATTGCCGTTGGTGTCATTAAAC
AAATGAATATAGAAGTTCCGTTATCCGCACTTTTAATCGTTTATTTTATTTATTATATTTTC
GAAAAGAACATTAATTTCAAAGCGTATGCGATTAAGTATTTAAATGGTTTTACATTAAGA
AAAATAATTTTTGAAAATTTTTCAAAAAAATGCACGTATTGGGTAACGTTAATCATAAGT
CAAATTCTTTTAACTACAAGTGTACTTTGGATATTAAATTACACGGGTAATTTAGATTTAT
TCATATTAAGATTAGTTCTGCTTTCATGTCTTTTCATTTTAACGATTAGTGTTATTAATTTA
TGGACTTTCTTAATGTTACTAAATTTAAATATTGCTAATATGATTAAAGGTAAGCAGCACT
TTAAAACAATTCGTTTTATTAATACAGTTTGTAAAAGTATTCTCTTAGTACTAATAGCTAG
TGTAATGATAGAAAATACTAGTGTTATTAAAGATTTGAATAAAATAAAGGAAACTGAGA
AATATTGGAATGTATTAGATGATTATTACACGATTGAATTTGCACCTTATCACGAAACGA
AACAAAGTTTGATTGATAATATGCTGCGATCAGAACAATTAGTAAAGGCTAGTGAAGCA
GAAAATAATGCGATTTTATTCAAACCAAAGGGTGACTCCGTTGACAATGACAACTTTTCG
CCTGATGAGGGGAATGTAATATTAGTAAATAATCAGTTCTGGTCGATTTATTACAAGCAG
TTTCAACCTGATATTCCGATAAAAAATCAAAAAAATAATGTCGAAGTAATTATTCCACAA
AAGTTTCATGCAATGCGTAATGAAATCAATCAAGCATATCATTCATGGTTTGAATTTGTAC
AAAATAAAAATAATAAAGAGAATAAGTTATCTATACAGTTTATCAACAAAAATGATTATC
GAATTTTTTCATTTGATGCACGAGATAGTCGCCATTTGTCATTTATAGAGGCGCCAATCAT
TGTGAATGTTCAGGCATCAGATTTATCGAATGATTTTTATTATGCCATGATCAGTCAAGGC
GGGTATTTATTCAAAAATTATGACGCGCTAGTAAAAAATATTGAAAATTATCATCTTGAT
GGGGAAATCAGTGGAATAACCAATTATAAAGATAGCGTGATGGAAATGTATCATGAAAA

CAATTTGAAATTAACAGTACTCAACTTTTCACAAATCATTATCGCAATCATTTTAATAATT
ATTATTTTATTTGATGTGAAATATTATTTTGAACAGCATCGAAAATTACTCGTAATCAAAA
AGCTATATGGTTATTCAACATTAAGAGCCAATTACCAATACTTATTAATAAATAATATAG
TTGTTGTTTTTATTGGAATATTGACGAATGTAATTTTACATTCTCAGTATATAATGATGAT
ATTTGCAACGATTCTTGTCGTTCAAATATTATTGCAGATTTGCAGCCTATACTATCATGGC
CGACGTTTTAATGAAGTTATCAAGGAGTTTTAA

> **yxeA**
Function: protein coding sequence; putative exported protein (core genome, variable)
Best match: yxeA_CC005_Mu50-VRSA_BA000017.4[1085632:1085952] (completely identical)
Position: 025-contig_192_RC: 133628 … 133949; Length: 321 bp
Sequence:
ATGAAATTTATCATTGCAATATTATTAGGATTAATCGTATCCATTACTATTGCTTTTACAA
TCATACATCATCCTATACTTGATCGTTTTAATCCTTTCTTAAAAACGGAGTATAGTTATGC
CAAAGTGCCAAAAGGTACGCAACAATATGTTAATATTACGGCTTATAGTGAAAGAGGGG
AAAAGCTTGATTATAAATTAACATTTAATGGATTTTCACCTAGTAGAACGTATGTCGAAA
TAAAGCATAAAGGGCAATATGTCATATCGATCACATATGTTGAAAAAGAGGATATACCA
AAAGAGGTAAGACAAGAATGA

> **Q2FZN0**
Function: protein coding sequence; ABC transporter/ATP-binding protein (core genome, constant)
Best match: Q2FZN0_CC005_Mu50-VRSA_BA000017.4[1085949:1086590] (completely identical)
Position: 025-contig_192_RC: 133945 … 134587; Length: 642 bp
Sequence:
ATGATAGAATTAAAAGATTTAACCATACAAAAAGGTAATATACATATTTTGAAGAAACTT
AATTTGAAATTTCAATGTGGAAAATCATATGCACTTATTGGTAAAAGTGGATGCGGTAAA
TCTACATTATTAAATACTATTGCTGGACTTGAAAAAACGGGGAAACAATATGTCTATTTT
AATGGTCAATTAGAACAATTTAAATCTAATTTTTACAGAGATAAATTAGGATATTTATTTC
AAAATTATGGATTAATCGATAATTTGACAGTAAATGAAAATTTAGATATTGGATTAGCAT
ATAAAAAAATAAGTAAGAAAGAAAAAGAACAATTAAAGATACGTTATATAGAACAGTTT
GGTCTGTCAAACAGTTTAAAAAGAAAAGTTCATACGCTAAGTGGAGGTGAACAACAACG
TGTCGCTTTAATTAGAATGATGTTAAAAGATCCGATTGTTATGTTAGCTGATGAACCAAC
GGGTGCGTTAGATCCTAAAACAGGACAGATGATTATTCAATCATTATTTGATTTGGTCGA
TGAAAATAAAGTGCTGATTTTAGCAACACATGATATGGCTATTGCAAATCAATGTGACGA
AATAATAGATTTAGAACAGTATAGTAAAGTAGCATCTATGTGA

> **tarM**
Function: protein coding sequence; alpha-GlcNAc appending glycosyltransferase (core genome, variable)
Best match: tarM_trunc_CC005_Mu50-VRSA_BA000017.4[1087050:1087246]
Position: 025-contig_192_RC: 135046 … 135243; Length: 197 bp
Sequence:
GTGATTTTATAGAAGTTAATAAAAATGGTTATTTAATAGAAAAACATAATATTAAAGACA
TGGCTGATAAAATACTAAAGCTTGTTAATAATGATGTTTTAGCAGAGGAGTTTGGTTCGA
AAGCGAGAGAAAACATTATAGAAAAATATTCAACGGAATCAATATTAGAAAAATGGTTA
AATCTTTTCAATAGCTAA

> **Q5HH47**
Function: protein coding sequence; transport system extracellular binding lipoprotein (core genome, variable)
Best match: Q5HH47_CC005_Mu50-VRSA_BA000017.4[1088179:1089138] (completely identical)
Position: 025-contig_192_RC: 136175 … 137135; Length: 960 bp
Sequence:
GTGAATAGGAATATCGTTAAATTAGTTGTGTTTATGCTAATCTTAGTTGTAGCAGTAGCGG
GTTGTGGTCAAAAAGATACTGAAGAGAAAACTGAAATGACGACAATAAAAGATGAATTA

GGAACTGAAAAAATTAAGAAAAATCCTAAACGTGTTGTTGTATTAGAATATAGTTTTGCT
GATTATTTAGCAGCATTAGATATGAAACCTGTTGGTATTGCAGATGATGGCAGCACTAAA
AATATAACAAAGTCAGTAAGAGATAAGATTGGGGCATATGAATCGGTTGGATCTAGACC
GCAACCGAATATGGAAGTGATAAGTAAATTAAAACCGGATTTGATCATTGCAGATGTTAG
CAGACATAAGAAAATCAAATCAGAATTGAGCAAAATTGCTCCGACAATCATGTTAGTTAG
CGGTACGGGAGATTATAATGCAAATATTGAAGCATTTAAAACAGTCGCTAAAGCAGTAG
GCAAAGAGAAAGAAGGCGAGAAGCGTCTGGAAAAGCATGATAAAATATTAGCGGAGAT
TAGAAAGAAAATTGAACAGAGTACGTTAAAATCTGCATTTGCATTCGGTATCTCAAGAGC
AGGTATGTTTATTAATAATGAAGATACATTTATGGGACAATTCTTAATTAAAATGGGTATT
CAACCTGAAGTCACAAAAGACAAAACTACGCATGTTGGTGAACGCAAGGGTGGTCCTTA
TATATATTTAAATAATGAAGAACTTGCCAATATCAATCCAAAAGTTATGATTTTAGCCACT
GACGGAAAAACGGACAAAAATAGAACGAAATTCATTGATCCTGCAGTTTGGAAATCATT
AAAAGCTGTGAAAGATAACAAAGTTTATGACGTTGACCGAAATAAGTGGTTGAAATCAA
GGGGGATTATCGCAAGTGAAAGTATGGCAGAAGATTTAGAAAAAATTGCAGAAAAAGCA
AAATAA

> **sbrA**

Function: protein coding sequence; sigma-B regulated protein A (core genome, constant)
Best match: sbrA_CC005_Mu50-VRSA_BA000017.4[1089184:1089300:r]RC (completely identical)
Position: 025-contig_192_RC: 137180 … 137297; Length: 117 bp
Sequence:
TTAATTATGTTTATTATCTGTCGGATGTGATGATTTACCTGAAAGTTTATTTCGAATAAAT
TTAATTACATAACCGACAAGGATTGTTTTAACAGTTCTTTTAATGAATTGGCGCAT

> **menA**

Function: protein coding sequence; 1,4-dihydroxy-2-naphthoate octaprenyltransferase
Best match: menA_CC005_Mu50-VRSA_BA000017.4[1090362:1091300:r]RC (completely identical)
Position: 025-contig_192_RC: 138358 … 139297; Length: 939 bp
Sequence:
TTAAATGCCTGCAAATAATGCACTAATATAAATACCTAATGCATATAATAAACCGAAAAA
TGTATTTGTTTTACCAGCAGCAGCCATTGCTGGCATCATTGTAGGCGGTGTATCATTCTTC
TTGAAACGTCTGATAACTTTAACAGGCATTGGGAATGATAACAACGCAAGTAAGTAAAAT
AATGAGCCACCAGGTTTAATAATGATCGTAAGTACAATAAAGGCATAAGCGATAAAGTA
CATGATTGCCATAAATGTTAAAGAAGCATTTTTACCTAATAGAATGGGTAAAGTTTTGCG
ACCACTTGCTTTATCTTTGACACGGTCGCGAATATTGTTAGCCATATTAATTAAACCGATA
GTGATTACTATAGGTACACTTAACCAAATTACATAACTTTGAATATTGCCAGTTTGAATAA
AGAATGCAATAACGATAATAAACATACCCATAAATACGCCTGAGAATAATTCACCGAAA
GGCGTCCATGAAATAGGGAAAGGGCCACCTGTATATAGGTAACCAACAGCCATACATAC
TAATCCAACTGGTAATAACCAAAATGAAGAGTTAGCAGCTAAAAACAAACCTAATATTG
CTGCTAAGATGTAAAATGCAATGGCTAATCGTAGCACAAGCTCTGGGCTCATACCGTTGC
GAACAATGGCACCACCAATGCCTACAGATTCATGATCATCAAGGCCTTTTTTATAATCAT
AGTATTCATTAAACATATTAGTTGCTGCTTGAATAAGTAAGCATGCTAGTAACATGGCAA
TGAATAGGCTGATTTTAATATGATCTTCGCTACCAAGAAAATATATTTTAGATGCTGCTGT
ACCAACTAAAACGGGTACTACGGAAGCAGTTAATGTATGAGGACGCATTAAATGCCAAT
ATTTCTTAACTGTAGAATATTGCTGATATTGATTACTCAT

> **menF**

Function: protein coding sequence; putative menaquinone-specific isochorismate synthase
Best match: menF_CC005_Mu50-VRSA_BA000017.4[1091482:1092843] (completely identical)
Position: 025-contig_192_RC: 139478 … 140840; Length: 1362 bp
Sequence:
ATGGCTACGGGCGTATTAGAGGACGATATTGTCAAAGAGATATATGGCAGCTCAAAGGA
ATGGGTTTCAGTTGAAGTGAAATTATCACAGTCACTAGACCCGAGCACATTATTTCATCTC
ACTGACAATGAGGCAGGAGATCGCTTTTATATGCGTTTGAATGATAATCGAACGTCATAT

TTTGGCTACAAAGCAATTCAATTATTCAAAAATAATTCTAAAAATAAACAATCTATTTTTA
AAGACTGGGAAAAATTAAAACATAACATCACATTTATACATCCGCAATCTGAGAAACATC
ATCTTCGAGTTGTTGGAGGGTTTCAATTTTCAAGTCATAAATCAGATGATGAATGGCGAG
AGTTTGGACTAAATCATTTTGTATTACCTGAAGTTTTAATTTCAACTGATAATAATGGGAC
ATTTTTAACTTATACAGTTAAAAGGGAAAGTTTTACTGTTGAGGCATTGAACGATTTAATG
GATTTGTTCAACAATATATCGGATATAGATGTGGACGAGCAAATTGGGGAAATTACTAGA
AATGAAGATATTTATAAAGATGACTGGCGTCAACTTGTAGTAGAAGCTATAGAATCTATT
AATAATGAAGAAAAAATTGTACTAGCACGTAGACGGTTAATAAAGTTCGATAAAGATAT
CAGTATTCCATATATTCTAAAGCAAGCATATTCTAAAGAAAAAAACAGTTATATATTCTT
GTTAGAATCACAAGATTCTATTTTCTTTTCACAAACACCTGAACAATTAATAAAGGTCAAT
AATAAAATACTATCGACTAAAGCTGTAGCAGGTACAATTAAACGTTCACAAGATGAGGA
CGAAGATACAAAAAATGTTGAAGCATTTTTAAAAGATAATAAAAACTTAATCGAACATC
GATTTGTTGTTGACAGTATTTTACATGATATTAAACCTTATATCACTGAATTACATTATGA
TAAGACGCCTAAAATTCTAAAAAATGATCATTTATATCACTTGTACACTGAAATAAAGGC
GCCACTGAAGGATGATTCGTATATTAGTTTAATTGATCATTTACATCCAACACCTGCTTTA
GGTGGCTATCCAAAAGAATTTGCGATGGATTTTATTGAACAGAAAGAATTTGGTACACGA
GGATTATATGGTGCGCCGGTTGGCTATATAGATATATATGATGATTGTGAATTTATTGTTG
CAATTCGTTCGATGCTTATTAAGAAAGCACAAGCAACTTTATTTGCTGGGTGTGGCATTGT
TAAAGATTCTGATCCAGATAGTGAATTGGCAGAAACGAACCTTAAGTTCACACCTATGAT
GAATGCATTAGGAGTCGATATGAATGGGAAATCATAA

> **menD**

Function: protein coding sequence; 2-succinyl-6-hydroxy-2/4-cyclohexadiene-1-carboxylic acid synthase/2- oxoglutarate decarboxylase
Best match: menD_CC005_Mu50-VRSA_BA000017.4[1092830:1094503] (completely identical)
Position: 025-contig_192_RC: 140826 … 142500; Length: 1674 bp
Sequence:
ATGGGAAATCATAAAGCAGCTTTAACGAAGCAAGTTTTTACATTTGCATCTGAGTTATAT
GCATACGGCGTAAGGGAAGTAGTTATCAGTCCGGGATCACGCTCAACGCCACTTGCACTT
GCATTTGAAGCACATCCAAATATTAAAACATGGATACACCCCGATGAGCGAAGTGCAGC
ATTTTTTGCAGTTGGGTTAATTAAAGGCAGTGAAAGACCTGTCGCTATATTATGTACGTCA
GGTACAGCAGCAGCGAATTATACGCCTGCAATTGCTGAAAGCCAAATTAGTAGAATTCCA
TTAATCGTTTTAACAAGTGACCGTCCGCATGAATTAAGAAGTGTAGGCGCACCACAAGCG
ATTAATCAAGTAAATATGTTTAATAATTATGTAAGTTATGAGTTCGATATGCCTATTGCGG
ATGATAGTAAAGAGACCATTAATGCAATTTATTATCAAATGCAAATTGCTAGTCAATATT
TATATGGACCACATAAAGGGCCAATTCATTTTAACTTGCCATTTAGAGATCCGTTAACAC
CTGATTTGAATGCAACAGAATTGTTAACTTCTGAGATGAAGATTTTACCGCACTATCAAA
AAAGTATAGATGCATCGGCATTAAGACACATTTTAAATAAGAAAAAAGGTTTAATTATTG
TAGGGGATATGCAGCACCAAGAAGTTGATCAAATACTAACGTATTCAACGATATATGATT
TGCCTATTTTAGCTGATCCTTTAAGTCATTTAAGAAAATTTGATCATCCGAATGTTATCTG
TACATATGATTTGCTGTTTAGAAGCGGCTTAGACTTAAATGTGGATTTCGTAATTCGTGTT
GGGAAACCAGTGATTTCTAAAAAGTTGAATCAATGGTTAAAGAAAACTGATGCATTTCAA
ATATTAGTGCAAAACAATGATAAGATTGATGTCTTTCCTATAGCGCCAGATATTTCATATG
AGATTTCTGCAAATGATTTCTTTAGATCATTAATGGAAGACACGACCATCAATCGCGTAA
GTTGGTTAGAAAAATGGCAATGCTTAGAGAAAAAAGGGCGTAAAGAAATTAAATGTTAT
TTGGAACAAGCTACAGATGAGAGTGCATTCGTTGGTGAATTGATTAAGAAAACATCTGAA
AAAGATGCATTATTTATTAGTAATAGTATGCCTATCAGAGATGTAGATAACTTGTTATTGA
ATAAAAATATAGATGTCTATGCGAATCGTGGTGCGAATGGTATTGATGGTATCGTTTCAA
CTGCACTGGGTATGGCTGTGCATAAACGAATAACATTATTGATAGGTGATTTGTCATTTTA
TCATGATATGAATGGACTATTAATGTCAAAATTAAATAATATTCAGATGAATATTGTATT
ATTGAACAACGATGGTGGCGGTATTTTTTCATATTTACCACAAAAAGAAAGTGCAACTGA
CTATTTTGAACGGTTGTTTGGCACACCGACGGGATTGGATTTCGAGTATACAGCTAAGTT
ATATCAATTCGATTTTAAACGTTTTAACAGTGTTTCAGAATTTAAAAATGCCACATTGTTA
TCTGAAACTTCGACGATTTATGAATTGATAACGAATCGCGAAGATAACTTTAAACAGCAT
CAAATTTTATATCAGAAATTGAGTGAAATGATTCATGACACATTATAA

> **A5IRT0**
Function:
Best match: A5IRT0_CC005_Mu50-VRSA_BA000017.4[1094490:1095293] (completely identical)
Position: 025-contig_192_RC: 142486 … 143290; Length: 804 bp
Sequence:
ATGACACATTATAAATACTATGAAGCAAACGTTGAGACCAATCAAGTTTTAGTATTACTG
CATGGTTTTCTTAGCGACAGTCGTACTTATTATAATCACATCGATAAATATACTGATATCT
GTCATGTCATCACTATAGACTTACCAGGCCATGGCGAAGATCAGTCTTCAATGGATGAAA
CGTGGAATTTTGATTATATTACGACGTTGTTAGACCGAATTTTAGATAAATATAAAGATA
AATCAATAACATTGTTTGGATATTCAATGGGTGGGCGTGTTGCATTATATTATGCAATTAA
TGGTCACATCCCTATATCTAATTTGATATTAGAAAGTACGTCACCAGGTATTAAAGAAGA
AGCAAATCAATTGGAACGCCGTCTTGTTGATGATGCACGTGCTAAAGTATTAGACATAGC
AGGTATTGAATTATTTGTTAATGATTGGGAAAAGTTGCCATTATTTCAATCGCAACTAGA
ATTACCAGTTGAAATACAACATCAAATAAGACTACAACGATTGTCTCAATCGCCACAGAA
AATGGCCAAAGCATTAAGAGATTATGGTACAGGTCAAATGCCAAACTTATGGCCGCGCCT
GAAAGAAATTAAAGTACCAACATTAATATTAGCTGGAGAATATGATGAAAAATTTGTAC
AGATTGCGAAAAAAATGGCAAATTTAATTCCTAATAGTAAATGTAAATTAATTTCTGCTA
CAGGTCATACAATTCATGTGGAAGATAGTGATGAATTTGATACAATGATATTAGGATTTT
TAAAGGAGGAGCAAAATGACTAA

> **menB**
Function: protein coding sequence; naphthoate synthase
Best match: menB_CC005_Mu50-VRSA_BA000017.4[1095286:1096107] (completely identical)
Position: 025-contig_192_RC: 143282 … 144104; Length: 822 bp
Sequence:
ATGACTAACAGACAATGGGAAACACTTAGAGAATATGATGAAATCAAATATGAATTTTA
CGAAGGGATTGCTAAGGTAACAATAAATCGCCCTGAAGTACGCAATGCGTTTACACCTAA
AACAGTTGCTGAAATGATTGACGCATTTTCACGTGCACGTGATGATCAAAACGTTTCAGT
TATCGTATTAACTGGTGAAGGTGATTTAGCATTCTGTTCTGGTGGTGACCAGAAGAAACG
TGGACATGGTGGTTATGTAGGTGAAGACCAAATCCCTCGCTTAAATGTATTAGATTTACA
GCGTTTAATTCGTATTATTCCAAAACCGGTTATCGCGATGGTAAAAGGTTATGCTGTAGGT
GGCGGTAATGTACTAAATGTTGTTTGTGACTTAACGATTGCTGCTGATAATGCTATTTTTG
GACAAACTGGTCCTAAAGTAGGTTCATTTGATGCAGGTTATGGTTCAGGATATTTAGCAC
GTATCGTTGGACATAAGAAAGCACGTGAAATCTGGTACTTATGTCGTCAATACAATGCAC
AAGAAGCTTTAGATATGGGCCTAGTAAATACAGTGGTACCTTTAGATAAAGTTGAAGATG
AAACTGTGCAATGGTGTAAAGAGATTATGAAACACTCACCAACAGCTTTACGATTCCTTA
AAGCAGCTATGAATGCTGACACAGATGGTTTAGCTGGTTTACAACAAATGGCTGGGGATG
CAACATTGCTTTATTACACAACTGATGAAGCGAAAGAAGGCCGTGATGCGTTTAAAGAAA
AACGTGATCCTGACTTCGATCAATTCCCTAAATTCCCATAA

> **sspC**
Function: protein coding sequence; staphostatin B (core genome, variable)
Best match: sspC_CC005_Mu50-VRSA_BA000017.4[1096345:1096674:r]RC (completely identical)
Position: 025-contig_192_RC: 144341 … 144671; Length: 330 bp
Sequence:
TTATACTAAGCGCTCATAAACGATTGGGCGCGAAGTGCCAATACCTTGTTTGGATGACGT
TTGCATGAGGATATGCTGTGTATCATCATAATCAAGAATATAAGTGATTTCTTCATTATCA
ATAGATGAAAATTTAATGCGTTGATGTGCCGTATCTATAAAAAGAATATGATATTGATTG
TGACTTGTATCATCGCCATGACGTATACAAATTGATTTTTGTAATTGATGATTACTCCAAT
TACCAATGAATAAATTGATATTGGTTTGTTCTAGATGTGTGAGTTTGGTTGTGTCGTAAAC
TAAATTTATAAATTGTAGTTGATACAT

> **sspB**
Function: protein coding sequence; staphopain B (core genome, variable)
Best match: sspB_CC005_Mu50-VRSA_BA000017.4[1096712:1097893:r]RC (completely identical)

Position: 025-contig_192_RC: 144708 … 145890; Length: 1182 bp
Sequence:
TTAGTAACCTATCATTGAACCATACCAGTTATAATCACGATTGAATGATAAATGTAATAG
GCTTGAATCTGCATCTTGGATTGATAATTCTGTATCCCAAGGATTCCAGTAAATAAGTTTT
TCTTGGTCATTAATTTTAGCATTACCAACAACTGCTAGGGCATGTCCTAAATGTGGATCAT
TAGGGTTTTGAGATACACTTTGTGCAAGAATCATAATTCCTACATTATCTTTTGTAAGTTG
ATCAACTTGTTCATATGATGGTACGCCTTCTTGATAATGAATATCTCTGCCTTGTGATTTA
CCGTATTCAATCATTTGATTAGGGAATGTTGAGCAATTAGGAAGGTCTTGCTCACTTACTT
CAGGGTATAATGTACGCATAATATCATGTGCATTATAAGTGTCTGTATTTTTAGTTGCATT
TAATAATGCTGCCATACTAAATCCTGCACACCATGAGTTATCGAATTGTTGTTCTCTAATT
TTGAAGTTTTTTAATGTATTTTCATATTGAACTTGATCTTCTTGTATCGCTTCGTTTTCTTCA
ACTTTAGTAGGTGTTACTGTATTTTTTAACTCTTGTTTCAATTTTGCTGAAACTGTTTTAGC
ACTTTCTTTTTCTTTTACATTACCAGGTAGTGGCGTAGCTTTTACTAATCTAACTTTGCCAT
CTTCTTCAAAATAAAACCCTTTTTCATCAGTAAGAACAGTGATATTTGAATTTTTATCTTT
AATTTGGTCTAAATCTTTAGCGATGAAGTTTGAAATTTTAACACTGTAATTCATGTCTTCT
TTTGATTTGTTTAAATCATCTTTATTTTTAGGGCTAAGTGTTAAAGTATAAACAATTTTACC
GTCTTTTATAACTGGATAATAGTAACTATTATCTTCTTCACCATTAAATTTATAAATTTTAA
AAGCTTCGCCAAGTTCATATTTACCAGTTTTTGCATTACTTTGTTTATCTAATGCTTTAGCA
TAACCAGCAAATTGTTGTTGTGCTAGATCTTTTACTTTTTGAGGTACTTTGTCACTCTTAAC
ATTAATTTCTAGCTGTTTAGAGTGTGAATCGGCTTTGGCTTTATTGTTATTAGCAAATGCG
CCTAGTGATAAAATAAGCATTGAAACCATTATGATGCTTATAATATTGAATACTCTAGAT
TTATATGAACTATTCAT

> **sspA**
Function: protein coding sequence; glutamyl endopeptidase chv
Best match: sspA-Mu50_part_CC005_MR1_ACZQ01000091.1[1:118]RC
Position: 025-contig_192_RC: 145971 … 146089; Length: 118 bp
Sequence:
TTATGCAGCGTCAGGGTTGTCTGAATTATTGTTATCGCCATTGTCTGGATTATCAGGGTTG
TTAGGGTTGTCAGGGTTATTTGGTTCATCTGGGTTGTTAGGGTTATCAGGATTGTTA

> **aspC**
Function: protein coding sequence; putative aspartate transaminase (core genome, variable)
Best match: aspC_CC005_Mu50-VRSA_BA000017.4[1099531:1100685:r]RC (completely identical)
Position: 025-contig_192_RC: 147581 … 148736; Length: 1155 bp
Sequence:
TTATTTAGTATTTAAATATTTAGCAAGTCTCTTCATACCTTCTTTTAAAACATCCATTTCAT
AAGCATAGGAAATCCTTACAAATCCTTTACCGAATTCTGTAAAGGATGAGCCTGGAACGA
TTGCTAAATGTGTTGATTCAAGTAAATCGACACAAAATTCGAAATCGTCATCGGTGATAT
GTTTAATACTTGGGAAAATATAAAACGCGCCTTCAGGTTGAGCGGTAATCTCAAAACCTA
ATTTAGTTAATTCAGATACTAAATAATTTCTTCGTTCTACATAAGCTTCGTTCATATATTTA
GGAGCTTCTAATCCTTCGTTAAGTGCTGTTATACATGCTATTTGAGCTGGAACATTGGCAC
AAATACAATTATAGGCGTGCATAAATGTTAATTTATCAATCAAATATTGAGGCCCTAATA
GAAAACCAATTCTTATTCCGGTTGCTGAGTGTGATTTACTTAAACCACCAATTAATATTAA
TTGATCACGAATGTCTTCAAATTCAGCGAAGGATACATGTTTACCACTAAATGTATTTTCA
GCATAAATCTCATCGCTAATAATAAATATCGGATATTTTTTTAATACATTTACGATATTTA
AAACTTCATTTCTTTTTAAAACTACGCCAGTTGGATTAGTCGGATAATTTAACAAGACAGC
TTTTGTCTTTGGAGAAATATGACTTTCTAATGCATCAGGTGTAATTTTGAATTGTGTTGCT
GTTGTATCAATATAAATTGGTTTACCACCTAGTACTTCGATGAGTGGTATGTAGCCTGCAT
AAATTGGTCCCGGTATTATAATTTCATCTCCAGGCTCTATGATAGAACGTAACGTTGTGTC
TATTGCTTCACTTGCTCCATTTGTCACAATAATTTCTTCAGGATCATAGGAAAAATGATAA
CGATTTTTGAAGTATTGACTAATTGCTTCGCGAGTTTCTAATAACCCTTTATTGTGAGAGT
ATGATGTCTTGTCATTGTTAATAGCATCAATATATGCCTTTTTCACAACATCAGGCATCGG
GAAGTCTGGTTGGCCAATAGTTAAATTAACACAATCATCCAAATTATTCATACGATTTGA
AAATTGGCGAATACTTGGTGCTCTTAAATATTTAGAATTAGAATTTAAAGAAAGTTTCAT

> **atl**

Function: protein coding sequence; autolysin (core genome, variable)
Best match: atl-Mu50_CC005_ED98-hen_CP001781.1[1037532:1041278:r]RC (completely identical)
Position: 025-contig_192_RC: 150719 … 154466; Length: 3747 bp
Sequence:
TTATTTATATTGTGGGATGTCGAAGTATTTGCCGACTTCGCCAATTTTATCATAGTAGCCT
TTGATGATTTTAGCATTGATGTTAGCCCAATCTACATCTGTAGCATATTGGTGTGTTCCTG
GATGTGCAGGATTCCATCTCATTTTGTAAAGTGTATTTTGACCAGCTTTTACATATGAGTT
GCCGATGAATTTAGCACCACCAACGATTGCTTTTGATACTGTGTCCCAACCAGCTTGTTTA
GCATATTTAATACCTTCACGTAAAGGATCGTTATCATATGCAGCAATACCAAATACGTTA
TGGTATTTCGTGTTTGAGTTAGTTACAACTTTGTTGTTCACTACATCTGCACCTTTCGCTAA
TTGAGAAGTACCGTTACCTGTTTCTAATAGGGCATGTGAGATAAGATAAACTTCATTAAT
GCCATACATTTGAGCAGCTTTGTTAAATGCAGCACCTTGGTTTTCTAATACACCTTTACCT
TTTAAGAATTGATTAATTTTATCAATAGAAATATTTTGTGGTTGGTCTAAGCGTAAGAATT
GATATTTTAATGCTGGATCTTGAGCTAAACGCTTCGTATCCATTGCATGCTTAACATCATT
AAAGTTAGCATCTGTCCACTTACCTGGTACACGTTGTACTTGTGGTTTATATTGTAAACCA
GCTTGTATTTGAGCAACTTGGTTTAATGTCATACCTGTTTGATTATACTTAATTAATTCTTT
AGCTAAATCAGTTGATTTAATCCATGCTAATTTACCGTTAGATAATTTACCATAGTACCAA
GTTTGTCCATTAATGACTTGTTCTTTAACAACTGCGAATGGTTGTTCATTAAATGCTTTTA
ATGAGTATTTAGCTGTATCAGAATTTGGTGTTACATAGTAATAACCATTACCATTTTTAAT
TACATAAGTGTAGTTATAATCTTTGGCAGCTGATGTAGTTGGTTTCACAGCAGTTGGTGCA
GTTAAATCTTTTGCATTTACCCAACCAGTGCGGTTATTAATAGTACCGTATAAATAAACAT
CTTTGCCTACAGATACTTGTTTCGTTGCATTAAATGTACCTTGAGCAATGTTATTGCCTGTT
AAAATGACTTGGTTTTTAGTACCCCAAGGAACCATTGATAAGCCGTTATTTGATTTATTAA
CAGTATATTTTTGAGTCGTTTTAACTTCTTTGCCTAAGTTTTGAACATTTAAGTCTTTTACA
TTGAACCAACCTAATGGGATGTTATGGCTTGTATTGTTTAATAATACATACGTTTCATTAC
CATGAGCACGCTCTTTTGTTACATAGAACGTACGGTCTGCATATTTCGCACCGTTTTTCGC
TGTTTTTTCATAAACAGAAGCACGAATACCAGTGTTGTTTGGTTTAACTTGAGCAATCTTG
CTAACTGTTTGAGTCGTTTGTGGTTTAGTAACAGTATAAGCTTTTACAGCTGTTTTTGGTT
GTGCTACTGCTTTTTTAGGTGCAGCAGGTACAGCTAAATATGCTTTACTTACCCAACCAGA
TTTACCATTTACAGTTCCAAATAAATAGATAGATTTATCAATTTGTTGTTGCTTAGTCGCT
TTAAAAGTTTGGTTACCTGTACCAGAAACTGCACCAGCTTCTTGTTTATAAGTGCCCCAAG
GTACTGAATATAATTTAGTGCCTGGTTTTACTGTATATGTTTGCATTACATTTACAGGTGA
TTTTGCATTGTTATAAATAACGTCACCTTGTTTAACCCAACCAATTAAAGTTGGACTATTG
TAATCTTTAACTAAGTAGAATTTGTTTCCACCTAAACTTGCTTCTTTTGTTACAGCAAATG
TTTTTTGAACTTCTTTCGTTGGCTTACCAGTTTTGTCATAAACTGTAGTGAATAAGCCATTG
TTTTTAGCATTAATTTGAGCAACACCGTTTAATGATGAAACTGTTAATTTATTATTTGTTGT
AGGTGTTGATGGCTTAGGTATTGGTGTAGGCGTAGGTTTAGCAGTATCAACTAAATATGC
TTTACTTACCCAACCAGATTTACCATTCACAGAGCCATATAAATAAATTGATTTATCAATT
TGTTGTTGCTTTGAAGCCTTAAATGTTTGGTTTCCAGAGCCAGACACGCTACCAGCAACTT
GTTTAGATGTACCCCAAGGTACTGTATAAAGTTTCGTACCAGATTTGATTGAATATGATTG
ATTTACATTTACAGGTGATTTAGCTGTGTTGTAAACCACATCGCCTTCTTTAACCCAACCA
AATTTATTACCAGAATTGTAATCTTGAACAAGATAGAATTTTTGATTACCTAATGTAGCTG
TTTTAGATACAGCAAATGTTTTTTGAACTTCATTAGTTGCTTTACCAGTTTTGTCGTAAAC
AGTAGTATATAAACCACTATTTGTTGGTTTGATTTGTGCGACACCATTGTTTGCTGCAACT
GTTAATTTACCAGTTGATGGTTTCGACGGTGTTGTTGGTTTTGATGGTGTAGTAGGGGTAG
TTGTAAATTGCGTACCCCATGGCGCCACTTTACCCATTTTTATTAAATATTTTTCATTAATT
AAGTCATATAATTGATCATAACTATAATTATGACTTCTTAAATATCCATGTGGATCGGCAT
GGTCCGTACCACCTAAATATTTACTTACAGCGTAGTGAGTCCATACTGTACCATTTCCATC
ATACTCAGCACTGTCTGGTTTTAAACCATAATATTGTAATTGTGTAGCTGCATAGTCAGCA
TAGTTATTCATTGAACGTGCAAATGAAGCATAGTCGTGTGTGTGTACGATTTCAACATTG
ATGAATCTAGGGTTACCGACTGCACCGACACCCCAAGATAAGTAATCCGTTGGTGCTGTT
TCGATTATACGATCCCCATCAACAAATGCATGTACGAATGCGTTTTGATAGTTATTTTTCA
TATAACTAATTTCACCATTTATCGTCGAACGATCATTAGCTGTATCATGAACTACGATACC
TTCAGGACGACCTACGCCGTTACGGTATGCGTATTTAGGGAAGTAAGATGTATAATCTTC

TTCAATTTTAGGTGCTTTTAAGTTATTTTTACGAATGTAATCGTTAATTGAAGAGTTTACTT
GTGGTTTATATTTTGGCAAACTCGTTTTTGGTGTTGCAGCAACTGATCTTGGTTGTGCTGA
AGCGCTAAAAGTAGTTACTTTAGGTGTCGCTTCAGTTTTAGCTTTAGGTGCTGCGGCTTTA
TATTGCGTTTCAAGAGCTGCAGGTTTAGCAGCTGATTTAATTAATTCTGGATTAATTTGAT
GTTCTGAATTATCATCTTCATCATCAACTAAACTATAACCAGCATTTGTAACATTAGTGTT
AGTTTTAGGTGCTGTAGTGCTTGTTGACTTTGCAACAGGCTGCGTATTATTTGTAGTCGCT
GATTGATTAGCACGAGTGTCACCATTTACTTGTGCAGTATCAACTTTTTGACTTACTTGAG
CATTGCCTGTTTTGTTATTTGCTGTTTTTGGTTGGACAATAGCAGGGTCTTGATATACTTGA
GTGCCAGAAATGTTTTGCGTTGGATTTTTTACCTCAGCTTTTGCTTGTTCAGTAGTTGCTTT
AACTTTATTACTATCTAAAACATTTTTATTAGTAGTTTGATCTTGTGTCGTCTCAGCTGCTT
GAACTTGATGTGCAGTGACTGCTGAACCTACAAGCGTTAATGCAACCATTGATGGTAGTT
TGTAATTGAATTTTTTCGCCAT

## > fmtA

Function: protein coding sequence; autolysis and meticillin resistant-related protein (core genome, variable)
Best match: fmtA_CC001_MW2-USA400_BA000033.2[1034813:1036006] (completely identical)
Position: 025-contig_192_RC: 157468 … 158662; Length: 1194 bp
Sequence:
ATGAAATTTAATAAAGTAAAACTAGTTATACATGCGTGTGTACTATTATTTATCATTATTT
CTATAGCGTTAATTTTTCATCGATTACAGACGAAGACACATTCTATAGACCCAATACATA
AGGAAACAAAATTATCAGACAATGAAAAATATTTAGTGGATCGTAATAAGGAAAAGGTT
GCGCCGTCTAAACTAAAAGAGGTATATAATAGCAAGGATCCTAAATATAAGAAAATTGA
CAAGTATTTACAAAGTTCATTATTTAACGGTTCAGTAGCTATATATGAAAATGGCAAATT
GAAAATGAGTAAAGGTTATGGATATCAAGATTTTGAAAAAGGTATTAAAAACACACCGA
ATACGATGTTTTTAATAGGTTCAGCTCAAAAATTTTCAACAGGGTTACTGTTAAAACAGTT
AGAAGAAGAACATAAAATAAATATCAATGATCCAGTAAGTAAATACCTTCCATGGTTTAA
AACATCTAAGCCTATCCCATTGAAAGATTTAATGTTGCATCAAAGTGGATTATATAAATA
TAAATCCTCAAAAGATTATAAAAATTTAGATCAAGCAGTTAAAGCGATTCAAAAACGTGG
TATTGATCCTAAGAAATACAAAAAGCATATGTATAACGATGGGAATTATTTAGTACTTGC
GAAAGTAATTGAAGAAGTTACAGGTAAATCTTATGCTGAGAATTATTATACAAAAATAGG
AGATCCTTTAAAACTTCAGCACACAGCATTTTATGATGAACAACCTTTTAAAAAATATCT
AGCAAAAGGTTATGCTTATAATAGTACAGGACTTTCATTCTTAAGACCTAATATTTTGGAC
CAATACTATGGTGCAGGTAATTTATATATGACACCAACAGATATGGGTAAATTAATTACT
CAAATACAACAATATAAATTATTCAGTCCTAAAATAACCAATCCATTATTACATGAGTTT
GGTACGAAACAGTATCCAGATGAATATCGATATGGTTTCTATGCTAAGCCAACATTGAAT
AGACTTAACGGGGGATTCTTTGGACAAGTCTTTACTGTTTACTATAATGATAAGTATGTAG
TTGTACTTGCATTAAATGTAAAAGGAAACAATGAAGTTCGAATCAAACATATTTATAATG
ATATTTTAAAACAAAATAAACCTTACAATACGAAGGGTGTTATTGTTCAATAA

## > tnpIS232

Function: protein coding sequence; transposase for IS232
Best match: tnpIS232_trunc_CC001_MW2-USA400_BA000033.2[1036343:1036384]
Position: 025-contig_192_RC: 158998 … 159040; Length: 42 bp
Sequence:
TGAGATTATCTTTAGACATAAATATGGATTTTGAAGTTACTA

## > qoxD

Function: protein coding sequence; quinol oxidase subunit 4 (core genome, constant)
Best match: qoxD_CC001_MW2-USA400_BA000033.2[1036543:1036833:r]RC (completely identical)
Position: 025-contig_192_RC: 159198 … 159489; Length: 291 bp
Sequence:
TTATAAGTGTGAAGAGTGACCGCCTTGCATAACCCAATATGTTCCGACAACGAAACAAAG
TGTAATTACAAGAGCAAAGATAACTTTGAATGTTTGTAAACGTCCATCTTTACCTTCAGTT

AAATGCATGAACATTAATAATTGAAGTCCTGCTTGGACGAATGCAAAGCCAAAGATAATT
GTCAACTTCGCGTGGAATGTTAATGACGTGTATAGTGTTACGTATACTGCTAAAAGCGTT
AATACGATAGATGCGATAAATCCTACAGTATGTTTCATTATTGTACTCAT

> **qoxC**

Function: protein coding sequence; quinol oxidase subunit 3
Best match: qoxC_CC001_MW2-USA400_BA000033.2[1036830:1037435:r]RC (completely identical)
Position: 025-contig_192_RC: 159485 … 160091; Length: 606 bp
Sequence:
TCATCCGCTATACACCATCCCTATCATATATACGGCAGTAAAGATGAAAACCCAAACAAC
ATCTAAGAAGTGCCAGTATAAACTTACTATAAATAATTTTGGCGCATTATATTTGTCTAAT
CCGCGTCGTTGGATTTGGATTAATAAACAAATGGCCCAAACGATACCTAGCGATACGTGA
CAACCATGCGTTCCTAATAGGATAAAGAAACTAGACCAGTAAGAACCAATTGTTGGGTTA
ACGCCTTCTGATGCATAGTGTGCGAATTCATAAATTTCGAATCCAACAAAGACTAAACCT
AAAAGTAACGTAATGATCATCCAAAACATCATTAACTTTTGTTTTTCTTGGCGCATGTAGT
AAATAGCAATACCACATGTGTAAGAACTGAATAATAATGCAAACGTCATTATTAAAACA
AGAGGCAATTCAAATAACTCAGTAGTCATTTTACCTGCATAATCGCCACCATGTTGCAAA
GTTAATAGTGTCGCAAATAGGGTACCGAATAACGCAAATTCGGCTGTAATGAAAATCCAA
AAGCCAAGCTTATTTAATTCGCCTTCATGTGTGCGTGAATCAATAGTGTTTGTATCATGAC
TCAT

> **qoxB**

Function: protein coding sequence; quinol oxidase subunit 1
Best match: qoxB_CC001_MW2-USA400_BA000033.2[1037425:1039413:r]RC (completely identical)
Position: 025-contig_192_RC: 160080 … 162069; Length: 1989 bp
Sequence:
TCATGACTCATGACTTACAGCCTCCCTTTCTTTAATACGCGCTTCTCTTAATCTAGCTTCAG
TTTCAGCAACTTCAGCAGCAGGGATATGATATCCGTGATCGATTTGGAAACTGCGATAAA
TCATAGTACCAAAAATACCGAATAAACAAATTAATGCTGGAATTACAGTTTCGAAAATTA
AGAAGAAACCGCCGATAGTCATAAAGATACCAATCCAGAATCCAACAGGAGTATTGTTT
GGCATATGAATGTCTTTGTAATTATGGTTGTCTAAGTAATGACGACCATGTTCTTTCATAT
CAACAAATGTGTCGTAGTCATTCCAATCTGGTGTAATGGCAAAGTTGTATTTAGGTGGAA
TTGCTGATGCAGTAGTCCACTCTAGAGTACGACCAAGGCCATCCCAGTTATCTCCAGTTG
CTTCACGTGGAGATTTGAAGTGACTGTATACGATACTAACAACAAGGAATAAGAATCCGA
TTGCCATTAATAATGCACCGATAGTTGAAATTAAGTTTAATAAGAACCAACCATCTGATG
GCATATAAGTGTATAAACGACGTGGCATACCATCTAATCCAAGAATGAATTGTGGTAAGA
AACAAACGTTAAATCCGATCATGAAGAACCAGAAGCACCATTTGTTTAATGTTTCGTTTA
ATTTGTAACCCATCATCTTTGGATACCAGAAGATTAAACCAGCTAAGCAGGCAAATACAA
CACCAGTAACCAATGTATAGTGGAAGTGAGCTACTAAGAAGTACGTATTGTGATATTGAT
AGTCAGCTGATGCCATTGCTAACATTACACCCGTAACACCACCTAAAAGGAAGTTAGGGA
TAAATGCTAATGAGAATAGCATTGGTGACTCAAATGTAATACGTCCTTTATATAATGTTA
ATAACCAGTTAAACAATTTCACACCAGTTGGAATACCAATCAGCATTGTTGAAATTGAGA
AGAATGAGTTGATTAACGCACCATTACCCATTGTGAAGAAATGGTGAACCCAAACTAAG
AAACTAAGGAACGCGATACCGGCAGTTGCCCATACCATACTTTGATGTCCGAATAAACGC
TTACGAGCGAATGTCGGGATAATTTCTGAGTAAATACCAAATGCTGGAAGGATAACGATA
TAAACTTCAGGGTGCCCCCATACCCAGAAGAAGTTAGCCCAAAGCATTGGCATACCGCCA
TGTGCAACTGTGAAGAATGCTGTGTCAAATATTCTATCAGTTGTCATTAATGCTAACGCTA
CTGTTAAAGGAGGGAAAGCAAGAATAACAATTAATGTAGTAATAAATGTTGTTACTGTAA
ACATTGGCATTTGCATAAACTTCATAGTTGGTGTTTTACATCTTAAAATTGTTACAAAGAA
GTTGATACCTGTAGCTAAGGTACCAAGCCCTGAAATTTGTATAGCTATTAAGTAATAGTT
AACACCCGGACCAGGACTGAATTCACCTGCTAGTGGCGCATAGTTTGTCCAACCAGCTGC
TGGTGAACCACCAATAATAAATGACAGGTTGAATAAAATCATACCTGCAAAGAATAGCC
AGAAACTTACGTTGTTTAATACTGGGAATGCAACATCACGTGCTCCAATTTGTAATGGAA

CAACGATATTCCATAAACCAAAGATAAATGGCATTGCCATGAAGATAATCATGATTACAC
CATGTGTACTAAAAATTTCGTTATAGTGGTTAGATTCTAAAAATTTGTTATCAGGTACTGT
TAATTGCGCACGAATAAGTAACGCATCAATACCACCACGGACGAACATTAATACGGCAC
AGATTAAATACATAATACCGATTTTCTTATGGTCTACAGATGTGAACCATTCTTTGTAAAG
ATATTTCCATAATTTAAAGTAAGTAATTACTGCGATTAAACCAATAACTAAGAATGGGGC
ACCAATTTGTGCCATTGTAATCATCCAGTTACCTTTAACTAGTAATTGATCCCATGGAAAA
TTCAT

> **qoxA**
Function: protein coding sequence; quinol oxidase subunit 2
Best match: qoxA_CC001_MW2-USA400_BA000033.2[1039413:1040513:r]RC (completely identical)
Position: 025-contig_192_RC: 162068 … 163169; Length: 1101 bp
Sequence:
TTAATGTCCACCTCCATGATCATCATTGTCTTGATCTTGCGCATCTTTTGAAATTTTCTTCA
TTTCTTTCGCATTTTTCGATTCATCTTTCTTGAACTCATTGTTATATGGTTCGTCATTTCCAA
GAATCATCAACTTCATACCATGTCGTTTATAGTTCGCATTTGTAATTTGAGCTTTACGAGC
AGGTATTAATGGTTTGTCTGATACATCTTTAAACATATTTTCTTCACTAGTGAAGTTTGGA
TCTTTCAATTCGAAATTGAAACGTTTATATGCATAGAAGATGTATTCTGGATCGGCTGCTG
GATCAACAAACGCCATATGTGTACCATTAAATTCTAAAGCTTTATTAGGTGTGCTTGGTA
ATAATTGTTTATCAAATGTATCTTGATCTAACGTTTTCTTACCTTTAACTTCTTTCACCCAT
TTGTCGTAGTCTTTTTGACTAACGGCATTTACTTTAAATGTTTGACGTGAGAATCCTTCAC
CATTGAAGTTAGAGTTACGACCTCTGAACGTACCAGTTTGAGATGCTTCTAACGTCCAATT
CATTGTCATGCCAGTCATGGCATATTTTTGACCACCTAATTGTGGAATCCAGAAACTTGTC
ATTGTATCCATAGCTTGAAGCTTAAATACAACAGGACGATCTTTAGGGATTGTTAATGTG
TTAACAGTCTCTATATGTTCATCTGGATAAGCAAAGAACCATTTGTATCCTGCACTTACTG
CATATACAACCATTGGATCTTTCTCACTCTTCGGTGGTTTTTCGTAATCGTATAAAGTTTTA
ACTGTAGGAATAGCTAAAGCAGCAACGATTATGATAGGTATTACAAACCATATTGTTTCA
ATGATGGCATTATGGTGCATCTTACCAGATTCGGCATTCTTATTATAACTATACTTGTAAA
TAAAAATGGCGAACATGCCAAGTACAACGAAACAAATAACAAGCATGAAGACGATTGAA
TAAAGAATCAAGAACTTCTGACTACTTGCTACTGGCCCTTTTGCGTTGAAAATTTCTATAT
TTGAACAACCACTAAGTAAAATTAGTGTGCCAAATAATAGAAGCAAAGACTTAAATTTTG
ACAC

> **iraE**
Function: protein coding sequence; chitinase B (core genome, constant)
Best match: iraE_CC001_MW2-USA400_BA000033.2[1041085:1041402:r]RC (completely identical)
Position: 025-contig_192_RC: 163740 … 164058; Length: 318 bp
Sequence:
CTATTTAACCTCAATATACCTAGTGGAAATTTTAATTGCACCATCTGAATCAGTGACATGA
TATTTAATTTGATATTTACCAGATTTAGATGTATCAATTTGGCCATCGACTTTAATTTTATC
GGTTAAATCTCCATCTTCTTTATCAAATGCACTTATGCCGTTTAGAAGGTTATAATCTTGA
CCTTTCTTAATAACGATATCATTAGCGCCTTTAATTTGTGGTGTAGTATTCGTCGTTGCATC
TGCATTTAAATTTGGTGTTACTAGTGTAGCAGAAACACCGAGGGCTGATAATGACTGTAA
TAGTTTATTCAT

> **txbi_1000-2**
Function:  bidirectional rho-independent terminator
Best match: txbi_1000-2_CC001_MW2-USA400_BA000033.2[1041997:1042075] (completely identical)
Position: 025-contig_192_RC: 164655 … 164734; Length: 79 bp
Sequence:
AAGAACAAAGTGAAATGCTCCCTTCAAAGCCTTCATTTATTCAATGTCTACTTTGAAGGG
AGCATTTGATCGATTAATG

> **tx_universal2**

Function: rho indendent terminator
Best match: tx_universal2_ST425_LGA251-cow_FR821779.1[1071185:1071224:r]RC
Position: 026-contig_248_RC: 6 … 36; Length: 30 bp
Sequence:
AAAAGCTTGTTACAAGCGAATTTTCGTTCA

> **folD**

Function: protein coding sequence; bifunct methylenetetrahydrofolate dehydrogenase and methenyltetrahydrofolate
Best match: folD_CC008_NCTC8325_CP000253.1[978261:979121:r]RC (completely identical)
Position: 026-contig_248_RC: 96 … 957; Length: 861 bp
Sequence:
TTACGAATCAATACCTCGACGCATTTTTTCTGCAAGCAAAGTATTATTTAATACCATAGTA
ATTGTTAATGGACCAACGCCACCAGGAACTGGTGTAATAGCTCCAGCAATTTCTTTAACC
GCATCATAATCAACGTCACCTTTTAATTTGCCATTTTCATCTGGCGTATTGCCAACATCGA
TAATTACTGCTCCTTCTTTGACCACATCTTTTGTTACTAAACCAGGCTTACCAACTGCACT
GACAATGACATCAGCATCTTTTAAATATGATGCCATATCTTTTGAACGAGAATGTAAGAT
TGTTACTGATGCATTTTTTTGAAGTAGTAACTTAGAAACTGGTTGTCCGACAATATGACTT
CGTCCAATTACAACTGCATTTTTACCTTCTAAATCAATATCAGCATGTTTTAATATTTCCAT
GATGCCGAGCGGTGTGCAAGGTACAAAAGTTTGTTCATCGATATATAATTTCCCTATATTT
ATTGGATGAAAACCGTCCACATCTTTTTCAGGATTGATTGCTTCTAATATTTTCTGTTCGCT
AACTTGTTTTGGTAATGGTACTTGTACCAAAATACCACTTACAGAATCATCATTATTTAGT
CTATTTAGTTCGTTTAATACTTCTTCTTCAGTAGCTGTTTCTTCCAAATGTACGATTTCTGA
AATCATACCAATTTTTTCAGCTGCTTTCTTTTTTGATCTAACATAACTTTGACTAGCGCCAT
CATTACCAACTAATATAACGGATAATTTAGGTGTAAAACCCTTTTCTTTTAGCGCTTCAAC
TTGATCTTGTAACCCCTGTCTGTAGTCTTTGGCAATTTGTTTACCATCTAAAATTTTAGCAA
CCAT

> **purE**

Function: protein coding sequence; phosphoribosylaminoimidazole carboxylase catalytic subunit (core genome, constant)
Best match: purE_CC008_NCTC8325_CP000253.1[979322:979804] (completely identical)
Position: 026-contig_248_RC: 1157 … 1640; Length: 483 bp
Sequence:
GTGAAAGTAGCAGTCATTATGGGCAGTTCTTCCGATTGGAAAATTATGCAAGAGAGTTGT
AACATGTTGGATTATTTTGAAATTCCGTACGAAAAACAAGTAGTATCCGCACATCGTACG
CCAAAAATGATGGTTCAATTTGCTTCTGAAGCGAGAGAAAGAGGTATAAACATTATCATT
GCAGGCGCTGGCGGTGCGGCACATTTACCAGGTATGGTTGCATCATTAACGACGCTACCA
GTTATTGGAGTGCCGATTGAAACAAAAAGTTTAAAGGGTATAGATTCTTTATTATCAATT
GTTCAAATGCCAGGAGGTATTCCGGTTGCAACGACTGCAATTGGTGCAGCAGGTGCTAAA
AACGCAGGTATACTTGCAGCAAGAATGTTAAGTATTCAAAATCCTTCTTTAGTTGAAAAA
CTAAATCAGTATGAATCTTCGTTAATTCAAAAAGTGGAGGACATGCAAAATGAACTTCAA
TAA

> **purK**

Function: protein coding sequence; phosphoribosylaminoimidazole carboxylase ATPase subunit (core genome, constant)
Best match: purK_CC005_Mu50-VRSA_BA000017.4[1118401:1119525] (completely identical)
Position: 026-contig_248_RC: 1626 … 2751; Length: 1125 bp
Sequence:
ATGAACTTCAATAAATTAAAGTTTGGTGCGACTATTGGCATTATTGGTGGTGGTCAGCTTG
GAAAGATGATGGCACAATCAGCTCAAAAAATGGGTTATAAAGTGGTTGTATTGGATCCTT
CTGAAGATTGTCCATGTAGATACGTTGCACACGAATTTATACAAGCCAAGTATGACGATG

AAAAGGCACTCAATCAATTAGGACAAAAATGTGATGTGATTACTTATGAATTTGAAAACA
TTTCAGCCCAACAATTAAAACTATTATGTGAAAAGTACAATATTCCGCAAGGTTACCAAG
CTATACAGTTATTACAAGACCGCTTAACTGAAAAAGAAACATTAAAAAGTGCTGGTACCA
AAGTTGTCCCGTTCATTTCAGTAAAAGAATCTACAGATATTGACAAAGCAATTGAAACAT
TAGGATATCCTTTTATTGTAAAAACTAGATTTGGTGGCTACGATGGCAAAGGTCAAGTTTT
AATTAACAACGAAAAAGACTTACAAGAAGGTTTTAAATTAATTGAAACTAGTGAATGCGT
AGCTGAAAAATATTTGAATATCAAGAAAGAAGTATCTCTTACTGTTACAAGAGGAAACA
ACAATCAAATCACTTTTTTCCCATTACAAGAAAATGAGCATAGAAATCAAATACTTTTCA
AAACAATTGTTCCAGCGAGAATAGATAAAACAGCTGAGGCGAAAGAGCAAGTTAATAAA
ATTATCCAATCGATTCATTTCATTGGAACATTTACAGTGGAATTTTTTATAGATAGTAACA
ACCAATTGTATGTGAACGAGATTGCACCAAGGCCTCACAATTCCGGACATTATTCAATTG
AAGCATGTGATTATTCACAATTTGATACTCATATTTTAGCAGTTACCGGACAATCATTACC
AAATTCAATTGAATTATTAAAGCCTGCAGTCATGATGAACTTACTAGGTAAAGACTTAGA
TTTATTGGAAAATGAATTTAATGAACATCCAGAGTGGCACTTACATATTTATGGTAAGTCT
GGGCGTAAAGATAGCAGAAAAATGGGGCATATGACTGTACTAACGAATGATGTAAACCA
AACTGAACAAGATATGTACGCTAAATTTGAGGGGAGTAATTAA

> **purC**

Function: protein coding sequence; phosphoribosylaminoimidazole-succinocarboxamide synthase (core genome, variable)
Best match: purC_CC001_MW2-USA400_BA000033.2[1044843:1045547] (completely identical)
Position: 026-contig_248_RC: 2754 … 3459; Length: 705 bp
Sequence:
ATGACATTATTATATGAAGGAAAAGCGAAGCGCATTTTCTCAACAAATCAAGAAAATGA
ATTAAGAGTTGAATATAAAGATGAAGTTACTGCTGGAAACGGGGCTAAGAAAGACACAA
TGGCAGGTAAGGGGCGATTAAATAATCAAATTACTTCTATTATATTTAAATATTTACAAG
AAAATGGAATAGAAAGTCACTTTATTAAACAATTATCTGAAACAGAACAATTAGTTAAGC
CTGTGAAAATAATTCCATTAGAAGTAGTTGTTCGTAATATTGCTAGTGGATCTATTACAAA
GCGTTTAGGTTTTGAAAATGGTGAAGTTTTTAGAGAACCACTTGTAGAATTTTTCTATAAA
AATGATGCGTTAAATGATCCGTTGATAACGGATGACCATGTTAAATTGCTCAATATAGCA
TCAGATGAAGATATTGAAATACTAAAATCCAAAGCATTAAAGATTAATAATGTGTTGAAA
CAATTAATGGATGCTATGAATTTAAAATTAGTAGATTTTAAAATCGAATTTGGAAAGACT
GAGACTGGTCAAATTTTGTTAGCGGATGAAATATCTCCAGATACATGTCGAATTTGGGAT
AAAGCTACCAATGCAAACTTTGATAAAGATGTATATAGAAATAACACTGGATCACTGATT
GAAACATATCAAATATTTTTAAACAAATTGGAGGATTTAAAATAA

> **purS**

Function: protein coding sequence; phosphoribosylformylglycinamidine synthase (core genome, constant)
Best match: purS_CC001_MW2-USA400_BA000033.2[1045547:1045810] (completely identical)
Position: 026-contig_248_RC: 3458 … 3722; Length: 264 bp
Sequence:
ATGAAAACAATTGAACTACATATCACATTACAACCACAAGTATTAGATACGCAAGGACA
AACGCTTACTCGAGCTGTACATGACTTAGGTTATGCACAAGTGAATGATATTCGTGTAGG
AAAAGTATTATATATGACAGTGGATGAGGTTAGTGATGAAAAGGTACACAACATTATTAC
AACTCTAAGTGAAAAATTGTTTGCAAATACAGTGATTGAAGAATATAGCTATAAAGTGTT
AGATGATGAAAAGGAGAATGCATAA

> **purQ**

Function: protein coding sequence; phosphoribosylformylglycinamidine synthase 1 (core genome, constant)
Best match: purQ_CC005_Mu50-VRSA_BA000017.4[1120498:1121169] (completely identical)
Position: 026-contig_248_RC: 3723 … 4395; Length: 672 bp
Sequence:

ATGAAATTTGCGGTTCTTGTTTTTCCAGGTTCGAATTGTGATAGAGACATGTTTAATGCTG
CTATTAAAAGTGGTGTTGAAGCGGAATATGTAGATTATAGAGAAACATCACTAAGTGGAT
TTGATGGCGTACTTATTCCTGGTGGATTTTCATTCGGGGATTACTTAAGATCTGGGGCAAT
GGCTAGTGTAGCGCCGATTATTTCGGAAGTTAAACGTCTTGCAACTGAAGGTAAGCCAGT
ATTAGGTGTTTGTAATGGGTTTCAAATTTTAACTGAAATAGGCTTATTACCTGGTGCATTA
TTGCATAACGATTCACATTTATTTATTAGTAGAAATGAAGAGTTAGAAATAGTGAATAAT
CAAACGGCATTTACAAATCTTTATGAGCAAGGTGAAAAAGTTATATATCCTGTAGCTCAC
GGTGAAGGTCATTATTATTGTACTGATGAAATATATCAACAATTAAAAGCTAACAATCAA
ATTATTCTGAAATATGTGAATAATCCGAACGGTTCATATGATGATATTGCAGGAATTGTT
AACGAAAAAGGCAATGTATGTGGCATGATGCCACATCCTGAAAGAGCTTTAGAAACGTT
GTTAGGTACTGATAGTGGTGTGAAATTATTTGAAGCGATGGTAAAAAGTTGGAGGGAAC
AACATGTCTAA

> **purL**

Function: protein coding sequence; phosphoribosylformylglycinamidine synthase 2 (core genome, variable)
Best match: purL_CC005_A9299_ACKH01000027.1[19557:21746:r]
Position: 026-contig_248_RC: 4387 … 6577; Length: 2190 bp
Sequence:
ATGTCTAAATTTATCGAACCAAGCGTTGAAGAAATTAAACTTGAAAAAGTATATCAAGAT
ATGGGATTAAGTGATCAAGAATATGAAAAAGTTTGCGATATTTTAGGCAGACAACCTAAC
TTTACAGAAACAGGTATCTTTTCTGTTATGTGGAGTGAACATTGCTCTTATAAACATTCTA
AACCGTTTTTAAAGCAATTTCCTACGTCAGGTGAGCATGTGCTTATGGGTCCAGGTGAAG
GTGCAGGGGTAGTCGATATAGGTGATAATCAAGCCGTAGTATTTAAAGTAGAGTCTCACA
ATCATCCATCAGCAATTGAACCATATCAAGGGGCTGCTACAGGCGTTGGTGGAATCATTC
GTGACATTGTTTCTATTGGGGCTAGACCTATTAATTTGTTAAACAGTCTTAGATTTGGAGA
ATTAGATAATAAACAAAACCAAAGATTACTTAAAGGGGTTGTAAAGGGTATCGGAGGTT
ATGGTAACTGCATTGGTATTCCAACAACTGCTGGTGAAATCGAATTTGATGAATGTTATG
ATGGCAATCCACTTGTTAATGCAATGTGTGTTGGTGTTATCAATCACGACATGATTCAAA
AAGGCACAGCAAAAGGTGTAGGTAATTCGGTCATTTATGTTGGTTTGAAAACTGGTCGAG
ATGGTATTCATGGTGCTACTTTTGCATCTGAAGAATTGACGGAAGAAAGCGAAAGTAAAC
GACCTTCTGTACAAATCGGTGATCCATTTGTAGGTAAAAAATTAATGGAAGCAACACTTG
AAGCAATTACATTTGATGAATTAGTTGGTATTCAAGATATGGGTGCTGCTGGTTTAACATC
TTCATCGTCTGAAATGGCGGCAAAAGGTGGTAGTGGGTTACATTTGAGATTAGAACAAGT
GCCAACACGTGAGCCAGGTATTTCTCCTTATGAAATGATGCTTTCAGAAACTCAAGAACG
TATGTTACTAGTTGTTGAAAAAGGTAATGAACAAAAAATTCTTAGATTTATTTGATAAGCA
CGAATTGGATAGTGCTGTTATAGGTGAAGTTACAGATACAAATCGTTTTGTTTTAACATAT
GATGATGAAGTTTATGCTGACATTCCAGTTGAACCACTAGCTGATGAAGCACCTGTATAT
ATTTTAGAAGGAGAAGAAAAAGATTATAATACTTCTAAAAATGATTATACACACATCGAT
GTTAAAGATACTTTCTTTAAATTACTTAAGCATCCGACTATAGCATCTAAACACTATTTAT
ATGATCAATACGACCAACAAGTTGGTGCCAATACGATAATTAAGCCAGGACTTCAAGCAT
CGGTAGTACGTGTGGAAGGCACAAATAAGGCAATTGCTTCAACAATTGATGGTGAAGCG
CGTTATGTATATAACAATCCATATGAAGGTGGAAAGATGGTAGTAGCTGAAGCTTATCGA
AATTTAATTGCCGTGGGTGCAACACCATTAGCAATGACAGATTGTTTAAATTATGGTTCTC
CTGAAAAGAAAGAAATCTATCAACAGTTGATAGATTCAACGAAAGGTATGGCAGAAGCA
TGCGACATTCTTAAGACACCAGTAGTTTCTGGAAATGTATCTTTATATAACGAAACGAAA
GGTACTTCTATTTTCCCAACACCAGTTGTTGGAATGGTAGGTTTGATTGAAAATGTAAATT
ATTTAAATGATTTTGAACCTCAAGTTGGAGATAAATTATATTTAATCGGTGATACTAAGG
ACGACTTTGGTGGTAGTCAACTTGAAAAGTTAATTTATGGCAAAGTTAATCATGAATTTG
AGTCATTAGATTTGAGTTCAGAAGTTGAAAAAGGTGAATCAATCAAGACCGCTATTCGTG
AAGGACTATTATCACATGTTCAAACAGTTGGTAAAGGTGGCTTACTGATTACTTTAGCTA
AACTAAGTGCGCATTACGGTTTAGGATTAAAGTCTTCAATAGATATAACAAATGCACAAT
TGTTTAGTGAGACGCAAGGCCGATATGTTGTTTCTGTTAAATCAGGTAAAACTTTAAATAT
TGATAATGCAATAGAAATTGGACTTTTAACAGATAGTGATAATTTCAAAGTAACAACACC
ATATACAGAGATTAGTGAAAATGTTTCAGATATTAAACAAATATGGGAAGGGGCAATTG
CTCAATGTTTAACTACTCAGGATTAA

## > **purF**

Function: protein coding sequence; phosphoribosylpyrophosphate amidotransferase (core genome, variable)
Best match: purF_CC007_USA300_TCH959_AASB02000224.1[105546:107030]
Position: 026-contig_248_RC: 6555 … 8040; Length: 1485 bp
Sequence:
ATGTTTAACTACTCAGGATTAAACGAAGAATGTGGCGTGTTTGGTATTTGGAATCATCCT
GAAGCAGCGCAACTAACATATATGGGACTTCATAGTTTGCAACATCGTGGTCAAGAAGGT
GCAGGTATAGTTGTTTCTGATCAAAATGAATTAAAAGGCGAGCGAGGATTAGGCTTACTA
ACTGAAGCGATTAAAGATGATCAAATGGAACGATTAAAAGGATATCAACATGCAATTGG
TCACGTCCGTTATGCTACTTCAGGTAATAAAGGTATTGAAAATATTCAACCGTTTCTGTAT
CACTTTTATGATATGAGTGTAGGTATTTGTCATAATGGTAACCTCATTAATGCTAAATCAT
TGCGTCAGAATTTAGAAAAACAAGGTGCTATCTTCCATTCGTCTTCTGATACTGAAGTCAT
TATGCATTTGATACGTCGAAGTAAAGCTCCTACTTTTGAGGAAGCGTTAAAAGAAAGTTT
GCGAAAAGTTAAAGGCGGTTTTACATTTGCGATATTAACTAAAGATGCGTTATATGGCGC
AGTAGATCCAAATGCTATCAGACCACTTGTTGTAGGTAAAATGAAAGATGGTACATACAT
CCTTGCAAGTGAAACATGTGCAATAGATGTGTTAGGTGCAGAATTTGTTCAAGATATTCA
TGCAGGTGAATATGTCGTGATTAACGATAAAGGTATTACAGTTAAATCTTATACACATCA
TACGACAACTGCAATTTCTGCGATGGAATATATTTATTTTGCTAGACCAGACTCAACAAT
AGCTGGTAAAAATGTCCATGCAGTACGTAAAGCTTCTGGTAAAAAATTAGCCCAAGAAA
GCCCTGTAAATGCTGATATGGTCATCGGTGTACCCAATTCATCGCTATCAGCTGCGAGTG
GTTATGCTGAAGAAATAGGTTTGCCATATGAAATGGGACTAGTTAAAAATCAATATGTTG
CAAGAACATTTATTCAACCAACTCAAGAATTACGTGAGCAAGGTGTGAGAGTGAAGTTAT
CTGCGGTAAAAGATATAGTAGATGGGAAAAATATCATTCTTGTTGATGATTCCATTGTTC
GCGGTACGACAATTCGACGCATTGTGAAAATGTTAAAAGATTCTGGTGCAAATAAAGTAC
ATGTGCGTATAGCATCACCGGAATTTATGTTCCCAAGTTTTTATGGAATCGATGTTTCAAC
TACGGCAGAATTAATTTCTGCAAGCAAATCACCTGAAGAAATTAAAGATTATATTGGCGC
TGATTCATTAGCATATCTATCTGTAGATGGGTTAATTGAATCAATTGGTTTAGATTATGAC
GCGCCATATAGTGGCTTATGTGTAGAAAGTTTCACTGGAGATTATCCTGCAGGGTTATAT
GATTATGAAGCAAATTATAAAGCGCATTTAAGTCATCGACAAAAGCAATATATTTCTAAA
AACAAACACTTTTTTGATAGCGAGGGAAATTTAAATGTCTAA

## > **purM**

Function: protein coding sequence; phosphoribosylformylglycinamidine cyclo-ligase (core genome, constant)
Best match: purM_CC005_Mu50-VRSA_BA000017.4[1124807:1125835]
Position: 026-contig_248_RC: 8032 … 9061; Length: 1029 bp
Sequence:
ATGTCTAAAGCATATGAACAATCTGGTGTAAATATTCATGCTGGTTATGAAGCTGTAGAA
AGAATGTCTAGTCATGTTAAACGTACGATGCGTAAAGAAGTTATCGGTGGTTTAGGTGGA
TTCGGTGCTACATTTGATTTATCACAATTAAATATGACAGCACCAGTTTTAGTTTCTGGAA
CAGACGGAGTAGGTACGAAATTAAAACTAGCTATCGACTATGGGAAACATGATTCGATA
GGTATCGATGCAGTCGCAATGTGTGTTAATGATATTTTAACGACAGGTGCAGAACCATTG
TATTTTTTAGATTACATCGCTACAAATAAAGTTGTTCCTGAAGTTATTGAACAAATTGTTA
AAGGTATTAGTGATGCATGCGTTGAAACGAATACTGCACTTATCGGTGGAGAGACTGCTG
AAATGGGTGAAATGTATCACGAAGGTGAATATGATGTAGCCGGATTTGCAGTTGGAGCA
GTTGAAAAGGATGACTATGTAGATGGTTCAGAAGTGAAAGAGGGACAAGTTGTTATAGG
GCTTGCGTCAAGTGGCATTCATTCAAATGGATATAGTTTAGTGCGCAAATTAATTAATGA
ATCAGGCATTGATTTGGCATCAAACTTCGATAATCGTCCATTTATAGATGTCTTTTTAGAA
CCAACTAAATTATATGTCAAACCTGTACTTGCTTTAAAAAAAGAAGTTTCTATTAAGGCA
ATGAATCATATTACTGGTGGAGGCTTTTATGAAAATATTCCACGTGCATTGCCAGCCGGA
TATGCTGCTAGAATTGATACTACATCATTTCCAACGCCAAAAATATTTGATTGGTTACAAC
AACAAGGCAATATAGACACAAATGAAATGTATAACATTTTTAACATGGGTATTGGCTATA
CGGTTATCGTTGATGAAAAAGATGCATCACGCGCTTTGAAGATTTTAGCAGAACAAAATG

TGGAAGCTTATCAAATTGGTCATATTGTGAAAAATGAGTCAACTGCAATTGAATTGTTGG
GGGTATAA

> **purN**
Function: protein coding sequence; phosphoribosylglycinamide formyltransferase (core genome, constant)
Best match: purN_CC001_MW2-USA400_BA000033.2[1051152:1051718]
Position: 026-contig_248_RC: 9063 … 9630; Length: 567 bp
Sequence:
ATGGTTAAAATTGCGATTTTTGCATCAGGTTCAGGAAGTAACTTTGAAAATATAGTTGAG
CATATTGAATCAGGAAAACTTGAAAATATTGAAGTTACGGCGCTATATACGGATCATCAA
AATGCGTTTTGTATAGATAGAGCAAAAAAGCACGGTATTCCTGTTTATATTAATGAACCA
AAACAATTTGATTCAAAAGCAGCGTATGAACAACATTTAGTAACACTATTAAATAAAGAT
AAGGTAGAGTGGATTATTTTAGCTGGCTACATGCGTCTAATAGGTCCAGACTTATTAGCA
TCATTTGAAGGTAAAATATTGAATATACATCCATCTCTATTGCCGAAATATAAGGGGATT
GACGCAATAGGCCAAGCATATCATAGTGGCGATACTATTACTGGTTCGACAGTACATTAT
GTTGATAGTGGTATGGATACGGGAGAAATTATTGAACAGAGAAAATGTGATATTAGACC
GGACGATTCAAAAGAACAATTAGAAGAGAAAGTAAAAAAATTGGAATATGAATTATATC
CAAGTGTTATTGCTAAAATTGTAAAATAA

> **purH**
Function: protein coding sequence; bifunct phosphoribosylaminoimidazolecarboxamide formyltransferase/IMP cyclohydrolase (core genome, constant)
Best match: purH_CC001_MW2-USA400_BA000033.2[1051733:1053211]
Position: 026-contig_248_RC: 9644 … 11123; Length: 1479 bp
Sequence:
ATGAAGAAAGCTATTTTGAGCGTATCAAATAAAACAGGTATTGTAGAGTTTGCTAAAGCG
TTAACGCAATTAAATTATGAATTATATTCAACAGGTGGTACTAAACGTATATTAGATGAA
GCAAATGTACCAGTTCGTTCTGTTTCAGACTTAACACATTTTCCAGAAATAATGGATGGCC
GTGTTAAAACATTACATCCGGCTGTTCATGGTGGCATTTTAGCTGATCGAAATAAACCGC
AGCATTTAAATGAATTATCAGAACAACATATAGATTTAATTGATATGGTAGTAGTTAATT
TATATCCATTCCAACAAACTGTTGCAAACCCTGATGTGACGATGGACGAAGCAATTGAAA
ATATTGATATTGGTGGTCCAACAATGTTACGTGCTGCAGCTAAAAACTATAAACATGTAA
CAACAATTGTACATCCGGCAGATTATCATGAAGTATTGACGCGATTAAGAAACGATTCGT
TAGATGAGTCATATAGACAATCATTAATGATTAAAGTTTTTGAGCATACTGCAGAATATG
ATGAAGCGATTGTACGTTTCTTTAAAGGGGATAAAGAAACTTTAAGATATGGAGAAAATC
CACAACAATCAGCGTATTTTGTGAGAACTTCGAATGCTAAGCACACGATTGCAGGCGCTA
AACAATTACATGGGAAACAATTAAGCTATAACAATATTAAAGATGCAGATGCTACACTA
GCTTTAGTTAAAAAGTTTGATACACCTGCTGCAGTTGCGGTTAAACACATGAATCCATGT
GGTGTTGGTATCGGTGACACGATTGAACAAGCATTTCAACATGCGTATGAAGCGGATAGT
CAATCAATATTTGGTGGAATTGTTGCATTAAACCGAGCTGTAACACCTGAGTTAGCAGAG
CAATTGCATAGTATCTTTTTGGAAGTCATTATTGCACCAAAATTTACAGATGAAGCATTAG
ATATTTTAAAACAAAAGAAGAATGTAAGATTATTAGAAATTGATATGACTATAGACAGTA
ACGAAGAAGAGTTTGTTTCAGTATCTGGCGGATATTTAGTTCAAGATAAAGACAATTATG
TCGTGCCAAAAGAAGAAATGAAAGTTGTTACAGAAGTAGCACCTACTGATGAACAATGG
GAAGCAATGTTATTAGGATGGAAAGTTGTACCATCAGTAAAAAGTAATGCAATTATTTTA
AGTAATAATAAACAAACTGTAGGTATAGGTGCTGGACAAATGAATCGTGTCGGTGCTGCT
AAAATTGCGTTAGAGAGAGCTATTGAAATCAATGATCATGTAGCGTTAGTATCTGATGGA
TTTTTCCCTATGGGAGATACAGTTGAACTTGCAGCACAACATGGTATAAAGGCAATTATC
CAACCGGGTGGTTCGATTAAAGATCAAGATTCAATTGATATGGCTAATAAACATGGTATT
GCAATGGTAGTCACAGGCACTCGACATTTTAAACACTAA

> **purD**
Function: protein coding sequence; phosphoribosylamine-glycine ligase (core genome, constant)
Best match: purD_CC010_D139_ACSR01000034.1[61660:62907]

Position: 026-contig_248_RC: 11144 … 12392; Length: 1248 bp
Sequence:
ATGAATGTATTAGTAATTGGTGCTGGTGGACGAGAACATGCACTTGCATATAAACTTAAT
CAATCGAATCTAGTTAAACAAGTGTTTGTCATTCCAGGTAATGAGGCAATGACACCTATA
GCTGAAGTACACACTGAAATTTCAGAATCTAATCATCAAGGGATACTAGATTTTGCTAAA
CAGCAAAATGTTGATTGGGTAGTTATAGGTCCAGAACAGCCGCTAATTGATGGATTAGCA
GACATTTTACGAGCTAATGGTTTCAAAGTGTTTGGTCCAAATAAGCAAGCAGCTCAAATC
GAAGGCTCAAAATTATTTGCTAAAAAGATAATGAAAAAATATAATATTCCAACTGCTGAT
TATAAAGAAGTTGAGCGAAAAAAGGATGCTTTAACATATATTGAAAACTGTGAATTGCCC
GTTGTTGTCAAGAAAGATGGGTTAGCTGCTGGGAAAGGCGTTATTATTGCAGATACTATT
GAAGCAGCCAGAAGTGCTATTGAGATTATGTATGGTGATGAAGAAGAAGGTACTGTTGT
ATTTGAAACGTTTTTAGAAGGTGAAGAGTTCTCGCTAATGACATTTGTTAATGGTGATTTA
GCAGTACCTTTCGACTGTATTGCACAAGATCATAAACGCGCATTTGATCATGATGAAGGA
CCAAATACTGGTGGTATGGGGGCTTATTGTCCAGTACCACATATTAGTGACGATGTTTTA
AAACTTACAAATGAAACAATTGCACAACCCATTGCAAAGGCAATGCTTAATGAAGGTTAT
CAATTCTTCGGTGTATTATACATTGGTGCTATTTTAACTAAAGATGGTCCAAAAGTAATAG
AATTTAATGCCCGTTTTGGTGATCCTGAAGCTCAAGTATTATTAAGTCGCATGGAAAGTG
ATTTAATGCAGCATATTATTGATTTAGATGAAGGAAAACGTACTGAATTCAAATGGAAAA
ATGAATCTATTGTAGGGGTCATGTTGGCATCAAAAGGATATCCTGATGCATATGAAAAAG
GGCATAAAGTAAGTGGCTTTGATTTAAATGAAAACTATTTTGTTAGTGGATTAAAGAAGC
AAGGTGATACCTTTGTTACTTCAGGTGGTAGAGTTATACTTGCCATCGGAAAAGGTGACA
ATGTACAAGATGCACAGCGAGACGCATACGAAAAAGTATCTCAAATACAAAGTGACCAT
TTATTCTATCGTCATGACATTGCGAATAAAGCACTACAACTTAAATAA

> **repeat_Sgenus**

Function: repeat repeat element alien/(mobile element)obile/
Best match:
Position: 026-contig_248_RC: 12401 … 12431; Length: 30 bp
Sequence:
AAAAATACTAAGATTAGCTATGAAGAAATC

> **ykoC**

Function: protein coding sequence; ATP binding cassette transporter/membrane protein (core genome, constant)
Best match: ykoC_CC005_ED98-hen_CP001781.1[1064296:1065102:r]RC (completely identical)
Position: 026-contig_248_RC: 12658 … 13465; Length: 807 bp
Sequence:
TTAACCTACACGACCAAGACGAACATCATCTATGCCCGTGATGGGTAAGGTGATTGAACA
ATAATATGCCATAGTAATAATGGCAATTAAAACTATAATAAAGATTATATCTTTATATGA
GAAAGGTACGTTGTAATAGTAAGTACGAGGACCATCTCTAAATCCTTTCGACTCCATCGC
AACTGATAATTGATGTGCCTTTCTAATATTTTGGCTTAATAGAGGTATAATTAAATGCTTA
AATCGCTTTAACCCTCTATAATTTGCTGCGTCTATCATCTGATAGCGCATTTTTAAAGATC
TGCGAAGCTGTATTAAAGAACTAATCATTAAAGGTATCATACGAATGGCAGCCATGAATG
CATAAGCAACTTTTGATTTAACCTTTAAATGTTGCATTAAACTATAAAATATCATGACAAC
CTGAGATGTAAGTGCGATTAAGATACCGAAAAATGAAATAGCAATGGTTCTTAATGATAC
ATGTAAACCACGAACTAAACTTTCTGTTGTAATATGGATAAATCCGAATTTCAAAATTGT
ATGGCTACCATTCCCGTATAAAATCATGAACAGGGAAGAGAGTAATGCAAAGCCAATAC
TTATAGTTATAAAAATTGCTGTAATTTTAAACTGAGTACCATTAAACATCAATAAGAAAA
CTAACATTAAGATAGTGATATAAAGCATAAAATCGAAATTATGCACAAATATAATAAAG
AAAAATAGTATAATTCCAAGAAATAGTTTCGTTATAATGTTGACATCATCAACAAATGAT
TGCCGAACTTTCCATTGCTCATACAT

> **ykoD**

Function: protein coding sequence; ABC transporter/ATP-binding protein (core genome, constant)
Best match: ykoD_CC005_Mu50-VRSA_BA000017.4[1130233:1131633:r]RC (completely identical)

Position: 026-contig_248_RC: 13457 … 14858; Length: 1401 bp
Sequence:
TCATACATTCGTATCACCATCACAATCTAGTAACGCACCATCTGAAATTTTAAGTCTTCTT
GATGGATAACGTTCAATTATTTCATCGTCATGTGTAACCATGACAATACTTTGTCCCAAAT
TAATTCGCTTTTGGAAAAGTTTGATCAACTGGAATGTATTATGGCTATCAAGTCCAAATGT
CGGTTCATCTAAAAAGATAATATCAGCTTTAGAACTTAGTGCGGTAGCTACGCTAAGGCG
TCGTTTTTGACCAATAGACAACTCATAAGGATGTTGATCTTTTACATTTTGTAAATCTAAA
AGTTTTAAAAGTTGTATCGTTTCATCATCACTTTGATCTTTAGAAAGGTGATTAAAATGAA
TGTTAATTTCATCATAAACCGAATTTGTTATAAATTGTAATTCTGGGTTTTGATAAACTAG
GTACATGTGTTTTGCTGCATGTTTAATTTTTGTTAAACGCTGATTTTCAAAATAAACATCA
CCTTGATATTTAATCAATTGCATAATTGATTCAAGCAAGGTTGTTTTACCACTACCATTTG
CCCCTGTAATTGTAATCCACTCACCTAGACCAATTTCTAAATCTGAGAATGAGAGCAATG
TTGATTTACCGCGAATAATACGTCCATTTTTAAATTGTAATAAGTGTGAGTTTGTTGTTGG
AAAGTCAACACGACTTGGTGCGAATTCCCATGCACGTGGATGCCACACACCATATTCACT
GAGTAAATGAACATACTTCTGTAATATGATTTCAGGACATTCATCGGCAATGATATTTCC
GTTATAATCCATCAAAATGACGCGGTCGACATGATTCCAGATGTGTTTAACTTTATGTTCA
ACGATTACAACAGTTTGATCTTCCCAAAGTTCAATTAGTTTAGTCCATAAATCTTCTGTTG
CTTGAACATCTAACATTGCTGTCGGTTCATCTAAAAACAATGTTTTTGATTGTTGAAGAAT
GGTTTCAACAATTGCCAATTTCTGTTTCATCCCGCCACTTAAATCTTTGATATACGTTTCA
GGGGTAACATTTAAATTGACCATATTTAAAGCATTGATAATTAACGCATCCATGTCTTCAC
GTGGTACTTGTCTATTTTCTAAAACGAATGCAAGTTCTTCGTATACTTTTGGCATACAAAA
CTGGCTATCAGGGTCTTGGAAAATAACGCCACTTAATGGGTCAACGATTAGTTCATCATA
TTTCATAGGTAATTCAATTAAATTAGGAACAATACCACTTAATACATTCAGAAGTGTACTT
TTACCGCAACCAGAAGGACCGAGTAAAAGTACTTTTTCTTTGTCTTGAATAGTGATATTTA
AATGATCGAAAATTTTACGCTGACCATTTGGATATTTTAATCGTAAATCACTTACTTTTAA
CAC

## > ykoE

Function: protein coding sequence; substrate-specific component of transporter for hydroxy-methyl-pyrimidine (core genome, constant)
Best match: ykoE_CC001_MW2-USA400_BA000033.2[1056959:1057534:r]RC (completely identical)
Position: 026-contig_248_RC: 14872 … 15448; Length: 576 bp
Sequence:
TTATAAGTTGTCGTAATCTTCTTTAGCAGCTGGTCTAAATAATTTTGTTACGCCTGTCTTAT
CTAAAGCTTTTACTAAAAGGTAAGATAGGACGCCGGCGACTACTGCACCACTAATTAATC
TAAATACGATGAATAATGTTAAGTTCCAACCTGCAACTTCATTTAAATAACCATAGAAAT
AATCTATAGGGAAAGCCGCGATTGCTGTACAAAAACCTGCTAACATAGCTACCATAACTG
AACGAGATTGATATTTAAAAATTGCAAAGACAAGTTCACACGCTAAACCTTGTATGAAAG
CGTAAACGATTGTCGGAATATCGAAACGACCCATAATGATAGTTTCGCCGGCACCTGCAG
CAAATTCAGCCAGTAAAGCAATACCTGGTTTTGGAATAATTAGATAGCAGACAATCGCTG
CCATGAACCAAACCCCGTTTGTTAATTGTTCGAGGTGAAGGCCTGTAGCTTGCACACCAT
TGTAAACAAACCACCATAAATTGTAAATAACTGCGAATACTACTGAAATAAGTACGGTTA
CTAGTATTTCAGATAGCTTTAAACCTTTTGACAT

## > TPP_riboswitch_L1

Function: noncoding RNA thiamine pyrophosphate binding riboswitch/locus 1 (core genome, constant)
Best match: TPP_riboswitch_L1_CC001_MW2-USA400_BA000033.2[1057611:1057711:r]RC (completely identical)
Position: 026-contig_248_RC: 15524 … 15625; Length: 101 bp
Sequence:
ATATGTATAGTAACACTTTCCTACGCTAGTTCAAGCTAGATCAGGTTCAAAGGGTTTGAG
GGCAAGCCTCATCTCAGTATAAAACACCCCTAGTGTGTGCG

> **graF**

Function: protein coding sequence; putative protein (core genome, constant)
Best match: graF_CC001_MW2-USA400_BA000033.2[1058055:1058189:r]RC (completely identical)
Position: 026-contig_248_RC: 16220 … 16355; Length: 135 bp
Sequence:
TTAATTTAGTGTATCTTGGATATCTTGTTTTGTTTGATTAATATCTTCTGTTTTTTCTTCTTT
TTTATCTTTTAATTTTTCTTCAACTTCTTTAGCTTTTTCTGCTGCTTTTTTATTTTGATTTTCA
TTAGACAT

> **ywbD**

Function: protein coding sequence; S-adenosylmethionine-dependent methyltransferase (core genome, constant)
Best match: ywbD_CC001_MW2-USA400_BA000033.2[1060061:1061233]
Position: 026-contig_248_RC: 18226 … 19399; Length: 1173 bp
Sequence:
ATGAAAATAGCAACTCTGAACAAAGGCAAAGAAACAAAATATTTTAATGGATATCCTTTA
ATTGAAGAAGAGGATATCTATTCACAAGATCATTTAAAAGAAGGAGATATTTTTCAAATT
GTGACTGATAAATCACAATATGTTGCAACGGCTTATGTTGGTCGCCAACATAAAGGATTA
GGTTGGGTTCTAACATACGATAAAGCTCAAGAAATCAACACAGCTTTCTTTGTGAAATTG
TTTAATACTGCATTAGCAGAACGTGATTATTATTTTAATATAGATGGAACAAATGCTTTTA
GATTATTTAATGCTGAAGGTGATGGTGTTGGGGGATTAACAATCGACAATTACGATGGTC
ATTTGTTGATTCAATGGTACTCAAAAGGTATTTATAAATTTAAATATGCCATTCTTGAAGC
GGTTAGAAAAGTATTTGATTATAAATCTATTTACGAAAAAGTAAGATTTAAAGACAGCGA
ATATAGTGGTGGTTTTGTTGAAGGCGATGCACCTGAGTTTCCAATTGTTATCGAAGAAAA
CTTCACATTTTATAATGTAGACCTTGAAGATGGTTTGATGACAGGTATCTTTTTAGATCAA
AAAGAAGTGCGCAAGAAATTAAGGGGTCAATATGCCAAAGAACGCCATGTTTTAAACTT
ATTTAGTTATACAGGTGCTTTTTCTGTAATAGCAGCAAGTGAGGCATCTTCAACAACAAG
TGTAGATTTGGCTAATCGTTCTCGTAGTTTAACTGAAGAAAATTTTGGATTAAATGCTATT
GATCCTAAATCCCAATATATTTATGTCATGGACACTTTTGATTTCTATAAATATGCTGCAC
GACATGGACATAGTTATGACACGATCGTGATTGATCCACCTAGCTTTGCACGCAACAAAA
AACGTACATTTTCAGTGCAAAAAGATTATGACAAATTAATTAATGGCGCCTTAAATATTT
TATCATCTGAAGGAACATTATTGTTATGTACAAATGCAAGTGTATATCCATTAAAGCAAT
TTAAAAATACTATTAAAAAGACGCTTGAAGAGAGTGGCGTTGATTATGAATTAACTGAAG
TTATGGGATTACCAAAAGATTTTAAAACGCATCCACATTATAAGCCATCTAAATATTTAA
AAGCTGTTTTTGTAAATATTAGACATTAA

> **ptsH**

Function: protein coding sequence; phosphocarrier protein HPr (core genome, constant)
Best match: ptsH_CC001_MW2-USA400_BA000033.2[1061983:1062249]
Position: 026-contig_248_RC: 20148 … 20415; Length: 267 bp
Sequence:
ATGGAACAAAATTCATATGTAATCATCGACGAGACTGGTATTCACGCTAGACCAGCAACA
ATGTTAGTACAAACAGCTTCAAAATTCGATTCTGATATTCAATTAGAATATAACGGTAAG
AAAGTAAATTTAAAATCAATCATGGGTGTTATGAGCCTTGGTGTTGGTAAAGATGCTGAA
ATTACAATTTATGCTGACGGTAGTGATGAATCTGACGCCATTCAAGCAATCAGTGACGTC
TTATCAAAAGAAGGATTGACTAAATAA

> **ptsI**

Function: protein coding sequence; phosphoenolpyruvate-protein phosphotransferase (core genome, constant)
Best match: ptsI_CC001_MW2-USA400_BA000033.2[1062252:1063970]
Position: 026-contig_248_RC: 20417 … 22136; Length: 1719 bp
Sequence:

ATGTCTAAATTAATTAAAGGTATTGCCGCATCTGATGGTGTCGCAATTGCTAAAGCTTATT
TATTAGTTGAGCCAGACTTAACATTCGACAAAAATGAAAAAGTCACTGATGTTGAAGGAG
AAGTTGCAAAGTTCAATAGCGCTATCGAAGCTTCTAAAGTTGAGTTAACTAAAATTAGAA
ATAATGCAGAGGTTCAACTAGGTGCTGATAAAGCTGCTATCTTTGATGCACATTTATTAGT
TTTAGATGACCCTGAATTAATTCAACCAATCCAAGATAAGATTAAAAATGAAAACGCTAA
TGCTGCTACAGCATTAACGGATGTAACAACACAATTTGTTACAATTTTTGAATCTATGGAT
AACGAATACATGAAAGAACGTGCGGCTGATATTCGCGACGTTTCTAAACGTGTGTTATCA
CATATTTTAGGTGTAGAATTACCGAATCCGAGTATGATTGATGAAAGCGTTGTTATTGTA
GGGAATGACTTAACGCCATCTGATACTGCTCAATTAAATAAAGAATTCGTACAAGGTTTT
GCTACAAACATTGGCGGAAGAACAAGTCACTCTGCAATTATGAGTCGTTCTTTAGAAATT
CCAGCAATTGTTGGTACAAAATCAATTACTCAAGAAGTTAAACAAGGCGACATGATTATC
GTAGATGGATTAAATGGTGATGTAATCGTTAATCCAACTGAAGATGAGTTAATCGCTTAT
CAAGATAAACGTGAGCGTTATTTTGCTGACAAGAAAGAATTACAAAAACTACGTGATGCT
GATACTGTTACAGTTGATGGTGTTCACGCAGAGCTTGCTGCAAATATTGGTACACCTAAT
GATTTGCCAGGTGTTATTGAAAATGGTGCACAAGGTATCGGCTTATATAGAACTGAGTTT
TTATATATGGGTCGTGACCAAATGCCTACAGAAGAAGAACAATTTGAAGCTTATAAAGAA
GTATTAGAAGCAATGGGCGGTAAACGTGTTGTTGTACGTACTTTAGATATAGGTGGAGAT
AAAGAATTATCATACTTAAACTTGCCTGAAGAAATTAATCCATTCTTAGGTTACCGTGCG
ATTCGTTTATGCCTTGCGCAACAAGATATTTTCAGACCACAGCTACGTGCATTATTACGTG
CATCAGTTTATGGTAAGTTAAATATCATGTTCCCAATGGTTGCAACAATTAACGAATTTAG
AGAAGCTAAAGCTATATTATTAGAAGAAAAAGAAAACCTTAAAAATGAAGGTCATGACA
TTTCGGATGATATAGAATTAGGAATCATGGTAGAGATACCTGCAACAGCAGCATTAGCTG
ATGTCTTTGCTAAAGAAGTAGATTTCTTCAGTATCGGTACAAATGATTTAATTCAATACAC
ATTAGCTGCTGACCGTATGTCAGAGCGTGTATCATATCTATACCAACCATATAACCCTTCA
ATCTTACGTTTAGTTAAACAAGTTATTGAAGCGTCACATAAAGAAGGTAAATGGACAGGT
ATGTGTGGTGAAATGGCTGGAGATGAAACAGCTATTCCATTATTGCTTGGTTTAGGTTTA
GATGAGTTCTCTATGAGTGCAACGTCTATTCTGAAAGCAAGAAGACAAATTAATGGTTTA
AGTAAAAATGAAATGACTGAACTTGCTAACCGTGCAGTCGACTGTGCAACGCAAGAAGA
AGTTATTGAATTAGTTAACAACTACGTAAAATAA

## > nrdH

Function: protein coding sequence; glutaredoxin (core genome, constant)
Best match: nrdH_CC005_Mu50-VRSA_BA000017.4[1139211:1139444:r]RC (completely identical)
Position: 026-contig_248_RC: 22372 … 22606; Length: 234 bp
Sequence:
TTAATCTTGGATATTTAATACTTTGTTGATTTCATCAAGATCAACATGGTACATTGGATTG
CCATTCAACAAAATAAACGGAGTTGAAAAAGCATCAAAATCTATCATTTCGTTTCGATAT
TGTTGATTGTTGATATTTCTCTCTTCAAAATCAATTTGATGCTCATTTAGATAATTTTTTAC
AAATGTACAAGGTGGACAATCATTCTGCGTATAAACGATTATTTCTGACAT

## > cydA

Function: protein coding sequence; cytochrome D ubiquinol oxidase subunit 1 (core genome, constant)
Best match: cydA_CC001_MSSA476_BX571857.1[1093424:1094785]
Position: 026-contig_248_RC: 22803 … 24165; Length: 1362 bp
Sequence:
ATGGATACAGTTGAAATCAGTCGGTTTTTGACAGCTATGACTTTAGCAGTTCATATCATTT
TTGCAACGATTGGTGTTGGTATGCCTTTAATGTTCGCAATTGCAGAATTTTTAGGTATTCG
CAAAAATGATCTTCAATATATAGCTATGGCCAAAAGGTGGGCTAAAGCTTATACAATTAC
TGTAGCAGTGGGAGTTGTTACAGGTACAATTATAGGACTTCAATTATCATTGATTTGGCCT
ACTTTTATGGAAATGGGTGGACACGTTATTGCACTTCCATTATTTATGGAAACATTTGCGT
TCTTCTTTGAAGCTATTTTCTTAAGTATATATTTATATACTTGGGACCGTTTTAAAAATAA
ATGGACACATTTCTTAATTAGTATACCAGTAATTTTTGGTGGCTCTTTCTCAGCATTCTTCA
TTACTTCAGTGAATTCATTTATGAATACGCCTGCAGGTTTTGAGTTGAAGAATGGAAAGA
TGGTCAATGTTCAACCTATAGAAGCGATGTTTAACCCATCGTTTATAGTTCGATCATTTCA

CGTAATTACTACAGCCGGTATGACGATGGCATTTGTTATTGCATCAATAGCAGCTTTTAAA
TTATTGCGTAATCGTCAACCTAAAGATACTGTCTACCATAAGAAAGCTTTGAAAATGTCT
ATGATAGTTGGATTCTTTTCAACTTTACTTTCTATGTTGGCAGGGGATTTATCTGCAAAAT
TTTTGCATAAATTCCAACCTGAAAAATTAGCAGCTTATGAATGGCATTTCGATACATCTTC
CCATGCTAAATTATTATTATTTGGTGTGTTAGATGAAAAGACTCAGCAAGTTAAAGGTGC
GATTGAATTACCTGGACTATTGAGTTTCTTAGCAGATAATAGTGTCAAAACTAAAGTGCA
AGGGTTAAATGATTTTCCAAAAAGTTTACATCCGCCTATGATTGTCCATTATTTCTTTGAT
TTAATGGTAACGATGGGAATTTTATGTTTTGTCATTTCAGGTGTTTATGTTTTAACTTTAAT
GTTTAAAAAGCTTAGAAAGTTTTCTACTCATAAATGGATGCTTTACGGAATATTATTAACA
GGACCAGCTTCAATGCTAGCTATAGAATTTGGATGGTTCTTAACAGAGATGGGTAGACAG
CCTTGGATTGTTCGTGGTTATATGCGCGTGGCAGAAGCAGCAACACAAGCAGGCGGAATA
ACCTTCGTTACAATTTTATTTGGCATATTGTACATCATTTTAATGTATACATGTGCATACGT
ATTAATTCGTATGTTTAAAAATAAACCGGCGTATGAAGATGTAAATCGTTTAGCCAAGAA
GCAAGGAGGAGAAATAGAAAAATGA

> **cydB**
Function: protein coding sequence; cytochrome D ubiquinol oxidase subunit 2 (core genome, constant)
Best match: cydB_CC001_MW2-USA400_BA000033.2[1065996:1067015]
Position: 026-contig_248_RC: 24161 … 25181; Length: 1020 bp
Sequence:
ATGATTTATGCATTTATAGGTATAACAGTGTTATGGTTGTTTTTATTTTGCTATATCATTAT
TGCTTCTATAGATTTTGGGGCAGGTTTCTTCGCATTGCATTCAAAGTTAACTGGTGATGAA
AAGAAAATTAATCACTTAATTTCACGTTATTTGAACCCAGTTTGGGAAGTTACGAATGTA
TTCTTTGTTTTCTTCTTCGTAGGATTCGTAGGTTTCTTTCCAGAGTCAATCAAATATTTAGG
CACGGTATTGTTAATACCAGGTTCAATAGCACTGATTATGATATCATTGAGAAACAGTTTT
TATGCATTTGAAAATTATGGTCAAGATACAAAATTAGCATGGATGATCATGTATGGGGTA
AGTGGATTATTAATTCCAGCTTCATTATCTACTGCTTTAACTATTACAGAAGGTGGCTATA
TTAATGTTCGAAACAATGTTATCGATCTAGATTGGGTGCAGTTACTATTAAGCCCATTTGC
TTGGTCTGTAGTATTCTTGGCAATTATTTCAGTTTTATATATTTCATCAGGATTTTTGACAT
ATTATGCTAAAAAAGCAAATGACGAACCAGCATATAATTTAACAAGACAATGGCACATA
TTTTTAGGGCCGCCGATGATTATCATTTGTTTATTCGTATTTCTATCATTACGCATTCAAAA
TTCTGAACATTTTTATTCAGCTGTTTTTGACTATTGGTGGATGTTTGCTATAAGTTTCTTAT
TCTTTGCATTAGCTTCATTGTTAACATTCTTTAAGAAAAAACATGGTTTGGCTTTTGTATTT
GTTATTTTACAAATGATGTTCGCGTTCTTTGGCTATGGAATTAGTAAATTGCCATACCTTT
TATATCCGTTTGTAAAAATTACAGATGCATATGTTAATCCAGAAATGGGCTGGACATTAG
TGATTGTCTTTATTTTAGGTTTACTTTTATTACTTCCATCGTTAATATTATTATTAAGATTA
TTTGTTTTCGACAAAGAATATGTTGAAGGAAAGAAATCATAA

> **tx_universal2**
Function: rho indendent terminator
Best match: tx_universal2_CC093_JKD6159-ST93_CP002114.2[1411399:1411438:r]RC
Position: 027-contig_249: 0 … 30; Length: 30 bp
Sequence:
AAAAGCTTGTTACAAGCGCATTTTCGTTCA

> **rnjA**
Function: protein coding sequence; ribonuclease J1 (core genome, constant)
Best match: rnjA_CC005_Mu50-VRSA_BA000017.4[1143060:1144757:r]RC
Position: 027-contig_249: 168 … 1866; Length: 1698 bp
Sequence:
TTATTTATTGTTTGATTCTTTTTGTTCGTTTACCTTCATAATGACTGGTAAAATCATTGGTT
TTCTAGCTGTTTTTTCAAATAAATAAGGTTGTAATGTTTCAATGATAGAAGATTTAATCTG
ATGCCATTGAATATCTTTATTTTGATTTAACTTACTAATAACATCAGTTTTGATTTTGCGTT
GTGCATCATAAATTAATTGACCTGATTCCCTCATATATACAAATCCTCGAGAAATAATGTC

TGGACCAGAAAGTAATTTATTTGTATTAAAATCAATACTAACAACAACGATAACTAAACC
TTCTTCAGATAATAGCTTACGGTCTCTTATTACAACATTACCGATATCACCGATACCACTA
CCATCAACAAGTACATTACCAGATGGAATGCGACCAGCTTTACGTGCTGAATCGTGTGTT
AAAGCTAAGACATCTCCAATATCAAAGATGAAGACATTATCTTCTTCAACGCCGCATTCA
ACACCAGTCTCACCATGTGCTTTTAACATACGGTATTCACCATGAATAGGTAAGAAATAT
TTCGGCTTGATTAATCGAAGCATTAATTGTTGATCACCTTGAGAACCATGCCCTGAAGTAT
GGATGTTAGAAATCTTGCTATGGATAACATCTGCACCAGCTTTATACAAGGAATTAATAG
TTCTGTTAATACTTTTTGTATTACCTGGGATAGGTGATGAACTAAATACAACGGTATCTTC
AGGTATAATTTTAATTTGCTTATGAGTACCATTAGCAATTCTAGATAATGCTGCCATTGGT
TCACCTTGTGAACCAGTACACAGTATCAATAACTCATGCTTCGGTACGGTATTAATTTTAT
TAGGTTCAATAAATGTTTCAGGTGGTGCTTTAATATAACCAAGTTCCATACCGATTTTAAT
ATTGTTTTCCATCGAACGACCGAACGTAACAATTTTACGGTTATTTTTGATAGCAGCTTCA
ACTGCTTGTTGAACTCGGTAAATATTAGAAGCGAAGGTAGCAAATATAATACGACCTTTA
CAATTACGGAAGATCTTATCTACGTTTTGACCAACTTCACGTTCGCTTAAAGTAAAATCAG
GCACAAGTGAATTTGTTGAGTCTGAAAGTAAACATAGAACGCCTTCTTCGCCTAATTGAG
CCATTTTAGCAATGTTTGCTGGTTTGCCTACAGGTGTAAAATCAAATTTAAAGTCACCGGT
ATGAACTACTTTTCCTTCAGGTGTATCTACGATGACGCCATAAGTTTCAGGAATACTATGC
GTAGTTAAGTAGAAAGAAATCGTAAAGTGCTTAGATTTAATCACACTGTCCTCATTGATT
TCATTTAGTTTAGCAGTACGTAATAAATGATGTTCTTCAAGTTTATTACGGATTAAACCTA
ATGCTAAAGGACCACCATAAATAGGTATATTAAGTTGTTTTAATAGGAAGGGCACACCGC
CTATATGGTCTTCGTGACCATGTGTTATAAATAGGCCAACAATTTTATCTTGGTTTTGAAC
TAGATATGTGTAGTCAGGTATAACATAATCAATCCCTAATAAGTTATCATCAGGGAATTT
GATACCTGCATCGATAATGACAATTTCGTCTTTATACTCAACTGCATAAGTATTTTTACCG
ATTTCACCTAGACCTCCAAGTGCATATACACCTACTTCATTTGGATGTAATTGTTTCAT

## > def1

Function: protein coding sequence; peptide deformylase (core genome, constant)
Best match: def1_CC001-ST772_118_AJGE01000009.1[193196:193747:r]RC (completely identical)
Position: 027-contig_249: 2565 … 3117; Length: 552 bp
Sequence:
TTAAACTTCTACTGCATCTGTATGTGGTTGTAATGGGTGGTCTTTGTCAATGTGATCATAG
AACATTACACCATTTAAATGATCAATTTCATGTTGGAAAACAATTGCTGGATATCCTTTTA
GTCGTAATTGTATATCATTACCTTCGATGTCTTTGGCTTTAATTGTAATTCTATTATGACGG
TGAACTAGACCAGCAACATTATCATCGACACTAAGGCAACCTTCACCAGTTGGTAAATAA
GCTTCTTGAACGCTATGACTTACAATTTTTGGGTTCACAAGCATATAGTCATAAGATTTGC
CACTGCCATCATCTGGTATTAAAACAGCAATCATACGTTTAGAAATATTAATTTGAGGTG
CAGCCAAACCAACGCCTGAACGTAAACCATATCGTTTCGCGATTTCCTCATCTTGACTATT
TACTAAAAACTCTCTCATGGCGATTAATGTTTCTTTTTCTTCTTTAGTTAATGGTAATTCTA
ACTCAGCTGCTTTTTGACGCAAAGTTGGATGACCATCTCTAATGATGTCTTTCATTGTTAA
CAT

## > pdhA

Function: protein coding sequence; pyruvate dehydrogenase E1 component/alpha subunit (core genome, constant)
Best match: pdhA_CC001_MW2-USA400_BA000033.2[1072269:1073381] (completely identical)
Position: 027-contig_249: 4278 … 5391; Length: 1113 bp
Sequence:
ATGGCTCCTAAGTTACAAGCCCAATTCGATGCAGTAAAAGTTTTAAATGATACTCAATCG
AAATTTGAAATGGTTCAAATTTTGGATGAGAATGGTAACGTCGTAAATGAAGACTTAGTA
CCTGATCTTACGGATGAACAATTAGTGGAATTAATGGAAAGAATGGTATGGACTCGTATC
CTTGATCAACGTTCTATCTCATTAAACAGACAAGGACGTTTAGGTTTCTATGCACCAACTG
CTGGTCAAGAAGCATCACAATTAGCGTCACAATACGCTTTAGAAAAAGAAGATTACATTT
TACCGGGATACAGAGATGTTCCTCAAATTATTTGGCATGGTTTACCATTAACTGAAGCTTT
CTTATTCTCAAGAGGTCACTTCAAAGGAAATCAATTCCCTGAAGGCGTTAATGCATTAAG
CCCACAAATTATTATCGGTGCACAATACATTCAAGCTGCTGGTGTTGCATTTGCACTTAAA

AAACGTGGTAAAAATGCAGTTGCAATCACTTACACTGGTGACGGTGGTTCTTCACAAGGT
GATTTCTACGAAGGTATTAACTTTGCAGCAGCTTATAAAGCACCTGCAATTTTCGTTATTC
AAAACAATAACTATGCAATTTCAACACCAAGAAGCAAGCAAACTGCTGCTGAAACATTA
GCTCAAAAAGCAATTGCTGTAGGTATTCCTGGTATCCAAGTTGATGGTATGGATGCGTTA
GCTGTATATCAAGCAACTAAAGAAGCACGTGACCGCGCAGTTGCAGGTGAAGGTCCAAC
ATTAATTGAAACTATGACATATCGTTATGGTCCTCATACAATGGCTGGTGACGATCCAAC
TCGTTACAGAACTTCAGACGAAGATGCTGAATGGGAGAAAAAAGACCCATTAGTACGTTT
CCGTAAATTCCTTGAAAACAAAGGTTTATGGAATGAAGACAAAGAAAATGAAGTTATTG
AACGTGCAAAAGCTGATATTAAAGCAGCAATTAAAGAGGCTGATAACACTGAAAAACAA
ACTGTTACTTCTCTAATGGAAATTATGTATGAAGATATGCCTCAAAACTTAGCAGAACAA
TATGAAATTTACAAAGAGAAGGAGTCGAAGTAA

## > pdhB

Function: protein coding sequence; pyruvate dehydrogenase (core genome, constant)
Best match: pdhB_AF235026.1[32:1009]
Position: 027-contig_249: 5394 … 6372; Length: 978 bp
Sequence:
ATGGCACAAATGACAATGGTTCAAGCGATTAATGATGCGCTTAAAACTGAACTTAAAAAT
GACCAAGATGTTTTAATTTTTGGTGAAGACGTTGGTGTTAACGGCGGTGTTTTCCGTGTTA
CTGAAGGACTACAAAAAGAATTTGGTGAAGATAGAGTATTCGATACACCTTTAGCTGAAT
CAGGTATTGGTGGTTTAGCGATGGGTCTTGCAGTTGAAGGATTCCGTCCGGTTATGGAAG
TACAATTCTTAGGTTTCGTATTCGAAGTATTTGATGCGATTGCTGGACAAATTGCACGTAC
TCGTTTCCGTTCAGGCGGTACTAAAACTGCACCTGTAACAATTCGTAGCCCATTTGGTGGT
GGCGTACACACACCTGAATTGCACGCAGATAACTTAGAAGGTATTTTAGCTCAATCTCCA
GGTCTAAAGGTTGTTATTCCTTCAGGCCCATACGATGCGAAAGGTTTATTAATTTCTTCTA
TTAGAAGTAATGACCCAGTCGTATACTTGGAGCATATGAAATTGTATCGTTCATTCCGTG
AAGAAGTGCCTGAAGAAGAATATACAATTGACATTGGTAAGGCTAATGTGAAAAAAGAA
GGTAATGACATTTCAATCATCACATACGGTGCAATGGTTCAAGAATCAATGAAAGCTGCA
GAAGAACTTGAAAAAGATGGTTATTCTGTTGAAGTAATTGACTTACGTACTGTCCAACCA
ATCGATGTTGACACAATTGTAGCTTCAGTTGAAAAAACTGGTCGTGCAGTAGTAGTTCAA
GAAGCACAACGTCAAGCTGGTGTTGGTGCAGCAGTTGTAGCTGAATTAAGTGAACGTGCA
ATCCTTTCATTAGAAGCACCTATTGGAAGAGTTGCAGCAGCAGATACAATTTATCCATTC
ACTCAAGCTGAAAATGTTTGGTTACCAAACAAAAATGACATCATCGAAAAAGCAAAAGA
AACTTTAGAATTTTAA

## > pdhC

Function: protein coding sequence; dihydrolipoyllysine-residue acetyltransferase component of pyruvate dehydrogenase complex (core genome, constant)
Best match: pdhC_CC001_MW2-USA400_BA000033.2[1074453:1075745]
Position: 027-contig_249: 6462 … 7755; Length: 1293 bp
Sequence:
GTGGCATTTGAATTTAGATTACCCGATATCGGGGAAGGTATCCACGAAGGTGAAATTGTA
AAATGGTTTGTTAAAGCTGGAGATACTATTGAAGAAGACGATGTTTTAGCTGAGGTACAA
AACGATAAATCAGTAGTAGAAATCCCATCACCAGTATCTGGTACTGTAGAAGAAGTTATG
GTAGAAGAAGGTACAGTAGCTGTAGTTGGTGACGTTATTGTTAAAATCGATGCACCTGAT
GCAGAAGATATGCAATTTAAAGGTCATGATGATGATTCATCATCTAAAGAAGAACCTGCG
AAAGAGGAAGCGCCAGCAGAGCAAGCACCCGTAGCTACTCAAACTGAAGAAGTAGATGA
AAACAGAACTGTTAAAGCAATGCCTTCAGTACGTAAATACGCACGTGAAAAAGGTGTTA
ACATTAAAGCAGTTTCTGGATCTGGTAAAAATGGTCGTATTACAAAAGAAGATGTAGATG
CATACTTAAATGGTGGTGCACCAACAGCTTCAAATGAATCAGTTGCTTCAGCTACAAGTG
AAGAAGTTGCTGAAACTCCTGCAGCACCTGCAGCAGTAACATTAGAAGGCGACTTCCCAG
AAACAACTGAAAAAATCCCTGCTATGCGTAGAGCAATTGCGAAAGCAATGGTTAACTCTA
AGCATACTGCACCTCATGTAACATTAATGGATGAAATTGATGTTCAAGCATTATGGGATC
ACCGTAAGAAATTTAAAGAAATCGCAGCTGAACAAGGTACTAAGTTAACATTCTTACCTT
ATGTTGTTAAAGCACTTGTTTCTGCATTGAAAAAATACCCAGCACTTAACACTTCATTCAA

TGAAGAAGCTGGTGAAATCGTTCATAAACATTACTGGAATATCGGTATTGCAGCAGACAC
TGATAGAGGATTATTAGTACCTGTTGTTAAACATGCTGATCGTAAGTCTATTTTCCAAATT
TCAGATGAAATTAATGAATTAGCTGTTAAAGCACGTGATGGTAAATTAACAGCCGATGAA
ATGAAAGGTGCTACATGCACAATCAGTAATATCGGTTCAGCTGGTGGACAATGGTTCACT
CCAGTTATCAATCACCCAGAAGTAGCAATCTTAGGAATTGGCCGTATTGCTCAAAAACCT
ATCGTTAAAGATGGAGAAATTGTTGCAGCACCAGTATTAGCATTATCATTAAGCTTTGAC
CACAGACAAATTGATGGTGCAACTGGCCAAAATGCAATGAATCACATTAAACGTTTATTA
AATAATCCAGAATTATTATTAATGGAGGGGTAA

> **pdhD**
Function: protein coding sequence; dihydrolipoyl dehydrogenase (core genome, constant)
Best match: pdhD_CC001_MW2-USA400_BA000033.2[1075749:1077155] (completely identical)
Position: 027-contig_249: 7758 … 9165; Length: 1407 bp
Sequence:
ATGGTAGTTGGAGATTTCCCAATTGAAACAGATACTATAGTAATCGGAGCAGGTCCTGGT
GGATACGTTGCAGCAATTCGTGCAGCTCAATTAGGACAAAAAGTAACAATCGTTGAGAA
AGGTAATCTTGGTGGTGTTTGCTTAAACGTAGGATGTATTCCTTCAAAAGCATTACTACAT
GCTTCTCACCGTTTTGTTGAAGCACAACATTCTGAAAACTTAGGTGTTATTGCTGAAAGTG
TTTCTTTAAACTTCCAAAAAGTTCAAGAATTCAAATCATCAGTTGTTAATAAATTAACTGG
TGGTGTTGAAGGCTTACTTAAAGGTAACAAAGTTAACATCGTTAAAGGTGAAGCATATTT
CGTAGATAACAATAGCTTACGTGTTATGGACGAAAAGAGCGCACAAACATACAACTTTA
AAAATGCAATCATTGCAACAGGTTCAAGACCAATTGAAATTCCTAATTTCAAATTCGGTA
AACGTGTTATCGACTCAACAGGTGCTTTAAACTTACAAGAAGTACCAGGTAAATTAGTTG
TAGTTGGTGGAGGATACATTGGATCAGAATTAGGTACAGCATTTGCTAACTTTGGTTCAG
AAGTAACCATCCTTGAAGGTGCTAAAGATATCTTAGGTGGCTTCGAAAAACAAATGACAC
AACCTGTTAAAAAAGGTATGAAAGAAAAAGGTGTTGAAATCGTTACTGAAGCTATGGCT
AAATCAGCTGAAGAAACAGATAACGGAGTTAAAGTTACTTATGAAGCTAAAGGCGAAGA
GAAAACAATCGAAGCTGATTATGTATTAGTAACTGTAGGTCGTCGTCCAAACACAGACGA
ATTAGGCCTAGAAGAATTAGGTGTTAAATTCGCTGACCGTGGATTATTAGAAGTTGATAA
ACAAAGCCGTACGTCTATCAGCAATATCTATGCAATTGGTGATATCGTTCCAGGTTTACC
ACTTGCTCACAAAGCTAGCTATGAAGCTAAAGTTGCTGCTGAAGCAATTGATGGTCAAGC
TGCTGAAGTTGATTACATTGGTATGCCAGCAGTATGCTTTACTGAACCAGAATTAGCTAC
AGTTGGTTATTCAGAAGCGCAAGCTAAAGAAGAAGGTTTAGCAATTAAAGCTTCTAAATT
CCCATATGCAGCAAATGGTCGTGCATTATCATTAGATGATACTAACGGATTTGTTAAACTT
ATTACACTTAAAGAAGATGATACTTTAATCGGTGCTCAAGTAGTTGGTACTGGTGCATCA
GATATTATCTCTGAATTAGGTTTAGCAATTGAAGCTGGTATGAATGCTGAAGATATCGCA
TTAACAATCCATGCACATCCAACATTAGGTGAGATGACTATGGAAGCAGCAGAAAAAGC
TATCGGATACCCAATCCATACAATGTAA

> **potA**
Function: protein coding sequence; spermidine/putrescine import ATP-binding protein (core genome, constant)
Best match: potA_CC001_MW2-USA400_BA000033.2[1078294:1079388]
Position: 027-contig_249: 10303 … 11398; Length: 1095 bp
Sequence:
TTGGAACCGTTATTATCATTAAAATCAGTTAGTAAAAGCTACGATGATCTTAATATCTTAG
ATGACATAGATATTGATATTGAATCAGGATACTTTTATACATTATTAGGTCCTTCAGGTTG
TGGTAAAACAACAATTTTAAAATTAATTGCAGGGTTTGAATATCCTGACAGTGGTGAAGT
GATTTATCAAAACAAACCAATTGGTAATTTACCACCAAATAAACGTAAAGTGAATACAGT
CTTTCAAGATTATGCATTATTTCCACACTTAAACGTCTATGATAATATCGCTTTTGGTTTG
AAATTAAAAAAATTATCAAAAACCGAAATTGATCAAAAAGTAACTGAGGCATTAAAATT
AGTAAAACTTTCAGGTTATGAAAAAAGAAATATTAATGAAATGAGTGGCGGACAAAAGC
AACGTGTTGCAATTGCACGTGCTATCGTAAATGAACCAGAAATATTATTGTTAGATGAAT
CTTTATCCGCATTAGATTTGAAATTGCGTACTGAAATGCAATATGAATTACGAGAATTGC
AATCTAGATTAGGTATTACATTTATATTTGTAACACATGATCAAGAAGAAGCGTTAGCAT

TAAGTGACTTTCTTTTTGTATTAAAAGATGGGAAAATTCAACAATTTGGCACACCAACAG
ATATATATGACGAACCAGTGAATCGATTTGTAGCTGATTTTATTGGAGAATCTAATATTGT
TGAAGGGCGCATGGTTAGAGATTATGTCGTGAATATTTATGGGCAAGATTTCGAATGTGT
CGATATGGGTATTCCTGAAAATAAAAAAGTAGAAGTCGTTATTCGACCAGAAGATATATC
ATTAATCAAAGCTGAAGAAGGATTATTTAAAGCAACTGTTGATTCTATGTTATTTAGAGG
GGTCCACTATGAAATATGTTGTATAGACAATAAAGGTTATGAATGGGTAATACAAACGAC
TAAAAAAGCTGAAGAAGGCAGTGAAGTTGGTCTTTATTTTGATCCTGAAGCCATTCATAT
TATGGTTCCTGGAGAAACAGAAGAAGAATTTGATAAACGTATTGAAAGCTATGAGGAAG
TAGACAATGCGTAA

> **potB**

Function: protein coding sequence; spermidine/putrescine ABC transporter (core genome, constant)
Best match: potB_CC001_MW2-USA400_BA000033.2[1079381:1080178]
Position: 027-contig_249: 11390 … 12188; Length: 798 bp
Sequence:
ATGCGTAATACTAATAAATTTCTCTTAATCCCGTATTTACTATGGATGGTTATATTTATTAT
TGTACCAGTTGTATTACTCATTTATTTTTCATTTTTAGATATCAATGGACATTTTAGTTTCA
CGAATTATCAACAAATTTTTACTACAAAATATTTGAAAATGTTTGCATATTCAATTTTATA
TGCCGCTTTAATAACAATTATTACCTTGACTATCAGTTATCCAGCTGCCTATTATATTACT
CGTTCGAAATTTCAAAATATCTTATTAATGATAATGATTATTCCAACATGGATAAATTTAT
TGTTAAAGACATATGCTTTTATAGGTTTATTAAGTCATGATGGCGTGATTAATCAATTTTT
CCACTTATTTAATTTACCATCATTCAATTTGTTATTTACAACTGGTGCGTTTTTAGTAGTGG
CAAGTTACATTTATATACCATTTATGATTTTACCTATATTTAATAGCATGAAAGCAATTCC
TAATAATTTATTGCAGGCCTCAAGTGATTTAGGTGCTAGTCCTTTCTATACTTTTAGAAAA
GTAATCATGCCGTTAACAAAAGAAGGTGTTATGACTGGAATTCAAGTAACCTTTATTCCA
TCACTTTCATTATTTATGATTACAAGATTAATTGCAGGTAATAAAGTCATAAATATAGGTA
CGGCAATAGAGGAACAATTTTTAACAATTCAAAATTATGGTATGGGATCAACTATAGCTA
TATTCTTAATTGTATTTATGGCATTCATTTTAATCATTACAAAATCATCTAATGGGAGAGG
GTGA

> **potC**

Function: protein coding sequence; spermidine/putrescine transport system permease (core genome, constant)
Best match: potC_CC005_Mu50-VRSA_BA000017.4[1155085:1155894] (completely identical)
Position: 027-contig_249: 12193 … 13003; Length: 810 bp
Sequence:
ATGAAATGGTATGGAAAGCTGTATATCGGGATACTTTTAGCGATTTTATACATCCCAATA
TTCTTTTTAATGTTCTATTCATTCAATTCGGCTGGTAATATGATTCACTTTGAACATTTTAC
ATTAGAGCATTATCAATCATTATTTCAAAATGATCATTTAATGTCGGTCATTTTTAATACG
ATAGCTGTAGCACTTTTAGCAGCCTCAATTTCTACAGTTATTGGTACATTTGGTGCCATTG
CTATTTATTATTTAAGAAATAAAAAGTTTAAAGTAACTTTACTAACATTGAATAATGTCTT
GATGGTATCATCCGACGTTGTCATAGGTGCATCATTCTTAATTATGTTTACAACGATTGGC
CATTTTACTGGTCTTGGTTTAGGATTTTGGACAGTTCTAATATCTCATATTGCATTTTGCAT
ACCTATAGTTGTGATTATCGTCTTACCACAACTGTATGAAATGAATAATAATATGTTAAAT
GCTGCAAGAGATTTAGGAGCGACTGAACCACAATTATTAAGCAACATTATTATTCCTAAT
ATTTTACCTTCTATTATAGGAGGTTTCTTTATGGCTTTAACTTATTCACTAGACGATTTCAC
AGTAAGTTTCTTCGTTACTGGTAATGGCTTTAGTGTGTTATCAGTTGAAGTTTATGCTATG
GCGAGAAAAGGAATTAGTATGGAAATTAATGCGATTTCAACATTATTGTTTGCTGTTATT
GTATTAGGAATACTAGGATATTATTTGATTCAATACGTGATAAATAAGAAAAAACTAATC
AAGCGAGGTGTAAAATAA

> **potD**

Function: protein coding sequence; ABC transporter extracellular binding protein (core genome, constant)
Best match: potD_CC001_MW2-USA400_BA000033.2[1080993:1082066] (completely identical)

Position: 027-contig_249: 13002 … 14076; Length: 1074 bp
Sequence:
ATGAAACGTTTTTTACAACTCATTATAGGTGCATTAGTTGTGGGTATGCTTTGTCTTACTTT
AAGTCATTGGTTTAAATCTAAAGAACAAGTGCATACAAATCAAAAAATTTACGTATACAA
TTGGGGCGAATATATTGATCCAGAGTTAATTAAGAAATTTGAAAAAGAAACTGGCATTCA
AGTCGTTTATGAAACTTTCGATTCAAATGAAGCGATGGAAGCCAAAATTCGCAATGGCGG
TACACATTATGATGTTGCTTTTCCTAGTGAATATACAGTTCAAAAATTGAAAAGAGATCA
TTTATTGTTACCAATAGATCATAATAAGGTACCTAATATTAAAAATTTAGATTCAGATTAT
ATGAATATGTCATTTGATAGAGGCAATAAATATTCTTTACCTTATTTCTTTGGAACTGTAG
GTATTTTATATAATAAAGAAAAGTATCCAAATGAATCATTTGATAGTTGGAAGTCATTGT
ATAATCCTAAATTTAAAAACCAAATTTTACTAGTTGACGGTGCTAGAGAGATTATAGGCA
TGAGTTTGAATAAACTTGGGTATAATCTTAATGACCGTAATTCGCACCATTTAAAAGAAG
CAGAGCGAGATTTAACCAAACTAGCACCACAAGTAAGAGGTGTCGTAGGTGATGAAATT
ACCATGATGCTTCAACAAAATGAAGGTAACATAGCGGTTGTTTGGAGTGGTGTTGCAGCA
CCTCTAGTGCAAGAAGGGGATAAATATAATTATGTTATTCCTAAAGAAGGATCGAATTTA
TGGTTCGACAATATGGTAATTCCAAAAACGGCACAAAATAAAGAGGGTGCATATAAATTT
ATGAATTTTTTATTAGATGCTAAAAATAACAAGCAAAATACAGAATTCGTAGGCTATGCA
ACGCCAAACAAGGCTGCTCGACAATTGTTGCCTAAAGAGATTAAAGACGACCATCGTTTT
TATCCGACTAAGAAAGAGCAAGAACGCCTTGAAGTTTATAAAGATTTAGGACCTGAAGTT
TTAAGTGAATACAATGAAAACTTTTTGAATTTCAAAATGTCATTAAAATAA

## > **mntH**

Function: protein coding sequence; manganese transport protein (core genome, constant)
Best match: mntH_CC001-ST772_118_AJGE01000009.1[206581:207933:r]RC (completely identical)
Position: 027-contig_249: 15950 … 17303; Length: 1353 bp
Sequence:
TTAACTTTGTAGTTCTTGGAATGTTTGAACGATTAAATAGACATTTAAAATACTTAGAATA
ATAATAAGTGTCCATGAAATGATATTAACCCATGTTTTATTATAGAAAGGTCCCATCAAG
TCTTTATTACTAGTAGCTAATTGTAATGGTATTAAACAGAATGGCAGAGCAATACTTAAA
AATACTTGAGAGAATACAAGTAGTTGCTCAATTTTAGCTGCATTACCTTTAAAAATGATT
AAGCATACGATAACAGGAATGACAGCAAGGGAACGTGTAATTAAACGTCTTAACCAATT
TGGTATGTGTAATCTTAAAAATCCTTCCATTACAATTTGTCCTGCTAAAGTACCAGTAATC
GTTGAATTTTGACCTGACGCTAATAATGCAACTGCAAATAATGTACTCATGATTGCACCC
ATTGTTGCACCTAGTACAGGTTCAGTTTTTAAGGCGTGATATAAATCATAGAAACCACCT
AAATCGTCAGCATTTGAGTTGAAAAATAGTGATGCTCCTAACACTAATAATAAGCAATTG
ACTACAAATGCGATTGATAACTGAATGTTCGAATCTATCGTAGCAAATTTAATCGCTTGC
GCCTTTTCTTCATTGTTATGTCTTGAGTATGTTCTAGATTGTACAATTGATGAATGTAAGT
ACAAATTATGAGGCATAATTGTAGCGCCAATAATACCTAATGCAATATAGAGAATGCCGT
TATTTGTAATGATTTCACTATGTGGTATAAATCCATTTAACACAGCATTCAACTGTGGTGA
TGAAATATAGACTTCAAATATAAAGATGAATAACACTGTGAAAATTAATGTACCAACAAT
AGCTTCAATTTTTCTAAAACCATATTTCATTATAAATAGTAGTAAAAATACATCAAGTACA
GTTATTAGTGCACCGACGATTAAAGGTATGTTAAATAGGAGATTAAGAGCAATAGCACTA
CCAATAACCTCAGCAATATCTGTAGCGATAATTGCTAGTTCTGCAATGATCCAAAAGATT
ATAGCAATAGGTCTTGATAAATAATGTCGTGTCATTTGAGCTAAGTCCATACCTGTTGCTA
TTCCTAATCTCACTGTCATGCTTTGAAGTAACATTGCTGATAAACTTGAAATAAGAATTAC
GAATAGCAAAGTATAGCCATATTGGGCGCCACCTTGCATTGATGTTATCCAGTTTCCGGG
ATCCATGTAACCAACAGCGACTAATAACCCAGGTCCAAGAAATGATAAAAATTTCTGTTT
ATTTGAACTGCGATGATCGAATTTAATTGTATTGTTTATTTCGTCTAAACTTAATTGTTCAT
TTGTTGAATGTCGTTTATTATTCAT

## > **typA**

Function: protein coding sequence; GTP-binding protein (core genome, constant)
Best match: typA_CC001-ST772_118_AJGE01000009.1[210163:212010] (completely identical)
Position: 027-contig_249: 19532 … 21380; Length: 1848 bp

Sequence:
ATGACTAATAAAAGAGAAGATGTCCGCAATATAGCAATTATTGCTCACGTTGACCATGGT
AAAACAACTTTAGTAGATGAGTTGTTAAAACAATCTGGTATATTCAGAGAAAATGAACAT
GTCGATGAACGTGCAATGGACTCTAACGATATCGAAAGAGAGCGTGGAATTACGATTCTA
GCCAAAAATACGGCTGTTGATTATAAAGGTACACGTATTAATATTTTGGATACACCAGGA
CATGCAGACTTTGGTGGAGAAGTAGAACGTATTATGAAAATGGTTGATGGGGTTGTCTTA
GTAGTAGATGCGTATGAAGGTACAATGCCTCAAACACGTTTTGTACTTAAAAAAGCGCTA
GAACAAAACCTGAAACCTGTTGTTGTTGTTAATAAAATTGATAAACCATCAGCACGTCCA
GAGGGTGTTGTAGATGAAGTTTTAGATTTATTTATTGAATTAGAAGCAAACGATGAACAA
TTAGAATTCCCTGTTGTTTATGCTTCAGCAGTAAATGGAACAGCTAGCTTAGATCCTGAAA
AACAAGATGATAATTTACAATCATTATATGAAACGATTATTGATTATGTACCAGCTCCAA
TTGATAACAGTGATGAGCCATTACAATTCCAAGTAGCATTGTTGGACTACAATGATTATG
TTGGACGTATTGGTATTGGTCGTGTATTCAGAGGTAAAATGCGTGTCGGAGATAATGTAT
CACTAATTAAATTAGACGGTACAGTGAAAAACTTCCGTGTAACTAAAATCTTTGGTTACT
TTGGATTAAAACGTTTAGAAATTGAAGAAGCACAAGCTGGAGATTTAATTGCTGTTTCAG
GTATGGAAGACATTAATGTTGGTGAAACTGTAACACCACATGACCATCAAGAAGCATTGC
CAGTTCTACGTATTGATGAGCCTACTCTTGAAATGACATTTAAAGTTAACAATTCTCCATT
TGCTGGCCGTGAAGGTGACTTTGTAACAGCACGTCAAATTCAAGAACGTTTAAATCAACA
ATTAGAAACAGATGTATCTTTGAAAGTTTCTAACACAGATTCTCCAGATACATGGGTAGT
TGCTGGTCGCGGTGAATTGCATTTATCAATCCTTATTGAAAATATGCGTCGTGAAGGTTAT
GAATTACAAGTTTCAAAACCACAAGTAATTATTAAAGAAATAGATGGTGTAATGTGTGAA
CCATTTGAACGTGTGCAATGTGAAGTGCCACAAGAAAATGCAGGTGCTGTTATTGAATCA
TTAGGTGCACGTAAAGGTGAAATGGTTGATATGACTACAACTGATAATGGACTTACACGT
TTAATCTTTAATGTACCGGCTCGTGGTATGATTGGTTATACGACTGAATTTATGTCAATGA
CAAGAGGTTACGGTATTATTAACCATACATTTGAAGAATTTAGACCACGTATTAAAGCAC
AAATTGGCGGTCGTCGTAATGGTGCATTAATTTCAATGGATCAAGGTTCTGCAAGTACTT
ATGCCATTTTGGGACTTGAAGATAGAGGTGTAAACTTCATGGAACCTGGTACTGAAGTTT
ATGAAGGTATGATTGTTGGTGAACATAATCGTGAAAATGATTTAACTGTTAACATCACTA
AAACAAAACATCAAACTAACGTACGTTCTGCAACGAAAGACCAAACACAAACAATGAAT
AGACCGCGTATTCTAACATTGGAAGAAGCGTTACAATTCATTAATGATGATGAACTTGTT
GAGGTTACACCAGAAAGTATACGTTTAAGAAAGAAAATTTTAAACAAAAATGTTCGTGA
AAAAGAAGCAAAGCGTATCAAACAAATGATGCAAGAAAACGAATAA

> **ftsW2**

Function: protein coding sequence; putative cell division protein (core genome, variable)
Best match: ftsW2_CC001-ST772_118_AJGE01000009.1[213531:214757] (completely identical)
Position: 027-contig_249: 22900 … 24127; Length: 1227 bp
Sequence:
ATGAAGAATTTTAGAAGTATTTTACGGTATATTGGTAAAACCTCAAAGTTTATTGATTATC
CGTTATTAGTTACATATATTGTATTGAGTTTAATTGGATTAGTTATGGTATATAGTGCAAG
TATGGTTCCAGCAACTAAAGGCACATTGACTGGTGGTATCGATGTTCCAGGAACGTATTT
TTACAACCGACAATTAGCATATGTCATAATGAGTTTTATAATTGTATTTTTTATTGCATTTT
TAATGAATGTTAAATTACTGAGTAATATTAAAGTGCAAAAAGGTATGATTATAACTATCG
TCTCACTATTATTACTGACGTTAGTAATAGGTAAAGATATTAATGGTTCTAAAAGTTGGAT
AAACTTAGGATTTATGAACTTACAGGCATCTGAGTTATTAAAAATTGCAATCATATTATAT
ATCCCATTTATGATCAGTAAAAAAATGCCTAGAGTATTAAGTAAACCAAAATTAATTTTA
AGTCCTATTGTATTAGCATTAGGTTGTACGTTTTTAGTTTTCCTACAAAAAGACGTAGGGC
AAACATTACTAATATTAATTATTTTAGTTGCGATCATTTTTTATTCAGGAATTGGGGTAAA
CAAAGTCCTAAGATTTGGTATACCAGCAGTGCTAGGATTCTTAGTAGTATTTGTCATTGCA
TTAATGGCTGGTTGGTTACCAAGTTATTTAACAGCCAGATTTAGTACGCTAACAGATCCAT
TCCAATTCGAATCAGGAACTGGATACCATATTTCCAATTCATTGCTTGCGATAGGTAACG
GTGGCGTATTTGGAAAAGGATTAGGAAATAGTGCAATGAAATTGGGCTATTTACCAGAAC
CACATACAGATTTTATTTTTGCAATTATTTGCGAAGAATTAGGTTTAATCGGAGGATTGCT
AGTTATTACTTTAGAGTTCTTTATTGTATATCGTGCCTTCCAGTTTGCAAATAAAACATCA
TCATATTTTTATAAACTTGTGTGTGTTGGGATTGCCACATACTTTGGAAGTCAAACGTTTG
TAAACATTGGCGGTATTTCGGCAACAATTCCATTAACTGGTGTGCCATTGCCATTTATCAG

CTTTGGTGGATCATCAATGATTAGTTTAAGTATTGCTATGGGATTACTTCTGATTGTAGGT
AAACAAATCAAAGTAGACCAGCAACGAAAGAAACAACAACAAAAAGTTGATATAAGAA
GACAATTTAATTAA

## > tx_A5IS00

Function: rho indendent terminator
Best match: tx_A5IS00_CC001_MW2-USA400_BA000033.2[1092211:1092273] (completely identical)
Position: 027-contig_249: 24219 … 24282; Length: 63 bp
Sequence:
GATGAGTTCAATAACGGAAGTTAGGCAACCAAACAACGCTTAACTTCCTTTTTGTTTTGAT
AT

## > pycA

Function: protein coding sequence; pyruvate carboxylase (core genome, constant)
Best match: pycA_CC001_MW2-USA400_BA000033.2[1092672:1096124]
Position: 027-contig_249: 24680 … 28133; Length: 3453 bp
Sequence:
TTGAAACAAATAAAAAAGTTACTTGTTGCTAACCGTGGAGAAATTGCAATTCGTATATTC
AGAGCGGCGGCAGAATTAGACATCAGCACAGTTGCAATTTATTCGAATGAAGACAAAAG
TTCATTACATAGATATAAAGCAGATGAATCCTATTTAGTTGGAAGTGATTTAGGTCCTGCT
GAAAGTTATTTAAATATTGAGCGTATCATTGATGTAGCAAAACAAGCGAATGTGGATGCG
ATTCATCCTGGCTATGGATTTTTAAGTGAAAATGAACAATTTGCGCGTCGTTGTGCAGAA
GAAGGAATTAAATTTATTGGTCCTCATTTAGAACATTTAGATATGTTTGGAGATAAAGTT
AAAGCTCGTACAACGGCTATCAAGGCAGATTTACCAGTTATTCCTGGTACAGACGGTCCA
ATTAAATCATATGAATTAGCAAAAGAATTTGCAGAAGAAGCTGGTTTCCCGCTAATGATT
AAAGCCACAAGTGGTGGCGGCGGTAAAGGTATGAGAATCGTTCGTGAAGAAAGTGAATT
AGAAGATGCTTTCCATAGAGCAAAATCAGAAGCTGAAAAATCATTTGGTAATAGTGAAG
TTTACATAGAAAGATACATTGATAATCCAAAGCATATTGAAGTACAAGTTATAGGTGACG
AACATGGAAATATCGTACACTTATTTGAACGTGATTGTTCAGTACAACGTCGTCATCAAA
AAGTTGTAGAAGTTGCACCATCAGTTGGATTATCACCAACATTACGTCAACGTATTTGTG
ATGCTGCAATTCAATTGATGGAAAATATTAAATATGTCAATGCAGGTACTGTTGAATTTCT
AGTATCTGGTGACGAATTCTTCTTTATAGAAGTTAACCCTCGTGTACAAGTTGAGCATACA
ATTACAGAGATGGTAACAGGAATTGATATTGTTAAGACACAAATTTTAGTTGCAGCAGGT
GCCGATTTATTTGGTGAAGAAATTAATATGCCGCAACAAAAAGATATTACAACATTGGGC
TATGCCATCCAATGTCGTATTACAACAGAAGATCCGTTAAATGATTTCATGCCGGATACT
GGAACAATCATTGCTTATCGTTCAAGCGGTGGCTTTGGTGTACGTCTCGATGCTGGAGAT
GGTTTCCAAGGTGCTGAGATATCACCTTATTATGATTCATTACTCGTAAAATTATCTACAC
ACGCGATATCATTTAAACAAGCAGAAGAAAAAATGGTACGTTCATTACGAGAAATGCGT
ATTCGTGGTGTTAAAACTAATATTCCATTCTTAATTAATGTAATGAAGAATAAAAAGTTC
ACAAGTGGTGATTACACAACTAAATTTATTGAAGAAACACCAGAACTTTTCGACATTCAG
CCGTCTCTAGATAGAGGTACTAAAACATTAGAATATATAGGTAATGTAACAATTAATGGT
TTCCCAAATGTTGAGAAACGTCCGAAACCAGACTATGAATTAGCATCAATTCCAACTGTA
TCTTCAAGTAAAATCGCTTCATTTAGTGGTACGAAACAATTGCTTGATGAAGTAGGTCCA
AAAGGTGTAGCTGAATGGGTTAAAAAGCAGGATGATGTCTTACTAACAGATACAACCTTT
AGAGATGCACACCAATCATTATTAGCTACACGAGTTAGAACTAAGGATATGATTAATATC
GCATCCAAAACAGCGGACGTATTTAAAGATGGTTTCTCACTTGAAATGTGGGGCGGTGCT
ACATTTGATGTGGCATATAATTTCTTGAAGGAAAACCCATGGGAACGACTTGAACGTCTA
CGTAAAGCTATTCCAAATGTATTATTCCAAATGTTGTTACGTGCTTCAAACGCAGTTGGTT
ATAAAAACTATCCTGATAATGTTATTCATAAATTCGTACAAGAAAGTGCTAAAGCAGGCA
TAGATGTCTTTAGAATTTTCGATTCTTTAAACTGGGTAGATCAAATGAAAGTTGCCAATGA
AGCAGTACAAGAAGCGGGCAAAATCTCTGAAGGTACTATTTGTTATACAGGTGACATTTT
AAATCCTGAGCGATCAAACATTTATACTTTAGAGTATTATGTCAAACTAGCTAAAGAGTT
AGAACGTGAAGGTTTCCATATTTTAGCGATTAAAGATATGGCAGGCTTATTAAAACCTAA
AGCCGCTTACGAATTGATTGGTGAGTTAAAATCAGCTGTAGATTTACCAATTCATCTTCAC

ACTCATGATACAAGTGGTAATGGTTTATTAACATACAAACAAGCAATAGATGCTGGTGTC
GATATCATTGATACTGCTGTTGCTTCAATGAGTGGTTTAACAAGTCAGCCAAGCGCCAAT
TCGTTATATTATGCATTAAATGGCTTCCCACGCCACCTTAGAACTGATATTGAAGGTATGG
AGTCACTTAGTCATTATTGGTCAACTGTACGTACTTATTATTCAGACTTTGAAAGTGATAT
CAAATCACCGAATACTGAAATTTATCAACATGAAATGCCTGGTGGACAGTATTCGAATTT
AAGTCAACAAGCTAAAAGTTTAGGTTTAGGCGAAAGATTTGATGAAGTCAAAGATATGT
ATCGCAGAGTGAATTTCTTATTTGGTGATATCGTAAAAGTAACTCCATCGTCTAAAGTAGT
TGGTGATATGGCACTTTATATGGTACAAAATGATCTTGATGAACAATCCGTGATTACAGA
TGGCTATAAATTAGATTTCCCAGAATCAGTAGTGTCGTTCTTCAAAGGCGAAATAGGACA
ACCTGTAAATGGTTTTAATAAAGATTTACAAGCGGTTATTTTAAAAGGCCAAGAAGCACT
AACAGCTCGTCCAGGTGAATATCTAGAGCCAGTTGATTTTGAAAAAGTCCGTGAGTTGCT
TGAAGAAGAGCAACAAGGTCCTGTTACGGAGCAAGATATTATTAGTTATGTATTATATCC
AAAGGTATATGAACAATATATTCAAACTAGAAATCAATACGGAAACTTATCGTTACTTGA
TACGCCTACATTCTTCTTTGGAATGCGTAATGGTGAAACAGTAGAAATCGAAATCGATAA
GGGTAAACGATTAATTATTAAACTAGAAACGATTAGTGAACCAGATGAAAATGGTAATA
GAACGATTTACTATGCGATGAATGGTCAAGCGAGACGTATTTACATTAAAGATGAGAATG
TGCATACAAATGCGAACGTTAAGCCAAAAGCAGATAAGAGTAATCCAAGTCATATCGGT
GCGCAAATGCCAGGTTCAGTAACTGAAGTCAAGGTTAGTGTAGGTGAATCTGTGAAAGCT
AACCAGCCGTTGCTAATTACTGAAGCTATGAAAATGGAAACAACAATTCAAGCACCATTT
GACGGTGTGATTAAACAAGTAACTGTAAATAATGGTGACACAATAGCGACAGGCGATTT
ATTAATCGAAATTGAAAAAGCAACTGACTAA

> **ctaA**

Function: protein coding sequence; heme A synthase (core genome, constant)
Best match: ctaA_CC001_MW2-USA400_BA000033.2[1096264:1097175:r]RC (completely identical)
Position: 027-contig_249: 28272 … 29184; Length: 912 bp
Sequence:
TTATTGCTTGTCACTTCTTACTGATCGTAACATTAGCATGATAAAGTATGTTGTCATACCA
AATAAATAAGTGATAAATAATGCATGGAAAAGTGCGATTATCAGGTTAACATTTGTCATA
ATAGATAATGCACCTGTGATAACTTGTAATATAACAAGTATAAATGCAGCTGTATAACCA
TAATGAACAGTACGGTTATTTGGATAATTTTTAACAGCGTGAATATAAGTAATCATAATA
ATCGTAAACACTATAAAAGCCATGATACGATGCGTGAGTTGAACCCAATCTTGTTCTGAA
TGTGGTACAAGATCGTGGAATGGCAATGGCCAACCACCATATGCTAAACTTGCATCCGCA
TGTCTCACTAGTGCACCAGTATAAACACCACAATAAATGATGATTGCCATTAACCATGTT
AAACGTCTTAATGGCTTTTTGATATATAATTCGTCAGCTTCATATTTTTGATCTATAGAGA
AAATAATCAATGTTATTAAAAATACAGATGAGAAACTGATTAATGATATACCAAAGTGCA
ATGCTAAAACGTAATCGTTTTGTTGCCAAATAACAGCAGCAGCTCCGATTAATGCTTGCA
ATAATAAGAATCCAACACTAATGATTGATAAAGGTTTAATTTCTTTAATATAGCCTATATG
TTTCCATGCAGTGATAACTAACCATAAGACCATTAATAAAGACAAAGCTGAAACGGCTCT
ATGACTTAACTCAATAATCGTATCAATAGGAAAGAATTCTGGAATCAACGCACCATGACA
TAGTGGCCAAGAAGAACCACAACCATCAGCTGATCCGGTTTTGGTAACTAAGGCTCCACC
AAGTTGTACAAATGTCATCATTAACGTTGCTACGACACCTAACCATTTTAAATTCTTTTTG
CCAAACAA

> **ctaB**

Function: protein coding sequence; protoheme IX farnesyltransferase (core genome, constant)
Best match: ctaB_CC001_MW2-USA400_BA000033.2[1097615:1098526]
Position: 027-contig_249: 29623 … 30535; Length: 912 bp
Sequence:
ATGAGCAAAGAGCATACTTTGTCACAAAATATTAGCAGAGTTAACTTCAAAGAATTGCAA
CAGATAATTAAAATGGGACTTGTTCAAGGTAACTTAATTCCGGCTTTTGCGGGCGCATGG
CTAGCAGTTGTAATGACAAATCATTCCTTCTTATCATCAATACCTCAAATTTTATTAATGC
TATTTGGATCTACTTTAATTATGGGTGGCGCATGTGCGTTAAATAATTATTACGACCAAGA
TATTGATCGTATTATGCCTAGTAAACAAAATAGACCAACTGTAAATAATAGAATTACAGA

TCAAAATTTATTACTATTAAGTTTTGGTATGATGTTAGTTGGAGAAATTTGTTTGTTTTTAT
TGAATATACCATCAGGCGTACTTGGTCTTATGGGGATTGTAGGTTATGTGTCTTATTACTC
AATATGGTCTAAAAGACATACAACATGGAACACAGTCATTGGGAGTTTTCCTGGAGCAGT
ACCACCACTAATTGGATGGGTTGCAATTGAAGGACAAATTAGTTTAACAGCGATTGCGCT
GTTTTTAGTTGTATTTTGTTGGCAACCAATTCATTTTTATGCCTTAGCTATTAAACGTAAAG
ATGAATATGCACTTGCAAATATTCCAATGTTACCATCAGTTAAGGGCTTTAAACGTACAC
GTGTCAGTATGTTTATCTGGTTGATTATTTTATTGCCAGTACCTTTATTACTAATAAATTTA
GGTGTCGTATTCGTAGTGTTAGCTACATTATTAAATTTAGGATGGATTGCATTAGGTTTAA
CAACATTTAAGAAAAATTCAGATCAAACAAAATGGGCAACACAAATGTTTATATATTCAC
TAAATTATTTAGTGATCTTTTTCGTGTTAGCTGTGATTGTTTCATTACTTACTTTGATCTAG

> **coaD**

Function: protein coding sequence; phosphopantetheine adenylyltransferase (core genome, constant)
Best match: coaD_CC001_MW2-USA400_BA000033.2[1103314:1103796] (completely identical)
Position: 027-contig_249: 35322 … 35805; Length: 483 bp
Sequence:
ATGGAACATACAATAGCGGTCATTCCGGGTAGTTTTGACCCCATTACTTATGGTCATTTAG
ACATTATTGAGAGAAGTACAGATAGATTTGATGAAATTCATGTCTGTGTTCTTAAAAATA
GTAAAAAAGAAGGTACGTTTAGTTTAGAAGAGCGTATGGATTTAATTGAACAATCTGTTA
AACATTTACCTAATGTCAAGGTTCATCAATTTAGTGGTTTACTAGTCGATTATTGTGAACA
AGTAGGAGCTAAAACAATCATACGTGGTTTAAGAGCAGTCAGTGATTTTGAATATGAATT
ACGCTTAACTTCAATGAATAAAAAGTTGAACAATGAAATTGAAACGTTATATATGATGTC
TAGTACTAATTATTCATTTATAAGTTCAAGTATTGTTAAAGAAGTTGCAGCTTATCGAGCA
GATATTTCTGAATTCGTTCCACCTTATGTTGAAAAGGCATTGAAGAAGAAATTTAAGTAA

> **Q4L5E0**

Function: protein coding sequence; putative protein (core genome, constant)
Best match: Q4L5E0_CC030_MRSA252_BX571856.1[1145912:1146469] (completely identical)
Position: 027-contig_249: 37132 … 37690; Length: 558 bp
Sequence:
ATGAAATGGTCAATTACGCAATTAAGGAAATATCAAGGTAAGCCATTTGAATTTGATCAA
ACGGTGAGTTTTGACAATTTAAAAGAATCATTAGATTTAATTGATTTATCTCCAATTACAA
TCCAAGGTCAGTTAACCATTAAGTCAACAGAAGTCGTTGCGGATATTCACATTACTGGAA
CGTATACAATGCCTTGTGCACGTACTCTTGTACCAGTTAAAGTCCCACTAGATGTAACTAC
TACAGAAGTATTTGATTTAGAAGGGTACAATCAGTATAACGATGATCAAGATGATGTAGA
TGAACACTATCACATTATTAAAGATGGTATGGTTAATCTTCAGGATATTGTCGAGGATAT
AGTTATTATTGAGAAACCAATGAGAGCTTATTCAGAGCAAAGTGACCAAATGTTGACAGT
AGGTAATGGTTGGGAAGTAATCGATGAGGATCAATTAGATGAGCTTGCTAAACAGCAAG
AACAAGATGATTCAGAATCACGACAAGTTGATCCAAGGCTTCAAAAATTACAACAATTAT
ATGATAAAGAGCAATAA

> **rpmF**

Function: protein coding sequence; 50S ribosomal protein L32 (core genome, constant)
Best match: rpmF_CC001_MW2-USA400_BA000033.2[1105761:1105934] (completely identical)
Position: 027-contig_249: 37769 … 37943; Length: 174 bp
Sequence:
ATGGCAGTACCAAAAAGAAGAACTTCTAAAACTAGAAAAAACAAACGTCGTACGCATTT
CAAAATTTCAGTACCAGGTATGACTGAATGCCCAAACTGTGGCGAATACAAATTATCACA
CCGTGTATGTAAAAACTGTGGTTCTTACAATGGCGAAGAAGTAGCAGCTAAATAA

> **tx_rpmF**

Function:
Best match: tx_rpmF_isdB_1_CC001_MW2-USA400_BA000033.2[1105942:1106003]
Position: 027-contig_249: 37950 … 37980; Length: 30 bp
Sequence:

TACTCAATATAAAAAGTCCCGCTTAAAATG

## > attB_phiSa7

Function: attachment site for int (mobile element)
Best match: attB_phiSa7_empty_CC001_MW2-USA400_BA000033.21105980:1106068:rRC
Position: 027-contig_249: 37988 … 38077; Length: 89 bp
Sequence:
AAGTGGGACTTTTTATATTGAGAAAAATAATTGGCGAACGAGGTAACTGGATACCTCATC
CGCCAATTAAAATTTGTTAATTTAATAAT

## > isdB

Function: protein coding sequence; iron-regulated cell wall-anchored protein (core genome, variable)
Best match: isdB_CC001_MSSA476_BX571857.1[1134874:1136811:r]RC
Position: 027-contig_249: 38096 … 39989; Length: 1893 bp
Sequence:
TTAGTTTTTACGTTTTCTAGGTAATACGAATGCAACGATGCTACTTAAAGCTAATAATGCC
ATTAATGGTAATGTCATATCTTTATTTGATTCTTCACCAGTTTGTGGTAATGATTTTGCTTT
ATTTTCTTGTGTATTTTTATTGTTTTGGCTTTGAGTGTGTCCATCATTTGTGTTTTTAATGTT
TGCTTTTTGTAATGGAGCACTATCTTTTGCTTCGCTAGAACCTGCTGAAGTTTGAACAACA
TCTTTTGTTGTTTTTGATGAAGCAGTTGTTGGTTTTGCAACATTTTGAGTCGTAGATACTAC
CTTAGTTGGAGTTGTACTACTTGATTCTACTTCACCTTTAGTTGGTTTTGTAGCAGGCGTTT
TGTCTTTACCTGACTCACTAGATGCGTCATTTTCTTTTTCAACACTTGGTAATTGTTTATTG
TCATCTTTTTGGCTGTCTTGTTTTTGTGATTCTTTTTCAACAGGTGATGGTGTTGGTTTGCT
AGGCGTAGCTGGAGTAGCTTCCTTCTTAGCTGAGTTATCTTGTTGTTCTTTTTTGTTAGATT
TATCGGTATTGGCTTTTGTAAATGCTTCTTTATCAACGATTCTGACATGGTATTGTCCATC
ATAATCAATCGTTTTTACGTGAACTTTAACGATAGCATCATATAGAGTTTTACCTTCAACA
TATGGGAAAATAATTGTTCTAGTATTATTTTTAGCATCTTTGCTTATAGTTCTAACACGTT
GACCTTCAACCATGAAATCTTTCCAGTAATCGTCATTAGTAGTTTCCATGACCATATATTT
TTTGCCGTTAAGCATACCTGTTTTAATAGGGTGTTTAACAAAAGTATCCATCATAGATTCG
TTATTCTCAACACTTTCATAAACAACATATTTTGTATCTTGTAAATCAGTCATTTTTTCATT
TGTTGGTTGTACATTTTGGAATTCAGTAATAGCTGATTTCACTTGCTCATCTAAAGCTTTCT
TTGTATCCTCTAATTTCTTCTTGTACTCAGCCTTTAATTTTTCAGGAAGTTTATCTTGAATT
TTATTTAATTCATAAACTTGTCTTTCTAGTGTTTTCGCTTTTTTATATGGCGCTAATAATTT
TTCAGCTTTATAATCTTCTTCAGTTTTGAATTTATCTGCACTGTTATAAATTGGTTGTGCGA
ATTCCATTAATGTGTAATCGTATTTTTCTTCTTTGTTATTGAAGTGTGTTGAACTAACAATT
TTAACAGCTTTTGTTCCATTTGATACAGAGAAGCGAATGTAAGCATAATCTTTAACAGTAT
CGTATGATACTAATTTAATTGGCAACTTTTTGTCACCTTCATAAACTTCAAATTTTCTCCA
AAATTGACCTGATTGTAATCCTAATTCAATTTCTGGTTTTGAATCAGTGAAAATAACTCTA
GCAGGTTTAACAGAACTTGCATAATGATAAAACTGTTGAGTTCCATCTTTCTTTTTCATTT
CAAAATCAATTGGACGAGAGTTTGGTGCGCTATGATCTTTGTCTTTTATTGCAGGGTTTTT
AATCGCTTCTCTAAGTTCCTGATTCAAAATAGGATATGTATTGTTAGTGGCTTTTGCTGCT
GGTTTAACTTCTTTTGTTTCAGAAGTAGGGGCCTCAACTTCTTTATTAGATACTGAGACAG
CATTAGCTACTGGTTTAGTTTCTGGAGCTTTTTCAGATGTTGTTGTTGGACTTGCAACTGCT
TCAGTTTTTGGTTGTGCTTCTGTATTTGTACCACCTGTTTCTTCAGCTGCTGCTTGTGCTTC
GCCATTTGACATTAATAATAAAAGTGTACTAATTGCTACAGATGCAACGCCTAGTGATGA
CTTTCTAATTGAATAAAATGATTTAAATTCTTTTTGCTGTTTGTTCAT

## > isdA

Function: protein coding sequence; heme-binding protein (core genome, variable)
Best match: isdA_CC001-ST772_118_AJGE01000009.1[230822:231874:r]RC (completely identical)
Position: 027-contig_249: 40191 … 41244; Length: 1053 bp
Sequence:
TTATTTAGATTCTTTTCTTTTGAAAAGTAATAAAGATAATGAACCTAAAAGGGCAAGTGTT
GCGAAGGCAACTGTGCTAATAAAGTTATCAACTGAAGTTAAACCAGTTTTTGGTAATTCT
TTAGCTTTAGATGCTTGTTTAGGCGTTTCGTTATGTTTTGTAACTTTGTTAGTTTGTTGTGA

TTTATTATCACTTACAGCTTGATTGTTGCTTTCAGATTTCGCTGTTGCAACATCTTTAACAG
GTGTTTGAACTTTATTTTGTTCTTGAGCAGTTTGTGCTGTTTTAACTGTATGAGCAGTTTGT
GTTTTAGTTTGATCTTTTGTTGTGTCAGTACTTACAACTTTAGTAGAGTGATTGTCTTCAAC
TTTGCTTGTTGTAGTTACAGTAGGTTTAACTTTTTCAACTTTAGGTTGAACTGGTTTAGTTT
GCTCAGTAGGTGTTTTAGGTTGAGCTGGTTTTGGTTGAACCGGTTTAACATTGTTTGGTTT
TGCTGCGTCAGCTAATGTAGGAATTGCTTTTTCAAATTCCAAATGCGTAGTATATCTATGA
TTGTAATTAATTTGTGGCACGACAATATGTACTTTAGTAGTTAAGCTCTTATATCCAGGTT
CAACTGCAACATTGATTGTTCTAGTATCCGCTTTTTTATTATCGTTAACAACAGTTGTTGCT
AATTCTTGATTGTTTGCATTGTAAAATTTGTATTCTTTCCAGAATGATGCATTGTTTAACAC
GGTTTGGAAATAATATTTATTATTTTGTTTAATTACTTTACCAGGGTGTTGCATATAGTCA
TCCATGTGTGACTTCTCTGAAGAGCCATCTTTTTGCACTTGGAAATTAATTGGTTGTGATG
TTGCTTGAGAAACTTGTGTGCTTTGATTATTAGTTGCGTTCGTAGCTTCTGTTGCCGCATTG
ACTTGTTGGCTGCCTGCGCCTATGTATACAAGGGAACCTAAAATGATAGATGCTGTACCC
ATTGTAATCTTTTTCATAGCTGATGAACGTTGTTCTGATTGATACTTACTGTTTAAATAAT
GTTTTGTCAT

## > **isdC**

Function: protein coding sequence; extracellular heme-binding protein (core genome, constant)
Best match: isdC_CC001_MW2-USA400_BA000033.2[1109489:1110172]
Position: 027-contig_249: 41452 … 42136; Length: 684 bp
Sequence:
TTGAAAAATATTTTAAAAGTTTTTAATACAACGATTTTAGCGTTAATTATCATCATCGCGA
CATTCAGTAATTCTGCAAATGCCGCAGATAGCGGTACTTTGAATTATGAGGTTTACAAAT
ACAATACCAATGACACGTCAATTGCTAATGACTATTTTAATAAACCGGCAAAGTACATTA
AGAAAAATGGTAAATTGTATGTTCAAATAACTGTCAACCACAGTCATTGGATTACTGGAA
TGAGTATCGAAGGACATAAAGAAAATATTATTAGTAAAAACACTGCCAAAGATGAACGC
ACTTCTGAATTTGAAGTAAGTAAGTTGAACGGTAAAATAGATGGAAAAATTGACGTTTAT
ATCGATGAAAAAGTAAATGGTAAGCCATTCAAATATGACCATCATTACAACATTACATAT
AAATTTAATGGACCAACTGATGTAGCAGGTGCTAATGCACCAGGTAAAGATGATAAAAA
TTCTGCTTCAGGTAGTGACAAAGGATCTGATGGAACGACTACTGGTCAAAGTGAATCTAA
CAGTTCGAATAAAGACAAAGTAGAAAATCCACAAACAAATGCTGGTACACCTGCATATA
TATATGCAATACCAGTTGCATCCTTAGCATTATTAATCGCAATCACATTGTTTGTTAGAAA
AAAATCTAAAGGCAATGTGGAATAA

## > **isdD**

Function: protein coding sequence; iron-responsive Surface Determinate (core genome, variable)
Best match: isdD_CC008_NCTC8325_CP000253.1[1046312:1047388] (completely identical)
Position: 027-contig_249: 42135 … 43212; Length: 1077 bp
Sequence:
ATGAGAAATGTTAAACAAATTGCTACAAAATCTATTATAGCTATTATTAGCTTAGGTATA
CTTACATATACAACAATGATTGGTAGCGTGTTGGCTGATGAGATAAAATATCCATCAGCC
AAATTTAATCAACCTGAAGCAAAAGATAAAACAGAATTAACTACATCAATTTTTGATGAA
AAGATAAAAGAGAATAAAGCGTTAGAGTTACTAATTTTTAATCAAGAAAATAAAAATGT
AACTGAGGAACAACAACTAGTTGACGAAAAGGCGCAATTGATTTCAGATATGACTGGTA
AAATTTACTTGCAAGTAAAGCTAAAAGGTCAAATAGATAAAGAACAACTTGTTTTTCAAA
ATGACAAAAATGAAGAATTTCCTTTTGTTATAAAAGATGAAAAGGATGACACAATAGTA
AGAATTTTAATTGAACAGCATATGGATAAAATCAATATGCATGTTAAAACGTTGGCTGAA
AAGAAAAATCTAGATAACAAAGAAATGGTGTATTCTATTCATTTTAAAGAGAAAAAAGT
ACAACATGATGATGCAAAAGAAGTGCCTTCAAAACATCAAAATCAAGAAAATAATCAAG
ATCAGCTTAAAAAAGATATTGATGACAAAAAAGATAGTCAAAAATCAGATACTAAGGAA
AGACGTACTAGCCTTTTTACTGAAAAAGGATTAAATGATATTCCTGTACAAAAAGATAAA
GTGCAACAAGACAGTAATAAAAAGATTGAAAATGAGCGACCTAAAGCATCAGGTACATT
AAAAGTTGAAAATAGCCCTCCAACAATAAAAAAGGTTGAAAATAATCACAAAGAGCAAC
CGAAACATAAAGATGAAAAATCAAAAAAGGAAAAGAAAAAAGTAGTTGAAAAAGAAAA
AGCGTTACCAGCTTTTAATAGAGATGATGATAGCAAGAATAGTAGTCAATTATCTAGTGA

TATTAAAGAACTTGATGAACCAAATCATAAAAAGCAATATATGTTATTTGCAGCTGGCAT
TGTGTTAGCAACTATTTTACTTATTTCGGCACATTTATACAGCAGAAAGAGAGGTAACCA
AGTTTGA

> **isdE**

Function: protein coding sequence; heme-binding lipoprotein of ATP-binding cassette transporter (core genome, constant)
Best match: isdE_CC005_JH9_CP000703.1[1234393:1235271]
Position: 027-contig_249: 43208 … 44087; Length: 879 bp
Sequence:
TTGAGAATCATAAAGTATTTAACCATTTTAGTGATAAGCGTCGTTATCTTAACCAGCTGTC
AATCTTCCAGTTCTCAAGAATCAACTAAATCCGGCGAATTCAGAATCGTACCAACAACTG
TTGCATTGACAATGACATTGGACAAATTGGATTTACCAATTGTCGGCAAACCCACGTCAT
ATAAGACATTGCCTAATCGTTATAAAGATGTACCTGAAATTGGTCAACCAATGGAGCCGA
ATGTTGAAGCTGTTAAAAAATTAAAACCAACACATGTTTTGAGTGTGTCAACGATTAAAG
ATGAAATGCAACCATTTTACAAACAATTAAATATGAAAGGCTACTTTTATGATTTTGATA
GTTTAAAAGGGATGCAAAAGTCGATTACACAATTAGGTGATCAATTTAATCGTAAAGCAC
AAGCAAAAGAATTAAATGACCATTTAAATTCTGTTAAGCAAAAAATTGAAAATAAAGCA
GCTAAACAAAAGAAACATCCCAAAGTATTAATATTAATGGGTGTACCGGGTAGCTATTTA
GTAGCAACTGATAAATCATATATTGGTGATTTAGTTAAAATAGCAGGTGGAGAAAATGTT
ATTAAAGTGAAAGATCGTCAATATATTTCGTCTAATACTGAAAATTTGTTGAATATCAATC
CAGATATTATTTTAAGATTACCACACGGAATGCCTGAAGAAGTTAAGAAAATGTTTCAAA
AAGAATTTAAACAAAATGATATTTGGAAACATTTTAAAGCTGTGAAAAATAATCATGTTT
ATGACTTAGAGGAAGTGCCATTCGGTATTACAGCAAATGTTGATGCTGATAAGGCAATGA
CTCAATTATATGATTTATTTTATAAGGATAAAAAATAG

> **isdF**

Function: protein coding sequence; iron transport permease protein (core genome, constant)
Best match: isdF_CC030_MRSA252_BX571856.1[1152956:1153921]
Position: 027-contig_249: 44099 … 45065; Length: 966 bp
Sequence:
ATGATAAAAAATAAAAAGAAACTACTATTTTTATGTTTGTTAGTCATTTTAATCGCAACG
GCTTATATTTCGTTTGTAACCGGTACAATTAAATTGTCATTTAATGACCTATTTACAAAAT
TTACAACTGGTAGCAATGAAGCAGTGGATTCAATCATTGATTTGCGATTGCCACGTATAT
TAATTGCATTAATGGTTGGCGCAATGTTAGCAGTTTCTGGAGCATTATTACAAGCAGCAC
TACAAAATCCTTTGGCAGAGGCGAATATCATTGGCGTTTCTTCAGGTGCACTTATAATGA
GAGCACTTTGTATGTTGTTTATTCCACAATTGTACTTTTACTTACCATTATTAAGTTTTATT
GGAGGTTTAATACCATTTTTAATAATTATATTGTTGCATTCTAAATTTAGATTCAATGCTG
TAAGTATGATATTAGTAGGTGTTGCGTTATTCGTATTATTAAATGGTGTTTTAGAAATTTT
AACTCAAAACCCTTTAATGAAAATTCCTCAAGGCTTAACAATGAAAATATGGAGCGACGT
ATACATATTAGCAGTATCAGCATTATTGGGATTAATATTAACATTACTATTGTCCCCTAAA
TTGAATTTACTAAATTTAGACGACATACAAGCGCGAAGTATCGGTTTTAATATTGATCGTT
ACAGATGGTTAACAGGTTTATTAGCAGTATTTTTAGCAAGTGCAACTGTTGCGATTGTTGG
ACAACTAGCCTTTTTAGGTATTATTGTGCCACATGTGGTTAGAAAGCTAGTTGGGGGCAA
TTACAGAGTACTTATTCCGTTTCTACAGTTATTGGTGCATGGCTATTGTTAGTGGCTGAT
TTATTAGGACGAGTGATACAGCCTCCTTTAGAAATTCCAGCCAATGCTATTTTAATGATTG
TCGGTGGTCCAATGCTAATTTACTTAATTTGTCAAAGTCAACGAAATCGAATCTAA

> **srtB**

Function: protein coding sequence; sortase B/NPQTN specific (core genome, constant)
Best match: srtB_CC001_MW2-USA400_BA000033.2[1113163:1113897]
Position: 027-contig_249: 45126 … 45861; Length: 735 bp
Sequence:
ATGAGAATGAAGCGATTTTTAACTATTGTACAAATTTTATTGGTTGTAATTATTATCATTT
TTGGTTACAAAATTGTTCAAACATATATTGAAGATAAGCAAGAACGCGCAAATTATGAGA

AATTACAACAAAAATTTCAAATGCTGATGAGCAAACATCAAGAACATGTGAGACCACAA
TTTGAATCACTTGAAAAAATAAATAAAGACATTGTTGGATGGATAAAATTATCAGGAACA
TCATTAAATTATCCAGTACTACAAGGTAAGACAAATCACGATTATTTAAATTTAGATTTTG
AGCGAGAACATCGACGTAAAGGTAGTATTTTTATGGATTTTAGAAATGAATTGAAGAATT
TAAATCATAATACTATTTTATACGGGCACCATGTCGGTGATAATACGATGTTTGATGTGTT
AGAAGATTATTTAAAGCAATCGTTTTATGAAAAACACAAGATAATTGAATTTGACAATAA
ATATGGTAAATATCAATTGCAAGTATTTAGTGCATATAAAACTACTACTAAAGATAATTA
CATACGTACAGATTTTGAAAATGATCAAGATTATCAACAATTTTTAGATGAGACAAAACG
TAAATCTGTAATTAATTCAGATGTTAATGTAACGGTAAAAGATAGAATAATGACTTTATC
AACGTGCGAAGATGCATATAGTGAAACAACGAAAAGAATTGTTGTTGTCGCAAAAATAA
TTAAGGTAAGTTAA

> **isdG**

Function: protein coding sequence; iron transport lipoprotein (core genome, constant)
Best match: isdG_CC005_Mu50-VRSA_BA000017.4[1188814:1189137] (completely identical)
Position: 027-contig_249: 45879 … 46203; Length: 324 bp
Sequence:
ATGAAATTTATGGCAGAAAATAGGCTGACGTTAACAAAAGGAACAGCAAAAGATATTAT
AGAACGATTTTACACGAGACATGGGATTGAAACATTAGAAGGCTTTGATGGCATGTTTGT
TACACAAACTTTAGAACAAGAAGATTTTGATGAAGTGAAAATTTTAACAGTTTGGAAATC
AAAGCAAGCTTTTACGGATTGGTTAAAATCTGATGTCTTTAAAGCAGCGCATAAACATGT
TAGAAGTAAAAATGAAGATGAAAGTAGCCCGATTATCAATAACAAAGTAATTACATATG
ATATAGGCTATAGTTACATGAAATAA

> **spoU**

Function: protein coding sequence; putatuve tRNA/rRNA methyltransferase (core genome, constant)
Best match: spoU_CC001_MW2-USA400_BA000033.2[1114623:1115363]
Position: 027-contig_249: 46586 … 47327; Length: 741 bp
Sequence:
ATGGAACAAATTACTTCTGCACAAAATAATAGAATTAAACAAGCGAACAAGCTAAAAAA
GAAACGTGAGAGGGATAAAACTGGATTAGCTTTAATTGAAGGTGTGCATTTAATTGAAGA
AGCTTATCAAAGTGGAATTGTAATTACACAATTATTTGCAATTGAACCGGCAAGATTAGA
TCAGCAAATTATCGCATACGCGCAAGAAGTTTTTGAAATAAACATGAAAGTTGCTGAATC
TTTATCAGGTACAGTGACACCACAAGGGTTTTTCGCAATCATTGAGAAGCCGCATTATGA
TATTTCTAAAGCACAACAAGTATTGCTCATCGATCGTGTTCAAGATCCTGGAAATTTAGG
CACATTAATTAGAACTGCGGATGCTGCTGGAATGGATGCTGTAATAATGGAGAAGGGTAC
GACAGATCCTTATCAAGATAAAGTGTTGCGAGCGAGTCAAGGTAGTGTTTTCCATTTGCC
AGTTATGATACAAGATCTCGATACGTTTATTACTCAATTTAATGGTCCTGTTTATGGTACA
GCACTTGAAAACGCAGTGGCATACAAAGAAGTTACTTCAAGTGATTCTTTTGCATTACTA
TTAGGTAATGAGGGAGAAGGTGTTAATCCTGAATTATTAGCACATACTACAAAAAATTTA
ATCATACCTATTTATGGTAGTGCTGAAAGTTTAAATGTAGCGATTGCAGGTAGTATTTTAC
TTTATCATTTGAAAGGTTGA

> **tbox04**

Function: noncoding RNA T-box leader element (core genome, constant)
Best match: tbox04_CC001_MW2-USA400_BA000033.2[1115444:1115655] (completely identical)
Position: 027-contig_249: 47407 … 47619; Length: 212 bp
Sequence:
TAAACCAATAAAAAGGCATGGACATTTATATAAATAATTGTTTTAGGGAGAATAATCGTG
ACTGCAAGTTATTCCAATTATTTAAAGTCTTTTCACCTTTTTGGTTACTTAAAGAGATTTA
AGTCGGAAAGACAATCCGTTATCAATATTAAACAAGTGTATGCTTAGGCATAAATTTGGG
TGGTACCACGGAAATGACTTTCGTCCCTTAT

> **pheS**

Function: protein coding sequence; phenylalanyl-tRNA synthetase alpha chain (core genome, constant)
Best match: pheS_CC001_MW2-USA400_BA000033.2[1115744:1116802]
Position: 027-contig_249: 47707 … 48766; Length: 1059 bp
Sequence:
ATGTCTGAACAACAAACAATGTCAGAGTTAAAACAACAAGCGCTTGTAGATATTAATGA
AGCAAATGATGAACGTGCACTGCAAGAAGTTAAAGTGAAATACTTAGGTAAAAAAGGGT
CAGTTAGCGGACTAATGAAATTGATGAAGGATTTGCCGAATGAAGAGAAACCTGCGTTTG
GTCAAAAAGTGAATGAATTGCGTCAAACAATTCAAAATGAATTAGATGAAAGACAACAG
ATGTTAGTTAAAGAAAAATTAAATAAGCAATTGGCTGAAGAAACAATTGATGTATCATTA
CCAGGTCGTCATATTGAAATCGGTTCAAAGCATCCATTAACACGTACAATAGAAGAAATT
GAAGACTTATTCTTAGGTTTAGGTTATGAAATTGTGAATGGATATGAAGTTGAACAAGAT
CATTATAACTTCGAAATGCTGAATTTACCTAAATCACACCCTGCACGTGATATGCAAGAT
AGTTTCTATATTACGGATGAAATTTTATTACGTACGCATACATCACCAGTGCAGGCACGT
ACGATGGAATCACGTCATGGTCAAGGTCCAGTTAAAATTATTTGCCCTGGTAAAGTGTAT
CGTCGTGACTCTGATGATGCGACACATAGTCATCAATTTACACAAATTGAAGGATTAGTT
GTTGATAAAAACGTTAAAATGAGTGATTTGAAAGGCACTTTAGAATTGTTAGCTAAGAAA
TTATTTGGTGCTGATCGTGAAATTCGTTTACGTCCAAGTTACTTCCCATTCACTGAACCTT
CTGTAGAAGTTGATGTGTCATGTTTTAAATGTAAAGGAAAAGGTTGTAATGTGTGTAAAC
ACACAGGATGGATTGAAATTTTAGGTGCTGGAATGGTACATCCTAATGTATTAGAAATGG
CTGGTTTTGATTCTTCAGAGTACTCTGGATTTGCATTTGGTATGGGACCAGACCGTATTGC
AATGTTGAAATATGGTATAGAAGATATTCGTCATTTCTATACTAATGATGTGAGATTTTTA
GATCAATTTAAAGCGGTAGAAGATAGAGGTGACATGTAA

> **pheT**

Function: protein coding sequence; phenylalanyl-tRNA synthetase beta chain (core genome, constant)
Best match: pheT_CC001_ATCC51811_ADVP01000010.1[55786:58188:r]
Position: 027-contig_249: 48765 … 51168; Length: 2403 bp
Sequence:
ATGTTGATATCAAATGAATGGTTGAAAGAATATGTAACAATCGATGATTCTGTAAGTAAT
TTGGCAGAACGTATTACGCGCACAGGTATTGAAGTGGATGATTTAATTGACTACACAAAA
GATATCAAAAATTTAGTTGTCGGCTTCGTTAAGTCAAAAGAGAAACATCCTGATGCTGAT
AAATTAAATGTTTGCCAAGTTGATATCGGAGAAGACGAACCTGTACAAATCGTATGTGGT
GCACCGAACGTTGATGCAGGACAATATGTCATTGTTGCTAAAGTAGGTGGCAGATTGCCT
GGTGGTATTAAAATTAAGCGTGCCAAATTACGCGGTGAACGTTCAGAAGGTATGATTTGT
TCGTTACAAGAAATTGGTATTTCAAGTAACTATGTACCAAAAACGTTTGAATCTGGTATCT
ATGTATTTAGTGAAGCCCAAGTTCCAGGAACAGATGCCTTACAAGCTTTATATTTAGATG
ATCAAGTAATGGAATTTGATTTAACGCCGAATCGTGCAGATGCTTTAAGTATGATAGGTA
CTGCTTATGAAGTTGCAGCATTATATAATACAAAAATGACTAAGCCAGACACAACATCAA
ATGAGCTTGAGTTATCTGCAAATGATGAACTGACTGTGACAATAGAAAATGAAGATAAA
GTACCATATTATAGTGCACGTGTTGTTCACGACGTGACAATTGAACCCTCGCCAATTTGG
ATGCAAGCACGCTTAATAAAAGCGGGTATACGTCCTATTAATAATGTTGTTGACATTTCA
AATTATGTATTATTAGAATACGGTCAACCATTGCACATGTTTGATCAAGATGCGATTGGTT
CACAACAAATTGTTGTTCGTCAAGCTAATGAAGGCGAAAAAATGACAACATTAGATGAT
ACAGAACGTGAATTATTAACGAGCGATATTGTCATTACTAATGGACAAACTCCAATTGCA
TTAGCTGGTGTTATGGGTGGCGATTTTTCAGAAGTTAAAGAACAAACATCAAATATAGTG
ATTGAAGGTGCTATTTTTGATCCAGTTTCAATTCGTCATACATCAAGGCGTTTAAATTTAC
GCAGTGAATCATCTAGTCGTTTTGAAAAAGGAATAGCTACTGAATTTGTAGATGAAGCAG
TCGACCGTGCATGTTATTTATTACAAACTTATGCAAACGGAAAAGTGCTAAAAGATAGAG
TGTCTTCAGGAGAACTTGGTGCATTTATTACACCAATCGACATCACTGCTGATAAAATTA
ATCGCACTATTGGATTTGATTTGTCACAAAATGATATTGTTACTATTTTTAATCAATTAGG
GTTTGATACAGAAATAAATGATGATGTTATTACAGTGCAAGTACCATCACGTCGTAAAGA
TATTACAATTAAAGAAGATTTAATTGAAGAAGTTGCACGTATATATGGTTACGACGATAT
TCCATCAACGTTACCTGTCTTCGAAAAAGTTACTAGTGGTCAGCTAACTGATCGCCAATAT
AAAACTAGAATGGTTAAAGAAGTGTTAGAAGGTGCTGGATTAGACCAAGCTATTACGTAT
TCGTTAGTATCTAAAGAAGATGCTACTGCATTTTCGATGCAACAGCGTCAAACAATTGAT

TTATTGATGCCAATGAGTGAAGCGCATGCGTCATTACGTCAAAGTTTATTACCACATTTAA
TCGAAGCGGCATCATATAATGTGGCACGCAAAAATAAAGATGTAAAATTATTTGAAATCG
GCAATGTCTTCTTTGCTAATGGAGAAGGTGAACTACCAGATCAAGTTGAATATTTAAGTG
GTATTTTAACTGGAGATTATGTAGTCAATCAATGGCAAGGTAAGAAAGAAACGGTTGATT
TCTATTTAGCAAAAGGTGTCGTGGATCGAGTATCTGAAAAGTTAAACCTTGAATTTAGTT
ATCGTCGTGCCGATATTGATGGATTACATCCAGGTCGTACAGCTGAAATCTTATTAGAAA
ATAAAGTTGTTGGTTTTATTGGTGAATTACATCCAACATTAGCAGCTGATAATGATTTAAA
ACGTACGTATGTTTTTGAGTTGAATTTTGATGCATTAATGTCTGTGTCGGTAGGTTACATT
AATTACCAGCCAATTCCGAGATTCCCAGGCATGTCTCGTGACATTGCATTAGAAGTAGAT
CAAAATATTCCAGCAGCTGATTTATTATCAACGATTCATGCACATGGTGGCAATATATTA
AAAGATACACTTGTCTTTGATGTATATCAGGGCGAACATTTAGAAAAAGGTAAAAAATCA
ATTGCAATACGTTTAAATTATTTAGACACAGAAGAAACATTGACAGATGAGCGCGTTTCA
AAAGTACAAGCGGAAATTGAAGCAGCATTAATTGAACAAGGTGCTGTTATTAGATAA

> **rnhC**
Function: protein coding sequence; ribonuclease HIII (core genome, constant)
Best match: rnhC_CC001_MSSA476_BX571857.1[1148225:1149163:r]RC
Position: 028-contig_257_RC: 193 … 1132; Length: 939 bp
Sequence:
TCAAAGAGGCTTTAAAATTTTTTGGGCTTTTTCACGATTTTTAAAATGCTTTTTTGAAATG
GTATCTAAACGTGAAAGACCGTATTTTTTTATAATTTTGGCGGCAATTACATCGACTTTAG
CACCGGCACCTTTAGGAATCGTCATATTAATATTTTTTGATATTTGATCCATATATGTAAC
AAATGCGTATCGAGAAATTATGCTTGCCACTGCAATGGCTAATGACTTCGATTCTCCTTTT
GTTTCAAATTTTGTTTTCTTTGGAAGTGGTATATCTGATAATGCGTAATGGCTATACACTT
CGCGTTTTGCGAACTGATCAATGACGATATAGTCTAATTGAGACGAATCAATTTTTTCAA
GTACATTTTTGATGGCTTCATTATGAAGAACAGCTTTCATTTTTACTTGAGTCCAGCCTTTT
GCTTGCTGAATATTATATTTTTCATTGTGTAGTGTTAATAGTGAATGTGGGATGAAAGTAA
CCAATTGCTCAGCAAGTTCTACAATTTTGGTATCGGTTAATTTTTTTGAGTCATCTACACC
CAAAGTTTTTAAAATAGGGACATGCTCTTTGGTAACGAAAGCAGCACACACAGTCAACGG
ACCAAAGTAATCACCACTTCCAGCTTCATCACTACCAATACAGTTAAATTGATCATACATT
AAAGTTTGTTCAAGAAAAGAATTAGCCATATTTTTCTTTTTAGTTTTATTAGTATTCAATT
GAGAATGCTGCGGTAGAAGTTCTTCAGACACAGCTTCTGCATGATTACCCTGAAACATGA
CTTTACCTGATTGGTAAATATTTACAGTTGTATTTTGATACTTTGCACGTGCTTTCATACCT
TGAGGTAAATTCTCAGTATCAAAAGAAATGCGTGACATTAATGTCGTTATGTCTTTATCCG
ACAATTTAAAAACGATATTCGCCAT

> **zapA**
Function: protein coding sequence; cell division protein (core genome, constant)
Best match: zapA_CC001_MW2-USA400_BA000033.2[1120747:1121013] (completely identical)
Position: 028-contig_257_RC: 1501 … 1768; Length: 267 bp
Sequence:
ATGGCACAGTTTAAAAACAAGGTAAATGTATCAATTAATGATCAGCTTTTTACAATTGTT
GGGGAAGATAACCCAGAGCACATACGATATGTAGCACATTTAGTTGATGATAAAATAAA
AGAATTAGGGTATAAAGCAGCAGGTTTAGATACTTCAAGAAAAGCAATACTAACTGCTGT
GAATATTATGCATGAAAAAGTACTACTAGAAGAAGAAAATCGACGTTTGAAACAACAAA
TTCACAAATTGCAGCAGCGTGAGCAATAA

> **polX**
Function: protein coding sequence; DNA-dependent DNA polymerase IV beta subunit (core genome, constant)
Best match: polX_CC005_04-02981_CP001844.2[1154963:1156675]
Position: 028-contig_257_RC: 2362 … 4075; Length: 1713 bp
Sequence:
ATGACAAAAAAAGATGTTATCAAACTATTAGAACAAATTGCTACTTATATGGAATTAAAA
GGGGAAAATACTTTTAAAATATCAGCGTATCGAAAAGCAGCTCAAAGTCTTGAATTAGAT

GAACGACCATTAGATGAAATATCTGATGTAACGGAGTTAAAAGGCATTGGTAAAGGTGTT
GCAGAAGTAATCAATGATTACCGTGAGACCGGTGAATCTCAGTATTTACAGCAATTACAG
GAAGAAGTTCCGGAAGGTCTTATTCCACTTTTGAAAATTCAAGGACTTGGAAGCAAGAAA
ATTGCTAAGCTATATAAAGAGTTGAATATTGTTGATAAAGCGTCACTTCAAGTTGCTTGTG
AAAATGGAAAAGTTAGTGAATTAAGCGGATTTGCTAAGAAAACGGAACAAAACATATTA
GAAGCTGTGAAACAACTTGGTGCTAAGAAAGATAGATATCCAATTGATCAAATGAGAAG
ACTTAATCAAGAAATCATTGATTATATAGATACATTAAATTATATCGATCAATATTCATCT
GCAGGAAGCTTCCGTCGTTTTAAAGAAATGAGCAAAGATTTAGATTTCATAATAAGTACC
GATAACCCAAAAGCAGTGCAGCAGCAATTATTAAATATTCCCAATAAAGTAAAAGAAGT
TGCAGTGGGGAACACAAAAGTTTCATTAGAATTAGCGTATGATGATGAAACGATTGGTGT
CGATTTTCGATTAATTGAACCAAGTGCTTTTTATCATACATTGCAGCATTTTACTGGGTCA
AAAGAACATAATATAAGAATTCGACAACTTGCTAAAGCACGTGATGAAAAAGTTAGTGA
ATATGGAATTGAACAAGCTGATGGTACATTAATTCAATATGATAGTGAAGCCAAGATATA
TGAACATTTTAATGTGAATTTTATACCACCTGCTATGCGAGAAGATGGTAGCGAATTTGA
TAAAGATCTAAGTAATATCATTACATTAGATGATATTAATGGTGATATTCATATGCATAC
AACGTATAGTGATGGTGCGTTTTCTATTCGAGACATGGTAGAAGCAAATATCGCAAAAGG
TTATAAATTCATGGTAATTACTGATCATTCACAAAGTTTACGTGTTGCTAATGGCTTACAA
GTGGAAAGACTTTTAAGACAAAATGAAGAAATTAAGGCTTTAGATAAAGAATATAGTGA
AATTGATATTTATTCAGGTACAGAAATGGATATATTACCTGATGGCTCGCTGGATTATGAT
GATGAAATTTTAGCACAACTTGATTATGTAATTGGAGCTATTCATCAAAGCTTTAACCAAT
CAGAAGAACAAATTATGGAACGATTAGCTAATGCATGTCGCAATCCATACGTGCGACATA
TAGCGCATCCAACAGGGCGTATTATAGGTAGAAGAGATGGTTATAAACCGAATATTGAA
CAATTAATGGCATTAGCTGAAGAAACGAATACAGTATTAGAAATTAATGCCAATCCACAT
CGACTGGATCTGAACGCTGATATCGTTCGTAAATATCCAAATGTGAAATTAACTATTAAC
ACTGATGCGCATCATACAAATCATTTAGATTTTATGAATTATGGCGTAGCAACTGCGCAA
AAAGGATTTGTAACAAAAGATAGAGTGATTAACGCATTATCGCGTGAAGCTTTTAAAGAC
TTTATTGAAAATAATATAAAACTTAAGAAATAG

> **mutS2**

Function: protein coding sequence; DNA mismatch repair family protein (core genome, variable)
Best match: mutS2_CC007_TCH959-USA300_AASB02000228.1[1340:3688]
Position: 028-contig_257_RC: 4084 … 6433; Length: 2349 bp
Sequence:
ATGAGACAAAAAACATTAGACGTCTTAGAATTCGAAAAAATAAAATCACTCGTTGCCAAT
GAAACTATTAGTGACTTAGGCTTGGAAAAGGTCAATCAAATGATGCCAGCTACTAATTTT
GAAACGGTTGTTTTTCAAATGGAAGAAACGGATGAGATTGCTCAAATCTATAATAAGCAT
CGTTTACCAAGCTTGAGTGGCTTATCTAAAGTATCAGCATTCATTCATCGCGCTGATATTG
GCGGCGTTTTAAATGTATCAGAGCTTAACTTGATAAAAAGATTAATTCAAGTACAAAATC
AATTCAAGACATTTTATAATCAATTGGTTGAAGAAGATGAAGGTGTTAAATACCCAATAT
TAGATGACAAGATGAATCAATTACCTGTGTTAACTGATCTTTTTCAACAAATAAATGAAA
CATGCGATACGTATGATTTATATGATAATGCGAGTTATGAATTGCAAGGGATTAGAAGTA
AAATTTCTAGCACGAATCAACGTATTAGACAAAATTTGGACCGTATTGTTAAAAGCCAAG
CAAATCAGAAAAAATTATCAGATGCTATTGTAACAGTTAGGAATGAAAGAAACGTTATA
CCTGTCAAAGCTGAATATCGACAAGATTTTAATGGGATTGTACATGATCAATCTGCTTCA
GGACAAACATTGTATATTGAGCCATCATCAGTTGTTGAAATGAATAATCAAATTAGTCGA
TTACGTCATGACGAAGCAATTGAAAAAGAACGCATTTTAACGCAACTAACTGGTTATGTG
GCTGCGGACAAAGATGCACTACTTGTGGCAGAACAAGTCATGGGTCAGTTAGATTTTTTA
ATCGCAAAAGCGAGATATAGTAGAAGTATTAAAGGAACAAAGCCGATATTTAAAGAGGA
CCGTACTGTATATTTACCTAAAGCATACCATCCATTATTAAATCGTGAGACTGTTGTAGCT
AATACCATCGAATTTATGGAAGATATTGAAACGGTAATTATTACAGGACCGAATACAGGT
GGTAAAACTGTAACATTAAAAACATTAGGTTTAATTATTGTTATGGCTCAATCAGGATTG
TTGATTCCCACACTTGATGGTAGTCAGTTAAGTGTATTTAAAAATGTATATTGCGATATCG
GAGATGAACAATCAATAGAACAATCATTATCAACTTTTTCATCTCATATGACGAATATAG
TTGAAATTTTAAAGCATGCAGACAAACATAGTTTAGTTTTATTTGATGAATTAGGTGCAG
GTACAGATCCGAGTGAAGGTGCTGCATTAGCAATGAGCATTTTAGATCATGTTAGAAAAA
TTGGTTCTCTAGTAATGGCAACGACGCACTATCCTGAACTTAAAGCATATAGTTATAATC

GAGAAGGCGTTATGAATGCGAGTGTAGAATTTGATGTAGATACTTTGAGTCCAACGTATA
AGTTATTAATGGGTGTGCCGGGTCGTTCAAATGCTTTTGACATTTCTAAAAAGTTAGGTCT
TAGTTTGAATATTATTAATAAGGCTAAGACGATGATTGGTACTGATGAAAAAGAAATAAA
TGAAATGATTGAATCATTAGAGCGTAATTACAAACGTGTAGAGACACAGAGGTTAGAAC
TGGACCGTCTTGTAAAAGAAGCGGAGCAAGTGCATGATGATTTATCTAAGCAGTATCAAC
AATTCCAAAATTATGAAAAGTCTCTAATAGAGGAAGCGAAAGAAAAAGCAAATCAGAAG
ATTAAAGCTGCAACAAAAGAAGCTGACGATATTATTAAAGACTTAAGACAATTGCGTGA
ACAAAAAGGTGCAGATGTTAAAGAACATGAATTGATTGATAAGAAGAAACGATTAGATG
ATCATTATGAAGCGAAATCTATAAAGCAAAATGTACAAAAGCAAAAAATACGATAAAATT
GTTGCTGGTGATGAAGTAAAAGTATTATCTTACGGTCAAAAGGGTGAAGTTTTAGAAATT
GTCAATGATGAAGAAGCAATTGTTCAAATGGGAATTATTAAAATGAAGTTACCTATTGAA
GATTTAGAGAAAAAACAAAAAGAAAAAGTTAAGCCAACGAAAATGGTTACACGTCAAAA
TCGTCAAACAATTAAAACTGAACTTGACTTACGAGGCTATCGTTATGAGGATGCTTTAAT
TGAACTAGATCAATATTTAGATCAAGCTGTTTTAAGTAATTACGAACAAGTTTATATCATT
CATGGTAAAGGTACAGGTGCACTTCAAAAAGGTGTACAACAACATTTGAAAAAGCATAA
AAGTGTTAGTGACTTTAGAGGTGGTATGCCAAGCGAAGGTGGATTTGGCGTTACCGTTGC
AACACTAAAATAA

## > **trxA**
Function: protein coding sequence; thioredoxin (core genome, constant)
Best match: trxA_CC001_MW2-USA400_BA000033.2[1125851:1126165]
Position: 028-contig_257_RC: 6605 … 6920; Length: 315 bp
Sequence:
ATGGCAATCGTAAAAGTAACAGATGCAGATTTCGATTCAAAAGTAGAATCTGGTGTACAA
TTAGTAGATTTTTGGGCAACATGGTGTGGTCCATGTAAAATGATCGCTCCGGTATTAGAA
GAATTAGCAGCTGACTATGAAGGTAAAGCTGACATTTTAAAATTAGATGTTGATGAAAAT
CCATCAACTGCAGCTAAATATGAAGTGATGAGTATTCCAACATTAATCGTCTTTAAAGAC
GGTCAACCAGTTGATAAAGTTGTTGGTTTCCAACCAAAAGAAAACTTAGCTGAAGTTTTA
GATAAACATTTATAA

## > **uvrC**
Function: protein coding sequence; ultraviolet response system protein C (core genome, constant)
Best match: uvrC_CC001_MW2-USA400_BA000033.2[1126489:1128270]
Position: 028-contig_257_RC: 7244 … 9026; Length: 1782 bp
Sequence:
TTGGAAGACTATAAGCAACGAATTAAAAATAAATTAAATGTCGTACCTATGGAACCAGG
CTGCTATTTAATGAAAGATCGTAATGATCAAGTGATATATGTTGGCAAAGCTAAAAAGCT
AAGAAATCGATTGAGATCATATTTTACGGGTGCTCATGATGCTAAAACAACGAGACTGGT
TGGTGAAATACGTCGCTTTGAGTTTATTGTCACGTCAAGTGAAACAGAGTCACTTTTACTA
GAATTGAATCTGATTAAACAATATCAACCAAGATATAATATATTATTAAAGGATGATAAA
AGTTATCCATTTATTAAAATTACGAAGGAGAAATATCCTAGACTACTAGTGACGAGAACT
GTAAAACAAGGTACTGGCAAATATTTCGGACCGTATCCGAATGCATATTCTGCTCAAGAA
ACTAAAAAGTTATTAGACAGAATATATCCATATCGCAAATGTGATAAGATGCCAGATAAA
TTATGTCTTTATTACCATATTGGACAATGTTTAGGACCATGTGTATATGACGTTGATTTGA
GTAAATACGCACAAATGACGAAGGAAATTACTGATTTTCTGAATGGGGAAGACAAAACA
ATTTTAAAAAGTTTAGAAGAGCGAATGTTAACTGCAAGTGAATCACTTGATTTTGAACGG
GCTAAAGAATATAGAGATTTAATTCAACATATTCAAAATCTGACAAACAAACAAAAAATT
ATGTCATCAGATAAAACGATTCGTGATGTCTTTGGTTATAGTGTTGATAAAGGATGGATG
TGTATCCAAGTTTTCTTTATACGACAAGGTAATATGATAAAGCGAGATACAACGATGATT
CCATTACAGCAAACAGAAGAAGAAGAATTTTATACATTTATTGGACAATTTTATAGCTTA
AACCAACATATTTTACCTAAGGAAGTTCATGTACCACGTAATTTGGATAAAGAAATGATT
CAATCTGTTGTGGACACTAAAATCGTTCAACCCGCGCGAGGTCCCAAAAAAGATATGGTT
GACCTAGCTGCACATAACGCTAAAGTATCCTTAAATAATAAATTTGAATTAATATCACGT
GATGAGTCAAGAACGATTAAAGCTATTGAAGAACTTGGAACACAAATGGGAATTCAAAC
ACCAATTAGAATTGAAGCATTCGATAATTCTAATATTCAAGGTGTGGATCCAGTGTCAGC

AATGGTTACATTTGTCGACGGTAAACCAGATAAGAAAAATTATAGAAAGTATAAAATCA
AAACGGTTAAAGGTCCAGATGATTACAAATCAATGAGAGAAGTAGTAAGACGACGATAT
TCTCGCGTTTTAAACGAAGGATTACCATTACCTGATTTAATAATAGTAGATGGTGGTAAA
GGACATATGAACGGGGTTATTGATGTGCTACAAAACGAATTAGGTCTTGATATCCCTGTT
GCAGGTTTGCAGAAAAATGATAAACACCAAACATCTGAATTATTATATGGCGCTAGTGCA
GAAATTGTACCACTGAAGAAAAATAGCCAGGCATTTTATTTGTTGCATCGTATCCAAGAT
GAGGTTCACAGATTCGCAATCACATTTCATAGACAAACACGTCAAAAGACAGGCTTGAA
ATCAATACTTGATGATATAGATGGTATCGGTAACAAACGTAAAACATTATTATTGCGTTC
ATTCGGTTCAATCAAGAAAATGAAGGAAGCTACACTTGAAGATTTTAAAAATATAGGTAT
TCCTGAAAACGTCGCAAAGAACCTACATGAACAATTGCATAAATAA

> **sdhC**

Function: protein coding sequence; succinate dehydrogenase cytochrome b-558 (core genome, constant)
Best match: sdhC_CC001-ST772_118_AJGE01000009.1[251389:252003] (completely identical)
Position: 028-contig_257_RC: 9349 … 9964; Length: 615 bp
Sequence:
TTGGCTCAATCAAAAAATGAATTTTATCTAAGACGTATTCACTCGTTATTAGGTATTATCC
CAATAGGTGCATTTTTGGTCGTTCATTTATTAGTGAATCACCAAGCAACACAAGGTGCTG
AAGCGTTTAATAAGGCATCTAACTTTATGGAATCATTACCATTTCTAATTATTGTAGAATT
TTTATTTATATACATTCCGTTGTTATATCACGGTTTGTTTGGTATACACATTGCATTTACAG
CAAAAGAAAATGTTGGACATTACTCGATTTTTAGAAACTGGATGTTCTTTTTCCAAAGAG
TGAGTGGTATCTTAACATTTATCTTTATTGGTATCCATTTATGGCAAACACGTTTACAAAA
AGCATTTTACGGCAAAGAAGTGAATTACGATTTAATGCACGAAACATTGCAACATCTTGG
ATGGGCAATATTTTATATTATTTGTATTATTGCTGTTGTGTTCCACTTTGCAAATGGCTTAT
GGTCATTCTTAGTTACTTGGGGTGGACTTCAATCTCCAAAATCACAACGAGTATTTACATG
GGTTTCATTAATCGTATTCTTAGTTATTTCGTATATTGGTGTTACTGCAATTATTGCCTTTA
TGTAA

> **sdhA**

Function: protein coding sequence; succinate dehydrogenase subunit A (core genome, variable)
Best match: sdhA_CC001-ST772_118_AJGE01000009.1[252055:253821] (completely identical)
Position: 028-contig_257_RC: 10015 … 11782; Length: 1767 bp
Sequence:
ATGGCAGAGAAACATCTTATTGTTGTCGGAGGTGGCCTAGCGGGCTTAATGTCAACAATT
AAAGCGGCAGAAAAAGGTGCACATGTAGATTTGTTCTCAGTTGTACCAGTAAAGCGTTCG
CACTCTGTTTGTGCCCAAGGTGGCATTAATGGTGCGGTCAATACTAAAGGGGAAGGCGAT
TCTCCTTGGATTCACTTTGATGATACAGTGTATGGTGGCGACTTCCTTGCAAACCAACCAC
CTGTTAAAGCGATGACAGAGGCAGCACCTAAAATTATTCATTTATTAGACCGTATGGGCG
TAATGTTCAATAGAACAAATGAAGGTCTATTAGATTTTAGACGTTTCGGTGGTACATTAC
ACCACAGAACAGCATATGCAGGGGCAACAACTGGACAACAATTATTATATGCATTGGAT
GAACAAGTTCGTGCATATGAAGTAGATGGATTAGTTACGAAGTATGAAGGATGGGAATT
CCTTGGCATAGTTAAAGGTGACGATGATAGTGCAAGAGGTATCGTTGCACAAAATATGAC
AACTGCTGAGATTGAAACATTTGGTTCAGATGCAGTTATTATGGCAACGGGTGGCCCTGG
TATTATTTTCGGTAAAACAACAAACTCAATGATTAATACAGGATCAGCGGCTTCCATTGTT
TACCAACAAGGCGCTATTTATGCTAATGGTGAGTTCATTCAAATTCATCCTACTGCAATCC
CTGGTGATGATAAACTGCGACTAATGAGTGAATCAGCACGTGGTGAAGGTGGACGAATTT
GGACATATAAAGATGGTAAGCCTTGGTACTTCTTAGAAGAGAAATATCCTGATTATGGTA
ACTTAGTACCTCGTGATATCGCAACGCGTGAAATTTTCGATGTATGTATTAACCAAAAATT
AGGTATAAATGGCGAAAACATGGTATATCTTGATTTGTCACATAAAGATCCACATGAGTT
AGATGTAAAACTAGGTGGTATCATTGAAATTTATGAAAAATTCACTGGTGATGACTCACG
CAAAGTACCAATGAAGATTTTCCCAGCTGTTCACTATTCAATGGGTGGTCTATATGTAGAT
TATGATCAAATGACAAATATTAAAGGGTTATTTGCAGCTGGAGAATGTGACTTCTCTCAA
CATGGTGGTAACCGCTTAGGTGCCAATTCATTGTTATCAGCGATTTATGGTGGTACAGTA
GCAGGTCCAAACGCGATTGATTATATTTCAAATATTGATCGATCATATACTGATATGGAC

GAAAGTATTTTTGAAAAGCGTAAAGCTGAAGAGCAAGAACGTTTTGATAAATTATTAGCT
ATGCGCGGTACAGAAAATGCCTATAAATTACACCGTGAACTTGGTGAAATTATGACAGCA
AATGTAACTGTTGTTCGTGAAAATGAAAAACTGTTAGAAACAGATAAAAAGATTGTTGAA
TTGATGAAACGTTATGAAGATATTGATATGGAAGATACTCAAACTTGGAGTAACCAAGCG
GTATTCTTTACTCGTCAATTATGGAACATGTTAGTACTTGCACGTGTTATTACGATTGGTG
CATATAACCGTAACGAATCACGCGGTGCCCATTATAAACCAGAATTCCCAGAGCGTAATG
ATGAAGAGTGGTTAAAAACGACAATGGCCTCATTCCAAGGCGCATTTGAAAAACCACAG
TTTACTTATGATGACGTCGATGTGAGTTTAATACCACCTCGTAAACGTGATTACACAAGTA
AGTCTAAAGGGGGTAAAAAATAA

> **sdhB**
Function: protein coding sequence; succinate dehydrogenase subunit B (core genome, variable)
Best match: sdhB_CC001-ST772_118_AJGE01000009.1[253821:254636] (completely identical)
Position: 028-contig_257_RC: 11781 … 12597; Length: 816 bp
Sequence:
ATGACTGAACAATCAGTGAAAAACACTCCACAACATGAAACACAATCTAAACCGAAACA
AAAAACAGTAAAATTAATTATTAAACGACAAGATACAAGTGATTCTAAGCCTTATGAAG
AAACATTTGAAATTCCATATCGTGAAAATTTAAACGTCATTGCTTGTTTAATGGAAATTAG
ACGTAACCCAGTTAATATTAAAGGTGAAAAAACAACACCTGTTGTCTGGGATATGAACTG
CTTAGAAGAAGTATGTGGAGCATGTTCTATGGTTATCAATGGTCGTGCAAGACAATCTTG
TTCTGCGATTGTTGATCAATTAGAACAACCTATTCGTTTAGAGCCAATGAATACTTTCCCA
GTTATCCGTGACTTACAAGTTGATCGTTCTAGAATGTTCGATAACTTAAAACGTATGAAA
GCATGGATCCCAATTGATGGAACGTATGATTTAGGTCCGGGACCACGTATGCCAGAGAAA
AAACGTCAAACAGCTTATGAATTATCTAAATGTATGACATGTGGTGTATGTTTAGAGGTT
TGTCCTAATGTTACTGAAAATAATAAATTCGTTGGTGCGCAAGCAATCTCGCAAGTTCGTT
TGTTTAATTTGCACCCAACAGGATCTATGACTAAAGATGAACGTTTAAATGCATTAATGG
GTACTGGTGGCTTACAGCAGTGTGGAAATTCACAAAACTGTGTTAATGCTTGCCCTAAAG
GTATTCCATTAACAACATCCATTGCAGCAATGAACAGAGAAACAACATTCCACATGTTTA
AATCATTCTTTGGTTCAGACCATGAAGTAGAATAA

> **murI**
Function: protein coding sequence; glutamate racemase (core genome, variable)
Best match: murI_CC001-ST772_118_AJGE01000009.1[254877:255677] (completely identical)
Position: 028-contig_257_RC: 12837 … 13638; Length: 801 bp
Sequence:
ATGAATAAACCAATAGGTGTAATAGACTCTGGTGTCGGAGGTTTGACAGTAGCTAAAGA
AATTATGCGTCAGTTGCCAAATGAGACGATTTATTACTTAGGTGATATTGGGCGATGCCC
ATATGGGCCAAGACCAGGAGAACAAGTAAAACAATATACAGTTGAAATCGCTCGTAAAT
TAATGGAATTTGATATAAAAATGCTCGTGATTGCTTGTAATACAGCAACTGCTGTAGCTTT
AGAATATTTACAAAAGACCTTATCAATCCCAGTGATTGGCGTAATTGAACCAGGTGCTAG
AACAGCAATAATGACTACTAGAAATCAAAATGTATTAGTACTAGGAACGGAAGGCACAA
TTAAATCTGAAGCATATCGTACGCATATTAAACGTATCAATCCACATGTAGAGGTACATG
GCGTTGCCTGTCCAGGTTTTGTGCCACTTGTAGAACAAATGAGATATAGTGATCCAACAA
TTACAAGCATTGTCATTCATCAAACACTGAAACGTTGGCGTAATAGTGAGTCTGATACTG
TCATTTTAGGATGTACCCACTATCCATTGCTCTATAAACCTATCTATGATTATTTTGGTGGT
AAAAAGACAGTGATTTCGTCTGGATTAGAAACGGCTCGTGAAGTTAGTGCATTGCTAACA
TTTAGTAATGAACATGCAAGTTATACTGAAAATCCAGATCATCGATTTTTTGCAACAGGT
GATCCTACTCACATTACTAACATTATCAAAGAGTGGTTAAATTTATCTGTCAATGTGGAAC
GTATATCAGTGAATGACTAG

> **ntpA**
Function: protein coding sequence; nucleoside-triphosphatase (core genome, variable)
Best match: ntpA_CC008_NCTC8325_CP000253.1[1068965:1069552] (completely identical)
Position: 028-contig_257_RC: 13649 … 14237; Length: 588 bp
Sequence:

ATGAAAGAGATTGTTATTGCATCGAATAATCAAGGGAAAATAAATGACTTTAAAGTAATA
TTTCCAGATTACCACGTAATAGGTATTTCAGAACTAATACCAGATTTTGATGTGGAAGAA
ACAGGATCAACATTTGAAGAAAATGCTATATTAAAATCAGAAGCTGCTGCAAAAGCATT
GAATAAAACGGTCATAGCTGATGACAGTGGACTAGAAGTTTTTGCATTAAATGGTGAGCC
AGGTATATACTCTGCACGTTATGCTGGTGAAAATAAAAGCGATGAAGCAAATATTGAAA
AATTATTAAATAAGCTTGGTAATACAACTGATCGTCGTGCGCAATTTGTTTGTGTCATAAG
TATGAGTGGCCCTGATATGGAAACAAAAGTATTTAAAGGTACTGTTTCAGGTGAAATTGC
AGATGGAAAATATGGCGAAAATGGTTTCGGATATGATCCGATATTTTATGTACCGAAATT
AGATAAAACCATGGCTCAACTTTCAAAAGAACAAAAAGGGCAAATTAGTCATAGACGAA
ATGCGATTAATTTACTTCAAGCTTTTCTTGAAGGTGATAAAAATGTCTAA

## > Q5HGT1

Function: protein coding sequence; putative phosphodiesterase (core genome, variable)
Best match: Q5HGT1_CC001_ATCC51811_ADVP01000010.1[41018:41521:r]
Position: 028-contig_257_RC: 14229 … 14733; Length: 504 bp
Sequence:
ATGTCTAAATGGATTATTGTGAGTGATAACCATACAGAATCAGGCGTTTTATATCAAATTT
ATGAAATGCACCCAGATGCAGATGTATATTTACATTTAGGAGATTCAGAATTCGCGTATG
ATGATACGGAACTTAGCTTATTTAATAGAGTAAAAGGCAATTGTGATTTTTACCCAGAAT
TTGAAAATGAAGCGGTCGCAAAATATAATGACGTGAAAGCATTTTATACTCATGGACATT
TATATCAAGTCAATCGAACAAGAGATTTATTAGCTGAAAAAGGACTTGAATTAGGTTGTT
TGTTTGCATTTTATGGACATACACATGTGGCAAAATATGAGTATATTAATGGTGTTCATGT
TATTAATCCTGGAAGTATATCTCAATCTAGAAGTTCAATGGAAGAAACATATGCTGAAGT
TATTATTGATGATCAAACTTTACATGGCACCATCAATTTCAAAAATCGACATCACGAAAC
AATCAGTCATACTACTTTTTAA

## > Q6GA61

Function: protein coding sequence; putative protein (core genome, variable)
Best match: Q6GA61_CC008-ST72_TCH130_ACHD01000022.1[4264:4431:r] (completely identical)
Position: 028-contig_257_RC: 14860 … 15028; Length: 168 bp
Sequence:
ATGAAAAGAATTGTAATTTTAGAGAATGTGATTGTTGATGACCCTATATGGTTGAATCAT
GTTGATTATTTGGTTAAAAGTGTATTATTAAAAAGTATAAAATTAAGATTCCAAGAAGTC
AGGAAATTTGAAATTTTAGTTAAAATCATGATTCGTATACATATTTAA

## > ecb

Function: protein coding sequence; extracellular complement-binding protein (core genome, variable)
Best match: ecb_CC022_HE681097.1[1102220:1102549]
Position: 028-contig_257_RC: 15221 … 15551; Length: 330 bp
Sequence:
ATGAAAAAGAATTTTATTGGGAAATCAATTTTAAGCATAGCTGCTATTAGTTTAACGGTA
TCAACGTTTGCCGGTGAATCTCATGCACAAACTAAAAACGCTGAAACTGTAAAAAAATAT
AACGAGTATCAAACAAACTTTAAAAAACAAGTAAATAAAAAAGTAGTGGACGCACAAAA
AGCTGTAAACTTGTTCAAACGTACAAGAACTGTTGCAACACACCGTAAAGCACAAAGAG
CTGTTAATTTAATTCATTTCCAACACAGCTATGAAAAGAAAAAATTACAAAGACAAATCG
ATCTAGTTTTAAAATATAATACTTTAAAATAA

## > flr

Function: protein coding sequence; formyl peptide receptor inhibitory protein (core genome, variable)
Best match: flr_CC007_TCH959-USA300_AASB02000228.1[13158:13559:r]RC [4_150END]
Position: 028-contig_257_RC: 16172 … 16322; Length: 150 bp
Sequence:
GTCCTTTTTAGCTTGATCTGCTAAATTTTTTGCGATTTCTAAACCTTTCCATTCATAACTAA
AGAAAGCTTTAGCATCATTAGTTTGAGTTGAAAGACCTGCAGCGATTACTGTAGATGCGA
TAATAGTTTTTGTGATATTTTTTTCAT

> **Q2YXB9**
Function: protein coding sequence; putative membrane protein (core genome, constant)
Best match: Q2YXB9_CC005_Mu50-VRSA_BA000017.4[1211324:1211830] (completely identical)
Position: 028-contig_257_RC: 16991 … 17498; Length: 507 bp
Sequence:
ATGATAATTAAAATTTTAACAATTCTATTACTACTTTGTATATTGAGCTATTTGGTTACAA
ATAGAAAGAAGCCTTTTCTGTTCTTAAAGACACTCTTTATGGGTGTGGTATTTATCTTTAT
AGGATATATTTCACTGGCAATATCTGCCGTAATTATTTATGGTATTATTCAATTTATCACA
ATTGATTTTGGTAGTTTTTTCTTAATGGGTATTATATTGATCTTGATTTCAAGTATATTCCA
ATTATTTATAGTTAGATTACTTTTTAGAAAAAAGAATGTCGATTTGACAGAGGTTGTCGTT
TTAGAGCATTTAATTCAATGGTTCTTAGTTTACTTTGCGATCTATCAAGCAGTAAATGAAA
AAATGGACATTAATGATATTAATATCGACAATTTCCAATCTGTCTTTTTTGACGTGTCTAA
TTTGAATTTAGTAATTCTACCAACGTTAATCATTAGCTGGGTCACAATATTTAACTATAGA
ATGAGAAGTTACAAATAA

> **efb**
Function: protein coding sequence; extracellular fibrinogen-binding protein (core genome, variable)
Best match: efb_CC001-ST772_118_AJGE01000009.1[259797:260294] (completely identical)
Position: 028-contig_257_RC: 17757 … 18255; Length: 498 bp
Sequence:
ATGAAAAATAAATTGATAGCAAAATCTTTATTAACAATAGCGGCAATAGGTATTACTACA
ACTACAATTGCGTCAACAGCAGATGCGAGCGAAGGATACGGTCCAAGAGAAAAGAAACC
AGTGAGTATTAATCACAATATCGTAGAGTACAATGATGGTACTTTTAAATATCAATCTAG
ACCAAAATTTAACTCAACACCTAAATATATTAAATTCAAACATGACTATAATATTTTAGA
ATTTAACGATGGTACATTCGAATATGGTGCGCGTCCACAATTTAATAAACCAGCAGCGAA
AACTGATGCAACTATTAAAAAAGAACAAAAATTGATTCAAGCTCAAAATCTTGTGAGAG
AATTTGAAAAAACACATACTGTCAGTGCACATAGAAAAGCACAAAAGGCAGTCAACTTA
GTTTCGTTTGAATACAAAGTTAAGAAAATGATATTGCAAGAGCGAATTGATCAAGTATTA
AAGCAAGGATTAGTGAGATAA

> **scc**
Function: protein coding sequence; similar to complement inhibitor (core genome, variable)
Best match: scc_CC001_MW2-USA400_BA000033.2[1137633:1137983]
Position: 028-contig_257_RC: 18726 … 18756; Length: 30 bp
Sequence:
AAAGAAATTGATGAAATTATAAATAGATAA

> **tx_universal2**
Function:  rho indendent terminator
Best match: tx_universal2_CC025_21193_AFEG01000027.1[76692:76730:r] (completely identical)
Position: 028-contig_257_RC: 19032 … 19071; Length: 39 bp
Sequence:
CTAAATGAAAATACGCTTGTAACAAGTTTTTTCAATACT

> **Q2FZB6**
Function: protein coding sequence; putative protein (genomic island)
Best match: Q2FZB6_CC001_MW2-USA400_BA000033.21138347:1138525 [4_50ANF10END]
Position: 028-contig_257_RC: 19119 … 19185; Length: 66 bp
Sequence:
TGCAAGTTGGCGGGGCCCCAACATAGAGAAATTGGATCACCAATTTCAACAGACAATGC
AAGTTGG

> **STAR**

Function: Staphylococcus aureus repeat element
Best match: STAR_CC001_MW2-USA400_BA000033.21138524:1138745:rRC
Position: 028-contig_257_RC: 19127 … 19349; Length: 222 bp
Sequence:
GGCGGGGCCCCAACATAGAGAAATTGGATCACCAATTTCAACAGACAATGCAAGTTGGG
GTAGGACATCGATAAAAAAATACTTTTTCTTTAGAAATTAGTATTTCNTATGCATGAGTTT
TACTCATGTATTCATTTTTTAAGTAGACATTAGCTACAGCTAATGATAAAGAACCACTACA
TAATAAATCATTAGTGGTTCTTTATCATTTCTATCTCACTC

> **A5IS45**
Function: protein coding sequence; putative protein (core genome, variable)
Best match: A5IS45_CC001_MW2-USA400_BA000033.2[1138748:1138933:r]RC (completely identical)
Position: 028-contig_257_RC: 19351 … 19537; Length: 186 bp
Sequence:
TTACTGGAAGAAAAAGTTTACGTTTGTAGAACATGCCACAATACCAAAAATAATTAAGAA
AAATAAGACGATAAGCATGATGACACTTTTCAAACAACCTCTATCAGTTTCTTCCGATTTT
CTTTGTTGAACCTTTTTATAATCTTCAAGTAGTTTTGTCGCTTTTTTATTTATATGTTTATTC
AT

> **Q6GHS5**
Function: protein coding sequence; putative uncharacterized protein (core genome, variable)
Best match: Q6GHS5_CC001_MW2-USA400_BA000033.2[1139140:1139388:r]RC (completely identical)
Position: 028-contig_257_RC: 19746 … 19995; Length: 249 bp
Sequence:
CTAGAATACATAAATGAATAGTAATGGTGCACAGTATAGAATAATTAAGGCTATATTAAG
TATAAATATCGTTAACTGTAAGCTATCTTTAGTTTTAATATAAACTATTAGGATAATCGAC
GTAAGAAGAATCATATATATTAATGATGAAGAAGTCCATACAAAATCCGCATCATTTGTT
GTTAATAATGGGACTATAATTAATCCGAAATTAATCATGCATGCTATATATACTATAATGT
TATACAC

> **A5IS47**
Function: protein coding sequence; putative protein (core genome, variable)
Best match: A5IS47_CC005_Mu50-VRSA_BA000017.4[1214660:1214893:r]RC (completely identical)
Position: 028-contig_257_RC: 20158 … 20392; Length: 234 bp
Sequence:
TTAATGCTCATCAACATTTGGATTTTGAATATTCAATTCAAAAACTTTATTAGCTACGTCA
ATTGTAAAATCAGAACCATAGTTGACATGAGCTACTTTTAATTTTCCATCTAAATAATAGA
TTGCGATTGCAACATCGTAAAATTCGTCAATGACAAATAAACTCTTTTCGTTTGTTACAAC
CTCATGCTCTCCTGAGTATACAACGTTAATTTCCCAATCATTAAAAACCAT

> **hla**
Function: protein coding sequence; haemolysin alpha (core genome, variable)
Best match: hla_CC001-ST772_118_AJGE01000009.1[262908:263867:r]RC
Position: 028-contig_257_RC: 20868 … 21828; Length: 960 bp
Sequence:
TTAATTTGTCATTTCTTCTTTTTCCCAATCGATTTTATATCTTTCTGAAGAACGATCTATCC
ATTTATCTTTAGTATTGGTACCTTTCCAATTTGTTGAAGTCCAATGCAATTGGTAGTCATC
ACGAACTCGTTCGTATATTACATCTATATTTGTTTGTTGTTTGGATGCTTTTCTATCCATAG
TAATAACTGTAGCGAAGTCTGGTGAAAACCCTGAAGATAATAGAGAACTTGCTTTGTTAG
GATCAAGGAAGTTATCTGCTGCTTTCATAGAACCATTTCTAGTTTTCATGAAAAGTTGATT
GCCATATACCGGGTTCCAAGAATCTCTATCATATGGTCCCCAATTTTGATTCACCATATTG
TTAAATATCACTTTCCAGCCTACTTTTTTATCAGTTGGGCTCTCTAAAATTGTTTTGAAATC

AGGTTGAACATATTTCAGTGTATGACCAATCGAAACATTTGCACCAATAAGGCCGCCAAT
TTTTCCTGTATCATCACCAGTAACATTACCGTTGAATCCATAAGTTAAAGTACTCATATAC
TCTTTTGTATCAATCGAATTTCTTGGATAGCAATCAGATATTTGAGCTACTTCATTATCAG
GTAGTTGCAACTGTACCTTAAAGGCTGAAGGCCAGGCTAAACCACTTTTGTTAGCACCTT
CTTCGCTATAAACTCTATATTGACCAGCAATGGTACCTTTCGTTCTAATAACTAGCAGTTT
TTTATTGTGATTTTTATCATCGATAAAACTATAAAATACTTTTTTGTGCATGCCATTTTCTT
TATCATAAGTGACTAAATCACCTGTTTTTACTGTAGTATTGCTTCCAATATCTGTAGTACC
GGTTTTAATATTAATATCAGAATCTGCGGCATTAGCGACAGGATTCATTAATATGGAACC
TAGCAATAGTGTTGTTGTTACTGAGCTGACTATACGTGTTTTCAT

## > A6U0Y3

Function: protein coding sequence; putative protein (core genome, variable)
Best match: A6U0Y3_CC005_Mu50-VRSA_BA000017.4[1218522:1218668] (completely identical)
Position: 028-contig_257_RC: 22500 … 22647; Length: 147 bp
Sequence:
ATGAAACTATTTTATATCGTATTTCTTATTATTATATGGCTGAATATATTTTTAGGAAATG
AAATTATCCATACACTGACTGTTTTAATAACAACATTGTATATTGTTAATTCAAGAAAGG
GGATTAAAAATGACAGAGTTGAATAA

## > Q2FZB4

Function: protein coding sequence; putative protein (core genome, variable)
Best match: Q2FZB4_CC005_Mu50-VRSA_BA000017.4[1218652:1218849] (completely identical)
Position: 028-contig_257_RC: 22630 … 22828; Length: 198 bp
Sequence:
ATGACAGAGTTGAATAATATTATAAACTCTCTTCAATCTTTGTTTGAGTCTGAATCAGGCT
ATAAAATTTCGAAAAATTCAGGAGTTCCATATCAAACAGTACAAGATTTAAGAAATGGG
AAAACCAAACTAGAAGATGCTAGATTTAGAACGATTATTAAACTTTATAGTTACTATGTC
TCATTAAAAGAACATTAA

## > txbi_universal1

Function:  bidirectional rho independent terminator
Best match: txbi_universal1_CC001_MW2-USA400_BA000033.2[1397234:1397306:r]RC
(completely identical)
Position: 028-contig_257_RC: 22895 … 22968; Length: 73 bp
Sequence:
TAAATCCCTAAAAAAACAGCAGTAAGATAATTTTCAATTAGAAAATATCTTACTGCTGTT
CTCTATTTATACA

## > tx_universal3

Function: bidirectional rho-independent terminator
Best match: tx_universal3_CC008_COL_CP000046.1[2415294:2415357:r]RC (completely identical)
Position: 029-contig_258_RC: 7 … 71; Length: 64 bp
Sequence:
ATACATATCAAAAAAGCTGATTTCTATCAAATAATTAATAGAAATCAGCTTTTTTACATTG
CCT

## > tnpIS1272

Function: protein coding sequence; transposase of insertion element IS1272
Best match: tnpIS1272_15653_EU272084.1[3547:5193]
Position: 029-contig_258_RC: 43 … 73; Length: 30 bp
Sequence:
AATAGAAATCAGCTTTTTTACATTGCCTAA

## > tx_universal2

Function:  rho indendent terminator
Best match: tx_universal2_CC001_MW2-USA400_BA000033.2[1142336:1142376] (completely identical)
Position: 029-contig_258_RC: 121 … 162; Length: 41 bp
Sequence:
CCGAATGAAAATGCCCATGTAACAAGCATTTTTCAATTATA

> **repeat_nySAgamma**
Function:  repeat element
Best match: repeat_nySAgamma_CC008_COL_CP000046.12325058:2325185RC [4_50ANF10END]
Position: 029-contig_258_RC: 162 … 230; Length: 68 bp
Sequence:
GGCGGGGCCCCAACATAGAGAATTTCAAAAAGAAATTCTACAGACAATGCAAGTTGGCG
GGGCCCCAA

> **Q2FZB6**
Function: protein coding sequence; putative protein (genomic island)
Best match: Q2FZB6_CC008_COL_CP000046.1[1182242:1182414]
Position: 029-contig_258_RC: 245 … 275; Length: 30 bp
Sequence:
AAAGTCAGCTTACAATAATGTGCAAGTTGG

> **setB3**
Function: protein coding sequence; staph enterotoxin-like toxin B (core genome, variable)
Best match: setB3_CC001_MW2-USA400_BA000033.2[1142778:1143494:r]RC
Position: 029-contig_258_RC: 450 … 1167; Length: 717 bp
Sequence:
CTAATCAAAGAGGACGTCAACAGTTATTTTATTAGGATTTTTAACATAAACATTTGCTAG
ATCTGAATGTAATCTTTTGCTTAAATCAATAGTGTAGTTATTACCGCCACCGGTGATCTTA
AGCTTACCTTTATTACGATTTTCGTTATATAATATTTTATTTTTTATTAACGCTTCTCGTGC
ACGGAAATCGATTTCTTTCAATGTTAATACTGGTTTATTGCCTTGGTATATTTTATGCGCA
CCAATAATCGTTTGTAGTTTATCTTTGTATTGCACAAAAAGATTATAAGTTTTATCAGAAG
GTTTTGCGGCTGGTGTAACGCCACCTGTAAATGTCTCTCTATAAGACCACCATAACTGATC
AGTATCTTTGTCTTTTAGTCCAAACACATCTACGTAACGATCTTTTAACTGATTAATATTTC
CCCAACTTTCAGCGCCCCATAAAGATATATGTGCTGACCATGAATATCTCTTAAGTTCAAC
ATAAATGTTTCCATTATCATATTGATATAGCCATTTATTTGAAAATGAAAAATGAGGCTGT
GTGTAATATTTAATTAATTCATTGATGTTAGTTTCATCTTGACCAATACTATAAGCTTTAG
CTTCAGAGTAAAAACTAAATACTGGTTTTTGATTTTGAGGTAATACAGTACCTAATAGTA
ATAATGTTGTCGTTAAAATTATATTTTTCGTGATGTTCTTACTCAT

> **setB2**
Function: protein coding sequence; staph enterotoxin-like toxin B (core genome, variable)
Best match: setB2_CC001_MW2-USA400_BA000033.2[1143602:1144327:r]RC
Position: 029-contig_258_RC: 1274 … 2000; Length: 726 bp
Sequence:
TTAGTTTGATTTTTCGAGGATAACTTCAATTTTTGCATTTTGAGGTTTTTTAACATATCTAT
TTGCATCAGTTGATGGCAACCTTTTACTTAAATCTATTGTGTAGTTATTGTCTGCACCTGTT
ATTTTAATTTGTCCTTTATTATAAGAATTATTATATAATTTTTTACTTTTAATTAATGTTTG
ACGAATACGAAAATCTAATTCTTTTAAAGTTAAAACAGGCTTATTGCCTTCATAAACTGG
AAATCCGCCAGTAAACGTTTCTGCTTTATCTTTATATGTTACATTCAGTTTATAGTGTTTAT
CGTTAGATGTTGCTGCAGGAGTAACACCACCAGTAAACGTTTCTTGAGATAATGCAAAAG
AATCAATGGTTTCTTGGTCTTTTATGCCAAAAATATCAACGCTTTTATTTCTTAATTGGTTG
ATATTGCCCCAACTTTCAGGTCCATAAACTTGAATATGACTATACCAAGAAAACTGTAAC
AACGTTGCATGAATCGTACCGTTATCTTTTTGCCATAACGTACTGTTTGAGAAGGTTAAAT
ATTTTTGCGAGTAATATTTAGTTAACTCATTTACGTTAGTTTCGTTTTGATTTATATAATAA

GCTTTCGCTTCAGATGAAGAATTGATAGGTGTATTAGGAAATTGTGTAGATGCTGTACCT
AATAGTAACAATGTTGTTGATAAAATAATTTTTTTCGTGATGTTATTGTTCAT

> **setB1**
Function: protein coding sequence; staph enterotoxin-like toxin B (core genome, variable)
Best match: setB1_CC001_MW2-USA400_BA000033.2[1144422:1145147:r]RC
Position: 029-contig_258_RC: 2094 … 2820; Length: 726 bp
Sequence:
TTAGTTAGATTTTTCGAGTATGACTTCAATTTGTGCATTTTTAGGATTTTTAACATAACGGT
TTGTGTCAGTTAATTTTAACTTTTTACTTAAATCAATCGTGTAATTATTTCCATCAGCAGTT
ATCTTAATTTGACCTTTATTAAATTCTCCGTTATATAACTTTTTATTCTTTATTAATGTTTG
ACGAATACGGAAGTCTAATTCTTTTAGAGTTAATACTGGTTTATTTCCTTTGTAAAATTCA
TGTCCACCGATGATAGTTTGTTGTTTATCACTATATTTTAAAAATAGTCTATAAGGTTTAT
CAGATGAAGTAGCTGCTGGCGTAACACCACCAGTAAATGTTTCATCATAAGTCCAGTAAC
CTTCAACTGTGTCCTCATCTTTAGTTCCAAATATATCAACGTATTTATTTCTTAACTGATTA
ATGTTTCCCCAACTCTCGGATCCAAACACTTGAATATGACTATACCAAACCCACGTTTGCA
ATGTTGCATGAATGCTACCATTGGGCTTTTGCCATAACCATTTATTTGATAGTGATAAATG
AGGCTGTGTATAATACTTTATTAACTCATTGATATTAGTCTCGTTTTCACTGATATTATAG
GCTTTTGCTTCAGATGAAAAACTGATTGGTGTTTTAGGAAGTTGTGTTGATGTGGTTCCTA
AAAGTAACAATGCAGTTGATAAAACTAATTTATTCATGATGTTCTTTTTCAT

> **arcB-L1**
Function: protein coding sequence; ornithine carbamoyltransferase locus 1 (core genome, constant)
Best match: arcB-L1_CC001_MW2-USA400_BA000033.21145585:1146586 [4_50ANF10END]
Position: 029-contig_258_RC: 3257 … 4259; Length: 1002 bp
Sequence:
ATGAAAAATTTACGAAACAGAAGTTTTTTAACTTTATTAGACTTTTCACGACAAGAGGTA
GAATTCTTATTAACACTCTCCGAGGATTTAAAACGTGCTAAATATATTGGCACTGAAAAG
CCTATGTTAAAAAATAAAAATATTGCACTGTTATTTGAAAAAGATTCTACAAGAACGCGA
TGTGCATTTGAAGTTGCAGCGCATGATCAAGGTGCAAATGTAACTTATTTAGGCCCAACT
GGATCACAAATGGGTAAAAAAGAAACAACTAAAGATACTGCACGTGTGCTTGGTGGAAT
GTATGATGGCATTGAATACCGTGGTTTTTCACAAAGAACAGTAGAAACTTTAGCTGAGTA
TTCAGGCGTACCAGTGTGGAATGGTTTAACTGATGAAGATCATCCTACTCAAGTTCTTGCT
GATTTCTTAACAGCAAAAGAAGTCTTAAAAAAAGATTATGCAGATATTAACTTTACATAT
GTTGGAGATGGTCGTAATAACGTTGCAAATGCATTAATGCAAGGTGCTGCCATTATGGGT
ATGAACTTCCATTTAGTTTGTCCAAAAGAATTAAATCCAACAGATGAATTATTAAATCGC
TGTAAAAATATTGCCGCTGAAAATGGTGGCAACATATTAATCACAGATAATATTGACCAA
GGTGTAAAAGGTTCGGATGTAATTTACACTGACGTTTGGGTATCAATGGGTGAACCTGAT
GAAGTATGGAAAGAACGACTTGAATTATTGAAACCATATCAAGTAAATAAAGAAATGAT
GGATAAAACTGGTAATCCAAATGTTATTTTTGAGCATTGCTTACCATCTTTCCATAATGCT
GATACGAAAATTGGTCAACAAATTTTTGAAAAATATGGAATTCGAGAAATGGAAGTTAC
AGATGAAGTATTCGAAAGTAAAGCTTCAGTTGTATTCCAAGAAGCTGAGAACAGAATGC
ATACAATCAAAGCAGTGATGATTGCTACATTGGGTGAATTTTAA

> **arcC-L1**
Function: protein coding sequence; carbamate kinase/locus 1 (core genome, constant)
Best match: arcC-L1_CC001_ATCC51811-ST1_ADVP01000010.1[27450:28382:r]
Position: 029-contig_258_RC: 4281 … 5214; Length: 933 bp
Sequence:
ATGGCGAAAATAGTAGTAGCATTAGGTGGTAATGCTTTAGGAAAATCACCTCAAGAACA
ACTCGAGCTTGTTAAAAATACTGCGAAATCATTAGTAGGATTAATAACAAAAGGACATGA
GATTGTTATTAGTCATGGTAATGGACCACAGGTTGGAAGCATTAATTTGGGACTTAACTA
TGCTGCAGAACATAACCAAGGTCCGGCATTTCCATTTGCTGAATGTGGCGCAATGAGTCA
AGCTTACATCGGCTATCAATTACAAGAAAGCTTACAAAATGAATTGCATTCTATTGGAAT
GGATAAACAAGTGGTAACACTAGTGACACAAGTTGAAGTTGATGAAAATGATCCGGCAT

TTAACAATCCTTCAAAACCAATTGGGTTATTTTACAACAAAGAAGAAGCTGAACAAATTC
AAAAAGAAAAAGGATTTATATTTGTTGAAGATGCTGGAAGAGGATATAGACGCGTTGTTC
CTTCACCACAACCCATCTCTATTATTGAATTAGAGAGTATTAAAACACTTATTAAAAATG
ATACACTCGTTATTGCTGCTGGCGGTGGAGGTATACCAGTAATTAGAGAGCAACATGATG
GTTTTAAAGGTATTGATGCAGTTATAGACAAAGATAAAACAAGTGCATTGTTGGGTGCTA
ATATTCAATGCGATCAATTGATTATTTTAACAGCAATTGATTATGTATATATTAATTTTAA
CACTGAAAACCAACAGCCTTTGAAAACAATAAATGTTGATGAATTAAAACGATATATAG
ACGAAAATCAATTTGCAAAAGGAAGTATGTTACCAAAAATTGAAGCAGCCATATCATTTA
TTGAAAACAATCCAAAAGGAAGCGTGCTTATAACATCATTAAATGAATTAGATGCTGCCT
TAGAGGGTAAAGTAGGTACTGTGATTAAAAAGTAA

> **arcD1**
Function: protein coding sequence; C4 transporter/TRAP family (core genome, constant)
Best match: arcD1_CC008_NCTC8325_CP000253.1[1083751:1085307] (completely identical)
Position: 029-contig_258_RC: 5385 … 6942; Length: 1557 bp
Sequence:
GTGGAAAATACAATTAATGAAAGTGAAAAGAAAAAACGATTTAAATTAAAAATGCCAGG
TGCATTTATGATTTTATTCATTTTAACGGTTGTTGCAGTTATAGCAACATGGGTTATTCCTG
CTGGTGCATATTCTAAACTTTCTTACGAACCTTCATCCCAAGAACTAAAGATAGTTAACCC
TCATAACCAAGTGAAAAAGGTTCCGGGTACGCAACAGGAACTAGACAAAATGGGGGTTA
AAATTAAGATTGAACAATTTAAATCAGGTGCAATTAATAAGCCGGTATCAATTCCGAATA
CTTATGAAAGATTAAAGCAACATCCAGCTGGACCAGAACAAATAACAAGTAGCATGGTT
GAAGGTACGATAGAAGCGGTCGATATCATGGTATTCATTCTTGTACTAGGGGGACTTATT
GGCGTAGTTCAAGCCAGTGGTTCTTTTGAATCGGGATTGTTAGCTTTAACGAAGAAAACA
AAAGGGCATGAATTTATGCTAATTGTGTTTGTATCAATACTAATGATTATCGGCGGGACA
TTATGTGGTATTGAAGAAGAAGCTGTAGCATTTTATCCGATTTTAGTCCCTATATTTATAG
CGTTAGGATACGATTCTATCGTTTCAGTTGGTGCCATATTCCTTGCCAGCTCTGTCGGTAG
TACATTTTCAACTATTAACCCGTTCTCGGTTGTAATTGCCTCTAATGCCGCTGGTACAACT
TTTACGGATGGCTTGTATTGGAGAATAGGTGCTTGTATTGTCGGTGCGATTTTTGTTATTA
GTTATTTATATTGGTACTGTAAAAAAATTAAAAACGATCCTAAAGCGTCATATTCTTATGA
AGACAAAGATGCTTTTGAACAGCAATGGTCTGTATTAAAAGATGACGATAGTGCCCATTT
TACTTTGCGTAAGAAGATAATCCTTACATTATTTGTACTACCATTTCCAATTATGGTATGG
GGAGTTATGACGCAAGGTTGGTGGTTCCCAGTTATGGCTTCAGCATTTTTAATATTTACAA
TTATAATAATGTTTATTGCTGGGACAGGTAAATCTGGATTGGGAGAAAAAGGAACTGTAG
ATGCATTTGTCAATGGTGCATCAAGTTTAGTAGGTGTATCTTTAATTATTGGTTTAGCTCG
AGGTATTAATTTAGTGTTGAATGAAGGTATGATTTCAGATACAATCTTACACTTTTCATCA
TCTTTAGTTCAACATATGAGTGGACCATTATTTATCATCGTATTACTATTTATTTTCTTCTG
TTTAGGTTTTATCGTGCCATCATCTTCTGGCTTAGCGGTATTATCAATGCCTATCTTTGCAC
CACTAGCTGATACAGTAGGTATACCAAGATTCGTCATCGTTACGACATATCAATTTGGTC
AATATGCGATGTTATTCTTAGCGCCGACTGGACTTGTTATGGCCACACTACAAATGTTAA
ACATGCGATATTCACATTGGTTCCGATTTGTATGGCCGGTAGTTGCTTTTGTATTGATTTTC
GGTGGCGGAGTACTAATTACGCAAGTACTAATTTATTCATAA

> **A5IS57**
Function: protein coding sequence; putative membrane protein (core genome, constant)
Best match: A5IS57_CC005_Mu50-VRSA_BA000017.4[1226137:1226364] (completely identical)
Position: 029-contig_258_RC: 7249 … 7477; Length: 228 bp
Sequence:
ATGACACATTTGACAAAGGTTTTAGATACACTAACTGGAATATGCGTAGTATTATTATTTA
GTAAATATTTTGTGGCGTATGCAAATATGGTGTTTGATTGGAATTTAAGATGGTATTTGCT
AGAAAACATACCACATTTGCCAATTATATTATTTATTCTGATGTTTATTTTCGGAGTACCT
TCTGAAATGATAAAAGATAGGCAAAGGAAAAATAACGGTGTTTAA

> **txbi_per**
Function:  bidirectional rho-independent terminator of per

Best match: txbi_per_CC008_NCTC8325_CP000253.1[1086063:1086126] (completely identical)
Position: 029-contig_258_RC: 7697 … 7761; Length: 64 bp
Sequence:
AGGCATTGATATACACTAAAAAGAGGCAAGATTACCTGCCTCTTTTTAGTTATTAAATAT
ACGT

> **per**

Function: protein coding sequence; putative permease (core genome, variable)
Best match: per_CC001-ST772_118_AJGE01000008.1[7719:8666:r]RC (completely identical)
Position: 029-contig_258_RC: 7781 … 8729; Length: 948 bp
Sequence:
TTACTGAGACGCATTACGAAGAAATTTATCTTGTTTTTCCTTTTTTAAAAAGAAGTGAAGA
TATCCTATAAAGACTCTAAGTACTATTACAGTGGCTAATAACACTTCGATAAACAAAAGA
CCTTTCCAAATGTCTGGAAACATAAGTACAGGCAAACTGTTCTTTAAAGCAGTTGCTGAG
ATTACTAAAGGGAATGTGAAAGCTGAAAATACGGGCGAAAAAGGTTCTTTTAGTAATTTA
GGCAATTGTATAATGATATAAAAATAAAATATTTGAGCTAATAGTAAAAATATAATTACG
ATAAAAGCATTCGCCTTAGGAAAAGCTATAACATATGCTGCAGCGACTAAAGAAAATGG
TGCGCAAATTGTCGATGTGTTCGGTTTGATTGACGTCTGCAATGGAAATGCTTTTAATCGC
TTGAAAACTATAGGTAAGACAATACAAGTAGCTACAAAGCCATATATTACTGTTAATTGA
CCTATGAAAAAATATCCGCTAACGGGTGCCGTCAATCCTGCGATAGCAATACCAATAAAA
AGTACAGTCCACGAAGGATAAACATTTTCAAGTGAAAAATCTTTTAAATATTTTATTGAA
AAAATAATCATATGCGTCATAATTCCCACAAGGCATAAAATCCAAATAGGCGTTATTAAG
CTATTGATAAAAGTTATGTTACTAAAAAATGTATTTAAATAAGTAGTACCTAAAAAGCCA
GACATGAAAAATGTTGTGAACACTGATGAAACTAGAGGACTGTTTAATTGTTCTTTCACA
TTATTAAAATATTTAATCATAGTACATAAAAGGTGAATCCAAATCAAGAAAGCAAAGATT
CCGCAAACAGCGTTTAAAGTAAGAGATAAGTCTTTTAATAGATTACCCAGGCCTAATAAT
CCTAAGACTAGTCCTGACGTTACTAGAGGTGCTTTTTGAAGTCTCAT

> **A6QG73**

Function: protein coding sequence; putative DNA-binding protein (genomic island)
Best match: A6QG73_CC001-ST772_118_AJGE01000008.1[8915:9103] (completely identical)
Position: 029-contig_258_RC: 8977 … 9166; Length: 189 bp
Sequence:
ATGAGAAATCAAATTCAAAAACTATTAGACAGTGATTTGAGCAGTTTACATATATCGAAA
CAAACAGGAGTTCCACAAAGCACAATACACAGAATGAGAAAAAATGAAAGATCATTAGA
TAATATGTCATTGAAAAACGCTGAACTGCTTTATAAATTTGCCAATAGTATATTTAGCAAT
GAAAATTAA

> **tRNAarg3**

Function: tRNA tRNA-Arg (core genome, constant)
Best match: tRNAarg3_CC001_MW2-USA400_BA000033.2[1151964:1152042:r]RC (completely identical)
Position: 029-contig_258_RC: 9635 … 9714; Length: 79 bp
Sequence:
TAAACGTCCTGGGAGGGATTCGAACCCCCGACCGATGGCTTAGAAGGCCATTGCTCTATC
CAGCTGAGCTACCAGGACA

> **psmB1**

Function: protein coding sequence; phenol soluble modulin beta 1 (core genome, constant)
Best match: psmB1_CC001-ST772_118_AJGE01000008.1[10014:10148] (completely identical)
Position: 029-contig_258_RC: 10076 … 10211; Length: 135 bp
Sequence:
ATGGAAGGTTTATTTAACGCAATTAAAGATACCGTAACTGCAGCAATTAATAATGACGGC
GCAAAATTAGGCACAAGCATTGTGAGCATCGTTGAAAATGGCGTAGGTTTATTAGGTAAA
TTATTCGGATTCTAA

> **psmB2**
Function: protein coding sequence; phenol soluble modulin beta 2 (genomic island)
Best match: psmB2_CC001_MW2-USA400_BA000033.2[1152596:1152730] (completely identical)
Position: 029-contig_258_RC: 10267 … 10402; Length: 135 bp
Sequence:
ATGACTGGACTAGCAGAAGCAATCGCAAATACTGTGCAAGCTGCACAACAACATGATAG
TGTGAAATTAGGCACAAGTATCGTAGACATCGTTGCTAACGGTGTGGGTTTACTAGGTAA
ATTATTTGGATTCTAA

> **Q1Y2B3**
Function: protein coding sequence; putative haloacid dehalogenase-like hydrolase (core genome, constant)
Best match: Q1Y2B3_CC005_Mu50-VRSA_BA000017.4[1229408:1230103] (completely identical)
Position: 029-contig_258_RC: 10525 … 11221; Length: 696 bp
Sequence:
ATGGGGAAATTGGGATATAAAAATATTTTGATAGACTTTGATGATACAATTGTTGATTTTT
ATGATGCAGAAGAATGGGCGTTTCACTATATGGCGAATGTTTTTAATCATAAAGCAACAA
AGGATGATTTTTTAACATTTAAAAAAATCAATCACCAACATTGGGAAGCTTTTCAACAAA
ATAAATTAACGAAGTCTGAAGTATTATCAGAACGATTTGTGAATTACTTCAAACATCATC
AAATGGAAGTTGATGGGCATCGTGCAGATGTGTTATTTAGAAATGGATTAGCAGAAGCTA
AAGTTAAATACTTTGATCAAACATTAGAAACAATTGTCGAATTATCGAAAAGACATGATT
TATATATTGTTACTAATGGTGTAACCGAAACGCAAAAGAGAAGGTTAAATCAGACGCCGT
TGCATAAATATATTAAAAAGATATTTATATCTGAGGAAACAGGATATCAAAAACCTAATC
CGGAATTTTTTAATTATGTTTTTAATGATATTGGTGAGGATGAAAGACAGCACTCGATTAT
AGTTGGAGATTCTTTAACATCTGACATTCTAGGTGGAATCAATGCGGGTATAGCTACTTG
CTGGTTTAATTTTAGAGGATTTGATCATAATCCAGGAATTATACCTGATTATGAAATTAAT
TCATGGAAACAACTAAATGATATTGTACGTTAA

> **Q931T2**
Function: protein coding sequence; N-acetyltransferase/GNAT family (core genome, constant)
Best match: Q931T2_CC001_MW2-USA400_BA000033.2[1153658:1154098:r]RC (completely identical)
Position: 029-contig_258_RC: 11329 … 11770; Length: 441 bp
Sequence:
TTATGGTTTCCACAATAAGACATCATGTCCCTCTATATTTTTAGCTTCCACATCTGTAAAT
CCATGATGTAAAAAGAAATCCTTAGAATCATTTCTGCCAATGGCTTTAATTGGCATATTG
AAACTCTTTGCAAAATCAATCAATTCTGAAGCGTACCCTCTGTTTTGATATTTTGGTAATA
CTTCTAACTTCCATAATAATATATAATCTTCAAAATCTGGGAAGTAGATTTCTTCGACATC
ACCTTTTTTTAGTAATGCCATTCTAGCTCCTAATTGATCTCCGACAAATATGCCATAAAAT
GGTGAATCTGAACTTGCATCAATCATTTGACCGTTTAACTCATTGACCATGTATAAGTCTT
TGTTGCCAAACGCTCTAAAGTTTTCGAATAATTCGTCAGTTTTGTAATTAATTTCAAGACG
TTTGATTTCACTCAT

> **Q5HGQ4**
Function: protein coding sequence; UPF0747 protein (core genome, variable)
Best match: Q5HGQ4_CC001_MW2-USA400_BA000033.2[1154293:1155906]
Position: 029-contig_258_RC: 11964 … 13578; Length: 1614 bp
Sequence:
ATGGACTGTAAAGTAGTTAGTTTAAATGAAAAAGATCAGTTTATACCAAAAATAAAGAG
CAGTGACCCTGTAATAACAGGATTATTTCAATATGATGCAGCTCAACAAACTAGTTTTGA
AAAAAGGATGTCTAAAGAAAATAATGGAAGAGAAGCGGCATTAGCGAATGTTATTCGTG
AATATATGAGTGATTTAAAGCTTTCTAGTGAACAAGAATTAAACATACAACATTTAGCTA
ATGGTTCAAAAGTTGTGATTGGTGGACAACAAGCAGGGCTTTTCGGGGGACCATTGTATA
CATTCCATAAAATATTTTCAATCATTACTTTATCTAAGGAATTAACGGATACACATAAGCA

ACAAGTAGTACCAGTTTTTTGGATTGCAGGAGAAGATCATGATTTCGATGAAGTGAATCA
TACATTTGTTTATAACGAAAATCATGGGTCGCTGCATAAGGTTAAATATCATACAATGGA
GATGCCAGAGACGACTGTCTCTAGATATTATCCTGATAAGGCTGAGTTGAAACAAACTTT
AAAAACGATGTTCATTCATATGAAAGAAACTGTTCATACACAAGGTCTACTGGAGATTTG
TGACAGAATTATTGACCAATATGACTCGTGGACTGATATGTTTAAAGCACTACTGCATGA
AACATTTAAAGCATATGGCGTTCTATTTATAGATGCGCAGTTTGAGCCGTTAAGAAAAAT
GGAAGCGCCTATGTTTAAAAAGATTTTGAAAAAACATCAGTTGCTTGATGATGCTTTTAG
AGCAACACAACAACGCACTCAAAATCAAGGCTTGAATGCGATGATACAAACAGATACAA
ATGTTCATTTATTCTTACATGATGAAAATATGCGTCAATTAGTTTCGTATGATGGTAAGCA
TTTTAAATTAAATAAAACAGATAAGACATATATAAAGGAAGAAATTATAAATATTGCGG
AAAATCAACCTGAATTATTTTCTAATAATGTAGTGACAAGACCATTAATGGAAGAATGGT
TATTTAACACGGTGGCATTTGTTGGAGGACCGAGTGAAATTAAGTACTGGGCTGAACTAA
AAGATGTATTTGAACTATTTGATGTTGAAATGCCTATCGTGATGCCAAGGCTTAGAATTA
CTTATTTAAATGACCGTATAGAAAAATTACTTTCGAAATACAATATTCCATTAGAAAAAG
TGTTAGTCGATGGTGTTGAAGGAGAAAGAAGTAAGTTTATTAGAGAACAAGCATCACATC
AATTTATTGAAAAGGTAGAAGGTATGATTGAACAACAGCGTCGTCTAAACCAAGACTTAT
TAGATGAAGTGGCGGGGAATCAAAATAATATTAACCTTGTGAATAAAAATAATGAAATT
CATATACAACAGTATGATTATTTGTTAAAACGTTATCTTTTAAACATTGAAAGAGAAAAC
GACATCAGTATGAAGCAATTTAGAGAAATTCAAGAAACACTCCATCCAATGGGAGGATT
ACAAGAAAGAATATGGAATCCACTTCAAATTTTGAATGATTTTGGGACAGATGTGTTCAA
GCCCTCCACCTATCCACCACTTTCTTACACTTTTGATCATATTATTATAAAACCTTAA

## > mraZ

Function: protein coding sequence; putative protein (core genome, constant)
Best match: mraZ_CC001_MW2-USA400_BA000033.2[1156050:1156481] (completely identical)
Position: 029-contig_258_RC: 13721 … 14153; Length: 432 bp
Sequence:
ATGTTCATGGGAGAATACGATCATCAATTAGATACAAAAGGACGTATGATTATACCGTCC
AAGTTTCGTTATGACTTAAATGAGCGTTTTATTATCACAAGAGGCCTTGATAAATGTTTAT
TCGGTTACACTCTAGACGAATGGCAACAGATTGAAGAGAAAATGAAAACCTTACCTATG
ACAAAAAAAGACGCACGTAAGTTTATGCGTATGTTCTTCTCTGGTGCTGTTGAAGTAGAA
CTTGATAAGCAAGGGCGTATTAACATCCCTCAAAACTTGAGGAAATACGCTAATTTAACT
AAAGAATGTACAGTAATCGGTGTTTCAAATCGTATTGAGATTTGGGATAGAGAAACTTGG
AATGATTTCTATGAAGAATCTGAAGAAAGTTTCGAAGATATTGCTGAAGATTTAATAGAT
TTTGATTTTTAA

## > rsmH

Function: protein coding sequence; ribosomal RNA small subunit methyltransferase H (core genome, constant)
Best match: rsmH_CC001_MSSA476_BX571857.1[1185191:1186105]
Position: 029-contig_258_RC: 14189 … 15104; Length: 915 bp
Sequence:
ATGTTAAACGAAACCATTGATTATTTAAATGTAAAAGAAAATGGTGTGTACATTGACTGT
ACGCTAGGTGGAGCGGGACATGCCCTTTATTTACTAAATCAATTAAATGACGACGGAAGA
TTAATAGCAATCGATCAAGACCAAACTGCAATTGATAATGCTAAAGAGGTATTAAAGGAT
CATTTGCATAAGGTGACTTTTGTTCATAGCAACTTCCGTGAATTAACTCAAATATTAAAAG
ACTTAAACATTGAAAAAGTAGATGGAATTTATTACGACTTGGGTGTTTCAAGTCCCCAAC
TCGACATTCCAGAACGAGGATTCAGTTATCACCATGACGCAACATTAGACATGCGTATGG
ACCAAACACAAGAACTAACAGCATATGAAATTGTTAACAATTGGTCATATGAAGCGTTAG
TGAAGATTTTTTATCGTTATGGCGAGGAGAAATTTTCAAAACAGATAGCTCGAAGAATCG
AAGCACATCGCGAACAACAACCAATAACAACAACATTAGAATTAGTTGACATTATAAAA
GAAGGTATTCCTGCAAAAGCAAGAAGAAAAGGCGGACATCCTGCAAAACGAGTATTTCA
AGCACTACGAATTGCAGTAAACGATGAATTGTCAGCTTTTGAAGATTCAATAGAACAAGC
GATTGAATTAGTGAAAGTAGACGGCAGGATTTCGGTAATCACTTTCCATTCTTTAGAAGA
TCGTTTATGTAAACAGGTGTTCCAAGAATATGAAAAAGGTCCAGAGGTACCAAGAGGATT

ACCAGTTATACCAGAAGCATATACACCTAAGTTAAAGCGTGTTAATCGTAAACCGATTAC
CGCTACAGAAGAAGATTTAGATGACAATAACAGAGCACGAAGCGCGAAATTACGTGTAG
CTGAAATACTTAAATAA

> **ftsL**
Function: protein coding sequence; cell division protein L (core genome, constant)
Best match: ftsL_CC001_MW2-USA400_BA000033.2[1157446:1157847] (completely identical)
Position: 029-contig_258_RC: 15117 … 15519; Length: 402 bp
Sequence:
ATGGCTGTAGAAAAAGTGTACCAACCATATGACGAACAAGTTTATAATAGTATACCGAA
GCAACAACCACAAACTAAGCCCGAAAAGAAGACTGTTTCGAGAAAAGTGGTTGTACAAT
TAACTAAATTTGAAAAAGTTTTATACATAACTTTGATTACTGTAATTGCTATGTTAAGTAT
TTATATGCTATCTTTAAAAATGGATGCGTATGATACGCGAGGAAAGATTGCAGATTTAGA
TTATAAAATAGATAAACAATCAAGTGAAAACAGTGCTTTACAATCTGAAATCAAAAAGA
ATTCTTCTTATGAACGCATATACGAAAAGGCTAAGAAACAGGGGATGAGCCTTGAGAAC
GATAATGTAAAGGTAGTGCGTAGTAATGGCGAAGCAAAAAATTAA

> **pbpA**
Function: protein coding sequence; penicillin binding protein A (core genome, constant)
Best match: pbpA_CC001_ATCC51811_ADVP01000010.1[14930:17163:r]
Position: 029-contig_258_RC: 15499 … 17734; Length: 2235 bp
Sequence:
ATGGCGAAGCAAAAAATTAAAATTAAAAAAAATAAAATAGGGGCAGTCCTACTTGTTGG
TTTATTCGGACTGCTCTTTTTTATATTGGTTTTAAGAATTTCATATATCATGATTACTGGAC
ATTCTAATGGTCAAGACTTAGTCATGAAGGCAAATGAAAAGTATTTAGTTAAGAATGCAC
AACAACCAGAACGAGGAAAGATATATGATCGTAATGGTAAAGTGCTAGCAGAAGATGTA
GAAAGATATAAACTTGTTGCAGTAATAGATAAAAAGGCGAGTGCCAATTCTAAAAAACC
TAGGCATGTAGTTGATAAAAAAGAGACTGCAAAGAAATTATCTACAGTCATTGATATGAA
GCCAGAGGAAATTGAAAAGAGACTTAGTCAAAAGAAAGCTTTCCAAATTGAATTTGGAC
GCAAAGGAACAAATTTAACGTATCAGGACAAATTGAAAATAGAGAAAATGAATTTGCCT
GGTATTTCTTTATTGCCTGAAACAGAACGCTTTTATCCAAATGGCAATTTTGCATCACACT
TAATTGGTAGAGCTCAGAAAAATCCGGATACTGGTGAACTTAAAGGTGCACTTGGAGTTG
AAAAGATTTTTGATAGTTATTTAAGTGGATCTAAAGGATCATTGAGATATATTCATGATAT
TTGGGGATATATCGCACCAAATACTAAAAAAGAGAAGCAGCCTAAACGTGGTGATGATG
TCCATTTAACAATCGATTCAAATATTCAAGTATTTGTTGAAGAAGCTTTAGATGGCATGGT
TGAAAGATACCAGCCGAAAGATTTATTTGCGGTTGTCATGGATGCCAAAACTGGAGAAAT
TTTAGCATACAGTCAGCGACCAACATTTAATCCTGAAACTGGTAAAGACTTTGGTAAAAA
GTGGGCAAATGACCTTTATCAAAACACATACGAGCCTGGATCAACATTTAAATCATATGG
GTTAGCAGCTGCTATTCAAGAAGGTGCTTTTGATCCTGATAAGAAATATAAATCTGGACA
TAGAGATATTATGGGTTCACGTATTTCAGACTGGAATAGAGTCGGTTGGGGTGAAATCCC
AATGTCACTCGGATTTACTTATTCATCTAATACATTGATGATGCATTTACAAGATTTAGTT
GGTGCAGACAAAATGAAATCTTGGTATGAACGATTTGGATTTGGAAAATCAACTAAAGGT
ATGTTTGATGGAGAAGCACCTGGTCAAATTGGATGGAGTAATGAGTTACAACAAAAAAC
GTCATCATTTGGTCAATCGACAACTGTAACACCTGTTCAAATGTTACAGGCGCAATCAGC
GTTCTTTAATGATGGTAATATGTTAAAACCATGGTTTGTGAATAGCGTTGAAAATCCTGTT
AGTAAAAGACAATTTTATAAAGGGCAAAAACAAATCGCAGGCAAACCAATAACAAAAGA
TACTGCTGAAAAAGTTGAAAAGCAATTGGATTTAGTTGTGAATAGTAAGAAGAGTCACGC
TGCAAACTATCGTATTGATGGTTATGAGGTCGAAGGTAAGACTGGTACAGCACAAGTCGC
TGCACCTAATGGTGGTGGATACGTTAAAGGTCCAAACCCATATTTTGTAAGTTTTATGGGT
GACGCACCGAAGAAAAATCCTAAAGTTATTGTATACGCTGGTATGAGCTTGGCACAAAA
AAATGACCAAGAAGCTTATGAATTAGGTGTTAGTAAAGCGTTTAAACCAATAATGGAAA
ATACTTTGAAATATTTAAATGTAGGTAAATCAAAAGATGACACATCTAATGCAGAGTATA
GTAAAGTGCCAGATGTTGAAGGTCAAGACAAACAAAAAGCTATTGATAATGTGAGTGCA
AAATCATTAGAACCAGTTACTATCGGTTCTGGCACACAAATAAAAGCACAATCTATAAAA
GCAGGGAATAAAGTCTTACCTCATAGTAAAGTACTGTTATTAACAGACGGAGACTTAACT

ATGCCTGACATGTCAGGATGGACGAAAGAAGATGTCATTGCTTTTGAAAACCTAACAAAT
ATTAAAGTAAATTTAAAAGGTAGCGGTTTTGTGTCCCACCAATCAATTAGTAAGGGACAA
AAACTTACTGAAAAAGATAAAATAGACGTAGAATTTTCATCAGAGAATGTAGACAGCAA
TTCGACGAATAATTCTGATTCAAATTCAGATGATAAGAAGAAATCTGACAGTAAAACTGA
CAAGGATAAGTCGGACTAA

> **mraY**
Function: protein coding sequence; phospho-N-acetylmuramoyl-pentapeptide-transferase (core genome, variable)
Best match: mraY_CC001_MW2-USA400_BA000033.2[1160354:1161319]
Position: 029-contig_258_RC: 18025 … 18991; Length: 966 bp
Sequence:
ATGATTTTTGTATATGCGTTATTAGCGCTAGTGATTACATTTGTTTTGGTACCTGTTTTAAT
ACCTACATTAAAAAGGATGAAATTTGGTCAAAGTATTCGAGAAGAAGGTCCACAAAGCC
ATATGAAGAAGACTGGTACACCAACGATGGGTGGACTAACATTTCTATTAAGTATTGTGA
TAACGTCTTTGGTGGCTATTATATTTGTGGATCAAGCTAATCCAATCATACTGTTATTATT
TGTGACGATTGGTTTTGGGTTAATTGGTTTTATAGATGATTATATTATTGTTGTTAAAAAG
AATAACCAAGGTTTAACAAGTAAACAGAAGTTTTTGGCGCAAATTGGTATTGCGATTATT
TTCTTTGTTTTAAGTAATGTGTTTCATTTGGTGAATTTTTCTACGAGCATACATATTCCATT
TACGAATGTAGCAATCCCACTATCATTTGCATATGTTATTTTCATTGTTTTTTGGCAAGTA
GGTTTTTCTAATGCGGTAAATTTAACAGATGGTTTAGATGGATTAGCAACTGGACTGTCA
ATTATCGGATTTACAATGTATGCCATCATGAGCTTTGTGTTAGGAGAAACGGCAATTGGT
ATTTTCTGTATCATTATGTTGTTTGCACTTTTAGGATTTTTACCATATAACATTAACCCTGC
TAAAGTGTTTATGGGAGATACAGGTAGCTTAGCTTTAGGTGGTATATTTGCTACGATTTCA
ATCATGCTTAATCAGGAATTATCATTAATTTTTATAGGTTTAGTATTCGTAATTGAAACCT
TATCTGTTATGTTACAAGTCGCTAGCTTTAAATTGACTGGAAAGCGTATATTTAAAATGAG
TCCGATTCATCATCATTTTGAATTGATAGGATGGAGCGAATGGAAAGTAGTTACAGTATT
TTGGGCTGTTGGTCTGATTTCAGGTTTAATCGGTTTATGGATTGGAGTGCATTAA

> **murD**
Function: protein coding sequence; UDP-N-acetylmuramoylalanine-D-glutamate ligase (core genome, variable)
Best match: murD_CC001_MSSA476_BX571857.1[1189994:1191343]
Position: 029-contig_258_RC: 18992 … 20342; Length: 1350 bp
Sequence:
ATGCTTAATTATACAGGGTTAGAAAATAAAAATGTATTAGTTGTCGGTTTGGCAAAAAGT
GGTTATGAAGCAGCTAAATTATTAAGTAAATTAGGTGCGAATGTAACTGTCAATGATGGA
AAAGACTTATCACAAGATGCTCATGCAAAAGATTTAGAATCTATGGGCATTTCTGTTGTA
AGTGGAAGTCATCCATTAACGTTGCTTGATAATAATCCAATAATTGTTAAAAATCCTGGA
ATACCTTATACAGTATCTATTATTGATGAAGCAGTGAAACGAGGTTTGAAAATTTTAACA
GAAGTTGAGTTAAGTTATCTAATCTCTGAAGCACCAATCATAGCTGTAACGGGTACAAAT
GGTAAAACGACAGTTACTTCTCTAATTGGAGATATGTTTAAAAAAAGTCGCTTAACTGGA
AGATTATCCGGCAATATTGGTTATGTTGCATCTAAAGTAGCACAAGAAGTAAAGCCTACA
GATTATTTAGTTACAGAGTTGTCGTCATTCCAGTTACTTGGAATCGAAAAGTATAAACCA
CACATTGCTATAATTACTAACATTTATTCGGCGCATCTAGATTACCATGAAAATTTAGAAA
ACTATCAAAATGCTAAAAAGCAAATATATAAAAATCAAACGGAAGAAGATTATTTGATTT
GTAATTATCATCAAAGACAAGTGATAGAGTCGGAAGAATTAAAAGCTAAGACATTGTATT
TCTCAACTCAACAAGAAGTTGATGGTATTTATATTAAAGATGGTTTTATCATTTATAAAGG
TGTTCGTATTATTAACACTAAAGATCTAGTATTGCCTGGTGAACATAATTTAGAAAATATA
TTAGCAGCTGTGCTTGCTTGTATTTTAGCTGGTGTACCTATTAAAGCAATTATTGATAGTT
TAACTACATTTTCAGGAATAGAGCATAGATTGCAATATGTTGGTACTAATAGAACTAATA
AATATTATAATGATTCCAAAGCAACAAACACGCTAGCAACACAGTTTGCCTTAAATTCAT
TTAATCAACCAATCATTTGGTTATGTGGTGGTTTGGATCGAGGGAATGAATTTGACGAAC
TCATTCCTTATATGGAAAATGTTCGCGCGATGGTTGTATTCGGACAAACGAAAGCTAAGT
TTGCTAAACTAGGTAATAGTCAAGGGAAATCGGTCATTGAAGCGAACAATGTCGAAGAC

GCTGTTGATAAAGTACAAGATATTATAGAACCAAATGATGTTGTATTATTGTCACCTGCTT
GTGCGAGTTGGGATCAATATAGTACTTTTGAAGAGCGTGGAGAGAAATTTATTGAAAGAT
TCCGTGCCCATTTACCATCTTATTAA

## > ftsQ

Function: protein coding sequence; cell division protein Q (core genome, variable)
Best match: ftsQ_AC027138.10[4092:5412]
Position: 029-contig_258_RC: 20357 … 21677; Length: 1320 bp
Sequence:
ATGGATGATAAAACGAAGAACGATCAACAAGAATCAAATGAAGATAAAGATGAATTAGA
ATTATTTACGAGGAATACATCTAAGAAAAGACGGCAAAGAAAAAGATCAAAGGCTACAC
ATTTTTCTAATCAAAATAAAGATGATACATCTCAACAAGCTGATTTTGATGAAGAAATTT
ACTTGATAAATAAAGACTTCAAAAAAGAACAAAGCAATGATGAAAATAATGATTCTGCT
TCTAGTCGTGCAAATAATAATAATATCGATGATTCTACAGACTCTAATATTGAAAATGAG
GATTATAGATATAATCAAGAAATTGACGACCAAAATGAATCGAATGGAATTGCATTCGAC
AACGAACAACCTCAATCAGCTCCTAAAGAACAAAATGGCGACTCGAATGATGAGGAAAC
AGTAACGAAAAAAGAACGAAAAAGTAAAGTAACACAATTAAAGCCATTAACACTTGAAG
AAAAGCGGAAGTTAAGACGTAAGCGACAAAAACGAATCCAATACAGTGTTATTACAATA
TTAATATTGTTGATTGCTGTTATATTAATTTACATGTTTTCACCACTTAGTAAAATTGCGCA
TGTAAATATAAATGGAAATAACCACGTTAGTACTTCAAAGATAAACAAAGTTTTAGGTGT
TAAAAATGATTCAAGGATGTATACGTTTAGTAAAAAAAATGCTATTAATGATCTCGAAGA
GGATCCATTAATCAAAAGTGTTGAGATACACAAGCAATTACCAAACACATTAAACGTAG
ATATCACAGAAAATGAAATTATTGCTTTAGTGAAATATAAAGGTAAATATTTACCTTTATT
AGAAAATGGTAAATTGCTTAAAGGTTCAAATGATGTCAAAATTAATGATGCACCTGTCAT
GGATGGTTTCAAAGGTACAAAAGAAGATGATATGATTAAGGCGTTATCTGAAATGACAC
CTGAAGTTAGACGATATATTGCCGAAGTGTCATACGCCCCAAGTAAAAACAAACAAAGC
AGAATTGAATTGTTTACGACAGATGGACTTCAAGTAATCGGTGATATTTCGACGATATCT
AAGAAAATGAAATATTATCCGCAGATGTCACAATCATTATCAAGGGATAGTTCGGGTAAA
CTAAAAACACGAGGCTATATTGATTTATCAGTCGGTGCTTCATTTATCCCATACCGTGGAA
ACACGTCTAGTCAATCAGAAAGCGATAAAAATGTGACTAAATCATCTCAAGAGGAAAAT
CAAGCAAAAGAAGAATTACAAAGCGTTTTAAACAAAATTAACAAACAATCAAGTAAGAA
TAATTAA

## > ftsA

Function: protein coding sequence; cell division protein A (core genome, variable)
Best match: ftsA_CC005_Mu50-VRSA_BA000017.4[1240666:1242078] (completely identical)
Position: 029-contig_258_RC: 21782 … 23195; Length: 1413 bp
Sequence:
ATGGAAGAACATTACTACGTAAGTATTGATATTGGATCATCAAGCGTAAAAACAATAGTA
GGCGAGAAATTTCACAATGGTATAAATGTGATAGGTACAGGACAAACCTACACGAGCGG
TATAAAAAATGGTTTAATTGATGATTTTGATATTGCGCGACAAGCAATCAAAGACACAAT
TAAAAAGGCATCAATCGCTTCGGGTGTTGATATTAAAGAAGTTTTCCTGAAATTACCTAT
CATTGGAACGGAAGTTTATGATGAATCAAATGAAATCGACTTTTATGAGGATACAGAAAT
CAACGGTTCACATATCGAAAAAGTATTAGAAGGTATTAGAGAAAAAAATGATGTGCAAG
AAACAGAAGTAATTAATGTGTTCCCGATTCGTTTTATAGTCGATAAAGAAAATGAGGTTT
CAGACCCTAAAGAATTAATTGCCAGACATTCATTAAAGGTTGAAGCAGGCGTAATTGCTA
TTCAAAAATCGATTTTAATTAATATGATTAAATGCGTAGAAGCATGTGGTGTTGATGTATT
AGATGTTTACTCTGATGCATATAACTATGGTTCAATCCTAACAGCTACTGAAAAAGAGTT
AGGTGCATGTGTCATTGATATTGGTGAAGACGTTACGCAAGTTGCTTTTTATGAACGCGG
TGAATTAGTAGATGCTGATTCTATCGAAATGGCAGGGCGTGATATTACAGACGATATTGC
ACAAGGATTAAACACTTCTTATGAAACTGCTGAAAAAGTTAAACACCAATATGGTCATGC
ATTCTATGATTCTGCTTCAGATCAAGATATCTTCACTGTTGAACAGGTTGATAGTGATGAA
ACAGTACAGTATACTCAAAAAGATTTGAGTGACTTTATTGAAGCGCGTGTAGAAGAAATA
TTCTTCGAAGTATTTGATGTTTTACAAGATTTAGGATTAACAAAAGTAAATGGTGGGTTTA
TTGTAACTGGTGGATCTGCAAACTTACTTGGCGTAAAAGAATTATTATCAGATATGGTAA

GTGAAAAAGTTAGAATTCACACGCCATCACAAATGGGAATTAGAAAACCTGAATTTTCTT
CAGCAATTTCTACAATTTCTAGTAGTATCGCTTTTGATGAGTTATTAGATTATGTTACAAT
TAATTATCATGATAATGAAGAAACTGAAGAAGATGTTATTGATGTGAAAGACAAAGATA
ACGAATCTAAATTAGGCGGATTTGATTGGTTTAAACGTAAAACAAACAAAAAAGATACTC
ATGAAAATGAAGTAGAGTCAACAGATGAAGAAATTTATCAATCAGAAGATAATCATCAG
GAACATAAACAGAATCATGAACATGTTCAAGACAAAGATAAAGATAAAGAAGAAAGTA
AATTCAAAAAACTAATGAAATCTCTATTTGAATGA

## > **ftsZ**

Function: protein coding sequence; cell division protein Z (core genome, constant)
Best match: ftsZ_CC001_MW2-USA400_BA000033.2[1165556:1166728] (completely identical)
Position: 029-contig_258_RC: 23227 … 24400; Length: 1173 bp
Sequence:
ATGTTAGAATTTGAACAAGGATTTAATCATTTAGCGACTTTAAAGGTCATTGGTGTAGGT
GGTGGCGGTAACAACGCCGTAAACCGAATGATTGACCACGGAATGAATAATGTTGAATTT
ATCGCTATCAACACAGACGGTCAAGCTTTAAACTTATCTAAAGCTGAATCTAAAATCCAA
ATCGGTGAAAAATTAACACGTGGTTTAGGAGCAGGAGCTAATCCTGAAATCGGTAAAAA
AGCTGCAGAGGAATCTCGTGAACAAATTGAAGATGCAATCCAAGGTGCAGACATGGTAT
TTGTTACTTCTGGTATGGGTGGCGGAACTGGTACTGGTGCAGCACCAGTCGTTGCTAAAA
TTGCAAAAGAAATGGGCGCATTAACTGTTGGTGTTGTAACTCGTCCATTTAGTTTTGAAG
GACGTAAACGTCAAACTCAAGCTGCTGCTGGAGTAGAAGCTATGAAAGCTGCAGTAGAT
ACATTAATCGTTATACCAAATGACCGTTTATTAGATATCGTTGACAAATCTACGCCAATG
ATGGAAGCATTTAAAGAAGCTGATAACGTGTTACGCCAAGGTGTACAAGGTATCTCAGAC
TTAATCGCTGTTTCTGGTGAAGTAAACTTAGACTTTGCAGACGTTAAGACAATTATGTCTA
ACCAAGGTTCTGCATTAATGGGTATTGGTGTTTCTTCTGGTGAAAATAGAGCGGTAGAAG
CTGCTAAAAAAGCAATCTCTTCTCCATTACTTGAAACATCTATCGTTGGTGCACAAGGTGT
GCTTATGAATATTACTGGTGGCGAGTCATTGTCATTATTTGAAGCACAAGAGGCTGCTGA
TATTGTCCAAGATGCTGCAGATGAAGACGTTAATATGATTTTCGGTACAGTTATTAATCCT
GAATTACAAGATGAGATTGTTGTAACAGTTATTGCAACTGGTTTTGATGACAAACCAACA
TCACATGGTCGTAAATCTGGTAGCACTGGATTCGGAACAAGCGTAAATACTTCTAGCAAT
GCAACTTCTAAAGATGAATCATTCACTTCAAATTCATCAAATGCACAAGCAACTGATAGT
GTAAGTGAAAGAACACATACAACTAAAGAAGATGATATTCCTAGCTTCATTAGAAATAG
AGAAGAAAGACGTTCAAGAAGAACAAGACGTTAA

## > **ylmD**

Function: protein coding sequence; putative protein similar to Bacillus subtilis YlmD (core genome, constant)
Best match: ylmD_CC001_MW2-USA400_BA000033.2[1166988:1167779] (completely identical)
Position: 029-contig_258_RC: 24659 … 25451; Length: 792 bp
Sequence:
GTGAATGATAATTTTAAAAAGCAACCGCATCATTTAATATATGAAGAGTTATTACAACAA
GGTATTACTCTAGGTATTACAACTAGAGGCGATGGTTTAAGTGACTATCCTAAAAATGCT
TTTAATATGGCGAGATATATTGATGATCGCCCATATAATATTACTCAACATCAATTGCAAT
TAGCTGAAGAAATTGCGTTTGATAGAAAAAATTGGGTGTTTCCCATTCAAACACATGAAA
ATAAAGTCGCTTGTATTACAAAGGATGATATAGGCACAAATATAGACACTTTAACTGATG
CGCTTCATGGTATTGATGCGATGTACACATATGATAGTAATGTCTTATTAACGATGTGTTA
TGCAGACTGTGTACCAGTATATTTTTATAGTACAAAACATCATTTTATTGCATTGGCGCAT
GCAGGTTGGCGTGGTACCTATACTGAAATTGTAAAAGAAGTGCTAAAACATGTGAACTTT
GATTTGAAAGACTTACATGTCGTTATTGGACCATCTACATCATCAAGTTATGAAATTAATG
ATGATATTAAAAATAAATTTGAAACATTGCCAATTGATAGTGCCAACTATATTGAAACTA
GAGGACGAGATCGTCATGGTATTGATTTGAAAAAAGCCAATGCTGCATTATTAAATTATT
ATGGTGTTCCTAAAGAAAATATTTATACGACAGCGTATGCTACATCTGAACATTTAGAAT
TATTTTTCTCTTATCGATTAGAAAAAGGTCAAACAGGACGCATGTTAGCATTCATTGGTCA
ACAGTAA

> **ylmE**
Function: protein coding sequence; putative protein similar to Bacillus subtilis YlmE (core genome, constant)
Best match: ylmE_CC001_MW2-USA400_BA000033.2[1167797:1168471] (completely identical)
Position: 029-contig_258_RC: 25468 … 26143; Length: 675 bp
Sequence:
TTGCGTGTGAAAGATAATTTACAACAAATCTCAACACAAATTAATGACAAAAGTGAAAA
AAATAATTTTTCAACAAAACCAAACGTGATTGCAGTTACAAAATATGTTACAATAGAGCG
AGCTAAAGAAGCGTATGAGGCTGGAATAAGACATTTTGGTGAGAATAGATTGGAAGGCT
TTTTACAAAAGAAAGAAGCATTACCATCAGATGCGGTGATCCATTTTATAGGATCATTAC
AATCTCGAAAAGTTAAGGGCGTTATAAACGACGTAGATTATTTCCATGCTTTAGATCGAT
TGAGCTTAGCCAAAGAAATTAACAAACGTGCAGAACATAAAATTAAATGTTTCTTGCAAG
TGAACGTTTCGGGAGAAGCTTCTAAACATGGTATTGCTTTAGAAGATGTTGATCAGTTTAT
AGATGATCTTAAAAAATATGACAAAATCGAAATTGTAGGTTTAATGACGATGGCACCATT
GACAGATGATGAAGCATATATTAGATCGTTATTTAAACAGTTACGTTTGAAAAAAGAAGA
AATACAACGACTCAATTTAGAATATGCGCCTTGTGATGAATTATCAATGGGAATGAGTAA
TGACTATCTTATTGCAGTTGAAGAAGGTGCGACGTTTGTTAGAATTGGGACTAAACTTGT
AGGAGAAGAGGAGTGA

> **sepF**
Function: protein coding sequence; cell division protein (core genome, constant)
Best match: sepF_CC001_MW2-USA400_BA000033.2[1168468:1169031] (completely identical)
Position: 029-contig_258_RC: 26139 … 26703; Length: 564 bp
Sequence:
GTGAGCCACTTGGCTTTAAAAGATTTATTTAGTGGATTTTTTGTAATAGATGATGAAGAG
GAAGTAGAAGTACCTGACAAACAACAACAGGTCAATGAAGCGCCAGCAAAAGAGCAGTC
ACAACAAACAACAAAACAAAACGCAATCAAATCAGTCCCTCAAAAATCTGCATCAAGAT
ATACAACAACGTCAGAAGAAAGGAATAACCGTATGTCTAATTATTCAAAAAATAATTCAC
GTAATGTTGTAACTATGAACAATGCTACACCAAACAATGCATCACAAGAAAGTTCAAAA
ATGTGTTTATTCGAACCACGTGTTTTTTCAGATACACAAGATATTGCTGATGAGCTTAAAA
ACCGCCGTGCGACACTTGTCAATTTACAACGTATTGATAAAGTATCAGCGAAAAGAATTA
TTGATTTTTTAAGCGGTACTGTTTATGCAATCGGTGGAGATATCCAACGTGTAGGTACTGA
TATTTTCTTATGTACGCCTGATAATGTGGAAGTAGCTGGAAGCATTACAGACCATATTGA
AAATATGGAACATTCATTCGACTAA

> **ylmG**
Function: protein coding sequence; putative protein (core genome, constant)
Best match: ylmG_CC001_MW2-USA400_BA000033.2[1169043:1169333] (completely identical)
Position: 029-contig_258_RC: 26714 … 27005; Length: 291 bp
Sequence:
ATGGATATAAATGTGCTAGCTACAATATTTAAATTTATCCTTTTTGTTGTTGAAATTTATT
ATTTCGGCATGATTATATATTTCTTTACATCTTGGGTACCAAGTATTAGAGAAACTAAGGT
AGGTTATTTTTTAGCGAAAATATATGAACCTTTCTTACAACCATTTAGAAAAGTAATTCCA
CCTATTGGAATTATCGACATATCATCAATCGCTGCAATTATCGTTTTAGTATTATTCCAAA
AAGGGTTACTCCAAATCTTTAATTGGATTTTAATTCAATTACAATAA

> **ylmH**
Function: protein coding sequence; putative protein similar to Bacillus subtilis YlmH (core genome, variable)
Best match: ylmH_CC008_21232_AGTY01000013.1[4773:5579:r]
Position: 029-contig_258_RC: 27087 … 27894; Length: 807 bp
Sequence:
ATGGTATTTTATAATTTTTTTAAGGTAGTGATTAACATAGATATTTATCAACACTTTAGAC
AGGAAGAATACGAATTAATTGATCAGCTAACGGATAAATGTGATCAAGCGGAACAGCAT
TATGCACCAGTATTAACGCATTTTTTAGATCCAAGAGAGCAATATATATTGGAAGTGATT

TGTGGCAGTTATGAAGATTTAAACGTATCTTTTTATGGTGGACCTAATGCTGAAAGAAAA
AGAGCAATCATTTCGCCGAACTATTATGAACCTAAAGAAAGCGACTTTGAATTAACTTTA
ATGGAAATAGATTATCCTGAAAAATTCGTCACTTTAAAACATCAACATATTTTAGGGACA
TTAATGTCTTTAGGTATCGAACGCGAACAAGTTGGAGATATAATTGTGAATGAACGAATT
CAATTTGTTTTGACAAGTAGATTGGAATCATTTATTATGTTAGAATTACAACGTATTAAAG
GCGCATCAGTTAAACTTTATACTATTCCAGTAACAGATATGATACAATCTAATGAGAATT
GGAAAAATGAAAGTGCAACAGTTAGTTCTTTAAGGTTAGATGTTGTTATTAAAGAAATGA
TACGTAAATCACGTACGATTGCGAAACAACTAATCGAAAAAAAACGTGTTAAAGTGAAT
CACACTATTGTTGATTCAGCAGATTTTCAATTACAAGCAAATGATTTAATATCCATCCAAG
GTTTTGGTAGAGCACACATTACTGACTTAGGTGGTAAAACTAAAAAAGATAAAACGCAC
ATTACCTATAGAACATTATTCAAATAG

## > Q7A131

Function: protein coding sequence; putative cell-division initiation protein DivIVA (core genome, constant)
Best match: Q7A131_CC005_21201_AFTO01000027.1[4638:5254:r]
Position: 029-contig_258_RC: 27917 … 28535; Length: 618 bp
Sequence:
ATGCCTTTTACACCAAATGAAATTAAGAATAAAGAGTTTTCACGTGTAAAGAATGGTTTA
GAACCTACTGAAGTTGCTAATTTTTTGGAGCAACTAAGCACTGAAATTGAACGTCTTAAA
GAAGATAAAAAACAACTTGAAAAAGTAATCGAAGAGAGAGATACTAATATTAAGTCTTA
TCAAGACGTGCATCAATCTGTAAGTGATGCTTTGATACAAGCTCAAAAAGCTGGTGAAGA
AACTAAGCAAGCTGCAGAGAAACAAGCTGAAGCGATTATAGCTAAGGCAGAAGCGCAAG
CTAATCAAATGGTTGGTGACGCGGTAGAAAAAGCACGCCGTTTAGCATTCCAGACTGAAG
ATATGAAACGCCAATCAAAAGTATTTAGATCGCGTTTCCGTATGTTAGTTGAAGCGCAAT
TAGACTTATTAAAAAACGAAGATTGGGATTACTTGTTGAATTATGATTTAGACGCTGAAC
AAGTGACGCTTGAAAATATTCATCATTTGCATGAAAATGATTTAAAGCCAGATGAAGTTG
CAGCAAATGCACAAAATAATGCATCAAATACACCAGACAATAATCAACAATCCAATGAT
TCAGAAACAACTAAGAAGTAA

## > tbox11

Function: noncoding RNA T-box leader element (core genome, constant)
Best match: tbox11_CC001_MW2-USA400_BA000033.2[1170869:1171041] (completely identical)
Position: 029-contig_258_RC: 28540 … 28713; Length: 173 bp
Sequence:
AAATAAAGACAGACGCGTAATATACATTTAACTTTTCACAGCGAATTAGGTAATGGTGAG
AGCCTAGTAAAAGCATGTATGTTATATCACTGGCTTTTTAATATTTAAATAATGTAATGAG
AGAACTCTAAGTTGAGTTAATAAGGGTGGTACCGCGAGCAATCGTCCCTTTT

## > ileS

Function: protein coding sequence; isoleucyl-tRNA synthetase (core genome, constant)
Best match: ileS_CC008_NCTC8325_CP000253.1[1107130:1109883]
Position: 029-contig_258_RC: 28755 … 31509; Length: 2754 bp
Sequence:
ATGGATTACAAAGAAACGTTATTAATGCCTAAAACAGATTTCCCAATGCGAGGTGGTTTA
CCAAACAAGGAACCGCAAATTCAAGAAAAATGGGATGCAGAAGATCAATACCATAAAGC
GTTAGAAAAAAATAAAGGTAACGAAACATTCATTTTACATGATGGCCCACCATACGCGA
ATGGTAACTTACATATGGGACATGCCTTGAACAAAATTTTAAAAGACTTTATTGTACGTT
ATAAAACTATGCAAGGGTTCTATGCACCATACGTACCAGGTTGGGATACACATGGTTTAC
CAATTGAACAAGCATTAACGAAAAAAGGTGTTGACCGAAAGAAAATGTCAACGGCTGAA
TTCCGTGAGAAATGTAAAGAATTTGCTTTAGAACAAATTGAATTACAGAAAAAAGATTTT
AGACGTTTAGGTGTTCGTGGTGACTTTAATGATCCATATATTACATTAAAACCTGAATACG
AAGCTGCACAAATTCGTATTTTTGGAGAAATGGCAGATAAAGGTTTAATTTATAAAGGTA
AAAAGCCAGTTTATTGGTCTCCTTCAAGTGAGTCTTCATTAGCAGAAGCAGAAATTGAAT
ATCACGATAAACGTTCAGCATCAATTTACGTTGCATTTGACGTTAAAGATGACAAAGGTG

TCGTTGATGCAGATGCTAAATTTATTATCTGGACAACAACGCCATGGACAATTCCATCAA
ATGTTGCAATTACCGTTCATCCTGAATTAAAATATGGTCAATACAATGTAAATGGCGAAA
AATATATTATTGCAGAAGCCTTATCTGACGCTGTAGCAGAAGCACTGGATTGGGATAAAG
CATCAATCAAATTAGAAAAAGAATACACAGGTAAAGAATTGGAGTATGTTGTAGCACAA
CATCCATTCTTAGACAGAGAATCGTTAGTGATTAATGGTGATCATGTTACTACAGATGCT
GGTACAGGTTGTGTACATACAGCACCAGGCCACGGGGAAGATGACTATATTGTTGGTCAA
AAATATGAATTACCAGTAATTAGTCCAATCGATGATAAAGGTGTATTTACTGAAGAAGGC
GGCCAATTTGAAGGGATGTTCTATGATAAAGCTAATAAAGCCGTTACTGATTTATTAACA
GAAAAAGGTGCACTATTAAAATTAGACTTTATTACACATAGCTATCCACACGACTGGAGA
ACAAAAAAACCTGTAATCTTCCGTGCTACACCACAATGGTTTGCCTCAATCAGTAAAGTA
AGACAAGATATTTTAGATGCAATCGAAAATACAAACTTCAAAGTAAATTGGGGTGAAAC
ACGTATTTACAATATGGTTCGTGACCGTGGCGAATGGGTTATTTCTCGTCAACGTGTGTGG
GGTGTACCGTTACCAGTATTTTATGCTGAAAATGGCGAAATTATCATGACGAAAGAAACA
GTGAATCATGTTGCTGATTTATTTGCAGAACACGGTTCAAATATTTGGTTTGAAAGAGAA
GCGAAAGACTTACTACCAGAAGGATTTACACATCCAGGCAGCCCTAACGGTACATTTACT
AAAGAAACAGACATTATGGACGTTTGGTTTGATTCTGGTTCATCACACCGTGGCGTGTTG
GAAACAAGACCGGAATTAAGTTTCCCAGCGGATATGTATTTAGAAGGTAGTGACCAATAT
CGTGGTTGGTTCAACTCTTCTATCACAACTTCAGTTGCTACAAGAGGAGTATCACCTTATA
AATTCTTACTTTCTCATGGTTTTGTTATGGATGGTGAAGGTAAGAAAATGAGTAAATCTTT
AGGTAATGTGATTGTACCTGACCAAGTGGTTAAACAAAAAGGTGCTGATATTGCGAGACT
TTGGGTAAGTAGTACGGACTATTTAGCTGATGTTAGAATTTCTGATGAAATTTTAAAACA
AACATCTGATGTTTATCGTAAAATCAGAAATACATTAAGATTTATGTTAGGTAACATTAA
CGATTTCAATCCTGACACAGATAGCATTCCTGAATCAGAGTTATTAGAAGTGGATCGTTA
CTTGCTAAATCGTTTACGTGAATTTACTGCAAGTACGATTAACAACTATGAAAACTTTGAC
TACTTAAATATTTATCAAGAAGTTCAAAACTTTATCAATGTTGAGTTAAGTAATTTCTATT
TGGATTACGGTAAAGATATTTTATATATTGAACAACGTGATTCTCATATCCGTCGTAGTAT
GCAAACAGTGTTATATCAAATTTTAGTTGATATGACGAAGTTGTTAGCACCAATCTTAGT
GCATACAGCTGAAGAAGTTTGGTCTCATACACCACATGTTAAAGAAGAAAGTGTTCACTT
AGCAGACATGCCTAAAGTTGTAGAAGTAGATCAAGCTTTATTGGATAAATGGCGTACATT
TATGAATTTACGTGATGATGTGAACCGTGCATTAGAAACTGCTCGTAATGAAAAAGTTAT
TGGTAAATCATTAGAAGCTAAAGTTACGATTGCTAGTAACGATAAATTTAATGCATCTGA
ATTCTTAACTTCATTTGATGCATTACATCAATTATTTATCGTGTCACAAGTTAAAGTTGTA
GATAAGTTAGACGATCAGGCAACAGCTTATGAACATGGTGATATTGTCATCGAACATGCA
GATGGTGAAAAATGTGAAAGATGTTGGAACTATTCAGAGGATCTTGGTGCTGTTGATGAA
TTGACGCATCTATGTCCACGATGCCAACAAGTTGTAAAATCACTTGTATAA

> **A5IS80**

Function: protein coding sequence; glyoxalase family protein (core genome, constant)
Best match: A5IS80_CC005_Mu50-VRSA_BA000017.4[1250683:1251480]
Position: 029-contig_258_RC: 31798 … 32596; Length: 798 bp
Sequence:
ATGTATCATAACAGTAACGCAAACTTTGTAAATGGTATCACTTTAAATGTGAGAGATAAG
AATGAATTAAAGCCATTTTATGAGGACGTATTAGGATTAAATATTATAAATGAGACATTA
ACATCGATACAATATGAAGTAGGTCAAAATAATCATGTCATTACACTTGTTGAATTACAA
AATGGACGTGAACCTTTAATGTCCGAAGCGGGACTGTTTCATATCGCAATTAAACTACCT
CAAATTAGTGATTTAGCTAATTTACTAATTCATTTAAGCGAATATGATATTCCAGTTAACG
GAGGTATACAGCCTGCTTCGTTATCATTATTTTTTGAAGACCCAGAAGGAAACGGTTTTA
AATTTTATGTTGATAAAGACGAAGCGCAATGGACGAGGCAAAATGATTTAGTAAAAATT
GATATTAGACCATTAAATGTACCGAGATTAGTGAGTCATGCAACAAAATTGTTATGGTTA
GGTATTCCAGATGACGCTATTATAGGTGCATTGCATATTAAGACAATTCATTTATCAGAG
GTAAAAGACTACTACCTCGATTATTTTGGACTAGAGCAATCGGCATATATGGATGATTAT
TCAATATTTTTAGCATCGAATGGCTATTATCAACATTTGGCCATGAATGATTGGGTATCAG
CAACGAAACGTGTAGAAAATTTTGATACGTATGGATTAGCAATTGTTGACTTTCATTATCC
TGAAACAACACATTTAAATTTACAAGGTCCGGATGGTATCTATTATCGCTTTAATCATATC
GAAGTTGAAGATTAG

> **txbi_universal1**

Function: bidirectional rho independent terminator
Best match: txbi_universal1_CC005_ED98-hen_CP001781.1[1188459:1188529] (completely identical)
Position: 030-contig_240: 207 … 278; Length: 71 bp
Sequence:
TGTATAAATAGAGAACAGCAGTAAGATAATTTTTAATTAGAAAATATCTTACTACTGTCT
TTTTAGGGATT

> **lspA**

Function: protein coding sequence; lipoprotein signal peptidase (core genome, constant)
Best match: lspA_CC001_MW2-USA400_BA000033.2[1175777:1176268] (completely identical)
Position: 030-contig_240: 487 … 979; Length: 492 bp
Sequence:
ATGCACAAAAAATATTTTATTGGCACTTCCATTTTAATAGCAGTATTTGTCGTTATATTTG
ACCAAGTTACTAAATATATTATAGCTACTACAATGAAAATTGGAGATTCATTTGAAGTGA
TACCGCACTTTTTAAACATAACATCACATCGAAATAATGGTGCTGCATGGGGAATATTGA
GTGGAAAAATGACATTTTTCTTTATTATTACCATTATTATATTAATAGCCTTAGTATATTTC
TTTATTAAAGATGCTCAATATAATTTGTTTATGCAAGTTGCTATTAGTTTACTTTTTGCAGG
TGCACTTGGAAACTTTATTGATAGAATTTTAACAGGAGAAGTTGTTGACTTTATTGATACA
AATATTTTTGGTTATGATTTTCCAATATTTAATATCGCAGATTCAAGTTTAACAATTGGTG
TAATATTAATTATTATTGCCTTATTAAAGGATACTTCCAATAAAAAGGAGAAGGAGGTTA
AGTAA

> **ylyB**

Function: protein coding sequence; ribosomal large subunit pseudouridine synthase (core genome, constant)
Best match: ylyB_CC001_ATCC51811-ST1_ADVP01000009.1[220028:220945:r]
Position: 030-contig_240: 978 … 1896; Length: 918 bp
Sequence:
ATGGAGACTTATGAATTTAACATTACAGATAAAGAACAAACAGGTATGCGTGTAGATAA
GTTGCTGCCTGAATTAAATAGTGATTGGTCTCGTAACCAGATACAAGATTGGATTAAAGC
AGGTTTAGTCGTTGCAAACGATAAAGTTGTTAAATCTAATTATAAAGTGAAACTTAATGA
TCATATAGTTGTCACTGAAAAAGAAGTGGTTGAAGCTGATATTCTACCTGAAAATTTAAA
TTTAGATATTTATTATGAAGATGACGATGTTGCAGTTGTATATAAACCGAAAGGCATGGT
AGTTCATCCATCACCAGGGCATTATACCAATACATTAGTTAATGGTTTAATGTATCAAATT
AAAAATTTGTCAGGTATTAATGGAGAAATTCGTCCAGGTATTGTTCACCGTATAGATATG
GATACTTCTGGTTTATTAATGGTTGCTAAAAATGATATTGCTCATCGTGGGCTTGTAGAAC
AATTAATGGATAAATCTGTTAAAAGAAAATATATCGCTTTAGTTCACGGGAATATTCCTC
ATGATTACGGTACAATCGATGCGCCAATTGGTAGAAACAAAAATGATCGTCAATCTATGG
CTGTTGTTGATGATGGTAAGGAAGCAGTGACACATTTTAACGTACTAGAACATTTTAAAG
ATTATACGCTTGTTGAATGTCAACTTGAAACAGGACGTACGCATCAAATCCGTGTTCACA
TGAAATATATTGGCTTCCCATTAGTTGGTGATCCAAAGTATGGACCGAAAAAGACATTGG
ATATTGGTGGTCAAGCTCTACATGCTGGACTTATTGGATTCGAACATCCAGTAACAGGTG
AATATATTGAAAGACATGCTGAATTACCACAAGACTTTGAAGATTTATTAGATACAATTC
GAAAAAGAGATGCATAA

> **pyrR**

Function: protein coding sequence; pyrimidine operon regulator and uracil phosphoribosyltransferase (core genome, constant)
Best match: pyrR_CC001_MW2-USA400_BA000033.2[1177585:1178112] (completely identical)
Position: 030-contig_240: 2295 … 2823; Length: 528 bp
Sequence:
ATGTCTGAACGTATCATAATGGATGATGCCGCAATACAACGTACAGTGACGAGAATCGCT
CACGAAATTTTGGAGTATAACAAAGGTACTGATAATTTAATTCTTTTAGGTATCAAAACA

AGAGGTGAATATTTAGCGAATCGTATACAAGATAAAATTCATCAAATTGAGCAACAACGT
ATACCTACTGGAACAATTGATATTACATACTTTAGAGATGATATAGAGCACATGTCATCA
CTTACGACAAAAGACGCAATAGACATCGACACAGATATTACAGATAAAGTAGTCATCATT
ATTGATGATGTGCTGTATACTGGTCGAACGGTTCGTGCTTCACTTGATGCTATTTTGCTAA
ATGCTAGACCTATTAAAATTGGTTTAGCTGCTTTGGTTGATCGAGGACATCGTGAGTTACC
AATTCGAGCAGATTTTGTTGGTAAAAATATACCTACTTCTAAAGAGGAAACGGTAAGTGT
CTATTTAGAAGAAATGGATCAAAGAAATGCAGTTATAATTAAATAA

> **pyrP**

Function: protein coding sequence; uracil-cation symporter (core genome, constant)
Best match: pyrP_CC001-ST772_118_AJGE01000002.1[210156:211463:r] (completely identical)
Position: 030-contig_240: 3040 … 4348; Length: 1308 bp
Sequence:
ATGCAAAATGATGAAATGTTTGAACGAACAGTAAAACCAGTACTAGATGTAAATGAAAA
ACCACAACCAGCGCAATGGGCATTTTTAAGCTTACAACATTTATTTGCGATGTTTGGCGC
AACAGTACTAGTACCATTCTTAACAGGACTACCAATATCCGCAGCGTTACTAGCTTCGGG
AATCGGTACATTACTTTATATCTTAATAACGAAGGCGCAAATACCAGCATACTTGGGATC
TAGCTTTGCATTTATCACACCAATTATCACGGGATTAAGTACGCATAGCTTAGGAGACAT
GCTTGTAGCATTATTCATGAGTGGTGTTATGTACGTCATCATCGGAATTCTAATCAAATTA
AGTGGGACAGCATGGTTAATGAAATTATTACCACCAGTTGTTGTTGGACCAGTCATCATG
GTAATTGGTTTAAGCTTAGCGCCTACTGCAGTCAATATGGCAATGTATGAAAATCCTGGA
GATATGAAAGGTTACAATATCAGTTTCTTAATTGTTGCAATGATAACCTTGCTTGTAACAA
TAGTCGTTCAAGGATTTTTTAAAGGATTCCTATCTTTAATACCAGTACTTGTAGGTATTAT
CGTAGGTTATGTGGTAGCGATTTTTATGGGGATTGTGAAATTTGATGCAATTATGTCAGCA
AAATGGATAGATTTCCCTCATATTTATCTGCCATTTAAAGATTATGTACCTTCATTTCACTT
AGGACTTGTACTTGTAATGATTCCGATTGTGTTTGTAACAGTAAGTGAACATATTGGGCA
CCAAATGGTATTGAATAAAATCGTAGGTAGAAACTTCTTTGAAAAGCCAGGACTTGATAA
ATCAATCATTGGTGATGGTGTTTCTACAATGTTTGCCAGTATTATTGGTGGACCACCAAGT
ACAACATACGGTGAAAATATCGGTGTATTAGCGATTACCAGAATATACAGTATTTACGTC
ATTGGTGGTGCAGCAGTTATAGCAATTGTTTTAGCATTCATTGGTAAGTTCACTGCATTAA
TTTCTTCTATACCTACACCAGTTATGGGAGGAGTATCTATATTACTTTTCGGAATTATTGC
AGCAAGTGGCTTAAGAATGTTAGTTGAAAGCAAAGTAGATTTTGCGAACAATCGAAATTT
AGTTATAGCTTCTGTAATTTTAGTTGTAGGTATCGGTAATTTAGTATTTAACTTAAAAGAA
ATTGGTATCAACCTTCAAATTGAGGGGATGGCATTAGCTGCACTTTCAGGAATTATTTTGA
ACTTAATCTTACCTAAAGAGAAAAAACAAAACAATTAA

> **pyrB**

Function: protein coding sequence; aspartate carbamoyltransferase (core genome, constant)
Best match: pyrB_CC001_MW2-USA400_BA000033.2[1179665:1180546] (completely identical)
Position: 030-contig_240: 4375 … 5257; Length: 882 bp
Sequence:
ATGAATCATTTATTATCAATGGAACATTTATCTACAGATCAAATATACAAACTTATCCAA
AAGGCAAGTCAATTTAAATCTGGTGAACGTCAACTACCAAACTTTGAAGGGAAATATGTC
GCAAATTTATTCTTTGAAAATTCTACTCGAACAAAATGTAGTTTTGAAATGGCAGAACTT
AAGCTAGGGTTAAAAACGATTAGCTTTGAAACATCAACATCATCTGTTTCAAAAGGTGAA
TCTTTATATGACACATGTAAAACTTTAGAAAGTATTGGCTGTGATTTATTAGTCATTAGAC
ATCCGTTTAATAACTACTATGAAAAATTAGCGAATATTAACATCCCAATTGCGAATGCTG
GTGATGGTAGTGGACAACATCCAACACAAAGTTTACTTGATTTAATGACGATATATGAAG
AATATGGATATTTTGAAGGCTTGAATGTATTGATTTGTGGAGACATTAAAAATTCACGTG
TCGCACGTAGTAATTACCATAGTTTAAAAGCATTAGGTGCAAACGTAATGTTTAATAGCC
CAAATGCTTGGATTGATGATTCTTTAGAAGCACCTTATGTAAATATAGATGATGTTATAG
AAACAGTAGATATAGTTATGTTATTAAGAATTCAACATGAAAGACATGGGCTTGCAGAAG
AAACTAGATTTGCAGCAGATGATTATCATCAAAAGCATGGCTTAAATGAAGTGCGCTATA
ACAAATTACAAGAACATGCTATTGTTATGCATCCGGCACCTGTGAATAGAGGAGTAGAAA

TACAAAGCGATTTAGTAGAAGCTTCAAAATCAAGAATTTTTAAGCAAATGGAAAATGGC
GTTTACTTAAGAATGGCAGTCATTGATGAATTATTAAAATAG

> **pyrC**

Function: protein coding sequence; dihydroorotase (core genome, constant)
Best match: pyrC_CC005_Mu50-VRSA_BA000017.4[1257091:1258365] (completely identical)
Position: 030-contig_240: 5274 … 6549; Length: 1275 bp
Sequence:
ATGAAATTAATTAAAAACGGTAAAGTATTACAAAATGGAGAATTACAACAAGCAGATAT
TTTAATTGATGGTAAGGTAATTAAACAAATTGCACCTGCAATTGAACCAAGCAATGGTGT
TGACATCATAGATGCGAAAGGTCACTTTGTGTCACCTGGATTTGTCGATGTTCATGTTCAT
TTACGTGAACCTGGTGGTGAATATAAAGAGACAATTGAAACTGGTACTAAAGCTGCTGCT
AGAGGCGGATTTACAACTGTATGTCCAATGCCTAACACAAGACCGGTACCAGATTCTGTA
GAACATTTTGAAGCTTTACAAAAATTAATCGATGACAATGCTCAAGTACGTGTATTACCT
TATGCTTCAATTACAACACGTCAATTAGGTAAAGAATTGGTTGATTTCCCAGCACTAGTA
AAAGAAGGTGCCTTTGCGTTTACAGATGACGGTGTAGGAGTACAAACTGCAAGCATGAT
GTATGAAGGCATGATTGAAGCTGCAAAAGTAAACAAAGCCATCGTAGCACACTGTGAAG
ATAATTCATTAATCTATGGTGGTGCAATGCATGAAGGGAAACGCAGTAAAGAGTTAGGTA
TACCAGGTATTCCAAACATTTGTGAATCTGTTCAAATCGCAAGAGATGTACTATTAGCTG
AAGCAGCAGGTTGTCATTATCATGTATGTCATGTTTCTACTAAAGAAAGTGTTAGAGTCA
TTCGTGACGCTAAACGCGCAGGCATTCATGTTACAGCTGAAGTTACACCACACCATTTAT
TGTTAACAGAAGATGATATTCCTGGTAATAATGCCATTTATAAAATGAATCCACCATTGA
GAAGTACTGAAGATAGAGAGGCTTTGTTAGAAGGGTTACTAGACGGTACAATTGACTGTA
TCGCAACAGACCATGCACCACATGCACGTGATGAAAAAGCACAACCAATGGAAAAAGCA
CCATTCGGAATTGTTGGTAGTGAAACAGCATTCCCATTATTATATACGCATTTTGTAAAAA
ATGGTGATTGGACATTACAACAATTAGTAGATTACTTAACAATTAAACCATGTGAGACAT
TTAATTTAGAATACGGCACATTAAAAGAAAATGGTTATGCAGATTTAACAATCATTGATT
TAGATAGTGAACAAGAAATTAAAGGAGAAGATTTCTTATCAAAAGCAGATAATACACCA
TTTATCGGCTATAAAGTTTATGGAAATCCGATCTTAACAATGGTTGAAGGCGAAGTTAAA
TTTGAGGGGGATAAATAA

> **pyrAA**

Function: protein coding sequence; carbamoyl-phosphate synthase short chain (core genome, constant)
Best match: pyrAA_CC005_Mu50-VRSA_BA000017.4[1258367:1259467] (completely identical)
Position: 030-contig_240: 6550 … 7651; Length: 1101 bp
Sequence:
ATGCAAAGCAAACGTTATCTAGTGTTAGAAGACGGTTCTTTTTACGAAGGCTACCGTTTA
GGATCTGATAACTTAACTGTAGGAGAAATTGTATTTAATACAGCTATGACAGGTTATCAA
GAAACTATTTCAGATCCATCATATACAGGTCAGATCATTACTTTTACGTATCCATTAATCG
GTAATTATGGTATCAATAGAGACGATTTTGAATCATTAGTACCTACATTAAACGGTATTGT
AGTGAAAGAAGCGAGTGCGCATCCAAGTAATTTTAGACAACAAAAGACACTTCATGACG
TTTTAGAATTGCATCAAATTCCAGGGATTGCAGGTGTTGATACAAGAAGTATTACGCGTA
AAATTCGACAACACGGTGTGTTAAAAGCTGGTTTTACTGATCGAAAAGAAGATATTGATC
AACTTGTCAAACATTTACAACAAGTAGAATTACCTAAAAACGAAGTAGAAATCGTTTCGA
CTAAAACACCGTATGTTTCGACAGGTAAGGATCTAAGTGTCGTACTTGTAGACTTTGGTA
AGAAGCAAAATATTGTTCGAGAATTAAACGTCAGAGGTTGTAACGTCACAGTTGTACCAT
ATACAACTACTGCCGAAGAAATTTTAGCAATGGCTCCAGATGGCGTTATGCTATCAAACG
GACCAGGTAATCCTGAAGTTGTAGAATGTGCGATTCCAATGATTCAAGGAATTTTAGGGA
AAATTCCGTTCTTTGGTATCTGTCTAGGACATCAACTTTTTGCATTATCTCAAGGAGCAAG
CTCATTTAAAATGAAGTTTGGTCATCGTGGTGCGAACCATCCAGTTAAAAATTTAGAGAC
TGGAAAAGTTGATATTACGAGTCAAAACCATGGATATGCAATAGATATAGATTCGTTAAA
AAGTACTGATTTAGAAGTTACTCATCTTGCATTAAATGATGGTACTGTAGAAGGTTTAAA
ACATAAAACATTACCAGCATTTTCTGTTCAATACCATCCTGAAGCAAATCCAGGACCGTC
AGATTCAAACTATCTATTTGATGATTTTGTAGCAATGATGACTAATTTTAAGGAAAAGGA
GCGTCATATCAATGCCTAA

> **pyrAB**
Function: protein coding sequence; carbamoyl-phosphate synthase large chain (core genome, constant)
Best match: pyrAB_CC001-ST772_118_AJGE01000002.1[203687:206860:r]
Position: 030-contig_240: 7643 … 10817; Length: 3174 bp
Sequence:
ATGCCTAAACGTAATGATATCAAAACAATTTTAGTAATAGGGTCTGGGCCAATTATCATA
GGTCAAGCAGCTGAATTTGATTATGCTGGAACACAAGCATGTCTAGCTTTAAAAGAAGAG
GGATATCGAGTTATTCTTGTAAATTCAAATCCAGCGACAATCATGACTGATAAGGAAATT
GCGGATAAAGTATATATCGAACCGTTAACTCATGATTTTATAGCGCGAATTATACGTAAA
GAGCAACCTGACGCTTTACTTCCAACTTTAGGTGGTCAAACAGGTTTAAACATGGCGATT
CAACTACACGAAAGTGGTGTGCTTCAAGATAATAACGTCCAATTATTAGGAACTGAGCTA
ACATCAATTCAACAAGCAGAAGACCGTGAAATGTTTAGAACATTAATGAATGATTTAAAC
GTTCCTGTACCAGAGAGTGACATTGTAAATACAGTAGAGCAAGCCTTTAAATTCAAAGAG
CAAGTGGGATACCCGCTAATTGTTAGACCGGCATTTACGATGGGTGGTACCGGAGGCGGT
ATTTGTCATAATGATGAAGAATTACATGAAATCGTCTCAAATGGTCTTCATTATAGTCCAG
CAACGCAATGTTTATTAGAAAAATCTATCGCAGGTTTTAAAGAAATCGAATACGAAGTAA
TGCGTGATAAAAACGATAATGCCATCGTTGTATGTAACATGGAAAATATTGATCCAGTTG
GTATTCATACAGGCGATTCAATTGTTGTGGCTCCTAGCCAAACATTATCAGATGTTGAGTA
TCAAATGTTACGTGATGTTTCATTAAAAGTTATTCGAGCTTTAGGTATCGAAGGTGGTTGT
AATGTTCAATTAGCATTAGATCCCCATTCATTCGATTATTATATTATAGAAGTAAATCCGC
GTGTATCACGTTCATCAGCGTTAGCTTCAAAAGCAACAGGATATCCTATTGCAAAATTAG
CTGCTAAAATCGCGGTTGGTCTAACATTAGATGAAATGTTAAATCCAATTACAGGAACAT
CTTATGCAGCGTTTGAACCAACTTTAGACTATGTGATTTCAAAAATACCAAGATTTCCTTT
TGATAAATTTGAAAAAGGAGAACGAGAGCTTGGCACACAAATGAAAGCAACAGGTGAAG
TTATGGCCATTGGTCGAACTTACGAAGAATCATTGTTAAAAGCAATTCGATCACTTGAGT
ATGGTGTGCATCACTTAGGATTACCAAATGGTGAAAGCTTCGATCTTGATTATATTAAAG
AACGTATTTCACACCAAGATGATGAACGATTATTTTTCATCGGCGAAGCAATTAGAAGAG
GCACAACATTAGAAGAAATTCATAATATGACTCAGATTGATTACTTCTTCTTACACAAGTT
CCAAAACATTATTGATATTGAGCATCAATTAAAAGAGCATCAAGGTGATTTAGAATATCT
TAAATATGCAAAAGATTATGGATTTAGTGATAAAACAATAGCGCATCGCTTTAATATGAC
GGAAGAAGAAGTATATCAATTGCGTATGGAAAATGATATTAAACCTGTTTACAAGATGGT
TGATACTTGCGCAGCTGAATTTGAATCTTCAACACCATATTATTATGGTACATACGAAACT
GAAAATGAATCCATAGTTACTGACAAAGAAAAAATCTTAGTATTAGGCTCTGGACCAATT
CGAATCGGCCAAGGTGTAGAATTTGACTATGCGACAGTTCACGCCGTTTGGGCAATTCAA
AAAGCAGGGTACGAAGCGATAATTGTGAATAACAATCCAGAAACAGTTTCAACAGACTT
CTCAATTTCTGACAAATTATACTTTGAACCTTTAACTGAAGAAGATGTGATGAATATCATT
AATTTAGAAAAACCTAAAGGTGTCGTTGTACAATTTGGAGGACAAACAGCGATTAATTTA
GCAGACAAATTGGCTAAACATGGTGTTAAAATACTTGGTACTTCACTAGAAAATCTAAAT
CGTGCTGAAGATAGAAAAGAATTTGAAGCACTATTAAGAAAAATTAACGTGCCACAGCC
ACAAGGAAAAACAGCTACATCACCTGAGGAAGCATTAGCGAATGCTGCAGAAATCGGAT
ATCCGGTTGTAGTAAGACCTTCTTATGTATTAGGTGGTCGCGCAATGGAAATTGTAGACA
ATGACAAAGAGTTAGAAAACTATATGACCCAGGCTGTAAAAGCGAGTCCGGAACATCCG
GTACTAGTCGATAGATATTTAACTGGTAAAGAAATTGAAGTTGATGCGATTTGTGATGGA
GAAACGGTCATTATTCCAGGAATCATGGAACATATTGAACGTGCTGGTGTGCATAGTGGT
GACTCAATCGCTGTATATCCGCCACAAACTTTGACAGAAGACGAGTTAGCAACACTTGAG
GACTATACTATAAAATTAGCTAAAGGTTTAAACATCATTGGCTTAATCAACATTCAATTC
GTTATAGCTCACGATGGTGTGTATGTTTTAGAAGTAAATCCACGTTCTAGTAGAACGGTA
CCATTCTTAAGTAAAATTACTGATATTCCAATGGCACAATTAGCTATGCGAGCAATCATT
GGGGAAAAACTAACAGATATGGGTTATCAAGAAGGGGTTCAACCATATGCTGATGGTGT
CTTTGTGAAAGCACCGGTATTTAGTTTTAATAAATTGAAAAATGTTGATATTACTTTAGGA
CCTGAAATGAAGTCAACAGGTGAAGTGATGGGGAAAGATACTACATTAGAAAAGGCGTT
ATTCAAAGGGTTAACAGGTAGTGGTGTTGAAGTTAAAGATCACGGTACAGTATTAATGAC
CGTCAGTGACAAAGATAAAGAGGAAGTTGTTAAATTGGCACAACGCTTAAATGAAGTTG
GCTATAAAATTTTAGCAACGTCTGGAACAGCTAATAAATTAGCTGAGTATGACATACCTG
CAGAAGTAGTAGGCAAAATTGGTGGCGAAAATGATTTATTAACACGTATTCAAAATGGTG

ATGTTCAAATCGTTATAAATACAATGACTAAAGGTAAAGAAGTAGAAAGGGATGGCTTC
CAAATTAGACGTACTACAGTTGAAAATGGTATTCCATGTTTGACATCTTTAGATACAGCT
AATGCCTTAACGAATGTAATTGAAAGTATGACATTTACAATGCGTCAAATGTAA

## > pyrF

Function: protein coding sequence; orotidine 5'-phosphate decarboxylase (core genome, constant)
Best match: pyrF_CC005_Mu50-VRSA_BA000017.4[1262743:1263435] (completely identical)
Position: 030-contig_240: 10926 … 11619; Length: 693 bp
Sequence:
ATGAAAGATTTACCAATTATTGCATTAGATTTTGAATCAAAAGAAAAAGTAAATCAATTT
TTAGATTTATTTGATGAATCATTATTCGTAAAAGTAGGTATGGAACTTTTTTATCAAGAAG
GTCCTCAATTAATTAATGAGATAAAAGAAAGAGGCCATGATGTATTTTTAGATTTAAAAC
TGCATGATATTCCTAATACAGTTGGTAAGGCGATGGAAGGACTAGCTAAATTGAATGTTG
ATCTGGTAAATGTTCATGCTGCTGGTGGCGTAAAAATGATGTCTGAGGCCATTAAAGGAT
TAAGAAAACATAATCAACATACAAAAATTATTGCAGTAACACAGCTTACGTCAACAACA
GAAGACATGTTACGACACGAACAAAATATACAAACATCGATTGAAGAGGCCGTTTTAAA
TTATGCCAAGTTAGCAAATGCAGCTGGTTTAGATGGCGTTGTTTGTTCACCTCTTGAAAGT
CGTATGTTGACTGAAAAGTTAGGTACATCATTTTTAAAAGTAACACCAGGTATTAGACCT
AAAGGTGCATCTCAAGATGACCAACACCGTATTACGACACCGGAAGAAGCAAGACAGCT
TGGTTCGACGCATATTGTAGTCGGTAGACCGATTACACAAAGTGACAATCCAGTCGAAAG
TTATCATAAAATTAAAGAAAGTTGGTTAGTATAA

## > pyrE

Function: protein coding sequence; orotate phosphoribosyltransferase (core genome, constant)
Best match: pyrE_CC001-ST772_118_AJGE01000002.1[202274:202885:r] (completely identical)
Position: 030-contig_240: 11618 … 12230; Length: 612 bp
Sequence:
ATGGCTAAAGAAATTGCAAAATCATTATTAGATATTGAAGCTGTAACATTATCACCAAAT
GATTTATATACATGGAGTTCAGGTATTAAATCACCGATTTACTGTGATAACCGTGTTACGT
TAGGTTATCCTTTAGTTCGAGGCGCAATCCGCGATGGTTTAATTAACTTAATTAAAGAAC
ACTTTCCTGAAGTAGAAGTTATTTCTGGTACTGCAACAGCTGGTATTCCACATGCAGCTTT
TATTGCTGAAAAATTAAAATTACCAATGAATTATGTTCGTTCATCAAATAAGAGTCATGG
TAAGCAAAATCAAATCGAAGGTGCTAAAAGTGAAGGTAAAAAAGTAGTTGTGATAGAAG
ATTTAATTTCGACAGGAGGATCTTCAGTCACAGCAGTTGAAGCCTTAAAACAAGCAGGTG
CAGAAGTATTAGGTGTTGTAGCTATCTTTACTTACGGTTTGAAAAAAGCAGATGATACAT
TTAGCAATATTCAACTACCTTTTTACACTTTAAGTGATTACAATGAATTAATTGAAGTAGC
TGAAAATGAAGGTAAAATTTCTAGTGAAGATATCCAAACATTAGTTGAATGGAGAGACA
ACTTAGCATAA

## > Q5HGM6

Function: protein coding sequence; putative protein (core genome, constant)
Best match: Q5HGM6_CC005_Mu50-VRSA_BA000017.4[1264076:1264288] (completely identical)
Position: 030-contig_240: 12259 … 12472; Length: 213 bp
Sequence:
ATGAATGACAAAACATCTAATGATTTATATGGGAAGATAAAACATTGTAACGAATTTATC
AATCATTCAAATGATTCCAATCTATCTAGTAGTCACGATGTCGACGAAAGTTCAACGAAG
CAAAAACATATAAAAAATAAAACAACTATAGATCATAATGATGATTTATTTAAACATGTA
AAGGATATATTACGTAAACAAGGACAAATTTAA

## > Q5HGM5

Function: protein coding sequence; putative protein (core genome, constant)
Best match: Q5HGM5_CC001-ST772_118_AJGE01000002.1[201194:201595:r] (completely identical)
Position: 030-contig_240: 12908 … 13310; Length: 402 bp
Sequence:

ATGGATATTCCAAAAATCACGACATTTTTAATGTTTAATAACCAAGCTGAAGAAGCTGTT
AAACTATACACAAGCTTATTTGAAGATAGTGAGATTATAACAATGGCTAAGTATGGTGAA
AATGGACCTGGTGATCCTGGGACTGTACAACACTCAATATTTACATTAAATGGACAAGTA
TTCATGGCAATTGATGCTAATAGTGGCACAGAATTACCAATGAATCCTGCGATTTCATTAT
TTGTTACAGTAAAAGATACTATTGAAATGGAACGACTATTTAATGGATTAAAAGATGAAG
GTGCCATTTTAATGCCAAAAACGAATATGCCACCATACAGAGAGTTTGCTTGGGTTCAAG
ATAAGTTTGGAGTAAGTTTTCAATTAGCATTACCTGAGTAA

## > fbpA

Function: protein coding sequence; putative fibronectin/fibrinogen-binding protein (core genome, constant)
Best match: fbpA_AC027138.10[30240:31936:r]RC
Position: 030-contig_240: 13572 … 15270; Length: 1698 bp
Sequence:
TTATGATTGTTTCATTTTTTGAATAAGTTCATAATCAGGTGTAGCATACAAAGTTTTTTGA
TTGTCATATGTTACAAACCCAGGCTTTGCACCTGATGGTTTATGCACATTTTTAATTAACG
TGTAATCAACAGGTATTTGTCCAGAATTACCAGCTTTTGAAAAATATCCTGCTAACATAG
CCGCTTCCTTGATTGTCGTATCACTTGGTGCATCATTAAATATAACGACATGTGAACCAGG
AATATCTTTTGTGTGTAACCATGTGTGAGTTTTTTTAGCTTTTTTATTTGTTAAATAATCAT
TTTGCTTGTTATTCTTGCCAACATATATATCGTCGCCATCAGTTGATACATAATGTTGTAA
TTGAATCTGCGCTTTCTTTTTCTTAGTTTGATTTTTACGCTGTTTCATAAAGCCTTGTTCTG
CTAATTCATCTCTAATTTCATCAATGTCATGGACAGAAATATGATGTAATTGTTGTTCGAT
TGTTGAAAAATAATCTATATTGTCTTTCGTCAATTGAATTTGATGTTGTAATTCACGTTCTC
TCGTTTTCATACGATTATATTGTTTATAATAATATTGAGCATTTGCTGATGGGGATTTTGT
AGGATTTAAAGGAATGACAACTTCTTCATTCGTATAATAATTCAATGCCGTCACTTCTTTA
TCGCCTTGCTTAATTCGATATATATTAGCAGTGATCAATTCACCATATAACTGTTCAGTAT
CTTTATTTTTAGACTGCTCATATTCTTCAATCAACTTCGCTAATTTATTTTGATATTTGTGC
AACTGCTGTTGAACAAATCGAACTAAATCATTCGCACGTTGTTTAACGCGTTCACGTTCGC
CACGCGCATCATAAAAACGGTCAAGTAAATCATTTAATGAATCGTATGTAACTGTATCAT
CATTAAATTGATTTAACTTTATAAAATAGAAATCCTCTTTACCTGTTTCATGATTTTTATGA
AAAATAGGAGTAGGTGGTAACTTGGTTTCTGCCATTACTTCGTCAAATGCTTCTGGTAATG
TTGATGAAGTCATAAATTGACGACGACTAACGATTTCATTCGTAATTAAAGGGCTAAATC
CTTCAAACTGATTCAATAATTGTTTAGCAATATTACCTGCGTTAAAATCGATATATTTCAA
CACCTCTGCACCTGTAATATCATACGGATTTATTTTGTGCTGAGTAGGTGGTGCTTCATAA
TTAAATCCTGGCATTACTGTACGATAGTGATTCGTATTTGGTGTTAAGTGTTTAAATCCTT
CAATTATTTTGCGATTTTCATCTACTAAAATTAAGTTACTATGTTTACCCATAATCTCAAG
GATGACAGTGCGGTAAATAGTATCGCCAATTTCATCTTTACTCTTTATATCGATTTCAATG
CGACGATCATTACCAATTTGCTTAATCGATTCGATAATACCACCTTCTAAGTGTTTTCTAA
AAACACGCGCAAACATGGGTGGATTAAATGGATTATCATATTTTTTAGTAGTTAATTGTA
ATCTTGAAAGGTTTGGATGGATTGACAATAACAATTGATGGTTTTGTCTATTTTGACGTAC
AACCATTAGTATCGTGTCATTATCAGGTTGATTGATTTTGTGAACGCGTCCTGTTGTTAAA
AATTGTAGAGACTCAACCATTTTCTTTGTAAATAAGCCATCATAAGCCAT

## > gmk

Function: protein coding sequence; guanylate kinase (core genome, constant)
Best match: gmk_CC001_MW2-USA400_BA000033.2[1190834:1191457] (completely identical)
Position: 030-contig_240: 15544 … 16168; Length: 624 bp
Sequence:
ATGGATAATGAAAAAGGATTGTTAATCGTTTTATCAGGACCATCTGGAGTAGGTAAAGGT
ACTGTTAGAAAACGAATATTTGAAGATCCAAGTACATCATATAAGTATTCTATTTCAATG
ACAACACGTCAAATGCGTGAAGGTGAAGTTGATGGCGTAGATTACTTTTTTAAAACTAGG
GATGCGTTTGAAGCTTTAATCAAAGATGACCAATTTATAGAATATGCTGAATATGTAGGC
AACTATTATGGTACACCAGTTCAATATGTTAAAGATACAATGGACGAAGGTCATGATGTA
TTTTTAGAAATTGAAGTAGAAGGTGCAAAGCAAGTTAGAAAGAAATTTCCAGATGCGCTA
TTTATTTTCTTAGCACCTCCAAGTTTAGAACACTTGAGAGAGCGATTAGTAGGTAGAGGA

ACAGAATCTGATGAGAAAATACAAAGTCGTATTAACGAAGCGCGTAAAGAAGTTGAAAT
GATGAATTTATACGATTACGTTGTAGTTAATGATGAAGTAGAACTTGCGAAGAATAGAAT
TCAATGTATTGTAGAAGCTGAGCACTTAAAAAGAGAGCGCGTAGAAGCTAAGTATAGAA
AAATGATTTTGGAGGCTAAAAAATAA

> **rpoZ**
Function: protein coding sequence; DNA-directed RNA polymerase omega subunit (core genome, constant)
Best match: rpoZ_CC030_MRSA252_BX571856.11233626:1233844 [4_50ANF10END]
Position: 030-contig_240: 16167 … 16386; Length: 219 bp
Sequence:
ATGTTAAATCCACCATTAAACCAATTAACGTCACAAATTAAATCAAAGTATTTAATTGCA
ACAACTGCAGCGAAAAGAGCGCGTGAAATTGATGAACAACCTGAAACTGAATTATTAAG
TGAATATCATTCATTTAAACCAGTTGGTAGAGCGTTAGAAGAAATTGCTGACGGTAAAAT
TCGCCCTGTTATTTCAAGTGATTATTATGGTAAAGAATAG

> **coaBC**
Function: protein coding sequence; bifunct phosphopantothenoylcysteine decarboxylase/phosphopantothenate-cysteine ligase (core genome, constant)
Best match: coaBC_CC007_USA300_TCH959_AASB02000232.1[16672:17871]
Position: 030-contig_240: 16601 … 17801; Length: 1200 bp
Sequence:
ATGAAGAAAATATTATTAGCCGTTACAGGTGGCATTGCGGCATATAAAGCGATTGATTTG
ACAAGTAAGTTAACACAATCTGGGTATGAAGTTCGCGTTATGTTAACGAATCACGCACAA
AAATTTGTGACACCATTAGCATTTCAAGCAATAAGTCGAAATGCTGTTTATACAGATACT
TTTATAGAAGAAAATCCTTCAGAAATACAGCATATTGCATTAGGTGATTGGGCAGATGCA
ATCATTGTTGCACCTGCAACGGCAAATACAATTGCAAAATTGAGTGTAGGTATTGCTGAT
GATTTGGTGACATCAACGTTGCTAGCAACAGAGACACCGAAATTTATTGCGCCTGCTATG
AATGTGCATATGTATGAAAATAAACGTACGCAGCAAAATATTAATATTTTAAAAGAAGAT
GGGTATCATTTTATCGAACCAGGAAGCGGATTTCTAGCATGTGGTTATGTTGCTAAAGGA
CGTATGGAAGAACCGCTTCAAATCGTTTCTGTTATTGATGCTCATTTTCAAAATAGTAATC
GTTTAGCTAATAGTTCATTTCAAGATAAACGCGCATTGGTTACAGCAGGACCAACTATTG
AAGTTATCGATCCAGTCAGATTTGTATCCAATCGTTCTTCTGGAAAAATGGGCTATGCAAT
AGCTGAAGCATTGCGAAATCGAGGAGCTATCGTGACGTTAGTTGCTGGTCCTACAACACT
AGAGGATCCAAAAGATATTGAAGTTATTCATGTTCAAAGTGCTGAAGAAATGTTTGAACA
AGTGACAAGCCGATTTGACGAACAAGATATTGTTGTAAAAGCAGCAGCCGTATCTGACTA
TACACCAGTTGATGTATTAGAACATAAGATGAAAAAGCAGGATGGTGATTTATCAGTATC
TTTTAAACGTACTAAAGACATTCTTAAATATTTAGGTGAACATAAAACATCACAGTATTT
AATAGGCTTTGCAGCAGAGACTGAAGATATTGAAAATTATGCACAACAAAAATTACGCA
AGAAAAATGCAGATGTGATTATTTCAAATAATGTTGGGGATATGTCTATCGGATTTAGTT
CTGATGATAATGAATTGACAATGCATTTTAAAAATAATGAAAAGGTAAATATCAAGAAA
GGAAAAAAAGTAGTATTAGCTGCACAAATTTTAGATGAACTAGAAACTAGGTGGCAATA
A

> **tnpIS431-06**
Function: protein coding sequence; transposase for IS431 (mobile element)
Best match: tnpIS431-06_circ_S-epidermidis_GQ900381.1[43799:43807]RC (completely identical)
Position: 030-contig_240: 17151 … 17160; Length: 9 bp
Sequence:
ATAGTTCAT

> **priA**
Function: protein coding sequence; primosomal protein N' (core genome, constant)
Best match: priA_CC001_MW2-USA400_BA000033.2[1193090:1195498]
Position: 030-contig_240: 17800 … 20209; Length: 2409 bp

Sequence:
ATGATAGCGAAAGTCATAGTCGATGTCGCGTCGAAGAGCGTTGACTATAAATTTGATTAT
ATAATTCCTGAACAACTCGAATCTGTCATCCAACCTGGTGTGCGTGTGATTGTACCTTTTG
GACCAAGAACGATTCAAGGTTATGTAATGGAAGTAACAGCAGAACCTGATGCACAACTT
GACGTTTCGAAGTTAAAAAAAATCATAGAAGTGAAAGATATACAACCTGAATTAACATC
AGAATTAATAGCTTTAAGTGAGTGGATGGGTTCAACTCATGTCATTAAACGTATTTCTATG
CTAGAAGTGATGCTTCCGAGTGCTATTAAAGCGAAGTATAAAAAAGCATTTAAGATGAA
AGATGACAAAGAGGTACCTTCAGCTTTATTACAAAAATTTGATAAGCATGGTTACTATTA
TTATAAAGATGCGCAAAAAAATAATGATATTCAATTGCTTATGAAGTTGTTAAAAGATGA
TATCGTTGAAGAAAAAACGATTCTCACACAAAATATAACTAAAAAAACCAAGCGTGCTG
TTCGTGTCATTGAAGGGTATCATCCTGATGAAGTATTAGCTAAGTTGGAGAAAGTTATTA
AACAATACGATTTGTATGCTTACTTGTCTGAAGAACAACATAAAACAATATTTTTAACTG
ATATTGAGGATATGGGCTTTTCAAAATCCAGTTTAGATGGACTTATCAAAAAAGGTTATG
TTGAAAAATATGACGCGGTTGTTGAAAGAGACCCATTTAAAGATCGTGTTTTCGAACAAG
AATCAAAACAGCAATTAACAGAAGACCAATATAAAGCATATGAAGCGATTAAAGCTAAA
ATTGTAAGCCAAGAGCAAGAAACATTTTTACTTCATGGTGTGACGGGATCAGGTAAAACA
GAAGTATATTTACAAACGATAGAAGATGTTTTAAGCCAAGGAAAACAGGCGATGATGTT
AGTTCCTGAAATCGCTCTAACACCGCAAATGGTTTTACGCTTCAAACGTCGATTTGGTGAT
GACGTTGCTGTATTACATTCTGGCTTATCTAATGGGGAACGTTATGATGAGTGGCAAAAA
ATTAGGGATGGTCGTGCGAGAGTAAGTGTTGGTGCAAGGTCAAGTGTGTTCGCACCTTTC
AAAAATTTAGGGTTAATCATCATTGATGAAGAACATGAATCTACATATAAGCAAGAAGAT
TATCCGAGATATCATGCTAGAGAAATTGCCCAATGGCGAAGTGAATATCATCACTGCCCA
GTCATTTTAGGAAGTGCAACACCATGTCTTGAAAGTTATGCACGAGCTGAAAAAGGCGTT
TATCATTTGCTATCATTACCAAACAGAGTGAACCAACAAGCTTTACCTGAAATTGATATA
GTAGATATGCGTGAAGAATTGAGTGAAGGTAATCGGTCAATGTTTTCAAAAGATTTACGT
GAAGCCATACAATTAAGATTAGATCGACAGGAACAAGTTGTTTTATTTTTAAATCGACGT
GGTTATGCATCGTTTATGTTATGTCGGGATTGTGGATATGTACCGCAATGTCCAAACTGTG
ATATTTCATTAACGTATCATAAAACGACAGACTTATTAAAATGTCACTATTGTGGTTACCA
AGAGACGCCACCGAATCAATGTCCAAATTGTGAGAGTGAACACATTCGACAAGTAGGTA
CTGGTACTCAGAAAGTTGAAGAACTATTGCAACAAGAATTTGAAGATGCGCGCATAATTA
GGATGGATGTAGATACAACCTCAAAGAAAGGTGCACATGAAAAGTTATTGACTGAATTC
GAAAAAGGTAACGGTGATATTTTACTAGGTACTCAGATGATTGCGAAAGGATTAGATTAT
CCAAATATTACTTTAGTTGGTGTGCTGAATGCAGATACAATGTTAAATTTACCTGATTTTC
GGGCGAGCGAACGTACTTATCAACTATTAACGCAAGTGGCTGGTAGAGCTGGTCGTCATG
AAAAGGCAGGTCAAGTCATCATTCAAACGTATAATCCGGATCATTATTCAATATTGGATG
TTCAAAAAAATGATTATTTAACATTTTATCGTCAGGAAATGGAATATCGTAAATTAGGAA
AGTATCCACCGTATTATTATTTGATTAATTTCACAATCTCACATAAAGAAATGAAGAAGG
TTATGGAAGCATCGCAGCATGTTCATAAAATTTTATTACAGCATTTAACAGAAAAAGCGC
TTGTACTAGGTCCATCTCCGGCAGCACTTGCGAGAATCAACAATGAATTTAGATTCCAAA
TTTTAGTGAAATATAAAAGTGAACCTGGATTATTACAAGCCATTCAGTTTTTAGATGACTA
TTACCATGAAAAATTTATAAAAGAAAAATTAGCATTGAAGATTGATATTGATCCACAGAT
GATGATGTAA

## > def2

Function: protein coding sequence; peptide deformylase homolog (core genome, constant)
Best match: def2_CC001_MW2-USA400_BA000033.2[1197619:1198107] (completely identical)
Position: 030-contig_240: 22330 … 22819; Length: 489 bp
Sequence:
ATGGCGATTAAAAAGTTAGTACCAGCATCGCATCCTATTTTAACGAAAAAAGCGCAAGCA
GTTATAAAATTTGATGATTCGTTAAAAAGATTATTACAAGATTTAGAAGATACAATGTAT
GCACAAGAAGCAGCTGGCTTATGTGCACCTCAAATTAATCAGTCATTGCAAGTGGCAATC
ATTGATATGGAAATGGAAGGATTATTACAACTTGTTAATCCGAAAATTATTAGTCAATCA
AATGAAACGATAACAGACTTAGAAGGTTCAATTACATTGCCAGATGTTTACGGCGAAGTG
ACAAGAAGTAAAATGATAGTTGTCGAAAGTTATGACGTCAATGGGAACAAAGTTGAACT
AACTGCACATGAAGATGTAGCAAGAATGATTTTGCATATTATAGATCAAATGAACGGTAT

CCCTTTTACAGAACGTGCGGACCGTATTTTAACAGATAAAGAAGTGGAGGCATATTTTAT
AAATGACTAA

> **fmt**

Function: protein coding sequence; methionyl-tRNA formyltransferase (core genome, constant)
Best match: fmt_CC001_MW2-USA400_BA000033.2[1198100:1199035] (completely identical)
Position: 030-contig_240: 22811 … 23747; Length: 936 bp
Sequence:
ATGACTAAAATAATATTTATGGGTACACCAGACTTTTCAACAACTGTTTTAGAAATGCTTA
TTGCAGAACATGATGTCATTGCAGTCGTAACGCAACCAGATCGACCTGTTGGACGTAAAC
GTGTTATGACACCACCACCAGTTAAAAAAGTTGCAATGAAATATGATTTACCTGTATATC
AGCCTGAAAAATTAAGTGGATCAGAAGAATTAGAACAATTGCTTCAATTAGATGTAGATT
TAATTGTAACTGCTGCTTTTGGACAATTATTACCTGAATCATTGTTGGCATTACCAAAACT
TGGGGCAATTAATGTACATGCATCATTGTTACCGAAGTATAGAGGTGGTGCACCAATTCA
TCAGGCAATTATCGATGGTGAACAAGAAACCGGCATAACAATTATGTATATGGTTAAAAA
ATTAGATGCGGGTAATATTATTTCGCAACAAGCAATTAAAATAGAAGAAAATGATAATGT
CGGTACGATGCATGATAAATTAAGTGTATTAGGGGCAGATTTATTAAAAGAAACTTTACC
ATCTATTATAGAGGGCACAAATGAAAGTGTACCTCAAGATGATACGCAAGCAACATTTGC
TTCCAATATTCGACGCGAAGATGAGCGAATTAACTGGAATAAACCAGGAAGACAAGTGT
TTAATCAAATTCGTGGATTATCACCATGGCCAGTTGCTTATACAACTATGGATGACACTA
ACTTGAAAATATACGATGCTGAACTCGTTGAGACTAATAAGATAAACGAGCCTGGAACC
ATTATAGAAACGACTAAAAAAGCCATTATTGTTGCTACAAATGATAATGAAGCTGTTGCA
ATTAAAGATATGCAATTAGCTGGGAAAAAGAGAATGTTAGCTGCCAATTATTTAAGTGGT
GCGCAAAACACACTAGTAGGGAAGAAACTTATATGA

> **sun**

Function: protein coding sequence; ribosomal RNA small subunit methyltransferase B (core genome, constant)
Best match: sun_CC007_TCH959-USA300_AASB02000232.1[23813:25120]
Position: 030-contig_240: 23743 … 25051; Length: 1308 bp
Sequence:
ATGATAGAAAACGTGAGAAGTCTTGCTTTTGACACGATTCAAGATATATTAAATGAAGGT
GCGTATAGTAACTTGCGTATTAATGAAGTGTTGTCAGAAAATGAATTAAATGCAATGGAT
AAAGCCTTATTTACAGAAATTGTCTACGGAACCGTTAAAAGAAAATATACGTTAGATTTT
TATTTAAAGCCTTTTGTGAAAACAAAGATTAAGGCATGGGTTAGGCAATTATTATGGATG
AGTATTTATCAATATGTTTATTTAGATAAAGTTCCAAATCATGCCATTATTAATGAAGCAG
TTGAAATAGCAAAAGAACGCGGTGGCTATCATAATGGTAATGTCGTAAATGGTATTTTAC
GTACAATGATGCGTAGTGACTTACCTGATTTTAATGAAATTGCAGATCCTAAAAAAAGAA
TGGCAATCGAATATAGTATGCCGAAGTGGATTATAGATCATTGGGCAACACATTATGGTC
TCGAAGAAACTGAAACAATTTTACAGTCATTTTTAGAAACGACATCAACAACTGTGCGTG
CCAACCTGACGCGAGCATCATTAGATGATATTATTGAAAAGTTGCAAGACGAAGGTTATG
ACGTTGAAAAAGATCATGACTTACCTTATTGTCTCCATATAGGTGGACAACCAATTATTC
ATTCTCGTTCATTTAAAGATGGATTCGTTTCAATTCAAGATAAAAGCTCAATGTTTGTTGC
ACACATTATGAATGTAGACCGACATGATCACGTATTAGATGCATGTAGTGCACCTGGCGG
TAAAGCTTGTCACATTGCTGAAGTTTTAATGCCAGAAGGGCAAGTTGACGCTTCAGATAT
ACATGATCACAAAATAGACTTAATTAATTTTAATATAAAAAAATTACGATTAACAAATAT
TAAAGCTTTTCAACATGATGCGACAAAACCTTATGATAAAACATACGATAAGATACTTGT
TGATGCACCATGTAGCGGATTAGGTGTAATGAGACATAAGCCGGAGATTAAGTATACTCA
AAGCAAACAACATATTGAGTCACTAGTTGAATTACAGCTTGAAATATTGGAAAATGTAAA
AAACAATGTAAAAATAGGTGGCGAAATCATCTATTCAACATGTACAATTGAGCAACTAG
AAAATGAAAACGTGATTTATACGTTTTTGAAAAATAATAAAAACTTCGAATTTGAACCGT
TTCAACATCCGATAACTGGAGAGTTGGTCAAAACGTTACAAATCATGCCGCAAGACTTTA
ATTCAGATGGATTCTTTATCACTAAGATAAAAAGAAAGGACAATTAG

> **rlmN**

Function: protein coding sequence; ribosomal RNA large subunit methyltransferase (core genome, constant)
Best match: rlmN_CC001-ST772_118_AJGE01000002.1[188356:189450:r] (completely identical)
Position: 030-contig_240: 25053 … 26148; Length: 1095 bp
Sequence:
ATGATAACTGCTGAAAAGAAAAAGAAGAATAAATTTCTTCCAAATTTTGACAAGCAATCA
ATATATTCATTGCGATTTGACGAAATGCAAAACTGGCTCGTTGAACAAGGTCAACAAAAA
TTTCGAGCGAAACAGATTTTTGAATGGTTATATCAAAAAAGAGTAGATTCGATTGATGAA
ATGACGAACTTATCGAAAGACTTACGACAGCTTTTAAAAGATAACTTTACTGTTACAACT
TTAACAACTGTAGTAAAACAAGAAAGTAAAGACGGTACAATTAAATTCTTATTTGAATTA
CAAGATGGCTATACAATTGAAACTGTTTTAATGAGACATGATTATGGAAATTCAGTATGT
GTAACGACACAAGTAGGTTGTCGTATCGGATGTACGTTTTGTGCTTCTACACTTGGCGGCT
TAAAAAGAAACCTTGAAGCTGGCGAAATTGTTTCACAAGTTTTAACAGTTCAAAAAGCCC
TTGATGCTACAGAAGAGCGCGTATCTCAAATTGTCATAATGGGTATCGGTGAACCATTTG
AAAATTATGATGAAATGATGGACTTTTTAAGAATCGTCAATGATGATAATAGTTTAAATA
TTGGTGCACGTCACATTACAGTATCAACATCAGGTATCATTCCTAGAATATACGACTTTGC
GGATGAAGATATCCAAATTAATTTTGCTGTAAGCTTACACGCCGCAAAAGATGAAGTGCG
ATCACGCTTGATGCCAATTAACCGTGCATATAATGTTGAGAAGTTAATCGAAGCAATTCA
ATATTATCAAGAAAAAACAAATCGTCGTGTTACTTTTGAATATGGTCTGTTTGGTGGTGTG
AATGACCAACTAGAACATGCAAGAGAATTAGCACATTTAATAAAAGGCTTAAACTGCCA
TGTTAACTTAATTCCTGTCAACCATGTTCCAGAAAGAAATTATGTGAAAACGGCTAAAAA
TGATATCTTTAAATTTGAAAAAGAATTAAAGAGACTAGGAATTAATGCCACAATACGTCG
TGAACAAGGTTCGGATATTGACGCAGCTTGTGGTCAATTAAGAGCAAAGGAACGACAAG
TAGAAACGAGGTAA

## > stp1

Function: protein coding sequence; serine/threonine protein phosphatase (core genome, constant)
Best match: stp1_CC001_MW2-USA400_BA000033.2[1201443:1202186] (completely identical)
Position: 030-contig_240: 26154 … 26898; Length: 744 bp
Sequence:
ATGCTAGAGGCACAATTTTTTACTGATACTGGACAACATAGAGATAAGAATGAAGATGCG
GGTGGTATTTTTTATAATCGAACTAATCAACAACTTTTAGTTCTGTGTGATGGTATGGGCG
GCCATAAAGCAGGAGAAGTTGCAAGTAAATTTGTTACAGATGAGTTGAAATCCCGTTTTG
AAGCGGAAAATCTTATAGAAGAACATCAAGCTGAAAATTGGTTGCGTAATAATATAAAA
GATATAAATTTTCAGTTATATCACTATGCACAAGAAAATGCAGAATATAAAGGTATGGGT
ACAACATGTGTTTGTGCACTTGTTTTTGAAAAATCAGTTGTGATAGCAAATGTCGGTGATT
CTAGAGCCTATGTTATTAATAGTAGACAAATTGAACAAATTACTAGTGATCACTCATTTGT
TAATCATCTTGTTTTAACGGGTCAAATTACGCCGGAAGAAGCATTTACACATCCACAACG
TAATATTATTACGAAGGTGATGGGCACAGATAAACGTGTGAGTCCAGATTTGTTTATTAA
GCGATTAAATTTTTATGATTATTTATTATTAAATTCAGATGGATTAACTGATTATGTTAAA
GACAATGAAATTAAGCGTTTGTTAGTAAAAGAAGGTACAATAGAAGATCATGGTGATCA
ATTAATGCAATTGGCATTAGATAACCATTCGAAAGATAACGTTACTTTCATACTCGCGGC
TATTGAAGGTGATAAAGTATGA

## > pknB

Function: protein coding sequence; serine/threonine-protein kinase (core genome, constant)
Best match: pknB_Y18639.1[1:48:r] (completely identical)
Position: 030-contig_240: 26894 … 26942; Length: 48 bp
Sequence:
ATGATAGGTAAAATAATAAATGAACGATATAAAATTGTAGATAAGCTT

## > engC

Function: protein coding sequence; ribosome small subunit-dependent GTPase A (core genome, constant)
Best match: engC_CC001_MW2-USA400_BA000033.2[1204405:1205280]

Position: 030-contig_240: 29116 … 29992; Length: 876 bp
Sequence:
TTGAAGACAGGTCGAATAGTGAAATCAATTAGTGGGGTATATCAAGTAGACGTTAATGGC
GAACGTTTCAATACAAAACCACGAGGATTATTTAGAAAGAAAAAATTTTCACCGGTAGTT
GGTGATATAGTGGAATTTGAAGTACAAAACATTAACGAAGGCTATATTCATCAAGTGTTT
GAGCGGAAAAATGAGTTGAAAAGACCACCTGTAAGTAATATAGATACACTAGTAATTGT
AATGAGTGCTGTCGAGCCAAATTTTTCAACGCAATTATTAGATCGATTTTTAGTTATTGCA
CATTCGTATCAGTTAAATGCGAGAGTTTTGGTGACTAAAAAAGATAAAACACCAATTGAA
AAGCAGTTCGAAATAAATGAGTTGTTGAAAATATATGAAAATATTGGCTATGAGACTGAA
TTTATTGGAAATGATGATGATCGAAAAAAAATTGTAGAAGCTTGGCCAGCTGGACTTATA
GTACTTAGTGGTCAATCAGGTGTCGGTAAGTCCACTTTCTTAAATCATTATCGTCCAGAAC
TTAATCTTGAGACAAATGATATATCAAAATCATTAAATCGAGGAAAGCATACTACAAGAC
ATGTCGAACTATTCGAACGTCAAAACGGTTATATTGCAGACACACCTGGATTCAGTGCTT
TAGATTTTGATCATATAGATAAAGATGAAATAAAAGATTATTTTCTTGAATTAAATCGAT
ATGGTGAAACATGTAAGTTTAGAAATTGTAATCATATCAAAGAACCTAATTGTAATGTTA
AGCATCAATTAGAGATAGGGAATATTGCGCAATTTAGATACGACCATTATTTACAACTAT
TTAATGAAATTTCAAATAGAAAGGTTAGATATTAA

> **rpe**
Function: protein coding sequence; ribulose-5-phosphate 3-epimerase (core genome, constant)
Best match: rpe_CC001-ST772_118_AJGE01000002.1[183867:184511:r] (completely identical)
Position: 030-contig_240: 29992 … 30637; Length: 645 bp
Sequence:
ATGACAAAACTATATCCATCATTATTATCTGTTGATTTTTTGGATTTACAACATGAATTAA
AACGACTTGAAGAAGCAGGTGTCGACGGAGTTCATTTTGATGTTATGGATGGTCAATTTG
TGCCTAATATATCTATTGGTTTACCAATATTAGATGCAGTAAGAAAAGGCACAACATTAC
CTATAGACGTACATTTGATGATTGAAAATCCAGAAAAGTATATTGCATCATTTGCAGAAC
ATGGTGCCGATATGATTTCAATTCATGTCGAATCAACGCCTCATATTCATCGTGCTATTCA
AATGATTAAACATTTAGATAAAAAAGCTGGTGTAGTAATTAATCCTGGTACACCAATATC
ACAAATTGAACCTATTTTAGACATTGTTGATTATGTACTAGTGATGACAGTTAACCCAGG
GTTTGGTGGTCAATCATTTATTGATCAATGCGTAGAAAAAATAGCGGGTCTTAATGCTATT
AAAATGGAACGTCAATTAAACTTTGATATTGAAGTTGATGGAGGCGTAAATACCGATACA
GCGAAAGTTTGTGTTGAAAATGGTGCTACAATGCTAGTAACAGGTTCATTTTTCTTTAAAC
AAGAGGATTATAAAAAAGTCACACAAGGATTGAAAGGTTGA

> **thiN**
Function: protein coding sequence; thiamine pyrophosphokinase (core genome, constant)
Best match: thiN_CC001-ST772_118_AJGE01000002.1[183219:183860:r] (completely identical)
Position: 030-contig_240: 30643 … 31285; Length: 642 bp
Sequence:
ATGCATATAAATTTATTATGTTCTGATCGACACTTGCCGCAAGATATTTGGGCCAAAAGT
AATGAAGGTAAATGGGGCGGCGTTGATAGAGGTGCTTTGATTTTATTGAAGCATCAAATT
ATCCCTTTTTTCTCAGTGGGAGACTTTGATTCAGTCAGTAAAGAAGAACGCCAACTTTTAA
CAGAACAGTTACAAATCAAACCAGTTCAAGCTGAAAAAGCTGATACGGATTTAGCTTTAG
CGGTTGATAAAGCTGTTGCACTTGGATTTGATAGTATTACAATTTATGGTGCAACAGGCG
GACGATTAGATCACTTTTTTGGGGCAATTCAGTTATTATTGAAAAAAGCATATTATAAAC
ATGATGTTCATATAGAAGTTATCGATCAACAAAATAAAATTGAATTATTGCCTAAAGGTC
AACATACAGTTGAAAAAGATAAGAGTTATCCGTACATTTCATTTATACCGATGACTGATG
ATGTAGAACTTTCTCTAGCAGGTTTTAAATATAATTTAGCTAGACAAATGCTTAATATAGG
TTCTACTTTAACTATTTCCAATGAAATTGAGTCTTTGCAAGCGAAAGTAACTGTACATGAT
GGGTTGATTTTGCAAATTAGAAGTACAGATTTAAATTAA

> **rpmB**
Function: protein coding sequence; 50S ribosomal protein L28 (core genome, constant)

Best match: rpmB_CC001_MW2-USA400_BA000033.2[1206954:1207142:r]RC (completely identical)
Position: 030-contig_240: 31665 … 31854; Length: 189 bp
Sequence:
TTAAACTCTAGTTACTTTACCAGATTTTAAAGCACGTGCAGAAACCCAAACTTTTTAGGT
TTACCGTCAACTAGGATTCTAACTTTTGAAGGTTAGCGTTCCATCTACGTTTAGTAGAGT
TTAAAGCGTGTGAACGTCTGTTACCAGTCGAAGCTTTACGACCTGTTACGAAACATTGTTT
ACCCAT

> **yloV**
Function: protein coding sequence; glycerone kinase (core genome, constant)
Best match: yloV_CC030_MRSA252_BX571856.1[1250143:1251789]
Position: 030-contig_240: 32685 … 34332; Length: 1647 bp
Sequence:
ATGATTAGCAAAATTAATGGTAAATTATTTGCCGATATGATTATACAAGGGGCACAAAAT
TTATCTAACAATGCAGATTTGGTAGATTCTTTGAATGTGTATCCAGTGCCAGATGGTGATA
CAGGAACAAATATGAATCTTACTATGACTTCAGGTCGCGAAGAAGTAGAGAATAATTTGT
CGAAAAATATCGGCGAATTAGGTAAAACATTCTCGAAAGGTTTACTAATGGGTGCAAGA
GGTAACTCTGGTGTCATCTTGTCACAATTATTCAGAGGATTTTGTAAAAATATTGAAAGTG
AATCTGAAATTAATTCAAAATTGTTAGCTGAAAGTTTTCAAGCTGGTGTTGAAACGGCAT
ATAAAGCTGTTATGAAACCAGTTGAAGGTACAATACTTACAGTTGCAAAAGATGCTGCGC
AAGCTGCAATAGAAAAAGCAAATAATACTGAAGATTGTATAGAATTAATGGAGTACATT
ATTGTAAAAGCCAATGAATCACTTGAAAACACACCAAACTTATTAGCTGTACTTAAAGAA
GTTGGTGTTGTTGATAGTGGCGGTAAAGGTTTGTTATGCGTTTACGAAGGATTCTTAAAA
GCGCTTAAAGGTGAAAAAGTTGAAGCCAAAGTTGCAAAGATAGATAAAGATGAATTTGT
ACATGATGAACATGATTTCCATGGTGTAATTAATACTGAAGATATTATTTATGGCTATTGT
ACTGAAATGATGGTTCGTTTTGGAAAGAATAAAAAAGCCTTTGATGAACAAGAATTCAGG
CAAGATATGAGTCAATTTGGTGATTCTTTATTAGTCATTAATGATGAAGAAATTGTGAAA
GTTCACGTGCATACCGAATACCCAGGTAAAGTGTTTAATTATGGTCAACAATATGGTGAA
TTAATTAAACTTAAGGTTGAAAATATGAGAGAACAGCATCGTGAAGTGATTCGAAAAGA
ACAGCACACAGCTAAACCGAAAATGGAAACGGTTGAAACAGCGATTATTACTATTTCTAT
GGGTGAAGGTATTTCAGAGATATTTAAATCAATGGGTGCCACACATATCATTAGTGGTGG
ACAAACGATGAATCCTTCTACAGAAGATATCGTTAAAGTCATTGAACAATCAAAATGTAA
ACGTGCAATTATTTTACCGAATAATAAAAATATCTTAATGGCAAGTGAACAAGCAGCGAG
TATTGTTGATGCAGAAGCAGTTGTTATTCCAACGAAATCTATTCCTCAAGGTATAAGCGC
ACTATTCCAATATGATGTGGACGCAACACTTGAAGAAAATAAAGCGCAAATGGCTGATTC
AGTAAATAACGTTAAATCTGGTTCATTAACGTACGCTGTTCGTGATACGAAAATTGATGG
CGTTGAGATTAAAAAAGACGCGTTTATGGGCTTGATTGAAGATAAGATTGTAAGCAGCCA
AAGTGATCAATTAACAACGGTTACTGAGTTGTTAAATGAGATGTTAGCAGAAGATAGTGA
AATATTGACTGTGATTATTGGTCAAGATGCAGAGCAAGCAGTTACAGATAACATGATAAA
CTGGATTGAAGAGCAATATCCAGATGTAGAAGTGGAAGTTCATGAAGGTGGACAACCAA
TTTATCAATATTTCTTTTCAGTAGAATAA

> **recG**
Function: protein coding sequence; ATP-dependent DNA helicase (core genome, constant)
Best match: recG_CC001-ST772_118_AJGE01000002.1[177922:179982:r] (completely identical)
Position: 030-contig_240: 34521 … 36582; Length: 2061 bp
Sequence:
TTGGCTAAAGTAAACTTAATAGAAAGTCCATATTCTCTTTTACAATTAAAAGGTATAGGT
CCTAAGAAAATAGAAGTATTGCAACAACTAAATATTCATACAGTGGAAGATCTTGTTCTT
TATTTGCCAACTAGATATGAAGATAATACAGTGATTGATTTGAATCAAGCAGAAGATCAA
TCTAACGTTACGATAGAAGGACAAGTATATACAGCTCCAGTAGTTGCATTTTTTGGAAGA
AATAAATCAAAATTAACCGTTCATTTAATGGTAAATAATATTGCTGTCAAATGTATTTTTT
TCAATCAACCGTATTTAAAAAAGAAAATCGAATTAAATCAAACTATAACTGTTAAAGGTA
AGTGGAATAGGGTTAAACAGGAAATTACTGGTAATAGGGTTTTCTTTAATTCACAAGGGA

CACAAACTCAAGAAAACGCAGATGTTCAATTAGAACCAGTCTATCGTATTAAGGAAGGT
ATTAAACAAAAGCAAATACGAGACCAAATTAGACAAGCGTTAAATGATGTGACAATTCA
TGAATGGTTAACTGATGAACTAAGAGAAAAATATAAATTAGAGACCTTGGACTTTACTTT
GAACACATTACATCATCCTAAAAGTAAAGAGGATTTATTACGTGCTCGTAGAACCTATGC
ATTTACTGAACTGTTTTTATTCGAATTACGTATGCAATGGCTAAATAGATTAGAAAAGTCA
TCTGACGAAGCAATTGAAATTGATTATGACATAGACCAAGTTAAATCATTTATTGATCGT
TTACCTTTTGAACTAACTGAAGCACAGAAATCCAGTGTTAATGAAATTTTTAGAGATTTA
AAAGCACCAATACGTATGCATCGATTACTTCAAGGTGATGTAGGTTCAGGAAAAACAGTA
GTTGCTGCAATTTGTATGTATGCGTTAAAAACGGCTGGTTATCAATCAGCATTGATGGTAC
CAACTGAAATTTTAGCAGAGCAACATGCTGAAAGTTTAATGGCTTTATTTGGAGATTCTA
TGAACGTTGCATTGTTAACTGGGTCAGTAAAAGGTAAGAAACGAAAGATACTTTTAGAAC
AACTTGAAAATGGTACGATTGATTGTTTAATTGGAACCCATGCTTTGATTCAAGATGATGT
GATTTTCCATAATGTTGGTTTAGTAATTACAGATGAACAACATCGATTTGGTGTGAATCAA
CGCCAGCTTTTAAGAGAAAAAGGTGCAATGACGAATGTGTTATTTATGACAGCAACGCCG
ATACCAAGAACACTAGCAATATCAGTTTTTGGTGAGATGGATGTGTCTTCAATTAAACAA
TTACCAAAAGGTCGTAAACCTATCATTACTACTTGGGCAAAGCATGAGCAATACGATAAA
GTTTTGATGCAAATGACCTCAGAGTTGAAAAAAGGTCGTCAAGCATATGTCATTTGCCCG
CTTATAGAAAGTTCTGAGCATCTCGAAGATGTTCAAAATGTTGTCGCATTGTACGAGTCTT
TACAACAGTATTATGGTGTTTCCCGTGTAGGGTTATTGCATGGTAAGTTGTCTGCCGATGA
AAAAGATGAGGTCATGCAAAAGTTTAGCAATCATGAGATAGATGTTTTAGTTTCTACTAC
TGTTGTTGAAGTAGGTGTTAATGTACCGAATGCAACTTTTATGATGATTTATGATGCGGAT
CGCTTTGGATTATCAACTTTACATCAGTTACGCGGACGTGTGGGTAGAAGTGACCAGCAA
AGTTACTGTGTTTTAATTGCATCCCCTAAAACAGAAACAGGAATTGAAAGAATGACAATT
ATGACACAAACAACGGATGGATTTGAATTGAGTGAACGAGACTTAGAAATGCGTGGTCC
TGGCGATTTCTTTGGTGTTAAACAAAGTGGATTGCCAGATTTCTTAGTTGCCAATTTAGTT
GAAGATTATCGTATGTTAGAAGTTGCTCGTGATGAAGCAGCTGAACTTATTCAATCTGGC
GTATTCTTTGAAAATACGTATCAACATTTACGTCATTTTGTTGAAGAAAATTTATTACATC
GTAGTTTTGACTAA

## > **fapR**

Function: protein coding sequence; fatty acid and phospholipid biosynthesis transcript regulator (core genome, constant)
Best match: fapR_CC001-ST772_118_AJGE01000002.1[177147:177704:r] (completely identical)
Position: 030-contig_240: 36799 … 37357; Length: 558 bp
Sequence:
TTGAAACTAAAGAAAGATAAACGTAGAGAAGCAATCAGACAACAAATTGATAGCAATCC
CTTCATCACAGACCATGAACTAAGCGACTTATTTCAAGTGAGTATACAAACAATTCGTTT
AGATCGCACTTATTTAAACATACCAGAATTAAGGAAGCGTATTAAATTAGTTGCTGAAAA
GAATTATGACCAAATAAGTTCTATTGAAGAACAAGAATTTATTGGTGATTTGATTCAAGT
CAATCCAAATGTTAAAGCGCAATCAATTTTAGATATTACATCGGATTCTGTTTTTCATAAA
ACTGGAATTGCGCGTGGTCATGTGCTATTTGCTCAGGCAAATTCGTTATGTGTTGCGCTAA
TTAAGCAACCAACAGTTTTAACTCATGAGAGTAGCATTCAATTTATTGAAAAAGTAAAAT
TAAATGATACGGTAAGAGCAGAAGCACGAGTTGTAAATCAAACTGCAAAACATTATTAC
GTCGAAGTAAAGTCATATGTTAAACATACATTAGTTTTCAAAGGAAATTTTAAAATGTTTT
ATGATAAGCGAGGATAA

## > **plsX**

Function: protein coding sequence; glycerol-3-phosphate acyltransferase (core genome, constant)
Best match: plsX_CC008_NCTC8325_CP000253.1[1148670:1149656] (completely identical)
Position: 030-contig_240: 37361 … 38348; Length: 987 bp
Sequence:
ATGGTTAAATTAGCAATTGATATGATGGGTGGCGACAATGCGCCTGATATCGTATTAGAA
GCCGTACAAAAGGCTGTTGAAGACTTTAAAGATCTAGAAATTATACTTTTCGGTGACGAA
AAAAAGTATAATCTGAACCATGAACGAATCGAATTTAGACATTGTTCTGAAAAGATTGAA
ATGGAAGATGAGCCTGTTAGAGCGATTAAACGTAAAAAAGATAGCTCAATGGTAAAAAT

GGCTGAAGCTGTGAAATCTGGTGAAGCAGATGGATGTGTGTCAGCAGGTAATACTGGTGC
TTTAATGTCAGCTGGTTTATTCATTGTTGGACGTATTAAAGGTGTAGCTAGACCGGCTTTA
GTAGTAACATTGCCAACGATTGATGGAAAAGGTTTTGTCTTTTTAGACGTTGGTGCAAAT
GCTGATGCTAAACCTGAACACTTATTACAGTATGCGCAACTAGGGGATATTTATGCTCAA
AAAATTAGAGGTATTGATAATCCGAAAATCTCATTATTAAATATAGGAACCGAGCCAGCT
AAAGGTAATAGTTTAACGAAAAAATCATATGAGTTATTAAATCATGATCATTCATTGAAT
TTTGTTGGGAATATTGAAGCGAAGACATTAATGGATGGCGATACAGATGTTGTAGTTACC
GATGGCTATACTGGGAACATGGTCCTTAAAAATTTAGAAGGTACTGCAAAATCAATCGGT
AAAATGTTAAAAGATACGATTATGAGTAGTACTAAAAATAAATTAGCAGGTGCAATATTG
AAGAAAGATTTAGCTGAATTCGCTAAAAAGATGGATTACTCAGAATACGGTGGTTCCGTA
TTATTAGGATTGGAAGGTACTGTAGTTAAAGCACACGGTAGTTCAAATGCTAAAGCTTTT
TATTCTGCAATTAGACAAGCGAAAATCGCAGGAGAACAAAATATTGTACAAACAATGAA
AGAGACTGTAGGTGAATCAAATGAGTAA

## > fabD

Function: protein coding sequence; malonyl CoA-acyl carrier protein transacylase (core genome, constant)
Best match: fabD_CC008-ST72_TCH130_ACHD01000019.1[135693:136619:r] (completely identical)
Position: 030-contig_240: 38340 … 39267; Length: 927 bp
Sequence:
ATGAGTAAAACAGCAATTATTTTTCCGGGACAAGGTGCCCAAAAAGTTGGTATGGCACAA
GATTTGTTTAATAACAATGATCAAGCAACTGAAATTTTAACTTCAGCAGCAAAGACGTTA
GACTTTGATATTTTAGAGACAATGTTTACTGATGAAGAAGGTAAATTGGGTGAAACTGAA
AACACGCAACCAGCTTTATTGACGCATAGTTCGGCATTATTAGCAGCGCTAAAAAATTTG
AATCCTGATTTTACTATGGGGCATAGTTTAGGTGAATATTCAAGTTTAGTTGCAGCTGACG
TATTATCATTTGAAGATGCAGTTAAAATTGTTAGAAAACGTGGTCAATTAATGGCGCAAG
CATTTCCTACTGGTGTAGGAAGCATGGCTGCAGTATTGGGCTTAGATTTTGATAAAGTCG
ATGAAATTTGTAAGTCATTATCATCTGATGACAAAATAATTGAACCAGCAAACATTAATT
GCCCAGGTCAAATTGTTGTTTCAGGTCACAAAGCTTTAATTGATGAGCTAGTAGAAAAAG
GTAAATCATTAGGTGCAAAACGTGTCATGCCTTTAGCAGTATCTGGTCCATTCCATTCATC
GCTAATGAAAGTGATTGAAGAAGATTTTTCAAGTTATATTAATCAATTTGAATGGCGTGA
TGCTAAGTTTCCTGTAGTTCAAAATGTAAATGCGCAAGGTGAAACTGACAAAGAAGTAAT
TAAATCTAATATGGTCAAGCAATTATATTCACCAGTACAATTCATTAACTCAACAGAATG
GCTAATAGACCAAGGTGTTGATCATTTTATTGAAATTGGTCCTGGAAAAGTTTTATCTGGC
TTAATTAAAAAAATAAATAGAGATGTTAAGTTAACATCAATTCAAACTTTAGAAGATGTG
AAAGGATGGAATGAAAATGACTAA

## > fabG

Function: protein coding sequence; beta-ketoacyl-ACP reductase (core genome, constant)
Best match: fabG_CC001_MW2-USA400_BA000033.2[1214548:1215282] (completely identical)
Position: 030-contig_240: 39259 … 39994; Length: 735 bp
Sequence:
ATGACTAAGAGTGCTTTAGTAACAGGTGCATCAAGAGGAATTGGACGTAGTATTGCGTTA
CAATTAGCAGAAGAAGGATATAATGTAGCAGTAAACTATGCAGGCAGCAAAGAGAAAGC
TGAAGCAGTAGTCGAAGAAATCAAAGCTAAAGGTGTTGACAGTTTTGCGATTCAAGCAA
ATGTTGCCGATGCTGATGAAGTTAAAGCAATGATTAAAGAAGTAGTTAGCCAATTTGGTT
CTTTAGATGTTTTAGTAAATAATGCAGGTATTACTCGCGATAATTTATTAATGCGTATGAA
AGAACAAGAGTGGGATGATGTTATTGACACAAACTTAAAAGGTGTATTTAACTGTATCCA
AAAAGCAACACCACAAATGTTAAGACAACGTAGTGGTGCTATCATCAATTTATCAAGTGT
TGTTGGAGCAGTAGGTAATCCGGGACAAGCAAACTATGTTGCAACAAAAGCAGGTGTTA
TTGGTTTAACTAAATCTGCGGCGCGTGAATTAGCATCTCGTGGTATCACTGTAAATGCAGT
TGCACCTGGTTTTATTGTTTCTGATATGACAGATGCTTTAAGTGATGAGCTTAAAGAACAA
ATGTTGACTCAAATTCCGTTAGCACGTTTTGGTCAAGACACAGATATTGCTAATACAGTA

GCGTTCTTAGCATCAGACAAAGCAAAATATATTACAGGTCAAACAATCCATGTAAATGGT
GGAATGTACATGTAA

> **acpP**
Function: protein coding sequence; acyl carrier protein (core genome, constant)
Best match: acpP_CC001_MW2-USA400_BA000033.2[1215716:1215949] (completely identical)
Position: 030-contig_240: 40427 … 40661; Length: 234 bp
Sequence:
GTGGAAAATTTCGATAAAGTAAAAGATATCATCGTTGACCGTTTAGGTGTAGACGCTGAT
AAAGTAACTGAAGATGCATCTTTCAAAGATGATTTAGGCGCTGACTCACTTGATATCGCT
GAATTAGTAATGGAATTAGAAGACGAGTTTGGTACTGAAATTCCTGATGAAGAAGCTGA
AAAAATCAACACTGTTGGTGATGCTGTTAAATTTATTAACAGTCTTGAAAAATAA

> **rnc**
Function: protein coding sequence; ribonuclease 3 (core genome, constant)
Best match: rnc_CC001_MW2-USA400_BA000033.2[1216065:1216796] (completely identical)
Position: 030-contig_240: 40776 … 41508; Length: 732 bp
Sequence:
ATGTCTAAACAAAAGAAAAGTGAGATAGTTAATCGTTTTAGAAAGCGCTTTGATACTAAA
ATGACAGAGTTAGGCTTTACTTATCAAAATATTGATTTATACCAACAAGCATTTTCGCATT
CGAGTTTTATTAATGATTTTAATATGAATCGTTTAGACCATAATGAGCGTTTAGAGTTTTT
GGGTGATGCGGTATTAGAATTGACGGTTTCACGATATTTATTTGATAAACATCCCAACTTG
CCAGAAGGGAATTTAACAAAAATGCGTGCCACTATTGTATGTGAGCCCTCACTTGTAATA
TTTGCGAATAAAATTGGATTGAACGAAATGATTTTACTTGGTAAAGGTGAAGAGAAAACA
GGGGGACGTACAAGACCATCATTAATATCAGATGCATTCGAAGCATTTATTGGGGCATTG
TATTTGGATCAAGGACTAGATATAGTTTGGAAATTTGCTGAGAAAGTCATTTTCCCACAT
GTAGAACAAAATGAGTTATTAGGCGTGGTAGATTTTAAAACACAATTCCAAGAATATGTG
CACCAGCAAAATAAAGGTGATGTAACCTATAATTTAATAAAAGAAGAGGGACCGGCACA
TCATCGTCTATTCACTTCAGAAGTTATTCTGCAAGGGGAAGCAATAGCTGAAGGTAAAGG
GAAAACGAAAAAAGAATCAGAACAACGTGCTGCTGAAAGTGCCTATAAGCAATTAAAAC
AAATTAAATAG

> **smc**
Function: protein coding sequence; chromosome segregation SMC protein (core genome, constant)
Best match: smc_CC001_MW2-USA400_BA000033.2[1216943:1220509]
Position: 030-contig_240: 41654 … 45221; Length: 3567 bp
Sequence:
ATGGTTTATTTAAAATCAATAGATGCCATTGGATTTAAGTCTTTTGCAGATCAAACCAATG
TTCAATTCGATAAAGGTGTAACTGCAATTGTTGGTCCAAATGGAAGCGGTAAAAGTAATA
TTACAGATGCTATTAAATGGGTGTTGGGTGAACAATCGGCTAAATCATTACGTGGCTCAA
AAATGGAAGATATTATCTTCTCAGGTGCAGAACATCGCAAAGCTCAAAATTATGCTGAAG
TACAGTTAAGATTAGATAATCATTCTAAAAAGCTCAGTGTTGATGAAAACGAAGTTATTG
TAACAAGAAGATTGTATCGAAGTGGTGAAAGTGAGTACTACATAAATAATGACCGTGCA
AGATTAAAAGATATTGCCGATTTATTTTTAGATTCTGGATTGGGAAAAGAAGCGTATAGC
ATTATCTCGCAAGGTAGAGTTGATGAAATACTAAATGCTAAACCAATTGATAGACGTCAA
ATTATTGAAGAATCGGCTGGTGTACTTAAATATAAAAAACGTAAGGCTGAATCATTAAAT
AAACTTGACCAAACAGAAGATAATTTAACGAGAGTAGAAGACATTTTATATGATTTGGAA
GGTCGCGTAGAACCTCTAAAAGAGGAGGCAGCTATAGCTAAAGAATATAAGACACTTTC
ACATCAAATGAAACATAGTGACATTGTAGTTACAGTGCACGATATTGATCAATATACAAA
TGACAATAGACAATTAGATCAACGTTTAAATGATTTACAAGGTCAACAAGCAAATAAAG
AAGCTGACAAGCAACGTTTAAGCCAACAAATTCAACAATATAAAGGTAAACGACATCAA
CTTGATAACGATGTTGAATCGCTTAATTATCAATTAGTAAAAGCTACGGAAGCCTTTGAA
AAATATACGGGACAATTAAATGTTTTAGAAGAACGTAAGAAAAATCAATCTGAAACAAA
TGCACGATATGAAGAAGAACAAGAAAATTTAATGGAGCTTTTAGAAAATATATCAAATG
AGATTTCTGAAGCTCAAGATACTTATAAGTCTCTGAAAAGTAAACAAAAAGAACTCAATG

CTGTCATTCGTGAACTTGAAGAACAACTATATGTTTCAGACGAAGCACATGATGAAAAAT
TGGAAGAAATTAAAAACGAATACTATACATTAATGTCAGAGCAATCAGATGTTAACAAT
GATATTCGTTTTTAAAGCATACTATAGAAGAGAATGAGGCTAAAAAATCAAGACTAGAT
TCTCGATTAGTTGAAGTTTTTGAGCAATTGAAAGATATTCAGGGTCAAATAAAAACGACA
AAAAAAGAATATCAACAGACCAACAAAGAACTTTCTGCTGTAGATAAAGAAATTAAAAA
TATAGAAAAATATCTCACTGATACAAAAAAAGCACAAAATGAATACGAAGAGAAATTGT
ATCAAGCATATCGATATACCGAAAAAATGAAAACACGTATTGATAGTTTGGCAACGCAA
GAGGAAGAATATACTTATTTTTTCAATGGCGTCAAACATATTTTGAAAGCTAAAAATAAA
GAATTAAAGGGTATTCATGGTGCAGTTGCGGAAATTATTGATGTGCCATCTAAATTAACT
CAGGCAATTGAAACAGCATTAGGTGCTTCATTACAACATGTCATTGTAGATTCAGAAAAA
GATGGACGCCAGGCTATTCAATTTTTAAAAGAACGTAATTTAGGTCGTGCGACGTTTTTA
CCATTAAATGTTATACAGAGTAGAGTGGTAGCGACTGATATTAAATCTATTGCTAAAGAG
GCAAACGGATTTATTAGTATCGCTTCGGAAGCAGTTAAAGTAGCACCAGAATATCAAAAT
ATTATCGGGAATTTATTAGGTAATACGATTATCGTTGATCATTTAAAGCATGCAAATGAA
TTGGCACGTGCGATTAAATATCGAACTCGTATTGTTACTTTGGAAGGTGATATTGTAAATC
CTGGTGGTTCTATGACTGGTGGTGGCGCTCGTAAGTCCAAAAGTATTCTGTCTCAAAAAG
ACGAGTTGACAACAATGAGACACCAATTAGAAGATTACTTGCGTCAAACAGAATCATTTG
AACAACAATTTAAAGAGTTGAAGATAAAAAGTGATCAATTAAGTGAACTGTATTTTGAAA
AAAGTCAAAAGCATAATACACTTAAAGAGCAAGTGCATCATTTTGAAATGGAGCTCGAT
AGATTAACTACACAAGAAACACAAATAAAAAATGATCATGAAGAATTCGAATTTGAAAA
AAATGATGGTTATACGAGTGACAAAAGTCGACAAACTTTGAGTGAAAAAGAAACTCATT
TAGAAAGTATTAAAGCATCTTTAAAACGACTAGAAGATGAAATTGAACGCTACACAAAA
CTTTCTAAAGAAGGTAAGGAAAGCGTAACTAAAACACAACAAACGTTACATCAGAAACA
ATCTGATCTTGCTGTGGTTAAAGAGCGTATTAAAACACAACAACAGACAATAGATCGATT
AAATAATCAAAGTCAACAAACTAAACATCAATTAAAAGATGTTAAAGAAAAAAATTGCAT
TCTTTAATTCGGATGAAGTGATGGGCGAACAAGCTTTTCAAAATATTAAAGATCAAATTA
ATGGTCAACAAGAAACGAGAACACGCTTATCTGATGAATTAGATAAATTGAAACAACAA
CGTATTGAGTTGAATGAACAAATCGATGCGCAGGAAGCTACACTACAAGTTTGTCACCAA
GATATTTTAGCTATCGAAAATCACTACCAAGATATTAAAGCTGAACAATCAAAGTTAGAT
GTATTAATTCATCATGCGATAGATCATTTAAATGATGAATATCAATTGACTGTTGAACGTG
CGAAATCTGAATATACGAGTGATGAATCGATTGACGCATTACGTAAAAAAGTTAAGTTAA
TGAAGATGTCGATTGATGAACTAGGTCCTGTAAACTTAAATGCAATTGAACAATTTGAAG
AGTTAAATGAACGTTATACATTTTTAAGTGAACAACGTACAGATCTTCGTAAAGCTAAAG
AAACATTAGAGCAAATTATAAGTGAAATGGATCAAGAGGTTACTGAAAGATTTAAAGAA
ACTTTCCATGCTATTCAAGGACATTTTACAGCTGTGTTCAAACAATTGTTTGGTGGAGGCG
ATGCAGAATTGCAATTAACTGAAGCCGATTATTTAACAGCTGGTATTGATATTGTGGTAC
AACCACCGGGTAAAAAGTTGCAACATTTATCGTTACTGAGTGGTGGTGAGCGTGCATTAA
CTGCTATTGCTTTACTATTTGCAATTTTAAAAGTAAGATCTGCACCTTTTGTTATATTAGAT
GAGGTTGAAGCTGCACTAGATGAAGCAAATGTTATTAGATACGCAAAATATTTAAATGAG
TTATCAGACGAAACACAATTCATTGTTATTACACACCGTAAAGGAACAATGGAATTTGCG
GATAGGTTATACGGTGTAACAATGCAAGAATCAGGTGTTACTAAACTTGTGAGTGTGAAT
TTAAATACAATAGATGATGTGTTGAAGGAGGAGCAATAA

> **ftsY**

Function: protein coding sequence; signal recognition particle-docking protein (core genome, constant)
Best match: ftsY_CC001_MW2-USA400_BA000033.2[1220509:1221759] (completely identical)
Position: 030-contig_240: 45220 … 46471; Length: 1251 bp
Sequence:
ATGAGCTTTTTTAAACGCTTAAAAGATAAGTTTGCAACAAATAAAGAAAATGAAGAAGTT
AAATCCTTAACAGAAGAACAAGGTCAAGACAAATTAGAAGATACACATTCTGAAAGTTC
AACGCAGGACGCAAATGATTTAGCAGAAAATGCTGAAGTGAAAAAGAAGCCACGCAAGT
TGAGTGAAGCGGATTTTGATGACGATGGCTTAATATCAATTGAAGATTTTGAAGAAATTG
AAGCTCAAAAAATGGGTGCTAAATTTAAAGCAGGACTCGAAAAATCTCGTCAAAATTTCC
AAGAACAATTAAATAATTTGATAGCGAGATATCGTAAAGTAGATGAAGACTTTTTTGAAG
CTTTAGAAGAAATGTTAATCACTGCAGACGTCGGTTTTAATACAGTGATGACGTTAACTG

AAGAATTACGTATGGAAGCACAACGACGTAATATTCAAGATACTGAAGATTTGCGTGAA
GTCATTGTTGAAAAAATCGTAGAGATTTACCATCAAGAAGATGATAATTCAGAAGCTATG
AACTTAGAAGATGGTCGTTTAAATGTCATTTTAATGGTTGGTGTGAATGGTGTTGGTAAA
ACAACAACAATTGGAAAATTAGCTTACCGATATAAAATGGAAGGTAAAAAAGTAATGTT
AGCTGCGGGCGATACTTTTAGAGCGGGTGCTATTGATCAATTGAAAGTTTGGGGCGAACG
TGTTGGTGTAGACGTAATTAGCCAAAGTGAAGGTTCTGATCCAGCTGCTGTTATGTATGA
TGCGATTAATGCCGCTAAAAACAAAGGTGTTGATATTTTAATCTGTGATACCGCTGGACG
TTTACAAAATAAAACAAATCTAATGCAAGAATTAGAAAAAGTTAAGCGTGTAATTAATCG
AGCAGTGCCAGATGCGCCTCATGAAGCATTACTATGTTTAGATGCTACAACTGGTCAGAA
TGCGTTGTCACAAGCTAGAAACTTTAAAGAAGTAACAAATGTTACAGGTATTGTATTAAC
GAAATTAGATGGTACAGCCAAAGGTGGTATCGTATTAGCCATTCGTAATGAATTGCACAT
TCCAGTTAAATATGTAGGTTTAGGTGAGCAATTAGATGACTTACAACCATTTAACCCTGA
AAGTTATGTCTACGGCTTATTCGCTGATATGATTGAACAAAATGAAGAAATAACAACAGT
TGAAAATGATCAAATTGTAACAGAAGAAAAGGACGATAATCATGGGTCAAAATGA

> **P67249**

Function: protein coding sequence; putative transcription factor (core genome, constant)
Best match: P67249_CC001_MW2-USA400_BA000033.2[1221746:1222078] (completely identical)
Position: 030-contig_240: 46457 … 46790; Length: 333 bp
Sequence:
ATGGGTCAAAATGATTTAGTTAAAACGTTACGAATGAATTATTTGTTTGATTTTTATCAAT
CCTTATTGACGAATAAACAACGTAATTATTTGGAATTATTTTATCTTGAAGATTATTCTTT
AAGTGAAATCGCAGATACTTTTAATGTGAGTAGACAAGCAGTTTATGATAATATAAGAAG
AACTGGCGATTTAGTTGAAGATTATGAAAAGAAATTGGAATTATACCAGAAATTTGAGCA
ACGCCGAGAAATATATGATGAAATGAAACAACATTTAAGTAATCCAGAACAAATACAAC
GTTATATTCAACAATTAGAAGACTTAGAATAG

> **ffh**

Function: protein coding sequence; signal recognition particle subunit ffh (core genome, constant)
Best match: ffh_CC001_MW2-USA400_BA000033.2[1222104:1223471] (completely identical)
Position: 030-contig_240: 46815 … 48183; Length: 1368 bp
Sequence:
ATGGCATTTGAAGGGTTATCAGAACGCTTGCAAGCGACGATGCAAAAAATGCGTGGTAA
GGGTAAACTTACTGAAGCTGATATAAAGATAATGATGCGTGAAGTAAGATTAGCGTTACT
TGAGGCTGACGTAAACTTTAAAGTGGTAAAAGAATTTATTAAAACAGTATCAGAACGCGC
ATTAGGTTCCGATGTAATGCAATCATTAACACCAGGGCAACAAGTTATTAAAATAGTTCA
AGATGAATTAACGCAGTTGATGGGTGGAGAAAATACGTCGATTAATATGTCAAATAAAC
CACCTACTGTTGTTATGATGGTTGGTTTACAAGGTGCTGGTAAAACAACAACTGCAGGTA
AATTAGCATTATTGATGCGTAAAAAATACAACAAAAAACCTATGTTAGTTGCAGCAGATA
TTTATCGTCCAGCAGCGATAAATCAATTACAAACAGTAGGGAAACAAATTGATATTCCTG
TATACAGTGAAGGAGATCAAGTAAAGCCACAACAAATTGTAACTAATGCATTAAAACAT
GCTAAAGAAGAACATTTAGACTTTGTAATCATTGATACAGCAGGTCGATTACACATCGAT
GAAGCATTGATGAACGAATTAAAAGAAGTAAAAGACATTGCTAAACCAAACGAAATTAT
GTTAGTTGTCGATTCAATGACGGGTCAAGATGCTGTCAATGTTGCAGAATCTTTTGACGAT
CAACTTGATGTCACAGGTGTTACCTTAACTAAATTAGATGGTGATACACGTGGTGGTGCA
GCTTTATCTATTCGTTCGGTGACACAAAAACCAATTAAATTTGTTGGTATGAGTGAAAAG
TTAGATGGTTTAGAGCTATTCCATCCTGAACGTATGGCATCACGTATTTTAGGTATGGGTG
ATGTGTTAAGTTTAATTGAAAAAGCGCAACAAGATGTGGATCAAGAAAAAGCAAAAGAT
TTAGAGAAAAAGATGCGTGAGTCATCGTTTACTTTAGATGATTTTTTAGAACAACTTGATC
AGGTGAAAAATCTAGGACCACTGGATGATATTATGAAAATGATTCCAGGTATGAATAAA
ATGAAAGGGCTAGATAAGCTTAATATGAGTGAAAAGCAAATTGATCATATTAAAGCGAT
TATCCAGTCAATGACGCCGGCTGAAAGAAACAATCCAGACACATTGAATGTATCACGTAA
AAAGCGTATTGCTAAAGGGTCTGGTCGTTCATTACAAGAAGTCAATCGTTTGATGAAACA
ATTTAACGATATGAAGAAAATGATGAAACAATTCACTGGTGGCGGTAAAGGTAAAAAAG
GTAAACGCAATCAAATGCAAAATATGTTAAAAGGTATGAATTTACCGTTTTAA

> **rpsP**

Function: protein coding sequence; 30S ribosomal protein S16 (core genome, constant)
Best match: rpsP_CC001_MW2-USA400_BA000033.2[1223906:1224181] (completely identical)
Position: 030-contig_240: 48617 … 48893; Length: 276 bp
Sequence:
ATGGCAGTTAAAATTCGTTTAACACGTTTAGGTTCAAAAAGAAATCCATTCTATCGTATC
GTAGTAGCAGATGCTCGTTCTCCACGTGACGGACGTATCATCGAACAAATCGGTACTTAT
AACCCAACGAGCGCTAATGCTCCAGAAATTAAAGTTGACGAAGCGTTAGCTTTAAAATGG
TTAAATGATGGTGCGAAACCAACTGATACAGTTCACAATATCTTATCAAAAGAAGGTATT
ATGAAAAAATTTGACGAACAAAAGAAAGCTAAGTAA

> **rimM**

Function: protein coding sequence; 16S rRNA processing protein (core genome, constant)
Best match: rimM_CC001_MW2-USA400_BA000033.2[1224369:1224872] (completely identical)
Position: 030-contig_240: 49080 … 49584; Length: 504 bp
Sequence:
ATGAGAGTTGAAGTTGGTCAAATTGTTAACACACATGGTATTAAAGGTGAAATTAAAGTA
AAATCCAATTCAGACTTTACAGACGTTCGTTTTCAACCCGGTCAAGTGCTGACAGTTGTGC
ATAACAATAACGACCTTGAATATACTGTTAAGTCACATAGAGTGCATAAAGGGCTTCATA
TGCTTACATTTGAAGGTATTAATAATATTAATGATATTGAGCATTTAAAAGGGAGTTCTAT
TTATCAAGAGCGTGATCATGAAGATATCGTACTTGAGGAAAATGAATTTTATTATTCAGA
TATTATAGGATGTACAGTTTTTGATGATCAAGAAACACCAATAGGTCGTGTAATTAATAT
ATTTGAAACAGGTGCGAATGATGTGTGGGTGATTAAAGGATCTAAAGAATATTTGATTCC
TTATATTGCTGATGTTGTAAAAGAAGTGGATGTTGAAAATAAAAAAATTATCATCACGCC
AATGGAAGGATTGTTGGATTAA

> **trmD**

Function: protein coding sequence; tRNA (guanine-N(1)-)-methyltransferase (core genome, constant)
Best match: trmD_CC001_MW2-USA400_BA000033.2[1224872:1225609] (completely identical)
Position: 030-contig_240: 49583 … 50321; Length: 738 bp
Sequence:
ATGAAAATTGATTATTTAACTTTATTTCCTGAAATGTTTGATGGTGTTTTAAATCATTCAA
TTATGAAACGTGCCCAAGAAAACAATAAATTACAAATCAATACGGTTAATTTTAGAGATT
ATGCAATTAACAAGCACAACCAAGTAGATGATTATCCGTATGGTGGCGGACAAGGTATG
GTGTTAAAGCCTGAACCTGTTTTTAATGCGATGGAAGACTTAGATGTCACAGAACAAACA
CGCGTTATTTTAATGTGTCCACAAGGCGAGCCATTTTCACATCAGAAAGCTGTTGAATTA
AGCAAGGCCGACCACATCGTTTTCATATGCGGACATTATGAAGGTTACGATGAACGTATC
CGAACACATCTTGTCACAGATGAAATATCAATGGGTGACTATGTTTTAACTGGTGGAGAA
TTGCCAGCGATGACCATGACTGATGCTATTGTTAGACTGATTCCAGGTGTTTTAGGTAATG
AACAGTCACATCAAGACGATTCATTTTCAGATGGGTTATTAGAGTTTCCGCAATATACAC
GTCCGCGTGAATTTAAGGGTCTAACAGTTCCAGATGTTTTATTGTCTGGAAATCATGCCAA
TATTGATGCATGGAGACATGAGCAAAAGTTGATCCGCACATATAATAAAAGACCTGACTT
AATTGAAAAATATCCATTAACTAATGCAGATAAGCAAATATTAGAAAGATATAAAATAG
GATTGAAAAAAGGTTAG

> **L19**

Function: noncoding RNA ribosomal protein L leader RNA (core genome, constant)
Best match: L19_leader_CC001_MW2-USA400_BA000033.2[1225650:1225692] (completely identical)
Position: 030-contig_240: 50361 … 50404; Length: 43 bp
Sequence:
ACTATGATCCGCTGCTATATATTTGTCGAGGCAAGAACATAGG

> **rplS**

Function: protein coding sequence; 50S ribosomal protein L19 (core genome, constant)
Best match: rplS_CC001_MW2-USA400_BA000033.2[1225712:1226062] (completely identical)
Position: 030-contig_240: 50423 … 50774; Length: 351 bp
Sequence:
ATGACAAATCACAAATTAATCGAAGCAGTAACTAAATCACAATTACGTACAGACTTACCT
AGTTTCCGTCCTGGTGATACTTTACGTGTACACGTACGTATCATTGAGGGTACTCGTGAGC
GTATCCAAGTATTCGAAGGCGTTGTAATTAAACGTCGTGGCGGTGGCGTTTCTGAAACGT
TTACAGTTCGTAAAATTTCATCAGGTGTTGGCGTGGAACGTACATTCCCATTACACACACC
AAAAATTGAAAAAATCGAAGTTAAACGTCGTGGTAAAGTACGTCGTGCTAAATTATATTA
CTTACGTAGTTTACGTGGTAAAGCTGCTAGAATCCAAGAAATTCGTTAA

## > tx_universal2
Function:  rho indendent terminator
Best match: tx_universal2_CC030_MRSA252_BX571856.1[2550413:2550453]
Position: 030-contig_240: 51013 … 51043; Length: 30 bp
Sequence:
CTGAACGAAAATACGCTTGTAACAAGCTTT

## > repeat_nySAgamma
Function:  repeat element
Best match: repeat_nySAgamma_CC705_RF122-cow_AJ938182.1[2700866:2700976]RC
Position: 031-contig_247: 6 … 36; Length: 30 bp
Sequence:
TGGCGGAAAGTCAGCTTACAATAATGTGCA

## > Q2FZB6
Function: protein coding sequence; putative protein (genomic island)
Best match: Q2FZB6_CC008_COL_CP000046.1[1182242:1182414]
Position: 031-contig_247: 12 … 42; Length: 30 bp
Sequence:
AAAGTCAGCTTACAATAATGTGCAAGTTGG

## > yfhO
Function: protein coding sequence; putative membrane protein (core genome, variable)
Best match: yfhO_CC001-ST772_118_AJGE01000002.1[160663:163269]RC (completely identical)
Position: 031-contig_247: 115 … 2722; Length: 2607 bp
Sequence:
TTATTTTTGTCTTGCCCATCTCGTGAAAATAATACTACATATAATGCCAAATATAGTAATT
GTTATAAGTAAATAATAGTATGGTGGGGCATAGCTCAATTGAATCTTTGTTATATTTTTAG
GTGCTTTAATGCCGGTCATAACACCATTTACTTGTTCAACTTTAAGACTTTGATCACCTGA
TGTCGCTTTCATACCTTGATTATATGCTGTTGGCAAAACAATATACCCAGATGAATTTTTA
TTTTTAGTAATAGTATAACCTTGCTTTGTCTTACTAACTTTGACAGCCTCTAATGAATTTGA
AGCGTCTTTAAGCGTGGTATAATCTTCGCCGTATATCCCTTTTAAATTTACACGATACTTA
CCTTTAGGCAATGATAATCTAATCCTATCTGAAGCTTTAATGCGTATTGTTACGGGTGTTA
CAAAGCGTCGATATTTATAAGTGAGTTTATTTCTTTCTTGTGTATATTCATTCACTTTAACA
TCATGAGCTTTATCCGGCGAAAGTAATTCTAAATCCATTTCAAAATACAAATCTTTAAATT
GATTAGAAACTGATTTTGGCAACTGTACAGTTAGACCACCATTATTTTGTTTAACTTGTAA
TAAATGTTTTGTAGGAGATTGCCAGGCTGCACTATTTAATTTAATTGTTGAATCTGATAGT
AAATTTTTATTGGCTTTAAAATGTGTATTAACATCTTTAGTCTTGTTAGAAACAATCCCTT
GCAACATTGCTTGTTCTTTATCTAATGGAGATTTTAATTCTTTATTGGAAAAGACCTTATTT
GTAATATGTGCACTTGGATAATGGATGGTATTTTTAGAATGAATCCAACGAACTTTATTGT
CTTTGTGTTCAGACTTAATTTTAAATCCATATGGTAAGTTGTCATCATGATTCACTCTAATT
CGATCATTAACATTCCAAAGTGATAGTAAATTTTGACGATTGCCAAGTAATCTATAAGTG
CTGTTTTTATCGATTGGCATATTAATTTGGAGTGTCTTGTCATAATATTTTAATATGTCTCC
ATTAAAAATACTAGAATATAATGAAATGCCATTATAATGATATATAAATGGTGAATTTAA

TGCATAGTCTGACATATAATCAATGCGATTAAATGGACCAGTTGCATTTTGATTTATCTTT
TTTATAAGCTGGTTTACATAGTTACTATGGTAATCGTGTTGTTTCAACGTTGATAATGATT
GTTGATAAGGTTTGATTGCCATATTTTTATTGTTATCTAAAATGACGATTTGTTGAATCAT
AACGATTAATACTAATATTGCAACGGTTAATTTTTTATAACGCCATAAACTAAATTTTAAA
ATAACGGCAAGCACCATTAGCAGGATAATACCTACTATAAGTGCAAGTGGGTGTGTCGGT
GATAGTAATACATAAAGTAATGCGATGATGCTTACTGGTATTGTTCTGATTAAATAATATT
TCATATTTAATGTTGATAAATGTTGAATAAACAATCCGCAAAGAGCGCTTGATGATAGTG
CTAAGATATACACCCAACGCCTTTCTGGAAATGAAAAACCATTAAAAGCACTGTCGAAAT
ACTGTGATAATGAACCAATAAATAATAACCATGTTACTATTGCGAAAAGTCTATAAAAGT
AAAAACGATACAGTTTGAATGACAATAATGCAACGATAGTAAGAATTGAAATCGTAATA
TAAAATCCATCGCTAAAGAAAAAATAATGGTAATCAAGTGGTGTCAAAAACGGTATATC
AACATTGGGATTTTGCTTTCTGTCATTTTCCAAAAACGCAGAAATGCCAGTGAATAAACC
AAATACACTTGATAACACACTCAAAACTGTAGCAGATATGACGCAAATTAATTTTTGTGT
TCTAGAGACAATGTCATATTTGTAAGTGAAAATGAGTCGATATAAATAGTAGCAACCTAT
AATAATAGCTTGATAATAACTGAAATAAAAATTGCTAAATAGTGTTAAGGCTATCGCAAC
AATGAAAATACCGATTTTGCGTTGTTGAAAATATCTTTCCAAACCAAGAATCGATAATGG
TAATAAATATAATAAACTTCCATAAAATGACCAAGTAAAATTAAAGTATATAACGACAGT
TGACATGCCGTATAAAATCGTAGCGATCATATTTGTTGAGCGTTTAAAGTGTAATATTTTA
AATAAGTAGAAGGTCACGACAAATGTTATGATAGCTCTTATCATGGCCATAATAAGTTGG
TTTGTCGGCCAAAAATGTATTGTTGTCGGATTAAATATACCAACCGTTTCTCCTATTTTAA
TGAATAGAAAATTTAGCCACATTAAAGGTGACAGCGAATAATAATATGATAGTCCTTTCA
TATAATCGCCACCTAATCCAAACGATGCATCATATAAACTAGAAAAACTACTTAAATGTT
CATACAAATACATTTGAAATGGCATCATTTGACGGAATCCATCTCCAGCCCCACTAAAAA
CAGTACCATTCACAATATAATCATAGATATGAGTAGAAAATAAAATAAGCGTTAATATTA
CACTAATGAAAGTTATAACAAAGAATTGTTTGACGTTTGAATTTAGCCACTTTTTTAACAC

## > rbgA

Function: protein coding sequence; putative GTP-binding protein (core genome, constant)
Best match: rbgA_CC001_MW2-USA400_BA000033.2[1229652:1230536] (completely identical)
Position: 031-contig_247: 3122 … 4007; Length: 885 bp
Sequence:
ATGGTTATTCAATGGTATCCAGGACATATGGCGAAAGCCAAAAGAGAAGTAAGTGAACA
ATTAAAAAAAGTAGATGTAGTGTTTGAACTAGTAGATGCAAGAATTCCATATAGTTCAAG
AAACCCTATGATAGATGAAGTTATTAACCAAAAACCACGTGTTGTTATATTAAATAAAAA
AGATATGTCTAATTTAAATGAGATGTCAAAATGGGAACAATTTTTTATTGATAAAGGATA
CTATCCTGTATCGGTGGATGCTAAGCACGGTAAAAATTTAAAGAAAGTGGAAGCTGCAGC
AATTAAAGCGACTGCTGAAAAATTTGAACGTGAAAAAGCAAAGGGACTTAAGCCAAGAG
CGATTAGAGCGATGATTGTTGGAATTCCAAATGTTGGTAAATCCACATTAATAAATAAAC
TGGCAAAGCGTAGTATTGCGCAGACTGGTAATAAACCAGGTGTGACCAAACAACAACAA
TGGATTAAAGTTGGTAATGCATTACAACTATTAGACACACCAGGGATACTTTGGCCTAAA
TTTGAAGATGAAGAAGTCGGTAAGAAGTTGAGTTTAACAGGCGCGATTAAAGATAGCAT
TGTGCACTTAGATGAAGTAGCTATATATGGATTGAACTTTTTAATTCAACATGATTTAGCG
CGATTAAAGTCACATTATAATATTGAAGTTCCTGAAGATGCAGAAATCATAGCGTGGTTT
GATGCGATAGGGAAAAAACGTGGCTTAATTCGACGTGGTAATGAAATTGATTACGAAGC
AGTCATTGAACTGATTATTTATGATATTCGAAATGCTAAAATAGGAAATTATTGTTTTGAT
ATTTTTAAAGATATGACTGAGGAATTAGCAAATGACGCTAACAATTAA

## > rnhB

Function: protein coding sequence; ribonuclease HII (core genome, constant)
Best match: rnhB_CC001_MW2-USA400_BA000033.2[1230520:1231287] (completely identical)
Position: 031-contig_247: 3990 … 4758; Length: 768 bp
Sequence:
ATGACGCTAACAATTAAAGAAGTTAAGCAGTTGATTAATGCGGTTAATACAATAGAAGA
ATTAGAAAATCATGAATGCTTTTTAGATGAGCGAAAAGGTGTTCAAAATGCCATAGCTAG
GCGCAGAAAAGCGTTAGAAAAAGAACAAGCTTTAAAAGAAAAGTATGTTGAAATGACTT

ACTTTGAAAATGAAATATTAAAAGAGCATCCTAATGCAATTATTTGTGGTATTGATGAAG
TTGGAAGAGGACCTTTAGCAGGTCCAGTCGTTGCATGCGCAACAATTTTAAATTCAAATC
ACAATTATTTGGGCCTTGATGACTCGAAAAAAGTACCTGTTACGAAACGTCTAGAATTAA
ATGAAGCACTAAAAAATGAAGTTACTGCTTTTGCATATGGTATCGCGACAGCTGAAGAAA
TAGATGAATTTAATATTTATAAAGCTACTCAAATCGCCATGCAGCGAGCTATTGATGGAT
TATCAGTACAACCAACGCATTTATTGATAGACGCGATGACGCTTGATAATGCACTGCCTC
AAGTATCTTTAATCAAGGGTGATGCAAGAAGTGTATCTATTGCAGCGGCAAGTATCATGG
CAAAGGTTTTTCGTGATGATTATATGACACAGTTATCTAAAGATTATCCTGAATATGGTTT
TGAAAAAAACGCGGGTTACGGTACCAAACAACATTTACTAGCAATCGATGATATTGGCAT
TATGAAAGAGCATAGAAAAAGCTTTGAACCTATAAAATCGTTACTGTAA

> **sucC**

Function: protein coding sequence; succinyl-CoA synthetase subunit beta (core genome, constant)
Best match: sucC_CC001_MSSA476_BX571857.1[1259891:1261057] (completely identical)
Position: 031-contig_247: 4866 … 6033; Length: 1167 bp
Sequence:
ATGAATATCCACGAGTATCAAGGTAAAGAAATATTTCGTTCAATGGGCGTTGCAGTTCCA
GAAGGACGAGTAGCATTTACTGCTGAAGAAGCGGTGGAGAAAGCAAAAGAATTAAATTC
TGATGTTTATGTTGTAAAAGCACAAATTCATGCTGGAGGTAGAGGTAAAGCAGGCGGAGT
AAAAATTGCTAAATCTTTATCTGAGGTAGAAACATATGCAAAAGAATTATTAGGGAAAAC
TTTGGTGACACATCAAACTGGTCCAGAAGGTAAAGAAATTAAGCGTTTATATATCGAAGA
AGGTTGTGCTATTCAAAAAGAATATTACGTTGGATTCGTTATTGATCGTGCGACTGACCA
AGTAACATTGATGGCGTCTGAAGAAGGGGGCACTGAGATTGAAGAAGTTGCTGCGAAGA
CTCCTGAAAAGATCTTCAAAGAAACTATCGATCCAGTAATCGGACTTTCACCATTCCAAG
CAAGACGAATTGCGTTTAATATTAATATTCCTAAAGAATCTGTTAACAAAGCAGCTAAAT
TCTTATTAGCACTTTATAATGTATTCATTGAAAAAGATTGTTCAATCGTAGAAATCAACCC
ATTAGTTACAACAGCTGATGGTGATGTATTGGCATTAGATGCTAAAATTAATTTTGATGAT
AATGCATTATTCAGACATAAAGATGTTGTAGAATTACGTGATTTAGAAGAAGAAGATCCG
AAAGAGATTGAAGCGTCTAAACATGATTTATCATACATTGCATTAGATGGTGACATCGGA
TGTATGGTTAATGGTGCAGGTTTAGCCATGGCAACAATGGATACGATTAATCATTTCGGT
GGAAACCCAGCCAATTTCTTAGATGCAGGCGGAAGCGCTACTAGAGAAAAAGTAACTGA
AGCATTTAAAATCATTTTAGGTGATGAAAATGTTAAAGGTATTTTTGTAAACATTTTCGGT
GGCATTATGAAATGTGATGTTATCGCAGAAGGTATCGTTGAAGCTGTAAAAGAAGTAGAT
TTAACTTTACCACTAGTTGTACGCTTAGAAGGTACAAATGTTGAGTTAGGTAAAAAAATC
TTAAAAGACTCAGGATTAGCAATTGAACCAGCAGCAACAATGGCTGAAGGTGCACAAAA
AATTGTTAAACTAGTCAAAGAAGCATAA

> **sucD**

Function: protein coding sequence; succinyl-CoA synthetase subunit alpha (core genome, constant)
Best match: sucD_CC001_MW2-USA400_BA000033.2[1232584:1233492] (completely identical)
Position: 031-contig_247: 6054 … 6963; Length: 909 bp
Sequence:
ATGAGTGTATTTATAGATAAGAATACTAAAGTAATGGTACAAGGTATTACAGGGTCTACT
GCCCTTTTCCATACAAAACAAATGCTTGATTATGGTACGAAAATAGTAGCAGGTGTGACG
CCTGGTAAAGGTGGTCAAGTTGTTGAAGGCGTTCCTGTTTTCAACACTGTTGAAGAAGCT
AAAAATGAAACCGGGGCAACGGTTTCAGTCATTTACGTTCCAGCACCATTTGCTGCAGAC
TCAATTTTAGAAGCAGCTGATGCAGACTTAGATATGGTTATTTGTATCACTGAACATATTC
CTGTATTAGACATGGTTAAAGTTAAACGCTACTTACAAGGTAGAAAAACACGTTTAGTTG
GTCCAAACTGTCCAGGTGTGATTACAGCAGATGAATGTAAAATTGGTATTATGCCTGGCT
ATATTCACAAAAAAGGTCATGTTGGTGTAGTATCTCGTTCAGGTACATTAACATATGAAG
CAGTGCACCAATTGACTGAAGAAGGTATTGGTCAAACTACAGCTGTTGGTATTGGTGGAG
ACCCAGTCAACGGAACAAACTTTATTGATGTTTTAAAAGCATTCAATGAAGATGACGAAA
CGAAAGCAGTTGTTATGATTGGTGAAATCGGTGGTACGGCTGAAGAAGAAGCAGCTGAA
TGGATTAAAGCGAATATGACAAAACCAGTTGTAGGCTTTATCGGTGGACAAACAGCACCT
CCTGGAAAACGTATGGGACATGCTGGTGCAATCATTTCAGGTGGTAAAGGTACTGCTGAA

GAGAAAATTAAAACATTAAATAGTTGTGGTGTGAAAACAGCGGCAACACCTTCAGAAAT
TGGTTCAACATTAATTGAAGCTGCTAAAGAAGCAGGTATTTATGAAGCATTATTAACTGT
TAATAAATAA

> **lytN**

Function: protein coding sequence; cell-wall hydrolase (genomic island)
Best match: lytN_CC001_MW2-USA400_BA000033.2[1233686:1234837] (completely identical)
Position: 031-contig_247: 7156 … 8308; Length: 1152 bp
Sequence:
ATGTTTGTATATTATTGTAAGGAGTGTTTCATCATGAATAAACAACAAAGTAAAGTACGC
TATTCAATTAGAAAAGTTAGTATTGGAATTTTGTCAATTTCAATAGGTATGTTTTTGGCAT
TGGGTATGTCGAACAAAGCATATGCAGATGAAATTGATAAATCTAAAGATTTTACAAGAG
GGTATGAGCAAAATGTATTCGCGAAATCAGAGTTAAATGCTAATAAAAATACGACAAAA
GACAAAATAAAAAATGAAGGTGCTGTTAAAACATCGGACACAAGTTTAAAGTTAGACAA
CAAATCAGCAATTTCAAACGGAAATGAAATTAATCAAGATATAAAGATTTCAAATACTCC
GAAAAACTCAAGCCAAGGTAACAATCTAGTTATTAATAACAATGAACCTACTAAAGAAA
TTAAAATTGCAAACTTGGAAGCTCAAAATTCTAATCAGAAGAAAACGAATAAAGTTACTA
ATAATTACTTTGGTTACTACAGTTTTAGAGAAGCTCCAAAAACACAAATCTATACTGTAA
AAAAAGGAGACACACTTAGTGCTATAGCATTAAAATACAAAACTACAGTTTCAAATATTC
AAAATACAAATAATATAGCAAATCCTAATTTAATATTTATTGGTCAAAAATTAAAAGTGC
CAATGACACCATTAGTAGAACCAAAACCAAAAACAGTGTCTTCAAATAATAAAAGTAAT
AGTAATAGCAGTACATTAAATTATTTGAAAACATTAGAGAATAGAGGATGGGATTTCGAC
GGTAGTTATGGATGGCAATGTTTCGATTTAGTTAATGTATATTGGAATCATCTTTATGGTC
ATGGATTAAAAGGATATGGAGCTAAAGATATACCATATGCAAATAATTTTAATAGTGAAG
CTAAAATTTATCACAACACACCAACTTTCAAAGCTGAACCTGGGGACTTAGTGGTTTTTA
GTGGAAGATTTGGTGGAGGATATGGTCATACAGCTATTGTCTTAAATGGTGATTATGATG
GAAAATTAATGAAGTTCCAAAGTTTAGATCAAAACTGGAATAATGGTGGATGGCGTAAA
GCAGAGGTTGCACATAAAGTTGTTCATAATTATGAAAATGATATGATTTTTATTAGACCA
TTTAAAAAAGCATAA

> **fmhC**

Function: protein coding sequence; endopeptidase resistance gene (genomic island)
Best match: fmhC_CC008_TCH1516_CP000730.1[1264248:1265492] (completely identical)
Position: 031-contig_247: 8335 … 9580; Length: 1245 bp
Sequence:
ATGAAATTTTCAACTTTAAGTGAAGAAGAATTTACCAACTACACCAAAAAGCACTTCAAA
CATTATACACAGTCTATAGAATTATATAATTATAGAAATAAAATAAATCATGAAGCACAT
ATTGTGGGAGTGAAGAATGATAAAAATGAAGTTATAGCTGCATGTTTATTAACAGAGGCA
CGAATTTTTAAATTCTACAAATATTTCTACTCTCATAGAGGTCCTTTACTTGATTATTTCGA
TGCTAAATTAGTTTGTTACTTTTTTAAAGAATTATCTAAATTCATTTATAAAAATAGAGGA
GTATTTATTCTTGTTGATCCATATTTAATAGAGAATTTAAGAGATGCAAATGGTAGGATA
ATAAAGAATTATAATAATTCAGTGATAGTAAAGATGCTAGGGAAAATTGGGTATCTCCAT
CAAGGTTATACAACAGGATATTCAAATAAAAGTCAAATTAGGTGGATTTCTGTATTGGAT
TTAAAAGATAAAGATGAGAATCAACTTTTAAAAGAAATGGAATACCAAACTAGAAGAAA
TATAAAAAAGACTATTGAGATTGGTGTTAAGGTTGAAGATTTATCTATTGAAGAAACAAA
TCGATTTTATAAATTGTTTCAAATGGCTGAAGAAAAACATGGTTTTCATTTCATGAATGAA
GATTATTTTAAACGAATGCAAGAAATATATAAAGATAAGGCAATGTTAAAGATAGCTTGT
ATAAATCTTAATGAATATCAAGATAAATTAAAAATACAATTATTGAAAATCGAAAATGAA
ATGATGACTGTGAACAGAGCATTAAATGAAAATCCAAATTCTAAAAAAAATAAATCAAA
ATTAAATCAGTTAAATATGCAATTATCTAGTATTAATAATAGAATTAGTAAAACCGAAGA
ACTAATATTTGAAGATGGACCTGTTTTGGATTTAGCTGCTGCTTTATTTATATGTACTGAT
GATGAAGTTTATTATCTATCAAGTGGATCAAATCCGAAATATAATCAGTATATGGGTGCA
TATCATCTACAATGGCATATGATAAAATATGCAAAATCACATAATATTAATAGGTATAAT
TTTTATGGAATAACAGGCGTCTTTAGTAATGAGGCGGATGATTTTGGTGTTCAACAATTTA

AAAAGGGTTTTAATGCACATGTTGAAGAATTAATTGGTGATTTCATCAAACCAGTAAGAC
CAATTCTATATAAATTTGCAAAACTTATTTATAAGGTTTAA

> **dprA**

Function: protein coding sequence; putative DNA processing protein/chain A (core genome, variable)
Best match: dprA_CC001_MW2-USA400_BA000033.2[1236282:1237154] (completely identical)
Position: 031-contig_247: 9752 … 10625; Length: 873 bp
Sequence:
TTGATTAAACTATTTTTGCTTAAGTTATACTGGGCACACTTTTCGACTAAACAAATTCATC
AATTTTTAATGGCATATCCTAATGTAATTAAAGAGGAGGGAAGAAAAAAAGATAGTTATT
TATGTGAATGGGTGAATAGGGAAGAAAATGTTCATTTATTACGTAAATACTATGCTTTTA
TAAAACTTGATCATAACGATATTATTAAAGAACTGCAGAAATTAAAAGTAAGTTACATTA
CATATATGGATTCTGAATACCCAGTGCTATTAAAAGAAATATATCAATTTCCATTACTTCT
TTTCTATAAAGGGAACATCAAATTAATAAATAATATGCATCATTTGGCAGTAGTAGGTGC
AAGAGATTCTACAAGTTATACCCAACAGTCTTTAGAATTTTTATTATCAAATGATAAAAG
CAAATATTTAACAATTGTTTCCGGCCTTGCTCAAGGAGCTGATGCAATGGCACATCAAAT
AGCTTTAAAATACAATCTCCCTACAATTGCAGTTTTAGCCTTTGGCCATCAAACACATTAT
CCCAAAAGTACATTAGCATTAAGAAATAAAATAGAAGAAAAAGGTTTAGTTATATCCGA
ATATCCACCACATACACCAATTGCTAAATATAGATTTCCTGAGCGCAATAGAATTATCAG
CGGTTTGTCAAAAGGGGTTTTAATTACTGAGGCTAAGGAACAAAGTGGCAGTCACATCAC
GATAGATTTTGCATTAGAGCAAAATAGAAATGTTTATGTTTTACCTGGATCTATGTTTAAT
CCTATGACAAAAGGTAATTTATTACGTATCCAAGAAGGTGCTAAGGTAGTATTAAACGCT
AATGATATATTTGAAGACTACTATATTTAA

> **topA**

Function: protein coding sequence; DNA topoisomerase 1 (core genome, constant)
Best match: topA_CC001_MW2-USA400_BA000033.2[1237334:1239403]
Position: 031-contig_247: 10804 … 12874; Length: 2070 bp
Sequence:
TTGGCAGATAATTTAGTCATTGTTGAATCGCCTGCAAAAGCAAAAACCATTGAAAAGTAT
TTAGGTAAGAAATATAAAGTTATAGCTTCAATGGGACACGTCAGAGACTTACCAAGAAGT
CAAATGGGTGTCGACACTGAAGATAATTACGAACCAAAATATATAACAATACGCGGAAA
AGGTTCTGTTGTAAAAGAATTGAAAAAACATGCAAAAAAAGCGAAAAACGTCTTTCTCG
CAAGTGACCCCGACCGTGAAGGTGAAGCAATTGCTTGGCATTTATCAAAAATTTTAGAGC
TTGAAGATTCTAAAGAAAATCGCGTTGTTTTCAACGAAATAACTAAAGACGCTGTTAAAG
AAAGTTTTAAAAATCCTAGAGAAATTGAAATGAACTTAGTCGATGCACAACAAGCGCGTC
GAATATTAGATAGATTGGTTGGCTATAACATCTCGCCAGTTCTATGGAAAAAAGTAAAAA
AAGGTTTGTCAGCGGGTCGAGTTCAATCTGTTGCACTTCGTTTAGTCATTGACCGTGAAAA
TGAAATTCGAAACTTTAAACCAGAAGAATATTGGACTATTGAAGGAGAATTTAGATACAA
AAAATCAAAATTCAATGCTAAATTCCTTCATTATAAAAATAAACCTTTTAAATTAAAAAC
GAAAAAAGATGTTGAGAAAATTACAGCTGCACTAGATGGAGATCAATTCGAAATTACAA
ACGTGACTAAAAAAGAAAAAACGCGTAATCCAGCAAACCCATTTACAACTTCTACATTAC
AACAAGAGGCGGCACGTAAATTAAACTTTAAAGCAAGAAAAACAATGATGGTCGCACAA
CAATTATATGAAGGTATAGATTTGAAAAAACAAGGTACGATTGGTTTAATAACATATATG
AGAACCGATTCTACACGTATTTCAGATACTGCCAAAGCTGAAGCAAAACAGTATATAACT
GATAAATACGGTGAATCTTACACTTCTAAACGTAAAGCATCAGGGAAACAAGGTGACCA
AGATGCCCATGAGGCTATTAGACCTTCAAGTACTATGCGTACGCCAGATGATATGAAGTC
ATTTTTGACGAAAGACCAATACCGATTATACAAATTAATTTGGGAACGATTTGTTGCTAG
TCAAATGGCTCCAGCAATACTTGATACAGTCTCATTAGACATAACACAAGGTGACATTAA
ATTTAGAGCGAATGGTCAAACAATCAAGTTTAAAGGATTTATGACACTTTATGTAGAAAC
TAAAGATGATAGTGATAGCGAAAAGGAAAATAAACTGCCTAAATTAGAGCAAGGTGATA
AAGTCACAGCAACTCAAATTGAACCAGCTCAACACTATACACAACCACCTCCAAGATATA
CTGAGGCGAGATTAGTAAAAACACTAGAAGAATTGAAAATTGGGCGACCATCAACTTAT
GCACCGACAATAGATACGATTCAAAAGCGTAACTATGTCAAATTAGAAAGTAAGCGTTTT
GTTCCTACTGAGTTGGGAGAAATAGTTCATGAACAAGTGAAAGAATACTTCCCAGAGATT

ATTGATGTGGAATTCACAGTGAATATGGAAACGTTACTTGATAAGATTGCAGAAGGCGAC
ATTACATGGAGAAAAGTAATCGACGGTTTCTTTAGTAGCTTTAAACAAGATGTTGAACGT
GCTGAAGAAGAGATGGAAAAGATTGAAATCAAAGATGAGCCAGCCGGTGAAGACTGTGA
AGTTTGTGGTTCTCCTATGGTTATAAAAATGGGACGCTATGGTAAGTTCATGGCTTGCTCA
AACTTCCCGGATTGTCGTAATACAAAAGCGATAGTTAAGTCTATTGGTGTTAAATGTCCA
AAATGTAATGATGGTGACGTCGTAGAAAGAAAATCTAAAAAGAATCGTGTCTTTTATGGA
TGTTCGAAATATCCTGAATGCGACTTTATCTCTTGGGATAAGCCGATTGGAAGAGATTGT
CCAAAATGTAACCAATATCTTGTTGAAAATAAAAAAGGCAAGACAACACAAGTAATATG
TTCAAATTGCGATTATAAAGAGGCAGCGCAGAAATAA

> **tnpIS431-06**

Function: protein coding sequence; transposase for IS431 (mobile element)
Best match: tnpIS431-06_circ_S-epidermidis_GQ900381.1[43799:43807]RC (completely identical)
Position: 031-contig_247: 12319 … 12328; Length: 9 bp
Sequence:
ATAGTTCAT

> **gid**

Function: protein coding sequence; tRNA uridine 5-carboxymethylaminomethyl modification enzyme (core genome, constant)
Best match: gid_CC001_MW2-USA400_BA000033.2[1239559:1240866] (completely identical)
Position: 031-contig_247: 13029 … 14337; Length: 1308 bp
Sequence:
ATGACTCAAACTGTAAATGTAATAGGTGCTGGTCTTGCCGGTTCAGAAGCGGCATATCAA
TTAGCTGAAAGAGGAATTAAAGTTAATCTAATAGAGATGAGACCTGTTAAACAAACACC
AGCGCACCATACTGATAAATTTGCGGAACTTGTATGTTCCAATTCATTACGAGGAAATGC
TTTAACTAATGGTGTGGGTGTTTTAAAAGAAGAAATGAGAAGATTGAATTCTATAATTAT
TGAAGCGGCTGATAAGGCACGAGTTCCAGCTGGTGGTGCATTAGCAGTTGATAGACACG
ATTTTTCAGGTTATATTACTGAAACACTTAAAAATCATGAAAATATCACAGTTATTAATGA
AGAAATTAATGCCATTCCAGATGGATACACAATTATCGCAACAGGACCACTTACTACAGA
AACCCTTGCGCAAGAAATAGTGGACATTACTGGTAAAGATCAACTTTATTTCTATGATGC
GGCTGCTCCAATTATTGAAAAAGAATCTATTGATATGGATAAAGTTTACTTAAAGTCCCG
TTATGATAAAGGTGAAGCTGCATATTTAAACTGTCCTATGACTGAGGATGAATTTAATCG
CTTTTATGATGCAGTATTAGAAGCTGAAGTTGCGCCTGTAAATTCATTTGAAAAAGAAAA
ATATTTCGAGGGTTGTATGCCTTTTGAAGTAATGGCAGAACGCGGACGCAAGACATTACT
ATTTGGACCAATGAAACCAGTAGGATTAGAAGATCCAAAGACTGGGAAACGTCCTTATG
CAGTGGTTCAATTAAGACAAGATGATGCTGCTGGCACACTCTACAATATTGTCGGCTTCC
AAACGCATTTAAAATGGGGAGCTCAAAAAGAAGTTATTAAATTAATTCCAGGTTTAGAAA
ATGTTGATATTGTTAGATATGGTGTGATGCATAGAAATACCTTCATTAACTCACCGGACGT
ATTAAACGAGAAATATGAATTGATTTCACAACCAAACATACAGTTTGCGGGACAAATGAC
TGGTGTTGAAGGTTATGTAGAAAGCGCAGCTAGCGGCTTAGTTGCAGGTATCAATCTTGC
GCATAAAATATTAGGCAAAGGTGAGGTAGTATTTCCTAGAGAAACGATGATTGGAAGTA
TGGCTTACTATATTTCTCATGCTAAAAATAATAAGAATTTCCAACCTATGAATGCTAACTT
CGGGTTATTACCATCTTTAGAAACTAGAATTAAAGATAAAAAAGAACGCTATGAAGCAC
AAGCTAATAGAGCTTTGGATTACTTAGAAAATTTCAAAAAAACTTTATAA

> **xerC**

Function: protein coding sequence; site-specific recombinase C (core genome, constant)
Best match: xerC_CC008_NCTC8325_CP000253.1[1176773:1177669] (completely identical)
Position: 031-contig_247: 14753 … 15650; Length: 897 bp
Sequence:
TTGAATCATATTCAAGATGCGTTTTTAAATACATTGAAAGTTGAACGGAATTTTTCGGAAC
ACACATTGAAATCATATCAAGATGACTTAATTCAGTTTAATCAATTTTTAGAACAAGAAC
ATTTAGAGTTGAATACTTTTGAATACAGAGATGCTAGAAATTATTTGAGCTATTTATATTC
AAATCATTTGAAAAGAACATCTGTTTCTCGTAAAATCTCAACGTTAAGAACTTTCTATGA

ATATTGGATGACGCTTGATGAGAACATTATTAATCCATTTGTTCAATTAGTACATCCGAAA
AAAGAAAAATATCTTCCGCAATTCTTTTACGAAGAAGAAATGGAAGCGTTATTCAAAACT
GTAGAAGAGGACACTTCAAAAAATTTACGGGATCGAGTTATTCTTGAATTGTTGTATGCT
ACAGGCATCCGTGTTTCGGAATTAGTAAATATTAAAAAACAAGATATAGATTTTTACGCG
AATGGTGTTACCGTATTAGGAAAAGGGAGCAAAGAGCGCTTTGTACCGTTTGGTGCTTAT
TGTAGACAAAGCATCGAAAATTATTTAGAACATTTCAAACCAATTCAGTCATGCAATCAT
GATTTTCTTATTGTAAATATGAAGGGTGAAGCAATCACTGAACGCGGTGTACGATATGTT
TTAAATGATATTGTTAAACGAACAGCAGGCGTAAGTGAGATTCATCCCCACAAGCTCAGA
CATACATTTGCAACGCATTTATTGAATCAAGGTGCAGACCTAAGAACAGTACAATCGTTA
TTAGGTCATGTTAATTTGTCAACAACTGGTAAATATACACACGTATCTAACCAACAATTA
AGAAAAGTGTATCTAAATGCACATCCTCGAGCGAAAAAGGAGAATGAAACATGA

> **hslV**

Function: protein coding sequence; heat shock protein/ATP-dependent protease (core genome, constant)
Best match: hslV_CC001_MW2-USA400_BA000033.2[1242176:1242721] (completely identical)
Position: 031-contig_247: 15646 … 16192; Length: 546 bp
Sequence:
ATGAGTAATACAACATTACATGCAACAACAATTTATGCTGTAAGACATAATGGGAAAGC
AGCTATGGCTGGAGATGGGCAAGTAACGCTTGGTCAACAAGTCATCATGAAACAAACGG
CAAGAAAAGTGCGACGTTTATATGAAGGTAAAGTGTTAGCTGGTTTCGCAGGTAGTGTAG
CAGATGCGTTTACGTTATTTGAAAAATTCGAAACAAAATTACAACAGTTTAGTGGTAACT
TAGAAAGAGCTGCTGTTGAATTGGCACAAGAATGGCGAGGCGATAAACAATTACGTCAA
TTAGAAGCTATGCTAATTGTAATGGATAAAGATGCTATTTTAGTTGTCAGTGGAACTGGC
GAAGTTATTGCCCCAGATGATGACCTTATCGCTATTGGATCAGGAGGCAACTACGCATTA
AGCGCAGGACGTGCATTGAAACGCCATGCATCGCATTTGTCTGCTGAAGAAATGGCATAT
GAGAGCTTGAAAGTAGCGGCTGATATTTGTGTCTTTACCAACGATAATATTGTTGTTGAA
ACACTATAA

> **hslU**

Function: protein coding sequence; heat shock protein/ATPase subunit (core genome, constant)
Best match: hslU_CC001_MW2-USA400_BA000033.2[1242787:1244190]
Position: 031-contig_247: 16257 … 17661; Length: 1404 bp
Sequence:
ATGGATACAGCTGGAATAAGATTAACTCCAAAAGAAATCGTATCTAAATTAAATGAATAC
ATCGTTGGACAAAATGATGCTAAACGTAAAGTGGCAATTGCCTTACGTAATCGATACAGA
AGAAGTTTATTAGATGAGGAATCAAAGCAAGAAATTTCACCTAAAAATATTTTGATGATT
GGACCAACTGGCGTTGGTAAAACTGAAATTGCAAGAAGAATGGCCAAAGTTGTCGGCGC
GCCATTTATAAAAGTAGAAGCTACTAAATTTACTGAGGTAGGTTATGTAGGACGAGATGT
TGAAAGTATGGTTAGAGATCTTGTTGATGTTTCAGTAAGATTAGTCAAGGCACAGAAAAA
ATCATTGGTACAAGATGAAGCAACAGCTAAGGCCAATGAAAAACTTGTTAAGTTATTAGT
TCCAAGTATGAAAAAGAAAGCGTCTCAAACGAATAATCCTTTAGAGTCACTTTTCGGAGG
TGCAATTCCAAATTTCGGACAAAATAACGAAGATGAAGAAGAACCACCTACTGAGGAAA
TTAAAACAAAACGTTCTGAAATTAAGAGACAGCTAGAAGAAGGCAAACTTGAAAAAGAA
AAGGTAAGAATTAAAGTCGAACAAGATCCTGGTGCTTTAGGTATGCTAGGTACAAATCAA
AATCAGCAAATGCAAGAGATGATGAATCAATTAATGCCTAAAAAGAAAGTTGAGCGAGA
AGTTGCTGTTGAGACGGCAAGGAAAATCTTAGCTGATAGTTATGCGGATGAACTAATTGA
TCAAGAAAGCGCTAACCAAGAAGCGCTTGAATTAGCAGAACAAATGGGTATCATCTTTAT
AGATGAAATCGACAAAGTTGCGACGAATAATCATAATAGTGGTCAAGATGTCTCAAGAC
AAGGTGTTCAAAGAGATATTTTACCTATACTTGAAGGTAGCGTTATTCAAACCAAATATG
GTACTGTGAATACTGAACATATGCTGTTTATAGGTGCTGGAGCTTTCCATGTATCTAAGCC
GAGTGACTTGATACCAGAATTGCAAGGTCGTTTTCCGATTAGAGTTGAACTTGATAGTTT
ATCGGTAGAAGATTTTGTAAGAATTTTGACAGAACCAAAATTGTCATTAATTAAACAATA
TGAAGCATTGCTTCAAACAGAAGAAGTTACTGTAAACTTTACCGATGAAGCAATTACTCG
CTTAGCTGAGATTGCTTATCAAGTAAATCAAGATACAGACAACATTGGTGCACGTCGACT

TCATACAATTTTAGAAAAGATGCTAGAAGATTTATCATTCGAAGCACCAAGTATGCCGAA
TGCAGTTGTAGATATTACCCCACAATATGTTGATGATAAATTAAAATCAATTTCAACAAA
TAAAGATTTAAGTGCATTTATTCTATAA

## > codY

Function: protein coding sequence; GTP-sensing transcript pleiotropic repressor (core genome, constant)
Best match: codY_CC008_NCTC8325_CP000253.1[1179705:1180478] (completely identical)
Position: 031-contig_247: 17685 … 18459; Length: 774 bp
Sequence:
ATGAGCTTATTATCTAAAACGAGAGAGTTAAACACGTTACTTCAAAAACACAAAGGTATT
GCGGTTGATTTTAAAGATGTAGCACAAACGATTAGTAGCGTAACTGTAACAAATGTATTT
ATTGTATCGCGTCGAGGTAAAATTTTAGGATCGAGTCTAAATGAATTATTAAAAAGTCAA
AGAATTATTCAAATGTTGGAAGAAAGACATATTCCAAGTGAATATACAGAACGATTAATG
GAAGTTAAACAAACAGAATCAAATATTGATATCGACAATGTATTAACAGTATTCCCACCT
GAAAACAGAGAATTATTCATAGATAGTCGTACAACTATCTTCCCAATTTTAGGTGGAGGG
GAAAGATTAGGTACATTAGTACTTGGTCGAGTACATGATGATTTTAATGAAAATGATTTG
GTACTAGGTGAATATGCTGCTACAGTTATTGGTATGGAAATCTTACGTGAGAAGCATAGT
GAAGTAGAAAAAGAAGCGCGCGATAAAGCTGCTATTACAATGGCAATTAATTCATTATCT
TATTCTGAAAAAGAAGCGATTGAACATATCTTTGAAGAACTTGGCGGTACGGAAGGCCTA
TTAATCGCATCAAAAGTTGCAGATAGAGTTGGTATTACTAGATCTGTAATTGTAAATGCA
CTACGTAAATTAGAAAGTGCTGGTGTAATTGAATCACGTTCTTTAGGAATGAAAGGTACT
TTCATTAAAGTTAAAAAAGAAAAATTCTTAGATGAATTAGAAAAAAGTAAATAA

## > rpsB

Function: protein coding sequence; 30S ribosomal protein S2 (core genome, constant)
Best match: rpsB_ref_CC001_MW2-USA400_BA000033.2[1245330:1246097] (completely identical)
Position: 031-contig_247: 18800 … 19568; Length: 768 bp
Sequence:
ATGGCAGTAATTTCAATGAAACAATTACTAGAAGCGGGTGTTCACTTCGGTCACCAAACA
CGTCGTTGGAACCCAAAAATGAAAAAATATATCTTCACTGAGAGAAATGGTATTTATATC
ATCGACTTACAAAAAACAGTGAAAAAAGTAGACGAGGCATACAACTTCTTGAAACAAGT
TTCAGAAGATGGTGGACAAGTCTTATTCGTAGGAACTAAAAAACAAGCACAAGAATCAG
TTAAATCTGAAGCAGAACGTGCTGGTCAATTCTACATTAACCAAAGATGGTTAGGTGGAT
TATTAACTAACTATAAAACGATCTCAAAACGAATCAAACGTATTTCTGAAATTGAAAAAA
TGGAAGAAGATGGTTTATTCGAAGTATTACCTAAAAAAGAAGTAGTAGAACTTAAAAAA
GAATACGACCGTTTAATCAAATTCTTAGGCGGAATTCGTGATATGAAATCAATGCCTCAA
GCATTATTCGTAGTTGACCCACGTAAAGAGCGTAATGCAATTGCTGAAGCTCGTAAATTA
AATATTCCTATCGTAGGTATCGTTGACACTAACTGTGATCCTGACGAAATTGACTACGTTA
TCCCAGCAAACGACGATGCTATCCGTGCGGTTAAATTATTAACTGCTAAAATGGCAGATG
CAATCTTAGAAGGTCAACAAGGCGTTTCTAATGAAGAAGTAGCTGCAGAACAAAACATC
GATTTAGATGAAAAAGAAAAATCAGAAGAAACAGAAGCAACTGAAGAATAA

## > tsf

Function: protein coding sequence; translation elongation factor Ts (core genome, constant)
Best match: tsf_CC008_NCTC8325_CP000253.1[1181770:1182651] (completely identical)
Position: 031-contig_247: 19749 … 20631; Length: 882 bp
Sequence:
ATGGCAACTATTTCAGCAAAACTTGTTAAAGAATTACGTGAAAAAACTGGCGCGGGTATG
ATGGATTGTAAAAAAGCGCTAACTGAAACTGATGGTGACATCGATAAAGCGATTGACTA
CCTACGTGAAAAAGGTATTGCTAAAGCAGCTAAAAAAGCAGACCGTATTGCGGCTGAAG
GTTTAGTACATGTAGAAACTAAAGGTAACGACGCAGTTATCGTTGAAATCAACTCTGAAA
CAGACTTTGTTGCTCGTAACGAAGGTTTCCAAGAGTTAGTTAAAGAAATCGCTAATCAAG
TATTAGATACAAAAGCTGAAACTGTTGAAGCTTTAATGGAAACAACTTTACCAAATGGTA
AATCAGTTGATGAAAGAATTAAAGAAGCAATTTCAACAATCGGTGAAAAATTAAGTGTTC

GTCGTTTTGCTATCAGAACTAAAACTGATAACGATGCTTTCGGCGCTTACTTACACATGGG
TGGACGCATTGGTGTATTAACAGTTGTTGAAGGTTCAACTGACGAAGAAGCAGCAAGAG
ACGTTGCTATGCATATCGCTGCAATCAACCCTAAATATGTTTCTTCTGAACAAGTTAGCGA
AGAAGAAATCAACCACGAAAGAGAAGTTTTAAAACAACAAGCATTAAATGAAGGTAAAC
CAGAAAACATCGTTGAAAAAATGGTGGAAGGACGTTTACGTAAATACTTACAAGAAATT
TGTGCTGTAGATCAAGACTTCGTTAAAAACCCTGATGTAACAGTTGAAGCTTTCTTAAAA
ACAAAAGGTGGAAAACTTGTTGACTTCGTACGCTATGAAGTAGGCGAAGGTATGGAAAA
ACGCGAAGAAAACTTTGCGGATGAAGTTAAAGGACAAATGAAATAA

> **pyrH**
Function: protein coding sequence; uridylate kinase (core genome, constant)
Best match: pyrH_CC001_MW2-USA400_BA000033.2[1247297:1248019] (completely identical)
Position: 031-contig_247: 20767 … 21490; Length: 723 bp
Sequence:
ATGGCTCAAATTTCTAAATATAAACGTGTAGTTTTGAAACTAAGTGGTGAAGCGTTAGCT
GGAGAAAAAGGATTTGGCATAAATCCAGTAATTATTAAAAGTGTTGCTGAGCAAGTGGCT
GAAGTTGCTAAAATGGACTGTGAAATCGCAGTAATCGTTGGTGGCGGAAACATTTGGAG
AGGTAAAACAGGTAGTGACTTAGGTATGGACCGTGGAACTGCTGATTACATGGGTATGCT
TGCAACTGTAATGAATGCCTTAGCATTACAAGATAGTTTAGAACAATTGGATTGTGATAC
ACGAGTATTAACATCTATTGAAATGAAGCAAGTGGCTGAACCTTATATTCGTCGTCGTGC
AATTAGACACTTAGAAAAGAAACGCGTAGTTATTTTTGCTGCAGGTATTGGAAACCCATA
CTTCTCTACAGATACTACAGCGGCATTACGTGCTGCAGAAGTTGAAGCAGATGTTATTTT
AATGGGCAAAAATAATGTAGATGGTGTATATTCTGCAGATCCTAAAGTAAACAAAGATG
CGGTAAAATATGAACATTTAACGCATATTCAAATGCTTCAAGAAGGTTTACAAGTAATGG
ATTCAACAGCATCCTCATTCTGTATGGATAATAACATTCCGTTAACTGTTTTCTCTATTAT
GGAAGAAGGAAATATTAAACGTGCTGTTATGGGTGAAAAGATAGGTACGTTAATTACAA
AATAA

> **frr**
Function: protein coding sequence; ribosome recycling factor (core genome, constant)
Best match: frr_CC001_MW2-USA400_BA000033.2[1248038:1248592] (completely identical)
Position: 031-contig_247: 21508 … 22063; Length: 555 bp
Sequence:
ATGAGTGACATTATTAATGAAACTAAATCAAGAATGCAAAAATCAATCGAAAGCTTATCA
CGTGAATTAGCTAATATCAGTGCAGGAAGAGCTAATTCAAATTTATTAAACGGCGTAACA
GTTGATTACTATGGTGCACCAACACCTGTACAACAATTAGCAAGCATCAATGTTCCAGAA
GCACGTTTACTTGTTATTTCTCCATACGACAAAACTTCTGTAGCTGACATCGAAAAAGCG
ATAATAGCGGCTAACTTAGGTGTCAACCCAACAAGTGATGGTGAAGTGATACGTATTGCA
GTACCTGCCTTAACAGAAGAGCGTAGAAAAGAGCGCGTTAAAGATGTTAAGAAAATTGG
TGAAGAAGCTAAAGTATCTGTTCGAAATATTCGTCGTGATATGAATGATCAGTTGAAAAA
AGATGAAAAAAATGGCGACATTACTGAAGATGAGTTGAGAAGTGGTACTGAAGATGTTC
AGAAAGCAACAGACAATTCAATAAAAGAAATTGATCAAATGATTGCTGATAAAGAAAAA
GATATTATGTCAGTATAA

> **uppS**
Function: protein coding sequence; undecaprenyl pyrophosphate synthetase (core genome, constant)
Best match: uppS_CC001_MW2-USA400_BA000033.2[1248965:1249735] (completely identical)
Position: 031-contig_247: 22435 … 23206; Length: 771 bp
Sequence:
ATGTTTAAAAAGCTAATAAATAAAAAGAACACTATAAATAATTATAATGAAGAATTAGA
CTCGTCTAATATACCTGAACATATCGCTATTATTATGGATGGTAATGGGCGATGGGCTAA
GAAGCGAAAAATGCCTAGAATTAAAGGTCATTACGAAGGTATGCAAACAATAAAAAAAA
TTACTAGGGTAGCTAGTGATATTGGTGTTAAGTACTTAACTTTATACGCCTTTTCCACTGA
AAATTGGTCAAGACCTGAAAGTGAAGTAAATTATATTATGAATTTGCCTGTCAATTTCTTA
AAGACATTCTTACCGGAACTAATTGAAAAAAATGTCAAAGTTGAAACAATTGGATTTACT

GATAAGTTGCCAAAATCAACGATAGAAGCAATTAATAATGCTAAAGAAAAGACAGCTAA
TAATACCGGCTTAAAATTAATATTTGCAATTAATTATGGTGGCAGAGCAGAACTTGTTCA
TAGTATTAAAAATATGTTTGACGAGCTTCATCAACAAGGTTTAAATAGTGATATCATAGA
TGAAACATATATAAACAATCATTTAATGACAAAAGACTATCCTGATCCAGAGTTGTTAAT
TCGTACTTCAGGAGAACAAAGAATAAGTAATTTCTTGATTTGGCAAGTTTCGTATAGTGA
ATTTATCTTTAATCAAAAATTATGGCCTGACTTTGACGAAGATGAATTAATTAAATGTATA
AAAATTTATCAGTCACGTCAAAGACGCTTTGGCGGATTGAGTGAGGAGTAG

## > cdsA

Function: protein coding sequence; phosphatidate cytidylyltransferase (core genome, constant)
Best match: cdsA_CC008_NCTC8325_CP000253.1[1185233:1186015] (completely identical)
Position: 031-contig_247: 23212 … 23995; Length: 783 bp
Sequence:
ATGAAAGTTAGAACGCTGACAGCTATTATTGCCTTAATCGTATTCTTGCCTATCTTGTTAA
AAGGCGGCCTTGTGTTAATGATATTTGCTAATATATTAGCATTGATTGCATTAAAAGAATT
GTTGAATATGAATATGATTAAATTTGTTTCAGTTCCTGGTTTAATTAGTGCAGTTGGTCTT
ATCATCATTATGTTGCCACAACATGCAGGGCCATGGGTACAAGTAATTCAATTAAAAAGT
TTAATTGCAATGAGCTTTATTGTATTAAGTTATACTGTCTTATCTAAAAACAGATTTAGTT
TTATGGATGCTGCATTTTGCTTAATGTCTGTGGCTTATGTAGGCATTGGTTTTATGTTCTTT
TATGAAACGAGATCAGAAGGATTACATTACATATTATATGCCTTTTTAATTGTTTGGCTTA
CAGATACAGGGGCTTACTTGTTTGGTAAAATGATGGGTAAACATAAGCTTTGGCCAGTAA
TAAGTCCGAATAAAACAATCGAAGGATTCATAGGTGGCTTGTTCTGTAGTTTGATAGTAC
CACTTGCAATGTTATATTTTGTAGATTTCAATATGAATGTATGGATATTACTTGGAGTGAC
ATTGATTTTAAGTTTATTTGGTCAATTAGGTGATTTAGTGGAATCAGGATTTAAGCGTCAT
TTCGGCGTTAAAGACTCAGGTCGAATACTACCTGGACACGGTGGTATTTTAGACCGATTT
GACAGCTTTATGTTTGTGTTACCATTATTAAATATTTTATTAATACAATCTTAA

## > rseP

Function: protein coding sequence; RIP metalloprotease (core genome, variable)
Best match: rseP_CC001-ST772_118_AJGE01000002.1[137892:139178:r] (completely identical)
Position: 031-contig_247: 24206 … 25493; Length: 1287 bp
Sequence:
GTGAGCTATTTAGTTACAATAATTGCATTTATTATTGTTTTTGGTGTACTAGTAACTGTTCA
TGAATATGGCCATATGTTTTTTGCGAAAAGAGCAGGCATTATGTGTCCAGAATTTGCGAT
CGGTATGGGGCCGAAAATTTTTAGTTTTAGAAAAAATGAAACACTTTACACTATTAGGTT
ATTGCCTGTTGGTGGATATGTTCGTATGGCAGGAGATGGCTTAGAAGAGCCACCAGTCGA
GCCCGGTATGAACGTTAAAATTAAACTTAATGAAGAAAATGAAATAACACATATCATATT
AGATGATCATCATAAGTTTCAACAAATTGAAGCGATCGAAGTTAAAAAATGTGATTTTAA
GGATGACTTATTCATAGAAGGTATCACTGCTTATGATAATGAAAGACATCATTTTAAAAT
TGCTAGAAAGTCTTTCTTTGTTGAAAATGGTAGCTTAGTTCAAATTGCTCCGAGAGACAG
ACAATTTGCACATAAAAAGCCATGGCCGAAATTTTTAACATTATTTGCGGGACCGTTATTT
AACTTTATATTAGCTTTAGTCCTATTTATTGGTCTTGCATATTATCAAGGCACGCCTACGT
CTACTGTAGAACAAGTCGCAGATAAGTATCCAGCTCAACAAGCAGGATTACAAAAAGGT
GATAAGATCGTCCAAATTGGCAAATATAAAATATCTGAATTTGATGATGTTGATAAGGCG
TTAGATAAAGTTAAAGATAATAAGACGACTGTTAAATTTGAACGTGATGGTAAAACAAA
GTCAGTTGAATTAACACCTAAAAAGACTGAAAAAAAACTGACTAAAGTAAGTTCAGAGA
CGAAGTATGTTCTCGGATTCCAACCAGCGAGTGAACATACACTTTTTAAACCAATTGTATT
CGGATTTAAAAGCTTTTTAATCGGTAGTACTTATATTTTTACAGCTGTAGTAGGTATGTTG
GCTAGTATATTTACGGGCGGATTCTCATTTGATATGTTAAATGGTCCGGTTGGTATTTATC
ATAACGTCGACTCAGTTGTTAAAGCGGGTATCATTAGCTTAATTGGTTACACTGCGTTATT
AAGTGTAAACTTAGGTATTATGAATTTAATTCCTATTCCTGCACTAGACGGTGGTCGTATT
TTATTTGTTATATATGAAGCGATTTTCAGAAAACCAGTTAATAAAAAAGCGGAAACAACG
ATTATTGCTATTGGTGCCATTTTCATGGTCGTTATAATGATATTAGTAACGTGGAATGATA
TTCGACGATATTTCTTATAA

> **proS**

Function: protein coding sequence; prolyl-tRNA synthetase (core genome, constant)
Best match: proS_CC008_NCTC8325_CP000253.1[1187558:1189261]
Position: 031-contig_247: 25512 … 27216; Length: 1704 bp
Sequence:
ATGAAGCAATCCAAAGTTTTTATACCAACGATGCGTGACGTGCCATCAGAAGCAGAAGC
ACAAAGTCATCGTTTATTATTGAAATCGGGTTTGATAAAACAAAGTACAAGTGGGATTTA
TAGTTATTTACCGCTAGCAACACGTGTGTTAAATAATATTACTGCAATTGTGCGACAAGA
AATGGAACGTATCGATTCTGTTGAAATTTTAATGCCAGCGTTACAACAAGCTGAATTATG
GGAAGAATCAGGACGTTGGGGTGCATATGGCCCAGAATTAATGCGTTTACAAGATAGAC
ATGGAAGACAATTTGCATTAGGTCCAACACATGAAGAATTAGTTACATCAATAGTAAGAA
ATGAATTGAAATCATACAAACAATTACCGATGACATTATTCCAAATTCAATCTAAATTCC
GTGATGAAAAGAGACCACGTTTTGGTTTACTTCGTGGGCGTGAATTTATTATGAAAGATG
CATATTCATTCCATGCTGACGAGGCATCATTAGATCAAACGTATCAAGATATGTATCAAG
CGTATAGCCGTATTTTTGAGAGAGTTGGCATTAACGCAAGACCAGTAGTTGCAGATTCAG
GTGCTATAGGCGGTAGCCATACACATGAATTTATGGCATTAAGTGCTATCGGTGAGGATA
CAATCGTTTACAGTAAAGAAAGTGATTATGCTGCTAACATCGAAAAAGCAGAAGTCGTTT
ACGAACCAAATCATAAGCATACTACTGTGCAACCTTTAGAAAAAATTGAAACACCAAAT
GTTAAGACTGCGCAAGAATTGGCAGACTTCTTAGGTAGACCAGTAGATGAAATCGTTAAA
ACGATGATTTTCAAAGTTGATGGCGAATATATTATGGTTTTAGTGCGTGGCCATCATGAA
ATTAATGACATTAAATTAAAATCTTATTTCGGCACAGATAATATTGAATTAGCAACACAA
GACGAAATTGTTAATTTAGTTGGTGCAAATCCTGGTTCACTAGGTCCTGTAATTGATAAA
GAAATCAAAATTTATGCAGATAATTTTGTGCAAGATTTAAATAATTTAGTTGTCGGTGCTA
ACGAAGATGGTTATCACTTAATTAATGTAAATGTAGGTAGAGACTTCAACGTTGATGAAT
ATGGCGATTTCCGTTTTATTTTAGAAGGCGAAAAGTTAAGTGATGGTTCAGGCGTTGCAC
ATTTTGCTGAAGGTATTGAAGTTGGTCAAGTATTCAAATTGGGTACTAAGTATTCAGAAT
CAATGAATGCTACATTCTTAGATAACCAAGGAAAAGCTCAACCTTTAATTATGGGTTGTT
ACGGAATTGGAATTTCTAGAACGCTAAGTGCGATTGTTGAACAAAATCACGATGATAATG
GAATTGTTTGGCCTAAATCAGTTACTCCGTTTGATTTACATTTAATTTCTATTAATCCTAAG
AAAGATGATCAACGAGAACTAGCAGATGCACTATATGCTGAATTTAATACTAAATTTGAT
GTGTTGTACGATGATCGTCAGGAACGTGCAGGTGTCAAATTTAATGATGCCGATTTAATT
GGTTTACCACTGCGAATTGTTGTTGGTAAACGTGCATCGGAAGGTATTGTAGAAGTTAAA
GAACGTTTAACAGGTGATAGCGAAGAAGTTCACATTGATGACTTAATGACTGTCATTACA
AATAAATATGATAACTTAAAATAA

> **polC**

Function: protein coding sequence; DNA polymerase III alpha chain (core genome, constant)
Best match: polC_CC030_MRSA252_BX571856.1[1295711:1300021]
Position: 031-contig_247: 27479 … 31790; Length: 4311 bp
Sequence:
ATGACAGAGCAACAAAAATTTAAAGTGCTTGCTGATCAAATTAAAATTTCAAATCAATTA
GATGCTGAAATTTTAAATTCAGGTGAACTGACACGTATAGATGTTTCTAACAAAAACAGA
ACATGGGAATTTCATATTACATTACCACAATTCTTAGCTCATGAAGATTATTTATTATTTA
TAAATGCAATAGAGCAAGAGTTTAAAGATATCGCCAACGTTACATGTCGTTTTACGGTAA
CAAATGGCACGAATCAAGATGAACATGCAATTAAATACTTTGGGCACTGTATTGACCAAA
CAGCTTTATCTCCAAAAGTTAAAGGTCAATTGAAACAGAAAAAGCTTATTATGTCTGGAA
AAGTATTAAAAGTAATGGTATCAAATGACATTGAACGTAATCATTTTGATAAGGCATGTA
ATGGAAGTCTTATCAAAGCGTTTAGAAATTGTGGTTTTGATATCGATAAAATCATATTCG
AAACAAATGATAATGATCAAGAACAAAACTTAGCTTCTTTAGAAGCACATATTCAAGAA
GAAGACGAACAAAGTGCACGATTGGCAACAGAGAAACTTGAAAAAATGAAAGCTGAAA
AAGCGAAACAACAAGATAACAACGAAAGTGCTGTCGATAAGTGTCAAATTGGTAAGCCG
ATTCAAATTGAAAATATTAAACCAATTGAATCTATTATTGAGGAAGAGTTTAAAGTTGCA
ATAGAGGGTGTCATTTTTGATATAAACTTAAAAGAACTTAAAAGTGGTCGCCATATCGTA
GAAATTAAAGTGACTGACTATACGGACTCTTTAGTTTTAAAAATGTTTACTCGTAAAAAC
AAAGATGATTTAGAACATTTTAAAGCGCTAAGTGTTGGTAAATGGGTTAGGGCTCAAGGT

CGTATTGAAGAAGATACATTTATTAGAGATTTAGTTATGATGATGTCTGATATTGAAGAG
ATTAAAAAAGCGACAAAAAAAGATAAGGCTGAAGAAAAGCGTGTAGAATTCCACTTGCA
TACTGCAATGAGCCAAATGGATGGTATACCCAATATTGGTGCGTATGTTAAACAGGCAGC
AGACTGGGGACATCCAGCCATTGCGGTTACAGACCATAATGTTGTGCAAGCATTTCCAGA
TGCTCACGCAGCAGCGGAAAAACATGGCATTAAAATGATATACGGTATGGAAGGTATGT
TAGTTGATGATGGTGTTCCGATTGCATACAAACCACAAGATGTCGTATTAAAAGATGCTA
CTTATGTTGTGTTCGACGTTGAGACAACTGGTTTATCAAATCAGTATGATAAAATCATCGA
GCTTGCAGCTGTGAAAGTTCATAACGGTGAAATCATCGATAAGTTTGAAAGGTTTAGTAA
TCCGCATGAACGATTATCGGAAACGATTATCAATTTGACGCATATTACGGATGATATGTT
AGTAGATGCCCCTGAGATTGAAGAAGTACTTACAGAGTTTAAAGAATGGTTGGCGATGC
GATATTCGTAGCGCATAATGCTTCGTTTGATATGGGCTTCATCGATACGGGATATGAACG
TCTTGGGTTTGGACCATCAACGAATGGTGTTATCGATACTTTAGAATTATCTCGTACGATT
AATACTGAATATGGTAAACATGGTTTGAATTTCTTGGCTAAAAAATATGGCGTAGAATTA
ACGCAACATCACCGTGCCATTTATGATACAGAAGCAACAGCTTACATTTTCATAAAAATG
GTTCAACAAATGAAAGAATTAGGCGTATTAAATCATAACGAAATCAACAAAAAACTCAG
TAATGAAGATGCATATAAACGTGCAAGACCTAGTCATGTCACATTAATTGTACAAAACCA
ACAAGGTCTTAAAAATCTATTTAAAATTGTAAGTGCATCATTGGTGAAGTATTTCTACCGT
ACACCTCGAATTCCACGTTCATTGTTAGATGAATATCGTGAGGGATTATTGGTAGGTACA
GCGTGTGATGAAGGTGAATTATTTACGGCAGTTATGCAGAAGGACCAGAGTCAAGTTGA
AAAAATTGCCAAATATTATGATTTTATTGAAATTCAACCACCGGCACTTTATCAAGATTTA
ATTGATAGAGAGCTTATTAGAGATACTGAAACATTACATGAAATTTATCAACGTTTAATA
CATGCAGGTGACACAGCGGGTATACCTGTTATTGCGACAGGAAATGCACACTATTTGTTT
GAACATGATGGTATCGCACGTAAAATTTTAATAGCATCACAACCCGGCAATCCACTTAAT
CGCTCAACTTTACCGGAAGCACATTTTAGAACTACAGATGAAATGTTAAACGAGTTTCAT
TTTTTAGGTGAAGAAAAAGCGCATGAAATTGTTGTGAAAAATACAAACGAATTAGCAGA
TCGAATTGAACGTGTTGTTCCTATTAAAGATGAATTATACACACCGCGTATGGAAGGTGC
TAACGAAGAAATTAGAGAACTAAGTTATGCAAATGCGCGTAAACTGTATGGTGAAGACC
TGCCTCAAATCGTAATTGATCGATTAGAAAAAGAATTAAAAAGTATTATCGGTAATGGAT
TTGCGGTAATTTACTTAATTTCGCAACGTTTAGTTAAAAAATCATTAGATGATGGATACTT
AGTTGGTTCCCGTGGTTCAGTAGGTTCTAGTTTTGTAGCGACAATGACTGAGATTACTGAA
GTAAACCCGTTACCGCCACACTATATTTGTCCGAACTGTAAAACGAGTGAATTTTTCAAT
GATGGTTCAGTAGGATCAGGATTTGATTTACCTGATAAGACGTGTGAAACTTGTGGAGCG
CCACTTATTAAAGAAGGACAAGATATTCCGTTTGAAACATTTTTAGGATTTAAGGGAGAT
AAAGTTCCTGATATCGACTTGAACTTTAGTGGTGAATATCAACCGAATGCCCATAACTAC
ACAAAAGTATTATTTGGTGAGGATAAAGTATTCCGTGCAGGTACAATTGGTACTGTTGCT
GAAAAGACTGCTTTTGGTTATGTTAAAGGTTATTTGAATGATCAAGGTATCCACAAAAGA
GGTGCTGAAATAGATCGACTCGTTAAAGGATGTACAGGTGTTAAACGTACAACTGGACA
GCATCCAGGGGGTATTATTGTAGTACCTGATTACATGGATATTTATGATTTTACGCCGATA
CAATATCCTGCCGATGATCAAAATTCAGCATGGATGACGACACATTTTGATTTCCATTCTA
TTCATGATAATGTATTAAAACTTGATATACTTGGACACGATGATCCAACAATGATTCGTAT
GCTTCAAGATTTATCAGGAATTGATCCAAAAACAATACCTGTAGATGATAAAGAAGTTAT
GCAGATATTTAGTACACCTGAAAGTTTGGGTGTTACTGAAGATGAAATTTTATGTAAAAC
AGGTACGTTTGGGGTTCCAGAATTCGGTACAGGGTTCGTGCGTCAAATGTTAGAAGATAC
AAAGCCAACAACATTTTCTGAATTAGTTCAAATCTCAGGATTATCTCATGGTACAGATGT
GTGGTTAGGCAATGCTCAAGAATTAATTAAAACCGGTATATGTGATTTATCAAGTGTAAT
TGGTTGTCGTGATGATATCATGGTTTATTTAATGTATGCTGGTTTAGAACCATCAATGGCT
TTTAAAATAATGGAGTCAGTACGTAAAGGTAAAGGTTTAACTGAAGAAATGATTGAAAC
GATGAAAGAAAATGAAGTGCCGGATTGGTATTTAGATTCATGTCTTAAAATTAAGTACAT
GTTCCCTAAAGCCCATGCAGCAGCATACGTTTTAATGGCAGTACGTATCGCATATTTCAA
AGTACATCATCCACTTTATTACTATGCATCTTACTTTACAATTCGTGCGTCAGATTTTGATT
TAATCACGATGATTAAAGATAAAACAAGCATTCGAAATACTGTAAAAGACATGTATTCTC
GCTATATGGATCTAGGTAAAAAAGAAAAAGACGTATTAACTGTCTTGGAAATTATGAATG
AAATGGCACATCGAGGTTATCGCATGCAACCGATTAGTTTAGAAAAGAGTCAGGCGTTCG
AATTTATCATTGAAGGCGATACACTTATTCCGCCGTTCATATCAGTGCCTGGGCTTGGCGA
AAACGTTGCGAAACGAATTGTTGAAGCTCGTGACGATGGACCATTTTTATCAAAAGAAGA

TTTAAACAAAAAAGCTGGATTATCTCAGAAAATTATTGAGTATTTAGATGAGTTAGGCTC
ATTACCGAATTTACCAGATAAAGCTCAACTTTCGATATTCGATATGTAA

> **rimP**
Function: protein coding sequence; ribosome maturation factor (core genome, constant)
Best match: rimP_CC001_MW2-USA400_BA000033.2[1258609:1259076] (completely identical)
Position: 031-contig_247: 32079 … 32547; Length: 468 bp
Sequence:
ATGAGTAAAATTACAGAACAAGTAGAAGTGATTGTTAAACCAATTATGGAAGACTTGAA
TTTTGAACTTGTAGACGTTGAATATGTCAAAGAGGGTAGAGATCATTTTCTTAGAATCTCT
ATTGATAAAGAAGGTGGCGTAGATTTAAATGATTGTACGCTAGCTTCTGAAAAAATAAGT
GAAGCTATGGATGCAAATGATCCTATTCCTGAAATGTATTATTTAGACGTAGCGTCACCT
GGTGCAGAACGTCCAATTAAAAAAGAACAAGATTTCCAAAATGCAATAACTAAACCTGT
ATTTGTTTCTTTATATGTACCAATTGAAGGTGAAAAGGAATGGTTAGGCATTTTACAAGA
AGTCAATAATGAAACAATTGTAGTACAAGTTAAAATCAAAGCAAGAACGAAAGATATAG
AGATACCGAGAGACAAAATAGCAAAAGCACGTCACGCAGTTATGATTTAA

> **nusA**
Function: protein coding sequence; transcription termination-antitermination factor (core genome, constant)
Best match: nusA_CC008_NCTC8325_CP000253.1[1194613:1195788]
Position: 031-contig_247: 32567 … 33743; Length: 1176 bp
Sequence:
GTGTCAAGTAATGAATTATTATTAGCTACTGAGTATTTAGAAAAAGAAAAGAAGATTCCT
AGAGCAGTATTAATTGATGCTATTGAAGCAGCTTTAATTACTGCATACAAAAAGAATTAT
GATAGTGCAAGAAATGTCCGTGTGGAATTAAATATGGATCAAGGTACTTTCAAAGTTATC
GCTCGTAAAGATGTTGTTGAAGAAGTATTTGACGACAGAGATGAAGTGGATTTAAGTACA
GCGCTTGTTAAAAACCCTGCATATGAAATTGGTGATATATACGAAGAAGATGTAACACCT
AAAGATTTTGGTCGTGTAGGTGCTCAAGCAGCGAAACAAGCAGTAATGCAACGTCTTCGT
GATGCTGAACGTGAAATTTTATTTGAAGAATTTATAGACAAAGAAGAAGACATACTTACT
GGAATTATTGACCGTGTTGACCATCGTTATGTATATGTGAATTTAGGTCGTATCGAAGCTG
TTTTATCTGAAGCAGAAAGAAGTCCTAACGAAAAATATATTCCTAACGAACGTATCAAAG
TATATGTTAACAAAGTGGAACAAACGACAAAAGGTCCTCAAATCTATGTTTCTCGTAGCC
ATCCAGGTTTATTAAAACGTTTATTTGAACAAGAAGTTCCAGAAATTTACGATGGTACTG
TAATTGTTAAATCAGTAGCACGTGAAGCTGGCGATCGCTCTAAAATTAGTGTCTTCTCTGA
AAACAATGATATAGATGCTGTTGGTGCATGTGTTGGTGCTAAAGGCGCACGTGTTGAAGC
TGTTGTTGAAGAGCTAGGTGGCGAAAAAATCGACATCGTTCAATGGAATGAAGATCCAA
AAGTATTTGTAAAAAATGCTTTAAGCCCTTCTCAAGTTTTAGAAGTTATTGTTGATGAAAC
AAATCAATCTACAGTAGTTGTTGTTCCTGATTATCAATTGTCATTAGCGATTGGTAAAAGA
GGACAAAACGCACGTCTAGCTGCTAAATTAACCGGCTGGAAAATTGATATTAAATCAGA
AACAGATGCGCGTGAAGCGGGTATCTATCCAGTAGTTGAAGCTGAAAAAGTAACTGAAG
AAGATGTTGCTTTAGAAGATGCTGACACAACAGAATCAACCGAAGAGGTAAATGATGTTT
CAGTTGAAACAAATGTAGAGAAAGAATCTGAATAA

> **infB**
Function: protein coding sequence; translation initiation factor IF-2 (core genome, constant)
Best match: infB_CC001_MW2-USA400_BA000033.2[1260896:1263013]
Position: 031-contig_247: 34366 … 36484; Length: 2118 bp
Sequence:
ATGAGTAAACAAAGAATTTACGAATATGCGAAAGAATTAAATCTAAAGAGTAAAGAGAT
TATAGATGAGTTAAAAAGCATGAATATTGAGGTTTCAAATCATATGCAAGCTTTGGAAGA
TGACCAAATTAAAGCATTAGATAAAAAGTTCAAAAAAGAACAAAAGAACGACAATAAAC
AAAGCACTCAAAATAATCACCAAAAATCAAACAATCAAAACCAAAATAAAGGGCAACAA
AAAGATAACAAAAAGAATCAACAACAAAATAATAAAGGCAACAAAGGCAATAAAAAGA
ATAATAGAAATAATAAGAAAAATAACAAGAATAATAAACCACAAAATCAACCAGCTGCT

CCAAAAGAAATACCATCAAAAGTGACATATCAAGAAGGTATTACATTAGGCGAATTTGC
GGATAAATTAAATGTTGAATCATCAGAAATTATCAAAAAATTATTCTTACTTGGTATTGTT
GCTAATATCAATCAATCATTAAATCAAGAAACAATCGAATTAATTGCCGATGATTATGGC
GTTGAGGTTGAAGAAGAAGTTGTGATTAATGAAGAAGATTTATCAATCTACTTCGAAGAC
GAAAAAGATGATCCAGAAGCAATTGAGAGACCAGCAGTTGTAACAATTATGGGACATGT
TGACCATGGTAAAACGACTTTATTAGATTCAATTCGTCATACAAAAGTTACAGCAGGTGA
AGCAGGCGGAATCACTCAACATATTGGTGCATATCAAATTGAAAACGATGGCAAAAAAA
TCACTTTCTTAGATACACCGGGACATGCTGCATTTACAACGATGCGTGCGCGTGGTGCAC
AAGTAACAGATATTACAATTTTAGTAGTTGCAGCTGACGATGGTGTTATGCCACAAACAA
TTGAAGCAATTAACCATGCTAAAGAAGCAGAAGTACCAATTATTGTTGCAGTAAATAAAA
TTGATAAACCAACTTCAAACCCTGATCGAGTTATGCAAGAATTAACTGAATATGGTTTAA
TTCCTGAAGATTGGGGCGGCGAAACAATTTTCGTTCCACTTTCTGCATTAAGTGGTGATGG
TATCGACGATTTATTAGAAATGATAGGATTAGTTGCAGAAGTTCAAGAACTTAAAGCAAA
TCCTAAAAACCGTGCTGTTGGTACAGTTATCGAAGCTGAATTAGATAAATCACGTGGTCC
TTCTGCATCATTATTAGTACAAAATGGTACATTAAATGTTGGTGATGCGATTGTAGTTGGT
AATACTTACGGCCGTATCCGTGCAATGGTTAATGACTTAGGTCAAAGAATCAAAACGGCT
GGTCCATCAACGCCTGTTGAAATTACAGGTATTAATGATGTGCCACAAGCTGGGGATCGC
TTTGTTGTATTTAGTGATGAAAAACAAGCTCGTCGTATTGGTGAATCAAGACACGAAGCT
AGCATTGTACAACAACGTCAAGAAAGTAAAAATGTTTCATTAGATAACCTGTTTGAACAA
ATGAAACAAGGTGAAATGAAAGATTTAAACGTTATTATTAAAGGTGATGTTCAAGGTTCT
GTTGAAGCTTTAGCTGCATCATTAATGAAAATTGATGTTGAAGGCGTAAATGTTCGTATC
ATTCATACAGCGGTTGGTGCAATTAATGAGTCAGACGTGACACTTGCTAATGCCTCAAAT
GGTATTATCATTGGTTTCAATGTTCGTCCAGACAGTGGTGCAAAACGTGCTGCAGAAGCT
GAAAATGTTGATATGCGTTTACACAGAGTTATTTATAATGTTATCGAAGAAATTGAATCA
GCGATGAAAGGTTTACTTGATCCAGAATTTGAAGAACAAGTTATCGGACAAGCTGAAGTT
CGTCAAACATTCAAAGTTTCTAAAGTTGGTACTATTGCTGGATGTTATGTTACTGAAGGTA
AAATTACGCGAAATGCTGGTGTACGTATTATTCGTGATGGTATTGTTCAATATGAAGGCG
AATTAGATACACTTAAACGTTTCAAAGATGATGCTAAGGAAGTTGCAAAAGGTTATGAAT
GTGGTATTACAATTGAAAACTACAATGACCTTAAAGAAGGCGATGTTATCGAAGCATTTG
AAATGGTTGAAATTAAGCGTTAA

> **rbfA**

Function: protein coding sequence; ribosome-binding factor A (core genome, constant)
Best match: rbfA_CC001_MW2-USA400_BA000033.2[1263399:1263749] (completely identical)
Position: 031-contig_247: 36869 … 37220; Length: 351 bp
Sequence:
ATGAGCAGTATGAGAGCAGAGCGTGTTGGTGAACAAATGAAGAAGGAATTAATGGATAT
CATCAACAATAAAGTCAAAGATCCTCGAGTTGGTTTTATTACAATTACAGATGTTGTTTA
ACAAATGATTTATCGCAGGCTAAAGTATTTTTAACTGTATTAGGTAACGATAAAGAAGTA
GAAAATACATTTAAAGCACTTGATAAAGCAAAAGGCTTCATTAAGTCTGAATTAGGTTCT
AGAATGCGATTACGTATTATGCCGGAATTAATGTATGAATATGATCAATCAATCGAATAT
GGTAATAAAATTGAACGAATGATTCAAGATTTACACAAACAAGATAGATAA

> **truB**

Function: protein coding sequence; tRNA pseudouridine synthase B (core genome, constant)
Best match: truB_CC001_MSSA476_BX571857.1[1292414:1293331]
Position: 031-contig_247: 37389 … 38307; Length: 918 bp
Sequence:
ATGTATAATGGGATATTACCAGTATATAAAGAGCGCGGTTTAACAAGTCATGACGTTGTA
TTCAAATTGCGTAAAATATTAAAAACTAAAAAAATAGGTCACACGGGTACGCTTGATCCC
GAAGTTGCAGGCGTGTTACCGGTATGTATAGGTAATGCAACGAGAGTTAGTGATTATGTT
ATGGATATGGGCAAAGCTTATGAAGCAACTGTATCGATAGGAAGAAGTACAACGACTGA
AGATCAAACGGGTGATACATTGGAAACAAAAGGTGTACACTCAGCAGATTTTAATAAGG
ACGATATTGACCGATTGTTAGAAAGTTTTAAAGGTATCATTGAACAAATTCCGCCGATGT
ACTCATCCGTCAAAGTAAATGGTAAAAAATTATATGAATATGCGCGTAATAATGAAACAG

TTGAAAGACCAAAGCGTAAAGTTAATATTATAGACATTGGGCGTATATCTGAATTAGATT
TTAAAGAAAATGAGTGTCATTTTAAAATACGCGTCATCTGTGGTAAAGGTACATATATTA
GAACGCTAGCAACTGATATTGGTGTGAAATTAGGCTTTCCGGCACATATGTCGAAATTAA
CACGAATCGAGTCTGGTGGATTTGTGTTGAAAGATAGCCTTACATTAGAACAAATAAAAG
AACTTCATGAGCAGGATTCATTGCAAAATAAATTGTTTCCTTTAGAATATGGATTAAAGG
GTTTGCCAAGCATTAAAATTAAAGATTCGCACATAAAAAAACGTATTTTAAATGGGCAGA
AATTTAATAAAAATGAATTTGATAACAAAATTAAAGACCAAATTGTATTTATTGATGATG
ATTCAGAAAAAGTATTAGCAATTTATATGGTACACCCTACAAAAGAATCAGAAATTAAAC
CTAAAAAAGTCTTTAATTAA

> **ribF**

Function: protein coding sequence; riboflavin biosynthesis protein (core genome, constant)
Best match: ribF_CC008_NCTC8325_CP000253.1[1200366:1201337]
Position: 031-contig_247: 38321 … 39293; Length: 972 bp
Sequence:
ATGAAAGTCATAGAAGTGACACATCCTATACAATCTAAACAGTATATTACAGAGGATGTT
GCAATGGCATTCGGATTTTTCGATGGCATGCATAAAGGTCATGACAAAGTCTTTGATATA
TTAAACGAAATAGCTGAGGCACGCAGTTTAAAAAAAGCGGTGATGACATTTGATCCGCAT
CCATCTGTCGTGTTGAATCCTAAAAGAAAACGAACAACGTATTTAACGCCACTTTCAGAT
AAAATCGAAAAAATTAGCCAACATGATATTGATTATTGTATAGTGGTTAATTTTTCATCTA
GGTTTGCTAATGTGAGCGTTGAAGATTTTGTTGAAAATTATATAATTAAAAATAATGTAA
AAGAAGTCATTGCTGGTTTCGATTTTACTTTTGGTAAATTTGGAAAAGGTAATATGACTGT
ACTTCAAGAATATGATGCGTTTAATACGACAATTGTGAGTAAACAAGAAATTGAAAATGA
AAAAATTTCTACAACTTCTATTCGTCAAGATTTAATCAATGGTGAGTTGCAAAAAGCGAA
TGATGCTTTAGGCTATATATATTCTATTAAAGGCACTGTAGTGCAAGGTGAAAAAAGGGG
AAGAACTATTGGCTTCCCAACAGCTAACATTCAACCTAGTGATGATTATTTGTTACCTCGT
AAAGGTGTTTATGCTGTTAGTATTGAAATCGGCACTGAAAATAAATTATATCGAGGGGTA
GCTAACATAGGTGTAAAGCCAACATTTCATGATCCTAACAAAGCAGAAGTTGTCATCGAA
GTGAATATCTTTGACTTTGAGGATAATATTTATGGTGAACGAGTGACCGTGAATTGGCAT
CATTTCTTACGTCCTGAGATTAAATTTGATGGTATCGACCCATTAGTTAAACAAATGAACG
ATGATAAATCGCGTGCTAAATATTTATTAGCAGTTGATTTTGGTGATGAAGTAGCTTATAA
TATCTAG

> **rpsO**

Function: protein coding sequence; 30S ribosomal protein S15 (core genome, constant)
Best match: rpsO_CC001_MW2-USA400_BA000033.2[1265937:1266206]
Position: 031-contig_247: 39407 … 39677; Length: 270 bp
Sequence:
ATGGCAATTTCACAAGAACGTAAAAACGAAATCATTAAAGAATACCGTGTACACGAAAC
TGATACTGGTTCACCAGAAGTACAAATCGCTGTACTTACTGCAGAAATCAACGCAGTAAA
CGAACACTTACGTACACACAAAAAAGACCACCATTCACGTCGTGGGTTATTAAAAATGGT
AGGTCGTCGTAGACATTTATTAAACTACTTACGTAGTAAAGATATTCAACGTTACCGTGA
ATTAATTAAATCACTTGGTATCCGTCGTTAA

> **pnpA**

Function: protein coding sequence; polyribonucleotide nucleotidyltransferase (core genome, constant)
Best match: pnpA_CC001_MW2-USA400_BA000033.2[1266574:1268670]
Position: 031-contig_247: 40046 … 42143; Length: 2097 bp
Sequence:
ATGTCTCAAGAAAAGAAAGTTTTTAAAACTGAATGGGCAGGAAGATCTTTAACGATTGAA
ACAGGGCAATTAGCTAAACAAGCAAATGGCGCTGTATTGGTTCGTTATGGAGATACAGTC
GTGTTATCGACGGCAACTGCATCAAAAGAACCTCGTGATGGAGATTTCTTCCCATTAACA
GTGAACTATGAAGAAAAAATGTACGCTGCGGGTAAAATTCCTGGTGGATTTAAAAAGAG
AGAAGGACGTCCTGGTGACGATGCAACATTAACTGCGCGATTAATTGATAGACCAATTAG
ACCTTTATTCCCTAAAGGATATAAGCATGATGTTCAAATTATGAACATGGTATTAAGTGC

AGATCCTGATTGTTCACCACAAATGGCTGCAATGATTGGTTCATCTATGGCGCTTAGTGTG
TCGGATATTCCATTCCAAGGGCCAATCGCCGGTGTAAATGTGGGTTATATTGACGGTAAA
TATATCATTAACCCAACAGTAGAAGAAAAAGAAGTTTCTCGCTTAGACCTTGAAGTAGCT
GGTCATAAAGATGCAGTAAACATGGTAGAGGCAGGCGCTAGTGAGATTACTGAACAAGA
AATGTTAGAGGCGATCTTCTTTGGTCATGAAGAGATTCAACGTTTAGTTGATTTCCAACAA
CAAATCGTCGACCACATTCAACCTGTTAAACAAGAATTTATTCCAGCAGAGCGTGATGAA
GCGCTAGTTGAACGTGTAAAATCTTTAACCGAAGAAAAAGGACTTAAAGAAACAGTTTTA
ACATTTGATAAACAACAACGAGATGAAAATCTTGATAACTTAAAAGAAGAAATCGTCAA
TGAATTTATCGATGAAGAAGATCCAGAGAATGAATTACTTATTAAAGAAGTTTATGCAAT
TTTAAATGAATTAGTGAAAGAAGAAGTTAGACGTTTAATTGCAGATGAAAAAATTAGACC
AGACGGCCGTAAACCTGATGAAATCCGTCCATTAGATTCTGAAGTTGGTATTTTACCTAG
AACGCATGGTTCAGGTCTATTTACACGTGGTCAGACTCAAGCACTTTCAGTTTTAACATTA
GGTGCTTTAGGCGATTATCAATTAATTGATGGTTTAGGACCTGAAGAAGAAAAAAGATTC
ATGCATCATTACAACTTCCCGAATTTTTCAGTAGGTGAAACTGGTCCAGTACGTGCGCCA
GGTCGTCGTGAAATTGGACATGGTGCGTTAGGTGAAAGAGCATTAAAATATATTATTCCT
GATACTGCTGATTTCCCATATACAATTCGTATTGTAAGTGAGGTACTTGAATCAAATGGTT
CATCATCTCAAGCGTCAATTTGTGGATCAACATTAGCATTAATGGATGCGGGCGTACCGA
TTAAAGCACCAGTTGCTGGTATTGCTATGGGCCTTGTTACACGTGAAGATAGCTATACGA
TTTTAACTGATATCCAAGGTATGGAAGATGCATTAGGTGATATGGACTTTAAAGTCGCTG
GTACTAAAGAAGGTATTACAGCAATCCAAATGGATATTAAAATTGACGGTTTAACGCGTG
AAATTATCGAAGAGGCTCTAGAACAAGCGAGACGTGGTCGTTTAGAAATAATGAATCAT
ATGTTACAAACAATTGATCAACCACGTACTGAATTAAGTGCTTACGCGCCAAAAGTTGTA
ACTATGACAATTAAACCAGATAAGATTAGAGATGTTATCGGACCTGGTGGTAAAAAAATT
AACGAAATTATTGATGAAACAGGCGTTAAATTAGATATTGAACAAGATGGTACTATCTTT
ATTGGTGCCGTTGATCAAGCTATGATAAATCGCGCTCGTGAAATCATTGAGGAAATTACA
CGTGAAGCGGAAGTAGGTCAAACTTATCAAGCCACTGTTAAACGTATTGAAAAATACGGT
GCGTTTGTAGGCCTATTCCCGGGTAAAGATGCGTTGCTTCACATTTCACAAATTTCAAAAA
ATAGAATTGAAAAAGTGGAAGATGTATTAAAAATCGGTGACACAATTGAAGTTAAGATT
ACTGAAATTGATAAACAAGGTCGAGTAAATGCTTCACACAGAGCATTAGAAGAATAA

> **rnjB**

Function: protein coding sequence; ribonuclease J2 (core genome, constant)
Best match: rnjB_CC001_MW2-USA400_BA000033.2[1268906:1270579]
Position: 031-contig_247: 42378 … 44052; Length: 1674 bp
Sequence:
TTGAGTTTAATAAAGAAAAAGAATAAAGATATTCGCATTATACCATTAGGCGGTGTTGGC
GAAATTGCTAAAAATATGTATATCGTTGAAGTAGACGATGAAATGTTTATGTTAGATGCT
GGACTTATGTTTCCAGAAGACGAAATGCTAGGTATTGATATTGTTATACCAGACATTTCAT
ACGTACTTGAAAATAAAGATAAATTGAAGGGTATATTCCTTACACACGGACATGAGCACG
CGATTGGTGCAGTGAGTTATGTTTTAGAACAATTAGATGCACCAGTATATGGATCTAAAT
TGACAATAGCGTTAATTAAAGAAAATATGAAAGCCCGTAATATTGATAAAAAAGTTCGCT
ACTATACAGTTAATAATGATTCAATTATGAGATTCAAAAACGTGAATATTAGTTTCTTTAA
TACGACACACAGTATTCCTGATAGTTTAGGTGTTTGTATTCACACTTCATATGGTGCCATT
GTGTATACAGGTGAATTTAAGTTTGACCAAAGTTTACATGGACATTATGCACCAGATATT
AAACGTATGGCAGAGATTGGTGAAGAAGGCGTATTTGTCTTAATCAGTGATTCTACTGAG
GCAGAGAAACCTGGATATAATACTCCGGAAAATGTGATTGAACATCATATGTATGATGCT
TTTGCAAAAGTGCGAGGTCGCTTGATAGTTTCATGTTATGCTTCGAACTTTATACGTATTC
AGCAAGTTTTAAATATTGCTAGCAAGCTAAATCGTAAAGTGTCATTTTTAGGAAGATCAC
TTGAAAGTTCATTTAATATTGCTCGTAAAATGGGGTATTTCGACATTCCTAAAGATTTGCT
AATTCCTATAACAGAAGTTGATAATTATCCTAAAAATGAAGTGATAATTATAGCTACTGG
TATGCAAGGAGAACCTGTAGAAGCCTTAAGTCAAATGGCGCAACATAAGCATAAAATTA
TGAATATCGAAGAAGGCGATTCTGTATTTTTAGCAATTACGGCTTCTGCTAATATGGAAG
TTATCATTGCGAATACATTAAATGAGCTTGTACGTGCTGGCGCACATATTATTCCAAATAA
CAAGAAGATTCATGCTTCAAGTCATGGTTGCATGGAAGAATTAAAAATGATGATTAATAT
TATGAAACCTGAATACTTTATTCCTGTACAAGGTGAATTTAAAATGCAGATAGCACATGC
GAAGCTAGCAGCTGAAGCAGGTGTTGCACCAGAAAAGATTTTCCTTGTGGAAAAAGGAG

ATGTCATTAATTACAACGGTAAAGATATGATATTAAATGAAAAGGTAAATTCAGGAAATA
TTTTAATAGATGGTATTGGTATTGGGGATGTAGGAAATATCGTGTTGAGAGACCGTCATC
TTTTAGCAGAAGATGGTATCTTTATTGCTGTAGTAACGTTAGATCCTAAAAATAGACGTAT
AGCTGCGGGACCTGAAATTCAATCTCGTGGGTTTGTATATGTACGTGAAAGTGAAGACTT
ATTACGTGAAGCAGAAGAGAAAGTACGTGAAATAGTAGAGGCTGGTTTACAAGAAAAAC
GCATAGAATGGTCTGAAATTAAGCAAAATATGCGTGATCAAATTAGTAAACTATTATTCG
AAAGTACAAAACGTCGTCCTATGATTATTCCAGTAATTTCTGAAATTTAA

## > **ftsK**

Function: protein coding sequence; cell division protein K (core genome, variable)
Best match: ftsK_CC001_MW2-USA400_BA000033.2[1270962:1273205]
Position: 031-contig_247: 44433 … 46677; Length: 2244 bp
Sequence:
ATGGTGTTGGGTGTTTTCCAATTAGGAATAATAGGTCGTCTAATTGACAGCTTCTTTAATT
ATTTATTTGGGTACAGTAGATATTTAACATATATTTTAGTACTCTTAGCAACTGGTTTTATT
ACATACTCTAAACGTATTCCTAAAACTAGACGAACGGCTGGTTCGATTGTATTGCAAATT
GCATTGCTATTTGTATCACAGTTAGTTTTTCATTTTAATAGTGGTATCAAAGCTGAAAGAG
AACCTGTACTTTCTTATGTGTATCAGTCATACCAACACAGTCATTTCCCAAATTTTGGTGG
CGGTGTATTAGGCTTTTATTTATTAGAGTTAAGCGTACCTTTAATTTCATTATTTGGTGTAT
GTATTATTACTATTTTATTATTATGCTCAAGTGTTATTTTATTAACAAACCATCAACATCGT
GAAGTTGCAAAAGTTGCACTGGAAAATATAAAAGCTTGGTTTGGTTCATTTAATGAAAAA
ATGTCGGAAAGAAACCAAGAAAAACAATTGAAGCGTGAAGAAAAAGCAAGACTTAAAG
AAGAACAAAAGGCACGTCAAAATGAACAGCCACAAATAAAAGATGTGAGTGATTTTACG
GAAGTGCCTCAAGAAAGAGATATTCCAATTTATGGGCATACTGAAAATGAAAGTAAAAG
CCAGAGTCAACCAAGTCGAAAAAAACGAGTGTTTGATGCAGAGAATAGTTCGAATAACA
TCGTAAATCATCATCAAGCAGATCAGCAAGAACAATTAACAGAACAAACTCATAACAGT
GTTGAAAGTGAAAACACTATTGAAGAAGCTGGTGAAGTTACGAATGTATCGTATGTTGTT
CCACCGTTAACTTTACTTAATCAACCTGCAAAACAAAAAGCAACATCTAAAGCTGAAGTG
CAACGTAAAGGACAAGTACTAGAGAATACATTAAAAGATTTTGGGGTAAATGCAAAAGT
GACACAAATTAAAATTGGTCCTGTAGTAACTCAATATGAAATTCAACCAGCTCAAGGGGT
TAAAGTGAGTAAAATTGTAAACTTGCATAATGATATTGCATTAGCTTTAGCAGCAAAAGA
TGTTAGAATCGAAGCGCCAATACCTGGTCGTTCTGCAGTAGGTATTGAAGTGCCAAATGA
GAAAATTTCATTAGTTTCACTAAAAGAAGTTTTAGATGAAAAATTCCCGTCTAATAATAA
ACTAGAAGTTGGATTAGGAAGAGATATATCAGGTGATCCAATTACTGTTCCACTAAATGA
AATGCCACACTTATTGGTGGCAGGATCGACGGGTAGTGGTAAATCTGTTTGTATAAATGG
TATTATTACAAGTATTTTATTAAATGCTAAGCCGCATGAAGTTAAACTTATGTTAATCGAT
CCGAAAATGGTTGAACTAAATGTTTATAACGGAATTCCACACTTATTAATTCCGGTTGTTA
CAAATCCTCATAAAGCTGCTCAAGCTTTAGAAAAAATTGTAGCTGAGATGGAAAGACGTT
ATGATTTATTCCAACATTCATCAACTAGAAACATTAAAGGTTATAACGAATTAATCCGTA
AGCAAAATCAAGAATTAGATGAGAAGCAACCAGAATTACCTTATATCGTTGTTATTGTAG
ATGAGCTTGCAGATTTAATGATGGTAGCTGGTAAAGAAGTTGAAAATGCGATTCAACGTA
TTACACAAATGGCACGTGCAGCAGGTATACATTTAATTGTAGCGACACAAAGACCTTCTG
TGGATGTAATTACAGGTATCATTAAAAATAATATTCCATCTAGAATAGCTTTTGCTGTGAG
TTCTCAAACAGATTCAAGAACTATTATTGGTACTGGCGGCGCAGAAAAGTTACTTGGTAA
AGGTGACATGTTATACGTTGGAAATGGTGACTCATCACAAACACGTATTCAAGGGGCGTT
TTTAAGTGACCAAGAGGTGCAAGATGTTGTAAATTATGTAGTAGAACAACAACAGGCAA
ATTATGTAAAAGAAATGGAACCAGATGCACCAGTGGATAAATCGGAAATGAAAAGTGAA
GATGCTTTATATGATGAAGCGTATTTGTTTGTTGTTGAACAACAAAAGGCAAGTACATCA
TTGTTACAACGCCAATTTAGAATTGGTTATAATAGAGCATCTAGGTTGATGGATGATTTA
GAACGCAATCAGGTAATCGGTCCACAAAAAGGAAGCAAGCCTAGACAAGTTTTAATAGA
TCTTAATAATGACGAGGTGTAA

## > **pgsA**

Function: protein coding sequence; CDP-diacylglycerol--glycerol-3-phosphate 3-phosphatidyltransferase (core genome, constant)

Best match: pgsA_CC001_MW2-USA400_BA000033.2[1278586:1279164]
Position: 031-contig_247: 52057 … 52636; Length: 579 bp
Sequence:
ATGAATATTCCGAACCAGATTACGGTTTTTAGAGTAGTGTTAATACCAGTTTTTATATTGT
TTGCGTTAGTTGATTTTGGATTTGGCAATGTGTCATTTCTAGGAGGATATGAAATAAGGAT
TGAGTTATTAATCAGTGGTTTTATTTTTATATTGGCTTCCCTTAGCGATTTTGTTGATGGTT
ATTTAGCTAGAAAATGGAATTTAGTTACAAATATGGGGAAATTTTTGGATCCATTAGCGG
ATAAATTATTAGTTGCAAGTGCTTTAATTGTACTTGTGCAACTAGGACTAACAAATTCTGT
AGTAGCAATCATTATTATTGCCAGAGAATTTGCCGTAACTGGTTTACGTTTACTACAAATT
GAACAAGGATTCGTAAGTGCAGCTGGTCAATTAGGTAAAATTAAAACAGCAGTTACTATG
GTAGCAATTACTTGGTTGTTATTAGGTGATCCATTGGCAACATTGATTGGTTTGTCATTAG
GACAAATTTTATTATACATTGGCGTTATTTTTACTATCTTATCTGGTATTGAATACTTTTAT
AAAGGTAGAGATGTTTTTAAACAAAAATAA

> **cinA**

Function: protein coding sequence; competence-damage inducible protein (core genome, constant)
Best match: cinA_CC001_MW2-USA400_BA000033.2[1279391:1280542]
Position: 031-contig_247: 52860 … 54012; Length: 1152 bp
Sequence:
ATGTCAATTGCCATTATTGCTGTAGGCTCAGAACTATTGCTAGGTCAAATCGCTAATACCA
ACGGACAATTTCTATCTAAAGTATTTAATGAAATTGGACAAAATGTATTAGAACATAAAG
TTATTGGAGATAATAAAAAACGTTTAGAATCAAGTGTACGTCATGCGCTAGAAAAATATG
ATACTGTTATTTTAACAGGTGGCTTAGGTCCTACGAAAGATGACTTAACGAAGCATACAG
TGGCCCAGATTGTTGGTAAAGATTTAGTTATTGATGAGCCTTCTTTAAAATATATTGAAAG
CTATTTTGAGGAACAAGGACAAGAAATGACACCTAATAATAAACAACAGGCTTTAGTAA
TTGAAGGTTCAACTGTATTAACAAATCATCATGGCATGGCTCCAGGAATGATGGTGAATT
TTGAAAACAAACAAATTATTTTATTACCAGGTCCACCGAAAGAAATGCAACCAATGGTGA
AAAATGAATTGTTGTCACATTTTATAAACCATAATCGAATTATACATTCTGAACTATTAAG
ATTTGCGGGAATAGGTGAATCTAAAGTAGAAACAATATTAATAGATCTTATCGATAAACA
GACTAATCCTACGATTGCGCCTTTGGCGGGAAGTCATGAAGTATATATTAGATTGACTGC
AAATGCCGACTCAAAAGAACAAGCACAATCATTGATTCAACCTGTTAAACAAGAAATTCT
TGATCGTATTGGAGAATATTATTATGGTTCAGATGACACATTAATTGAGCAAGCTGTAAT
AAAGAAAATTCATGAACCTTTTGTAATATATGATGGTATTACTAATGGTGCTTTATATCAT
CGATTGAAAGAAGTGGATTTAAACGATGTTCTAAAGGGTATGATTAATCACAATGAAAAC
TTTGTTGATATTAATAAACCTATTGAGCAGCAATTAAAAGATGCAGTGCAATTTGTTAAT
AAATTGTTTAATGTGTCATCAGCAATTATTCTATTAGAGTATGATGGTGTAGTCCATATAG
GCTATGATAATAACTTAGAATTTAAAACTGAGCAATTTAAAATGTCTAAATCTAGAAATT
TATTAAAGAACAGAAGTCAAAATTATGCGCTCATAAGATTATTAAATTGGCTTAGAACAA
CAAATTAA

> **recA**

Function: protein coding sequence; recombinase A (core genome, constant)
Best match: recA_CC007_TCH959-USA300_AASB02000234.1[54337:55380] (completely identical)
Position: 031-contig_247: 54176 … 55220; Length: 1044 bp
Sequence:
TTGGATAACGATCGTCAAAAAGCTTTAGATACAGTAATTAAAAATATGGAGAAATCTTTC
GGTAAAGGTGCCGTAATGAAGTTGGGTGACAATATAGGTCGCCGAGTTTCAACTACATCA
ACTGGTTCAGTTACATTAGATAATGCGCTAGGTGTAGGTGGCTATCCTAAAGGACGAATT
ATTGAAATTTATGGTCCTGAAAGTTCTGGTAAGACAACAGTAGCGCTTCACGCTATTGCT
GAAGTACAAAGTAATGGCGGGGTGGCAGCATTTATCGATGCTGAACATGCTTTAGATCCA
GAATATGCTCAAGCATTAGGCGTAGATATCGATAATTTATATTTATCGCAACCGGATCAT
GGTGAACAAGGTCTTGAAATCGCCGAAGCATTTGTTAGAAGTGGTGCAGTTGATATTGTA
GTTGTAGACTCAGTTGCTGCTTTAACACCTAAAGCTGAAATTGAAGGAGAAATGGGAGAC
ACTCACGTTGGTTTACAAGCTCGTTTAATGTCACAAGCGTTACGTAAACTTTCAGGTGCTA
TTTCTAAATCAAATACAACTGCTATTTTCATCAACCAAATTCGTGAAAAAGTTGGTGTTAT

GTTCGGTAATCCAGAGACTACACCAGGTGGACGTGCATTAAAATTCTATAGTTCAGTAAG
ACTAGAAGTACGCCGTGCAGAACAGCTTAAACAAGGACAAGAAATTGTAGGTAATAGAA
CTAAAATTAAAGTCGTTAAAAATAAAGTGGCACCACCATTTAGAGTAGCTGAAGTTGATA
TTATGTATGGACAAGGTATTTCTAAAGAGGGTGAACTTATTGATTTAGGTGTTGAAAACG
ACATCGTTGATAAATCAGGAGCATGGTATTCTTACAATGGCGAACGAATGGGTCAAGGTA
AGGAAAATGTTAAAATGTACTTGAAAGAAAATCCACAAATTAAAGAAGAAATTGATCGT
AAATTGAGAGAAAAATTAGGTATATCTGATGGTGATGTTGAAGAAACAGAAGATGCACC
AAAGTCATTATTTGACGAAGAATAG

> **rny**
Function: protein coding sequence; ribonuclease Y (core genome, constant)
Best match: rny_CC001_MW2-USA400_BA000033.2[1282104:1283663]
Position: 031-contig_247: 55573 … 57133; Length: 1560 bp
Sequence:
GTGAATTTATTAAGCCTCCTACTCATTTTGCTGGGGATCATTCTAGGAGTTGTTGGAGGGT
ATGTTGTTGCCCGAAATTTGTTGCTTCAAAAGCAATCACAAGCTAGACAAACTGCCGAAG
ATATTGTAAATCAAGCACATAAAGAAGCTGACAATATCAAAAAAGAGAAATTACTTGAG
GCAAAAGAAGAAAACCAAATCCTAAGAGAACAAACTGAAGCAGAACTACGAGAAAGAC
GTAGCGAACTTCAAAGACAAGAAACCCGACTTCTTCAAAAAGAAGAAAACTTAGAGCGC
AAATCTGATCTATTAGATAAAAAAGATGAGATTTTAGAGCAAAAAGAATCAAAAATTGA
AGAAAAACAACAACAAGTAGATGCAAAAGAGAGTAGTGTTCAAACGTTAATAATGAAGC
ATGAACAAGAATTAGAACGCATCTCCGGTCTCACTCAAGAAGAAGCTATTAATGAGCAA
CTTCAAAGAGTAGAGGAAGAACTGTCACAAGATATTGCAGTACTTGTTAAAGAAAAAGA
AAAAGAAGCTAAAGAAAAAGTTGATAAAACAGCAAAAGAATTATTAGCTACAGCAGTAC
AAAGATTAGCAGCAGATCACACAAGTGAATCAACGGTATCAGTAGTTAACTTACCTAATG
ATGAGATGAAAGGTCGAATCATTGGACGAGAAGGACGAAACATCCGCACACTTGAAACT
TTAACTGGCATTGATTTAATTATTGATGACACACCAGAAGCGGTTATATTATCTGGTTTTG
ATCCAATAAGAAGAGAAATTGCTAGAACAGCACTTGTTAACTTAGTATCTGATGGACGTA
TTCATCCAGGTAGAATTGAAGATATGGTCGAAAAAGCTAGAAAAGAAGTAGAGGATATT
ATTAGAGAAGCAGGTGAACAAGCTACATTTGAAGTGAACGCACATAATATGCATCCTGA
CTTAGTAAAAATTGTAGGGCGTTTAAACTATCGTACGAGTTACGGTCAAAATGTACTTAA
ACATTCAATTGAAGTTGCGCATCTTGCTAGTATGTTAGCTGCTGAGCTAGGCGAAGATGA
GACATTAGCGAAACGAGCTGGACTTTTACATGATGTTGGTAAAGCAATTGATCATGAAGT
AGAAGGTAGTCATGTTGAAATCGGTGTAGAATTAGCGAAAAAATATGGTGAAAATGAAA
CGGTTATTAATGCAATCCATTCTCATCATGGTGATGTTGAACCTACATCTATTATATCTAT
CCTTGTTGCTGCTGCAGATGCATTGTCTGCGGCTCGTCCAGGTGCAAGAAAAGAAACATT
AGAGAATTATATTCGTCGATTAGAACGTTTAGAAACGTTATCAGAAAGTTATGATGGTGT
AGAAAAAGCATTTGCGATTCAGGCAGGTAGAGAAATCCGAGTGATTGTATCTCCTGAAG
AAATTGATGATTTAAAATCTTATCGATTGGCTAGAGATATTAAAAATCAGATTGAAGATG
AATTACAATATCCTGGTCATATCAAGGTGACAGTTGTTCGAGAGACTAGAGCAGTAGAAT
ATGCGAAATAA

> **porA**
Function: protein coding sequence; pyruvate ferredoxin oxidoreductase alpha chain (core genome, constant)
Best match: porA_CC005_Mu50-omega_BABM01000001.1[1362657:1364417]
Position: 031-contig_247: 58755 … 60516; Length: 1761 bp
Sequence:
ATGAAACCACAATTATCGTGGAAAGTTGGCGGTCAACAAGGCGAAGGTATTGAATCAAC
TGGGGAAATCTTCGCTACGGCTATGAATAGAAAAGGATATTATTTATATGGATATAGACA
TTTTTCAAGTCGTATCAAAGGTGGACATACGAATAATAAAATTAGAGTTTCTACGACGCC
TGTTCATGCAATTAGTGATGATTTAGATATTTTGATTGCATTTGACCAAGAAACAATTGAT
GTTAACCATCATGAAATGAGAGAAGACAGTACTATTTTAGCTGATGCCAAGGCTAAACCT
GTGAAACCAGAAGGATGTCATGCACAGCTTATTGAATTACCTTTTACAGCAACCGCTAAA
GAATTAGGTACAGCATTAATGAAAAACATGGTTGCAATAGGTGCTACTAGCGCATTGATG

AATTTGAATACAAATACATTTGAAGAACTTATTACTAATATGTTTTCTAAAAAAGGTGAC
AAGGTAGTTGAAGTCAATATCCAAGCATTAAACGAAGGTTATCAATTAATGCAATCCCGC
TTACCTGAAATCGACGGGGACTTTGAATTAGAGTCAACAGATGCACTACCACATCTATAT
ATGATTGGTAACGATGCCATTGGATTAGGTGCAATTGCTGCAGGTTCACAATTTATGGCG
GCATATCCTATTACACCTGCGTCTGAAGTTATGGAATATATGATTGCCAATATATCTAAAG
TAAACGGAGCGGTTATTCAAACAGAAGATGAAATTGCTGCTGTAACTATGGCTATTGGTG
CAAATTATGGTGGAGTCAGAGCGTTTACGGCTAGTGCTGGTCCAGGTTTATCTTTAATGAT
GGAAGCAATTGGATTATCTGGTATGACTGAAACGCCATTAGTCATTATTAATACTCAACG
AGGTGGACCTTCTACTGGATTACCTACGAAACAAGAACAGTCAGATTTAATGCAAATGAT
TTATGGTACACATGGTGATATTCCAAAAATAGTTGTAGCACCAACTGATGCAGAAGATGC
ATTTTATTTAACTATGGAAGCCTTTAATTTAGCAGAACAATATCAATGCCCTGTTATAGTT
TTAAGTGATTTGCAATTATCTTTAGGTAAACAAACTGTTGAAAAATTAGATTATAATCGC
ATTGAAATTAAACGTGGTGAAATCATTCAATCTGATATTGAACGTGAAGAAGATGATAAA
GGTTATTTCAAGCGTTATGCGTTAACATCCAATGGTGTTTCTCCTAGACCTATCCCCGGTG
TTAAAGGAGGTATTCATCATATAACTGGTGTGGAACACAATGAAGAAGGTAAACCTAGT
GAATCTGCGTCAAATAGACAACAACAAATGGAAAAACGAATGCGTAAAATTGAGCAGTT
ACTAATTGAATCGCCAGTAGAAGCTAACTTACAACATGAGGATGCAGATATTCTTTATAT
CGGTTTTATTTCTACAAAAGGTGCAATTCAAGAAGGTAGTAACCGTTTGAATCAACAAGG
CATAAAAGTTAACACTATACAAATTAGACAATTGCATCCATTCCCAACAAGCGTTATTCA
AGATGCAGTTAATAAAGCGAAGAAAGTCGTTGTAGTGGAGCACAATTATCAAGGACAAT
TGGCTAGTATTATAAAAATGAATGTCAATATTCATGATAAGATTGAAAATTATACAAAGT
ATGATGGGACACCTTTCCTACCACATGAAATCGAAGAAAAAGGCAAAATAATTGCTACTG
AAATAAAGGAGATGGTATAG

> **porB**

Function: protein coding sequence; 2-oxoglutarate ferredoxin oxidoreductase subunit beta (core genome, constant)
Best match: porB_CC705_RF122-cow_AJ938182.1[1252184:1253050]
Position: 031-contig_247: 60516 … 61383; Length: 867 bp
Sequence:
ATGGCGACATTTAAAGATTTTAGAAATAATGTTAAGCCTAACTGGTGCCCTGGATGTGGC
GATTTCTCAGTACAAGCTGCAATTCAAAAAGCAGCCGCAAATATAGGGTTAGAACCTGAA
GAAGTAGCTATCATCACCGGTATAGGATGTTCTGGCCGTCTTTCAGGATATATTAATTCTT
ATGGCGTTCATTCTATTCACGGACGTGCATTACCTTTAGCTCAAGGTGTAAAAATGGCGA
ATAAAGATTTAACTGTTATTGCATCTGGAGGAGATGGTGATGGTTATGCTATAGGTATGG
GGCATACAATCCATGCTTTAAGAAGAAATATGAACATGACGTATATAGTCATGGATAATC
AAATTTATGGTTTGACAAAGGGACAAACATCGCCGTCATCAGCAGTAGGATTTGTTACTA
AAACAACGCCAAAAGGTAATATAGAAAAAAATGTTGCGCCTTTAGAATTAGCATTATCAT
CTGGTGCCACATTTGTAGCCCAAGGTTTTTCAAGTGATATTAAAGGATTAACAAAACTAA
TTGAAGATGCAATTAATCATGATGGATTTTCATTCGTTAATGTCTTTTCACCATGTGTGAC
TTATAATAAAATTAACACATACGATTGGTTTAAAGAACATTTAACAAGTGTTGATGACAT
TGAAAATTATGATTCTACAGATAAACAATTAGCGACTAGAACTGTTATTGAACATGAATC
TTTAGTAACTGGTATTGTTTATCAAGATAAAGAAACACCATCATATGAATCTCAAATTAA
AGAGTTAGATGATACACCACTTGCTAAAAGAGATATCAAAATTACTGAAGACACGTTCAA
TGCATTAACTGAACAATTTATTTAA

> **miaB**

Function: protein coding sequence; (dimethylallyl)adenosine tRNA methylthiotransferase (core genome, constant)
Best match: miaB_CC001_MW2-USA400_BA000033.2[1288434:1289978]
Position: 031-contig_247: 61903 … 63448; Length: 1545 bp
Sequence:
GTGAACGAAGAACAAAGAAAAGCAAGTTCTGTGGATGTTTTAGCTGAGAGAGATAAGAA
AGCAGAAAAAGATTATAGTAAATATTTTGAACATGTTTATCAACCGCCTAATTTAAAAGA
AGCGAAAAAAAGAGGTAAACAAGAAGTTCGTTATAATAGAGATTTCCAAATTGATGAAA

AATATCGCGGTATGGGGAACGAGCGTACATTTTTAATTAAAACATATGGATGTCAAATGA
ATGCACATGACACTGAGGTCATTGCTGGTATACTTAAAGCATTAGGCTATCAAGCAACGA
CTGATATTAACACTGCAGATGTTATTTTAATTAATACATGTGCGATTAGAGAAAATGCCG
AGAACAAAGTGTTTAGTGAAATAGGTAATTTGAAGCATTTGAAAAAAGAACGACCTGAT
ATTTTAATCGGTGTTTGTGGTTGTATGTCACAAGAAGAGTCAGTAGTGAATAAAATTTTA
AAATCGTATCAAAATGTAGATATGATATTTGGTACACATAATATTCATCATTTACCAGAA
ATTTTAGAAGAAGCATACTTATCTAAAGCAATGGTTGTTGAAGTATGGTCTAAAGAAGGA
GACGTTATTGAAAATCTTCCAAAAGTCCGTGAAGGCAACATTAAAGCATGGGTCAATATT
ATGTATGGTTGTGATAAGTTTTGTACATATTGTATTGTTCCATTTACAAGAGGTAAAGAAC
GAAGCCGTAGACCTGAAGACATTATAGATGAAGTACGTGAACTTGCTCGTGAAGGTTACA
AAGAAATAACGCTTTTAGGTCAAAATGTAAATTCTTATGGTAAAGATTTACAGGATATAG
AATATGACTTAGGAGATCTTTTACAAGCAATTTCTAAAATAGCGATTCCAAGAGTTCGTTT
CACAACAAGTCATCCTTGGGACTTTACAGATCACATGATTGATGTTATTTCAGAGGGTGG
TAATATCGTTCCGCATATCCACTTGCCAGTTCAATCTGGAAATAATGCAGTATTAAAAAT
AATGGGTAGAAAATATACACGAGAAAGTTATTTGGATTTAGTAAAACGAATCAAAGATA
GAATTCCTAATGTAGCATTAACTACAGATATTATTGTAGGGTATCCAAATGAATCAGAGG
AACAATTTGAAGAAACTTTAACTCTGTATGATGAAGTTGGTTTTGAACATGCATATACGT
ACTTGTATTCACAACGTGATGGTACGCCTGCTGCTAAAATGAAAGATAATGTACCTTTAG
ATGTCAAAAAGGAACGATTGCAACGTTTGAATAAAAAAGTTGGTCATTATTCACAAATAG
CTATGAGTAAGTACGAAGGACAAACTGTAACAGTACTTTGTGAAGGTAGTAGTAAAAAA
GATGATCAGGTTCTTGCTGGCTACACTGATAAAAATAAGCTAGTTAATTTCAAAGCGCCT
AAAGAAATGATTGGTAAACTAGTGGAAGTACGAATAGATGAAGCTAAACAGTATTCATT
AAATGGCAGTTTTGTAAAGGAAGTAGAGCCGGAAATGGTGATTCAATAA

> **thiW**

Function: protein coding sequence; ThiW family protein (core genome, constant)
Best match: thiW_CC001-ST772_118_AJGE01000002.1[99053:99544:r] (completely identical)
Position: 031-contig_247: 63840 … 64332; Length: 492 bp
Sequence:
ATGAAATCAAGAAAACTGGCTATAACTGCACTTTTAATTGCAATAAATGTTGTATTAAGC
AGTATTATCATCATTCCTCTAGGACCAGTTAAGGCAGCACCAGTACAGCATTTTGTAAAT
GTATTAAGTGCGGTGATAGTAGGTCCTTGGTATGGATTAGCTCAAGCGCTTATATCATCA
ATTTTAAGAGTTCTTTTTGGTACTGGTACAGCTTTTGCATTTCCGGGTAGTATGATTGGAG
TTTTATTGGCTAGTATGTTTTACATATATCGTAAACATATATTTATGGCCGCGGTCGGTGA
AGTACTTGGAACTGGTGTCATCGGAAGTTTAATTTGTATACCATTAGCATATTTCCTTGGG
CTTCAAGACTTCTTCATTAAACCGTTAATGATTACGTTCATAGTCTCAAGTGCTATCGGAT
CTATTATAAGTTATTTCTTATTAATTACTCTAAAAAAACGTGGTATTCTTCAAAGGTTTAT
AAAATAA

> **mutS**

Function: protein coding sequence; DNA mismatch repair protein (core genome, constant)
Best match: mutS_CC001_MW2-USA400_BA000033.2[1291165:1293783]
Position: 031-contig_247: 64634 … 67253; Length: 2619 bp
Sequence:
ATGTCTAATGTTACACCAATGATGCAGCAATATTTAAAAATAAAATCAGAATACCAAGAT
TGCTTATTATTTTTTAGACTAGGTGATTTCTATGAAATGTTTTATGAAGATGCCAAGGAGG
CATCACGTGTACTTGAAATTACTTTAACTAAAAGAGATGCTAAAAAAGAAAATCCAATTC
CGATGTGTGGTGTTCCGTATCATTCTGCAGATAGTTATATAGATACACTTGTTAATAATGG
ATATAAAGTAGCTATTTGTGAACAGATGGAAGATCCGAAACAAACGAAAGGTATGGTTA
GACGTGAGGTAGTAAGAATTGTGACTCCAGGAACTGTGATGGAGCAAGGTGGTGTAGAT
GATAAACAAAATAACTATATTTTAAGTTTTGTTATGAATCAACCTGAAATTGCGCTTAGTT
ACTGTGATGTTTCTACTGGCGAATTAAAGGTTACACATTTTAATGATGAAGCGACTTTATT
AAATGAAATTACGACGATAAACCCTAACGAAGTTGTTATCAATGACAATATTTCCGATAA
TTTAAAAAGACAAATTAATATGGTGACAGAAACAATAACAGTCAGGGAAACGTTATCAT
CAGAAATCTATAGTGTGAATCAAACTGAACATAAATTAATGTATCAAGCGACACAATTAT

TGCTAGATTATATTCATCATACACAAAAACGTGATTTATCGCATATCGAGGATGTTGTTCA
ATATGCAGCTATAGATTATATGAAAATGGATTTTTATGCTAAGAGAAACCTTGAGTTAAC
GGAAAGCATTCGATTAAAATCAAAAAAAGGAACGCTACTTTGGCTAATGGACGAAACGA
AAACACCAATGGGAGCACGCCGCTTAAAACAATGGATAGATAGACCACTAATAAGTAAA
GAACAAATTGAAGCACGATTAGATATCGTTGATGAATTTAGTGCTCATTTCATAGAAAGA
GACACCTTAAGAACATATCTTAATCAAGTGTATGATATTGAACGTCTTGTTGGGCGTGTTA
GTTACGGAAATGTTAATGCGAGAGATTTAATTCAACTTAAACATTCCATTTCTGAAATAC
CGAATATTAAAGCATTACTAAATTCTATGAATCAGAATACTCTTGTACAAGTTAATCAAC
TAGAACCCCTTGATGATTTACTTGATATATTAGAACAGAGTTTAGTAGAAGAACCACCAA
TTTCAGTTAAAGATGGCGGACTATTCAAAGTTGGTTTTAATACGCAATTAGATGAATATCT
TGAAGCTTCAAAAAACGGAAAAACATGGTTAGCAGAATTACAAGCCAAAGAAAGACAAC
GTACAGGAATAAAATCATTGAAAATAAGCTTTAATAAAGTGTTTGGTTATTTTATAGAAA
TAACACGTGCCAACTTGCAAAATTTTGAACCAAGTGAATTTGGTTATATGAGGAAGCAAA
CGTTATCGAATGCTGAACGTTTTATAACTGATGAACTTAAAGAAAAAGAAGATATCATTT
TAGGTGCGGAAGACAAAGCCATCGAATTAGAATATCAATTATTTGTTCAGCTACGTGAAG
AAGTTAAAAAATATACTGAACGTTTACAACAACAAGCTAAAATTATTTCAGAGCTAGATT
GTTTACAGAGCTTTGCAGAAATTGCTCAAAAATATAATTACACTAGGCCTTCATTTAGTG
AAAATAAAACATTAGAATTAGTGGAATCTAGGCACCCAGTAGTGGAAAGAGTAATGGAT
TATAATGACTATGTGCCTAATAATTGTCGATTAGATAATGAAACATTTATATATTTAATTA
CAGGTCCGAATATGTCTGGTAAATCGACATATATGAGACAAGTTGCCATAATTAGTATAA
TGGCCCAAATGGGAGCTTATGTCCCTTGTAAAGAGGCAGTGTTACCTATATTTGATCAAA
TATTCACTAGAATAGGTGCGGCAGATGATTTGGTTTCAGGTAAGAGTACGTTTATGGTAG
AAATGCTAGAAGCACAAAAGGCATTAACTTATGCAACAGAGGATAGTTTGATTATTTTCG
ATGAAATTGGACGTGGTACTTCAACGTATGACGGTTTAGCTTTAGCGCAGGCAATGATAG
AGTATGTAGCTGAAACATCGCATGCTAAAACGTTATTTTCAACACATTATCATGAATTGA
CAACATTAGATCAAGCATTACCAAGTCTAAAAAATGTTCACGTCGCTGCTAATGAATATA
AAGGTGAACTTATATTCTTGCATAAAGTCAAAGATGGTGCAGTTGACGATAGTTATGGTA
TTCAAGTTGCGAAATTAGCTGATTTACCTGAAAAAGTTATTAGCAGAGCACAAGTGATCC
TAAGCGAGTTTGAAGCGTCTGCTGGTAAAAAATCATCGATATCAAATTTAAAAATGGTCG
AAAATGAACCTGAAATTAATCAAGAAAATTTAAACTTAAGTGTTGAAGAAACAACTGAT
ACTTTATCCCAAAAAGACTTTGAACAAGCATCATTTGATTTGTTTGAAAATGATCAAGAA
AGCGAGATTGAACTACAAATTAAAAATTTGAATTTATCTAATATGACACCAATTGAGGCA
TTGGTGAAGTTAAGTGAATTACAAAATCAATTAAAATAG

## > **mutL**

Function: protein coding sequence; DNA mismatch repair protein (core genome, constant)
Best match: mutL_CC001_MW2-USA400_BA000033.2[1293796:1295805]
Position: 031-contig_247: 67265 … 69275; Length: 2010 bp
Sequence:
ATGGGGAAAATTAAAGAACTCCAAACCTCATTAGCAAATAAAATCGCAGCAGGTGAAGT
AGTTGAAAGACCGAGTTCTGTTGTGAAAGAACTGTTGGAAAATGCTATAGATGCAGGCGC
TACAGAAATAAGCATTGAAGTAGAGGAATCTGGCGTCCAATCTATTCGCGTAGTCGATAA
TGGAAGCGGAATTGAAGCGGAAGACTTAGGATTAGTATTTCATAGACATGCGACTAGTA
AATTAGATCAAGATGAAGATTTATTTCATATTAGGACATTAGGATTCCGTGGTGAAGCAC
TAGCCAGTATTTCATCAGTTGCTAAAGTAACATTGAAGACTTGCACGGATAATGCTAATG
GAAATGAAATATATGTAGAAAATGGTGAAATATTAAATCATAAGCCTGCAAAAGCGAAA
AAAGGAACAGATATACTTGTAGAATCATTATTTTATAATACACCAGCACGTTTAAAATAT
ATTAAAAGTTTATACACTGAACTAGGTAAAATAACAGATATTGTCAACAGAATGGCAATG
AGCCATCCGGACATTCGAATAGCACTCATTTCAGATGGCAAAACAATGTTAAGTACAAAT
GGTTCAGGACGAACTAATGAAGTGATGGCAGAGATTTATGGGATGAAAGTTGCACGAGA
TTTAGTACATATATCTGGAGATACAAGTGATTATCACATTGAAGGTTTTGTTGCAAAGCCT
GAACATTCTAGAAGTAATAAGCACTATATTTCTATTTTTATTAATGGACGATACATTAAAA
ACTTTATGCTAAATAAAGCGATTTTAGAAGGCTATCATACACTCTTAACAATAGGTAGGT
TCCCGATTTGTTATATTAATATTGAAATGGATCCAATCTTAGTAGACGTAAATGTTCATCC
AACAAAACTAGAAGTGCGTTTATCAAAAGAAGAGCAACTATACCAATTGATTGTGAGCA
AAATACAAGAAGCATTTAAAGACCGTATATTAATTCCTAAAAATAACTTGGATTATGTGC

CGAAAAAAAATAAAGTGTTACATTCATTCGAACAACAAAAAATCGAATTTGAACAAAGA
CAAAACACAGAGAATAATCAAGAGAAGACGTTTTCATCTGAAGAAAGTAACAGTAAGCC
ATTTATGGTAGAAAATCAAAACGATGAAATAGTTATAAGAGAAGATTCATATAATCCATT
CGTAACGAAAACGTCTGAAAGTTTAATAGCTGATGATGAATCTTCCGGTTATAATAATAC
ACGTGAAAAAGATGAAGACTACTTCAAAAAGCAACAAGAAATTCTACAAGAAATGGATC
AAACATTTGATTCGAATGACGATACATCTGTGCAAAATTATGAGAATAAAGCGTCTGATG
ATTATTATGATGTAAACGATATTAAAGGAACAAAAAGTAAAGACCCTAAACGAAGAATT
CCATATATGGAAATTGTTGGCCAAGTACATGGAACGTATATTATTGCTCAAAATGAATTT
GGCATGTACATGATTGACCAGCATGCAGCTCAAGAAAGAATAAAATATGAATATTTTCGA
GATAAAATAGGTGAAGTTACCAATGAAGTACAAGATTTATTAATCCCGTTAACATTTCAT
TTTTCAAAAGATGAACAATTAGTCATTGATCAATATAAAAATGAGCTTCAACAAGTAGGT
ATCATGTTAGAACATTTTGGTGGTCATGATTATATTGTAAGTAGCTATCCAGTTTGGTTCC
CTAAAGATGAAGTAGAAGAAATTATTAAAGATATGATTGAGCTAATTTTGGAAGAGAAA
AAAGTAGATATCAAAAAATTACGTGAAGATGTAGCAATCATGATGTCATGTAAAAAATCT
ATTAAAGCGAATCATTATTTACAAAAACATGAAATGTCTGATTTAATTGATCAATTAAGA
GAAGCGGAAGATCCATTTACATGTCCACATGGTCGTCCAATTATCATTAATTTTTCAAAAT
ACGAATTAGAAAAATTATTTAAGCGTGTGATGTAG

> **glpP**

Function: protein coding sequence; glycerol uptake operon antiterminator regulatory protein (core genome, constant)
Best match: glpP_CC001_MW2-USA400_BA000033.2[1295820:1296353] (completely identical)
Position: 031-contig_247: 69289 … 69823; Length: 534 bp
Sequence:
GTGAATAACAACATATTGCCTGCCATAAGAAACATTAAAGATTTAGAGAAACTGATTAAA
ACAGATTATAAAATGTGTGTGCTTCTAGATATGCATATAGGACATATAAAAAGTATTATG
GAATTGCTGAAGCAAAATCATATAGAGTGTTTTATTCATATAGATTTGATAAAAGGTTTA
AGCCACGATGAATTTGCAAGTGAATTTATTATTCAGCAATACAAGCCAAAAGGTATCGTA
TCGACTAAATCTAAAGTAATAAAAAAAGCTAAATCATTAAATACTTTAACGATTTTTAGA
GTATTTATTATTGATAGTCAAGCATTGAAACGCAGTATAGATTTGATAAAAAAAGTTGAA
CCTGATTTTGTTGAAGTACTTCCAGGTGTTGCGAGTAAAGCGATTCATCATATTCAGAAA
GAAACAAACACACAAGTCATTGCAGGTGGCCTAATTAATACAATAGATGAAGTCAATGA
AGCTGTTAAAAATGGAGCGAAATATGTAACAACTAGTTATGATAAACTTTGGTAA

> **glpF**

Function: protein coding sequence; glycerol uptake facilitator (core genome, constant)
Best match: glpF_CC001_MW2-USA400_BA000033.2[1296828:1297646] (completely identical)
Position: 031-contig_247: 70297 … 71116; Length: 819 bp
Sequence:
ATGAATGTATATTTAGCAGAATTCCTAGGAACTGCAATCTTAATCCTTTTTGGTGGTGGCG
TTTGTGCCAATGTCAATTTAAAGAGAAGTGCTGCGAATGGTGCTGATTGGATTGTCATCA
CAGCTGGATGGGGATTAGCGGTTACAATGGGTGTGTTTGCTGTCGGTCAATTCTCAGGTG
CACATTTAAACCCAGCGGTGTCTTTAGCTCTTGCATTAGACGGAAGTTTTGATTGGTCATT
AGTTCCTGGTTATATTGTTGCTCAAATGTTAGGTGCAATTGTCGGAGCAACAATTGTATGG
TTAATGTACTTGCCACATTGGAAAGCGACAGAAGAAGCTGGCGCGAAATTAGGTGTTTTC
TCTACAGCACCGGCTATTAAGAATTACTTTGCCAACTTTTTAAGTGAGATTATCGGAACA
ATGGCATTAACTTTAGGTATTTTATTTATCGGTGTAAACAAAATTGCCGATGGTTTAAATC
CTTTAATTGTCGGAGCATTAATTGTTGCAATCGGATTAAGTTTAGGCGGTGCTACTGGTTA
TGCAATCAACCCAGCACGTGATTTAGGTCCGAGAATTGCACATGCGATTTTACCAATAGC
TGGTAAAGGTGGTTCAAATTGGTCATATGCAATCGTTCCTATCTTAGGACCAATTGCCGGT
GGTTTATTAGGTGCAGTGGTATACGCTGTATTTTATAAACATACATTTAATATTGGTTGTG
CAATTGCAATTGTTGTAGTTATTATTACTTTGATTTTAGGTTACATTTTAAATAAATCATCA
AAAAAAGGTGATATCGAATCAATTTACTAA

> **glpK**

Function: protein coding sequence; glycerol kinase (core genome, constant)
Best match: glpK_CC001_MSSA476_BX571857.1[1326270:1327766]
Position: 031-contig_247: 71244 … 72741; Length: 1497 bp
Sequence:
ATGGAAAAATATATTTTATCTATAGACCAAGGAACAACAAGCTCAAGAGCGATTTTATTC
AATCAAAAAGGGGAAATTGCAGGGGTAGCACAACGTGAGTTTAAGCAATATTTTCCACA
ATCAGGTTGGGTTGAACATGATGCAAATGAAATTTGGACATCTGTGTTAGCTGTAATGAC
GGAAGTAATTAATGAAAATGATGTTAGAGCTGATCAAATTGCAGGAATCGGTATTACAA
ACCAACGTGAAACAACGGTTGTTTGGGACAAACATACTGGCCGCCCAATTTATCACGCAA
TTGTTTGGCAATCACGTCAAACACAATCAATTTGTTCAGAGTTAAAACAACAAGGATATG
AACAAACATTTAGAGATAAGACAGGATTACTTTTAGATCCGTATTTTGCAGGTACAAAAG
TTAAATGGATTCTAGACAATGTTGAAGGTGCACGAGAAAAAGCAGAAAATGGCGATCTA
TTATTTGGAACGATTGATACTTGGTTAGTATGGAAATTATCAGGAAAAGCTGCGCATATT
ACTGATTATTCAAATGCGAGTCGTACATTAATGTTTAATATCCATGATTTAGAATGGGAC
GATGAGTTATTAGAACTACTTACAGTACCTAAAAATATGTTGCCAGAAGTTAAAGCTTCG
AGTGAAGTATATGGTAAGACAATTGATTACCACTTCTATGGTCAAGAAGTACCAATCGCT
GGAGTAGCTGGTGATCAACAAGCAGCATTATTTGGACAAGCTTGCTTCGAACGTGGTGAC
GTGAAAAACACATATGGAACTGGTGGCTTCATGTTAATGAATACAGGTGACAAAGCGGTT
AAATCTGAAAGTGGTTTATTAACAACAATTGCTTATGGTATTGATGGAAAAGTAAATTAT
GCGCTTGAAGGTTCCATCTTTGTTTCGGGTTCAGCAATCCAATGGTTACGTGATGGATTAA
GAATGATTAATTCAGCACCACAATCAGAAAGTTATGCGACACGAGTTGACTCTACTGAGG
GTGTTTATGTTGTTCCAGCTTTTGTAGGTTTAGGAACACCATATTGGGATTCTGAAGCACG
TGGTGCGATTTTTCGGTTTAACACGTGGAACTGAAAAAGAGCACTTTATCCGTGCAACTTT
AGAATCACTATGTTACCAAACTCGTGACGTTATGGAAGCAATGTCAAAAGACTCTGGTAT
TGATGTCCAAAGTTTACGTGTCGATGGTGGTGCAGTTAAAAATAACTTTATTATGCAGTTC
CAAGCAGACATTGTTAATACTTCTGTTGAAAGACCTGAAATTCAAGAAACTACAGCTTTA
GGTGCTGCATTTTTAGCAGGTTTAGCAGTTGGATTCTGGGAGAGTAAAGATGATATCGCT
AAAAACTGGAAATTAGAAGAAAAATTCGATCCGAAAATGGATGAAGGCGAAAGAGAAA
AATTATATAGAGGTTGGAAAAAAGCTGTTGAAGCAACACAAGTTTTTAAAACAGAATAA

> **glpD**

Function: protein coding sequence; aerobic glycerol-3-phosphate dehydrogenase (core genome, constant)
Best match: glpD_CC001_MW2-USA400_BA000033.2[1299381:1301102]
Position: 031-contig_247: 72850 … 74572; Length: 1722 bp
Sequence:
ATGTGGGACAAGTCTCTCGTTTTTACATTTTTTAGGAGGCGTTTTGGAATGGCATTGTCTA
CTTTTAAGAGAGAACATATTAAAAAGAATTTAAGAAATGATGAATATGATTTAGTAATTA
TTGGTGGCGGTATTACAGGTGCAGGTATTGCACTAGACGCGAGTGAAAGAGGAATGAAA
GTTGCATTAGTTGAAATGCAAGACTTTGCACAAGGAACAAGCTCAAGATCTACAAAATTA
GTCCATGGTGGTTTGCGTTACTTAAAACAATTCCAAATTGGAGTAGTTGCCGAAACTGGT
AAAGAACGTGCGATTGTTTATGAAAATGGGCCTCATGTTACGACTCCAGAGTGGATGCTT
TTACCAATGCATAAAGGTGGAACATTTGGTAAATTCTCAACATCAATTGGTTTAGGAATG
TATGATCGTTTAGCAGGTGTTAAGAAGTCTGAACGTAAAAAAATGTTATCTAAAAAAGAA
ACTTTAGCTAAAGAACCATTAGTTAAAAAAGAAGGTCTAAAAGGCGGCGGTTACTATGTT
GAATATCGTACTGACGATGCGCGTTTAACTATTGAAGTTATGAAGCGTGCTGCTGAAAAA
GGCGCAGAAATTATCAACTATACTAAATCTGAACACTTCACTTATGATAAAAATCAACAA
GTAAATGGTGTTAAAGTTATAGATAAATTAACTAATGAAAATTATACAATTAAGGCTAAA
AAAGTGGTTAATGCAGCAGGTCCATGGGTTGATGATGTTAGAAGTGGTGATTATGCACGC
AATAATAAAAAATTACGTTTAACTAAAGGTGTACATGTTGTTATTGATCAATCAAAATTC
CCATTAGGTCAAGCAGTATACTTTGATACTGAAAAAGATGGAAGAATGATTTTTGCAATT
CCACGTGAAGGAAAAGCGTATGTAGGTACTACAGATACATTCTATGACAATATCAAATCT
TCACCATTAACTACACAAGAAGACAGAGACTATTTAATCGATGCGATTAATTACATGTTC
CCTAGTGTTAATGTTACAGATGAAGATATTGAATCAACATGGGCAGGAATTAGACCATTA
ATTTACGAAGAAGGCAAAGACCCTTCTGAAATCTCTCGTAAGGATGAAATTTGGGAAGGT
AAATCAGGTTTATTAACTATTGCAGGTGGTAAATTAACAGGCTATCGTCACATGGCTCAA

GACATTGTTGATTTAGTATCTAAACGCTTGAAAAAAGACTACGGTTTAACATTTAGTCCAT
GTAATACAAAAGGTCTGGCAATTTCAGGTGGCGATGTAGGTGGTAGCAAGAACTTTGATG
CGTTTGTAGAGCAAAAAGTAGATGTAGCTAAAGGATTCGGCATTGATGAAGATGTTGCAA
GACGTTTAGCATCTAAATATGGTTCAAATGTTGATGAATTGTTCAACATTGCGCAAACAT
CTCAATACCATGATAGCAAGTTACCATTAGAAATTTATGTAGAACTTGTTTATAGTATTCA
ACAAGAAATGGTATACAAACCTAACGATTTCTTAGTTCGTCGTTCTGGTAAAATGTATTTC
AATATTAAAGATGTATTAGATTATAAAGATGCTGTCATCGATATTATGGTAGATATGCTT
GATTACTCTCCAGCTCAAATTGAAGCATATACTGAAGAAGTTGAGCAAGCAATTAAAGAA
GCGCAACATGGAAATAATCAACCAGCAGTTAAAGAATAA

> **miaA**

Function: protein coding sequence; tRNA Delta(2)-isopentenylpyrophosphate transferase (core genome, constant)
Best match: miaA_CC001_MW2-USA400_BA000033.2[1302184:1303119] (completely identical)
Position: 031-contig_247: 75653 … 76589; Length: 936 bp
Sequence:
ATGAATAAAAATAAGCCTTTTATTGTAGTAATTGTGGGGCCAACTGCTTCAGGTAAAACA
GAGCTTAGCATAGAACTCGCGAAGCGTATCAATGGTGAAATCATAAGCGGTGATTCTATG
CAAGTTTACAAACATATGAATATTGGAACTGCAAAAGTAACACCTGAAGAAATGGATGG
TATTCCACATCATTTAATTGATATCTTGAATCCTGATGATACATTTTCAGCATATGAATTC
AAGCGATTAGCAGAAGATTTAATTACTGATATAACGAATAGAGGTAAAGTTCCAATCATA
GCAGGTGGAACAGGCTTATATATTCAATCATTAATATATAATTATGAATTAGAAGATGAA
ACAGTTACACCTGCACAATTATCCATAGTTAAACAAAAGTTATCTGCATTAGAACATTTA
GATAATCAGCAACTACACGATTATTTAGCTCAATTTGATGCGGTTTCTGCAGAAAATATTC
ACCCTAACAACCGCCAAAGAGTGTTGCGCGCTATTGAATATTATTTAAAAACAAAAAAAC
TTTTGAGTAATCGCAAGAAAGTGCAACAATTTACTGAAAATTATGATACATTATTATTAG
GGATTGAAATGTCGCGTAAAACATTATATTCAAGAATAAATAAACGTGTTGATATTATGT
TGGATCACGGATTATTTAGAGAAGTGCAACAACTTGTTGAACAAGGCTATGAATCTTGCC
AAAGTATGCAAGCTATTGGATATAAAGAATTAATACCTGTGATTAACGGACAAATGATTT
ATGAAGATGCTGTCAATGATTTAAAGCAACATTCACGCCAATATGCAAAACGACAAATG
ACATGGTTCAAGAATAAAATGAGTGTTCATTGGTTAGATAAAGAAAATATGTCACTTCAA
ATGATGTTAGATGAGATTACAACCCAGATTAAGTAA

> **hfq**

Function: protein coding sequence; RNA chaperone Hfq (core genome, constant)
Best match: hfq_CC001_MW2-USA400_BA000033.2[1303134:1303367] (completely identical)
Position: 031-contig_247: 76603 … 76837; Length: 234 bp
Sequence:
ATGATTGCAAACGAAAACATCCAAGACAAAGCACTAGAGAATTTTAAAGCAAACCAAAC
TGAAGTAACTGTATTCTTTCTAAACGGTTTCCAAATGAAAGGTGTTATTGAAGAATACGA
CAAGTATGTCGTAAGCTTAAATTCTCAAGGCAAACAACACTTGATTTACAAACATGCGAT
CAGCACTTATACAGTAGAAACTGAAGGTCAAGCATCTACTGAAAGTGAAGAATAA

> **gpxA-L1**

Function: protein coding sequence; glutathione peroxidase locus 1 (core genome, constant)
Best match: gpxA-L1_CC008_NCTC8325_CP000253.1[1239095:1239571:r]RC (completely identical)
Position: 031-contig_247: 77058 … 77535; Length: 477 bp
Sequence:
TTATAATAATTTTTCTATTTCTCTTTCGATTTGAACAGGTTTTTTTTGAGGTGCAAATCGTT
TAACAACGTTACCTTCGCGATCCACTAAAAACTTAGTGAAATTCCATTTGATTTTCTCATT
AAAGAATCCGTGTTGTGCCGCAGTCAAATATCTAAATAAAGGTAATTGATGTTCCCCTTTT
ACGTCTATTTTTTGATGCATAGGGAAGGTAACACCATAGTTTAATTTACAGTTTTGAGCTG
CTTCTTCGCCTGAACCAGGTTCTTGGCCACCAAATTGATTACAAGGGAAACCTAGAATTA
CAAACCCTTGATCTTTGTATTTCTCGTATAATGATTGCAAACCTTCAAATTGTGAAGTAAA

GCCACATTCGCTAGCTGTATTAACAATTAGCATAACGTCACCCTTATATGCATCTAATTTG
TAAGTAACACCTTTATTTGTTTCTACTACAAAATCATAAATTGTCTCCAT

### > **ynbA**

Function: protein coding sequence; GTP-binding proteinase modulator (core genome, constant)
Best match: ynbA_CC008_NCTC8325_CP000253.1[1239683:1240921] (completely identical)
Position: 031-contig_247: 77646 … 78885; Length: 1239 bp
Sequence:
ATGGCTCAGCAACAAATTCATGATACTAAAAATAAACTAGAAAAAGCTGTCTTAGTCGGT
GTACATGCTCAAGATGATAAGCAATTTAATTTTGAGTCTACAATGGAGGAATTATCATCT
TTATCAGAGACTTGCCAACTTGAAGTGTTGGGTCAAATTACTCAAAACAGAGATCGTGTA
GATCGCAAATATTATGTTGGTAAAGGTAAAATTGAAGAAATTCAAGCATTTATTGAGTTC
AAAGATATTGATGTAGTCATCACAAATGATGAATTAACGACTGCACAATCCAAATCACTA
AATGAAGCTTTAGGTGTAAAAATTATTGATAGAACTCAGTTGATTCTTGAAATATTTGCAT
TAAGAGCAAGAAGTAAAGAAGGTAAATTGCAAGTAGAGCTAGCACAACTTGATTATTTA
TTACCTAGATTGCAAGGCCATGGTAAAAGCCTTTCTCGTTTAGGTGGCGGTATTGGAACT
AGAGGCCCTGGTGAAACGAAGTTAGAGATGGATCGCAGACATATTCGAACTCGTATGAA
TGAAATTAAACATCAATTGCGGACGGTAGAAGAACATCGCGAAAGATATCGAAATAAAA
GAAATCAAAATCAGGTGTTTCAAGTAGCTTTAGTTGGTTATACAAATGCTGGTAAATCAT
CATGGTTTAATGTTTTAGCAAATGAAGAGACGTATGAAAAAGATCAATTATTTGCAACGT
TAGATCCTAAAACACGACAAATTCAAATAAATGATGGATTTAATTTAATTATTTCAGATA
CTGTTGGTTTTATACAGAAACTACCTACGACGTTAATTGCAGCTTTTAAATCAACTTTAGA
AGAGGCTAAAGGTGCAGATTTATTAGTACATGTCGTAGATAGTAGCCATCCTGAATACCG
TACGCAGTATGACACAGTTAATGATTTAATCAAACAATTAGATATGAGTCATATTTCTCA
AATAGTTATTTTTAATAAAAAGGACTTATGTGATCATGCATCAAATCGTCCAGCAAGTGA
TTTGCCTAATGTTTTTGTTTCTTCTAAAAATGATGGTGATAAATTACTTGTTAAGACGTTAT
TTATTGATGAAATCAAAAGGCAATTAACTTATTATGATGAGACAATTGCGACGAATAATG
CAGATCGATTATATTTTCTAAAACAACATACATTAGTGACTGAACTTAAATATGATGAAA
TTGAAAATGTTTATCGTATAAAAGGATTTAAAAAAATAA

### > **glnR**

Function: protein coding sequence; glutamine synthetase repressor (core genome, constant)
Best match: glnR_CC001_MW2-USA400_BA000033.2[1306916:1307284]
Position: 031-contig_247: 80384 … 80753; Length: 369 bp
Sequence:
ATGATATCGAATGATGCAATCAGACGAAATATGGCTGTCTTCTCTATGAGTGTAGTAAGT
AAGTTAACGGATTTAACACCAAGGCAAATACGTTACTATGAAACACATGAACTCATCAAA
CCTGAAAGAACAGAAGGTCAAAAACGTCTGTTCTCACTCAATGATTTGGAAAGATTACTA
GAAATTAAATCATTATTAGAAAAAGGATTTAATATCAAAGGGATTAAACAAATCATTTAT
GACTCACAAGAGCATTTAACAACAGATGAACAAGAGATAAGAAAAAAGATGATTGTAGA
TGCCACGCAAAAGCCTATTGGAGAAACTTTGCCAATAAATCGTGGTGATTTATCCCGATT
TATTAAATAA

### > **glnA**

Function: protein coding sequence; glutamine synthetase/type I (core genome, variable)
Best match: glnA_CC007_TCH959-USA300_AASB02000234.1[80932:82272] (completely identical)
Position: 031-contig_247: 80771 … 82112; Length: 1341 bp
Sequence:
ATGCCAAAACGTACTTTCACTAAAGACGACATTCGTAAATTTGCAGAAGAGGAAAATGTA
AGATATTTAAGATTACAATTCACTGATATTTTAGGAACAATTAAAAATGTTGAAGTGCCT
GTAAGCCAATTAGAAAAAGTACTTGATAACGAAATGATGTTTGACGGTTCTTCTATCGAA
GGTTTCGTACGTATCGAAGAATCAGATATGTACTTACATCCAGATTTAGATACTTGGGTA
ATCTTCCCATGGACTGCTGGACAAGGTAAAGTTGCACGTTTAATTTGTGATGTATATAAA
ACAGATGGAACACCATTTGAAGGGGATCCTCGTGCAAACTTAAAACGTGTATTAAAAGA
AATGGAAGATTTAGGCTTCACAGACTTTAACCTAGGGCCTGAACCAGAATTCTTCTTGTTT

AAGTTGGATGAAAAAGGGGAACCAACTTTAGAACTTAATGATGATGGTGGATATTTCGAT
TTAGCACCTACAGATTTAGGTGAAAACTGTCGTCGTGATATTGTTTTAGAATTAGAGGAT
ATGGGCTTCGATATTGAAGCTAGTCACCATGAAGTTGCCCCTGGTCAACATGAAATTGAC
TTTAAATATGCAGATGCTGTTACAGCATGTGATAATATCCAAACATTTAAATTGGTTGTTA
AAACAATCGCACGTAAACATAATTTACACGCAACATTTATGCCTAAACCATTATTCGGTG
TGAATGGTAGCGGTATGCACTTTAACGTTTCATTATTCAAAGGTAAAGAAAATGCATTCT
TTGATCCAAATACTGAAATGGGCTTAACGGAAACTGCATATCAATTTACAGCAGGTGTGC
TTAAAAATGCACGCGGATTTACTGCTGTATGTAACCCGTTAGTAAACTCATATAAACGTTT
AGTACCTGGTTATGAAGCACCATGTTATATTGCATGGAGTGGTAAAAACCGTTCACCATT
AATCCGTGTACCATCTTCAAGAGGATTATCTACTCGTATCGAAGTACGTTCAGTAGATCC
AGCTGCAAACCCATACATGGCGTTAGCTGCAATCTTAGAAGCTGGACTAGATGGTATTAA
AAATAAATTAAAAGTTCCAGAACCAGTTAACCAAAATATTTACGAAATGAACCGTGAAG
AACGTGAAGCAGTAGGCATTCAAGACTTACCTTCAACACTTTATACTGCATTAAAAGCAA
TGCGTGAAAATGAAGTTATTAAAAAAGCTTTAGGAAATCATATCTATAATCAATTTATTA
ATTCAAAATCAATTGAATGGGATTACTACAGAACTCAAGTATCTGAATGGGAAAGAGATC
AGTACATGAAGCAATATTAA

## > A6U1C8

Function: protein coding sequence; putative protein (core genome, constant)
Best match: A6U1C8_CC001-ST772_118_AJGE01000002.1[80583:80780:r] (completely identical)
Position: 031-contig_247: 82604 … 82802; Length: 198 bp
Sequence:
ATGAATTGCTATGATGAAATATTCAATACAATCAAAAAGTTGATAGAAAACAAAGAGAT
ATCGAGTTATCAAATTAATAAAGATACTGGGATAAGTTACGGTAATATTAATGCTATGCG
CCGTGGAGAAAGAAGAATAGAAAATTTAAGCTTAAAGAATTCAAAAATCTTATATGAAT
ATGCGAAAAAGGTATTATAA

## > Q2FYY4

Function: protein coding sequence; putative protein (core genome, variable)
Best match: Q2FYY4_CC001-ST772_118_AJGE01000002.1[79669:79893:r] (completely identical)
Position: 031-contig_247: 83491 … 83716; Length: 225 bp
Sequence:
TTGACAAAAATAATGAACCAGTTTAAAGAAATTTATAATACAATAGAAAAATTACTAAAT
GATAAAACAATATCTAATTATAGAATTAATCAAGACACTGGTGTTTCTTATGGTGGTATA
AGTGAATTAAGAAGCGGGAAAAGAAAAGTGAATAATTTAACTTTAGAAACAGCGGAAAA
ACTCTATAATTACCAAAAACAATTAGAAATAATGATTGAAGATTAA

## > A5ISJ3

Function: protein coding sequence; putative protein (core genome, variable)
Best match: A5ISJ3_CC001_MW2-USA400_BA000033.2[1310546:1310752] (completely identical)
Position: 031-contig_247: 84012 … 84219; Length: 207 bp
Sequence:
ATGAATGAGATTGAAACTATTATAAGTGAAATAGAAAAGTTATTAACTAACAATACACCA
TATAGTATTTCAAAAAACTCAGGTGTACCACGTCAAACAGTTACTGATTTAAAGGTAGGT
AAAACTAAAATAAAAGAAGCTAAATTTAAAACGATAATCAAGTTATATGAATATCAAAG
AACATTAGAAAATAAAACAGAATGTTAA

## > Q2YXQ4

Function: protein coding sequence; putative protein (core genome, constant)
Best match: Q2YXQ4_CC001-ST772_118_AJGE01000002.1[77742:77927:r] (completely identical)
Position: 031-contig_247: 85457 … 85643; Length: 186 bp
Sequence:
ATGTCAGAATACAAGAAAAAGATAATTGAATTAATTGAAAGTAATTTAACAGGATATGA
AATTTCTAAAAAAACTGGAGTTTCTCAATATGTACTTTCACAATTAAGACAGGGCAAACG

CGAAGTAGATAATCTAACCCTTAATACAACAGAAAAATTATATGAATATGCCAATAAAGT
TTTGTAA

> **txbi_1000-3**
Function: ter bidirectional rho-independent terminator
Best match: txbi_1000-3_CC001_MW2-USA400_BA000033.2[1312393:1312470]
Position: 031-contig_247: 85860 … 85890; Length: 30 bp
Sequence:
TGGATCGTCTTGAAATGCTCCCTTCAAAGT

> **Q2YXQ3**
Function:  putative protein (genomic island)
Best match: Q2YXQ3_trunc_CC001_MW2-USA400_BA000033.2[1312686:1312726]
Position: 031-contig_247: 86154 … 86195; Length: 41 bp
Sequence:
ATGTTCAAAGTGAATTATTCGATTTTAAGTTATTATCCAGA

> **A6QGL3**
Function: protein coding sequence; putative protein (genomic island)
Best match: A6QGL3_CC093_JKD6159-ST93_CP002114.2[1298443:1299024]
Position: 031-contig_247: 86684 … 86714; Length: 30 bp
Sequence:
ATGCTTTTTTAACTATTTACTGAAATAGCA

> **Q8NWW7-L2**
Function: protein coding sequence; putative protein (genomic island)
Best match: Q8NWW7-L2_CC030_58-424_ACUT01000044.1[91:613]
Position: 031-contig_247: 86843 … 86873; Length: 30 bp
Sequence:
ATGATACCAGGTGTGAATGCGCCACCTATG

> **A6QGL6-Sepi**
Function: protein coding sequence; putative protein
Best match: A6QGL6-Sepi_CC705_RF122-cow_AJ938182.1[1280590:1281533]
Position: 031-contig_247: 86936 … 86966; Length: 30 bp
Sequence:
GAGCGTGAAGGTAAATATCAAGTAGAAGAT

> **Q8NWX1**
Function: protein coding sequence; putative protein (genomic island)
Best match: Q8NWX1_ref_CC001_MSSA476_BX571857.1[1342141:1342575] (completely identical)
Position: 031-contig_247: 87113 … 87548; Length: 435 bp
Sequence:
TTGCTTAAAGATGCCAAAGAAAACAATGATAGCAATGAAGTAGCTTATTTATTAAAAGAT
GGTAAAGTTACAAAAGTATATGGTGATCAAGATAGTGTATCTTTTGCACCAGGGGAAAAA
GCAACAGAATTGTTATTTAACAGTAAACCGAATTCAATTGTTATGTTACATAACCATCCTG
GGCAGTCTAGTTTTTCTCTTACAGACTTGTATTTATTTATATTTAATAATTCTATTAAAACA
CTGACAATTGTTACAAATAAAGGTCAAACAAAGTACTTAACTAAGACAAAAGAATATTG
CAAATCAACTTGTATTGATTGTATTAAAAAATATAATAAAAATAAGAATATAAAAAAATT
CAATCATAAGGATATTGATATGATTCTAAAGAGATTATATAATAGTGGTAACATAATATA
TAAAGTTAGGTGA

> **A6QGL8**

Function: protein coding sequence; aromatic amino acid beta-eliminating lyase/threonine aldolase (core genome, variable)
Best match: A6QGL8_CC001-ST772_118_AJGE01000002.1[72308:73333]RC (completely identical)
Position: 031-contig_247: 90051 … 91077; Length: 1026 bp
Sequence:
TTAGATTAATTCTAATAGTTTATTTAAATTTTCTTCGGTTGTCGCCCAGCTGGTTGCGAATC
TAACAACACGATGTTGATCATCGTATTTTTCCCAAACAGCAAATTTAACTTTTTGTTCTAA
CTCTGCTATTTTCTCGTTACTTAAAATAAAAAATTGTTGATTGGTTGGAGAATCAAAGTAA
AGACGATAGCCTTTATTTTTAAACCCGTCTTTCATCTTATTTGCCATTTCGATAGCATGTCT
GCTTATATTAAAATATAAATTGTCCGTAAATAATTCTAAAAATTGTATGCCTGTTAACCGT
CCTTTTGCTAAAAGGGCACCGTGGTGTTTGATTCGAGTGGTAAATTGTTTTGGTTCATTAT
TTTTTGTGAAAACAATAGCTTCTCCGCAAAGTGCACCAATCTTCGTACCACCTATATAAAA
TACATCACAATATTTAGCGATGTCTTTAATAGTCATATCTGATTGATCACTCATTAATGCA
TATCCTAAACGTGCACCATCCATAAATAATGGAAGCTTATATTGCTTACATACTTTACATA
ACTCTTTCAATTCTGATTTAGAGTATAATGTGCCATATTCTGTAGGATGAGAAATATATAC
CATTCCTGGGAATACCATATGGTCCTTTTTAAAATCACTTTTAAATGTCTCCATGTAAGTT
TCAACATCTGAAGCACTAACTTTTCCTTCGTTAGATGCTAACGTAATTACTTTATGACCAC
TAAATTCAATAGCACCGCCTTCATGTACAGCAACATGTCCCGTATCCGCTGACAATACAC
CTTCGTAGCTTTCTAACATTGAATTAATAACAACCTGATTGGTTTGCGTTCCACCTACTAA
AAAACGGATAGTAGCATTTGGACAATCAATTGTATCTTTAATCTTTTCAATTGCCTGAGCT
GTGAATTGATCAAAGCCATATCCCGAAGCTTGTACAAGATTTGTATCTACTAATCGTTTTA
ATACTTTTTCATGAGCACCTTCTAAATAATCATTTTCAAATGAGATCAC

## > A5ISJ9

Function: protein coding sequence; putative protein (core genome, variable)
Best match: A5ISJ9_CC001_MW2-USA400_BA000033.2[1317882:1318079] (completely identical)
Position: 031-contig_247: 91346 … 91544; Length: 198 bp
Sequence:
ATGGAACAAATTAAACTTAAAACTTTTACAGCTGAGACTTTAGAGTTATTAGAAAAAAAC
ATTAATGCTTTTTTAAGTTCTGAAGAAGCTACAAATTTAAAATTAGTAAATATTACTATTA
AAGAAATAGAAGAAAGAACATTCCCAAATAATGAAGAAGAATTCAATGCAATTTTAACT
TTATCTGTGAATAAATAA

## > cls1

Function: protein coding sequence; cardiolipin synthases 1 (core genome, variable)
Best match: cls1_CC005_ED98-hen_CP001781.1[1328794:1330275]
Position: 031-contig_247: 91600 … 93082; Length: 1482 bp
Sequence:
ATGCGATATACATTTTCAAATGATTTAGGAACACTTTTTACCATTATTTTAGCCATTGGAT
TCATCATTAACTTAGTATTGGCTTTTGTTATTATCTTTTTAGAAAGAAATAGGCGTACAGC
GAGTTCAACTTGGGCATGGCTATTTGTACTTTTTGTCTTACCATTGATTGGTTTTATTCTTT
ACTTGTTTTTTGGTAGAACCGTTTCGGCACGCAAATTGAATAAAAACAATGGTAACGTGT
TAACGGATTTCGATGGACTTTTAAAACAACAAATAGAAAGCTTTGATAAAGGTAATTATG
GTACTGATAACAAACAAGTTCAAAAACATCATGATTTAGTACGTATGCTTTTGATGGATC
AAGATGGTTTTTTAACTGAAAATAATAAAGTTGATCATTTCATTGATGGAAATGATTTATA
TGATCAAGTTTTAAAAGATATTAAAAATGCAAAAGAATATATCCATTTAGAGTACTATAC
TTTCGCTTTAGATGGTTTAGGTAAAAGAATTTTACATGCTTTAGAAGAAAAATTGAAACA
AGGTCTAGAAGTAAAAATATTATATGATGATGTTGGATCTAAAAATGTTAAGATGGCAAA
TTTTGATCACTTTAAATCATTAGGTGGCGAAGTTGAAGCATTTTTTGCTTCTAAATTACCC
TTATTAAATTTCCGTATGAATAACAGAAATCATAGAAAAATCATCGTAATCGATGGTCAA
CTAGGTTATGTCGGAGGATTTAACATTGGTGATGAATATCTAGGATTAGGTAAACTAGGG
TATTGGAGAGATACGCATTTACGTATACAAGGGGATGCGGTTGATGCACTGCAGTTGCGA
TTTATTTTAGACTGGAATTCGCAAGCGCACCGTCCACAATTTGAATATGATGTTAAGTATT
TCCCTAAAAAGAACGGACCATTGGGCAATTCACCAATTCAAATAGCTGCAAGTGGCCCAG
CTAGTGACTGGCATCAAATTGAATATGGTTATACAAAAATGATTATGAGCGCTAAGAAGT

CGGTATATTTGCAATCGCCTTATTTCATTCCGGATAACTCATATATAAATGCCATTAAAAT
TGCAGCTAAATCAGGTGTAGATGTTCATCTAATGATTCCATGTAAGCCAGATCATCCTTTA
GTTTATTGGGCGACATTTTCAAATGCCTCTGACTTATTATCAAGTGGTGTTAAAATTTATA
CGTATGAAAATGGATTTATACATTCTAAAATGTGCTTAATTGATGATGAAATCGTATCAG
TGGGCACAGCAAATATGGACTTTAGAAGTTTTGAATTAAATTTTGAAGTAAATGCCTTTG
TATATGATGAAAATCTTGCTAAGGATTTAAGGGTGGCTTATGAACATGATATTACAAAAT
CAAAACAACTAACCAAAGAATCATATGCCAATAGACCGCTGTCTGTTAAATTCAAAGAAT
CGTTAGCAAAATTAGTTTCGCCAATTTTATAA

## > A6QGM1

Function: protein coding sequence; ABC transporter ATP-binding protein (genomic island)
Best match: A6QGM1_CC001_MW2-USA400_BA000033.2[1319801:1320700]
Position: 031-contig_247: 93265 … 94165; Length: 900 bp
Sequence:
TTGATTCAAATATCTAATATCAACAAGTCATTTAATAAAAGATGTGTTCTAAAAAATATTT
CGTTCGATATTGAACAAGGTAAATGTATCGCTTTAATTGGAAAAAATGGTGCTGGAAAGT
CAACGTTAATTGATATATTAATTGGTAATGTTAATGCTAATTCTGGTGAGATATTTGATAA
AGACAAGTTATTACAAAGTGAAAATCGCAGTATAATGTTCCAAAAAACGATGTTTACAGA
TCAATTAAAAGTTATTGAGATTATCAACTTATATCAATCATTTTACGAAAATCCATTACCA
TTGGAAGAAATAATAGAACTGACGAAATTTGATTCTAGTCAACTGAACCAATTTGTAAAT
AAACTTTCTGGTGGTCAACAACGATTACTCGATTTTGTATTATCTTTAATCGGACAACCAC
AATTGATCTTATTAGATGAACCAACATCGACTATGGATATAGAAATTAGAGAATATTTTT
GGTCAATTATTGAAAATTTAAAAGAAGATAATCGAACGATACTCTATACATCGCACTATA
TTGAAGAAGTCGAACGTATGTCAGACAAAATTATTCTCATTGAAAATGGAGAAATAATAC
TTAATGATTCAACGTCACATATTAGAACCAATCAGCAATCTCAGATTACGTTATCCGATG
AATATATAAGAAAGTTAAAACTAGATAAAGATGATTTAGTTATTCAAAAAAATCATAATG
GCACTATCAAAATTATTACTTCAAATGTAAATGATACGATTTTATATCTTCAACAACTTCA
TATTAATTTGGATGATATTGAAATACAAAAAGTCTCAATTGTTGATTCATACTTCAACAAT
AAAAAGCAAAGGGGATCTAATTATGATACTAAGTTACTTGAAAATCGAATTTAA

## > Q5HGA1

Function: protein coding sequence; putative ABC transporter/permease protein (genomic island)
Best match: Q5HGA1_CC008_NCTC8325_CP000253.1[1256491:1257222] (completely identical)
Position: 031-contig_247: 94133 … 94865; Length: 732 bp
Sequence:
ATGATACTAAGTTACTTGAAAATCGAATTTAAAGTTATAATGCGTAAAAAAACAACATTA
ATATTATCTATTTTATTTCCTGTTATATTCTATATATTATTTACTTCGATATTGGAATTGCC
GGAAGATGTTAAACCTAAATTTTATAAAGAGTATATGTATAGTATGACGGTTTATAGTTT
GTTAAGTTTTAGTTTACTAACTTTTCCATTAGATATTATTAATGAAAAACAAAATGAATGG
CGCCAAAGATTAATGGTAACACCATTTACTTTTACTAGTTATTATATTTCAAAAGTAGTGA
AAACTATGCTGCAATTTGCAATAGCGATATTAGTTATTTTTATGGTTGGACATTTTTATAA
AGGTGTTGCAATGAGTGCAGTTCAATGGTTAGAGTCAGGAATATTTTTATGGTTAGGTGC
GTCTCTATTAATAACTTTTGGCATATTATTTTCTTTGTTAAATGATATTCAAAAAACAAGT
GCTTTAGCTAATATCGTAACAATTGGTTTAGCAGTATTAGGTGGATTGTGGTTTCCGATAA
ACACATTTCCAAATTGGCTTCAACATGTTGCTCATGTTTTACCGAGCTATCATTTGCGTAA
ACTAGGTGTAGATATTGCTTCAAATCATCATATCAATTTAATATCATTTGCTATAATACTC
TTGTATGCTTTAGGGAGTATAATAGCAGTATATTGTATTAGTCATTTTAAAAGGGCGGAA
TAA

## > A5ISK3

Function: protein coding sequence; putative sensor kinase protein (genomic island)
Best match: A5ISK3_CC001_MW2-USA400_BA000033.2[1321404:1322495] (completely identical)
Position: 031-contig_247: 94868 … 95960; Length: 1092 bp
Sequence:

ATGAAATTTTTAAAAGATACTTCAATTGCTGAAATATCGTCTATACTTTATCTGATTTTTC
CTATTGCCGGTATATTTTTTAATGAAGTATATGGTCCCAAATGGTTGTATATTATATCAGT
CATTGTCTTTTCGTTGTCGTATCTTATATTAGTTATAGTAAATAATAGACTTAATACATTA
ATGTTTTACATTTTGTTGATTATTCATTATTTTATTATTTGTTATTTTGTTTTCAGTGTACAT
CCAATGCTAAGTTTGTTTTTCTTTTATAGTGCTTTTGCCGTTCCATTTACTTTTAAAAATAA
TGTTAAAAAAACGGCAACTAATCTTTTCATACTAACAATGATTATATGTACAATAATAAC
GTACTTATTGTATAACAACTATTTTGTTGCAATGATGGTTTATTATGTCGTTATATCGTTAA
TAATGCTAGATAATTTTAAAAAAATGAAAAACCGTGAATATCAAAAAGAAATAGCAGAA
AAAAATAGACATATTAATACATTAATTGCTGAACAAGAGCGACATAGAATTGGTCAAGA
CTTACATGATACGTTAGGGCATGTGTTTGCAAGTTTATCATTAAAATCAGAATTAGCTTAT
AAACTAATAGATGCTGATGTAGAAAAAGTAAAAGCTGAATTATTAGCAATTAATAAATTA
TCTCGTGAATCATTGAACAAAGTTCGAGAAATTATTGATGATGTAAAATTACCATCATTT
ATTGAAGAGATTGATAGTATACGTAAAGTTTTAAAAGATGCTGATATTGATTTTACATTTG
AAAATAAAGAATTAGCGCAAGTATTAAGTCCTACTAAACAATCTATGTTAGTTATGATTA
CGCGTGAAGCGATAAATAATGTTATTAAACATGCAAATGCTTCAAAAGTTCATGGTAAAT
TAAAAACTGTAAACAATCATAAATTACTGCTTATGATTGAAGATGATGGCAAAGGTATCG
ATAGTGATTGTGAGGTGAAAAGTATTTCACAGCGTGTACAACATTTAAATGGAACTTTAG
CAGTCGACTCAACAAATGGAACTAAAATAATCATTGAAATCTCAACAGGAGGAATAGCA
TGA

> **A5ISK4**
Function: protein coding sequence; two component transcript regulator/LuxR family (genomic island)
Best match: A5ISK4_CC001_MW2-USA400_BA000033.2[1322492:1323094] (completely identical)
Position: 031-contig_247: 95956 … 96559; Length: 603 bp
Sequence:
ATGACATCTTTAATTATTGCAGAAGATCAAAATATGTTACGACAGGCAATGGTTCAATTA
ATTAAACTACATGGTGATTTTGAAATTTTAGCAGATACTGATAATGGTCTCGATGCAATG
AAACTTATTGAAGAATATAATCCTAACGTTGTTATTTTAGATATAGAAATGCCAGGCATG
ACTGGACTTGAAGTTTTAGCGGAAATTAGAAAAAAGCATTTGAATATTAAAGTGATTATT
GTAACAACTTTTAAAAGACCGGGATACTTTGAAAAAGCAGTTGTGAATGATGTGGATGCA
TATGTTTTAAAAGAACGTTCTATAGAAGAATTGGTGGAAACCATTAATAAAGTAAATAAC
GGAGAGAAAGAATATAGCGCCACATTGATGACTTCATTTTTTGTAGATAAAAACCCATTA
ACGCCCAAAGAACAAATTGTATTAAGGGAAATTGGCAATGGTTTAAGTAGTAAAGAAAT
AAGTGAAAAATTATTTTTGACAGATGGAACAGTTAGAAATTATACATCTGTTATAATTGA
TAAATTATTTGCAGATAATCGTTTTGATGCTTGGAAAAAGGCAAATGAAAAAGGCTGGAT
CTAA

> **Q99UF3**
Function: protein coding sequence; putative protein (core genome, constant)
Best match: Q99UF3_CC001_MW2-USA400_BA000033.2[1323207:1323395:r]RC (completely identical)
Position: 031-contig_247: 96671 … 96860; Length: 189 bp
Sequence:
CTAATTTTTTCGCTTATTATAAAACGAAACAATCAAGTAAACGATAAAGCCTACAAAGAT
ACCCAATAAAATAGATAGTACTGCCGTCACTATTAATGGTAATTTGAAAAATATTTGTAG
GAAAATACCAATGATAATTGCGATAATTACTGCAATTAATGTGACTGTATTTTCATTTGAA
ATGTTCAT

> **nuc2**
Function: protein coding sequence; nuclease family protein (core genome, constant)
Best match: nuc2_CC001_MW2-USA400_BA000033.2[1323534:1324067] (completely identical)
Position: 031-contig_247: 96998 … 97532; Length: 534 bp
Sequence:
ATGAAGTCAAATAAATCGCTTGCTATGATTGTGGTAGCCATCATTATTGTAGGTGTATTAG
CATTTCAATTTATGAATCATACGGGTCCTTTCAAAAAGGGGACGAATCATGAAACTGTAC

AAGATTTAAATGGTAAAGATAAAGTACATGTTCAAAGAGTTGTGGATGGTGATACATTTA
TTGCAAATCAAAATGGTAAAGAAATTAAAGTTAGGCTTATAGGGGTTGATACGCCAGAA
ACGGTGAAACCGAATACGCCTGTACAACCATTTGGCAAAGAAGCATCAAATTATAGTAA
GAAGACATTAACAAATCAAGATGTTTATTTAGAATATGATAAAGAAAAACAAGATCGCT
ATGGTAGAACATTGGCGTATGTATGGATAAGTAAAGATCGTATGTACAATAAGGAATTAG
TGGAAAAGGGACTTGCTAGAGAGAAGTATTTTTCACCAAATGGCAAATATAGAAATGTAT
TTATAGAAGCACAAAATAAAGCTAAACAACAGAAATTAAATATTTGGAGTAAATAA

> **Q5HG96**

Function: protein coding sequence; putative protein (core genome, constant)
Best match: Q5HG96_CC001_MW2-USA400_BA000033.2[1324210:1325064:r]RC (completely identical)
Position: 031-contig_247: 97674 … 98529; Length: 855 bp
Sequence:
TTACTTGAATATAGCTGTGATTTGATCAACCTGTTCTTTATTTAAAGGGTGTTTAGATAGC
TCTATGGCTTCGCTAATAATACGTCGTAACGAAGATTTTGTTGAAACTGTATCAACATATT
TATCTCTTTCTTCAGAACCTTGAATGATATTAACTGTACCATCACTAAAGTAAATAACACC
GGTACTTTGGACTTTAAAGTTAAAGTTTTGTTCAATATGATCCTTTAATGCTTTCGCATTAT
TTGCTGCTAATTGATACGGATCATAATCATAAAAATCATAAATAACACGATTTGGTTGAA
CTGTTTCTGTAAAAGTATAGATAGACTTTCTTGATGAATTAAACTGATCATGATATTGCTG
GCTAATGTAATGACCGACAACTTTTTCAATATTACTATTGCTTATTTCTGTATCATGCTCAT
CAGGTACATCAAAGTGATAAAATGTTTTTTCACCCCAGCTTTTTACATCAACGTTTATTAA
ACCGATATCTGAAACAATGATAAAATCAAATGAACGTGCATACTCGAAAAACGGATGTTT
AGTAGCTAAATTGCTTGTAACGATAATATCGTAATATTTTAATTTACCATTTTCGAGATAG
CTATCTAAAATCTTGCGTAATTCTCTTTTAGCATTTTTGTATGCATGGTGGCCAACATCATT
ATTAGAACTCAGCATTTGACTTCTTAATTCAGCATTTTCTGCACTTAATGCTTTGTTCTTTT
TAATAAGTTGCTTTCTTGCATAAACTTCGGTATCTATTTTACTATTACTATACCTTTGATTT
AAAACTAATATACCAATTAATGCTACAATGATAATGATAAGTACAACATAAAAAGACAT

> **Q5HG95**

Function: protein coding sequence; putative protein (core genome, constant)
Best match: Q5HG95_CC001_MW2-USA400_BA000033.2[1325380:1325970]
Position: 031-contig_247: 98844 … 99435; Length: 591 bp
Sequence:
TTGAGAGTGAATGAAATGAATGCTAAAGAACAATTAGTGGACAATTTAATGAAAACATC
ATCGCAATTATTTAAATTTCACGGTGAAGTTGCCATGCAGCTTTTCTTAAATGATGAATTA
AAATTACCTTCTATTGTTGAAATATGCGTAGAACGTAAGCGTTTAAGTGATATTGTGAAA
GTTATTCCGCAATCATATGCGTTACTATACATAGATAAGCAAGATCAAGCAATAGCTAAA
GAAGATTTATCACTTTCAAAAATTGCAAAAGTTTATGTGCAATATGATGATACAACAATA
ATGAGTATTTTCGTTTATGATGTAGTAAACGATGAATGGATTTTTAGATTGGATCCGAATA
TACGTATACCTAAGAGTAACATATACTTCCATAGTTTAAATTGGGATGTGGATTATATTAA
ACCGGAGATCGTTCTAATGTATGATCTAATGCAACACCATCAGTATCATCATTATTCCAAT
TATAAACGAGTCATAGATGCATTAAGCTACTATCAATTTTTTATTTTAAAATTTGTAGTAG
GTGAGCAACGTATTAAGGATGCAATCCAGAGAACAATAAATAATTAA

> **txbi_thrA**

Function:  bidirectional rho-independent terminator of thrA
Best match: txbi_thrA_CC001_MW2-USA400_BA000033.2[1325962:1326031:r]RC (completely identical)
Position: 031-contig_247: 99426 … 99496; Length: 70 bp
Sequence:
AATAATTAAGAAAAAGCAATTCATAACGCAGTTGAATACATGTGTTACGAATTGCTTTTA
TATTAGTTTT

> **thrA**

Function: protein coding sequence; aspartokinase (core genome, constant)
Best match: thrA_CC001_MW2-USA400_BA000033.2[1326024:1327406:r]RC
Position: 031-contig_247: 99488 … 100871; Length: 1383 bp
Sequence:
TTAGTTTTTATCACACAAGTTTTTTAATGCAACCCCGTGATAGCAAAACTCATATGTAGAT
AATACAGCTTTTTCAGCATCATCTACATGAATTCCAAACATCATCGAGATTTCCGAAGCA
CCTTGGTTAATCATTTTTAAGTTAATTTTTGATTCAGCTAAGGCATGTGTAATTTTATTTGC
AGTACCGATGACTTTATTCATACCTTCGCCGACAATCATTAATATTGCTAAATCATGCTCA
ATACTTAGCTCATCAACATCACATTTTTGACGAATTTCATTTAATACTTTTGTTTCTTTATT
TTGAATTTGTTTTGAGCGCATAACGATACTGATAGTATCAATACCTGAAGGCATATGATC
AAATGAAATATTATTATCCTCTAAGACACCTAATATCTTTCTAGTAAAGCCGACTTGTCTA
TTCATTAAATACTTTTTGATATTAATAACGGTAAAATCTTTATCACAACTTATACCGCTAA
TCACATTTTTCGCATTTATTTCTCTATCATGCACTATAAATGTACCTTTATCTTGAGGGCGG
TTCGTATTTTTAATCACCACAGGGATGCGATCTTTATAAAGTGGTTGTAAGGCTTCATCAT
GGAAAACACTAAAACCAGCATAAGATAATTCACGCATTTCTCGATAAGTGATTTCTTCGA
TTAATTCAGGATCTTTGATGATATTTGGATTAGCTTTATAAATACCAGACACATCGGTGAA
ATTTTCATAAATTGTAGCTCTAACACCACTTGATATGATGGCGCCAGTTATATCTGATCCG
CCACGTGGAAATGTAACTATATATCCTTCATGAGATACGCCGAAAAATCCTGGGATAATT
AGTTTCTCATCATAATCTCTTAATTTTTTAATTTCAGAGTAAGCACTATCTAATATTTGTGC
TTCTTGTGGGACGTCAGTAACAAAAATACCCGCTTCCTTCGGTGATATATATTTTGTTGGT
ATACCTTGACTATTATTATATAAAGCTATCAATTGCGCATTAAAATCTTCACCACAAGAA
AGTAATGCATCTAATAGTCTCTTTGGTTCATTTTTTAATTGATTAATATAATGTTCCAAAG
TCACATCTATCGTCCGTAAAATACTTTCATCCATTTGCAATTCTTTTACAATATCATCATA
ACGCTGAATAATTTCTCTTTTTTTATCATGATAATCAAGATGATTAATGACCTTTTCATAT
AATCTGATTAACAAATCAGTTGTTTTAATATCATTATCATGTCTTTTACCTGGAGCAGAAA
CGATAACAATCTTTCGCTCTGGATCAGAATTAACAATATTTAAAACCTTTTTAATTTGAGT
AGCATTGGAGACGGAGCTACCACCGAATTTGGAAACTTTCAT

> **metL**
Function: protein coding sequence; homoserine dehydrogenase (core genome, constant)
Best match: metL_CC001_MW2-USA400_BA000033.2[1327597:1328877]
Position: 031-contig_247: 101061 … 102342; Length: 1281 bp
Sequence:
ATGAAAAAATTAAATATAGCATTATTAGGATTAGGTACTGTCGGATCTGGTGTTGTTAAA
ATCATCGAAGAGAACCGACAGCAAATTCAAGATACATTAAATAAAGATATTGTCATAAA
GCATATTCTTGTTCGAGATAAATCTAAAAAGAGACCGCTAAATATTAGCCAATATCATTT
AACTGAAGATGTTAATGAAATTTTAAATGATGATTCATTAGATATTATCGTTGAAGTCAT
GGGAGGAATTGAACCAACTGTAGATTGGTTAAGAACAGCACTTAAAAATAAAAAACATG
TTATTACCGCAAATAAAGATTTATTAGCAGTACATCTTAAACTTTTAGAAGATTTAGCAG
AAGAAAATGGTGTAGCTTTAAAGTTTGAAGCGAGTGTAGCAGGTGGTATTCCGATCGTAA
ATGCCATAAATAATGGTTTGAATGCGAATAATATTTCAAAATTTATGGGAATTTTAAATG
GTACCTCTAATTTTATTTTATCTAAAATGACTAAAGAGCAAACGACATTTGAGGAAGCAC
TTGATGAAGCGAAAAGACTTGGTTTTGCTGAAGCGGATCCAACTGATGATGTAGAAGGG
GTAGATGCAGCGCGTAAAGTTGTCATTACATCATATTTATCATTTAACCAAGTCATTAAAT
TAAACGACGTTAAACGAAGAGGAATTAGTGGCGTAACTTTAACTGATATTAATGTAGCCG
ATCAACTGGGGTATAAAATTAAATTGATTGGTAAGGGAATATATGAAAATGGCAAAGTT
AATGCATCGGTAGAACCAACGTTAATTGATAAAAAGCATCAATTAGCAGCTGTAGAGGA
TGAATATAACGCGATTTATGTCATTGGTGATGCTGTTGGTGACACGATGTTTTATGGAAA
AGGAGCAGGCAGTTTAGCAACAGGTAGTGCCGTTGTCAGTGATTTATTGAATGTAGCATT
ATTCTTTGAATCAGATTTACACACATTGCCACCACATTTTGAATTAAAGACAGATAAAAC
ACGGGAAATGATGGATTCAGATGCAGAAATTAATATTAAAGAAAAATCCAATTTCTTTGT
AGTAGTGAATCATGTCAAAGGTTCAATTGAAAATTTTGAAAATGAGTTAAAGGCAATATT
ACCATTTCACCGATCATTAAGAGTTGCAAATTACGATAATCAATCATATGCCGCTGTTATA
GTTGGATTGGAATCATCACCGGAAGAATTAATCACTAAGCATGGATACGAAGTTGACAA
AGTATACCCAGTAGAAGGAGTTTAA

## > thrC

Function: protein coding sequence; threonine synthase (core genome, constant)
Best match: thrC_CC008_NCTC8325_CP000253.1[1264705:1265766] (completely identical)
Position: 031-contig_247: 102347 … 103409; Length: 1062 bp
Sequence:
ATGAGAAGATGGCAAGGATTAGTAGAAGAGTTTAAAGCACATTTACCAGTAAATGAAAA
TACACCAAAATTAACATTGAACGAGGGAAATACACCACTCATTCATTGTGAAAATATGTC
TAAAATACTAGGCATAGATTTATATGTGAAGTATGAAGGTGCCAATCCGACAGGTTCATT
TAAAGATCGCGGTATGGTAATGGCTGTGACAAAAGCAAAAGAGCAAGGTAAGAAAATTG
TAATATGCGCTTCGACTGGAAATACATCAGCGTCTGCAGCAGCATATGCAGCGAGAGCAG
GTTTAAAAGCTATCGTCGTAATACCAGAAGGTAAAATTGCATTAGGTAAATTGTCGCAAG
CAGTAATGTATGGTGCAGAAATCGTTTCTATTGAAGGAAACTTTGATGAAGCTTTAGAAA
TTGTAAAAGAAATTGCAAAAAGTGGCGAAATCGAGCTTGTAAACTCTGTCAATCCATTTA
GAATCGAAGGACAAAAGACAGGCTCATTTGAAATTGTACAACAATTAGACGGTGAAGCA
CCTGATATTTTAGCGATTCCTGTAGGTAATGCAGGTAATATTACTGCATATTGGAAAGGCT
TTAAAGAATATCATGAAGCTAAAGGATCACAATTGCCGAAAATGTTTGGCTTCCAAGCTG
AAGGCGCATCACCAATTGTTCAAAATAAAGTCATTAAAAATCCTGAAACGATTGCAACTG
CTATTCGAATTGGTAATCCTGCTAGTTGGGATAAGGCGACTAATGCTCTTAAAGAATCAA
ATGGATTAATAGATAGTGTTACTGATGATGAAATTCTAGAAGCATATCAGTTAATGACAA
CTAAAGAAGGTGTCTTTAGTGAACCAGCGAGTAATGCTTCTATTGCAGGTTTAATTAAAT
TGCATAGACAAGGTAAATTACCTCAAGGTAAAAAAGTAGTTGCTATTTTAACTGGTAATG
GATTAAAAGATCCTGATACTGCTATTTCACTACTAGATAATCCGATAAAGCCATTGCCAA
ATGATAAAGATAGCATTATCGATTATATTAAAGGAGCTTTATAA

## > thrB

Function: protein coding sequence; homoserine kinase (core genome, constant)
Best match: thrB_CC001_MSSA476_BX571857.1[1358442:1359356]
Position: 031-contig_247: 103410 … 104325; Length: 915 bp
Sequence:
ATGTCGAATGTTTTGGAGTTAACAATTCCTGCATCAACAGCCAACCTTGGAGTTGGCTTTG
ATTCTATAGGTATGGCTTTAGATAAATTTTTGCATCTGTCTGTAAAGGAAACATCAGGGA
CAAAATGGGAATATATTTTCCATGATGATGCATCTAAGCAATTGCCTACTGACGAAACAA
ACTTTATTTATCATGTAGCACAACAAGTTGCTTCTAAATATAGTGTTGACTTGCCTAATTT
ATGTATCGAAATGAGAAGTGATATTCCATTAGCAAGAGGGTTAGGTTCGTCAGCTTCTGC
TTTAGTAGGAGCTATATATATCGCAAATTATTTTGGTGATATCCAACTGTCTAAACATGAG
GTATTACAATTAGCGACTGAAATCGAAGGACATCCTGATAATGTTGCGCCGACCATTTAT
GGTGGTTTAATCGCTGGATATTATAATGATGTCTCGAAAGAAACGTCAGTTGCACATATC
GACATACCAGACGTGGATGTGATTGTAACGATACCAACTTATGAACTAAAAACAGAAGC
ATCAAGACGTGCTTTACCACAAAAATTAACACATAGTGAAGCGGTTAAAAGTAGTGCAAT
TAGTAATACAATGATTTGTGCATTAGCACAGCACAATTATGAATTAGCAGGTAAACTCAT
GCAACAAGATGGCTTTCATGAACCGTATCGTCAGCATTTAATTGCTGAATTTGATGAAGT
GAAAACAATTGCTATTCAACATAATGCCTATGCAACTGTAATTAGTGGTGCTGGACCAAC
TATTTTAATATTTAGTCGTAAAGAAAATAGTGGGGAATTGGTTCGCTCTTTAAATAGTCAG
GTAGTATCATGCCATTCTGAATTGGTCGATATTAATATCAGTGGTGTTAAAGAACGAATT
GTATACCAATAG

## > Q5HG87

Function: protein coding sequence; putative amino acid permease (core genome, constant)
Best match: Q5HG87_CC001_MW2-USA400_BA000033.2[1332546:1334000:r]RC
Position: 031-contig_247: 106010 … 107465; Length: 1455 bp
Sequence:
TTATTTTTGATTGTCATAATCGTGTGGTTTTAAATTTATTGTTTCTAGCTTTACAAATTTTG
TTTTGTGAATGATTTTATGAATAAAGTAAATCAACGCTAGAATGATTAAAGGTAAAAAGT
TTTTAAAAGCATTTAACCATTGATCTTTTAAAATATATTCAACTGAGCCACCAAATAGCAA
GAATAATAGTGTAGTGATGACAATGATTGGTCCTAATGGATAAAAAGGTGCTTTATATGG

TAGGACCTTATTAGGGTCTTGACCTTGTTTTTAATAGCTTGTCGCAATCGTATTTGTGAC
CAAATGCTTGATCCCCAAACAACTATAATCATTGAACCAATAATTTCAAGTAAATTAAAA
ACGGCATTTGAATTAAAGTTTGCATAAATAATAACAATAACAACGACTGCATAAGTAGTT
AATAATGCTCTTAAAGGTAACTTAGTTGTCTTGTTTAATTTACTTAAAAATTGGGGTGCTT
TTTTGTCTGAACTTAAGGAATACAACATTCTGCCTGTTGTATAAACACCTGAATTTGCAGC
GGATAATAATGAAGTTAAAATAACCGCGTTGATTACTGATGCTGCAAAGGCTATGCCTAC
TCTATCGAATACAATTGTAAATGGGCTTTGACTTATTGAACTACTTGCTCTTAATAATGAT
GGATCTGTGTACGGAATAATTGCACCAATTACTGCAATTGATAAGACATAGAATAAAAGA
ATACGCCAAAATACTTGTTTAATTGCCTTAGGCATAGACTTTTTAGGGTCATCTGATTCAC
CAGCAGTTACTGCTACTACTTCTGTACCACCAACCGAAAATCCGGCGACTAATAATACGC
CTAAGAAACCAGAGATACCACCAACAAACGGTGCTTGGCCTTTTGTATAGTTTTCAAATC
CATATGTATGACCACCTAAGATACCGAAAATCATTAAAAAGCCAAAAATAACGAATACG
ATAATTGTTAACACTTTAATCAATGATAACCAAAACTCAGTTTCTCCAAATGATTTTACAG
AAAAAATGTTTAATAATAGTAAAATTGTAATAAAGATTAAGCTCCAAGTAATGGGGTGG
AAAAATTTAAATGTGTCCCAGAAATAAAGCACATTTGACGCTACTATGACATCAACACTT
GTAACTAATGACCACAATGCCCAATACAACCATCCCATGGTAAAGCCAAGAGATGAGTC
AATAAAGCGTGTTGAATAAGAGCTGAATGAACCTGATACTGGATAAAATGTTGCCAACTC
TCCAATTGATGACATTAAGAAATATAGCATGACACCAATAACAAGATAAGCGAGTATAG
CGCCTCCAGGACCAGCTTGAGAAATGATATTACCAGTAGCTACAAATAGACCAGTCCCAA
TTGCACCACCTATAGCAATCATGGAAATGTGTCTTGAGTTAAGACTACGGTTCATTTTATT
ATCTTCCAT

## > katA

Function: protein coding sequence; catalase A (core genome, variable)
Best match: katA_CC001_MW2-USA400_BA000033.2[1334204:1335721] (completely identical)
Position: 031-contig_247: 107668 … 109186; Length: 1518 bp
Sequence:
ATGTCACAACAAGACAAAAAGTTAACTGGTGTTTTTGGGCATCCAGTATCAGACCGAGAA
AATAGTATGACAGCAGGGCCTAGGGGACCTCTTTTAATGCAAGATATTTACTTTTTAGAG
CAAATGTCTCAATTTGATAGAGAAGTAATACCAGAACGTCGAATGCATGCCAAAGGTTCT
GGTGCATTTGGGACATTTACTGTAACTAAAGATATAACAAAATATACGAATGCTAAAATA
TTCTCTGAAATAGGTAAGCAAACCGAAATGTTTGCCCGTTTCTCTACTGTAGCAGGAGAA
CGTGGTGCTGCTGATGCGGAGCGTGACATTCGAGGATTTGCGTTAAAGTTCTACACTGAA
GAAGGGAACTGGGATTTAGTAGGGAATAACACACCAGTATTCTTCTTTAGAGATCCAAAG
TTATTTGTTAGTTTAAATCGTGCGGTGAAACGAGATCCTAGAACAAATATGAGAGATGCA
CAAAATAACTGGGATTTCTGGACGGGTCTTCCAGAAGCATTGCACCAAGTAACGATCTTA
ATGTCAGATAGAGGGATTCCTAAAGATTTACGTCATATGCATGGGTTCGGTTCTCACACA
TACTCTATGTATAATGATTCTGGTGAACGTGTTTGGGTTAAATTCCATTTTAGAACGCAAC
AAGGTATTGAAAACTTAACTGATGAAGAAGCTGCTGAAATTATAGCTACAGATCGTGATT
CATCTCAACGCGATTTATTCGAAGCCATTGAAAAAGGTGATTATCCAAAATGGACAATGT
ATATTCAAGTAATGACTGAGGAACAAGCTAAAAACCATAAAGATAATCCATTTGATTTAA
CAAAAGTATGGTATCACGATGAGTATCCTCTAATTGAAGTTGGAGAGTTTGAATTAAATA
GAAATCCAGATAATTACTTTATGGATGTTGAACAAGCTGCGTTTGCACCAACTAATATTA
TTCCAGGATTAGATTTTTCTCCAGACAAAATGCTGCAAGGGCGTTTATTCTCATATGGCGA
TGCGCAAAGATATCGATTAGGAGTTAATCATTGGCAGATTCCTGTAAACCAACCTAAAGG
TGTTGGTATTGAAAATATTTGTCCTTTTAGTAGAGATGGTCAAATGCGCGTAGTTGACAAT
AATCAAGGTGGAGGAACACATTATTATCCAAATAACCATGGTAAATTTGATTCTCAACCT
GAATATAAAAAGCCACCATTCCCAACTGATGGATATGGCTATGAATATAATCAACGTCAA
GATGATGATAATTATTTTGAACAACCAGGTAAATTGTTTAGATTACAATCAGAGGACGCT
AAAGAAAGAATTTTTACAAATACAGCAAATGCAATGGAAGGCGTAACGGATGATGTTAA
ACGACGTCATATTCGTCATTGTTACAAAGCTGACCCAGAATATGGTAAAGGTGTTGCAAA
AGCATTAGGTATTGATATAAATTCTATTGATCTTGAAACTGAAAATGATGAAACATACGA
AAACTTTGAAAAATAA

## > rpmG2

Function: protein coding sequence; 50S ribosomal protein L33/locus 2 (core genome, constant)
Best match: rpmG2_CC001_MW2-USA400_BA000033.2[1335812:1335961] (completely identical)
Position: 031-contig_247: 109276 … 109426; Length: 150 bp
Sequence:
GTGCGCGTAAACGTAACATTAGCATGCACAGAATGTGGCGATCGTAACTATATCACTACT
AAAAATAAACGTAATAATCCTGAGCGTATTGAAATGAAAAAATATTGCCCAAGATTAAA
CAAATATACGTTACATCGTGAAACTAAGTAA

> **rpsN**
Function: protein coding sequence; 30S ribosomal protein S14 (core genome, constant)
Best match: rpsN_CC001_MW2-USA400_BA000033.2[1336415:1336684] (completely identical)
Position: 031-contig_247: 109879 … 110149; Length: 270 bp
Sequence:
ATGGCTAAGAAATCTAAAATAGCAAAAGAGAGAAAAAGAGAAGAGTTAGTAAATAAAT
ATTACGAATTACGTAAAGAGTTAAAAGCAAAAGGTGATTACGAAGCGTTAAGAAAATTA
CCAAGAGATTCATCACCTACACGTTTAACTAGAAGATGTAAAGTAACTGGAAGACCTAGA
GGTGTATTACGTAAATTTGAAATGTCTCGTATTGCGTTTAGAGAACATGCGCACAAAGGA
CAAATTCCAGGTGTTAAAAAATCAAGTTGGTAA

> **guaC**
Function: protein coding sequence; GMP reductase (core genome, constant)
Best match: guaC_CC001_MW2-USA400_BA000033.2[1336841:1337818]
Position: 031-contig_247: 110305 … 111283; Length: 978 bp
Sequence:
GTGAAAATATTTGATTACGAAGATATTCAATTAATACCTAATAAATGCATAGTTGAAAGT
AGGTCTGAATGTGATACAACTATCCAATTTGGTCCGAAAAAATTCAATCTACCTGTAGTT
CCTGCAAATATGCAAACAGTTATGAATGAGAAATTAGCGAAATGGTTTGCTGAAAATGAT
TACTTTTATATCATGCATCGTTTTGATGAAGAAGCAAGAATACCTTTTATAAAACATATGC
AAAATTCAGGCTTATTTGCATCTATTTCAGTTGGTGTAAAGAAAGCGGAATTTGATTTTAT
TGAAAAGTTAGCTCAAGAAAAATTAATCCCCGAATATATTACAATAGATATTGCGCATGG
TCACTCAGATTCAGTGATAAACATGATTAAACATATAAAAAACCATATACCTGATAGTTT
TGTTATTGCTGGTAATGTTGGTACGCCAGAAGGTGTTAGAGAATTAGAAAATGCTGGTGC
TGATGCTACCAAAGTCGGTATAGGTCCTGGTAGAGTTTGTATTACAAAGATTAAAACAGG
TTTTGGTACTGGTGGTTGGCAGTTAGCGGCATTAAACATATGTAGTAAAGCAGCTCGTAA
ACCTTTGATTGCTGATGGTGGTATAAGAACGCATGGCGACATCGCTAAATCAATTAGATT
TGGTGCATCAATGGTCATGATTGGTTCATTATTTGCGGCACACGAAGAATCACCTGGTGA
AACTGTAGAACTTGATGGTAAACAGTATAAAGAATATTTTGGTAGTGCATCTGAATTTCA
AAAAGGCGAACATAAAAATGTGGAAGGTAAAAAAATGTTTGTAGAACATAAGGGTTCAT
TAATGGATACCTTAAAAGAAATGCAACAAGATTTACAAAGCTCAATTTCATATGCCGGTG
GAAAAGACTTGAAATCATTACGTACTGTAGATTATGTTATTGTTAGAAACTCTATTTTCAA
CGGTGATAGAGATTAA

> **lexA**
Function: protein coding sequence; DNA damage-inducible LexA repressor (core genome, constant)
Best match: lexA_CC007_TCH959-USA300_AASB02000234.1[113984:114607:r]RC
Position: 031-contig_247: 112700 … 113324; Length: 624 bp
Sequence:
TTACATTTCGCGGTACAAACCAATTACTTTCCCAATTACAGCAACATTGTCGAGGTAAATT
GGCTCCATTGTACTATTTTCAGGTTGTAATCGATAACGATTTTTTTCTTTATAGAAGCGTTT
GACAGTTGCTTCATCTTCCTCAGTCATAGCAACAATAATGTCTCCATTTTCTGCTATGGTT
TGACTGCGAACAATTACTTTGTCTCCGTCTAATATACCAGCCTCAATCATACTGTCGCCTA
CGACGTTTAATATGAATATGTCGCTATTGTGTGTCGATGTTAAGTGTTCAGGTAATGGAA
AATATTCTTCAATATTTTCTACTGCGGTAATAGGAACACCTGCTGTGACTTTACCAATAAC
TGGCACATGAATCGTTTCTTCCATATTAATATTTTCATTTGTTTGATCACTTACAATTTCTA
TAGCACGTGGTTTCGTCGGATCTCTTCTTATATAGCCTTTTTCTTCAAGACGTGAAAGGTG

ACCATGAACAGTTGAACTGGATGCTAAGCCAACTGCTTCACCAATTTCGCGAACACTAGG
CGGATAACCTTTCATTTGAACAACTTGTTTAATATAGTTATATATTTCGCTTTGTCGTTTTG
TTAATTCTCTCAT

> **tkt**

Function: protein coding sequence; transketolase (core genome, constant)
Best match: tkt_CC001_MW2-USA400_BA000033.2[1340731:1342719]
Position: 031-contig_247: 114195 … 116184; Length: 1989 bp
Sequence:
ATGTTTAATGAAAAAGATCAATTAGCTGTTGATACGCTGCGTGCACTAAGTATCGACACA
ATCGAAAAAGCGAATTCTGGTCATCCAGGATTACCTATGGGAGCTGCCCCAATGGCTTAC
ACTTTGTGGACACGTCATCTGAATTTTAATCCACAATCTAAAGATTACTTCAATAGAGACC
GTTTCGTATTATCTGCAGGGCATGGTTCAGCATTATTGTATAGCTTGTTACATGTTTCTGG
TAGTTTAGAATTAGAAGAATTAAAGCAATTTAGACAATGGGGTTCTAAAACACCAGGTCA
TCCTGAATACAGACATACAGATGGTGTAGAAGTTACTACCGGACCACTTGGACAAGGTTT
TGCTATGTCAGTAGGATTAGCTTTAGCAGAAGATCACCTAGCAGGGAAATTTAATAAAGA
AGGATATAATGTTGTAGATCATTACACATATGTATTAGCTTCTGACGGTGATTTAATGGA
AGGTATTTCGCATGAAGCAGCTTCATTTGCTGGACATAATAAATTAAGTAAATTAGTTGTT
TTATACGATTCAAATGATATTTCATTAGATGGCGAATTAAACAAAGCTTTTTCTGAAAAC
ACAAAAGCTCGTTTTGAAGCATATGGTTGGAATTACTTACTAGTTAAAGATGGTAATGAT
TTAGAAGAAATTGATAATGCGATTACTACTGCTAAATCTCAAGAAGGACCAACAATTATC
GAAGTTAAAACAACAATCGGATTTGGTTCACCGAATAAAGCAGGAACTAATGGTGTTCAT
GGGGCACCTTTAGGTGAAGATGAAAGAAAATTAACATTTGAAAATTACGGTTTAGATCCT
GAAAAACGTTTTAATGTTTCAGAAGAAGTATACGAAATTTTCCAAAATACTATGTTAAAA
CGTGCTAATGAAGATGAATCTCAATGGAATTCATTATTAGAAAAATATGCAGAAACATAT
CCTGAATTAGCAGAAGAATTTAAATTAGCAATTAGTGGTAAATTGCCTAAAAATTATAAG
GATGAATTACCACGTTTTGAACTTGGTCATAATGGTGCATCTCGTGCTGATTCTGGTACTG
TTATTCAAGCAATCAGTAAAACTGTTCCTTCATTCTTTGGTGGATCAGCAGACCTTGCTGG
TTCAAACAAATCCAATGTAAATGATGCAACTGATTATAGTTCTGAAACACCTGAAGGTAA
AAATGTGTGGTTTGGTGTACGTGAATTTGCTATGGGTGCTGCTGTAAATGGTATGGCTGC
ACATGGAGGTTTACATCCATATGGTGCAACATTCTTCGTATTTAGTGATTATTTAAAACCA
GCGTTACGTTTATCATCAATTATGGGATTAAATGCAACGTTCATCTTCACACATGATTCAA
TTGCAGTAGGTGAAGATGGTCCGACTCATGAACCAATTGAGCAATTAGCTGGATTAAGAG
CCATTCCAAATATGAATGTTATCCGTCCTGCTGATGGTAATGAAACAAGAGTAGCATGGG
AAGTTGCCTTAGAATCTGAATCTACACCTACTTCATTAGTATTGACACGTCAAAACTTACC
GGTATTAGATGTACCAGAAGATGTAGTTGAAGAAGGCGTTCGAAAAGGTGCCTATACAG
TTTATGGCTCTGAAGAGACACCAGAATTCCTATTATTAGCTTCAGGTTCAGAAGTTAGTCT
TGCAGTTGAAGCTGCTAAAGATCTTGAAAAACAAGGTAAATCAGTACGTGTTGTTTCAAT
GCCTAACTGGAATGCATTTGAACAACAATCTGAAGAATATAAAGAATCAGTTATTCCATC
AAGCGTAACAAAACGTGTTGCGATTGAAATGGCTTCACCGCTTGGATGGCATAAATATGT
AGGTACTGCAGGTAAAGTTATTGCTATTGACGGCTTTGGCGCAAGTGCACCTGGCGATTT
AGTAGTTGAAAAATATGGATTTACTAAAGAAAATATCTTAAACCAAGTTATGAGCTTATA
A

> **ccdC**

Function: protein coding sequence; membrane protein involved in cytochrome C biogenesis (core genome, constant)
Best match: ccdC_CC001_MW2-USA400_BA000033.2[1343418:1343885] (completely identical)
Position: 031-contig_247: 116882 … 117350; Length: 468 bp
Sequence:
GTGCTCTATTTAATATTTTCAATCATTGTAGCTTTATTTATGGGAACTATAGTTATAGTTAT
TCGTATGAAAGCTCAAAATTATCCGGTAAATGAGAAAAAAATAGTTTTGCCACCGTTTTT
TATGGCGACCGGTGCATTGATGTACGTCGTTCCATATTTTAGGCTAACAGGATCGGAAAT
GCTAGAAGCCTTTATAATTGGTTTGCTTTTTTCTACAGTTCTAATTTGGACTTCTCGATTTG
AAGTCAAAGGTACAGAAATTTATATGAAACGATCTAAAGCATTTCCAGTTATTTTGATTT

CATTACTTATCATTCGTACTGTGATGAAAATATTCATTAGTAATGAAATAGATCCTGGAG
AATTAGGCGGCATGTTCTTTTTATTAGCATTCTGTATGATTGTTCCTTGGAGAGCAGCAAT
GCTATATAAATACAAAAAACTAAAGAAAACATTAATCAATTAA

> **sbcD**

Function: protein coding sequence; nuclease SbcCD subunit D (core genome, variable)
Best match: sbcD_CC001-ST772_118_AJGE01000002.1[44790:45911:r] (completely identical)
Position: 031-contig_247: 117473 … 118595; Length: 1122 bp
Sequence:
ATGAAAATTATACATACAGCAGACTGGCACTTAGGGAAAATATTAAATGGCAAACAGCT
TTTAGAAGATCAAGCGTATATTTTAGATATGTTCGTAGAAAAAATGAAAGAAGAAGAAC
CCGATATCATTGTGATAGCTGGAGATTTATATGACACAACATATCCAAGTAAAGATGCAA
TCATGTTATTAGAACAAGCGATTGGAAAGTTAAATTTAGAACTGCGTATACCAATAATTA
TGATTAGTGGAAATCACGATGGTAAAGAGAGGTTAAACTATGGGGCGAGTTGGTTTGAA
CATAATCAGTTATTTATAAGAACAGATTTTACATCGATTAATTCACCAATAGAGATAAAT
GGGGTTAATTTTTATACACTCCCTTATGCTACTGTGAGCGAAATGAAACACTACTTTGAAG
ATGACACCATTGAAACACATCAACAGGGAATTACGCGCTGTATTGAAACAATAGCACCG
GAAATTGATGAAGATGCCGTCAATATTTTAATTAGTCATCTGACTGTTCAAGGTGGAAAG
ACATCTGATTCTGAAAGACCATTAACTATTGGAACGGTTGAATCAGTTCAGAAAGGTGTT
TTTGATATATTTGATTATGTCATGCTAGGTCACTTGCATCATCCATTTAGTATAGAAGACG
ACAAAATTAAATATAGTGGCTCCTTATTGCAGTATTCATTTTCGGAAGCGGGTCAAGCTA
AAGGGTATAGACGTGTAACAATTAATGATGGCATTATTAACGATGTATTTATTCCTCTTAA
GCCACTTAGACAATTGGAAATTATCTCAGGCGAATATAATGATGTTATTAATGAAAAAGT
TCATGTGAAAAATAAAGATAATTATTTACATTTTAAACTTAAAAATATGTCTCATATTACT
GATCCAATGATGAGTTTAAAACAAATTTATCCTAATACTTTAGCGCTGACGAATGAAACT
TTTAATTACAATGAAGAAAATAATGCTATAGAAATAAGTGGAAAAGATGACATGTCAATT
ATCGAAATGTTTTATAAACATATAACTGATAAAGAATTATCGGATATCCAATCTAAAAAG
ATAAAAAATATTTTAGAAAACGAATTGAGAAAGGAGGATTAA

> **sbcC**

Function: protein coding sequence; nuclease SbcCD subunit C (core genome, constant)
Best match: sbcC_CC001_MSSA476_BX571857.1[1373630:1376659]
Position: 031-contig_247: 118598 … 121628; Length: 3030 bp
Sequence:
ATGAAACCATTACATTTAAAGTTGAATAATTTCGGCCCCTTTTTAAAAGAAGAAATTGAT
TTTTCTAAAATTGATAATAATGAATTGTTTTTAATAAGTGGTAAGACTGGATCGGGTAAA
ACAATGATTTTTGATGCAATGACTTATGCCTTGTTTGGTAAAGCATCAACTGAACAAAGA
GAAGAAAATGATTTGAGAAGTCATTTCGCTGATGGTAAACAGCCGATGTCAGTAACATTT
GAATTTCAATTAAATCATCGAATTTATAAAGTGCATAGACAAGGCCCTTATATCAAAGAA
GGTAATACAACAAAAACGAACGCTAAATTTGATGTATTTGAGATGGTGGATGGCAAGTAT
GAAATTAGAGAAAGTAAAGTAATTTCAGGTACCCAATTCATTATTGAATTATTAGGAGTA
AATGCAGATCAATTCCGACAATTGTTTATTTTGCCTCAAGGTGAATTCAAACGCTTTTTAA
TATCAAACAGTCGTGAAAAGCAAGGGATATTAAGAACACTGTTTGACAGTGAAAAATTT
GAAGCTATACGAGAAATATTAAAAGAAGAAGTAAAAAAAGAAAAAGCTCAAATCGAGA
ATAGATATCAACAAATTGACCTTTTATGGCAAGAAATTGAATCATTTGATGATGACAATA
TAAAAGGCTTATTAGAGGTTGCCACTCAACAGATAGACAAATTGATTGAAAATATACCAC
TTTTACAAGCTAGGTCGAAAGAAATACTAGCATCTGTAAATGAAAGTAAAGAAACTGCTA
TTAAAGAATTTGAAATAATAGAAAAGAAAACATTAGAAAATAATATATTAAAAGATAAT
ATTAATCAACTCAACAAAAATAAAATTGATTTCGTTCAATTGAAAGAACAACAACCTGAA
ATAGAGGGAATTGAAGCTAAGTTAAAGTTGTTACAAGATATTACAAACCTATTGAATTAT
ATTGAAAATAGAGAAAAAATTGAAACTAAAATTGCTAATAGCAAAAAAGATATTTCTAA
AACCAATAATAAAATATTGAATCTTGATTGTGATAAGCGAAACATAGACAAAGAGAAAA
AAATGTTAGAAGAAAACGGAGATTTAATTGAAAGTAAAATCTCTTTTATTGATAAAACTA
GAGTATTATTTAACGATATTAATAAGTATCAACAAAGTTATCTCAATATTGAAAGCTTGA
GAACTGAGGGTGAACAATTAGGTGATGAATTAAATAATCTAATTAAAGGTTTAGAAAAG

GTCGAAGATTCAATAGGTAATAACGAAAGTGATTACGAGAAAATTATCGAACTAAATAA
TGCGATAACTAACATAAATAATGAAATTAATATAATTAAAGAAAATGAAAAAGCTAAAG
CTGAATTAGATAAACTATTAGGTAGTAAGCAAGAGTTAGAGAATCAAATTAATGAAGAA
ACAACTATAATGAAGAATCTCGAAATAAAATTAGATCACTACGATAAATCAAAATTGGA
CTTAAATGATAAAGAAAGCTTTATAAGTGAAATTAAATCTGCTGTAAAGATTGGAGATCA
ATGTCCGATATGTGGTAATGAAATTCAGGATTTAGGGCATCATATTGATTTTGACAGTATT
GCTAAACGTCAAAATGAAATTAAAGAAATTGAAGCAAATATCCACGCAATAAAATCGAA
TATTGCTGTGCATAATTCTGAAATTAAATTTGTTAATGAAAAAATATCGAATATTAATATT
AAAACGCAAAGTGATTTTTCACTCGAAGTATTGAATAAGCGTCTGCTAGAAAATGAAAAT
GCATTGAATAATCAAAGAGATCTTAATAAATTTATAGAACAAATGAAAGAAGAAAAAGA
TAATCTAACGTTGCAAATTCATAATAAACAATTGCGACTAAATAAAAATGAATCTGAGTT
GAAATTATGTCGAGATCTCATCACTGAATTTGAAACACTCTCGAAATATAATAATATCAC
TAATTTTGAGGTGGATTATAAGAAGTATATTCAAGATGTGAACCAACATCAAGAACACTC
AAAGGAGATTGAAGATAAGTCAATGCAATTGTCTCAAAGAAAGTTAATTGAGCAAAATA
ATCTAAATCACTATGAAAATCAACTAGAAACTTACAATAATGACTTAGAATTGAATGAAA
AATCTATTGAAATGGAAATGTCGAGGCTGAATTTAACTGACGACAATGATATAAATGAAA
TAATAGCCTGGAGGGGCGAGCAAGAGGAATTAGAGCAGAAAAGGGATACTTATAAAAA
ACGTTATCATGAATTTGAAATGGAAATAGCTAGGTTAGAATCATTAACCAAGGATAAAGA
GTTATTGGACTCTGATAAATTAAAAGATGAATATGAGCAAAAAAAAGAAAAGATGAATA
CACTGATAGATGAATACTCTGCTGTTCATTATCAATGTCAAAATAATATTAATAAAACAC
AATCTATAGTTTCGCATATTAATTACTTAAATCAAGAATTAAAGGATCAACAAGAAATTT
TTCAATTGGCTGAAATTGTCAGTGGTAAGAATAACAAAAATCTTACATTGGAAAACTTTG
TCTTAATTTACTATTTAGATCAAATTATTGCCCAAGCAAATCTGAGATTAGCAACAATGTC
AGATAATCGATACCAACTAATTAGGCGAGAAGCGGTTTCTCATGGTCTTAGTGGCCTAGA
AATTGATGTATTTGATTTGCATTCAAATAAGTCTAGACATATTAGCTCGTTATCAGGTGGA
GAAACTTTCCAATCGTCGCTTGCATTAGCTTTAGGGTTAAGCGAAATTGTACAGCAGCAA
TCAGGAGGTATTTCACTAGAATCAATATTTATTGATGAAGGATTCGGTACATTAGATCAA
GAAACGCTTGAAACAGCGTTAGACACTTTATTAAATCTTAAATCAACTGGTAGAATGGTT
GGGATTATTTCACATGTGAGCGAATTGAAAAATAGAATACCTTTAGTTTTAGAAGTGAAA
TCAGATCAATATCAGAGTTCAACAAGATTCAAAAGAAATTAA

## > **mscL**

Function: protein coding sequence; large-conductance mechanosensitive ion channel (core genome, constant)
Best match: mscL_CC001_MW2-USA400_BA000033.2[1348234:1348596:r]RC
Position: 031-contig_247: 121698 … 122061; Length: 363 bp
Sequence:
TTATTTTTTCTCACGTAATAAATCTCTGATTTCAGTTAATAACACAACATTTTCTTCCACAA
CTGCTTCTTCTTCGGCTTCTTCTTTCTTCATTAATGTATTTGCAATCTTAACAAAGATGAAT
AAAGCAAACGCGATGATAATAAAGTCGATAACAGATTGGATAAATAAACCGTATTTAAT
ACCCCAGAATGACCATTCTTTAGCAAAATCAACTGATCCGAAAATTTTACCAATTAATGG
CATGATGATATTTTCTACTAATGAAGATATAATCTTGTTGAAAGCTGCACCCATCACAAC
AGCAATTGCTAAATCTAAGACGTTACCTTTTAAGGCGAACTCTTTGAATTCTTTTAACAT

## > **opuD1**

Function: protein coding sequence; glycine betaine transporter 1 (core genome, constant)
Best match: opuD1_CC001_MW2-USA400_BA000033.2[1348800:1350446]
Position: 031-contig_247: 122264 … 123911; Length: 1647 bp
Sequence:
ATGAATTCTTCTTCACCAGAGAATCCAAATGGAAAGAAGTATTCACCAGTCTTCATCTAT
AGTGCAATTGTTGTTGCTATAGTCGTATTACTTGGTGCATTTTTACCTGAACAATTCAACT
ATGTTACCAATAATATTAAAATGTGGATTACAGAAAAGTTAGGTTGGTATTATCTTATTCT
TACTACGATTATCGTGTTCTTCTGTATATTCCTTATTTTTAGTCCTATTGGAAAACTTAAAC
TAGGTAAACCAAATGACAAACCTGAGTTTAATACAATTTCATGGTTTGCTATGTTGTTTAG
TGCTGGTATGGGGATAGGTTTGGTGTTTTATGGTGCAGCTGAACCGATGGCGCACTTTGCT

ACGCCACCTACAGCAGATCCCAAAACTACTGAAGCTTATACTGAAGCTCTACGTTCAACA
TTTTTCCATTGGGGATTCCATGCTTGGGCTGTTTATGGTGTTGTTGCGTTAGCGTTGGCAT
ATTCGCAATTCCGTAAAGGTGAACCAGGTTTATTATCTAGAACTTTACGTCCTCTTTTAGG
TGATAAAGTAGAAGGTCCTATTGGGATTTTTATTGACGTTTTATCTGTATTTGCGACAATC
GTTGGGGTAGCCGTTTCGTTAGGTATGGGTGCTCTACAAATTAATGGTGGTTTACATTACT
TGTTCAATGTTCCAAATAATACGTTTGTACAAGCGATTATCATCATTGTTGTTACTATCTT
ATTTATAGCAAGTGCATGGTCTGGATTAAGTAAAGGTATTCAATACTTAAGTAACTTGAA
CATTGGTTTAGGTACTATTTTAATGGTAGCTGCTTTAATTGTTGGACCAACTGTTCTTATTT
TAAATATGTTAACTAGCTCTACGGGTAGTTTACTAAACACATTCTTGTTTAATAGTTTTGA
TACAGCAGCTTTAAATCCTCAAAAACGTGAATGGATGTCTTCATGGACACTTTATTACTG
GGGTTGGTGGTTAAGTTGGAGCCCATTCGTTGGAGTGTTTATTGCACGAGTTTCAAAAGG
ACGTTCAATTAGAGAGTTCATTTCTGGTGTCTTGCTAGTTCCAGCAATTGTTAGTTTTGTTT
GGTTTAGTGTCTTTGGTGTATTAGGCATCGAGACAGGTAAGAAACACAAAGAAATTTTTG
ATATGACTCCTGAAACACAGCTATTTGGAGTGTTTAATCATGTGCCATTTGGCATTGTTTT
ATCGTTGATTGCATTATTATTAATTGCATCATTCTTTATTACATCTGCTGACTCAGCAACAT
TTGTATTAGGAATGCAAACAACATTTGGTTCATTAAATCCATCTAGTATGGTAAAAGTTGT
TTGGGGAATTTCACAGGCCTTAATAGCATTTGTACTTTTATTAGCTGGTGGCGGTAACGGC
GCTGAAGCTTTAAATGCGATTCAAAGTGCTGCAATTATAAGTGCATTCCCATTCTCCTTTG
TCGTCATACTCATGATGGTAAGTTTCTACAAGGATGCGAACCAGGAACGTAAATTCCTAG
GTTTAACATTGACTCCGAATAAACATCGCTTACAAGAATATATCAAGAGTCAACAAGAAG
ATTATGAATCTGACATTCTTGAAAAGCGTCAGTCACGTAGAAATATAGAGAAAAAAGAT
AACTAA

> **acnA**

Function: protein coding sequence; aconitate hydratase (core genome, constant)
Best match: acnA_CC001_MW2-USA400_BA000033.2[1351280:1353985]
Position: 032-contig_259_RC: 390 … 3096; Length: 2706 bp
Sequence:
ATGGCTGCAAATTTTAAAGAGCAATCAAAAAAACATTTTGACTTGAATGGCCAAAGTTAT
ACTTACTATGATTTAAAAGCTGTAGAAGAGCAAGGTATTACTAAAGTTTCCAATTTACCTT
ATTCAATTCGTGTTTTGTTAGAATCTTTACTTCGTCAAGAAGATGATTTTGTAATTACAGA
CGATCATATTAAAGCTTTAAGTCAGTTTGGAAAAGATGGAAATGAAGGCGAGGTACCATT
TAAACCTTCTCGTGTTATTTTACAAGATTTCACAGGTGTACCAGCCGTAGTTGATTTAGCT
TCTTTACGTAAAGCAATGGATGACGTTGGGGGAGATATTACTAAAATTAATCCAGAAGTA
CCGGTGGATTTAGTTATTGACCACTCAGTTCAAGTGGATAGCTATGCAAATCCAGAAGCT
CTTGAACGTAATATGAAATTAGAATTTGAACGTAACTATGAACGTTATCAGTTTTTAAATT
GGGCAACGAAAGCATTTGATAATTACAATGCAGTTCCTCCTGCAACTGGAATAGTTCACC
AAGTTAACTTAGAATATTTAGCAAGTGTTGTACATGTTCGTGATGTAGATGGTGAAAAAA
CTGCATTTCCGGATACATTAGTTGGTACTGATTCACATACAACAATGATAAATGGTATTG
GCGTACTAGGATGGGGTGTTGGTGGTATTGAAGCTGAAGCTGGAATGCTTGGACAACCTT
CTTATTTCCCAATTCCAGAGGTTATTGGTGTACGACTAGTAAATTCATTACCACAAGGCGC
AACAGCAACTGATTTAGCGTTAAGAGTAACTCAAGAGCTACGTAAAAAAGGTGTTGTTGG
TAAATTTGTAGAGTTCTTTGGTCCAGGTGTACAACATTTACCACTAGCAGACCGTGCTACA
ATTGCAAACATGGCACCAGAGTATGGAGCAACTTGCGGATTCTTCCCAGTTGATGATGAA
TCTCTTAAATATATGAAGTTAACTGGTAGATCAGACGAACATATCGCGCTAGTAAAAGAA
TATTTGAAACAAAACCATATGTTCTTTGATGTTGAGAAAGAAGATCCTAATTATACAGAT
GTTATCGAATTGGATTTATCAACAGTTGAAGCATCGCTTTCAGGACCAAAACGTCCTCAA
GATTTAATTTTCTTAAGTGATATGAAATCATCATTTGAAAATTCTGTAACAGCTCCAGCAG
GCAACCAAGGACACGGTTTAGATAAAAGTGAATTTGATAAGAAAGCTGAAATTAACTTT
AAAGATGGATCAAAAGCTACAATGAAAACAGGTGATATTGCAATAGCAGCAATTACATC
ATGTACAAATACATCTAACCCTTATGTAATGTTAGGTGCAGGTTTAGTTGCTAAAAAAGC
AGTTGAAAAAGGCTTGAAAGTTCCTGAATACGTTAAAACTTCTCTAGCACCAGGATCAAA
AGTTGTTACCGGATATTTAAGAGATGCTGGCTTACAACCTTATTTAGATGATTTAGGCTTC
AACTTGGTTGGTTATGGATGTACAACTTGTATCGGTAATTCAGGTCCTTTATTACCAGAAA
TTGAAAAAGCGATTGCTGATGAGGACCTATTAGTGACATCTGTATTATCTGGTAACCGTA
ACTTTGAAGGTCGTATCCATCCTCTTGTTAAAGCCAATTACCTAGCTTCACCACAGTTAGT

TGTTGCTTATGCATTAGCTGGAACGGTTGATATTGATTTACAGAATGAACCTATTGGTAAA
GGTAATGACGGTGAAGATGTATATTTGAAAGATATTTGGCCATCAATTAAAGAAGTTTCA
GATACCGTTGATAGTGTTGTAACACCTGAATTATTTATTGAAGAATATAATAACGTATAC
AATAACAACGAATTATGGAATGAGATTGATGTAACTGATCAACCTCTATATGACTTTGAT
CCTAATTCAACATACATTCAAAATCCATCATTCTTCCAAGGATTATCTAAAGAACCGGGT
ACGATTGTTCCATTAAATGGTTTACGTGTTATGGGTAAATTCGGTGATTCTGTGACAACTG
ACCACATCTCTCCAGCAGGTGCAATTGGTAAAGATACGCCAGCTGGTAAATATTTACAAG
ATCATCAAGTGCCTATTCGTGAATTTAATTCATATGGTTCAAGACGTGGTAATCACGAAG
TAATGGTTCGAGGTACGTTTGCTAATATACGTATTAAAAACCAATTAGCGCCAGGTACTG
AAGGTGGTTTTACAACTTATTGGCCAACAAATGAAGTAATGCCTATCTTTGATGCTGCAA
TGAAATATAAAGAAGATGGTACAGGTTTAGTTGTATTAGCTGGTAACGATTATGGTATGG
GTTCATCTCGTGACTGGGCAGCAAAAGGTACAAACTTATTAGGTGTTAAAACAGTTATTG
CACAAAGTTATGAACGTATCCATCGTTCAAATTTAGTTATGATGGGTGTATTACCATTAGA
GTTTAAAAAAGGTGAATCAGCTGATTCTCTTGGTCTAGATGGTACAGAAGAAATTTCTGT
TAATATTGATGAAAATGTTCAACCACATGACTACGTCAAAGTTACTGCTAAGAAGCAAGA
TGGTGATTTGGTAGAATTTGACGCTATGGTTCGTTTTGACTCACTTGTTGAAATGGATTAC
TATCGTCACGGTGGAATTTTACAAATGGTTTTAAGAAATAAATTAGCGCAATAA

> **grlB**

Function: protein coding sequence; DNA topoisomerase IV/subunit B (core genome, constant)
Best match: grlB_CC001_MW2-USA400_BA000033.2[1356324:1358315] (completely identical)
Position: 032-contig_259_RC: 5435 … 7427; Length: 1992 bp
Sequence:
ATGAATAAACAAAATAATTATTCAGATGATTCAATACAGGTTTTAGAGGGGTTAGAAGCA
GTTCGTAAAAGACCTGGTATGTATATTGGATCAACTGATAAACGGGGATTACATCATCTA
GTATATGAAATTGTCGATAACTCCGTCGATGAAGTATTGAATGGTTACGGTAACGAAATA
GATGTAACAATTAATAAAGATGGTAGTATTTCTATAGAAGATAATGGACGTGGTATGCCA
ACAGGTATACATAAATCAGGTAAACCGACAGTCGAAGTTATCTTTACTGTTTTACATGCA
GGAGGTAAATTTGGACAAGGCGGCTATAAAACTTCAGGTGGTCTTCACGGTGTTGGTGCT
TCAGTTGTAAATGCATTGAGTGAATGGCTTGAAGTTGAAATCCATCGAGATGGTAATATA
TATCATCAAAGTTTTAAAAACGGTGGTTCGCCATCTTCTGGTTTAGTGAAAAAAGGTAAA
ACTAAGAAAACAGGTACCAAAGTAACATTTAAACCTGATGACACAATTTTTAAAGCATCT
ACATCATTTAATTTTGATGTTTTAAGCGAACGACTACAAGAGTCTGCGTTCTTATTGAAAA
ATTTAAAAATAACGCTTAATGATTTACGCAGTGGTAAAGAGCGTCAAGAGCATTACCATT
ATGAAGAAGGAATCAAAGAGTTTGTTAGTTATGTCAATGAAGGAAAAGAAGTTTTGCAT
GACGTGGCTACATTTTCAGGTGAAGCAAATGGTATAGAGGTAGACGTAGCTTTCCAATAT
AATGATCAATATTCAGAAAGTATTTTAAGTTTTGTAAATAATGTACGTACTAAAGATGGT
GGTACACATGAAGTTGGTTTTAAAACAGCAATGACACGTGTATTTAATGATTATGCACGT
CGTATTAATGAACTTAAAACAAAAGATAAAAACTTAGATGGTAATGATATTCGTGAAGGT
TTAACAGCTGTTGTGTCTGTACGTATTCCAGAAGAATTATTACAATTTGAAGGACAAACG
AAATCTAAATTGGGTACTTCTGAAGCTAGAAGTGCTGTTGATTCAGTTGTTGCAGACAAA
TTGCCATTCTATTTAGAAGAAAAAGGACAATTGTCTAAATCACTTGTGAAAAAAGCGATT
AAAGCACAACAAGCAAGGGAAGCTGCACGTAAAGCTCGTGAAGATGCTCGTTCAGGTAA
GAAAAACAAGCGTAAAGACACTTTGCTATCTGGTAAATTAACACCTGCACAAAGTAAAA
ACACTGAAAAAAATGAATTGTATTTAGTCGAAGGTGATTCTGCGGGAGGTTCAGCAAAAC
TTGGACGAGACCGCAAATTCCAAGCGATATTACCATTACGTGGTAAGGTAATTAATACAG
AGAAAGCACGTCTAGAAGATATTTTTAAAAATGAAGAAATTAATACAATTATCCACACAA
TCGGGGCAGGCGTTGGTACTGACTTTAAAATTGAAGATAGTAATTATAATCGTGTAATTA
TTATGACTGATGCTGATACTGATGGTGCGCATATTCAAGTGCTATTGTTAACATTCTTCTT
CAAATATATGAAACCGCTTGTTCAAGCAGGTCGTGTATTTATTGCTTTACCTCCACTTTAT
AAATTGGAAAAAGGTAAAGGCAAAACAAAGCGAGTTGAATACGCTTGGACAGACGAAG
AGCTTAATAAATTACAAAAAGAACTTGGTAAAGGCTTCACGTTACAACGTTACAAAGGTT
TGGGTGAGATGAACCCTGAACAATTATGGGAAACGACGATGAACCCAGAAACACGAACT
TTAATTCGTGTACAAGTTGAAGATGAAGTGCGTTCATCTAAACGTGTAACAACATTAATG
GGTGACAAAGTACAACCTAGACGTGAATGGATTGAAAAGCATGTTGAGTTTGGTATGCA

AGAGGACCAAAGTATTTTAGATAATTCTGAAGTACAAGTGCTTGAAAATGATCAATTTGA
TGAGGAGGAAATCTAG

> **grlA**

Function: protein coding sequence; DNA topoisomerase IV/subunit A (core genome, constant)
Best match: grlA_CC001_MSSA476_BX571857.1[1386869:1389271]
Position: 032-contig_259_RC: 7426 … 9829; Length: 2403 bp
Sequence:
GTGAGTGAAATAATTCAAGATTTATCACTTGAAGATGTTTTAGGTGATCGCTTTGGAAGA
TATAGTAAATATATTATTCAAGAGCGTGCATTGCCAGATGTTCGTGATGGTTTAAAACCA
GTACAACGTCGTATTTTATATGCAATGTATTCAAGTGGTAATACACACGATAAAAATTTC
CGTAAAAGTGCGAAAACAGTCGGTGATGTTATTGGTCAATATCATCCACATGGAGACTAC
TCAGTGTACGAAGCAATGGTCCGTTTAAGTCAAGACTGGAAGTTACGACATGTCTTAATA
GAAATGCATGGTAATAATGGTAGTATCGATAATGATCCGCCAGCGGCAATGCGTTACACT
GAAGCTAAGTTAAGCTTACTAGCTGAAGAGTTATTACGTGATATTAATAAAGAGACAGTT
TCTTTCATTCCAAACTATGATGATACGACACTCGAACCAATGGTATTGCCATCAAGATTTC
CTAACTTACTAGTGAATGGTTCTACAGGTATATCTGCAGGTTACGCGACAGATATACCAC
CACATAATTTAGCTGAAGTGATTCAAGCAACACTTAAATATATTGATAATCCGGATATTA
CAGTCAATCAATTAATGAAATATATTAAAGGTCCTGATTTTCCAACTGGTGGTATTATTCA
AGGTATTGATGGTATTAAAAAAGCTTATGAATCAGGTAAAGGTAGAATTATAGTTCGTTC
TAAAGTTGAAGAAGAAACTTTACGCAATGGACGTAAACAGTTAATTATTACTGAAATTCC
ATATGAAGTGAACAAAAGTAGCTTAGTAAAACGTATCGATGAATTACGTGCTGACAAAA
AAGTCGATGGTATCGTTGAAGTACGTGATGAAACTGATAGAACTGGTTTACGAATAGCAA
TTGAATTGAAAAAAGATGTGAACAGTGAATCAATCAAAAATTATCTTTATAAAAACTCTG
ATTTACAGATTTCATATAATTTCAACATGGTCGCTATTAGTGATGGTCGTCCAAAATTGAT
GGGTATTCGTCAAATTATAGATAGTTATTTGAATCACCAAATTGAGGTTGTTGCAAATAG
AACGAAGTTTGAATTAGATAATGCAGAAAAACGTATGCATATCGTTGAAGGTTTGATTAA
AGCGTTGTCAATTTTAGATAAAGTAATCGAATTGATTCGTAGCTCTAAAAACAAGCGTGA
CGCTAAAGAAAACCTTATCGAAGTATACGAGTTCACAGAAGAACAGGCTGAAGCAATTG
TAATGTTACAGTTATATCGTTTAACAAACACTGATATAGTTGCGCTTGAAGGTGAACATA
AAGAACTTGAAGCATTAATCAAACAATTACGTCATATTCTTGATAACCATGATGCATTAT
TGAATGTCATAAAAGAAGAATTGAATGAAATTAAAAAGAAATTCAAATCTGAACGACTG
TCTTTAATTGAAGCAGAAATTGAAGAAATTAAAATTGACAAAGAAGTTATGGTGCCTAGT
GAAGAAGTTATTTTAAGTATGACACGTCATGGATATATTAAACGTACTTCTATTCGTAGCT
TTAATGCTAGCGGTGTTGAAGATATTGGTTTAAAAGATGGTGACAGTTTACTTAAACATC
AAGAAGTAAATACGCAAGATACCGTACTAGTATTTACAAATAAAGGTCGTTATCTATTTA
TACCGGTTCATAAATTAGCAGATATTCGTTGGAAAGAATTGGGACAACATGTATCACAAA
TAGTTCCTATCGAAGAAGATGAAGTGGTTATTAATGTCTTTAATGAAAAGGACTTTAATA
CAGATGCATTTTATGTTTTTGCGACTCAAAATGGCATGATTAAGAAAAGTACAGTGCCTC
TATTTAAAACAACGCGTTTTAATAAACCTTTAATTGCTACTAAAGTTAAAGAAAATGATG
ATTTGATTAGTGTTATGCGCTTTGAAAAAGATCAATTAATTACCGTCATTACTAATAAAGG
TATGTCATTAACGTATAATACAAGTGAACTATCAGATACCGGATTAAGGGCAGCTGGTGT
TAAATCAATAAATCTTAAAGCTGAAGATTTCGTTGTTATGACAGAAGGTGTTTCTGAAAA
TGATACTATATTGATGGCCACACAACGCGGCTCGTTAAAACGTATTAGTTTTAAAATCTTA
CAAGTTGCTAAAAGAGCACAACGTGGAATAACTTTATTAAAAGAATTAAAGAAAAATCC
ACATCGTATTGTAGCTGCACATGTAGTGACAGGTGAACATAGTCAATATACATTATATTC
AAAATCAAATGAAGAACATGGTTTAATTAATGATATTCATAAATCTGAACAATATACAAA
TGGCTCATTCATTGTAGATACAGATGATTTTGGTGAAGTAATAGACATGTATATTAGCTA
A

> **alsT**

Function: protein coding sequence; sodium-alanine symporter family protein (core genome, constant)
Best match: alsT_CC001_MW2-USA400_BA000033.2[1360967:1362427] (completely identical)
Position: 032-contig_259_RC: 10078 … 11539; Length: 1461 bp
Sequence:

TTGAAAGATTTCGATAGTTTAATTCCTGGATGGTTTAAAGAATTTGTCCATGTTGGTACCG
ATTTAATATGGTCTCAATATTTAATTGGTCTATTATTGACAGCTGGATTCTTCTTTACAATT
AGTTCTAAATTCGTCCAATTACGAATGTTACCTGAAATGTTTAGAGCTTTAGTAGAACGTC
CAGAAACTTTAGAAGATGGTAAGAAGGGTATTTCGCCATTCCAAGCATTTGCGATTAGTG
CTGGTTCGAGAGTTGGTACTGGTAATATTGCTGGTGTTGCGACTGCGATTGTTTTAGGCGG
TCCAGGTGCAGTGTTTTGGATGTGGGTTATTGCATTTATAGGTGCAGCGAGTGCATTTATA
GAAGCGACTTTGGCTCAGGTTTATAAAGTACATGATAAAGATGGTGGATTCCGTGGTGGT
CCAGCTTACTATATTACTAAAGGTTTAAATCAAAAATGGCTAGGTATCGTATTTGCGATTT
TAATTACAATTACATTTGCATTTGTATTTAACACAGTGCAATCTAATACAATTGCGGAGTC
GTTAAATACGCAATATAATATTAGTCCAGTAATCACAGGTATTATTTTAGCAATCGTAAC
AGCTATTATTATATTTGGTGGTGTACGTAGTATTGCTACGTTATCTTCGTTAATTGTACCG
ATTATGGCTATCATTTACATTGGTATGGTTTTAGTAATATTGCTATTTAATTTAGATCAAA
TTGTTCCTATGATAGGTACGATTATTAAAAGTGCATTTGGTATCGAACAAGTAACTGGTG
GCGCTGTAGGTGCTGCGGTTCTTCAAGGTATCAAACGTGGTTTATTCTCTAACGAAGCTG
GTATGGGTTCTGCGCCGAATGCAGCGGCAACTGCTGCCGTACCACACCCTGTTAAGCAAG
GTTTAATCCAATCATTAGGTGTGTTCTTTGATACAATGTTGGTTTGTACAGCAACTGCAAT
CATGATTTTACTATATTCAGGACTGAAATTTGGTGATAACGCACCTCAAGGTGTTGCAGTT
ACTCAATCAGCACTTAATGAGCATTTAGGTTCTGCTGGAGGTATTTTCTTAACAATAGCAG
TTACACTGTTTGCATTTTCATCTGTGGTAGGTAATTACTATTACGGTCAATCTAATATTGA
ATTTTTATCAACAAACCGTGTAATATTATTTATCTTTAGATGTCTTGTTGTAGTACTTGTCT
TTGTCGGTGCAGTTGTAAAAACAGAAACAGTATGGAATACGGCAGACTTATTTATGGGCT
TAATGGCAATTGTAAACATTATTTCCATTATAGGACTGTCCAATGTAGCTTTTGCATTGAT
GAAAGATTATCAAAAGCAGAAAAAAGAAGGCAAGAACCCTGTCTTTAAACCTGAAAACT
TAGAAATTAACTTATTTGGAATTAGTGCTTGGGGCGCTAACAAATATAAGAACTCTGATA
AATAA

> **tx_alsT**

Function: rho-independent terminator of alsT
Best match: tx_alsT_CC001_MW2-USA400_BA000033.2[1362455:1362520:r]RC (completely identical)
Position: 032-contig_259_RC: 11566 … 11632; Length: 66 bp
Sequence:
TAAAAATCAAATAAACCCTGAGGTTTTCAAATTAAAGCGAAAATCTCAGGGTTTTAAATT
TGTACT

> **glcT**

Function: protein coding sequence; transcript antiterminator (core genome, constant)
Best match: glcT_CC001_MW2-USA400_BA000033.2[1362927:1363778] (completely identical)
Position: 032-contig_259_RC: 12038 … 12890; Length: 852 bp
Sequence:
ATGGGAGAATATATTGTTACTAAAACATTGAACAACAATGTCGTAGTATGTACTAATAAT
GATCAAGAAGTTATTTTAATCGGTAAAGGTATTGGTTTTAACAAAAAAGAGGGAATGGCG
TTAAACGACCAAACTATTACAATAGAGAAAATTTATAAATTAGAGAGTGAGCAACAAAA
AGCACATTATAAAAGTTTAGTTGAAATCGCTGATGATAATGTATTACAAGTAATTATTGA
TTCGTTGAATTTTATTTCTAATACTGCGATGAATGTTGATTCAAAACAACTTGTAGTTTCA
TTAACGGATCATATTATATTTGCTTATAAACGCTTAAAACAAAATCAAGTTATTAGCAATC
CATTTGTTATGGAAACTATGCAGTTATATAGTGATGCATATCATATTGCTAAACAGGTGAT
TGATCAGTTAAATGCAGCATTAGATGTACATTTTCCTGAAGATGAGATAGGATTTATTGC
ATTACATATTGCATCTAATACAGAAGATTTATCTATGCATGAGATGACCTTGATCAATAAT
GTTATTAAAAAAGGTATAGATATCATTGAATCAGACCTTGTGACAACTGTTGATAAGGAA
TCATTACAATACCAACGTTTTATAAGGCACGTACAATTTTTAATTCGCCGATTAAGAAGA
AAAGAATATATACATGCACAAGATGATTTTGTGTCTATGATTAAAAATCACTATCCGATT
TGCTATAACACAGCATATAAAATTTTAACTATGATACAAAAACAATTTGATGTTAATATC
AGTGAGTCTGAAATTATATATTTAACATTACACATTCATCATTTTGAAGAAAGGATTAATC
AATCCTAA

## > mprF

Function: protein coding sequence; lysylphosphatidylglycerol synthetase (core genome, variable)
Best match: mprF_CC001_MW2-USA400_BA000033.2[1365602:1368124] (completely identical)
Position: 032-contig_259_RC: 14713 … 17236; Length: 2523 bp
Sequence:
ATGAATCAGGAAGTTAAAAACAAAATATTTTCAATCTTAAAAATTACGTTTGCTACAGCT
TTATTTATTTTTGTAGTAATCACATTGTATCGGGAGTTATCTGGTATTAACTTTAAAGATA
CGTTGGTTGAATTTAGTAAGATTAACCGTATGTCCTTAGTTTTACTATTTATTGGTGGTGG
GGCATCGCTTGTTATTCTATCAATGTATGATGTGATTTTATCTAGAGCTTTAAAAATGGAT
ATATCCTTAGGCAAAGTTTTAAGAGTAAGTTATATCATCAATGCATTGAATGCGATTGTA
GGTTTCGGTGGCTTTATTGGTGCAGGCGTTAGAGCTATGGTTTATAAAAACTATACGCAT
GATAAAAAGAAATTAGTTCACTTTATATCCTTAATACTTATTTCAATGTTGACAGGTTTAA
GCTTATTATCATTGTTAATTGTATTCCATGTTTTCGATGCATCTTTAATTTTAGATAAGATT
ACATGGGTAAGATGGGTATTATATGTAGTGTCATTTTTCTTACCATTATTCATTATTTATTC
AATGGTTAGACCACCTGATAAAAACAATCGTTTTGTAGGATTGTACTGCACTTTAGTGTC
GTGTGTTGAATGGTTAGCAGCTGCAGTTGTATTATATTTCTGTGGTGTAATTGTTGACGCT
CATGTATCATTCATGTCCTTTATTGCAATATTTATCATTGCTGCATTATCAGGTTTAGTCAG
CTTTATTCCTGGTGGTTTCGGCGCTTTCGATTTAGTTGTATTACTAGGATTTAAAACTTTAG
GTGTCCCTGAGGAAAAAGTATTATTAATGCTACTTCTATATCGTTTTGCGTACTATTTTGT
ACCGGTAATTATTGCATTAATTTTATCATCATTTGAATTTGGTACATCAGCTAAGAAGTAC
ATTGAGGGATCTAAATACTTTATTCCTGCTAAAGATGTTACGTCATTTTTAATGTCTTATC
AAAAGGATATTATTGCTAAAATTCCATCATTATCATTAGCAATTTTAGTATTCTTTACAAG
TATGATCTTTTTTGTAAATAACTTAACGATTGTTTACGATGCTTTATATGATGGAAATCAC
TTAACGTATTATATTCTATTGGCAATTCATACTAGTGCTTGTTTATTACTTTTACTGAATGT
AGTTGGTATTTATAAGCAAAGTAGACGTGCCATCATCTTTGCTATGATTTCAATTTTATTA
ATCACAGTGGCGACATTCTTCACTTACGCTTCATATATTTTAATAACATGGTTAGCTATTA
TTTTTGTTCTGCTTATTGTAGCTTTCCGTAGAGCACGTAGGTTGAAACGCCCAGTAAGAAT
GAGAAATATAGTTGCAATGCTTTTATTCAGTTTATTTATTTTATATGTTAACCATATATTTA
TTGCTGGAACGTTATATGCATTAGATATTTATACGATTGAAATGCATACATCTGTATTGCG
CTATTACTTCTGGCTTACGATTTTAATCATCGCTATCATCATAGGTATGATTGCATGGTTG
TTTGATTATCAATTTAGCAAAGTACGCATTTCTTCTAAAATTGAAGATTGCGAGGAGATTA
TTAATCAGTATGGCGGTAATTATTTGAGTCACTTGATATATAGTGGTGACAAGCAGTTTTT
CACTAATGAAAATAAAACAGCATTTTTAATGTATCGTTATAAAGCAAGTTCATTAGTGGT
TCTTGGAGATCCGTTAGGTGATGAAAATGCCTTTGATGAATTGTTAGAAGCATTCTATAAT
TACGCTGAGTATTTAGGCTATGATGTTATATTCTATCAAGTTACAGATCAACACATGCCTT
TATATCATAATTTCGGTAACCAATTTTTCAAATTAGGTGAAGAAGCAATTATTGATTTAAC
GCAATTTTCAACTTCAGGTAAAAAACGCCGTGGATTTAGAGCGACTTTAAACAAATTCGA
TGAACTTAATATTTCATTCGAAATTATTGAACCACCGTTTTCAACTGAATTTATAAATGAA
CTTCAACATGTAAGTGATTTATGGCTAGATAATCGTCAGGAAATGCATTTCTCTGTGGGTC
AATTTAATGAAGAATACTTATCTAAAGCGCCAATTGGTGTAATGCGAAATGAAGAAAATG
AAGTAATTGCATTTTGTAGTTTAATGCCAACATACTTTAATGATGCCATTTCAGTCGATTT
AATTAGATGGTTGCCAGAGTTAGATTTACCATTAATGGATGGTCTATACTTGCATATGTTA
CTTTGGAGTAAAGAACAAGGTTATACAAAATTTAATATGGGTATGGCAACGTTATCGAAC
GTTGGTCAATTGCATTATTCATATTTAAGAGAACGACTTGCAGGCCGTGTCTTTGAACATT
TCAACGGTCTATATCGTTTCCAAGGATTACGTCGTTATAAATCTAAATATAATCCGAATTG
GGAACCACGCTTTTTAGTTTATCGTAAAGATAATTCGCTTTGGGAATCACTTTCTAAAGTA
ATGCGTGTAATACGTCACAAATAA

## > msrA1

Function: protein coding sequence; peptide methionine sulfoxide reductase A1 (core genome, constant)
Best match: msrA1_CC001_MW2-USA400_BA000033.2[1368327:1368836:r]RC (completely identical)
Position: 032-contig_259_RC: 17438 … 17948; Length: 510 bp

Sequence:
TTATTGCTTATTTTTGTATTCTTGGCGTATTTTTTGTTCTTCTGCATAGCGCTCTGGATTTTT
CTTATAAAAATCTTGGTGATAGTCTTCGGCTTTGTAAAATTGTGACGCGGGTAATATTTTT
GTTGCAATTGCCTTATCAGCATTAATCGTATTTTTAAGCTGCTCGATATAAGTCTCAGCGA
GTTCTTTTTGATGATCATTAGTGTAGAAAATAGCTGTTTGATATTGAGGACCACGGTCTTG
ATATTGACCCCCTGTATCTAATGGGTCAATGACTGAGAAAAATATTTCTAATAACTTATTG
TATGAGAATAATGCAACATCATATTGAATTTCAACAGTTTCTAAATGACCACTCGTACCT
GATTTTACTTGTTCGTAAGTAGGATTTTCAATATGTCCGCCCATATATCCAGAAGTTACTT
TTTCTATGCCGTCAAAGGTGTCAAATGGTTTCGTCATACACCAAAAGCAACCTCCGGCAA
AATAAGCTGTATTAATATTCAT

## > msrR
Function: protein coding sequence; regulatory protein msrR (core genome, constant)
Best match: msrR_CC001_MW2-USA400_BA000033.2[1368973:1369956] (completely identical)
Position: 032-contig_259_RC: 18084 … 19068; Length: 984 bp
Sequence:
ATGGATAAAGAAACTAATGACAACGAATATAGACGTCAAAGTGAACATCGCACTTCGGC
GCCTAAGCGAAAAAAGAAGAAGAAAATTAGGAAATTACCTATCATTCTTCTGATTGTTGT
AATTTTACTTATCGCATTAGTTGTATATATTGTGCATAGTTACAATAGCGGTGTAGAATAT
GCCAAGAAACATGCGAAAGATGTTAAAGTACATCAATTTAATGGACCAGTAAAAAATGA
TGGTAAAATTTCTATTCTTGTACTCGGTGCAGATAAAGCACAAGGTGGACAATCAAGAAC
AGATTCTATCATGGTTGTTCAATATGACTTTATCAATAAAAAGATGAAAATGATGTCTGTC
ATGCGTGATATTTATGCAGATATTCCAGGATATGGAAAACACAAAATTAATTCAGCATAC
GCTTTAGGTGGTCCAGAGCTACTTAGAAAAACACTTGATAAAAATTTAGGAATTAATCCT
GAATATTATGCAGTAGTTGATTTTACTGGATTTGAGAAAATGATTGATGAATTAATGCCA
GAAGGTGTACCAATTAATGTCGAAAAGGATATGTCGAAAAATATTGGTGTATCTTTGAAA
AAGGGTAACCATAGGTTGAATGGTAAAGAATTACTTGGTTATGCAAGATTCCGTCACGAC
CCTGAAGGTGACTTCGGACGCGTGCGACGTCAGCAACAAGTGATGCAAACATTGAAAAA
AGAAATGGTTAATTTTAGAACAGTTGTTAAATTACCAAAAGTTGCAGGTATTTTAAGAGG
CTATGTGAATACAAACATTCCTGATTCAGGGATTTTCCAAACAGGTTTGAGTTTTGGTATC
CGAGGTGAAAAAGATGTTAAGTCATTGACTGTGCCAATCAAGAACTCATACGAAGATGTC
AATACAAATACTGATGGTAGTGCATTACAGATTAATAAAAACACAAATAAACAAGCTATT
AAAGACTTTTTAGATGAAGATTAA

## > txbi_msrR
Function:  bidirectional rho-independent terminator of msrR
Best match: txbi_msrR_CC001_MW2-USA400_BA000033.2[1369949:1370017] (completely identical)
Position: 032-contig_259_RC: 19060 … 19129; Length: 69 bp
Sequence:
AAGATTAAAAATAAACAAGGCGATTTCTATCATACTCAGATAGAAGTCGCCTTGTTCGTT
TTCAAAGAA

## > dmpI
Function: protein coding sequence; 4-oxalocrotonate tautomerase (core genome, constant)
Best match: dmpI_CC001_MW2-USA400_BA000033.2[1370044:1370232:r]RC (completely identical)
Position: 032-contig_259_RC: 19155 … 19344; Length: 189 bp
Sequence:
TTATTGATCTGACTTTCTTACGCCAGCCACACCATAATGGTTTGGTTTCATTTCTTCTATAA
CAACGTGAATTGCTTGTCTATTTGCCCCCGTTGTTTTTTCTACGGCGTCAGTTACTTCGCTA
ACTAAATTTTTTAATTGTTCATCCGAACGACCTTCTAATAATTTTACATTGACGATTGGCA
TCAT

## > Q99UB7

Function: protein coding sequence; DNA-damage repair protein (core genome, constant)
Best match: Q99UB7_CC001_MW2-USA400_BA000033.2[1370377:1371639] (completely identical)
Position: 032-contig_259_RC: 19488 … 20751; Length: 1263 bp
Sequence:
GTGTATAATTATCATTTATTAGAAGATAGGGATGTTCTATGTATTGACCAAAAAAGTTTTT
TTGCGAGTGTTTCTTGTATTGAAAAGGGGCTAGATCCATTAGAAACAAAGCTAGCTGTTG
TTGCAGATACTAAGCGTCAGGGTTCTGTAATATTGGCTGCGACACCTAAATTAAAAGAAT
TAGGCATCAAGACAGGGTCGCGATTGTTTGAAATACCACATAGAAATGATATTTACATTA
TCAATCCAAGTATGCGTAAATATCTTAATGTTTCAGTTGCTATTTCTAAGATTGCATTGCG
TTATATTCCACCTGAAGATTTACACCAATATAGTATTGACGAATTTTTTATGGATGTTACT
GATAGCTATCATAGATTTAGTTCTACAGTACATGCATTTTGCGAAAGACTTAAACGTGAA
ATTTATGAAGAAACAGGCATTTATTGTACTGTGGGCATTGGTTCTAATATGTTATTAAGTA
AAATTGCTATGGATGTTGAAGCGAAGCATAGTCAAAATGGTATAGCTGAATGGCGATATC
AAGATGTACCAACGAAATTATGGCCAATTCAGCCCTTGCGAGATTTTTGGGGTATTAATC
GTCGAACAGAAGCCAAATTGAATAAAAGAGGAATTTTTACTATAGGAGATTTAGCGAAA
TATCCATATAAATTTTTAAAAAAAGAGTTCGGTATTTTAGGTGTTGATATGCATCTACATG
CGAATGGGATAGATCAGAGTAAAGTACGTGAAAAGCACAAGATCAGCAATCCATCGATA
TGCAAAAGTCAAATATTAATGAGAGATTATCATTTTGATGAAGCAAAAGTAGTAATGCAA
GAGTTAATTGAAGATGTTGCTAGCAGAGTTCGAGCAAGAAAAAAAGTGGCAAGAACGAT
ACATTTTGCCTTTGGCTATAGTGATGAAGGCGGTGTACATAAGCAATATACTTTGAAAGA
TCCAACAAACTTAGAAAAAGATATTTATAAAGTAGTAATGCATTTCGCAGATAAATTATG
TAATAAACAAGCACTATATCGTACGCTAAGTATATCTTTGAGTCAATTTATTAATGAGGA
TGAGCGACAGTTAAGTCTGTTTGAAGATGAATACCAACGCAAACGTGACGAATGTCTAGC
TAAAACGATAGACCAATTACATTTGAAATACGGCAAAGGTATTGTGTCCAAAGCAGTATC
GTTTACAGAAGCAGGTACAAAACACGGCAGATTAGGTTTAATGGCTGGACATAAAATGT
AA

## > tyrA

Function: protein coding sequence; prephenate dehydrogenase (core genome, constant)
Best match: tyrA_CC001_MW2-USA400_BA000033.2[1371775:1372866:r]RC (completely identical)
Position: 032-contig_259_RC: 20886 … 21978; Length: 1092 bp
Sequence:
TTATTGGATATAACAATCAAAATCACTCAATGCTTGCATACCGCGTTCTCGGTCAGTAGG
GTTTTTGAAACTAATTTTTAAAGCACCGTATATATCTTCGCGTACTTCTAAGATTCTTAAG
TTGCTTATAGATATGTTATGTAAACTCAGGATATAAGTCACTTTACTTATCATACCTGATT
CATCCGGAATGTCTACATATAGATCATACGCAGTATTTAGTCCACCTAGTTGTTTAGCGGG
TAGTGCGTCGCGATACGATTTAGCTTGGGCAAAAAATGATAACAATTTTTCAGAATCATT
GCTTTCAATTAGTCTTTCTAAATCTTGAAACTGACTTTTTAGCTGTCGAATCATTTCTAAA
ATATACGTTTTATTACTCAAGGTGATATCTTTCCACATTTGTGCATTACTACTAGCTATAC
GAGTGATATCACGAAAACCACCAGCTGCAAGTTTATTAACTAAATGATGTTCTTGACCGT
TCTTTTGACTAACATGAACTAAACTAGATGCAACGATATGAGGTAAATGACTTACGACGC
TTGTTACGTAGTCGTGTTCTTCAGCAGTAGTTACAATAAATTTAGCAAGAGTAGGTGATA
ACAGTTCTTTTAACGTGTTTGCTGCTTGCTCATTTCTTGGCTCATTGTAGACTAAAATATA
ATAAGCGTTTTCAAATAAGTGCTTTTTAGCATTTAGTACACCAGATTTATGACTACCAGCC
ATTGGATGACCACTGACTAAATGAATATTATGCTTTAATAAATTGCATTCGTGTTGCTGTA
TCATTGCTTTAGTACTACCAGTATCAGAAACAATAACACCAGGTTTAGTTGGCATATCTAT
AAGCTCGCTAAGATATTTATTTGTGATAGCAACAGGTGTTGCATAAATAATTACATCGGC
TTTTTTAATAGCTTCACTATAATTTAAACATTTTTCATTAATAATGCCGATTGATTTAGCTT
TATCTAACTGAGAAGTATCTGCATCGTATGCAATAATATTAGTATTAGGGTTATGGTATTT
TATATTGCTAGCAAGACTTCCACCAATTAATCCAAGCCCAACAAATAAAACTGTTGTCAT

## > tnpIS431-06

Function: protein coding sequence; transposase for IS431 (mobile element)

Best match: tnpIS431-06_circ_S-epidermidis_GQ900381.1[43799:43807] (completely identical)
Position: 032-contig_259_RC: 23214 … 23223; Length: 9 bp
Sequence:
ATGAACTAT

## > tbox05

Function: noncoding RNA T-box leader element (core genome, constant)
Best match: tbox05_CC001_MW2-USA400_BA000033.2[1374124:1374297] (completely identical)
Position: 032-contig_259_RC: 23235 … 23409; Length: 174 bp
Sequence:
AATTTTCTAAAGAAATAGTAGCAGATATGAAACGTAGCAAATAGAAAGCTAATGGGTGA
TGGGAATTAGCACGCCATATCTTGTGAATTGGACTTTGGAAAACAATTGAATGAGTTTTG
AAAGTGAACATGAATTATGTTAACTAAGGTGGCACCACGGTAACGCGTCCTTACA

## > tbox06

Function: noncoding RNA T-box leader element (core genome, constant)
Best match: tbox06_CC001_MW2-USA400_BA000033.2[1374325:1374511] (completely identical)
Position: 032-contig_259_RC: 23436 … 23623; Length: 187 bp
Sequence:
ATTTAGACAAAATGTAGTAGTTAATTAAAGGTAGCAACAGAAAGTTAGTGGATGATGTG
AACTAACACCGAGATTAATGAAATTGGGTTTTGTCTGCAACAGAAAAATTATATATAGTA
AAGAGTGAACTATGAATATTTCGAATATTCGGTTAATTTAGGTGGTACCACGCGTCAGCG
TCCTTTAT

## > trpE

Function: protein coding sequence; putative anthranilate synthase component I (core genome, constant)
Best match: trpE_CC005_Mu50-VRSA_BA000017.4[1448894:1450300]
Position: 032-contig_259_RC: 23665 … 25072; Length: 1407 bp
Sequence:
ATGGATATATTTTATAAAAAAATAAAAGCAAATGTAACGCCCGAAGTTTTAGCACAACTT
CATTCCAAGAAGATCATTTTGGAAAGTACAAATCAACAACAAACTAAAGGTCGCTATTCA
GTTGTTATTTTTGATATTTATGGCACTTTAACTTTAGATAATGATGTATTATCAGTAAGTA
CTTTAAAAGAATCGTATCAAATCACTGAAAGACCGTACCATTATTTAACGACTAAAATAA
ATGAAGACTACCATAATATTCAAGATGAGCAACTTAAGTCATTACCATTTATATCTGGAT
ATGTTGGGACGTGTAGCTTTGATTTAGTAAGACATGAATTTCCTAAATTGCAATCAATAC
AATTAGAAGATCACAAGCAGCACGATGTAAGGTTATATATGGTTGAACAAGTTTATGTAT
TTGACCATTACAAAGATGAGTTATATATCATCGCGACGAATCAATTTTCAAATTCAACAA
AATCAGATCTTGAGAATCGAGTTATTAAGTCTATCGAAGACTTAACTAAAATCCAACCAT
TCATGCCTACACAAGATTTTGATTTTAAAACTAAAGAAATTCAATCAAACATTTCTGAAG
AAAGATTTATCGAAATGATTCAGTATTTCAAAGAGAAAATAACAGAAGGGGATATGTTCC
AAGTTGTGCCATCAAGAATTTACAAATATGCGCATCATGCTAGTCAGCATTTAAATCAAC
TTTCGTTTCAACTGTATCAAAATTTAAAACGACAAAACCCAAGTCCATATATGTATTATCT
TAATATCGATCAACCATATATTGTCGGTAGTTCTCCCGAAAGTTTCGTAAGTGTCAAAGAT
CAAATTGTAACAACTAATCCTATTGCAGGTACGATTCAACGTGGTGAGACGACACAAATA
GATAATGAGAATATGAAACAACTACTTAATGATCCAAAAGAATGCAGCGAACATCGTAT
GCTAGTTGATTTAGGACGTAATGATATTCATAGAGTAAGTAAAATCGGTACCTCAAAAAT
TACTAAATTAATGGTTATTGAAAAATATGAACATGTTATGCATATCGTAAGTGAAATCAC
AGGTAAAATAAATCAAAATTTATCACCAATGACAGTTATTGCGAATTTATTACCAACAGG
TACCGTTTCAGGTGCACCAAAATTACGTGCAATTGAAAGAATATATGAACAATATCCACA
TAAACGAGGCGTTTATAGTGGTGGTGTTGGATACATAAATTGTAATCATAACTTAGATTTT
GCTTTAGCAATTCGAACGATGATGATAGATGAGCAGTATATCAACGTAGAAGCTGGTTGT
GGCGTTGTATATGATTCTATTCCTGAAAAAGAACTGAATGAAACGAAATTGAAAGCTAAA
AGCTTATTGGAGGTGAGGCCATGA

> **trpG**

Function: protein coding sequence; anthranilate synthase component II (core genome, constant)
Best match: trpG_CC001_MW2-USA400_BA000033.21375957:1376523 [4_50ANF10END]
Position: 032-contig_259_RC: 25068 … 25635; Length: 567 bp
Sequence:
ATGATCTTAGTTGTAGATAATTATGATTCCTTTACATATAACCTAGTGGATATTGTTGCTC
AACATACTGACGTCATTGTTCAATATCCTGATGATGATAATGTGCTGAATCAATCGGTGG
ACGCTGTTATTATATCTCCTGGTCCAGGGCATCCATTAGACGATCAACAGTTAATGAAAA
TCATATCAACCTATCAACACAAACCCATTTTAGGTATTTGCCTAGGTGCTCAGGCACTGAC
TTGTTATTACGGTGGAGAAGTCATTAAAGGCGACAAGGTTATGCACGGAAAAGTTGATAC
ACTAAAGGTTATATCGCATCATCAACATCTGTTATATCAAGATATACCAGAACAGTTTTC
AATTATGAGATATCATTCATTAATAAGTAACCCTGACAATTTTCCAGAAGAATTGAAAAT
TACTGGACGTACCGAAGATTGTATACAGTCATTTGAGCATAAAGAAAGACCGCATTACGG
TATTCAGTACCATCCTGAATCATTTGCTACAGACTATGGTGTCAAAATAATTACAAATTTT
ATTAATCTAGTGAAGGAAGGATGA

> **trpD**

Function: protein coding sequence; anthranilate phosphoribosyltransferase (core genome, variable)
Best match: trpD_CC001_MW2-USA400_BA000033.2[1376529:1377527]
Position: 032-contig_259_RC: 25640 … 26639; Length: 999 bp
Sequence:
ATGACATTACTAACAAGAATAAAAACTGAAACTATATTACTTGAAAGCGACATTAAAGA
GCTAATCGATATACTTATTTCTCCTAGTATTGGAACTGATATTAAATATGAATTACTTAGT
TCCTATTCGGAGCGAGAAATCCAACAACAAGAATTAACATATATTGTACGTAGCTTAATT
AATACAATGTATCCACATCAACCATGTTATGAAGGGGCTATGTGTGTGTGCGGCACAGGT
GGTGACAAGTCAAATAGTTTCAACATTTCAACGACTGTTGCTTTTGTTGTAGCTAGTGCTG
GCATAAAAGTTATAAAACATGGTAATAAAAGTATTACCTCAAATTCAGGTAGTACGGATT
TGTTAAATCAAATGAACATACAAACAACAACTGTTGATGATACACCTAACCAATTAAATG
AAAAAGACCTTGTATTCATTGGTGCAACTGAATCATATCCAATCATGAAGTATATGCAAC
CAGTTAGAAAAATGATTGGAAAGCCTACAATATTAAACCTTGTGGGTCCATTAATTAATC
CATATCACTTAACGTATCAAATGGTAGGCGTCTTTGATCCTACAAAGTTAAAGTTAGTTGC
TAAAACGATTAAAGATTTAGGCAGAAAACGTGCAATCGTTTTACATGGTGCAAATGGTAT
GGATGAAGCAACACTATCTGGTGATAATTTGATATATGAATTGACTGAAGATGGAGAAAT
CAAAAATTACACATTAAATGCGACTGATTATGGTTTGAAATATGCACCGAATAGTGATTT
TAAAGGTGGTTCACCTGAAGAAAACTTAGCAATCACACTGAATATTTTAAATGGTAATGA
TCGCTCAAGTCGACGTGATGTGGTTGTCTTAAATGCAGGTTTAAGCCTATATGTTGCAGA
GAAAGTGGATACCATCGCAGAAGGCATAGAACTTGCAACTACATTGATTGATAATGGTG
AAGCATTGAAAAAATACCATCAAATGAGAGGTGAATAA

> **trpC**

Function: protein coding sequence; indole-3-glycerol phosphate synthase (core genome, variable)
Best match: trpC_CC005_ED98-hen_CP001781.1[1388305:1389087]
Position: 032-contig_259_RC: 26640 … 27423; Length: 783 bp
Sequence:
ATGACGATTTTAGCAGAAATTGTTAAATATAAACAGTCACTTTTACAAAATGGCTATTAT
CAAGACAAACTTAATACCTTAAAAAGTGTGAAGATTCAGAATAAAAAATCTTTTATAAAC
GCAATTGAGAAAGAACCAAGGCTAGCAATTATTGCAGAAATTAAATCGAAGAGTCCTAC
AGTTAATGACTTACCTGAACGAGATTTATCGCAACAAATCTCAGATTATGACCAATATGG
TGCAAATGCCGTGTCCATTTTAACTGATGAAAAGTACTTTGGTGGTAGTTTTGAAAGATTA
CAAGCATTGACGACAAAAACAACATTACCCGTATTATGCAAAGACTTTATTATAGACCCG
CTTCAAATTGATGTTGCCAAACAAGCTGGTGCATCTATGATTTTATTGATCGTTAACATCT
TATCGGATAAACAATTGAAAGATTTATATAACTACGCTATATCGCAAAATTTAGAAGTGT
TAGTTGAAGTACATGATCGCCATGAATTAGAACGTGCCTATAAGGTTAATGCCAAATTGA
TTGGTGTAAATAACAGGGACTTAAAACGATTTGTCACAAATGTGGAACATACAAATACTA
TTTTAGAAAATAAAAAACCAAATCATTATTATATTTCTGAAAGTGGTATTCACGATGCAT

CTGATGTAAGAAAAATCTTGCATAGTGGTATCGATGGCTTACTAATAGGTGAGGCGCTTA
TGCGTTGTGACAATCTATCTGAATTTTTACCACAACTGAAAATGCAAAAGGTGAAGTCAT
GA

> **trpF**

Function: protein coding sequence; N-(5'-phosphoribosyl)anthranilate isomerase (core genome, variable)
Best match: trpF_CC001_MW2-USA400_BA000033.2[1378311:1378943] (completely identical)
Position: 032-contig_259_RC: 27422 … 28055; Length: 633 bp
Sequence:
ATGAAATTGAAATTTTGTGGCTTTACATCAATAAAGGATGTTACAGCGGCCAGTCAATTA
CCTATTGATGCGATAGGTTTCATCCATTATGAAAAAAGTAAAAGGCATCAAACAATTACC
CAAATAAAAAAGTTAGCGTCTGCTGTTCCAAATCATATCGATAAAGTATGTGTCATGGTA
AATCCTGATTTAACAACAATTGAACACGTATTAAGCAATACGTCAATTAACACAATACAG
TTACACGGCACAGAATCTATTGATTTTATACAGGAAATTAAAAAGAAATATTCAAGCATT
AAAATCACTAAAGCTTTAGCTGCAGATGAAAACATAATCCAAAACATAAATAAATATAA
AGGGTTCGTAGATTTATTTATTATCGACACACCCTCAGTGTCGTATGGTGGTACCGGTCAA
ACATATGACTGGACTATTTTGAAGCACATAAAAGACATACCTTATTTGATAGCAGGAGGC
ATTAACTCTGAAAATATTCAAACAGTTAATCAACTTAAATTATCACATCAAGGTTATGAT
CTTGCATCAGGTATAGAAGTAAATGGGCGAAAAGATATAGAAAAAATGACAGCAATTGT
AAATATTGTGAAAGGAGATAGAGACAATGAATAA

> **trpB**

Function: protein coding sequence; tryptophan synthase beta chain (core genome, constant)
Best match: trpB_CC005_Mu50-VRSA_BA000017.4[1453276:1454490]
Position: 032-contig_259_RC: 29112 … 29262; Length: 150 bp
Sequence:
GAAAGTGCACATGCACTGAGTTATGTTGAAAGACTAGCGCCTACGATGTCGAAAGAAGA
TATTATTGTAGTAACTATTTCTGGACGTGGCGATAAAGATATGGAAACAATTAGACAATA
TATGGCAGAGCGAGGTCTTGCAAATGACTAA

> **trpA**

Function: protein coding sequence; tryptophan synthase alpha chain (core genome, variable)
Best match: trpA_CC001_MW2-USA400_BA000033.2[1380143:1380871]
Position: 032-contig_259_RC: 29254 … 29983; Length: 729 bp
Sequence:
ATGACTAAATTATTTATACCTTATATTATGGGCAATAAAGATTTGATTGAAAATGCAACA
TTGTTGAGTGAAAATGGTGCAGATATAATTGAAATTGGTGTACCTTTCTCTGATCCGGTTG
CTGATGGTCCAGTTATCATGGAAGCAGGTCAACAAGCGATTAAACAAGGCATAACGATA
GATTATATTTTCGAGCAATTAGAAAAACACGGTAATCAAATTAAGTGTAACTATGTATTA
ATGACGTATTATAATATTATTTGTCATTATGGAGAACAAGCGTTTTTTGAAAAATGTCGAG
ATACTGGTGTCTACGGCTTAATTATTCCTGATTTACCATATGAATTATCGCAGCGTTTAAA
ACAACAATTTAGTCACTATGGCGTCAAAATCATATCGTTAGTTGCGATGACTACTGATGA
CAAACGTATAAAAGATATCGTATCCCATGCGGAAGGCTTTATTTATACTGTGACGATGAA
TGCGACAACAGGGCAAAACGGTGCGTTTCATCCAGAATTAAAACGAAAAATTGAGTCAA
TTAAAGCGATAGCCAATGTGCCAGTTGTCGCAGGATTTGGTATAAGAACACCACAACATG
TTGCAGATATAAAAGAGGTTGCAGATGGCATTGTCATTGGTAGCGAAATCGTTAAGCGAT
TTAAATCTAACACGCGTGAGGAAATCATTAAATATTTACAATCTATCCAACAAACATTGA
ATAATTAA

> **femA**

Function: protein coding sequence; factor essential for meticillin resistance (core genome, variable)
Best match: femA_CC001-ST772_118_AJGE01000002.1[7407:8669:r] (completely identical)
Position: 032-contig_259_RC: 30304 … 31567; Length: 1263 bp
Sequence:

ATGAAGTTTACAAATTTAACAGCTAAAGAGTTTGGTGCCTTTACAGATAGCATGCCATAC
AGTCATTTCACGCAAACTGTTGGCCACTATGAGTTAAAACTTGCTGAAGGTTATGAAACA
CATTTAGTGGGAATAAAAAACAATAATAACGAGGTTATTGCAGCTTGCTTACTTACTGCT
GTACCTGTTATGAAAGTGTTCAAGTATTTTTATTCAAATCGCGGTCCAGTGATCGATTATG
AAAATCAAGAACTCGTACACTTTTTCTTTAATGAATTATCAAAATATGTTAAAAAACATC
GTTGTCTATACCTACATATCGATCCATATTTACCATATCAATACTTGAATCATGATGGCGA
GATTACAGGTAATGCTGGTAATGATTGGTTCTTTGATAAAATGAGTAACTTAGGATTTGA
ACATACTGGATTCCATAAAGGGTTTGATCCTGTGCTACAAATTCGTTATCACTCAGTGTTA
GATTTAAAAGATAAAACAGCAGATGACATCATTAAAAATATGGATGGACTTAGAAAAAG
AAACACGAAAAAAGTTAAAAAGAATGGTGTTAAAGTAAGATATTTATCTGAAGAAGAAC
TGCCAATTTTTAGATCATTTATGGAAGATACGTCAGAATCAAAAGCTTTTGCTGATCGTGA
TGACAAATTTTACTACAATCGCTTAAAATACTACAAAGATCGTGTGTTAGTACCTTTAGCG
TATATCAATTTTGATGAATATATTAAAGAACTAAATGAAGAGCGTGATATTTTAAATAAA
GATTTAAATAAAGCATTAAAGGATATTGAAAAACGTCCTGAAAACAAAAAAGCGCATAA
CAAGCGAGATAACTTACAACAACAACTTGATGCAAATGAGCAAAAGATTGAAGAAGGTA
AACGTCTACAAGAAGAACATGGTAATGAATTACCTATCTCTGCTGGTTTCTTCTTTATCAA
TCCATTTGAAGTTGTTTATTATGCTGGTGGTACATCAAATGCATTCCGTCATTTTGCCGGA
AGTTATGCAGTGCAATGGGAAATGATTAATTATGCATTAAATCATGGCATTGACCGTTAT
AATTTCTATGGTGTTAGTGGTAAATTTACAGAAGATGCTGAAGATGCTGGTGTAGTTAAA
TTCAAAAAAGGTTACAATGCTGAAATTATTGAATATGTTGGTGACTTTATTAAACCAATT
AATAAACCTGTTTACGCAGCATATACCGCACTTAAAAAAGTTAAAGACAGAATTTTTTAG

> **femB**

Function: protein coding sequence; aminoacyltransferase (core genome, constant)
Best match: femB_CC001-ST772_118_AJGE01000002.1[6129:7388:r] (completely identical)
Position: 032-contig_259_RC: 31585 … 32845; Length: 1260 bp
Sequence:
ATGAAATTTACAGAGTTAACTGTTACCGAATTTGACAACTTTGTACAAAATCCATCATTG
GAAAGTCATTATTTCCAAGTAAAAGAAAATATAGTTACCCGTGAGAATGATGGCTTTGAA
GTAGTTTTATTAGGTATTAAAGACGACAATAACAAAGTAATTGCAGCAAGCCTTTTCTCT
AAAATTCCTACTATGGGAAGTTATGTTTACTATTCGAATCGTGGTCCAGTAATGGATTTTT
CAGATTTAGGATTAGTTGATTATTATTTAAAAGAGTTAGATAAATATTTACAGCAACATC
AATGTTTATATGTTAAATTAGATCCGTATTGGTTATATCATCTATATGATAAAGATATCGT
GCCATTTGAAGGTCGCGAGAAAAATGATGCCTTAGTAAACTTGTTTAAATCACATGGTTA
CGAGCATCATGGCTTTACAACTGAGTATGATACATCGAGCCAAGTACGATGGATGGGCGT
ATTAAACCTTGAAGGTAAAACACCCGAAACATTGAAAAAGACATTTGATAGTCAACGTA
AACGTAATATTAATAAAGCGATAAACTATGGTGTTAAAGTCAGATTCCTTGAACGTGATG
AGTTCAATCTTTTCTTAGATTTATATCGTGAAACTGAAGAGCGTGCTGGATTTGTATCAAA
AACAGATGATTATTTTTATAACTTTATTGACACATATGGAGATAAAGTATTAGTACCATTA
GCATATATTGACCTTGATGAATATGTGTTAAAGTTGCAACAGGAATTGAATGACAAAGAA
AATCGTCGTGATCAAATGATGGCGAAAGAAAACAAATCAGATAAGCAAATGAAGAAAAT
TGCAGAATTAGATAAGCAAATTGATCATGATCAGCATGAATTATTGAATGCAAGTGAATT
GAGCAAAACGGACGGCCCAATTCTAAACCTTGCTTCTGGCGTTTATTTTGCAAATGCATA
TGAAGTGAATTATTTCTCTGGTGGTTCATCAGAAAAATATAATCAATTTATGGGACCATA
CATGATGCATTGGTTTATGATTAACTATTGCTTCGATAATGGCTATGATCGTTATAATTTC
TATGGTTTATCAGGTGATTTTACGGAAAACAGTGAAGATTATGGCGTATACCGCTTTAAA
CGTGGATTTAATGTACAAATCGAAGAATTAATAGGGGATTTCTATAAACCAATTCATAAA
GTGAAATATTGGTTGTTCACAACATTGGATAAATTACGTAAAAAATTAAAGAAATAG

> **tx_femB**

Function:  rho-independent terminator of femB (core genome, constant)
Best match: tx_femB_CC008_COL_CP000046.1[1423390:1423448] (completely identical)
Position: 032-contig_259_RC: 32845 … 32904; Length: 59 bp
Sequence:
ATGTAAATGATGTTAGGGCTTATAGTTATTGATACTATAGGCTCTTTTTATATGTTTT

> **cof**
Function: protein coding sequence; Cof-like hydrolase (core genome, variable)
Best match: cof_CC001-ST772_118_AJGE01000002.1[4685:5451:r] (completely identical)
Position: 032-contig_259_RC: 33522 … 34289; Length: 767 bp
Sequence:
ATGAGATTTGTCTTTGATATTGATGGTACGCTTTGTTTTGACGGCCGATTAATTGACCAGA
CTATTATTGATACATTGTTACAATTACAACATGCTGGTCATGAACTTATATTTGCATCAGC
ACGTCCGATTCGTGATTTGTTGCCAGTTTTACCATCAGTATTTCATCAGCACACATTAATT
GGCGCAAATGGTGCTATGATTTCACAGCAATCAAAGATTTCTGTTATCAAACCAATTCAT
ACAGATACATATCACCATATCTTAAAAATAATTCAAAAGTATGAGTTAGATTATATTATT
GATGATGATTGGAATTATGCTGCACAACTTGACGCTGAGAACGCGATTTTTGAGCGTTTA
GATCCACATAAGCTGGCAGTTTGTATTGATGTTGCAAATATCGACACACCAATCAAGATC
ATTTTATAAATATAGACCCGGCACAAATTACAACTATATTAGACGAGCTAAATAAATACC
ATCAAGAATTGGAAATGATTCACCATTCAAATGAGTATAACATTGATATAACAGCGCAAA
ATATTAATAAATATACTGCATTACAATATATATTTGATGCAGATGTTAAATATATAGCATT
TGGTAATGACCACAATGATATTGTCATGTTACAACATGCTAGTAGTGGCTATATTATAGG
ACCATCAGAAGCATACACACACGCAATATTGAAACTTGATAAAATCAAACACATCAATA
ATAATACACAAGCTATTTGCAAAGTCTTAAAATCATATAAATAA

> **Q5HG42**
Function: protein coding sequence; putative protein (core genome, variable)
Best match: Q5HG42_CC001_MW2-USA400_BA000033.2[1385765:1386238:r]RC (completely identical)
Position: 032-contig_259_RC: 34336 … 34810; Length: 474 bp
Sequence:
TTAATAAAATTCGTCCTCGAACATTTCTTCCTCTTCATCTAATCCAAATAATTCTGCCATTT
CTCCATGTTCAATTAACATGTTTAAATATGCATCGCGGAGTTCTTCTTCACTCATATCATT
AATCATTTCTTTAAGACTATCAATCCACATATTTCTGCGTAATTGATAGTCTTCTTCAACTT
CGTTTAACATCATTATATGTTTATTTGCTGCTTCTGGACTAGCTGTAAAGAGTAATGCAAT
CATATGTTTACATATCACTCGTCTTCCATCAGCATGAGGACAATTACATTTGGATTTTCTA
GGATGTTCCATATCAATATAACAACGATATACTTTGTTGCCACTGCCCTTTACTTCAGCCT
CATGCTGCGTTTCTGAAAATGATTTTAAGTTAATGACGCATTCACTTTGATAATAATTAAA
GCCTCTTTCTATAGAACGAATACTTGCAATATCAAGTAATCCCAT

> **opp2F**
Function: protein coding sequence; oligopeptide transport ATP-binding protein (core genome, variable)
Best match: opp2F_CC001-ST772_118_AJGE01000002.1[3339:4040]RC (completely identical)
Position: 032-contig_259_RC: 34933 … 35635; Length: 702 bp
Sequence:
CTAATATGAAAATGCTTGCACTAATTCTTTTGTATAAGGGTGTCTATCAACATTAAATAAT
TCCTCTATTGCAAAATCATCGACTATCATGCCATCCTTAAGAACGATAATTCTATTAACTA
AGCGTTGTAACACGGATAAATCATGAGAAATAACGATAAAATGATTTAAGTTCGTAATCG
TTTGCGCTTTTAATATATTGATTACATTTTGTTCAGCTATAACATCTAAATTTGAAGTTATC
TCATCACATATTAAAACGCGAGGCTGTGCTAATAACGAACGCATGACATTAAATCTTTGT
AATTGTCCGCCACTCACTTCGCTTGGTAATTTAGTCAATAATTGCGCGTTTAACTCAAAAG
TAGATAAATGTTGTAATAATAATTGATCCTGAGCAGTATTATCAGTTAGGCCTCTGTAATA
ATATAACGCTTCTTTTAATGAGGTCTCAATCGTCCAATCAGGGTTAAAGCTAGTTAAAGG
GTGTTGGAAAATCGGTAACACAGCATTGTCACTTAAGTAAATTTCTCCTTTAACAGGTTTA
AACAAGCCAAGAACCAATGAAGCGAGCGTACTTTTACCACAGCCACTTTCGCCTAAAATA
CCAACATTTTCTCCATCGGGTATAGTAATATTGATATCTTGTAGCACCATCTGCTTTTTATT
ATAACCAAAAGTCACATGTTTTAATTCAATCAT

> **opp2D**

Function: protein coding sequence; oligopeptide transport ATP-binding protein (core genome, variable)
Best match: opp2D_CC001-ST772_118_AJGE01000002.1[2573:3346]RC (completely identical)
Position: 032-contig_259_RC: 35627 … 36401; Length: 774 bp
Sequence:
TCAATCATAGTAATCCCTCTTTAATTGTGTTCTATATTTAATTAGACGTTCAGTATACGGA
TGCAAATGCTCATACTTGAAATGATTAATATTACCTCGTTCAATGATTTGACCTTCTTTTA
AAACATAAATGTACTGACAATATTTCAATACATGACTTAAGTTATGTGTGATAATAAATA
ATGTTTGACCATGTTCTAATACAATATGCTGTAATAAATCCATCACTTGATTACCGTTCAA
AGCATCCAATGATGCAACTGGTTCGTCTGCAATGATTAATTTAGGCTCCAACATGAGAAC
GCTTGCTATGTATACGCGTTCAAGTTGGCCCCCAGAAAGTTGGAAACTATATTTATTTAAT
ATATCTTTGCTTTGTAAATTAACCCACGACAAAGCCTTATCAACTTTGGACAAAGCCTCTT
CTTTACTACCTTTATAATGCTTACGATAAATCGCAGTTAACTGTTTACCTAATTTAGTATG
GTCGTTAAAACTTTCTGCATAATTTTGGGAAATATAGCCAATTGTATGACCATAATATTGA
CTCAATCTACTAACATTTTCTCCATCAAATTGGTATGAATCATACGTGCAGGTTAAATCAA
ATGGTAAATATTCAAGTAAGGCTTTAGCAATCAAACTTTTTCCAGCGCCGCTCTCTCCAAT
CAAGGCATTAATCTGTTGACTAAAAATTTTCAAATCAATCCCTTTAATAAGTGATTTCTCA
CTAGTATTCTTTATTGTTAAATTTTGTATATCAATGAGACTCAT

> **opp2C**
Function: protein coding sequence; oligopeptide transport system permease protein (core genome, variable)
Best match: opp2C_CC001-ST772_118_AJGE01000002.1[1756:2586]RC (completely identical)
Position: 032-contig_259_RC: 36387 … 37218; Length: 831 bp
Sequence:
TCAATGAGACTCATCATATTCACCCCGTTGTTTCAGCAATCTATCTCTTAGCGCATCTCCG
GTTAAATTAAAAATTAAAATAGTTATAGCAATGACTGAAGCAGGTGCAATCAACATAATT
GGATGAGACGAAATAAAATCACGACCTTGTTGCAACATAGCGCCCCACTCTGGTGTTGGC
GGTTGTGCACCTAACCCAATAAATGATAGTGAACTTATATATAGAATGATTTTACCGAAA
TCAACGACCATCAAAACGATAATAGCCGGTATAATTTTAGGTGTTAAATGACGTATTAAT
ATTGTTCTTGTAGGTACATGAAATAATTGTGCCATTTTTATATAAGGCTTATTCATTTCGCT
ATTAACTATACTTCTAGTCAACCTTGTGTAATTCATCCATTTTATTACTGTAATTGAGATA
ACTAAATTCCATAAAGATGGTTGAAAAAAGCTTGCTAAAGCAATCATGATGATAAATTCT
GGAATACTTAGACCAACATCAATAAACCTTAACACTAATCGTTCAATCCACCCTTTTTTAT
ATCCGGCAAATAGACCTAGTGTAACACCTATGACAACGATAGCTATTAATGTTAAAACAG
TAACAAACAATGTTGAACGTGCACCGATAATAATTCGGGTAAATAAATCTCTCCCATAAT
CATCAGTTCCTAATAAATGCAACCAACTAATAGGTTCAAAAGTTTGTGATAAATTGACTT
TGGTTGCATTTTCACTACTGACAAAGAATTGCAGTACAATTACCACAAAAATAAATGCAA
CGAATACAAAAAATATCAGGTTATTTTTTGAAAATATTTTATGCAT

> **opp2B**
Function: protein coding sequence; oligopeptide transport system permease protein (core genome, variable)
Best match: opp2B_CC001-ST772_118_AJGE01000002.1[777:1763]RC (completely identical)
Position: 032-contig_259_RC: 37210 … 38197; Length: 987 bp
Sequence:
TTATGCATGACGGTCACTACTTTCTGATATCAATGGTGTATTGGTTTTGTTTTTTGGATTTC
CTAATTGTAAACGCTGCTTCGGATCAAGTAATAACGTTAATAAATCAGCAATCGTATTGA
TAATAACAACGAAGAAGCCAATAAATAACACGCATCCTTGAATAACAGGATAATCTCGA
GATTTAATACTATCCATTAATAGATAACCAATACCAGGTATATCAAATAAATTTTCAATC
ACTACAGTACCACCTATTAGACTGCCAAGTGAAATCCCTAGTAATGGGATAATCGGCAAA
ATTGTTGGTTTTAGTAAATCATGAATTAAAATATAACGTTCATTCATACCGCGTAATCTTG
ATGCTTGTACGATATTACTTTGCAATAACATCAATAAATTAGAACGCACTAAACGAATGA
TGTATGCACACATACCTAAAGATAGCGTGATTACAGGTAATATAAACTGACTTAGTATAA
CGCTATCTATATTCATTAAATTTGTGACAATAAATAATAAAATAATACCGATAAAGAACG

CTGGTAAACTAATCGATAGTGTTGAGATCACTCTAATCACTTTATCCGTCCACTTATGAAA
TCGTTTGGCTGCTATAATGCCGAGCGGTATAGATATGCATAACGACACTACTAATGTTGA
ACAAGATATGAGTAATGTTATGGGTGCATAGTTGAATAATATCTGTGTTACCGGTTCTTTT
GATTCAAAACTTTTTCCTAAATTAAAATGTAATAAATGATTCATCCAATGCCACCACTGTA
CCAATAAAGAATCATTTAATCCCAATTTATCTTTGGTTGCATTTATTTGTTCCGTCGACAC
TTGTGCTACATCAAGATGTAATATTTTATCAACAGGATTGCCTGGTGATAATTTCATTAAA
ATGAATGTAAGTGTAGAAATAACAAATAAAACAACTATCATTTGCATCAGTCTATACAAC
ATAGACTTAATTATGAACAT

## > Q5HG37

Function: protein coding sequence; putative protein (core genome, constant)
Best match: Q5HG37_CC001-ST772_118_AJGE01000002.1[129:473]RC (completely identical)
Position: 032-contig_259_RC: 38500 … 38845; Length: 345 bp
Sequence:
TTATTTTTTATCTTTACGGCGAAGTTCAGCGCCCTCATAGCCGTATTTTTCAATTTGCTTTT
CTAATTTACGCGCTTTTCTTTCTTTACGCCAATTTCTAGTAAAATACCATAATAGAAAACT
AATTAATAAACTCATGATCGCTAAAAATGCAGCGTATCCTAATAATGGTTGATATTTTAT
ATCTTGAAAATTTGGAATAAAAAATGCAAGCACACCTAATATAACAAATGTAATTACTGC
AGATACAAACCATTTATTTAAAACTAAGCAACAGAATATTGTTAATAAAATCATTATTAA
TGTTGTGATCCATAAATAATTAGGTATATCGAATAATGTCAT

## > tnpIS431-06

Function: protein coding sequence; transposase for IS431 (mobile element)
Best match: tnpIS431-06_circ_FM207105.1[6418:7045]RC
Position: 033-contig_261_RC: 18 … 48; Length: 30 bp
Sequence:
AGATCTGAAGAGACCTGCGGTTCTTTTTAT

## > pepF2

Function: protein coding sequence; oligoendopeptidase F/M3 family (core genome, variable)
Best match: pepF2_CC005_Mu50-VRSA_BA000017.4[1464866:1466680] (completely identical)
Position: 033-contig_261_RC: 197 … 2012; Length: 1815 bp
Sequence:
ATGTCTCAAGGTTTACCTTTAAGAGAAGATGTTCCTGTTTCAGAAACATGGGATTTAGTA
GACTTATTTAAAGATGATCAACAATATTATGAAAGTATTGACGCTCTAGTACAACAAGCA
AATCAATTTCATCATACATATGCAACAACATTAAATTCAATCGAACAAATTAATACTGCT
TTAGCTGAATTAGAAAATATTTTAATTGCCTTAGATCGCTTAAGTAATTATGCAGAACTAC
GTTTAAGTGTAGATACTAGTAATATCGAGGCACAAGTATTGAGCGCTAAATTATCTACTA
CATACGGTAAAATTGTTAGCCAATTATCATTTGTAGAGTCAGAAATACTTGAATTACCAG
AAGAAATACTTCAACAATTAGAAGAATCATGTCCATATCAACACTATATTAAACAGTTAA
TAAAACAAAAGCCATTCCAATTATCTGCGTCGGTAGAACAAGTATTAGCAACTTTATCAC
CTACGCTAAACAGTCCTTACGATTTATACGGCACGACAAAAATGCTAGATATTACATTCG
ATTCATTTGAACATGATGGTACAACGTACCCTGTCGACTATGCTACGTTTGAAAATGATTA
TGAAGATAATAAAGATCCTGAGTTTAGACGTAAAAGTTTCAAATCGTTTAGCGATGGGAT
TCGAAAATATCAGCATACTACCGCGGCTACATATAATATGCAAGTACAACAAGAAAAAA
TTGAAGCTGATTTACGTGGATTTGAATCAGTCATCGATTATTTATTACATAGTCAAGAAGT
AACGCGTGATATGTTTGACCGTCAAATCGATATGATTATGCGTGACTTGGCACCAGTTAT
GCAGAAATATGCTAAACTTTTACAACGTATTCACGGATTAGATAACATGCGTTTTGAAGA
CTTGAAGATTTCTGTAGACCCTGATTATGAACCAGAGATTTCAATTGAAGACTCAAAAAA
TTATATTTTCGGTGCGTTAAGTGTTTTAGGTGATGACTATACAAACATGTTACGTGAAGCA
TACGATCAGCGATGGATTGATTTTGCACAAAATAAAGGTAAAGATACAGGCGCATTTTGT
GCAAGTCCATACTTTACACATTCATATGTGTTTATTTCTTGGACTGGTAAAATGGCTGAAG
CATTTGTCTTAGCACATGAATTAGGTCATGCAGGTCATTTTACATTAGCTCAAAAACATCA
ACCATATCTTGAATCAGAAGCATCAATGTACTTTGTTGAAGCCCCTTCTACAATGAATGA
AATGTTGATGGCCAATTATTTATTTAACACAAGTGATAATCCAAGATTTAAGCGTTGGGTT

ATTGGCTCAATTTTATCTAGAACATATTATCATAATATGGTTACCCATTTATTAGAAGCTG
CTTATCAACGTGAAGTGTATCACAAAGTAGATCAAGGTGAATCTTTAAATGCGCCGACAT
TAAATGAAATAATGCTAAATGTTTATAAACAATTTTTTGGAGATGCAGTAGACATGACTG
AGGGTGCTGAATTAACATGGATGCGTCAACCTCATTACTATATGGGATTATATTCGTATA
CGTATTCTGCTGGCTTAACAATCGGAACTGTCGTTTCTCAAAAGATTAAAAATGAAGGCC
AACCAGCTGTTGATGCTTGGTTAGAAACATTGAAAAAAGGTGGTAGTGTATCACCTGTCG
AACTTGCAAACATTGCAGGTGTAGACATTACTACAGAACAGCCACTTAAATCTACAATTC
AATATATTTCTGATTTAGTCGATGAAGTTGAAAAATTAACAGATGAAATTGAGCAAGCAA
ATAACTAA

## > **txbi_pepF2**

Function:  bidirectional rho-independent terminator of pepF2 and phoU
Best match: txbi_pepF2_CC005_Mu50-VRSA_BA000017.4[1466699:1466764] (completely identical)
Position: 033-contig_261_RC: 2030 … 2096; Length: 66 bp
Sequence:
GTAGAACAATATAAACCAAGATTTTCACATTACTAATTGTGAGAATCTTGGTTTTTTTGAG
GGTTT

## > **phoU**

Function: protein coding sequence; phosphate transport system regulatory protein (core genome, constant)
Best match: phoU_CC001_MW2-USA400_BA000033.2[1392431:1393072:r]RC (completely identical)
Position: 033-contig_261_RC: 2151 … 2793; Length: 642 bp
Sequence:
TTATTGTTCGTAATGTGTACCTGTTAAATAAAAATAAACACTTTCAGCGATGTTAATAATA
TGATCACCAATACGTTCTAAATGTCTTGCTGCTAAATGAGCTTGTGCAGCGACAAATGGA
TCGTTATCAATAAGATACGTTGCGTTAATAATATGACTATATAAGTCATCGATATCTTCAT
CACGCTCAATTATTTCTCTTATTAATACGGTATCTTTCTTTTTAAATGCTTGATCTAAGTCC
TTTAACATTAACATAGCTAATTTACCCATTGTCTTTAAACGGGTTAACACATAATCATCTG
TAATCTTTGTACGCAATCGAATATTGGCAATACTCGATGCATTATCTCCTATTCTTTCTAA
ATCGGAGGCGATTTTTAATGAAGAAATCATCATACGCAAATCACTCGCAATGGGCTGTTG
CTTTGTAATTAACATGATAACTCGCTCATTAATATCATAATTTAATTGATTGATATGTTTA
TCGTTTTTAACTGTTTGTCGTGCAAAGCCTCTATCGTCAATACTTAATGATTTTATACCATT
TTCAATACTCACATAGACATTTGCACCTAACCGACGTAATTCCTTTATTAAATCATCAAGT
TGCTCCTGATATCGCTGTCTAATTATTGCCAT

## > **pstB**

Function: protein coding sequence; phosphate import ATP-binding protein (core genome, constant)
Best match: pstB_CC001_MW2-USA400_BA000033.2[1393079:1393930:r]RC
Position: 033-contig_261_RC: 2799 … 3651; Length: 852 bp
Sequence:
TCAACCAAACCTTCCTGAAATATAATCTTCTGTTTTCTTGTTTGATGGGTTAGAGAAAATT
TTATCAGTATCATCATATTCATTGACATAACCATTTAAGAAAAATGCAGTTTTATCTGATA
CACGAGCTGCTTGTTGCATATTATGTGTAACCATAATAATTGTATACTTTTCTTTTAGTTCT
TGAACCAACTCTTCTACTCTTAATGTTGAGATTGGATCTAATGCTGATGTCGGTTCATCCA
TTAAAATAACTTCAGGTTCAATTGCTAAACAACGCGCGATACAAACACGTTGTTGTTGCC
CACCGGATAAACTATATGCATTTGTGTGCAACCTATCCTTTAATTCATCCCAAATTGCAGC
GCCACGTAATGATTTCTCAACGATTTCATCAAGAACTTTTTTATTTTTAATACCGTGAATC
TTTGGACCGTAAGTAATATTATCGTATATTGATTTTGGAAATGGATTAGGTTGTTGAAAGA
CCATGCCCACATTTGTACGTAATTGTTCTTTAGAATATTTTTGATCAAAAATGTCTTGATC
TCGATATAATATTTTACCAGCTGTTTTCACAGAAGGTACTAACTCAACCATTCGATTCAAA
GTTTTAATATATGTTGATTTGCCACAACCAGATGGACCTATAATGGCAGTAATTTGGTTTT
CATAAATATCTAAATTAATATTTTGTAATGCATGATTTTCGCCATACCATAAGTCTAAATT

TTGTGTTGAATATATAACAGAATGTGAGTTTGTATCATCTGGTGTTTTATGATGACTTTGT
GAGACATCAAACGTATGACTTTGAGATATTTGTTTAGTTTGTGCAAGTGTTTGCGCCAT

> **pstA**

Function: protein coding sequence; phosphate ABC transporter/permease subunit A (core genome, variable)
Best match: pstA_CC001-ST772_118_AJGE01000027.1[3642:4559:r]RC (completely identical)
Position: 033-contig_261_RC: 3697 … 4615; Length: 918 bp
Sequence:
TTAGAATTTTTTACTAAATTTGTTACGTAAAATAATCGCAACGCCATTCATTAAGATTAAG
ATAACTAGTAAAACGATAATGCCTGCCGATGCAACATTCTGGAATTCTTCTTGAGGCATT
TTCGCCCAAGTAAATATTTGGATTGGTAATGCTGAAAATTGATCCAATATACTTCTAGGTG
TTGCCAATAATATAGTCGGTATACCGATTAGCACAAGTGGCGCTGTTTCTCCCAGTGCTCT
TGAAAGAGACAAAATGAATCCAGTTAAAATACCAGGTAACGCTGCTGGTAAGACAACAC
GTCTTATCGTTTGCCATTTATTAGCACCTAAACCATAAGAAGCTTCGCGTACTGAGTTAGG
TACAGCTCTAATTGCTTCCTGACTTGAAACAATAATAATTGGTAATATCAGTAAGGTCATT
GTTAGCGCTGCTGCCAATATACTGTTACCCATTTTCAAGGCTTCAATCCCCGCACCACCAA
CGAACAAAGTGTAACCTAATAACCCAAATACAACTGATGGTACACCAGCTAAATTGGAA
ATACTGATTTTAACAAACTGAGTAAATTTGTTGTTTTTCGCATATTCTTCTAAGTATATAG
CTGTACCTATTCCTAGGATGATTGATAATGGAATGATACTTAACATTAACCAAAGTGAAC
CGATTAACGCGCCTTTAACGCCAGCCATAGATGGTGTTGAAGAAGAAAAATTAGTGAAA
AATTGTAAATTTAAATGACTTACCCCTTTAATCAATGTTTGAGTTAACAACGCAATAAGTA
CGACAAGTCCTAATAATGTACATGCTAAAAATATGAGTTTGAACACTTTATTTTTAACCGT
TCTGGATGATAAATGTTTTTGGACAAGTTGTTGATCGACGAGTGATTGTCTATTATTATCT
GTCGTTTCCAT

> **pstC**

Function: protein coding sequence; phosphate ABC transporter/permease subunit C (core genome, variable)
Best match: pstC_CC001_MW2-USA400_BA000033.2[1394896:1395822:r]RC (completely identical)
Position: 033-contig_261_RC: 4616 … 5543; Length: 927 bp
Sequence:
TTAATACTCCTCCCTAAAACGCTTAGAAATCCACTGAGAAAGTAAATTCATGATTAAGGT
AAAGATAAATAGTGTGAACCCTACAGCATAAATACTGTAATAAATATTTGATCCAAATGT
TGCATCACCTGTCGCTATCTCAACAATATATCCAGTCATTGTTTGAATCGAACTTGTTAAA
CTTAATGAAGCTGTTGGCGAACTACCTGCCGCTAATGATACAATCATCGTTTCTCCAATTG
CTCTTGAAATCGCGAGAACGATTGAAGCTACAATACCTGATGTTGCTGCGGGAAGTACGA
CTTTAGTTGCTACTTCTAATTTAGTTGCTCCAAGTCCATAGGCACCTTCTCGAATTTTATTT
GGTACAGATGCCATTGCATCCTCACTCAAACTTGTGATGAGAGGGACAATCATAATACCG
ACAACTAAGCCGGGACTTATAGCATTAAACTCTCCAAGACCTGGTATGAAAGATCTTAAT
ACTGGTGTAACAAAGGTTAATGCAAAGAAACCAAACACAATTGTTGGTATTCCTGCTAAA
ATTTCTAATATCGGTTTAATTATGCGTCGTGCACGGTCACTTGCATATTCACTTAAATAAA
TTGCTGCACCAAGCCCGACTGGAACTGCAAATATAGTCGCAATAACTGTGATTTTTAAAG
TCCCTATTATCAATGCCCAGATACCAAACTTAGGGTCTGAACCGGTAGGATTCCAAGTAG
TAGAAAATAGAAATTCAGTTATTGGAATTCTGGTGAAAAAAGTGATGGTTTCTAAAAGCA
ATGTGATTAATATACCTAGTGTTGTTAAAATGGAAATCGCTGAAATTGCGGCTAAAATAA
CTGGTATAATTTTGTCATTATGCTTCCCTTTTTTATTATTATTTTTTTCGATTAAAGCTTTAA
CATTAGTAGATGAAGTCAT

> **pstS**

Function: protein coding sequence; phosphate ABC superfamily ATP binding cassette transporter/binding protein (core genome, constant)
Best match: pstS_CC001_MW2-USA400_BA000033.2[1396013:1396996:r]RC
Position: 033-contig_261_RC: 5733 … 6717; Length: 984 bp

Sequence:
TTATTTTTTGTCTTCAGACTTTTTATCATCAGATTTCTTGTCGTCTGATTTTTGATTTTTATC
AATAAATGCTTTTAAATCATCTAATTGTGATTTGTATGTTTTCTCTGGTGCTGCTACATATC
CAGCTTCTTCAGCTGCTTTACCTTTATCTTCTAAGACGAATTTGATAAATTCTGACATTACT
TTATTATCTTTCAATGCTTTTTCATTTACATAAATGAATAATGGTCTACTTAATGCATAAG
AGTTATCTTGAATTGTTTTTTTCGTAGGCTCTGTTGCTTTACCATTTTCATCTTTGATTTTAA
CTTCTTTTAATTTATCTTTATTTTGAACGTAGAAGTTATATCCAAAGTATCCGATTCCCTCT
TTGTTTTTCGTTACAGAAGAAACGATAGCATTTGTATCAGCATTTTTTTCTGCTTTAATATC
TTCTTTATTCATTACTTCATTTTCAAAGAAGTCATAAGTACCATGACTTGAGTTTGGTGAT
ACAGCATTTATTTTTTTATCTGGCCATTTACTATTAACATCTTTCCATGTTTTAGCTTTTCC
AGAATAAATTGCTTTTAATTGCTGTTTGTCTAATTCATCCACAAAATCATTTTCTTTATTTA
TAGCAACCGTTACACCATCTTGCGCAATTTTGAATTCTTTGTATTTGATATTCTTATCTTGT
AATTTTTGCTTCTCTTCATCTTTAATTGGTCTAGAAGCATCAGCGAAGTCGATATCTCCTG
CAATGAATTTTTGGAAACCAGCACCTGTACCAGCTTGTCCTGCTGAGATTTTAGCATCCGA
GTGATCTTGAGCCCATTTTTCATTTAATTTCTCCACAATTGGTGCTACTGTTGATGAGCCA
TCACCTTTAGCTTCCCCTTTTAAATCACTATTACCACTGCCACCATTACCGCCACCACAAG
CACCTAATAATAGTGTTGCACCTAAAGCTGTAGTACCAACAAATTGCCATTTTTTCAT

## > txbi_universal1
Function:  bidirectional rho independent terminator
Best match: txbi_universal1_CC001_MW2-USA400_BA000033.2[1397234:1397306:r]RC (completely identical)
Position: 033-contig_261_RC: 6950 … 7023; Length: 73 bp
Sequence:
TAAATCCCTAAAAAAACAGCAGTAAGATAATTTTCAATTAGAAAATATCTTACTGCTGTT
CTCTATTTATACA

## > tnpIS1272
Function: protein coding sequence; transposase of insertion element IS1272
Best match: tnpIS1272_trunc_CC075_MSHR1132_FR821777.2[2351735:2352717]
Position: 034-contig_264_RC: 7 … 37; Length: 30 bp
Sequence:
TAAACGAGAGCTAGGTTTTGTATTAATGGC

## > tx_universal3
Function:  bidirectional rho-independent terminator
Best match: tx_universal3_CC001_MW2-USA400_BA000033.2[1397772:1397835:r]RC (completely identical)
Position: 034-contig_264_RC: 83 … 147; Length: 64 bp
Sequence:
ATACATATCAAAAAAGCTAATTTCCATCAAATAATTAATAGAAATCAGCTTTTTTACATTG
CCT

## > cvfB
Function: protein coding sequence; conserved virulence factor B (core genome, constant)
Best match: cvfB_CC005_Mu50-VRSA_BA000017.4[1472360:1473262:r]RC (completely identical)
Position: 034-contig_264_RC: 284 … 1187; Length: 903 bp
Sequence:
TTATTCTTTTGAGTCAATTCGACTCCAACCTTTTTTAGTTAAAGTGATTTTACCTGTTTCTA
TATTAATAATCTTCTGTTTATATAAGTGACCGATTGCACGTTTGAATGAACCTTTACTCAT
ATTGAATACTTCTTTAATCGCTTCAGGGCTTGATTTGTCCCAGAATGGTAATTCACCATCA
TATTCAACTAGTAAATCAAAGATGACTTGGCCGTCATCGTCTAAACGTTCATGTGCAAGT
GGTAAAAATGAACCATTTAACTCACCTTTATCATTATGCCCGATAATTCTAACTTGAACAG
ATTCACCTAATCTTGGTTCAGCTTTACGTTCTGATTCATGTACGAAAATTTTGTAACCTGA
TTCGCTTAATAAGAAGCTACCAATTCGTAATACGCGGTAAGGTTTGGCTTCAATGACTTC

GTTTTTTAAATTATCATCGTGTACAGGTGTAAACATATTTTCTACAACAGATTCACTCGCT
AAACGTCCATACATATGATTCTCACGGTCAATTCGTAATGTAACTAGCAAATGATCACCA
GGTTGTGGCCATAGTGATTTCACTTTTGGTAAATCTTCCCATGGTACTAACACTTCACGGG
GTAAACCAACATCTATACGTGCCCCATCGCGATCCGTTTTAAGTACTTTAGCAAAATCAT
ATTTATCTTTCGTAATATCAGGCATATTTTGAGTTGCAAATAATTCACCTGAACGGTTTGG
ATAAATGAAGAAACTATATTCTTCACCTACTTCTAATTCATCATCATCGTTCATTTCTGAT
TGGTTTAACTTTACGTTTTCACCGTTTGGTCCTTTTAAAAGGTAAGTTGAGCCTTGTAACC
CTACTACTTCAAGGAATTCTATAGAACCTACTATATCTTTGTCTAATGCCAT

## > **ykpA**

Function: protein coding sequence; ABC transporter/ATP-binding protein (core genome, variable)
Best match: ykpA_CC001-ST772_118_AJGE01000012.1[28773:30374:r] (completely identical)
Position: 034-contig_264_RC: 1333 … 2935; Length: 1602 bp
Sequence:
ATGTTACAAGTAACTGATGTGAGTTTACGTTTTGGAGATCGTAAACTATTTGAAGATGTA
AATATTAAATTTACAGAAGGTAATTGTTATGGATTAATTGGTGCGAATGGTGCAGGTAAA
TCAACATTCTTAAAAATATTATCTGGTGAATTAGATTCTCAAACAGGACATGTTTCATTAG
GGAAAAATGAACGTCTAGCTGTTTTAAAACAGGACCACTATGCTTATGAAGATGAACGCG
TGCTTGATGTTGTAATTAAAGGTCACGAACGTCTTTATGAGGTTATGAAAGAAAAAGATG
AAATCTATATGAAGCCAGATTTCAGTGATGAAGATGGTATCCGTGCTGCTGAACTTGAAG
GTGAATTTGCAGAAATGAATGGTTGGAATGCTGAAGCTGATGCTGCTAACCTTTTATCTG
GTTTAGGTATCGATCCAACTTTACACGATAAAAAAATGGCTGAATTAGAAAACAACCAAA
AAATTAAAGTATTATTAGCGCAAAGTTTATTCGGTGAACCAGACGTACTATTACTGGATG
AGCCTACTAACGGTCTCGATATTCCAGCAATCAGTTGGTTAGAAGATTTCTTAATTAACTT
TGATAATACTGTTATCGTAGTATCGCATGACCGTCATTTCTTAAATAATGTATGTACTCAT
ATCGCTGATTTAGACTTCGGTAAAATTAAAGTTTATGTTGGTAACTATGATTTTTGGTATC
AATCTAGTCAGTTAGCTCAAAAGATGGCTCAAGAACAAAACAAGAAAAAAGAAGAAAA
AATGAAAGAGTTACAGGACTTTATTGCACGTTTCTCAGCTAACGCTTCTAAATCTAAACA
AGCAACAAGTCGTAAAAAACAACTTGAGAAAATTGAATTAGATGATATTCAACCATCATC
AAGAAGATATCCTTTCGTTAAATTCACGCCTGAGCGTGAGATTGGTAACGACTTATTAAT
CGTTCAAAATCTTTCTAAAACAATTGACGGCGAAAAAGTATTAGATAATGTATCATTCAC
AATGAATCCAAATGATAAAGCGATTTTAATTGGAGATAGTGAAATTGCAAAAACAACATT
ACTTAAAATATTAGCTGGCGAAATGGAACCAGACGAAGGTTCATTTAAATGGGGTGTTAC
TACATCATTAAGTTACTTCCCTAAAGATAACTCAGAGTTCTTTGAAGGTGTAAATATGAAT
CTCGTTGATTGGTTAAGACAATATGCTCCTGAAGATGAACAAACAGAAACATTTTTACGT
GGTTTCTTAGGTCGTATGTTATTTAGTGGTGAAGAAGTTAAGAAAAAAGCTAGTGTGCTT
TCAGGTGGAGAAAAAGTACGTTGTATGCTAAGTAAAATGATGTTATCAAGTGCGAATGTA
CTTTTACTTGACGAACCTACTAACCACTTAGACTTAGAAAGTATTACTGCTGTCAATGATG
GTCTTAAATCATTTAAAGGTTCTATCATCTTTACTTCTTATGACTTCGAATTTATCAACACG
ATTGCAAACCGTGTTATCGATTTAAATAAACAAGGCGGCGTTTCAAAAGAAATTCCATAT
GAAGAATACTTGCAAGAAATCGGCGTTTTAAAATAA

## > **RF00168**

Function: noncoding RNA lysine riboswitch (core genome, constant)
Best match: RF00168_CC008_COL_CP000046.1[1440344:1440519] (completely identical)
Position: 034-contig_264_RC: 3537 … 3713; Length: 176 bp
Sequence:
ATATTTTGATGAGGCGCATCAATCATGAGTAAAGTTTAGATTACTGTCTGCTAACAGCTA
AATTTGAAAGGGTGCGATGCCGAAGCAATTATAATAGCAGTTATAATTTGTTGGACTTTT
TGGTTAAGAGCTGAGAGTTTGTCATTATTTAAAAATAATGGAGTGCATCACTTGTA

## > **lysC**

Function: protein coding sequence; aspartokinase
Best match: lysC_CC005_JH1_CP000736.1[1524197:1525402]
Position: 034-contig_264_RC: 3794 … 5000; Length: 1206 bp

Sequence:
ATGGTAACAAGAAGTGTGTTGAAATTTGGCGGATCATCCGTCAGTGATTTTACAAAAATA
AAAAGGATCGCTGAAATGTTAAAGGAGCGAGTCAATCAAGATGAACAATTAATTGTCGT
TGTAAGTGCTATGGGTAACACAACAGATCAATTAATGACGAATGTATCGACCTTGACTAA
AGCACCAAAACAACAAGAACTGGCATTATTATTGACAACCGGAGAGCAACAAACTGTAT
CTTATTTATCAATGGTATTAAATGATATCGGTGTGAATGCCAAAGCAATGACTGGCTATC
AAGCAGGTATTAAAACCATTGGCCATCATTTAAAAAGTAAAATTGCTCAAATTAATCCTC
AAACATTTGAACAAGCCTTTCAAGAAAACGATATTTTAGTAGTTGCTGGATTTCAAGGCA
TCAATGAACATCAGGAATTAACAACTTTAGGCAGAGGCGGTTCTGATACGACCGCTGTGG
CACTTGCTGTTAGTAATCAAATACCTTGTGAAATTTATACCGACGTTGATGGTGTGTATGC
CACTGACCCAAGACTTTTACCAAAAGCTAAACGACTAGACATCGTCTCATATGAAGAAAT
GATGGAAATGAGCGCTTTAGGTGCTGGTGTACTTGAAACAAGAAGTGTTGAATTAGCTAA
AAACTATAATATCCCTTTATATTTAGGAAAAACTTTATCGAACGTGAAAGGAACATGGAT
TATGTCAAATGAAGAAATATTAGAGAAAAAAGCAGTTACTGGTGTGGCTTTGGATAAAC
ATATGATGCATGTAACAATTAGTTATCCCCTACCTGACAATCAGCTACTTACCCAACTATT
TACGGAACTTGAAGAAGGTGCTGTAAATGTTGATATGATTTCACAAATCGTCAACTTGGA
TGGACTACAACTATCCTTCACGATTAAAGATAGTGATTTTCATCAAATTTCTATGATTCTT
GAAACATTAAAGAATCAATATGAAGCATTAGCTTATAAAATCAATGAGCATTATGTCAAA
ATTTCATTAATTGGCTCAGGCATGCGTGATATGTCAGGTGTGGCATCAAAAGCATTTTTGA
CATTAATTGAAAATAATATACCTTTCTACCAAACAACAACATCTGAAATAAGTATTTCAT
ACGTCATTGATGATTTTAATGGGCAACAAGCGGTAGAAAAACTATATGACGCATTTAACA
TTTAA

> **asd**
Function: protein coding sequence; aspartate semialdehyde dehydrogenase (core genome, constant)
Best match: asd_CC005_Mu50-VRSA_BA000017.4[1477341:1478330]
Position: 034-contig_264_RC: 5063 … 6053; Length: 990 bp
Sequence:
ATGACAAAGTTAGCAGTTGTGGGTGCAACAGGATTAGTAGGCACAAAAATGTTGGAGAC
ATTAAATCGTAAAAATATTCCTTTCGATGAATTAGTATTATTTTCATCAGCACGTTCTGCA
GGGCAAGAAGTTGAATTTCAAGAAAAAACATATACAGTACAAGAATTAACTGATGCTCG
TGCTAGTGAACATTTCGATTATGTATTAATGAGTGCTGGTGGCGGTACAAGCGAACACTT
TGCCCCACTCTTTGAAAAAGCTGGTGCAATCGTTATAGACAATTCAAGTCAATGGCGTAT
GGCAGAAGATATTGATTTAATCGTTCCGGAAGTCAATGAACCTACATTTACAAGAGGTAT
CATTGCCAATCCAAACTGCTCTACGATTCAATCTGTTGTGCCATTAAAAGTATTGCAAGAT
GCTTATGGTTTAAAACGAGTGGCATATACAACATATCAAGCTGTATCAGGTTCAGGGATG
AAAGGTAAGAAAGATTTAGCTGAAGGTGTAAATGGTAAAGCACCAGAAGCATATCCACA
TCCAATTTATAATAATGTGTTACCGCATATTGATGTGTTTTTAGAAAACGGATATACAAAA
GAAGAACAAAAAATGATTGATGAGACGAGAAAAATTTTAAATGCGCCAGACTTAAAAGT
AACAGCAACATGCGCACGTGTGCCTGTTCAAGATAGTCATAGTGTTGAAATTGATGTAAC
GCTTGACAAAGAAACGACAGCAGAAGATATTAAAGCGTTATTTGATCAAGATGACCGCG
TTGTTTTAGTAGACAATCCAGAGAACAATGAATATCCAATGGCAATTAATTCCACTAATA
AAGATGAAGTGTTTGTTGGCCGTATACGTAGAGATGATTCATTAGAAAATACTTTCCATG
TATGGTGTACATCAGACAATTTATTAAAAGGTGCTGCATTAAATGCTGTACAAGTATTGG
AACAAGTTATGCGTTTAAAAGGAGCGAATTAA

> **dapA**
Function: protein coding sequence; dihydrodipicolinate synthase (core genome, variable)
Best match: dapA_CC001_MW2-USA400_BA000033.2[1403749:1404636] (completely identical)
Position: 034-contig_264_RC: 6054 … 6942; Length: 888 bp
Sequence:
ATGACACATTTATTTGAGGGTGTTGGCGTTGCACTTACAACCCCTTTTACAAATAACAAA
ATTAATATTGAAGCTTTGAAAACACACGTTAATTTTTTACTAGAAAATAATGCCCAAGCA
ATCATCGTTAATGGAACTACTGCTGAGAGCCCTACTTTAACAACAGATGAAAAAGAACGC
ATTCTAAAAACAGTTATTGATCTTGTAGATAAACGTGTTCCTGTCATAGCAGGAACTGGC

ACTAATGATACTGAAAAGTCAATCCAAGCTTCAATCCAAGCTAAAGCCTTAGGGGCTGAT
GCAATTATGTTAATTACGCCCTACTACAACAAAACGAATCAACGTGGTTTAGTCAAACAC
TTTGAAGCGATTGCAGATGCTGTGAAATTACCAGTCGTGCTGTACAATGTTCCTTCAAGA
ACGAACATGACAATTGAACCAGAAACTGTAGAAATATTAAGTCAACATCCTTATATAGTT
GCTTTAAAAGATGCTACGAATGATTTTGAGTATTTAGAAGAAGTGAAAAAGCGAATTGAT
ACAAATTCATTTGCATTATATAGTGGCAATGATGACAACGTCGTTGAATACTATCAACGT
GGCGGTCAAGGGGTTATCTCTGTTATTGCCAATGTCATTCCTAAAGAATTTCAAGCGTTAT
ACGATGCTCAACAAAGTGGATTAGATATTCAAGATCAATTTAAACCAATCGGCACACTGT
TATCAGCCCTATCAGTTGATATTAACCCAATTCCTATTAAAGCTTTAACAAGTTATTTAGG
ATTTGGAAATTATGAATTACGTTTACCATTGGTTAGCCTAGAAGATACAGATACTAAAGT
GCTTCGTGAAGCATATGACACATTTAAAGCGGGTGAAAATGAGTGA

> **dapB**

Function: protein coding sequence; dihydrodipicolinate reductase (core genome, variable)
Best match: dapB_CC001_MW2-USA400_BA000033.2[1404633:1405355] (completely identical)
Position: 034-contig_264_RC: 6938 … 7661; Length: 723 bp
Sequence:
GTGAAAATATTACTAATTGGCTATGGTGCAATGAATCAACGCGTTGCTAGATTAGCAGAA
GAAAAAGGACATGAAATCGTTGGGGTCATTGAAAATACACCGAAAGCAACAACGCCATA
TCAACAATATCAACATATTGCAGATGTTAAAGATGCCGATGTTGCAATAGATTTTTCAAA
TCCAAATCTGCTTTTCCCTTTATTAGATGAAGAGTTTCATTTGCCATTAGTTGTGGCAACA
ACTGGCGAGAAAGAAAAACTACTTAATAAGTTAGATGAATTGAGTCAAAATATACCTGT
ATTTTTCAGCGCGAACATGAGTTATGGCGTTCATGCACTGACTAAAATTTTAGCAGCTGCT
GTTCCCCTACTTGATGAATTCGACATCGAATTAACTGAGGCACATCATAATAAAAAAGTA
GATGCACCAAGTGGTACGTTAGAAAAATTGTATGATGTGATCGTATCTTTGAAAGAAAAT
GTAACACCTGTGTATGATAGACATGAATTAAATGAAAAACGCCAGCCACAAGATATTGGT
ATACATTCTATTCGTGGAGGTACGATTGTCGGTGAACATGAAGTTCTATTTGCTGGCACTG
ATGAAACGATTCAAATCACGCATCGTGCACAATCAAAAGATATTTTTGCGAATGGTGCAA
TACAAGCAGCAGAACGCTTAGTTAATAAACCAAACGGCTTTTATACGTTTGACAACTTAT
AA

> **dapD**

Function: protein coding sequence; 2,3,4,5-tetrahydropyridine-2,6-dicarboxylate N-acetyltransferase (core genome, constant)
Best match: dapD_CC005_Mu50-VRSA_BA000017.4[1479965:1480684] (completely identical)
Position: 034-contig_264_RC: 7687 … 8407; Length: 720 bp
Sequence:
ATGGTACAACATTTAACAGCTGAAGAAATTATTCAATATATAAGTGATGCTAAAAAGTCT
ACACCAATAAAAGTATATTTAAATGGTAATTTTGAAGGCATCACATACCCAGAAAGTTTT
AAAGTATTTGGTTCAGAACAATCTAAAGTAATCTTTTGTGAAGCGGATGATTGGAAACCT
TTTTACGAAGCATATGGTAGTCAATTCGAAGATATAGAAATTGAAATGGATCGTCGCAAT
TCTGCCATTCCATTAAAAGACTTAACAAATACGAATGCACGTATTGAACCAGGTGCATTT
ATTAGAGAACAAGCCATTATTGAAGATGGTGCTGTCGTTATGATGGGCGCAACAATTAAT
ATTGGCGCAGTCGTTGGCGAAGGTACAATGATTGATATGAATGCTACTCTCGGTGGTCGT
GCTACAACTGGTAAAAATGTACATGTAGGGGCTGGCGCAGTATTGGCAGGTGTGATTGAA
CCCCCTAGTGCATCACCAGTTATAATCGAGGATGATGTATTAATCGGTGCAAATGCAGTT
ATTTTAGAAGGTGTACGTGTTGGTAAAGGTGCTATTGTTGCAGCTGGCGCGATTGTGACA
CAAGATGTACCAGCTGGTGCAGTTGTTGCTGGTACACCTGCAAAAGTGATTAAGCAAGCT
TCTGAAGTACAAGATACTAAAAAAGAGATTGTAGCAGCATTAAGAAAACTGAATGACTA
G

> **hipO**

Function: protein coding sequence; hippurate hydrolase
Best match: hipO_CC001_MW2-USA400_BA000033.2[1406244:1407395]
Position: 034-contig_264_RC: 8549 … 9701; Length: 1152 bp

Sequence:
ATGAATGAATTAGAATTTGTTACGAAACATCGCCGTCATTTACATCAACATCCTGAATTA
AGCTTACATGAATTTGAAACAACTGCTTATATTAAAGCGTTTTTAGATAGTTTAAATATTA
AATACGATTGCCCATTGGAAACTGGCGTCATTGCATACTTAGAAGGTAATGGCTCACATA
CGATAGCGTATAGAGCTGATATTGATGCGTTACCTATTTTAGAGGAAAATGATGTGCCTT
ATCGCAGTCAATCTGATCATGTGATGCATGCTTGTGGACATGATGGTCATACAACTGCAT
TAATGCTTTTTGTACAACGTTGCAAAGACATGCAAGACGCAGGTCAATTACCGCAAAATG
TCGTTTTCATTTTCCAACCTGCAGAAGAAACTGGTGGCGGTGCAAATCGATTAATAAAAG
CCGGTGCCTTTGATAAGTATCCAATTGAAGCGGTATTTGGTATTCATGTTAACCCATTTGC
TGATGAAGGCATTGCAGTGATAAGAGATGAAGAAATTACGGCCAGCGCAACAGAGTATC
GCTTTTTCTTAACAGGCCTGTCAAGTCATGTTGCTGATAAAGAACAAGGTCATTCTTGTGG
TGAAGCATTACAACATGTATTAACTCAAATATCACAAATTCAACAATTTCACCTTAACGG
TTTGAAACGAAATATTGTTCATATTGGTCATTTTAAAGCTGGTGAAGCGATTAACACTGTA
CCAAGTAATGGTTATTTAGAAGGTACAATTCGTACATATGATATTGATGATTTAACAATC
GTTAAAAATCAAATGCACAAGATAGCAGAAAGTGTCAAGCTTCTGTTTAATGTAGAATGT
GAAGTTAAATTTGCAGAAGGTTATCCCCCTACAATCAATAGTCCGAAATTACGTACTCAA
ATAGAGGACGCCTTAATAAAAGCTGATTTAAATGTCTATGACAAACCAACGCCATTCTTA
TTTGGGGAAGATTTTAGTTTTTATGGTCAACAACTAGCTCCAGCTTACTTTGTTTTTATAG
GAACACGAAATGAAGATAAAGGTTTTGTAACTGGTTTGCACACATCACATTTAAATTTTG
ATGAAAAAGTGTTAATAAACGTGGTTAATTTTTACGAAAATTTATTAAATAATTACAAAG
AGGTGTAA

> **alr2**

Function: protein coding sequence; putative alanine racemase/locus 2 (core genome, variable)
Best match: alr2_CC001_MW2-USA400_BA000033.2[1407400:1408485]
Position: 034-contig_264_RC: 9705 … 10791; Length: 1086 bp
Sequence:
TTGACAGCAACATGGTCTGTAAATAAGAAAATATTTTTACAAAATGCAATCACAGTCAAA
AACAATCAGCCATTAATGGCAGTTGTTAAAAATAATGCATATCACTATGACCTAGAATTT
GCTGTAACTCAGTTTATCCATGCAGGTATAGATACATTTAGCACAACATCACTACGAGAA
GCAATTCAAATTAGACAACTTGCTCCAGATGCAACAATCTTTTTAATGAATGCAGTATAC
GAATTTGATTTAGTCCGTGAACATCAAATACACATGACTTTGCCGTCGTTGACATATTACT
ATAACCACAAAAATGATTTAGCTGGTATTCATGTTCACTTAGAATTTGAAAATTTATTACA
TCGGTCTGGATTTAAAGATTTAAACGAAATTAAAGAAGTATTGAAGGATCACCATCAAAA
TCAAAATGCAAAAATGATTATTAGTGGTTTATGGACCCATTTTGGATATGCTGATGAATTC
GATGTGTCAGATTATAATGTTGAACGTTCACAATGGATGGAAATTGTTGAATCACTTTTAT
CTGAAGGTTATCAGTTCGACCTAATCCATGCACAAAATAGTGCGAGTTTTTATCGGGAAG
GACAAATATTACTACCCCACCATACACATGCGCGTGTAGGTATTGCGTTATACGGTTCAA
GACCATATAGTTCACTGAATCAACATGATATTGTTCAGTCATTGACTGTAAAAGCACATG
TTATTCAAGTGCGCGAAGTGCAAGCTGGTGATTATTGCGGTTATAGCTTTGCCTTTGAAGT
GACTAAAAACAATACAAAATTAGCTGTAGTTGATATCGGTTATGGCGATGGAATTTTAAG
AACACGTGCTAAACATGAAGCACTTATCAATGGTAAACGCTACCCGATACGTGCATTAAT
GATGAGCCATATGTTTGTTGAAGTAGATGGCAATGTACATGCACAAGATGAAGTTATTCT
TTATAATAATGATATCCGCATCGATGAATATACCTTTAAAGGTGTTGGTGCAAATTCTGA
ACAATTAAGTGCTATGAATCATGATTCTTTAAAAAAGGAGTACATTTCAAATGACTGTTA
A

> **lysA**

Function: protein coding sequence; diaminopimelate decarboxylase
Best match: lysA_CC005_Mu50-VRSA_BA000017.4[1483058:1484323] (completely identical)
Position: 034-contig_264_RC: 10780 … 12046; Length: 1266 bp
Sequence:
ATGACTGTTAAATATAATCAAAATGGCGAATTAACAATGGATGGTATTAGTTTAAAAACG
ATTGCACAAAGCTTTGGTACACCTACCATTGTTTATGATGAACTACAAATTAGAGAACAG
ATGCGCCGTTACCATCGCGCATTTAAAGATAGTGGATTAAAATACAATATTTCATACGCC

TCAAAGGCATTTACTTGCATTCAAATGGTCAAACTTGTAGCTGAGGAAGATTTACAGTTA
GATGTTGTTTCTGAAGGTGAATTATATACAGCTTTAGAAGCAGGTTTTGAACCGAGTCGC
ATCCATTTCCATGGTAACAATAAAACGAAACATGAAATTAGGTATGCTTTAGAAAATAAT
ATCGGTTATTTTGTTATAGATTCATTAGAAGAAATTGAATTAATAGACCGCTATGCTAATG
ATACGGTTCAAGTTGTATTACGAGTTAATCCAGGTGTTGAAGCACATACACACGAATTTA
TTCAAACTGGGCAAGAAGATAGTAAGTTTGGATTATCAATTCAATATGGCTTAGCTAAAA
AAGCAATTGACAAAGTCCAACAATCTAAACACTTAAAATTAAAAGGTGTACATTGTCATA
TTGGTTCACAGATTGAAGGTACAGAAGCATTTATTGAAACTGCTAAAATTGTTTTACGTTG
GCTTAAAGAGCAAGGCATTCAAGTTGAATTATTAAACCTTGGTGGTGGCTTTGGTATTAA
ATATGTTGAAGGTGACGAAAGTTTCCCTATCGAAAGTGGTATTAAAGATATTACAGACGC
AATAAAATCCGAAATTAAAGTTCTAGGTATAGATGCACCAGAAATAGGTATTGAACCGG
GACGATCAATTGTAGGTGAAGCTGGCGTTACTTTATATGAAGTTGGAACCATTAAAGAAA
TTCCAGAGATTAATAAATATGTTTCAATCGATGGCGGTATGAGTGATCATATCAGAACTG
CACTTTATGACGCAAAGTATCAAGCATTGCTTGTTAATAGAAATGAAGAAGCAGATGACA
GTGTAACTATAGCTGGAAAATTATGTGAGTCTGGTGATATCATTATTAAAGACGCTAAAT
TACCTTCATCAGTCAAACGTGGAGACTATCTTGCTATATTATCAACTGGTGCATATCATTA
CTCTATGGCATCCAATTACAATCAAATGCAAAAGCCTTCTGTGTTTTTCTTAAAAGATGGC
AAAGCACGTGAAGTTATAAAGCGACAATCGTTAAGACAACTCATTATTAATGATACAAA
ATAA

> **msa**
Function: protein coding sequence; modulator of sarA
Best match: msa_CC005_Mu50-VRSA_BA000017.4[1484563:1484964:r]RC (completely identical)
Position: 034-contig_264_RC: 12285 … 12687; Length: 402 bp
Sequence:
TTAGATTTTATGCTTTACTTGCTTTTTCAAATAATATAAAACGATAAGAATTAACAACAAA
TATAATGTATAAAACAGAATTTCATCAAATCCAAAATCTTTACTTGTAATTAATACATCAA
TATCTTCTAATTCCATCATCTCTGCTTTAATATAATTATTAAACTTTGGCAAAGTCATTAA
ATGTACATTGTAAATATAGTAATAAAAGTCGATAAATATGATGTTAAAAAATATGTATGT
TTTATCAATATTAGTTTTGAAAAATAGTATCCCAGAAATCATTATCGGAATCACTAATACA
ATCATCATTGCTGCTAAAATATTAATATTTAATCCAATTGCAGATAATACACCGAAAACT
AATAAATTAGCAACTAAACTAAGGATAAGATATTTCAT

> **cspA**
Function: protein coding sequence; cold shock protein A (core genome, constant)
Best match: cspA_CC001_MW2-USA400_BA000033.2[1410578:1410778:r]RC (completely identical)
Position: 034-contig_264_RC: 12883 … 13084; Length: 201 bp
Sequence:
TTATAGTTTAACAACGTTTGCAGCTTGTGGACCGCGGTCGCCTTCAACTACTTCAAACTCA
ACAGCTTGACCTTCTTCTAATGATTTGTAACCATCTTGGTTAATTGCTGAAAAATGTACGA
ATACGTCATTTTCTCCTTCAACTTCGATAAAGCCGAATCCTTTTTCAGCGTTAAACCATTT
AACTGTACCTTGTTTCAT

> **tnpIS431-06**
Function: protein coding sequence; transposase for IS431 (mobile element)
Best match: tnpIS431-06_circ_S-epidermidis_GQ900381.1[43799:43807]RC (completely identical)
Position: 034-contig_264_RC: 13347 … 13356; Length: 9 bp
Sequence:
ATAGTTCAT

> **xpaC**
Function: protein coding sequence; 5-bromo-4-chloroindolyl phosphate hydrolysis protein (core genome, constant)
Best match: xpaC_CC001_MW2-USA400_BA000033.2[1411708:1412337] (completely identical)

Position: 034-contig_264_RC: 14013 … 14643; Length: 630 bp
Sequence:
ATGACAGTGAGATATAATATTTCTCATATATTTGGGGTGTTAGTGGGAATTCCTGTAGCGT
TTTTAACAAGCATATTTGGGATGATTGCACTTGATGTATCTTTTTTAATTGATATGTCTATT
GGTATTGTTGGCTTTTTAATGACATACCTACCGATACAAAAACTCACTTCACGCAAATATT
TAAACGAAATTGGTTTGACTAGAAAAGACTATCGCTATATTCGAAATCAGTTAAATCATA
CACACCAAAAACTTAGAGGTATTTTAAAAACGTATGTCAATATAAGATCAATTAAAGATT
TTAGGCAGATTAATGATATATACCAAATTTCACGTTCTATTTATACGACAGTTAGACAGA
GACCTGCATCATTTTATAAAGTTGAAGGCTTTTTTTATTCTCATATTGATAATGCTTTAAAT
TTGGTTGATGCATATACACGTCTAGCAAAAATGCCCAAAAAATCAATTAATGAACAGCAA
AAGTTAGAACAAACACGAATTACTTTGGATGAGGTCAAACGAACATTAATCGCTGATTTA
AAGCGTCTCAACGAAGATGATTATGAACGTTTAGATATTGAAATGGAATTAAATAAGTTA
CATCAAAAACATCATCAAGATTGA

## > terA

Function: protein coding sequence; putative tellurite resistance protein (core genome, constant)
Best match: terA_CC008_NCTC8325_CP000253.1[1347708:1348844] (completely identical)
Position: 034-contig_264_RC: 14674 … 15811; Length: 1137 bp
Sequence:
ATGACTGAAAATAAAAGTTTCAAAGAAAGCCATCCACTAGATGATTTTATAAGCGATAAA
GAATTATCGAATACTACTATTCAAAAAGAAAAGTTAACAATTGAACAACAAAAACAGGT
AGACACAATCAGTAAACAAATTAACCCTTTAGACAATGAAGGTTTATTAGCGTTTGGTTC
TGATTTACAGAAACAAATGTCTCAATTTTCACATCAAATGTTGGATGAAGTACAAAGTAA
AGATGTTGGTCCTATTGGAGATACTTTGTCAGATCTAATGTCAAAACTAAAGTCAGTTAA
TCCAAATGAGTTAAATACTGATAAACCATCTATGTTAAAAAGAATTTTTAGCAGAGCAAA
GTCGTCTATCAATGAAATCTTTTCAAGAATGCAATCAGTTAGTGCTCAAGTCGATCGCAT
AACGATTCAACTGCAGAAACATCAAACACATTTAACAAGAGATATTGAATTATTAGATAC
GCTATATGATAAAAACAAACAATACTTTGATGACTTATCATTGCATATCATTGCTGCACA
GCAAAAAAAGTTGCAATTAGAAAATGAAAAGCTACCACAATTGCAACAGCAAGCGCAGC
AATCCACTAATCAAATGGATATTCAACAAGTTGCAGATATGCAGCAATTTATAGATAGAC
TAGATAAACGCATATATGACTTACAGCTTTCAAGACAAATAGCTTTGCAAACTGCGCCAC
AAATTCGTATGATTCAAAATGTTAATCAAGCACTTGCCGAGAAGATACAAAGTTCAATTT
TGACAAGTATTCCACTATGGAAAAATCAAATGGCCATTGCGCTTACATTAATGAGACAGC
GTAATGCAGTTGCTGCACAACGAGCTGTCACTGATACAACTAATGATTTATTAACAGCAA
ATGCTGAAATGTTGAAACAAAATGCGATTGAAACTGCAACAGAAAATGAGCGTGGCATT
GTTGATCTTGATACATTGAAACGTACACAGCGTAACATTATTGAGACAATTGAAGAAACA
TTAATTATTCAACAACACGGTCGCGAAGAACGACAATTAGCTGAAAAAGAATTACAACA
ATTAGAACAAGATTTAAAGTCACATTTAGTGAACATCAAAGGACCGAATAAACAATCAT
AA

## > braB

Function: protein coding sequence; branched-chain amino acid transport system II carrier protein (core genome, constant)
Best match: braB_CC005_Mu50-VRSA_BA000017.4[1488161:1489504:r]RC (completely identical)
Position: 034-contig_264_RC: 15883 … 17227; Length: 1344 bp
Sequence:
TTATTCCTGTTGATATTTAATTGGATCTTGTTTTACAAATATGCCGACTAGATAGCCTAAT
ATCGTTGCAATAATTGCTACTGGGAACCACTCTAAAGAATACGCTCTTAGAGGCAATGAT
TCTATAAAGTTAATTTTCAGCCAACCTAACTTACTAATAACACTGAAAATCGACAATATA
AATACGATAATAACTGGAATTTGTTGTGAAATGCGTTTTGTCGGTATGAATTTGGCAATTA
AAATTAATAATACAACAGTTATTGCTACTGGGTATACAATGCTTAATACCGGAATTGACA
TTGAGATAACAGCATTTAAACCTTGGTTAGCAATAATAAAACTCATTAAAATGAAAACTA
ATACAAATGCTTTGTATGATACTTTAGGTACGATTCTATGGAAATATTCAGAAACTGCAA
CAATAAGCCCGCATGCTGTAGTTAGACATGCCAGCGCCACAATGATGCCCAATAAATATT
TTCCGAATGAACCAAATCCTGTTGAAGCCATTGTCGTTAATAAATATGTCCCAATGTTTCG

ATCTTTGGATTTCAATTGATCTAACGTCATGTCACTTACTGGCATATGATTACCAATATAA
CCTAATGAAATATATATGAAAATTAAAGCTACGGCTGCAATTAAACCAGCAGTCAAAGTT
TGTTTGAATATTTGATTTGTTTTAGTAATGCCTGTTAGTTTTACTGCATTAACAACAATCAT
TGAAAAAGCAATTGCTGCAATGGCATCCATTGTTAAATAGCCTTGTGTAAAGCCTTCAGC
AAAACTTGAAAAATTAGAATGATATAGTGCTTCATTGCCCTTTCCAGCACTATTACCGCTA
AAGTCTAAGTATCCTTTAATAATCATCGCTAAAATAGTAATCAATAATAATGGTGTTAAT
AATGAACCAATACGATCGATTAACTTAGATGGATTTAAACAAATATACAAAACGACTATG
AAGTAGATAATCGTAAATATAAATAAAGCGATACTACTATTGCTATGTATAATTGGTGTA
ATTGTCATTTCAAAAGATGTAGATGCAGTTCTAGGTATTGCGAAAAGTGGTCCTATAGTC
AAATAAATGATGATTAAGAACAATATTGAAAATTTAGGTGAAATTTTATTTAATGCGCCA
ATATATCCTTCTTTATCAAGTGCACCTACAATCACACCTAATAATGGTAAACCAATCCCAG
TTAGAACAAACGCTAAAATGGCGGCCAGAAAAATTGACCACTATCCAATCCGAGATTA
GGAGGAAAAATTAGATTGCCTGCGCCAAAAAACATAGCGAAGAGCGTAAACCCAATGAC
CCATGTATTTTTATTCAT

> **norD**

Function: protein coding sequence; nitric oxide reductase activation protein (core genome, constant)
Best match: norD_CC008-ST72_21259_AFTS01000032.1[24894:26774]RC
Position: 034-contig_264_RC: 17451 … 19338; Length: 1887 bp
Sequence:
TTATAAAGATTTAAGTAGTAATTTTTTTAATAATGGAGAGAGATGTCCAGGTAAATGCGC
AACACCTTCAACGAAAATAGCATATTGACCATAAATATTATGAATTGTTTGTTCAACATCT
TCAGTTATTGGATCTTGACTCAAAAACACATTAAATACTTCAATGCCAAATTTACGTGAC
ATTTCTACAGCTTCATACGTATCAATAATACCATCTTGACTATAATTAAATGCAGACGGTT
CGCCGTCTGAAAATACGATTAAAAATCGTTGATGTTGATTTCGACGCATTAATCGTTCACT
TGCAACTCTAATAGCAACACCATCACGATTATCATCTTGAGGTTCAAGTGCCATAATACG
TGGGCCATCTTTTTCAAAGGTTGAGTAATCATAGTTAATAATTTCATTAATGATATTTGGT
TGTGCATGATCGTCTGAATCAAAGGCATCCTCACTGAATGATAAAATTTCATGTTTAATGT
TCAATGCTTTTAACGTCTCGTGGAATAACACTACACCTTTCTTCGTTTCAGCCATTTTATCA
TGCATACTTGCTGACGCATCAATTAACAATGTAAACGTCGCATCAAATGATTTACTTAAA
TCTTGTTTTTTGTAAAACAATTTATATTGATCGTCGATAAACCAATTAATTAAATCCTTTTG
TAATCGTCCTTTTGTTAAATTAAAACGTGCATCTCGTTGTTCTCGTTCAATCGTTTTCTTTA
TAATTTGAATTAAGTCTTTGATTTCATATTGTACGTCTTGTTTCGATTCTTGATATTCTAAA
ACATATTGTGGCTCAATTTCAGGAATTTGCCATTTTATTTCTACATTTTTGTTCACACCATC
AAGTTGAAATGCTTGGCTTTGACCAACAGCATCGCCTTCTTCACGATTTAGCGTATCGTTT
GAACCTTTGCCCTTTTTGGTCATCATATCAGTCATATCATCTGTAGCATCGCCTTCACGTG
CTTCATCATTACCCAATGTTTCACTATTTTGTCCTTCGTGAAGTTCCATTTCTAAATAGGCG
CCACCTTTTGACTCACTATCTGCAGATTTAGAGTCAGCTTTTTCACTTCCGTTATCATCATC
TTCTGAAGTATCATCTTGCCCATCGACTTGACTTGCATCAGTTCTCTTTAAATCATCAAAC
TCTGGACTAGCTAATGTATTATATAGTGTTTTAGGTAAGTAATAATATTCGTTTAGCATAT
CTTCTTTTAAAATATCATCTACTTGATACATAATTCTTTGAGCCAGATACATATTATCTTCA
GAATTTTGATTTTGGAAAAAATTAGGTAAATATAAGAACATATTAACTACAATATCATCT
AAATCTGAATGTATAGATGGTATATCAAAGAAATCTTGGCTTAAAAATGCATGTTCTAAA
TACAAAAATAATAAATCTGTATATTGTGTTTTAGTACGATACACTTTAATTTGAGATTCCG
TATATGATATACGTGTATCTAAGCGAAGATCAATTAATTTAGCAGTACTTGGGCGCTCAA
CTTTAATAGAATTTAATACGCGCATATCTTCTAATAATTTAAAAAGTTGTTGATAAAATTT
AGGGTGTTTAAAAGTTTTATCTTGTACTACTTCATTTACAATTTGTACATCCATCATATGA
TAACCGTAAGCAGCTAACATAACATCTGTTTTTAAACCAGCCATTTCGATATGGCTTTGTC
GATGTGACCAAAACCAACTCGTAATCAAAACATTTTGAACAGGATTATAGTAAGGGAATT
TTTGGATTTTAACTTGTGTTTGTTCATTTTTTAATAAGAGTCGAGCTAAGTCTTGCAACATC
ATTACTTGCTTGGCATCTAACTGTTCGTCGTTAAATTTTATGAAACGATCACTCAT

> **txbi_odhB**

Function:  rho independent terminator of odhB
Best match: txbi_odhB_CC005_Mu50-VRSA_BA000017.4[1494112:1494182:r]RC

Position: 034-contig_264_RC: 21833 … 21904; Length: 71 bp
Sequence:
TATAAAAATAAAGTACAGCAGTAAGATATTTTGACTAGAAAATAATCTTACTGCTGTTTG
TTATTGATTTG

> **odhB**
Function: protein coding sequence; dihydrolipoyllysine-residue succinyltransferase (core genome, constant)
Best match: odhB_CC001-ST772_118_AJGE01000012.1[8495:9763]RC (completely identical)
Position: 034-contig_264_RC: 21944 … 23213; Length: 1269 bp
Sequence:
TTAAGATTCTAATAATAAGTCTTCTGGGTTTTCAATTAATTCTTTAATTGTTTTTAAGAATC
CAACTGCTTCTTTACCGTCAATAATTCTATGATCATAGCTTAATGCAATATACATCATTGG
ACGATTTTCGATTGTATCTTGATCAATCGCAATTGGTCTTGTAATAATTGAATGCATGCCT
AAGATTGCAGCTTGATTACCATTGATAATTGGCGTACTCATCATTGATCCAAAAATACCG
CCATTTGTAATCGTAAATGAACCATTAACCATATCATCTAAGCCAAGTTTTTCTCTCGTG
CTTTAACTGCTAAATTAGCAATTTCTGCTTCGATTTCTGCAAAATTCTTTTTATCACAATCT
CTTACAAATGGTACTAATAATCCATCATCTGTAGAAACAGCTACACCAATATCATAATAT
TGTTTCGTAATCATGTCGTCGCCGTCGATTTCTGCATTAACTTCTGGATACTTTTTCAAAGC
TGCTACAGAAGCTTTAGTAAAGAATGACATAAATCCTAATTTAGTACCATCATGATCTTTC
ATAAATTGTTCTTTCTTACGTTTACGCAATTCCATAACATTTGTCATGTCAACTTCGTTAAA
TGTTGTTAACATAGCTGTATTATTAGATACCTCTAATAATTTTTTGGCAGCTGTTTTCTTTC
TGCGTGACATTTTTTTCACGAATCACTGGTTTTGTAGGATATTGATTGTATTTTTTCTCTTCT
TTTGCAGGTGCTTGTTGTGTTGTTTGTGTTGATGCCGGTGCCTGTTGTTTCTTATCAATATC
TTCTTTACGAACCACATCATTTGTTTTCGGACTTACTTCAGCAAGATTCACACCATTTTCA
CGAGCATATCGACGCGCAGAAGGCGTAGCATTAATACGTTGCTGATTGTCATCATTTGCT
TGATTGACTTCAGCTTTATCTACCGAATTATTTGTTGTTTCTTCTTTTTTATTATTTGTTTCT
TCATTTTGTTGTGGAGTATTGTCGTTACTATTTTCTTTAGAAGCATTGCCACTACCTTCGCC
GATGACAGCAATTGCTTGTCCAACTTCTACAGTGTCGCCTTCACTTGCAAGTTGTTCAGAT
AATACACCTGCTTCTTCAGATACAACTTCGACATTAACTTTATCAGTTTCTAATTCAAGAA
TAGCTTCACCTTTTTCTACGCTATCCCCTACGTTTTTCAACCATTCTGCAATGGTACCTTCT
GTAATAGATTCTGCTAATTCTGGAACTTTAACCTCTGGCAT

> **odhA**
Function: protein coding sequence; 2-oxoglutarate dehydrogenase E1 component (core genome, variable)
Best match: odhA_CC001-ST772_118_AJGE01000012.1[5683:8481]RC (completely identical)
Position: 034-contig_264_RC: 23226 … 26025; Length: 2799 bp
Sequence:
CTAGTTATTTTTTAATGCATTTTCTATAATTTTATTTTGAACAAGTTTATGAATTTCTCCAT
CGCCTTCAGCTGGAGCAGCCCTTTGAATTCTGCCATGATAACTTAAATCATATTTATCTGC
AACTAGCACTTTAACATATGGATAGACATATAACCATGCACCTTGATTTTTAGGTTCTTCT
TGTACCCATGACACTTCTTCAAGGTTTGGCAATTGTGCTAGTAATGCTTCAATCTCTTCCT
CTGGGAATGGATACAATCTTTCAATCGCAACGAGTAATACTGATTCGTCTGGATTTTTAGC
TAATGCTTCTTTTAAATCAATGAACATTTTACCAGTCGCTAAAATAACTTTTGTAACCTTA
TCCGATTGATATGATTCTGTCAAAATTGGCTCAAATCCACCAGAAGTAAATTCATCAATT
GGTTTTGCAACTGTTTTATTTCTCAATAAGCTTTTTGGTGACATAACAACCAATGGTCGCA
TTTGTTCAGAATCTAAACTAGCCGCTTGTGCACGCAATAAGTGGAAATAATTACTTGAAC
TAGATAAGTTGACAACTGTGCAATTATTTTCAGCAGCTAATTGTAAAAACCTCTCTAATCT
TGCTGATGAATGTTCAGGACCTTGACCCTCATATGCATGAGGTAAGAATAATGTTAATCC
TGAACGTTCTCCCCATTTTGAGCGAGAACTGAATAAGAAGTTGTCAAAAATCATTTGTGA
CATATTTGCAAAATCACCATATTGCGCTTCCCAAATATTGAAGCTTTTTTTGTTTTCCACAT
TATAGCCGTATTCAAAACCAACTACTGCTGCTTCTGAAAGCGGAGAATTGTGTATATCAA
ATGTCGCTTTTTGATCAGGAACATGATGTAAAGGTGTATATGTTTCACCTGTTTGCTCATC
ATGTAACACGGCATGCCTATGACTGAATGTACCACGTTCACTATCTTGACCAGTTAAGCG

AATCGGTGTACCATCTTGTAAAATTGTCGCAAATGCAAGTTGTTCTGCTTGTGCCCAATCA
ACTAAGCCATCTTCTTTATTAAACGGCTCATGACGCTTCTCAAGAACTTTGTTTAACTTTTT
CAAAATGTTAAAGCCATCCGGATATGTTAACAATGCATCATTTATTTCTTTCAAATGATCA
AACGTAAATGATTGTTCGTCTGCTTGTAACGGTAATGCAAGTTCTGCAGGTTTTTCCATAT
CTGGATTATCCATTTTATCAGCTTTATTAATTTTATCATGAGCTTGTCTTAGTTCCTTTTGG
ACTTGTTCTATAAATGAATGCATTTCATCTTCTGAAATGACACCTTCATTAACAAGCTTTT
TACCAAACACATATTCAACAGAGTCATGTTTGCGAATATTCTGATAAGGAACTGGATTAG
TAATTGATGGTTCATCCATTTCGTTATGTCCGAAACGACGATAACCTACTAAATCAATAAC
GACGTCTTTATGAAACTCTTTTCTAAATTCCATTGCAATATCAATTGCTTCAATAGTAGCT
TCAACGTCATCAGCATTGACATGGAATATTGGCACATCATAACCTTTGGCCACATCTGTA
GAATAAGTTGTTGAACGTGCATCAATTGGTTCTGTAGTAAATCCAATTCTATTGTTAGTAA
TAATATGCAATGAACCACCCGTAGAATAGCCTTTCAAGTTTCCTAAGTTCATTGTTTCGAA
GTTAATTCCTTGACCAGGATAAGCAGCATCGCCATGTATAATAATTGGCATTGCTTTATGA
TGATCAGTCGTCGGAGCCCCAGCACGTTGTGTATCATCTTGTGCTGCTCTCGTACGCCCCT
CAACAACAGGTGCAACAATTTCCAAGTGACTTGGATTGTTAGCCAGTGCAATACGCTGCA
TTGTACCGTATGAATCAGTAGTTTTAATGCCACCAAGGTGATATTTCACATCACCAGTCCA
TCCAGCAGTTAACTGCAAGCTACCATCTTCAGGTAAGAATTTCATTGGATCTGTATGCATA
AATTCTGAAATCATCATTTCGTACGGTTTTTCTAAGACATGCGTTAAAACGTTTAAACGTC
CACGGTGAGCCATGCCTATTTGTATATTTTTAATACCTTCTTTCGCAGCAATCGTAATAGT
ACGTTGTAACATCGGTACAAGTGCGTCTACCCCTTCAATTGAAAAACGCTTTGCACCAAC
GAAGTTTTTATGAAGATATTTTTCAAACCCTTCAACATACGCTAATTGTTTGAATAGTGCC
CTTTTTTCGTTATTATTTAACGTTACTTTATATGGCGTTTCAATTCTTCTTTTTAACCAACC
ACGTTCGGTATTGTTATTAATATGTGTATACTCAAATGCAATTGGTCCTTTGTAACGTTTTT
CCATTCTTAAAATTGCTTCATAAGCATTATCATAAATGTCGGCAAAGTGATCTGAAACAA
TTCCTGCTGATATACCTTCCAAAGTCTGTTGATCTAAATCAAAGTCTTCAATCTCTAATTT
AGGTACATGTTTCCTTTTTGGAGGATTTACAGGATAAATATCGGCTTTAAGATGCCCGTAT
TGGCGAATATTATCAATTAAACGCATGACACGCTTAATTGTGCCGTCGCTATTTTGGCTAC
TTGTACTTTTTAAAGCTGGTACAATTGAGTCATCATTCTTAATTGTGCTGAATAAGACTTG
TAAATCTTCTGGTACAGATGATGGATCTTGTAAAAAGTCATCATATAGATCTAACATTAG
ACCTAAATTCGCACCGAAGTTTACAGGAGCCTCTGAAACTTCTTTTCTTTCGTTAGTCAT

> **arlS**

Function: protein coding sequence; signal transduction histidine-protein kinase (core genome, constant)
Best match: arlS_CC001-ST772_118_AJGE01000012.1[4044:5399]RC (completely identical)
Position: 034-contig_264_RC: 26308 … 27664; Length: 1356 bp
Sequence:
TTAAAATATGATTTTAAACGTTGTTCCTTTGTTAATTTCACTTTTAATTTTAATCGATCCTC
CGTTTAATTGAATGATTTTTTGAGCAATAGATAATCCAAGTCCATTACCGCCTTGACTTCT
TGAACGAGATTTATCCACTCGATAAAAGCGATCAAAAATGAAATCTTGATCTTCCTCTGG
AATACCAATTCCATGATCTGTAATTTCAATTATTTTTTGCTTATTTTTTAACCTTGTCTTAA
CTTTAATTTTCTTATTCTTCACATCATATTTGATTGCATTATCAATAAAGATTAAAAATAAT
TGTTCGAATTGATGAGGTTTCATTTTAATTTCTAGATTTTTAGATGTCAGATCCGTATCAA
ATTGATAATCAGGATGCAATTGTTTTAATGAGTGTATTCGCGAACGAATTTCATCATTAAT
ATGCACGGTCTGTGCTTCAGAAGAAATGTCATTTACATCTCCTTTAGTCAATTCAAGTAAT
TCTTCGACTAATTTTATGATACGATTCATTTCTTCAATAGAAATATTTAACGATTCTTCTAA
TACTGCTGGGTCTTTTTTTCCCCATCGCTGAATCAAATTTAAATGACCTTGAATAATTTGT
AATGGTGTTCGTAATTCATGTGACGCATCTTCAACAAATTGTCTTTGTTGATTAAATGATT
CTTCAATTTGGCTCATCATCTCATTAAACGTATTTGCTAAATTATCTATTTCTTCATAATTT
GTATTTAATTGCAATTTATTTTGAAAACCATCTCGTCGAATCTCAATCATTTTATTTGATA
AACTGACAAGCGGTTTAGTAATTTGTGTTGAAAATACATAACTGATTGTGGCAGTTATAA
TTGTTGCAATCACTCCAAATGCCAGCGCAATGATATACAATGATTTTACGATGTTATCATA
ATTTTCTAGTGAATGAATTAACAAGCTATACCCTTTGAAATCTTGCGTTGTAATTGGTTCT
TTAATAATTAAATATTCAATGCCTTTATAGCGTTTTTTTATTACGCGGTCAAAATAACGGT
GTTCATAACCTGGTTCAACTCTCACTGTGTTATCATTCGATGTCTCAAATAATTTATTATTA
TGCTCATCATAAATAATTATCTCTTGAAAATTACCTAAAGATGCATTCAAGTCTAATGCAG

ATATATCTTTAACAGGCTTAGAATGAAATAAATTATTAATATCGCTTGAGCTTCGTTCTGC
ATCATCAAGCTCACTATTATGCAGTGTATCTTTCAAGAAAAAAATAATAATTAAACAAAA
CAAAAATATCGTGACAAACGTAATCATTGTGGTAACAATAATCCAGTTATTGCGCAATTT
ACGTTTTGTCAT

## > **arlR**

Function: protein coding sequence; response regulator
Best match: arlR_CC005_Mu50-VRSA_BA000017.4[1499939:1500598:r]RC (completely identical)
Position: 034-contig_264_RC: 27660 … 28320; Length: 660 bp
Sequence:
TCATCGTATCACATACCCAACGCCACGAACTGTTTCAATCATTTTGTCACGATCGTATGGT
TTTAACTTGTTTCGTAAATATCTTATATAAACATCTACGACATTTGTTTCTACTTCACTATT
ATAACCCCATACATGATTTAAAATTTGTTCCCGTTGCATAACATGGTTTTTATTTTCAGCT
AGAAGATATAGTAAATCATACTCTGTTTTTGTTAATTCAATTTCTGCGCCATTTACCGTCA
CTTTAAAAGCGTTCTTATCAATTGTAATACCGTTGACATCGATAATATCCTTTTGTGGCTG
ACGACGTAAAATTGCACGAATTCTTGCTAAAAGTTCTTCAATATCAAACGGCTTAACTAT
ATAATCGTCTGCACCGTAATCAAGCCCAGCAACTTTGTCATACGTATCACTTTTCGCTGTA
ATTATAATGATAGGTGTAGATTGTTGTTGTCTAATTTTGCGACAAATTTCTAAGCCATTAA
TTGACGGCAACATTAAATCTAATATGATTAAATCATAGTAATGGCTAAGCGCTTTATCTA
AACCGTCTTGTCCATCATACTCTGTGTCCACATTGTAATTTTCATGTGTGAGTTCCAATTC
AAGAAATCTTGCTAAGTTTTGTTCATCTTCTACTATTAAAATTTGCGTCAT

## > **murG**

Function: protein coding sequence; undecaprenyldiphospho-muramoylpentapeptide beta-N-acetylglucosaminyltransferase (core genome, constant)
Best match: murG_CC001-ST772_118_AJGE01000012.1[900:1970]RC (completely identical)
Position: 034-contig_264_RC: 29738 … 30809; Length: 1071 bp
Sequence:
TTAATTCAATGCGTCTTTAATCATCTTATCAAATAAAGCTTCTTTCGTATAACTTTGTTCAT
ACGATTTCATATTATTGATAATTCGAGTTCTTTCCTGTTCCATTTCATTTAGTTCTTGTAAT
AAAATTTGTGCTGTTAATTGTTCTTCATCAATCGTTTTAGCATAACCTTTATCAGCAAAAT
GATTTGCATTGTCAATTTGGTCGCCTCGGGATTGATCTAAACCTAATGGTACTAATAACAT
TGGTATACGTAATGTTAAGAACTCATAAATCGCATTTGATCCAGCTCTACTTATTACTGTG
TCCGTAATTGCTAATAAATCTGTTAAATCCTCTTTAACAAATTCATATTGTATATATCCTG
ATTTTTTAACTTGAGCATCTTTTAATCCTTTACCAGTTAAATGTATCACTTGATATTGTTGT
AATAATGCATCTAAGTTTTCGCGAATAATGCTATTTAATTTTTTACTTCCTAAGCTTCCAC
CCATAACGAGTAAAACTTTTTTATTTTCATTAAAGCCTGTTAATTGATAACCATTATGTGC
ATTACCATTTTTTAAATCTTCTCGAATTGTTGCTCCAATAAAATCAGCTTTCTCTTTAGGTA
GGTAGTTTAGCGTTTCTTCAAATGTTGTATATATTTTCTTGGCAAATTTAAGTGCTATCTTA
TTCGCTAATCCTGGTGTTAAGTCAGATTCATGAATAATAGTTGGTATATTTAATGATTTGG
CTGCAATAACAACAGGCACAGATACAAATCCACCTTTTGAAAATAATAGATCAGGTTTTT
CTTTTTTCAAAACTTTACGAGCATCAAGAATACCTTTCAATACTTTAAATACGTCTTTGGC
ATTTTCTAAAGAAATATATCTTCTTAATTTACCACTCGAAATAGGATAATACTTAATTTCT
GGTAGTTGTGATTCAATCATTTCTCTTTCAATACCATTTTTAGAACCAATATAAAGCGCTT
CATAACCTTGTGATAATGCAGTTGGAATTAAACTTAAATTTACTGATACGTGTCCAACTGT
TCCCCCTCCGGTAAATGCGATTTTCGTCAT

## > **txbi_universal1**

Function:  bidirectional rho independent terminator
Best match: txbi_universal1_CC001_MW2-USA400_BA000033.2[1430297:1430367:r]RC (completely identical)
Position: 034-contig_264_RC: 31663 … 31734; Length: 71 bp
Sequence:
TACATCCCTAAAAAAAGCAGTAAGATAATTTTCAATTAGAAAATATCTTACTGCTGTTCTC
TATTTATACA

> **tnpIS1272**
Function: protein coding sequence; transposase of insertion element IS1272
Best match: tnpIS1272_trunc_CC001_MW2-USA400_BA000033.2[1430518:1431031]
Position: 035-contig_268_RC: 8 … 38; Length: 30 bp
Sequence:
AACACTATATCGTGACTTAGTTGAAGAAAA

> **tx_universal3**
Function:  bidirectional rho-independent terminator
Best match: tx_universal3_CC001_MW2-USA400_BA000033.2[1397772:1397835:r]RC
Position: 035-contig_268_RC: 57 … 87; Length: 30 bp
Sequence:
TTAATAGAAATCAGCTTTTTACATTGCCT

> **ctpA**
Function: protein coding sequence; carboxy-terminal processing protease (core genome, constant)
Best match: ctpA_CC001-ST772_118_AJGE01000010.1[7960:9450]RC (completely identical)
Position: 035-contig_268_RC: 157 … 1648; Length: 1491 bp
Sequence:
TTATTTTAAAATATTAATCAATTTATCGAGAACATCATCATGTTTATTAGCTTTTTCAACTA
ATAACTCAGTAAATTTATTATTCGTTTCTTTATTAAATTCACCAGTTACCTCGAGTTTATTC
GCTTGTTGGAAAGCTTTAACTTGATTTTCTAAAGCTTGATCAAATTGCGTTGTTTCATTAT
CAACTTTATAACCTAAAGCTGACAAACCAATTTTAATAGTTTTAATATTTTTATCATCGTC
TCCAACTTTAAATGTTTTCGTATTAGGAATGACATTTAAAGATTGATATTTAGGTGTGTCA
ATAGTAACGTCTGGTTTAATGCCTTTACCGTGAATATAATGACCATCTGGCGTTAACCATT
TCATTTCAGTATATTTTAACAATGAACCATCCTTAAACTCTCTTGTAGTTTGTACGACACC
TTTGCCGAATGTTTTTGACCCATAAACTTTAGCTTTATTATAGTCTTTTAGCGCACCAGTA
AACACTTCAGAAGCGCTAGCTGAACCTTCATTCACTAAGATGGATATATCCATGTCTTTCG
CTTCTTTTAACGCATCATTAGAAGTTTGAATTGCTTCAGTATCTTTACCTTTTTCTAGTTTA
ACAACAGTTTTTCCTTTATTGATAAAAATATTTGCCATTTTAACAGCTTCATCTAGTAGTC
CACCTGGATTATTTCTTAAATCTAAAACAATCTTTTTCAAACCATCTTTGTGAGCTTTTAG
AACTGCATCTTTCAATTCACCTGATGTATCATTCTGGAATTTATTAATAGTAATAACTCCA
ACTTTACCTTTTTTCTTATACTCAACACTTTTAACATGAATTTTTTCACGTTTAATCTTAAC
GTCTTTTTCTTCACTACCTCGTTGAACAGTTAAAGTGACTTCAGTGTTTTCTTTACCACGAA
CATCTTTGACAACTTCATCTAATGCTTTTCCTTTAATTGATTTTCCATTTACTTTAGTAATG
ACATCTTTAGGACGAATGCCAGCACGTTCTGCTGGAGATCCCTTCATAGGACTAGTAACC
ATAATTTGATCATTTTTCTTTTGCATTTCTGCACCAATACCTACAAAATCACCTGAAACAC
CTTCATTAAAGGATTTCGTTTGTTCTTTTGTTAAATATTCAGAATAAGGATCTTTTAATTCT
TTGACCATGCCATCAATTGCAGCTTTACTTAACTTGTCAGAGTCCTGTTTTTTGTAATAAT
CACTATTTAAGATTTTATACACATTTTCAATTTTATTTAAGTTTGCTTGATCAGTTTTGTTT
AAACCACTTATTTTTTGATTTATAAAAATATATGCAACAACTGTGATGACAGCTGTTATTA
GGATTGTACCAATTAATATTGATATGAATTGCCAACGTTTTAAGTGAACGCGTTTCGATG
AATTAGTTTCTTGGTCTTGATTGCTTGTTGCAATTTCAGCGCGTTCATCATCGGATGAAGA
TGTGTGTTGCTTATCATCCAT

> **ptsIIA**
Function: protein coding sequence; PTS system/glucose-specific IIA component (core genome, constant)
Best match: ptsIIA_CC008_COL_CP000046.1[1471380:1471880:r]RC (completely identical)
Position: 035-contig_268_RC: 2060 … 2561; Length: 501 bp
Sequence:
TTAGTTCATTGTCACATCAATCACTTTTGTTTCACCTTTAATCACAGCATTTTCATCATAAA
TATTAATTGAAGCTGCTTGATCAGTGTTAGTAATTATAATTGGTGAAATTACAGATTTAGC
GTTATTATTAATATATTCAAGGTTGAATCTTACTAATGGATCTCCGACGTTAACTTCGTCA

CCACTAGACACTAACACTTCAAATCCTTCACCGTCTAATTGAACTGTGTCTAAACCGATAT
GAACTAATAATTCTAATCCGTTATCTGCTTTTAACCCAATCGCATGCTTAGTTGGAAAGAC
ATTGTCAACACGTCCTGCAATTGGAGACACAACTTCTCCTTCAGTTGGATTAATACCAAA
ACCTTCGCCCATCATTTTTTGTGCGAATACAGGATCTGGAATATCTTCAATTTTCACGAAT
TCTCCAGTTAATGGTGCATAAATTGCGATATCTTTCTGAACTTCTTTGCCTTTTCCGAATA
ATTTTTTAAACAT

> **mrsB**

Function: protein coding sequence; peptide methionine sulfoxide reductase (core genome, constant)
Best match: mrsB_CC001_MW2-USA400_BA000033.2[1433566:1433994:r]RC (completely identical)
Position: 035-contig_268_RC: 2572 … 3001; Length: 429 bp
Sequence:
CTACTTATCAAAATGTGATATTAAATCGCCATAACCCAATTCTTCTAACTTTTCATATGGA
ATAAATTGAATTGCAGCGGAATTGATACAGTATCTTAAGCCGCCACTTTCTTTAGGTCCAT
CATTAAAGACATGTCCTAAATGACTATTTGATTCTTCTGAACGCACTTCAGTTCTCAACAT
ACCAAATGATTTGTCGACTAATTCTATAATTTCATCGTCATCAAGCGCTTTGGAAAAGCTA
GGCCATCCACATTCAGAATGAAACTTTTCTTCAGATGTAAATAAAGGTTTACCAGAAATT
TTATCTACATAAATTCCTTTAGCAAAATGATTCCAATATTCATTCATAAATGGTGGTTCAG
TGCCGTTTTCTTGTGTAACAATATATTCTATATCTGTTAGTTCACTTTTATCTTTTTTAAGC
AT

> **msrA2**

Function: protein coding sequence; peptide methionine sulfoxide reductase A2 (core genome, constant)
Best match: msrA2_CC001-ST772_118_AJGE01000010.1[6081:6614]RC (completely identical)
Position: 035-contig_268_RC: 2993 … 3527; Length: 534 bp
Sequence:
TTAAGCATTTTGATTCCCCCAATGTGATTCTATAAACGCTTTTCTACCTGAACCACGTTGA
TATTGGTAATAATGTACCGGGTTCTTTTTATAATAATCTTGATGGTAGTCTTCAGCTGGAT
AGAAATTTTTATATGGTTTAATAGGTGTAATCACTGGTTTCTTGAAAATACCTTGTTCATT
TAATTGTTGCTTTTTAAACTCAGCAGCCTTTTTCTGATGTTCATCATGATAGAAAATGACT
GGTTGATAGCTTTCGCCTCTATCGAAAAATTGCCCTTGATCATCAGTTGGGTCAAATGTTT
TGAAATATATGTCTAATATATTTTCAAAGGAAGTAACCTCTGGATCAAACGTAATTTGTA
CTGCTTCGACATGGCCGGTTTGATTCGTACATACCTGTTCATAAGTTGGGTTGTCAACATG
ACCGCCACTATAACCAGATACGACTGACTTGATGCCTGGATATGATGTAAATGGTTTAAC
CATGCACCAGAAACATCCTCCTGCTAATGTTGCATATTCTTTTGTCAT

> **degV**

Function: protein coding sequence; DegV domain-containing protein (core genome, variable)
Best match: degV_CC001_MW2-USA400_BA000033.21434606:1435445:rRC
Position: 035-contig_268_RC: 3612 … 4452; Length: 840 bp
Sequence:
TTACTTCTTAAGGACTACTAGGCCAATCGCACCTTGACCAGTATGTGCAGAAATAACTGG
TGTAGTTACATTTATATCGTAATTATTCACATGAAAAGCTTCATTAAAAACTTTCTTCAAT
TTATCAACATATTCAATGACGTTAGCATGTGCGACACCAACGGATTTGATTTCATGATCTC
CTATAAATTCAGCAATTTCCTTTTTCAAGTATTGGATACTAGAATTTTGAGTTCTCGCATT
GTGCACAAGCTCTAAGCGACCATCATCTAGTGTACCAATTGGTTTAATTTTCATAAGATTA
CCAATCAAACCTTTTGTTTTACTAATTCTGCCACCTTTAATTAATTGATTCAATTGCCCTAT
AACTACAAATAATTTAATGTTTTCTCTTAAATGATTTAACTTTTTAACTATTTCAGAAGTT
GAGACACCTTCTTTTACAAGCTCTACTAGGTGTTGTATTTGATACCCTAAACCAAAAGAA
ATAGATTTTGAATCAATAACAGTTACATTAGCATCTACCATTTGACTTGCTTGGTAAGCAG
TGTTATATGTACCACTTAATCCTGAAGAAAGATGAATACTTATGATTTCAGAGCCATCTTT
TCCTAGTTCTTCATAAGCAGATATAAATTCACCTATGGCTGGCTGACTTGTCTTTACATCT
TCATCATTTTCAATATGATTAATAAATTCTTCTGATGTAATATCTACTTGGTCAACGTATG

AAGCTCCTTCAATAGTTAAACTTAAAGGAATTACATGAATGTTGTTTGCTTCTAAGTATTC
TTTAGATAAATCGGATGTTGAGTCTGTTACTATAATCTGTTTTGTCAT

## > folA

Function: protein coding sequence; chromosomal dihydrofolate reductase (core genome, constant)
Best match: folA_CC008-ST72_21259_AFTS01000032.1[7714:8193]RC (completely identical)
Position: 035-contig_268_RC: 4466 … 4946; Length: 480 bp
Sequence:
TTATTTTTTACGAATTAAATGTAGAAAGGTATGTGGAATTGTATTTTTCTCATCTAGTTTA
CCTTCAACTGAAGAGGCAATTTCCCAGTCTTCAAATGTATAAGGTGGAAAGAACGTATCA
CCACGGAATTTACCTTCAATAACAGTAATATACATGTCGTCCACTTTATCAATCATTTCTT
CAAATAATGTTTGCCCTCCAAATATGAAAACATGGCCCGGTAGTTGGTAAATATCTTCAA
TAGAGTGAATTACATCAACGCCCTCTACGTTGAAACTTGTATCTGAAGTAAGTACAACAT
TTCGACGATTCGGTAGTGGTTTACCAATCGATTCAAATGTCTTACGACCCATTACTAAAGT
ATGACCTGTTGATAATTTTTTAACATGCTTCAAATCATTTGGTAGGTGCCAAGGTAATTGA
TTTTCAAAACCAATTACTCGTTGCAAGTCATGTGCAACTAGAATGGATAAAGTCAT

## > thyA-chr

Function: protein coding sequence; thymidylate synthase (core genome, constant)
Best match: thyA-chr_CC005_Mu50-VRSA_BA000017.4[1508996:1509952:r]RC
Position: 035-contig_268_RC: 5145 … 6102; Length: 957 bp
Sequence:
CTACACTGCTATTGGAGCTTTTATTGCTGGATGTGATTCATAGCCAACAATTTCCAAATCT
TCATAATTTATGTCGAAAATAGACTTGTCACTGTTAATTTTTAATGTTGGAGGATTGAAAC
TTTCACGTGCTAATTGTGTTTGAATCGCATCAATATGATTTGAATAAATATGTGCATCTCC
AAATGTATGCACAAATTCTCCTACTTCAAGTCCACATTCTTTGGCAATAAGGTGTGTCAAT
AAAGCGTAGCTTGCGATATTAAATGGCACACCTAAAAAGATATCTGCGCTACGTTGGTAT
AACTGGCAACTTAACTTACCATCTTGGACATAAAACTGGAACATGGTATGACAAGGCGGA
AGTGCCATTGTATCAATTTCTGTTGGATTCCATGCAGATACGATGTGTCGTCTTGAATCTG
GATTATGCTTAATTTGTTCAATTACTGTTTTAAGTTGATCAAAATGATTACCATCTTTATCA
ACCCAATCTCGCCATTGTTTACCATAAACATTTCCTAAATCACCGAATTGCTTCGCAAAAG
TATCATCTTCAAGAATACGTTGCTTAAATTGTTTCATTTGTTCTTTATATTGTTCGTTAAAT
TCAGGATCACTCAATGCACGATGCCCGAAATCTGTCATATCTGGACCTTTATACTCGTCTG
ATTTGATATAATTTTCAAAAGCCCATTCGTTCCATATATTATTATTATATTTTAATAAGTAT
TGGATGTTTGTATCTCCTTTAATGAACCATAATAATTCGGTTGCTACTAATTTAAAAGAAA
CTTTCTTTGTCGTTAATAGTGGAAATCCTTTAGATAAGTCAAAGCGAAGTTGATGACCAA
ATTTTGAAATCGTACCTGTATTTGTGCGATCATTTCGTGTATTTCCTATTTCTAAAACTTCT
TCACAAAGACTGTGATATGCTGCATCAAATGAATTCAACAT

## > cvfC3

Function: protein coding sequence; conserved virulence factor C operon/protein 3 (core genome, variable)
Best match: cvfC3_CC001-ST772_118_AJGE01000010.1[1505:2629]RC (completely identical)
Position: 035-contig_268_RC: 6978 … 8103; Length: 1125 bp
Sequence:
TTAAATTGTTCTGTTTGCCATTTGTTTCCATACTGAACCAAGTGCCTCATCTCCGTTTTCAA
TACGAGATATGGCCATTTCAATTTGTAATTTAACTTCAAACGCATTGTCATTAATATGGGC
TTTTAGTGCGGGAAGCTGCTCTGCATTACCTTCATCAAAGATAAACATAGCAGCACGCCA
CCTAACGATTTTCTGTGGATCATCTAATAGTAGCACCATTTCTGGTAGTGCCTCTGGATAC
CCTAAATCGCTTATGCAATCCCCTGCTGTTCTTCTTACAGCAGGACTTTTATCACGAAGCC
CCTTATATAAATACGGTAAAATTTCTTTACTTTCAATCATACCTAATAATACAATCGCTTG
ACGTCTAACCGGTACTTTTTCATCAGATAAAGCTAAATCAAGCAGCGGTATATCTTCAAA
AGTCGGCTTTGGAAAATGGTTTAACATTCGTAATCGAGTCTTCCAATTATCAGTCGCATGA
TATTCATCCAAAGAGACATGTCGATAGAAATGATAATTATTAGTTACGTGATTTTCTTCTA
AAGCATGCTTTACCAATACAGGTAACTGTGATTCTGGATAGGTAGCTAGCACTTCTTCTA

GGACACCATCCATTACTTCTTCAATATTTCCATAGCGATTTCCTAAATCTAGCCATTTACG
CATAAAAACAATATTGTCATGTTCTGTTTGCGCTTGAGTCATATAATCAACATATGTTTGT
GGTAATTGTTCTCTTAATTCTTGGTCAGCAGAAGTTAGCTTAATTTGATACGGTATACCCT
TAAAGGTTAATAGTTCAGCTTTAATTTCACCAAAATGATTGTCAATTTGAGCTTCATTTAC
AGATTCTAAAACCTGATTCCCATCAGAAAAAACAGCTTTAATATCAGGTAATATGACTTC
CCAATCAGCTTTTGGCGCCTTATCAACAGCTAAGAAGTTCATGACATGAAAAATAGAAGT
GATACCGTCTATAGATAACAACTGATTTATAAATCTTGGTTGTGTTTCTTCTACTTTTTTAT
AAGTATTAGATAACTTGTCTTTTCTTGTAAATGACAAAACAACTTTCATTGTATTTGGACT
TGGCGTTGGCTCTATACGTAAAATTTCCAT

## > cvfC4

Function: protein coding sequence; conserved virulence factor C operon/protein 4 (core genome, constant)
Best match: cvfC4_CC001_MW2-USA400_BA000033.2[1437519:1437956:r]RC (completely identical)
Position: 035-contig_268_RC: 6525 … 6963; Length: 438 bp
Sequence:
CTATTTACAATTTTCGTCAAAGGCATCCTTTAAGTCCATTGCAATGTCATTAATATCTCTA
CCTTCGATAAATTCTCTAGGCATAAAATAAACTAAATCTTGACCTTTGAATAAAGCATAC
GAAGGACTAGATGGTGCTTGCTGAATGAATTCTCGCATTGTAGCAGTTGCTTCTTTATCTT
GCCCAGCAAAAACTGTAACTGTATTTGTAGGTCTATGTTCATTTTGTGTTGCAACTGCTAC
TGCAGCTGGTCTTGCTAATCCAGCTGCACAGCCGCATGTAGAGTTAATAACTACAAAAGT
AGTGTCATCAGCATTTACTTGGTTCATATACTCCGATACTGCTTCGCTCGTTTCTAAACTT
GTAAAACCATTTTGAGTTAATTCGCCACGCATTTGTTGCGCAATTTCTTTCATATAAGCAT
CATATGCATTCAT

## > cvfC2

Function: protein coding sequence; conserved virulence factor C operon/protein 2 (core genome, variable)
Best match: cvfC2_CC705_RF122-cow_AJ938182.1[1401081:1401332:r]RC (completely identical)
Position: 035-contig_268_RC: 8140 … 8392; Length: 252 bp
Sequence:
TTATTCGAATACAGCCTCTACTTTTGGCAATACTGTTTCCCAATTTGCGTCATTTTCTTTAT
CTACTGAAATAAAGTCCATAACATGGAAAATTGATTTAACACCTTCAACCTTTAAGATGT
CATTAATAAATGCTGGCTGTGAATCATCAACTTTAGTATACGTATCTGATGTCATACCTTC
TCTGCTTTCACTAAGTGTAATCTTCATTGTGTTGTGGTTCGGTGTTTCTGATATAGATATAA
TTTGCAT

## > cvfC1

Function: protein coding sequence; conserved virulence factor C operon/protein 1 (core genome, constant)
Best match: cvfC1_CC001_MW2-USA400_BA000033.2[1439397:1439591:r]RC
Position: 035-contig_268_RC: 8403 … 8598; Length: 195 bp
Sequence:
CTATTGAAGTTCATTTCCTAATTGTTTTATTTCTTTCCCTATTGAACACTCATTGATACAAA
AATGATGGGCCTTCGTTTTCCCCTCTAATTTACGCAGACGAGTTTTGATTGGACATTGATT
GCAATAAGTATTCATTAAATCATCTATTTTCGCGAGTGCATTTTGCTCTGAAAAAGTTAAA
ATACGCGACAC

## > Q5HFZ1

Function: protein coding sequence; putative membrane protein (core genome, constant)
Best match: Q5HFZ1_CC005_Mu50-VRSA_BA000017.4[1512693:1513397]
Position: 035-contig_268_RC: 8842 … 9547; Length: 705 bp
Sequence:

ATGTATAATGAAATTTTAGGACTAGTTACGTTTATTGCAACGTTCGTACTTATGGTACTTA
TGTATCGCTTTTTTGGTAAACAAGGTTTAATTGCATGGGTTGCAATTGGCACAATCATTGC
CAACATACAAGTGATTAAAACTGTAGAAATCTTTGGTATTTCAGCAACTTTAGGTAATGT
CATGTTTGCTTCTATTTATTTAGCAACAGATATTTTAAATGATATTTATGGGCGTAGAGTT
GCAAAAAGAGCAGTTTGGTTAGGCTTTTCATCAACATTAATTATGATTATTGTTATGCAAT
TGTCATTACATTTTATTCCTGCACCAGAAGATATGGCACAAAAAGCATTACACGCAATCT
TTGATGTTGTGCCACGTATTGCTTTAGGCTCAATCGTCGCATATATTATTGGTCAACATAT
TGATGTATTTATCTTTTCACTAATTAAAAAAGTATTTAGTTCTGATAAAACGTTTTTCATCC
GTGCATATGGTAGTACATTTTTAAGCTCAATAATTGATACAGCTTTATTTGTAGCTATCGC
TTTTATCGGAAGTTTACCTGGTACAGTTGTATTTGAAATATTTATTACAACTTACGTATTA
AAATTAGCTTCAACAGTTTTCAATGTACCATTTGGATATATTGCTAAATCATTTTATCGTA
AAGGTAAGATTGATAAGTTAGATCAAGGCTATTAA

> **STAR**
Function:  Staphylococcus aureus repeat element
Best match: STAR_CC005_CF-Marseille_CABA01000081.1[16587:16794]
Position: 035-contig_268_RC: 9637 … 9667; Length: 30 bp
Sequence:
CATTGTCTGTAGAAATTGGGAATCCAATTT

> **tx_universal2**
Function:  rho indendent terminator
Best match: tx_universal2_CC001_MW2-USA400_BA000033.2[1440798:1440838:r]RC
Position: 036-contig_189: 2 … 32; Length: 30 bp
Sequence:
AAAGCTTGTTACAAGCGCATTTTTGTTCAG

> **rnhA**
Function: protein coding sequence; ribonuclease HI-like protein (core genome, variable)
Best match: rnhA_CC008_NCTC8325_CP000253.1[1375631:1376032] (completely identical)
Position: 036-contig_189: 273 … 675; Length: 402 bp
Sequence:
ATGGCGAAAATAAATTTTGATGCTGCGACGAAAGGAAATCCAGGCATAAGTACATGTGC
CATTGTAATCAAAGAAGATGAGCAGCATTATACATATACACATGAGTTAGGCGAAATGG
ATAACCACACTGCAGAATGGGCTGCATGTATTTATGCACTAGAACATGCACGTGAATTAA
ATGTTCAAAACGCACTATTATATACAGACTCAAAGCTAATTGCAGATAGCATTGAAGCTG
GTTATGTGAAAAACGCAAATTTCAAACCTTATTTTGATCAAATAGAAATATTTGAAAAAG
ATTTTGATTTATTATTTGTTAAATGGATACCGAGAGAACAAAACAAAGAAGCGAATCAAC
ACGCTCAACAGGCATTGTATAAATTAATTAAAAAGAATAAATAA

> **tx_rnhA**
Function:  rho indendent terminator
Best match: tx_rnhA_ebh_CC001_MW2-USA400_BA000033.2[1441469:1441533] (completely identical)
Position: 036-contig_189: 662 … 727; Length: 65 bp
Sequence:
AAAGAATAAATAAAAGGAGCACCGACTGATGTGATGTCAATCGGTGCTCCTCTATTTATC
AAAGA

> **ebh**
Function: protein coding sequence; (genomic island)
Best match: ebh-Mu50_CC005_04-02981_CP001844.2[1472718:1504353:r]RC
Position: 036-contig_189: 733 … 32377; Length: 31644 bp
Sequence:

TTATTTTTTATTTTTCTTAGATTTCTTTTTCTTCTTTTTAGCTGCTACCTTTTTAGAAGTATTT
TTTGATGCTGACTTTTTGTCTTTGGATTGGTTATCTTTTTGATTTTTCTTTTTAGCCAATAAT
AAAGGAACGTCCTCATCTTTAGATTCAATACTAGTTGTTGTTTCAACATCTTCCTCTTTATC
TTTGCGTCGTTTTGCGAATAAGAGTGGTGAATGTTCGTTATCTTTCAACACTTTTTCATCTG
TGTTTTCATTTGTAACTTCCACATCTTCTTCATCTTCTTTACGACGACGTTTTGGTAAGAAG
AACGGCGTATGTTTAACTTTATCGAGTGACTCGCCATTATTTAGCGAATCTTTTTCTTCAA
CAGTAACATCTTCTTCATCTTCTTTTCTGCGACGTTTCGCAAATAAAAGTGGTAAATGTTT
TGTATCGTCTAAAGTCTCTTTTATTGAATCTTTATTATTATCTCTTATTTCTAATTCTTCCTC
TTCATCTTCTTTACGACGACGTTTGGCAATGAAGAACCAGAAACTAGCTAGTAAGCCAGA
GATACCCACCACACCAATAGCGTTTTTAATAACATTACCGAAATTATTACTAAAGTGACG
CATATGAAGTGGATCAATGTCATCATCTTCATCAATTTTCTTTTTATGTCCAATAGTCGAA
CTGTTAAATGGATGATTTGCAGTTCCATAACCAATTGTTAAATGGCTATTAGATTCATTTC
CAGTACTTGACGATTGTTTAGCACGATCAGATATTACAATTTGTACTGCTGAAATTCGAGC
AATACCATTGTTCAATGCAGCCTCAACTTGCTGTAATGTATGCGCATTATTAATATCTCTA
ATTGTTTCAAGCACAATTTCGTTAATTTGATTCATTGCTGCTTGTTTTTCATCAGTTGTGCC
AATTTCACTATTTCGAATACTATTTATTTTTTCATTTGAAAAGTCTTTAATTCTACTAATTG
CTTCTTGCTTGCGTTTAGCTAGTTCTTCTGCTGTTGGATTAGCTGCTTTCATTTGAGCTTTA
AATTGTTCCAATTGCTCAGTTATTTCATCGATGCTTTGCGCACGTTGAATCGCTTGAATTG
CTTGTTCTTTTAATTGATTTAACTTAGCAATAGCTTCTTGCTTCTCTTTATCTGTTAGATCA
GTACGAGCTTTGATTTCATCAATCATATGTTGAATTGCATCTTCAATCAATTTAATTGCAT
TTGATTTTGCTTGTTCAATCGTAAATTGTTCTGGATTAAATTGTTCAATATGCGCTTGTTCT
TGTTGTTGAATTTCTTCAACTGAATGAACATCAGGTGCATTGTTAATATGATCAATCGCTT
GTTGTTTAAGCTTATTAACCTCTGCAATTGCAGCTTCTTTTTGTTCCAGTGTTAATGTCACA
CTATTATTGATTTCATTAATCTTTTGTTGTGCTGCATTTTCGATTGATTCAATTGCCTGTTG
TTTGACTACTGACAATTCTTTTTCTACAACTTTTACAGGAACATTAACAATCACTTTTGAT
CCATCAAGCAATGTAACTTCAACTTTTGCTGTTAAGCTATCAGAAATCGTTGCAGTTGATG
GTGTATCAATAACTTCTGCTGAAAGCTGATCAATTAAGTTTATGTGTGCAAGAATATCTTG
TTCTGTAATGATGTCATCACGATGTACAATGAGTTCACCATTAACTAGGATTTGCTCAGGT
GTTGCTTCAAAAATTTCATTTACAGCAGGTTGTTCATCAACCTCCCATACATGTGTATTTC
TAATGTCATCTAAACCTAAGTTTAATCCTCGATTTAATTGATCTATAGTTTGAGCATTCTC
AACGTTTTGTAGTGCTCGTTTTTCAGCTTCGTCAATTTCTTTGAGCGCTTTTGCTTTTTGCT
CAGGTGTTAAATCTGGATTTGAATTGATTTGATCACGCTTCGCATTAGCTAAGGCTTTTAT
TGCATTTTTCGCATCTTCTTTAGCTTTCACTAAATCTTTGATGTCTTGCAATGCTTGTGCAA
GTTGTGCTTTGGCTTGTTCAATTTCTTCTTTAGTCATCGCATTGTTAATGCCGTTATGACCT
TGTTGAAGTATTTGATTAATTCGATCTTTAAGTGCTTGTTTTTCCTTATCTGTTAGATTTGG
ATTTTGATCGATTTCGTCAATTAAAGCTTGTACACGTTTATCAATATCTTGTTTCGCATTTT
CTTTAGCTTTCACTAAATCTTTGATGTCTTGTAATGCTTGTGCAAGTTGTGCTTTGGCTTGT
TCAATTTCTTCTTTAGTCATCGCATTGTTAATGTCGTTATGACCTTGTTGAAGTATTTGGTT
AATTCTATCTTTAAGCGCTTGTTTTTCCTTATCTGTTAGATTTGGATTTCGATCAATTTCGT
CAATTAATGCTTGAACTTGCTTGTCAACATCTTTATTCGCATCAATTTTAGCTTTCGGTATT
TCATTGGCATGCACTTGTTCAATCGCATGGTTGCCAGCTGTTTGAACTTGAGATACATCCT
GATTACTTGTTGCTTTATTAATGTTGTTGATGATGCTGTTTGCCAATTCTTCTGCTTTATTT
TTCGCAATAAGCTTGTCTTGATCCGTCGCATTTGAAGCTTCGATTTCTTTTAGCTTATTAGC
TAAAGCTTGATTAATAGATTGAATTGCCTTTTCTTTAGCATCTTGTAGTCGTTGATCACCA
TTAAGATTATGGATTGCATCATTGACTGCTTGGATTGCGCCATTGATATCATTCACATTTG
TGTTATCACTATTTAGCAATGTATTTGCTAGACGTTTGGCATCATCGAAGTTTGTTTTAGC
ATTATCGTCAGCGTTTTGGTAATTGACAGTTTGCTCTGCATTTGGAATTTCATTGTCAACT
AAATGTTTCAATGTTTCCATTGCATCATTTAAGTCAATTTGATTATTAACAATATCTGTTA
CATCTGATACAGTATCGGCATTGTTAATTGCTTTATGTGCAAGATCTTGTTGCTGTTGATT
TAATCCATTTAACGAATTAACAAACGCATTTGCTTTATCTTTGGCATTTGCAAGGTTTTGG
TCTCCATTTAATGCATTTTGAGCATCGATAATATTTTGTTTCAATTGCTCTGCTTCAGCTTT
TGCAATTGCATTACCTTGCACTTTATCTAACTCATGTGCTGCATTCGCAATTGCATTATCA
TAATTTGCTTTCAAATTGTCATCTGCATTGATGTAATTTGTGCTGCTGATAACACCAAGGT
GCTGTTCGTCAATATATTGTTGTACTGCACCCATTGCATTATCTAATTTAGTTGCTCTATTA
ATTGCTCGAGATGCTTTATTTAGCGTTTCAGCATTATTAATTTGTTGGATTGCTAATTGTCT
TTGAGCATTGTTTAGATGTGTTAATTGATCCAATCTTTGTAATGCTTCAGCTTTACGATTAT

TAAGTCTTTCTTCACCATTTAATGCTTTCTTAGCTGCAGTGACTGCATCATTTAATTTGATA
ACTTGTTCTGCAGTTAAGTTTTGCCCAGACGATTTATCAAGTGCTTGTTTCGCTTTATCAA
CCGCTTCATCATATACTTGTTTGACTTGTGTATCTGCATTGACATAGTTCGTGCTACCTTTC
GTGCGTCCTTCATTGCCAGTGATTTCTTGTGACAATTGATCCATTGCACGATTAAGTGCCT
TAGCCTCATCTACAATTTGTTGGATTTGATTTAAATCGTTTGATTGATCGATGCGACCTTT
AAATCCATCTTGTTGAGCATTGTTTAATGCATTCAATTGGTCGATGTCATGTTTACCATTT
GTTTTAGCAAGTGCTACACGTTCATCACCATTTAATGCTTGTTTTGCATTTAAAATATTTTG
AAGTGCTTGATCCACTTGTTGCTTATTCGCATTTTGTTTCAATACATTTTCAGCATCAGCA
ATCGCTTGTTTATAAGCCTTTTGCTTATCTGAATCTGCTTGTGTGTAATCTATAGTTTGCTC
AACGTTAGCATGTTCATTAACTGCTTGTTGTAATTGATCCATTGATTGGTTCAATTGCGTT
GCTTGATCTACTAATTCAGCGATTGGTTGAAGTTTCGTCGCTTGATCAATATTTTGTTTAG
CAGTTGCAATTTGATCAGCATTTAAATGTGTTAATTGGTCAATAGTTTGTTTCGCTTGTGT
TTTAGCTTCAGCGACTCTCTCATTACCATTTAACTCATTCACTTTTTCTTGAAGCTTAGTTA
ATGCTTGTTCTACAGCGTCTTTATTCGCATTTGAACCATTAGTTGGATCTGTAATGCTTTGT
GCAGCTTGTAACGCTTGATCTACTGCTTCTTTTTTATCAGTTGACGCTTCAGTGTAATTTGG
TTGAGCCTTATCTGTATTCACTTGATCAACTTTATTTTTCAATGTTTCCATCGCGTGATCAA
GTTCAGTAGCAGTTTGAACATGTTGTGCAACCTCTGTTCTTGTAGGCGCTGCATTTATAGC
ATCAGTTAATGTTTGTTGTTGTGCATGATTTAAGTTTGGCAATGCATTTACAGTTGTTACT
GCTTGTTGTTGATCACGAGCAAGTTTTTGATCACCATGTAGATTATCTTTTGCAGTTGTTA
CTGCTTGTGTTAATTGTTCTACTTGTGATTTGTCGAGTGTTGGATTACCTGTTTGGTTAATT
AAATCTTTTGCATTTTGAACTGCTGCTTGGTAAGCATCTTTTTGCGGTTTATCTTCATTGAT
AAACTTGCTACCCGCTTCCGTTTGTTGTTGATCTTGAATACTATTTCGTAATGCTTGCATTG
CTTGATCAAGCGCTTTTGCTTCAGCTAATTTTTGCGCTACTTCGCCACGAGTTGCTGCGTT
GTTTATTTGGCTTTCAAGTGCTTGACGTTGCGGATTATTCAAACCATTTAATTGATTTAAA
TCAGTAACCGCATGTTGTTTATCGTCTGCAAGTTTTTGATCACCGTGTAGGTTATCTTTAG
CTTGTTTAAAAGCATGTGTCAATTGTTCAACTTGTGCTTTATCAAGCGTTGGATTACCTGT
TTGGTTAATTAAATCTTTTGCATTTTGAACTGCTGCTTGGTAAGCATCTTTTTGCGGTTTAT
CTTCATTAATAAACTTGCTACCAGATTCTGTTTGTTGTTGATCTTGAATGCTATTACGTAA
AGCTTCCATTGCTTGGTTAAGTGCTTGTGCTTCAGTTAATTTTTGTGCTACTTCACCACGA
GTTGCTGCATTATTGATTTGATTTTCAAGTGCTTGACGTTGTGGTGTATTCAAGTTAGACA
AGTTATTTAACGTTTCTGTTGCACGTTGCTTATCTTGAGCTAGTTTTTGATCACCATGTAAA
TTGTTTTTAGCATCAGTCACTGCCTGTGTCGCTTGATCAATGATTGATTTAGCTAATGTAG
GATCAGTTGTTTTGTTAATCAAATCTTTCGCGTGTTGTACTGCTTGCGTATATGCATCTTTT
TGCGCTTGATCCTCGTTAATAAATTTACTACTTGCTTCAGTTTGTGGTTGATCCTTAATACT
TTCTTTTAATGCTTTCATCGCTTCATTTAATGCTTGCGCTTGTGCAATGATTTCAGCCACTT
TATCACGAGTTGTTGCATTATTAATTTGATTTTCTAGCGCTTGTTGTTGAGCTGGTGTTAAT
TGATCTAAATGATTTAGAGAATTTCCAGCAGTTTGCTTATCTTGAGCTAATCGTTCAACAC
CATCTAATGCATTTTTAGATGATATTACTGCTTGAGTCGCACTTGATACATTACCTTTATT
GATAGTTGGATTATTTAATCCTGCAATGATAGACTCAGCATTTTGAACTGCTTCATCATAG
GCTTGTTTTTTATTCGGTTCTGCATTGACATATGCACTGCTCGCACGTGTTGCATCTTTGTC
AGCAATACTTTGTTGTAATGCATCCATAAGTTGATCTAATGCTTGTACTTCAGTCAAATCT
TGCTTAACTGCTGTTCTAGTTGTTTCACTATCAATTAACGTATCTTCCATATGTTTTTGTGC
ATTGTTTAGATGTGCTAATTGGCTAACCGTTTGTTTAGCATGATCTTTATCATTTTGTAATT
TCACATCACCATGTAATGCTGCTTTCGTTGTATTCACAGTTGCCGCTACTTGATTAATCGC
ATTATTATCTAATGTTGCAGTTTGTTCATTGATAATATTATTTGCGGCTTGAACAGCTTGA
TCATAGTTTTGTTGCTCTGGTTGATCTTCGTTGATATATTTGCTATTTGCTTTAGTTGCTGC
GTTATCTTGAATGCTCTGTCTTAAACCATGCATTGCTTGGTTAAGCGTATTCGCATTTTGTT
CAATTTGATGAACTGCAGTTACTAAAGTTGCAGCTGTAACTTGGTCTTTTAATGCTGTTTT
TTGAGGATCATTTAAGCTTGTTAAAGCATCAATTGCTTGCGTCGCATTTGTTTTAGCAACT
TGTAAATTATGATCTCCATTTAACGCTTGAAGTGTTGTTTGAACTTTAGTAATAGCTTGTT
CTACTTCTGTTTGGTTCGCATTTTGACCAGTTTGTTTGTTAATCATTGCTGCTGCTTCATTT
ACCGCTGTATTATAAGCAGTTTGTTTATCAGTATCAGCATTGATGAAGTTACCACGTTGTT
CAACGGCTTCATGATCTGCAATCGCATTAATTAAGTTACCCATCGCAGTATTTAAAGATTC
TGCTGTTTGTTTGTGACCATTAACTGCAGCAATATCAGATGATTGCGTAATTTGTGATTGT
AACTGTTGCTTTTGCGCATTATTCAAATGATCTAATTGATCAAGTCTGTTGTTTGCTTGTTG
TTTAGCTGCTGCAAGGTTTTCATCACCATCTAATGCAACTTTCGTATTATTCACTTGTGTCG

TTGCACCATTAACATCATCTGGATTCATCGTAGGCGATGTAGTTTGATTAATGATATTATT
AGCAGCTGTTACAGCTTTGTTATAGTCATTTTGTCTTTGCGCAGTTGCATCATGATAATCT
TCAGACGCTAATGTCGTTTGATTATCCGCAACACTATTTCTTAATAATTCCATCGCATGAT
CTAACGTTTGTGCTTTTTGATTAATCGTAGTGACATCTGTAACAAGTGGCGCTTGTGTAAT
TTGACTTTCAAATGATTGTTTTTGTGCATCATTTAAGTGTGTTAAGTTCGCCAATTGTTGTT
TTGCTTGTTGTTTTGCATTTGCCAAATTGTCATTACCATTTAATGCTTGTTTCGCTGCGTTC
ACTTCATTAGTGATGCTTTCAACTTGTGCTTGTACTTCATTAGTACCACTTTGTTTATTCAA
TAATGCTTCAGCTTTTGCTACTGCCTGTTTATACGCATTTTGTTTGTCAGGATCTGCATTTA
AGTAATTACCGTCCGCTAATATTTGATCTTTATCATTAATAGCTTGTGATAATTGATCCAT
TGCTTGATCTAGGCTCGTTGCTGTTTGTTTAACTTGGTTCACTGTAGCAATTTCAGGCGAT
TGATCGATTTGACCTTTTAATGCTTGTTTTTGAGCATCGTTTAAATGCGTCATTCCACTTAC
GGCATCTTTAGCATTTTGCTTTTCAGTGGCTAATTTAGCTTCACCGTTTAAGCTATTTTTAG
CATCAGTCACTTGAGTTAATGCACGAGTAATGTCATCTGGGTTAAGCGTTGGATTTGTCGT
TTGATTGATAATACCTTCCGCTTGAGACACTGCATTTGTATATGCTGTTTGCTTATCGACA
TCAGCATCGTGGTAGTTCTCACTTGCTTTCACAGTATCTTTATTTGCAATACCTTGTTTCAA
GTTACCCATTGCTGTATTCACATTTTGTGCATTTTGTTTAGTTTGTTCAACTGTAGCTAAAG
CTTGTGCTTGATTGATTTGGTTTCGTAATGCATCACGTTGTGGTTGATTTAAGTCACGTAA
CGTATCAAGATTTGCTAAAGCATCTTGTTTCGCTTGCGCTAATTTTTCATCACCGTTTAAT
GCATCTTTCGCTTGATTCATAGTTGTAACTGCTTTTGTTACTGTATCAGGCGCCAATACAG
GTGTTGGTGTGCCATTTGCGATTTGTTGCGCTTGGTTAGCTGCATTGTTATAAGCTTGTTGT
TTGTCTTGATCCGCTTGTGTAAAGTCAACTGATTGTGGTACTTGACTATTCGCTTGAATTT
GTTGTTTCAACGTACCCATTGCATTATTTAACGCATCAGCATTTTGCTTAATAGCATTAAC
ACCTGTAACAAGTTCTGCATGCGACACTTGGTCTTTTAATGCTGTTTTTTGTGGTTGATTTA
AGTTTGGTAACTGATCAATGGCTGTATTTGCATTGTCTTTAGCAACTTGTAAGTTATGGTT
ACCGTTTAAATCACCCTTAGCTTGATTCACTTGTTGTAACGCTTGAGCCACTTGTTGTGGA
TCCACGTTTGCATTTGGTGTACCACTAATGATTTGTTCAGCATGCGCTACCGCATTGTTAT
ATGCATTTTGCTTATCAGTATCAGCATTGACAAAGTTACCATTTGCTTTAGTTTGGTCTTTA
TCTGCAATACCTTGTTTCAATTGTGTCATTGCTTGATTTAAATCTTGTGCTGTTTGCTTAAT
CGTGTTTACATTTGCAACAGTTTGCGCAGCATCGACTTGTTGTTTTAATGCATCTTTTTGCG
CTTGATTTAAATCATTTGCATTTGTAATTACTTGTTTCGCTGCATCTTTTGCACGTTGTACA
TTGTCATTACCATTCAATGCTTGTTTCGCTTCGTTCACACGTTGCATCGCTTGTTCCACTTC
AGTTTGACTTGCATTGCTGCCATTTGCTTTAGATAAAATACCTTCTGCATGTGAAACCGCA
TTAGTATAATCATCTTTCTTAGCTTGATCTGCATCAGTAAAGTTAATGCTATTTAATGTCTC
AGTTTTATCTTGTAATGCATTGTTTAAGTTAGTCATAGCATTATTCAGATTTTGAGCATTTT
GCTTAACATTATTAACTGTTGCAATATCTGGTGCTTGTTCAACTTGAGTTGTTAGCGCTTG
TTTTTGAGCATCATTTAAATGATCTAGTGTACCAAGTGTTGTCTTAGCATCTTGTTTGGCA
TCAGCTAATTTTTGATTACCGTTAAGTGCTTGATTTGTGTCATTCACTTTAGTTAATGCTTG
TTGAATTGTATTTGGATCCATTGTTGGATTTGTTGTTTGTTTCAACAATTCTTCTGCTTTTG
TTACTGCATTATCATATGCTGTTTGCTTGTCAGTATCAGCATCGTGATAGTTCTCGCTACCT
TTAATTTGTGCTTTATTCGCAATACCTTGTTTCAATTGTGTCATCGCGTCATTAAGCGTTGT
TGCCGCTTGTTGAATAGCATTGACGTTTGGTACAAGTGTTGCTTGCGTGATTTGTTTGCTG
TATTCATCACGTTGCGCTTGGTTTAAGTTTGGCAATTGATCAATAGCATGTTGAACATTTT
GTTTCGCCGTTGCTAAGTTTGTATTACCATTTAAATCATTTTTAGCTTGCGTAACTTTATTT
AACGCTGCAGTAATTTCGCTAGGTGTAACGACAACATCAGGCGTACCACTAATTAATGCT
TCAGCTTTCGCTACTGCTTGATTATATGCATTTTGCTTGTCAGAATCTGCATTGACAAAGT
TACCATCAGCTTTTGTTTGTTCTTTATCTGCAATGCCTTGTTTTAATTGTGTCATCGCATTG
TTTAACTCTTGCGCCGTTTGTTTAACATTGTTTACACCAGCTACAGTAGTTGCATTTTGTAC
TTGTTGTTTTAATGCATCTTTCTGTGCTTGGTTAAGATCATTAGAGTTATTAATTAATGCTG
TTGCTTCGTCTTTTGCATGTTGAACGTTGGCATTACCATTTAATGCTTGTTTTGCTGCATTT
ACTTGTTGGATTGCTTGTTCAACTTCAGTTTGTGTTGCATTACCACCATTAGCTTTTGAAAT
GATATTTTCAGCATTTGTAACTGCAGTGTTATATGCTTGTTGTTTTGCTTGATCTGCATCAG
TGAAGTTAACTGTTGCTTTCGTAGCATCTTTATCAGCTATCGCTTGTTTCAAGTTACCCATC
GCAGTATTTAAGTTTTGCGCATTTTGCTTAATTTGATTTGCTTCATCAACAGTATGCGCGC
CATTAATTTGATTAGTAACAGCTTGTTTTTGCGCATTATTTAAGTTGTCTAATGAACCTAA
AGATTGCGTTGCTTGTTGCTTCGCTGCCTCTAAGTTTCATTACCATTTAAAGCATTTTTAG
ACGTGCTTACTTGTCCAGCAGCTTGATTGATAACAGTCGGATCTAATGAAGGGTTTGTAG

TTTGATCAATAATACCTTGTGCAGTTGTGACAGCATTATTGTACGCATCCTTTTTATTCGG
ACTTGCATCAGTATAATTTTGGTTTTGTTTTGTTGTCGCATTATCTGCAATACTTTGACGTA
ATTTGTCCATTGCTGCATCAACATTGTTTGCTTTTTGTTCATTACCTTGTGCTTCTGCAACA
GTAGTCGATTGTTGTACCAATTGTTTTAATGCCTCTTTTTGTGCATTTGTTAAATGGCTTAA
ACCGTCAATTGCTGTATTTGCGTGTTGTTTCGCTTTTTCAAGGTTTTGAGTACCATTAAGC
GCTGCTTTTGTAGTATTCACTTGATTCATAGCTGCTTCAACTTGATCTTTAGGCACGTTCGT
ACCTGTAGATTTATTTAAAATATTTTCAGCATTACGAACCGCTTCATTGTATGCATTTTTCT
TCTCTGGATCTGCATCTGCAAAGTTTTGACTGCCAAGTGTAGTGTCTTTATCATTCAAGCT
ATTTTTCAAGTTACCCATCGCTGTATTCAGTTCCTGAGCGGTTTGAATGGCTTGCGTTACT
TCAGCTACAGTGTGCGCTTGATTGATGTTTCCAGTAACAGCACCTTTTTGTGCATTGTTTA
AGTTATCTAACGTAGATAATGTTTGGTTTGCATTGTTTTTAGCATCTGTTACTTTTCTATCA
CCATTCAACGCTTGTTTAGTAGTTGTTACAGTTGAAAGTGCTGCTTCAACTTGTGATTGGT
TCGCATTTGTACCATTTGCTTGATTAATAATATTTTCAGCAGCAGTTACCGCATTGTTGTA
TGCTGTTTGTTTTTCTGGATCTGCATCTGTAAAGTTAACACTTTGTTTCACTTGATCTTTGT
CTTGGATTCCGTTGATTAAACGTTCCATTGCTTGATCTAATTGTTCAGCTTTAGTTTTCACT
TGATTTACTGTGCTAATATTTGGTGCATTTGTCACTTGTGTATTTAAATCTTGCTTTTGAGC
ATTGTTTAAATGACTTAATCGGTTGATGTTAGCAGTTGTTTCTTGCTTTTTATTTGCTAAGT
TTTGCGCACCATTAAGCGCAGTTTTGTTAGTGTTCACTTGATTAGCTGCTTGTGTTACTGC
AGATGGATCAAGTGTCGGCTGACTCGTTGCATTAATGATATTTTCAGCACTTGTAACTGCT
GTATTGTATGCATTTTGTTTATCTGTATCTGCATCAACATATGGTTGACTCGCTTTTACTTC
ATCTTTATTCGCAATACTATTTCGAAGTTGATTCATTGCATTGTCAAGATTTGTTGCATTGT
CTTTCACACTTTGTACACCAGTTACTTGTGTCGCGCTATCAATTTGACCAGTAATACTTTG
TTTTTGTGCATCAGATAAATGTGTCATTTGACGTAATGCATCTTTAGCTTGTTGCTTCGCA
GTTGCTAAGTTTGTATCACCATTTAAACTTGTTTCAGCATTATTGACACGTTGTGTTGCCG
CAGTAACATCTGCTGCATTTGTAATCACATTAGGTGAACCATTTACGATATTCTTCGCAGC
ATTATATGCATCAGTGTAAGCACCTTGTTTATCAGGACTTGCGTTAGTGTAATTACCACCA
GCTACAATTGTGTCATGATCACCAATTGCTTGTTTTAATTGATCCATCGCATTATTAAGTG
ATGTAGCACTATTTTTCAGATCAGTTACACCTGATACGCGTTGTGCAGCATTCACTTGTTG
TTTCAATGCTGTTTTTTGCGGTGTATTCAATTGTGTCAAGTTATCGATAGCTTGTGTAGCTT
CAGTTTTAGCGCGTTGTAAGTTCGCGTCACCGTTCAAAGCATTTTCAGTAGACGTTACACT
TTGAATTGCTTGTTCAACAGCTTGCTTATCTTTATTCGTACCATGATCTTTATTTAAGTAAG
ATTCAGCTTGTGTGATGGCATTATCAAATGCTGTTTTCTTAGTTGGATCTGCGTCAATGTA
GTTTTCGCTCGCTTTAGTTTGATCTTTATTTGCGATTGCTGACTCTAATCGTTGCATTGCAC
CATCTAAGTCTTGTGCTTTCGTTTTAACACTATTTACACCGTTGACTGTTGTTGCACCTTCG
ATTTGTGATGTTAAATCTTGTTTTTGTGCATTATTTAAGTGTGTTAATGTACCTAAGTTTGC
TTTCGCATCTGATTTTGCTTGTCTTACATTTGCATCACCGTTTAAAGCATTCTTAGCAGTCG
TTACTTGATTTAATGCAGCTTCAACTTGTGCTTTTGTCATATTTTGACCGTTTGCTTTATCT
AAAATACCTTGAGCATTCGTAACAGCATTTGTGTAAGCATCTTTCTTACCTTGGTCAGCAT
CTGTAAAGTTCACTTGTTGTTTAACAGTGTTTTGGTCATTAATACCATTTTGAAGTTGACC
CATTGCTGTATTTAATTCATTAGCAGTATTTTGTGCAGCAGTTACGTCTGCTACTGTCGTT
GCATTGTCAATTTGACTAGTTGCTGCAGTTGATTGCGCGTTATTCAAGTTACTATATGAAG
CTAATGCACGTTTTGCTGTTTCTTTCGCTTGAGCAACTTTATTATCACCATTCAAGTCGTTT
TTAGCTTGATTCAATTGGTTAAGCGCTGCTTGAACTTGTGTTTCATTCGCGTTACTGCCATT
TGCATTAGTAATTGCTTCTGCTTGTGTAACTGCAGTATCATACGCTTGTTGTTTTGGTTGAT
CCGCATCTGTGTAATTGACACTACGTTTCGTATTATCCTTTTCAGCAAGTGCATGTTTCAA
ATTACCCATCGCTGTGTTTAACGATGTTGCTTTATTTTTAGCCGCTGTGACAGCTGCAAGA
TTTGGTGCTTGATCCACTTCAGCATTTGCCGCAGTTCGTTGTGCATTGTTCAAGTCTGTCA
AACGACCGATATCTGATTTCGCAGTTTGTTTTGCTGCTGCTAATTTTTCATCACCGTTCAAT
GCAGACTTCGCACTGTTCACTTGGCTCGCTTTTTGGTTAATTGTATCAGGATTCATTTCAG
GGTTATTCGTTGCACTAATAATACCTTCAGCATTAGTTACCGCATCATTGTATGCATTTTG
TAAATCTGCATTCGCGTCTTGATAATCTTCGCTTGATTTAGTCGCATCTTTAGAAGCAATA
CTTTGTCTTAATTGATTCATTGCTTGATTTAAAGTACCAGCATTTGCTTGGATGCCTTGAA
CACCAGCAACAGTTGTACCACGTTCAATTTGTTCTGTTAAGTTTGCTTTTTGAGCATTAGT
TAAATGTGACATTGTCGCTAATTGTTGTTTAGCTGTGTTCTTCGCTTCATTTAATCGCGCGT
CACCATTTAATGCTGTTTTAGCAGTATTCACACGTTGTAATGCTTGTTCAACGGCTACTTT
ATTGTCATTTGAACCAGCTGTTTTATCTAATAACGTTTTAGCTGCTGTTACAGCATCATTG

TATGCAGTTTGTTTATCTCTATCTGCTTCAGTATATTTCTGAGCTGCTTTTGTAGCTGCTTC
GTCATTAATACCACGTTGTAAGTTGCTCATTGCAGTATTTAATTCAGATGCCATTGTAGAC
ACTTGATTTACACCTGCAACTGTTGTCGCACCTTCAATTTGTGATTTCAATGCTGCTTTTTG
TGCATTATTAATTGATGTAAGGTTATTCAATGCATTTTTCGCAGTTGTCTTAGCGTTCGCA
ACATTTTGATTACCGTTCAATTCGTTTTTAGCACGTTGTACATTTTGTAACGCAGTTTCGAC
ACCGTCTTTTGCAGTATTTGGACCTTGTGCTTTATTTAAAATTTGTTCAGCTTGCGTCACTG
CATTTGTATATGCATTACGTTTCGCTTCATCTGCATCAGTGAAGTTAACACCTTGCTTAAT
CGTATTTTGATCTTGAATACCATTTTTCAAGTTCGTCATAGCTGTATTTAATGCGTCCGCAT
TATTTTGCGCTTGTGTTACTTCATTAACATGCGTTGCACCTTCGATTTGGCGTTTCGCTGCA
TCTTTTTGTGCATTAGTTAAGTCACTTAAGCCATTCAAATGTTGCTTCGCATTTGTTTGCGC
AGTTCTTAAGTTTTCTTGACCGTTTAACGCTTGTTGTTTAGCTGTCACTTGGTCTTTCGCTT
GATTAATTTCTTGCGCAATCATTGATGGACTAGTTGTTTGACCAATGATTGCTTTTGCTGC
AGTGACTGCATTGTTGTAGTCATTTTGTTTGTTTTGACTTGCATCTGTGTAGTTTTGACCTG
CTTTAATCGTTGCTTCATTCGCAATGCTATCTCGTAGACCTTTCATTGCAGTGTTTAATGTT
TGTGCATTATCACGAACAGTTTGAACATTTGGCAACGTCGTCGCTTGACCCACTTGTTCTT
TAAGTTTTGCTTTTTGCGGACCATTTAAAGAAGTTAAACCATCAATTGCTGTGTTAGCATT
TTGTTTCGCTACTTCTAAATTATGATTACCGTTTAACTGCGTCTTCGCATTCGTTACTTGCG
TTGCTGCATTTGTAACATCTGATGGTGTTAACGTTGGTGTTGGTGTACCACTAACGATATG
TTCTGCAGCTGTCACTTTTTCATCATATGCTTGGCGTTTATTCGCATCAGCATTGACATAAT
TACCACTAGCTTTTGTGTCAGCTTTATCAGCAATGGCACGTTTTAAAGCTGTCATCGCAGT
ATTTAATTCAGTCGCAGTATGTTCAACACCATTTGCTGCAGATACGCGTCCTGCACTTGTT
ACTTGTGCTTTCAATGCTTCTTTTTGTTTTGTATTTAAGTCCGAAGCATTTGTAATCGCTGT
GTTCGCAGCCTGTTTCGCACGTTCTAAGTTTTGAATACCGTTTAATGCAGTATTGGCTTGT
GTAACAGCTTGCATTGCTCTTTCGACATCTGCTTTAGGTGTATTTCCTCCAGCTGTTTTATT
TAAAATAGTTGCTGCTGCATTTACTGCTTGAGAATAAGCCGTTCGTTTAGCATCATCAGCA
TCTTGATAATTTTGACTTTGTAACGTCGTGTCTTTATCACGAATTGATGTTTCTAATTGACC
CATAGCACCATCTAATTGTTGCGCTTTGGCTTTAACTGTATTAACACCTTCAACATTTGTT
GCTTGTGTAATTTCATTATCTAACGCATTACGTTGTGCATTATTAATGTGTGTTAATGTAC
CTAACGTTTGTTTCGCAGCAGCTTTAGCTTCATTTAATTTCGCATCACCGTTCAACGCCGT
CTTCGTACTGTTCACATTTTGTAATGCTTGTTCAACTGCTGCTTTTTCTACATTTTGACCAC
TAGCTTTTGTTAAAATTGCTTTTGCTGCATTTACTGCTTGATCATAAGCTGATTTCTTACTT
GGCTCAGCATCTAGGTATTTCTGAGTTTGTTTTGTTTGTGTCTCATCATTGATACCATTTTG
TAAACTGTGCATTGCGTTATTTAATTCTGTTGCTTTTGCAGTTTCTTGATTTACACCAGCTA
CTGTAGTTGCACCATCAATGTTACGCGTTAACGCATCTTTTTGTGCATTGTTAATTGATGT
TAAGTTATTCAAGTTGTTTTTCGCTGTTGTCTTAGCTTGAGCTAAGTTTTGCGCACCATTTA
ATGCATGTTCTTTAGTTGTCACTTGTGATGTTGCTTGCGTAATAGTATTTGGTTCCATCGTT
GGGTTCGATGTTTGATTAATGATTGCTTTTGCTGCAGTAACTGCGCTGTCGTACTCGTTAC
GATTATTTGGACTTGCGTCAGTGTAGTTTTGACCTGCTTTAATCGTTGCTTCATTCGCAAT
ACTATCTCTTAAGCCTTTCATCGCTGTATTCAACGTTTGAGAACTATTTTTAACAGTTTGA
ACACCATCTAATGTAGTTGCACTTTGAACTTGTTCTTTTAATTTTGCTTTTTGTACATTATT
CAATTGTGCTAAGCCGTCAATTGTATTGTTAGCATGCTCTTTCGCTACACGTAAGTTGTTA
TCACCATTTAATGCTGTTTTAGCCGCATTGACTTGTGATGCCGCACTATTTACGTCATTGA
CTGTTAATGTCGCATTTGGTGTACCATTAATGATATTTTCAGCGTTAGTCACTTTTGAATC
ATACGCTTGACGTTTTTCTTGATCAGCATTGACATAATTACCGCTTGCTTTTGTTCTTTCTT
TATCCGCAATAGCTGCTTTAAGTGCTGTCATCGCACCGTTCAATTCAGTAGCTGTATGTTG
AACGTTATTTGCATCAGATACACGTTGTGCATTGTTAACTTGTGTTTTTAATGCATCTTTTT
GTTTTTGGTTTAAGTCTTGCGCGTTGTTAATTGTGTTCGTCGCATTTGTTTTAGCTTGAGCT
AAGTTTTGTGCACCATTTAACGCTGCTTTTGTTGTATTGACTTGATTTGCCATGTCATTAAT
CGCATTAGCATCCATATTTGGATTGTTCGTTGCATTAATAACACCATTTGCATTATTTACA
GCAGTATTGTAATTATTTTTGTTGCTATCAGATGCATCAAGATAATTTTGACTTGTTTTCGT
CGTATTATCATTTTGGATACTTGTACGTAATGCACCCATGGCAGTATTTAATGTATTACCT
TTATCTTTAACACCATTTACACCAACTACATTTTGCGCTTGTTCAACTTGATGCTTCAAGTT
GTCTTTTTGTAATTGTGTTAAGTTAGGTAAACCATTAATCGTATTTGTTGCTGAAGTTTTCG
CATTTCTTAAGTTTTCAGCACCATTTAAAGCCGCTTTCGCTCTTGTAACTGCATTTAATGC
ATTATCAACGTCTGCTTTAGATGTGTTACCACCTGTTTGTTTATTTAAAATGCCTTCAGCC
GCTGTGACAGCTTGCGTATATGCATTTCGTTTTGATTCATCTGCATCAAGATAATTTTGAT

TTCTTAATGTCGCATCTTTATCATTGATTGCACCTTGTAAGCTATTCATAGCGCCGTCTAAT
GTATTGGCATTTGTTTTTACAGTATTAACGCCATCAACAGTCGTCGCTTGATTGATTTGAC
CTTCTAACGCAGTACGTTGTGCATTCGTAATATGGTTTAAAGTACCTAAGTTTTGTCTAGC
TGCCGCTTTCGCTTCTGCCAGTTTAGCATCCCCATTCAATGCATCTTTCGTACTTGTTACTT
GTTGTAATGCACGGTCAACTGCTGCTTTATCTGAATTTGAACCTGTTTGTTTATTTAAAAT
TGCTTTCGCAGCAGCTACAGCTTGATCATAAGCAGTTTTCTTACTTTGTTCAGCGTCACGA
TATTTTTGAGTTTGTTTTGTTGCATTTTCATCATCAATACCATGTTGTAATTGACCCATAGC
TGTATTAAGTTCATTTGCAGTTTGTTGGATACTTGTTACATTTGCAACACGTTGCGCACTT
GTCACTTGTGCTTTCAACGCATCTTTTTGCGCTTTATTTAAGTTAGTAGCACCATCGATGG
CATTTGTTGCTGTTTGTTTCGCTTGCGTTAAATTATGTGTACCATCTAATGCATTTTTCGTT
GATGTCACTTGTGTAGCGATTTGGTTAATTGCATTAGCATCCATATTTGGATTGCTTGTTG
CATTAATGACACCATTAGCACTATCAACAGCATTGTTATAGTTTGTTTTGTTACTTCCTGT
AGCATCAAGATAGTTTTGGCCATTTTTCGTAGCTGTGTTATCTTGAATGCTATTTCTTAAC
GTACCCATAGCACCATTTAATGTGTTGGCACTTGATTTAACAGTATCTACACCTGCAACAT
TTTGTGCTTGTTGCACTTGTTGTTTCAATTTATCTTTTTGAGCAGTTGTTAAATGTTGAAGT
CCGTTTATAGTAGTGTTTGCATTTGATTTAGCTTGCTCTAAATTATGGTTACCATTTAGGG
CACCTTTCGCATTAGCAACTTGTGACAACGCGTTTTCAACGGCAGCTTTATCTAAATTTGA
TCCACTATTTTTATTTAAAATATTTTCTGCGTTCGCAACGGCTTGAGTATAAGCAGTTTTCT
TATCTGAATCAGCATCATGATAGTTTTCACTTGCTAGTGTATTTGCTTTATCATTAATACC
ATTTTGCAAGTTAGCCATCGCTGTATTCAATTGTGTACCTGTATTAGATACAGCTTCAACA
CCAGCTACAGTTGTTGCTTGATCAATTTTAGTTGTTAAGTCACGTTTTTGTGCATTATTAAT
TGACGTTAAGTTATTTAATGCATTTTTAGCTGCATTCTTCGCATTTGTAACGTTTTGATCAC
CATTCAATGCATTTTTAGCACTTGAAACATTTTGAATAGCCGCTTCAACATCTTGTTTAGA
CGTATTTGCACCTTGCGTTTTATTCAGAATTGCTTCAGCTCTGCTTACCGCATTTGTATACG
CATCACGTTTAGCTTTATCTGCATCAGTGAAGTTAACACCTTGTTTAACTGTGTTTTGATC
ATGGATAGCTTGTTCCAAGTTGCCCATTTGCGTGTTTAACTCAGTTGCTGCATTTTTAGCA
GCTGTTACCTCACTCACATGACCTGCACGGTCAATTTGTGATGAGATGGCAGATTTTTGGT
TATTTGTTAAGTGTGATAACGTATTTAAGTTTTGCTTAGCAGTGTTTTGTGCATTTCTTAAG
TTTTCAGCACCGTTTAAACCATTTTTAGCATTATTCACTTGTGTTGCAGCTTGGGTAATCG
CACTAGCATCCATAGTCGGATTGTTAGTTTGATTAATGATACCTTTCGCATTTGACACAGC
GCTATTATATGTTGATTGGTTATTCGGACTTGCGTCTGTATAGTTTTGACTTGCTTTGACTG
TTGTTTCGTTAGCAATACTATCTCTTAAGCCTTTCATTGCATTGTTCAATGCTTGTCCATTT
GTTTGAACAGATTGTACGTCTTCTAATCTATTGGCTTGTCCCACTTGTTCTTTTAATTTAGC
TTTTTGAGGTGTATTTAATTGTGTTAATGCATCAATAGCAGTATTGGCGTTTTGTTTTGCTT
CACGTAATCTTTCGTCACCATTTAATTCTTGTTTCGCGCTGTTTACTTGATTAGCTGCAGCT
GTTACTTCTGAAGGTGTCGTAACAACCGTTGGCGTACCGCTAATAATATGTTCAGCATTG
GTAACTTTAGTTGTGTAAGCATTTTGTTTAGTGCTATCGGCATTAACATATTTACTGCTTG
CTAACGTATTCGTCTTATCTGCGATGGCATGTTTTAATGTGCCCATTGCCGTGTTCAGTTC
AGTCGCATTGTGCTGTACATCTTGTGCATTAGATACGCGTTGAGCACCATTAGCTTGTGCT
TTTAATGCATCTTTTTGTTTTTGATTTAAATCAGATGCGCCATTGATTGCTGTAATCGCTGC
TTGTTTCGCATTATTTAAGTTTTGCGTACCATTTAATGCATGTTTCGCACTATTAACATTAT
TAAGTGCTTGTTCAACCGCTGTTTTCGCTGTATTCGGTCCAGTTTGTTTATTTAAAATGGTT
TCGGCAGCTGATATAGCTTGATTGTAAGCATTACGTTTTTGCTCATCAGCATCCAAGAAGT
TTTGGCTCGCTAATGTTCCTGACTTATCGTTGATAGCCGTTTGTAAGTTACCCATTGCGCC
ATCTAAACTATTTGCACTTTGTTTAACTGATTCAACACCAGCTAAGTTTGTAGCTTGTGAA
ATTTGATTTGTTAAATCATTGCGTTGTGCTGTAGTGATATGCGTTAAAGTACCTAAATGTT
GTTTCGCTGCGTTTTGAGCTGCAATTAATTTTGCATCACCATTCAATGCATCTTTCGCAGT
ATTAACACGTTGCAATGCTGCTTCAACTGCATTTTGCGCAGTGTTTGGACCTGTCGATTTA
TTTAAAATCGCTTTCGCTGCAGTAATAGCATTATCATACTCTTGTTGTTTATTTGTATCAGC
ATCACGATATTTCTCAGATGATTTCACTTGAGATTCGTTGTTAATACCATTTTGTAAGTTA
GTCATAGCTTGATCTAGATGTTGTGCATTTTGTTTTACAGTATCAACACCACTCACTCTTG
TCGCACTAGTAATTTGACTAATCAAATTGTTCTTTTGAGCATCTGTAATACTCGTTAATGT
GTTTAAGTACGTTTTCGCATTTTGTTTTGCCGTAGCTAAGTTTTCATCACCGTTAAGTGCCG
TTTTAGAACTATTCACTTGCTCTGCTTTTTGTGTAATCGTACTTGGATTCATTTCTGGATTA
CTATTTGCATTAATAATCGTTTCAGCAGCAGCTACTGCGTTGTTATATGCTGTTTGCTTATC
ATTATTAGCATCTACGTAATCTTCACTTGCTTTAGTCGCATCTTTGTTGGCAATACTTTGTC

TTAACGTATTCATGGCTTGATCTAATGTATTGGCATTTGATTGAACCGTATGAACACCAGC
GACAGTAGTACCACTATTAATTTGGTTTGTTAAATTCGTTTTTTGTGCAGTTGTTAAATGC
GTCATATTATTTAACTGCTGTTTTGCTGTTTGCTTCGCTTGATCTAATAAACGCGTACCATC
TAAGTTATTTTTAGCAGAATTCACTTGATTCATCGCTTCAGTAACTTGATCTTTCGTTTTAT
TTTGACCATTTGATTTATTTAAAATATCTTTCGCAGCTTGTACCGCATTTGTGTATGCTGTT
TTCTTACTTGGTGTCGCATCTTGATAATTTTGACTATTAAGTGTCGTTTGTTCATCATTGAT
CGCACCTTGCAAGTTACCCATCGCTGTATTTAAATCTGTACCTTGTTGTTTAACAGTATTT
ACGCCAGCAATATTTGATGCAGCATTAATTTTAGATTTAACATCTGCTTTTTGTGCATTAT
TTAAATGTGGTAATGCATCAATTGCTCTTGCAGCATCTGTTTTAGATTGTGCTAATTTTTC
ATCACCATTTAATGCATTTTTATTAGTAGTAACAGCTGAAGTTGCTCGATTAACATCATCA
ACAGACATTGTCGGATTAGCTGTTTGATTAATAATTGTTTCAGCACTTGAAACTGCACTAT
TATATGCATTTTGTTTAGCTGTATCTGCATCCGCATAATCTTCTGTACGTTTCACTTGATCT
TTATCTGCAACAGCTTGTCTTAAGTTACCCATTGCACTATTTAAGTTAGTTGCATTTTGCTT
AATCGTATTAACTGTCTCGATTTGATGCGCACCATTAATTTGCGATTGTAAGTTTTGACGT
TGTGCATTATTCAAGTTATTTAATTGACCTAATGCAGTGTTCGCTTCTTGTTTGGCAGCATT
TAATTTTGTATTACCATTCAATGCTTGTTCTTTACTTGTGACATTTGATAAAGCATTGTTAA
CATCACTTGGTGTTATAACTGGATGTTGTGCATTACCATTAATAATGTCATTCGCATGGTT
GTATGCATTGTTGTAATCATTTTTCTTATTAGTATCTGCGTTGACATAATTATCACTTTGTA
CGACTTGGTTATGATTAGCAACGCCACGTTTTAAACCTGTCATAGCAGTATTTAAGCTTTG
AGTCGTTTGTTTAATATCATTTACTGCTTGCACGTTTTGTGCACTATTCACTTGTTGTGTTA
ATGCATTCTTTTGTGCTTGGTTTAAATCACTTGCATGCGTAATCGCATTTGTTGCTTCTGTT
TTCGCACGTTGTAAGTTTTGTTGACCATCTAAAGCATCTTTCGTCGATTTAACAGATGCTG
CTTTTTGGTTCACTGTGTTAACATCCATCGTTGGATTAGTCGTTTCTCCAATGACACCTTTA
GCCGCATTAACTGCATTATCATACGCTTGTTTATTAGCTGGTGTTGCGTCAGTGTAATTTT
GACCTGATTTAATTGTATTATTATCCGCAACACTGTCTTTTAATTTCGTCATCGCAGTATTT
AAAGCCGTAATGTTAGACTTAATTGTTTCAAGCGCAGCATGATTTTGAGCAGCATTAATTT
GTTGCGTGAAATTATTTTGTTGTGCTTGGTTTAAGTTAGATGCACCATGTAATGTTGTTAA
CGCTGGCTGTCTATCTAATGTTAAACCTTGACGTGCTGTATTTAATTGATCCTTAGCTTGG
TTTGCTTCTGCCACTTTATCATTGATATTTTGGACAGTTGGGTTGCCATTCAACACTTGATT
AATTTCAGTTAACTTTTGACGCGCTGCTTGTAATTTTTGGTTGTACGCATTTAACGAAGCG
GTCGTCATACCTGTTGTATCCGTTGGTTGATTAATGCTTTGTTCTAATTGCGTTTTCGCAGT
TTGAAGCGATGCTTTATCTGGTGTTAAAGCTTGACGTGCATGATCCAAATCAGATTTAGCT
TGATTTGCTGCAGACGTATTTGTATTAATTTGATCTACTGTTGGTGAACCTGCTAATACTT
GATTGATTTGTTGAATCTTATTACGTGCAGCCGTTAACTTCGCATTGTATGCATTTACAGA
GTCTTGCGTCATACCAGTTGTACTTGTTTGCGTATCAATACTTTGTTGTAATTGAGCTTTCG
CATTTTCTAAAGGCGCTTTATCGACTGTTAAGCCATTACGTGCTTGATCAAGTGCTGATTT
AGCGGCATTCGCTGCTGTCACGTTTTGACGAATCGTTGCAACATCTGGATGAGAAGCCAG
TACGCGATCAATTTCTTGAATTTTAGTTCTAGCTGCTGAAAGTTTGTCATTAAATGCAGCA
ACAGATGCGCTTGTCATACCAGTCGTACTCGTTGGCTGATCAATATCATTTTGCAACTGAG
TTTTTGCAGTTTGTAATGGTGCCAAGTCTGGTGTTAATCCAGCAATTGCTTGTTTTAAGCT
ATTATATTTTTCTTCTACTTTTGTTTTTTCTGCGGCAATTTGTTGGTCTGTCGCGTCACCATT
GTTAATAACATTTTGTGCATTTGTTGATTCTGTTTGACCTGCACGTTTAGCATTTTCATATG
CTTGGATTGATGATTGTGTCATACCATCAGTAGTTACTGATTTATTGATTTCTTCATCAAG
TTTCGTCTTAGCAGTTCTTAAAGCACTATTATCAGCTAAAGGTACTAATTGATTAATTGCT
TGCGTTAATCGCTCATTGACACGATTTACATTTGTTAACGCAGATTGTACCTCTTGCACTG
TTCTTATTGGCTTCTGAATGATAGCATTAGCGCTATTTTTAGCACTTGTTAAGTCACTTTGA
AGTGCACGAATCGAATTATTGTAAGCAGTAATACTTGCCGGCTTCTTACCAGTCGTTGTAC
CTGTGCGATTCAATTGTTGCACTGCTTGCTCTAAGGCATGTGTATCTGCAGTTAAATCATG
TTTCGCTTGGTTTAATGCTGTTAATGCGTTATCAACACGATGTTTTTCATCTGAAATTTGTT
GTGCAGTTGCATCGCCATTGTCAATAACACGTTGAGCTGCAGTTATTTCAGTTTCTGCTTC
ACGCTTCTTCGCATTATAGTTATCAATACTTTGTTGCGTCATACCAGCAGTTGATGGTACT
TGGTTCACAGAACTTTGTAAGTTATTTTTAGACGTTACTAATTGGCTATTATCTTCTTTATT
TTGAAGTAATGCTTTAGCTTGATCAATCTTAGTTTGTGCTGCACGAACTTTAGTTAGTGCG
TCAGAAACTTGTTGTGGTGTTGCACGCTCATTATTAATCACTTGTTGTGCTTCTGTTTTCGC
AGTATTGATTTGTTGTTGCGCATTATGCATTGCATTATTGTACTGCGTAATTGTACCTGGC
TTTTTACCTTCAGTGCTTACTGGATCATCTAAATGATTTTTAGCTGTGATTAACTCACGTTT

ATCCGCTTTTGTGAAAATGGACTCTTGTACTTCTTCAGTACTACCATCATTGTAAGTTACT
GTCACAGGAATCGTCGTTGTGCTACCACCAGCTAAATTAGTAGGCATTGCTGTGCCATTTT
TAATCGTTGCAGTACGTTTATTAGCAACTTGAACTGCATTGTTAATTTCAGCTGCTGTTAC
ATTTGAACCATAATCTTTCACAATTTCAGTTGTGTTGACAGTATGTGCTGCCGGTGCAGTT
AATGTACTTGGATTACTACTTACTGAGTGACCTGTACCTGCTTTCGGAGTAATTGTGATTG
ATGAATTTGGTTTTATAGTATTGGCATTGAACGTCACTTTACCAGTTTGTGCATCTAACGT
TACATAGTCAGGCTTATTCGCAATTGTCCATTGATTATTCTGACCACGAACAACATTAATT
GTCTTACTATGTTCTGCACCATTACCCACTTTTTCAGTGTAAGCAATATCCATTGCTTGTGT
TGGATTAATTAAATGTCCTGATGGATTATTAGGCGTGATATCAATATGACCATTTTGCCAA
ATCTTAGTAGTCGCTTGGTTCGGTTGTGGTGCGACAACTGTGAAATCATCACTACGTTGCT
CATCACTGATTGTTTCACCACTACCTTGTGTTGCAACAACTTGAATTGTATCAGCAGGATT
GAAAGTACCTGCTGCAACAGTAATACCATTATTAGTTCCAGCAATACCTGCTACAGTTGC
TGCAGATGCTTCTTTCACCCATGGACTTGTATTATTGCGACGTGTAAATGTTGTCACGACA
CTGCTGTTACGTTTAATAACTAATTTATCAGCATACGTCGTTACGTTACCGGCATGTGTAT
TCACTGTTTGGTTTGCTCCAGGTGCAATTGTAATCGCTCCTGCCGCTGTTTCAGTCACAGT
TGGTTTCGCTGGTTGCACATCTTTTACTACAAATTTCGCTGGTAAAGATGTTGCAAAAGTA
TGTCCGTTATAGATGACGTCATATTTTGCGTTAACGACTTTAGCCATATTAGGTTTATTCA
TAGCTGCCCAGTTTGCATCGTTTGTACCAGTTGCTGCATTATTCCATTTATACGTAAATCC
ATCTGTTGGTAAACCATTAGCGTTTTGCATATGTGCATATCCTGATGCTTGCGTACCACTT
GCTAAAGTGCCACCAACTGTTGTTGTATAAGTAGTTTGAGGGAATTCAAATTGATATACG
TTCACAGTTACAGGAACTCGTTTAGCAGCTGAAATACCTGGATATGTGACATCGACATTT
AAATGTTGAACGCCTGCTTGCTGGTTATTTGGTTGTTGTCTATTTGCCCATGCTGCTGTAA
TACCATTCGTATTAGTATTTTGATCAAATGTAATGTAATCAATAGCGTTTGTACCATGTGT
CAAATTTTGACCTTTCACATCACGTGATGGCGCCTTAGCATTAGCAACTGGATAAACTTTG
ACTGGAACTTCAACATTACGCGTACCTTGACCACTAGGTAATGTTACAACCGCAGTTTTAT
GAGTGTTACCGACTGTATTCTTCCATGTATCTGGACTATCATGCCATGCAACCGTTGCCCC
ATGTGGCGGGTTTTGAATAAATCTTTCTACGTGTCCGAAATCAAAACCGTCGCCACCTTTA
ATAAATACAGCGCCTTCAGTAGTTGCTTGTAATTGTGGTGTCACTGTTACTGTTGCACTGT
CATTTGAATCAACAGATTCATTTCTTGTTACATCTATCGCTCGACCATGTTCATCTTGAGT
AGTGTACGTCACATTATTCATTGTGATAGAAGACCTTGCCTTAATCACACCATTTGGTAAC
GCATCACTTACCGTTACAACTGCAGTATTACCACTACCATAAGTCGTGTTTGTAATCGTTA
ATGGCGTATTATCTGCTTTAAATAATTTAATAGATGAACTACTTAACACATTATTAATTTT
AATTTGTTGGTTAGTAAGACCTGCTTTATAAGTCACAGAGTTGCCATCAATTCGCGGTGG
ATCTGGTTTAACTTTTGCTAAAATCTTGATAATTTGCGTTTGGTGATTATCATAAGTAACT
CGAATATCCCTTGTTGAAATACCAACCACATCTTTACTTGGTCCATATGAATTTGTAAATT
GGAAATTCATACGTTGCGCATTTTGTGTCCAAGAATCATTAACTGGTTTAACATATTGTTT
CGCATCATACACATCAGATACACCTGGGAACACAGCGCCTCTATTCGTCTCAAAGACATG
CTTCGGTACTGTACTTACTACTTTATATGTTGCTGTTTTCGTAATCGGCGTTGTTTCACCAT
CAATTAAGATATGTGCAGTTACTGTTATATCTTCACCAATACGTGTATTATCTTTATTTGG
CGCTTGACCACTTACCCAAGTAATCGTCGCATCTGGAATGGCACTACCATTAGATAACTT
AAAGTAATCTGATGCACTAGAACCATTACCTGCTGGGAAGTCTTGGCCTTGTACAGTGTA
ATGCGAATGTGCAACGATTTCTGGAATGACATGCTTTACAGGTACAGTCACTGTTGATGT
TGTTCCATCTTGATAAGTAGCAGTTACTGTAACATTGGCATTATTTCCAGTACGACTGACA
TTACTAACTGTTTTACTAGTGATTTCATTCGCACTTGCTCTTGCATAACTTCTATTTGGTAC
TGTTTCAGTAAACGTTAATGAATTAATAATTGCTGTTTGATCAGCTTGTGATACTGTCGCA
TTATTCGAAATATTGGCAATTCTCACAGGATTAGCAGCCGTTGATGAAGTACCAACTCGA
TATTTATCACGCAAAGGTTTCACTGTTACATTGAACGAAGTTGTAGCTGTATTGCCGCTTG
TATCAGTTGCTAATAAATTGATTGTCTTATTAGTTGCTGATGTCACATTTGGTGCCGTTGC
AGAAACATGTTGATGGTTATTATCAACAGTACCTGTAATTTGTGATGTATTTGGTACAGTT
ACAGATTGCACACCAAAGTTATCATTCGCTGTAATAGTATTTGTAAATGTTTCACCTGAGA
ACACTTGATGATTAGTGTAATTACCTACTGAAATTGTAGGATTAACTAAGTCACTTGGTAC
AAAATAAATGTTAATAGCGTCAGTAGTATTTCCTGTATTTTGATTTAAATGTTCAACATAT
TGTTTTGGACCATATGGCGTTAAGTATAACTGTGCTTTATAAACTGCATCACTTGCATTAT
GTGTAGAATTACTTTTTACAACGTGGTCAATTGTAAATGCGCCAGCACGATTAGCTGCCG
GTTCGTTTACGTTAACAACAGTTGAGTTACTATGGTTTGCACGAGTATTTGAATTTGTAAC
AGGTTGCCCACCATAACCGTTTGAAGGGTTGATGATGTCTAACACTTGAATCACTTGACC

ATTCAACGTAAATTGACGTTGACCATCAGTTGTCGCATTTGATTGTGAATAACCAGTCGTT
CTAAAGTTAGGTCTTCCGCCAGCTTCTGCTTGTGTTTTTCATTACCAGTAATATTATTACG
CAATCCAGGCACTGTAGTAGTCGCTTTTAGCATCGTTAAAATATCATTAGTTGTAATTTGT
GTGCCTAATGTAGCATCATAACGATAAATTAAAGATTTATGGTCATCAGACCATGATAAA
CCACCATCTGTTGGTCTGTTTGCAATTTTTGCATTCGTCCATGTCACTGTATAATCTTGCGG
GAAATTAACCCAACGCAAGAAATTCATATTAGCTAGGTTTGACGCGAATGATTTCGTTAA
TCGACCTTTATTAATATTTACTGTAATAGTACTTACATTAGCACCATTAGGTGTATTTGTA
ACTGAAATATCACCCTCGGCAAGCGTAATTACATCTCTATTTGCATTAATAAAAGCTTGTT
TTACTTTTGAGATTTCATCAGTTGTTAAATCATATGTTGACGTACGATACACACGTTGATC
GCCTTCAGGTTGTTTCACATCAACGATTTTTAAACTAATATAGAAATCTTCAGTGTCTTTA
TGATATGCATTTGAAGCAACTATATGATATAACTGCGTGCCTGCATTCATTTGACTAACTT
CTTGTGCAGTTGGAATAGTTCTATCAGATGTAATATTTGTAAATGTGCCACTTGGAATAGC
TTGTCCATTACTTAAAGTAAAATATTGTTTAATATCACCAATACTAAATGATTGTCCTTTA
GCAATCGTTACCGTTGCAGTTTTAGCTGCATTGGCAGTTTGATATTCAGATTTCACAACTG
GTTCGTATGTCATCACATTTGTAGCATCAAGCGTTGTTGATGAGCCATCACGGTAATGTAA
TGTGACATTACCATTATTATCGACAGTTACTGGATCAGTTGATGCTAAATATCCTCTTATA
TTTTGGTTTTTATTCATAAAGGCAGTAATTATGCTTTGCTTTTCATCATTAGATACAGCAGT
CGGATTGACTACTACAACTTTCTCAGTATTTCCTAATACAATCGGATGAGCATCTTCACTA
ATTTTACCTACATGAATTGACACATGTTTAGATTGACTATCATTTGTCGTATTATTGACAT
TGTCTGTCGCTGATACTTTAAATGTAATATCACTGTTAAATGCCTGATTCATAGAGACTCT
ACCTGTAATAGCTAATGAGCCGTTTTTGTTGTCGGATTTAGTTAAATTTGATGTCCAACCA
CTTGGCAATGTCGTAATAGTTGTACTTTTTAATCCAGAATGTCTATCTGAAACCCCCATAG
TAAAGCTAACTTCATCACCACGATAATATTTGGCATTTATTCCAACAGGATTATTGATTGT
TGGTGGAATGTCATCTGGCAAAATAGTAACAGCATTACTCATATTAGATGCAACTACTTT
ATTATAATAAACTACTGCTCTAATTTGTTTACCTACTGGTGGTATGAATACAACATAATCT
TTTGGTTGTATAGTAATTGTGTTATGGACCCCATCAGGTGTACCAGTCGCAAGTATCGTAT
AACCAGATGGTATTTGATCTGCCTCACTACCACCATTCGTAGTTTGATTTGTTGGTGCAAC
TAAATAAACCAATGCAGATGGATCCAACGGTATTCCCGAAATATTAACAGGCACTTTTTG
TAATGCTGTACCTCTTAATTGCTCAGCTGTCGTTGTAATTGTTGGTGTATGTGGTTTAACA
CGAATTTCGAATAATTGTTCTTTACGTCCAAAACGTATCGTACCAAAGTAATCCCCTGGTA
ATAAATTACCAACAAGTTCAATGTTACGTTCTCTAGCATTATTAGTTCTATTTGTCGAGTT
TTCAATGTGTAATGTACGCGGAATACCTGTAATTTGCCAATCTTTATCTGTAAAATTCTCA
TTACTTGTTAAATTAATACGTAATGGTTGAATAGATGTTACGCTACCACCTAAATTCACGG
AAGCATTCCCTTGGTTACCTGCATATACATAGAAAACTGGATGCCCAGCTTGTTTTGAAA
ATGGTTTTTCAGCAATATTTTGAGTATCGTCATTTTTAAAGAATTTAATATTATGTTGGATT
TCAGGTAATACCGTTAAGTTAACAGGAACTAAAGTTTCACTATTACCATAACCATCAGGA
TAGTCGAAACCAATCATTAATCTTATTGGGTTTCTAGTTCTTGCTGCTTCTTGTAATTTTTG
ATCAGTAAATAATTGACTTAATTGATATTCATTTAATTCTTGAACGCCCCCCGGTCTAAAA
GCGTTAGTATTTGCATTAGTTGGGTAAAAACCTAACACTTTATTACTCGGCGTCTTAACAT
AACTTAAAGGATCATCAACAAAATCATCTATATTTAGGTTTGTACTTTCTAATACCTTTTT
CGTTTGTACATTTCCTTGACTCACTTCTACACGTTTAGTTGTACCTATGCGATATGTACGAT
TAACCACTTGTCTTAAACCATTAAGACGTTGTTGATGGTCTTGCGTTGATTCGACGTATAG
TTGACTGTACATAAATAATTGATTGTACTCTAAAGCACGCCCACCTGCTGCATAATACAA
TTTATTAACAGTTGGACCTTGTGTTTTAAATGAAATTGTATAACTTGCATCACCTTGACCG
ATGAAAGTATAAACTTTAGGAACACCCGGTGATTCAGCAGGTTTTTGACCATTAATATAT
TCAAAATTTTGTCTACCAAATGCTTTAGTAGCAGCTGTTGCTCCAGCTTCACTAAAATAAT
CACTCGCTCTAGATAAATCTTCTAAAGAATAAATATGGACAGTTGGCCAGTCATATATTA
CTTGGCCACTTCTATTTCTTATTTTAAAGTCATGTGAACGATGAGGATCATTTACTCCATA
TTCCCACATTTGTTGAAGTGGTTTATTTGCACCTGCTCCTGCTCCATGACTCCATTGTACAT
TTGTTCCATCTGAATTAACTGTTACAAAGTCAGTTCTTCCTACTGGCACTTGGTCTGCTGG
TAATGCAAACCAGAAAATCATATTTTGATGTGGAGCATGTCCTTTATTAAACTTAATAAC
CCATTGGTACCCGTCTCCAGTTTGTTTCGCTGTCATAAGAGCAATTTCCTTTGAGTCAGAG
GCCTTCAATGTATTCCATGGTGTAGTTGGTAAATTATATGGCGCCCCTTTACTAGAAGCAT
TGATATATGTACCAACAGGATAGCCCGAACCATCATTAGCATTTTCAACATAATATGATG
AATTTGCATTTTGATAGTCTGATACTGATCTAGGCTCTGCAGCTCTTGATTCAACCGAACG
CGTTTGAATTCTATTTGAACGTCGGCTAGTCTGTTGACGTTGAGGCATTGTACGAATTAAA

GCAACAGCTTGTTCCAATTCTTTGTATGCAAGTTGCAAGTCTTTATTGTTTATTGGATCATT
ACTGCCTAATAACGTATTAATCTTATCAAAAATGCCTTTTACATAGTTAAATGTGCCAGAA
TCTGCATTTTCACGGTCAATAAAGCGATAATCATGATAATTTGCATCAAAAAATGCTTGT
AATTCATTTCGGTCATGTTGTACGTTACCATTATCAGATGAATCTGAAGCTGTTACTACAT
TTGCTTCATGATTTTCTACATGAGCCGCTCTATTGTCGTTTCCATTTTTATTAGCAGATTGA
CTTTCGTTTTGTTTATGCTTACTTTGTTCTTTATCTGGTTGTGTTGTCGCAGCCGTTGCCGA
ATCTTTCTTTGTATTTACATTTTGAGATGCTGGTTGTTCATCATTAGTAGTAGATGATTGCG
CTACTTTTTGATCTACTAAATTAGCTTGACTAATATTTGGTTGCTCATTTTCATGAGTAGCA
GATAATGATTGACCATTTTGTGAATTTTGAGAATTTTGTACTTGAGATTCTCGATTCTCAG
TCTGTTCATTATTACTTTGTTGTTTCTGTTTAACCACGCTTGCTGGTTGATTTGTTTCAGCA
GCATGTGCTTGTGATGTATTGAATCCTAAAAATACCAATGTCGCAATGACAGTTGAAAAT
GTACCAACTGTATATTTACGAATACTAAACTTTTGAATTTTATCACGATAATTCAC

> **tx_norB-var1**

Function: rho indendent terminator (core genome, variable)
Best match: tx_norB-var1_CC001-ST772_118_AJGE01000058.1[120245:120317]RC (completely identical)
Position: 036-contig_189: 32658 … 32731; Length: 73 bp
Sequence:
AAGAGTAATAAAAAACCAATCCCTATGTTCATTTTAAATAATGACACATAAGAATCGGTT
TATATTTTTGTTT

> **norB**

Function: protein coding sequence; multidrug efflux pump (core genome, variable)
Best match: norB_CC001-ST772_118_AJGE01000058.1[118810:120201]RC (completely identical)
Position: 036-contig_189: 32774 … 34166; Length: 1392 bp
Sequence:
TCATAATTGAGTGTCGTTTTGTTTAGGCACAAGTAACAAAATGATAACGAATGATAATAT
TCCCATACCTGCATTTAACCATAATGCAATCATTGCACCTGTATAAATGTTTGTCATATTT
GATACGATTGCATATACTGCACCACTCAATGCGACGCCAAATGCTCCACCTAATGCAGAA
GCCATTTTATAGATACCTGCAGCAACGCCTACTTTTTCTAACGGTGCATTTGCAATTGCTG
TATCTGTTGATGGTGTAGCATATATCCCTAGTCCTAAACCAAAGAATAAATAACCTATAA
TACAACAAATGACATACAATATTTCTGGCAAGAAAGTTAATGAAATGAGACATTCTCCGA
CAATAAGAACTCCTGTTCCAATTAACATTGGTTTCTTGCATCCGAGTGTTTGAAGTAACTT
TTCACCAACACGAATCATAATTAGTACCATTACTAAATAAGTGATTGATAAACTTCCTGCT
TGCAATGAAGAATATCCTAAACCTCTTTGAACAAATGTGTTGGCTACTATTAATGTTCCTG
CAACACCATTTAACAAAAAGTTTGAAGCTGTTGCACCTGTGTAAGCTTTATTTTTAAATAA
TTTAAAATCGATTAAAGGATTTGTAGCACGCTTTTCAAGAACTATAAATAAACTAAAAGA
TCCAATTGCAATAGCTAATAAAGTAATAAAAAGAAGTGAGGTTACACCTAATTCTGATCC
TTTAGTAATTAAAATATTTAAACTGAGGAGCATAATGACTAAAAGAACCAGACCTTTAAT
GTCAAATTTATTTAGAGAAATCGATTTAGATTTAGTTTCAGGTGTGCCTTTAATAAGAAAC
AGTGCAATTAATGAAATTATAATTGATAGGATGAAAATCCAACGCCAACCTAAAAGCGTT
GCAACTGCACCTCCAAAAAATGAACAAACACCAGAGCCGCCCCATGAGCCAATTGACCA
ATAACTTAAAGCGCGTTGTCTATCTTTCCCAATGTAATATGACTTAATAATAGACAAAGTT
GCAGGCATAATACATGCTGCTGAAAGTCCTTGAATTAATCTTCCTATAATAAGTAATAAA
GGAATATTTGAAATAATGATTAATAATGAACCTAATATATTTAAGATAATACCAATGTTC
GTGAGTTTAATTCTGCCATATTTATCAGCAAGACCACCTGCTCCTACTACAAACATTCCTG
AAAATAAAGCAGTTATACTAACGGCGATATTAACCGTTCCAATATCTGTATTGAAACTAT
CTTCAAGTATTGGTACAACATTAACCAATGATTGTGCAAATAGCCAAAACGTTATTACAC
TTAAAACAATTCCTATTAACAACTTATTATTGCCTTCAAATGCCTCTCTTGACGGCTTTTCC
AT

> **Q5HFY6**

Function: protein coding sequence; amino acid permease (core genome, variable)

Best match: Q5HFY6_CC001-ST772_118_AJGE01000058.1[117332:118654]RC (completely identical)
Position: 036-contig_189: 34321 … 35644; Length: 1323 bp
Sequence:
TTATGCTGCTTTTTGTTTCTTCTTATAGTAATAAACTGGTATACCAAGTGCTGTTATTAGAA
TTCCAATGATTGCTAATATAAATTGTGTAAACAGTGTATTAATTAATACAAAAGATCCTG
CCAAAATAGCAATTAAAGGTATGATCGGATATAACGGTACTTTATATGGACGCTCCATAT
TTGGTTCACGTTTTCTTAAAATTATTACAGCAACAAATGACATACAATAGAACAACCAAA
TAACAAAGATTAACATGTTTGTAATTGTATCAAATGCTCCCATTGACATCATGATGATAG
CGATTATAAGTTGTATAATTGCGCCAAACCATGGTGCACCAGATTTTGTTAATTTTGCGAA
TAAGTGGCTAAATGGCAATAATTTTCTTTCAGCCATTGCATATGGTACGCGCATACCAGTC
ATAGTATAGCCATTGATCGTACCATAAACAGAAATTAATATACCGATTGTAATAATCTTA
CCGCCATTTTCACCAAATAATATTTTTGATGTATCTGAAGCTGCATTTAAATTACCAGCAA
GTAGTTCTATTGGCAACGTTAATAAGAATGTAGCGTTAATTAATAAATACACAGCCATAA
TACAACCGATACCAACTGAAATCGCTAAAGGTAAATCGCGTTTAGGATTTTTAAGTTCCC
CCGCAACATTTCCTACATGAATCCAACCATCATAAGCAAACATAGTTGCTAATAAACCAC
TACCAATTGCTGTAAAGAAACCATTTCCTGAATTACCTGTAGTTGGAATTAATGAAAAAG
TGATATCTCCAGATTGAAAAATACCAAAAATTACAATAACGATGATTGGAATCAGTTTAA
TTACTAAAGTAACTGATTGTAAAATTCCGCCTGCTTTTGAACCTAGGAAATTTATCAACAC
AATAGATAACGCAGATGCGATTGCTATTGGTATTAACGAACCTATAGATAAATGGAATAA
ATTAATTAGCTGTGTCGCAAATACGATAGACAATGCTGCTACGTTAGCTGGAAAATAAAT
AAATGATTGCGCCCAACCTGATAGGAAGCCCCAGAAATCACCGTATGTATATTCTATATA
CTTCGTTAAGCCACCTGTTTCAGGGATTGCAGCAGCAAGTTCTGCTGCTGTTAACCCCGCA
CAAATGGTAATGATGCCGCCTAGGAACCATACAAACAAGGCCATTCCTGCTGTTCCTGTT
ACTTCTGTTACGTTTGATATTTTAAAGAATACTCCTGAACCAATAACTGTCCCCATAACAA
TAGCAAACGCTGAGAAGAAACCTATATTTTTTTGTAATTCTTTACCATTTGACAT

## > **ilvA1**
Function: protein coding sequence; threonine dehydratase locus 1 (core genome, variable)
Best match: ilvA1-CC45_ref_CC045-agrI_A9635_ACKI01000001.1[48611:49651]RC
Position: 036-contig_189: 35674 … 36715; Length: 1041 bp
Sequence:
TTAACCTACCACACCCTTGCTTGTATCTGCAATATTCAGTCCATGTTCAATGACACCTGAA
ACTCTAGTTAAGTCAACATTCCCGCCTGAAACTAAAGCAACAACATTTTTATCTTCAAGCC
ATTTATTGTTTATTTTTCCACTTAAAATTGCAGCTGTTGGTAATGCGCCTGCACCTTCAGTA
ATAATTTTGGCACGCTGCATTAAATCTTTCATAGCATGTTCAATTTCTTCTTCAGTAACAA
GAATAAATTCATCTACTAAATGTTTAACTACTTCATATGTTTGTTCACCAGGAACTTTTAC
ATCACAACCATCTGCTATTGTGCTATCCACTCGATGTTCAGTTAAATCTCTCTTATAGAAA
GACTCAGCCATACCATGAACATTCTCAGATTGAACACCGATAATATGAATTGAAGGGTTA
AATGATTTTAATGCGGTGGCAATACCTGCAATTAATCCTCCACCGCCAACTGGTACGATG
ACTGTATTCACATTCCAAATATCATCTAAAATTTCTAAACCAATTGTTCCTTGGCCTGCCA
TTACAAACTTATCGTCATATGGATGAACGATTGTCATGCCATTTTCTTTCGCTAATTCTTCC
ATATAAAGTCTAGTTTCGTTAAAGTTTTTACCTTTTAAAATGACTTTTGCACCATATCCTTT
GGTAGCATTCTGTTTAGCAATTGGTGCTGTTTCTGGCATCACAATCGTTGCATCAATACCT
AATAGTTTTGCTGTCAAAGCTACACCTTGTGCATGATTTCCGGCTGATGCGCCAATAATAC
CTTTAGCTTTTTGTTCATCTGACAAATGATTAATTTTATTGCTAGCACCTCTAAATTTAAAT
GAACCTGTAAACTGCATATTTTCAAGTTTTAAATATACGTTACCTTTAGTGATATTTTGAC
TTAAATACATAGATTTAATTAGTGGTGTTCGACGAATAAATGGTTTAATGCTTGCTTTAGC
TTCTTCGATATCTCCTAAACTTACAATATGTGCTGTTTGTAATGTAACTGTGTTGGTTGTCA
T

## > **ald1**
Function: protein coding sequence; alanine dehydrogenase 1 (core genome, variable)
Best match: ald1_CC001_MW2-USA400_BA000033.2[1475688:1476806:r]RC
Position: 036-contig_189: 36809 … 37928; Length: 1119 bp

Sequence:
CTATTCGATAACATTTAATATTTCTTTATAATCTAGGTCATGTGATGAAGCTAATCCTTGA
TTTGTCACTTGTCCTTGGTAAATGTTTACACCAGTACTTAAGGCTTCATTATCTTTAATTGC
TTGTTCTAAGCCTTTGTCACAAATTTCTAATATATAATCAATATTTCCTTGTGCTAATGCCA
TTGTTGAAGTTCTTGGGACTGCTCCTGGTTGATTTGGTACACCATAATGAATCACACCTTC
TTCTTCATACACTGGATCAGAAATTGTAGTTGGTCTAATTGTTTCAATAGTTCCACCTTGG
TCAATAGCTATATCGATTAATACTGAACCTTTTTTCATTGATTTAACCATCTCACGAGTAA
CCAATTTTGGCGGTTTCGCACCTGGAATTAAAATTGTAGAAATAAATACATCTGCTTTCTT
AATTTGTTCTGCTAAATTTTCTGGTGTTGATTTGACTACTGTGACATCTTTTTCTGCATACA
TATCTTCAAGATATTTAATGCGGTCATCGTTTAACTCGATAATGATTACTTTAGCATATAG
TCCCAAGGCAACATTTGCTGCATTTGTTGCTGCTACGCCACCACCGAAAATCACATATGT
ACTACCAGGTATATCCACATTTTCATGTACACCAGTCACTAAAGTACCTTGACCACCATGT
TGTGCTTCAGAGTAGTAAGCTCCCATAATTGCTGAGCGTTGACCTGCTATAGCACTCATTG
GCGCTAATAATTCTGCTTTTCCATTTTTTATAATGGTTTCACCACTAATCGCAGTTACACC
AACTTCTTGCATTTTTTCTACTATTTCTTTTGAAGATGCTAGATGTAAAAATCCCCAGATA
ATTTGATTCTTTTTGAAATATTGATATTCGCTTTCATGAGGTTCTTTTACTTTGATAACAAG
ATCAGCTTCCCATGCTTGTTCGTGAGTTACGATCTTAGCGCCTTCTTTTTCATACATATCGT
TAGAAAATCCTGAACCAATGCCAGCATTTTTTTCAACAATTACTTTATGTCCTGCATCCGT
TAACTTACGCACATTTTCGGGTGTGCAAGCTACACGTCCTTCACCTTGTTTTAATTCTTTG
ACTACTGCAACTAACAT

> **tnpIS431-06**

Function: protein coding sequence; transposase for IS431 (mobile element)
Best match: tnpIS431-06_circ_S-epidermidis_GQ900381.1[43799:43807]RC (completely identical)
Position: 036-contig_189: 38563 … 38572; Length: 9 bp
Sequence:
ATAGTTCAT

> **Q2FGZ8**

Function: protein coding sequence; putative protein (core genome, variable)
Best match: Q2FGZ8_CC001_ATCC51811_ADVP01000007.1[49342:52782]RC
Position: 036-contig_189: 39300 … 42741; Length: 3441 bp
Sequence:
TTAATATAAAATAGTTTTCAGTTGTTGATGTTTATCTTTCAATTGATTGATAAGTGTTGTAT
CTAAATCAAATGCTAATAAATCGTTTATTTGCGTTTGAATATTAGCTTCAAAACTTTCAAA
TTGCGATTCAGCTTCTACAGCCATTTGCTTAACATATAATTCAAGTTGTTGCCTCAATAAT
CCCATTTGTGGTTGTAATAATCCTAACGTACTTTGACAAATTGATTCATGTATATCTCTTT
GCCCATTTGGATTCAAAATTTTACGTTTTGTTAATTGTTTAGGCAATGCATTTAGCATATC
GTTGAAATCGATGTGCAATAATGGTTGCTCTATATTAGCTGATTCAAAGTTAAACTGAGG
ATTAATAATGACATGTAAATCTGCTAATTGTTGAACGATTGGTGCAATTTGCTCAGTGAGT
TGCTTATTAAAGTATTTTTTTATACGTTCTGTAATTAAAGATTGCTCTAAAAACAATCGTT
GATGAATTTGATCTAAGTATACTCTCGTAGACACTTTCTTTTCTTCATTAAAATCACTATTT
TGCGTCATTTGAGAATTAAACACTGATTTAACATCGTCAAGTAGTTGTAGTTTTAAACGG
GCATTTAAATGATAAACTTGTTCTTCAACTTCGTTGTCTGTATGTTGTAACGTCGCATCAA
TTAATTGATGTTGTAAACGCTGTTTATCTTTATAATCTGTTAACCTTTGTTGGCGACGTGA
AATATCAGCTTTATTTGTTTCAAATTCTGTAATCATCTCTACATAAGAACGATCCATTTGT
TGAAGCTGATGAATCATTTGTTGTTCTAAAATTGATTTAGATTCAACATCAACAAATTGTT
GTATGCTTTGTTTTAATTGATCAATGCCCTTATCTTCAGCTTGCAATGCATTTCGACTTGAT
ACAGCAAAAATGTCTGATTGTAAGTGTACTTGTCTTAATGCATCTGATACATATGTTTCAA
CTGCTTCAAGATCATCTTGACTTTCTGCTAAATCAGCAGCATTAATTACCATTTTAAATGC
TTGGTTTTCATTCAACTGGTTCATATCTTTCATGTGTTCTATAAACGCTTTGTCATTATCAG
TAAATGAATGATTAAAATAACTTACATACAATATTAAGTCTGAAGAAGTTAAAATTTGTT
CGGTTTCATTTGTATGCCTTTGGTTATTTGAGTGTAGCCCTAATGAATCAACAATTATTTT
ACCTTTTAACCAATCATGCATTAATGCAATGTGTACTGTTTTAACAAATGTTGCGTATTCA
TCTTCTGCACTCCACTTTTTCAATTCTTGTTGATTAATGGCATGTTTTTCACCATTTTCCAA

CATATTGACATACAAATTATAATGTTTTTCAACTGCATGTACAAAAGCGAGTTGATTTTTA
TTTAAATGTGATTTTAACTTTTCTAAATCTGAATTAATAAAGTCTTCTATAGTTGAAAAAG
ACATATCTTGGTATTCAACTACTGCATTAATTTCATCTAATAATTGCGATTGTGATTTTAG
CGTTATATAACTCTCGTCTCCATAAGATATTTCTGTAGTAGCTGCTGTAGTAGGATTTGGA
GAACTGACTAAAATCTGCTCGCCTAATAATGCATTTATCAAACTACTTTTACCAGCACTAA
ATGTTCCAAATACACCTATTTTTATTAATTTATTATCTATACGTGTTAATGTTTCGTGGATA
TCTTGCTTTGTACGCTTGAACAAAGGCACATCCGAAATTATATCAAGCGCTTTTTGAATAT
CTATAGACATATTTGTTGTAGCTGTATTTTGATTTAGCTGCTGATTATCACGATTATCCTG
AGTATTATCAGTAGCCACTTGATATGTTGTCTCTTGTCGACCTATTAATTTATCTAGAGAT
TCATCTAAATGAATATAGTAGTGACGATAATTCTTAGTCGTCAATGACTGACGCAGCTCA
TTTAATTCTGTATAACGTTGATATTCTTTTAAATCATCACTTTCTTCTGTTGGTAATTCATC
TGCCTGTACATTTTCTATTATTTCTTTAAAAATTGGTGTTGATTGTTGTTCAACATATTTCT
TAATGGCTTTAACCACTTCGTCTGAAAATGTAAGTACATAAGTATTGCTGATTGATGTTTG
TGGTTGATATAAATCTTCAATCATTTCCGGCTTAACGTCATAATGCTGATTTAATACTTTA
TCTGAAGCTTCTTTTTTATTGATAAAACGCGTTACAAATGACATATCTTCTCGCATTGGTT
GACGAATTTGTTGATTAACATGTTCTTGTAACGCATCTGTCGCTGTTAATAATCGCTGTTG
TTGAATTTGTAGCTTTTTCTTCTTTTTATTAAAAAACCCACCAACATTAAAGTCTTGAGAC
ATACTTTCCAAATAACTTCGTAACATTTCTCTCATATTATGCGGCATAATGTATGCATTTT
CTAAAATATTTTTACGCTTATTCTTTAAAAATGCCATTAATTCATCTGGATTATTTAAAAG
TTGTGCCTCTTCACTAATTGCTTGATGTTGTTGACTATTTAAAAATGCTTGTTCAAACTCCG
CTTCTTCGATACCTAAATCTTCTAGTACTTCCTGAATTTCAGACTGAATGTAATCTAGCTG
AGCTTCGGTAATGTATTCAACCGTTCTTGATGTATAATCCTCTATTGTCTCTCTATGTTGAT
CTAATGAAATTAGATAACTTGATAATACTTCAAGTTCATTTTCAGGGTGATCAAATTTAGA
TACATAAAAGGTGCGTTCTAATTTAATGCCCCAATCTGCAATTGATTTTTCAACTCGTGAT
TTAAACGTAGAGAATGACAATTCATCGTCTTGATGCTTGTCAATTTGATTAATGATAAAC
ACAACAGGTATTCCAACATCATTTATATGCTTCATAAACTTAAAGTTAAGTTCAGATTGA
ACGTGGTTATAGTCAACCGTATAAAATATCATATTACTTGTATACATATATTGTTCTGTTA
TTGACTGATGTGATGCAACATTTGAATCAACACCTGGTGTATCTTGCAACGTAAACCCATT
TTCAAATTTAGCTGATTGAAAATTAATTTCTACAGATTCAACGTCGACATTTTGGCGATTC
ATTTCCCTTACTTCATCATAATTAGATAATTTGGCATACGTTTGATTCGGCAAATTAGCAA
TAATATCGTGATTGTCTGAAACTGACACAATAGCAGTATTACTTGTCGTTGGTACAGGAG
AACTTGGTAAGATATCTTGTTCAATTAATAAATTTATCAGTGTCGATTTACCTGCAGAAAA
ATGTCCAACGAACGAACATGTATATTGCTGCAAATATACTTTCTTAATTACTTGGTTAATT
GTATGTAAAAGTGCTTCATTTCGCGACTTTTCAACTTCTTTTTTTAATTTATATAAAAGATC
TAATTGTTCTTTATTAATCAT

> **piuB**

Function: protein coding sequence; iron-regulated membrane protein (core genome, variable)
Best match: piuB_CC001_MW2-USA400_BA000033.2[1481924:1483264]
Position: 036-contig_189: 43044 … 44385; Length: 1341 bp
Sequence:
ATGAAAAATACATTTAATCCATTACAAAGATTACATTTCTATGCAGCAATATTTATTGCTC
CACTGTTAATCACTTTAACCATTTCGGGCATTGGTTACTTGTTCTTTCCAGAAGTTGAAAA
TAATATTTATAAGAATGAGTTTTTTGGTGACAGTGATGTAAAAACGCATCAAACATTAAA
TGATGCAGTACATCAAGTTGAACAACAATATGAAGGATTCTTTGTAAGTAAAGTTAGCAT
ACTTGATGAACCATATAACAAACGAATTACACTGAGTGATATGGTAGGAAATCAACGTTA
CGTCTTTCTAGATCATAACAATCAATTTGTTGCAGATCAAAATGCGAAACATACGTATTCT
AATGTGATGCGAAGTATACATAGTTCTTTGTTTACTGAAAATACTATTATTAATTATTTAG
TAGAGTTAACCGCATGTTGGACGATATTCATGATTTTATCTGGTACTTATTTACTCATTAA
GAAGCATTTAATTTCTAACAAAAGTAAGGCACTTCGTTGGCAAAAGTGGCACGCAATGAT
TGGAGTTATCATTGCAATTCCAGTATTTGTATTAGTTTTAACTGGATTGCCATGGTCTGGT
TTTATGGGCAGTAAAATTGCCGGTATGATGGACACAAACGGTGACCTTGGTCAAGGTGAA
TTAGCGATTAATCCACCTAAATCAGATTTGAACGAATTACCTTGGGCTACACGTAAAAAT
AAACAGCCAGCTTCATCCGAAAAAGGTTCAAGTGGTCATCATGGTAATGCAGCAATGCCT
CAAACCAAATTAGATTATCAAATATCTATTGATAAGGTCGTTGAACAGGCGCAAAAATCT
GGTATTAAAAAGCCGTTTTCAATCGTATATCCAAGTGATAAAAATGGTGCCTTTATTGTAT

CTAATACTAGTAATTCAGGTGTTACTGGGCTAGATGTATCACCTTACAAGGAACAAACAC
TTTATTTCGATCAATATAGCGGTAAAAAGCTAGGTACGATTAAATATGATGACTACGGTA
TTATTGCTAAATGGTTTACATGGGGCATTCCGCTTCACGAAGGTCATTTATTCGGCATTTT
AAATAAAATCATTAATTTATTTGTATGTATCGCTTTATTAGTAGCCATTGGCATGGGGTTT
GTCTCTTGGATAAAGCGTACAAAAAATACTGCAGTAAAAGTACCACATCGCGTAAAAAA
ACCAGCATCTATATCACTCATAATATGTTTAATTGTATTAGGATTATTAATGCCATTATTT
GGATTATCACTTATCCTTGTATTTATAATTGAATTAATATTATATATTAAAGATCGTCGTG
CTAAACAATAA

> **ypsC**
Function: protein coding sequence; site-specific DNA-methyltransferase (core genome, constant)
Best match: ypsC_CC008-ST72_TCH130_ACHD01000016.1[132658:133803]RC
Position: 036-contig_189: 45006 … 46152; Length: 1146 bp
Sequence:
TTAGTTTTCAATAGTTTTACGTTCTGTCTTTTTACCCCAGTATTGATAATAAGTACATTCTA
TATATCCATTAAATAACTTACGTCGCTTTGTTGCTTTACGATCTACTAAATATTCAAATTCT
TTATTACTTGTTAAAATGTATGTAGATAAAAATGGATGTTGTTTCATTAGTTTACCAATAT
AACGGTACATTTCTTCAACTTCTTCACGATCACCAATACGTTCACCATATGGAGGATTTCC
AATTAACGCCACCGGTTCTTCTGTATCAATTGTTAATGTATTGACATCTTTTACACTAAAT
TTAATAATATCAGACAACCCAACTTCTTCAGCGTTACGCTTAGCAATCTCTACCATTTCTG
GATCGATATCAGAAGCATATACTTCGATTTCTTTATCATAATCAGCCACCTTATCCGCTTC
ATCACGGTAATCATCATAAATATTTGCTGGCATGATGTTCCATTGCTCTGATACGAACTCG
CGATTAAAACCAGGTGCGATATTTTGAGCAATTAAACAAGCTTCTATAGCTATTGTACCC
GAACCGCAAAATGGATCAATTAAAGGTGTATCACCTTTCCAGTTTGCAAGACGGATTAAA
CTTGCTGCCAACGTTTCTTTAATTGGTGCTTCACCTTGTGCTAATCTATATCCACGTCTGTT
CAAACCAGAACCTGATGTATCGATAGTCAATAATACATTATCTTTTAAAATGGCAACTTC
AACGGGGTATTTCGCACCTGATTCATTTAACCAACCTTTTTCGTTATATGCGCGACGTAAT
CGTTCAACAATAGCTTTCTTAGTTATCGCCTGACAATCTGGCACACTATGTAGTGTTGATT
TAACGCTTCTACCTTGAACTGGGAAGTTACCCTCTTTATCAATTATAGATTCCCAAGGGAG
CGCTTTGGTTTGTTCGAATAATTCGTCAAACGTTGTTGCGTTAAAACGTCCAACAACAATT
TTGATTCGGTCTGCTGTGCGCAACCATAAATTTGCCTTTACAATTGCACTTGCGTCTCCTT
CAAAAAATATACGACCATTTTCAACATTTGTTTCATAGCCTAATTCTTGAATTTCCCTAGC
AACAACAGCTTCTAATCCCATTGGACAAACTGCAAGTAATTGAAACAT

> **rnpB**
Function: noncoding RNA ribonuclease P RNA component (core genome, constant)
Best match: rnpB_CC001_MW2-USA400_BA000033.2[1485649:1486045:r]RC (completely identical)
Position: 036-contig_189: 46346 … 46743; Length: 397 bp
Sequence:
ACTAGTAGTGATATTTCTATAAGCCATGTTCTGTTCCATCGTACTCATCACGTGCACTAGT
CACACTGGTACTCAGGTGATAACCATCTGTCTACACCACTTCATTTCGCGAAGTGTGTCTC
GTTTATACGTTGAATTCCGTTAAACAAGTGCTCCTACCAAATTTGGATTGCTCACTCGAGG
GGTTTACCGCGTTCCACCTTTTATATTTCTATAAAAGCTACGTCACTGTGGCACTTTCAAA
TTACTCTATCCATATCGAAAGACTTAGGATATTTCATTGCCGTCAAATTAATGCCTTGATT
TATTGTTTCATCAAGCACGAACACTACAATCATCTCAGACTGTGTGAGCATGGACTTTCCT
CTATATAATATAGCGATTACCCAAAATATCA

> **gpsB**
Function: protein coding sequence; cell cycle protein (core genome, constant)
Best match: gpsB_CC001_MW2-USA400_BA000033.2[1486105:1486449:r]RC (completely identical)
Position: 036-contig_189: 46802 … 47147; Length: 345 bp
Sequence:

TTATTTACCAAATACAGCTTTTTCTAAGTTTGAAATACGTTTTAAAATATCTACATTATTTG
AAGATGTATTTGTTGTTGTATTATTCGAAGAAAAACTTTTATTGTCCTGAGGTCTTGATGT
TGCTACACGTAGTCTTAATTCTTCTAATTCTTTTTTAAGTTTATGATTCTCTTCTGATAATT
TTACAACTTCATTATTCATATCGGCCATTTTTTGATAATCAGCAATAATGTCATCTAAAAA
TGCATCTACTTCTTCTCTTCTATAGCCACGAGCCATCGTTTTTTCAAAATCTTTTTCATAAA
TATCTTTTGCTGATAATTTCAATGAAACATCTGACAT

## > UPF0398

Function: protein coding sequence; unknown protein fold 0398 (core genome, constant)
Best match: UPF0398_CC001_MW2-USA400_BA000033.2[1486463:1487026:r]RC (completely identical)
Position: 036-contig_189: 47160 … 47724; Length: 564 bp
Sequence:
TTAGAAACTTTGATCTTCAGACCACTGTAAGTCATTGATGAATGCTGTTAATTCATCGAAC
GTCACAATATCACAAGTATAGTTTGTTTTATCCATAAAATCAACTAACATCTGCTTGAAGA
ACTTAGGACTTGCCTCTTGTTCCTCATCATAAATGAGCAATGTTTGATCCGAATGTTCAAG
CATAAATTGATCTGCTTGTTTAAATTGAAAAGGACCCTGATACGACGTATGAAAAATACT
ATCAACATAATCTGCATGCTTAATTATGTTGGCATACTTACTTTGATTATGTTCATTCCACT
TTTCTGTATGTCCTTGGAACGGTGTAATTACAGCAAACTTTAACGAATCATATGTTCGTTG
TAATTCAATAACAACTTCTGCAGTCCATAATTCTATACCCATTTGCCCTTGTATTAACACC
CATTCTAATCCTTCATCCAACAGTTGTTCAATTTTATGTTTTATAAATTGTTTTAAATAATG
TACTTCAGGTGCGTCATCTTTAAAAATGTTTAATTCGAATGATTTGTAACCTGTTACATAA
ACTGTTTTAACCAT

## > recU

Function: protein coding sequence; recombination protein U (core genome, constant)
Best match: recU_CC001_MW2-USA400_BA000033.2[1487908:1488534] (completely identical)
Position: 036-contig_189: 48606 … 49233; Length: 627 bp
Sequence:
ATGAATTATCCAAATGGTAAACCATATCGTAAAAATAGTGCTATAGACGGAGGGAAAAA
GACCGCTGCCTTTAGTAATATTGAGTATGGTGGACGTGGTATGTCACTTGAAAAAGATAT
CGAACATTCAAATACGTTTTATCTTAAAAGCGACATTGCAGTTATTCACAAAAAGCCTAC
GCCAGTACAAATAGTTAATGTCAACTATCCTAAGCGGAGTAAAGCTGTGATTAACGAAGC
TTATTTTCGTACACCTTCAACAACTGATTACAACGGCGTTTATCAAGGTTATTATATTGAT
TTTGAAGCAAAGGAAACTAAAAACAAGACGTCCTTTCCTTTAAATAATATTCATGACCAT
CAAGTCGAACATATGAAAAATGCATATCAACAAAAAGGTATTGTGTTTTTAATGATTCGT
TTTAAAACGCTAGATGAAGTTTATCTTTTACCCTATTCAAAATTCGAAGTATTTTGGAAGA
GATATAAAGATAATATTAAAAAGTCTATAACAGTTGATGAAATACGAAAAAATGGTTAC
CATATTCCTTATCAGTATCAACCAAGATTAGACTATCTAAAAGCAGTTGATAAGTTGATA
TTAGATGAAAGTGAGGACCGCGTATGA

## > pbpB

Function: protein coding sequence; penicillin binding protein B (core genome, constant)
Best match: pbpB_CC001_MW2-USA400_BA000033.2[1488531:1490714]
Position: 036-contig_189: 49229 … 51413; Length: 2184 bp
Sequence:
ATGACGGAAAACAAAGGATCTTCTCAGCCTAAGAAAAATGGTAATAATGGTGGGAAATC
CAACTCAAAAAAGAATAGAAATGTGAAGAGAACGATTATTAAGATTATTGGCTTCATGAT
TATTGCATTTTTCGTTGTTCTTTTACTAGGTATCTTATTGTTTGCTTATTATGCTTGGAAAG
CACCTGCTTTTACCGAAGCTAAATTACAAGATCCGATTCCTGCAAAGATATATGACAAGA
ACGGAGAACTTGTTAAAACATTAGATAATGGCCAAAGACATGAGCATGTAAATTTAAAA
GACGTGCCGAAATCAATGAAAGACGCAGTACTTGCAACTGAAGACAATCGTTTCTACGA
ACATGGCGCACTTGATTATAAACGTTTATTCGGTGCAATTGGTAAGAACTTGACTGGTGG
ATTTGGTTCTGAAGGTGCCTCAACATTAACACAACAAGTTGTTAAAGATGCATTTTTATCA
CAACATAAATCTATTGGACGTAAAGCTCAAGAAGCATACTTATCATATCGTTTAGAACAA

GAGTATAGTAAAGATGATATCTTCCAAGTATATCTAAACAAAATTTACTATTCTGATGGC
GTAACAGGTATTAAAGCTGCTGCTAAGTATTACTTTAATAAAGATTTAAAAGATTTAAAC
TTAGCGGAAGAAGCTTATTTAGCCGGTTTACCTCAGGTTCCAAACAACTATAATATTTATG
ATCATCCAAAAGCTGCTGAAGATCGTAAAAACACTGTTTTATACTTAATGCATTATCATA
AACGCATTACAGATAAACAGTGGGAAGATGCTAAGAAAATCGATTTAAAAGCGAACTTA
GTAAATCGTACTCCTGAAGAACGTCAAAACATTGATACAAATCAAGATTCTGAGTATAAT
TCATACGTTAACTTTGTGAAATCTGAATTAATGAATAATAAAGCATTCAAAGATGAAAAT
TTAGGTAATGTATTACAAAGTGGTATTAAAATTTATACAAATATGGATAAAGATGTTCAA
AAAACATTACAAAATGATGTTGATAATGGAAGCTTCTACAAGAATAAAGACCAACAAGT
TGGTGCAACGATTCTTGATAGTAAAACTGGTGGTTTAGTTGCTATATCTGGTGGACGTGAT
TTCAAAGACGTCGTTAACAGAAACCAAGCAACAGATCCTCACCCTACTGGTTCATCTTTA
AAACCTTTCTTAGCGTATGGACCTGCCATTGAAAATATGAAATGGGCAACAAACCATGCG
ATTCAAGATGAATCTTCATATCAAGTTGACGGTTCTACATTTAGAAACTATGATACGAAG
AGTCACGGTACTGTATCTATTTATGATGCTTTACGACAAAGTTTCAATATCCCAGCTTTAA
AAGCTTGGCAATCAGTTAAGCAAAATGCTGGTAATGATGCACCTAAGAAATTCGCTGCCA
AACTTGGCTTAAACTACGAAGGCGATATTGGTCCATCTGAAGTACTTGGTGGTTCTGCTTC
AGAATTCTCACCAACACAATTAGCATCAGCATTTGCTGCAATCGCTAACGGTGGTACTTA
TAACAACGCGCATTCAATTCAAAAAGTAGTTACTCGTGATGGTGAAACAATCGAATACGA
TCATACTAGCCATAAAGCGATGAGTGATTACACTGCATACATGTTAGCTGAGATGCTAAA
AGGTACATTTAAACCATATGGTTCTGCATATGGCCATGGTGTATCTGGAGTAAATATGGG
TGCTAAGACAGGTACTGGTACTTACGGTGCTGAAACTTATTCACAATATAATTTACCTGAT
AATGCAGCGAAAGACGTGTGGATTAACGGCTTTACACCTCAATACACTATGTCAGTGTGG
ATGGGCTTCAGTAAAGTTAAACAATATGGTGAAAACTCATTTGTGGGACATAGCCAACAA
GAATATCCACAGTTCTTATATGAAAATGTGATGTCAAAAATTTCATCTAGAGATGGCGAA
GACTTTAAACGTCCTAGCTCAGTAAGTGGTAGTATCCCATCAATCAATGTTTCTGGTAGTC
AAGATAACAACACTACAAATCGTAGTACACACGGTGGTAGTGACACATCAGCAAACAGC
AGTGGTACTGCACAATCAAATAACAATACTAGATCTCAACAATCTAGAAACAGCGGTGG
ATTAACAGGTATATTCAACTAA

> **Q1YBU7**

Function: protein coding sequence; putative protein (core genome, constant)
Best match: Q1YBU7_CC001_MW2-USA400_BA000033.2[1491278:1491619:r]RC
Position: 036-contig_189: 51978 … 52320; Length: 342 bp
Sequence:
CTATACCTGCTTATTCTTTAACCTTGTTTGATAACTAGCATTCATTTTAATCAATTCAGTTT
TAAAGGTCTTCATTTGTCGATAGCCCATAAAATGATATTTACGTTCAATCATATAATCATA
TCTTTCTTTAAAATTCATTGGCACAACAGGATATTGATTTAAATTATCAAAATCAATATTG
CCAGTTATGTCATTAACCTGCTTAAAATAATCAATAATTTTACTATGGTATTCATCCAAGT
AGGGCTTTGCTTCACTAGAACCTAATGTCTTATTTTTGGCAAATACATCAATCTCTTGTTC
TAAATTATCAAATACCTCTTTTGTAATATTTGTCAT

> **nth**

Function: protein coding sequence; putative endonuclease III (core genome, constant)
Best match: nth_S-epidermidis_AGTZ01000030.1[432903:433562:r]RC (completely identical)
Position: 036-contig_189: 52324 … 52984; Length: 660 bp
Sequence:
TCACGCTTCTTTCAAACTAGCTTTATAACGTTTTTGTCCTTCTCTACAATCTTCTAATAATG
GACAAATATCGCATTTAGGTTTTCGGGCTAAACAGTGGTATCTTCCAAAGAAAATGAGTT
GATGATGGCTCCTATTCCATCTATCTCTAGGTATGACAGAACATAAACGGTCTTCTACCTG
TCTCACATTATCTTTCCAACGATTAATACCTAAGCGTTTAGAAACACGTTCTACATGCGTA
TCAACAGCTAATGAAGGTTCATCAAATGCTACACTCATGACTACATTAGCTGTTTTACGTC
CTACACCTGCTAAACTTTCTAATTCCTTATGTGTTTGTGGTATTTCTCCATTAAATTGATCA
ATCAAAGATTGACAAAGTTTCTTAATATTCTTAGCTTTGTTACGATACAAACCGATAGAA
CGAATATCATTCATAAGTTCTTCATCACTGACTGCCAAATAATCTTCAGGCGTTTTGTATT
TTTTAAACAGCTCAGTTGTTACTCTATTTACTAGAACGTCTGTACATTGCGCTGACAATAA

TACAGCAATAGTTAATTCGAACGGATTATCATGTTTTAATTCACATTCTGCATCCGGAAAC
ATATTTGCTATAACATCAACCATTTCTAATGCTTTTTTCTTACTTACCAT

> **dnaD**

Function: protein coding sequence; primosome/dnaD subunit (core genome, constant)
Best match: dnaD_CC001_MW2-USA400_BA000033.21492273:1492959:rRC
Position: 036-contig_189: 52973 … 53660; Length: 687 bp
Sequence:
TTACTTACCATCAAGGTTCTCCCCATTTAACCAATCAAATTTAGGTACCGTTTTAACTGTG
TGCGTCATTTTCGGTTTATTGAATTTTTCTCTTATTTTTCTAGAATCGTCAATTGTTTTGAC
ATTGTTTTTCTTCCAATTAAGTAAAATACGATCTATATATTTAAAGCTAAGTTTATTCAAA
CTATTCGCCTCGTCTAATGCCGCTTGTATAATTGCAGTATCGTGTTTATCAACATCAATCC
ATTGATTTAACGTTTCTATTTCATATGGAGATAACGGCCTTGCAAATGTATCCTCTAAAAC
TCTAAATAATTGTTTAAATTTTTCTTTACTATTTTGCTCTTTCGTTTCCATACTTTGTTGCTT
CAATATATGACTTAATTTTTCGAAAAAAGGATCTAGATTCATATATTCGGTAAATCTACCT
TCTTCATCTTTTTGAACTTGTAATTCTAGCAATTCACGTTGTATCAAATTTTGAATAACCAT
TGTAATATCACGTGGTTGCATAGTTGAGCCCTTCTGAAGTAATTCAATTGAAGGCTGTTTA
TTTGATGTTTCGGAAGCATAAATCAATTTAAGCAAAATGACTAAATCTTGCTCATCTAAA
CCTAAGTCACTGTAATGGTCTAATAATTCTCTTCGTATCACTACAGGTCTTGCTTTTAATT
GATATTTATCCAT

> **asnS**

Function: protein coding sequence; asparaginyl-tRNA synthetase (core genome, constant)
Best match: asnS_CC001_MW2-USA400_BA000033.2[1493287:1494579:r]RC (completely identical)
Position: 036-contig_189: 53987 … 55280; Length: 1293 bp
Sequence:
TTATGGATATAAACGGTTTAATAATCTTGGGAATGGCGCTGTTTCACGAACGTGTTCAAC
ACCAGAAATCCATGCTACTGTACGTTCTAAACCTAAACCAAATCCACAGTGTGGCACACT
ACCATAACGACGTAAGTCTAAGTAGTAACTATATGCTTCTTCGTCTAATCCATGTTCTTTA
ACGCGTTGTTCTAACAATTCTAAGTCATCCACACGTTCAGATCCACCAATAATTTCACCGT
ATCCTTCAGGTGCAATTAAGTCTGCACATAATACAGTTTCTTCATTTTCAGGATTTGGTTG
CATATAGAAAGGCTTAATTTTAGTTGGATAATTAGTAATAAACACCGGTAAATCATAATG
ATTAGCAATGGCTGTTTCATGTGGCGCACCAAAATCTTCGCCCCATTCAATATCATCAAA
GCCTTCTGATTTTAAGAATTCAATTGCATCATCATATGAAATTCTAGGGAATGGTGTCGCA
ACTTTTTCAAGTTTTGATGTATCACGCTCTAAAATTTTCAACTCTAGTTTACAATTTTCTAA
AACTGATTTTACAACATGTGTTACATATTGTTCTTGAATTTCTAAACTTTCAGCATGATTT
GTGAAAGCCATTTCCCCTTCAATCATCCAGAACTCGATCAAGTGTCTACGTGTTTTTGATT
TTTCAGCTCTGAAAGTTGGACCAAATGAAAATACTTTTCCGTGTGCCATTGCTGCAGCTTC
TAAGTATAACTGACCACTTTGAGATAAAAACGCATCTTGATCAAAGTATTTAGTATGGAA
TAATTCACTTGTACCTTCTGGCGCACTTGCTGTCAAAATTGGTGGATCAACCTTTGTAAAT
CCATCTTTGTTGAAAAATTCATACGTTGCACGAATAACTTCATTTCTAATTTTCATTACAG
CATGTTGTTTTTTAGAACGTAACCATAAATGACGGTGATCCATTAAGAATTCTGTACCATG
ATTTTTAGGTGTAATCGGATAGTCATGCGCTTCTGAAATAACTTCAATTGATTTCACTTGC
ATTTCGTATCCTAAGTCAGAACGATTATCTTCTGTAATTGTGCCTGTAACGTATAGAGATG
ATTCTTGAGTAATTTCTTTCGCAAGTTTGAATACCTCTTCATCAACTTCTGATTTAACTACT
ACGCCTTGCATAAAGCCTGTTCCATCACGTAATTGTAAAAAGGCGATTTTACCACTTGAA
CGTTTATTTGTTAGCCAAGCACCAATTGTAACGTCTTGGTTTAAATGATCTTTCGCTTGTTT
AATCGTTGTTTTCAT

> **dinG**

Function: protein coding sequence; single-stranded DNA and RNA nuclease (core genome, constant)
Best match: dinG_CC001_MW2-USA400_BA000033.2[1494901:1497594:r]RC
Position: 036-contig_189: 55601 … 58295; Length: 2694 bp
Sequence:

TCACTTTTTCTTTTTTTGAATTTGTCTTAATAATTTTCCAAACTGTTGAATGTCGCCTTTTTT
CTGACGATAATTTTCAAGTGTTTGTTCAAAAAAGTTTTTATAATTACTGTTTATAAGTCGA
TCATCAAATGAAACTATTATGCCGCGATCATTTTCACTTCTAATTAATCTTCCAAGTCCTT
GTCTAAAACGTGTAACTGCATCAGGTAATACATATTCCTTGAAAGTTGAAGTGAATTCAG
AATCCATAAGCCAATATTTTGCATTATGCTTGTTCATAAACGGTAACTTCGCTATCATCAC
ACATTTAATACCATTTGCTTGAAAATCAAAACCTTCAAAAAATGTTGACGTACCAAGCAG
TATGGCCTTATCAAAATTATTAAACTGTTGTACTATTTTATAATTTTGGTTCTGCTGTTGTG
TTAATACAACATAATCTTCAAATTCTGGCAATTCATTTAGCATATCTTGTACCATATGCAT
CATTTTATAACTCGTAAATAAGACTAAACATTTTGATGACGTTATAGTCGTATATTCAATA
ATATAGCTTACAATCGATGCTACATACTCATCTATATTTTTATATTGATAAGATGCTACAT
CACTCGGTATAAATACACTTGTATTTTTTGCACTTTGTAACGACGTGTTAACTTCAAATGT
ATTAAAGTGAACATCTTTGTTGAATAACTGTTTAAAAGCTTCAAACGAATGATTAAATTTT
AAAGTACCAGATATAAAAATGAGTGATTTAAATTTTTCCAATACTTGTTTCGTTAATACAT
CTTTTACAGCGTAATCTTTCACATATAAACGTATTGTTGATTTTTGAGATAAATTTTTAATC
GAAATGAAACTAGTATGTCCAGCTTTTAAACTTTGTTCGATATTTTTAAATTTATCTTTTA
AATACAACAACTGTTTCCGTAATGATTTAACTGTTTTATGACTAATGCCATTGAATATTTC
TAGCGTTTTATTTAACTTATCGATAATCGCATGTAAATCCTTCAAAATGTCTTTTGTTTCAA
AAGTAAATACATTATGGAAGCGATGAATATCATCATCATAAACATCAGAATCATTGATAA
TCGTAAATATCGTTGAGAACAATTGCTCATTTAACTCATGAATCTCATTCATACTAGCCTT
CAAGCCAAAAATATCAATTGGTGCAATATCTAATTTTTCCAAAATTCGCTGCTTTTCCAGT
TGATCAATTGCCTTTAACAATTTTTCATTTTCGTTTTTACCAATCAAACCAAGCTGATATTT
AATATCAGCATAACTCAACTCATTTGTCACTTGATTTAAGGCATAGTCTGGTAAGCGATGT
GCTTCATCCACTATACAATCATCAAACAATTGATATATTGAATTTTCAACATCAGAATGA
ATTAAATGTGCATGATTTGTAATACCAATTTGAATGTTCTGTGCATTTCGCTTAATAAAAT
TATAATAATGAACATCGTGACGTGCCGGTACATATGTTTCAATTTTCTGGTCAAAATACAT
CTTTTGACCACCTTTTAAATTTAATTCCTGTATATCTCCGGACGGCGTCTCTGTAATCCAA
ATCAACAATTGCATTTTCAAGATATTCACTTCGTAATTACTTGTGTCATCTTTTAAAATTTG
ACTAATAAGTCCCAATGAAATGTAATCACTTTTACTTTTAATCAATAGTGCATTAATTTTA
AAATTCAACGCTTCATTCATTGCTGGAATATCTTTTTCTAACAATTGACTTTGCAGTAATT
TAGTATTGGTAGAAATCATGACATGCTTCCCAGTTTCAATATTATACATCAAGGCCGCAA
GTAAATATGCTAATGATTTACCACTGCCTAGTGATGCTTCAATCATTGCTTTTTCACTATG
CATGAGCTGATCTAATATAGTTTCCGCTAAATATAATTGTTGCGGTCGATATGTTAAGCCA
AGTTGATCTACAGCTTTGCTATATAAAGACTTCAAGCTGCCATTATAATTTGTTGTCGGCT
TTTTAAAATCAACTTGCTTACGATAGATAATCTGTTCGAACTTTTCGTACGATTTATCCAA
TGGCTTTGCATCATATTGCCTAACCATCTCAAAGAAAATATCATACAAATCGTATTTCAAC
TGTTTACTTAAATAATATAATTGCTTTAAAGTATCTAACGGTAACTTTTCAAATTTTTCAA
AAGCTAATATCATCAATTTAGCAGTAGTAGCGGCATCTTCGTCAGCTCGATGGGCATTTG
CTAAGGTAATACCATGTGCCTCTGCTAATTCACTTAATTGATAGCTTTTATCTGTAGGAAA
AGCTATTTTAAAGATTTCTAGTGTATCTATAACTTTTTTGGGACGATATTGAATATTACAA
TCTTTAAATGCCTTTTTAATAAAATTCAAATCAAAATCTACATTATGAGCGACAAAAATG
CAATCTTTTATCTTATCGTAGATTTCTTGTGCAACTTGATTAAAATATGGCGCTTGTTGTA
GCATATTTTCTTCAATGGATGTTAACGCTTGAATGAACGGCGGAATCTCTAAATTTGTTCT
AATCATAGAATGATATGTATCAATAATTTGGTTATTGCGCACAAACGTTATACCAATTTG
AATGATATCGTCAAAATCTAATTGGTTGCCTGTTGTTTCCAAATCCACAACGGCATAGGTT
GCCATACCCAT

## > birA

Function: protein coding sequence; BirA bifunct protein (core genome, constant)
Best match: birA_CC001_MW2-USA400_BA000033.2[1497618:1498589:r]RC
Position: 036-contig_189: 58318 … 59290; Length: 972 bp
Sequence:
TTAAAAATCTATATCTGCACTAATTAAACGGTGTGATTCACCCGCTTCATCTCTAACAATT
AGATAGCCATCGTAATCTAAATCAATTGCTTGTCCTTTAAACTGTTTATCATTTTCTGTAA
ATAGCAACGTTCTATTCCAAATATTAGAAGCTGCAATATATTCTTCACGAATTTCAGAAA
AAGGTAACGTTAAAAATTGATTATATCTTTTTTCAATTTCTTGAAGTAATCTCTCTAAAAA
TTGATATCTATCTAATTTATTTTTATCATGTAATTGTATACTTGTTGCTCTATGTCTAATAC

TTTCATCAAAGTCTTCTAGTTGTTGCGTCAAATTAATACCTATACCACATATTATTGCTTCT
ATACCATCATTATTAGCAACCATTTCAGTTAAGAAACCACACACTTTACCATTATCAATAT
ATATATCATTCGGCCATTTCACTTTGACTTCATCTTGACTAAAATGTTGAATCGCATCTCTT
ATCCCTAATGCAATAAATAAATTAAATTTAGATATCATTGAGAATGCAACGTTAGGTCTT
AACACGACAGACATCCAAAGTCCTTGCCCTTTTGAAGAACTCCAATGTCTATTAAATCGC
CCACGACCTTTCGTTTGTTCATCACTCAAGATAAAAAATGAAGATTGATTTCCAACAAGT
GACTTTTTCGCAGCAAGTTGTGTAGAATCTATTGAATCGTATACTTCACTAAAATCAAACA
AAGCAGAACTTTTTGTATATTGGTCTATTATACCTTGATACCAAATATCTGGGAGTTGTTG
TAATAAATGCCCTTTATGATTTACTGAATCAATTTTACATCCCTCTAACTTTAATTGGTCA
ATCACTTTTTTTACTGCAGTGCGCGATATATTAAGTGATTCCGCAATGCTTTGTCCAGATA
TATAATTCGGTTTATTTTTATAGAGTAATTGAAGTACATCTTGACTATATTTTGACAT

## > **papS**

Function: protein coding sequence; tRNA CCA-pyrophosphorylase (core genome, constant)
Best match: papS_CC001_MW2-USA400_BA000033.2[1498576:1499778:r]RC
Position: 036-contig_189: 59276 … 60479; Length: 1203 bp
Sequence:
CTATATTTTGACATGATTATCCACCCATTTCAAAATTTCAGTTTCTTCGTTGCTTACTTTAC
CTGTTACAATCGCTATCTCAATTTGTCTTAGCACATCTTTTAACCACGGACCACTTTTGGC
ATTTAAATGTGCCATAAGTACACCGCCATTAACCATCATGTCTTTTCTATTATGCATAGGT
AAACGATGCAATGTTTCATCAATCGTTTGAAGGTTAACGATTAATGGTTCATGTCCTTGAA
TATCATTTGCCTTTAACACGTCTGCTGCAACCATTACATTTTTAATGAGATGCGTATCATA
ATCATAAACAAACATTTTTAATTGTTCTTTTGTAATAATACTTGGTAATGCATTCATAATT
TGAATATATTGATTGATATCTTTTACTTGTCGGTTACTTAGCTTTAAAGGCTTCAATGAGT
AATTAATATCAAATTTAACTGACACTATAGCAATCAACAATTCTAAATCAATTGGTTCAG
TTACATTAATTTGATTCATATCAAGTTGTTCGAAATATGGCATATAATTAAATGCTTTCAG
CGATTTTAAATGAATAAAACTCTCTTCAACATTAATACCTCGCATTAATTTAGTTAGTTCA
ATCACTATACGCTCAATTGATAAAAAATTTAATATCTGCCATTTGTGTACGCATCGCTTCAA
ATGTTTCCGTTGCAATATCAAATGATAATTGTGACTGGAATCTTAAACATCGAATCATAC
GTAAAGCATCTTCTTGGAATCGTTCCTCAGCTATACCTACAGTTCTTATTATTCGATTATT
AATATCTTGTTGACCATCAAAATAATCATACAATTTGTATGCTGTATCCATTGCTATCGCA
TTCATCGTGAAATCTCTTCGTTGCAAATCTTCGTATAAATCCCGAACAAATGTAACACCAC
TTGGTCTACGGTGATCGACATAATCTTCTTCAGCCCGGAATGTTGTCACTTCATAATTTTC
ATTATTAAAAACTACATTTATGGTGCCATGTTCTTTACCTACAGGTATCGTATGACTAAAG
ATAGATTCTATTTCATCCGGCGTTGCACTTGTTGTGATATCTATATCATGAATATTTCTTCC
CATGACATAATCTCTTACAGAGCCACCTACATAATATGCTTCAAAACCATTGTCTTGAATT
TGTTCTAATATAGGCCTTGCCTGTTCAAATAATGATTTATCCAT

## > **bshA**

Function: protein coding sequence; N-acetyl-alpha-D-glucosaminyl L-malate synthase (core genome, constant)
Best match: bshA_CC001_MW2-USA400_BA000033.2[1499783:1500925:r]RC
Position: 036-contig_189: 60483 … 61626; Length: 1143 bp
Sequence:
TTACTCGCCTTTACTTTTGTTATGCTCATTTAGCATTTTTTGATAATAATACTCATATTGAT
CTGTAATAAGTTCTGATCCAAAACGTTCAGCAATATCTGCTAGCATGTTTTTCTGAAGTTT
GTTGTATAACACCTTATCTTCAAGTAATCGGATAGCATAGTCACTCGCTGAATCACAATC
ACCCACATCTACGACAAATCCAGTTTCACCATGTTTAATAACCTCTTTAATTCCACCGGCA
TTTGAACCAATTGGAACGACGCCTGTTTTCATAGCCTCAAGTAAAGTTAGTCCAAAGCTTT
CTTTTTCACTTAATAATAATACTAAGTCAGATAATTGGTAAAATTCACTTACGCAATCTTG
TTTCCCTAAAAATAAAACATCCTCTTCTACGTTTAACTCTTTCGTCAATTGACGCATTGGC
ACTAATTCAGGACCATCTCCAAGTAAAATTAATTTACTAGGTATCTTTTCACGTACTTTTG
CAAATGTTTCTATAATAGTATCTATGCGTTTTACTTGTCTAAAATTCGATACATGTATTAA
CACTTTTTCATCTGGTGCTATACCAAATTGTGATTTTAGTGCTGTGTTATGTTTAGTTGGAA
ACTCATTTTCACGTACAAAATTATAAATCGGTATAATTTCTTTGTTAGTTTCAATAATTTC

ATGTGTTTCTTGTGCTAAAGATTTACTCACACTTGTCACAATATCACTTTTTTCAATGCCA
AATTTAATTGCACCTTGGAGTGAATGATCATAGCCCAAAACAGTAATATCAGTACCGTGT
AGCGTTGTCATAATTTTTATATCTTTACCTGACATCTCACGAGCTAAAATCCCACAAATTG
CATGAGGTACAGCATAGTGCATATGCAACAAATCAAGATCATATTCTTTAATAACTTCAG
CGATTTTAGTACTTAACGTAATATCATACGGTGGATACTGAAATACTGCATATTGATTCAC
TTCAACTTGATGAAAAATCATATTCGGTAATGGTTTTCTTATTCTAAACGGGATATTTGAA
GTGATAAAATGTACTTCGTGACCTCGCTCTGCTAATTTAATTCCTAATTCTGTGGCAATAA
TTCCAGAACCACCCATGGACGGGTAACATGTTATACCTATCTTCAT

## > ypjD

Function: protein coding sequence; MazG nucleotide pyrophosphohydrolase (core genome, constant)
Best match: ypjD_CC001_MW2-USA400_BA000033.2[1501167:1501484:r]RC (completely identical)
Position: 036-contig_189: 61867 … 62185; Length: 318 bp
Sequence:
TCACTTTCTTTCGAATCGATTTTTATCTCTTGTATTAAACTTTTCCATAGTTTCATTAAAGC
TCTCTGTCATATCTATTCCCATTGAATTCGCTAAACATAACAACACAAATAAATTATCACC
TAATTCTGCTTTAATCGTATTTGCTTCCTCTGAATCTTTCTTCTTTTTTTCACCATAGGTATG
ATTTATTTCACGTGCAAGTTCGCCCACTTCTTCAGTCAATCTAGCTAAGTTAGCTAATGGT
GAAAAATATCCTGTTTTAAATTGTCCAATATATTCATCAACTTCACGTTGCATTTCTACCA
TTGATTTCAT

## > ypiA

Function: protein coding sequence; putative protein (core genome, constant)
Best match: ypiA_NCTC8325-4_L05004.11384:1735RC
Position: 036-contig_189: 65331 … 65684; Length: 353 bp
Sequence:
TTATCAATAGCAACTTCCATCATATTAATTTGTTGATATAAATCTGCTTCTAACATCAACT
TTTCAGGTGATGGTTCAACATAACTTAAATATTCTAACGCTTCGTCAGTTTGATTTTCAGA
CATTAAACCTTCAATAAAATAAATCAGCAATTCACTTTCGTCTGGATATTTTTGATATAAC
ACGCGGAATACTTCCAAACCTTGTGGCATTAATCCAAAATTGTAAAGTGTCTCTCCTAGA
ATAAATAATGCGTCATCGTTGTCAGTAGTTATTGCTTCATTAACACGAGAGTCTAAATTTT
CTAGTTTTTGTAGATTGATATCGTCTATTAATTTATAGATATCTTCCAT

## > aroA

Function: protein coding sequence; 5-enolpyruvylshikimate-3-phosphate synthase (core genome, variable)
Best match: aroA_CC001_ATCC51811-ST1_ADVP01000007.1[24732:26030]RC
Position: 036-contig_189: 65690 … 66989; Length: 1299 bp
Sequence:
TTATCCCTCATTTTCTAAAAGCTTTAGTTTTGGTAAAAATCCTGGAAATGATACATTTACA
GCATCAAATTGTTTGATTTTGACAGGCTCGCTTGAAAGTAGAGAAGCAACTGCAAGCATC
ATTCCTATTCGATGATCAGTTAAACTATCAACTGTTGCATTTGTTTTAAATTCTGACGGAT
GAATAATCAATCCATCATTAGTTGGTTGTAATTCAAACCCTAACAAGTTTAACATATCAG
CCGTTGTATCAATTCTATTTGTTTCTTTTACTTTTAATTCCTCGGCATCTTTAATTGTACTCG
TGCCAACTGCTTGTGTACAAAGTAATGCTATTACAGGCAGTTCATCAATTGCTTTTGGAAC
TAATTCTCCTTCGATTGTTATTGGTTGAAGCATTGGTGTGTATTGAATACGAATAGAAGCA
GTAGGTTCAGCACCAGTTGTTTGATTGAAAAGTTGGATATTACCGCCCATTTTTTCAACAA
TATCAATAATACCTGAACGTGTTGGATTGATTCCAACATTATGAATTGTTACATCACTTCC
TGGTGTGATAAGTGCTGCAACAATAAAGAACGCTGCAGATGAAATATCGCCAGGAACAT
GAAAATCTGCAGGTTTAATGTATCGAATTGCTTCAGGGGTTGTATTAATTGATAACCCTTC
TGCTTCAATTGGAATATTAAAATGTTTGAACATCGTCTCAGTATGATTTCGACTTACATCT
AATTCTTTAATGATGGTCGGTTCCTTAGAAAACAAACTTGCAAATAAAATGGCACTTTTTA
CTTGTGCACTTGCAACTCCCATTTGATAATTTATACCTTTTATGACAGATGGCTTAATAAT
TAATGGTGTATAATTATCTTCAATACCTTCAATATTCGCATCCATAAGTTTCAATGGTCTC

AAGACACGATCCATTGGCCTTTTACCAATTGAAACATCGCCAGACAAAACACTTTCAATA
CCTAAACCACTTAACAAACCAGCTAATAATCGTGTTGTCGTACCAGAATTACCTGTATAC
AATACTTGATGTGGCGTGTTAAAAGATTGATATCCTGGGGAAGTCACAACTAATTTTTCA
TCATCTTCTTTGATTTCTACACCTAACAGTCGGAAAATGTCCATCGTACGACGACAATCTT
CGCCAAGTAGTGGCTTATATATAGTAGATACACCTTCAGCTAGCGACGCCAACATGATTG
CACGGTGTGTCATTGACTTATCGCCCGGCACTTCTATTTCGCCCTTTAACGGACCTGAAAT
ATCAATGATTTGTTCATTTACCAT

> **aroB**

Function: protein coding sequence; aroB (core genome, variable)
Best match: aroB_CC005_Mu50-VRSA_BA000017.4[1580838:1581902:r]RC
Position: 036-contig_189: 66998 … 68063; Length: 1065 bp
Sequence:
CTACTTAAAATATGTTTTTAGTTGTTCACACGCATGTTGCAGTGTTGTTTGATCAATGTGTT
GCACTACAATATCGCCAAAATGTTTAATCAATACCATTTGAACTCCTTGTTTGTCGTTCTT
TTTATCACTTAGCATGTATTGATATATCGTTTCGAAATCTATGTCGGTTATTGTTTCTAAAG
GATAACCTAATTTCGTTAAATAATTAATATAATGTTGGATATCGTGATTAGAATTGAACA
ATATATTCGCAACAATAAATTGATATATTATGCCTATCATTACGGCATGACCATGAGGTA
TTTTATGATAGTATTCAACAGCATGCCCAAATGTATGACCTAAATTTAAAAATTTACGTAC
ACCTTGTTCTTTTTCATCTGCAACAACAATATCCAGCTTCGTTTCAATACCTTTAGCAATAT
ATTTATCCATACCATTTAATGACTGTAATATCTCTCTATCTTTAAAGTGCTGTTCGATATCT
TGCGTCGTTGATTCACCATTCAATAACGCATGCTTATAAACTTCTGCATAGCCACTTAATA
TTTGCTCAAATGGTAACGTCTTTAAAAAATCTAAATCATAAATCACAGCAGTTGGACGAT
AAAATGCACCGATAAGGTTTTTACCTTGTTTTGAGTTAATACCCACTTTACCGCCAACACT
AGAATCATGCGCCAAAATCGTTGTAGGAACTTGTATAAAATGGACACCTCTTAATAGTGT
TGCTGCTACAAATCCTGCAAAATCTCCTGTCGCACCACCACCAACAGCTATAATCGCTGT
ATTACGCGTTACATGATGTGACAAAATGTATTCTAATGTTTCTTGATATTGCTCAAATGTT
TTCGTCTTTTCACCAGCTGGAATAATGACTTTATGCACATTTTCATATGATAAAATATCAT
CAAATTTATCAGCAAAATATTGATTTACATGCTCGTCAATTAATATAAAACTTTGATCAAA
CTGATCAATATACGTGCTAATATGGTCAATTGCACCATGTTCAACAAATATTGGATAATT
GTTTGAAGGATAGGTTGTTTGTAATTTCAT

> **aroC**

Function: protein coding sequence; aroC (core genome, constant)
Best match: aroC_CC005_Mu50-VRSA_BA000017.4[1581928:1583094:r]RC
Position: 036-contig_189: 68088 … 69255; Length: 1167 bp
Sequence:
TTAAAACTCAACATTTAATTGTCTGCGGTCAGCAATTTGTTGTTGAAGTTGTTCAATATGG
TTTGATTGAAACTCTTCAAGTAATGCTTTCGCTATTTCAAATGCAACAACGTGTTCACACA
CTATACTTGCTGCAGGTACAGCACAACTATCAGAACGTTCAATTGTTGCCTTAAAGTCTTC
TTTAGTATTTATATCAACTGAATTTAATGGTTTATATAACGTAGGAATAGGTTTCATTACA
CCATTTACAATAATTGGCATACCATTTGACATACCACCCTCTAAACCACCTAAATGATTAG
ATCCACGATAATAACCAATTTCACTATTATATAGAATTTCATCTTGAATCTCACTACCTGG
CTTTTCAGCTGCTTTAAATCCTTCACCAAAGCTTACACCTTTAAAAGCATTTATGCTGACA
ACACCTTGTGCAATCTTACCATCTAACTTACGATCATAATGCACATAACTACCAACACCA
ACAGGCATATTTTCAACTACAACTTGAACGACACCGCCAATTGAATCTCCTTCATTTTTAG
CTTCGTCAATTTTATCTCGCATTGCTTGTGCGATACTGTCATCAATTACACGAACATCATT
ACGATCAAGATTTGCTTTAAATGTTTCTGAATCATAAAAATCTTTATCTTTAATTCCACCT
ATTTCAACAACACGACTGTATATATCGATATCTAACTGTTGTAATAACACTTTACATAAGG
CACCGACTGCAACTCGAGCTGCTGTTTCTCTAGCAGATGATCGCTCTAGCACATTTCGTAA
ATCACGATGATTATATTTCATACCTCCAACCAAATCTGCATGACCAGGTCTTGGTTTTGTA
ATAGTACGTTTCATATTTTCACGTTCTTCTTCACTTATTGGAGCTGCTCCCATAATTTTTCT
CCAATGCGTAAAGTCATCATTGGTTACAACCATAGTAATTGGACTACCTAATGTATAACC
ATTTCTAACGCCTGATACTATTTCTACTGTATCTTTCTCAATTTGCATGCGTCGGCCACGA
CCATAACCCCCTTGACGCTTGAACATTTCTTTATTAATATCTTCAACTTTAATTTCTAAATT

TGCTGGTATACCTTCGACAATAACTGTTAATTGAGGTCCATGTGATTCTCCTGAAGTTAGA
TATCTCAT

## > Q2FYG8

Function: protein coding sequence; putative protein (genomic island)
Best match: Q2FYG8_CC001_MSSA476_BX571857.1[1534588:1534788:r]RC (completely identical)
Position: 036-contig_189: 69641 … 69842; Length: 201 bp
Sequence:
TTAAGCGCTTTTGTTATACTTTGACTTCAAAAATATAATCAATTCTTTTACTAATACATAA
AAAATATAAATGAGAGTCAGTATAAAAATAATTTTTGTTATAGGATTAAGTGGTTTATCT
AAAAATATAGCACCATATAGTAATTCCATTAATTTTAAGAACATTAAAATCCCCAAAATA
ATCGAAATAATATTCTTCAT

## > ndk

Function: protein coding sequence; nucleoside diphosphate kinase (core genome, constant)
Best match: ndk_CC001-ST772_118_AJGE01000058.1[82476:82925]RC (completely identical)
Position: 036-contig_189: 70050 … 70500; Length: 450 bp
Sequence:
TTATTCATATAACCATGCATCACGTGGTGAAGCATAGCTAGTAATTTCATTTTCATTAAAC
CATAGATTAATTTCACGTTCAGCAGACTCTAATGAATCTGAACCGTGAATGATATTTCTAC
CAACAGTTAAACCTAAATCACCTCTAATTGATCCTGGTGAAGCTTCTGAAGGATTGGTGC
TGCCAATAATATGTCTAGATACATTAACTGCATCTTCACCTTCAACTACCATTGCGAACAC
TGGTGCTGATGTAATAAATGAAATTAAATCATTATAAAACGGTTTGCCTTGGTGTTCACC
ATAATGTGTTTCAGCAAGTTCCATTGGTACTTGCATTAATTTACCACCGACAAGTTTTAGT
CCTTTTCTTTCAATTCTTGAAATTACTTCACCAATTAGATTTCTTTGTACTGCATCTGGTTT
AATCATTAAAAATGTACGTTCCAC

## > gerCC

Function: protein coding sequence; heptaprenyl diphosphate syntase component II (core genome, constant)
Best match: gerCC_CC001_MW2-USA400_BA000033.2[1509891:1510850:r]RC
Position: 036-contig_189: 70591 … 71551; Length: 960 bp
Sequence:
CTACGTGTTTCTTGAACCCATTTTTTTCGTCAAACTTAAAAGTAGTGATTTCGGATGTCCA
TCTGGTAACTCAGAAATCAAATCCAAAGCTTTACTTAAATACTTCGAACTTACTGCTTTAG
ATTCATTAATACTGTCAGACTTTCTAATAATTTGAATACATTCTTCAAATTCTTTTCGATCG
CTGTCACGTCGTAATTGCTCAATTTTCAATTTGAAGTCTGGGTTTTTACGCATTTCTAATA
AAATCGGTAACGTAATATGACCATTAAGCAAATCACTTCCGACCGGCTTACCTAATTTCTT
TTCGGTACTTGTGAAGTCTAATACATCATCAATGATTTGGAAGCTCATACCTATATAATGA
CCAATCATTTTCAATTTTCGTACAGTCTCTTTATCAGATTGAGATGTAATTGCACCAACTT
CAGTTGATAATTGAATTAACAGTGCTGTTTTGCGATTGATACGTCGTAAATAATTGATAAT
TGTCTGTTGACTGTTAAATTGGTCTTGAAATTGGAAAAGTTCCCCTCTACAAACATCAACG
ATAGATTCAGATATCAATTGATGTACACGATTATCTTTAACGGCCATTAAGTGTTCAAGTC
CTAATGCCAATAAAAAATTCCCAGTTAAAATAGCCGTTGTCTGATCCCATTTCTTTGATAT
GGTTAACTTGCCTCGACGCTTGTCGCTTTTATCAATAACGTCATCATGAACAAGTGTTGCC
ATATGAATTAACTCTAATGCGACTGCAACATGATACGTTTGTTCAGACGTTTGTTCATCTT
TACCAAATTGGCTACTCAGAATCACAAATGCAGGTCGTACACGTTTTCCACCAGAAGACA
ATAAATGTAAAGAAGCTTGTTCTAGAACAGAATCTTTACTTTTTATTGCCTTTTCAAGTCG
TTGTTCCACTTTCTTAATTTCATTGTTCATGTTTAACTTTGCCAC

## > ubiE

Function: protein coding sequence; menaquinone biosynthesis methyltransferase (core genome, constant)
Best match: ubiE_CC001_MW2-USA400_BA000033.2[1510852:1511577:r]RC

Position: 036-contig_189: 71552 … 72278; Length: 726 bp
Sequence:
TTAATCACCTTTGGTATTATCTTTTTCTTTATAGCCAAGGTGCATTGCAGCAACGCCCCCT
GTAAAACTACGTACTCTTACATTTATGAAACCCGCTTCTTCAAACATGCGCTTCAACTCTT
CTTTTCCAGGAAAATTAAACGTAGATTGCTGTAACCATTCATATTCTTCTTTTGATTTTGC
AAATAATTTTCCAAAAATAGGCATAACAAATTTAAAGTATAATGCATACATTTGTTTAAA
GACTGGCAAAGTTGGTTGGCTCGTTTCAAGACATACCACCATACCACCTGGTTTAAGTAC
TCTATTCATTTCTTTTAACGCGACTAAATAGTCTGGCACATTTCTTAATCCAAACCCAATT
GTTACATAATCAAAAGAATTGTCTTCAAACGGCAATTCCATTGCATCACCATGAACAAGT
TTAACATTTTCCATTGAAGCAGTTTTTTCTTTTCCTACTTCTAACATATTCTCACTAAAGTC
AATACCAGTAACTTCACCTGTTGGTCCTACAGCTTTGCTTAATGCGATTGTCCAATCACCA
GTACCACAACAAACATCTAATGCTTTCGTCCCTTTTCTAACACCCATGTCTTTCATGACGC
GTTTTCTCCATACTTTATGCTGCTCAAAACTAATAATATTATTTAATCTATCATATTTTTTT
GAAATATTTTGAAAAACGCGATGTACTTGCTCTTTATTTGCTTTATTGTCAGCCAT

> **gerCA**
Function: protein coding sequence; putative protein (core genome, constant)
Best match: gerCA_CC001_MW2-USA400_BA000033.2[1511580:1512152:r]RC
Position: 036-contig_189: 72280 … 72853; Length: 573 bp
Sequence:
TTAATTACCTCTACTTTTTAAATAACTTTTTTGGATATCGTGTAAGTAATGCTTTACTTCAC
TTTGATTATATTTCTTGAAGTATGATGGATAGTAATCAGACATATCTTCAAATAAATAATT
ATATATTTCCGACTCATCAATATTGATACCGAAATGAGATAACGTAATATATGGGAAAAG
TGTTTCAATTTTTACTATTGCTTGAGAAATTTCATAATCATTTAAAGCTTGATGATGTAAT
GAAGATTTCAATTCATTAATTTCAACAATTGCTTTACTAATTTCATTTTGAAATGATAAAT
CATTGATTTCTGCTAGTAGCGTATAAAAATGTGCACTAATTAAATCTCCAATCAAAATGG
AATGTTTAGACAAATGATTATATGTAATGTCATCAAGGTGTCTCATTGATGTGTCAATTGT
GAGGCATGCCACTTTGGCAACATCTGGAATATCATATGAATCAAGTAACTTACCTAACCG
ATGATTAATATTAATGGATTCATATTCTGATACGCCCTTTAATCTTTCTTCTATTTGTCTTT
CCAATTTGCTAACAGTTGTTTCCAT

> **hup**
Function: protein coding sequence; DNA-binding protein HU (core genome, constant)
Best match: hup_CC001_MW2-USA400_BA000033.2[1512583:1512855:r]RC (completely identical)
Position: 036-contig_189: 73283 … 73556; Length: 273 bp
Sequence:
TTATTTTACAGCATCTTTTAATGCTTTACCAGCTTTGAATGCTGGAACTTTACTTGCTGGG
ATATCAATTTCTTTACCAGTTTGAGGGTTACGACCTTTACGTGCAGCACGTTCACGTACCT
CAAAGTTACCGAAACCAATTAATTGTACTTTTTCACCTTTAGCAAGTGAGTTTTGGATTGA
TTCGAATACAGCATCTACTGCTGAACCAGCTTCTTTTTTAGTTAAATCAGCTTGCTCTGCA
ACTGCATTGATTAAATCTGTTTTGTTCAT

> **gpdA**
Function: protein coding sequence; glycerol-3-phosphate dehydrogenase (core genome, constant)
Best match: gpdA_CC001_MSSA476_BX571857.1[1538674:1539672:r]RC
Position: 036-contig_189: 73726 … 74725; Length: 999 bp
Sequence:
TTATTCAGATTTTTTATCGCGCTCCATTAAATCTTTTACGCATTCTTTTACTGAGATATTTT
CAAATAATACTCTATATAATGCATTTGTAATTGGCATATCCACATTTTTTTCTTTAGCTAA
ATGATAAACTGATTTAGTTGTATAAATACCTTCAACAACCATATTCATTTCAGATAATGCT
TGATCCATTGATTCACCTTGTCCAAGTTTATATCCTAATGTGAAATTTCGAGAATGTGTTG
ATGTGCAAGTAACGATTAAGTCACCGATACCACCTAAACCTAGAAATGTCATAGGATCGG
CACCTAACTTTTCACCTAATCTACTAATTTCCGCTAAACCACGAGTCATTAATGCAGCTTT
TGCATTATCACCGTAGCCAATTCCAGCTACGATACCACTTGCTACTGCGATGATATTTTTC
AATGCACCACCAAGTTCAACACCAATCAAGTCATCATTCGTGTACACACGTAAATAATCA

TTCATAAATAAATCTTGCGTTAATTTACTTACATTTTTATCTTTTGATGAAGCAGCAACTG
TAGTTGGTTGCTTGACTACAACTTCTTCCGCATGACTTGGCCCGGACAACACGCCAATACC
TGCATTATATTCAGGTGAAATAGAATCTTCAATCATTTCTGACACACGTTTAAATGTCCCA
TTTTCAATACCTTTAGCAACATGTATAAAAGTCTTTTTAGATGTCAGCTTATCATTAATTT
GAGTAGCAACTTCTCGCATTGCTTTAGTAGGTAAAGCCATTAAGTAAATATCTGCAAATT
GAATTGCTTTGGTCATATCTGAAGTAGCGATGATGTTAACATCTAATTTCGCGTATTTTAA
ATACTTTTTATTTGTATGACATGTATTTAATTCATCAACAGCATCTTGATTTTTACCCCACA
TCAAAACATCATGTCCATTTTCTGCAAGAACATTGGCAAGGGCTGTCCCAAAACTTCCCA
TACCAAAAACGGTAATTTTAGTCAT

> **engA**

Function: protein coding sequence; GTP-binding protein engA (core genome, constant)
Best match: engA_CC001_MW2-USA400_BA000033.2[1514041:1515351:r]RC
Position: 036-contig_189: 74741 … 76052; Length: 1311 bp
Sequence:
TTAATTTCTCTTTCGAGCTATAATATGAATTGGTGTACCTTCAAAACCAAAAGCGGCACG
GATTTGATTCTCTAAATAGCGTTTATAAGAAAAATGCATTAATTCTACATCATTAACAAAT
ACAACAAATGTCGGTGGTTCTATAGCAACTTGTGTTGCATAAAAGACATTCAAACGTCTA
CCTTTGTCTGTTGGTGTAGGGTTCATGGAAATTGCATCAGTAACGACTTCATTTAAAGTTG
AACTTTGAACACGTTTTTTATGGTTTTCACTTGCTTCATTAATGTAAGGGAATAATGTACG
TAATCTTGTGCGTTCTTTAGCAGACACAAAAGCAATTTGTGCATAATCTAAAAATTGGAA
TTCTTTACGTACTTCATCTTCAAATTTCTTCATCGTTTTACTATCTTTTTCCACAGTATCCCA
TTTATTTACGACAATCACGACTGCTTTACCTTGTTCATGTGCATATCCTGCAACGCGTTTA
TCTTGTTCAATGATGCCTTGTTCTGCATCAATGACCACTAAAACAACATTTGAACGTTCAA
TCGCTTTTAAAGCTCTTAGTACTGAATATTTCTCAGTTGATTCATATACTTTTCCTTTTTTA
CGCATACCAGCAGTATCGATTAAAACATAATCTTGTCCATCATAACTATACTCTGTATCAA
TAGCGTCTCTCGTTGTCCCTGCAACATTAGAAACGATAACGCGATCTTCACCTAAAATAG
CATTTACTAAACTTGATTTACCTACGTTTGGTCGTCCAATAATGGATAGTCGAATTGTATC
TTCATCATAAGGATCTTCTTCCTCTTCACCAAAATGAGAAACAACTGCATCTAACAAGTC
ACCAAGACCTAAACCATGTGATCCTGATATCGGATACGGTTCACCAAATCCTAATGAATA
GAAATCATACACGTCTGTACGCATTTCCATATTATCTACTTTGTTAACCGCTAATACGACC
GGTTTTTTAGATTTGTATAAAATTTGAGCGACCATTTCATCGCTTTGTGTCAATCCTTCAC
GCACGTTAACCATAAAAATAATAACATCCGCTTCATCTATGGCGATTTCTGCCTGCGCTCT
AATTTGTGTTTGGAATGGTGCATCACCAATTTCAATACCACCTGTATCAATAATATTGAAA
TCATGTGTTAACCATTCACCTGAAGAATAAATACGATCTCGTGTTACACCTGGCGTGTCTT
CCACAATCGAAACACGTTCTCCAACTATTCTATTAAAAATTGTAGATTTACCTACATTAGG
CCTACCTACAATAGCTACTATAGGTTTAGTCAT

> **rpsA**

Function: protein coding sequence; 30S ribosomal protein S1 (core genome, constant)
Best match: rpsA_CC001_MW2-USA400_BA000033.2[1515573:1516748:r]RC
Position: 036-contig_189: 76273 … 77449; Length: 1176 bp
Sequence:
TTATAGTTTAAGATTTTTAAGTTTATCACCAATCATATCGCCAATTGTTGGATTATCTTCTT
CTTCGTTTTCTAAGTACGCCTTAGTCGTAGAAGGATCACTTTCAACAACATCTTCGTTTGG
TAATGTTGCTTTAATAGATAGTGATACTCTTTCATTCTCTTCATCAATACCTAATATTTTAA
CATTTACTTGTTGACCAGGTTCTAACACTTCACCTGGCGTACCAATGTGTTTGTGTGCAAT
TTCAGAAATATGTACAAGTCCTTGTACACCTGGTGCAATTTCAACAAATGCACCAAAGTT
TGCCAATCTTACTACGACACCTTCAATGACATCATTTTCGTGGAATTGACCTTTAATATTT
TCGAAAGGTGTTGGTAACGTATCTTTGATTGATAATGAAATACGTTCTGTATCTCTATCAA
TAGATTTAATTTTAACTTTAACATCTTGACCAATTGAAACTACTTCTTCTGGTGTTTGAAC
ATGTTCGTGAGAAAGTTCAGATACATGCACTAAACCATCAACACCACCAATGTCTATAAA
TGCACCAAATTGAGTTAAACGCGCTACTTTACCATCAATAACATCGCCTTCATTTAAAGAT
TGTAATAATTGATCTTTTTTAGCATCGTTTTCTTCTTGTTCAACTGCTTTACGGCTTAAAAT
GACTCTATTATTTTCAGGATCCAATTCTTCAACTTTAATACGAATTGTTTGTCCATCAAAC

ACAGAGAAATCCTCAATGAAGTCTGTTGAAATTAGTGAAGCCGGAACAAAACCTCTTTGT
CCTACATCAACAACCAAACCACCTTTAACAACTTCTGTTACTTTCGCTTCGATGATTTCAT
TATTATCTAATTTTTCTTGTAAATAACTATAAGACTTCTCAGTTTCAAGTTGTCTTCTAGAT
AAGATGTAAGCTCCAGTTTCATTTTCTTCATCAAACTCAACTTTAGTGACATATGCTTCAA
CTTCGTCGCCCTCTTTTACAACTTCACTTGGGCTATCAATATGATGCGTAGATAGTTGACT
AATAGGAATAATCCCATTAAATTTACCACCGTTGATATGAACAACAACTTGCTTGTCTTCA
ACTTGTTGTACCTCGCCAGTGACTTTGTCACCTTCTTTAATATCGTTAATCATTGATTCATT
GAATTCTTCAGTCAT

## > Q2FYF8

Function: protein coding sequence; putative protein (core genome, variable)
Best match: Q2FYF8_trunc_CC001_MW2-USA400_BA000033.2[1517005:1517102:r]RC
Position: 036-contig_189: 77705 … 77803; Length: 98 bp
Sequence:
TCATTGTTCTCTACAAGATATATTATAGTAAAATTTTATACTTTTATAAAACAAAAAGCCA
CGTAATTGAGAAACTTTTACATTTTGTTAGTAAGAAG

## > cmk

Function: protein coding sequence; cytidine monophosphate kinase (core genome, constant)
Best match: cmk_CC001-ST772_118_AJGE01000058.1[74170:74829]RC (completely identical)
Position: 036-contig_189: 78146 … 78806; Length: 660 bp
Sequence:
TTATTTAATTTGACTCACCATCGCTAAAATTTCGTCAGTAACTTCTTCAATCGACTTGCCT
GTCGTATCTAATGTCACTGCATCATCTGCTTTTCTTAATGGTGATATTTCACGGTTCATGTC
ATATTGATCACGAGCTTCAATATCACGTTTTAAATCTTCAAAATTTGATTCGATACCTCTT
AATTGATTATCTTTATATCTTCTTTCTGCTCGCTCTTCAACTGATGCAATCATATATACTTT
TAAATCTGCATCTGGTAGCACTACAGTTCCGATATCGCGACCATCCATTACGATACCTTTT
TCTGCAGCTAACTCTTTTTGTTTTTAACGGCGAATGAACGTACTGGCTCTTTAGATGCAA
CGTATGAAACATGTTGCGTCACATCATTATTTCTTAAAAAGTCTGTTACATCTTCGTTATC
TAAAATGACACATTGACCTTTATCTGCTTTATAAGTTAAATCTAATGTTGTTTGGTCAACT
AGTTTTGCAAAGTCCTCAGTTTTGTTTAATTTTAAATATTTGTATGTTAATGCACGATACA
TTGCTCCTGTATCGACATAAATCATTGATAGTTCACTGGCTACACGTTTTGCAATTGTACT
TTTTCCGGCAGCAGCTGGACCATCTAATGCAATATTAATGGCTTTCAT

## > ansA

Function: protein coding sequence; putative L-asparaginase (core genome, constant)
Best match: ansA_CC001_MW2-USA400_BA000033.2[1518182:1519150]
Position: 036-contig_189: 79701 … 79851; Length: 150 bp
Sequence:
TACGCATACGATGGTGGTGGTTACCAACTCGCACAACAAGGTTTTATTTTTTCTAACGGTT
TGAATGGTCCAAAAGCAAGATTAAAATTATTAGTTGCGTTAAGCAACAATTTAGATAAAG
CTGAAATCAAATCATATTTTGAATTATAA

## > A5IT07

Function: protein coding sequence; FAD-dependent pyridine nucleotide-disulphide oxidoreductase (core genome, constant)
Best match: A5IT07_CC001_MW2-USA400_BA000033.2[1519265:1520251:r]RC (completely identical)
Position: 036-contig_189: 79965 … 80952; Length: 987 bp
Sequence:
TTATGATTCTAACGGCGTTTGTTTCTTAGCTAGCATGCTTTGAGCAATAATGCCACCGTGG
AATTTACCATTTTCAATAAAAATGGTATTCGCATCATTCCCTGCAGCAATCACACCTGCAA
TGTAGCAATTTTCGATATTTGTTTCGTATGTCTCTTTATTATACATAGGCGCTGTTCCAAAT
TCATTTGTATTAATTTGAATACCTACAGATTTTAAAAATTCATAATCGGGATGATAACCAA
TCATCGCAAAAACATAATCATTGTGTATCGTTTTACTTTCACCATTTACTTCATAAGTCAC

AGTATCTTCAGTTATTTGGGTAACATTAGCATTAAATTCCATGTCAATTTTTTCATGATTTA
CTAATGCTGTGAAATTTGGAAGTATCCACGGTTTAATTGAAGGCGAATAATCTCCACCAC
GATATAGAACCGTCACGTTAGCACCAGCTTTTTCCAACTCCAAAGCAGCATCGATAGCCG
AATTCTTACCACCAATAATAACAACATCTTGATCAAAATACGGATGTGCCTCTTTAAAAT
AATGGAACACTTTAGGTAAATCCGCACCTTCAACCTCTAATGTATTATGCTGACCATAAT
AGCCTGTCGCGATTGTTAAAAATCGACATTCATAAACATCTTTCGTCGTAGTAATTGTAAA
TTTATTATTCATTTTTTTAACAGTTAATACTTCTTCAAATGCATTTACTTTTAATTGATGAT
GTTTTACAACTTCTCGGTAATAAACTAGCGCTTGATTACGTCTTGGTTTACTTTCTTCAAC
GATAAACGGTACGTCCCCAATACTTAATTTATCACTTGATGAGAAAAAATGTTTGGTGAGT
AGGATAATTATATATTGATTCAACGACATTACCCTTTTCAATAATTAAGGTATCAATACCT
TTTCTTTTTTGTTCAATAGCCGCACTTAATCCGCATGGCCCTCCACCAATTATGATACTTTC
AACTTTTTGCAT

> **ebpS**
Function: protein coding sequence; cell surface elastin binding protein (core genome, variable)
Best match: ebpS_CC001_MW2-USA400_BA000033.2[1520653:1522113:r]RC (completely identical)
Position: 036-contig_189: 81353 … 82814; Length: 1461 bp
Sequence:
TTATGGAATAACGATTTGTTGACCGTTTCTAATATTGTTACCACTTAAACCATTGGCACGT
CTAATTTTTTCAACATTTTCCGGTGAACCTGAACCGTAGTATTGAATTGCGATACGGTATA
AGTTTCTTGACCATTCACTGTATGTCTTTGGCCACCACCTTGACGTTGTTGTTGCTGTTGT
TGATTTTGATTAGCTTGTTGTTGATTTTGATTATTTTGTGCTTGATTGTTCGCTTGATTAGC
GTTGTTTTGATCATTATCAGATTCATCTTTAGTCGCTTTGTCTTGATCCTCTTTTGATTTATC
ACTGTCTGTAGATTTTGATTTATCTTTAGAAGCGTCTTTAGATGTGTCTTTGTCTTTACTTT
CATCAGCATTATTTTTATTTGTATTCGCGATTTTATTTTCTTTTGTACCATTATTATGATTGT
TTAATGCCATGCCTCCAAATATCGCTAATGCACCGATAATTAGTACAGCTGCAATTAATG
GTAACAATACTTTGGCCATGCCACCTTTTTTACGTTCTTTATCTCTGTCATGATTGTCATGT
TCATCATGGTTTTGGCTTGCATTATTAGAGGCATGTGGTTTTGAAGCGGCAGAAGCACTTT
TACTTGCTGCGCCTCCAGCCAAACCTGCTGTTCCAGCACCGATCGCTGCACCTTTTTTGCC
ATTATGATGATCTTTAGACTTATCTTGAGACACTTTATCCTCAGTCGAGTTATTCGACTTG
TCAGAATTACTTTTGTTTTGAGCGTCATTTGAATGTTTCTTAGCTTTAGAAACACCCATTG
CACCAGCTGCACCTGCAACACCCGCTGTTCCAGCACCAATTGCTGCGCCTTTTTTACCACT
ATGATGATCTTTAGATTCATCTTTGTCTTGCTTAACAGTTACATCATCATGTTTATCTTTTG
ATGTATTTGCTTGGTTAGCACCTGTTGCAAAATATGGTTTAGGTTGCTGAGATTGTTCAGC
TTCACTCTTATCAGAAACTGTTGAATGCTCAGTGTTATTTTCTGCTTCTTTAATAGTCTCGT
GTTTATCATTGTCTTCGATTGGTTCTGGATGTGATTTATCCATTGCAAAAGCATTCTTATTA
TAATATTCCTCTTCATGTTGAGGTGTACTGTCTTGATGGCTAGGTTCTTGACTTTCAGTACT
GTGTGATGATTCGACTTGACGATCATCTATTGTGCCAGCCTCATTTTGAACATTGTCTTCA
GATGTTTGTGATTCATTGTGAACTTGTTTATTATGATTCGTTGCTAAATCACGGCGTCTTTT
TCTTCTTTGGGCATTTCTTGGCGGAAACTGTTGCTCCGTATTCTCTATTGTATCCTGATGTT
CTAATTCTGATTGGTCTTTTTCAACATCTTCCGTATGGTCTTGATGTGAATTTGTGTCTATC
GATTGACGATTTTTTTCAAAGTCATCTTTAAAATTATTAGACAT

> **recQ2**
Function: protein coding sequence; ATP-dependent DNA helicase (core genome, variable)
Best match: recQ2_CC001_MW2-USA400_BA000033.2[1522266:1523645:r]RC
Position: 036-contig_189: 82966 … 84346; Length: 1380 bp
Sequence:
CTATCTGAGAAATAAATTTTGCAACTTTTCATCAAATCCAATACTTCTAATTACCTTCTTCT
TATTTAAAATTGCGATATCAGTTATATTAGAATCATTGTCACAACATCGATCTTGTGCCGG
TGGATATTCACCGAAAAATTCTAATAAATACTTCCGTCTACATTGATCCAATTTGCAATAG
CCAATCATGCGAAAGAATCCTAATTGCTTTCGTTTAAATGATTGCTTAAATATCTGTTTCA
AGGCGCCGATACTATAGAATGAATGCAACGTTGTCAAAACGTCTTGTTTATCGGGAGCTA
AAAATTCTCCTATTTCGAAATTTTGTACATCTTCTTCTGTTATCATATCTGCAAATAATAAC

GTTTCTAAAATATATTTATCGTCCGGTTGGAATAAACTAATTGCCTGACTTAGTTCACCAT
CGCGACCCGCACGGCCAATTTCTTGAATGTAGTTAGAAGGACTTGTTGAAAGATGAAAGT
GAATGATTGTGCGAATATCTTTTTTATTAATTCCCATACCAAAAGCACTCGTTGCGACTAT
AATCGGAATATCATTATTTAAAAATTGTTGTTGAACTGTGTGTCGCTCTTGATAATTCATA
TCACCATGATAAATACCTGTAAGAAAACCTGAATCATAAATAAGTTGCGCTAAATTCAGA
CACATCTTTTTCGATGAGACATAAATAATCGTTGGTCCCGACTGTTGTAGAAACGGCAGC
AACCATTCAATTTTATCTTCATCATCATGAAAATTAAGATGCTTAAAGCTTATGTTTGGGC
GATTCATTGTAGTTTTAATAACATTGAATTGAATCGCTAACATTTCCGTCAAATCATCTTG
TAAATGCGGTGGTGCAGTTGCTGTCAATGCTAAGACAACCGCTTCTTTAAAATGCTTTGTT
ACTTTTCCTATTAGAGCATAATGTGGTCTGAAATCATATCCCCATTCAGATAGGCAATGTG
CTTCATCTAGAACAATCATGCCAAAGTCTATCATAGATATTAATTTAAAATTTGACGGTTG
CAGGAGAAATTCTGGACTTAGAAAGATGAAGCGGCTATGTCGTAAACATTTAATATTATG
CTTTTTCTCAATTTCATCCATACCAGAGTGAATAGATGTTACATGTTTTTCTCCATTTATTT
TCAACTGCATAACTTGGTCATCCATTAAAGATATTAACGGTGAGATAATTAATGTCGGCT
TACCTGATAAATACGTAGGTATTTGATAACACAAACTCTTTCCACTTCCAGTTGGAAGAA
TACCTAGAGTGTGCTGTTGAGACATTATACTTTCTATAATTTCCTGTTGTCCCGGTTTAAA
ACTCTCGAAGCCAAATTTGTTTCGTAAAATATCATGCAACAT

> **A5IT10**
Function: protein coding sequence; putative protein (core genome, constant)
Best match: A5IT10_CC001_MW2-USA400_BA000033.2[1523635:1524588:r]RC
Position: 036-contig_189: 84335 … 85289; Length: 954 bp
Sequence:
TCATGCAACATTCAGATCACCTCTTTCAAATCCAACGATTAATACTTTTAATTGAAAATAT
GATAACGTGTCAAATTGTTCTTTGTAAAATTTTAATCGTTCGCCACGATGTTGTTGATAAA
AATTCAAAAATTGCAGTTGATTTTCTTGTTCAACATAATCATCGTAATTAGACATGTAACC
TTTGATTAAGATTTCAAGTACATGATCTTCGATAGTATTGATTTTAACTTGTTGTTGAGCC
GCTATATCTTCCATCGTGAATTGTTCAAGCAATCTTGTATAAGTAATATAAGTTTGGTTTA
ATAATGTAGGTTTCATTATTATTTTTGATAAAATAGTATATTCTCCACTTTCTAATTCAAAC
ATCATTATCACTAAATTATTCATTTCGTATTCAAACAATTGCTGTTGAGATAGTTTTTCTAT
TAAACTAACTTGTTGTCTCGTATACATTGGTTCATCATAGCCTTGCAAATAATAATGTAAG
TAACATGGCCCTTTTAAAGTTATTGCTTTAAACAAGTTATGTAGTTCATTTTCAAAATCAA
TTTGTAGCTTGTTTTCTTTAATGTAGTTATAGACAATCTTTACAGTTTCTTGTATCTTATTA
TTTTGAGAGATTGGTACAAAATGAAAAACATGTTGTTTGGTATTAGACATGGTTTGCACT
AATAGTTGAATTGCTTGAAATGTTTGCCCCATACTTTCAAATGTATATCTAGGATGCAACA
TGATTTCCATTTCAGCATTAAATTCAGTTATATTTTCTAAAAATAGCTCAAAAGACGGATA
TTTTAATAGTGGTAAACTGTGATATAATGACAACTGTTGTTGACTACAAGCGTCAAAAAA
GGTTTGGTGAGATTTCTTACCAACTAAGATATTGTAAATACTTTTATTTGTTTTATAGTTA
AATGTTTGTTGTAATGCTGTTTTTATAATGTGTTGCAA

> **fer**
Function: protein coding sequence; ferredoxin (core genome, constant)
Best match: fer_CC001-ST772_118_AJGE01000058.1[67331:67579:r] (completely identical)
Position: 036-contig_189: 85396 … 85645; Length: 249 bp
Sequence:
TTGGCAAAATATACAATCGTTGATATGGATACTTGTATTGCATGTGGTGCATGCGGTGCA
GCAGCACCAGATATATATGATTACGACGACGAAGGTATTGCTTTCGTAATCCTTGACGAT
AACCAAGGTACTGCAGAAGTACCTGAGGAATTATATGAAGATATGGAAGATGCAATTGA
TGGATGCCCTACAGATTCTATTAAAATTGCAGACGAATCATTTGATGGGGACGCTTTAAA
ATTTGAATAA

> **A5IT12**
Function: protein coding sequence; putative membrane protein (core genome, constant)
Best match: A5IT12_CC001-ST772_118_AJGE01000058.1[66680:67225]RC (completely identical)
Position: 036-contig_189: 85750 … 86296; Length: 546 bp

Sequence:
TTAAATTCTTTTCAAGAAATTCGCAAGCCTTCTATATAGTAAAATAAATACAATAGAAAT
GACGATACCTTTAATAATATTGAATGGTATAATTCCTGAAACAATGATTACTTTAAGATTA
TTTGCGATATCAGCTAAGTTAAATATCATACCGTACAAAGGTAATAGAACGAAATAGTTC
AAGATACTCAACACGATAGTCATAACGATTGTTGCAATGATTAATCCAGTAATCAAAGAT
TTTGTTGAACGTTTATTTTTATAGATGGCGTAAGCAGTTAATAAGAAACTTGCGCCTGCTA
AAAAGTTAGCAAATGGTCCAACTGGATCGCCCATACTAAATAAGTAGTTCAATAAATTTT
TAACCAGTGCAACTACGATACCGGCAACTGGTCCAAACGTAAATGTAGCTAGTAGTGACG
GTACATCACTAAAATCTAAAGTTAAGTATGGTGGCAAAAATGGTATAGGAAACTTGATAA
AAGTTAACACAAACGCAATCGCGCTCAACATACTTATTGTGATAAGACGTTTATTTTGTTG
CAT

## > fmn-L1

Function: noncoding RNA FMN riboswitch/locus 1 (core genome, constant)
Best match: fmn-L1_CC001_MW2-USA400_BA000033.2[1525657:1525791:r]RC (completely identical)
Position: 036-contig_189: 86357 … 86492; Length: 135 bp
Sequence:
CTCCATCTTCTCCCATCCAGACTATACTGTCGGCTCTAGAATCTCACTAGATCAGCCACTA
ATATGATACATACTAGCAGGTCGCAGGCTTAATTTACTGCCGGTTGGGAATTACACCCGA
CCCCGAAGATGAAT

## > graD04

Function: protein coding sequence; putative lipoprotein (core genome, variable)
Best match: graD04-MW2_ref_CC001_MW2-USA400_BA000033.2[1526005:1526919:r]RC (completely identical)
Position: 036-contig_189: 86705 … 87620; Length: 915 bp
Sequence:
TTATTCATGATTAAATACACCTACTTCTATTACATCCTTCTTACTATAATTAGGTCTTGTTG
TATTTATATATTTATTAGAAATTTGAAGAGAAACATTCAAATCATGAGGAATATTCTTTTT
CTTTTTTAGCACTTCAGAAAAATCAATCATCTCGTCTATTGTATTATCTTTAGTAAAATTAT
CTTTAGTACTAAATAAAGTTGTAACAACATATGGACGTGATTTATCTTTAAGTTGTTTTTT
TGAGTTTCTCATACCGTCCCTAAAAGACTTATCATCCTCTTTGATTAAGGGTTGAAAATAC
TTTTTATATTCATCTAAAGTATATGGAATTGCAGTCAAATAATAATACTCATTTCTAAAAC
CACTATTCTGCGTTTTCATAATTGCATTTTCAGTATAACCAGTATATTTATATTTACTTTTA
TATTCTTTTAAAACTTCTGTTAAATTATCCAACTCTTCCTTATGTGCTCGATATTCAAAGCC
ACTCAACACTGTACCAACTAAAGTACTCATATCATCGCCTTTATCCTCACTTCTTAATGAG
CTATCACTGTCAATAATTGATTTGTCAAATGGAATACTCGCATTAAATACGATATCGTGGT
CATCACAATGCACGTATACTTCTACGCCGTCGCCACTACCTACAACATTCGTAGCTTTAAC
TTTTAGACCGAAGTTATCCATAAAAAAATTGTTCGCCACGTTTAGCAATTTTATCTTTATGC
TTCTTCGCAAATTCAATCGCATCTTTTTCTGCAGGTGGTTGGAAGCCTTGACCTACATATT
TTGAAGCTTCCATTTCTTCTGGTACTGATTTTGTTTCTTTATTAGATTCGTTATTGGTAGTT
GAACATCCTGATAGTATTATCGTTGCTATTAAGATTAGTTTAATTCTTTTAAACAT

## > graD03

Function: protein coding sequence; (core genome, variable)
Best match: graD03-Mu50_CC005_21172_AFEF01000006.1[385055:385951:r]RC
Position: 036-contig_189: 87590 … 87620; Length: 30 bp
Sequence:
TATTAAGATTAGTTTAATTCTTTTAAACAT

## > graD02

Function: protein coding sequence; (core genome, variable)
Best match: graD02-COL_CC008_NCTC8325_CP000253.1[1461363:1462280:r]RC
Position: 036-contig_189: 88564 … 88594; Length: 30 bp

Sequence:
TATTAAGATTAGTTTAGTTCTTTTAAACAT

## > A5IT17, disrupted - upstream fragment
Function: protein coding sequence; putative protein: disrupted - upstream fragment (core genome, variable)
Best match: A5IT17-CC1_ref_CC001_MSSA476_BX571857.1[1554595:1556757:r]RC#[2_150ANF&END]#A5IT17#[CDS:putative protein]#[cv:54.602:54.2126] MANUAL OVERRIDE
Position: 036-contig_189: 89647 … 89782; Length: 136 bp
Sequence:
TTAGTATACAGCTAGTTTTTCTAATTGTTCTTTAACTTGAATTAAGTTTGACCGTATTAGA
GAGGCAGATTGATCCATCGTTTGAATTGCTTGTCCTTCATTTTCGTTCAAGCCATTACAAA
CAACTTCAAACTGT

## > attB_phiSa2-1
Function:  attachment site of bacteriophage (mobile element)
Best match: attB_phiSa2-1_occupied_CC001_MW2-USA400_BA000033.2[1529083:1529151] (completely identical)
Position: 036-contig_189: 89783 … 89852; Length: 69 bp
Sequence:
TGTGCCATTTGATCAAGACGCGCATGAGCTTGTGTGTTTAAAATAAACATATCGTCATAA
TGTGATGGT

## > tx_lukF-PV
Function:
Best match: tx_lukF-PV_ref_CC001_MW2-USA400_BA000033.2[1529274:1529329:r]RC (completely identical)
Position: 036-contig_189: 89974 … 90030; Length: 56 bp
Sequence:
TTTGCCCTGAAAAAATAACCACGTCAATTAAGACGTGGTTACCCTAATATAGAAAG

## > lukF-PV
Function: protein coding sequence; Pantone-Valentine leukocidine F (mobile element)
Best match: lukF-PV_ref_CC001_MW2-USA400_BA000033.2[1529381:1530358:r]RC (completely identical)
Position: 036-contig_189: 90081 … 91059; Length: 978 bp
Sequence:
TTAGCTCATAGGATTTTTTTCCTTAGATTGAGTATCTATTAATTTAACTGTATGATTTTCCC
AATCAACTTCATAAATTGATGTATGAGTTGCTCTATTTTCATCTTTATAATTATTACCTATC
CAGTGAAGTTGATTCCAAAAGTTTGTATATCTATCCATTTCTCTTTGATAAGTAACAGTAA
TTTTTGATTTTTTTGCAGCGTTTTGTTTTCGAGATAGGACACCAATAAATTCTGGATTGAA
GTTACCTCTGGATAACACTGGCATTTTGTGATATTCCAAGAAGTTTTGTCCAGCATTTAAG
TTGCTTTGTCTTGAGCCTAAAAACATTTCATTACCATAAGTTGAATGATAACTATCTCTGC
CATATGGTCCCCAACCATTATTCATAATTTTATGTGCTTCAACATCCCAACCAATTTTTTTG
AAATTAGTTCTTTTATCTAAGCTAGTTCTATAGCTTTCTTGTTTATAGTTAATTGTCTCTGA
AAAAGATTTTGAACCATTACCTCCACCTGATAAGCCGTTAGAGATATTAATATCTCCACC
ATAAGAATAACCTACCGTTTGTTGTACTTGAAATTCTTCATTTTGATTTTTAGGTGCATAA
TCTACAACGTTTACTGAGTCATTAGAATCTGAATTAATTGAAATGTTGTACTTAGAACCCC
AATAAAATTGAGAACTAATAGTGTCTTTTGGATTTGGCTTTGTATAGCCAGAATAAATGTT
TCCAGCAGCTTTGAGTATTAATGTATCTTTATCATAACTTTTATCTTTAATAAAATTAAAA
GTTAAAATCTGAGAAATTTTTAACTTATCGGAATCTGATGTTGCAGTTGTTTTGTACAAAG
TAATTTTATCATCAACCTTTTTCTCACTTACAGGTGTGATATGTTGAGCTGCATCAACTGT
ATTGGATAGCAAAAGCAATGCAATTGATGTAACAACTGATGATTTGACTATTTTTTTCAT

## > **lukS-PV**

Function: protein coding sequence; Pantone-Valentine leukocidine S (mobile element)
Best match: lukS-PV_CC001_CIGC128_AHVY01000005.1[221466:222404:r]RC (completely identical)
Position: 036-contig_189: 91060 … 91999; Length: 939 bp
Sequence:
TCAATTATGTCCTTTCACTTTAATTTCATGAGTTTTCCAGTTCACTTCATATTTAACTGTGT
AATTTCTGTTTACAAATGCGTTGTGTATTCTAGATCCTTCTAAATAACTATTGCCATAGTG
TGTTGTTCTTCTAGTAGCATGAGTAACATCCATATTTCTGCCATACGTTATTTCAAATTCA
CTTGTATCTCCTGAGCCTTTTTCATGAGAAACAGTTGCAATAAATGAAGGATTGAAACCA
CTGTGTACTAATGGGGGTAATTCATTGTCTGGCACAAAATAGTCTCTCGGATTTTGACTAT
ATGGTTTATATCCAACAAATAAATTTGGATCATGTCCAGACATTTTACCTAATGATGTGAT
AAATGAATTAGCTTTTATTCCCCATTGAACACTTTTTGAATTTTGATGTTCTACTTCACTGA
TATAGTTTTGTTGATTATAACTAATTGTTTTTGAATAATTAAATGAACCATTACCTCCTGTT
GATGGACCACTATTAAAATTACCACCTATGTTATAACCTAATGTTTGACTAACATTTACTG
AATCTATTTTATTTTTAGGTAGATAATTTATTAAATCTACATTGGGGTCATTTGTTTTGAGA
CCAATATTGTATTGGAAAGGCCACCTCATTGCTTTTATATGATCTGTGTTTTTGTAATTGT
AATAAGTAGTCTTTGAATTGATAAAACCTTGCATTTTTAAAATCAAAGCGTCTTTGTTATA
CTTTTTATCTTTAACAAAATCAAACTGAATATTTTGTGTGACCCCCCACTTATCGCTACTT
GTATCTTCTGTTCTTTTGACTACCTCAGCGCCATCACCAATATTCTCAATATTGTTATCAGC
TTTAGATTCATGAAACGAAGTAGCAATAGGAGTGATTATTCCTAACGACAATGTTGCAGC
TAATAGTCTTTTTTTGACCAT

## > **O80065**

Function: protein coding sequence; putative protein directly upstream of lukS-PV - leader-peptide of lukPV (mobile element)
Best match: O80065_pseudo_CC001_CIGC128_AHVY01000005.1[222512:222644:r]RC (completely identical)
Position: 036-contig_189: 92106 … 92239; Length: 133 bp
Sequence:
CTATAAATGTGTTTTTTTACTATTATTTCGAAAAAAACCCGTTCATTACCAAACAAATTAT
AAATAATTTATATTTTGATTGATAATAAACATAAAACCACGATATAGTTGACCATAACAA
CTTTTTAATCAT

## > **tx_amidase1-phiSLT**

Function:
Best match: tx_amidase1-phiSLT_CC008_TCH1516_CP000730.1[1562363:1562418:r]RC (completely identical)
Position: 036-contig_189: 92327 … 92383; Length: 56 bp
Sequence:
ATTAATTTTAAATAATAGGCAAGTACCGAAGTACCTGCCTAAGATTCATCATATAC

## > **amidase1-phi12**

Function: protein coding sequence; bacteriophage amidase (mobile element)
Best match: amidase1-phi12_CC001_MW2-USA400_BA000033.2[1531688:1533142:r]RC
Position: 036-contig_189: 92388 … 93843; Length: 1455 bp
Sequence:
TCAAACTGCACTAAACTTACCAAAACTGCTTATTCTATTACCTGCCTTGTCTACCTCTCCT
GTAGCAATATAACGACGTTGTCCACTATTAGCAATATAAGTAATCCATCTATACCCATTG
ATGCAATATGCGCCGTCATATTTGATTGTTGCGTTATTAGGTAATACACCTGTAATTCTTG
AATTAGTTGAATAGCCATCCCTCACGTTATTACCTTTAACATTGGCAACTGTGTAATAACC
AGTTTCTTTTTTATACGGTACATTATTTTTATCGGGTGTATAACCTGCTGGCACTGGCGGA
TTCTTTTCGTTTTTAACTGGTGTTTTAACCTCACTAGATATCACACCACCGATAGGCTTACC
ATGAATCGCACCGGCTATTAATTTAGAATACAAGTCATAGTTTTTCTTAATCCAATCCATA
TCATTTTTATTAGTGATAAAACCTAATTCAGATAAGCGATAATTTATATTTATTTCTGCTG

ATACGTTAACATTTAGTAAATCGTTACGAGGTGTTACACCTCTTATTTGTCCTAAATTATT
TTTAATAACATCTTGTATACTTTTATCAATAGTATCTGCATTGAATTGACTTGAGATAATA
ACATGCCCACCACTTGCGCTTTCTCCTGCTGCGTCTAAATGTATTTCTAGAACAATGTCAT
ACCCCTGTGATTTAACCCAATATAAGCCATAATCTTTTTTATTACCTACATTAACACCGTA
TGCTGTATCTTGATACATGTCTTGTGATTGACTTGAGCCACCATATAATGCGACTTCATGA
CCGGCATGTCTTAAATACTTAGCGATATTTGGCGTTATATATTTACGTATAAAATCGCGTT
CGTTTGTTCCGTTTCCTACTGCTCCAGGATCGTTATAACCATGACCGGCTACAAGCATAAT
TTTTTTAGGTTTAATTACTGCTTGCTTTTTGGCAGTTGCTTGCTTAATAATGCTTTTAGCTT
TATCCCCAACACTTACTTTGTCAGGGAAATTTAATCTAATAAAATACATTGGGTCATCGTA
ATAATGAACATGTCTTGTAACAGTTTCAGGACCCCAACCAGGTTGCACAACGCCATTTGT
CCAACCTTTACCATTCCAATTTTGGCCAAACGATGTGAAAGTGTTTAAATTTGCGCTCTCA
ATAATTTCAACGTGTCCAGCTCCGCCACCATACTTCGACGGGAAAACGACAATGTCCAAC
TTTTGCGGTAAAAAGCTATCATAGTTTTTAATTATTTGCCCGTATTTTTCAATCCTTGCTTT
ATTATCAAATGGAATATTATAAGCGTATAAACCTTGTAACCTTTCGCCTGTTGCTATCATA
AAAAACATATTTGCGTAATCGTAACACTGAAATCCATAAAACAAATCAGGATTGAACTGC
TTCCCTAATGAATTATCAAACCATTTTTCTGCTTGGTTTTTTGTTATCAACAT

## > holin1-phiL54a

Function: protein coding sequence; bacteriophage holin (mobile element)
Best match: holin1-phiL54a_CC001_MSSA476_BX571857.1[1023464:1023766]RC +MANUAL OVERRIDE
Position: 036-contig_189: 93853 … 94156; Length: 303 bp
Sequence:
CTACCCTAAATCATTTGTGTCGTTCATATTCGTAGGTGTCATTACTTCTTTAATTGGCGCTT
GCCCTGTTGCTTTTCTATACTTGTTTCAGCTTTATATTTCTTTAGCTTTTGATTTGCCCATT
TACCTTCTTGAGATGTTGGATTATCTTTATATGTAGTATATAAAGCAACAACTGTTAAGAT
AATCGATGAAACACTTTCTTCATCTACTGGTATCGGACTTATACCTTTATTCGCTAAAAAC
TGATTGACTAACGCTAAAATTAATACTATATATCTTGTTATTACTTTTGCATTCAT

## > sprFG

Function: noncoding RNA small pathogenicity island RNA F and G (mobile element)
Best match: sprFG_CC005_ED98-hen_CP001781.1892742:892952:r [4_50ANF10END]
Position: 036-contig_189: 94155 … 94366; Length: 211 bp
Sequence:
TTTGTTTGCTCCTTTTATCCAAAATAAAAAGACGACTAATAAGCCGTCTATTTGATATTTA
TATTATGGTGTGTTAATTTATATTTAGAAAAAGGGCAACATACGCAAACATGTTACCCTA
ATGAGCCCGTTAAAAAGACGGTGGCTATTTTAGATTAAAGATTAAATTAATAACCATTTA
ACCATCGAAACCAACCAAAGTTAGCGATGG

## > rli28

Function: noncoding RNA Listeria sRNA rli28 homolog, locus 2 (core genome, variable)
Best match: rli28_L2-1_CC001_MW2-USA400_BA000033.2[2051199:2051377:r]RC (completely identical)
Position: 036-contig_189: 94395 … 94574; Length: 179 bp
Sequence:
TTGATTACTAGACCTATCAATGCAATAAGGAATAAACCAAACTGCAACATGGTACTAATT
GTAATCATTAGGCGTCTCCTTTCTAAAGATTTCAGTAATGCCACCATAGGCACCACCTCCT
TATACTCAGATAGCCACCATCTATCCAACTTGCTCAAAGCATATTATAGCACATATAC

## > txpA-phage

Function: protein coding sequence; toxin involved in plasmid maintenance
Best match: txpA-phage_ref|BA000033|MW2-USA400 BA000033.2[2051171:2051305:r] MANUAL OVERRIDE
Position: 036-contig_189: 94400 … 94535; Length: 135 bp
Sequence:

TATTTTTTTATTGCTTAATTCAATAAGCTTGATTACTAGACCTATCAATGCAATAAGGAAT
AAACCAAACTGCAACATGGTACTAATTGTAATCATTAGGCGTCTCCTTTCTAAAGATTTCA
GTAATGCCACCAT

> **entA**

Function: protein coding sequence; enterotoxin A (mobile element)
Best match: entA-FRI281A_CC001-ST772_118_AJGE01000058.1[57550:58323]RC (completely identical)
Position: 036-contig_189: 94652 … 95426; Length: 774 bp
Sequence:
TTAACTTGTATATAAATATATATCAATATGCATGTTTTCAGAGTTAATCGTTTTATTATCTC
TATATATTCTTAATAGTGTATTTGAATTCTGTCCTTGAGCACCAAATAAATCGTAATTAAC
CGAAGGTTCTGTAGAAGTATGAAACACGATTAATCCCCTCTGAACCTTCCCATCAAAAAC
ATCAGAGTTATATAAATTATATTTTTCCTGTAAATAACGTCTTGCTTGAAGATCCAACTCC
TGAACAGTTACATTTTTCTTATTCGTTTTAACCGTTTCCAAAGGTACTGTATTTTGTTTACC
GTCTAGCCATAAATTGATTGGCACTTTTTTCTCTTCGGTCAATCGATTATTATCATGTAAC
GTTACACCACCATACATGCAAGCTGTTTTGTTTGGTGTACCACCCGCACATTGATAACCAT
AATAAGCACCATATAAGTCTACTTTTTTCCCTTTATATTTATCAACAATATCCTTTGAATC
AAAATCTACTAATAAATCGTTATACCATGAATGATTTGTAAAAAAGCCTTTAAACAATAT
AGTATGCTGTAAAAATTGATCGTGACTCTCTTTATTTTCAGTTTTAGCTTTTTCATTGTAAT
AATAGATTTGTTTAAGATTGCCTAAAGCTGTTCCCTGCAATTCAGACTTTTTTCGCAAATC
TTTTTCATTTATTTCTTCGCTTTTCTCGCTACCATTTACAAGTGGACTTGTTGTCCACGTTA
GGGCAATGAATAAAAGTAGTATAAATGCTGTTTTTTTCAT

> **minor-phiNM3**

Function: protein coding sequence; bacteriophage minor structural protein (mobile element)
Best match: minor-phiNM3_CC001_TCH70-ST1_ACHH02000016.1[79909:83694]RC
Position: 036-contig_189: 96751 … 100537; Length: 3786 bp
Sequence:
CTATCATTCATTTATTAATTCCTCCAGTTTTTCGATTTTTGATTGCTGGTTTTTAATAATAG
GTATTAAATGAACCCAAAGTCTATCGTATGCGATACCTTCGATTTCTCCGTTGTCATCATA
AATAACAAATTCATTAAATCCTAATTCTTCAACCTCTTCCGCTATTAAGCCAGTATGTCGA
CTAAGTTTAAAAGTATCATCAGATAACTTTTTACCACTTTCCAATTCTTTAGCCATTATTTC
CGATTCATATTTGTCAAACCATGTACGAATTGGAAGCTTTAAAATCTCTTTTGAATGACTG
AACTGATCGTCTTCATTGATGTATTGGTTTTCGATTGAAATTTTATACTTTTTGGCAGAAG
TAGCACGCCCAATTGTTCCAGCAGAAGTAATGTGTAAGTTAGCAGGTGCTGAATAAGTAC
GCTTATAAATAGAATTAGAAGCGACTCTATCGCCAGCATTATCAGAACCTACCGCCAATA
AATCATAACTTTGAATGCTAACGTAACTATTTCCATCTCTTCGTTTAACTAAGTTGAATTT
GCCCATACCTGATTCAATTGTAGTGTCTCCGCCTGTAGCATAGTCACCATTAACGACTTGA
ACCAATCCTTTATTGCTACGTTTAGAAAATCTTAATCCAGCACCGTACTTATAGTTTTCAT
CTGAACCAAACATGATATAACCGTCAGTTTCGTATGCACTATCAGCGTTTGATAATGTGA
ATGCGAATCGGTTTAAACCAGGTTTATTTTTGGTGTTCGGAGATAAATATATCGGTGCTTC
TCTACTTTCAATATTAGCTGAAGCATATGAATCGATAATAATTCGGTTGTAGTCAGATGTT
AAAGCGACTACACCGCCATAAGAATTGATTGTTATGCCATTCATACCACTATCACTGTAA
GTTTTATCCCACCATTGAATAGTACCGGATGAACCTCCGTCTTCGCCTTCTCCATCAATAT
ATGTTGAAATACCAAAATGTGACATATAAAGTGAACCGCCTGCGGTATTATTTCTAAACC
TTAGATGTCCATCTTTAAGACGTGTGAATATATCATCGGTTGATCGTTTGCCTTTCCAAGT
TCGTTGTACGATTCCGCCAAGTTCAATAAAATCATTTTGAACTTGTACATAACGGTTAGCA
TTTCCGCCTTTAATACCAATTCTATTCACATTGATGTCAAGACCTTCATTTGATAAGTTTA
GGCTATTAACAATGTCATTTTTACCAACTTTGTTATTAATATTATTTGCGACTACATTAAA
TTCTCTATTCGCTGTTATATCAACTTTATCGCCACTAATTTTCACGCCATCTTTATCAATAG
TATGTGAAGTAATCGCCCCATTTTCGTCATACCTTAAATAGATGCCTTTCATAGCATTTAC
AGTGATGTCTGCTAACACTCTTGATAGTGTTCTTTTAGAAGCATTAAACTCTTCTTTAGTA
GTTCTTAATTTGATTTCCTTACCATTTTGTATAATTTGAGAACCATAGCGAGTCAATGTTTT
CCTCTGTGCATCTGTGCTTTCTTTGACCTTGTTGTCTGTATAAGCATTAGCTTTCTTTTCAG

CGTTTCTAGCCTTTAGTTCTGCGTTTTGTTTTGCCTCTTCAAGTTTAGCTTGAGCATCTTGT
ATAGCGCGTTGCTCTTCTTCCGAAATTTTACCATCAGCATACGCTTGCGATTCCTTCTCTTT
AAGATCATCTTGAGCATCAATGTATGATTTTAAAGCTTCTTGCGCTTCTTGATTTGCTTGTT
CAATACTTGCTTTAATCTCAGGATTATTGGACAAATCACTTAACTGGTCATCAGTATATTG
TTTTTGTTCTTCCAATCCGTTTCGATATTCGTTTAACGTAACTTTATCTTTGATTTCACCTTT
TAAAGTCGTTCTCTCAGCTTCAGCAGTATCTAAACGTTCAACAATACCGTCTTTGTCTGTT
TTATAGTCCGATGTTTTTACATAGTCACGTAATTGTTCTTTTGTGGATTCTCTAGCTGCTTC
AATAGCTGTTTTAACAACATTAGGTTCTCCGACTAACTGCAAATCTTCATTCACCGTTAAA
CCAAATTTTGTTGCTATTATTTCCAACGCTTCTTTATATTTTTCATCAGTGTATTGTGACTG
TAATAATTTAAATCTATCTGAAATGGCGATTTTAACATCTTCTACATCTGTATAAACATCT
TGTAATTTCTTTCTATACTCAAGAAATAAAGCTTGTGTATCTACCAACCGACCAATCGTTG
CAGTTTCGGGTGTCATAGATTCTAAATTATTTTTAATTTGATTATAAACATCAATCACAGC
GTCTAAACTTGCTTGTAAGTCCGCTTTCAAATCATTATCTACTAAGTACTCGCTATTCAGT
AATTCTGTAGCTTCTGACAAAAGACTAGCGTGTTGTATAGATAAATTAATAAAAATATTG
TTTAATTCACTGAATAGCGCTTTCTCTCTTGTTATACCACCTAATTTTTCAACATCATTTGG
TGTTGCTTCAATCCATCGACCATTCCAATATCTACGCAAGACAGCAACATCAGGGTTACTT
GTATCATACCAAAGCATATCATTGACTGGATTTTCTGGCGGTGTATCACTTTTGTGTATTT
TGCGTTCAAAGTATTCTAATTCACCATCTACAACATCTTTAACTATAGTGTTGATATTGCT
AATATTATCGTTTAACTTTTGATGTATTATGTTCAATCGCTTGTTAAACTCTTCTCGTAATT
CTGATTCTTTGAACTCTTTAGGTTGACCGAATGTATATGTGCTATTTTCTGAAATTATGTTA
TATTCTTCGGCAATAACTTCTGCCTCTACATACAATGGCGGGTTAAAATCTCTATGTTTTA
CTCTGACTGTATCACCAATTGATATAATCTCGTGCGGATACGTAACTTCCAAATCAGTAG
AAGTAATCTCATATGACATAACTGCCGACTTACGTTTATTTAACTCTGTTTTGGCTAAAGA
ACGCAACCGTGTTTCATTCATATTTTGATCATCTGATTGAGGTTCGTATATTCCCCAAATA
TAACGGGTAGGTAAGTTGAATTGACTTTGTGCTTCGTCATCAGTCACAACTAACTCTAAA
CGCTTTCCTTTGTCATTTTCAGGTCCCACAGCAATTAATGCTGTTTTGATTTCTGACATATC
AATCTTCCTAGTTAACCCAACCAAATCTTTACCATACTCAATTTCTTTACCTTTGAATAAG
CTGTTTTTCTTTTTGAGTACCACATATCTACCTTTGACGGTATTAGAACTAAGCTCTATATA
AAAATCCAATACCATTTTATAGGTTGTACATAATTGCTTTAAAACTTCATATCTAGTTTGA
TAAGAAGTCCATGACGTAGTACGTAAGCCATCGTATTCGGTTTGTTCAGAAACTTCCCAA
CCTGTATCGCTCAACACATCTTTCAATGCTTCTGAAGTTGTCTTTTTCTCAAATTTGCCTGG
TGCATACGGTTTAGCTGTTGTTATATCAGCAAGATAAGACGCTATACATTCTATCTCTGTG
TAGCCGTCCATCGTATCTTGAACCCAGTTAATAATAAATTCACGCCATTGTTTGTTTGAAT
CCCTTATAATAACACGATGTCGTTCACGGAACTTTTCAGCTCTTTCTGATGATATGAGCAG
TTCAAGCATTTCTGAATTGTCATTAACATTACGTTTATGAATCGCTCTAACTAAGGAAGGG
TCATCAGTAGAAAGGAAATCTATAATCTTGTCGTTAAAATCTAAAACATGTATCAC

> **Q8SDK3-phiNM3**

Function: protein coding sequence; putative bacteriophagal protein (mobile element)
Best match: Q8SDK3-phiNM3_CC001-ST772_118_AJGE01000058.1[50939:52423]RC (completely identical)
Position: 036-contig_189: 100552 … 102037; Length: 1485 bp
Sequence:
CTATAAATATCTATCTTGCCATTTAACCGTCGTATCAAAGACGTTTTCAGGTTGTATGATT
AATTCACTGTACCCAGAATCAACATTGAAATAATTACTTCCAAACGATTTCTCGCTCAAC
ATTGGTTCCTCATTGATGACAACACTTTTTGCTTGCATATCTATTTTTACTAAATCACCTTT
TTGTATAATGACATCCCTTGCGCCTTTCGGTTTTGGTAGAATCTCCGTATTGAATGAACCT
AATCCATTCATCTCCATCCACTTATAACCATTATACTTCGCACTATAGATAGCTATGATAG
AAGCTGGACGCTGATAAAACTTACCGCCATCTATCCACTCTTTCTCATCCATATCAATAGG
TTTACGTCTATCTGGGTCTTTAATGTGATCAAATTTCCAAGTTTTAATAGAAAATTTATTA
CCTACTCTTCTGAGCCGCATATAAACAACGATTCTGTCCAAGTTATACATTATCGGTTTAT
TCTGATAGTCGTATATCTTTTTGGGGTCTCCTTTTTGGTTATACAACGTAACAACAATATG
TCCTATTTTTCTATCATGATATTTATTTTCATAACCAATAGAAGCAAGTAACTTACCATCA
CTATCATAAATATGTTGTGCTGTTCTTCCGGCACCTTTACCTTTTTGTTCAACAATACATTT
ATAGGTAATTTGAAAATCTGTCATCGCTTTAGGGAGCCCTCGTTTCGTGCCAGCACCAAC
CCAACCTTTTGCATCAGGAAAATTAGTTGCTTTATATCCTTCGCCAAGATTGGATATCACA

AAGTCACCGCCGACCTTACCACCTAAATCATTACTTGGAATATCTTCAGTAATCATCTTAG
TCCAACCTTTGAAATCACGAAACTCACTATGATAAACAGGAGGCATGTAATCCTTAACTT
CTTTGGTTACCTCATCATCACCAACCATAAAATAATCTTCATCATTTTTAGTGATCATAAA
GTAACTAGATGGTTTAATTGCTCGGGCTTCAACAATTAAAGGAGTGTCAGCAGTCCCACT
ATTTACAACTGAAACTTGGTCTGAAATCGCAGTATTTTTATTTCCTGTTACTGAATATTTG
TAAGGGTCTGTTAGTACTACTTTGATAGTGAACTTAACAGGTATTGTAAATTCTTTGTGCA
GCTTTATTGGTCCTTCAAAATAAGCGTTCCAGTACCAATCTTTAGATTTGAATTGTAATTT
AACTTGTTCCTCGTAGTTAAAAAACTTTACTAATTCATTCAAGACGTCATCATATGTTTTA
ATGCCGTTGTGAGATAAATAGTCATTACGTACCACTAAAGGTATATCAAAACTATAAGAT
TCAAGCCTACGCCCTTTATATATAGCCCCCGAACGTCCATCTACATTTCTGTTTTTAAAA
CATAATTAAAAGAGGGTATTTCAAACCCTCTTTCGACATACAACCAAGGAATTGTTTTGTT
GTTCACTTTAATAGTGTCTATCAT

> **measure1-phiNM3**

Function: protein coding sequence; bacteriophage tail length tape measure protein (mobile element)
Best match: measure1-phiNM3_CC005_04-02981_CP001844.2[2051791:2056320:r]RC
Position: 036-contig_189: 102033 … 106578; Length: 4545 bp
Sequence:
TCATTGAATAGCAATTCCTCCTTTTCTAAACTTTACTTTTGTTGATTCTTGCCTTTCTCGCTT
TTCTATAGACGCGTTCACCTTTTTATCAAAAGCGTATTCGTCAATAATCGGCTGATAATCT
TTATCTGCAATCACATCGTTTGATTGCGCTATTTTCAGTAATAAAGCTATTTGTTGTTGCTG
TTGTTCAATCATTTTCAATAATAAGCTTGGGTCATCAAACCCATTTACACTAGACAATTGA
CTAGGACGCTTATTTTTACTCGCTTTTCTCCCTCTTACTTCTGCTGCTGCATAATGTAACAT
CTTCATTGCTTCGTTTTTACGTGCAGGATCAGTAGGAATAACCCACTCTGGATATCCTTCT
TCTCCTAAGTGGTACAATCCGTTGTAGACTTTGCCACCAGTAGCATATGCGTAATCACCA
GCGCGTTTGAACGCAGCTCTCCATGAGCCCGTTCTTGGTACCCATTTACCCACAATATATC
TCATAGCCGATATAGCTTGATGAGTTGGGTTAAGAGGATTATTGTAACCCGACTTTGCGT
ACGCTCTAAATGAAGGATCTATCATTTGGAACATACCTCTTGAAGGTGTACCAGCTCTTG
CGTTGCTATCCCAATTATTAACTGCATTAGCTGTATAATTGGATTCACGTCTTGCTACACG
CATCATTTCGTGTGTAATCCAGCTAGCTTTGTATTGACCTCCAAGTATATTTTGAGCTGTT
CTAATCGCTCTGCGCGCATTTTCAGAACCACTCCCTCCAGGTGAATTCTTTCCACCAGTTT
TGTCGTTTTTCCGTAACCAAGGAATAGGGTCTGTCGAATACCTATTGGACTCTCCGCCTTG
ATTGACTTGGAAATGTAAATGGCGGTAATTAGTCATAGAACCTGTATTACCTGATTTACC
AATTAATTGACCAGCTTTAATTTGTTCACCTGTCCTACGCAATTGTTCAGATAAGTGCATG
AACCACAAAAATGTTCGACCTTTTTGAACAGTAATTGCTTTACCGCCACCATAGTTGTCAT
ACCAACTTCTAACACGTCCACCCATTGGCGTACGTATAGGGGTACCGGTCGGCGTATCAT
AGTCAACACCATGATGAACGCCTCCGTTAAATGGATAATTGGGGTTAGGAGGTTTTGGCG
GTGCTGAATAAGGTTGTAGTATTCTGAAACTATCAAACACAGAACCATCTCCCGCTTGGC
TCTCTAATCCTTCTTTTATCCAATTAATCGCCTTACTTTTAATCTTATTCCAAGACGCTTTT
GTTATATCGCCAACAATACCCATACCTTTAGTTAGAGAGCTAAAGTCAACACCAAACGCT
TTGAGCACATAATTTAAAAGCTTACCCGGATTATCAATAAAGTCCATTACATCGCCAACT
TTATCGCCAAGCCATTTGGTACCTTTACCTATTTGATCTTTTGTCCAGTTAAATGCCAATG
ATGCACTAGATTTAATATCTTTCCACATAGTAGTACCGAAATGAAATCTCGGAAGCGTTC
CGTTTAACATTGAATAAGTTTGTGCACCGTTGTATACTTTTGAGCCTTTAGGTAAATAAGC
AGTAGTGTCTGTATTAGGTGTGATTACACGTTTACCGTTAGGGAATTCAATCATTTCATTT
CTAAAACCATTTGGACCATTTCCACGTCCTTTATCCCCAACTGTAGCGAATGTATCACGTG
CAATCTTACCGTTCTTAACTAATCTTGTAGTAGTATGTGTGTGCTCTGTACCAGTGTGTAA
CCTAGGTATTTCATCCATACCTAACTTACCACCGACCCAGTTTAAGCCTTCAATTAATTTA
TTAAGTCCTTTTTTAATAGCATCTACCATACCGCCGATATGATCTTTAATTTTACCAATGA
TAGATTTTAAACCGTCACGCATGTTTCCGAAGATATTACGTACTTTATCCCACAAACGACC
AGCTATACCTACCGTGTTATCTTTAATAGAGTTCCAGATGTTTGACATCCAATTTCTTAAT
TTAGTAAATATATCTTTCGTCGCATTCCATAAACTTGTGAATTTAGACCTTACACCCGTAA
ATAACGAATGAGCCTTGCCGACGGTATTGCTTTTGATATTATTCCACGCATTAGATAACCA
GTTTTTCATATTAGTGAAAATAGATTTAACAATATTGTATAAGAAACCAAAAATACTTTTC
GTTGCATTCCAAATTGCAGATAATGATTTTGTGAAAATACCTTTGACAACACCCCAGATA
CCGGATATTAAACCTTTAAGCAATCCACCAAAGTATCTAACAACACCTAGAATCTTACCT

ACAAACCACAGTTGTATTAAATTCCAAATTAACTGCACAGTGCCTTTTAGTATCATCACA
ATACCGTCCCAAACACCTCGCCAATTACCAGTGAATAAACTTGAAAAGAACTTAATAAAG
CCAAGTATGATATTTAAAGCACCTTGTATTACTCCTTTTATATTCTCCCAAGTACTGACAA
TCAAGGCTTTAACCGCCGGCCAAATAAATTGCATCACTTGCCAAATCGCAAACATGATTG
GTTTAATTACAAAATTTAAGATAAATTCAAATATAGCTTTGATAAAATTGCATATATTTG
AAGCGCTTGAACAATAGAAATTCCGTTTTCATTAAAGAATCCATTAATTTGACTCCAAAT
ATCTTTAGCGAAATCAACGATTGCTGAAACCGCTTGTTTAAAGACGTTTTTAACGGAATC
AATGAAAGGTTGGATAAATTGAATGAAATTATTAAACGTTTGTTTAACACTGTTAATTGC
ACCATTAACAAAATTTCTGAATGTTTCAGATTTCTTATAAGCTATTGTAAATGCGACTGCT
AAACCAGCCAGTACACCTAACACGATACCAATTGGACCAGTTAATGCTGTGAAGACTGTT
CCTAAAATAGGCACTTTAGTTGATAAAAAACTAATCAATCCGTCAGCCTTTGCAATACTA
GCTAATAATGGAGCTAATACAGTTACTGCGTTGCCAACTGTGCTTATGAATGCACCTAAT
CCAAAAACTACAGGACCAATTGCAGCAGCAATACCACCGAATATAACAATCGACCTTTTA
GATCCATCACTTAAACTTGAAAACCAATCAACTGCTACAGATAGCTTTTTGATTAATTCTT
CCATGACTGGAGCAAACGCACTTTCAATAGAAGCCCATACATCAGCACCTACTAATTTAA
GTTTATTCATTGCTACTTTAAATCTTTCGGAGCCACTTTCAGAATCTTTAAATGTCTGATTG
ACCGTTCCTTGCGAATCTTCGATAGTTTTTAAGAACTCTTGGTAACTAAAGCGACCGCCTT
TAATAGCATCTGCTAAATCAGGACCTGCTTTTGCACCAAATGCTTCAATCGCTAAACTTGT
TGCGCTAGCTATATCCGGTGTCCTTTCAATTTCTGCTAATGTCTTCTTAAATTCTTCTCTTG
GGTCTTTACCCGCTTTACCCCAATTGGATATAGCTTTTTTCAAACCATTGAAGGCTATTTC
AGTATTAACACCTGATTTCTCCCATTGAGAGAATAAAGCGATTGATTCTTTCATCTCAAAG
CCCATAGCCCTCATTGGAGCACCGTATTTAGTAATGCTATCAGCTAATGTATCAACACTTA
TACCGCTAGCCTGTGCTGCTTTCGCTACCATATCAAGTACACTTTGATACTCATCAGCTTC
AATACCTGCATCACCCATTGCACGCGTAATTAATTGAACGGCTTGTACGCCGTCAGAACC
TGTTATGTGACTAAATTTCAAGAATGACTCTGTGGCACTCTCAAGTTCTTTGCCAGTGAAA
CCTAACCTTGTGTTAACTTCCCCTAAAACACCGCCTACAGTCTCAGCGTCTTCTGGAAAGT
TGCCATAAACATCTTTAAATGAATTCTGCAACTTCTTAAGCTCTCCGCCGGTTGCTCCTGT
TGCTTGGGTAACTGTATCTAAACCTTTATCAACTTCTGCAAAAGCTTTCCTGATGCTGCT
GCAATACCTAAAACAGGTGCAGTTACACCAATCATCAAACCTTTACCAATGGATTTTAAA
CCATCACCCATTTTTGTTAATTTAGGTCCCATACTTTCAAAAACTTTACTGGTTTTTCCCCA
GCCACTTTCTGCCATTCTTTGAGCTTCAACTTGAGCTTTTTTGAACTCTTCAAACTCAGTTG
TTGTTTTTTCTAGTTCTTTTTCTAAAAAATTCAGCTCATTTGCTTGTTTGTTATATTCTTGTC
GTAATTTTTGAGCTTCCGCGCTGTTTTCGCCCTGTTCTTGAGATACCTTGCCATATTGCTTG
GCTAAATCATCAACGTTTTTCTTATAACCTGTGATAGTTCCATCAAGTTCTTTAATCCTTTG
TTTGTAACTATGAGTTGATTTTTCGGTATATTTGAAGTTGTTACCGGTTAACTTTAAGTCA
GAATTTAAAGTTTTAAAGTTTCGTTTGATTTCTGCAAATGATCTATTTAAATTTGCTGCAT
CTAAATCCAAACCTATAGATAAACCTTTTATTCTTTCTCCCAT

> **tailL-CC5**

Function: protein coding sequence; bacteriophage major tail protein (mobile element)
Best match: tailL-CC5_CC005_N315_BA000018.3[2024201:2024845:r]RC (completely identical)
Position: 036-contig_189: 107488 … 108133; Length: 645 bp
Sequence:
TTACAAAGTTTCTTCGTTACCCTCTGTCACGTTTCCAGTATATTCTTCGCCTAAAATTTTCT
TTAAGAAAGCCTCTTCGCCTTTTTCACCGTCTCCATCATGATTTGTCATGTTAGCTGAATC
AAAGATATACTTACGTACTGACTTTTTATTATCAACTAAAGGGAAAAGTGCCTCACCTTC
AACCTCTTCACTTGAGAAATCCCAATCTTTCTCAGCCGTTTCTCCATCGATTTTAGGATTT
GTAAACATAACTTTAGGTAATAAAACTGTTCTAAATGTACCGTCTCTACGCTCTTGTCTGA
ACCATACAGCTACGTAATTGTTTTGTTTACCTTGTTTCTCTTCGTAAACGCCATCTTCATCA
TAATCTTCATTAAAAACAATTTTGCGAATCTCTTTAGGGAACGCATGCATTTGTAATGAGA
TTTTACCTTCTCCGTCTGTATTCCCTGATTCAATTGGACCGCCATCAGCATAAGCTGTTTTT
AGTTCTCCACCAGTTTCAACACCAATTTTTTGTAATCCTCTTGTTTTTGTAATATCACTATA
TTTTAATTCCGCGCCTTCTTTCGTTAATTTAGCGAAACCTAAACCAGTAATGTTAATATAC
GCCTTTGGCGCACTTGCATGTTTTACTGCCAT

> **A0EWZ3**
Function: protein coding sequence; bacteriophage major capsid protein (mobile element)
Best match: A0EWZ3-phiNM3_CC005_Mu50-VRSA_BA000017.4[2103511:2104656:r]RC (completely identical)
Position: 036-contig_189: 109862 … 111008; Length: 1146 bp
Sequence:
TTATAGTGTTTCTTCGGTACCTTCTAAAGCTGGCTTATGTCCTTTTAAATCTAATTTCCAAA
CAGCAGCAACTTTATTATCTTTCGCTTTGCCGTAAGCAAATTGTTTTGCAGTGTATAAATC
CATATCATCTAACGCAAGTGTTTCTTTAAATTTCTGAACATTAATACCACCAGCTAAATAA
CCATCATATAAACCTTTAACGTACGTTAAAACCTTACCTGCTTCTTGGACTGTAGACTCGA
TAACATTCAAATTAAATGGTAAAGCAGTAACATATACGCCATTTGCATTTAAATGTGTAT
ACTGTGCTTGAACCTCAAAAGCATCGGACGGATTAACAACCATTGTTACATTACCTTTAA
CCGCTACTGATTTACCTTTCTCGTTAGTTGAGTGGTATTTAAACACTTGCGTCAATTCATT
AACTGTAGCGCGCGGATTAGCAAATGTAAGCGTACCTTGTTCTTCTTTCTCTGGATAAGCA
CCCTCAGTTACCGATACACCTTTTTGTACTTGACGGTTTAAGCCGATTGGTTGGTCTTTAC
CAGTACCTTTTAAGAACGCAGTTTCAAGCGCCACTGCAAATGCTTCTTCGATTTGAACAC
GAACAAATCTTTCAATCCACGCAGGACCAAAATCATTTAAATCTTTTGGTAAAACAACAA
ACGCTGTCAATTTATTTTGAATTGCTGTTTCTTCACTGAACGCAGCATCTAATTGACCTTT
AATTTCACCATAGATTTTACCCCAAACGGCTACGCCAGAAGTTTCAGATTTTAAGAACTTC
AAACGCAAACCAGCGTTTTTAATACCTAAATCAGCTAATAACGGATGATTCGTCGTCAAA
TCTTCAAAAATTCTATCAATTGTTTCTTCTGGCAAAAGTTTTTCTTCTTTATAGTTAACGTT
TTTATTGATATCCATGAAGAAACTTCTTTGGTTTGCACTCAAAGATTGTGCTGATTTAGGT
AAACTAGAAACTCTTTCAGCTTCTGCTTTTGCTTGTAATTTAGTTTCTTCAAATAGTTGGTT
AATCATGTCACCGTACAATTCATTTTGTCTTTCTTGCGGTTCACCGTTGTTTACTGCATTAA
TAAATTCGTTTTTCGCATTTGCGAATGTTTCCGATAAATTTATAGTCAT

> **Q6GF91**
Function: protein coding sequence; putative protease (mobile element)
Best match: Q6GF91_CC005_A9299_ACKH01000012.1[7311:8048]RC
Position: 036-contig_189: 111031 … 111769; Length: 738 bp
Sequence:
TTAAAAAAGGAATCTTGAAAATCCATTTGCTGATACTTTACTATCTGCAACATCGATTTCT
GATTCCTTTTCTTTCATATTTATTTTTTCAATTACTTTATTTGCTATTGCGTCAATATCAATG
TTAACCTCTGGCGTTTTACTTACCAAAGCTGTTACACGATTTAATACATCTTTCGATAACA
CTTGTGTATTGCTTGCTACAATTTGCATATTGTCGTTTTCAAACATTTTACTATCCGCAAAA
CCTTGTTCAATGGCTTCATCAGCATTTAGCCACGTTTCCCTAGCCATCATTTCTACAAGTT
CTTGTTTGTTTTTACCAGCTCTAACCGCATATGCCGCAGCCATTATTTGACCAACATGTTC
TAATGTTTCTGCAGCATGATTTAGATCTTTCGCTTCTCCTTGCGCAATACTTGAAGGATTG
TGAATCATCATTCTAGCAACCGGACTCATTTCGATGTGGTCACCAGCCATTGCGATAAGC
GATGCCGCACTTGCTGCTATTGCTGTGATACGAACATTCACTTTGCCTTTATGAGCTCTTA
AATGTGTATATATTTCACTACCAGCTACTAGGTTACCACCATTTGAGTTAATTATAATATC
AACATCTTCATCACTAAATTCTAGTTGTGTTAAAACATCTTTAGGACAAGTCGAATCCATA
CCAAGCATTTCGTAAACCCATTTATCTTCGTTGGAAACGATGACGCCTTTAATCTCCGCTT
TCAT

> **portal1-phiNM3**
Function: protein coding sequence; bacteriophage portal protein type 1 (mobile element)
Best match: portal1-phiNM3_CC005_Mu50-VRSA_BA000017.4[2105401:2106588:r]RC
Position: 036-contig_189: 111752 … 112940; Length: 1188 bp
Sequence:
TTAATCTCCGCTTTCATCTTCATCACCACCTTTCAAAGTGTTTTCATCTTTTTCTTTTTCATC
ATTTTCACCACTGTTAGCTTTTTCGTAGTTTTTAGTAATCAGGTATTCGTCTAATTCAGGAT
TGTCTGATGATTCTTCACCTAACATAATCCGCACCTCATTCCTTGTAAATGAACCAGAACT
TACAAGTTTGTCAATTGCTTCAGCATATTGAAGTGGGTCTTTTTTATTCACACCGACAATT
TCTATTCTTGTATCTTTCAAATACATACTTTGTGTTATGAGTTTCGCGTTTAATTCGTTCTG

AATCTTTTTTAATAAAGGTGTTAAACAGAACTTCTCAAATACAAGCGTGTTTTTTTCCAAA
TCAGCTGTTTCTCCGTAAATCAAACCTGGAGGTATACCAATCATCAACGCAACATTTTTTA
TTGCATCTCTCATTAGCTCACTCAATTCAGAAAAAGGCATGTTACTATTCTTACCACCATT
AGATAATTCCTCATAATCAAAACCTTCTATCAAAGGCGCGATTGCTAGTTGATTTTTATTA
AAAGTATTGAATAATTTATTTGTGAACGCTTGTAATTTTTCTATATTCTTTTCGTCATATGC
GCTAGAGGCAGATTTCAAAATCCCTCTTATTTGATAGTTTTTAATTGTGCACCTATCATT
CTTCCGAATATTTTCCCGTAATCTTCGAATAGACTTTCTACAAAGTGTGTCACTTTATTGTT
GTTGTACTTTAAATATATGACCTCTTGCATTGTGAAAGTACGTTGATAAGTATAATCTTTA
ACCGTTACATCTTTGAATATATCATCATACAAAGCGTACTCTTCTCTGTAAAAGCTATCTG
CGATAAGTAATTCTTTGCTGTCACTTACTACGATTAAAACCTCGTTATCATAAATTAGTTT
ATATATAACTTGTTGCCAAAAACTATCGCTTGATAAGTCAGTATTTGGTTTTATATTTAAC
TTGTAGTAAACATCATTCTTTTGAATTCTATTACCTTCCAATACTTTAAAATGACTTTGAG
CGACAGCTCGCGCAACAAATTCAATACAACTATCAATCGCTAAACGTTTCACATACGCTT
GTTGTGATAGATCTTCTATCATATCTAAATCAAGCATATATGTTATATCTTTCCTAGTTTTA
AATATCTTTTCTAGAATACTCAT

> **terminase-phiNM3**
Function: protein coding sequence; bacteriophagal terminase (mobile element)
Best match: terminase-phiNM3_CC005_Mu50-VRSA_BA000017.4[2106604:2108265:r]RC +MANUAL OVERRIDE
Position: 036-contig_189: 112955 … 114617; Length: 1662 bp
Sequence:
TTAGAAATCTATACTCATTAATGCATCAAGCGCTTTAGACATGTCTTTGTCTACTATATCG
TCTGCTCTATATAATGCGTGAACAAAAGCCATGAACCCATCGGTTTTTCTTCTATTTTCAT
CTTTTTTAATATATTCTTTATTACCATCGGGTTTAACCTTTACTGCAACATTATTAGTAAAC
CAACGCATCAAAGGATTGTCTCCATATATTACGTTATGTTTCGCAAACATTGTATCGATAC
GTGGTGCAAGTAATCCATGTATTGCTTTTGGATTTCTAAGTACTTCAAGTTTTATGCCAGC
ATCCTCAAACGCACGTCTTACAATATCAGTTCTATAATTATCAGCTATGACTTTTTCAAGC
CCATATTTTTCTCTAGCCTTTAAAAACCAATCAACTATATATTCAATTTCAATGACATCAT
CATCGACAATGGTCAATAATCCCATTTTTTCCCATTCTTTAATAGGAGGTTCTAATTTGAC
ATCATCCAAAAACCCTTGTCTTACAAACGAATGTCCTAACCAAATGTAATCATCGTTTTTT
CGGAATAATAGCCCTACACTTGCAAAATCTCGAATGTTTGCAAAGTCTAAACCACCAATA
CACATTTGATTATCTAAATTTGGTATCTCTCTATTAGTCGCTAGTATTTCTTTCCATGGTGC
TATTACTTTTTCAAGGTCAACTTCAGGCAAATTCATTCGCTTAGTCATGAATTCGGGCTTA
TTTGAACGGTTGAATGGTAAATCGTTATATTCTTCTTCAATCGTACTTAGCAGTGTTTTAG
CGTATTCTGATAACGGTTTATGTAACATTGGGTTCGCCTTTTCCCACGTCTGTCTGTCATC
AACTTCTTTTGGATCGTCTAACTTACAATAAAAAGCAAACAATCTACTATTTTTAACCTTG
CCACTTAATACACTTGCAATTTTGTGCTTCATTGCATCGATATAACCCTCTCTAACAAAAC
CATCAGTACTTATATAAAACGTTCTTCTATTTTTCTTTTTACCTAATCCACCACGTTTGACG
TTTACCATTTCAGGACCAAAGAAATAATGAATTTCATCAAAAATAACACACCCCTCACGT
CCACCGTCTTTGGTTTTTGTGTTTGATGTGTTATATCGAATAACCGATTTAGTTGCACGGTT
TATTATTTTTGCTTTACTAACTTCATAAGGAGCTTTTGGCGTTTTACCCGTCTTATTTCGTT
TGTTATCCATTAAAACGGTTCTGATTTCATCAAACGATGTTTTTGCTTGATCTTCACTATTA
GCAACAATGGAGATGTGATATTCTTTAACTCCGTGTAAGGGCGTAGAAAGAAAATCACTA
ATAGCACTTATTAGACCGTTTTTCCCGCCTCCACGTCCCATGAAAATAGCAAATTCTGTAA
AGAAAGCTTCATCTGTATTTTTATCTATAAGAAATATATTAGCTATGATAAACCTTTGAAA
TGGTAATGTTGGAAAATACCATTTTTCAATAAATTTGATACAATCCTCGATTTTCTGTTCA
TCAAAATATACATCATCTCGTGAATATATATGTTTTTGTAGATAATTAAAGAGATCAATTC
TTTCTTTATTTAAAATTATCTTTCCTTGTTTCCACAAATTTATATATTCATCAACGTATTTA
TTACTTATCAT

> **rinB-phiPVL**
Function: protein coding sequence; bacteriophage transcript activator B (mobile element)
Best match: rinB-phiPVL_CC008_TCH1516_CP000730.1[2111630:2111779:r]RC (completely identical)

Position: 036-contig_189: 116263 … 116413; Length: 150 bp
Sequence:
TCACTCACTTACCTCCGCTCGAAAGACGTAATCACTCGGCGCCTCTACATCATCATTAGCC
GTCATCATAATATATACTTGCTCAGTTACATACTTACCTAACTCATACATCGCTAGTAAGA
ATAATAATCTTAGTATTTGCTTAATCAT

## > dut-phi

Function: protein coding sequence; bifunct protein-dUTP pyrophosphatase and derepression of GI (mobile element)/phage
Best match: dut-phi12_CC030_MRSA252_BX571856.1[2152883:2153425:r]RC
Position: 036-contig_189: 117277 … 117820; Length: 543 bp
Sequence:
TTACACTCCGCTACTTCCGAAGCCTTTTGCTCCACGTTCTGAAACACTCTCGAATTCCTCC
ACTTGCTTTAGTTCAGGTGTCCATATAGGCACGATAACCAGTTGTGCTAGTTTGTCGCCTT
TGTTGATTTGGTAAACTCTTCTTATGCCTCTTCCATCTTGTACATAGTTACCTTTTATATCT
AAAATGCTTATTAATCCATCTTCTAATTCAGCGTCTATATCATCATATAAAAAGGGTATTC
CATCACGTTCTTCATCATTCTTAATATTAATCCCTAAATTGCCGTGATATCCGGCGTCTAT
CTTGCCTGTTTCAATCACTAAATGTGTTTTACTACTTACACCACTACGGCTAGTTAATAGC
CCGACATAGCCCTCTGGTATGCTTACAGCTACATCTGTTTTAATCACTGCCTTTTCTTGTG
GCTCAAGTACGACAGTTTCAGCTGAGAATATGTCATAACCTGCATCCGTCTTATGATTTCG
TTCGGGCATTCTAGCATTTTCTGATAATAGCCTTACTTGTAATGTGTTAGTCAT

## > sri

Function: protein coding sequence; staphylococcal replication inhibitor (mobile element)
Best match: sri_CC008_Newman_AP009351.1[1110173:1110331]RC (completely identical)
Position: 036-contig_189: 120465 … 120624; Length: 159 bp
Sequence:
TTAATATTCGACGATAGCGGGGCGTGTATGACGTTCTGCAAGTTTTTGGATAAATAGGTC
GTACAACCTATTTTCATCGCCCTGTGCCTCATCTATGAGTTTCTGAGCGTACATATCTGAA
CACTCAAGTTTAGTTTTTAAAAATTCTTTGGTTACCAT

## > istB

Function: protein coding sequence; replicative DNA helicase (mobile element)
Best match: istB-phiPV83_phage_FJ713816.1[12631:13404]RC AND istB-phi53_CC005_04-02981_CP001844.2[886487:887266]RC:
Position: 036-contig_189: 120617 … 121397; Length: 780 bp
Sequence:
TTACCATGTATCTCGCTCCCTGAAATCGTCTCCGATTACTCTTACTTTTCTTGCATTGTGTT
TCATTCTTGAATTGATACGTTGCCAGTTCATATTTTGATTTAGTTCTTTATCACTAAAGTTA
GTTGTAAAGATGTTGTTTTTACCTACTCTGTTATCAACAATGCTGAAAAGTTTATTTAAAG
TGTGCTCTGTGTTTTCTACACCCATATCATCTAGTACAAGTAAATCAATATCACTTAGCAA
TCTGACTAGCTCGTCTGTAGTCTCTACTGCATTTTTGTTGTATGTCGCTTTGATACGATCCA
TCAACATTGGTATGTGCATAAAAGCAACCGTATGCCCTTTAGCTTTAACTGCTTTTGCGAT
AGCGTATGCTAGGTGGCTTTTACCAGTTCCGTATGAACCTTGCAATATTAATGATTTTGGC
TCTTTTGTAGAGAAGCCTTGAACGTACTCTATTGCTGTTTGTTTAGCGTGTACTTGTTTTTC
ATTTTGTGGCTTGTAGTTTTTGACTGTTGCATCTCTTAAAGACGGATTAACGTTTGATTGA
TTGAATATGTTGTTTATCTTCCGTTGCTTGTTTCGCTTATATTCCTCATAGATTTCACATTT
GCAACCGTCTTTATACTCGTAACCATTCGGGTGTTTTTAGTAGGAGCAAACTTATATAAG
TCGTATTCACTTCCACATCTCTCACATTTCAATCCTTTTTCGACATGAGTAGGTTGATATTT
TTTCAAGCTTTCGTTTATCTTTTCGCTGAATAGTGGTTTCAT

## > Q4ZAK4

Function: protein coding sequence; putative bacteriophagal protein l(mobile element)
Best match: Q4ZAK4-phiSLT_CC005_04-02981_CP001844.2[885707:886477]RC (completely identical)

Position: 036-contig_189: 121406 … 122177; Length: 771 bp
Sequence:
CTAATCCCAATAACTTTCGTCGTACTTCATGCGTTCCAATTGATTCGTGCCAGTTGGTTGT
ATTTTTTGATTGAGGTACCCCTCAAATTTACTGCCAAAAAGTGTTTCTGGTCTAAGGTATT
TATCGCTATCCGTGTTTAACCATTCAGCTGTTTTGATATCAATCACCTTTTTAAAATCCTCC
AACCTAAAATCTTGATTCCATCTTGCTTTAATAAAATCTTTTGTTTTAGCTGTATTATGTTT
AAAATGCTTTCCTGCTTTTTTATTTAAGTATTCGATAATTTCTTTATAGGGAATGGAAGAC
ACCGTCGGGTTGCCCGACAATATACTTCCTTCATTATTAGTATTGTTATTATTAGTTAAAT
CATTATTAGTACTATTATTATTAGTAGTATGCGATTTACCATTAACGGTTTTTCCATTGTTG
GTTTTACCGTTAACGGTTTTTCCAACGTTGGAAAATCGAATGTGGTGCGGTTGCTCATATA
CTAAGTACTCATAACCATTTAACCTACCACTTTTATCACGTTTTCTACTACGTTGAATGTA
TCCAATTTCTTCCAGTTCCTTGATTCCACTCTTTAAACCGCTAAGTCCATCAGTTGAATGTT
GCTCTAGTTCTGTTTCGTAAATTTGCCAGTTATCAGGTCGACTTAACAAATAAAGTAGAAT
ACCTTTAGCCTTCCAACTTATATTAGAATCATGTATAAAATCTTTGTGTACTGTGACAAAG
TTACCTGATTCTTTGTAAACTCTAAATGTTGCCAT

> **ssb3-phage**

Function: protein coding sequence; putative DNA binding protein (mobile element)
Best match: ssb3-phiPVL_CC008_NCTC8325_CP000253.1[2065949:2066500:r]RC (completely identical)
Position: 036-contig_189: 123346 … 123898; Length: 552 bp
Sequence:
TTAAAACGCTAAATCTTGGTCGTCATATCCAAATTGGCCACTGCTTTCAAATGGATTGCTT
TGTTGAGACATTGATGTTTGTTGTTGTGCCCCGTTATTTTCTTCAGCTTTTTGCTTATCTGT
CTTCGGAATAGGTTTGTTAACAACATCATCGCCCTTTTTGTAAGGTTTAATAAATGAAAAA
TCCGTAAAATACTTACCTTCATCTTCATTGAATTTCCATTTCAATACCAAGTGACAAAACT
TACCAATAAGATCATTGGTATCAAAATCTAAGCTAGGAAGATTTAACTTAATACCTAATC
GAGTAACTAATTCAATCAATTGTTTTTCTTGGAAATCATATTTATACGGCGGTACAAATTG
ATTATGTTTATATTGTTTGCCTTCATCATTTTCAAATACGATTGTGAAATATCTATTTTCTC
TATCATTGAATTCAATATTTTTAACTTTCACTGTGAATTCTCCAGCTTGAAACCCTGCTGA
GCCGTTATAAAACTTTTCTTGATTTGTTTCTTTAGTAAATTGCGCTTGTCCTGTGATTTTCA
T

> **DUF2483-phi80a**

Function: protein coding sequence; putative bacteriophagal protein (mobile element)
Best match: DUF2483-phi80a_CC010_H19-ST10_ACSS01000056.1[6024:6245:r]RC (completely identical)
Position: 036-contig_189: 124707 … 124929; Length: 222 bp
Sequence:
TCAGTCATGTTCTACCTCCTCATACTCAATTGTTTCTGTCACTGTTTTCTTGATTGCTTTGT
GCTTAGACATATCAATAACAGTTTTGTCTAGTCCGTCGAATTCTCTTGCGTCTCGCATATC
AGTTGAATACTTCACTGTATCGTTCACTTCGGTTGGTCGGTTTGTAATAAATAGATTTTCA
TCTTTATGCTTGATTAGATAAGTTACAGTCTGCTTCAT

> **ant-phiPV83**

Function: protein coding sequence; bacteriophage antirepressor (mobile element)
Best match: ant-phiPV83_phage_AY508486.1[26092:26889]RC
Position: 036-contig_189: 127131 … 127929; Length: 798 bp
Sequence:
TTAAGATGTTTGTTTTTCTCCTAAAAACTTATTAACAAAGTATTGTTGTCCTTTGCCTGTTA
CTTTTGGTGTACGTGATACTTTACTTGAACCATCTGGATTATTAATTATTCGTTTTTTGATA
TCCAAGATTTTTAGATCCATACTCTTTTGAGTTGGTAAGTTATAACTTTCTCCACTCTTTTT
AATGAGATATCCATTATTTCTTAACCATTTGAATAATCTGTTTTGTCCTATATCCACACCG
TTTTGTTTAAGTATTTTCGCAAGCTCTCCGACGAGTATTGAATTGTCGCTACCAGCTACTG
AGTCAGCGAATAATACTTTTGGTTTGTTAACTTCTACTTGCTGTTGTAAAAGTAAGTTTTG

CTCTTTTTCTTTCTTATACTCAGTCAACACTGTAATGATGTAGTCTGGATCTTTTAATGTTT
GTTCAATTACATTGTCTGTTGCGTAGATACCGTGTTTGCGAATAGCTGGTAGGACATCTGA
TGTTACCCAGCGTTTGAATTTTCTAGCGTTTTCTCTAATTTTTTCGTTTTGCTTTGTTTAGA
AGCATCGAAGATTAGACTGTATAATCCTGATTCGTTGATAATGATCATATTTCTGTTTTGA
CCTGATGCACTAAATTGGTGCGTCAGCTTGTCCTCGCTATCAACATGATTTCTGATGGCAT
TGTCTGTCCTTGCATATCCTAAAATCTCAGCAATATCTTTTCCTACAAAATAAGGTTCGTT
TTCAATTTCCACTGTTCTTACTGGTAGCTCTTTAAAATTAAATGTTTGTAATGCTTGCAT

> **repressor-var3**

Function: protein coding sequence; bacteriophage repressor (mobile element)
Best match: repressor-var3_phage_AP001553.1[2342:3070:r] [4_50ANF10END]
Position: 036-contig_189: 128338 … 129067; Length: 729 bp
Sequence:
GTGTTCAGAAATATGAACAGTTTTAAGGATAGATTAAAGCAAATTATGTCTGAACGGAAG
ATATCTCAATCAGAGCTATCAAGAAGGACTGGTATTGGCAGAAACTCAATTAGCGATTAT
TTAAATGGAAAATATGAAGCGAAACAAGACAAAGTCTTTGAACTAGCAAAGGCTTTAAA
CGTTAACGAAGCGTGGCTTATGGGTTTTGATATTTCTAAGAATAGAAAAATTGAAAATAA
CGACATCACTTCCATATACAACAAACTCACACCTCCCCGCCAAAAAAACGTACTTAACTA
TGCAAATGAACAATTGGAAGAACAGAATTCTAAAGGAGATAACGTTGTAGATATTAATTC
ATATAAACAGGAGAAAACTCCAGTTAACGTCAATGGTTGCGTCTCTGCTGGTGTAGGAGA
ACGTTTACACGATGAAACGCTATTTACTGAAATGGTTAAAGGACCTATCCCCACACACGA
TTTAGCGTTAAAAGTAAATGGTGATTCTATGGAACCTATGTTTAAAGACGGTGAAATTAT
ATTCGTAGAGAAAACTCACAATATAAAGAATGGACAAATTGGTATATTCATCATTGAAGA
AGAAGCGTACGTTAAGAAAGTCTTTGTTGAAGATGATAGATTGACTCTAGTTTCACTAAA
TAAAGAATACGATGATCTACACTTTTACAGGAACGAAAGTGTGAGGTTAATTGGAAAAGT
TATTTTATAG

> **int1-phiPVL01**

Function: protein coding sequence; bacteriophage integrase (mobile element)
Best match: int1-phiPVL01_CC001-ST772_118_AJGE01000058.1[20637:21842:r] (completely identical)
Position: 036-contig_189: 131132 … 132338; Length: 1206 bp
Sequence:
ATGTGGATTGAAAAATTTAAAAACAAAAATAACGAAACTAAATACAGATACTACGAGAA
GTACAAAGATCCATACACAGATAAATGGAAACGTGTAAGTATTGTCTTGAACAAAAATA
CAAAACAATCACAAAAAGAAGCTATGTTCCGTTTAGAAGAAAAAATAAAAGAAAAACTA
AACAACAAGTCGTCAAGCGAATTAAAAACTTTGACTTTTCACGCGCTATTAGATGAATGG
CTTGAATATCATATAAAAACATCAGGTTCAAAGTTGACTACTCTTAATAATATAAAAATA
AGAATTAAAAACATTAAACGATACTGCTCTGAGAACTTGCTTTTAAACAAACTTGATACA
AAATATATGCAGATATTTATTAATAAATTATCAGATATCTATTCTCAAAATCAAGTAACCC
GTCAACTCGGAGATATGAAAGGAGCTATTAAATATGCAGTTAAATTTTACAATTATCCAA
ACGAATATTTGTTAACTAATGTCAAAATACCTAAAAGAAGAAAAACAATAGAAGATATC
GAAAAAGATGAATCTAAAATGTACAACTATTTAGAAATGAACCAAGTCCTACAGATACG
TGATCATATACTAAATAATAATAAGTTACACAAGCGAAATCGCATTTTAATTGCCAGTAT
CTTAGAAGTACAGGCTTTAACTGGTATGCGCATAGGAGAATTACAAGCACTTCAGGAAAA
AGATATAGATTTATTAAACAAAACTATCAATATAACAGGTACAATTCACCGCATTAAATA
TGAAGAAGGATTCGGATACAAAGATACTACAAAGACTATAAGTTCAAAAAGAAGTATCA
GCATCAATTCTAGAACCGTAGAAATTTTTAAAAAGATAATACTGGAAAACAAAATGTTGA
AAAGATGGAATTCTAGCTATGTTGACAGAGGGTTCATATTCACAACAAAAAAAGGAAAT
CCTTTATGTAATAATCAAATTGCCAGTGTGCTTAAGAAAACTACAAAAGCTTTAAATATG
AATAAGAAAGTTACCACGCACACATTTAGACATACACACATAACTTTATTAGTAGAAATG
AATGTTTCTTTAAAAGCAATTATGAAAAGGGTAGGACATGTAGATGAAAAAACAACAAT
TCGCATATATACTCATGTAACTGAAAGAATGGATAGAGAACTAACTCAAAAACTCGAAA
ACATTCCAAGTTAG

## > A5IT17, disrupted - downstream fragment
Function: protein coding sequence; putative protein: disrupted - downstream fragment
Best match: A5IT17-CC1_ref_CC001_MSSA476_BX571857.1[1554595:1556757:r]RC#[2_150ANF&END]#A5IT17#[CDS:putative protein]#[cv:54.602:54.2126] MANUAL OVERRIDE
Position: 036-contig_189: 132339 … 134408; Length: 2070 bp
Sequence:
CTTAAATCTGCCCTTTTTTTGCCCTTATATTTTTTATAAGCTTTATAAAATGCTTGAAAACA
CTGGCATTAAAGATTTTCTTGGACTAATTATATCATCATAATGTGATGGTTCAAATATCAT
TTGTACAATCAAAGGCTTCATGTTCTTAACAATATCATCTAAATGGTTATCTAAAATTGGT
GACACTGCTTTTAAATCATTAAGAAAAGGCTCCCATTTGCCTAAAGTATTATCTAATTCTT
CTAATTTAGTTTTAATATAATTACAAGTTACATTAGGAATCAGGGACAAAAATTCTTTCTT
TTTTACATTTAACATTTCAATTGCATGTCTTAAATTCTTACGTATTTTGGGAATTGTATTAA
TCAAATATTTTATTACATCGACAATTTTCGATGCATATTCATCATATATACCTTGAACATA
GTCTGCTATTTTTTTTAATACCATCATCGATATGGTCTTTTAATATTTTCATTTTTCTTCCTAA
ATAATTAGAAGGTAAAGCTAGACCCTGCACCATATTTTCACCGCTATAATTAATTTGATA
ATTTCCATCTAAAATTGTTGCATCTTGTTGCTTCATAATACTTCTAATATCTGCAATTTGCC
TACCATAAATATCATTTTGATTTTTTATTTGCTGAATATTCTGTTTTACTACTTTCAAATGT
TTCATCATTTCTTCAGATACTCCATCCTTGAAGTCATGATCAATATTTTTGAAAATTTCTAA
AATTTCATTATCTATACTATCATACACTTTTTCTATAAAAGATTTTATACCTTTAAACAACT
CATTAATTCTTTCTTTTAATGCATCCAATGCAAAATCAGGTAATAAGTGTTTAACAGCACT
AATACTTTCTATTGTTTCATCTGCAACTTCTTCAAGTGCGTTTATTTTACTAATTAAAGTTC
TTTCCATTTCTTCTAATTGAAATAAGTTAATCTTATCCTTAAATCCTTCTGATAATTGTTTC
TTTCTATCTGCAAAATTTTTATTTTCATTTTCTGAGATGTTAAAACTTTCATTTAAAAAGAT
TACGCATTCTGCTAACATACCACTTGTTTCACCAGTAATCAGTTTACTCAACGCATCAAGA
TTTTCTAAATTAAGTTTAATTAAAGTTCCTTTTCCGGAACGTGCAATCGAATCTCCTGTCC
AAACATTTATTGGAATTCGCCCATCCATATCTAATGTTATGTTAATAGTCTTTTTTACTTTT
TTTCCATTTTTAATTTCTGTATCTTTTACCGACTTAATTTTGATTAGTGGTACAGTATCGTA
TGTGTTATCTTTTCTATTTAACTTCCTTTTATAACCTACATGGCTGTCTATTAAAGCATCTA
ACCTGGGCACACCATCACTAATGTTAACGCGTTTTCCTGGCATATCTTTGATGAATGGATC
TTGTAACCATGTTAATAAATCGTTGGTACTATTAAAACTAATCATATTATCAAAGCGTGGT
CTAGCAAATTTCTGCCAAGCAGCATAAGGAATCATTGCTGAGTCAGTAGCAACAACTTTT
TCATTCGGATGTTTCGCTCCTTGATATTTTGCTCCTGCACCACCTTCCGAATTACCACCATC
CGCCACAATTGTTTTGTTTTTGTAATTATAAGGTTTAGTTTTGTATTTTCTTCTAAATTCAT
TTTCATCAAGAACAGAAGCATCTTTAATTTTTTGTCGATAACTATTTGCGAATTCTTCTAC
TTGCTTTAAATAATCTGTGCTTTCATTATCATTATTCATTAATTTAGCATTTTGGAGCCAAT
CATCTCCAAAACCCGATGATTTTAATGGATTATTTGGGTTAATTGCCTCATTAGATGTTCC
TTGATAAATTATTGTCTGTTGACCAGTTGGATTCCCCTGATCATTAAGTAACTCGTATGTT
TTAATATCAGCAGCACCATTCATATTATCATTACTATTATCATTATAATTATCTATTTGTTT
GAACCGTTTTCCATTTACTTTAAAATCTTTTTTTTCATTTATATCTTGATAAACCCAATAAC
TGCTCAATTCTGCTATGTCCCTATCATTTATTTTATTCAC

## > graD01
Function: protein coding sequence; size of CDS enlarged with respect to SACOL1533 (core genome, variable)
Best match: graD01-MW2_CC001_MW2-USA400_BA000033.2[1577011:1577919:r]RC
Position: 036-contig_189: 134419 … 135328; Length: 909 bp
Sequence:
TTACCCTTCAATTGGATTTATATCGTCATAAAATGGTTTTTTAGTATTTATACTAGATTTTC
CTAATTGAATAGTAACTTGAGATTTTTGAGGCATATTGGGTTTTTCTTTTAATTTATTACTC
AATTCAATCACATCATCTACTGTATTGTCTTTAGTAAAGTTTTTCTTAGTTGAAAAAAGTG
TAGAAACTGCATCAGTATTGGCAGTATAATCTAACTCTTTTCTAGCTTGTTGCATACCTTC
TTTAAATTCTTTATCATTTTTATGAATCAACGGTTCGTAATATTTACGATATTCTTTTAAGT
TTCTTGATAAATATGTGATATAAAAGTATTCATTTTGATATCCAACGTTTTGTGTCTTGTTA
ATTGCCTCTTTTGTAAAGCCTGTATATTGATATTTCTTTTCATTTTCTTTGAAGAATTTATA

TAAGTTATCATACTTTTCTTTTTGCGCTCGATATTCAAAGCCACTCAGCACTGTACCCACC
ATCATACTCATATCATCACCATTGTCATTACTGCGCATTGATCCTTTTTGATGGATGGCAT
CTTTGTACAAAGGTAGACTTGCATTAAATACAATGCCATGATCTTCACAATGCACATAAA
CTTCTATACCATCATCTTTACCTACAACATTGGTAGCCTTTACCTTCAGTCCAAAGTTGTCT
TTAAAGAATTGTTCCCCTACTTTTTCAAATTCTTTACGATGCTTCTTCGCAAATTCAATCGC
ATCTTTTTCTGCAGGCGGTTGGAAGCCTTGGCCTACATATTTTGAAGCTTCCATTTCTTCTG
GTACAGATTTTGTTTCTGTGTTTGTGTCTTTATTTGATTCATTTTCCATCGCGGAACATCCC
CCTAAAATTAATGTCGTGGTTAAAACTGATCCAATGAACTTTTTCAT

## > srrB

Function: protein coding sequence; staph respiratory response protein B-sensor histidine kinase (core genome, constant)
Best match: srrB_CC008_NCTC8325_CP000253.1[1511734:1513485:r]RC (completely identical)
Position: 036-contig_189: 135572 … 137324; Length: 1752 bp
Sequence:
TTATTCTGGTTTTGGTAGTTTAATAATAAATGTTGTGCCTTTCCCTAATTCGCTTTTAACAT
CTATGGAACCACCATGCTCTTCGATAATCATTTTACAAATGAACAAACCTAAACCGGTAC
CTTGTTTACCTCGCGTTCTCGCTGCATCAACTTTATAAAAACGATCAAATACTTGTTGTAA
ATGTTCTGGTGCAATGCCTGTACCTGTATCTTTAATGTATAAAATATCTTCGCTTTCATTTT
CATCACAAGTAATTGCAATTTCATCTCCAGGTTTCGTATAACGTGATGCATTATCAATTAA
GTTCGTTAGTACTTGGTCCATGCGATCCATATCATAACTCCAAACACGCTTCTTACAATAA
TTAAAAGTCATATTTAGACCTAAATCATCAGCTTGTTGGCGATACTTAATTTTCATCTTAT
CTAGTAACGCTGCAATAGGCTGAACTTCTTTATTTACGGATAACCCTTCAGCATCCATGCG
TGCGACATTTAACAATTCATTAACTAAACGATTTAAACGTTTCGATTCATCAAGGACAAT
GGCAAGCGATTCTTTTATTTCATCCGGTTCTGTAACAATACCATCTACAATTGATTCAGTA
TAACCTTGAAGTAATGATATCGGTGTACGTAATTCATGTGATACATTAGCAATGAAATCT
TTCTTCATTTGATCTAGATTGTGCTCATTAGTCATATCACGAACTGTCACAACAACACCAC
TTTTACCTCCCTGTTCAATCTTGTCGATATAGCTTGTGGTCACAACAAAGAATCGTGCATT
CATTTCTAAATCGCGCATTTCAGTTTGTTTTGATTTAAAAGTATCTTCTATTTGTCTTAATA
AGAAAGCTTTAGCATCTTCATCAATATTGTCCATAATATCATTCGCCATCTTATTAGATAA
GATAATTTGTCGACTCTCATTAATACCTAGGACACCTTCTACCATAGAGTTAATTAAGCTG
TCTCTAATATTTTTAGATGTGGATAATGCGTCGACATGCTCTTCGATTTCTGTACTCATCTG
ATTAAATGCCTGCGATAATTGACCAATTTCATCTTTCGTTGTGACAGAAGGTTTATAAGAG
TAATCCCCTTCAGATACACGTGTAGCTTGGTCTCTTAAACGTCTTAAAGGTTTTGTAATTC
TTGACGATAAGAAAAACGCAAAGACTGTTGTAATTGTTAAGAAAATAACAGCCGTAATT
ATGGTGATAATCGTAATAGCATTATTTGTATCTTCGATTGATTTCAAGTCTTTATATATAA
AGACTCCACTATATTTGCTATGACTATTCTTCTGTGCTTTTGTTGGATAGCCTAACAAAAT
ATATGTTTGAGATGAGCCCTTTTCTTTAATCGTTACATTTCGAGTAACAGATTTACCTTTAT
CAAACACATCGTCAAAATGGTCGTTGTTGACTACTTCATTCAACATTTGCTTTTTAATATT
AGAAAGTGAAGCCGTTGATTGACGATGTTTATTATTTATAATCATCAACCCACCAGGATT
TTCAATTAATGTTTGACTATATTTTATTGCTTCTTCTTTATTATGTGATTGTTCGACCAGTG
AACTTATACGTCTAGCATCTTCTCTTATGGCATTTTCGGTTTCTTGTGTGAAATAGTATTGC
ATAAAGGTAATTAAAGCAATACTTAATAAAATTAAAACTGTCGTCACTATTAAAATAATA
GTTAACCACAGTTTAATTACGACACTATTTAGCCGGCTCATCAT

## > srrA

Function: protein coding sequence; staph respiratory response protein A-DNA-binding response regulator (core genome, constant)
Best match: srrA_CC005_Mu50-VRSA_BA000017.4[1608983:1609708:r]RC (completely identical)
Position: 036-contig_189: 137304 … 138030; Length: 726 bp
Sequence:
CTATTTAGCCGGCTCATCATTAGATTTAACCTCAAATTTATACCCAACGCCCCAGACTGTT
TGAATCATATGCGCAGCTTCGCTAGACACACGATTTAACTTTTCTCTAAGTCGTTTAACAT
GAGTATCAACTGTTCTTAAATCACCATAGAATTCATAATGCCAAACTTCTTTTAATAATTG
TTCACGGTCAAATACTTTATTTGGTGTTTTAGCTAAATATATTAATAATTCGTACTCTTTAG

GAGTCAAATTAACTTCTTGATTATCAGCAAGTACGCGATGTGCATCATTATCTATTTCTAA
ATGTTTAAATTCAATCACATCACGTGCGTGAGGTTCGCTTTGTTCTACAGTTGTAGATTGC
GTTCTTCTTAGAAGTGCTTTAACTCTTAAGACTACTTCTCTTGGTGAAAATGGTTTGACGA
TATAATCATCTGCACCAGATTCAAAACCTTCAACACGGTTTGTTTCTTCACCTTTAGCAGT
CAACATAATAATCGGTGTTTGTTTATGTTCACGCAATTTAGTTGCCACCTGGATACCATCC
ATTTCAGGCAACATTAAATCTAGTAGTATGCAAGCATAATTATTCTCCATTGCAAGTTCAT
AAGCCTCTTGGCCATTACTTGCTTCATGGATTTCAAAAGATTCTCTTTCTAAATACATTTT
AAGTAATCTTCTGATTCTATCCTCATCATCTACGATAAGTATTTCGTTCGACAT

> **rluB**

Function: protein coding sequence; RNA pseudouridine synthase (core genome, constant)
Best match: rluB_CC008_NCTC8325_CP000253.1[1514324:1515061:r]RC (completely identical)
Position: 036-contig_189: 138162 … 138900; Length: 738 bp
Sequence:
TTATTTCCCATGTTCTGCTAAATGACGCATCACTTTTACTTCATGAGGCGTCAATACACGT
CCTTCACCAGCATTCAAACCGACAACATTTAAAGGCCCATATTCAATACGAGACAGTTTC
GTCACTTGATGACCAAAATGTTCGAACATTCTTCTGACTTGGCGATTACGACCTTCTGTAA
TTGTAATTTCAACCAATGTTGTGTTTTTATCTTTATCTTGTTTCTTAACTTTCACTTCAGCC
GGTTGCGTCATACCATCTTCTAATTCAATACCTTTTTCTAGCGCTTTCACTTCTTCTCTCAT
TAAATAACCTTTTAATTTCGCAACATATTTTTTCTTAATTTGATATCTTGGATGTGTCATTA
AATTAGTAAATTCACCATCATTTGTGAGTAATAACAATCCAGAAGTATCATAGTCTAAAC
GACCAACAGGATAAATACGTGCTTCTATATCTTTAAAGTAATCTGTAACCACTGTACGTC
CTCTATCATCAGATACACTTGTTATCACTTGAGTTGGTTTATGGAATAAAATGTAAATTTT
GTCTTCTAGTTCTATTTTAATACCTTCAACTTCAATCGTATCTGATGGCTTCACTTTTGTTC
CTAATTCAGTGACAGTCGTACCATTCACTTTCACTTTTCCTTCAGAAATTAAAGTTTCTGC
CTTACGTCTTGAAGTATAACCGCTATTAGCTATACGTTTTTGTAATCGTTCTAATTCTTTAG
TCAT

> **scpB**

Function: protein coding sequence; chromosome segregation and condensation protein B (core genome, constant)
Best match: scpB_CC005_Mu50-VRSA_BA000017.4[1610571:1611113:r]RC (completely identical)
Position: 036-contig_189: 138892 … 139435; Length: 543 bp
Sequence:
TTAGTCATTATTTTCTCCTTTTTGATTGACTAGATTACTGAAAAAAGCATCCATTTCTTCAT
CGTCTTCTTCAGTTGTCGGCAAATCTTCTATATTGGAAATACCAAATACATTTAAAAATAA
ATCAGTAGTAATTAACTGTTGGCTACGCTGTTCATTAACCACTTTAGCCTCAACTAGTCCT
TTGGCAATCAATGTCTTAACTGCACCATCTGAATTGATACTACGAATTAATTCAATATCAC
TTCTTGATAATGGCTGGTTATAAGCAATAATTGATAGTACTTCCATTGCTGCTTGTGATAA
TTTCATTTGTGACTTTTGTTCAATTAATTGTTCAATATACGTTGCCGCTTCTTTTTTAGTCG
TTAAAACATACGTCGTTCCAAATCGTTGTATCATTAATCCATGTGATGAATAATTTTCAAT
TAATTCAACGAGTTGGTCTTTCGACATATCTAATATTTCTAATAGTTGTTTTTCATCTAAAC
CTTCATCGCCAGCTGTAAATAAAAGCGACTCTAATATACCATGATTATCCAA

> **scpA**

Function: protein coding sequence; chromosome segregation and condensation protein A (core genome, constant)
Best match: scpA_CC001_MW2-USA400_BA000033.2[1582019:1582750:r]RC
Position: 036-contig_189: 139427 … 140159; Length: 732 bp
Sequence:
TTATCCAAAATGGTAGTTCACTCCTCTAATAATGTTAATATCTTCAAAATTACGTTGTTGC
TCAATATTAATTATTCCTGCTTTTGACATCTCTAAAATAGCTAAAAAGTGAGTGACTACTT
GTTCAATTGGCTCAGAAAACGTAAACAGACTAAAGAAGTTAAAATGATCTTTATCTTTCA
ATCTCGATGTCACTTGTTCTGTAGCTTGTTGAATGGTAAATGTCTCTTTTCGGATTTCAAC
AGATTTAGGTGTATTTAACTCAACTCTATTTTTAACTCTTTGATAAGCTACAATTAATTCA

GTTAAATCAATCGTATGATTTGGATTCCAAGATTCATCTGTTTCCAAATGAGATAAATCTG
TCGGTCTTTTTGTAAAATAAAAATCTCTTTCTTCTTTCATGTCATTTAAAATAGCAGTATAT
TCTTTATAATTTTGATATTCTATTAAACGCCCAACTAAATCTTCCCGTGGGTCATCATCAA
CATCCATATCTGATGTTGATTGTGGTAATAGCATCTTACTTTTAATCATTAAGAGTTCTGA
CGCTAATACTAGGTATTCACTTGCAATATTAATTTCAAGCTGTTTCATTGCATGAACGTAC
TGCATATACTGCTCTGTTAATGCTTGCATAGGAATATCATAAATATCTATTTCAAATTTTT
GGATAAGATGCAGTAATAAATCTAATGGTCCATTGAAAGCATCTAATTTAACTTCATACA
T

## > Q2FY75

Function: protein coding sequence; putative protein (core genome, constant)
Best match: Q2FY75_CC001_MW2-USA400_BA000033.2[1582842:1583348]
Position: 036-contig_189: 140250 … 140757; Length: 507 bp
Sequence:
ATGCAACAGGCACTGATAAATTTCTATTATCAATTTCATACTAAGCAACATTATTTCCTAT
GTCATGACATTTTAGAAGATGCATGGAAAGCTGAAAATAACTACAGTAAGCAAGATGCA
GTTGTTAGTTTAATCTTGTTTGCAACTGCTTGTTACCATTATCGCCGCAATAATTTAAAAG
GCGCCTATAAGTCTTTTAATAAATCTAAAGAAATAATACAAAATGCTAAAGACAGAGATA
CGTTATACCTTAATTTAAATGATTATCAGTTGTTAATTGAGCAACAGATTGCAAAATTGAA
TGCAGCAAAGCCTTTTTCATCTGTTATATTACCAATAACTCCAGACTTTGAACGCATCATC
AAGGCCAACTATCCAGACTATGATTATAATCAGGAAACATCAACAGATCCTTTTATTGTA
GACCATCACATGCGGCGTGATCGTTCAGAAGTGATTGCAGCAAAAGAAGAAGCGATTCA
ATTAAGAAAACATAGACGTAATTAA

## > xerD

Function: protein coding sequence; chromosomal tyrosine recombinase D (core genome, constant)
Best match: xerD_CC001_MW2-USA400_BA000033.2[1583426:1584313:r]RC
Position: 036-contig_189: 140834 … 141722; Length: 888 bp
Sequence:
TTATGCTCTAGGATGAAATTGGTTATACATTTTTCTAATTTGAGATTTTGAAACATGTGTA
TAGAGTTGCGTAGTAGATATGTCAGAGTGACCTAACATCTCTTGCACTGCTCTTAAATCTG
CGCCATTTTCCAATAAATGTGTCGCAAAAGAGTGGCGTAACGTATGTGGCGTTAACGTCT
TTTTAATGTTTGCCTTTACACCATTTTGTTTAATCATTTTCCATATTGCTTGTCGTGATAAA
GGTTTACCATGCATATTTAAAAATAAGACTTCGGTAACAGTCTTTTTTAAAAGTTGCGGTC
TAATCGTTTCAATATAAGTAGTTAAGTACTCAATGACTGCGTCGCCTAATGGCACAATTCT
TTCTTTATCGCCTTTACCAAATACGCGTACAAACCCCATTATTAAGTTCACGTTCTCTAAC
TCTAAATGTATCAATTCAGATACACGCATTCCCGTTGCGTACAGAAGTTCTAACATCGTAC
GATCACGATAACCATTAATTTTATTTAAATCCGGCGTTTCTAATAAAGCCAACACTTCGTC
AACATTTAAAACGTCAGGCAATTTTTTGTCATATTTTGGTGAATCTAATAATACCGTTGGA
TCTTTCGCCGCATATTTTTCTCTTATAGCAAATTGATGAAAACTACGGATTGTTGAAATAA
ATCGCGCAATAGATTTAGCAGATTGCCCTTGGTCTATTAAATGCCCCAAACACTCTTGAAT
TAATTGTCGATCTATAAAATCAATATGCGAGATATGATGTTCAGTCATATAATCTTGATAC
TTTTTCAAATCACGTCTATAAGCACCAATTGTGTTAGAACTTAGTCCTTTTTCAATTTGTAT
AAAACGCAAATATTCTTCAATAATTGTTTCCAT

## > fur

Function: protein coding sequence; ferric uptake regulator (core genome, constant)
Best match: fur_CC005_Mu50-VRSA_BA000017.4[1613449:1613898:r]RC
Position: 036-contig_189: 142070 … 142220; Length: 150 bp
Sequence:
TTTGTCTTTTACTTTCAGATATACGTCTTCAGCACTTAGATGATCTTTTTCATTTTCAATTA
GAACTCTAACAGTAGCTTCGCGTTGTGGCGTTAGCTTATATGATGATTGTTGTAATTGTTG
CTTAACGCGATTTAATCGTTCTTCCAA

## > nudF

Function: protein coding sequence; NUDIX hydrolase (core genome, constant)
Best match: nudF_AF118839.1[1:169]RC (completely identical)
Position: 036-contig_189: 142324 … 142493; Length: 169 bp
Sequence:
TTATTTAGAATGATTATAATTTAATAATAGGTGTTGCAATGCAATGATAGTCTTCGCATCT
TCTATTTCTTTATTCATTAGCATCGATTTAACATTCTCAATCGGAACTTTAATGACTTCGAC
AAATTCATCTTCATCTAAGTGAACTGTACCTTCTTCTAAATTATCC

> **Q5HFS2**
Function: protein coding sequence; putative oxidoreductase (core genome, constant)
Best match: Q5HFS2_CC005_Mu50-VRSA_BA000017.41614627:1615535 [4_50ANF10END]
Position: 036-contig_189: 142948 … 143857; Length: 909 bp
Sequence:
ATGCAAAAAAATATATTAAAAAGTGGTATTGCATTATCTGAATTAGGTCTAGGTTGCATG
AGTTTAGGTACAGATTTAAAAAAAGCCGAACAAATTATAGATTGTGCTGTTGAAAATGGT
ATCACTTATTTTGATACAGCAGACATGTACGATAAAGGTATAAATGAATCAGTTGTTGGT
AAGGCACTTCTTAAATATCAACAACGCGATGATATTGTCATTGGTACAAAAGTAGGCAAT
CGTTTAACAAAAGATGGCAGTACAACATGGGATCCGAGTAAATCCTATATTAAAGAGGC
AGTTAAAGGTTCACTAAAGCGTTTAGGTATCGATCATATCGATTTATATCAACTTCATGGC
GGAACCATTGATGACCCATTAGACGAAACAATAAGCGCATTTGATGAATTGAAACAAGA
AGGAATTATACGTGCTTACGGTATTTCTTCTATTCGCCCAAATGTAATTGATTATTATTTA
AAACATAGTCAAATCGAAACGATAATGTCTCAATTCAATTTGATTGATAATCGTCCAGAA
TCATTATTAGATGCAATCCACACCAATGATGTTAAAGTATTGGCAAGAGGACCTGTGTCT
AAAGGATTATTAACTTCAAACAGTGTTAATGTGCTCGACAATAAATTTAAAGATGGTATT
TTTGATTATTCTCATGATGAATTGGGTGAAACAATAGCCTCTATTAAAGAAATTGAAAGT
AATTTATCTGCATTGACATTTAGTTATTTAACATCACATGACGTGCTTGGTTCCATCATTG
TAGGTGCAAGTAGCGTCGACCAATTAAAAGAAAATATTGAAAACTATCATACTAAAGTTA
GTTTAGATCAGATTAAAACAGCAAGAGCTCGTGTAAAGGATTTGGAATATACCAACCATT
TAGTGTAG

> **tx_Q5HFS2**
Function:  rho independent terminator of Q5HFS2
Best match: tx_Q5HFS2_CC001_MW2-USA400_BA000033.2[1586456:1586530]
Position: 036-contig_189: 143909 … 143939; Length: 30 bp
Sequence:
GCCTTACTGCTGTTGTTTATGTACAATTCG

> **proC**
Function: protein coding sequence; pyrroline-5-carboxylate reductase (core genome, constant)
Best match: proC_CC001-ST772_118_AJGE01000058.1[6926:7741:r] (completely identical)
Position: 036-contig_189: 145233 … 146049; Length: 816 bp
Sequence:
ATGAAACTCGTATTTTATGGAGCTGGTAATATGGCACAAGCTATATTTACAGGAATTATT
AACTCAAGCAACTTAGATGCCAATGATATATATTTAACAAATAAATCTAATGAACAAGCT
TTAAAAGCATTCGCTGAAAAACTAGGTGTTAACTATAGTTATGATGATGCGACATTATTA
AAAGATGCAGATTATGTTTTTTTAGGTACCAAACCGCATGACTTTGATGCTCTAGCAACA
CGCATCAAACCACATATCACAAAAGACAATTGCTTCATTTCAATTATGGCAGGTATTCCG
ATTGATTATATTAAACAACAATTAGAATGCCAAAATCCAGTTGCTAGAATTATGCCAAAC
ACAAATGCGCAAGTCGGACACTCTGTTACTGGCATTAGTTTTTCAAACAACTTTGACCCTA
AATCTAAAGATGAAATTAACGATTTAGTTAAAGCATTTGGTTCTGTAATTGAAGTATCAG
AAGATCATTTACATCAAGTAACAGCTATCACCGGAAGCGGCCCAGCATTTTTATATCATG
TATTCGAGCAATACGTTAAAGCTGGTACGAAACTTGGTCTAGAAAAAGAACAAGTTGAA
GAATCTATACGCAACCTTATTATAGGTACAAGTAAGATGATTGAACGTTCAGATTTGAGC
ATGGCTCAATTAAGAAAAAATATTACCTCTAAAGGTGGTACGACACAAGCTGGCCTTGAT

ACATTGTCACAATATGATTTAGTATCTATTTTCGAAGATTGTCTAAATGCTGCCGTCGACC
GTAGTATTGAACTTTCTAATGTAGAAGACCAATAA

> **rnz**
Function: protein coding sequence; ribonuclease Z (core genome, constant)
Best match: rnz_CC001_MW2-USA400_BA000033.2[1588743:1589663:r]RC
Position: 036-contig_189: 146151 … 147072; Length: 921 bp
Sequence:
TTAGACTTTAAAACTATCAAAATCTTTTACAAAATTAAAATTAGGTGTATCTTCATTTTGT
ATCAATGTTTGATAAATTTCATTTATATCTTCTGTATTATAGCGATTGCTCAAATGTGTAA
TCAACGTACGTTTAACATTGGCTTCTTTTATCAATGCAAATACGTCTTCAATATGGCTATG
ATGATAATTGTTGGCTAAATGCTTTTCACCATCTATATAGGTCGCTTCATGTACCATCACA
TCAGCATCTCTAGAAATCACACGTTCATTAGAACATGGTTTTGTATCACCAAAAATTGCTA
CAACTGGACCCTGTTTGGACTCACCTCTAAAATCTTTTGATTGATAAACTTGACCATTATG
TTCAAATGTATCATGAGATTTTACTTCTTGATATTTAGGACCTGGTTCAAGACCAATGTTT
TTTAACGCTTCAACATTGATTGTACCTGTAGTTTCAGGTGCCATTACTCTATATCCATATG
ATGGAATACCATGATTAAGTAAATGCGCCTCTACAGTAAAACCATCATGATGATATGTCA
GATGATCATCGATTTCAATATATGTAATTGGATAGTTTAAATGTGACTCTGATAAATTCAT
AGACATTTCCACATATGCTTTAATTCCTTTTGGTCCAACCAATGTAAGCGGCTTCTGTTCA
CCGCCCTGAAAAGAACGACTAGAAAGTAATCCTGGCAAACCAAAAATATGATCGCCATG
CATATGAGTAATAAATATATGTGTCACTTTTCCTAATTTAATTGCATGATGTAAAATTTGG
TGCTGTGTACCTTCACCAACGTCGAAAAGCCATATGGAATTGGAATATGGTTCTAAATTT
AAGGCGATTGCTTGTGTATTTCTCTCTTTTGTAGGCAAACCTGCACTCGTTCCAAAAAATG
TAACTTCCAT

> **zwf**
Function: protein coding sequence; glucose-6-phosphate 1-dehydrogenase (core genome, constant)
Best match: zwf_CC001_MW2-USA400_BA000033.2[1589976:1591460]
Position: 036-contig_189: 147384 … 148869; Length: 1485 bp
Sequence:
TTGAGTACTAAAAACAAACACATCCCTTGTTTAATCACAATCTTTGGTGCAACTGGTGACT
TAAGCCATCGTAAGTTGTTTCCATCAATATTCCATCTCTACCAACAAGACAATTTAGATGA
ACATATTGCCATCATCGGTATTGGACGTCGTGACATTACTAATGATGATTTCCGTAATCAA
GTAAAATCATCAATTCAAAAGCACGTAAAAGATACAAACAAAATTGACGCGTTTATGGA
ACATGTCTTCTATCATAGACATGATGTTAGTAATGAAGAAAGCTATCAAGAATTACTAGA
TTTTAGTAATGAATTAGATAGCCAATTTGAATTAAAAGGTAATCGACTATTCTATTTAGCA
ATGGCACCACAATTCTTTGGCGTTATTTCTGATTATCTAAAATCTTCTGGTCTTACTGATA
CAAAAGGATTTAAACGCCTTGTTATCGAAAAACCATTCGGTAGTGATTTAAAATCAGCCG
AAGCATTAAACAATCAAATTCGTAAATCATTTAAAGAAGAAGAAATTTATCGTATTGACC
ACTATTTAGGAAAAGACATGGTTCAAAATATCGAGGTATTACGTTTTGCGAATGCGATGT
TTGAACCATTATGGAATAACAAATATATTTCAAACATCCAAGTTACATCTTCTGAAATACT
AGGTGTTGAAGATCGTGGTGGTTATTATGAATCAAGTGGTGCGCTAAAAGATATGGTGCA
AAACCACATGTTACAAATGGTTGCATTATTGGCTATGGAAGCACCTATTAGTTTAAATAG
TGAAGATATCCGTGCCGAGAAAGTAAAAGTACTTAAATCACTGCGTCATTTCCAATCTGA
AGATGTTAAAAAGAACTTTGTTCGTGGTCAATATGGCGAAGGCTATATCGATGGTAAACA
AGTTAAAGCATACCGTGATGAAGATCGCGTTGCAGATGACTCTAACACACCTACCTTTGT
TTCAGGTAAATTAACAATTGATAACTTTAGATGGGCTGGCGTACCATTCTATATTCGTACT
GGTAAACGTATGAAATCTAAAACAATTCAAGTTGTCGTTGAATTTAAAGAAGTACCAATG
AACTTATACTATGAAACTGATAAACTGTTAGATTCAAACCTATTAGTAATCAATATCCAA
CCTAATGAAGGTGTATCTTTACATCTAAATGCTAAGAAAAATACACAAGGTATCGAAACA
GAACCTGTCCAATTGTCTTACTCAATGAGCGCTCAAGATAAAATGAATACTGTAGATGCA
TATGAAAATCTATTATTCGATTGTCTTAAAGGTGATGCCACTAACTTCACGCACTGGGAA
GAATTAAAATCAACATGGAAATTTGTTGATGCAATTCAAGATGAATGGAATATGGTTGAT
CCAGAATTCCCTAACTATGAATCAGGTACTAATGGTCCATTAGAAAGTGATTTACTACTT
GCTCGTGATGGTAACCATTGGTGGGACGATATTCAATAA

> **graE**
Function: protein coding sequence; putative transcription regulator/AraC/XylS family (core genome, constant)
Best match: graE_CC001-ST772_118_AJGE01000058.1[3010:3876:r] (completely identical)
Position: 036-contig_189: 149098 … 149965; Length: 867 bp
Sequence:
TTGGACGTTATCAAGCAAATACAACAGGCAATTGTTTATATTGAAGATCGTTTATTAGAG
CCTTTCCATTTGCAAGAATTAAGTGATTACGTTGGTCTTTCGCCATACCATCTTGATCAAT
CATTTAAAATGATTGTCGGCTTATCTCCAGAAGCTTATGCACGCGCGCGTAAAATGACAC
TCGCTGCAAATGATGTGATTAATGGTGCTACACGACTTGTAGATATCGCTAAAAAATATC
ACTATGCAAATTCAAATGATTTTGCAAATGATTTAGTGATTTTCACGGCGTATCACCTAT
TCAAGCTTCTACTAAAAAAGATGAATTACAAATTCAAGAGCGATTATATATCAAATTATC
AACTACTGAGAGAGCACCCTATCCATACAGATTAGAAGAGACAGATGATATTTCATTGGT
TGGATATGCACGATTTATAGACACTAAGTATTTGTCACATCCTTTTAATGTTCCGGATTTT
TTAGAAGACTTGCTCATTGATGGTAAAATTAAAGAGTTACGACGATATAATGACGTTAGT
CCATTTGAACTATTTGTTATTAGTTGTCCTCTTGAAAATGGTTTAGAAATATTTGTAGGTG
TACCAAGTGAACGTTATCCTGCACACTTAGAAAGCCGATTTTTACCTGGCAAACATTGTG
CGAAATTCAATTTACAAGGTGAAATTGATTATGCAACTAATGAAGCTTGGTACTATATTG
AATCAAGTTTGCAGTTAACATTGCCATATGAACGAAATGATTTATATGTTGAAGTGTACC
CTCTCGATATTTCATTTAATGACCCATTCACTAAAATTCAGCTTTGGATTCCTGTTAAACA
GAGTCCTTATGACGAAGTTTAA

> **malA**
Function: protein coding sequence; alpha-D-1,4-glucosidase (core genome, constant)
Best match: malA_CC001-ST772_118_AJGE01000058.1[1279:2928]RC (completely identical)
Position: 036-contig_189: 150046 … 151696; Length: 1650 bp
Sequence:
TTAATTTAGTTCGATAACACATGCTTCATATGGACGTAACTGTTTTAAATTAACTTTGGCA
TCATAATTAAATAGCTTTACTTCTCCATGGCTTAAATCAAATGGTACAGTTAATTCTGCTT
CGTGGTTAGTAAGATTACCTACAATAAGAACTTGCTTTTCATTTAATGTTCTCGTGTACGC
AAAAACTTGTGAATTTTCAGCATCTACTAAATCAAATTGACCATATACGTATACATCATTA
GACTTTCTTAATTGAATTAAATCTTTATAAAATTGTAATACTGAATGCTCATCTTCTAATT
GTTGTGCAACATTGATAGTTTTATAATTCGGATTCACTAGGAACCACGGTTCACCATTTGT
AAATCCTCCATTTAACGTATCATCCCATTGCATTGGTGTGCGAGAATTATCTCGGTTCTCA
TCTTTATATTTCGCAAGTAAAGCGTCTACATCTCCACCTTGAGCTTTCACTATTTGATAGT
CATTTTTAACAGCAACATCGTTAAACGTTTCAATACTTTCAAATGGATAATTCGTCATACC
AATTTCTTGACCTTGATAAATGAATGGCGTACCTTGTTGCAAGAAATAAACAGCTGCATG
ACTTGTTGCTGATTCATACCAATACTTGTCATCGTCACCCCACGTCGATACACGTCGTGGT
TGGTCGTGATTTTCAATAAACAACGCATTCCAACCTTTATTTTCAAGTTGTTTTTGCCATCT
ATTTAATACAGATTTATACGAATTTACATCAAAGTGAGAATCACCACTATTCCACAGTCC
CAAATGTTCAAATTGGAATATCATATTAAATTTACCATTTTCTTCCCCGACCCAGTCATCA
GCATCATCAGGACTTACACCATTCGCTTCACCAACAGTCATAATGTCATACTTACTTAATG
AGCGATCTTTCATCTCTTGTAACCAAGTTTGTATACCTGGCTGATTCATATCTACATCAAA
TGCTGGAGCATATGTTTTATCCTCAGGTACAGGTAAGTCACCCGCTTCAAACGTCTTTTTA
ATATGCGTAATTGCATCTACTCTAAATCCATCAATGCCTTTATCAAACCACCAGTTCATCA
TTTCAAATACAGCATCTCTAACTTCTGGATTAGCCCAATTCAAATCAGGTTGTTTTTTACT
GAATAAATGGAAATAATATTGCTCAGTATTAGCATCATATTCCCATGTAGATCCATTAAA
GATACTTTCCCAGTTGTTAGGTTCAGAGCCATCTGGCTTTGGATCTTGCCAAATATACCAA
TCGCGCTTTGGATTGTCCTTACTAGACTTAGATTCTATAAACCAAGGATGTTCATCAGATG
TATGATTTACAACTAAATCTAAAATAAGCTTCATGCCTCTATCATGAACACCTTTTAATAA
ACGATCAAAGTCTTCCATCGTTCCAAATTCATCCATAATCTCTTGGTAGTCACTAATATCA
TAACCATTGTCATCATTAGGTGATTTAAACATTGGACTGAGCCAAATGACATCGATACCT
AAATCTTTTAAGTAGTCCAATTTATCAATCATTCCAGGTAAATCCCCAATACCATCGTGAT

TACTATCATTAAAACTTCTTGGATATACTTGATATGCTACTGCTTCTTTCCACCATTGCTTA
TTCAT

## > malR
Function: protein coding sequence; maltose operon transcript repressor (core genome, constant)
Best match: malR_CC001-ST772_118_AJGE01000058.1[244:1263]RC (completely identical)
Position: 036-contig_189: 151711 … 152731; Length: 1020 bp
Sequence:
CTATCGCTGTGTTGATTTTCTTATTTTTAATTCTATATCTATAATGACGAGTTCAATAACAT
CCTGTGCTTTATTTTTTAATATATTTAAAATTGCTGCACCAGCCTGTTGACCTAACATTCG
AGGCTTGATGTCAATACTGGTTTGAGGTGGTGACGCAATTTCGGTTAAATAAGAATCATT
GAACGTTGCTGTCATTACATCTTTCGGAATTTCAATATTAAGTTCATATAGGACACTTAAA
ATCGCTAAATGTAACACAGCATCTAACGAAATGATTGCCTGTTTAATATTTGGGTCCTTCA
AACGCGTATGTAGATTTTGCATGTAATTTAAAATAACTTCTCTTTCATTACTAGTCTCAAT
AATTTGATAATTAATTTTATTTTGAGAAGCTATCGTTTCAAATCCTTGAATCCTGTCTTTCG
AAACTTCAAAATCGCCTTTTTCTGTAATAAATATTAATTCATCTACACCTTGTTCAATAAC
ATGTCGTGTCAAATTTTCAGAAGCTAATATATTATCATTATCTATATGTGTAAATTGATGA
TCTATATCCGATGTAGGCTTACCAATCACAATAAATGGCATGCTTTCATCAATTAACATTT
GTTTAATCGGATCATTTTCTTTTGAATAGAGCAGTATAAACGCATCAACCATTCGTTGTTT
AATCATTTTATAAACTTCATCCATTAAATCATTCATATTATTTGAGACTGTCGTTTGTGTAC
CATAGCCATGCTGGTTACATGTTTCAGAAATCCCTAGCAATACATTGATGTAGAATGGAT
TCAGTCGAATAGGCTCCTCAGACCCTTTTAACACTAAACCAATTTTATATGTTTGGTTAGT
AATTAAAGTTCTAGCAGCAGTATTAGGAAAATAATTCAATTCTTCCATAACTTTCTTCACT
TTTGAAATTGTCGCTTCGCTAATACGTTCATTTCCTTTTATAACTCTTGAAACTGTCGAAG
GAGAAACACCGGCTTTTAGTGCAACATCTTTAATCGTAACCAT

## > repORI-px
Function:  origin of replication of plasmids
Best match: repORI-pUB110-2_CC705_RF122-cow_AJ938182.1[1270028:1270090:r] (completely identical)
Position: 036-contig_189: 152988 … 153051; Length: 63 bp
Sequence:
AAGCGCAATTATCTCTATAATTAAAAGTGCCTAAACAAATAACTAAAGGAGATAATGCGC
CTA

## > tnpISSha1-2
Function: protein coding sequence; putative transposase
Best match: tnpISSha1-2_CC010_D139-ST145_ACSR01000081.1[34410:35725]
Position: 036-contig_189: 153050 … 154366; Length: 1316 bp
Sequence:
ATGAATAATGTTATACTAAATACCTTTGATTTTAAAGATAAAAATATTATTTTTGACGGTA
AACTTGAGAAAATTGAGTATAAAGGGAGAAAGTGTTTCTTTTATTACGCAAAACTAATCT
ATACACCTGAGGTGTGCCCAAACTGCAATTGTAAAAACATTAATGGTAATTTAATCAAAA
ATGGATCTAAGACTTCTAGAATCACTATGCCTAAAATCTCCGAATATCCAACTTATATCAT
GTTGCGTAAGCAACGTTTTAAATGTAAAACGTGCGAACGATACTTTACAGCCGAAACACC
TGAAGTTGATAAATACTGTTTTATATCAAAAAAACACGATCAGCTGTACTTAATAAAGCA
GCAGAAATTCGTTCTGAAAAATCTATAGCGAAATCCTGCTCTGTGTCAGCTACCACAGTT
TCTCGTATCATTGATGAGGCCGCTCAATCGCTTCATCAATCGCCGTATTCTGCACTTCCTG
AACATATCATGATGGATGAATTTAAAAGTTTTAAAAATGTTTCTGGGAAAATGAGTTTTA
TATACGCTGATGCACAAACGCATCATATTATTGATATTGTTGAGGATCGTAGATTAAGTG
AGTTGAAAAAGTATTTTTATCGTTTTTCACTTAAAGCTAGAAAACGTGTAAAGACTGTTTC
CATTGATATGCATGAAGGCTATATGACATTGATTAAAGAGATGTTTCCGAACGCTAAATT
AGTTATCGATAGATTCCATATTGTTCAATTATTAAATCGTGCTTTAAATGGTATTCGAGTC
GCTGTTATGAATGAATTAAGAACGAAGAATCAACCTTTATATAATAAGTTTAAAAGATAT
GCGAAATTACTATTAAAACCTGGAGAAGATTTAGAGGCATTTGAATATCGTAAAGTAGCT

TTATTTAAGGAATGGAAGACACAAAAGGGTATCGTCAAGTATTTATTAGATCAAGATGAT
TCGTTAAATGACGCCTACCAATATATCAATCAGCTACGATTCAAACTTAAACATAATGAT
TATGAAGGCTTTATTCACGAATTAAAACATATGCCATTATCCCAAACGCATTCAGTTGTCC
AACGAGCAATTAAGACACTTAACAAACATGAATACTTTATAAAAAATACATTTGATTATT
ACAATTTATCAAATGGTCCTTTAGAAGGTATAAATAACAAAATAAAATTAATTAAACGTA
CATCTTTTGGTTATGGAAGTTACAATCATTTGCGCAATCGAATATTATTATGTTCAAAACT
TTACGCTCCAAAAAGTAAAAAGGAAGTTAAGCAATGTTTAGTTGCCTAA

> **tx_sprA**
Function:  rho-independent terminator of sprA
Best match: tx_sprA_ref_CC030_MRSA252_BX571856.1[1972968:1973028]RC (completely identical)
Position: 036-contig_189: 154324 … 154385; Length: 61 bp
Sequence:
CCAAAAAGTAAAAAGGAAGTTAAGCAATGTTTAGTTGCCTAACTTCCGATATTGAACGCA
T

> **gnd**
Function: protein coding sequence; 6-phosphogluconate dehydrogenase/decarboxylating (core genome, constant)
Best match: gnd_CC010_D139_ACSR01000049.1[127014:128420:r]RC (completely identical)
Position: 036-contig_189: 155019 … 156426; Length: 1407 bp
Sequence:
TTATTCTTCAATCCATTGTGTATGGAATACGCCTTCTTTATCTTTTCTTTCGTACGTGTGAG
CACCGAAGTAGTCACGTTGTGCTTGAATTAAGTTTGCAGGTAAATCAGCTGCACGGTAAC
TATCATAGTAATTAATACTTGATGAGAAACCAGGTGTTGGTACACCATTTTGAACACCAG
TTGCGACAACATCACGTAACGCATCTTGATATTCAGTAACGATGTTTTTAAAGTAAGGAT
CTAGCAATAAGTTTTGTAATCCTGGATTATTATCGTAAGCATCTTTGATCTTTTGTAAGAA
TTGTGCACGGATAATGCAACCTTCTCTCCAAATCATAGCTAAATCACCAAGTTTTAAATTC
CATTCATTATCTTCACTTGCTTTACGCATTTGCGCGAAACCTTGTGCATAAGAACAAATTT
TACTCATATATAATGCTTTACGAATTTTTTCTAAAAAGTCTTTCTTGTCACCATCAAATGA
TGCTTTTGGACCATTTAATTCTTTAGAAGCATTTACGCGCTCTTCTTTGATTGAAGAGATA
AAACGTGCAAATACAGATTCAGTAATGATTGTTAATGGAATACCTAATTCTAATGCGTTA
ATTGAAGTCCATTTTCCTGTACCTTTTTGACCTGCAGTATCAAGAATTTTTTCAACTAATG
CTTCTTTATTTTCATCTAATTTCATGAAAATATCACCAGTGATTTCAATTAAATAACTTTCT
AATTCACCAGCATTCCAGTCTTTGAACGTTTGAGCAATGTCTTCATGAGACATGCCTAATA
ATTCTTTCATCATAGCATAACTTTCTGCAATTAATTGCATGTCAGCATATTCGATACCATT
GTGTACCATTTTCACATAGTGTCCAGCACCATTAGGTCCAATATAAGTAACACATGAAGC
ACCGTCTTTTGCCTTTGCAGCAATTGCATCAAGAATATCTGCAACTTTGTTATAAGCTTCT
TCTTGTCCACCCGGCATTAATGACGGACCAGTTAACGCTCCAATTTCACCACCAGAAACG
CCCATACCAATAAAGTTGATTGCACTTTGTGCTAATGCTTTATTACGTCTGATAGTATCTT
GATAGTTTGTATTACCACCATCAATTAAAATATCTCCATCATCTAATAAAGGTAACAAAC
TATCAATCGTTGCATCCGTAGCTTTACCTGCTTGAACCATTAATAAAATTTTACGTGGTTT
TTCTAAAGAATTAACAAATTCTTCCAATGAGTACGTTGGATGAATATTTTTCCCTTTTGAT
TCTTCAACCATTAAATCAGTTTTTTCACTTGAGCGGTTAAATACAGATACACTATATCCGC
GTGATTCAATATTCCAAGCTAGGTTTTTACCCATAACGGCTAAACCAATAACTCCAATTTG
TTGTGTCAT

> **yqjA**
Function: protein coding sequence; YqjA like protein (core genome, constant)
Best match: yqjA_CC001-ST772_118_AJGE01000022.1[78435:79415]RC (completely identical)
Position: 036-contig_189: 158195 … 159176; Length: 981 bp
Sequence:
TTAAGCTACTTCACTATGCATTTTCAATGAACCAAATTGCGATTTGATTTGTAAATATTCT
TCTAATTCATTTAATATTTGAATAATACTTGCTCTCGAGTTAAGCGCTTTGTGTGTTGTTGG

CAATGGAAGTTCATCCAATTTCAAACGCGTCTCATACAAATTGTGTAAACGCATTGCTGT
ATAGTCATTACTATTCACATTTAGACCAATTTCTTTCAGCAGTGACGCAACATCATTTAAA
AGTGGATCTTTATGACAGATACTTTCGATGAGCGGTTTCATTCTCATTAACAATTCCACTT
GCTCTTCTCGCATATCAAAATAATGATAGTATGAATTTTCGTTTCTAACAAAATGATTTTT
AACATCTCGGAACGCGATAGACTTCGCCTTTTTAATATTTAAAAGTAACACTTCAAATTCA
ATCGCAATGGTATCTTCATATTTTTCACAAATATAACTATATTTACTAAAAATATCAGCAA
TTTGTTGCTCAATTTTACATTTGTATTCGTCTAGTTGTTTGTCTAAACTTGGCATCATTAAA
TTCATTGTAAATGCAATGCTTAGTCCAATTAACAGTAATAATGTTTCATTAACAATTAAAT
GTGCATCAATTGATTTTGCATTAAAAACATGAAGTAATATAACGCAACTCGTAATGACAC
CTTCTTGTACTTTTAATACGACAGTTAATGGTATAAATAACAATACGATAATACCGAGTA
CAATTGGACTCTGACCTAATAAACTAAATATTGCTGAACCTAAAAACAATACTAAAAAAC
ATGATACTAATCTTGAAATAATCGCTTGTAGCGAATGTACTTTTGTATGTTTAATACATAA
TACGACTAATATGGCGCTTGAAGCATAATTATCTAAACCTAACAGCTTACTAATAATTAC
ACCTAAAGTCATACCCACTGCTGTTTTTATTGTTCTAAATCCAATCTTGTAAGGATTTAAC
TTTAACAT

> **bfmBB**

Function: protein coding sequence; branched-chain alpha-keto acid dehydrogenase E2 (core genome, variable)
Best match: bfmBB_CC025_21193_AFEG01000018.1[25570:26844:r]RC (completely identical)
Position: 036-contig_189: 159977 … 161252; Length: 1275 bp
Sequence:
CTAATATATATTTGTATTTTCTAAAGTATACTGTTCGATACGCTGTTTAATATGATTCATA
AATTTACCTGTTTGTAAACCATCTAAAATACGATGATCAATTGAAATACATAAATTAACC
ATGTTACGAATTGCAATCATATCATTAATTACTACTGGCTTTTTAACGATTGATTCTACTT
GTAAAATCGCTGCTTGTGGATGATTTATAATACCCATTGATGATACTGAACCAAATGTAC
CAGTATTATTTACCGTAAATGTACCGCCCTGCATATCTTCAGCTGTCAATTGCTTATTACG
CGCTTTCATTGCTAAAGTATTAATTTCTCTAGCTATACCTTTGATTGACTTTTCGTCTGCAT
GCTTAATCACAGGTACGTATAATTTATTTTCATCAGCAACAGCAATTGAAATATTAATGTC
TTTATGTAAGACAATTTCATTTCCTTGCCAGCTACTATTTAATAAAGGATATGCTTTTAAA
GCATCTGCTACAGCTTTTACAAAGAAAGCAAAGAACGTTAGATTATATCCTTCTTTATTTT
TAAAGCTGTTTTTATAATGATTTCTCGTATTCACAAGATTTGTAGCATCTACTTCAATCAT
CATCCATGCATGTGGAATCTCTGTTACACTATTAACCATATTTTGCGCAATTGCTTTACGC
ACACCATTTACTGGTATTGTGCTGTTTTCACTATTGTCTTCAGATGATTGGTTACTTGATGT
ATCTACTGATGTTGATTTTGTTTGAACTTGTTTGTCAGATTGAGCTGTGGTACCACCATTTT
CAATAACTGACATTATATCCTTCTTAGTTACACGACCTTCAAATCCACTACCTACAACTTG
TGATAAATCAATGTCATGCTCTGAAGCGAGTTTAAATACAACAGGTGAAAAGCGACCATT
ATTACGTGGTTGATTTTGTTTAGCAGTAGATGTCTGTTCCACTGTTGCACTAGCTTTTTTAG
TAGATTTCTGAGTATGCTCATCCACTTTTGCTTGTATCTCTTCAGTTGTTTCATTTGTCTTTT
CATCAGCAGTTTCAATTTTACAGATAATTGTATCAATAGCTACTGTCTGCCCCGCTTCAAC
TAAAATTTCTGTAATTGTTCCTGATATCGTGGAAGGGACTTCAGCTGTCACTTTATCTGTA
ATAACTTCACATAATGGTTCATATTCATCAATATGATCACCAACAGAAACTAACCATTGTT
CAATGGTACCTTCATGAACACTCTCACCTAACTTAGGCATTGTTATTTCCAT

> **bfmBAB**

Function: protein coding sequence; 2-oxoisovalerate dehydrogenase beta subunit (core genome, variable)
Best match: bfmBAB_CC001-ST772_118_AJGE01000022.1[75363:76346]RC (completely identical)
Position: 036-contig_189: 161264 … 162248; Length: 984 bp
Sequence:
CTAGAATTCTGCTAATTCACGCATTTTATTTAAGATTTTTTCTGGATTCATCATAATTTCAT
TTTCTAATACAGGTGAAAATGGCATAGATGGTACATCTGGAGCAGCTAAACGCATGATTG
GTGCATCTAAATCGAACAAGCAATGCTCTGCAATAATCGCTGACACTTCTGACATAATAC
TACCTTCTAAATTATCTTCAGTTACAAGTAAAACTTTACCTGTATGTTTAGCACGATCAAT
AATTGTTTCTTTATCTAATGGATAAACAGTTCGTAAATCTACGACTTCAACATTGATACCG

TCTGCAGCTAAAATATCAGCGGCTTGTAAGCAATAATTGACCATTAATCCATAACAAAAT
ACTGTTAAATCTTCACCTTCACGTTTAACATCTGCTTTTCCTAAAGGTACAGTGTAATATT
CTTCTGGCACTTCTTCCTTTAAGAAACGATAAGCTTTTTATGCTCAAAGTACAATACTGG
GTCATTTGATTCGATAGATGATAATAAAAGCCCTTTAGCATCATACGGTGTGGAAGGAAT
AACAATTGTTAAACCTGGTGATGAAGCAAATATACTTTCAATACTTTGTGAATGATATAG
TCCTCCGTGAACACCGCCACCAAATGGTGCACGAATCGTTAATGGGCATTGCCAATCATT
ATTTGAACGATAACGCATTTTCGCAGCTTCACTAATAATTTGATTTGTCGCAGGTAAAATA
AAATCTGCAAATTGAATTTCTGCAATTGGTCTTTTACCAACCATAGCTGCGCCAATAGCTG
TTCCGACAATATTTGACTCAGCTAATGGTGTATCGATAACTCTGTCCTCACCATATTGTTG
TTGCAGTCCTTGAGTAGTACCAAATACGCCACCTTTTTACCAACATCTTCACCAAGAATA
AATACATCTTTATTTTGTTGTAATGCTAAGTCTTGTGCCTGGCGAATCGCCTCTAAATAAG
ATAATTTAGCCAT

## > bfmBAA

Function: protein coding sequence; branched-chain alpha-keto acid dehydrogenase E1 (core genome, constant)
Best match: bfmBAA_CC130_O11-CC130_AEUQ01000006.1[5327:6319:r]RC
Position: 036-contig_189: 162247 … 163240; Length: 993 bp
Sequence:
TTAGTTAAGACTCCCTTCTTCGTACACAAATGCATAGGCTTCTTCGACACTTGGATATGGC
GCGTCTTCAGCAGCCTTTGTCGCTTTATTGATAATGTCTTTATGTTCTGCTTCTATTTCTGC
CAACCAAGCATCATCGATAATGCCAGCTGAAAGCAACTCTTTTTTGAACTTTTCATTGCAG
TCTGCTTTTTTAAGCGCTTCACGCTCTTCTTTCGTACGATATTGATCGTCATCATCTGATGA
ATGAGCTGTCATACGACTTGTTACTGCTTCAATCAAAGTTGAACCTTGACCAGAAATAGC
TCGATCTCTTGCTTCTTTCATCGCTTTATACATTGCTAATGGATCATTACCATCTACTTGTT
CACCATGTATACCGTAACCAAGTGCTCTATCCGATAATTTTTCAGCTGCGTATTGTAATGA
ATCAGGTACTGAAATTGCATATTTATTATTTATAATGACACATACAAAAGGAAGTTTGTG
TACACCCGCGAAGTTTAAACCTTCATGGAAGTCACCTTGGTTTGAGCTACCTTCACCAAC
AGTTGCTGTTGCAATTTTCTTCTTACCATCCATTTTTATAGCTAAAGCAGCACCAACAGCA
TGGGGTATTTGAGTTGCTACCGGTGAACTTTGTGACAAAATATTCTTAGCTCTACTACTAA
AGTGTGATGGCATTTGTTTTCCACCAGAGTTAACATCGTCTTTCTTTCCAAACGCTGATAA
AAACGTATCATACGCTGAGATACCCATATAAGTAACGAAAGCTAGATCTCTATAATAAGG
CGCTGTAATATCACCTTCTTCTAATGCGTATGCCATCCCAATCTGAGTTGCTTCTTGTCCTT
GACCACTTACAACAAATGGAATTTTACCTGCACGGTTCAATAACCACAGTCTTTCATCTAT
TTTTCTACCTAAATCCATCCATTTATATATTACTTTTAGGTCTTCTTCGCTAAGGCCTAATG
ATTTATAATCAATCAT

## > lpdA

Function: protein coding sequence; dihydrolipoyl dehydrogenase (core genome, constant)
Best match: lpdA_CC007_TCH959-USA300_AASB02000244.1[30668:32089:r]RC
Position: 036-contig_189: 163255 … 164677; Length: 1422 bp
Sequence:
TTATACGTGAATAGCTCTACTTTCTGCTTTCAATCCTAATTCCATCAACACTTCAGAGATG
GAAGGATGTGCGTGTGTTGTTAGTCCTAATTCTAATGCCGAGCCATTCATGAACTGTAAC
AGTGATGCCTCATTAATCAATTCTGTTACATGTGGACCAATCATATTAATACCCACAATTT
CTTCAGTTGATTGATCAATCACCATTTCGCTATACCCTTCGTTTGCGTCATGGCTATCAAT
CACTGCTTTACCAATTGCTTTAAATGGTACTTTAAAACTTTTAACTTTCATTCCCTCTGCCT
TTGCTTGTTCAATGTTTAAACCGATAGAAGCAATTTCAGGTTGTGAATAAATACACTTAG
GCATCATGTTATAGTTTACTGGGATTGGGTTCCCCTCAAACATATGATCAACAGCCACAA
CACCTTCTTTTGATCCAACATGTGCCAATTGTAATTTTCCTATACAATCACCAGCTGCATA
AATATGTTTATCTTCAGTTTGTTGAAATTCGTTCGTTAAAATATGTCCTGATGTTGAAAGT
TTTATTTTAGTGTTGTTTAAACCAATATCTGATGTGTTAGGTTTTCTACCAATCGATAGCA
ACACTTTATCTACTTTAATTATGTCTGAGGAAATTTCAAACGTAACACCATCTTCGTTAAC
ATTTATATCATTTTCAGAAAGTTTTATTCCCTCATAGAATTTAACACCACGTGCTGACAAT
GATTTTTTTAATAGTTGTGAAGCTTGTTTACTTTCAGTTGGTAAAATTCTTTCACCTGCTTC

TATAACTGTTACGTCAACACTTAAATCTATCATCAATGATGCAAATTCCATTCCGATAACA
CCACCACCAATAATACCAATACTTGATGGTAACGTCTTTAATGATAATATATCATCGCTA
GATAAAATTTTATCATGATCAAATGATAAGAATGGCAACTCTGCAGGCGAAGAACCAGTT
GCAATTAATACAAATTGGTTGGGTAATAAGTCTGATTCACCATCTTCATATTCGACAGAA
ATTGTGCCACTTTGAGGTGAAAATATAGATGTACCTAGAATACGTCCCGTGCCATTATAA
ATGTCAATGTGATTGTGTTGCATTAAATGCTTTACACCTTGATACATTTGATTAATAATGT
CTTCTTTTCGTGCCAACATATTTTCAAAATTAACATTAGCATCTTTGACATCAACGCCAAA
CATTGCTGCCTGTTTTACTGTTTGAAATACTTCAGCAGATTTAAGCAGCGATTTAGTAGGA
ATACAACCTTTATGGAGACAAGTACCTCCTAATAGTTGTCGTTCTACTATTGCCACTTTTT
TACCTAATTGAGACGCACGTATCGCAGCAACATATCCTGCAGTACCTCCACCGAGAACGA
CTAAATCATATTGTTTCTCTGACAT

> **recN**

Function: protein coding sequence; DNA repair protein N (core genome, variable)
Best match: recN_CC705_RF122-cow_AJ938182.1[1515551:1517230:r]RC
Position: 036-contig_189: 164827 … 166507; Length: 1680 bp
Sequence:
TTATCTACGTCTTTGATTGTGTTGTATCATTTCTCTTGCATTTTCTCGAGTTAAATCAGTAA
CACTTGCACCTGAAATCATTCGTGCAATTTCATCTACTTTATCATCGCCAATTAACTCTTG
AACTTGTGTTGTTGTACGATCATCTTTTGATGACTTCGAAATTAATAAATGATGGTCGCTC
ATCGATGCAACTTGTGGCAAGTGAGAAATACAAATAACTTGTATATATTCTGCTATATCT
CGCATTTTCTCTGCCATTTTTTGTGCGGCTTGTCCAGATACACCTGAGTCAACCTCATCGA
ATAAAATTGCAGTTTGACCTCTCGATTTAACAAAAATACTTTTTAACGCTAACATAATTCT
AGAAAGTTCTCCACCTGACGCAATTTTATTTAAACTTTTTAATGGTTCCCCTTTATTTGGA
CTGATTAAAAATTCTACAAATTCGATTCCATCAATATTCGGTTCTTCTAATTTTTTAAATG
AAATCTCAAGATTTGCGTCTTTCATTTGTAAGTTTTGAATTTCCGATACAATGTGGTCTCTT
AACTCCCTAGCGACTATACGACGCTGCTTTGATAACGCTTGTCCAACCTCTATAACTTGAT
TATACAATGCATTTATTTCTTCTCGTAACTGCGATGTACTTTGTTCATAGTTTTCAATTTTA
TTGATTTCATTATTAAGCTTTTCTTGATATGCGATTAATTCTGAAATATCTTTTCCATATTT
ACGTTTTAAATTATTCAGCAAATTCATACGAGATTCATACTCGTTTAACACTTGTTCGTCG
AATTCTGTATTAGCCATTTCATCATATAACTCATGTTTTGCATCTTCTAAAATGTAATAAA
ATTGATCAATATCTTCTTTTAATTTGTCATATTTGTTTGGAACTATATCGTTTATTGTTAAC
AAATGGTTGCTTAGTTCATACAAACGATCAGTGATAGCATTTTCATCCGTTAATGTCATAT
GTGCGTTATTAAGCGCTAAGCTTAATTTTTCAGAGTTTTGAATGCGTTTAATATCTATTTC
AAGTTGCTCTATTTCGCCTTCTTTTAGATGTGCTTCAGACAATTCTTCTAATTGGAATTTCA
TTAAATCTAAACGCTGCAGCAATGCTTGGTCTGCTGATTCTAAATCTTCTAACTCTTGCTT
TTTGGCTTTATAATTTTGAAAAGTTTGATGATATTTATCCAACAAATCTTGATAACGTGAT
TCTGCGTAATTATCCAATAATGTTAAATGGTATTTTTGTTTCAACAAAGACTGCGTTTCAT
GTTGGCCATGAATATCTAATAATTCTTGCATAACTTTTCGTAAATCTTGTAAAGTAACTGT
TTGATTATTAATTTTACAAAGACTTTTACCAGAGCTGAAAATTTCCCGTTTAACTAATAAA
AAATCTTCATCTACATCAATATCCATATTTTTCAATATATGTATAGCATCTTTACTCTCGTC
AATATCAAATATACCTTCGATGACAGCCTTTTTTTCACCATGTCTTACAAAATCAGATGAA
GCTCTCATTCCAATTAATTGTCCAATTGCATCTATAATAATTGACTTACCTGAACCCGTTT
CACCACTTAAAACAGTTAAACCATCAGAAAATTGAATTTCTAACTCTTCAATAATAGCAA
ATTGCTTGATTGATAAGGTTTGTAACAT

> **ahrC**

Function: protein coding sequence; arginine repressor (core genome, constant)
Best match: ahrC_CC005_Mu50-VRSA_BA000017.4[1638477:1638929:r]RC (completely identical)
Position: 036-contig_189: 166522 … 166975; Length: 453 bp
Sequence:
TTATAACAAATTGAAAATTCTTGACTTGATTTCATCACTTGCCTCTTTGCTTCGACAAATA
ATTAAACAAGTATCATCACCACAAATTGTGCCTAGTACTTCTTCCCAATTGATTTGGTCTA
ATATAGCTCCAATAGATTGTGCATTACCAGGTAATGTTTTTAGAACAAGTAAATTATCAG
TACCATCTATATTAACAAAGGAATCCATTAAATAACGTCCCAATTTTTCTAAAGGATGGA

ATTTTCTATCATTTGGTAAACTATAAACATATTGACCTGAAGGTATAGGTACTTTAATAAG
TTGTAGTTCTTTAATATCACGAGAAACAGTTGCTTGAGTGACATTTAAATCATAATCGTTT
AATCGTTTAACTAATTCATCTTGTGTCTCTATCTGTTCATTTGAAATAATTTCTCTAATTTT
TATATGCCTAACCGATTTTTTGGGCAC

> **ispA**
Function: protein coding sequence; geranyltranstransferase (core genome, constant)
Best match: ispA_CC005_Mu50-VRSA_BA000017.4[1639361:1640242:r]RC (completely identical)
Position: 036-contig_189: 167406 … 168288; Length: 882 bp
Sequence:
TTAGTGATCCCTGCTATAAAATAAATCAACGATTTCTAATAAGTGTTTTGTATTGAATTGT
TCATCAATTTGCGTTAGTTCATCCACTGCTGCGTCTCTATGATAAGTCAATTTATCTTCTGC
GCCATCTTTCCCTAATAAACTCACGTACGTACTTTTATTATTTTCAAGATCGCTGCCCACTT
TTTTACCTAACTTTGCTTCATCACCATAGCAGTCTAATAAATCATCTTTAATCTGGAACAT
CATACCTAAATGATAACTATAACTTTCTAAATGTTCTTTAGTTGCATCATCGACATTAGCG
ATATCTGCTGCACTCATAACCGCAAAAGTTAATAATGCTCCTGTTTTTGTTTTGTGTATCA
TTTCCAAAGTTTCAAGATCAATTGGTTGGCCTTCGCTTTGCATATCTAACATTTGACCGCC
GACCATTCCAACATGACCACTTGCTATTGACAGCCGTTGTAGAACTTTTATTTTTACTTCA
TCAGTTAATCTATCATCACTTGAAATAAGTTCAAATGCTTTAGTTAATAAAGCATCACCTG
CTAATATCGCAGTCCACTCACCATATACTTTATGATTTGTTAATTTCCCTCGTCGATAATC
ATCATTATCCATCGCTGGTAGGTCATCATGAATAAGTGAATATGTATGAATCATTTCTAGT
GCAATTGCGCTCTTCACACCTAACTCATACTCGGTATTTAGTGAATCTAAAGTGAGTAATA
ACAGAACTGGTCGGATGCGTTTACCTCCAGCATTTAATGAATACAACATGCTTTCTTCTAG
CTGAGTATCCATTACTGATTTATTTATCGCAACCGATAATTCATTATTGACTTCATCTATTA
ATTTATTCATCGGTAGATTCGTCAT

> **xseB**
Function: protein coding sequence; exodeoxyribonuclease 7 small subunit (core genome, constant)
Best match: xseB_CC001_MW2-USA400_BA000033.2[1609352:1609582:r]RC (completely identical)
Position: 036-contig_189: 168265 … 168496; Length: 231 bp
Sequence:
TTATTCATCGGTAGATTCGTCATTTTTTACATCCTCAGCTTCTTCTTTTATTAAGTCATTCA
CCTTTTTTTCGGCATTTTTTAAAGTTGTGTCACAAGCTGCTGATAGTTTCATACCACGTTGA
TATAAATCTAATGATTCCTCTAAAGATACTGTTTCATTATCTAATTTTTGAACAATTTGCTC
TAATTCTTGCATCATTTCTTCAAAACTTTGCGTTTCTTTAGTCAT

> **xseA**
Function: protein coding sequence; exodeoxyribonuclease 7 large subunit (core genome, constant)
Best match: xseA_CC005_Mu50-VRSA_BA000017.4[1640443:1641780:r]RC (completely identical)
Position: 036-contig_189: 168488 … 169826; Length: 1338 bp
Sequence:
TTAGTCATTATTACACCTTACTTTCGTAACTTTTGCATCTACTAAGCCATCTTTCATTGTTA
ACGTCAATTGATCATTTTCTGTTAAATCTTTAGTACTCGTAATGACTTCGTCTTTTTTATTA
ACAATTGCATATCCACGCAACATTGTATTAGTTGGACTTAAATTGTTTAAGTTTTCTACTT
TATTTTTCAAATCATTTTTATAACTTAATATCTTAGAATTCAATAATTTAACAAGTTGGTTT
GTCAATTGAAGATTATTTTGTTGTTCTTGATTAACACTACTTAGTAATGCTTTTAAATTATA
ACGTTGTTGCAACAGCATTAATCGATGTCTCTGTTGTTCAAAAGTTGCCTGAATTTGTTGT
TTCAGTCTCTTTTCTAAATCATCTCGACGTTGTATCTGTTGATCATACAATAAAGTTGGTT
GTTTAAACTTGTAATACGATGACAAATGTTCAACATGTTTACGTTGTTGTTCTAAATGTTT
CTTGATGAAACGAGTCAATGTAAACTGATATTGCTGTATTTGTTGCAGCAATTCATATTGG
TCTGGTGTTGCAATAACAGCAGCTTGAGTTGGAGTCGCAGCTCTGATGTCTGCAGCAAAA
TCACTTAATGTAAAGTCTGTTTCATGACCTACTGCTGATATAATCGGTGTCTTACAATTAT
ATATTGCACGGACGACAGCTTCTTCGTTGAAATTCCATAAATCTTCTATGGATCCACCACC
TCGACCTACAATAATGGTATCTACACCTAAACTATCTGCATATTCAATTTTTTCAATAATG

TCGTCTTTTGCTTTTTCACCTTGAACCAAAGTACTAATTTGTATTTGTTCAGCTAATGGAA
AACGACTATTTATCGTTGAATGGATATCTCGAATTGCGGCACCTGTACTCGCTGTTAAAAC
TGCAATTTTTTTAGGAAACTTAGGTATTGATTTCTTATTCGCTTTATCAAAACAACCTTCTT
CAGTTAATTTTTTCTTTAATGCTTCTAATTTTTGATATAAGTTCCCTATACCATCTAATTGC
ATTTTATTTACATAAATTTGATAGTTTCCACGACGTTCAAAAACAGAAACACGTGCTTCTA
ATAAGACTTCATCTCCTTCTTTAGGTTCGAAGTTTAATTTAGAAGCACTACCTTTGAACAT
CATGGCACTTATAACGCTCTCTTTATCTTTCACATTAAAGTATAAATGACCACTTGAATGC
TTTTTGAAATTTGAAAGCTCACCTTTAATCAATACAGATTGGAGATGTGGATCTTGATCAA
ATTTATATTTAATATATTTCGTTAAAGCTGAAACACTTAAATAATCTGACAT

> **nusB**
Function: protein coding sequence; N utilization substance protein B (core genome, constant)
Best match: nusB_CC005_Mu50-VRSA_BA000017.4[1641797:1642186:r]RC (completely identical)
Position: 036-contig_189: 169842 … 170232; Length: 390 bp
Sequence:
TTATTTTTTTATATTACTCAATACACCATTTATAAATTTATAATGATCATCATCACTGAATT
GTTTTGTTAATTCAACTGCTTCATTCATTACGACTTTAGCAGGTGTATCACTGTGTAATATT
TCATATGTTGCCATTCTTAAAATAATACGATCCGTTTTTAATAAACGTGCAATAGTCCAAT
CTTTTAAATAAGGACTAATTGTCTCGTCTAATACAGGTTCGTGATCTTTAACGCCAGAAAC
TAGCCAATGAATAAATTCAAAGTCTAAATCTGGATTATCGTCTTTAATAAAACTTATCGCT
TCATTTATCGTTAAATCACTGTCCTTCATTTCTAATTGAAATAAAGTTTGAAAAGCTTGCA
CTCGGGATTCTTTACGACTCAT

> **yqhY**
Function: protein coding sequence; putative protein (core genome, constant)
Best match: yqhY_CC001_MW2-USA400_BA000033.2[1611378:1611740:r]RC (completely identical)
Position: 036-contig_189: 170291 … 170654; Length: 363 bp
Sequence:
TTACTTTTCAATAACGATTTGTGTAATGTGAATATTAATTTGCTTAGGTTCTATCGCTGTCA
TATTAGAAATTGAATTAAAAATTGACGTTTGAATTTTGTTTGCAGTTTTTGAAATATTAAC
ACCATGTTTTAATGCACAATATACATCTATATATATGCCATCTTCTTTACTCTCGATTTTTA
AATCACGGCTTAAATTTTTACGACTAACTTTTTCTAAATTTGTTTCTTTTAATTCAGCAAAA
TGGCCAGTGATGCCTTCGACTTCCGAAGTAGCTATACTTGCAATAACAGATAGCACTTCT
GGCGCTATTTCTACTTTACCTAATTTTGAATTTGAATAATCAGTTACTTTGACCAT

> **accC**
Function: protein coding sequence; acetyl-CoA carboxylase/biotin carboxylase (core genome, constant)
Best match: accC_CC001_MW2-USA400_BA000033.2[1611755:1613110:r]RC (completely identical)
Position: 036-contig_189: 170668 … 172024; Length: 1356 bp
Sequence:
TTAACCTTCATCATTCATAATGCTATTTTGCTCTAAAAAGTTTGTATTAAATTTACCGCTTC
TAAATATATCGTTATTCAATAATTTAATATGGAATGGAATAGTTGTATCAATACCAAGAA
CCACAAATTCACTTAATGCACGAATGCCAGCCATAATCGCTTCATCTCGTGTCGGTTCATG
TATGATTAATTTCGCTACCATCGAATCATAATATGGCGGTATCGTATAATTAGTATAACAT
GCTGACTCTATTCGAACACCATATCCACCTGGTGCAAGATATTGCTCAATTTTACCTGGTG
ATGGCATAAAGTTCTTGTAAGGATTTTCAGCATTAATTCTAAATTCAATTGCGTGTCCTGT
TAATTTAATATCTTCTTGTTTATACGGTAACACGTCACCCATAGCAACCTGTAATTGTAAT
TTAACTAAATCAATTCCTGTTACCATTTCAGTTACAGGATGTTCTACTTGAATACGTGTAT
TCATTTCCATAAAATAAAATTTATTATCATTTAAATCATATATAAACTCAATTGTTCCCGC
ATTTTCATAATTTACAGCTTTCGCTGCACGCACTGCGGCATTTCCCATTTCACGACGTGTT
TCATCATCTAAAATTGGGGAAGGTGCTTCTTCCACTAATTTCTGCATACGTCTTTGAATTG
TACAATCACGTTCTCCTAAATGAATTACATTACCATAGCTGTCCCCAACAATTTGGATTTC

AATATGGCGGAAGTTTTCGATGAATTTCTCCATATAAAGTCCACCATTACCAAATGCAGT
TTGAGCTTCTTGTTCTGTCATTCGGAAGCCAGTTTCAAGTTCTTTTTCATCACGAGCAACA
CGAATACCTTTTCCGCCACCACCAGCAGTAGCTTTAATGATGACCGGATAGCCAATTTTTT
TAGCGATTTTCTTAGCTTCTGAGACGTCTTTCATTAAACCATCACTACCAGGAACAACTGG
AACATTGGCTTTGATCATTTCTGCCTTAGCAACATCTTTGATACCCATTTTTTGGATAGATT
GATAACTTGGTCCAATGAACTTCAATTGGCATGCTTCGCATAATTCTGCAAAGTCAGCATT
TTCAGCTAAAAAGCCATAACCCGGATGAACGCCATCACAACCTGTAGAAGTTGCAATAG
ATAAGATGTTCGGAATATTTAAATATGAATCTTTAGACAAAGTGGGACCTACGCAATATG
CTTCATCAGCAATTTGAGTATGTAGCGCATCTTTATCCCCTTCAGAATAGATTGCAACAGT
TTGGATGCCTAAATCACGACAAGCGCGAATAATCCTAACTGCGATTTCACCGCGGTTTGC
AATTAAAACCTTTTTCAT

> **accB**

Function: protein coding sequence; acetyl-CoA carboxylase/biotin carboxyl carrier protein (core genome, constant)
Best match: accB_CC001_MW2-USA400_BA000033.2[1613110:1613574:r]RC (completely identical)
Position: 036-contig_189: 172023 … 172488; Length: 465 bp
Sequence:
TTATTTCACCTTAAATAACGGTTGGCCATACTCTACCATTTGTCCGTCTTCTACTAAGATTT
CAACAATTTCACCTGAAATTTCTGCTTGAATTTCATTAAATAGTTTCATTGCCTCTAAAAT
ACACACTGTTGTTTCATTTGAAACAGTGTCCCCAACTTGCACATATGCTTCTTCGTCTGGA
GATGGCGATTTGTAAAATGTACCTACCATAGGTGCATTAATTGTTTTGTGATTATCTGAAG
TTGGCTTTGGAGCTTCAGTTTTATTGCTATCAGTTGATTGTACTTGAGGCATAGGCATTGC
CGCAGCTTCAACTGGCATTTGTGAGATTTGTGGCGTGATAATCTCAGTTTCTTTTTCTTTCT
TAAGCGTCACTTTGCCTTTAGTATCTTCAATATTGATTTCCGTTAAAGTTGATTTATCCAG
AATTTCAATTAATTCTTTGATTTCTTTAAAGTTCAT

> **efp**

Function: protein coding sequence; translation elongation factor P (core genome, constant)
Best match: efp_CC005_Mu50-VRSA_BA000017.4[1644916:1645473:r]RC (completely identical)
Position: 036-contig_189: 172961 … 173519; Length: 558 bp
Sequence:
TTATCCTCTTGAAATGTAGCTTCCATCACCAGTGTTGATAATTAAAACGTCACCTTCGTTT
ACAAATAAAGGTACATTTAATGTATAACCAGTTTCAACAGTTGCCGATTTAGTGGCACCA
GTTGCAGTATCACCTTTAATACCAGGTTCTGTTTCAGTTACTGTTAATTCAACAGTTTTAG
GTAATTCAACACCGATAGTTTCACCTTCGTATGTTTGAATTTGTACTTCCATACCTTCTTTT
AAGTAATTCAATTCTTCTTTTAAGTAATCACTTGAAAGTTCTGTTTGTTCAAAGCTTTCATT
ATCCATAAATACATGATTATCTCCGTCAGCATATAAATATTGCATGCGACGATTTTCAATC
ATTGCTGGTTCAACTTTTTCACCAGCTCTAAACGTTTTCTCTTGAATTGCACCAGTTCTTAA
ATTACGTAATTTTGAACGAACGAATGCTGAACCTTTACCAGGCTTTACATGTTGGAAGTCT
ATAACTTTCCAAATAGCGTTATCAACAGAAATTGTTAAACCTGTTTTAAAATCATTAACCG
AAATCAT

> **A5IT58**

Function: protein coding sequence; proline dipeptidase (core genome, constant)
Best match: A5IT58_CC025_21193_AFEG01000018.1[39138:40199:r]RC (completely identical)
Position: 036-contig_189: 173544 … 174606; Length: 1062 bp
Sequence:
TTATGTTAAAACTATAAGGTCTTTTGTGCATTTAGTAAAGACTTGACAACCATTTTCTGTA
ATTAAAATATCATCTTCTATTCTTATACCGCCCAAACCTTCTATATAAACACCAGGTTCTA
CTGTAACACAGTTGTTAACTTGAAGTTTATCTTGTATCGTACGAGCCAGCATTGGCCCTTC
ATGGATTTCTAAACCAATACCATGTCCTAGTGAATGTCCAAATTCTTTTCCATACCCTTTT
GACTCTAAATAGTTTCTTGAAATGGCATCAGCTTCTGCACCAGTCATGCCAGGTCTAATCT
CATTAATTGCTTTCATTTGAGATTCAAGTACTATTTGATATATTTCTTTCAGTTTAGGATCT

GGTTCTCCAATAGCAAATGTTCTAGTAATATCTGAACAATAGCCGTTATAATATGCGCCA
AAATCTAATGTAATCATGTCGCCTTTTTCAATAATTTTGTCACTTGCAACACCATGTGGTA
ATGCACCTCTATGACCAGATGCTACAATCGTATCGAATGATGGTCCATCTGCTCCTAATTC
TAGCATTTTACTTTCTAATATTGCCTTTAATTCTTTTTCAGTCATACCTGCTTTTACAACAG
TTAAAATATATTCATATGTTTCATCAACAATATTAGCTGCTTTTTGAATTAAAGCAATTTC
GTCAGCATCTTTGACGTCTCTAATTTTATCTACAGTATTAGAAATGCTTATTAATGATATA
CGACTTTTATTTAATTCAAGGTATGTATCATAACTTACATGATGCCCCTCAAAACCTACAT
TTTCAAAATTTTCTTGGTGTAGCAATTCTTTAATCTCACCAATAATAGTAGATTTACGATT
AATAATTTCATAATTTGGCGCTTGCTTAGTTGCTTGATCAATATATCTAAAGTCTGTTATC
AAATATTGTTTATCTTTAGATATGATAAGTGCTCCACTGGTACCAGTAAAACCTGATAAAT
ATCTTCTATTGTAATCCGAAAGAATGATAATCGCATCTAAATGTTTTTGTTCTAAAATACG
ATGCACTTGTGTTATTCTGCTCAT

## > **gcvPB**

Function: protein coding sequence; glycine cleavage system P/subunit 2 (core genome, constant)
Best match: gcvPB_CC001_MW2-USA400_BA000033.2[1618416:1619888:r]RC
Position: 036-contig_189: 177325 … 178798; Length: 1473 bp
Sequence:
TTATTTTTCCTGTTTAAGATTTTCAAACTTTAATATTGGTTTACGAGCAGCTGTAGCTTCGT
CTAATCGATCAATCACAGTTGTATGTGGTGCTTCTAGCACTTTATCAGGATCATTTTTAGC
TTCTTCAGCAATACTAATTAATGTATCGATAAAATAATCAAGTGTTTCTTTAGACTCTGTC
TCAGTCGGTTCAATCATCATACCTTCTTCAACATTTAATGGGAAGTATATTGTTGGTGGAT
GTACACCGAAATCTAATAATCGCTTAGCCATGTCTAAAGTACGTACACCAAATTCTTTTTG
ACGCACACCACTTAACACAAACTCGTGTTTACAATATTGTTTATAAGGTATTTCAAAGTGT
TCAGATAAACGTGCTTTAATATAATTCGCATTAAGAACCGCTGCTTCAGAAACCTCTTTAA
GTCCAGTTGCTCCCATAGTTCGAATATACGTATAAGCTCTTAAGTAAATACCAAAGTTAC
CATAAAATGGTTTTACACGTCCGATAGAATTTTTAATGTCATTATCATATTTAAATTTGTC
GCCATCTTTAATAACCATTGGCTTTGGTAAGTAACTTGCTAGTTCTTTTACTACACCGACT
GGACCTGAACCAGGACCGCCACCACCATGTGGACCAGTAAATGTTTTATGCAAGTTTAAA
TGAACAGCATCAAATCCCATATCTCCTGGGCGAACTTTGTCCATAATAGCGTTTAAATTCG
CACCATCATAATATAATAGACCACCAGCATTATGGACGATTTCACGGATTTCCATAATAT
TTTTTTCGAAAATACCTAAAGTGTTTGGATTAGTTAACATAATAGCTGCTGTATTTTCATT
TACAACACGTTTCAAGTCATCAATATCAACTTCGCCACGTTCGTTTGATTTTACAGTAACT
GATTTAAATCCTGCAAATGAAGCTGAGGCTGGATTCGTACCATGCGCAGAATCTGGCACA
ATGACTTCATCACGATGACCTTCACCATTATTCTCATGGTAAGCTTTAAATATCATCAATG
CAGTCCATTCACCATGTGCGCCAGCAGCTGGTTGTAATGTCACCTCATCCATACCAGTAAT
TTCTTTTAATTCTTCTTGCAAACTATAAATAATTTCTAATGAACCTTGAACTTGATCTTCAT
CTTGTAATGGATGTGATTCACTAAATCCTGGTATTCTAGCAACCTTTTCATTAATTTTAGG
GTTATACTTCATCGTACATGAACCCAATGGATAAAATCCGTTGTCTACACCGAAATTTTTA
TTTGAAAGTTCAGTATAATGACGTACTAAGTCTAGTTCAGCAACTTCAGGAAACTCCGCT
TTGTTTTTACGAATAAATTTATCATCTAACAATGACTCAACAGAATTTGTTTTAATATCAC
TTTTTGGTAATGAATATGCATATCTGCCTTCACGAGATCTTTCAAAAATTAATGGACTTGA
TTTACTAGTCAT

## > **gcvPA**

Function: protein coding sequence; glycine cleavage system P/subunit 1 (core genome, constant)
Best match: gcvPA_CC001_MW2-USA400_BA000033.2[1619881:1621227:r]RC
Position: 036-contig_189: 178790 … 180137; Length: 1347 bp
Sequence:
CTAGTCATTTAACTCACCAGCCTTTTCTACAAATGTATCGATTTCATCTTTTGTTCTTAATT
CAGTTACAGCTATTAACATGTGATTTTTAAAGTCGTCTGAAACAACACCTAAATCAAAAC
CACCGATAATATTGTACTTCACTAATTCCTCGTTAACTTGTTGAATTGGTTTGTCAAATTT
GACTACAAACTCATTGAAAGATGTACCATCTAATACTTCAAAACCTTTTTTAATAAATTGT
TGTTTAGCATAGTTAGCATGTTCTATATTTTGAACTGCAATATCATAGATACCTTGTTTAC
CAAGTGCTGACATTGCAATTGATGACGCTAAAGCATTTAATGCTTGGTTAGAACAAATAT

TAGATGTCGCTTTATCGCGTCGAATATGTTGTTCACGTGCTTGTAATGTTAATACAAAGCC
ACGATTACCTTCATCATCTTGTGTTTGACCGACTAATCTACCTGGCACTTTACGCATTAAC
TTTTTCGTCGTTGCAAAATATCCACAATGTGGCCCACCGAATTGAGCAGGAATTCCGAAT
GGCTGAGTATCACCTACAACAATATCTGCACCAAATGAACCTGGAGGTGTAAGTAATCCC
AATGCTAATGGATTTGCATATACGATAAATAATGCTTTTTATCTTCAATAAAGCTATGAA
TCTTTTCAAGATCTTCAATTGAACCGTAAAAGTTTGGATATTGTACTGCAAAAGCTGCTGT
TTCATCATCCACTGCTGCTTCTAATTTTTTCAAATCTGTAACAGTGCCATCTAAATCGATTT
CCATTACTTCGAATTCCTTACGCGTCTTAGCATAAGTATGAAGTACTTGTAATGCTTGATA
ATGTAAACCTTTTGAGACTACAATTTTATTTTTCTTTGTTTGACTAAATGCTAAGATACAT
GCTTCAGCAAAGCTAGTCATCCCATCATACATAGAAGAATTTGCTACATCCATATCTGTTA
ATTCACAAATTAAAGTTTGGAACTCAAAAATGGCTTGTAATTCACCTTGAGAAATTTCCG
GTTGATATGGCGTATATGCTGTGTAAAATTCTGATCTTGAAATCATAGCATCCACAACTG
ATGGCGCGTAATGATCATAAACACCAGCACCCAAAAATGATGTATGCGTTTCTTTAGTGA
TATTCTTGCTTGCAATGCGATTTAATCTTCTAAGTAACGTTGTTTCCGCTTCGCCTTCAGCA
ATATTTAAATCTCTATTTAATAAAATGTCACTTGGTACATCACCGAATAATTCTCCTATAG
ATTTTGCACCAATTGTTTGTAACATTTCTTGCTTGTCTTTTTCAGTTAAAGGTATATAACGA
TGACTCAC

> **gcvT**

Function: protein coding sequence; glycine cleavage system T/protein component (core genome, constant)
Best match: gcvT_CC001_MW2-USA400_BA000033.2[1621247:1622338:r]RC
Position: 036-contig_189: 180156 … 181248; Length: 1092 bp
Sequence:
TTATTTATCAATTTGATTTTTCTTAACAATTTTCGCTTTTAATTGACGCTTACGAACTTGAA
CAAGTAACTCTCTACCCATTTCAAACTCATCTCTTTTTATCATTGCAAGTGCAATTGATTTT
CCTGATGATGGAGACTGTGTTCCTGAAGTTACTTCTCCAATAATATTTCCATCTAAATCCA
TAACTTCATAACCAGTTCTTGCAATTCCTTTTTCAAGTAATTCTAATCCCACTGTTCTTCTT
GGTGCACCATTTTCTTTTTGATCTTTTAATACAGATTTACCAATAAAATCAGTATCAATTA
ATGGTTTACTTGCAAAAGCGATACCACCTTCATATGGTGTAATTGATTCAGTTAAATCTTG
TCCATGTAATGGCAATCCAGCCTCTAATCTTAATGTATCACGAGCGCCTAACCCACATGG
CATAACATTATACTCTAATAAACCATCCCAAATTTTTTCAGTATCATCGATATTACAATAA
ATTTCAAACCCATCTTCACCTGTGTAACCTGACTGAGATAAAATGACGTTTGCTCCAAATA
ATTTGAAACCCTGTTTAAATTCAAACATTTTCATTTCAGTTACATCTTCATCAACTAATTG
ATTAATTAAATCTCTAGCTTTTGGTCCTTGTATTGCTAATTGACCATATTGGTTTGATACAT
TTTGTACTTCAACATCAAATTTCTCTTTGTGTTTTAAAATCCAATTAAAATCTTTTTCAGTA
TTAGCAGCATTAACAACTAATAAATAATTGTCGTCAGCTAATTTATATATTACTAAATCAT
CAATAATACCGCCTTCTTCATTACATAAAGCAGTATATAATGCTTTTGAAGTAGTTAAATT
ATCAGTATCATTTGATAATAAATATTGCACAAACTGACTAGCATCTTTACCTGTTACTTCA
ATTTCACCCATATGACTAACATCAAACAGGCCAATTTCGTATCGAACAGCATTATGCTCCT
CTTTAATACTTGAAAATTGAACAGGCATCGCCCATCCTCCGAATTCCACAATTTTTGCACC
TCTATCAACATAATTTTGATATAAAGGTGTTTGTTTTAAATCACTTGACAT

> **aroK**

Function: protein coding sequence; shikimate kinase (core genome, constant)
Best match: aroK_CC001_MW2-USA400_BA000033.2[1622497:1623021:r]RC
Position: 036-contig_189: 181406 … 181931; Length: 525 bp
Sequence:
CTAATACTGATCACTCGCTTTTATTAAATTTAGCAATTCATAATATATTTCTGAAATTGAT
AGCAAATGACTATCAAATTTCTTGAATGCGATTTCATTATATCTTAAATTCCGCGAGCAAT
ACAAGTCATTTAACTGCTTGATTGTCTTATTATTTGCATTAGGTCGATGTGGGTCATCATT
GATTCGACTATATATAATATCAATATTACAATCTAACCAAATAATGTTTTTTTGATTTTTC
AAAAAATTAAATGCCTCTTCACTTTCAATTATACCACCACCAGTAGCAATTATATCTGCAG
TGTTAATACATTCTTGCAAACATGTGAACTCTAAATTCCTGAAATATTGTTCACCATGTTT
ACTAAATATTTCTGGTATTGTTAACTTATACTTCTCTTCGATATATGAATCTATATCAATA

AATGATAAATTTTGCTCATCTGCAACGTATTTACCAATCGTAGATTTACCGGTACCCATGA
AACCAATTAAAATTATTGGTGATTTATCATGATTCAT

> **comGF**
Function: protein coding sequence; late competence protein F (core genome, constant)
Best match: comGF_CC001_MW2-USA400_BA000033.2[1623254:1623751:r]RC
Position: 036-contig_189: 182163 … 182661; Length: 498 bp
Sequence:
TTATACATAAATAGTTTTAGTCTGCACATTTGTACCGACTTTAACTGTTATCGTTATTTTAA
TAATGGATTTATAGTAGATATTTGCAGTAAATGCTGTAACATTATTAATCATTGTTATATT
TCCTCTGTCATTTACAACTTTAATAATTTTATTATTTATTAATTTGTATTCGATCATTTCTTC
ACCTTTATGTAAAATGATACGCTGTTGCCTAATTTCAATATCGTTTCTATCTACTCCTTTAA
AATCCTCTAGAATATCTCTTGAGAAAAATTCGAAATCTACAGTTGTTAATTCCCTACTTTC
AATTAGAAAAGTTTTGCTAAGTCTAATTAAGTCTGGAACAATTAGTAAAGTTATACTTAT
AACCATCATCGCTACTAACATTTCAATGAGCGAAAAAGCTTTGACATTAATACTGTATAC
ATGTCTTTTGATAAGAAGTGGTATTTTTTGAAATAGCACAAATTTGTTGGTCACTTTGCTT
AATATCAT

> **comGE**
Function: protein coding sequence; late competence protein E (core genome, variable)
Best match: comGE_ST425_LGA251-cow_FR821779.1[1602358:1602657:r]RC (completely identical)
Position: 036-contig_189: 182578 … 182878; Length: 300 bp
Sequence:
TTAATACTGTATACATGTCTTTTGATAAGAAGTGGTATTTTTTGAAATAGCACAAATTTGT
TGGTCACTTTGCTTAATATCATACTTCCCTATAGTTACCCCCTTCTTAAGTTCTTCTTTATT
AATTTTAGATACAGTCGTCAAAATTACTTTAGAAGCATCAATTGTTTGTAGTTTATGGTTT
ATACTCGCTTGCATTTGATTCATCATTGGTATCAATAGTAATGTAATCAATCCAATTAGCA
AAAATCCAGCCATACTATCTATTAAGAATGATCCTTTACACTTATAGCTTTTCAT

> **comGD**
Function: protein coding sequence; late competence protein D (core genome, constant)
Best match: comGD_CC001_MW2-USA400_BA000033.2[1624000:1624446:r]RC
Position: 036-contig_189: 182864 … 183311; Length: 447 bp
Sequence:
TTATAGCTTTTCATAACGAATTCTTCCTTTTTCAATATGGAATATTATTCTATAAATTGAAT
TGTTATTGTCAATTGTTATACTACCAAATTTATTGATATTCCCTTTTTTATCAAAGGCAATA
ATATCAACTTTTGCAACATTAATTATTTTGCCTACTTTTAATTTTAGAAATCGTATTTTATT
ATTCTCTATTACTTTAATAGTGTCACTGTTTTCATAAAATCTAACATTGATATATCCTTGAT
TTGCTATAGCTTGCGACTTAATATAATTCAATTCAGTAATAAAAGAAATGATATTTGCCTC
ATCATCTATTACTCTAAGATTGCTTAATCCTTTAGATGTCATTGTCAAAAGTAGAAATATA
CTGATTAACATCATTACCACAAGCATCTCAATCATAGTAAATGCTGACTGCTTTCTAATTT
GCAACTGCTTCTCCAT

> **comGC**
Function: protein coding sequence; late competence protein C (core genome, constant)
Best match: comGC_CC005_Mu50-VRSA_BA000017.4[1655244:1655555:r]RC (completely identical)
Position: 036-contig_189: 183288 … 183600; Length: 312 bp
Sequence:
CTAATTTGCAACTGCTTCTCCATTACTAATTGTTATTGTCTCTCCTGATTTACATGTCTTTT
GTGCTTCTTTTATAAAACCATCTGCAATTAAGTCTTCAATAGACGATGGATTTCTATTATG
TTTCAATGCATACGCTTCAATTTGACTATTAACCATTTTTACCTGTGCATTACAACCTGTTG
ATTGTATGTGAGCAGTTTGTTTAGCAATATTTGGAATGATTAAAATTAATAATAAACTGAT

GATTAATAACACTAATAGCATCTCTATCAATGTAAACGCTTGAGTTTTCTTAAGAAATTTA
AACAT

> **comGB**
Function: protein coding sequence; late competence protein B (core genome, variable)
Best match: comGB_CC001-ST772_118_AJGE01000022.1[52927:53997]RC (completely identical)
Position: 036-contig_189: 183613 … 184684; Length: 1071 bp
Sequence:
TTATTTTATACTTTGCATCATTTGAAACATTGGTAACATAATTACTAAATAAATTGCGACA
ATAAATAAACCTAAAATCAAAAATAAAATAGGCTGTAAAAACTGAGTCTGTTTTATCGCT
TTATCTTCTATTTGTTTTACTAATATTTGCGAATATAACTTTAGTTCTACTTCTAGCTTCCC
TCTCTTTTCACCTTGTAGCACAAACTTAATTAATTGAGGCTTAAAGCATTTTAGTTTTTCTA
AAATTTGAGGCAAACCATATCCCATTTCTGAATAAGTTAATAAGTATTTACCTAGAAACT
GTCTAAATGGATCACTACTATGGTTAATATAAACGTCTACTATTGATTGAAGTGTAATACC
ATTTTTATAAAACAACACTAATTCATTAGTTACAAAATAAGTTTTAAATAATTGGAAATA
GCCTGATATTAGCGGTAGTTTCATCACAAAGTTTATCTTATTGAGCATATTTAAATTGTTA
TAAATTAATTTCATAATAATAGCCAACATAGATACTATTATGAGCATTACTACAATTATA
GTAGGTAAGCTGGTAATGAAAAAAGACAATGTTTTTGAAAAGAGGATAGTTGAATATTC
ATAGAAGTATATAATTGTTGAAACTGTGGAATTACTGTGAGGTTTAATATAATAATCATA
GCAATAAAAATAGAAACTAGTATTAAGGGGTATTGCAGTGTCTTTAACAATCGTTGTTCT
GACTTTCTATTCACTTTCATATAATTTACGGTTTCTTCTAGAACGTCTATAATATTACCAAA
TCTTTCTGCCAAATATACTTGCATGACGATAGTATCGCTATAACCTATCAGTGATAATATC
TGATTGCATGGTGCACCATTTGAAATTTCACTTAGAATGGTGGCACCTAATTGCTTATTTT
TATATGCCATTTGAAGATTTAAAAATTGAAAACTTTGATACAGAGTGAAACCATATTTCA
ACAAATTACATAAATTTGAAAGTAAGTCGATTTGTTGGGCCTTACTTAATTGTCGCTTCTT
AGAATGTAGTTTAAATGTATTTATCCATTGTAGTTTCAC

> **comGA**
Function: protein coding sequence; late competence protein A (core genome, constant)
Best match: comGA_CC001-ST772_118_AJGE01000022.1[51981:52955]RC (completely identical)
Position: 036-contig_189: 184655 … 185630; Length: 975 bp
Sequence:
TTAAATGTATTTATCCATTGTAGTTTCACAAATGACACCTGCTTTTGTCATATCATCAAGT
TTATCTTCTAAGTTCTTAAATGATGATGGTAATGAATGATTATGGGAAAAGAAATATCGG
AGTTGTTGCTGAGATAGAATTTCACATACTAATTGTCGCTGTTGCTTAATAGTAGTTACAA
GTCGTTGGTTTATAATTAAGTTAGTTGCCTGTATCAATTCTTGTACAGAAATGCCCATTTC
TAATAGCCTTAAAATAGCACCTTTACAATCAGTTGCATGCAATGTAGTCAGAACAAGGTG
ACCACTTAAACTAGCCTGTATAACACACTTGGCAACATCTTTATCTCTGATTTCACCTATT
AAAATAACATCAGGATCACATCTTAAAATAGCTTTAAACGAATTTACATAGTTTATGCCA
GCTTTATCATTCACATTAATTTGGACGATACCAGGAATTTGCATCTCTACAGGATCCTCTA
TAGAAATTACATTTAAATTCAAGGCTTTATTCGCGTATGAGACCATTTGATACATTAATGT
ACTCTTTCCTGAACCAGTTGGTCCACTAAACAATAGTAATCCTTGTTTCTTATTCATGAGG
TGTTTAAAATCATTGAATTTATAAGTTGATTTCTGTGGTTGAAAAAATTGAGGTACAATTC
TGATAACACAACTTTCTTGGCCAAGTGACAATGGTAAAGTTGATATTCTCAAAAAATATA
TTTTATTGAAAAGGTAACTATATCGACCGCTCTGTGCGACTTGCTGTGTAGAAACATCAA
GCCCAGCTTGAAACTTCATATAAACTAATAACTTTTGATAAATGCTATTCCCAATTTGTTC
ATACTGCTCCAAGTTATCATTAATTCTAAATTTAATACTTACTTCATTTTTAACTGGAATA
AAATGTACATCACTCGCTTTCATTTCTATCGCTTTATTAATTATTTCTTGAAATAGAATCTT
CAA

> **glk**
Function: protein coding sequence; glucokinase (core genome, constant)
Best match: glk_CC001_MW2-USA400_BA000033.2[1627766:1628752:r]RC (completely identical)
Position: 036-contig_189: 186630 … 187617; Length: 987 bp
Sequence:

TTATTTTACCCCCTCTTTATCTAATACATAGGTCTTGATTAATCCTGCTGCTCCTGTAATAC
CTGCATCATTACCTAATTTCGCTTGTACAATTTCAGTTTCAAATTGAGCAGGTGCAAATGT
TAAATTATGATATTCTGTTTTAATATTTTCAATTAAAATAGGTCCTGCAGTAGACATTCCT
CCACCTAGAACGATATATTTCGGATTACTTGTAACACTAATAATACTACATAAATATCCA
ATATAGTTTGCAACCTTTTCAGTAATGAAAATACAGAATTGGTCACCAGCTTTTGCCGCAT
CAAAAACAGCTTTTGCTGTAACCTTATTTTCTTTAATCAATTCTAATATAGAAGATCTAAA
CGTCAACTTCGGATAGTAGAAGTTAACTAAGTTAACAACGCCTGTCGCTGAAGCAACTGT
TTCAATACATCCAGAACGACCACAATTACATTTAAATCGTTGATCGAAGTCTGCTCTAAA
ATGACCTATTTCTGCGCCAGAGCCATTATGACCATGTACGATTTCACCATTTGAAATAATT
CCTCCACCTAGACCTGTACCAAGTGTGATGGCAACAACATCATCGGCACCTTCACCAGCA
CCTTTGTGTTTCTCCCCTAAAGCAGCTATGTTAGCATCATTATCTACATACACTGGACAAT
CAACGAATTGTTCAAAAATCTCACGTACATTAACTTTTTCTGGCCAATATAAGTTTACTGC
TCCATTTACTGTACCTTTTTCAAAGTCAACAGGACCTGGTACACCAATACCTACGCCAAGT
ACATTTGAAAAATTATAATTATTTTCATTTACTTTTTCAACAAACGAATCATAAATTCCTT
TCAAAAGTGTATATCCTGTACTATCAGATGTATCAGTGTGAATAGACCATTTATGTAATTG
TTCTAATTCAGGTGTGAAAATACCTAATTTACAAGTCGTCCCGCCTACATCAGCTGCTAAA
ATAATTTTTGTCAT

## > **rpmG1**

Function: protein coding sequence; 50S ribosomal protein L33/locus 1 (core genome, constant)
Best match: rpmG1_CC001_MW2-USA400_BA000033.2[1631156:1631305:r]RC (completely identical)
Position: 036-contig_189: 190020 … 190170; Length: 150 bp
Sequence:
TTATTTTGTTTCACGGTGTAAAGTTTGTTTGTTTTCACGTGAACAGAATTTCTTCATTTCAA
CACGTTCTGGATTATTTCTTTTGTTCTTAGTTGTAATGTAGTTTCTGTCACCACATTCCGTA
CAAGCTAAAGTTACGTTTACGCGCAT

## > **pbpC**

Function: protein coding sequence; penicillin binding protein C (core genome, constant)
Best match: pbpC_CC005_Mu50-VRSA_BA000017.4[1662238:1664313:r]RC (completely identical)
Position: 036-contig_189: 190282 … 192358; Length: 2076 bp
Sequence:
TTATTTGTCTTTGTCTTTATTTTTATCATCTTTACCTAACTGCTTAAAGTAGTAGTTAATTA
CATCTCTACCTAAGTCTCCACCTGTTAACCATGGTGGTGGTACAGGCTGATTTGTATATAC
AATTGAAAACGCTAATTTTGGATCATCAATTGGCGCGTATCCTATATAAGTAGAGTTAAC
TCTTGGCTCTCCGTTTTGGAACACTTCAGCGGTACCCGTTTTACCAGCAGTAGGTACTACT
GTATCTTTAAAACTAACATATCCAGTACCATCTTTATCATTAAATGCCATTTTGAATCCTT
CTTGAATTTGTTTGATTTCCTTTTCAGTATTATTAACCTTGTTCAAGACAGTGCCATTAATT
TTCTTCTTGAGTGGACCAACCTCATCTTTATTAGTTGATTCATGAATCGTTAATCCAATGT
GTGGCTGTATTCTATAACCATCATTCGCTATAGTTGAAACATATTGTGATAATTGTAATGG
TGTATAGGTATCATATTGACCAATTGATAAATCTAGATAATTACCTGGATTATTTGTTAAT
GGTTCGATTTGACCTCTTGTTTCATTTGGTAAATCTATCCCTGTTTTCACACCTAAGCCTAC
TTGATTTAATCCTCTTCTAAGCTTTTGGGCAGGTGAACTTATATCTGAAGGTAAAGCCATA
CCAGAATAATAAGGGTCTCCCGCTAATTTTAATGCTGTTTTAAACATATATACGTTTGATG
AATGCATCAAAGCTTGCTTATCATTAATAGATACATGCCCGTTTTTATTGAAGTATGATCG
TTTTGTCAAACCACCTTGGAAATGTAATGGTTCATCGACCATTGTTTCTCCAACTTTGATA
GCTTTATTCTGATAACCAGCTAATAATGTTCCACCTTTTACAGAAGATCCAACCGCAAATT
GAGAAGTAAACGTACCAATGTCATAATCAGTCATTTTACCACTCTTATTAATCTGCTTTCC
GGCAAGCGCAAGAATGTCTCCATTTTTAGGATTTTGTACAACCATCATAGCATTATCCATA
TCTTTTGCACCTTGGCTGCGAAGCTTCTTAATTTGTTTATCTAATAATGCTTCTACTTCTTT
TTGAAGATCTATATCGATCGTTAATTTCAAATCTTGACCGCGAGCGCCAGGATTTAACACT
TCTGAAGATGTAACTTTACCAGATTTGTCCGTTGTGTATTTCATTTCTTTCTTCTTACCACG
CAATACATCTTCATATTGATATTCTAGGTAAGATTTTCCAACACGATCATTGCGTGAATAT
CCTTTGGATAAGTAATGTTCTGTCAATTCTTTTGGAATACCTTCAGCAGGTGTCGATACAT

CTCCGAATATACCTCTTAAAGTATCGCCATATGGATATTTTCTATCCCAATCCATAGACGT
GTTAACACCTGGTAATTTGGAAAGTTGCTGAGAAACTGCTGCATACTCTTTTTCACTGACA
TCTTCATTTTTTATCATTTGTGGATCTAAAACTGTTCCTGCATTCATCTCTCGAAAAATAGC
TAAAACTTGTAAATCTTTAGAAGACAATTCATCTAATTGTGATTTTCCGATTTTCGATAAC
AGTTGTTTATCATATTGATCTTGTTTAATACTTCCATCTGCTAACATAGCTTGTTCTTTTGT
CATCATTGCTTTTGCTTTTTTAGGATGCAACTGAATCCAGAAATCTTTCTTATCACGTTCTG
TAATTTTCTTAGTATCCATCTTGATTAGCTTTGATAACTTTTCAGCCGTATCCAACATTTCC
GATTGTGTTGTTTTTCGACCCCTAGTATATGTAATAGCCATTTTAGAAGCATTATCAACTA
AAACTTTCCCATTTCTGTCTAAAATACGACCTCTTGGCACAGACTCATTCACTGTAATGTT
TTCATCATTTTTTATAATTTGTTTATAATGTGAGCCTTGTGCGATTTGTAAATAACCTAAAC
GTAGTACTAGTACTGCAAAAATAAATACAATCACACCAAATATAAAGTTAATTCTCTTGT
TAATTGTATTTTGAACGATTTCATCATTTGATTTTTCTTTTAGTCTTTTTAACAA

> **sodA-L2**

Function: protein coding sequence; superoxide dismutase/locus 2 (core genome, constant)
Best match: sodA-L2_CC005_Mu50-VRSA_BA000017.4[1664434:1665033:r]RC (completely identical)
Position: 036-contig_189: 192478 … 193078; Length: 600 bp
Sequence:
TTATTTTGTTGCATTATATAATTCGTCAACTTTTTCCCAGTTAACTACATTCCAAAATGCGC
CAATGTAGTCAGGGCGTTTGTTTTGATATTTTAGGTAATAAGCGTGTTCCCATACGTCTAA
ACCTAAAATAGGTGTTTTACCCTCAGTTAATGGATTATCTTGGTTTGGTGTAGTCACAATT
TCTAACTGGCCATTGTTTACGACTAACCAAGCCCAACCTGAACCAAAGCGTGCAGCTGCT
TTGTCAGCAAATTCTTTTTTAAATTCTTCTAAAGAACCCCATTGTTCTTTAATTTTTTCTAC
TACAGTACCTTTTTCTTCTGAGTTTGGTGAAAGTAACTCCCAGAATAATGAATGGTTTAAA
TGTCCACCGCCATTATTACGTACAGCAGTTTGGATGTTAGCTGGTACACTGTCTAAATTAG
CAACAATTTCTTCAATAGATTTAGATTCTAAATCTGTACCTTCTACTGCAGCATTTAATTT
CGTAACATACGTGTTATGATGTCTGTCATGGTGAATTTCCATAGTTTCTTTGTCAAAATGT
GGTTCTAATGCATCAAATGCGTATGGTAATTTTGGTAATTCAAAAGCCAT

> **zur**

Function: protein coding sequence; zinc uptake regulator (core genome, constant)
Best match: zur_CC008_COL_CP000046.1[1641975:1642385:r]RC (completely identical)
Position: 036-contig_189: 193353 … 193764; Length: 411 bp
Sequence:
TTAATCTTGGCAAGACTCACATACACCATAAACTTCAAGTTTGTGTTTGTGAATATTAACA
CCAGGTAGTGATAATTTTATCTGATCTATTGGACAATAATCTATTACCTTTGTATCTCCAC
ACTTTTCACAGATAAAATGATGATGATGATGGTTTGTACAAGCGATTCTAAACTTCATTTC
ACCATCAAGTTCTGTATTTTCAATAATTCCTAAATCTTTAAATAAGTGCAGGTTTCTATAT
ATTGTGTCGAATGAAATTCCAGGATAATTTTCATCCATAACTTGTTGTATATACTTTGCGT
TTATATACTTATCTTCTTCGACAAAAATATCTAACATATCTTTACGTTTATCTGTATATTTT
AAACCGTTCTCTTTTAAAATTTTAATAGCATCATTTGTATTCAT

> **mreB**

Function: protein coding sequence; ABC transporter/permease protein (core genome, constant)
Best match: mreB_CC001_MW2-USA400_BA000033.2[1634886:1635719:r]RC (completely identical)
Position: 036-contig_189: 193750 … 194584; Length: 834 bp
Sequence:
TCATTTGTATTCATTGATATTAGCTCCCTTTTTAAACTTCATTCGCATTTTCTGATAAGCCA
TTGTAATCATAAGTAAAATAACAAGTAGAACTACAATTACACCACCCGGAGAAATGTCCA
TATAGAAAGCTAGGACTAAGCCTAATATTACTGATAATTCACCTAAAAATACACTTAGTA
ATATCAATTGCTTAAAACTTTTTGTTATTCGCATACTTATTGCAATTGGTAACGTGATTAA
CGCACTTACTAACAGTATCCCTACAACACGCATTGAGGCAGAAATAACCATCGCTACAAT
AACAATAAATAAAAATTGAATCCATTTAGGAATGCCAATGACTTTACTATATTCCTCATC

AAATGACAATATAAATAATTCTTTATAAAACAATGTAATAAACAGAACAACTATGATGGC
AATGACAATAATCGTTGTTAAATCACTTATATTCACTGCGCTTATTGAGCCAAATAGCAAT
CCAACAATTTCTTGATTGAACCCATCAGCTAATGAAATGAAGATTGCACTCAAGGCGATA
CCAGCACTCATTATAATTGGAATAGCAATTTCTTGGTAAGCAGTGTATGACGTTCTTAATT
TTTCAATTAGAAGCGCACCTACTATTGCGAATAAGATTCCAAACCACATTGGATTAATAA
ATACTAGTGTTGGCATAATAGTAAGTAAAAACATACCGAAAGATATACCACCTAAAGTTA
CATGACTTAGAGCATCAGCTATAAGTGATAGTCGTCTAACAACGATAAAAGCACCGATTA
GAGGCGCAATAAAACCTATCAAGATACCACTAATTAAAGAGTACCTCAT

## > mreA

Function: protein coding sequence; zinc ABC transporter/ATP binding protein (core genome, constant)
Best match: mreA_CC001_MW2-USA400_BA000033.2[1635791:1636576:r]RC
Position: 036-contig_189: 194655 … 195441; Length: 786 bp
Sequence:
TTAATTACAACATTCTCGATTATGCTGATGATCGACAAAACGTACAGGATGTCCATAAAT
TTTTGAAATTTCAACTTCATCAAGTGATTTAAACTCATCAGTTGTACCATGGAAATGCAAA
TGCTTATTTAAACATGCTACTTCAGTAGCAGTATCTGCTACAACACCGATATCATGAGTAA
CTATGATAATGGTGATACCTTCTTGTTTTAATTGATCTAAAGTATTATAAAATTCACTTAC
ATGTTTTGCATCAATACCATTCGTTGGTTCATCAAGTACTAATACTGCAGGTTCTGAAATC
AATGCTCGAGCAATCATTACACGTTGTTGTTGACCACCTGATAATTCTGCTATATTTTTAT
GAATTAAATCACTTATATTCAGTCTTTCTAGTACTTTAATCACTTTTTCATTATCTTTGCTA
TTAAATGTTTGGAAAAGACGTTTTGTCTTTGTTAATCCGCTTAAAACAACTTCTTTAACAC
TTGCTGGGAAACCTGAATTAAAGGCATTTGCTTTTTGTGATACATAGCTTAATTTAATTGA
TGTTTTCTTATTTTTAAAATCAATACCTTCAACAAAAATCTCACCACTTTGTAAAGGTAAT
AACCCTAGAATCAACTTCAATAATGTTGATTTACCAGCACCATTTGGTCCAACAATTGCTA
AAAATTCACCTTTATTTATTTTAATGTTTATATTTCTAACACTTTTTATGATCATAGTAG
TAATTGACATTTTTCAATTCAAAGACTGGTGTCATCGTATTCTCACCTCGCAT

## > nfo

Function: protein coding sequence; endonuclease IV (core genome, constant)
Best match: nfo_CC008_NCTC8325_CP000253.1[1570227:1571117:r]RC (completely identical)
Position: 036-contig_189: 195566 … 196457; Length: 891 bp
Sequence:
TTATTGTTGCATAACCTTATTTTTTAATTCTGGGTCAAATTGCTGTTGTTTTAACATTTCAA
TTTCAAGTTTATATGGCGGTTTTTATTTTTCTTATCTTCACCAACATAAGGTGTTTCTAAG
ATTTTCGGAATATCTTTAAAACTATCATGATGCACAATGTAATTTAATGCATCAAAACCA
ATGTAACCGAAGCCAATATTTTCATGTCGGTCTTTTTGAGCGCCACGGTCATTTTTAGAAT
CATTGACATGAACAACTTTGATTCTGTCGACTCCAATGATTTTATCAAATTCATTTAATAC
GCCATCAAAGTCCTCTTTAACATTATATCCAGCATCATGCGTATGACATGTATCAAAACAT
ACTGATAAACGTTCGTTATTATGAACTCCATCAATAATACGTGCTAACTCTTCAAATGAGC
GACCAATCTCTGTACCTTTACCTGCCATCGTTTCAAGCGCAATACGTACATTATTGTCATT
CGTTAAAACTTCATTTAATCCTTCAATAATCTTATTAATTCCGGCATCAACACCAGCTCCA
ACATGCGCACCTGGATGTAATACAATATCTTTAGCCCCTATAGCTTGCGTTCTTTCAATTT
CTTGTTGCAAGAAATCTACACCAAGATTAAACGTTTCTGGTTTGGTTGTATTTGCAATATT
AATGATGTATGGTGCATGAACAACAATATTAGATAAACCATATTTTTCCATCACTTCATG
ACCTTTAGTTATATTTAAATCTTCAATACTTTTACGGCGCGTGTTTTGAGGTGCACCAGTA
TAAATCATAAATGTTGTTTCACCATATTCATGCGCTTCTATAGCAGAACCTTCTAACATCT
TTTTACCACTCATTGAAACATGTGATCCTAATAACAT

## > cshB

Function: protein coding sequence; DEAD-box ATP dependent RNA helicase (core genome, variable)
Best match: cshB_CC001-ST772_118_AJGE01000022.1[39798:41144]RC (completely identical)
Position: 036-contig_189: 196466 … 197813; Length: 1347 bp
Sequence:

CTAACCTTTTTTGTTTTGCTTACGTTTTTGTCTATTTTGCTGCTTACTAAATTGCTTACGCTC
TTGACGTTTCATTTTTTCAACTTCTTGTTTAAATTTCTTCTTATAACCTGGTTTAACTTTGTT
TTTAATTTTACTTCGAACTTTGTTCTTCACTTGATTAGTTAAATGGTCATCTTTGCGCATTC
TTGCTTGACGCTGATTGTGCGCTTTAACTTCTTTTAACTCACCATCTTTAATATCAACAGTA
TTGAATACAAAACCGCGATCTTCTATTAATGAAATATTGTGTTCTTCATCAGGACTATAAA
GCGTAATTGCTACACCTTTATAATTCCCACGACCAGTTCGTCCAACTCTATGCGTAAAGAA
GTCAATATCATTTGGCACATCAAAATTGATGACATGACTAACACCTTCAATATCAATACC
ACGAGATGCTAAATCGCTGGCAATAACGTATTGGAATTCTAAATTACGTATACGTTTCAT
TTGTTGTTTACGTTCACGTGGCGTTAAACCACCATGAATCATACCAACTTTAATACCAGCT
TCATTTAGTGAACGTGCTAAATCATTTGCATTATCTCTACTATTACAGAAAATAATACATA
AGTATGGATTTAGTATATCAATTAAATTTAAAGTTTTTTCAACTTTAGCTGCACCTTTAGT
AGGTATTAAAAAGAATTCGATGTTCTTTTTATTTTGTTTTTTACTGTCGACAGCTACATATT
CTGGATGACTTAAATATTTATTTAAAAATGGTTGTAACTGTTGTGGAATTGTAGCACTAAA
CACCGCAATATTTGCATTATCTTCCAATCTTGCAGCAATGTAATCTACATCTTCAATTAAT
CCTAAGTCAATCATAAGATCCGCTTCATCAATAACTAAATATGATGCTAAGTGCACATGT
AAATGTCCCGTTTTAGCTAAGTCATTAATTCTAGTAGGGGTGCCTATAATCAATTGTGGTT
GTGCATTACAACGTTGTCTATCTTTCTCTATATCTGTACCACCAATAAAAACTTTAACTGA
AACACCAGCTTTAAATTGGCTTAAATGGTTCGCTGCATCGTATAGTTGTTGTGCAAGTTCT
CTTGTTGGTGCAACTACGATTGCTTGTGGTTCTTTTATTTCACTATCAATTAACTGCATTAA
TGGTAATAAAAATGCATGAGATTTCCCTGTACCCGTTTGAGATTGACCAATTAAATTTGTT
CTCTTTAGTATTCTTGGAATAATTCGATTCTGAATTTCAGTTGGTTTTTCAAAATTAAGGTC
TTTCACAGCGTCAATTAAACTAGATTCTAGATTAAATTGTTCGAATGGATGTTTTGCCAT

## > **sigA**

Function: protein coding sequence; sigma factor A of RNA polymerase (core genome, constant)
Best match: sigA_CC005_Mu50-VRSA_BA000017.4[1671793:1672899:r]RC (completely identical)
Position: 036-contig_189: 199837 … 200944; Length: 1107 bp
Sequence:
TTAATCCATAAAGTCTTTCAAACGTTTACTACGACTTGGATGTCTTAATTTTCTAAGTGCT
TTTGCTTCAATTTGTCGAATACGTTCACGTGTAACACCGAAAACTTTACCAACTTCTTCAA
GTGTTCTTGTTCTGCCGTCATCAAGACCAAATCTTAATCGTAATACATTTTCTTCTCTATCA
GTTAATGTATCAAGCACATCTTCTAATTGCTCTTTTAATAATTCATAAGCAGCATGATCTG
AAGGACTTTGTGCTTCCTGATCCTCAATAAAGTCTCCTAAATGACTATCATCTTCTTCACC
AATTGGTGTTTCTAATGAAACAGGTTCTTGCGCAATTTTTAAAATTTCACGAACTTTTTCT
GCTGGTAAATCCATTTCTTCACCAATTTCTTCTGGTGCTGGATCTCGACCTAAGTCCTGTA
ATAATTGACGTTGAACACGAATTAATTTATTAATTGTTTCTACCATATGCACAGGGATACG
AATCGTACGTGCTTGGTCAGCAATTGCACGAGTGATTGCTTGTCTAATCCACCATGTTGCA
TATGTTGAAAACTTAAATCCTTTGTTAAAGTCAAATTTTTCAACAGCTTTAATAAGACCCA
TATTACCTTCTTGGATTAAATCAAGGAATAACATACCACGACCTACGTATCTTTTAGCAAT
ACTTACAACTAAACGTAAGTTCGCTTCTGCAAGTCTTGATTTTGCTACTTCATCACCTTGT
TCAATACGTTTGGCTAATTCGATTTCTTCTTGTGCACTTAATAAGTTAACACGCCCAATTT
CTTTAAGGTACATACGAACTGGGTCATTTATTTTAACACCTGGAGGGGCACTAAGATCAC
TTGGATTCAGTTTCTCGTCAGTATCTGAACTATCTTTTTCATTAACTAGTGAAATATCATTA
TCATTTAATTGATCAAAGAAATCATCCATTTGATCAGAGTCGATATCAAAATTCTGAAGTT
TTTCAGCAATTTCTTCATGACTTAAATGACCCTCTTTTTTACCTTTTTCAATTAATTGCTTC
TTAACATCTTCTAATGTTAATGTCGGATCAATTGTTTGTTTTTTAATTTTAACTGTGTTATC
AGACAT

## > **dnaG-var1**

Function: protein coding sequence; chromosomal DNA primase/variant 1 (core genome, variable)
Best match: dnaG-var1_CC001-ST772_118_AJGE01000022.1[34644:36443]RC (completely identical)
Position: 036-contig_189: 201167 … 202967; Length: 1800 bp
Sequence:

CTACATGCGTTCTTTATTCTTAGCAACAATTTGCTGTAAATAGTATTTTTGTAATTCTACAT
CGCCAATCCTTGTAGCTTCCCTTAATTTATGATTCAATGACTCAATTGTTTCTTGTCCTTTT
TCATTAATAACATTGACATAATCATCAATTTCATTTTCATATGGTTCGTCATTCAAATTAT
ATTGTTCTAAGCTAATTAGTGTTTCTCTCAACTCATTTGAATTAACATACTGCACAGCATC
ACTGATATTATATTGATCATTTTCCGCATAAAAATCATGTAAGACTTCGAATACATATTTA
AAATGCTGATTTGTGAAGTTATCCTTATCAACACTTTCATAATAATTTAAAAATGTATCTT
TATCTCTCATTAAATGTTTTAAAAATGCTCGCTCCGCTTTTTCTTGACGGCTCAAATTGTCA
AATTGTGCCATACCAATTGGCTCAGGTTCAATATAACCGCCATACTCATCTTCTGGATAAT
AATTGGCTGGTGCTTGATTGAATTGTATTTCGTTAGCTAACTGCTCAGGACTAACATTGAA
AAATGGCGCAACATCATTTATAGCCTTTTGTTGCAAAATCGATGACTTCATAAGTGAAAT
GTCATGACTCAGTTCTTTCAAATAACGTTCATATGAAAGGTCATTATGTGCAATTTCATCT
TTTAATATACTTACTTTATAATGTGCAAATGACTTTTTGTCATTTTTTACAAAAGTAGTAA
ATGCGTCGTTGCCATACTTACCAATGTATTCATCCGGATCCATGCCAGATGGCAATTGTAT
AACAAATACATTTAGCCCTTGCTGTAACAAATTTTGACCTGTTTTAAGTGTTGCTTCACTA
CCCGCAAAATCCCCATCAAACATTAATGTTATATTTGATGTTAACTTTCGTATAAAAGTAA
TATGTTCATCTGACAACTGTGTCCCCATTGTTGCAACAACGTTTTTCAAGCCAGCAGTATC
AGATTTTATAACATCCATAAAACCTTCTAGTAATACGATTTCATCTAATTTTCTAATTGAT
TTACGCGCTTTATCTAAGTTGTATAACAACTTTCTTTTTTGAAAGATAGGTGTTTCAGGAC
TATTTAGGTATTTTGGTTCTTGACCGGTATATGTTCGACCTGAATATCCAACAATTCTTCCT
TGCGCATTTTTCAAAGGAAACATAATACGATTTCGAAATCTATCGTAATAACTGAAATTTT
CTTCGTTACGTGATAATAATCCGGCTTCATATGCTAATTCAATATCGTAACCCTTTTTTTG
AAGAAAATCATGACAAAAATGTGAGCTATCGGGTGCAAAGCCAATGCCTCGCTCTTTAAT
AAGCGCATCTGTAAAACCACGTTCTTGTAAGTATGTTAATGCTTGTTCGCCTTCGACTGTC
TTTGTTAAAGCGTAATAATAAAATTCTTGTATTAACTCATGCATTTCAATCATTTGTAAAT
CATCAGAAGCAATTTGAACATTTGAGTTAGATTGTGTTGCCTCAATATCTACAGCAACATT
AACTCTATCACCTAATTCTTTAACCGCTTCAACAAATGATATGTCTTTAATTTCTTGAGTA
AATTGAAAAACATTGCCACCTTTTTTACAACCAAAACAATGACAGATTTGTTTATCTTCAG
AAACTGTAAATGAAGGTGTCTTTTCATCATGAAAAGGACACAAACCTATATAATTGCGTC
CTCTCTTTTCTAGTTTTACATATTCACTTACCAAGTCTAAAATGTCGGTTTTATCTTTTATT
TCATTAATGATCGATTGATCTATTCGCAA

> **ccpN**
Function: protein coding sequence; catabolite control protein N (core genome, constant)
Best match: ccpN_CC001_MW2-USA400_BA000033.2[1645010:1645633:r]RC
Position: 036-contig_189: 203856 … 204480; Length: 624 bp
Sequence:
CTATTCTTTATATAATGCTACTAATAACTTAGCTATTGTTGTTTTGGAAATTCTTCCAATTA
CTTCATACTTTTGATTATCTTTTTTTCTTACAATTGGAATCGAATCAATTTCTTTTTCAATC
ATTCTATCTGCTGCGTATATGACTAATTCGCTTTCCTCTAAATAAGTGACATTAGGCATAC
GTGTCATATTTACACTAATAGGTACTGTATGAATATCTGCTCCAATCATTGAAGCTCTTAA
TAAATCTTTTCTTGAACACACACCAACAAAATCGTTATCTTCATTAATAATAAATAATGTA
CTTACATCTTCTAAAAAAATTGTACAAATAGCGTCATAAACTGTTGTATTCTCTCTTAGCA
CAACAGGTTGAGACATATAGTCCTTAACCTCAAATTGTCGAAGTTTTTCATTAAAAAATTT
ACCTTTTGATTTACCTGAATAATAATATCCAACTCGGGGACGCGCTTCTAAAAAACCTGA
CATTGTTAATATCGCTAAATCTGGTCTGAGCGTTGCTCTTGTTAAATTCAACTTATCTGCT
ATTTGTTCACCAGTAATGGGTCCTTGAGTTTTAACAATTTCGATGATTCGTTCTTGTCTTTG
ACTGAGTTCTAT

> **glyS**
Function: protein coding sequence; glycyl-tRNA synthetase (core genome, constant)
Best match: glyS_CC001-ST772_118_AJGE01000022.1[31405:32796:r] (completely identical)
Position: 036-contig_189: 204814 … 206206; Length: 1392 bp
Sequence:
ATGGCAAAAGATATGGATACAATTGTTTCATTAGCAAAACACAGAGGTTTTGTGTTCCCT
GGTAGTGATATTTACGGTGGTTTATCAAACACATGGGATTATGGTCCTTTAGGTGTTGAAT

TAAAGAATAATGTTAAAAAAGCTTGGTGGCAAAAATTCATTACACAATCACCGTTTAACG
TTGGTATCGACGCTGCAATCTTAATGAATCCAAAAGTATGGGAAGCTTCAGGACACTTAA
ACAACTTCAACGACCCAATGATTGATAATAAAGATAGTAAAATTCGATATCGCGCTGATA
AATTAATTGAAGATTATATGCAAGATGTTAAAGGTGATGAAAACTTCATTGCCGATGGTT
TAAGTTTTGAACAAATGAAAAAAATTATTGACGATGAAGGTATTGTTTGTCCTGTAAGTA
AAACTGCTAACTGGACTGAAATTCGCCAATTCAATTTAATGTTTAAAACATTCCAAGGTG
TAACTGAAGATTCTACAAATGAAATTTTCTTACGTCCTGAAACAGCACAAGGTATTTTTGT
AAACTATAAAAACGTGCAACGTTCAATGCGTAAAAAATTACCATTTGGTATCGGTCAAAT
TGGTAAATCATTCCGTAATGAAATCACTCCAGGTAATTTCATTTTCAGAACAAGAGAATT
TGAACAAATGGAACTTGAATTCTTCTGTAAACCTGGAGAAGAAATCGAATGGCAAAATTA
TTGGAAAACTTTTGCAAGTGACTGGTTAACAAGCTTAAATATGAGCAGTGAAAATATGCG
TTTACGTGATCATGATGAAGATGAATTATCTCATTACTCAAATGCAACAACTGATATTGA
ATATAAATTCCCATTTGGTTGGGGTGAGTTATGGGGTATCGCAAGTCGTACAGACTTCGA
CTTACGTAAACATGCTGAACACTCTGGTGAAGATTTCAGATACCATGATCCAGAAACGAA
CGAAAAATATATTCCATATTGTATCGAGCCATCACTTGGTGCAGATCGTGTAACATTAGC
TTTCTTATGTGATGCATATGATGAAGAAGGCGTTGAAGGTAGTAAAGATGCACGTACAGT
TTTACACTTCCATCCTGCATTAGCACCATATAAAGCAGCGATTTTACCTTTAAGTAAGAAA
TTATCTGGCGAAGCGATTAAGATTTTTGAGCAATTAAGTTCTAAATTCTCAATCGATTTCG
ATGAATCACAATCTATCGGTAAAAGATACCGTCGTCAAGATGAAATCGGTACACCTTATT
GTGTAACATTCGACTTTGATTCATTAGAAGATAATCAAGTTACAGTACGTGACAGAGATT
CAATGGAACAAGTTCGTATGCCAATCTCAGAGTTAGAAGCTTTCTTAACTGAAAAAACAA
AATTCTAA

> **tx_glyS**
Function: ter ho-independent terminator
Best match: tx_glyS_CC001_MW2-USA400_BA000033.2[1647392:1647453] (completely identical)
Position: 036-contig_189: 206238 … 206300; Length: 62 bp
Sequence:
TATAATTTATAAAAAGAGATACTGGTTAGTAAAATTGCCAGTATCTCTTTTCTATTCGAAA
A

> **txbi_recO**
Function: ter bidirectional rho-independent terminator of recO
Best match: txbi_recO_CC001_MW2-USA400_BA000033.2[1647439:1647509:r]RC (completely identical)
Position: 036-contig_189: 206285 … 206356; Length: 71 bp
Sequence:
TTTTCTATTCGAAAAAGGTATTCAAATAAACACATCTATGTGGCTACTTGAATACCTTTTA
TTATTTTATG

> **recO**
Function: protein coding sequence; DNA repair protein O (core genome, variable)
Best match: recO_CC001-ST772_118_AJGE01000022.1[30502:31254]RC (completely identical)
Position: 036-contig_189: 206356 … 207109; Length: 753 bp
Sequence:
TTATTGTTCCAATCTTTTTAATTGGTTGATTAGTTTCTGACTTTTAAAAAACATACCTGCAT
ATTCACGATATAACATTAAAATGATATCTGACATTTCATCAATAATTTCTTGATGGATATT
CAATGAATTCATTTTATCTATTGGTAATTTTTGTAATACATCTAATAAATATAGTGTTTTAT
TCGATAATATAACTGCATGTACATCTTTAGAAGCCTCTTGCCTTGAAATCGCACCGTCAAA
CTTAAAACTATAACCTATTAAATCTGCTTGTGTGTCATTACCACTCACAGCACAGCGATTA
AATGATGCAGTAAAACCAAATCGTTTCATACACTTTAACATAACTACGACTGACATTAAC
TGTGCAGATGTACCTGATTCTATTTTTTCAAGAACAAATTGTAATAATTGATAGTTATATG
GTGCAATGTCACCTTCATCCATTGAGCGCTCAATAGTTTCAGCTGCCAGAGAGGCATAAC
TGCTTACGTAAAGGTCCATTTGTAATTTATAATGTTGACTAATAACATCTACAGAATTTAA
CGTTCCCATACCTCGCCACTGATTGTAAATAAACAAACCATAAACAAACAATTGCGTTTG

CGCTTGTAAACCCGTCTTAACTTTTTTAGCACGCCTTGCCATAAGTGGTACTTTTGCACCA
TGCTCATTTAAAATCGTGATAATTTTATCAGATTCACCATAATCAACTGCTTTGATGATAA
TCCCTTTTTGGCGCATTAACAA

> **era**
Function: protein coding sequence; GTP-binding protein (core genome, constant)
Best match: era_CC008_TCH1516_CP000730.1[1679178:1680077:r]RC (completely identical)
Position: 036-contig_189: 207130 … 208030; Length: 900 bp
Sequence:
TTAATCTTGGTCTTCAACATAACCAATTTGGCGAATAAAGTTAACTTTGTTTCGCCAGTCT
CTTTGAACTTTGACCCATAATTCTAAGTAAACTTTAGAGCCTAGAAGCATTTCTATATCAC
GTCTCGCACGTTTTCCTACTTCTTTTAACTTTTTACCGCCTTTTCCAATGACAATTCCTTTTT
GCGAATCTCTTTCAACATATATAGTTGCTTCGATATGAACACGATCTTCGCTTTCTTTAAC
CATACGGTCCACATTAACACCAATCGCATGAGGGATTTCTTCACTTGTAAGATGAAGGAT
TTTTTCACGAATGATTTCACCCACTACAAATTGTTCAGGATGGTCTGAAATTTGATCATCT
GGATAATATTTAGGTCCTTCGGGTAAATACGTCTTTAAAACATCAATAAAATGATCGACA
TTTAGCCCTTCTAATGCTGAAATAGGTACAATCTCTGTAAAGTCCATATAACTTTGATATT
CTTCAATCTTTGGCATTAATTCATCTGGATGCACTAAATCTATTTTATTTAATACTAAAAA
TACTGGTGTCTTAACATTTTTCAACATTTCTATAATATATTCATCGCCTCGTCCAATTTCCT
CATTGGCATTAACCATAAACATGATTGCATCTATCTCAGATAATGTATTTTTAGCGACTTT
CATCATATAGTCACCTAATTTGTGTTTAGGTTTATGAATACCTGGCGTATCAATGAATATA
ATTTGCGCGTCATCTCTTGTCATAACACCTTGAATTTTATTTCTAGTTGTTTGAGCTTTATC
GGACATGATTGCTATTTTATGGCCGATCACTCTATTAACAAATGTTGACTTTCCTACATTT
GGTCTACCTATAATTGAAACAAATCCTGATTTATGTTCTGTCAT

> **cdd**
Function: protein coding sequence; cytidine deaminase (core genome, constant)
Best match: cdd_CC008_NCTC8325_CP000253.1[1582690:1583094:r]RC (completely identical)
Position: 036-contig_189: 208030 … 208435; Length: 405 bp
Sequence:
TTATTCTAAATCCTTTCCTGAAAATCCAAATGGTAGTAACTCTGCGACTGTCATCATAACC
ATATCTCCTTTATGATTTGTCATATACACAGGCATATCATCATCACATAATTCCTTCAAAA
CTTGACGACATGCACCACAAGGTGATGACGGTTTATCTGCATCTACGGTTACAGTTATTG
ATTCAAAATCACCTGGTCTGTATCCTTGAGAAATTGCCGATACCAAACTAGCTCGTTCAG
CACATATCGATAATGGATAAGAAGCATTTTCTACATTGGTACCATAAAAAGTTCTACCGT
CTTTCGTTTTTAAATAAGCCCCTACTTTAAATTGACTGTATGGCGAATATGATTCTTGTTGT
GCTTTTCTAACTTCTTGAAAATAATGAGGTTGATAACTCAT

> **dgkA**
Function: protein coding sequence; diacylglycerol kinase (core genome, constant)
Best match: dgkA_CC008_NCTC8325_CP000253.1[1583105:1583449:r]RC (completely identical)
Position: 036-contig_189: 208445 … 208790; Length: 345 bp
Sequence:
CTAAAATAACGCTATAAAATGTGGTAAAAATACTATTAAACCTATAATAAATGCTAATAT
TGAAACTATAAGTACACTAAAAGCCGCAATATCTTTAGCGTATTTAGCTAAATCATGATA
TTCAACGGTCACTAAATCGACAACATATTCAATAGCAGTGTTTAAAGCTTCAACAGTGAG
AACTAATGCAATAGCAATGAGTATAAATATCCACTCAATCCGATTAATATTTAGTACGAG
ACCAAAGACAATAGCAACAATCATTGCAAACACATGTAAAAGAAATTTATAGTCTTTTTG
AATTAAGATTTTCAGCCCATCAAGTGCATATTTAAACCTTTTCAT

> **phoH**
Function: protein coding sequence; phosphate starvation-induced protein (core genome, constant)
Best match: phoH_CC005_Mu50-VRSA_BA000017.4[1681233:1682153:r]RC (completely identical)
Position: 036-contig_189: 209260 … 210181; Length: 921 bp
Sequence:

TTAATTCTCTCCTTCATAATGTTCAATGATCTTACTTACCAATGGATGTCTTACTACATCGC
TCTGATCTAATTTCAATATACTTATACCTTTAACGTTGTGTAACCTACTGACCGCTTCCTTA
AGTCCACTTTTAACACCTTTAGGTAAATCGATTTGAGTTTGGTCACCAGTAACTACCATTT
TTGAGCCAAAACCTAGTCTTGTTAAAAACATTTTCATTTGCGCATGTGTCGTATTCTGCGC
CTCATCAAGAATTACAAATGCATCTTCTAATGTTCGTCCGCGCATATATGCAAGTGGCGCT
ATTTCGATAATGCCTCTTTCAATAAATCGCTCTGTTTGTTCACGCCCAAGAACAGTATATA
GACCATCATATAAAGGTCTTAAATATGGATCTACCTTTTCTTTCAAATCTCCTGGTAAAAA
TCCAAGTGACTCTCCTGCTTCAACAGCAGGTCTTGTTAATACAATACGTTTAACAGCACCT
TTACGGAGTTGCTTTGCTGCATAAACTACAGCTAAGAATGTCTTACCTGTACCAGCAGGA
CCTATACCAAATACTAAATCATTATTTTTCATGGCATTAACATATATACGTTGCCCCATCG
TTTTCGCACGAATCGTCTTACCAAATGCATCTTTAGTTATCTCTTCATCATATAAATCTAA
CAGATGTTGAATTGTGTTATTATGCGCCATTTTAATAGCTGCTTCAACATCTTTAATTGTA
ATATTATTACCTAAATCAATAACCTTCAGCAAATTGATTAATACTGATTCCGCTTTTTCTA
CGTTTTCTATTTTTGTACCTTTAACGGCAACTTCTTGTCCTCTTGCATGGATGACAACATCG
AAACTCTCTTCAATTGCTTTTAAATGTTCATCATTATTTCCAATTAAAGCTTGAGATTGGTT
CAT

> **rpsU**
Function: protein coding sequence; 30S ribosomal protein S21 (core genome, constant)
Best match: rpsU_CC001_MW2-USA400_BA000033.2[1654314:1654490:r]RC (completely identical)
Position: 036-contig_189: 213160 … 213337; Length: 177 bp
Sequence:
TTATTTGAATTTACGTTTACGTGCAGCTTCTGATTTCTTTTTACGTTTTACGCTTGGTTTTTC
GTAAAATTCACGTTTACGTACTTCTTGGATTGTTCCACTTTTAGAAACTGAACGTTTAAAT
CTACGTAACGCATCTTCAAGTGATTCATTTTTACGTACTACTGTTTTAGACAT

> **prmA**
Function: protein coding sequence; ribosomal protein L11 methyltransferase (core genome, constant)
Best match: prmA_CC005_Mu50-VRSA_BA000017.4[1687709:1688647:r]RC (completely identical)
Position: 036-contig_189: 215736 … 216675; Length: 939 bp
Sequence:
TTATTCACTCACTTTCTGGCCAACAAGACAAACCCAACCATTGTCATGTTGTTCTGAAATA
ATTTTAAAACCTACACGCTCCATATGTGACTGTATACCTTCATACTTCTCTTTTATAATACC
AGAAGTAATAAAATAACCGCCTTCATTTAGAGTATTATAAGCATCTTCAATCATTTCATCA
ATAATATGCGCTAAAATATTTGCTATTACAATATCAAATTTTTCTGTTTCGTCTTTCAATA
AGTTACCTGGAACAGCTTCAATTAACGTTTCACAATGATTTCTTCTGAAGTTTTCTTTAGC
TACACTCACTGCCATTTCATCAATATCCAACGCTTTAATACGTTTTACACCGATTAGATGA
CTTGCAATACTTAATATACCTGAGCCAGTACCAACATCAATTACTGAATGCTGTGGCAAT
ACATATGTTTCTATTGCCTTCAAACACATACTTGTAGTCGGATGATCGCCTGTTCCAAAAG
CCATACCTGGGTCGAGCTCAATGCAAAGCTCTTCATCCGCTTCTTTAGCATATGTTTCCCA
ACTAGGAACTATTGTGAACTTCTTCGACGCTCGGAATGGATGGAAATAGTTTTTCCATTCA
TTTTCCCAATCCGTCTCTGCAATAATTTGCTCACTGAATTGAATGTTATGTTGATCAAGTT
CATCTAAATTTAATAACTCATCTTTAATTTGCTGTCGCAACTTATCATCATAAGTCATTTC
ATTAAAATAGGCTTTCAATCTTACTCCCTTATCTGGATAATCCTCTTTTTTCAAAGCGTAA
ATTTCACCGTATTTATCTTCTGGTTGGTTAATTAAATCATCTGAATCTTCTATCACGACACC
ATTTGATCCATGATTTTCAAGTATATTGGTAGCCAATTCTACTGCTTCATGATTAATAATA
ATTGAAAGCTCTGTCCAGTTCAT

> **dnaJ**
Function: protein coding sequence; chaperone (core genome, variable)
Best match: dnaJ_CC001_MW2-USA400_BA000033.2[1657832:1658971:r]RC (completely identical)
Position: 036-contig_189: 216678 … 217818; Length: 1140 bp
Sequence:

TTATTCTCCCTTAAAGAATCTTTTTGCTCTATCTTTAAAATTCGAAGGTTGTTCATTAATTT
CTTCACCATTTAATTGGGCAAATTCTTTCATTAGTTCTTTTTGTCTATCTGTTAATTTAGTA
GGCGTTACTACTTTAATATCAACATATAAATCTCCGTATCCATAGCCATGAACATTTTTTA
TACCCTTTTCTTTTAAGCGGAATTGCTTACCTGTTTGTGTACCAGCAGGGATTGTTAACAT
AACTTCATTATTTAATGTTGGTATTTTTATTTCATCGCCTAAAGCTGCTTGTGGGAAGCTA
ACATTTAATTTGTAATAAATATCATCACCATCACGTTTAAATGTTTCAGATGGTTTAACTC
TAAATACTACGTATAAATCACCAGCAGGTCCTCCATTCACGCCTGGAGAGCCTTCACCAG
CTAATCTAATTTGTTGTTCATTGTCGACACCTTCAGGTACTTTCACTTCTAATTTAACTGTT
TTATTTTCAGTACCTTTTCCGTGACATGTTGGACAAGCTTCTTCAAATTCTTGACCACTTCC
ATTACATTTAGGACAAACTTGTTCAGTACGAACTCTACCTAAAATTGTGTTTTGTTCTACA
GCTACATGACCAGCGCCATTACAGTAACTACAAGTCTTTTTACTTGTTCCAGGCTTTGCAC
CATCACCATGACATGTTTCGCATGTTACATCTTTACGGATTGAAATTTCTTTTGTTGTACC
AAATACCGCTTCTTCAAATGTTAATGTCATTGTATACTGAAGATCATCACCTTTTTGCGGT
GCATTTGGATCTCTTTGTCTGCCGCCACCGAAGAAAGAGCTAAAGATATCTTCAAAACCG
CCGCCACCGAAGCCACTAAAACCGCCAAAGTCAGAGCCATTGAATCCTTGTCCACCAAAA
CCTTGTGGACCATCATGTCCAAATTGATCATAGCTTGCGCGTTTATTATCATCACTTAAAA
CTTCATAGGCTTCAGAAATTTCTTTAAACTTTTCATCTGCACCTTCTTCTTTGTTAATATCT
GGATGATATTTTTTCGAAAGCTTTCGATACGCTTTTTTGATTTCATCTTTTGAAGCATCTTT
ACTAATGCCTAAAACTTCATAATAGTCTCTTTTGGCCAC

> **dnaK**

Function: protein coding sequence; chaperone (core genome, constant)
Best match: dnaK_CC001-ST772_118_AJGE01000022.1[17825:19657]RC (completely identical)
Position: 036-contig_189: 217953 … 219786; Length: 1833 bp
Sequence:
TTATTTTTTGTCGTCGTCTTTTACTTCTTTAAATTCAGCATCTTCTACAGTACTATCGTTGTT
TTGACCAGCATTTGCACCTTGTGCTTGTTGTTGCTGTTGAGCCGCTTGCTCATATACTTTTG
CTGATAATTCTTGAATCACTTTTTCAAGTTCTTCTTTTTTAGATTTAATATCTTCTATATCTT
GACCTTCTAAAGCAGTTTTAAGAGCGTCTTTTTTCTCTTCAGCAGATTTTTTATCTTCTTCA
CCAATATTTTCGCCTAAATCAGTTAAAGTTTTTTCAACTTGGAATACTAGACTGTCAGCTT
CGTTTCTTAAGTCTACTTCTTCACGACGTTTTTTATCTGCTTCAGCGTTAACTTCAGCATCT
TTTACCATACGGTCGATTTCTTCGTCTGATAATGAAGAACTTGATTGAATTGTAATTCTTT
GTTCTTTATTTGTACCTAAGTCTTTTGCAGTTACATTTACAATACCGTTTTTATCGATATCA
AACGTTACTTCAATTTGAGGTTTACCACGTTCAGCTGGTGGAATATCAGTCAATTGGAATC
TACCAAGTGTTTTATTATCCGCAGCCATTGGACGTTCACCTTGTAATACGTGTACATCTAC
TGATGGTTGATTATCTACTGCTGTTGAATAGATTTGAGATTTAGATGTAGGAATCGTAGTG
TTACGTTCAATTAACGTATTCATACGTCCACCTAAAATTTCAATACCTAAAGATAGTGGTG
TTACGTCTAATAATACTACGTCTTTAACGTCACCTGTGATAACGCCACCTTGGATTGCAGC
TCCCATTGCCACTACTTCGTCCGGGTTTACACCTTTGTTAGGTTCTTTACCGATTTCTTTTT
TGACAGCTTCTTGTACTGCTGGAATACGAGTTGATCCACCAACTAAGATAACTTCATCGA
TATCTGAGTTTGTTAAGCCAGCGTCTTTCATTGCTTGGCGTGTAGGTTCCATTGTTCTTCTA
ATTAATGAATCTGATAATTCTTCAAATTTAGAACGAGTTAAGTTTACTTCTAAGTGTAATG
GACCGTTTTCACCAGCTGAGATAAATGGTAATGAGATTTGAGTTTGTGATACACCTGATA
AGTCTTTTTTAGCTTTTTCAGCAGCATCTTTCAAACGTTGTAATGCCATTTTATCTTGAGAT
AAGTCTACGCCATTTTCTTTTTTGAATTCTGCAACTAGGTAGTCAATAATTACTTGGTCAA
AATCATCACCGCCAAGTTTGTTGTCACCGGCTGTTGATAGTACTTCGAATACACCGTCACC
TAATTCTAGGATAGATACGTCAAATGTACCGCCACCTAAGTCAAAAACAAGAACTTTTTC
ATCTTTATCAGTTTTATCTAAACCATATGCTAATGCTGCAGCTGTTGGTTCATTAATGATA
CGCTCAACTTCTAAACCAGCAATTTTACCAGCATCTTTAGTTGCTTGACGTTCAGCATCGT
TAAAGTATGCAGGTACTGTAATTACAGCTTTGTCAACTTTCTCACCTAAATAGCTTTCAGC
TGTATTTTTTAAGTTTTGTAAAATCATAGCTGAGATTTCTTGTGGTGTGTATGATTTACCTT
CAATATCTACTTTATAATCAGTACCCATATGACGTTTAATAGATTGAACAGTGTTTGGGTT
TGTAATAGCTTGACGTTTAGCTACTTCACCAACTTGAGTTTCTCCATTTTTGAAAGCTACA
ACAGATGGTGTTGTACGTGAACCTTCAGGGTTTTGAATTACTTTTGGCTCATCGCCTTCTA
ATACTGTTACACATGAATTTGTTGTACCTAAGTCTATACCAATAATTTTACTCAT

### > grpE

Function: protein coding sequence; DnaK cofactor (core genome, constant)
Best match: grpE_CC001_MW2-USA400_BA000033.2[1661008:1661634:r]RC
Position: 036-contig_189: 219854 … 220481; Length: 627 bp
Sequence:
TTATTGGTTTACTTTGACCATTGATGGTCTTAATACTCTATCTTTAAGCTTGTATCCTTTTT
GTAGTTCTTGAGTGATTTCGCCAGATTCAAAATCAGGGTTATCATCTTGAACTACAGCTTG
GTGAATATTTGGATCAAATGCTTCACCTTCAGTTTTAATAACTTCAAGACCATTATCTTTT
AGTGCGTTAATCAAACTTTCATGCATCATTTGTACACCTTTTTGAAGAGATTTAAAAGTCT
CATCATCACCTTCAATTTGAAGTGCACGTTCTATATTGTCTATTGCTGGTAAAATATCTGT
TAACACACGTTGTGCTTGATATGTTTTATTTATTTCATTTTCTTTTTGAATTCTACGCTTAT
AATTTTCAAACTCAGCGTAGAGCCTTAAATATTTCTCTTCGTTTTCATCTGCTAATTGTTGA
AGTTCATTAATTTTTTGATCTTTTGGATCTATTTCTTCAATAACATTCTCGTCAGACGTTTC
TTCTATTGCTTCATCTTGTAAATGACCTTTACTTTCTTCAGCTTGTTCAACTGAATCATCAA
TATTTTGTTTGACGTTTGTTTCTTCAACTGTTGATTCAGTGTTTTTTTCAACTGATTCGTCTT
TATTTGTCAT

### > hrcA

Function: protein coding sequence; heat-inducible transcription repressor (core genome, constant)
Best match: hrcA_CC001_MW2-USA400_BA000033.2[1661666:1662643:r]RC (completely identical)
Position: 036-contig_189: 220512 … 221490; Length: 978 bp
Sequence:
TTACCAAATTCTATTTAATAATTGAATGACATTTTGATAATGCATAGCTGTAGGTCCAATC
ACAGCGATTTGACCTTTTAACGTTTCATCAAAATGATATTGACTTGTTACAATTGAAATAT
CACTTAAGCTGTCATCAATTTCATTACCAATTTTTACATTAATATTTGGTGAAGATATATC
TTGTAATAATTCTGCAATTCTATTTGATTCTATATATTGTAGAATGGGCTGAATTGAAGAT
ACATTACTTTCATTCAATGCATCAATAAGTTTAACCTTTCCACCCATATAAATGCTATTAC
TTTGATTAGAAATATGATTATTCATCGTATTTAACAATTTATTGATAAATATTTCTTCCTGC
TCTGATTGAACAAAAGAGACAATATCATCTTGTAAATTCTGATTAAACTCAGTTAGTTTGT
TTGTAACAAAATTTGATATTGTATTTAGTTTGTCATTATTAAACGGTATGTCTGAAGCAAG
ATGTACATGCTCAACATGACCTGATGAAAATACGATAACCATTATAACTAAATTAGGATT
AGCACGAATCAAGTGTACATTATTGATAATATCTTGTTTATGATTAGGATGAACAACTAA
AGTTGTATATTGAGATATATTTGATAATTCATCTGCAAAATATGTCAATGCTGATGATACA
TCATATTGATTCTCAACTAACAATTGATTTAATCGTCTTAATTTATTTGTTTTTTGATGAGA
TGTTTGTTCAAGTAAACGATTGACATAATACCTAAAACCTAATTGTGATGGCGAACGCCC
TGAAGAACTATGTGTCTTTTCGATATAGTTTAAATCTTCAAGCTGTTTCATCTCATTTCTAA
TTGTAGCAGGACTAACATTCAAGTTATGTCGCTCAATTAGTGTTTTAGAACCAACGGGTT
GTCCAAAATCAACATAATCCTCAACAATTGCGTTTAATATACTCAATTGCCTATCTGTAAT
CAT

### > hemN

Function: protein coding sequence; oxygen-independent coproporphyrinogen III oxidase (core genome, variable)
Best match: hemN_CC001_MW2-USA400_BA000033.2[1662744:1663901:r]RC (completely identical)
Position: 036-contig_189: 221590 … 222748; Length: 1158 bp
Sequence:
TTAATCATTTATTAGGAAAGCTTCAAAAACCTCATTACCTATGACTTTCCCTCGCTTTGTT
AGTGCAATCGCATCGTTCTTTTCTACAATTAATTCCTTCTCTTTTAAATTATTTATTGTTTG
ACCAAAGACACTTTCAATAGATTGGTCAAACTTCTTTTTGAACCTACTACTACTCACACCT
TCATTTAAACGCAAACCAAGAAACATTTCTTCTTCCATTCTCTCAGTCAAAGAAGGTTTAT
TTGATACTAAAATTGCTTTACTTTCTTTATTTATAGCTTTGATATAATGATTCACTGGATTG
ATATTCGTATAACGCACACCATCTACATAACCACTTGCACCTGCTCCAAATCCATAATATT
CCTCATTAAACCAGTAAACCTTATTATGTTCTGATTCATGGCCATCTAATGCAAAATTAGA

TATTTCGTATTGATGGAAAGGAGATTGTTCTATCTTAGACATCAGCAACTGATACATGTCA
GCACCTAAATCCTCATTAGGAAGTTTAAGCAACCCTTTTCTATACATATTATAAAATTGGG
TTTTAGGTTCAAGTATTAAGCCGTAACTCGAAATATGTTGAATATCCATATCTAAAGCTAG
ATCTAAACTTTGTTCAAAATCTTCAATCGTCTGTTTCGGTAAATGATACATTAAATCTAAA
CTGATTGATTTAATACCTGCGTTTTTAGCATTTAACACCGAAGTGTAAATATCTTCAGTAT
TGTGCGTTCTACCTAAAACAGACAATAACTCCGGCTTGAATGTTTGAACGCCCATTGAAA
TTCTGTTTACTCCATATTTTTCTAATAGTTGGACTTTCTCTTTAGTTAACTCATCAGGATTT
GCTTCAAATGTATACTCGCCTGTGATTGTAAACGTATCACGTATTGCTTTAAGTAATCTTT
CCAACTGATTAATAGAAAGGGCCGTTGGTGTGCCGCCACCTACATACATGGTCTTTAAGT
TCCTATATTTTGCTGTAGACATTTCTGTTATTAGTGCATCTAAGTACTCATCTACAGGTTG
ATTCTGTATAAAATATTTATTGAAATCACAATATGTACATATTCTTACACAAAATGGAATA
TGTATATATGCACTTTGTACCGTCATTTTAATGCCCCGCTTCTCAGACTGTTTATCCAA

## > **STAR**

Function: Staphylococcus aureus repeat element
Best match: STAR_CC001_MSSA476_BX571857.1[1643721:1643947] (completely identical)
Position: 036-contig_189: 222857 … 223084; Length: 227 bp
Sequence:
GGGAGTGAGATAGAAATAATAAAGAACCACTAATGATTTATTATGTAGTGGCTCTTACAC
ATTAGCCAGATGTAATATGTCCATAAAAATAAAATTACATGAGTAAAACTCATGCATAAG
GGAGTGGGACAGAAATGATATTTTCGCAAAATTTATTTCGTTGTCCCACCCCAACTTGCAT
TGCCTGTAGAATTTCTTTTCGAAATTCTCTATGTTGGGGCCCCACC

## > **repeat_nySAgamma**

Function: repeat element
Best match: repeat_nySAgamma_CC001_MW2-USA400_BA000033.2[1841748:1841853:r]
Position: 036-contig_189: 223047 … 223077; Length: 30 bp
Sequence:
AATTTCTTTTCGAAATTCTCTATGTTGGGG

## > **Q2FZB6**

Function: protein coding sequence; putative protein (genomic island)
Best match: Q2FZB6_CC008_COL_CP000046.1[1182242:1182414]RC
Position: 036-contig_189: 223084 … 223114; Length: 30 bp
Sequence:
CCAACTTGCACATTATTGTAAGCTGACTTT

## > **lepA**

Function: protein coding sequence; elongation factor 4 (core genome, variable)
Best match: lepA_CC001_MSSA476_BX571857.1[1644144:1645967:r]RC (completely identical)
Position: 036-contig_189: 223337 … 225161; Length: 1824 bp
Sequence:
TTATTCGTCATCCATTTTCAATACAGCCAAGAAAGCATCCTGTGGAATTTCAACATTACCA
ACTGCTTTCATCTTAGCTTTACCTGCTTTTTGTTTTTCAAGTAATTTACGTTTACGGCTTAT
GTCACCGCCATAACATTTAGCTAAAACGTTTTTACCCATTGATTTAATATTTGTACGCGCT
ACAATTTTTTGTCCTATTGCAGCCTGTACAGGTACTTCAAATTGCTGTCTTGGAATTAACG
TTTTAAGTTTTTCAACTAATGCTTTACCACGTTCATATGCAAAATCTCTATGAACTATGAA
GCTTAGCGCATCCACTTTATCACCGTTTAATAAAATATCCATCTTAACTAAATTACTTTCT
TTATTTTCGATGAATTCATAATCAAATGATGCATATCCTTTAGTATTAGATTTAAGTTGAT
CGAAGAAATCAAATACAACTTCAGCTAAAGGTAATTCATAAACAATATTTACACGAATAT
CATCTAAATAGTCCATATTTATAAATTGTCCACGTTTACGTTGACATAATTCCATTACTGC
ACCTACATAGTCATTTGGAACCATCATAGTTGCACGAACATATGGCTCAAATATTTTATCA
ATTTTATCACGATCTGGCATTTGTGCTGGGTTATCAACCGTCACTTCTGAACCGTCCCTTA
AAATACATTGATAAATTACAGATGGTGCTGTTGCAATTAATTCAATGCCAAATTCTCTTTC
AATTCTTTCTTGAATTATTTCCATGTGTAACATACCTAAGAAACCAGTTCTATAACCAAAA

CCTAATGCTTGTGACGATTCAGGCTCAAATTCTAATGATGCATCATTCAATTGTAATTTTT
CTAATGCTTCTCTTAAATCATTATAATTTTTGTTATCTATTGGGAACAGTCCGCAATATAC
CATTGGATTCATTTTCTTATAACCTTGCAATGGTTCTGATGCAGGTCTACTAGCTAATGTG
ATGGTGTCACCAACCCTAGAATCATCAACATTTTTAATACTTGCAATAATATAACCAACA
TCACCAACTGTTAATTCATCAACTGGAAGCTGCTTAGGTGTATTAATTCCAACTTCTGTTA
CTTCGAACTCTTTACCAGTCGCCATCATTCGAATTTTATCTCCGGCTTTAACAACACCGTC
TACAATTCTTATCGATGAAATTACCCCTCTATATGGATCATACTCAGAATCAAATATTAAC
GCTTTTAGTGGTGCTTCTGGGTCGCCATCTGGAGCTGGCACAACTTCAACTATTTTCTCTA
GTATCTCTTCAATTCCAATGTTAGATTTAGCACTTGCTAAAACAACATCGTCTTGGTCTAA
ACCTATCATATCTTCAATTTCTTGTTTCACGCGTTCAGGTTCTGCAGCAGGTAAATCAATT
TTGTTAATAACAGGCAATAACTCTAACTCATTATCTAATGCTAAATAAACATTTGCTAATG
TTTGTGCTTCGATACCTTGAGCCGCATCTACTACTAAAATCGCGCCCTCACAAGCTGCCAA
AGAACGTGACACTTCATATGTAAAATCGACGTGTCCAGGCGTATCGATTAAATGGAATGT
ATAAGTATTTCCATCTTTAGCTTCGTACTTTAAACGAACTGCGTTTAATTTGATTGTAATA
CCACGTTCTCTTTCTAAATCCATTGAATCTAGTAACTGATCTTGCATATCTCTTGTTTCAAC
TGATTTGGTATTTTCTAAAATTCTATCAGCCAATGTAGATTTTCCGTGGTCAATATGTGCT
ATAATCGAGAAATTCCTTATATTCTCTCTTCTTTTTAAGCGTTGCTCATTATCCAT

## > **rpsT**

Function: protein coding sequence; 30S ribosomal protein S20 (core genome, constant)
Best match: rpsT_CC001_MW2-USA400_BA000033.2[1666661:1666912] (completely identical)
Position: 036-contig_189: 225522 … 225774; Length: 252 bp
Sequence:
ATGGCAAATATCAAATCTGCAATTAAACGTGTAAAAACAACTGAAAAAGCTGAAGCACG
CAACATTTCACAAAAGAGTGCAATGCGTACAGCAGTTAAAAACGCTAAAACAGCTGTTTC
AAATAACGCTGATAATAAAAATGAATTAGTAAGCTTAGCAGTTAAGTTAGTAGACAAAG
CTGCTCAAAGTAATTTAATACATTCAAACAAAGCTGACCGTATTAAATCACAATTAATGA
CTGCAAATAAATAA

## > **holA**

Function: protein coding sequence; DNA polymerase III/delta subunit (core genome, constant)
Best match: holA_CC001-ST772_118_AJGE01000022.1[10818:11792]RC (completely identical)
Position: 036-contig_189: 225818 … 226793; Length: 975 bp
Sequence:
TTATAAAGATAGAATGAATAATTCCAGTATTAACTGTTTATCCATATATGATGATTTAAGT
TTATAATCAGTTTCCGCACAAGCATCTATAATATTCAATAATTCATCAAGTTGATAATGTC
TTACTTGTCCTAACGCTAATTTTACTCTGTATGGATGTACGCCTATTGTTTTAGCAATTTGC
TGTCCACTATATCCTTTTTGACTCAAAATCTTACATTGATAAAATAATCGGTAATTACTTG
TGATTAGTGCAAGTAATTTAATAGGTTCTTCTTTCATAGTTATTAAATCTTTTACTAAATG
AATTGCTTGTTCTTTCTTTCTTTTCTGAATGTATTCAGTCAGTAAAAATACATTTTGCTCTA
AACTTCTATTAATAATTTGGTTAACATCCTGCTTATTAATTGTTAGTCTATCGCCTAAAAA
TAAAATCAACTTTTCTATCTCTTGTGAGACAATATTAAAGTTAATACCTGTCAACTCAATA
AATAAATCTAATGCATCTCTTTTGATATCTTTGAAATTCTCATTTAATTTACTTTGAATCCA
TTTTTTTATTTCTTCTTCAGACATTTGCTCTATTTTTTTAAGCCTTGCATGCTTTTTTAGAGT
TTTAGTTAACTTTTTTCTTTCATCAAGTTTATTTTGATATATCTCAAAGACAATCAAATTTT
CGCCATCATATTTTTCAATAAATTCTATTAATTGATTTACATTATGAGCCATATCTTTTGGC
GCTTTTTCACCTGTAAATATATATGCATTTTTAACCAAAATTGCTTTTTTATCTGAAAAGA
AAGGCAATGTTAATGTTTCTTCAACAATTGGTGCAATCTCTGTTTCGTATAAATTATATTT
CACAAAGTTAAAGTCATCTCTATCACTTTTCAAAAATTGTGATATAATTTCTGCACTTTGT
TTTTCAACCAATTCAGGCACATCTCCATAAATAGCTACAATATTGTCGCTCAT

## > **comEC**

Function: protein coding sequence; DNA internalization-related competence protein (core genome, constant)
Best match: comEC_CC001-ST772_118_AJGE01000022.1[8560:10761]RC (completely identical)

Position: 036-contig_189: 226849 … 229051; Length: 2202 bp
Sequence:
CTATAAACCACTTGCATTTCCATAAGAGTTTGAATCAACTTTTAAATTATCATCTAAGTCA
ATTGTAACTTGACCGTTTTGTTGACTATTGTAAATGCGACTGCGAATCCTTTGCAATCGTT
TAACAACTTCTATATTAGGAAGATGATACATATTGTTCTTCCCAGAAGAAATCAAACTTA
TTTTAGGCTTAATCATCTCTATAAATTCTTTAGAACTACTTGTCTTGCTCCCATGATGACCT
ACTTTTAAAATATCAATCTCCGGCAAGTTATATTTTTTTAGTAGTAAAGATTCATTATTTTT
ACTAGCATCGCCCATTAATAAAACTTTTTTATTTTGATATGTAATCATAGTAATAATCGAA
TACTCATTTTTATCTCGGCTATTTGGAATAAAACTATCAAAAAATAGAAAACTACTATCTC
CAAGTTTAAAACTACTAACTTGTCTTACATCTATAAGTTTAATGTTGTACTTATGGCTTAA
TTTCGATAATAACATCAATGTATTACTACTATATCCCTTATTGTATATCACTATATGTTTAA
TTTTAATATGACTAATAATATATTCCACTTCACCAATATGGTCATTGTGTGGATGTGTTAA
AATTAGATACTCTAATTCATTTATCCCTCTTTCATTTAGCGTTGGTAAAATATGATATTTA
GAAATTGAATAACTAGGTTGTTTAGTATCATCAATCACTTTCCCACCTGTATCAATCAAGA
CATTTTGGTTCTTACCGCCTTCATATAAAATACTGTCTCCCTGCCCCACATTTAACATTGTA
ATTTTGTGATGTGAATTTGTTGGAAACGTTATTAATAATGTCAGAATAATTATAGTCCAAA
ACGTAACCAATATATATTTACGTTTAGCCAATAACCAAAATATGTAATAAACAGAAATTA
TAAATATTATAAATATCCAATCATTAAAATTGGGAACAGAAAAATGTGATTGCTTAATTC
TTGTGAATAGGTCTAGTAACCAGTCATGAAAATTAAAACTTAGGTCAACCAAGTAATTTA
GCGGCGTTAATCCCACAATAAAATGACTTGTAATAAAGAATAAAATAGATAGCGGAAAC
AATATAATCGAATAGTACGGTACAAAAATCAAATTAGATAAAAATCCCACCCATTGAAGT
TGATGAAAGTTTGGAATGGCAACGATAAATGAAGCTAATTGTGCAATAAACGTAATTATG
AATAATGATTGTAACTTTGACAATTGCTGTAAAAAAGGAAAAAGTAGCATAATAAAAAA
TGAAATGATGAATGAAAATTGAAATCCAATATCATAAACAACTAGTGGATTTAAAATAA
ACATAATTATAAATGCAAATGCTAATAGCTGAATACCCTTTATTTTAATTTGCTTAGTAAT
AAGCAGTACAAGAGTTGTCATTATTATAGCTCTTACAGCACTAGGTGCATAATTTGTGTAT
TGAGCAAATAAAGCTAATACAATGATTGTAATTCCTTTAATGACAAATAAAGGTAAATTT
AATCGTTTTAAAGGTTTGTAAATTAAGAATACAATTGCAGCTATATGCGAGCCACTAACT
GCCAGCAAATGATATATACCTATCTCTTTAACACGTTCCTTAAATTGCTCATTAATTTCTTT
TACGTCACCAGTAATCAATGCCATAATACGATCCGGAAACTTAATACCCGAATCATAAAT
TCGATTCATTATAAACTGTTTATGTTTCTCAATTAAATTAGACCGGTTCGATTCTAGGCAG
CTTTGTACAACTATACTTTGTAATTTAAGAGTTACAAACTTATTGTCATTAACTTTGAATT
GTCCTTTAACAATACAATTACGACTTTCAATTTTCTTTAAATCAAAATTCTTTTTATTTGTT
AAAAAGAAAGGATATATTTCATTTTTTAAACTCAACCTACCTTTATATGTGTCACTACCTT
GACGTTGAATTTGGATTACTTGAGCACGCTCATTAAACTGAGAATTACGTTCAATATAATT
GATATAATTAAATATTGCTTGTTGTGAATAATGTAAATACCATGAGGAGAAAATGATTAA
AAAGAGAGAAATAGGGGCATAAACGATTTTATTTTTACGATAAGTAATATACAAAAGTA
AAATGAAAAGAAATGTAGAGAGCACTTTGCTAGAATTCCAAAGCACTCCTACAATCATTG
ATAACGCGACATACAGCAA

> **comEB**

Function: protein coding sequence; competence protein EB (core genome, constant)
Best match: comEB_CC001-ST772_118_AJGE01000022.1[8124:8555]RC (completely identical)
Position: 036-contig_189: 229055 … 229487; Length: 432 bp
Sequence:
TTAACCTTTAGTCAGATATTTAGCAACATATTCTGGTGAAAATGGAATTTTTTTAAATTCA
ATACCAGATTGTTTGAGTAATTTAGTTGCATATTCATGGTTATGATAATCTTCTGCATAGT
AGATACGCTTTATACCTGCTTGAATAATTGACTTTGTACAATTTAGGCATGGAAAATGAG
TAACATAGATTGTTGCACCTTCAGTAGATACACCTTGTTTTGCACATTGTAATAAAGCATT
CATTTCTGCATGTATCGTTCTGATACAATGTCCATCTTCAATTAAACATCCTTCATCTATAC
AATGCACCTCGCCAGCTACAGAGCCATTATACCCACCAGCAATAATACGATTATCCTTAA
CAATCGTTGCACCTACAGATAATCTTTGACAAGTTGAACGTAATGCTAGCAAATGACTTT
GTGCCAT

> **comEA**

Function: protein coding sequence; competence protein EA (core genome, variable)
Best match: comEA_CC007_TCH959-USA300_AASB02000245.1[63067:63753:r]RC (completely identical)
Position: 036-contig_189: 229608 … 230295; Length: 687 bp
Sequence:
TTATGTCGTGAAATAAGATTTCAGTTTATCAAAAGTTTTACTTCCAAAACCTTTTACTTTTT
TCAAATCGTCAATTTCTTGAAATGCACCTTGTTGGTTGCGATATTCAACAATTGCATTAGC
TTTAGCTTGCCCTACTCCAGGAACAGACATCAATTCTGATACAGATGCCGTATTTAAATTT
ACTTTAGTATTATTTGTGTTCCCATTTTTTACGTGCACACTGTTTACTTCAATTTGTGGTTC
AACATTCTTTTGTCCTTTATGAGGTATGAAAATCATTTTTTGATCTGTTAATTTTTCAGACA
AATTAATTCGACTTACATCTGCATCATCCAATAATTGTGCTTTATCAAGTAAATCAACTAC
TCTATCCTTAGATGTCATTTTATAAACATTAGGATGTTTAACAGCACCTTTTACATCGACA
TATACAGGACCTTTATTTTTGGAATTATCTCCATCTTTGACCTGGACATCTTCTAATTTGGA
CAAACTATTATTTTCACTAGTGCTTTGTTTCAGAGCAGTATCTTTATTTTCAAAATTTCTTG
AAGTATAATCATCTTGTCTCCAGAATATAAAACCAATTAATACCATAATTAAAACAACAG
CACTTATAATATATAACTTCCACTGAGTTAAAAAATCTTTATAGCGTAATAAAAATTGAT
ACAATAAAACCAC

## > nadD

Function: protein coding sequence; nicotinate-nucleotide adenylyltransferase (Deamido-NAD(+) pyrophosphorylase) (Deamido-NAD(+) diphosphorylase) (core genome, constant)
Best match: nadD_CC001_MW2-USA400_BA000033.2[1673120:1673689:r]RC (completely identical)
Position: 036-contig_189: 231981 … 232551; Length: 570 bp
Sequence:
TCAATGTTCATATAATCCTTCCCCCTTAATATAGTTTTCAACGGATTTAGGAACAAGAACT
TGGATAGATTTCCCTTCACTAACTCTTTGTCGAATCATTGTCGAACTTATATCTACCCTAG
GTATCTGAATTGCAATCATAGCATTTTCAACATTTTGACTATTTTTGTCTCGATTTACAACT
ACAAAAGTAACCATTTCTTTTAAGTATTCAATTTGATACCATTTCTCTAGTTGGTTATACT
GATCCGTCCCAATAACAAAGTACAACTCACTGTCTTTGTGTTGCTCCTTGAATGCCTTGAT
CGTGTCATAGGTATAACTCTGACCACCACGTTTAATTTCATCGTCGCAAATATCTCCAAAA
CCAAGCTCGTCGATAATCATCTGTATCATTGTTAATCTGTGCTGAACATCTATAAAATTAT
TGTGCTTTTTCAATGGAGACATAAAACTAGGTAAAAAATAAAATTCATCTGGCTGTAATT
CATGAAATACTTCGCTAGCTACTATCATATGTGCAGTATGGATAGGGTTAAACTGACCGC
CGTAAAGTACTATCTTTTTCAT

## > aroE

Function: protein coding sequence; shikimate 5-dehydrogenase (core genome, constant)
Best match: aroE_CC001_MW2-USA400_BA000033.2[1673986:1674792:r]RC (completely identical)
Position: 036-contig_189: 232847 … 233654; Length: 807 bp
Sequence:
TTATTCTCCTTTTAATTGTTGTAAAACTGCTGTTTTCATAGAATTAATATCAGCATCTTTAT
TAGTCCAAATTTTAAAGCTTTCCGCACCTTGGTAAACAAACATATCTAAGCCATTATAAAT
ATGGTTTCCCTTGCGCTCTGCTTCCTCTAAAATAGGTGTTTTATACGGTATATAAACAATA
TCACTCATTAAAGTATTGGGAGAAAGATGCTTTAAATTAATAATACTTTCGTTATTTCCAG
CCATACCCGCTGGTGTTGTATTAATAACGATATCGAATTCAGCTAAATACTTTTCAGCATC
TGCTAATGAAATTTGGTTTATATTTAAATTCCAAGATTCAAAACGAGCCATCGTTCTATTC
GCAACAGTTAATTTGGGCTTTACAAATTTTGCTAATTCATAAGCAATACCTTTACTTGCAC
CACCTGCGCCCAAAATTAAAATGTATGCATTTTCTAAATCTGGATAAACGCTGTGCAATC
CTTTAACATAACCAATACCATCTGTATTATACCCTATCCACTTGTCATCTTTTATCAAAAC
AGTGTTAACTGCACCTGCATTAATCGCTTGTTCATCAACATGATCTAAATACGGTATGATA
CGTTCTTTATGAGGAATTGTGATATTAAAGCCATCTAATTCTTTTTTCGAAATAATTTCTTT
AATTAAATGAAAATCTTCAATTGGAATATTTAAAGCTTCATAAGTATCATCTAATCCTAA

AGAATTAAAATTTGCTCTATGCATAACGGGCGACAAGGAATGTGAAATAGGATTTCCGAT
AACTGCAAATTTCAT

> **mtnN**
Function: protein coding sequence; 5'-methylthioadenosine/S-adenosylhomocysteine nucleosidase (core genome, variable)
Best match: mtnN_CC005_CF-Marseille_CABA01000038.1[1580:2267]RC
Position: 036-contig_189: 235315 … 236002; Length: 687 bp
Sequence:
TTATAATTGAGACACTAATGCTTCAACAGTTTGACTTGATGATACAGCTGCTTTTTCTAAA
AATGCTTCGAAGCTCATTTCCGCTTCTCCATTTGCTAAGTCTGAAACTGCACGAACTACAA
CAAATGGTACATTAAATTGATAACATGTTTGTGCAATTGCAGTTGCTTCCATTTCAACCGC
CATCGCATTTGGAAATGCTTTTTTAATTTTTTGGCGTTGTTCAACACTACCGATAAAGCTA
TCACCACTTACAATTAAGCCTACTTTAGCTGTTAATTGTTGTTGTTGTACAACTTGAGATA
CTTTTTCTATTAAAGGTTTACTTGATTGAAATGCTACCGGCATCTGTGGTATTTGTCCATAT
TCATAACCAAATGCTGTTGCATCTGCATCATGATATTTTACATCATCACTTATAAGAACGT
CACCTACATTTAAACTTTCATCTAAAGCTCCAGCAGAACCTGTATTAATAATGACGTCCG
GTTTAAACTTATTAATTAATAATGTCGTAGAAATTGCAGCATTAACTTTTCCAATGCCACT
TTGGGTAATCACTACTTCTCTATCTTTTAAAATGCCAGTATAAAATTTAACATGTGCAACT
GAAATTTCGCTTAATTGTGTTAATTTATTTTTTAATATTGTTACTTCTTCTTCCATGGCACC
AATTATACCAATCAT

> **entX**
Function: protein coding sequence; putative enterotoxin homolog (core genome, variable)
Best match: entX_CC001-ST772_118_AJGE01000022.1[120:824]RC (completely identical)
Position: 036-contig_189: 236786 … 237491; Length: 705 bp
Sequence:
TTATGATTTGAATAAATAGATATCTAAATGCAATTTGTCACTATTGATAGTTTTATTATCA
GCATACATTTTTAATACCTCATAACCATACTCACCATTTAAATTATAAAAATCATATTCTA
CATTTGTTCATCATCATTATGATATTTAACATACCCTTTAACAATGTCGCCACCTTGTTCG
TACAACTTATACTTCTCATTCAAATAATTTCTTAATTGAACATCTAATTCTTGAATGGTAA
CAATTTTCTTTTTCGTTTTTACTGCTGTTAATTCTATTTCTGCTTGATGTCCGTCAATCCATA
AATTACAAGGTATATTCTTATAATCATCGTATTTATTATTGTCACTTAAAGTAACACCACC
ATAACTACATTGCGTTTTATTGGTTGCTCCCCCATGACATTCATAGCTATAGCTTCCAGCA
AATATATCGATATCCTTATTTATAAATTTCTTTGAAAGTGCCTCATTTTCAAATTCAACTTT
AAAATCCTTTTTCGAAGCAACATTTACGAACATGTCATGAAATAACAAATCATGCTTCAA
CAGTCTATCGTTTGTGTTTTTATTTTCAAGCTGAGTTGGATTGATGAATGACATCTTAGCA
TTACTTAGACGCTTTGAATCAAACTTACTTTTATGATGTAAGTCTGAATATTCGATTGCAC
TCGCTGAATTTGTAAAAATTCCTTCTAACAA

> **srtA**
Function: protein coding sequence; sortase A/LPXTG specific (core genome, constant)
Best match: srtA_CC008_NCTC8325_CP000253.1[2611952:2612572:r] (completely identical)
Position: 036-contig_189: 238737 … 239358; Length: 621 bp
Sequence:
ATGAAAAAATGGACAAATCGATTAATGACAATCGCTGGTGTGGTACTTATCCTAGTGGCA
GCATATTTGTTTGCTAAACCACATATCGATAATTATCTTCACGATAAAGATAAAGATGAA
AAGATTGAACAATATGATAAAAATGTAAAAGAACAGGCGAGTAAAGATAAAAAGCAGC
AAGCTAAACCTCAAATTCCGAAAGATAAATCGAAAGTGGCAGGCTATATTGAAATTCCA
GATGCTGATATTAAAGAACCAGTATATCCAGGACCAGCAACACCTGAACAATTAAATAG
AGGTGTAAGCTTTGCAGAAGAAAATGAATCACTAGATGATCAAAATATTTCAATTGCAGG
ACACACTTTCATTGACCGTCCGAACTATCAATTTACAAATCTTAAAGCAGCCAAAAAAGG
TAGTATGGTGTACTTTAAAGTTGGTAATGAAACACGTAAGTATAAAATGACAAGTATAAG
AGATGTTAAGCCTACAGATGTAGGAGTTCTAGATGAACAAAAAGGTAAAGATAAACAAT

TAACATTAATTACTTGTGATGATTACAATGAAAAGACAGGCGTTTGGGAAAAACGTAAAA
TCTTTGTAGCTACAGAAGTCAAATAA

> **ldhD**
Function: protein coding sequence; D-lactate dehydrogenase (core genome, constant)
Best match: ldhD_CC001-ST772_118_AJGE01000055.1[24720:25712]RC (completely identical)
Position: 036-contig_189: 242259 … 243252; Length: 993 bp
Sequence:
TTAATTTAAACGTGTTTCACATGTACCAGTGTTAATGACAGATAATGCTGCGTTTAAACCA
CCTTCAACAAGGTTTTGTACTGCTTCATCAGAGAAGAAAGCAATATGTGGCGTTACTAAA
ATTCTTTCATGTTCGATTAACTCTAATAATGTTTTATCGTCAATGTCTTTATTAGTCCAGTC
ATTTGTGAAGTATGCAGCTTCATTTTCATAAGTATCAATCGCAGCACCTAACAAAGTACC
ATCGTTCACTGCAGCGATTAAATCAGGTGTATTGATGACTGCACCACGTGCTGCGTTAAC
TAAGATTGCACCTTTTTTCACATGATCAAACATTGCTTTATCGAATAAATGATAGCTTTCT
TTGTTCGCTGGAACATGTAAAGAAATAATATCGGCATCTTTAATAGCTTCTTTAACACTAT
CTTTATAAGTTAAAAAGTCTAAATCTTTATTAGGATAAGCGTCATAAGCTGTAATTGTAGC
ACCAAATCCTGCATATATTTTAGCTGTAGCAGCACCGATACGACCCGTACCGATAATTGC
AACAGTCATATTTTTAACTGGTTTAGACATGATTTCTGCTTGCCAAGTAAAATCATGTGCT
TGTACACGGCGTTCAATATCTGGGAAGCGACGCACTAATTGTAGGGCGATAGAAACAGA
ATACTCTGCAATTGTTTCAGGTGAATAACTAGGAACGTTAGATATCACAATATTGTGTTTT
TTAGCTAAATCTAAATCATACATATCAAATCCAGCAGTACGTTGTGCAATTTGTTTAATAC
CGTAAGATTCTAATTTAGGATAAACGTCATTTTCTAACTTACCAAATTGCATTGTAGTTAC
GCCATCGTAATCTTTTAATTGATCGACTGTAGCACTTGATAATAGCTCTTTAGAAGTAGTT
ACTTCGACATTATTCTTTTTCCCCCAATTTAATGCCATCTCTTTCTCATAATCACGCGTACC
AAAGAACATAATTTTTGTCAT

> **frp**
Function: protein coding sequence; NAD(P)H nitroreductase (core genome, constant)
Best match: frp_CC001_MW2-USA400_BA000033.2[2605185:2605856:r]
Position: 036-contig_189: 243521 … 244193; Length: 672 bp
Sequence:
ATGAGCAATATGAATCAAACAATTATGGATGCATTTCATTTCAGACATGCGACTAAGCAA
TTCGATCCACAAAAGAAAGTTTCGAAAGAAGATTTTGAAACAATATTAGAGTCAGGTAG
ATTGTCTCCAAGTTCTCTTGGGTTAGAACCTTGGAAGTTTGTCGTGATTCAAGATCAAGCG
TTACGTGATGAATTAAAAGCGCACAGTTGGGGCGCAGCAAAACAATTAGATACAGCGAG
CCATTTTGTGCTAATTTTTGCGCGTAAAAATGTAACGTCAAAATCACCGTATGTACAACAT
ATGTTAAGAGATATTAAAAAATATGAGGCACAAACGATTCCAGCTGTTGAACAAAAATTC
GATGCATTCCAAGCAGATTTCCATATTTCTGATAATGATCAAGCCTTGTATGACTGGTCAA
GTAAACAAACGTATATCGCATTAGGCAATATGATGACGACAGCCGCATTGTTAGGTATTG
ATTCATGTCCGATGGAAGGTTTTAGTCTGGATACAGTGACAGACATTTTAGCAAATAAAG
GTATCTTAGATACTGAGCAATTTGGTTTATCAGTGATGGTTGCATTTGGCTACAGACAACA
AGATCCACCGAAAAATAAAACACGCCAAGCATATGAAGATGTTATTGAATGGGTTGGAC
CAAAAGAATAA

> **Q5HD32**
Function: protein coding sequence; acetyl-CoA binding protein (core genome, variable)
Best match: Q5HD32_CC005_Mu50-VRSA_BA000017.4[2665096:2665380]RC (completely identical)
Position: 036-contig_189: 245290 … 245575; Length: 285 bp
Sequence:
TTAACCAAGATATACATCTTGATATGAATCTTCTTTTTCTAACATATGTTTGGCAAATGAA
CATGAGGCAATAATTTTTAAATGATTTTCTCGAGCGTGTTCAACAACTGCTTTAACTAGTT
TTTTGCCAACACCTTGACCACCAAGTTCATCAGATACGCCTGTATGATCAATGTTAATTTC
ATTATTATCCACAAAACGGTATGTGATTTCAGCTAAAGCATTATTTTCATCATCACCAATA
TAGAATTTGTTCTCGCCTTGTTTGATTTCAAGGTTACTCAT

> **Q1Y5K1**
Function: protein coding sequence; putative phospholipase/carboxylesterase (core genome, variable)
Best match: Q1Y5K1_CC001-ST772_118_AJGE01000055.1[20076:20669:r] (completely identical)
Position: 036-contig_189: 247302 … 247896; Length: 594 bp
Sequence:
ATGGAACATATTTTTAGAGAAGGACAAAATGGTGCGCCAATACTAATATTATTGCATGGT
ACAGGTGGTGATGAGTTCGATTTATTACCGTTAGGCGAAGCATTGAATGAAAATTATCAC
TTGTTAAGTATTAGAGGACAAGTTTCAGAAAATGGGATGAACCGTTATTTCAAACGTCTT
GGTGAAGGTGTTTATGATGAAGAAGATTTGGCATTTCGTGGACAAGAATTGTTAACGTTC
ATTAAAGAAGCTGCTGAACGTTATGATTTTGATATTGAAAAAGCAGTACTTGTTGGATTTT
CAAATGGATCAAATATAGCGATTAACTTAATGTTGCGTTCAGAAGCACCATTTAAAAAAG
CATTGTTATATGCACCGTTATACCCAGTTGAAGTAACGTCAACAAAGGATTTATCAGATG
TCAGTGTGTTGCTTTCTATGGGGAAACATGATCCAATTGTGCCATTAGCTGCAAGTGAAC
AAGTCATTAACTTGTTTAATACACGTGGGGCACAAGTCGAAGAAGTTTGGGTGAAGGGCC
ATGAAATTACAGAAACTGGATTAACGGCTGGTCAACAAATACTTGGGAAATAA

> **Q1Y5K2**
Function: protein coding sequence; putative membrane protein (core genome, variable)
Best match: Q1Y5K2_CC001-ST772_118_AJGE01000055.1[18892:19971]RC (completely identical)
Position: 036-contig_189: 248000 … 249080; Length: 1080 bp
Sequence:
TTAATCGATATGAGATTTGCCGGTAATACGCTTAATTAAACCTTTATAGAGTACAGGTAT
GAGTAAGATGAAACCGAACAATCCCATAATAGGGAATACTTTTCCAATTAATGAAATGA
AACCGATAAATGTACTAATATAAGTGATGACAGCCATTGTAATAATAATGATGAAGTAAC
GTCTGCTGAATGGAACGCTGAAACGTGACGCAAATGCATACATTAATCCAACAACAGTAT
TGTAGATGACAAGTATCATAATGACAGACATAATAATACCAATTGACGGAGACATTTGTG
TCGCTAATTTTAATGTAGGTAGATCTACGTGTTTAATTTTATCGAATTGAGAAATTAAACC
TAGATTAATCATCATGAGTAAAAATGTAATGATTAAACCGCCAATCAAGCCCCCGTATAA
CGTTGAGTCACGATATTTAACTTTACTACCCATCACTGATAAGAAGCTGAAGGCAGCAGC
AATTTGCAAGCTTGCATAGTTAATCGCATCAAACCACCATCCAGGTGATAATGATTTCTG
CTTATGAATCTGAGCATCATTATTAGCGGCAGTAAAATCAAGATGACTTGTTGTGAAATA
GTAGACCGCAATCATAATGACAATCGCAATTAAAAATGGGGTAACACCGCCAAGCACAG
CAATTAAACGATCGAATTTTAGATACAGTGTTGCTAAAATAAAGGCGACTAATATGAGTG
CGCTCAGCCAATACGGTAAGTTGAAACTTTGATGAATGGTTGACGCACCACCTGCAGTCA
TAATAATAGCTAAAGACAACATAAACATTGTTAAAATAATATCAAAACCTCTTGCAATAG
AGGGGTATAAGAAATAGTTAATTGAATCAGAATGATTTCTGGACTTTAGATGATGACCTG
TATGCATGACAACCATTCCACCTAAAGTAATCAATAGTCCTGTTACAATAATGCCTGAAA
TGCTATATGCGCCATGACTTGTGAAAAACTGGAAAATTTCTTGACCAGTAGCAAAGCCGG
CACCAACGACAACACCAACAAAGGCAAATGCCACAATAATGGACTCTTTTAAGATACGC
AT

> **sprA-L5**
Function: noncoding RNA small pathogenicity island RNA A/Locus 5 (core genome, variable)
Best match: sprA-L5_CC008_COL_CP000046.1[2589877:2590091]RC (completely identical)
Position: 036-contig_189: 249274 … 249489; Length: 215 bp
Sequence:
ATAGATACACCAATCCCCTCACTACTCGCAATAGTGAGGGGATTTTTTCGGTGTAGCTA
GGTCGCCTATTTATCATCGTGTTTGCGTAGCCAATGCGTAAACACAGTACCACTAAATAA
GTGCACGATACATGCATCAAATGTCGTCTTTAGTCTAAGTAACGATCATGCATTAACATTT
TCAAAATATCTATTTGAGCTTGAAGATCTTTACC

> **DR**
Function: direct repeat
Best match: DR_rsaJ_trunc_CC001_MW2-USA400_BA000033.2[2600053:2600100:r]

Position: 036-contig_189: 249275 … 249323; Length: 48 bp
Sequence:
TAGATACACCAATCCCCTCACTACTCGCAATAGTGAGGGGATTTTTTT

### > fstAT

Function:  Fst antitoxin sRNA, locus 3
Best match: fstAT_L3_CC008_COL_CP000046.1[2589989:2590065:r] (completely identical)
Position: 036-contig_189: 249300 … 249377; Length: 77 bp
Sequence:
CGCAATAGTGAGGGGATTTTTTTCGGTGTAGCTAGGTCGCCTATTTATCATCGTGTTTGCG
TAGCCAATGCGTAAAC

### > fbp

Function: protein coding sequence; fructose-bisphosphatase (core genome, variable)
Best match: fbp_CC001-ST772_118_AJGE01000055.1[16584:18548]RC (completely identical)
Position: 036-contig_189: 249423 … 251388; Length: 1965 bp
Sequence:
TTAGTCTAAGTAACGATCATGCATTAACATTTTCAAAATATCTATTTGAGCTTGAAGATCT
TTACCAATATTGGTATCACGAATCTTCTTACGTTGTAATTCTTTATCTACGACGCGCTTTAT
AGAAAGTTCATCGATACCTTCGGAAAGTATTTTTTCTTTAGCGTTAAATTGTTGGTGTGCA
ACGAGTTGCATACCGAATGAATTATACAATAGTGTATAGCCTGCAATGCCAGTTGTTGAC
TGATAAGCTTTTGAAAAGCCACCATCAATGACAAGCATCTTTCCATCAGCCTTGATAGGA
TCTTCGCCATTGATTTCTTTCACTGGTGTGTGACCATTAATAATGCGTCCTTCATCTGGATT
TAATCCGAAATCACTGAGCATTTTACGAACCATATTCACATCTTCACGAAGATGATAGTA
CGGATTCTTTTCTTCTTTATGAGAAGCTTTATCTGCAATAAAGTATCGCTCAAACGTAGTC
ATGGCACGTTTACCAAATAGTGACGAATATTTCCCAGTCCATAAATACCAAACTAAATCC
GTCGATAAGTCATCAGTATTTTCTTTTTCATCAAATGATTTACGGACATGATACTCAAACA
CATCTAATAATTCTTGGCCGCTGTAAGTATGACCATCAATTTCAAATGATTCCATCTCACC
ATTTTCATCAACTGGAATACAACCATGAATGAGTAAATTACCATTATATGGTAAGTAAAG
CGAACCTTTACGCATCAAGAAAGACATATGACGACGTAATTTTTCAGATTGTTGGAATGA
CAATAATAGTTTATTCATGACTTCTTCTTCTTCAGGTAGTAATTCTGCTGGATTATTACGAT
TGATAGTTTGGAAACATGTGTCTTTCAACGGGTATGTATTACCATAAACTGTAATTTCATT
TGTATCATAATTAACCTTTTCAAGCACAAGACGTTCTTCCATTTCGAAATTTGGACGACGT
TTAATAATTGGTATTTCTAACTTGAATTGAATCATCGCAATAGCTTGATGAATTTTAGTAA
TTTGACTTTCTTCACGTTGAGTTAAACGTTCGTGTTTGTCAGGTCTTTTTTTAGGCTTAAAA
GCAGGATTATCTGCGTCATAGTATTTTTCAGCTAAAGTAAGCAGTGGTCTTAAATTAATGC
CATAAGCGTCTTCGATAATATCTAAATTATCATAGCGTGCACAAATTCGAAGTAAGTTTG
CTAAGCATACTTTTGACCCAGCATAGGCTCCAACCCAAAGCACATCATGATTACCCCATT
GAATATCTAGGGAATGATAATTAATCAGTGTATCCATAATTTTATCTGGTTGTGGTCCACG
ATCATAAATATCACCGACAACGTGTAAATGATCGACGACTAAGCGTTGTACGGAATAAGC
GAGTCCAATAATTAAATCATCTGCCTGTTTAAGTTCAATTACTTGGTTAACAAGTGTTTCG
TAATATGATTTTTTATTTTGATATTCATTACTTTTATACAGTAGTTCCTCAATAATATAAAC
GTATTGTTTTGGCAATGCTTTACGCAGTTTTGAACGCGTATATTTTGAAGAACAATATTTA
ATCAACTCAATTAAATGTTCGATTGTTGTGATATACCAGACATTAAGTTGACCGCAACTTT
GGAAATCACTTTTAATCAATTTTAATTTGTCTTCTGGATAGTAGACAAGAGCAGTTAAATC
ATTAAGCTCCTTAGTTGAAAGTCTCTCTTTGAAAATATCATTGATTTTCGCTCGCACATTC
CCAGAACCGTTGCGTAATACGTGTTGGAAAGCTTCGTATTCACCATGTAAATCGCTGACG
AAATGTTCCGTACCTTTAGGTAATTCTAAAATTGATTCTAAATTGATAATTTCAGTTGCAA
GTTTTTCTGGAGTATCAAAATTTTGGGATAGTAAATCTAAATACTTCTTTTTTAATTCTTTT
TCAGTAATTTGAGTCAT

### > smp

Function: protein coding sequence; putative membrane spanning protein (core genome, variable)
Best match: smp_CC005_Mu50-VRSA_BA000017.4[2658142:2658912]RC (completely identical)
Position: 036-contig_189: 251760 … 252531; Length: 771 bp

Sequence:
CTAAATATTTTTCTTCTTGAAAATAAATACTACAAGTATTAAGAAAATAGAAATATAAGC
AATATTACCAATAAACAATTCATGAAGTTCTAATTTCGTCGACTTACTAAAGCCTTTTTCA
ACTGTTTGAAGCATAATATTCATCATATTAATAGGATTCCACTTTAGCCAGTCTATTTTTT
CTAAAAGTGCCGTTTGAATAACTGCTAAAATAGAACTTGCAAAATAAAAAACAATACCTA
CAGCAATGGCTACACCCGTTGAATTAGTTGCAGATGATAATAGCAACGTTAAGCTTAAAA
CTAACCAAACGCCAACAAATGTACCTAAACTAACTAATAATAATTGATTCAATAAAGATA
ATTGATTACCGCTACTTTCAAATATATTTAAATCATTAAAGAATAAAGACCCAATAACAA
TTGAAGCAATAATTGTAATAACAAAATAAATTAAAGAAATAATAAATAATGTGATGATTT
TGCTAACAATCATAGTTGTTCTTGAATATTCACGATAGAGTAAATTTTTAATCGTACCGTA
ATGAAATTCCATTGAAATGATTGTACTTGCTTGAATAATTAATAAAAATGCAAACCATGA
AAAACCATTATAAGCAGATGTGAAAGATTCCTGTGGCGTAAAAATTTCATTGTATTTTGT
AGCGATATAACCTTGAGCAACCATTAGTAGAATGAAGACAATAGGTGCGATAAAAGTTG
ATTTCTTTTTAAATAATTTGAAACATTCTTGTTTAATTAAAGTTCCCAT

> **stp**
Function: protein coding sequence; putative ABC transporter (core genome, variable)
Best match: stp_CC005_Mu50-VRSA_BA000017.4[2657445:2658140]RC (completely identical)
Position: 036-contig_189: 252532 … 253228; Length: 696 bp
Sequence:
TTATGCTTGGTCCTCCCTTTCTATGATTTCTAGTAATACATTTTCTAAATCATTATCTTTAA
ATTGTTTAAGTTCTTCTTCCGATGTTTCTGTAACAATTTTACCTTTGTTAATAATAAGGATA
GAGTTTGTGATTTTAACTAACTCACTTAAAATATGACTCGAAATTAAGAAAGTAACACCT
TCTTGCGACTTTTGGACAATCAATTCACGTACATCTCGCACAGCTTTTGGATCTAAGCCGT
TCATTGGTTCATCTAAGATAATGAATTGAGGTTCATTTAAAAATGCTATAGCAATTCCTAA
TTTTTGTTTCATACCAAGAGAATACGTTTTAGCTTTTTTATGAATGTATTCATCCATATGAA
GTTGTGAGACAATTTTATCGATATCTTGAGTGTTTTTTGATTCATTCAATAACTTCAAGTTT
TCATATCCAGACATAAAAGGATATATTCCTGGATTTTCAATCAATGCACCGATATTGCTTT
TGCTGTCTTTGCTGTTAATAACATTAAAATTTCCGCTTTGGAAACTAGAGTAACCTAATAT
AACTTTCATTAAAGTTGTTTTACCTGCACCATTCGCTCCAACGAGACCAACTATTTGTCCG
CGTTTCAGCTTAAATGATACATCTTCGAGAATCGTTTTGTTGCCTATCTTCTTTGTTAAATG
TTCTATTGTTAAAACACCCAT

> **yvmA**
Function: protein coding sequence; putative transporter (genomic island)
Best match: yvmA_CC009_21334_AGTW01000007.1[14685:15848:r]
Position: 036-contig_189: 253618 … 253648; Length: 30 bp
Sequence:
GGTGACTTTCAGCTAAAGTATGCCAAATAA

> **dedA**
Function: protein coding sequence; DedA family protein (core genome, variable)
Best match: dedA_CC001-ST772_118_AJGE01000055.1[13615:14226]RC (completely identical)
Position: 036-contig_189: 253745 … 254357; Length: 612 bp
Sequence:
TTATTTTACTCTCTTACGTTTGCCAATGTAACGTATAGCAAAGTAAATGACTGCGATAATG
ATGACAACATACATAATACGTGAATATGTATGAAGACCAGTCATCAACATACCAAAGCTA
TCGCTCAACGTGCGTCCTAGTAAAATTAAACCGAAATTCCAAATTGTTGTACCTATTAATG
AAATAACGGTAAATGTCACAACATTCATGCGGTTTACACCGGCTGGAATGGTAATCAATA
CACGTAATACAGGTATGAAACGACAGATAAATACAGCCCATACGCCATACTTTTTAAACC
AATCATTTGCTCGCTTTAAATCCTTACTTTTCAATTTAATCCACTTACCGTGTCGATCAATA
AAACGATATAGACGTTCTTCTGAAATCAAACGGCAGATATAATATAAAATTAACAGCCCT
ATAAACGATGCGATGGTTGCAATAATAAATAAGGTTAAAATTGATAAATGTGATTTAACA
GACATAAGGCCAGCAAATGTCAGAATAATTTCCGATGGTACGATAGGTAAAACATTTTCT

AATAAAATTAATATAAAAATGGCTGCATACCCAAAACGACTAATAAATTCAGTGATAATT
TGTTCCAT

> **ywaC**
Function: protein coding sequence; GTP-pyrophosphokinase (core genome, variable)
Best match: ywaC_CC005_Mu50-VRSA_BA000017.4[2650714:2651406:r] (completely identical)
Position: 036-contig_189: 259266 … 259959; Length: 693 bp
Sequence:
ATGTATGTAGATCGAAAACCATCACTATATTTAGAGGATTTGCGACATGATTTTAAAAAT
AGTTTAAGTAAATTTGAAAATGGTGATGAAGCATTTGATACGTTATTAGGTTTCGTAGAG
TTAGATCATATTTATTCGTCAGCACTAAAGGAAATAAGCACTAAACTGAGTATTTTAGAT
GACAATTTCAATCACATTTATAAACACAATCCTATACATCATATGGAGCGACGTGTGAAA
GAAATGCGTAGTTTAATAGAAAAGCTTAATCGTAAAGGATTACAGATTAGTGCAGAAACT
GCCAAAGAACACATACTGGATATTGCGGGAATTCGCGTAGTATGTAATTACTTAGATGAT
ATTTATTTGATTGAAGAGATGTTGCTTAAACAAGAAGACGTACAATTGATAAAACGTAAA
GATTATATTCAGCACCCTAAAGAAAATGGTTACCGCAGTTTACATATCGTTGTATCCATTC
CAGTCTTTTTAGCAGAACGCGTTGAGGTATTGCCTGTTGAAATTCAAATTAGAACGATAG
GTATGGATATGTGGGCAAGTTTAGAACATAAAATACGTTATAAAAACAATGCAGAGACG
GAAAAGTATCGAGATTTACTGAAAGAATGTGCGACAGAGATTACTGAAGTTGAAGATAA
ATTACAACAAATTCATTCTGAAATAACAGAGTAG

> **gntR**
Function: protein coding sequence; gluconate operon transcript repressor (core genome, constant)
Best match: gntR_CC005_Mu50-VRSA_BA000017.4[2648952:2649632:r] (completely identical)
Position: 036-contig_189: 261040 … 261721; Length: 681 bp
Sequence:
ATGATGTATGGATATCCAGAGAAATGGTTGGAAGGTATGACAACTGGAGAAGGTATCGC
GGCAGAATTACGCTTAGGTATTGTGAATGGTCACATAGCTGAAGGTACGTTACTCACTGA
AAATCAAATGGCAAAGCAATTTAATGTGAGTCGTTCGCCAATTCGAGATGCATTTAAATT
ATTGCAACAAAATCAACTCATCCAATTAGAAAGAATGGGTGCACATGTGTTGCCGTTTGG
GGAACAAGAAAAGAAAGAAATGTACGATTTGCGACTGATGCTAGAGTCATTTGCATTTTC
AAGAGTTAAAAATCAAGAGAGACTACCTATCGTAAAAGAAATGAAGAAACAACTTGAAA
TGATGAAAGTGGCAGTAAAATTTGAAGATGCAGAATCATTTACGAAGCATGACTTTGAAT
TTCATGAAACATTAATCAAAGCATCTAACCACCAATATTTAAACTCATTTTGGAGTCATTT
AAAACCAGTAATGATGGCACTCGTTTTAACATCCATGCGACAACGTATGCAACAGAACCC
GCAAGATTTTGAACGCATACATCATAATCATCAAGTGTTTATTGATGCAGTGGAACAATA
CGACAGTCAAATTTTGAAGGAAGCGTTTCATTTAAATTTTGACGATGTTGGTAAAGATAT
TGAAGGATTTTGGTTGAATTAA

> **gntK**
Function: protein coding sequence; gluconate kinase (core genome, variable)
Best match: gntK_CC005_Mu50-VRSA_BA000017.4[2647375:2648928:r] (completely identical)
Position: 036-contig_189: 261744 … 263298; Length: 1554 bp
Sequence:
ATGAAATATATGATTGGTGTCGACATTGGAACGACGAGTACCAAGTCAGTCTTATATGAT
GAAAATGGAGCTTTTATCATGAAACATCAAATCGGCTATGATTTACACACACCAAACGTT
GATGTCTCAGAAGAAAACCCAGATGAATTATTTGATGCGGTATTAATGACAATTAAATAC
ATAATGAGGGAATCGAAAGTTAATCAAGATGATATTAAATTTGTGTCATTTAGTGCGCAA
ATGCATAGCTTGATTGCGATGGATCAGCAACATCAAAGATTAACAAATAATATTACTTGG
GCAGATAACCGCGCTGCAAAATATGCAACAGTAATAAATGAAGTGCATGATGGCAATGC
GATTTATCAGCGAACAGGTACGCCTATTCATCCTATGTCGCCTTTAGTGAAAATTTTTTGG
ATGAAACATGAATGGCAAGATGTATTTCAACGTACTGCTAAGTTTGCAGATATTAAAACA
TACATTTTCTATCATTTATTTGATACATATATCATTGATTATTCAATGGCTTCCGCAACAG
GGATGTTTAATTTAGAAACATTAGATTGGGATGTTGGGGCATTAGAATTGCTTGGTATTTC
CAAGGAAATGTTCCCGGAATTAGTGCCAACAACGTACGTAATGAAAGGCATGAAAGAAC

GTTATGCAACATTAATGGGGCTTAATGAAGATACACCGTTTGTTATTGGTGCGAGTGATG
GGGTCCTTTCTAATTTGGGTGTCAATAGTGTTGGTAAAGGAGAAGTTGCTGTCACAATCG
GTACATCTGGCGCGATTCGTACTGTGATAGATAAACCACGTACTGATTACAAAGGTAGAA
TATTTTGTTATGTCTTAACAGAGGACCACTACGTCATCGGAGGTCCTGTAAATAATGGTG
GTGTCGTATTGAGATGGTTGCGCGACGAGTTGCTAGCGAGTGAAGTCGAAACTGCGAAAC
GTCTCGGTGTTGATCCTTATGATGTCTTAACTCAAATTGCAAAGCGTGTTAAACCAGGTGC
AGATGGTTTAATATTCCATCCCTATTTAGCTGGAGAACGTGCGCCGCTTTGGAACGCAAA
TGCAAGAGGTTCATTCTTCGGTTTAACTTTATCTCATAAAAAAGAACATATGATTCGCGCT
GCATTAGAAGGTGTTCTTTACAATTTGTATACCGTCTACCTTGCACTCATTGAAGTAATGA
ATGAAACGCCTAATATGATAAAAGCAACAGGTGGTTTTGCGAAAAGTGAAGTATGGCGT
CAAATGATGTCAGATATATTTGACACAGAGTTAGTGGTTCCTGAAAGTTATGAAAGTTCA
TGCTTAGGTGCCTGCGTGCTTGGACTTAAAGCTGTAGGTGACATTGAAGATTTTTCAATCG
TTTCATCGATGGTCGGTGCTACAAATAATCATACGCCGTTTGAAGAAAATGTCGCTGTTTA
CCAAGAGCTCGTATCCATTTTTATCAATTTAAGTCGTTCTTTAACAGAGAATTATGAACAA
ATTGCAGATTTTCAACGCCAACATATGGCTGAAAATAAAACACAATAA

> **gntP**

Function: protein coding sequence; gluconate permease (core genome, variable)
Best match: gntP_CC001_MW2-USA400_BA000033.2[2584171:2585529:r] (completely identical)
Position: 036-contig_189: 263414 … 264773; Length: 1359 bp
Sequence:
ATGTTTAACGAAATATGGCCGTTAATCAGTGTTGTTTTAGGTATCGTTATTTTATTAGTAT
TAATTATTGGGTTTAAATTAAATACATTTATTTCATTAATTATCACATCGATGATTACAGC
GTTAATGCTAGGTATACCATTGACTAAAATTATGGAGACGATTGAGAAAGGGATGGGCA
GTACGCTCGGCCACATTGCACTAATATTCGGTTTAGGTGCCATACTTGGGAAATTACTTGC
TGATGGTGGTGGTGCAACTCGAATTGCAGACACGTTGATTCAAAAATTTGGTCAAAAACA
TGTACAATGGGCAATGCTTGTTGCAGCATTTATCGTCGGTATTGCATTGTTCTTTGAAGTA
GGTTTAGTCTTACTGATTCCATTAGTATTTACAGTAGCAAAACGCGCAAATGTTTCAGTGT
TAAAACTAGGACTACCTATGGTAACAGCTTTATCTGTGACACATGGCTTTTTACCACCACA
TCCAGGACCGGTAGTCATCGCAAAAGAATTAAAGGCAAATGTTGGAGATGTATTACTGTA
CGGTATGATTATTGCCATTCCAGTTACACTCATTGCAGGACCTATATTTAACAAAGTTGCA
CAAAAAATGATTCCGTCTGCGTATACACGAGAAGGCGATATTTCAGCGTTAGGTGCACAA
AAAGAATTTACGGATCAAGAGATGCCAGGATTTGGTATGAGTTTATTAACTGCAACATTA
CCAGTCATATTAATGTTAGTGTCTACGATAACGCAACTTGTAACTGGGCACGACAAACCT
ACAAATCTATTTGAATCTATCATTTATATGATAGGAACAGCAGGGACAGCCATGCTAATA
GCAGTATTATTTGCAATCGTTACGATGGGATTAATGAGAAAACGTAAGATGAATCATATT
ATGGAATCAGTGACGAATGCGATTTATCCAATCGGAATGATGTTATTGATTATTGGCGGT
GGCGGTACATTTAAACAAGTATTAATTGACGGTGGTGTCGGAAATACAATTGCTAAAATG
TTTGAAGGTACAGAGATGTCTCCAATTTTACTAGCATGGATTGTTGCAGCTGTGCTACGTA
TCGCATTAGGTTCGGCTACAGTAGCTGCGATTTCAACTACAGGTATTGTCTTACCATTATT
ACAATCATCAGATGTAAATGTTGCATTAGTTGTACTTGCGATAGGTGCAGGTAGTGTGAT
TTTGTCTCATGTAAATGACGCAGGATTTTGGATGTTTAAAGAATATTTTGGTTTAACTGTA
AAAGAAACATTCTTAACATGGTCATTATTAGAAACGATTATTTCCGTTTCAGGTATCATCT
TCATTCTATTCATCAGCTTATTTGTTTAA

> **Q2G207**

Function: protein coding sequence; putative protein (core genome, variable)
Best match: Q2G207_CC001_MW2-USA400_BA000033.2[2583837:2584037:r] (completely identical)
Position: 036-contig_189: 264906 … 265107; Length: 201 bp
Sequence:
ATGAAAGGCGTTTCAAGACATGTTTTTGTGCGTATTGTACAGGCGATAATTATGAAACAC
TTAGTATATTGTTTTAAATTAGATAATGATGAATTTAATTTGAAAAATAAGTATAAAAAA
TACAAGCCTTGTGTGACAAGGGTTTATGATGACTTGAATACAATTTATAGGTATATTTCAA
ATAATAAAATTATCAATTAA

> **fnbA**
Function: protein coding sequence; fibronectin-binding protein A
Best match: fnbA-COL_CC008_D30-ST8_ABFB01000035.150:2835:r [4_50ANF10END]
Position: 036-contig_189: 265241 … 268087; Length: 2846 bp
Sequence:
GTGAAAAACAATCTTAGGTACGGCATTAGAAAACATAAATTGGGAGCAGCATCAGTATT
CTTAGGAACAATGATCGTTGTTGGGATGGGACAAGATAAAGAAGCTGCAGCATCAGAAC
AAAAGACAACTACAGTAGAAGAAAATGGGAATTCAGCTACTGATAATAAAACAAGTGAA
ACACAAACAACTGCTACTAACGTTAATCATATAGAAGAAACTCAATCATATAACGCAACA
GTAACAGAACAACCGTCAAACGCAACACAAGTAACAACTGAAGAAGCACCAAAAGCAGT
ACAAGCACCACAAACTGCACAACCAGCAAATGTAGAAACAGTTAAAGAAGAAGAGAAA
CCTCAAGTTAAGGAAACGACACAACCTCAAGACAATAGCGGAAATCAAAGACAAGTAGA
TTTAACACCTAAAAAGGTTACACAAAATCAAGGGACAGAAACACAAGTTGAAGTGGCAC
AGCCAAGAACGGCATCAGAAAGTAAGCCACGTGTGACAAGATCAGCAGATGTAGCGGAA
GCTAAGGAAGCTAGTGACGTTTCAGAAGTTAAAGGCACAGATGTTACAAGTAAAGTTAC
AGTAGAAAGTGGTTCTATTGAGGCACCTCAAGGAAATAAAGTAGAGCCACATGCTGGTC
AACGTGTCGTATTGAAATACAAATTGAAATTCGCAGATGGATTAAAAAGAGGAGATTATT
TTGATTTTACATTATCAAATAATGTAAATACTTATGGGGTTTCAACAGCTAGAAAGGTAC
CAGAGATTAAAAATGGCTCAGTTGTAATGGCTACAGGTGAGATCTTAGGGAATGGTAAC
ATAAGATATACATTTACTAACGAAATTGAACACAAGGTAGAGGTAACAGCTAATTTAGA
AATCAACTTATTTATTGACCCTAAAACTGTACAAAGCAATGGAGAACAAAAGATTACTTC
TAAATTAAATGGTGAAGAAACAGAAAAAACAATACCAGTTGTTTATAATCCAGGTGTTAG
CAATAGTTATACAAATGTAAATGGATCAATTGAAACATTTAATAAAGAATCTAATAAATT
TACACATATAGCTTATATTAAGCCAATGAATGGAAACCAGTCAAACACTGTATCAGTAAC
AGGGACGTTGACTGAAGGTAGTAATTTAGCTGGTGGACAACCTACTGTTAAAGTATATGA
ATATCTAGGGAAAAAAGATGAATTGCCACAAAGTGTTTATGCAAATACATCAGATACTAA
CAAATTCAAAGATGTAACAAAGGAAATGAATGGAAAATTGAGTGTGCAAGACAATGGTA
GTTACTCATTGAATTTAGATAAGTTGGATAAAACGTATGTCATTCATTATACAGGTGAAT
ATTTGCAAGGGTCAGATCAGGTTAATTTTAGAACTGAATTATATGGGTATCCAGAACGAG
CATATAAATCTTACTATGTTTATGGGGGATATCGTTTAACTTGGGATAATGGTTTAGTTTT
ATATAGCAATAAAGCTGACGGCAATGGTAAAAATGGACAAATTATTCAAGATAATGATTT
TGAATATAAAGAAGATACTGCAAAAGGAACTATGAGCGGGCAGTACGATGCCAAGCAAA
TTATTGAAACAGAAGAAAATCAAGACAATACACCGCTTGACATTGATTACCACACAGCTA
TAGATGGTGAGGGTGGTTATGTTGATGGGTATATTGAAACAATAGAAGAAACGGATTCAT
CAGCTATTGATATCGATTACCATACTGCTGTGGATAGTGAAGCGGGTCACGTTGGAGGAT
ACACTGAGTCCTCTGAGGAATCAAATCCAATTGACTTTGAAGAATCGACACATGAAAATT
CAAAACATCACGCTGATGTTGTTGAATATGAAGAGGATACAAATCCAGGTGGTGGCCAA
GTAACAACTGAGTCTAACTTAGTTGAATTTGACGAAGAGTCTACAAAAGGTATTGTAACT
GGCGCAGTGAGCGACCATACAACAATTGAAGATACGAAAGAATATACGACTGAAAGTAA
TCTGATTGAACTAGTAGATGAACTACCTGAAGAACATGGTCAAGTACAAGGACCAATCG
AGGAAATTACTGAAAACAATCATCATATTTCTCATTCTGGTTTAGGAACTGAAAATGGTC
ACGGTAATTATGGCGTGATTGAAGAAATCGAAGAAAATAGCCACGTTGATATTAAGAGT
GAATTAGGTTACGAAGGTGGCCAAAATAGCGGTAACCAGTCATTCGAGGAAGACACAGA
AGAAGACAAACCTAAATATGAACAAGGTGGCAATATCGTAGATATCGATTTCGACAGTG
TACCTCAAATTCATGGTCAAAATAAAGGTGACCAGTCATTCGAAGAAGATACAGAGAAA
GACAAGCCTAAATATGAACATGGCGGTAATATCATTGATATCGACTTCGACAGTGTGCCA
CAAATTCATGGATTCAATAAGCATAATGAAATTATTGAAGAAGATACAAACAAAGATAA
ACCTAATTATCAATTCGGTGGACACAATAGTGTTGACTTTGAAGAAGATACACTTCCAAA
AGTAAGCGGCCAAAATGAAGGTCAACAAACGATTGAAGAAGATACAACGCCGCCAACGC
CACCGACACCAGAAGTACCGAGTGAGCCGGAAACACCAATGCCACCGACACCAGAAGTA
CCGAGTGAGCCGGAAACACCAACGCCACCAACACCAGAGGTACCAAGTGAGCCGGAAAC
ACCAACACCACCGACTCCGGAAGTACCAAGTGAGCCGGAAACACCAACACCACCGACAC
CAGAAGTGCCGAGTGAGCCAGAAACACCAACACCGCCAACACCAGAGGTACC

> **STAR**
Function: Staphylococcus aureus repeat element
Best match: STAR_CC130_O46-CC130_AEUR01000011.1[50936:51159:r]RC
Position: 037-contig_188_RC: 40 … 70; Length: 30 bp
Sequence:
AGTGGCTCTTTATCATTTCTGTCCCACTCC

> **greA**
Function: protein coding sequence; transcription elongation factor (core genome, constant)
Best match: greA_CC001_MW2-USA400_BA000033.2[1686770:1687246:r]RC (completely identical)
Position: 037-contig_188_RC: 7798 … 8275; Length: 477 bp
Sequence:
TTATTGGATATTAACAATTTTTACGTTCATTTCGCCACCATTAGGTAGTGGAACACGAACT
TCATCATCTAAACCTTTACCAATTAACGCTTTAGCCATTGGTGATTCATTTGAAATCTTAC
CATTAAATGCATCTGATTCAGCTGAACCAACGATTTGATAACTTTCCTCTTCATCACCTGG
TAATTCTACAAACGTTACTGTTTTACCAATTTTAACAACGTTGTTATCTCCAGTATCTTCA
ATGATTAATGCATTTCTTAACATATGCTCAATTCTTTGAATATCTTGTTCGATGAATCCTTG
TTCATCTTTTGCTGCATCATACTCAGAGTTCTCTGATAAGTCACCAAATGAACGTGCAACT
TTAATTTTCTCTACAACTTCAGGACGCTTAACTGTTTTTAATTCTTCAAGTTCACGCTCTAA
TTTTTCAAAACCTTCTTGAGTCATTGGATATTGCTTTTGATTTTCCAT

> **udk**
Function: protein coding sequence; uridine and cytidine kinase (core genome, constant)
Best match: udk_CC001_MW2-USA400_BA000033.2[1687274:1687897:r]RC (completely identical)
Position: 037-contig_188_RC: 8302 … 8926; Length: 624 bp
Sequence:
CTATTGCTTGCTAACTAAAGACTGAATTTTTGTTGTCATAATATCTATTGCAACTTTATTGC
TCCCACCTTCAGGAATAATTATATCAGCATATTTCTTAGTCGGTTCAATAAATTGGTCATG
CATAGGTCTAACAACACTTAAATATTGATTGATAACAGAGTCCATTGAACGCCCACGCTC
TTTAGTATCTCGTGTTAAACGGCGTAATATTCTCAAGTCTGCATCTGTATCAACATATATT
TTAACATCCATCATATCACGTAATACCTTATTTCTAAAGCGAAAATACCTTCTACGATAA
TAACATCTTTAGGTTTAAAATCAATGGTAATGTCACTTCTTGTATGACTAGCATAATCATA
TGTCGGTACTTCTACTGCTTTACCATTTTTCAAGTCTTTAAGATTTTCAATTAATAAATCAT
TATCGAATGCAAATGGATGGTCATAATTGGTTTCTAGGCGCTCGTCGAAAGTCAAGTGCT
TTTGATCTTTATAATAGTAATCTTGAGCAAGTAAAGCGACACTATGACCTTCTAAGTTTTT
CATAATTTCGTTAGTTACAGTTGTTTTTCCTGAGCCAGATCCACCAGCTATGCCAATGATT
GTAGTAGCTTTCAT

> **alaS**
Function: protein coding sequence; alanyl-tRNA synthetase (core genome, constant)
Best match: alaS_CC005_Mu50-VRSA_BA000017.4[1722925:1725555:r]RC (completely identical)
Position: 037-contig_188_RC: 13132 … 15763; Length: 2631 bp
Sequence:
TTATAGATTTTTAATGTAATCTTTAATAAAGCTTAATGATTTTGAGATATTTTCAGGTTGT
GTACCGCCACCTTGAGCCATATCTGGACGACCGCCACCTTTACCACCAACGATTGGTGCC
ATTTGTTTGATAAGATCACCGGCTTTAACGTTATTTGTTAAAGATTTAGGGACAGTTGCAA
CCATCGATACTTTATCATCAACATTACTTGCAAGAATGATAATTGTATCTTGTAGTTTAGA
TTTAAAATCATCCATTGTCGAGCGAATTGCTTTCGCATTTGGTACATCCACTTCAGTAACC
AATACTTTATAGCCATTGATTTCTTCAACTTGATCTTCAATATTACCCATTTTAAGTGATGT
GATTTCTTTGTCACGTTGCTCTAATTGTTTTAATAATGCTTTTTCTTCATCTTGTAATTGTGT
TAACTTTTCGACTACTTGATCATCAGATTTCACTTTCATCTGTGATTTCATCGTATTAAATT
TCTCTTGAATATCTTCTAAATATAAGAAAGCTGCTTTACCTGTTAATGCTTCAATACGACG
CACACCAGCTCCTGTACCTGACTCACTTACTATTTTGAATAAGCCAATTTCAGAAGTATTG
CGGACATGAATACCACCACATAATTCAATTGAAAATGGTGCCATATTTACTACACGCACA

ACATCACCATATTTTTCACCGAATAATGCCATTGCGCCCATTTCTTTAGCTGAAGCAATAT
CCATTTCTTGAATGTTAACGTCAATACCTTTCCAAATTTCTTCATTTACTAAGCGTTCAACT
TGATCAATTTCATCATTAGTCATTGGACCAAAATGAGAGAAATCAAAACGTAAACGATCT
GCTTCTACTAGTGAACCAGCTTGGTTAACATGATCACCCAGTACTGATTTCAACGCTGCAT
GTAATAAATGTGTTGCACTATGGTTCTTTTGAATGTCACGTCGATCATTTTGGTTCACTTC
AGCAGACACTGTAGCGCCAACATTTACTTGGCCAAATTGTACTACTCCTTTATGCAAGTTT
TGACCATTTGGTGCTTTGGTTACTTCACTAACAGCAATTTCAAAATTGTCATTATAAACAA
TACCTGTATCCGCAACTTGTCCACCACTGACTGCATAAAATGGTGTTTCCGTTAACATGAA
GTATACTGTTTCACCCGCTTCAACTTGTGAAACTTCTTCACCATTATATATCAAGTGTGTT
AGTGTTGTTTGAGCTGTCGCAGTATCATAACCAACAAAAGTACTTGCAGATGTAATATTTT
TCAATACTTCACTTTGAACTTGCATTGATTGAGAATTTTGACGTGCTTGACGTGCACGATC
ACGTTGTTGTTGCATTTCTGACTCAAATGTTGTCATATCAACTTTCAATCCTGCTTGCACTG
CTATTTCTTCAGTTAATTCAATTGGGAACCCGTACGTATCATACAATTTAAATGCATCTTT
CCCATTAATTTCATTTGTTGTCGCTTTAGCTTTTTTAATTAATTCATTTAAAATCGCTAAAC
CATCTTCTAATGTTTCATGGAATCGTTCTTCTTCAGACTTTATAACACGCTTAATGAAATC
TGCTTTTTCCTTAACATTTGGATAATATGGTTCCATAATGTCTGCAACAATATCAACAAGT
TTGTACATAAATGGCTCATTGATTCCTAACGTTTGACTAAAACGAACGGCACGACGTAAC
AATCGACGTAATACATATCCTCTACCTTCATTGGCAGGTAATGCACCATCAGAAATTGCA
AATGCAATCGTACGAATGTGGTCAGCAATTACTTTAAATGCCACATCTTGTTCGTTGTTTA
CTAAATATTGTTTACCTGATACTTTTTCGATTTCATTCATTATAGGCATAAATAAATCTGTT
TCATAGTTAGTACGTACATTTTGAGAAACTGAGGCCATACGCTCAAGCCCCATGCCGGTA
TCAATATTTTTATTAGGTAATGGTGTGTAACTATGATCTTTATTATGATTGAATTCACTAA
ATACTAAGTTCCATACTTCAAGATAGCGTTCATTTTCTCCACCTGGATACATTTCTTCTGC
CGGATCGTCTTGTCCATATGCTTCTCCGCGATCATAGAAAATCTCAGTGTTCGGTCCTGAA
GGCCCTTCACCAATATCCCAGAAGTTACCTTCAATGCGAATAATACGACTTTCTTCAAGCC
CAATATCTTTATGCCAAATGTTATATGCTTCCATATCTTCCGGATGAATCGTAACGTACAA
TTTATCTGGCTCCATACCCATCCATTTATCACTCGTTAAAAATTCCCAAGCAAATTCAATC
GCTTCTTGTTTAAAATAATCACCAATTGAGAAGTTACCTAACATTTCAAAGAATGTATGGT
GACGCGCTGTGAAACCAACATTTTCAATATCATTTGTACGAATAGCTTTTTGAGAGTTTAC
AATTCTTGGCTTTTTAGGTGTTTCACGTCCATCAAAATATTTCTTTAATGTTGCTACACCTG
AATTAATCCATAATAATGTATCATCATCAATTGGCACTAATGGTGCAGAAGGTTCAACCA
TATGTCCTTTTTCAACAAAGAAATCTAGATATTTTTGTCTAATTTCACTCGCTTTTAACTTT
TTCAT

## > tbox07

Function: noncoding RNA T-box leader element (core genome, constant)
Best match: tbox07_CC001_MW2-USA400_BA000033.2[1694866:1695043:r]RC (completely identical)
Position: 037-contig_188_RC: 15894 … 16072; Length: 178 bp
Sequence:
AAAAGGGACGAACGTTATCGCGGTACCACCCTAGTTATAAATGCAATTCAACACATTTAT
CACTTTAAATCGACTATACAGTTGTGCATAAAGTAGCGTTCACTAATGTTTGTTGTACTTT
TCACCAACCAGTACATCTCTGATAAACAAATCATTAACTACTCATCTTTATACGAAT

## > recD

Function: protein coding sequence; ATP-dependent DNA helicase/RecD/TraA family (core genome, variable)
Best match: recD_CC005_Mu50-VRSA_BA000017.4[1725898:1728375:r]RC (completely identical)
Position: 037-contig_188_RC: 16105 … 18583; Length: 2478 bp
Sequence:
TCAACGTTCTATAAAGTCATACGGCGTGATTTCTCCCATATTAATCATTGGGTCAATTTTA
AACATTGTAGCTTCCGTTAATACATTTATATCTGTTTTTGTTGAATCAGACGTAACTTCTCC
ACTATCATTCGATGACATTGGCGCTTCTACTTGATCATCTATTGTCGTTTGTGAAGCTCCT
GTATCATTAGTTGCTGTGTTTTCCAGCATTTCTTCATCTTCTGAATTAAAATAATTTTTCAA
CAATGTACATAATTGTGTTAAACGCGCTTGACCATTTGTTTTTAATCCAATATCAAATGCT

TCTGGATCACCAAGTAGAACTAAACTCGTTTTCGCTCTAGTTAAACCAGTATATAATATCG
GTCTTTGTAACATTCTAAAATACTGTTTAACAATAGGCATGATAACAATAGGAAATTCTG
AACCTTGTGATTTATGGATTGATGTACAATAAGCATGTGTTAATTCCATCATATCTTGTTT
CGTAAATGTAATTTCATTACCTTCAAAATCCACAACAAGTACATCTTTATTAAGGGCATTT
TCTTTCGCCCAAAAAATACCAACAATAACTCCTATGTCACCATTGAATATGTTATCATTTG
GCCTATTAACAAGTTGTAATACTTTGTCACCTTTTCTAAAGACTACATCACCAAACTCAAT
TTCTCGTGTGTCTTTCTTTTTAGGGTTTAAAATATCTTGTAAAACTTGATTTAAACGTTTAA
TACCGGCATTTCCTTTATACATTGGTGCAAGCACTTGAATATCAGCCATAGTATACCCTTT
ATTAACAGCACTAGTAACTACCTTCTCAACAACTGTTGGTATTTGGTTTGCCTGACAGTTA
ATAAAACTTCTATCATGAAAACGCTGTGTAATATCAATTTTCTGACCCAATTTCATTCGAT
GTGCTAATTCTATAATGCTTGAACCATCTTGTTGACGATATACTTCAGTCAGATTAACTCG
TGGTATAGCTTTCGATTCAATTAAATCTTTAAATACTTGACCAGGACCTACAGAAGGCAA
TTGGTCCTCATCACCTACAAATATCAATTGTGCATCTAAAGGAACTGCACTTAAAAATTG
GTGAAACAACCAAGTATCTACCATAGACATCTCATCAATGATTATGAGTCGTGCGTTTAT
TTCATTTTCTAATATATCCTCTGGCTTTGTGTCTTGATTCCAACCTATTAAACGATGAATCG
TCATTGCTTCTAATCCAGTTGACTCTTGTAGTCTCTTAGACGCTCTTCCTGTTGGCGCTGCT
AATACAACTGGATAATCATCATTGACATAATCATCATAATCTAATGATAAGCCATGAATC
TCAGCATATAATTCTACAATACCTTTAATTACTGTCGTCTTTCCTGTTCCCGGTCCACCGGT
TAATAGCATCACCTTAGAATTGATAGCCGTTTGCAAAGCTTCTTTTTGTGAAGCTGCATAG
TTCACTTGATTCGCATCTTCTATTTCACCAATATGCATTTGTAAATCTGACTGTTCAATTTC
CGTAAGTTTATTTGTATGCGTCTTTATTCTGAATAAGTTTTGAACACTTTTGATTTCAGAAT
AATACAAACTTGGAATTGCAACTTGTTCATTGTCAATAATTAGTCGTTTTTCCTCATTTAA
GTATTGCAACATTTCGTCTAATTTTTCAGGTTCGATGACCTCTTCATCTTGATAATTTAATA
CATCAACCGTTAAATCTATAACAACATTGATAGGCAAATATGTATGTCCCTGTTTAATAC
ATTCTTCTTCTAACGTATAGAGCAACGCAGCTTTTAATCGTTCATTATCGTTATAAGCGAT
ACCAATATTTCTAGCAAGTTGATCTGCTTTATTAAAACCAATACCTTTAATATCATAAATC
AATTGATATGGATTTCGATCTAAAATAGTCAGTGTATCGCCGAGATAAAACTGATAAATT
GCCATTGAAAGTTTAGGACCAAACCCTAAATCATGTAAACGAATCATTATTTTTTCAGATT
CTTGATTTGCTGAAATTTGTTCTGCAATTTGTTTCTGTTTCTTTTTAGATAATCCCGAAACT
TTTTCTAGCACTGAATGGTCATCTAATATATCATTTATCGCATTGTCACCTAATGTATTAA
CAATATTTTGAGCTGTCTTTTTACCTACACCTTTAAACAAATCACTAGATAAATAACTTAT
AATTGCTTCTTTCGTTTGTGGCATTTCTTTTTCAAAAGTCTCTGCTTTTAATTGTTTACCAT
AACGTGGATGATCAACAACTTGCCCTTTAAATGTGTAGACATCGCCTTCAACAATATTCG
GAAGAAACCCTACAACAGTTGGCATTGTATCAAAGTCTTCATTTGTTTCAATAGTATCTAC
TTTAAGCACTGTATAAAAATTATCACTGTTTGAAACAATATCGCTTCAACAGTACCTTTG
ATCATTGAATAATCAAATAGTGTAGGGTCTGACAT

> **mnmA**

Function: protein coding sequence; tRNA-specific 2-thiouridylase (core genome, constant)
Best match: mnmA_CC005_N315_BA000018.3[1653329:1654447:r]RC (completely identical)
Position: 037-contig_188_RC: 19833 … 20952; Length: 1119 bp
Sequence:
TTATACAACATAATTTAATTGACCTTCATTTTTGAACACATCGTCAATTGTTGCACCACCA
AGACACACATCACCTTGATAAAAAACAACTGCTTGTCCAGGTGTGATTGCTCTTACTGGC
TCAGCAAAAGTAACACGTAGTGCATGGTCGTTTTCACGTTTCACAAAAACTTTCGTATCTT
TTTGGCGATATCTAAATTTAGCTGTACATTCAAAACCTTGATCTAAGTCATTATCTTCTGG
ATTTACAAATGAATAGTCTGAAGCAATTAAGTAATCACTGTATAATGCATCGTGATGGAA
TCCTTGTTCTACATATAAAACATTATCTTTTAGGTTTTTACCGACAACAAACCAAGGATCG
CCATCTCCACCTATACCTAATCCATGTCTTTGTCCTATTGTGTAATACATCAAACCACTAT
GTTTACCCATTTTCTTACCATCAAGTGTTATCATATCACCCGGTTGTGCAGGTAAATATTG
TGATAAAAATGTTTTAAAGTTTTTTTCGCCGATAAAACAAATGCCTGTAGAATCTTTTTTC
TTAGCAGTAACAAGTCCTTGTTCTTCAGCAATTCGACGCACTTCACTCTTTTCGATGTCGC
CAATTGGGAACATCACTTTTGAAAGTTGTTGTTGAGATAATTGATTCAAGAAGTATGTTTG
ATCTTTATTATTATCTACACCACGTAACATTTCAACATGACCATCTTCATGACGATGTATG
CGTGCGTAATGTCCTGTTGCTACATAATCTGCACCTAAATTCATCGCATGATCTAAAAAG
GCTTTAAACTTAATTTCTTTATTACACATAACGTCTGGATTTGGAGTACGACCTTTTTTGTA

TTCATCTAAGAAATACGTAAAGACTTTATCCCAATATTCTTTTTCAAAATTAACAGCGTAA
TATGGAATGCCAATTTGATTACACACTTCAATAACATCGTTGTAATCTTCAGTTGCAGTAC
ATACGCCATTTTCGTCAGTGTCATCCCAGTTTTTCATAAATATGCCAATGACATCATAACC
TTGTTCTTTTAAGACGTGGGCTGTTACAGAACTATCTACACCGCCTGACATACCAACGAC
AACACGTATATCTTTATTTGACAA

> **csd1**

Function: protein coding sequence; cysteine desulfurase (core genome, constant)
Best match: csd1_CC005_Mu50-VRSA_BA000017.4[1730859:1732001:r]RC (completely identical)
Position: 037-contig_188_RC: 20952 … 22095; Length: 1143 bp
Sequence:
TTATGACTCCTCCTTAAATTTAAAATATATTTTATGAATTTCAGCTACAATTGCATTAATTT
CATTTTCAGTAGTCAATTCGTTAAAACTAAATCGAATCGAATGATTTGATCGCTCCTCATC
TTCGAACATTGCATCTAAAACATGCGACGGTTGTGTAGAGCCTGCTGTACATGCAGATCC
AGACGACACATAGATTTGTGCCATATCCAACAATGTTAACATCATTTCAACTTCAACAAA
CGGAAAATATAGATTTACAATATGGCCTGTAGCATCCGTCATTGAACCATTTAATTCAAA
TGGAATCGCTCTTTCTTGTAATTTAACTAAAAATTGTTCTTTTAAATTCATTAAATGAATA
TTGTTATCGTCTCGATTCTTTTCTGCTAATTGTAATGCTTTAGCCATCCCGACAATTTGTGC
AAGATTTTCAGTACCTGCACGGCGTTTCAATTCTTGTTCACCGCCAAGTTGAGGATAATCT
AATGTGACATGGTCTTTAACTAATAATGCACCGACACCTTTTGGTCCGCCAAACTTATGTG
CAGTAATACTCATTGCGTCGATCTCAAATTCGTCAAACTTAACATCAAGATGTCCAATTGC
TTGAACCGCATCAACATGGAAATATGCATTTGTCTCAGCAATAATATCTTGAATATCATA
AATTTGTTGCACTGTGCCAACTTCATTATTTACAAACATAATAGATACTAAAATCGTCTTA
TCTGTAATTGTTTCTTCAAGTTGATCTAAATCAATAGCACCTGTATCATCAACATCTAGAT
ATGTTACATCAAAACCTTCTCGCTCTAATTGTTCAAAAACATGTAACACAGAATGATGTTC
AATCTTCGATGTGATAATGTGATTACCCAATTGTTCATTTGCTTTTACTATGCCTTTAATTG
CCGTATTATTCGATTCTGTTGCACCACTCGTAAATATAATTTCATGTGTATCTGCACCAAG
TAATTGTGCAATTTGACGTCTTGACTCATCTAAATATTTACGCGCATCTCTTCCCTTAGCA
TGTATTGATGATGGATTACCATAATGCGAATTGTAAATCATCATCATCGCATCTACTACTT
CAGGTTTTACTGGTGTGGTCGCAGCATAATCTGCATAAATTTCCAT

> **csbD-L2**

Function: protein coding sequence; stress response protein/locus 2 (core genome, constant)
Best match: csbD-L2_CC001_MW2-USA400_BA000033.2[1702814:1702996:r]RC (completely identical)
Position: 037-contig_188_RC: 23842 … 24025; Length: 183 bp
Sequence:
TTATTTTTTAAGTTTATCAATTGCATCAGTTATTTTGTTTTTAGCATTTTCAACAACTTCTTT
TGCTTTACCAGTCGCTTTATCTTGCTGACCTTCTTTTTCTAATTCTTTGTTATCAGTAACGT
TACCTACTGTTTCTTTAACATTTCCTTTAAATTGATCGAACTTACTTTCGTCTGCCAT

> **cymR**

Function: protein coding sequence; HTH-type transcript regulator (core genome, constant)
Best match: cymR_CC005_Mu50-VRSA_BA000017.4[1734032:1734454:r]RC (completely identical)
Position: 037-contig_188_RC: 24124 … 24547; Length: 423 bp
Sequence:
TTAAATATAAAACATGTATCCGTCTAAATCTTCACTTGTATCTACATATTCCGCTAAATAT
TTCAATGTTGTATTATCTAAAACATCTCTCACTGCATCTCTCATGCGAATCCATAGTTGTTT
TTGCGCAGGTGGTTCTGATTCAATACTTTCAACAAATGTAATTGGACCTTCTAACAGTCTT
ATAATATCCCCTGCTGAGATTTCTTCCGCTGGTACTCTTAATTGGTATCCACCTTTAGCAC
CGCGTACACTTCGAATTAACCCCGCATTTCTTAAAGGACCTACAAGCTGTTCTAAATATA
AATCACTCAAATTATTTTCTTCAGCAATTGACTTTAATGATATACATCCTTGCCCCTCTTTT
TTAGCAAGAGAAATCATCAATGTAAGTCCATATCTCCCTTTAGTAGAAATTTTCAT

> **ssrS**

Function: noncoding RNA 6S-RNA (core genome, constant)
Best match: ssrS_CC001_MW2-USA400_BA000033.2[1705971:1706202:r]RC (completely identical)
Position: 037-contig_188_RC: 26999 … 27231; Length: 232 bp
Sequence:
ATAAAAATACGCAAGACAAAGTCTTGCGTATCGATAGAGTCCGTATTGCCGTAGTTATAA
TAGCTTGATCATTCGGCCTGTTATATACAGGTGGGTGCCCTGTTTCTTGTTTTGTACGTCCT
TCATATAAGGCGTGTACGCTGCAAGAAAACCCATTGGGCTCCCTTGATCAAAGAGTGTTA
GGCCCAAATTAAAAAGCAAACTTACGAACAACTCAGATGACTATCTTATG

> **aspS**
Function: protein coding sequence; aspartyl-tRNA synthetase (core genome, constant)
Best match: aspS_CC005_N315_BA000018.3[1660796:1662562:r]RC (completely identical)
Position: 037-contig_188_RC: 27300 … 29067; Length: 1767 bp
Sequence:
TTAGTGACGAATTCGCAAAGAAAGTTCTTCTAATTGTTTATCAGAAACTTCACCAGGCGC
ATTCGTTAATAAACATGTAGCAGATGCTGTTTTAGGGAATGCGATTGTATCTCTCAAGTTT
GTTCTATTAGTCAATAACATGACTAATCGGTCTAATCCTAATGCAATACCGCCATGTGGTG
GTGCACCATATTTAAATGCATCTAGTAAGAAGCCGAACTGTTCTTGTGCTTGTTCTTTAGT
AAATCCAAGAACTTCGAACATTTTTTCTTGTAACTCACCATCATGAATTCTGATTGAACCG
CCACCTAATTCATAACCATTTAATACTATGTCATAAGCATTTGCCTCAGCTTCTTCTGGCG
CAGTGCCAAGCTTAGCAATATCAGCTTCTTTTGGAGATGTAAATGGATGATGTGCTGCAA
CGTAACGTTTCGCATCTTCATCATATTCTAATAATGGCCAATCTGTCACCCATAAGAAGTT
TAATTTTGTTTCATCGATTAAACCTAATTCTTTAGCTAATTTGACACGTAATGCACCTAAA
CTTTGTGCAACGACATTTGGTTTGTCTGCAACAAACATTACTAAGTCACCAGCTTCAGCAC
CAGTTAATGTAAGTAATGTTTCAACATTTTCTGTTTCAAAGAAACGTCCAATTGGACCTGT
CAAACCATCTTCCACAACTTTAACCCACGCTAATCCTTTAGCACCATAGATGTTTACAAAT
TCTGTTAAAGCATCCATATCTTTACGAGTATATTGTTCAGCTGCACCTTTAGCGACAATTG
CTTTAATTTCACCATCATTTTCAACAGTATCTTTAAATACTTTAAAGTCCATATCACGTCCT
AATTGAGAAACGTCAATTAATTCCATTTCAAAACGTGTATCTGGTTTATCAGAACCATAG
CGACGCATCGCTTCTTTATATGTCATGCGTGGGAAAGCGCCATTAATTTCAACGCCTTTAA
CTTCTTTAACAACTTTTTTAAGCATTTCTTCACCCATTTGCATCACATCTTCTTGATCTACA
AAACTCATTTCAATATCGACTTGTGTGAATTCAGGTTGACGATCTGCACGTAAATCTTCGT
CACGGAAGCATTTTACGATTTGGTAGTATTTGTCAAATCCACTAATCATCAATAATTGCTT
AAATAATTGTGGTGATTGTGGTAATGCATAAAATTCACCATCATGAACACGAGATGGTAC
TAAATAGTCACGTGCACCCTCAGGTGTTGACTTCGTTAGTACTGGTGTTTCGATGTCAAAG
AACCCTTCATCATCCAAATATTGACGAATAGAACGTGTAATTTGATGTCTCATTTTAAATG
TTTGCGCTAACTCTTGACGACGTAAATCTAAATAACGGTATTTTAATCGAATATTTTCATC
AACGTTAACATTTTCTTCATTTATAGAAAATGGTGGTGTCTCAGATTTATTAATCACTTTA
ATATTTGTAACTTGTACTTCAACTTGGCCAGTTTTAATTTTAGGATTAACTGTTTCAGGGT
CACGCTTCGTAACTGTACCTTGAACTTCTACAACATATTCAGAACGTACTGTTTCAGCAAT
TTTCAATGCCTCTTCTGAAAATGCAGGATTAAACACGACTTGTACAATTCCTTCTCTATCT
CTTAAATCAACGAAAATCAATCCACCTAGGTCACGACGATTGTTAACCCATCCTTTTAAT
GTAATTTCTTGTCCTAAAAATGCTTCAGTAACTAATCCACAATAAGTTGTTCTCTTACTCA
T

> **hisS**
Function: protein coding sequence; histidyl-tRNA synthetase (core genome, constant)
Best match: hisS_CC005_Mu50-VRSA_BA000017.4[1738990:1740252:r]RC (completely identical)
Position: 037-contig_188_RC: 29082 … 30345; Length: 1263 bp
Sequence:
CTACTTCTTAAAATATTCGACTAATGCGTCTAATTCAATTGTTTCAGATTCACCAGTTGTC
ATATTTTTAACATCGATTTTATTATTTTCTAATTCTTGATCACCAATAACGATTGTAAACTT
GGCACCTAAACGGTCTGCTTGTTTCATTTGTCCTTTAATTTTACGCTGTAAGTAGTCTTTAT
CTGCTTTAATACCATTATGTCTCAAATGATTTAATAACTTCACAGCATATCGATCTGCTTG

ATCACCCATTGTAACAATGAATAAATCTAAGTTTTCTTCAATATCTAATTCGATACCTTCT
TCTTCAAGTGCAAGCAATAATCGTTCTATACTTAGCGCAAAACCAATACCTGTTTCACTTG
GACCATCTAGCAATTCTAATAAACCATTATAACGGCCACCACCACAAAGCGTTGTAATGG
CACCATCATAGTTAGGATTATCCATCATTAATTCAAATGCTGTATGTGTATAATAATCCAA
TCCACGAACTAAGTTAGGATCTTCAATATATGGAATACCTAAATCATCTAAATAAGCTTTT
ACTTGTTCATAATATGCCTTAGATTCCTCATTTAAGAAATCAGTGATTCTAGGTGCAGTCT
TAATCGCTTCTTTATCACGGTCAACTTTACAATCCAAAATTCGCATCGGATTTGTATGCAA
ACGTGATTGACAATCTGAACAAAATTCATGAATTACTGGTTCAAAGTGTTTCACTAACGC
TTCGTTATATTCTTTTCGAGACGCCATATCCCCTACACTATTAATAACAAGCTTTAAATGT
TTTAATCCAAATGATTGATAAATATGCATAACCATAGCTAATACTTCTGCATCTACGCTAG
GATTTTCAGCACCAATAGCTTCTACACCAAATTGATTAAATTGACGATAGCGCCCTTTTTG
CTTACGTTCATATCTAAACATCGGTCCATTGTAATAAAGTTTAATTGGTTGGTTTGGATTA
CCTTGCATTTTATGTTCAATATATGAACGCACAACTGCAGCTGTTCCCTCAGGTCTTAATG
TAATACTTCTATCGCCTTTATCTTTAAATGTATACATTTCTTTTTGTACGACATCGGTTGAA
TCACCAACACCTCTTGCAAAAAGATCTGTACTTTCAAAAATTGGTGTTCTTATTTCTTTAT
AATTATAAAATGTCATTAATTCATCTAATTGATTTTCAATGTAACGCCATTTCTTTGAATC
TTCAGGTAAAATATCCTGCGTCCCTCTAGGTATTTTAATCAT

## > **lytH**

Function: protein coding sequence; N-acetylmuramoyl-L-alanine amidase (core genome, variable)
Best match: lytH_CC005_04-02981_CP001844.2[1698852:1699727:r]RC
Position: 037-contig_188_RC: 30805 … 31681; Length: 876 bp
Sequence:
CTACGCAGAAAAATAAATTTTAAGTCCATCAACAATTGCTTGTTCTAAAATTTGTCTATGT
AATTGATCTTTAATCATCGTTTCATCAGTTGGGTTACTAATATAACCTAATTCTAATAAAA
CAGCAGGAACTTTTGTTTGTCTTAACACTTGATAATTTTCTTGTCTTGAACCGCGATTAGA
AAGTAAACCTTTCTTCTGAATCGTAGCGTCTAACGTATCTGCTAAAGCTCTTTGATTATCA
TGATACCAATAAACTGTCATTCCATTTGCATTAGATGATTCTAATGCATCATTATGTATAC
TCAAATAGGCATCGCCTTTGATATCACGATTTTCTAGTGAAACATATGTATCGTCTGTTCT
TGTCATCTTAACAGTTGCGCCTTCTTTTTCTAAAGTACGCTGCAATTCTTTTGCTGTTTTCA
ACGTATAGTCTTTTTCTAAACTTTTATATTTAGTATTGCTTGAAGCACCCTGGTCACTACCT
CCATGACCAGGATCAAGCACTATTGTTTTACCTTGCAAAGGATTTTTCTCCTTCGTATTAT
CCGCGACAATATCTAAATTTGTGTGCCATCCAGCTATCCAACCTTTTTCATTACTGGATGT
ATCTTCAACTTCAATCCATTTACCTACTTTACCAATCTTTTTAAAATGGTCACCTTTCTCAA
CTTTATATATGACTGGATACGCAGCGTTTGGACCTGTACGTAATTCAGTATTTTCAGTTAT
CGTGATGTTCCCACTATCTTCACTATTGCTATTCAGCAATAAAAATAAAAAGATGATAAA
TAAGACAAAGGCAATCACTACTATTAGAGTACGTTTATTTTTAAGACCCTTTTTAGATAAC
CATGCCTCTATTTTTTTCAT

## > **dtd**

Function: protein coding sequence; D-tyrosyl-tRNA(Tyr) deacylase (core genome, constant)
Best match: dtd_CC001_MW2-USA400_BA000033.2[1710649:1711101:r]RC (completely identical)
Position: 037-contig_188_RC: 31677 … 32130; Length: 453 bp
Sequence:
TCATTGAATTTTGCCGTCCTGACTTTCATAAATAATAGTGACTGGACCATCATTATTTATG
CTAACATTCATGTGTGTTCCAAATTCACCTGTTTTCACAGTAAGACCATACGCTCGTAGCG
CATCATTAAAATACTCATAAATTTTTACCGCTTGATCAGGATTTTTAGAATTTGAGAAACC
TGGACGGTTACCTTTTTTTACATCTGCATAGAGAGTAAATTGTGAAACTGATAGTATTTCA
CCATTCATTTGTTGGATATTAAAGTTTAATTTATTATTGTCATCTTCAAATAATCTTGCATT
AGCAATTTTCTTTGCAATTACATCTGCATCTTGCTCTGTAGAGTTCTGACCGATACCGACT
AATAAACAATATCCTTTTTTGATTTGATTATTTAATGTATCATTCGTCACCGATGCTTCTTT
AACTCTTTGTACAACTACTTTCAT

## > **relA2**

Function: protein coding sequence; GTP pyrophosphokinase (core genome, variable)

Best match: relA2_CC001_MW2-USA400_BA000033.2[1711113:1713302:r]RC (completely identical)
Position: 037-contig_188_RC: 32141 … 34331; Length: 2190 bp
Sequence:
CTAGTTCCAAACTCTTGTTACTGTATAAACATCACCAAGTTGTTTGATCTTTTCTACCACA
CGATAAACATCATTCACGTTTTTCACCATGACACTAATATTTATTATTGCATTTTTATCAAT
ATCTGAACGTCCTGAAACTTTAATTAAATTGCCGGCTGTCGAGCTAACAGCTTGTAGTACT
TCATTCAACAAGCCATTTCGGTCATACGCAGTTACCTCTAAATCAACCTGATATTTTTGAG
TTGCGTCTTTTGATTTTACCCATTCAACATTAATTAGTCGTTCAGTTTCGTTCTTAATATTT
GGGCAATCAGTGCGATGTACTTTAATACCGTGACCTTTGGTGATATAACCTACAATATCA
TCACCTGGTATAGGATTACAACATTTTGACAACTTGATAAGTACATTTTCTAAACCTTCTA
CATAGACACCACTATCAGTAATGATGTTGTCTTTAATAGGCAATGATTTCGTAACTTCTTG
TGCTTCATTTAAAGCACGTTGTTTATCTAAAATACGTTGTCTTTCAGTTAATTTATTAACA
ATCTGTAAGGATGTCACGCCGCCAAATCCTACAGCTGCGAATAAATCATCTTCATTTGCA
AAGTTATATTTTTCATTAACAACCTGAATATTTTTCTCTGTCAAAATATCTTCGACTCTAA
ATCCTTGCTCTTTTATTTCAACTTCAACCATCATTCGGCCTTTTTCAATATTAGATGAACGA
TCTTGTTTTTTGAAGAAACTTTTAATTTTACCTTTGGCACTAGACGATTTAACAATTTTCAA
CCAATCACGACTTGGTCCATATGAATGTTTACTAGTACGTATTTCAACAATATCGCCTGTT
TGTAAAATATAGTCAATTGGTACAATTTTGCCATTCACCTTGGCACCAATCATCTTATTAC
CTACTTCACTGTGAATCGCATAAGCAAAATCAATCGGCACAGCACCATATGGCAACTCAA
TAACATCACTCGCTGGGGTAAATGCGTATACTTTGTCACTCTGTAAGTCATATTTTAAGGT
TTCCATAAATTCTTGAGCGTCAGACGATGTATGATCCGCTTCAGCTAATTCTTTTAACCAA
TTTAACTTATTTTGATAAGTTTGATCTTTTTCACTTACTTTTTTACCTTCTTTGTAAGCCCAG
TGTGCTGCAACACCATGCTCAGCAATTTCGTGCATATCAAACGTTCGTATTTGGATTTCGA
GCGGGTCTCCATTTGGACCTACTACTGTAGTATGCAATGACTGATACAAATTTTGTTTAGG
CATTGCAATATAATCTTTAAAACGTCCTGGCATCGGTTTCCATAACGTATGCACCAACCCA
AGTATCGCATAACAATCATTAATAGAATTGACAATAACACGTATCGCCAACAAATCAAAA
ATTTGATCAAATTGTTTTTTCTGCTTCATCATTTTCCGATAAATACTGTAAATATGTTTAGG
TCTACCATTTATATCGCCTTCGATATTCATTCGGTCCATTTCAGTACGTATTCTATCAATAG
CCGTTTCGATATACGCTTCACGTTCACTACGTTTCTTCTTCATTAAATTGACTATTCTAAAA
TATTGCACATTATCAATATAACGAAGAGCCGTATCTTCTAGTTCCCATTTAATTGTATTAA
TACCAAGACGATGTGCTAAAGGTGCATAAATTTCTAATGTTTCTCGAGAAATTCTAATTTG
TTTTTCACGCGGCATGGCTTTCAAGGTACGCATATTATGTAATCTGTCTGCTAATTTCACC
AAAATTACGCGTACATCTTTGGCAATCGCAATAAATAACTTGCGATGATTTTCAGCTTGTT
GTTCTTCTTTTGAGCGGTATTTTACTTTTTTAAGCTTCGTCACACCATCAACAATTCGAGCA
ACTTCTTCATTGAACATTTCTTTTACATCTTCAAATGTATACGGTGTATCTTCAATTACATC
ATGCAAAAAAACCTGCGACAATCGTCGGTCCGTCTAATCGCATTTCTGTTAAAATACCTGC
AACTTGTATAGGATGCATAATGTATGGTAATCCGTTTTTTCGGAACTGACCTTTATGTGCT
TCATAAGCAATATGATAGCTTTTTAAAACATACTCATATTCATCTGCTGACAAATATGATT
TTGCTTTGTGAAGAACTTCATCTGCACTATATGGATATTCGTTGTTCAT

## > apt

Function: protein coding sequence; adenine phosphoribosyltransferase (core genome, constant)
Best match: apt_CC001_MW2-USA400_BA000033.2[1713730:1714248:r]RC (completely identical)
Position: 037-contig_188_RC: 34758 … 35277; Length: 519 bp
Sequence:
TTATTCGTCGTATGAGATTAAACTCATAACATCGTAATCTTTAATTTTTTCAATACCATTTA
AATATTTCAATTCAATTATAAATGCAATACCTACTACGATACCGCCTAATTTTTCAACTAA
TTTTATTGCTGCTTCAATCGTACCACCAGTAGCTAATAAATCATCTGTAATTAACACACGT
TGACCTGGTTTAATTGCATCTTTGTGCATTGTTAAAACATTTGTACCATATTCTAGGTCAT
ACTCATAACGAATGACTTCACGAGGTAATTTCCCTTCTTTTCTAACAGGTGCAAAGCCAAT
CCCCATTGAATAAGCTACAGGACAGCCAATGATAAAGCCACGCGCTTCAGGTCCTACAAC
GATATCAACATCTCTGTCTTTTGCGTATTCTACAATTTTATCTGTTGCATAGCCATATGCTT
CACCATTATCCATAATTGTAGTAATATCCTTGAAACTAACACCTGGTTTCGGCCAATCTTG
AACTTCTGATACGTATTGCTTTAAATCCAT

> **recJ**
Function: protein coding sequence; single stranded DNA-specific exonuclease (core genome, variable)
Best match: recJ_CC005_Mu50-VRSA_BA000017.4[1745207:1747480:r]RC (completely identical)
Position: 037-contig_188_RC: 35298 … 37572; Length: 2274 bp
Sequence:
TCACGACAATTGTGACTTTATCCAATTTTTTATTTCTGAAAAATCTTGATATAATAATTGC
TTTTCAACATCCATACGTTGTTGTCTTAATTGATATACTTTGCTGGAATCAATCGATCTTTT
ATCAGGTTGTTGATTGATTCGAATTAAACCATCTTCTTGTGTTACAAATTTTAAGTCTAAG
AAAACTTTCAACATGAATTTAAGTGTATCTGGTTTCACACTTAAATGTTGACACAATAAC
ATACCCTCTTTCTGGATATTTGTTTCTTGTTTAGTTATTAATGCTTTATAACACTTTTTAAA
AATATCCATATTAGGTATACCATCGAAGTAAATCGAATGATTATGTTGCAAAACTATATA
AAGTTGAGAAAATTGCAGTTGTTGCAAGGAATTAGACAAGTCTTCCATTGACGTTGGTAA
ATCTCTTAATACTACTTTATCAGTTTGTTGTTTAATTTCTTCACCATAATAATATTCATTCG
CATTTACTTTATCACTTTTAGGATGAATAAGCACGACAATATTTTCATCATTTTCTGTAAA
AGGTAAACTTTTTCGCTTACTTCTATAATCTAATATTTGCTGTTCATTCATCGCAATATCTT
GAATAATTAATTGCGGTGATTGATTACCATTCCATTCGTTGATTTGAACAGATCCTAATAT
ATTAATTGGCTGTTCATCTTGTAACTCAGGTTCTAAGTGTCCATTTTGCCAAAATAGCGCG
GCGATATTACTTTCACCAAGTGTCAATTTTAGATGATTTTTTTGTTGACCGATCGCCTTAA
CTGAAGAAACTGATAAATCATCCATTTCAAAAATAGGTCTAGAAAAATCTGTTCCGAAGG
GTCTTAAACGATTCATATCACGAATATTTTTAATCGTTATATCATTTTCTGTTAATAATAC
GTCTACTGGCTTTACGGGATCTAACGAAGTTGTTTTAGATAATTCTTTCATCCATTTATTTA
AACCTTCAGCTAACGATTCTATATTTTCAATATCCATCGTCATACCTGCAGCCATATGATG
GCCGCCAAATTTAGCGATTAACTCTTGATGTGCTGATAGTATTTCAAACATCGACACTTGA
TCAATTGATCTGGCGGAACCTTTTGCATGATTTTGCTCCCTATCAATATTTAAAATTAATG
TTGGCAAAGCAAATGTTTCGACAATTTTCGAAGCAACAATACCTAAGACACCTTCATGCC
AATTTTCTTTTGCTAAAAGTAAAAATAAATCTCCCTTTTTAACTTTCGTTTCTGCCATAGCC
ATTGCTTCTTCTGTGATAGTTGCTACAATATCTTTTCTTTCACGGTTAAAATGTTCAACTTG
TTCTGCTAAAAATGCAGCTTCTTCTTCGTCGTCAGTCATCAACAATTCGCAAGCTAATGAT
GCGTCATCTAAACGACCTACAGCATTAAGTCTAGGTCCAATAATAAAACCAATTGTTTCT
TCATCAATATTGTCATTGTATCCCGCTTCTTTTAGCAATGCTTTAACAGAGGTCGGACATT
GATCATTTAAGACTTTTAATCCTTGTTTCACTAATGATCGATTTTCATCAGTTAAGGATAC
TAAATCCGCAATGGTACCTATCGCAACTAATGCTTTAAAATAATCAGGTACATTTTCAATC
AATGCTTGTGCTAATTTGTATGCAACACCTGCACCACACAATTGTTGGAACGGATAATTA
AACGATGGATGCATTGGATGTACGATTGCATATGCTTCTGGTAATGTACTACCAATTTCAT
GATGATCAGTTACAATGACATCAACTCCTAAATCTTGAACCATTTTAATTTCATTATGACC
TTGTATGCCATTATCAACAGTTATGATTAATGTTATGCCTTCATCATGAGCATTTCTAAAT
GCTAGTTCGTTTGGTCCATATCCTTCGGTAAAGCGGTTAGGAATATGCCATCCTACTTGTG
CACCTAAAAGTTGTAATGTTGTCACTAAAATTGTAGTTGAGGTAACACCGTCGGCATCGT
AATCACCATAAACTAGGATTTTCTCATCATTCGCTATCGCTCTTTTAATTCTTTCAATAGCC
TTAGTCATATCGCTCAATTGCAATGCATCATGATTGACATCTGTGTCTGAAATGATGGATT
CAATTGCTTGTTCATCAATAATCGATTTACTTTCTAATATTTTTTTTACGATTGGCGTTAAC
TTTAATTTTGATGTTAATTCATCACTTATGTATTCAGCTGGTTTAGTTAATTTCCACTTATA
CTTCGGTTTAATCAT

> **secF**
Function: protein coding sequence; protein translocase subunit D and F (core genome, variable)
Best match: secF_CC001-ST772_118_AJGE01000020.1[37615:39894:r]RC (completely identical)
Position: 037-contig_188_RC: 37774 … 40054; Length: 2280 bp
Sequence:
TTAAACTAAAATCTTTTCATCGTTCGATTTCTTTTCTTTATATACAACTAATTTGTGTTTCG
GCGATTTTTTCAACTGACGTTTTTTCATTATTCCCCATAGCGGAACGGCAATGAAGATTGA
AGAGAATACACCAGAAATCAATCCGATAAATAACGCTAAAGTAAAGTTGAATATCGTAG
GAGCACCGAAGAATAGTATAGCAACTACTACTACAATAACTGTTAATACTGTATTAATTG
AACGTGTCATTGTCTGTCTAATTGATCTATTAACGATATCATCAATTTGTTCTGTTGTCGTA
ATCACTTTAACCTTTTGTAAGTTTTCACGTACACGGTCAAACGTTACGATTGTATCATTAA

TTGAATAACCGACAATTGTTAATACAGCGGCGATAAATGTTAAATCTACTTCAATTCTAA
ATAAACTGAAAATCGCTACTATAATGAATACATCATGTAATAATGCCAATACAGATGAAA
GACCCATGCGCCATTCAAATCGTAATGATACATAGATGATGATACCTATCGATGCATAGA
TTAATGCAAGCATTGCATTTTTTGCTAATTCCTGTCCAATAATTGGTGATACAGTATTAAT
TTGAGGTGTGTCACCGAATTTCGATTTAATCTTATCACTCAATTTATTATCTTGAGCACGC
GTTAAATCGTCTTTAAATTGAACAGTTGCTACTTTATTATCTTTACCATTGATTTGAATTTG
ATCCGCTTTAAGTCCACTATCTTTTACAACTTGCTCAACCTTTTGTTGAGTAATTGCTTGTT
TAGATTGGAAATCTACACGTGTACCACTTGAGAAATCAATTCCTAAGTTTAACTTGAAGA
TATAAAGAATAACTAAACCGACAACTACAATTAAAATACTTACTCCAATTAATGGCTTAG
CTAATTTAACAAAATTCCATTTCTCGAATGAAGTTTTAAGGTCATGAACATCTACACCTTC
ATTAATATCATGTCGTTTATTCTTTTTAACACCAAATAACCAAAATTGATTTTTGAATATA
TTTGATGAAACAAGTAATGATAATAAGAATCTTGATAAGAACACGGCTGTAACAAAGAT
CATTAGAATACCTAATAATAACATTGTCGCGAAACCTTTAACTGAACTTTCACCGAAGAA
GAATAATACTGCTGCGGCGATAACTGTTGTTAAGTTAGAATCAAAAATTGTTAGGAATGA
ACTTTTGTTTGCTTTAGAAAAGGCTTGCTTTATCGTTCTACCTATTCGAAGTTCATCCTTAA
TACGCTCATACATGATAATATTGGCATCTACAGCCATACCTACACCTAATACCAACGCCG
CTAATCCTGGTAAAGTTAGAACCCCGGAAATGAAATTAAATGCTACTAACGTTAGATAGA
TATAAGTTGTCAATGCAATAATCGCTACTAAACCAGGTAATCGGTAGAATCCAAGCATGA
ATAAATAAATTAATGCTACACCAATAAACGATGCAAACACAGTTTTATCTAATGCATCTT
GACCAAATTGGGCACCTACTGAGTTTGAATAAATTTCTTTCAAGTCAACTGGTAAAGAAC
CTGCATTTAACAATTCGGCGATTTGTTTTGCTTTTTTAACGCCTTCTTGTCCTTTAAATCCA
CCCGAGATTTCTACGCTATCAGAATTGATTGGTTGATCAACACTTGCTGCAGAAATAAAT
TTAGGGTTTTTCTTTTGTGCTTCTTTTTTATAGCTGTCACCTTTTTTGAAATCTAACCAAAC
AACCATGACATTATCACGTTTCTTAGAGATTTCTTCCGTTACTTTTTTAAATTTGTTTTTGT
CTTTTACTTTAAAAGTAACTGTAGGCTGGTTTGTTTCCTGTTTAAATTCTTGTTTGGCAGAT
CCCTGTTTAATATCAGAACCGCTTAATTTTACTTTATCTTCTGCATCGCGAATTGTTAAATT
AGCTTGAGAAGATAAAATTTTACGTGCTTCATTCTGGTCTGTTACACCAGCAAGTTGAACT
CTAATTCTATTAGGTTCTTCAACTTGAATTTTAGGTTCCGAAACACCTAAAACGTTAACAC
GATTTTCTAATGTTTGCGCTGTTGATTGTAAGGCTTTTTTATCTATTTTGTCGCCTTTATTT
AAAGGATCGACTTGATAAAGCACCTCAAATCCACCTTGCAAATCAAGTCCTAAATTGACA
TTCTTTATAACACTTTTATAAGTTGCAGCCATTCCGGCAAACAACAATACGACTAAAAGC
AAGAACGCAATTATTCTACTACTTTTCTTCAC

> **yajC**

Function: protein coding sequence; protein translocase subunit yajC (core genome, constant)
Best match: yajC_CC001_MW2-USA400_BA000033.2[1719300:1719560:r]RC (completely identical)
Position: 037-contig_188_RC: 40328 … 40589; Length: 261 bp
Sequence:
TTATGAAGGGTCAACTTGTTTAATAGCAGGTTTTTCGAAAGTTAATTCAGTACCATGACCA
TTAACTGTAATAACAACAGTTGTTTCATCTACTGCTTTAACAGTACCTTTAATACCACCAA
TAGTTGTAATTCTTTGACCAGATTGAATGTTATTAATCAACTCACGATGCTGTTTCGCACG
TTTTTGTTGTGGTCTGATCATCAAGAAATACATAACCGCAAAAATTACGACTATATATATT
AGTAATGAAAATTGCAT

> **tgt**

Function: protein coding sequence; queuine tRNA-ribosyltransferase (core genome, constant)
Best match: tgt_CC001_MW2-USA400_BA000033.2[1719579:1720718:r]RC (completely identical)
Position: 037-contig_188_RC: 40607 … 41747; Length: 1140 bp
Sequence:
TTAAAAGTTTTTTGGGTTCTCAACATTTAATCCATATTGCTCGAAGAATTCTTCTTTGAAA
TCTAAAAGACGATCTTCTCGAATGGCTTGTCTTATATCTTCCATTAATTTTAGCAGAAAAT
GTAAATTATGAATAGTAGTAAGACGAATACCAAAAGTTTCCTCTGCCTTGATTAAATGAC
GTATATACGCTCTTGAATAGTTTTGACATGTATAACAGTCACAATTCTCATCTAACGGTCT
TAAATCATCTGCAAATTTTGCATTTTTAATAACTAAACGACCTTGCGATGTCATACAAGTA

CCATTTCTGGCAATACGTGTCGGTAAGACACAATCAAACATATCCATGCCGCGAATACTA
CATTCGATTAACGCATCTGGAGATCCTACACCCATTAAATATCTTGGTTTATCTTTAGGCA
TAAACTGCTCTGTATGTTCAACCATTTTATACATAACCGGTTTAGGTTCACCAACTGACAA
ACCGCCGATTGCATAACCAGGAAAATCTAATTCTACTAAATCCTTTGCACTTTGTTCTCTT
AAATCTTCATATTCGCCACCTTGTATAATGCCGAACAATGCTTGATCTTCAGGTCTTTGGT
GTGCATCTAGACATCTTTTCGCCCAACGTGTTGTACGTTCAATAGATTTTTTACATAATC
ATATTCAGCAGGCATCGGTGGACATTCATCAAATGCCATCATAATATCAGATCCTAAATC
ATTTTGAATTTGCATTGATTTCTCAGGACTCAAAAATAATTTAGACCCATTAGTATGATGT
CTAAATTCCACGCCTTCTTCTGTAATTTTACGTAAATTACTTAAACTAAACACTTGGAAAC
CGCCTGAATCTGTAAGAATCGGACCATCCCAATTCATGAATTTATGTAATCCCCCAGCGT
GTTTGATAATATCATTTCCGGGTTGTAACCACAAATGATATGTGTTGCCCAAAATGATTTT
TGCTTCAATTTGTCTTAACTCTTCTGGACTCATTGTTTTAACGGTTGCTTTAGTACCAACTG
GCATAAACATAGGTGTTTCAAATGAACCGTGTGGTGTGTGCACGATACCTAAACGCGCAC
CTGATTGTTTACAAGTTTTAATGTGTTCGTATGTTACTGCAGGCAT

> **queA**

Function: protein coding sequence; S-adenosylmethionine-tRNA ribosyltransferase-isomerase (core genome, variable)
Best match: queA_CC001_MW2-USA400_BA000033.2[1720741:1721766:r]RC (completely identical)
Position: 037-contig_188_RC: 41769 … 42795; Length: 1026 bp
Sequence:
TTATATAATTAACATTGCATCGCCAAAACTAAAGAATCTATATTCTAAATTTACTGCTGTT
TTATAAGCATTCAGAACATTTTCACGAGTACTAAACGCTGATACTAGCATAACTAATGTT
GATTTTGGTAAATGAAAATTAGTAATCTGGCCATCAATTGCTTTAAAATCAAATCCTGGA
TAAATAAATATATTAGTCCAGCCACTCGTTTCAACAAATTTATCATGATCGCGTCGAATTG
TTTCAAGTGTACGTGTTGAAGTTGTACCAACTGATATAATGCGATGTCCTTTGGACTTAGT
ATCATTTAATAAATCAGCTGTTTCTTGTGTCATTTGATAATATTCACTATGCATTTCGTGGT
CATTCACATCATCGACGCTCACCGGTCTAAACGTACCTAACCCAACATGTAATGTAACAA
ATGCGATATTAACGCCTTTATTTTTAATTTCAGTTAATAACTCATCAGTAAAATGTAATCC
TGCTGTTGGTGCTGCCGCTGAACCACTTTCTTTAGCGTAAACTGTTTGATAACGATCTGGA
TCATCTAAACGTTCTTTGATGTATGGTGGCAGTGGCATTTCCCCTAATTCATCTAATCTTTC
TTGTAAAATACCTTCATAATGTAAACGCATGATGCGTCCACCTTGATCCATTTCTTTTATG
CACTCAGCTATAATTTTGCCATTACCAAAATTCAATTTATTACCAACTTTAATACGCTTAG
CTGGTTTCAGTAAGACTTCCCAATCATTACCTTCAATTTGAGTTAACATTAACATTTCAAC
TTTTGCACCAGTTTCTTCTTTTAAACCAAAAAGTCTAGCTGGCATTACTCGCGTATCGTTA
AGCACTAATGTATCACCAGGTCTAAAATACTCAATGATATCTTTGAAATGTAAATGTTTC
ATTTCACCAGTTTCTCTATCCATGACTAATAAACGACTATGATCACGATCTTTTAAAGGCG
TTTGAGCAATTAATGATTCTGGTAAGTCATAGTCAAATTCTTCAATATTCAC

> **ruvB**

Function: protein coding sequence; Holliday junction ATP-dependent DNA helicase subunit B (core genome, constant)
Best match: ruvB_CC001_MW2-USA400_BA000033.2[1721768:1722772:r]RC
Position: 037-contig_188_RC: 42796 … 43801; Length: 1005 bp
Sequence:
TTATCCTCTCTCCTCATTCGACTTTGCAAAATGTTCATAAGCTAATGGTGTTGCTTTTCTGC
CACGTGGCGTACGTTCTAAAAAGCCTTTCTGAATAAGAAATGGCTCATAAACGTCCTCAA
TTGTAATACGTTCTTCACCAATTGTTACGGCAATCGTATCTAAACCAACAGGTCCGCCATT
ATACTGCTTAATAATACAGTTCATCATTTTATGATCAATGTAATCTAGTCCGTGTTGATCA
ACTTGAAGTAAACCTAATGCGTGCTTCGTTGTTTCAATGTATATTTGTTCATCTTCATTCAC
TTGCTGGAAGTCTCTTACCCGCTTCAATAGTCGATTTGCTACTCTTGGAGTCCCTCTAGAA
CGTTTAGCAAGTTCAATGGCACTTTCTTCATCAATACCTGTGCCTAAAACCTCAGCTGTTC
TAATAATGATTTCTTTTAAATCTGATTCATTATAATATTCTAATCTTAAGTGCACACCAAA
TCGATCCCTTAGTGGACCTGTTAAGCTGCCAGCTCGCGTTGTTGCACCTACCAAAGTGAAT

GGAGGTAAGTCGATACGGATACTTCTAGCCTCATCGCCTTTACCAATGATAATATCTAAA
AAGAAATCTTCCATTGCAGGGTATAACACTTCTTCAACAACACTACTCAGTCTGTGTATTT
CATCAATAAACAAAACATCTCCAGGTTGAAGTCCTGATAAAATTGCAGCCAAATCACCAG
GTCTTTCTAATGAAGGCCCTGATACTGTACGTATATTAACTTCCATTTCATTGGCAATGAT
ATTAGATAATGTTGTCTTACCTAATCCAGGGGGGCCAAAAAGCAATACATGATCTAATGG
TTCATGACGAAGTTTAGCCGCTTTAATAAATACTTCTAAATTACTTTTTATTGAATTTTGA
CCAATATATTGTCGTAATCTCGTAGGTCTAAGCGACAATTCGAAATCAGTTTCTTCACTAT
GCATTGATTGATCAACCATACGCTCATTCAT

## > ruvA

Function: protein coding sequence; Holliday junction ATP-dependent DNA helicase subunit A (core genome, constant)
Best match: ruvA_CC008_NCTC8325_CP000253.1[1655840:1656442:r]RC
Position: 037-contig_188_RC: 43832 … 44435; Length: 603 bp
Sequence:
TTAAGATACAACTAATTGAAGACCTGCCTTAACAGCTTCATCAACTGAGTCATATTTATTT
TTATTTAACGTTTTCTCAACTTTTGCAAGCTCTCGTTTAGAATAACCTAATGCTTCTAACGC
TAACATTGCTTCTTGCACGAATTGATCTTGCACCGTCGAAGTAGCGTCTACTTGTAATAAT
GAATCGCTATCTTCTTCAGTGATTTTCACTTTACCTTTTAAATCTAAGACAATCTGTCTTGC
CGTTTTCTTACCAATTCCTGGGAATTTAGTTAAATACGTATCATTTTCATTTTCAATGGCAC
GTTTTACTTCATTAGGCGTACTTGTCGCTAAAATAGCTAAAGCTGATTTCGGACCAATACC
AGTAACTTTAATTAAACTCAAGAACATATCTTTCTCTTCTTCACTACTAAATCCATACAAT
AATTGTGCATCTTCACGAACAATTAAAGATGTATGAATTAAAACTTCATGATCTAGATGC
TTTTGAAAACGATAAGAATTTGGTGTTTGAATTTCATAACCAACACCAGCAGTTTCAACA
ACTACGTGTGTAGGATATAAATGTGTTAACTTACCTTTGACATACGCGTACAT

## > pheB

Function: protein coding sequence; ACT domain-containing protein (core genome, constant)
Best match: pheB_CC030_MRSA252_BX571856.1[1788403:1788861:r]RC (completely identical)
Position: 037-contig_188_RC: 44448 … 44907; Length: 459 bp
Sequence:
TTACATACTCATACTAATTAATTCTACTTTTGATACATAATCTAAATTTCTCAAAGCGCCA
ATAACATCTTCTACTGAAGTTTCTTTAGATTTAGCATTCAGTGATAATGTTATTGTTGCTTT
TTCTTCCATTGGAATACTTTGATGAATCGTTAATACAGATAGTTCTAACTTTGATATAACA
TCTAGTACACGTGCCAACATACCCACAATATCAGTTACATATAAAATTAATGTAAATTCT
CGATGGTCAAGCATTTTATCGTCTACTGGAAATATCGTTTCTCTATATTTATAAAAAGCAC
TTCTAGATAGATCAAACTGTTTAACGGCATCATAAATGGACAATGTCGGATCACTTTTTA
AGGCATCTTTAATCTTCAATGTTTTAACCACGGATTCAGGCAAGACATCTTCTCTAATTAA
ATAAAACTTTTTATAATCTTTATTGTCCATCAT

## > obgE

Function: protein coding sequence; GTPase obg (core genome, variable)
Best match: obgE_CC010_D139_ACSR01000051.1[25341:26633:r]RC
Position: 037-contig_188_RC: 44916 … 46209; Length: 1293 bp
Sequence:
CTATTCAACGAATTCAAATTCTCCTCCAAGAATTCTAACGATATCACCATTTTTACAACCA
CGTTCTCTAAGCGCATCATCAATACCCATCGAACGCATTTGACGAGCAAATCGACGTACT
GCTGGATCACTGTTAAAGTCAGTCATTTTAAACATTCTTTCAATAGCATTACCACTTACCA
CATAAGCACCATCATCATCTCTTGAAATTGTAAATTTATCTTGTGACGGTGTATGTTTATA
TAATACTCGGTTAATGCCAACCGACTCCTCTTCTTCAACTGTGAAGTCAACATCTTTATAT
TCTTCTAATTTATCTGCTATTGCATATAATAATTGATCAATATTATCACGCGTTATTGTTGA
AACTGGAATAACTGGCACATCTTCGCCAATTTCTTCTTTAAACAAGATTAAATTATCTTGT
GATTCAGGTAAATCCATCTTGTTAGCTACTACGATTTGAGGTCTATCTTCTAAACGTTGCT
CGTACGCGGCTAATTCTTGATTAATGACTTTATAATCTTCAATAGGTTCTCTACCTTCAGA
ACCACTCATATCAATCATGTGAACAATAACTTTTGTTCTCTCTACATGTCTTAAAAATTGA

TGTCCTAATCCAACGCCATCAGATGCACCTTCAATTAAACCTGGTAAATCTGCCATAACA
AAACTTCGTTGATCAGGTGTTGAAACAACACCTAGATTTGGTTTAATCGTTGTAAAATGA
TATGCCCCAATTTTAGGCTTAGCTTTTGAAACGATAGATAATAAAGTCGATTTACCCACAC
TAGGGAAACCTACTAATCCTACATCAGCTAATAATTTCAATTCTAAAGATACATCTAATTC
CTCACCTGGTTCACCTTTTTCACTGAAGTCAGGTGCAGGGTTTCTAGGTGTTGCAAAACGT
GAATTACCTCGGCCACCTCGACCGCCCTTCGCTACTACAGCTCTTTGACCATCTTCAACAA
GATCTGCTAACACTTCGTCTGTTTCAACATTTTTAATAATTGTACCAGGTGGAACTTTTAA
TACTAAATCTTCCGCATTTTTACCATGCATATTGCTACTTTGGCCATTTTCACCTTTGCTTG
CTTTAAAATGACGTTGATATCTAAAATCTAATAACGTTCTTAAACCTTCATCCACTTCAAA
TACGACTGAAGCACCTTTACCACCGTCACCGCCAGCTGGTCCACCAAATGGTACATATTT
TTCTCTTCTGTATGCGGTAATACCATTACCACCATCACCGGCTTTAAGAGATATTTTGACT
TGATCGACAAACAT

## > **rpmA**

Function: protein coding sequence; 50S ribosomal protein L27 (core genome, constant)
Best match: rpmA_CC001_MW2-USA400_BA000033.2[1725665:1725949:r]RC (completely identical)
Position: 038-contig_319_RC: 311 … 596; Length: 285 bp
Sequence:
TTATTCAGCTACTGCATATACAGAAACTTGTTTTTTGTCGCGACCTTTACGTTCGAATTTA
ACAACGCCGTCGATTTTAGCGAATAATGTATCATCGCCACCACGACCTACATTTTCACCA
GGGTAAATTTTAGTACCACGTTGGCGATATAAAATTGAACCACCTGTTACGAATTGACCG
TCAGCACGTTTAGCACCTAAGCGTTTTGATTCAGAGTCACGTCCGTTTTTTGTAGAACTTA
CCCCTTTTTTAGATGCGAAGAATTGTAAGTTTAATTTTAACAT

## > **DUF464**

Function: protein coding sequence; putative protein (core genome, constant)
Best match: DUF464_CC001_MW2-USA400_BA000033.2[1725961:1726281:r]RC (completely identical)
Position: 038-contig_319_RC: 607 … 928; Length: 321 bp
Sequence:
TCACTTATAATTTAATCTAATATTCTCATTATATTCTTCTTCAATAGTTTGTAAAGACACAA
GCATTGTTTGAAGAATTAGTTGCGCTTCATCGTTATTTGTATCAACGCTTCTTATATGAAA
ATGACCACCATTGTCGTCATAATTGATATCTGGTCTCTCAGATGTCAATCCTATAATCGCA
TTAACACTACCAAACAATACAGCTGAAGCTCCAGCACAAACGATATCATGACCATATTCA
CCATGGTCAGCATGGCCATCCATAATAACGTCTGTTACTTTGCCTTCATCATTAACTGTAA
TATCAACAGTAATCAT

## > **rplU**

Function: protein coding sequence; 50S ribosomal protein L21 (core genome, constant)
Best match: rplU_AF349567.1[184:492]RC
Position: 038-contig_319_RC: 933 … 1242; Length: 309 bp
Sequence:
TTACGCGTTGATTTTATCGATTGTTAATTTAGTGTATGGTTGACGATGGCCTTTTTTACGTT
TTGAATTTTTACGACGTTTGTATGTGAATACAGTAATTTTTTTACCGCGACCTTGTTTATTA
ACAGTAGCAGTAACTGTTGCACCTTCAACTGTTGGCGCTCCAACTTTAACTGAATCTCCAC
CTACAAATAATACTTTATCAAATGTAAAAGTATCTCCTTCGTTTACGTCTAATTTTTCAAC
GAAGATTTCTTGACCTTCTTCTACTTTGATTTGTTTTCCACCTGTTTCAATAATAGCAAACA
T

## > **L21**

Function: noncoding RNA ribosomal protein L leader RNA (core genome, constant)
Best match: L21_leader-var2_CC025_21193_AFEG01000006.1[87810:88063:r]RC (completely identical)
Position: 038-contig_319_RC: 1254 … 1508; Length: 254 bp

Sequence:
CTGTATAATAAGTCACGCCATACATAGGTGACATTTCGTTATTGAGTCAAGCGTTTGCACT
CTACGTTGTTTCGGTCGCAACTCGAGTCATTTATAAACATAAACTCTTCTCGTTTCTTCCTT
CAACGCCTTGATTGACAATCGCTTTCTTTCAATAACTTCTGCAAATGAGTCAAGCGTTTGC
ACTCTGTGGGAAAATCGCTTTCTTCATTAAAAATGTTCTTGAACCTATATTTGAGCGGTTG
TATGTAGCT

> **mreD**
Function: protein coding sequence; cell shape determinant D (core genome, constant)
Best match: mreD_CC008_NCTC8325_CP000253.1[1659668:1660198:r]RC (completely identical)
Position: 038-contig_319_RC: 1534 … 2065; Length: 531 bp
Sequence:
TTACCATTGACGACGTTTCATGTCAATGTCATTGTTTGTTTTTTTAAGGAACTTTATAATCA
ACGGATAAAGCATAATCAACAGTACAAAATTCATAATTAATGTTGGCAATAATCTAAAGA
CTACAAAATGAATAATATCAAATTGAATGAATCCTAACATACCGTATATTAATGCCACAT
AGACTTCTAATAATAAGGTGCTGGCTAATATAATAATGAATAACATCGAATGATCTTTGT
AAAAAATTTTAAAGAATCGATCTATAAGTGCTAAAAACAATATATAGCCAAATAAGTAC
ACTCCATAAATACTACCAAAGTATACATCAGTCATTACGCCTAAAAATATGCTGAGCAAT
AATGATACGCCAAAGCCACGATACACTACCATCATTAAAATATACATAAATGTAAGGTGT
GGTACAAATACAAGTTCAAACTTACCTATGTGCATTGGAATAAGAAGCCCAATTGCAGTA
TCTATATAAAATAGTAAAATACCTATCAAAAAATAATACAGTGTACGCAT

> **mreC**
Function: protein coding sequence; similar to rod shape-determining protein (core genome, constant)
Best match: mreC_CC188_21340_AGTX01000037.1[31211:32053]RC (completely identical)
Position: 038-contig_319_RC: 2064 … 2907; Length: 843 bp
Sequence:
TTATTTATCCCTGCTTTCATCATCAGGAATTGTTTTAGGATCTCTTTTTGCAACATAAACAT
GACTCAAATCTGTTAAGTCTGCACCAGTCTTAACCCTAACTTCTTTAGCTAAGCCGTATTG
ATCATTTTGAACCTTAGTCACTTCTCCTATATATAAATTACTTGGTAGTTGATCAGCTAAT
CCACTTGTAACGACTTTATCACCTTTTGAGATATTATCTCTATTATTAATGTCACTAATTAC
AAGTTCTGAGTTCTTTTCATCATAACGATCAATTAAACCAAATATATTTTTAGAACCGTGT
TGTATATTTACAGATAATTTACCCGCACGTGTATTAGTTGAGATTAAATCAACTTGTGAAG
AAAATTTATTAACTTTAGTAACTCTTCCAACAAAACCTTGTGATGTCATCACAGCCATATT
TGAAGTTATACCTGATTTAGATCCCTTATCAATTACAATTGTATTCATCCACTGATCCGGA
TTTCTTGCCAAAACCGTAGTAGAAATAGGATCAAATTTTGAAATATCTTTTAAATCAAGCT
CTTTTTTTAATTTTTCATTTTCCGCTTCTAATTGTTGGTTCTTAGATTCTAACTGGCTAATCT
TATTTTTAGATTCTTTAGAATCTCCTTTTTTAAAAAAGTCCCCAATCGTACCAGCAACAAA
ATTAACTGGATAACTCACAACTCGTTGTCCAAAAGACACAGAATCACCTATATATTGTTC
AGGAGGTGATTGAGATTGTGAACGTATGGACAGCCCAATTAATGCAATAAAAACGATAA
TTGCACATAAAACAACAATTAATTTGTTATTTTTAAAAAACTTAAGCAC

> **Q5HFB3**
Function: protein coding sequence; putative protein (core genome, constant)
Best match: Q5HFB3_CC001_MW2-USA400_BA000033.2[1728721:1729194:r]RC (completely identical)
Position: 038-contig_319_RC: 3298 … 3772; Length: 474 bp
Sequence:
CTATTGATAAGCATTTTCGGGTTTTAGTTGTAAATTTTGCCCTAATTCATTTAAGTCTTGTT
GCATTTCAAATTCAGTATTGTAAACACGCATTGACTCATCTCCAAATTTATAAAGAATAA
ATTCGTCTCCTCTTTGACCTATAATATATTGATCATTATAAGCCATGCGATTCATTCCAGA
CACAGCCATAAACTCTTGTTTATCTATCATTTTAAATACATTTTTAATTTGGCTTAATGGTA
CATTCTTTATTAAATCATTTTCTTTTAATGAATATTTCTCACCACTTACATAAACATGTTGG
GCATCATTAGTGGGTTGCGTAACTTGATTCATTTGATCATTATCGTGATTTACAGGATTCC

AATCTTGTTCTTTTAAAAAAGGTGCGTATTTCAAGGCGATATAAACAATTATCACAATTGC
TATTACTGCTATAATGTTTTTAATCATACTAAATATCAATCTCAT

> **Q5HFB2**
Function: protein coding sequence; putative protein (core genome, variable)
Best match: Q5HFB2_CC001_MW2-USA400_BA000033.2[1729668:1729952:r]RC
Position: 038-contig_319_RC: 4245 … 4530; Length: 285 bp
Sequence:
TTACTTACGCCCAAATGCGTTAGATAAAAATCGTATCAGCGCGTAGCTACCACTTAGTAA
AAGGCCCCAAACGATTCCAGTAAAAATTGAGCCAATTGCTACACTTAATAAAGGAAAAG
CTTGTTTCGTAACGATCACACCTGCTAACGCTGTTACTAAAATACCTAACGTAGATAAAG
CTAATGTTATTAATAGATTGCGCTTTGTTACAGTTTCATTTTTAATATTTAACTGCATGGTA
TAAACCATTGGCACGAGAACCCCGATAATAATAAATAGAGTCAC

> **radC**
Function: protein coding sequence; DNA repair protein radC homolog (core genome, constant)
Best match: radC_CC001_MW2-USA400_BA000033.2[1730020:1730706:r]RC
Position: 038-contig_319_RC: 4597 … 5284; Length: 687 bp
Sequence:
TCAATCATTTTCATCAAAGTAACCCGCTTCTACAAGACTGGTAAATCTATTATCACCGATT
ATAATATGATCCAACAAATCTATCCCTAAAATCAAACCACACTCCTTCAACCTCATTGTTG
TTATGATATCTTCTTGTGAGGGCGTTACATCACCGGATGGATGATTATGAACTGCGATAAT
TGCATTGGCATTTTCTCTCACCGCAATACTAAAAATTTCACGTGGATGTACAATCGAACTA
TTTAATGTACCTTTAAAAACACAGGTTTCTTTAATCACTACATTTTTTGAATTTAACAATA
AAATGACAAAATGTTCTTGTGTTAAATCTTTCATTGTTGGAATCATATAATCAGCAACATC
ACTTGGTTGCGTTATTTTTATACGATTATTTTCAGCTCTTCTCCCCATCCTTTCCCCTAGCT
CAAATGCTGCTTTTAAAGTAATTGCTTTTTGTAATCCAATCCCTTTAACTTGTATCAAATC
GTTAATTGAAGATTTTTTCAATTCATTCAGATTCGAAGCAGATTTAAGCAGTTCATTACTA
ATGTCTATGCTCGAGAATCCTTTTCTTCCGGTGTTAATTAATATAGCTAATAATTCTGTATT
CGAAAGACTTTTTGCACCATGGCTTAACAAACGTTCTCTTGGCATTTCTGAAGTTACCATT
TCTTTAATTTTCAA

> **Q5HFB0**
Function:
Best match: Q5HFB0_BM3327_AF186237.2[9:715]RC
Position: 038-contig_319_RC: 5280 … 5988; Length: 708 bp
Sequence:
TCAAAAATATACGCCTCCTAAAAATTGATGGATATCATTATAAAAAAGTGAATTGATAAA
AAAGGAAATAAATATAAATGGAACAAGGGGTAATAGTTTAATCGGCTTAAATATCATGG
TAATTAAAGCAACTAAACCAGCAATGACAAATGTAAATAAAATGACATAAATAGTGAAT
TGGAGAGGGAAAAACAAAGAAAGTGCAGATATTAGTAAAACGTCACCATAACCAATATA
TGCCCGAAATAAAAAGTAGAATATATGCGTGGTCATACTAATAATGAGAAAAGCTACTG
GATAAATCATACTTAACGAGAGAGAAACGATACAATAAATTATAATTAAACGACAATCT
AACATTAAAGAAGTGATATCGGTCATAGTAAAAATAAGCAGAAAAACATATGTAGTTAT
AAATAGCGTAGCATTTACGTATGTGAAATCATACTTAATAAAGACGATAGGTATTAAAGC
AAAGGTTTCCCCTAAGAAATGTGTTAGGGAAATACGCTTTCGACAGTTTCGACATCGCCC
TTTTAATAATAAAAAACTAATAATCGGCATTAATTCATACCATTTAAGTGACGAATTACA
ATAATCACATTTCGATCTTCTATGTAAATAATCAAATGACGTTTCTTCTATAGATATAAAT
TGATATAGAAAACTAAAAATACAACTGCAACTATAAGATAACAATACTACCAA

> **folC**
Function: protein coding sequence; folylpolyglutamate synthase/dihydrofolate synthase (core genome, constant)
Best match: folC_CC001_MSSA476_BX571857.1[1711332:1712603:r]RC
Position: 038-contig_319_RC: 6257 … 7529; Length: 1272 bp

Sequence:
TTATAATGCTTCAAAGTCTAATTTTGATTTAACTTCACTTATGAAATACAGACTACCGGTA
ATTACTAATGTATCACCTTGATAATTTTTATAAATTCAACGTAGTCATCTACTAATTGTA
TTTCATCATTTTCAATACTACCTACAATTTCTTCTTTGCGTAACGCTTTCGGAAAATCAAAT
TCAGTTGCATAAAACGTATGCGCAATTAAACTTAAATGTTTGACCATCTCGTTAATCGGTT
TTCCGTTTATTGCTGAGAACAAAATATCTACTTTTTCTTTATCATGGTACTGTTTAATTGTA
TCAATTAGAGCATCTATACTCTCTGAATTATGTGCGCCATCCAAAATGATTAAAGGTTTGT
CATGCACCTGCTCAATACGTCCAGTCCAACGAACTGATTCAATACCGTCTATCATCTTATT
GAAATCTAATTCAATTAATCCTTGTTCATTTAATTCAATAAGAGCTGTTATGGCTAATGCA
GCATTTTGTTTCTGATGTTCACCTAGCATACTTAAAATGATTGTTTCTAATTCATAATCTTT
ATAACGGTAAGTAAATTCATCATTTTGCGATACAACAACAATTTCTCTATCTAATTCAATT
GGCTTCGCATGTTGTTCAATTGCGCGTTCACGAACATATTTTAATGCATCTTCATTTTTAA
CAGCATATATCACTGGAACGTTAGGCTTTATAATCGCGCCTTTATCCCTAGCAATATCTAG
ATAAGTACCACCTAAAATATCTGTATGGTCTAGACCGATACTAGTTAAGATTGATAAAAC
CGGTGTAAAGACATTTGTCGAATCGTTCTTTATACCCAATCCAGCCTCAACAATGACAAA
ATCAACAGGATGTATTTCACCAAAATATAAAAACATCATCGCTGTGATTATTTCGAATTC
AGTTGCAACACCTAAATCTGTTTCACGTTCCATCATTTCACTTACTGGTTTAATACGTGAT
ACTAATTCTACAATAGCGTCATTTGATATTGGCACACCATTTAGACTAATTCGTTCATTAA
ATGTTTCAATAAACGGCGACGTAAATGTACCTACTTCATAACCATTTTCAACTAAAGCTGT
TCTAAGGTAAGCAACTGTAGAGCCTTTACCATTTGTGCCACCTACATGAATACCCTTAATG
TTATTTTGAGGATTATTAAATTGTGCTAGCATCCATTCCATACGTTTAACACCTGGTTTGA
TGCCAAATTTAGTTCTTTCGTGTATCCAATACAAGCTCTCTAGGTAATTCAT

> **valS**

Function: protein coding sequence; valyl-tRNA synthetase (core genome, constant)
Best match: valS_CC001_MW2-USA400_BA000033.2[1732964:1735594:r]RC
Position: 038-contig_319_RC: 7541 … 10172; Length: 2631 bp
Sequence:
CTATGCTTTTAATTGTTCAATTCTTGCCTTCACACCATCATATTTTTCTTGATAATCTTGTTT
TTTACGTTTTTCTTCATTTATAACCTTTTCAGGTGCTTTACTTACAAAGTTTTCATTAGAGA
GCTTTTTATCTACTCTATCTAATTCGCTTTGAAGTTTAGCTAATTCTTTTTCCAAACGGCTG
ATTTCCTTATCCATATCAATTAGCCCTTCTAATGGTAATACCACTTTACCTGCAATTACAA
CTGATGTCATTGCTTTCTCAGGAATTTCCACGTCAGTGCTAATATTTAAGGTACTAGGATT
ACAGAATTTGATTAAATAATCTTTGTTTTGTGATAAAGTTGTTTCAATTTCTTTATCTTTAG
CTTGAATTAAAATAGGTATTTCTTTAGACAATGGCGTATTTACTTCTACACGTGATTGTCT
TACAGATTTAATGATTTCAACAAGTTGTTGCATTGTTTGTTTACTTTCTTCAAAAATCAAT
GATTCACGCACTTCTGGCCATGAAGCTTTAACAATTGTGTCACCTTCATGTGGTAAACTTT
GCCATATTTTCTCTGTTACAAATGGCATGAATGGATGTAGCATTCTCATAATATTGTCTAA
AGTATAACTCAATACTGAACGTGTAACTTGTTTTTGTTCTTCATCATTACCATTCATTGGA
ATTTTACTCATTTCAATGTACCAATCACAGAAATCATCCCAAATGAAATTATATAATGCAC
GTCCAACTTCGCCAAATTCATATTTGTCACTTAGATCAGTAACTGTTGCAATCGTTTCATT
TAAACGTGTTAGAATCCATTTATCTGCTAATGATAAGTTACCACTTAAATCGATATCTTCA
ACTTTAAAGTCTTCACCGATATTCATTAAACTGAAACGTGCCCCATTCCAGATTTTATTGA
TAAAGTTCCACACTGACTCAACTTTTTCAGTTGAGTATCTTAAATCATGTCCTGGAGATGA
ACCTGTTGCTAAGAAGTAACGCAAGCTATCAGCACCGTATTCGTCAATAACATCCATTGG
ATCCACACCATTACCTAATGATTTACTCATCTTACGCCCGTCTTCAGCACGAACTAAACCG
TGTAATAATACATCATTAAATGGACGACGATCTGTAAATTCTAAGCCTTGGAATATCATG
CGTGCTACCCAGAAAAAGATAATATCGTAACCTGTAACTAAGGCATTTGTTGGGTAGTAT
CGTTTAAAGTCTTCACTTTCTAAATCAGGCCAACCTAACGTAGAGAAAGGCCATAAAGCA
CTTGAGAACCACGTATCTAATACATCTTCATCTTGTTGCCAATTTTCAATATCAGTTGGCG
CTTCTTCTCCAACATATATTTCGCCTGTTTCTTTATGATACCAAGCCGGAATTTGATGACC
CCACCATAATTGTCTTGAAATCGTCCAATCTCTAATATTTTCCATCCATTGGTTAAATGTA
TGTTCGAAACGTTGCGGATAAAAATCAATACGATCATCTGTTTTTTGGTTATCTAATGAAC
GTTTCGCTAAGTCTTCCATGCGCACAAACCATTGTGTTGATAAATATGGTTCAACAACAG
CGCCAGATCGTTCTGAATGACCTACAGAATGAACATGATCTTCAATCTTGATAACTAAAT
CTTGTTCTTTTAAATCTTTAACTAGCTGTTTACGACAATCAAAACGGTCCATACCTTCGTA

TTTACCCGCTTTGTCGTTCATTTTACCATTTTCATCCATAACGATAATATTTTCTAATTGAT
GTCTTTGACCAATTTCAAAATCATTAGGGTCATGTGCTGGTGTCACTTTCATAGCACCAGA
ACCGAAGTCTATATCAACATACTCATCTGCTAAAATAGGCAGTTCGCGTCCTACGATTGG
TAATATAACAGTTTTACCGATTACATCTTTGTATCGTTCGTCATTAGGGTTAACAACAATC
GCTGTATCACCTAACATCGTTTCTGGTCTTGTTGTTGCAATTTCAATAAAACCTTCACCAT
CAGCGTAAGGATATTTAAAATGATAAAACGCACCTTGAACATCTTCATGTATTACTTCAA
TATCAGATAAAGCTGTACGTGCTTTAGGATCCCAATTTATAATACGTTCGCCACGATAAA
TAATTCCTTTATTGTATAAATCAACAAAAACTTTTTTAACTGCTTTACTTAAACCTTCATCT
AAAGTAAAACGTTCTCTACTATAATCTAAACCTAGACCTAATTTAGCCCATTGCGCACGA
ATAAATGACGCATACTCTTCTTTCCAATCCCATGCCTGTTCTAAAAACTTTTCACGACCAA
GATCATATCTAGTTATTCCTTGTTCATTTAATTTAGCTTCTACCTTTGCCTGTGTCGCAATA
CCAGCATGATCCATACCTGGTAAGTATAACGTATCGTATCCTTGCATACGTTTCATACGTG
TAATGATATCTTGTAAAGTCGTATCCCATGCATGTCCTAAATGTAATTTACCAGTTACATT
TGGTGGCGGGATAACAATTGTATATGTTTCTTTTGATTTATCTTCTGACGGTTTAAAATAA
CCATTCTTTACCCATTCTTCATAACGTCCCGCTTCAACTTCACGAGGATCATATTTTGGTTT
CATTTCCAT

> **tbox08**

Function: noncoding RNA T-box leader element (core genome, constant)
Best match: tbox08_CC008_NCTC8325_CP000253.1[1668350:1668651:r]RC (completely identical)
Position: 038-contig_319_RC: 10215 … 10517; Length: 302 bp
Sequence:
AAATAGGACGGATATTCCGTGGTACCACCTATATTCAAGAAGGATGATTAATATCAAATT
CACTCTTTTAACATAATTGGAATAATCATACCAATACTATCATCGTGAAATTTGAAATGCT
TCATCTCTTCAAGCACTCTAGATTATGATTAACGCTCAAACACGTCTTAGCCTACTATTAA
TCACGTTCAGCTAAGATACTCTGTGGGCTACCTTCAGTAAAAATCATTTACATACTCACAC
CAAATCATATGCTCTCTTTAAAATAATTTGAACTTACTCTTCCCAAATCCTATATTAAA

> **tag**

Function: protein coding sequence; DNA-3-methyladenine glycosidase (core genome, constant)
Best match: tag_CC001_MW2-USA400_BA000033.2[1735997:1736557]
Position: 038-contig_319_RC: 10574 … 11135; Length: 561 bp
Sequence:
ATGAATGAATGCGCATTTGGTACTAAAGATCCAGTCTACTTAGACTATCATGATCATGTA
TGGGGACAACCGCTCTATGATAGCAAGGCATTGTTTAAACTTTTAGCATTAGAATCACAA
CATGCTGGGCTATCTTGGTTAACTATTTTAAAAAAGAAAGAAGCCTATGAAGAAGCATTT
TATGATTTCGAACCAGAAAAGGTAGCACAAATGACCGCTCAAGATATCGACCGCTTAATG
ACTTTTCCAAATATCGTTCATCATCGTAAAAAATTAGAAGCAATTGTTAATCAAGCTCAA
GGGTATTTAAAAATTGAACAAGCATATGGTAGTTTTAGTAAATTTTTTATGGTCATATGTAA
ATGGTAAGCCTAAAGATTTGCAGTATGAACATGCTTCTGATCGTATCACAGTTGATGATA
CTGCAACACAACTATCTAAAGATTTAAAACAATACGGGTTTAAATTTTTAGGTCCAGTAA
CAGTATTTTCGTTTTTAGAAGCAGCCGGTTTATATGATGCACATTTAAAAGATTGTCCATC
AAAGCCTAAACACAATTAA

> **abrB**

Function: protein coding sequence; putative ammonia monooxygenase (core genome, variable)
Best match: abrB_CC005_Mu50-VRSA_BA000017.4[1774152:1775225:r]RC
Position: 038-contig_319_RC: 11357 … 12431; Length: 1074 bp
Sequence:
CTAATCTTCACGCTTATTCGAACGATATTTTAAAAAGTAATTAATTCCTGGTGCAATAATA
AATAGGATGAAAAAGATTCGGAAAATATGATAACTCGTAATCATAGCAACATCGGCACC
AGTAGCTAATGCAACTAAAACTATCTGATTAACCCCTCCTGGTGCTGCACCAAGAAACAA
TTCATTAATAGGATTATTATCAAAGAAATGTATGACATAAACCATGATTAGCGCACCAAT
TATCAACATAATATTTTGAATTGTAATTGCGATTGCTAGTCTACCTTTTAAATCTGACAAT
AAATGCGCAATTTGAACTCCAATTCTAATCATATATATTAGTTGTGCCATGTTCAACAACC

AATGATCTAGTGTAAATGTTAAACCTGTAGAAAAATTCCAAACAATTAATACAATGAGTG
GTGCTAATAATTGAAATGTTGGAAACTTTATTTTAGACATAATTAGATAAACTATAAAGA
TAGCTATCGCTAAAATAACTATTTGCCTTATGTTTAATACTTGTGATAAAGGCAAGACTTT
TGTTAACTTTCCATTCGCATGCATGTTACCATCATGAAAAAAATATGAAATGAACGGTAC
TAAAACAACAACAAATATAATTCGTGATGTTTGCGTTAAGCTAACAACTAACAAATTAGC
ACGTTTGTCTTGTTCAGCCATGACCAGCATTTGTGTTAGTGCTCCTGGTATAACACTTAAA
ATAGCTGTTTCCGTATTAATACGTGCAATTTTTTTAAAAATAAATGCCATTACTATTGCAA
TTAATAATATCGAAATAGATACAACAATAATCGAAAGCCAATTGTCTTTAATATCCATAA
CGACATTTTTCGTAAACGTTGATCCGATTTGCACCCCTAATAGTACAATCCCTAATTCACT
AAGTAAGAATGGCCATTTAATATCAAGTTTGAAAACTTTCACACAAATGATTGATGCGAT
AATAGGACCAAACATAAATGGGAGTAATACGTGCGATGAATACAATATAATACTAATAA
AAAATGATAAAACGAACACAATGAAATTATTTCTATATATCATTGTCAT

> **tx_hemL1**
Function:  rho-independent terminator
Best match: tx_hemL1_CC001_MW2-USA400_BA000033.2[1737994:1738046:r]RC (completely identical)
Position: 038-contig_319_RC: 12568 … 12621; Length: 53 bp
Sequence:
ATATACATTTAAAAAGAGCCTGAACAAAGTTCAGGCTCTCAATTTGTCCGTAT

> **hemL1**
Function: protein coding sequence; glutamate-1-semialdehyde 2,1-aminomutase/locus 1
Best match: hemL1_CC001_MSSA476_BX571857.1[1717701:1718987:r]RC (completely identical)
Position: 038-contig_319_RC: 12623 … 13910; Length: 1287 bp
Sequence:
TTATTTTACAATACGACTTAAAGCCGTATCAAATGCTTGAATCGTTTTTCAATATCTTCTT
TCGTGTGTGCCGTAGATAGGAATGTACCTTCAAATTGAGATGGTGGTAAAAACACACCTT
CTTTTGCCATTTCTCGATACATTTCTGCAAATAATTTCAAATCACTTTTATTCGCTTGTTCA
AAATTAGTTACAGGTCCTTCATTTAAGAAATAACCAATCATTGAACCTGCTCTATTTACAG
TTATTGGTACATTGTGTTTAGCAAATACACGCTTTAAACCGTCTTCAAGTATATCGCCTAA
CATATTAAAATACTCATATGTCTCTGGCGTTAATTGGCTTAACGTTTCATAACCACTTGTC
ATTGCAAGAGGATTTCCTGATAACGTACCCGCTTGATAAATATTTCCTAATGGTGCTATAT
GATCCATGATTTCTTTTTTACCACCAAAAGCACCTACAGGTAGTCCTCCACCGATAACTTT
TCCTAAGCAAGTTAAATCTGGTGTCACACCAAAGTAACCTTGTGCACAATGATAACCGAC
TCTGAAACCAGTCATTACTTCATCGAAAATTAGCAATGCGCCGTATTCAGTCGTAATATTT
CTTAATCCCTGTAAAAAACCTTCAATCGGCGGTACGACACCCATATTACCAGCAACAGGT
TCTACGATTACACCAGCAATATCGTCTCCAAATTTTTCGAAAGCGATTTTAAGTGCATCTA
AATCATTGTATGGAACTGTAATTGTATTTTTAGCAATACCTTCAGGCACACCAGGAGAAT
CCGGCAATCCTAATGTTGCCACCCCAGAACCAGCTTTGATTAATAACGAATCACTATGAC
CATGATAGCAACCTTCAAATTTCACAATTTTATTTCTTCCAGTATAACCACGTGCTAATCT
TAAAGTATCCAATGTAGCTTCTGTACCAGATGACACCATACGCACTTTTTCTATTGAAGGT
ACTCGGTCAATAACGAGCTGCGCCAATTTATTTTCAAGTAATGTTGATGCACCAAAACTT
GTACCTTTATCAATTGCTTCATGTAAATGACTAATAACTTGAGGGTCTCTATGTCCTAAAA
TAAGTGGCCCCCAACTTAGTACATAGTCGATATACTCGTTACCATCGATATCATAAATTTT
TGAACCTTTACCGTGATCCATAAAAATTGCTGGTGTATCTACTGATTTAAATGCGCGTACT
GGACTATTTACACCACCAGGCATTAAAGTTTCAGCAACCTTCATTGCTTCTTCTGATTTCG
TATATCTCAT

> **hemB**
Function: protein coding sequence; porphobilinogen synthase
Best match: hemB_CC001_MW2-USA400_BA000033.2[1739383:1740357:r]RC
Position: 038-contig_319_RC: 13957 … 14932; Length: 975 bp
Sequence:

TTATTTATCTAAATAGCGACAAATGTCCTTTGCAAAATACGTAATAATCATATCAGCACCT
GCACGTTTCATTGAAACCATTTGTTCCATAACGACACGTTCTTCATCTATCCAACCATTTT
GTGCCGCTGCTTTAGTCATACTATATTCTCCACTCACATTATATGCAACAACTGGAACATT
CGTATGATTTTTAACATCTCGAACTATGTCTAAATAACTTAGAGCAGGTTTAACAATCATC
ATGTCGCACCCTTCTTTAAGATCACTTTCTAATTCACGAAGTGCTTCCAAACGGTTAGCAG
GGTCCATCTGATACGTTTTTCTATCCCCAAATGATGGCGCTGAATCTGCTGCATCTCTAAA
AGGTCCAAAGAAACTTGATGCATACTTGACACCATAACTCATTATAGGAATATTGTAATA
GCCGGCTTCATCTAATCCACGACGAATTTCAGCAACAAAACCATCCATCATATTACTTGG
CGCAATAATATCAGCACCAGCTTCCACTTGAGAAATTGCTGTTTTAACAAGTAGTGGCAA
TGATTTATCATTGTCAACGTCATGTGTATGGTCATCAATCACACCACAATGACCATGATCA
GTATATTCACATAAACAAGTGTCTGCAACAATTAATAAGTCATCATACATTTTTTTAGCAA
TACGTGTTGCCTGTTGAATAACACCATCGTGAATGTATGCACCAGTACCTATATCATCTTT
TGAGTTTGGAACACCGAAAAACATAATGGCACGTATGCCTAAGTCATAAGCTTCTTTTAA
TTCACTTTCAAGTAAATTCAAACTGATTTGGTATACACCTGGCAATGACTTAATTTCTTTT
TTCACATCGTCTTTTTCAACTACAAAAATTGGATATATTAAATCTTCTTTTCTTACATGATT
CTCTCTAACCATATCTCTCATTGTCGCTGATGATCTCAATCTTCTATGTCTATCAAATTTCA
T

## > hemD

Function: protein coding sequence; uroporphyrinogen III synthase
Best match: hemD_CC001_MW2-USA400_BA000033.2[1740360:1741028:r]RC (completely identical)
Position: 038-contig_319_RC: 14934 … 15603; Length: 669 bp
Sequence:
TTAGCCCCTACTTTCTAAAATCTTTTCAATTAGTGATTCGAGTGTTTGAATTTCTGCAATTG
TTACTGGTTGTTGATATGATTTAATGGTCCGTGCTGTTTGTTCTCCAATAGCAAAATACGA
CTTGAATTTTGGTACAAATCCTTCATTAAAATAATAACGTACTGCCGACGAACTTGAAAA
TGTTAATGCATCGATTTGTTGATGTTCTATCATTTCTTTAACATCTTGTATATTTTGTTTGTT
AGGCACTGAAGTATATAAATCTATTTTAACAACTTCATTATCTTTAGATAACGCTGCTAAT
AACAATGGTCTCGCCAATTCACTCGAAGGCAAAAGTATTTTTTGGTTAGTTTGATTAAATG
ATTTTAAAAATCCTTCTTGAGAAAAGTCGTTTGGCATAAAATCAACTCGAATGCCAAGTG
ATTCACAATATTGCGCTGTCTTACTTCCTATCACAGCAATGTTATCAACATTAATTCCTTTT
AGATATTTATAAAAGAATTTCACAGCATTTTTAGATGAAAAAATAAGCCAGTCATAGCGT
TGATTTAACAAATGAATATCAAAATTTAGTGGCTTTATATCAATAAAGGGTTTGTGAATA
ATTGATACTAAATCACTTTGCATGTCATTTGTTTGTGTCATAACTACAACTGGCTTCAT

## > hemC

Function: protein coding sequence; porphobilinogen deaminase
Best match: hemC_CC001_MW2-USA400_BA000033.2[1741050:1741976:r]RC
Position: 038-contig_319_RC: 15624 … 16551; Length: 927 bp
Sequence:
TTAATGTTGTTCATTTAAGCGTTTTATAATTTCATAAGCACCTTGCTCTTTTAATTTGTTAC
TCACTGTTTTGCCTAACTCAACCGGATCTGTTCCGTTCATTGTATATTCAAATCGTTCTTTA
CCATCTGGGGTCATAATTAAACCTGTAAATTCGATTTCGTTTTGATCTGAGATTGTAGCAT
ATCCTGCGATTGGCACCTGACAACTACCATCCATTTCTGCTAAAAACGTTCGTTCAGCAGT
CACACATTTTGCAACCTCATCATTATGTACTTTGCTTAATAATGTTAATAGTTCTTCATCGT
CACTACGACATTCTATCCCTAAAGCACCTTGTCCGATTGCAGGTAACAATGTATCTCTATC
AAGATAAGATGTTACAATATCATCTGACCAGCCCATTCTTCTTAAACCAGCTGCAGCTAA
AATAATCGCATCATAATCTTCAGTTTGTAACTTTTCTAATCGTGTATCTATATTACCTCTAA
TCCATTTAATCTCTAAATTAGGATACTTAGATAATATTTGTGCACCACGACGTAATGAACT
AGTACCAATAATACTGCCTTCTGGCAATTGGGATAGTGGTGTATGTGTTTTAGAAATATA
CGCATCAAAAGGTAATTCTCTATCAGGGATACAACCTAATGTTAAACCTTCCGGAATTAC
ACTTGGTACGTCTTTAAGCGAGTGTATTGCCATATCGATATTTTTTTCAAAAAGTTCATGT
TGTATTTCTTTAACAAATAAGCCTTTGCCTCCGACTTTAGACAATTGCTTATCTACTATAC
GATCGCCTTTCGTGACAATTTCTTTAATTTCAATTTCTAGATTTGGCTCGACAGCTTTTAAT

TTATCAATAAATTGCTGGCTTTGTGTTAAAGCTAATTTACTTCTTCTGGAGCCAACGACTA
ATTTACGCAT

> **hemX**

Function: protein coding sequence; membrane uroporphyrinogen III methylase
Best match: hemX_CC001_MSSA476_BX571857.1[1721670:1722485:r]RC
Position: 038-contig_319_RC: 16592 … 17408; Length: 816 bp
Sequence:
TCAATTCACAAAATGTGTTGCAAAAAATAAATTAATCATATTTAAGCAAAATAAAATAAT
GTTATAGTATATTAAATACCTTGAATTCAACCATTTGTTGATTCTAAGCAATATATAACTT
CCATATAATATTGTAATAATTGAAGAGAGTATTACCTTCGGGTCAATGAATATACGTTCA
CCAACTGAAATTACACCCCACTGTGTACCTAAAATAATACTAAATATGAGAATTATCCAC
CCACTTAACGTTGAGTAAAACACAATTGATTCAAGTGTAGCAACGCTACCTATTCTAAAG
TATTTTTGATCAAAACGTTTTTCCTTCAAATTACGGTATTGCATGATATACAGTAATGCAT
TGACAAAAGCTAAGGCAAAGAAGACATAACTTAACACAGCTAGACCGATATGGACTAAC
AGTAACTCGTCTACAACAGCAATTTTCTGAACCTTATTAGTATAATGTGTCGGTTGAAATG
TATTCATCCCTAAAAGTGTTAACCCTATTAAATTCAAGAAAAACACAGAGAAATTCAATA
CTTTGATAAGGTTAAGAATTAAAGAGATTGAAATAATCAGCCAACTTAAAGTATAGAATA
CATCTGAAATAGACCCTAATGGAATATGTCTAGTTTGTATAATAAAAATAGATAAAGAGA
TTGTTTGTAAAACCCAAACAATCCCCAATAAATATATGCCTAAACTTCTAATCTTATGACT
TTTTTGGACAAAATCATAAAAATAGCAAATGATACTGATTAAGTATATTAATAATATAAT
TTCATTGAATCGAATAAACAGGTTTTCTTGCAT

> **hemA1**

Function: protein coding sequence; glutamyl-tRNA reductase (core genome, variable)
Best match: hemA1_CC001-ST772_118_AJGE01000003.1[45337:46683]RC (completely identical)
Position: 038-contig_319_RC: 17429 … 18776; Length: 1347 bp
Sequence:
TTATTCAAAACTAAAGATACGTCGCGCTGAAATTTCCTTGACCTTGCTTTCTTTTTGTTGCT
TCGCTTGCTCATGAGGACATTCAGCTTCGATATCAAATATATTTTGAAATAGCTCTAATTT
TTCATTACTTTTCTTATCACTACTTAATTCTTTGGCCTGTTTAATAGGATCTTTCAACATTT
GATTGATGATACTTTTTGTATGTTTAGATATAATTTTACGTTCTCTTTCGCTTAACCCTGGC
AATTTACGATCAATACTGTCCATCGTTTCTGCTTGAATTGTCATAGCTTTTTCACGTAAAG
CTCTAATCACTGGAACAACACCCAACATACTAATCCACTCATTGTGTGCATGTATTTCTGC
AGGAATTTGTTCCGAAATTGTTGCAGCCGCTAATTGTCGCTCACGTAAGTTTGCATCAACT
AAACCTTTTAAGTCATCAACATCATAATTAAAGATGTTTGTGATGGCACTAATACCAGGT
TCAATATCTCGAGGAACTGCAATATCAATCAATACTAGTGAATCTTGCTTTCTATTTTCTG
CAATTCTTTCTATCATTTCATTTGTAATGATATAAGATTGTGCACTCGTTGAACTAATCAC
AATATCTGCACTTTCAAGTAAATTTGGTAATGATGATAGTTCATCATATTTCACTTGATGC
TTTGCTGCTAATTTCATAGCATTTTCAATTGTTCTATTTACTACTGTAATATCAGTAATTCC
AGAACCAAGAAGATTTAATAGTGATAATTCACTCATTTCCCCTGCACCAATAATGATAGC
TTGCTTACTTTTCAATTTGCCAAATACTTTTTTCGCCAACTCGACCGCAGCATAAGACACA
CTTACAGCATTATCAGCTATATCTGTTTCATTATGTGCTCTTTTTGCAAAAGTAATTGCCTG
TTTAAATAGATGATTAAAAATTGTTCCTGTCGTACCTGTGCTTTGCGCTAAGAAAAATGCA
TCTCTTATTTGACCTAAAATTTGAGTTTCTCCAAGTACGATTGAATCTAAACCAGAAGTGA
CACGCAATAAATGTTCTACTGCTTCGTCCCCCACTTTTACTTCTGACATTGCTTTAATATCA
TCTACTTCAAATCCAAATGCACGAGCTAGAAATCGTTGAATATAGTAACGACCTGTGTGA
ATTTGATCAACAACAGCATATACTTCAGTTCGATTACATGTTGATAATATGACATTTTCTA
AAATAGATTTAGTTTCATATAAATCTTCATGGGCAATTCGTAAGGCATCATCTCTAAAAG
CAACTTGCTCTCTTAGTGCAACATCAGCTGTGCGATGATTTATACTAATTGCAATAAAATG
CAT

> **tnpIS431-06**

Function: protein coding sequence; transposase for IS431 (mobile element)
Best match: tnpIS431-06_circ_S-epidermidis_GQ900381.1[43799:43807]RC (completely identical)

Position: 038-contig_319_RC: 18076 … 18085; Length: 9 bp
Sequence:
ATAGTTCAT

> **engB**
Function: protein coding sequence; GTP-binding protein
Best match: engB_CC001_MSSA476_BX571857.1[1724070:1724660:r]RC (completely identical)
Position: 038-contig_319_RC: 18992 … 19583; Length: 591 bp
Sequence:
CTATGAAATATACGGTTCAATTAAATTCCATATTTGTTGTTGTTTATTATTTTGAATTGATG
AATAACTTACAATTGTATCGTCTGGGTCCATATCTAATTGTGTCTTAATATTTTTAATATG
CTTTTGAACTTTACCTTTTGGAATTTTGTCTTCTTTAGTGCATATAACTAAAGTAGGAATAT
CAAAATGTTTCAAATAATTGTACATTAAGATATCATCTTGTGTTGGATCATGTCTTAAATC
AACTAATTGAATAACTAATTGCAAATTCTCTCTCTTAGTTATATATTCCTCAATCATTTTCC
CAAATTTTTCACGTTGTGTTTTACTTACTTTAGCATATCCATACCCTGGAACATCCACAAA
AATAAGTTGTTCATCTATATTATAAAAATTTAACGTTTGCGTTTTGCCGGGTTGCTGTGAT
GTACGTGCCATATTTTTTCTGCCAATCATACTATTGATAAATGTAGACTTACCTACATTAG
ATCGACCGCTCAGTGCAACTTCAGACAATTCTGTTTCTGGATATTGTTCTTCTTTTACTGC
ACTAATGATTAATTCTATATTATTAGGATTAACTTTCAT

> **txbi_clpX**
Function:  bidirectional rho-independent terminator of clpX
Best match: txbi_clpX_CC001_MW2-USA400_BA000033.2[1745075:1745150:r]RC (completely identical)
Position: 038-contig_319_RC: 19649 … 19725; Length: 76 bp
Sequence:
ATACATTATAAAAAAGCTCCGATCAAAGTTAAACTATTTGAGCGTCAACTTTGATTGGAG
CTTTTCACTTTTATAA

> **clpX**
Function: protein coding sequence; ATP-dependent chaperone protease/ATP-binding subunit
Best match: clpX_CC001_MW2-USA400_BA000033.2[1745162:1746424:r]RC
Position: 038-contig_319_RC: 19736 … 20999; Length: 1263 bp
Sequence:
TTAAGCTGATGTTTTACTATTATTAATTAAATTGCCTTCTGCGTCGTATAGTTCTGGTTCAG
TTTCTTCATTAATTGTTTGTGCTGTAATAACTACCTTCGTTACATTTTCGTTAGAAGGCACA
TCAAACATAATATCGATTAACGATTCTTCTATGATTGAACGTAAACCACGCGCACCTGTTT
TTCTTTCAATTGCTTTTTCACTAATTGCTGATAAAGCTTCTTCAGTGAACTCTAAATCCACA
TCATCTAATTCCAGCATTTTAGTATATTGTTTCACAAGTGCATTTTTAGGTTGCGTTAAGA
TGTTTTTCAACGCAGTTACATCTAATGTTTCTAAATTAGCTACAATTGGCACACGTCCGAT
AAATTCAGGAATCAAACCATAGGCTTGCAAATCTTCTGGGCGAATTTGTGCTAATAATGC
TTGTTCGTCATATTTATCAGCTTCATTGCTTGAGAAACCAATAACTTTTTCACCAAGACGG
CGCTTAATCACTTCTTCAATACCATCAAAGGCACCACCAAGAATAAATAAGATATTTGTT
GTATCAATTTGAATCATTTCTTGGTTTGGATGTTTGCGTCCCCCTTGTGGCGGAACACTTG
CAGTCGTACCTTCTAAGATTTTAAGCAATGCTTGTTGAACACCTTCACCTGAAACGTCACG
TGTTATAGATGTGTTTTCAGATTTACGTGCAATTTTATCAATTTCATCTACATAAATAATA
CCTTTTTCGGCTTTATCAATGTCAAAGTCAGCTGCTTGAATTAATCTCAACAAGATATTTT
CAACATCATCGCCTACATAACCAGCTTCAGTTAAACTTGTCGCATCTGCAATTGCAAATG
GTACATTCAACGTCTTGGCTAATGTTTGAGCTAATAATGTTTTACCACTACCTGTTGGCCC
AATTAATGCAATGTTACTTTTTTGTAATTCAACATCATCTTCTTTTGGTCCTAATTGTTGAA
TACGCTTATAGTGGTTATAAACAGCTACAGCTAAAGATTTTTTAGCTTTTTCTTGACCAAT
AACATATTCGTTTAAATGATCCATAATTTCTTTAGGAGTAGGTAATTCTGTCATCGCTTCA
GAAGTGTTTTGAGCTAATTCTTCTTCGACGATTTCTGAGCATAATTCAATACACTCATTAC
AAATATATACACCACTTCCTGCTACAAGTTTTTTACTTGATCTTGGTCTTTTCCGCAGAA
AGAGCATTTCAAATTTTCTTCATCTTCATTGAATTTAAACAT

> **tig**
Function: protein coding sequence; trigger factor (core genome, constant)
Best match: tig_CC001_MW2-USA400_BA000033.2[1746575:1747876:r]RC
Position: 038-contig_319_RC: 21149 … 22451; Length: 1302 bp
Sequence:
TTAATCTTCTTTAGTTCCTTCAACGAACTTTGCGTTATCTCTTAATAAATCGATAACTTTTT
GGATACGAACATCATTTTTAATGATATCAGTATTACCTAAAGTATTTTTGATATCTTCAAC
TGAGATATTAAATTGTTTACTCATTTTTTCTAATTCTTTATCGATATCTTCATCAGTAGCTT
CGATTTTTTCAGCTTCAGCGATCGCAGTTAAAGTTAAGTTAGTTTTAACACGTTGTTCTGC
ATCGTCTTTCATTTGCTCTCTTAATTGAGTTTCATCTTGACCTGAGATTTGGAAGTACGTTT
GTAAATCTAAACCTTGTTGTTGAATTCTTTGTGCAAATTCAGACACCATACGATCTAATTC
AGTATTAACCATTGCTTCAGGAATATCGATTGTTGTATTATCAGTAGCTTTTGTAATCGCT
TCTTCTTTTTCAACATTTTCAGCATCTGTAGCTTTTTGTTCAGCTAAACGTTTACGTAAGTT
TTCTTTGTACTCGTCTACTGTATTTGCTTCTGCATCTAATTCATTAGCAATTTCATCTGTTA
ATTCTGGAACTTCTTTAAATTTAATTTCGTTAACTTTTGTTTTGAAAGTTGCTTCTTTACCA
GCTAATTCTTCAGCATGGTATTCTTCTGGGAATGTTACGACAACATCTTTTTCTTCGTCAA
CTTTCATACCTTCTAATTGCTCTTCGAAACCAGGTATGAATGAACCTGAACCGATTTCTAA
ATCGTAACCTTCAGCTTGTCCACCTTCGAATTCTTCTCCGTCAACTGAACCACTAAAGTCG
ATGTTAACTGTGTCGCCATTTTCAACAACACCATCTTCTTTAACTACCATTTCAGCTAAAT
GTCCTAAGCTGTGGTCAATCGCTTCTCGTAACTCATCATCAGATAATTCAGTTTCTTGTTTT
TCAATTTCAAGACCTTTATAGTCTCCTAATTTAACTTCTGGCTCAACTATAACTGTTGCTTC
AAAAATGAAATCTTTACCTTTTTCAATTTGAGTAACACTTACTTCTGGTTGTGCAACTGGT
TTAATATCAGTTTCGTCAATTGCTTCACCATAAGCATCTGGTAATAAAATGTCGATAGCAT
CTTGATATAATGCTTCTACACCAAAGCGTTGTTCAAAAATTGGACGTGGCACTTTACCTTT
ACGGAATCCAGGTACGTTAATTTGTTTAACCACTTTTTTGAATGCTTGATCTAACGCTTTG
TTTACTTTTTCTGCAGGAACAGTAACAGTTAATAAACCTTCGTTACCTTCCTTTTTTTCCCA
AGTTGCTGTCAT

> **rplT**
Function: protein coding sequence; 50S ribosomal protein L20 (core genome, constant)
Best match: rplT_CC001_MW2-USA400_BA000033.2[1749737:1750093:r]RC (completely identical)
Position: 038-contig_319_RC: 24311 … 24668; Length: 357 bp
Sequence:
TTATTTTAAAGCATCTTTAGCTTTAGTTACTAATTGAGCAAATGCTTTTTCGTCAGAAATT
GCGATTTCTGATAACATTTTACGGTTAATGTCGATACCAGCTTTTTTCAAACCGTTCATTA
AACGTGAGTAGCTCATTTCATGTTGACGAGCTGCTGCGTTGATACGTGTAATCCATAATTT
ACGGAAGTCACGTTTACGTTGACGACGGTCACGGAAAGCATATTGACCTGATTTCATTAC
TTGTTGCTTAGCTACTTTGTATAATGTATGTTTTGAACCGAAGTAACCTTTAGCTAATTTA
ATCGTTTTTTTACGACGCGCTCTTGTTACTGTTCCACCTTTAACTCGTGGCAT

> **rpmI**
Function: protein coding sequence; 50S ribosomal protein L35 (core genome, constant)
Best match: rpmI_CC001_MW2-USA400_BA000033.2[1750140:1750340:r]RC (completely identical)
Position: 038-contig_319_RC: 24714 … 24915; Length: 201 bp
Sequence:
TTATTTTTTGTATGCTAATAATTGTTTTACACGTTTCATATCGCTCTTAGACACTAATCTAG
CTTTACGTAATTGACGTTTTTGTTTAGTGCTCTTGTTTGCGAATAAGTGAGATGTGAAAGC
TCTTGAACGTTTTAATTGACCTGAAGCAGTTCTTTTAACACGTTTAGCTGCTCCGCGGTGA
GTTTTCATTTTTGGCAT

> **infC**
Function: protein coding sequence; translation initiation factor IF-3 (core genome, constant)

Best match: infC_CC001_MW2-USA400_BA000033.2[1750369:1750896:r]RC (completely identical)
Position: 038-contig_319_RC: 24943 … 25471; Length: 528 bp
Sequence:
TTATTTTTCAGCTGTTGGCGCTAACATGATAAACATTTGACGCCCGTCCATTTTAGGTTTTT
GTTCAACTGTTGCTATATCTTTGCATTCATCTGCATATTTTTCTAGCACACGTTGACCAATT
TCCTTATGCGTAATGGCACGCCCTCTGAAACGAATAGATACTTTACATTTATCGCCTTTAG
TTAAGAATTTACGTCCGTTTTTCAACTTAGTTTGGAAATCATGTTCCTCAATTGTTGGACTT
AAACGAATTTCTTTAACATTGATAATTTTTTGTTTCTTTTTCATTTCTTTTTCTTTTTTCTGT
TGTTCGAATTTGAATTTACCGTAATCCATAATTCTTGCAACTGGTGGTTTCGCATTCGGTG
CAACGACCACTAAGTCTAAATCTACACGTTCAGCCATTTCTAAAGCTTCACGCTTTGATTT
AACACCAATTTGTTCACCATCTTGACCGATTAAACGTAATTCTTTTGCACGAATTTTGTCA
TTGATTTGAGTTTGATCTTTTGCTATGGTTGACAC

## > **L20**

Function: noncoding RNA ribosomal protein L leader RNA (core genome, constant)
Best match: L20_leader_CC001_MW2-USA400_BA000033.2[1750925:1751050:r]RC (completely identical)
Position: 038-contig_319_RC: 25499 … 25625; Length: 126 bp
Sequence:
AGCAAAAAGGAAGAGCAGGTATAAAATACCCGCTCTTCCTTATACACAGTTATGTGTAAT
GTGATTAACCTGCCAACTGCTTTATGCGTCGCTACAGGTGAGAAGCGGGTGCTTCTACTT
GGTTCG

## > **lysP**

Function: protein coding sequence; lysine specific permease (core genome, constant)
Best match: lysP_AC078832.7[17642:19135:r]RC
Position: 038-contig_319_RC: 25699 … 27193; Length: 1494 bp
Sequence:
TTAACCTTTTATTTCATCCATTGACACGTCTTGACGTAAATCTACTTGTTCTAATGGAATTT
TTTTCGTTTTATATCGAAGCTTATGATAAATAAAGAATGCTAAAAATACTGGGATTCCCAT
ATACGTAATTAAGAATCGACTAAAATTAAAATCTCCTGTTTTAATAAAGTCAACATCTTG
ACCAATAATTACTACAATACATAAAAAGCCAGCAAATAATGGTCCGAATGGGAATAATTT
AGCAGTATATTTTAATTTAGATTTGTCATAATTTTGTTTATCAAATGCTCTTCTAAATCGAT
AATGACTTACTGCTATACCTACCCAAGCAATAAAACCAGTTAAACCACTTGCTGCAACGA
TATATTCGTATGCACCTTTTGTAAGGCTTTGTAATACGAAAATTATCACTACAATGATTGC
TGTAACGAGTAACGACATATATGGCACACCGTTTTTATTTGTTTTACCAAATGCTTCAAAC
GCTAATTTATCTTTACTCATTGAATATAGCATTCGAGTTGAAGCATACATACCTGAGTTAC
CTGCTGATAACACAGACGTTAAAATGACTGCATTCATAAATGATGCTGCAAACGCAAATC
CAGCATTTTTAAACACTAATGTGAATGGAGACGTTGCTACATTATCACTACCACCCATTA
ATGCACTACTATCATAAGGAATTAACATACCGATAACAAAAATGGCTAAAATGTAAAAT
AATAAAATTCTCCAGAATACTTGTTTAATTGCTTTCGGCACAGCACGTTCAGGATTTTCTG
ATTCACCAGCCGTAATACCAATTAACTCAGTACCTTGGAATGAGAAACCAGCGATTAAGA
ATACACCTAGAATTGATAATAAACTTCCTCCTAAGTTGCCACCAAGAATAGGACCTTCAC
CTTTATTAAATATTTCGAATCCTACAACATGACCACCCATGATTCCGACAATCGTTAATAA
ACCAATTGCAATGAAAACAATAACTGTAACCACTTTTATCAATGCCAACCAGTATTCACT
TTCACCATAGACGCGAACTGATAACGAATTCAGACTAAAAATTATAACTAAGAACAACG
CACTCCATGCCCAAGCGGGTATGCCTTGCAATGGTGTCCAATATTGAATGACTTGTGCTG
CAATCGTAATATCTGCTGCTACAGTTACTACCCAGTTAAACCAATAGTTCCAACCAAGCG
CAAACCCTAAAGATGGATCAACAAATCTTGTAGCATATGTACTAAATGAACCTGATACTG
GCAAATACGTAGCCATTTCGCCAAGTGACGTCATTAAGAAAAATACCATTATTCCGATAA
TTGCGTATCCTATTAATGCACCCAAAGCACCTGCATCATGAATTGCTCCACCAGAAGTTA
CAAATAAACCTGTACCAATACAACCCCCAATCGCAATCATAGAAATATGACGATCTTTAA
GTCCCCTTTTGACAACATTGTTACTTTCATTTTGAACTTTTGACAT

> **Lys_riboswitch**
Function: noncoding RNA lysine riboswitch (core genome, constant)
Best match: Lys_riboswitch_CC001-ST772_118_AJGE01000003.1[36600:36775]RC (completely identical)
Position: 038-contig_319_RC: 27337 … 27513; Length: 176 bp
Sequence:
ATATAAGGTAGCACATCACATTCTGTGACAGTACAGTTCCTATTCGAAAACTGTCCCAAT
AGATATAATTTATGGTTTATATCTATTTCGGCATCTTTACCTTTCACTTGTTCAACTTAGGT
ACCATAAATACTTCTGACAAGTTACTAATTAAACATGCAACCTCTAACTCAATT

> **thrS**
Function: protein coding sequence; threonine--tRNA ligase (core genome, variable)
Best match: thrS_CC005_JH1_CP000736.1[1838653:1840590:r]RC
Position: 038-contig_319_RC: 27618 … 29556; Length: 1938 bp
Sequence:
CTATCTATGTTTTTTCAAACGAATTTCATCAACTAGATTCCAGATAAATTCATCTTTTTCAA
CTGTTTCTTGGTCTTGCGATCCATATTGACGCACATTCACTTGATTATTTTCAACTTCCTTA
TCCCCAACTACGATTTGATAAGGTATTTTTTGCATTTGAGCTTCTCTAATTTTATAACCCAT
TTTTTCATTACGGTCATCAATACTTACACGAACACCTTGAGATTTCAATTCATCTTGCAAT
TGGCGCGCATAATCATAATGTAAATCAACGTTAACTGGAATGATTTGAACTTGTTTTGGC
GCTAACCAAGTTGGGAATGCACCTTTAGTTTCTTCAGTTAAAAATGCTACAAATCGTTCCA
TTGTTGATACAACACCACGATGAATCACAACTGGACGATGATGTTCACCATCTTGACCAA
TATAAGTTAAATCAAAACGTTCTGGTAATAAGAAATCAAGTTGTGCTGTTGATAATGTCT
CTTCTTTACCCATCGCTGTTTTAACTTGAACATCTAGTTTCGGACCATAGAATGCCGCTTC
ACCAATCGCTTCTTCGTACGATAAGCCAAGCTCATCCGCTGCCTCTTTAAGCATATTTTCA
GCTTTATTCCACATGTCATCATCATCAAAGTACTTTTCTTTATCTTCAGGGTCTCTATAACT
TAATCTAAAGCTATAATCCTCGAAACCAAAGTCTTTATACACATCAATAATCATGTTTACA
ACGCGTTTGAATTCTTCTTTAATTTGATCAGGTCGAACAAAGATATGTGAATCATTTAAAG
TCATACCACGAACACGTTGTAATCCTGATACAGCACCACTTGCTTCATATCTATGCATCGT
TCCTAGCTCAGCGATACGGATAGGTAATTCACGATATGAATGTGGTTTATTCGCATAAAT
CATCATATGATGTGGACAGTTCATTGGACGTAATACCATAGATTCAGTTTCATCTAACTGC
ATTGGTGGGAACATATCTTCTTGATAGTGATCCCAGTGACCAGATGTTTTGTATAAATCAA
CATTAGCAAGTACTGGTGTATAAACGTGGTCATATCCCATGCTAACTTCTTTATCAACAAT
GTAACGTTCAATTTCACGTCTAATTGTTGCACCGTTAGGTAACCATAATGGCAAACCAGC
ACCAACTAATTGGCTATTTGTGAATAGTTCTAACTCTTTACCAATTTTACGATGATCACGT
TCTTTACGCTCTTCTAACATTTGTAAATGTGCTTTCAATTCTTTTTTATCAAAGAAAGCAGT
ACCGTATATACGTTGTAACATTTTGTTGTTACTATCTCCACGCCAGTATGCACCTGCTGTA
GATAATAGTTTAAACTCTTTAATTTTAGCTGTTGATGGAACGTGAACTCCACGACATAAAT
CAGTAAAATCACCTTGACTATATAATGTTACATTTTCATCTTCAGGAATCGCGTCGATTAA
TTCTAATTTGTATTCATCATTGCTGAATAACTCTTTCGCTTCATCTCGTGAAACCACTTTTC
GTTCGATTTTCATATTTTCGTTAACGATTTGTTTCATTGTTTTTTCAATTTGTTCAAAGTCA
TCAGATGAGATGTTTTGGTCAATGTCGAAGTCATAGTAGAATCCACCTTCTATTACAGGA
CCTACACCAAATTTAACATTACCATATAACCTTTTAATCGCGTGTGCCATTAAATGTGCAG
TAGAATGACGTAATACCTCTAACGCTTCTTCACTACCTGGTGTCACAATTTCAATTGATCC
ATCAGTTTCAAGCGGTTTAGTTAAATCTACAAGTTGCCCGTTAAATTTGCCGGCAACAGCT
TTTTTACGTAATCCAGGACTAATTGATTGTGCTATATCTTCAGTAGTAGTACCTTTATCAA
ACGCCTTTTTATTACCATCTGGAAATTGAATATTAATTTGTTCCAT

> **tbox09**
Function: noncoding RNA T-box leader element (core genome, constant)
Best match: tbox09_CC008_NCTC8325_CP000253.1[1687854:1688065:r]RC (completely identical)
Position: 038-contig_319_RC: 29716 … 29928; Length: 212 bp
Sequence:
ACAAGGGACGAGATCGTCGTGGTACCACCCTAGTTATCTAATACAATTTATGTATTAAAT
ATCTCTGCTTAAGATAACGGTCTTGATCCGGGTATTCATTACAAATACCATAAATGAAGT

AGTAATCATCTAATTTATTAACCATATTCTCATCAAACAATGGCTTTCTGTGTAATATTGA
TTAGATCATCTTGTCTTCAATCATTTAAACG

> **dnaI**
Function: protein coding sequence; primosomal protein (core genome, constant)
Best match: dnaI_CC008_NCTC8325_CP000253.1[1688106:1689026:r]RC (completely identical)
Position: 038-contig_319_RC: 29968 … 30889; Length: 921 bp
Sequence:
TCAATTGTTTCTGAAATTTTCTCCTGATAAAAAGTATGGTGTTGACAAAGATTTGACACGT
TCAATAATACGTGCTGCTTTAGTCTTCTCTTCACCATCACGAGTCATCGCTAAATGATGTT
CCAATTCACTATAGTCAAAATTAGAACTAAAGAATGTTGGTAATTCATGAACCATTCGAT
AATGTAGCAAAGGTCCAATTACCTCATCTCTCACCCATGGAGTCACTTCTTCAGCCCCAAT
ATCATCAAGCATTAAAATGTTTGCTTCTCTTACGCGATGTAATTTCTTTTCAAAAGAACCA
TCTTTAAAGCCACCTTTTAATGTTCTAATAAATTCCGGTAAATAAATAATTGTCGAACGTA
CCTTCTTAGATTTGAGCTGATTCGCAATTGCACCTAGAATAAAAGATTTACCTGTCCCAAA
TGGACCATAAAGGTAAAGGCCTTTCACTTGTTCCCCATTAGTTATTGCTGTACAAATATCA
TCTGCTGCCATAGCTACATCAAGACGGTCTCGATGATTCATATAAATATCTTTCAATTTGG
CATTTAAAGTATCTCGTTGCATATGATGAGATGTAATTAGCTCAGCTTCAAAGCGTTCTTC
GTCGTACTTGATTTTACATGGGCATTGTAAATAGCGTATTTTAATTCGGTTATTATCAACA
TATAACTCAGGCACATGCCCCTTTACGAAATTTGGACAATCAGCAAATTTATGACCGTCA
TAATGTTTTTGTTGATCTTTATACTCTTGTAACACATTTAAGTCTTCATCAATCATAGCATT
CGTTAATTCAGCTCGATGCGCTTCCAAAAATTGCTTAACATCTGGGTCATTGATTACTTCT
TTTTTATCTTTTCTATTCTTTTTTCAAAGTCCTGCGACGTGTTAATTATACTTTTAAATTGC
TTCAT

> **dnaB**
Function: protein coding sequence; chromosomal replication initiation and membrane attachment protein (core genome, variable)
Best match: dnaB_CC001-ST772_118_AJGE01000003.1[31824:33224]RC (completely identical)
Position: 038-contig_319_RC: 30888 … 32289; Length: 1401 bp
Sequence:
TTATTGACTGTCCTCCTCCCATTTTTTAGATAATTTATCTAGAAATGCTTGTCGATCTTGCT
CTAATTGTTGATCATCTACGCTATTATCTTTAGCCGAATCTTCTTCACTAGGTTTATCTCTA
TTTTCTAACCATTTAGGTGTTTTTTCTTTTGAAATACGATTACGCTGCCCATAGTATGAACC
ACGCTTTTGGTAATTTCCGCTAGAACCCTCATTTTTAGATTGATTAACTTTTTTAGCGTAAT
TATATGCTTCTTTAGCTGTCTTAATACCTTTTTTCTTCCAATTTGATGCTATTTCCAAAATA
TACGCTTTAGGAAGTTTCATATCTTCTTTTAACATGACAAATTGCAACAAAATATTAATGA
CGCCAAAAGACATATTTTCACGTTCAATTAATTCTTCAACCATTGTCTTTTGCGATATAGT
TGGTTCTGATTTAGACCAAGAAGCTAACATATCAATTGGACTCGTTTGTTCAAGTAACTCA
AACCATTCATCACTTTGTGGCTTTGGATTCACTTCTGAAGATTTACCCGTCGAAGATGATG
TAGCAGGAGATTTCACCTGTAATTTAGGCATTTGATTTTCGTGTTCCATTAAGTAATACGA
GCGTGCTTGTTTACGCATTTCTTCAAAGGATAACTGTTGTCCACTTGTAATTGAATTTAAA
ATAACATGCTTCATGCCATCTGCTGTTAAACCATATAAAGTCGCGAGTTGTGTAATTAAA
CGCTTTGCATCTTTGGTAACAATGTCTTGACTAATAAAATGTTTACCTAACATTTGTCTCA
ACATTTCAAAGTCAAAAGATTCATTTGATAAATCGATACCTTGGTACGGTTCATTAATCG
GAATATCACTTGTATCGATATCTATTTTTGTAGACGGCACTTTAAAAACATCAGTAAATTG
TCTTGTTACCTGTTTAAATTCACTCAAATCAATTTGTTGATACTCAAAGTATTTCTTCAACT
CATGAAATCGACGATGCTCGACTTCACTATATAAAAAGATTGACAACATTGGATCATTAA
AAAATAAATGTGCTGAAGGCGGTTGAATTAATTGGTAAACAAATTGTGTTTCTTGTTCAT
CATGTTTGACAAACGCCTTTAACAATCCAATCGCTTCAAGTAAGTCCATTTGTTGTCTAAA
CTCTAGTAAATTAATTTTAAGTTCATTCATAAAAATATAATGAGAAAGAATCAATGTTTC
ATTATGACTTTCTTTAACGAATTGAGTCATAAAATGATATAAACCCACTGCTTGCGTTCCA
ATTAGCGGTGTATACAGTCGATTCAATACCTCTAAATGATTCGTATTTAAATCAAAGTGTT
GCATAACTTTGAATTGATCCTTTGGTCTTAAGCCGAATTCGAAGGCTTGTCGTCCCAT

> **nrdR**

Function: protein coding sequence; transcript repressor of nrdD/nrdG operon (core genome, constant)
Best match: nrdR_CC005_Mu50-VRSA_BA000017.4[1795087:1795557:r]RC (completely identical)
Position: 038-contig_319_RC: 32289 … 32760; Length: 471 bp
Sequence:
TTAAGCATCACTCCGTTTGTTTTCGCTTAAAATCCCTTGCATCGATGCTAACAATTGATCA
ACATCTTTAAATTCTTTATAGACTGATGCAAATCTAACATATGAAACTTGATCAACATGCA
TTAACAAGTTCATAACGTGTTCACCTATATCTCGTGAAGACACTTCCGTATGACCTTCATC
TCGTAATTGCCATTCAACCTTGTTTGTTATGTCTTCAAGTTGTTGATATCTAACTGGTCGTT
TCTCACAAGAACGCACAAGTCCATTAAGTATTTTTTCTCTTGAAAACTGCTCTCTTGTGCC
ATCTTTTTTCACAACTATAAGCTGACTAACTTCGATATGTTCAAATGTAGTGAAACGTGTT
CCACAATTTTCACATTCTCTTCGTCTTCGAATGGCATTTAATTCATCGGCATGCCTTGAAT
CTACAACTTTAGATTGTGTAGAATTACATTTCGGGCATTTCAT

> **gapB**

Function: protein coding sequence; extracellular glyceraldehyde 3-phosphate dehydrogenase/locus 2 (core genome, constant)
Best match: gapB_CC001-ST772_118_AJGE01000003.1[16775:17800]RC (completely identical)
Position: 038-contig_319_RC: 32970 … 33996; Length: 1026 bp
Sequence:
TTAACTTGCACTTACAGTTTCTTTTGATGTCAAAAGTGCTCCAATTTGCTCAGCAACATCT
ACAACTCTATTTGAATAACCCCATTCATTATCATACCAAGCAATAACTTTTACTTTATTCC
CTGACATGACCATTGTTGATTTTGCATCAATAATAGCTGAATTTGGATTAGTATTAAAATC
AACAGACACTAGTGGTTGATGTTCGACTTCTATGATACCTTCTAAACCTGCATTTTCAAAA
GCTTGGTTTACTTCTTCTGCAGTTACTTCTTTTTCTAAATCAACAACTAAATCAACGAGCG
ATACATTCTTTGTTGGTACACGTAATGCCATGCCGTGTAATTTACCTTCTAATTCTGGTAA
TACTTCTTTTAAAGCTTTCGCCGCACCAGTAGAAGTAGGAATAATGCTTTCATTACATGAG
CGTGCACGTCTTAAATCTTTATGTGGATTATCAATATTTTTTTGGTCATTTGTAATAGCGTG
AACAGTAGTCATTAAACCATTAACTATTCCAAACTGATTATTTAAAACTTTTGCAACTGGA
CCAATGCAATTAGTAGTACATGAAGCATTACTAAAAATGTCAAATGCTTCTATATCTAAT
TGGTTATCATTTACGCCTTTAACTACCATTTGAACATGTCCACCTTTTGAAGGACCAGTTA
ACAATACTTTTTTGGCACCTGCTTTAATATGTGCGATGGCTTTATCACCATGATTAAATTT
ACCAGTTGCATCTATAGCAATATCGATATCTAATTCTTTCCATGGCAAGTTTTCAGGATTG
CGATCAGCAACCAATTTAATTTTATGATCACCAACTTGCAATCCATTTTCAATCGGTTCAA
CTTTTAGATTATATTTTCCATGTGTTGTATCGTAATTGATTAAATGTGCAATTGTTTCGGGT
GGATAACTAGCATTTATCGCTACTACATTTAAATTTTTATTTTGTAATGCAATACGTAATA
CCATTCTTCCAATTCTACCCATACCATTAATTGCAATATTCGTTGACAT

> **coaE**

Function: protein coding sequence; dephospho-CoA kinase (core genome, variable)
Best match: coaE_CC001_MW2-USA400_BA000033.2[1759590:1760213:r]RC
Position: 038-contig_319_RC: 34639 … 34789; Length: 150 bp
Sequence:
TACTTGAGCTAAACCTTTACTCCCCTTTTTAACAGCTTCCCTGGCTGCTTTATCAGCATCTA
CTACTTTAAAACCGAATACGGATAAGAGTTCTGATACTGTTGATTTTCCTGAGGCGATTCC
ACCTGTTAGACCAATAACTTTCGGCAT

> **mutM**

Function: protein coding sequence; DNA glycosylase (core genome, constant)
Best match: mutM_CC001_MW2-USA400_BA000033.2[1760229:1761101:r]RC
Position: 038-contig_319_RC: 34804 … 35677; Length: 873 bp
Sequence:
TTATTTTTGACATACTGGACAATAATGACTATTTCTTGTCGCGATGATTTTTGTTTCAATTT
GACTTCCACACACTTTGCATACCGGCTGCTTATATACATTAAGATGCAATTGCATCTCACC
AGTTTTTCCATCAGCATGACGATAATCTGAAATACTTGTACCGCCATATTTAATACCTTCT

TCTAGTACTTCTCTAACATAATAAAAAACCATTTCTTGTTGTTGGTGTGTTAAGTCTTTTAC
TTTTTTATCTGGTAAAACACCTGCACGAAACAACGCTTCACATGCGTAAATATTTCCACAA
CCTGCGATTACTTTATGATCCAAAATCACTTGTTTGATTGGTTTATTCTTATTAGACTGTTG
ATGAATTCGATTTAAATAATACATCAATGCTTCATTTGAAAAAGGTTCAGGCGCTATTTCT
AAAAATGAAGGATAAGATGCTACAGACGCAACATTTCTAATTTCTCCAAAACGACGTATA
TCTGAATAAATTAACTTTTTGTCATTTGACAACTCAAAAATAACATGCCAATGCTTACGAT
AATTAGGTATCATAATATCTTCAAGTTCATCTACAATGAAAAAACCACCCGCCATACCTA
AATGACTAATTAATGTACGTTGTTCTCGTTTATTATCTAGCTGAAAAACGATATATTTACT
TCTTCGTTCTACATTAGTAATGGTATAGCCTTCCGATAAAGTTTTAAAAGTATCTAATTCA
ATTCCTTTTATAATTGTTTCCTTGCCTTGAGCTTTACCTTCGATTACTTTATCCGAAAATTT
AACGTGTTCAATTTTTTGATTTATAACGTAGGGTTCAATTCCTCTTTTTACATGTTCTACTT
CTGGTAATTCGGGCAT

> **polA**

Function: protein coding sequence; DNA polymerase I (core genome, variable)
Best match: polA_CC009_21334_AGTW01000036.1[30951:33581:r]RC (completely identical)
Position: 038-contig_319_RC: 35692 … 38323; Length: 2631 bp
Sequence:
TTATTTTGCATCATACCAAGTTGCACCATAACTTGAGTCTACTTTTAATGGAACATCTAAT
TGCAATGCATTTTCCATTATTTCTTCTACAAATTCACTAAATGAATCTACTTCTGACTTAG
GTACTTCAAAAATTAATTCATCGTGTACTTGTAATAATAGTTTAGCTTGATATGTTGTCTC
TTTCATTTTTTGTGCAAATTTAACCATTGCCAGTTTAATGATATCTGCAGCACTGCCTTGTA
TTGGCGTATTCATAGCAGTACGTTCAGCAAAGCCGCGTAAATTAAAGTTACGACTCGTAA
TATCAGGAATATAGCGTCGACGATGTAGCAATGTTTCCACATAACCTAAAGCTTTGGCAT
CTTTTACAATATCAGACATATATTGTTTTACACCTGGGAAACTAGCTAAATAATCATCAAT
GAATGCTTTTGCTTTTTTACGAGTAATACCTAAACTTTGACTTAAACCATAATCACTTATC
CCATAAACAATTCCAAAGTTAACCGCTTTTGCTTGACGACGCATTAAACTATCGACTTGAT
CAGCTTCTACACCAAATACTTTCATAGCAGTTGCTGTATGAATATCATCGCCGTTGATAAA
TGCTTCTTTCATACTCTCATCTTGTGTAATGTGTGCTAATACACGCAATTCAATTTGAGAA
TAATCTGCTGATAATATAACGCTATCTTTTGAAGTTGGTTTAAAGGCTTTTCTTATTTTACG
CCCTTCTTCAAGTCTAACCGGAATATTTTGTAAATTAGGATCTACACTTGATAAACGTCCA
GTTTGCGCTAAAGTTTGGTTAAAACGTGTATGGATACGTTGATCATCACTAATTACCTTTT
GCAATCCTTCAACATAAGTAGACTGTAACTTAGATAATTGACGATATTCTAAAATATAAT
CAATGATAGGATGTTCACCTTGCAATTGCTCTAAGACATCTACAGCAGTAGAATATCCTG
TTTTCGTCTTTTTAATAACAGGTAATTGTAATGTCTCAAACAATACAACACCTAATTGCTT
AGGAGAATTTATATTAAAATCTTCACCAGCTGCATCATGGATATTTCGAATCAAGACGTC
TAATTTTTCTTGAATTTCTTTTTCCATTTCTTCTAAATCATGAACATCTGTAAATATACCAA
TTTCTTCCATTTCACTTAAAATCTTAGCTAGCGGTAGCTCTAAATCAGCTAAGAGTTCTAC
CTGATTGTATTCTTCTAATTGTTTATCCATATTTGGTTTCGCAAAGTAAATTGCATCAGTA
ATAGAAGCAACATATGGATTTAAAACATCATCTTCAGGTACCTTAAATTTCTTACCTTTTC
CATATATACTCACATCGTCTTTCACAAAACTTTGACCGTACAATGAAACAACTGATTGAA
CATCACTAATCGTACGAGATGGATCAATTATATAACTGGCCAACATAATATCGAAAGAAA
TATTTTGAATATCAATTCCCAATCTATGTGATGCTACATATGTTTTTTTAGCATCATATACG
ACTTTTTTCGTATTCGGATTTTCTAACCATGAAACTAGTTCGGCATAATTATTTATGTCATC
CGCATTAATTACAATATGTTTCTCACCTGTAAATAAAGAGAATTTCAAAATATTATTTCGC
AAATAGTTACCACCGTCTAATTCGAAATGGATGGCCGCTTCTTTCAATAAAGTAAAATCA
ATATTATCAAAAGACGTTTCAATTTCAAATGTCTTTTCTATTGCATCTTCAACGCTTGCTG
ATTGATCAATGTCAGCCAACAATTGTTTGAATTCTAACTTCTTAAACAATTCGATTTTTTC
TTGTTGTTCATCTTGATGAGTCATTAACGTATCTTCAAGTTTTACTTCAATCGGACTATCTA
CATTAATCGTTGCTAATTCTTTACTCATTAATGCATCTTCTTTGCTATTTTGAAGTTTTTCTT
TTAACTTTTTACCTGAAATTTCATCTAAATGTTCATAGACACCTTCTACTGTGTCAAATTG
GTTTAGCAATTTTATTGCTGTTTTCTCTCCAACACCTGCAACACCTGGTATATTATCAGAA
GTATCTCCCATTAATCCTTTCATATCAATAATTTGATTAGGTGTTAACCCGTTGTATTTTTC
CGCAATAAAGTCAGGTGTATAATGATCAACATCAGTAACACCTTTTTTAGTGTAATAAAT
GGTTACATTATCCGTTGCAAGTTGTGTTAAATCTCGGTCTCCCGTAATAATAATTGTCTGA
AAGCCCGCTTTATCTGCTTCTTTACTTAAAGTTCCGATAATATCATCTGCCTCATAGTTATC

TAATTCATAACGTTTAATATGATAAGCATCTAATAATTGGCGAATATAAGGAAATTGCTC
ACTTAGTTCAGGCGGCGTTTTCTGGCGTCCACCTTTATATTCACTATATTTTTCATGTCTGA
AAGTCGTTTTACCTGCATCAAACGCTACTAAAAAATGATTTGGCTTTTCTTCTTTTAAAAT
CTTCTCTAGTAACATTGCAAAACCATATACTGCATTGGTATGAATGCCTGCTTTGTTTGAT
AACAAAGGTAATGCATAAAAAGCTCTAAAACTTAAGCTATTACCATCGATTAATACTAAT
TTATTCAC

## > Q2YTE9

Function: protein coding sequence; putative membrane protein (core genome, variable)
Best match: Q2YTE9_CC001_MSSA476_BX571857.1[1743693:1745180:r]RC
Position: 038-contig_319_RC: 38616 … 40104; Length: 1488 bp
Sequence:
TTATGAATCGTCTGAACCAAATGGAATATATTTAACTTTGGTCATTTTCGGTAATTCTTCA
TAATTATTAAACCACTCTTGGTAATTTTTATTGATTGGTGACGGATGATGCATATAATGTT
CAAACGGCAATGATTCCACTGTATAAACCTCTTGTCGTGGATGATCTTTAATTTGTTGTTT
TAATAACTGAACTCTTTTTTCATGTTCATAATGAACATAAATAAATGCACTAAGATATATC
ACAGCTAAAACTAGTGATGCACCTTTAATAAAATTAACATTGATTGACTTATATTTCCGA
AATTCTTTTAATAAAATAATTAAAATGATTACATGTATCGTATAAACAATCAAAAAATTA
CCTGGTTCTATTGGAGTAACAATGACTAGTGTCGACGCCGAAACACATATTGCAATAAGT
AAGGAATATAAAGTGATTTGTGTTTTACGATCATTAATAGACAAATAAATACCTACAAAT
ATCGAAAACGCAAAGTAATCACATACAATTACGTTCACAAAACCAACCAATCCAATATCT
GTATTTTTATTTAATAAAAACTGATTGTAAAATAGTAAATAATAAAGCGGTAAAGTGATA
AATCCTATCATAATGATACGACGCTTTAATATTGTTAAATGTACATACCGATCACTTCTAA
GCAGTAGATAAATAATAAGCGCTGCAATCATACTTAAAATTATAATCTGACTGAAAATGA
CACCATATGGAAGTGACGTTGATATCATTTCTGCAAATTTTGAAAAGATACCTTGATTATT
TGAAACTTGTTGATATTCTGATCCTTCAAATAAAATTTTGCGATAATTTGGATTGGAAAAC
ATAATGATTGTACCTATAGTAGCAATCATAAATGAAAACAATAATTTATAATTAAGTGTT
CGATTTGCAGTAAATTCATATAGAAAAGCTAAAATTAAAATGATACAGTGGAATAATGTC
ACATTCTCCATAAATAATTGACCGAAGAAGCATAAAACATAGAATAGTACAGTAACACTT
ACCGGCTGCTTTTCTTTATATATAATTGCATTAATACAATAATAAATAATAAAGAGTGAA
ATTAGTGTTGATGTCGCATAATTATAAAATCCTGCAAACCAGCCATATGTATCTGCATAA
ATAGCACTTGGTAAAATTAACATTAAAGAAAATGCTAACAAATAATAGCTTGTCCACGCT
TTACAACGTGTAATGTGCATAATCATCCAAATGATGCCCATACTAATGAGACCATATGAA
AGCCAACGTAACCAGCTTACATGTACAGCTATAATTTCAAAGATATTTCCGATATAGCGA
CCATTAAGTGATGCAAATCCTACTTTTAAAATATCAGTATTATAATTGCTAAACCATTGTA
AATCATCGTGCATGAGTGGTAGTAAGATACCCATAAAAGTATAAAACAATAATATCGCA
ATTAATATCAAAGTTGTCTTGTGTAATTGAATTGTTTTCAC

## > phoR

Function: protein coding sequence; multi-sensor signal transduction histidine kinase precursor (core genome, constant)
Best match: phoR_CC001_MSSA476_BX571857.1[1745679:1747340:r]RC
Position: 038-contig_319_RC: 40602 … 42264; Length: 1662 bp
Sequence:
TTATTCTTTATAATCTTTTAGAATAACTTTGAACGTTGAGCCTTTGCCAACTTGACTATTCA
CTTCAATATTGCCTTGGTGGGCCTCTACTATATGCTTTGTAATTGATAATCCAAGACCTGT
CCCACCAGAATCTCTACTTCGTGCTTTATCTACTCTATAAAATCTTTCAAAAATACGTTGT
TGGTCTTCTAATTTAATACCTATACCAAAATCTTGTACTTCGAAAATGACACGAAAGTCAT
CTCGATACACACGAACGTTAATATCTCCATCTTCATAAGAATAGTTAATTGCATTCGTTAA
CAAATTCGTGATAACTTGAGCAATTTTACTTTCTTGTGCTTTAACAATGACATCTTTTTCA
ATATCAGTATGAATGGAAATATTTTTTTGATTGGCTTGAGTCATCATATTATCAATAATAC
GCCGCGTTAAATCTGATAAATTCATATAGTCTGTATCTAACTCTGTATGTTGCTCAATATG
CGATAAATCTAACAAATCTGTTACTAAAGATTCTATTCGATTTGATTCTTTTAAAATTATG
TTTAAAAACATGTCTAATGATTCCGCATCATTTTTTGCACCATCAATAAGCGTTTCAGCAA
ATCCCTTAATTGAAGTAATAGGTGTTTTTAATTCATGTGAAACATTTGCTACAAACTCACG

TCTTAGATTTTCAAGTTGTTTCAGATTTGTTATGTCATGCATCACAACTAAAATCCCTTGC
AAACTTTTTTGAGACCTAGTTAAAATCGGAACGCATGAAATATCAAAGTACTTGGCATGG
ACTTGGTTTATTGCAACTTCCAATTGTTCATAAATAGGTTTTTCAACTTTAAAACTTTCTAA
AATTAATTGCTCAATTTCAGTATTAACATAGCCGTGATAGCCTACTTGTTCAATATTATGC
GAGATGTTGAACTGTTCATAATACGCTTTATTTGCAACAACGATTTTTCCATTTCGATCTA
TCATTAAAATAGCACTTGGAATATTTTCAATCGTTGTTTTTAAACGGTTGGATTGAATTTT
TTGCTCATTATTAAGCTTTTGAAGGCGTCGTGCTAAATCATTGGTAGACACAAAAAGCGC
TTTAGTTTCTACAACATTACTTTCAGGTACACGTATGTGATAATAACCATTTGCCAACAAT
TGTGTTGCATAAGTAACTTCTTGAATGGGACGGATTAATGTACGCTTAAAACTACGGCTT
GCAAAATACAGACAAAGGAGTACAACTAAACATGTCAAAATAAGATATTTCCACAACGT
CCAATGCATTTCTGTAATATCGTTATTGTAACCTTTAATCCATACATGATAACCGTTAACC
TTCTTATTAAAAATAAAAACGTCCCTTTTTTGAATATAGCTATCACTATTTGGGATAGCTT
TCAGTTTGCCACTACTAAACACCTTTTTATCATGTTTAGTTATCAATAAATCTATATTTTGC
TGTTTTACAATTTCTTTAACTTTATCAATCTCATTATCTTGGACTAAATAAATATATGATCT
TGCATCTGTTGCTAGAGCTTGTTCGTGTTTTTCTGATAAAACATATGTGATGGAAGCGTGA
ATAATAATGCCTAATGTAACAAAACTGATAATTAATATACTGCTTATCAATAACATTAAG
CGGTGGTGAAACTTCAT

> **phoP**

Function: protein coding sequence; alkaline phosphatase synthesis transcript regulatory protein (core genome, constant)
Best match: phoP_CC008_COL_CP000046.1[1774360:1775064:r]RC (completely identical)
Position: 038-contig_319_RC: 42263 … 42968; Length: 705 bp
Sequence:
TCATTGTTCTTTAGGTCTTTCCAATTTATAGCCTAAGCCACGCACAGTTTTAATAAGTTGT
GGCTTCTTAGGATTATCTTCTAATTTATCTCTTAAATGACTGATATGTACATCAACAATTC
TTGAGTCTCCTGCAAATTCATAATTCCATACCGTATTTAACATATGCTCTCTCGTAATGAC
TCTGCCTTGTCTTTCTATCAAATAAAGCAAGAGTTCAAATTCTTTCGGTGTTAGCTCCAAT
AACTCATTATGCTTATATACTTCAAAATAATCGGGTCTAATACGTATCGATCCGATGGTAA
TATCATCGTCAATCTCTTCTACTTCTTTTACAAATTGAGAACGTCTTAAAATGGCTTTCAC
ACGGGCAACAACTTCTCTAGGTGAAAAAGGCTTAGTCATATAGTCATCGGCACCTAATTC
TAAACCTAATACCCGATCAAATTCATCATTTTTCGCCGTTAACATTAATATAGGGACTAAA
TTTTTATTTGTTCTTACAGTCTTACATACGTCAATGCCATCTTTTTTAGGTAGCATAACATC
TAAAATAATTAAATCTGGCTGTTCACTTTCTACCTTTTCTAAAGCCTCATCACCATCAAAT
GCGACAACAACTTCATAACCAGCTGTTTCTAAGTTATATTTAAGTAATGTTACGATTGAAT
GTTCGTCATCTACTACCAACACTTTTTGCGACAT

> **citC**

Function: protein coding sequence; Isocitrate dehydrogenase NADP (core genome, variable)
Best match: citC_CC080_11819-97_CP003194.1[1798190:1799458:r]RC (completely identical)
Position: 038-contig_319_RC: 43357 … 44626; Length: 1269 bp
Sequence:
TTATTTTAAATTTTTAATCAATTCATCTGCAAATGCTGATGTAGAAACTTCTTCAGCACCA
TCCATTAAACGGGCAAAGTCATAAGTAACAACTTTTGAAGCAATTGTATCTTCAATTGAA
TCTGTAATCTTATCTGCCGCTTCTTGCCATCCTAAATGTTCTAACATTAATACAGAACTTA
AAATTACTGAAGATGGATTCACTTTATTTAAACCTGCATATTTTGGAGCTGTACCATGTGT
TGCTTCAAAAATAGCATGACCTGTTTCATAATTAATGTTTGCACCTGGCGCAATACCAATA
CCACCAACTTGTGCAGCTAAAGCATCTGAAATATAGTCACCATTCAAGTTCATAGTTGCT
ACAACATCATGCTCAGCTGGACGAGTTAAAATTTGTTGTAAGAAAATGTCAGCAATAGAA
TCTTTAATGATAATCTTGCCTTCTTTTTCAGCTTTTTCTTGAGCAGCATTAGCAGCATCTCT
GCCTTCTTTTTCAACAATTTCGTCATATTGTTGCCAAGTGAATACTTGATCACCAAATTCA
GATAATGCTAAATCGTAACCCCACTGCTTAAATGAGCCTTCTGTAAATTTCATAATATTAC
CTTTATGAACTAAAGTAACTGATTTACGGTTATTATCGATAGCATATTGTATAGCTGCTCT
AACTAATCGCTCAGTTCCTTCTTTAGAAACTGGTTTAATACCAATACCTGAAGTTTCTGGG
AATCGAATGTTTGTCGCACCCATTTCGTTTTGTAAGAAGTCAATTACCTTTTTAACTTCTGT

TGTACCTTCTTTAAATTCAATACCAGCATAAATGTCTTCAGTATTTTCACGGAAAATAACC
ATATCAACATCTTGTGGACGTTTAACAGGTGATGGTACTCCTTTAAACCAACGTACCGGT
CTTAAACAAGTAAATAAATCTAATTCTTGGCGTAAAGCCACATTTAATGATCTAATACCA
CCACCAATTGGTGTTGTTAAAGGTCCTTTAACAGCAATTAAATATTCTTTAATTGTATCAA
GTGTTTCTTGAGGTAACCATTCACCAGTTGTATCAAATGCTTTTTGGCCAGCTAGCACTTC
TTTCCATTCAATGCGTTTTTCGCCATTATAGGCTTTCTCAACAGCAGCATCTATAACTCGG
CTTGCTGCCTTCCAAATATCCGGTCCAATTCCATCACCGATAATAAATGGGATAATTGGTT
CATTAGGTACGTTTAATCCTTCAGTTCCTTGAGTAATTTTTTCTGCAGTCAT

> **citZ**

Function: protein coding sequence; 2-methylcitrate synthase (core genome, constant)
Best match: citZ_CC001_MW2-USA400_BA000033.2[1770098:1771219:r]RC (completely identical)
Position: 038-contig_319_RC: 44674 … 45796; Length: 1122 bp
Sequence:
TTATTTTCTTTCTTCAAGCGGGATATACTTACGATTCGTTTCGCCAATATATTTCGCTCTAG
GACGCATAATTCTATTATCTTTATATTGTTCTAAAATATGAGCAATCCATCCTGCAGAACG
ACTTACAGCAAAGATTGGCGTGAATAAGTCATGAGGTATTTCCATACAGTGATAAACACT
CGCACTATAAAAATCAACATTAGGAATTAATCCTTTTTCTTCTGCCATACGTTTTTCCATTT
TCACTGACATTTCAAATAATTCTTCACGACCAGCGTCTTTCGTAATTTGACGGCTCATTTC
TCTTAAATATTTCGCTCTAGGATCACCATCTTTATATACACGATGACCGAAGCCCATTACT
TTATCTTTATTAGCAAATTTTTCATCTAAGTAAGCATCAACATTTTCAATTGACCCAATCT
CAGATAACATCGTCATAACTTGTTCGTTTGCACCACCATGTAATGGCCCTTTCAGAGAACC
TACAGCTGCTACAATACCTGAGTACATATCTGACAATGATGATACCGCACAACGTGCTGT
AAATGCAGATGCGTTCAACTCATGATCAGCGTGTAAAATAAGTGCTTTATTGAAGGCTTC
TACTTCTATATCTGTTGGTAATTCCCCACGTAACATATATAGGAAGTTTGCCGCATAACTT
AAGTCAGGATTAGGCTTAAGTGGTTCTTTATCTTGTCTTACTCGAGCAAACGCTGTAACTA
ATGATGCTACTTTAGCCTGTATACGCATTGCTCTTTCATAACGATTTTCATCTGATTCATTT
TCAGCATCAGGATCGAAATGTGCAATATATGATAATGACGTACGTAATGCTGTCATTGGA
TGCACGTGATCTGTAACATACTCCTCAAAATGTGTATACACACGAGGATTTAATGTCATG
TATTGATTTAATTTCCCTTTTAGATGAGCAAGCTCTTCTTCGTTTGGCAATCTATAGTTCCA
TAATAGGAAAATAACTTCTTCAAATTGCGCATTTTCAGCTAGATCATCAATATCATAGCC
GGCATAAGTCAATTGACTTTCAATAATTGAACTTATTTTAGTCTCCGCTGCGATAACCCCT
TCTAAACCTCTTTGTAATTCTGCCAT

> **aapA**

Function: protein coding sequence; amino acid permease-associated region (core genome, variable)
Best match: aapA_CC001_MW2-USA400_BA000033.2[1771567:1772928] (completely identical)
Position: 038-contig_319_RC: 46143 … 47505; Length: 1362 bp
Sequence:
ATGGCTGAAAAATTACAAAGGGAACTGAGCAATCGCCACATACAATTAATTGCAATTGG
CGGTGCAATTGGTACAGGCTTATTCTTAGGTGCTGGTCAAACGATTGCATTAACCGGCCC
TTCAATTCTATTAACATACATCATTATAGGATTTATGTTATTTATGTTTATGCGAGGTTTAG
GAGAAATCATTATACAGAATACTGAATTTAAATCTTTCGCAGATGTAACCAATACATATA
TTGGGCCTTTTGCAGGATTTGTTACCGGATGGACATACTGGTTCTGTTGGATTATTACAGG
TATGGCTGAAGTAACGGCTGTGGCAAAATATGTTAGCTTTTGGTTCCCAGAAATTCCAAA
CTGGATAAGTGCACTATTTTGTGTACTGTTATTAATGTCATTCAACCTACTTAGCGCAAGA
CTTTTCGGAGAATTAGAATTTTGGTTCTCTATCATTAAAATAGCGACAATTATTGGTTTAA
TAGTAGTTGGTTTCGTCATGATTCTATTTGCATTTAAAACTCAATTCGGGCATGCCAGTTT
CACAAATTTATATGAACACGGCATATTCGCTAAAGGTGCTTCTGGATTCTTTATGTCTTTC
CAAATGGCACTATTCTCATTTGTAGGAATTGAAATGATTGGTGTTACAGCTGGGGAAACA
AAAGATCCAGTTAAAACAATTCCAAAAGCAATTAACAGTGTACCCATTAGAATTTTAATA
TTTTACGTTGGGGCGTTAGCGGTTATCATGTCTATTATCCCTTGGCAGCAAGTTGATCCTG
ATAACAGTCCATTCGTAAAATTATTCGCATTGATCGGTATTCCGTTTGCTGCGGGCTTGAT
TAATTTTGTAGTATTAACCGCTGCTGCTTCATCATGTAATAGTGGTATATTCTCAAATAGC
CGTATGCTTTTCGGTTTATCAAGTCAACAACAAGCACCTCCGAACTTTTCTAAGACGAATA

AATATGGCGTTCCACATGTTGCAATCTTTGCTTCATCAGCATTATTACTTGTGGCAGCATT
ACTAAACTATATTTTCCCAGATGCGACAAAAGTATTTACGTATGTGACTACCATCTCTACA
GTGTTATTTTTAGTTGTATGGGGTCTGATTATCATTGCATATATCAATTATAGTCGTAAAA
ACCCAGATCTACATAAAAATGCTACGTACAAACTATTAGGTGGTAAATATATGGGCTACT
TAATATTTGTATTCTTCATTTTTGTGTTCGGGTTATTATTTATTAATGTTGATACAAGACGT
GCAATTTATTTTATTCCGATTTGGTTTATACTTTTAGCATTTATGTACTTAAGATATAAACG
TATCGCTGCTAAATCAAATAAATAA

## > **pfkA**

Function: protein coding sequence; 6-phosphofructokinase (core genome, constant)
Best match: pfkA_CC001_MW2-USA400_BA000033.2[1775055:1776023:r]RC (completely identical)
Position: 038-contig_319_RC: 49631 … 50600; Length: 969 bp
Sequence:
TTATATAGATAACTTGTTAGCAAGTTCATATAGACTATAATCAAATTTATGATCTTTACCA
TCAAAAATTTCATCAAAAGATGTTGCTACAATTTTATTGTTCTTAATTCCAACACCCTTAG
CTGTTTCACCTTGCATTAATAAGTCTACCGCATATCCACCTAAACGTGATGCTAAAACTCT
ATCCGCACCTGTTGGGCTACCACCACGTTGAACGTGACCTAACACAGACACTCTATTATC
AACATTGATGTATTGTGATAATTCTTTTTGACAATCTTGCGCAGTCATACAACCTTCTGCT
ACAAGAACGATTGAGTGTTTCTTACCACGTTTAATACCTTGTTCAATTTTATCAGCTATTT
CTTTAATATCTGTTTTCACTTCTGGAACTACAATTGTCTCAGCACCAACTGATAATCCAGC
CCATAATGCTAGATCTCCACAATCACGGCCCATTGCTTCAATGATAAATGTTCGTGCGTG
ACTTGACGCAGTGTCTCTAATTTTGTCGACTAAGCCAATAATCGTATTTAATGCTGTGTCA
AATCCAATTGTAAAATCAGTACCATTGATATCATTGTCAATCGTACCAGGAATACCGATA
GTTTGAATTTCTTTGCATTCCTCACTGATGCGTTGTGCACCGCGATAACTACCGTCACCAC
CAATAACTACAAGGCCCTCAATCCCTCTTTTACGTAAGTTTTCGATTGCAACTTTACGTAC
TTCTTGCTCCTTAAACTCTGGACATCTTGCTGAATACAAGAATGTACCTCCACGCTGAATC
GTATCCCCAACTGATCCTAATTCAAGTTTATGAATATCATCATTTAACAATCCTTGGTAAC
CATGATACACACCATAAACTTCAATTTCATTGTAAATTGCTGTACGAACAACTGCTCTTAC
GGCAGCATTCATTCCAGGTGAATCTCCACCACTAGTTAAAACTGCAATTTTCTTCAT

## > **pykA**

Function: protein coding sequence; pyruvate kinase (core genome, constant)
Best match: pykA_CC001_MW2-USA400_BA000033.2[1773276:1775033:r]RC
Position: 038-contig_319_RC: 47852 … 49610; Length: 1758 bp
Sequence:
TTATAGTACGTTTGCATATCCTTCAAAGATTTTACCTTGAGCAGCATCAATCGTAACTAAC
ATGTTATTGCTTATGTTTTTAACAGCTTTTTCTACACCTACAACTGTTGGAATACCTTTTTC
TAAACCAACAATTGCACTTGGTGATGTAATACCATTTTCTTCTGTAATTAAGCCTAAAGCT
TTTTCTACATAAGGTACAAACGTTTCATCGATTGAGTTAGTAACGATAACTTTGTCAGATA
AATCTTTACCTTCTAAATCTTTAACAGTTTCAGCAACTAACGTAGTACCAACAACTGATCC
ACGTCCAATACCTTGACCATTAGCAATTTCGTCACCAACTAGGTGGATTTTCATCATATTA
GTAGTTCCAGTTTCACCAGTTGGTACACCAGCAGTAATAATGATTAAATCACCATTAGAT
ACTCTACCAGTTTCAACAGCTGTTGCAACTGCATTGTTTAACAATGCATCTGTACTCTTAC
GTCCTTTTTTAACTACAGGTTGAACTCCCCAAACAATTGAACATTGACGTGCAGTTTCTTC
ACTTGGAGTCACCGCAATAATGTCTGAATGTGGACGATATTTAGAGATAGTACGTGCCGT
TGAACCACTTTCAGTAGCAGCTACAATTGCTTTAACATTTAAGTTTAAAGCTGTATGTGCA
ACCGAAATACCGATAGCATTCACTAATGAAGTTTCAACTAATTTAGTACGATCTGACAAT
AACTTTTTGTAATCTTGGGCTGCTTCAGCTGATACAGCAATATTTCTCATTGTTTTAACAG
CTTCTTCAGGATATAAACCAGCAGCAGTTTCACCAGATAACATTACTGCATCTGTACCATC
ATAGATTGCGTTGGCAACGTCACTAGCTTCTGCACGTGTAGCACGTGGGTTACGTTGCAT
AGAATCTAACATTTGTGTAGCTGTAATAACTGGTTTACCTAATTTGTTACATTGTCTGATT
AAATCTTTTTGAACCATTGGTACTTTTTCAGGTGGAATTTCAACACCCATGTCACCACGTG
CAACCATTAAACCATCAGACACTTCAAGAATTTCCGCAATATTATCAATACCTTCTTGATT
TTCAATTTTAGGGAATACTGAAATGTTAGCTTTTTGTTCTTCTAAAATTTCACGAATTTCTA

AAACATCACTAGGACGACGTACGAAACTTGCTGCAATGAAGTCAACATTTTCTTTAATAC
CGAAACGGATATCTTCAGCATCTTTTTCTGTAATACCAGGTAAACTTACTCTTACGCCAGG
TAAGTTAACACCTTTTTTGTTTTTAAGCTCACCAGAGTTTAAAATATCACATTTAACTTCTT
TTTTAGCATGGTCAATATCTTTAACTTGTAATTCAATTAAGCCATCATCAAGTAAAATGTA
TGAACCTACTTGAACATCGTTAATTAAGTTTTCATATGTTACTGAGAACTTTTCAGGCGTT
CCTTCAACTTCATTCATGCTAACAATAACTTCGTTACCACGTTCAAGTTCAATGATACCGT
CTTTCATATTATGCGTACGAATTTCTGGACCTTTTGTATCTAATAAAATTGCTACAATTTTG
TCTAATCTTTTAGCTACTTTACGAATTGTATCAATTCTACCTTTATGCTCTTCATGACTACC
ATGTGAAAAGTTTAATCGTGCAACGTTCATACCAGCATTGATTAATTTCTCAATCATTTCT
TCTGATTCTGAAGCTGGTCCAATTGTACATACAATTTTAGTTTTTCTCAT

> **accA**

Function: protein coding sequence; acetyl-coenzyme A carboxylase carboxyl transferase subunit alpha (core genome, constant)
Best match: accA_CC005_Mu50-VRSA_BA000017.4[1815186:1816130:r]RC
Position: 039-contig_321: 160 … 1105; Length: 945 bp
Sequence:
TTATTCTATATAAGAACCGATATTTCTGAATTTTTCAAAGCGATCATTAGCAATTTCATCA
CGTGATAATGACTCAAGTGAATCTAACTGTGCAACAAACGCTGATTTAATAGCTAAAGCT
TGCTGTTCAACATCTTTATGTGCACCGCCAAGTGGTTCAGAAATGACATCATCTATAATAC
CTAATTGCTTAATATCATGGGCAGTAATTTTCATTGTTTCAGCTGCAATTTTAGCCAAATT
ACTGTCTTTCCATAATAATGCCGCTGCACCTTCAGGAGATATAACAGAGTAAGTACTATT
CTCTAACATCAATACTTTATTGGCAATACCAATACCTAGAGCACCTCCACTGCCACCTTCA
CCAATGACAATCGCAATAACTGGTACTTTTAGTGAAGCCATCTCAATCAAATTTGTTGCG
ATAGATTCACTTTGTCCACGTTCTTCCGCAGCTTTACCAGGATATGCACCTTTTGTATCTAT
AAATGTAAAGATAGGACGATTGAATTTTTCAGCTTGTTTCATTAAACGTAATGCTTTTCGA
TAACCTTCTGGATGCGCCATACCAAAATTTCGATAAATATTATCTTTTGTATCTTTTCCAC
GTTGTTGTCCAATAACTGTAACAGCACGACCATTTAAAAAGCCAATACCACCAATCATTG
CTGGATCATCTCTAAAATTACGATCACCATGTAGTTCCATAAACGAATCAAAGATATATG
GAATATAATCTAGGGTCGTAGGTCTTTCTTGCAAACGCGCAATTTGCACACGATCCCATG
GTTTTAGATTTGTATATATTTTTTTAGTTTCTCGTTCCAATGACGCTTCAAGCATGTCAATT
TCTTCTTGTAAATCCACATCATTTTTATCTTGAGATTCTTTTAAAGATTCAATTTTATTTCG
AATTTCAAAAAGTGGTTTTTCAAAATCTAACAT

> **accD**

Function: protein coding sequence; acetyl-CoA carboxylase/carboxyl transferase/beta subunit (core genome, constant)
Best match: accD_CC005_Mu50-VRSA_BA000017.4[1816130:1816987:r]RC (completely identical)
Position: 039-contig_321: 1104 … 1962; Length: 858 bp
Sequence:
TTATTTAGTCACCTCTTGATGGATTTTTAGAATTTCAGACAATGTTTGACGCATATCATTA
CGATGTACAACTTTATCCAATTGTCCATGCTCTAATAAAAATTCTGCAGTTTGGAAATCAT
CTGGCAATTTTTCGTTTATTGTCTGTTCAATAACTCGACGACCTGCAAAACCTATCAACGC
TTTTGGCTCACTTAAATTTATATCACCAACTGATGCAAAACTTGCAGATACACCACCAGTA
GTTGGATGTGTTAAATATGATATATATAATAGTCCAGCGTCAGAATGACGTTTTAAAGAT
ACACTGGTTTTACCCATTTGCATCAAGGAAATAATACCTTCTTGCATACGTGCACCACCAC
TTGCAGAGAAAAGAATAAATGGTAAACGGTTCTCAGTGCAGTAATCAATGATGCGACAT
ATCTTTTCACCGATAACCGATCCCATACTTCCCATTCTAAAACGTGAATCCATGACAGCAA
CGCCAAATTTCATACCATCTAGTTGCGCTGTACCAGTCACAACTGCTTCTTTAAGACCTGT
CTTTTGTTGGTCCTTTTCAATTTTTTCTAAATAACTTGGAAAATCTAATGGATTCGCAGAG
GTCATTCCCTTATCGAATTCTGTAAATGATCCTTCATCAGAAATTGCTTCTATACGTTTAT
ACGCAGTTAAAGCAATATGATGATCACAATTAAAGCACACATTTAAATTTTCAGCTAATT
CTTTTGTGTACATAATTTTCTTACACTTTGGACACTTAGTCATAATACCTGCAGGCACATC
ATTATTTTTAGAGTCTTGTACTGTAAGATATTTCTTTTTCTTTGTTCGATTAAAAAAATCTT
TAAACAT

## > **maeB**

Function: protein coding sequence; malate dehydrogenase (core genome, variable)
Best match: maeB_CC001_MW2-USA400_BA000033.2[1778288:1779517:r]RC (completely identical)
Position: 039-contig_321: 2156 … 3386; Length: 1230 bp
Sequence:
TTATTGTAAATCTGTAAGTTTCATTGTTTTATCATACACATCTTGCGGATCAACTTCAATCC
TAGCTACTCCAGATTCCATTGCCGCTTTAGCAACATTACGAGCAACTGATGGCGCTACAC
GTTTATCAAACGGTCCTGGGATACAGTAGTCTTCATTTAATTCAGAACTATCGATTAAATC
AGCAATCGCTTCTACAGCTGCCTTTTTCATTTCTTCATTTATATGTGTAGCTTCAACCTCTA
ATGCACCTCTAAAAATACCAGGGAAAGCTAATACATTATTAATTTGGTTAGGATAGTCTG
AACGTCCTGTACCAACAACTCGTGCACCTGCCGCTTTGGCATCATCAGGTATTATTTCAGG
ATTTGGATTAGCCATTGCAAATATAATTGGATTATCTGCCATACTCTTAACCATATCTTGT
GACAGCGCATTAGCTACAGAAACTCCGATAAATACATCTGCGTCTTTTACGACTTCTTCTA
AAGACCCTTCAATCTTATCTTTATTTGTCCATTTAGCTACAACATCTTTCGTAGGATTCATA
CCATATGAACGTCCTTCAAAAATTGCGCCTCTTGAGTCACACATAACCATATTTCTTACAC
CATACGCGTATAGTAATTTAACAATGGCTATTCCTGCTGCACCAGCACCATTTAGTACAA
CTTTTATTTTAGCAATATCTTTGTTAACAACTCTCAATGCATTTACCAAACCTGCCATTGTT
ACAATTGCTGTACCATGTTGATCGTCATGGAATACCGGAATATTAGTTTCTTTTTTCAATC
GTTCTTCAATTTCAAAACAACGTGGTGCCGAAATATCCTCTAAATTAATACCACCATAATT
AGGTTCTAACAACTTAACTGTTTTAATGATTTCTTCGGTATCAGTTGTATTTAACGCAATA
GGCACCCCATTGATACCAGCGAAGCTTTTGAATAATACTGCTTTACCTTCCATTACAGGA
ATACTTGCTTCAGGTCCAATGTTACCTAAACCTAATACCGCTGTTCCATCAGTAATAACTG
CAACTGTATTTCCTTTAATTGTGTAATCATATACTTTTCTTTTATCTTCATAAATATCTTTA
CACGGTTCAGCAACGCCAGGTGAGTATGCTAAACTTAATTCCTCTTTATTAGTAACTTTTA
CATTTGGTTTAACTTCTAATTTACCTTGATTACGTTTGTGCATTTCCAATGCTTCATCTCTT
AATGACAT

## > **dnaE**

Function: protein coding sequence; DNA polymerase III subunit alpha (core genome, variable)
Best match: dnaE_CC007_TCH959-USA300_AASB02000248.1[20452:23649:r]RC
Position: 039-contig_321: 3835 … 7033; Length: 3198 bp
Sequence:
TTATATAAGCCTAATATCACTAGGGTTAAAGGATTGTATAAAATTATTAAACATACTATCT
TTTTGATTAATATAGCCTATAGTAGTCATTTGTTTAATCGTTTCATCATAAAAGGATAACA
TAACATCATTAGCATTCTCTTTCGTAGCTTTAATCATCTCTTCAAACATATCTATTTGTGAT
TTATTTCTAATTATAATTTGTTTGGCAAATGCTAATTTTTGTTCTTCAAAAGTGGCTAATGT
TTGAATCTCATTTATAATTAGTTGCCGTTGTTGCTTTCTATGGTCAAATTTCCCGCTAACTA
TAAACAAGTCATTATGTGATAACAACTCTTCGTACTTTTTAAACTGATTAGGGAAAATCA
CACCATCTAAAGTTTCAATGCCATCATTTAATATGACGAATGCCATATTTTGACCATTTTT
AGTTCGAATTTGTTTAACTTTATCAAACTGTACTAATATAGGTTTATTATTCTGCGCGTTA
CTCAATTTAAATATCGTTAAATATTGTTTGGCAACAAACTTTTTATCTACTGGGTGTTGCG
AAACATAAAATCCTAAATATTCTTTTTCGTACTGACTAATAAGTGCATCAGGCAATTCTTC
TTTATCTTCATACATCTGTTTTGGCGTTAAAATATCAAATAAAAAACCATCTTGTTCAATG
TTTAAATCGCCATCCAACACTTGATCAATAGCTTGCAACAACGTTGAACGTGTTTTACCAA
AAGCATCAAACGCTCCCACTAAAATCAGTGCTTCAAGTAACTTTCTCGTTTTGACTCTCTT
CGGTATACGTCTAGCAAAATCAAAGAAATCTTTATATTTGCCGTTCTGATAACGTTCATCA
ACAATCACTTTCACACTTTGATAACCAACACCTTTAATTGTACCAATTGATAAATAAATGC
CTTCTTGGGAAGGTTTATAAAACCAATGACTTTCGTTAATGTTCGGTGGCAATATAGTGAT
ACCTTGTTTTTTTGCTTCTTCTATCATTTGAGCAGTTTTCTTCTCACTTCCAATAACATTACT
TAAAATATTTGCGTAAAAATAATTTGGATAATGGACTTTTAAAAAGCTCATAATGTATGC
AATTTTAGAATAGCTGACAGCATGTGCTCTAGGAAAACCATAATCAGCAAATTTCAGAAT
CAAATCAAATATTTGCTTACTAATGTCTTCGTGATAACCATTTTGCTTTGCACCTTCTATA
AAATGTTGACGCTCACTTTCAAGAACAGCTCTATTTTTTTTACTCATTGCTCTTCTTAAAAT

ATCCGCTTCACCATAACTGAAGTTTGCAAATGTGCTCGCTATTTGCATAATTTGCTCTTGA
TAAATAATAACACCGTAAGTATTTTTAATATCGGTTCTAAATGCGGATGTAAATATTGA
ACTTTGCTTGGATCATGTCTTCTTGTAATGTAAGTTGGAATTTCTTCCATTGGACCTGGTCT
ATACAAAGAAGTTACAGCCACAATATCTTCAAAGTGTTCCGGCTTTAATTTTTTTAATACA
CTTCTTACACCGTCAGACTCTAATTGGAATATGCCAGTCGTATCTCCTTGCGACAACAATT
CAAACACTTTTTGATCATCGAACGGAATCTTTTCGATATCAATATTAATACCTAAATCTTT
TTTGACTTGTGTTAAGATTTGATGAATAATCGATAAGTTTCTCAAACCTAGAAAATCTATT
TTTAATAACCCAATACGTTCGGCTTCAGTCATTGTCCATTGCGTTAATAATCCTGTATCCC
CTTTCGTTAAAGGGGCATATTCATATAATGGATGGTCATTAATAATAATTCCTGCCGCATG
TGTAGATGTATGTCTTGGTAAACCTTCTAACTTTTTACAAATACTGAACCAGCGTTCATGT
CGATGGTTTCGATGTACAAACTTTTTAAAATCGTCAATTTGATATGCTTCATCAAGTGTAA
TTCCTAATTTATGTGGGATTAAACTTGAAATTTCATTTAATGTAACTTCATCAAACCCCAT
AATTCTTCCAACATCTCTAGCAACTGCTCTTGCAAGCAGATGACCGAAAGTCACAATTCC
AGATACATGTAGCTCGCCATATTTTTCTTGGACGTACTGAATGACCCTTTCTCGGCGTGTA
TCTTCAAAGTCAATATCAATATCAGGCATTGTTACACGTTCTGGGTTTAAAAAACGTTCAA
ATAATAGATTGAATTTAATAGGATCAATCGTTGTAATTCCCAATAAATAACTGACCAGTG
AGCCAGCTGAAGAACCACGACCAGGACCTACCATCACATCATTCGTTTTCGCATAATGGA
TTAAATCACTTACTATTAAGAAATAATCTTCAAAACCCATATTAGTAATAACTTTATACTC
ATATTTCAATCGCTCTAAATAGACGTCATAATTAAGTTCTAATTTTTTCAATTGTGTAACT
AAGACACGCCACAAATATTTTTTAGCTGATTCATCATTAGGTGTCTCATATTGAGGAAGT
AGAGATTGATGATATTTTAATTCTGCATCACACTTTTGAGCTATAACATCAACCTGCGTTA
AATATTCTTGGTTAATATCTAATTGATTAATTTCCTTTTCAGTTAAAAAATGTGCACCAAA
ATCTTCTTGATCATGAATTAAGTCTAATTTTGTATTGTCTCTAATAGCTGCTAATGCAGAA
ATCGTATCGGCATCTTGACGTGTTTGGTAACAAACATTTTGAATCCAAACATGTTTTCTAC
CTTGAATCGAAATACTAAGGTGGTCCATATATGTGTCATTATGGGTTTCAAACACTTGTAT
AATATCACGATGTTGATCACCGACTTTTTTAAAAATGATAATCATATTGTTAGAAAATCGT
TTTAATAATTCAAACGACACATGTTCTAATGCATTCATTTTTATTTCCGATGATAGTTGAT
ACAAATCTTTTAATCCATCATTATTTTTAGCTAGAACAACTGTTTCGACTGTATTTAATCC
ATTTGTCACATATATTGTCATACCAAAAATCGGTTTAATGTTATTTGCTATACATGCATCA
TAAAATTTAGGAAAACCATACAATACATTGGTGTCAGTTATGGCAAGTGCATCAACATTT
TCAGACACAGCAAGTCTTACGGCATCTTCTATTTTTAAGCTTGAATTTAACAAATCATAAG
CCGTATGAATATTTAAATATGCCACCAT

> **repeat_nySAgamma**
Function:  repeat element
Best match: repeat_nySAgamma_CC001_MW2-USA400_BA000033.2[1041811:1041923]RC
Position: 039-contig_321: 8227 … 8257; Length: 30 bp
Sequence:
GGGGCCCCAACACAGAGAATTTCGAAAAGA

> **uspA**
Function: protein coding sequence; universal stress regulator (core genome, constant)
Best match: uspA_CC001_MW2-USA400_BA000033.2[1786083:1786496] (completely identical)
Position: 039-contig_321: 10008 … 10422; Length: 414 bp
Sequence:
ATGTATAAAAATATATTACTTGGTGTAGACACTCAGTTAAAAAATGAAAAAGCACTAAAA
GAAGTGTCTAAATTAGCTGGCGAAGGTACAGTCGTAACAGTTTTAAACGCAATCAGCGAA
CAAGATGCTCAAGCATCAATTAAAGCAGGTGTTCATTTAAACAAACTTACTGAAGAACGA
AGCAAGCGATTGGAAAAAACACGCAAAGCTTTAGAAGATTATGGTATTGATTATGACCA
AATAATTGTTCGTGGTAATGCAAAAGAAGAACTATTAAAACATGCTAATAGCGGTAAATA
CGAAATTGTTGTTTTAAGTAACCGTAAAGCAGAAGACAAAAAGAAATTTGTACTTGGAAG
TGTCAGCCACAAAGTAGCAAAACGTGCGACTATCCCTGTATTAATCGTTAAATAA

> **pepQ**
Function: protein coding sequence; Xaa-Pro dipeptidase (core genome, variable)

Best match: pepQ_CC005_Mu50-VRSA_BA000017.4[1826417:1827472]
Position: 039-contig_321: 11590 … 12646; Length: 1056 bp
Sequence:
ATGACAAAAATATCAAAAATAATAGACGAACTGAACAATCAACAAGCTGATGCAGCATG
GATTACAACACCGTTGAATGTATATTATTTTACTGGATACCGTAGCGAACCCCATGAAAG
ATTATTTGCATTATTGATTAAGAAAGATGGTAAACAAGTACTATTTTGTCCAAAAATGGA
AGTCGAAGAAGTCAAAGCATCACCTTTCACAGGTGAAATCGTTGGATATTTAGACACTGA
AAACCCTTTCTCACTTTATCCTCAAACAATCAATAAATTACTAATTGAAAGCGAGCACTTA
ACAGTAGCACGCCAAAAACAATTAACCTCTGGTTTCAATGTCAATTCATTCGGAGATGTT
GATTTAACAATCAAACAATTGAGAAATATTAAATCCGAAGATGAAATTAGCAAAATACG
TAAAGCTGCTGAGTTAGCAGATAAGTGTATCGAAATAGGTGTTTCTTATTTAAAAGAAGG
TGTGACTGAACGTGAAGTAGTCAACCATATTGAGCAAACTATCAAACAATATGGCGTCAA
TGAAATGAGTTTTGATACGATGGTTTTATTTGGAGATCATGCCGCATCACCTCATGGCACA
CCAGGAGATCGCAGATTAAAAAGCAATGAATATGTACTATTTGATTTAGGTGTAATTTAT
GAGCATTATTGTAGCGATATGACACGTACCATTAAATTTGGTGAACCTAGCAAAGAAGCA
CAAGAAATTTATAATATTGTATTAGAAGCAGAAACATCTGCAATCCAAGCAATTAAACCT
GGAATACCTTTAAAAGATATCGATCATATCGCTAGAAATATTATTTTAGAAAAAGGTTAT
GGTGAATATTTCCCTCATCGCTTAGGTCATGGTTTAGGATTACAAGAACATGAATATCAA
GATGTTTCAAGTACTAATTCTAATTTGTTAGAAGCTGGCATGGTTATTACAATCGAACCAG
GTATTTATGTACCAGGTGTTGCAGGTGTAAGAATTGAAGATGACATACTTGTCACTAATG
AAGGATATGAAGTATTAACACATTACGAAAAATAA

> **ald2**

Function: protein coding sequence; alanine dehydrogenase 2 (core genome, constant)
Best match: ald2_CC008-ST72_TCH130_ACHD01000037.1[70695:71813]RC (completely identical)
Position: 039-contig_321: 12961 … 14080; Length: 1119 bp
Sequence:
TTATAATTGTAATGCTTCTTCTACAGATTTATATTCCATTTCAAATGCCTCTGCAACGCCTT
TATTGGTTACATGACCTTTGTAAGTATTTAAACCTAATGATAATGGTTGATTTGATTTAAA
TGCTTCTCTATACCCTTTATTAGCTAGCATGAGCGCATAAGGTAGCGTAGCATTATTTAAA
GCTAACGTCGAAGTACGCGGTACTGCACCTGGCATATTTGCAACTGCATAATGAACCACA
CCATGCTTAATATATGTAGGATCATCATGTGTCGTAATTTTATCAGTTGTTTCAAAAATAC
CGCCTTGATCAATAGCAATGTCAATAATAACTGACCCATTTTTCATTTGTTTAATCATGTC
TTCTGTTACAAGTCTTGGCGCTTTAGCACCTGGAATTAAAACTGCACCTATTACTAAATCA
CTTTGTTTAACATACAACTCAATATTCAACGGATTTGACATAATTGTATGTACACGTCCAC
CGAATAAATCATCTAATTGTTGTAAACGCTTTGGATTAACATCTAAAATCGTAACATCTGC
ACCTAGTCCTAGTGCAATTTTAGCTGCATTTGTTCCTGCTTGACCACCACCGATAATAGTT
ACTTTACCTTTAGGTACTCCTGGAACACCACCTAGTAGAATTCCCATACCACCATTGAGTT
TTTGTAGGAACTCTGCACCAACTTGAGCTGACATTCTTCCTGCTACCTCACTCATTGGTGA
TAACAATGGTAAAGATCGGTCTGGTAACTGCACAGTCTCATATGCAATACTAATTACTTTT
CTATCTATCAAAGCTTGTGTTAATTTTTCTTCATTTGCTAAATGAAGATAAGTGAATAATA
CAAGCCCTTCTTTAAAATATGGATATTCAGATTCAAGTGGTTCTTTAACTTTAATAACCAT
ATCCACATCCCAAACTTTTGCTTGTTCAGCAACAATCTCAGCACCTGCTTCTTTGTAATCT
ACATCTTCAAAGAACGATCCTGAACCCGCATTTGTTTCCACTAAAACAGTATGCCCACTTT
CTACTAAAGCGTGCACACCACTTGGTGATAAACCAACACGATTTTCATTATTTTTAATCTC
CCTTGGTATACCAATTTTCAT

> **ackA**

Function: protein coding sequence; acetate kinase (core genome, variable)
Best match: ackA_CC005_JH1_CP000736.1[1876510:1877712:r]RC
Position: 039-contig_321: 14966 … 16169; Length: 1203 bp
Sequence:
TTATTTTAAACCACCGAATGTCATAACATCACGGGCAATCATACTTTCTTCATCTGTTGGA
ATAACGACAACTTTAACTGGTGAATGAGGATAGTTAATAAATCCTTCTTTACCACGTAGT
AAGTTTTCATTTTTCTTAGGATCCCAGTAAACACCCATAAATTCTAAGCCTTCAAGAACTT

TCGCACGAATTTCTACTGAGTTTTCACCGATACCTGCTGTAAATACGATAACATCAACACC
ATGCATTCTCGCAGCATATGATCCAATATATTTGTGAATTTTAGAAGCAAATACATCTAA
AGCCATTTGTGAACGTGCTTTACCTGATTCAGCTTCTTCTGATAAGTCACGTAAATCACTA
GATGTACCTGATAATCCTAATAAACCTGATTCTTTGTTTAAGATTTCCAATACTTGTTCAG
CAGTTTTACCTGTTTTTTCCATAATAAATGGAATTAAAGCAGGGTCAATATTACCAGAAC
GAGTACCCATTGTTACACCAGCAAGTGGTGTGAAGCCCATTGATGTATCAATAGATTTAC
CGCCATCGATAGCTGCAATTGATGCTCCATTACCAATGTGACATGAAATAATACGTAAAT
CTTCAATTGGCTTATCTAACATTTCTGCCGCTCTTTGTGATACAAATTTATGGCTTGTACCA
TGGAAACCATACTTACGAATGCCATAATCTTTATAATAATGATATGGCAAGCTGTATAGA
TATGCTTTTTCAGGCATTGTTTGATGGAATGCTGTATCAAAAATTGCCACATGAGGGATAT
TTGGTAATAATTTACGGAAAGCACGAATACCCATCAAGTTAGCTGGGTTGTGAAGCGGTG
CTAATTCGCTTAATTCTTCAATTTCCTTTTCAACCTCATCAGTAATAGCTACTGATTCAGG
GAATTTTTCACCACCATGTACAACACGGTGACCTGTTCCATCGATATCGTTAATATCATTA
ATAATATTGTGCGCTTTAAAAGCATCCAACATGATATCAACTGCCTCAACGTGATCCTTG
ATATCTTGTACTGTTTTAACTTTTTCCCCGTTGACTTCAATTGTAAAAATTGAATCCTTCAA
TCCGATTCTTTCTATTAAACCTTTTGTTACTAATTCCTCTTCAGGCATTCTAATTAATTGAA
ATTTTAATGATGAACTACCAGCATTGATAGCCAAGATTAATTTTGACAT

> **tpx**

Function: protein coding sequence; thiol peroxidase (core genome, constant)
Best match: tpx_CC001_MW2-USA400_BA000033.2[1793401:1793895:r]RC (completely identical)
Position: 039-contig_321: 17326 … 17821; Length: 495 bp
Sequence:
TTAAATATTTTTGTATGCAGCTAAAGCAGCATCAAAATCTGGGAAATCAGTACCTTCACT
AACGATTTCTTTATAAACAACTTTATTATCTGCATCTAATACAAATACTGCACGAGCTAAT
AAGCGAAGTTCTTCCATAACAACGCCATAGTTTTCACCAAATGATAAGTCACGGTGGTCA
CTTAATGTAATGACATTGTCTAAACCTGCTGAAGCGCACCATCTTTTTTGTGCGAATGGTA
AGTCTGCTGAAATTGTAAGCACAATCCCCTCTTCTTTAGAAGCATCAGAGTTGAATTTGCG
AGTCTGCTGATCACAAACACCTGTATCAATTGATGGTACCACACTAATTAATTTCTTTTTA
CCAGCATAATCTGCTAATGTTACTTGATTTAAGTCATTATCTAACACTGTAAAATCAGGTG
CAAAATCACCTTCATTAATTTGTTGACCTTTTAAGTGGATTGGTCCACCTTTGAATGTTAT
TTCAGTCAT

> **thiI**

Function: protein coding sequence; thiamine biosynthesis protein (core genome, variable)
Best match: thiI_CC001_MSSA476_BX571857.1[1774460:1775683:r]RC
Position: 039-contig_321: 18732 … 19956; Length: 1224 bp
Sequence:
TTATAAAAAGTCATTTATTAATTGGTTTGTTTGCTGTTCTTTAATAGTTTTATAATCACTAG
TTATTTCAAGTGTTTCAATATTTTCAACAGCACGATTAATCATCTCTTCAAAATCAAAGAC
GCTTTCATATTGAACTACCTTATCAAAGTTTGGTTCGGTTACTGGATTTTTAGGGGTGAAA
ATTGTACAACAATCTTCAAATGGTTGAATAGATGTTTCAAATGTACCAATTTCTTTCGATT
TAATAATAATTTCTTCTTTATCGTAAGTTAATAAAGGACGTAATACAGGAGTAGAAGTTA
CATTATTAATTGCATACATGCTATGAAGTGTTTGACTGGCTACCTGCCCTAGGTTTTCACC
ATTTACAATAGCTAAAGCCCCTATTTGATGTACTAATTTATCAGCAACACGCATCATCATA
CGTCTCGTTGAAGTCATTGTATATCTTGGATGTACAACTTTATTTACCTGTTTTTGCAATTC
TGTAAATGGTACAATATGCAATTTAATTGGTCCAACACGTTCAGCTAAAATACGTGTCAA
TTCAATAACTTTTTCTTTTGCTTGATCACTTGTAAATGGTGGACTATGGAAATGAATCGCT
TCAATTGTTACGCCACGTCTCATCACTTCCATCCCAGCAACTGGTGAGTCTATACCGCCTG
AAAGCATTAGTAACGTCTTACCACCAGTACCAACTGGTAATCCACCTGAACCCGGAACAA
CTTCTTCATACATATAAATTGCATCTAATCTAACTTCCACTCGAATTTCATGATCTGGACG
TTTGACATTCACTGAAATATTGTCGAAGTGCTTCAATACTGCACCACCCAATTCACGCTGT
AATTCATACGTATCCATTGGGAAATTTTTATCGGCACGCTTCACATCAATTTTAAATGTGC
TGTTTTCTTCAAATTGCTGCGCAAATTTAATTGCCGCTGCACTCATTGCCTCTATTGTTTTT
TCTACTTTTAATACTGGACTAATAGATTTAATACCGAAAATTTTTGATAATCGATATGTTA

TTTCATTTATATCTGCATGGTCTTCAAGTTCAATATACATACGATCTCGTTTGCCTTTAACG
ACAAACCCATCAAGTCCTTTTAATGACTTATTTACATTATTTCTTAATTGATTTACAAATTT
TTTTCTATTTGAACCCTTTAATGTTAACTCCCCGTATCTAACAAGCAAGTGATCATACTTC
AT

> **csd2**

Function: protein coding sequence; cysteine desulfurase 2 (core genome, variable)
Best match: csd2_CC001_MW2-USA400_BA000033.2[1796030:1797169:r]RC (completely identical)
Position: 039-contig_321: 19955 … 21095; Length: 1140 bp
Sequence:
TTATTTTAGCAACTCCTTAATTTCCTCATAAATGATGATAAATATTTCTTTAAACCTTGCTA
TATCTTCTTTAGTTGTAGTAGCCCCAAATGATAATCTTATACTACCTTCAATAGATTTGTCT
GATAATCCCATTGCAGCCAATACTTCATTTAATTTATTACGTTTAGATGAACAAGCACTCG
TCGTAGATATCATAATGTCATATTTTGAAAAAGCATTAACTAATACTTCACCTTTTACGCC
AGGAAAACTAATATTTAAAACGAATGGTGAACCAGAAGTTGAAGAATTAATATAAACTC
CATGATATTTATTTAAAAATTGACGGACGTCATTATTTAACACAGTAACAAATGCATTCA
ATGCTTCAAAGTTTTCATTAGCTATCTTCATCGCTTTAACCATTGCAATATCATTTGGCAA
ATTAACTGTTCCACTTCTAACGCCATATTCTTGACCACCACCATGGACAGTTGGTTCAACA
TTTTGAATGTGATTTACAAGTAAGACGCCTTGGCCTTTTAAACCATTAAACTTGTGTCCAC
TTAAACTAATACTATCTATGTTATTGAGATCCATTGAAATTTTGCCGAATGCTTGAACCGC
ATCTACATGAAAATGTGCCTTAGGATAATTTTTTATAACTTTAGCCATTTGTGGAATAGGC
TGTATTTGTCCAGTTACATTATTTACATACATACATGTTACTAAACCGACTTTGTCTGACA
TTAATTCTTTGAAGTGTTCTAAGTTAATACTGCCATCTTTCTTTACATCAACATATTTAACT
TTAAATCCTTCGTGTGCTTCCAAATATCTTACAACCTCTAATACGGACGGATGCTCTAACA
CGGATGTAATTATTTCCTTCGCTGTATCAAATTTACGATAGGCAATACCTTTTAAAGCAAG
ATTATTGGATTCAGTTGCACCACTAGTGAATACAACATCATAATTTGTTTTTGAATTAATC
ATTGCATTAATTTGGGCTTTTGCTTGTTGTAGTAATTGATTTGCCTGCAAACCAGCTTTAT
GCGGACTATTCGGATTATAATACATTGATTGATTTACTTTTAAATAAGTATCTAACACTTC
TTCAAATGCTTTCGTCGTTGCCGCATTATCTAGATATATCAA

> **ezrA**

Function: protein coding sequence; septation ring formation regulator (core genome, constant)
Best match: ezrA_CC001_MW2-USA400_BA000033.2[1797541:1799235:r]RC (completely identical)
Position: 039-contig_321: 21466 … 23161; Length: 1695 bp
Sequence:
CTATTGCTTAATAACTTCTTCTTCAATATGTTTAGTAACACCTGGCTCAACACTTTCAAGA
GCTTGCTCTGCAATTTCAATCGCACGCTTATAGCGATTATTTTTAAATAATCGTTCAGCTT
CATTTAAGCTCTTATCAACATTGCTATAGTCCTTACGATATCTATTTCCATATTGAATTAAT
TTCTCTGCATAAACAGCATTAACAAGAACATCATTTGCTTCATCTTCAAATGTATTCATTT
GAATCACAATTTTAGACACTTTATCTTTTAACTGTTTAACGTGTATTGGACGTTCACTAAA
TTGTTCGTTAACATCACGAACTTCATGATCAATTTCATTTTTCATGATGATAAACCTTTCA
GGAACGCTTGTTAAGTTAGAAGCAAGTAATCGACGATACACTTCTTCTTTCTTCGATTGGA
CTCGTAGCAGATTGTCTTCTGCTTCTGCTTCATCTTCACGCAATTGAATCAGATGATTTTGT
AGCTTTTCTTGTTTGTCATTAATAACTGTGACATGATCTTCTAAATATTGTAAATTATCCTG
AACCTCGCTATATCGCACAGCAGATTTAGACATTTCTTTTAAAATATCATCATATACAGAA
ATTAAACTTTGAATTTCATTTTCAAATTGACGAACACTTTGAGCATCAGATTCATTTATAT
AGTAGTTTTCACGTACATATTCAATTTCTGTTTGCAATGTATAATTCATGTCTTTAGCTTTG
AATAAGTTATCCGTAATGATATCTTTTGTTTCTTCGACATCATTTTTAGCTTTAACTTCATG
TTCAATTAAATCATACATGTCATCTAACTTATCATTGATATTAGCTAGTTTATCATTAGCTT
CTTCTAATTCTAAGCGGCTAATTAATGGTTCAACGAAACTAAGCTCTGTTTTTAAGCTTTG
TAATGTACTGTCTACTTTTACGTGATCCAGATCATACCCTTCAACTTTAAGATCACGGCAA
CCATATTTTAAATCTTGGAATTGACCAGGTAATTCTTTTTGAGTTTCTCTAATTAATTCTGG
TATTTCTTCCATATAAGATCTTAGCTGTTTCATTTGTTCATTCAAGGCAGCTATATGGTTGT

GCGCTTGTACATAATTACCATCAGCTTTTAGTACTTCATATTGCTCTAACCTTGGCTCAAA
CTTTTCAATTTCAGTTTCAAGTAGACTTGCTGCCTCACCAAATTGATGACGATTTGCTAAA
ACATCACGTTTCATTTCACGATAATCAACCTTACATTTGTCATATAATTCATCATTATCTTT
GTATAACGCAATAATTTCATTTACATCTTCTAATTGTTGCTGATAGCTTTGTTCGTAACTAT
CCATCAACTCATTTGCATCATCAATTTCACTTTGAGATGCGTTGAAACTAAATTTATCTAA
TAAAGCCTCAGCATTATGGATTTTTTCTTCCACAGGAGCTAGATACTTATTTGTACTTTCT
ACGTTGTCCTTTTTCATTGCATCGTATTTCGTTTTTGTTTCACCTTTTAAATTCAACTTAGA
TAATTGTGCAAGGTTTTGATCAAAAGGTAACGTCTCAATTTCATTTTTACGTTCGATTGCT
TTTTCAATAATTTGTCGTTTATTTGAACGTAAATAGAATAATACACCTACAGCAATCAATA
TAATCACAATTATTGCCAAAATGATATATAACACCAT

> **rpsD**
Function: protein coding sequence; 30S ribosomal protein S4 (core genome, constant)
Best match: rpsD_CC001_MW2-USA400_BA000033.2[1800080:1800682] (completely identical)
Position: 039-contig_321: 24005 … 24608; Length: 603 bp
Sequence:
ATGGCTCGATTCAGAGGTTCAAACTGGAAAAAATCTCGTCGTTTAGGTATCTCTTTAAGC
GGTACTGGTAAAGAATTAGAAAAACGTCCTTACGCACCAGGACAACATGGTCCAAACCA
ACGTAAAAAATTATCAGAATATGGTTTACAATTACGTGAAAAACAAAAATTACGTTACTT
ATATGGAATGACTGAAAGACAATTCCGTAACACATTTGACATCGCTGGTAAAAAATTCGG
TGTACACGGTGAAAACTTCATGATCTTATTAGCAAGTCGTTTAGACGCTGTTGTTTATTCA
TTAGGTTTAGCTCGTACTCGTCGTCAAGCACGTCAATTAGTTAACCACGGTCATATCTTAG
TAGATGGTAAACGTGTTGATATTCCATCTTATTCTGTTAAACCTGGTCAAACAATTTCAGT
TCGTGAAAAATCTCAAAAATTAAACATCATCGTTGAATCAGTTGAAATCAACAATTTCGT
ACCTGAGTACTTAAACTTTGATGCTGACAGCTTAACTGGTACTTTCGTACGTTTACCAGAA
CGTAGCGAATTACCTGCTGAAATTAACGAACAATTAATCGTTGAGTACTACTCAAGATAA

> **ugpQ2**
Function: protein coding sequence; chromosomal glycerophosphodiester phosphodiesterase (core genome, constant)
Best match: ugpQ2_CC008_NCTC8325_CP000253.1[1735395:1736138:r]RC (completely identical)
Position: 039-contig_321: 24823 … 25567; Length: 744 bp
Sequence:
TCACTTAATAAATAATTTCGGATTATCTGTAATTAATCCATCTACACCCATTTGTCTTAATT
TTTCTCCAGTTTTCAATTTATTAACTGTGTATGGCATAACCTGTAATTGATGATGATGTGC
TTTATCAACAAATTTTCTGGTCACTAATGCATAATTAGGATTAACATAACTAGCAATTTGA
GCAATTCTTGAAAAGTTTGGCTTTTTATACCAATAATTACGTTTACTACAAAGCACACCTA
ATTCATATATACTGCCCAATGTGTTTAACTTTTCGATGCATTCGATATCAAAAGATTGTAT
AACTACTTGTGTCGCATCGACTTTCTTTTCTTCCAAAAATGCCAGCAATTTACATTCTATTT
CTGGATATAAATTCGGACTTTTCAATTCAATCAATAGCTTTTTATCATACTTTAAGCATAA
TGAAAGTACTTCATCTAACGTGGGTATTCGTTCTCCCTTAAAAGCAACATCTTTATAACTA
CCAAAATCAAATGATTTTAATTGCGATAATGTGTAATCAGCAATACGCCCCTTACCATCC
GATGTTCTATCAATTGTTTCATCATGTATCACAACAAAATGTTGGTCTTTGGTCAAATGAA
CATCTATTTCTAACATAGCAACATTGAGCCCCATTACCTCTCGATAACCGACCATTGTATT
TTCAGGAAAATCACTCGGCAATCCACGGTGCGAAACAATTTGTAATTCATCTTTCAGTTTA
TTCAGAGTCAT

> **serA**
Function: protein coding sequence; D-3-phosphoglycerate dehydrogenase (core genome, constant)
Best match: serA_CC005_Mu50-VRSA_BA000017.4[1843700:1845304] (completely identical)
Position: 039-contig_321: 27353 … 28958; Length: 1605 bp
Sequence:
ATGAAGCAATTTAATGTACTCGTTGCAGATCCCATATCAAAAGATGGTATCAAAGCATTA
TTAGATCACGAACAATTCAATGTAGATATTCAAACTGGCTTGTCCGAAGAAGCATTAATC
AAAATTATACCTTCATACCATGCTTTAATCGTTCGTAGTCAAACTACGGTTACTGAAAATA

TCATAAATGCTGCTGATTCTTTAAAAGTAATCGCACGCGCCGGTGTTGGTGTAGATAATA
TTAATATTAATGCTGCAACATTAAAAGGTATTTTAGTTATTAATGCCCCAGATGGTAATAC
GATTTCAGCTACTGAACATTCACTGGCAATGTTATTATCAATGGCTCGAAATATTCCGCAA
GCACACCAATCACTTACAAATAAAGAATGGAATCGAAATGCATTTAAAGGTACTGAGCTT
TATCATAAAACATTAGGTGTCATTGGTGCTGGTAGAATTGGTTTAGGTGTTGCTAAACGT
GCGCAAAGTTTCGGAATGAAAATACTAGCTTTTGACCCTTACTTAACGGATGAAAAAGCA
AAATCTTTAAGTATTACGAAGGCAACAGTTGATGAGATTGCCCAACATTCTGATTTCGTT
ACATTACATACACCACTAACACCTAAAACAAAAGGCTTAATTAATGCTGACTTTTTTGCC
AAAGCAAAACCTAGTTTGCAAATAATCAATGTGGCACGTGGTGGTATTATTGATGAAAAG
GCGCTAATAAAAGCATTAGACGAAGGACAAATTAGTCGGGCAGCTATCGATGTGTTTGA
ACATGAACCTGCAACTGATTCGCCTCTTGTTGCACATGATAAAATTATTGTTACACCTCAT
TTGGGTGCATCAACAGTCGAAGCTCAAGAAAAAGTGGCAATTTCTGTTTCAAATGAAATC
ATCGAAATTTTAATTGATGGTACTGTAACGCATGCAGTGAATGCACCTAAAATGGACTTA
AGCAATATAGATGATACTGTAAAATCATTCATCAATTTAAGCCAAACAGTTGGTGAATTA
GCTATTCAATTAATGTACAATGCACCAAGCTCTATTAAAATTACGTACGGTGGCGACTTA
GCCTCTATTGATAGTAGTTTATTAACACGTACAATTATTACTCATATTTTAAAAGATGATC
TTGGTCCTGAAGTCAATATTATCAATGCTCTAATGTTGTTAAATCAACAACAAGTGACATT
AAATATTGAAAATAATAAAGCAGAGACAGGTTTTAGTAACTACTTAGAGGTAGAACTATC
AAACGATAGCGATTCCGTTAAAGTTGGCGCTTCTGTCTTTACAGGTTTCGGTCCAAGAATT
GTTAGAATTAATAATTTTTCTGTAGACTTAAAGCCAAATCAATATCAAATTGTGTCATATC
ATAATGATACTCCAGGTATGGTAGGAAAAACTGGCGCATTGTTAGGTAAATACAATATCA
ACATTGCATCTATGACTTTAGGTAGAACTGAAGCGGGCGGAGATGCGCTAATGATTTTAT
CCGTTGATCAACCTGTTTCAAACAATATAATTGATGAACTTAAACAAGTTGGTGAATACA
ATCAAATTTTCACAACTGAATTGACGGTACAGTCATAA

## > tnpIS1272

Function: protein coding sequence; transposase of insertion element IS1272
Best match: tnpIS1272_15653_EU272084.1[3547:5193]RC
Position: 039-contig_321: 29013 … 29043; Length: 30 bp
Sequence:
TTAGGCAATGTAAAAAAGCTGATTTCTATT

## > tx_universal3

Function:  bidirectional rho-independent terminator
Best match: tx_universal3_CC001_MW2-USA400_BA000033.2[1397772:1397835:r]
Position: 039-contig_321: 29015 … 29045; Length: 30 bp
Sequence:
AGGCAATGTAAAAAAGCTGATTTCTATTAA

## > txbi_universal1

Function: bidirectional rho independent terminator
Best match: txbi_universal1_CC001_MW2-USA400_BA000033.2[1397234:1397306:r] (completely identical)
Position: 040-contig_241_RC: 10 … 83; Length: 73 bp
Sequence:
TGTATAAATAGAGAACAGCAGTAAGATATTTTCTAATTGAAAATTATCTTACTGCTGTTTT
TTTAGGGATTTA

## > nagE

Function: protein coding sequence; PTS system/N-acetylglucosamine-specific IIBC subunit precursor
Best match: nagE_CC001_MW2-USA400_BA000033.2[1806419:1807885:r]RC
Position: 040-contig_241_RC: 1433 … 2900; Length: 1467 bp
Sequence:
TTATAAATGTTTTTCAATTTCATCAGCAACCTGCTGTACGTGTGTACCGACAATAACTTGA
GTTGAATGTTTACCATTAACAGTAACACCAACTGCACCGGCGTTTTAATCTTCTGTTGAT

CAATAATAGATGTGTCTTTTAACTCTAGACGCAACCTTGTTGCACAATTGGTTAAATTAAC
AATATTCTCTTGACCGCCTAAACCTTCTAATATTTGTATAGCATGTTGATGATATTTACTTT
GTTTAATATCATTTTCACCAGGAGCAATATTATCTTTTACAACTGTTGGATCAACTAATTC
ATTTTCACCTCTACCAATCGTATTCAAGTTAAATACTTGGATTACTACACGGAAAATCACA
TAGTATAAGATGAAAAATACAACACCTTGAACAAGCAACATCAATGGATGATTTGATACT
GGATTAATTAGTGATAACACATAATCTATCAAACCTGCACTAAATGAAAATCCAGCTGTC
CAATGGAATGTAGCTGCGATAAATAAAGATAATCCTGTTAATAACGCATGAACAACATAT
AAGATTGGCGCAACAAACATAAATGCAAACTCAATCGGCTCTGTAACACCAACGAAAAA
TGCTGCAACTGAACTCGCTAGGAACCAACCGTAAACTTGTTTTTTCTGAGTCGTTTTAGCT
GTATGATACATTGCTAACGCAGCCGCTGGAATACCGAACATCATGATTGGGAAGAATCCC
GCTTGATAGCGTCCTGTAATACCTTTTATAGCATCTTTGCCACTTTGGAATTTACCAATAT
CATTAATACCAATCGTATCAAACCAGAACACACTATTCAGTGCATGATGTAATCCTGTAG
GAATTAATAATCTATTGGCAACACCATATATGAAAGCTCCAAACGATCCTAAACCGACTA
TAGATTCACCAAATTTTACAATCCATGAATAAAGTAGTGGCCATAAGAATAACAATATGA
CAACTAAAAATGTACAGTAAAATGCAGTCATAATTGGAACTAGACGTTTACCACTAAAAA
ATGATAATGCTAATGGTAATTCTGTTTCACTAAACTTATTGTATGCATAGGCTGCTATTAA
ACCTATTACAATACCAACAAAGACATTGCCATTATTCATCTTTTCAAAAGCTGAATTTATT
TCCGAAGCTTTCATTCCTAATAAAGGCGCTAATTTCATTGGTGATAATACAACTGTAACTA
AAAAATATCCTAACGTAGCTGCAAGCGCGACTGCACCATCATTTTTCTTTGCCATTCCTAT
AGCTACACCAATTGCAAATAAAATACCTAATTGCTCTAAAATCGTAGTACCTACCGTAGT
AAAGAACATTGCGATTTTCGGCGTCGCATGAAGTGCATTTAACGTATTACCAATTCCGGC
AATAATTGCTGCAGCCGGTAAAATGGCAACTGGTAACATTAACGAACGCCCTAAATTTTG
GAAAAATTTATACAT

> **tyrS**

Function: protein coding sequence; tyrosyl-tRNA synthetase (core genome, constant)
Best match: tyrS_CC001_MW2-USA400_BA000033.2[1810170:1811432:r]RC
Position: 040-contig_241_RC: 5184 … 6447; Length: 1263 bp
Sequence:
TTATTGATAGTTAACCATGAAGTATTTTTCTTACCGCGACGAATAATCGTAAATTCGCCA
TCAATTTTATCTTCTGGTGCTAAAGCATAATTAACATCTTGTTGTCTCTCACCATTAATATA
AATCGCACCATTGTTAACATCTTCACGTGCTTGTCGTTTAGAAGGAGAAATGCCTGTTTCA
ATAAGGACTTCAACGATATTTGTTGTGTCATTTGATAATGTCACTTGAGGCACATCTTTAA
ATCCATCTTTTAATTCTTTCGCTGATAATGATTTTAAATCACCACTAAATAATGCTTGTGA
AATACGGATTGCATCATTTAATGCATCTTCACCATGAATAAATTTAGTTACTTCTTCAGCT
AATGTTTTTTGAGCTTCACGTAAATGCGGTGCTTCATTTTTAGATTGTTCTAAGCGATCAA
TTTCTTCTTTTCCTAAGAAAGTAAAGTATTTTAAGAATTTAATTACATCTTCGTCTGATTGA
TTAATCCAGAATTGATAAAATTCATAAGGACTTGTTTTTTCAGCATCTAACCAAACAGCA
CCTGACTCAGACTTACCAAATTTCTTACCATCTGATTTAGTTACAAGCGGAATAGTTAAAC
CGTATGCGTCTGTTTGACCATACATACGACGCATTAATTCAATACCACTTGTGATATTACC
CCATTGATCTGATCCACCTACTTGAATCTTACAATTCAATTCTCTATTCAAATGACCGAAA
TCAATAGCTTGTAAAATCGTGTATGTGAATTCTGTATATGAAATACCATGTTCTAAACGAC
TTTGGATTGAATCTTTACCTAACATGTAATTAACGCCGACGTGTTTACCATAGTCACGTAA
AAAGCTAATTAATGAAATTTGTCCTAACCAGTCTCTATTATTAACAAGCACTGCACCATG
GTCTGTTCCAAATTCAAAAATATTGTGCATTTGTTTACTAATACCTTCGATATTTTTATCTA
CTTGTTCTTCTGTTTGTAGCACACGTTCTTCTGATTTACCTGATGGATCACCAATCATACCT
GTACCACCACCAATTAAAACGATAGGACGATGTCCATGTTCTTGAAAACGTCTTAATGTT
AAGAATGGTAGTAAGTGACCAATATGTAAACTATCTGCCGTTGGATCGGCACCGCAGTAT
AACGTCACTTGTTCTTTATTTAATAAATCTTCAATACCTTGTTCATCAGTTTGTTGATAAAT
AAGACCTCTCCATTTTAAATCTTCAATTAATACATTCGTCAT

> **tbox10**

Function: noncoding RNA T-box leader element (core genome, constant)
Best match: tbox10_CC001_MW2-USA400_BA000033.2[1811524:1811725:r]RC (completely identical)

Position: 040-contig_241_RC: 6537 … 6739; Length: 202 bp
Sequence:
TGTAAGGGCGCGATTGCACGTTACCACCAAACTTAAACATAATCATAAGATAATGTTCAC
TCTATTAATGATACGTTCATTAATAAACGTAGGACATGTTAGTTATAAAGGTGTATTCATA
TTATTAAAAACACTAGTTCACAGCGACCACTAGCTCTCTGATGATTTCAAATAATATTACT
TGTCCTTTTATCCTATTCTT

> **sgtA**
Function: protein coding sequence; monofunctional transglycosylase A (core genome, constant)
Best match: sgtA_CC001-ST772_118_AJGE01000048.1[6764:7669] (completely identical)
Position: 040-contig_241_RC: 6827 … 7733; Length: 906 bp
Sequence:
ATGACGAATCAAGACAACAATCATCAATTGAATCATCGTATATATCATTTTGAAAAGATA
TATAAAGCTATCAAACATGTCATTGTTTTATATTTATGATTTTCATTGCCATCGTTGCTAT
CGCTGTGATTGCGATGTCTTTATATTTTCATCATTTAACTAAAACGTCCGACTCATTATCA
GATGATGCTTTAATAAAAAAAGTTCGACAAATACCTGGCGATGAATTATTAGATCATAAT
AACAAAAATTTATTATATGAGTATAACCATTCTCAAAACTCACTCATTATAGGCCCTAAA
ACATCAAGTCCAAATGTCATTAAAGCATTAACGTCATCTGAAGACACTTTATTTTATAAA
CATGATGGCATCTTACCAAAGGCGATTTTAAGAGCAATGATACAAGATATTTTTAATACT
GATCAAAGTTCAGGTGGTAGCACAATTACACAACAACTTGTTAAAAATCAAGTTCTTACC
AACGAAAAAACATATAGTAGAAAAGCAAATGAACTTCGCCTAGCAATTAGATTAGAACA
CCTACTCTCAAAAGATGAAATTATATATACATATTTAAATATAGTTCCCTTCGGTAGAGAT
TATAATGGCGCTAATATTTCCGGAATTGCATCCGCTTCATATAGTTTGTTTGGTAATTCAC
CAAAAGATTTATCAATTGCACAATCTGCATACCTTATCGGTTTGTTGCAAAGCCCTTATGG
CTATACACCCTACGAAAAAGATGGAACGTTAAAATCGGATAAAGATTTGAAATATAGTAT
TCAAAGACAACATTATGTATTAAAACGTATGTTAATCGAAGATCAAATCACTGAAAAAGA
ATACAACGACGCATTAAAATATGATATTAAATCACATTTGTTAAATCGAAAAAAGCGTTA
A

> **isdH**
Function: protein coding sequence; haptoglobin-binding surface anchored protein (core genome, variable)
Best match: isdH_CC007_TCH959-USA300_AASB02000248.1[53213:55894:r]RC
Position: 040-contig_241_RC: 7938 … 10620; Length: 2682 bp
Sequence:
TTATTTAGATTCTTTTCTGAATTTAGGAATGAGTAAAGCTAACATACCTAATAACGCATAT
AAGCCCCACCATGATTGGCTTGAAGTTGTTTCTCCAGTTTTTGGTAGCATTTTAGATTGAC
TAAGTTTGTTTTCTTTCGATGGTGTGCCGGCCTTTTCTTTTGTTTTCACTGTTTTGTCCACA
GTTTTATGAATATCACTCGGCAGATGTTCAGTTGCTTTTTTGTCAGTAACTTTGATAGTAC
TGATAGGATGATATTTCATTGTATCAATGTTTGTTTCAGTTCCAGTATTATTAACTTTATTT
TTATCGGCAATATGAGCAAGCTGTATGACTTTGTCTTTATTTTTATTAGAGTCTTTATCAGT
ATCGACATTCGATGACATACCAACGCTATTATCAACACCCTTATCAGTGTCTTTAGCAATT
TGAGTATCCATTTCTTTTAAATCATACTTATCGAAGTGATTGTTATCCAACATATCGGATA
AATGATCAACATCATGTTGCACATCTTTGTCAACACTACTATCAGTATCTTTAACCATGTC
AGACTCTGGTTCTATCACATCTGCTTTATCAGACGCATCTTTAGGATTTGTTGCAGTGCTG
CTATTTTCAGCTACATCTGTATCAGCAACCTTATCTTCTTGTCCTGTTTGTACATTTAGCGG
TTCACTCGTGTTATTTTGTGATATATCATCATCTTTTGTATTGATATCCTGATTTATAATTC
TGACATGATATTGACCTTCATAACCAATGTTTGCCACAACGACTTTAACAATCGCATTGTA
AACTGCTTTGTCAGGTATATATGGGAAAATCAGCGTTCTAGAATTATTTTTAGGATCTTTA
GAAACAGTAGTGACACGTTTACCTTCTACAATTAAATCTTTCCAGTAACTGTCATCCTTTG
TTTTCATCACTACATATTTTTGACCATTTAAAGTTGCTGTATAGAATGGATGTTCAACAAA
GCCGTCCATAACTGACTCACTATTTTCTTCACTTTCAAAAACAACAAAATGCGCTTCTTGT
AAATCTGTTAATTGATCATTTGTAGGTGTAACATTTTCAAATTCCGTCACTGCTGATTTAA
CTTGATCAGCTAACTCTACTCTAGTTTGATCTAATTTCTTTTTATATTCCGCCTTATATTTTT
CTGGCAATTTCTCTTGTAATTTTTCTAATTCATAAACTTGTCTTTCTAACGTTTTAGCTTTG

TGATACGGAGCTAATAATTTTTGTAAATTGTATGTTTCTTCATCCACATAGTCGTCTGGGT
TATTAGTAATAGGCTGTGCAAAGACCATTAGCGTATAATCATAGTCTTCATGGATGTTCTC
ACCATATTCAATAGATGACACAATTTTAACTTCTCTCGTACCATTAGATACTGGGAAACG
AATATAGGCATAATCTTTATCAGAATCATATGATACTAATTCGACTGGTAACTTTTTGTCA
CCTTCATAAACTTCAAATTTCTTCCATGTTGAAGCTGTCTTTAAACCTAATTCAATTATTG
GTCCTGTTTTTGTAAAAATGACAGTTGCTGGTTCAACAGTACTAGCATAATGATAGAACT
GTCTTTCACCTTTATCATTTTTCATTTGAAAATCAATTGGTCGCCAATTATCAGCTGTATGT
TCTTTATCGATGATAGCCGGGTTTTTAATTGCATCTTGTAGTGATTCATCTGCTGGTGGAT
ACTGTTGATTAACATCCGACTGATTACTTGACTCATTCGTTTTATCGTTAGTATTACCATTA
GAATTTGTTTCATGAGCTGGTTGGCTTGACGCTTGATCTGCATTCGTTGACGATTTTGGTT
GTGCAGGTTGACTCATATTGGTCGTTGCCTGCGGTTGATTATTAGCATTGTTGATCATTGA
TGTATTTTGATTAGATGTATTCGTGTTTGTTTGATTACTTGCGACTGAACTTGATTGATCAT
TCGTTACTACTGCATCATTTGTATCTGATTTTACAAGTGAAGGATCGTTATAAATAGGTTT
AGCAAATACTAATTTAGTATAATCATAATTTGTTTCTTCTCCATCATCAATTTGAGTCGAA
GAAACAATTTTCAATTCTTGTGTGCCATCTGAAACTGGGAATCGAATATAGGCATGGTCT
TCTGGTACAGGACTATATGATACAAGTCTCACTGGCAGTTTTTGATTGTTTTCATAGACTT
CAAACTTCTTCCATGTTGAAGCAGTATTGATGTCTAATTCAACTTCTGCTTTCTTTTTAGTG
TAATACACATCTGCTGGATCTTTGATGCTGAAAAAGTGATAGTACTGCGTTTCATTGTTTT
TATCTAATAACTGGAAATTGACTTGTTCTCTTGGACCTATATCATGTTCTTTATTTTCTAAT
GCAGGATCTTTAATTGCATCTTTAAGTGATTCATCCGCTGCAGGATAGTTTTTCGCAGTGT
TTGCTGGTTGCGTAGCAGGTTGTGTTTGATTTGTATCCTTAGGTGGCTGAGTTGTAGTTGC
ATTATTATTTTGATTTTCCGAGATTTTATCTGAAGTATTTGTATTTTCTGCTGCTTGTGCTT
GATGTTGAGAAGTAATTAAAAATAGTGTACTGACAATGACCGATGCAACGCCTAGAGTTG
ATTTTCTAATAGAATAGAAAGACCTTAATTTTGGGTGATGTTTGTTCAT

## > furbox-isdH

Function:  binding site of ferric uptake regulator next to isdH
Best match: furbox-isdH_CC001_MW2-USA400_BA000033.2[1815633:1815668] (completely identical)
Position: 040-contig_241_RC: 10643 … 10679; Length: 36 bp
Sequence:
TATATTTAATTGATAATAATTATCAATATCAATTAT

## > rsaB

Function: noncoding RNA ncRNA of Staphylococcus aureus B (core genome, variable)
Best match: rsaB_CC008_COL_CP000046.1[1824119:1824178] (completely identical)
Position: 040-contig_241_RC: 10673 … 10733; Length: 60 bp
Sequence:
AATTATTAGTGTTAATTGTAGCGCATCCCAATTAAATATTGCAACCTATTTTATTAATTT

## > fhs

Function: protein coding sequence; formyltetrahydrofolate synthetase
Best match: fhs_CC001_MW2-USA400_BA000033.2[1815943:1817610:r]RC
Position: 040-contig_241_RC: 10952 … 12620; Length: 1668 bp
Sequence:
TTAGAATAACCCAATTGCATGACCATCATCAGTAACATCCATGTTTAATGCTGCTGGTTTT
TTAGGTAAACCAGGCATAGTCATGATTGCACCTGTCAACGCTACGATAAATCCTGCACCT
GTTTTCGCTTCTAATTCACGAATTGTAATTTCAAATCCTGATGGTGCACCTAACAACGTTT
GATCATCTGAGAATGAATATTGTGTTTTCGCCATACATACTGGGTAATTATCCCAACCATT
TTCTTTAAATTGTTTTAATTGTTTTTGCGCTTTACTGCTAAACGTTACTTTTGAACCGCCAT
AGATTTCAGTCACAATCTTTTCAATCTTTTGCTCTAATGGTAATTCTAATTCATATAAAGG
TTTAAATGAATTAGGTTGATCAATGACTTCTAATACTTCATTTGCTAAGTCAACGCCACCT
TTACCACCTTTTTCCCAAACTTCAGTTAAGGCAATTCGTACGTTATTTTCTTTAGCCCAAG
ATTTTACATATTCTACTTCTGCATCGGTATCATGTATAAATGCATTAATTGCAACAACCGG
TTCTACACCGAATTTTTTAATATTATTAACATGACGCTCTAAATTAACAATTCCTGCTTTTA

CTGCTTCTACATTTTCTTCTTTTAAATTATCTTTCGCTACACCACCATGCATTTTTAACGCA
CGAATTGTCGCAACAACAACGACAGCTGCCGGATCAAATCCTGCTTCACGCGCTTTAATA
TCCATGAATTTTTCAGCGCCTAAGTCTGAACCAAATCCAGCTTCCGTTACAACGATATCAG
CTAAATCACGTGCTGTTTCAGTTGCTAAAATTGAGTTACAACCGTGTGCGATATTCGCAA
ATGGTCCACCATGAACTAATGCAGGTGTCCCTTCAATTGATTGTACTAAGTTTGGTTTTAT
TGCATCTTTTAAAATCATTGCAAGTGCACCTTCCACTTTTAAATCTGCAACTGTAACTGGC
TTGCGATCTCTAGTGTAACCAATAGTAATACGACTAATTTTATCTTTTAAGTCTTTAATAC
TTCTACTTAAACATAAAATCGCCATAATTTCAGACGCTACTGTAATATTAAAGCCATCTTC
ACGTGGTACACCATTTGTAGGTCCACCTAACCCAACGTTTACATGTCTAAGTGCACGATC
ATTCATATCTAATACACGTTTCCACTCAATACGTCTTTGATCGATTCCTAATTCGTTACCTT
GGTGAATATGATTATCGATAAACGCAGACAATGCATTATTTGCAGTTGTAATCGCATGGA
AATCTCCGTTGAAATGTAAGTTGATATCTTCCATAGGTAAGACTTGCGCATAACCACCAC
CAGTCGCACCACCTTTGATACCAAATGTTGGTCCTAAAGCAGGCTCTCTTAATGCAACCA
TAACGTTTTTATTTAACTCATGGAATGCATCAGCTAAACCAACTGTAACCGTTGATTTACC
TTCACCAGCTGGTGTTGGGCTCATCGCAGTTACTAAAACAACTTTCCCTTTGTTTTCTCTTG
GCGTAATTTTATTAATGTCGATTTTAGCTTTGTAATGACCATAAGGTTCTAATGCATCCTC
TGAAATACCTACTGATGCAGCAATATCCTTAATTGGTTGTAGTGTTGATTGATTCGCAATA
TCTAAATCTGATAAATGAGTCAA

> **acsA1**

Function: protein coding sequence; acetyl-coenzyme A synthetase
Best match: acsA1_CC005_JH1_CP000736.1[1903505:1905211:r]RC
Position: 040-contig_241_RC: 13059 … 14766; Length: 1707 bp
Sequence:
TTATTCCATTGTACTTAAATCCCCAGCATCTAAATTTAATTCCCAAGCTTTTAATACACGT
CTCATAATTTTACCTGACCGTGTTTTAGGTAATTTATCTTTAAATTCGATTTCACGTGGTGC
TGCATGTGCCGACAAACCTTCTTTAACAAATATACGAATGTCTTCTTTTAATTCGTCTGTT
GGTTCATATCCTTTTCTCAGTGCAACAAACGCCTTAATTATTTCACCGCGAACCGGATCTG
GTTTACCAATAATTCCTGCTTCGGCAACTGCTTCGTGTTCAACCAATTTAGACTCAACCTC
AAATGGTCCAACTCGTTCACCAGCTGTCATAATTACATCATCAACACGTCCTTGGAACCA
GAAGTAACCATCTTCATCTTTATATGCCGAATCACCAGATACATACCAGTCTCCAATAAA
ATATGATTTATATTTTTCTGGATTCTTCCAGATACGATACATCATTGATGGCCAGCCTTTTT
TTATAGCAAGGTTGCCCATTCGATTTGGTGGTAATTCATTACCTGCATCATCGATAATTGC
AGCTTGAATACCAGGTAATGGTTTGCCCATTGAGCCAAGCTTGACGTCCATCGTTGGATA
GTTAACAATCATATGTCCACCTGTTTCTGTCATCCACCAAGTATCTAACACCGTTAAACCG
TATACTTTTTTCGCCCATTTTATAACTTCAGGATTTAAAGGCTCACCTACTGATAGAATCG
AACGTAACGATGACAAGTCATATTTCTCAACAATATCGTCACCAGCACTCATTAACATTC
TTAAAGCTGTTGGTGCCGTATACCAAATCGTCACTTTAAAATCTTCAATCATACTATACCA
CTGTTCTGGTGAAAAGCGACCACCAGCTATACAATTTGTAGCGCCATTTAACCATGGTGC
AAAAATACCATAAGATGTTCCTGTAACCCAACCTGGATCTGCTGTACACCAATAAACATC
ATCTTCTTGTAAATCTAATACATATTTTCCAGAAATATAGTGCACTAACATTGCTTGTTGA
ACATGCAATACACCTTTAGGTTGCCCAGTAGAACCTGATGTATAATGTAAAATCAAACCA
TCATCCGACTTTAACCATTCAATGTCAAATTCATCGCTAGCAGTTTCCATCAAACTAATGA
AGTCTATGTAATTGTCTTCTACATCCTCATCTACGACAACAATTTTTTTCAAGTTCGGTAA
TTTATCTACAGGTACTCGAGGTAACAATGCCTTATTAGTAATTAACACTTTAGCTTCACTG
TTCTCTAATCTATCCGCAACTGCCTTTTCCATAAATGCTTCAAATAACGGCCCAACAATTG
CACCAATTTTTAAAACACCTAACAACGCAAAATATAGTTCAGGTGTACGCGACATAAATA
TAAATACTCTGTCACCTTTGTCAACTTCTGCATGTTCAGACAAAACATTCGCTGCTTTATT
AGATAATCGTTGCATATCTTTATAAGTATACGATTCTTTTCTGTACTCATCTTTGTAATTTA
ACGCTATTTTATCCCCTAATCCTTGATCTACATGGCGATCTATACATTCATATGCCATGTT
CATTTTTCCAGTTTCACTCCAAGAAAATGCTTGTTCTACGTCTTTCCAATCAAAAGTATTA
TATGTTTCTTCATAATCTTTAAGGTTATGTTTACCTTGCGCTCCTTTATAAACTTCGACTTT
CAT

> **acuA**

Function: protein coding sequence; acetoin utilization protein A
Best match: acuA_CC001_MSSA476_BX571857.1[1799578:1800210]
Position: 040-contig_241_RC: 14934 … 15567; Length: 633 bp
Sequence:
ATGAATCATTTAAAGACGTATCAATCCGAAGATTATTACATTCATGACAAGCAATTTGTT
ATTGAAGGTCCTTTAACATACGAATATTTGAAAGCGCTTACTTTCGATGCGCATTTAACCG
CATTTAGAGATGCTGAAGATCAGTATGAAGCTTTGTTAGAAATTACAACATTACCAGAAG
GTAGAATTTATGTTGCTCGCCAAGATCAACTCATTGTGGGTTATGTCACTTTCCACTATCC
TGATGAAATTGAGCGCTGGTCTACAGGTAACCTTCCATATTTAATCGAATTGGGGGCAAT
TGAAGTCAGCATCAATTTTAGGCAATTACATCTTGCAGAAAAGCTAATACAACTTAGCCT
TTCTACACCAGAATTCGAGGATTATATCGTTATAACTACTGAATATTACTGGCATTGGGAT
TTAAAAAATTCAAAGTTAGATGTATTTGACTATAAAAAATTAATGCAGCGGTTAATGGCA
ACTGGTGGACTTGAAATATTCGCTACAGATGATCCAGAAATAACAAGTCATCCAGCTAAT
TGTTTAATGGCAAGAATTGGCAAAAATATTACATTAGAACAGCAACAAGCGTTTGATGAT
ATTCGTTATATGAATCGGTTTTTCTTTTAA

> **acuC**

Function: protein coding sequence; acetoin utilization protein C
Best match: acuC_CC001_MW2-USA400_BA000033.2[1820582:1821751]
Position: 040-contig_241_RC: 15591 … 16761; Length: 1170 bp
Sequence:
ATGCAACAACATTCATCAAAAACTGCATATGTTTATTCAGATAAGTTATTACAATATCGA
TTTCATGACCAACATCCCTTCAATCAAATGCGTTTAAAATTAACAACAGAGCTACTTTTGA
ATGCAAATTTATTGTCTCCAGAACAAATAGTACAACCTAGAATTGCAACAGATGACGAAT
TAATGTTAATTCATAAATATGATTACGTCGAAGCAATTAAGCATGCTTCACATGGCATTAT
CAGTGAAGATGAGGCTAAGAAATATGGATTAAATGATGAAGAGAATGGTCAATTTAAGC
ATATGCACCGCCATAGTGCCACAATTGTTGGAGGCGCTTTAACTTTAGCAGATCTTATTAT
GTCAGGCAAAGTATTAAATGGTTGTCACTTAGGTGGTGGATTGCATCACGCTCAACCTGG
TCGAGCTAGTGGTTTTTGTATATACAATGATATTGCAATTACCGCACAATACTTAGCTAAA
GAATACAATCAACGCGTTTTAATCATAGATACCGATGCACATCATGGAGATGGTACACAA
TGGAGTTTCTATGCCGATAACCATGTTACTACTTATTCTATCCATGAAACCGGAAAATTTC
TTTTCCCAGGCTCTGGTCACTATACTGAGCGCGGTGAAGATATCGGCTATGGACACACTG
TAAATGTCCCACTTGAACCGTATACAGAAGATGCATCATTTTTGGAGTGTTTTAAATTAAC
AGTTGAGCCTGTCGTAAAGAGTTTTAAACCTGATATTATTCTAAGCGTAAATGGTGTCGA
TATACATTATCGTGATCCACTAACTCATCTAAATTGTACGTTACATTCATTATATGAAATT
CCATATTTTGTAAAATATTTAGCTGATTCTTATACGAATGGAAAGGTAATTATGTTTGGTG
GCGGAGGCTACAATATTTGGAGAGTCGTACCACGTGCATGGAGTCATGTATTCTTAAGTT
TAATTGATCAACCAATTCAAAGTGGTTATTTACCGTTAGAATGGATTAATAAATGGAAAC
ATTATTCATCTGAATTATTACCTAAAAGATGGGAAGATCGTTTAAATGATTATACCTATGT
CCCCCGCACAAAAGAAATTAGTGAAAAAAATAAAAAATTAGCTTTACATATAGCGAGTT
GGTACGAATCTACTCGTCAATAA

> **ccpA**

Function: protein coding sequence; catabolite control protein A
Best match: ccpA_CC001_MW2-USA400_BA000033.2[1821849:1822838:r]RC
Position: 040-contig_241_RC: 16858 … 17848; Length: 990 bp
Sequence:
TTATTTTGTAGTTCCTCGGTATTCAATTCTGTGAGGTAAAACTACATTTGGTTCTTCTATCT
TTTCATCGTTCATATATTTTGTTAATAAGCGCATCCCTACTGCACCGATATCATATAATGG
TTGAATAACACTAGAAAGTTGTGGTCTAACCATCTCAACTAAACGTGTATTATTGAAACT
AATAATTTGTAATTCCTCTGGAACTTTAATACCAGCATCCATTGCACTATGCATAATACCA
ATAGCTTCTTCGTCACTGATACATAATATAGCATCTGGAAGATTACCTTTCATTTTGGCAA
AAGCTTTTACGCCTTCTTTATAACTTTCAGCACCAGAACAATTCAATGTATCACCTAATTG
AAGACCATTTTTATTTAACACTTCAGTTAAACCTTCTAAAACATCTTCTTGAGCTTTTTTAG
AATGTTCTCCACCTACTAAAGCAAATGATTTAGCGCCTTTTTCAATTAATTCTCCCGTAAT

TTCTTTCGCAGCTTCAGTAAAATCAATATTAACTGATGCTATATGTGCATCCTTACCATTT
GTTCCTGATACTACTACAGGTACAGATGATTGATTTATCAATTCTTTCATTTCTTCAGTAA
TTGTACCACCAAGGAAAATAATACCATCAACTTGTTTACTTAATAAGTTATTAAAAATTTC
TTTTTCTTTTTCAGGATCGTTATCTGAATTTGAAATAATTGAGTGATATTTATACATTGTTG
CAATATCTTCAAGTCCACGAGCAAGTTGTGAATAATAGATATTAGATATATCTGGAATGA
TCACACCTACTGTTGTTGTCTTTTTACTAGCTAAACCTCTAGCAACAGCATTTGGACGATA
ATTCAAACGCTTAATGACTTCGTTAACTTTATTTTTAGTTTCTGCTTTAACATTTTGGTTCC
CATTAACAACACGCGACACTGTGGCCATAGAGACACGCGCTTCTCTTGCTACATCATATA
TAGTAACTGTCAT

> **aroA2**
Function: protein coding sequence; bifunct chorismate mutase/phospho-2-dehydro-3-deoxyheptonate aldolase (core genome, variable)
Best match: aroA2_CC001_MW2-USA400_BA000033.2[1823378:1824469:r]RC
Position: 040-contig_241_RC: 18387 … 19479; Length: 1092 bp
Sequence:
TTATTTTAACTTTTTAGCGTTATATAAATCAGCTAAAGGCTTTAATTCATCATAAAATGCT
TGGAATTCATCTAAATCCATTTGTTGACCCGCATCACTAAGTGCAACAGATGGATCTGGA
TGCACCTCAGCCATAACACCATCAGCACCTACTGCTAATGCTGCTTTCGCAGTTGGTAAC
ATGATATCTTTACGACCTGTACTATGCGTAACATCTACCATGACTGGTAAGTGTGTACCTT
GTTTTAAAATTGGTACTGCTGAAATATCTAAAGTGTTACGTGTCGCCTTTTCATAAGTTCG
GATTCCGCGTTCACATAAAATAATGTTTTGATTACCTTGTGAAGCAATGTATTCAGCTGCA
TAAACAAACTCTTCGATTGTAGCAGATAAACCACGTTTTAATAGAATAGGCTTTTTCGTAC
GCCCAGCTTCTTTTAATAACTCAAAGTTTTGCATATTACGTGCACCAATTTGGAATACATC
TAAATATTCATCGGCTACTTCAAAATCATTAGGATTTACAATTTCACTAACTACATTTAAA
TCGTATTTATCTTTAATCTGTTTAAGTATTTTAAGTCCTTCAACACCTAGGCCTTGGAAGTC
ATAAGGCGATGTACGTGGTTTAAAAGCACCGCCACGAATAAACTTTTCACCTTTGGCATG
TAAATTTTTAGCTACCGCTTCTACTTGTTCAAATGATTCAACAGAGCATGGTCCAAATACA
AATGATTTGTTGCCATCTCCAATAATACCCCCATTTTCAAATGTTACAATCGTATCCTCAG
GTTTCAACTTACGAGATACGTATAAATGTTTTTCATTTTCAGATTTTTGTAAATCTGTAGA
GGCTTTGAAAATTTCTTTAAATAATTGTTTAATAGTATTATCGTTGAATGGTCCTTTGTTAC
TATCGATTAAGTCGTTAAGCATTTCTTTTTCACGTTGTGGATCATAGATACGTGTACCTTG
TTTTAATTTTTCTTCCCCAATTTTTTGTGCTAGTTCACCACGTTTAGATAATAAGTCTAAAA
TTTGATGATTCAGTGATACAATCTCACTTCTGTATGATTCTAATTTATTACTCAT

> **tx_universal3**
Function:  bidirectional rho-independent terminator
Best match: tx_universal3_CC001_MW2-USA400_BA000033.2[1397772:1397835:r]RC
Position: 041-contig_187_RC: 19 … 49; Length: 30 bp
Sequence:
TTAATAGAAATCAGCTTTTTTACATTGCCT

> **Q99TC6**
Function: protein coding sequence; putative protein
Best match: Q99TC6-CC1_CC001_MSSA476_BX571857.1[1804822:1805868:r]RC
Position: 041-contig_187_RC: 149 … 1355; Length: 1206 bp
Sequence:
TTAGTCATTGAATGTACGTTTCTCTATTTTACTATTAGCTTTTTCAACTTTAGCATTTGGCG
TTTTTGATGCATTGGATGATTTAGCATTTGTTGTTTTTGAATTCTTATTACTAGTTTGATTA
TTCTTTTGAGTTGACTTACTTGAACTACTTTGTTTCTTTGCACCTTGTGATGATTTCTTATT
ATGTTGCTTTTTCTGACCTGAAGTTTTATTTGTAGATGCTTTTGATGCATTTCGTTTATTTG
AAGGTGTTGTTTTTTTAGATTGTTTACCTGATTTCTTGTCAACAGCTGTATTATTAGTTGTT
TTTTCATCAGCTTTACGTGTAATAACACCATTTTCAAATTTAGCTTCTTTTTGTTTAACAGT
GTCTTTATTATCTATATTAACTGTCTCTGTCTCTGATACATCATTCTTTTTAGTAACTAATT
GAGGAATTTCTTTCAAGTCATTTTTAGCAACTGGTTTTTCTGCAAATAACGCTTCAGTTAA

TTGACTCTCTTTTGAACCTGGTGTTAATTTAGCTTGCTTCTGTTTTGCAGCGTTTGCTAATC
TTTCAGCTTGTGACTCTTCTTTCGATACCTTTAATGCCACTGCTTTTGATTCTTTATTTGAT
ACTTCTGCACTTTTATCTGTTTCTGCTTGTGCTTCTTTTTTAGCTTCTTGAATCTCTTGTGCC
TCTTGTGATGTATCACTCAAATTATTTGCACTTGCTTCTTCTTTTATTGCTGCTTGTTGTGC
TTTTAATGCCACTGGTTTTGATTCTTTATTTGATACTTCCGCACTTTTATCTGTTTCTGCTTG
TGCTTTTTTCTTCGCTTCTTGAATCTCTTGTGCCTCTTGTGATGTATCACTTAAATTATTTGC
ACTTGCTTCTTCTTTTATCGCTGCTTGTTGTGCTTTTAATGCCGCTTGCTCATTTTTAGATTT
GTTTAAAAATCCTTCAACACGTTCCTTTGTAAAGGCAACCGTTTCTTCAAGTTGCGTTTTT
CTTTCTTCAAACTTTTGCGACAGTTCTTGTTCTTTGACTTTTAAATCATCTGCTTTTTGATA
AACTTTATTTTTAAAATACAAACCTAAAGCTGAACCAACAAGCGCGCCAGTTATAAAACT
AACAACAAAATCTTTACGGTTGGGTAATGGTTCATTTTGATAAGTGTGTTTATTTTTTAAT
GTTCTTTTATTATTTTGTTGTTGCGTCAT

> **yoxC**
Function: protein coding sequence; general stress protein (core genome, constant)
Best match: yoxC_CC001_MW2-USA400_BA000033.2[1826607:1827098:r]RC (completely identical)
Position: 041-contig_187_RC: 1428 … 1920; Length: 492 bp
Sequence:
TTATTTATCTACTCGAGAAGTATAGCTATGATTTGCATCAGTTGCTACATTATTAGCTTTG
TAATTTGCACTTCCACGACGGTAGTGTCTATTTTGCCATTTGTCTGCAATTTCCATTGCAA
CATTTGACCATTGAACAACTTGTGAGATTTTATCTTCATTTTGAGAAATATTATGTGTAAT
TGAATTTGTTACACGATCTACAGAGCTGTTTAACGTTTGTACTGAGTCACCGATACCTTTA
ACAGCATCTACAACTGAGTTTAAACGATCTACTTTACCTTGGATATCCTCAGTTAAACGGT
TTACTTTATGAAGTAAATCTGTTGTTTCACGAGTAATACCTTGAACTTGACCTTCTACACC
GTCAAGTGTTTTTGCAACATAATCTAAGTTTTTCTTAACAGAATTTAATACAGCTACGATA
CCGATACATAAAATTAAGAATGCAATCGCAGCGATAATTCCAGCAATTGGTAAAATCCAA
TCCAT

> **murC**
Function: protein coding sequence; UDP-N-acetylmuramate--L-alanine ligase (core genome, constant)
Best match: murC_CC001_MW2-USA400_BA000033.2[1827172:1828485:r]RC
Position: 041-contig_187_RC: 1993 … 3307; Length: 1314 bp
Sequence:
TTAAAACGCATTTTTCATGCCTAATTTATCTAAATATGCATTTTGTAATTTTTGAATATCAC
CTGCACCCATAAATAAAACAACAGCATTATCAAATTGTTCTAATACATTAATAGAATCTT
CATTAATTAACGATGCACCTTCAATTTTATCAATTAAATCTTGTATCGTTAATGCGCCAGT
ATTTTCTCTAATTGATCCAAAAATTTCACATAAGAATACACGATCTGCTTTACTTAAACTT
TCTGCAAATTCATTTAAAAATGCTTGTGTTCTAGAGAAAGTGTGTGGTTGAAATACTGCA
ACAACTTCTTTATGTGGATATTTCTTTCGTGCTGTTTCAATTGTAGCACTAATTTCTCTTGG
ATGGTGTGCATAATCATCTACAATAACTTGATTTGCAATTGTAGTTTCATTGAAACGACGT
TTAACACCACCGAACGTTTCTAATGCTTCTTTAATATTTGTAACATCTAGCTTCTCTAAAT
AACTAATCGCAATTACAGCTAATGCATTTAAAACTGTATGGTCACCATATTGTGGAGACA
GGAAGTGATCATAAAACTCACCATCCACATACACATCAAAAGCAGTACCTTTATCCGTAA
TTTGAATATTTTGAGCATAAATGTCATCCGAATCTTTAAATCCATAGTAATAAATTGGAAC
ATCTGCTTCAATTTTACGTAGATGTTCATCATCACCCCAAGCAATAATACCTTTTTTAACA
TTATGTGCCATTTCTTGGAATGCATCAAAAACATCATTAATATCTTTAAAATAATCAGGAT
GATCGAAATCAATATTTGTCATAATTGCGTAATCAGGTTTATAACTTAAAAAGTGACGTC
TATATTCACATGCCTCAAAAGCGAAATAATCACTTTCAGGCAATCCCATACCTGTGCCAT
CACCAATTAAAAATGAAGTTTTTTTATCACCATTCATAACATGTGATAATAAACCTGTTGT
AGAAGTTTTACCATGTGCACCAGTTACAGCTACTGAAGTATATTGATCAATAATCTGTCCT
AAAAAATCATTATAACTTACAACATCTAATTTCAATTGATGTGCACGTACTATTTCTTCAT
GGCTACTCGCGAATGCATTACCTTGTATAACTACCATATCTTCTTTTATGTTATTAGCATC
AAATGGTAATATTTTTATCCCCTTATTTCTAAGAGCAACTTCTGTAAATACGTAGTTCTCA

ATATCCGATCCTTGAACTTCATGTCCTAAATCATGCATGATTTGTGCTAATGAACTCATGC
CAGAACCTTTAATTCCGACAAAATGATAGTGTGTCAT

## > Q5HF33

Function: protein coding sequence; cell division protein (core genome, variable)
Best match: Q5HF33_CC001_MW2-USA400_BA000033.2[1828509:1832333:r]RC
Position: 041-contig_187_RC: 3330 … 7155; Length: 3825 bp
Sequence:
TTATTCTTTATTTAAATCTGCTTCCGTAACATAAACATCCCTTGGTTTTGAACCATTAGCAC
TCGAAACATAACCGAGTTGCTCTAATTGATCGATAATTCTTGCTGCTCTATTATAGCCAAT
TTGGAAATGTCTTTGGATTAATGATGTTGAAATATGTCCTTCATTAACCATAAATGCACAA
ACATCATCAAATAATTCATCTTGTGATTGTGTTTGTGTTTTTTTCAACAATTCTTTTTCTTC
AAATAGATAGTCCGGTTCTCTTTGTTGTTTGATAAAATCAACAACATCATCAATTTCGTCA
TCAGAAACAAATGTACCTTGAACTCTAATCGGTTTATTCATACCGCTACCAAGATATAAC
ATATCGCCATATCCTAACAAGCGTTCTGCTCCACCACTGTCTAATATCGTTCTCGAATCTA
CACTTGATGATACCATAAATGCAATTCTTGTTGGTATGTTGGCTTTAATTAAACCTGTAAT
TACATTGACAGATGGTCTTTGCGTAGCTACTAACATATGAATACCACATGCTCTCGCTTTT
TGAGCAATTCTAGCAATAGACTGCTCAACTTCTTGCGGAGCCATCATCATTAAATCAGCC
AACTCATCAATTACAATGACAATTTTTGGCATTCTTTCATCATATGGTGCTTTTTTGTTAAA
TGCTGTTATATTACGTACATGGTAATGTGCAAATAACTTATAACGTCGTTCCATTTCTTCT
ACGGCCCATTTTAAACTCTGTGTAGCTGCTTTGACATCTGTAATTACCGGTGCAACTAAAT
GTGGCAAACCATTATAAGGAGCTAATTCAACCATTTTTGGATCGATAAGTAATAATCTTA
ATTCCTCAGGGTGATTTTTATATAGTAAAGACATCAAAATACTATTGATACAAACTGATTT
CCCTGATCCAGTTGCACCTGCAATTAGTGCGTGTGGCGTTTTAGCAATATCCATAAGTAAT
GGTTCATTATTAATTCTATACCCCATCGCAACTGTTAATTTAGATTCAGCATTTTTAAAAC
TTGGAGATTCAATAATAGAACGTAAGTTGACTGTCGTTGGATTTTGGTTCGGAACTTCAAT
ACCAACACGACTAGTTCCTGGAATAGGCGCTTCTATACGAATATCTTTCGCTGCCAATGC
CATTTTAATGTCATCTTGTAATGCCGTAATTCTTGAAACTTTAACACCTTTTTCAACTGATA
ATTCAAATCTTGTAACACTTGGACCTTCAGTTACATCTTGTACTTCTGCAGGTACATTAAA
GTAAAATAATGCGTCATTCAGTTCTTTCTTTTTATCTGTAATCCAGTCCTCGTCCGACTCA
ATAACTTGTGGTTCTTCTAGTAATGAAACACTTGGCAATTTAATATTTGGGCCTTTACGAA
TCATCGGCTTAGATGTTTGTACAGCTTGATTTGTTGCAACATCTTGTTGGTTCGTCCGAGG
TCTATTTTCATTAGCTGTATCTTCATTTTGATTTGAAAATGAAGGCTGTAAATCTTTTTGTT
GATCATCATTATCTTGTTGTCCACTAGTATTGTTTGGATGTGTTGTTTCTTCGCTTTCTTCA
GTTATGTCGCTTACTTCTGAAACTGAAGACGAAGTTGATTGATCAACTTGCTCATTTTCCA
ATTGATTATCATCTTGGCTTTCTTCAAATATTTCCGTTTGAGTTGAATCAGCAGAAGGTTT
CTGCTCTGAATATTGTTGTGCATTTTGATAATCATTTTCTGTTTCTGCATGACCAATAAGTT
GATTGTTCTCAACATTATTTGATGTCATATTATTTATTTTATATGCATTTGTATTTGACTCT
TGTGAATAAGTGCTTGATGATGGTGTGGCTTGACTCGCAGTCTTGCTTTCACTCACAGCTT
GCTTACTTTGTACAGGCTTCAATTCAGGCACATTGACTTTTGAATGCTTTTTACGATCCAT
CATACGCTTTTTATCAGATGGCGTCATGACAACATTAAATGGTCTTTTACTTAAAGTTGAA
ACCTTTTCAGTCTGTTTCTTTGGATTTACGTTTTCAATAGTTTTCTCTTCGACATTTTGTTCA
CTTTCAGCTGTTTCGTTTTGCACATTTTCTGTAATTTCTGCGTTATGCTTTTCTATTTCATTC
ATGCTTGAATCTTCCACAACATGACCATCATTCACAATATTCGTTTTCAATGAGTTGACAC
TTAAGTTTGTTTGGTCATCTTTTTTATGACGCGGTGCTAATTCAATTTCATTATCAACCGTT
TTATCGATATTATTTACTTGAGATTCAGTTACTGCGTGCTCTTGCGTTTTAGGAGCGTCTA
CATGTATTTCAGACGCGTTTTTATTGGTATTATCTTCGATATTTGAAGTAGTATCCTCATCT
ACTTCTGTATCATTTAATTCATAAAATTCTGCATCATCGATTTGACTTCCTGAATATTCTTC
TTTATTCGTTGCTCCATCTTCTTCTGAATCAGCAATTAATCTGGATTGATTTTTTAGTTCAT
CTTTATCATTTACTTCAACGTTATGTTGTAGTGCTGATTGATGTTGAGACGTTACATTCGA
AACCGTAACTTCATCATCTGACAATTGTTTTGTTGTCGATACTTGATTCAAACTAACTTCC
TCATAATTGTATGGACTTTCATCTACATGAGAGTCATTAGTGTCTTCATTTTGTATTGACG
CTTGTTTATTTTCATCATTTGATACAGTTTCATTCTCTATGCCATTTGTATGAAGCTGACTA
GCATCTGTACTATTATCTGTTAAGTCACTATCATCATTTAATGAACTATCACCAACATAGC
GTTCCGCTTGTTTAGCATACATTTCATCAATTGCACGTTGTATTGCATCTTGCTCTTCGTTT
TTATGTTCTTCACGCTTTTGTTGCAACGCTTTTTTAAATCGACGCTTTTGAAGCACTTTACG

TTCTCGTTCACGTCTAATTTCTTCAACAATTTGTGAAGCATAAATATTTTCAATTTTGATAG
TATTATCAACTTTTGAATAATTAGGCATTGATTTTGTTGTTGATGATACATTTTCATCAGAT
GATTTAGATGCAGTTCCTTGTTTGACAACACTATCATTTTGTTTTTCTTGTTCTAATTGTTT
ATTTTGAGACGTTTGCGTCTTAGCTACATATTTATCATATTTTTGTTTATCTGACTCAACTT
TTTCTGAAGGTTTACTTACAGGGATACGACCATTTTCTAACTTTTTAGGTTTCATTGTGCC
AAAAATAGCTGACGGTACCTCTGAAGTCTTGAAACTTTCTTTGTGATAATCTGGAGTTGA
ATCTTTAGCACGATGATTTGTATTATTAGAATACATATTATGTGTTTTTGACTTGTGCGTTT
CCTTCTCAATACCATTAATTGCAGAAACATATGTACCTGGCTTATTCGTATGGTAATGTGA
GTGATCTTTATATTTTATACTTTGCTGTGATATTTTAGAATTCCCACGTTGTTCACTATAAT
TTTGTTCTTCAGTTGTTTGATTTCTTCTACGGCGATGTCGTTTTTGTGAACGAGAATCGTGG
CTTTGTTTGCGATAGTCTCGATGGTATTGTTCTTTTTCATCTGAAATAATATCTGCAGATTG
TTCAACATTTTCATTTTCATAAGCTACGCTCATAGGAAAACGGAATTTTCCCCTCGGACGA
CTATAAATATCATTATTTTGAGGCAGTAATGAGTCATGATCGTTATCTATATTTTGTGATT
CTTGACGTCTTTTTTTCTTTCTATGAATCAAGTCATCATTTGAATCATTATCTTCGCCGAAT
AATTTATCAAACCAGCTCAT

> **tnpIS431-06**

Function: protein coding sequence; transposase for IS431 (mobile element)
Best match: tnpIS431-06_circ_S-epidermidis_GQ900381.1[43799:43807] (completely identical)
Position: 041-contig_187_RC: 6128 … 6137; Length: 9 bp
Sequence:
ATGAACTAT

> **pheT2**

Function: protein coding sequence; tRNA-binding domain protein (core genome, variable)
Best match: pheT2_CC008-ST72_TCH130_ACHD01000037.1[27922:28518]RC (completely identical)
Position: 041-contig_187_RC: 7175 … 7772; Length: 597 bp
Sequence:
TTATTCAAAAAATGCTTGTCCAATTTCATAGCTGTCATTTAATACCATAATACCTTTTTCTT
CAGGTGCATTAGGTAAATTCAATTCTTTCATTGAACAAATCATACCGCTTGAGGCAACAC
CACGTAATTCAGCATCTTTAATTACCATACCGCTAGGCATCACTGCACCTACTTTAGCAAC
AACAACTTTCTGTCCAGCTTCAACGTTAGGCGCGCCACATACAATTTGTAATGTGTCATTT
CCAACGTTTACATTTAGTACACTTAATTTATCTGCATCAGGATGTTTGTCTTTAGTTTCAAC
GTAGCCAACTACAAATTTCGGTGATAGATCAGCATTTAATTTATAATCAAAACCAGCTTC
TGAAATACGCTTTTGGAATACATTTACAAGTTCATCAGTTAATTTAATATGACCTTTTTCT
TCAATTGTTATATCTTTTGAGATTTCAAAAATATTATAACCTACAACATTACCTTCATTAG
TAATTTCAACAACATTACCTTTTTTATTGTAGTTTAATTCACCTTCAACTGGTTCAATTTGT
AAAAATGCGACATCTCCTACATATTTAGGATTGTAAAATAAATTCAT

> **Q5HF31**

Function: protein coding sequence; putative protein (core genome, constant)
Best match: Q5HF31_CC001_MSSA476_BX571857.1[1812314:1813171:r]RC (completely identical)
Position: 041-contig_187_RC: 7800 … 8658; Length: 858 bp
Sequence:
CTATTTATCTTTATTAAATTTACGACGATTTGCTTCTAAACGCTGAATCACGTTTGGATCTC
TTTTTTGTTTATTATTTTTACCTAAAATAAATATCGGTTCAAGATGACCCTGTTTATATCCA
AAGGATAATGATGTAATTGGAACTAGACCTTTAGTGAAAAATTCCATTGTTAAATGTGCC
ATCACATCATATCCTGTTTTATTGCGTATATCTGCAATAATTAACACATCTTGGTGTGGCA
CTGCTACGAGCATTTCGCCTTGACATTGTGCCTCAATTTCATTTAAAAATGCAGTATTTAG
TATCCTACTTGCATCATACCCGTCATTTGAGTTAATAAAATAAAAAATATTACCTTTTACT
TCATCAGTCGTATATGAATTTGACAATTTTCTAACATTAAACAGAGACATTTCTCTTATTT
GTTGTTCAGTTAACTTCAAATCCTCTAACATGCTTTCGTCAATTAGACGATATGATTTCCC
TAAATCGACTGCATAATAAACTGCTGTTTCTGCAGTATGCTCATCATAGATAAAAGGAAC
ACCTTGTTTAGTTTTTTTATCAAAGCTAGTCGCTCTAATGACAGGCATAATTTGACTAGAT

GATATACTCTCAAGGGTTTTATCTGCCATTTGTGCAATAGCTTCATCAACGTAATAAACAA
TTTCATCTACAATTTTTTCTTTTTTATCTTCATATTTTGCGACTATAGCGTTAAGTTTAATC
GTGATACCTTTGTTATTATCTGTTCGATAAATACGCAAAGTTTCTTCTTCACGATTAAATTT
AAAATCAACGTCTAAATGGCTTAAACGTTCCTTTAATTTATCTCTCATTTGAAAGGTATTC
AT

## > pepA1

Function: protein coding sequence; glutamyl aminopeptidase/locus 1 (core genome, constant)
Best match: pepA1_CC001_MW2-USA400_BA000033.2[1834312:1835388:r]RC
Position: 041-contig_187_RC: 9133 … 10210; Length: 1077 bp
Sequence:
TTATTTATATTGTAATGTTTCTATTTGATTATTATCTAAATTACAAATGGCTTCTGAAAGTA
AAGATCTAGCTGCAAAATAGTCTCTTATATCAAATACTGAGTCTGTACTATGAATATATC
GTGCACATACACCAATAACTGCAGTCGGAATACCAATATTAGCTTTATGAATTTCTCCAC
CATCTGTTCCACCTGGTGACATATAGTATTGATGTTCAATGTCATGTGCTTCTACTAACTT
TAATAAATAGTCTCTAAATACAGGCTTTAAAATCATTGTACCGTCTTTTATGCGAATTAAC
GTCCCTTTACCAAGTTCACCAGATAATGGTTGGTTTCCTTTAACGTCATTGGCAGGTGAAC
AATCAACTACAAATGCAACGTCTGGGTCTATCATCTCTGCAGATGCTTTCGCACCTCGTAA
TCCAACCTCTTCTTGAACATTTGCGCCAACATACAAGTCTACATCTAATTCTATATCTTTT
AATAATTCTAGTATTTCAATTGCCAAGACACAACCATAACGATTATCCCATGCTTTAGCAC
TATATCGATGTTCAGATAACTGTGTGAATGGCGTGTGAGGTACAATTGTATCTCCTATATC
TATTCCGCGCTCACGCACCTCATCTTCATTTTGAGCACCTATATCTAATGTTAAATCTTTA
ATTTCCGGTGCACCTTCACTACCAGTACGAAAATGTTTAGGTATATTAGAAACAACACCG
ATAATTTTATCGCCATTTCTATTTTTAATTACTAAGCGTTGTCCTTGCCAAATATCATTTGC
AACACCACCTAAATTTGTGAATTGAATCATTCCATTTTTAGTGATATTTGTAATCATAAAT
CCGATTTCATCCATATGTGCTGCAATCATTACACGTTTTGCATTTGGATTTTTAGATTTTTT
CACACCAAAAAATCCACCCATACGATTTTCAATAAATTCATCTACGTACAGCGCCATTTG
CTGAGTCATATAATTTTTTACTTCTTCTTCAAAACCTGGTGCCCCATGAAGCTCAGTTAAA
GTTTGAATTCGTTGTAATGTTACTTTTTTATTTATGTTCAT

## > Q6G8I0

Function: protein coding sequence; Metallo-beta-lactamase superfamily protein (core genome, constant)
Best match: Q6G8I0_CC008_COL_CP000046.1[1844520:1845362:r]RC
Position: 041-contig_187_RC: 10732 … 11575; Length: 843 bp
Sequence:
TTAGTTATTATCAACTAATGTTTCACGTAAAATATATGCATCTATGTTTTCACCATCATCG
CTGTATTTTACAGCAAAGTAGTTTTCATCATGATAAAACAAGAACCAATATTGTTGCTGA
ATAAAATATGGTATCATGCGTTCTTTTTCACGAATCGATTGCATAGGATAATCATCATATG
CCGTTACCCATAGAGGATTTTTATGTGCAGTAGTTGGGAATATATCACCCATATGAACTG
CTTTATCTCCTTGACTTTCAATCGTAATAATCGTGTGGCCAAAGCTATGACCTCCACTATG
TTGCATCTTGATACCCGGAACCGGTTCAAAATGTTTTTCGAATAAAATCAACTTGTTACTA
TAATCGCCTTTATTCTTATCCCAGTAAGTTGATTTACTTCTTATATTAGGTGCAATAAACTC
ATGCCACTCATCTTGTTGCACAACATGAATCGCATTTTCAAAAATTGCATGTCCCGCTTGA
TCAGTCAAACCGGCAGCATGATCAAAATGCATATGTGTCATTAGCACATAATCAATATCC
TTTGGCGTTAAATTATAATTTGCCAAATCAGCAATAATATGACTTTCTTCATCTACTCCAA
AATTACGTAATTGCTTTTCAGATAATTTACCATTACCAATACCCGCATCTATAATCAAATT
ATATTGAGCCGTTTGAATCAAAATTGGATGTGTCGGTAAATTGATTTGATTTCGTTCATTT
GCATTGTATTGCTTTGACCACAACGGCTTCGGAACAACACCAAACATTGCACCGCCATCC
ATTTTTGTATTGCCACCATTTAGATAATGAATAGATATATCCCCGATTTTCAT

## > tnpISSha1-2

Function: protein coding sequence; putative transposase
Best match: tnpISSha1-2_CC705_RF122-cow_AJ938182.1[1268707:1270028:r]RC
Position: 041-contig_187_RC: 11804 … 11834; Length: 30 bp

Sequence:
ATCAAAGGTATTTAGTATAACATTGTTCAT

> **repORI-px**
Function:  origin of replication of plasmids
Best match: repORI-pUB110-2_CC005_Mu50-VRSA_BA000017.4[1877259:1877321:r]RC (completely identical)
Position: 041-contig_187_RC: 11833 … 11896; Length: 63 bp
Sequence:
TAGGCGCATTATCTCCTTTAGTTATTTGTTTAGTCACTTTTAATTATAGAGATAATTGCGCT
T

> **txbi_trmB**
Function:  bidirectional rho-independent terminator of trmB
Best match: txbi_trmB_CC008_COL_CP000046.1[1845779:1845844:r]RC (completely identical)
Position: 041-contig_187_RC: 12001 … 12067; Length: 66 bp
Sequence:
TATAGTACGAAAAAACACTTATGATTGTATGTGTACATACCCTCATAAGTGCTCTATTTTT
GTGAA

> **trmB**
Function: protein coding sequence; tRNA (guanine-N(7)-)-methyltransferase (core genome, variable)
Best match: trmB_CC008_930918-3_ABFA01000009.1[4364:5008]RC
Position: 041-contig_187_RC: 12054 … 12699; Length: 645 bp
Sequence:
CTATTTTTGTGAATGGAATTTCGCTTCCATACGATAAATACGTGACCCTTTATCCGAAAAT
TTCTTTTCATATTCTGTTAAAATATTACTGCCATCGTCTTCTTGATGTAAATTTAGATTTAT
TTTTGTAAAATACATTCCAAATTGAGACATACTTTCTAAACTGTAGGCAAATAGTCCTCTG
TTATCAGTTTTAAAATGTAAATCTCCTTCATCATTTAAGATTTGTTGATACAACGCTAAAA
ACGTATGATACGTTAAACGTCGTTTTGCATGACGATTTTTTGGCCATGGATCTGAAAAGTT
CAAATAAATACGCGAAACTTCGCCGTCTTTAAAATATTCATTTAATTCAATGGCGTCATTA
CAAATAATCTTTAAATTTGTTAAACCCATCTCTTTAACTTTATCCAATACTTTATAAACGA
TACTTTTCTCACGTTCCATTGAAATATAGTTAATATGAGGATTTTGAGCAGCTAATGTTGT
AATAAACTGCCCCATACCCGAACCAATTTCAATGTGTATCGGTTGCGTTTTATCAAACCAT
TCAGTCATTTTCCCTGCATGTTGACCGTCCATGTCAACCAATTCAGGATGACCTTTTAAAT
AATCTTCAGCCCATGGTTTGTATCGAACTCTCAT

> **daaA**
Function: protein coding sequence; D-alanine aminotransferase (core genome, constant)
Best match: daaA_CC008_NCTC8325_CP000253.1[1773873:1774721:r]RC (completely identical)
Position: 041-contig_187_RC: 14054 … 14903; Length: 849 bp
Sequence:
TTAAATACTGTGTGACTCTATATACTTTTCAAATCCTTCTTGTAGTTGACGTGTAATTGGG
CCAACTTTACCATCATTAACTGGTTCACCATCTAATTTAATAACAGGTGTAACCTCAGCTG
AAGTACTTGAAACAATAACTTCATCTGCGTTTTTCAAGAAATCTACAGTAAACGTTTCTTC
TTTAAATGGGATGTTATAGTCTTCGGCAATTTTTTTAATTACAATTCGTGTAATACCATTA
AGAATATAGTTGTTAATCGGATGTGTATAAATCACACCGTCTTTAATTGCATAAGCATTAC
TTGAAGATCCTTCAGTTACAGTTTCACCTCGATGTTGAATTGCTTCAACTGCATTATATTT
CACAGCATATTCTTTTGCTAATACATTTCCTAATAAGTTCAAGCTTTTAATGTCGCAACGT
AACCATCGGATATCTTCAACGGTAACACCATTCACACCATTTTCTAAATGATCATAAGGA
CGATCATAACTCTTTGTATAAGCAACAATTGCTGGTTCTACTTCAGGTGTCGGGAAGCTAT
GATTCCTTTCAGCTACACCACGCGTTGCTTGAATATAAATTGCCCCAGTTTCAATTTGATT
CATATCAACTAATTTACGAGATAGTTCAATTAATTCTTCTACAGAATAATTTAAATCTAAA
CCAATCTCATTGGCACTACGTAAAAATCTTTCATAATGTTCTGTTACTGTAAATAACTTAC

CATTATATACTCGAATGTATTCATAAATACCATCGCCAAATACGTATCCTCTGTCGTTGTA
TGAAACCTTTGCTTCACTTGGACTTACAAACTCACCATTTAAAAAAATTTTTTCCAT

> **repeat_nySAgamma**
Function:  repeat element
Best match: repeat_nySAgamma_CC008_COL_CP000046.11850350:1850460:rRC [4_50ANF10END]
Position: 041-contig_187_RC: 16572 … 16798; Length: 226 bp
Sequence:
CGGGGCCCCAACATAGAGAATTTCGAAAAGAAATTCTACAGACAATGCAAGTTGGCGGG
GCCCCAACATAGAAGCTGGCCAATAGTCAGCTTTCAATAATGTGAAAGTTGGGGTAAGG
GCCCCAACACAGAAGCTGGCCAATAGTCAGCTTTCAATAATGTGCAAGTTGGCGGGGCCC
CAACACAGAAGCTGGCCAATAGTCAGCTTTCAATAATGTGCAAGTTGG

> **tx_universal2**
Function:  rho indendent terminator
Best match: tx_universal2_CC001_MW2-USA400_BA000033.2[1842046:1842086] (completely identical)
Position: 041-contig_187_RC: 16990 … 17031; Length: 41 bp
Sequence:
CTGAATGAAAATACGCTTGTAACAAGCTTTTTCAATTCTA

> **rsuA**
Function: protein coding sequence; pseudouridine synthase (core genome, constant)
Best match: rsuA_CC001_MW2-USA400_BA000033.2[1842616:1843311:r]RC
Position: 041-contig_187_RC: 17560 … 18256; Length: 696 bp
Sequence:
TTATTTATAATTTAATTTGTCAAAATCATTTTCAGTTAATAAACGATATTCTCCTGAATCTA
GATTGCTGTCCAATTCTAAATCAGCAATTTTGATACGTCTTAAATGTAATACCTCATTTTG
AATGCTATGAAACATTCGTTTAACTTGATGATATTTTCCTTCATAAATTGTTACGTGTGAC
GTTTGATTATCAATATAAGTTAAAATTGCAGGCTTAACCTTGCCATCAGACAGTGTTACAC
CCTCTTTAAAAGCTTGAATGTCGTCTTCAGTGATAGGATTTGCTGAAATAACTTCATATTT
TTTAGAAACATGTTTGTTTGGACTCATTAATTCATGATTAAAATCACCATCATTCGTTATC
AATAAAAGCCCTTCTGTATCTTTATCAAGACGACCAACCGGAAAAATATTTAGATGTTGG
TATTCAGGTATTAAATCAATAACGGTTTTTGAATGATGATCTTCAGTTGCCGATATATAAC
CTTTTGGCTTATTTAACATAATATAGACATTTTCAACGTATTCTATTAATTCTCCACGAACT
GTTATCTTATCGTTTTCTGGTTCTATATGTGTTTTTGGTGATTTAATTACTTGTTCGTTGAC
ATTTACAAGGCCTTTTTTAAGTAACTGTTTGACCTCATTACGTGTACCGACGCCCATATTT
GCTAAAAATTTATCTATTCTCAT

> **Q1Y9Y5**
Function: protein coding sequence; NAD(FAD)-utilizing dehydrogenase (core genome, variable)
Best match: Q1Y9Y5_CC001_MW2-USA400_BA000033.2[1845376:1846644] (completely identical)
Position: 041-contig_187_RC: 20320 … 21589; Length: 1269 bp
Sequence:
ATGTATCAAACAATTATTATCGGAGGCGGACCTAGCGGCTTAATGGCGGCAGTAGCTGCA
AGCGAACAAAGTAGCAGTGTGTTACTCATTGAAAAAAAGAAAGGTCTAGGTCGTAAACT
CAAAATATCTGGTGGCGGTAGATGTAACGTAACTAATCGATTACCATATGCTGAAATTAT
TAAGAACATTCCTGGAAATGGGAAATTTTTATATAGTCCCTTTTCAATTTTTGATAATGAA
TCCATCATAGATTTTTTTGAGTCTAGGGGTGTTAAATTAAAAGAAGAAGATCACGGGCGT
ATGTTTCCAGTTTCCAACAAAGCACAAGACGTGGTTGATACATTAGTGACAACTATCGAA
CGCCAACATGTAACGATTAAAGAAGAAGAAGCTGTTAGTAGAATCGAAGTTAATACAGA
CCAAACTTTCACTGTACATACTCAAAATAATAGTTATGAAAGCCATTCGCTAGTGATTGCT
ACAGGTGGTACAAGTGTCCCTCAAACTGGTTCAACTGGTGATGGTTATAAGTTCGCACAA

GATTTAGGTCATACCATTACTGAGTTATTCCCGACCGAAGTTCCAATTACATCAGCTGAAC
CTTTCATCAAATCCAATCGTCTAAAAGGTTTAAGTTTAAAAGATGTTGAATTGTCAGTACT
TAAGAAAAATGGTAAAAAACGCATCAGTCATCAAATGGATATGTTATTTACTCATTTTGG
TATCAGTGGTCCAGCTGCATTAAGATGTAGTCAGTTTGTTTATAAAGAACAAAAAAATCA
AAAGACACAGCACATTTCTATGGCAATCGATGCATTTCCTGAATTAAACCATGAACAATT
AAAACAACACATCACATCATTATTATCGGACACACCAGATAAAATCATTAAAAACAGTTT
GCATGGTCTAATTGAAGAGCGCTACTTACTGTTCATGCTGGAACAAGCAGGAATCGATGA
AAATACCACATCACATCACTTATCAAATCAACAATTGAACGACTTAGTAAATATGTTTAA
AGGGTTTGTATTTAAGGTGAACGGGACATTACCTATAGATAAGGCATTTGTCACAGGTGG
TGGTGTGTCACTTAAAGAAATTCAACCTAAAACAATGATGTCTAAATTAGTTCCGGGATT
ATTTTTATGTGGTGAAGTATTAGATATACATGGTTATACTGGTGGTTATAATATTACAAGT
GCACTCGTAACAGGACATGTCGCTGGATTATATGCCGGACATTACTCACATGCATCAATG
GAATAA

> **sasC**
Function: protein coding sequence; LPXTG-sorted surface protein C (core genome, variable)
Best match: sasC-COL_CC008_NCTC8325_CP000253.1[1781582:1788142:r]RC
Position: 041-contig_187_RC: 21705 … 28266; Length: 6561 bp
Sequence:
TTATGATTCTTTTTCGTTTTTAGTACGTCTTCTAGCTAACAAAGCCGCACCTGTAATCAGT
GCAAATTCTTTCAATGGTAAATCCATTCCTTCAGAACCTGTATTTGGAAGTTCTTTTTCAA
CTTTGCGCGATTCATGTGTCTCTTCTTTTTTAATAGGCGTACAAACTTTTGGAGCTGGCTG
AATTTCTTTTGGTGATACTTTCGTCGCTTCAGCTGGTAATTTAATTGCTAAAATTTCATCAA
CAATGAATTGCGTGTGTTGTTTGATGTCATTTAATGTCGCATCTTCATCAATCATTCTATTG
CCATCTGCAACATATTGATCAATTAATACTTTTACTTTAGCTAATTGTTCTGGTGTTGCGA
TCGCTTTGAATTTCGCATATGTTTGTTGAGCAATGTTATCAATTCGCAGTAAGCTATTTTCT
TTTTCAGTAATTACTGCTTCTATATCGCTTAATGCAACATTAAATCGTTTTAAAACTGCAT
CAACATCAGCATTAGTGCGTGCTGTTTTTAATTCTTCATCCATTTGTAATTTTAAAGCAGT
TATAGCTTTTAATGCATCAGCCTTATTACGATTACTTACTTTTCGATAATTTTGCACTAAA
GCAGTGACGCGTGCAAGATCATCATTAATCGTTTTTTCAGCATCTGGCTTTTTAATAGGAT
GTACATCTAAATCATGTATTGTTTGTAGATTTAATGATGCTGTTTTATCAACTTGTGCATT
GCTACGATCTTGATCAATTTGTCCAATAGCAGTGTCATAAATATTTTGTAACTGTGCTAAT
ATACTATTTCTTTCTTCTACCGTTGCTTGAATATTCGCTTCAATTGCTTGTTTTTTATCGTTG
AATAATGTTGTCAATTGTTCTCGAGCAGACGCCTTTCTGTTAATAACAGGTTCGATTTCAC
GAATTTCGTTTTTCTCATCATGCAATAAATATGCCACATCAGCATTAGTCACTGCACTAGC
AATTTGTTGTTTAGCTTTAATTAACTCTTTTTCAACTTGTGCTATTGCAATATTTTGTTCTTC
ATCTGTCGCTTCGTTATTTGCTTTAATTAAATTAATTTTATTTGTAGCGATATTTTGAATTT
GTTGTAATGCTGTTGCTTTAACTGTTGTCGCTGGTTTAATTTTTGAAATAATATTTTGAGCA
TTTATACTATCTTGATTAACTTGGGCAGTCTTATCTGCATGATTGATCTGATCAATAGCCT
GATTAAGTGCTTGTTCTACTAAATGTTTAGCAGCTAGTCTTTCTTCTTCAGTTGATAAATC
GCTTTGATCGATTAGTGCATTTTGAGCTTCGGCTTTTACACCAACAGATTGACGCGCTGCT
GGTTTAACTTGAACTTTAGGTAAAATCACTTTGATGTTGTCGTTGCCATCAGTCTCAGTTC
GATCCACTTCTGCATTCGTTTTGTTTTGTGCAATGTCATTTTTAATTGTATTTACAATTTTA
TTTAAAGTATCAATAGCAACATCTCTTTCATCTTGAGTAGTATCCAACGTATTTCGGATTG
CATCGAGTTGATTTTTATTATTTTCTTCAATGCTATCAAGCGCAGCTCGTTTACGTCTTACT
TTAGGTTTTATTTGCTCTATTGCCTTGATTGTTTGATTTCTAACATCAGTAACAGCAGCATC
TTGATTTGTATTTTCTATTTCTTGTTGCGCTTGTTTGAGTGTGTCGCTAATTAATTGATTCG
CTTCATCTAATTCATCAACTGTTGCATGTGGTGTATCTTTTATTGATTCTACTTGATTTTCT
GCACTTGCTTTTATTGCTTGTTGTGCTTCAGGCTTAATTACAATATGAGGTTGTACACCTTT
TAGTGTAGCAATGCCATTTGTTTCAACACGTTTCACATCATTATTCGCTATTGCTTGATCG
ATGTTTTGTAATGCACGTTTTTCATTATTCGCTAATTGATTTAAAGCAACTTGTTTTTCTTC
ATCAGTCGCATGTTCCGCTTGCTCAATTTCGCGCTTTTTAGCTTCATATTGTTGCTTAACTG
CATCTCTAGCAGCTGCTCTAACAATATGGTCAGGCGTCACTAAAGCAATGCTATCAAGCG
CTTGACTTGTTGTATCATCAACTTGTTGATTTGTTCTATTATTCGTAATATCTGTCATGGCT
TGATTTACAAATTCATTGATTTTATCTAGTGCTACTTGTCTTTCTTCTGCTGTTGCTTCTTTA
TCCTGATTAATCTTAGCACGTAATTCATTCGCTTTTTGATTGATTTTTTCACGTGCAGCTGG

TTTAACTTTTGTTTCAGGTTGAATAATTTTAATCGCTGATACACCATTTGTTGCTGCTTGAT
TCACCTGACTATTCGTTTGAGCTTGGTCAATAGCTGCAAGTGCTTTTTCTTTTTCTTTTGCT
AATGCTTGTGAAGCAACTTCTTTCTCATTATCTGTTGAATCAAGACTATTATCAATTTGCT
GTTGCTTTTCTTTAACAGCTTTTTCAATATCTGCAATTGCCTTTGGTTTAATTACTACTTCA
GCTTCAACATTATCTATAGCATTTACCGCTTGATTTGTAGTTGCGTCTACCTGATCATTTGT
TTGGTTTTGATTAATTTGATTAAATGCTTGATCTTTAAGTTGATTGATTTGATTAACAGCA
GCCTGCTTTTCTTCGTCAGTTGCATTAGGTGTTTGTTTAACCGCTTCAATACGCTTCGCCAC
TTCAGCAGTGATTTTATCTCGCGCTGCTTGTTTTTTTACTACGTCAACTTGAACAGCATCG
ATATTATTCTCTGCTACTGTCGCAGCTTGGTCTACTTCTGCATTTGTGTTAGCTTGTTTAAT
ACTTTCAATAGCTTGTTGTCTGTCTTGATTTAAAGTATTGATCGCAGCATTTTTCTCATCAT
TCGTTGCATCTGGTGTAGCATTGATTTCAGCTAATTTAGCATTATAATGCTGATTGATTTG
TGCTAATGCTGCAGGTTTTTTAACAATATTTGGCTGAATCGCATTAATTGCTTTTGTACCT
AATTGTTGCGCTTGATCTACTTCCGCATTTGTATCAGCTTGATTTATATTATTAATTGCCGT
TGCTAACTCTTGATCCACTTGATTTAAAGCCACTTGCTTTTCTTCAGTTGTTGCATTTGTGT
TTTGATTAATTTCTTGCTTTTTAGCAGTTGCTAAATCATTTAATACACCTGTAGCAGTTTGT
TTCTTCGTTACATGCGGTTGAACTGCGCCGATTTGATTGACTGCATCATCTCTAATACTAT
TGACCATCGCAGTAGTAGACGTCACACCAATATCAGTTAATGCTCTATTTTTAAGTGTATT
GACACGATTTATCGCTTCTTGTTTCTCTTCTCGAGTCGCGCCAGGTGTTGCATTGATATTTG
TTATCGCCTCTCGCGCTTTATCATTTACAACATTGCGAGCATCCGTTTTAACTTGTGTTGCC
GGTTGAATCACAACTATATTTTGTGTGCCCTGATCTTTTGCTTGTGCAACTTCTTGATTCGT
ATCTGCTGCATTAATATTTTGCTTCGCTGTGGCTACAGCTTGATCAAGTAATTGTTGTGCT
GCTTGTTGTTCTTCGAGCGTCGCATCATTATTATTAAATATCGTATTATGTTGCGTTTCCGC
ACTTTTATCAATGGCATTTTTTGCATCTGTTTTTACTTTTGTAGCTGGTGTAATTGCTTGTA
TTCCTTGTATCGCATTTGTCTTCACTTGTTCCACTTCAGCATTGGTATTAGCGTTTAAAATG
TTTGTGTTTGCTGCAGTTACAGCAGCATTCACTTTGTCAATTGCTGCTTGTCTTTCTTCTTG
AGTCGCATCAGGATTAGCATTGATCTCTGCGATATGTTGTTGTGCATCATGTGATACAGCA
TCCCTTGCAGCTTGCTTAACAACAGTTACAGGAGCAATTGGATTAATGGCATTTAGACCA
TCTCCTTTGGCGTTATCAACATCAGCATTTGTTGTTGCTTGATTGATTTGTTCTAAAGCATG
GTTGGTTGCTTGAGTTAATTCGTTCAATGCTGCATTTTTCTCTTCCTGAGTTGCTTCTCTAT
TACTATTTATTTGTTGTCTTTTCGTTGCTGTTGCTTGGTTAATTGCATCTCTTGCAGCTTTTT
TATGAGTTACTTGAGGAACAACTGCTCCAATAGTATTGATGCCATTATTTTTAGCTGTTTC
AACGTCAGCATTTGTAGTAGCATTCGTAACATTATCAATAGCATCTGTTTCATCCGTAGCT
AATTGATTTATTGCATCTTGAATCTCGTCTTCAGTAGCATCTGGTGTTGCATTGATAATTTC
CCTTTGTTTCGTTGCTTTATCACGTATTGCTTTTTTAGCATTTGGTTTAACAACCGGTGTTG
CAGTATCCCCACTTAAGGTCTGTATACCTTGATCTTTGATTCTAGTAACGCCATCATCAGT
CGTTTGATCACCAATATCACCAATAATATTGCCTTTATGTTCCTCGATAACTTGTATTGCT
GCTTGTTTTTCTTCATCTGTCAGTTCATCATTTTGATTAACTAAATCTTCCATTTGATCAGC
TTTTTGATTTACTGCATTTTCAGCATCAACACTTCGAATTAAAGTATGTTGCATCTGATTA
GTTAATGTATCAATATCTGCTTGAGATACTCTTTTATTTAATGGTACATTGTTACGATTTTC
AGCTAAAATTGTTTGTGCACGTTTTTTAAGATCATTAAAGATATCTAATGATGCAAATGTA
TAATCAGCTTGTTGAATACGTCTGTCAACTTCGGCTTGTAATGCATCTTTATTCATGATGA
TATCGATAGTATATGGATTTGTACTTACAGTATGACTTTCTGCAGCTGAATTAATGAAATC
TTGTGTATATGTTTTATACGTTAATGTATCATTAAATGTTACTGTTCTTGGTGTCGGTACAT
TATTAACACGCAACTTATACTTTAAATCCAATATTTTATCAGGCATTAGTCGTGCAGGTGA
ATTCGTTGTGCCACCACCAGTGCTTTTAATTGTTATCACTCGATTCGCCGCATCATATGTA
ACATTCATATCATTAACATCAACGCCTGAATTGTTACTTGGAAAATCTTTAGTCAATGAAT
TGTTCACATATTCTACCCCTTCAGGTAATTGAATTTTATAAACAAATTCATCTGTGTCTAA
AGAAGCACCAGAATTACCATTATTTTTTAGTGATGTTGTAACTGTAAATTCTTTATTATTC
GTTGCTGTGGGTTCCATTGTTCGTCTTTCAACATAAACATGTGACCCAGAATGAAGTCCGA
TAGAGTCAACAAAGCTATAGTATTTGTAACCATCTTTTAGTTGATAAATGCCACGCGCAT
CTGTTATTGCGTCATTTTTAGGTACAAATTGAATTTTGAGATTTCTCACATTATCAGGTAC
TTTAAATAAACGCAAAGTTGGACCGCCTTCAACAGTCTTTTCAGCAATCGTATCATTAGTA
TCAGCATTTTTGATAATAACATTTGTTGCGCCTTGACCATTTTTAGTTGGCATTGTATTAA
ATTCAAAGATTAATTCAGAATTTGGATTTACTGTTAAAGATTTTTCAATACCATTAAAATC
TCCATGATTACTAGCGTCCGTCCCGCTTATACGTCCTAATGCAAGTACATTGCCTTGTGCC
TGATAGGTTCTGTTGCTCCCAGCCTCAAACATACTTGTTCTAACCATCGCATGACTTAGCA

CACCTACTTTACCACCATTGATAAGTGTAAAGCCTGGTAAATTATCAACAACTGTTACTG
ATGGTACAGAACGGTTGGTACTTGGTCTAATACTATTGTCATCAAATGACAGCACTTCGTT
AGGTGCATTTTGTCCTGCATTATTGGCATTAGGATCAGTTGTAGGCGTGTATGGCGCTGTA
ATTGCAACTGGTGCACCACCGTTTCCTGCCGCTGCAGCCCCTGGATCTGCTGGAGTTGCAT
TAGGATCTGCCGGTGCCGCACGTCTACTTCTCTTTTTCGGTCTATTAGATGCTGGTTCAGC
AATTGCAACTAGCTCTGGTTTATCTGAAGAATGACGAACATCTTCTTGAATTTCCTTTAAA
GTTAGATGTCCTCCTGAACCATTTTCATTTGTTTTATTTGGTACATTTAATGTGGTATTATT
CGACGCTACATCATTATTATTAGCGTTTGACACTGTCTCAGTTGCTGTTGTATTGCCATCT
GTATGATTATTGTTTTGTGCTGAAGGCGTACTTGATTGCACTGATGTTGGCGTAGCTTGAT
TTACTATACTACCATTATTATGATTAACCAATGCTTGATTCGTTGCTTGATTGGTTGTTGCA
GATTGATTAGGTGTATTCTGCGCACTATTTGCTAAACCTCTATTATTAGCAACATCTTTAT
CTTGTGAATTTACAGGTGTTGCTTGATTAGTATCGCTTTGTACATTATTATCCGTAGTTAA
GGCTTGTGCACCATTTGGGTTTGAAAGTAATAAAACTGTTCCGATTAAAGTAGAGAATAT
GCCTACTTTATACTTCCTAATACTATATTTATTTTTCTTTAACAAATTCAT

> **leuS**

Function: protein coding sequence; leucyl-tRNA synthetase (core genome, variable)
Best match: leuS_CC001_MW2-USA400_BA000033.2[1853980:1856400:r]RC (completely identical)
Position: 041-contig_187_RC: 28924 … 31345; Length: 2421 bp
Sequence:
TTATTTAGCTACAATATTGACTAATTTTTGAGGAACAGCGATGACTTTCATGATGTCTTTA
CCTTCAATACTCGCTTTAACATTGTCATTAGATAAGGCAATTTCTTGCATTTCTTCTTTTGA
TGTATCTTTAGCAATTTTAATTTTAGCTCTCAATTTACCATTTACTTGAACAACGATTTCTA
CTTCATCATCTACAAGTAGTGCTTCGTCATAAGTTGGCCAAGGTTGGTACGTAATAGACTC
TTCATGTCCTAATTTTGACCATAATTCTTCACCGATATGTGGTGCAATAGGTGCTAACATT
TTAACGAAGCCTTCAATGTAAGGTTTATAAACTTCATCAACTTTATAACACTCATTAATAA
ATACCATTAATTGACTAATAGCAGTATTAAATCCTAATGTTTCAAAGTCTTCTGTTACCTT
TTTAACAGTTTGGTTATAAACTTTATCTAAAGATTTATTATTTGTAGTTACAATTTTTGAAC
TCAATGTTCCATCTTCATTTACCATTAAACGCCATACGCGATCTAAGAATCGACGAGACC
CATCTAATCCTTTTTCACTCCATGCAATTGCAGCATCTAAAGGTCCCATAAACATTTCGTA
AAGACGTAAAGTATCTGCACCATGAGACTGTACTATATCATCAGGATTGATTACATTTCC
TTTAGATTTACTCATCTTCTCATTACCTTCTCCTAAAATCATACCTTGGTTAAATAATTTTT
GGAAAGGTTCTTTAGTAGGTACGATACCCAAATCATAAAGGACTTTATGCCAAAATCTTG
CATATAATAAGTGAAGAACCGCATGTTCTACTCCACCGATATATAAATCAACAGGTAACC
AATGTTTTAATTTTTCAGGATCTGCTAACATATTTTCATTTTTAGGATCGATGTAACGTAA
ATAATACCAACAACTACCTGCCCATTGTGGCATTGTATTTGTTTCACGACGTCCTTTCATA
CCTGTTTTTTCATCTACAACATTTACAAATGAATCAATATTAGCTAGTGGAGACTCACCAG
TCCCTGATGGCTTGATTTCATCTGTTTCAGGTAACAACAATGGTAGCTCTTCTTCAGGAAC
AGTTGTCATTGTTCCATCTTCCCAATGAATGACAGGAATTGGTTCGCCCCAATAACGCTGA
CGACTGAATAACCAATCTCTTAATTTGTAATTAACTTTCTTTTCGCCAGCACCTTTTTGCTC
TAATAATTGAATAGCTTTAGTAATTGCCGCTTCATTTTCTAAACCATCAAGTTCACCAGAA
TTAATATGTTTACCTTCACCAGTGTATGCTGCTTCTTCAACATTTCCACCTTCGATGACTTC
AATGATTGGCAAATCAAACTTTTTAGCAAATTCATAATCTCTGTCATCATGCGCTGGTACT
GCCATAATTGCTCCAGTACCATATGTTGATAATACATAATCAGCAATCCAAATTTGTACTT
TTTCACCAGATAAAGGATTAATTGCATATGCACCAGTAAACACACCTGATTTATCTTTTGC
TAAATCTGTACGTTCTAAATCTGACTTTTTAGAAGCTTCTGTTTGATAAGCTTTTACTTTTT
CTTTATATTCATCAGTTGTAATTGAATTAACTAATGCATGTTCAGGACTTAAGACTAAGAA
TGATGCACCATAGATTGTATCTGGTCTAGTCGTAAATACTTCTACTTTTCCTTCCGCTTCCG
TATTATCTACATCAAATGAAACTTTGGCCCCTTCAGAACGTCCAATCCAATTGCGCTGCAT
ATCTTTTAAAGATTCAGGCCAATCTAAATCATCTAAATCTGCTAATAATTGATCTGCATAT
TCTGTGATTTTAAGTACCCATTGTTTCATCGGCTTACGATAAACTGGATGTCCACCACGTT
CAGAGACACCATCAATCACTTCTTCGTTAGATAAAACAGTGCCTAATGCTGGACACCAGT
TAACTGCAACTTCATCAACGTATGCTAAACCTTTGTTATATAACTGTATGAAAATCCACTG
TGTCCATTTATAGTATTCTGGATCTGTTGTATTAACTTCACGATCCCAATCATAACTGAAC
CCTAATTCTTTAATTTGTCGTTTAAAAGTTTGGATATTTTTCTTTGTAAATTCACGTGGGTC

GTTGCCAGTGTCTAAAGCATATTGCTCTGCTGGTAATCCGAATGCATCCCACCCCATCGG
ATGTAATACATTATATCCTTGCATTCTTTTATATCTTGAAATGATATCTGTTGCTGTATAGC
CCTCAGGATGTCCAACATGTAAACCAGCACCTGATGGATATGGAAACATGTCTAAAGCAT
AAAATTTCTTTTGACCTAAGTTATCATTTGTTTTAAATGTTTTATTTTCGTCCCAATAGTCT
TGCCATTTCTTTTCAATTTGATTGTGGTTGTAATTCAA

> **rot**
Function: protein coding sequence; repressor of toxins (core genome, variable)
Best match: rot_CC001-ST772_118_AJGE01000022.1[107171:107671:r]RC (completely identical)
Position: 041-contig_187_RC: 34559 … 35060; Length: 501 bp
Sequence:
TTACACAGCAATAATTGCGTTTAAACTATTTTGCATTGCTGTTGCTCTACTTGCAATCGCA
TCACTGATGAAATTCAACAACTCTACTTTCTCTTGTTGTAACTTTTCATTGAAATGAATAA
TAACTGTTCTTTCATCGTCAACAGGACGCTCTTTGTAAATCCATTCTAATTCAACTAAATT
ATTATACGTTCTCGTACGCTTATACGGTTTAACTTCAACAAATCTGTCCATTTCTTTAAGC
GTCATAGAACCTTTTTGCCATAAAGTTAGTAAAATTAAAATTTCTTCTCTAGACATTTTGT
ATTCGCTTTCAATCTCGCTGAAAATTGAGTTAATGTCACCCAAAAGTGTTTCTAATTGCAA
AATCCCAAATACAGTGTCGTTATTTACTTTTTTCATGCTAAACATCTCCCAATTAATAATC
ACATATTTAGCTACCATACAAGTATTAACAAACATCCCAACAATCCCAAAACTTGTATGT
GCTAACTTATGCAT

> **tnpIS1272**
Function: protein coding sequence; transposase for IS1272 variation TCH (mobile element)
Best match: tnpIS1272-COL_trunc_CC008_NCTC8325_CP000253.1[1795407:1796080:r]RC
Position: 041-contig_187_RC: 35518 … 35548; Length: 30 bp
Sequence:
ATCTTTCAGTTCTTTTTACGGGTCTGTTT

> **txbi_universal1**
Function: bidirectional rho independent terminator
Best match: txbi_universal1_CC001_MW2-USA400_BA000033.2[1175005:1175076:r] (completely identical)
Position: 042-contig_273_RC: 6 … 78; Length: 72 bp
Sequence:
TGTATAAATAGAGAACAGCAGTAAGATATTTTCTAATTGAAAATTATCTTACTGCTGTTTT
TTAGGGATTTA

> **pldB**
Function: protein coding sequence; lysophospholipase (core genome, constant)
Best match: pldB_CC008_NCTC8325_CP000253.1[1796427:1797254:r]RC (completely identical)
Position: 042-contig_273_RC: 99 … 927; Length: 828 bp
Sequence:
TTAAATTTCTACAATTTCGTCATCTTCAACAATAAAGCCCATTGTATTGACGCTGTTATTT
AAGAAAGTCAGAATATAACGCATTACTTCATCACGTTCTGGCTCATTGTGAACCTCGTGG
TAAAAACCTTGCCAAGCTTTAAAATATAATTCAGGTGTTTGATATTTTTCTTTAAACTCAT
CAATTGCCCTAGTATCAACAATTAAATCCTTCGTTCCATACATTAATAGCGTTGGCATTGG
TTGAATGTCATGAATATGAGCCATCGTATCTTTCATCGTCTCATTAATTGTATTATACCAA
TGATACGTTGCTTTTTTTAACATTAAACCATCGTTAACTGTTTCTTCAACAATTTCTAAATT
ACGTGTTAAATCTTTTGGTTCTACACCAACATTAAAACGTGTGTCTTTTGAAATTTTACCT
ATATTTGAAACAAGTTTATCTTTACGATTTTTTCCATTCTTTTGAAGTTCTAGCATAGGAG
AAATTAACATCATCCCCTCGATTGGCAATTCTACTTTTTCAAGTAAATTTAATAAAATCAA
ACCGCCAAGTCCTACCCCTAATACATAAGTAGGAATTTTATATTCATTAGCTATCTTTAAC
CAGTCTAGCAAACTTTCGTGATACGTTTGAAAGTTTTCAATTTGTCCTTTATTAGCTCTTG
AAGTTTGACCTTGACCAGGCAAATCTCCCATAATCACATGATAGCCATTTCTTCTTAACAT

CGTAATAACATATGCATATCTTCCCGTATGTTCTAATATATTATGAGCAATAACAACGAC
GCCTTTCGCATCATTTTCAGCTTCCCACTTCCACAT

> **putA**
Function: protein coding sequence; proline dehydrogenase (core genome, constant)
Best match: putA_CC008_NCTC8325_CP000253.1[1797488:1798489] (completely identical)
Position: 042-contig_273_RC: 1160 … 2162; Length: 1002 bp
Sequence:
ATGGCACTATTAAAGAATTTTTTTATCGGATTATCTAATAATAGTTTTTAAACAACGCAG
CAAAAAAAGTGGGCCCACGTTTGGGCGCCAATAAAGTCGTTGCCGGAAATACAATTCCA
GAGTTAATTAATACAATCGAATACTTAAATGACAAGAATATCGCTGTTACGGTAGACAAT
TTAGGGGAATTTGTCGGTACAGTTGAAGAAAGTAATCATGCTAAAGAACAAATTTTAACA
ATTATGGACGCGCTTCATCAACATGGCGTAAAGGCACATATGTCTGTTAAATTGAGTCAG
TTAGGTGCAGAATTCGACTTAGAATTAGCTTACCAAAATTTAAGAGAGATTTTACTTAAA
GCAAATACTTACAACAATATGCATATAAATATTGATACTGAAAAATATGCTAGCCTGCAA
CAAATTGTTCAAGTTTTAGATCGCTTAAAAGGCGAATTTAGAAATGTTGGTACTGTAATTC
AAGCATATTTATACGATAGCCACGAATTAGTTGATAAGTACCAAGATTTACGATTACGTT
TGGTTAAAGGTGCATATAAAGAAAACGAATCAATTGCATTTCAATCTAAGGAAGACGTA
GATGCAAATTACATCAAAATAATTGAACAACGTTTGTTAAACGCACGCAATTTCACTTCA
ATTGCAACACATGACCATCGCATCATTAATCATGTAAAACAATTTATGAAAGAAAATCAC
ATTGAAAAAGATCGTATGGAATTCCAAATGCTCTATGGTTTTAGATCAGAGTTAGCAGAA
GAAATCGCAAATGAAGGCTATAATTTCACTATTTATGTACCTTATGGCGATGATTGGTTTG
CGTATTTTATGAGAAGATTAGCAGAACGCCCACAAAACCTATCTCTTGCTGTAAAAGAAT
TTGTGAAACCTGCTGGCTTAAAACGTGTTGGCATAATTGCAGCTTTAGGAGCTACAGTTA
TGTTAGGTTTAAGTACAATTAAAAAATTATGCCGTAAATAG

> **ribH**
Function: protein coding sequence; 6,7-dimethyl-8-ribityllumazine synthase (core genome, constant)
Best match: ribH_CC008_NCTC8325_CP000253.1[1798611:1799075:r]RC (completely identical)
Position: 042-contig_273_RC: 2283 … 2748; Length: 465 bp
Sequence:
CTATGCTTTTATAGATTTTAATAAATTAGCCATTTCAATTGCACTTACTGCTGCTTCAGCA
CCTTTATTGCCAGCTTTCGTACCTGCTCTTTCCACAGCTTGTTCAATACTTTCAGTCGTTAA
AATACCAAATATGACTGGTACATTAGTTTGATCATTCACTTTAGAAACACCTTTCGCGACT
TCATTACAAACATAATCATAATGAGACGTAGCACCGCGAATTACGCATCCTAATGTAATT
ACTGCATCATAATTTCCTGATGAGGCTAATTTTTTAGCTACTAAAGGAATTTCAAACGCAC
CTGGCACAAATGCTACATCAATATTGTCTTCATTAACATCATGTCGAATCAAAGTATCTTT
TGCACCTTCAAGTAATCTTCCAGTGATAAAATCATTAAATCGACTAACTACGATTGCAAC
TTTCAAATCTTTTCCAATTAATTTACCTTCAAAATTCAT

> **ribA**
Function: protein coding sequence; bifunct 3,4-dihydroxy-2-butanone-4-phosphate synthase/GTP cyclohydrolase II (core genome, variable)
Best match: ribA_CC001_MW2-USA400_BA000033.2[1863251:1864432:r]RC
Position: 042-contig_273_RC: 2760 … 3942; Length: 1182 bp
Sequence:
CTATATTAAATGACCCATTTTTATTTTTTTCGTTTCCATATAATCATGATTATGTACCGTTT
CTGGTACGATAACTTCAATTCTTTCTGCAATATCAATGCCATATTGTTTTAATCCCTCAAA
TTTACTTGGATTATTACTTAATAAATTGATATGTTCGATGTTAAAATATTTTAAAATCTGT
GCAGCAATATGATAATCTCGTAAATCTTCATCAAAACCTAATGCTAAATTTGCAGTTACT
GTATCATATCCTTGCTCAATTAATTCATATGCGCGTAATTTGTTTAACAATCCTATGCCAC
GACCTTCTTGAGGTAGATAAATAATCATGCCACCATGTTCATTGATATACTTCATAGACG
ATTCAAGTTGAGCACCACAATCACAACGTTGACTATGGAAAATATCGCCTGTAAGGCACG
CAGAATGTAAGCGTACATTTTCATGTTGTCGAATTGCACCTTTTGTCAGTACAACTATCTC
TTCATCTGTGTATGTCGCTTTAAAACCATACATATCAAATGTTCCGAAATCTGTAGGCATT

TTCACTTTTGCCTTAAATTCAATTTCTGGTTCTAATTTTTTACGATATTCAATTAAATCATC
AATCGTAATCATCTTTAATTGATGTTTTTCTTTAAACTTTTGTAAATCTTGTCCTTTCGCCA
TCGTGCCGTCATCATTCATAATCTCACAAATGACACCAGCGGGCTTGGCACCAGTAAGTT
TAGCTAAATCAACAGCCGCTTCTGTGTGTCCATTTCTAGCTAATACGCCTTTATCTTGTGC
TACTAATGGAAATAAATGACCAGGACGATTAAAATCTTTAGCTTCACTACTAGGATCAAT
GAGCTTTTTGGCAGTCAATGTACGTTCATAAGCACTAATTCCTGTTGTTGTATCTACATGA
TCAATACTCACTGTAAATTGCGTACCAAAGATGTCGGAGTTATCATCAACCATTTGTACC
AAATCCAAACGTTGTGCAATATCTTTAGACACTGGTGCGCATATTAATCCCCTTGCTTCTT
TCGCCATAAAATTAATGGTATTATCGTTCATCCATTCAGTGACCGCTACTAAATCACCTTC
ATTTTCACGATTCTCATCATCTACTACAATAATTGTTTCTCCATTTTTTAAAGCCATTAAAG
CACTGTCAATATTATCGAATTGCAT

## > ribB

Function: protein coding sequence; riboflavin synthase subunit alpha (core genome, constant)
Best match: ribB_CC001_MW2-USA400_BA000033.2[1864443:1865075:r]RC
Position: 042-contig_273_RC: 3952 … 4585; Length: 633 bp
Sequence:
CTAAAAACCAAATGCTCTTAATTTATCTACAGATAATTGGTCTTTATCTTTATTTAAAATA
TTTTCAACATATTTAAACAAAACGTCTGTTTCTAAATGTACTTTATCTCCTAATTTTTTGGA
TGATAAAATCGTTGAACGCCTCGTTTCTGGAATAAGATGAATGTCAAAACTGTTATCATG
CTTATCAAATACCGTTAGACTTACACCATCCACAGTAATAGACCCTTGCTTAACTAACTGA
TTATTAATATGTTGGCTACATTGAATCGTAATAATTTTGGCATTGGCTGTTTCATTTATTTT
TGAAACTGTTCCTAGTTCATCTACATGACCGAGGACAAAATGTCCACCAAACCTACCGTT
ACCACCCATGGCACGCTCTAAATTTACTTCTGATTGTCGCTTAACATCTGCTAAATAGGTT
TTATTTTCAGTGCCTTTAATTACTTGAACAGTAAAAGATGTCTGATTAAAATCAATCACTG
TTAAACATGCACCATTAACACTGATGGAATCACCAATATGCATATCTGCCGTAATCTTAT
GTGCTTCGATTTCAATCGTCCTGACTGATTGACGAATTTGAACACTTTTAACGACACCTAT
TTCTTCAACGATGCCAGTAAACAT

## > ribD

Function: protein coding sequence; riboflavin specific deaminase (core genome, constant)
Best match: ribD_CC001_MW2-USA400_BA000033.2[1865082:1866125:r]RC
Position: 042-contig_273_RC: 4591 … 5635; Length: 1044 bp
Sequence:
TCACTTCTTTCGTAAAGTTAATTTAACATTTTGATTTAATAACTCGGAATGAACAATTTCA
AATTGGTTCGCATCTGGTATCTCAATCACATCATTTGTTTGATAAAATTGATAATTTCCAG
ATCCGCCAATTAATTTCGGGGCATAATAGAGAATAAATTCATCTATATAATTAGATTGGA
GAAATTCTGAAGTAGTGGTTGGACCTGCCTCGACTAGCAAAGTTCCAACTCCTCTTTTATA
TAAATTGTGAAGAATTGTTGTTAAATCGCAAGACTTCAAGTAAATAATTTCAATATGTGTT
TGATTGGTTGTTAAATTTGGATTTTCAGTATATATCCAAATTGGTGTTGATTCATCTTGAT
AAATTTGCTGATTAAAATGAATATTCCCAGACTTAGACAATATTACTTTTATAGGGTTTTT
TCCATCTTGAATACGTGTAGTATATTGTGGATCATCTAATTCAACTGTACGTCTTCCAGTT
AACACTGCGTCGTGTCGATTTCTTAACTTATAGACATCTTGTTTAACCTCTTTGTTAGTAAT
CCATTGACTTTGTCCATTATCATTCGCTTGTTTACCATCTAAACTTGCAGATACTTTCACTG
TAATTTGTGGCAGTTGCTTTGCTTTTGCTTTAAAAAAGGCTTGGTATAATTGTGATGCCCG
TTCATCATCAACGCATTCAACCTCAATACCGTGAGCCCGTAACGTCTCATCACCATGTGTG
TCTAACGAATTGTCTTTTGTTGCGTATACTACTTTTGCTATCTTACAATCAATTATTTTGTT
AACACAGGGTGGTGTTGAACCAAAATGACTACATGGCTCTAACGTAATATAAATCGTCGC
ACCTTCAGCATTTTGTTGTGCCATATCAAGTGCTTGAACTTCCGCATGCTTGTCACCTTTTC
TCAAGTGTGCACCAATACCAACAATCCTACCTTCTTTAACTACAACAGCGCCAACGGGTG
GATTAACACCTGTTTGACCTTGTACCATATTTGCAAGTTGAATCGCATAATCCATAAATTG
ACTCAA

## > fmn-L2

Function: noncoding RNA FMN riboswitch/locus 2 (core genome, constant)

Best match: fmn-L2_CC001_MW2-USA400_BA000033.2[1866254:1866385:r]RC (completely identical)
Position: 042-contig_273_RC: 5763 … 5895; Length: 132 bp
Sequence:
ATTCTTTCTCCCATCCAGACTTTAACTGTCGGCTCTAGAATCTCACTAGATCAGCCACTAA
TATGAAACATATTAGCAGGTCGCAGGCTTTATTTACTGCCGGTTGGGAATTTCACCCTGCC
CCGAAAGAAT

> **Q5HF04**
Function: protein coding sequence; pyridine nucleotide-disulfide oxidoreductase (core genome, constant)
Best match: Q5HF04_CC009_21334_AGTW01000033.1[40471:41973:r]RC
Position: 042-contig_273_RC: 6115 … 7618; Length: 1503 bp
Sequence:
TTATTGATTGTCACTGCGCTGTTGTTGCTCATTCGATTCTAAAGCATCATATAATTGAGAT
ACTGTATGCGCAACTTGTTCTACTATCATTTTCACACCATTTCGTAGTTTATTAACACCGTT
TGTCATTTGACCTATCGCAATCATATTTGTTAATGTTCCAAACCTTGGACTAATAACTTGA
TTGGTTTCCGGTATGATTTGTATGCCTCCCATTGGGTGTGCTTGTACAATTTGTCTATTTTC
AAGATTTTTAATTAATTGATCATCTTGATCCAATTCATTTAAATGACTTTTTGCACCTGTCG
CGTTAATGACAACATTATATATGTCTACTGATTCTTGGTTTTTGTATGAAAAATAATACAA
CTTGCCATCATGTTTCACATCTTCTAAATCTTTTTTCAAAATTAAAGACTTATTTTCGATTA
ATTCAATAATTAGTTCAGCAGTTCTTGGAGGCATTGGATTTGAATTTAATTGAATCATCTT
TGAGTATTTTTGATTAAATTGATGTTGGTCTTCAATACTTAAGCTATTCCATATCCAATTTA
AATTCTCTTTCAAATGTTCAATCATACTTTGGAAAATGCCCATTTCTGTTGGACGCGCTAA
ATCATACTTCAAATCTGCAATATGATTTCCTGTACGTCTATGTACTAATTTTTTAAAATCA
ATGTCATATTCAGCACATTCTTTTAAAAATAAAGAAACTAAAGTATCAAGCGGTGCATTG
CCGAAATGATGTTTTTTAATGTCATTTAATTTGTCTTTAGTTAAGTACTTGAATGTCACGTC
TATCATTGTACCTCTTACACTTGGTAAATGAGCAGAACGACTCGTCATAGTAATTGGTAAT
TTCGGATGATGAGCAGCAACATAACGGACAACATCTAAACTGGCAAGGCCTGTACCAAT
AATCGCAATATCGTCCAGTTCATTTACTTCGTCTAACGTATTATATGTTGGATAAGGCGTA
GCGATATATCCTTTTTTACCCTTTAAGTTATATGGATCATGGTAGGCAAATGTACCACATG
TTAAAAATACATAATCGTACGCTTGCCATGATTGTTCTGAATTTGTAGTACATATGTAATA
AGTTAAATTCGTTTCATCGATATTAGAATTTGTATAAATCTCTTGAACTTTATTATAATTA
GTTGATATATTTGGATATTTTTTCGTGAACATAGATAAATAAGATTTCATATAATGTCCGA
ATACAAATCTAGGTAAATATGCAGGTTCATCAAAATTAAAATCAGTTTGTTGTTTATACC
ACTTCCAAAATTCAGTCTCATCATCTAAATTTAAACTCATCTTTTTCGAAGGCATATTAAT
TAGCAGCTCAGAACTATCATTTTGAAATGGTACGCCCTGTCCCATATTTACTTTATCATCG
TATAAATCTATATCTAATTGATTAAACTTCGGGTGCTTAACTAACTCTCTCAATACACTTA
CACCAGCAGTTCCCATGCCTATTATTGCTACACGCAT

> **arsR**
Function: protein coding sequence; repressor of arsenic resistance operon
Best match: arsR_CC001_MW2-USA400_BA000033.2[1868631:1868945]
Position: 042-contig_273_RC: 8140 … 8455; Length: 315 bp
Sequence:
ATGACGTATAAAGAACTAGCAACATTTTTAAAAGTTTTATCAGATTCAAGCAGATTAGAA
ATACTAGATTTACTTTCTTGTGGAGAGTTATGCGCTTGTGATTTGTTAGCACATTTTCAATT
CTCTCAACCTACACTTAGCTATCATATGAAAGCATTAGTAAAATCCAACTTAGTTACGAC
ACGAAAAAATCGGAAATAAACATTTATACCAGCTCAATCATAATATTTTTGAGTCCGTAAT
TAATAACTTGTCTAAGGTTCATACCTCTAATCAACGATGTATTTGTCATAACCTTAAGACT
GGTGAATGCTAA

> **arsB**
Function: protein coding sequence; arsenical pump membrane protein (core genome, variable)
Best match: arsB_AF422190.1[3069:4361:r]

Position: 042-contig_273_RC: 8454 … 9747; Length: 1293 bp
Sequence:
ATGATGACAACTTTAGCGACACTCATTTTTCTAGTAACTTTATTATTTGTATTATGGCAAC
CTAAAGGCCTAGATATTGGCATTACCGCATTAACTGGTGCCTTTATTGCTGTTATTACTGG
TGTTGTAAGTTTTTCCGATGTTTTAGAAGTAACAGGTATTGTTTGGAATGCTACTTTGACT
TTTGTCTCAGTCATTCTTATTTCATTAATATTAGATAAAGTTGGATTATTCGAATGGTCAG
CTATTCACATGCTTCATGCTTCAAAAGGCAATGGTTTAAAAATGTTCGTTTATATCATATT
ATTGGGTGCCCTTGTTGCTGCATTTTTCGCAAATGATGGCGCAGCGTTAATCTTAACGCCT
ATTGTATTAGCAATGGTTAAAAATATTGGATTTAATAAGCGCGCAATATTACCATTTATTA
TTGCAAGTGGTTTTATAGCTGACACAACTTCTTTACCTTTAATCGTGAGCAATCTAGTGAA
TATTATATCTGCTGATTATTTTCATGTAGGATTCGTTCGATATTTTAGTAGAATGATTATAC
CTAATTTATTCTCACTTTTAGCAAGTATTATAGTATTGTGGTTATATTTTAGAAAAGCGAT
ACCTAAAACGTTTGATGATAATAATATAAAGCATCCTAAAGATGCCATTAATGATTTAAA
GCTATTTAAAATTTCATGGATTGTTCTAGTTATATTACTTTTCGGCTATCTAATCAGTGAAT
TTACTAAAATTCCGGTATCAATTTTCACTGGAATCATTGCTTTTATTTTTCTAATGTTGGCT
CGTAAATCAAATGCTGTAAATATTAAGCAAGTCATTAAGGGCGCACCTTGGAATATAGTA
TTATTTTCAATTGGTATGTATATCGTCGTATTCGGCTTAAGAAATGCTGGCATTACTTTAA
TATTGGCTAAAATATTAGAATATATTTCCAATTACGGTCTATTTAGCACTATTTTGGGAAT
GGGCTTCATTTCAGCGTTTTTATCATCAATAATGAATAATATGCCTACAGTTTTAATAGAT
GCGATTGCTATTGGTCAATCAAATGTCCATGGCATGTTAAAAGAAGGCCTAATTTATGCG
AATGTTATCGGTTCTGATTTAGGTCCAAAAATTACACCGATAGGCTCTTTAGCTACATTAC
TGTGGTTACACGTCTTAACACAAAAAGATGTTAAGATTTCTTGGGGCACATACTTTAAAA
CTGGTATCATCATTACAATTCCAGTACTATTTATAACCCTCATAGGGTTGTATCTAACACT
TATCATATTTTAA

## > arsC
Function: protein coding sequence; arsenate reductase
Best match: arsC_CC007_TCH959-USA300_AASB02000250.1[22664:23059]
Position: 042-contig_273_RC: 9764 … 10160; Length: 396 bp
Sequence:
ATGACTAAAAAAACAATTTATTTTATATGTACAGGCAACTCATGTCGAAGTCAAATGGCT
GAAGGTTGGGCTAAACAAATCTTAGCAGATGATTGGAATGTATATTCTGCTGGTATCGAA
ACACACGGTGTTAATCCCAAAGCGATAGAAGCTATGAAAGAAGTAGGCATTGATATATC
AAATCATACATCAGATTTAATCGATAATAATATTATTAAAAATTCAAATTTAGTTGTTACA
TTATGTAGTGATGCAGACGTAAATTGCCCTTCTTTACCAACAAATGTTAAGAAAGAACAT
TGGGGATTTGATGATCCTGCAGGCAAGCCTTGGTCAGAATTCCAACGTGTAAGAGATGAA
ATTAAAATCGCAATTGAAAATTTCAAATCACGATGA

## > gad
Function: protein coding sequence; mannosyl-glycoprotein endo-beta-N-acetylglucosaminidase (core genome, constant)
Best match: gad_CC008_NCTC8325_CP000253.1[1806782:1807636:r]RC
Position: 043-contig_278: 64 … 919; Length: 855 bp
Sequence:
TTACTTATTCAAATGTTTACTGTCATCTTTATACACAAAGTATTTGAAGTATTTTCCTTCAG
TCTTCATGTTCTTATAAAAGTCAGCGATAATTGTTGCATTACTTTCTGCCCACTTAATATCT
GTAGCATATTGATGTTCTCCTGGATTTTTTGGATTCCATCTCATACTATACAATGTATTTTG
ATCTGTGCTTGATAAGAAGTGCTTATGAATGAAATCAGCACCGCCTGAAATAGCTTTTTC
AGGTGTATCCCAACCATGCTTTTTAGCATATTCTGCACCTGTTTTAATTGGGTCTTTATCA
AGGGCTCCTACTCCATAGAAATTGTAGTATTTTTTGCCATCAATTTCGACTCCATTAGCTA
ATTCACTTTTAACTGCGCCAGTTTCTAATAATGCATGTGAAATTAAATAAACTTCGTTAAC
GTGCTTATCTTTAGCAGCTTTTAAGAAATCATCCGTATGTTTCAATAACGTTGGTCTATCT
ACTAACATACGTTTAATTCTATTTTTATCAATCCCTTGATACTTTGATAAATCTAAAAATT
GATATTTTTGCTTTTCATTATCGATAAAAGTACCGCTATCCATTGCACTTTTAATTTCAGTT
GCAGATGCATCTCTCCATGCATCATTCTTTTTATTTGATACCTGTTGACTCGTATAATTATT

TATTTGTTTCTTTGCTGCATCGTTTAATGTAACATTTAACTTTTCAATCTTAATGTCGGATT
TAACATGTTTGAAAAATATCTGATCGGATATCATTGAGAAAAATAAAAATGAGACAACA
GCAAATATGACAACAAGTCCTATTATTCCAAAAATAGAACCTTTCTTGTGTTTATTCAT

> **sigS**

Function: protein coding sequence; RNA polymerase sigma factor S (core genome, variable)
Best match: sigS_CC005_Mu50-VRSA_BA000017.4[1911863:1912333]
Position: 043-contig_278: 1617 … 2088; Length: 471 bp
Sequence:
TTGAAATTTAATGACGTATACAACAAACACCACAAAATCATACACCATCTTTTAAAAAAA
TATAATATTAGCTATAATTATGATGAGTATTATCAACTACTCTTGATAAAAATGTGGCAAT
TGAGTCAGATATATAAACCCTCAAGCAAGCAATCTTTATCCTCTTTTTATTCACTCGATT
ACATTATTACCTTATCGATTTATTCAGACAACAAAATCAATTAAAAGATGTCATTTTATGT
GAGAATAATTCACCAACATTAACTGAACAACCAACATACTTTAATGAACATGACCTTCGA
TTACAAGATATCTTCAAGCTTTTAAATCATAGAGAAAGACTATGGCTCAAACTATACCTT
GAAGGATACAAGCAATTTGAAATTGCTGAAATCATGTCATTATCGCTTTCAACGATTAAA
TTAATTAAGATGTCCGTTAAGCGTAAATGCCAACATAATTTTAATTAG

> **tal**

Function: protein coding sequence; transaldolase (core genome, constant)
Best match: tal_CC001_MW2-USA400_BA000033.2[1874704:1875417:r]RC (completely identical)
Position: 043-contig_278: 4443 … 5157; Length: 714 bp
Sequence:
TTATAGAATAGAAAGACCTGAAGATTGAATATCTTTCGCAAAGCCTTTAACTGTATCTAC
TGATAATTCGTTAATATCGCGACCTAAGTTTGTATTCACTTTTTTCACAACATCTGCAGGG
CATGTAATAATATCTGCACCAATTTCATCAGCTTGAATCACATTGAATAATTCGCGGCAA
CTTGCCCATAATAATTTAACGCCGTCTTTACTATGCGTAACTTTTACAGCCTCTTTCATTAA
TGGTAATGGATCTACGCCTGTATCTGCAATACGTCCTGCAAATACTGAAACATATGTTGG
CACACCTTCAGTTACTGCTTCAGTTATTTCTTTAACTTGTTCAATTGTGTAAACAGCCGTA
ACGTTTAATCTCACATTGTCAGCTGAAAGCTTTTTAATTAAAGGAATCGTTGATTCACCTT
TTGTATTTACAATAGGAATTTTAACAAATACATTTTCGCCATATTGTTTTAGAATTGCTGC
TTCTTTTTCCATAGTTTCTAAATCGTCTGCAAATACTTCAAATGAAATTGAAGCATCTGGA
ATTTCTTTCACAGCTTCTTCAGCAAAAGCTTTGTAATCTGTTACGCCCGCTTTCGCCATTA
AACTAGGATTTGTTGTAAAACCATCCACTTGTTTGTTTTTATAAGCTGCTTTCATTTCTTCA
ATATCTGCACCGTCCGCAAATACTTCTACATTTAGTTTAGCCAT

> **crcB1**

Function: protein coding sequence; protein CrcB homolog 1 (core genome, constant)
Best match: crcB1_CC005_Mu50-VRSA_BA000017.4[1916143:1916586]
Position: 043-contig_278: 5896 … 6340; Length: 444 bp
Sequence:
ATGCATCGACAATTTTTGTCGTCGTGTTGCCAAAACCTCTTTTTTAAATTCAAACTACTTCT
TTTCGAGGTGAACCAAATGCAATATGTATATATTTTTATCGGTGGTGCTTTAGGCGCTTTA
TTACGTTACCTCATTTCTTTTCTGAATACTGACGGAGGTTTTCCAATCGGAACACTGATAG
CCAATTTGACTGGTGCCTTTGTAATGGGATTGCTAACAGCCTTAACAATTGCATTTTTTTC
AAACCATCCGACCCTAAAAAAAGCTATTACGACTGGTTTTCTTGGTGCTTTAACGACTTTT
TCAACATTTCAATTAGAATTAATACATATGTTTGATCATCAACAATTTATAACTTTACTAC
TATATACTGTAACAAGTTATGTCTTTGGTATTTTGTTATGTTACGTCGGTATAAAACTAGG
TGGTGGTTTATCATGA

> **crcB2**

Function: protein coding sequence; protein CrcB homolog 2 (core genome, variable)
Best match: crcB2_CC001-ST772_118_AJGE01000029.1[22014:22367:r] (completely identical)
Position: 043-contig_278: 6336 … 6690; Length: 354 bp
Sequence:

ATGATATCAATCATTTTAGTCATGATTGGCGGCGGTTTCGGCGCAATTGCTAGAAGTGCC
ATTACTGATTATTTTAATCATAAATTTACTTCAAAGTTACCTATCGCAACATTGATAGTAA
ATCTAGTTGGTAGTTTTTTAATTGGATTAACTATAGGCTTATCAATTTCAATCTCATGGTTC
CCTGCGTTCTTTGTTACCGGTTTTTTAGGTGGCTTAACAACTTTCTCAACGTTAGCTAAAG
AACTTACACTAATGATGACGCCAAAATTTAATATTAACCTTTTTCTCAATTATTCACTTTT
ACAATTCATCATTGGATTTATAGCTTGTTATATTGGCTATCATATTTAA

## > tx_universal2

Function: rho indendent terminator
Best match: tx_universal2_CC007_TCH959-USA300_AASB02000023.1[4549:4580:r]RC (completely identical)
Position: 044-contig_322_RC: 0 … 32; Length: 32 bp
Sequence:
AAAAAGCTTGTTACAAGCGCATTTTCGTTCAG

## > metK

Function: protein coding sequence; S-adenosylmethionine synthetase (core genome, constant)
Best match: metK_CC001_MW2-USA400_BA000033.2[1879411:1880604:r]RC
Position: 044-contig_322_RC: 2210 … 3404; Length: 1194 bp
Sequence:
TTATTTTACTGCGTCTTTTAATTCTTCCACTTTGTCTAATTTTTCCCATGGGAATAAGACAT
CTGTACGTCCAAAATGACCATAAGCAGCAGTTTGTTTGTAAATTGGTTGTTTCAAATCAA
GCATTTTAATAATACCAGCAGGTCTTAGGTCAAAGTGTTTTCTAACTGCTTCAACAAGTTG
CCCTTCAGAAACTTTACCTGTTCCAAATGTATCAATTGCAATTGACACTGGTTCTGCAACA
CCAATCGCATATGCCAATTGTACTTCACATTGATCTGCTAAACCTGCTGCAACAATATTTT
TAGCCACATAACGTGCAGCGTATGCAGCTGAACGGTCTACTTTTGTAGGATCCTTACCAC
TGAAGCATCCGCCACCATGACGTGCATAGCCACCGTACGTATCAACAATGATTTTACGTC
CTGTTAATCCTGCATCACCTTGAGGTCCACCGATTACAAAGCGTCCTGTAGGATTGATGTA
GAATTTAGTTTGTTCATTAATCAAGTTTTCTGGAACAGTTGGATAAATGACATGTGCTTTA
ATGTCTTCTTGAATTTGTTCAAGTGTCACATCCTCAGCATGTTGTGTTGATACGACAATCG
TATCAATACGTACTGGGTTATCATTTTCATCATATTCAACAGTGACCTGAACTTTACCGTC
TGGTCGTAAATAATTTAACGTACCATCTTTACGCACATCTGATAAACGTTTTGCCAATTGA
TGTGATAAATAAATTGCTAGAGGCATATACGTCTCTGTTTCATTCGTTGCGTAACCAAACA
TTAAACCTTGGTCACCTGCACCTGTTGCTTCAATTTCTTCTTCGCTATCTTTATCACGATAC
TCTAATGCATTATCCACGCCTTGTGCAATGTCAGGTGATTGTTCATCAATCGCAGTTAAAA
TTGCCATTGTTTCATAATCATAACCATATTTTGCTCTTGTGTATCCAATTTCTTTAATTGTT
TCTCTAACAACTTTCGGAATATCAACATATGTTGTTGTAGAAATTTCGCCGGCGATCAATG
CCATACCTGTTGTAACAGTTGTTTCACAAGCTACACGTGCATTTGGGTCGTCTTTTAAAAT
AGCATCTAATATTGCATCTGACACTTGGTCAGCGATTTTATCTGGGTGTCCTTCTGTAACA
GACTCTGAAGTAAATAATCGTTTGTTATTTAACAT

## > sam-L3

Function: noncoding RNA SAM riboswitch locus 3 (core genome, constant)
Best match: sam-L3_CC001_MW2-USA400_BA000033.2[1880710:1880821:r]RC (completely identical)
Position: 044-contig_322_RC: 3509 … 3621; Length: 112 bp
Sequence:
CTCTTATCGTTCAGACCTATTTGTCTGCAAACGGTTTGGCACCTTTCTTTTATAAAAAAAG
AGGTTGCTGGGTTTCATTGGGTCCATGTCCCTCCACCACTCAGGATAAGAG

## > pckA

Function: protein coding sequence; phosphoenolpyruvate carboxykinase ATP (core genome, constant)
Best match: pckA_CC001_MW2-USA400_BA000033.21880976:1882568
Position: 044-contig_322_RC: 3775 … 5368; Length: 1593 bp
Sequence:

ATGTCAGTAGACACATACAATGAAACAACTAAAATTGACAAATTACTGAAAAAACCAAC
GTCACATTTTCAACTTTCGACGACACAACTTTATAATAAAATCTTAGACAATAACGAAGG
GGTATTAACAGAACTTGGTGCTGTTAATGCAAGTACTGGAAAATATACTGGTCGTTCGCC
TAAAGACAAATTTTTTGTCTCTGAACCTTCATATAGAGATAACATTGATTGGGGAGAAAT
TAATCAACCTATCGATGAAGAAACTTTCTTGAAGTTATACCATAAAGTACTAGACTATTT
AGATAAAAAAGATGAACTATACGTATTTAAAGGCTACGCTGGTAGCGATAAAGATACAA
TGTTAAAACTTACAGTCATCAATGAATTAGCATGGCATAATTTATTTGCTAAAAATATGTT
TATTAGACCTGAATCAAAAGAAGAAGCTACAAAGATTAAACCTAACTTCACTATCGTTTC
TGCACCACATTTTAAAGCAGATCCAGAAGTTGATGGTACTAAATCTGAAACCTTTGTCAT
TATTTCATTTAAACACAAAGTCATTTTAATCGGCGGTACTGAATACGCTGGTGAAATGAA
AAAAGGTATCTTCTCTGTAATGAATTATCTCTTACCGATGCAAGATATTATGAGCATGCAT
TGCTCAGCAAACGTTGGTGAAAAAGGCGATGTTGCATTATTCTTTGGTCTATCTGGCACTG
GTAAAACAACCTTATCGGCTGACCCACACCGTAAACTAATCGGTGATGATGAACACGGAT
GGAATAAAAACGGGGTCTTTAATATCGAAGGTGGCTGCTATGCAAAAGCAATTAATCTTT
CCAAAGAAAAAGAACCACAGATTTTTGACGCAATCAAATATGGTGCAATTTTAGAGAAC
ACTGTAGTTGCAGAAGATGGTTCAGTGGACTTTGAAGACAATCGTTATACAGAAAACACG
CGTGCCGCTTATCCAATTAATCACATTGACAATATTGTAGTACCATCAAAAGCAGCACAT
CCAAATACAATTATTTTCTTAACTGCGGATGCATTTGGTGTTATTCCACCGATTTCAAAGT
TAAATAAAGACCAAGCAATGTATCATTTCTTGAGTGGTTTCACTTCTAAATTAGCTGGTAC
AGAGCGTGGTGTGACAGAACCTGAACCATCATTCTCAACATGTTTCGGTGCACCGTTCTT
CCCGTTACACCCTACTGTTTACGCTGATTTATTAGGTGAACTTATCGATTTACATGATGTT
GATGTTTATCTTGTTAATACTGGATGGACTGGCGGAAAATATGGTGTAGGACGTAGAATC
AGCTTACATTACACACGTCAAATGGTAAACCAAGCGATTTCTGGCAAATTGAAAAATGCA
GAATATACAAAAGATAGTACGTTTGGTTTAAGCATTCCTGTAGAAATTGAAGATGTACCG
AAAACAATTTTAAATCCAATTAATGCTTGGAGCGACAAAGAGAAATATAAAGCACAAGC
AGAAGATTTAATTCAACGTTTTGAAAAGAACTTCGAAAAATTTGGTGAAAAAGTTGAACA
TATTGCTGAAAAAGGTAGCTTCAACAAATAA

> **tnpIS431-06**
Function: protein coding sequence; transposase for IS431 (mobile element)
Best match: tnpIS431-06_circ_S-epidermidis_GQ900381.1[43799:43807] (completely identical)
Position: 044-contig_322_RC: 4085 … 4094; Length: 9 bp
Sequence:
ATGAACTAT

> **menC**
Function: protein coding sequence; O-succinylbenzoic acid synthetase (core genome, constant)
Best match: menC_CC001_MW2-USA400_BA000033.2[1884490:1885491:r]RC
Position: 044-contig_322_RC: 7164 … 8166; Length: 1002 bp
Sequence:
TTAATATGGTTGTAATTGAGTTATATCCACTAAAGGGGGGCGAAATTCGAGTCGCCCCTC
TTTTAATATGCCTGAATGCGCCACCACATCTTGTTCAAAATAATAACCTGCTGGTGTAACA
TCTCCTGGATAATCACCTTTACGAGCAAGCATCGCTGTAAAATAGCGGCTTAAACCATAT
TCGTACATGCCGCCAATAACCACTTTTGCACCATGACTTTTCAAAGTATCAATTGCCGTTT
GCACTTTATCAATGCCACCTAGACGAAATGGTTTTAATACAACAACTTTCACATTGTATAA
TTCTATCAAATTAATTATGTCCGACAATGATGTCGCCTTTTCATCTAGAGCAATCGGAGGT
ATTGTTCCATCCACTACTTCATCAAGCATGGAGATATCTTTAAATGGCTCTTCGATATAAA
GAACCTGTTCACGCGCTAATAACTGTAACTGTGTGAAATCTTGACGATTCAAGGACTCAT
TTGCATCTATAACCAATTGAAAGTGAAAGTCTAATTCTCGTAACACTCTAATTTGATCCAT
GATTTGAGGTGTCCATTTTAATTTAATTCTAGTCGGCTTTGTTGCTTTCAATGACTCTAGTT
GTTTATTTGATAAGCCGCTCGCTGTCGCTCCATATGCTACTGAAAATGAAGGCAGTACAT
GAAACATTTGATACAATGCCATGACAATAGTTGCCCTTGCAGAAGGCGTATTTTCCAATG
AATCTACTAATTTTAGTGCTGCTTCATACGTTTCAAATGATTTATTTCTATTATCTTCGAAC
CATTGCTCAATTACATGTTTCACTGAAGCAATTGTTTCATGATCATACCAATCTGTTTGAA
AAGCGTTACACTCCCCGAAATATGCATTTCCCTTATCATCAATCAATTCGATAAACAAAC

AATCACGATGCATTAATGTGACTTTCGGTGTTACAATTTGTGCCTTAAATGGCTCACTATA
TTTATAAAAATGCAAAGCTGTCAACTTCAT

> **menE**
Function: protein coding sequence; O-succinylbenzoic acid CoA ligase (core genome, constant)
Best match: menE_CC005_Mu50-VRSA_BA000017.4[1927096:1928574:r]RC
Position: 044-contig_322_RC: 8170 … 9649; Length: 1479 bp
Sequence:
TCATCCTCTATACAACTTATTTCTCTGTAATTTACCCGTTGATGTATAAGGTAAAGTATCA
ACCTTTTCAAAGTGTTTCGGTACTTTATATTTCGCTAAATGTTTTGATAAATATGCAATCA
ATTGTGCCTTTGAAATGTCACTTTCACTGACAAAATATAATTTAGGCACTTGGCCCCAAGT
ATCATCAGGATGCCCTACACATACTGCGTCACTGATACCTGGAAATTGCTTCGCTACCGTT
TCAATTTGATATGGATAAATATTTTCACCGCCACTAATAATTAAATCTTTACGTCGGTCAT
AAATCATGACATAACCTTCATAATCTATTTCAGCAATGTCACCCGTATTAAAATAACCATT
TTCAAACGTATCCGTTAAATCTGTTGGATACAAATATCCATTCATCACATTGGCGCCTTTA
ATCATTAATTCTCCATGACCTTCTTTATTAGGATTTTTAACTTTTACATCAACATTGGCACT
TGGCATACCAACAGTGTCAGGACGTGCATGCAACATTTCCGGTGTTGCTGTTAAAAATTG
CGAACATGTCTCAGTCATACCAAATGAATTATAAATTGGCAGGTTATATTGTAATGCCGA
CTCTATCATAGTGGCAGATAATTTAGCACCGCCGAGTAATATTTTTTGTAAATTATAAGGT
TCATGTAAACCTTGTTGCATAAGCCAATTTAAAGTTTGTGGCACAAGCGAAATGTGCGTG
ATTCGTTCATTTTTAATCATCGTTAAAATTTGTTCGGCATTGAATTTATCAACAATGCGCA
CAGTAAAACCTTCAATAACAGCTCTTAAAAGTACACTGAGACCCGAAATATGATAAATCG
GCAAGACAGATAGCCAATTAGTGTCACGATCAAATCCCAAGCTCTCTTTACATCCGATTG
CACTGGCATAATGATTACGAAACGTTTGTGGCACCGCTTTTTGAGGGCCCGTTGTCCCTGA
TGTAAACATAATTGATGCAATGTCATCTAAATTAAATGATGTATTTAATATGTTGGACGG
CGACTCTTTCGGCACCACAGTTTCATTCGATGTATCATATTGGATACCCATTGTGTTGTCC
AACAAACCGTTCGTTGTAATATCCCTTCCAGCGAATTCAATATCATCCAGCGATACAATTT
GAAACCCTCGCAATTCCAGTGGCAAGGTACAAAAAATCAATTGTACATCGATTGACCTCA
TCTGATTCGTCATCTCATTAGGTGTCAACCTTGTATTAATCATCGCAATTTCAATATTTGCC
AACCAACATGCATGTATTAAAATGATCGATTGAATCGAATTATCTATGTATAGCCCGACA
CGAGATTGTTGATAAGCCTTGAATCTTTTAGCCAATAGACTCGCTTCACAGTATAAATTTT
GATAAGTATAAGATTCTTGACCGTCTGTTATCGCAATATGATGTCCATTTTGTTGTGCTTG
TTTATATAACCAAAAGTCCAT

> **Q5HEY1**
Function: protein coding sequence; lipoprotein (core genome, variable)
Best match: Q5HEY1_CC005_Mu50-VRSA_BA000017.4[1928733:1929215:r]RC
Position: 044-contig_322_RC: 9807 … 10290; Length: 483 bp
Sequence:
TCAGTTATTTTTATTTAATTTTAGTGTCATTCTGTCATTTTGATGTGGTGATTTACCCATTG
TTGCCACATCATCTGCAATGTCAATTGGTATACGATTCATGTCTTGTAATGCACTTAAATG
GAATACTTCATCATCTAAATTTTCAATGAGATATACATAATATGTTACCTTGTCCTTTTTAT
ATTTTAACGTTTTCCAAAAGTCCGGCTTGCAATTCAATACATTATCCGGAATATATTCAAT
AAATAAGTAACGTTTGCTGCCTACTTTGTCTACGAAATATTTTACAGTGCCTTTTTCTATA
CCTCTTATATGTGCATAGTCTGCTGAAAAGTAAATACTACCTATTGTTTCATTATGTTGTT
GTATTTCAAATCGTTGGCCTACTATTTTATTATTTGTGCCACAACTACTTAAAAAAATCAG
TAGATATAGCATTAAACATATTTTCATCCCCTTGAATTTTAAAAACTTTTTCAA

> **Q5HEX8**
Function: protein coding sequence; putative protein (core genome, variable)
Best match: Q5HEX8_CC705_RF122-cow_AJ938182.1[1799182:1799806]
Position: 044-contig_322_RC: 12366 … 12991; Length: 625 bp
Sequence:
ATGAAATTCAAAGCTATCGTTGCAATCACATTATCATTTTCACTATTAACTGCCTGCGGTG
CTAATCAACATAAAGAAAATAATAGTAAATCAAATGACACTACTAAAAAGACGCAAGAA

ACTGACAACACTACACAGTCAAATACAGAAAAGCAAATGACACCACAAGAAGCCGAAGA
TATCGTTCGAAACGATTACAAAGCAAGAGGTGCTAACGAAAATCAAACATTAAATTATA
AAACAAATCTTGAACGGAGTAATGAACATGAATATTATGTTGAACATCTAGTCCGCGATG
CAGTTGGCACACCTTTAAAACGTTGCGCTATTGTTAATCGACACAATGGTACGATTATTA
ATATTTTTGATGATATGTCAGAAAAAGATAAAGAAGAATTTGAAGCATTTAAAAAGAGA
AGCCCTAAATACAACCCAGGTATGAGTGATCATGATAAAACAGATGGTGAGTCAGAAGA
CATTCAACATCATGACAATGATAATAACAAAGCCATTCAAAATGATACACCAGATCAAA
AAGCCGTTGATAAAAACGATAAAAATGCTGTTAATAAAGAAGAAAAACACGATAACCGT
GTAAATAAATCAGCAGAAACTAAAGTTAAAT

## > DUF955-L1

Function: protein coding sequence; putative bacteriophagal protein (genomic island)
Best match: DUF955-L1_CC008_NCTC8325_CP000253.1[1830347:1830916:r]RC
Position: 044-contig_322_RC: 14673 … 15231; Length: 558 bp
Sequence:
TCAAATATGTATTCCTAAATTATACAGCCTTAATCCAGCAGCTACTTTCGAAACTTCCAAC
TTAATTGATATAAGATTTAATAGTTTGTTTCGTTCTTTTTCAGATAAACCAGAATTTAAAT
TGATATTATTGACTTCATAAAAATTATAGACTAATGCCTCTATTTGCTTTTTAGGCATAAG
TAAGTCGACTGAAAACTGATTTACGTCGCTTTCATAAATCATTTCATGTAAATTCTTTAGA
CTATTATCGTTGTTATCTTTATATTTGTATAAATAACGACCGAGTTCACGAGCTATTGCAA
ATCTTGTATTATTAATCGAGTGATTATTATTGATATAAATCGTTCTTCCACTTAAATAACC
CGAAAAATTACCCTCTATTTTAATATATTTAACTTTTAAATTAAGTTGAAATAATAGCTTG
TCTATGTCAATAGCAAAATGTTCAGAAGTAATAAAAAGTTGATCCATTTTGTCCTTTATAA
ATGGCTGAAATAATCGAACTATTTTTGGTTCTAAAATATCTTCATAATGAACTTTCTCAAT
AACTTTCAA

## > DUF1433

Function: protein coding sequence; DUF1433 family protein (genomic island)
Best match: DUF1433-var1_CC007_TCH959-USA300_AASB02000250.1[47620:48063:r]RC
Position: 044-contig_322_RC: 15831 … 15861; Length: 30 bp
Sequence:
AACTACCACAAATACATATCTCTTTTTCAT

## > sprA-L1

Function: noncoding RNA small pathogenicity island RNA A/Locus 1
Best match: sprA-L1_CC001_MW2-USA400_BA000033.2[1893970:1894188] (completely identical)
Position: 044-contig_322_RC: 16261 … 16480; Length: 219 bp
Sequence:
AGTCGCCTATCTCTCAGGCGTCAATTTAGACGCAGAGAGGAGGTGTATAAGGTGATGCTT
ATTTTCGTTCACATCATAGCACCAGTCATCAGTGGCTGTGCCATTGCGTTTTTTTCTTATTG
GCTAAGTAGACGCAATACAAAATAGGTGACATATAGCCGCACCAATAAAAATCCCCTCA
CTACCGCAAATAGTGAGGGGATTGGTGTATAAGTAAAT

## > ldr_fst-L1

Function:  hemolytic peptide
Best match: ldr_fst-L1_CC001_MW2-USA400_BA000033.2[1894021:1894116] (completely identical)
Position: 044-contig_322_RC: 16312 … 16408; Length: 96 bp
Sequence:
GTGATGCTTATTTTCGTTCACATCATAGCACCAGTCATCAGTGGCTGTGCCATTGCGTTTT
TTTCTTATTGGCTAAGTAGACGCAATACAAAATAG

## > fstAT

Function:  Fst antitoxin sRNA, locus 1

Best match: fstAT_L1_CC005_Mu50-VRSA_BA000017.4[1934451:1934510:r]RC (completely identical)
Position: 044-contig_322_RC: 16435 … 16495; Length: 60 bp
Sequence:
CCCCTCACTACCGCAAATAGTGAGGGGATTGGTGTATAAGTAAATACTTATTTTCGTTAT

> **tx_sprA**
Function: rho-independent terminator of sprA
Best match: tx_sprA_CC005_Mu50-VRSA_BA000017.4[1934606:1934666]
Position: 044-contig_322_RC: 16590 … 16620; Length: 30 bp
Sequence:
ATGAGTTCAATATCGGAAGTTAGGCAACTA

> **hsdS-L1**
Function: protein coding sequence; type I restriction-modification system site-specificity determinate-locus unknown
Best match: hsdS-CC25_ref_DQ309452.1[1:1224]RC
Position: 044-contig_322_RC: 20862 … 20892; Length: 30 bp
Sequence:
TGGCAAATTTTTCTTTTGTGTATTACTCAT

> **hsdM-Lx**
Function: protein coding sequence; type I restriction-modification system DNA methylase unknown locus (genomic island)
Best match: hsdM_CC009_21334_AGTW01000033.1[72697:74253:r]RC
Position: 044-contig_322_RC: 20884 … 21034; Length: 150 bp
Sequence:
TTACTCATCTTTCAACACCCCAAGTTCTTTCAGGTATGCATTGATTTCTTGTTCAACTTGTG
CGATTTCTTTGTCGATATTTTTCAAATCTTGTTGGACTTGATCTAAATCAATTGGCGCTTCT
TCTTCGAATGTATCGACATACCTCGG

> **ear2**
Function: protein coding sequence; putative protein (genomic island)
Best match: ear2-118_ref_CC001-ST772_118_AJGE01000001.1[12657:13238:r] (completely identical)
Position: 045-contig_58: 319 … 901; Length: 582 bp
Sequence:
ATGAATACAAAATTTTTAGGTAAAACATTAGTAGCAAGTGCTTTAGTATTAACAACATTG
GGAACAGGATTACATTCTTCATATTTAGGATTAGATACAAACAAAGTTGTTAAAACAGCA
AAAGCAGAAGAAAATATGACAGATGGTCAGTTGTGGAAAAAAGTTAAAGATTCATTAAT
TGATTCAGATATTATTAGTGGTAACCGAGATGAAGAAATAAAAGTAACTTATTCACTTAA
AGATGGTAATTCGCCTAGTGTGACTGCACCTGGAAATGATAGCGGCGATAATTTTAATCA
AAAAATTGATTTTGGTAGTTTAAAACAAGTTGATATAAGTAAACAAAATATTGGTGAAGA
AGATTTTAACAAAAGATTAGATGCGAATAAAACTTGGAACACTTTTACTCAAAAATTAAA
GGATGCTGGTTTATTGAAAAATGGTCAAAAAGTAACCATTCAAACTACTAATCCAACAAC
ATCTTCGACAAACAGTATTACCGGCACAGTAGGTGAAACATTGAATGATAAAAATGCTGA
TCCATTAGAAAAACGCTTAATCAACAAAATCACAATTGAATAA

> **sprB**
Function: noncoding RNA small pathogenicity island RNA B (genomic island)
Best match: sprB_CC010_D139_ACSR01000053.1[6042:6510:r]RC
Position: 045-contig_58: 905 … 935; Length: 30 bp
Sequence:
TAAACTCAAATTTCCTTAATTCATAAAACC

> **A5ITY2**
Function: protein coding sequence; putative protein (genomic island)
Best match: A5ITY2_CC005_N315_BA000018.3[1867608:1868081]
Position: 045-contig_58: 1746 … 2220; Length: 474 bp
Sequence:
ATGCATTTGAAACTGAACGATGAAGATTTAAATTATTTAGTAAATATAAGTAAAGAAATA
CTTGAACAATACAATATTAAATTAGACGAAACTTGTAAGGGAGATATTAACATAACATCA
TGTAATTCTGAATACTTTTTCACTTTAAGCTATTTTTTAAAGCCTGGAAAAGCAACTATAA
ATTTCAGAGAATGCCGTTACAACTATAGCTTGTTACGTTTAAATCTTAATGACGGTTTTCA
TAAAAACTCTAATAATGAGAAAATAAGGGGAAATAGAATTAATATATTTTCTGAAAATG
AATTTATACAAAAAGCTGATGGCGCAACCCATATGAAAGCATATCCTTTACCTTATAATA
TATTTGACGATAGCTCTGATTTTGTAAAACAATTATTTACTTTGCTTGAATATACAAAAAC
TAATCATAATGATAATATTAAAATTGAAACTAATCTATTCATGAGTTGGTGA

> **A5ITY3**
Function: protein coding sequence; putative protein (genomic island)
Best match: A5ITY3_CC005_N315_BA000018.3[1868086:1868877]
Position: 045-contig_58: 2224 … 3016; Length: 792 bp
Sequence:
ATGCATGACAACACAATTAAATCTATTAGCAAAGACTATTTTGATTACTTACAAACAGAA
TCAAAGTTTATCCCATTAGAAAATGAAACTATAGAATTTTATTCACCTATCGTCGATTATT
TTGGTGATTCTATTTCTGTAAATATATCTTTTTCAGGGGATCGATATAAACTAACTGATCA
TGGTGAAACTTTGTGGAACATGGAACAATTTGGTTTAGATCTAACTAATCATAAAAAACA
AAAAAAGTATCAACTTCTCAATAATATTATACAAAATTACGGTCTAGTCATTGAAAATAA
GCACATAAACTTTTATACAAATAGAATAGACTTATCTCAAGCCATTCATGATTATGTTTTG
GCTATTTCCGAGATTAGTTATTTGGGAATATTAAAGAAAGAAAATATTAAGTCATTATTT
AAAGACGAAGTGATGCAATATTTTCTATCAAACAGAAAGATTTATCCTAATATATTTCCC
GAATTTAAGGTAGAGGGGAAATCTAAGTTAATTCATAGTTTCGAAGCAGTATTTCCAGGA
GAAGCTACAGAGTATGTTAAAACAATTAAACGAATCGATAAAAACAATGCAAAAAATGT
CCTTTTCGATTGGAATGATGTTGAAGTACATAGAAATAAAAATTTTGATTCTAATGCTAG
ACTTAATATCATTACAGAACAACAAGGCGATATTAACGAAGCAGTATCAACAATGTTGTC
TCAATATAATGTAGAGGTATTTTCATTTGAAAACAAAAAACAACTTGAAGAAAAGTTTAG
TAACGTATAA

> **sprC1**
Function: noncoding RNA small pathogenicity island RNA C (genomic island)
Best match: sprC1_CC001-ST772_118_AJGE01000001.1[9842:10244]RC (completely identical)
Position: 045-contig_58: 3313 … 3716; Length: 403 bp
Sequence:
AAAAAATTAAATGCTATGATTTAGCCGAATTAAAAACATATATACTTATTTACTATGAAC
TAAAAGTTTATCCTAAAAATAAAAAACCATGATTTCGAAGTCTTCATAAACTGAAGCTTC
TACTCTCATGGCAATTTATACTATGATATTCAATTATAATGCCTAGTCTCATAGTAAACGG
CTACTACATTCGCATGTACTTCGTTTTACTGTCCACGCATGTTCGTTGACTTATTTATATTA
TAATATAAATATTTTGTTTTTCAATACTTAATTTTAAAAATCCCCTATGAAAATGTTGATG
AGCTGCATCTTTAAATCTTATTATACATTGTATGAAATTATATTGCGAGGTACTAAATTGA
TAATATACACTTTTGGTTGTGTATGCATCTCACCTATC

> **Q2FXA9**
Function: protein coding sequence; putative bacteriophagal protein (mobile element)
Best match: Q2FXA9_CC001_MW2-USA400_BA000033.21921296:1921751
Position: 045-contig_58: 3786 … 4242; Length: 456 bp
Sequence:
ATGGCTAAACTCGATTTAAATAGTCTTGACGACGAGCACGTAAAATTATTAATAAATGAA
TTAAAATATCCAGAAACTCATATCGATGTAAATGAATTAAAAACAATAGTTGCTAGTCGA
ATAAATGAAAGGCAAGAAATAATAAGTTTTAAGTTAGGAATAAAGTACTTATTAACAAT

AAAAAGAGGGAACATAGAAAAAGATAGGTTTTCAATTTCAATCATTTTTAAAGATACCTA
TCACACCCTAGTTAGAATAGATATTAACGGTGGTACTCACGATAATCCAGATGGAACATT
CGCTCCGAAAAGTCATATTCACATATATAATGATAAGTATGATAAAAAGGATAGGTTTGC
TTATGAAATTAACTTGAAAGATTTCCCCGACATCTATAACTTGTATAATGTGTATATGTCG
TTTTTAGAGTATAATAATATAAAAGACCTTGAATAA

## > **Q2FXA8**

Function: protein coding sequence; putative protein (mobile element)
Best match: Q2FXA8_CC001-ST772_118_AJGE01000001.1[8515:9303:r] (completely identical)
Position: 045-contig_58: 4254 … 5043; Length: 789 bp
Sequence:
ATGACTACATTTGATGCTAAAAAATTAAAAAAAGAATATCTTGATTGGTATAATCAGACC
TTAGAGTTTTCTAATTTATCAAACAATGTAGTAAGAATAGATACTCCTTTTAAAGATAATT
CTTTAGATAATTTAATAATTTACGCTTTATACGATCAGTCCAGAGACATGATTACACTGAC
AGATGACGGCTATACTATATTTGATTTAGAAAATAATGGTATTTTTTTAAATAAATCAAA
AAAACATAAAAAGATTTTTGAAGAGCACCTTTCAGCTTACGGTATTAAATATAACGATAA
AACTCACGAAATTTTTGTTCAAACTAACTTTAAAAATTTTAATAAATCGAAACATAATTTA
TTACAGTGCCTTATATTTGTTAATGATATGTACTTACTTTCTAATCCTAAGTCACAGAACA
TATTTACAGAAGATGTTGCAAACAAATTGGATGAACATAACATTTATTACGGAAGAGATT
TACCTATTATAGGAAGCAGTGGTGTTGTTCATAATTTCGACTTTTTTATTAGCGCTAAGAA
AAATCAAAAAGAAAAATTTATCAATGCTATTTCTAACCCTAATAATTCTATGATTATTAA
GTCGAAAATAACGGATGCTATGCAAGCAAAAAAAATAAAAAGACACAGGCAAAATGAG
TTTATTTTTATTTTAAATGACTCAAAAAAAGAAATAAATGAACATAATAAAAACCTTCTTC
ATGAAAACTATATTAGTACTATAGATTATAGCGAATTAGACGAAAAGATAGGTTTATTGA
TTTAA

## > **Q99T51**

Function: protein coding sequence; putative protein (genomic island)
Best match: Q99T51_CC005_N315_BA000018.3[1874153:1874647]
Position: 045-contig_58: 5876 … 6371; Length: 495 bp
Sequence:
ATGAGGAGATTACTCAGTCTATTACTAGCTAGTACAATGATTTTAGTCGCATGTGGGAAT
GCTAACAATGAAAATAAGAAAAAGGAAGACGAAAAAAAATCAGAAGTAAAAAAAGAAG
CTAAGAAAAATAATGATAAACCAAAGAACGAAAAGAAGAATCAAGATATAAATAAAAA
CAATAATGAGCAAGTTCAAAATGTCAATAATAAACAACCAGTCTTAAATAATAATAGTAA
TCATAACAATCAACAACAAAGCAATAAAAACAATTACACAGTTATAGAAAACGGGAATA
CAGTTACAGAAATTTATAATGGACAATCACACACAACTACAAATAACCCTATTAGAGAAG
AATATGTAGAGGGAGAAACAGACACGATATATAAAAGAGAGTATAAGCATTTTTATACA
CCTGAAGAAGCTCAAAGAGCACAAGAAAATAGCGACCAAATTTTAAGAGAAATGGGTCT
CGAACCAGAAAGATATGAATAA

## > **A6U2S7**

Function: protein coding sequence; putative protein (genomic island)
Best match: A6U2S7_CC005_Mu50-VRSA_BA000017.41952518:1952754 [4_50ANF10END]
Position: 045-contig_58: 6448 … 6685; Length: 237 bp
Sequence:
ATGATTAGGTTCGATGTTAATGGTAGTGATCATGCAAATCCACCAAATAATGAAAGAATA
CCTACGCCACATATACACATATATACTGAAGAATATAATAATGGTGGTATTGCGATTCCA
TTAAAAGATATTGAAGATTTAGAATTAACAGATGAAATTATAGAATCATTAGACTTCTTT
ATGAAGTATACTAATATAAAACACGATAATGTTATAATAGAACAAAGATTACTGTAA

## > **Q7A4X2**

Function: protein coding sequence; putative protein (core genome, variable)
Best match: Q7A4X2_CC001-ST772_118_AJGE01000001.1[6076:6852:r] (completely identical)
Position: 045-contig_58: 6705 … 7482; Length: 777 bp

Sequence:
GTGAAGACAATCGAAGAAAGAATGAACGAATATTTCAACTGGTTAAAGCAAAATTATAT
CTTCAAAGAATTAGATAGTTCTACAGAAATTACTACTCCCTTTAAGAACCATTTGAATGAT
TTTATTAGAATATATGCTGATACACTACCTAATAATGAAATTTGTCTATCAGATGACGGAT
TGACGATTAATGAATTAGAAATGTTGGGTATTGATATAAACACAAAAACGAGAACTAAA
CTTATACAAAATATTTTAAATCAATTTAATTTAAAATTAGTTGATAAAGAAATTACAGCG
GATGTTAAAAATGAGAGTTTTGCTCAGTCTAAACATAATTTAATTCAAGGCATATTGAAA
ATTTATGACCTTACTTTAACTACTAAATCTAACGTTACAAACATATTCTATGAAGAAGTAT
TTGAATTCTTATATGACCAAGAAATAAGAGGTTTAGCACAAGTTTCTGTTTCTGGAGAAT
CAGGTTTAAAATATTCTATTGATTATATTGTTTCTGAAACAAAATCACAACCAGAAAAAC
TAGTTAACTTTACAAACAACTTAGATTTCAACAAAATAACTAATGAAGCTTATATTTACC
GTGATATCAAACCAAACAGACCATCCAGAAACAAATTAGAACCTATCATGTTGATAATTG
TAAATGATGTAGATCATCCGATAAATGAAAGAGCACAAACTGTAGCAGAACATGAAAAC
CTTGAGATATTAAAATGGTCTAATAAATCAAAAATCATTGAAACTTTAACCTCATAA

## > **entG**

Function: protein coding sequence; enterotoxin G (genomic island)
Best match: entG_CC001-ST772_118_AJGE01000001.1[4748:5524]RC (completely identical)
Position: 045-contig_58: 8033 … 8810; Length: 777 bp
Sequence:
TCAGTGAGTATTAAGAAATACTTCCATTTTAATACTCTTAGAATCAACAACTTTATTATCT
CCGTAAATATTTAAAAACTTATATGGAACAAAAGGTACTAGTTCTTTTTTAGGAAATAAG
TCAAACCAAAAACTTGTATTGTTCTTTTCAGTAAATTTTATATATCCAGATTCAAATGCAG
AACCATCAAACTCGTATAGCTTTTTTTCTTTAGTGAGCTAGTGTCTTGCTTTGTAATCTAGT
TCCTGAATAGTAACCATATTCTTATTTGTAGTTATTGTAAATCCAAGTGATTGTCTATTGT
CGATTGTTACCTGTACAGTAATTAATTTATCTCTTTCATTTTCTGAACTATTAAATGTAAG
ACCACCATACATACAACAACCTCCAAAATTTTGGTTTATATCCGGTTCAGATTTAGGTATT
ATACATGTATAAAAATATGGAACGCCAAAAATGTCTACTTTTTTATCTTTATAATTGTTAG
CTAATTCTGTATTTTCTAATTCAGTTTTAACCTCATTATAACTCTTATACTCAATTGGAAAA
ATTAAATCATGAGATAAAAACTGTCTAGAATTAATAACTCCTCTTCCTTCAACAGGTGGA
GACGTATAAAGATTCATTACATTACCCATAGTTCCCTTATTATTTTTATAATCACTTACTTT
ATTTAGTTCGTCTAATTTAGGATCGGGTTGAGCATTCACATAATTCATATTATGAAAAACT
ATTTCTAGAATCAAAATAATAATTACAGTAGATAATTTCTTCAT

## > **entN**

Function: protein coding sequence; enterotoxin N (genomic island)
Best match: entN_CC005_Mu50-VRSA_BA000017.4[1955162:1955917:r]RC (completely identical)
Position: 045-contig_58: 9092 … 9848; Length: 756 bp
Sequence:
TTAATCTTTATATAAAAATACATCAATATGATAATTAGATGAGCTAACTGTTCTATTATCA
CTATAAAATTGAAAAAACTCTGCTCCCACTGAACCTTTTACGTTATATAAATCATAATAA
AATGATTGATCTTGATGATTATGAGAATGAAAGAAAATGCATCCTTTTTGTATGTTACCG
GTATCTTTATTGTATATTTTATATAAATTTTCTAATTTAAATCGAACTTTAGTGTCTAATTC
TTGTACTGTTACTTTAGCCTTTTTGGTTTTTATAACAAAACCTTCTTGTTGGACACCATCTT
TAAATACATTAACGCCTATAACTTTCTCTTCATCTAATTGATTTCCATCATGTATCGTAACT
CCTCCGTATAAGCATGATGTTTTTTCTTCAGTTAAGCCTACACATTTATTTCCAAAATACA
GTCCATAAATATCTATATTTTTTCCTTTAAATTGATTTGCTAAATCTGATGAGTTAAACTC
AACTTTCAAACTAGAAGTTTTAAGTACGGATATATCAATATTTTTTAATATTATAGTATTA
TTCAGTAGTTGATCTGTACTAATTTTATTTGACTCGTCTAATTGCCACGTTATATCAGTATA
ATAGCTTGTTAAATTAAATAACTTACTACTATCTAGATCAGATTTTTTCTTTAAATCTTTTT
TGTCTACTTCAGCATTAACATAATTATTATTAATAAGACATAATAAAGTTATTATAATTGC
AGCTATGTAGAACAATCTCAT

## > **entU**

Function: protein coding sequence; enterotoxin U/enterotoxin Y (genomic island)

Best match: entU_CC001-ST772_118_AJGE01000001.1[2901:3671]RC (completely identical)
Position: 045-contig_58: 9886 … 10657; Length: 771 bp
Sequence:
TTATTTTTTGGTTAAATGAACTTCTACATTAATAGATTTACTCTCAACTGTTTTATTATCAT
TATAAATTAGTAAATATTTAGTCGGATAAAATTTTTTACCAGATTCAGGCATCAAATCATA
CCAAAAAGAATGACCACTTCCTTCGATAAACTTTATATAGCCAGTCTCATAAGGCGAACT
GTTAAATTCATATAAATTTTTATGCTTAAGTAAATAGTTTCTAACTTTATAATCTATTTCTT
GTGCTGTTATGTTTTTCTTATTAGTAGGTATATCAAAAGATAATGAATTTCTCTCGTTTTCA
TAAACTTTAATTAAGATATTATGAGAGTTATCAGTTGAATTATTTTTATCTATTTGATTTCC
ATCATGCTCGGTCACACCGCCATACATACACGTTTTTTCAATTAATCTACCATCATTTAAT
TCCATATTATCCGATGAAAAATAGCATTGATTATAATAGTTTGTCCCAAACAAATCTACGT
TTTTATTTTTATATTTATCCGAAAGGCTTTTATTATTAAATTCTGTTTTTAAAATTTTAGAA
CCATTTATTTTAAATAATAAATCATGTTGTAAAAACTTTTCTTGGGATTTAATGTTTGTTTC
TGATACGTGTTTATCATCATACAAATACCTCATATTATCCATTAGACCAGTGAATTCACTC
GCTTTATTCAATTGTTCTGGTTTAGGATTGCCATTTAACATAAATAGTAAGCTGCAAGACT
TAACACATATGAAGATAAAAGCAAATAACTTCAT

> **entI**

Function: protein coding sequence; enterotoxin I (genomic island)
Best match: entI_CC001-ST772_118_AJGE01000001.1[2019:2747]RC (completely identical)
Position: 045-contig_58: 10810 … 11539; Length: 729 bp
Sequence:
TTAGTTACTATCTACATATGATATTTCGACATCAAGATGAAATTTTTCAGATTCTATTATTT
TATTATCTTCATAAATTTTCAAAAAACTTACAGGCAGTCCATCTCCTGTATAAAACAAATC
ATATGAAAATGATTTTTCATTATTTAAATGAAATAAAACTTTCCCATTATTAAAACCTGAA
TAAAATTTAGATTTATATCCATATTCTTTGCCTTTACCAGTGTTATTATGACCATATATATT
GTATTCTTCTTGAAGATATCTCCTTAATTTAACATCAATTTCTTGAGCTGTTACTAGTTTTT
TATTAGTTGCTATTTTGTCAGTAGAAATTGTTTTATGTTTGCCATTAACCCAAAGATTAAT
AGGGATTTTTCTAGCAGAATTTAAGTATTGTCCTGATAAAGTGGCCCCTCCAAACATGTAT
TTAGATTTACAAGGACCATTATAATCAATGCCAAAAATATCCAGTTTCTTTCCTTTAAATT
TACTTATTTCGTCCCAATTATTAGATTCTGAGATCAAATCATTGGTACCTGTTGAAAATTC
GAGTTGATTTGCAATAGGTAGATTTTTATCTGTGACGCCTTTTAAATCTATATAATCATGT
TTTGTATAGAAATTTCTTAAGTTACCTACACCAATATCACCTTGAGCATACGTAAGATCTT
TAATGTTAAAAAGTAATATAAAAACTAATATAAAACTATATTTAAATTTTTTCAT

> **entM**

Function: protein coding sequence; enterotoxin M (genomic island)
Best match: entM_CC001-ST772_118_AJGE01000001.1[1265:1984]RC (completely identical)
Position: 045-contig_58: 11573 … 12293; Length: 720 bp
Sequence:
TCAACTTTCGTCCTTATAAGATATTTCTACATCTAAATGGAATTTTTCAGTTTCGACAGTTT
TGTTGTCATTATATATTTTTAAGAAACTTTCAGCTTGTCCTGTTCCAGTATCAAATAAGTC
ATAAGAAAATGATGAACCATCATTCAAATGAAATAATATTTTTCCTGCATTAAATCCAGA
ACTAAATTTTGACTTATTACCATAATTTCTTCCTTTATTTGTATCATTAAAGCCATAAATAT
TATATTCTTCTTGTAGATATCTTCTTAATTTAGTATCAATTTCTTGAGCTGTTACTAACTTT
TTATTAGTTGATACTTTGTCAGTAGATATAGTTTGATGTTCTCCATTAACCCAAAGATTAA
TAGGAATACGTCTAGATTTCTCTAAATAATCACCTGCTAATGTAACTCCACCGTATATATA
TTTGTTTTTCAAACAATATCCACTATAACTTAGACCATATACATCTACAGCATGATTTTTG
AATTTCTTTACATCATCAACGTTCTTAAATTCAGCTGTTATTGTCGAATTATCCATAGAAA
AAACTAATTGATGCGAAAGATGATTATTTTCAGGATTAATACTTTGGTGGTCTTCAATTGG
ATAGCTACCATAATAGTTCCTAAGATTCAAAACTCCGACATCAGCGGTTGCGATATGATT
TTGCGAATAGCAAAACAATAAAACAACAATGATAAGTATTCTTTTCAT

> **entO**

Function: protein coding sequence; enterotoxin O (genomic island)

Best match: entO_CC005_Mu50-VRSA_BA000017.4[1958644:1959408:r]RC (completely identical)
Position: 045-contig_58: 12573 … 13338; Length: 765 bp
Sequence:
TTATGTAAATAAATAAACATCAATATGATAGTCTGATGAATCTATTGTTTTATTATCATTA
TAAATTTGCAAATATTGATCTGGTAAATTTCCTTTAATATAAAATAAATCATAATAAAATG
ATTCTTTATGCTCCGAATGAGAATGAAATTTAATATAACCTTTTTGTACATCACTAGTCAT
TCTGTCATACAATTTATATGCATTATTTAATTTTGTTCTTACTTTAATATCAAGTTCTTGTG
CAGTAACTTTCTTTTTATCTGTAGTAACGATAAATGTATTAACATTTACATTATCCTTATAC
ACAGCTACTCCTATATTTTTAGGCTCGCTTAATTTATTATTTTCATGAGGTGTTACCCCACC
ATATGTACAGGCAGTATCCACTTGATGCTCACCATGACAATGTGCTTTATAGTAAACACC
ATAAATATCAACAGTCTTTCCTAGAAATTCTTTAGAAATCGCTGATGAGCTAAATTCCACT
TTTAAATCTTTAAATTCAGCAGATATTCCATCTAACCAATTAATACTTTTAAATAATAAAT
CGAAGTCTAAGAATTTTTCTGTAGTTGATACAATTGATTTTACTGTCGTAAATCGATTATT
TATATAATCATCATTAATATTATGTAAAGCAATAGGGTCTACACTTGACTTCTTACACAAA
CTCTCTATTTTAGGATCTTCTTCATTTGCATATGCATTGTTTACACTACATATTGCAATTAA
ATTTAAAATTAATAATAATACATTTAACAT

## > tx_tRNAser3

Function:  rho indendent terminator
Best match: tx_tRNAser3_1_CC010_H19-ST10_ACSS01000051.1[11343:11405:r]RC
Position: 045-contig_58: 13516 … 13546; Length: 30 bp
Sequence:
AAAAACCTTAAAAATAGCGCTTAAAATGGG

## > tRNAser3

Function:  tRNA serine
Best match: tRNAser3-Mu50_CC005_N315_BA000018.3[1881844:1881935:r]RC (completely identical)
Position: 045-contig_58: 13566 … 13658; Length: 92 bp
Sequence:
TAACGGAGAGTGAGGGATTCGAACCCTCGAGACGCTTGTGGCGCCTACACACTTTCCAGG
CGTGCTCCTTCGGCCAACTCGGACAACTCTCC

## > tRNAhis

Function: tRNA histidine
Best match: tRNAhis_CC001_MW2-USA400_BA000033.2[1924191:1924266:r]RC
Position: 046-contig_53_RC: 44 … 74; Length: 30 bp
Sequence:
CCGATGTGTTAACCACTTCACCACAACCGC

## > tRNAphe

Function:  tRNA phenylalanin
Best match: tRNAphe_CC001_MW2-USA400_BA000033.2[1924277:1924352:r]RC (completely identical)
Position: 046-contig_53_RC: 84 … 160; Length: 76 bp
Sequence:
GAATGGTTCAGGACAGAGTCGAACTGCCGACACATGGAGCTTCAATCCATTGCTCTACCA
ACTGAGCTACTGAACC

## > tRNAasp

Function:  tRNA asparagine
Best match: tRNAasp_CC001_MW2-USA400_BA000033.2[1924369:1924444:r]RC (completely identical)
Position: 046-contig_53_RC: 176 … 252; Length: 76 bp
Sequence:

TGGCGGTCTCGACGGGAATCGAACCCGCGATCTCCTGCGTGACAGGCAGGCGTGTTAACC
GCTACACTACGAGACC

> **tRNAmet_ini**
Function:  tRNA methionine initiator
Best match: tRNAmet_ini_CC001_MW2-USA400_BA000033.2[1924466:1924539:r]RC (completely identical)
Position: 046-contig_53_RC: 273 … 347; Length: 74 bp
Sequence:
TTGCGGGAGGCGGATTTGAACCACCGACCTTCGGGTTATGAGCCCGACGAGCTACCGAAC
TGCTCCATCCCGCG

> **tx_universal2**
Function:  rho indendent terminator
Best match: tx_universal2_CC001_MW2-USA400_BA000033.2[1924855:1924893:r]RC (completely identical)
Position: 046-contig_53_RC: 662 … 701; Length: 39 bp
Sequence:
AGAAATTATAAAAGCTTGTTACACCGCATATTCTTTCAG

> **A6QI12**
Function: protein coding sequence; putative protein (core genome, variable)
Best match: A6QI12_CC001-ST772_118_AJGE01000032.1[31477:32031]RC (completely identical)
Position: 046-contig_53_RC: 818 … 1373; Length: 555 bp
Sequence:
CTATCGTGTCATGTAATCTTGCATCCGATCTTGCAACGCTGTAAATGTTTCGAAGCCATCC
TCTTCTAAGAAGTGCCCTCCATCTTCCACGATTCGCAAGTTCCCCTCTAATGCATTCATTA
AACGCTGGGTTTCTTTATAAGAAACGTATTTGTCATTTTTAGAACTCAATCCGTAAAAATT
GTCAACTTTCTTTTTAATATTATCGTAATCAATGGTTACATTACTTAAATCAATATCTAAA
TCTATATTTTCTGCATCTTCTTTAAAGCCCGCTATACTAAAAAAGCCTTCAATCGGCTGAT
CAATCATTTCAATATATTTTAAAGCTGTGATTGAACCTAAACCATGTGTTACAAAATATGT
ATCCTTTTTGCGTACATTAATTTGTTTCGTCATAGCTTCAATCCACTGATCCACTGTCTTCG
CTTCAGGGGATTCAAAATTAAATAATGTTACGTCATATCCTTCTAAAGTTAAGTTATGCTC
CAACCACTGATACCAATGATTTCTACTATTTCCATGCATAGAATGTACAATAATTACATCT
GTCAT

> **hemY**
Function: protein coding sequence; protoporphyrinogen oxidase (core genome, variable)
Best match: hemY_CC001-ST772_118_AJGE01000032.1[29756:31156]RC (completely identical)
Position: 046-contig_53_RC: 1693 … 3094; Length: 1401 bp
Sequence:
TTACAACTCTGCGATTACTTCTTCAGCAGCAACTTTACCTTGCGTAATACAATCAGGTAGT
CCAACCGCTTCAAAAGATGCACCAGTTACTCTAAGTCGTGGATATGTTTGTTTAATATGTG
CTTGAATCTGTCTAATTTGTTGAATATGACCGACATGGTACTGTGGCATACTTTTCGGCAA
ACGATTGACAATTGTAAATTCAGGATCACCTTTAAATGTCATCATTTGACTTAAATCTCTA
CGTACAATCGATACTAATTCATTATCTGTATGATCATCAACCACAGTATCACCTGGTTTAC
CTACATACGCACGAATCAAAACCTTACCTTCTGGTGTAGTAAATGGCCATTTTTTCGATGT
CCAAGTACATGCGGTAATGTCTGTATCACTTGTTCTCGCAATCACGAAGCCAGTACCATC
ATAAGTATTTTCAATGTCTTTTTCATCAAATGCCAATACAACAGTTGCAACAGTCGTACTA
TCCATCGTTTTAAAGTAATCAAATGCTGGATCTTGCCCGAACCAATTCAAAAAGACTTGA
TGTGGTGTTGTCACTAATATTCCATCGAATACATCTTCTTGTTGATTACTGTAGACAATTTT
ATATTGCTTTTGAGATGTAATAATATCATCCACTGACGTATTGTAGCGTATTGTCACACCT
TTATTTTTAACATCTTGTTCTAATGCTTCAATAAATGAGCTTAAACCATGCTTAAATTGTTT
GAATTGTCCTTTTGGTGCGCCAGGATATAATTGTCTTTGTTTCAGACGCTTATTTTTCTCAT
CCTTCATACCTTTTATCAGACTTCCGAATGCCTCTTCTTTTTCTTTAAAATTAGGAAACGTA

CTCATCAAACTTAATTTATCAATATCGGTACCATAAATACCACCCATTAAAGGCTCTATTA
AATTCTCAAGTACCTCATTACCTAATCTTGCTCTGAAAAATGCACCAACAGAAATGTCAC
CATTCTGCATTTGAGTAGGTTTTTTAATAAATCAAACCCTGCTCTTAACTTTCCAAATGG
TGAAATTAATTTAGTTGTCACAAACGGTTTGATATCTGTCGGAATTCCCATAATTGATCCA
CCTGGAATAGGATACAATTTGTTTTTCGCAAAAATATAAGATTGTCCAGTCGTATTTGTAA
AAATATCTTGTTCTAATCCAATATCTTTCGCTAATTCTGTCATGATTGTTTTTCTACCTAAA
TAAGATTCAGGCCCTAGTTCAATCATATAACCATCTTTACGATACGATTGAATCTTTCCCC
CCGGACGATTCGATGCTTCAAAGATTGTTATGTCAATATTAGGATCCTGCTGTTTTAAAAA
ATATGCACTTGATAAACCTGTGATCCCCGCTCCTATTATAGCCACTGATTTAGTCAC

> **hemH**

Function: protein coding sequence; ferrochelatase (core genome, constant)
Best match: hemH_CC001-ST772_118_AJGE01000032.1[28809:29732]RC (completely identical)
Position: 046-contig_53_RC: 3117 … 4041; Length: 924 bp
Sequence:
TTAAAATATGGACTTTATTTCATCAACAATTGCACCGATAAATAATGGATGTGTATTCGGC
ATTTCCGGTCGATAATAATTCGCACCAATATCATCGCAAACAACTTTACATTCATAATCAT
TGTCATAAAGCACCTCTAAATGCTCACATACAAAACCTACTGGTGTATATATAAAGTTTTT
ATACTGATGTTTTTCATATAAATCACGTGTTAAATCTTGTACATCTGGCCCTAACCAAGGT
GTACCTGTATTACCTTCAGATTGCCAACCAATCGCGATATGTTCAATATTAGATTGTTCTT
TAATTAAAAGTGCAGTATGTTCTAGTTCTTGTGGATATGGATCATTATTCTTTTCGATTAA
ACCTTTTGGCAAACTATGTGCCGAAACAACTAATACCGTGTCTTTATGTTCCTCTTCCGGT
ATTTGAGCTAATGTTTCGTTGACTTTATTCGTCCAATATTCAATAAATTTAGGTTGTTCATA
ATAATGTTTCACATGTGTAAGTTGAATACCATATTTTGCAGCTTCTTCATCAGCACGTTTG
TCATATGATCCTACTGAAAATGAAGAATAATGTGGTGCTAGTACTACAGTAATTGCTTCA
GTAATGCCATCATTGTGCATTTGTTCAACCGCATCTTCGATAAATGGTGAAATGTGTTTTA
ATCCTAAGTATAGTTTAAATTCAACATCTGCATATGCTTTATTTAATGCTGAAACTAGCGC
ATCAGCTTGGTCATCTGTTGTACCTGCTAATGGTGATAAACCACCTATAAATTCATATCTA
TCTTTCAAATCTTGAAGTTCTTCTTCAGATGGACGTTTACCATGTCTAATATCTGTATAAT
ATGGCTCTATGTCACTTTCTTTATAAGGTGTGCCATAAGCCATAACTAATAATCCCATTTT
TTTAGTCAT

> **hemE**

Function: protein coding sequence; uroporphyrinogen decarboxylase (core genome, constant)
Best match: hemE_CC001-ST772_118_AJGE01000032.1[27714:28751]RC (completely identical)
Position: 046-contig_53_RC: 4098 … 5136; Length: 1038 bp
Sequence:
TTATCTTTGTGTATATGTGTGTACGAATTCGCTTACTTTACGTAACGTCTCTGGTTGCACTT
CTGGGAAAACACCGTGTCCTAAATTAAAGATGTGTTTACCGTTCTCCATACCTTGATCTAA
TATTGGTTTCAATCTCTCTTCAATGACATTCCATGGTGCTAATAAAATTGATGGATCTAAA
TTCCCTTGTAATGTTTTAGTAACGCCTAATTGTTGAGCCTGATTAATAGACGTTCTCCAAT
CTAGGCCTAATACATCAATCGGTAAATCATTCCATTCATTGATTAAATGACTGGCACCTAC
ACCGAATAAAATTACCGGCACATCATGTTTTTCTTTAACCTCACTGATTAATCGAATCATA
TGTGGTTTAATGTAACGTCTGTAATCCTCGACATTTAATGCACCTACCCATGAATCGAAAA
TTTGAATCAATTCGGCGCCTGCTTCGACTTGAGCTGTTACATATTTAACAGATACATCAAC
TAAATGATTCATTAAAGCAAACCATGTTGCTTCATCTCTATACATCATCGCTTTTGTAAAA
TTGTAATTTTTCGATGGTCCGCCTTCAATCATATATGACGCTAATGTAAATGGTGCCCCAG
TAAATCCTATTAGCGGCACATTTAACTTTTCTTCTGTTAAAAGTTTAATTGTATCTAATAC
ATATGGTACATCTCGTTCGGGGTCTATTTGAGAAAGTTTCTCAACATCTTGAATTGTTTTG
ATTGGATTATGAATCACTGGACCAATACCCGATTTAATTTCTACATCGACACCAATTGGCT
TTAATGGTGTCATAATATCTTTGTATAAAATTGCTGCATCTGTATGATAATTATCAACTGG
TAAATGTGTTACATAAGCGCACAACTCCGGCTGATGTGTAATATCGAATAGTGAATATTT
TTCTTTCAATTTTCGATATTCTGGTTGCGAACGGCCAGCTTGTCGCATAAACCAAACAGGT
GTATGTGATGTTTCTTCACCTTTGATCATTTTTAAAATTGTATTGTTTTTATTATGCACCTT

> **traP**
Function: protein coding sequence; signal transduction protein (core genome, variable)
Best match: traP_CC008_NCTC8325_CP000253.1[1870108:1870611] (completely identical)
Position: 046-contig_53_RC: 5398 … 5902; Length: 504 bp
Sequence:
ATGAAGAAACTATATACATCTTATGGCACTTATGGATTTTTACATCAGATAAAAATCAAT
AACCCGACCCATCAACTATTCCAATTTTCAGCATCAGATACTTCAGTTATTTTTGAAGAAA
CTGATGGTGAGACTGTTTTAAAATCACCTTCAATATATGAAGTTATTAAAGAAATTGGTG
AATTCAGTGAACATCATTTCTATTGTGCAATCTTCATTCCATCAACAGAAGATCATGCATA
TCAACTTGAAAAGAAACTGATTAGTGTAGACGATAATTTCAGAAACTTTGGTGGCTTTAA
AAGCTATCGTTTGTTAAGACCTGCTAAAGGTACAACATACAAAATTTATTTCGGATTTGCT
GATCGACATGCATACGAAGACTTTAAGCAATCTGATGCCTTTAATGACCATTTTTCAAAA
GACGCATTAAGTCATTACTTTGGTTCAAGCGGACAACATTCAAGTTATTTTGAAAGATAT
CTATACCCAATAAAAGAATAG

> **hit**
Function: protein coding sequence; histidine triad cell-cycle regulator (core genome, constant)
Best match: hit_CC001_MW2-USA400_BA000033.2[1932307:1932729] (completely identical)
Position: 046-contig_53_RC: 8115 … 8538; Length: 423 bp
Sequence:
ATGTCAGAAACAATTTTCGGCAAAATTTTAACTGGAGAAATTCCTAGCTTTAAAGTATAT
GAAGACGATTATGTCTATGCCTTTTTAGATATATCACAAGTTACTAAAGGACATACGTTAT
TAATTCCTAAAAAAGCTTCTGCTAATATCTTTGAAACTGATGAAGAAACAATGAAACATA
TCGGTGCAGCATTACCTAAAGTAGCAAATGCTATTAAGCGTGCATTTAATCCTGATGGTTT
AAACATTATTCAAAATAATGGTGAGTTTGCAGATCAATCTGTATTTCATATTCATTTCCAC
TTAATTCCTCGATATGAAAATGATATTGATGGATTTGGTTATAAGTGGGAAACACATGAA
GACATTTTAGATAACGATGCAAAACAACAAATTGCTGAACAAATTCAAGCACAATTTTAA

> **gvpP**
Function: protein coding sequence; gas vesicle protein-like protein (core genome, constant)
Best match: gvpP_CC001_MW2-USA400_BA000033.2[1932871:1933236] (completely identical)
Position: 046-contig_53_RC: 8679 … 9045; Length: 366 bp
Sequence:
ATGAAAGCATCACGCATTCTATTCGGTATCGGTGTTGGCGTAGCAGCTGGTTTTGTAGTTG
CACTTCAAGGACGAGACGACAAAAGTGTCAAGAACAACACGATCGATCGTACTGCCCCT
ACTGGTTCAAAATCAGAACTACAACGTGAATTTGAAACGATTAAACAAAGTTTTAATGAC
ATTTTAAACTATGGTGTTCAAATTAAAAACGAAAGTGCGGAATTTGGTAGTTCAATTGGT
GGTGAAATTAAGTCATTACTTGGAAACTTCAAATCTGACATTAATCCTAATATTGAACGTT
TACAGTCACACATCGAAAATTTACAAAATCGTGGCGAGGATATTGGAAACGAAATTTCTA
AGTAG

> **prsA2**
Function: protein coding sequence; peptidyl-prolyl cis-trans isomerase (core genome, constant)
Best match: prsA2_CC001_MW2-USA400_BA000033.2[1934731:1935693]
Position: 046-contig_53_RC: 10539 … 11502; Length: 963 bp
Sequence:
ATGAAGATGATAAACAAATTAATCGTTCCGGTAACAGCTAGTGCTTTATTATTAGGCGCT
TGTGGCGCTAGTGCCACAGACTCTAAAGAAAATACATTAATTTCTTCTAAAGCTGGAGAC
GTAACAGTTGCAGATACAATGAAAAAAATCGGTAAAGATCAAATTGCAAATGCATCATTT
ACTGAAATGTTAAATAAAATTTTAGCTGATAAATATAAAAATAAAGTTAATGATAAGAAG
ATTGACGAACAAATTGAAAAAATGCAAAAGCAATACGGCGGTAAAGATAAATTTGAAAA
GGCCCTTCAACAGCAAGGTTTAACAGCCGATAAATATAAAGAAAATTTACGTACTGCTGC
TTATCATAAAGAATTACTATCAGATAAAATTAAAATCTCTGATTCTGAAATTAAAGAAGA
CAGCAAGAAAGCTTCACATATTTTAATTAAAGTTAAATCTAAGAAAAGCGACAAAGAAG
GCTTAGATGATAAAGAAGCGAAACAAAAAGCTGAAGAAATTCAAAAAGAAGTTTCAAAA

GATCCAAGTAAATTTGGTGAAATCGCTAAAAAAGAATCAATGGATACTGGTTCAGCTAAA
AAAGATGGCGAATTAGGTTATGTTCTTAAAGGACAAACTGATAAAGATTTTGAAAAAGC
ACTATTTAAGCTTAAAGATGGTGAAGTATCAGAGGTTGTTAAATCAAGCTTTGGATATCA
TATTATTAAAGCTGATAAACCAACAGACTTTAACAGTGAAAAACAAAGCCTGAAAGAAA
AATTAGTCGATCAGAAAGTACAAAAAAATCCAAAATTATTAACTGATGCATACAAAGAT
CTATTAAAAGAATACGATGTTGACTTTAAAGATCGTGATATTAAATCAGTTGTCGAAGAT
AAAATCTTAAACCCTGAAAAACTTAAACAAGGTGGCGCACAAGGCGGACAATCCGGCAT
GAGCCAATAA

## > **cbf**

Function: protein coding sequence; replication enhancer (core genome, constant)
Best match: cbf_CC001-ST772_118_AJGE01000032.1[20286:21227]RC (completely identical)
Position: 046-contig_53_RC: 11622 … 12564; Length: 942 bp
Sequence:
TTAATCGAGTGATTCAGGATTGTAGAATCTACGATTTTCAAGACCAAATATTTTATCTGTA
AACTGACCCTTGTCAGTTTTTTTATATGCCTTTTCAAACATATTCATTCTAGCATCGATATT
ATCGATATAGCATAAAATTTCTGCTTCTTTTAAGTATGGCAGTTTTGGCGAACCATACTCT
AACTTACCATGATGAGATAAAATCATATGTCTTAACAACATGATTTCTTCTCCTTCAATGT
TCAATTCACGAGCTGCTTCAACTACTTCATCACTCGCAATCGAGATGTGTCCTAATAAGTT
ACCTTCGACTGTATACGACGTCGCAACAGGACCACTCAATTCTCTAACTTTACCAATATCA
TGCAAAATAATACCACTATATAACAAACTTTTGTTTAACAATGGATAAATGTCACAAATT
GATTTTGCAATACGTAACATCGTTAATACATGATAGCTTAAGCCACTCGCAAAGTTATGA
TGATGAGAACTAGCAGCTGGATATGTGTAAAATCGTTCTTGATATTTTTTCAATAAATGAC
GTGTGATACGTTGTAAATTAGCATTTTCAATATCTAGCAAATAATGAGAAATCTCTTCTTG
TATTTCTGCCGGTGATAAAGGTGCACCATCTACAAATTGTTCTGTTTTTAATTGATCTTCA
GTTGTCGCTAGTCTAATTTGGTTGACTTTCATCTGTTTATTTCCGCGATAGTTTATGATGTC
ACCTTTAACATGTACAATTTCTTCAGGCTTGATTGTTGCCATATCATTTTTTGTAGCCGTCC
AAAATTTCGCTTCAATTTCACCACTTTTATCTTGCAAATGTAATGTCATATAATCTTTACCT
TGTGCTGTTACACCCTGTGTAGCTTTATGCACTAAGAAAAAGTGATCAACTGAATCTCCG
GGATTTAGATTCTCTATATTTCTCAT

## > **Q5HET2**

Function: protein coding sequence; DNA double-strand break repair rad50 ATPase (core genome, constant)
Best match: Q5HET2_CC005_04-02981_CP001844.2[1927769:1930705:r]RC
Position: 046-contig_53_RC: 12560 … 15497; Length: 2937 bp
Sequence:
TCATCGTTTCCCGCCTTCCTCTATTTTGTTTAATGTAATCACTTCTTTTGATGGAACAATAT
TATCTTTTACACATGTAAAGTATAGTACTTGATAGTGTTCTGATAATGATCGTAAATAATT
CAACATTTTTTCAGTACGCTTTTTATCAAAATGAACAAATGCATCATCAACAATTAATGGG
AACGGATAATATGGTCTTAGTACCTTAATTAAACTGATACGTAAAGCTACATAAAGTAAT
TCTTTTGTAGATTGACTTAGTTCAACAGGATCATATAATTGACCATTAACATGTTTAACCG
TAATTGAATCTTCATTATAGTTAATCATCGTATATCTGCCATCTGTTAAATGCTTCAATATT
TCTACCGCTTCATTAATAACTTGAGGCAAACGTTTATCTTTAATTTGTTTAATGTGTTCATC
AACTAAACTTTGTAAATAACTTAAACTTGCCCAATCTTTTGCGATATCATTAAGTTGATTT
TTAAGACTGTGATATTCATGTCTTAAATTAGCAAGCGTTGTATCAGTTTCCATGTGATTGA
TTTGTGCACTTAAATCACTGACTTGTGCTTGCATTTCAAGATATTGCTCATTATATTCGTCA
ACTTGAGTAGCCAATAAATGATCTTCTTCTTCAAGTTGTGCAGTTGTTTTTTCACTTAAAC
TAGAACTTAATTCATAAGAATAGTTTTGGTTCTCAAGATATTTAGTTAAATCATTAAAACG
ACTCAAATTACTAGTATAAGTTTGGTAATCTTCATGATGTTGGTAAAAATCTTCTTCAGTA
CCAACATTGATAAAATCGAATAGTGCTGTAATTTCTTTATTATTTTCTTCTAATTGAGCAT
TTAAATGATTTAATTCATTTGTAACAAGTTTGGTATTTTCAGCATTAATACGCCATTTTTCA
TTCGTGTCTTCAGCTGATTTCAACCATTGTTGCACATCGTGGAATAAAGATAATTTGTTGA
AATAAACAAATTGTGATTTTGTAACAGCTTCAGCATGATTGTAGAATGTATCTAATTCTTG
AACCAATTGCTGGCGTTGTTGATTTAAATCACTGATATGTTGATCTAATGCTTTAATATTC

GCCATTGTAGAAATACTATCAACAATTAAATCATTTGAAATTTTAGATGATAAGTATAAT
TCATCCTTAACGTTTTCAACTGTCGATTGTAATTCATCATGACGCCCTTTCGCATCATTTAA
ACGACCTTCAATATACTGACGTTTCTCTTCTAAAATATCTTTATTTTTCAATGCCTGTTGCC
AGTGATCACGAATGCGATATTGCTCATCAAGATCAAAATCTAAGTCATAGTTTTCATCTA
AAATGGCTAGTTGTGCTTTAATTTCTTCGATTTCATCTGTGATGGCCTCGCTATAATCTACT
TCTTTTGATTTAGACATGATGATACCGATAACAAATACTAAAGTTAATACTGCGAAAATA
ATACCAAACAGCATGTTGTTTGAAATAAATGAGAAGGCAGTTAAACCAATACCTACTAAT
GTTAAAAGAATAAACGTTGTTCGTAACAATTTTTGACGTTTTTGTTTTTCTTGTTGCTCAAT
TTCGAAACGTTCTTTCAATTTGCTATACAAGTTCTCTTTTTCGTTTAATTCAATGACTTGTT
GTGAGTATTCTTTTTTCTTTTCAAAAGTCTCTTCAGGAACTATTTTTTCTTCAACAGAATCT
AGTTCGCTATGAACCGCATTATCTTCGATTTTATTTTCTTCTAAACTACGTTCTAATTGTTT
AATGTATGCAGCTTGTTCTTGTTTATTCTTGATTTGCTCACTGACATAACTTTTCATTGCCT
CTGAACTATCTACGTCATGATGCGTTTCAGACCAACCAATATTTGCTTGCAATTCATCTTT
ATCACGTTGTTTATTCGCAATATCCTTTTCGATTGCAGTAAGTTCAAATTCTTTATTTTTAA
TTTCATTTTCTTGTTGATTCAAACTAATAAAGGCGTCAATATCAGATTGCTTAACTGGCTC
TAATTGAGTCGCTTCTTCTTTAAGTTGAGCTAAACGCTCATTTCTTAAACCAATATCTCTTT
CTAACGATTGCTTATGCGCTCGTGCTTTTTCGTAACGATCCACACCTTTTTCTGGGAATGT
GATTGGCTCAATATTTAACTGTTGTTCTAGAGACTTCCATTCTTGTGAATGATCATGTAAA
GCAACCTCTTTTTGTTTTTCTTCATGCATTTTTGATAATTGATTTAAATTATGCTTTAAATT
CTCTAATCGACGTGATGATTTATCACGATCATCTACTAAGCGATGATATGTTTCTAGTTTA
GCTTCTTCTTCACGAATTTGACTTTCTAGTTGTTTTAATTGCTCAATTTGTTGATTAATGAT
CGGATTTTTACCTGATTTTTTATATAATTCATCTTTTTTACGATTAATCACTTCGCGCATTG
ACGTGAATTCAGTTGATCCTAAAGCCCCTGCTTGTAATAAATAATCTTGCAATTGTTTTTC
ATTTAGATTTCTATGAATGTCTTGAAGCCCTAGTACATCAAATGAAAAGATACCTTGATAT
GTCTTTTTAGAAATATAATTAAGTTTCTTTTGTAACCAAGCATCATCACGCACAGCACCAT
TAGGTAAATATACTTTCACATCACCTTGAGCACTGCCTTTAATTCGTTCAACTTCAATCTC
TAAGCCATCATCAAGAATAAGTACTAATTTACCACCGTATTGGTTACCTAGACGTGGTTCT
AGTCTTGGCTCTTTAGACTTTTTAGTTGGAAAACCAAATAATATCGAATGGATGAATGCTT
GAATCGTCGATTTACCCGCTTCATTTTCACCAAAAAATTTCAGTGAAGTTTTTATTAAATTC
AATTTTACGTTGAACAAATTGACCGTAACCATAAATTTCAAGTGATTTAATTATCAT

## > **Q5HET1**

Function: protein coding sequence; DNA repair exonuclease (core genome, constant)
Best match: Q5HET1_CC001_MW2-USA400_BA000033.2[1939678:1940874:r]RC
Position: 046-contig_53_RC: 15486 … 16683; Length: 1197 bp
Sequence:
TTAATTATCATTTTGTTCACCTCTCATTTCAGCTTTTAATATTTCTTCAGCACGATTAACTA
ATGCTGTATGGTCGAATGTTCCATAATCGTCTAGGAACTTTGATGCCCTTGGATTTAAATA
TAAATCTGACATCGCTTTATCAAAAACAGTTTGATCGACTAATAATTCCGCTGAAAATTC
ATTAACTAAAGGTGACTCATCATTTTGTGCATATTGTATTTTTAACTCATCAATATATACA
AATTGATTTTCGTTTTCTTCATAATCTGTAATCATTTCTTCAACTTGTAATAAATCTTGAGG
TGAAATTAATGTCTCACTATTAATAACAAGCGTTAAACGATAAAAGGCTTTTCCTTCTTCT
CTCACTTGTTCTTTAAAGTTTTGAATGACCTCGTATAAACCTTGCTTAGATGTCTTATCCGT
TTCAATAGTTGCTTCTTCAAATCTAATATACTGTGTAGGATAAAATTTAGTCTTTAATTTT
AAGTGGTCACCCTCGATTAATAAGCAACCTTTTTCACCTTGCTCATTAAAATGTCTACCTT
GAATATTACCTGAATAGTTAATTACAGGCATATCACTTAATTGTTGACGTTCATGTATATG
ACCTAAAGCCCAATAATGATACAATTTACTGTTTAAATCTTCTAAAATGAACTCGGTATAT
CTTTCGTTAACTGAAGATTTACTATACGTACCATGCAAGACACCAATATGTATGCCTTTTT
GGCCTTGACTTGATGGATATTCATCAATCTTGTTCTCATAACTTGCTCTATTTTCATAACTA
AATCCGTGAATATAAATTGTTTCACCAGATTTAGTAATTGCTTCATACGTCTCAACTTTAT
TTGAAAATACAGAAACATTATCTGGCCAGTTTGATGAAATCTTTGATGATAATGGGTCGT
GATTACCATGACAAACATAAACAAAGATTTGTTCATTTTGTAAACGTTCAAATTGCTGTTT
TAAGAAAATTTCAGCACGTAATGTTCTGTTTTCACTATCAAATAAATCACCCGCGATAATT
ACAAAATCAACATCTTGTTGTAATGCAATATCTACAATATTTTTAAAACTTTCATAAGCAC
TTTTTTGAACATCCTCAAAAATTTTAGGGCTTATATGACTCTTAGATTTGAAAGGACTATC
TAAATGTAAATCCGAACAATGAATAAATTTAACCAT

> **tx_SACOL1902_1**
Function:  rho independent terminator
Best match: tx_SACOL1902_1_CC001_MW2-USA400_BA000033.2[1941460:1941522:r]RC (completely identical)
Position: 046-contig_53_RC: 17268 … 17331; Length: 63 bp
Sequence:
TTCAAAATGCAAAAAGGAAGTTAGGACACGTTTACTAGCCTAACTTCCGTTATCAAGCTC
ATC

> **tx_SACOL1902_2**
Function:  rho independent terminator
Best match: tx_SACOL1902_2_CC001_MW2-USA400_BA000033.2[1941668:1941728:r]RC (completely identical)
Position: 046-contig_53_RC: 17632 … 17693; Length: 61 bp
Sequence:
TCAAAACTAAAAAAACTACCCACAAAGTCTCGGACTCTGTCGGCAGTCTATTCCTCTATTT

> **xdrA**
Function: protein coding sequence; DNA binding helix-turn-helix protein (core genome, constant)
Best match: xdrA_CC001_MW2-USA400_BA000033.2[1943472:1943936:r]RC (completely identical)
Position: 046-contig_53_RC: 19436 … 19901; Length: 465 bp
Sequence:
CTAGATATGTACTAATTCTTCTTTAGCATTTCTGTTAAAGTATGTTTCAGCTTCTCTCATTT
TAGAAGTACCGAAAATTGGTTGGTTATCTGGATTTAATACACGATAAATATTGCTTACTTT
GTTACTTTGTAAAATGTACCCATTACGAGTCTCAATGTTATTCCAATAAATATCACTTGTT
GGTGCATGGTTTGGATATGCACAGTGATTTCTTGAAATTGTTTGAACGATTTCTAATGGAT
CACAGCCAAATGTACTGTTTAATACTTCAGAGTCTCTGAATAATGCACAAATTGAAATAC
AAGTTGTCCAGTTTGGTAATACTCTCTCTTTTTCGATTTGTACTAAAGTCTTTTTAGAAAGT
CCAATTGTTTGCGCCATAGTATCTTGCGTATAACCAGCCTCTATACGAACCATTTTAAATT
TTGTTTGAATTAAATCTGTAAAACTCTGTCTATCCAT

> **yhcR**
Function: protein coding sequence; DNA-binding response regulator (core genome, constant)
Best match: yhcR_CC001_MW2-USA400_BA000033.2[1944292:1944915:r]RC
Position: 046-contig_53_RC: 20256 … 20880; Length: 624 bp
Sequence:
CTAAATCAACTTATTTTCCATTGCATAAATTGCTGCTTGTGTACGATCGCTAACTTGTAAT
TTACTAAATATATGACTGACATGTGTTTTAATTGTTTTTTCAGATACAAATAAAGTTTCTG
CAATCTCTTTATTTGTTTTACCTTTAACCATTTCACGTAACACTTCAATTTCTCTCTTTGAC
AACTTATTCGTGTAGTGTGGTTTTTGGCTAACTGTTTCGAATACATCTTGTGCCTTAGGAT
GTATCATTTTTTCACCGTTCATAACTCGTCTAATAGTTTCAATTAATTGCTGAGGCTCAAC
GTCTTTCATTTCATAACCATCAGCACCTTTATTAATTGCTGAAATTACATGTTCATCATCA
ACATAACTTGTTAATACCAAAACTTTAATATCCGGATAATGTGCCTTAATATATTCCGTAA
TTTCAATACCATTCATGCCAGGCATCACTAAATCTAATAGCACAATATCAGGGTGCTCAT
GCTCTTTTAAATATTCTAAAAATGTTTCTCCATCTGCAAAGTCTTGTAAAACTTCTATGTTT
TCAATCGTGGATAATAAAAATCGCAATCCTTGTCGCACAATATAATGGTCATCTACTAAT
ATTACTTTGTTCAT

> **yhcS**
Function: protein coding sequence; sensor histidine kinase (core genome, variable)
Best match: yhcS_CC008_NCTC8325_CP000253.1[1885345:1886457:r]RC (completely identical)
Position: 046-contig_53_RC: 20901 … 22014; Length: 1113 bp
Sequence:

CTATTTTATAGGAATTGTGAATTGTATTTGTGTACCCTTTGTTGGCTGAGAATGAAAGGTC
ACTTTACCTCTTAATAATTTAACTCTTTGTTTTATGTTATTAATACCGTGTGATGAAGCTAT
CTGAACATTATCGATCTCAAATCCTTGACCATAATCAATCACGTCAATATATAGTATATCG
TTCATTTGTTTTAATGTAAGATCCATTTTATTCGTATCAGCATGTTTCTTAACATTATTAAT
ACACTCTTGTAATGCTCTGTATATGTTTTCTTCGATTTCATTAGATAAATCGATTAAACCTT
CTACATTTACATTTAATTGTATATGCATTAATTTACTATATGCTGTCAAAGCATGAATTAA
ACCTTGCTCAAGTCCAACTGGCTTAAGTTGCCAAATCAATGCACGCATTTCATTAACGGC
ATTTTGACTCGTTTCCTCAATCGTCTTGAATGCTTGTTTAGCGATGGATTCGTTTGACATGC
CATACGCAGCATGTGCTGTTAGTTTTACAGAAAATAACATTTGATTTACTGAATCATGTAA
ATCTCTAGCTAAACGATTACGTTCATTAATTTTTGCCGCTTCTTTTTCACGGTCTGTTAAAT
AAATACGTTTGATGGCTGACCCTAATTGAAATGCGACAGACTCTAGCAACTCTAAATCTT
CATCGCTATATATTTCAGTATTTGGAGACGCTACATTTAAAATGCCGAATTGTTCTTGACC
CGATTTAAGTGGTACCGTTGCATGATGTGTAATATTGTCATTTTGGCTAGGAAATGCTTTA
GAGGCTAAGTTAATACGAGAACAATTGACGATATTCGACGCTTTCATTAGCCTACGTTGA
TTAAATGCTTTCACACACCAACAAGACCCATCTTTAATATAGTGACAGTGGTCTGCTGTCA
AAGATTGTGGTAGAGCCACATGTGATACAAGTTCATGTTCACCTACGCTATTGATGAAAA
ATATCCAGCCTGTCGTGAAATTACTGCCCTCAATTAAATATTTTAACGCACCTTGGGTCAT
GCTATACATTTCAGTTTCTTCGTTTAAAAATTCGGCAATCTCTTTTAATAAAGCTAGTCGC
GTCCTTTGTTCCAT

> **rluA2**
Function: protein coding sequence; RNA pseudouridylate synthase (core genome, constant)
Best match: rluA2_CC005_Mu50-VRSA_BA000017.4[1982040:1982861] (completely identical)
Position: 046-contig_53_RC: 22176 … 22998; Length: 822 bp
Sequence:
ATGAAATTTAAAATACCAGAAAACTTTAATGACTTAAGTTTACGAGATATTTTCCAACAA
CTTAAGGTACCTAAAAAAGATTTACATCATTTAAATATGTCTAAAGATATTACTATTAATG
ATAAACCTGCGCGATTAATGGATAAAGTGCATACTGGCGACGATGTATTTGTTCCAACCA
TCGATGAAAAAAGTAATTATGTTCCAAGTTATCGTTATGCACAAATTAAATACGAAGACG
ATGATATGGCAATCGTAATGAAACCTAAAGGTGTTAAGACTCACCCTAATGATTTAAAAG
AAAGCAATACTTTAATGAATCATGTGATTTACACTATTGATAGTGACTATGTCGAACCAA
TTCATCGACTGGACCAGGAAACAGTAGGATTATTAATTGTTGCTAAAAATCCTTTAATGA
AAAAAATTCTTGATCGCATGTTAGAAGACAATGATATTACGCGGATATACAAAGCAAATG
TTAAGGCACTTTTACCTTTAAAACCACAAACGATTGATATGCCAATTGGTAAAGATAAAT
TCCATTCGAATAAACGACGTGTGTCTCCTACTGGACAGCGTGCAATTACACACATTTTAA
CTTCAAAAATGATAAAAGAAGCTGTGTGCCAACTTGAAATCAAGTTGGATACTGGACGTA
CTCATCAAATTCGTGTGCATTTAGCTGAAATTGGTCACCCTGTTATTGGTGATCCTTTATA
TGGTGATTCAACGTTAAGACAATTAGAACTTGAAAGTTACAAAATAGAGTTTGTGCATCC
CTTGACTAAGGAAGTCATTTCCGTTTCTTTGGATGACTAA

> **fumC**
Function: protein coding sequence; fumarate hydratase class II (core genome, variable)
Best match: fumC_CC008_NCTC8325_CP000253.1[1887904:1889289:r]RC
Position: 046-contig_53_RC: 23456 … 24842; Length: 1386 bp
Sequence:
TTAATGAGGATCTACCATATCTTCTGGTTTAATCCATGCTTCAAATTGTTCTTCTGTAACAT
ATCCAGTTTGAATTGCAGATTCTTTTAAAGTTAAACCTTCTTTATGGGCTTTCTTAGCAATT
TGAGCTGCTTTTTCATAACCAATATGTGGATTTAATGCAGTAACTAACATTAATGATTGAT
TTAAATAATTATCAATATTCTCTTCGATTGGTTCAATGCCCACTGCACAATTGTTATTAAA
TGTTTCCATACCATCAGCTAAAAGGTAAATTGATTGTAGTGTATTATGCATAATAACTGGT
TTATAAACATTCAATTCAAAGTTACCTTGTGAACTTGCGAAGCCAACAACTGTATCATTAC
CCATTACTTGGACTGCAACCATTGTTAACATTTCACATTGTGTAGGATTAACTTTACCAGG
CATAATTGATGAACCTGGTTCATTTTCAGGGATGGAAATTTCTGCCAAACCAGCTCGTGG
CCCTGAAGCCAACCATCTCACATCATTAGCAATTTTCATTAAGTCTCCTGCTAGCGCCTTC
AATGTTCCATGTAGTTGTACTACTTCATCATGTGCTGTTAATGCATGGAATTTATTTTCAG

AAGATACAAATGGATAACCCGTATTTTCTGAAATATAATGTGCCACTTTATCACCAAATT
CAGGATGCGCATTAATACCAGTACCAACAGCCGTACCACCGATGGCAAGATTTAAAATGT
GCTTCTTAGATTCAGATAACATTATTTCGCAACGGTCAAGCATATAACGCCAGCCACTAA
TCTCTTGTCCTAGTTTGATCGGCGTTGCATCTTGTAAATGTGTACGACCAATTTTAATAAT
TGAATCAAATTTGTCTTCTTTTTCTTTCAAAGTATTTCTTAAAAGTTTTAATGCAGGTTCTA
ATTTTGTTTCAACCTCTTGATATAATGCAACGTGCATAGCAGTTGGGAATGTATCATTCGA
ACTTTGTGATTTATTTACATCATCATTTGGGTGGATACTTTCATCAATTTGATGATCTTTTA
AATACATATTAGCAACATAACTTACTACTTCGTTCACATTCATATTACTTTGTGTACCGCT
TCCTGTTTGCCATACAACTAGTGGGAAGTGTTCATCTAATTCACCTGATAAAATTTGATCA
CATGCGTATACAATGGCATCTTTCTTTGCCTCGCTTAATTTTCCTAAATCAAAATTAGCTA
ATGCTGCTGCACGCTTTAGTTGTGCAAAACCATAAACTACTTCGATTGGCATACGCTCTTT
ACCAACTGGGAAATTACGTTTACTTCTTTCTGTTTGAGCACCCCAATATTTATCTGCAGGT
ACTTCTATTTCTCCAAAAGTATCATGTTCAATTCTTACTGACAT

> **glnQ**

Function: protein coding sequence; glutamine transport ATP-binding protein (core genome, variable)
Best match: glnQ_CC001_MW2-USA400_BA000033.2[1952764:1953492:r]RC (completely identical)
Position: 046-contig_53_RC: 29011 … 29740; Length: 729 bp
Sequence:
TTATAATACTCTTGCTAAGAAATTTTGTGTTCTTTCATGTTGCGGTTGTTCAAATATTTCGA
CTGGTGTGCCTGACTCTACGACAACGCCATCTGCCATAAATATGACTTTGTCACTTACATC
TTTGGCAAATCCCATTTCATGTGTCACAACCACCATGGTCATACCTTCTTTGGCTAGGTCT
TTCATTACTTTTAATACATCACCAACTACCTCAGGATCTAATGCTGAAGTTGGTTCATCGA
ATAAAATAACATCTGGATGCATTGCTAAAGCTCTTGCAATTGCTACCCTTTGCTTTTGACC
ACCTGATAATTGATTCGGATATACATCTGCTTTTTCTTTTAATCCCACTTTATCTAATAACG
ACAATGCTTCCTTATGTAATTCATCGTTATTATCTTTCTTTAATAATTTAGGAGCTAAAAT
AATATTATCGACAACTTTTTTATGTGGAAATAGGTTGAAGTTTTGAAATACCATACCCATT
TTTTGACGTAGTTTATCTACTTGTGTCCCTTTTTCCGTTAAGTCATTGCCTTCAAAAATCAC
TTGACCTTTAGTGGGTACTTCTAATAAATTCATACATCTTAACAATGTACTTTTACCACTA
CCAGATGGACCTATTATTGCTACTACTTCCCCTTGATTGATTTCAAGATTGATATCTTTTA
AAACTTCATTATCTCCAAAAACTTTATTAAGATTGTTTATTTTAATCACTGGCAT

> **Q1Y9N4**

Function: protein coding sequence; phosphotransferase system (PTS)/EIIC domain protein (core genome, constant)
Best match: Q1Y9N4_CC001-ST772_118_AJGE01000032.1[502:1563]RC (completely identical)
Position: 046-contig_53_RC: 31442 … 32504; Length: 1062 bp
Sequence:
TTATTGTTTTGGAACTTCAAATTTATAAATATCATTAGAATATATAGGAATGAATTTTTTA
CACAATTCATGAACAATAAATGCCGATACAAATGGTATGACAAAATAACCAAGTGATAA
TAAAATAATATTCATTGTTGGATCACCTGACATTCGGTTAAATGCATTTATTGGGCCAACT
AATCCTGTATATCCAAATCCAGCAGACAATGGTGTTCCTTTAACCTGAAGAACGTAAGCA
ATGATTCCAGTAATTATTCCATTTATAGTCAATGGTATCGAAATAATTAAATTTTTCAAGT
AAACTGGGATCATCATTTTTGCTGCTCCTATGAGTAAAACTGCATTTACACCAATAGAATT
GACACGCAATGAGCCAAATAAGAAAGTAACACAGGCAGCCACTATACCTAGGTTTGCTG
CTCCACTTCCTAGTCCGTTTAAACTAATCGCAGTTGCAATCGCTACTAACGATATTGGTGT
TACCATTAATAATGAAAATGCCACACTAATAAGTATAGACATTAACAACGGATTTAAGTC
TGTAAAAGAATGAATTACATTTCCAATTGCTTGAGTAATTTTTCGAATGTAAGGTAATGTG
ATTAGACCGATACCCCCACTAACGATAGGTACTAAAACTGGTAATATAATTAATTCAAAA
GATCCAAGTTTGTTTTGTAATACCATATATATAAGACATGCAATAATAACAACCAAACTC
GTATTTATAATGTCACCTATACCTTTTAACATAAAACTATTATTGCTATATACAACAGCAC
CTGAACCAATCATAGCTGATGTACCTACTATAGCAGCACCTGCGCCATTAAATTTAAATT
GATGAGCAGCTAAAACCCCAATAATAAATGCCATAAATGATTGAATTAGTATCACTAACT
GATACGTTAATTCTAAAATTTCATTACCACTTTTAAATATTTTTAATACTTCACCTAATAA

AGCATTCGGAACAAGTGCAATAACAACACCAGCACCAATAGAATTTAAAATCTTACTGA
AAAACTGTTTATTATCAGCATTCTTTGCGTTACTCAT

> **tx_tRNAleu1**
Function:  rho independent terminator
Best match: tx_tRNAleu1_1_CC030_MRSA252_BX571856.1[2033795:2033847:r]RC (completely identical)
Position: 046-contig_53_RC: 32987 … 33040; Length: 53 bp
Sequence:
AGAGAGAAATAAAAAGAAACCTTGTTTTACAAGGTTTCTAGTACGTTATGTTA

> **tRNAleu1**
Function: tRNA tRNA leucine
Best match: tRNAleu1_CC001_MW2-USA400_BA000033.2[1956800:1956886:r]RC (completely identical)
Position: 046-contig_53_RC: 33056 … 33143; Length: 87 bp
Sequence:
TTATACCGGTGGTCGGGGTCGAACCGACACTCCACAAGTGGAACGGGATTTTGAGTCCCG
CGCGTCTGCCAATTCCGCCACACCGGC

> **tRNAgly2**
Function: tRNA tRNA glycin
Best match: tRNAgly2_CC001_MW2-USA400_BA000033.2[1956925:1956999:r]RC (completely identical)
Position: 046-contig_53_RC: 33181 … 33256; Length: 75 bp
Sequence:
TGGAGCGGAAGATAGGATTTACACCTATACCTCGTTCCGGGAAGGAACGTGTTCTAAAAG
TTGAACTACTCCCGC

> **tRNAcys**
Function: tRNA tRNA cysteine
Best match: tRNAcys_CC001_MW2-USA400_BA000033.2[1957185:1957258:r]RC (completely identical)
Position: 046-contig_53_RC: 33441 … 33515; Length: 74 bp
Sequence:
TGGAGGCGGCAACCGGATTTGAACCGGTGATAAAGGTTTTGCAGACCTCTGCCTTACCAC
TTGGCTATGCCGCC

> **tRNAtrp**
Function: tRNA tRNA tryptophan
Best match: tRNAtrp_CC001_MW2-USA400_BA000033.2[1957422:1957495:r]RC
Position: 047-contig_203: 50 … 80; Length: 30 bp
Sequence:
ACCTCTATTCTACCGTTGAACTATGCCCCT

> **tRNAtyr**
Function: tRNA tRNA tyrosine
Best match: tRNAtyr_CC001_MW2-USA400_BA000033.2[1957508:1957591:r]RC (completely identical)
Position: 047-contig_203: 92 … 176; Length: 84 bp
Sequence:
AAATGGAGGGGGGCAGATTCGAACTGCCGAACCCGAAGGAGCGGATTTACAGTCCGCCG
CGTTTAGCCACTTCGCTACCCCTCC

## > tRNAthr
Function: tRNA tRNA tryptophan
Best match: tRNAthr_CC001_MW2-USA400_BA000033.2[1957597:1957672:r]RC (completely identical)
Position: 047-contig_203: 181 … 257; Length: 76 bp
Sequence:
TGGTGCCGGCCAGAGGACTTGAACCCCCAACCTACTGATTACAAGTCAGTTGCTCTACCA
ATTGAGCTAGGCCGGC

## > tRNAphe
Function: tRNA tRNA phenylalanin
Best match: tRNAphe_CC001_MW2-USA400_BA000033.2[1957677:1957752:r]RC (completely identical)
Position: 047-contig_203: 261 … 337; Length: 76 bp
Sequence:
AAATGGTTCAGGACAGAGTCGAACTGCCGACACATGGAGCTTCAATCCATTGCTCTACCA
ACTGAGCTACTGAACC

## > tRNAasp
Function: tRNA tRNA asparagine
Best match: tRNAasp_CC001_MW2-USA400_BA000033.2[1924369:1924444:r]RC (completely identical)
Position: 047-contig_203: 355 … 431; Length: 76 bp
Sequence:
TGGCGGTCTCGACGGGAATCGAACCCGCGATCTCCTGCGTGACAGGCAGGCGTGTTAACC
GCTACACTACGAGACC

## > tRNAmet_ini
Function: tRNA tRNA methionine initiator
Best match: tRNAmet_ini_CC001_MW2-USA400_BA000033.2[1924466:1924539:r]RC (completely identical)
Position: 047-contig_203: 440 … 514; Length: 74 bp
Sequence:
TTGCGGGAGGCGGATTTGAACCACCGACCTTCGGGTTATGAGCCCGACGAGCTACCGAAC
TGCTCCATCCCGCG

## > tRNAser1
Function: tRNA tRNA serine
Best match: tRNAser1_CC001_MW2-USA400_BA000033.2[1957944:1958036:r]RC
Position: 047-contig_203: 528 … 558; Length: 30 bp
Sequence:
TGGCGGAGGAAGAGGGATTCGAACCCCCGC

## > tRNAile
Function: tRNA tRNA isoleucin
Best match: tRNAile_k2CAT_CC001_MW2-USA400_BA000033.2[1958257:1958333:r]RC (completely identical)
Position: 048-contig_124_RC: 14 … 91; Length: 77 bp
Sequence:
ATATGGACCTTGCAGGACTCGAACCTGCGACCGAACGGTTATGAGCCGTTAGCTCTAACC
AACTGAGCTAAAGGTCC

## > tRNAmet_elo
Function: tRNA tRNA methionine

Best match: tRNAmet_elo_CC001_MW2-USA400_BA000033.2[1958359:1958435:r]RC (completely identical)
Position: 048-contig_124_RC: 116 … 193; Length: 77 bp
Sequence:
TAGTGGCGGTGGAGGGGATCGAACCCCCGACCTCACGGGTATGAACCGTACGCTCTAGCCAGCTGAGCTACACCGCC

> **tRNAala**
Function: tRNA tRNA-Ala
Best match: tRNAala_CC001_MW2-USA400_BA000033.2[1958457:1958532:r]RC (completely identical)
Position: 048-contig_124_RC: 214 … 290; Length: 76 bp
Sequence:
TGGTGGAGACTAGCGGGATCGAACCGCTGACCTCCTGCGTGCAAAGCAGGCGCTCTCCCAGCTGAGCTAAGCCCCC

> **tRNApro**
Function: tRNA tRNA prolin
Best match: tRNApro_CC001_MW2-USA400_BA000033.2[1958547:1958623:r]RC (completely identical)
Position: 048-contig_124_RC: 304 … 381; Length: 77 bp
Sequence:
ATATCGGGAAGACAGGATTCGAACCTGCGACCCCTTGGTCCCAAACCAAGTGCTCTACCAAGCTGAGCTACTTCCCG

> **tRNAarg**
Function: tRNA tRNA-Arg (core genome, constant)
Best match: tRNAarg_ICG_CC001_MW2-USA400_BA000033.2[1958630:1958706:r]RC (completely identical)
Position: 048-contig_124_RC: 387 … 464; Length: 77 bp
Sequence:
TAACGCGCCCGATAGGAGTCGAACCCATAACCTCTTGATCCGTAGTCAAACGCTCTATCCAATTGAGCTACGGGCGC

> **tRNAleu2**
Function: tRNA tRNA leucine
Best match: tRNAleu2_CC001_MW2-USA400_BA000033.2[1958725:1958813:r]RC (completely identical)
Position: 048-contig_124_RC: 482 … 571; Length: 89 bp
Sequence:
TGGTGCCGAGGACCGGAATCGAACCGGTACGGTGATCACTCACCGCAGGATTTTAAGTCCTGTGCGTCTGCCAGTTCCGCCACCCCGGC

> **tRNAgly3**
Function: tRNA tRNA glycin
Best match: tRNAgly3_CC001_MW2-USA400_BA000033.2[1958824:1958898:r]RC (completely identical)
Position: 048-contig_124_RC: 581 … 656; Length: 75 bp
Sequence:
TGGAGCAGAAGACGGGATTCGAACCCGCGACCCCAACCTTGGCAAGGTTGTATTCTACCGCTGAACTACTTCTGC

> **tRNAleu3**
Function: tRNA tRNA leucine

Best match: tRNAleu3_CC001_MW2-USA400_BA000033.2[1958899:1958983:r]RC (completely identical)
Position: 048-contig_124_RC: 656 … 741; Length: 85 bp
Sequence:
ATATGCGGGTGAAGGGAGTCGAACCCCCACGCCGTAAGGCGCTAGATCCTAAGTCTAGT
GCGTCTGCCAATTCCGCCACACCCGC

## > tRNAlys

Function: tRNA tRNA lysine
Best match: tRNAlys_CC001_MW2-USA400_BA000033.21958987:1959062:rRC
Position: 048-contig_124_RC: 744 … 820; Length: 76 bp
Sequence:
TGGTGAGCCATAGAGGATTTGAACCTCTGACCCTCTGATTAAAAGTCAGATGCTCTACCA
ACTGAGCTAATGGCTC

## > tx_perR

Function: rho independent terminator of perR
Best match: tx_perR_CC005_Mu50-VRSA_BA000017.4[2002305:2002361:r]RC (completely identical)
Position: 049-contig_251_RC: 623 … 680; Length: 57 bp
Sequence:
TAAGCATAAAAAAGAAGTTAAACAACTTTCATGTCGTTTAACTTCTTAAATATTACA

## > perR

Function: protein coding sequence; repressor of peroxide operon
Best match: perR_CC001_MW2-USA400_BA000033.2[1965203:1965649:r]RC (completely identical)
Position: 049-contig_251_RC: 737 … 1184; Length: 447 bp
Sequence:
TTATTTATCTTGGCATTCTTTACAAACTCCATAAATTTCCATTCGATGATGTGTTACGTCAA
AGTCAGTCATATGCTGAGCTAATCTTTCAATTTCATTTAACTGTGGATATTGAAAATCAAC
AATCTTACCACATTGTTCACATATAATATGATAATGATTATGTGTATTAAAGTCGAATCGA
CTTGATGAGTCTCCATATGTTAATTCTTTTACAATTCCAATATCTTTAAACACTCTTAAGTT
ATTATATATTGTCGCAACACTTATATTTGGAAAATCAGGTGAAAGTGCTTGATAAATTTCA
TCAGCTGTTGGATGAGTATGTGAAGAAATTAAATAACGTAATATTGCTTGTCTTTGAGGT
GTAATTCTTACGCCTGCTTGTCGCAATGATGCAATTGATTCTTCTAGTTCATGTTCAATTG
ATTCTATTTCAACACTCAT

## > bcp

Function: protein coding sequence; bacterioferritin comigratory protein (core genome, constant)
Best match: bcp_CC001-ST772_118_AJGE01000047.1[2167:2622:r]RC (completely identical)
Position: 049-contig_251_RC: 2236 … 2692; Length: 456 bp
Sequence:
TCACCCCAAAATGTTTTTAAGTTCTTCTATTTGTGTTTTAACCTTAACCTTCTCGATAACAT
CTAATACTTTACCTTGTTCATCTATTATAAAAGTCGTTCTTACAATGCCCATACTTTCTTTG
CCAAATGATTTTTTCAACTGATATACGCCAGTTTCTTTAGCTAATTTAAAATCTTCATCTA
CTAATAAATCGAAATTCAATCCGTGTTTCTCAATAAAATTTTGGTGTTTTTTCTTTGAATC
ACCGCTTATACCATATACTGCAACATCTAAATCATTGAACATTTCTAAATTGTCTCTAAAG
TCACAAGCTTCTGTGGTACAAGTAGGTGTATTATCTCTAGGATAAAAATATATAATCGCC
TTTTTACCTTTTAATGTATCATTTGTAATGACAGTTCCGTCTTGATTTTCTAATTTAAATAT
TGGAAATTGTTCTCCTTTTTGCAACAT

## > hemL2

Function: protein coding sequence; glutamate-1-semialdehyde 2,1-aminomutase/locus 2
Best match: hemL2_CC001_MW2-USA400_BA000033.2[1967238:1968527]

Position: 049-contig_251_RC: 2772 … 4062; Length: 1290 bp
Sequence:
ATGAATTTTAGTGAAAGTGAACGTTTACAACAACTTTCAAACGAATATATTCTAGGCGGT
GTCAATTCCCCTTCTCGTTCTTATAAAGCTGTAGGAGGCGGTGCACCTGTTGTTATGAAAG
AAGGACACGGTGCATATTTATATGATGTCGATGGCAATAAATTTATTGATTACCTTCAAG
CATACGGTCCAATTATTACGGGGCATGCACATCCTCATATTACTAAAGCAATTCAAGAAC
AAGCAGCCAAAGGTGTTTTATTTGGTACACCGACTGAACTAGAAATTGAATTCAGCAAAA
AATTACGTGATGCAATTCCATCTCTTGAGAAAATTCGCTTTGTAAATTCTGGAACAGAAG
CAGTCATGACAACAATTCGTGTTGCACGTGCATATACTAAAAGAAATAAAATTATAAAAT
TTGCTGGATCTTATCATGGCCATTCTGATTTAGTATTGGTTGCAGCAGGTAGTGGCCCATC
TCAGCTCGGTTCTCCAGACTCAGCTGGTGTTCCAGAAAGCGTCGCACGTGAAGTCATTAC
TGTACCTTTCAATGATATTAACGCATATAAAGAAGCAATTGAATTTTGGGGTGATGAAAT
TGCCGCAGTATTAATAGAACCAATTGTTGGTAACTTTGGAATGGTAATGCCTCAACCTGG
ATTTTTAGAAGAGGTTAATGAAATTTCGCATAACAATGGGACACTAGTGATTTATGATGA
AGTAATTACTGCATTCCGTTTCCATTACGGTGCCGCTCAAGATTTATTAGGTGTTATCCCT
GATTTAACTGCATTTGGTAAAATTGTTGGCGGTGGTTTACCAATTGGAGGCTATGGTGGA
CGTCAAGATATTATGGAACAAGTAGCACCTCTAGGACCTGCATATCAAGCTGGTACAATG
GCTGGTAACCCGTTATCTATGAAAGCAGGTATTGCATTACTCGAAGTACTAGAACAAGAC
GGTGTTTATGAAAAATTAGACAGCTTAGGCCAACAACTAGAAGAAGGTTTACTTAAATTA
ATCGAAAAACATAATATCACAGCTACAATTAATCGTATTTATGGATCTTTAACATTGTACT
TTACAGATGAAAAAGTCACACATTATGATCAAGTTGAACATTCTGACGGCGAAGCGTTCG
GTAAATTTTTCAAATTAATGTTAAATCAAGGTATCAATTTAGCACCTTCTAAGTTTGAAGC
TTGGTTCTTAACAACTGAACATACAGAAGAAGATATTCAACAAACTTTAAAAGCTGCAGA
CTATGCTTTTAGTCAAATGAAATAA

## > Q7A4T3

Function: protein coding sequence; putative ABC transporter ATP-binding protein (core genome, constant)
Best match: Q7A4T3_CC001_MW2-USA400_BA000033.2[1970106:1971842:r]RC
Position: 049-contig_251_RC: 5639 … 7376; Length: 1737 bp
Sequence:
TTATAAGTTTTGAATGCTATATAAATGCTCGTAAGCACCTTGTTTTGCAATCAATTCACGA
TGCGTACCTGTTTCAACAATATGTCCATTTTCAATTACGACAATTTTGTCAGCATGTGTAA
TAGTGGACAAGCGATGCGCTACGATAAGTGTCGTTCGATCTTTACTCAACACATCTAATG
CTTCTTGAATAATGGATTCACTTTCTAAATCAAGTGCACTTGTTGCTTCATCCAAGATAAG
AATTGGCGGATTATTTAAAAATATTCTAGCAATCGATAATCTTTGTTTTTGACCACCTGAT
AATTTAACACCTCGTTCACCTACTTCAGTGTCATATCCCTGTGGCAAGTTCATAATAAAGT
CATGTGCATTAGCCATTTTCGCCGCTTCAACTACTTCTTCATCTGTTGCTGTTGGACGACC
AAGTAAAATATTTTCCTTAACTGTATCAGAGAATAAAATATTATCCTGTTGCACCAATCCT
ATTTGATTTCTTAAACTTCCCGTTAAAAAATCTTTAATATTGTGACCATCTATTAAAATTT
GCCCAGAAGTTACATCGTAAAATCTCGGTATTAAGTTAATTAATGTTGATTTACCACCACC
ACTCATACCTACGAAAGCAACTGTTTCTCCTTTTTCAATACTCAAATTAATATCTTTTAAA
ATTGGATCTTCGTTATCGTTATATTGAAAACTAACATGATCAATATCAATACGACCTTGTT
TAATTTCAATAGGTTGAGCACCAACACCATTTTTAATGTCATAATCTTCGTCAATTAATTG
GAATACACGGTCCATTGAAGCAAAACTTTGCGTTAAAGTTGTAAATGATGCGACTAAACG
ACGTAAAGGCCCGAACAATAACTCTAAGTATCCAACAAATGCTGCAAGTGTACCTACTGT
GATTGATCCAGAAATAGCAAGATATGCACCAACACCGATGACAATAATTGGTCCAATATC
TGTAACTGTATTAATTGCGGCAAAGGAATAGGCATTCCATCTTGTATGTTTCAACGCACGT
GTTAAGAAATTAGTATTCTTTTTATCAAAGTTTTTCGCTTCATTGTCTTCAATCGCAAAACT
TTTAACGACTGAAATACCTTGAACACGTTCATGCAAGAATCCTTGAACCTCAGCTAATGC
TTGAGATCTTTCACGTGTCAATTTTCTTAATCTTCCAAAGAAAACGTACACCGTTAAAATG
TAAAATGGGAAGATAAACAGTGCTGCTAAAGTCAATTTCACATCTAAAAAGAACATTATG
GATAGTGCAATAATAATTGTTATACAATCTAACCAAATATTCATTAACCCGGTTAAAATG
AAATCTTTTGTTTGTTCAACATCATTAATCACTCTAGATATTACTTGACCTACTTGATTATT
AGCATAAAATCTCGCACTTAAAGCTTGTAAATGGTTATATAACTTTTTACGTATATCATAC
AATATTTTATTACTTGTCCATTGCGCCAAATATTGACGTATAAATTCAATTGGTGGTCTCA

CTATTACAAAAATAAATAATGCGATACCAATGGCAATAGTTAAATGATGAACTTTTTCAT
CAGTCGTTAATGCGTGGTTATTAATCACGCCATCTATTGCATATTTAATTAAAAGTGGTAT
AAGCATTGGTATACCAAACTTAATTATTCCAACAATAATCGTTGCAAAAATACGATATTT
GTATGGCTTAACAAATTGCAAATATCGTTTAATCAT

> **mutY**

Function: protein coding sequence; A/G-specific adenine glycosylase (core genome, variable)
Best match: mutY_CC001_MW2-USA400_BA000033.2[1972977:1974014:r]RC
Position: 049-contig_251_RC: 8511 … 9549; Length: 1038 bp
Sequence:
TTACCCATTTATAAATTGATAAATCTTTGACATAGGTACAGGGAATGTATATTGGTCTCGA
TCACTTAAATTAAACCAAATCATGTCATCTGGTAATGTTTCAATGTTAATTGCTCCTGAAA
CGGCGTATACTTTAATCTTCCATGTTAAATGAGTAAATTGATGCTTCAACTCAAAAATAG
GTGTTTCTACTGGTTGAATGTCATGACCGATTTTTTCAGTCATTTTACGTCTAGCATGCTCA
CTTTCAAACATAGGAAATTGCCACATACCATGCAATAATTTTTCGCTACGCTTTTGCAACA
GATATTGACCTTGATTATTTCTAATTAAAAAGACGGATTGCTCAATTACTTTTTTACTTAC
ATTTTTAGATTTAACAGGTAACTTTTCAAATGTACCTTTATCAAATGCCTCACAGTTTTCTT
GAACTGGACAAAATAAGCATAATGGATTTTTTGGTGTACAAATTAACGCCCCTAATTCCA
TCATAGCTTGATTAAACGTTCCAGCTTCTGTAGTAACATACGGTAATAATTCTTGTTCGTA
CGATTTCCTCGTCGATTGTAATTTAATATCTCGATAGTCATCATTCAATCTAGACCATACT
CGAAAAACATTTCCGTCTACAGTTGCTAGTGGTACATTATATGCAATGCTCATTACTGCAG
CTTGTGTGTATGGGCCAACACCTTTTAACGCTTTAAATTGATCAGGATCTTTGGGAACTAA
GCCTTCATATTTATCATGAACTTCTTTAATCGCCGTATGAAAATTTCGAGCTCTACTATAA
TATCCTAAGCCTTCCCAATACTTTAACACTTCATCTTCCGAAGCTTGACTCAAAACTTCCA
CAGTTGGAAATCGTTCAACAAAACGATGATAATAGTCAATAACTGTTTTAACTTGTGTCT
GTTGTAACATGACCTCACTTAACCAAATATAGTACGGATTGGTCGTTTGTCGCCATGGCAT
TTCTCTTTGATTTTCATCAAACCAGTGTATCAAATTTTCTTTAAAACTAGACTGCTGATAC
AT

> **recX**

Function: protein coding sequence; regulator of recombination (core genome, constant)
Best match: recX_CC007_USA300_TCH959_AASB02000001.1[13505:14323:r]RC (completely identical)
Position: 049-contig_251_RC: 13607 … 14426; Length: 819 bp
Sequence:
TCACTCAATTTCTTCTGTTCCATCGGCAATACCACTTTCTTCTAATTTAGCTTTAATTTTAT
CATATTTATATCCTTTTCTCATAAGGCCTTCGATAGTTTTTGAAATTAATTTCTGTTGCGTG
TACTTCTTTCGATTTTTATTATAAATTTTTTCTAAATCTCGTTGTAATAAATCGTCTAAAAC
CGCTTCATCTTGTGTAAAATCCATTTCATTCAATACAGCATGAATGGTTTCCATTTCAAAC
CCTTTTTGAATTAAAGATTGCATTACTTTTTCTTTAACCTTATTTTGTGGCCCTTTTTTTGTT
TTAGATATTTTTTCAGCAATTTGGATAATATCATCAAGTTCCTGTTGTTCTCTATAAAGTTC
TGTAAACATTTCAATGATATTTGGTTCTATACCAAGTTGATATAGTTTTTGTTGATAAATT
TTAGGTCCTTTATCTGTCGTGCGAATCATTGTATTTTTTAAACTTTCCGCATAATCTTGATG
GTCGATTAACTTTTCGCGATAACAATATTCAATCACTTCAGAAATCGCTTGCTCTGATATC
TCTTCTTTTTGTAAATATTGTATAACTTCTTTTTCAGTTCTCTTTTTATATGATAAATATTGG
ATTGCTTTGTTTAAACCTATGCGATAATGATCATACTTTTGAATCTCTGCCATGTCAGCAG
CTTCAAGTTGTTGCCCTTTTTTTAAATTAAATTTGACTAATGTATCGATATCTATACCCATT
TCAAATTGTTCGTCTAAAAAAAGATTAAAACGTTCTTTGTTTTTCTTTTGAACTTCTATTTT
AGTAATCTTCGGCAC

> **sgtB**

Function: protein coding sequence; monofunctional glycosyltransferase B (core genome, constant)
Best match: sgtB_CC001-ST772_118_AJGE01000047.1[14617:15426:r]RC (completely identical)
Position: 049-contig_251_RC: 14686 … 15496; Length: 810 bp
Sequence:

TTAACGATTTAATTGTGACATAGCCTGTTGATATTGTGTTTCATTGATATAATTTTGTTGCT
TCATTTTTTCTAAGTTCGTGCTTACACGTTGCGTAAAATTCTCTGACATATTATTGATATTA
TATACGCTAGGTGCATTGACTTTACTAGCTAAAATAGCGCTTTGTAAAACTGTTATGTGAG
ACATTGTTGTACTATTTTTATTCACGGTTGTTCCAAAGTAATGGTTTGCTGCGCCCTCAAG
CGTATATTGATTATCCCCAAAGTAAATATTATTTAAATAAAAGCTTAAAATTTCGTTCTTA
TTATATTGTTTTTCAACTCGATGAGCTACAAATAATTCTTTTACTTTTCTAGTAAATGAAC
GATCATTATCATAAAAATAATTTTTGACAACTTGTTGTGTAATGGTACTACCACCTTGCAC
ATCTCTGTCGCTAATCGTTGAAAATAAAGCTCTAGTTGTACCTTTCAAATCGAATCCATGA
TGATTGTAGAATCGTTCATCTTCCATTGAAATAAAGGCACCTTTAACATACTCTGGCATGT
TATCAGCTGACACAAAACTACTTTTATTTTCAATTTTTCTTAGTTCATCCACATTATCGCGT
GTAGATAAAAAATACATGATACCAATAAACAATGCGATAATGATTAGAATGGTTAATAA
TATTTTTAATAGTATTCGTTTACTTTTTTTCTTTTTCGGCGGTTTGCCAACTGGTTGATAAT
ACGTATTATAGTGAGGTTCGTGTTTCATATGCTCAAAATGTTCATTTGAGTTTGAGTACTT
ATCGCTTCTTTTCAT

> **pfpI**
Function: protein coding sequence; intracellular protease (core genome, variable)
Best match: pfpI_CC001-ST772_118_AJGE01000047.1[15767:16282:r]RC (completely identical)
Position: 049-contig_251_RC: 15836 … 16352; Length: 516 bp
Sequence:
CTATTGTAATTGTTTAACGATTTCTCGATTAAAATCATCTAAATCGTCTGGTACTCGACTT
GTTACAATATTGTTGTCTACAACTACTGACTCATCAACTACATGTGCGCCTGCATTTGATA
AATCTTTGCGCACATTTAATACTGCTGTTAACGTACGACCTTTTAAATCGTCTGTATCTAT
TAGTATTTGTGGCCCATGACAAATGGCAAATGTTGGTACATCATTTTTAGTAAAGTATTTA
GCAAATGTGCCATATCGACCTTCTGTATCTCCACGTAAATGATCTGGTGAAAATCCTCCA
GGAATTAATAATGCATCATAATCTTCTGGTTTAGCTTCTGCAATGCCTACATCGACAGTAA
CTTTTTCACCGTGTTTACCAACAACTTCACTATTTGCAGTATCTCCAATCACTACAGTATT
AAAGCCTGCATTCTCTAATGCCTCTTTAGGGCTTGAATATTCTATATCTTCAAATTCGTTT
GCTAGAATAATTGCTACTTTTTTAGTCAT

> **Q5HEP8**
Function: protein coding sequence; putative protein (core genome, constant)
Best match: Q5HEP8_CC001_MW2-USA400_BA000033.2[1980946:1981107:r]RC (completely identical)
Position: 049-contig_251_RC: 16482 … 16644; Length: 162 bp
Sequence:
TTACTTAGATACTAATTTAACCTTTTCTTCAAGCTGATCTAACAATCCAATCCATTCATCT
ATATCTTCAACACGTACTTCATCAGGATTTACATGATCGATATCCTCAATAAACTTATTTA
AACGCGCTTTTATCTGTTCGATTGTTTGCTGTTCATTCAT

> **attB_phiSa6**
Function: attachment site for int6-phi11 (mobile element)
Best match: attB_phiSa6_empty_CC001_MW2-USA400_BA000033.2[1981330:1981425] (completely identical)
Position: 049-contig_251_RC: 16866 … 16962; Length: 96 bp
Sequence:
GATGAATAGCGTCAAATTGAAGCAACCTGTTAGCATTTACAATGATCCATGGGAATCATA
TAACGATGTTAAAGAACATGGCCAATTAACTTTAAG

> **A5IU43**
Function: protein coding sequence; putative protein (core genome, variable)
Best match: A5IU43_CC001_MW2-USA400_BA000033.2[1981331:1982482] (completely identical)
Position: 049-contig_251_RC: 16867 … 18019; Length: 1152 bp
Sequence:

ATGAATAGCGTCAAATTGAAGCAACCTGTTAGCATTTACAATGATCCATGGGAATCATAT
AACGATGTTAAAGAACATGGCCAATTAACTTTAAGTAACATCGAATTTACAACTACAAAT
CTTTGTAATATGCGTTGTAGCCACTGTGCAGTTGGTTATACTTTACAAACTGTCGACCCCG
AGCCTTTAGATATGGACTTAATTTATCGTAGACTTGATGAAATTCCAAATCTGCGAACGA
TGTCAATTACAGGTGGCGAACCAATGTTTTCTAAAAAGTCTATTAGAAATGTTGTTAAAC
CTCTATTAAAGTATGCACATCATCGAGGTATATATACACAAATGAATTCAAACCTAACAT
TGCCTCAAGATCGTTATTTAGATATTGCTGAATATATCGATGTTATGCATATCTCACATAA
CTGGGGAACAACTGATGAATTCGCAAATGTTGGCTTTGGCGCAATGAAGAAGCAACCAC
CGTTAAAAGCTAAGTTAAAATTATATGAACAAATGATTTCGAATGCACGTACATTATCAG
AACAAGGAATGTTTGTATCTGCGGAAACAATGCTCAATCAAAGTACGCTACCACATTTAC
GAAAAATACATCAAGAAGTCGTTCATGATATGAAATGTAGCAGACACGAGATTCACCCT
ATGTATCCAGCTGACTTTGCAAGTCAATTAAATGTGTTAACTCTAGCGGAAATGAAAAAG
ACAATTCATGATATATTGGATTTCAGAGATGAAGATATTTGGATGTTATTTGGTACTTTGC
CTGTGTTTCCATGCTTAAAAGATGATGAAGATCAAAAGTTACTATCACGTTTAAGAACTG
CTAAAAATGTAACGACTAGAAATGACCCGGATGGCCGTAGTCGTTTAAATGTCAATGTAT
TTACAGGTAATGTAATCGTAACTGATTTCGGAGATGAAACAGGTACAATTTCGAATATAC
AAAAAGATAAATTAACAGATGTATTTGATAAATGGTTATCCTCTGATCTTGCTAAATCATT
AAATTGTCATTGTTCCGAGTTTAGTTGTTTAGGACCAAATGTTCTTGTTAAAAATATGTAC
TATCCGAATATGGATTTTAAAGATAATGAGCGTCATATGCACAAACAACCACAAATTATA
CAATTTTAA

> **A5IU44**

Function: protein coding sequence; acyl-CoA thioester hydrolase (core genome, constant)
Best match: A5IU44_CC005_N315_BA000018.3[1942162:1942692:r]RC (completely identical)
Position: 049-contig_251_RC: 18279 … 18810; Length: 531 bp
Sequence:
TTAGTCTCTAATATGTTGCACTTGAGCTAAATATTCAATTGTTTGTTTACTTTCAATGCGAC
GTGCTTTTCTTCGTTCAACACGTTGTGGTGCTGTATCATAAAACCATTTTTCAACATCATCT
TCTGGATATACACCAGGTACATGTTTAGGTTTGCCTTCATCATCTAACGCAACAAATGTTA
AGTAACTTAATGCAGCTAAATCATGCTTGTTATTAAATACATCGTCAATTCTAATTTGAAC
GACCACTTCCATTGAACTAGTCCCAGCGTATGAAACCATCGCTACGTATTGTAATATGTCC
CCTGTTTTAATCGGCTTTAAGAAATCTACTGAGTCTGTAGATGCGGTAACTACTTGCGCAC
CAGCATGTTTCATAGCTGTGATTGCTGCAATTTCATCAATATTAGCCATCAATGTACCACC
AAACATTGTATGATGGTGATTCGTATCTTGAGGGAAAACTTGTCTATTTTTATAACATTTT
GATTCTGACATAGATTTCATTGGTCTGTCCTGATTTGTCAT

> **pepS**

Function: protein coding sequence; aminopeptidase (core genome, variable)
Best match: pepS_CC008_NCTC8325_CP000253.1[1968999:1970246:r]RC
Position: 049-contig_251_RC: 18899 … 20147; Length: 1248 bp
Sequence:
TTAAAATGTTGATGCCCAATTTCCATTTTCAAATACTAGTTCTTTTGAACCATCTTCGAAT
ATGCCATAAATAGTCAAATCACTACTACCAATCATGAAATCGACATGTACATTTGAATCA
TTTAATCCGCTTGCAATCTTTTCCTCAACAGTCATTTCCGTTCCACCTTGAATATTAAAGG
CGTAAGCAGATCCAATCGCTAAATGACATGCTGCATTTTCATCAAATAAAGTATTGTAAA
AAATGGTATTACGATTTGATATCGGTGAATCATCAGGTACTAATGCTACTTCACCTAATCT
TCTTGAACCTTCATCAGTATTGATTAAATCTTTCAATACCGTTTCACCTTTTTCAGCTGAAA
AATCAATAATCTCTCCATCTTTAAACATCAATTTAAATTGATCGATAATTGTGCCATTATA
ACTTAAAGGCAATTTATTAGTAACATATCCATCTACTCTATTTCGATCTGGTGCTGTAAAT
ACTTCTTCAGTTGGTATATTAGCAATAAAAGCTTGTTCCTTGCCGTTAACATAACTTGTAG
CATCTTCCCAGATATGATTTTTCGCTAAACCTACCGTTAAATCAGTACCTTCAGATACATA
ATGCAACGCATGGTAATTTTTTTGTTGTAACTTTTGAGCGTAAACACTTAAATTAGCGATG
TGTTGACGCCAATTTTCAACTGGATCGTTACCATCAATTCGCACAATATCGAATACCTCGT
CAATAAACTTAATATATGCTTCTTCTACAGATAGTTCAGGATACACTCTCTTCGCCCAAGC
TTTAGATGGAAAAGCAGCTACGACCCATGGAAATTGATTTTTTTGTACACTTTCCATATAT

CCTTTAAATGCGCGTGCGTTTTGTTGTTGAAATGCTTGTAATTTTTGGCTATCGATACCAT
CCATTAAATCAGGATCTTCACTAATAAGTGCTAAATTAGCAGCGCCACGTTTAACGTAAT
CCATTCTCGCTTCAACATCATATGATTTTAACTCATGATTAGCAAAATGTTCCACAGATTC
ATTTTCAAATTTTAAGCGTTTTAAGGTTGGATCGCTATATACAACACGTACATCTGAAGCG
CCACAATGATATGCTTCTTCTACAATTAAATGCGTTAATTCTAATGTTTCTACTGATGATC
TAATAAATACTGGCTGTTTTGGTTGAACATTCATACCAACCTTAACTAATAGTTCAGCGTA
TTGTTGTAACTTTTCTTTGTAATTAGTCAA

> **rbn**

Function: protein coding sequence; ribonuclease BN (core genome, variable)
Best match: rbn_CC001_MW2-USA400_BA000033.2[1985991:1987202]
Position: 049-contig_251_RC: 21529 … 22747; Length: 1218 bp
Sequence:
ATGTCGAAAAAGGATCACTCTTCTTCAAAATACCTTAATTCTGTTAAGGAAGCGCAAGAG
GAGTCAAAAAAGAAAAATAAAAGTAATCCCAAAATTGATGTTGATCGTACATATATTGA
ACCTCAACAATTCCAATCTAAGAAACCTAAAAAAGATGATCAGGTTTTCTTCTTATCAAG
ATTAAATAAACCTGCAAAATATAAGAAAGACTCTAATTTCTTATCATATCTCATCTATCGC
ATAGGAAAAGATGATGCCTCAGGACTAGCAGCACAAATGACTTACCATTTCGTACTTGCT
ATGTTCCCTATGTTGCTTTTCCTATTAACATTATTACCATTTTTCAATATTAAGCAGAGTCA
AATTACTAATATGTTAAGCAATGCACCCGCTGAAACATCTACTCTAATTAAGAGTGTAAT
TGGTGATATAACTCAAAACTCCAGTGGTGGCTTATTATCTATCGGTTTGATTTTAGCAATT
TGGTCAGCTTCAAATGGAATGACTGCAATTATGAATTCTTTCAATGTTGCTTACGATGTAG
AAGATAGCCGTAATGGAATCGTATTAAAACTACTAAGTGTTGTCTTCACTGTAGTTATGG
GCGTTGTGTTTGTAGTTGCTCTAGCATTACCAACGCTTGGTTCTGTAATTAGTCATTTCCTA
TTCGGTCCACTTGGATTTGACGAACAAGTGAAATGGATTTTTAACCTTATTAGAATTGTGT
TACCAATCATTATTATATTTATCGTATTTATCGTGTTATATTCGGTTGCACCTAACGTTAAA
ACGAAGCTTAAGTCAGTATTACCAGGTGCAGTATTTACTTCAATTATTTGGTTAGCTGGTT
CATTTGGTTTTGGTTGGTATATTTCAAATTTTGGTAACTATTCTAAAACATATGGCAGTAT
CGCGGGTATCATCATTTTGTTACTATGGTTATATATCACAAGTTTTATTATAATTGTCGGT
GCTGAAATCAATGCAATCATTCATCAGCGTAGTGTAATTAAAGGTAAAACACCTGAAGAA
GCAGCATTAGAACATGATGACAATAATAAAAATCATTATAATGAGAACACTAGGTACGA
ATATGATGAGGATAATAATGCAACACATCAACGTACGTATCATGTTGATGAACATCCTAG
TGATACCTATCCAGAAGATGATAAAAATATAAAAGACAAAATCGTTGATAAGCTTAAAA
AAGACTAA

> **tnpIS431-06**

Function: protein coding sequence; transposase for IS431 (mobile element)
Best match: tnpIS431-06_circ_S-epidermidis_GQ900381.1[43799:43807] (completely identical)
Position: 049-contig_251_RC: 22853 … 22862; Length: 9 bp
Sequence:
ATGAACTAT

> **vraR**

Function: protein coding sequence; response regulator protein (core genome, constant)
Best match: vraR_CC005_Mu50-VRSA_BA000017.4[2024537:2025166:r]RC
Position: 049-contig_251_RC: 22858 … 23488; Length: 630 bp
Sequence:
CTATTGAATTAAATTATGTTGGAATGCATAGATGACAGCTTGTGTTCTATCTTGCACTTCT
AACTTACTTAAAATGTTACTCACATGTGTCTTAACCGTTTTAATAGTAATATGCGATGCAC
TAGCAATTTCTTGATTTGAGTAACCTTTCGCAATCAATAATAATATTTCCATTTCTCGTTCT
GTAAGCATTTCATATAACTCTGCGCGCTTTTTCATACGGTTACGCATTTTCACTAAAACTT
CCGGTTCAAAAACAGATTCTCCTCTAGAAGTTTTACGAACTGCATCGGCGATATCTTTTGC
ACTTGTTGTTTTTAAAATGTAACTATCGACACCTGCATCTAATGCACGATATACCTCTTTA
TCTTCAATAAAACTAGTTAATATTAATACTTTAATTTGCGGTAAATCTTTTTTAATCTGAG
TCGTCGCTTCTACACCATCCATGTCATCCATAAGTAAATCCATTAAAATTAAATCTGGCTT

CAACTCATGGGCTTTGGCAATTGCTTCTTTACCAGAAGCGCCTTCACCAACTACTTCAATA
TCACTTTGCGTTGATAGATAACTTGAAATTCCTATACGTACCATTTCATGATCATCCACAA
ACAATACTTTAATCGTCAT

## > vraS

Function: protein coding sequence; sensor protein (core genome, constant)
Best match: vraS_CC008-ST72_21259_AFTS01000001.1[23550:24593:r]RC (completely identical)
Position: 049-contig_251_RC: 23477 … 24521; Length: 1044 bp
Sequence:
TTAATCGTCATACGAATCCTCCTTATTTAAAGGTGCTTTCACCTCGATACGTGTACCTGAA
TCTGGCAATGATACAATATGGAACGTTGCACCAATTTCCAAAGCTCTTTCACGCATATTTT
TAAGTCCATAACTTTGTTCTAATTTTTCATCAACATTAAAACCTTTACCATTATCTTGAATT
CTCAACAATAAATAATCGTCTTTATTAAACAATTCTACTGTCACTTTTGTACCGTTTGAAT
GACGCAATGTATTCGAAATTGCTTCCTGTGTAATTCTGAACAAATGATCTTCAATACCTTT
AGGCACTTTAAAATCTTGTATTTCATGCACAACTTTCATTGGCACTTTTTTTTGTAAATCA
ATAACTAAATCTTTAATACCCTCACCTAAAGATTTGTCTTTTAAACCAAGCGGTCTTAAAT
GTAACAGCAAAGCACGCATTTCTAACTGCGAATCTTGAACCATTTTCTCTAAAATAGGAA
TTTGTTGGTCTAATGGTGGTTCTAACTTCGTTTCTTTGATAGCAGATAGCATCATACTTGC
CGCAAAAAGTTGCTGACTAACAGAATCGTGAAGTTCTCGTGCTAGTCTTTGACGTTCATCT
TCAATAATCTTTTTAACTTTCACATCATTAATATTATAATTTTCATTGGTTAAGTTTTGAGT
TTTAAGTCGCAACTTATGCAATTCTTGATTTAAAGGTACGAGTGTATGGTATAAATCTAAC
GTTTCACTATATATTTCTATATTTTGATCATTAATGCCAACTGTTTCGCCTTCCATTGAACG
CTCAATTTGCGTCTTAATCCAATCATTTTGCTGATTGATTTTGTAAGCGAGTACCGAACCA
ACAATAATACACAATAATATGATGATGAGATTTAAAAATAAAAAGACTGGTATTCCGAAT
ATTTGTGTATAAAACATACCTTGAAAATAGATGATATTTACAAAAACTTTATCGATGAAC
AGAAATGCAGCTAGCATGCTATATACTAAGATGAGCATTGAACCAATTGTTCTAATGTAG
TGGTTCAT

## > ampM

Function: protein coding sequence; methionine aminopeptidase type I (core genome, constant)
Best match: ampM_CC001_MW2-USA400_BA000033.2[1990292:1991050:r]RC
Position: 049-contig_251_RC: 25836 … 26595; Length: 759 bp
Sequence:
CTATTCTTCTTCAATTTTTGTCGTTAAAATCGGACCATCCTTAGTCACGATAACCGTATGC
TCAATTTGAGCAACAAAACTTTTATCGCTCGTTTCAAAAGCCCATTCATTTTTACCTTCTG
TAACAAATGATGCATTTGATGAGATAAACGGTTCAATAGCTAATACCATACCTTCAGTTA
ATAATGTTTTGTCTTTTGGATCAAAGTAATTAAGTACATGTGCTGGTGCTTCATGTAATGA
TAAACCAACACCATGACCTGTTAAGTTTTTAATGACTTTCAAATCATTTTGTCTAGCTGTA
TTATGCACCGCTTTACCAATGTTACTTAACTTAGTACCCGGTTTTACTTTTGCAATTGCATT
CTCAAATGCCATCGTTGCTACGTCACATACTTTTTGTTTCATTGGATCATCTGATTCTCCAA
CGACAAATGAAATACCTGTATCTGCATAATAGCCATTCTTCAAAGCCGATACATCAATAT
TTACTAAATCTCCTTCACGAATGACACGCTTACTTGGAATCCCATGTGCCACCTCTTCATT
GACACTAATACACGTTTGACCAGGAAAATTTTCATCATGAATTGGCGCAGAAATAGCACC
GTATTCTTCAAATAACTCTTTCGCAATATTATCAAGCTCTTTCGTAGTGATACCTGGTTTG
GTTGCAGCTTGCATTGTATTGCGCACTTTAGCGCATATGTATCCAATTTCTTTTAACGCTT
GTAATTCTTCTTCTGTTTTTACAATCAT

## > Q2FZB6

Function: protein coding sequence; putative protein (genomic island)
Best match: Q2FZB6_CC030_MRSA252_BX571856.1[2068297:2068469:r]RC
Position: 049-contig_251_RC: 26731 … 26761; Length: 30 bp
Sequence:
ACCCCAACTTGCATTGCCTGTAGAAATTGT

## > tx_universal2

Function:  rho indendent terminator
Best match: tx_universal2_CC001_MW2-USA400_BA000033.2[1991364:1991404:r]RC (completely identical)
Position: 049-contig_251_RC: 26849 … 26890; Length: 41 bp
Sequence:
TATAATTGAAAAATGCTTGTTACAAGTGCATTTTATTTCAG

> **tx_universal2**
Function: rho indendent terminator
Best match: tx_universal2_CC001_MW2-USA400_BA000033.2[1067981:1068021:r]RC (completely identical)
Position: 050-contig_246_RC: 9 … 50; Length: 41 bp
Sequence:
TAGAATTGAAAAAAGCTTGTTACAAGCGCATTTTCGTTCAG

> **furbox-ftnA**
Function:  binding site of ferric uptake regulator next to ftnA
Best match: furbox-ftnA_CC001_MW2-USA400_BA000033.2[1995858:1995894:r]RC (completely identical)
Position: 050-contig_246_RC: 3042 … 3079; Length: 37 bp
Sequence:
ATAATTAAAAATGATATTTATTCTCAATTAAATATCG

> **ftnA**
Function: protein coding sequence; ferritin (core genome, constant)
Best match: ftnA_CC001_MW2-USA400_BA000033.2[1996034:1996534] (completely identical)
Position: 050-contig_246_RC: 3218 … 3719; Length: 501 bp
Sequence:
ATGTTAAGTAAAAATTTATTAGAAGCATTAAATGATCAAATGAACCATGAGTACTTTGCA
GCACACGCATATATGGCAATGGCAGCATACTGTGATAAAGAATCGTACGAAGGATTTGC
AAACTTCTTCATTCAACAAGCTAAAGAAGAACGTTTCCATGGACAAAAGATTTATAACTA
TATTAACGACAGAGGTGCACATGCAGAATTCAGAGCAGTTTCAGCACCAAAAATTGACTT
TTCAAGCATACTAGAAACTTTCAAAGACAGCTTATCTCAAGAACAAGAAGTAACAAGAC
GTTTCTATAACTTATCTGAAATCGCTCGTCAAGATAAAGATTATGCAACTATCTCATTCTT
AAACTGGTTCTTAGATGAACAAGTCGAAGAAGAATCAATGTTTGAAACTCACATCAATTA
TTTAACTCGTATCGGCGATGACAGCAATGCATTATATCTTTACGAAAAAGAACTTGGCGC
TCGTACATTCGACGAAGAATAA

> **dinB**
Function: protein coding sequence; DNA polymerase IV (core genome, variable)
Best match: dinB_CC001-ST772_118_AJGE01000047.1[32901:33971:r]RC (completely identical)
Position: 050-contig_246_RC: 4729 … 5800; Length: 1071 bp
Sequence:
TTATATAAAGTCATATATCGTCATATTTTTATAAGTTGATTGTTCTAAATTACCTACAGTG
ACACCAATAAGTCGAATTGGTACATCAGGGTCTTTTAAATCGTTATAAAGTAAATATGCA
ATATTATAAATATCTTCTTCAGAACTAACCGAATCTCTTAAACTCATCTGTTTAGATAGCG
TTTCAAATTGATAAGTTTTAATTTTAACCGTTACAGTTTTAGCTGACTTCTGTAATTTATTT
AGACGTTCAGCTGTTTTACCTGACAATTCCCATACTTTTCTTAAAATCTCTTCATCATCATT
CACGTCTGTTGCAAATGTGCGTTCAGTCCCTACTGATTTTCTTACTCTTGATGATTTCACTT
CACTATGGTCAATACCGCGTGCCTTGTTATATAAACCCCGACCTCTTTTTCCAAACAAACG
TATTAATTCAAATTCCGTTTTCTCATATAAATCTCTACCGTTAAAAATACCATTATCATGC
ATTACTTTTTTGGAAGCTTTACCTACGCCTGGAAAATCTCCAATATCCAATGTCATCAAAA
TATCATGGACATTTTGATAATCAATCACAGTCATACCATCAGGTTTATTCATACCACTCGC
TAATTTAGCTAAAAATTTGTTATAAGAAACACCTGCAGATGCTGTTAAATGTGTCTGCTCT
AGAATATCTTTTCTAATATACTGAGCAATTTTCGAAGCAGGAAGGTCTGGTCTCACTAATT

CTGTAATATCTAAATACGCTTCATCCAATGACATCGGTTCTACCTTATCTGTATAACTTCG
GAAAATAGACATAATCTGCGCAGATGTTTCTCGGTAAGCACCAAAATTACTTGTGACAAA
GTATCCATTTGGACATAATTTATGCGCTTGTGACATAGGCATTGCTGAATGGACGCCGTAT
TTTCGTGCTTCATAAGATGCCGTAGAGACAACACCCCTACTGCTTGCTTTACCACCAACAA
TGACTGGTTTCCCTTTCAATTTGGGGTTATCTCTCATTTCGACTTGTGCAAAAAAATAGTC
CATATCTATATGAATAATTCGTCTCTCAGTCAA

## > Q2FZB6

Function: protein coding sequence; putative protein (genomic island)
Best match: Q2FZB6_CC705_RF122-cow_AJ938182.1[1965427:1965599:r]RC
Position: 050-contig_246_RC: 9236 … 9266; Length: 30 bp
Sequence:
CTACCCCAACTTGCATTGTCTGTAGAATTT

## > repeat_nySAgamma

Function:  repeat element
Best match: repeat_nySAgamma_CC030_MRSA252_BX571856.1[2078922:2079031]
Position: 050-contig_246_RC: 9299 … 9329; Length: 30 bp
Sequence:
CCAACTTGCACATTATTGTAAGCTGACAGA

## > gatB

Function: protein coding sequence; aspartyl/glutamyl-tRNA amidotransferase/subunit B (core genome, variable)
Best match: gatB_CC001_MW2-USA400_BA000033.2[2002605:2004032:r]RC (completely identical)
Position: 050-contig_246_RC: 9847 … 11275; Length: 1428 bp
Sequence:
TTATCTTTTATCTAATTCTTGTTTTAATAGTTGATTTACTAATTGTGGATTAGCTTGACCTT
TAGACGCTTTCATAATTTGACCAACTAAGAAGCCCATAGCTTTGCCTTTACCATTTTTGTA
ATCTTCAACTGATTGTTCGTTATTGTCTAATGCTTCATTTACAAATTTTAGAAGTGTTGCTT
CATCAGAAATTTGAACTAAGCCATTATCTTCCATAATCTGTTTAGCATTACCACCTTTAGC
TGCTAACTCTGGGAAGACTTTCTTCGCAATTTTACTGCTCATTGTTCCGTCTTCGATAAGTT
TAATCATACCTGCTAAATTTTCTGGTGTTAATTTAGTATCTAATAATTCTACTTGATTTTTA
TTTAAATATTCGTTTACGCCACCCATTAACCAGTTAGATGTTAATTTAACATCTGCACCGT
GTTCAATTGTTGATTCAAAGAAATCTGACATTTCTTTAGTCAATGTTAATACGTGTGCATC
GTATGCAGGTAAACCTAATTCATTTACATACTTAGCTTTACGTTCATCTGGTAATTCAGGA
ATTGTCTGACGAACACGCTCTTTCCAAGCATCATCAATATATAAAGGTACAATGTCAGGC
TCTGGGAAGTAACGGTAATCATCAGAACCTTCTTTAACACGCATTAAAATTGTTTTACCTG
TAGATTCATCAAATCGACGTGTTTCTTGTCCGATTTCTCCACCATTTAACAATTCTTCTTCT
TGGCGTTTTTCTTCATATTCTAAACCTTTACGTACATAGTTAAATGAGTTTAAGTTTTTCAA
TTCGGCTTTAGTACCAAATTTTTCTTGACCATATGGACGTAAAGAGATGTTAGCATCACAA
CGTAAAGATCCCTCTTCCATCTTAACGTCTGATACACCAGTGTATTGAATAATTGAACGCA
ATTTTTCTAAATATGCATATGCTTCTTTAGGTGAACGAATATCTGGTTCAGATACGATTTC
AATTAGCGGTGTACCTTGACGGTTCAAGTCAACTAATGAATACTCACCTTTATGTGTTGAC
TTACCAGCATCTTCTTCCATGTGAAGACGAGTAATACCGATTCGTTTTGTTTCACCGTCGA
CTTCGATATCGATATATCCATTTTCACCAATTGGTTGATCAAATTGAGAAATTTGATATGC
TTTTGGATTATCTGGATAGAAATAGTTCTTACGGTCAAACTTAGATTCTGTTGCGATTTCC
ATATTTAGTGCCATTGCAGCACGCATTGCCCAGTCTACTGCACGCTTATTAACAACTGGTA
AGACACCTGGATATGCTAAGTCGATAACATTTGTATTTGAGTTAGGTTCTGCTCCAAAAT
GCGCTGGTGATGGAGAAAACATTTTTGAGTCCGTTTTTAACTCTACGTGAACTTCAAGTCC
TATAACTGTTTCAAAATGCAT

## > gatA

Function: protein coding sequence; aspartyl/glutamyl-tRNA amidotransferase/subunit A (core genome, variable)
Best match: gatA_CC001-ST772_118_AJGE01000047.1[39459:40916:r]RC (completely identical)
Position: 050-contig_246_RC: 11287 … 12745; Length: 1458 bp
Sequence:
TTATAATTTTTCATAAACGTCATGTAAATTGTATTGTGTTTCATATTGATAAGCGACACGA
TATAACGTTTTTTCATCGAATGGTTTACCAATGAACTGTAAACCGATTGGTCGGCCATTTG
ATTGTCCACAAGGAACAGAAATACCAGGTAATCCAGCTAAGTTTACTGGTGTTGTTAATA
AATCATTGGCATACATTGTTAATGGATCATCAATTTCTTCACCTAAATTAAACGCAGTTGT
AGGCGCTGTTGGACCAACTACTACATCATAATTTTCGAATACTTTATCAAAGTCATTTTTA
ATCAATGTTCTAACTTTTTGAGATTTTTTATAGTAAGCATCATAGTAACCTGAACTTAATG
CAAATGTACCTAAGAAAATACGACGTTTTACTTCTTTACCGAAACCTTCAGATCTTGACAT
TTTATATAATTCTTCTAATGAATGAGCTTCTTTAGAATGATAACCATAACGAATTCCGTCA
AAACGAGAAAGGTTTGACGAAGCTTCTGATGATGCAATCACGTAATATGATGGAATACC
AAATTTAGTATTTGGCAATGATACTTCCTCAACGACAGCACCTAAAGATTTTAAAGTTTCT
ACAGCGTTTTGAACTGCTTCTTTTACGTCATCAGCTACACCTTCACCTAAGTATTCTTTAG
GTAATGCAACTTTTAATCCTTTAATATCTTTACCAATTTCAGATGTAAAGTCTACATCATC
AACTGGTGCACTTGTAGAGTCATTAACATCTGCACCAGAAATAGCTTCTAATACGATTGC
ATTATCTTTTACATTTCGAGTCAATGGACCAATTTGGTCTAATGAAGATGCAAAAGCAAC
TAATCCAAATCGAGATACACGACCGTATGTTGGTTTCATACCGACAACGCCACAATATGC
AGCCGGTTGTCTAATTGAACCACCTGTGTCTGAACCTAAGCTAAATGGTACTAAGCCAGC
TGCAACTGCTGCTGCAGATCCACCTGATGAACCACCTGGTACTGCTTTATGGTCAAATGG
GTTAACTGTTTTTTTGAAATAAGATGTTTCTGTTGAACCACCCATTGCAAACTCATCCATG
TTTAATTTACCGATTAAAACGGCATTCTCTTTATGTAGTTTTTCCATTACAGTAGATTCGTA
AATTGGCACAAAACCTTCTAACATTTTACTTGCACATGTTGTTTCTAATCCGTTTGTAATA
ATGTTATCTTTTATACCCATTGGAATACCAAATAATTTGCCATCCATTTGATCTTTTGCTTG
TAATTCATCCAATTCTTGCGCTTTTTTGATTGCATTTTCTTTATCCAGCGCTAGAAAAGACT
TAATTGTTGGATCAGTCTCTTCAATTGCATCATATATATCTTTAACAACATCAGATGGTTT
GATTTTTTTGTCTTTTATTAAAGTTAATAAATTCTCAACCGATTCGTAGCGAATGCTCAT

## > **gatC2**

Function: protein coding sequence; aspartyl/glutamyl-tRNA amidotransferase/subunit C (core genome, constant)
Best match: gatC2_CC001_MW2-USA400_BA000033.2[2005504:2005806:r]RC (completely identical)
Position: 050-contig_246_RC: 12746 … 13049; Length: 303 bp
Sequence:
TTACGCGTCCTCCTCATTCATGATTGTAGGCACTTTAAATTGTCCATCTTCTGTTTCTTTGG
CATTTTTCAAAGCTAATTCTTGCGGAATACCTTTAATTGCTTTATCTTCACGTAAAACGTTT
TGTAAATCTAAAACGTGATATGTAGGTTCAACGCCTTCTGTATCAGCGCTATCATTTTGTT
TTGCAAAATCTAAAATGCTTTCTAATGTGTTGGCCATTTCTTCCGTTTCTTCAGGAGAAAT
TTGAAGTCTTGCAAGATTCGCGATATGCTCAACTTCTTCACGTGTTACTTTTGTCAT

## > **putP**

Function: protein coding sequence; proline permease (core genome, constant)
Best match: putP_CC005_Mu50-VRSA_BA000017.4[2043451:2044989] (completely identical)
Position: 050-contig_246_RC: 13415 … 14954; Length: 1539 bp
Sequence:
ATGCTTACAATGGGGACAGCATTAAGTCAACAAGTAGATGCCAATTGGCAAACTTATATT
ATGATTGCCGTCTACTTCTTGATACTAATCGTTATTGGCTTTTACGGTTACAAGCAAGCAA
CTGGTAACCTAAGCGAGTACATGTTAGGTGGACGTAGTATTGGACCGTATATTACTGCAT
TATCAGCTGGAGCTTCAGATATGAGTGGATGGATGATTATGGGGCTACCTGGTTCTGTCT
ATAGCACTGGTCTATCAGCTATGTGGATTACAATCGGTTTAACATTAGGTGCTTATATAAA
TTACTTTGTTGTTGCTCCTAGACTTCGTGTTTATACCGAATTAGCTGGAGATGCAATTACA
TTACCAGATTTCTTTAAAAATCGTTTAAACGATAAAAATAATGTGTTAAAGATTATTTCTG

GATTGATTATCGTAGTATTCTTTACATTATATACACATTCTGGTTTCGTATCTGGTGGTAA
ATTATTTGAAAGTGCTTTTGGATTAGATTATCATTTCGGTTTAATATTAGTTGCTTTCATTG
TCATTTTCTATACTTTCTTTGGTGGATATTTAGCTGTATCAATTACAGATTTCTTCCAAGGT
GTCATTATGTTAATTGCGATGGTTATGGTCCCTATTGTTGCTATGATGAATTTAAACGGCT
GGGGAACGTTTCATGATGTAGCAGCTATGAAACCTACAAATTTAAATTTATTTAAAGGGT
TATCATTTATAGGAATTATCTCTCTATTTTCATGGGGATTAGGTTATTTCGGTCAACCTCAT
ATCATTGTAAGGTTTATGTCTATTAAATCACACAAGATGCTACCTAAAGCTAGACGTTTA
GGTATTAGCTGGATGGCTGTTGGTTTATTAGGCGCTGTGGCTGTTGGTTTAACAGGTATTG
CATTCGTACCTGCTTATCATATTAAACTAGAAGATCCTGAGACATTATTCATCGTGATGAG
TCAAGTACTCTTCCATCCTCTTGTAGGTGGTTTCTTACTTGCTGCGATTCTAGCTGCAATTA
TGAGCACGATTTCTTCACAATTACTTGTAACATCTAGTTCACTAACGGAAGACTTTTATAA
ATTAATTCGTGGTGAAGAAAAAGCTAAAACGCACCAAAAAGAATTTGTTATGATTGGAA
GATTATCTGTATTAGTTGTAGCAATTGTTGCCATCGCGATTGCATGGAATCCAAACGACA
CAATTCTAAACTTAGTAGGTAACGCTTGGGCCGGATTTGGTGCATCGTTCAGTCCACTTGT
GCTATTTGCACTTTACTGGAAAGGTTTGACACGTGCCGGTGCTGTAAGTGGAATGGTTTC
AGGTGCCTTAGTCGTTATCGTTTGGATTGCGTGGATTAAACCATTGGCACATATCAACGA
AATATTCGGCTTATATGAAATTATTCCTGGATTTATTGTAAGTGTAATCGTTACATATGTT
GTAAGTAAACTTACTAAAAAACCTGGTGCATTTGTTGAAACTGACTTAAACAAAGTTCGT
GACATCGTTAGAGAAAAATAA

> **camS**

Function: protein coding sequence; bacterial pheromone cAM373 (core genome, constant)
Best match: camS_CC001-ST772_118_AJGE01000047.1[43214:44419:r]RC (completely identical)
Position: 050-contig_246_RC: 15042 … 16248; Length: 1206 bp
Sequence:
TTAATTACTGTAAATATGAACTTGCGGTTCTTTGTCATCTTTTGTCTTACTAATTAAAGCAC
GTGGTTGGTTACCATCTTTAATACGAATTTCATAGTTATCGATTTTATCGAAATATTTATTC
GCTTGTTCAGTAACGTACTGTGTAATACCAATTGTTTCAGCTTGTCCATAGTAATCGATTG
GTAAATCTACTACTAATCGTTGTGGCTTTTTATCAACAAATTTAACTTTCCCTACTGCTTGT
GTGAAATTAGAAAAATATGATTGCAAATTATCATTAAATTGCTTGAAATTATTATTTAAAT
TTTCATCATAATCTGCTGCTGTTGAAGAAGGTAATAAAGCTGTTTTTTCATTGATATTATG
CCATTCATTAAGCTTTGTTTGACTCTTTTCTGCAGTCGCTTGAGTGATAAATTCACCTGGT
GTGATTGAATCTTCACTTGATTGCTTATAAATTGCAAAATGAATTGGTATATCTTTTAAAT
CATCATTTTCACGTAACCTTGATAATATCTCACTAGCCATTTGTTTACCTTGCTTTTTAACT
TCGCTATCATCTAGTTTTTTACTAAAAGTCGGTCCATCTTTTTCTTTTTTATAGTAATAAAC
ACTATTCATAGCTAAACCAATCGTCATACCTTTAATATTCTTACCTTTTGTATCTCCACCAC
CATAAAAATCTTGCTCTAAAATGTTAGATAAATAGGCTGGTGATTTTTCTGCAATCTTTTC
AGGATTTGTTTCACCTTCGTGTGATGGATTAAGTCCTAAATTTTCATTCGCTTTCTTGTCTT
TTTTATCTTTTTCAGACATCTTATCGATTTCACGTTTTGTATACTTAGGATTTAAATAGGCA
TTAATTGTTTTCTTGTCCAAAAATTGACCATCTTGATACAAATATTTATCTGTTGGAAATA
CTTCTTTACTTAAGTTCAATAAACCATCTTCAAAGTCGCCGCCATTATAACTATTTGCCAT
GTTATCTTGTAAAAGTCCTCTTGCCTGGCTTTCTTTAAATGGTAACAATGTACGATAGTTA
TCACCTTGTACATTTTTATCCGTAGCAATTTCTTTTACTTGATTTGAACTATTGTTATGTTT
TTGATTATCTTTTCCAGCCTGGTCATCCTTATGGTTACCACAAGCAGCGAGTATAAAGATA
GCTGTAATCAATAATACTAATGTACGCTTCATCGACAT

> **ligA**

Function: protein coding sequence; NAD-dependent DNA ligase (core genome, constant)
Best match: ligA_CC001-ST772_118_AJGE01000047.1[44426:46429:r]RC (completely identical)
Position: 050-contig_246_RC: 16254 … 18258; Length: 2004 bp
Sequence:
CTAACTATTTAATTCATTTTGCTTATCTACAAATTGTTGCTCTGTCCAAATTTCAATACCTA
AACTTTGTGCTTTTGTTAATTTTGAACCTGCATCTTCACCAGCAATAACGACATCTGTATT
TTTAGTAACGCTACTTGTAACTTTAGCACCTTGTGATGCAAGCCATTTAGATGCTTCATTG
CGTGTCATTTGATGTAGCTTACCAGTCAGTACTATCGTTTTACCACTAAATTCAGGATGTC

CTTCAATATCTGATGTTTTGATACCTTTATAAATCATATTAACATGTTTATCTTTTAATTTT
TGAATTAAAGCACGAATATCTTCATTTTCTAAATAAGTAACTACAGATTGTGCTACTTTAT
CACCTATATCATGAATTTCTACTAATTCCGCTTCAGTTACCGTTAGTAATCGATCTATCGT
TTCATATTTTTCTGCTAACACTTGGCTCGCTTTAACACCTAAATGCCTAATACCTAGACCA
AATAATAAATTTTCTAAAGAGTTGTCCTTAGCTTGTTGAATGGCAGCTAATAAATTATCAA
CTTTTTTCTGCCCCATTCTATCTAAAGGTAATAAATCTTCTTCTGTTAAATAGAAAATATC
AGCAACATCTTTAATTAATTCGCTTTGATAAAGCTGTTGAATAATTTTAGTGCCTAAACCA
TCAATATTCATGGCTTGTCTTGATACAAAGTGAATTAATCCTTCAACAAGTTGTGCTTGGC
ATTTTGGATTAATGCAACGAAGTGCTACTTCGCCTTCAATACGTACTAATTCATGTCCACA
ACTTGGACAATGGGTTGGCATATGATATGTGACAGCATCCTCAGGTCTACGTTCTGGAAT
ACTACGTACAACTTCAGGTATGATGTCACCTGCTTTTTTCACTACAACACTATCACCAATT
CGAATATCTCTGTCATGAATTAAATCCTCATTGTGCAAAGATGCTCTTGATACAGTTGTAC
CAGCTACTTTTACTGGTTCTAAAATAGCAGTAGGTGTGACTACACCTGTTCGTCCAATACT
TAATTCAATATCTAATAATTTAGTTACTACTTCCTCAGCTGGAAATTTATAAGCAATGGCC
CATCTAGGAGATTTTTGTGTGAATCCCATCTCATCCTGTTGATCTAAATCATTAACCTTAA
TAACAATCCCATCAATATCATAAGGTAATGACTCTCTTTGGCTTGTCCATTTTTCAATATA
CTCTAAAACACCATCGATATTATTTACACGCGCTCTATTTTTATTCGTTGTAAAACCTAAT
TTATCTAACTCATCTAATGCTTCACTTTGCGAACGCGCATTGAAATCAGTGAAATCATTGA
CACTATATATAAATACGCTTAGCTTTCGTTTTGCCGTTAATTTAGAATCTAACTGTCTTAA
TGATCCCGCAGCAGCGTTTCTTGGATTTGCAAATAACTGCTCATCATTTTTTTCTTTTTCTT
CATTTAATCGTAAAAATGAACGTCTCGGCATATATGCTTCACCACGAACTTCTACATTTAA
TGGTTCTTTCATTTTCAAAGGTATCGCATGAATTGTTTTTAAATTTTCGGTAATATCTTCAC
CTGTTGTTCCATCACCACGTGTTAAACCTTGAACGAAGTATCCATCAACATATTTCAATGA
TACTGCTAAGCCATCAATTTTTAATTCGCACATATATTCAACGTTGCCAATTTGTTCACGT
ATGCGTTGGTCGAATTTTCTCAAATCATCTTCATTAAATGCATTCCCTAAACTTAACATTG
GCGTGTCATGGTTGACTTTATTGAAAGAGGCTTGGGCTTCACCGCCAACTCTAACTGTTGG
AGAATCTACAGTCTTATACTCAGGATGCTCCTCTTCTATTTTAATCAGTTCATGAAGTAAT
TTGTCATATTCACTATCTGGTACAGATGGATTATCCTCTACATAGTATTCATAACTGTATT
GATTTAATAAATCATGTAACTCGTTCACACGAGACGATAAATCAGCCAT

## > pcrA

Function: protein coding sequence; ATP-dependent DNA helicase (core genome, constant)
Best match: pcrA_CC001_MW2-USA400_BA000033.2[2011019:2013211:r]RC (completely identical)
Position: 050-contig_246_RC: 18261 … 20454; Length: 2193 bp
Sequence:
TTAATCCTCCTTTTTTTCAATTGGTGCAAATTGCGCTAACAAACGTTTTGGCCCTTGTGATT
TAAAGATAATATCTAGTTCGATTGAGCCATTTTTCTCGTTTACATTACTCACCATGCCTTCT
CCCCAGGCTTTATGCATCACTTTGTCACCTACATTCCAATCAGATGACAATACTTGTTTTT
TCGTTGACGTTGTTCGTTGACTAAATCCGCGTTTAGCAAAAGGTTTTGCCTTAGGTTGTAT
CGTTTGTCGTTTGCCACTTGAATGATTTTCTAATAGTGATTCTGGAATTTCCTTTAAAAATC
TGGATGGCATATTTGACTGAGGGCGACCAAATAACATTCTTGATGTCGCATGAGTGATAT
ATAACACCTCTTCAGCCCTTGTAATTGCTACATAACAAATACGACGTTCTTCTTGCATTTC
ATGATCATCTTCACTCTTAATCGCTCTAATATGTGGGAATAAAGATTCTTCCATCCCCATT
ATAAAGACAATTGGAAATTCAAGGCCCTTAGCCGAGTGCATCGTCATTAGTGTTACGCCA
TTTTCAGTATCTGCCTCATCAATATCAGCTACTAACGATAAATCCGTTAAAAAGTTAATTA
ATGACTGTTCTTCTAATGGGGTATTTTCCTCATAGTCTTTTGGTACTGACATAAATTCATC
GATGTTTTCTAATCTACTTCGAGATTCTAATGTATTTTCACGTTCAAGCATTTCTCGATAGC
CAGACTTTTGTAATACTTCATCAACTATTTCATGAATTTCTAAAAATTCTTGTTCTTTTATC
AAGCTTTGGATTAACTCGTAAAAATTAAGACACTCTTGTGTCACCTTTTTTGACAATCCGA
TAAAATCAGCTTCTCCAAGTGCATCAAACATACTGATATTGTTTTGAAGTGCATAGTTTTG
AACTTTTTCAACAGATGAAGGACCTACACCTCTTTTTGGAACATTAATAATACGTTGCAA
ACTAATGTCATCATTACTATTGGCAATTATACGCAAATAACTTAATAAATCTTTGATTTCT
TTACGGTCATAGAACTTTTGGCCACCAACCATTGTGTATGGCATATTAGATTTCATGAATG
TTTCCTCAAGTACACGTGATTGTGCATTCGTTCTATATAAAATTGCCATATCTTGATATTTC
TTACCATTACGTTGATGCTTCATAATTTCTCGTATTACAAATTCCGCTTCATCACGTTCCGT

CATTGCTTCATAGTAATGAATTTTCTCACCATTCGTATTTGCAGTCCACAGTCCTTTTGGCT
TACGTTCAGAATTATTTTTAATCACTTCGTTAGCCGCATTTAAAATCGTTTTCGTCGAACG
ATAATTTTGCTCTAAAAAGATTGTATTCGCTTCTGGATAGTCTTTTTCAAATGATAAGATA
TTTTGAATATCAGCACCACGCCAACCATAAATTGACTGATCTGAGTCACCTACAACACAT
AAGTTTTTAAACTTACTTGCTAATAATTTAACTAATGTGTATTGTGCTTTATTAGTATCTTG
ATACTCATCTACATGAATATATTGGAATTTGTTCTGATAATATTCTAGAACTTCTGGTACA
CGCTCAAATAAGTTAATCGTTGTCATAATAAGGTCATCAAAATCTAACGCTTCATTACGT
GACAATTGGCGTTGATATCCACTATAAACCGTTGCTACCATTTGCGAGTGATAATCTGTG
GCTTCTTTTTGAGCATCTGCAGGTGTTTTAAGTTCATTTTTCAAATTACTGATCGCACCGAT
AAACATACGAGGTTCAAACTTTTTACTATCAATATTTTCATTTTTTAAGACGTCTTTAATA
ACAGATTTTTGGTCTGTAGGATCAATTATCGTAAAATTGCGTTCTATACCAATTCGATCTG
CATCACGACGTAAAATACGAACACACATTGAGTGGAATGTTGACATCCAAATAACTTCTG
CTTGATCACCTACTAATTTTTGAACACGTTCTTTCATTTCTCTTGCAGCTTTATTTGTAAAA
GTAATAGCCAAAACATTGTATGGTGAGACATCTTTTTCGTCTAATAAATAAGCAATTCTAT
GTGTTAAAACACGTGTCTTCCCTGAACCAGCACCTGCCATAATTAACAATGGTCCTTCTGT
TGTCTTTACAGCTTCACTTTGCTCTGTATTCATATGATTTAATAACGCATTCAT

## > **pcrB**

Function: protein coding sequence; heptaprenylglyceryl phosphate synthase (core genome, constant)
Best match: pcrB_CC001_MW2-USA400_BA000033.2[2013208:2013900:r]RC (completely identical)
Position: 050-contig_246_RC: 20450 … 21143; Length: 693 bp
Sequence:
TCATTTACTAGACTCCTTTATTTTTACTGTTTTTAAAGCTTTTTTAATATCTTTATAAATAA
TATCACCGACGATAATTGTATCTGCAATAGCTGCCATCTCTGTCGCTTGTTGTTCTGAGGA
AATACCGCCACCATAAAAAAGTTGCGTTTCTGTTAGATGTTCTGAGACAGCTTGAACCTTT
GATACGTCGCCATAAATGCCACTATACTCTATATACATAACCGGTAATCGATACATATGA
TTGACCATTTGGGCATATGCTTCTAAATCTTCTGTTGTTAAATCTGTATTTGCTTTGGTATG
TTTTGCCACTTTGCTATCAGCATTGCACACGACATACCCTTCAAATATTACTTCCTCAAAA
TCTATACTATGTCCATATGTTTTAAGCGCTTCTAATAATGTACCATTGTGAAATACAACAT
CTGTACTGTTCAGTACTGTAGGTACAAAATAAAAATCAAAACCAGGCATTACACTTTCGA
TGTTTGATATTTCAAGTACTAAAGGCAATGGGTATCTTCTTACTCTGCTCATTAAATGAAT
GACATTATCTTCAGTAACGTCATCAGTTCCACCAATCATAATTGCATCTGTTTGAGACATA
CAAATCGCATCTAAATCATCATCTGAAATATGTTTAGCTGGGTCTAATTTAAAAATATGG
CGCCATTTTTTAATGTCATACAT

## > **purB**

Function: protein coding sequence; adenylosuccinate lyase (core genome, constant)
Best match: purB_CC001-ST772_118_AJGE01000047.1[49897:51192:r]RC (completely identical)
Position: 050-contig_246_RC: 21725 … 23021; Length: 1296 bp
Sequence:
TTATGCTAATCCAGCGCGTTCGAAAATAGTGTCAACTTGATTCAAATGATGTTTAGGATC
GAAACATTCATCCAATTCTTCTTTTGTTAAAACACTTGTAATAGACTCATCTTGTTCGATT
AATTCACGGAACGGTGTTTTCGTTTCCCAAGATATCATCGCTTTTGGTTGAACTTTGTCGT
ATGCTTCTTCACGAACCATACCTTTATTAATTAATGCTAATAAGACACGTTGTGAGAAAAT
CAGACCAAATGTTTTATCTATGTTATTACGCATATTATCTTCAAATACAGTTAAACGGTCC
ACAATATTTGTGAAACGATTCAATGCATAATCTAGTGCTATTGTAACATCTGGTAACATA
ATACGCTCAGCAGAAGAATGAGAAATATCTCTTTCATGCCATAATGGCACATTCTCATAA
GCTGTAGTAATATAACCACGAATGACTCTTGAAATACCTGTGATATTTTCAGAACCAATT
GGATTTCGTTTATGAGGCATTGCAGATGAACCTTTTTGGCCTTTTGCAAATGCTTCTTCAA
CTTCTCTCGTTTCGGTTTTTTGAAGGTTACGTATTTCAACGGCAAATTTTTCTAGTGATGTC
GCGATTAATGCTAATGTCGCAATATAGTATGCATGTCGATCGCGTTGCAATGTTTGCGTTG
ATACAGGCGCTGTGCCAATACCTAAATGTTTACACACATAACTTTCTATTTCAGGAGGAA
TGTTAGCAAAAGTACCTACTGCACCACTCATTTTCCCTACTTCAATTTCTTCTCTTACTTGT
TTGAAACGTTGTAAGTTACGTTGCATTTCCGTGTACCACAATGCCATTTTGACACCAAATG

TAGTTGGTTCTGCATGCACTCCATGTGTACGTCCCATCATCAATGTATATTTATAATTTTTT
GCTTTTTCAGCTAAAACGTCGATAAATCTTTCTAAATCTTTTTCAATAATGTCATTTGCTTG
TTTAATAACGAAACTTAAAGCTGTATCTACAACATCAGTAGAAGTTAAACCATAATGTAC
CCACTTACGTTCTTCACCTAGCGTTTCAGAAACTTGTCTAGTAAAGGCTACAACATCATGG
CGCGTTTCTTGTTCAATTTCTTGTGCACGTTCGACATTTACCTTTGCGTTTTGACGAATTTT
TTGTACGTCAGCTTTCGGTATATGTCCTAATTCACTCCATGCTTCACATGCTAAAATTTCC
ACTTCTAACCATGCTTCATAGCGATTTTGATCAGTCCAAATATTAGACATTTCTTCTCTAG
AATAGCGTTCAATCAT

> **sspP**
Function: protein coding sequence; staphopain A (genomic island)
Best match: sspP_CC001-ST772_118_AJGE01000047.1[52053:53219] (completely identical)
Position: 050-contig_246_RC: 23881 … 25048; Length: 1167 bp
Sequence:
ATGAAAAGAAACTTTCCAAAATTAATAGCTTTAAGTTTAATATTTTCTTTAAGTGTTACCC
CTATTGCAAACGCTGAGAGCAATTCAAATATCAAAGCAAAAGATAAGAAGCATGTGCAA
GTTAATGTCGAGGACAAGAGTGTTCCTACTGACGTACGTAATTTAGCGCAAAAAGACTAT
CTTTCTTATGTTACTTCACTTGATAAAATCTATAATAAAGAGAAAGCCTCGTATACACTTG
GTGAACCTTTTAAAATTTATAAATTCAACAAAAAGAGTGATGGTAATTATTATTTTCCAGT
TTTGAACACTGAAGGTAATATCGATTATATAGTTACAATTTCACCAAAAATCACTAAGTA
TTCTAGTTCATCTTCTAAATACACAATCAATGTTTCTCCATTTTTATCTAAAGTCTTAAATC
AATATAAAGATCAACAAATTACTATTTTGACAAACTCAAAAGGTTACTATGTGGTTACTC
AAAATCATAAAGCTAAACTAGTACTAAAAACACCACGTCTAGAAGATAAAAAGTTAAAA
AAGACTGAGAGTATACCTACTGGCAATAATGTAACGCAACTAAAGCAAAAAGCTTCTGTC
ACTATGCCAACTAGTCAATTTAAAAGTAATAATTATACTTACAACGAGCAATATATAAAT
AAACTTGAGAATTTCAAGATAAGAGAAACTCAAGGTAACAATGGTTGGTGTGCAGGCTA
TACGATGTCTGAATTACTAAATGCGACTTATAATACTAACAAATATCATGCAGAAGCTGT
GATGAGATTTTTACACCCAAACTTACAAGGACAACGATTCCAATTTACTGGTTTAACGCC
AAGAGAGATGATTTATTTTGGACAAACTCAGGGAAGAAGTCCACAACTTCTTAATAGAAT
GACTACATATAATGAAGTTGACAATCTTACTAAAAATAATAAAGGAATTGCTGTTTTAGG
TAGTAGAGTAGAATCAAGAAATGGTATGCACGCAGGGCATGCCATGGCAGTTGTTGGGA
ACGCTAAATTAGATAATGGACAAGAGGTTATAATTATATGGAATCCATGGGATAATGGTT
TTATGACTCAAGATGCTAAAAACAATGTTATTCCAGTATCCAATGGCGACCATTATCAAT
GGTATTCATCTATTTATGGTTATTAA

> **sspS**
Function: protein coding sequence; staphostatin A (genomic island)
Best match: sspS_CC001_MW2-USA400_BA000033.2[2017836:2018162] (completely identical)
Position: 050-contig_246_RC: 25078 … 25405; Length: 327 bp
Sequence:
ATGGAACAATTTGAATTATTTAGTATTGATAAATTCAAATGTAATTCAGAGGCTAAGTAT
TATCTTAATATTATTGAGGGAGAATGGCATCCTCAAGATTTAAATGATAGTCCTTTAAAAT
TTATTCTCAGTACCTCAGACGATTCTGATTACATTTGCAAATATATAAATACAGAACACA
AACAACTCACATTATATAATAAAAATAATAGCTCAATTGTTATTGAAATATTTATACCAA
ATGATAATAAAATACTACTAACAATTATGAACACAGAAGCTTTGGGAACTTCTCCTAGAA
TGACTTTCATTAAGCAAAAGTCATAA

> **Q5HEL1**
Function: protein coding sequence; putative protein (core genome, variable)
Best match: Q5HEL1_CC001_MW2-USA400_BA000033.2[2018454:2018627:r]RC (completely identical)
Position: 050-contig_246_RC: 25696 … 25870; Length: 174 bp
Sequence:

TTATTGGATTTTCTTACCTACAAAAATGATTTCTTGATGTGATACTTCAACCGTGCCATCTT
TTTGCTCTTGAAACACGGGTTTTTCAATACGTTTTACCGGCATATACCCTTGCATTTTCATT
CGTGACAAACAATCAGCAATTGTTTCATTCTCATTTACTTTGAATTTCAT

## > UPF0316

Function: protein coding sequence; uncharacterised protein family 0316 (core genome, constant)
Best match: UPF0316_CC001_MW2-USA400_BA000033.2[2018608:2019210:r]RC (completely identical)
Position: 050-contig_246_RC: 25850 … 26453; Length: 603 bp
Sequence:
TCATTTACTTTGAATTTCATGTTCTACTACACTTTCCAGTTCTTCTGGTTCATAATCTTTAA
GCTTTCTACGACGAATGCCTTTAGTCCAGAATCCACCATGTATGTTTCGAGGTTCATACGC
AATGATAAATGCTTTCGGATCTAAATTTTTTATCGTATCCATCAATTTACGTTCATATTTTC
TTGGTGTTAAAATTTGCATCACCATACGACTACCATCTCTACCAAACGCAGCATAGTGCG
TAACGCCATATCCTAAATTTCGAAGTTCATTCGGTAAATCTAACTCATATTCTGCTGAAGT
TACATTTACAACTGTATAACCTAATGCCAGTTTTTCTTCTATTTTCATACCAACAATGATA
CCTATTGAAAAACCAAATGCGTAGGCAATAATATTTTGAATATGGTCTAAATTAGACATA
ACCAAACCTAAACCAACGATATAAACTAATACTTCTAAAAAACTAACTGATGCAGCAATA
TAACGATAACCTTTCAACGTTAAAATTGTTCGCATCGTTAAAAACGTTACATAACAAACG
TTAATGATAAATATAGTTAGTACCATTAACCATGGATTTTCTGTTACGAATGACAT

## > nadE

Function: protein coding sequence; NH(3)-dependent NAD(+) synthetase (core genome, variable)
Best match: nadE_CC001-ST772_118_AJGE01000047.1[54895:55716:r]RC (completely identical)
Position: 050-contig_246_RC: 26723 … 27545; Length: 822 bp
Sequence:
TTAGGATTTTGGCCACGTGTATCTTGTATATGCAAGTTCACGTTTGTGTGCATTTCGTATA
TAATGATTTTCAATTACTTTTTGTTCTTCTGGCGTAACTGGCTTACCTTCTAAATAATTATC
AATCGCCTCATAAGTTACACCTAATGCATCTTCATCTGGAAGCTGTGGTTTATCATCTTCT
AAATCAGCAGTTGGCGTTTTTTCATATAATTCCTTTGGCGCACCAAGATACGCTAATAATT
GACGACCTTGTCGTTTATTCAAACCAAATATAGGTGCGATATCTGCAGCACCATCACCGT
ACTTCGTATAAAAACCAGTTATATTTTCAGCTGAATGATCTGTTCCTACTACAATACCTTG
TCGGTTTGAAGCAATTGAAAATTGTACTTTCATACGTTCACGCGCTTTTTCATTTCCTTTTT
GGAAATCTGTAAGAACAATACCGGCTTCTTTTAATGATTGTACACTTTGATCAACTGCAG
GCTTAATATTGACTGTTACTATTTCATCTGGTTCAATGAATCGCAAAGCTTGGTCAACTTC
ATCAGCATCTTTTTGAACTCCATAAGGTAATTTAACTGCAATAAACGTACAATCAATGCCT
TCTTCACGTAATTCGTTAACAGACATTTGTACTAATTTTCCAACTAATGTAGAATCTTGTC
CTCCTGAAATACCTAACACTAAAGATTTTATAAATGAATGTGATTGTACATAATTTTTTAT
AAATTGCTTTAATTCCATAATTTCTTCAGCACTATCGATACGCTTTTTCACTTTCATTTCTT
GTACAATAACGTCTTGTAATTTACTCAT

## > nadC

Function: protein coding sequence; putative nicotinate phosphoribosyltransferase (core genome, variable)
Best match: nadC_CC001-ST772_118_AJGE01000047.1[55709:57178:r]RC (completely identical)
Position: 050-contig_246_RC: 27537 … 29007; Length: 1470 bp
Sequence:
TTACTCATTATCTTCTTCCATCTCCTTAACGTGTTCCGCAACTTCAAAAATACGTTTATGTT
TATTATCCCAACATGCCTTGCTTAAATCGACTGGATATTCTTGTGGATTAAGGAAACGCTT
ATTTTCATCCCAAATAGATTGTAATCCTTGTGCTAAATATTCACGTGATTCATCTTCTGTTG
GCATTTGATATACTAATTTACCATTTTCATAAATATTATGATGCAAATCAATGGCTTCGAA
AGATTTTATAAATTTCATTTTATAAGTATGCACTGGATGGAATAATTTTAAAGGTTGTTCA
TCGTATGGATTTTCATTTTCCAAAGTAATATAATCGCCTTCTGCCTTACCTGTTTTCTTGTT
TATAATGCGATATACATTTTTCTTACCTGGCGTCGTAACCTTTTCAGCGTTATTTGATAATT
TAATACGATCACTATATGAACCATCTTCATTTTCAATAGCTACAAGTTTATATACTGCACC

TAATGCTGGTTGATCGTATCCTGTAATCAGCTTTGTACCAACGCCCCAAGAATCTACTTTT
GCACCTTGTGCTTTCAAACTCGTAATCGTTTCTTCATCCAAATCATTAGACGCGATAATTT
TAGTTTCAGTAAATCCTGCTTCATCAAGCATACGTCTTGCCTCTTTAGATAAATAAGCGAT
ATCTCCAGAATCTAATCGAATACCTACAAAGTTAATTTTGTCACCTAATTCTTTTGCAACT
TTTATTGCATTTGGCACGCCAGATTTTAAAGTATGGAATGTATCTACTAGGAACACACAA
TTTTTATGTCTTTCAGCATATTTTTTGAAGGCAACATATTCGTCTCCATAAGTTTGGACAA
ATGCATGTGCATGTGTACCAGACACAGGTATACCAAATAATTTCCCCGCCCTAACATTAC
TTGTAGAATCAAAGCCCCCGATGTAAGCAGCTCTAGCGCCCCACAATGCTGCATCAATTT
CTTGCGCACGACGTGTACCAAACTCCATTAATTTATCATTTGATGCAATTTGACGAATTCT
GCTAGCCTTTGTTGTAATTAGTGTATGGAAATTTACAATGTTTAATAAAATTGTTTCTATT
AATTGCGCTTGAATCAATGGTGCTTCTACGCGTAACAATGGTTCGTTACCAAAGCATAAT
TCGCCTTCTTGCATCGAACGGATGCTGCCTGTGAATTTTAAATCTTTTAAATATGATAAGA
AATCATCCTTGTAGCCAATAGACTTTAAATATTCCAAATCAGATTCTGAAAATCCAAAAT
GTTCTATAAAATCAATGACGCGTTTTAAACCATTAAAAACAGCATAGCCACTATTAAATG
GCATTTTTCTAAAATACAATCAAATACAGCCATTTTTTCATGAATATTATCATTCCAATA
ACTTTCAGCCATATTTATTTGATATAAGTCATTATGTAACATTAAACTGTCGTCTTCTAATT
GGTACAC

> **nos**

Function: protein coding sequence; nitric oxide synthase oxygenase (core genome, variable)
Best match: nos_CC001-ST772_118_AJGE01000047.1[57362:58438] (completely identical)
Position: 050-contig_246_RC: 29190 … 30267; Length: 1077 bp
Sequence:
ATGTTATTTAAAGAGGCTCAAGCTTTCATAGAAAACATGTATAAAGAGTGTCATTATGAA
ACGCAAATTATCAATAAACGTTTACATGACATTGAACTAGAAATAAAAGAAACTGGGAC
ATATACACATACAGAAGAAGAACTTATTTATGGTGCTAAAATGGCTTGGCGTAATTCAAA
TCGTTGCATTGGTCGTTTATTTTGGGATTCGTTAAATGTCATTGATGCAAGAGATGTTACT
GACGAAGCATCGTTCCTATCATCAATTACTTATCATATTACACAGGCTACAAATGAAGGT
AAATTAAAGCCGTATATTACTATATATGCTCCAAAGGATGGACCTAAAATTTTCAACAAT
CAATTAATTCGCTATGCTGGCTATGACAATTGTGGTGATCCTGCTGAAAAAGAAGTTACA
CGCTTAGCAAATCACTTAGGTTGGAAAGGAAAAGGTACTAATTTTGACGTGTTACCACTG
ATTTACCAATTACCTAATGAGTCAGTTAAATTTTACGAATATCCTACTTCATTAATTAAAG
AAGTACCTATTGAACATAATCATTATCCAAAATTAAGAAAATTGAACTTAAAATGGTATG
CAGTCCCTATCATTTCCAATATGGACTTAAAAATCGGTGGCATTGTATATCCAACTGCACC
CTTTAACGGTTGGTATATGGTAACTGAAATTGGCGTACGTAACTTTATTGATGATTACCGT
TACAATTTACTAGAAAAAGTTGCAGATGCGTTTGAATTTGATACACTTAAAAATAATTCA
TTTAATAAAGATCGAGCACTTGTTGAATTGAACTATGCTGTGTATCATTCCTTTAAAAAAG
AAGGCGTATCAATTGTCGATCATTTGACCGCTGCAAAGCAATTCGAACTATTCGAACGTA
ACGAAGCACAACAAGGTCGTCAAGTTACCGGAAAATGGTCTTGGCTAGCACCGCCATTAT
CTCCAACATTGACGTCAAATTATCATCACGGATATGACAATACAGTAAAAGATCCAAACT
TTTTCTATAAAAAGAAAGAATCAAATGCTAACCAGTGCCCTTTCCATCATTAA

> **pheA**

Function: protein coding sequence; prephenate dehydratase (core genome, variable)
Best match: pheA_CC005_Mu50-VRSA_BA000017.4[2060321:2061115] (completely identical)
Position: 050-contig_246_RC: 30286 … 31081; Length: 795 bp
Sequence:
ATGCAATTATATTATTTAGGCCCTAAAGGTACATTTTCATATTTGGCTTGTCGTCAGTATT
TTTCTGAAAATGAAGCAACTTTTCAACCAAAGTCAAATTTATTTGAAGTCATCAAGGCAG
TTGCAGATGATGACACATCGATTGGTGTTGTCCCTATTGAAAATTCTATAGAAGGAACAA
TCAACATTGTGGCAGATGCACTTGCACAGCAAGATGTCTTTGCACACGGTGAAATACGTT
TAGATATTAACTTTGCATTATATGGTAATGGAACAGATTCCATTTCTGATATTAAAAAGT
GTATTCTATTGCACCAGCAATCAGTCAAACAACAAATTACATACATCAACATCAATTTGA
CTATGATTATGTCGACAGTACAATTCAAAGTTTGACTAAAATTGAAAATGGCGTTGCAGC
AATTGCACCTTTAGGTAGTGGCGAAGCTTATGGTTTTACACCTATTGATACACATATTGAA

GACTATCCACATAACGTGACACGTTTTTTAGTTATAAAAAATCAGCAGCAATTTGATCAA
AATGCGACATCATTGATGTTTTTAATTACACCAATGCATGATAAACCTGGGCTGTTAGCA
AGTGTGCTTAATACATTTGCTTTGTTTAATATTAATTTATCCTGGATTGAGTCTCGTCCTTT
AAAAACACAATTAGGTATGTATCGATTTTTCGTTCAGGCTGATTCTGCAATTACAACCGAT
ATAAAGAAAGTCATTGCTATTTTAGAAACCTTAGATTTCAAAGTAGAAATGATTGGTGCT
TTTAATTAA

> **sdcS**

Function: protein coding sequence; sodium-dependent dicarboxylate transporter (core genome, variable)
Best match: sdcS_CC005_Mu50-VRSA_BA000017.4[2061305:2062867:r]RC (completely identical)
Position: 050-contig_246_RC: 31270 … 32833; Length: 1563 bp
Sequence:
CTATTTCAATGGCAGTGGTTGATTTATATCAATACCTAAAACTATAGGCATTACATAATAC
ACGACTAAAATAATAATTATTGCACTGATTAAGTTTACCCAGAATCCTACTGATGCCATTT
GTTTGATAGATATTTTACCTGAACCAAAGATAATTGCATTCGGTGGTGTCCCTACTGGTAA
CATGTATGCACAGTTAGCCGCCATAGCTGCAGGTGCCATAAGTAGTAATGGATGCACTCC
AACAGCAACAGACAACGTTGCTAAAATCGGTAAAATCATCGTTGCAGTTGCAGTATTAGA
TGTCACTTCAGTTAAAAATAAGACAAAGATTGTTATGACAATTACAATAAGAATCGGACT
AACACCATTTAATGATTTCAACTGTTCGCCTAACCATTTTGCTAAACCACTTTCAGAAATA
CCTTTCGCTAATGCTAAACCGCCACCAAATAAAATTAATACACCCCAAGGGAGCTCTTTT
GCAACTTCCCAGTCAATGATACGGCGATGTTTTTCAGTATTTTTAGCTGGAATAATAAATA
ATAATATTGATATAAAAATAGCAATCGTACCATCTGCAACAGATGACGTAACTTCCCATT
TTTTCAGAAGAAACTCTCTTGTAATCCATAATAAGCTGGCAAGTACAAAGATAGTTTGTA
CTACCTTTTCTTCATACTTCATTTTGCCAAGCTCGTCTAACTTTTGTTTAATTAACGTCTGA
CCACCAGGTAAATATTTCAAATCATGTCTAAACGCAACATATCTTAAATAGAGCCAAGTA
ATACCTAACAAAACAATGACCGTTGGAATCCCTACAATCATCCATTTAGCAAAACTAATT
TCATGTCCAAAATGTTGCATGTATTGTCCTTTTAAAATAATTAATGGCGGGGTTCCGATTA
ATGTACCCAAGCCACCAATCGTACCTGCATAGCCAATTGCTAAAACTAGAGATTTTTCAA
ACTTTTGAATACTTGTTTGATTCGTATTGGCTTCTTGTAAATCATGTGCTTCCTTAATAATT
GCTAAACCAATCGGAATCATAATCATTACAGCTGCAGTGTTCGATACAAACATAGATAAG
AATCCTGTTGCCACCATGAATCCAAGTAATATTTTTGAAGTGCTTGCACCAATTAAATTAA
TAATAGTCAAAGCTACTCTCGTATGTAAATTCCATCTTTCCATTGCAATTGCCAAAATAAA
TCCACCTAAAAACAAAAAGATAATATCATTGCCATATTCGGATGATACTTGTTCTGGTGT
AAGTATGTGACCTAATGGTAATAACACAATTGGTAATAAGCTCGTTGCTGCAATAGGAAT
TGCTTCAGTAATCCACCAAGTCGCAATCCATAAAGTAATCGCTAAGACATATACGCCTTT
CCAAGGTAAGTCTTGTGGATGAAAGAACAATAATGTTAATAGGAAAAGTAATGGACCTA
ATATTAGTCCAATAAGTTGTCCCGCACTAAACGGTTTCTTTTTCTTTGATTTTGAAAAGAA
TGTTAAATACCTTTTCGATATCATTGATTGATGTTGATTGAAATAAGCCAT

> **ppaC**

Function: protein coding sequence; manganese-dependent inorganic pyrophosphatase (core genome, variable)
Best match: ppaC_CC001-ST772_118_AJGE01000047.1[63291:64220] (completely identical)
Position: 050-contig_246_RC: 35119 … 36049; Length: 930 bp
Sequence:
ATGGCTAAAACATATATTTTCGGACATAAGAATCCAGACACTGATGCAATTTCATCTGCG
ATTATTATGGCAGAATTTGAACAACTTCGAGGTAATTCAGGAGCCAAAGCATACCGTTTA
GGTGATGTGAGTGCAGAAACTCAATTCGCGTTAGATACATTTAATGTACCTGCTCCGGAA
TTATTAACAGATGATTTAGATGGTCAAGATGTTATCTTAGTTGATCATAACGAATTCCAAC
AAAGTTCTGATACGATTGCCTCTGCTACGATTAAGCATGTAATTGATCATCACAGAATTG
CAAATTTCGAAACTGCTGGTCCTTTATGTTATCGTGCTGAACCAGTTGGTTGTACAGCTAC
AATTTTATACAAAATGTTTAGAGAACGTGGCTTTGAAATTAAACCTGAAATTGCCGGTTT
AATGTTATCAGCAATTATCTCAGATAGCTTACTTTTCAAATCACCAACATGTACACAACA
AGATGTTAAAGCAGCTGAAGAATTAAAAGATATTGCTAAAGTTGATATTCAAAAGTACG

GCTTAGATATGTTAAAAGCAGGTGCTTCAACAACTGATAAATCAGTTGAATTCTTATTAA
ACATGGATGCTAAATCATTTACTATGGGTGACTATGTGACTCGTATTGCACAAGTTAATG
CTGTTGACCTTGACGAAGTGTTAAATCGTAAAGAAGATTTAGAAAAAGAAATGTTAGCTG
TAAGTGCACAAGAAAAATATGACTTATTTGTACTTGTTGTTACTGACATCATTAATAGTGA
TTCTAAAATTTTAGTTGTAGGTGCTGAAAAAGATAAAGTCGGCGAAGCATTCAATGTTCA
ATTAGAAGATGACATGGCCTTCTTATCTGGTGTTGTTTCTCGAAAAAAACAAATCGTACCT
CAAATCACTGAAGCATTAGCAAAATAA

> **aldH**

Function: protein coding sequence; carotinoid aldehyde dehydrogenase (core genome, variable)
Best match: aldH_CC001-ST772_118_AJGE01000047.1[64638:66017] (completely identical)
Position: 050-contig_246_RC: 36466 … 37846; Length: 1380 bp
Sequence:
ATGAATATCATTGAGCAAAAATTTTATGACAGTAAAGCTTTTTTCAATACACAACAAACT
AAAGATATTAGTTTTAGAAAAGAGCAATTAAAGAAGTTAAGCAAAGCTATTAAATCATA
CGAGAGCGATATTTTAGAAGCACTATATACAGATTTAGGAAAAAATAAAGTCGAAGCTT
ATGCTACTGAAATTGGCATAACTTTGAAAAGTATCAAAAATGCCCGTAAGGAACTTAAAA
ACTGGACTAAAACAAAAAATGTAGACACACCTTTATATTTATTTCCAACAAAAAGCTATA
TCAAAAAAGAACCTTATGGAACAGTTTTGATCATTGCACCATTTAACTATCCTTTTCAACT
AGTATTCGAACCTTTAATCGGTGCTATTGCAGCAGGTAATACGGCAATTATTAAACCATC
TGAGTTGACACCAAATGTTGCACGAGTGATTAAACGATTAATCAATGAAACATTTGATGC
AAATTACATTGAAGTTATTGAGGGAGGAATTGAAGAAACGCAAACGTTAATTCACTTACC
TTTTGACTATGTCTTCTTTACAGGAAGTGAAAATGTAGGCAAAATCGTTTATCAAGCTGCC
AGCGAGAATTTAGTTCCTGTGACATTAGAAATGGGCGGTAAATCTCCAGTCATCGTTGAT
GAAACAGCGAATATTAAAGTTGCTAGTGAGCGCATTTGTTTTGGGAAATTCACTAATGCC
GGCCAAACATGTGTTGCACCAGATTACATTTTAGTACACGAATCTGTAAAAGATGATTTA
ATCACAGCCCTATCAAAAACGTTGCGTGAATTTTATGGTCAAAATATACAACAAAGTCCA
GATTATGGCCGCATTGTAAACCTTAAACATTATCATCGTCTGACTTCATTACTTAACAGTG
CACAAATGAATATTGTATTTGGTGGTCATAGTGATGAGGATGAACGTTATATAGAACCAA
CATTGTTAGATCACGTTACAAGTGATTCAGCAATTATGCAAGAAGAAATTTTTGGTCCTAT
CTTACCGATTTTAACGTATCAGTCATTGGATGAAGCAATAGCCTTTATTCACCAAAGACC
AAAACCTTTGAGTTTATATTTATTTAGCGAAGATGAAAATGCTACACAACGTGTAATAAA
CGAGCTATCATTTGGCGGCGGCGCTATTAATGATACATTGATGCACCTAGCGAATCCTAA
ATTACCATTTGGTGGTGTTGGCGCCTCAGGTATGGGACGCTATCATGGTAAATATTCATTC
GACACTTTTACACATGAAAAAAGCTACATTTTCAAATCTACACGATTAGAATCAGGTGTC
CATTTACCACCATATAAAGGTAAATTTAAATACATCAAAGCTTTCTTTAAAAAATTAA

> **tnpA**

Function: protein coding sequence; transposase A (mobile element)
Best match: tnpA_CC398_CC001-ST772_118_AJGE01000047.1[67721:68833] (completely identical)
Position: 050-contig_246_RC: 39549 … 40662; Length: 1113 bp
Sequence:
ATGAAAATAGTAGAAGTAAAATCTAAGAATGGTATCAATTTTATGATTTTAGATGGTAAT
AATGAACCTATAGTAGATGCAGTAAGATATTTGAAGTATCTGGATAGTGTTAATAAAAGT
TTAAATACCAAGAAAACCTATGCCTATGCACTAAAAAAATTTTTTGTTTACTTAGAAAGT
AAAAAGATATGCTATAAAGAAGTTAGTTTTGATAACTTTGTTGATTTTATAAGATGGATG
AAAACACCTTTTGAATATGAGAATGTCCTCTCTTATCACCGAAAAGGAAAAAGCATTAGT
CCTAAGACAATTAATCTGACTATTACTGTAGTATCTAATTTTTATGATTATCTCTATAGGA
GCAAAAAATTAGATGTTAATTTCTATGATTTTATACATACGGAAAGTAAATACTCTAAAA
AATATAAAAGTTTCATGCATCACGTAAACAAGGACTATGGAATGTTGAAAAATATTTTGA
AAGTAAAAGAACCAAAGAAAAAACTAGAAGTGTTAACCAATGTTGAGGTTAAGAAATTA
TTAGGAGCAGCTAATAATATTAGAGATAAATTCTTAATACAATTATTATATGAAACAGGA
TTACGTATAGGTGAGGTATTATCATTACGCATTGATGATATCAAATTTGATTTTAGAAAAG
GTCACCAAATAGTTTTGAAAAATAGATTTAATAGCAATGGTACTTATTTAAAAACTGGTG

AAAGAAAAATATTTATTTCTCAATCGTTAATTGATTTATATGACGATTATGTGTACGAAAT
AATTGACGAACTTTCCATATGTTCTGATTATCTTTTTGTAAAAATAAAGGGTAGAAATGTA
GGAGAAGCTATGCATTATAGTGATATATATTCATTATTTAAAAGGCTAAAACATAAAACA
TCTATAAATGTTCACCCCCATTTATTTCGCCATACACATGCAACTGTTTTTTATAATGAAA
CTAAGGATATTAAACAAGTTCAAGAAAGACTCGGTCATAGTAATATACAAACGACTATTA
ATTTATATGTTCATCCCACTGAAGAAGATATACGTGAAGATTGGAATAAAGTTAAGCATC
AATTTCAAGTTTTTAATAAAGGGGAATAA

> **tnpB_CC398**

Function: protein coding sequence; site-specific recombinase/phage integrase family (mobile element)
Best match: tnpB_CC398_CC001-ST772_118_AJGE01000047.1[68836:70899] (completely identical)
Position: 050-contig_246_RC: 40664 … 42728; Length: 2064 bp
Sequence:
ATGTCAATACCAATTAATTTGCCTACAAACAGTACTATGATTAATGAATTATGTACATTAC
AAAGTAGAACTATAAACATAAAAGGAGAAGTGCTAATAACTGAAATATATGATGATTAC
TTTTTAAAAAATGATGAATGGCACATTTCAGCTTTTAACAAGTTCGAGCAATTTCAAGAT
AGTATCAAGAACTATAAAGACAAAAGAAAAAATATTTTCTTTAGGATTAAAAGTGAAAA
TTTGAATTTAGAATTTAAATACCTTTTTTTGAAACTTATAGTTAAAGAGGATTGGTCTTTA
TCTAATGTATTTAATACTGGAGCAGTGAAACTTAACAAGATTGCTGAGTTCTTTAATGAA
GTTTATCCAAATTTAAATTCTTTACTAGATTGTGATATTAATATGTTAGAAAAGTACTGGT
TTAGTTGGTTGACTAAAAATAATATACCTATAAAAAGAAAATCATCAACAATAGTTTTTG
GTGATTATGAACATAAAAGTGGACTAGCTTCTTTTTTAAAGAATATGTATATTAATCTAAA
TAAATTTATTGATAAGCGAGAAGAATGGGAAAAAGATAAATGGGATATTAGAAATTTAG
AAAAATATGGATTGAGTTATAATAAAACACTAACTGGTAATTATTTGAACTTTGAAAAAA
TTGAGTCAATTAAAATGCGAGAATTAGCAAAAAAATATTTAAAAAATCGTTTGATAACTG
GTGATATAGCATTTGCTACAGCAAGATTTTATATAAGAGTTTTGACTAGGTTTTTTCAAAA
TATATCTAAAAATAAAGAAACAAGGAATAGTTTAAATGAACTAGATAGATGTCATATCG
AAGCATATATAGAATTTTTATTTGAATATGCTAATAGTAAAAATGTGCAAAGTTCTAGGA
ACTTCGTTAGAGAAGAATTAAAAGCAATTAGAAGGTTCTTAAATGATATTGCTAGTCAAA
ATTATGATATAGCCCCCTCTGAAGATATACGTTTTTTAATTTATCCTCAAGACTTTCCTAA
GCGCGAAAAGAAAAATAGTAGCCAAATTGATTATATCCCAGATTTTGTATTGGAACAGCT
TTTTGAGCATATAAATGATTTGCACAAAGATTTAATACCTGTAGTTTGGATAGCTTTTAAA
ACAGGACTAAGAATTTCTGATGTATTAACATTAAAAAATAATTGTCTTGCAAAAGTTAAT
GGAAAGTTTTCAATAATTACTGATATTGCTAAAACTTTTGTTAAAGGACATAGGATACCA
ATCGATAATGAATTAGCTGATATAGTAGCAGTTTTAATAGCCAATTCTAAAATTAAAAGT
ACAAAAGATAACAACCCTAATAATTATATTTTTGCTATTCATAAAGGAAAAAGAAAAGG
AATGCCCTTTACACAGCATATGGTCAGAGCACACTTAAATCATTTAGCAAAAACAAAAAA
TATTCTTGATGAACAAGGTGAAGTCTTCCATTTCAAAACTCATCAGTTTAGACATACTTAT
GCAGTGAAATTGTTAAATGGTGGAGCAGATATATTAACCATACAAGAATTATTAGCACAT
TCCTCACCAGAAATGACACTCAGATATGCTAAGTTACTTGATGATACAAAAAGAAAGGCT
TTTGAATCAGTTATTGATCAAGGGGCTTTTAGTTTTGATGTTGACGGTAAAATTAAAAATA
TACAGCATAGTAGTGAATTATCAGAAAAAGCATTGAATTCTTTATGGCAAGAACATAAAT
TAAACGCAATGGACAATCCTTATGGTACTTGTCATGCTAGATTGAGCGGTGATTGTCCAT
ATATGGAAGCTCCGCCATGCTTAACTTGTAATTCTGGGAAACCATGTAAGGATCTAGCTA
TTGGATTTTCTGATTTAGATGTTGAAAAATATGAATTACATATCAAATCAACGGTCAAATC
CATAGAATTAGCTAAAAATAATAATAGACAAGATATAGTTGAAAAACATACTAATATATT
AAATAAATATGAAGAAATATTAGGTAATATAAAAGATGGAAACATTATATTCGGGAGAA
GTAATAGAATAAAAGTATAG

> **tnpC_CC398**

Function: protein coding sequence; transposase C (mobile element)
Best match: tnpC_CC398_CC001-ST772_118_AJGE01000047.1[70908:71273] (completely identical)
Position: 050-contig_246_RC: 42736 … 43102; Length: 366 bp

Sequence:
ATGGGAAAATTTAATAGACAAGAACAATTAAAACAATTACATGCAGAACGAAAAGTTAA
AACCGAGCAAAAAGTTTCTAAAGCAATAAATGAATTGGTTCGAAAAAATGAAGAAATTA
ACTTTAATATTGTTGCTAAGCATTCAAATGTATCTAAAGCTACTTTATATAATAATAGTAA
AATTCGAAAGAGAATCGAAGAGTTACGTGAACAAAGTAAAGACATATTTGTACATAAAA
ATAACAGTGACGGTAAAGACGCATTAATTTCTTCATTAAAAAGGAGAGTAAAATCTTTAG
AAAATGAGAAAAAGAGGCTCAAAGATGAAATAAACACATTATATAATAAAATATATGAA
GATATTTAA

## > **blaZ**
Function: protein coding sequence; beta-lactamase (mobile element)
Best match: blaZ-3_CC001-ST772_118_AJGE01000047.172026:72871:rRC [4_50ANF10END]
Position: 050-contig_246_RC: 43854 … 44700; Length: 846 bp
Sequence:
TTAAAATTTGTTTATTACATTCTTGGCGGTTTCACTTATCAATTTATCATTAGGTTTATCAC
TTTTATTGTCTTTATTCGTAAAAATGACTAAAATAATAGGTTCAGATTGGTTCTTAGGATA
AATAAAAGCTACATCATTTCTAGAAGCATATGTTATTGCTTGACCACTTTTATCAGCAACC
TTATAGTCTTTTGGAACACCATCTTTAATTAAAGTGTCTCCGTTTTTATTATTTAACATTAA
ATCAAGTAAGAAATTTTTATTTTTTTTGCTTAATTTTCCATTTGCGATAAGTTTATTTAAAG
TCTTGCCGAAAGCAGCAGGCGTTGAAGTATCTTTTTTGCTCTTTGGTGAATAGTAATTTAA
TTCTATTTCATATCTAACTGGATTTGTTACTTTATCTCCCAATTTTTTTAAACGTTTTTTAAT
TTTTTTGATTCCACCGATTTCGTTTATAATTTTATTGTTTGCTGTATTATCACTATACTTCAT
TGAAGCTTCAATAAGTTCTTTTAAAGTGATATCTTTTCCTACATATTTTTCTAAAATAGGA
GAATAAGCAACTATATCATCTTTGTTAATATGTACTTTTTTATTTAACTTATTATAAGGTA
CTTGTTCTAACAAAATAGCACTATTTATCGCTTTTGAAGTTGAAGCATAGGCAAATCTCTT
ATCGGCATTAAATTTTACTTCCTTACCACTTTTAGTATCTAAAGCATAAACACCAATATGA
GCATTATATTTTTTTTCTAAATCATTTAACTCTTTGGCATGTGAACTGTTTGAATTACATGC
ACTTAAAACTAAAGCAATTGCAATTAAAAATATTAACTTTTTCAA

## > **blaR-pUB101**
Function: protein coding sequence; beta lactamase regulatory protein (mobile element)
Best match: blaR-pUB101_part_S-xylosus_DQ016060.1[1:241:r]
Position: 050-contig_246_RC: 44806 … 45047; Length: 241 bp
Sequence:
ATGGCTAAATTACTAATAATGAGCGTAGTAAGCTTTTGTTTTATATTTCTATTGTTAGTAT
TTTTTAGGTATATATTAAAACGCTATTTTAATTATAGTTTAAATTATAAAGTTTGGTATCT
AACTGTGCTTGCTGGATTAATTCCTTTTATCCCAATTAAATTCTCTTTTATAAAATTTAATA
ATGTGAATAATCAATCGCCCACAGTTGAAAGTAGGTCACACGACTTGAACCATAACA

## > **blaI**
Function: protein coding sequence; beta lactamase repressor inhibitor (mobile element)
Best match: blaI_CC008-ST72_TCH130-ST72_ACHD01000263.1[102996:103376] (completely identical)
Position: 050-contig_246_RC: 46553 … 46934; Length: 381 bp
Sequence:
ATGGCCAATAAGCAAGTTGAAATATCTATGGCTGAATGGGATGTTATGAATATAATATGG
AATAAAAAATCAGTATCAGCTAATGAAATTGTAGTTGAGATTCAAAAAAATAAAGAAGT
TAGCGATAAGACGATTAGAACATTAATTACAAGACTATATAAAAAAGAGATTATAAAAC
GATATAAATATAATAATATTTATTTTTACTCATCAATTATTAAAGAAGATGATATTAAAAT
GAAAACTGCTAAAACCTTTCTTAATAAATTGTATGGAGGGGATATGAAAAGTTTAGTGCT
TAATTTTGCGAAAAATGAAGAATTAAATAACAAAGAAATTGAAGAATTGCGAGACATTTT
AAATGATATTAGTAAAAAATAA

## > **tnpIS1272**
Function: protein coding sequence; transposase of insertion element IS1272

Best match: tnpIS1272_trunc_CC398_SO385-pig_AM990992.1[2480969:2481165:r]RC
Position: 050-contig_246_RC: 47356 … 47386; Length: 30

TATGCATATAAGATGATTTTTAACATCATT

## > **txbi_universal1**
Function: bidirectional rho independent terminator
Best match: txbi_universal1_CC022_HE681097.1[2006687:2006756:r]
Position: 051-contig_201: 35 … 65; Length: 30 bp
Sequence:
AAATTATCTTACTGCTGTTTTTTAGGGATT

## > **A6U330**
Function: protein coding sequence; acyl-coenzyme A 6-aminopenicillanic acid acyl-transferase (core genome, constant)
Best match: A6U330_CC001-ST772_118_AJGE01000011.1[593:1705] (completely identical)
Position: 051-contig_201: 640 … 1753; Length: 1113 bp
Sequence:
ATGACATTCAATATACATACGATTACTATTTATTTACAATATATTACAACTAAAAAGAAA
GGGGCCGATGATATGCAACAAGTGACATCAGATATCATGACTTTTAGAGGTTCACATTTC
GAGTTAGGTGTTAAAACTGGTAAATGGCTTCAACAAACACCTCTTCTAAAAAATCGAGAA
AAAGAATGGAAAAAACGTGTCCCACGTTTCGATATAGATGTAAATGAAACTTATCAAATA
TTCCAAACATACGCACCTCAAATATGGGAAGAATTAATGGGACTACAAAGTATACTCAAA
ATGCCAACACGCCAAATTATTTTAAACTTTGGACATTATCGTTTTACCGATTTAAAAGAAA
GTGGTTGCACGGTCTTTCAAGGCAAAGACTTCATGGTTAGAAATTATGATTACCACCCTG
CAACTTATGATGGTCGCTACTTACTTTATCAACCTACAGATAGCGGTTTAGCTCAAATTGG
TCCTGTATCAAGGGTAACTGGAAGAATGGATGGTATGAATGAGTCAGGATTAACTATGGG
CTATAACTTTATGCACCGGAAGAAACCAGCAAATGGTTTTGTATGTTATATGATTGGTCGT
CTAATTCTTGAAAATTGCCGAAATGTAACAGAAGCAATCCAATTATTGAAAGAAATACCA
CACAGAAGCTCATTTAGCTACATACTGATGGACAAATCATTGAACCATGCAATTGTTGAA
GTGACGCCGAGATCAATAGATGTTCGTTATGATAATATATGTACAAATCATTTTGAAATA
TTAACACATGAAAATCGCAATTATACAAAAGAATCTAAAGAAAGACTGGCGCGTACCAT
TTCACAAACTAATGATAATTTAGATATGACTACAGCATTTAAGTTATTCAATAATCCTCAG
TATGAAATTTACAGTAAACTATTCAAAAGCTGGTCTGGCACCATCCATACATCAATGTAT
CACCCTGAAACATTAACTGCATATTTTACATTGGGAGAAAATGCGCCACCAGAAATAATA
GATTTCAAATCATGGTTAGATGGTCAAGACCTTAATATCACTCACTTTACTGGCAATATAG
ATACCGACTTAACTTTTGCCAACAAATAA

## > **Q5HEJ0**
Function: protein coding sequence; ABC-2 type transport system permease (core genome, variable)
Best match: Q5HEJ0_CC001-ST772_118_AJGE01000011.1[4492:5232:r]RC (completely identical)
Position: 051-contig_201: 4539 … 5280; Length: 741 bp
Sequence:
TTAAATGTCTTTTTTAGCGACATATAAGATATTCAAAATGAAGAATATCACTATAGAACC
TAATGAAATTACCCATTGCCAATTGCTAAAATCAAAGTTAGAGCTAATCATTTTATCAGTT
AAGTAAGCAAAAGGGATATAATCAAAAGCATCTAAAACTTTTTCACCGTATCTTGGGATA
AATGTAATAAAAGGTTTAATAAACGGTACAATGAGTACTAAAAATACACCTAATGAAAA
TATCATTGCTGTTTTTTGTGATGCAAGTGTGATTAAGAATAAAAACAAGCCAAAGAATAA
TAAAAAGATTAGGAAGAACCATAAATTATCTACATATTTAGAAAACGCCAAATTTTTTCC
ATTACTAGATGCCTGCACAACAATCAAAATCACATAATGAATTAATGCAAATAATATTGA
AATTAAAATAATTGAAATTGTTTTCGAAATAATGTATCCAACGCGTGTTTTAACTTTACTC
ATATATAGTTGAATTGTACCTTGTGAAAAATCACGCGTAATTGTCTTAACAACAAACATT
AATCCAATTATTAACAATAACCATTTTGCTGCAGCAAATACAGATCCATAGACAATAATA
TGTTTCGGGTTATCCATCGAATTTGCCATAAGTATACTTTGAGTTGCAATCAAACTAGATA

CGACTAGTATCGCTAAATATGTTAAAGGACTTTTAAATATACTATAAAAATCATACTTAA
CTAAATTTAAAATTCTCAT

## > nodI2

Function: protein coding sequence; nodulation ABC transporter ATP-binding protein (core genome, variable)
Best match: nodI2_CC001-ST772_118_AJGE01000011.1[5232:6104:r]RC (completely identical)
Position: 051-contig_201: 5279 … 6152; Length: 873

TTATTTATCACCTCTTTGATTTATATTGAAGTACGTATCACGCAATGAACTTTTACGTGTTT
CAATATATTTCGGATAAATATCTAATTCAGATAATGCTTTTAGTATAACTTGATAATTTTT
TTGTGCATTAATCATTAAATGTCCGTCTGCTTTGTCGACATTTTGTAATTGGAAATGCTCT
GCAAGATATTTTTCAGTTCTATCAAAGTCTTTATGATCAACAGTAACTATCGTTGTGTCAG
ATGCAACACCTTCCTCCATGTTTACATCTTGAACAAAATGTCCGTCTCTTAAAAATACAGC
TCTATCACAAATTAATTCAATATCTTCTAACTTATGACTTGATATTAGAATTCTCATATCA
AGTTCATTTACTAAAGACTTAATTGTAGTCAGCACATCAATTGAGCCATCTGGATCCATAC
CATTTGTAGGCTCATCCAAGATTAAAAATTTAGGTTTATTCATTAAAGATACTGCAATTGC
TAACTTTTGCTTCATCCCCATTGAATATTTCTTAACTTTCTTTTTAATATAAGGTCTCATAC
CAAATGCATCTATAATTTTGTCTGTGTATGCTTTATCAAAACCCTTACCTAATACTTGTGC
AAATAATTTCAAGTTATACAATCCTGATTTATTATCATATAATTTAGGATGCTCAATTAAA
AAACCGATATTATCTGCATTATCAATGTCTACTTTTCCATCAAATTTAATAATATTACCAT
TCATCACTTTCATAACGGTTGTTTACCAACACCGTTTTTCCTATTAATCCGACAATTCTA
CTATCGCCAAAGTCAAAATCAATATCATTTAAAACGACATTTGAGCCGTATTTTTTGTAA
TATGTTCTAATTTCAT

## > nodI1

Function: protein coding sequence; nodulation ABC transporter ATP-binding protein (core genome, variable)
Best match: nodI1_CC001-ST772_118_AJGE01000011.1[6782:7678:r]RC (completely identical)
Position: 051-contig_201: 6829 … 7726; Length: 897 bp
Sequence:
TTAAAAACCTTCTTCCATCAATTTGATGTTTTCATGATATTTAGGTTTTCTCTTTTCAAGAT
AAACCATTAAGTTCTCAATAGAAGGTGTAGTGATATTCACTTTATGACCTAAAATTTCTTT
AAATACTTGCGCATGTTCTGTTAATCCTATAAATCCCGTACGCTTATGTTCTTCATAAATA
AGTAGAGATGCTAATTCATCATCAAGGTCCTCAATAGCACCACTCACAACTTGATATCTTT
GTAACAGTTGTTCTTTTGAACCATTTAAGATAACTTCACCGTCACTTAAGTGAATGATATA
ATCAGCTATTTTTTCTAAATCTGAGATAATGTGTGTCGACATGAATATTGTTTTATTTTCAT
CAATTAATTCTTGCTGAATTATCTCCAATAGCTCATTTCTAGCTAAGGGATCAAGACCTGA
AGTAGGCTCATCAAAAATATACAATTCAGCATGATGTGAAAATGCAACTGCTAGTGATAA
TTTCATTTTCATGCCAGTCGAAAATGTTTTTATTGATTTATTGATTGGTAAATCGAACTTTT
CAAGATAAAATTCAAATACTTGATGGTCCCATTTACGATAAAAAGGGGTAATCATTTTTT
CAAGTTGCTTCGTAGTCCATCTCTCATTAAAATAATTTTCAGAGTAAACAAACCCAATTCT
ATTCTTTAACTCTATCGGATTGAGAGCCATATCCTCTTCTAATACTCTAATAACACCAGTT
TGTGGTTGATACAAATCCATAATTAACCTTATTATTGTGGTTTTACCAGCGCCATTTCTAC
CAATAAAACCAGTGACGAATCCTTGAGGAACTTTGAAAGAGATATTCTTTAAATTAAACT
GATCACTTGAATAATTAACATTACTTAATTCTATGGCATTCAT

## > Q5HEI3

Function: protein coding sequence; aspartate transaminase (core genome, constant)
Best match: Q5HEI3_CC001_MW2-USA400_BA000033.2[2041987:2043273] (completely identical)
Position: 051-contig_201: 9078 … 10365; Length: 1287 bp
Sequence:
ATGAATCCTTTAGCCCAAAGCTTAAATGAACAACTTCAACAGTCAAATGCAACTGCCTTT
GCAATGTTATCTGACTTAGGTCAAAATATGTTTTATCCAAAAGGCATTTTATCTCAATCTG
CTGAAGCAAAGAGTACAACATATAATGCAACTATAGGTATGGCGACAAACAAAGACGGA

AAAATGTTTGCATCATCTTTAGATGCAATGTTTAATGATTTAACTCCAGATGAAATATTCC
CTTATGCGCCACCACAAGGCATCGAAGAATTACGTGATTTATGGCAACAAAAAATGTTGC
GTGACAATCCAGAGCTATCAATCGACAACATGTCACTACCAATTGTTACGAATGCATTAA
CACATGGTTTATCTTTAGTTGGCGATTTATTTGTAAATCAAGGTGACACTATCTTGTTACC
AGATCATAATTGGGGTAATTACAAACTTGTTTTCAATACGAGAAATGGTGCAAACCTTCA
AACATATCCTATCTTTGATAAAGACGGGCATTATACTACTGATTCACTTGTAGAAGCTTTA
CAATCATACAATAAAGATAAAGTCATTATGATTTTAAATTATCCTAATAATCCGACAGGT
TACACACCTACGCATAAAGAAGTGACTACCATCGTCGATGCAATTAAAGCATTAGCTGAT
AAAGGTACAAAAGTTATAGCTGTTGTGGATGACGCATACTATGGTTTATTCTATGAAGAT
GTGTATACTCAATCATTATTTACTGCATTATCTAATTTAAATTCAAATGCAATATTACCTG
TTCGTTTAGATGGTGCAACAAAAGAATTTTTCGCATGGGGATTCCGTGTTGGGTTTATGAC
ATTTGGAACGTCTGATCAAACAACTAAAGAAGTATTAGAAGCCAAAGTAAAAGGTCTTAT
ACGAAGTAACATTTCTAGTGGACCTCTTCCAACACAAAGCGCTGTTAAGCATGTATTAAA
AAATAATAAACAATTCGATAAAGAAATCGAACAAAATATTCAAACATTAAAAGAACGCT
ATGAAGTCACTAAGGAAGTTGTCTATGCTGATCAATATCATTCACATTGGCAAGCCTATG
ACTTTAACTCTGGATACTTTATGGCAATAAAAGTTCATGATGTTGACCCTGAAGAACTTCG
TAAACATTTAATTGAAAAATATTCAATTGGTGTTATTGCACTTAATGCAACAGATATTCGT
ATTGCGTTTAGCTGTGTAGAAAAAGAAGATATCCCACATGTATTTGATTCAATTGCTAAA
GCGATTGATGACTTAAGATAA

> **tx_map**
Function: rho-independent terminator of map
Best match: tx_map_ref_CC001_MW2-USA400_BA000033.2[2043540:2043593:r]RC (completely identical)
Position: 051-contig_201: 10631 … 10685; Length: 54 bp
Sequence:
TCTACAAATGTAAAAGGCACCCCACGATTGTGAGATGCCTTATTATAAAATTAA

> **map**
Function: protein coding sequence; major histocompatibility complex class II analog protein (core genome, variable)
Best match: map-MW2_part_CC007_TCH959-USA300_AASB02000005.1[6846:7192:r]RC
Position: 051-contig_201: 10688 … 11035; Length: 347 bp
Sequence:
TTAGAATTTAAGTTCAATATCTACCTTTTTAATGTCAGTTGCTTTAAATACTCTTTCATCTT
TATACTCAGTTTTTAAGTCTACATATTTTGACGAACCATTTTTAAAATACACTTTATACAC
TGCGCGTTCAGCAAGTCCTATGTCATGTTTAGTTATCCCTTGATCATTTGCCATTGCTGATT
TAAGTTTTATACTTAAATCATTAATTGTAATTTTGCTTGCATTATAGAACACAAATTCATT
TGAAACTATGTTTTCAAATTTATTTATTGTTACTGGGAATTTCACATTATTAGATTTATTTA
TTTTTTTTTTGATTTAGTGTATTGTTTTACATCAATATC

> **map**
Function: protein coding sequence; major histocompatibility complex class II analog protein (core genome, variable)
Best match: map-MW2_part_Z262_LCL_10042.1[45974:47138]RC
Position: 052-contig_193_RC: 0 … 1165; Length: 1165 bp
Sequence:
ATAAAATTGGTGTTGATGTGCCATTTACTGCAATTGTGTATGGTACATATCTGTCCGCTTT
CGCTTTTGACCCTGTTTTAACAGTAATTTCAATTCTCTTAACATCTTTCGCATGAACTAAGT
TTGCTGTGTAATTTTTTGAATTTAACTGTAAGATTCTTTTACCACCATTTTTAAAATAAACA
GTATAATAAGCTTTCTTAGCATATTTTAAATCTTGTTCACTAATACCTCTATCATGCTTCAG
TACTGATTTTATTTGACTAGTTAAATTTTTGTAATTTGTCCAAGGTTTATTTGAAAATGAA
AGATTCGATAAAATGTTTGTAGATGTACCATTTAAATTAATTGAATATGGAACTTGATAG
TTTCTTTTAGCTTTATTTCGATATGTTTTTTTGTATCTACGTTAATATTGATACTTTTAATA
TCACTTGAATTGATTAAATTCGCTGTGTAAATACCTGATTTCAAATCTATAACTTTCTTCG

TTCCATTTTTAAAATTAACTGTATATTTCGCTTGCTTCGAAAGTCTTAAATCAACATCAGT
AATACCTCTATTTGATTCTAAAACTGATTTAACTTTACCCTCTAAATCTTTGTAACTAATAT
TTTGATTTTTATTAAATGTTAGGTTTGATAAAATGTTTTGGCTTGTGCCGTTCACTGTGATT
GTATATGGCACTTGAACATTTGCTTTAGCTTTATCTTTAGGTTGCTTTTTAGTATCTACGTT
AACACTGATAGCTTTAATGTCACTTGTATTGATTAAGTCAGCTGTGTAGATACCTGATTTC
AAATCGATAACTCTTTTTGTTCCATTTTTAAAATGAACCGTATATTCCGCTTGCTTTGAAA
GTCTTAAATCGATATCACTAATACCTCTATTAAAGTATAAAACTGATTTAACTTTATTCTC
TATATCTTTATAACTAATATTTTGATTCTTATTAAATGTTAAGCTTGATAAAATGTTTTGGC
TTGTGCCGTTCACAGTAATTGTATATGGAATCTGGATGTTAGAATGTCCATGGTGTAATGT
ACTTGATGATTTATCTAATGGCTTAGCTGCGGCAGATGCTTCGTTAGTATTAAAGTTTGCT
CCTGTTGATGCTATAACGCCTAATGCTAATGTTGTTGTAATCAATGACTTAAATTTCAT

## > hlb, disrupted - upstream fragment

Function: protein coding sequence; hemolysin beta, disrupted - upstream fragment (core genome, constant)
Best match:
hlb_CC008_COL_CP000046.1[2063726:2064718]#[2_150ANF&END]#hlb#[CDS:hemolysine beta]#[cc:72.8598:73.1883] MANUAL OVERRIDE
Position: 052-contig_193_RC: 1554 … 2326; Length: 772 bp
Sequence:
ATGGTGAAAAAAACAAAATCCAATTCACTAAAAAAAGTTGCAACACTTGCATTAGCAAA
TTTATTATTAGTTGGTGCACTTACTGACAATAGTGCCAAAGCCGAATCTAAGAAAGATGA
TACTGATTTGAAGTTAGTTAGTCATAACGTTTATATGTTATCGACCGTTTTGTATCCGAAT
TGGAGACTTTTAACATAAAATTACTTATCATTCAAAAAGTAAAACAGCATAATATCAAGG
TTTATAACTTTATCATTATCAATAATACCCCATATAAAATAAAATTTTAGGGACTTTTTAG
GGACTTTAAATTTAAAATTACAAGTTTAATAGAAACATCAAAATAATCACATGTTTGTGT
GGAATGTACACCCCAAAAGCTAGACTGAAAAATCTATTTTTTGAGGTGTATTTTTATAGG
TAAATATAATAAATTAGAGTAGACAACTCAGAATTCCAATTTTATAATAATATTGCTTGA
CATATCAAACTAGATAGTACTATTTTGAATATATTATTATAATCAAAAATTCATTGTAAAC
TTTTAGACAAAAGGAAGTAATAAAAACGTGAAATTTAAAACAACTAAAGAATGTAAAAG
TAATAATATCTTTAAAAGAAGTCAAGAAATTAATAATAGAGAAAGTGAAAAGGGTTGTTT
ATGGGGCATTAGCATGTTGATTCTACTTTTCTTATTGATTCTGTTTGGAATAACTGCTTGTT
CATCAAGCATTCACTTTATTAATTAGATTTTTTTACTTGGAGGTATCAT

## > Q6GFB6

Function: protein coding sequence; putative membrane protein (mobile element)
Best match: Q6GFB6_CC008_NCTC8325_CP000253.1[2032513:2032821] (completely identical)
Position: 052-contig_193_RC: 2326 … 2635; Length: 309 bp
Sequence:
GTGAAGAACCATACAAATATAATTAATATCTTATTAGTTATAGTCAACTCATTAACTCATT
TTCTAACTCTAAACACCTCATTTTTTAATAGTTCAGCATCGGATTTCTGTTTTATCATAGGG
GCTATATTTTTCTTGATCGGAATTTTTGTTGCAATATACGGTATGAAGCGAGCAACATATT
GGTTAAACTTATTGATTTTATTTACCAATATTTTTTATTTTCTACACTTCTGTGTTTTACTTT
TGTTAAAATATATAGGATTTAAATTATTTATTTATGAAGGGTGTGTATTGTTATTTACCTA
A

## > scn

Function: protein coding sequence; staph complement inhibitor (SCIN) (mobile element)
Best match: scn_part_phage_U01872.1[3690:3944:r]RC
Position: 052-contig_193_RC: 2924 … 3179; Length: 255 bp
Sequence:
TTTTAATATTTACTTTTTAGTGCTTCGTCAATTTCGTTATAAATCTTTTGAAGTTGATATTT
TGCTTCTGACATTTTCTTAAAGTCTTTTGACTTAAGAGCATACATTGCTTTTAGACCTGAA
ATTTTTATAGTTCGCTTATAATAAGTGTTTAAACTTCCAGTAGCTAATTCATTAACATTTA

GTTCATCTAATAACGATTTTAATTCATTAGCTAACTTTTCGTTTTGATATTCATTCGATGTT
GGCAAGCTT

## > sprD

Function: noncoding RNA small pathogenicity island RNA D (mobile element)
Best match: sprD_CC001_MW2-USA400_BA000033.2[2047917:2048058:r]RC (completely identical)
Position: 052-contig_193_RC: 3435 … 3577; Length: 142 bp
Sequence:
GGTAAGCACCGAAATGCTTACCTACTTCCCATAAACAATATAACACATATACATTGATTT
GGAAAGCGCAAAAATAAATTTAATATACTAGCCCGAAGGGGAGCAATACATAAAAAATG
AAAGGCGCTCCTTGAAAACGCCC

## > hlb, disrupted - downstream fragment

Function: protein coding sequence; hemolysin beta, disrupted - downstream fragment (core genome, constant)
Best match:
hlb_CC008_COL_CP000046.1[2063726:2064718]#[2_150ANF&END]#hlb#[CDS:hemolysine beta]#[cc:72.8598:73.1883] MANUAL OVERRIDE
Position: 052-contig_193_RC: 3578 … 4713; Length: 1136 bp
Sequence:
AAGGTAATATTAACATAAAATGGCTACTATTGCATTATCTAATTTTATTTAATTAAAACAA
ATATATAGCAAAAAAAAACTAGCCCGAAGGCTAGCTTATGAATAGATGAAAATTTGAAC
ACATTGCTGTGTCTAAAATGATTATAGCATAATTTTAAACTATTCTATAATAAATTAATTA
ACTTCATATGAAACTTTACACACTTTTTTTCAAACTGCACTAAACTTACCAAAACTGCTTA
TTCGGTTGCCTGCTATGTCTACCTCTCCGGTCGCAATATAGCGACGTTGTCCACTATTAGC
AATATAAGTAATCCATCTATGGGGGGCAATATAAACGCGCTGATTTAATCGGACAATCTT
CTTATATTAAAAATAATGATGTCGTAATATTCAATGAAGCATTTGATAATGGTGCATCAG
ACAAATTATTAAGTAATGTGAAAAAAGAATATCCTTACCAAACACCTGTACTCGGTCGTT
CTCAATCAGGGTGGGACAAAACTGAAGGTAGCTACTCATCAACTGTTGCAGAAGATGGT
GGCGTAGCGATTGTAAGTAAATATCCTATTAAAGAAAAAATCCAGCATGTTTTCAAAAGC
GGTTGTGGATTCGATAATGATAGCAACAAAGGCTTTGTTTATACAAAAATAGAAAAAAAT
GGTAAGAACGTTCACGTTATCGGTACACATACACAATCTGAAGATTCACGTTGTGGTGCT
GGACATGATAGAAAAATTAGAGCTGAACAAATGAAAGAAATCAGTGACTTTGTTAAAAA
GAAAAATATCCCTAAAGATGAAACGGTATATATAGGTGGCGACCTTAATGTTAATAAAG
GCACTCCAGAGTTCAAAGATATGCTTAAAAACTTGAATGTAAATGATGTTCTATATGCAG
GTCATAATAGCACATGGGACCCTCAATCAAATTCAATTGCGAAATATAATTACCCTAATG
GTAAACCAGAACATTTAGACTATATATTTACAGATAAAGATCATAAACAACCAAAACAAT
TAGTCAATGAAGTTGTGACTGAAAAACCTAAGCCATGGGATGTATATGCGTTCCCATATT
ACTACGTTTACAATGATTTTTCAGATCATTACCCAATCAAAGCCTATAGTAAATAG

## > lukX

Function: protein coding sequence; leukocidin/hemolysin toxin family protein (core genome, variable)
Best match: lukX_CC001-ST772_118_AJGE01000025.1[41511:42527]RC (completely identical)
Position: 052-contig_193_RC: 4950 … 5967; Length: 1017 bp
Sequence:
TTATTTCTTTTCATTATCATTAAGTACTTTTACAAACTTCACATTATGTGTCTTCCAATCAA
CTTCATATAATGCTGATAATTTTTCTTCTTTTTTATCTACATGGTTTTCACCAGACCAATAG
CCCCAGAAACCATGACGATTCCAATCTATTTTAAACTCATCCATTGATCTTTTATAATGAA
CAACAAATTTTGATTTACCTTCATCTTTTTTATCATGTGACATAACAGCTAAAAATTCTGG
ATTAAACCCTTCAGAAACAGTTACAGGCATCTTATTCTTAGGCGTAAAATTATCTTTCGCC
CATAAATTTCCATTTCGTGTTAAAGAAAAGATTTCACTTTTAGTTCTATTATCACTATCATT
AGTTAATTGTCTCGTATGGTCATGTCCCATATTATTTATCAAATGTGCTTCTACTTTCCAAC
CTACACCTTTATGTGACGTAGATTGATCAAGTAATGTACGATACGATGGTTGTTGATAACT
AATCGTCTCTGAATAATTACTCTCTTTTGTAATATTTCCAGTTAACCCTCCACGATTAATC

GAAAAATCTCCACCTGTTTTATAACCATACGTATATTTAACTTCTCTTGATTCATCCTGATT
TTTTGGTGCAAAGTCAGTCACATTTGTATTGTTGTTGTCATCAACATTTTGAATTGAAACT
GAATAAGATCCAGGCCATCTTAATGTACTATTCCAATAACCATTTGGGTCTAGAATTCTTA
AACCACTACCTATTGTTCCTTTCGCTTTAATAAATACTGTTTCTTTATCATAATTAGGTTCA
GTTAAGAAGTTAAATTGTAAGCTTTGAGTAATATTTTTTTGACTATCACTTGTTGTAGCTG
TACGTGTATACATTTTAGTATCACCATCAAGATTCTTCTCAGAAACAGCTTTAATTTCAGA
ATTAATTTTTGCATAAGAAGTAGCTGGTAATACAGTGAAAGTAGTCGATAGTGCTAACGT
ACAAATTGTGATATTTTTATATAGTTGTTTAATCAT

> **lukY**

Function: protein coding sequence; leukocidin/hemolysin toxin family protein (core genome, variable)
Best match: lukY-MW2_Z262_LCL_10042.1[40098:41150]RC (completely identical)
Position: 052-contig_193_RC: 5988 … 7041; Length: 1053 bp
Sequence:
TTATCCTTCTCTAAATGATTTATTATCAGAGTATTTATCAACGACTTTAACTGTTTTATTTT
TCCAATCAACTTCATAAGTGACAATTAATCTTTGACCATCTTTATTTTTTTCTAAAATTGG
AGGTGCATAATGTATTCCAGGTCTGTTTTTCAAAATATCTTGATTTCGTGTGTATGTTACTT
CAAATTGCGTTTTCTCATTTGACTTTTCATTAGATAAATAAGTTAAAAATTCTGGATTAAA
GCCACTTCTTACTAATGCTGGGTATCTATATTTTGAAGCAAAGCTTAGTTCAGGGTTTTCT
ACAGTAGCAATTCTCGTATTTCTATAGAATAATAATTCATCATTTCTATTTTTCACTTCTCC
ACCATACTTCAAGTCATTCGCAATAACTGACCAGTGTACATGCCAGTTATTATTTTTACCG
CTGGCAATTGTGTCATAATTTTGCTGATTATAACTAATCGTTTTGGAGTAGCTATTTGATG
AAGTTCGTCCAATACCTTTCGTTGAATCGAATTTACCACCTGAGCTATATGAAAATGTACT
GTCTACTTTCGCAGTTGAAATTTTATTTTTCGGCAATTGGTCTAATATTTCAGTTTTACGAT
TTCTTTTTACTTGAAAATCTACATGGTACTCACTTGGATACTTTAACCAATTTGAATTTTTT
TCTTCTTTATGAGATTCAAACTTTAAATTTGAATGAATTGAGCCTTGTTTTTTAACAAGTA
ATACATTCTTGTCATAAGTTGGATCATCGATAAAGTCGAATTGTAAATTTTGGAGTATATT
TGTTTTCTCATCATATACTGTTTCAGTTCGTTTTGTGATTTTACCGTTTTTCCCAATATCATC
AGGTACTGTTGAATTTTTATCTTTATTAGTAACATTACGTTTGTCTTTTTGTTGAGACTTAT
CAACATGTTCTTTCTTATTTTGATCTTGCGAGTCTTTATGAGCTGAATTTGCTTGAGTCGTT
GCTGCTGATAACAATAAAATTGCACATGATAATGATGACGCTATTAAAACACGTTTTTTA
TTTTTCAT

> **dapE**

Function: protein coding sequence; succinyl-diaminopimelate desuccinylase (core genome, variable)
Best match: dapE_CC001-ST772_118_AJGE01000025.1[38779:40002:r] (completely identical)
Position: 052-contig_193_RC: 7475 … 8699; Length: 1224 bp
Sequence:
ATGACAACTTTTAGTGAAAAAGAAAAAATTCAATTACTAGCAGATATTGTTGAACTACAA
ACTGAAAATAATAATGAAATAGACGTTTGTAATTATTTAAAAGATTTATTCGACAAGTAC
GATATTAAATCTGAGATTTTGAAAGTTAATGAACACCGCGCCAATATCGTTGCAGAAATC
GGTAACGGCTCACCTATACTCGCATTGAGTGGTCATATGGATGTTGTTGATGCAGGAAAT
CAAGATAATTGGACTTATCCCCCTTTTCAACTCACAGAAAAAGATGGCAAATTATATGGT
CGAGGCACAACAGATATGAAAGGCGGTCTGATGGCTTTGGTCATCACACTCATTGAATTA
AAAGAACAAAATCAATTACCTCAAGGAACGATAAGATTACTAGCTACTGCTGGCGAAGA
GAAAGAACAAGAAGGTGCCAAATTATTGGCTGATAAAGGCTATTTAGACGATGTAGATG
GCTTAATTATTGCTGAACCAACTGGATCAGGAATTTATTATGCACATAAAGGGTCTATGT
CATGTAAAGTAACTGCAACTGGTAAAGCTGTCCATAGCTCAGTTCCATTTATTGGTGACA
ATGCAATTGATACACTGCTTGAATTTTATAATCAATTTAAAGAAAAATATTCAGAGCTTA
AAAAACATGATACTAAACATGAATTAGATGTTGCACCCATGTTCAAATCATTGATAGGAA
AAGAAATTTCTGAAGAGGATGCAAATTATGCATCTGGTCTTACAGCTGTATGTTCGATTA
TAAATGGCGGCAAACAATTTAACTCTGTACCAGATGAAGCTTCACTTGAATTTAACGTAA
GACCAGTTCCTGAGTATGATAACGACTTTATAGAATCGTTTTTCCAAAATATCATTAATAA
TGTGGATAGCAATAAGCTTTCACTCGATATTCCAAGCAATCACCGACCTGTAACAAGCGA
TAAAAATAGCAAATTAATTACTACGATTAAAGATGTAGCTTCTAGTTATGTAGACAAAGA

CGATATATTTGTTTCGGCGCTTGTAGGTGCAACAGATGCCTCTAGTTTCTTAGGTGATAAT
AAGGACAATGTTGATTTAGCTATTTTGGACCAGGTAATCCATTAATGGCACATCAAATC
GATGAATATATTGAAAAAGATATGTATCTGAAATATATTGATATTTTTAAAGAAGCTTCC
ATTCAATATTTAAAAGAGAAATAA

> **repeat_nySAgamma**
Function: repeat element
Best match: repeat_nySAgamma_CC001_MW2-USA400_BA000033.2[819195:819303]
Position: 052-contig_193_RC: 8952 … 8982; Length: 30 bp
Sequence:
TTCTTTTCGAAATTCTCTGTGTTGGGGCCC

> **STAR**
Function: Staphylococcus aureus repeat element
Best match: STAR_CC005_Mu50-VRSA_BA000017.4[1694829:1695052]
Position: 052-contig_193_RC: 9011 … 9041; Length: 30 bp
Sequence:
TGGGAATCCAATTTCTCTGTGTTGGGGCCC

> **A1JUG1**
Function: protein coding sequence; putative sodium transport protein (core genome, constant)
Best match: A1JUG1_CC001-ST772_118_AJGE01000025.1[36969:38276:r] (completely identical)
Position: 052-contig_193_RC: 9201 … 10509; Length: 1308 bp
Sequence:
ATGAACAAAGTCAACAAACCTTTATATTTTTATTTAATGCTTTTCTTCTCTACAACTATCAT
AGGTGCTCTTTTATTATATTTACCTTTCACTGGTAAAAAGCCAATATCTTTTTTAGATGCCC
TATTTATAGCTTCAAGTGCATTTACAGTTACTGGCCTATCCCCAGTTGATATAGGATCTCA
GTTTAATATACTTGGTGAAATAGTAATACTATTATTAATTCAAATAGGTGGTCTGGGTATC
GTGACCGTAACCCTATTGACACTAGTATTTTTAAATAGAAAGATATCAATGAAAAATAGA
TTCTTGATTATGGTTACATGGAATATTGACGAACCTGGTGGTGTTATTAAGCTAATTAAAC
ACTTGGCTATTTATAGTTTAGTCACTGAATTAATTGGTATGATTTGTTTGTGTTTATCTTTT
ATACCAAAATTTGGTATAGGCAAAGGTTTATTTTTAAGCTTATTCACATCAGTATCAGCTT
TTAATAATGCTGGATTTGCCCTTTTTAAGAATAACTTAATAGATTATTCTAGTGATCCAAT
TGTCATTATTACAATCTCAATACTCATAATATTTGGAGGTATTGGACATTTTGTCGTGATA
GACTTTATTAATTGTAAAAAATTGAGTAAATTATCTTTACATTCTAAATTAGTCTTAACTA
CAACTAGTATCCTAATAATTATAGGAGCTATTACATTCTTTTTATTAGAACAGTTTAATAC
TATGCAACATATGGGACTAGTTGAAAAAATCGGAAATTCTTTTTTCCAATCAGTAACAAC
ACGAACAGCGGGTTTTAACAGTATAGATATAGCAAGCATTAACAAATCTACCGCATTAAT
GTTAATGCTACTTATGTTTATTGGTGGTGCCCCTCTCAGTGCAGCTGGAGGAATTAAAATA
ACTACTTTTGCAGTTGCGTTTATTTTTGTACTAAATTATATACGTAAAGAAAATAATGTTT
CAGTATTCAATAAAGAAATATCTGACAAACATATAAAACTATCTATTGTTACCATTAATA
TCTCATTTCTATTTATCAGCATCATTACTTTTATATTATCGATAATTAATCCGAACATATCA
TTAATCAAGTTATTATTCGAAGTGGTTTCTGCATTCGGAACAGTAGGGTTAAGTATGAAC
CTTACCACAGAATATCATGGTATTACTAAAATAATTATTATATTCGTTATGCTTTGCGGTA
AAGTAGGACTATTAACTTTATTAAGAACATTTATACCACCAAAAAGTCCTAAAAATTACC
GCTACACTAAAGGACAAATTTATCTATAA

> **txbi_A1JUG1**
Function: bidirectional rho-independent terminator of A1JUG1
Best match: txbi_A1JUG1_CC001_MW2-USA400_BA000033.2[2097322:2097367] (completely identical)
Position: 052-contig_193_RC: 10554 … 10600; Length: 46 bp
Sequence:
ATTACATTAAAAAAACTACCTAAACAGGTAGTTTTTATTTATTTT

> **attB_nySa4-var2**
Function:  attachment site of genomic island nySa4 (mobile element)
Best match: attB_nySa4-var2_Z262_LCL_10042.1[36037:36137:r] (completely identical)
Position: 052-contig_193_RC: 11001 … 11102; Length: 101 bp
Sequence:
GTGCATAATGTAATTTTCTAGTTAGTCAGAACTATAAACAGTTTTACATCATTCTTGGCAT
GCTACCCATGTTAGGTTGGTCATTATTTTTTTCTGGAATT

> **groL**
Function: protein coding sequence; 60 kDa chaperonin GroEL (core genome, constant)
Best match: groL_CC001-ST772_118_AJGE01000025.1[34817:36433]RC (completely identical)
Position: 052-contig_193_RC: 11044 … 12661; Length: 1617 bp
Sequence:
TTACATCATTCTTGGCATGCTACCCATGTTAGGTTGGTCATTATTTTTTTCTGGAATTGATG
CTACAACCGCTTCAGTCGTTAAGAACATTGCTGCAACACTTGCTGCATGTTGTAATGCTGA
GCGTGTTACTTTAGTTGGATCAACGATACCTGCTTCTAACATATTAACCCACTCGTTTGTA
GCAGCGTTAAAACCAACACCTGGTTCTGCATTTTTCAAGCGTTCAACGATGACAGAGCCT
TCTAATCCTGCATTTTCAGCAATTTGGCGAACTGGTGCAGTTAATGCTTTAAGTACAATAT
TCACACCTGTTTCAATGTCGCCTTCAGCTTCAATTTCACTTACTTTTTGGTAAACATTTACT
AGTGCAGTACCACCACCTGCAACAATACCTTCTTCAACTGCTGCACGTGTAGAGTTTAAA
GCATCTTCAATACGTAATTTACGTTCTTTAAGTTCTGTTTCACTTGCTGCACCTACCTTGAT
AACTGCAACACCGCCTGCTAATTTAGCTAAGCGCTCTTGTAATTTTTCACGATCAAAGTCA
GATTCAGTTTCTTCAATTTGAGATTTCAATTGGCTAACACGTGCATCAATGCTGTTTTCGT
CACCGTCACCATCAACAACAGTGGTATTATCTTTAGTTACTTCTACTTTACTTGCAGTACC
TAACATATCAATTGATGCATCTTTTAAATCTAAGCCTAAATCATCAGTAATCACTTGCGCA
CCAGTTAAAATAGCTAAATCTTCAAGCATCGCTTTTCTACGATCACCAAAACCAGGTGCT
TTTACTGCAACAGCTGTAAATGTACCACGCATGCGGTTTAGCACGATATTTGTTAACGCAT
CGCCTTCAACTTCATCAGCTACAATTAAGATTGGACGATTAGATTGAACCACTTGTTCTAA
TAAAGGTAAGATATCTTGGAAAGACGAGATTTTCTTATCTGTTACTAAAATGTATGGGCG
TTCTAATTCAGCAACCATTTTATCTGAATCAGTAACCATATATGGTGATTGATAACCACGA
TCAAATTGCATACCTTCAACCACTTCTAGTTCAGTGTTTAGTCCATTTGATTCTTCAATTGT
AATGACACCATCGTTACCTACTTTTTCCATAGCTTCAGAAATATAACGTCCAATTTCTTCA
TCTGCTGCTGAAATCGCACCTACTTGCGCTATTTCATTTTTATTTTCAACTTTTTGAGAATT
TTCATGTAATGCTTCTACAGCAACTTTAACTGCTTTGTCGATACCTTGACGTAAACCAACT
GGGTTCGCACCACTTGTAACATTTTTCAAGCCTTCTTGAATCATTGCTTGAGCTAATACTG
TTGCAGTTGTCGTACCGTCACCAGCAATTTCATTTGTCTTATTTGCAACTTCTTGAACTAGT
TTAGCCCCCATATTTTCATATGGATCTTCTAATTCGATTTCTTTAGCAATCGTTACACCATC
GTTCGTAATTAAAGGTGCTGTAAACTCTTTATCTAATACAACATTACGTCCTTTAGGACCA
ATCGTTACTTTAACTGCATTTGCTAATTGGTCTACACCACGTAACATTGCTTGACGTGCAT
CTTCAGAGAATTTCAATTGTTTAACCAT

> **attB_nySa4-var1**
Function:  attachment site of genomic island nySa4 (mobile element)
Best match: attB_nySa4-var1_empty_CC005_04-02981_CP001844.2[2089669:2089769]
Position: 052-contig_193_RC: 11072 … 11102; Length: 30 bp
Sequence:
TTAGGTTGGTCATTATTTTTTTCTGGAATT

> **groS**
Function: protein coding sequence; 10 kDa chaperonin GroES (core genome, constant)
Best match: groS_CC005_Mu50-VRSA_BA000017.4[2150463:2150747:r]RC (completely identical)
Position: 052-contig_193_RC: 12736 … 13021; Length: 285 bp
Sequence:
TTATTCAATTACCGCTAAAATATCTTCTTCATTTAATACTAGATATGTTTCATTATCTCGTT
TAACTTCTGTACCAGCATATTGTTGGAACACGACACGGTCCCCTTCTTTCACTTCAGGAGT

CACTCTTGTACCATCATTTAATAGACGTCCAGTTCCTACTGCAACGATAACGCCTTCGTTT
GATTTTTCTTTAGCACTATCAGTTAAAACAATACCACTTTTAGTTGTTTGTTCTTGTTCTTT
TTTCTCAATAATCACACGATTTCCAATTGGTTTTAGCAT

## > Q1Y5R7

Function: protein coding sequence; putative membrane protein (core genome, constant)
Best match: Q1Y5R7_CC005_Mu50-VRSA_BA000017.4[2150922:2151665] (completely identical)
Position: 052-contig_193_RC: 13195 … 13939; Length: 744 bp
Sequence:
ATGACAAGATTATGGGCATCATTGCTAACTGTTATTATTTATATATTGTCTCAATTTTTAC
CGCTTCTCATTGTAAAAAAATTACCATTTGTACAATATAGTGGCATAGAACTGACTAAAG
CAGTCATTTACATACAACTTGTTCTATTTTTAATCGCCGCCACGACGATTATTTTAATTAA
TTTAAAAATTAAAAATCCAACAAAATTAGAATTAGAAGTTAAAGAACCTAAAAAATATA
TCATTCCATGGGCATTGCTTGGATTTGCATTGGTAATGATTTATCAAATGGTAGTGAGCAT
TGTATTAACGCAAATTTATGGTGGACAACAAGTAAGTCCTAATACAGAAAAGCTAATTAT
TATTGCTCGAAAAATACCTATATTTATCTTCTTTGTATCTATTATCGGTCCTTTATTAGAAG
AATACGTATTCAGAAAAGTAATCTTTGGAGAATTATTTAATGCGATTAAAGGTAATCGTA
TCGTAGCATTTATTATTGCTACAACAGTAAGTTCATTAATATTTGCATTAGCACATAATGA
TTTCAAATTTATTCCAGTTTATTTTGGTATGGGCGTCATTTTTTCATTAGCATATGTATGGA
CAAAACGGCTTGCTGTTCCAATTATTATCCATATGTTACAAAACGGATTTGTCGTTATATT
CCAATTACTTAATCCTGAAGCTTTGAAAAAAGCCACGGAACAAGCGAATTTTATATATCA
CATTTTTATTCCATAA

## > txbi_Q2YUD5

Function:  bidirectional rho-independent terminator of Q2YUD5
Best match: txbi_Q2YUD5_CC005_N315_BA000018.3[2075230:2075293:r]RC (completely identical)
Position: 052-contig_193_RC: 13933 … 13997; Length: 64 bp
Sequence:
CCATAATATAAAAAACGAATCACACACGGATTATCGTCGCTGTGATTCGTTTTTATTTTTC
TTT

## > Q2YUD5

Function: protein coding sequence; membrane anchored KP/DP repeat protein (core genome, variable)
Best match: Q2YUD5_Z262_LCL_10042.1[31916:33175]RC (completely identical)
Position: 052-contig_193_RC: 13963 … 15223; Length: 1260 bp
Sequence:
TTATCGTCGCTGTGATTCGTTTTTATTTTTCTTTTTATTTCGCTTTACAAATAAGAATAGCA
TGATTGAAAATACCACCATCATAGAACATACAATTGCTAGCCAACTTTTACTATATGCAG
TTGACGCACTTTTCTTATTATCTTCTTGTTTTTTAACTACTGTAAAATCGTCTTTTTCATGC
GGTTCAAATGAGCGTTGTTTTGAATCCGGCTTATCTTCGGGCTTTTTCAAATCTGGCATTG
ATCCAATTTCACCAGTAATTAATGCGAGTACTTGTTCATCTAAATTACGATAGTACCTTTC
TGATTTCAATGGATTGAAATATTGGAATCTAAAGTAATTCGATTGCTGTTGTAATCCATTT
AAATATGCATTTCCGCTGAAATTTTGTTTTCGAATAATATTATAAATGTCTTCATCAGTAA
CTTCTCCATAATCTTTGCCCAACTTATTAATTTGATTTAAAATATACGGATTATACTTGTA
AGCCCCATTTGCCAAGGCTAAAAATCGTTGGGATACAAAATCATTACCAGTAGGATTTTG
TGAGTTTCCTTGATTCCCATTTGGTTGCCATTGACCTTGATTAGATCCATCTGAAGCATTT
GGGTTCCATGTTCCCCCATTTTTCGAGCCACCAGAATGATTGGAATCCCCAGGTTGATCGG
GATCTGGTGACGGGTTAGGATTTGGCTTATTAGGGTCTGGTTTTGGATTCGGATTTGGCTT
TGGTTTATCTGGATCTGGTTTTGGATCCGGATTTGGCTTTGGTTTATCTGGGTCTGGTTTTG
GATCTGGATTTGGTTTCGGGTTATCTGGATCTGGCTTTGGATCCGGATTTGGCTTTGGTTT
ATCTGGGTCTGGTTTTGGATCCGGTTTTGGTTTCGAGTTATCTGGATCTGGTTTTGGATTCG
GACTTGGGTTTTGGTTATTTGGGTCTGGTTTTGAATCTGTACTTGGGTTTTGGTTGTTTGAG
TCTGGCTTCACATTATTCGAATCAGGATTAGCATCACGACTATTCGAACCTGTTCCTTCAT
TACCCGTTTGATTTGATTCGTGAGGTGTCCCAGTCTCATTATTATTCATTTCACTATTTACT

GATGGTGAAGTTATATTATGATTCAAATTAGTAGTTGGTTTTTCTCCATTTAAGTTATCCTT
TGCTTCAACTGTGTGCGTTGCAAGCCCTAAACTACTACTACTTAAAATTAAAATACTTGTT
GAAAGTAACATTTTCTTAAACCAATGATATGACAT

## > **Q1XYY6**

Function: protein coding sequence; putative nitroreductase family protein (core genome, constant)
Best match: Q1XYY6_CC001_MW2-USA400_BA000033.2[2105308:2105934]
Position: 052-contig_193_RC: 15419 … 16046; Length: 627 bp
Sequence:
ATGGGCTTATTTAAAAAAGATAAAAAAGCAATGACTTTTGACAATGCAATGGAAGAAAG
ACGTTCAATATATAATTTGAAAGATTCGATATCAATTAGCGATGACGAATTAGAATCAAT
GATTGCACATGCAGTTAAACATGTTCCTTCATCATTTAATTCTCAATCAACTAGAATTGTT
CTCTTGTTAAATGACAACAATAATAAATTTTGGGATAACACAAAAGCAATTTTAAAAGAA
GTCATGGGAGAGAATCGTGATTTCGAGCCAACTGAACAAAAAATTGATAACTTCAAACAT
TCATACGGTACAATTTTGTTTTATGAAGATCAAGATGTTGTAAGTGGTTTACAAGAACAA
ATGCCAAACTACTATGATAATTTTGCAATATGGTCAACTCAAACAAATGCCATGCATCAA
TTTGCAATTTGGACTGCATTAGCTACTAAAGGCATAGGTGCTTCATTACAACACTATAATC
CACTTGTCGATGAGATGACTTCAAATGAATTTAATATTCCTAAAAGTTGGAAACTTATTGC
TCAAATGCCATTTGGTGATATTCGTGAAGCTGCTGGCGAAAAAACATTTAATCCAGTGGA
AGATCGTTTCGTAATAAAAAAATAA

## > **P55177**

Function: protein coding sequence; putative C-N hydrolase (core genome, constant)
Best match: P55177_CC005_Mu50-VRSA_BA000017.4[2154121:2154906] (completely identical)
Position: 052-contig_193_RC: 16406 … 17192; Length: 786 bp
Sequence:
ATGAAAGTCCAAATTTATCAATTACCGATTATTTTTGGAGATAGTTCTAAAAATGAAACT
CAAATAACGCAATGGTTCGAAAAAAATATGAATGCTGAAGTAGATGTAGTCGTTTTACCT
GAAATGTGGAATAATGGCTATGATTTAGAGCATTTAAATGAAAAAGCAGATAATAATCTA
GGACAAAGTTTTTCATTTATTAAACATTTAGCTGAAAAATATAAAGTCGATATCGTAGCT
GGGTCAGTTTCTAACATTAGAAACTATCAAATATTTAATACAGCTTTTAGTGTGAATAAA
AGTGGCCAGCTGATTAATGAATACGACAAAGTTCATTTAGTTCCTATGTTAAGAGAGCAT
GAATTTTTAACCGCTGGCGAAAACGTAGCTGAACCCTTTCAATTGTCTGACGGTACTTATG
TGACCCAACTTATCTGTTATGATTTACGTTTTCCAGAATTATTAAGATATCCTGCTCGTAG
TGGTGCTAAAATTGCATTTTATGTGGCGCAATGGCCTATGTCACGTCTTCAACATTGGCAT
TCATTACTAAAGGCACGTGCAATTGAAAATAATATGTTTGTCATTGGAACTAATAGCACT
GGATTCGATGGTAACACAGAATATGCTGGTCATTCGATAGTTATAAACCCAAATGGAGAT
TTAGTTGGAGAATTAAATGAATCCGCAGATATTTTGACTGTAGATTTAAACTTAAATGAA
GTAGAACAGCAACGCGAAAATATACCTGTATTTAAAAGTATTAAATTAGATTTGTATAAA
TAA

## > **rnaIII**

Function:  regulatory RNA III (core genome, constant)
Best match: rnaIII_CC005_Mu50-VRSA_BA000017.4[2155157:2155670:r]RC (completely identical)
Position: 052-contig_193_RC: 17442 … 17956; Length: 514 bp
Sequence:
AAGGCCGCGAGCTTGGGAGGGGCTCACGACCATACTTATTATTAAGGGAATGTTTTACAG
TTATTTTTTCAATCTATTTTTGGGGATGTTATTAATTATGAAAAAAATTTTATTTATCAAAT
AATGATAAATCGATGTTGTTTACGATAGCTTACATGCTAGAAATAATTATCTGTAATGAT
AATTAAGAAAAATACATAGCACTGAGTCCAAGGAAACTAACTCTACTAGCAAATGTTACT
CACTTGCTAATTTTACATGGTTATTAAGTTGGGATGGCTTAATAACTCATACTTTTAGTAC
TATACGAAGATAACAAATTTACAATGAAAGTAATTAATTATTCATCTTATTTTTTAGTGAA
TTTGTTCACTGTGTCGATAATCCATTTTACTAAGTCACCGATTGTTGAAATGATATCTTGT
GCCATTGAAATCACTCCTTCCTTAATTAAGATAAAAATTCTTAAAATTAAACAACTCATCA
ACTATTTTCCATCACATCTCTGTGATCT

> **hld**
Function: protein coding sequence; hemolysin delta (core genome, constant)
Best match: hld_CC005_N315_BA000018.3[2079076:2079210:r]RC (completely identical)
Position: 052-contig_193_RC: 17791 … 17926; Length: 135 bp
Sequence:
TTATTTTTTAGTGAATTTGTTCACTGTGTCGATAATCCATTTTACTAAGTCACCGATTGTTG
AAATGATATCTTGTGCCATTGAAATCACTCCTTCCTTAATTAAGATAAAAATTCTTAAAAT
TAAACAACTCAT

> **agrB**
Function: protein coding sequence; accessory gene regulator B (core genome, variable)
Best match: agrB-II_CC001-ST772_118_AJGE01000025.1[28753:29316:r] (completely identical)
Position: 052-contig_193_RC: 18161 … 18725; Length: 564 bp
Sequence:
TTGAATTATTTTGATAACAAAATTGACCAGTTTGCCACGTATCTTCAAAAGAGAAATAAC
TTAGATCATATTCAATTTTTGCAAGTACGTTTAGGGATGCAAATTATAGTTGGTAATTTTT
TTAAAATACTTGTAACATATAGTATTTCTATATTCTTAAGTGTATTTTTGTTTACCTTAGTT
ACACACTTATCCTATATGTTAATACGCTATAATGCACATGGTGCTCATGCAAAGTCTTCGA
TACTTTGTTACATACAAAGTATTTTAACATTTGTATTTGTACCTTATTTTCTAATTAATATA
GACATTAATTTTACTTATTTACTGGCCTTATCTATTATAGGATTGATTTCCGTTGTTATTTA
TGCACCTGCAGCAACGAAAAAGCAACCTATACCAATAAAATTAGTAAAACGTAAAAAGT
ATTTATCTATAATTATGTATTTATTAGTTTTGATTCTATCCTTAATAATACATCCATTTTAT
GCTCAATTTATGTTATTAGGAATTTTAGTAGAATCTATTACATTATTACCTATTTTTTTCCC
TAAGGAGGATTAA

> **agrD**
Function: protein coding sequence; accessory gene regulator D (core genome, variable)
Best match: agrD-II_CC005_Mu50-VRSA_BA000017.4[2156442:2156585] (completely identical)
Position: 052-contig_193_RC: 18727 … 18871; Length: 144 bp
Sequence:
ATGAATACACTTGTTAATATGTTTTTTGATTTTATAATTAAATTAGCTAAAGCAATCGGAA
TTGTCGGTGGCGTAAACGCTTGCAGCAGTTTATTTGATGAACCTAAAGTACCCGCTGAAT
TAACGAATTTATACGACAAATAG

> **agrC**
Function: protein coding sequence; accessory gene regulator C (core genome, variable)
Best match: agrC-II_CC005_Mu50-VRSA_BA000017.4[2156615:2157898] (completely identical)
Position: 052-contig_193_RC: 18900 … 20184; Length: 1284 bp
Sequence:
ATGGAAACAATAAACAACTTAGCTATGGCCACTTTTCAATTAGTAATACTTTTTACAGTA
GCCAAATTCATTTCCTTTGTTAAATTTAATTTAAGGGATTATTTTATTATAGTTGGAATTAT
AATTCCAACCATGTTTTTGTATTACTTCTATGGAAGTAGAGCCGTATTGATTCCAACTTTT
TCATCTATCATATTTTTATTCTTTAAATTAAAGTACTATGCAATAGTAACAATTTTAGTGA
CCATGATCATAATGTATTTGAGTAATTTTGCAACAGTAGGTTTGTTTTTAACTTTAAGAAA
ATACACTACTGATCCTGCAATACTCTTACCATTATATATACTTTCTTTTTCATCAGTTTCAT
TATTAGCGACATATTTGGTAAGAATATCTCTAAAGAAATTTAAAAAATCATACTTATCTTT
AAATAAAACTTATATGATTATAATTTCATTCGTATTATTTGCAACATTTGCATTTTTTTATA
TTTATTCTACAAATACAAGTTCAAACGGTGATAGCTTAATTCCATATGCACTTGTATTTAT
AGGATTAATAATTTTTATCTCTGTTGTAATACTTATAATGTCGCTTTTCACCCTTAAAGAG
ATGAAATACAAACGAAATCAAGAAGAAATTGAGACCTATTACGAATACACATTAAAGAT
TGAAGCAATTAATAATGAAATGCGCAAGTTCCGTCATGATTATGTCAATATCTTAACGAC
ACTTTCAGAATACATTCGCGAAGATGACATGATTGGTCTACGCGCTTATTTCAATAAAAA
TATTGTACCTATGAAAGATAATTTGCAGATGAATGCTATTAAATTAAATGGTATCGAGAA
TCTTAAAGTACGTGAGATAAAAGGCTTAATTACTGCGAAAATTTTACGTGCACAAGAAAT

GAATATTCCAATTAGTATCGAAATACCCGATGAAGTAAGTAGCATTAACTTGAATATGAT
CGATTTAAGTCGCAGTATTGGTATTATTCTTGATAATGCAATTGAGGCATCAACTGAAATT
GATGACCCTATCATTCGCGTTGCATTTATTGAAAGTGAAAATTCAGTAACGTTTATTGTTA
TGAATAAATGCGCTGATGATATACCACGCATTCATGAATTGTTCCAAGAAAGTTTTTCTAC
TAAAGGTGAAGGTCGTGGTTTAGGTCTATCAACTTTAAAAGAAATTGCTGATAATGCAGA
CAATGTCTTATTAGATACAATTATCGAAAATGGTTTCTTTATTCAAAAAGTTGAAATTATT
AACAACTAG

## > **agrA**

Function: protein coding sequence; accessory gene regulator A (core genome, variable)
Best match: agrA_CC001_MW2-USA400_BA000033.2[2110092:2110808] (completely identical)
Position: 052-contig_193_RC: 20202 … 20919; Length: 717 bp
Sequence:
ATGAAAATTTTCATTTGCGAAGACGATCCAAAACAAAGAGAAAACATGGTTACCATTATT
AAAAATTATATAATGATAGAAGAAAAGCCTATGGAAATTGCCCTCGCAACTGATAATCCT
TATGAGGTGCTTGAGCAAGCTAAAAATATGAATGACATAGGCTGTTACTTTTTAGATATT
CAACTTTCAACTGATATTAATGGTATCAAATTAGGCAGTGAAATTCGTAAGCATGACCCA
GTTGGTAACATTATTTTCGTTACGAGTCACAGTGAACTTACCTATTTAACATTTGTCTACA
AAGTTGCAGCGATGGATTTTATTTTTAAAGATGATCCAGCAGAATTAAGAACTCGAATTA
TAGATTGTTTAGAAACTGCACATACACGCTTACAATTGTTGTCTAAAGATAATAGCGTTG
AAACGATTGAATTAAAACGTGGCAGTAATTCAGTGTATGTTCAATATGATGATATTATGT
TTTTTGAATCATCAACAAAATCTCACAGACTCATTGCCCATTTAGATAACCGTCAAATTGA
ATTTTATGGTAATTTAAAAGAACTGAGTCAATTAGATGATCGTTTCTTTAGATGTCATAAT
AGCTTTGTCGTCAATCGCCATAATATTGAATCTATAGATTCGAAAGAGCGAATTGTCTATT
TTAAAAATAAAGAACACTGCTATGCATCGGTGAGAAACGTTAAAAAAATATAA

## > **frk**

Function: protein coding sequence; kinase/pfkB family (core genome, constant)
Best match: frk_Z262_LCL_10042.1[24892:25851]RC (completely identical)
Position: 052-contig_193_RC: 21287 … 22247; Length: 960 bp
Sequence:
TTAAAATGCTTGTTCAACCTCTTGTAATGTTGGCAAACTATTAATGGCACCATACTTCGTT
GTCACTATTGCAGCAACGCGATTGCTAAACGCTAAAATTTCCTCACCTTCATTTTCAAATA
ACTGAGTTAAATTTAACACATCGGTTGCTAAAATCCTGCTAATAACTGCACCAATAAAAG
CATCCCCTGCCCCAGTTGTATCAACAGGTTTTACCTTATAGCCACTATGATAATGATTAAT
ACCATTTTTCAAATATACGGCAGCACCATCTGCTCCCTTCGTATAAATGACTACAGTAACA
TTACCTGTAAATAATGATTGAATGGCTTCATTTTCATCATGAATACCTGTAATAAATTCTA
ATTCTTCATCTGAAACTTTTACAATATGCGCCAATGGCAAAAAGGTATGAATAGTTTGTCT
TAAATCTTCAGCGTTATCCCATAATGGCAATCTTACATTAGGATCAAATACGACAGTACC
ATTTGCATTTAATGTTTTGGTAATCAATTGATAATGTGCATCTCTCATCGGACTATCGACC
AAATCAACAGAACAAAAATGTACTACATCATTTTCATTCACATCAATATCATTCACAAAA
CTTGGTTCAAATAACATATCAGCAGAGGGCTTACGATAAAAAGAAAAATCTCGCTCGCCC
GCTTCAGTCAAACTCACAAAAGCTAACGCAGTGTTTGCTTCATTCGTGCGATACACTTTTG
ATACATCTACACCAATACTTGATATCGTTTCAATAATGCTATCTCCAAATGCATCATTACC
TAATTGTGTAATCATATACGCTTGTTGTCCTAACTTTTGAACTGTACAAGCTACATTGCAT
GGCGCGCCACCAATTTGCTTCGTAAATGTTTGAACATCTTTTAATTTCGCATTTGTAACAT
TTGGAATAAAATCAATTAACGCTTCTCCTATTGAAAATAGACGTCTCAT

## > **scrB**

Function: protein coding sequence; sucrose-6-phosphate hydrolase (core genome, constant)
Best match: scrB_CC005_Mu50-VRSA_BA000017.4[2159961:2161445:r]RC (completely identical)
Position: 052-contig_193_RC: 22243 … 23728; Length: 1485 bp
Sequence:
TCATTTATGTTCATCCTTTAAATCATATTTAGTAAATTGTAAATATACTTGCCCTGATTCTG
TTGATGTTTTAATACCTAGTGCGTCTTCTGTCGGGAAAATTCGAGATGTCAAAACACGTTC

CCCATCATTACAGAAAATTTCTATACTAGATGTATCAACAAAAATTTGTAATTGCTTTAAT
GGCGTGTCTAATATCGTACTACGCGTCGTACCTTCAACATTTGTCGGCAATAAACCACTGT
CGCTGCGATCTAATGTTATTTTATTTTCACGCTTGTTATAAGCAATTAATGTTGAAGAAGT
CTTAGATGTACGTAATTCAAAGTACACTTCGGTAGCATCATTATCCAAAATATCTATGATT
AATTCATACTGTTTACCTTCATACGGATGTAATTTTCGAGTAAATTTATTTGCGTAGCCTA
ATGCTGTCTCTTTATTGTGACGTAATTTTTCCAACGCTGGATATGGACGTTGCTTAAGTTG
ACCATTTTCTACATTTAATACACGAGGAATTGTTAAGCAATGGGCCCATCCTTCATTATCA
GTAGGATATTCGATTTCCGGTAACCCCATCCATCCAATTAGTACTCGTCGGCCTTTTTCAT
CTAAAAATGTTTGAGGTGCATAGAAATCAAAGCCATTATCAAGCTCGACAAAATTTTCAT
GTTCATATGTTAACTTTTCAATATCAAACTTTCCAAGTATATAACCACTTTGATAAATATT
TTTGAACTGATTGCCTTTTGGTTCAATACCTTGTGGACAAATAAGTATGACATCTTGATTA
TCTAAATTAAAGTAATCTGGGCATTCCCACATATATCCAAAATCATCTAACTCTGTATTTA
TTTCACCTAAATAATGCCAATTAATTATATCTTCAGTATTATAAAGTAATAATCGACCTTG
CTGATCATTATTTTGTGCGCCAATGATTGCATAATATTTCTCACCATATTTAAAAACTTTA
GGATCTCTAAAATGACTTGTATATCCTTCTGGTTGCTGGCTAATTACTGGCTTTGGAAACT
TTTCAACTGAACCGTCTTCTTTCAATCGTGCGATCATCTGACTCGCATGTCGTTGCCAATG
ATTATCTCGATGATTTCCTGTGTACATATAATATAAATGCCCGTTATATTCAAAAGCGCTA
CCGCTATAAACACCATGGCTGTCATATTTAGTATCTGGATTTAATATTGGCCCTTCAGGTT
TAAAGTTTATTAAGTCATCACTCGTGTAGTTATACCAATACTTTAAGCCATGTACTGCGCC
TAATGGGAACCATTGATGTGAAACATAATACTTCCCTTTATAAAAAATAAGTCCGTTGGG
ATCATTTAATAAACCTGTTTCTGGTTGTATATGAAATTGTTGACGAAATTTTGATTGATCA
ACTTGTTGTTTTAATGTTTTAAAATACTCAGTATCAACGTCCTCGATTCGTTGATAACGTT
CTTCTCTAGTCCATTCGGTCAT

> **scrR**

Function: protein coding sequence; sucrose operon repressor (core genome, constant)
Best match: scrR_CC005_Mu50-VRSA_BA000017.4[2161594:2162544:r]RC (completely identical)
Position: 052-contig_193_RC: 23876 … 24827; Length: 951 bp
Sequence:
CTAAATATTAACATCTACTGTGACGCTATATGGCATATCTTGCTTTTTAAGCATCTGTTGT
ATCTCTTCCATCGCGCATTGGCCAGCTTCAAAATAATTATAATGAATTGTTTTTATCGATG
GAGACACTAATTGTGTCATTGGGTCACCACCAAAACCATATATTTGATGTGGTTTCATAA
CATCATTTTTATCAGAATAATATTTATAGGCAGCTAATGCAATCGTATCAGTTGCTCCAAC
AACCGCATCTACTTGCTCCACATTTTCCAAAACATTTGCAACATCTTTTTGTGCTTCCACA
TAAGTAAAATTTGTTTCATGTATATTAGGTTTAATTTGGTATTTAGCTAACTGGTCAAGTA
AACCACGTTTTCTATGTATACCAACTGCAATATCTTTTTCACTTACACTAAACACTTCAAC
TTGTTGATATCCCTGTTGACCAATCCATTCGCCTATAATTTGACCTGCTTTATAATCATCAT
GCACAATACTATGAAGTTGTTCATGTTGTTGACCAACAATAACGATTGGTACATTCATTTT
ATTAATGACTTCAATATGTCTCTCTGTTATGTCTGTAGCCATTAAAACAATACCATCTACT
TTACTGCGTGCTAATGTTTCAAGCGCTTGTATTTCTGCTTCGATATTTAAACCTGTGTAATT
TAAAATTAATTGTGATTCATATTTTTGGCATTGTTTTGCCAATCCTTTGATTGTTTCATCTA
CTGCATATGAATTCATTCTAGGTATAATGGCACCAATAAGGTGTGTTTGTCTCGCTCTTAA
ACTTTGAGCAAATTGATTCGGTTGATAGTCATGTTCTGCTATAATTCTTGTTAATTTTTCAC
TTGTTTTTTTACTGACAGATCCATTATTTAAAAATCTAGATACTGTGCTTTTTGAAACGCCT
GCCAATTTGGCAATATCAGATATATTTTTCAT

> **nrgA**

Function: protein coding sequence; putative ammonium transporter (core genome, variable)
Best match: nrgA_CC005_Mu50-VRSA_BA000017.4[2162727:2163977:r]RC (completely identical)
Position: 052-contig_193_RC: 25009 … 26260; Length: 1251 bp
Sequence:
CTAATATCGAATATGTTTATTGAATCTATTTAGCTCACCTTCAAAGTAAGCATGTTCACCG
TGAACAATCTTGTCTAATCCTATATTCGTTTCTTGTTCCGTAACAGATAATGGTGTAATTA
ATTTAATTACTTTCGCAATAATAAACGTCATGACGATACTAAAAATTACAACTGCTGTTAC
ACATAATATTTGTACAAGTATAATATGTATGTCACCAGTATAAATAAAGCCATTCTCAAT

GTCAGGATTGGCTTTTTTACTTTGGAAAACTGCTGTTAAAACAGCACCAATAATACCACC
AACACCATGAATACCAAATGCATCTAATGCATCATGATATTTTAGTTTTACCTTGATGTAA
TTAATGACAATATAACAACAGATACCTCCTATTAAAGCCATTATTGTTGCACTAAGATAT
GTTACATATCCTGCTGCAGGAGTAATAACAACTAATCCTGCTAATGCACCGAGTAAAAGT
CCAAGTAAACTTGTCGTCTTTTTAAAAATATATTCTAAAATTAACCAACCTATAGCACCTG
CACTGGCTGAAATGACAGTATTTGTAAATGCAAGCATCGCAATATTATCAAATGTAAAAG
CACTACCTACATTAAATCCATACCAACCAATCCACACGAATATACCGCCAATCAACGTAA
TGATAAGATTATGTGGTGTTGATTCAGAATGTTTGTTTCCTTTTCCAATCATAATAGCTAA
TACTAAACCAGAAACACCTGATGTAATATGAACAACCGTACCTCCAGCGAAATCTAATAC
ACCGAGTTTATTAATCCAACCGCCGCCCCAAACCCAATGTGCTACTGGACTGTATACAAG
AGCAGTCCATATTACTACGAATAATAAATAAGGAATAAACTTCATTTTCTCAGCGATTGA
ACCAGATAAAATAGAAATTGCAATCGTACAAAACATCATTTGAAATAACATAAACAAAG
CGAAAGGAATATGTGGGCTAATATCTTCTTGAGTCGCAAAACCTACATGATTAAGAAAAG
TATATTCCCAATTTCCGAACCATAAATTCCCATTCCCAAAACTAATTGTAAAACCAACTGT
TATCCATACAAATGTAACAAGCACAATTGCTGCCATACTTTGCATGACAGTATTAAGCGC
ATTTTTAGATTGAACTAACCCACCATAAAATAAACTTAATCCTGGTGTCATTAACCAAACT
AATAATGTACACAAAAACATAAATATCGTATCGTTAAGATTCAT

## > **Q5HEF7**

Function: protein coding sequence; putative protein (core genome, variable)
Best match: Q5HEF7_CC005_Mu50-VRSA_BA000017.4[2164185:2164409:r]RC (completely identical)
Position: 052-contig_193_RC: 26467 … 26692; Length: 225 bp
Sequence:
TTAAACTTTTTGAATTGTAATTGTCCATGAAGCATTATCAATTTGTTCATAGTTTGTTATAG
GATAACCATTTTCTGCAGCCCAATTTGGAATGGCTTCCGTCGCTTGCGTGCAATCAAAATC
AATTTTTAATTCATCTCCAGATTGCAATGTTGCCATTTTCTTTTGCGCTTCAATTAACGGAA
ATGGACATACCATTCCTACTGTACCTAATTCGTGTATCAT

## > **DUF395**

Function: protein coding sequence; domain of unknown function 395 (core genome, constant)
Best match: DUF395_CC001_MW2-USA400_BA000033.2[2116641:2117720:r]RC (completely identical)
Position: 052-contig_193_RC: 26751 … 27831; Length: 1080 bp
Sequence:
CTATACTATTTGCGTTTGCTGTTTAACCTTTGCAGATTGTTGTTGTACTTTTTTCATTGGAC
GAACAAAGATAAAATGACTCATTGTCCATACACCAACTATCATTGATGCTAGCGCAATCC
ATCCTTGCCAAGTCATCGTTGCTGTTTCAACCAAACCGTTACCGATAGAACAACCACCAG
CAACTGACGCACCAAATCCCATACATATGCCACCAATAGCACTGTTTCGTATTGTAATCTT
ATCTGGCAATCGCCATTTAAATTCTCTTGATCCTCTAGCTGCAATATATGAACCAATGAAA
ATTCCTAGAACTAAAAAGACACCCCAATCAATAAATTTAGTTTCACCTGTAATCAAAAAG
TGTACTAAATTTGCTGATGGCGTTGTTATACCTAAACCGTCATTTCTTCCAGTTGATGCAC
TCATTGGCCAAGCTAAGAGTGCGATAAGTCCAATTACAATTGCTGCAATAAATGGATGGT
ATCGTTTTTCGAAAAGGTAATATCTAATACCTGTATAACGTTGCTTTAGTTTTGGAACAGC
AACTCGAACTTTCTTATTATTGAGTGTTCTTACAACTAGAAAAAATGGTGATTATAGTTAAT
ATAACGACTAATCCCCAAAACGGAATGCCAGTTGTTTGAGACATATCACTATTTACATTC
GTTGGTTGATTAATTTTATCCATTACTGGCTTTAAAATCCCTGTTTTAGTGATTGCTGCAGT
AACAGCATATAATACTAATGCAATCCAACTACCAATTAGCCCTTCACCAGCGCGATACCA
AGTACCTGTTGCACACCCTCCAGCCAATACTATTCCAATTCCAAAAATAAAAGAACCTAT
AATTGTTCCCAATATTGGAAAACTATGTGCAGGAATTTTTAAAATATCTGTTGCCGTCAAA
ATCAATAATCCTATACTTTGAATAGTAATAGCGATTAATAATGCATAGAACATCTTATTAT
TCTTTTGCACATACATATCTCGAAAGCCACCTGTTAAACAAAATCTTGTTCTTTGCATGAC
AAAACCTAAGAGTCCCCCGACAATTAAGCCACTAAAAATCATCCACAT

## > **rex**

Function: protein coding sequence; redox-sensing transcript repressor (core genome, constant)
Best match: rex_ST425_LGA251-cow_FR821779.1[2052020:2052655:r]RC (completely identical)
Position: 052-contig_193_RC: 28134 … 28770; Length: 636 bp
Sequence:
TTATTCACTGTAATTTTTCATAAAGAATAATAACGACTGTAATTCTATACCTAAGTCAATT
TGATGTACTTGCACATCTGAAGGCGTATTAATTCTACCAGGAGTGAAGTTTAAAATACCT
TTCACACCAGCTTGGACGAGTTCATCTGCAACTTTCTGTGCAACTCTTTCTGGTGTAGTTA
GAATCACAACATCTATTTCTTCCTTCTTCAATGTTGTTATTAATTCATCGTTATCTTTTACA
ATAACGTTCCCTATTTTCTGGCCAATAACATCTTCTTTTACGTCAAACGCTTCTGTAATCGT
CATATCGTCATGTATTGAAAAGTTATATGTGAGCAAAGCTTTCCCTAGGTTCCCAACTCCG
ACAATTGCGATTTTGATCATGTCACTCTCGCTTAGTTCAGATTTAAAGAAATCCAATAAAC
TATCTATATTATATCCGTACCCTTTTTTACCTAATTCGCCAAAATATGAAAAGTCACGACG
AATTGTTGCCGAGTCAATTTGTAACGCATCGCTAATCGCTTTTGAATTTACACGATCTATA
CCTTTAGATTTTAATGAACTGACAAATCTATAATATAACGGCAAACGTTTTAAAGTTGCTC
GAGGAATTTTAACTTGGTCACTCAT

## > **Q1Y630**

Function: protein coding sequence; ABC transporter/ATP-binding protein (core genome, constant)
Best match: Q1Y630_Z262_LCL_10042.1[16188:18116:r] (completely identical)
Position: 052-contig_193_RC: 29022 … 30951; Length: 1929 bp
Sequence:
ATGATACTTTTACAACTCAATCATATATCAAAATCGTTCGATGGTGAAGATATATTTACTG
ATGTTGATTTTGAAGTAAAAACTGGTGAACGAATTGGTATCGTAGGAAGAAATGGCGCCG
GTAAATCAACATTGATGAAAATTATAGCTGGCGTAGAAAACTATGATTCTGGAAATGTGT
CAAAAATTAAAAACTTAAAACTAGGTTATTTAACTCAACAAATGACTTTAAATTCTAATG
CAACAGTTTTTGAAGAAATGTCAAAACCATTTGAACATATTAAACGAATGGAAAGCTTAA
TCAAAGAAGAAACAGATTGGTTATCAAAACATGCAAATGATTACGATAGTGATACATAT
AAAACACATATGTCCCGTTATGAATCTTTATCGAATCAATTTGAACAATTAGAAGGATAT
CAATATGAAAGTAAAATTAAAACAGTACTTTACGGGTTAAATTTTAGTGAAGAAGATTTC
AATAAACCTATCAATGATTTTAGCGGTGGCCAAAAAACACGTTTATCTTTAGCTCAAATG
CTATTAAACGAACCTGATTTATTACTTTTAGATGAACCTACTAACCACTTAGATTTAGAAA
CGACAAAGTGGCTTGAAGATTATCTACGTTATTTTAAAGGTGCAATCGTCATCATCAGCC
ATGATCGTTACTTTTTAGATAAAATAGTTACTCAAATTTATGATGTGGCTTTAGGTGATGT
CAAACGCTATGTTGGTAATTACGAGGAATTTATACAGCAACGGGATTTATATTATCAAAA
ACGAATGCAAGAATATGAAAGTCAACAAGCAGAAATAAAACGATTAGAAACTTTTGTTG
AGAAAAATATTACCCGTGCTTCAACAAGTGGAATGGCAAAAAGTAGACGTAAGATTTTA
GAAAAAATGGAACGCATTGATAAACCAATGTTAGATGCCAAAAGTGCAAATATTCAATTT
GGCTTTGACCGGAATACAGGTAATGACGTCATGCATGTAAAAAATTTAGAAATCGGTTAT
CAAACTGCAATTACCAAACCTATCAGTATAGAGGTCTCTAAAGGCGATCATATAGCAATC
ATTGGGCCAAATGGTATTGGAAAATCGACCTTAATTAAAACTATTGCTAATCAACAAAAA
GCGCTTAATGGCGATATTACTTTCGGCGCAAATTTACAAATTGGTTATTATGATCAAAAG
CAAGCAGAATTTAAATCTAGTAAAACGATTTTAGATTATGTGTGGGATCAATATCCGTTA
ATGAATGAAAAAGATATTCGAGCAGTTCTTGGACGTTTCTTATTTGTACAAGACGATGTT
AAAAAGATAATTAATGATTTATCTGGTGGTGAAAAAGCACGTTTACAACTAGCACTACTT
ATGTTGCAGCGTGACAATGTACTTATTTTAGATGAACCTACCAACCACCTCGATATAGATT
CAAAAGAAATGTTAGAGCAAGCACTCCAACATTTTGAAGGAACAATACTATTTGTTTCCC
ATGATCGTTACTTTATTAACCAATTAGCAAACAAGGTATTTGATTTAACAATTGATGGCG
GAAAGATGTATTTAGGAGATTATCAATATTACATTGAAAAAGTTGAGGAAGCGGAAGCG
TTGAAAGCGCACCAAGAAGAACAGTCAGTTAACGTACAAGCACATAAAAAGTCTATGGA
ACAATCGTCGTATCATAACCAAAAAGAACAAAGACGTGAACAACGCAAACTTGAACGAC
AAATATCCGAGTGTGAAAATGAAATAGAAACTTTAGAGACTACTATCTTACAAATAGACG
AGCAATTAACCCAACCAGAAGTGTATAACAATCCACAAAAAGCAAACGAATTAGCTATC
CAAAAACAAGATAGCGAACAAAAATTAGAACACGCTATGTCTAAATGGGAAGAATTACA
ACAAAAACTATAA

> **mutS3**
Function: protein coding sequence; DNA mismatch repair protein domain-containing protein/locus 3 (core genome, variable)
Best match: mutS3_Z262_LCL_10042.1[14215:15825:r] (completely identical)
Position: 052-contig_193_RC: 31313 … 32924; Length: 1611 bp
Sequence:
ATGAGCACAAATCAAACATTTTTAATATTTGTTATAGCAATTATTATACTTACATCGGTAA
TAGGAATTGTTGGACGATACATGAGTCGTCAACGTCTTTTAAAATCTATGGAAACATTAT
GGCAAACGATTTCTCCATTAGAAGCTTTTATCAGACCGAACTCACATTTCGACTATGAGT
ATAAGCTCTACAAGGAAAAATTTGAATCACATTCACTAGTTGATGATAAAACTTGGTCCG
ACTTAAATATGAATGCAATCTTTCATAAGATGAATTATAATTTAACAGCTATTGGTGAAA
TGAAGCTATATGCCTGTTTACGTGGAATGCTTTCAATTACGAACAAATCATTACTTAGTTT
ATTTAATGATAATGCTGAATTTAGAAAAAACGTAACATATCATTTAGCTTTGATTGGTAA
AACTGTTTATCCAACATTTCCAGACCAAATCACACCGGTAAAACGTCAAAATATATTGTT
TCTATGCCCGTTTTTACCAGTTATCTCATTTGCAGTTATATTTATTAATTCACAAGTTGGTA
TTTTATTATTTCTTATGAGTTGCCTATTTAATATCATTTTATCAGCAACTTTAAAGCGTACA
TATGAGGATGACTTAAAATCTATCTTTTATGCATCCAATGTTTTAAAACAAGGCTATACTA
TCTCAAAAATTAAACATGCTCCACAACCCGAAGTCAATTTTAAGCAATTTAGAACAGCAC
GTCATTTAACGAGTGTTTTAGCTGAAGTTAATGATGAAGATATCGGAGCTATGGTAATTA
AACTAGTTAAACTTATTTTCATGCTTGATTATGTTTTATTTCATTCAATTCAAAAAAGTTAT
ACAACACATATGAATGAGCTTAAAAATTGTTTCGATTATATAGCTGAATTAGATAATCAT
TATGCATTAGCAATGTATCGTAGAACGCTAGAATGCTATACTGAACCACAAATTGATGAT
TCAAATGATGGCATAGTATTCTCTGAGTTAACACATCCACTCATAGCAGATGCAGTAGCC
AATGATTTCTCACTATCACAAAATATACTGTTAACTGGGTCTAATGCTTCGGGTAAATCAA
CATTTATGAAATCCATAGCAATAAACATTATTCTTGCATCGGCTATACAAACAGTTACCG
CAAGCAAATTTGTATATCAACCTGGAATTGTGTTCACGTCAATGGCAAATGCCGATGATG
TGTTATCAGGCGATAGTTATTTTATGGCTGAATTAAAATCTATTAAGCGTATTGTTGAAAT
TCCAGATAATCAAAAAAATATACTGCTTTATAGATGAAATTTTTAAAGGTACCAACACAAC
TGAACGAATTGCCGCTTCAGAATCAGTACTATCATTTTTACATGAAAAATCTAACTTTAGA
GTTATTGCAGCAACACATGATATTGAGTTAGCTGAACTCTTAAAACAACGTTATGAAAAT
TATCATTTCAATGAGGTAATAGAAAATAATAACATACATTTTGATTACAAAATTAAGCCT
GGCAAAGCAAATACTCGTAATGCCATCGAATTATTAAAAATCACTTCATTTCCAGCAAAA
ATATATGAACGAGCAAAAGATAATGTTTCCAATGGTTAG

> **tx_universal2**
Function:  rho indendent terminator
Best match: tx_universal2_CC045-agrI_21194_AGTU01000022.1[11729:11767] (completely identical)
Position: 052-contig_193_RC: 32985 … 33024; Length: 39 bp
Sequence:
CTAAATGAAAATGCGCTTGTAATAAGTTTTTTCAATACT

> **Q2FZB6**
Function: protein coding sequence; putative protein (genomic island)
Best match: Q2FZB6_CC008_COL_CP000046.1[1182242:1182414]
Position: 052-contig_193_RC: 33055 … 33085; Length: 30 bp
Sequence:
AAAGTCAGCTTACAATAATGTGCAAGTTGG

> **gcp**
Function: protein coding sequence; O-sialoglycoprotein endopeptidase (core genome, constant)
Best match: gcp_Z262_LCL_10042.1[12875:13900]RC (completely identical)
Position: 052-contig_193_RC: 33238 … 34264; Length: 1026 bp
Sequence:

TTATTCTGCAGAATACTCTTCTAAATCTATATTGCTGTGCCCATTTAATGCTAAATCAGCA
AATCGACCTTGCTGATACAAATAGTGGCCGGCAACGCCTATCATTGCAGCATTATCTGTG
CATAATTTAGGACTTGGGATAGTTAATTGAATGTCATTGACTTTGCATTGATCCGCTAAAG
ATTGACGTAATCCTTTATTACTCGCCACGCCACCAGCAACAATTAATCGCTGAACACCAT
ATTCTTTACAAGCTTGAATAGCTTTAAACGTAAGCACCTCTACAACACTGTTTTGAAAGCT
CGTTGCTACGTTAGCTTCAATGATTGGAATATTTTTTTGTCGTTGATTGTGAAGTTGATTG
ATTACGGCACTTTTCAACCCACTAAAACTAAAATCATAACTATCTTTATCCAACCAAACA
CGAGGGAATGAATAAGTATCTTCACCTTCAGCAGCCAACCGATCAACTTGTGGACCACCT
GGATAATTTAAACCAATTGTTCGTGCCACTTTATCATAAGCCTCACCTACTGCGTCATCTC
GTGTTTCACCAATGACTTCAAATGATAAATGATCCTTCATATAAACTAATTCAGTATGTCC
ACCTGAAACAATAAGTGCAATTAGCGGGAATGTTAATGGCTCTTCTATGTGATTAGCATA
TATATGTCCTGCAATATGATGAACAGGAATAAGTGGCTTATCGTAAGCAAATGCCAATGC
TTTGGCTGCATTAACACCTATTAGTAACGCACCAATTAGTCCAGGGCCTTCTGTAACCGCT
ATGGCATCAATATCTTCTATTGATACATCGGCATCCACTAGAGCCTCGTTTATTGTTGTTG
TTATACCTTCAACGTGATGTCTACTTGCCACTTCGGGAACGACACCGCCAAATCGTTTATG
ACTTTCAATCTGACTTAAAACTGTATTTGATAAAATATCTCTGCCATTTTTTATAACACTA
ACGCTTGTTTCATCACAACTTGTTTCAACAGCTAGTATTAATATATCTTTAGTCAT

> **rimI**

Function: protein coding sequence; ribosomal-protein-alanine acetyltransferase (core genome, constant)
Best match: rimI_CC001_MW2-USA400_BA000033.2[2123932:2124396:r]RC (completely identical)
Position: 052-contig_193_RC: 34256 … 34721; Length: 465 bp
Sequence:
TTAGTCATTTAAATTCACCCACATAACCATTGCGTCCTCACCTTCACCATAATAATTTTTA
CGTTTACCACCATATTGAAATCCTAAATTTTCATATACATGTTGTGCCACTTTATTATTAA
CTCTTACTTCTAAACTCATCACATCACAAGTGTGACTTGCATAGTTTATTCCGTATTTTAA
AAGCATTTGACCTAAACCATAGCCTCTATAATTATCATCGATTGCAACTGTTGTAATTTGA
GCTTGATCGATAACAATCCATAAACCTAAATAACCAATAATTTGTTGTTCAAATTCTAAG
ACAAAATATTTCGCAAAGTTATTTTGCTCTATTTCATGATAAAATGCGTCAATTGTCCAAG
AACTGTCATTGAAACTCCGACGCTCAATATCAAAGACTTGTGGCACATCTTCTTTAGTCAT
CTCTCTAATGTTTAATTGTTCTTTTGACTGTTGATCCAA

> **ilvD**

Function: protein coding sequence; dihydroxy-acid dehydratase (core genome, variable)
Best match: ilvD_CC001-ST772_118_AJGE01000025.1[9480:11168:r] (completely identical)
Position: 052-contig_193_RC: 36309 … 37998; Length: 1689 bp
Sequence:
ATGCGAAGCGACATGATCAAAAAAGGAGATCACCAAGCACCAGCAAGAAGTCTTTTACA
TGCCACGGGCGCGCTAAAAAGTCCAACTGATATGAACAAACCATTTGTAGCTATTTGTAA
CTCTTATATTGATATTGTTCCTGGACATGTTCATTTAAGAGAGCTTGCAGATATAGCTAAA
GAAGCAATTAGAGAAGCCGGTGCCATTCCATTTGAATTCAATACAATTGGTGTTGATGAT
GGAATAGCTATGGGACATATCGGAATGCGATATTCTCTACCATCACGTGAAATTATTGCA
GATGCAGCTGAAACTGTAATTAACGCTCATTGGTTTGACGGCGTATTTTACATTCCTAATT
GTGACAAGATTACACCCGGTATGATTTTAGCAGCCATGAGGACAAACGTACCAGCTATCT
TTTGCTCTGGTGGACCAATGAAAGCTGGCTTATCTGCACATGGAAAAGCATTAACACTTT
CATCAATGTTTGAAGCAGTCGGCGCATTTAAAGAAGGATCGATTTCTAAAGAAGAATTTT
TAGATATGGAACAAAATGCCTGCCCTACTTGTGGTTCATGTGCTGGGATGTTTACTGCAA
ATTCAATGAACTGTTTGATGGAAGTTTTAGGTCTAGCATTACCATACAACGGTACTGCACT
TGCAGTCAGTGATCAGCGACGCGAAATGATTCGCCAAGCAGCTTTTAAATTAGTTGAAAA
TATTAAAAATGATTTAAAACCACGTGATATCGTTACTCGCGAAGCAATTGATGATGCATT
TGCACTTGATATGGCTATGGGTGGTTCAACAAACACGGTACTGCATACGTTAGCCATTGC
CAATGAAACTGGTATTGATTATGACTTAGAGCGCATTAATGCTATTGCCAAACGCACACC
ATATTTATCAAAAATAGCACCTAGTTCATCGTATTCAATGCATGATGTGCATGAAGCTGG

TGGCGTCCCAGCAATTATTAATGAATTGATGAAGAAAGATGGCACGTTACACCCAGATAG
AATCACAGTTACTGGCAAAACGTTACGTGAAAATAACGAAGGCAAAGAAATTAAGAACT
TTGATGTCATTCACTCTCTTGATGCACCATATGATGCACAAGGCGGTTTATCTATCTTATT
TGGTAATATCGCCCCTAAAGGCGCAGTTATTAAAGTTGGCGGCGTTGATCCATCTATCAA
AACATTTACTGGGAAAGCAATTTGTTTCAATTCGCATGATGAAGCTGTTGAAGCAATAGA
CAATCGTACCGTTCGTGCAGGCCACGTCGTTGTCATTAGATATGAAGGACCTAAAGGTGG
ACCAGGTATGCCTGAAATGTTAGCACCTACTTCCTCTATTGTTGGTCGCGGCTTAGGTAAA
GATGTTGCATTAATTACTGATGGGCGTTTTTCCGGTGCCACAAGAGGTATTGCAGTTGGTC
ATATTTCCCCTGAAGCTGCATCTGGTGGACCAATTGCCTTAATTGAAGATGGAGATGAGA
TTACTATTGATTTAACAAATCGTACATTAAACGTAAACCAGCCTGAAGATGTTCTAGCGC
GTCGCCGAGAATCTTTAACACCATTTAAAGCGAAAGTAAAAACAGGTTATCTAGCTCGTT
ATACTGCCCTAGTAACTAGCGCAAATACAGGTGGCGTCATGCAAGTCCCTGAGAATTTAA
TTTAA

## > **ilvB**

Function: protein coding sequence; acetolactate synthase/large subunit
Best match: ilvB_CC001_MW2-USA400_BA000033.2[2127700:2129469]
Position: 052-contig_193_RC: 38025 … 39795; Length: 1770 bp
Sequence:
ATGTCTAAAACTCAACATGAAGTAAACCAAAATATTGACCCTTTAAAAATGGCTGAATCA
CTTGAACCTGAACAACTAAATGAAAAAACTTTAAATGATATGCGTTCAGGATCAGAAGTG
CTAGTAGAAGCTCTACTTAAAGAAAATGTGGATTATTTATTCGGTTATCCTGGTGGTGCCG
TACTACCTTTATATGACACGTTTTATGATGGTAAAATCAAACATATTTTAGCAAGACACG
AACAAGGTGCTGTTCATGCTGCAGAAGGTTATGCACGTGTATCTGGTAAAACTGGCGTCG
TTGTAGTTACAAGCGGTCCAGGTGCAACTAATGTAATGACAGGTATTACGGATGCACATT
GCGACTCTTTACCTCTAGTTGTATTCACTGGACAAGTTGCTACACCAGGCATTGGTAAAG
ATGCATTCCAAGAAGCGGATATTCTATCTATGACTTCACCAATTACAAAACAAAATTATC
AAGTGAAACGTGTTGAAGATATCCCTAAAATCGTACACGAAGCTTTCCATGTAGCTAATT
CTGGACGCAAAGGTCCTGTAGTGATTGATTTTCCAAAAGATATGGGTGTTTTAGCTACAA
ATGTGGATTTATGCGACGAAATCAATATTCCAGGTTATGAAGTTGTTACAGAACCAGAAA
ATAAAGACATTGACACTTTCATCTCACTTTTAAAAGAAGCGAAAAAGCCTGTCGTATTAG
CCGGCGCAGGTATTAATCAATCAAAATCAAATCAATTATTAACACAGTTTGTTAATAAAC
ATCAAATCCCAACAGTTACTACTTTACTAGGTCTAGGTGCTGTACCTTATGAGGATACACT
ATTTTTAGGTATGGGAGGTATGCATGGTTCTTATGCTAGTAACATGGCATTAACTGAGTGT
GATTTACTCATTAATTTAGGTAGCCGCTTCGATGATAGATTAGCAAGCAAACCTGATGCC
TTTGCACCTAACGCCAAAATTGTACATGTAGATATTGATCCTTCAGAAATCAATAAAGTT
ATTCATGTAGATTTAGGTATTATTGCAGACTGTAAAAGATTTTTAGAATGTTTAAATGATA
AAAATGTTGAGACTATAGAACACAGTGACTGGGTTAAACATTGTCAAAATAATAAGCAG
AAACACCCATTTAAACTTGGTGAAGAAGATCAAGTATTTTGTAAGCCACAACAAACAATC
GAATATATCGGCAAAATTACAAATGGTGAAGCAATTGTTACTACAGACGTGGGACAACA
TCAAATGTGGGCAGCTCAATTTTATCCATTTAAAAATCACGGACAATGGGTTACAAGCGG
TGGTTTAGGAACAATGGGATTCGGTATTCCTTCGTCAATTGGTGCCAAATTAGCTAATCCT
GATAAAACAGTCGTATGTTTCGTCGGTGACGGTGGTTTCCAAATGACAAACCAAGAAATG
GCACTTTTACCCGAATATGGTTTAGATGTCAAAATCGTACTAATCAATAATGGAACATTA
GGTATGGTTAAACAATGGCAAGATAAGTTCTTTAATCAACGCTTCTCACACTCAGTATTTA
ATGGTCAACCTGATTTTATGAAAATGGCAGAAGCATATGGCGTCAAAGGTTTCTTAATCG
ATAAGCCAGAACAACTGGAAGAACAATTAGATGCAGCGTTTGCTTATCAAGGACCAGTTT
TAATTGAGGTTCGTATTTCCCCTACTGAAGCTGTAACCCCAATGGTTCCGAGTGGCAAATC
AAATCATGAAATGGAGGGCTTATAA

## > **ilvH**

Function: protein coding sequence; acetolactate synthase isozyme III small subunit
Best match: ilvH_CC001_MW2-USA400_BA000033.2[2129469:2129723]
Position: 052-contig_193_RC: 39794 … 40049; Length: 255 bp
Sequence:

ATGACAAGAATTCTTAAATTACAAGTTGCGGATCAAGTCAGCACGCTAAATCGAATTACA
AGTGCTTTTGTTCGCCTACAATATAATATCGATACATTACATGTTACACATTCTGAACAAC
CTGGGATTTCTAACATGGAAATTCAAGTCGATATTCAAGATGATACATCACTTCATATATT
AATTAAAAAATTGAAGCAACAAATTAATGTTTTAACGGTTGAATGCTACGACCTTGTTGA
TAACGAAGCTTAA

## > **ilvC**

Function: protein coding sequence; ketol-acid reductoisomerase
Best match: ilvC_CC705_RF122-cow_AJ938182.1[2067727:2068731]
Position: 052-contig_193_RC: 40185 … 41190; Length: 1005 bp
Sequence:
ATGACAACAGTTTATTATGATCAAGATGTAAAAACGGACGCTTTACAAGGCAAAAAAATT
GCAGTAGTAGGTTATGGATCACAAGGCCACGCGCATGCACAAAACTTAAAAGACAATGG
ATATGATGTAGTCATCGGTATTCGCCCAGGTCGTTCTTTTGACAAAGCTAAAGAAGATGG
ATTTGATGTGTTCCCTGTTGCAGAAGCAGTTAAGCAAGCTGATGTAATTATGGTGCTATTA
CCTGATGAAATTCAAGGTGATGTATACAAAAACGAAATTGAACCAAATTTAGAAAAACA
TAATGCGCTTGCATTTGCTCATGGCTTTAACATTCATTTTGGTGTTATTCAACCACCAGCT
GATGTTGATGTATTTTTAGTAGCTCCTAAAGGACCGGGTCATTTAGTTAGACGTACATTTG
TTGAAGGTTCTGCTGTACCATCACTATTTGGTATTCAACAAGACGCTTCAGGTCAAGCAC
GTAATATTGCTTTAAGTTATGCAAAAGGTATTGGTGCAACTCGTGCAGGTGTTATTGAAA
CAACATTTAAAGAAGAAACTGAGACAGATTTATTTGGTGAACAAGCAGTACTTTGCGGTG
GTGTATCGAAATTAATTCAAAGTGGCTTTGAAACATTAGTAGAAGCGGGTTATCAACCAG
AATTAGCTTATTTTGAAGTATTACATGAAATGAAATTAATCGTTGATTTGATGTATGAAGG
CGGTATGGAAAATGTACGTTACTCAATTTCAAATACTGCTGAATTTGGTGACTATGTTTCA
GGACCACGTGTTATCACACCAGATGTTAAAGAAAATATGAAAGCTGTATTAACTGATATC
CAAAATGGTAACTTCAGTAATCGCTTTATCGAAGACAATAAAAATGGATTCAAAGAATTT
TATAAATTACGCGAAGAACAACATGGTCATCAAATTGAAAAAGTTGGTCGTGAATTACGC
GAAATGATGCCTTTTATTAAATCTAAAAGCATTGAAAAATAA

## > **leuA1**

Function: protein coding sequence; 2-isopropylmalate synthase/locus 1 (core genome, constant)
Best match: leuA1_CC001-ST772_118_AJGE01000025.1[4729:6258:r] (completely identical)
Position: 052-contig_193_RC: 41219 … 42749; Length: 1530 bp
Sequence:
ATGAGTAGTCATATTCAAATTTTTGATACGACACTAAGAGACGGTGAACAAACACCAGGA
GTGAATTTTACTTTTGATGAACGCTTGCGTATTGCATTGCAATTAGAAAAATGGGGTGTA
GATGTTATTGAAGCTGGATTTCCTGCTTCAAGTACAGGTAGCTTTAAATCTGTTCAAGCAA
TTGCACAAACATTAACAACAACGGCTGTATGTGGTTTAGCTAGATGTAAAAAATCTGACA
TCGATGCTGTATATGAAGCAACAAAAGATGCAGCGAAGCCGGTCGTGCATGTTTTTATAG
CAACATCACCTATTCATCTTGAACATAAACTTAAAATGTCTCAAGAAGACGTTTTAGCAT
CTATTAAAGAACATGTCACATACGCGAAACAATTATTTGACGTTGTTCAATTTTCACCTGA
AGATGCAACGCGTACTGAATTACCATTCTTAGTGAAATGTGTACAAACTGCCGTTGACGC
TGGAGCTACAGTTATTAATATTCCTGATACAGTCGGCTACAGTTACCATGATGAATATGC
ACATATTTTCAAAACCTTAACAGAATCTGTAACATCTTCAAATGAAATTATTTATAGTGCT
CATTGCCATGACGATTTAGGAATGGCTGTTTCAAATAGTTTAGCTGCAATTGAAGGCGGT
GCGAGACGAATTGAAGGCACTGTAAATGGTATTGGTGAACGAGCAGGTAATGCAGCACT
TGAAGAAGTCGCGCTTGCACTATACGTTCGAAATGATCATTATGGTGCTCAAACTGCTCTT
AATCTCGAAGAAACTAAAAAAACATCGGATTTAATTTCAAGATATGCAGGTATTCGAGTG
CCTAGAAATAAAGCAATTGTTGGCCAAAATGCATTTAGTCATGAATCAGGTATTCACCAA
GATGGCGTATTAAAACATCGTGAAACATATGAAATTATGACACCTCAACTTGTTGGTGTA
AGCACGACTGAACTTCCATTAGGAAAATTATCTGGTAAACACGCCTTCTCAGAGAAGTTA
AAAGCATTAGGTTATGACATTGATAAAGAAGCACAAATAGATTTATTTAAACAATTCAAG
GCCATTGCGGACAAAAAGAAATCTGTTTCAGATAGAGATATTCATGCGATTATTCAAGGT
TCTGAGCATGAGCATCAAGCACTTTATAAATTGGAAACACTGCAACTACAATATGTCTCT
AGCGGCCTTCAAAGTGCTGTTGTTGTTGTTAAAGATAAAGAGGGGCATATTTACCAGGAT

TCAAGTATTGGTACTGGTTCAATCGTAGCAATTTACAATGCAGTTGATCGTATTTTCCAGA
AAGAAACAGAATTAATTGATTATCGTATTAATTCTGTCACTGAAGGTACTGATGCCCAAG
CAGAAGTACATGTAAATTTATTGATTGAAGGTAAGACTGTCAATGGCTTTGGTATTGATC
ATGATATTTTACAAGCCTCTTGTAAAGCATACGTAGAAGCACATGCTAAATTTGCAGCTG
AAAATGTTGAAAAGGTAGGTAATTAA

> **leuB**

Function: protein coding sequence; 3-isopropylmalate dehydrogenase
Best match: leuB_CC001_MSSA476_BX571857.1[2111551:2112597]
Position: 052-contig_193_RC: 42751 … 43798; Length: 1047 bp
Sequence:
ATGACTTATAACATTGTTGCCCTACCTGGTGATGGAATCGGTCCAGAAATTTTGAACGGA
TCTCTATCATTGCTTGAAATTATAAGTAATAAATATAACTTTAATTATCAAATAGAGCACC
ACGAATTTGGTGGTGCCTCTATTGATACATTCGGCGAACCTTTAACTGAGAAAACCTTAA
ATGCGTGTAAAAGAGCAGATGCTATTTTACTGGGTGCAATCGGTGGACCTAAATGGACAG
ATCCTAACAATCGACCAGAACAAGGATTATTAAAATTGCGTAAATCCTTAAATTTATTTG
CAAATATACGCCCCACTACCGTTGTCAAAGGCGCTAGTTCTTTATCACCTTTAAAGGAAG
AACGCGTTGAAGGCACAGATTTAGTTATAGTCCGTGAATTGACAAGTGGTATTTATTTTG
GAGAACCTAGACATTTTAATAATCACGAGGCCTTAGATTCTCTTACTTATACAAGAGAAG
AAATAGAACGCATTGTTCACGTAGCATTTAAATTGGCCGCTTCAAGACGAGGAAAACTAA
CATCAGTTGATAAAGAAAATGTATTAGCTTCTAGTAAATTGTGGCGCAAAGTCGTAAATG
AAGTAAGTCAATTATATCCAGAAGTAACAGTAAATCACTTACTTGTTGATGCTTGTAGTA
TGCATTTAATCACAAATCCAAAACAATTTGACGTCATCGTATGTGAAAACTTATTTGGCG
ATATTTTAAGTGATGAAGCTTCAGTGATTCCTGGTTCACTTGGTTTATCACCTTCTGCTAG
TTTTAGTAACGATGGTCCAAGATTGTATGAGCCTATTCATGGATCAGCACCAGATATTGC
AGGTAAAAACGTTGCCAATCCATTTGGAATGATTCTATCTTTAGCGATGTGTTTACGTGAA
AGCTTAAATCAACAAGATGCTGCAGATGAATTAGAACAACATATTTATAACATGATTGAA
CATGGGCAAACGACAGCAGATTTAGGCGGCAAATTGAATACTACTGATATTTTCGAAATT
CTATCTCAAAAATTGAATCACTAA

> **leuC**

Function: protein coding sequence; 3-isopropylmalate dehydratase/large subunit
Best match: leuC_CC001_MW2-USA400_BA000033.2[2133486:2134856]
Position: 052-contig_193_RC: 43811 … 45182; Length: 1371 bp
Sequence:
ATGGGTCAAACATTATTTGACAAGGTGTGGAACAGACATGTGTTATACGGGAAATTGGGC
GAACCGCAACTATTATACATTGATTTACACCTTATACATGAAGTTACTTCTCCTCAAGCAT
TTGAAGGACTTAGGCTTCAAAACAGAAAATTAAGACGCCCAGATTTAACATTTGCAACAC
TCGATCACAATGTTCCTACTATTGATATTTTCAATATTAAAGATGAAATTGCAAACAAAC
AAATCACAACATTACAAAAAAACGCCATAGATTTTGGGGTGCATATTTTTGATATGGGCT
CTGATGAACAAGGTATTGTTCACATGGTAGGACCTGAGACAGGACTTACACAGCCTGGCA
AGACAATCGTTTGTGGTGACTCTCACACAGCAACACATGGAGCATTTGGTGCTATTGCAT
TTGGAATTGGAACAAGTGAAGTTGAACATGTTTTCGCAACTCAAACGCTATGGCAAACAA
AACCCAAAAACTTAAAAATCGATATTAATGGTACCTTACCAACAGGCGTCTATGCTAAGG
ACATTATTCTGCATTTAATTAAAACGTATGGTGTTGACTTTGGTACAGGCTATGCTTTGGA
ATTTACTGGCGAAACAATTAAAAACCTTTCAATGGACGGTCGAATGACTATTTGTAACAT
GGCTATCGAAGGTGGTGCCAAATACGGCATAATCCAACCTGATGATATAACATTTGAATA
TGTTAAAGGGAGACCATTTGCCGATAACTTCGCTAAATCAGTTGATAAGTGGCGTGAGCT
ATATTCTGATGACGACGCGATATTTGATCGTGTAATTGAACTTGATGTTTCAACATTAGAA
CCACAAGTGACATGGGGAACTAATCCTGAAATGGGTGTTAATTTCAGTGAACCATTCCCT
GAAATCAATGATATCAACGATCAACGTGCGTATGATTATATGGGGTTGGAACCAGGTCAA
AAAGCTGAAGACATCGACTTAGGGTATGTTTTCCTCGGTTCATGTACAAATGCTAGACTA
TCAGATTTGATTGAAGCTAGTCATATTGTTAAAGGAAATAAAGTTCACCCAAATATTACA
GCTATTGTCGTACCAGGTTCTCGTACAGTAAAAAAAGAAGCAGAAAAATTAGGTCTAGAT
ACTATCTTTAAAAATGCAGGATTTGAATGGCGTGAACCAGGATGTTCAATGTGTTTAGGC

ATGAATCCTGACCAAGTACCTGAGGGCGTACATTGTGCATCTACAAGTAATCGAAACTTT
GAAGGACGACAAGGTAAAGGTGCAAGAACACATTTAGTATCCCCTGCTATGGCAGCAGC
AGCAGCTATTCATGGTAAATTTGTGGACGTAAGAAAGGTGGTTGTTTAA

## > **leuD**

Function: protein coding sequence; 3-isopropylmalate dehydratase/small subunit
Best match: leuD_CC008_NCTC8325_CP000253.1[2120679:2121251]
Position: 052-contig_193_RC: 45182 … 45755; Length: 573 bp
Sequence:
ATGGCAGCAATCAAACCTATTACAACATATAAAGGTAAAATAGTCCCTCTCTTCAACGAC
AATATCGATACAGACCAAATCATTCCTAAGGTACACTTAAAGCGTATTTCAAAAAGTGGC
TTTGGTCCATTTGCTTTTGATGAATGGCGGTACTTACCTGATGGTTCAGATAATCCTGATT
TCAATCCAAATAAGCCTCAATATAAAGGTGCATCCATATTAATTACTGGAGATAATTTTG
GATGTGGTTCAAGTCGTGAACATGCAGCTTGGGCACTTAAGGACTATGGTTTTCATATTAT
TATTGCAGGAAGTTTCAGTGACATTTTTTATATGAATTGTACAAAAAATGCAATGTTGCCT
ATCGTTTTAGAAAAAAATGCACGTGAACATCTTGCTAAATATGTTGAAATTGAGGTCGAT
TTACCAAATCAAACTGTGTCATCACCAGACAAGCGTTTCCATTTTGAAATTGATGAAACTT
GGAAGAATAAACTTGTAAATGGCTTAGATGACATTGCAATCACCCTACAATATGAATCAT
TAATAGAAAAATATGAAAAATCACTTTAA

## > **ilvA2**

Function: protein coding sequence; threonine dehydratase locus 2 (core genome, variable)
Best match: ilvA2-CC30_CC010_D139_ACSR01000065.1[40724:41992]
Position: 052-contig_193_RC: 45769 … 47038; Length: 1269 bp
Sequence:
ATGACAGTCAAAACAACAGTTTCTACGAAAGATATCGATGAAGCATTTTTAAGACTTAAA
GATATTGTCAAAGAAACACCTTTACAATTAGACCATTACTTATCTCAAAAGTATGATTGT
AAAGTCTATTTAAAACGAGAAGATTTACAATGGGTACGTTCTTTTAAATTAAGAGGTGCT
TACAACGCTATTTCTGTTTTATCAGATGAAGCTAAAAGTAAAGGTATTACATGTGCGAGT
GCAGGTAATCATGCTCAAGGTGTTGCCTATACAGCTAAAAAACTTAATTTAAACGCTGTT
ATCTTTATGCCAGTCACTACACCTTTACAAAAGGTAAATCAAGTAAAGTTCTTTGGAAAT
AGTAACGTTGAAGTTGTACTCACTGGTGATACATTTGATCACTGTTTAGCTGAAGCTTTAA
CTTATACAAGTGAACATCAAATGAACTTTATAGATCCATTCAATAATGTTCATACAATTTC
TGGACAAGGTACGCTTGCTAAAGAAATGCTAGAACAATCAAAGACTGACAATGTTAACTT
TGATTATTTATTTGCCGCTATTGGTGGTGGTGGATTAATTTCAGGTATTAGTACTTACTTTA
AAACCTATTCACCTACTACGAAAATTATAGGTGTTGAACCTTCAGGTGCAAGTAGTATGT
ATGAATCTGTTGTCGTAAATAATCAGGTAGTCACATTGCCTAATATCGATAAATTTGTGG
ACGGTGCATCTGTAGCTAGAGTTGGCGATATTACATTTGAAATTGCAAAAGAAAATGTAG
ATGATTACGTTCAAGTAGATGAAGGTGCAGTTTGTTCTACGATTTTAGACATGTATTCAAA
ACAAGCAATTGTAGCAGAACCTGCTGGCGCATTAAGTGTGAGTGCTCTTGAAAACTACAA
AGACCACATTAAGGGTAAAACTGTTGTTTGTGTCATTAGTGGTGGTAATAATGATATTAA
TCGTATGAAGGAAATTGAAGAACGTTCTTTATTATATGAAGAAATGAAACATTATTTTAT
TTTAAACTTCCCTCAACGCCCTGGTGCATTAAGAGAATTTGTAAATGATGTTTTAGGCCCA
CAAGACGATATTACAAAATTTGAATACTTGAAAAAATCTTCTCAAAATACAGGTACTGTC
ATTATTGGTATTCAACTTAAAGATCATGATGATTTAATACAACTCAAACAACGTGTAAAT
CATTTCGATCCTTCCAATATTTATATTAATGAAAATAAGATGTTATATTCATTGTTAATTT
AA

## > **txbi_ilvA_rrf**

Function:  bidirectional rho independent terminator of ileV and rrf
Best match: txbi_ilvA_rrf_CC001_MW2-USA400_BA000033.2[2136714:2136784:r]RC (completely identical)
Position: 052-contig_193_RC: 47039 … 47110; Length: 71 bp
Sequence:

ACATAGTAAGAAAAACAGTCATAAATTGATTTCTAATTGAAATCATCTTATGACTGCTTTT
TATTATACTT

> **tRNAgly1**
Function: tRNA tRNA glycin
Best match: tRNAgly1_CC001_MW2-USA400_BA000033.2[1924102:1924175:r]RC (completely identical)
Position: 053-contig_55_RC: 1 … 75; Length: 74 bp
Sequence:
TGGAGCGGGTGATGGGAATCGAACCCACAACATCAGCTTGGAAGGCTGAGGTTTTGCCAT
TAAACTACACCCGC

> **tRNAleu4**
Function: tRNA tRNA leucine
Best match: tRNAleu4_CC001_MW2-USA400_BA000033.2[2142599:2142684:r]RC (completely identical)
Position: 053-contig_55_RC: 93 … 179; Length: 86 bp
Sequence:
TGATGCGGCCGAGAGGACTTGAACCTCCACGGGATTTCTCCCACTAGGCCCTCAACCTAG
CGCGTCTGCCATTCCGCCACGACCGC

> **sprL**
Function: protein coding sequence; metallopeptidase/SprT family (core genome, variable)
Best match: sprL_CC005_ACKJ01000004.118768:19223RC
Position: 053-contig_55_RC: 825 … 1281; Length: 456 bp
Sequence:
TTATTTCAATTGTTTTTTCATTCTTAATTTTCCATTGCAATGTCCGCAACGCATACGATTCG
TATCAACTTTACGGATACGTATATATTTTGCATGACATTTAGTACAATAATATTCATAATT
CGCCCGTTGTTGATAGCTTTCAATGCTGTTACAAAATCTAGGCGCTCCAACCTGTTGGCTC
AATCGTTTAAAATCTTGATCTTTATGTTGATAACCTTTACCAGCAATATGCAAGTGATAAT
GACACAATTCGTGCAGTATAATTTTTACAACAGCATCTTCTCTATAATGCTCATATTGTTT
TGGATTAATTTCAATATCATGGGACTTTAAAAGATAACGTCCGCCTGTTGTACGTAACCTT
TTATTAAAATATGCACAATGTCGAAACGTACGTCCAAATTTTTCTTCCGAAAGGTTCTCAA
CCATTCGCTGAAGTTTGTCATTATTCAT

> **yhgF**
Function: protein coding sequence; RNA binding S1 domain protein (core genome, constant)
Best match: yhgF_CC008-ST72_TCH130_ACHD01000014.1[15203:17353]RC
Position: 053-contig_55_RC: 1273 … 3424; Length: 2151 bp
Sequence:
TTATTCATGTGGATCAATCATCGTTAATGATACTTTGTCTTTATTTTTGTCAATACTGTAAA
TCCAAACGTCAACGATATCACCGACACTGACAATATCCATTGGATTTTTTACGAACTTCTT
AGAAAGTTTCGAAACATGGACAAGTCCATCTTGTTTAACACCTATATCTACAAATGCTCC
AAAATCTACTACATTTCTAACTGTGCCACTCAATTTCATTCCTTCTTGTAAATCTTCAATTG
ATAGCACATCGGATTTAAGGATTGGTGTTTCAAACTCGTCCCTTGGATCTCGATTAGGTGC
TTTCAAGGATTTAATAATATCCTCTAATGTAGGTACACCGACTTGTAATTCAATCGCCAGT
TCATCCATATCCAATGAATTTAATTTATCTTTTAATGCATCACTACCTAAATCATTGCCAC
CAAATCCGAGTTTATCTAATAACTGATACGTTACTTTATAGCTTTCGGGATGAATCGATGT
ATTATCCAATGGCTCTGAACCGTCTACTATTCTTAAAAATCCTATACTCTGCTCAAAAGTT
TTAGCACCCAAACGCTTAATCTTGCTTAACTCTTTATTATGCTTTATTGCGCCATTTTCTTC
TCTATATGCAATTATATTTTTAGCAATTTGCGAACTTAGACCAGACACATATTGTAATAAT
GATGATGATGCTGTATTGACATCAACACCTACTTGGTTAACTGCTGTTTCAACGACGAAA
GTTAATGCATTTTCTAGTGCTTTTTGATTAACATCATGCTGGTATTGTCCGACACCAATAG
ATTTAGGATCGATTTTAACTAATTCACTTAATGGATCTTGAACGCGTCGTCCAATTGATAC
GGCACTTCTTTCTTCCACTTGAAAATCAGGAAATTCATCTCTAGCAATTTCTGATGCTGAG

TATACTGAAGCGCCCGCTTCATTGACAATGATGAATTGTACTGGCAACTGATGCTTTTTAA
TTAAATCTGCAACAAATTGTTCTGTTTCACGACTTGCAGTACCATTGCCAATTGCAATTAA
TTGCACATCATACGCTTTAACCATTTGTACAAAATCCTTCTCTGCTGCCTCTTTTTTAGAAA
CTGGTGGATGCGGATAAATCACACCTTTTGCTATAAAAGTACCGAATGGGTTAATGACTG
CTAATTTACAACCTGTTCTAAATGCTGGATCTACGCCTAATATTTGTTTACCTTTCATTGG
AGGTTGCAGTAATAGATTTCTTAAGTTTTCACTAAAAACATCTATTGCATGATTTTCAGCT
TTTTCAGTTAAATCAGCATGGATTTCACGCTCTATCGAAGGGACAATTAAGCGTTTCAAG
CTATCTTTAATCGCCTCTAAAATATAACTGCGATTTACATTATTATGATTGATTTCTTGAC
GTGCAATGAAATCCTCTACTGATGTCGTATCGAATTCAAACTTTACAGATAATACTTTCTC
TTTTTCACCACGATTAACAGCTAAAACTCTATGATTAGCAATGCGTTTAATTGGCTCACTA
TATGCATAGTACATTTCAAAAATACCTTTTTCATCTTCAGCATTTTTCTTTTTAGATGTAGT
TAACACACCTTGATGATACATATCTTTTAAAATTTTTGTTCTATATTTAGGATTATCTGAA
ATTTGTTCCGCAATAATATCTTGTGCACCTTTGATAGCATCTTCAACCGATTGCACTTCTTC
ATTTATAAATTGTTGTGCTTTTTCTTCAATTGAGACTTCATGTTTACGTGCCTTCATCCATA
TCGCTAATGGCTCTAACCCTTTACGTTTCGCCTCAGTTGCCCTTGTCTTTTTCTTTTGTTTA
AAAGGCCTATATAGGTCTTCAACACGTTGTAATTTGTTCTGTTTTAAAATATCGTTCTTTA
ATTCCTCAGTAAGTAATCCTTGCTGTTCTATATTTTTGATAACTTCTTCTTTACGTTTTTGT
AAATTGACCATATATTGGTATTCGTCATCAATTTGCTTTATTTGAACTTCATCTAGTCCAC
CAGTTTGCTCTTTTCGATACCTCGCAATAAATGGTACTGTATTTTTTTCTTCTAGCAGTGTT
AATACTGCTTCAATTTGTTTTTTACTAAATTGATATTTCTCTATGATTGAATTAATCAATTG
ATTGTCCAT

> **sigB**

Function: protein coding sequence; sigma factor B of RNA polymerase (core genome, constant)
Best match: sigB_AJ920004.1[2472:3242]RC
Position: 053-contig_55_RC: 3857 … 4628; Length: 771 bp
Sequence:
CTATTTATGTGCTGCTTCTTGTAATTTCTTAATTGCCGTTCTTTGAAGTCGTGATACATGCA
TTTGACTTAAACCGATACGCTCACCTGTCTCTTTTTGACTCAAACCTTCAATAAACGTACA
TTGTATGATTTCTCGTTCGCGATCAGATAATATAGGTAATATTTTTTCTAAAATCATACGT
TTTTCAGTTAAGTCATAATGGTCATCTTGTTGTCCCATAATATCTAATAGCGTAACAGTTG
AACCATCTTTATCAGCTTCAATGGAATGATCAACACTTAACGCATTATAACTTTGTCCCAT
TTCCATTGCTTCTAACACTTCTTCTTCTGAAACTTCTAATCGATCAGCTATTTCACTGATAG
AAGGTGAACGCTCTAATTCAGCGGTTAGTTCATCGCTCACTTTTTTGATTCTTGGCCCAAT
TTCTTTAATACGTCTCGGAACATGTACACTCCAAGTTTTATCTCGTAGATATCTTTTGATTT
CACCGATTACAGTAGGTACTAAAAAGGCTTCAAACTTCCGTTCAAAGGACATATCGAATC
TATTTATGGCACCTATTAAACCAACCATACCAACTTGAACTAAATCTTCGTGATGTGATTG
TCCTTTAGAATATTTATATGCCAATGACTCAATTAGTTTTTGGTAATGTTTAACTAACTTAT
CCTGTGCATCTGTATTCTTATTTTCTTGGTGTTCTTTAATCCATTGGTTAATTTGCTCAGGT
GAAATTTCATTAGCTGATTTCGACTCTTTCGCCAT

> **rsbW**

Function: protein coding sequence; serine-protein kinase (core genome, constant)
Best match: rsbW_CC005_Mu50-VRSA_BA000017.4[2195328:2195807:r]RC (completely identical)
Position: 053-contig_55_RC: 4602 … 5082; Length: 480 bp
Sequence:
TTAGCTGATTTCGACTCTTTCGCCATTATTTCGCACCTGCTCTTTTTTTATATACTTAGTCA
TACTGATTGTCACACCAGATTCTTTATATACTGTGACTTCATCCATTAAAGATTCGATTAA
AAATAAACCTAGGCCACCTTCGCGTAAAAAGTCTATATTTTCGTCTTTATCGTAAGGACCT
ATTTTTGATTTAGTTGTTTCATAATCAAAACTGTCACCTTTATCAGAAATAACAATTTTAA
TTTTATCTTCTAAAATTTCAAAATATATGTTAATAATGCCCACATTGTTATTTTCTTTGTAT
GCATGTTTAACTGCATTTGTCACAGCTTCACTAACTGCAATCTTGGCATCTTCAATATCAT
CATATGTAGCACCAGCTCTCGAAAAAACGCCAGAAAGTGTTAAACGAATTAAACTTACAT
ACTCTGCCGATGCTGGCACGCGCATTTCGATAAAATCTTCTTTAGATTGCAT

> **rsbV**
Function: protein coding sequence; anti-sigma-B factor antagonist (core genome, constant)
Best match: rsbV_CC001_MW2-USA400_BA000033.2[2147588:2147914:r]RC (completely identical)
Position: 053-contig_55_RC: 5083 … 5410; Length: 327 bp
Sequence:
TTATTCGACCTCCGTTCCTTCATTAACATGCATTAAATCCTTAAGACCAGTAATTTCAAAT
AGTCTACCGATACGATCTGACACACCTAAAATGTATAGTTCTTTATCATTTTGGTTTAATG
CTTTTAATGTACCTACGAATAAACCTAAACCTGTCGAATCCATATAACTCACATTTTCTAA
ATTAACATAAATATCACGAGTTCCATCTTGTCTCATAGGTGTTAAAACCTCTTCTAATTCA
GGCACAGTATAAACATCTAATTCTCCACCGACTTTAACTTCGTAAAATTTATCTTGAGTGG
TTGTTTCTATATTAAGATTCAT

> **rsbU**
Function: protein coding sequence; serine/threonine-protein kinase (core genome, constant)
Best match: rsbU_CC001_MW2-USA400_BA000033.2[2148033:2149034:r]RC (completely identical)
Position: 053-contig_55_RC: 5528 … 6530; Length: 1002 bp
Sequence:
TTAATTTACTCTTTTTATAATCAAAATAGTCATATCATCTTTTTTATTTGGGTTTTGAAGCT
TTAAAATTGCTTCATAGATAATTTGAACAATATCTTGTGGGTGCATATGTTTATGTTTTTT
AATATATTCTAAAAGTTTTTGTTTATCTATAAAGGTACCTTCACTATTTCTAGCTTCAGTCA
CACCATCCGTTAAAATGATAATTAAATCATCAAGGTATATTGGAATTTCTTGTTGTTGATA
TCGTGTTTGTGAACTGATTCCTAACACTCTACCTCTAACTGAAATTTCTTCGAATTCTTCTT
TTTCAGCGCGATAAATATATCCAGGCTCATGACCAGCTGAACTACAATACAATAAATGGT
TCATTTCTTCATATAAACCATAAAACATTGTGACGAACATATTTTGATTAATATTCTTTTC
AACAACACGATTTAAACGTTTTAAACCATCACTCGGTAATTGTGAGTGTCCATAAGAATC
CATGCCAAACTTTATCATACTCATTGCTAAAGCAGCTGGTATACCTTTTCCAATGACATCT
GCAACAGCAAAGCTCATTGTGCCATCGTTATGGTCAATTAAATTAAAATAATCTCCACTT
ACTTTTTGTGCCGCCACTGAAATAACGCCAATTTGAATACTATCAAATTGTGGAATATCTG
TTTTAAGCATTGTTTGTTGTAAGCTAGAAGCTAAGTCTATCTCTTTATCGTGAACTTGAAG
TTTATCTACCAATCTTTGATAATCTCGATAACTATAACCAAAGCCTTTAACGATTTCTTGT
AAGACATCTAATGTCTTGAACACATCTTCACGCGTTAAGTTTAACGTCAATATATAGTTTT
TGTGAATATCGACAATGTCTTCAGGCAAGACGTCCTTACGAATCACTTCGTCAGTGTATTT
CTCACACTTTTTTATCAATTCTACTTTATCTTGGCACGTTAAACTTTCATCAATTAAACCCT
TATAATGTTGCTTAAATTCTTCCAC

> **mazF**
Function: protein coding sequence; mRNA interferase (core genome, constant)
Best match: mazF_CC001_MW2-USA400_BA000033.2[2149382:2149744:r]RC (completely identical)
Position: 053-contig_55_RC: 6878 … 7241; Length: 363 bp
Sequence:
CTAATTTTTCTGGTGAGCTACTGCATTCAGCCCTAAACTAATCATTAGTGCATTATCTACT
TCTTTCATTTTATCATCGGATAAGTACGTCAGTTTTTCTTTCAATCGTTTTTTATCAAGTGT
ACGAATTTGTTCTAATAATATAACTGAGTCTTTATCCAACTTATACTTTTTCTTTTCAATCT
CTACATGTGTCGGTATTTTCGCTTTATTAATCCTACCAGTTATTGCCGCAACAATAACTGT
AGGACTATATTTATTACCAGTATCATTTTGAATTATGACTACAGGTCTGACTCCCCCTTGT
TCAGATCCCTGTACTGGTGATAAATCTGCTAAATAAACATCTCCTCGTCTAATCAT

> **mazE**
Function: protein coding sequence; antitoxin (core genome, constant)
Best match: mazE_CC001_MW2-USA400_BA000033.2[2149741:2149911:r]RC (completely identical)
Position: 053-contig_55_RC: 7237 … 7408; Length: 171 bp

Sequence:
TCATTCATTCGTTGAATTAGAAGATAAATATGTTTCGTTGCAATCGCATGCTTCACACTCT
ATCGGAAAAGCTTCGTTCGCTAGGGAGAGATTTAAATCAGCCATTTGTGAATATCCTTCTT
TTAAAGATTGTTCTAAGCTATGACTTCTATTTTGACTAAAAGATAACAT

## > alr1

Function: protein coding sequence; alanine racemase/locus 1 (core genome, constant)
Best match: alr1_CC080_11819-97_CP003194.1[2171700:2172848:r]RC (completely identical)
Position: 053-contig_55_RC: 7492 … 8641; Length: 1149 bp
Sequence:
TTATTTTAACAATTCATTTGTTACAAAACGTTGATCACCATCATGATAGATTCGCGGCAAA
CGTCTCGACAAGTTACAAAGCACTTCATAATTAATAGTATGTTGCTTTTCAGCTACCACCT
CTACCGACTGTGGACTTTCTCTATGATTATCTATTAAAATCACCGAATCTCCAGCTTTAAC
TTGATCTGGCACTTTAACAATTGTCTGATCCATACATACGCGACCAATAACTTCGCATTGA
TGACCATTTACATTTACAAAGCTACCTTGCATTATGCGTAAATAGCCATCTGCATATCCAA
TAGGTAACAAAGCTATTGTAGTTGGGTCAGTAGCTGTATAAGTTGCACCATAACTTACAG
ACTCACCCGCTTGTAGCGTCTTTGTTTGAACTACATTAGCAATTAATTGCACACTTGGTTT
AAGGTGTACTTTAACTTTTTGCTGTACATACTCTGATGGATAATATCCATAAAGGGAAATT
CCTGGTCTTATTGCATTACAGAATTGGCAATCCATTAATAGAGAGCCTGCTGAGTTCTGAC
AATGTATATATTCAGGTTTAATTGCTTCATTGACCATATCTTTAAAACGTTGATATTGTTC
AGTTGTCATATCTCCTGGTTCGTCAGCACAGGCAAAGTGTGTAAACACGCCTTCAAATAC
AAGTTGCTCATATTGTTGAATGATTTCAATCACTTCTTGATACGTATTAGTATCTTTAATA
CCTAAACGTCCCATTCCTGTATCTAATTTAATGTGCAACCATAACTTTTTCTCTTGCTCACC
AGAAATGTTTTTAATTGCTTCTTTCAACCACTGTTTAGACGGAACCGTTAAGGCAACTCGG
TGTTGTATCGCTTTATCAATATCTTTAGCTGGTAACACACCTAAGACTAAAATTTTAGCAG
TAATCCCATGCATTCTAAGTTCTATCGCTTCATCTAACGTTGCTACAGCAAAAAATGTGGC
GCCATTTTCCATTAAATGACGTGCTACTTTAACACTACCTAGTCCATAGGCATTGGCTTTA
ACGACAGCCATCACTGTTTTATTTGGATGCAATGTACTGAATACTTTGAAATTTGATGCAA
CAGCGTTTAAATCTACATTCATATACGCAGATCTATAATATTTATCCGACAT

## > acpS

Function: protein coding sequence; holo-acyl-carrier-protein synthase (core genome, constant)
Best match: acpS_CC001_MW2-USA400_BA000033.2[2151210:2151569:r]RC (completely identical)
Position: 053-contig_55_RC: 8706 … 9066; Length: 360 bp
Sequence:
TTAAAATGCTGACTTTTCTAAAACAACTTGGCTCATCGCATAATGCTCAGTGTGTGAGATA
CTAACATGTACGATGAACCCTTCGTAATCAATCTTTGGTTTGCCAAGTTCGTCGTTGTAAC
AGTCTATATCGTTAAAAGCTACATGTTTTCCTAAGCCTGTGCCTAATGCTTTACTGAACGC
TTCTTTTGTAGCAAACCTGCCAGCTAAAAATTCAATTTTACGTTGCTCATGTGTGAAATTG
TTGAATTTGTGCTGTTCATTTTTAGTTAAAATCCGCTCAACCAATTTTGGCTGCTTACTATA
TAACACTTTTATTCGATCGATTTCGATTAAATCTACACCAATTCCATGTATCAT

## > A5IUL8

Function: protein coding sequence; putative protein (core genome, variable)
Best match: A5IUL8_ref_CC001_MW2-USA400_BA000033.2[2151573:2152064:r]RC (completely identical)
Position: 053-contig_55_RC: 9069 … 9561; Length: 492 bp
Sequence:
TTATTCCTCATTTTTATTGTTTTCTAAATGTCTCTCGTTATTTAACCATCGTTTCAATCTACG
TGCTTCACTTCTATCTATTAATTCAATTTCAGCTTGACCACCCGCTGTATAAAGGGTCAAA
GTTGCTAATCGATAGCGTCTCATTATAGGACCAACATCGATATCAATATTTTGAATACGA
AAATATGGTATTACTTTTTCATCCAAAAATAAAATGCCGTTTCGTACACGCAAATGGTGTT
TTTCAAATGCATATCTGCAGTGCTTATATCGATATACTGGCGCTATGACAAGCGTGAAAA
CAGCAACAAGTAATATTATAATCACACTACTAACAATGGATATATTGTTATCTAATATCTC

CCAAAATAGCCAGTTCAAAATATTAAATGCGATTAAAAGTACAAGCGCTATGGGTATCCA
AAACAGCACAGCACTTAACCTCATCACTTTTTAGCGTGTGGTGACATAAAATTATAATC
CCTCAT

## > **Q9ZAH8**

Function: protein coding sequence; putative membrane protein (core genome, constant)
Best match: Q9ZAH8_CC001_MW2-USA400_BA000033.2[2152051:2153634:r]RC
Position: 053-contig_55_RC: 9547 … 11131; Length: 1584 bp
Sequence:
TTATAATCCCTCATCATTTTCACCTCTTAAATACCATGATTTCAATTTATTTGCATCTTCAC
TTCTTGCGTATTTTAGGTTAATCGATTGGGCTCCCACACCTTTAGCAACAATAAAGCTAAA
ATTATTTAAATTGTTGCGTTTAAGTAATGTATGTTGCCAAGTGTCAAATCCTATAATGTGA
TGCGCTTTAAAATAATAAATATTTCGTTTCAATAGCTCGAAATTCTGGATAGTAATTTGTT
CTTCTGTCATTTTAAAACCCGCATGTTTGACATAAAGATATCCTTTGATCACAAATAAACC
AATAATGACTATTGTTATAATCGTAAATAACAATAATAATTGATTCCAAAAATAACAGCC
AATACCTGCCATAGCTATGACAATAATACTAGGTATTAAAAAGTGTCTGTGGAAACCTGA
CAAAGGCATACCTTCATTAACTTGTTGATAAGATAAATCTGGTACTAAATTCTGGATAATT
TGATATGCTTTGTCTCGTTTAATAAACGGCAATATCGGCACACTACCTGAATCATTGTCAT
CATCTTCGATTTTTTTATTTTCAAAATCACTTGTGATGATAAAATGTATTGCTGTGTAGCC
AAATAATCTTCGTATATATGACTGACGTTCAACAACAGCTTGCAATCGTTTCGTTGGTACA
GTTACTTTCTTCTTGTTAAAGAGACCATATTCAATTTTAAGTTGCTCATTTTCAAGCATCAC
CGAAAAGCCATAAAATCTTATCATCGTTATAATCGTTCCAATAATATATGCCACTATTAAT
ACTAGTAAAATGATGATTAATACTGAAATACTTACAATTTGAACCCATTGACTAATTTCAT
GATTTAGCTTCGACCATGGGATCAACTCTCTTACAGCCCCGTAAATCGGTACTAAAGCTG
CTAACGTTACACCAATGGCGCCACTGGTCATTGCCATAAATAGTGATTCTTTAAAATTCAT
CTGATATATAGGAATGCGTTTATTTTTCTGATTAAGCATACTATCAGTGTTCTGCACTTCA
TCTAAGCGACCTTCTGCGATGTCTTCCACATTACCTTCAATGTCATGATTACAGTTGTCAT
TCTTCTCAGCACTAGACTTTTGCGCCACTTCTGTCTTCAACTCTGTTTGCAATTGATCAATA
TAGCGTTCAAGATATTCACCTTGTTTTTTCGAAATAACACTTAAGACAATACCATCACTTG
GTGTTTTGATTTGTAAATCCACACCACCAATAATTTGATTGACGACCCCTTGTGTCATATC
AACGGACTGAATACGTTTTATATTTAATTCTTTACGCTTTTTATTAAAAATACCTGTTGTTA
AAATGAAATAATTATCCTCAATCCAATATCGCGTGTTCATAATTCCGACAATTTGAGAAA
TGTATGATATTAAAAAGAATACAAATACAATACCTATCCATAAATATGATTCGGGATTCG
CATAATCAAAATCTTTCAATTGAAAGATAATGAAAATAAAAAAGACGACTATGTTTTGTT
TGATAGCATTGATTATGCCATTAAAATATGAAATCGGATGTAATTTTTGAGGTTCAGACA
T

## > **Q9ZAH9**

Function: protein coding sequence; putative protein (core genome, variable)
Best match: Q9ZAH9_CC015_CIGC93_AHVD01000025.1[4311:4790:r]RC (completely identical)
Position: 053-contig_55_RC: 11123 … 11603; Length: 480 bp
Sequence:
TCAGACATCACTTTCAACCCCTCTCAAATTCGACATAGTTCTCGCTTCGATTATTTTAACA
TCGTCATGAGACATCATCGGTAAATAAATAGTATGACCTGCAGTCATAAATCCAACTTTA
TACAAATTAAGCACTTTACTAATTGGATTAGATTTAATCGACAAGTATTGTAAACGTTCA
ATTCGACTCGTTTCTTCTTTATATATAAAAAATGATGTACGATATTGTACACTTAGTTGAT
CAACTTTATAAAAGCGACAATGATATTGCCATAAAGGCTTAATAAATAATTTTAATGTAC
TCAGAGCACCTAAAACCAACAAAATATAAAGTAAGTAATGTGGCCATTCAAATCTTAACC
ATATAAAATAAAAAATGACATACACAGCTACACTCAATATAAATTCTAAGCCATTCGTAA
TGTAGTAATACAACAATGCTGACTTAGGACTCTTAGACAACTTAGTATAATCTGACAT

## > **kdpC-chr**

Function: protein coding sequence; potassium-transporting ATPase/C subunit (core genome, constant)
Best match: kdpC-chr_CC001_MW2-USA400_BA000033.2[2154315:2154875:r]RC
Position: 053-contig_55_RC: 11811 … 12372; Length: 561 bp

Sequence:
TCAATCTTTCATCTTATCAAGAGCAATATTTAGTTCAAGCACATTCACATAATCATTTGTT
AACACACCACGCTGCTTACGATGTTGAATCAAGTCGGCCACTCTTGAAGTAGATATATGA
CGAGCATCAGCAATACGAGGTGCTTGCTTCAATGCATTTTCAACCGTAATATGCGGATCT
AAGCCCGACCCAGAACTTGTTGCAGCATCTATTGTTACATTTGAATTCCCAAATTTAACAT
GATGTTTCATGCGTGCTATTAATTCGGTGTTTCCATTCGATTCATTACTTCCACCTGATGAT
ACGCCGTTTTTATATAATTTTTCAGGATTCATATTATAATCAACTGCACTCGGTCTCCCGT
GAAAATATCGTGTCTCTGTCCAGTGCTGTCCAATCAATTTTGATCCAACTATACGATTGTC
ATACGTAATTAAACTGCCATTTGCTTGTTGATAAAAAAATATTTGACCAATTAACGTGAT
AGCTAACGGGAATAAAAATCCACATAATACCATAGTTATTATCGTTAAACAAATACTATT
TCTTATCGTATTCAT

## > **kdpB-chr**

Function: protein coding sequence; potassium-transporting ATPase B chain (core genome, constant)
Best match: kdpB-chr_CC001_MW2-USA400_BA000033.2[2154895:2156922:r]RC
Position: 053-contig_55_RC: 12391 … 14419; Length: 2028 bp
Sequence:
TTACACAAAAAATTGTACAATCATATCTATTAATTTAATGCCTAAAAACGGGACGATTAA
TCCACCTAATCCATAAATCAACATATTATTTATAAAGATTCTATCAATGCTGTAACCCTTT
ACTTTTACACCTTTCATGGCAATTGGAATTAAGGCAACAATGATTAATGCATTGAATATC
AAAGCTGATATAATTGCAGATTTAGGTGACGATAAATGCATAATATTTAGTGAAGTCATT
TCTGGAATTGTTGACATCATTAGTGCAGGTAAAATTGCAAAGTATTTTGCTACGTCATTAG
CCAAACTAAATGTCGTTAATGCACCTCTCGTCATTAATAATTGTTTGCCTATTTTTACAAC
CTCTATTAACTTTGTAGGATTCGAATCTAAATCAATTAGATTAGCTGCCTCTTTAGCACTA
ATTGTCCCTGAGTTCATTGCTAATCCTATATTCGCTTGTGCTAGCGCAGGTGCATCATTTG
TACCATCTCCTGTCATCGCAACAATATGGCCTTTCGCTTGTTCATCTTTGATGACTTTAATT
TTATCTTCGGGTTTACACTCTGCAACAAATCTATCAACCCCGGCTCCTTTTGCAATTGTAG
CTGCTGTTAAAGCATTATCACCTGTACACATAACTGTTTCAATCCCCATTTTTCTCAATTC
AGTAAATCGTTCTACAAGACCATCTTTAATCACATCTTTTAAATAAATCACGCCAAGCAT
GACATTGTTTTCAATGACTATTAATGGTGTGCCACCTTTACTCGATACATCCATACAGAGA
GACTCAATGTTAAGAGGAATATTGCCTTGTTGTTGTTTGACAAGATTTATCATACTATTAG
GTGCACCTTTGAATACCGATATTTCATTTGTAATGATTCCGCTCATTCTAGTTTCAGCTGT
AAAAGGCTTATATGTGCCATCAATGTCTTTAGGCAGCTCATTTATATACATCTGCTTCGCT
AATCGTACAATACTTTTTCCTTCTGGCGTATCATCGTAGATTGATGACATATAAGCAGCGA
CTATCAATTTTTCCCACATTTGTTGATTCACTGGTAAAAATTCACTAGCGATTCGATTGCC
ATAAGTGATTGTCCCTGTCTTGTCTAAAATCATTACATCGACATCTCCACATACTTCTACA
GCACGCCCACTTTTCGCTAATACATTGAATTGAGTAACACGATCCATGCCTGCAATACCA
ATCGCCGATAACAAACCACCGATTGTCGTTGGTATTAAACATACTGTTAACGCAATGAGC
ATCGCAATAGGTAAAATTAAATGCAGGTAAGATGCTATTGGATATAACGTTACAATAACG
ACTAAAAATATAATTGTTAACGTTGTTAATAATGTAAAAAGTGCAATTTCATTTGGTGTTT
TATTTCTTTCCGCCCCTTCAACTAAGGCAATCATTTTATCTAAAAAAGATGTACCCGCTTC
ACTCTCAACACGTATTTCTAACCAATCAGATGTTACAAGTGTACCGCCAATGACTCCATC
AAAATCGCCACCTGATTCTTTTATCACAGGTGCAGACTCACCAGTAATTGCAGATTCATC
AACGGTTGCTAATCCATTTATTACAACGCCATCAGCAGGGATTGTTTCTCCATTTTCTACC
CGAATATTTTGTCCGGCTTTTAACTCTGTGGCGTTCACTATCCGATACGCACCATTTTCTTC
TATCAATCGAGCAGTTAAATTTGATTGTGCTTGTCTTAAACTATCAGCTTGCGCTTTTCCA
CGACCTTCAGCAAAGGCTTCTGAAAAATTAGCAAACAATATAGTTATTAATAATATGATA
AAAATTGTAATCAAATAACCTCGCGATAGATAGCTAGTTCCAAATATGTCAGGAAAACAT
ATTAATATCAACGTTAAAATCATTCCAACCTCAACGACAAACATTATCGGATTTTTTATTA
ATTGTTTAGGATTCAGCTTATAAAAACTCATTTTCAAAGCTTCTATCACCATTGTTTGATT
GAAATATTTATTTACATGATGCAT

## > **kdpA-chr**

Function: protein coding sequence; potassium-transporting ATPase A chain 1 (core genome, constant)
Best match: kdpA-chr_CC005_JH1_CP000736.1[2234952:2236628:r]RC

Position: 053-contig_55_RC: 14437 … 14587; Length: 150 bp
Sequence:
TTATTTTAAAGTTAAAAATTCACCAATAGGACCAAGTAATAGTACTGGAATAAATGTCAA
ACCACTTAGTAAAACGATAAATACGATTAGTGATACGCCAAAATAAGGTTTATCAATCGC
TATTGTATATTTATCTTGATGGTATGATTT

## > kdpD-chr

Function: protein coding sequence; sensor histidine kinase (core genome, constant)
Best match: kdpD-chr_CC001_MW2-USA400_BA000033.2[2158889:2161546]
Position: 053-contig_55_RC: 16385 … 19043; Length: 2658 bp
Sequence:
ATGTCAAACACTGAATCGCTAAACATAGGAAAAAAGCGTGGATCTTTAACAATTTACATC
GGTTACAGTCCAGGTGTTGGTAAAACATTTGAGATGCTTTCAAATGCCATTGAACTATTTC
AAAGTAACGTTGATATTAAAATAGGATATATTGAACCGCATCAGCGTGATGAAACAAAT
GCATTAGCTGAACAATTACCTAAAATCACAACCAATTTTACTAAACATGGTAGTCATCAT
TTTCAATATTTAGATGTCGACCGCATAATCGAAGAATCGCCGACAATAGTACTTATCGAT
GAGTTAGCACATACGAATATTTCTAGAGATCGTCATGAGAAACGATATATGGATATTGAA
GAAATTTTAAATCATGGTATCGATGTTCATACCACTTTGAACATTCAACATATTGAAAGTT
TAAGTAGTCAAATTGAACTGATGACCGGTGTACATGTTAAAGAACGTGTACCCGACTATT
TCATAATGAGCGCCGATGTATTAGAAGTCGTAGATATCTCACCTGAACAATTAATTAAAC
GCTTAAAAGCTGGCAAGGTATATAAAAAGGATAGGCTAGATGTAGCATTTAGTAATTTCT
TTACGTATGCCCACCTAAGCGAACTGCGTACATTGACGTTAAGAACAGTTGCCGACTTGA
TGAGTGATAAAGAAAAAGTCCGACACAATCATAAAACGTCACTCAAACCTCATATTGCTG
TGGCAATTAGTGGGAGCATTTATAATGAAGCAGTAATTAAAGAGGCATTCCATATTGCTC
AAAAAGAACATGCGAAATTCACTGCTATTTATATAGATGTATTCGAAAAAAACAGACAAT
ATAAAGATAGTCAAAAGCAAGTGCATCAACATCTCATGCTTGCAAAATCATTAGGAGCA
AAAGTAAAAGTAGTTTATAGCCAAACCGTTGCATTAGGATTAGACGAATGGTGTAAAAAT
CAAGATGTAACCAAATTAATTATCGGACAACATATTAGAAATAAGTGGCAAGACTTTTTC
AATACACCTTTAATTGACCATTTAATGTCCTTTGAACATAGCTATAAAGTCGAAATCGTTC
CAATCAAACAAATACCTGTTGAATTGAAAATGAACAAATCACCCTATCGTCCTAAAGGCA
AACGTTTCGCCATAGATATGTTAAAAATGATTTTGATTCAAATAATTTGTGTAATGATGGG
ACTGTGGATTTATCAACTTGATAAGCATGAGTCTAGTACGATTATTTTAATGATTTTTCTC
ATCGGCATCATTTTGTTATCCATTTGGACGCGATCCTTCATCATTGGCTTTTTAGCAGCAA
TTATTAATGTATTTGTTTTTAATTATTTTTTTACGGAACCTAGATATACATTTGAAGTATAT
CGCTTTGACTATCCTATTACATTTATCGTTAGCATTTTAACAAGTATTTTAACGAGTGCTTT
ATTAAAACAAATTAAATTCCAATACTCCATTACTAAAAAGCAACTTTATCGTACAGATTT
ATTATTTCAATTTAATGATTCGATTAAACAAACTTATACAGTTGAAAACTTACTAATAAAT
GCTGGATACCAAATTAATCAATTATTGCAACAATCAATTACTATATACGTTATCAATCAAT
CGAAAGTAATTAAAACAATACCATTGCAAAACCATATCGATAATACGACTCAACAGCAT
GAACAAGCATTAAGCTGGGTAATTAAAAATGAAAGACAAGCTGGTGCAACAACAGATAC
ATTTCCAGGCATTAATAAATGGTTGATACCAATTGGCACATCTCCGATTAAAGGCATTCT
AGCCATTGATTACCAAAGTTCTCAAGTGATTAATCCATATGATGCATCAATATTAGAATC
AATGTTAAATGAACTTTCTCTCGCCGTTGAAAATGTGACGTTACTTAAACAAACGAGAGA
ATCCATGTTACAGGCTGAACGTCAACTGACACATTCAAATTTTTTAAGATCAATTTCGCAT
GACATACGTACACCGTTAACAACAATTATGGGAAATTTAGATATTTTAGTATCACATAGT
AAAGATATGTCTATCATAGAAAAGGAACAATTACTTGTGCATAGTTTTCAAGAAAGTCAG
TATTTATATCTATTAGTGACAAATATTTTATCATTAACAAAATTACAGTCATCCAATGTTC
AGATAAAATTGCAACCTTATCTCGTTAGTGAATTAGTAGAAGAAATCGATATGATTTTAG
AACGTCGCCATTTAAAAAAGCGTATAACTGTATCATCCTCAGTAGATTTACAGTTTATAC
ACATTGACAGTAAGTTGATTTTACAAGCTTTATTCAATTTGATTGAAAATGCAGTTAAACA
CACTTCTACTGACACCAAAATCAATTTATCTATTCGTTATGCTAGCGATGAACAAATCGA
ATTTGCCGTTATAGACGAAGGACCTGGAATTAGTTTAGAAGAACAACAAAAGATATTCGA
GCCTTTTTACACAGGATCAAATAAATATTTTAAAGATAACCAAAAAGAAAGCATGGGACT
AGGCTTATATTTAGTACAGACTATCCTACACAAACATCAATCAAACTTACAATATAAACC
GAACCAACCACACGGCAGTATATTCTATTTCAATATAGATACAGACTTTAATGAAGGAGA
CGTATAA

> **kdpE-chr**
Function: protein coding sequence; KDP operon transcript regulatory protein KdpE (core genome, constant)
Best match: kdpE-chr_CC001_MW2-USA400_BA000033.2[2161546:2162241]
Position: 053-contig_55_RC: 19042 … 19738; Length: 696 bp
Sequence:
ATGCAATCTAAAATATTGATAATTGAAGATGATCACGCAATCACACATTTACTTGATGTT
GCATTAACTTTAGATTATTACAATGTAACTACAGCCGACAATGCCACACAAGCACACTTT
AAAATTCAAATTGATAAACCAGATGTCATTTTATTAGATTTAGGTTTACCAGATAAAGAT
GGATTATGTTTGATTTCAGAAATCAGGCAACATACTGACATTCCTATCATTGTAATAAGTG
CAAGACAAGAAGAACAAACAATTATTCAAGCTTTAGATAACGGTGCGAATGACTATATG
ACTAAACCTTTTAATGTTGATGAGCTTCGGGCACGAATACGAGTCATCGAGAGAATCGCT
AAGTCACATCAAGAAACTAACATTGTATTTACTAACGGTTTGCTAAGCATCGACTTTGGC
TCCAAATCTGTTGTTATTAATAATCAGGAGGTACATTTGACTCCAAATGAATTTAGCTTGT
TAGAACTATTGTCAAATCATAAAGGAAAAGTACTCACTTATGAGATGATATTAAAACGAA
TATATGGCTATGTTAATAAGACTGAAATGCCTAGTTTAAGAGTGCATATGACATCATTAA
GACAAAAACTTTCACAATGTCATGAGGACGCAAAAGATATTATTAAAACACATCCACGC
ATTGGTTATCAAATGTTGCAGTGGAAAGAGAAATAA

> **cshA**
Function: protein coding sequence; ATP-dependent RNA helicase (core genome, constant)
Best match: cshA_CC001-ST772_118_AJGE01000006.1[51248:52768]RC (completely identical)
Position: 053-contig_55_RC: 20107 … 21628; Length: 1521 bp
Sequence:
TTATTTTTGATGGTCAGCAAATGTGCGACCTTTCATAGGTCTGCTTCCACCGCTGCTCTTCT
CTTTACGGTCGAATTTTTTTGTACTTTTCTTTTTACTTGAGTATCCTTTTGAACGTTTACTCT
TACTGTCAAATTTAGGATTACCACGCTTACTATTTCTGTTACGAGAACCGCTTGGTTTACC
ATTACGGCCTTTGCGAGATAATGGTTTTTCAAAAGTTAATTGAACTTCAACTTCATCGTTC
GCTTCTACTAACTCTTGTAAAAGTGCAGCAACTAAATCAACATCGTTATATTCATTTAACA
ACTCTGTAGAAATACGTTTCAAGCGTGATTCTGACTCTTTAGACATCCAGTTTTCAACTTT
TTCTTTGATGTCATCTTCACGTGCTTGAAGTACTTCTTTACGATGAGGTGGACGAAGTGCA
CTCATTTTTCTACCATTTGCATCTTCAATTTGTCTGATATAATCCATTTCGATTGGATTAAC
AAACGTTACAGCGATACCTTCTTTACCAGCACGACCCGTACGACCAATACGGTGAGTATA
GCTTTCAGTATCTTGAGGTATATCAAAGTTATAAACATGACTCACACCAGAAATATCTAG
TCCTCTTGCTGCTACATCAGTAGCGACTAAAATATTAATTTGGTCATTTTTAAATTTCTTTA
ATACTTCTAAACGTTTCGCTTGTGTAATATCACCATGTAAACCTTCAGCTTTATATCCTTTA
GAAATCAAAGCACTTGTTAATTCATCAACACGACGTTTTGTACGTCCGAATACGATTGCT
AATTCAGGTTGATGAACATCTAGGAAATTTGTAAATGTATCAAATTTCTCTAATTCTTTAA
CAATTGTATAGAATTCTTCGATTTGTGGATCAGACATTTCATTATTCATTGTCTTAATGATT
TTTGGTGATTTCATAAATTGTTGTACTAAAGCTTGGATTGCTTTAGGCATTGTAGCTGAGA
ACAACATTGTTTGACGTTGTACTGCTGGAATTTTATCCATAATAAATCTCATATCATCGAT
GAATCCCATATTCATCATTTCATCAGCTTCATCTAAAATCAAAGTATGAATTCCGTCAGTT
TTTAATGTGCGACGATTTAAATGGTCGATAACACGCCCAGGTGTTCCGACTACGATTTGT
GGGCCTTTTTTCAAGGCTTTAATTTGGCGTTCGATAGGCATACCACCGAATACAGTAACA
ACTTGGACACCTTGTCCACGGCTAAATTCTCTTAATTGTTCAGCTACCTGCATTGCCAATT
CTCTTGTAGGTGCTAAAATCAACGATTGAACCCCTTGTTTCCCTACTACTTTCTCAATTAA
AGGAATACCGAATGCTCCTGTTTTACCTGTACCGGTTTGAGCTTGCCCAAGGATATCAATT
CCTTGTAACGCATAAGGGATACTGTCTTTTTGGATAGGTGTCGGCTCTTTAAATCCCATTG
ATTCAAGTGACTGAACCGTATTATCCGAAATCCCTAGTTCTTTAAAATTTTGCAA

> **murF**
Function: protein coding sequence; UDP-N-acetylmuramoyl-tripeptide--D-alanyl-D-alanine ligase (core genome, constant)
Best match: murF_CC005_Mu50-VRSA_BA000017.4[2214371:2215729:r]RC

Position: 053-contig_55_RC: 22144 … 23503; Length: 1359 bp
Sequence:
CTATGAAATTAAAGCATTTACAACTTCTTCTAACTTCATACCACGTGATCCTTTAACTAAT
ACACGGTCATGCGCTTTTAAATCGCTTGTTAAAACTTCTATCATATCGTCTTTAGAATTGA
AGTGTTGTGCTTTTTCGACATGTTGCTGGCCCGAATCATAAATATACTTCGCTTCATTACC
AAACGTATACAACACATCTATATGCTTTTCTTCTAAATAATTACCTACACCGATATGCATT
TCTTTGCTATTTTCACCTAATTCTAAAACATCTCCTAAAATTAGAATGCGACGCCCTGTCA
AAGTACTCAGTGTATCAATAGCTGCTCTCATACTTGTAGGACTTGCATTATAGGCATCATT
TATCACAGTAATATCATTTTCTAATGTATGTTGCTCCATACGCATACCAGTTAAGCTGACA
TTTTTTAAATTTTGATAGATTGTGTTATATGTCAAACCTAATTCATGACCAACCGCAATGG
CAATCGTCGCATTTTTCATATTATGCTTTCCTAATATTGGTAGATCATAATGTTCTTTATTA
TTAATCGTAAATGAAATACCTGTAGTATCTCTATCATCAACAGAACAAACTAATGCATTA
TCAGTAGCAACACCGATACTAATACATTTTGCATTTTCAACTTCTTTAACATGCGGTTTCA
ATAATGGTTCATCGCCATCATATATAAACGTACCATTATCTTTTAGACCTATTGTAATTTC
AGATTTAGCTTTAGCAATCCCCTCGCGCGAACCTAAATCTTGCATATGTGACTCACCAATA
TTAGTTATAACTGCAATATCTGGTTGAGCGAGGTTTGACAGAAATTCAATTTCATGGAAA
CCTGACATCCCCATCTCCAATATTGATATTTCAGTATCATTATCTAATTCCAAAATAGTTA
AAGGTAAACCAATTTCATTATTGTAATTACCTTGCGTTTTCTTAACTTTAAATTCGGTATG
CAATACACTTTCAATCATATCTTTAGTCGTTGTTTTACCATTAGACCCTGTGACGGCAATT
ACTTTAGGGTTTACATGTCTCAAGTAAGCTTGTGCCAATTGTTGTAATGCCGTTAATGTGT
CTTCAACCCATATAATAGGCCCGCTTACATTTTCATCTATAGGTGTTCCTCTTTGATAAAA
AGCAGCTCCAGCACCATCTTGTAATGCTTTAGAGACAAAGCGATGACCGTCAACATTTTC
ACCTTTAAATGGTATAAATAACATATTTTTAGAAATTGCTCGTGAATCAATTGTGACTCCA
TTTATCACTTGATTTAAAAATTGATCTTCAATTTCACAAGGAATCCATGATTGAATTTGCT
TTAATGTAACATTAATCAT

## > ddlA

Function: protein coding sequence; D-alanine--D-alanine ligase (core genome, constant)
Best match: ddlA_CC001_MW2-USA400_BA000033.2[2166020:2167090:r]RC
Position: 053-contig_55_RC: 23517 … 24588; Length: 1071 bp
Sequence:
TTAGTCAATTTTGTATTTATTTTTCTGTTTATCCTGGTGACGTTCTTTAGCAAGCTCGATAA
GTTTTGTAATCAGTTCTGGATAAGATAAGCCCATATTTTCCCATAACTTTGGATACATACT
GAAAGCCGTAAATCCAGGCATTGCATTTGTTTCATTAATATATATTTGGTTGTCTTCTGTT
ACAAAGAAATCAGCACGGACTAAACCAGAACAATCTGTCGCTTTGAATGCCTCTAATGCC
ATATTTCTAAGCGTTAATTGAACATCTTCGTCTAAGTCAGCTGGAATTTGTAATTGAACCT
TACCATCTTTATATTTTGATTTGTAATCGTAAAACGCGACATCTTTTACGACTTCACCTGG
CCATGTCGCTTCAGGATAGTCATTTCCTAAAACTGCTACTTCAATTTCACGTGCGTTAACG
CCTTGTTCTATAACAAGCTTACGGTCAAATTGGAATGCTTCTTTAATACCTTCTTTAAGTT
CCGCTTCATTATTACATTTACTGATACCTACACTTGACCCTAAGTTAGCAGGTTTAACAAA
GACTGGGTAATTTAATTTATCATTTACTAATTTTAAAATGTTATGTTCATATTTTTCATATT
CAGAACGTAAGAAACTAATATAAGGTAACTGTGGTAACCCTCGATGTTCAAATAATTGTT
TCATTACAAGTTTGTCCATAGAACTTGCAGCTGACAATACACCATTTCCTACATATGGTAC
ATCCAAAACTTCAAAAAGTCCTTGAATCGTGCCATCTTCACCATTAGGACCATGTAATAA
TGGGAATACTGCATCGTATGGTTGTCCTGAACTACTTTCTTTCAATAGCTGTGAAATCTCA
AGCGCCTCTCCATTTTCTAAATGAAGCTCATCAGTAGATTTAATTTCAGCTGTAATATTAT
TTTGCTTTCTCCAATCACCATCATTGGTAATATAAATGATATCAACATGATATTTGTCTTT
ATCTATTGCATTTAATACATTTTGTGCTGTCAGAATCGATACTTCGTGTTCTGCACTTTTCC
CTCCAAAAACGATACAAATATTTTCTTTTGTCAT

## > ftsW

Function: protein coding sequence; cell division protein
Best match: ftsW_CC005_Mu50-VRSA_BA000017.4[2217132:2218334] (completely identical)
Position: 053-contig_55_RC: 24905 … 26108; Length: 1203 bp
Sequence:

ATGAATTATTCATCTCGTCAACAGCCGGATAAGCATTGGCTTCGCAAAGTAGACTGGGTA
TTAGTAGCCACTATAGCTGTTTTAGCAATTTTCAGTGTTCTGCTTATTAACTCGGCAATGG
GCGGTGGTCAATACAGTGCTAATTTCGGTATCAGACAAATTTTTTATTACATTTTAGGTGC
AATTTTTGCAGGTATCATCATGTTTATTTCACCTAAAAAGATTAAACATTATACATATTTA
TTGTATTTCTTAATCTGTCTATTATTAATAGGCTTGCTCGTTATTCCTGAGTCACCTATTAC
ACCTATTATCAATGGTGCCAAAAGTTGGTACACGTTTGGCCCTATCAGTATTCAGCCATCT
GAATTCATGAAAATTATTTTAATTTTAGCATTAGCGCGTGTCGTTTCTAGACATAATCAAT
TCACATTCAATAAATCATTCCAAAGTGATTTGTTATTATTTTTCAAAATTATTGGTGTCTC
GTTAGTACCAAGTATTTTAATATTACTGCAAAATGACCTAGGAACTACATTAGTATTAGCT
GCTATTATTGCAGGTGTGATGTTAGTAAGTGGTATAACATGGCGTATCTTAGCACCTATCT
TTATTACAGGTATTGTTGGTGCAATGACAGTCATTTTAGGTATTCTATATGCACCCGCATT
AATTGAAAATTTATTAGGTGTCCAACTGTATCAAATGGGACGAATCAATTCATGGCTTGA
CCCCTATACATATAGTAGTGGTGATGGCTATCATTTAACTGAATCACTTAAAGCTATCGGT
TCTGGACAGTTACTAGGTAAAGGATACAATCACGGTGAAGTTTATATACCTGAAAATCAT
ACTGACTTTATCTTTTCAGTGATTGGAGAGGAACTTGGCTTTATCGGTTCTGTCATATTGA
TCTTAATATTTTTATTTTTAATCTTCCATCTAATAAGATTAGCTGCGAAAATTGAAGATCA
ATTTAACAAAATCTTTATCGTTGGTTTCGTCACTTTACTTGTGTTCCATATTTTACAAAATA
TTGGTATGACAATTCAGTTGTTACCAATCACTGGTATTCCATTACCATTTATTAGTTATGG
TGGTAGTGCGCTATGGAGTATGATGACTGGAATAGGTATAGTCTTATCAATTTATTATCAT
GAACCAAAACGATATGTCGATTTATACCATCCAAAAAGTAATTAA

## > Q9XBC0-chr

Function: protein coding sequence; putative protein (core genome, constant)
Best match: Q9XBC0-chr_CC005_Mu50-VRSA_BA000017.4[2219084:2219377:r]RC (completely identical)
Position: 053-contig_55_RC: 26857 … 27151; Length: 294 bp
Sequence:
TTAGTCTTTAATCAATTTTTGAAAAGTCACTAATAACTCTTCCATTGCCTCTTCCTGAGCA
CCTTGATTAACTTTATTCATAATACAACTTTTCATATGTTGTTCTAATAACTTTATCGCAAC
ACTGTTTAACGCCGAACGTGTCGCTCTTATTTGCGTAAGGACATCATCACAATAGACATCT
TCTTCAATCATGCGATTAATCGCTCTCACTTGTCCTTCAATTCGATTTAAACGTGATTTAA
GATTCGTTTTTATTTGTTCAGAATGATGTGCATTATCTTGTTCAGTCAT

## > cls2

Function: protein coding sequence; cardiolipin synthases 2 (core genome, constant)
Best match: cls2_CC005_Mu50-VRSA_BA000017.4[2219543:2221027] (completely identical)
Position: 053-contig_55_RC: 27316 … 28801; Length: 1485 bp
Sequence:
ATGATAGAGTTATTATCCATTGCACTCAAGCATTCTAATATTATTTTAAATTCAATATTTA
TTGGTGCATTTATTTTAAACTTATTATTCGCCTTTACCATTATTTTCATGGAAAGACGTTCT
GCCAATTCTATCTGGGCTTGGTTACTAGTCTTAGTTTTCTTGCCTTTATTCGGCTTCATTTT
ATACTTACTATTAGGACGACAAATTCAACGTGACCAAATTTTCAAAATTGATAAGGAAGA
TAAAAAAGGATTAGAGTTAATCGTTGATGAGCAATTAGCTGCTTTAAAAAATGAAAACTT
TTCAAATTCCAATTATCAAATTGTAAAATTTAAAGAAATGATTCAAATGTTGTTATATAAT
AACGCAGCATTTTTAACAACAGACAACGATTTAAAAATATACACAGACGGCCAAGAAAA
ATTTGATGACCTAATACAAGACATCCGTAATGCTACTGATTATATTCATTTTCAGTACTAT
ATTATTCAAAATGATGAATTAGGTCGTACCATTTTAAATGAACTTGGTAAAAAAGCGGAA
CAAGGTGTAGAAGTTAAAATTCTTTATGATGACATGGGTTCTCGTGGACTGCGTAAAAAA
GGCTTACGCCCGTTTCGCAATAAAGGTGGACATGCTGAAGCATTTTTCCCATCAAAATTA
CCTTTAATTAACTTGCGTATGAACAATCGAAACCATCGAAAAATTGTTGTAATAGATGGG
CAAATTGGATATGTTGGTGGTTTTAATGTTGGTGATGAGTACTTAGGTAAATCAAAAAAA
TTCGGCTATTGGCGAGATACGCATTTACGAATTGTCGGGGATGCAGTGAATGCATTGCAA
TTACGATTTATTCTAGATTGGAATTCACAAGCCACACGTGACCACATCTCCTATGATGATC
GTTATTTCCCAGATGTAAATTCTGGTGGAACAATTGGCGTTCAAATAGCTTCTAGTGGTCC
TGACGAAGAATGGGAACAGATTAAATACGGCTATTTGAAAATGATTTCATCTGCTAAAAA

ATCGATTTATATTCAATCTCCCTATTTCATACCTGATCAAGCCTTTTTAGATTCTATTAAAA
TTGCGGCATTAGGTGGTGTTGATGTCAATATCATGATTCCTAATAAACCTGACCATCCGTT
TGTTTTTTGGGCTACTTTAAAAAATGCAGCATCCTTATTAGATGCCGGTGTTAAAGTATTT
CACTACGACAATGGCTTTTTACACTCAAAAACACTTGTTATAGATGATGAAATTGCAAGT
GTGGGAACAGCTAATATGGACCATCGCAGTTTCACATTGAATTTCGAAGTCAACGCTTTT
ATTTATGACCAACAAATTGCCAAAAAATTAAAACAAGCTTTTATAGATGATTTAGCAGTA
TCTTCTGAATTAACAAAAGCACGTTATGCTAAGCGAAGTCTTTGGATTAAATTTAAAGAA
GGTATTTCACAATTATTGTCACCTATCTTATAA

## > A5IUN5

Function: protein coding sequence; metal-dependent phosphohydrolase HD subdomain protein (core genome, constant)
Best match: A5IUN5_CC008_NCTC8325_CP000253.1[2156888:2157535] (completely identical)
Position: 053-contig_55_RC: 28828 … 29476; Length: 648 bp
Sequence:
ATGCAACAATCAGACGTCATTAGTGCTGCCAAAAAATATATGGAATCTATTCATCAAAAT
GATTATACAGGCCATGATATTGCGCATGTATATCGTGTCACTGCTTTAGCTAAATCAATCG
CTGAAAATGAAGGTGTTAATGATACTTTAGTCATTGAACTCGCATGTTTGCTTCATGATAC
CGTTGACGAAAAAGTTGTAGATGCTAACAAACAATATGTTGAATTGAAGTCATTTTTATC
TTCTTTATCACTATCAACCGAAGATCAAGAGCACATTTTATTTATTATTAATAATATGAGC
TATCGCAATGGCAAAAATGATCATGTCACTTTATCTTTAGAAGGTCAAATTGTCAGGGAT
GCAGATCGTCTTGATGCTATAGGCGCTATAGGTGTTGCACGAACATTTCAATTTGCAGGA
CACTTTGGTGAACCTATGTGGACAGAACATATGTCACTAGATAAGATTAATGATGATTTA
GTTGAACAGTTGCCACCATCTGCAATTAAACATTTCTTTGAAAAATTACTTAAGTTAGAAT
CTTTAATGCATACAGATACGGCGAAGATGATTGCTAAAGAACGTCACGACTTTATGATGA
TGTACTTGAAACAGTTTTTTACGGAATGGAATTGTCACGACTAG

## > txbi_universal1-var1

Function:  rho independent terminator of oxaA
Best match: txbi_universal1-var1_CC001_MW2-USA400_BA000033.2[2172406:2172478:r]RC
Position: 053-contig_55_RC: 29762 … 29792; Length: 30 bp
Sequence:
TATTTCAAATAAAAACAGCAGTAGGATGAC

## > txbi_oxaA

Function:  rho independent terminator of oxaA
Best match: txbi_oxaA_CC005_Mu50-VRSA_BA000017.4[2222431:2222513:r]RC (completely identical)
Position: 053-contig_55_RC: 29863 … 29946; Length: 83 bp
Sequence:
TATTGAAGCGAAAAATAATGTGCGCCCTATAAAGTTAAAATTATCTTCAACTTTTAGGGT
GCACATTATTTGGACTTGCTAAG

## > oxaA

Function: protein coding sequence; membrane protein (core genome, constant)
Best match: oxaA_CC008_NCTC8325_CP000253.1[2158348:2159220:r]RC
Position: 053-contig_55_RC: 29947 … 30820; Length: 873 bp
Sequence:
TTATTTCTTTTTCTTTTTAGACACAACTTGTGTGTTTTTGCCTTTTTTATTGCTGCCGCCGTT
GTGCTCTCTTTCATACGCTTCAATGAAAGGTTGTACTTCTTTTTTAGCGACTTTTTCATAAT
AAATGTTCGCAAAGTGTGTTTGAACTACAAGGAACGCCGCACTGACTGACCAGTACAAAC
CAAGTGCTGATGCTGAGCTTAATGAAATCCAGATAATCATAATTGGTGAAATGACCATCA
TCATGTAACCCATTTGACGTTGTTCGTCTGGCATCGTTTTACTTGATACATATGCTTGGAT
AAAGTATAAAACACCGGCAATAATTGTAATCCAAATATCAGGACGTCCTAAATCGAACC
ATAAGAAGTGTGGATATTTAAACAAACCATCTACAAGTTGGTCTTTAAGTACAAAGTATA

ATCCCATGATGATTGGCAATTGGATTAGCATTGGTAAACAACCCAACATACTCTTAATCG
GGTTCATGTCATACTTTTTATATACTTGCATTAATTCTTGGTTTGCAGCCATTTTTTCTTCTT
GTGTACGCGCACGTTTCACTTTTTCTTGAATTTTTTCAACTTCTGGCTTTGCAACTTTCATT
TTTTGACGCATCATATGACTATTTTTATAGTTTGACAACATGAATGGTAATAAAATAATAC
GAATTACCAATACAAGGATAATAATAGCTAAACCATAATTGTCGTTTAATAAGTTATTTC
CCAACCAATCCAATACATTTTTCATTGGATCTACGAATGTATTGTAGAAAAACCCACTAC
GTTTTTCAGGTTTAGAATAGTCACAACCAGCCAAAAAGACCATAATACCTAAAAATAATG
GTAGTAACGCTTTTTTCTTCAT

> **thiM**
Function: protein coding sequence; hydroxyethylthiazole kinase (core genome, variable)
Best match: thiM_CC008_NCTC8325_CP000253.1[2159949:2160740:r]RC (completely identical)
Position: 053-contig_55_RC: 31548 … 32340; Length: 792 bp
Sequence:
TTATTCCACCTCTTGAATACGAATCCGTTGTTGATAGGTTGTTTCATTTAAATGATATAAC
GTATCAAGCAACAATGGTGAAAACGTACCAGGACCACCACAATTTTCATTTTCAGCAGCT
ACCTCAGCAGCAATATTAAATACGCTTACCGCTTCAATTAACGCTTCTATGTCTGGTTCTG
TTTCTCTAAATAAAAATCCAGCAATAACGCCTCCTAATAAACAACCAGCTCCAGTTACTC
GTGCTAATAATGGAGATCCATTAGCTAATACGAAGGCTTTATTATCTTGAACAATAACGT
CCTCTTTACCTGTGATTACTATTGCAGTTTTATATGTTGCGTAAGCCTTTTTCGCTATTGCA
ACCGCATCAAGATTAGCATCACTATCTGTACCTTTCATAGTTGCTGTATCATCAATCAACG
CTAATATTTCAGATGCATTGCCTTTAATTACTGATACTTTCGCTGATTTCAATAATAATTTA
CAAAATTGCTTTCGATATGTAGAAGCACCAACAGCTACAGGGTCAAATACAATAGGTAA
GCCTGCCTCATTTGCCGTTTGAGCAATCGCAATAATATCTTGTTCATTTTCTGCTGTTAAA
GTACCGATATTGATTAATAGCGCTTGTGCAACTTTGTAAAATTCTTCAGCTTCTTCGGGAG
CTTCACTCATTGCAGGGCTAGCACCAATACTTAATAAACCATTCGCTGTAAAATTTTTAAC
TACATCGTTTGTATAACAAATTGTTAATGGGTTTTCAATACGTATTTTATTTAGATAATTC
AT

> **thiE**
Function: protein coding sequence; thiamine-phosphate pyrophosphorylase (core genome, constant)
Best match: thiE_CC001_MW2-USA400_BA000033.2[2173549:2174190:r]RC
Position: 053-contig_55_RC: 30905 … 31547; Length: 642 bp
Sequence:
CTAATTATTAAAAAAATCTTTGAATCGATTAACAGTCTTTTCAATATTTTCACTTTTAGAA
ATGGCTGAAATGACTGAAATTCCATTGGCACCTGCTTCTACAATCGGCGCCACATTATTA
GTATTGATACCGCCAATAGCTACAATCGGTAGTTGCGGATTCATTTCTTTAAACGTTGCAA
TCATTTTTGGACCTACTGGTATATGCGCGTCATGCTTCGACGGCGTAGGATAGATTGGTCC
AACACCTATATAATCAACATGAGTTAAATCAGATTTTGCATACTCATCTAAATCACTAAT
ACTAAGTCCAATAATTTTATCAGTGAAATATTGTGCTATCTCTTTGACTTTCGCATCATCTT
GACCGACATGTATACCATCCGCGTTAATTTCTTTTGCCAAGGATACATCATCATTAACGAT
AAAAGGCACATCATATTGATGACAGAGATGCTGTAATTCTTTAGCTAATACAAGTTTATC
GTTTCCTTTTAAAGCTGATTCACCCTTTTCTCGAAATTGGAAAAGTGTAATCCCTGCCTTT
AATGCTGTTTCTAATACTTCATGAATAGTTCGATGACTCGGAACATCGGAGGTCCCACAA
ATAAAATACACATTTAGATACGATTGGTTAAACAT

> **thiD2**
Function: protein coding sequence; phosphomethylpyrimidine kinase (core genome, constant)
Best match: thiD2_CC001_MW2-USA400_BA000033.2[2174967:2175797:r]RC
Position: 053-contig_55_RC: 32323 … 33154; Length: 831 bp
Sequence:
TTATTTAGATAATTCATCGTCTAATCCCTCTTTCTTTAAATATGCAAAATGATTCACTGGA
CCTCTACCACGGCCGATTTCAGGCGTATATTGTATACTCATTGAAATAAACTTTTTAGCCT
TGTGTACAGCCTCAAATAATCGTCTACCTTTTGCAAGTTCTGCCGTTATAACTGCTGAAAA
TGTACACCCTGTTCCATGCGTATGTTTTGTTTTAAATCGTTCATTTTCAAATGTTTGAACAC

CTTCGTTAGTAAATAAATAATCTTTTGCTATATCAGTATCATTTGAATGACCGCCTTTAAT
GATGACACCTTTACTACCAATCTCATTAATAAAGATGCGGCCAGCCTGCATAATTTTTTCT
TCACTATCAATGGTTAGTCCCGTTATTTCTTCAGCTTCTGGTAAATTCGGTGTTACTATGTC
AGCTAATGGCAATAACCTATGCTGCAAGTTTTGCTTTGTGTCATTATCCATTAATGAATCA
CCGCTTTTCGCCAACATAACAGGATCAATTACATATGGAATAGATTCATGTTGCATTAAA
TAATGACGAATCGTTTCCATAGTATCTGCTGTAGCAATCATCCCCGTTTTAATAGCATGCG
GTAAGGTATCGTTGAAGACACTATCAAGTTGTTCATCTACCCATTGATGATTTAAATTATG
AATATGTTGTACGCCCAATGTATTTTGAGCAACAATACTTGTAACGACGCCCATACCATA
TACACCACATGAATGAAATGATTTTAAATCAGCCATTACGCCGGCACCACCTGTTGGATC
AGTACCTGCAATGGTTAATGCTATTTTAGGTTTAATCAT

> **tenA**
Function: protein coding sequence; TENA/THI-4 family protein (core genome, variable)
Best match: tenA_CC001-ST772_118_AJGE01000006.1[39040:39729]RC (completely identical)
Position: 053-contig_55_RC: 33146 … 33836; Length: 690 bp
Sequence:
TTAATCATTTACTTTTCCTCCAAATTCCCATTGTTCTAGTGTCATAGCCATATTGAAAAATC
GACGCTCATGTATGCAACTTTCTAAAAATACTTGCTTTACTTGTTCCAATTCCTTGTCAGA
CATTGATTCGGCTAATTTATTCATTAATGCCTCAAATACGTTAATTATGTCATCCATTTCA
GTACTATAAAAATCAAACCATTTTGCCGTATCTTTTTCTCTATTAAGCTTATGATCACTTTG
AGAGCGCTTAGCTAATTCTGCATAAATATATGGACAAGGTGCCATTGCAGCAATTGTATA
AATAGCATTTTCACGACTATGCGCTTGGAAATACATATGTTTAATGTAATGGTCGCCACTT
GGAGGCCAAACTTTTGTTTTAATGATTTCTTCGTATGATTCACCAACAATTTGCGCTAAAA
TATCATGCGCAAGTACTTCACCTTCAACCATAAATTCTATTTGCTCTACTAAAAATTTTAC
GTCATTCATGCTATTCATTTTTGGAATTAACAAAGCATATAAGTTTGTGAATTCTTTTAAA
TAAGCTGCATCAGCTTGTAAATAGTGACGTAATGCATCAGCTTGTATATTGCCTAATAAC
ATTTTTTGAATAAAGTCATCCTCATAAATATCATTAATGATTGGCTTTGCAGCTTGGTACA
ATTTTTGTGAAAATTCCAT

> **TPP_riboswitch_L2**
Function: noncoding RNA thiamine pyrophosphate binding riboswitch/locus 2 (core genome, constant)
Best match: TPP_riboswitch_L2_CC001_MW2-USA400_BA000033.2[2176559:2176661:r]RC (completely identical)
Position: 053-contig_55_RC: 33915 … 34018; Length: 103 bp
Sequence:
TCACTTCAATAACTACTTTCCTACGTTGGTACTAACCAAATCAGGTCATAAGGGTCTGAAC
AATTCATCTCAGCCATATCATTAGGCTCCCCTAGTAGTTCCT

> **sceD**
Function: protein coding sequence; transglycosylase (core genome, constant)
Best match: sceD_CC001-ST772_118_AJGE01000006.1[37958:38653]RC (completely identical)
Position: 053-contig_55_RC: 34222 … 34918; Length: 696 bp
Sequence:
TTATGCAGTAACCCAATGTCCAGCGCCACCAGTGTTATATAATTTTACTGCTGCGGCATCT
TGAACACTTTCAGGAGCATTTGCTGGTGATACACCTTTATATTTAGCAGGTGCTACTGAAT
CCCAAGTTGATTGTAAGAATTGATACTTACCAGCTGCACCTGATGTTGGATTTACAGCAT
GAATATTACCACCTGATTCACGTTGAGCAATTTGTTTTAGATGAGCATTCACATTTACTGA
TGAACCTTCTGATGCTTTTGATTCAGTTGGTGTTGCAGTAACTTGTGAATTGTTTGATATT
GATGCTTGTGGTTGTTGAGTTTGAGCATTTTGTGGTGCTTCAACTTCTTGTGATTGTACTTG
ATTAGCTTGAACAGCTGATGGTGCAACATTATTAGTTGCAGGTGCTTGTGCACTCATGTCT
GCTCCATTAGCACCTGTTGCATGGTAATTCCAAGCAAAGTGTGTACCATCTGATTCAAAG
TGATAAGTAAACCCTTCATAGTCAAATGTATAATTATAAGCCCCAGCTTCAATTGGTTTTT
GATTTAATGTTTGATCATTTGATTGCGCCATTTGCGCTAAAGATGCTTTATTTAAGTCCGC

TTCACTTGCATGGGCTTCGTGACCTGCATTTCCTGCTACGATTCCTAAACCTACTGCTAAT
GATGATGCGAGTAATGTTTTCTTCAT

> **ywpF**
Function: protein coding sequence; putative protein (core genome, constant)
Best match: ywpF_CC001_MW2-USA400_BA000033.2[2178676:2179116] (completely identical)
Position: 053-contig_55_RC: 35895 … 36336; Length: 441 bp
Sequence:
ATGAAAACTTTTAAAGCAGTAAGGTTTCAAATTGTGAATGAACACGGCAGAATTATAGAA
TATGAATTAGAAGATGGCGTAATAATTAATAAAGAGGAAAGTGGCACGGGTTGGTTGCTT
GAAATTGTCATTTCTAATGAGCATTATGAAACATTTAAGGAATATCAAGACAATGAACAG
TTATTGGATATTCGTGTCGTCATCACGCGTCCAGCCAATGATCCAGCTTTATTTGAATCTA
CTGTGAAAAGTATTAAAAATTTTAAAACAACAATGTCTATCGTATTTGAATGTCATATTTA
CACTTTACGTCAACAGTATGCTGAAAGTTTATTAGAACAACTTATTGATGATGGATTATCT
GGTGAAGAATTAAAAAAATCATTTAATCGAATGATGCAGTCTAAACCAAAGTTAAAGGA
TGAAAAATTAGAAGAGTAA

> **fabZ**
Function: protein coding sequence; beta-hydroxyacyl-(Acyl-carrier-protein) dehydratase
Best match: fabZ_CC001_MW2-USA400_BA000033.2[2179174:2179614:r]RC (completely identical)
Position: 053-contig_55_RC: 36393 … 36834; Length: 441 bp
Sequence:
TTATTTTACATCTTGAATTGCAAATGTAAGTTCACAACTACAAGCAAGTTGACCATCGAC
AGTAGCTTTAGCATTACCTTTACCGATTGGTCCTTTAATTTTAGTGATTTCTACTTCCAACG
TTAAAGTATCACCAGGTACTACTTGACGTTTAAAACGACATTTATCAATACCAGCAAATA
AAGCGATTTTACCTTTATTTTCTTCACTATTTAAAATAGCTACCGCACCTGTTTGAGCTAA
CGCTTCAGTAATTAATACGCCTGGCATTACCGCATACTCAGGAAAATGCCCTTGAAAGAA
TGGTTCGTTTCCTGATACTTGTTTAATAGCCACACAACGTTGACCTTCTTCATATTCAACT
ACTTTATCAATTAATAAAAATGGCTGTCTGTGAGGTATAATTTGTTTAATTTGGTTATAAT
CAAAAATTGTTTCCAT

> **murA**
Function: protein coding sequence; UDP-N-acetylglucosamine 1-carboxyvinyltransferase 1
Best match: murA_CC005_Mu50-VRSA_BA000017.4[2229436:2230701:r]RC (completely identical)
Position: 053-contig_55_RC: 36867 … 38133; Length: 1266 bp
Sequence:
TTAATCGTTAATACGTTCAATGTCTGCACCTAATTGCTTCAATTTACCGTGTAAGTCAACA
TAGCCTCTATCTAGGTGCGTTAATTCAGTAACGCTTGTTTTACCATCAGCAACTAATCCAG
CTAAAATTAAGGCTGCTGCTGCTCTTAAATCAGTCGCTTTAACTTGTGCACCTTGCAATTG
ACTTTTACCTTCAAGTTTAGCACTACGACCTTCTACATTGATATTAGCATTCATACGTTTG
AACTCTGCAACATGCATAAAACGGTTTTCAAAAACAGTTTCGGTTACGACTTTATGACCA
TTTGCCGTTAATAACAATGCCATCATTTGTGATTGCATATCAGTCGGGAATCCAGGATGTG
GTAGTGTTTTGATGTCTACAGGTTGTAATTCCCCTTCAGCACGTACACGAATACCATCTTC
TTGATAGTCCAATTCAACGCCCATTTCTTCTAGTTTATAGACTAAACTCGCCATATGTTCT
TTGATTGCACCACGTACAAAAATATCACCACGCGTTATAGCACCAGCGATTAGTAATGTG
CCTGCTTCAATTCTATCTGGAATGATAGCATGTTCTACACCATGTAATGATTCTACACCAT
TGATTGTAATTGTGTCTGTACCAGCACCAGTAATTCTACCACCCATTTCATTAATGTAGTT
TGCTAAATCGACAATTTCAGGTTCTTTAGCTGCATTTTCAATTAAAGTCTTACCCTTAGCT
AATGATGCTGCCATAATAATATTTTGTGTTGCTCCTACACTTGGAAAATCTAAATGAATTG
ATGTACCTTTTAATCCATCTTTAGCATTAGCATAAATATTACCATTTTCAAGATGAATTTC
TGCGCCTAAAGCTTCAAAACCTTTAATGTGTTGCTCAATCGGTCTACTTCCAATTGCACAA
CCACCAGGCAATGCAACAATAGCATGTCCTAGTCTTGCTAAAAGAGGTCCCATAACTAAA
ATACTTGCACGCATTTTACTAACATATTCATATGGTGCCTCTTCATTTAGAGTCTTTGTTGC
ATCAACGACAACAGCATTTTCGTCCTTTTTGTATGTAACGTCAGCATTTAAAGTTGTTAAT

ACATTATTTATTGTTTCTACATCACTTAAAGCTGGAACATTAACTAATTTACTCGGTTTAT
CAGAAGCTAATAAAGATGCTGTCAATATTGGTAATACTGCATTTTTAGCACCTTCTACTTT
AACTTCACCCGTTAATTTATTTCCACCTTTGATTACTATTTTATCCAT

## > **STAR**

Function:  Staphylococcus aureus repeat element
Best match: STAR_CC008_TCH1516_CP000730.1[801877:802102:r]RC
Position: 053-contig_55_RC: 38791 … 38821; Length: 30 bp
Sequence:
TGCGGGGCCCCAACATAGAGAAATTGGAAC

## > **Q2FZB6**

Function: protein coding sequence; putative protein (genomic island)
Best match: Q2FZB6_CC030_MRSA252_BX571856.1[2068297:2068469:r]
Position: 053-contig_55_RC: 38793 … 38823; Length: 30 bp
Sequence:
CGGGGCCCCAACATAGAGAAATTGGAACAC

## > **atpE**

Function: protein coding sequence; ATP synthase F1 sector subunit epsilon (core genome, constant)
Best match: atpE_CC001_MW2-USA400_BA000033.2[2181819:2182223:r]RC (completely identical)
Position: 053-contig_55_RC: 39095 … 39500; Length: 405 bp
Sequence:
CTATTTTAATTCAGCCACACGCAAACGGTTATTTGCTCTCTCTAAAGCTCTTTCGGCTCTA
TGAATATCAGTATTGTCGTCATCATTTTCCAAGTGAGACTCTGCTCTTGCTTTGGCTAATTT
AGCTCTTTCAACATCAATTTCTCTTGCAGTTTCTGCAGTCTGAACAATGATTGAAACTTTA
TCTTTTCTAACTTCAACAAAGCCATCGCTTACAGCAATATATTCAGTTCCATCGTGAAATT
TCACTTTTACAAAGCCTGTTTTTAAAGCAGCTACAGTTGGAATATGTCCACTCATGACACC
TATCTCACCAGCTGTTGTTTGCATAACAACGAGTTCAACATTATCACGATTGTAAACAGA
ACCATTAGGAGTGACAATATCTAGGTTTAATGTATTCAT

## > **atpD**

Function: protein coding sequence; ATP synthase F1 sector subunit beta (core genome, constant)
Best match: atpD_CC001_MW2-USA400_BA000033.2[2182243:2183655:r]RC (completely identical)
Position: 053-contig_55_RC: 39519 … 40932; Length: 1413 bp
Sequence:
TTATACTTCAACACCCATATCTTTAGCTTTTGCAATAACATCATCCATGCTACCAACTAAA
CGGAAAGCATCTTCTGGAATATGGTCATATTTACCATCTAAGATATCTTTAAAGTTTGCAA
CTGTTGTCTTAACAGGTACATAAGAACCTTTTTGACCAGTAAATTGTTCCGCTACGTGGAA
GTTTTGAGATAAGAAGAATTGAATTCTACGTGCGCGTTCAACTGTTTGTTTATCTTCATCA
GATAATTCGTCCATACCTAAGATAGCAATGATATCTTGTAATTCACGGTATTTTTGAAGTG
TTGATTGTACATCACGAGCTACTTCATAATGTTCTTGACCTACAATTGATGGTTCCAATGC
TCTTGATGTAGACGCTAATGGATCCACGGCTGGATAAATACCCATTTCAGTTAATTTACGT
TCTAAGTTTGTAGTTGCATCTAAATGGGCAAACGCTGTCGCAGGCGCTGGGTCAGTATAG
TCATCGGCAGGTACGAATACCGCTTGAATAGAAGTAACTGATCCTTTTGTTGTAGACGTA
ATACGTTCTTGTAATTGTCCCATTTCAGTAGCAAGTGTTGGTTGGTAACCTACTGCAGAAG
GCATACGACCTAATAATGCAGATACCTCAGAACCAGCTTGTGTAAATCTGAAAATGTTAT
CGATGAATAATAATACGTCTTGACCTTGTTCGTCACGGAAATATTCAGCCATTGTTAAACC
AGATAATGCAACACGCATACGTGCACCAGGTGGCTCATTCATTTGCCCGAATACCATGGC
TGTTTTCTTAATTACACCACTGTCACTCATTTCGAAGTATAAATCGTTACCTTCACGAGTA
CGTTCACCTACACCGGCGAATACAGAAATACCACCGTGCTCTTGAGCGATGTTGTTAATT
AATTCTTGGATTAATACTGTTTTACCTACACCGGCACCACCGAACAATCCGATTTTACCAC
CTTTAATATAAGGTGCTAGTAAATCTACTACTTTAATACCTGTTTCTAAAATTTGAACTTC

TGTTGAAAGTTCATCGAATGCTGGTGCTTGACGATGGATAGGATCGCGGCGAACAGAATC
ACTAATTTCTTCTTTAAGGTCAATTGTTTCACCTAGTACATTAAATACACGACCTAATGTT
TCGTCACCAACAGGTACACTAATTTCTTTGCCTGTATCTTTTACATCCATGCCTCTTTGGAC
ACCATCAGTTGAATCCATCGCAATTGTACGAACAACGTCGTCACCTAATTGCAGCGCAAC
TTCTAATGTTAGTTGTATTGTACCTTCTTCTTTAGGCACATCAATAACCAAGGCGTTATTA
ATTTTAGGAACTTCGTTATGTTCAAATCGAACATCAATTACAGGACCCATAACTTGAGTTA
CACGGCCAATTCCCAT

## > **atpG**

Function: protein coding sequence; ATP synthase gamma chain (core genome, constant)
Best match: atpG_CC001-ST772_118_AJGE01000006.1[31056:31922]RC (completely identical)
Position: 053-contig_55_RC: 40953 … 41820; Length: 867 bp
Sequence:
TTATTCAAGCGCTGCGGAACCACCAACAATTTCAGTAATTTGTTGCGTAATTTCTGCTTGT
CTCGCTCTGTTATATTCTAATGATAAGTCATCAATAAGTTCAGTTGCATTATCAGTGGCAT
TTTTCATCGCAGTCATACGTGTCGCATGCTCACTTGCTTTTGCGTCTAATATTGTTCCGTAA
ATCAAACTCTCAACATATTGAGGCAAGATTACACTTAAGATAGATTCTTTATCTGGCTCA
AATTCATAAGAAGACAAATGACCATGCCCCTTACTAGAATCCTCTTGAGATAATGGTAAT
ACTTGTCTAGATGTAGGCTTGTTTTCAAGAACGCTGACATAATGACTATAGTATATATTTA
ATTCATCAATTTCTTCTTCACTGTATAAGTCTATAGCATGGTTAGCTAGTGCTTGAACAGA
TTTGAAAGAAGGTTGATCAGGTACGTCTACTTGAGAATACTCAATGTCATAACCTCTATTT
TTTAGGAAATCAACACCTTGTTGCCCTAAAACTACAATACTGTATTCGCTACTATCTTGAT
GTTTCGCTTCAATATCAGTAATCAATTTTTTAAGCACGTTTGCACTATATGCACCTGCTAA
ACCTTTATCACTCGTGATAACTAAATATCCACTTCTAGTAATTTTACGAGGTCTTAGCATT
GGATGGTTTGTATTGCTACTTGCCCCTGCAACTGCAGTAATTGCATCTTGCATTTTATCCA
TATATGGTGTGAATTGTTTTGTATTTTTTTCAGCTCTACGAAGTTTTGAACTTGATACCATG
TTCATCGCTTTCGTAATCTGCTTCATTTTTTTGGTTGATTTTATTCGAGTATCTATTTCTTTA
AGAGAAGCCAC

## > **atpA**

Function: protein coding sequence; ATP synthase subunit alpha (core genome, constant)
Best match: atpA_CC001_MW2-USA400_BA000033.2[2184574:2186082:r]RC (completely identical)
Position: 053-contig_55_RC: 41850 … 43359; Length: 1509 bp
Sequence:
TTATTCAGATTTGCTAAAGCTTTTTTTGAATTCGTTAATTGCTGTGTCAAACTTCTCAGCAT
CTGGTAAGCCACCAGTTTCTCTGATTTCATTTAACAGTTCAGTAGCATTTGATTCTGCCCA
GTGGTTTAACTCATCTTCAAAACGTGTGATATCTACAACTGGAATATCATCTAAATATCCT
TTTGTTAATGCATAAATAATCAACACTTGGTGTTCGACTGGTAATGGTTTGTTTTGATCTT
GTTTTAAGACTTCAACAGTACGTTTACCACGTTCTAATTTACTTGCAGTAAATTCATCAAG
GTCTGAACCGAATTGTGCAAATGATTCAAGTTCTCTGTATGACGCTAAGTCAAGACGTAA
CGTACCAGCAACTTTCTTCATTGCTTTAATTTGTGCAGATCCACCAACACGAGATACAGAT
TGTCCAGCATTAATCGCTGGTCTTACACCTGAGAAGAATAAATCAGATTGTAAGAAGATT
TGTCCATCTGTAATTGAAATAACGTTTGTTGGTACATAAGCTGAAATATCACCAGCTTGTG
TTTCGATAATTGGTAATGCAGTAATTGAACCGCCACCTAAGTCATCGTTTAATTTTGCTGC
TCTTTCTAATAATCTACTATGTAAGTAGAATACGTCACCTGGGTATGCTTCACGACCTGGA
GGTCTACGTAATAATAATGATAACTCACGATAAGCTGCTGCTTGTTTAGTTAAATCATCAT
AAACGATTAAAACATGTTTACCGTTAAACATGAATTCTTCACCCATTGTTACACCTGAATA
TGGTGCAATATATAATAATGGAGAAGGTTCAGAAGCTGATGCTGCTACAACAATAGTGTA
GTCTAAAGCGCCTGCTTGTCTTAACTTTTCAACATTTGCTCTTACTGTTGAATCTTTTTGAC
CAATAGCAACATAGATACAAATCGTACCTTGATCTTTTTGGTTCAAAATTGTGTCAATTGC
AATTGTTGTTTTACCTGTTTGACGGTCACCGATGATTAACTCACGTTGACCTCTACCAATT
GGTACTAAAGCATCAATTGCTTTGATACCTGTTTGTAATGGCTCATCTACTGATTTACGAT
CCATTACACCAGTAGCTTTTTTCTCTACTGGACGTGTTTTAGTTGTGTTAATCGGTCCTTGT
CCATCAATAGGTTGTCCTAATGGATTAACAACTCTTCCGATTAGTTCTTCACCTACTGGTA

CTTCCATGATACGACCAGTACGTTTAACTTCGTCACCTTCAGTAATACCTGTGTATGGTCC
TAAAATAACCACACCCACGTTTGACTCTTCAAGGTTTTGGGCTAAACCAAGTACGCCGTT
ATGGAATTCTACTAGCTCACCAGCCATAACGTCATTTAATCCGTGAATTAATGCAATACC
ATCACCAATTTGTAATACAGTACCTACATCAGTTACGGACATTTCTGACTCATAATTTTCA
ATTTGTGAGCGAAGTAATGCACTGATTTCTTCAGCTTTTATGGCCAT

> **atpH**

Function: protein coding sequence; ATP synthase subunit delta (core genome, constant)
Best match: atpH_CC001_MW2-USA400_BA000033.2[2186104:2186643:r]RC (completely identical)
Position: 053-contig_55_RC: 43380 … 43920; Length: 540 bp
Sequence:
TTAATTAACTCTTCTAAATTTTCTTTGTAATTGGACAAGATCATTTCTAACACTACCATCTA
ATACAGTTGTGCCGACTTTAACTCTAAATCCACCAATTAAATCTGGATTAATTTTTGTATC
TACAATAACTTTAGATAACTTCGTTTGTTGAGTTACTAGTTTGACAATCTTATCTAACTCTT
CTTGACTCAATTCATATGTTGACTCAATTGTTGCAAAATCTTGATTGTAGTGTCCGTTATA
TAAGCTTTGGAACGCCTTGAATACGTCAGCTATTAATGAGATATGTCTATTATCTGCTAAC
ACGTACATCATATTTTTAATGTATGGATTAATGTCAGTAAATACACCATTAATCAACTCAC
GACGTTGTTCTGCTGTTTGAGTTGGATTACTGTCAACCATTTTCAATTGCTCAATTTTATCT
TTTACTGCTTCATTTATAACTGTTAATTCTTCATTAATAGTCTCTAAATTATTTGTATCTAA
TGACACGTCAAATAATGCTTTAGCATACTTGTTAGCTACTTTTACCAT

> **atpF**

Function: protein coding sequence; ATP synthase subunit b (core genome, constant)
Best match: atpF_CC001_MW2-USA400_BA000033.2[2186643:2187164:r]RC (completely identical)
Position: 053-contig_55_RC: 43919 … 44441; Length: 522 bp
Sequence:
TTATTTATCGCCTGCCTCTTTTAGATACTTGTCAACCAATGCTTTTTGGTCTTGTTCAGAAA
TTTCTTTTCTAAGAACTTTAGAAGCAATTAACACTGATAGTTCAGATACTTGATTATTAAT
ATCTGCAATGGCACGTTCTTTTTGGCTATTGATTTCACTTTGTGCTGTTTCAATCATACCGT
TTGCACGTACGTTTGCTTCATGAATAATTTGTTCTTGCTGTTGACGTGCTTGAACCTTAGC
ATCTTCTAAAATCTTTTGAACTTCTTCTTGTGTTTCTTTAAGTTTTTGTTTATTTTCTTCTTC
AAGTTTCTGTGCATTTAACTTAGCTTGTTCTGCGTCATCGATATCTCTGTTAATATCTCTTT
CACGTTTATCCATTACATCTTTCAATGGACCCCATGCGAACTTTTTAAGTAACGCAAGTAA
CACGATGAAAGTTAGGACCTGTACAATCACAGTACCCCACTCAACGCCTCCAGCTGCACC
AAGAACGAATAAGTTAGCTGTTTCAGTCAC

> **atpL**

Function: protein coding sequence; ATP synthase subunit c (core genome, constant)
Best match: atpL_CC001_MW2-USA400_BA000033.2[2187361:2187573:r]RC (completely identical)
Position: 053-contig_55_RC: 44638 … 44851; Length: 213 bp
Sequence:
TTATCCAGCAAATGTCATGAATGCAATTACTACACCGATGATAGGTAATGCCTCAACTAA
ACCTACACCAATGAACATGATACCCATTAATTGACCACGTGCTTCTGGTTGACGTGCTAC
ACCTTCAACTGTTCTTGAAACGATTAAACCGTTACCGATACCTGCTCCTAATGCTGATAAA
CCAATTGCGATTGCTGCTGCGATTAAATTCAT

> **atpB**

Function: protein coding sequence; ATP synthase subunit a (core genome, constant)
Best match: atpB_CC001-ST772_118_AJGE01000006.1[27254:27982]RC (completely identical)
Position: 053-contig_55_RC: 44893 … 45622; Length: 729 bp
Sequence:

TTAGTGTTCATCTGCCACTTTATGTGACATATAAACCATCGAAAGCATAATAAAGATATA
TGCTTGGATTGTTCCTACAAATATTGAAAATGCTTGCCAAACAATTAATCCTGGGATACTA
ATAATCCAACCCCATGCTGGTTCGTTAAAGAATAAGCCAGCAAGTAATGTTAAAAGTATC
TCACCTGCAAATATGTTACCGTACAAACGCAGACCAAGCGTTAATGTTGAAGTGAACTCT
TCAAAAACATTAATAATTGCCAATGGCCAAAACGGCTGTACATAACCTTTAAGATATTGT
TTCGTACCACGCATTTTAATTCCATAAAAGTGAGTTAACAGTATTATCGTTGTAGACAACG
TTAAAGTCACTGTTGCATCAGCTGTCGGTGATTTCCACCACAATGTGTGATCTTTCGTTAC
TATAGAAAACGGAAGACCTAACATATTAGCTATAAAAATGTACAGAATCAGCGTTACTGC
TAAGAAGTGGAATTGACCACCTTTTTTCCAAGCCATGTTACCTTCAATGATTCCCCTCACG
AAATCAAAAATCCATTCAACGAAATTTTGTTTGCCAGTTGGTCTTTTTTTCAAATTACGCG
TACAAATGATAGCAAGTAGAAAAACAAGAAACGCCGTAACAAGTATCGTCAATATACTT
GATAAATTGAAAACGATATCAAAACCGAATAAATTCCAACTCACGAGCGGGGATTTGTG
ATCCAT

> **atpI**
Function: protein coding sequence; membrane protein related to F-ATPase (core genome, variable)
Best match: atpI_CC001_MW2-USA400_BA000033.2[2188365:2188718:r]RC (completely identical)
Position: 053-contig_55_RC: 45642 … 45996; Length: 354 bp
Sequence:
TTATTTGCTCACCTTTAGTAAAGGACGTATGATAACTACAACATATGAAATCATTAAACC
AATAATTATACCGATGATACTTACATGCGCTTTATTAAGGTACCAAATCATACAGGCAAT
AATTGCAACTAAGTATCGCCACATATTTCCAGTTGAAATATGCATAGTGTCTGGTCTTTTA
GCTTTAGCTAAATAGCATTCAAAAATACATGTGTTGATTGCAGAACCAGTAACACCAATG
ATTAATCCTAAGATAAATGCATGGTGTGTATAAAGGTATAATCCTCCCAATATTATTATTA
GATAAAAATAATATTGAATGAACTGTTTAAAAATGTTGTAGAAACGACTCAT

> **mnaA**
Function: protein coding sequence; UDP-N-acetylglucosamine 2-epimerase (core genome, variable)
Best match: mnaA_CC001-ST772_118_AJGE01000006.1[25583:26713]RC (completely identical)
Position: 053-contig_55_RC: 46162 … 47293; Length: 1131 bp
Sequence:
TCAGAAATCGCTTGGTTTTTCAGTGATTAAATTCAAATAATACTTAATATGATTAACTATT
CTTTCTGAAGCAAATCCATCACCGTAAGGATTAGATGCTTCAGACATTTGATGATATAAT
CTCTCATCATCGATTAATTCTTTTGCAGCTTGATACACATTTTGCTTATTTGTTCCAATGAC
TTTTAATGTGCCAGCTTCAACACCTTCAGGACGTTCTGTAACGCTTCGCAAAACTAAAACT
GGCTTATTAAATGATGGCGCTTCTTCCTGAATTCCACCTGAATCTGTCAAAATAAAATAA
GATTTTTTAGCAAAATTATGGAAATCTACTACGTCCAAAGGTTCAATCAATTCAATTCTGT
CATGACTACCTAAAATCTTTTGAGCCACCTCTCGAACTTTCGGGTTTTTATGCATTGGATA
TACTAGTGCTAAATCAGTATACTCATCTATTAAGCGTCTAACCGCTTTAAATATATTTTCC
ATGGGTTGCCCGATATTTTCTCGTCGGTGTGCTGTCATAAGAATGAATTTTTTGTCATGGT
ATTTATCCATGATGTTAGATTTATAATTGTCATCAACTGTATATTTCATAGCATCAATCGC
TGTATTACCAGTGACAACAACACTTTCTGAATATTTCCCTTCACTTAACAAATGCGATGCA
GCATTTTTAGTAGGTGCAAAATGTAAGTCAGCTAATACACCAACTAATTGTCTATTCACCT
CTTCCGGAAAAGGTGAATATTTATCATAACTTCTAAGCCCTGCTTCAACGTGTCCAATCGG
CACTTGGTTATAAAATGCCGCTAAACCACCTGCAAATGTCGTCATCGTATCACCATGTAC
AAGTACCATGTCTGGTTTTTCTAATTGAACCACTTGTTCTAATTGAGTGATTGATTTAGAA
GTTATCTCAGAAAGTGTCTGTCCTGATTTCATAATATTCAAATCGTATTTTGGTTTGATTTC
AAAGGTACTTAATACTGAATCAAGCATTTCTCTATGCTGTGCTGTAACAACAACAATTGG
CTCGAGCATTTTTTCTTGTTCCAAAGCTTTAACTAAAGGAGCCATCTTTATGGCTTCGGGT
CTTGTTCCAAATATGGTCATAATCTTTTTCATCAA

> **upp**
Function: protein coding sequence; uracil phosphoribosyltransferase (core genome, constant)
Best match: upp_CC005_Mu50-VRSA_BA000017.4[2239825:2240454:r]RC (completely identical)
Position: 053-contig_55_RC: 47313 … 47943; Length: 630 bp

Sequence:
CTATTTAGTACCAAACAATCTATCTCCAGCGTCGCCTAACCCTGGTGTGATATATGCTTTA
TCATTTAGCTTTTCATCAAGTGCAGCAATATAAATATCTACATCCGGATGTGCTTCATGCA
TCTTTTCTACGCCTTCTGGTGCTGCAATTAAACACATGAAGCGAATATTTTTAGCGCCACG
TTTCTTCAATGAAGTAATAGCTTCAATTGCTGATGCGCCTGTTGCTAACATAGGATCAACA
ACAATGATTTGTCTTTCAGTAATATCTTGAGGTAACTTAGCAAAATACTCTACAGCCTTTA
ATGTTTCGGGATCTCGATATAAACCGATATGTCCAACTCTGGCTGCAGGTACTAAACTTA
AAATACCATCAGTCATACCTAAACCAGCTCTTAAAATTGGAACGATAGCTAATTTTTTAC
CAGCTAATCGTTTAGCCGTCATTTTAGTTACAGGCGTTTCAATATCAACATCCTGAAGCTC
TAAGTCTCTAGTTACTTCATATGCCATCAACATACCAACTTCGTCTACAAGTTCTCTAAAT
TCTTTAGTACCTGTATTTACATCTCTAATATAGCTTAGTTTGTGTTGAATTAATGGATGATC
GAAAACGTGTACTTTACTCAT

## > glyA

Function: protein coding sequence; serine hydroxymethyltransferase (core genome, constant)
Best match: glyA_CC001_MW2-USA400_BA000033.2[2190693:2191931:r]RC (completely identical)
Position: 053-contig_55_RC: 47970 … 49209; Length: 1239 bp
Sequence:
TTATTGATATAGAGGATATTCAGCTGTTAATTTCGCAACGCGTTCTTTAGCTTGTTGTAAT
TTTTCTTCATCTTTACTATTTTTCAATGCTAAACTGATGATTTTTGCAACTTCCTCAAAAGC
TTTTTCATCAAATCCACGCGTTGTTGCAGCAGGTGTACCTAAACGTATACCACTCGTTACA
AAAGGTTTTTCTTGATCGAACGGAATGGTATTTTTGTTACATGTGATACCAACTGAATCTA
AAGTCTCTTCAGCTTCTTTACCAGTAAGTCCTATAGACCCTTTTACATCAACAGCTACTAA
GTGATTATCTGTACCGCCAGAAACAATTCTAAATCCTTCATTAATTAATGCTTCTGCAAGA
ACTTTTGCGTTTTTAACCACTTGTTGTTGATACGTTTTGAAATTATTTTCTAACGCTTCTCC
AAAAGCAACTGCTTTTGCTGCAATAACATGCTCAAGAGGTCCACCTTGAATACCAGGGAA
AATTGTTTTATCTATGTCTTTTTTATATTCTTCCTTACATAAAATCATACCACCACGTGGTC
CGCGTAATGTTTTGTGTGTTGTAGTTGTTACAAAATCAGCATATTCTACTGGATTTGGATG
TAAACCTGCCGCTACTAATCCTGCAATATGTGCCATGTCTACCATTAACTTAGCGTTTACT
TCATCTGCGATTTCTTTAAACTTTTTGAAGTCAATTGTTCTTGAATATGCTGATGCTCCTGC
CACAATAAGCTTAGGCTTATGCTCTAACGCTAATTTACGAACTTCATCATAATTGATTCGT
TCTGTGTCTTTATCTACTCCATATTCAACGAAATTGTAGAATTTACCACTAAAATTAACAG
GCGCTCCATGTGTCAAGTGACCACCATGACTCAAATTCATACCTAAAACTGTGTCGCCCA
TTTCTAATGCAACTAAGTAAACAGCCATGTTCGCTTGTGAACCTGAATGTGGTTGAACATT
GACATGTTCAGCTCCAAACAATGCTTTAGCACGATCAATTGCGATGCTTTCAGTAACATCT
ACAAACTCACAGCCACCATAATATCGGCGTCCTGGATAGCCTTCAGCATACTTATTAGTC
AACACTGAACCTTGTGCTTCCATAACCGCTTCCGATACAAAATTTTCCGATGCGATTAACT
CTATGTTGCTATTTTGTCTCTGAAATTCTCTCTCGATTGCTTCTGCGATAACTTTATCTTGC
TTGGTGATATAAGACAT

## > sua5

Function: protein coding sequence; YrdC/SUA5 multi-domain translation factor protein (core genome, variable)
Best match: sua5_CC001-ST772_118_AJGE01000006.1[21538:22593]RC (completely identical)
Position: 053-contig_55_RC: 50282 … 51338; Length: 1056 bp
Sequence:
TCATAGTTCACATCCTTTAATAATGTGATTACCTGCAGCTTTTAACATGCGATTCATAATT
GCTTCTGTATTATCATTCAGCTCAAAGCCGTATATATACGCCGCTGAAATATTTTCATTTT
CATCAAGTGAATGTAACACATCATAAAGATTATGACTCGCTTGTTTAACATCATTGTCATC
CTGACATAATTGAATGAATTGCGCTTCACTTGGTATAAACGCCACCTTATTACTCGGCACA
ATAAAAGCTATAGAAGACCAATCTTTACCGTCATTTCCAATTTTGCTCTCAATATCTGTAA
TAATTGTAAGTGGTGTATTGGGTGAGTAATGCTTATACTTCATACCTGGTGCAATTGGCTG
TTCAGTATCATTATAATCAGCATGGGCGATACTATTCGGAAGTATTTCTGTAATCATTGCT
GCTGTTATAGAACCAGGTCTTGCAATTTTATAAGGAAAAGATGTGCAATCTAAAACCGTA

CTTTCTAATCCTTCTTCACTTTGTTCAGCTTGAACAATACCATCGATACGGCCATTCAAAT
CTTGATATACATGATTGAAAGTTGTTGGTGAAGGTCTACCACTTAAATTAGCACTTGGAG
CAGCTAGAGGTTCATTTATGATTTGTAATAATTGTCTACCTACAGAATGGCTTGGCATTCT
AACAGCAACTGATGATAAACCTCCAGAAACTTTTCGACATAGATAGCCTGGCTTTAACGG
CAATATAAACGAAATAGGGCCCGGCCAGAATGCCTGCATTAACTTTTCTACGCGTGGATC
CAAAGTATATGTAAAATCTTTTAATTGACCTTTACTGTGTATATGAACAATAAGCGGATTG
TCAGATGGACGGCCTTTAGCTTCATATATTTTAGCTACAGCTTCTTCATCTGTCGCATTTG
CTGCAAGTCCATAAACTGTTTCAGTTGGTAAACCTATTAAACCACCGTTTAAAACAATGT
CTTTTATTTCATTAATTTTAGGATATTGCTGTAAATCTTCATTATATTCTCTAACATCCCAA
ATTTTAGTATCCAACTTAATCAC

> **prfA**

Function: protein coding sequence; peptide chain release factor 1 (core genome, constant)
Best match: prfA_CC001_MW2-USA400_BA000033.2[2194958:2196034:r]RC (completely identical)
Position: 053-contig_55_RC: 52235 … 53312; Length: 1077 bp
Sequence:
TTATAATTCACCATTATTAAGTTCTTTCAATTTATCTGTCTGCTCTGATAAAGTCAGTGCAT
CTATAATTTCTTCTAAATGGCCTTCCATAATTTGCCCTAATTTTTGAAGCGTTAGACCTAT
ACGATGGTCTGTTACACGGCTTTGTGGATAATTATAAGTTCGAATACGTTCTGAACGATC
ACCAGTACCGACTGCTGATTTACGTTGTGACGCATACTTTTGTTGTTCTTCTTGAACTTTCA
TATCGTATAAACGTGCTTTTAACACTTTCATTGCTTTTTCACGGTTTTGAATTTGAGACTTC
TCAGAAGATGTTGCAATGACACCAGTTGGTAAATGGGTAATACGTACTGCAGAGTCAGTT
GTGTTTACGTGCTGACCACCTGCACCACTTGAACGATACGTGTCGATTTTTAAATCTTCAT
TTCTAATTTCAATTTCTACATCTTCAACTTCTGGTAAAACTGCCACTGTAGCTGTTGAAGT
ATGAATACGTCCACCTGATTCTGTTTCAGGCACACGTTGAACGCGGTGCGCACCATTTTCA
AATTTCAATTTACTATACGCGCCATTACCAGAAACTGAGAAACTAATTTCTTTGTAACCAC
CATGGTCACTTTCAGACGCTTCTACTATTTCAGTTTTGAATCCTTGTGATTCAGCATACTTT
GAATACATACGCATTAAATCACCAGCAAAAATCGCAGCCTCATCACCACCTGCTGCTGCT
CTTATTTCTACAATAACGTCTTTGTCATCATTAGGATCTTTAGGAATCAATAATATTTTAA
GCTCTTCTTCAAGATTTGGAAGTTCAGCTTTAATACCATTACTCTCCTCTTTTAACATTTCT
ACTTCTTCTTTATCATCAGTCTCACTTAACATTTCTTCAATATCAGCTAATTCTTCTTTTTTA
GCTTTATAGTTACGATAAACATCTACAGTTTTTTGTAAATCAGCTTGCTCTTTAGAATATT
TACGTAATTTATCTGAATCATTTACAACATCTGGGTCACTTAACAGTTCATTTAACTGTTC
GTATCTTTCTTCTACAATATCTAATTGATCAAACAC

> **tdk**

Function: protein coding sequence; thymidine kinase (core genome, constant)
Best match: tdk_CC005_N315_BA000018.3[2169392:2169991:r]RC (completely identical)
Position: 053-contig_55_RC: 53312 … 53912; Length: 600 bp
Sequence:
TTATAATTCCTCCTTATTATTATCACTAGGTGCTACGATATGGTGCGCGCGACAACGTGGC
TCATAACTTTCATTGGCACCTACTAAGATAATCGGATCATCGATTTTAGCTGGTTTACCAT
TTATTAATCGTTGCGTTCTACTAGATGAAGAACCACAAACAGCACAAACTGCTTGAAGTT
TCGTTACTTGTTCACTGACAGCCATCAATTTAGGCATTGGTTCGAACGGTTCGCCCCTAAA
ATCCATATCTAATCCAGCAACAATAACACGGTGTCCATCTGCTGATAGTTTTTCTACTATA
CTTACAATTTCATCGTCAAAAAATTGCACTTCGTCTATTCCTATAACATCAACATTAGTTA
AGTTGTGCGTCATAATTTCACTTGCTTTAGAAATATTAATCGCTTCAATGGCATTACCATT
ATGAGAGACCACTTTTTCTTTATGATATCGATCATCAATCGCCGGTTTAAATACAACGACT
TTTTGTTTAGCGTATATACCCCTTCTTAGACGTCTTATTAGTTCTTCGGATTTACCGCTAAA
CATACTACCTGTAATACATTCTATCCAACCGGAATGGTAAGTTTCATACAT

> **rpmE2**

Function: protein coding sequence; 50S ribosomal protein L31 type B (core genome, constant)

Best match: rpmE2_CC001_MW2-USA400_BA000033.2[2196981:2197235:r]RC (completely identical)
Position: 053-contig_55_RC: 54258 … 54513; Length: 255 bp
Sequence:
TTAGTTGTTTGATTTAAGACCAAACTTTTTGTTGAAACGTTCCACACGTCCATCCGCAGCA
GCGAATTTTTGACGTCCAGTGTAGAATGGATGTGAATCAGATGAAATATCTAAACGAATA
ACTGGGTATTCTTTTCCATCTTCCCACTCCATCATTTCTGAAGATGTTTTTGTAGAACCGCT
TAAGAATTTGAAGTTTGTAGTAGTATCTAAAAAGATTACTTGGTGATACTCAGGATGAAT
TCCTTGTTTCAT

> **rho**
Function: protein coding sequence; transcription termination factor (core genome, constant)
Best match: rho_CC005_Mu50-VRSA_BA000017.4[2247142:2248458:r]RC (completely identical)
Position: 053-contig_55_RC: 54630 … 55947; Length: 1317 bp
Sequence:
TTAAATTATAGGTCGACCCGTTTTAGTACTTTCTTCTGCAGACTTTTGTAGCTGCTTGAAG
AAATCTTCATTATTCTTAGACCTTTTAAGTTTGCGAATAAATCTTTCAGTAAAGTCAGTTG
AGTCAGTGAATAGATTTCTTAATTGCCATAATGTGTCTAATTCAGATTTACTTATTAACAA
TTCTTCTTTACGCGTTGAACTTCTGCCAATATCAATTGCAGGGAAGATACGACGTTCAGAC
AATTTACGATCTAAATGTAACTCCATGTTACCTGTTCCTTTAAATTCTTCGTAAATCATAT
CGTCCATACGTGAACCCGTATCAACTAATGCAGTTGCAAGTATTGTTAAACTTCCACCCG
CTTCAATATTTCTCGCTGCACCGAAGAATGCTTTTGGTTTGTGTAAAGATGCAGGATCTAA
ACCACCTGATAATGTACGACCACTTGGTGGAATAACTAAGTTATAAGCGCGTGCTAATCT
CGTTATAGAATCCATTAAAATAATGACATCTTCCCCAATTTCTACTAAACGCTTTGCACGT
TCAAGTAATAATTCAGCTACTTTAACATGGTGTTCTGGTGGTTCGTCAAACGTTGAATGAA
CGACTTCAGCAGCTTCTACTGAGCGTTCTAAATCTGTCACCTCTTCAGGACGCTCGCCAAC
TAACAAAATAAATAGCTTTGCATCTGGTTTGTTCGTACTGATTGCATTCGCTATTTCTTTTA
ATAACGATGTTTTACCTGCTTTAGGTGGCGCCACTATTAAACCACGTTGACCTAAACCAAT
CGGTGTTACTAAATCCATGATGCGCGTTGAATAATTTTGTGTTTCTGTCTCTAATTTAATA
CGCTCATCTGGATAAAGTGGTGTCAAAGCTTGGAAATGCGGACGTTTCTTCACTTCTTCTG
CGTTATGGTCATTGACAAAGTCAACTTGTAATAAGCCATAATATTTTTCGTTATCTTTAGG
TTTTCTAACTTTCCCAGTTACTTTATCCCCACGTTTAATTTCAAAACGACGAATTTGGCTA
GCAGATATATAAATATCTTTTTCCCCTTTAGAATAGTTCACTGTTCTTAAAAAACCATAAC
CATCTGGTTGTATATCATCTAAGATACCTTCCATATAATAGTTACCATCTTTTTCCATTTGT
GCTTCCATAATAGCTAGAACAAGTTCTTTTTTATTTAATTTACTATAGTTCGTCAACTTAA
GAGTTTTAGCTTTTTGAGTGAGTTCCTTGGTAGTATAGTTCTTGTACAATTCGTGGAATGA
TTCATACTGAGGAGATGTACGTTCTCTTTCAGGCAT

> **aldA3**
Function: protein coding sequence; aldehyde dehydrogenase/locus 3 (core genome, variable)
Best match: aldA3_CC005_Mu50-VRSA_BA000017.4[2248705:2250132:r]RC (completely identical)
Position: 053-contig_55_RC: 56193 … 57621; Length: 1428 bp
Sequence:
TTATTTAAAATATCCAGCTATAGATTTCACTTCTAAGAACTCTTCAATACCATAATCGCCC
CATTCACGACCTAAACCAGATTGTTTATAGCCACCAAATGGTAAATCTGGCTTTCTACCTG
CTTCGTTTATTTCTACTGTACCTGCTTCAATAGAACGAGCTACTTTATGCAATGTTTCTTTG
TCCTTACCAATAACATATCCTGCCAAACCATATTTTGTATCATTTGCAATTTGAATCGCTT
CATCTAAATCGTTATAAGTGATAACTGACATTACTGGCCCAAAAATTTCTTCTTGTGCTAT
CGTCATTTGATTATCTACATTAATAAAAATTGTCGGACGTGCAAAGTATCCTTTTTCAAGT
CCTTCTGGTTTACCAGGACCACCATAAAATAATTCAGCACCTTCTTCAATACCTTTATTAA
TATAATTTTGTACTTGATCAAATTGTTTTTTACTAATGATAGGGCCTACTTGTGTACCATCT
TCTCTTGGATTACCGACACGCACTTGGCTAAATTGTTCTTTTAATTCAGCTAAGAATGCAT
CTTTAATTTTGTTAGGCACTAAAACACGTGTACCAGCTGTACATACTTGACCAGTATTATT
AACAACTTTGCCTGTTGTTGCTTTAGCCGCTTCTTTAATATCTACGTCATCTAGGACGATA

TATGGTGATTTGCCACCAAGCTCTAATGATACCTTTTTAAAATCTTTAGCGGCTTTTTCCA
TAATTTTAGAACCAGTAGGGCCTGATCCTGTGAATGACATCATGCGTACTTTAGGATGTTC
AGATAAAGGATTCCCAACACCAGCACCATCACCATTAACAAGGTTAAATACACCTTTAGG
AACACCGACTTTATCAAAAATCTCAGCTAAAATAACAGCTGCAAATGGTGTTTCTTCAGA
TGGTTTAAGTACAACTGGACTACCAGCCGCAAATGCTGCTGCTAATTTTAATGATGTTTGG
TTTGTAGGGAAGTTCCACGGTGTAATTAATCCAGATACACCGATTGCTTCTTTAACAACTA
AATCATCTCCGCGGCGTTCTTCAAATTCGTAGTTATCTAATGCGTCTCTCGCTGCAACAAA
ATGGTTTAGTCCCATTTGATAATGGACACGCTCAGATAATGATAAAGGAGCACCTAATTC
ATCCGTAATAGCTTGTACAATATCGTCTTTTCTGTTTTCATATTCTTTTACAATTTTATCTA
ATAACGCTTGTCTTTCTTTCACAGATGTATGACGGAACTCTAAATAAACATCGTCTGCCGC
CTCGACGGCTTTATCAACATCAGCTTTATTACCTTTAGCAACTTTCCCGATTACTTCTTCGG
TTGCTGGATTTATAACTTCTATCGTTTCATTACTATTACTTTCTACCCATTCGCCATTAATG
TATTGCTTTGTGTAGTCTCTCAT

> **murZ**

Function: protein coding sequence; UDP-N-acetylglucosamine 1-carboxyvinyltransferase 2 (core genome, constant)
Best match: murZ_CC005_Mu50-VRSA_BA000017.4[2250794:2252053:r]RC (completely identical)
Position: 053-contig_55_RC: 58282 … 59542; Length: 1260 bp
Sequence:
CTATACAGTTTCCGTCCAAATATCTGCACCTAAAGCTTTTAAGTGTTCTACAATATCTGTA
TAACCTCTATAAATATGTTTTACATTGTAAATTGTAGTTACACCCTCAGCAATTAAACCAG
CAATAATTAAACAAGCTCCTGCTCTTAAATCGCTAGCATAAACTTCAGCACCATGTAATG
TTGATGGTTTTATCGTTGCTGTGCCTTCGTCAACTTCAATATTTGCACCCATGCGCTTTAAT
TCTTCAACATGTTTAAAACGCTCCGGATAAATCGTATCAGTTACAAATGAAGGACCATTT
GCCATAAATAATAATGGTGTAATAGGCTGTTGCAAATCAGTAGCAAAACCTGGATATACT
AGTGTTTTAATATCAACAAATTGATATGGCGCATTATTATTGATGCGAATTCTTTCGTCTC
TTACATCAACATTCACACCTAATTCACTAAATTTAGCAGTTAATGTTTCTACATGTTTCGG
AACAATATTATTTAATATAACATTTTCTCCACATGCTGCAGCGATACACATATATGTGCCT
GCTTCAATTCTATCAGGTATAACTTGATACTCAGAACCATGTAATTCTTTGACACCATTGA
TTTTAATTGTTGATGTGCCCGCTCCCTTAATATTAGCTCCCATACTTGTTAAGAAGTTAGC
AACATCAACTACTTCCGGTTCTTTAGCAGCATTTTCAATTACAGTTTGTCCTGTTGCATAA
ACTGCAGCTAGCATAATGTTAATTGTTGCACCTACGCTAACCATATCTAGAAAAATATGC
GCACCTTTTAATTCTTTAGCTTCAATTTTCATTGATGTCGTGCTTGATTCATCAATTTCAGC
ACCTAAAGCTTTAAATCCTTTAATATGTTGATCAATTGGACGTGGCCCAAGTGGACATCCT
CCCGGTAAACCAATCACACATTTTTTAAATCTACCTAACATGGCACCCATCATATAGTATG
AAGCACGTAATGATTCAACTTTATTATTTGGTAATGCAGCATTTTGTATTTCAGTTGTATC
AACTTCTAATTCCGTACCATTTAGTGATGCCTTAATATTTAAATCTTCTAATAAACTCACT
AAAGTTTTAACATCAGAGATTTGCGGTAACCCTTCTAATTTCACATGTCCTTGAGCTAATA
ATGTTGCAGGAATGATAGCTACAGCACTGTTTTTCGCGCCACTGATATTAACTTCCCCATT
TAGTGTGCGTCCACCTCTTATTTTTATTACCTCTTGAGCCAT

> **fbaA**

Function: protein coding sequence; fructose-bisphosphate aldolase (core genome, constant)
Best match: fbaA_CC005_Mu50-VRSA_BA000017.4[2252519:2253379:r]RC (completely identical)
Position: 053-contig_55_RC: 60007 … 60868; Length: 861 bp
Sequence:
TTATTTAGCGCGGTTTGAAGTACCGAACTCTTTAATTTTACCTTTAACTGTTTCTTTGATGG
CTTCACGTGCAGGTCCTAAGTATTTACGAGGATCGTAAACTTCTTTGTCGTTATTTAAAAC
GTCACGAACTGCTTTTGCTGAAGCGATTTGGTTTTCAGTGTTTACGTTAATTTTAGCTGTA
CCAAATGGAATTGCTTTTTGGATATCTTTAGTCGGGATACCAGTACCACCGTGTAATACTA
ATGGTAAACCTGTAGATAAACCGATTTCTTCCATTTCTTTAAATCCTAATTTTGGTTCACC
TTTGTATGGACCATGAACTGAACCTAATGCTGGCGCTAATGCATCAATACCAGTTTTTTCA
ACTAGTTCTTGACATTCTTTAGGATCAGCATAAATGATGCCGTCTGCTACAACATCATCTT
CTTGTCCACCAACAGTACCTAATTCAGCTTCTACAGAAACACCTTTTTCATGAGCGTATTC

AACAACTTTTTTAGTTGTTGCTACGTTTTCTTCGAATGGGCTGTGTGAAGCATCGATCATT
ACTGATGTGAAACCAGCATCGATAGCTTCTTTACATTTTTCAAAGCTTGAACCATGGTCTA
AATGGATTGCTACAGGAATAGTGATGTTTAAGTCATGCATTAACCCTTCAACCATTTTAAC
AATTGTGTAGAAACCGCTCATGTAACGAGCAGCACCTTCAGAAACACCTAAAATTACAGG
TGCATTTTCTTCTTGTGACGCTTCTAAAATTGCTTGAGTGAATTCTAGGTTATTAATATTGT
ATTGACCTACCGCATAACCATTTTCTTTTGCATCAATTAACATTTCTTTCATTGAAACTAA
AGGCAT

> **pyrG**
Function: protein coding sequence; CTP synthase (core genome, constant)
Best match: pyrG_CC005_Mu50-VRSA_BA000017.4[2254227:2255837:r]RC (completely identical)
Position: 053-contig_55_RC: 61715 … 63326; Length: 1611 bp
Sequence:
TTATTTATTTTGTTGATATTTTAATGAAGCTTCAATAAATGATTTAAAAATCGGGTGCGGA
CGATTTGGTCTAGATAAGAATTCTGGGTGGAATTGACAAGCAATAAAGAAATCATTTGTC
GGAATCTCTACCATTTCTACTAAACGTCCATCTGGACTTGTACCAGAAATTACCATACCAT
TTGCTTCTAATTGTTCTCTATAGTCATTATTAAATTCATAACGATGACGATGTCTTTCTTCA
ATTTCCGCTTTACCATAAACATCTTGTGCCAATGTGCCTTCTTTAATAGAACATGGATATA
AGCCTAAGCGTAATGTACCACCTAAATCTTCGATATCTTTTTGTTCTGGTAATAAATCTAT
AATTGGGTATGGTGTTGCTGGGTCTAATTCAGCTGAATGTGCGCCTTCAAGTCCTAATACG
TTTCTTGAAAATTCAACTGTTGCAAGTTGCATTCCTAAACAAATACCAAAGAATGGTACA
TTGTTTTCTCTAGCATACTTAATTGCACTAATTTTACCTTCACTTGCACGGAAACCAAATC
CACCTGGTACTAAAATACCGTCGACATCTGCAAGGTATTCGGCTGCATTTTCATCTGTTAC
TTCACTTGAATCAATCCATCTAATGTCAATATCTTTGGCAAAAGGATATCCAGCATGTTTC
AATGATTCAACAACTGATAAATATGCATCTTGTAAGCTAACATATTTACCTACTAAACCA
ATTGTAATTTTACCATCTAAATTATTAACGATATCTAACAACTGTTTCCATTCATCAAGCT
GTGTTTCATATTTCGCGTTTAATTGTAAACGTTTAATAACGATATCATCCATATTTTGTTGG
CTTAATTGTAATGGAATTTCGTATAAAGAGTCTGCATCACGACATTCAATAACACTTTCTT
TATTAATGTCACAGAATAATGCAATTTTATCTTTTAAATCTTGTGTCATTTCATATTCAGTT
CTTACAACGATTAAGTCTGGTTGAATACCTAAGCCTCGTAATTCTTTAACACTATGTTGTG
TTGGCTTCGTTTTCATTTCTCCAGCAGCTTTAATATAAGGCAGTAATGTACAGTGAACATA
CATAACATTTTCTCTACCTAAATCGCTACGAATTTGACGAATCGCTTCAATAAACGGTAAT
GACTCAATATCACCTGTTGTACCGCCAATTTCAGTGATAACAACGTCTGCATTCGTACTTT
CCCCTGCAAGTAATAAACGTTCTTTAATTTCATTTGTAATATGCGGAATAACTTGAACTGT
TCCGCCTAAGTAATCACCACGACGTTCTTTTTTCAATACGTGTGAATACACTTTACCGGCT
GTCACATTTGAAAACTTGTTTAAATTAATATCAATAAATCTTTCGTAATGTCCTAAGTCTA
GGTCAGTTTCTGCACCATCATCCGTTACGAATACTTCACCATGTTGATAAGGACTCATTGT
ACCTGGGTCAACATTTAAGTATGGATCGAATTTTTGAATTGTTACATTTAGACCTCTATCT
TTTAATAATCTACCTAGAGAAGATGCTGTAATACCCTTCCCTAATGATGAAACTACGCCA
CCTGTTACAAAAATAAATTTTGTCAT

> **rpoE**
Function: protein coding sequence; DNA-directed RNA polymerase delta subunit (core genome, variable)
Best match: rpoE_CC005_Mu50-VRSA_BA000017.4[2256173:2256703:r]RC (completely identical)
Position: 053-contig_55_RC: 63661 … 64192; Length: 531 bp
Sequence:
TTAATCGTTGAAGTCTTCTTCGTCTTCAAACACTTCTTCGTCTTCGTCTAGTTCATCTTCAT
CTTCTTCAATGACTATATCCGAATGATTGATTTCTTCTTCAACCTGCTCATCTTCTGGATCA
TTTAGTTCTTCTTGATCATCTGTTTGAGCTGGAATATCATCGTCGTCATCCATTTCATCTTC
GCCCAATAATTTTAAGTTTTGATCTTCTTCATCATCTGCATCCAGAATATCGAATTTTTGA
ATAGTTGGTGCGATTTTCTCTTCAATATCATCTACCGAATACCAATCACGTAATCCCCATA
AATTTTCTCCAACATTTAAAAAACGACCATCTGTGTTTAAATCTGTGTAAAATTGTACAAC
GCGATTTTCAATTTCTTCGTACTCATAATCACCTAACGCTCTAAACTCATCGATGATATCA

TATAAGTTCATAGTTTCGCCTTTATCATTCAATAATGTATAAGCCATATCAATAAATGATT
TTTCATCAACCATTTGTTTTGTATAATCTTGAATTTTCAT

> **coaW**
Function: protein coding sequence; pantothenate kinase type II
Best match: coaW_CC005_Mu50-VRSA_BA000017.4[2258100:2258903] (completely identical)
Position: 053-contig_55_RC: 65588 … 66392; Length: 804 bp
Sequence:
ATGAAAGTTGGCATTGACGCTGGCGGTACACTAATTAAAATCGTTCAAGAGCAAGATAAC
CAACGTACTTTTAAAACTGAATTAACTAAAAATATTGATCAAGTTGTGGAATGGTTAAAC
CAACAGCAAATTGAAAAATTATGCTTAACTGGAGGTAATGCAGGTGTCATTGCTGAAAAC
ATCAACATTCCTGCACAAATTTTTGTAGAGTTTGATGCTGCATCTCAAGGACTAGGTATTT
TGTTAAAAGAGCAAGGCCATGACTTAGCAGATTATATTTTTGCAAATGTTGGTACCGGTA
CGTCACTACATTATTTCGACGGCCAATCACAACGTCGTGTAGGCGGTATCGGTACAGGTG
GAGGCATGATTCAAGGACTCGGTTATTTACTAAGTCAAATCACTGATTATAAACAGTTAA
CTGACATGGCACAACACGGTGATCGTAACACTATCGATTTAAAGGTTAGACATATTTATA
AAGATACCGAACCACCCATTCCCGGTGATTTAACAGCAGCTAATTTTGGTCATGTACTAC
ATCATTTAGATGCTGATTTTACACCTAGCAATAAATTAGCAGCCGTTATTGGTGTCGTTGG
CGAAGTTGTTACGACAATGGCCATTACTGTTGCACGTGAATTTAAAACTGAAAATATCGT
TTATATCGGTTCTTCATTCCATAATAACGCTTTATTACGCAAAGTTGTGGAAGATTATACT
GTGTTACGCGGTTGCAAACCGTACTATGTTGAAAACGGCGCATTTTCAGGTGCTATTGGT
GCATTATATTTAGGTAAATAG

> **txbi_coaW**
Function:  bidirectional rho-independent terminator of coaW
Best match: txbi_coaW_CC005_Mu50-VRSA_BA000017.4[2258894:2258970] (completely identical)
Position: 053-contig_55_RC: 66382 … 66459; Length: 77 bp
Sequence:
AGGTAAATAGAAAAAGACCATTTATATGCGCGTTGTTTTAAATAAACGCTATATAAATGG
CCTTTTATTTTATGGTC

> **tx_A5IUS7**
Function:  rho-independent terminator of coaW
Best match: tx_A5IUS7_CC001_MW2-USA400_BA000033.2[2210230:2210272:r]RC (completely identical)
Position: 053-contig_55_RC: 67509 … 67552; Length: 43 bp
Sequence:
CTAGAATCGAAAAAAGCTTGTTACAAACGCATATTCAGTCAAC

> **A5IUS7**
Function: protein coding sequence; putative protein (core genome, constant)
Best match: A5IUS7_CC001_MW2-USA400_BA000033.2[2210313:2211506:r]RC
Position: 053-contig_55_RC: 67592 … 68786; Length: 1194 bp
Sequence:
CTAATAGTGTTCAAGTTCAGCTAAAAATGCGCGTTCATTCTCCAGTACTGTTTCTTTTGAA
TCACCATGCCAAATACAGCCAAACCTTGATTTAACTTTATCTTCACCGTACCAATCACCAT
CATAATAAGATAACGGGTATAAACTACCTTCTTTGACATATTTCAAAATCGTATTGAAGA
AATGTGTGTTATCACCTTTTGAATGATAACTATAAAACTTTTGATATCCTGAATTCAACTC
GTTAGCAAGTAATAACATGCTCGTTGAACCATTTTGTCTAAAGTTTAAATCAATCGCATA
AACGTTATCATCCTCATCGACGAGTAGGTCAAAACCAGCTACACCAAAGAATCCTTGATT
CACACCGTTTTCCATAATTTGTCTACCTGCTTCTATAACGTGCTCTGGTACATTAGTAGTA
TTTTCATTTCCGTTATAAAATCCGTACTTATCCGTTAATTGCGTTGCCGCACCAAGATATT
GAATGCCTAGCGATTCCGAGTATGCAAACTGAACGCAATAATTCGCTTTTTCTTCAATCTT
CTGTTCGATAATAAGCGAATTCGTTTCAGCAGTTGCCTCTTTAATACGCGTTATCGCTTTT

TGTAAATCTGCATCATGATAACAAATCATAACGCCATAGCCACCTGCTGTCGGAAGATCA
TCTCCCGGCTTAATTACTAACGGGAATTCTCAATTCTTAATCTCGTTTTCGAATTGCTCAA
TTTTTACAACTTTTCTTTTTGGTAAAAACTTCCCATTTGTCCATTCAGGTATTCTTGCTTTA
TTATTTAAAGCAACAAATAACGTTTTATCTAATGCATAATATTGCTGATTCAAGATTGTTT
CATCATGAATATATTGAAAATAAATCTTTTTATTTTCCTTATGTGCCAATTGTTTGATCAA
GTTTTCGTAAGATTGCTGATTGTTAAATGTAAAAATTGAGTTCGGTACTTCCTTACCAATA
ACTTGAAATAGCTGATGCAATTTGTCTGTCGCACTAGCTTCGTGAACAATAACAGGTAAT
TGATTTGCTATTAATAACTCCCTACCAGTTAAAAAATTAGATTGATGTTCGTCCGGTTTCA
ACCATGGATTCGATATATACGAAGGTCTTGACGTATAGACAACATCTTTGTCATATAAAT
CACTTAACGTTAAGTTCGGCTCATTACCATTATTTGTCAT

> **hmrA**

Function: protein coding sequence; putative peptidase
Best match: hmrA_CC005_Mu50-VRSA_BA000017.4[2261297:2262481:r]RC (completely identical)
Position: 053-contig_55_RC: 68785 … 69970; Length: 1185 bp
Sequence:
TTACTTCCCATTCCCTTTCAAATGCGCATGCTCTTCAATAATGTCTTGATAAACGTCTTGAT
TTGTAATTAACTCTAACCCCATCAACGCCATTATTTTAGCACCTTTAATTAATGCTTCATC
ACCATGTACACTCGCAGCCGCCTCTCTAAATCTATGCGTATGTCCTACTAAATTACGTGAT
CCTATTTTAATATGAGGATGTATTGTTGGCACAACATGACTTACGTTCCCTGTATCCGTAG
AGCCATAACCAAAATCATCATCAATAACTGCTTCACCAACTTCTTCAGCATATTTAGCAA
ATAAATCATCTAATTTCGGCGTTTTAATGAATTCATTCACACCGTTTTGAATTGGACCAAA
TTCATAATCACAACCAGTCTGTATCGCAGCTCCACGTGCGATTTGATTTACTTTTTCTGTT
AATATATCCAATTCTTTACGCGTCATTGCTCTAGTATAAAAACGAGCATGTGTATAGTCTG
GAATAATATTAGCTGCTTTCCCGCCATCTAAAATCACACCATGCACACGTTGATCTTTTTT
AATATGTTGTCGTAGTTGTGCTACACCATTAAAATAACTAATCATAGCGTCTAATGCATTT
AACGCTTCATCTGCATTTTCAGAGGCATGAGCACTTTTTCCGTAAAATTTAACATCTAAAA
CATCCACTGCCAAAGTATCAATCGTTTTATAAGTTTCATTTCCCGGATGAATCATTAAGGC
AATGTCTATTTGATCAATCACACCAGCCTTGACATAAGAAGCTTTAGCGCTACCATTTTCC
CCACCTTCTTCAGCTGGACATCCAAGAACGACTACTTTACCACCAATTTGGTCAATCACTT
GCTTCAAACCAATTGCACCAAGCACACTTGCAGTTCCAATGATATTATGACCACAAGCAT
GACCCAACCCTGGCAAAGCATCGTATTCTGCTAAAAAACCTATAGCTGGCCCGTCAAGAC
CTGAATCATATGTCGCTATAAACCCAGTTGCATGCCCAGCAATCTCGGTTTCAATTTCAAA
ATCATGCTCTTTCAAACGATCTATTAAAGTTCGAGACGCAAATATTTCTTCATTACCAAGT
TCAGGACGTTCATGAATTCTATGACTGATTTCGATATAACTATATTTATTTGTTTCTATATA
ATCGAGAATTTGTTGTTTTTCACTCAT

> **luxS**

Function: protein coding sequence; putative autoinducer-2 production protein (core genome, constant)
Best match: luxS_CC005_Mu50-VRSA_BA000017.4[2262792:2263262] (completely identical)
Position: 053-contig_55_RC: 70280 … 70751; Length: 471 bp
Sequence:
ATGACAAAAATGAATGTTGAAAGTTTCAATTTAGATCATACTAAAGTGGTTGCCCCATTT
ATTAGATTAGCGGGAACGATGGAAGGATTAAACGGAGATGTCATTCACAAATACGACAT
TCGTTTCAAACAACCAAACAAAGAACATATGGATATGCCAGGACTACATTCATTAGAACA
TTTAATGGCTGAAAATATTAGAAATCATAGTGACAAAGTTGTTGATTTAAGTCCTATGGG
TTGCCAAACTGGTTTCTATGTATCATTTATTAATCATGATAATTATGATGATGTATTAAAT
ATTGTTGAAGCAACTTTAAATGATGTGCTAAATGCTACTGAAGTGCCTGCTTGTAATGAA
GTACAATGTGGCTGGGCAGCAAGTCATTCATTAGAAGGTGCTAAAACTATCGCTCAAGCA
TTTCTAGACAAACGAAACGAATGGCATGATGTTTTCGGTACAGGAAAATAA

> **qrrB**

Function: noncoding RNA antisense RNA (core genome, constant)
Best match: qrrB_CC001_MW2-USA400_BA000033.2[2213319:2213663:r]RC (completely identical)

Position: 053-contig_55_RC: 70598 … 70943; Length: 345 bp
Sequence:
AATGATGTGCTAAATGCTACTGAAGTGCCTGCTTGTAATGAAGTACAATGTGGCTGGGCA
GCAAGTCATTCATTAGAAGGTGCTAAAACTATCGCTCAAGCATTTCTAGACAAACGAAAC
GAATGGCATGATGTTTTCGGTACAGGAAAATAAATCTTAGTCAATCAAGTTAATCAGAAA
AGCAGTCGAACAATGATGTTACAATCGCCATTGTCCAACTGCTTTTTATTATGCTTCAAAG
TCAAAAAATCGAACAAATGAAAAAGTAAAATCTTTAACATTTGTCCGATTTATTTGAGAA
CCACTATAATTTCTTAATTAGTCCCATTAACACGAACTGCATAG

> **pdp**
Function: protein coding sequence; pyrimidine-nucleoside phosphorylase (core genome, constant)
Best match: pdp_CC005_N315_BA000018.3[2187346:2188647:r]RC (completely identical)
Position: 053-contig_55_RC: 71266 … 72568; Length: 1302 bp
Sequence:
CTACTCTGTAATGATTTTATGAATTAATGTAGGCGATACAACATGATCAGCAATTGTTATG
CTTGAATCTAATTTTTTAACAACATCGTCTACATCTTGGCGATTACTGTGAATCGTTAATA
ATGATTCTCCTTCTTCTACTTTATCACCAATTTTTTTATTTAAAACAATACCAACCGCTAAA
TCAATATCATCCTCTTTTGTTAAACGTCCCGCTCCTAACATCATCGAAGCGACACCTATAT
CGTTAGAGACTAATTCAGTCACATAACCTGATTTTTTAGCTTTATATTCAATTTGATATTG
AGCTTGTGGCAAACGCTCTGGATGGTCAATAACAGTTTCGTCGCCACCTTGGTTTTTAATA
AATGTTTTGAATTTTTCTAATGCTGCACCTGAATTAATTGCCTCAATTAGCAACGCTCTCG
CTTCTTCAAGCGTTTCAGCTTTGTTTGCAAGTACAACCATTTGAGAACCTAATGTTAATAC
AAGTTCTGTTAAATCTTTCGGACCTTGTCCTTTCAACGTATCAATTGCTTCTTGTAACTCAA
GCGCATTGCCAATCGCACGTCCAAGTGGCTGATTCATATCAGAAATAATCGCCATCGTAT
TACGTCCCACATTATTACCAATACGTACCATTGCGTGCGCTAATGCTTCAGCATCTTCTAA
TGTTTTCATAAATGCACCGCTACCAGTTTTTACATCTAATACAATTGCATCTGCACCAGCA
GCAATCTTTTTACTCATAATTGAAGAGGCAATTAATGGTATTGAATTGACAGTACCAGTA
ACATCCCTTAAGGCATATAATTTTTTGTCTGCAGGAGTTAAATTTCCTGATTGTCCTACAA
CTGCCACTTTATTTTCATTAACCAATTTCACAAATGTTGCTTCATCTATTTCAACATGAAA
ACCATCAATTGCTTCTAATTTATCAATCGTACCACCTGTATGACCTAATCCACGCCCACTC
ATTTTTGCAACAGGAACATCTACAGCTGCTACTAATGGTGCTAAAACCAATGTAGTTGTA
TCTCCTACACCACCTGTTGAGTGCTTATCTACTTTGACACCTTTAATATCACTCAAATCTAT
CATATCACCAGAATTAACCATAGCCATCGTTAATGCTGCACGCTCATCATCATTCATATCT
TGGAAATAAATCGCCATTGCTAAACTTGATGCTTGGTAATCAGGAATATCCCCTTTAACA
TAGCCGTCAATAAAGAAATTAATTTCTTCCGTTGTTAGTGTATGACCGTCACGCTTTTTCT
CAATAATGTCTATCATTCTCAT

> **deoC-L2**
Function: protein coding sequence; deoxyribose-phosphate aldolase locus 2 (core genome, constant)
Best match: deoC-L2_CC005_Mu50-VRSA_BA000017.4[2265358:2266020:r]RC
Position: 054-contig_56_RC: 87 … 237; Length: 150 bp
Sequence:
TAGTCGCTCTGCTGCATATTTAACATGCGTTGGATTCACACATACAGATTTAAAATGGTAT
GCTTTCGCTTCATCGATGATTTGATCGATTTGCGTACGTGTTGACTCAGGCTTCAATAAAG
TGTGATCAATCAATTTTGCACTATTCAT

> **deoD-L2**
Function: protein coding sequence; purine nucleoside phosphorylase locus 2 (core genome, constant)
Best match: deoD-L2_CC001_MW2-USA400_BA000033.2[2216546:2217256] (completely identical)
Position: 054-contig_56_RC: 550 … 1261; Length: 711 bp
Sequence:
ATGACAAAAGGTACACCACATATTCAACCAAATGGAGTAAAAATTGCTAAAACAGTATT
AATGCCTGGCGATCCGCTACGCGCAAAATATATTGCTGATAATTTTTTAGAAAATGTTGA
ACAATTTAACGATGTACGTAACATGTTTGGTTACACTGGTACATATAAAGGTAAAGAAGT

TTCTGTAATGGGTTCTGGTATGGGTATTCCAAGTATTGGTATTTACTCATATGAGTTATAC
AACTTCTTTGATGTAGATACAATCATTCGTATCGGTTCTTGTGGCGCATTACAAGAAAATG
TTAACTTATACGATGTTATTATTGCACAAGCTGCATCAACTAATTCAAATTATGTAGATCA
ATACAATATTCCAGGTCATTTCGCGCCTATCGCTGACTTCGAGTTAGTAACTAAAGCTAA
AAATGTCGCTGACCAAATCGGTGCTACTACACACGTAGGTAACGTATTATCTTCTGATAC
ATTTTACAATGCCGATCCAACATTCAATGATGCTTGGAAAAAAATGGGTATTTTAGGTAT
CGAAATGGAATCAGCTGGTTTATATTTAAATGCGATTCATGCTGGTAAAAAAGCACTTGG
TATTTTCACAGTAAGTGATCATATTTTACGTGACGAAGCTACTACACCTGAAGAACGTCA
AAATTCATTTACACAAATGATGGAAATCGCTTTAGAAATCGCAGAGTAA

## > **dpsA**

Function: protein coding sequence; general stress protein 20U (core genome, constant)
Best match: dpsA_CC001_MW2-USA400_BA000033.2[2217377:2217820:r]RC (completely identical)
Position: 054-contig_56_RC: 1381 … 1825; Length: 444 bp
Sequence:
TTAGCTTAAGTAAGATTTAAACATCCAATTATGTTTATCTACTGATGTTTGCATACCTATA
AACATATCTTCTGATACATCATCGCCAGCATTACCAGCAATTTCGATTGCGTTTTCTAATT
GTTTTGAGATATTTGTGAAGTCTTGTGATAATTCTTCAACCATTTGTTCTGCAGAGTAACC
TTTCGCAGCTTCTTTAACAATTGATTGCTCTAAGCATTCAGTTAATGTACCTACAGGGTTT
CCTCCTACCGCTAAAATTCTTTCAGCTAATTCGTCTACATATTGGCTTGCTTCATTATATAA
TTCTTCAAATTTAACGTGTAATGAGAAGAAGTTAGGTCCTTTCACATACCAGTGGAAATT
ATGTAGCTTTGTGTAAGCTACTGTCCAGTTTGCTACTTGTTGATTCAATTCTTTTACAACAT
CTTGTTGATTACTCAT

## > **manA1**

Function: protein coding sequence; mannose-6-phosphate isomerase/locus 1 (core genome, constant)
Best match: manA1_CC005_N315_BA000018.3[2194552:2195490:r]RC (completely identical)
Position: 054-contig_56_RC: 5200 … 6139; Length: 939 bp
Sequence:
TTACACATAGCTAATCATCAATGTGAAATCGCCTTCAAAGACACTATCCAAATCTTCAGA
AGTCAAAATAAAGTTTGTACCAGTAGTCAGTTTGAAAATTTCACCATCGACAATCATTTG
TCCTTCACCTTCCAACACTGTAACTAAACAGAACTCTCTAGGCTTCATATAATTTAACGTG
CCAGAAATTTCCCATTTAACCAATGTAAAGAAATCATTCGATACAATGTGTGTACACTTA
TGGTTTTCAATAATTTCACTTTCAGGCAAAATATTAGGTAATGGTGCATTGTACTGAATAA
CGTCTAAAGCTTTTTCAATATTTAACGGTCTATCATTATATTGATTATCTTGACGATTGAA
ATCATAAAGTCTATATGTAATGTCTGACGATTGCATCGTTTCGTATGCTAATATTCCTGAA
GAAATAGTATGTACTGTTCCTGCTGGAATAAAATAGAATTCTCCAGGTTTTACTTTAATAT
ATCTAAGTATCGACTCTATCGTTCCGTGTTGAACATGATTCGCAACTTCTTCTCTAGACTC
TGCTAATGTCCCTATAACTATTTCTGCATCTTCTTCTGCATCTATAATATACCAACATTCAG
ATTTGCCATATTGCCCGTTTTCATGCTCATAAGCATAAGAATTATCAGGGTGCACATGAAT
AGAAAGTGATTCTCTTGCATCCACTATTTTAGTTAGAAGCGGAAAATCTTTGCTTGGGAA
ATCACCAAACAATTCACGATGTTCTGACCAAATACGGTCTAATGTTTGACCTTGATATGGT
CCATTAATAATCTCGCTCGTACCATTTGGATGTGCTGACACACACCAACATTCCCCAGTTG
TATCATTGTCTAATTGATATCCAAACTCACTTAGACGTTGACCGCCCCATAATTTTGTTTTT
AAAATTGGTTGTAAAAATAATGGCAT

## > **Q1Y8Q1**

Function: protein coding sequence; putative protein/contains epimerase domain (core genome, constant)
Best match: Q1Y8Q1_CC001_MW2-USA400_BA000033.2[2222571:2223236:r]RC (completely identical)
Position: 054-contig_56_RC: 6574 … 7240; Length: 666 bp
Sequence:

TTAATCCTTTTCAATTTCAAATTGAGTTAAAGCATCTTTAATGTCCTGCTCACCACTAATA
ATTTGAAACTCTTGGTGATTAAAATGATTGGATGTGACAATTTCTTTTAATACTGTCGCAA
CATCTTCTCTAGGAATTTCACCTTTACCATCAAAATATTGTGCAGCTTCTATCTTTCCAGAT
CCTGCTGCATTTGTAAGTGCCCCTGGATGTAAAATTGTATAATTCAAACCTGAACGTCTTA
AATAGTCATCAGCGTAATGTTTAGCTATTGTATATGGCTTTAAATCACCGCTATCATCAAA
AGCCTGACGTCTCGAATCATATGTTGAAACCATGACATAGTGTTTAATATTAGCCTCTTTA
CTCGCAATCATTGATTTAACAGCACCATCTAAATCGACAATAATTGTTTTATCTGCACCCG
TGTTCCCTCCAGAACCTACTGAAAAGATAACTTTATCGAATGGTTTAAACGTCTCAGTTAA
AGTCTCTATTGAATCATTTTCAACATCAACAAGAATTGCTTTCATACCTTGTGATTTTAAC
GCATTAAGTTGATCTGATTGCCTAACACCAGCAGTAAATGGTACATTTTCTTTTGCTAATT
GTTGCACTAGTAACGAACCTACACCGCCATTAGCACCTATAACCAAAATATTCAT

> **czrA**

Function: protein coding sequence; regulator of zink and cobalt transporter protein (core genome, constant)
Best match: czrA_CC001_MW2-USA400_BA000033.2[2223388:2223708] (completely identical)
Position: 054-contig_56_RC: 7391 … 7712; Length: 321 bp
Sequence:
ATGTCAGAACAATATTCAGAAATAAATACAGATACATTAGAACGCGTAACTGAAATTTTC
AAGGCATTAGGCGATTACAATCGAATACGTATCATGGAATTGTTATCAGTCAGTGAAGCA
AGTGTTGGTCACATTTCACATCAATTGAATTTATCTCAATCAAATGTCTCGCACCAATTAA
AATTACTTAAAAGTGTGCATCTTGTGAAAGCAAAACGACAAGGCCAATCAATGATTTATT
CATTAGATGACATCCACGTAGCAACTATGTTAAAGCAAGCCATACATCACGCGAATCATC
CTAAAGAAAGTGGGTTATAA

> **czrB**

Function: protein coding sequence; zink and cobalt transporter protein (core genome, constant)
Best match: czrB_CC005_Mu50-VRSA_BA000017.4[2273497:2274474] (completely identical)
Position: 054-contig_56_RC: 7713 … 8691; Length: 978 bp
Sequence:
ATGTCTCATTCACATCATCATGACCATATGCATAGTCATGTAACTACAAATAATAAGAAA
GTATTGTTTATATCGTTTTTAATAATCGGTCTATATATGTTTATCGAAATCATCGGCGGTCT
CCTTGCTAACAGCTTGGCATTACTATCTGACGGTATCCATATGTTTAGCGACACATTCTCA
TTAGGTGTTGCACTTGTCGCATTTATTTATGCTGAAAAGAATGCCACAACTACAAAAACA
TTTGGTTATAAACGTTTCGAAGTACTCGCAGCGTTATTTAACGGTGTAACGCTTTTTGTAA
TAAGTATTTTGATTGTTTTTGAAGCGATTAAACGTTTCTTTGTTCCTTCTGAAGTTCAATCA
AAAGAAATGTTAATCATTAGTATTATCGGTTTAATTGTCAATATCGTTGTTGCATTCTTTA
TGTTTAAAGGCGGCGACACTTCACACAATTTAAATATGCGTGGTGCTTTTCTACATGTTAT
CGGAGACTTATTAGGTTCAGTTGGCGCCATTACTGCAGCTATTTTAATTTGGGCATTTGGA
TGGACAATCGCCGATCCTATCGCAAGTATTTTAGTTTCCGTTATTATTTTAAAAAGTGCTT
GGGGTATCACAAAATCTTCAATTAACATTTTAATGGAAGGCACACCAAGTGATGTTGATA
TAGATGAAGTTATAACTACTATTAAAAAGGATTCACGAATACAAAGTGTGCATGATTGCC
ATGTTTGGACAATTTCAAATGATATGAATGCATTGAGTTGTCATGTTGTTGTAGACCATAC
ATTGACAATGAAAGAATGTGAATTATTATTAGAAAACATTGAGCATGATTTATTACATTT
AAATATTCACCATATGACTATTCAATTAGAAACGCCTAATCACAAACATGATGAATCGAT
TATATGTTCAGGAACACATAGTCATTCACATAACCATCATGCTCATCATCACGCGCATGT
ACATTAA

> **Q9ZB00**

Function: protein coding sequence; lytic regulatory protein (core genome, variable)
Best match: Q9ZB00_CC005_Mu50-VRSA_BA000017.4[2274740:2275831] (completely identical)
Position: 054-contig_56_RC: 8956 … 10048; Length: 1092 bp
Sequence:
TTGTTTACTTACTTTAAATCAGCATTTAAAAATGCTAAACCACAATTATTAATAACATTGA
TATATGCACTTATAGCATTTGCTGTAATTGCAGTTGTATATTTACTTGCTAATTTCCAGCTT

GCTAAGTATGCACAAACTATTGCAATTTATTCACAATTTGGGCAAAAACCACCAGTAGAT
GCATATTTAAAAGTTATCGCTGTATTACTTATAGCAGCTGTAGTTTCACTGTTTGTTTTAGT
TCAAATTTTTATCGGAATTACAAATGTTATGAAACGAGCAATGAGTCATGAAAAAGTAAA
ATTTACCGATTTATTTATTGCATTTAAAAAAGGTAATTATTTAAAGAGCGTTCTGATCGGT
CTTGTATCTATAGCAATGATTATCGTTCTTTCACTTCTGACATCATTACTATACAAATTATT
TTCTCCAGTATCAGAAATGATTATGAATTCAGTACAATCATCATATGCAGATAGTACACA
CCTGATTGGTATTGCTATTACAACTCAAAGCATCATAATCATTGTTGTTTTATTGATTAAA
GCAATTATTACTTGGTTATTGTTAATACCTATTTTCAACTTTATGACTAGTTTCGTTGAATC
AACTAACGATAAAGTAAAAACACACTTAGCTAATGGATTTAAAGCAATGAAAAATGGTC
AAAAAACATTTTTCAAATTTTTCATTGGAATACTATTATTAAACTTAATTATTATTTTATTT
AAAACACCTGTTGGTTACTTGATTTCATTTAATACGCAATCACTCTCTCAAAGTGTTGCAG
AAAATATCATAAGAGTTTATACAGTAATGACAATCATTTTATTCGTTGTTATTCATGCAAT
CATTTTAATGGGTATCGTTCAATATTACTTAAAACGTGGTCAAAAAATTACTAAAGATAA
AGTAAAAACAGCTGACAAGGATAAAAAAGTTGTCACTGAACCAAAAAATACAAAAGTAG
AAAATGGTAAAGTGACAACATCTGTTGAAACAAAAACAGAAAAAGCACAAGATTCATTA
AATGATAATACAACAAAAACGATGACTTCAGATAAACCAGAAGACAATCAACCAAAATA
A

> **A8YYB5**
Function: protein coding sequence; putative protein (genomic island)
Best match: A8YYB5_ref_CC001_MW2-USA400_BA000033.2[2226480:2226560:r]RC (completely identical)
Position: 054-contig_56_RC: 10480 … 10561; Length: 81 bp
Sequence:
TTATTTTTTATGGTCTTGAGTACTAATCAATACTAAACCAATGATTGTTAATACAACCATC
AGCATTTCAAAATCAGACAT

> **rli28**
Function: noncoding RNA Listeria sRNA rli28 homolog, locus 3 (core genome, variable)
Best match: rli28_L3_CC001_MW2-USA400_BA000033.2[2226487:2226667:r]RC (completely identical)
Position: 054-contig_56_RC: 10487 … 10668; Length: 181 bp
Sequence:
TTATGGTCTTGAGTACTAATCAATACTAAACCAATGATTGTTAATACAACCATCAGCATTT
CAAAATCAGACATGCTAGTCCTTTCTAGGAGTAACAACTATAACCATAGGCATCACCCCT
TTATTAAGAGATTAGCCACCATCTATCCAACTTGCTCAATAAATATTATAACATGCACAC

> **res**
Function: protein coding sequence; plasmid resolvase pla
Best match: res-COL_trunc_CC005_JH1_CP000736.1[2306356:2306519:r]RC
Position: 054-contig_56_RC: 10682 … 10846; Length: 164 bp
Sequence:
TCATAGTATAAGTTTTTTCATTTCTTAATCAACTCGATAAACAGTTGAGTGACATACCCTT
GTCTAACTAGCTATTTCTTCCCCTGTTATCGTTTGTTCATCATATAAAAATTTAATTTCTCA
TTTTTTATTCTCTGATAAAGAAGAGCACCAACCTTTTCGGC

> **tnpIS232**
Function: protein coding sequence; transposase for IS232
Best match: tnpIS232_trunc_CC001_MW2-USA400_BA000033.2[2226890:2227200]
Position: 054-contig_56_RC: 10890 … 11201; Length: 311 bp
Sequence:
AAAGAGAAATCGTCAGTCTATTATTGATAAGTATTATCCTATCCTCGAAAAATTACTTTCA
GACACTAGTGAACAAAAATTTTACTATAAGCGTATACTTTGGCAATATTCGAAGGAAAAA
CATGGATTAACCTGTGCATATTATACATTTAGAGCTTATATACTTAAACATGATGAATTTA
CTCCTTATATTATGAAATGTTATCAATAGATGTCGCCTAAAGATACAACAAGATTCGAGA

CAAAACCTGATCATCAAGCACAGTTTGACTGGAAAGAAAGCATTAACTTTAAAACGAAA
GATAATCAAA

## > Q6GES5
Function: protein coding sequence; haloacid dehalogenase-like hydrolase (core genome, variable)
Best match: Q6GES5_CC005_Mu50-VRSA_BA000017.4[2277864:2278733:r]RC (completely identical)
Position: 054-contig_56_RC: 12080 … 12950; Length: 870 bp
Sequence:
TTAATCAGCTAAAAAATGCTTTGCTAAATATTTTGCTGCGCCATCCTCTTCATTCGTGTAT
GCCGTTACATCTGAAGTTAATGCTTGGATTTCAGGGCGTGCATTTTTCATAGCAACTGTAT
AATGACCAAATTCAAACATTGCTCTATCATTGTCGCTATCTCCAATAACTAACGTTTCTTC
TTGATGAATACCAAAATGTGCAATCATTTCTTTAATGCCTGTACCTTTATCAGTTTGATAA
GCCATTGTTTCCGCATTAAATCTTGATGAATTTGAAACACTAATCTGTAGTTGCACATGAT
TTTGCTTTAATTCATCTCTAAATGCTGTTATTTTTTCTAAATTAGAACTGAATAGATAAATT
TTTGAATATTCACCTTCAGGAAACTTAGTTACCCAATCTATCTTACCAGCAAGCGCATCTT
GTCTTGAAGACCATTCACTATGACTTACGCCATTAATAGGATCTTGACTACGAATCATATC
TCGCATCCATGTTTCATCTTCTTTTAAAGAAACTCTATTACCTTCAAAAGGAAATACCTCA
TAATAAATTTGTTGGCGCTTAGCTAAATTAGTAATTTGTTGCACTTGAGCCAATGATAAAC
CATGCTTGAAGATAATTTCTCCATCTACTTCTCCAATTGTTCCATTTGAACTAATGATGCC
ATTAACCGCAAAATCTTGAGGTACAAGTTGATGTATTTCAGAATGCGAACGTCCTGTTGC
CAAAAATACTTTATATCCTTTCTCTCTCAATTGATTAATGACATCTTTCGTATACGTTGATG
CTTGATTATTTTCATGTAAAATTGTTCCATCCATGTCCAAAAATATTGCTTTTACGTTATCC
ATATTGCCCTCCAC

## > A5IUU4
Function: protein coding sequence; putative ABC transporter ATP-binding protein (core genome, variable)
Best match: A5IUU4_CC005_Mu50-VRSA_BA000017.4[2278789:2279571:r]RC (completely identical)
Position: 054-contig_56_RC: 13005 … 13788; Length: 783 bp
Sequence:
TTACTCTAACATTGCCATAGAAAATCGATTATTCCATCTTTGAACTGCTACATTTTTCTGG
AAAAATCGTGACATGTTTTCAGATGTCAAAATATCATCTATTAATCCTTGTTGTACACTTT
CCCCATCTTTTAATAGCAAAATTTTAGTAAAGTTACCAGTAATTTCTTCAATAAAGTGCGT
CACATAAATCATCGCAAGCGTTGGATATGAATCTGACAATGAGTCAAGTATATTCAATAG
TGACTCACGAGCAATAAAGTCTAAACCAGCTGCTGGCTCATCTAAAATTAAAACCTGAGG
TTGCCCCATTAAAGCTCGTGCAATCATCACTCGTTGTTTTTCACCTGTAGATAAATAACCA
ATATATTGTTGCGCTTTAGCAGATATTCCAACTAATTTAAGTAAATGATGTGCTTCATTAC
GTACCTCATCATCAATATCTTGATAAACACCAATTGATTTAAAAGCACCGCTTATCACCAC
ATCGATTACTCTTTCACCCTCTTGAAACTTTTCCAGTAAACTATGAGATACAAAACCTATA
TGTTGTCGTACAGTCTCTGCAGAATACCCTACCTTGCCTGGCATTTTACCAAATAGATTAA
CACCGCCAGTTGTCGCAGGCTCATATGCATTTAATATATTTAATAGCGTTGTCTTACCAGC
ACCATTCAACCCATATAATATCCATTTATCACCTTTAGCAATTTGCCAAGAAATATTTTTT
AAAATTGTTTTTCCTTGCTTCATTCGCCCAATTTGATCTAATTGAATTAACAT

## > glmS
Function: protein coding sequence; glucosamine--fructose-6-phosphate aminotransferase (core genome, variable)
Best match: glmS_CC005_Mu50-VRSA_BA000017.4[2279803:2281608:r]RC (completely identical)
Position: 055-contig_54: 86 … 1892; Length: 1806 bp
Sequence:
TTATTCTACAGTAACTGATTTAGCAAGGTTACGTGGTTTATCCACATCTAAATCTCTGTGT
AATGCTGCATAATATGAAATCAATTGTAATGCAACTACTGAAACTAATGGTGTCAATAAT
TCATGTACATGAGGAATGACATAAGTGTCGCCTTCTTTTTCAAGACCCTCCATAGAAATA

ATACATGGATGTGCACCACGTGCTACTACTTCTTTAACGTTACCACGAATTGATAAATTAA
CGTTTTCTTGAGTTGCTAAAGCAACAACTGGTGTGCCATCTTCGATTAATGCAATCGTACC
ATGTTTAAGTTCGCCACCAGCAAAACCTTCTGCTTGAATGTAAGAAATTTCTTTAAGTTTT
AACGCACCTTCTAAACTTACGTTATAGTCAATAGTACGTCCGATAAAGAATGCATTGCGT
GTTGTTTCTAAGAAATCTGTTGCAATTTGTTCCATAATTGGTGCATCGTCAACAATTGCTT
CTATTGCTGTTGTTACTTTTGCTAATTCTCTCAATAAATCAATATCTGCTTCACGACCGTGC
TCTTTTGCAACGATTTGAGACAAGATTGATAATACTGCAATTTGTGCAGTATATGCTTTTG
TAGATGCAACTGCGATTTCAGGACCCGCATGTAATAACAATGTGTGGTCTGCTTCACGTG
ATAAAGTTGAACCTGCAACATTAGTGATTGTTAATGATTTATGACCTAATTTATTAGTTTC
AACTAATACTGCACGGCTATCTGCTGTTTCACCTGATTGAGAAATATAAACGAACAATGG
TTTTTCAGATAATAATGGCATGTTGTAGACAAACTCTGATGCAACGTGTACTTCAGTTGGT
ACGCCAGCCCATTTTTCTAAAAATTCTTTACCTACTAAACCTGCATGGTAGCTTGTACCTG
CTGCAATAACGTAAATGCGGTCTGCTTCTTTAACATCATTGATGATGTCTTGATCAATTTT
CAAGTTACCTTCTGCATCTTGATATTCTTGAATAATACGACGCATTACTGCTGGTTGTTCA
TGAATTTCTTTTAACATGTAGTGTGCATAAACACCTTTTTCAGCATCTGATGCATCAATTT
CAGCAATATATGAATCACGTTCTACAACGTTTCCATCTGCATCTTTAATAATAACTTCATC
TTTTTTAACAATAACGATTTCATGGTCATGGATTTCTTTATATTCGCTTGTCACTTGTAACA
TTGCAAGTGCGTCTGATGCGATAACATTGAAACCTTCACCAACACCTAATAATAATGGTG
ATTTATTTTTAGCAACATAGATTGTGTCTTTGTCTTCAGCATCTAATAAACCTAATGCATA
TGAACCATGTAATAATGACACAACTTTTGTAAATGCTTCTTCAGTTGAAAGTCCTTGATTT
GAAAAGTATTCAACTAATTGAACGATAACTTCTGTATCTGTTTCTGAAATGAATGATACA
CCTTGTAAGTATTCACCTTTTAACTCTTCATAGTTTTCAATAACACCGTTATGAACTAGAG
TAAAACGGCCATTTGATGATTGATGTGGATGAGAGTTTTCATGATTCGGTACACCGTGTG
TTGCCCAACGTGTGTGACCGATTCCAACAGGTCCATCAAAATCGCTACTATCAGCAACTT
TACGTAATTCTGCAATACGACCTTTTTCTTTAAATACAGTTGTATTATCATCATTTACTACC
GCGATACCTGCAGAGTCATAACCTCTGTATTCTAATTTTTCTAAACCTTTTAATAATAATT
CTTTGGCATTATCATAGCCAATATAACCAACAATTCCACACAT

## > **RF00234**

Function: noncoding RNA glucosamine-6-phosphate activated ribozyme (core genome, constant)
Best match: RF00234_CC001_MW2-USA400_BA000033.2[2232027:2232243:r]RC (completely identical)
Position: 055-contig_54: 2093 … 2310; Length: 217 bp
Sequence:
TTCTGGCGATCTTATTAACTTTGTCCATTAAGTCACCCTAATGTTTTACTTAATAAGCTAA
CGAATGAGCCACATCCGGGATAGCATCCGCCGATCTATTCGATCACTATCCTCCTCGTCTA
CAAATACATATATTGCACTCTATAAAGGCCACTCATATATTAACCTTTAATCTTCAAATAC
AAATATTTATTTGCACAGGCGCTTTAACTGTACT

## > **mtlA**

Function: protein coding sequence; PTS system mannitol-specific EIICB component (core genome, variable)
Best match: mtlA_CC001-ST772_118_AJGE01000074.1[16584:18122:r] (completely identical)
Position: 055-contig_54: 2572 … 4111; Length: 1539 bp
Sequence:
ATGTCGCAAACTGAAGAGAAAAAAGGAATTGGTCGTCGCGTTCAAGCATTCGGATCATTT
TTGAGTAGTATGATTATGCCAAACATTGGTGCATTCATCGCTTGGGGTTTCATCGCGGCCA
TTTTTATAGATAATGGATGGTTACCTAACAAAGATTTAGCTACTTTAGCAGGACCAATGA
TTACTTATTTAATCCCATTATTGATCGCATTTAGTGGTGGTCGTTTAATTTATGATTTACGT
GGTGGTATCATCGCAGCAACAGCAACTATGGGGGTCATCGTTGCATTACCTGATACACCA
ATGTTACTTGGTGCAATGATTATGGGTCCACTTGTTGGTTGGTTAATGAAGAAGACCGAC
CAATTGATTCAACCTAGAACGCCACAAGGTTTTGAAATGTTATTTAATAACTTTTCTGCTG
GTATTTTAGGATTTATCATGACGATTGCAGGATTTAAAATTTTAGCACCACTTATGAAATT
TATTATGCATATTTTATCAGTGGCAGTTGAAGCATTAGTACATGCACACCTACTTCCACTT
GTAAGTATTTTAGTAGAGCCTGCAAAAATTGTATTTTTAAACAATGCGATTAACCATGGT

GTATTCACACCGCTTGGTGCAGATCAAGCTGCAAAAGCTGGTCAATCAATTTTATACACA
ATTGAATCTAACCCTGGACCAGGTTTAGGTATCTTACTTGCTTATATGATTTTTGGAAAAG
GCACTGCAAAAGCAACGTCATATGGTGCTGGAATTATCCACTTCTTAGGTGGTATTCATG
AAATTTATTTCCCATATGTATTAATGCGTCCTTTATTATTTATCGCTGTTATTTTAGGTGGA
ATGACTGGTGTTGCAACTTATCAAGCAACAGGATTTGGATTTAAAAGTCCCGCGTCACCA
GGTTCATTTATAGTTTACTGTTTAAATGCACCTAAAGGTGAATTCTTGCACATGTTGCTCG
GTGTCTTCCTTGCTGCACTTGTATCATTCGTTGTAGCTGCTTTAATTATGAAGTTCACTAGA
GAACCAAAGCAGGATTTAGAAGCTGCGACAGCTCAAATGGAAAATACTAAAGGGAAAAA
ATCAAGCGTTGCTTCTAAGTTAGTATCTTCTGATAAAAATGTTAATACAGAAGAAAATGC
TAGTGGTAATGTTAGTGAAACATCTTCATCAGATGATGATCCTGAAGCGCTATTGGATAA
TTACAACACTGAAGATGTTGATGCACACAATTACAATAATATAAATCATGTTATTTTTGCC
TGCGATGCGGGTATGGGTTCTAGTGCAATGGGTGCAAGCATGTTACGTAATAAATTTAAA
AAGGCGGGCATTAATGATATTACAGTTACAAATACTGCGATTAATCAATTGCCAAAAGAT
GCTCAATTAGTTATCACTCAGAAAAAACTAACTGATCGTGCTATTAAACAAACACCAAAT
GCCATCCATATTTCAGTGGATAATTTCCTTAATTCACCAAGATATGAAAAACTTTTAAATA
ATCTAAAAAAAGATGATCAAGCATAA

## > **mtlR**

Function: protein coding sequence; mannitol operon transcript antiterminator (core genome, constant)
Best match: mtlR_CC005_Mu50-VRSA_BA000017.4[2283862:2285994] (completely identical)
Position: 055-contig_54: 4145 … 6278; Length: 2133 bp
Sequence:
ATGTTATTGAGTACACGTGAAAAAGAAATGATAGCCCTATTGATTAAGTACCACAGTCAA
TATATCACTATACACGACATTGCTCAGCAACTTGCGGTGTCCTCTCGTACTATTCACCGTG
AATTAAAAGGTGTTGAAGCATATTTAACTTCATTTTCATTAACTTTAGAACGCGCAAACA
AAAAAGGGCTACGCATTGCTGGCGCAGATTCTGATTTAAACGATTTGAAGCAATCGATTG
CACAACATCAAACCATTGACTTATCTGTTGAAGAGCAGAAAGTAATTATTATATACGCTT
TGATACAAGCCAAGGAGCCAGTTAAACAATATAGTTTAGCGCAAGAAATTGGCGTTTCTG
TCCAGACTTTAGCAAAGATGTTAGATGATTTAGAGCTTGATTTAAATAAGTACCAACTAT
CTTTATCTCGAAAGCGTGGCGAAGGCATTTACTTGGTAGGTACTGAATCAAAGAAACGTG
AATTTTTAAGTCAATTAATGGTGAATAACTTAAATAGTACTAGCGTTTATTCAGTAATTGA
AAATCATTTTGTCTTTCATTCATTAAATCAAATCCACAAAGACTTTGTTGACTTAGAGCGC
ATTTTTAATGTTGAAAGACTATTAATGGACTACCTAAGTGCCTTACCCTACCAACTTACCG
AATCAAGTTATTTAACTTTAACTGTCCATATCGTGCTATCCATTTCACGTATAAAAAATGG
AGAGTATGTCGCATTAAACGATGATATTTATGATTCTGTACAAAACACATTTGAACACAA
AGTAGCAAGCGAACTTGCTGATAAACTTGGTCAAATATATGACGTCACGTTTAATCAGGC
AGAAATTGCTTTCATTACTATCCATTTACGTGGAGCTAAACGAAAAAATCTTAATGATAC
ATCATTAAATAATCGTTGTGAAGAAAACAAAATTAAAGCGTTTGTTAACAAAGTAGAAAT
GATTTCCGGTATGACATTTGCAGATTTGGATACTTTAGTAGATGGACTGACGCTACACCTT
AATCCTGCAATCAATCGTTTGCAAGCTAATATCGAGACCTATAATCCGTTAACAGACATG
ATTAAGTTCAAATATCCAAGACTATTTGAAAATGTAAGATTAGCTTTAAATGATTGTTGG
CCTGATTTGATTTTTCCAGAGAGTGAAATTGCTTTTATAGTTTTACACTTTGGTGGCTCGA
TTAAAAACCAAGGTAATCGATTTTTAAACATATTAGTCGTTTGCAGCAGTGGTATGGGAA
CTAGTCGTCTATTATCAACTCGTCTAGAGCAAGTTTTTAGTGAGATTGAGCGTATTACACA
AGCATCAGTCAGCGATTTGAAGTCACTAGATTTAAGTCAATATGATGGCATTATTTCTACT
GTGAATTTAGACATCGACTCCCCCTATTTAACGGTAAACCCATTATTACCAGATAGTGAT
ATCAGTTATGTCGCACAGTTTTTAAATACAAAGTCTACGTTCCAAGAGACGCATGATAAA
TCATCAAACATGATTGATAAGGATGATGTTCATGTTGAAACGAAAGATGTTGATGGCAAC
ACATCTTTTGAAAATGAACAAACTTCATACTTAACTTCAGTTTTCGAAAAACATTTAAGTG
ACGAAAAATCAGAACAATTATTGCATCATATGCGTTCGGGTTTAACTTTGCTTGATTCAGT
GAAAATAGTTAGTACCGAAGTTAAACAGTGGCAAACATATATCGCAGATTATCTATATCA
ATGCGATGTAATAAACGATCCAACGTCATTCGCTGAACTACTAGAGCAACGATTGATTGA
CAATCCAGGATGGATATTAAGTCCATATCCTGTTGCAATACCACACCTAAGAGACAATAT
GATTAAACACCCTATGATTCTAATCACAGTTTTAGAAGAACCGTTAACATTGCCTAGTATT
CAAAATGACAATCAAACAATTAAATATATGATTTCCATGTTTATTCCTGACAATGATTTTA
TGGCATCACTGGTAAGTGACTTGTCCGAATTTTTAAGTTTGAAATTAGAATCTATTGATAC

TTTTATGGAAAATCCACAGGAACTTGAAACATTATTAAGAAACAAATTTTTAGAACGAAT
TAAAAAACAATTTATTTAG

> **mtlF**
Function: protein coding sequence; mannitol-specific phosphotransferase enzyme IIA component (core genome, variable)
Best match: mtlF_CC005_Mu50-VRSA_BA000017.4[2286006:2286440] (completely identical)
Position: 055-contig_54: 6289 … 6724; Length: 435 bp
Sequence:
ATGAGCGAATTATTTAGTAATGACAATATCTTTTTAAATGTAAATGTTAACAGCCAAAAT
GAAGCAATTGAAAAAGCAGGTAAAGCCTTAGTTGATAGTGGTGCTGTAACAGATGCTTAT
ATTCAAGCAATGAAAGATCGTGAGCAAGTTGTATCAACATTTATGGGAAATGGCTTAGCA
ATTCCTCATGGCACAGATGAAGCTAAAACAAATGTGATTCACTCAGGTTTAACATTATTA
CAAATCCCTGAAGGCGTTGACTGGGATGGCGAAGTAGTTAAAGTTGTAGTGGGAATCGCT
GGTAAAGATGGCGAACATTTAGACTTGTTATCTAAAATTGCAATTACATTTAGCGAAGAA
GAAAATGTGGATCGTATCGTTCAAGCAAAATCTGCAGAAGAAATTAAACAAGTATTCGA
GGAGGCAGATGCATAA

> **mtlD**
Function: protein coding sequence; mannitol-1-phosphate 5-dehydrogenase (core genome, variable)
Best match: mtlD_CC005_Mu50-VRSA_BA000017.4[2286440:2287546] (completely identical)
Position: 055-contig_54: 6723 … 7830; Length: 1107 bp
Sequence:
ATGAAAGCAGTTCACTTTGGTGCTGGTAACATAGGTCGTGGTTTCATTGGTTATATTCTTG
CAGACAACAATGTTAAAGTAACATTTGCAGACGTCAATGAAGAAATCATTAATGCTTTAG
CTCATGATCATCAATACGATGTTATTTTAGCTGATGAGTCTAAAACAACGACGCGCGTGA
ATAATGTTGATGCAATTAATTCAATGCAACCTTCTGAAGCGTTGAAACAAGCAATTCTAG
AAGCTGATATTATTACAACAGCTGTTGGTGTTAACATACTACCTATTATTGCTAAATCTTT
TGCGCCTTTCTTAAAAGAAAAAACAAACCATGTTAATATTGTTGCTTGTGAGAATGCTATT
ATGGCAACTGATACATTGAAAAAAGCAGTACTTGATATTACTGGCCCTCTTGGTAACAAT
ATTCATTTTGCTAACTCAGCAGTTGATAGAATTGTACCATTACAAAAGAATGAAAATATA
TTAGACGTTATGGTTGAGCCATTTTACGAATGGGTTGTTGAAAAAGATGCATGGTATGGT
CCAGAACTAAACCATATTAAATATGTTGATGATTTAACACCATATATTGAGCGTAAATTA
TTAACTGTGAATACAGGACATGCATATTTAGCGTATGCTGGTAAATTTGCAGGTAAAGCT
ACCGTATTAGACGCTGTAAAAGATAGTTCAATTGAAGCTGGCTTACGCCGTGTTTTAGCT
GAAACAAGTCAATATATTACTAATGAATTTGATTTTACTGAAGCGGAACAAGCTGGTTAT
GTTGAAAAAATAATAGACCGTTTCAACAATTCTTATTTATCTGATGAAGTGACACGTGTC
GGACGAGGTACGTTACGTAAAATTGGCCCTAAAGATAGAATTATAAAACCATTAACATAT
CTTTATAATAAAGATTTAGAACGCACTGGTTTATTAAATACAGCTGCATTGTTATTGAAGT
ATGATGATACAGCAGACCAAGAAACTGTTGAGAAAAATAATTACATTAAAGAACACGGT
TTAAAAGCGTTTTTAAGCGAATATGCTAAAGTTGACGATGGCTTAGCCGATGAAATAATT
GAAGCGTACAATTCACTTTCATAA

> **sasB**
Function: protein coding sequence; LPXTG-sorted surface protein B chv
Best match: sasB-CC133_ref_CC133_ED133-shep_CP001996.1[2253619:2259735:r]RC
Position: 055-contig_54: 8148 … 15594; Length: 7446 bp
Sequence:
TTATTCTTCAGTTTGTTGGTCTTTCTTAGTGAATCTTCTAATTAAGAATGCCATACCTGCAC
CTAGAGCTAATTCAGCATATGGTAAATCGTCATTATGTGACATACCAGTATCTGGTAAAG
TTTTAGCTTGTTGTTTAGGCTTATTAACTTTTCCTTGTTGGTCTGATTTTGCATTTGCTTTGT
TTTCTTCAGGCTTAGTTACATTAAGCATATCTTGATGCATACTATGACTACCATTTGAAAC
TGTTGCTGGAGATGCATTGGCACCGTCGTTTTGCGTAGCTTTATTGTTTGCAGCTGAACCA
ACTGATTTTTGCGTATCATTAGTTTGCATATCTTTAGCTTTATTGTTTGCAACTGGGCCAAC
TGATTTTTGCGTATCATTAGTATCTGCTGTTGCCGTATCATCTTTTTGGCTAACATTAGTTG

AAGTCATTTTATCTTTTGCTTCAGAAGATGCAGATGTTGATGGTTTATTCGAAACTTCAGT
ATCAGCTTTGCTTGGCGATTTATCTGCTTCGTTAGATGCAACGTTAGTTTCAGACTTAAGT
TGTCCTGCATCAGTTTGATTTGTCGTACTTTCTTCTTTATCTTTTGATGTATTAGAAGGTAC
ATTTGGTTCTGTTATGTCTGCTGAAGGCAATGTTTCAGTTGTTGATTCAACCATACTTTGA
TTTGTTGAATCTCTACCATCTTTTTCTGCCTTAGCTTTATTTTCAGATTTTGGTTGTGCAAC
CTTGCCATTAGTTGATTGAGATTCAGCACTATTATTTACTTCAGCATTTTGTTTTGAATCAT
TTACAGATGCATTATCTTTACTATCAGCAGATGATGCTGCTTCTGTGCTCGCAGTTGTTGG
AGCCGTTGCTGTTGATCCTGTTGGTGCATTCTCGTTTGTTGCTGTAGTTGTACTATTGTTAT
TTGTTGTGCTTTCTGCTGGCGTTGCATTATCTGTTTCTGTTACAGGTTTATCAGTTGTGCCG
TTATTAGTTGATTCTACTTCTGGTTTACTAGTTACATCGTTATCCATTGTCGGACTGTTTGT
TGATGCATCTGCACTAGAATTGTTATTAGCTTGCGGTTTATCCTTTGCACCATCAGTTGCT
GATGTTACTGTTGTTTCACCTGTTGCCGCATCACTATTATTTGGTGTTGTCGGAGAAGCGT
CTGCTTTGCCATTATCTGTCGTCTCAGATACGTTAGGTTGTCCAGTGTTTTCTGGTGTTGCA
TTAGCATTTGAATTTGCTGTTGCATCATTATTATCTGTACCATTATTAGTATCATTAGCATC
TGGATCATTCTGAGGCACAATCGCTTCAATTGCAGGTATCGTTACATTTTGTAATTCAGCA
ACCTCTGTATTTGTTTGTGTTTTATCTAATTTATCAGCAAATCTGTCAAAATATCTACCTAA
ATCTGTACGTGCAATTTCTTTCGCCGATGCATCTGCATCTGCATTTTTAATTATTTCTATTT
GCTTGTTAACCACTTCTCTAATTGCTTCCAAAGCATTTTTCTTAACTACTGGATTAATACGT
TGTGCTTTAAGTTGTTCAAGCGCACTATTTTTGACAGTAGCGATTTCTGCATTTGTAGTTT
GATCAGAAATATCTTCAGTTGCTTTTGATAAAATGTCTTCTAAAGCATTCGTAAACGCTTC
TTTTTCTTCAGTTGTAGCATCAGCGTCGACATTTACACCTGCTTCAATCTGGTCTAGTGCA
GTTTCTAATTCTTCGATAGCTTTTTGTTTTTCTGTTGAGTCAATTTGAATGTTATCAAATGC
CTCAAGTCCTTGAGCTTTCGCTTTTTCAACTTCAGCAGTTGTTGTTGCATCAGTAATACCTT
GTTTAGCTTGATCTGTAATTTGTTTAATCATTGCTAATGCTTCAGTTTTTTCTTCAGCAGTT
AACTGGTCACTATGATCAATTGATTCTTTCGTATCTTCTGCTTTAGCTTCAATAGCTTGGTT
CGCTTTAGGTTTAACAGTAGCATCTACTTGAATAGTATCAATTGCTGCTTTACCTTGTGTT
AATGCATCATCAACGTCACCATTATCCACACCATTATTAATGTTTTCTAATGCAGTTTGAA
CATTTTGGTCAACTTGCTTAATTGCTTGTTGCTTTTCATCTTGTGTTGCATTAGTGTCAGCT
GAAATATTATTTTTCTTCTGATCTGCATAAGCATATAAATCTGTTGTAGCTGATTCTTTTTT
ACCTGTTGGAATTGTGTAATCGTTAATATTATCTAAGTCATTATGAATTTGAACTTCAATG
TCATCTTTAGAAGTAGCTTGATTAACATTTTGATCCGCAGTTTGTTTTAATTCAGCAAGTT
TTTGTTTCGCTTCAGCAATTTCACTTGAAGTCGATGCGTTAGAGTTATCCGCTTCGTTTACT
TTAGCATTATATGCATCTTCAATTTTAGCTAAAGCATCTTTTTTGTACTCACTAAATGTTTT
AACTGCATTAATTTTAGCTTTTCCTTCTTTAACTGCATTATCAACATATTCATTTGTTGATG
ATTGATCGACATTTGTTTTCGCTTGATTTAATTCAGTATCAACTTCTTGTTTTGCATCATTA
ATTTCTTGTTGTGATGCATTTGGTGTTTGTTCTATTTGTGTTTTCTTATCAGCTGCAGCTTG
ATCTAATTCTTTTTTACCTGCTGGTTTCTTAACAGGATTTGCATGAAGTTGTTGAACTTTTT
GTACTGCTGTATCTTTAGCAGTAGTTACATCACCTGTAGTAGTTGCTGCATTAATATTATT
TAAACCTTCTTCATATGCTGCTCTAACTGGTCCAATATCGTTACCTTTTTCTTCATTAGTAG
TCTCATTATTATTAAGTATTTCAGTTATTTTATTTTGCATTTCAGTTAGCAATTCTGCTCTT
GCATTCGTTTTAACATCTGTTGATGCTTGAATTGGGTCAATTGCTTGAATTGCATTATCTTT
TGCAGTGTTTACATCATCGATTGACTGTGCATTTTCAATATTTTGATTACCTTGTGTTAATT
GTGCGTCTACTTGTTGATTTGCTTGTTCTTTTTCTTCAGTAGTCGCATCTGCAGTTTGTGCA
ATAAGCGCTTTTTGTTCGTTTGCTTTTGTTGCTAATTCATCTTTCGCAACATCTTTAATTGT
TGTATCTGCAGTAATACCTTGAATATCAGCAACTGCTTGATCTTTAATTTGCGTAACATCA
TTAGTTGTTTGTGCATTTAAGATATCTTGATACGCTTTTTCTTTAGCTTTTAAAACTAAATC
TTTTGCTGCATTTTTCTCTTCAGTTGTAGCGCCAGTTGTATTATCAATTGCTTGATTTTGAG
TTGTCACAGCTTGATCAACTTCATTTTTCGCATTCGATTTAACTGCTGTTGCTGGTTGTGTG
CTTTGAATTGAATTCTTTCCAGCGTCTTTCGCTGTATCTACACCATTATCATCAGTTGCAGC
TGTAATATTATTTTTCGCGTCTGTAACTGCTGTTGCTAATTGTTGAATAGCTGCTTCTTTTT
CTTCATTTGTAGCGTTCTGATCATTATTGATAACATTTGTTTGCGTTGCTTGTAATTGATCA
ATCTCATCTTTAGCCGCTTGTTTCTTAACAACTTTTGGTGTTACCGCATTAATTGCTGCTTC
TGCATTAGCTTTCGCTTCATCAACTTGTGCGTTAGTAGTTGCTGCTGAAATGGCTTGATTT
GCTTTACCATTTTCAGTATTTGCTTCTGCATCAGCTGCTTGTTTTTCTTCATCTGTTGCATCT
GGTGTAGCTTGAATCTCTTGCAATTTGTTATTTAAAATTGCTGTGATTTCATTACGTGCAG
TTGCTTTTTTATTAACTGTTGGTGTTACTTGATCAATACTAGTTTCACCTGTCGTTTTCGCT

TGGTCTACATCATTTTGACTATTAGCAGCTTCAATGTTGCTATTTGCTTGTGTCACAGCATT
ATCTACGTCCGCATTAGCCGCTGCAATTTCTTCAGCAGTAATATCTTGCGTTTGAGCGATT
GCTGTTTTACGTTCATTCGCTTTCGTAGCAATTGCTTGTTTCGCATTATCTTTAGTTGTTGT
TGCTGGCTGAATCGCTTCAATTTTAGCAATTTCTGCATTTTTAGCCGCTTCAACTTCCACAT
TTGAATGAGCTGCATCTATTGCTGCATCAGCAGCCGTTTTTTCAGTTTGAACTTGTTGTTT
AGCAGCTTCTTTTTCTTCTGTTGTTGAACCGTTATTAGCATCAATTGCTGTTTCTTGAGCTT
GTACTTTATCTGCAATTGCTTGTTTTGCTGCTGGTTTAACATTTGCATCTGGCGTAATTGCA
GCTATTGTAGCTTCATTAGTAGTTTTTGCATTATCTACATCTGTATTTGCTGTAGCATTATC
TATATCAGCGTTTGCAGTAACTACTGCTTGATCAACTTTATCTTTTGCAGCTTGTTGTTCTT
CAGTAGTCGAATCATTCATTGCTTCAATTGCAGTTTTACGTTCACTTGCTTTTTGAGCGATT
TCCGCTTTAGCATCCGATTTCTTAGTTGTGGCAGCTTGCACTTGATTAATTGCAGCAATAC
CATTGTCTTTAGCTGTTGTTACATCACTGTTTGTATTTGCAGCATCAAGATTTGTTCTTGCT
TCTTGCTTTTTAGCATCTAATTCTGTATATGCAGCTTCTTTTTCTTCTGTAGTTGAAGCATT
GCTATTTTGGATTTCTTGTTTACGTGTATTATATGCGTTTTCTACTTCTGTATCAGCTGCAG
GTTTAACTTTTGCTTGTGTTTGAATTGCATTGATTTTATCTAATACTTTTGCTTTAGTATCA
GCCACTTCTTGTTGTGATTTAACAACTTGGATGTCATGTAAACCTTCTGTTTGAGCTGCAT
CTACTGCTGCATTTGCCTCTGCTACTTCTTCATCAGTTGCATTTGAAACTGTAGCATTTTGC
GCTTTTCTAGCTGTAGCTGCTTGTCTAACTTCTTTATAAGCATCCATTTTAGTTTGAGTTGA
ATCCGTAATATTCTCAATTTTGAATATTTCTTCGTTTTTAACGCGTTCTAAATCTTGTGCAG
TCGTTGTCGCTTCAATTGCTTTAACACCAGAAACTTTAGCTGCATTAATACGTTCAATAGC
TTCCGCTACTTCTTCATTTGTTGTATCAGGATTTAAAGGTGCTTGATCAATTTGTGCTTGAA
CAACTTCGTCAAATTCTTCAAGAGCTGCTGCTTTAGCTGTTGTAGCAGGTGTAGTTTGATT
AATATCATTGATTGCTTTTGTTTTAATGGCTTCTACTTGTGCATTTGTTGTTGCTGCATCAA
TATCTTTAATTGCCTTTGTTTCAATTTTACCAATTTTATCATTTGCTACATCTTTTTCATCTT
GTAATGATGCATTTGACTTTTTAATTTGTTGTTTACGAGTTGTAACTGCTTGGATAATATCT
TGTTTCGCTTGAGGTTTAACTGTTGGTGTAATCACATCTTGTTCTAACACTGCGATACCAT
TATCTTTTTCAGTTGTGACACCTTGTGATGTTGTTTGTTTATTAATTTCTGCGATTGCATTA
TTTTTATCATTGTTAATTTTAGTCACAAGCGCTGCAACTTCATCTTGCGTAACTTTTTTACT
TTGTTGTGCTGCTGTAACCTTTTCTTGAGCTTTAGTATCTAATTCAGCAATTGCTGCTTGAT
TATCAATTAATGCAGCTTGTAATTTAGTTACTAAACCGTCAATATCCGCTTGAGATGCACG
ATTTGCAGTTTCAACGTGATTCGCATCTTCATTTAAAATAGTGTCTGCTTGTTGTTTAAGTT
TATTGTATTCTGCAATTGATGCTGTTGTGTAATGACTATTATCAACTTGTGAGTTTACTTGT
TGTTGCAACGCATCTTTGTTCATTTCAACCGCTACTGAAAATGGATCTGCAGTTAGTGTAA
CTTCTGGTTGCGCATTATTAATTACAACATCTGAAGCAGTACGATATGTTAATTTTTCATT
AAAATCAATATTTTTAGGTGTATCGATATTCGCAACATTAACTTTATATGATAATTTTAAA
GATTTATCTGGGAATAAAACTTCTTTAGTGTGTGTACCACGTGCCGTTGTCACACCTTGGC
TTGTAAATGTAACTTTATTTGCAGTTTGATCATAATTAACAGTCATATTTTTCAATACTGT
ACTGTCTTCATTACCATTAGGGAATGTTGTAGTTAATGAATCATTAACGTAAGTTACACCT
TGTGGTAATGTTACTTCATATTTAAATTGATCTGCTTTTAATGAAGCACCAAAATTACCAT
TGTTACCGATTTCAGTATTAATAGTATATTCTTTATTGTTAGTTGCTTTCGGTGCTAAATCT
CTATTTTTGACATATAAATGTGATCCTGAGAATAGACCTACATTATCAATGAATGAATAG
TATTTATAACCATCTTTATTTGTTGTAATTCTTGAAGCATCAGCAACTGCTGTATTGTCAG
GAATAAATTGTAAATCTAAACGATCAGCATCAGTTGGTACTTTAAATAAATGTGCAGTAC
CAGTCTTAGCAACATTTACAGTTGCTAATTCAGTATTTTTCTTAGCATCTTTAATAACTAA
ATTTGTTGCACCTTGCTTACTATTCGTTTGCATAGTAGTAAAGTTAATAATTAATTCTGAA
TCTGGTTTTACATTTACAGTTTTTGTAATACCGTTAAAGTTACCATGATCTGTAGAATCAT
TTGCATTCACACGACCTAATGCAGCCACGTTTCCTTTAGCTTGATAGTTTTGAGGGTTATT
CTTATCAAACATATCGCTTCGTCTTAATTCTGAGTTAACGAAACCAATCTTACCGTTGTTA
ATTAATGAATAACCATTTACTTTATCTGTAACAGTTACAGTTGGATCCTGTCTATTCTCAT
CTGTTGATATGGCAGGATCATCAAATGTTAATGTCGTATTAATACTGCCTTCACCAGTATT
GCTAGCATTTGGATCTTGAGTTTGTGCGTTTGCTGCTACAGGTGCTGCTGGTTGCGCTGCT
GCTGGAGCATTCGCTGGCTGTGTTTGATTTGCCGGTGTTGCATTATTATTAGGTGTTGCTT
GGTTATTTCCTTGACCTGCTTGGTTTGCCGGTGCTGCTTGATTTCCAGGTTGTGCTTGTGCA
GCGTTATTCGGATCAGCTTGATTACCTTGTCCAGCTGGTTGTGCTGCTCCTCCTGCTGGAT
TAGCCTGTCCACCTTGGTTTGCTGGCTGTGCTGCAGGATTAGCTTGAGCACCAGCATTTGC
GTTAGGCTGTGTATTGGCATCAGCTGGTTGTGCTGGTTGATTTTGTGCAGGCTGATTTTGC

TCTGCTGCAGACGCTGTTGTCGGGTTAGTAGATATAAAAGTAACAGTGGCAATTAAAGCT
GAAAAAATACCGACATTAAATTTTCTGATACTAAATTTTTGTTGTCTGAATAAATTCAT

## > **glmM**

Function: protein coding sequence; phosphoglucosamine mutase (core genome, constant)
Best match: glmM_CC005_Mu50-VRSA_BA000017.4[2295588:2296943:r]RC (completely identical)
Position: 055-contig_54: 15871 … 17227; Length: 1356 bp
Sequence:
TTATTTATCTAATCCCATTTTATCTTGAACCACATCAGCTATTTGTTGTGCAAATCTTTCAG
CATCTTCATCAGTTGCTGCTTCAACCATGACACGAACTAATGGTTCTGTTCCAGAAGGTCT
TACTAAAATTCGACCTTCTCCATTCATTTCTACTTCTACTTTAGTCATAACTTCTTTAACGT
CAACATTTTCTTCAACACGATATTTATCTGTTACGCGTACGTTAATTAATGATTGTGGATA
TTTTTTCATTTGTCCAGCTAATTCACTTAGTGATTTACCAGTCATTTTTATTACAGAAGCTA
ATTGAATACCAGTTAATAAACCATCACCAGTTGTATTGTAATCCATCATAACGATATGCC
CAGATTGTTCTCCACCTAAGTTATAATTACCGCGACGCATTTCTTCTACTACATATCTGTC
GCCAACTTTAGTTTTATTAGATTTAATTCCTTCTTGTTCAAGCGCTTTGTAAAAACCTAAA
TTACTCATAACAGTAGAAACAATCATGTCATTATTCAATTCTTGATTTTTATGCATTTCTTG
ACCAATAATAAACATAATTTGGTCACCGTCAACGATTTGACCATTCTCATCTACTGCTATG
ATTCTGTCTCCATCGCCGTCAAATGCTAACCCAAAATCACTTTCAGTTTCAACTACTTTTT
CAGCTAATTTTTCAGGATGTGTAGAGCCACATTTCTCATTGATATTATATCCATCAGGACT
ACATCCAATTGTTTCAGTATCTGCTTCTAAATCACCAAATAAGAATGGCGCTAGTGATGA
TGTTGAACCATTTGCACCATCTAAAACAATTTTCAAACCTTCAAAGTTAACATCTACTGTT
GATTTTAAATAGCTCAAATATTTTTGTGCCCCTTCAAAGTAATCTGAATAATGTACAATAT
CATTGCCAACTGGTCTTGGTAATTCTGGATTTTCTTGATCCAATAATGCTTCAATTTCATTT
TCTTGTTCATCTGATAGTTTAAAACCATCTGATCCAAAGAATTTAATACCATTATCTGCAA
CTGGATTATGAGAGGCTGAAATCATTACACCTAACTCTGCACCCATATCGCGTGTTAAAT
ATGCAACACCTGGTGTTGAAATAATACCTAATCGCATCACTTCTGCACCAATTGAAATCA
AACCAGCTATTAATGCTGATTCTAACATTTCACCTGAAACTCTAGTATCGCGACCTACAA
GTACACGTGGGTGTTTTTCACCTTTATTATGTGCTAGAACATAGCCACCGTATCTTCCTAA
TTTAAATGCCAATTCAGGTGTTAGTTCTTGGTTTGCGACACCTCTTACTCCGTCTGTACCA
AAATATTTTCCCAT

## > **dacA**

Function: protein coding sequence; cyclic di-AMP cyclase (core genome, constant)
Best match: dacA_CC005_Mu50-VRSA_BA000017.4[2297904:2298713:r]RC (completely identical)
Position: 055-contig_54: 18187 … 18997; Length: 810 bp
Sequence:
TTATTTCACACCTTTCTTTTGAAAGCGTGTGCCAAACCAATGTTCAGCAAGCAACTCTTCA
AAAATTTCGTTTGAAATGTCTCGTCGTAATTTTCCATCAAATGTTACCGAAATATCACCAG
TTTCTTCAGATACAATAACGGTAAATGCATCAGATACTTCTGAAATACCAACCGCAGCTC
TATGTCTTGTACCCAAACTTTTAGATATCTTAGGACTATCAGACAATGGCAAATAACTTGC
TGCTGCTGCAATCTTAGTGCCTTGAATAATCATTGCACCATCATGTAAAGGTGTGTTAGGT
ATAAAGACATTAATTAAAAGTTCTTGCGAAATATTTGAATCCATTGCAATACCTGTTTCAA
TATAATCTTGAAGACCTGTTTCTTTTTCAAAGACAATTAATGCACCTATACGTCTTTTAGC
CATATATTGCACAGCCTTTGAAACCGATTGAATCAATTTCTCTTCATCTTTACTATACGTA
TTAGAAGTATAGCGTTTTAAAAAGCTACCTCTACCAAGTTGTTCTAACGCACGTCTAATTT
CTGGTTGGAATATTACTATTAAAGCTAATACCCCCCATTGAATAACGATATCGAATAATTT
AGATGTTGCAGTCAAGTTCAATATCATACTTATCTGCTGACCAATAACAATTACTAATATC
CCTTTAAGTAATTGTATCGCTTTAGTTCCCTTAAAGACCGTGATGAGAAGATAAAGTACA
TACCAAACTATCAGTAAATCAAGGATACTCGTTACAATTTTTAACGTACTGAGGTTTTGA
AAAAAGTTGGAAAAATCCAT

## > **argI**

Function: protein coding sequence; arginase (core genome, constant)

Best match: argI_CC005_Mu50-VRSA_BA000017.4[2298902:2299810:r]RC (completely identical)
Position: 055-contig_54: 19185 … 20094; Length: 909 bp
Sequence:
TTATAATAAAGTTTCACCAAAAAATGTTCCAACTAATGAAACCGCTTGTTCAGCAGTATG
ATTATTACTGTCAATCAATGGATTTACTTCAACTAAATCCATTGAGGAAATTAAATGTGAT
TGATGCAGTAATTCCAATGCAAAATGGCTTTCTCTATAACTAAGACCACCCAAAACTCTA
GTACCAGTGCCTGGCGTTTCAAGCGGATCTAAAGCATCAACATCTAAAGATAAATGAACG
CCATCAACATTGCGTGACTTCAAATATTCTATTGTATTTTCAATTACTTCCTTTATCCCTAA
TTTATCAATATCTGACATAGTAAATGTTTTAATATTATGATCTTTGATAAATTGTCTTTCAC
CTTTATCTAAATCTCTCATACCAATTAGTACGATGTTTTCTGGCTTGATTACATTACTATTT
AATTCTAAAAGTTCTTTGGGACCTTCGCCTGTCAAAATCCTTAGAGGCATACCATGAATAT
TTCCACTTGGTGACTCTTCAGGTATATTTAAATCACCATGTGCATCATACCAAATAACACC
TAAATTATTATAATGTTTACTTATTGCTGATACTGAACCTACCGCAATAGAATGATCACCA
CCAAGAACTAGAGGAAATCTGTTATTTTCAATTGATGCTGAAACCTCTTTATTTAATTTTT
GATTAACATCTATAATTTCATCATAATTTCTTAATCCTTTTTGTTCACTATGAAATTTTTCA
ATGTTCACAGCAGGTACCTTAATATCCCCCTTGTCATATACATCAAGGTCTAATTGCTTTA
ATCTTGAAATTAATCCAGCATATCTAATTGCTGTTGGTCCTAAATCAACACCTAATTTTCT
TTGTCCAAATGTTGATGGTGCACCTATAATATCAATTGCTTTTGTCTTTGTCAT

> **tRNAlys**
Function: tRNA tRNA lysine
Best match: tRNAlys_CC001_MW2-USA400_BA000033.2[2250231:2250306:r]RC (completely identical)
Position: 055-contig_54: 20228 … 20304; Length: 76 bp
Sequence:
TAGTGAGCCATAGAGGATTCGAACCTCTGACCCTCTGATTAAAAGTCAGATGCTCTACCA
ACTGAGCTAATGGCTC

> **Q5HDM5**
Function: protein coding sequence; LysR family transcript regulator (core genome, variable)
Best match: Q5HDM5_CC005_N315_BA000018.3[2388202:2389086]RC (completely identical)
Position: 056-contig_190_RC: 88 … 973; Length: 885 bp
Sequence:
TTAAAGAGCCTCTAAAAGTCCATATAAATAATCTTGGCATTGTTGTACGAATGTATGTACT
[truncated: 388,255 more chars]
